# Illuminating the bacterial microbiome of Australian ticks with *16S* and *Rickettsia*-specific next-generation sequencing

**DOI:** 10.1016/j.crpvbd.2021.100037

**Published:** 2021-06-11

**Authors:** Telleasha L. Greay, Kimberly L. Evasco, Megan L. Evans, Charlotte L. Oskam, Paola A. Magni, Una M. Ryan, Peter J. Irwin

**Affiliations:** a College of Science, Health, Engineering and Education, Murdoch University, 90 South Street, Murdoch, Western Australia, 6150, Australia; b Western Australian State Agricultural Biotechnology Centre, Murdoch University, 90 South Street, Murdoch, Western Australia 6150, Australia; c Executive Consultant, EpiSeq, PO Box 357, Kwinana, Western Australia, 6966, Australia; d A/Senior Scientific Officer, Medical Entomology Unit, Department of Health, 1A Brockway Road, Mount Claremont, Western Australia, 6010, Australia; e Cardio Respiratory Sleep, Level 1, 52-54 Monash Avenue, Nedlands, Western Australia, 6009, Australia; f Centre for Biosecurity and One Health, Harry Butler Institute, Murdoch University, 90 South Street, Murdoch, Western Australia 6150, Australia; g Murdoch University Singapore, Kingʼs Centre, 390 Havelock Road, Singapore, 169662, Republic of Singapore

**Keywords:** Ticks, Microbiome, NGS, *16S*, Novel species, Australia

## Abstract

Next-generation sequencing (NGS) studies show that mosquito and tick microbiomes influence the transmission of pathogens, opening new avenues for vector-borne pathogen control. Recent microbiological studies of Australian ticks highlight fundamental knowledge gaps of tick-borne agents. This investigation explored the composition, diversity and prevalence of bacteria in Australian ticks (*n* = 655) from companion animals (dogs, cats and horses). Bacterial *16S* NGS was used to identify most bacterial taxa and a *Rickettsia*-specific NGS assay was developed to identify *Rickettsia* species that were indistinguishable at the V1-2 regions of *16S*. Sanger sequencing of near full-length *16S* was used to confirm whether species detected by *16S* NGS were novel. The haemotropic bacterial pathogens *Anaplasma platys*, *Bartonella clarridgeiae*, “*Candidatus* Mycoplasma haematoparvum” and *Coxiella burnetii* were identified in *Rhipicephalus sanguineus* (*s.l*.) from Queensland (QLD), Western Australia, the Northern Territory (NT), and South Australia, *Ixodes holocyclus* from QLD, *Rh. sanguineus* (*s.l.*) from the NT, and *I. holocyclus* from QLD, respectively. Analysis of the control data showed that cross-talk compromises the detection of rare species as filtering thresholds for less abundant sequences had to be applied to mitigate false positives. A comparison of the taxonomic assignments made with *16S* sequence databases revealed inconsistencies. The *Rickettsia*-specific *citrate synthase* gene NGS assay enabled the identification of *Rickettsia* co-infections with potentially novel species and genotypes most similar (97.9–99.1%) to *Rickettsia raoultii* and *Rickettsia gravesii*. “*Candidatus* Rickettsia jingxinensis” was identified for the first time in Australia. Phylogenetic analysis of near full-length *16S* sequences confirmed a novel *Coxiellaceae* genus and species, two novel *Francisella* species, and two novel *Francisella* genotypes. Cross-talk raises concerns for the MiSeq platform as a diagnostic tool for clinical samples. This study provides recommendations for adjustments to Illuminaʼs *16S* metagenomic sequencing protocol that help track and reduce cross-talk from cross-contamination during library preparation. The inconsistencies in taxonomic assignment emphasise the need for curated and quality-checked sequence databases.

## Highlights

• Bacterial pathogens identified in ticks from companion animals with *16S* NGS.
• Sanger sequencing confirmed novel *Coxiellaceae* gen. sp. and *Francisella*.
• “*Candidatus* Rickettsia jingxinensis” was identified with *Rickettsia*-specific NGS.
• Comparison of taxonomic assignments in *16S* sequence databases revealed errors.
• Modifications to the *16S* metagenomic library protocol (Illumina) are provided.

## Graphical abstract

**Figure:**
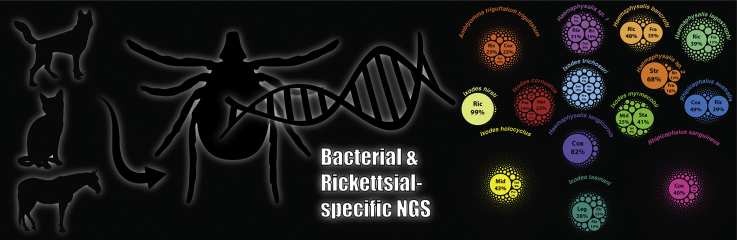

## 1 Introduction

Hard ticks (Arachnida: Ixodoidea, Acari: Ixodidae) transmit pathogens to companion animals, livestock and humans (Dantas-Torres et al., 2012). An understanding of the taxonomic complexity and community structure of a tickʼs internal microbiome is essential for the future development of microbial manipulation strategies to potentially reduce the transmission of tick-borne pathogens (TBPs). Tick microbiome studies have been performed since 2011 (Andreotti et al., 2011) using several technologies and methodologies, e.g. amplicon sequencing with the Ion Torrent (Thermo Fisher) and MiSeq (Illumina) platforms, and shotgun sequencing with the Ion Torrent (Thermo Fisher) and HiSeq (Illumina) platforms (Narasimhan and Fikrig, 2015; Greay et al., 2018a). Most next-generation sequencing (NGS) studies on tick microbiomes have been performed in Asia, Europe and North America, while in Australia such studies are more recent and limited in number. However, since the review by Greay et al. (2018a), additional tick NGS studies have been published.

Barbosa et al. (2017) studied the diversity of trypanosomes in *Ixodes holocyclus*, the eastern paralysis tick (Barker et al., 2014), and *Ixodes tasmani*, the common marsupial tick, using amplicon NGS with the MiSeq (Illumina) platform and identified co-infections of *Trypanosoma* species (Barbosa et al., 2017). RNA NGS (platform not specified) was performed on the salivary glands of *I. holocyclus* by OʼBrien et al. (2018) to screen the salivary gland virome for novel viruses. A novel (+)ssRNA *Flavivirus* species, Ixodes holocyclus iflavirus (IhIV), was identified (OʼBrien et al., 2018). Harvey et al. (2019) used metatranscriptomic sequencing on the shotgun sequencing platform Hiseq2500 (Illumina) to identify viral, bacterial and eukaryotic species. A novel dsRNA *Coltivirus* species (family *Reoviridae*), and (+)ssRNA *Flaviviridae* species, including IhIV, were also found in *I. holocyclus*. Additionally, Harvey et al. (2019) used the RNA transcript data to identify other species based on the cytochrome *c* oxidase subunit 1 gene (*cox*1) including the ticks, fungi, bacteria (“*Candidatus* Midichloria mitochondrii”, *Francisella persica* and *Kluyvera intermedia*) and a protozoan parasite (*Trypanosoma* sp.) (Harvey et al., 2019). More recently, Egan et al. (2020) targeted the V1-2 regions of *16S* with NGS on the MiSeq (Illumina) platform to detect bacteria in ticks collected from 27 wildlife species. Potentially novel bacterial species were identified belonging to the genera *Neoehrlichia*, *Anaplasma*, *Ehrlichia* and *Francisella* (Egan et al., 2020).

In Australia, the study of microorganisms in ticks has increased in response to human patients reported to have locally acquired Lyme disease-like illness (Brown, 2018). Whether these patients acquired local infections of *Borrelia burgdorferi* (*s.l.*) species has been widely discussed (Heath and Hardwick, 2011; Beaman, 2016; Chalada et al., 2016; Collignon et al., 2016; Graves and Stenos, 2017; Dehhaghi et al., 2019). A comprehensive study dating back to the 1990s on 12,000 ticks from New South Wales (NSW) found no evidence of *B. burgdorferi* spirochaetes with microscopy, culture, immunohistochemical and PCR methods (Russell et al., 1994). The cause(s) of Lyme disease-like illness in people residing in Australia remains unclear as there is no published evidence that *B. burgdorferi* (*s.l*.) species occur in Australian ticks. To date, non-endemic ticks that vector *B. burgdorferi* (*s.l.*) have not been identified in Australia.

NGS technologies have allowed the discovery of “*Candidatus* Borrelia tachyglossi”, “*Candidatus* Ehrlichia occidentalis”, “*Candidatus* Ehrlichia ornithorhynchi”, “*Candidatus* Neoehrlichia arcana” and “*Candidatus* Neoehrlichia australis” in Australian ticks that bite native fauna, humans and companion animals (Gofton et al., 2015a, 2016, 2017; Loh et al., 2017, Gofton et al., 2018). Besides the use of NGS, more recent studies have used conventional molecular methods (Sanger sequencing) to identify novel species of *Babesia*, *Hepatozoon*, *Theileria* and Sarcocystidae gen. sp. in Australian tick parasites of humans, companion animals and other animals (Greay et al., 2018b; Loh et al., 2018a, b; Storey-Lewis et al., 2018; Greay et al., 2019). Despite the recent discoveries, further basic research of tick-borne microorganisms in Australia is required. Notably, there are only two confirmed TBP of companion animals in Australia, *Babesia vogeli* (Irwin, 1989; Irwin and Hutchinson, 1991), formerly *Babesia canis vogeli* (Penzhorn, 2020) and *Ehrlichia canis* (The Department of Primary Industries and Regional Development, 2020). These infect dogs and are transmitted by *Rhipicephalus sanguineus* (*s.l.*) (brown dog tick) (Groves et al., 1975; Irwin, 1989; Irwin and Hutchinson, 1991).

A previous Australian study analysing ticks recovered from dogs, cats and horses showed that primers broadly targeting Apicomplexa were pivotal for the identification of novel parasites. In the study by Greay et al. (2018b), eight novel parasites and a novel genus and species (Sarcocystidae gen. sp.) were identified that may infect both companion animals, humans or other hosts. Despite the use of conventional molecular methods (conventional PCR (cnPCR) and Sanger sequencing), the approach allowed the identification of an exotic TBP, *Hepatozoon canis* in *I. holocyclus* infesting a Maremma Sheepdog living at Sarina, Queensland (QLD) (Greay et al., 2018b). In a follow-up investigation, the dog was also found infected with *H. canis* (Greay et al., 2018c). It remains to be determined whether this TBP is endemic to Australia. The growing discoveries of novel and exotic TBPs demonstrate the need for ongoing microbiological surveillance in ticks using state-of-the-art technology.

The present study used *16S* amplicon NGS with MiSeq (Illumina) to explore the composition and diversity of the bacterial microbiome of *Amblyomma triguttatum triguttatum*, *Haemaphysalis* spp., *Ixodes* spp. and *Rhipicephalus* spp., with a special focus on bacterial pathogens and novel species. Furthermore, a comparison of the taxonomic assignments of tick-associated zero-radius operational taxonomic units (ZOTUs) with the popular *16S* sequence databases Greengenes, RDP Classifier and SILVA was performed. To confirm whether short (~300 bp) bacterial ZOTUs represented novel bacterial species or genotypes, near full-length *16S* sequences were phylogenetically analysed. As Spotted fever group *Rickettsia* (SFGR) have highly conserved *16S*, *Rickettsia*-specific NGS assays were developed to identify SFGR and potential co-infections. Lastly, based on the caveats encountered with the *16S* metagenomic sequencing library preparation protocol from Illumina, modifications to the protocol have been proposed in the discussion. These recommendations will improve the accuracy of future microbiome studies that use the MiSeq platform for research or diagnostic purposes.

## 2 Materials and methods

### 2.1 Tick collection and identification

Ticks were collected from companion animals (cats, dogs and horses) during a nationwide tick survey between 2012 and 2015 (Greay et al., 2016). Individual specimens were stored in 70% ethanol at 4 °C before and after morphological identification based on taxonomic keys (Roberts, 1970; Barker and Walker, 2014). Specimens of *Ixodes* and *Haemaphysalis* that could not be confidently identified based on morphological keys were sequenced for species identification *via cox*1 analyses (methods are described in *Sections* 2.7, 2.8). Forceps and all other instruments used to handle the ticks were sterilised with DNA AWAY™ (Molecular Bio-Products Inc., San Diego, CA, USA) between samples. Collection locations included all Australian states and territories, except for the Australian Capital Territory. The sample metadata considered information such as collection location, ecoregion (Department of Agriculture, Water and the Environment, 2020), tick species, instar/sex, host and feeding status (unfed, fed and “pale”). “Pale” ticks refer to female ticks that were at advanced stages of egg development, potentially due to low haem content in eggs or a colour polymorphism (Perner et al., 2016; Pekár et al., 2017). The metadata are deposited in the National Centre for Biotechnology Information (NCBI) Sequence Read Archive (SRA) under the BioProject accession number PRJNA640465.

### 2.2 DNA extraction

Genomic DNA (gDNA) was used from individual ticks, most of which had been previously extracted and screened for apicomplexan parasites (Greay et al., 2018b). As a summary of the procedure, ticks were first washed with 10% sodium hypochlorite, followed by a 70% ethanol wash and finally rinsed in sterile water [250 μl of this water was added to the extraction reagent controls (ExCs)]. An ExC (*n* = 21) was included alongside each batch of gDNA extractions, and gDNA was extracted using the QIAGEN DNeasy® Blood & Tissue Kit (Qiagen, Hilden, Germany) following the manufacturerʼs recommendations, with minor modifications as described in Greay et al. (2018b). Purified gDNA was stored at −20 °C.

### 2.3 NGS library preparation and sequencing

**Fig. 1:**
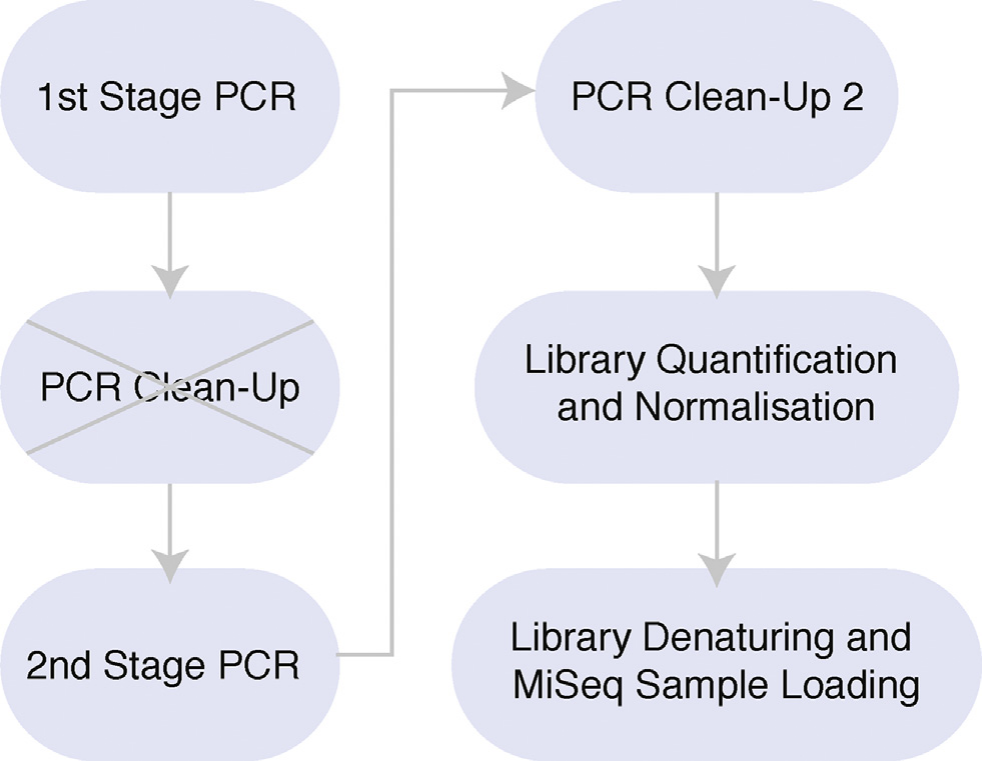
Diagram of *16S* NGS workflow with modifications used in this study. The original *16S* metagenomic sequencing library preparation protocol is from Illumina (Part # 15044223 Rev. B; Illumina, USA). The first PCR clean-up step with Agencourt AMPure XP Beads (Beckman Coulter Inc., CA, USA) was removed from the workflow (indicated by a grey cross).

*16S* NGS was used to sequence V1-2 regions of *16S* in the samples outlined in Table 1. Replicates of samples that were suspected of cross-talk or had inadequate sequencing depth when assessed during preliminary bioinformatic analyses were sequenced in subsequent NGS libraries (Table 1). The V1-2 region of *16S* has insufficient hypervariability for SFGR differentiation. Therefore, samples that were positive for rickettsial *16S* were screened using cnPCR with *Rickettsia*-specific 17 kDa (17 kDa common antigen gene), *gltA* (citrate synthase gene), *ompA* (outer membrane protein A gene) and *ompB* (outer membrane protein B gene) primers. The *16S* NGS *Rickettsia-*positives were prepared for *Rickettsia*-specific NGS to identify species and co-infections. The NGS libraries were prepared and sequenced following the *16S* metagenomic sequencing library preparation protocol from Illumina (Part # 15044223 Rev. B; Illumina, USA), with modifications to the first stage PCRs and first PCR clean-up (Fig. 1). For the first stage cnPCRs, the primers that were used for the amplification of bacterial *16S* and rickettsial 17 kDa, *gltA*, *ompA* and *ompB* genes are summarised in Table 2. A “*Ca.* Midichloria” blocking primer (MidBlocker) was used in the *16S* cnPCRs to reduce the amplification of “*Ca.* Midichloria spp.” in *I. holocyclus* samples as previously described by Gofton et al. (2015b). To note, minor modifications to the final concentration of the MidBlocker primer were applied: 3 μM final concentration was used for all samples as higher concentrations were inhibiting bacterial amplification. First stage PCR primers were modified to include Illumina MiSeq adapter sequences (Part # 15044223 Rev. B; Illumina, USA) and the cnPCRs were carried out as described by Gofton et al. (2015b). The first PCR clean-up step was avoided to reduce the risk of cross-contamination of unindexed first stage PCR amplicons (Fig. 1). Other measures employed to minimise cross-contamination of unindexed first stage PCR amplicons included the use of individually capped PCR tubes with hinged lids rather than 96-well PCR plates and not conducting gel electrophoresis of unindexed first stage PCR amplicons. No-template controls (NTCs, *n* = 25) were included in the first stage PCRs and second stage PCR NTCs [referred to herein as index controls (ICs)] were included during index PCR setup. *16S* and *Rickettsia*-specific gene libraries were sequenced using paired-end sequencing on the Illumina MiSeq platform (Illumina, San Diego, CA, USA) with MiSeq v2 500-cycle (Illumina, SanDiego, CA, USA) and MiSeq v3 600-cycle kits.

**Table 1:** Summary of the number of ticks and individual sample numbers screened with *16S* NGS from dogs, cats and horses

Tick species	Common name	Dogs		Cats		Horses		Total S no.	Total S and R no.
		S^a^ no.	R^b^ no.	S no.	R no.	S no.	R no.		
*Amblyomma triguttatum triguttatum*	Ornate kangaroo tick	5	0	0	0	12	0	17	17
*Haemaphysalis bancrofti*	Wallaby tick	1	0	1	0	2	0	4	4
*Haemaphysalis lagostrophi*^a^	–	0	0	0	0	1	0	1	1
*Haemaphysalis longicornis*	Bush tick or Asian longhorned tick	46	0	0	0	1	0	47	47
*Haemaphysalis* sp. genotype 1^b^	–	0	0	0	0	3	0	3	3
*Haemaphysalis* sp. genotype 2^c^	–	0	0	0	0	1	0	1	1
*Ixodes cornuatus*	Southern paralysis tick	4	0	0	0	0	0	4	4
*Ixodes hirsti*	Hirstʼs marsupial tick	0	0	1	0	0	0	1	1
*Ixodes holocyclus*	Eastern paralysis tick	188	19	124	28	22	12	334	393
*Ixodes myrmecobii*	–	4	0	1	0	0	0	5	5
*Ixodes tasmani*	Common marsupial tick	48	1	9	0	1	0	58	59
*Ixodes trichosuri*^d^	Possum tick	2	0	1	0	0	0	3	3
*Rhipicephalus australis*	Australian cattle tick	1	0	0	0	2	0	3	3
*Rhipicephalus sanguineus* (*s.l.*)	Brown dog tick	174	0	0	0	0	0	174	174
Grand total		473	20	137	28	45	12	655	715

*Note*: The number of sample replicates sequenced in subsequent *16S* NGS assays suspected of cross-talk or with inadequate sequencing depth during preliminary bioinformatics analyses are also included.

*Abbreviations*: S, sample; R, replicate; –, no common name.

a GenBank accession number: MN686569.

b GenBank accession numbers: MN686564-MN686566.

c GenBank accession number: MN686567.

d GenBank accession numbers: MN686562, MN686563 and MN686568.

**Table 2:** Summary of PCR primers and properties

Target gene	Target organism	Primer name	Primer sequence (5′-3′)	Expected amplicon length (bp)	T_ann_ (°C)	Reference
*16S* NGS						
*16S*	Bacteria (V1-2)	27F–Y	AGAGTTTGATCCTGGCTYAG	300	58	Gofton et al. (2015b)
		338R	GGATCACTCGATCGGTAGGAG			Turner et al. (1999)
	“*Ca.* Midichloria spp.”	MidBlocker	TGCTGCCTCCCGTAGGAGT	na	62	Gofton et al. (2015b)
*Rickettsia*-specific NGS						
17 kDa	*Rickettsia* spp.	Rr17kDa1^a^	GCTCTTGCAACTTCTATGTT	435	57	Williams et al. (1992)
		Rr17kDa2^a^	CATTGTTCGTCAGGTTGGCG			
*gltA*	*Rickettsia* spp.	RpCS.877	GGGGGCCTGCTCACGGCGG	380	48	Regnery et al. (1991)
		RpCS.1258n	ATTGCAAAAAGTACAGTGAACA			
*ompA*	SFGR	Rr190.70p	ATGGCGAATATTTCTCCAAAA	530	48	Regnery et al. (1991)
		Rr190.602n	AGTGCAGCATTCGCTCCCCCT			
*ompB*	*Rickettsia* spp.	120-M59	CCGCAGGGTTGGTAACTGC	555	50	Roux and Raoult (2000)
		ompBr	GAGGAGCTTTTTGAGTTGTAG			Owen (2007)
PCR assays targeting bacterial taxa						
*IS1111a*	*Coxiella burnetii*	IS1111aF	GTTTCATCCGCGGTGTTAAT	na	64	Banazis et al. (2010)
		IS1111aR	TGCAAGAATACGGACTCACG			
		IS1111aP^b^	CCCACCGCTTCGCTCGCTAA			
*16S*	*Coxiella* spp.	QR-F0	ATTGAAGAGTTTGATTCTGG	1,450	48	Masuzawa et al. (1997)
		QR-R0	CGGCCTCCCGAAGGTTAG			
	*Francisella* spp.	Fr153F0.1	GCCCATTTGAGGGGGATACC	1,170	60	Barns et al. (2005)
		Fr1281R0.1	GGACTAAGAGTACCTTTTTGAGT			
	Legionellales sp.^d^	8F	AGAGTTTGATCCTGGCTCAG	1,400–1,500	54	Edwards et al. (1989)
		1492R	GGTTACCTTGTTACGACTT			Stackebrandt and Liesack (1993)
	“*Ca*. Neoehrlichia spp.”	EC12A	TGATCCTGGCTCAGAACGAACG	1,460	48^c^	Paddock et al. (1997)
		EC9	TACCTTGTTACGACTT			Anderson et al. (1991)
		A17a	GCGGCAAGCCTAACACAT	1,265	54^c^	Kawahara et al. (2004)
		IS58-1345r	CACCAGCTTCGAGTTAAACC			
PCR assays targeting tick taxa						
*cox*1	*Haemaphysalis* spp.^e^	cox1F	GGAACAATATATTTAATTTTTGG	750	55	Chitimia et al. (2010)
		cox1R	ATCTATCCCTACTGTAAATATATG			
	*Ixodes trichosuri*	HCO2064	GGTGGGCTCATACAATAAATCC	850	48	Song et al. (2011)
		HCO1240	CCACAAATCATAAAGACATTGG			

*Abbreviation*: na, not applicable.

a Originally named “primer 1” and “primer 2” by Williams et al. (1992).

b Dual labelled probe; 5′ labelled with 6-FAM™ and 3′ labelled with BHQ®-1.

c Methods used from Gofton et al. (2016).

d The 8F/1492R primers are universal primers that amplify the *16S* gene of bacteria and eukaryotes (Galkiewicz and Kellogg, 2008).

e Primers also amplify *Argas persicus*, *Dermacentor marginatus* and *Ixodes ricinus* (Chitimia et al., 2010).

### 2.4 Bioinformatics analysis

Paired-end reads for each gene (*16S*, 17 kDa, *gltA*, *ompA* and *ompB*) were merged (minimum 50 bp overlap), trimmed of primers and distal bases, quality filtered (maximum expected error threshold of 1.0) and singletons were removed with USEARCH v10.0 (Edgar, 2010). Reads were denoised into ZOTUs (Edgar, 2018b) with the UNOISE3 algorithm (Edgar, 2020), which claims to also correct sequencing error and remove chimeras. Taxonomic assignment of *16S* ZOTUs was performed in QIIME 2 v2018.2 (Bolyen et al., 2019) using the QIIME2 feature classifier plugin (Bokulich et al., 2018), and taxonomic assignments by three different *16S* sequence databases were compared: the August 2013 release of the Greengenes sequence database (McDonald et al., 2012), SILVA v132 (Quast et al., 2013) and RDP Classifier v2.11 (Wang et al., 2007). Taxonomy assigned with the *16S* sequence databases was cross-checked by using the Basic Local Alignment Search Tool (BLAST) command line tool (BLAST+) using the blastn search application to compare ZOTU sequences with nearest matches from the NCBI non-redundant nucleotide (nr/nt) database. Taxonomy was assigned to 17 kDa, *gltA*, *ompA* and *ompB* ZOTUs also using BLAST+ with the blastn search application and the NCBI nr/nt database. A complete list of the *16S* ZOTU sequences is provided in Additional file 1.

In order to control for *16S* sequence laboratory and reagent contaminants, cross-contaminants and cross-talk, the proportion of reads for each ZOTU identified in the NTCs was removed from the respective ZOTU sequences in the samples and ExCs. Similarly, the proportion of reads for each ZOTU in the ExCs was removed from the respective ZOTU reads in the samples that the ExCs were extracted alongside. Further explanation of the data filtering technique used in this study is provided in Additional file 2. The tick-associated bacterial sequences (TABS) detected in the controls are provided in Additional file 3.

To assess whether sequencing depth was adequate for the samples, alpha rarefaction plots were generated with the R package *vegan* (Oksanen et al., 2018) using R software (R Development Core Team, 2013).

The scripts used to process the NGS datasets have been made available on the GitHub repository https://github.com/Telleasha-Greay/Illuminating-the-bacterial-microbiome-of-Australian-ticks-USEARCH-amplicon-NGS-pipeline.

### 2.5 Statistical analysis

For bacterial *16S* NGS, alpha and beta diversity metrics were produced using QIIME2 v2019.4 (Bolyen et al., 2019). Alpha diversity indices included the observed number of ZOTUs, the abundance-based coverage estimator (ACE) metric (Lee and Chao, 1994) and the Chao1 index (Chao, 1984). Beta diversity was assessed *via* principal coordinate analysis (PCoA) with the Bray-Curtis dissimilarity based on the fraction of overabundant ZOTUs (Sorensen, 1948). As the data did not meet the assumption of normality for parametric testing, the nonparametric Kruskal-Wallis and permutational multivariate analysis of variance (PERMANOVA) tests were used to compare alpha and beta diversity metrics, respectively, for tick species, instar/sex, feeding status, host species and ecoregion. Alpha and beta diversity plots were produced with the R package *phyloseq* (McMurdie and Holmes, 2013) using R software (R Development Core Team, 2013). The calculation and application of thresholds used to estimate the number of samples that were positive for ZOTUs is outlined in Additional file 2. The 95% confidence intervals (CIs) calculated for prevalence estimates were based on the methods by Rozsa et al. (2000).

### 2.6 *Coxiella burnetii*-specific real-time PCR assay

A real-time PCR (qPCR) assay was carried out on all samples to assess for the presence of the insertion sequence (IS) element of *Coxiella burnetii*. Primers and a dual-labelled probe that targeted the IS element 1111a (*IS1111a*) were used following the previously described methodology (Banazis et al., 2010) (Table 2). *Coxiella burnetii* DNA isolated from the Q-Vax™ vaccine (CSL, Parkville, Australia) and NTCs were included in each qPCR assay.

### 2.7 cnPCR for *COI* of *Ixodes* and *Haemaphysalis* ticks and *16S* of “*Ca.* Neoehrlichia”, *Coxiella*, *Francisella* and Legionellales bacteria

cnPCR assays were performed to generate amplicons for Sanger sequencing of *cox*1 of *Ixodes* nymphs that had been tentatively identified as *I. cornuatus* (Greay et al., 2016) and *Haemaphysalis* males and females that did not match morphological descriptions (Greay et al., 2018b). A longer region of *16S* was targeted using conventional molecular methods cnPCR and Sanger sequencing for potentially novel bacterial species and genotypes. Potentially novel bacteria were indicated by sequence dissimilarity of ~1% or greater when *16S* ZOTUs were compared to NCBI nr/nt database sequences. These included “*Ca.* Neoehrlichia sp.” ZOTU 104, *Coxiella* sp. ZOTU 82 and *Francisella* spp. ZOTUs 13, 42, 70, 97, 230 and 11,745. cnPCRs were performed in 25 μl reaction volumes with 1× KAPA Taq buffer (Sigma-Aldrich, St. Louis, Missouri, USA), 1 mM dNTPs, 0.04 mg BSA (Fisher Biotec, Perth, Western Australia (WA), Australia), 400 nM of each forward and reverse primer, 0.02 U KAPA Taq DNA Polymerase (Sigma-Aldrich) and 1 μl of neat gDNA. For the “*Ca.* Neoehrlichia” assays, 1 μl of primary PCR (EC12A/EC9) product was used as template DNA for the nested PCR (A17a/IS58-1345r). The final MgCl_2_ concentrations, annealing temperatures (T_ann_) and thermal cycling conditions for each cnPCR assay were carried out according to the studies cited in Table 2. NTCs were included alongside each cnPCR. PCR products were electrophoresed in 1% agarose gel containing SYBR Safe Gel Stain (Invitrogen, Carlsbad, California, USA) and visualised with a dark reader trans-illuminator (Clare Chemical Research, Dolores, Colorado, USA).

### 2.8 Sanger sequencing

PCR products of the expected amplicon size were excised from agarose gels with sterile scalpel blades and purified for Sanger sequencing with a filtered pipette tip method (Yang et al., 2013). Purified PCR products were sequenced in forward and reverse directions independently on a 96-capillary 3730xl DNA Analyzer (Thermo Fisher Scientific, Waltham, Massachusetts, USA) using an ABI Prism™ BigDye v3.1. Cycle Sequencing kit (Applied Biosystems, Foster City, California, USA) according to the manufacturerʼs instructions.

### 2.9 Sanger sequencing data and phylogenetic analyses

Forward and reverse sequence chromatograms were aligned and merged to generate consensus sequences and were trimmed of primers and low-quality bases using Geneious v10.2.2 (58). BLAST compared the consensus sequences to the NCBI nr/nt database. For phylogenetic analyses of Legionellales and *Francisella* spp., *16S* sequences available from GenBank for these genera were imported into Geneious v10.2.2 (Kearse et al., 2012) and aligned using the MUSCLE alignment tool (Edgar, 2004). Nucleotide alignments were imported into the program PhyML (Guindon et al., 2010) and assessed for the most appropriate nucleotide substitution model based on Bayesian Information Criterion (BIC). Bayesian phylogenetic trees were constructed using the MrBayes v3.2.6 plug-in (Ronquist et al., 2012) for Geneious v10.2.2 (Kearse et al., 2012).

## 3 Results

### 3.1 NGS statistics summary

Approximately 50 million paired-end V1-2 *16S* reads were obtained for all samples, sample replicates and controls with *16S* NGS (*n* = 818). After the reads were pre-processed (merged, quality filtered, singletons and chimeras removed) and post-processed [the proportion of ZOTU TABS in the controls filtered from the samples (Additional file 2 and Additional file 3)] a total of ~30.5 million sequences for the samples and sample replicates were obtained (average (avg) 42,460; standard deviation (SD) ± 30,235; range 381–268,998) (Table 3). The alpha rarefaction plots indicated that adequate sequencing depth for ZOTU diversity was obtained for most samples, with one notable outlier for an *A. t. triguttatum* sample, which had an unusually large number of ZOTUs (Additional file 4). There were ~4 million unprocessed paired-end *Rickettsia*-specific reads obtained at the 17 kDa, *gltA*, *ompA* and *ompB* loci (*n* = 101), but only 25% of the reads passed merging, quality filtering, singleton (majority of the reads removed were singletons) and chimera removal steps. After the reads were post-processed (non-*Rickettsia* sequences and primer dimer removed), there were 82,150 17 kDa (avg 8,215; SD ± 9,473; range 2–28,087), 237,557 gltA (avg 3,443; SD ± 4,432; range 0–23,687) and 1,578 *ompA* (avg 175; SD ± 353; range 0–1,093) sequences in the tick samples. Only three *Rickettsia ompB* sequences were obtained from tick samples (Table 4). *16S* and *Rickettsia*-specific read totals, sequence compositions and prevalence estimates for samples are provided in Additional file 5.

**Table 3:** Bacterial *16S* NGS statistics

Sequence statistic	Raw (unprocessed) reads	Pre-processed sequences^a^	Processed *16S* sequences^b^				
	Grand total (*n* = 818)		Tick S and R (*n* = 715)	ExCs (S, *n* = 24, S and R, *n* = 73)	NTCs (*n* = 25)	ICs (*n* = 5)	Grand total (*n* = 818)
Average	64,949	43,093	42,460	27,728	27,860	35	39,965
SD	48,248	31,379	30,235	22,661	49,408	16	17,728
Minimum	40	6	381	7	5	10	5
Maximum	310,998	270,639	268,998	133,691	206,724	46	268,998
Total	49,988,584	33,043,873	30,401,633	1,607,983	681,510	146	32,691,272
No. of ZOTUs	na		11,474	484	265	38	11,474

*Abbreviations*: S, sample; R, replicate; na, not applicable.

a Merged, quality filtered sequences with singletons and chimeras removed.

b Merged, quality filtered sequences with singletons, chimeras and proportions of ZOTU TABS removed from samples (Additional file 2).

**Table 4:** *Rickettsia*-specific NGS statistics

Sequence statistic	Raw (unprocessed) reads	Pre-processed^a^	Processed *Rickettsia*-specific sequences^b^						
	Grand total (*n* = 101)		17 kDa S (*n* = 10)	*gltA* S (*n* = 69)	*ompA* S (*n* = 9)	*ompB* S (*n* = 8)	NTCs (*n* = 4)	IC (*n* = 1)	Grand total (*n* = 101)
Average	39,545	12,922	8,215	3,443	175	1	na	na	3,278
SD	39,627	17,291	9,473	4,432	353	1			5,161
Minimum	44	2	2	0	0	0	2	2	0
Maximum	306,412	132,382	28,087	23,687	1,093	2	2	2	28,087
Total	4,033,630^c^	1,266,447	82,150	237,557	1,578	3	2	2	321,292
No. of ZOTUs	na		10	23	3	1	1	1	37

*Abbreviations*: S, sample; na, not applicable.

a Merged, quality filtered sequences with singletons and chimeras removed.

b Merged, quality filtered sequences with singletons, chimeras and non-*Rickettsia* sequences removed.

c The *Rickettsia*-specific libraries were pooled with other NGS libraries on the v3 kit.

### 3.2 Dominant bacterial *16S* sequence compositions

**Fig. 2:**
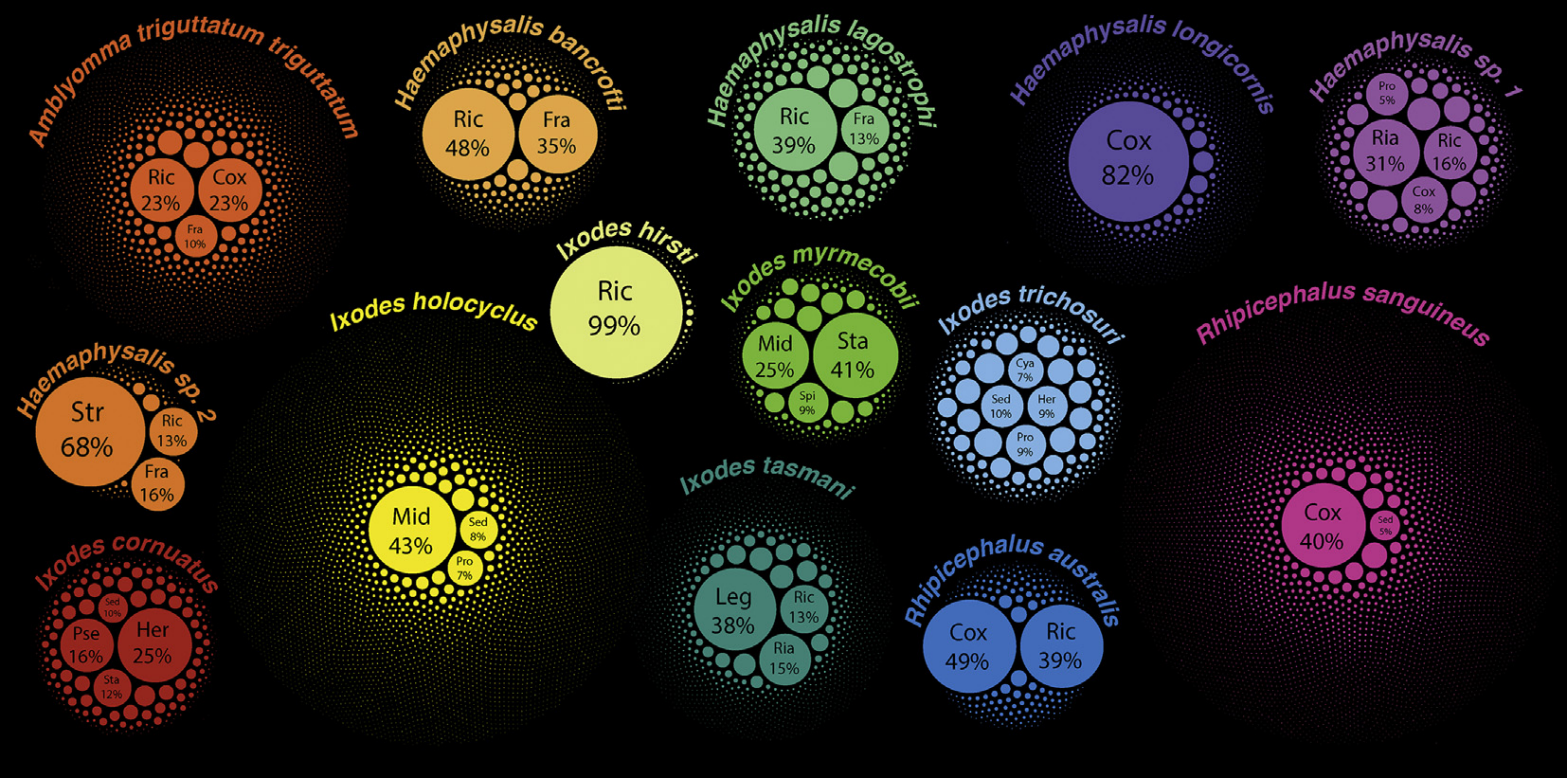
A circle packing graph of ZOTUs detected with *16S* NGS in different tick species. Genera and ZOTUs are nested according to tick species and weighted based on *16S* sequence composition. Genera with sequence compositions of ≥ 15% are labelled as follows: Cox, *Coxiella*; Fra, *Francisella*; Her, *Herbaspirillum*; Leg, Legionellales fam. gen.; Mid, “*Ca.* Midichloria”; Pse, *Pseudomonas*; Ria, *Rickettsiella*; Ric, *Rickettsia*; Sta, *Staphylococcus*; and Str, *Streptococcus*. ZOTUs with sequence compositions of < 15% not labelled. The graph was generated using RAWGraphs software (Mauri et al., 2017).

The most abundant *16S* sequences for bacterial genera in each tick species were determined based on the overall percent composition of sequences for each ZOTU and the total number of sequences derived from each tick species. Fig. 2 provides a visual representation of the sequence composition for abundant and less abundant sequences for each ZOTU, with abundant (> 15% sequence composition) genera pooled if > 1 ZOTU of the same genus had a high sequence composition. The following genera had the most abundant sequences: “*Ca.* Midichloria” in *I. holocyclus* and *Ixodes myrmecobii*; *Coxiella* in *A. t. triguttatum*, *Haemaphysalis longicornis*, *Rhipicephalus australis* and *Rh. sanguineus* (*s.l.*); *Francisella* in *Haemaphysalis bancrofti* and *Haemaphysalis* sp. genotype 2; *Herbaspirillum* in *Ixodes cornuatus*; Legionellales fam. gen. in *I. tasmani*; *Pseudomonas* in *I. cornuatus*; *Rickettsiella* in *Haemaphysalis* sp. genotype 1 and *I. tasmani*; *Rickettsia* in *A. t. triguttatum*, *H. bancrofti*, *Haemaphysalis lagostrophi*, *Haemaphysalis* sp. genotype 1, *Ixodes hirsti* and *Rh. australis*; *Staphylococcus* in *I. myrmecobii*; and *Streptococcus* in *Haemaphysalis* sp. genotype 2 (Fig. 2). A *16S* sequence composition plot for bacterial taxa with sequence compositions ≥ 1% is provided in Additional file 6.

The average *16S* sequence composition for each dominant ZOTU (≥ 5% average sequence composition) among tick samples for each tick species is presented in Table 5. *Coxiella* sp. (ZOTU 4) had the greatest average sequence composition of 82.4% (SD ± 30.0%) in *Haemaphysalis longicornis* (*n* = 47) (Table 5).

**Table 5:** Average *16S* sequence composition of dominant taxa in tick species

Tick species	Dominant ZOTU^a^ (GenBank ID)	Avg sequence composition (%) ± SD	Range of avg sequence composition (%)
*Amblyomma triguttatum triguttatum* (*n* = 17)	*Coxiella* sp. ZOTU 4 (MT914306)	28.4 ± 34.9	0.0–97.8
	*Rickettsia* sp. ZOTU 14 (MT914316)	11.5 ± 24.3	0.0–80.7
	*Rickettsia* sp. ZOTU 9 (MT914311)	6.1 ± 15.9	0.0–55.5
	*Francisella* sp. ZOTU 13 (MT914315)	6.0 ± 17.2	0.0–60.1
	*Francisella* sp. ZOTU 42 (MT914326)	5.7 ± 11.8	0.0–38.7
*Haemaphysalis bancrofti* (*n* = 4)	*Rickettsia* sp. ZOTU 14 (MT914316)	26.3 ± 30.4	0.0–53.3
	*Rickettsia* sp. ZOTU 9 (MT914311)	22.4 ± 27.9	0.0–57.6
	*Francisella* sp. ZOTU 42 (MT914326)	17.7 ± 21.4	0.0–43.0
	*Francisella* sp. ZOTU 13 (MT914315)	16.8 ± 33.0	0.0–66.2
*Haemaphysalis lagostrophi* (*n* = 1)^b^	*Rickettsia* sp. ZOTU 14 (MT914316)	38.6	na
	*Francisella* sp. ZOTU 42 (MT914326)	8.3	
	*Francisella* sp. ZOTU 70 (MT914331)	5.2	
*Haemaphysalis longicornis* (*n* = 47)	*Coxiella* sp. ZOTU 4 (MT914306)	82.4 ± 30.0	0.0–99.7
*Haemaphysalis* sp. genotype 1 (*n* = 3)	*Rickettsia* sp. ZOTU 33 (MT914322)	10.8 ± 18.8	0.0–32.5
	*Rickettsiella* sp. ZOTU 17 (MT921652)	14.1 ± 24.4	0.0–42.2
	*Coxiella* sp. ZOTU 82 (MT914333)	5.5 ± 6.8	0.0–13.0
*Haemaphysalis* sp. genotype 2 (*n* = 1)^b^	*Streptococcus equi* ZOTU 95 (MT921653)	67.9	na
	*Rickettsia* sp. ZOTU 14 (MT914316)	12.7	
	*Francisella* sp. ZOTU 42 (MT914326)	9.2	
	*Francisella* sp. ZOTU 70 (MT914331)	6.7	
*Ixodes cornuatus* (*n* = 4)	*Herbaspirillum* sp. ZOTU 10 (MT914312)	22.1 ± 13.5	2.3–31.4
	*Staphylococcus* sp. ZOTU 23 (MT914318)	15.7 ± 31.4	0.0–62.8
	*Pseudomonas* sp. ZOTU 24 (MT914319)	11.3 ± 8.1	0.4–19.5
	“*Ca.* Neoehrlichia arcana” ZOTU 40 (MT914325)	6.7 ± 13.4	0.0–26.8
	*Chitinophagaceae* gen. sp. ZOTU 5 (MT914307)	5.9 ± 4.4	0.0–10.8
	*Burkholderiaceae* sp. ZOTU 37 (MT914324)	5.4 ± 3.6	0.5–8.2
*Ixodes hirsti* (*n* = 1)^b^	*Rickettsia* sp. ZOTU 171 (MT914338)	30.5	na
	*Rickettsia* sp. ZOTU 182 (MT914339)	28.3	
	*Rickettsia* sp. ZOTU 223 (MT914340)	21.5	
	*Rickettsia* sp. ZOTU 224 (MT914341)	18.6	
*Ixodes holocyclus* (*n* = 334)	“*Ca.* Midichloria sp.” ZOTU 1 (MT914303)	22.7 ± 26.2	0.0–98.5
	“*Ca.* Midichloria sp.” ZOTU 2 (MT914304)	14.9 ± 25.7	0.0–91.2
	*Chitinophagaceae* gen. sp. ZOTU 5 (MT914307)	8.7 ± 13.0	0.0–63.1
	*Cutibacterium* sp. ZOTU 6 (MT914308)	7.3 ± 9.0	0.0–53.8
*Ixodes myrmecobii* (*n* = 5)	“*Ca.* Midichloria sp.” ZOTU 2 (MT914304)	18.9 ± 35.9	0.2–82.5
	*Staphylococcus* sp. ZOTU 23 (MT914318)	17.5 ± 39.1	0.0–87.5
	*Staphylococcus* sp. ZOTU 65 (MT914330)	8.4 ± 18.9	0.0–42.2
	*Cytophagaceae* gen. sp. ZOTU 12 (MT914314)	7.8 ± 12.9	0.0–29.9
	*Staphylococcus* sp. ZOTU 78 (MT914332)	7.3 ± 16.3	0.0–36.4
*Ixodes tasmani* (*n* = 58)	*Coxiellaceae* sp. ZOTU 7 (MT914309)	42.9 ± 47.4	0.0–99.9
	*Rickettsia* sp. ZOTU 11 (MT914313)	13.5 ± 20.8	0.0–80.6
	*Rickettsiella* sp. ZOTU 28 (MT914320)	6.3 ± 8.7	0.0–23.6
*Ixodes trichosuri* (*n* = 3)	*Herbaspirillum* sp. ZOTU 10 (MT914312)	17.2 ± 15.0	0.0–27.4
	*Pseudomonas* sp. ZOTU 24 (MT914319)	8.6 ± 7.6	0.0–14.6
	*Cutibacterium* sp. ZOTU 6 (MT914308)	6.8 ± 6.2	0.4–12.8
	*Chitinophagaceae* gen. sp. ZOTU 5 (MT914307)	6.6 ± 7.1	0.0–27.4
*Rhipicephalus australis* (*n* = 3)	*Rickettsia* sp. ZOTU 9 (MT914311)	52.7 ± 48.0	0.0–94.0
	*Coxiella* sp. ZOTU 82 (MT914333)	31.1 ± 53.8	0.0–93.3
*Rhipicephalus sanguineus* (*s.l.*) (*n* = 174)	“*Ca.* Coxiella massiliensis” ZOTU 3 (MT914305)	45.2 ± 29.1	0.0–99.2

a ≥ 5% average *16S* sequence composition.

b Average *16S* sequence composition not applicable (na) as *n* = 1.

### 3.3 Prevalence of tick-associated and haemotropic pathogens

**Fig. 3:**
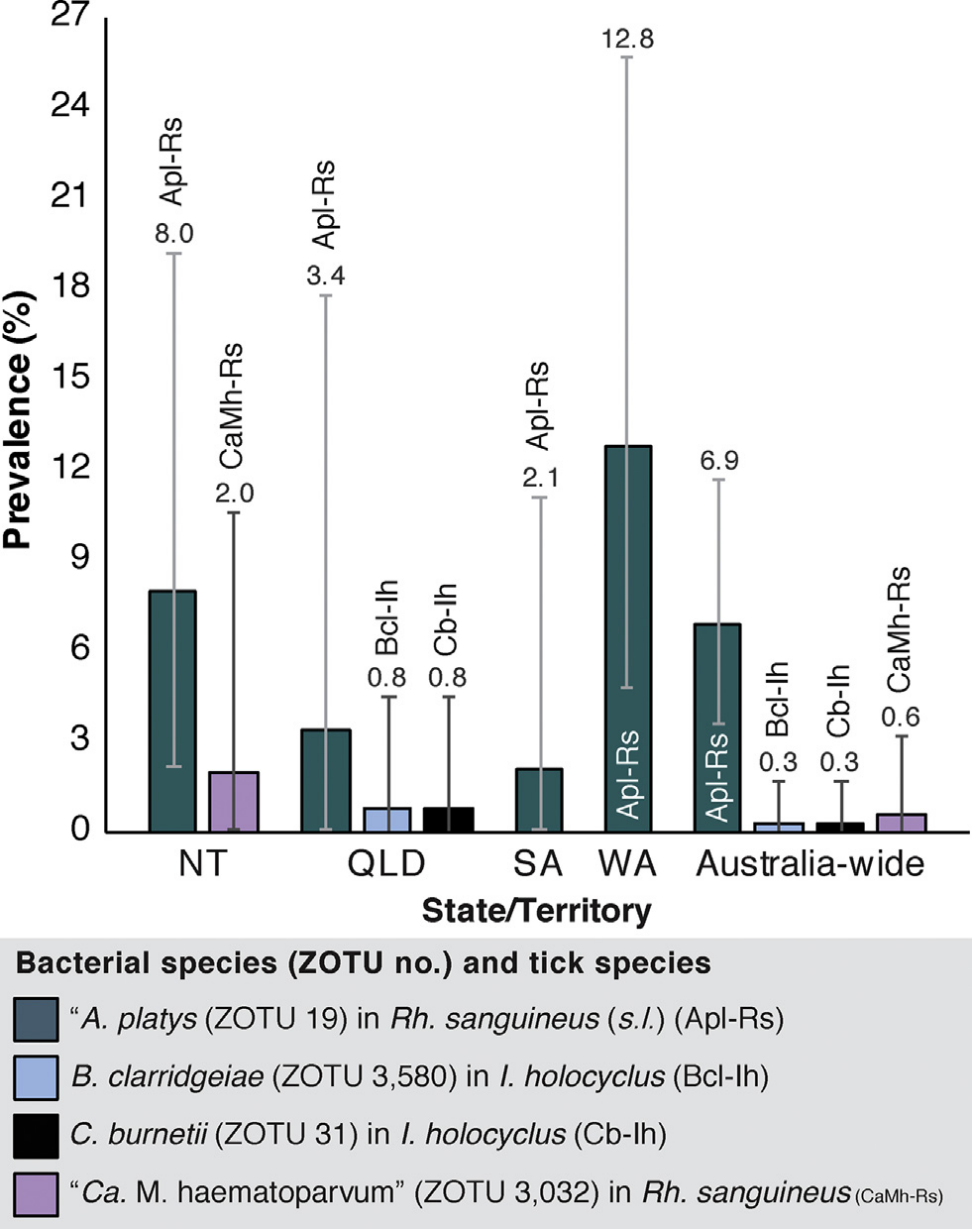
Prevalence of tick-associated and haemotropic pathogens. Prevalence for *Anaplasmaplatys* and “*Candidatus* Mycoplasma haematoparvum” estimated for *Rhipicephalus sanguineus* (*s.l*.), and *Coxiella burnetii* and *Bartonella clarridgeiae* prevalence estimated for *Ixodes holocyclus*. Error bars represent 95% confidence intervals.

The overall prevalence of *Anaplasma platys* (family *Anaplasmataceae*) (MT914317) in *Rh. sanguineus* (*s.l*.) larvae, nymphs, males and females was 6.9% (12/174; 95% CI: 3.6–11.7%) (Fig. 3). The *Rh. sanguineus* (*s.l.*) ticks positive for *A. platys* were collected from dogs in the Northern Territory (NT) (8.0%, 4/50; 95% CI: 2.2–19.2%), Queensland (QLD) (3.4%, 1/29; 95% CI: 0.1–17.8%), South Australia (SA) (2.1%, 1/48; 95% CI: 0.1–11.1%) and WA (12.8%, 6/47; 95% CI: 4.8–25.7%). “*Candidatus* Mycoplasma haematoparvum” (family *Mycoplasmataceae*) (MT914408) was also identified in a *Rh. sanguineus* (*s.l.*) larva removed from a dog in the NT (2.0%, 1/50; 95% CI: 0.1–10.6%). *Bartonella clarridgeiae* (family *Bartonellaceae*) (MT914409) and *C. burnetii* (family *Coxiellaceae*) (MT914321) were both detected in 0.3% (1/334; 95% CI: 0–1.7%) of *I. holocyclus* removed from cats in QLD (0.8%, 1/122; 95% CI: 0–4.5%, for each pathogen) (Fig. 3). The feeding status was not recorded for three of the *A. platys*-positive *Rh. sanguineus* (*s.l.*), but all other ticks positive for tick-associated bacterial and haemotropic pathogens had fed on their hosts (Additional file 5 and metadata provided in NCBI SRA for BioProject PRJNA640465).

### 3.4 Prevalence of dominant *Anaplasmataceae*, “*Candidatus* Midichloriaceae”, *Coxiellaceae*, *Francisellaceae* and *Rickettsiaceae* species

**Fig. 4:**
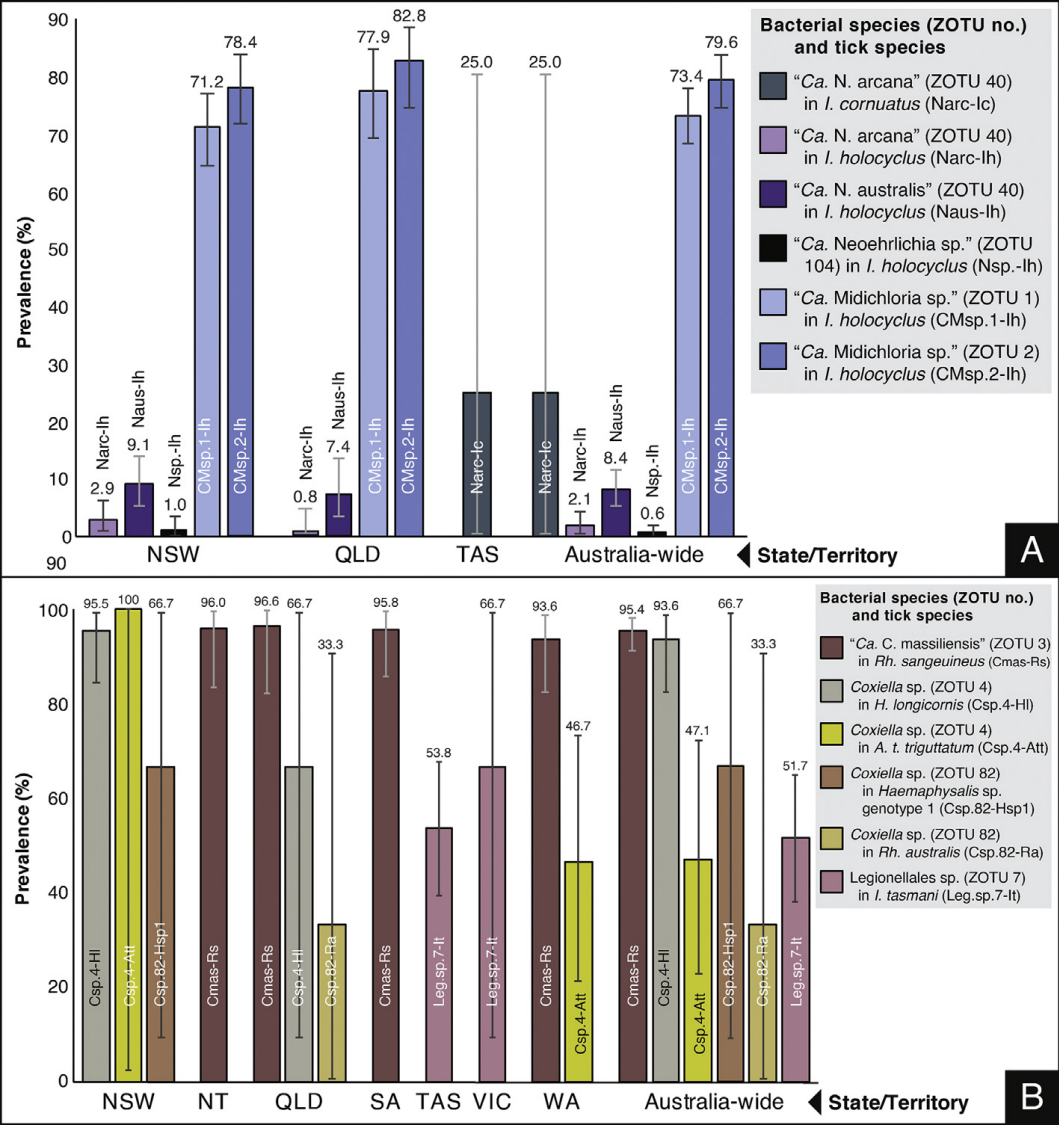
Prevalence of *Anaplasmataceae* and *Coxiellaceae* species. **A***Anaplasmataceae* species prevalence tick species. **B***Coxiellaceae* species prevalence tick species. Error bars represent 95% confidence intervals.

Other members of the family *Anaplasmataceae* were identified, including “*Ca.* N. arcana”, “*Ca.* N. australis” and “*Ca.* Neoehrlichia spp.“. “*Candidatus* Neoehrlichia arcana” (MT914325) was detected in a female *I. cornuatus* collected from a dog in Tasmania (TAS) (25%, 1/4; 95% CI: 0.6–80.6%) and in 2.1% of *I. holocyclus* collected Australia-wide (7/334; 95% CI: 0.8–4.3%) (Fig. 4A). The prevalence of “*Ca.* N. arcana” in *I. holocyclus* was 2.9% in NSW (6/208; 95% CI: 1.1–6.2%), whereas the prevalence was 0.8% in QLD (1/122; 95% CI: 0–4.5%). However, the difference in the prevalence of “*Ca.* N. arcana” in *I. holocyclus* in the two regions assessed with the Fisherʼs exact test (minimum expected count = 2.59) was not statistically significant (*P* = 0.267). *Ixodes holocyclus* positive for “*Ca.* N. arcana” were collected from horses, cats and dogs in NSW, and from a dog in QLD. The overall prevalence of “*Ca.* N. australis” (MT914310) in *I. holocyclus* was 8.4% (28/334; 95% CI: 5.6–11.9%), and was higher than the prevalence of “*Ca.* N. arcana” in *I. holocyclus* (χ^2^ = 11.7, *df* = 1, *P* = 0.0006). *Ixodes holocyclus* positive for “*Ca.* N. australis” were collected from dogs, cats and horses in NSW (9.1%, 19/208; 95% CI: 5.6–13.9%) and from dogs and cats in QLD (7.4%, 9/122; 95% CI: 3.4–13.5%). There was no statistically significant difference between the prevalence of “*Ca.* N. australis” in *I. holocyclus* from NSW and QLD (χ^2^ = 0.306, *df* = 1, *P* = 0.580). Most “*Ca.* N. arcana”-positive instars had fed on their hosts, but two male and five female *I. holocyclus* from NSW and QLD were unfed. “*Candidatus* Neoehrlichia sp.” (ZOTU 104; MT914336) had the closest sequence similarity (99.3%) to a “*Ca.* Neoehrlichia sp.” (KT203914) sequence isolated from *I. holocyclus* in Australia and was 99.0% similar to “*Ca.* N. arcana” (MT914325) and 94.5% similar to “*Ca.* N. australis” (MT914310). “*Candidatus* Neoehrlichia sp.” (ZOTU 104) had a prevalence of 1.0% (2/208; 95% CI: 0.1–3.4%) in an unfed nymph and unfed female *I. holocyclus* from cats in NSW (Fig. 4A). *Wolbachia* sp. ZOTU 58 (MT914329) was 100% similar to *Wolbachia* sp. (Z49261) isolated from *Dirofilaria immitis* (heartworm) in Italy and 13.8% (4/29; 95% CI: 3.9–31.7%) of female *Rh. sanguineus* (*s.l.*) from dogs in QLD were positive (Additional file 5). The feeding status of the *Wolbachia*-positive *Rh. sanguineus* (*s.l.*) ticks was not recorded.

Two “*Ca.* Midichloria sp.” ZOTUs with 3.8% sequence dissimilarity, ZOTU 1 (MT914303) and ZOTU 2 (MT914304), were 96.9% and 97.9% similar, respectively, to “*Ca.* M. mitochondrii” (AJ566640) isolated from *Ixodes ricinus* in Italy. “*Candidatus* Midichloria sp.” ZOTU 1 was 100% similar to “*Ca.* Midichloria sp.” isolate Ixholo1 (FM992372) and “*Ca.* Midichloria sp.” ZOTU 2 was 100% similar to “*Ca.* Midichloria sp.” isolate Ixholo2 (FM992373), both isolated from *I. holocyclus* in Australia. Overall, 73.4% (245/334; 95% CI: 68.3–78.0%) of *I. holocyclus* nymphs, males and females collected from dogs and cats were positive for “*Ca.* Midichloria sp.” ZOTU 1, while 79.6% (266/334; 95% CI: 74.9–83.8%) of *I. holocyclus* nymphs, males and females collected from dogs, cats and horses were positive for “*Ca.* Midichloria” sp. ZOTU 2 (Fig. 4A). A single *I. holocyclus* female that was removed from a dog in WA was positive for “*Ca.* Midichloria” sp. ZOTU 2 (Additional file 5). However, as *I. holocyclus* is not known to occur in WA, this dog may have been infested with *I. holocyclus* on the eastern coast within its distribution range prior to travelling to WA.

Almost all *Rh. sanguineus* (*s.l.*) collected from dogs were positive for “*Candidatus* Coxiella massiliensis” (ZOTU 3; MT914305) (95.4%, 166/174; 95% CI: 91.1–98.0%) (Fig. 4B). *Coxiella* sp. ZOTU 4 (MT914306) was 100% similar to *Coxiella* sp. isolated from *H. longicornis* in Korea (AY342036), and *A. t. triguttatum* and *H. longicornis* were positive for *Coxiella* sp. ZOTU 4 with an overall prevalence of 47.1% (8/17; 95% CI: 21.3–73.4%) and 93.6% (44/47; 95% CI: 82.5–98.7%), respectively. *Haemaphysalis* sp. genotype 1 from NSW (66.7%, 2/3; 95% CI: 9.4–99.2%) and *Rh. australis* from QLD (33.3%, 1/3; 95% CI: 0.8–90.6%) were positive for *Coxiella* sp. ZOTU 82 (MT914333), which was 98.6% similar to *Coxiella* sp. isolated from *Rhipicephalus turanicus* in Israel (JQ480818). Legionellales sp. (ZOTU 7) (MT914309) was most similar (95.1%) to a “*Coxiellaceae* bacterium” sequence previously isolated from *I. tasmani* in Australia (EU430251), and overall, 51.7% (30/58; 95% CI: 38.2–65.0%) of *I. tasmani* were positive (Fig. 4B).

**Fig. 5:**
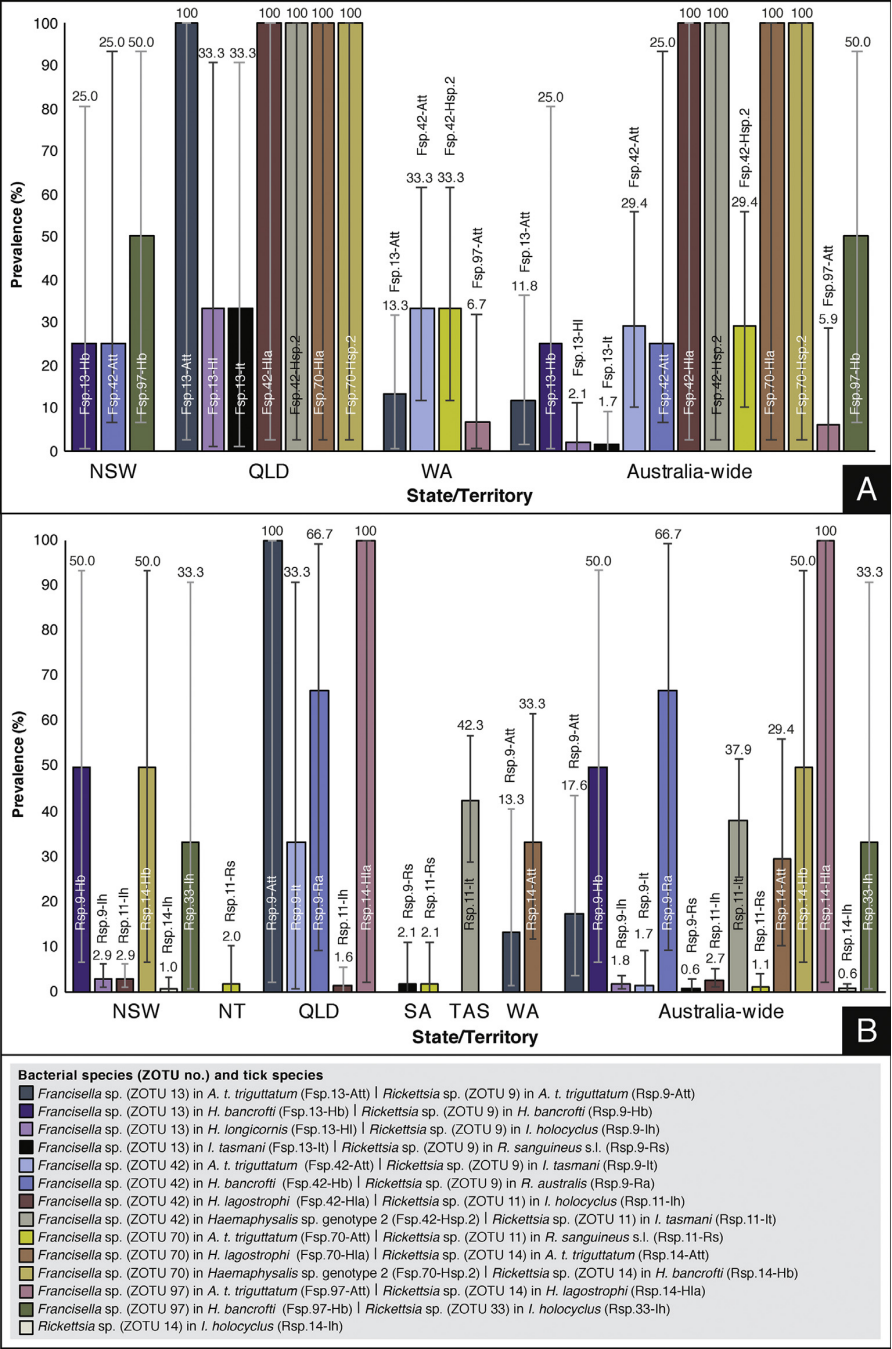
Prevalence of *Francisellaceae* and *Rickettsiaceae* species. **A***Francisellaceae* species prevalence tick species. **B***Rickettsiaceae* species prevalence tick species. Error bars represent 95% confidence intervals.

*Francisella* sp. ZOTU 13 (MT914315) was 99.3% similar to *Francisella* sp. isolated from *Dermacentor nitens* in Ecuador (AY375401). *Amblyomma triguttatum triguttatum* (11.8%, 2/17; 95% CI: 1.5–36.4%), *H. bancrofti* (25.0%, 1/4; 95% CI: 0.6–80.6%), *H. longicornis* (2.1%, 1/47; 95% CI: 0.1–11.3%) and *I. tasmani* (1.7%, 1/59; 95% CI: 0–9.1%) were positive for *Francisella* sp. ZOTU 13 (Fig. 5A). *Francisella* sp. ZOTU 42 (MT914326) was 97.3% similar to *Francisella* sp. isolated from *Ornithodoros moubata* (AB001522; location not specified). *Amblyomma triguttatum triguttatum* (29.4%, 5/17; 95% CI: 10.3–56.0%), *H. bancrofti* (50%, 2/4; 95% CI: 6.8–93.2%), *H. lagostrophi* (100%, 1/1; 95% CI: 2.5–100%) and *Haemaphysalis* sp. genotype 2 (100%, 1/1; 95% CI: 2.5–100%) were *Francisella* sp. ZOTU 42-positive. *Amblyomma triguttatum triguttatum* (29.4%, 5/17; 95% CI: 2.5–100%), *H. lagostrophi* (100%, 1/1; 95% CI: 2.5–100%) and *Haemaphysalis* sp. genotype 2 (100%, 1/1; 95% CI: 2.5–100%) were also positive for *Francisella* sp. ZOTU 70 (MT914331). *Amblyomma triguttatum triguttatum* (5.6%, 1/18; 95% CI: 0.1–27.3%) and *H. bancrofti* (50%, 2/4; 95% CI: 6.8–93.2%) were positive for *Francisella* sp. ZOTU 97 (MT914335) (Fig. 5A).

*Rickettsia* sp. ZOTU 9 (MT914311) was 100% similar to *Rickettsia rickettsii* (U11021) and *Rickettsia slovaca* (L36224). 17.9% of *A. t. triguttatum* (3/17; 95% CI: 3.8–43.4%), 50.0% of *H. bancrofti* (2/4; 95% CI: 6.8–93.2%), 1.8% of *I. holocyclus* (6/334, 95% CI: 0.7–3.9%), 1.7% of *I. tasmani* (1/58; 95% CI: 0–9.2%), 66.7% of *Rh. australis* (2/3; 95% CI: 9.4–99.2%) and 0.6% of *Rh. sanguineus* (*s.l.*) (1/174; 95% CI: 0–3.2%) were positive for *Rickettsia* sp. ZOTU 9 (Fig. 5B). *Rickettsia* sp. ZOTU 11 (MT914313) was 99.7% similar to *Rickettsia massiliae* (GQ144453). 2.7% of *I. holocyclus* (9/334; 95% CI: 1.2–5.1%), 37.9% of *I. tasmani* (22/58; 95% CI: 25.5–51.6%) and 1.1% of *Rh. sanguineus* (*s.l.*) (2/174; 95% CI: 0.1–4.1%) were *Rickettsia* sp. ZOTU 11-positive. *Rickettsia* sp. ZOTU 14 (MT914316) was detected in multiple tick species (Fig. 5B), but *Rickettsia* sp. ZOTU 33 (MT914322), which was 99.7% similar to *Rickettsia raoultii* (KJ410261), was only detected in *Haemaphysalis* sp. genotype 1 (33.3%, 1/3; 95% CI: 0.8–90.6%).

**Fig. 6:**
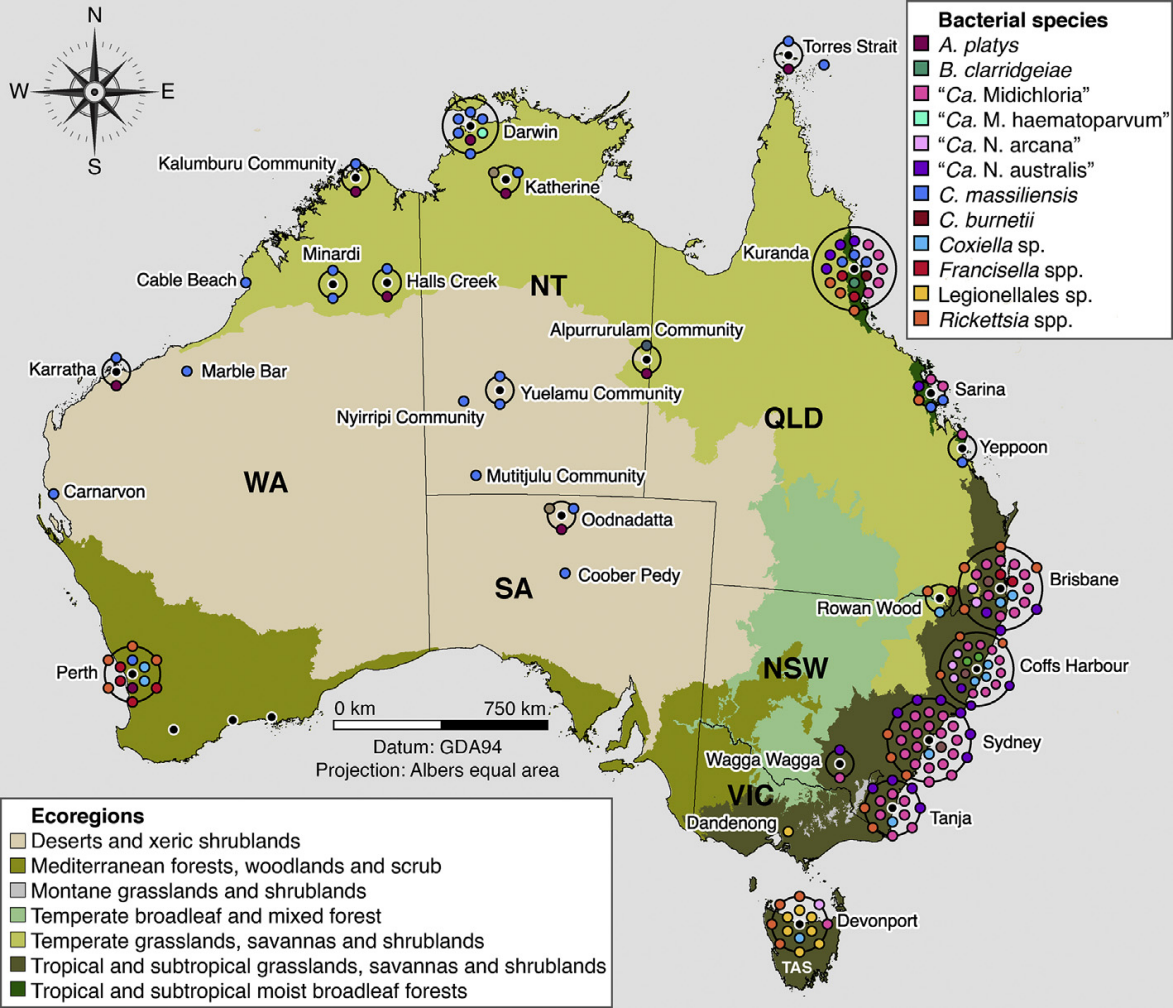
Sample collection localities of ticks positive for pathogens and tick-associated bacteria. These include “*Candidatus* Midichloria”, *Coxiella* sp. (ZOTU 4), *Francisella* spp., Legionellales sp. (ZOTU 7) and *Rickettsia* spp. The concentric rings (black) indicate that sample collection localities were displaced; displaced and non-displaced collection localities are represented by a black point encircled by a white stroke and are labelled with the city, town or Aboriginal Community that is closest to the point. QGIS3 v3.4 software was used to map points and perform concentric ring displacement and the map was overlaid with terrestrial ecoregions in Australia (Department of Agriculture, Water and the Environment, 2020).

CIs, type of instars, and hosts for all prevalence estimates that have been reported are provided in Additional file 5 and in the SRA (PRJNA640465). The sample collection locations of ticks and the samples positive for TABS, and tick-associated bacterial and haemotropic pathogens are summarised in Fig. 6.

### 3.5 *Bartonellaceae*, “*Candidatus* Midichloriaceae”, *Coxiellaceae*, *Francisellaceae*, Mycoplasmatales and *Rickettsiaceae* with low *16S* sequence compositions

The vast majority (99%) of ZOTUs identified in samples had sequence compositions of ≤ 1%. Other members of *Bartonellaceae* that had low sequence compositions were identified, including *Bartonella* sp. ZOTU 8459 (MT914444) and *Bartonella* sp. ZOTU 8612 (MT914350) that were 100% similar to two different *Bartonella apis* genotypes (CP015821 and CP015625, respectively) isolated from *Apis mellifera* (Western honey bee) in Switzerland. *Bartonella* sp. ZOTU 8459 was detected in a *Rh. sanguineus* (*s.l.*) female from a dog in QLD (2.0%, 1/50; 95% CI: 0.1–10.6%) and *Bartonella* sp. ZOTU 8612 was detected in a *H. longicornis* female from a dog in NSW (2.3%, 1/44; 95% CI: 0.1–12%) (Additional file 5). Other ZOTUs with low sequence abundances of note were three Mycoplasmatales sp. ZOTUs [3592 (MT914346), 10463 (MT914416) and 12798 (MT914355)] assigned to the *Mycoplasmataceae* family by SILVA and Greengenes. Mycoplasmatales sp. ZOTU 3592 (MT914346) was detected in a *H. lagostrophi* female that had fed on a horse in QLD and was 98.0% similar to an “uncultured bacterium clone” isolated from a horse (EU463716; location unspecified). Mycoplasmatales sp. ZOTU 10463 (MT914416) was 96.9% similar to an “uncultured bacterium clone” isolated from *Equus africanus asinus* (donkey) in the USA (EU473607) and was detected in a female *I. holocyclus* (feeding status not recorded) from a horse in NSW. Mycoplasmatales sp. ZOTU 12798 (MT914355) was most similar (98.0%) to an “uncultured bacterium clone” isolated from *Achatina fulica* (giant African snail) in the USA (EU473607) and was identified in an *I. trichosuri* nymph that had fed on a cat in TAS, and in a *Rh. sanguineus* male that had fed on a dog in WA (Additional file 5).

One *I. holocyclus* nymph from a dog in Turramurra, NSW was positive for *Wolbachia* sp. ZOTU 952 (MT914406), family *Anaplasmataceae*, which was 99.7% similar to *Wolbachia* sp. (LC370586) isolated from *Meimuna opalifera* (Walkerʼs cicada) in Japan. Other *Wolbachia* sp. sequences that were detected included ZOTU 6044 (MT914443) and ZOTU 14275 (MT914424) (99.0% and 97.9% similar, respectively, to *Wolbachia* sp. (AJ575104) isolated from *Mesaphorura italica* (springtail) in France) in a fed *A. t. triguttatum* from a horse in Gidgegannup, WA (6.7%, 1/15; 95% CI: 0.2–31.9%). *Wolbachia* sp. ZOTU 9835 and ZOTU 14686 (96.9% and 98.5% similar, respectively, to *Wolbachia* sp. (Z49261) isolated from *Dirofilaria immitis* in Italy) were detected in female *Rh. sanguineus* (*s.l.*) from dogs in QLD; 17.2% (5/29; 95% CI: 5.8–35.8%) were positive for ZOTU 9835 and 13.8% (4/29; 95% CI: 3.9–31.7%) were positive for ZOTU 14686 (Additional file 5).

ZOTUs with low sequence numbers for genera that are usually associated with ticks included 67 “*Candidatus* Midichloria” ZOTUs, 44 *Coxiella* ZOTUs, 2 *Francisella* sp. ZOTUs (43 and 11745), 34 *Rickettsiella* spp. ZOTUs and 14 *Rickettsia* spp. ZOTUs (Additional file 5).

### 3.6 Alpha diversity

Alpha diversity estimates of ACE, Chao1 and observed ZOTU metrics had significantly different distributions for tick species (Kruskal-Wallis test for all groups; *P* < 0.05) (refer to Additional file 7 for test statistics and *P-*values). Tick species that had small sample sizes (*n* < 5) were excluded from the diversity analyses. The distribution of ACE, Chao1 and observed ZOTU metrics was not significantly different for pairwise comparisons of *A. t. triguttatum* (*n* = 17) with *Rh. sanguineus* (*s.l.*) (*n* = 174), *H. longicornis* (*n* = 47) with *I. myrmecobii* (*n* = 5), *I. holocyclus* (*n* = 334) with *I. myrmecobii* (*n* = 5) and *I. myrmecobii* (*n* = 5) with *I. tasmani* (*n* = 58), with *P-*values ranging from *P =* 0.170 to *P* = 0.903 (refer to Additional file 1 for diversity plots). Additionally, the ACE and Chao1 metrics did not differ in distribution for *H. longicornis* compared with *I. holocyclus* (*P* = 0.708 and *P* = 0.943, respectively). *Ixodes holocyclus* and *Rh. sanguineus* had sufficient sample sizes for comparisons of alpha diversity estimates for different ecoregions. The distribution of all alpha diversity indices was significantly different for *I. holocyclus* from temperate broadleaf and mixed forests (TBMF) (*n* = 238) compared with *I. holocyclus* from tropical and subtropical moist broadleaf forests (TSMBF) (*n* = 91) (*P* < 0.05). However, only *Rh. sanguineus* from deserts and xeric scrublands (DXS) (*n* = 82) compared with *Rh. sanguineus* from tropical and subtropical grasslands, savannas and shrublands (TSGSS) (*n* = 78) had significantly different distributions for the ACE (*P* = 0.026) and Chao1 (*P* = 0.035) metrics. *Haemaphysalis longicornis* (all from TBMF) instars (females, *n* = 23; and nymphs, *n* = 24) had different distributions for all alpha diversity metrics (*P* < 0.05). *Ixodes holocyclus* from TBMF had different distributions for ACE and Chao1 metrics (*P* < 0.05) for females (*n* = 163) compared with males (*n* = 44) (*P* = 0.010 for ACE and *P* = 0.022 for Chao1) and for males (*n* = 44) compared with nymphs (*n* = 31) (*P* = 0.010 for ACE and *P* = 0.020 for Chao1), but there was no difference between females (*n* = 163) and nymphs (*n* = 163) (*P* < 0.05). The only other statistically significant difference in alpha diversity metrics for instars was for *I. tasmani* (all from TBMF) that had a difference in observed ZOTUs between females (*n* = 45) and nymphs (*n* = 6) (*P* = 0.019). There were also statistically significant differences in alpha diversity metrics for different host species for *I. holocyclus* females from TBMF (*P* < 0.05) for dogs compared with cats, and cats compared with horses, but *P* > 0.05 for dogs compared with horses. As the alpha diversity metrics were not statistically significantly different for *I. holocyclus* females and nymphs from TSMBF, females and nymphs from this ecoregion were assessed together for host differences for dogs (*n* = 39) and cats (*n* = 50), and there was a difference for all three metrics (*P* < 0.05). Alpha diversity was assessed for blood meal (feeding status) for *I. holocyclus* and *I. tasmani* females from different hosts (*n* > 5 for each group). There was only a statistically significant difference in the distribution of alpha diversity metrics for unfed (*n* = 7) and fed (*n* = 33) *I. tasmani* from dogs (*P* < 0.05) (Additional file 7).

### 3.7 Beta diversity

**Fig. 7:**
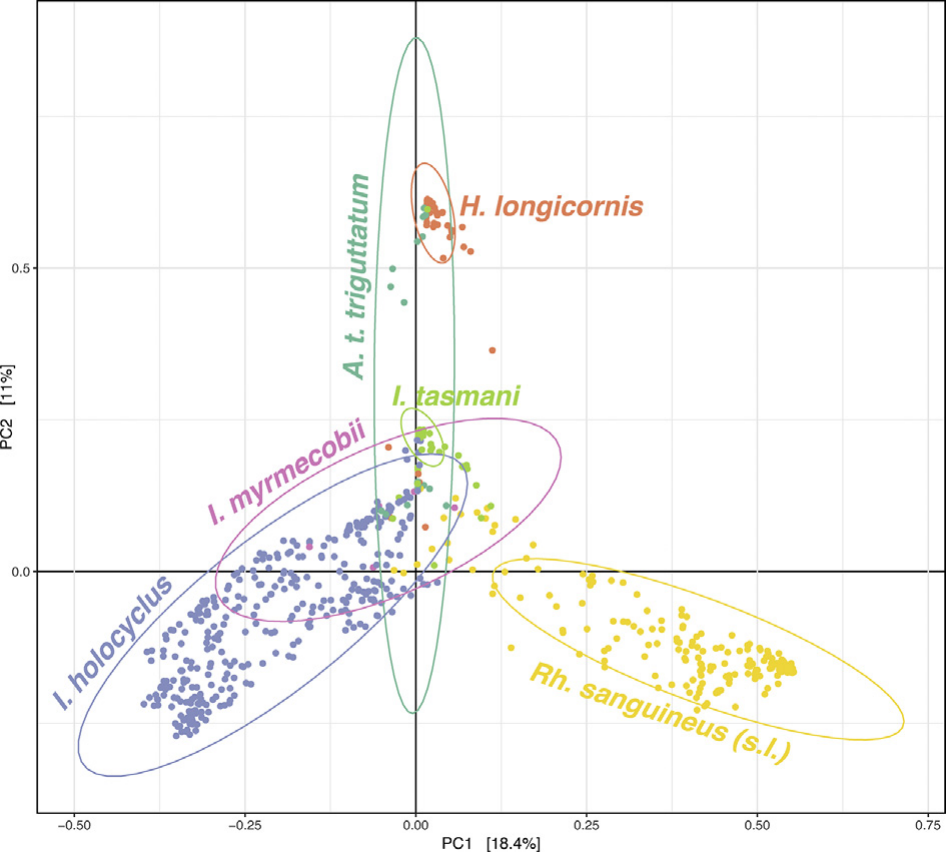
Principal coordinates analysis ordination plot based on Bray-Curtis distances between tick species. There was statistically significant separation of the Bray-Curtis distances for tick species (pseudo-*F* statistic, 45.4; *P* = 0.001). Clustering of *Ixodes holocyclus* compared with *Rhipicephalus sanguineus* (*s.l.*) had the highest pseudo-*F* statistic (111.2), indicating greater cluster separation, while clustering of *Ixodes myrmecobii* compared with *I. holocyclus* had the lowest pseudo-*F* statistic (2.1), indicating low cluster separation. Ellipsoids represent 95% confidence intervals for each group.

PERMANOVA was used to test whether Bray-Curtis distances were different between tick species and other variables that could influence ZOTU diversity, including ecoregion, instar, host species and feeding status. Tick species (*P* = 0.001), ecoregions for *I. holocyclus* and *Rh. sanguineus* (*P* = 0.015), *I. holocyclus* instars from TBMF and TSMBF, *Rh. sanguineus* instars from TSGSS (*P* = 0.001) and hosts of *I. holocyclus* instars from TBMF and TSMBF (*P* = 0.001) had a statistically significant separation of Bray-Curtis distances. A principal coordinates analysis (PCoA) plot for tick species is presented in Fig. 7. All PERMANOVA test statistics, *P-*values and PCoA plots for other groups are presented in Additional file 7 and Additional file 8.

### 3.8 Comparison of bacterial *16S* taxonomic assignment with RDP classifier, SILVA and Greengenes

For the tick-associated bacterial taxa that were compared in Table 6, Greengenes had the lowest number of incorrect taxonomic assignments at the family and genus levels (91.0% and 100.0% accuracy, respectively), but assigned 100% (4/4) of the ZOTUs to the incorrect species. Overall, SILVA had a higher percentage of correct assignments across all taxonomic levels (81.5%, 22/27), but had the lowest percentage of correct family level taxa (76.9%, 10/13) compared with Greengenes and RDP Classifier (83.3%, 10/12). RDP Classifier assigned only 41.7% (5/12) of the taxa to the appropriate genus (Table 6). SILVA did not provide species names for most ZOTUs, which was the appropriate option in most cases as many of the TABS ZOTUs do not have species names. However, the pathogens *A. platys*, *B. clarridgeiae*, *C. burnetii* and “*Ca.* M. haematoparvum” were only assigned at the genus level by SILVA and Greengenes. The family *Anaplasmataceae* had the correct taxonomic assignments made by SILVA, but RDP Classifier only assigned *A. platys* to the appropriate genus, and “*Ca.* Neoehrlichia” sequences were assigned the incorrect species (“*Candidatus* Neoehrlichia mikurensis”) with the Greengenes database with high confidence levels (CL) (0.96–1.00). Additionally, all six *Wolbachia* ZOTUs were assigned to the family *Rickettsiaceae* instead of *Anaplasmataceae* in Greengenes (CL 0.71–1.00). For *Bartonellaceae* ZOTUs, the family was assigned correctly by Greengenes, but SILVA and RDP Classifier misassigned the ZOTUs as *Rhizobiaceae*, except for *B. clarridgeiae* (ZOTU 3580), which was assigned correctly by RDP Classifier. However, SILVA assigned the three *Bartonella* ZOTUs to the correct genera, while Greengenes and RDP Classifier only provided the genus name *Bartonella* for *B. clarridgeiae* (ZOTU 3580). For 67 “*Ca.* Midichloria” ZOTUs, SILVA assigned 67.1% with the correct taxa, whereas Greengenes did not assign taxonomy at the family, genus or species level and RDP Classifier assigned 0% to the appropriate family and genus. All three databases performed taxonomic assignments well for *Coxiella* spp. and *Rickettsia* spp., with 0% assigned incorrect family or genus level taxonomy. Taxonomy could not be confidently determined for Legionellales sp. ZOTU 7 based on comparisons with NCBI nr/nt submissions and required further phylogenetic analysis. RDP Classifier was the only database that determined the correct family level taxonomy for Legionellales sp. ZOTU 7 (Table 6). Overall comparisons between the taxonomic assignments of all ZOTUs made with Greengenes, RDP Classifier, SILVA and the NCBI nr/nt BLAST results are provided in Additional file 9. Comparisons to the NCBI nr/nt database can be viewed in Additional file 5.

**Table 6:** Percent of correct taxonomic assignments with Greengenes, RDP Classifier and SILVA at family (F), genus (G) and species (S) levels

Family (no. of ZOTUs)		Greengenes				RDP Classifier			SILVA			
	A/U^a^	F (%)	G (%)	S (%)	Overall (%)	F (%)	G (%)	Overall (%)	F (%)	G (%)	S (%)	Overall (%)
***Anaplasmataceae* (*n* = 11)**	**A**	**45.5**	**100**	**0**	**72.7**	**100**	**9.1**	**54.5**	**100**	**100**	**–**	**100**
	**U**	**–**	**–**	**63.6**	**21.2**	**–**	**–**	**–**	**–**	**–**	**100**	**33.3**
*A. platys* (*n =* 1)	A	100	100	–	100	100	100	100	100	100	–	100
	U	–	–	100	33.3	–	–	–	–	–	100	33.3
“*Ca.* N. arcana” (*n* = 1)	A	100	100	0	66.7	100	0	50.0	100	100	–	100
	U	–	–	–	–	–	–	–	–	–	100	33.3
“*Ca.* N. australis” (*n* = 1)	A	100	100	0	66.7	100	0	50.0	100	100	–	100
	U	–	–	–	–	–	–	–	–	–	100	33.3
“*Ca.* Neoehrlichia spp.” (*n* = 2)	A	100	100	0	66.7	100	0	50.0	100	100	–	100
	U	–	–	–	–	–	–	–	–	–	100	33.3
*Wolbachia* spp. (*n* = 6)	A	0.0	100	–	50.0	100	0	50.0	100	100	–	100
	U	–	–	100	33.3	–	–	–	–	–	100	33.3
***Bartonellaceae* (*n* = 3)**	**A**	**100**	**50.0**	**0**	**57.1**	**33.3**	**33.3**	**33.3**	**0**	**100**	**–**	**50.0**
	**U**	**–**	**33.3**	**33.3**	**22.2**	**–**	**–**	**–**	**–**	**–**	**100**	**33.3**
*B. clarridgeiae* (*n* = 1)	A	100	100	–	100	100	100	100	0	100	–	50.0
	U	–	–	100	33.3	–	–	–	–	–	100	33.3
*B. apis* (*n* = 2)	A	100	–	–	100	0	0	0	0	100	–	50.0
	U	–	100	100	66.7	–	–	–	–	–	100	33.3
**“*Ca.* Midichloriaceae” (*n* = 70)**	**A**	**–**	**–**	**–**	**–**	**0**	**0**	**0**	**100**	**100**	**1.4**	**67.1**
	**U**	**100**	**100**	**100**	**100**	**–**	**–**	**–**	**–**	**–**	**90.0**	**30.0**
*“Ca.* Midichloria spp.” (*n* = 70)	A	–	–	–	–	0	0	0	100	100	0	66.7
	U	100	100	100	100	–	–	–	–	–	90.0	30.0
***Coxiellaceae* (*n* = 49)**	**A**	**100**	**100**	**–**	**100**	**100**	**98.0**	**99.0**	**98.0**	**98.0**	**–**	**98.0**
	**U**	**2.0**	**2.0**	**100**	**34.7**	**–**	**–**	**–**	**–**	**–**	**100**	**34.7**
*C. burnetii* (*n* = 1)	A	100	100	–	100	100	100	100	100	100	–	100
	U	–	–	100	33.3	–	–	–	–	–	1.0	33.3
*Coxiella* spp. (*n* = 47)	A	100	100	–	100	100	100	100	100	100	–	100
	U	–	–	100	33.3	–	–	–	–	–	100	33.3
Legionellales (ZOTU 7)^b^ (*n* = 1)	A	–	–	–	–	100	0	50.0	0	0	–	0
	U	100	100	100	100	–	–	–	–	–	100	33.3
***Mycoplasmataceae* (*n =* 1)**	**A**	**100**	**100**	**–**	**100**	**–**	**–**	**–**	**100**	**100**	**–**	**100**
	**U**	**–**	**–**	**100**	**33.3**	**100**	**100**	**100**	**–**	**–**	**100**	**33.3**
“*Ca.* M. haematoparvum” (*n* = 1)	A	100	100	–	100	–	–	–	100	100	–	100
	U	–	–	100	33.3	100	100	100	–	–	100	33.3
***Rickettsiaceae***	**A**	**100**	**100**	**–**	**100**	**100**	**100**	**100**	**100**	**100**	**–**	**100**
	**U**	**–**	**–**	**100**	**33.3**	**–**	**–**	**–**	**–**	**–**	**100**	**33.3**
*Rickettsia* spp.	A	100	100	–	100	100	100	100	100	100	–	100
	U	–	–	100	33.3	–	–	–	–	–	100	33.3
Grand total (*n* = 13)^c^	A	91.0 (10/11)	100 (10/10)	0 (0/4)	80.0 (20/25)	83.3 (10/12)	41.7 (5/12)	62.5 (15/24)	76.9 (10/13)	92.3 (12/13)	50.0 (1/2)	81.5 (22/27)
	U	15.4 (2/13)	23.1 (3/13)	76.9 (10/13)	38.5 (15/39)	7.7 (1/13)	7.7 (1/13)	7.7 (2/26)	0 (0/13)	0 (0/13)	100 (13/13)^d^	33.3 (13/39)

*Note:* Bacterial family percentages are presented in bold typeface.

a A, Percent of taxa assigned; U, Percent of taxa unassigned.

b This represents *Coxiellaceae* gen. sp. genotype ZOTU 7, refer to Table 8.

c ZOTUs grouped that belong under the same species name.

d Seven “*Candidatus* Midichloria sp.” ZOTUs were classified at the species level with the isolate name “*Ca* Midichloria sp. Ixholo1”.

### 3.9 *Rickettsia* species identification with NGS

All samples that were positive for *Rickettsia 16S* sequences were screened for *Rickettsia* 17 kDa, *gltA*, *ompA* and *ompB*, and only samples that were positive by cnPCR were sequenced with NGS. The *Rickettsia gltA* NGS assay outperformed the other assays, producing the largest number of *Rickettsia* ZOTUs (*n* = 23) and products had a length of ~336 bp. The 17 kDa NGS assay had ten *Rickettsia* ZOTUs and products of ~388 bp, but the *ompA* and *ompB* NGS assays underperformed with only three and one *Rickettsia* ZOTUs detected, respectively (Table 4). 28 of the samples screened with *gltA* NGS had low numbers of *Rickettsia* reads (0–9), and the *Rickettsia* reads present in these samples may be attributed to cross-talk, therefore are not presented in Table 7; refer to Additional file 5 for read totals. The ZOTUs that had the most abundant sequences for *gltA* are summarised in Table 7. “*Candidatus* Rickettsia tasmanensis” ZOTU 1 (MT914482) had a high sequence composition in most (19/20) *I. tasmani* ticks, but only 0.3% of “*Ca.* Ri. tasmanensis” (ZOTU 1) sequences were found in one *I. tasmani*, which was mostly composed of “*Candidatus* Rickettsia antechini” ZOTU 48 (MT914483) reads (Table 7). The predominant “*Ca.* Ri. tasmanensis” sequence (ZOTU 1) that was found in *I. tasmani* was 100% similar to “*Ca.* Ri. tasmanensis” (GQ223391) isolated from *I. tasmani* in TAS. “*Candidatus* Rickettsia antechini” (ZOTU 48) that was detected in one *I. tasmani* collected from a horse in QLD was 100% similar to “*Ca.* Ri. antechini” (DQ372954) isolated from ectoparasites of *Antechinus flavipes* (yellow-footed antechinus) in WA. “*Candidatus* Rickettsia jingxinensis” ZOTU 8 (MT914489) was identified in all *Haemaphysalis* ticks tested: four *H. bancrofti*; one *H. longicornis*; two *Haemaphysalis* sp. genotype 1; and one *Haemaphysalis* sp. genotype 2. “*Candidatus* Rickettsia jingxinensis” (ZOTU 8) was 100% similar to “*Ca.* Ri. jingxinensis” (MH500217) isolated from *H. longicornis* in China. *Rickettsia gravesii* ZOTU 4 (MT914486), 100% similar to *Ri. gravesii* (DQ269435) isolated from *A. t. triguttatum* in WA, was identified in all 11 *A. t. triguttatum* that were cnPCR *gltA-*positive. *Rickettsia gravesii* (ZOTU 4) was predominantly found in seven *A. t. triguttatum*, with sequence compositions ranging from 99.8 to 99.9%, while four *A. t. triguttatum* had lower *Ri. gravesii* (ZOTU 4) sequence compositions (27.7–87.4%). The dominant *Rickettsia gltA* sequences in one *I. holocyclus* were *Ri. gravesii* (ZOTU 4). The four *A. t. triguttatum* that had lower *Ri. gravesii* (ZOTU 4) sequence compositions were co-infected with other *Rickettsia* genotypes that had sequence compositions ranging from 1.0 to 29.2%, and these genotypes were most similar (97.9–99.1%) to *Ri. raoultii* (MH267733) and *Ri. gravesii* (DQ269435) isolates (Table 7).

**Table 7:** NCBI nr/nt BLAST results for *Rickettsia* species identified at the 17 kDa, *gltA* and *ompA* loci with NGS

Species (ZOTU no.)	GenBank ID	Top match NCBI nr/nt database	GenBank ID	Similarity (%)	Tick species	Sample ID (*x*/*n*)	Sequence composition (%)	No. of *Rickettsia* sequences
17 kDa								
*Rickettsia* sp. (ZOTU 2)	MT914472	*Ri.raoultii*	MH212173	99.7	*A. t. triguttatum*	NoMBA2; DT4P1D6; DT3P2F1 (3/3)	56.6–99.8	8,657–28,087
*Rickettsia* sp. (ZOTU 3)	MT914477	*Ri.raoultii*	MH212173	99.5	*I. tasmani*	NoMBB1 (1/2)	98.2	13,902
*Rickettsia* sp. (ZOTU 5)	MT914473	*Rickettsia* sp.	MH177454	100	*H. longicornis*	DT3P1D8 (1/1)	33.3	9
*Rickettsia* sp. (ZOTU 20)	MT914478	*Ri.raoultii*	MH212177	99.0	*A. t. triguttatum*	DT3P2F1 (1/3)	21.3	28,087
*Rickettsia* sp. (ZOTU 25)	MT914474	*Ri.sibirica*	MF002549	99.5	*I. tasmani*	DT1P1F5 (1/2)	96.3	2,561
*Rickettsia* sp. (ZOTU 29)	MT914479	*Rickettsia* sp.	KY576906	99.0	*A. t. triguttatum*	DT4P1D6 (1/3)	21.6	8,657
*Rickettsia* sp. (ZOTU 32)	MT914475	*Ri.raoultii*	MH212177	99.0	*A. t. triguttatum*	DT3P2F1 (1/3)	8.0	28,087
*Rickettsia* sp. (ZOTU 36)	MT914480	*Ri.raoultii*	MH212177	99.2	*A. t. triguttatum*	DT3P2F1 (1/3)	6.4	28,087
*Rickettsia* sp. (ZOTU 75)	MT914476	*Ri.raoultii*	MH212177	99.2	*A. t. triguttatum*	DT4P1D6 (1/3)	6.3	8,657
*Rickettsia* sp. (ZOTU 81)	MT914481	*Rickettsia* sp.	KY576906	98.7	*A. t. triguttatum*	DT4P1D6 (1/3)	2.8	8,657
*gltA*								
“*Ca.* Rickettsia tasmanensis” (ZOTU 1)	MT914482	“*Ca.* Ri. tasmanensis”	GQ223391	100	*I. tasmani*	(19/20)^a^	99.7–99.9	1,655–23,687
						P2E11 (1/20)	0.3	5,989
*Rickettsia gravesii* (ZOTU 4)	MT914486	*Ri.gravesii*	DQ269435	100	*A. t. triguttatum*	(7/11)^a^	99.8–99.9	878–9,272
						P1E12; P1H6; P2B3; P2E10 (4/11)	27.7–87.4	3,228–8,872
					*I. holocyclus*	P2E9 (1/1)	90.4	7,312
“*Ca.* Rickettsia jingxinensis” (ZOTU 8)	MT914489	“*Ca.* Ri. jingxinensis”	MH500217	100	*H. bancrofti*	(4/4)^a^	99.1–99.4	1,854–11,024
					*H. longicornis*	P2C6 (1/1)	99.4	5,612
					*Haemaphysalis* sp. genotype 1	P1F3; P2E3 (2/2)	99.2	3,063
					*Haemaphysalis* sp. genotype 2	P1F5 (1/1)	99.2	5,590
“*Ca.* Rickettsia antechini” (ZOTU 48)	MT914483	“*Ca.* Ri. antechini”	DQ372954	100	*I. tasmani*	P2E11 (1/20)	99.6	5,989
*Rickettsia* sp. (ZOTU 111)	MT914493	*Ri.raoultii*	MH267733	98.2	*A. t. triguttatum*	P2E10; P1E12 (2/11)	6.9–37.9	4,164–6,973
*Rickettsia* sp. (ZOTU 177)	MT914490	*Ri.raoultii*	MH267733	99.1	*A. t. triguttatum*	P1E12; P1H6; P2B3; P2E10 (4/11)	1.6–29.2	3,228–8,872
*Rickettsia* sp. (ZOTU 318)	MT914484	*Ri.gravesii*	DQ269435	99.1	*A. t. triguttatum*	P2E10; P1E12 (2/11)	6.8–7.0	4,164–6,973
*Rickettsia* sp. (ZOTU 320)	NS^b^	*Ri.raoultii*	MH267733	99.1	*A. t. triguttatum*	P1E12; P1H6; P2B3; P2E10 (4/11)	1.2–4.4	3,228–8,872
*Rickettsia* sp. (ZOTU 430)	MT914491	*Ri.gravesii*	DQ269435	97.9	*A. t. triguttatum*	P2E10; P1E12 (2/11)	2.8–8.1	4,164–6,973
*Rickettsia* sp. (ZOTU 469)	NS^b^	*Ri.gravesii*	DQ269435	98.8	*A. t. triguttatum*	P2B3; P1H6 (2/11)	2.8–8.5	3,228–8,872
*Rickettsia* sp. (ZOTU 479)	MT914485	*Ri.raoultii*	MH267733	98.8	*A. t. triguttatum*	P2E10; P1E12 (2/11)	1.6–4.7	4,164–6,973
*Rickettsia* sp. (ZOTU 503)	MT914494	*Ri.gravesii*	DQ269435	98.2	*A. t. triguttatum*	P2E10; P1E12 (2/11)	1.7–2.5	4,164–6,973
*Rickettsia* sp. (ZOTU 742)	MT914488	*Ri.gravesii*	DQ269435	98.8	*A. t. triguttatum*	P2E10; P1E12 (2/11)	1.6–3.8	4,164–6,973
*Rickettsia* sp. (ZOTU 999)	MT914495	*Ri.gravesii*	DQ269435	98.5	*A. t. triguttatum*	P2E10; P1E12 (2/11)	1.1–1.5	4,164–6,973
*Rickettsia* sp. (ZOTU 1029)	MT914492	*Ri.raoultii*	MH267733	98.2	*A. t. triguttatum*	P2E10 (1/11)	1.0	6,973
*ompA*								
*Rickettsia* sp. (ZOTU 13)	MT900476	*Rickettsia* sp.	KT835150	99.4	*A. t. triguttatum*	ompAB1; ompAE4; ompAE5 (3/3)	33.9–100	59–1,093
					*I. tasmani*	ompAE1 (1/3)	23.4	231
*Rickettsia* sp. (ZOTU 20)	MT900477	*Rickettsia* sp.	KT835145	98.8	*I. tasmani*	ompAE1 (1/3)	76.6	231
					*A. t. triguttatum*	ompAE4; ompAE5 (2/3)	22.6–66.1	59–137
“*Ca.* Rickettsia tasmanensis” (ZOTU 26)	MT900478	“*Ca.* Ri. tasmanensis”	GQ223392	100	*I. tasmani*	ompAC7; ompAD6 (2/3)	92.3–100	13–43

a Refer to Appendix B, Electronic File B.4 or SRA (PRJNA640465) for Sample IDs.

b Not submitted to GenBank as error detected in translated protein sequence.

### 3.10 Near full-length *16S* sequence analysis of *Coxiella* sp., Legionellales and *Francisella* spp.

**Fig. 8:**
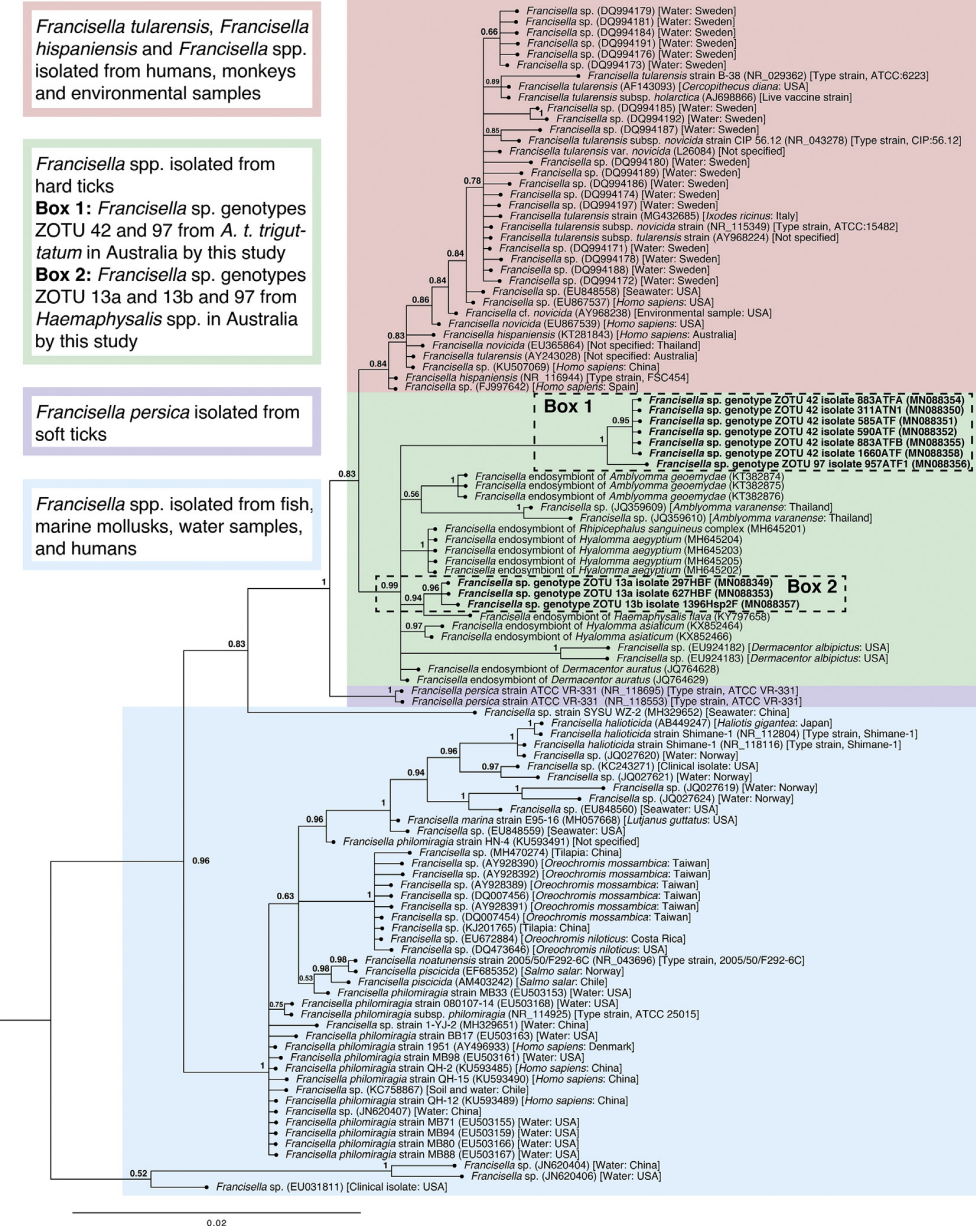
Bayesian phylogenetic tree of *16S* sequences of *Francisella* species. The alignment (including gaps) is 1,088 bp. The tree was built using the following parameters: HKY85 + G + I model; 1,100,000 Markov chain Monte Carlo (MCMC) length; “burn-in” length of 10,000; subsampling frequency of 200. The tree was rooted with the outgroup sequence *Legionella pneumophila* strain Philadelphia 1 (NR_074231) (not shown). The scale-bar indicates the number of nucleotide substitutions per site. Sequences from this study are in bold typeface in Boxes 1 and 2.

A 1,365 bp *Coxiella* sp. *16S* sequence (MN088359) was obtained *via* Sanger sequencing, and when compared to the *Coxiella* ZOTUs, was most similar [99.7% sequence similarity, one single nucleotide polymorphism (SNP)] to *Coxiella* sp. ZOTU 82 (MT914333) over 287 bp. The closest NCBI nr/nt match to *Coxiella* sp. ZOTU 82 had 98.6% similarity to *Coxiella* sp. (JQ480818) from *Rh. turanicus* in Israel, but the ~1.3 kb *Coxiella* sp. *16S* sequence (MN088359) was 99.8% similar to *Coxiella* sp. (KP994830) isolated from *Rh. australis* in New Caledonia (Table 8). Ten *Francisella 16S* sequences 1,066–1,085 bp in length were obtained from *H. bancrofti*, *Haemaphysalis* sp. genotype 2 and *A. t. triguttatum*. The ~1 kb *Francisella* sequences were compared to the *Francisella 16S* NGS ZOTUs (genetic distances presented in Additional file 10). *Francisella* isolates from two *H. bancrofti* (MN088349 and MN088353) and one *Haemaphysalis* sp. genotype 2 (MN088357) were 100% identical to ZOTU 13 (MT914315) across 152–154 bp. The ~1 kb *Francisella* sequences from *Haemaphysalis* spp. had 0.2% sequence dissimilarity. Six of the ~1 kb *Francisella* isolates from *A. t. triguttatum* were 100% similar to ZOTU 42 (MT914326) across 152–154 bp, and five of the ~1 kb *Francisella* isolates (MN088350-MN088352, MN088354, MN088355 and MN088358) were 100% similar to each other. *Francisella* genotype ZOTU 97 (MN088356) was 99.9% similar to the other five *Francisella* isolates from *A. t. triguttatum* and was 100% similar to *Francisella* sp. ZOTU 97 (MT914335) across 154 bp. When the ~1 kb *Francisella* isolates were compared with NCBI nr/nt submissions, *Francisella* sp. genotypes ZOTU 13a (MN088349 and MN088353) and 13b (MN088357) were most similar (99.5–99.7%) to *Francisella* sp. (JQ764629) from *Dermacentor auratus* from Thailand, *Francisella* sp. genotype ZOTU 42 (MN088350, MN088352, MN088355, MN088358, MN088351 and MN088354) was most similar (97.8–97.9%) to *Francisella* sp. (JQ764629) and *Francisella* sp. genotype ZOTU 97 (MN088356) was most similar (97.7%) to *Francisella* sp. (JQ764629). *Francisella* sp. genotype ZOTU 13a and 13b were 2.2–2.3% dissimilar to *Francisella* sp. genotype ZOTU 42 and 2.4–2.5% dissimilar to *Francisella* sp. genotype ZOTU 97, and *Francisella* sp. genotype ZOTU 42 was 0.6–0.7% dissimilar to *Francisella* sp. genotype ZOTU 97 (Additional file 10). Phylogenetic analysis of *Francisella* sequences > 1 kb showed that the *Francisella* sequences obtained in this study were distinct from a clade of *Francisella tularensis* and *Francisella hispaniensis* sequences. They grouped with a clade of *Francisella* endosymbionts of hard ticks with strong support (posterior probability (pp) = 0.99) (Fig. 8).

**Table 8:** Top NCBI nr/nt database hits to near full-length *Francisella*, Legionellales sp. and *Coxiella* sp.

Species	Closest *16S* ZOTU no. match	GenBank ID	Tick species, instar/sex (sample ID)	Tick host and collection location	Top NCBI nr/nt database match (GenBank ID)	Percent identity (%)	Query cover (%)
*Coxiella* sp. genotype ZOTU 82	82	MN088359	*Rh. australis*, nymph (H20RAN)	Dog, Sarina, QLD	KP994830	99.8	88
*Francisella* sp. genotype ZOTU 13a	13	MN088349	*H. bancrofti*, female (297HBF)		JQ764629	99.6	100
		MN088353	*H. bancrofti*, female (627HBF)			99.7	
*Francisella* sp. genotype ZOTU 13b		MN088357	*Haemaphysalis* sp. genotype 2 (1396Hsp2F)			99.5	
*Francisella* sp. genotype ZOTU 42	42	MN088350	*A. t. triguttatum*, nymph (311ATN1)		JQ764629	97.9	100
		MN088352	*A. t. triguttatum*, female (590ATF)				
		MN088355	*A. t. triguttatum*, female (883ATFB)				
		MN088358	*A. t. triguttatum*, female (1660ATF)				
		MN088351	*A. t. triguttatum*, female (585ATF)			97.8	
		MN088354	*A. t. triguttatum*, female (883ATFA)				
*Francisella* sp. genotype ZOTU 97	97	MN088356	*A. t. triguttatum*, female (957ATF1)		JQ764629	97.7	100
*Coxiellaceae* gen. sp. genotype ZOTU 7	7	MN088348	*I. tasmani*, nymph (1628ITN)	Dog, Northdown, TAS	*Coxiellaceae* bacterium (EU430251)	97.9	60

**Fig. 9:**
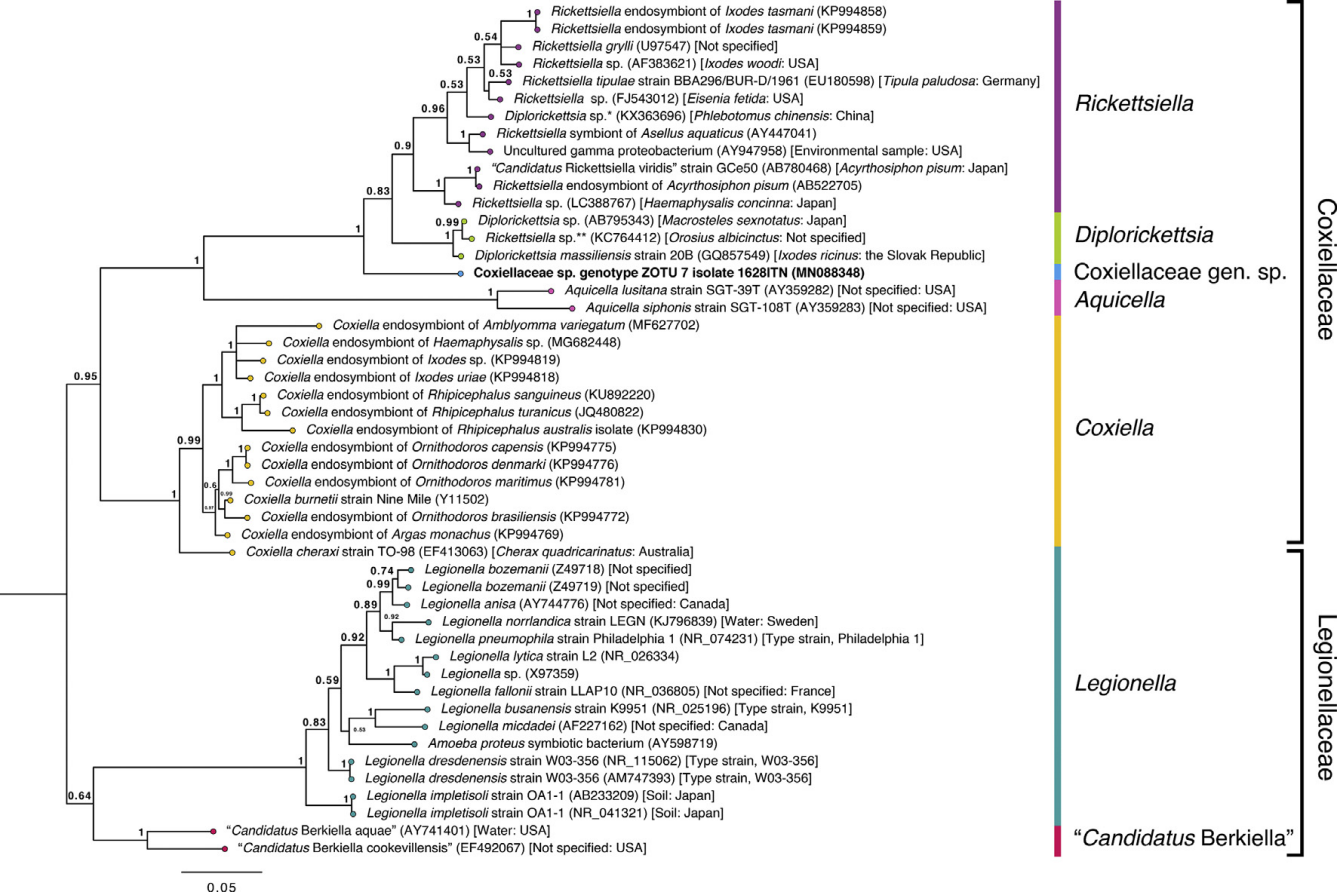
Bayesian phylogenetic tree of *16S* sequences of Legionellales species, including *Coxiellaceae* gen. sp. The alignment was 1,075 bp (including gaps) in length. The tree was built using the following parameters: GTR + G model; 1,100,000 MCMC length; “burn-in” length of 10,000; subsampling frequency of 200. The tree was rooted with the outgroup sequence *Francisella tularensis holarctica* strain FSC 257 (AY968231) (not shown). The scale-bar indicates the number of nucleotide substitutions per site.

The 1,501 bp sequence obtained from *I. tasmani* (MN088348) was 100% similar to Legionellales sp. ZOTU 7 (MT914309) across 326 bp, and when compared with NCBI nr/nt submissions, was most similar (97.9%) to a *Coxiellaceae* sp. (EU430251) previously isolated from *I. tasmani* collected from *Sarcophilus harrisii* (Tasmanian devil) in TAS (Table 8). A phylogenetic tree (Fig. 9) constructed of ~1 kb *16S* sequences of Legionellales species supported the grouping of the ~1.5 kb sequence from *I. tasmani* [*Coxiellaceae* gen. sp. genotype ZOTU 7 (MN088348)] in the family *Coxiellaceae* (pp = 0.95). There was also strong support (pp = 1.0) for the paraphyletic grouping of *Coxiellaceae* gen. sp. genotype ZOTU 7 to *Diplorickettsia* and *Rickettsiella* species (Fig. 9). *Coxiellaceae* sp. (EU430251) from *I. tasmani* was not included in the phylogenetic tree to allow for improved taxonomic resolution as the sequence was < 1 kb. Genetic distances are presented in Additional file 10.

## 4 Discussion

### 4.1 Pathogens

The haemotropic bacterial pathogen *A. platys* was identified in 6.9% (12/174; 95% CI: 3.6–11.7%) of *Rh. sanguineus* (*s.l.*) ticks removed from dogs in the Torres Strait (QLD), northern WA, Perth (WA), northern and central NT and northern SA. *Anaplasma platys* causes canine infectious cyclic thrombocytopenia (CICT) and is a zoonotic agent (Maggi et al., 2013b; Arraga-Alvarado et al., 2014; Breitschwerdt et al., 2014). This pathogen occurs in dogs from the NT (Martin et al., 2005; Brown et al., 2006; Barker et al., 2012; Shapiro et al., 2017) (77.8%; 7/9), NSW (Brown et al., 2006; Barker et al., 2012; Shapiro et al., 2017) and southeast QLD (Hii et al., 2012), but has not been previously reported from Perth or from the Torres Strait. *Rhipicephalus sanguineus* (*s.l.*) is a suspected vector of *A. platys* as DNA of *A. platys* has been detected in *Rh. sanguineus* (*s.l.*) ticks by many other studies outside of Australia (Sanogo et al., 2003; Kamani et al., 2013; Latrofa et al., 2014; Ramos et al., 2014; Estrada-Pena et al., 2017). However, experimental demonstration of vector competency is required (Simpson et al., 1991; Snellgrove et al., 2020). Similarly, the haemoplasma “*Ca.* M. haematoparvum”, which was identified in a *Rh. sanguineus* (*s.l.*) tick from a dog in the NT (2.0%, 1/50; 95% CI: 0.1–10.6%), is zoonotic (Maggi et al., 2013a) and may be vectored by *Rh. sanguineus* (*s.l.*) in Australia.

An engorged *I. holocyclus* collected from a cat was positive for *B. clarridgeiae* in QLD (0.8%, 1/122; 95% CI: 0–4.5%). *Bartonella clarridgeiae* occurs in QLD cats (Barrs et al., 2010), and its suspected vector is the cat flea (*Ctenocephalides felis*) (Bouhsira et al., 2013). Only one engorged *I. holocyclus* (0.3%; 1/334; 95% CI: 0–1.7%) was positive for the zoonotic pathogen *C. burnetii*, the causative agent of Q fever in humans. Coxiellosis infections, or evidence of exposure to *C. burnetii* with serological tests, have been found in dogs, cats and horses in Australia (Cooper et al., 2011, 2012; Kopecny et al., 2013; Tozer et al., 2014; Shapiro et al., 2016). *Coxiella burnetii* can be transmitted *via* exposure to infected animals (Potter et al., 2011; Cooper et al., 2013) and their infected by-products (Tozer et al., 2014), or *via* inhalation of aerosolised particles (Schneeberger et al., 2014). Companion animals (cats and dogs) can be a source of *C. burnetii* infection for humans (Cann et al., 1996) and cats have caused Q fever outbreaks amongst veterinary staff in NSW and QLD (Maywood and Boyd, 2011; Malo et al., 2018). *Ixodes holocyclus* and *H. humerosa* (bandicoot tick) have been implicated as vectors for *C. burnetii* (Smith, 1940, 1942). The low prevalence of *C. burnetii* detected in *I. holocyclus* collected from Q fever endemic areas in the present study (0.3%, 1/334; 95% CI: 0.1–10.6%) suggests that the pathogen either occurs at a low prevalence in *I. holocyclus*, or that *I. holocyclus* may not be a vector of *C. burnetii*. To note, a limitation of this study is that the ticks found positive for pathogens were either engorged with host blood or the feeding status was not recorded, therefore it is not possible to ascertain whether the ticks or the hosts were infected.

### 4.2 Endosymbionts

Accordingly, with other *16S* NGS studies on tick microbiomes (Andreotti et al., 2011; Carpi et al., 2011; Lalzar et al., 2012; Hawlena et al., 2013; Budachetri et al., 2014; Ponnusamy et al., 2014; Qiu et al., 2014; Zhang et al., 2014; Gofton et al., 2015b), this study found a dominant sequence composition and a high prevalence of bacterial endosymbionts (Fig. 2 and Table 5). “*Candidatus* Midichloria mitochondrii” and “*Ca.* C. massiliensis” have been previously found in *I. holocyclus* and *Rh. sanguineus* ticks, respectively, from Australia (Beninati et al., 2009; Gofton et al., 2015b; Oskam et al., 2017, 2018). Of note, “*Candidatus* Midichloria sp.” ZOTU 1 and “*Ca.* Midichloria sp.” ZOTU 2 have not been referred to as “*Ca.* M. mitochondrii” in this study as the sequences were 2.1–3.1% dissimilar to “*Ca.* Mi. mitochondrii”. Furthermore, there was 3.8% sequence dissimilarity between “*Ca.* Midichloria sp.” ZOTU 1 and “*Ca.* Midichloria sp.” ZOTU 2. Therefore, it is likely that these two different “*Ca.* Midichloria” ZOTUs are two different species, originally sequenced from *I. holocyclus* by Beninati et al. (2009) for two reasons: (i) interspecific distances of full length *16S* sequences from bacteria can be < 1% (Janda and Abbott, 2007); and (ii) “*Candidatus* Midichloria spp.” have not been observed in the ovarian cell mitochondria in *I. holocyclus* (Beninati et al., 2009), unlike “*Ca.* Mi. mitochondrii” originally described in *I. ricinus* in Europe (Sacchi et al., 2004). The pathogenicity of “*Ca.* Midichloria spp.” and their role in the tick microbiome is yet to be investigated.

Bacterial endosymbionts play important roles in ticks, such as promoting tick survival (Zhong et al., 2007; Clayton et al., 2015; Smith et al., 2015) and can influence the acquisition, colonisation and transmission of TBPs (Dib et al., 2008; Telford and Wormser, 2010; Ahantarig et al., 2013; Narasimhan et al., 2014; Gall et al., 2016). *Coxiella* sp. ZOTU 4 found in *H. longicornis* from Australia was most similar (100%) to the *Coxiella* endosymbionts detected from *H. longicornis* from Japan (AB001519), Korea (AY342035; AY342036), China (JN866564) and from *Haemaphysalis lagrangei* and *Haemaphysalis* sp. in Thailand (KC170756; KC170757). The *Coxiella* endosymbiont of *H. longicornis* in China has a beneficial role in tick survival by promoting tick reproduction and development (Zhang et al., 2017). “*Candidatus* Coxiella massiliensis” may have a similar role in *Rh. sanguineus* (*s.l.*), and has also been linked to human infections (Angelakis et al., 2016). Studies are required to determine the roles of endosymbionts in tick microbiomes in Australia, whether they can be transmitted to hosts by ticks and if they have any pathological effects.

### 4.3 Novel species

This study demonstrated the utility of *16S* amplicon NGS with the MiSeq platform for the discovery of novel bacteria. Many tick-associated ZOTUs detected showed < 99.0% similarity to sequences in the NCBI nr/nt database (Additional file 5). However, as only short (~300 bp) *16S* V1-2 regions were sequenced, further sequencing of near full-length *16S* and other loci is required for species novelty confirmation and description. For example, *Coxiella* sp. ZOTU 82 obtained from *Rh. australis* in this study appeared to be a novel species, with 98.6% similarity to its nearest GenBank match, *Coxiella* sp. (JQ480818) from *Rh. turanicus*. However, the > 1.3 kb *Coxiella* sp. sequenced obtained from *Rh. australis* by Sanger sequencing in this study had 99.8% similarity to a *Coxiella* endosymbiont previously isolated from *Rh. australis* that only spanned 123 bp of *Coxiella* sp. ZOTU 82 and did not appear in the top 60 BLAST results. Sanger sequencing and phylogenetic analysis of > 1 kb *16S* sequences provided evidence of a novel *Coxiellaceae* genus and species, and novel *Francisella* species and genotypes (Fig. 8, Fig. 9, Table 8, and Additional file 10). *Rickettsiella* species from the family *Coxiellaceae* are associated with pathological effects in arthropods (Cordaux et al., 2007). *Rickettsiella* species have been previously identified in *I. tasmani* in VIC (Vilcins et al., 2009a) and a variety of *Rickettsiella* species were also detected in the present study (Additional file 5). The genus *Diplorickettsia*, recently described from *I. ricinus* in the Slovak Republic (Mediannikov et al., 2010), is also part of the family *Coxiellaceae* and *Diplorickettsia massiliensis* has been isolated from humans in France (Subramanian et al., 2012). *Coxiellaceae* gen. sp. ZOTU 7 (MT914309) was identified in 51.7% (30/58; 95% CI: 38.2–65.0%) of *I. tasmani* females, nymphs and males (Additional file 5) with average *16S* sequence compositions of 42.9 ± 47.4% (Fig. 2 and Table 5).

Aside from the present study, there has been only one other published study that has characterised near full-length *16S* of *Francisella* (currently misnamed as *Rickettsia* sp. on GenBank) in ticks from Australia (*Amblyomma fimbriatum* collected from reptiles in the NT) (Vilcins et al., 2009b). Between the three different *Francisella* sp. genotypes obtained by that study, EU283840-2 were most similar (97.5–99.4%) to *Francisella* sp. genotype 13a (MN088349 and MN088353) from *H. bancrofti*. The *Francisella* sequences from *A. t. triguttatum* and *Haemaphysalis* species in this study were distinct from the clade that includes the zoonotic pathogen *Francisella tularensis*, which occurs in the Northern Hemisphere (but has also been detected in Australia, discussed below) and can be transmitted to humans by ticks, flies, mosquitoes, direct contact with infected animals, ingestion of contaminated food or water or *via* inhalation of infective aerosols (Kingry and Petersen, 2014). *Francisella tularensis* subsp. *novicida*-like (Whipp et al., 2003) and *Francisella hispaniensis* (Aravena-Román et al., 2015), first isolated from human blood in Spain (Huber et al., 2010), have been diagnosed in Australian patients (Whipp et al., 2003). *Francisella tularensis* subsp. *holarctica* biovar *japonica* was recently identified in ringtail possums from Sydney, NSW (Eden et al., 2017). All *Francisella* ZOTUs obtained in the present study were distinct from these isolates (Fig. 8). The novel *Francisella* species in *A. t. triguttatum*, novel *Francisella* genotypes in *Haemaphysalis* spp. and *A. t. triguttatum* and novel *Coxiellaceae* gen. sp. in *I. tasmani* identified by this study require further investigation of their genetic and phenotypic characteristics, their role in ticks, and whether their transmission cycle occurs outside of ticks. Unfortunately, as no live ticks were received, bacteria could not be cultivated by this study. This is the next logical step in research on tick-associated microbes that may impact human and animal health.

### 4.4 Bacterial diversity

Several studies have shown that a combination of factors including tick species, geography, climate, host species and blood-feeding have an influence on the microbiome of ticks (Carpi et al., 2011; Lalzar et al., 2012; Hawlena et al., 2013; Menchaca et al., 2013; Ponnusamy et al., 2014; Qiu et al., 2014; Williams-Newkirk et al., 2014; Zhang et al., 2014; Rynkiewicz et al., 2015; Van Treuren et al., 2015; Trout Fryxell and DeBruyn, 2016; Zolnik et al., 2016; Abraham et al., 2017; Gurfield et al., 2017; Swei and Kwan, 2017). The present study was able to demonstrate that the bacterial microbiome diversity was unique to each tick species (Fig. 7) and was affected by other variables considered (ecoregions, instars and host species). However, there was a lack of statistical support regarding the feeding status (Additional file 7 and Additional file 8), which may be due to host influences on the bacterial composition of ticks. Despite rinsing with bleach and vigorous vortexing, the hostʼs skin microflora could be retained within grooves and crevices on the exoskeleton of the tick.

### 4.5 *Rickettsia*-specific NGS assay

Tick-associated pathogens of humans in Australia include the SFGR species *Rickettsia australis*, *Rickettsia honei* and *Rickettsia honei marmionii*. These pathogens cause Queensland tick typhus, Flinders Island spotted fever and Australian spotted fever, respectively. There is also evidence of exposure of dogs and cats to SFGR in Australia (Sexton et al., 1991; Izzard et al., 2010). *Ixodes holocyclus* and *I. cornuatus* may transmit *Ri. australis* and *Ri. honei*, respectively, to companion animals (Domrow and Campbell, 1974; Graves et al., 1993, 2016). However, this study did not detect these SFGR pathogens in ticks (Table 7). The most dominant rickettsial sequences identified were “*Ca.* Ri. tasmanensis” in *I. tasmani*, *Ri. gravesii* in *A. t. triguttatum* and “*Ca.* Ri. jingxinensis” in *Haemaphysalis* spp. (Table 7). Interestingly, “*Ca.* Ri. antechini” has been previously reported in ectoparasites from the yellow-footed antechinus in WA (DQ372954), but in this study “*Ca.* Ri. antechini” was detected in an *I. tasmani* female that had fed on a horse from Kuranda, QLD (Table 7). The yellow-footed antechinus is distributed in QLD and *I. tasmani* is known to feed on the small marsupial (Roberts, 1960). Therefore, it is hypothesised that the *I. tasmani* tick fed on the marsupial that hosted “*Ca.* Ri. antechini” as a larva and/or nymph before feeding on the horse as an adult.

To the authors’ knowledge, this research is the first to report “*Ca.* Ri. jingxinensis” in *Haemaphysalis* spp. in Australia. “*Candidatus* Rickettsia jingxinensis” has been reported in *H. longicornis* and *Rh. microplus* ticks, and a human from China, although the pathogenicity of this *Rickettsia* species is not yet confirmed (Liu et al., 2016; Guo et al., 2018). Overall, the rickettsial *gltA* NGS assay, which targeted nucleotide positions 797–815 to 1,178–1,157 relative to the open reading frame, was a useful tool to identify rickettsial species that could not be distinguished at *16S* and was able to identify *Rickettsia* co-infections in *A. t. triguttatum* and *I. tasmani*, and potentially novel *Rickettsia* species or genotypes most similar (97.9–99.1%) to *Ri. raoultii* (MH267733) and *Ri. gravesii* (DQ269435) isolates (Table 7). However, the NGS assays for the 17 kDa, *ompA* and *ompB* loci require further NGS optimisation.

### 4.6 Prevalence estimates with *16S* NGS

One of the major caveats of the MiSeq platform for multiplexing samples is the occurrence of cross-talk, which can be as high as 10% and results in false-positives (Sinha et al., 2017). There are many ways that cross-talk can occur when using the MiSeq platform: (i) during the first stage PCR, the adapter sequences can bind to indices incorporated into amplicons from previous MiSeq assays, which emphasises the importance of a unidirectional workflow for NGS library preparation; (ii) the clean-up step for primer dimers after the first stage PCR is prone to cross-contamination of unindexed amplicons with MiSeq adapter sequences (and was therefore removed from this studyʼs library preparation procedure); (iii) index hopping (MacConaill et al., 2018), index or amplicon cross-contamination and PCR error in the indices during the second stage PCR; and (iv) sequencing error of the indices during the sequencing assay. A number of methods have been proposed in recent years to aid in mitigating cross-talk, including quality filtering of index reads (Wright and Vetsigian, 2016), bioinformatic algorithms (Edgar, 2018a), and for shear ligation library building methods, a unique molecular identifier (UMI) (MacConaill et al., 2018). However, the application of such methods does not overcome the issue of cross-contamination of amplicons that can occur during the first and second stage PCR setup.

In this study, the prevalence of cross-talk reads (due to cross-contamination of amplicons and indices during the first and second stage PCR setup) was estimated to be in a range between 0.012 and 1.8%, based on the proportion of TABS identified in the ExCs and NTCs (Additional file 2, Table S1 and Additional file 3). The reads identified in the ICs were low in number, e.g. for *16S* NGS the read totals ranged between 10 and 46 (Table 3). These ICs had indices added to them, but no DNA. The largest number of reads in each of these samples were from the most abundant TABS in the library, including *Coxiella*, *Coxiellaceae* gen. sp., “*Ca.* Midichloria” and *Rickettsia* (Additional file 3 and Table 5). This is likely due to PCR error incorporated into the indices during the second stage PCR or sequencing error, and the percent of misassigned sequences due to this was very low, 4.5 × 10^−6^% (146/32,691,272) (Table 3). This study could not bioinformatically remove all the ZOTUs found in the ExCs, NTCs or ICs from the samples for contaminant filtering because tick-associated bacteria and pathogen ZOTUs were found in the controls. This would cause tick-associated ZOTUs to be removed from the samples and grossly underestimate prevalence. Also, sequences present in low abundance (e.g. the sequences that made up <1% of the dataset) could not be removed as this would cause less abundant sequences from pathogens, such as *B. clarridgeiae* and “*Ca.* M. haematoparvum”, to be removed as well. The use of filtering thresholds to reduce false positives for prevalence estimates by this study was only tested for accuracy for *C. burnetii* by qPCR. Other pathogens and endosymbionts detected by this study should be assessed by future studies with qPCR or single PCR to determine the overall accuracy of the thresholds applied to control for false positives.

The prevalence of “*Ca.* N. arcana” and “*Ca.* N. australis” was estimated by this study to be 2.1% (7/334; 95% CI: 0.8–4.3%) and 8.4% (28/334; 95% CI: 5.6–11.9%), respectively. This is similar to the prevalence of “*Ca.* N. arcana” and “*Ca.* N. australis” *16S*, *groESL* and *gltA* sequences assessed by Gofton et al. (2016) with nested cnPCR, which was 3.1% (12/391; 95% CI: 1.6–5.3%) and 8.7% (34/391; 95% CI: 6.1–11.9%), respectively (Gofton et al., 2016). Previous NGS studies of “*Ca.* Neoehrlichia spp.” in *I. holocyclus* in Australia have reported a prevalence of 7.7% (15/196) using the Ion Torrent PGM platform (Gofton et al., 2015b), and 88.9% (248/279) with the MiSeq platform (Gofton et al., 2015a), with the latter high prevalence likely due to cross-talk that was not considered for mitigating false positives. The lower prevalence of “*Ca.* Neoehrlichia spp.” with the Ion Torrent (Thermo Fisher) platform is likely due to the use of fusion primers that are incorporated in the first PCR library building step (Ion Amplicon Library Preparation, Fusion Method for use with Ion Torrent Personal Genome Machine® System, Part 4468326 Rev. C 07/2012).

### 4.7 Notes on the *16S* sequence databases and bioinformatics analyses

Inconsistencies in taxonomic assignments with Greengenes, RDP Classifier and SILVA (Table 6) highlights the need for validation of taxa with more comprehensive databases such as NCBI nr/nt. Future studies on tick microbiomes with *16S* amplicon NGS platforms would benefit from a curated and quality-checked database of tick-associated *16S* and *18S* sequences. Although the UNOISE3 algorithm (Edgar, 2020), USEARCH v10.0, was used to denoise sequences, correct for sequencing error and remove chimeric sequences from the NGS datasets, more chimeric sequences were detected with the UCHIME2 algorithm (Edgar, 2016) that is implemented by NCBI SRA to check for chimeras in OTUs and ZOTUs prior to submission to GenBank. Therefore, it is recommended to check for chimeras with additional chimera detection software, such as UCHIME2, rather than relying on the chimera filter integrated into the UNOISE3 algorithm in USEARCH v10.0 prior to further data analysis. A list of the *16S* ZOTUs that were then processed with UCHIME2 is available in Additional file 11.

### 4.8 Recommendations for future amplicon NGS studies

Modifications to the Illuminaʼs *16S* metagenomic sequencing protocol are necessary to reduce the amount of cross-talk resulting in false positives. Such errors can impact the accuracy of microbiome studiesʼ prevalence estimates and can lead to a misdiagnosis in clinical settings. The inclusion of 6–8 bp UMIs between the primers and MiSeq adapters for the first round PCR will enable amplicons that are cross-contaminated prior to indexing to be demultiplexed back to the correct sample. Likewise, including UMIs in the indices will also improve the identification and control for index cross-talk, and can be used as a quality control procedure for assessing the decontamination of amplicons from previous NGS assays if the same sequence of UMIs are not reused in the same laboratory. As an alternative, other platforms that include barcode sequences during the initial amplicon PCR (e.g. Ion Torrent PGM) could be used.

## 5 Conclusions

This study has demonstrated that amplicon NGS is a vital tool for comprehensive bacterial surveillance for tick-borne or tick-associated pathogens (*A. platys*, *B. clarridgeiae*, “*Ca.* M. haematoparvum” and *C. burnetii*), endosymbionts and novel taxa (*Coxiellaceae* gen. sp., and *Francisella* spp.). Amplicon NGS is more than an identification method, enabling assessments of bacterial diversity, influential factors of the microbiome, co-infections and prevalence. Although the critical approaches used by this study detected issues of cross-talk in the data and limitations in *16S* sequence database taxonomic assignments, solutions to overcome these caveats have been proposed that will aid future amplicon NGS studies that use the MiSeq (Illumina) platform.

## Funding

This study was funded by the 10.13039/501100000923Australian Research Council (Linkage Project 130100050), Bayer Australia Ltd and Bayer AG (Germany).

## Ethical approval

The opportunistic removal of ticks from animal hosts was sanctioned by the Murdoch University Animal Ethics Committee (Permit No. 2011/005).

## CRediT author statement

TLG designed the molecular experiments and bioinformatic approaches, acquired, analysed, and interpreted the NGS data, produced and analysed the phylogenetic trees, contributed to the conceptualisation of *16S* NGS, conceived the idea for *Rickettsia*-specific NGS and wrote the majority of the manuscript. KLE and MLE acquired, analysed and interpreted the Sanger sequencing data for bacterial and tick identification, respectively. KLE also contributed to writing the manuscript and used her skills in clinical microbiology to analyse the taxonomic NGS datasets. CLO directed laboratory work that provided preliminary data for the grant proposal that funded this study, and this contributed to the conceptualisation of the study. PAM substantively revised the manuscript. UMR substantively revised the manuscript and contributed to the conceptualisation of the grant proposal that funded this study. PJI conceived the overarching idea for the study. All authors read and approved the final manuscript.

## Data availability

Sequences generated by Sanger sequencing from this study were submitted to GenBank under the accession numbers MN088348-MN088359 and MN686562-MN686569. The ZOTU sequences with confirmed taxonomy generated from this study were submitted to GenBank and have the following accession numbers: MT900476-MT900478, MT914303-MT914469, MT914472-MT914495 and MT914472-MT914495. Raw NGS sequence files and metadata were deposited in the NCBI SRA under the BioProject accession number PRJNA640465, https://www.ncbi.nlm.nih.gov/bioproject/PRJNA640465/. The bioinformatic analysis pipeline used to analyse the NGS data is available in GitHub, https://github.com/Telleasha-Greay/Illuminating-the-bacterial-microbiome-of-Australian-ticks-USEARCH-amplicon-NGS-pipeline.

## Declaration of competing interests

The authors declare that they have no known competing financial interests or personal relationships that could have appeared to influence the work reported in this paper.

## Appendix A Supplementary data

Multimedia component 1 **Additional file 1.** List of *16S* NGS ZOTUs denoised with UNOISE3 in USEARCH v10.0. Multimedia component 1

Multimedia component 10 **Additional file 10.** Distance matrices for *Francisella* and Legionellales alignments. Multimedia component 10

Multimedia component 11 **Additional file 11.**List of *16S* NGS ZOTUs denoised with UNOISE3 in USEARCH v10.0 with chimeras removed using UCHIME2. Multimedia component 11

Multimedia component 2 **Additional file 2.**Summary of *16S* NGS control data, filtering methods applied, and calculation of sequence thresholds for prevalence estimates. Multimedia component 2

Multimedia component 3 **Additional file 3.**Tick-associated bacterial *16S* sequences in ExCs, NTCs and ICs and Rickettsia-specific sequences in NTCs and ICs. Multimedia component 3

Multimedia component 4 **Additional file 4.**Alpha rarefaction plots of the number of *16S* ZOTUs detected and the sequencing depth of each tick species. Multimedia component 4

Multimedia component 5 **Additional file 5.***16S* and *Rickettsia*-specific read totals and prevalence estimates for samples. Multimedia component 5

Multimedia component 6 **Additional file 6.**Bacterial *16S* sequence composition for ≥ 1%. Multimedia component 6

Multimedia component 7 **Additional file 7.**Alpha and beta diversity statistics. Multimedia component 7

Multimedia component 8 **Additional file 8.**Alpha and beta diversity plots. Multimedia component 8

Multimedia component 9 **Additional file 9.**Comparison of Greengenes, RDP Classifier, and SILVA taxonomy. Multimedia component 9

## References

[bib1] Abraham N.M., Liu L., Jutras B.L., Yadav A.K., Narasimhan S., Gopalakrishnan V. (2017). Pathogen-mediated manipulation of arthropod microbiota to promote infection. Proc. Natl. Acad. Sci. U.S.A..

[bib2] Ahantarig A., Trinachartvanit W., Baimai V., Grubhoffer L. (2013). Hard ticks and their bacterial endosymbionts (or would be pathogens). Folia Microbiol..

[bib3] Anderson B.E., Dawson J.E., Jones D.C., Wilson K.H. (1991). *Ehrlichia chaffeensis*, a new species associated with human ehrlichiosis. J. Clin. Microbiol..

[bib4] Andreotti R., Pérez de León A.A., Dowd S.E., Guerrero F.D., Bendele K.G., Scoles G.A. (2011). Assessment of bacterial diversity in the cattle tick *Rhipicephalus* (*Boophilus) microplus* through tag-encoded pyrosequencing. BMC Microbiol..

[bib5] Angelakis E., Mediannikov O., Jos S.-L., Berenger J.-M., Parola P., Raoult D. (2016). *Candidatus* Coxiella massiliensis infection. Emerg. Infect. Dis..

[bib6] Aravena-Román M., Merritt A., Inglis T.J.J. (2015). First case of *Francisella* bacteraemia in western Australia. New Microbes New Infect..

[bib7] Arraga-Alvarado C.M., Qurollo B.A., Parra O.C., Berrueta M.A., Hegarty B.C., Breitschwerdt E.B. (2014). Molecular evidence of *Anaplasma platys* infection in two women from Venezuela. Am. J. Trop. Med. Hyg..

[bib8] Banazis M.J., Bestall A.S., Reid S.A., Fenwick S.G. (2010). A survey of Western Australian sheep, cattle and kangaroos to determine the prevalence of *Coxiella burnetii*. Vet. Microbiol..

[bib9] Barbosa A.D., Gofton A.W., Paparini A., Codello A., Greay T., Gillett A. (2017). Increased genetic diversity and prevalence of co-infection with *Trypanosoma* spp. in koalas (*Phascolarctos cinereus*) and their ticks identified using next-generation sequencing (NGS). PloS One.

[bib10] Barker S.C., Walker A.R. (2014). Ticks of Australia. The species that infest domestic animals and humans. Zootaxa.

[bib11] Barker E.N., Langton D.A., Helps C.R., Brown G., Malik R., Shaw S.E., Tasker S. (2012). Haemoparasites of free-roaming dogs associated with several remote Aboriginal communities in Australia. BMC Vet. Res..

[bib12] Barker S.C., Walker A.R., Campelo D. (2014). A list of the 70 species of Australian ticks; diagnostic guides to and species accounts of *Ixodes holocyclus* (paralysis tick), *Ixodes cornuatus* (southern paralysis tick) and *Rhipicephalus australis* (Australian cattle tick); and consideration of the place of Australia in the evolution of ticks with comments on four controversial ideas. Int. J. Parasitol..

[bib13] Barns S.M., Grow C.C., Okinaka R.T., Keim P., Kuske C.R. (2005). Detection of diverse new *Francisella*-like bacteria in environmental samples. Appl. Environ. Microbiol..

[bib14] Barrs V.R., Beatty J.A., Wilson B.J., Evans N., Gowan R., Baral R.M. (2010). Prevalence of *Bartonella* species, *Rickettsia felis*, haemoplasmas and the *Ehrlichia* group in the blood of cats and fleas in eastern Australia. Aust. Vet. J..

[bib15] Beaman M.H. (2016). Lyme disease: why the controversy?. Intern. Med. J..

[bib16] Beninati T., Riegler M., Vilcins I.M., Sacchi L., McFadyen R., Krockenberger M. (2009). Absence of the symbiont *Candidatus* Midichloria mitochondrii in the mitochondria of the tick *Ixodes holocyclus*. FEMS Microbiol. Lett..

[bib17] Bokulich N.A., Kaehler B.D., Rideout J.R., Dillon M., Bolyen E., Knight R. (2018). Optimizing taxonomic classification of marker-gene amplicon sequences with QIIME 2's q2-feature-classifier plugin. Microbiome.

[bib18] Bolyen E., Rideout J.R., Dillon M.R., Bokulich N.A., Abnet C.C., Al-Ghalith G.A. (2019). Reproducible, interactive, scalable and extensible microbiome data science using QIIME 2. Nat. Biotechnol..

[bib19] Bouhsira E., Ferrandez Y., Liu M., Franc M., Boulouis H.J., Biville F. (2013). *Ctenocephalides felis* an *in vitro* potential vector for five *Bartonella* species. Comp. Immunol. Microbiol. Infect. Dis..

[bib20] Breitschwerdt E.B., Hegarty B.C., Qurollo B.A., Saito T.B., Maggi R.G., Blanton L.S., Bouyer D.H. (2014). Intravascular persistence of *Anaplasma platys, Ehrlichia chaffeensis*, and *Ehrlichia ewingii* DNA in the blood of a dog and two family members. Parasit. Vectors.

[bib21] Brown J.D. (2018). A description of ‛Australian Lyme diseaseʼ epidemiology and impact: an analysis of submissions to an Australian senate inquiry. Intern. Med. J..

[bib22] Brown G.K., Canfield P.J., Dunstan R.H., Roberts T.K., Martin A.R., Brown C.S., Irving R. (2006). Detection of *Anaplasma platys* and *Babesia canis vogeli* and their impact on platelet numbers in free-roaming dogs associated with remote Aboriginal communities in Australia. Aust. Vet. J..

[bib23] Budachetri K., Browning R.E., Adamson S.W., Dowd S.E., Chao C.-C., Ching W.-M., Karim S. (2014). An insight into the microbiome of the *Amblyomma maculatum* (Acari: Ixodidae). J. Med. Entomol..

[bib24] Cann B., Buhariwalla F., Marrie T.J. (1996). A dog-related outbreak of Q fever. Clin. Infect. Dis..

[bib25] Carpi G., Cagnacci F., Wittekindt N.E., Zhao F., Qi J., Tomsho L.P. (2011). Metagenomic profile of the bacterial communities associated with *Ixodes ricinus* ticks. PloS One.

[bib26] Chalada M.J., Stenos J., Bradbury R.S. (2016). Is there a Lyme-like disease in Australia? Summary of the findings to date. One Health.

[bib27] Chao A. (1984). Nonparametric estimation of the number of classes in a population. Scand. J. Stat..

[bib28] Chitimia L., Lin R.Q., Cosoroaba I., Wu X.Y., Song H.Q., Yuan Z.G., Zhu X.Q. (2010). Genetic characterization of ticks from southwestern Romania by sequences of mitochondrial *cox*1 and *nad*5 genes. Exp. Appl. Acarol..

[bib29] Clayton K.A., Gall C.A., Mason K.L., Scoles G.A., Brayton K.A. (2015). The characterization and manipulation of the bacterial microbiome of the Rocky Mountain wood tick *Dermacentor andersoni*. Parasit. Vectors.

[bib30] Collignon P.J., Lum G.D., Robson J.M. (2016). Does Lyme disease exist in Australia?. Med. J. Aust..

[bib31] Cooper A., Hedlefs R., Ketheesan N., Govan B. (2011). Serological evidence of *Coxiella burnetii* infection in dogs in a regional centre. Aust. Vet. J..

[bib32] Cooper A., Goullet M., Mitchell J., Ketheesan N., Govan B. (2012). Serological evidence of *Coxiella burnetii* exposure in native marsupials and introduced animals in Queensland, Australia. Epidemiol. Infect..

[bib33] Cooper A., Stephens J., Ketheesan N., Govan B. (2013). Detection of *Coxiella burnetii* DNA in wildlife and ticks in northern Queensland, Australia. Vector Borne Zoonotic Dis..

[bib34] Cordaux R., Paces-Fessy M., Raimond M., Michel-Salzat A., Zimmer M., Bouchon D. (2007). Molecular characterization and evolution of arthropod-pathogenic *Rickettsiella* bacteria. Appl. Environ. Microbiol..

[bib35] Dantas-Torres F., Chomel B.B., Otranto D. (2012). Ticks and tick-borne diseases: a One Health perspective. Trends Parasitol..

[bib36] Dehhaghi M., Kazemi Shariat Panahi H., Holmes E.C., Hudson B.J., Schloeffel R., Guillemin G.J. (2019). Human tick-borne diseases in Australia. Front. Cell. Infect. Microbiol..

[bib37] Department of Agriculture, Water and the Environment (2020). https://www.environment.gov.au/land/nrs/science/ibra/australias-ecoregions.

[bib38] Dib L., Bitam I., Tahri M., Bensouilah M., De Meeus T. (2008). Competitive exclusion between piroplasmosis and anaplasmosis agents within cattle. PLoS Pathog..

[bib39] Domrow R., Campbell R.W. (1974). Rickettsioses in Australia: isolation of *Rickettsia tsutsugamushi* and *R. australis* from naturally infected arthropods. Trans. R. Soc. Trop. Med. Hyg..

[bib40] Eden J.-S., Rose K., Ng J., Shi M., Wang Q., Sintchenko V., Holmes E.C. (2017). *Francisella tularensis* ssp. *holarctica* in ringtail possums, Australia. Emerg. Infect. Dis..

[bib41] Edgar R.C. (2004). MUSCLE: multiple sequence alignment with high accuracy and high throughput. Nucleic Acids Res..

[bib42] Edgar R.C. (2010). Search and clustering orders of magnitude faster than BLAST. Bioinformatics.

[bib43] Edgar R. (2016). UCHIME2: improved chimera prediction for amplicon sequencing. bioRxiv.

[bib44] Edgar R.C. (2018). UNCROSS2: identification of cross-talk in 16S rRNA OTU tables. bioRxivorg.

[bib45] Edgar R.C. (2018). Updating the 97% identity threshold for *16S* ribosomal RNA OTUs. Bioinformatics.

[bib46] Edgar R. (2020). https://drive5.com/usearch/manual/cmd_unoise3.html.

[bib47] Edwards U., Rogall T., Blocker H., Emde M., Bottger E.C. (1989). Isolation and direct complete nucleotide determination of entire genes. Characterization of a gene coding for *16S* ribosomal RNA. Nucleic Acids Res..

[bib48] Egan S.L., Loh S.M., Banks P.B., Gillett A., Ahlstrom L., Ryan U.M. (2020). Bacterial community profiling highlights complex diversity and novel organisms in wildlife ticks. Ticks Tick Borne Dis..

[bib49] Estrada-Pena A., Roura X., Sainz A., Miro G., Solano-Gallego L. (2017). Species of ticks and carried pathogens in owned dogs in Spain: results of a one-year national survey. Ticks Tick Borne Dis..

[bib50] Galkiewicz J.P., Kellogg C.A. (2008). Cross-kingdom amplification using bacterial-specific primers: complications for studies of coral microbial ecology. Appl. Environ. Microbiol..

[bib51] Gall C.A., Reif K.E., Scoles G.A., Mason K.L., Mousel M., Noh S.M., Brayton K.A. (2016). The bacterial microbiome of *Dermacentor andersoni* ticks influences pathogen susceptibility. ISME J..

[bib52] Gofton A.W., Doggett S., Ratchford A., Oskam C.L., Paparini A., Ryan U., Irwin P. (2015). Bacterial profiling reveals novel “*Ca*. Neoehrlichia”, *Ehrlichia*, and *Anaplasma* species in Australian human-biting ticks. PloS One.

[bib53] Gofton A.W., Oskam C.L., Lo N., Beninati T., Wei H., McCarl V. (2015). Inhibition of the endosymbiont “*Candidatus* Midichloria mitochondrii” during *16S* rRNA gene profiling reveals potential pathogens in *Ixodes* ticks from Australia. Parasit. Vectors.

[bib54] Gofton A.W., Doggett S., Ratchford A., Ryan U., Irwin P. (2016). Phylogenetic characterisation of two novel *Anaplasmataceae* from Australian *Ixodes holocyclus* ticks: “*Candidatus* Neoehrlichia australis” and “*Candidatus* Neoehrlichia arcana”. Int. J. Syst. Evol. Microbiol..

[bib55] Gofton A.W., Waudby H.P., Petit S., Greay T.L., Ryan U.M., Irwin P.J. (2017). Detection and phylogenetic characterisation of novel *Anaplasma* and *Ehrlichia* species in *Amblyomma triguttatum* subsp. from four allopatric populations in Australia. Ticks Tick Borne Dis..

[bib56] Gofton A.W., Loh S.M., Barbosa A.D., Paparini A., Gillett A., Macgregor J. (2018). A novel *Ehrlichia* species in blood and *Ixodes ornithorhynchi* ticks from platypuses (*Ornithorhynchus anatinus*) in Queensland and Tasmania, Australia. Ticks Tick Borne Dis..

[bib57] Graves S.R., Stenos J. (2017). Tick-borne infectious diseases in Australia. Med. J. Aust..

[bib58] Graves S.R., Stewart L., Stenos J., Stewart R.S., Schmidt E., Hudson S. (1993). Spotted fever group rickettsial infection in south-eastern Australia: isolation of rickettsiae. Comp. Immunol. Microbiol. Infect. Dis..

[bib59] Graves S.R., Jackson C., Hussain-Yusuf H., Vincent G., Nguyen C., Stenos J., Webster M. (2016). *Ixodes holocyclus* tick-transmitted human pathogens in north-eastern New South Wales, Australia. Trav. Med. Infect. Dis..

[bib61] Greay T.L., Gofton A.W., Paparini A., Ryan U.M., Oskam C.L., Irwin P.J. (2018). Recent insights into the tick microbiome gained through next-generation sequencing. Parasit. Vectors.

[bib60] Greay T.L., Oskam C.L., Gofton A.W., Rees R.L., Ryan U.M., Irwin P.J. (2016). A survey of ticks (Acari: Ixodidae) of companion animals in Australia.. Parasit. Vectors.

[bib62] Greay T.L., Zahedi A., Krige A.-S., Owens J.M., Rees R.L., Ryan U.M. (2018). Endemic, exotic and novel apicomplexan parasites detected during a national study of ticks from companion animals in Australia. Parasit. Vectors.

[bib63] Greay T.L., Barbosa A.D., Rees R.L., Paparini A., Ryan U.M., Oskam C.L., Irwin P.J. (2018). An Australian dog diagnosed with an exotic tick-borne infection: should Australia still be considered free from *Hepatozoon canis*?. Int. J. Parasitol..

[bib64] Greay T.L., Zahedi A., Krige A.-S., Owens J.M., Rees R.L., Ryan U.M. (2019). Response to the letter to the editor by Harris. Parasit. Vectors.

[bib65] Groves M.G., Dennis G.L., Amyx H.L., Huxsoll D.L. (1975). Transmission of *Ehrlichia canis* to dogs by ticks (*Rhipicephalus sanguineus*). Am. J. Vet. Res..

[bib66] Guindon S., Dufayard J.F., Lefort V., Anisimova M., Hordijk W., Gascuel O. (2010). New algorithms and methods to estimate maximum-likelihood phylogenies: assessing the performance of PhyML 3.0. Syst. Biol..

[bib67] Guo W.-P., Huang B., Zhao Q., Xu G., Liu B., Wang Y.-H., Zhou E.-M. (2018). Human-pathogenic *Anaplasma* spp., and *Rickettsia* spp. in animals in Xi’an, China. PLoS Negl. Trop. Dis..

[bib68] Gurfield N., Grewal S., Cua L.S., Torres P.J., Kelley S.T. (2017). Endosymbiont interference and microbial diversity of the Pacific coast tick, *Dermacentor occidentalis*, in San Diego County, California. PeerJ.

[bib69] Harvey E., Rose K., Eden J.S., Lo N., Abeyasuriya T., Shi M., Doggett S.L., Holmes E.C. (2019). Extensive diversity of RNA viruses in Australian ticks. J. Virol..

[bib70] Hawlena H., Rynkiewicz E., Toh E., Alfred A., Durden L.A., Hastriter M.W. (2013). The arthropod, but not the vertebrate host or its environment, dictates bacterial community composition of fleas and ticks. ISME J..

[bib71] Heath A.C., Hardwick S. (2011). The role of humans in the importation of ticks to New Zealand: a threat to public health and biosecurity. N. Z. Med. J..

[bib72] Hii S., Kopp S., Thompson M., OʼLeary C., Rees R., Traub R. (2012). Canine vector-borne disease pathogens in dogs from south-east Queensland and north-east Northern Territory. Aust. Vet. J..

[bib73] Huber B., Escudero R., Busse H.J., Seibold E., Scholz H.C., Anda P. (2010). Description of *Francisella hispaniensis* sp. nov., isolated from human blood, reclassification of *Francisella novicida* (Larson et al., 1955) Olsufiev et al. 1959 as *Francisella tularensis* subsp. *novicida* comb. nov. and emended description of the genus *Francisella*. Int. J. Syst. Evol. Microbiol..

[bib74] Irwin P.J. (1989).

[bib75] Irwin P.J., Hutchinson G.W. (1991). Clinical and pathological findings of *Babesia* infection in dogs. Aust. Vet. J..

[bib76] Izzard L., Cox E., Stenos J., Waterston M., Fenwick S., Graves S. (2010). Serological prevalence study of exposure of cats and dogs in Launceston, Tasmania, Australia to spotted fever group rickettsiae. Aust. Vet. J..

[bib77] Janda J.M., Abbott S.L. (2007). *16S* rRNA gene sequencing for bacterial identification in the diagnostic laboratory: pluses, perils, and pitfalls. J. Clin. Microbiol..

[bib78] Kamani J., Baneth G., Mumcuoglu K.Y., Waziri N.E., Eyal O., Guthmann Y., Harrus S. (2013). Molecular detection and characterization of tick-borne pathogens in dogs and ticks from Nigeria. PLoS Negl. Trop. Dis..

[bib79] Kawahara M., Rikihisa Y., Isogai E., Takahashi M., Misumi H., Suto C. (2004). Ultrastructure and phylogenetic analysis of “*Candidatus* Neoehrlichia mikurensis” in the family *Anaplasmataceae*, isolated from wild rats and found in *Ixodes ovatus* ticks. Int. J. Syst. Evol. Microbiol..

[bib80] Kearse M., Moir R., Wilson A., Stones-Havas S., Cheung M., Sturrock S. (2012). Geneious Basic: an integrated and extendable desktop software platform for the organization and analysis of sequence data. Bioinformatics.

[bib81] Kingry L.C., Petersen J.M. (2014). Comparative review of *Francisella tularensis* and *Francisella novicida*. Front. Cell. Infect. Microbiol..

[bib82] Kopecny L., Bosward K.L., Shapiro A., Norris J.M. (2013). Investigating *Coxiella burnetii* infection in a breeding cattery at the centre of a Q fever outbreak. J. Feline Med. Surg..

[bib83] Lalzar I., Harrus S., Mumcuoglu K.Y., Gottlieb Y. (2012). Composition and seasonal variation of *Rhipicephalus turanicus* and *Rhipicephalus sanguineus* bacterial communities. Appl. Environ. Microbiol..

[bib84] Latrofa M.S., Dantas-Torres F., Giannelli A., Otranto D. (2014). Molecular detection of tick-borne pathogens in *Rhipicephalus sanguineus* group ticks. Ticks Tick Borne Dis..

[bib85] Lee S.M., Chao A. (1994). Estimating population size *via* sample coverage for closed capture-recapture models. Biometrics.

[bib87] Loh S.M., Gillett A., Ryan U., Irwin P., Oskam C. (2017). Molecular characterization of “*Candidatus* Borrelia tachyglossi” (family *Spirochaetaceae*) in echidna ticks, *Bothriocroton concolor*. Int. J. Syst. Evol. Microbiol..

[bib86] Liu H., Li Q., Zhang X., Li Z., Wang Z., Song M. (2016). Characterization of rickettsiae in ticks in northeastern China. Parasit. Vectors.

[bib88] Loh S.-M., Egan S., Gillett A., Banks P.B., Ryan U.M., Irwin P.J., Oskam C.L. (2018). Molecular surveillance of piroplasms in ticks from small and medium-sized urban and peri-urban mammals in Australia. Int. J. Parasitol. Parasites Wildl..

[bib89] Loh S.M., Paparini A., Ryan U., Irwin P., Oskam C. (2018). Identification of *Theileria fuliginosa*-like species in *Ixodes australiensis* ticks from western grey kangaroos (*Macropus fuliginosus*) in Western Australia. Ticks Tick Borne Dis..

[bib90] MacConaill L.E., Burns R.T., Nag A., Coleman H.A., Slevin M.K., Giorda K. (2018). Unique, dual-indexed sequencing adapters with UMIs effectively eliminate index cross-talk and significantly improve sensitivity of massively parallel sequencing. BMC Genomics.

[bib91] Maggi R.G., Compton S.M., Trull C.L., Mascarelli P.E., Mozayeni B.R., Breitschwerdt E.B. (2013). Infection with hemotropic *Mycoplasma* species in patients with or without extensive arthropod or animal contact. J. Clin. Microbiol..

[bib92] Maggi R.G., Mascarelli P.E., Havenga L.N., Naidoo V., Breitschwerdt E.B. (2013). Co-infection with *Anaplasma platys, Bartonella henselae* and *Candidatus* Mycoplasma haematoparvum in a veterinarian. Parasit. Vectors.

[bib93] Malo J.A., Colbran C., Young M., Vasant B., Jarvinen K., Viney K., Lambert S.B. (2018). An outbreak of Q fever associated with parturient cat exposure at an animal refuge and veterinary clinic in southeast Queensland. Aust. N. Z. J. Publ. Health.

[bib94] Martin A.R., Brown G.K., Hugh Dunstan R., Roberts T.K. (2005). *Anaplasma platys*: an improved PCR for its detection in dogs. Exp. Parasitol..

[bib95] Masuzawa T., Sawaki K., Nagaoka H., Akiyama M., Hirai K., Yanagihara Y. (1997). Identification of rickettsiae isolated in Japan as *Coxiella burnetii* by *16S* rRNA sequencing. Int. J. Syst. Evol. Microbiol..

[bib96] Mauri M., Elli T., Caviglia G., Uboldi G., Azzi M. (2017). Proceedings of the 12th Biannual Conference on Italian SIGCHI Chapter.

[bib97] Maywood P., Boyd R. (2011).

[bib98] McDonald D., Price M.N., Goodrich J., Nawrocki E.P., DeSantis T.Z., Probst A. (2012). An improved Greengenes taxonomy with explicit ranks for ecological and evolutionary analyses of bacteria and archaea. ISME J..

[bib99] McMurdie P.J., Holmes S. (2013). phyloseq: an R package for reproducible interactive analysis and graphics of microbiome census data. PloS One.

[bib100] Mediannikov O., Sekeyova Z., Birg M.L., Raoult D. (2010). A novel obligate intracellular gamma-proteobacterium associated with ixodid ticks, *Diplorickettsia massiliensis*, gen. nov., sp. nov. PloS One.

[bib101] Menchaca A.C., Visi D.K., Strey O.F., Teel P.D., Kalinowski K., Allen M.S., Williamson P.C. (2013). Preliminary assessment of microbiome changes following blood-feeding and survivorship in the *Amblyomma americanum* nymph-to-adult transition using semiconductor sequencing. PloS One.

[bib102] Narasimhan S., Fikrig E. (2015). Tick microbiome: the force within. Trends Parasitol..

[bib103] Narasimhan S., Rajeevan N., Liu L., Zhao Yang O., Heisig J. (2014). Gut microbiota of the tick vector *Ixodes scapularis* modulate colonization of the Lyme disease spirochete. Cell Host Microbe.

[bib104] OʼBrien C.A., Hall-Mendelin S., Hobson-Peters J., Deliyannis G., Allen A., Lew-Tabor A. (2018). Discovery of a novel iflavirus sequence in the eastern paralysis tick *Ixodes holocyclus*. Arch. Virol..

[bib105] Oksanen J., Blanchet F.G., Kindt R., Legendre P., Minchin P., O’hara R. (2018). https://cran.ism.ac.jp/web/packages/vegan/vegan.pdf.

[bib106] Oskam C.L., Gofton A.W., Greay T.L., Yang R., Doggett S., Ryan U.M., Irwin P.J. (2017). Molecular investigation into the presence of a *Coxiella* sp. in *Rhipicephalus sanguineus* ticks in Australia. Vet. Microbiol..

[bib107] Oskam C., Owens J., Codello A., Gofton A., Greay T. (2018). Rethinking *Coxiella* infections in Australia. Microbiol. Aust..

[bib108] Owen H.C. (2007).

[bib109] Paddock C.D., Sumner J.W., Shore G.M., Bartley D.C., Elie R.C., McQuade J.G. (1997). Isolation and characterization of *Ehrlichia chaffeensis* strains from patients with fatal ehrlichiosis. J. Clin. Microbiol..

[bib110] Pekár S., Petráková L., Corcobado G., Whyte R. (2017). Revision of eastern Australian ant-mimicking spiders of the genus *Myrmarachne* (Araneae, Salticidae) reveals a complex of species and forms. Zool. J. Linn. Soc..

[bib111] Penzhorn B.L. (2020). Don’t let sleeping dogs lie: unravelling the identity and taxonomy of *Babesia canis, Babesia rossi* and *Babesia vogeli*. Parasit. Vectors.

[bib112] Perner J., Sobotka R., Sima R., Konvickova J., Sojka D., Oliveira P.L.d. (2016). Acquisition of exogenous haem is essential for tick reproduction. Elife.

[bib113] Ponnusamy L., Gonzalez A., Van Treuren W., Weiss S., Parobek C.M., Juliano J.J. (2014). Diversity of rickettsiales in the microbiome of the lone star tick, *Amblyomma americanum*. Appl. Environ. Microbiol..

[bib114] Potter A.S., Banazis M.J., Yang R., Reid S.A., Fenwick S.G. (2011). Prevalence of *Coxiella burnetii* in western grey kangaroos (*Macropus fuliginosus*) in Western Australia. J. Wildl. Dis..

[bib115] Qiu Y., Nakao R., Ohnuma A., Kawamori F., Sugimoto C. (2014). Microbial population analysis of the salivary glands of ticks; a possible strategy for the surveillance of bacterial pathogens. PloS One.

[bib116] Quast C., Pruesse E., Yilmaz P., Gerken J., Schweer T., Yarza P., Peplies J., Glöckner F.O. (2013). The SILVA ribosomal RNA gene database project: improved data processing and web-based tools. Nucleic Acids Res..

[bib117] R Development Core Team (2013). https://www.R-project.org/.

[bib118] Ramos R.A., Latrofa M.S., Giannelli A., Lacasella V., Campbell B.E., Dantas-Torres F., Otranto D. (2014). Detection of *Anaplasma platys* in dogs and *Rhipicephalus sanguineus* group ticks by a quantitative real-time PCR. Vet. Parasitol..

[bib119] Regnery R.L., Spruill C.L., Plikaytis B.D. (1991). Genotypic identification of rickettsiae and estimation of intraspecies sequence divergence for portions of two rickettsial genes. J. Bacteriol..

[bib120] Roberts F.H.S. (1960). A systematic study of the Australian species of the genus *Ixodes* (Acarina: Ixodidae). Aust. J. Zool..

[bib121] Roberts F.H.S. (1970).

[bib122] Ronquist F., Teslenko M., van der Mark P., Ayres D.L., Darling A., Höhna S. (2012). MrBayes 3.2: efficient Bayesian phylogenetic inference and model choice across a large model space. Syst. Biol..

[bib123] Roux V., Raoult D. (2000). Phylogenetic analysis of members of the genus *Rickettsia* using the gene encoding the outer-membrane protein rOmpB (ompB). Int. J. Syst. Evol. Microbiol..

[bib124] Rozsa L., Reiczigel J., Majoros G. (2000). Quantifying parasites in samples of hosts. J. Parasitol..

[bib125] Russell R.C., Doggett S.L., Munro R., Ellis J., Avery D., Hunt C., Dickeson D. (1994). Lyme disease: a search for a causative agent in ticks in south-eastern Australia. Epidemiol. Infect..

[bib126] Rynkiewicz E.C., Hemmerich C., Rusch D.B., Fuqua C., Clay K. (2015). Concordance of bacterial communities of two tick species and blood of their shared rodent host. Mol. Ecol..

[bib127] Sacchi L., Bigliardi E., Corona S., Beninati T., Lo N., Franceschi A. (2004). A symbiont of the tick *Ixodes ricinus* invades and consumes mitochondria in a mode similar to that of the parasitic bacterium *Bdellovibrio bacteriovorus*. Tissue Cell.

[bib128] Sanogo Y.O., Davoust B., Inokuma H., Camicas J.L., Parola P., Brouqui P. (2003). First evidence of *Anaplasma platys* in *Rhipicephalus sanguineus* (Acari: Ixodida) collected from dogs in Africa. Onderstepoort J. Vet. Res..

[bib129] Schneeberger P.M., Wintenberger C., van der Hoek W., Stahl J.P. (2014). Q fever in The Netherlands - 2007–2010: what we learned from the largest outbreak ever. Med. Maladies Infect..

[bib130] Sexton D.J., Banks J., Graves S., Hughes K., Dwyer B. (1991). Prevalence of antibodies to spotted fever group rickettsiae in dogs from southeastern Australia. Am. J. Trop. Med. Hyg..

[bib131] Shapiro A.J., Norris J.M., Heller J., Brown G., Malik R., Bosward K.L. (2016). Seroprevalence of *Coxiella burnetii* in Australian dogs. Zoon. Publ. Health..

[bib132] Shapiro A.J., Brown G., Norris J.M., Bosward K.L., Marriot D.J., Balakrishnan N. (2017). Vector-borne and zoonotic diseases of dogs in north-west New South Wales and the northern territory, Australia. BMC Vet. Res..

[bib133] Simpson R.M., Gaunt S.D., Hair J.A., Kocan K.M., Henk W.G., Casey H.W. (1991). Evaluation of *Rhipicephalus sanguineus* as a potential biologic vector of *Ehrlichia platys*. Am. J. Vet. Res..

[bib134] Sinha R., Stanley G., Gulati G.S., Ezran C., Travaglini K.J., Wei E. (2017). Index switching causes “spreading-of-signal” among multiplexed samples in Illumina HiSeq 4000 DNA sequencing. bioRxivorg.

[bib135] Smith D.J.W. (1940). Studies in the epidemiology of Q fever 3. The transmission of Q fever by the tick *Haemaphysalis humerosa*. Aust. J. Exp. Biol. Med. Sci..

[bib136] Smith D.J.W. (1942). Studies in the epidemiology of Q fever. 10. The transmission of Q fever by the tick *Ixodes holocyclus* (with notes on tick-paralysis in bandicoots). Aust. J. Exp. Biol. Med. Sci..

[bib137] Smith T.A., Driscoll T., Gillespie J.J., Raghavan R. (2015). A *Coxiella*-like endosymbiont is a potential vitamin source for the lone star tick. Genome Biol. Evol..

[bib138] Snellgrove A.N., Krapiunaya I., Ford S.L., Stanley H.M., Wickson A.G., Hartzer K.L., Levin M.L. (2020). Vector competence of *Rhipicephalus sanguineus sensu stricto* for *Anaplasma platys*. Ticks Tick Borne Dis..

[bib139] Song S., Shao R., Atwell R., Barker S., Vankan D. (2011). Phylogenetic and phylogeographic relationships in *Ixodes holocyclus* and *Ixodes cornuatus* (Acari: Ixodidae) inferred from COX1 and ITS2 sequences. Int. J. Parasitol..

[bib140] Sorensen T.A. (1948). A method of establishing groups of equal amplitude in plant sociology based on similarity of species content and its application to analyses of the vegetation on Danish commons. Biol. Skar..

[bib162] Stackebrandt E., Liesack W., Goodfellow M., O’Donnell A.G. (1993). Handbook of New Bacterial Systematics.

[bib141] Storey-Lewis B., Mitrovic A., McParland B. (2018). Molecular detection and characterisation of *Babesia* and *Theileria* in Australian hard ticks. Ticks Tick Borne Dis..

[bib142] Subramanian G., Mediannikov O., Angelakis E., Socolovschi C., Kaplanski G., Martzolff L., Raoult D. (2012). *Diplorickettsia massiliensis* as a human pathogen. Eur. J. Clin. Microbiol. Infect. Dis..

[bib143] Swei A., Kwan J.Y. (2017). Tick microbiome and pathogen acquisition altered by host blood meal. ISME J..

[bib144] Telford S.R., Wormser G.P. (2010). *Bartonella* spp. transmission by ticks not established. Emerg. Infect. Dis..

[bib145] The Department of Primary Industries and Regional Development (2020). https://www.agric.wa.gov.au/ehrlichiosis.

[bib146] Tozer S.J., Lambert S.B., Strong C.L., Field H.E., Sloots T.P., Nissen M.D. (2014). Potential animal and environmental sources of Q fever infection for humans in Queensland. Zoon. Publ. Health.

[bib147] Van Treuren W., Ponnusamy L., Brinkerhoff R.J., Gonzalez A., Parobek C.M., Juliano J.J. (2015). Variation in the microbiota of *Ixodes* ticks with regard to geography, species, and sex. Appl. Environ. Microbiol..

[bib148] Trout Fryxell R.T., DeBruyn J.M. (2016). The microbiome of *Ehrlichia* infected and uninfected lone star ticks (*Amblyomma americanum*). PloS One.

[bib149] Turner S., Pryer K.M., Miao V.P., Palmer J.D. (1999). Investigating deep phylogenetic relationships among cyanobacteria and plastids by small subunit rRNA sequence analysis. J. Eukaryot. Microbiol..

[bib150] Vilcins I.-m.E., Old J.M., Deane E. (2009). Molecular detection of *Rickettsia, Coxiella* and *Rickettsiella* DNA in three native Australian tick species. Exp. Appl. Acarol..

[bib151] Vilcins I.M., Fournier P.E., Old J.M., Deane E. (2009). Evidence for the presence of *Francisella* and spotted fever group rickettsia DNA in the tick *Amblyomma fimbriatum* (Acari: Ixodidae), Northern Territory, Australia. J. Med. Entomol..

[bib152] Wang Q., Garrity G.M., Tiedje J.M., Cole J.R. (2007). Naive Bayesian classifier for rapid assignment of rRNA sequences into the new bacterial taxonomy. Appl. Environ. Microbiol..

[bib153] Whipp M.J., Davis J.M., Lum G., de Boer J., Zhou Y., Bearden S.W. (2003). Characterization of a *novicida*-like subspecies of *Francisella tularensis* isolated in Australia. J. Med. Microbiol..

[bib154] Williams S.G., Sacci J.B., Schriefer M.E., Andersen E.M., Fujioka K.K., Sorvillo F.J. (1992). Typhus and typhuslike rickettsiae associated with opossums and their fleas in Los Angeles County, California. J. Clin. Microbiol..

[bib155] Williams-Newkirk A.J., Rowe L.A., Mixson-Hayden T.R., Dasch G.A. (2014). Characterization of the bacterial communities of life stages of free living lone star ticks (*Amblyomma americanum*). PloS One.

[bib156] Wright E.S., Vetsigian K.H. (2016). Quality filtering of Illumina index reads mitigates sample cross-talk. BMC Genomics.

[bib157] Yang R., Murphy C., Song Y., Ng-Hublin J., Estcourt A., Hijjawi N. (2013). Specific and quantitative detection and identification of *Cryptosporidium hominis* and *C. parvum* in clinical and environmental samples. Exp. Parasitol..

[bib158] Zhang X.-C., Yang Z.-N., Lu B., Ma X.-F., Zhang C.-X., Xu H.-J. (2014). The composition and transmission of microbiome in hard tick, *Ixodes persulcatus*, during blood meal. Ticks Tick Borne Dis..

[bib159] Zhang C.M., Li N.X., Zhang T.T., Qiu Z.X., Li Y., Li L.W., Liu J.Z. (2017). Endosymbiont CLS-HI plays a role in reproduction and development of *Haemaphysalis longicornis*. Exp. Appl. Acarol..

[bib160] Zhong J., Jasinskas A., Barbour A.G. (2007). Antibiotic treatment of the tick vector *Amblyomma americanum* reduced reproductive fitness. PloS One.

[bib161] Zolnik C.P., Prill R.J., Falco R.C., Daniels T.J., Kolokotronis S.-O. (2016). Microbiome changes through ontogeny of a tick pathogen vector. Mol. Ecol..

