# Supplementary material for: Illuminating the bacterial microbiome of Australian ticks with 16S and Rickettsia-specific next-generation sequencing
Source: Curr Res Parasitol Vector Borne Dis. 2021 Jun 11;1:100037. doi: 10.1016/j.crpvbd.2021.100037 (PMC8906098; doi:10.1016/j.crpvbd.2021.100037)
Supplement: Multimedia component 2 — Additional file 2.Summary of 16S NGS control data, filtering methods applied, and calculation of sequence thresholds for prevalence estimates. [file mmc2.docx]

### **Additional file 2.** Summary of *16S* NGS control data, filtering methods applied, and calculation of sequence thresholds for prevalence estimates.

# 16S NGS control data summary

Approximately 1.6 million (average = 27,728), 680,000 (average = 27,860), and 146 (average = 35) reads were obtained from the ExCs, NTCs and ICs, respectively. The proportions of the most abundant *16S* ZOTU sequences (≥0.1%) that were detected in the controls, and the proportions of positive samples are presented in Figure S1. The most abundant species detected in the ExCs was *Herbaspirillum* sp. (ZOTU 10), with an average (replicate ExCs were averaged) of 2,540 sequences (13% of overall ExC sequences) in 92% (22/24) of ExCs. The other most prevalent species detected in 92% of the ExCs were *Pseudomonas* sp. (ZOTU 24; 1,046 average sequences/sample), *Cupriavidus* sp. (ZOTU 37; 625 average sequences/sample, and ZOTU 74; 113 average sequences/sample), *Cutibacterium* sp. (ZOTU 6; 577 average sequences/sample), *Pseudomonas* sp. (ZOTU 60; 291 average sequences/sample), *Delftia* sp. (ZOTU 29; 133 average sequences/sample) and *Methylobacterium* sp. (ZOTU 107; 100 average sequences/sample). For the NTCs, Chitinophagaceae gen. sp. (ZOTU 5) had the most abundant *16S* sequences (139,518; 20% of all sequences detected in the NTCs), and was identified in 60% of the NTCs. However, the most prevalent ZOTUs in the NTCs were *Cutibacterium* sp. (ZOTU 6; 72,759 total sequences, 11% of sequence total in NTCs), *Ralstonia* sp. (ZOTU 34; 6,266 total sequences in NTCs, 0.9% of sequence total in NTCs) and “*Ca*. Midichloria sp.” (ZOTU 1; 167 total sequences in NTCs, 0.02% of sequence total in NTCs), which all occurred in 64% of the NTCs. For the ICs, “*Ca*. Midichloria sp.” had the most abundant sequences overall (ZOTU 1; 28 total sequences in ICs, 19% of sequence total in ICs), and was the most prevalent, found in all ICs (ranging from 1-13 sequences/sample).

### **Figure S1.** Proportion of bacterial *16S* sequences for the most abundant ZOTUs detected in the ExCs, NTCs, and ICs. ZOTUs that constituted ≥0.1% of the total *16S* sequences from the ExCs (A), NTCs (B), and ICs (C) were included in the plots.

Tick-associated bacterial sequences (TABS) that were identified in the controls are summarised in Additional File 3. *Anaplasma platys*, “*Ca.* Neoehrlichia spp.”, *Coxiella* spp., Coxiellaceae gen. spp., *Rickettsiella*, “*Ca.* Midichloria spp.”, *Rickettsia* spp., and Rickettsiaceae spp. were detected in the ExCs (Additional file 3). The lowest proportion of ExCs positive for TABS was 0.04 (1/24) for Coxiellaceae gen. sp., *Rickettsiella* spp. and *Rickettsia* spp., and the highest proportion of ExCs positive for TABS was 0.8 (20/24) for Coxiellaceae gen. sp. The TABS were identified in only one of the replicate ExCs in most cases (81.1%). The number of TABS in two or more replicates ranged from 1-105, and a higher number of TABS was not observed with a greater sequencing depth in all cases. For example, the sequencing depth was 6,449 for E11.1 with 105 “*Ca.* Midichloria sp.” ZOTU 1 sequences, but the duplicate of this sample, E11.2, had a greater sequencing depth of 36,080, but only two “*Ca.* Midichloria sp.” ZOTU 1 sequences were detected. To assess the relationship between the number of TABS identified in ExCs/NTCs and sequencing depth, a non-parametric Spearman's rank-order correlation was performed. There was a weak positive correlation between the number of TABS in ExCs/NTCs and sequencing depth, *r_s_*(109) = 0.291, *p* < 0.01.

The greatest number of TABS identified in the NTCs was found in NTC 2, which had 146 *Coxiella* sp. ZOTU 3 sequences (0.1% sequence composition), and this NTC had a large sequencing depth of 123,060 sequences. The NTC with the highest composition of TABS was found in NTC 10 that had a composition of 32% (8/25) for *Coxiella* sp. ZOTU 4, but also had a low sequencing depth. The number of different TABS identified in the NTCs ranged from 1-9 (M = 4). In the ICs, “*Ca.* Midichloria sp.” ZOTU 1 had the most sequences in IC2 (13/46), and the number of different TABS in the ICs ranged from 3-5 (M = 3).

# Controlling for contaminant sequences

To reduce the number of false positives for prevalence estimates of tick-associated bacteria and pathogens based on the *16S* NGS dataset, the highest proportion of TABS for each ZOTU in the ExCs and NTCs were used as a threshold. The samples were considered positive for tick-associated bacteria and pathogens if the proportion of TABS in the samples were > the highest proportion of TABS for respective tick-associated bacteria and pathogens in the ExCs and NTCs. The proportion of TABS in the controls are provided in Additional File 3, and the thresholds used for each tick-associated bacteria and pathogen ZOTUs are provided in Table S1. All samples were screened for *Coxiella burnetii* with *C. burnetii*-specific qPCR, and the only qPCR-positive sample had 48,105 reads for *C. burnetii* in the *16S* NGS assay. There were 11 other samples that had 1-3 *C. burnetii* reads detected by *16S* NGS, indicating that these low-level reads were a result of cross-talk that was not identified in the NTCs, ExCs, or ICs. The highest proportion of cross-talk *C. burnetii* sequences was 0.00015, therefore, samples with a proportion of ≤0.00015 TABS were considered to be false positives.

**Table S1.** Sequence proportion thresholds for tick-associated bacteria and pathogen ZOTUs set for samples to be considered positive.

| **Family** | **Species** | **ZOTU** | **ExC/NTC ID** | **No. TABS** | **ExC or NTC sequencing depth^a^** | **Highest proportion of TABS** | **TABS proportion threshold applied** |
| --- | --- | --- | --- | --- | --- | --- | --- |
| Anaplasmataceae | “*Candidatus* Neoehrlichia arcana” | 40 | E6.1 | 1,166^b^ | 63,186 | 0.01845 | 0.01845 |
|  | “*Candidatus* Neoehrlichia australis” | 8 | E11.1 | 46 | 6,449 | 0.00713 | 0.00713 |
|  | *Anaplasma platys* | 19 | E9.1 | 2 | 32,957 | 0.00006 | 0.00015 |
| “*Ca.* Midichloriaceae” | “*Ca.* Midichloria sp.” | 1 | E11.1 | 105 | 6,449 | 0.01628 | 0.01628 |
|  | “*Ca.* Midichloria sp.” | 2 | NTC9 | 35 | 5,834 | 0.006 | 0.006 |
| Coxiellaceae | Coxiella sp. | 3 | E1.2 | 26 | 3,444 | 0.00755 | 0.00755 |
|  | Coxiella sp. | 4 | E11.2 | 334 | 36,080 | 0.00926 | 0.00926 |
|  | Coxiellaceae sp. | 7 | E19.2 | 5 | 1,312 | 0.00381 | 0.00381 |
|  | *C. burnetii* | 31 | ND | ND | ND | ND | 0.00015 |
|  | Coxiellaceae sp. | 57 | E16.1 | 3 | 84,234 | 0.00004 | 0.00015 |
|  | Coxiellaceae sp. | 115 | NTC3 | 2 | 25,547 | 0.00008 | 0.00015 |
| Francisellaceae | *Francisella* sp. | 13 | E11.2 | 1 | 1,093 | 0.00091 | 0.00091 |
|  | *Francisella* sp. | 42 | E20.4 | 2 | 5,834 | 0.00034 | 0.00034 |
| Rickettsiaceae | *Rickettsia* sp. | 9 | E2.2 | 1 | 3,287 | 0.0003 | 0.0003 |
|  | *Rickettsia* sp. | 11 | E11.1 | 7 | 6,449 | 0.00109 | 0.00109 |
|  | *Rickettsia* sp. | 14 | NTC9 | 9 | 5834 | 0.00154 | 0.00154 |
|  | *Rickettsia* sp. | 33 | E24 | 3 | 2,827 | 0.00106 | 0.00106 |
|  | *Rickettsia* sp. | 51 | E17.1 | 2 | 4,623 | 0.00043 | 0.00043 |
|  | *Rickettsia* sp. | 182 | NTC25 | 1 | 25,549 | 0.00004 | 0.00015 |
|  | *Rickettsia* sp. | 223 | E22.2 | 2 | 1,541 | 0.0013 | 0.0013 |
|  | *Rickettsia* sp. | 224 | E22.2 | 2 | 1,541 | 0.0013 | 0.0013 |
|  | *Rickettsia* sp. | 252 | E16.1 | 3 | 25,547 | 0.00012 | 0.00012 |
|  | *Rickettsia* sp. | 4,336 | E11.1 | 1 | 6,449 | 0.00016 | 0.00016 |
|  | Rickettsiaceae sp. | 4,746 | E1.2 | 1 | 123,060 | 0.00001 | 0.00015 |
|  | Rickettsiaceae sp. | 10,457 | E18.1 | 10 | 40,270 | 0.00025 | 0.00025 |

^a^Controls were considered for TABS proportion thresholds if sequencing depth was >1,000 reads as the majority of samples had a sequencing depth of >1,000.

^b^Contamination of the genomic DNA of this ExC is unlikely as no ZOTU 40 sequences were identified in duplicates of this ExC (E6.2 and E6.3). Rather, this is indicative of cross-contamination during the MiSeq library preparation process.
