# Supplementary material for: Illuminating the bacterial microbiome of Australian ticks with 16S and Rickettsia-specific next-generation sequencing
Source: Curr Res Parasitol Vector Borne Dis. 2021 Jun 11;1:100037. doi: 10.1016/j.crpvbd.2021.100037 (PMC8906098; doi:10.1016/j.crpvbd.2021.100037)
Supplement: Multimedia component 4 — Additional file 4.Alpha rarefaction plots of the number of 16S ZOTUs detected and the sequencing depth of each tick species. [file mmc4.pdf]

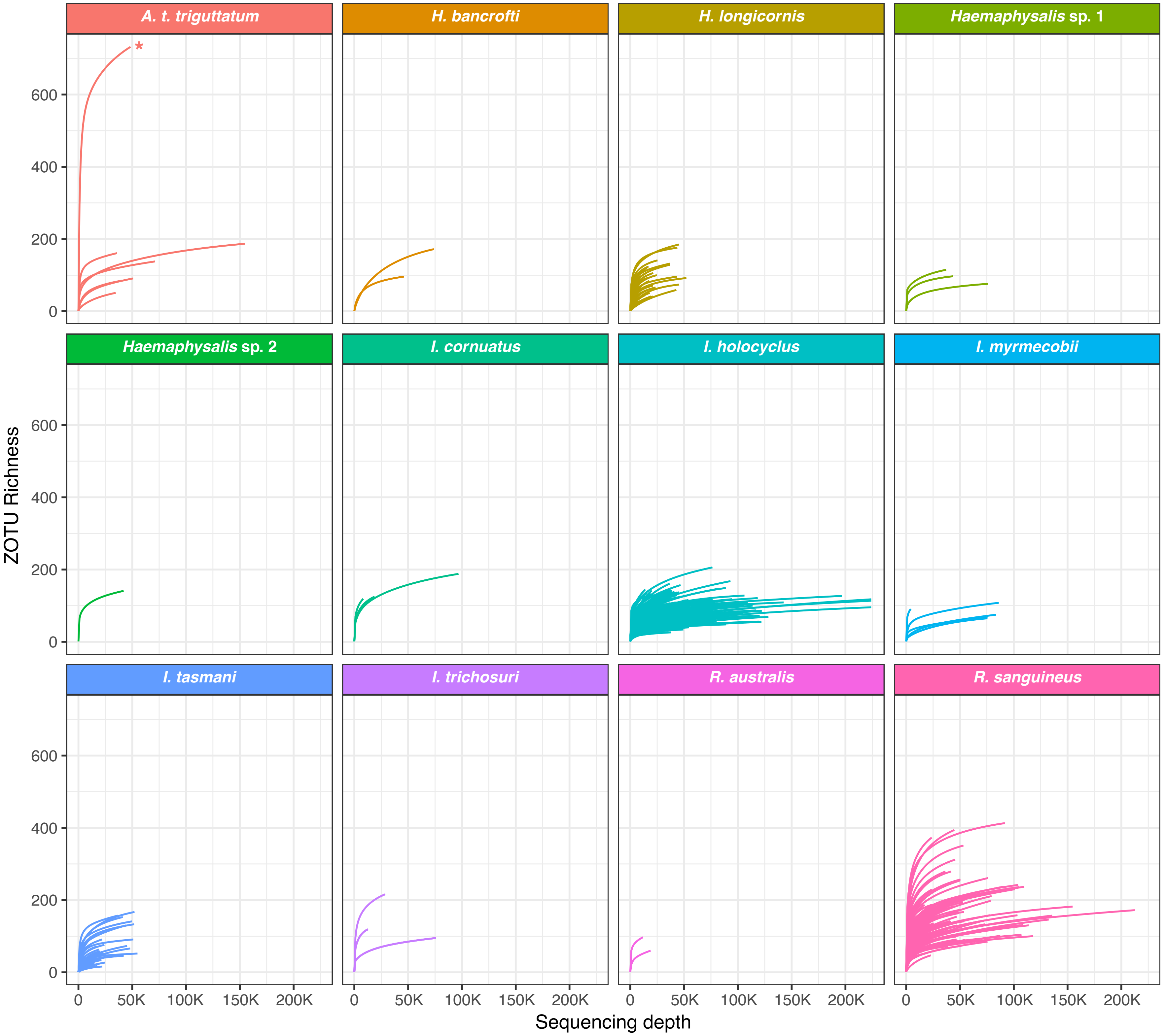

Additional file 4. Alpha rarefaction plots of the number of *16S* ZOTUs detected and the sequencing depth of each tick species.
