# Supplementary figures and images for: Illuminating the bacterial microbiome of Australian ticks with 16S and Rickettsia-specific next-generation sequencing
Source: Curr Res Parasitol Vector Borne Dis. 2021 Jun 11;1:100037. doi: 10.1016/j.crpvbd.2021.100037 (PMC8906098; doi:10.1016/j.crpvbd.2021.100037)

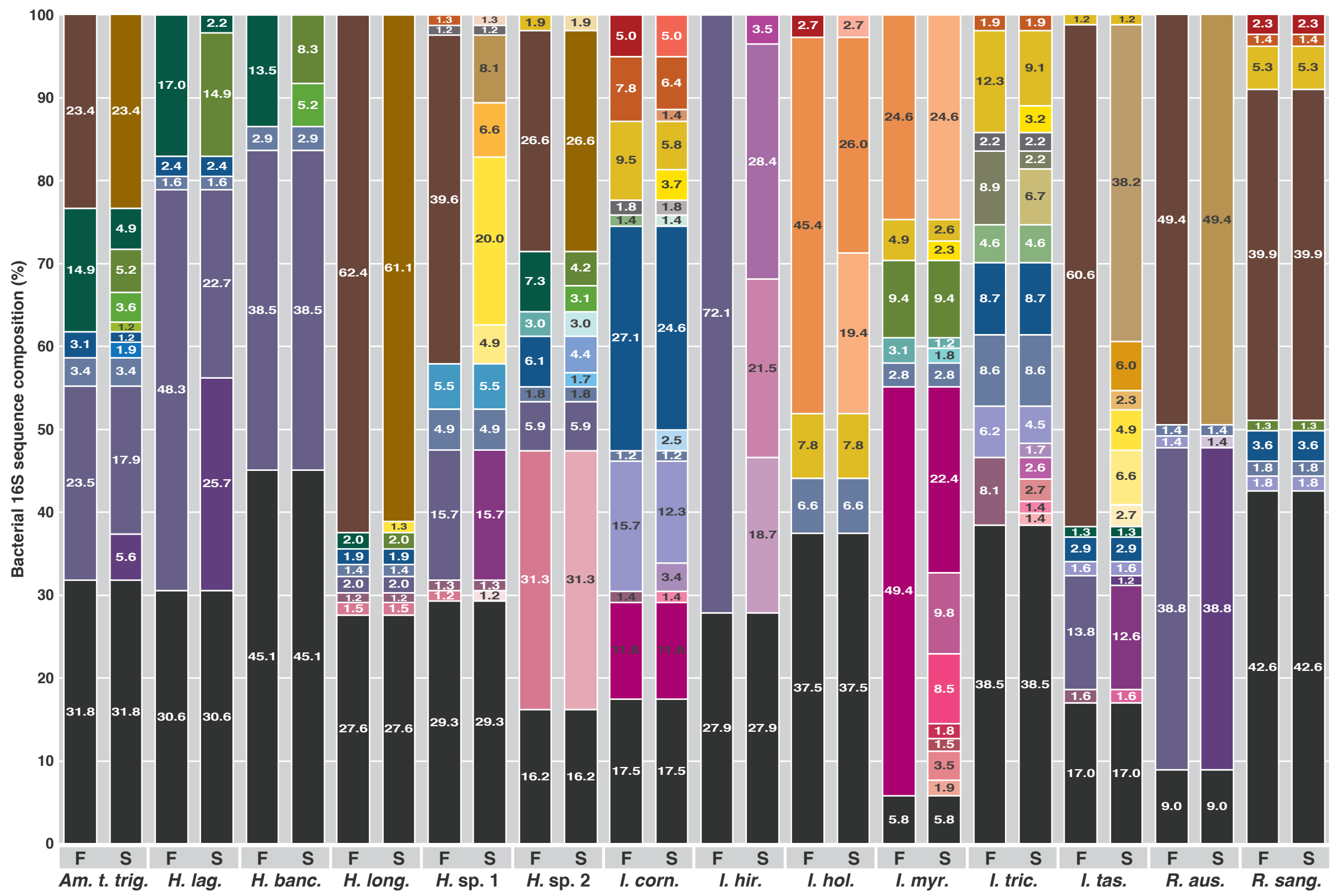

Additional file 6. Bacterial 16S sequence composition for > 1%.

Supplement: Multimedia component 6 — Additional file 6.Bacterial 16S sequence composition for ≥ 1%. [file mmc6.pdf]

## Additional file 8. Alpha and beta diversity plots

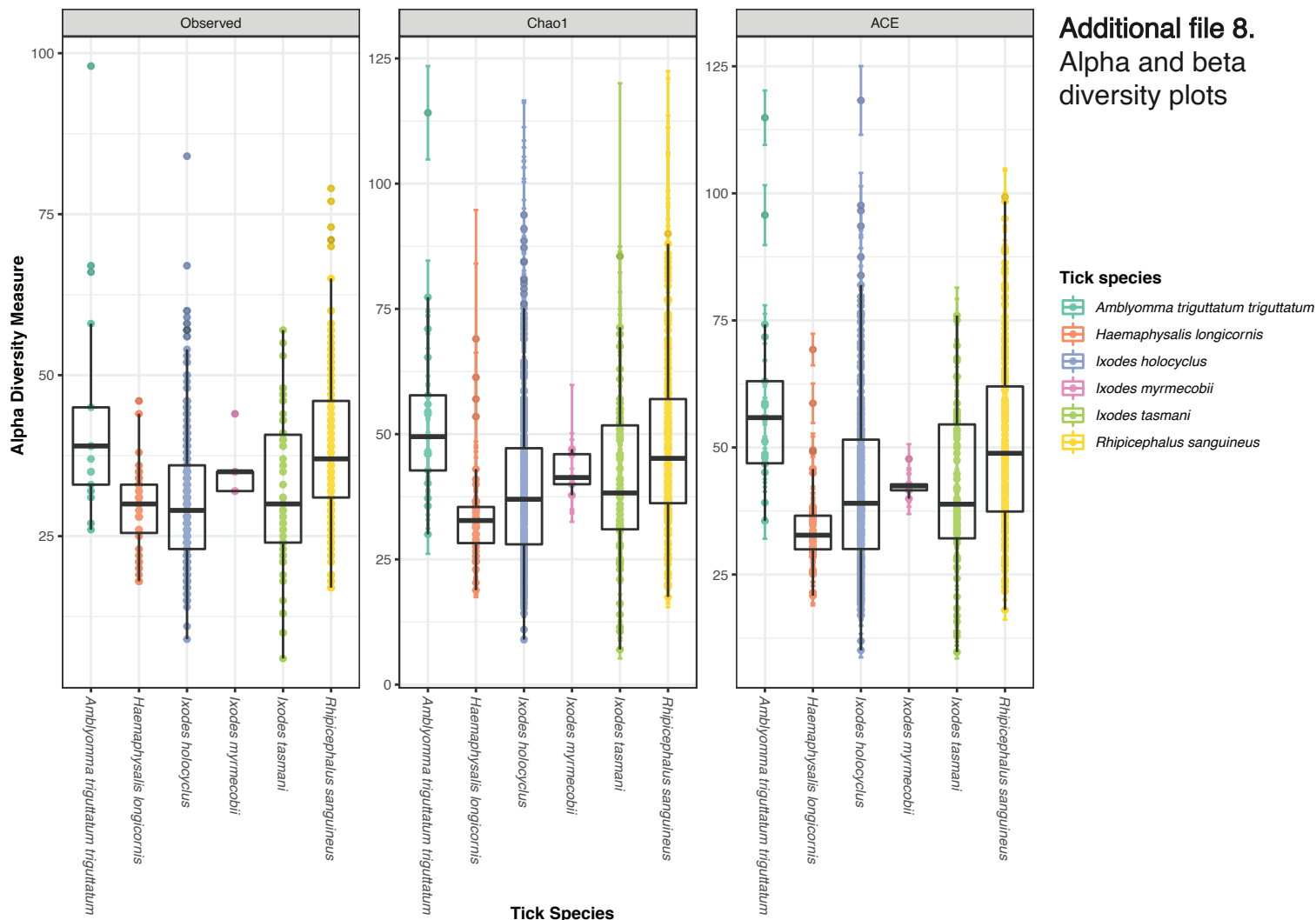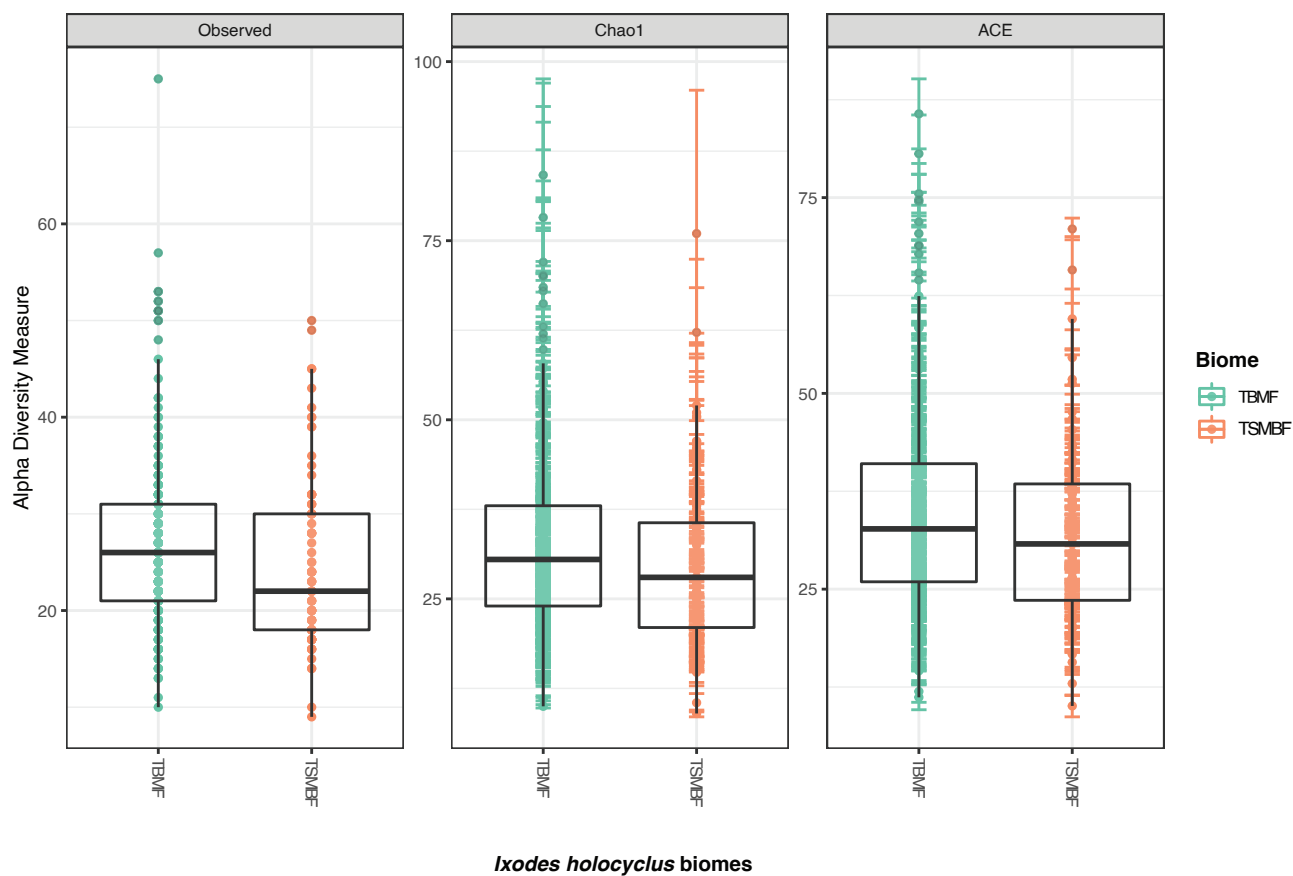

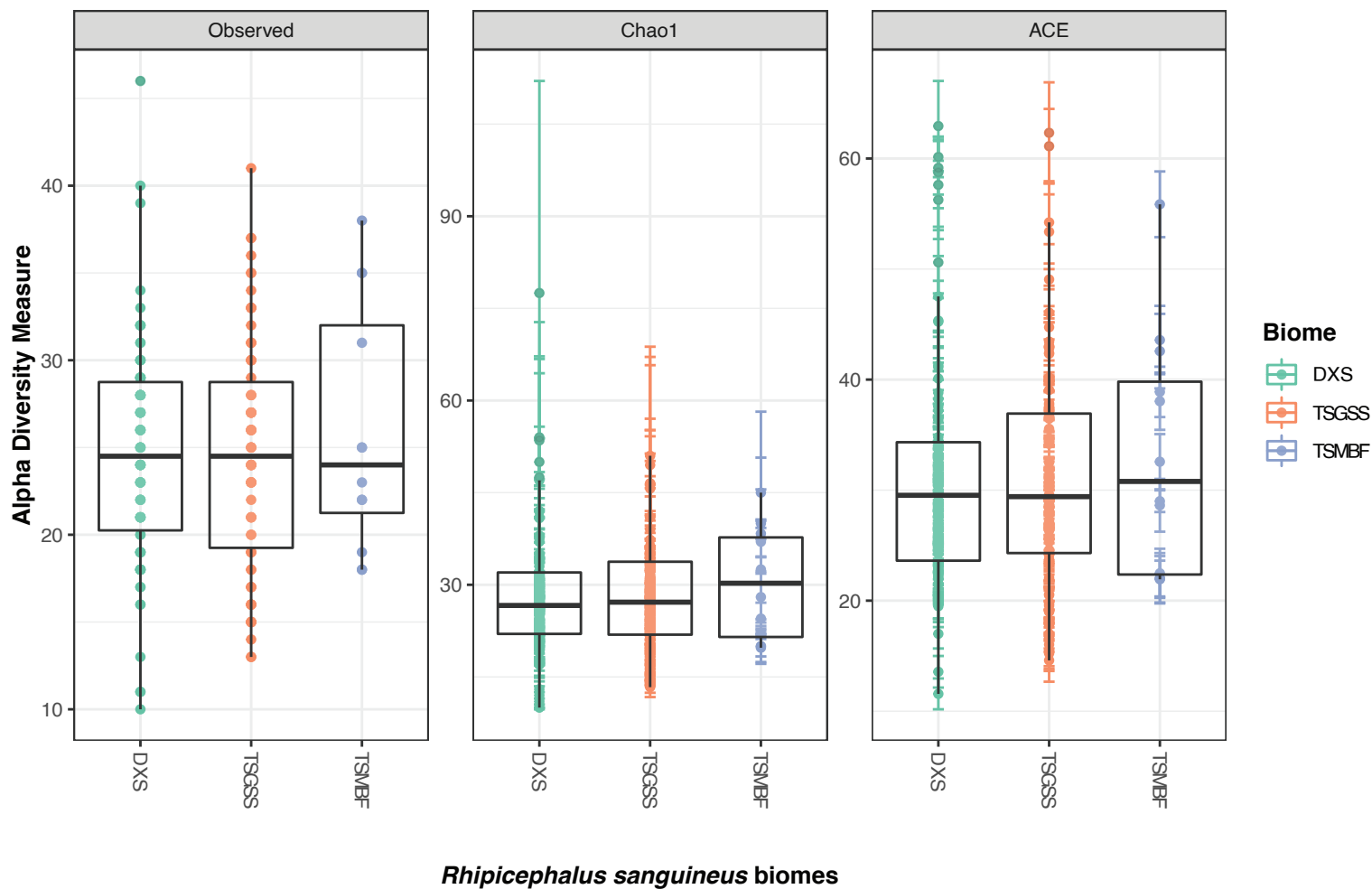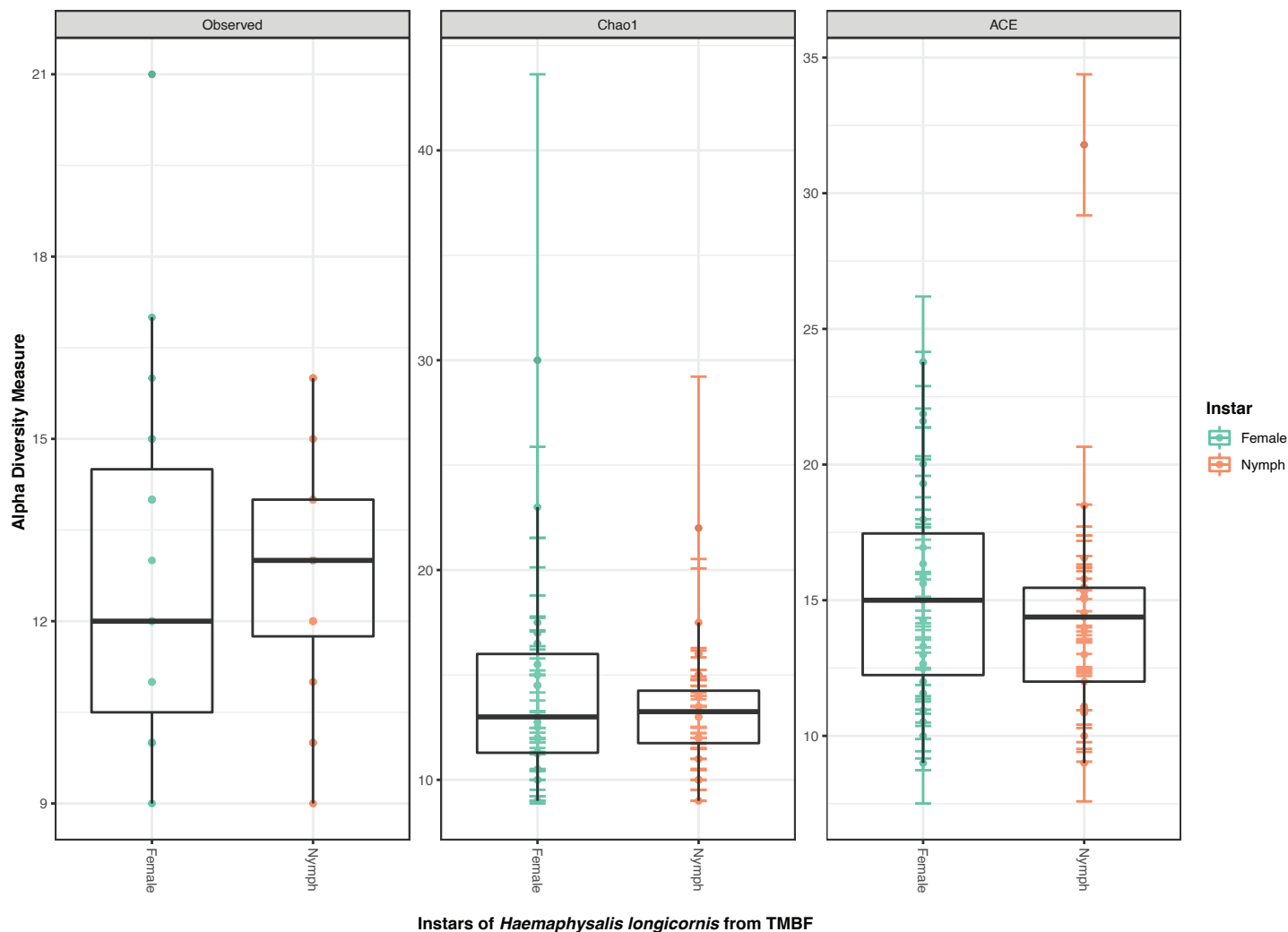

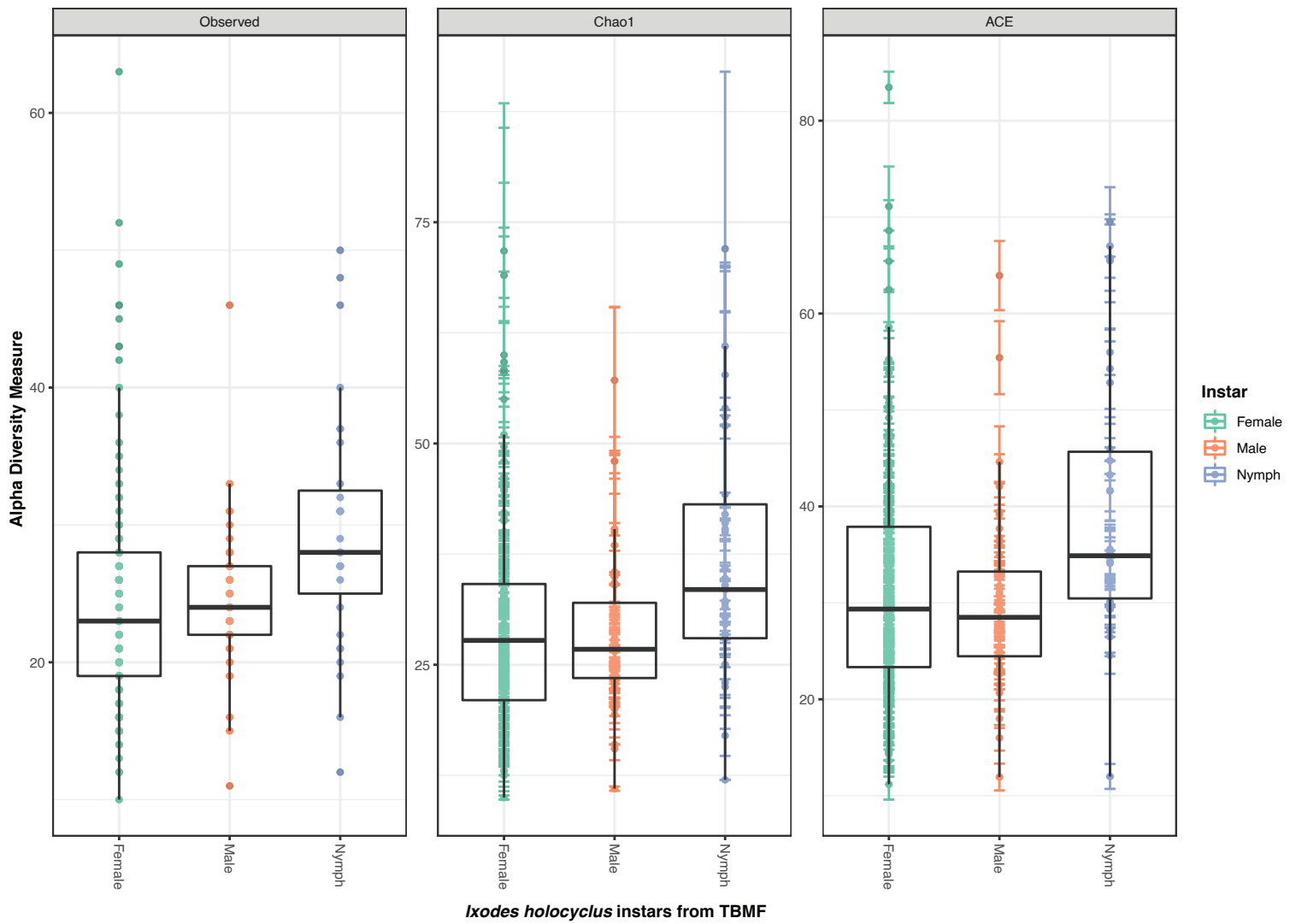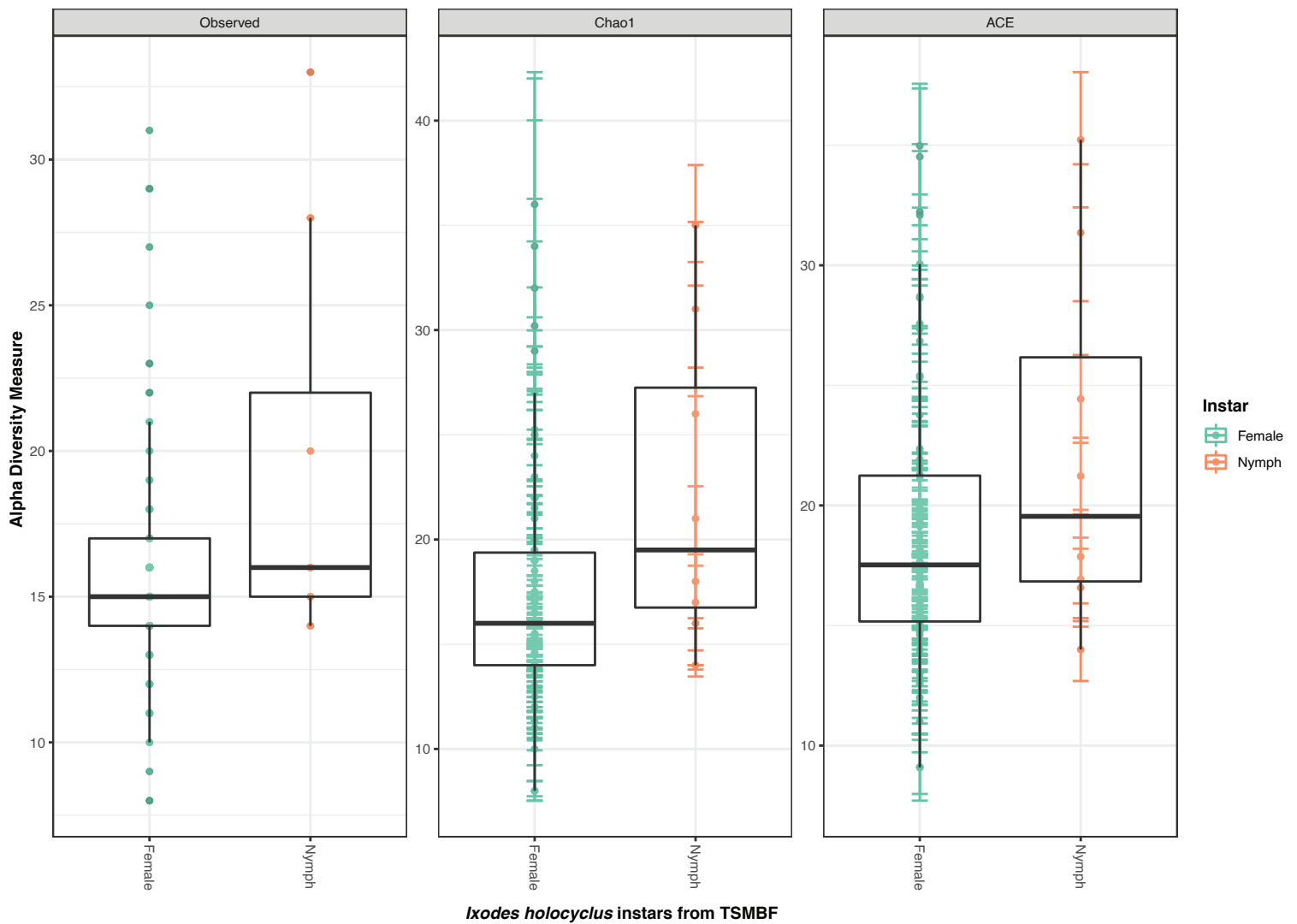

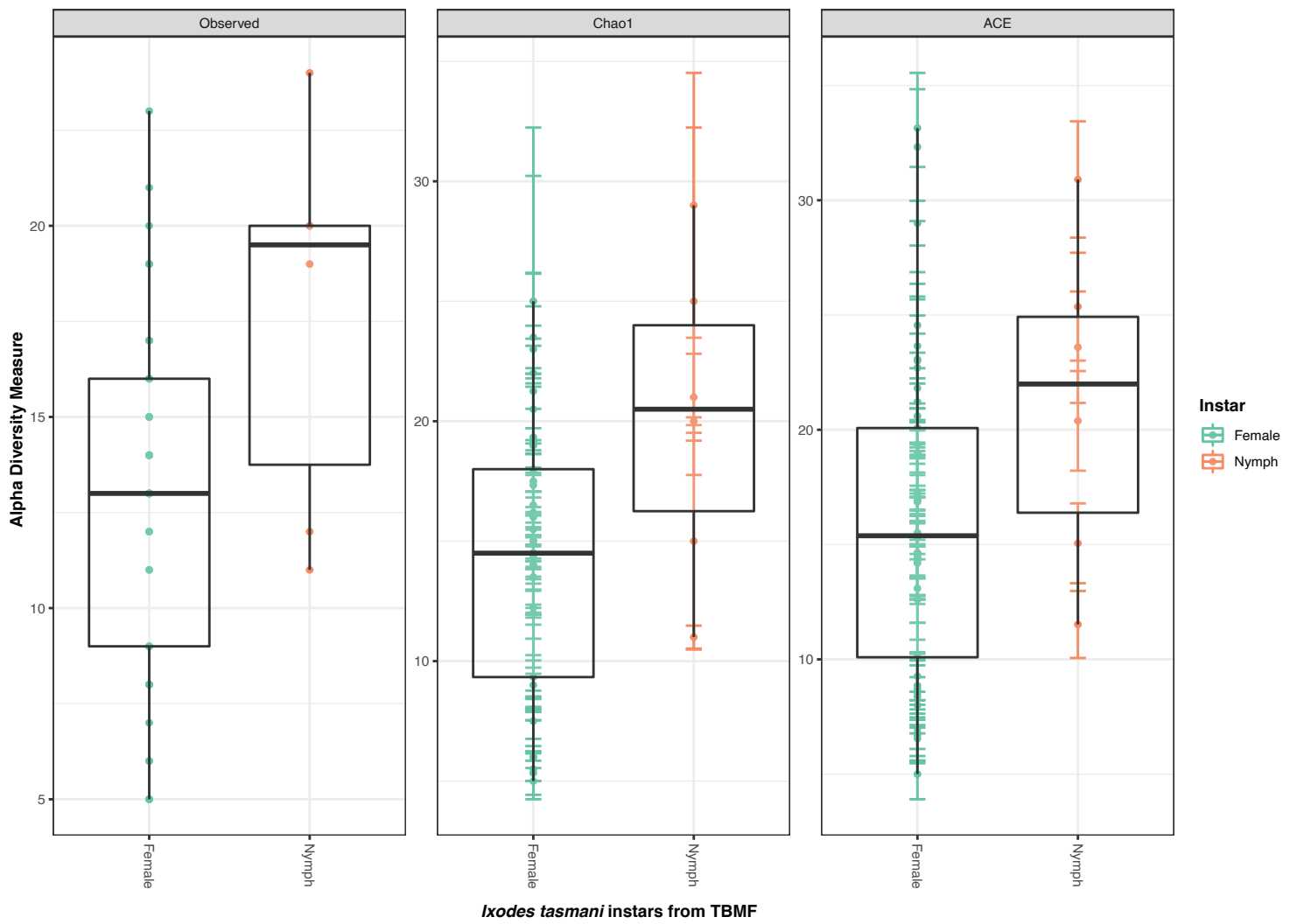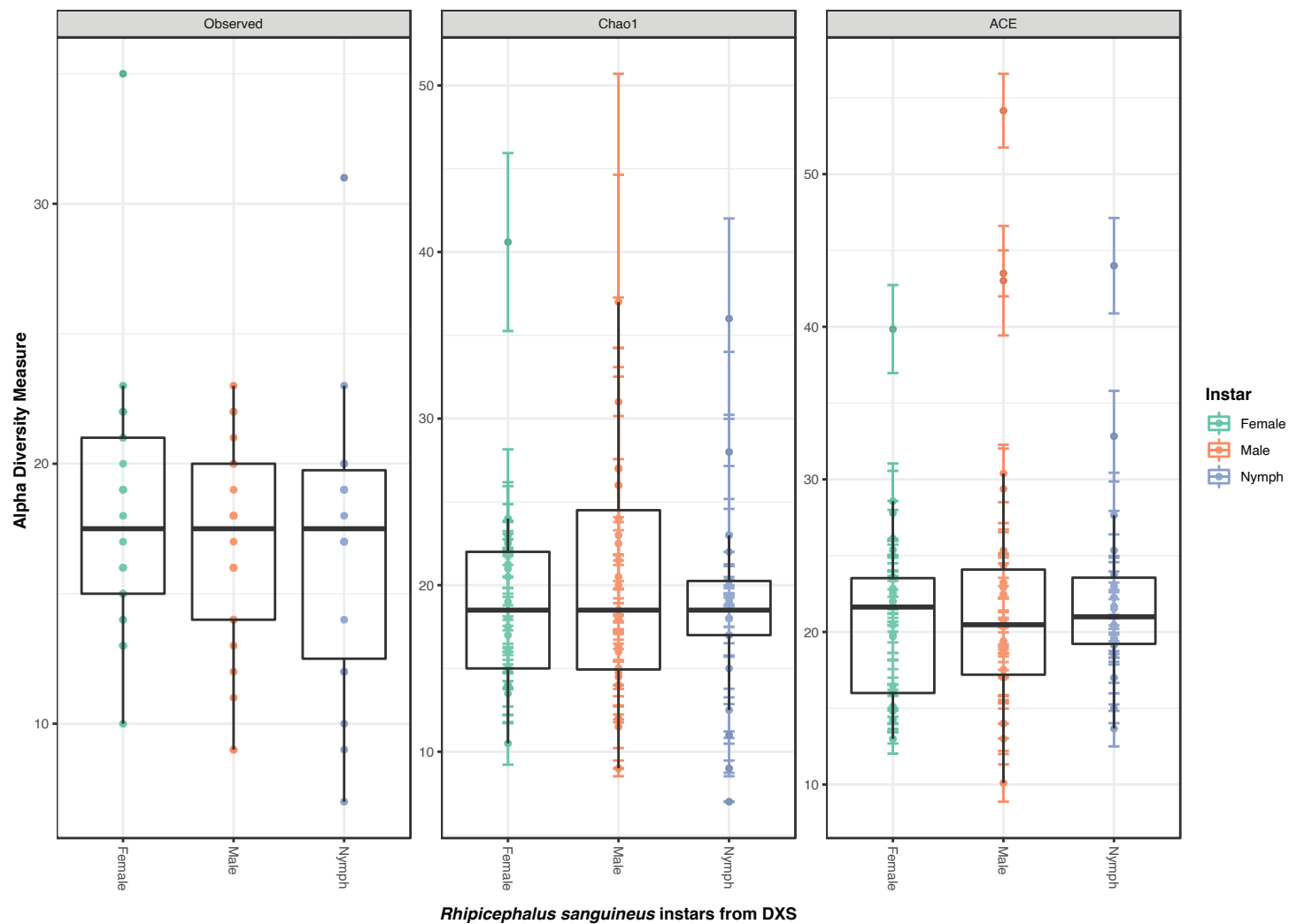

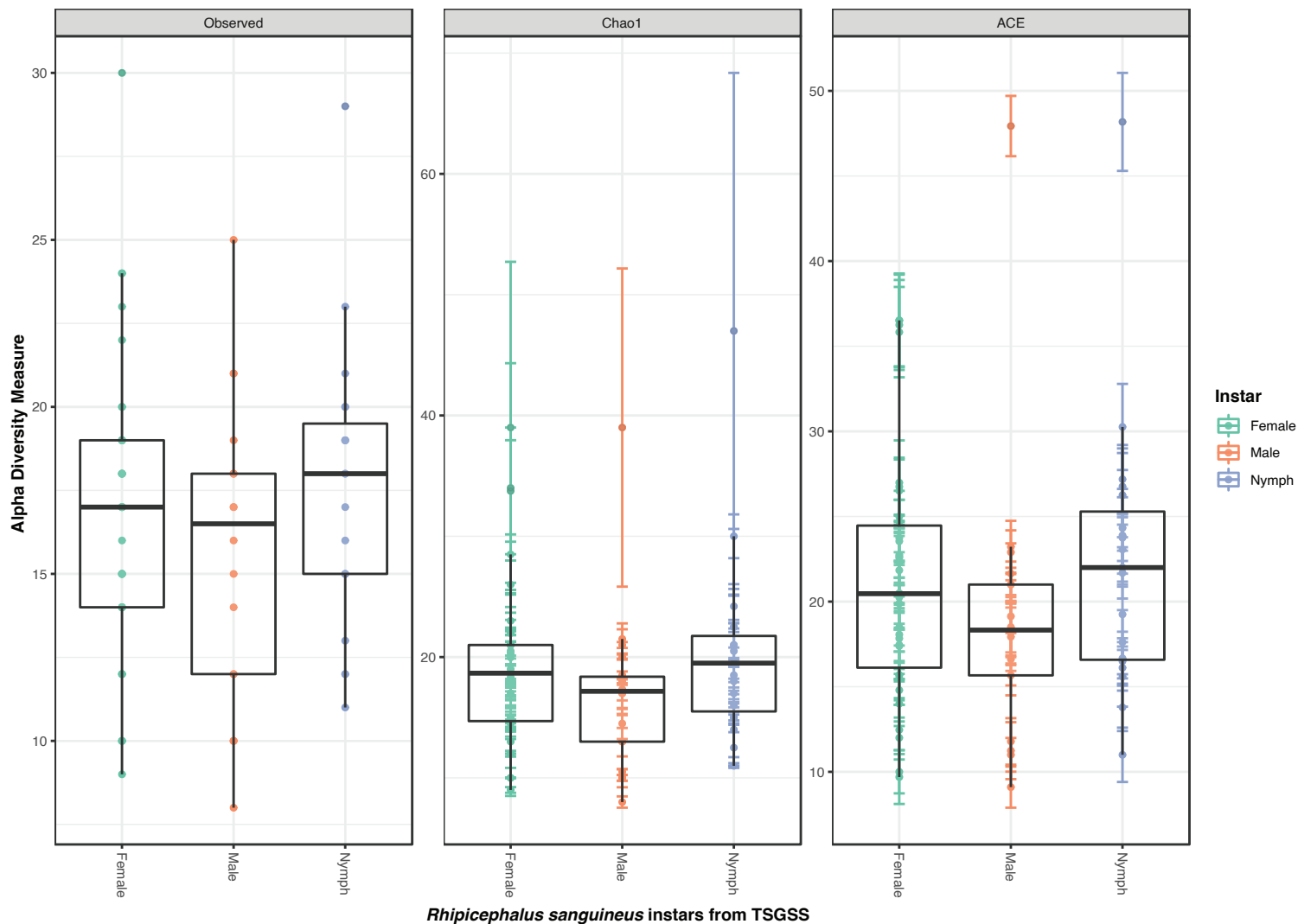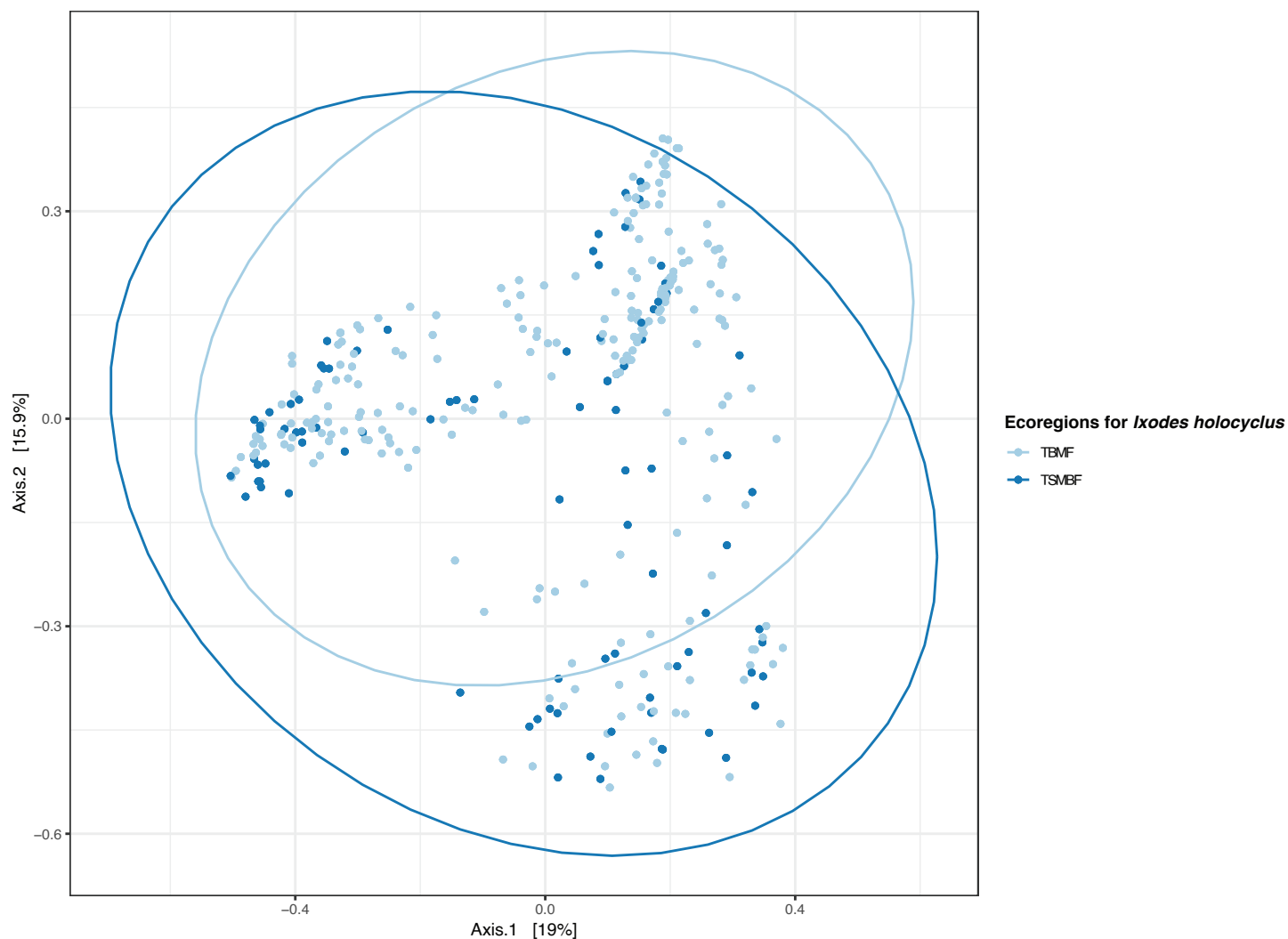

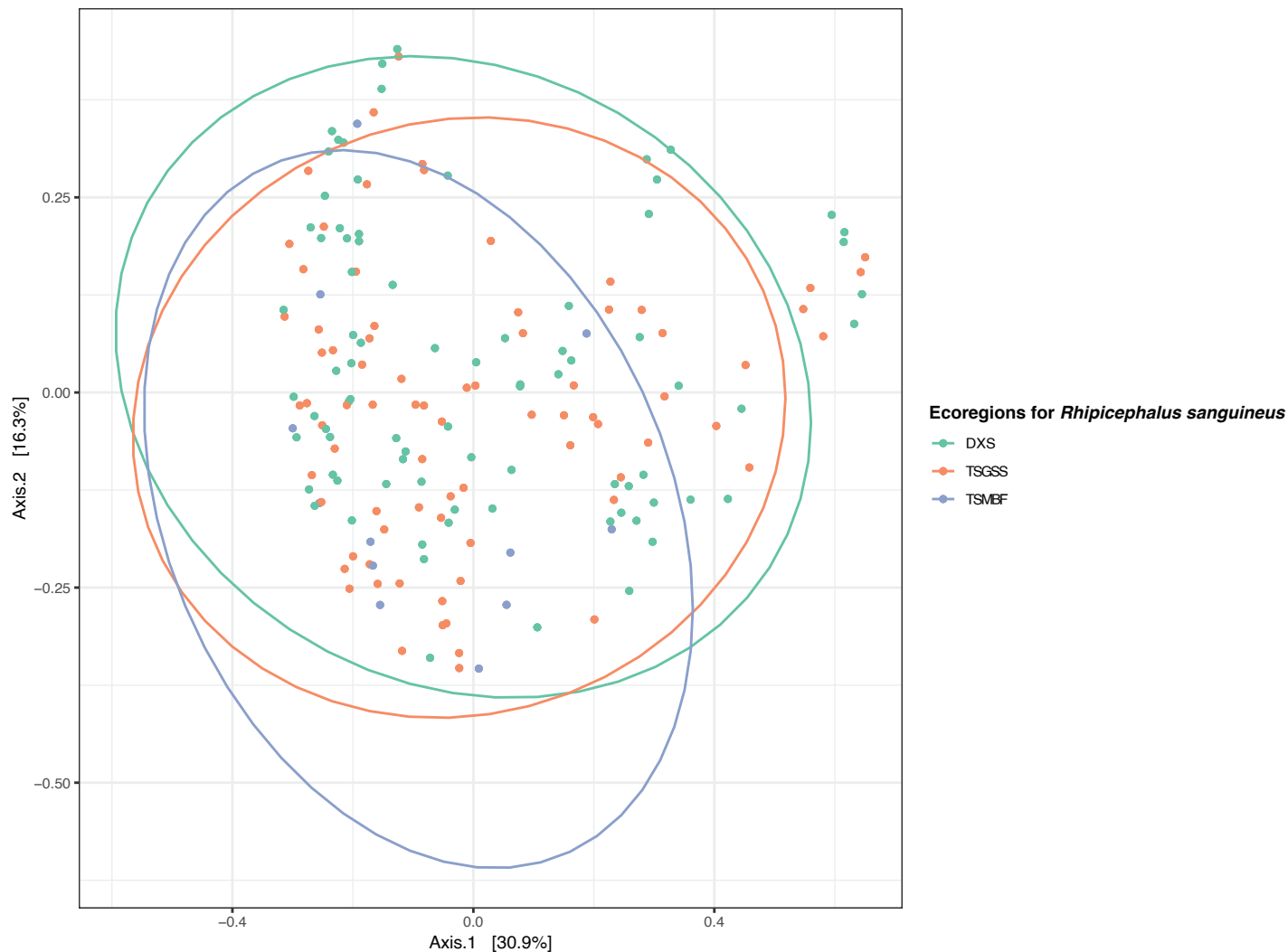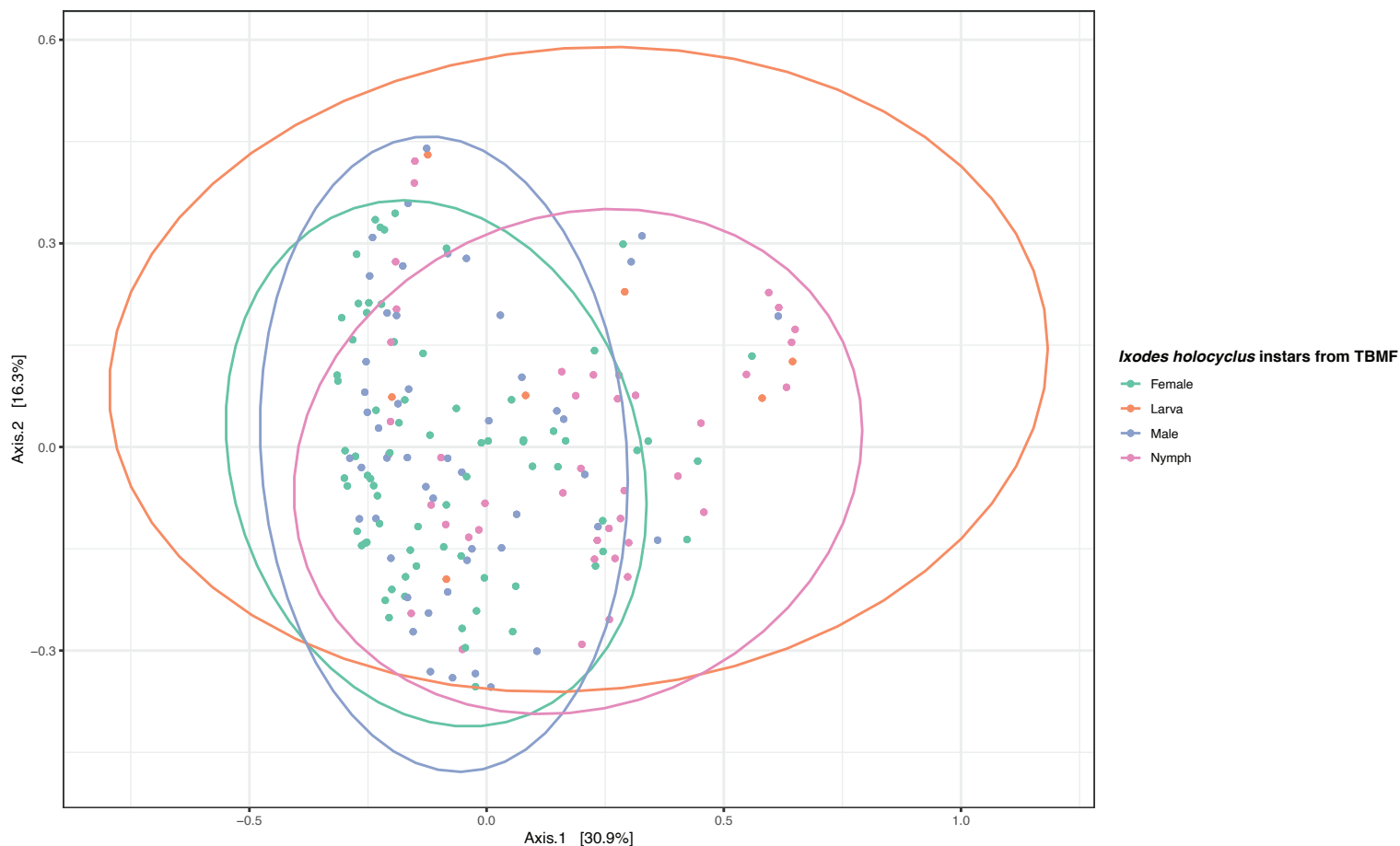

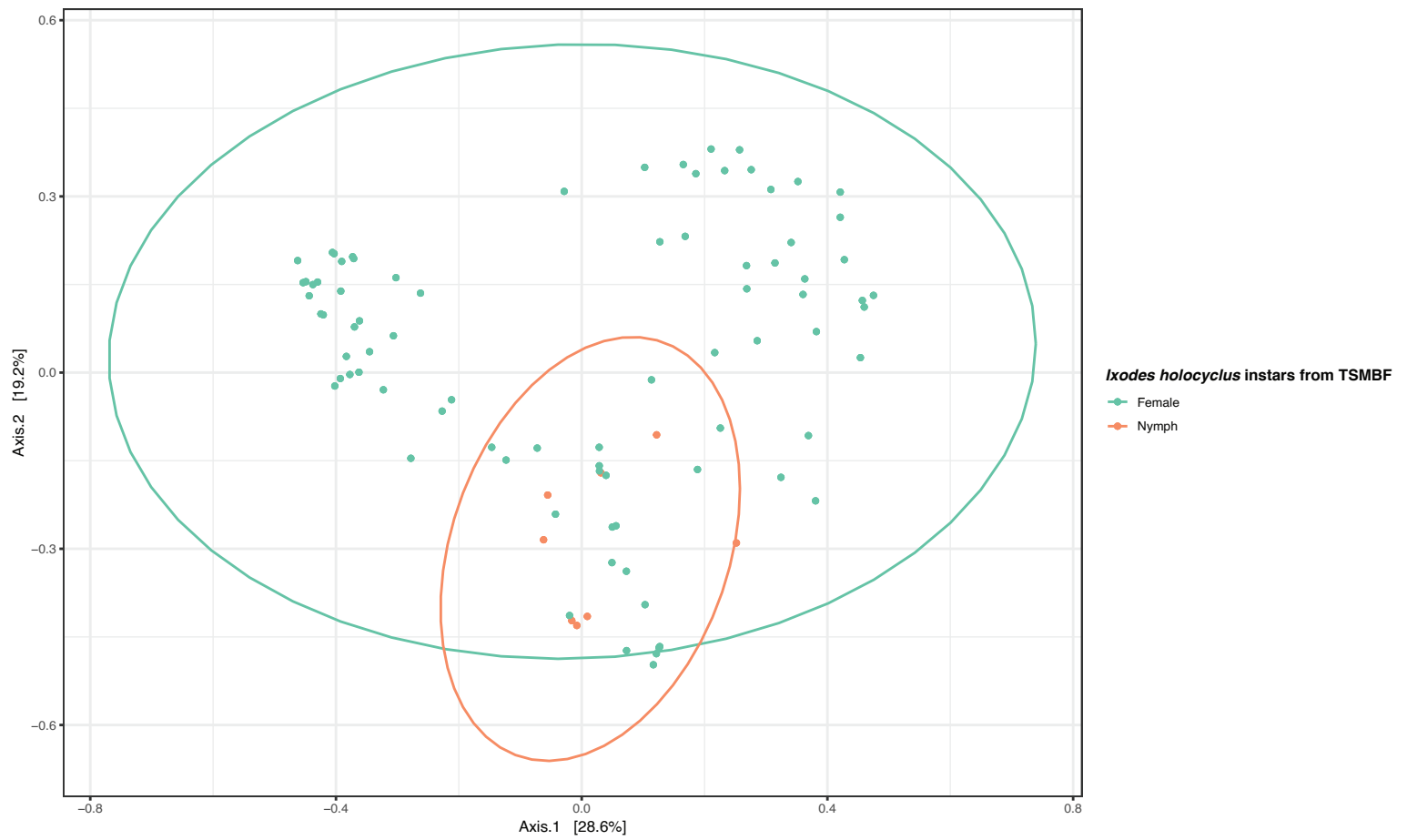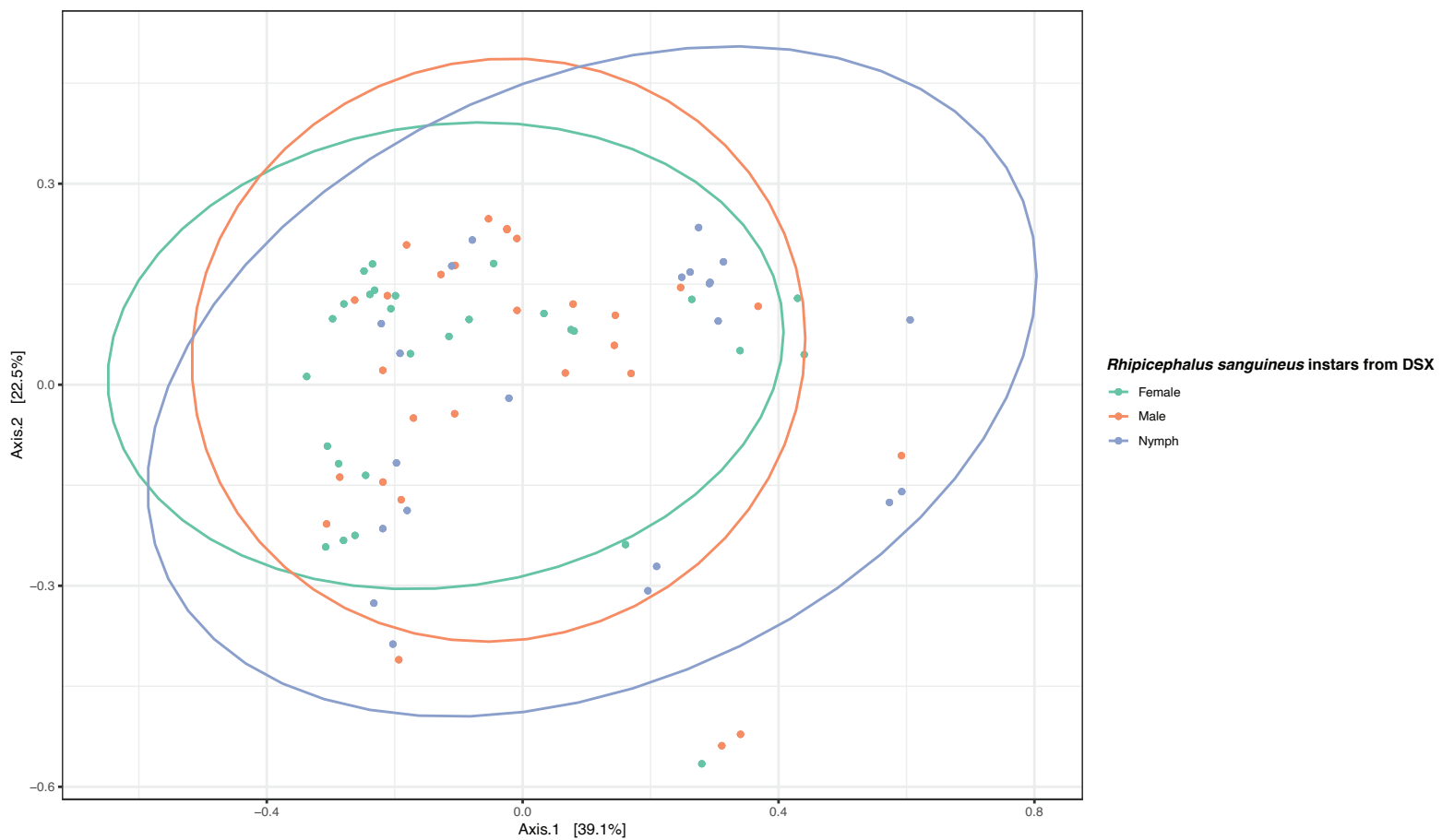

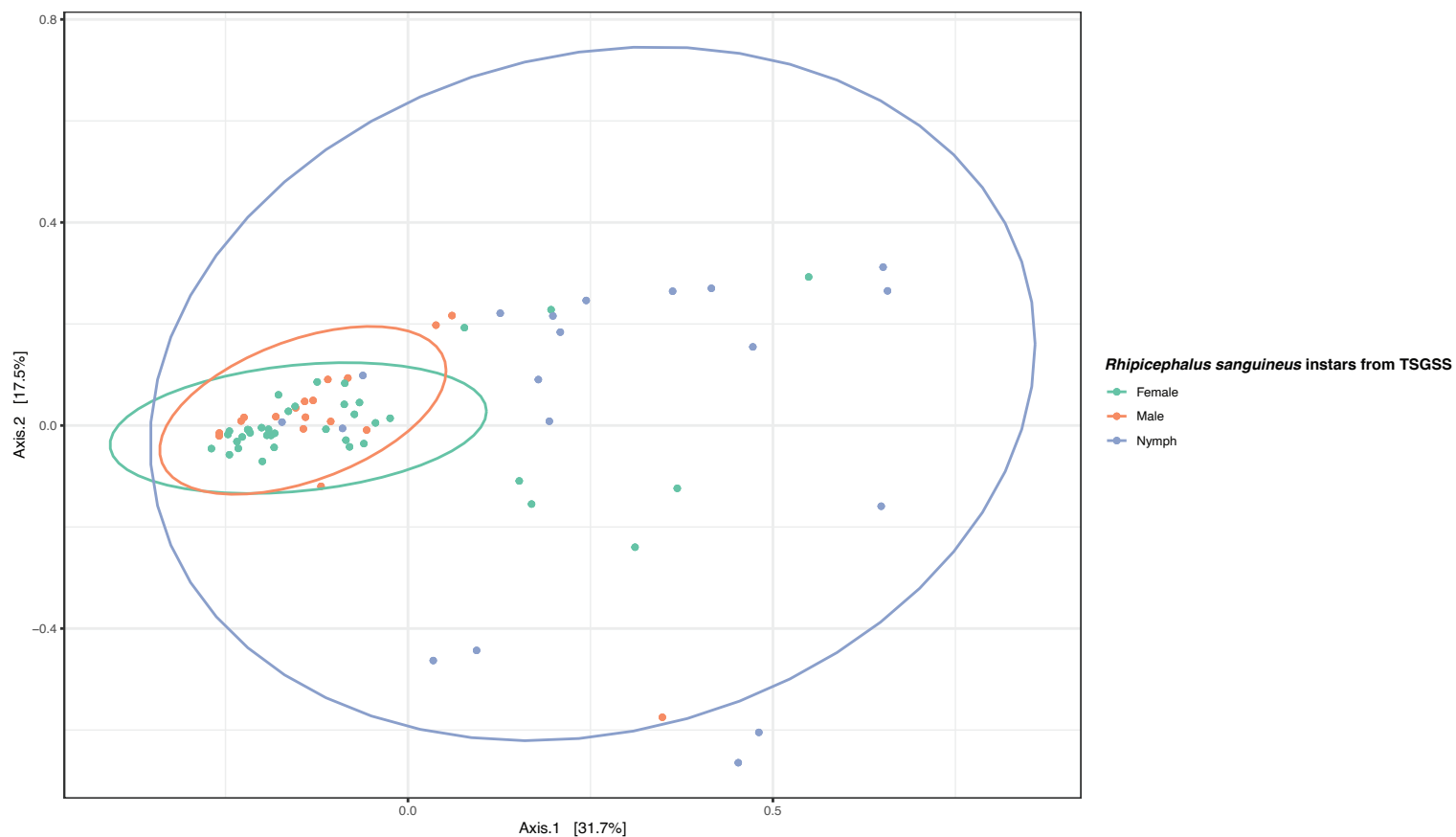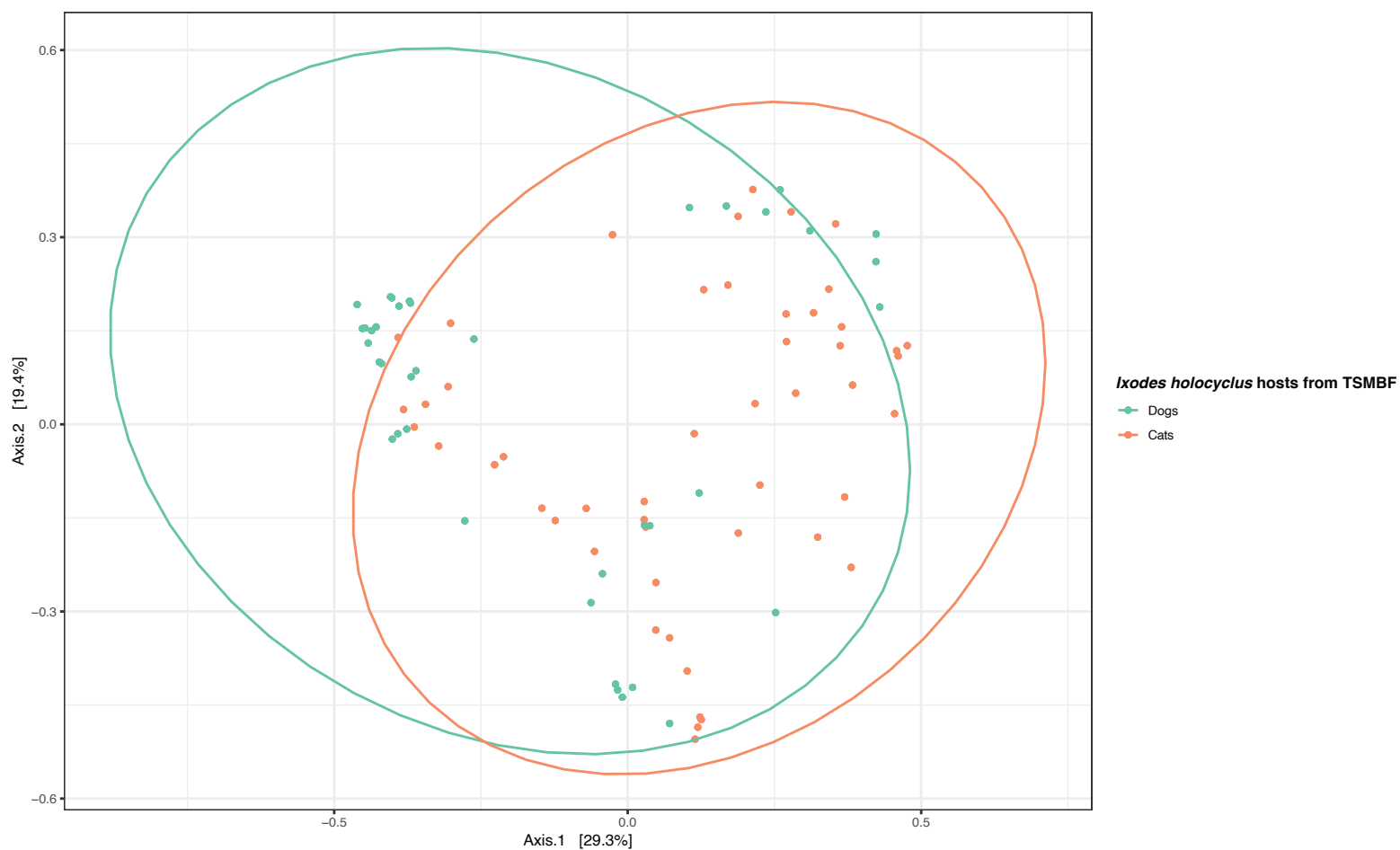

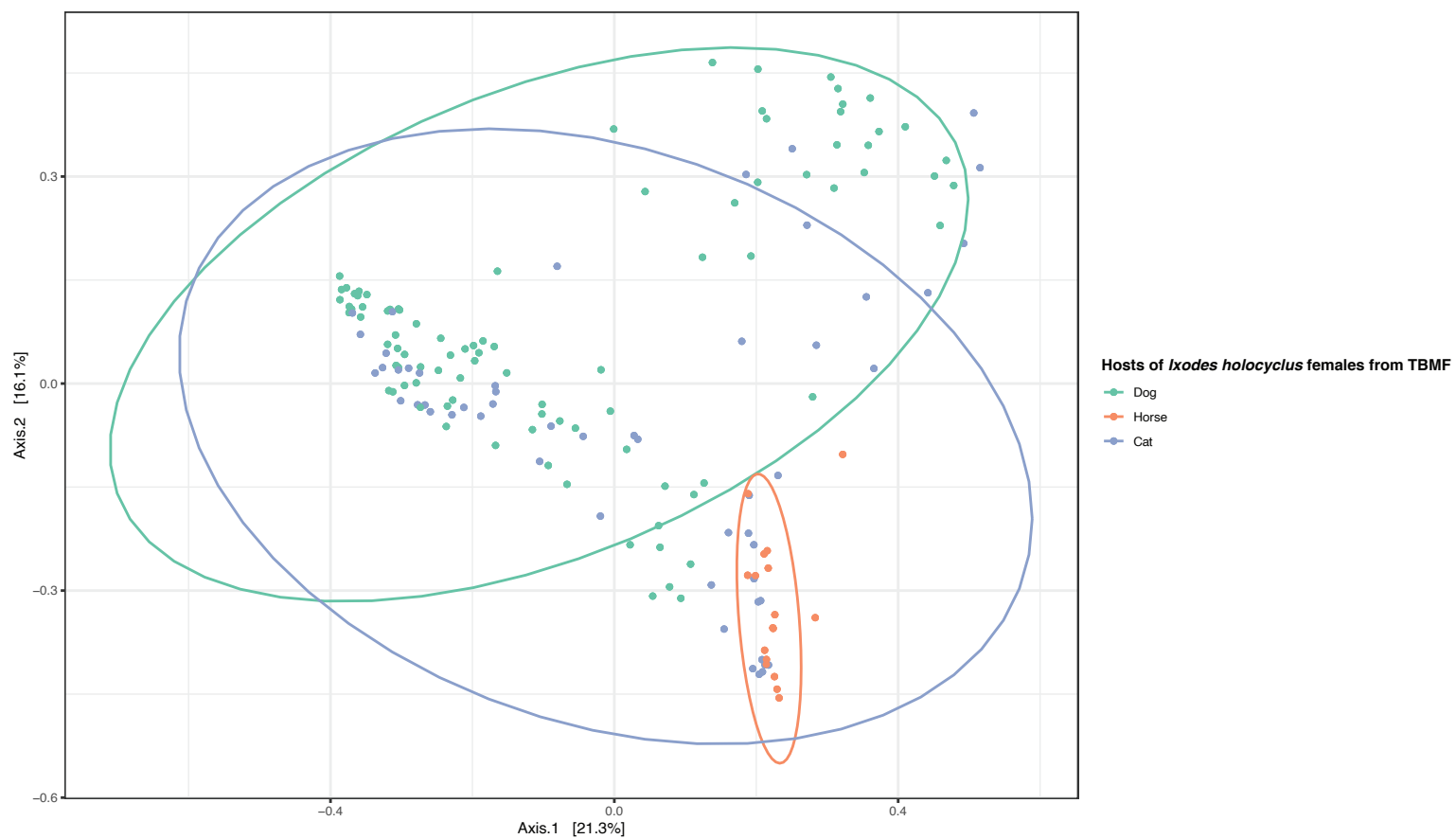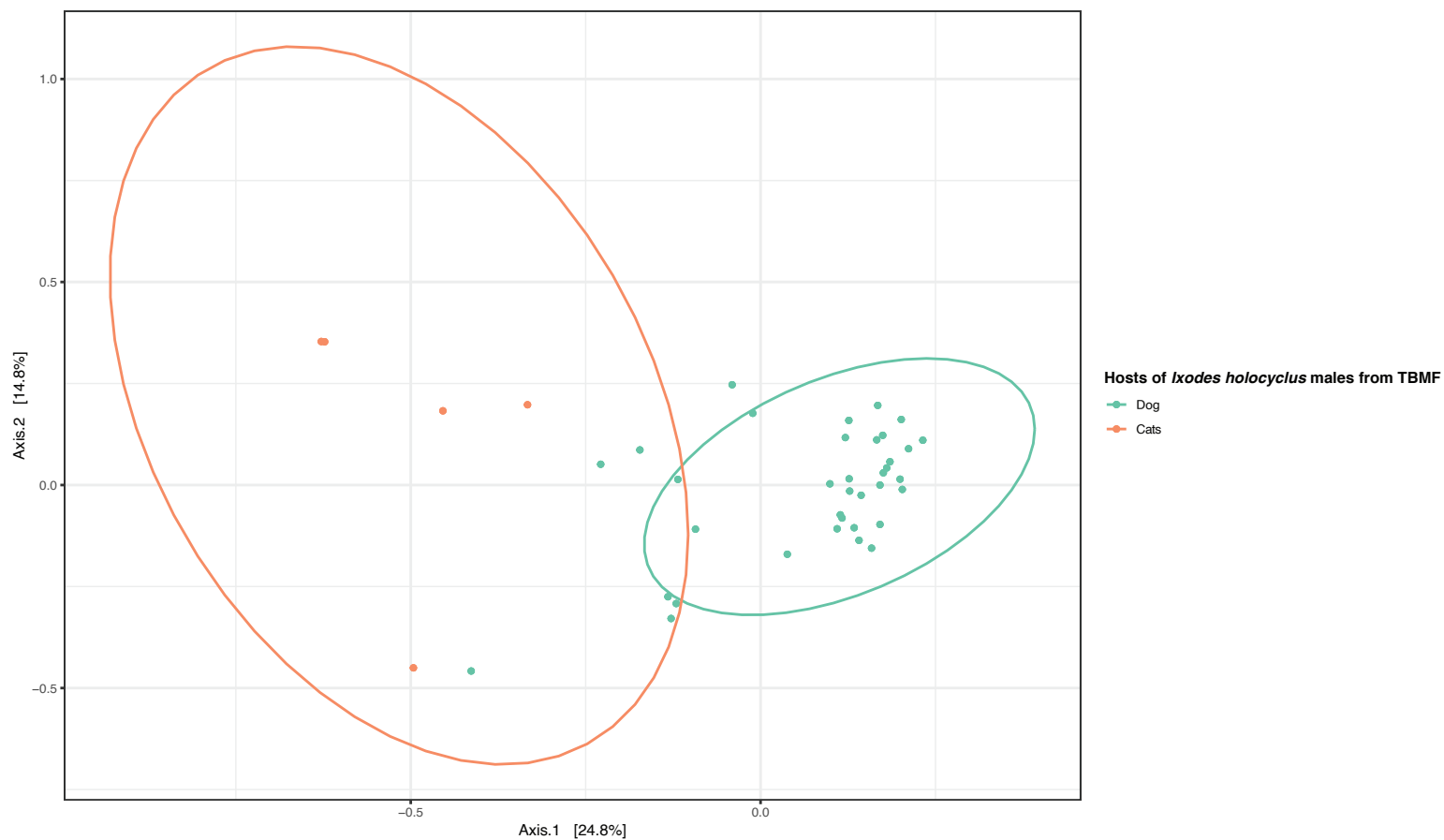

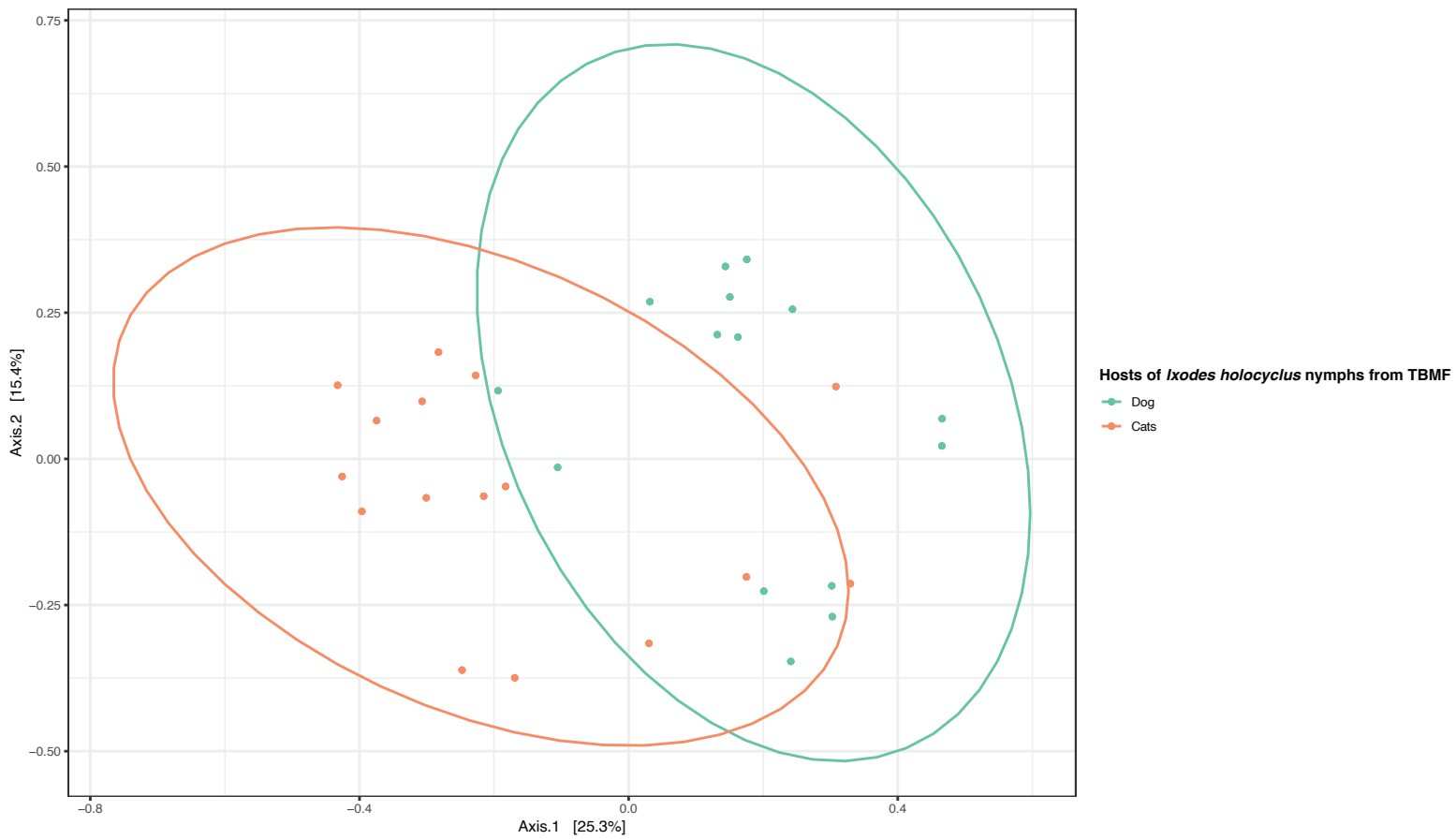

Supplement: Multimedia component 8 — Additional file 8.Alpha and beta diversity plots. [file mmc8.pdf]
