# Supplementary material for: Illuminating the bacterial microbiome of Australian ticks with 16S and Rickettsia-specific next-generation sequencing
Source: Curr Res Parasitol Vector Borne Dis. 2021 Jun 11;1:100037. doi: 10.1016/j.crpvbd.2021.100037 (PMC8906098; doi:10.1016/j.crpvbd.2021.100037)
Supplement: Multimedia component 1 — Additional file 1. List of 16S NGS ZOTUs denoised with UNOISE3 in USEARCH v10.0. [file mmc1.pdf]

Additional file 1. List of 16S NGS ZOTUs denoised with UNOISE3 in USEARCH v10.0

>Zotu1
GAACGCTGGCGGCGTGCTTAACACATGCAAGTCGAACGAGCAAGAGCTGTAGCAATACAGGGTTCTGC
TAGTGGCAGACGGGTGAGTAATACATGGGAATCTACCTTGCAGTGCGAAATAACAGTTGGAAGCAATT
GCTAATGTCGCATATAGCCGAGAGGTGAAAGGTTTACTGCTGCAAGATGGGCCCATGCAGGATTAGCT
AGTTGGTGAGGTAATGGCTCACCAAGGCAACGATCTTTAGTTGGTCTGAGAGGATGATCAGCCACACT
GGAACTGAGACACGGTC
>Zotu2
GAACGCTGGCGGTGTGCTTAACACATGCAAGTCGAACGGGCAAGAGCTGTAGCAATACAGAATTCTGC
TAGTGGCAGACGGGTGAGTAATACATGGGAATCTACCTTGCAGTGCGAAATAACAGTTGGAAGCAATT
GCTAATGTCGCATATAGCCGAGAGGTGAAAGATTTATCGCTGCAAGACGAGCCCATGCAGGATTAGCT
AGTTGGTGGGGTAATGGCTCACCAAGGCGACGATCTTTAGTTGGTCTGAGAGGATGATCAGCCACACT
GGAACTGAGACACGGTC
>Zotu3
GAACGCTAGCGGCATGCCTAACACATGCAAGTCGAACGGCAGCGGGGGGAGCTTGCTCCCTGACGGCG
AGTGGCGGACGGGTGAGTAACACGTAGGAATCTACCTTAATTATAATAGTTAGTGGGGGATAACCCGG
GGAAACTCGGGCTAATACCGCATAATCTCTTCAAAGCAAAGCGGGGGATCTTCGGACCTCGTGCTATG
AGATGAGCCTGCGTCGGATTAGCTAGTTGGTAGGGTAATGGCCTACCAAGGCGACGATCCGTAGCTGG
TCTGAGAGGACGATCAGCCACACTGGGACTGAGACACGGCC
>Zotu4
GAACGCTAGCGGCATGCTTAACACATGCAAGTCGAACGGCAGAGCGGGGAGTTTATGCTCCCTGGCGG
CGAGTGGCGGACGGGTGAGTAATACGTAGGAATCTACCTTATAGAGGGGGACAACCCGGGGAAACTCG
GGCTAATACCGCATGATCTCTATGGAGTAAAGCGGGGGATCTTCTGACCTCGCGCTATAAGATGAGCC
TATGTCGGATTAGCTTGTTGGTGGGGTAATTGCCTACCAAGGCGACGATCCGTAGCTGGTCTGAGAGG
ATGATCAGCCACACTGGGACTGAGACACGGCC
>Zotu5
GAACGCTAGCGGCAGGCTTAATACATGCAAGTCGTGGGGCAGCATGAGTAGCAATACTTGATGGCGAC
CGGCAAACGGGTGCGGAACACGTACACAACCTTCCGTCAAGCGGGGAATAGCCCAGAGAAATTTGGAT
TAATACCCCATAATATATTTGATAAGGCATCTTATTAATATTAAAGCTTCGGCACTTGATGATGGGTG
TGCGGCTGATTAGATAGTTGGTGAGGTAACGGCTCACCAAGTCTACGATCAGTAGCTGATGTGAGAGC
ATGATCAGCCACACGGGCACTGAGACACGGGC
>Zotu6
GAACGCTGGCGGCGTGCTTAACACATGCAAGTCGAACGGAAAGGCCCTGCTTTTGTGGGGTGCTCGAG
TGGCGAACGGGTGAGTAACACGTGAGTAACCTGCCCTTGACTTTGGGATAACTTCAGGAAACTGGGGC
TAATACCGGATAGGAGCTCCTGCTGCATGGTGGGGGTTGGAAAGTTTCGGCGGTTGGGGATGGACTCG
CGGCTTATCAGCTTGTTGGTGGGGTAGTGGCTTACCAAGGCTTTGACGGGTAGCCGGCCTGAGAGGGT
GACCGGCCACATTGGGACTGAGATACGGCC
>Zotu7
GAACGCTGGCGGTATGCTTAACACATGCAAGTCGAACGGCAGCATGTTCCTCACTCAAATCACTTTAA
TTTCTTTTTGGAAAAGAAATACGGAAAGTGATTTGAGTGAGGAATGATGGCAAGTGGCGGACGGGTGA
GTAATAGGTAGGAATCTGTCCTAGAGAGGGGGATAACTTTCGGAAACGAGAGCTAATACCACATGATA
TTGAAAAATCAAAGGCAGGGGACCTTTCGAGGCCTTGCGCTCTAGGGTGAGCCTGCATCGGATTAGCT
AGTTGGTAGGGTAAAAGCCTACCAAGGCGACGATCCGTAGCTGGTCTGAGAGGATGACCAGCCACACT
GGGACTGAGACACGGCC
>Zotu8
GAACGCTGGCGGCAAGCTTAACACATGCAAGTCGAACGAATTACAATTATAGCTTGCTACAATTGTAG
TTAGTGGCAGACGGGTGAGTAATGTATAGGAATCTGCCTAGTAGTATGGAATAGCTGTTAGAAATGAC
AGGTAATACCGTATAATCCCTACGGGGGAAAGATTTATCGCTATTAGATGAGCCTATATTAGATTAGC
TAGTTGGTGAGGTAATGGCTTACCAAGGTTATGATCTATAGCTGGTCTGAGAGGACGATCAGCCACAC
TGGAACTGAGATACGGTC
>Zotu9
GAACGCTATCGGTATGCTTAACACATGCAAGTCGAACGGACTAATTGGGGCTTGCTCCAATTAGTTAG
TGGCAGACGGGTGAGTAACACGTGGGAATCTACCCATCAGTACGGAATAACTTTTAGAAATAAAAGCT
AATACCGTATATTCTCTGCGGAGGAAAGATTTATCGCTGATGGATGAGCCCGCGTCAGATTAGGTAGT
TGGTGAGGTAATGGCTCACCAAGCCGACGATCTGTAGCTGGTCTGAGAGGATGATCAGCCACACTGGG

ACTGAGACACGGCC
>Zotu10
GAACGCTGGCGGCATGCCTTACACATGCAAGTCGAACGGCAGCATAGGAGCTTGCTCCTGATGGCGAG
TGGCGAACGGGTGAGTAATATATCGGAACGTGCCCTAGAGTGGGGGATAACTAGTCGAAAGACTAGCT
AATACCGCATACGATCTACGGATGAAAGTGGGGGATCGCAAGACCTCATGCTCCTGGAGCGGCCGATA
TCTGATTAGCTAGTTGGTGGGGTAAAAGCTCACCAAGGCGACGATCAGTAGCTGGTCTGAGAGGACGA
CCAGCCACACTGGGACTGAGACACGGCC
>Zotu11
GAACGCTATCGGTATGCTTAACACATGCAAGTCGAACGGACTAATTGGGGCTTGCTCCAATTAGTTAG
TGGCAGACGGGTGAGTAACATGTGGGAATCTACCCATCAGTACGGAATAACTTTTAGAAATAAAAGCT
AATACCGTATATTCTCTACGGAGGAAAGATTTATCGCTGATGGATGAGCCCGCGTCAGATTAGGTAGT
TGGTGAGGTAATGGCTCACCAAGCCGACGATCTGTAGCTGGTCTGAGAGGATGATCAGCCACACTGGG
ACTGAGACACGGCC
>Zotu12
GAACGCTAGCGGCAGGCCTAATACATGCAAGTCGAACGGTTCGCAAGGACAGTGGCAAACGGGTGCGT
AACGCGTAAGCAACCTGCCTCATACTGGGGGATAGCCCGGCGAAAGCTGGGGTAAACCCGCACGGTCC
AGTGTCATCACCTGATGATATTGGTAAACATTTATGGGTATGAGAGGGGCTTGCGTCTGATTAGCTAG
TTGGGGGGGTAACGGCCCACCAAGGCGATGATCAGTAGGGGTTCTGAGAGGATTGGCCCCCACATGGG
TACTGAGATACGGAC
>Zotu13
GAACGCTGGTGGCATGCTTAACACATGCAAGTCGAACGGTACAGGACTAGCTTGCTAGTTGCTGACGA
GTGGCGGACGGGTGAGTAACGCGTAGGAATCTGCCCATCTGAGGGGGATACCAGTTGGAAACGACTGT
TAATACCGCATAGTATCTGTGGATTAAAGGTGGCTTTTAGGCTGTCGCAGATGGATGAGCCTGCGTTA
GATTAGCTAGTTGGTGGGGTAAGGGCCTACCAAGGCTACGATCCATAGCTGATTTGAGAGGATGATCA
GCCACATTGGGACTGAGACACGGCC
>Zotu14
GAACGCTATCGGTATGCTTAACACATGCAAGTCGAACGGACTAATTGGGGCTTGCTCCAATTAGTTAG
TGGCAGACGGGTGAGTAACACGTGGGAATCTGTCCATCAGTACGGAATAACTTTTAGAAATAAAAGCT
AATACCGTATATTCTCTACGGAGGAAAGATTTATCGCTGATGGGTGAGCCCGCGTCAGATTAGGTAGT
TGGTGAGGTAATGGCTCACCAAGCCGACGATCTGTAGCTGGTCTGAGAGGATGATCAGCCACACTGGG
ACTGAGACACGGCC
>Zotu15
GAACGCTAGCGGCAGGCTTAATACATGCAAGTCGTGGGGCAGCACAGGGTAGCAATACTTGGGTGGCG
ACCGGCAAACGGGTGCGGAACACGTACACAACCTTCCTACAAGTGGGGAATAGCCCAGAGAAATTTGG
ATTAATACCCCGTAACATATAGAGATGGCATCATTTTTATATTATAGCTACGGCGCTTGTTGATGGGT
GTGCGGCTGATTAGGTAGTTGGCGGGGTAACGGCCCACCAAGCCTTCGATCAGTAGCTGATGTGAGAG
CATGATCAGCCACACGGGCACTGAGACACGGGC
>Zotu16
GAACGCTGGCGGCATGCCTTACACATGCAAGTCGAACGGTAACAGGTTAAGCTGACGAGTGGCGAACG
GGTGAGTAATGTATCGGAACGTGCCCAGTCGTGGGGGATAACTGCTCGAAAGAGCAGCTAATACCGCA
TACGACCTGAGGGTGAAAGCGGGGGATCGCAAGACCTCGCGCGATTGGAGCGGCCGATATCAGATTAG
GTAGTTGGTGAGGTAAAGGCTCACCAAGCCGACGATCTGTAGCTGGTCTGAGAGGACGACCAGCCACA
CTGGGACTGAGACACGGCC
>Zotu17
GAACGCTGGCGGCATGCCTAACACATGCAAGTCGAACGGCAGCACAGTAAAGATTTCGGTCTTTACGG
GTGGCGAGTGGCGGACGGGTGAGTAAAGCGTAGGAATCTGTCCTGGAGAGTGGGATAACCCGAGGAAA
CTCGGGCTAATACCGCATGATATCGAGAGATCAAAGCAGGGGACCCTTCGGGGCCTTGCGCTCTAGGA
TGAGCCTACGTCGGATTAGCTAGTTGGTGGGGTAAAGGCCTACCAAGGCGACGATCCGTAGCTGGTCT
GAGAGGATGACCAGCCACACTGGGACTGAGACACGGCC
>Zotu18
GAACGCTAGCGGGAGGCCTAATACATGCAAGTCGAACGGTAACAGGGTAGCAATACTGCTGACGAGTG
GCGCACGGGTGAGTAACACGTACACAACCTGCCTTATACTGGGGCATAGCCTTGGGAAACTAAGATTA
ATTCCCCATAACATTGCGAAATGGCATCATTTTGCAATTAAAATTCCGATGGTATAAGATGGGTGTGC
GTCTGATTAGCTAGTTGGCGGGGTAACGGCCCACCAAGGCAACGATCAGTAACTGGTCTGAGAGGATG

ATCAGTCACACGGGCACTGAGACACGGGC
>Zotu19
GAACGCTGGCGGCAAGCTTAACACATGCAAGTCGAACGGATTTTTGTCGTAGCTTGCTATGATAAAAA
TTAGTGGCAGACGGGTGAGTAATGCATAGGAATCTACCTAGTAGTATGGGATAGCCACTAGAAATGGT
GGGTAATACTGTATAATCCCTGCGGGGGAAAGATTTATCGCTATTAGATGAGCCTATGTTAGATTAGC
TAGTTGGTAGGGTAAAGGCCTACCAAGGCAGTGATCTATAGCTGGTCTGAGAGGATGATCAGCCACAC
TGGAACTGAGATACGGTC
>Zotu20
GAACGCTAGCGGGATGCCTTACACATGCAAGTCGAACGGCAGCACGGACTTCGGTCTGGTGGCGAGTG
GCGAACGGGTGAGTAATGTATCGGAACGTGCCTAGTAGCGGGGGATAACTACGCGAAAGCGTAGCTAA
TACCGCATACGCCCTACGGGGGAAAGCAGGGGATCGCAAGACCTTGCACTATTAGAGCGGCCGATATC
GGATTAGCTAGTTGGTGGGGTAACGGCTCACCAAGGCGACGATCCGTAGCTGGTTTGAGAGGACGACC
AGCCACACTGGGACTGAGACACGGCC
>Zotu21
GAACGCTAGCGGGATGCCTTACACATGCAAGTCGAACGGCAGCACGGACTTCGGTCTGGTGGCGAGTG
GCGAACGGGTGAGTAATGTATCGGAACGTGCCCAGTAGCGGGGGATAACTACGCGAAAGCGTAGCTAA
TACCGCATACGCCCTACGGGGGAAAGCAGGGGATCGCAAGACCTTGCACTATTGGAGCGGCCGATATC
GGATTAGCTAGTTGGTGGGGTAACGGCTCACCAAGGCGACGATCCGTAGCTGGTTTGAGAGGACGACC
AGCCACACTGGGACTGAGACACGGCC
>Zotu23
GAACGCTGGCGGCGTGCCTAATACATGCAAGTCGAGCGAACAGATAAGGAGCTTGCTCCTTTGACGTT
AGCGGCGGACGGGTGAGTAACACGTGGGTAACCTACCTATAAGACTGGAATAACTCCGGGAAACCGGG
GCTAATGCCGGATAACATGTTGAACCGCATGGTTCTACAGTGAAAGACGGTCTTGCTGTCACTTATAG
ATGGACCCGCGCCGTATTAGCTAGTTGGTGGGGTAACGGCCTACCAAGGCGACGATACGTAGCCGACC
TGAGAGGGTGATCGGCCACACTGGAACTGAGACACGGTC
>Zotu24
GAACGCTGGCGGCAGGCCTAACACATGCAAGTCGAGCGGATGAGAGGAGCTTGCTCCTGGATTCAGCG
GCGGACGGGTGAGTAATGCCTAGGAATCTGCCTGGTAGTGGGGGACAACGTTTCGAAAGGAACGCTAA
TACCGCATACGTCCTACGGGAGAAAGCAGGGGACCTTCGGGCCTTGCGCTATCAGATGAGCCTAGGTC
GGATTAGCTAGTTGGTGAGGTAATGGCTCACCAAGGCGACGATCCGTAACTGGTCTGAGAGGATGATC
AGTCACACTGGAACTGAGACACGGTC
>Zotu25
GAACGCTGGCGGCAGGCTTAACACATGCAAGTCGAGCGGGCGTAGCAATACGTCAGCGGCAGACGGGT
GAGTAACGCGTGGGAACATACCTTTTGGTTCGGAACAACACAGGGAAACTTGTGCTAATACCGGATAA
GCCCTTACGGGGAAAGATTTATCGCCGAAAGATTGGCCCGCGTCTGATTAGCTAGTTGGTGAGGTAAC
GGCTCACCAAGGCGACGATCAGTAGCTGGTCTGAGAGGATGATCAGCCACATTGGGACTGAGACACGG
CC
>Zotu26
GAACGCTGGCGGCATGCCTAACACATGCAAGTCGAACGAGCCCTTCGGGGTTAGTGGCGCACGGGTGC
GTAACGCGTGGGAATCTGCCCTTGGGTTCGGAATAACTCGCCGAAAGGCGTGCTAATACCGGATGATG
TCGAAAGACCAAAGATTTATCGCCCAAGGATGAGCCCGCGTAAGATTAGGTAGTTGGTGAGGTAAAGG
CTCACCAAGCCGACGATCTTTAGCTGGTCTGAGAGGATGATCAGCCACACTGGGACTGAGACACGGCC
>Zotu27
GAACGCTAGCGGCAGGCTTAATACATGCAAGTCGAGGGGCAGCACGGTATAGCAATATATGGGTGGCG
ACCGGCAAACGGGTGCGGAACACGTACACAACCTTCCGGTGAGAGGGGGATAGCCCAGAGAAATTTGG
ATTAATACCCCATACTATAATGGTCAGGCATCTGATTATTATCAAAGGCTTCGGCCGCTTATTGATGG
GTGTGCGTCTGATTAGGTAGTTGGCGGGGTAGAGGCCCACCAAGCCTACGATCAGTAGCTGATGTGAG
AGCATGATCAGCCACACGGGCACTGAGACACGGGC
>Zotu28
AAACGCTGGCGGCATGCCTAACACATGCAAGTCGAACGGCAGCACAGTAAAGATTTCGGTCTTTACGG
GTGGCGAGTGGCGGATGGGTGAGTAAAGCGTAGGAATCTGTCCTGGAGAGTGGGATAACCCGAGGAAA
CTCGGGCTAATACCGCATGATATCGAGAGATCAAAGCAGGGGACCCTTCGGGGCCTTGCGCTCTAGGA
TGAGCCTACGTCGGATTAGCTAGTTGGTGGGGTAAAGGCCTACCAAGGCGACGATCCGTAGCTGGTCT
GAGAGGATGACCAGCCACACTGGGACTGAGACACGTCC

>Zotu29
GAACGCTGGCGGCATGCCTTACACATGCAAGTCGAACGGTAACAGGTCTTCGGACGCTGACGAGTGGC
GAACGGGTGAGTAATACATCGGAACGTGCCCAGTCGTGGGGGATAACTACTCGAAAGAGTAGCTAATA
CCGCATACGATCTGAGGATGAAAGCGGGGGACCTTCGGGCCTCGCGCGATTGGAGCGGCCGATGGCAG
ATTAGGTAGTTGGTGGGATAAAAGCTTACCAAGCCGACGATCTGTAGCTGGTCTGAGAGGACGACCAG
CCACACTGGGACTGAGACACGGCC
>Zotu30
GAACGCTGGCGGCATGCCTAACACATGCAAGTCGAACGGCAGCACAGTAAAGATTTCGGTCTTTACGG
GTGGCGAGTGGCGGACGGGTGAGTAAAGCGTAGGAATCTGTCCTGGAGAGTGGAATAACCCGAGGAAA
CTCGGGCTAATACCGCATGATATCGAGAGATCAAAGCAGGGGACCCTTCGGGGCCTTGCGCTCTAGGA
TGAGCCTACGTCGGATTAGCTAGTTGGTGGGGTAAAGGCCTACCAAGGCGACGATCCGTAGCTGGTCT
GAGAGGATGACCAGCCACACTGGGACTGAGACACGGCC
>Zotu31
GAACGCTAGCGGCATGCTTAACACATGCAAGTCGAACGGCAGCGCAGGGAGCTTGCTCCCTGGCGGCG
AGTGGCGGACGGGTGAGTAATGCGTAGGAATCTACCTTGTAGTGGGGGATAACCTGGGGAAACTCGGG
CTAATACCGCATAATCTCTTTGGAGCAAAGCGGGGGATCTTCGGACCTCGTGCTATAAGATGAGCCTA
CGTCGGATTAGCTTGTTGGTGGGGTAATGGCCTACCAAGGCGACGATCCGTAGCTGGTCTGAGAGGAC
GATCAGCCACACTGGGACTGAGACACGGCC
>Zotu32
GAACGCTGGCGGCGTGCTTAACACATGCAAGTCGAACGGAAAGGCCCTGCTTGCAGGGTACTCGAGTG
GCGAACGGGTGAGTAACACGTGGGTGATCTGCCCTGCACTTCGGGATAAGCCTGGGAAACTGGGTCTA
ATACCGGATAGGAGCCATTTTTAGTGTGATGGTTGGAAAGTTTTTTCGGTGTAGGATGAGCTCGCGGC
CTATCAGCTTGTTGGTGGGGTAATGGCCTACCAAGGCGGCGACGGGTAGCCGGCCTGAGAGGGTGGAC
GGCCACATTGGGACTGAGATACGGCC
>Zotu33
GAACGCTATCGGTATGCTTAACACATGCAAGTCGAACGGACTAATTGGAGCTTGCTCCAATTAGTTAG
TGGCAGACGGGTGAGTAACACGTGGGAATCTACCCATCAGTACGGAATAACTTTTAGAAATAAAAGCT
AATACCGCATATTCTCTACGGAGGAAAGATTTATCGCTGATGGATGAGCCCGCGTCAGATTAGGTAGT
TGGTGAGGTAATGGCTCACCAAGCCGACGATCTGTAGCTGGTCTGAGAGGATGATCAGCCACACTGGG
ACTGAGACACGGCC
>Zotu34
GAACGCTGGCGGCATGCCTTACACATGCAAGTCGAACGGCAGCATGATCTAGCTTGCTAGATTGATGG
CGAGTGGCGAACGGGTGAGTAATACATCGGAACGTGCCCTGTAGTGGGGGATAACTAGTCGAAAGATT
AGCTAATACCGCATACGACCTGAGGGTGAAAGTGGGGGACCGCAAGGCCTCATGCTATAGGAGCGGCC
GATGTCTGATTAGCTAGTTGGTGAGGTAAAGGCTCACCAAGGCGACGATCAGTAGCTGGTCTGAGAGG
ACGATCAGCCACACTGGGACTGAGACACGGCC
>Zotu35
GAACGCTGGCGGCAGGCTTAACACATGCAAGTCGAGCGCCCCGCAAGGGGAGCGGCAGACGGGTGAGT
AACGCGTGGGAATCTACCTTTTGCTACGGAACAACAGTTGGAAACGACTGCTAATACCGTATGTGCCC
TTCGGGGGAAAGATTTATCGGCAAAGGATGAGCCCGCGTTGGATTAGCTAGTTGGTGAGGTAAAGGCT
CACCAAGGCGACGATCCATAGCTGGTCTGAGAGGATGATCAGCCACACTGGGACTGAGACACGGCC
>Zotu36
GAACGCTGGTGGCATGCTTAACACATGCAAGTCGAACGGTAACAGGACTAGCTTGCTAGTTGCTGACG
AGTGGCGGACGGGTGAGTAACACGTAGGAATCTGCCCATCTGAGGGGGATACCAGTTGGAAACGACTA
TTAATACCGCATAGTATCTGTGGATTAAAGGTGGCTTTTGAGCTGTCGCATATGGATGAGCCTGCGTT
GGATTAGCTAGTTGGTGAGGTAAGGGCCTACCAAGGCTACGATCCATAGTTGATTTGAGAGGATGATC
AGCCACATTGGGACTGAGACACGGCC
>Zotu37
GAACGCTGGCGGCATGCCTTACACATGCAAGTCGAACGGCAGCGCGGACTTCGGTCTGGCGGCGAGTG
GCGAACGGGTGAGTAATACATCGGAACGTACCCTGTTGTGGGGGATAACTAGTCGAAAGATTAGCTAA
TACCGCATACGACCTGAGGGTGAAAGCGGGGGACCGTAAGGCCTCGCGCAGCAGGAGCGGCCGATGTC
TGATTAGCTAGTTGGTGGGGTAAAGGCCCACCAAGGCGACGATCAGTAGCTGGTCTGAGAGGACGATC
AGCCACACTGGGACTGAGACACGGCC
>Zotu38

GAACGCTGGCGGCATGCCTAACACATGCAAGTCGAACGAGACCTTCGGGTCTAGTGGCGCACGGGTGC
GTAACGCGTGGGAACCTGCCCTTAGGTTCGGAATAACTCCCCGAAAGGGGTGCTAATACCGGATAATG
TCTTCGGACCAAAGATTTATCGCCTTTGGATGGGCCCGCGTTGGATTAGCTAGTTGGTAGGGTAAAAG
CCTACCAAGGCGACGATCCATAGCTGGTCTGAGAGGATGATCAGCCACACTGGGACTGAGACACGGCC
>Zotu39
GAACGCTGGCGGCATGCCTTACACATGCAAGTCGAACGGCAGCACGGGAGCAATCCTGGTGGCGAGTG
GCGAACGGGTGAGTAAAGTATCGGAACGTGCCCAGTCGTGGGGGATAACGTAGCGAAAGCTACGCTAA
TACCGCATACGATCTATGGATGAAAGCGGGGGATCGCAAGACCTCGCGCGATTGGAGCGGCCGATATC
AGATTAGGTAGTTGGTGGGGTAAAGGCCTACCAAGCCGACGATCTGTAGCTGGTCTGAGAGGACGACC
AGCCACACTGGGACTGAGACACGGCC
>Zotu40
GAACGCTGGCGGCAAGCCTAACACATGCAAGTCGAACGAATTATCTTTATAGTTTACTATAAGGATAA
TTAGTGGCAGACGGGTGAGTAATGCATAGGAATCTACCTAGTAGTATGGAATAGCTGTTAGAAATGAC
AGGTAATACCGTATAATCCCTGAGGGGGAAAGATTTATCGCTATTAGATGAGCCTATGTTAGATTAGC
TAGTTGGTGAGGTAATGGCTTACCAAGGCTGTGATCTATAGCTGGTCTGAGAGGACGATCAGCCACAC
TGGAACTGAGATACGGTC
>Zotu41
GAACGCTGGCGGCAGGCTTAACACATGCAAGTCGAGCGCCCCGCAAGGGGAGCGGCAGACGGGTGAGT
AACGCGTGGGAATCTACCCATCACTACGGAACAACTCCGGGAAACTGGAGCTAATACCGTATACGTCC
TTCGGGAGAAAGATTTATCGGTGATGGATGAGCCCGCGTTGGATTAGCTAGTTGGTGGGGTAATGGCC
TACCAAGGCGACGATCCATAGCTGGTCTGAGAGGATGATCAGCCACACTGGGACTGAGACACGGCC
>Zotu42
GAACGCTGGTGGCATGCTTAACACATGCAAGTCGAACGGTAACAGGACTAGCTTGCTAGTTGCTGACG
AGTGGCGGACGGGTGAGTAACACGTAGGAATCTGCCCATCTGAGGGGGATACCAGTTGGAAACGACTA
TTAATACCGCATAGTATCTGTGGATTAAAGATGGCTTTTAGGCTGTCGCGGATGGATGAGCCTGCGTT
GGATTAGCTAGTTGGTGAGGTAAGGGCCTACCAAGGCTACGATCCATAGTTGATTTGAGAGGATGATC
AGCCACATTGGGACTGAGACACGGCC
>Zotu43
GAACGCTGGTGGCATGCTTAACACATGCAAGTCGAACGGTAACAGGACTAGCTTGCTAGTTGCTGACG
AGTGGCGGACGGGTGAGTAACACGTAGGAATCTGCCCATCTGAGGGGGATACCAGTTGGAAACGACTA
TTAATACCGCATAATATCTGTGGATTAAAGGTGGCTTTTGAGCTGTCGCAGATGGATGAGCCTGCGTT
GGATTAGCTAGTTGGTGAGGTAAGGGCCTACCAAGGCTACGATCCATAGTTGATTTGAGAGGATGATC
AGCCACATTGGGACTGAGACACGGCC
>Zotu44
GAACGCTAGCGGGAGGCCTAACACATGCAAGCCGAGCGGTATTGTTTCTTCGGAAATGAGAGAGCGGC
GTACGGGTGCGTAACACGTGTGCAACCTGCCTTTATCTGGGGGATAGCCTTTCGAAAGGAAGATTAAT
ACTCCATAATATATTGAACGGCATCGTTTAATATTGAAAGCTCCGGCGGATAGAGATGGGCACGCGCA
AGATTAGCTAGTTGGTGAGGTAACGGCTCACCAAGGCGATGATCTTTAGGGGGCCTGAGAGGGTGATC
CCCCACACTGGTACTGAGACACGGAC
>Zotu45
GAACGCTGGCGGCGTGCCTAATACATGCAAGTCGAGCGAACAGACGAGGAGCTTGCTCCTCTGACGTT
AGCGGCGGACGGGTGAGTAACACGTGGATAACCTACCTATAAGACTGGGATAACTTCGGGAAACCGGA
GCTAATACCGGATAATATATTGAACCGCATGGTTCAATAGTGAAAGACGGTTTTGCTGTCACTTATAG
ATGGATCCGCGCCGCATTAGCTAGTTGGTAAGGTAACGGCTTACCAAGGCAACGATGCGTAGCCGACC
TGAGAGGGTGATCGGCCACACTGGAACTGAGACACGGTC
>Zotu46
GAACGCTGGCGGCATGCCTAACACATGCAAGTCGAACGGCAGCACAGTAAAGATTTCGGTCTTTATGG
GTGGCGAGTGGCGGACGGGTGAGTAATGCGTAGGGATCTGTCCTGGAGAGAGGGATAACCCGAGGAAA
CTCGGGCTAATACCTCATGATATTGAGAGATCAAAGGCAGGGGACCGAAAGGCCTTGCGCTCTGGGGT
GAGCCTACGTCGGATTAGCTAGTTGGTGGGGTAAGAGCCTACCAAGGCGACGATCCGTAGCTGGTCTG
AGAGGATGACCAGCCACACTGGGACTGAGACACGGCC
>Zotu47
GAACGCTGGCGGCGGGCCTCATACATGCAAGTCGAGCGCGAAAGTCCGCAAGGATGAGTAAAGCGGCG
CACGGGTGAGTAACACGTGGATAATCTACCCTTTGGATTGGAATAACCTGTCGAAAGACGGGCTAATG

CCGAGTAAGCTCTCGGGAGCGTGAAGCTCTTGCGAGGAAAGATGGCGCAAGCTATCGCCGATGGATGA
GTCTGCGTCCCATTAGTTAGTTGGTGAGGTAACAGCTTACCAAGACGATGATGGGTACCTGGTCTGAG
AGGATGATCAGGCACACTGGAACTGAAACACGGTC
>Zotu48
GAACGCTGGCGGCGTGCTTAACACATGCAAGTCGAACGGAAAGGCCCTTTCGGGGGTACTCGAGTGGC
GAACGGGTGAGTAACACGTGGGTGATCTGCCCTGCACTTCGGGATAAGCTTGGGAAACTGGGTCTAAT
ACCGGATAGGACTACGGACTGCATGGTCTGTGGTGGAAAGCTTTTGCGGTGTGGGATGGGCCCGCGGC
CTATCAGCTTGTTGGTGGGGTGATGGCCTACCAAGGCGACGACGGGTAGCCGGCCTGAGAGGGTGTCC
GGCCACACTGGGACTGAGATACGGCC
>Zotu49
GAACGCTGGCGGCATGCCTAACACATGCAAGTCGAACGATTGCCTTCGGGTGATAGTGGCGCACGGGT
GCGTAACGCGTGGGAATCTGCCCTTAGGTTCGGAATAACAGCTGGAAACGGCTGCTAATACCGGATGA
TATCGCGAGATCAAAGATTTATCGCCTGAGGATGAGCCCGCGTTGGATTAGGTAGTTGGTGGGGTAAA
GGCCTACCAAGCCGACGATCCATAGCTGGTCTGAGAGGATGATCAGCCACACTGGGACTGAGACACGG
CC
>Zotu50
GAACGCTGGCGGCATGCCTTACACATGCAAGTCGAACGGCAGCATGATCTAGCTTGCTAGATTGATGG
CGAGTGGCGAACGGGTGAGTAATACATCGGAACGTGCCCTGTAGTGGGGGATAACTAGTCGAAAGATT
AGCTAATACCGCATACGACCTGAGGGTGAAAGTGGGGGACCGCAAGGCCTCATGCTATAGGAGCGGCC
GATGTCTGATTAGCTAGTTGGTGGGGTAAAGGCCCACCAAGGCGACGATCAGTAGCTGGTCTGAGAGG
ACGATCAGCCACACTGGGACTGAGACACGGCC
>Zotu51
GAACGCTATCGGTATGCTTAACACATGCAAGTCGAACGAACTAATTGGGGCTTGCTCTAATTAGTTAG
TGGCAGACGGGTGAGTAACACGTGGGAATCTGCCCATCAGTATGGAATAACTTTTAGAAATAAAAGCT
AATACCGTATATTCTCTACGGAGGAAAGATTTATCGCTGATGGATGAGCCCGCGTCAGATTAGGTAGT
TGGTGAGGTAATGGCTCACCAAGCCTACGATCTGTAGCTGGTCTGAGAGGATGATCAGCCACACTGGG
ACTGAGACACGGCC
>Zotu52
GAACGCTGGCGGCGTGCCTAATACATGCAAGTAGAACGCTGAAGGAGGAGCTTGCTCTTCTGGATGAG
TTGCGAACGGGTGAGTAACGCGTAGGTAACCTGCCTGGTAGCGGGGGATAACTATTGGAAACGATAGC
TAATACCGCATAATAGTAGATGTTGCATGACATTTGCTTAAAAGGTGCAATTGCATCACTACCAGATG
GACCTGCGTTGTATTAGCTAGTTGGTGAGGTAACGGCTCACCAAGGCAACGATACATAGCCGACCTGA
GAGGGTGATCGGCCACACTGGGACTGAGACACGGCC
>Zotu53
GAACGCTGGCGGCGTGCCTAATACATGCAAGTAGAACGCTGAAGGAGGAGCTTGCTTCTCTGGATGAG
TTGCGAACGGGTGAGTAACGCGTAGGTAACCTGCCTGGTAGCGGGGGATAACTATTGGAAACGATAGC
TAATACCGCATAATAGTAGATGTTGCATGACATTTGCTTAAAAGGTGCAATTGCATCACTACCAGATG
GACCTGCGTTGTATTAGCTAGTTGGTGAGGTAACGGCTCACCAAGGCAACGATACATAGCCGACCTGA
GAGGGTGATCGGCCACACTGGGACTGAGACACGGCC
>Zotu54
GAACGCTGGCGGCATGCTTAACACATGCAAGTCGGACGGGAAGTGGTGTTTCCAGTGGCGGACGGGTG
AGTAACGCGTAAGAACCTGCCCTTGGGAGGGGAACAACAGCTGGAAACGGCTGCTAATACCCCGTAGG
CTGAGGAGCAAAAGGAGGAATCCGCCCGAGGAGGGGCTCGCGTCTGATTAGCTAGTTGGTGAGGCAAT
AGCTTACCAAGGCGATGATCAGTAGCTGGTCCGAGAGGATGATCAGCCACACTGGGACTGAGACACGG
CC
>Zotu55
GAACGCTGGCGGTAGGCCTAACACATGCAAGTCGAACGGCAGCACAGAGGAGCTTGCTCCTTGGGTGG
CGAGTGGCGGACGGGTGAGGAATACATCGGAATCTACTCTGTCGTGGGGGATAACGTAGGGAAACTTA
CGCTAATACCGCATACGACCTACGGGTGAAAGCAGGGGATCTTCGGACCTTGCGCGATTGAATGAGCC
GATGTCGGATTAGCTAGTTGGCGGGGTAAAGGCCCACCAAGGCGACGATCCGTAGCTGGTCTGAGAGG
ATGATCAGCCACACTGGAACTGAGACACGGTC
>Zotu56
GAACGCTGGCGGCATGCCTAACACATGCAAGTCGAACGAAGGCTTCGGCCTTAGTGGCGCACGGGTGC
GTAACGCGTGGGAATCTGCCCCTTGGTTCGGAATAACAGTTAGAAATGACTGCTAATACCGGATGATG

ACTTCGGTCCAAAGATTTATCGCCGAGGGATGAGCCCGCGTAGGATTAGGTAGTTGGTGGGGTAAAGG
CCTACCAAGCCGACGATCCTTAGCTGGTCTGAGAGGATGATCAGCCACACTGGGACTGAGACACGGCC
>Zotu57
GAACGCTGGCGGTATGCTTAACACATGCAAGTCGAACGGCAGCATGTTCCTCACTCAAATCACTTTAA
TTTATTTTTGGAAAAGAAATACGGAAAGTGATTTGAGTGAGGAATGATGGCAAGTGGCGGACGGGTGA
GTAATGGGTAGGAATCTGTCCTAGAGAGGGGGATAACTTTCGGAAACGAGAGCTAATACCACATGATA
TTGAAAAATCAAAGGCAGGGGACCTTTCGAGGCCTTGCGCTCTAGGGTGAGCCTGCATCGGATTAGCT
AGTTGGTAGGGTAAAAGCCTACCAAGGCGACGATCCGTAGCTGGTCTGAGAGGATGACCAGCCACACT
GGGACTGAGACACGGCC
>Zotu58
GAACGCTGGCGGCAAGCCTAACACATGCAAGTCGAACGGGGTTATATATAGCTTGCTATAGTGTAGCC
TAGTGGCAGACGGGTGAGTAATGTATAGGAATCTACCTAGTAGTACGGAATAATTACTGGAAACGGCA
ACTAATACCGTATACGCCCTATGGGGGAAAGATTTATTGCTATTAGATGAGCCTATATTAGATTAGCT
AGTTGGTAGGGTAATGGCCTACCAAGGCTATGATCTATAGCTGATCTGAGAGGATGATCAGCCACACT
GGAACTGAGATACGGTC
>Zotu60
GAACGCTGGCGGCAGGCCTAACACATGCAAGTCGAGCGGATGAAAGGAGCTTGCTCCTGGATTCAGCG
GCGGACGGGTGAGTAATGCCTAGGAATCTGCCTGGTAGTGGGGGACAACGTTTCGAAAGGAACGCTAA
TACCGCATACGTCCTACGGGAGAAAGCAGGGGACCTTCGGGCCTTGCGCTATCAGATGAGCCTAGGTC
GGATTAGCTAGTTGGTGAGGTAATGGCTCACCAAGGCGACGATCCGTAACTGGTCTGAGAGGATGATC
AGTCACACTGGAACTGAGACACGGTC
>Zotu61
GAACGCTGGCGGCGTGCCTAATACATGCAAGTAGAACGCTGAAGAGAGGAGCTTGCTCTTCTTGGATG
AGTTGCGAACGGGTGAGTAACGCGTAGGTAACCTGCCTTGTAGCGGGGGATAACTATTGGAAACGATA
GCTAATACCGCATAACAATGGATGACACATGTCATTTATTTGAAAGGGGCAATTGCTCCACTACAAGA
TGGACCTGCGTTGTATTAGCTAGTAGGTGAGGTAATGGCTCACCTAGGCGACGATACATAGCCGACCT
GAGAGGGTGATCGGCCACACTGGGACTGAGACACGGCC
>Zotu62
GAACGCTGGCGGCATGCCTAACACATGCAAGTCGAACGGCAGCACAGTAAAGATTTCGGTCTTTACGG
GTGGCGAGTGGCGGACGGGTGAGTAAAGCGTAGGAATCTGTCCTGGAGAGTGGAATAACCCGAGGAAA
CTCGGGCTAATACCGCATGATATCGAGAGATCAAAGCAGGGGACCCTTCGGGGCCTTGCGCTCTAGGA
TGAGCCTACGTCGGATTAGCTAGTTGGTGGGGTAAAGGCCTACCAAGGCGACGATCCGTAGCTGGTCT
GAGAGGATGACCAGCCACACTGGGACTGAGACACGTCC
>Zotu63
GAACGCTGGCGGCGTGCCTAATACATGCAAGTAGAACGCTGAGGACTGGTGCTTGCACCGGTCCAAGG
AGTTGCGAACGGGTGAGTAACGCGTAGGTAACCTACCTCATAGCGGGGGATAACTATTGGAAACGATA
GCTAATACCGCATAAAAGTGTTTAACACATGTTAAACATTTAAAAGATGCAACTGCATCACTATGAGA
TGGACCTGCGTTGTATTAGCTAGTTGGTGAGGTAACGGCTCACCAAGGCGACGATACATAGCCGACCT
GAGAGGGTGATCGGCCACACTGGGACTGAGACACGGCC
>Zotu64
AAACGCTGGCGGCATGCCTAACACATGCAAGTCGAACGGCAGCACAGTAAAGATTTCGGTCTTTACGG
GTGGCGAGTGGCGGACGGGTGAGTAAAGCGTAGGAATCTGTCCTGGAGAGTGGGATAACCCGAGGAAA
CTCGGGCTAATACCGCATGATATCGAGAGATCAAAGCAGGGGACCCTTCGGGGCCTTGCGCTCTAGGA
TGAGCCTACGTCGGATTAGCTAGTTGGTGGGGTAAAGGCCTACCAAGGCGACGATCCGTAGCTGGTCT
GAGAGGATGACCAGCCACACTGGGACTGAGACACGGCC
>Zotu65
GAACGCTGGCGGCGTGCCTAATACATGCAAGTCGAGCGAACAGATAAGGAGCTTGCTCCTTTGACGTT
AGCGGCGGACGGGTGAGTAACACGTGGATAACCTACCTATAAGACTGGGATAACTTCGGGAAACCGGA
GCTAATACCGGATAACATATTGAACCGCATGGTTCAATAGTGAAAGGCGGCTTTGCTGTCACTTATAG
ATGGATCCGCGCCGTATTAGCTAGTTGGTAAGGTAACGGCTTACCAAGGCAACGATACGTAGCCGACC
TGAGAGGGTGATCGGCCACACTGGAACTGAGACACGGTC
>Zotu66
GAACGCTGGCGGCGTGCTTAACACATGCAAGTCGAACGGAAAGGCCCTGCTTGCAGGGTACTCGAGTG
GCGAACGGGTGAGTAACACGTGGGTGATCTGCCTCGTACTTCGGGATAAGCCTGGGAAACTGGGTCTA

ATACCGGATAGGACCGGCCTTTAGTGTGGTTGGTGGAAAGTTTTGTCGGTACGAGATGAGCCCGCGGC
CTATCAGCTTGTTGGTGGGGTAATGGCCTACCAAGGCGACGACGGGTAGCCGGCCTGAGAGGGTGTAC
GGCCACATTGGGACTGAGACACGGCC
>Zotu67
AAACGCTGGCGGCATGCCTAACACATGCAAGTCGAACGGCAGCACAGTAAAGATTTCGGTCTTTACGG
GTGGCGAGTGGCGGATGGGTGAGTAAAGCGTAGGAATCTGTCCTGGAGAGTGGGATAACCCGAGGAAA
CTCGGGCTAATACCGCATGATATCGAGAGATCAAAGCAGGGGACCCTTCGGGGCCTTGCGCTCTAGGA
TGAGCCTACGTCGGATTAGCTAGTTGGTGGGGTAAAGGCCTACCAAGGCGACGATCCGTAGCTGGTCT
GAGAGGATGACCAGCCACACTGGGACTGAGACACGGCC
>Zotu68
GAACGCTAGCGGCAGGCTTAATACATGCAAGTCGTGGGGCAGCATGAGTAGCAATACTTGATGGCGAC
CGGCAAACGGGTGCGGAACACGTACACAACCTTCCGATAAGTGGGGAATAGCCCAGAGAAATTTGGAT
TAATACCCCGTAACATATAGAGTAGGCATCTTCTTTATATTATAGCTTCGGCGCTTATTGATGGGTGT
GCGTCTGATTAGGTAGTTGGCGGGGTAACGGCCCACCAAGCCTACGATCAGTAGCTGATGTGAGAGCA
TGATCAGCCACACGGGCACTGAGACACGGGC
>Zotu69
GAACGCTGGCGGCGTGCCTAATACATGCAAGTCGAGCGAACAGACGAGGAGCTTGCTCCTCTGACGTT
AGCGGCGGACGGGTGAGTAACACGTGGATAACCTACCTATAAGACTGGGATAACTTCGGGAAACCGGA
GCTAATACCGGATAACATGTTGAACCGCATGGTTCAACAGTGAAAGACGGTCTTGCTGTCACTTATAG
ATGGATCCGCGCCGCATTAGCTAGTTGGTAAGGTAACGGCTTACCAAGGCAACGATGCGTAGCCGACC
TGAGAGGGTGATCGGCCACACTGGAACTGAGACACGGTC
>Zotu70
GAACGCTGGTGGCATGCTTAACACATGCAAGTCGAACGGTAACAGGACTAGCTTGCTAGTTGCTGACG
AGTGGCGGACGGGTGAGTAACACGTAGGAATCTGCCCATCTGAGGGGATACCAGTTGGAAACGACTAT
TAATACCGCATAGTATCTGTGGATTAAAGATGGCTTTTAGGCTGTCGCGGATGGATGAGCCTGCGTTG
GATTAGCTAGTTGGTGAGGTAAGGGCCTACCAAGGCTACGATCCATAGTTGATTTGAGAGGATGATCA
GCCACATTGGGACTGAGACACGACC
>Zotu71
GAACGCTGGCGGCGTGCCTAATACATGCAAGTCGAGCGAACAGATAAGGAGCTTGCTCCTTTGAAGTT
AGCGGCGGACGGGTGAGTAACACGTGGGTAACCTACCTATAAGACTGGGATAACTTCGGGAAACCGGA
GCTAATACCGGATAACATTTAGAACCGCATGGTTCTAAAGTGAAAGATGGTTTTGCTATCACTTATAG
ATGGACCCGCGCCGTATTAGCTAGTTGGTAAGGTAACGGCTTACCAAGGCGACGATACGTAGCCGACC
TGAGAGGGTGATCGGCCACACTGGAACTGAGACACGGTC
>Zotu72
GAACGCTGGCGGCATGCCTAACACATGCAAGTCGAACGAACACTTTCGGGTGTTAGTGGCGCACGGGT
GCGTAACGCGTGGGAATCTGCCCTTAGGTTCGGAATAACAGCGAGAAATTGCTGCTAATACCGGATGA
TGGCTTCGGCCCAAAGATTTATCGCCTAGGGATGAGCCCGCGTAAGATTAGGTAGTTGGTGTGGTAAA
GGCGCACCAAGCCGACGATCTTTAGCTGGTCTGAGAGGATGATCAGCCACACTGGGACTGAGACACGG
CC
>Zotu73
GAACGCTGGCGGCGTGCCTAATACATGCAAGTAGAACGCTGAAGGAAGGAGCTTGCTCTTTCCGGATG
AGTTGCGAACGGGTGAGTAACGCGTAGGTAACCTGCCTGGTAGCGGGGGATAACTATTGGAAACGATA
GCTAATACCGCATAACAGTAGATATTGCATGATATCTGCTTGAAAGGTGCAATTGCACCACTACCAGA
TGGACCTGCGTTGTATTAGCTAGTTGGTGAGGTAACGGCTCACCAAGGCGACGATACATAGCCGACCT
GAGAGGGTGATCGGCCACACTGGGACTGAGACACGGCC
>Zotu74
AAACGCTGGCGGCATGCCTTACACATGCAAGTCGAACGGCAGCGCGGACTTCGGTCTGGCGGCGAGTG
GCGAACGGGTGAGTAATACATCGGAACGTACCCTGTTGTGGGGGATAACTAGTCGAAAGATTAGCTAA
TACCGCATACGACCTGAGGGTGAAAGCGGGGGACCGTAAGGCCTCGCGCAGCAGGAGCGGCCGATGTC
TGATTAGCTAGTTGGTGGGGTAAAGGCCCACCAAGGCGACGATCAGTAGCTGGTCTGAGAGGACGATC
AGCCACACTGGGACTGAGACACGGCC
>Zotu75
GAACGCTGGCGGCATGCCTTACACATGCAAGTCGAACGGCATCGCGGGAAAGCTTGCTTTCCTGGCGG
CGAGTGGCGAACGGGTGAGTAACACATCGGAACGTACCGGCTAGTGGGGGATAACCCGTCGAAAGACG

GGCTAATACCGCATACGCTCTTCGGAGGAAAGCAGGGGACCTTTGGGCCTTGCGCTAGCCGAGCGGCC
GATGTCCGATTAGCTAGTTGGTGGGGTAAAGGCCTACCAAGGCGACGATCGGTAGCGGGTCTGAGAGG
ACGATCCGCCACACTGGGACTGAGACACGGCC
>Zotu76
GAACGCTGGCGGCAGGCTTAACACATGCAAGTCGAGCGCCCCGCAAGGGGAGCGGCAGACGGGTGAGT
AACGCGTGGGAATCTACCCATCTCTACGGAACAACTCCGGGAAACTGGAGCTAATACCGTATACGTCC
TTATGGAGAAAGATTTATCGGAGATGGATGAGCCCGCGTTGGATTAGCTAGTTGGTGGGGTAATGGCC
TACCAAGGCGACGATCCATAGCTGGTCTGAGAGGATGATCAGCCACACTGGGACTGAGACACGGCC
>Zotu77
AAACGCTGGCGGCATGCCTAACACATGCAAGTCGAACGGCAGCACAGTAAAGATTTCGGTCTTTACGG
GTGGCGAGTGGCGGACGGGTGAGTAAAGCGTAGGAATCTGTCCTGGAGAGTGGGATAACCCGAGGAAA
CTCGGGCTAATACCGCATGATATCGAGAGATCAAAGCAGGGGACCCTTCGGGGCCTTGCGCTCTAGGA
TGAGCCTACGTCGGATTAGCTAGTTGGTGGGTGGGGTAAAGGCCTACCAAGGCGACGATCCGTAGCTG
GTCTGAGAGGATGACCAGCCACACTGGGACTGAGACACGTCC
>Zotu78
GAACGCTGGCGGCGTGCCTAATACATGCAAGTCGAGCGAACAGATAAGGAGCTTGCTCCTTTGACGTT
AGCGGCGGACGGGTGAGTAACACGTGGATAACCTACCTATAAGACTGGGATAACTTCGGGAAACCGGA
GCTAATACCGGATAAGATTTTGAACCGCATGGTTCAATAGTGAAAGACGGCCTTGCTGTCACTTATAG
ATGGATCCGCGCCGTATTAGCTAGTTGGTAAGGTAACGGCTTACCAAGGCAACGATACGTAGCCGACC
TGAGAGGGTGATCGGCCACACTGGAACTGAGACACGGTC
>Zotu79
GAACGCTGGCGGCATGCCTAACACATGCAAGTCGAACGGCAGCACAGTAAAGATTTCGGTCTTTACGG
GTGGCGAGTGGCGGACGGGTGAGTAAAGCGTAGGAATCTGTCCTGGAGAGTGGGATAACCCGAGGAAA
CTCGGGCTAATACCGCATGATATCGAGAGATCAAAGCAGGGGACCCTTCGGGGCCTTGCGCTCTAGGA
TGAGCCTACGTCGGATTAGCTAGTTGGTGGGTGGGGTAAAGGCCTACCAAGGCGACGATCCGTAGCTG
GTCTGAGAGGATGACCAGCCACACTGGGACTGAGACACGTCC
>Zotu80
GAACGCTGGCGGCATGCCTAACACATGCAAGTCGAACGAGACCTTCGGGTCTAGTGGCGCACGGGTGC
GTAACGCGTGGGAACCTGCCCTTAGGTTCGGAATAACTCCCCGAAAGGGGTGCTAATACCGGATAATG
TCTTCGGACCAAAGATTTATCGCCTTTGGATGGGCCCGCGTTGGATTAGCTAGTTGGTGAGGTAAAAG
CTCACCAAGGCGACGATCCATAGCTGGTCTGAGAGGATGATCAGCCACACTGGGACTGAGACACGGCC
>Zotu82
GAACGCTAGCGGCATGCCTAACACATGCAAGTCGAACGGCAACGGGGGGAGCTTAGCTCCCTGACGGC
AAGTGGCGGACGGGTGAGTAATACGTAGGAATCTACCTTATAGTGGGGGATAACCTGGGGAAACTCAG
GCTAATACCGCATAATCTTTCTTTAAAGCAAAGCGGGGGATCTTCGGACCTCGTGCTATAAGATGAGC
CTGCGTCGGATTAGCTAGTTGGTAGGGTAATAGCCTACCAAGGCAACAATCCGTAGCTGGTCTGAGAG
GACGATCAGCCACACTGGGACTGAGACACGGCC
>Zotu83
GAACGCTGGCGGCATGCCTAACACATGCAAGTCGAACGGCAGCACAGTAAAGATTTCGGTCTTTACGG
GTGGCGAGTGGCGGATGGGTGAGTAAAGCGTAGGAATCTGTCCTGGAGAGTGGGATAACCCGAGGAAA
CTCGGGCTAATACCGCATGATATCGAGAGATCAAAGCAGGGGACCCTTCGGGGCCTTGCGCTCTAGGA
TGAGCCTACGTCGGATTAGCTAGTTGGTGGGGTAAAGGCCTACCAAGGCGACGATCCGTAGCTGGTCT
GAGAGGATGACCAGCCACACTGGGACTGAGACACGTCC
>Zotu84
GAACGCTGGCGGCAGGCCTAACACATGCAAGTCGAGCGCCCTTTCGGGGGAGCGGCGGACGGGTGAGT
AACGCGTGGGAATATGCCCTTCTCTACGGAATAGCCTCGGGAAACTGAGAGTAATACCGTATACGCCC
TTAGGGGGAAAGATTTATCGGGGAAGGATTAGCCCGCGTTGGATTAGGTAGTTGGTGGGGTAATGGCC
TACCAAGCCGACGATCCATAGCTGGTTTGAGAGGATGATCAGCCACACTGGGACTGAGACACGGCC
>Zotu85
GAACGCTGGCGGCGTGCTTAACACATGCAAGTCGAACGGAAAGGCCCTTTCGGGGGTACTCGAGTGGC
GAACGGGTGAGTAACACGTGGGTGATCTGCCCTGCACTTCGGGATAAGCTTGGGAAACTGGGTCTAAT
ACCGGATAGGACTACGAATCGCATGGTTTGTGGTGGAAAGCTTTTGCGGTGTGGGATGGGCCCGCGGC
CTATCAGCTTGTTGGTGGGGTGATGGCCTACCAAGGCGACGACGGGTAGCCGGCCTGAGAGGGTGTCC
GGCCACACTGGGACTGAGATACGGCC

>Zotu86
GAACGCTGGCGGCAGGCTTAACACATGCAAGTCGAGCGGGCGTAGCAATACGTCAGCGGCAGACGGGT
GAGTAACACGTGGGAACGTACCTTTTGGTTCGGAACAACTGAGGGAAACTTCAGCTAATACCGGATAA
GCCCTAACGGGGAAAGATTTATCGCCGAAAGATCGGCCCGCGTCTGATTAGCTAGTTGGTGAGGTAAT
GGCTCACCAAGGCGACGATCAGTAGCTGGTCTGAGAGGATGATCAGCCACACTGGGACTGAGACACGG
CC
>Zotu87
GAACGCTGGCGGCAGGCCTAACACATGCAAGTCGAACGGTAACAGGAAACAGCTTGCTGTTTCGCTGA
CGAGTGGCGGACGGGTGAGTAATGTCTGGGAAACTGCCTGATGGAGGGGGATAACTACTGGAAACGGT
AGCTAATACCGCATAACGTCGCAAGACCAAAGAGGGGGACCTTCGGGCCTCTTGCCATCGGATGTGCC
CAGATGGGATTAGCTAGTAGGTGGGGTAACGGCTCACCTAGGCGACGATCCCTAGCTGGTCTGAGAGG
ATGACCAGCCACACTGGAACTGAGACACGGTC
>Zotu88
GAACGCTGGCGGCAGGCTTAACACATGCAAGTCGAGCGCCCCGCAAGGGGAGCGGCAGACGGGTGAGT
AACGCGTGGGAATCTACCCTTTTCTACGGAATAACGCAGGGAAACTTGTGCTAATACCGTATGTGTCC
TTCGGGAGAAAGATTTATCGGGAAAGGATGAGCCCGCGTTGGATTAGCTAGTTGGTGGGGTAAAGGCC
TACCAAGGCGACGATCCATAGCTGGTCTGAGAGGATGATCAGCCACATTGGGACTGAGACACGGCC
>Zotu89
GAACGCTGGCGGCAGGCTTAACACATGCAAGTCGAACGGCACTTCGGTGCTAGTGGCAGACGGGTGA
GTAACACGTGGGAACGTACCTTTCGGTTCGGAATAATTCAGGGAAACTTGGACTAATACCGGATACGC
CCTTCGGGGGAAAGATTTATCGCCGATAGATCGGCCCGCGTCTGATTAGCTAGTTGGTGAGGTAATGG
CTCACCAAGGCGACGATCAGTAGCTGGTCTGAGAGGATGATCAGCCACATTGGGACTGAGACACGGCC
>Zotu90
GAACGCTGGCGGCGTGCTTAACACATGCAAGTCGTACGGTAAGGCCCTTTCGGGGGTACACGAGTGGC
GAACGGGTGAGTAACACGTGAGTAACCTGCCCACAACTTTGGGATAACGCTAGGAAACTGGTGCTAAT
ACTGGATATGTGCTCCTGCTGCATGGTGGGGGTTGGAAAGCTCCGGCGGTTGTGGATGGACTCGCGGC
CTATCAGCTTGTTGGTGGGGTAGTGGCCTACCAAGGCGGCGACGGGTAGCCGGCCTGAGAGGGTGACC
GGCCACATTGGGACTGAGATACGGCC
>Zotu91
GAACGCTGGCGGCAGGCTTAACACATGCAAGTCGAACGCGTGTAGCAATACACGAGTGGCGCACGGGT
GAGTAACGCGTGGATATCTGCCTTTTGGTTCGGAATAACCCCGGGAAACTGGGGCTAATACCGGATGG
TTCCTTCGGGATAAAGATTTATCGCCAAAAGATGAGTCCGCGTACGATTAGCTAGTTGGTGAGGTAAT
GGCTCACCAAGGCGACGATCGTTAGCTGGTCTGAGAGGACGATCAGCCACACTGGGACTGAGACACGG
CC
>Zotu92
GAACGCTGGTGGCATACTTAACACATGCAAGTCGAACGGTAACAGGACTAGCTTGCTAGTTGCTGACG
AGTGGCGGACGGGTGAGTAACGCGTAGGAATCTGCCCATCTGAGGGGGATACCAGTTGGAAACGACTG
TTAATACCGCATAGTATCTGTGGATTAAAGGTGGCTTTTGGGCTGTCGCAGATGGATGAGCCTGCGTT
GGATTAGCTAGTTGGTGGGGTAAAGGCCTACCAAGGAAGGCTACGATCCATAGCTGATTTGAGAGAAT
GATCAGCCACATTGGGACTGAGACACGGCC
>Zotu93
GAACGCTGGCGGCGTGCTTAACACATGCAAGTCGAACGGAAAGGCCCTTCGGGGTACTCGAGTGGCGA
ACGGGTGAGTAACACGTGGGTGATCTGCCCTGCACTCTGGGATAAGCCTGGGAAACTGGGTCTAATAC
CGGATAGGACCACACACTTCATGGTGAGTGGTGCAAAGCTTTTGCGGTGTGGGATGAGCCCGCGGCCT
ATCAGCTTGTTGGTGGGGTAATGGCCCACCAAGGCGACGACGGGTAGCCGGCCTGAGAGGGTGACCGG
CCACACTGGGACTGAGATACGGCC
>Zotu94
GAACGCTGGCGGCGTGCTTAACACATGCAAGTCGAGCGGTAAGGCCTTTCGGGGTACACGAGCGGCGA
ACGGGTGAGTAACACGTGGGTGATCTGCCCTGCACTTCGGGATAAGCCTGGGAAACTGGGTCTAATAC
CGGATATGACCTCCTATCGCATGGTGGGTGGTGGAAAGATTTATCGGTGCAGGATGGGCCCGCGGCCT
ATCAGCTTGTTGGTGGGGTAATGGCCTACCAAGGCGACGACGGGTAGCCGACCTGAGAGGGTGACCGG
CCACACTGGGACTGAGACACGGCC
>Zotu95
GAACGCTGGCGGCGTGCCTAATACATGCAAGTAGAACGCAGAGGTCAGGTGCTTGCACTGGACTGATG

AGTTGCGAACGGGTGAGTAACGCGTAGGTAACCTACCTGATAGCGGGGGATAACTATTGGAAACGATA
GCTAATACCGCATAAAAGCCAATGACCCATGTCATTGGCTTGAAAGAAGCAAACGCTTCACTATGAGA
TGGACCTGCGTTGTATTAGCTAGTTGGTAGGGTAAAGGCCTACCAAGGCGACGATACATAGCCGACCT
GAGAGGGTGAACGGCCACACTGGGACTGAGACACGGCC
>Zotu96
GAACGCTGGCGGCGTGCTTAACACATGCAAGTCGAACGCTGAAGCTTGGTGCTTGCACTGGGTGGATG
AGTGGCGAACGGGTGAGTAATACGTGAGTAACCTGCCCTTGACTCTGGGATAAGCCTGGGAAACTGGG
TCTAATACTGGATATGACCTCCTGTCGCATGGTGGGGGTGGAAAGGGTTTGTACTGGTTGTGGATGG
GCTCACGGCCTATCAGCTTGTTGGTGGGGTAATGGCCTACCAAGGCGACGACGGGTAGCCGGCCTGAG
AGGGTGACCGGCCACACTGGGACTGAGACACGGCC
>Zotu97
GAACGCTGGTGGCATGCTTAACACATGCAAGTCGAACGGTAACAGGACTAGCTTGCTAGTTGCTGACG
AGTGGCGGACGGGTGAGTAACACGTAGGAATCTGCCCATCTGAGGGGGATACCAGTTGGAAACGACTA
TTAATACCGCATAGTATCTGTGGATTAAAAATGGCTTTTAGGCTGTCGCGGATGGATGAGCCTGCGTT
GGATTAGCTAGTTGGTGAGGTAAGGGCCTACCAAGGCTACGATCCATAGTTGATTTGAGAGGATGATC
AGCCACATTGGGACTGAGACACGGCC
>Zotu98
AAACGCTGGCGGCATGCCTAACACATGCAAGTCGAACGGCAGCACAGTAAAGATTTCGGTCTTTACGG
GTGGCGAGTGGCGGACGGGTGAGTAAAGCGTAGGAATCTGTCCTGGAGAGTGGAATAACCCGAGGAAA
CTCGGGCTAATACCGCATGATATCGAGAGATCAAAGCAGGGGACCCTTCGGGGCCTTGCGCTCTAGGA
TGAGCCTACGTCGGATTAGCTAGTTGGTGGGGTAAAGGCCTACCAAGGCGACGATCCGTAGCTGGTCT
GAGAGGATGACCAGCCACACTGGGACTGAGACACGGCC
>Zotu99
GAACGCTGGCGGCATGCCTAACACATGCAAGTCGAACGCATCGCAAGATGAGTGGCGCACGGGTGAGT
AACGCGTGGGAACCTGCCCTATGGTGCGGAATAACACCGGGAAACTGGTGCTAATACCGCATGTGTCC
TTCGGTCGTGGAGATCGGAGGAGAAAGATTTATCGCCATAGGAGGGGCCCGCGTCCGATTAGGTAGTT
GGTGGGGTAATGGCCTACCAAGCCTTCGATCGGTAGCTGGTCTGAGAGGATGATCAGCCACACTGGGA
CTGAGACACGGCC
>Zotu100
GAACGCTGGCGGCGTGCCTAATACATGCAAGTCGAGCGAACAGACGAGGAGCTTGCTCCTCTGACGTT
AGCGGCGGACGGGTGAGTAACACGTGGATAACCTACCTATAAGACTGGGATAACTTCGGGAAACCGGA
GCTAATACCAGATAACATGTTGAACCGCATGGTTCAACAGTGAAAGACGGTCTTGCTGTCACTTATAG
ATGGATCCGCGCCGCATTAGCTAGTTGGTAAGGTAACGGCTTACCAAGGCAACGATGCGTAGCCGACC
TGAGAGGGTGATCGGCCACACTGGAACTGAGACACGGTC
>Zotu101
GAACGCTGGCGGCGTGCCTAATACATGCAAGTAGAACGCTGAAGGAGGAGCTTGCTCTTCTGGATGAG
TTGCGAACGGGTGAGTAACGCGTAGGTAACCTGCCTGGTAGCGGGGGATAACTATTGGAAACGATAGC
TAATACCGCATAAGAGTAGATGTTGCATGACATTTGCTTAAAAGGTGCAATTGCATCACTACCAGATG
GACCTGCGTTGTATTAGCTAGTTGGTGAGGTAACGGCTCACCAAGGCAACGATACATAGCCGACCTGA
GAGGGTGATCGGCCACACTGGGACTGAGACACGGCC
>Zotu102
GAACGCTGGCGGCATGCCTTACACATGCAAGTCGAACGGCAGCGCGGGAGCAATCCTGGCGGCGAGTG
GCGAACGGGTGAGTAATACATCGGAACGTGCCCAATCGTGGGGGATAACGCAGCGAAAGCTGTGCTAA
TACCGCATACGATCTACGGATGAAAGCAGGGGATCGCAAGACCTTGCGCGAATGGAGCGGCCGATGGC
AGATTAGGTAGTTGGTGAGGTAAAGGCTCACCAAGCCTTCGATCTGTAGCTGGTCTGAGAGGACGACC
AGCCACACTGGGACTGAGACACGGCC
>Zotu103
GAACGCTGGCGGCATGCTTTACACATGCAAGTCGAACGGCAGCGCGGGGCAACCTGGCGGCGAGTGGC
GAACGGGTGAGTAATATATCGGAACGTACCCAAGAGTGGGGGATAACGTAGCGAAAGTTACGCTAATA
CCGCATACGATCTAAGGATGAAAGTGGGGGATCGCAAGACCTCATGCTCCTGGAGCGGCCGATATCTG
ATTAGCTAGTTGGTGAGGTAAAGGCTCACCAAGGCGACGATCAGTAGCTGGTCTGAGAGGACGACCAG
CCACACTGGGACTGAGACACGGCC
>Zotu104
GAACGCTGGCGGCAAGCCTAACACATGCAAGTCGAACGAATTATCCTTATAGTTTACTATAAGTATAA

TTAGTGGCAGACGGGTGAGTAATGTATAGGAATCTACCTAGTAGTATGGAATAGCTGTTAGAAATGAC
AGGTAATACCGTATAATCCCTGAGGGGGAAAGATTTATCGCTATTAGATGAGCCTATGTTAGATTAGC
TAGTTGGTGAGGTAATGGCTTACCAAGGCTGTGATCTATAGCTGGTCTGAGAGGACGATCAGCCACAC
TGGAACTGAGATACGGTC
>Zotu106
GAACGCTGGCGGCATGCTTAACACATGCAAGTCGAACGGGAAGTGGTGTTTCCAGTGGCGAACGGGTG
AGTAACGCGTAAGAACCTGCCCTTGGGAGGGGAACAACAACTGGAAACGGTTGCTAATACCCCGTAGG
CTGAGGAGCAAAAGGAGAAATCCGCCCAAGGAGGGGCTCGCGTCTGATTAGCTAGTTGGTGAGGTAAT
AGCTTACCAAGGCGATGATCAGTAGCTGGTCCGAGAGGATGATCAGCCACACTGGGACTGAGACACGG
CC
>Zotu107
GAACGCTGGCGGCAGGCTTAACACATGCAAGTCGAGCGGGCCCTTCGGGGTCAGCGGCGGACGGGTGA
GTAACGCGTGGGAACGTGCCTTCTGGTTCGGAATAACCCTGGGAAACTAGGGCTAATACCGGATACGC
CCTTTTGGGGAAAGGTTTACTGCCGGAAGATCGGCCCGCGTCTGATTAGCTAGTTGGTGGGGTAACGG
CCTACCAAGGCGACGATCAGTAGCTGGTCTGAGAGGATGATCAGCCACACTGGGACTGAGACACGGCC
>Zotu108
GAACGCTGGCGGCAGGCTTAACACATGCAAGTCGAACGCCCCGCAAGGGGAGTGGCAGACGGGTGAGT
AACGCGTGGGAACATACCCTTTCCTGCGGAATAGCTCCGGGAAACTGGAATTAATACCGCATACGCCC
TACGGGGGAAAGATTTATCGGGGAAGGATTGGCCCGCGTTGGATTAGCTAGTTGGTGGGGTAAAGGCC
TACCAAGGCGACGATCCATAGCTGGTCTGAGAGGATGATCAGCCACATTGGGACTGAGACACGGCC
>Zotu109
GAACGCTGGCGGCAGGCTTAACACATGCAAGTCGAGCGGGCGTAGCAATACGTCAGCGGCAGACGGGT
GAGTAACGCGTGGGAACGTACCTTTTGGTTCGGAACAACACAGGGAAACTTGTGCTAATACCGGATAA
GCCCTTACGGGGAAAGATTTATCGCCGAAAGATCGGCCCGCGTCTGATTAGCTAGTTGGTAGGGTAAT
GGCCTACCAAGGCGACGATCAGTAGCTGGTCTGAGAGGATGATCAGCCACATTGGGACTGAGACACGG
CC
>Zotu110
GAACGCTGGCGGCGTGCTTAACACATGCAAGTCGAACGGAAAGGCCTCTTTCGGGGGGTACTCGAGTG
GCGAACGGGTGAGTAACACGTGAGTAACCTGCCCTTGACTTTGGGATAACTTCAGGAAACTGGGGCTA
ATACCGGATAGGAGCTCCTGCTGCATGGTGGGGGTTGGAAAGTTTCGGCGGTTGGGGATGGACTCGCG
GCTTATCAGCTTGTTGGTGGGGTAGTGGCTTACCAAGGCTTTGACGGGTAGCCGGCCTGAGAGGGTGA
CCGGCCACATTGGGACTGAGATACGGCC
>Zotu111
GAACGCTGGCGGCATGCCTAACACATGCAAGTCGAACGAAGGCTTCGGCCTTAGTGGCGCACGGGTGC
GTAACGCGTGGGAATCTGCCCTTGGGTTCGGAATAACAGCGAGAAATTGCTGCTAATACCGGATAATG
TCGCAAGACCAAAGATTTATTGCCCAAGGATGAGCCCGCGTTGGATTAGCTAGTTGGTGGGGTAAAAG
CCTACCAAGGCGACGATCCATAGCTGGTCTGAGAGGATGATCAGCCACACTGGGACTGAGACACGGCC
>Zotu112
GAACGCTGGCGGCGTGCCTAATACATGCAAGTAGAACGCTGAAGGAGGAGCTTGCTTCTCCGGATGAG
TTGCGAACGGGTGAGTAACGCGTAGGTAACCTGCCTGGTAGCGGGGGATAACTATTGGAAACGATAGC
TAATACCGCATAACAGTAGATGTTGCATGACATTTGCTTAAAAGGTGCAATTGCATCACTACCAGATG
GACCTGCGTTGTATTAGCTAGTTGGTGGGGTAACGGCTCACCAAGGCGACGATACATAGCCGACCTGA
GAGGGTGATCGGCCACACTGGGACTGAGACACGGCC
>Zotu113
GAACGCTGGCGGCGTGCCTAATACATGCAAGTCGAGCGAACAGACGAGGAGCTTGCTCCTTTGACGTT
AGCGGCGGACGGGTGAGTAACACGTGGATAACCTACCTATAAGACTGGGATAACTTCGGGAAACCGGA
GCTAATACCGGATAACATGTTGAACCGCATGGTTCAACAGTGAAAGACGGTCTTGCTGTCACTTATAG
ATGGATCCGCGCCGCATTAGCTAGTTGGTAAGGTAACGGCTTACCAAGGCAACGATGCGTAGCCGACC
TGAGAGGGTGATCGGCCACACTGGAACTGAGACACGGTC
>Zotu114
GAACGCTGGCGGCGTGCTTAACACATGCAAGTCGAACGGTGAAGTCCCTTCGGGGGCGGATCAGTGGC
GAACGGGTGAGTAACACGTGGGCAATCTGCCCCTCACTGTGGGATAACTCCGGGAAACCGGGGCTAAT
ACCGCATACGAAGCCTGGCGGCATCGCCGGGTTTGGAAAGTTTTTCGGTGAGGGATGAGCCCGCGGCC
TATCAGCTTGTTGGTGAGGTAATGGCTCACCAAGGCGACGACGGGTAGCCGGCCTGAGAGGGCGACCG

GCCACACTGGGACTGAGACACGGCC
>Zotu115
GAACGCTAGCGGCATGCTTAACACATGCAAGTCGAACGGCAGCGCAAGGGGCTTTCTCCTGGCGGCGA
GTGGCGGACGGGTGAGTAATGCGTAGGAATCTACCTTAAATAGTGGGGGATAACCTGAGGAAACTCGG
GCTAATACCGCATGATCTCTTCGGAGCAAAGCGGGGGATCGTTAGACCTCGTGCTATAAGATGAGCTT
ACGTCGGATTAGTTTGTTGGTGGGGTAAAGGCCTACCAAGGCGATGATCCGTAGCTGGTCTGAGAGGA
CGATCAGCCACACTGGGACTGAGACACGGCC
>Zotu116
GAACGCTGGCGGCGTGCCTAATACATGCAAGTAGAACGCTGAAGAGAGGAGCTTGCTCTTCTTGGATG
AGTTGCGAACGGGTGAGTAACGCGTAGGTAACCTGCCTTGTAGCGGGGGATAACTATTGGAAACGATA
GCTAATACCGCATAACAATGAATGACACATGTCATTTATTTGAAAGGGGCAATTGCTCCACTACAAGA
TGGACCTGCGTTGTATTAGCTAGTAGGTGAGGTAACGGCTCACCTAGGCGACGATACATAGCCGACCT
GAGAGGGTGATCGGCCACACTGGGACTGAGACACGGCC
>Zotu117
GAACGCTGGCGGCGTGCTTAACACATGCAAGTCGAACGATGAAGCCCAGCTTGCTGGGTGGATTAGTG
GCGAACGGGTGAGTAACACGTGAGTAACCTGCCCTTAACTCTGGGATAAGCCTGGGAAACTGGGTCTA
ATACCGGATAGGAGCGTCCACCGCATGGTGGGTGTTGGAAAGATTTATCGGTTTTGGATGGACTCGCG
GCCTATCAGCTTGTTGGTGAGGTAATGGCTCACCAAGGCGACGACGGGTAGCCGGCCTGAGAGGGTGA
CCGGCCACACTGGGACTGAGACACGGCC
>Zotu118
GAACGCTGGCGGCGTGCCTAATACATGCAAGTCGAGCGAACTGAAGAGGAGCTTGCTCCTTTGACGTT
AGCGGCGGACGGGTGAGTAACACGTGGGTAACCTACCTATAAGACTGGAATAACTCCGGGAAACCGGG
GCTAATGCCGGATAATATGTTGAACCGCATGGTTCAACAGTGAAAGACGGTTTTGCTGTCACTTATAG
ATGGACCCGCGCCGCATTAGCTAGTTGGTGAGGTAACGGCTCACCAAGGCGACGATGCGTAGCCGACC
TGAGAGGGTGATCGGCCACACTGGAACTGAGACACGGTC
>Zotu119
GAACGCTGGCGGCAGGCCTAACACATGCAAGTCGAGCGGATGAGGAGAGCTTGCTCTCTGATTCAGCG
GCGGACGGGTGAGTAATGCCTAGGAATCTGCCTGGTAGTGGGGGACAACGTTTCGAAAGGAACGCTAA
TACCGCATACGTCCTACGGGAGAAAGTGGGGGATCTTCGGACCTCACGCTATCAGATGAGCCTAGGTC
GGATTAGCTAGTTGGTGGGGTAACGGCTCACCAAGGCGACGATCCGTAACTGGTCTGAGAGGATGATC
AGTCACACTGGAACTGAGACACGGTC
>Zotu120
GAACGCTGGCGGCAGGCCTAACACATGCAAGTCGAACGGTAACAGGAAGCAGCTTGCTGCTTCGCTGA
CGAGTGGCGGACGGGTGAGTAATGTCTGGGAAACTGCCTGATGGAGGGGGATAACTACTGGAAACGGT
AGCTAATACCGCATAACGTCGCAAGACCAAAGAGGGGGACCTTCGGGCCTCTTGCCATCGGATGTGCC
CAGATGGGATTAGCTAGTAGGTGGGGTAACGGCTCACCTAGGCGACGATCCCTAGCTGGTCTGAGAGG
ATGACCAGCCACACTGGAACTGAGACACGGTC
>Zotu121
GAACGCTGGCGGCATGCCTAACACATGCAAGTCGAACGAAGGCTTCGGCCTTAGTGGCGCACGGGTGC
GTAACGCGTGGGAATCTGCCCTCAGGTTCGGAATAACAGCGAGAAATTGCTGCTAATACCGGATGATA
TCGCGAGATCAAAGATTTATCGCCTGAGGATGAGCCCGCGTAGGATTAGCTAGTTGGTGTGGTAAAGG
CGCACCAAGGCGACGATCCTTAGCTGGTCTGAGAGGATGATCAGCCACACTGGGACTGAGACACGGCC
>Zotu122
GAACGCTGGCGGTGTGCTTAATACATGCAAGTCGAACGAAGAGGCCCTTTCGGGGGTACTTCAGTGGC
GGACGGCTGAGTAATGCATAGGTAACGTGCCCTTAACTGGGGGATAACACCGGGAAACTGGTGCTAAT
ACCGCATAAGTGCCAGGTTGAAATACCTGTCATTAAAGAATTTCGGTTAAGGATCGGCCTGTGTCGGA
TTAGCTTGTTGGTAGGGTAATGGCCTACCAAGGCGACGATCCGTAGTTGGTCTGAGAGGATGACCAGC
CAGAGTGGGACTGAGAACGGCC
>Zotu123
GAACGCTGGCGGCGTGCCTAATACATGCAAGTCGAACGCCTTTTCTTTCACCGGAGCTTGCTCCACCG
AAAGAAAAGGAGTGGCGAACGGGTGAGTAACACGTGGGTAACCTGCCCATCAGAAGGGGATAACACTT
GGAAACAGGTGCTAATACCGTATAACAATAGAAACCGCATGGTTTCTATTTGAAAGGCGCTTTTGCGT
CACTGATGGATGGACCCGCGGTGCATTAGCTAGTTGGTGAGGTAACGGCTCACCAAGGCAACGATGCA
TAGCCGACCTGAGAGGGTGATCGGCCACATTGGGACTGAGACACGGCC

>Zotu124
GAACGCTGGCGGCGTGCCTAATACATGCAAGTAGAACGCTGAAGGAGGAGCTTGCTTCTCTGGATGAG
TTGCGAACGGGTGAGTAACGCGTAGGTAACCTGCCTGGTAGCGGGGGATAACTATTGGAAACGATAGC
TAATACCGCATAAGAGTAGATGTTGCATGACATTTGCTTAAAAGGTGCAATTGCATCACTACCAGATG
GACCTGCGTTGTATTAGCTAGTTGGTGGGGTAACGGCTCACCAAGGCGACGATACATAGCCGACCTGA
GAGGGTGATCGGCCACACTGGGACTGAGACACGGCC
>Zotu125
GAACGCTGGCGGCGGGCCTCATACATGCAAGTCGAGCGCGAAAGTCCGCAAGGATGAGTAAAGCGGCG
CACGGGTGAGTAACACGTGGATAATCTACCCTTTGGATTGGAATAACCTGTCGAAAGACGGGCTAATG
CCGAGTACGCACTCGAGGGCGTGAAGCTCTTGAGTGGAAAGATGGCGCAAGCTATCGCCGATGGATGA
GTCTGCGTCCCATTAGTTAGTTGGTGAGGTAACGGCTTACCAAGACAATGATGGGTACCTGGTCTGAG
AGGATGATCAGGCACACTGGAACTGAAACACGGTC
>Zotu126
GAACGCTGGCGGCAGGCTTAACACATGCAAGTCGAGCGGGCGTAGCAATACGTCAGCGGCAGACGGGT
GAGTAACGCGTGGGAACGTACCTTTTGGTTCGGAACAACACAGGGAAACTTGTGCTAATACCGGATAA
GCCCTTACGGGGAAAGATTTATCGCCGAAAGATCGGCCCGCGTCTGATTAGCTAGTTGGTGAGGTAAC
GGCTCACCAAGGCGACGATCAGTAGCTGGTCTGAGAGGATGATCAGCCACATTGGGACTGAGACACGG
CC
>Zotu127
GAACGCTGGCGGCGTGCCTAATACATGCAAGTCGAGCGAACTGACGAGGAGCTTGCTCCTTTGACGTT
AGCGGCGGACGGGTGAGTAACACGTGGGTAACCTACCTATAAGACTGGAATAACTCCGGGAAACCGGG
GCTAATGCCGGATAACATATCGAACCGCATGGTTCGATAGTGAAAGACGGTCTTGCTGTCACTTATAG
ATGGACCCGCGCCGTATTAGCTAGTTGGTGAGGTAACGGCTCACCAAGGCGACGATACGTAGCCGACC
TGAGAGGGTGATCGGCCACACTGGAACTGAGACACGGTC
>Zotu128
GAACGCTAGCGGCAGGCTTAATACATGCAAGTCGTGGGGCAGCATGTCCCGACGCAAGTCGGGATGAT
GGCGACCGGCAAACGGGTGCGGAACACGTACACAACCTTCCTTTAAGTGGGGAATAGCCCAGGGAAAC
TTGGATTAATACCCCGTAATATTATGAAGTGGCATCATTTTATAATTATAGCTCCGGCGCTTGAAGAT
GGGTGTGCGGCTGATTAGGTAGTTGGCGGGGTAACGGCCCACCAAGCCTACGATCAGTAACTGGTGTG
AGAGCACGACCAGTCACACGGGCACTGAGACACGGGC
>Zotu129
CAACGCTGGCGGCGCGCCTAACACATGCAAGTCGAACGAGAAAGTGGAGCAATCCATGAGTAAAGTGG
CGTACGGGTGAGTAACACGTGGATCATCTACCCTCTAGTGGGGAATACCGCTGGGAAACCGGCGACAA
TACCGCATAAGCTCGAGAGAGGAAAGCAGCAATGCGCTGGAGGAGGAGTCCGCGGTCGATTAGTTAGT
TGGCAGGGTAAAGGCCTACCAAGACTGTGATCGATAGCCGGCCTGAGAGGGCACACGGCCACACTGGC
ACTGAAACACGGGC
>Zotu130
GAACGCTGGCGGCGTGCCTAATACATGCAAGTCGTACGCAATTGATCCACCGAGTGCTTGCACTCACC
GGATCGTTGAGTGGCGCACGGGTGAGTAACACGTGGGTAACCTGCCCACCAGAGGGGGATAACAGTCG
GAAACGATTGCTAATACCGCATAGGTTGAACGACCGCATGGTCGCTCAAGGAAAGACGGCTTTGGCTG
TCACTGGTGGATGGACCCGCGGCGTATTAGTTAGTTGGTGAGGTAACGGCTCACCAAGACAATGATAC
GTAGCCGACCTGAGAGGGTGATCGGCCACATTGGGACTGAGACACGGCC
>Zotu131
GAACGCTAGCGGCAGGCCTAATACATGCAAGTCGAACGATGCTTAGTAGCAATACTAAGTGTAGTGGC
GCACGGGTGCGTAACACGTATGCAACCTACCTTTAACAGGGGGATAGCCCGGGGAAACCCGGATTAAT
ACCCCGTATGGTGTGATATTGGCATCAGTATTACATTAAAGTTACGATGGTTAAAGATGGGCATGCGT
CTGATTAGTTAGTTGGTGAGGTAACGGCTCACCAAGACGATGATCAGTAGGGGACCTGAGAGGGTGAT
CCCCCACACTGGCACTGAGATACGGGC
>Zotu132
GAACGCTGGCGGCATGCTTTACACATGCAAGTCGAGCGGCAGCGCGGGGGCAACCCTGGCGGCGAGCG
GCGAACGGGTGAGTAATGCATCGGAACGTATCCTGTAGTGGGGGATAACCCAGCGAAAGTTGGGCTAA
TACCGCATGAGCTCTGAGGAGGAAAGCGGGGGACCTGAAAGGGCCTCGCGCTGCCGGAGCGGCCGATG
TCCGATTAGCTAGTTGGTGGGGTAAAGGCTTACCAAGGCGACGATCGGTAGCTGGTCTGAGAGGATGA
TCAGCCACACTGGGACTGAGACACGGCC

>Zotu133
GAACGCTGGCGGCATGCCTTACACATGCAAGTCGAACGGCAGCACGGGGGCAACCCTGGTGGCGAGTG
GCGAACGGGTGAGTAATACATCGGAACGTGTCCTGTAGTGGGGGATAGCCCGGCGAAAGCCGGATTAA
TACCGCATACGCTCTACGGAGGAAAGGGGGGGATCTTAGGACCTCTCGCTACAGGGGCGGCCGATGGC
AGATTAGCTAGTTGGTGGGGTAAAGGCCTACCAAGGCGACGATCTGTAGCTGGTCTGAGAGGACGACC
AGCCACACTGGGACTGAGACACGGCC
>Zotu134
GAACGCTAGCGGCAGGCTTAATACATGCAAGTCGTGGGGCAGCGCAGGTAGCAATACTGGGCGGCGAC
CGGCAAACGGGTGCGGAACACGTACACAACCTTCCTATAAGTGGGGAATAGCCCAGAGAAATTTGGAT
TAATACCCCGTAACATAATGATGTGGCATCACATTGTTATTATAGCTTCGGCGCTTATTGATGGGTGT
GCGGCTGATTAGATAGTTGGCGGGGTAACGGCCCACCAAGTCTACGATCAGTAGCTGATGTGAGAGCA
TGATCAGCCACACGGGCACTGAGACACGGGC
>Zotu135
GAACGCTGGCGGCGTGCCTAATACATGCAAGTCGAGCGGACGAATCGAAAGCTTGCTTTTGATTCGTT
AGCGGCGGACGGGTGAGTAACACGTGGGCAACCTGCCCTGTAGACGGGGATAACACCGAGAAATCGGT
GCTAATACCGGATAACACGAAATGTCGCATGACGTTTCGTTGAAAGACGGCGCAAGCTGTCGCTACAG
GATGGGCCCGCGGCGCATTAGCTAGTTGGTGAGGTAACGGCTCACCAAGGCGACGATGCGTAGCCGAC
CTGAGAGGGTGATCGGCCACACTGGGACTGAGACACGGCC
>Zotu136
GAACGCTAGCGGGAGGCCTAACACATGCAAGCCGAGCGGTATTTGTTCTTCGGAACAGAGAGAGCGGC
GCACGGGTGCGGAACACGTGTGCAACCTGCCTTTATCTGGGGGATAGCCTTTCGAAAGGAAGATTAAT
ACCCCATAATATATTGAGTGGCATCATTTGATATTGAAAACTCCGGTGGATAGAGATGGGCACGCGCA
AGATTAGATAGTTGGTGAGGTAACGGCTCACCAAGTCAATGATCTTTAGGGGGCCTGAGAGGGTGATC
CCCCACACTGGTACTGAGACACGGAC
>Zotu137
GAACGCTGGCGGCAGGCCTAACACATGCAAGTCGAACGGTAACAGGAAGAAGCTTGCTTCTTTGCTGA
CGAGTGGCGGACGGGTGAGTAATGTCTGGGAAACTGCCTGATGGAGGGGGATAACTACTGGAAACGGT
AGCTAATACCGCATAACGTCGCAAGACCAAAGAGGGGGACCTTCGGGCCTCTTGCCATCGGATGTGCC
CAGATGGGATTAGCTAGTAGGTGGGGTAACGGCTCACCTAGGCGACGATCCCTAGCTGGTCTGAGAGG
ATGACCAGCCACACTGGAACTGAGACACGGTC
>Zotu138
GAACGCTGGCGGCGTGCCTAATACATGCAAGTCGAGCGAACAGACGAGGAGCTTGCTCCTTTGACGTT
AGCGGCGGACGGGTGAGTAACACGTGGGTAACCTACCTATAAGACTGGGATAACTTCGGGAAACCGGA
GCTAATACCGGATAATATTTCGAACCGCATGGTTCGATAGTGAAAGATGGCTCTGCTATCACTTATAG
ATGGACCTGCGCCGTATTAGCTAGTTGGTAAGGTAACGGCTTACCAAGGCAACGATACGTAGCCGACC
TGAGAGGGTGATCGGCCACACTGGAACTGAGACACGGTC
>Zotu139
GAACGCTAGCGGGAGGCCTAACACATGCAAGCCGAGCGGTAGAGTTTCTTCGGAAACTTGAGAGCGGC
GTACGGGTGCGTAACACGTGTGCAACCTACCTTTATCTGGGGGATAGCCTTTCGAAAGGAAGATTAAT
ACCCCATAATATAATGATTGGCATCAATTATTATTGAAAACTCCGGTGGATAGAGATGGGCACGCGCA
AGATTAGATAGTTGGTGAGGTAACGGCTCACCAAGTCAATGATCTTTAGGGGTCCTGAGAGGGAGATC
CCCCACACTGGTACTGAGACACGGAC
>Zotu140
GAACGCTAGCGGGAGGCCTAACACATGCAAGCCGAGCGGTAGAGTTCCTTCGGGAACTTGAGAGCGGC
GTACGGGTGCGTAACACGTGTGCAACCTACCTTTATCTGGGGGATAGCCTTTCGAAAGGAAGATTAAT
ACCCCATAATATAATGATTGGCATCAATTATTATTGAAAACTCCGGTGGATAGAGATGGGCACGCGCA
AGATTAGATAGTTGGTGAGGTAACGGCTCACCAAGTCAATGATCTTTAGGGGTCCTGAGAGGGAGATC
CCCCACACTGGTACTGAGACACGGAC
>Zotu141
GAACGCTGGCGGCGTGCTTAACACATGCAAGTCGAACGGAAAGGCCCAAGCTTGCTTGGGTACTCGAG
TGGCGAACGGGTGAGTAACACGTGGGTGATCTGCCCTGCACTTCGGGATAAGCCTGGGAAACTGGGTC
TAATACCGGATAGGACGATGGTTTGGATGCCATTGTGGAAAGTTTTTCGGTGTGGGATGAGCTCGCGG
CCTATCAGCTTGTTGGTGGGGTAATGGCCTACCAAGGCGTCGACGGGTAGCCGGCCTGAGAGGGTGTA
CGGCCACATTGGGACTGAGATACGGCC

>Zotu142
GAACGCTGGCGGCGTGCCTAATACATGCAAGTCGAGCGAACAGACGAGGAGCTTGCTCCTTTGACGTT
AGCGGCGGACGGGTGAGTAACACGTGGGTAACCTACCTATAAGACTGGGATAACTTCGGGAAACCGGA
GCTAATACCGGATAATATTTCGAACCGCATGGTTCGATAGTGAAAGATGGCTTTGCTATCACTTATAG
ATGGACCTGCGCCGTATTAGCTAGTTGGTAAGGTAACGGCTTACCAAGGCAACGATACGTAGCCGACC
TGAGAGGGTGATCGGCCACACTGGAACTGAGACACGGTC
>Zotu143
AAACGCTGGCGGCATGCCTAACACATGCAAGTCGAACGGCAGCACAGTAAAGATTTCGGTCTTTACGG
GTGGCGAGTGGCGGACGGGTGAGTAAAGCGTAGGAATCTGTCCTGGAGAGTGGAATAACCCGAGGAAA
CTCGGGCTAATACCGCATGATATCGAGAGATCAAAGCAGGGGACCCTTCGGGGCCTTGCGCTCTAGGA
TGAGCCTACGTCGGATTAGCTAGTTGGTGGGGTAAAGGCCTACCAAGGCGACGATCCGTAGCTGGTCT
GAGAGGATGACCAGCCACACTGGGACTGAGACACGTCC
>Zotu144
GAACGCTGGCGGCGTGCCTAATACATGCAAGTAGAACGCTGAAGGAGGAGCTTGCTCTTTCCGGATGA
GTTGCGAACGGGTGAGTAACGCGTAGGTAACCTGCCTGGTAGCGGGGGATAACTATTGGAAACGATAG
CTAATACCGCATAACAGTAGATATTGCATGATATCTACTTGAAAGGTGCAATTGCATCACTACCAGAT
GGACCTGCGTTGTATTAGCTAGTTGGTGAGGTAACGGCTCACCAAGGCGACGATACATAGCCGACCTG
AGAGGGTGATCGGCCACACTGGGACTGAGACACGGCC
>Zotu145
GAACGCTGGCGGCATGCCTTACACATGCAAGTCGAACGGTAACAGGTTAAGCTGACGAGTGGCGAACG
GGTGAGTAATATATCGGAACGTGCCCAGTCGTGGGGGATAACTGCTCGAAAGAGCAGCTAATACCGCA
TACGACCTGAGGGTGAAAGCGGGGGATCGCAAGACCTCGCGCGATTGGAGCGGCCGATATCAGATTAG
GTAGTTGGTGGGGTAAAGGCCCACCAAGCCAACGATCTGTAGCTGGTCTGAGAGGACGACCAGCCACA
CTGGGACTGAGACACGGCC
>Zotu146
GAACGCTGGCGGTCTGCTTAACACATGCAAGTCGAACGGTGTCTTCGGACACAGTGGCGGACGGGTGA
GTAACGCGTGAGAATCTACCCTCAGGTCGGGGATAACTCCTGGAAACGGGAGCTAATACCGGATGTGC
CGAAAGGTGAAAGGTTAACTGCCTGGGGAGGAGCTCGCGTCTGATTAGCTAGTTGGTGGGTTAAAAGC
TTACCAAGGCGACGATCAGTAGCTGGTCTGAGAGGATGAGCAGCCACACTGGGACTGAGACACGGCC
>Zotu147
GAACGCTGGCGGCGTGCCTAATACATGCAAGTAGAACGCTGAAGGAGGAGCTTGCTTCTCTGGATGAG
TTGCGAACGGGTGAGTAACGCGTAGGTAACCTGCCTGGTAGCGGGGGATAACTATTGGAAACGATAGC
TAATACCGCATAAGAGTAGATGTTGCATAACATTTGCTTAAAAGGTGCAATTGCACCACTACCAGATG
GACCTGCGTTGTATTAGCTAGTTGGTGGGGTAACGGCTCACCAAGGCGACGATACATAGCCGACCTGA
GAGGGTGATCGGCCACACTGGGACTGAGACACGGCC
>Zotu148
GAACGCTGGCGGCATGCTTAACACATGCAAGTCGGACGGGAAGTGGTGTTTCCAGTGGCGGACGGGTG
AGTAACGCGTAAGAACCTGCCCTTGGGAGGGGAACAACAACTGGAAACGGTTGCTAATACCCCGTAGG
CTGAGGAGCAAAAGGAGGAATCCGCCCGAGGAGGGGCTTGCGTCTGATTAGCTAGTTGGTGAGGTAAT
AGCTTACCAAGGCGATGATCAGTAGCTGGTCCGAGAGGATGATCAGCCACACTGGGACTGAGACACGG
CC
>Zotu149
GAACGCTGGCGGCGTGCTTAACACATGCAAGTCGAACGAAGAGCGATGGAAGCTTGCTTCTATCAATC
TTAGTGGCGAACGGGTGAGTAACGCGTAATCAACCTGCCCTTCAGAGGGGGACAACAGTTGGAAACGA
CTGCTAATACCGCATACGATCTAATCTCGGCATCGAGGATAGATGAAAGGTGGCCTCTACATGTAAGC
TATCACTGAAGGAGGGGATTGCGTCTGATTAGCTAGTTGGAGGGGTAACGGCCCACCAAGGCGATGAT
CAGTAGCCGGTCTGAGAGGATGAACGGCCACATTGGGACTGAGACACGGCC
>Zotu150
GAACGCTGGCGGCAGGCCTAACACATGCAAGTCGAACGGTAGCACAGAGGAGCTTGCTCCTTGGGTGA
CGAGTGGCGGACGGGTGAGTAATGTCTGGGAAACTGCCCGATGGAGGGGGATAACTACTGGAAACGGT
AGCTAATACCGCATAACGTCGCAAGACCAAAGAGGGGGACCTTCGGGCCTCTTGCCATCGGATGTGCC
CAGATGGGATTAGCTAGTAGGTGGGGTAACGGCTCACCTAGGCGACGATCCCTAGCTGGTCTGAGAGG
ATGACCAGCCACACTGGAACTGAGACACGGTC
>Zotu151

GAACGCTGGCGGCGTGCTTAACACATGCAAGTCGAACGGAAAGGCTCCAGCTTGCTGGAGTACTCGAG
TGGCGAACGGGTGAGTAACACGTGGGTAATCTGCCCTGCACTCTGGGATAAGCCTGGGAAACTGGGTC
TAATACTGGATATGACCTTTCTCCTTATGGGGTTTGGTGGAAAGTTTTTCGGTGTAGGATGAGCCCGC
GGCCTATCAGCTTGTTGGTGGGGTGATGGCCTACCAAGGCGACGACGGGTAGCCGGCCTGAGAGGGTG
ATCGGCCACATTGGGACTGAGATACGGCC
>Zotu152
GAACGCTGGCGGCGTGCTTAACACATGCAAGTCGAACGGTGACGAAGAGCTTGCTCTTCTGATCAGTG
GCGAACGGGTGAGTAACACGTGAGCAACCTACCCTTGACACTGGGATAACAGTTGGAAACAGCTGCTA
ATACCGGATATGAACTCAGCAGGCATCTGCCGAGTTGGAAAGCTCCGACGGTCAAGGATGGGCTCGCG
GCCTATCAGCTCGTTGGTGAGGTAACGGCTCACCAAGGCGACGACGGGTAGCCGGCCTGAGAGGGCGA
CCGGCCACACTGGGACTGAGATACGGCC
>Zotu153
GAACGCTGGCGGCGTGCTTAACACATGCAAGTCGAACGCTGAAGCTTGGTGCTTGCACTGGGTGGATG
AGTGGCGAACGGGTGAGTAATACGTGAGTAACCTGCCCTTGACTCTGGGATAAGCCTGGGAAACTGGG
TCTAATACTGGATACGACATGTCACCGCATGGTGGTGTGTGGAAAGGGTTTTACTGGTTTTGGATGGG
CTCACGGCCTATCAGCTTGTTGGTGGGGTAATGGCTCACCAAGGCGACGACGGGTAGCCGGCCTGAGA
GGGTGACCGGCCACACTGGGACTGAGACACGGCC
>Zotu154
GAACGCTGGCGGCGTGCTTAACACATGCAAGTCGAACGAAGAGCGATGGAAGCTTGCTTCTATCAATC
TTAGTGGCGAACGGGTGAGTAACGCGTAATCAACCTGCCCTTCAGAGGGGGACAACAGTTGGAAACGA
CTGCTAATACCGCATACGATCCAATCTCGGCATCGAGGATGGATGAAAGGTGGCCTCTATTTATAAGC
TATCACTGAAGGAGGGGATTGCGTCTGATTAGCTAGTTGGAGGGGTAACGGCCCACCAAGGCAATGAT
CAGTAGCCGGTCTGAGAGGATGAACGGCCACATTGGGACTGAGACACGGCC
>Zotu155
GAACGCTAGCGGCAGGCCTAACACATGCAAGTCGAGGGGCAGCACAAGTAAAGCTTGCTTTATTGGTG
GCGACCGGCGCACGGGTGAGTAACGCGTATGCAACCTACCTTGTACAGGGGGATAGCCCGAAGAAATT
CGGATTAATACCCCATAGTATTATTTAACTGCATAGTTTGAATAATTAAAGTTTCGGCGGTACAAGAT
GGGCATGCGTCCTATTAGCTAGATGGTGAGGTAACGGCTCACCATGGCAACGATAGGTAGGGGGCCTG
AGAGGGTGGTCCCCCACACTGGTACTGAGACACGGAC
>Zotu156
GAACGCTGGCGGCATGCTTAACACATGCAAGTCGAACGGCAGCACAGTAGAGCTTGCTCTATGGGTGG
CGAGTGGCGGACGGGTGAGTAATGCATCGGGACCTACCCAGACGTGGGGGATAACGTAGGGAAACTTA
CGCTAATACCGCATACGTCCTACGGGAGAAAGCGGGGGATCGAAAGACCTCGCGCGGTTGGATGGACC
GATGTGCGATTAGCTTGTTGGTGAGGTAACGGCTCACCAAGGCGACGATCGCTAGCTGGTCTGAGAGG
ATGATCAGCCACACTGGGACTGAGACACGGCC
>Zotu158
GAACGCTGGCGGTATGCTTTACACATGCAAGTCATACGAGGGTTTTTAACTCAAGTGGCGGACGGGTG
AGTAACACGTAAGAATTTGCCTTTAGGAGGGGGATAACAACCACAAAAGGTTGCTAATACCCCATATG
CTTTCGAGTGAAACGGATTTTTTCCGCCTAGAGAGAAGCTTGCGGCTGATTAGCTTGTTGGTGAGGTA
ATGGCTTACCAAGGCGACGATCAGTATCTGGTTTGAGAGGACGATCAGACACACTGGAACTGAGACAC
GGTC
>Zotu159
GAACGCTGGCGGCATGCCTAACACATGCAAGTCGAACGAGCCCTTCGGGGTTAGTGGCGCACGGGTGC
GTAACGCGTGGGAATCTGCCCTTTGCTTCGGAATAACTTCGGGAAACTGAAGCTAATACCGGATGATG
TCCTCATTAGAGATATTGGGGACCAAAGATTTATCGGCAAAGGATGAGCCCGCGTAGGATTAGGTAGT
TGGTGGGGTAAAGGCCTACCAAGCCGACGATCCTTAGCTGGTCTGAGAGGATGATCAGCCACACTGGG
ACTGAGACACGGCC
>Zotu160
GAACGCTGGCGGCATGCTTTACACATGCAAGTCGAACGGCAGCGCGGGGCAACCTGGCGGCGAGTGGC
GAACGGGTGAGTAATATATCGGAACGTACCCAAGAGTGGGGGATAACGTAGCGAAAGTTACGCTAATA
CCGCATACGATCTAAGGATGAAAGTGGGGGATCGCAAGACCTCATGCTCCTGGAGCGGCCGATATCTG
ATTAGCTAGTTGGTGAGGTAAAGGCTCACCAAGGCGACGATCAGTAGCTGGTCTGAGAGGACGACCAG
CCACACTGGAACTGAGACACGGTC
>Zotu161

GAACGCTGGCGGCATGCCTAACACATGCAAGTCGAACGAAGGCTTCGGCCTTAGTGGCGCACGGGTGC
GTAACGCGTGGGAATCTGCCCTTGGGTTCGGGATAACAGTGAGAAATTACTGCTAATACCGGATGATG
ACTTCGGTCCAAAGATTTATCGCCCAGGGATGAGCCCGCGTTGGATTAGCTAGTTGGTGGGGTAAAGG
CCTACCAAGGCGACGATCCATAGCTGGTCTGAGAGGATGATCAGCCACACTGGGACTGAGACACGGCC
>Zotu162
GAACGCTGGCGGCGTGCTTAACACATGCAAGTCGAACGGAAAGGCTCCTGCTTGCAGGGGTACTCGAG
TGGCGAACGGGTGAGTAACACGTGGGTGATCTGCCCTGCACTTCGGGATAAGCCTGGGAAACTGGGTC
TAATACCGGATAGGACGATGGTTTGGATGCCATTGTGGAAAGTTTTTTCGGTGTGGGATGAGCTCGCG
GCCTATCAGCTTGTTGGTGGGGTAATGGCCTACCAAGGCGTCGACGGGTAGCCGGCCTGAGAGGGTGT
ACGGCCACATTGGGACTGAGATACGGCC
>Zotu163
GAACGCTGGCGGCGTGCCTAATACATGCAAGTCGAGCGAATGGATTGAGAGCTTGCTCTCAAGAAGTT
AGCGGCGGACGGGTGAGTAACACGTGGGTAACCTGCCCATAAGACTGGGATAACTCCGGGAAACCGGG
GCTAATACCGGATAACATTTTGAACTGCATGGTTCGAAATTGAAAGGCGGCTTCGGCTGTCACTTATG
GATGGACCCGCGTCGCATTAGCTAGTTGGTGAGGTAACGGCTCACCAAGGCAACGATGCGTAGCCGAC
CTGAGAGGGTGATCGGCCACACTGGGACTGAGACACGGCC
>Zotu164
GAACGCTAGCGGCAGGCTTAATACATGCAAGTCGTGGGGCAGCGCAGTGTAGCAATACATGGGCGGCG
ACCGGCAAACGGGTGCGGAACACGTACACAACCTTCCTACAAGTGGGGAATAGCCCAGAGAAATTTGG
ATTAATACCCCGTAACATATAAAGATGGCATCATTTTTATATTATAGCTACGGCGCTTGTTGATGGGT
GTGCGGCTGATTAGGTAGTTGGCGGGGTAATGGCCCACCAAGCCTGCGATCAGTAGCTGATGTGAGAG
CATGATCAGCCACACGGGCACTGAGACACGGGC
>Zotu165
GAACGCTGGCGGTGTGCCTAATACATGCAAGTCGAGCGGCAGCACGAATAGTTTACTATTTGGTGGCG
AGCGGCGGACGGCTGAGTAACACGTAGGAACATACCCCAAAGTGAGGGATAACTGCTCGAAAGAGTAG
CTAATACCGCATGTGATCTAAGGATTAAAGCTTCGGCGCTTTGGGAATGGCCTGCGTTCGATTAGCTA
GTTGGTAAGGTAAAAGCTTACCAAGGCGACGATCGATAGCTGGTCTGAGAGGATGATCAGCCAGACTG
GGACTGAGACACGGCC
>Zotu166
GAACGCTAGCGGCAGGCTTAATACATGCAAGTCGAGGGACAGCACAGGTAGCAATACTGGGTGGTAAC
CGGCAAACGGGTGCGGAACACGTACACAACCTTCCTTTAAGTGGGGGATAGCCCAGAGAAATTTGGAT
TAATACCCCGTAACATAGTGAACTGGCATCAGTTTATTATTATAGCTACGGCGCTTAATGATGGGTGT
GCGTCTGATTAGATAGTTGGCGAGGTAACGGCTCACCAAGTCTACGATCAGTAGCTGATGTGAGAGCA
TGATCAGCCACACGGGCACTGAGACACGGGC
>Zotu168
GAACGCTGGCGGCGTGCTTAACACATGCAAGTCGAACGGAAAGGCCCAGCTTGCTGGGTGCTCGAGTG
GCGAACGGGTGAGTAACACGTGGGTGATCTGCCTCTAACTTCGGGATAAGCTTGGGAAACTGGGTCTA
ATACCGGATAGGACAATCGTTTAGTGTCGGTTGTGGAAAGTTTTTTCGGTTAGGGATGAGCCCGCGGC
CTATCAGCTTGTTGGTGGGGTAATGGCCTACCAAGGCGTCGACGGGTAGCCGGCCTGAGAGGGTGGAC
GGCCACATTGGGACTGAGATACGGCC
>Zotu169
GAACGCTGGCGGCGTGCCTAATACATGCAAGTAGAACGCTGAAGGAGGAGCTTGCTTCTCCGGATGAG
TTGCGAACGGGTGAGTAACGCGTAGGTAACCTGCCTGGTAGCGGGGGATAACTATTGGAAACGATAGC
TAATACCGCATAACAGTAGATATTGCATGATATCTGCTTGAAAGGTGCAATTGCATCACTACCAGATG
GACCTGCGTTGTATTAGCTAGTTGGTGGGGTAACGGCTCACCAAGGCGACGATACATAGCCGACCTGA
GAGGGTGATCGGCCACACTGGGACTGAGACACGGCC
>Zotu170
GAACGCTTGCGGCGTGCCTAAGAAATGCAAGTCGAGCGGGGGAGCAATCCCTAGCGGCGAACGGTCGC
GTAACACGTAGGCAACCTGCCCCAGAGACCGGGACAACACTCCGAAAGGGGTGCTAATACCGGATGCG
GCCGGCGGAAGACCTCTTCTGCCGATTAAAGCCCTTCGGGGGCTCTGGGAGGGGCCTGCGGCCTATCA
GCTAGATGGTGGGGTGATAGCCTACCATGGCGACGACGGGTAGCTGGTCTGAGAGGATGGTCAGCCGG
ACTGGGACTGAGATACGGCC
>Zotu171
GAACGCTATCGGTATGCTTAACACATGCAAGTCGGACGAATTAATTAGGGCTTGCCCTAATTAATTAG

TGGCAGACGGGTGAGTAACACGTGGGAATCTACCCATCAGTACGGAATAACTTTTAGAAATAAAAGCT
AATACCGTATATTCTCTGAGGAAAGATTTATCGCTGATGGATGAGCCCACGCCAGATTAGGTAGTTGG
TGAAGTAATGGCTCACCAAGCCAACGATCTGTAGCTGGTCTGAGAGGATGATCAGCCACACTGGGACT
GAGACACGGCC
>Zotu172
GAACGCTGGCGGCGTGCCTAATACATGCAAGTCGAGCGAACAGATAAGGAGCTTGCTCCTTTGACGTT
AGCGGCGGACGGGTGAGTAACACGTGGGTAACCTACCTATAAGACTGGAATAACTCCGGGAAACCGGG
GCTAATGCCGGATAACATTTAGAACCGCATGGTTCTAAAGTGAAAGATGGTTTTGCTATCACTTATAG
ATGGACCCGCGCCGTATTAGCTAGTTGGTAAGGTAACGGCTTACCAAGGCAACGATACGTAGCCGACC
TGAGAGGGTGATCGGCCACACTGGAACTGAGACACGGTC
>Zotu173
GAACGCTGGCGGCGTGCCTAATACATGCAAGTAGAACGCTGAAGGAGGAGCTTGCTCTTCTGGATGAG
TTGCGAACGGGTGAGTAACGCGTAGGTAACCTGCCTGGTAGCGGGGGATAACTATTGGAAACGATAGC
TAATACCGCATAACAGCAAATGTTGCATGACATTTGCTTAAAAGGTGCAATTGCATCACTACCAGATG
GACCTGCGTTGTATTAGCTAGTTGGTGGGGTAATAGCTCACCAAGGCGACGATACATAGCCGACCTGA
GAGGGTGATCGGCCACACTGGGACTGAGACACGGCC
>Zotu174
GAACGCTGGCGGCGTGCCTAATACATGCAAGTAGAACGCTGAAGAGAGGAGCTTGCTCTTCTTGGATG
AGTTGCGAACGGGTGAGTAACGCGTAGGTAACCTGCCTGGTAGCGGGGGATAACTATTGGAAACGATA
GCTAATACCGCATAAAATTGATTATTGCATGATAATTAATTGAAAGATGCAATTGCATCACTACCAGA
TGGACCTGCGTTGTATTAGCTAGTTGGTGAGGTAACGGCTCACCAAGGCGACGATACATAGCCGACCT
GAGAGGGTGATCGGCCACACTGGGACTGAGACACGGCC
>Zotu175
GAACGCTGGCGGCGTGCCTAATACATGCAAGTCGAGCGGACCGGACGAGAGCTTGCTCTTGTTTGGTC
AGCGGCGGACGGGTGAGTAACACGTGGGCAACCTGCCCGCAAGACCGGGATAACTCCGGGAAACCGGA
GCTAATACCGGATAACACCAAAGACCGCATGGTCTTTGGTTGAAAGGCGGCTTCGGCTGTCACTTGCG
GATGGGCCCGCGGCGCATTAGCTAGTTGGTGAGGTAACGGCTCACCAAGGCGACGATGCGTAGCCGGC
CTGAGAGGGTGACCGGCCACACTGGGACTGAGACACGGCC
>Zotu176
GAACGCTGGCGGCGTGCTTAACACATGCAAGTCGAACGATGATCCGGTGCTTGCACCGGGGATTAGTG
GCGAACGGGTGAGTAACACGTGAGTAACCTGCCCTTAACTCTGGGATAAGCCTGGGAAACTGGGTCTA
ATACCGGATATGACTCCTCATCGCATGGTGGGGGGTGGAAAGCTTTTGTGGTTTTGGATGGACTCGCG
GCCTATCAGCTTGTTGGTGAGGTAATGGCTCACCAAGGCGACGACGGGTAGCCGGCCTGAGAGGGTGA
CCGGCCACACTGGGACTGAGACACGGCC
>Zotu177
GAACGCTGGCGGCGTGCCTAATACATGCAAGTAGAACGCTGAAGAGAGGAGCTTGCTCTTCTTGGATG
AGTTGCGAACGGGTGAGTAACGCGTAGGTAACCTGCCTTGTAGCGGGGGATAACTATTGGAAACGATA
GCTAATACCGCATAACAATGGATGACACATGTCATTTATTTGAAAGGGGCAATTGCTCCACTACAAGA
TGGACCTGCGTTGTATTAGCTAGTAGGTGAGGTAACGGCTCACCTAGGCGACGATACATAGCCGACCT
GAGAGGGTGATCGGCCACACTGGGACTGAGACACGGCC
>Zotu178
GAACGCTGGCGGCGTGCCTAATACATGCAAGTAGAACGCTGAAGGAAGGAGCTTGCTCTTTCCGGATG
AGTTGCGAACGGGTGAGTAACGCGTAGGTAACCTGCCTGGTAGCGGGGGATAACTATTGGAAACGATA
GCTAATACCGCATAACAGTAGATATTGCATGATATCTGCTTGAAAGGTGCAAATGCATCACTACCAGA
TGGACCTGCGTTGTATTAGCTAGTTGGTGAGGTAACGGCTCACCAAGGCGACGATACATAGCCGACCT
GAGAGGGTGATCGGCCACACTGGGACTGAGACACGGCC
>Zotu179
GAACGCTGGCGGCGTGCTTAACACATGCAAGTCGAACGATGAAGCGGTGCTTGCACCGTGGATTAGTG
GCGAACGGGTGAGTAACACGTGAGTAACCTGCCTTCCACTCTGGGATAAGCGCTGGAAACGGCGTCTA
ATACTGGATATGACATTGCCTCGCATGGGGTGGTGTGGAAAGATTTTTTGGTGGTAGATGGACTCGCG
GCCTATCAGCTTGTTGGTGGGGTAATGGCCTACCAAGGCGACGACGGGTAGCCGGCCTGAGAGGGTGA
CCGGCCACACTGGGACTGAGACACGGCC
>Zotu180
GAACGCTGGCGGCGTGCCTAATACATGCAAGTCGAGCGAACAGATAAGGAGCTTGCTCCTTTGACGTT

AGCGGCGGACGGGTGAGTAACACGTGGGTAACCTACCTATAAGACTGGAATAACTCCGGGAAACCGGG
GCTAATGCCGGATAATACACAGAACCGCATGGTTCTGTGATGAAAGACGGTTTTGCTGTCACTTATAG
ATGGACCCGCGGCGTATTAGCTAGTTGGTAAGGTAACGGCTTACCAAGGCAACGATACGTAGCCGACC
TGAGAGGGTGATCGGCCACACTGGAACTGAGACACGGTC
>Zotu181
GAACGCTGGCGGCGTGCTTAACACATGCAAGTCGAACGGAAAGGCCCAGCTTGCTGGGTGCTCGAGTG
GCGAACGGGTGAGTAACACGTGGGTGATCTGCCCCTAACTTCGGGATAAGCTTGGGAAACTGGGTCTA
ATACCGGATAGGACAATCGTTTAGTGTCGGTTGTGGAAAGTTTTTCGGTTAGGGATGAGCCCGCGGC
CTATCAGCTTGTTGGTGGGGTAATGGCCTACCAAGGCGTCGACGGGTAGCCGGCCTGAGAGGGTGGAC
GGCCACATTGGGACTGAGATACGGCC
>Zotu182
GAACGCTATCGGTATGCTTAACACATGCAAGTCGGACGAATTAATTAGGGCTTGCCCTAATTAATTAG
TGGCAGACGGGTGAGTAACACGTGGGAATCTACCCATCAGTACGGAATAACTTTTAGAAATAAAAGCT
AATACCGTATATTCTCTAAGGAGGAAAGATTTATCGCTGATGGATGAGCCCGCGCCAGATTAGGTAGT
TGGTGAAGTAATGGCTCACCAAGCCAACGATCTGTAGCTGGTCTGAGAGGATGATCAGCCACACTGGG
ACTGAGACACGGCC
>Zotu183
GAACGCTGGCGGCATGCCTAATACATGCAAGTTGAACGATGATGTTCTCTGCTTGCAGAGAATTGAAG
AGTAGCGAACGGGTGAGTAACACGTGGGGAACCTGCCCATGAGAGGGGGATAACATTCGGAAACGGAT
GCTAATACCGCATAATGCTTTGGACCGCCTGGTCCAGAGACGAAAGACGGCTTTTGCTGTCACTCATG
GATGGCCCCGCGCCGTATTAGCTAGTTGGTGAGGTAATGGCTCACCAAAGCTGTGATACGTAGCCGAC
CTGAGAGGGTGATCGGCCACACTGGGACTGAGACACGGCC
>Zotu184
GAACGCTGGCGGCGTGCCTAATACATGCAAGTAGAACGCTGAAGGAGGAGCTTGCTTCTCTGGATGAG
TTGCGAACGGGTGAGTAACGCGTAGGTAACCTGCCTGGTAGCGGGGGATAACTATTGGAAACGATAGC
TAATACCGCATAAGAGTAGATGTTGCATGACATTTGCTTAAAAGGTGCAATTGCATCACTACCAGATG
GACCTGCGTTGTATTAGCTAGTTGGTGAGGTAACGGCTCACCAAGGCAACGATACATAGCCGACCTGA
GAGGGTGATCGGCCACACTGGGACTGAGACACGGCC
>Zotu186
GAACGCTGGCGGCGTGCTTAACACATGCAAGTCGAACGGAAAGGCCCTGCTTGCAGGGTGCTCGAGTG
GCGAACGGGTGAGTAACACGTGGGTGATCTGCCCCTTACTTTGGGATAAGCCTGGGAAACTGGGTCTA
ATACTGGATAGGACCATGCTGTAGGTGGTGTGGTGGAAAGATTAGTTTCGGTAAGGGATGAGCTCGCG
GCCTATCAGCTTGTTGGTGGGGTAATGGCCTACCAAGGCGTCGACGGGTAGCCGGCCTGAGAGGGTGG
ACGGCCACATTGGGACTGAGATACGGCC
>Zotu188
GATAATAAGCAAACAAAGAGTAAAAGAATCCCTGAATCAGCCGACGGTAATTCCATGACGTGGGTCAG
CGTTAAATTATTGACCAAAGCGATTGATCGCTGTATCTGGCGTTATATATGAAATGAACATAACAAAC
ATCAAGTCGCTGTTTGGCAAGATGAGAAGCAGAACTTGAAGTCACATCCTGATTCGTTCTTTAAGGTG
GGGGT
>Zotu189
GAACGCTGGCGGCATGCCTAACACATGCAAGTCGAACGATGCTTTCGGGCATAGTGGCGCACGGGTGC
GTAACGCGTGGGAATCTGCCCTTGGGTTCGGAATAACAGTGAGAAATTGCTGCTAATACCGGATGATG
TCTTCGGACCAAAGATTTATCGCCCAGGGATGAGCCCGCGTAGGATTAGCTAGTTGGTGGGGTAATGG
CCTACCAAGGCGACGATCCTTAGCTGGTCTGAGAGGATGATCAGCCACACTGGGACTGAGACACGGCC
>Zotu190
GAACGCTGGCGGCGTGCCTAATACATGCAAGTCGAACGAACAGACTTGGAAGCTTGCTTCCAATGACG
TTAGTGGCGCACGGGTGAGTAACACGTGGAGAACCTACCCTTTAGCGGGGGATAACAGTCGGAAACGA
CTGCTAAGACCGCATAGAACGTTAAGTCGCATGACAAAACGAGGAAAAATGGCTTCGGCTATTACTAA
AGGATGGCTCCGCGGTGCATTAGTTAGTTGGTAAGGTAACGGCTTACCAAGACAGTGATGCATAGCCG
ACCTGAGAGGGTGATCGGCCACATTGGGACTGAGACACGGCC
>Zotu191
GAACGCTGGCGGCGTGCCTAATACATGCAAGTCGAGCGAACGGACGAGAAGCTTGCTTCTCTGATGTT
AGCGGCGGACGGGTGAGTAACACGTGGATAACCTACCTATAAGACTGGGATAACTTCGGGAAACCGGA
GCTAATACCGGATAATATTTTGAACCGCATGGTTCAAAAGTGAAAGACGGTCTTGCTGTCACTTATAG

ATGGATCCGCGCTGCATTAGCTAGTTGGTAAGGTAACGGCTTACCAAGGCAACGATGCATAGCCGACC
TGAGAGGGTGATCGGCCACACTGGAACTGAGACACGGTC
>Zotu193
GAACGCTGGCGGCGTGCCTAATACATGCAAGTCGAGCGGATTGAACGGGAGCTTGCTTCCGTTCGGTT
AGCGGCGGACGGGTGAGTAACACGTGGGCAACCTGCCCGTAAGACCGGGATAACTTCGGGAAACCGGA
GCTAATACCGGATAACACCGAAGACCGCATGGTCTTCGGTTGAAAGGCGGCTTTGGCTGTCACTTACG
GATGGGCCCGCGGCGCATTAGCTAGTTGGTGAGGTAACGGCTCACCAAGGCGACGATGCGTAGCCGAC
CTGAGAGGGTGATCGGCCACACTGGGACTGAGACACGGCC
>Zotu194
GAACGCTAGCGGCAGGCCTAATACATGCAAGTCGAACGGTGTCTAGCAATAGGCATAGTGGCGCACGG
GTGCGTAACACGTAACTAACCTACCTATAACTGGGGGATAGCCCGCCGAAAGGCGGATTAATACCGCA
TAAGCTAGTTGAACGGCATCGTTTGATTAGTAAAGATTTATTGGTTATAGATGGGGTTGCGGGTCATT
AGCTAGTTGGTAAGGTAACGGCTTACCAAGGCGACGATGACTAGGGGAGCTGAGAGGCTGGTCCCCCA
CACGGGCACTGAGATACGGGC
>Zotu195
GAACGCTGGCGGCGTGCCTAATACATGCAAGTCGAGCGAATGGATTAAGAGCTTGCTCTTATGAAGTT
AGCGGCGGACGGGTGAGTAACACGTGGGTAACCTACCCATAAGACTGGGATAACTCCGGGAAACCGGG
GCTAATACCGGATAATATTTTGAACTGCATAGTTCGAAATTGAAAGGCGGCTTCGGCTGTCACTTATG
GATGGACCCGCGTCGCATTAGCTAGTTGGTGAGGTAACGGCTCACCAAGGCGACGATGCGTAGCCGAC
CTGAGAGGGTGATCGGCCACACTGGGACTGAGACACGGCC
>Zotu196
GAACGCTGGCGGCGTGCCTAATACATGCAAGTCGAGCGAACGGATAAGGAGCTTGCTCCTTTGAAGTT
AGCGGCGGACGGGTGAGTAACACGTGGGTAACCTACCTATAAGACTGGAATAACTTCGGGAAACCGGA
GCTAATGCCGGATAACATTTGGAACCGCATGGTTCTAAAGTAAAAGATGGTTTTGCTATCACTTATAG
ATGGACCCGCGCCGTATTAGCTAGTTGGTAAGGTAACGGCTTACCAAGGCAACGATACGTAGCCGACC
TGAGAGGGTGATCGGCCACACTGGAACTGAGACACGGTC
>Zotu197
GAACGCTGGCGGCGTGCCTAATACATGCAAGTAGAACGCTGAAGGAGGAGCTTGCTTCTCTGGATGAG
TTGCGAACGGGTGAGTAACGCGTAGGTAACCTGCCTGGTAGCGGGGGATAACTATTGGAAACGATAGC
TAATACCGCATAATAGCAGTTATTGCATGATAACTGTTTGAAAGGTGCAATTGCACCACTACCAGATG
GACCTGCGTTGTATTAGCTAGTTGGTGGGGTAACGGCTCACCAAGGCGACGATACATAGCCGACCTGA
GAGGGTGATCGGCCACACTGGGACTGAGACACGGCC
>Zotu198
GAACGCTGGCGGCGTGCCTAATACATGCAAGTCGAGCGAACAGATAAGGAGCTTGCTCCTTTGACGTT
AGCGGCGGACGGGTGAGTAACACGTGGATAACCTACCTATAAGACTGGGATAACTTCGGGAAACCGGA
GCTAATACCGGATAAGATTTTGAACCGCATGGTTCAATAGTGAAAGACGGCCTTGCTGTGACTTATAG
ATGGATCCGCGCCGTATTAGCTAGTTGGTAAGGTAACGGCTTACCAAGGCAACGATACGTAGCCGACC
TGAGAGGGTGATCGGCCACACTGGAACTGAGACACGGTC
>Zotu199
GAACGCTGGCGGCGTGCTTAACACATGCAAGTCGAACGGAAAGGCCAGTGCTTGCACTGGTGCTCGAG
TGGCGAACGGGTGAGTAACACGTGGGTGATCTGCCCTGCACTTTGGGATAAGCCTGGGAAACTGGGTC
TAATACCAGATAGGACCATGCTTTGGATGGTGTGGTGGAAAGTTTTTTCGGTGTGGGATGAGCTTGCG
GCCTATCAGCTTGTTGGTGGGGTAATGGCCTACCAAGGCGTCGACGGGTAGCCGGCCTGAGAGGGTGT
ACGGCCACATTGGGACTGAGATACGGCC
>Zotu200
GAACGCTAGCGGGAGGCCTAACACATGCAAGCCGAGCGGTATTGTTTCTTCGGAAATGAGAGAGCGGC
GTACGGGTGCGGAACACGTGTGCAACCTGCCTTTATCTGGGGGATAGCCTTTCGAAAGGAAGATTAAT
ACTCCATAATATATTGAACGGCATCGTTTAATATTGAAAGCTCCGGCGGATAGAGATGGGCACGCGCA
AGATTAGCTAGTTGGTGAGGTAACGGCTCACCAAGGCGATGATCTTTAGGGGGCCTGAGAGGGTGATC
CCCCACACTGGTACTGAGACACGGAC
>Zotu201
GAACGCTGGCGGCATGCCTAACACATGCAAGTCGAACGAGATCTTCGGATCTAGTGGCGCACGGGTGC
GTAACGCGTGGGAATCTGCCTTGGGGTTCGGAATAACTCCCCGAAAGGGGTGCTAATACCGGATGATG
TCGAAAGACCAAAGATTTATCGCCCTGAGATGAGCCCGCGTAGGATTAGCTAGTTGGTGTGGTAAAGG

CGCACCAAGGCGACGATCCTTAGCTGGTCTGAGAGGATGATCAGCCACACTGGGACTGAGACACGGCC
>Zotu202
GAACGCTGGCGGCGTGCCTAATACATGCAAGTCGAGCGGACCAAATCGGAGCTTGCTCTGGTTTGGTC
AGCGGCGGACGGGTGAGTAACACGTGGGCAACCTGCCCGCAAGACCGGGATAACTCCGGGAAACCGGA
GCTAATACCGGATAACACCGAAGACCGCATGGTCTTTGGTTGAAAGGCGGCCTTTGGCTGTCACTTGC
GGATGGGCCCGCGGCGCATTAGCTAGTTGGTGAGGTAACGGCTCACCAAGGCGACGATGCGTAGCCGG
CCTGAGAGGGTGACCGGCCACACTGGGACTGAGACACGGCC
>Zotu203
GAACGCTGGCGGCGTGCTTAACACATGCAAGTCGAGCGGTAAGGCCCTTCGGGGTACACGAGCGGCGA
ACGGGTGAGTAACACGTGGGTGATCTGCCTCGCACTCTGGGATAAGCCTGGGAAACTGGGTCTAATAC
CGGATATGAACATGGTCTGCATGGACTGTGTTGGAAAGGTTTACTGGTGCGAGATGGGCCCGCGGCCT
ATCAGCTTGTTGGTGGGGTAATGGCCTACCAAGGCGACGACGGGTAGCCGACCTGAGAGGGTGACCGG
CCACACTGGGACTGAGACACGGCC
>Zotu204
GAACGCTGGCGGCAGGCCTAACACATGCAAGTCGAGCGGATGAGTGGAGCTTGCTCCATGATTCAGCG
GCGGACGGGTGAGTAATGCCTAGGAATCTGCCTGGTAGTGGGGGACAACGTTTCGAAAGGAACGCTAA
TACCGCATACGTCCTACGGGAGAAAGTGGGGGATCTTCGGACCTCACGCTATCAGATGAGCCTAGGTC
GGATTAGCTAGTTGGTGAGGTAAAGGCTCACCAAGGCGACGATCCGTAACTGGTCTGAGAGGATGATC
AGTCACACTGGAACTGAGACACGGTC
>Zotu205
GAACGCTGGCGGCGTGCTTAACACATGCAAGTCGAACGCTGAAGCTCAGCTTGCTGGGTGGATGAGTG
GCGAACGGGTGAGTAACACGTGAGTAACCTGCCCCCTTCTTTGGGATAACGCCCGGAAACGGGTGCTA
ATACTGGATATTCACTGATCTTCGCATGGGGGTTGGTGGAAAGGTTTTTCTGGTGGGGGATGGGCTC
GCGGCCTATCAGCTTGTTGGTGGGGTGATGGCCTACCAAGGCTTTGACGGGTAGCCGGCCTGAGAGGG
TGACCGGTCACATTGGGACTGAGATACGGCC
>Zotu206
GAACGCTGGCGGCACGCCTAATACATGCAAGTCGAGCGCGTGAAGCTTCCAGAAGCCTTCGGGCGGAC
GGGAGTGGATCGAGCGGCGGACGGGTGAGTAACACGTAGGCAACCTGCCTGTAAGACCGGGATAACCC
CGGGAAACCGGGGCTAATACCGGATAGGACCTTCGGTCGCATGACCGTTGATTGAAAGGTGGCCGCAA
GGCTACCGCTTACAGATGGGCCTGCGGCGCATTAGCTGGTTGGTGAGGTAACGGCTCACCAAGGCGAC
GATGCGTAGCCGGCCTGAGAGGGTGACCGGCCACACTGGGACTGAGACACGGCC
>Zotu207
GAACGCTGGCGGCGTGCTTAACACATGCAAGTCGAACGGAAAGGCCCAAGCTTGCTTGGGTACTCGAG
TGGCGAACGGGTGAGTAACACGTGGGTGATCTGCCCTGCACTTCGGGATAAGCCTGGGAAACTGGGTC
TAATACCGGATAGGACCACATTTTGGATGGTGTGGTGGAAAGTTTTTCGGTGTGGGATGAGCTCGCGG
CCTATCAGCTTGTTGGTGGGGTAATGGCCTACCAAGGCGTCGACGGGTAGCCGGCCTGAGAGGGTGTA
CGGCCACATTGGGACTGAGATACGGCC
>Zotu208
GAACGCTGGCGGCAGGCTTAACACATGCAAGTCGAACGGGTGTAGCAATACATCAGTGGCAGACGGGT
GAGTAACACGTGGGAACCTTCCTAGAGGTACGGAACAACGCAGGGAAACTTGCGCTAATACCGTATAA
GCTCTTGCGAGGAAAGATTTATCGCCTTTAGACGGGCCCGCGTAGGATTAGGTAGTTGGTGGGGTAAC
GGCCTACCAAGCCTACGATCCTTAGCTGATCTGAGAGGATGATCAGCCACACTGGAACTGAGACACGG
TC
>Zotu209
GAATGCTGGCGGCGTGCCTAACACATGCAAGTCGAGCGGTAACAGGAGAATTTATTCTTGCTGACGAG
CGGCGGACGGCTGAGTAACGCGTGGGAACATGCCCCAAAGTGAGGGATAAGCTCCAGAAATGGAGTCT
AATACCGCATATGATCTTCGGATTAAAGGATTTATCCGCTTTGGGAGTGGCCTGCGTACGATTAGATA
GTTGGTGAGGTAATGGCTTACCAAGTCTACGATCGTTAGCTGGTCTGAGAGGATGACCAGCCAGACTG
GAACTGAGACACGGTC
>Zotu210
GAACGCTGGCGGCGTGCTTAACACATGCAAGTCTTAGGGGATTTATTCTTTCGGGAATAAGTTCACTG
GCGCACGGGTGCGTAACGCGTAAGTAATCTGCCCTAGAGACTGGCACAACTCGTCGAAAGACGGGCTA
ATTCCAGATGATACTGAAAGGTCAAAGGTTTCGACCGCTCTAGGATGAGCTTGCGTCTGATTAGCTAG
TTGGTGAGGTAATGGCTCACCAAGGCTACGATCAGTAGCCGGTCTGAGAGGATGATCGGCCACATTGG

AACTGAGACACGGTC
>Zotu211
GAACGCTAGCGGGAGGCCTAACACATGCAAGCCGAGCGGTAGGGTTTCTTCGGAAACCTGAGAGCGGC
GCACGGGTGCGGAACACGTGTGCAACCTACCTTTATCAGGGGGATAGCCTTTCGAAAGGAAGATTAAT
ACCCCATAATATATTAGATGGCATCATTTGATATTGAAAACTCCGGTGCATAGAGATGGGCACGCGCA
AGATTAGATAGTTGGTGAGGTAACGGCTCACCAAGTCAGTGATCTTTAGGGGGCCTGAGAGGGTGATC
CCCCACACTGGTACTGAGACACGGAC
>Zotu212
GAACGCTGGCGGCGTGCCTAATACATGCAAGTCGAGCGGACCAAATCGGAGCTTGCTCTGATTTGGTC
AGCGGCGGACGGGTGAGTAACACGTGGGCAACCTGCCCGCAAGACCGGGATAACTCCGGGAAACCGGA
GCTAATACCGGATAACACCGAAGACCGCATGGTCTTTGGTTGAAAGGCGGCCTTTGGCTGTCACTTGC
GGATGGGCCCGCGGCGCATTAGCTAGTTGGTGAGGTAACGGCTCACCAAGGCGACGATGCGTAGCCGG
CCTGAGAGGGTGACCGGCCACACTGGGACTGAGACACGGCC
>Zotu213
GAACGCTGGCGGCGTGCTTAACACATGCAAGTCGAACGGTGAAGGGATCAGTTTTCTGGTTCTGGATG
AGTGGCGAACGGGTGAGTAACACGTGAGTAACCTGCCCCCTTCTTCTGGATAACCGCATGAAAGTGTG
GCTAATACGGGATATTCTGCGCCTGCTGCATGGTGGGTGTGGGAAAGATATTGTTTCGGTGGGGGATG
GGCTCGCGGCCTATCAGCTTGTTGGTGGGGTGATGGCCTACCAAGGCGGTGACGGGTAGCCGGCCTGA
GAGGGTGGACGGTCACACTGGGACTGAGACACGGCC
>Zotu214
GAACGCTGGCGGCGTGCTTAACACATGCAAGTCGAACGGGATTTAGTAAACAGAAGCCTCGGTGGAAG
ATTACTAATGAGAGTGGCGAACGGGTGAGTAACGCGTGAGCAACCTGCCTATGACAGTGGGATAGCCT
CGGGAAACCGGGATTAATACCGCATAAAATCGTAGAAACACATGTTTCAACGGTCAAAGATTTATCGG
TCATAGATGGGCTCGCGTCTGATTAGCTAGTTGGTGAGATAACAGCCCACCAAGGCGACGATCAGTAG
CCGGTCTGAGAGGATGAACGGCCACATTGGAACTGAGACACGGTC
>Zotu215
GAACGCTAGCGGCAGGCCTAACACATGCAAGTCGAGGGAGAAGCCCTTCGGGGCGGAAACCGGCGCAC
GGGTGCGTAACGCGTATGCAACCTACCTTTCACAGGGGGATAGCCCGGAGAAATTTGGATTAATATCC
CATAATATTGTTGGATGGCATCATTTGGCAATTAAAGTTACGATGGTGGAAGATGGGCATGCGTCCTA
TTAGCTAGTTGGCGAGGTAACGGCTCGCCAAGGCGACGATAGGTAGGGGTCCTGAGAGGGAGATCCCC
CACACTGGTACTGAGACAGGGAC
>Zotu216
GAACGCTGGCGGCGTGCCTAATACATGCAAGTCGAGCGAACAGACGAGGAGCTTGCTCCTTTGACGTT
AGCGGCGGACGGGTGAGTAACACGTAGGTAACCTACCTATAAGACTGGGATAACTTCGGGAAACCGGA
GCTAATACCGGATAATATTTCGAACCGCATGGTTCGATAGTGAAAGATGGCTTTGCTATCACTTATAG
ATGGACCTGCGCCGTATTAGCTAGTTGGTAAGGTAACGGCTTACCAAGGCAACGATACGTAGCCGACC
TGAGAGGGTGATCGGCCACACTGGAACTGAGACACGGTC
>Zotu217
GAACGCTGGCGGCATGCCTAATACATGCAAGTTGAACGATGATATTCTCTGCTTGCAGAGAATTGAAG
AGTAGCGAACGGGTGAGCAACACGTGGGGAACCTGCCCATGAGAGGGGGATAACATTCGGAAACGGAT
GCTAATACCGCATAATGCTTTGGACCGCCTGGTCCAGAGACGAAAGACGGCTTTTGCTGTCACTCATG
GATGGCCCCGCGCCGTATTAGCTAGTTGGTGAGGTAATGGCTCACCAAAGCTGTGATACGTAGCCGAC
CTGAGAGGGTGATCGGCCACACTGGGACTGAGACACGGCC
>Zotu218
AAACGCTGGCGGCATGCCTAACACATGCAAGTCGAACGGCAGCACAGTAAAGATTTCGGTCTTTACGG
GTGGCGAGTGGCGGATGGGTGAGTAAAGCGTAGGAATCTGTCCTGGAGAGTGGAATAACCCGAGGAAA
CTCGGGCTAATACCGCATGATATCGAGAGATCAAAGCAGGGGACCCTTCGGGGCCTTGCGCTCTAGGA
TGAGCCTACGTCGGATTAGCTAGTTGGTGGGGTAAAGGCCTACCAAGGCGACGATCCGTAGCTGGTCT
GAGAGGATGACCAGCCACACTGGGACTGAGACACGGCC
>Zotu219
GAACGCTGGCGGCGTGCTTAACACATGCAAGTCGAACGGAAAGGCTCCTGCTTGCAGGGGTACTCGAG
TGGCGAACGGGTGAGTAACACGTGGGTGATCTGCCCTGCACTTCGGGATAAGCCTGGGAAACTGGGTC
TAATACCGGATAGGACCACATTTTGGATGGTGTGGTGGAAAGTTTTTCGGTGTGGGATGAGCTCGCGG
CCTATCAGCTTGTTGGTGGGGTAATGGCCTACCAAGGCGTCGACGGGTAGCCGGCCTGAGAGGGTGTA

CGGCCACATTGGGACTGAGATACGGCC
>Zotu221
GAACGCTGGCGGCGTGCTTAACACATGCAAGTCGAACGATGAAGCTCCAGCTTGCTGGGGTGGATTAG
TGGCGAACGGGTGAGTAATACGTGAGTAACCTGCCCTTGACTCTGGGATAAGCCTGGGAAACTGGGTC
TAATACTGGATACTACACATTCTCGCATGGGGGTGTGTGGAAAGGGTTTTACTGGTTTTGGATGGGCT
CACGGCCTATCAGCTTGTTGGTGGGGTAATGGCCTACCAAGGCGACGACGGGTAGCCGGCCTGAGAGG
GTGACCGGCCACACTGGGACTGAGACACGGCC
>Zotu222
GAACGCTGGCGGCATGCCTAACACATGCAAGTCGAACGAACCCTTCGGGGTTAGTGGCGCACGGGTGC
GTAACGCGTGGGAATCTGCCCTTTGCTTCGGAATAACTCAGGGAAACTTGTGCTAATACCGGATGATG
TCTTCGGACCAAAGATTTATCGGCAAGGGATGAGCCCGCGTAGGATTAGGTAGTTGGTGGGGTAAAGG
CCTACCAAGCCGACGATCCTTAGCTGGTCTGAGAGGATGATCAGCCACACTGGGACTGAGACACGGCC
>Zotu223
GAACGCTATCGGTATGCTTAACACATGCAAGTCGGACGAATTAATTAGGGCTTGCCCTAATTAATTAG
TGGCAGACGGGTGAGTAACACGTGGGAATCTACCCATCAGTACGGAATAACTTTTAGAAATAAAAACT
AATACCGTATATTCTCTAAGGAGGAAAGATTTATCGCTGATGGATGAGCCCGCGCCAGATTAGGTAGT
TGAAGTAATGGCTCACCAAGCCAACGATCTGTAGCTGGTCTGAGAGGATGATCAGCCACACTGGGACT
GAGACACGGCC
>Zotu224
GAACGCTATCGGTATGCTTAACACATGCAAGTCGGACGAATTAATTAGGGCTTGCCCTAATTAATTAG
TGGCAGACGGGTGAGTAACACGTGGGAATCTACCCATCAGTACGGAATAACTTTTAGAAATAAAAGCT
AATACCGTATATTCTCTAAGGAGGAAAGATTTATCGCTGATGGATGAGCCCGCGCCAGATTAGGTAGT
TGGTGAAGTAATGGCTCACCAAGCCAACAATCTGTAGCTGGTCTGAGAGGATGATCAGCCACACTGGG
ACTGAGACACGGCC
>Zotu225
GAACGCTAGCGGCAGGCCTAATACATGCAAGTCGGACGGGATTGGAGAGCTTGCTTTCCATGAGAGTG
GCGCACGGGTGCGTAACACGTATGCAACCTACCTTTGTCAGGGGGATAGCCTCTCGAAAGAGAGATTA
AGACCGCATAACATTATTGAGTGGCATCATTTAATAATCAAATATTTATAGGACAAAGATGGGCATGC
GGAACATTAGCTAGTTGGTGCGGTAACGGCGTACCAAGGCTACGATGTTTAGGGGATCTGAGAGGATG
ACCCCCCACACTGGTACTGAGACACGGAC
>Zotu226
GAACGCTGGCGGCGTGCTTAACACATGCAAGTCGAACGGGATTTAGTAAACAGAAGCCTCGGTGGAAG
ATTACTAATGAGAGTGGCGAACGGGTGAGTAACGCGTGAGCAACCTGCCTATGACAGTGGGATAGCCT
CGGGAAACCGGGATTAATACCGCATAAAATCGTAGAAACACATGTTTTAACGGTCAAAGATTTATCGG
TCATAGATGGGCTCGCGTCTGATTAGCTAGTTGGTGAGATAACAGCCCACCAAGGCGACGATCAGTAG
CCGGTCTGAGAGGATGAACGGCCACATTGGAACTGAGACACGGTC
>Zotu227
GAACGCTGGCGGCGTGCTTAACACATGCAAGTCGAACGGGATCCGGGCTTTCGGGTTTGGTGAGAGTG
GCGAACGGGTGAGTAACACGTGAGTAACCTGCCCCCTTCTTTGGGATAACGTCGGGAAATCGGCGCTA
ATACTGGATATTCTTGCTGCATCGCATGGTGTGGTTTGGAAAGGGTTTCTGGTGGGGGATGGGCTCGC
GGCCTATCAGCTTGTTGGTGGGGTGATGGCCTACCAAGGCTTTGACGGGTAGCCGGCCTGAGAGGGTG
TCCGGCCACACTGGGACTGAGACACGGCC
>Zotu228
GAACGCTGGCGGCGTGCCTAATACATGCAAGTAGAACGCTGAAGGAGGAGCTTGCTTCTCTGGATGAG
TTGCGAACGGGTGAGTAACGCGTAGGTAACCTGCCTGGTAGCGGGGGATAACTATTGGAAACGATAGC
TAATACCGCATAACAGTAGATGTTGCATGACATTTGCTTAAAAGGTGCAATTGCATCACTACCAGATG
GACCTGCGTTGTATTAGCTAGTTGGTGGGGTAACGGCTCACCAAGGCGACGATACATAGCCGACCTGA
GAGGGTGATCGGCCACACTGGGACTGAGACACGGCC
>Zotu229
GAATGCTGGCGGCGTGGATAAGTCATGCAAGTCAAGGGGGCCCGCAAGGGCAACCGGCAGACGAGGTA
GTAACACGTAGGTAGATCCCCCGAAGTCGGGCATAGCCAGTCGAAAGACTGGGTAATTCCCGATAGTC
CCGCAAGGGTAAAGGTTTTTCGCTTCGGGAGTTGCCTGCGTAGTATCAGCTAGTTGGTAGGGTAACGG
CCTACCAAGGCTACGACGCTTAGGGGAGCTGAGAGGCTGACCCCCACCGATGGAACTGCGACACGGTC
>Zotu230

GAACGCTGGTGGCATGCTTAACACATGCAAGTCGAACGGTACAGGACTAGCTTGCTAGTTGCTGACGA
GTGGCGGACGGGTGAGTAACGCGTAGGAATCTGCCCATCTGAGGGGGATACCAGTTGGAAACGACTGT
TAATACCGCATAGTATCTGTGGATTAAAGGTGGCTTTTGGGCTGTCGCAGATGGATGAGCCTGCGTTG
GATTAGCTAGTTGGTGGGGTAAGGGCCTACCAAGGCTACGATCCATAGCTGATTTGAGAGGATGATCA
GCCACATTGGAACTGAGACACGGCC
>Zotu231
GAACGCTGGCGGCGTGCCTAATACATGCAAGTCGAGCGAACAGACGAGGAGCTTGCTCCTCTGATGTT
AGCGGCGGACGGGTGAGTAACACGTGGATAACCTACCTATAAGACTGGGATAACTTCGGGAAACCGGA
GCTAATACCGGATAACATGTTGAACCGCATGGTTCAACAGTGAAAGACGGTCTTGCTGTCACTTATAG
ATGGATCCGCGCCGCATTAGCTAGTTGGTAAGGTAACGGCTTACCAAGGCAACGATGCGTAGCCGACC
TGAGAGGGTGATCGGCCACACTGGAACTGAGACACGGTC
>Zotu232
GAACGCTGGCGGCGTGCTTAACACATGCAAGTCGAACGGAAAGGCCCTGCTTGCAGGGTGCTCGAGTG
GCGAACGGGTGAGTAACACGTGGGTGATCTGCCCCTTATTTGGGATAAGCCTGGGAAACTGGGTCTA
ATACTGGATAGGACCATGCTGTAGGTGGTGTGGTGGAAAGATTAGTTTCGGTAAGGGATGAGCTCGCG
GCCTATCAGCTTGTTGGTGGGGTAATGGCCTACCAAGGCGTCGACGGGTAGCCGGCCTGAGAGGGTGG
ACGGCCACATTGGGACTGAGATACGGCC
>Zotu233
GAACGCTAGCGGCAGGCCTAATACATGCAAGTCGGACGGGATTCAGGGAGTAGCTTGCTACGAACTGA
TGAGAGTGGCGCACGGGTGCGTAACGCGTGAGCAACCTACCCATATCAGGGGGATAGCCTCTCGAAAG
AGAGATTAACACCGCATAACATCATTAACCGGCATCGGTTTTTGATCAAATATTTATAGGATATGGAT
GGGCTCGCGTGACATTAGCTAGTTGGAGAGGTAACGGCTCACCAAGGCTACGATGTCTAGGGGCTCTG
AGAGGAGAATCCCCCACACTGGTACTGAGACACGGAC
>Zotu234
GAACGCTGGCGGCAGGCCTAACACATGCAAGTCGAGCGAGCATTTCGGTGCTAGCGGCGGACGGGTGA
GTAACGCGTGGGAACGTGCCCTTTTCTGCGGAATAGTCCTGGGAAACTGGGGGTAATACCGCATACGC
CCTTTTGGGGAAAGATTTATCGGAGAAGGATCGGCCCGCGTTGGATTAGGTAGTTGGTGGGGTAATGG
CCCACCAAGCCGACGATCCATAGCTGGTTTGAGAGGATGATCAGCCACACTGGGACTGAGACACGGCC
>Zotu235
CAACGCTGGCGGCGCGCTTAACACATGCAAGTCGCACGAGAAAGGGGAGCAATCCCTGAGTAAAGTGG
CGTACGGGTGAGTAACACGTGGATGATCTACCTTCTAGTGGGGAATAACCCTGGGAAACCGGGGCTAA
TACCGCATAAGCTCGAGAGAGGAAAGCAGCAATGCGCTGGAAGAGGAGTCCGCGGCCGATTAGCTAGT
TGGCAGGGTAAGAGCCTACCAAGGCAGAGATCGGTAGCCGGCCTGAGAGGGCACACGGCCACACTGGC
ACTGAAACACGGGC
>Zotu236
AAACGCAAGAGACGTGCCTAACACATGCAAATTAAATAGTTAATAGTAACTATAGTGAATTGGTGAGT
AATATATGAAAATTTAAATTTAGATTAGAAGTAATCCTAATAAATTAGGAGTAATCTCTTATAAGTGC
ATAAGTGCTTTTAAAAGTTAATACTGTCTAAAGAAAAGTTCATATCTGATTAGCTAGTTAGTAGGGTA
AAGGCCTACTAAGGCGAAGATCAGTAGCTACCCTGAGAGGGGGAATAGCCACATTGGGATTGAAATAC
AGCC
>Zotu237
GAACGCTAGCGGCAGGCCTAATACATGCAAGTCGAACGGGATTGAAGAGCTTGCTTTTCATGAGAGTG
GCGCACGGGTGCGTAACACGTATGTAACCTACCTTAATCTGGGGGATAGCCTCTCGAAAGAGAGATTA
ACACCGCATAACATTATCGGATAGCATTTTCTGATAATCAAATATTTATAGGATTAAGATGGGCATGC
GGAACATTAGCTAGTTGGTAAGGTAATGGCTTACCAAGGCTACGATGTTTAGGGGATCTGAGAGGATG
ACCCCCCACACTGGTACTGAGACACGGAC
>Zotu238
GAACGCTGGCGGCGTGCCTAATACATGCAAGTAGAACGCTGAAGGAGGAGCTTGCTTCTCTGGATGAG
TTGCGAACGGGTGAGTAACGCGTAGGTAACCTGCCTGGTAGCGGGGGATAACTATTGGAAACGATAGC
TAATACCGCATAAGAGTAGATGTTGCATGACATTTGCTTAAAAGGTGCAATTGCATCACTACCAGATG
GACCTGCGTTGTATTAGCTAGTTGGTGAGGTAACGGCTCACCAAGGCGACGATACATAGCCGACCTGA
GAGGGTGATCGGCCACACTGGGACTGAGACACGGCC
>Zotu239
GAACGCTAGCGGCAGGCTTAATACATGCAAGTCGTGGGGCAGCGCAGGTAGCAATACTGGGCGGCGAC

CGGCAAACGGGTGCGGAACACGTACACAACCTTCCTACAAGTGGGGAATAGCCCAGAGAAATTTGGAT
TAATACCCCGTAACATAGCGATGTGGCATCACATTGCTATTATAGCTTCGGCGCTTGTTGATGGGTGT
GCGGCTGATTAGATAGTTGGCGGGGTAACGGCCCACCAAGTCTACGATCAGTAGCTGATGTGAGAGCA
TGATCAGCCACACGGGCACTGAGACACGGGC
>Zotu240
GAACGCTGGCGGCAGGCTTAACACATGCAAGTCGAGCGCCCCGCAAGGGGAGCGGCAGACGGGTGAGT
AACGCGTGGGAACGTACCTTTTGGTTCGGAACAACACAGGGAAACTTGTGCTAATACCGGATAAGCCC
TTACGGGGAAAGATTTATCGCCGAAAGATCGGCCCGCGTCTGATTAGCTTGTTGGTGAGGTAACGGCT
CACCAAGGCGACGATCAGTAGCTGGTCTGAGAGGATGATCAGCCACATTGGGACTGAGACACGGCC
>Zotu241
GAACGCTGGCGGCATGCCTTACACATGCAAGTCGAACGGTAACAGGTCTTCGGATGCTGACGAGTGGC
GAACGGGTGAGTAATACATCGGAACGTGCCCGAGAGTGGGGGATAACGAAGCGAAAGCTTTGCTAATA
CCGCATACGATCTCAGGATGAAAGCAGGGGACCGCAAGGCCTTGCGCTCACGGAGCGGCCGATGGCAG
ATTAGGTAGTTGGTGGGATAAAAGCTTACCAAGCCGACGATCTGTAGCTGGTCTGAGAGGACGACCAG
CCACACTGGGACTGAGACACGGCC
>Zotu242
GAACGCTGGCGGCAGGCCTAACACATGCAAGTCGAACGGCAGCACAGTAAGAGCTTGCTCTTATGGGT
GGCGAGTGGCGGACGGGTGAGGAATACATCGGAATCTACTCTTTCGTGGGGGATAACGTAGGGAAACT
TACGCTAATACCGCATACGACCTACGGGTGAAAGCGGAGGACCTTCGGGCTTCGCGCGATTGAATGAG
CCGATGTCGGATTAGCTAGTTGGCGGGGTAAAGGCCCACCAAGGCGACGATCCGTAGCTGGTCTGAGA
GGATGATCAGCCACACTGGAACTGAGACACGGTC
>Zotu243
GAACGCTGGCGGCATGCCTAACACATGCAAGTCGAACGAGACCTTCGGGTCTAGTGGCGCACGGGTGC
GTAACGCGTGGGAATCTGCCTTTCGGTTCGGAATAACTCGGGGAAACCCGTGCTAATACCGGATGATG
TCGCAAGACCAAAGATTTATCGCCGAAAGATGAGCCCGCGTCGGATTAGCTAGTTGGTGGGGTAAAGG
CCTACCAAGGCGACGATCCGTAGCTGGTCTGAGAGGATGATCAGCCACACTGGGACTGAGACACGGCC
>Zotu244
GAACGCTGGCGGCGCGCTTAACACATGCAAGTCGAGCGAGGCCCCGGGCTTGCCCGGGTGTCCTAGCG
GCGAACGGGTGAGTAACACGTGAGGAACCTGCCCCGAAGACCGGGATAACACCGGGAAACCGGTGCTA
ATACCGGATGCCCTCATTGGATCGCATGGTCCGATGAGGAAATGGATTCCGCTTCGGGATGGTCTCGC
GGCCTATCAGCTTGTTGGTGGGGTAACGGCCTACCAAGGCAGCGACGGGTAGCTGGTCTGAGAGGATG
TCCAGCCACACTGGGACTGAGACACGGCC
>Zotu245
GAACGCTGGCGGCAGGCTTAACACATGCAAGTCGAGCGCCCCGCAAGGGGAGCGGCAGACGGGTGAGT
AACGCGTGGGAACATGCCCTTCGGTTCGGAATAACTCAGGGAAACTTGAGCTAATACCGGATACGTCC
GTAAGGAGAAAGATTTATTGCCGAAGGATTGGCCCGCGTCCGATTAGCTAGTTGGTGAGGTAACGGCT
CACCAAGGCGACGATCGGTAGCTGGTCTGAGAGGATGATCAGCCACACTGGGACTGAGACACGGCC
>Zotu246
GAACGCTGGCTGCGTGCTTAACACATGCAAGTCGAACGCTGAAGCCTGGTGCTTGCACCGGGTGGATG
AGTGGCGAACGGGTGAGTAACACGTGAGTAACCTGCCCCTGACTTCGGGATAAGCCCGGGAAACTGGG
TCTAATACCGGATACGACCGTCGGACGCATGTCTGGTGGTGGAAAGTTTTTTCGGTTGGGGATGGGCT
CGCGGCCTATCAGTTTGTTGGTGAGGTAATGGCTCACCAAGACGACGACGGGTAGCCGGCCTGAGAGG
GCGACCGGCCACACTGGGACTGAGACACGGCC
>Zotu247
GAACGCTGGCGGCGTGCTTAACACATGCAAGTCGAACGGTGAACACGGAGCTTGCTCTGTGGGATCAG
TGGCGAACGGGTGAGTAACACGTGAGCAACCTGCCCCTGACTCTGGGATAAGCGCTGGAAACGGCGTC
TAATACTGGATATGTGACGTGACCGCATGGTCTGCGTCTGGAAAGAATTTCGGTTGGGGATGGGCTCG
CGGCCTATCAGCTTGTTGGTGAGGTAATGGCTCACCAAGGCGTCGACGGGTAGCCGGCCTGAGAGGGT
GACCGGCCACACTGGGACTGAGACACGGCC
>Zotu248
GAACGCTGGCGGCGTGCCTAATACATGCAAGTAGAACGCTGAAGAGAGGAGCTTGCTCTTCTTGGATG
AGTTGCGAACGGGTGAGTAACGCGTAGGTAACCTGCCTGGTAGCGGGGGATAACTATTGGAAACGATA
GCTAATACCGCATGAAATTGCTTATCGCATGATAATTAATTGAAAGATGCAATTGCATCACTACCAGA
TGGACCTGCGTTGTATTAGCTAGTTGGTGAGGTAACGGCTCACCAAGGCGACGATACATAGCCGACCT

GAGAGGGTGATCGGCCACACTGGGACTGAGACACGGCC
>Zotu249
CGTCAGGTCCCAGTGCGCTTGGTTAATTACACCTTAGAAGACCTCTCCCTGCCGCGAAGAAGGGCAGT
AGCGCAATTCATCCCGGCGAGCATTACAGAAATCGGACAAACGGAGAGTATCGGACTAACAGGA
>Zotu250
GAACGCTGGCGGCGTGCTTAACACATGCAAGTCGAACGATGAAGCCCAGCTTGCTGGGTGGATTAGTG
GCGAACGGGTGAGTAACACGTGGGTGATCTGCCCTGCACTCTGGGATAAGCCTGGGAAACTGGGTCTA
ATACCGGATATGACCTCTTGCTGCATGGCGAGGGGTGGAAAGTTTTTCGGTGCAGGATGAGCCCGCGG
CCTATCAGCTTGTTGGTGGGGTAATGGCCTACCAAGGCGACGACGGGTAGCCGGCCTGAGAGGGCGAC
CGGCCACACTGGGACTGAGACACGGCC
>Zotu251
GAACGCTGGCGGCATGCCTAATACATGCAAGTCGAGCGAACAGATGAAGTGCTTGCACTTCTGACGTT
AGCGGCGAACGGGTGAGTAACACGTAAGGAATCTACCTATAAGCGGGGGATAACATTCGGAAACGGGT
GCTAATACCGCATAATATCTTCTTCCGCATGGAAGAAGATTGAAAGACGGCTCTGCTGTCACTTATAG
ATGACCTTGCGGTGCATTAGTTAGTTGGTGGGGTAATGGCCTACCAAGACGATGATGCATAGCCGACC
TGAGAGGGTGATCGGCCACATTGGGACTGAGACACGGCC
>Zotu252
GAACGCTATCGGTATGCTTAACACATGCAAGTCGAACGGACTAATTGGGGCTTGCTCCAATTAGTTAG
TGGCAGACGGGTGAGTAACACGTGGGAATCTACCCATCAGTACGGAATAACTTTTAGAAATAAAAGCT
AATACCGTATATTCTCTACGGAGGAAAGATTTATCGCTAATGGATGAGCCCGCGTCAGATTAGGTAGT
TGGTGAGGTAACGGCTCACCAAGCCGACGATCTGTAGCTGGTCTGAGAGGATGATCAGCCACACTGGG
ACTGAGACACGGCC
>Zotu253
GAACGCTGGCGGCATGCTTAACACATGCAAGTCGGACGGGAAGTGGTGTTTCCAGTGGCGGACGGGTG
AGTAACGCGTAAGAACCTGCCCTTGGGAGGGGAACAACAGCTGGAAACGGCTGCTAATACCCCATAGG
CTGAGGAGCAAAAGGAGGAATCCGCCCAAGGAGGGGCTCGCGTCTGATTAGTTAGTTGGTGAGGCAAT
GGCTTACCAAGGCGACGATCAGTAGCTGGTCCGAGAGGATGATCAGCCACACTGGGACTGAGACACGG
CC
>Zotu254
GAACGCTGGCGGCAGGCCTAACACATGCAAGTCGAACGAACTCTTCGGAGTTAGTGGCGGACGGGTGA
GTAACACGTGGGAACGTGCCTTTAGGTTCGGAATAACTCAGGGAAACTTGTGCTAATACCGAATGTGC
CCTTCGGGGGAAAGATTTATCGCCTTTAGAGCGGCCCGCGTCTGATTAGCTAGTTGGTGAGGTAAAGG
CTCACCAAGGCGACGATCAGTAGCTGGTCTGAGAGGATGATCAGCCACATTGGGACTGAGACACGGCC
>Zotu255
GAACGCTAGCGGCATGCTTAACACATGCAAGTCGAACGGCAGTGCGGGGAGCTTGCTCCCTGGCGGCG
AGTGGCGGACGGGTGAGTAATGCGTAGGAATCTACCTTGTAGTGGGGGATAACCTGGGGAAACTCGGG
CTAATACCGCATGACAGAAGACAGAAGACAGAGGACCGAAGACAGATCATTGTGTCGTTTCGCGACAA
ATTTTCAAAATTTGTCCGCAGTCACACACAATCAGCTGTCTTCTGTCTTCTGTCTTCTGTCTTCTGAA
AGCGGGGGATCTTCGGACCTCGCGCTGTAAGACGAGCCTACGTCGGATTAGCTTGTTGGTGGGGTAAT
GGCCTACCAAGGCGACGATCCGTAGCTGGTCTGAGAGGACGATCAGCCACACTGGGACTGAGACACGG
CC
>Zotu256
GAACGCTGGCGGCGTGCTTAACACATGCAAGTCGAACGATGAACCTCCTTCGGGAGGGGATTAGTGGC
GAACGGGTGAGTAACACGTGGGCAATCTGCCCTTCACTCTGGGACAAGCCCTGGAAACGGGGTCTAAT
ACCGGATACGACTACCGACCGCATGGTCTGGTGGTGGAAAGCTCCGGCGGTGAAGGATGAGCCCGCGG
CCTATCAGCTTGTTGGTGGGGTGATGGCCTACCAAGGCGACGACGGGTAGCCGGCCTGAGAGGGCGAC
CGGCCACACTGGGACTGAGACACGGCC
>Zotu257
GAACGCTGGCGGCGTGCCTAATACATGCAAGTCGAGCGAACGGATAAGGAGCTTGCTCCTTTGAAGTT
AGCGGCGGACGGGTGAGTAACACGTGGGTAACCTACCTATAAGACTGGGATAACTTCGGGAAACCGGA
GCTAATACCGGATAACATTTAGAACCGCATGGTTCTAAAGTGAAAGATGGTTTTGCTATCACTTATAG
ATGGACCCGCGCCGTATTAGCTAGTTGGTAAGGTAACGGCTTACCAAGGCGACGATACGTAGCCGACC
TGAGAGGGTGATCGGCCACACTGGAACTGAGACACGGTC
>Zotu258

GAACGCTGGCGGTATGCTTAACACATGCAAGTCGAACGGTGCTTTAGGGCACAGTGGCGGACGGGTGA
GTAACGCGTGAGAATCTGGCTTCAGGTCTGGGACAACCACTGGAAACGGTGGCTAATACCGGATGTGC
CGAGAGGTGAAAGGTTAACTGCCTGAAGATGAGCTCGCGTCTGATTAGCTAGTTGGAAGTGTAATGGA
CTCCCAAGGCGACGATCAGTAGCTGGTCTGAGAGGACGATCAGCCACACTGGGACTGAGACACGGCC
>Zotu259
GAACGCTAGCTACAGGCTTAACACATGCAAGTCGAGGGGCAGCGGGAAGCAAGCTTGCTTGCTTTGCC
GGCGACCGGCGTACGGGTGCGTAACGCGTACCGAACCTACCTCACACTCCGGGATAGCCCTGCGAAAG
CAGGATTAATACCGGGTAGCCTCACTATATCGCATGATATTATGAGTAAAGATTTATTGGTGTGAGAT
GGCGGTGCGTCCCATTAGTTAGTTGGCGGGGTAACGGCCCACCAAGACGACGATGGGTAGGGGTTCTG
AGAGGAAGGTCCCCCACATTGGAACTGAGACACGGTC
>Zotu260
GAACGCTAGCGGCAGGCTTAACACATGCAAGTCGAGGGGTATAGTTCTTCGGAGCTAGAGACCGGCGC
ACGGGTGCGTAACGCGTATGCAATCTACCTTTTACAGAGGGATAGCCCAGAGAAATTTGGATTAATAC
CTCATAGTATGTGAGAATCGCATGGTTTTCACATTAAAGTCACAACGGTAAAAGATGAGCATGCGTCC
CATTAGCTTGTTGGTAAGGTAACGGCTTACCAAGGCAACGATGGGTAGGGGTCCTGAGAGGGAGATCC
CCCACACTGGTACTGAGACACGGAC
>Zotu261
AAACGCTGGCGGCATGCCTAACACATGCAAGTCGAACGGCAGCACAGTAAAGATTTCGGTCTTTACGG
GTGGCGAGTGGCGGATGGGCGAGTAAAGCGTAGGAATCTGTCCTGGAGAGTGGGATAACCCGAGGAAA
CTCGGGCTAATACCGCATGATATCGAGAGATCAAAGCAGGGGACCCTTCGGGGCCTTGCGCTCTAGGA
TGAGCCTACGTCGGATTAGCTAGTTGGTGGGGTAAAGGCCTACCAAGGCGACGATCCGTAGCTGGTCT
GAGAGGATGACCAGCCACACTGGGACTGAGACACGGCC
>Zotu262
GAACGCTGGCGGCGTGCCTAATACATGCAAGTCGAGCGGATGAATCGAAAGCTTGCTTTTGATTCGTT
AGCGGCGGACGGGTGAGTAACACGTGGGCAACCTGCCCTGTAGACGGGGATAACACCGAGAAATCGGT
GCTAATACCGGATAACACGAAATGTCGCATGACGTTTCGTTGAAAGACGGCGCAAGCTGTCGCTACAG
GATGGGCCCGCGGCGCATTAGCTAGTTGGTGAGGTAACGGCTCACCAAGGCGACGATGCGTAGCCGAC
CTGAGAGGGTGATCGGCCACACTGGGACTGAGACACGGCC
>Zotu263
GAATGCTGGCGGCGTGGATAAGGCATGCAAGTCAAGGGGGCCGCAAGGCAACCGGCAGACGAGGTAGT
AACACGTAGGTACGTACTCCAGAGTCGGGCATAGCCAGCCGAAAGGTTGGGTAATTCCCGATGGTCCC
GCAAGGGTAAAGGTTTTTCGCTCTGGAAGCGGCCTGCGCAGTATCAGCTAGTTGGTAAGGTAATGGCT
TACCAAGGCTACGACGCTTAGGGGAGCTGAGAGGCTGACCCCCACCGATGGGACTGAGAGACGGCC
>Zotu264
GAACGCTGGCGGCGTGCTTAACACATGCAAGTCGAACGGAAAGGCCCTTCGGGGTGCTCGAGTGGCGA
ACGGGTGAGTAACACGTGGGTGATCTGCCCTGCACTTTGGGATAAGCCTGGGAAACTGGGTCTAATAC
CGAATATGATCATCGGCTTCATGGTCGGTGGTGGAAAGCTTTTGCGGTGTGGGATGGGCCCGCGGCCT
ATCAGCTTGTTGGTGGGGTAATGGCCTACCAAGGCGTCGACGGGTAGCCGGCCTGAGAGGGTGTCCGG
CCACACTGGGACTGAGATACGGCC
>Zotu266
GAACGCTGGCGGCGTGCTTAACACATGCAAGTCGAACGATGAACCACTTCGGTGGGGATTAGTGGCGA
ACGGGTGAGTAACACGTGGGCAATCTGCCCTTCACTTCGGGACAAGCCCTGGAAACGGGGTCTAATAC
CGGATATCACTTCCATTCGCATGGGTGGGGGTTGAAAGCTCCGGCGGTGAAGGATGAGCCCGCGGCCT
ATCAGCTTGTTGGTGAGGTAATGGCTCACCAAGGCGACGACGGGTAGCCGGCCTGAGAGGGCGACCGG
CCACACTGGGACTGAGACACGGCC
>Zotu267
GAACGCTGGCGGCGTGCCTAATACATGCAAGTCGAGCGGACCGAACGAGAGCTTGCTCTTGTTTGGTC
AGCGGCGGACGGGTGAGTAACACGTGGGCAACCTGCCCGCAAGACCGGGATAACTCCGGGAAACCGGA
GCTAATACCGGATAACACCAAAGACCGCATGGTCTTTGGTTGAAAGGCGGCTTCGGCTGTCACTTGCG
GATGGGCCCGCGGCGCATTAGCTAGTTGGTGAGGTAACGGCTCACCAAGGCGACGATGCGTAGCCGGC
CTGAGAGGGTGACCGGCCACACTGGGACTGAGACACGGCC
>Zotu268
GAACGCTGGCGGCGTGCTTAACACATGCAAGTCGAACGATGAAGCCTTAGCTTGCTTTGGTGGATTAG
TGGCGAACGGGTGAGTAACACGTGAGCAACGTGCCCAGGACTCTGGAATAACCGCCGGAAACGGTGGC

TAATACTGGATAGGTGCCAAGGCCGCATGGTCTTTGGTTGGAAAGATTTTTCGGTTCTGGATCGGCTC
GCGGCCTATCAGCTTGTTGGTGGGGTAATGGCCTACCAAGGCGACGACGGGTAGCCGGCCTGAGAGGG
TGACCGGCCACACTGGGACTGAGACACGGCC
>Zotu269
GAACGCTGGCGGCATGCTTAACACATGCAAGTCGAACGCAGTACTCAAGCTTGCTTGAGTAGCTGAGT
GGCAGACGGGTGAGTAACACGTGGGAACTTACCTTTTAGACTGGGACAACCACTGGAAACGGTGGCTA
ATACCGGATACGCCCTGAGGGGGAAAGCAGAAATGCGCTAAAGGAGAGGCCCGCGGCGGATTAGGTAG
TTGGTGGGGTAAAGGCCTACCAAGCCGATGATCTGTAGCTGGTTTGAGAGGATGATCAGCCACACTGG
GACTGAGACACGGCC
>Zotu270
GAACGCTGGCGGTGTGCTTCACACATGCAAGTCGAACGGGGTAGCAATACCCAGTGGCGGACGGGTGA
GTAACACGTGGGAATCTACCATTAGATGGGGGATAGCAGGCCGAAAGGCCTATTAATACCGCATATGT
AGGCATCTGGACACAGGTGTTTATGAAAGGAGTAATCCGTCTGATGATGAGCCCGCGCCCGATTAGCT
AGTTGGTAGGGTAAAAGCCTACCAAGGCGACGATCGGTAGGTGGTCTGAGAGGATGATCACCCACAAT
GGGACTGAGACACGGCC
>Zotu271
GAACGCTAGCTACAGGCTTAACACATGCAAGTCGAGGGGAAACGGCATTGAGTGCTTGCACTCTTTGG
ACGTCGACCGGCGCACGGGTGAGTAACGCGTATCCAACCTTCCCATTACTGTGGGATAACCTGCCGAA
AGGCAGACTAATACCGCATAGTCTTCGATGACGGCATCAGATTTGAAGTAAAGATTTATCGGTAATGG
ATGGGGATGCGTCTGATTAGCTTGTTGGCGGGGTAACGGCCCACCAAGGCAACGATCAGTAGGGGTTC
TGAGAGGAAGGTCCCCCACATTGGAACTGAGACACGGTC
>Zotu272
GAACGCTGGCGGCAGGCTTAACACATGCAAGTCGAACGGGCGTAGCAATACGTCAGTGGCAGACGGGT
GAGTAACACGTGGGAACGTACCTCTTGGTTCGGAACAACACAGGGAAACTTGTGCTAATACCGGATAA
GCCCTTACGGGGAAAGATTTATCGCCGAGAGATCGGCCCGCGTCTGATTAGCTAGTTGGTGAGGTAAC
GGCTCACCAAGGCGACGATCAGTAGCTGGTCTGAGAGGATGATCAGCCACATTGGGACTGAGACACGG
CC
>Zotu273
GAACGCTGGCGGTATGCTTTACACATGCAAGTCGTACGAGAATTTTTAATTCAAGTGGCGGACGGGTG
AGTAACACGTAAGAATTTGCCTTTAGGAGGGGGATAACAACTGGAAACGGTTGCTAATACCCCATATG
CTTTCGAGTGAAATGGAGTTTTCCGCCTAAAGAGAAGCTTGCGGCTGATTAGCTTGTTGGTAAGGTAA
TGGCTTACCAAGGCGACGATCAGTATCTGGTTTGAGAGGACGATCAGACACACTGGAACTGAGACACG
GTC
>Zotu274
GAACGCTGGCGGCGTGCTTAACACATGCAAGTCGAACGATGAAGCTTGGTGCTTGCACTGGGTGGATT
AGTGGCGAACGGGTGAGTAACACGTGAGTAACCTGCCCTTTACTTCGGGATAAGCTTGGGAAACTGGG
TCTAATACCGGATATTCTGCACGGTCGGATGGTTGTGTAGGAAAGATTTTTTGGTAAGGGATGGGCTC
GCGGCCTATCAGTTTGTTGGTGGGGTGATGGCCTACCAAGACGACGACGGGTAGCCGGCCTGAGAGGG
CGACCGGCCACACTGGGACTGAGACACGGCC
>Zotu275
GAACGCTGGCGGCGTGCCTAATACATGCAAGTCGAGCGGACCGAACGAGAGCTTGCTCTTGTTTGGTC
AGCGGCGGACGGGTGAGTAACACGTGGGCAACCTGCCCGCAAGACCGGGATAACTCCGGGAAACCGGA
GCTAATACCGGATAACACCAAAGACCGCATGGTCTTTGGTTGAAAGGCGGCTTCGGCTGCCACTTGCG
GATGGGCCCGCGGCGCATTAGCTAGTTGGTGAGGTAACGGCTCACCAAGGCGACGATGCGTAGCCGGC
CTGAGAGGGTGACCGGCCACACTGGGACTGAGACACGGCC
>Zotu276
GAACGCTGGCGGCGTGCTTAACACATGCAAGTCACGGGGGCCCGCAAGGGTAACCGGCGAACGGGTGC
GTAACACGTGAGCAATCTGCCGATGTCTGGGGGATAGCCGGCCCAACGGCCGGGTAATACCGCATACG
ATCGCTCGGGGGAGTCCCTGGGCGATGAAACCTCCGGGGGACATCGAGGAGCTCGCGCCCTATCAGCT
AGTTGGTGAGGTAATGGCTCACCAAGGCTACGACGGGTAGCTGGTCTGAGAGGATGGCCAGCCACATT
GGGACTGCGACACGGCC
>Zotu277
GAACGCTGGCGGTGTGCTTAACACATGCAAGTCGAACGGTAAGGCCCTTTCGGGGGTACACGAGTGGC
GAACGGGTGAGTAACACGTGAGTAACCTGCCCTCATCTCTGGGATAACAGTCGGAAACGGTTGCTAAT

ACTGGATATGAGCTTGCAAGGCATCTTGTGGGTTGGAAAGATTTTTTTGGTTGGGGATGGACTCGCGG
CCTATCAGCTTGTTGGTGAGGTAGTGGCTCACCAAGGCTTCGACGGGTAGCCGGCCTGAGAGGGCGAC
CGGCCACATTGGGACTGAGATACGGCC
>Zotu278
GAACGCTGGCGGCATGCCTAACACATGCAAGTCGAACGAAGGCTTCGGCCTTAGTGGCGCACGGGTGC
GTAACGCGTGGGAATCTGCCCCTCGGTTCGGAATAACAGTTAGAAATGACTGCTAATACCGGATGATG
ACGTAAGTCCAAAGATTTATCGCCGAGGGATGAGCCCGCGTAGGATTAGCTAGTTGGTGTGGTAAGAG
CGCACCAAGGCGACGATCCTTAGCTGGTCTGAGAGGATGATCAGCCACACTGGGACTGAGACACGGCC
>Zotu279
GAACGCTAGCGGCAGGCCTTACACATGCAAGTCGAGGGGTAACAGGGAAAAGCTTGCTTTTCCGCTGA
CGACCGGCGCACGGGTGCGTAACACGTATGCAACCTACCTCTTACTGAGGAATAGCCCAGAGAAATTT
GGATTAATACCTCATAGTATCACCAGTTCGCATGTTCAGGTGATTAAAGGTTACGGTAAGAGATGGGC
ATGCGCCCCATTAGCTAGTTGGTGAGGTAATGGCTCACCAAGGCAACGATGGGTAGGGGGTCTGAGAG
GACTATCCCCCACACTGGTACTGAGACACGGAC
>Zotu280
GAACGCTGGCGGCGTGCTTAACACATGCAAGTCGAACGGAAAGGCCAGTGCTTGCACTGGTACTCGAG
TGGCGAACGGGTGAGTAACACGTGGGTGATCTGCCTTGTACTCTGGGATAAGCTTGGGAAACTGGGTC
TAATACCGGATAATAACTGCCTTTAGTGTGGTGGTTGGAAAGTTTTTTCGGTGCAAGATGAGCTCGCG
GCCTATCAGCTTGTTGGTGGGGTAATGGCCTACCAAGGCGTCGACGGGTAGCCGGCCTGAGAGGGTGT
ACGGCCACATTGGGACTGAGATACGGCC
>Zotu281
GAACGCTGGCGGCGTGCCTAATACATGCAAGTCGAGCGGACCGAATAGAAGCTTGCTTCTGTTCGGTT
AGCGGCGGACGGGTGAGTAACACGTGGGCAACCTGCCCGTAAGACCGGGATAACTTCGGGAAACCGGA
GCTAATACCGGATAACACCGAAGACCGCATGGTCTTCGGTTGAAAGGCGGCTTTGGCTGTCACTTACG
GATGGGCCCGCGGCGCATTAGCTAGTTGGTGAGGTAACGGCTCACCAAAGCGACGATGCGTAGCCGAC
CTGAGAGGGTGATCGGCCACACTGGGACTGAGACACGGCC
>Zotu282
GAATGCTGGCGGCGCGCCTAACACATGCAAGTCGAGCGGAAAGGCCCTTCGGGGTACTCGAGCGGCGG
ACGGCTGAGTAACGCGTGGGAACATGCCCCAAAGTGAGGAATAACTGCCCGAAAGGGTAGCTAATGCC
GCATATGATCTTAGGATTAAAGGATTTATCCGCTTTGGGAGTGGCCCGCGTACGATTAGGTAGTTGGT
GAGGTAAAGGCTCACCAAGCCCACGATCGTTAACTGGTCTGAGAGGATGATCAGTCAGACTGGAACTG
AGACACGGTC
>Zotu283
GAACGCTGGCGGCGTGCCTAATACATGCAAGTCGAGCGAACAGATAAGGAGCTTGCTCCTTTGACGTT
AGCGGCGGACGGGTGAGTAACACGTGGGTAACCTACCTATAAGACTGGAATAACTCCGGGAAACCGGG
GCTAATGCCGGATAATATTTAGAACCGCATGGTTCTAAAGTGAAAGATGGTTTTGCTATCACTTATAG
ATGGACCCGCGCCGTATTAGCTAGTTGGTGGGGTAATGGCTTACCAAGGCAACGATACGTAGCCGACC
TGAGAGGGTGATCGGCCACACTGGAACTGAGACACGGTC
>Zotu284
GAACGCTGGCGGTCTGCTTAACACATGCAAGTCGAACGGGCTCTTTCGGGAGCTAGTGGCGGACGGGT
GAGTAACGCGTGAGAACCTGCCTTCAGGTCGGGGACAACAGAGGGAAACTTCTGCTAATACCGGATGA
GCCGAAAGGTAAAAGATTTATCGCCTGAAGAGGGGCTCGCGTCTGATTAGCTAGTTGGTGTGGTAAGA
GCGCACCAAGGCGACGATCAGTAGCTGGTCTGAGAGGATGACCAGCCACACTGGGACTGAGACACGGC
C
>Zotu285
GAACGCTGGCGGCGTGCTTAACACATGCAAGTCGAACGATGAAAGAGGAGCTTGCTCCTCTGGATTAG
TGGCGAACGGGTGAGTAACACGTGAGCAATCTGCCCCTGACTCCGGAATAACTACGGGAAACTGTAGC
TAATACCGGATATGCGCCGAACAGGCATCTGTATCGGTGGGAAAGAATTTCGGTCAGGGATGAGCTCG
CGGCCTATCAGCTTGTTGGTGAGGTAACGGCTCACCAAGGCGACGACGGGTAGCCGGCCTGAGAGGGT
GACCGGCCACATTGGGACTGAGACACGGCC
>Zotu286
GAACGCTGGCGGCATGCTTTACACATGCAAGTCGAACGGCAGCGCGGGCTTCGGCCTGGCGGCGAGTG
GCGAACGGGTGAGTAATGCATCGGAACGTGCCCAGTAGTGGGGGATAGCCCGGCGAAAGCCGGATTAA
TACCGCATACGACCTGAGGGTGAAAGCGGGGGACCGAAAGGCCTCGCGCTATTGGAGCGGCCGATGTC

GGATTAGCTAGTTGGTGGGGTAAAGGCTTACCAAGGCGACGATCCGTAGCTGGTCTGAGAGGACGACC
AGCCACACTGGGACTGAGACACGGCC
>Zotu287
GAACGCTGGCGGCGTGCTTAACACATGCAAGTCGAACGGAAAGGCCCAAGCTTGCTTGGGTACTCGAG
TGGCGAACGGGTGAGTAACACGTGGGTGATCTGCCCTGCACTTCGGGATAAGCCTGGGAAACTGGGTC
TAATACCGGATAGGACCGCATTTTGGATGGTGTGGTGGAAAGTTTTTCGGTGTGGGATGAGCTCGCGG
CCTATCAGCTTGTTGGTGGGGTAATGGCCTACCAAGGCGTCGACGGGTAGCCGGCCTGAGAGGGTGTA
CGGCCACATTGGGACTGAGATACGGCC
>Zotu288
GAACGCTGGCGGCGTGCTTAACACATGCAAGTCGAACGGTAAGGCTCCAGCTTGCTGGGGTACACGAG
TGGCGAACGGGTGAGTAACACGTGGGTGACCTGCCCTGCACTTCGGGATAAGCCTGGGAAACTGGGTC
TAATACCGGATAGGACCATGGTGTGGATACTGTGGTGGAAAGTTTTTTCGGTGTGGGATGGGCCCGCG
GCCTATCAGCTTGTTGGTGGGGTAATGGCCTACCAAGGCGGCGACGGGTAGCCGGCCTGAGAGGGTGG
ACGGCCACATTGGGACTGAGACACGGCC
>Zotu289
GAACGCTGGCGGCATGCTTTACACATGCAAGTCGAACGGCAGCGCGGGGCAACCTGGCGGCGAGTGGC
GAACGGGTGAGTAATATATCGGAACGTACCCAAGAGTGGGGGATAACGTAGCGAAAGTTACGCTAATA
CCGCATACGATCCAAGGATGAAAGCAGGGGACCGCAAGGCCTTGTGCTCCTGGAGCGGCCGATATCTG
ATTAGCTAGTTGGTGAGGTAAAGGCTCACCAAGGCGACGATCAGTAGCTGGTCTGAGAGGACGACCAG
CCACACTGGAACTGAGACACGGTC
>Zotu290
GAACGCTGGCGGCAGGCTTAACACATGCAAGTCGAACGCGTGTAGCAATACACGAGTGGCGCACGGGT
GAGTAACGCGTGGATATCTGCCTTTTGGTTCGGAATAACCCCGGGAAACTGGGGCTAATACCGGATGG
TTCCTACGGGATAAAGATTTATCGCCAAAAGATGAGTCCGCGTACGATTAGCTAGTTGGTGAGGTAAT
GGCTCACCAAGGCGACGATCGTTAGCTGGTCTGAGAGGACGATCAGCCACACTGGGACTGAGACACGG
CC
>Zotu291
GAACGCTGGCGGCAGGCCTAACACATGCAAGTCGAGCGGATGAGAAGAGCTTGCTCTTCGATTCAGCG
GCGGACGGGTGAGTAATACCTAGGAATCTGCCTGGTAGTGGGGGACAACGTTTCGAAAGGAACGCTAA
TACCGCATACGTCCTACGGGAGAAAGCAGGGGACCTTCGGGCCTTGCGCTATCAGATGAGCCTAGGTC
GGATTAGCTAGTTGGTGAGGTAATGGCTCACCAAGGCTACGATCCGTAACTGGTCTGAGAGGATGATC
AGTCACACTGGAACTGAGACACGGTC
>Zotu292
GAACGCTGGCGGTATGCTTAACACATGCAAGTCGAACGGTAGCAGGCCTTCGGGCGCTGACGAGTGGC
GGACGGGTGAGTAACGCGTAGGAATCTGCCTCATAGTGGGGGACAACGTGGGGAAACTCACGCTAATA
CCGCATACGCCCTACGGGGGAAAGCGGGGGATCTTCGGACCTCGCGCTATGAGATGAGCCTGCGTTAG
ATTAGCTAGTTGGTGGGGTAAAGGCCTACCAAGGCGACGATCTATAGCTGGTCTGAGAGGACGATCAG
CCACACTGGGACTGAGACACGGCC
>Zotu293
GAACGCTAGCGGCAGGCATAATACATGCAAGGCGAAGGGGCAGCAATGTCACTGTCGTACGGGTGCGT
AACGCGTATGCAACCTACCTATCACTGGGGGATAGCCCGGGGAAACCCGGATTAATACCGCATAAAAC
AGGGGCACCGCATGGTGATATTTGTTAAAGATTTATTGGTGATAGATGGGCATGCGTTCGATTAGCTA
GTTGGCGGGGTAACGGCCCACCAAGGCGACGATCGATAGGGGAGCTGAGAGGTTGGTCCCCCACACGG
GCACTGAGATACGGGC
>Zotu294
GAACGCTGGCGGCGTGCTTAACACATGCAAGTCGAACGATGAAGCGGTGCTTGCACCGTGGATTAGTG
GCGAACGGGTGAGTAATACGTGAGTGACCTACCTTTGACTCTGGGATAAGCCTGGGAAACTGGGTCTA
ATACCGGATACGACCAATCTCTGCATGGGGTGTTGGTGGAAAGCGTTATGGAGTGGTTTTAGATGGGC
TCACGGCCTATCAGCTTGTTGGTGAGGTAATGGCTTACCAAGGCGACGACGGGTAGCCGGCCTGAGAG
GGTGACCGGCCACACTGGGACTGAGACACGGCC
>Zotu295
GAACGCTGGCGGTATGCTTAACACATGCAAGTCGAACGGTAGCAGGCCTTCGGGCGCTGACGAGTGGC
GGACGGGTGAGTAACGCGTAGGAATCTGCCTCATAGTGGGGGACAACGTGGGGAAACTCACGCTAATA
CCGCATACGCCCTACGGGGGAAAGCGGGGGACCTTCGGGCCTCGCGCTATGAGATGAGCCTGCGTTAG

ATTAGCTAGTTGGTGGGGTAAAGGCCTACCAAGGCGACGATCTATAGCTGGTCTGAGAGGACGATCAG
CCACACTGGGACTGAGACACGGCC
>Zotu296
GAACGCTGGCGGCGTGCTTAACACATGCAAGTCGAACGCTGAAGCTCAGCTTTTGTTGGGTGGATGAG
TGGCGAACGGGTGAGTAACACGTGAGTAACCTGCCCTCTTCTTTGGGATAACGCCCGGAAACGGGTGC
TAATACTGGATATTCACTGGCCTTCGCATGGGGGTTGGTGGAAAGGTTTGTTCTGGTGGGGGATGGGC
TCGCGGCCTATCAGCTTGTTGGTGGGGTGATGGCCTACCAAGGCTTTGACGGGTAGCCGGCCTGAGAG
GGTGACCGGTCACATTGGGACTGAGATACGGCC
>Zotu297
GAACGCTGGCGGCGTGCCTAATACATGCAAGTCGAACGCTTTTTCTTTCACCGGAGCTTGCTCCACCG
GAAGAAAAGGAGTGGCGAACGGGTGAGTAACACGTGGGTAACCTGCCCATCAGAAGGGGATAACACTT
GGAAACAGGTGCTAATACCGTATAACAATAGAAACCGCATGGTTTCTATTTGAAAGGCGCTTTTGCGT
CACTGATGGATGGACCCGCGGTGCATTAGCTAGTTGGTGAGGTAACGGCTCACCAAGGCAACGATGCA
TAGCCGACCTGAGAGGGTGATCGGCCACATTGGGACTGAGACACGGCC
>Zotu298
GAACGCTAGCTACAGGCTTAACACATGCAAGTCGAGGGGAAACGGCATTTAGTGCTTGCACCGAATGG
ACGTCGACCGGCGCACGGGTGAGTAACGCGTATCCAACCTTCCCATAACTAAGGGATAACCTGCCGAA
AGGCAGACTAATACCTTATGTAATCCTTTGATGGCATCAGATAAGGATAAAAGATTTATCGGTTATGG
ATGGGGATGCGTCTGATTAGCTTGTTGGCGGGGTAACGGCCCACCAAGGCAACGATCAGTAGGGGTTC
TGAGAGGAAGGTCCCCCACATTGGAACTGAGACACGGTC
>Zotu299
GAACGCTGGCGGCGTGCCTAATACATGCAAGTCGAACGAACAGCGACTTGAACTTGTTCAAGTCAATG
TTAGTGGCGCACGGGTGAGTAACACGTGGAGAACCTACCCTTAAGCGGGGGATAACCATTGGAAACGA
TGACTAATACCGCATAGAATCACAGATCGCATGATCAATGAAGGAAAGACGGCTTCGGCTGTCACTTA
AGGATGGCTCCGCGGTGCATTAGCTAGATGGTGGGGTAAAGGCCTACCATGGCAATGATGCATAGCCG
ACCTGAGAGGGTGATCGGCCACATTGGGACTGAGACACGGCC
>Zotu300
GAACGCTGGCGGCAGGCCTAACACATGCAAGTCGAGCGGACCCTTCGGGGTTAGCGGCGGACGGGTGA
GTAACGCGTGGGAATATGCCCTTCTCTACGGAATAGTCCTGGGAAACTGGGGGTAATACCGTATACGC
CCTTTGGGGGAAAGATTTATCGGGGAAGGATTAGCCCGCGTTGGATTAGGTAGTTGGTGGGGTAATGG
CCTACCAAGCCGACGATCCATAGCTGGTTTGAGAGGATGATCAGCCACACTGGGACTGAGACACGGCC
>Zotu301
GAACGCTGGCGGCGTGCCTAATACATGCAAGTCGAGCGAATGGATTGAGAGCTTGCTCTCAAGAAGTT
AGCGGCGGACGGGTGAGTAACACGTGGGTAACCTGCCCATAAGACTGGGATAACTCCGGGAAACCGGG
GCTAATACCGGATAACATTTTGAACCGCATGGTTCGAAATTGAAAGGCGGCTTCGGCTGTCACTTATG
GATGGACCCGCGTCGCATTAGCTAGTTGGTGAGGTAACGGCTCACCAAGGCAACGATGCGTAGCCGAC
CTGAGAGGGTGATCGGCCACACTGGGACTGAGACACGGCC
>Zotu302
GAACGCTGGCGGAATGCTTTACACATGCAAGTCGAACGGCAGCACGAGAGCTTGCTCTTGGTGGCGAG
TGGCGAACGGGTGAGTAATATATCGGAACGTATCCAATAATGGGGGATAACTAATCGAAAGGTTGGCT
AATACCGCATACGCCCTACGGGGGAAAGCAGGGGATCTTCGGACCTTGCGTTAATGGAGCGGCTGATA
TCTGATTAGCTAGTAGGTGAGGTAAAGGCTCACCTAGGCGACGATCAGTAGCTGGTCTGAGAGGACGA
CCAGCCACACTGGAACTGAGACACGGTC
>Zotu303
GAACGCTGGCGGCGTGCCTAATACATGCAAGTCGAGCGAACAGATAAGGAGCTTGCTCCTTTGACGTT
AGCGGCGGACGGGTGAGTAACACGTGGGTAACTTACCTATAAGACTGGAATAACTCCGGGAAACCGGG
GCTAATGCCGGATAACATGTTGAACCGCATGGTTCTACAGTGAAAGGCGGCTTTGCTGTCACTTATAG
ATGGACCCGCGCCGTATTAGCTAGTTGGTGAGGTAACAGCTCACCAAGGCAACGATACGTAGCCGACC
TGAGAGGGTGATCGGCCACACTGGAACTGAGACACGGTC
>Zotu304
GAACGCTGGCGGCGCGCCTAACACATGCAAGTCGAGCGAGGTTCAAAGCTTCGGTTTTGAACCTAGCG
GCGGACGGGTGAGTAACGCGTGAGCAACCTGGCCTTCAGAGGGGGATACCGTCTGGAAACGGACGTTA
ATACCGCATAACATGTCGGGAGGGCATCTTCCTGACATCAAAGGAGAAATTCACTGAAGGATGGGCTC
GCGTCCGATTAGGTAGTTGGTGAGGTAACGGCTCACCAAGCCCGCGATCGGTAGCCGGACTGAGAGGT

TGGCCGGCCACATTGGGACTGAGACACGGCC
>Zotu305
CAACGCTGGCGGCGCGCTTAACACATGCAAGTCGAACGAGAAAGTGGAGCAATCCATGAGTAAAGTGG
CGCACGGGTGAGTAACACGTGGATCATCTGCCCTCTTGTGGGGAATACCGCTGGGAAACCGGCGACAA
TACCGCATAAGCTCGGAAGAGGAAAGCCGAAAGGCGCAGGAGGAGGAGTCCGCGGTCGATTAGTTAGT
TGGCAGGGTAACGGCCTACCAAGACTGTGATCGATAGCCGGCCTGAGAGGGCACACGGCCACACTGGC
ACTGAAACACGGGC
>Zotu306
GAACGCTGGCGGCGTGCCTAATACATGCAAGTCGAACGCTTTTTCTTTCACCGGAGCTTGCTCCACCG
AAAGAAAAGGAGTGGCGAACGGGTGAGTAACACGTGGGTAACCTGCCCATCAGAAGGGGATAACACTT
GGAAACAGGTGCTAATACCGTATAACAATAGAAACCGCATGGTTTCTATTTGAAAGGCGCTTTTGCGT
CACTGATGGATGGACCCGCGGTGCATTAGCTAGTTGGTGAGGTAACGGCTCACCAAGGCAACGATGCA
TAGCCGACCTGAGAGGGTGATCGGCCACATTGGGACTGAGACACGGCC
>Zotu307
GAACGCTGGCGGCAGGCTTAACACATGCAAGTCGAACGGGCACCTTCGGGTGTCAGTGGCAGACGGGT
GAGTAACACGTGGGAACGTACCCTTCGGTTCGGAATAACTCAGGGAAACTTGAGCTAATACCGGATAC
GCCCTTTTGGGGAAAGGTTTACTGCCGAAGGATCGGCCCGCGTCTGATTAGCTTGTTGGTGGGGTAAC
GGCCTACCAAGGCGACGATCAGTAGCTGGTCTGAGAGGATGATCAGCCACACTGGGACTGAGACACGG
CC
>Zotu308
CAACGCTGGCGGCGTGCCTAACACATGCAAGTCGCACGAGAAAGGGGCTTCGGCCCTGAGTAAAGTGG
CGTACGGGTGAGTAACACGTGACTAACCTACCCTCGAGTGGGGAATAACCTAGAGAAATCTGGGCTAA
TACCGCATAACACTCATGAGTCAAAGCAGCAATGCGCTTGAGGAGGGGGTCGCGGCAGATTAGCTAGT
TGGTGAGGTAATGGCTCACCAAGGCGATGATCTGTATCCGGCCTGAGAGGGCGCACGGACACACTGGA
ACTGAAACACGGTC
>Zotu309
CAACGCTGGCGGCGCGCTTAACACATGCAAGTCGCACGAGAAAGGGGAGCAATCCCTGAGTAAAGTGG
CGTACGGGTGAGTAACACGTGGATGATCTACCTTCTAGTGGGGAATAACCCTGGGAAACTGGGGCTAA
TACCGCATAAGCTCGAGAGAGGAAAGCAGCAATGCGCTGGAAGAGGAGTCCGCGGCCGATTAGCTAGT
TGGCAGGGTAAAAGCCTACCAAGGCAGAGATCGGTAGCCGGCCTGAGAGGGCACACGGCCACACTGGC
ACTGAAACACGGGC
>Zotu310
GAACGCTGGCGGCAGGCCTAACACATGCAAGTCGAGCGCGCCCTTCGGGGTGAGCGGCGGACGGGTGA
GTAACGCGTGGGAACGTGCCCTTCTCTGCGGAATAGCCTCGGGAAACTGAGAGTAATACCGCATACGC
CCTTTGGGGGAAAGATTTATCGGGGAAGGATCGGCCCGCGTTGGATTAGGTAGTTGGTGGGGTAATGG
CCCACCAAGCCGACGATCCATAGCTGGTTTGAGAGGATGATCAGCCACACTGGGACTGAGACACGGCC
>Zotu311
GAACGCTGGCGGCGTGCCTAATACATGCAAGTAGAACGCTGAAGGAGGAGCTTGCTTCTCCGGATGAG
TTGCGAACGGGTGAGTAACGCGTAGGTAACCTGCCTGGTAGCGGGGGATAACTATTGGAAACGATAGC
TAATACCGCATAATAGTAGATGTTGCATGACATTTACTTAAAAGGTGCAATTGCATCACTACCAGATG
GACCTGCGTTGTATTAGCTAGTTGGTGGGGTAACGGCTCACCAAGGCGACGATACATAGCCGACCTGA
GAGGGTGATCGGCCACACTGGGACTGAGACACGGCC
>Zotu312
GAACGCTGGCGGCGTGCCTAATACATGCAAGTCGAGCGAGCGGAACTAACAGATTTACTTCGGTAATG
ACGTTAGGAAAGCGAGCGGCGGATGGGTGAGTAACACGTGGGGAACCTGCCCCATAGTCTGGGATACC
ACTTGGAAACAGGTGCTAATACCGGATAAGAAAGCAGATCGCATGATCAGCTTTTAAAAGGCGGCGTA
AGCTGTCGCTATGGGATGGCCCCGCGGTGCATTAGCTAGTTGGTAAGGTAAAGGCTTACCAAGGCGAT
GATGCATAGCCGAGTTGAGAGACTGATCGGCCACATTGGGACTGAGACACGGCC
>Zotu313
GAACGTTAGCGGCACGCCTAACACATGCAAGTCGCACGAGAAAGGGGCAACCTGAGTAAAGTGGCGCA
CGGGTGCGTAACACGTAGATAACCTGCCCTTCGAAGGGGGATAACGTTCCGAAAGGAGCGCTAATACC
GCATACAGCATCATCCGGTTCGCCGGGAGATGGCAAAGGTAGGGACCGGCAACGGCCTATCGTCGAAG
GAGGGGTCTGCGGCCCATCAGCTAGTTGGGGAGGTAATGGCTCACCAAGGCAGAGACGGGTAGCTGGT
CTGAGAGGATGATCAGCCACACTGGTACTGGAACACGGAC

>Zotu314
GAACGCTGGCGGCGTGCCTAATACATGCAAGTCGAGCGAATGGATTAAGAGCTTGCTCTTATGAAGTT
AGCGGCGGACGGGTGAGTAACACGTGGGTAACCTGCCCATAAGACTGGGATAACTCCGGGAAACCGGG
GCTAATACCGGATAACATTTTGAACCGCATGGTTCGAAATTGAAAGGCGGCTTCGGCTGTCACTTATG
GATGGACCCGCGTCGCATTAGCTAGTTGGTGAGGTAACGGCTCACCAAGGCAACGATGCGTAGCCGAC
CTGAGAGGGTGATCGGCCACACTGGGACTGAGACACGGCC
>Zotu315
GAACGCTGGCGGCGTGCTTAACACATGCAAGTCGAACGGAAAGGCCCAGCTTGCTGGGTACTCGAGTG
GCGAACGGGTGAGTAACACGTGGGTGATCTGCCCTGCACTCTGGGATAAGCCTGGGAAACTGGGTCTA
ATACCGGATATGACCATGCCATGCATGTGGTGTGGTGGAAAGCTTTTGCGGTGTGGGATGGGCCCGCG
GCCTATCAGCTTGTTGGTGGGGTAATGGCCTACCAAGGCGACGACGGGTAGCCGACCTGAGAGGGTGA
TCGGCCACACTGGGACTGAGACACGGCC
>Zotu316
GAACGCTGGCGGCGTGCTTAACACATGCAAGTCGAACGGAAAGGCCCTTCGGGGTACTCGAGTGGCGA
ACGGGTGAGTAACACGTGGGTGATCTGCCCTGCACTTCGGGATAAGCCTGGGAAACTGGGTCTAATAC
CGGATATGACCGCGCATCTCATGGTGTGTGGTGGAAAGCTTTTGCGGTGTGGGATGGGCCCGCGGCCT
ATCAGCTTGTTGGTGGGGTAATGGCCTACCAAGGCGTCGACGGGTAGCCGGCCTGAGAGGGTGTCCGG
CCACACTGGGACTGAGATACGGCC
>Zotu317
GAACGCTAGCGGCAGGCCTAATACATGCAAGACGAACGGCAAGAATGATAGCAATATTGTTCCTAGAG
TGTCGTAAGGGTGCGTAACACGTATGCAATCTACCCGAAACTGGAGCATAGCTCCCCGAAAGGGGAAT
TAACACTCCATAAGATCATAGGATGGCATCATCTAGTGATTAAAACTGAGGTGGTTTCGGATGAGCAT
GCGTTTGATTAGTTAGTTGGTGAGGTAACGGCTCACCAAGACGATGATCAATAGGGGAACTGAGAGGT
TGATCCCCCACACTGGCACTGAGATACGGGC
>Zotu318
GAACGCTAGCGGCAGGCTTAATACATGCAAGGCGAAGGGGCAGCAATGTCACTGTCGTACGGGTGCGT
AACGCGTATGCAACCTACCTATCACTGGGGGATAGCCCGGGGAAACCCGGATTAATACCGCATAAAAC
AGGGGCACCGCATGGTGATATTTGTTAAAGATTTATTGGTGATAGATGGGCATGCGTTCGATTAGCTA
GTTGGCGGGGTAACGGCCCACCAAGGCGACGATCGATAGGGGAGCTGAGAGGTTGATCCCCCACACGG
GCACTGAGATACGGGC
>Zotu319
GAACGCTGGCGGCATGCTTAACACATGCAAGTCGAACGGGAAGTGGTGTTTCCAGTGGCGAACGGGTG
AGTAATGCGTAAGAACCTGCCCTTGGGAGGGGAACAACAGCTGGAAACGGTTGCTAATACCCCATAGA
ATTTAGTACCTCGTAGAGGTAGGCTGAGGAGCAAAAGGGAGGAATCCGCCCAAGGAGGGGCTCACGTC
TGATTAGCTAGTTGGTGAGGCAATGGCTTACCAAGGCTCCGATCAGTAGTTGGTCTGAGAGGATGATC
AGCCACACTGGGACTGAGACACGGCC
>Zotu320
GAACGCTGGCGGCGTGCTTAACACATGCAAGTCGAACGCTGAAGCCCAGCTTGCTGGGTGGATGAGTG
GCGAACGGGTGAGTAACACGTGAGTAACCTGCCCCCTTCTTTGGGATAACGCCCGGAAACGGGTGCTA
ATACTGGATATTCACTGGCCTTCGCATGGGGGTTGGTGGAAAGGTTTTTCTGGTGGGGGATGGGCTC
GCGGCCTATCAGCTTGTTGGTGGGGTGATGGCCTACCAAGGCTTTGACGGGTAGCCGGCCTGAGAGGG
TGACCGGTCACATTGGGACTGAGATACGGCC
>Zotu321
GAACGCTGGCGGCAGGCTTAACACATGCAAGTCGAACGCATCCTTCGGGGTGAGTGGCAGACGGGTGA
GTAACGCGTGGGAACGTGCCCTTCAGTTCGGAATAACCCAGGGAAACTTGGGCTAATACCGGATACGT
GCGAGAGCAGAAAGATTTATCGCTGAAGGATCGGCCCGCGTCTGATTAGCTAGTTGGTGGGGTAATGG
CCCACCAAGGCGACGATCAGTAGCTGGTCTGAGAGGATGATCAGCCACATTGGGACTGAGACACGGCC
>Zotu322
GAACGCTGGCGGCAGGCTTAACACATGCAAGTCGAGCGGGCCCTTCGGGGTCAGCGGCGGACGGGTGA
GTAACGCGTGGGAACGTGCCTTCTGGTCTGGAATAACCCTGGGAAACTAGGGCTAATACCGGATACGC
CCTTTTGGGGAAAGGTTTACTGCCGGAAGATCGGCCCGCGTCTGATTAGCTAGTTGGTGGGGTAACGG
CCTACCAAGGCGACGATCAGTAGCTGGTCTGAGAGGATGATCAGCCACACTGGGACTGAGACACGGCC
>Zotu323
GAATGCTGGCGGCGTGCCTAACACATGCAAGTCGAGCGGTAACATGAATAATTTATTATTTGATGACG

AGCGGCGGACGGCTGAGTAACGCGTGGGAACATGCCCCAAAGTGAGGAATAAGCTCCAGAAATGGAGT
CTAATGCCGCATATGATCTTCGGATTAAAGGATTTATCCGCTTTGGGAGTGGCCTGCGTACGATTAGA
TAGTTGGTGAGGTAATGGCTTACCAAGTCTACGATCGTTAGCTGGTCTGAGAGGATGACCAGCCAGAC
TGGAACTGAGACACGGTC
>Zotu324
GAACGCTGGCGGCGTGCCTAATACATGCAAGTCGAACGCTTCCTACCGGTGCTTGCACCGGGAAAGAA
GAGTGGCGGACGGGTGAGTAACACGTGGGTAACCTACCTACAAGCGGGGGATAACATTCGGAAACGGA
TGCTAATACCGCATAGATCTTTTTGTCTCCTGGCAGAAAGAAGAAAGACGGTTTCGGCTGTCACTTGT
AGATGGACCCGCGGCGCATTAGTTAGTTGGTGAGGTAATGGCTCACCAAGACGATGATGCGTAGCCGA
CCTGAGAGGGTGATCGGCCACACTGGGACTGAGACACGGCC
>Zotu325
GAACGCTAGCGGCAGGCTTAATACATGCAAGTCGAGGGGCAGCACGAATAGCAATATTTGGTGGCGAC
CGGCGCACGGGTGCGTAACACGTATACAATCTACCTTCAACAGGGGGATAGCCTTTCGAAAGAGAGAT
TAATACCCCGTAATATATGAGATGGCATCATTTTATATTGAAAGTTCCGACCGTTGAAGATGAGTATG
CGTCTGATTAGCTAGTAGGTGAGGTAACGGCTCACCTAGGCTACGATCAGTAGGGGATCTGAGAGGAC
TAACCCCCACACTGGTACTGAGACACGGAC
>Zotu326
GAACGCTAGCTACAGGCTTAACACATGCAAGTCGAGGGGAAACGATGAGAGAGCTTGCTCTCTTAGGC
GTCGACCGGCGCACGGGTGAGTAACGCGTATCCAACCTGCCCATTACAGGGGAATAACCTTGCGAAAG
TAAGACTAATGCCCCATGGTATTCTCAGCAGACATCTAATGAGGATTAAAGATTTATCGGTAATGGAT
GGGGATGCGTCTGATTAGCTTGTTGGCGGGGTAACGGCCCACCAAGGCGACGATCAGTAGGGGTTCTG
AGAGGAAGGTCCCCCACATTGGAACTGAGACACGGTC
>Zotu327
GAACGCTGGCGGCAGGCCTAACACATGCAAGTCGAACGAACCTTTCGGGGTTAGTGGCGGACGGGTGA
GTAACACGTGGGAACGTGCCTTTAGGTTCGGAATAACTCAGGGAAACTTGTGCTAATACCGAATGTGC
CCTTCGGGGGAAAGATTCATCGCCTTTAGAGCGGCCCGCGTCTGATTAGCTAGTTGGTTGAGGTAATG
GCTCACCAAGGCGACGATCAGTAGCTGGTCTGAGAGGATGATCAGCCACATTGGGACTGAGACACGGC
C
>Zotu328
GAACGCTGGCGGCGTGCTTAACACATGCAAGTCGAACGATGAAGCCCAGCTTGCTGGGCGGATTAGTG
GCGAACGGGTGAGTAACACGTGAGTAACCTGCCCTTGACTCTGGGATAAGCCCGGGAAACTGGGTCTA
ATACCGGATAGGAGCGCCTGTCGCATGACGGGTGCTGGAAAGATCTTTCGGTCATGGATGGACTCGCG
GCCTATCAGCTTGTTGGTGAGGTAACGGCTCACCAAGGCGACGACGGGTAGCCGGCCTGAGAGGGTGA
CCGGCCACACTGGGACTGAGACACGGCC
>Zotu329
GAACGCTGGCGGCGTGCTTAACACATGCAAGTCGAACGATGAAGGAGGAGCTTGCTCCTCCGGATTAG
TGGCGAACGGGTGAGTAATACGTGAGCAATGTGCCCTTGACTCTGGGATAAGCGCTGGAAACGGCGTC
TAATACCGGATACGACGATCGGAGGCATCTCCTGGTCGTGGAAAGAACTTCGGTCAAGGATCAGCTCA
CGGCCTATCAGCTAGTTGGTGAGGTAACGGCTCACCAAGGCGACGACGGGTAGCCGGCCTGAGAGGGT
GACCGGCCACACTGGGACTGAGACACGGCC
>Zotu330
GAACGCTAGCGGCAGGCTTAATACATGCAAGTCGAGGGGCAGCAGGATAGCAATATTGCTGGCGACCG
GCGCACGGGTGCGTAACACGTATACAATCTACCTTTAACAGGGGGATAGCCCGAAGAAATTCGGATTA
ATACCCCATAATAGTTTTCTTGGCATCAAGAGGATTTGAAAGCTCCGGCGGTTAGAGATGAGTATGCG
GTCTATTAGCTAGTAGGTGAGGTAACGGCTCACCTAGGCTACGATAGATAGGGGATCTGAGAGGACTA
CCCCCCACACTGGTACTGAGACACGGAC
>Zotu331
GAACGCTGGCGGCAGGCCTAACACATGCAAGTCGAACGAACCTTTCGGGGTTAGTGGCGGACGGGTGA
GTAACACGTGGGAACGTGCCTTTAGGTTCGGAATAACTCAGGGAAACTTGTGCTAATACCGAATGTGC
CCTTCGGGGGAAAGATTTATCGCCTTTAGAGCGGCCCGCGTCTGATTAGCTAGTTGGTTGAGGTAATG
GCTCACCAAGGCGACGATCAGTAGCTGGTCTGAGAGGATGATCAGCCACATTGGGACTGAGACACGGC
C
>Zotu332
GAACGCTGGCGGCGTGCTTAACACATGCAAGTCGAACGGAAAGGCCCTTTCGGGGGTACTCGAGTGGC

GAACGGGTGAGTAACACGTGGGTGATCTGCCCTGCACTTCGGGATAAGCTTGGGAAACTGGGTCTAAT
ACCGGATATGATCACGGGCTTCATGGTCTGTGGTGGAAAGCTTTTGCGGTGTGGGATGGGCCCGCGGC
CTATCAGCTTGTTGGTGGGGTAATGGCCTACCAAGGCGACGACGGGTAGCCGGCCTGAGAGGGTGTCC
GGCCACACTGGGACTGAGATACGGCC
>Zotu333
GAACGCTGGCGGCATGCCTAACACATGCAAGTCGAACGAAGCCTTCGGGCTTAGTGGCGCACGGGTGC
GTAACGCGTGGGAATCTGCCTCTTGCTTCGGAATAACAGTTAGAAATGACTGCTAATACCGGATGATG
TCTTCGGACCAAAGATTTATCGGCAAGAGATGAGCCCGCGTAGGATTAGGTAGTTGGTGGGGTAAAGG
CCTACCAAGCCGACGATCCTTAGCTGGTCTGAGAGGATGATCAGCCACACTGGGACTGAGACACGGCC
>Zotu334
GAACGCTTGCGGCGTGCCTAAGAAATGCAAGTCGAACGGATAGTCCAGCAATGGTCTATTAGTGGCGA
ACGGTCGCGTAACACGTAGGCAACCTGCCCGGAAGTGGGGGACAACAGCCTGAAAGGGCTGCTAATAC
CGCATGTGGCCGCCTGGTGGCATCACCGGGTGTCTAAAGCTTTATGCGCTTCTGGATGGGCTTGCGGC
CTATCAGGTAGTTGGTGGGGTAATGGCCTACCAAGCCGACGACGGGTAGCTGGTCTGAGAGGACGATC
AGCCGGATTGGGACTGAGATACGGCC
>Zotu335
GAACGCTAGCTACAGGCTTAACACATGCAAGTCGAGGGGCAGCATTATCGAAGCTTGCTTTGATAGAT
GGCGACCGGCGCACGGGTGAGTAACGCGTATCCAACCTTCCCATCACTCAGGGATAGCCCGCCGAAAG
GCGAATTAATACCTGATGGTCTCCTTTGCAGACATCTAACGAGGAGTAAAGATTTATTGGTGATGGAT
GGGGATGCGTCTGATTAGGTAGTAGGCGGGGTAACGGCCCACCTAGCCGACGATCAGTAGGGGTTCTG
AGAGGAAGGTCCCCCACATTGGAACTGAGACACGGTC
>Zotu336
GAACGCTAGCGGCAGGCTTAACACATGCAAGTCGAGGGGTAGAGTTAGCTTGCTAACTTGAGACCGGC
GCACGGGTGCGTAACGCGTATGCAATCTACCTTTTACAGAGGGATAGCCCAGAGAAATTTGGATTAAT
ACCTCATAGTATAATGACTCGGCATCGAGATATTATTAAAGTCACAACGGTAAAAGATGAGCATGCGT
CCCATTAGCTAGTTGGTGTGGTAACGGCACACCAAGGCAACGATGGGTAGGGGTCCTGAGAGGGAGAT
CCCCCACACTGGTACTGAGACACGGAC
>Zotu337
GAACGCTGGCGGCGTGCCTTAAGCATGCAAGTCGTATGATCGCAAGATAATGGCGGACGGGTGAGTAA
CGCGTGAGAATCTGCCTTAAAGTGGGGCATAGCCCTCCGAAAGGAGGGGTAATACCGCATGTGATAGC
AATATCAAAGCAGCAATGCGCTTTAAGATGAGCTCGCGTAAGATTAGCTAGTTGGTGGGGTAAAGGCC
TACCAAGGCGACGATCTTTAGCTGGTCTGAGAGGATGGTCAGCCACAATGGGACTGAGACACGGCC
>Zotu338
GAACGCTGGAAATGTGGATACCGGCATGCAAGTCAAGGGGCCGCAAGGCACCGGCGAACGGCTTAGTA
ACACGTAGATACATACCCAAAAGTGGGGCATAGCTCGTCGAAAGACGGGGTAATTTCCCATGTTCCCG
AAAGGGCAAAGCAGCAATGCGCTTTTGGAATGGTCTGCGGTCTATCAGCTAGTTGGTGAGGTAATGGC
TCACCAAGGCTATGACGGATAGGGGGCGTGAGAGCGCGACCCCCAAGAATGGCACTGCGATACGGGC
>Zotu339
GAACGCTAGCGGGAGGCCTAACACATGCAAGCCGAGCGGTAGAGTTTCTTCGGAAACTTGAGAGCGGC
GCACGGGTGCGTAACACGTGTGCAACCTACCTTTATCTGGGGGATAGCCTTTCGAAAGGAAGATTAAT
ACCCCATAATATAATGATTGGCATCAATTATTATTGAAAACTCCGGTGGATAGAGATGGGCACGCGCA
AGATTAGATAGTTGGTGAGGTAACGGCTCACCAAGTCAATGATCTTTAGGGGTCCTGAGAGGGAGATC
CCCCACACTGGTACTGAGACACGGAC
>Zotu340
GAACGCTGGCGGCGTGCTTAACACATGCAAGTCGAACGGAAAGGCCCGCTTGCGGGTACTCGAGTGGC
GAACGGGTGAGTAACACGTGGGCAACCTGCCCTCAGCTCTGGGATAACTCCGGGAAACCGGTGCTAAT
ACTGGATATCGACTTCTGCCTCGCATGGGGTGTTGTTGAAAGTTTTTCGGCTGGGGATGGGCCCGCGG
CCTATCAGCTTGTTGGTGGGGTGATGGCCTACCAAGGCGACGACGGGTAGCCGGCCTGAGAGGGCGAC
CGGCCACACTGGGACTGAGACACGGCC
>Zotu342
GAACGCTGGCGGCAGGCTTAACACATGCAAGTCGAGCGGGCATAGCAATATGTCAGCGGCAGACGGGT
GAGTAACGCGTGGGAACGTACCTTTTGGTTCGGAACAACACAGGGAAACTTGTGCTAATACCGGATAA
GCCCTTACGGGGAAAGATTTATCGCCGAAAGATCGGCCCGCGTCTGATTAGCTAGTTGGTGAGGTAAT
GGCTCACCAAGGCGACGATCAGTAGCTGGTCTGAGAGGATGATCAGCCACATTGGGACTGAGACACGG

CC
>Zotu343
GAACGCTGGCGGCGTGCCTAATACATGCAAGTAGAACGCTGAAGACTTTAGCTTGCTAGAGTTGGAAG
AGTTGCGAACGGGTGAGTAACGCGTAGGTAACCTGCCTACTAGCGGGGGATAACTATTGGAAACGATA
GCTAATACCGCATAATAGTATTTAACACATGTTAGAGACTTGAAAGATGCAATTGCATCACTAGTAGA
TGGACCTGCGTTGTATTAGCTAGTTGGTGGGGTAACGGCCTACCAAGGCGACGATACATAGCCGACCT
GAGAGGGTGATCGGCCACACTGGGACTGAGACACGGCC
>Zotu345
GAACGCTGGCGGCAGGCCTAACACATGCAAGTCGAGCGGTAGAGAGAAGCTTGCTTCTCTTGAGAGCG
GCGGACGGGTGAGTAATACCTAGGAATCTGCCTGATAGTGGGGGATAACGTTCGGAAACGGACGCTAA
TACCGCATACGTCCTACGGGAGAAAGCAGGGGACCTTCGGGCCTTGCGCTATCAGATGAGCCTAGGTC
GGATTAGCTAGTTGGTGAGGTAATGGCTCACCAAGGCTACGATCCGTAACTGGTCTGAGAGGATGATC
AGTCACACTGGAACTGAGACACGGTC
>Zotu346
GAACGCTGGCGGCAGGCTTAACACATGCAAGTCGAGCGGGGAAGGGTAGCTTGCTACCTGACCTAGCG
GCGGACGGGTGAGTAATGCTTAGGAATCTGCCTATTAGTGGGGGACAACATTCCGAAAGGAATGCTAA
TACCGCATACGCCCTACGGGGGAAAGCAGGGGATCTTCGGACCTTGCGCTAATAGATGAGCCTAAGTC
AGATTAGCTAGTTGGTGGGGTAAAGGCCTACCAAGGCGACGATCTGTAGCGGGTCTGAGAGGATGATC
CGCCACACTGGGACTGAGACACGGCC
>Zotu347
GAATGCTGGCGGCGTGCCTAACACATGCAAGTCGAGCGGTAAGGCCCCTTCGGGGGTACACGAGCGGC
GAACGGCTGAGTAACGCGTGGGAACATGCCCCAAAGTGAGGAATAAGCTCCAGAAATGGAGTCTAATG
CCGCATATGATCTTCGGATTAAAGGATTTATCCGCTTTGGGAGTGGCCTGCGTACGATTAGATTGTTG
GTAAGGTAATGGCTTACCAAGTCTACGATCGTTAGCTGGTCTGAGAGGATGACCAGCCAGACTGGAAC
TGAGACACGGTC
>Zotu348
GAACGCTGGCGGCGTGCCTAATACATGCAAGTCGAGCGAACAGATAAGGAGCTTGCTCCTTTGACGTT
AGCGGCGGACGGGTGAGTAACACGTGGGTAACCTACCTATAAGACTGGAATAACTCCGGGAAACCGGG
GCTAATGCCGGATAACATGTTGAACCGCATGGTTCTACAGTGAAAGGCGGCTTTGCTGTCACTTATAG
ATGGACCCGCGCCGTATTAGCTAGTTGGTGAGGTGACGGCTCACCAAGGCAACGATACGTAGCCGACC
TGAGAGGGTGATCGGCCACACTGGAACTGAGACACGGTC
>Zotu349
GAACGCTAGCGGCAGGCCTAATACATGCAAGTCAAGGGGTAGCAATACACCGGCGGACGGGTGCGTAA
CGCGTACACAACATACCTTTAAGCGGGGGATAGCATTGGGAAACTGGTGGTAATACCCCATAACATTG
CGGGTTGGCATTAACTTGCAATTAAAGTTTCGGCGCTTAAAGATTGGTGTGCGTCTGATTAGCTAGTT
GGCGGGGTAACGGCCCACCAAGGCGACGATCAGTAGGGGGTGTGAGAGCATGACCCCCCACATGGGTA
CTGAGACACGGAC
>Zotu350
GAACGCTGGCGGCGTGCCTAATACATGCAAGTCGAACGAACGGATAAAGAGCTTGCTCTTTTGAAGTT
AGTGGCGGACGGGTGAGTAACACGTGGGTAACCTGCCTCACAGCTGGGGATAACATCGAGAAATCGAT
GCTAATACCGAATGTGCTGAACATCATAAGATGTTCAAGTGAAAGACGGTTTCGGCTGTCACTGTGAG
ATGGACCCGCGCTGGATTAGCTAGTTGGTAAGGTAATGGCTTACCAAGGCGACGATCCATAGCCGACC
TGAGAGGGTGATCGGCCACATTGGGACTGAGACACGGCC
>Zotu351
GAACGCTGGCGGCGTGCTTAACACATGCAAGTCGAACGGTGAACTCCGCTTGCGGGGGGATCAGTGGC
GAACGGGTGAGTAACACGTGGGCAACCTGCCCCTGGCTCTGGGATAACTCCAAGAAATTGGGGCTAAT
ACCGGATATGACCGCTGACCGCATGGTCTGGTGGTGGAAAGATTTATCGGCTGGGGATGGGCCCGCGG
CCTATCAGCTTGTTGGTGGGGTAGTGGCCTACCAAGGCGACGACGGGTAGCCGGCCTGAGAGGGTGAC
CGGCCACACTGGGACTGAGACACGGCC
>Zotu352
GAACGCTGGCGGCGTGCTTAACACATGCAAGTCGAACGATGAAGCCAGCTTGCTGGTGGATTAGTGGC
GAACGGGTGAGTAACACGTGGGCAACCTGCCCTCAGCTCTGGGATAACTCCAAGAAATTGGTGCTAAT
ACCGGATATTCTTCTTTCACCGCATGGTGGTTGGAGGAAAGTTTTTCGGCTGGGGATGGGCCCGCGGC
CTATCAGCTTGTTGGTGGGGTGATGGCCTACCAAGGCGACGACGGGTAGCCGGCCTGAGAGGGTGACC

GGCCACACTGGGACTGAGACACGGCC
>Zotu353
GAACGCTGGCGGCAGGCTTAACACATGCAAGTCGAACGAGTGTAGCAATACACTAGTGGCGCACGGGT
GAGTAACACGTGGATATCTGCCTTTTGGTTCGGAATAACCCTGGGAAACTAGGGCTAATACCGAATGG
TTCCTACGGGATAAAGATTTATCGCCAAAAGATGAGTCCGCGTCCGATTAGCTAGTTGGTGAGGTAAT
GGCTCACCAAGGCGACGATCGGTAGCTGGTCTGAGAGGACGATCAGCCACACTGGGACTGAGACACGG
CC
>Zotu354
GAACGCTGGCGGCGTGCCTAATACATGCAAGTCGAGCGAACAGAGAAGGAGCTTGCTCCTTTGACGTT
AGCGGCGGACGGGTGAGTAACACGTGGGCAACCTACCTTATAGTTTGGGATAACTCCGGGAAACCGGG
GCTAATACCGAATAATCTGTTTCACCTCATGGTGAAATATTGAAAGACGGTTTCGGCTGTCGCTATAG
GATGGGCCCGCGGCGCATTAGCTAGTTGGTGAGGTAACGGCTCACCAAGGCGACGATGCGTAGCCGAC
CTGAGAGGGTGATCGGCCACACTGGGACTGAGACACGGCC
>Zotu355
GAACGCTGGCGGCGTGCTTAACACATGCAAGTCGAGCGATGAAGCACCTTCGGGTGTGAATTAGCGGC
GAACGGGTGAGGAACACGTGAAGAATCTGCCTTCAACTCTGGGATAACTCCGGGAAACCGGGGCTAAT
ACCGGATATGAGCCTTGATCGCATGATCGGGGCTGGAAAGTTTTTCGGTTGAAGATGACTTCGCGGCC
TATCAGCTTGTTGGTGAGGTAATGGCTCACCAAGGCGACGACGGGTAGCCGGCCTGAGAGGGCGACCG
GCCACACTGGGACTGAGACACGGCC
>Zotu356
GAACGCTAGCGGCAGGCCTAATACATGCAAGTCGAGGGGTAGGGTAGCAATACTTGAGACCGGCGCAC
GGGTGCGTAACGCGTATGGAACTTGTCCGATCCTCTGGGATAGCCCCCGGAAACGGGGATTAATACCG
GATGGCCCGTGGTTGCGGCATCGCAGCCACGGTAAAGCTCAGGCGGGATCGGGTGGCCATGCGTTCGA
TTAGCTAGTTGGCGGGGTAACGGCCCACCAAGGCGACGATCGATAGGGGTCCTGAGAGGGAGATCCCC
CACACTGGTACTGAGACACGGGC
>Zotu357
GAACGCTGGCGGCGTGCCTAATACATGCAAGTCGAACGAGAGCGACCGGTGCTTGCACTGGTCAATCT
AGTGGCGAACGGGTGAGTAACACGTGGGTAACCTGCCCATCAGAGGGGGATAACATCCGGAAACGGAT
GCTAAAACCGCATAGGTCTTCGAGCCGCATGGCTTGAAGAGGAAAAGAGGCGCAAGCTTCTGCTGATG
GATGGACCCGCGGTGCATTAGCTAGTTGGTGAGGTAACGGCTCACCAAGGCCGTGATGCATAGCCGAC
CTGAGAGGGTGATCGGCCACATTGGGACTGAGACACGGCC
>Zotu358
GAACGCTAGCGGGAGGCCTAACACATGCAAGCCGAGCGGTATTGTTTCTTCGGAAATGAGAGAGCGGC
GTACGGGTGCGGAACACGTGTGCAACCTGCCTTTATCTGGGGGATAGCCTTTCGAAAGGAAGATTAAT
ACTCCATAACATATTGAACGGCATCGTTTAATATTGAAAGCTCCGGCGGATAGAGATGGGCACGCGCA
AGATTAGCTAGTTGGTGAGGTAACGGCTCACCAAGGCGATGATCTTTAGGGGGCCTGAGAGGGTGATC
CCCCACACTGGTACTGAGACACGGAC
>Zotu359
AAACGCTGGCGGCATGCCTAACACATGCAAGTCGAACGGCAGCACAGTAAAGATTTCGGTCTTTACGG
GTGGCGAGTGGCGGACGGGTGAGTAAAGCGTAGGAATCTGTCCTGGAGAGTGGGATAACCCGAGGAAA
CTCGGGCTAATACCGCATGATATCGAGAGATCAAAGCAGGGGACCCTTCGGGGCCTTGCGCTCTAGGA
TGAGCCTACGTTGGATTAGCTAGTTGGTGGGTGGGGTAAAGGCCTACCAAGGCGACGATCCGTAGCTG
GTCTGAGAGGATGACCAGCCACACTGGGACTGAGACACGTCC
>Zotu360
GAACGCTGGCGGCATGCTTTACACATGCAAGTCGAACGGCAGCGCGGGGCAACCTGGCGGCGAGTGGC
GAACGGGTGAGTAATATATCGGAACGTACCCAAGAGTGGGGGATAACGTAGCGAAAGTTACGCTAATA
CCGCATACGATCTAAGGATGAAAGCGGGGGATCGCAAGACCTCGTGCTCCTGGAGCGGCCGATATCTG
ATTAGCTAGTTGGTGAGGTAAAGGCTCACCAAGGCGACGATCAGTAGCTGGTCTGAGAGGACGACCAG
CCACACTGGAACTGAGACACGGTC
>Zotu361
GAACGCTGGCGGCATGCCTTACACATGCAAGTCGAACGGTAACAGGTTAAGCTGACGAGTGGCGAACG
GGTGAGTAATATATCGGAACGTGCCCAGTCGTGGGGGATAACTACTCGAAAGAGTAGCTAATACCGCA
TACGACCTGAGGGTGAAAGCGGGGGATCGCAAGACCTCGCGCGATTGGAGCGGCCGATATCAGATTAG
GTAGTTGGTGGGGTAAAGCCCACCAAGCCAACGATCTGTAGCTGGTCTGAGAGGACGACCAGCCACA

CTGGGACTGAGACACGGCC
>Zotu362
GAACGCTGGCGGCATGCCTTACACATGCAAGTCGAACGGTAACAGGTCTTCGGATGCTGACGAGTGGC
GAACGGGTGAGTAATACATCGGAACGTGCCTAGTAGTGGGGGATAACTACTCGAAAGAGTGGCTAATA
CCGCATGAGATCTACGGATGAAAGCAGGGGATCGCAAGACCTTGTGCTACTAGAGCGGCCGATGGCAG
ATTAGGTAGTTGGTGGGATAAAAGCTTACCAAGCCGACGATCTGTAGCTGGTCTGAGAGGACGATCAG
CCACACTGGGACTGAGACACGGCC
>Zotu363
GAACGCTGGCGGCGTGCCTAATACATGCAAGTCGAGCGAACAGACGAGGAGCTTGCTCCTTTGACGTT
AGCGGCGGACGGGTGAGTAACACGTGGGTAACCTACCTATAAGACTGGGATAACTTCGGGAAACCGGA
GCTAATACCGGATAATATTTCGAACCGCATGGTTCGATAGTGAAAGATGGCTCTGCTATCACTTATAG
ATGGACCTGCGCCGTATTAGCTAGTTGGTAAGGTAACAGCTTACCAAGGCAACGATACGTAGCCGACC
TGAGAGGGTGATCGGCCACACTGGAACTGAGACACGGTC
>Zotu364
GAACGCTGGCGGCGTGCCTAATACATGCAAGTCGAGCGAGCGGAACCAACAGATTTACTTCGGTAATG
ACGTTGGGAAAGCGAGCGGCGGATGGGTGAGTAACACGTGGGGAACCTGCCCCTAAGTCTGGGATACC
ATTTGGAAACAGGTGCTAATACCGGATAATAAAGCAGATCGCATGATCAGCTTTTGAAAGGCGGCGTA
AGCTGTCGCTAAGGGATGGCCCCGCGGTGCATTAGCTAGTTGGTAAGGTAACGGCTTACCAAGGCGAC
GATGCATAGCCGAGTTGAGAGACTGATCGGCCACATTGGGACTGAGACACGGCC
>Zotu365
GAACGCTGGCGGCGTGCTTAACACATGCAAGTCGAACGATGATGCCCAGCTTGCTGGGTGGATTAGTG
GCGAACGGGTGAGTAACACGTGAGTAACCTGCCCCTGACTCTGGGATAAGCGTTGGAAACGACGTCTA
ATACTGGATATGATCACTGGCCGCATGGTCTGGTGGTGGAAAGATTTTTTGGTTGGGGATGGACTCGC
GGCCTATCAGCTTGTTGGTGAGGTAATGGCTCACCAAGGCGACGACGGGTAGCCGGCCTGAGAGGGTG
ACCGGCCACACTGGGACTGAGACACGGCC
>Zotu366
GAACGCTGGCGGCGTGCTTAACACATGCAAGTCGAACGATGATCCGGTGCTTGCACCGGGGATTAGTG
GCGAACGGGTGAGTAACACGTGAGTAACCTGCCCTTAACTCTGGGATAAGCCTGGGAAACTGGGTCTA
ATACCGGATATGACTGATCATCGCATGGTGGTTGGTGGAAAGCTTTATTGTGGTTTTGGATGGACTCG
CGGCCTATCAGCTTGTTGGTGAGGTAATGGCTTACCAAGGCGACGACGGGTAGCCGGCCTGAGAGGGT
GACCGGCCACACTGGGACTGAGACACGGCC
>Zotu367
GAACGCTGGCGGCGTGCCTAATACATGCAAGTCGAGCGCCCTGCACTTTGCCCATCAGCCCAAACAAC
GGAGCTGGTGAGCAGCGTGTAGGGAGCGGCGGACGGCTGAGTAACACGTAGGAACGTACCCCAAAGTG
AGGCATAAGCACCAGAAATGGTGTCTAATACCGCATGTGCTCTTAGGAGTAAAGCTTCGGCGCTTTGG
GAACGGCCTGCGCATGATTAGCTTGTTGGTGAGGTAAAAGCTCACCAAGGCGACGATCATTAGCTGGT
CTGAGAGGATGACCAGCCAGACTGGGACTGAGAACGGCC
>Zotu368
CAACGCTGGCGGCGTGCCTAATGCATGCAAGTCGAGCGGGGTCGTTCTTCGGAGGGGCTTACGCGGCG
GACGGGTGCGTAACACGTGGGGAACCTGCCCTGGGGTGGGGGATAGCCGAGGGAAACCTTGGGTAATT
CCGCATACGCTTGCGTACCGGAAGGGTACGTGAGGAAAGGGTCTTTCGGGACTCGCCTTGGGAGGGGC
CTGCGGCCGATTAGCTAGACGGTGGGGTAACGGCTCACCGTGGCGATGATCGGTAGCTGGTCTGAGAG
GACGGCCAGCCACACGGGGACTGAGAGACGGCC
>Zotu369
GAACGCTAGCGGGAGGCCTAACACATGCAAGCCGAGCGGTATTTGTTCTTCGGAACAGAGAGAGCGGC
GCACGGGTGCGGAACACGTGTGCAACCTGCCTTTATCTGGGGGATAGCCTTTCGAAAGGAAGATTAAT
ACCCCATAATATATTGAGTGGCATCACTTAATATTGAAAACTCCGGTGGATAGAGATGGGCACGCGCA
AGATTAGATAGTTGGTGAGGTAACGGCTCACCAAGTCAATGATCTTTAGGGGGCCTGAGAGGGTGATC
CCCCACACTGGTACTGAGACACGGAC
>Zotu370
TCGACAGCATCGCTTGCTGCTTATAAAGTGAACAGCTCTTGGGAAAGCCTTTGGTTTTGCAGGAACCC
CACGAAATCAACGCTTGCCATAGAGGCACGCACTTTAATGATGTGGGCGCGGACGGCACACAGCAGCA
CATCGTGC
>Zotu371

GAACGCTGGCGGCAGGCTTAACACATGCAAGTCGAGCGGGCCCTTCGGGGTCAGCGGCAGACGGGTGA
GTAACGCGTGGGAACGTGCCCTTCGGTTCGGAATAACTCAGGGAAACTTGAGCTAATACCGGATACGC
CCTTTTGGGGAAAGGCTTGACTGCCGAAGGATCGGCCCGCGTCTGATTAGCTAGTTGGTGAGGTTATG
GCTCACCAAGGCGACGATCAGTAGCTGGTCTGAGAGGATGATCAGCCACACTGGGACTGAGACACGGC
C
>Zotu372
GAATGCTGGCGGCGCGCCTAACACATGCAAGTCGAGCGGTAACAGGTCTAGTTTACTAGATGCTGACG
AGCGGCGGACGGCTGAGTAACGCGTGGGAACATGCCCCAAAGTGAGGAATAACTGCCCGAAAGGGTAG
CTAATGCCGCATATGATCTTAGGATTAAAGGATTTATCCGCTTTGGGAGTGGCCTGCGTACGATTAGG
TAGTTGGTGAGGTAATGGCTCACCAAGCCTACGATCGTTAACTGGTCTGAGAGGATGATCAGTCAGAC
TGGAACTGAGACACGGTC
>Zotu373
GAACGCTAGCGGCAGGCTTAACACATGCAAGTCGAGGGGCAGCAGGTCTTCGGACGCTGGCGACCGGC
AAACGGGTGCGGAACACGTACGCAACATACCTTTATCTGGGGCATAGCCCGAAGAAATTCGGATTAAC
ACCCCATAAGATTATAGAGTGGCATCACTTAGTAATTAAAAATTCATTGGATAGAGATTGGCGTGCGT
CCTATTAGCTAGTTGGCGGGGTAAAGGCCCACCAAGGCGACGATAGGTAGCTGATGTGAGAGCATGAT
CAGCCACACGGGCACTGAGACACGGGC
>Zotu374
GAACGCTGGCGGCGTGCTTAACACATGCAAGTCGAGCGAGAAGTTTTGAAAAGATTCTTCGGATGAAA
TTTAAAATGGAAAGCGGCGGACGGGTGAGTAACGCGTAGGCAACCTGCCCCATACAAAGGGATAGCAT
TTGGAAACGAATATTAATACCTTATGAAACTTAACTAGTGCATGCTAGGTAGGTCAAAGATTTATCGG
TATGGGATGGGCCTGCGTCTGATTAGCTAGTTGGTGAGGTAACGGCTCACCAAGGCGACGATCAGTAG
CCGACCTGAGAGGGTGAACGGCCACATTGGAACTGAGACACGGTC
>Zotu375
GAACGCTGGCGGCAGGCCTAACACATGCAAGTCGAGCGGTAGAGAGAAGCTTGCTTCTCTTGAGAGCG
GCGGACGGGTGAGTAATGCCTAGGAATCTGCCTGGTAGTGGGGGATAACGTTCGGAAACGGACGCTAA
TACCGCATACGTCCTACGGGAGAAAGCAGGGGACCTTCGGGCCTTGCGCTATCAGATGAGCCTAGGTC
GGATTAGCTAGTTGGTGAGGTAATGGCTCACCAAGGCGACGATCCGTAACTGGTCTGAGAGGATGATC
AGTCACACTGGAACTGAGACACGGTC
>Zotu376
GAACGCTAGCGACAGGCTTAACACATGCAAGTCGAGGGGCATCATGATTTGTAGCAATACAAATTGAT
GGCGACCGGCGCACGGGTGAGTAACGCGTATGCAACCTACCTATCAGAGGGGGATAGCCCATTGAAAG
ATGGATTAATACCGCATAAAACAGGGGTCCCGCATGGGAATATTTGTTAAAGATTTATTGCTGATAGA
TGGGCATGCGTTCCATTAGCTGGTTGGTAAGGTAACGGCTTACCAAGGCATCGATGGATAGGGGTTCT
GAGAGGAAGGTCCCCCACACTGGTACTGAGACACGGAC
>Zotu377
GAACGCTGGCGGCGTGCTTAACACATGCAAGTCGAACGAGACCCTTCCTTCGGGGAGGTGTTCTAGTG
GCGAACGGGTGAGTAACACGTGGGCAACCTGCCCCTGGCTCTGGGATAACTCCAAGAAATTGGGGCTA
ATACCGGATATGACCGCTGACCGCATGGTCTGGTGGTGGAAAGATTTATCGGCTGGGGATGGGCCCGC
GGCCTATCAGCTTGTTGGTGGGGTAGTGGCCTACCAAGGCGACGACGGGTAGCCGGCCTGAGAGGGTG
ACCGGCCACACTGGGACTGAGACACGGCC
>Zotu379
GAACGCTGGCGGCAGGCCTAACACATGCAAGTCGAACGGTAACAGGAAGAAGCTTGCTTCTTTGCTGA
CGAGTGGCGGACGGGTGAGTAATGTCTGGGAAACTGCCTGATGGAGGGGGATAACTACTGGAAACGGT
AGCTAATACCGCATAACGTCGCAAGACCAAAGAGGGGGACCTTCGGGCCTCTTGCCATCGGATGTGCC
CAGATGGGATTAGCTTGTTGGTGGGGTAACGGCTCACCAAGGCGACGATCCCTAGCTGGTCTGAGAGG
ATGACCAGCCACACTGGAACTGAGACACGGTC
>Zotu380
GAACGCTGGCGGCATGCCTTACACATGCAAGTCGAACGGTAACAGGTCTTCGGATGCTGACGAGTGGC
GAACGGGTGAGTAATACATCGGAACGTGCCCGATCGTGGGGGATAACGAGGCGAAAGCTTTGCTAATA
CCGCATACGATCTACGGATGAAAGCGGGGGATCTTCGGACCTCGCGCGGACGGAGCGGCCGATGGCAG
ATTAGGTAGTTGGTGGGATAAAAGCTTACCAAGCCGACGATCTGTAGCTGGTCTGAGAGGATGATCAG
CCACACTGGGACTGAGACACGGCC
>Zotu381

GAACGCTAGCGGCAGGCTTAATACATGCAAGTCGAACGGTTACAGCAATGTGACGAGTGGCGCACGGG
TGAGTAACGCGTACATAACATACCTTTAACTGGCGAATAGCCTTGGGAAACTGAGAGTAATACGCCAT
AACATTGTGATTCGGCATCGGATTACAATTAAAGTTCCGGCGGTTAGAGATTGATGTGCGTTGGATTA
GGTAGTTGGTGAGGTAACGGCTCACCAAGCCTTCGATCCATAGGGGGCGTGAGAGCGTGGTCCCCCAC
ACGGGTACTGAGACACGGAC
>Zotu382
GAACGCTGGCGGCAGGCTTAACACATGCAAGTCGAACGCGTGTAGCAATACACGAGTGGCGCACGGGT
GAGTAACGCGTGGATATCTGCCTTTTGGTTCGGAATAACCCCGGGAAACTGGGGCTAATACCGGATGG
TTCCTACGGGATAAAGATTTATCGCCAAAAGATGAGTCCGCGTACGATTAGCTAGTTGGTGAGGTAAA
GGCTCACCAAGGCGACGATCGTTAGCTGGTCTGAGAGGACGATCAGCCACACTGGGACTGAGACACGG
CC
>Zotu383
TAATGTTGGCAGTGTGCCTAATACATGCAAGTCGAGCGATATAGCGGCGGACGAGAGAGTAATAGGAA
GGAACATGCCCCGAACTCAAGGATAGCCACCCGAAAGGGTGGGTAATCCTGGATAGTCCCGCAAGGGT
AAAGCTTTATGCGGTTCGGGAGTGGCCTTCCTCCTATCAGCTTGTTGGTGAGGTAATAGCTCACCAAG
GCGATGACGGGTAGCTGGTCTGAGAGGATGACCAGCCTCGATGGGACTGAGACACGGCC
>Zotu384
GAACGCTGGCGGCGTGCCTAATACATGCAAGTAGAACGCTGAAGGAGGAGCTTGCTTCTCCGGATGAG
TTGCGAACGGGTGAGTAACGCGTAGGTAACCTGCCTGGTAGCGGGGGATAACTATTGGAAACGATAGC
TAATACCGCATAATAGTAGATGTTGCATGACATTTGCTTAAAAGGTGCAATTGCATCACTACCAGATG
GACCTGCGTTGTATTAGCTAGTTGGTGGGGTAACGGCTCACCAAGGCGACGATACATAGCCGACCTGA
GAGGGTGATCGGCCACACTGGGACTGAGACACGGCC
>Zotu385
GAACGCTGGCGGCAGGCTTAACACATGCAAGTCGAACGGGTGTAGCAATACATCAGTGGCAGACGGGT
GAGTAACACGTGGGAACCTTCCTCGTTGTACGGAACAACTCAGGGAAACTTGAGCTAATACCGTATAC
GTCCGAGAGGAGAAAGATTTATCGCAATGAGACGGGCCCGCGTCGGATTAGCTAGTTGGTGGGGTAAT
GGCCTACCAAGGCGACGATCCGTAGCTGATCTGAGAGGATGATCAGCCACACTGGGACTGAGACACGG
CC
>Zotu386
GAACGCTGGCGGCATGCCTAATACATGCAAGTCGAGCGAACAGATGAAGTGCTTGCACTTCTGACGTT
AGCGGCGAACGGGTGAGTAACACGTAAGGAATCTACCTATAAGCGGGGGATAACATTCGGAAACGGGT
GCTAATACCGCATAATATCTTCTTCCGCATGGAAGAAGATTGAAAGACGGCTCTGCTGTCACTTATGG
ATGACCTTGCGGTGCATTAGTTAGTTGGTGGGGTAATGGCCTACCAAGACGATGATGCATAGCCGACC
TGAGAGGGTGATCGGCCACATTGGGACTGAGACACGGCC
>Zotu387
GAACGCTGGCGGCGTGCCTAATACATGCAAGTCGAGCGAATCAGATGGGAGCTTGCTCCCGGATGATT
AGCGGCGGACGGGTGAGTAACACGTGGGCAACCTGCCCGTAAGATTGGGATAACTCCGGGAAACCGGT
GCTAATACCGAATAGTTTCTGACACCTCATGGTGTCAGAGGGAAAGTTGGCTTCGGCTAACACTTACG
GATGGGCCCGCGGCGCATTAGCTAGTTGGTGAGGTAACGGCTCACCAAGGCGACGATGCGTAGCCGAC
CTGAGAGGGTGATCGGCCACACTGGGACTGAGACACGGCC
>Zotu388
GAACGCTGGCGGCGTGCCTAATACATGCAAGTCGAGCGAGGAGCTTTTAGCTCCTAGCGGCGAACGGG
TGAGTAACACGTGGGCAACCTGCCCGTAAGACTGGGATAACTTCGGGAAACCGGAGCTAATACTGGAT
AATCTTGATCTTCGCATGAAGGACAAGTAAAAGATTTATCACTTACGGATGGGCCCGCGGTGCATTAG
CTTGTTGGTGGGGTAATGGCCTACCAAGGCGACGATGCATAGCCGACCTGAGAGGGTGACCGGCCACA
CTGGGACTGAGACACGGCC
>Zotu389
GAACGCTGGCGGCGTGCCTAATACATGCAAGTCGAGCGAACAGATAAGGAGCTTGCTCCTTTGACGTT
AGCGGCGGACGGGTGAGTAACACGTGGGTAACTTACCTATAAGACTGGAATAACTCCGGGAAACCGGG
GCTAATGCCGGATAACATGTTGAACCGCATGGTTCTACAGTGAAAGGCGGCTTTGCTGTCACTTATAG
ATGGACCCGCGCCGTATTAGCTAGTTGGTGAGGTAACGGCTCACCAAGGCAACGATACGTAGCCGACC
TGAGAGGGTGATCGGCCACACTGGAACTGAGACACGGTC
>Zotu390
GAACGCTGGCGGCGTGCCTAATACATGCAAGTCGAGCGAAGTTTTTCTGGTGCTTGCACCGGAAAAAC

TTAGCGGCGAACGGGTGAGTAACACGTAAAGAACCTGCCTCATAGACTGGGACAACTATTGGAAACGA
TAGCTAATACCGGATAACAGCATTAACTGCATGGTTGATGTTTAAAAGTTGGTTTTGCTAACACTATG
AAATGGCTTTGCGGTGCATTAGCTAGTTGGTGGGGTAAAGGCCTACCAAGGCGACGATGCATAGCCGA
CCTGAGAGGGTGATCGGCCACACTGGGACTGAGACACGGCC
>Zotu391
GAACGCTGGCGGCGCGCCTAAAACATGCAAGTCGAACGAGAAGCCCGGGCTTGCCCGGGTGGACAGTG
GCGAACGGGTGAGTAACACGTGAGGAACCTGCCCCGAAGACCGGGACAACACCGGGAAACCGGTGCTA
ATACCGGATACCTTCACCGGATCGCATGGTCTGGTGAAGAAATGGATTCCGCTTCGGGATGGCCTCGC
GGCCTATCAGCTAGTTGGTGAGGTAACGGCTCACCAAGGCGACGACGGGTAGCTGGTCTGAGAGGATG
TCCAGCCACACTGGGACTGAGACACGGCC
>Zotu392
GAACGCTGGCGGCGTGCCTAATACATGCAAGTCGAGCGAACTGACGAGGAGCTTGCTCCTTTGACGTT
AGCGGCGGACGGGTGAGTAACACGTGGGTAACCTACCTATAAGACTGGAATAACTCCGGGAAACCGGG
GCTAATGCCGGATAACATATCGAACCGCATGGTTCGATAGTGAAAGACGGTCTTGCTGTCACTTATAG
ATGGACCCGCGCCGTATTAGCTAGTTGGTGAGGTGACGGCTCACCAAGGCAACGATACGTAGCCGACC
TGAGAGGGTGATCGGCCACACTGGAACTGAGACACGGTC
>Zotu393
GAACGCTGGCGGCGTGCCTAATACATGCAAGTAGAACGCTGAAGCTTGGTGCTTGCGCCGAGCGGATG
AGTTGCGAACGGGTGAGTAACGCGTAGGTAACCTGCCTGGTAGCGGGGGATAACTATTGGAAACGATA
GCTAATACCGCATAATAGTAGATGTTGCATGACATTTGCTTAAAAGGTGCAATTGCATCACTACCAGA
TGGACCTGCGTTGTATTAGCTAGTTGGTGAGGTAACGGCTCACCAAGGCAACGATACATAGCCGACCT
GAGAGGGTGATCGGCCACACTGGGACTGAGACACGGCC
>Zotu394
GAACGCTGGCGGTGTGCTTCACACATGCAAGTCGAACGAGGTAGCAATACCTAGTGGCGGACGGGTGA
GTAACGCGTGGGAATCTGCCTTTAGATGGGGGATAACGGTCCGAAAGGATCGCTAATACCGCATATGC
CGAGAGGTGAAAGGAGTAATCCGTCTAAAGATGAGCCCGCGTCCGATTAGCTAGTTGGTGGGGTAAAG
GCCTACCAAGGCGACGATCGGTAGCTGGTCTGAGAGGATGATCAGCCACAATGGGACTGAGACACGGC
C
>Zotu395
GAACGCTGGCGGCGTGCTTAACACATGCAAGTCGAGCGGTAAGGCCTTTCGGGGTACACGAGCGGCGA
ACGGGTGAGTAACACGTGGGTGATCTGCCCTGCACTCTGGGATAAGCTTGGGAAACTGGGTCTAATAC
CGGATATGACCACAGCATGCATGTGTTGTGGTGGAAAGATTTATCGGTGCAGGATGGGCCCGCGGCCT
ATCAGCTTGTTGGTGGGGTAATGGCCTACCAAGGCGACGACGGGTAGCCGACCTGAGAGGGTGACCGG
CCACACTGGGACTGAGACACGGCC
>Zotu396
GAACGCTGGCGGCATGCCTTACACATGCAAGTCGAACGGCAGCACGGGTGCTTGCACCTGGTGGCGAG
TGGCGAACGGGTGAGTAATACATCGGAATGTACCTTGTAGTGGGGGATAGCTCGGCGAAAGCCGGATT
AATACCGCATACGCTCTGAGGAGGAAAGCGGGGGACCTTCGGGCCTCGTGCTACAAGAGCAGCCGATG
TCAGATTAGCTTGTTGGTGAGGTAAAAGCTCACCAAGGCGACGATCTGTAGCTGGTCTGAGAGGACGA
CCAGCCACACTGGGACTGAGACACGGCC
>Zotu397
GAACGCTGGCGGCGTGCTTAACACATGCAAGTCGAGCGAAGCGGCTGAATTGATTCTTCGGATGATTT
TCAGTTGACTGAGCGGCGGACGGGTGAGTAACGCGTGGGTGACCTGCCCCATACCGGGGGATAACAGC
TGGAAACGGCTGCTAATACCGCATAAGCGCACAGAGCTGCATGGCTCCGTGTGAAAAACTCTGGTGGT
ATGGGATGGGCCCGCGTCTGATTAGGCAGTTGGCGGGGTAACGGCCCACCAAACCGACGATCAGTAGC
CGGCCTGAGAGGGCGACCGGCCACATTGGGACTGAGACACGGCC
>Zotu398
GAACGCTAGCGGCAGGCCTAATACATGCAAGTCGAACGCGATTATCCAGCTTGCTGGATATGAAAGTG
GCGCACGGGTGCGTAACGCGTGAGCAACCTACCTCTGTCAGGGGGATAGCCCGGCGAAAGTCGGATTA
ACACCGCATGACATTAAGATGTGGCATCACATTATAATCAAATATTTATAGGACAGAGATGGGCTCGC
GTGACATTAGCTAGTTGGAGAGGTAACGGCTCACCAAGGCAACGATGTCTAGGGGCTCTGAGAGGAGA
ATCCCCCACACTGGTACTGAGACACGGAC
>Zotu399
GAACGCTGGCGGCGTGCTTAACACATGCAAGTCGAACGGAAAGGCTCCTGCTTGCAGGGGTACTCGAG

TGGCGAACGGGTGAGTAACACGTGGGTGATCTGCCCTGCACTTCGGGATAAGCCTGGGAAACTGGGTC
TAATACCGGATAGGACGATGGTTTGGATGCCATTGTGGAAAGTTTTTTCGGTGTGGGATGAGCTCGCG
GCCTATCAGCTTGTTGGTGGGGTAATGGCCTACCAAGGCGTCGACGAGTAGCCGGCCTGAGAGGGTGT
ACGGCCACATTGGGACTGAGATACGGCC
>Zotu400
GAACGCTGATAGAATGCTTAACACATGCAAGTCTTTGGCGAAGCTGTGCTTGCATAGCCTAGCCAAGG
CGGACGGGTGAGTAACGCGTAAGGAACTTGCCCTGCAGTCAGGGATAACAGACGGAAACGACTGATAA
GACCTGGTAAAGTCAGGCGGACTCATGTCCAGCCTGATGAAAAGAGATGCTGCAGGAGAGCCTTGCGT
CCTATTAGCTTGTTGGTGGGGTAACGGCCCACCAAGGCGATGATAGGTAGCCGGCCTGAGAGGGTGGA
CGGCCACAAGGGGACTGAGATACGGCC
>Zotu401
GAACGCTGGCGGCGTGCTTAACACATGCAAGTCGAACGGAAAGGCCAGTGCTTGCACTGGTACTCGAG
TGGCGAACGGGTGAGTAACACGTGGGTGATCTGCCCTGCACTTCGGGATAAGCCTGGGAAACTGGGTC
TAATACTGGATAGGACCACTTCTTGGATGGTGTGGTGGAAAGTTTTTTCGGTGTGGGATGAGCTCGCG
GCCTATCAGCTTGTTGGTGGGGTAATGGCCTACCAAGGCGTCGACGGGTAGCCGGCCTGAGAGGGTGT
ACGGCCACATTGGGACTGAGATACGGCC
>Zotu402
GAACGCTGGCGGCAGGCTTAACACATGCAAGTCGAGCGGAGATGAGGTGCTTGCACCTTATCTTAGCG
GCGGACGGGTGAGTAATGCTTAGGAATCTGCCTATTAGTGGGGGACAACATTCCGAAAGGAATGCTAA
TACCGCATACGTCCTACGGGAGAAAGCAGGGGATCTTCGGACCTTGCGCTAATAGATGAGCCTAAGTC
GGATTAGCTAGTTGGTGGGGTAAAGGCCTACCAAGGCGACGATCTGTAGCGGGTCTGAGAGGATGATC
CGCCACACTGGGACTGAGACACGGCC
>Zotu403
GAACGCTGGCGGCAGGCCTAACACATGCAAGTCGAGCGGATGAGGGGAGCTTGCTCCCTGATTTAGCG
GCGGACGGGTGAGTAATGTATAGGGAGCTGCCCGATAGAGGGGGATACCAGTTGGAAACGACTGTTAA
TACCGCATAATGTCTACGGACCAAAGTGTGGGACCTTCGGGCCACATGCTATCGGATGCACCTATATG
GGATTAGCTAGTTGGTGGGGTAACGGCTCACCAAGGCGACGATCCCTAGCTGGTTTGAGAGGATGATC
AGCCACACTGGAACTGAGACACGGTC
>Zotu404
GAACGCTAGCGGCAGGCTTAACACATGCAAGTCGAGGGGTATAGGTTTTCGGATCTAGAGACCGGCGC
ACGGGTGCGTAACGCGTATGCAATCTACCTTTTGCAGAGGGATAGCCCAGAGAAATTTGGATTAATAC
CTCATAGTATATAGAGTTGGCATCAACATTATATTAAAGTCACAACGGCAAAAGATGAGCATGCGTCC
CATTAGCTTGTTGGTAAGGTAACGGCTTACCAAGGCTACGATGGGTAGGGGTCCTGAGAGGGAGATCC
CCCACACTGGTACTGAGACACGGAC
>Zotu405
GAACGCTGGCGGCGTGCTTAACACATGCAAGTCGAACGGGAAACCTTTTATTGAAGCTTCGGCAGATT
TAGCTGGTTTCTAGTGGCGGACGGGTGAGTAACGCGTGGGTAACCTGCCTTATACAGGGGGATAACAG
TCAGAAATGACTGCTAATACCGCATAAGCGCACAGAGCTGCATGGCTCGGTGTGAAAAACTCCGGTGG
TATAAGATGGACCCGCGTTGGATTAGCTAGTTGGTGAGGTAACGGCCCACCAAGGCGACGATCCATAG
CCGGCCTGAGAGGGTGAACGGCCACATTGGGACTGAGACACGGCC
>Zotu406
GAACGCTGGCGGCGTGCCTAATACATGCAAGTCGAGCGAACAGATAAGGAGCTTGCTCCTTTGACGTT
AGCGGCGGACGGGTGAGTAACACGTGGGTAACCTACCTATAAGACTGGGATAACTTCGGGAAACCGGA
GCTAATACCGGATAACATTTGGAACCGCATGGTTCTAAAGTGAAAGATGGTTTTGCTATCACTTATAG
ATGGACCCGCGCCGTATTAGCTAGTTGGTAAGGTAACGGCTTACCAAGGCGACGATACGTAGCCGACC
TGAGAGGGTGATCGGCCACACTGGAACTGAGACACGGTC
>Zotu407
GAACGCTGGCGGCGTGCCTAACACATGCAAGTCGAACGGAGATTTCAGCAATGGAATCTTAGTGGCGA
ACGGGTGAGTAACGCGTAGACAACCTACCTTTTAGATGGGGACAACACTGCGAAAGTGGTGCTAATAC
CGAATGTTGCATTTTGGTTGCATAACCGAAATACTAAAGATGGCCTCTATTTATAAGCTATCGCTAAA
AGATGGGTCTGCGTCTGATTAGCTAGTTGGTGAGGTAATGGCTCACCAAGGCGACGATCAGTAGCCGG
TCTGAGAGGATGAACGGCCACACTGGGACTGAGACACGGCC
>Zotu408
GAACGCTGGCGGCGTGCCTAATACATGCAAGTCGAGCGAAGTTTTTCTGGTGCTTGCACCGGAAAAAC

TTAGCGGCGAACGGGTGAGTAACACGTAAAGAACCTGCCTCATAGACTGGGACAACTATTGGAAACGA
TAGCTAATACCGGATAACAGCATTAACTGCATGGTTGATGTTTAAAAGTTGGTTTTGCTAACACTATG
AGATGGCTTTGCGGTGCATTAGCTAGTTGGTGGGGTAAAGGCCTACCAAGGCGACGATGCATAGCCGA
CCTGAGAGGGTGATCGGCCACACTGGGACTGAGACACGGCC
>Zotu409
GAACGCTGGCGGCAGGCTTAACACATGCAAGTCGAGCGAAGGCTTCGGCCTTAGCGGCGGACGGGTGA
GTAACGCGTGGGAACGTGCCCTTTTCTTCGGAATAGCCCAGGGAAACTTGGATTAATACCGGATACGC
CCTTCGGGGGAAAGATTTATCGGAGAAGGATCGGCCCGCGTTGGATTAGGTAGTTGGTGGGGTAATGG
CCTACCAAGCCGACGATCCATAGCTGGTTTGAGAGGATGATCAGCCACACTGGGACTGAGACACGGCC
>Zotu410
GAACGCTAGCTACAGGCTTAACACATGCAAGTCGAGGGGAAACGGCATTAAGTGCTTGCACTTTTTGG
ACGTCGACCGGCGCACGGGTGAGTAACGCGTATCCAACCTTCCCATGACTAAGGGATAACCTGCCGAA
AGGCAGACTAATACCTTATGGTCTTCACTGACGGCATCAGATGTGAAGTAAAGATTTATCGGTTATGG
ATGGGGATGCGTCTGATTAGCTTGTTGGCGGGGTAACGGCCCACCAAGGCAACGATCAGTAGGGGTTC
TGAGAGGAAGGTCCCCCACATTGGAACTGAGACACGGTC
>Zotu411
GAACGCTGGCGGCATGCCTAATACATGCAAGTTGAACGATGATATTCTCTGCTTGCAGAGAATTGAAG
AGTAGCGAACGGGTGAGTAACACGTGGGGAACCTGCCCATGAGAGGGGGATAACATTCGGAAACGGAT
GCTAATACCGCATAATGCTTTGGACCGCCTGGTCCAGAGACGAAAGACGGCTTTTGCTGTCACTCATG
GATGGCCCCGCGCCGTATTAGCTAGTTGGTGAGGTAATGGCTCACCAAAGCTGTGATACGTAGCCGAC
CTGAGAGGGTGATCGGCCACACTGGGACTGAGACACGGCC
>Zotu412
GAACGCTGGCGGCATGCTTAACACATGCAAGTCGAACGGGAAGTGGTGTTTCCAGTGGCGAACGGGTG
AGTAACGCGTAAGAACCTGCCCTTGGGAGGGGAACAACAACTGGAAACGGTTGCTAATACCCCGTAGG
CTGAGGAGCAAAAGGAGAAATCCGCCCAAGGAGGGGCTCGCGTCTGATTAGCTAGTTGGTGAGGCAAT
AGCTTACCAAGGCGATGATCAGTAGCTGGTCCGAGAGGATGATCAGCCACACTGGGACTGAGACACGG
CC
>Zotu413
GAACGCTGACAGAATGCTTAACACATGCAAGTCAACTTGAACTTCGGTTTGGGTGGCGGACGGGTGAG
TAACGCGTAAAGAACTTGCCTCACAGCTAGGGACAACATTTGGAAACGAATGCTAATACCTGATATTA
TGATTATATGGCATCGTATAATTATGAAAGCTATATGCGCTGTGAGAGAGCTTTGCGTCCCATTAGCT
AGTTGGAGAGGTAACGGCTCACCAAGGCGATGATGGGTAGCCGGCCTGAGAGGGTGATCGGCCACAAG
GGGACTGAGACACGGCC
>Zotu414
GAACGCTGGCGGCGTGCCTAATACATGCAAGTCGAGCGAACAGATGAGGAGCTTGCTCCTTTGACGTT
AGCGGCGGACGGGTGAGTAACACGTGGGTAACCTACCTATAAGACTGGAATAACTCCGGGAAACCGGG
GCTAATGCCGGATAATACACAGAACCGCATGGTTCTGTGATGAAAGACGGTTTTGCTGTCACTTATAG
ATGGACCCGCGGCGTATTAGCTAGTTGGTAAGGTAACGGCTTACCAAGGCAACGATACGTAGCCGACC
TGAGAGGGTGATCGGCCACACTGGAACTGAGACACGGTC
>Zotu415
GAACGCTAGCGGCAGGCCTAATACATGCAAGTCGAACGGGCGGTAGCAATACCGTTAGTGGCGCACGG
GTGCGTAACGCGTAACCAACCTACCTGGATCTGGGGGATAGCCCGCCGAAAGGCGGATTAATACCGCA
TAAGCTACGTGACCGGCATCGGTTGGGTAGTAAAGATTTATTGGATTCAGATGGGGTTGCGTAGCATT
AGCTAGATGGCGGGGTAACGGCCCACCATGGCGACGATGCTTAGGGGACCTGAGAGGGTGATCCCCCA
CACTGGCACTGAGATACGGGC
>Zotu416
GAACGCTGGCGGCGTGCCTAATACATGCAAGTCGAGCGAACAGATAAGGAGCTTGCTCCTTTGAAGTT
AGCGGCGGACGGGTGAGTAACACGTGGGTAACCTACCTATAAGACTGGGATAACTTCGGGAAACCGGA
GCTAATACCGGATAACATTTAGAACCGCATGGTTCTAAAGTGAAAGATGGTTTTGCTATCACTTATAG
ATGGACCCGCGCCGTATTAGCTAGTTGGTAAGGTAATGGCTTACCAAGGCGACGATACGTAGCCGACC
TGAGAGGGTGATCGGCCACACTGGAACTGAGACACGGTC
>Zotu417
GAACGCTGGCGGCGTGCCTAATACATGCAAGTTGAGCGCTGAAGGTTGGTACTTGTACCGACTGGATG
AGCAGCGAACGGGTGAGTAACGCGTGGGGAATCTGCCTTTGAGCGGGGGACAACATTTGGAAACGAAT

GCTAATACCGCATAAAAACTTTAAACACAAGTTTTAAGTTTGAAAGATGCAATTGCATCACTCAAAGA
TGATCCCGCGTTGTATTAGCTAGTTGGTGAGGTAAAGGCTCACCAAGGCGATGATACATAGCCGACCT
GAGAGGGTGATCGGCCACATTGGGACTGAGACACGGCC
>Zotu418
GAACGCTGGCGGCGTGCTTAACACATGCAAGTCGAGCGACAGTGTTCTTCGGAACACATGGAGCGGCG
AACGGGTGAGTAACACGTGGGCAACCTGCCCTCAGCTCTGGGATAACTCCGGGAAACCGGGGCTAATA
CCGGATACGACCTTCCCTCGCATGGGGGTTGGTGGAAAGTTCTTCGGCTGAGGATGGGCCCGCGGCCT
ATCAGCTTGTTGGTGGGGTGATGGCCTACCAAGGCGACGACGGGTAGCCGGCCTGAGAGGGCGACCGG
CCACACTGGGACTGAGACACGGCC
>Zotu419
GAACGCTGGCGGCGTGCCTAATACATGCAAGTAGAACGCTGAAGAGAGGAGCTTGCTCTTCTTGGATG
AGTTGCGAACGGGTGAGTAACGCGTAGGTAACCTGCCTGGTAGCGGGGGATAACTATTGGAAACGATA
GCTAATACCGCATAAAATTGATTATTGCATGATAATTAATTGAAAGATGCAACTGCATCACTACCAGA
TGGACCTGCGTTGTATTAGCTAGTTGGTGAGGTAACGGCTCACCAAGGCGACGATACATAGCCGACCT
GAGAGGGTGATCGGCCACACTGGGACTGAGACACGGCC
>Zotu420
GAACGCTAGCGGCAGGCTTAACACATGCAAGTCGAGGGGCAGCAGGGACTTCGGTCCGCTGGCGACCG
GCAAACGGGTGCGGAACACGTACGCAACATACCTTAAACTGGGGCATAGCCTTCTGAAAAGGAGATTA
ACACCCCATAGTATCTTTAGGTGGCATCACCTGTTGATTAAAAATTCATTGGTTTAAGATTGGCGTGC
GTCCTATTAGCTAGTTGGCGGGGTAAAGGCCCACCAAGGCGACGATAGGTAGCTGATGTGAGAGCATG
ATCAGCCACACGGGCACTGAGACACGGGC
>Zotu421
GAACGCTGGCGGCGTGCCTAATACATGCAAGTCGAGCGAGCTGAATTCAAAGATTCCTTCGGGATGAT
TTGTTGGACGCTAGCGGCGGATGGGTGAGTAACACGTGGGCAATCTGCCCTAAAGACTGGGATACCAC
TTGGAAACAGGTGCTAATACCGGATAACAACATGAATCGCATGATTCAAGTTTGAAAGGCGGCGTAAG
CTGTCACTTTAGGATGAGCCCGCGGCGCATTAGCTAGTTGGTGGGGTAAAGGCCTACCAAGGCAATGA
TGCGTAGCCGAGTTGAGAGACTGATCGGCCACATTGGGACTGAGACACGGCC
>Zotu422
GAACGCTGGCGGCGTGCTTAACACATGCAAGTCGAACGATGAAGCCTAGCTTGCTAGGTGGATTAGTG
GCGAACGGGTGAGTAATACGTGAGTAACCTACCTTTAACTCTGGGATAAGCCTGGGAAACTGGGTCTA
ATACCGGATACGACCAATCTCCGCATGGGGTGTTGGTGGAAAGCGTTATGTAGTGGTTATAGATGGGC
TCACGGCCTATCAGCTTGTTGGTGAGGTAACGGCTCACCAAGGCGACGACGGGTAGCCGGCCTGAGAG
GGTGACCGGCCACACTGGGACTGAGACACGGCC
>Zotu423
GAACGCTGGCGGCATGCTTAACACATGCAAGTTGAACGCAGGATAAGCCTTCGGGTTTATTGCTGAGT
AGCAAACGGGTGAGTAACGCGTGGGAATCTGCCTTTATGTGGGGGATAACTAAGGGAAACTTTAGCTA
ATACCGCATACGCTCTACGGAGGAAAGACTTCGGTCGCATAAAGAGGAGCCCGCGTCAGATTAGGTAG
TTGGTAGGGTAAAGGCCTACCAAGCCAATGATCTGTAGCTGGTCTGAGAGGATGATCAGCCACACTGG
GACTGAGACACGGCC
>Zotu424
GAACGCTGGCGGCGTGCTTAACACATGCAAGTCGAACGATGATGCCCAGCTTGCTGGGCGGATTAGTG
GCGAACGGGTGAGTAATACGTGAGTAACCTGCCCTTGACTCTGGGATAAGCCTGGGAAACTGGGTCTA
ATACTGGATACTACCTCTTGCCGCATGGTGGGTGGTGGAAAGGGTTTTACTGGTTTTGGATGGGCTCA
CGGCCTATCAGCTTGTTGGTGGGGTAATGGCTCACCAAGGCGACGACGGGTAGCCGGCCTGAGAGGGT
GACCGGCCACACTGGGACTGAGACACGGCC
>Zotu425
GAACGCTAGCGGGAGGCCTAACACATGCAAGCCGAGCGGTAGAAGATCTTCGGATCTTTGAGAGCGGC
GTACGGGTGCGGAACACGTGTGCAACCTGCCTTTATCAGGGGGATAGCCTTTCGAAAGGAAGATTAAT
ACCCCATAATATATTGATTGGCATCAGTTAATATTGAAAACTCCGGTGGATAGAGATGGGCACGCGCA
AGATTAGATAGTTGGTAGGGTAACGGCCTACCAAGTCAGTGATCTTTAGGGGGCCTGAGAGGGTGATC
CCCCACACTGGTACTGAGACACGGAC
>Zotu426
GAACGTTGGCGGCGTGCTTAACACATGCAAGTCGAGCGAGAAAGTTCCTTCGGGAATGAGTACAGCGG
CGAACGGGTGAGTAACACGTGAGAATCTACCCTTTAGTGAAGAATACCTTCGAGAAATCGAAGTTAAT

GCTGCATAAGTCCCTTCGGGGAGAAAGATGGCCTCTTTGCTGTCGCTAAAGGATGAGCTTGCGTACTA
TTAGCTAGTTGGTGGGGTAAAGGCCTACCAAGGCGATGATGGTTAGCCGGCCTGAGAGGGTGTACGGC
CACACTGGAACTTAGACACGGTC
>Zotu427
GAACGCTAGCGGCAGGCTTAATACATGCAAGGCGAGGGGGCAGCAATGTCACCGTCGTACGGGTGCGT
AACGCGTATGCAACCTACCTATCACTGGGGGATAGCCCGGGGAAACCCGGATTAATACCGCATAATAC
ATTCGGGCCTCATGGCCTGATTAGTTAAAGATTTATTGGTGACAGATGGGCATGCGTTCGATTAGCTA
GTTGGTGCAGGTAACGGCTCACCAAGGCGACGATCGATAGGGGAGCTGAGAGGTTGATCCCCCACACG
GGCACTGAGATACGGGC
>Zotu428
GAACGCTGGCGGTGTGCTTCACACATGCAAGTCGAACGGGGTAGCAATACCCAGTGGCGGACGGGTGA
GTAACGCGTAGAAATCTGCCTTGAGATGGGGGATAACAGTCCGAAAGGACTGCTAATACCGCATATGT
ATGTATCTCGACAGAGGTATATATGAAAGGAGTAATCCGTCTTAAGATGAGTCTGCGTCCGATTAGCT
AGTTGGTAGGGTAAAGGCCTACCAAGGCGACGATCGGTAGCTGGTCTGAGAGGATGATCAGCCACAAT
GGGACTGAGACACGGCC
>Zotu429
GAACGCTGGCGGCGTGCTTAACACATGCAAGTCGAACGGAAAGGCCCTTTCGGGGGTACTCGAGTGGC
GAACGGGTGAGTAACACGTGGGTGATCTGCCCTGCACTTCGGGATAAGCTTGGGAAACTGGGTCTAAT
ACCGGATAGGACCATGCCATGCATGTGGTGTGGTGGAAAGCTTTTGCGGTGTGGGATGGGCCCGCGGC
CTATCAGCTTGTTGGTGGGGTGATGGCCTACCAAGGCGACGACGGGTAGCCGGCCTGAGAGGGTGTCC
GGCCACACTGGGACTGAGATACGGCC
>Zotu430
GAACGCTGGCGGCGTGCTTAACACATGCAAGTCGAACGGAAAGGCCCTTTCGGGGGTACTCGAGTGGC
GAACGGGTGAGTAACACGTGGGTGATCTGCCCTGCACTTCGGGATAAGCTTGGGAAACTGGGTCTAAT
ACCGGATAGGACTACGCACTGCATGGTGTGTGGTGGAAAGCTTTTGCGGTGTGGGATGGGCCCGCGGC
CTATCAGCTTGTTGGTGGGGTGATGGCCTACCAAGGCGACGACGGGTAGCCGGCCTGAGAGGGTGTCC
GGCCACACTGGGACTGAGATACGGCC
>Zotu431
GAACGCTGGCGGCGTGCTTAACACATGCAAGTCGAACGATGAAGCCCTTTCGGGGGTGGATTAGTGGC
GAACGGGTGAGTAACACGTGGGCAATCTGCCCTGCACTTCGGGACAAGCCCTGGAAACGGGGTCTAAT
ACCGGATATGACCATCGGGTGCATGCTCTGATGGTGGAAAGCTCCGGCGGTGCAGGATGAGCCCGCGG
CCTATCAGCTTGTTGGTGGGGTGATGGCCTACCAAGGCGACGACGGGTAGCCGGCCTGAGAGGGCGAC
CGGCCACACTGGGACTGAGACACGGCC
>Zotu432
GAACGCTGGCGGCAGGCTTAACACATGCAAGTCGAACGCCCCGCAAGGGGAGTGGCAGACGGGTGAGT
AACACGTGGGAACGTACCTTTTGGTTCGGAATAACTCAGGGAAACTTGAGCTAATACCGGATAAGCCC
GCAAGGGGAAAGATTTATTGCCGAAAGATCGGCCCGCGTCTGATTAGCTAGTTGGTGAGGTAACGGCT
CACCAAGGCGACGATCAGTAGCTGGTCTGAGAGGATGACCAGCCACACTGGGACTGAGACACGGCC
>Zotu433
GAACGCTGGCGGCATGCCTAACACATGCAAGTCGAACGAAGGCTTCGGCCTTAGTGGCGCACGGGTGC
GTAACGCGTGGGAATCTGCCCCTTGGTTCGGAATAACAGTTAGAAATGACTGCTAATACCGGATGATG
ACGTAAGTCCAAAGATTTATCGCCGAGGGATGAGCCCGCGTAGGATTAGCTAGTTGGTGTGGTAAGAG
CGCACCAAGGCGACGATCCTTAGCTGGTCTGAGAGGATGATCAGCCACACTGGGACTGAGACACGGCC
>Zotu434
GAACGCTGGCGGCAGGCTTAACACATGCAAGTCGAACGGTCCTTCGGGATAGTGGCGGACGGGTGAGT
AACACGTGGGAACGTACCCTTAGGTTCGGAACAACACAGGGAAACTTGTGCTAATACCGGATGAGCCC
TTCGGGGGAAAGATTTATCGCCTTTGGAGCGGCCCGCGTCTGATTAGCTAGTTGGTGAGGTAATGGCT
CACCAAGGCGACGATCAGTAGCTGGTCTGAGAGGATGGCCAGCCACACTGGGACTGAGACACGGCC
>Zotu436
GAACGCTAGCGGGAGGCCTAACACATGCAAGTCGAACGGGATTGGGAGCTTGCTCCCATGAGAGTGGC
GCACGGGTGAGTAACGCGTATGCAACCTACCTTTAACAGAGGGATAGCCTATAGAAATGTGGATTAAT
ACCTCATAGTTCTGAGAAGCCGCATGGTTTTTCAGATAAAGCTCCGGCGGTTAAAGATGGGCATGCGT
GACATTAGCTAGTTGGTAAGGTAACGGCTTACCAAGGCTACGATGTCTAGGGGTTCTGAGAGGATGAT
CCCCCACACTGGTACTGAGACACGGAC

>Zotu437
GAACGCTAGCTACAGGCTTAACACATGCAAGTCGAGGGGCATCATGAGGTCAGCTTGCTGACTTTGAT
GGCGACCGGCGCACGGGTGCGTAACGCGTATCCAATCTACCGCATACTCGGGGATAGCCTTGCGAAAG
TAAGATTAATACCCGATGTTGTTTGTTATTCGCATGAATGATAAACCAAAGAATTTCGGTATGCGATG
AGGATGCGTCCCATTAGGTAGTTGGTGAGGTAACGGCTCACCAAGCCTCCGATGGGTAGGGGTTCTGA
GAGGAAGATCCCCCACACTGGAACTGAGACACGGTC
>Zotu438
GAACGCTGGCGGCGTGCCTAATACATGCAAGTCGAGCGAACGGATAAGGAGCTTGCTCCTTTGAAGTT
AGCGGCGGACGGGTGAGTAACACGTGGGTAACCTACCTATAAGACTGGGATAACTTCGGGAAACCGGA
GCTAATACCGGATAACATTTAGAACCGCATGGTTCTAAAGTGAAAGATGGTTTTGCTATCACTTATAG
ATGGACCCGCGCCGTATTAGCTAGTTGGTAAGGTAATGGCTTACCAAGGCGACGATACGTAGCCGACC
TGAGAGGGTGATCGGCCACACTGGAACTGAGACACGGTC
>Zotu439
GAACGCTGGCGGCATGCTTTACACATGCAAGTCGAACGGCATCGGGGAGGAGCTTGCTCCTCTGCCGG
CGAGTGGCGAACGGGTGAGTAATACATCGGAACGTGTCGAGAAGAGGGGGATAACCATCCGAAAGGGT
GGCTAATACCGCATAAATTCTGAGGAAGAAAGCTGGGGACCTAGCGATAGGCCTAGCGCTTTTTGAGC
GGCCGATGGCGGATTAGCTAGTTGGTGAGGTAAAGGCTCACCAAGGCGAAGATCTGTAGCGGGTCTGA
GAGGATGATCCGCCACACTGGGACTGAGACACGGCC
>Zotu440
GAACGCTAGCGGGAGGCTTAACACATGCAAGTCGAGGGGTACAGTAGCAATACTGGAGACCGGCGCAC
GGGTGAGTAACGCGTATGCAACCTGCCCTTTACAGGAGGATAGCCCGCTGAAAGGCGGATTAATACTG
CATAAGATTGTACTGCTGCATGGCAGAGCAATAAAAGCTGAGGCGGTAAAGGATGGGCATGCGTGACA
TTAGCTAGTTGGTGGGGTAACGGCCCACCAAGGCAACGATGTCTAGGGGTTCTGAGAGGAAGGTCCCC
CACACTGGTACTGAGACACGGAC
>Zotu441
GAACGCTAGCGGCAGGCCTAATACATGCAAGTCGGACGGGATTCCGTTGTAGCTTGCTATGACGGATG
AGAGTGGCGCACGGGTGCGTAACGCGTGAGCAACCTGCCCATGTCAGGGGGATAGCCCGTTGAAAGAC
GGATTAACACCGCATAATACATTATGCCCGCCTGGGTATTCTGTTAAATATTTATAGGACATGGATGG
GCTCGCGTGGCATTAGCTTGTTGGTAAGGTAACGGCTTACCAAGGCTATGATGCCTAGGGGCTCTGAG
AGGAGAATCCCCCACACTGGTACTGAGACACGGAC
>Zotu442
GAACGCTGGCGGCGTGCCTAATACATGCAAGTCGAGCGAATGGATTAAGAGCTTGCTCTTATGAAGTT
AGCGGCGGACGGGTGAGTAACACGTGGGTAACCTGCCCATAAGACTGGGATAACTCCGGGAAACCGGG
GCTAATACCGGATAATATTTTGAACTGCATGGTTCGAAATTGAAAGGCGGCTTCGGCTGTCACTTATG
GATGGACCCGCGTCGCATTAGCTAGTTGGTGAGGTAACGGCTCACCAAGGCAACGATGCGTAGCCGAC
CTGAGAGGGTGATCGGCCACACTGGGACTGAGACACGGCC
>Zotu443
GAACGCTGGCGGCGTGCTTAACACATGCAAGTCGAACGATGATCCCAGCTTGCTGGGGGATTAGTGGC
GAACGGGTGAGTAACACGTGAGTAACCTGCCCTTGACTCTGGGATAAGCCTGGGAAACTGGGTCTAAT
ACCGGATATGACTCCTCATCGCATGGTGGGGGGTGGAAAGCTTTTGTGGTTTTGGATGGACTCGCGGC
CTATCAGCTTGTTGGTGGGGTAATGGCCTACCAAGGCGACGACGGGTAGCCGGCCTGAGAGGGTGACC
GGCCACACTGGGACTGAGACACGGCC
>Zotu444
GAACGTTAGCGGCGCGCTTAACACATGCAAGTCGCACGAGAAAGGGCTTCGGCCCCGGTACAGTGGCG
CACGGGTGAGTAACACGTGGGTAATCTTCCCTCTGGCGGGGGATAACCTTCCGAAAGGAGGGCTAATA
CCGCATAAGACCACGACTCCGAAAGGAGACGAGGTTAAAGCTGGCCTCTATACATAAGCTAGCACCAG
AGGATGAGCCCGCGGCCCATCAGGTAGTTGGTAGGGTAACGGCCTACCAAGCCTAAGACGGGTAGCTG
GTCTGAGAGGATGATCAGCCACACTGGAACTGAGACACGGTC
>Zotu445
GAACGCTTGCGGCGTGCCTAAGAAATGCAAGTCGAACGGGCACCTTCGGGTGCTAGTGGCGAACGGTC
GCGTAACACGTAAGAAACCTGCCCCGGAGTGGGGGACAACGGCCCGAAAGGGCCGCTAATACCGCATG
TGGTCGGTGGATCGCCTGGTCCTCCGTCCAAAGCTTAATGCGCTCCGGGATGGTCTTGCGGCCTATCA
GGTAGATGGTGGGGTAACGGCCTACCATGCCGACGACGGGTAGCTGGTCTGAGAGGACGATCAGCCGG
ATTGGGACTGAGATACGGCC

>Zotu446
GAACGCTGGCGGCATGCCTAATACATGCAAGTCGAACGCTGAAGCACTCTGCTTGCAGAGTGCGGATG
AGTGGCGAACGGGTGAGTAACACGTGGGAAATCTGCCTATAAGAAGGGGATAACATTCGGAAACGGAT
GCTAATACCGCATATATTCTTGGACCGCCTGGCCCTTGAAAGAAAGACGGCTTTGCTGTCGCTTATAG
ATGATCCCGCGGCGTATTAGTTAGTTGGTGGGGTAAAGGCCTACCAAGACAATGATACGTAGCCGACC
TGAGAGGGTGATCGGCCACACTGGGACTGAGACACGGCC
>Zotu447
GAACGCTGGCTGCGTGCTTAACACATGCAAGTCGAACGCTGAAGCCTGGTGCTTGCACCGGGTGGATG
AGTGGCGAACGGGTGAGTAACACGTGAGTAACCTGCCCCTGACTTCGGGATAAGCCCGGGAAACTGGG
TCTAATACCGGATACGACTGCCGGACGCATGTCTGGTGGTGGAAAGTTTTTTCGGTTGGGGATGGGCT
CGCGGCCTATCAGTTTGTTGGTGAGGTAATGGCTCACCAAGACGACGACGGGTAGCCGGCCTGAGAGG
GCGACCGGCCACACTGGGACTGAGACACGGCC
>Zotu448
TAACGCTAGCGGCAGGCATAATACATGCAAGTTGAACGAGATAAGTTGTAGCAATATGACTTTGAAAG
TGGCGCACGGGTGCGTAACACGTATGCAACCTGCCCATATCTGGGGAATAGCCTCTCGAAAGAGAGAT
TAATACCCCATACCACAATGTTAAGGCATCTTAATATTGTGAAAGCTCAGGCGGATATGGATGGGCAT
GCGGCTGATTAGCTAGTTGGTAGGGTAACGGCCTACCAAGGCTACGATCAGTAGGGGGTCTGAGAGGA
TGTCCCCCCACACTGGTACTGAGATACGGAC
>Zotu449
GAACGCTGGCGGCGTGCTTAACACATGCAAGTCGAACGGGCTTCTGGGGTTCTGGCTTTTTTGGTTGG
GGTTCTGGGGGTTAGTGGCGAACGGGTGAGTAACACGTGAGTAACCTGCCCTTCACTTCTGGATAACC
GCTTGAAAGGGTGGCTAATACGGGATGTTTCTGGGTCTGTCGCATGGCGGGTTCGGGAAAGATGGCAC
TGTTTGGTGTTTTTGGTGGGGGATGGGCTCGCGGCCTATCAGCTTGTTGGTGGGGTGATGGCCTGCCA
AGGCTTTGACGGGTAGCCGGCCTGAGAGGGTGGGCGGTCACACTGGGACTGAGATACGGCC
>Zotu450
GAACGCTGGCGGCAGGCTTAACACATGCAAGTCGAGCGCCCCGCAAGGGGAGCGGCAGACGGGTGAGT
AACGCGTGGGAATCTACCCTGATCTGCGGGATAGCTCCGGGAAACTGGAATTAATACCGCATGTGCCC
TGAGGGGCAAAGATTTATCGGATTGGGATGAGCCCGCGTCGGATTAGCTAGTTGGTGGGGTAAAGGCC
TACCAAGGCGACGATCCGTAGCTGGTCTAAGAGGATGATCAGCCACATTGGGACTGAGACACGGCC
>Zotu451
GAATGCTGGCGGCGTGCTTAACACATGCAAGTCGAGCGGTAACATGTTTCTCACTGTCTTCTTCTTTT
TATAAGGAGGGAACTGTGAGAAATGATGACGAGCGGCGGACGGCTGAGTAACGCGTGGGAACATGCCC
CAAAGTGAGGAATAAGCTCCAGAAATGGAGTCTAATGCCGCATATGATCTTCGGATTAAAGGATTTAT
CCGCTTTGGGAGTGGCCTGCGTACGATTAGGTAGTTGGTGGGGTAATAGCCTACCAAGCCTACGATCG
TTAGCTGGTCTGAGAGGATGACCAGCCAGACTGGAACTGAGACACGGTC
>Zotu452
TAACGCTAGCGGCAGGCCTAATACATGCAAGTCGAACGGCAGCACGAATAGCAATATTTGGTGGCGAG
TGGCGCACGGGTGCGTAACGCGTATGCAATCTACCTATAACTGGGGAATAACCCCGAGAAATCGGGGC
TAATACCGCATAATACTACTGAACGGCATCGCTTGGTAGTCAAATCTCCGGAGGTTATAGATGAGCAT
GCGTGACATTAGCTTGTTGGTGAGGTAACGGCTCACCAAGGCTACGATGTCTAGGGGGTCTGAGAGGA
TGATCCCCCACACTGGTACTGAGACACGGAC
>Zotu453
GAACGCTGGCGGCGTGCTTAACACATGCAAGTCGAACGATGATGCCCAGCTTGCTGGGTGGATTAGTG
GCGAACGGGTGAGTAATACGTGAGTAACCTGCCCTTGACTCTGGGATAAGCCTGGGAAACTGGGTCTA
ATACTGGATACTACCTCTCATCGCATGGTGGGTGGTGGAAAGGGTTTTACTGGTTTTGGATGGGCTCA
CGGCCTATCAGCTTGTTGGTGGGGTAATGGCTCACCAAGGCGACGACGGGTAGCCGGCCTGAGAGGGT
GACCGGCCACACTGGGACTGAGACACGGCC
>Zotu454
GAACGCTGGCGGTATGCCTAAGACATGCAAGTCGCACGATCTGTTTTCGGACGGGTAGTGGCGGACGG
GTGAGTAACACGTGGGTGATCTGCCCAGAAGTCCGGGACAACTAGGCGAAAGCTTAGCTAATCCGGGA
TGTGGACCGAGATTTTGATCTTTGTCTAAAGCTTCGGCGCTTCTGGATGAGCCTGCGGCGCATCAGCT
GGTTGGCGGGGTAACGGCCCACCAAGGCGACGACGCGTAGCTGGTCTGAGAGGACGACCAGCCACAGG
GGTACTGAGACACGGAC
>Zotu455

GAACGCTGGCGGTCTGCTTAACACATGCAAGTCGAACGGGATGCTTTCGGGCATCTAGTGGCGGACGG
GTGAGTAACGCGTGAGAATCTGCCCTTGGGAGTGGGACAACGACTGGAAACGGTCGCTAACACCGCAT
GAGCCGAGAGGTAAAAGTATTTATAGCCCAAGGATGAGCTCGCGTCGGATTAGCTAGTTGGTAGTGTA
ATGGACTACCAAGGCGACGATCCGTAGCTGGTCTGAGAGGATGACCAGCCACACTGGGACTGAGACAC
GGCC
>Zotu456
GAACGCTAGCGGCAGGCTTAACACATGCAAGTCGAGGGGTAGAAGCAGCTTGCTGCTTTGAGACCGGC
GCACGGGTGCGTAACGCGTATACAATCTACCTTTTACAGAGGGATAGCCCAGAGAAATTTGGATTAAT
ACCTCATAGTATTTTCGAATGGCATCATTTGATTATTAAAGTTCCAACGGTAAAAGATGAGTATGCGT
CCCATTAGCTAGATGGTAAGGTAACGGCTTACCATGGCAACGATGGGTAGGGGTCCTGAGAGGGAGAT
CCCCCACACTGGTACTGAGACACGGAC
>Zotu457
GAACGCTGGCGGCAGGCCTAACACATGCAAGTCGAGCGGATGAGAGGAGCTTGCTCCTCGATTCAGCG
GCGGACGGGTGAGTAATGCCTAGGAATCTGCCTAGTAGTGGGGGACAACGTTTCGAAAGGAACGCTAA
TACCGCATACGTCCTACGGGAGAAAGTGGGGGATCTTCGGACCTCACGCTATTAGATGAGCCTAGGTC
GGATTAGCTAGTTGGTAGGGTAAAGGCCTACCAAGGCGACGATCCGTAACTGGTCTGAGAGGATGATC
AGTCACACTGGAACTGAGACACGGTC
>Zotu458
GAACGCTGGCGGCGTGCCTAATACATGCAAGTCGAGCGGACGAATCGAAAGCTTGCTTTTGATTCGTT
AGCGGCGGACGGGTGAGTAACACGTGGGCAACCTGCCCTGTAGACGGGGATAACACCGAGAAATCGGT
GCTAATACCGGATAACACGAAATGTCGCATGACGTTTCGTTGAAAGACGGCGTAAGCTGTCGCTACAG
GATGGGCCCGCGGCGCATTAGCTAGTTGGTGAGGTAACGGCTCACCAAGGCGACGATGCGTAGCCGAC
CTGAGAGGGTGATCGGCCACACTGGGACTGAGACACGGCC
>Zotu459
GAACGCTAGCGGCAGGCCTAATACATGCAAGTCGAACGGTCTCTAGCAATAGGGATAGTGGCGCACGG
GTGCGTAACGCGTAACCAACCTGCCCTGAACTGGGGGATAGCCCGCCGAAAGGCGGATTAATACCGCA
TATACCCGCAGCCTGGCATCAGGCAACGGGGAAAGATTTATTGGTTCAGGATGGGGTTGCGTGACATT
AGCTAGTTGGCGGGGTAACGGCCCACCAAGGCGACGATGTCTAGGGGACCTGAGAGGGTGATCCCCCA
CACTGGCACTGAGATACGGGC
>Zotu460
TAACGTTGGCGGCGTGCATAACACATGCAAGTTGAGCGTGGCTTTAGTTTACTAAAGTCTAGCAGCGG
ACGGCTGAGTAATGCACAGGAACGTACCCGGAAGTGGGGAATAGCCCATCGAAAGATGGGGTAATACC
GCATGTGCTCGCAAGAGTAAAGCCGCAAGGCGCTACCGGAACGGCCTGTGTCCTATCAGGTTGTTGGT
GGGGTAAAAGCCTACCAAGCCTATGACGGGTAACTGGTCTGAGAGGATGACCAGTCAGATCGGGACTG
AGACACGGCC
>Zotu461
GAACGCTGGCGGCGTGCCTAACACATGCAAGTCGAGCGATTCTCTTCGGAGAAGAGCGGCGGACGGGT
GAGTAACGCGTGGGTAACCTGCCCTGTACACACGGATAACATACCGAAAGGTATGCTAATACGAGATA
ATATGCTTTTATCGCATGGTAGAAGTATCAAAGCTCCGGCGGTACAGGATGGACCCGCGTCTGATTAG
CTAGTTGGTAAGGTAACGGCTTACCAAGGCGACGATCAGTAGCCGACCTGAGAGGGTGATCGGCCACA
TTGGAACTGAGACACGGTC
>Zotu462
GAACGCTGGCGGCATGCTTTACACATGCAAGTCGAACGGCAGCACGGGTGCTTGCACCTGGTGGCGAG
TGGCGAACGGGTGAGTAATGCACCGGAACGTATCCAATAATGGGGGATAACGCACCGAAAGGTGTGCT
AATACCGCATACGCCCTGAGGGGGAAAGCGGGGGATCGAAAGACCTCGCGTTAATGGAGCGGCCGATG
TCGGATTAGCTAGTTGGTGAGGTAAAGGCTCACCAAGGCGACGATCCGTAGCTGGTCTGAGAGGACGA
CCAGCCACACTGGAACTGAGACACGGTC
>Zotu463
GAACGCTGGCGGCATGCCTAATACATGCAAGTCGAACGCTGAAGCACTCTGCTTGCAGAGTGCGGATG
AGTGGCGAACGGGTGAGTAACACGTGGGGAATCTGCCTATAAGTGGGGGATAACATTCGGAAACGGAT
GCTAATACCGCATATATTCTTGGACCGCCTGGTCCTTGAAAGAAAGACGGCTTTGCTGTCGCTTATAG
ATGATCCCGCGGCGTATTAGTTAGTTGGTGGGGTAAAGGCCTACCAAGACAATGATACGTAGCCGACC
TGAGAGGGTGATCGGCCACACTGGGACTGAGACACGGCC
>Zotu464

GAACGTTGGCGGCGTGCCTAATACATGCAAGTCGAACGCGGTCTCCTAATTGAAAAACCGTGCTTGCA
CGGGTTGGATTTTAGATCGGACCGAGTGGCGAACTGGTGAGTAACACGTGGGTAACCTGCCCAGAAGT
AGGGGATAACACCTGGAAACAGATGCTAATACCGTATAACAACTAAAACTACATGGTTTTAGTTTGAA
AGCTGGCCTTGGCGCTAGTGCTTTTGGATGGACCCGCGGCGTATTAGCTAGTTGGTGAGATAATAGCT
CACCAAGGCGATGATACGTAGCAGACCTGAGAGGGTAATCTGCCACAATGGGACTGAGACACGGCC
>Zotu465
GAACGCTGGCGGTAGGCCTAACACATGCAAGTCGAACGGCAGCACAGTAAGAGCTTGCTCTTATGGGT
GGCGAGTGGCGGACGGGTGAGGAATACATCGGAATCTACCTTTTCGTGGGGGATAACGTAGGGAAACT
TACGCTAATACCGCATACGACCTTCGGGTGAAAGCAGGGGACCTTCGGGCCTTGCGCGGATAGATGAG
CCGATGTCGGATTAGCTAGTTGGCGGGGTAAAGGCCCACCAAGGCGACGATCCGTAGCTGGTCTGAGA
GGATGATCAGCCACACTGGAACTGAGACACGGTC
>Zotu466
CGTCAGGTCCCAGTGCGTTTGGTTAATTACACCTTAGAAGACCTTTCACTGCCGCGAAAAAGGGTAGT
AGCGCAATTCATCCCGGCGAACATTACAGAAAGCGGACAAACAGAAAGTATCGGACTAACAGGA
>Zotu467
GAACGCTGGCGGCAGGCTTAACACATGCAAGTCGAACGCACCTTTCGGGGTGAGTGGCAGACGGGTGA
GTAACGCGTGGGAATCTACCCTTCTCTACGGGATAGCTCCGGGAAACTGGAATTAATACCGTATACGT
CCTGAGGGAGAAAGATTTATCGGGGAAGGATGAGCCCGCGTTGGATTAGCTAGTTGGTGGGGTAAAGG
CCTACCAAGGCGACGATCCATAGCTGGTCTAAGAGGATGATCAGCCACATTGGGACTGAGACACGGCC
>Zotu468
GAACGCTGGCGGCGTGCCTAATACATGCAAGTAGAACGCTGAAGGAGGAGCTTGCTCTTCTGGATGAG
TTGCGAACGGGTGAGTAACGCGTAGGTAACCTGCCTGGTAGTGGGGGATAACTATTGGAAACGATAGC
TAATACCGCATAATAACAGTTGTTGCATGACAACTGTTTGAAAGGTGCAATTGCATCACTACCAGATG
GACCTGCGTTGTATTAGCTAGTTGGTGGGGTAACGGCTCACCAAGGCGACGATACATAGCCGACCTGA
GAGGGTGATCGGCCACACTGGGACTGAGACACGGCC
>Zotu469
GAACGCTGGCGGCATGCCTTACACATGCAAGTCGAACGGCAGCACGGGGGCAACCCTGGTGGCGAGTG
GCGAACGGGTGAGTAATACATCGGAACGTGTCCTGTAGTGGGGGATAGCCCGGCGAAAGCCGGATTAA
TACCGCATACGATCTGTGGATGAAAGCGGGGGATCCTTCGGGACCTCGCGCTACAGGGGCGGCCGATG
GCAGATTAGCTAGTTGGTGGGGTAAAGGCCTACCAAGGCGACGATCTGTAGCTGGTCTGAGAGGACGA
CCAGCCACACTGGGACTGAGACACGGCC
>Zotu470
GAACGCTAGCGGCAGGCTTAACACATGCAAGTCGAGGGGCAGCGCAGGTAGCAATACTGGGCGGCGAC
CGGCAAACGGGTGCGGAACACGTACAGAATCTACCTTGATCTGGGGCATAGCCCGGAGAAATCCGGAT
TAACTCCCCATAACATATATGAGTGGCATCACTTGAATATTAAAAATTTATTGGATCGAGATGACTGT
GCGTCCCATTAGCTAGTTGGTAGGGTAACGGCCTACCAAGGCGACGATGGGTAGCTGGTGTGAGAGCA
CGACCAGCCACACGGGCACTGAGACACGGGC
>Zotu471
GAACGCTAGCGGCAGGCTTAACACATGCAAGTCGAGGGGCAGCATATAAATTGCTTGCAATTTATGAT
GGCGACCGGCGCACGGGTGCGTAACGCGTATGCAACCTACCTGTAACAGGGGGATAACACTGAGAAAT
TGGTACTAATACCCCGTAATATTACGAGAGGCATCTTTTGTAGTTGAAAGTTCCGGCGGTTACAGATG
GGCATGCGTTGTATTAGATAGTTGGTGAGGTAACGGCTCACCAAGTCGACGATACATAGGGGGACTGA
GAGGTTAACCCCCCACATTGGTACTGAGACACGGAC
>Zotu472
GAACGCTGGCGGCATGCCTAACACATGCAAGTCGAACGAGACCTTCGGGTCTAGTGGCGCACGGGTGC
GTAACGCGTGGGAATCTGCCCTTGGGTGCGGAATAACTTCTCGAAAGGGATGCTAATACCGCATAATG
TCGTAAGACCAAAGATTTATCGCCCAAGGATGAGCCCGCGTAAGATTAGCTAGTTGGTGGGGTAAAAG
CCTACCAAGGCGACGATCTTTAGCTGGTCTGAGAGGATGATCAGCCACACTGGGACTGAGACACGGCC
>Zotu473
GAACGCTGGCGGCAGGCCTAACACATGCAAGTCGAGCGCATCCTTCGGGGTGAGCGGCGGACGGGTGA
GTAACGCGTGGGAATATGCCTTTCGGTACGGAATAGTCTCGGGAAACTGGGGGTAATACCGTATACGC
CCTATTGGGGAAAGATTTATCGCCGAAAGATTAGCCCGCGTTGGATTAGGTAGTTGGTGGGGTAATGG
CCCACCAAGCCGACGATCCATAGCTGGTTTGAGAGGATGATCAGCCACACTGGGACTGAGACACGGCC
>Zotu474

GAACGCTGGCGGCAGGCCTAACACATGCAAGTCGAACGGATCCTTCGGGATTAGTGGCGGACGGGTGA
GTAACACGTGGGAACGTGCCCTTTGGTTCGGAACAACTCAGGGAAACTTGAGCTAATACCGGATGTGC
CCTTCGGGGGAAAGATTTATCGCCATTGGAGCGGCCCGCGTCTGATTAGCTAGTTGGTGGGGTAAAGG
CCCACCAAGGCGACGATCAGTAGCTGGTCTGAGAGGATGATCAGCCACATTGGGACTGAGACACGGCC
>Zotu475
GAACGCTGGCGGCATGCTTAACACATGCAAGTCGAACGGCAGCATATTGTGTAGCAATACACAGTGAT
GGCGAGTGGCGGACGGGTGAGTAAAGCATAGGAATCTGCCGAGAAGAGGGGGATAACTGAGGGAAACT
TCAGCTAAGACCACATAATGTCTACGGACCAAATCAGGGGACCTTCGGGCCTTGAGCTTTTTGATGAG
CCTATGCCGGATTAGCTAGTTGGTAGGGTAAGAGCCTACCAAGGCGAAGATCCGTAGCTGGTCTGAGA
GGATGATCAGCCACACTGGGACTGAGACACGGCC
>Zotu476
GAACGCTGGCGGCGTGCTTAACACATGCAAGTCGAACGGAAAGGCCCTTCGGGGTACTCGAGTGGCGA
ACGGGTGAGTAACACGTGGGTGATCTGCCCTGCACTTTGGGATAAGCCTGGGAAACTGGGTCTAATAC
CGAATATGATCACGCTTCTCATGGGGTGTGGTGGAAAGCTTTTGCGGTGTGGGATGGGCCCGCGGCCT
ATCAGCTTGTTGGTGGGGTAATGGCCTACCAAGGCGACGACGGGTAGCCGGCCTGAGAGGGTGTCCGG
CCACACTGGGACTGAGATACGGCC
>Zotu477
GAACGCTGGCGGCATGCCTTACACATGCAAGTCGAACGGCAGCACGGGTGCTTGCACCTGGTGGCGAG
TGGCGAACGGGTGAGTAATATATCGGAACGTGCCCTAGAGTGGGGGATAACGTAGCGAAAGTTACGCT
AATACCGCATACGATCTACGGATGAAAGTGGGGGACCGCAAGGCCTCACGCTCCTGGAGCGGCCGATA
TCTGATTAGCTAGTTGGTGGGGTAAAAGCCTACCAAGGCGACGATCAGTAGCTGGTCTGAGAGGACGA
CCAGCCACACTGGGACTGAGACACGGCC
>Zotu478
GAACGCTGGCGGCGTGCCTAATACATGCAAGTCGAACGCGAGCGATTGGAGCTTGCTCTAATCAATCG
AGTGGCGAACGGGTGAGTAACACGTGGGCAACCTGCCCATCAGCCTGGGATAACCACGGGAAACCGTG
GCTAATACCGGATAGACCGAGTGACAGAAGTCACAATGGAGAAAGTCGGTTTTACTGACACTGATGGA
TGGGCCTGCGGCGCATTAGCTAGTTGGAGAGGTAGTGGCTCCCCAAGGCAACGATGCGTAGCCGACCT
GAGAGGGTGAACGGCCACACTGGAACTGAGACACGGTC
>Zotu479
GAACGCTGGCGGCGTGCCTAACACATGCAAGTCGAGCGAACCCTTCGGGGTGAGCGGCGGACGGGTGA
GTAACGCGTGGGTAACCTGCCCTGTACACACGGATAACATACCGAAAGGTATGCTAATACGAGATAAT
ATGTAATGATCGCATGGTCGATACATCAAAGCTCCGGCGGTACAGGATGGACCCGCGTCTGATTAGCT
AGTTGGTGAGGTAATGGCTCACCAAGGCGACGATCAGTAGCCGACCTGAGAGGGTGATCGGCCACATT
GGAACTGAGACACGGTC
>Zotu480
GAACGCTAGCGGCAGGCCTAATACATGCAAGTCGAACGCCCAGCAATGGGAGTGGCGCACGGGTGCGT
AACGCGTATGCAACCTACCTTAAACTGGGGGATAGCCTTCCGAAAGGAAGATTAATACCCCATAGTAT
CACGAGAGGGCATCTGATTGTGATTAAAGATTTATCGGTTTAAGATGGGCATGCGTGACATTAGCTAG
TTGGTGAGGTAACGGCTCACCAAGGCTACGATGTCTAGGGGTTCTGAGAGGATGATCCCCCACACTGG
TACTGAGATACGGAC
>Zotu481
GAACGCTGGCGGCGTGCTTAACACATGCAAGTCGAACGGTGATGCGGAGCTTGCTCCGCGGATCAGTG
GCGAACGGGTGAGTAACACGTGAGTAACCTGCCCCTGACTCTGGGATAAGCGTTGGAAACGACGTCTA
ATACCGGATACGACCCATGAGGGCATCTTCGTGGGTGGAAAGAATTTCGGTTGGGGATGGACTCGCGG
CCTATCAGCTTGTTGGTGAGGTAATGGCTCACCAAGGCGACGACGGGTAGCCGGCCTGAGAGGGTGAC
CGGCCACACTGGGACTGAGACACGGCC
>Zotu482
GAACGCTAGCGGCAGGCCTAATACATGCAAGTCGAACGGTAAATATTCAGCAATGGATATGAGAGTGA
CGTACGGGTGCGTAACACGTATGCAACCTACCCTTTACAGGCGGATAGCTCGGGGAAACTCGGATTAA
CCCGTCATAATATAATTGGATGGCATCATTTGATTATTAAATCTACGGAGGTAAAGGATGGGCATGCG
TCTGATTAGTTAGTTGGCGGTGTAACGGACCACCAAGACGATGATCAGTAGGGGAACTGAGAGGTTGA
TCCCCCACACTGGCACTGAGATACGGGC
>Zotu483
GAACGCTGGCGGCGTGCCTAATACATGCAAGTCGAGCGCATGGAGATAGAACTTGTTCTATTGAAGTG

AGCGGCGCACGGGTGAGTAACACGTGGGAAACCTACCCTTTAGCGGGGGATAACACTGGGAAACGAGT
GCTAATACCGCATAGAGCATCTGGTTGCATGACCGGATGAGGAAAGACGCTACGGCGTCACTAAAGGA
TGGTCCCGCGGTGCATTAGCTAGTTGGTAAGGTAGCGGCTTACCAAGGCGATGATGCATAGCCGACCT
GAGAGGGTGATCGGCCACATTGGGACTGAGACACGGCC
>Zotu484
GAACGCTGGCGGCGTGCTTAACACATGCAAGTCGAACGGGGTGTTTATTTCGGTAAACACCAAGTGGC
GAACGGGTGAGTAACGCGTAAGCAATCTACCTTCAAGATGGGGACAACACTTCGAAAGGGGTGCTAAT
ACCGAATGAATGTAAGAGTATCGCATGAGACACTTACTAAAGGAGGCCTCTGACATAAGCTTCCGCTT
GAAGATGAGCTTGCGTCTGATTAGCTAGTTGGTGAGGGTAAAGGCCCACCAAGGCGACGATCAGTAGC
CGGTCTGAGAGGATGAACGGCCACATTGGGACTGAGACACGGCC
>Zotu485
GAACGCTGGCGGCGTGCTTAACACATGCAAGTCGAACGGAAAGGCCCTTCGGGGTACTCGAGTGGCGA
ACGGGTGAGTAACACGTGGGTGATCTGCCCTGCACTTCGGGATAAGCCTGGGAAACTGGGTCTAATAC
CGGATATGATCATCGGCTTCATGGTCGGTGGTGGAAAGCTTTTGCGGTGTGGGATGGGCCCGCGGCCT
ATCAGCTTGTTGGTGGGGTAATGGCCTACCAAGGCGTCGACGGGTAGCCGGCCTGAGAGGGTGTCCGG
CCACACTGGGACTGAGATACGGCC
>Zotu486
GAACGCTGGCGGCAGGCTTAACACATGCAAGTCGAACGCCCGAGCAATCGGGAGTGGCAGACGGGTGA
GTAACGCGTGGGAACATACCCTTTGGACTGGAATAACTGGGGGAAACTTCAGCTAAGACCGGATACGT
GCGAGAGCAGAATGATTTATCGCCGAAGGATTGGCCCGCGTCCGATTAGCTAGTTGGTGTTGGTAATG
GCTCACCAAGGCGACGATCGGTAGCTGGTCTGAGAGGATGACCAGCCACATTGGGACTGAGACACGGC
C
>Zotu487
GAACGCTGGCGGCGTGCTTAACACATGCAAGTCGAGCGGAAAGGCCACTTCGGTGGTACTCGAGCGGC
GAACGGGTGAGTAACACGTGAGTAATCTGCCCCTGGCTTTGGGATAGCCACCGGAAACGGTGATTAAT
ACCGGATATGACAGCTTCTCGCATGAGATGGTTGTGGAAAGTTTTTCGGCCAGGGATGTGCTCGCGGC
CTATCAGCTTGATGGTGAGGTAATGGCTCACCATGGCTTCGACGGGTAGCCGGCCTGAGAGGGTGACC
GGCCACACTGGGACTGAGACACGGCC
>Zotu488
CAACGCTGGCGGCGCGCTTAACACATGCAAGTCGAACGAGAAAGGGAGCAATCCTGAGTAAAGTGGCG
TACTGGTGAGTAACACGTGGATATCTACCTCTTGGTGGGGAATAACCTGGGGAAACCTGGGCTAATAC
CGCATAAGTTCGAGAGAAGAAAGGCGAAAGTCGCCGAGAGAGGAGTCCGCGGTCGATTAGGTAGTTGG
CAGGGTAAAGGCCTACCAAGCCGAAGATCGATAGCCGGCCTGAGAGGGCACACGGCCACACTGGCACT
GAAACACGGGC
>Zotu489
GAACGCTAGCGGGATGCTTTACACATGCAAGTCGAACGGCAGCGCGGACTTCGGTCTGGCGGCGAGTG
GCGAACGGGTGAGTAATGTATCGGAACGTGCCCAGTAGCGGGGGATAACTACGCGAAAGCGTAGCTAA
TACCGCATACGCCCTACGGGGGAAAGCAGGGGATCGTAAGACCTTGCACTATTGGAGCGGCCGATATC
GGATTAGCTAGTTGGTGAGGTAACGGCTCACCAAGGCGACGATCCGTAGCTGGTTTGAGAGGACGACC
AGCCACACTGGGACTGAGACACGGCC
>Zotu490
GAACGCTGGCGGCGTGCCTTATACATGCAAGTCAAACGGGTGTAGCAATACACCAGTGGCGAACGGGT
GAGTAATACGTAGGTACTTTGCTTAAGTTGGGCATAACTAGCCGAAAGGTTAGCTAATTCCCGATGTG
ATCGTAAGATCAAAGTCGCAAGACGCTTAAGAATAGGCCTGCGTCCTATCAGCTTGTTGGCGGGGTAA
AAGCCCACCAAGGCTATGACGGGTAGCTGGTCTGAGAGGATGATCAGCCACAATGGGACTGCGACACG
GCC
>Zotu491
GAACGCTAGCGGTGCGCCTAACACATGCAAGTCGAGCGGGTTTATTTGTGACACAGAGTAGGGAAGCA
ATTCCACATTCTGTGTCACAGGTAATCTAGCGGCAAACGGGTGCGTAACACGTTGATATCTCCCCCGA
AGTCAGGGATAGCCTCAAGAAATTGAGAGTAATACCGGATGGTCCCGTAAGGGTAAAGGAGCAATTCG
CTTCGGGAAGAGTCTGCGGCCTATCAGCTAGTTGGCGGGGTAATAGCCCACCAAGGCGATGACGGGTA
GCTGGTCTGAGAGGACGGCCAGCCACAATGGAACTGAGACACGGTC
>Zotu492
GAACGCTGGCGGCGTGCCTAATACATGCAAGTCGAGCGAACAGATGAGAAGCTTGCTTCTCTGATGTT

AGCGGCGGACGGGTGAGTAACACGTGGGTAACCTACCTATAAGACTGGGATAACTCCGGGAAACCGGG
GCTAATACCGGATAATATTTTGAACCGCATGGTTCAATAGTGAAAGACGGTTTCGGCTGTCACTTATA
GATGGACCCGCGCCGTATTAGCTAGTTGGTAAGGTAACGGCTTACCAAGGCGACGATACGTAGCCGAC
CTGAGAGGGTGATCGGCCACACTGGAACTGAGACACGGTC
>Zotu493
GAACGCTAGCGGCAGGCTTAATACATGCAAGTCGAGGGGCAGCGCAGTGTAGCAATACATGGGCGGCG
ACCGGCAAACGGGTGCGGAACACGTACAGAACCTTCCTTCGAGCGGGGAATAGCCCAGAGAAATTTGG
ATTAATACCCCATAGTATAACGGAGTGGCATCACTTTGTTATTAAAGATTTATCACTTGAAGATGGCT
GTGCGGCTGATTAGGTAGTTGGCGGGGTAACGGCCCACCAAGCCTTCGATCAGTAACTGGTGTGAGAG
CACGACCAGTCACACGGGCACTGAGACACGGGC
>Zotu494
GAACGCTGGCGGCATGCCTAACACATGCAAGTCGAACGAGACCTTCGGGTCTAGTGGCGCACGGGTGC
GTAACGCGTGGGAACCTGCCCTTAGGTTCGGAATAACTCCTCGAAAGGGGTGCTAATACCGGATAATG
TCTTCGGACCAAAGATTTATCGCCTTTGGATGGGCCCGCGTTGGATTAGCTAGTTGGTGAGGTAAAGG
CTCACCAAGGCGACGATCCATAGCTGGTCTTAGAGGATGATCAGCCACACTGGGACTGAGACACGGCC
>Zotu495
GAACGCTGGCGGCGTGCCTAATACATGCAAGTCGAGCGGACAGAAGGGAGCTTGCTCCCGGATGTTAG
CGGCGGACGGGTGAGTAACACGTGGGTAACCTGCCTGTAAGACTGGGATAACTCCGGGAAACCGGAGC
TAATACCGGATAGTTCCTTGAACCGCATGGTTCAAGGATGAAAGACGGTTTCGGCTGTCACTTACAGA
TGGACCCGCGGCGCATTAGCTAGTTGGTGGGGTAATGGCTCACCAAGGCGACGATGCGTAGCCGACCT
GAGAGGGTGATCGGCCACACTGGGACTGAGACACGGCC
>Zotu496
GAACGCTGACAGAATGCTTAACACATGCAAGTCAACTTGAATTTGGGTTTTTAACTTAGGTTTGGGTG
GCGGACGGGTGAGTAACGCGTAAAGAACTTGCCTCACAGGTAGGGACAACATTTGGAAACGAATGCTA
ATACCTAATATTATGATAATAGGGCATCCTATAATTATGAAAGCTAAAAGCACTGTGAGAGAGCTTTG
CGTCCCATTAGCTAGTTGGAGAGGTAACGGCTCACCAAGGCGATGATGGGTAGCCGGCCTGAGAGGGT
GATCGGCCACAAGGGGACTGAGACACGGCC
>Zotu497
GAACGCTGGCGGTAGGCCTAACACATGCAAGTCGAACGGCAGCACAGTAAGAGCTTGCTCTTATGGGT
GGCGAGTGGCGGACGGGTGAGGAATACATCGGAATCTACTTTTTCGTGGGGGATAACGTAGGGAAACT
TACGCTAATACCGCATACGACCTACGGGTGAAAGCAGGGGATCTTCGGACCTTGCGCGATTGAATGAG
CCGATGTCGGATTAGCTAGTTGGCGGGGTAAAGGCCCACCAAGGCGACGATCCGTAGCTGGTCTGAGA
GGATGATCAGCCACACTGGAACTGAGACACGGTC
>Zotu498
GAACGCTGGCGGCAGGCCTAACACATGCAAGTCGAGCGGATGATGGGAGCTTGCTCCCGGATTCAGCG
GCGGACGGGTGAGTAATGCCTAGGAATCTGCCTGGTAGTGGGGGACAACGTTTCGAAAGGAACGCTAA
TACCGCATACGTCCTACGGGAGAAAGCAGGGGACCTTCGGGCCTTGCGCTATCAGATGAGCCTAGGTC
GGATTAGCTAGTTGGTGGGGTAAAGGCCTACCAAGGCGACGATCCGTAACTGGTCTGAGAGGATGATC
AGTCACACTGGAACTGAGACACGGTC
>Zotu499
GAACGCTGGCGGCGTGCTTAACACATGCAAGTCGAGCGGAAAGGCCCTTCGGGGTACTCGAGCGGCGA
ACGGGTGAGTAACACGTGAGTAACCTGCCCTGGACTTTGGGATAACCCTCGGAAACGGGGGCTAATAC
CGGATACGACACGTTCCTGCATGGGATGCGTGTGGAAAGATTTTTTGGTCTGGGATGGGCTCGCGGCC
TATCAGCTTGTTGGTGGGGTGATGGCCTACCAAGGCGACGACGGGTAGCCGGCCTGAGAGGGCGACCG
GCCACACTGGGACTGAGACACGGCC
>Zotu500
GAACGCTGGCGGCGTGCTTAACACATGCAAGTCGAGCGGAAAGGCCCTTTCGGGGGTACTCGAGCGGC
GAACGGGTGAGTAACACGTGAGTAATCTGCCCTTCACTTTGGGATAGCTCCCGGAAACGGGGATTAAT
ACCGGATATGACCATGGGTCGCATGATCTTGTGGTGGAAAGTTTTTTCGGTGGGGGATGTGCTCGCGG
CCTATCAGCTTGTTGGTGGGGTAATGGCCTACCAAGGCTTCGACGGGTAGCCGGCCTGAGAGGGTGAC
CGGCCACACTGGGACTGAGACACGGCC
>Zotu501
GAACGCTAGCTACAGGCTTAACACATGCAAGTCGAGGGGCAGCATGACCTAGCAATAGGTTGATGGCG
ACCGGCGCACGGGTGAGTAACACGTATCCAACCTACCGGTTATTCCGGGATAGCCTTTCGAAAGAAAG

ATTAATACCGGATAGTATAACGAGAAGGCATCTTTTTGTTATTAAAGAATTTCGATAACCGATGGGGA
TGCGTTCCATTAGTTTGTTGGCGGGGTAACGGCCCACCAAGACATCGATGGATAGGGGTTCTGAGAGG
AAGGTCCCCCACATTGGAACTGAGACACGGTC
>Zotu502
GAACGCTGGCGGCATGCTTAACACATGCAAGTCGAACGGCAGCACAGCAGAGCTTGCTCTGTGGGTGG
CGAGTGGCGGACGGGTGAGTAATGCATCGGGACCTACCCAGACGTGGGGGATAACGTAGGGAAACTTA
CGCTAATACCGCATACGTCCTACGGGAGAAAGCGGGGGATCTTCGGACCTCGCGCGGTTGGATGGACC
GATGTGCGATTAGCTAGTTGGTGAGGTAACGGCTCACCAAGGCGACGATCGCTAGCTGGTCTGAGAGG
ATGATCAGCCACACTGGGACTGAGACACGGCC
>Zotu503
GAACGCTGGCGGCGTGCCTAATACATGCAAGTAGAACGCTGAAGAGAGGAGCTTGCTCTTCTTGGATG
AGTTGCGAACGGGTGAGTAACGCGTAGGTAACCTGCCTGGTAGCGGGGGATAACTATTGGAAACGATA
GCTAATACCGCATAAAATGGATTATCGCATGATAATTCATTGAAAGGTGCAAATGCATCACTACCAGA
TGGACCTGCGTTGTATTAGCTAGTTGGTGGGGTAACGGCTCACCAAGGCGACGATACATAGCCGACCT
GAGAGGGTGATCGGCCACACTGGGACTGAGACACGGCC
>Zotu504
GAATGCTGGCGGCGCGCCTAACACATGCAAGTCGAGCGGTAACATGGACTTCGGTCTGATGACGAGCG
GCGGACGGCTGAGTAACGCGTGGGAACATGCCCCAAAGTGAGGAATAAGCTCCAGAAATGGAGTCTAA
TGCCGCATATGGTCTTCGGATTAAAGGATTTATCCGCTTTGGGAGTGGCCTGCGTATGATTAGATAGT
TGGTGAGGTAATGGCTCACCAAGTCTACGATCATTAACTGGTCTGAGAGGATGATCAGTCAGACTGGA
ACTGAGACACGGTC
>Zotu505
GAACGCTGGCGGCAGGCTTAACACATGCAAGTCGAGCGAGACCTTCGGGTCTAGCGGCGGACGGGTGA
GTAACGCGTGGGAACGTGCCCTTCTCTACGGAATAGCCCCGGGAAACTGGGAGTAATACCGTATACGC
CCTTTGGGGGAAAGATTTATCGGAGAAGGATCGGCCCGCGTTGGATTAGGTAGTTGGTGGGGTAATGG
CCCACCAAGCCGACGATCCATAGCTGGTTTGAGAGGATGATCAGCCACACTGGGACTGAGACACGGCC
>Zotu506
GAACGCTGGAAACGTGGATACCGGCATGCAAGTCGAGCGGACCCGCAAGGGTCAGCGGCGAACGGCTT
AGTAACACGTGGGTACATACCCTAAACTGGAGGACAGCTCGTCGAAAGACGGGGTAATACTCCATAGT
CTCGCAAGAGTAAAGTCGTAAGACGGTTTAGGAATGGCCTGCGGCCTATCAGATAGTTGGCGAGGTAA
TGGCTCACCAAGTCTATGACGGGTAGGGGGCGTGAGAGCGCGACCCCCAAGAATGGCACTGCGACACG
GGC
>Zotu507
GAACGCTGGCGGCGCGCTTAACACATGCAAGTCGAGCGATGAAACTTTAACAGAACCCTTCGGGGCGA
AGATAAAGTGGATTAGCGGCGGACGGGTGAGTAACACGTGAGTAACCTGCCTTAGACATTGGGATAGC
CTCGGGAAACCGGGATTAATACCAAATAAAATCATAATGACACATGTCATAACGATCAAAACTCCGGT
GGTCTAAGATGGACTCGCGTCCCATTAGCTAGTTGGTAGAGTAAAAGCCTACCAAGGCGACGATGGGT
AGCCGACCTGAGAGGGTGAACGGCCACATTGGAACTGAGAAACGGTC
>Zotu508
GAACGCTGGAAATGTGGATACCGGCATGCAAGTCAAGGGGCCGCAAGGCACCGGCGAACGGCTTAGTA
ACACGTAGATACATACCGTAAAGTGGGGCACAGCTCGTCGAAAGACGGGGTAATTTCCCATGTGACCG
CAAGGTTAAAGGCGCAAGCCGCTTTATGAATGGTCTGCGGTCTATCAGCTAGTTGGTGAGGTAATGGC
TCACCAAGGCTATGACGGATAGGGGGCGTGAGAGCGCGACCCCCAAGAATGGCACTGCGACACGGGC
>Zotu509
GAATGCTGGCGGCGTGGATAAGTCATGCAAGTCAAGGGGGCCCGCAAGGGCAACCGGCAGACGAGGTA
GTAACACGTAGGTACGTCCCCCGAAGTCGGGCATAGCCATCCGAAAGGATGGGTAATTCCCGATAGTC
CCGCAAGGGTAAAGGTTTTTCGCTTCGGGAGCGGCCTGCGTAGTATCAGCTAGTTGGTAAGGTAATGG
CTTACCAAGGCTACGACGCTTAGGGGAGCTGAGAGGCTGACCCCCACCGATGGAACTGCGACACGGTC
>Zotu510
GAACGCTGGCGGCGTGCTTAACACATGCAAGTCGAACGGAAAGGCCCTTCGGGGTACTCGAGTGGCGA
ACGGGTGAGTAACACGTGGGTGATCTGCCTCGTACTCTGGGATAAGCCTGGGAAACTGGGTCTAATAC
CGGATAGGACCTCTGGTCGCATGGCTGGTGGTGGAAAGTTTTTCGGTACGAGATGGGCCCGCGGCCTA
TCAGCTTGTTGGTGGGGTAATGGCCTACCAAGGCGACGACGGGTAGCCGGCCTGAGAGGGCGACCGGC
CACACTGGGACTGAGACACGGCC

>Zotu511
GAACGCTGGCGGCGTGCTTAACACATGCAAGTCGAACGATGAAGCACCAGCTTGCTGGTGTGGATTAG
TGGCGAACGGGTGAGTAACACGTGAGTAACCTGCCCCTCACTCTGGGATAAGCCTGGGAAACTGGGTC
TAATACTGGATACGACCGATCTCCGCATGGAGTGTTGGTGGAAAGTTTTTGTGGTGGGGGATGGACTC
GCGGCCTATCAGCTTGTTGGTGGGGTAATGGCCTACCAAGGCGACGACGGGTAGCCGGCCTGAGAGGG
CGACCGGCCACACTGGGACTGAGACACGGCC
>Zotu512
GAACGCTGGCGGCGTGCTTAACACATGCAAGTCGAACGGTGAAGCTCCAGCTTGCTGGAGTGGATCAG
TGGCGAACGGGTGAGTAACACGTGAGCAACCTGCCCCTGACTCTGGGATAACAGCCGGAAACGGCTGC
TAATACCGGATATGCACCATGAACGCATGTTCTGTGGTGGGAAAGATTTTTCGGTTGGGGATGGGCTC
GCGGCCTATCAGCTTGTTGGTGAGGTAATGGCTCACCAAGGCGTCGACGGGTAGCCGGCCTGAGAGGG
TGACCGGCCACACTGGGACTGAGACACGGCC
>Zotu513
GAACGCTGGCGGCGTGCTTAACACATGCAAGTCGAACGGTGACGGAGAGCTTGCTCTCCTGATCAGTG
GCGAACGGGTGAGTAACACGTGGGCAACCTGCCCCAGACTTCGGGATAACGCTTCGAAAGAGGTGCTA
ATACCGAATACGAACTCCTTGGGCATCCTTGGAGTTGGAAAGTTTTTCGGTCTGGGATGGGCCCGCGG
CCTATCAGCTTGTTGGTGAGGTAATGGCTCACCAAGGCGACGACGGGTAGCCGGCCTGAGAGGGCGAC
CGGCCACACTGGGACTGAGACACGGCC
>Zotu514
GAATGCTGGCGGCGTGGATAAGTCATGCAAGTCAAGGGGGCCCGCAAGGGCAACCGGCAGACGAGGTA
GTAACACGTAGGTAGATCCCCAGAAGTCGGGCATAGCCAGTCGAAAGACTGGGTAATTCCCGATAGTC
CCGCAAGGGTAAAGGTTTTTCGCTTCTGGAGTTGCCTGCGTAGTATCAGCTAGTTGGTAGGGTAATGG
CCTACCAAGGCTACGACGCTTAGGGGGGCTGAGAGGCCGATCCCCACCGATGGAACTGCGACACGGTC
>Zotu515
GAACGCTGGCGGCGTGCTTAACACATGCAAGTCGAACGATGAACTCCAGCTTGCTGGGGGGATTAGTG
GCGAACGGGTGAGTAACACGTGAGTAACCTGCCCTTGACTCTGGGATAAGCGCTGGAAACGGCGTCTA
ATACCGGATACGAGCTTCAGTCGCATGGCTAGGAGTTGGAAAGAATTTTGGTCAAGGATGGACTCGCG
GCCTATCAGCTTGTTGGTGAGGTAATGGCTCACCAAGGCGACGACGGGTAGCCGGCCTGAGAGGGTGA
CCGGCCACACTGGGACTGAGACACGGCC
>Zotu517
GAACGCTGGCGGCGTGCCTAACACATGCAAGTCGAACGGAGCACCTTCGGGTGCTTAGTGGCGAACGG
GTGAGTAACGCGTGAGAAACCTGCCTTTCAGTGGGGGACAACAGTTGGAAACGACTGCTAATACCGCA
TAATGTCATTTGGGGGCATCCCTGAATGACCAAAGATTTATTGCTGAAAGATGGTCTCGCGTCTGATT
AGCTAGTTGGTGAGGTAACGGCTCACCAAGGCGACGATCAGTAGCCGGTCTGAGAGGATGAACGGCCA
CATTGGGACTGAGATACGGCC
>Zotu518
GAACGCTGGCGGCGTGCTTAACACATGCAAGTCGAACGGGAAACCTTTTATTGAAGCTTCGGCAGATT
TAGATTGTTTCTAGTGGCGGACGGGTGAGTAACGCGTGGGTAACCTGCCTTATACAGGGGGATAACAG
TCAGAAATGACTGCTAATACCGCATAAGCGCACAGAGCTGCATGGCTCGGTGTGAAAAACTCCGGTGG
TATAAGATGGACCCGCGTTGGATTAGCTAGTTGGTGAGGTAACGGCCCACCAAGGCGACGATCCATAG
CCGGCCTGAGAGGGTGAACGGCCACATTGGGACTGAGACACGGCC
>Zotu519
GAACGCTGGCGGCAGGCTTAACACATGCAAGTCGAACGCTCCGCAAGGAGAGTGGCAGACGGGTGAGT
AACACGTGGGAACGTACCCTTTACTACGGAACAACTCCGGGAAACTGGAGCTAATACCGTATGTGCCC
TTCGGGGGAAAGATTTATCGGTAAAGGATCGGCCCGCGTTGGATTAGCTAGTTGGTGGGGTAAAGGCC
TACCAAGGCGACGATCCATAGCTGGTCTGAGAGGATGATCAGCCACATTGGGACTGAGACACGGCC
>Zotu520
GAACGCTGGCGGCGTGCTTAACACATGCAAGTCGAACGGAAAGGCCCTTCGGGGTACTCGAGTGGCGA
ACGGGTGAGTAACACGTGGGTGATCTGCCCTGCACTTCGGGATAAGCCTGGGAAACTGGGTCTAATAC
CGGATATGACCTCAGGATGCATGTTCTGGGGTGGAAAGCTTTTGCGGTGTGGGATGGGCCCGCGGCCT
ATCAGCTTGTTGGTGGGGTCACGGCCTACCAAGGCGACGACGGGTAGCCGGCCTGAGAGGGTGACCGG
CCACACTGGGACTGAGATACGGCC
>Zotu521
GAACGCTAGCGGGAGGCCTAACACATGCAAGCCGAGCGGTAGAGATCTTTCGGGATCTTGAGAGCGGC

GTACGGGTGCGGAACACGTGTGCAACCTGCCTTTATCTGGGGGATAGCCTTTCGAAAGGAAGATTAAT
ACCCCATAATATGTTGGATGGCATCATTCGACATTGAAAACTCCGGTGGATAGAGATGGGCACGCGCA
AGATTAGATAGTTGGTGAGGTAACGGCTCACCAAGTCTGCGATCTTTAGGGGGCCTGAGAGGGTGATC
CCCCACACTGGTACTGAGACACGGAC
>Zotu522
GAACGCTTGCGGCGTGCCTAAGAAATGCAAGTCGAGCGGGGAGCAATCCCAGCGGCGAACGGTCGCGT
AACACGTAAGCAACCTGCCCGAAAGACCGGGATAACGTTTCGAAAGAGGCGCTAATACCGGATGTGAC
CGCCGGGAGACATCTCCTGGTGATTAAATCATTTATGGCTTTCGGAGGGGCTTGCGGCCTATCAGCTA
GATGGCGGGGTAACGGCTCACCATGGCGTCGACGGGTAGCTGGTCTGAGAGGATGGTCAGCCGGACTG
GGACTGAGATACGGCC
>Zotu523
GAACGCTGGCGGCGTGCTTAACACATGCAAGTCGAACGGTTTGCGCAAGTAAACAGTGGCGCACGGGT
GAGTAACACGTAGCTAACCTGCCCTCGAGCGCGGGATAACGTTCCGAAAGGAATGCTAATACCGCATA
AGCTCACGGTCTGTAGAGGATGGTGAGAAAAGGCTTAGGTCACTTGAGGAGGGGGCTGCGACCCATCA
GCTCGTTGGCGAGGTAACGGCTCACCAAGGCGACGACGGGTAGGGGGCCTGAGAGGGTGGCCCCCCAC
ACTGGGACTGAGACACGGCC
>Zotu524
GAACGCTAGCTACAGGCTTAACACATGCAAGTCGAGGGGCAGCATGGAAGAAGCTTGCTTCTTCTGAT
GGCGACCGGCGCACGGGTGCGTAACGCGTATCAAACCTGCCTCATACTCGGGGATAGCCTTGCGAAAG
TAAGATTAATACCCGATGTTGTTATGTTACCGCATGGTGATATAACCAAAGATTTATCGGTATGAGAT
GGTGATGCGTCCGATTAGGTAGTAGGCGGGGTAACGGCCCACCTAGCCTTCGATCGGTAGGGGTTCTG
AGAGGAAGGTCCCCCACACTGGAACTGAGACACGGTC
>Zotu525
GAACGCTAGCGGCAGGCTTAATACATGCAAGTCGAGGGGCAGCACGAATAGCAATATTTGGTGGCGAC
CGGCGCACGGGTGCGTAACACGTATACAATCTACCTTCAACAGGGGGATAGCCTTTCGAAAGAGAGAT
TAATACCCCGTAATATATTTGCTGGCATCAGTGAATATTGAAAGTTCCGACGGTTGAAGATGAGTATG
CGTCTGATTAGCTAGTTGGTGAGGTAACGGCTCACCAAGGCGACGATCAGTAGGGGATCTGAGAGGAC
TAACCCCCACACTGGTACTGAGACACGGAC
>Zotu526
GAACGCTGGCAGCGTGCTTAACACATGCAAGTCGAACGGTGAAGCCAAGCTTGCTTGGTGGATCAGTG
GCGAACGGGTGAGTAACACGTGAGCAACCTGCCCCGGACTCTGGGATAAGCGCTGGAAACGGCGTCTA
ATACCGGATATGAACCACAGCCGCATGGTTGGTGGTTGGAAAGATTTTTTGGTTCGGGATGGGCTCGC
GGCCTATCAGCTTGTTGGTGAGGTAATGGCTCACCAAGGCGTCGACGGGTAGCCGGCCTGAGAGGGTG
ACCGGCCACACTGGGACTGAGACACGGCC
>Zotu527
GAACGCTGGCGGCGTGCCTAATACATGCAAGTCGAACGAACCGCGACTAGGTGCTTGCACTTGGTCAA
GGTGAGTGGCGAACGGGTGAGTAACACGTGGGTAACCTACCTCATAGTGGGGGATAACAGTCGGAAAC
GACTGCTAATACCGCATAGGACATGGAATCACATGATTCAGTGAGGAAAGGTGGCGCAAGCTATCGCT
AAGAGATGGACCCGCGGTGCATTAGCTAGTTGGTAGGGTAAAGGCCTACCAAGGCGATGATGCATAGC
CGACCTGAGAGGGTGATCGGCCACATTGGGACTGAGACACGGCC
>Zotu528
TAACGTTGGCAGCGTGCCTAATACATGCAAGTCGCGCGATATAGCGGCGGACGAGAGAGTAATAGTAA
GGAACTTACCCCTGAGTGAAGGATAGCCATCCGAAAGGATGGGTAATCCTGCATAATCCCGCAAGGGC
AAAGCCGCAAGGCGCTTAGGGAGAGGCCTTACTCCTATCAGCTTGTTGGTGAGGTAAAGGCTCACCAA
GGCGATGACGGGTAGCTGGTCTGAGAGGATGACCAGCCTCGATGGGACTGAGACACGGCC
>Zotu529
GAACGCTGGCGGCAGGCCTAACACATGCAAGTCGAACGCTGTAGCAATACAGAGTGGCAGACGGGTGA
GTAACGCGTGGGAACATGCCCTTCGGTTCGGAATAACTCAGGGAAACTTGAGCTAATACCGGATACGC
CCTTAGGGGGAAAGATTTATTGCCGAAGGATTGGCCCGCGTCCGATTAGCTAGTTGGTGAGGTAATGG
CTCACCAAGGCGACGATCGGTAGCTGGTCTGAGAGGATGATCAGCCACACTGGGACTGAGACACGGCC
>Zotu530
GAACGCTGGCGGCATGCCTAACACATGCAAGTCGAACGAAGGCTTCGGCCTTAGTGGCGCACGGGTGC
GTAACGCGTGGGAATCTGCCCTTAGGTTCGGAATAACAGCTGGAAACGGCTGCTAATACCGGATGATA
TCGCGAGATCAAAGATTTATCGCCTGAGGATGAGCCCGCGTTGGATTAGGTAGTTGGTGGGGTAAAGG

CCTACCAAGCCGACGATCCATAGCTGGTCTGAGAGGATGATCAGCCACACTGGGACTGAGACACGGCC
>Zotu531
GAACGCTGGCGGCGTGCTTAACACATGCAAGTCGAACGATGAAGCGGTGCTTGCACCGTGGATTAGTG
GCGAACGGGTGAGTAACACGTGAGTAACCTGCCTTCCACTCTGGGATAAGCGCTGGAAACGGCGTCTA
ATACTGGATATGACATTGCCTCGCATGGGGTGGTGTGTAAAGATTTTTTGGTGGTAGATGGACTCGCG
GCCTATCAGCTTGTTGGTGGGGTAATGGCCTACCAAGGCGACGACGGGTAGCCGGCCTGAGAGGGTGA
CCGGCCACACTGGGACTGAGACACGGCC
>Zotu533
GAACGCTAGCGGGAGGCCTAACACATGCAAGCCGAGCGGTAGAAGATCTTCGGATCTTTGAGAGCGGC
GCACGGGTGCGGAACACGTGTGCAACCTGCCTTTATCTGGGGGATAGCCTTTCGAAAGGAAGATTAAT
ACCCCATAATATATTTTACGGCATCGTAAGATATTGAAAACTCCGGTGGATAGAGATGGGCACGCGCA
AGATTAGTTAGTTGGTGAGGTAACGGCTCACCAAGACGATGATCTTTAGGGGGCCTGAGAGGGTGATC
CCCCACACTGGTACTGAGACACGGAC
>Zotu534
GAACGCTGGCGGTGTGCCTAATACATGCAAGTCGAGCGGCAGCACGAATAGTTTACTATTTGGTGGCG
AGAGGCGGACGGCTGAGTAACACGTAGGAACATACCCCAAAGTGAGGGATAACTGCTCGAAAGAGTAG
CTAATACCGCATGTGATCTAAGGATTAAAGCTTCGGCGCTTTGGGAATGGCCTGCGTTCGATTAGCTA
GTTGGTAAGGTAAAAGCTTACCAAGGCGACGATCGATAGCTGGTCTGAGAGGATGATCAGCCAGACTG
GGACTGAGACACGGCC
>Zotu535
GAACGCTGGCGGCGTGCTTAACACATGCAAGTCGAGCGGTAAGGCTCCTTCGGGAGTACACGAGCGGC
GAACGGGTGAGTAACACGTGAGCAATCTGCCCTTCACTTCGGGATAACTCCGGGAAACCGGTGCTAAT
ACCGAATACGACCACTTCACGCATGTGATGGTGGTGGAAAGTTCCGGCGGTGGAGGATGAGCTCGCGG
CCTATCAGCTTGTTGGTGGGGTAATGGCCCACCAAGGCAACGACGGGTAGCCGGCCTGAGAGGGTGAC
CGGCCACACTGGGACTGAGACACGGCC
>Zotu536
GAACGCTGGCGGCATGCTTAACACATGCAAGTCGAACGAGATAAAATTCACTTTAACACATGTTAAAG
TGAGCTTTATCTAGTGGCGGACGGGTGAGTAACGCGTAAGAACCTACCCTTAGGAGGGGGACAACAAT
TGGAAACGATTGCTAATACCCCATATGCTGAGGAGTAAAACGGTAATAACCGCCTAGGGATGGGCTTG
CGTCTGATTAGCTTGTTGGTGAGGTAAATGCTTACCAAGGCAATGATCAGTAGTTGGTCTGAGAGGAT
GACCAGCCACACTGGGACTGAGACACGGCC
>Zotu537
GAACGCTGGCGGCGTGCTTAACACATGCAAGTCGAACGATGAAAGAGAATCCGATCTCTTCGGAGTGA
AGAATCTCTGGATTAGTGGCGGACGGGTGAGTAACGCGTGAGCAATCTGCCTTACACAAAGGGATAGC
CTCGGGAAACCGGGATTAATACCATATGATATCTATTAACCGCATGGCCTATAGATTAAAGAATTTCG
GTGTAAGATGAGCTCGCGTCCCATTAATTAGTTGGTGAGGGTAACGGCCCACCAAGATAGCGATGGGT
AGCCGATCTAAGAGGATGACCGGCCACATTGGAACTGAGAAACGGTC
>Zotu538
GAACGCTGGCGGCGTGCTTAACACATGCAAGTCGAACGGTAAGGCCCTTCGGGGTACACGAGTGGCGA
ACGGGTGAGTAACACGTGGGTGATCTGCCCTGCACTTCGGGATAAGCCCGGGAAACTGGGTCTAATAC
CGGATAGTACTGCGCGGTGCATGCCGTGTGGTGGAAAGATTTATCGGTGCAGGATGGGCCCGCGGCCT
ATCAGCTTGTTGGTGGGGTAATGGCCTACCAAGGCGACGACGGGTAGCCGGCCTGAGAGGGCGACCGG
CCACACTGGGACTGAGACACGGCC
>Zotu539
GAACGCTAGCGGGAGGCCTAACACATGCAAGCCGAGCGGTATTGTTTCTTCGGAAATGAGAGAGCGGC
GTACGGGTGCGGAACACGTGTGCAACCTGCCTTTATCTGGGGGATAGCCTTTCGAAAGGAAGATTAAT
ACCCCATAATATACTGAATGGCATCATTCGGTATTGAAAACTCCGGTGGATAGAGATGGGCACGCGCA
AGATTAGATAGTTGGTGAGGTAACGGCTCACCAAGTCTGCGATCTTTAGGGGGCCTGAGAGGGTGATC
CCCCACACTGGTACTGAGACACGGAC
>Zotu540
GAACGCTGGCGGCGTGCTTAACACATGCAAGTCGAACGCTGAAGCTTGGTGCTTGCACTTAGGTGGAT
GAGTGGCGAACGGGTGAGTAATACGTGAGTAACCTGCCCTTGACTCTGGGATAAGCCTGGGAAACTGG
GTCTAATACTGGATATGACCTCCTGTCGCATGGTGGGGGGTGGAAAGGGTTTGTACTGGTTGTGGATG
GGCTCACGGCCTATCAGCTTGTTGGTGGGGTAATGGCCTACCAAGGCGACGACGGGTAGCCGGCCTGA

GAGGGTGACCGGCCACACTGGGACTGAGACACGGCC
>Zotu541
GAACGCTGGCGGCGTGCTTAACACATGCAAGTCGAGCGGTAAGGCCCCTTCGGGGGTACACGAGCGGC
GAACGGGTGAGTAACACGTGAGTAATCTGCCCTTCAGTTCGGGATAACCACCGGAAACGGTGGCTAAT
ACCGGATACGACCAGCCTCGGCATCGGGTGCTGGTGGAAAGTTTTTTCGCTGGAGGATGTGCTCGCGG
CCTATCAGCTTGTTGGTGAGGTAACGGCTCACCAAGGCTTCGACGGGTAGCCGGCCTGAGAGGGTGAC
CGGCCACACTGGGACTGAGACACGGCC
>Zotu542
GAACGCTGGCGGCGTGCTTAACACATGCAAGTCGAACGGGAACCTTTATTTCGGTAAAGGTGAGAGTG
GCAAACGGGTGAGTAACACGTAGGCAACCTGCCGACAGGATGGGGACAACATTCCGAAAGGAATGCTA
ATACCGAATGGAGTCCGGGGATGGCATCATCCCTGGATAAAAGATGGCCTCTGAATATGCTATCGCCT
GTCGATGGGCCTGCGTCTGATTAGCCAGTTGGCGGGGTAACGGCCCACCAAAGCGACGATCAGTAGCC
GGTCTGAGAGGATGAACGGCCACATTGGGACTGAGACACGGCC
>Zotu543
GAACGCTGGCGGCGTGCTTAACACATGCAAGTCGAGAGGTAAGGCCTTTCGGGGTACACGAGCGGCGA
ACGGGTGAGTAACACGTGGGTGATCTGCCCTGCACTTCGGGATAAGCCTGGGAAACTGGGTCTAATAC
CGGATATGACTTCAGGTTGCATGACTTGGGGTGGAAAGATTTATCGGTGCAGGATGGGCCCGCGGCCT
ATCAGCTTGTTGGTGGGGTAATGGCCTACCAAGGCGACGACGGGTAGCCGACCTGAGAGGGTGACCGG
CCACACTGGGACTGAGACACGGCC
>Zotu544
GAACGCTGGCGGCGTGCTTAACACATGCAAGTCGAACGGTGAACTCCGCTTGCGGGGGGATCAGTGGC
GAACGGGTGAGTAACACGTGGGCAACCTGCCCTCAGCTCTGGGATAACTCCAAGAAATTGGGGCTAAT
ACCGGATATGACTGCTGACCGCATGGTCTGGTGGTGGAAAGATTTATCGGCTGGGGATGGGCCCGCGG
CCTATCAGCTTGTTGGTGGGGTAGTGGCCTACCAAGGCGACGACGGGTAGCCGGCCTGAGAGGGTGAC
CGGCCACACTGGGACTGAGACACGGCC
>Zotu545
GAACGCTAGCGACAGGCCTAACACATGCAAGTCGAGGGGCAGCACGGTGTAGCAATACACGGGTGGCG
ACCGGCGCACGGGTGAGTAACGCGTATGCAACCTGCCTACTACAAGGGGATAACCCGTTGAAAGACGG
ACTAATACCCTATAACACAGGGGATCCGCATGGATATATTTGTTAAAGATTTATCGGTAGTAGATGGG
CATGCGTTCCATTAGCTTGTTGGTGAGGTAACGGCTCACCAAGGCAACGATGGATAGGGGAACTGAGA
GGTTGATCCCCCACACTGGAACTGAGACACGGTC
>Zotu546
GAACGCTGGCGGCAGGCTTAACACATGCAAGTCGAACGCGTGTAGCAATACACGAGTGGCGCACGGGT
GAGTAACACGTGGATATCTGCCTTTTGGTTCGGAATAACCCTGGGAAACTAGGGCTAATACCGAATGG
TTCCTACGGGATAAAGATTTATCGCCAAAAGATGAGTCCGCGTCCGATTAGCTAGTTGGTGAGGTAAT
GGCTCACCAAGGCGACGATCGGTAGCTGGTCTGAGAGGACGATCAGCCACACTGGGACTGAGACACGG
CC
>Zotu547
GAACGCTGGCGGCGTGCTTAACACATGCAAGTCGAACGGTGAAACAGGAGCTTGCTCCTGCGGATCAG
TGGCGAACGGGTGAGTAACACGTGAGAAATCTGCCCTGGACTCTGGGATAAGCGTTGGAAACGACGTC
TAATACCGGATACGAGCTGCGAAGGCATCTTCAGCAGCTGGAAAGAACTTCGGTCCAGGATGATCTCG
CGGCCTATCAGCTAGTTGGTGAGGTAACGGCTCACCAAGGCGACGACGGGTAGCCGGCCTGAGAGGGT
GACCGGCCACACTGGGACTGAGACACGGCC
>Zotu548
CGCAAGCCTTGGGGAACTGTGCAGCTGGTTTGATCGGGAACGCGAGTACCGCCCAGGTAGCTCAGGCT
AGTGTCATGACCGACTGTCCAGCACGAGAACAGCTCAACGGCATCTTCTTCCTTCGCCGCCCCACAAT
ATACGCGCGTAACCGGCCGCGCACTTCAAATAGCATAAATTCCTATCACAATA
>Zotu549
GAACGCTGGCGGCAGGCCTAACACATGCAAGTCGAGCGCGCCCTTCGGGGTGAGCGGCGGACGGGTGA
GTAACGCGTGGGAACGTGCCCTTTTCTACGGAATAGCCTCGGGAAACTGAGAGTAATACCGTATACGC
CCTTTGGGGGAAAGATTTATCGGAAAAGGATCGGCCCGCGTTGGATTAGGTAGTTGGTGGGGTAATGG
CCTACCAAGCCGACGATCCATAGCTGGTTTGAGAGGATGATCAGCCACACTGGGACTGAGACACGGCC
>Zotu550
GAACGCTAGCGGCAGGCTTAATACATGCAAGTCGAGGGGCAGCGGGGGTAGCAATACCCGCCGGCGAC

CGGCAAACGGGTGCGGAACACGTACAGAACCTTCCTTTAAGCGGGGGATAGCCCATAGAAATGTGGAT
TAATACCCCATAGTAATATTTGGTGGCATCATTAGATATTTAAAGATTTATCACTTAAAGATGGCTGT
GCGGCTGATTAGGTAGTTGGTGTGGGTAACGGCCCACCAAGCCAGCGATCAGTAACTGGTGTGAGAGC
ACGACCAGTCACACGGGCACTGAGACACGGGC
>Zotu551
GAACGCTGGCGGCGTGCTTAACACATGCAAGTCGAGCGGGGCACTTCTGGTGTCCAGCGGCGAACGGG
TGAGTAACACGTGGGCAACCTGCCCTCAGCTCTGGGATAAGCCGGGGAAACCCGGTCTAATACCGGAT
ATGACATGCTCTCGCATGGGGGTGTGTGGAAAGATTTATCGGCTGGGGATGGGCCCGCGGCCTATCAG
CTTGTTGGTGGGGTGATGGCCTACCAAGGCGACGACGGGTAGCCGGCCTGAGAGGGCGATCGGTCACA
CTGGGACTGAGACACGGCC
>Zotu552
GAACGCTGGCGGCATGCTTTACACATGCAAGTCGAACGGTAACAGGTCTTCGGATGCTGACGAGTGGC
GAACGGGTGAGTAATACATCGGAACGTGCCTAGTAGTGGGGGATAACTACTCGAAAGAGTAGCTAATA
CCGCATGAGATCTACGGATGAAAGCAGGGGACCTTCGGGCCTTGTGCTACTAGAGCGGCTGATGGCAG
ATTAGGTAGTTGGTGGGGTAAAGGCTTACCAAGCCTGCGATCTGTAGCTGGTCTGAGAGGACGACCAG
CCACACTGGGACTGAGACACGGCC
>Zotu553
GAACGCTAGCGGCAGGCCTAATACATGCAAGTCGAGGGGTAGAGTAGGCTTGCTTACTTGAGACCGGC
GCACGGGTGCGTAACGCGTATGCAACCTACCTTTATCTGGGGGATAGCCCGGAGAAATCCGGATTAAT
ACCGCATAAAATCACAGAATGGCATTATTCAATGATCAAACATTAATGGGATAGAGATGGGCATGCGT
GTCATTAGCTAGTTGGCGGGGTAACGGCCCACCAAGGCGACGATGACTAGGGGATCTGAGAGGATGAC
CCCCCACACTGGTACTGAGACACGGAC
>Zotu554
GAACGCTGGCGGCGTGCCTAAGACATGCAAGTCGAGTGGGTTTAGACCCACGGCGAACGGGTGAGTAA
TACCTAGGAACGTTTTTCAAGTGGAGCATAACCAGTCGAAAGATTGGATAATACTCCATGTGACCGCA
AGGTTAAAGTCGCAAGACGCTTGAAGTGCGGCCTGGGCACCATCAGCTAGTTGGTAAGGTAATGGCTT
ACCAAGGCTATGACGGTTAGGGGCCCCGAGAGGGGAGACCCCCACACTGGAACTGCGACACGGTC
>Zotu555
GAACGCTAGCGGCAGGCTTAATACATGCAAGTCGTGGGGCAGCAGGAGTGGTAGCAATACCACTTGCT
GGCGACCGGCAAACGGGTGCGGAACACGTACGCAACTTACCCTTAAGTGGGGGATAGCCCGGGGAAAC
CCGGATTAATACCTCGTAATACTTAGGTGTGGCATCACACTTTAGTTATAGCCTTCGGGCGCTTAAGG
ATGGGCGTGCGTCCCATTAGGTAGTTGGTTGAGGTAACGGCTCACCAAGCCTGCGATGGGTAACTGGT
GTGAGAGCACGACCAGTCACACGGGCACTGAGACACGGGC
>Zotu556
GAACGCTAGCGGCAGGCTTAACACATGCAAGTCGAGGGGTAGTCTAGCAATAGATGAGACCGGCGCAC
GGGTGCGTAACGCGTATGCAATCTACCTTTTACAGGGGAATAGCCCAGAGAAATTTGGATTAATGCCC
CATGGTATTATCGAGTGGCATCATTTGATAATTAAAGTTCCAACGGTAAAAGATGAGCATGCGTCCTA
TTAGCTTGTTGGTAAGGTAACGGCTTACCAAGGCTACGATAGGTAGGGGTCCTGAGAGGGAGATCCCC
CACACTGGTACTGAGACACGGAC
>Zotu557
GAACGCTAGCGGCAGGCTTAATACATGCAAGTCGTGGGGCAGCATGATGTAGCAATACATTGATGGCG
ACCGGCAAACGGGTGCGGAACACGTACACAACCTTCCTACAAGTGGGGAATAGCCCAGAGAAATTTGG
ATTAATACCCCGTAACATATTTGAGATGGCATCATCTTAATATTATAGCTTCGGCGCTTGTTGATGGG
TGTGCGGCTGATTAGATAGTTGGCGGGGTAACGGCCCACCAAGTCTACGATCAGTAGCTGATGTGAGA
GCATGATCAGCCACACGGGCACTGAGACACGGGC
>Zotu558
GAACGCTGGCGGCGCGCTTAACACATGCAAGTCGAGCGAGAACCGGGGCTTCGGCCCCGGGGACAGCG
GCGAACGGGTGAGTAACACGTGGGTAATCTGCCCTCGGTTCTGGGATAGCCCGGGGAAACCCGGATTA
ATACCGGATAGCCTCTCCAGCCTTCGGGCCGGCGAGAAAAGGTAGCTTCGGCCTCCGACCGAGGATGA
GCCCGCGGCGGATTAGCTTGTTGGCGGGGTAACGGCCCACCAAGGCGACGATCCGTAGCTGGTCTGAG
AGGACGATCAGCCACACTGGGACTGAGACACGGCC
>Zotu559
GAACGCTGGCGGCGTGCTTAACACATGCAAGTCGAACGGTAAGGCCCTTTTGGGGGTACACGAGTGGC
GAACGGGTGAGTAACACGTGAGTAACCTGCCCTTGACTCTGGGATAATTCTTCGAAAGAGGTTGTAAT

ACTGGATATGAGCTTCTGCCGCATGGTGGGGGTTGGAAAGTTCTGGCGGTCAGGGATGGGCTCGCGGC
CTATCAGCTTGTTGGTGAGGTAGTGGCTTACCAAGGCGACGACGGGTAGCCGGCCTGAGAGGGCGACC
GGCCACACTGGGACTGAGATACGGCC
>Zotu560
GAACGCTAGCGGCAGGCTTAACACATGCAAGTCGAGGGGTATAGTAGCAATACTAGAGACCGGCGCAC
GGGTGCGTAACGCGTATGCAATCTACCTTTTACAGAGGGATAGCCCAGAGAAATTTGGATTAATACCT
CATGGTATAACGAGTCCGCATGGATTTGTTATTAAAGTCACAACGGTAAAAGATGAGCATGCGTCCCA
TTAGCTTGTTGGTAAGGTAACGGCTTACCAAGGCTACGATGGGTAGGGGTCCTGAGAGGGAGATCCCC
CACACTGGTACTGAGACACGGAC
>Zotu561
GAACGCTGGCGGCGTGCTTAACACATGCAAGTCGAACGATGAACCTCACTTGTGGGGGGATTAGTGGC
GAACGGGTGAGTAACACGTGAGTAACCTGCCCTTGACTCTGGGATAAGCCTGGGAAACCGGGTCTAAT
ACTGGATACGACCTTCTGGCGCATGCCATGTTGGTGGAAAGCTTTTGTGGTTTTGGATGGACTCGCGG
CCTATCAGCTTGTTGGTGGGGTAATGGCCTACCAAGGCGACGACGGGTAGCCGGCCTGAGAGGGTGAC
CGGCCACACTGGGACTGAGACACGGCC
>Zotu562
GAACGCTGGCGGCGTGCTTAACACATGCAAGTGGAGCGACGAAACGGGCTTCGGCCCGTGGCAAAGCC
GCGAACGGGTGAGTAACACGTGGGTAACCTGCCCCGATGACCGGGACAACCCGAGGAAACTCGGGCTA
ATACCGGATGTTCCCCCATCACACCAGTGGTGGGAGGAAAGGTAGCTTCGGCCTCCGCATCGGGATGG
GCCCGCGGCCCATTAGCTTGTTGGTGGGGTAATGGCCTACCAAGGCTGCGATGGGTAGCTGGTCTGAG
AGGACGATCAGCCACACTGGGACTGAGACACGGCC
>Zotu563
GAACGCTAGCGGCAGGCCTAATACATGCAAGTCGAGGGGTAGAGGTTGCTTGCAATCTTGAGACCGGC
GCACGGGTGCGTAACGCGTATGTAACCTACCTTTATCTGGGGGATAGCCCGGAGAAATCCGGATTAAT
ACCGCATAAAATCACAGTATTGCATAATACAATGATCAAACATTTATGGGATGAAGATGGGCATGCGT
GTCATTAGCTAGTTGGCGGGGTAACGGCCCACCAAGGCGACGATGACTAGGGGATCTGAGAGGATGAC
CCCCCACACTGGTACTGAGACACGGAC
>Zotu564
GAACGCTGGCGGCGTGCTTAACACATGCAAGTCGAACGGTAAGGCCCCTTCGGGGGTACACGAGTGGC
GAACGGGTGAGTAACACGTGAGCAACCTGCCCTTGACTTCGGAATACCAGCTGGAAACAGCTGCTAAT
ACCGGATATGACACTTGCCCTCATGGGTGGGTGTGGAAAGCTCCGGCGGTCAGGGATGGGCTCGCGGC
CTATCAGCTTGTTGGTGAGGTAATGGCTCACCAAGGCTTCGACGGGTAGCCGGCCTGAGAGGGCGACC
GGCCACACTGGGACTGAGATACGGCC
>Zotu566
GAACGCTGGCGGGATGCTTTACACATGCAAGTCGAACGGCAACGCGGACTTCGGTCTGGCGGCGAGTG
GCGAACGGGTGAGTAATGTATCGGAACGTACCCAGTAGAGGGGGATAACTACGCGAAAGCGTAGCTAA
TACCGCATAAGCCCTAAGGGGGAAAGCGGGGGATCTTCGGACCTCGCGCTATTGGAGCGGCTGATATC
AGATTAGCTAGTTGGTGGGGTAAAGGCCTACCAAGGCGACGATCTGTAGCTGGTTTGAGAGGACGACC
AGCCACACTGGGACTGAGACACGGCC
>Zotu567
GAACGCTGGCGGCGTGCTTAACACATGCAAGTCGAACGGTGAAGCCCAGCTTGCTGGGTGGATCAGTG
GCGAACGGGTGAGTAACACGTGAGCAATCTGCCCCTGACTCTGGGATAAGCGCTGGAAACGGCGTCTA
ATACTGGATATGAACCGTGGAGGCATCTTCAACGGTTGGAAAGATTTTTTGGTCAGGGATGAGCTCGC
GGCCTATCAGCTTGTTGGTGAGGTAATGGCTCACCAAGGCGTCGACGGGTAGCCGGCCTGAGAGGGTG
ACCGGCCACACTGGGACTGAGACACGGCC
>Zotu568
AAACGCTGGCGGCGTGCATGAGACATGCAAGTCGAACGAACGAGCTTGCTCGTTAGTGGCGGACGGCT
GAGTAACGCGTAGGTGACCTGCCCCGAAGTGGGGGATAACTGCCCGAAAGGGTGGCTAATACCGCATG
TGGCGACACTTTTATTGGTGTCATCAAAGCCGCGAGGCGCTTCGGGAGGGGCCTGCGTCCGATTAGCT
CGTTGGTGGGGTAATGGCTCACCAAGGCGACGATCGGTAGGTGGTCTGAGAGGACGATCACCCAGACT
GGGACTGAGACACGGCC
>Zotu569
GAACGCTGGCGGCGTGCTTAACACATGCAAGTCGAACGATGATGGTGGTGCTTGCACTGCCTGATTAG
TGGCGAACGGGTGAGTAACACGTGAGTAACCTGCCCTTCACTTCGGGATAACCTCGGGAAATCGTGGC

TAATACCGGATATGAGCTCCTGCCGCATGGTGGGTGTTGGAAAGATTTATCGGTGAGGGATGGACTCG
CGGCCTATCAGTTTGTTGGTGAGGTAATGGCTCACCAAGGCGATGACGGGTAGCCGGCCTGAGAGGGC
GACCGGCCACACTGGGACTGAGACACGGCC
>Zotu570
GAACGCTGGCGGCGTGCCTAATACATGCAAGTCGAGCGAACTGAAAAGGAGCTTGCTCCTTTGACGTT
AGCGGCGGACGGGTGAGTAACACGTGGGTAACCTACCTATAAGACTGGAATAACTCCGGGAAACCGGG
GCTAATGCCGGATAATATGTTGAACCGCATGGTTCAACAGTGAAAGACGGTTTTGCTGTCACTTATAG
ATGGACCCGCGCCGCATTAGCTAGTTGGTGAGGTAACGGCTCACCAAGGCGACGATGCGTAGCCGACC
TGAGAGGGTGATCGGCCACACTGGAACTGAGACACGGTC
>Zotu571
GAACGCTGGCGGCGTGGATGAGGCATGCAAGTCGAATGGGTTTAGACCCATGGCAGACGAGGTAGGAA
CAAGTAGGTACGTACCCCAAAGTCAGGGATAACCCGGAGAAATCCGGAATAATACTTGATGGTCTCGC
GAGAGTAAAGATTTATCGCTTTGGGAACGGCCTGCTTTCTATCAGCTTGTTGGTAAGGTAACGGCTTA
CCAAGGCTACGACGGATAGGGGAGGTGAGAGCCTGACCCCCACCGATGGAACTGCGACACGGTC
>Zotu572
GAACGCTAGCGGCAGGCCTAATACATGCAAGTCGAGGGGTAGAGTTAGCTTGCTAACTTGAGACCGGC
GCACGGGTGCGTAACGCGTATGCAACCTACCTTTATCTGGGGGATAGCCCGAAGAAATTCGGATTAAC
ACCGCATAAAATCACAGTATCGCATGATACAATGATCAAATATTTATAGGATAAAGATGGGCATGCGT
GTCATTAGTTAGTTGGCGGGGTAACGGCCCACCAAGACCACGATGACTAGGGGATCTGAGAGGATGAC
CCCCCACACTGGTACTGAGACACGGAC
>Zotu573
GAACGCTGGCGGCGTGCCTAATACATGCAAGTCGAGCGAACAGATAAGGAGCTTGCTCCTTTGACGTT
AGCGGCGGACGGGTGAGTAACACGTGGATAACCTACCTATAAGACTGGGATAACTTCGGGAAACCGGA
GCTAATACCGGATAAGATTTTGAACCGCATGGTTCAATAGTGAAAGGCGGCTTTGCTGTCACTTATAG
ATGGATCCGCGCCGTATTAGCTAGTTGGTAAGGTAACGGCTTACCAAGGCAACGATACGTAGCCGACC
TGAGAGGGTGATCGGCCACACTGGAACTGAGACACGGTC
>Zotu574
GAACGCTGGCGGCGTGCCTAATACATGCAAGTCGAGCGAACGGACAAGGAGCTTGCTCCTTTGAAGTT
AGCGGCGGACGGGTGAGTAACACGTGGGTAACCTACCTATAAGACTGGAATAACTCCGGGAAACCGGG
GCTAATGCCGGATAACATGTTGAACCGCATGGTTCAACAGTGAAAGACGGTCTTGCTGTCACTTATAG
ATGGACCCGCGCCGTATTAGCTAGTTGGTGGGGTAACGGCCTACCAAGGCGACGATACGTAGCCGACC
TGAGAGGGTGATCGGCCACACTGGAACTGAGACACGGTC
>Zotu576
GAACGCTGGCGGCGTGCCTAATACATGCAAGTAGAACGCTGAAGCTTGGTGCTTGCACCGAGCGGATG
AGTTGCGAACGGGTGAGTAACGCGTAGGTAACCTGCCTCTTAGCGGGGGATAACTATTGGAAACGATA
GCTAATACCGCATAAAAGTCGATATCGCATGATGTTGACTTGAAAGGTGCAATTGCATCACTAAGAGA
TGGACCTGCGTTGTATTAGCTAGTTGGTGAGGTAACGGCTCACCAAGGCGACGATACATAGCCGACCT
GAGAGGGTGATCGGCCACACTGGGACTGAGACACGGCC
>Zotu577
GAACGCTGGCGGCGTGCCTAATACATGCAAGTCGAGCGGACCGGATCGGAGCTTGCTCTGGTTTGGTC
AGCGGCGGACGGGTGAGTAACACGTGGGCAACCTGCCCGCAAGACCGGGATAACTCCGGGAAACCGGA
GCTAATACCGGATAACACCGAAGACCGCATGGTCTTTGGTTGAAAGGCGGCCTTTGGCTGTCACTTGC
GGATGGGCCCGCGGCGCATTAGCTAGTTGGTGAGGTAACGGCTCACCAAGGCGACGATGCGTAGCCGG
CCTGAGAGGGTGACCGGCCACACTGGGACTGAGACACGGCC
>Zotu578
GAACGCTGGCGGCATGCCTAACACATGCAAGTCGAACGAAGGCTTCGGCCTTAGTGGCGCACGGGTGC
GTAACGCGTGGGAATCTGCCCTCAGGTTCGGAATAACAGCGAGAAATTGCTGCTAATACCGGATGATA
TCGCGAGATCAAAGATTTATCGCCTGAGGATGAGCCCGCGTAGGATTAGCTAGTTGGTGTGATAAAGG
CGCACCAAGGCGACGATCCTTAGCTGGTCTGAGAGGATGATCAGCCACACTGGGACTGAGACACGGCC
>Zotu579
GAACGCTGGCGGCGTGGATAAGGCATGCAAGTCAAGCGGGTCCCTCACCTTTCGCAAGAAAGGTGAGG
GATACCGCGGCGAACGAGTTAGTAACACCTTGGTATCTGCCTCAAAGACGGGCATAACCAGTCGAAAG
ATTGGCTAATTCCCGATGGTCCCTTACGGGTAAAGGAGCAATCCGCTTTGAGAGGAGCCTAGGTCCTA
TCAGCTTGTTGGCGGGGTAACGGCCCACCAAGGCTATGACGGGTAGGAGGTGTGAGAGCACGACCTTC

AACGACGAAACTGAGATACGGTT
>Zotu580
GAACGCTGGCGGTGTGCTTCACACATGCAAGTCGAACGGGGTAGCAATACCTAGTGGCGGACGGGTGA
GTAACGCGTGGGAATCTGCCCAAGGGTGGGGGATAACTAACCGAAAGGTTAGCTAATACCGCATATGC
CGTGAGGTAAAAGGAGTAATCCGCCTTTGGATGAGCCTGCGTCCGATTAGCTTGTTGGTGGGGTAAAA
GCCTACCAAGGCTACGATCGGTAGCTGGTCTGAGAGGATGATCAGCCACAATGGGACTGAGACACGGC
C
>Zotu581
GAACGCTGGCGGCATGCCTTACACATGCAAGTCGAACGGCAGCACGGACTTCGGTCTGGTGGCGAGTG
GCGAACGGGTGAGTAATACATCGGAACGTGTCCTGTAGTGGGGGATAGCCCGGCGAAAGCCGGATTAA
TACCGCATACGCTCTACGGAGGAAAGGGGGGGATCGCAAGACCTCCCGCTACAGGGGCGGCCGATGGC
AGATTAGCTAGTTGGTGGGGTAAAGGCCTACCAAGGCGACGATCTGTAGCTGGTCTGAGAGGACGACC
AGCCACACTGGGACTGAGACACGGCC
>Zotu582
TAACGCTGGCAGAGTGCATAACACATGCAAGTCGAGCGGGACTGTTTGAGATTACATCAAGAGCAGTT
TAGCGGCAGACGAGTGAGTAACACGTAAGCAATCTACCCCCGAATGGCGAACAACCAGCCGAAAGGCT
GGCTAATACGCCGTATTCTCCTTGGAAGGCATCTTCCGAGGAGGAGAGACCCGCAAGGGTCGTTCAGG
GAGGAGCTTGCGGCCCATCAGCTAGTTGGCGGTGTAACGGACCACCAAGGCTACGACGGGTACCCGGG
CTGAAAGGCCGGACGGGCACGATGGCACTGAGACACGGGC
>Zotu583
GAACGCTGGCGGCAGGCTTAACACATGCAAGTCGAACGAGTGTAGCAATACACTAGTGGCGCACGGGT
GAGTAACACGTGGATATCTGCCTTTTGGTTCGGGATAACCCTGGGAAACTAGGGCTAATACCGAATGG
TTCCTACGGGATAAAGATTTATCGCCAAAAGATGAGTCCGCGTCCGATTAGCTAGTTGGTGAGGTAAT
GGCTCACCAAGGCGACGATCGGTAGCTGGTCTGAGAGGACGATCAGCCACACTGGGACTGAGACACGG
CC
>Zotu584
GAACGCTAGCGGCAGGCCTAATACATGCAAGTCGAACGGGGCCGCAAGGCCCAGTGGCGCACGGGTGC
GTAACGCGTAGGCAATCTGCCTATCTCTGGGGGATAGCCCGCCGAAAGGTGGATTAATACCGCATGAG
ACCATAACACGGCATCGTGTAGTGGTTAAAGAATTTCGGAGATAGATGAGCCTGCGTGCCATTAGCTA
GTTGGCGGGGTAAGGGCCCACCAAGGCGACGATGGCTAGGGGAGCTGAGAGGCCGATCCCCCACACTG
GCACTGAGATACGGGC
>Zotu585
GAACGCTGGCGGCGTGCTTAACACATGCAAGTCGAACGGTAAGGCCCCTTCGGGGGTACACGAGTGGC
GAACGGGTGAGTAACACGTGAGTAACCTGCCCTTGACTCTGGGATAACAGCTGGAAACAGCTGCTAAT
ACCGGATATCAGCCTCGATGGCATCTTCGAGGCTTGAAAGCTCCGGCGGTCATGGATGGACTCGCGGC
CTATCAGCTTGTTGGTGAGGTAACGGCTCACCAAGGCGACGACGGGTAGCCGGCCTGAGAGGGCGACC
GGCCACATTGGGACTGAGATACGGCC
>Zotu586
GAATGCTGGCGGCGCGCCTAACACATGCAAGTCGAGCGGAAAGGCCCTTCGGGGTACTCGAGCGGCGG
ACGGCTGAGTAACGCGTGGGAACATGCCCCAAAGTGAGGAATAACTGCCCGAAAGGGTAGCTAATGCC
GCATATGATCTTAGGATTAAAGGATTTATCCGCTTTGGGAGTGGCCCGCGTACGATTAGGTAGTTGGT
GAGGTAAAGGCTCACCAAGCCGACGATCGTTAACTGGTCTGAGAGGATGATCAGTCAGACTGGAACTG
AGACACGGTC
>Zotu587
GAACGCTAGCGGCAGGCCTAATACATGCAAGTCGAGCGGCAAGTCGAGGAGTAATCTAAGACCTAGAG
CGGCGTACGGGTGCGTAACGCGTATGCAACCTACCTCATACAGGGGGATAGCCCGGAGAAATCTGGAT
TAATACCCCATGGCACAGCACAAGGGCATCTGCGAGCTGTTAAAGATTCATCGGTATGAGATGGGCAT
GCGTCTGATTAGCTAGTTGGCGGTGTAACGGACCACCAAGGCGACGATCAGTAGGGGTTCTGAGAGGA
AGGTCCCCCACACTGGCACTGAGATACGGGC
>Zotu588
GAACGCTGGCGGCGTGCTTAACACATGCAAGTCGAACGATGAAGCCCAGCTTGCTGGGTGGATTAGTG
GCGAACGGGTGAGTAACACGTGAGTAACCTGCCCTTGACTCTGGGATAAGCCTGGGAAACTGGGTCTA
ATACCGGATAGGAACGTCCACCGCATGGTGGTTGTTGGAAAGATTTATCGGTCATGGATGGACTCGCG
GCCTATCAGCTTGTTGGTGAGGTAATGGCTCACCAAGGCGACGACGGGTAGCCGGCCTGAGAGGGTGA

CCGGCCACACTGGGACTGAGACACGGCC
>Zotu589
GAACGCTAGCGGCAGGCTTAATACATGCAAGTCGAGGGGCAGCAGGGCTGTAGCAATACAGTTGCTGG
CGACCGGCAAACGGGTGCGGAACACGTACGCAACTTACCCAGAACTGGGGCATAGCCCTCCGAAAGGA
GGATTAACACCCCATAAGATAGTAGAGTGGCATCACTCAGCTATTAAAACTCCGGTGGTTTTGGATGG
GCGTGCGGCCCATTAGATAGTTGGTGAGGTAACGGCTCACCAAGTCTGCGATGGGTAACTGGTGTGAG
AGCACGACCAGTCACACGGGCACTGAGACACGGGC
>Zotu590
GAACGCTGGCGGCATGCTTTACACATGCAAGTCGAACGGTAACAGGTCTTCGGATGCTGACGAGTGGC
GAACGGGTGAGTAATACATCGGAACGTGCCTAGTAGTGGGGGATAACTACTCGAAAGAGTAGCTAATA
CCGCATGAGATCTACGGATGAAAGCAGGGGACCTTCGGGCCTTGTGCTACTAGAGCGGCTGATGGCAG
ATTAGGTAGTTGGTGGGGTAAAGGCTCACCAAGCCTGCGATCTGTAGCTGGTCTGAGAGGACGACCAG
CCACACTGGGACTGAGACACGGCC
>Zotu591
GAACGCTGGCGGCATGCTTTACACATGCAAGTCGAACGGCAGCGCGGGGCAACCTGGCGGCGAGTGGC
GAACGGGTGAGTAATATATCGGAACGTACCCAAGAGTGGGGGATAACGTAGCGAAAGTTACGCTAATA
CCGCATACGATCTAAGGATGAAAGTGGGGGATCTTCGGACCTCATGCTCCTGGAGCGGCCGATATCTG
ATTAGCTAGTTGGTGAGGTAAAGGCTCACCAAGGCGACGATCAGTAGCTGGTCTGAGAGGACGACCAG
CCACACTGGAACTGAGACACGGTC
>Zotu592
GAACGCTGGCGGCATGCCTTACACATGCAAGTCGAACGGTAACAGGTCTTCGGATGCTGACGAGTGGC
GAACGGGTGAGTAATACATCGGAACGTGCCCGATCGTGGGGGATAACGAAGCGAAAGCTTTGCTAATA
CCGCATACGATCTACGGATGAAAGCAGGGGACCGCAAGGCCTTGCGCGAACGGAGCGGCCGATGGCAG
ATTAGGTAGTTGGTGGGATAAAAGCTTACCAAGCCGACGATCTGTAGCTGGTCTGAGAGGACGACCAG
CCACACTGGGACTGAGACACGGCC
>Zotu593
GAACGCTGGCGGCGTGCTTAACACATGCAAGTCGAGCGGTAAGGCCTTTCGGGGTACACGAGCGGCGA
ACGGGTGAGTAACACGTGGGTGATCTGCCCTGCACTCTGGGATAAGCTTGGGAAACTGGGTCTAATAC
CGGATATGACCGCAGCATGCATGTGTTGTGGTGGAAAGATTTATCGGTGCAGGATGGGCCCGCGGCCT
ATCAGCTTGTTGGTGGGGTAATGGCCTACCAAGGCGACGACGGGTAGCCGACCTGAGAGGGTGACCGG
CCACACTGGGACTGAGACACGGCC
>Zotu594
GAACGCTGACAGAATGCTTAACACATGCAAGTCAACTTGAATTTGGGTTTTTAACTTAGGTTTGGGTG
GCGGACGGGTGAGTAACGCGTAAAGAACTTGCCTCACAGGTAGGGACAACATTTGGAAACGAATGCTA
ATACCTAATATTATGATAATAGGGCATCCTATAATTATGAAAGCTATAAGCGCTGTGAGAGAGCTTTG
CGTCCCATTAGCTAGTTGGAGAGGTAACGGCTCACCAAGGCGATGATGGGTAGCCGGCCTGAGAGGGT
GATCGGCCACAAGGGGACTGAGACACGGCC
>Zotu595
GAACGCTGGTAACGTGGATACCGGCATGCAAGTCACGGGGGCCGCAAGGCAACCGGCGAACGGCTTAG
TAACACGTGGGTACATACCTTAGAGTGGAGCATAGCTCGTCGAAAGACGGGGTAATTCTCCATAGCAT
CGCAAGATTAAACCCGTAAGGGGCTCTAAGAATGGCCTGCGGCCTATCAGTTAGTTGGCGAGGTAATA
GCTCACCAAGACGATGACGGGTAGGGGGCGTGAGAGCGCGACCCCCAAGAATGGCACTGCGACACGGG
C
>Zotu596
GAACGCTAGCGGGAGGCCTAACACATGCAAGCCGAGCGGTATTGTTTCTTCGGAAATGAGAGAGCGGC
GTACGGGTGCGGAACACGTGTGCAACCTGCCTTTATCTGGGGGATAGCCTTTCGAAAGGAAGATTAAT
ACTCCATAATATATTGAACGGCATCGTTTAATATTGAAAGCTCCGGCGGATAGAGATGGGCACGCGCA
AGATTAGCTAGTTGGTGAGGTAACGGCTCACCAAGGCAATGATCTTTAGGGGGCCTGAGAGGGTGATC
CCCCACACTGGTACTGAGACACGGAC
>Zotu597
GAACGCTGGCGGCGTGCTTAAGACATGCAAGTCGAACGGGCATCTTCGGATGCTAGTGGCGCACGGGT
GAGTAACGCGTAACTGACCTACCCAGAAGTCTGGAATAACGGGTCGAAAGATCCGCTAATACTGGATG
TGCAGTGTCGCTGTGGCGACACTGTAAAGATTGATCGCTTGTGGATGGGGTTGCGTTTCATCAGCTGG
TTGGTGGGGTAAAGGCCTACCAAGGCGACGACGAATAGCCGGCCTGAGAGGGTGGCCGGCCACAGGGG

CACTGAGACACGGGT
>Zotu598
GAACGCTGGCGGCGTGCCTAATACATGCAAGTCGAGCGAGCTGAATTCAAAGATCCCTTCGGGGTGAT
TTGTTGGACGCTAGCGGCGGATGGGTGAGTAACACGTGGGCAATCTGCCCTAAAGACTGGGATACCAC
TTGGAAACAGGTGCTAATACCGGATAACAACATGAATCGCATGATTCAAGTTTGAAAGGCGGCGCAAG
CTGTCACTTTAGGATGAGCCCGCGGCGCATTAGCTAGTTGGTGGGGTAAAGGCCTACCAAGGCAATGA
TGCGTAGCCGAGTTGAGAGACTGATCGGCCACATTGGGACTGAGACACGGCC
>Zotu599
GAACGCTGGCGGCGTGCTTAACACATGCAAGTCGAACGGAAAGGCCTGCAGCTTGCTGCGGGTACTCG
AGTGGCGAACGGGTGAGTAACACGTGGGTGATCTGCCCTCAACTTTGGGATAAGCCTGGGAAACTGGG
TCTAATACCGGATATGACCTTTCTTTAGTGTGGAAGGTGGAAAGCTTTTGCGGTTGGGGATGAGCTCG
CGGCCTATCAGCTTGTTGGTGGGGTAATGGCCTACCAAGGCGTCGACGGGTAGCCGGCCTGAGAGGGT
GTACGGCCACATTGGGACTGAGATACGGCC
>Zotu600
GAACGCTGGCGGCGTGCTTAACACATGCAAGTCGAACGGTGAAGCCCTTCGGGGTGGATCAGTGGCGA
ACGGGTGAGTAACACGTGAGCAACCTGCCCCTGGCTTCGGGATAACTCCGGGAAACCGGGGCTAATAC
CGAATATGACACGCTCTCGCATGGGATGTGTGTGGAAAGTTTTTCGGCTGGGGATGGGCTCGCGGCCT
ATCAGCTTGTTGGTGGGGTGATGGCCTACCAAGGCGACGACGGGTAGCCGGCCTGAGAGGGCGACCGG
CCACACTGGGACTGAGACACGGCC
>Zotu601
GAACGCTGGCGGCAGGCTTAACACATGCAAGTCGAGCGGGCGTAGCAATACGTCAGCGGCAGACGGGT
GAGTAACGCGTGGGAACGTACCTTTTGGTTCGGAACAACACAGGGAAACTTGTGCTAATACCGGATAA
GCCCTTACGGGGAAAGATTTATCGCCGAAAGATCGGCCCGCGTCTGATTAGCTAGTTGGTAGGGTAAC
GGCCTACCAAGGCGACGATCAGTAGCTGGTCTGAGAGGATGATCAGCCACATTGGGACTGAGACACGG
CC
>Zotu602
GAACGCTAGCGGCAGGCCTAATACATGCAAGTCGGACGGGATTGGAGAGCTTGCTTTCCATGAGAGTG
GCGCACGGGTGCGTAACACGTATGTAACCTACCTTATCCAGGGGGATAGCCTCTCGAAAGAGAGATTA
ACACCGCATAACATTGAGACACAGCATTGTGATTCAATCAAATATTTATAGGGATAAGATGGGCATGC
GGGACATTAGCTAGTTGGCGGGGTAACGGCCCACCAAGGCGACGATGTCTAGGGGATCTGAGAGGATG
ACCCCCCATACTGGTACTGAGACACGGAC
>Zotu603
GAACGCTGGCGGTGTGCTTAACACATGCAAGTGGTGCGACGAACCAGAGCTTGCTCTGGGGCAAAGCC
GCGAACGGGTGAGTAACACGTGGGTAATCTACCCCGATGACCGGGACAACCCGAGGAAACTCGGGCTA
ATACCGGATGTGGTACACAGGCTTAAGCCTGTTTACTAAAGGTAGCTTCGGCTTCCGCATTGGGAGGG
GCCCGCGGCCCATTAGCTTGTTGGTGGGGTCAAGGCCTACCAAGGCAACGATGGGTAGCTGGTCTGAG
AGGACGATCAGCCACACTGGGACTGAGACACGGCC
>Zotu604
GAACGCTGGCGGCGTGCCTAATACATGCAAGTAGAACGCTGAAGGAGGAGCTTGCTTCTCCGGATGAG
TTGCGAACGGGTGAGTAACGCGTAGGTAACCTGCCTGGTAGCGGGGGATAACTATTGGAAACGATAGC
TAATACCGCATAAGAGTAGATGTTGCATGACATTTGCTTAAAAGGTGCAATTGCATCACTACCAGATG
GACCTGCGTTGTATTAGCTAGTTGGTGGGGTAACGGCTCACCAAGGCGACGATACATAGCCGACCTGA
GAGGGTGATCGGCCACACTGGGACTGAGACACGGCC
>Zotu605
GAACGCTGGCGGCAGGCTTAACACATGCAAGTCGAGCGCATCCTTCGGGGTGAGCGGCAGACGGGTGA
GTAACGCGTGGGAATCTACCTTTTGCTACGGAATAGCTCCGGGAAACTGGAATTAATACCGTATGTGC
TCTTTGGCGGTGGACGCTGGAGAGGAAAGATTTATCGGCAAAGGATGAGCCCGCGTTGGATTAGCTAG
TTGGTGGGGTAAAGGCCTACCAAGGCGACGATCCATAGCTGGTCTGAGAGGATGATCAGCCACATTGG
GACTGAGACACGGCC
>Zotu606
CAACGCTGGCGGCGCGCTTAACACATGCAAGTCGAACGAGAAAGGGGAGCAATCCCTGAGTACAGTGG
CGTACGGGTGAGTAACACGTGGATGATCTACCTTTTGGTGGGGAATACCTCTGGGAAACCGGAGACAA
TACCGCATAAGCTCGGAAGAGGAAACCCGCAAGGGGCCGAAAGAGGAGTCCGCGGCCGATTAGCTAGT
TGGTGGGGTAAAAGCTCACCAAGGCAAAGATCGGTAGCCGGCCTGAGAGGGCACACGGCCACACTGGC

ACTGAAACACGGGC
>Zotu607
GAACGCTGGCGGCAGGCTTAACACATGCAAGTCGAACGCCCCGCAAGGGGAGTGGCAGACGGGTGAGT
AACACGTGGGAACGTACCCTTTTCTACGGAATAACCCAGGGAAACTTGGACTAATACCGTATGTGCCC
TTCGGGGGAAAGATTTATCGGAAAAGGATCGGCCCGCGTTGGATTAGCTAGTTGGTGGGGTAAAGGCC
TACCAAGGCGACGATCCATAGCTGGTCTGAGAGGATGATCAGCCACATTGGGACTGAGACACGGCC
>Zotu608
GAATGCTGGCGGCGTGGATAAGTCATGCAAGTCAAGGGGGCCCGCAAGGGCAACCGGCAGACGAGGTA
GTAACACGTAGGTACGTCCCCCGAAGTCGGGCATAGCCATCCGAAAGGATGGGTAATTCCCGATAGTC
CCGCAAGGGTAAAGGTTTTTCGCTTCGGGAGCGGCCTGCGTAGTATCAGCTAGTTGGTAGGGTAATGG
CCTACCAAGGCTACGACGCTTAGGGGAGCTGAGAGGCTGACCCCCACCGATGGAACTGCGACACGGTC
>Zotu609
GAACGCTGGCGGCGTGCCTAACACATGCAAGTCGAGCGGTAACAGGTGTAGCAATACATGCTGACGAG
CGGCGAACGGGTGAGTAATGCTTCGGAATCTACCTTATGGTGGGGAATAACTAGTCGAAAGATTAGCT
AATACCGCATACGTCCTACGGGAGAAAGCAGGGGATCGTAAGACCTTGCGCCAAAAGATGAGCCGAAG
TCGGATTAGCTAGTTGGTAGGGTAACGGCCTACCAAGGCGACGATCCGTAGCTGGTCTGAGAGGACGA
CCAGCCACACTGGAACTGAGACACGGTC
>Zotu610
GAACGCTGGCGGCAGGCCTAACACATGCAAGTCGAACGCCTTAGCAATAAGGAGTGGCAGACGGGTGA
GTAACACGTGGGAACATACCCTTCGGTTCGGAATAACTCAGGGAAACTTGTGCTAATACCGGATACGT
CAGTGATGAGAAAGATTTATCGCCGAAGGATTGGCCCGCGTCTGATTAGCTAGTTGGTGAGGTAATGG
CTCACCAAGGCGACGATCAGTAGCTGGTCTGAGAGGATGACCAGCCACATTGGGACTGAGACACGGCC
>Zotu611
GAACGCTAGCGGCAGGCTTAACACATGCAAGTCGAGGGGTAGAAGCAGCTTGCTGCTTTGAGACCGGC
GCACGGGTGCGTAACGCGTATACAATCTACCTTTTACAGAGGGATAGCCCAGAGAAATTTGGATTAAT
ACCTCATAGTATTTTCGAATCGCATGATTTGATTATTAAAGTTCCAACGGTAAAAGATGAGTATGCGT
CCCATTAGCTAGATGGTAAGGTAACGGCTTACCATGGCAACGATGGGTAGGGGTCCTGAGAGGGAGAT
CCCCCACACTGGTACTGAGACACGGAC
>Zotu612
TAACGCTGGCGGCGTGCATAACACATGCAAGTCGAACGATGAAACTTAAAAGATTTCTTCGGAATGAC
CTTAAGTGGATTAGTGGCGAACGGGTGAGTAACGCGTGAGTAACCTGCCTTACACAAGGGGATAGCCT
CTGGAAACGGAGAATAATACCCTATGAAACTATCTTGTCGCATGAAAAGATAGTCAAAGTGTATTAGC
GGTGTAAGATGGACTTGCGTCTGATTAGCTAGTTGGTGAGATAACAGCCCACCAAGGCAACGATCAGT
AGCCGGCTTGAGAGAGTGTACGGCCACATTGGGACTGAGACACGGCC
>Zotu613
GAACGCTGGCGGCGCGCCTAATACATGCAAGTCGAACGGGTACCTCTTCGGAGGTGCCAGTGGCGGAC
GGGTGAGTAACACGTGGGTGACCTGCCCTGTGGTGGGGAATAACTCTGGGAAACCAGGGCTAATACCG
CATACGCTCAGCGTTTCATTACGCTGAGGAAAGATTGATCGCCATAGGAGGGGCCTGCGCTGGATTAG
CTGGTCGGTGGGGTAATGGCCTACCGAGGCTGCGATCCATAGCTGGTCTGAGAGGACGACCAGCCACA
CTGGGACTGAGACACGGCC
>Zotu614
GAACGCTGACAGAATGCTTAACACATGCAAGTCAACTTGAACTTCGGTTTGGGTGGCGGACGGGTGAG
TAACGCGTAAAGAACTTGCCTCACAGATAGGGACAACATTTGGAAACGAATGCTAATACCTGATATTA
TGATTTTAGGGCATCCTAGAATTATGAAAGCTATATGCGCTGTGAGAGAGCTTTGCGTCCCATTAGCT
AGTTGGAGAGGTAACGGCTCACCAAGGCGATGATGGGTAGCCGGCCTGAGAGGGTGATCGGCCACAAG
GGGACTGAGACACGGCC
>Zotu615
GAACGCTGGCGGCATGCCTAATACATGCAAGTTGAACGATGATGTTCTCTGCTTGCAGAGAATTGAAG
AGTAGCGAACGGGTGAGTAACACGTGGGGAGCCTGCCCATGAGAGGGGGATAACATTCGGAAACGGAT
GCTAATACCGCATAATGCTTTGGACCGCCTGGTCCAGAGACGAAAGACGACTTTTGCTGTCACTCATG
GATGGCCCCGCGCCGTATTAGCTAGTTGGTGAGGTAATGGCTCACCAAAGCTGTGATACGTAGCCGAC
CTGAGAGGGTGATCGGCCACACTGGGACTGAGACACGGCC
>Zotu616
GAACGCTGGCGGCGTGCCTAATACATGCAAGTCGAACGCACAGCGAAAGGTGCTTGCACCTTTCAAGT

GAGTGGCGAACGGGTGAGTAACACGTGGACAACCTGCCTCAAGGCTGGGGATAACATTTGGAAACAGA
TGCTAATACCGAATGAAACTTAGTGTCGCATGATACGAAGTTAAAAGGCGCTTTGGCGTCACCTAGAG
ATGGATCCGCGGTGCATTAGTTAGTTGGTGGGGTAAAGGCCTACCAAGACAATGATGCATAGCCGAGT
TGAGAGACTGATCGGCCACATTGGGACTGAGACACGGCC
>Zotu617
GAACGCTGACAGAATGCTTAACACATGCAAGTCATTGGCGAAGCTGCGCTTGCGCAGTCTAGCCAAGG
CGGACGGGTGAGTAACGCGTAAGGAACTTGCCCTGCAGACAGGGATAACAGACGGAAACGACTGATAA
CACCTGGTATTGTCAGGCTGGCGCATGCCGGCCTGATGAAAGGAGACGCTGCAGGAGAGCCTTGCGTC
CTATTAGCTAGTTGGTGGGGTAACGGCCCACCAAGGCGATGATAGGTAGCCGGCCTGAGAGGGTGAAC
GGCCACAAGGGGACTGAGATACGGCC
>Zotu618
GAACGCTAGCGGCAGGCTTAACACATGCAAGTCGAGCGGTAGATGGACTTCGGTTCATTGAGAGCGGC
AAACGGGTGCGGAACACGTACGCAACATACCCTTCTCTGGGGGATAGCCTTCTGAAAAGGAGATTAAT
ACCCCATAAGATAATAGAGTGGCATCACTTAGTTATTAAAGATTTATTGGAGAGGGATTGGCGTGCGT
CCTATTAGCTAGTTGGTAAGGTAATGGCTTACCAAGGCGACGATAGGTAGCTGGTGTGAGAGCACGAC
CAGCCACACGGGCACTGAGACACGGGC
>Zotu619
GAACGCTGGCGGCGTGCTTAACACATGCAAGTGGAGCGACGAACCGGGGCTTGCCCCGGGGCAGAGCC
GCGAACGGGTGAGTAACACGTGGGTTACCTGCCCCAATGACCGGGACAACCCGAGGAAACTCGGGCTA
ATACCGGATGTGGTCTTCGGACGAAAGGAAGCTTCGGCCTCCGCATTGGGATGGGCCCGCGGCGCATT
AGCTTGTTGGTGGGGTAACGGCCTACCAAGGCGACGATGCGTAGCTGGTCTGAGAGGACGATCAGCCA
CACTGGGACTGAGACACGGCC
>Zotu620
GAACGCTGGCGGCGTGCCTAAAACATGCAAGTCGAACGGGGGGTAACCCCAGTGGCGAACGGCGGAGT
AATACATAAGCAACGTGCCCTAAAGATCGGGATAGTCAGTGGAAACGCTGGGTAATACCGGATGTGAC
GGAAAGAAGGCATCTTCATTCCATTAAATGGTTTTTCGCTTTAGGAGCGGCTTATGGCCTATCAGGTA
GTTGGTGAGGTAACGGCTCACCAAGCCGACGACGGGTAGCGGGTCTGAGAGGACGACCCGCTCGAGTG
GGACTGAGACACGGCC
>Zotu621
GAACGCTAGCGGCAGGCCTAATACATGCAAGTCGAACGGTGATATTTATATCATAGTGGCGCACGGGT
GCGTAACACGTATGCAACCTACCTATTATTGGGGGATAGCCTTTGGAAACGGAGATTAATACCGCATA
ATACGATTAACTGGCATCGGTTGATTGTTAAAGCATTTGCGATAATAGATGGGCATGCGTCCAATTAG
TTAGTTGGTGTGGTAATGGCTCACCAAGACGATGATTGGTAGGGGATCTGAGAGGATAATCCCCCACA
CTGGCACTGAGATACGGGC
>Zotu623
GAACGCTGGCGGCAGGCTTAACACATGCAAGTCGAGCGGGCGCTTTCGGGCGTCAGCGGCGGACGGGT
TAGTAACGCGTGGGAACGTGCCTTCTGCTCTGGAATAGCCCTGGGAAACTGGGGGTAATACCGGATGT
GCCCTGAGGGGGAAAGATTTATCGGCAGGAGATCGGCCCGCGTCTGATTAGGTAGTTGGTGGGGTAAT
GGCTCACCAAGCCGACGATCGGTAGCTGGTTTGAGAGGATGATCAGCAACACTGGGACTGAGACACGG
CC
>Zotu624
GAACGCTGGCGGCATGCTTTACACATGCAAGTCGAACGGCAGCGCGGGGCAACCTGGCGGCGAGTGGC
GAACGGGTGAGTAATATATCGGAACGTACCCAAGAGTGGGGGATAACGTAGCGAAAGTTACGCTAATA
CCGCATACGATCTAAGGATGAAAGTGGGGGATCGCAAGACCTCATGCTCCTGGAGCGGCCGATATCTG
ATTAGCTAGTTGGTGGGGTAAAAGCTCACCAAGGCGACGATCAGTAGCTGGTCTGAGAGGACGACCAG
CCACACTGGGACTGAGACACGGCC
>Zotu625
GAACGCTAGCGGCAGGCCTAATACATGCAAGTCGAACGAGATTAACCAGCTTGCTGGTTATGAAAGTG
GCGCACGGGTGCGTAACGCGTATGCAACCTACCTTATTCAGGGGGATAGCCCGAAGAAATTCGGATTA
ACACCGCATAAAATCACAGTATAGCATTATACAATGATCAAATATTTATAGGAATAAGATGGGCATGC
GTGTCATTAGCTAGTTGGCGGGGTAATGGCCCACCAAGGCGACGATGACTAGGGGATCTGAGAGGATG
ACCCCCCACACTGGTACTGAGACACGGAC
>Zotu626
GAACGCTAGCGGCAGGCTTAATACATGCAAGTCGAGGGGTAACAGGGGTAGCAATACCTGCTGACGAC

CGGCAAACGGGTGCGGAACACGTACGCAACCTTCCCAAAACTGGTGAATAGCCTTCCGAAAGGGAGAT
TAATACACCGTAACTTAATGAAGTGGCATCACTTTATTAATATAGCTCCGGCGGTTTTGGATGGGCGT
GCGCCTGATTAGGTAGTTGGCGGGGTAACGGCCCACCAAGCCTGCGATCAGTAACTGGTGTGAGAGCA
CGACCAGTCACACGGGCACTGAGACACGGGC
>Zotu627
GAACGCTGGCGGCGTGCTTAACACATGCAAGTCGAACGATGATGCCCTGCTTGCAGGGTGGATTAGTG
GCGAACGGGTGAGTAACACGTGGGTGATCTGCCCCGCACTTCGGGATAAGCCTGGGAAACTGGGTCTA
ATACCGGATACGACCCCCTGGTAGGTCAGGGGGTGGAAAGATTTATCGGTGTGGGATGAGCCTGCGGC
CTATCAGCTTGTTGGTGGGGTAATGGCCTACCAAGGCGTCGACGGGTAGCCGGCCTGAGAGGGTGATC
GGCCACATTGGGACTGAGACACGGCC
>Zotu628
GAACGCTAGCGGCAGGCCTAACACATGCAAGTCGAGGGGTAGAGTTAGCTTGCTAACTTGAGACCGGC
GCACGGGTGCGTAACGCGTATGCAATCTACCTTATACTGAGGGATAGCCCGGAGAAATCCGGATTAAT
ACCTTATAGTATATTAAAGTGGCATCATTTTGATATTAAAGATTTATTGGTATAAGATGAGCATGCGT
CCCATTAGTTAGTTGGTGTGGTAACGGCACACCAAGACGATGATGGGTAGGGGTCCTGAGAGGGAGAT
CCCCCACACTGGTACTGAGACACGGAC
>Zotu629
GAACGCTGGCGGCAGGCTTAACACATGCAAGTCGAACGGGCGTAGCAATACGTCAGTGGCAGACGGGT
GAGTAACACGTGGGAACGTACCTCTTGGTTCGGAACAACACAGGGAAACTTGTGCTAATACCGGATAA
GCCCTTACGGGGAAAGATTTATCGCCGAGAGATCGGCCCGCGTCTGATTAGCTAGTTGGTAGGGTAAT
GGCCTACCAAGGCGACGATCAGTAGCTGGTCTGAGAGGATGATCAGCCACATTGGGACTGAGACACGG
CC
>Zotu630
GAACGCTAGCGGCAGGCTTAATACATGCAAGTCGAGGGGTAACATGGGTAGCAATACCTGATGACGAC
CGGCAAACGGGTGCGGAACACGTACGCAATCTTCCCGAAACTGGTGAATAGCCCTCCGAAAGGAGGAT
TAATACACCGTAAGATAACGAGGAGGCATCTCCTTGTTATTATAGTAGCAATACGGTTTTGGATGAGC
GTGCGCCTGATTAGGTAGTTGGCGGGGTAACGGCCCACCAAGCCTACGATCAGTAACTGGTGTGAGAG
CACGACCAGTCACACGGGCACTGAGACACGGGC
>Zotu631
GAACGCTGGCGGCAGGCCTAACACATGCAAGTCGAGCGAGACCTTCGGGTCTAGCGGCGGACGGGTGA
GTAACGCGTGGGAATATGCCCTTTTCTACGGAATAGTCCTGGGAAACTGGGGGTAATACCGTATACGC
CCTTTGGGGGAAAGATTTATCGGAGAAGGATTAGCCCGCGTTGGATTAGGTAGTTGGTGGGGTAATGG
CCTACCAAGCCGACGATCCATAGCTGGTTTGAGAGGATGATCAGCCACACTGGGACTGAGACACGGCC
>Zotu632
GAACGCTGGCGGCGTGCTTAACACATGCAAGTCGAACGGTCGCAAGACAGTGGCGCACGGGTGAGGAA
CACGTAGCTTATCTGCCCCGAAGTGTGGGATAACGCCCCGAAAGGGGTGCTAATACCGCATAAGCTCC
CGGACTATAGAGGTCTGGGAGAAAAGGCGCAAGTCGCTTTGGGAGGAGGCTGCGGCCCATCAGGTAGT
TGGTGCGGTAACGGCGCACCAAGCCAACGACGGGTAGGGGACCTGAGAGGGTGATCCCCCACACTGGG
ACTGAGACACGGCC
>Zotu633
GAACGCTAGCGGGAGGCCTAACACATGCAAGCCGAGCGGTAGAAAGTAGCTTGCTACTTTTGAGAGCG
GCGTACGGGTGCGTAACACGTGTGCAACCTGCCTTTATCTGGAGAATAGCCTTTCGAAAGGAAGATTA
ATGCTCCATAACATATTGAATGGCATCATTTAATATTGAAAGCTCCGGCGGATAGAGATGGGCACGCG
CAAGATTAGTTAGTTGGTGAGGTAACGGCTCACCAAGGCGATGATCTTTAGGGGGCCTGAGAGGGTGA
TCCCCCACACTGGTACTGAGACACGGAC
>Zotu634
GAACGCTAGCGGCAGGCTTAATACATGCAAGTCGAGGGGCAGCATGACTGTAGCAATACAGTTGATGG
CGACCGGCAAACGGGTGCGGAACACGTACGCAATCTACCCAAAACTGATGAATAGCCCTCCGAAAGGA
GGATTAATACATCGTAACTTAATGAAGTGGCATCACTTTATTAATATAGCTCCGGCGGTTTTGGATGA
GCGTGCGCCTGATTAGGTAGTTGGCGGGGTAACGGCCCACCAAGCCTGCGATCAGTAACTGGTGTGAG
AGCACGACCAGTCACACGGGCACTGAGACACGGGC
>Zotu635
GAACGCTGGCGGCGTGCTTAACACATGCAAGTCGAGCGGAAAGGCCCTTCGGGGTACTCGAGCGGCGA
ACGGGTGAGTAACACGTGAGTAATCTGCCCTGTGCTTTGGGATAGCCACCGGAAACGGTGATTAATAC

CGGATATGACCAGGTACCGCATGGTGTTTGGTGGAAAGTTTTTCGGCGCAGGATGTGCTCGCGGCCTA
TCAGCTTGTTGGTGGGGTAATGGCCTACCAAGGCTTCGACGGGTAGCCGGCCTGAGAGGGTGACCGGC
CACACTGGGACTGAGACACGGCC
>Zotu636
GAACGCTGGCGGCGTGCTTAACACATGCAAGTCGAACGGTCGCAAGACAGTGGCGCACGGGTGAGTAA
CACGTAGCTAACCTGCCTTCGAGTGCGGAATAACGCTCCGAAAGGAGTGCTAATACCGCATAAGCTCA
CGAGAAGTAGATGCTTGTGAGAAAAGGGGAAACTCGCTTGAAGAGGGGGCTGCGACCCATCAGGTAGT
TGGTGTGGTAAAAGCGCACCAAGCCGACGACGGGTAGGGGGCCTGAGAGGGTGGTCCCCCACACTGGG
ACTGAGACACGGCC
>Zotu637
GAACGCTGGCGGCACGCCTAACACATGCAAGTCGAACGGCAGCACGGGGTAGATTTATCTATTTGGGT
GGCGAGTGGCGGACGGGTGAGTAACGCGTAGGAATATGCCTTTAAGACTGGGATAGCCTTGGGAAACT
GAGGGTAATACCGGATGAGCTCTGCGGAGGAAAGCTGGGGACCAGAGATGGCCTGGCGCTTAAAGAAT
AGCCTGCGTCTGATTAGCTAGTTGGTGGGGTAAAGGCCTACCAAGGCGACGATCGGTAGCTGGACTGA
GAGGTTGGACAGCCACACTGGGACTGAGACACGGCC
>Zotu638
GAACGCTAGCTACAGGCTTAACACATGCAAGTCGAGGGGCAGCATGGAAGAAGCTTGCTTCTTCTGAT
GGCGACCGGCGCACGGGTGCGTAACGCGTATCAAACCTGCCTCATACTCGGGGATAGCCTTGCGAAAG
TAAGATTAATACCCGATGTTGTTATGTTGCCGCATGGTGATATAACCAAAGATTTATCGGTATGAGAT
GGTGATGCGTCCGATTAGGTAGTAGGCGGGGTAACGGCCCACCTAGCCTTCGATCGGTAGGGGTTCTG
AGAGGAAGGTCCCCCACACTGGAACTGAGACACGGTC
>Zotu639
GAACGCTAGCGGCAGGCCTAATACATGCAAGTCGAACGATACTTTCCAGCTTGCTGGGGAGGAAAGTG
GCGCACGGGTGCGTAACGCGTATGCAACCTACCTTAATCAGGGGGATAGCCCGAAGAAATTCGGATTA
ACACCGCATAAAATCACAGTACAGCATTGTACAATGATCAAATATTTATAGGATTAAGATGGGCATGC
GTGACATTAGCTAGTTGGCGGGGTAACGGCCCACCAAGGCTACGATGTCTAGGGGATCTGAGAGGATG
ACCCCCCACACTGGTACTGAGACACGGAC
>Zotu640
GAACGCTGGCGGCATGCCTAACACATGCAAGTCGAACGAAGGCTTCGGCCTTAGTGGCGCACGGGTGC
GTAACGCGTGGGAATCTGCCCTTGGGTTCGGAATAACAGTTAGAAATGACTGCTAATACCGGATGATG
ACGAAAGTCCAAAGATTTATCGCCCAGGGATGAGCCCGCGTAGGATTAGGTAGTTGGTGGGGTAAAGG
CCTACCAAGCCGACGATCCTTAGCTGGTCTGAGAGGATGATCAGCCACACTGGGACTGAGACACGGCC
>Zotu641
GAACGCTGGCGGCATGCCTAACACATGCAAGTCGAACGAGATCTTCGGATCTAGTGGCGCACGGGTGC
GTAACGCGTGGGAATCTGCCTTGGGGTTCGGAATAACTCCCCGAAAGGGGTGCTAATACCGGATGATG
TCGAAAGACCAAAGATTTATCGCCCTGAGATGAGCCCGCGTAGGATTAGCTAGTTGGTGTGGTAAAGG
CGCACCAAGGCGACGATCCTTAGCTGGTCTAAGAGGATGATCAGCCACACTGGGACTGAGACACGGCC
>Zotu642
GAATGCTGGCGGCGTGGATAAGTCATGCAAGTCAAGGGGGCCGCAAGGCAACCGGCAGACGAGGTAGT
AACACGTAGGTAGATCCCCCGAAGTCGGGCATAGCCGGTCGAAAGACCGGGTAATTCCCGATAGTCCC
GCAAGGGTAAAGTTTTTTCGCTTCGGGAGTTGCCTGCGTAGTATCAGCTAGTTGGTAGGGTAATGGCC
TACCAAGGCAACGACGCTTAGGGGGGCTGAGAGGCCGATCCCCACCGATGGAACTGCGACACGGTC
>Zotu643
GAACGCTGGCGGCGTGCTTAACACATGCAAGTCGAACGAAGAGCGATGGAAGCTTGCTTCTATCAATC
TTAGTGGCGAACGGGTGAGTAACGCGTAATCAACCTGCCCTTCAGAGGGGGACAACAGTTGGAAACGA
CTGCTAATACCGCATACGATCTGACCTCGGCATCGAGGATAGATGAAAGGTGGCCTCTATTTATAAGC
TATCACTGAAGGAGGGGATTGCGTCTGATTAGCTAGTTGGAGGGGTAACGGCCCACCAAGGCGATGAT
CAGTAGCCGGTCTGAGAGGATGAACGGCCACATTGGGACTGAGACACGGCC
>Zotu644
GAACGCTGGCGGCAGGCTTAACACATGCAAGTCGAGCGCCCCGCAAGGGGAGCGGCAGACGGGTGAGT
AACACGTGGGAACGTACCCTTTGGTTCGGAACAACCCAGGGAAACTTGGGCTAATACCGGATAAGTCC
GTAAGGAGAAAGATTTATCGCCAAAGGATCGGCCCGCGTCTGATTAGCTAGTTGGTAGGGTAATGGCC
TACCAAGGCTACGATCAGTAGCTGGTCTGAGAGGATGATCAGCCACATTGGGACTGAGACACGGCC
>Zotu645

GAACGCTGGCGGCGTGCCTAATACATGCAAGTCGAACGAACAGCGACTTGAACTTGTTCAAGACAATG
TTAGTGGCGCACGGGTGAGTAACACGTGGAGAACCTACCCTTAAGCGGGGGATAACCATTGGAAACGA
TGACTAATACCGCATAGAATCACAGATCGCATGATCAATGAAGGAAAGACGGCTTCGGCTGTCACTTA
AGGATGGCTCCGCGGTGCATTAGCTAGATGGTGGGGTAAAGGCCTACCATGGCAATGATGCATAGCCG
ACCTGAGAGGGTGATCGGCCACATTGGGACTGAGACACGGCC
>Zotu646
GAACGCTGGCGGCATGCTTAACACATGCAAGTCGCACGGGCAGCAATGTCAGTGGCGGACGGGTGAGT
AACGCGTAGGAACATGTCCATGAGGTGGGGGACAACCCTGGGAAACTGGGGCTAATACCGCATATCGG
CTGAGGCCGAAAGCCTTCGGGCGCCTTTGGAGTGGCCTGCGTTCGATTAGCTTGTTGGTGGGGTAATG
GCCTACCAAGGCTACGATCGATAGCTGGTCTGAGAGGACGACCAGCCACACTGGGACTGAGACACGGC
C
>Zotu647
GAACGCTGGCGGCAGGCCTAACACATGCAAGTCGAACGGTAGCACAGAGGAGCTTGCTCCTCGGGTGA
CGAGTGGCGGACGGGTGAGTAATGTCTGGGAAACTGCCTGATGGAGGGGGATAACTACTGGAAACGGT
AGCTAATACCGCATAACGTCGCAAGACCAAAGAGGGGGACCTTCGGGCCTCTTGCCATCAGATGTGCC
CAGATGGGATTAGCTAGTAGGTGGGGTAATGGCTCACCTAGGCGACGATCCCTAGCTGGTCTGAGAGG
ATGACCAGCCACACTGGAACTGAGACACGGTC
>Zotu648
GAACGCTGGCGGCAGGCCTAACACATGCAAGTCGAACGGCAGCACAGAAGAGCTTGCTCTTTGGGTGG
CGAGTGGCGGACGGGTGAGTAATGTCTGGGAAACTGCCCGATGGAGGGGGATAACTACTGGAAACGGT
AGCTAATACCGCATAACGTCGCGAGACCAAAGTGGGGGACCTTCGGGCCTCACACCATCGGATGTGCC
CAGATGGGATTAGCTAGTAGGTGGGGTAATGGCTCACCTAGGCGACGATCCCTAGCTGGTCTGAGAGG
ATGACCAGCCACACTGGAACTGAGACACGGTC
>Zotu649
GAACGCTGGCGGCGTGCCTAATACATGCAAGTAGAACGCTGAAGAGAGGAGTTTGCTCTTCTTGGATG
AGTTGCGAACGGGTGAGTAACGCGTAGGTAACCTGCCTGGTAGCGGGGGATAACTATTGGAAACGATA
GCTAATACCGCATAAAATGGATTATCGCATGATAATTCATTGAAAGGTGCAAATGCATCACTACCAGA
TGGACCTGCGTTGTATTAGCTAGTTGGTGGGGTAACGGCTCACCAAGGCGACGATACATAGCCGACCT
GAGAGGGTGATCGGCCACACTGGGACTGAGACACGGCC
>Zotu650
GAACGCTGGCGGCGCGCCTAACACATGCAAGTCGAACGATTAAAGCACCTTCGGGTGTGTATAAAGTG
GCGAACGGCTGAGTAACACGTGGGCAACCTGCCCCTCTCATTGGGATAGCCACGGGAAACCGTGGATA
ATACCGAATACTTCGAGTCTCCCGCATGGAAGACTCGAGAAAGCTCCGGCGGAGAGGGATGGGCCCGC
GGCCTGTTAGCTTGTTGGTGGGGTAACGGCCTACCAAGGCAATGATGGGTAGCTGGGTTGAGAGACCG
ACCAGCCAGATTGGGACTGAGACACGGCC
>Zotu651
GAACGCTGGCGGCGTGCCTAACACATGCAAGTCGAGCGAGGTCCATCCGGTGGCAACACCGGGGAAGA
CCTAGCGGCGAACGGGTGAGTAACACGTGAGAAACCTGCCCCGAAATCTGGGATAACCCAAGGAAACT
CGGGCTAATACCGGATGTCCTTCATTGATCGCATGGTCGGTGAAGGAAAGAATTTCGTTTCGGGAGGG
TCTCGCGGCCTATCAGCTAGTTGGCGGGGTAACGGCCCACCAAGGCATCGACGGGTAGCTGGTCTGAG
AGGACGATCAGCCACACTGGGACTGAGACACGGCC
>Zotu652
GAACGCTGGCGGCGTGCCTAAGACATGCAAGTCGAGTGGGTTTAGACCCACGGCGAACGGGTGAGTAA
TACCTAGGAACTTTCTTTAAGTGGAGGATAACCAGTCGAAAGATTGGATAATACTTCATGTGGCAGTA
ATGCTAAAGCAGCAATGCGCTTAAAGTGAGGCCTGGGCACCATCAGCTAGTTGGTAAGGTAATGGCTT
ACCAAGGCTATGACGGTTAGGGGTCCCGAGAGGGATAACCCCCACACTGGAACTGCGACACGGTC
>Zotu653
GAACGCTGGCGGCATGCCTAACACATGCAAGTCGAACGAAGGCTTCGGCCTTAGTGGCGCACGGGTGC
GTAACGCGTGGGAATCTGCCCTTGGGTTCGGAATAACAGTTAGAAATGACTGCTAATACCGGATGATG
TCGTAAGACCAAAGATTTATCGCCCAAGGATGAGCCCGCGTAGGATTAGCTAGTTGGTGAGGTAAAAG
CTCACCAAGGCGACGATCCTTAGCTGGTCTGAGAGGATGATCAGCCACACTGGGACTGAGACACGGCC
>Zotu654
GAACGCTGGTGGCGTGCTTAACACATGCAAGTCGAGCGAGGCGTCCGCTTTCGGGTGGATAGTCGAGC
GGCGAACGGGTGAGTAACACGTGAGTAATCTTCCCTGGGCTCGGGGATAACCTCGGGAAACTGAGGGT

AATACCGGATGTGCGTTTTCTGGCGCCTGCTGGGAGACGGAAAGGTTCCATCTCGTGAGGGGTGGTTC
CGGCCTTGGATGAGCTCGCGGCCTATCAGCTTGTTGGTGCGGTGAGGGCGTACCAAGGCGACGACGGG
TAGCCGGCCTGAGAGGGTGACCGGCCACACTGGGACTGAGATACGGCC
>Zotu655
GAACGCTGGCGGCGTGCTTAACACATGCAAGTCGAACGCGCCTGTCCTCTGGTTTTCTGGGGGGTGGG
TGAGTGGCGAACGGGTGAGTAACACGTGAGTAACCTGCCCCCTTCTTCGGGATAACGGTCTGAAAGGG
CTGCTAATACCGGGTATTCACTGGTCTCCGCATGGGGTCTGGTGGAAAGGTTTTTTTCTGGTGGGGGA
TGGGCTCGCGGCCTATCAGCTTGTTGGTGGGGTGATGGCCCACCAAGGCTTTGACGGGTAGCCGGCCT
GAGAGGGTGACCGGTCACATTGGGACTGAGATACGGCC
>Zotu656
GAACGCTGGCGGCGTGCCTAATACATGCAAGTCGAGCGGACCGAATAGAAGCTTGCTTCTGTTCGGTT
AGCGGCGGACGGGTGAGTAACACGTGGGCAACCTGCCCGTAAGACCGGGATAACTTCGGGAAACCGGA
GCTAATACCGGATAACACCGAAGACCGCATGGTCTTTGGTTGAAAGACGGCTTTGGCTGTCACTTACG
GATGGGCCCGCGGCGCATTAGCTAGTTGGTGAGGTAACGGCTCACCAAGGCGACGATGCGTAGCCGAC
CTGAGAGGGTGATCGGCCACACTGGGACTGAGACACGGCC
>Zotu657
GAACGCTGGCGGCAGGCCTAACACATGCAAGTCGAACGGTAACAGGAAACAGCTTGCTGTTTCGCTGA
CGAGTGGCGGACGGGTGAGTAATGTCTGGGAAACTGCCTGATGGAGGGGGATAACTACTGGAAACGGT
AGCTAATACCGCATAACGTCGCAAGACCAAAGAGGGGGACCTTCGGGCCTCTTGCCATCGGATGTGCC
CAGATGGGATTAGCTTGTTGGTGGGGTAACGGCTCACCAAGGCGACGATCCCTAGCTGGTCTGAGAGG
ATGACCAGCCACACTGGAACTGAGACACGGTC
>Zotu658
GAACGCTGGCGGCGTGCTTAACACATGCAAGTCGAACGAAGAGCGATGGAAGCTTGCTTCTATCAATC
TTAGTGGCGAACGGGTGAGTAACGCGTAATCAACCTGCCCTTCAGAGGGGGACAACAGTTGGAAACGA
CTGCTAATACCGCATACGATTTAATCTCGGCATCGAGGATAGATGAAAGGTGGCCTCTACATGTAAGC
TATCACTGAAGGAGGGGATTGCGTCTGATTAGCTAGTTGGAGGGGTAACGGCCCACCAAGGCGATGAT
CAGTAGCCGGTCTGAGAGGATGAACGGCCACATTGGGACTGAGACACGGCC
>Zotu659
GAACGCTAGCTACAGGCTTAACACATGCAAGTCGAGGGGCAGCACGTTGTAGCAATACGATGGTGGCG
ACCGGCAAACGGGTGAGTAACACGTATCCAACCTGCCTGTTACTTGAGGATAGCCTCCTGAAAGGGAG
ATTAATACTCAATGTGGTACATTTTTCACATGCTTAATGTACTAAAGATTTATCGGTAACAGATGGGG
ATGCGTCCCATTAGGTAGTCGGCGGGGTAACGGCCCACCGAGCCGATGATGGGTAGGGGTTCTGAGAG
GAAGGTCCCCCACATTGGAACTTAGACACGGTC
>Zotu660
GAACGCTGGCGGCATGCCTAATACATGCAAGTCGAACGAGATCTTCGGATCTAGTGGCGCACGGGTGC
GTAACGCGTGGGAATCTGCCCTTGGGTTCGGAATAACTTCTGGAAACGGAAGCTAATACCGGATGATG
ACGTAAGTCCAAAGATTTATCGCCCAAGGATGAGCCCGCGTAGGATTAGCTAGTTGGTGAGGTAAAGG
CTCACCAAGGCGACGATCCTTAGCTGGTCTGAGAGGATGATCAGCCACACTGGGACTGAGACACGGCC
>Zotu661
GAACGCTAGCGGGAGGCCTAACACATGCAAGCCGAGCGGTAGGGTTTCTTCGGAAACCTGAGAGCGGC
GCACGGGTGCGGAACACGTGTGCAACCTACCTTTATCAGGGGGATAGCCTTTCGAAAGGAAGATTAAT
ACCCCATAATATATTAGATGGCATCATTTGATATTGAAAACTCCGGTGGATAGAGATGGGCACGCGCA
AGATTAGATAGTTGGTGAGGTAACGGCTCACCAAGTCAGTGATCTTTAGGGGGCCTGAGAGGGTGATC
CCCCACACTGGTACTGAGACACGGAC
>Zotu662
AAACGCTGGCGGCGTGCGTAACACATGCAAGTCGAACGAGAATGTGCTGACAGATCCTTAGGGTGAAG
ATAGCACAGGACAGTGGCGGATGGGTGAGTAACGCGTAAGAAACCTGCCTTTCACACCGGGATAGCAG
CTGGAAACGGCTATTAATACCGGATGACACTTTTTCCCCGCATGAGGAAGAGGTTAAAGAATTTCGGT
GAAAGATGGTCTTGCGTCTGATTAGCTAGTTGGTGGGGTAACGGCCTACCAAGGCGACGATCAGTAGC
CGGCCTGAGAGGGTGTACGGCCACATTGGGACTGAGACACGGCC
>Zotu663
GAACGCTGGCGGCGTGCTTAACACATGCAAGTCGAACGAGGTCCAACTGATGGCAACATCAGGGAAGA
CCTAGTGGCGAACGGGTGCGTAACACGTGAGGAACCTACCCCGGTCTCTGGGATAACAGTTGGAAACG
ACTGCTAATACCGGATGCCGTCGCAGCATCGCATGGTGCAGTGACGAAAGGCCTGCCGGACCGGGAGG

GCCTCGCGGCCTATCAGCTTGTTGGTGGGGTAATGGCCTACCAAGGCATCGACGGGTAGCTGGTCTGA
GAGGATGATCAGCCACACTGGGACTGAGACACGGCC
>Zotu664
GAACGCTGGCGGCGTGCCTAATACATGCAAGTCGAGCGAACAGATAAGGAGCTTGCTCCTTTGACGTT
AGCGGCGGACGGGTGAGTAACACGTGGGTAACCTACCTATAAGACTGGAATAACTCCGGGAAACCGGG
GCTAATGCCGGATAATATTTAGAACCGCATGGTTCTAAAGTGAAAGATGGTTTTACTATCACTTATAG
ATGGACCCGCGCCGTATTAGCTAGTTGGTGGGGTAATGGCTTACCAAGGCAACGATACGTAGCCGACC
TGAGAGGGTGATCGGCCACACTGGAACTGAGACACGGTC
>Zotu665
GAACGCTGGCGGCGTGCTTAACACATGCAAGTCGAACGGAAAGGCCAGTGCTTGCACTGGTACTCGAG
TGGCGAACGGGTGAGTAACACGTGGGTGATCTGCCCTGCACTTCGGGATAAGCCTGGGAAACTGGGTC
TAATACCGGATAGGACCGCATTTTGGATGGTGTGGTGGAAAGTTTTTCGGTGTGGGATGAGCTCGCGG
CCTATCAGCTTGTTGGTGGGGTAATGGCCTACCAAGGCGTCGACGGGTAGCCGGCCTGAGAGGGTGTA
CGGCCACATTGGGACTGAGATACGGCC
>Zotu666
GAACGCTGGAAACGTGGATACCGGCATGCAAGTCGAGCGGCCAGCAATGGCAGCGGCGAACGGCTTAG
TAACACGTAGGTACATACCCCAAAGTGGAGGACAGCTCGTCGAAAGACGGGGTAATACTCCATAGCAT
CGCAAGATTAAAGTAGCAATACGCTTTGGGAATGGCCTGCGACCTATCAGATAGTTGGCGAGGTAATA
GCTCACCAAGTCTATGACGGGTAGGGGGCGTGAGAGCGCGACCCCCAAGAATGGCACTGCGACACGGG
C
>Zotu667
GAACGCTGGCGGCAGGCCTCAAACATGCAAGTCTAGGGGGTGTAGCAATACATCACCGGCGGACGGGT
GCGTAACACGTGGGAATATACCTTTTGGTTCGGAACAACGCAGGGAAACTTGCGCTAATACCGAATGT
GCCCTTACGGGGAAAGATTTATCGCCAAAGGATTAGCCCGCGTAGGATTAGCTAGTTGGTGAGGTAAT
GGCTCACCAAGGCTCCGATCCTTAGCTGTTCTGAGAGGAAGATCAGCCACACTGGGACTGAGACACGG
CC
>Zotu668
GAACGCTAGCTACAGGCTTAACACATGCAAGTCGAGGGGCAGCATGGTGGATGCTTGCATTCACCGAT
GGCGACCGGCGAATGGGTGAGTAACGCGTATCCAACCTGGCGTGCACTCCGGCACAGCCCCTGGAAAC
GGGGATTAATTCCGGATGGTGTGTTATCAGCGCATGCTGTTATCACTAAAGGTCACGGTGCACGATGG
GGATGCGTTCCATTAGATTGTTGGCGGGGTAACGGCCCACCAAGTCATCGATGGATAGGGGTTCTGAG
AGGAAGGTCCCCCACACTGGAACTGAGACACGGTC
>Zotu669
GAACGCTGGCGGCGTGCCTAATACATGCAAGTCGAGCGGGCCGAACGAGAGCTTGCTCTTGTTCGGTC
AGCGGCGGACGGGTGAGTAACACGTGGGCAACCTGCCCGCAAGACCGGGATAACTCCGGGAAACCGGA
GCTAATACCGGATAACACCAAAGACCGCATGGTCTTTGGTTGAAAGGCGGCTTCGGCTGTCACTTGCG
GATGGGCCCGCGGCGCATTAGCTAGTTGGTGAGGTAACGGCTCACCAAGGCGACGATGCGTAGCCGGC
CTGAGAGGGTGACCGGCCACACTGGGACTGAGACACGGCC
>Zotu670
GAACGCTGGCGGCGTGCTTAACACATGCAAGTCGAACGGAAAGGCCCAAGCTTGCTTGGGTACTCGAG
TGGCGAACGGGTGAGTAACACGTGGGTGATCTGCCCTGCACTTCGGGATAAGCCTAGGAAACTGGGTC
TAATACCGGATAGGACCACTTCTTGGATGTTGTGGTGGAAAGTTTTTTCGGTGTGGGATGAGCTCGCG
GCCTATCAGCTTGTTGGTGGGGTAATGGCCTACCAAGGCGTCGACGGGTAACCGGCCTGAGAGGGTGT
ACGGCCACATTGGGACTGAGATACGGCC
>Zotu671
GAACGCTGGCGGCGTGCTTAACACATGCAAGTCGAGCGGAAAGGCCCTTTTGGGGGTACTCGAGCGGC
GAACGGGTGAGTATCACGTGAGTAATCTGCCCCCCTCTTTTGGATAACCCTCGGAAACGGGGGCTAAT
ACGGAATATTCAGTGCTGCCTGCATGGGTGGTGTTGGAAAGGATTCGTTTGGTGGGGGATGAGCTCGC
GGCCTATCAGCTTGATGGTGGGGTAATGGCCTACCATGGCTTTGACGGGTAGCCGGCCTGAGAGGGTG
TCCGGCCACACTGGGACTGAGACACGGCC
>Zotu673
GAACGCTGGCGGCGTGCCTAATACATGCAAGTCGAACGAATCTTCTTCGGAAGATGAGTGGCGAACGG
GTGAGTAATACATAGGTAACCTGCCCCTGTGCGGGGGATAACAGGAGGAAACTCCTGCTAATACCGCA
TAGCCATGAGCACCGCATGGAGCTCATGCCAAATATCCTTTCGGGGATAGCGCAGGGATGGACCTATG

GCGCATTAGCTTGTTGGCGGGGTAACGGCCCACCAAGGCAACGATGCGTAGCCGGCCTGAGAGGGCGG
ACGGCCACATTGGGACTGAGACACGGCC
>Zotu674
GAACGCTGGCGGCAGGCCTAACACATGCAAGTCGAACGCCCCGCAAGGGGAGTGGCAGACGGGTGAGT
AACGCGTGGGAACCTACCTTACGGTCGGGAATAGCTCAGGGAAACTTGGGGTAATACCGGATACGCCC
TTACGGGGAAAGATTTATCGCCGTTAGATGGGCCCGCGTAGGATTAGCTAGTTGGTGAGGTAACGGCT
CACCAAGGCGACGATCCTTAGCTGGTCTGAGAGGATGATCAGCCACACTGGGACTGAGACACGGCC
>Zotu675
GAACGCTAGCGATAGGCTTAACACATGCAAGTCGAGGGGCAGCAGGGACTTCGGTCTGCTGGCGACCG
GCGCACGGGTGCGTAACGCGTATGCAACTTGCCTTATAGAGGGGGATAACCCGTTGAAAGACGGACTA
ATACCGCGTATTCTTATTTAGTGGCATCATTAGATAAGGAAAGATTAATTGCTATAAGATAGGCATGC
GTCCCATTAGCTAGTTGGTGAGGTAACGGCTCACCAAGGCGACGATGGGTAGGGGAACTGAGAGGTTG
AACCCCCACACTGGTACTGAGACACGGAC
>Zotu676
GAACGCTGGCGGCATGCCTAATACATGCAAGTCGAACGCTGAAGCACTCTGCTTGCAGAGTGCGGATG
AGTGGCGAACGGGTGAGTAACACGTGGGAAATCTGCCTAAGAGTGGGGGATAACATTCGGAAACGGAT
GCTAATACCGCATATATTCTTGAACCGCATGGTTCTGGAAAGAAAGACGGCTTTGCTGTCGCTTTTAG
ATGATCCCGCGGCGTATTAGTTAGTTGGTGGGGTAATGGCTTACCAAGACCATGATACGTAGCCGACC
TGAGAGGGTGATCGGCCACACTGGGACTGAGACACGGCC
>Zotu677
GAACGCTAGCTACAGGCTTAACACATGCAAGTCGAGGGGAAACGACATTGAAGCTTGCTTCGATGGTC
GTCGACCGGCGCACGGGTGAGTAACGCGTATCCAACCTGCCTCTGACCAAGGGATAACCCGTCGAAAG
TCGGACTAATACCTTATGCAGTCGTCGGAAGTCATCTGATGACGACGAAAGATTTCATCGGTGAGAGA
TGGGGATGCGTCTGATTAGCTTGTTGGTGGGGTAACGGCCCACCAAGGCGACGATCAGTAGGGGTTCT
GAGAGGAAGGTCCCCCACATTGGAACTGAGACACGGTC
>Zotu678
GAACGCTGGCGGCAGGCCTAACACATGCAAGTCGAACGCTCGTAGCAATACGGGAGTGGCAGACGGGT
GAGTAACACGTGGGAACGTACCCTTCGGTTCGGGATAACTCAGGGAAACTTGAGCTAATACCGGATAC
GTCCGAGAGGAGAAAGGTTTACTGCCGAAGGATCGGCCCGCGTCTGATTAGCTAGTTGGTGAGGTAAC
GGCTCACCAAGGCGACGATCAGTAGCTGGTCTGAGAGGATGATCAGCCACATTGGGACTGAGACACGG
CC
>Zotu679
GAACGCTGGCGGCGTGCCTAATACATGCAAGTCGAGCGAACAGATAAGGAGCTTGCTCCTTTGACGTT
AGCGGCGGACGGGTGAGTAACACGTGGGTAACCTACCTATAAGACTGGAATAACTCCGGGAAACCGGG
GCTAATGCCGGATAACATGTTGAACCGCATGGTTCTACAGTGAAAGGCGGCTTTGCTGTCACTTATAG
ATGGACCCGCGCCGTATTAGCTAGTTGGTGAGGTAACGGCTCACCAAGGCAACGATACGTAGCCGACC
TGAGAGGGTGATCGGCCACACTGGAACTGAGACACGGTC
>Zotu681
GAACGCTGGCGGCAGGCTTAACACATGCAAGTCGAACGCCCCGCAAGGGGAGTGGCAGACGGGTGAGT
AACGCGTGGGAATCTACCCAGATCTTCGGAACAACTGAGGGAAACTTCAGCTAATACCGGATACGCCC
TACGGGGGAAAGATTTATCGGTTCTGGATGAGCCCGCGTTGGATTAGCTAGTTGGTGAGGTAATGGCC
CACCAAGGCGACGATCCATAGCTGGTCTGAGAGGATGATCAGCCACACTGGGACTGAGACACGGCC
>Zotu682
GAACGCTGGCGGCGTGCTTAACACATGCAAGTCGAACGGTGAAAGGGAGCTTGCTCTCTGGATCAGTG
GCGAACGGGTGAGTAACACGTGAGCAATCTGCCCTTGACTCTGGGATAAGCGTTGGAAACGACGTCTA
ATACCGGATACGAGCTGTGAAGGCATCTTCAACAGCTGGAAAGAACTTCGGTCAAGGATGAGCTCGCG
GCCTATCAGCTAGTTGGTGAGGTAATGGCTCACCAAGGCGTCGACGGGTAGCCGGCCTGAGAGGGTGA
CCGGCCACACTGGGACTGAGACACGGCC
>Zotu683
GAACGCTAGCGGCAGGCCTAATACATGCAAGTCGGACGGGATTGAGGAGCTTGCTTCTCATGAGAGTG
GCGCACGGGTGCGTAACACGTATGTAACCTACCTTAGTCAGGGGGATAGCCTCTCGAAAGAGAGATTA
ACACCGCATAACATAATGATATGGCATCATATTATTATCAAATATTTATAGGACTAAGATGGGCATGC
GGAACATTAGCTAGTTGGTAAGGTAACGGCTTACCAAGGCTACGATGTTTAGGGGATCTGAGAGGATG
ACCCCCCACACTGGTACTGAGACACGGAC

>Zotu684
GAACGCTGGCGGCGTGCCTAATACATGCAAGTCGAACGGTCTTTTCTATGGAAGCTTGCTTCCACTGA
GAAGATAGTGGCGAACGGGTGAGTAACACGTGGGTAACCTGCCCATAAGAGGGGGATAACATCCGGAA
ACGGGTGCTAATACCGCATAGTTTTCTTGATCGCATGATCGGGAAAGGAAAGACGGCCTTTGTGCTGT
CGCTTATGGATGGACCCGCGGCGTATTAGTTAGTTGGTGAGGTAACGGCTCACCAAGACGATGATACG
TAGCCGACCTGAGAGGGTGATCGGCCACATTGGGACTGAGACACGGCC
>Zotu685
GAACGCTGGCGGCGTGCCTAATACATGCAAGTTGAGCGATGAAGATTGGTGCTTGCACCAATTTGAAG
AGCAGCGAACGGGTGAGTAACGCGTGGGGAATCTGCCTTTGAGCGGGGGACAACATTTGGAAACGAAT
GCTAATACCGCATAACAACTTTAAACATAAGTTTTAAGTTTGAAAGATGCAATTGCATCACTCAAAGA
TGATCCCGCGTTGTATTAGCTAGTTGGTGAGGTAAAGGCTCACCAAGGCGATGATACATAGCCGACCT
GAGAGGGTGATCGGCCACATTGGGACTGAGACACGGCC
>Zotu686
GAACGCTGGCGGCGTGCCTAATACATGCAAGTCGAGCGCCTGCACAGTGCTCATTAACTAAACTTAGT
GAGTAATGTGCAGGAGCGGCGGACGGCTGAGTAACGCGTAGGAACGTACCCCAGAGTGAGGGATAAGC
CCGAGAAATCGGGTCTAATACCGCATAATCTCTTCGGAGCAAAGCTTTATGCGCTTTGGGAACGGCCT
GCGTATGATTAGCTTGTTGGTGAGGTAATGGCTCACCAAGGCAATGATCATTAGCTGGTCTGAGAGGA
TGATCAGCCAGACTGGGACTGAGACACGGCC
>Zotu687
GAACGCTGGCGGCGTGCTTAACACATGCAAGTCGAACGATGAAGCACTTCGGTGTGGATTAGTGGCGA
ACGGGTGAGTAACACGTGAGCAACCTGCCCCTTTCTCTGGGATAACCGCCGGAAACGGTGGCTAATAC
CGGATATTCACTTTTCCGTGCATGCGGTTTGGTGGAAAGATTTTTTGGTGGGGGATGGGCTCGCGGCC
TATCAGCTTGTTGGTGGGGTAATGGCCTACCAAGGCTTCGACGGGTAGCCGGCCTGAGAGGGCGACCG
GCCACACTGGGACTGAGATACGGCC
>Zotu688
GAACGCTAGCGGCAGGCCTAATACATGCAAGTCGAACGGGTGTAGCAATACACTAGTGGCGCACGGGT
GCGTAACGCGTAACCAACCTACCGACAACTGGGGGATAGCCCGCCGAAAGGCGGATTAATACCGCATA
TACCGGCAGCCTGGCATCAGGTAACTGGGAAAGATTTATTGGTTGTCGATGGGGTTGCGTAGCATTAG
CTAGATGGCGGGGTAACGGCCCACCATGGCGACGATGCTTAGGGGACCTGAGAGGGTGATCCCCCACA
CTGGCACTGAGATACGGGC
>Zotu689
GAACGCTGGCGGCGTGCTTAACACATGCAAGTCGAACGGTGAACACGGAGCTTGCTCTGTGGGATCAG
TGGCGAACGGGTGAGTAACACGTGAGCAACCTGCCCCTGACTCTGGGATAAGCGCTGGAAACGGCGTC
TAATACTGGATATGTGACGTGACCGCATGGTCTGCGTTTGGAAAGATTTTTCGGTTGGGGATGGGCTC
GCGGCCTATCAGCTTGTTGGTGAGGTAATGGCTCACCAAGGCGTCGACGGGTAGCCGGCCTGAGAGGG
TGACCGGCCACACTGGGACTGAGACACGGCC
>Zotu690
GAACGCTGGCGGCGTGCTTAACACATGCAAGTCGAACGGTGATGCTCCAGCTTGCTGGAGTGGATCAG
TGGCGAACGGGTGAGTAACACGTGAGCAACCTGCCCCAGACTCTGGAATAAGCGCTGGAAACGGCGTC
TAATACTGGATACGAAACTGGCCCGCATGGGCATGGTTTGGAAAGTTTTTCGGTTTGGGATGGGCTCG
CGGCCTATCAGCTTGTTGGTGAGGTAATGGCTCACCAAGGCGACGACGGGTAGCCGGCCTGAGAGGGC
GACCGGCCACACTGGGACTGAGACACGGCC
>Zotu691
GAACGCTGGCGGCGTGCCTAATACATGCAAGTCGAGCGGCAGCACGGTCTTCGGACTGGTGGCGAGCG
GCGGACGGCAGAGTAACGCGTAGGAATATACCCCAAAGTGAGGGATAAGCTCCAGAAATGGAGTCTAA
TACCGCATATGGTCTTCGGATTAAAGCCTTCGGGCGCTATGGGAATAGCCTGCGTTCGATTAGCTAGT
TGGTGAGGTAATGGCTCACCAAGGCGACGATCGATAGCTGGTCTGAGAGGATGATCAGCCTGACTGGA
ACTGAGATACGGTC
>Zotu692
GAACGCTGGCGGCGTGCTTAACACATGCAAGTCGAACGGACCCTTTGGGGTTAGTGGCGAACGGGTGA
GTAACACGTGGGTGATCTGCCCTGCACTTTGGGATAAGCCTGGGAAACTGGGTCTAATACCGGATAGG
ACTACGCACTGCATGGTGTGTGGTGGAAAGCTTTTGCGGTGTGGGATGGGCCCGCGGCCTATCAGCTT
GTTGGTGGGGTGATGGCCTACCAAGGCGACGACGGGTAGCCGGCCTGAGAGGGTGACCGGCCACACTG
GGACTGAGATACGGCC

>Zotu693
GAACGCTGGCGGCGTGCCTAATACATGCAAGTCGAACGGTTTAGTTCCAAGAGGCTTGCCTTTTGGAA
CTAGACAGTGGCGGACGGGTGAGTAACACGTAGGCAACCTACCTGTCAAACCGGGATAACCTCGGGAA
ACCGAAGCTAAACCCGGATAAGCTCCTTTTTCGCATGAAGGGGGAGTGAAAAGGTGCGACCTGATGAC
AGATGGGCCTGCGGCGCATTAGCTAGTAGGTAGGGTAACGGCCTACCTAGGCGATGATGCGTAGCCGA
CCTGAGAGGGCGTACGGCCACACTGGGACTGAGATACGGCC
>Zotu694
GAACGCTGGCGGCGTGCTTAACACATGCAAGTCGAACGATGAAGCCCTTCGGGGTGGATTAGTGGCGA
ACGGGTGAGTAACACGTGGGCAATCTGCCCTTCACTCTGGGACAAGCCCTGGAAACGGGGTCTAATAC
CGGATAACACTCTGTCCCGCATGGGACGGGGTTGAAAGCTCCGGCGGTGAAGGATGAGCCCGCGGCCT
ATCAGCTTGTTGGTGGGGTAATGGCCTACCAAGGCGACGACGGGTAGCCGGCCTGAGAGGGCGACCGG
CCACACTGGGACTGAGACACGGCC
>Zotu696
GAACGCTAGCGGCAGGCCTAATACATGCAAGTCGAACGATAGTAGGGAGCTTGCTCTCTACGAAAAGT
GGCGCACGGGTGCGTAACGCGTATGCAACCTACCTTAATCAGGGGGATAGCCCGAAGAAATTCGGATT
AACACCGCATAAAATCACAGTATAGCATTATACAATGATCAAATATTTATAGGATTAAGATGGGCATG
CGTGACATTAGTTAGTTGGTGAGGTAACGGCTCACCAAGACCACGATGTCTAGGGGATCTGAGAGGAT
GACCCCCCACACTGGTACTGAGACACGGAC
>Zotu697
GAACGCTGGCGGCATGCTTTACACATGCAAGTCGAACGGCAGCGCGGGGCAACCTGGCGGCGAGTGGC
GAACGGGTGAGTAACATATCGGAACGTACCCAAGAGTGGGGGATAACGTAGCGAAAGTTACGCTAATA
CCGCATACGATCCAAGGATGAAAGTGGGGGATCGCAAGACCTCATGCTCCTGGAGCGGCCGATATCTG
ATTAGCTAGTTGGTGAGGTAAAGGCTCACCAAGGCGACGATCAGTAGCTGGTCTGAGAGGACGACCAG
CCACACTGGAACTGAGACACGGTC
>Zotu698
GAACGCTGGCGGCATGCCTAACACATGCAAGTCGAACGAATTTTCAGCTTCGGTTGGAAGTTAGTGGC
GCACGGGTGCGTAACGCGTGGGAATCTGCCCCTTGGTTCGGAATAACAGTTAGAAATGACTGCTAATA
CCGGATGATGACGAAAGTCCAAAGATTTATCGCCGAGGGATGAGCCCGCGTAGGATTAGCTAGTTGGT
GGGGTAAAGGCTCACCAAGGCGACGATCCTTAGCTGGTCTGAGAGGATGATCAGCCACACTGGGACTG
AGACACGGCC
>Zotu699
GAACGCTAGCGGCAGGCTTAACACATGCAAGTCGAGGGGTAGAAGTTTTCGGAATTTGAGACCGGCGC
ACGGGTGCGTAACGCGTATGCAATCTACCTTTTACAGAGGGATAGCCCAGAGAAATTTGGATTAATAC
CTCATAGTGTTATAGAGTGGCATCACTTTATAACTAAAGTCACAACGGTAAAAGATGAGCATGCGTCC
CATTAGCTAGATGGTAAGGTAACGGCTTACCATGGCTACGATGGGTAGGGGTCCTGAGAGGGAGATCC
CCCACACTGGTACTGAGACACGGAC
>Zotu700
GAACGCTGGCGGCGTGCCTAACACATGCAAGTCGAACGGATGGTGTAGCAATACATCGTTAGTGGCAA
ACGGGTGAGTAATATATAGGTAATCTGCCCTTTAGTCTGGGATAACTTGCCGAAAGGTGAGCTAATAC
TGGATACGTTCCTAGGGTCGCATGGCTCCGGGAAGAAAGATTTATCGCTAAAGGAAGAGCCTATATCT
GATTAGCTAGTTGGTGGGGTAATGGCCTACCAAGGCGACGATCAGTAGCTGGTCTGAGAGGACGAACA
GCCACACTGGGACTGAGACACGGCC
>Zotu701
GAACGCTGGCGGCATGCCTAACACATGCAAGTCGAACGAGATCTTCGGATCTAGTGGCGCACGGGTGC
GTAACGCGTGGGAATCTGCCTTGGGGTTCGGAATAACTCCCCGAAAGGGGTGCTAATACCGGATGATG
TCGAGAGACCAAAGATTTATCGCCCTGAGATGAGCCCGCGTAGGATTAGCTAGTTGGTGTGGTAAAGG
CGCACCAAGGCGACGATCCTTAGCTGGTCTGAGAGGATGATCAGCCACACTGGGACTGAGACACGGCC
>Zotu702
GAACGCTGGCGGCGTGCTTAACACATGCAAGTCGAACGGAAAGGCCCTGCTTGCAGGGTACTCGAGTG
GCGAACGGGTGAGTAACACGTGGGTGATCTGCCCTGCACTTCGGGATAAGCTTGGGAAACTGGGTCTA
ATACCGGATAGGAGCCATCTTTAGTGTGGTGGTTGGAAAGTTTTTTCGGTGTAGGATGAGCTCGCGGC
CTATCAGCTTGTTGGTGGGGTAATGGCCTACCAAGGCGGCGACGGGTAGCCGGCCTGAGAGGGTGTAC
GGCCACATTGGGACTGAGATACGGCC
>Zotu703

GAACGCTGGCGGCATGCCTAACACATGCAAGTCGAACGAGATCTTCGGGTCTAGTGGCGCACGGGTGC
GTAACGCGTGGGAATCTGCCCTTTGGTTCGGAATAACAGTTGGAAACGACTGCTAATACCGGATGATG
ACGTAAGTCCAAAGATTTATCGCCAGAGGATGAGCCCGCGTAGGATTAGCTAGTTGGTGTGGTAAGAG
CGCACCAAGGCGACGATCCTTAGCTGGTCTGAGAGGATGATCAGCCACACTGGGACTGAGACACGGCC
>Zotu704
GAACGCTGGCGGCGTGCTTAACACATGCAAGTCGAACGGTGAAGGAGAGCTTGCTCTCTGGATCAGTG
GCGAACGGGTGAGTAACACGTGAGCAATCTGCCCCTGACTCTGGGATAAGCGCTGGAAACGGCGTCTA
ATACCGGATACGAGCTGCGAAGGCATCTTCAGCAGCTGGAAAGAACTTCGGTCAGGGATGAGCTCGCG
GCCTATCAGCTAGTTGGTGAGGTAACGGCTCACCAAGGCGTCGACGGGTAGCCGGCCTGAGAGGGTGA
CCGGCCACACTGGGACTGAGACACGGCC
>Zotu705
GAACGCTGGCGGTGTGCTTCACACATGCAAGTCGAACGAGGTAGCAATACCTAGTGGCGGACGGGTGA
GTAACGCGTGGGAATCTGCCTTTAGATGGGGGATAACGGCCGAAAGGTCCGCTAATACCGCATATGC
CGCAAGGTGAAAGGAGTAATCCGTCTAAAGATGAGCCCGCGTCCGATTAGCTAGTTGGTAGAGTAAAA
GCCTACCAAGGCGACGATCGGTAGCTGGTCTGAGAGGATGATCAGCCACAATGGGACTGAGACACGGC
C
>Zotu706
GAACGCTGGTGGCGTGCTTAACACATGCAAGTCGAGCGAGGCGTCCGCTTTCGGGTGGATAGTCGAGC
GGCGAACGGGTGAGTAACACGTGAGTAATCTTCCCTGGGCTCTGGGATAACCTCGGGAAACTGAGGGT
AATACCGGATGTGCGTTTCCTGGCGCCTGCTGGGAGACGGAAAGGTTGCGTGCCGTGAGGTGCGTTTC
CGGCCTTGGATGAGCTCGCGGCCTATCAGCTTGTTGGTGCGGTGATGGCGTACCAAGGCGACGACGGG
TAGCCGGCCTGAGAGGGTGACCGGCCACACTGGGACTGAGATACGGCC
>Zotu707
GAACGCTGGCGGCGTGCTTAACACATGCAAGTCGAGCGGTAAGGCCCTTCGGGGTACACGAGCGGCGA
ACGGGTGAGTAACACGTGAGCAATCTGCCCTTCACTCTGGGATAGCCTCGGGAAACTGAGATTAATAC
CGGATACGACCACTTCGGGCATCCGATGGTGGTGGAAAGTTCCGGCGGTGAAGGATGAGCTCGCGGCC
TATCAGCTTGTTGGCGGGGTAATGGCCCACCAAGGCTTCGACGGGTAGCCGGCCTGAGAGGGTGACCG
GCCACACTGGGACTGAGACACGGCC
>Zotu708
GAACGCTGGCGGCGTGCTTAACACATGCAAGTCGAACGGAGTTTGTATGGAATGAGTCTTCGGACAAA
TGAAATATAAACTTAGTGGCGGACGGGTGAGTAACGTGTGGGTAACCTACCCTATACAGGAGGATAAC
AGTAAGAAATTATTGCTAAAACTGCATAAGATTACGAGTTCGCATGAACAAGTGATAAAAACTTTAGT
GGTATAGGATGGGCCCGCATCTGATTAGGTAGTTGGTGGAGTAAAAGCCTACCAAGCCGACGATCAGT
AGCCGATCTGAGAGGATGACCGGCCACATTGGGACTGAGACACGGCC
>Zotu709
GAACGCTAGCTACAGGCTTAACACATGCAAGTCGAGGGGCAGCGGGAAGCAAGCTTGCTTGCTTTGCC
GGCGACCGGCGTACGGGTGCGTAACGCGTACCGAACCTACCTCACACTCCGGGATAGCCCTGCGAAAG
CAGGATTAATACCGGGTAGCCTCACTATATCGCATGATATTATGAGTAAAGATTCATTGGTGTGAGAT
GGCGGTGCGTCCCATTAGTTAGTTGGCGGGGTAACGGCCCACCAAGACGACGATGGGTAGGGGTTCTG
AGAGGAAGGTCCCCCACATTGGAACTGAGACACGGTC
>Zotu710
GAACGCTGGCGGCGTGCTTAACACATGCAAGTCGAACGGAAAGGCCCTTCGGGGTACTCGAGTGGCGA
ACGGGTGAGTAACACGTGGGTGATCTGCCCTGCACTTTGGGATAAGCCTGGGAAACTGGGTCTAATAC
CGAATACACCCTGCTGGTCGCATGGCCTGGTGGGGGAAAGCTTTTGCGGTGTGGGATGGGCCCGCGGC
CTATCAGCTTGTTGGTGGGGTGATGGCCTACCAAGGCGACGACGGGTAGCCGGCCTGAGAGGGTGTCC
GGCCACACTGGGACTGAGATACGGCC
>Zotu711
GAACGCTGGCGGCATGCTTAACACATGCAAGTCGGACGGGAAGTGGTGTTTCCAGTGGCGGACGGGTG
AGTAACGCGTAAGAACCTGCCCTTGGGAGGGGAACAACAGCTGGAAACGGCTACTAATACCCCATAGG
CTGAGGAGCAAAAGGAGGAATCCGCCCAAGGAGGGGCTCGCGTCTGATTAGTTAGTTGGTGAGGCAAT
GGCTTACCAAGGCGACGATCAGTAGCTGGTCCGAGAGGATGATCAGCCACACTGGGACTGAGACACGG
CC
>Zotu713
GAACGCTGGCGGCGTGCCTAATACATGCAAGTCGAGCGAATCTTTTGGGGGCTTGCTCCCAAAAGATT

AGCGGCGGACGGGTGAGTAACACGTGGGCAACCTGCCTATAAGACTGGGATAACTTCGGGAAACCGGA
GCTAATACCGGATATGTTCTTTTCTCGCATGAGAGAAGATGGAAAGACGGTTTCGGCTGTCACTTATA
GATGGGCCTGCGGCGCATTAGCTAGTTGGTGGGGTAATGGCTCACCAAGGCGACGATGCGTAGCCGAC
CTGAGAGGGTGATCGGCCACACTGGGACTGAGACACGGCC
>Zotu714
AAACGCTGGCGGGGTGCATGAGACATGCAAGTCGAACGAGGTGCCATCGGGCTTGCTCGATGGCTACC
TAGTGGCGGACGGCTGAGTAACGCGTAGGTGACCTACCCGGAAGTGGGGAATAACTGCCCGAAAGGGT
GGCTAATACCGCATGTGGTCGACGATTCATTTCGTCGTCTAAAGGAGCAATTCGCTTCCGGAGGGGCC
TGCGTCCGATTAGCTAGTTGGTGGGGTAACGGCTCACCAAGGCGACGATCGGTAGCTGGTCTGAGAGG
ATGATCAGCCAGACTGGGACTGAGAACGGCC
>Zotu715
CAACGCTGGCGGCGCGCTTAACACATGCAAGTCGCACGAGAAAGTGGAGCAATCCATGAGTACAGTGG
CGTACGGGTGAGTAACACGTGGATCATCTGCCTCTTGATGGGGAATAACCCTGGGAAACCGGGGCTAA
TACCGCATAAGCCTGAAAAGGGAAAGCAGCAATGCGTCGAGAGAGGAGTCCGCGGCCGATTAGCTAGT
TGGTAGGGTAATGGCCTACCAAGGCAATGATCGGTAGCCGGCCTGAGAGGGCACACGGCCACACTGGC
ACTGAAACACGGGC
>Zotu716
CAACGCTGGCGGCGTGCCTAACACATGCAAGTCGAACGAGAAAGTGGAGCAATCCATGAGTAAAGTGG
CGCACGGGTGAGTAACACGTGACTAACCTACCTTTGAGCGGGGGATAACCTAGGGAAACCTGGGCTAA
TACCGCATAATACCTACGGGTCAAAGCGAGCAATCGCACTTAAAGAGGGGGTCGCGGCAGATTAGCTA
GTTGGTGAGGTAATGGCTCACCAAGGCGATGATCTGTATCCGGCCTGAGAGGGCGCACGGACACACTG
GAACTGAAACACGGTC
>Zotu717
GAACGTTGGCGGCGTGCCTAATACATGCAAGTCGAACGCGGTCTCCTAATTGAAAAACCGTGCTTGCA
CGGGTTGGATTTTAGATCGGACCGAGTGGCGAACTGGTGAGTAACACGTGGGTAACCTGCCCAGAAGT
AGGGGATAACACCTGGAAACAGATGCTAATACCGTATAACAACTAAAACCACATGGTTTTAGTTTGAA
AGCTGGCCTTGGTGCTAGTGCTTTTGGATGGACCCGCGGCGTATTAGCTAGTTGGTGAGATAATAGCT
CACCAAGGCGATGATACGTAGCAGACCTGAGAGGGTAATCTGCCACAATGGGACTGAGACACGGCC
>Zotu718
GAACGCTAGCGGCAGGCTTAATACATGCAAGTCGAGGGGCAGCGGGAGTAGCAATACTTGCCGGCGAC
CGGCGCACGGGTGCGTAACACGTATACAATCTACCCTTTACAGGGGGATAGCCCTCCGAAAGGAGGAT
TAATACCCCATAATAAATTGAATGGCATCATTTGATTTTGAAAGATTTATCGGTAAAGGATGAGTATG
CGTCTGATTAGCTAGTTGGAGAGGTAACGGCTCCCCAAGGCGACGATCAGTAGGGGATCTGAGAGGAC
TACCCCCCACACTGGTACTGAGACACGGAC
>Zotu719
GAACGCTGGCGGCGCGCTTAACACATGCAAGTCGAGCGATGAAATCTTAACAGAACCCTTCGGGGCGA
AGATAAGACGGATTAGCGGCGGACGGGTGAGTAACACGTGAGTAACCTGCCTTTTACACAGGGATAGC
CTCGGGAAACCGGGATTAAAACCTGATGAAACTATCAAATCACATGATTAAGAAAGTTAAAACTCCGG
TGGTAAAAGATGGACTCGCGTCCCATTAGCTAGTTGGTGAAGGTAACGGCCCACCAAGGCAACGATGG
GTAGCCGGCCTGAGAGGGTGAACGGCCACATTGGAACTGAGAAACGGTC
>Zotu720
GAACGCTAGCGGCAGGCCTAATACATGCAAGTCGAACGGTGAGTAGCAATACTCATAGTGGCGCACGG
GTGCGTAACACGTAGGTAACCTACCTTTTACCGGGGGATAACGATGGGAAACCATCGCTAATACCGCA
TAAGATAGCAGAGGGGCATCTCTTAGTTATTAAAGATTTATTGGTAGAAGATGGGCCTGCGTCCGATT
AGCTAGTTGGTGGTGTAACAGACCACCAAGGCGATGATCGGTAGGGGTTCTGAGAGGAAGATCCCCCA
CACTGGCACTGAGATACGGGC
>Zotu721
GAACGCTGGCGGCGTGCTTAACACATGCAAGTCGAACGGAAAGGCCCTTCGGGGTACTCGAGTGGCGA
ACGGGTGAGTAACACGTGGGTGATCTGCCTCGTACTCTGGGATAAGCCTGGGAAACTGGGTCTAATAC
CGGATAGGACCACTGGTCGCATGGCTGGTGGTGGAAAGTTTTTCGGTACGAGATGGGCCCGCGGCCTA
TCAGCTTGTTGGTGGGGTAATGGCCTACCAAGGCGACGACGGGTAGCCGGCCTGAGAGGGCGACCGGC
CACACTGGGACTGAGACACGGCC
>Zotu722
GAACGCTGGCGGCATGCTTTACACATGCAAGTCGAACGGTAACAGGGAGCTTGCTCCGCTGACGAGTG

GCGAACGGGTGAGTAATATATCGGAACGTGCCCAAGAGTGGGGGATAACGTAGCGAAAGTTACGCTAA
TACCGCATACGATCTATGGATGAAAGCAGGGGACCTTCGGGCCTTGTGCTCCTGGAGCGGCCGATATC
TGATTAGCTAGTTGGTGAGGTAAAGGCTCACCAAGGCGACGATCAGTAGCTGGTCTGAGAGGACGACC
AGCCACACTGGAACTGAGACACGGTC
>Zotu723
GAACGCTGGCGGCGTGCCTAATACATGCAAGTCGAACGAACCGCGACTAGGTGCTTGCACTTGGTCAA
GGTGAGTGGCGAACGGGTGAGTAACACGTGGGTAACCTACCTCATAGTGGGGGATAACAGTCGGAAAC
GACTGCTAATACCGCATAGGACATAGGATCACATGATCCAGTGAGGAAAGGTGGCGCAAGCTATCGCT
AAGAGATGGACCCGCGGTGCATTAGCTAGTTGGTAGGGTAAAGGCCTACCAAGGCGATGATGCATAGC
CGACCTGAGAGGGTGATCGGCCACATTGGGACTGAGACACGGCC
>Zotu724
GAACGCTGGCGGCGTGCCTAATACATGCAAGTAGAACGCTGAAGGAGGAGCTTGCTTCTCTGGATGAG
TTGCGAACGGGTGAGTAACGCGTAGGTAACCTGCCTGGTAGCGGGGGATAACTATTGGAAACGATAGC
TAATACCGCATAAGAGTAGATGTTGCATGACATTTGCTTAAAAGGTGCAATTGCATCACTACCAGATG
GACCTGCGTTGTATTAGCTAGTTGGTGAGGTAACGGCTCACCAAGGCGACGATACATAGCCGACCTGA
GAGGGTGATCGGCCACACTGAGACTGAGACACGGCC
>Zotu725
GAACGCTGGCGGTGTGCTTCACACATGCAAGTCGAACGAGGTAGCAATACCTAGTGGCGGACGGGTGA
GTAACGCGTGGGAATCTGCCTTTAGATGGGGGATAGCAGGCCGAAAGGCCTATTAATACCGCATATGC
CGTAAGGTAAAAGGAGTAATCCGTCTAAAGATGAGCCCGCGTCCGATTAGCTAGTTGGTGAGGTAAAG
GCTTACCAAGGCTACGATCGGTAGGTGGTCTGAGAGGATGATCACCCACAATGGGACTGAGACACGGC
C
>Zotu726
CAACGCTGGCGGCGCGCTTAACACATGCAAGTCGAACGAGAAAGGGGAGCAATCCCTGAGTAAAGTGG
CGCACGGGTGAGTAACACGTGGATCATCTACCCTCTTGTGGGGAATACCGATGGGAAACCGTCGACAA
TACCGCATAAGCTCGAGAGAGGAAAGCCGCAAGGCGCAGGAGGAGGAGTCCGCGGTCGATTAGTTAGT
TGGCAGGGTAAAAGCCTACCAAGACTGTGATCGATAGCCGGCCTGAGAGGGCACACGGCCACACTGGC
ACTGAAACACGGGC
>Zotu727
GAACGCTGGCGGCGTGCTTAACACATGCAAGTCGAACGATGATGCCCAGCTTGCTGGGTGGATTAGTG
GCGAACGGGTGAGTAACACGTGAGTAACCTGCCCCTGACTCCGGGATAAGCGTTGGAAACGACGTCTA
ATACTGGATACGACGGCCGATCGCATGGTCTGGTCGTGGAAAGAATTTTGGTTGGGGATGGACTCGCG
GCCTATCAGCTTGTTGGTGAGGTAATGGCTCACCAAGGCGACGACGGGTAGCCGGCCTGAGAGGGTGA
CCGGCCACACTGGGACTGAGACACGGCC
>Zotu728
GAATGCTGGCGGCGTGCCTAACACATGCAAGTCGAGCGGCAGCGAGGGGTGCTTGCACCCTGTCGGCG
AGCGGCGAACGGCTGAGTAACGCGTAGGAATTTGCCCCAAAGTGAGGAATAACTGCCCGAAAGGGTAG
CTAATGCCGCATATGGTCTTCGGATTAAAGGATTTATCCGCTTTGGGAGAAGCCTGCGTTGGATTAGG
TAGTTGGTAGGGTAATGGCCTACCAAGCCGACGATCCATAGCTGGTCTGAGAGGATGATCAGCCAGAC
TGGAACTGAGACACGGTC
>Zotu730
TAACGCTGGCGGCGTGCATAACACATGCAAGTCGAACGATGAAACTTAGTAGACTTCTTCGGAAAGAC
ACTAAGCGGATTAGTGGCGAACGGGTGAGTAACGCGTGAGTAACCTGCCTTACACAAGGGGATAGCCT
TTGGAAACGAAGAATAATACCCTATAAAACTATAAAAGCACATGCTAGAATGGTCAAAGTGCTAGCGG
TGTAAGATGGACTTGCGTCTGATTAGCTAGTTGGTGAGATAAAAGCTCACCAAGGCAACGATCAGTAG
CCGGCTTGAGAGAGTGTACGGCCACATTGGGACTGAGACACGGCC
>Zotu731
GAATGCTGGCGGCGTGCCTAACACATGCAAGTCGAGCGGCATCGCGGTCTTCGGATTGGCGGCGAGCG
GCGGACGGCTGAGTAACGCGTGGGAACATACCCCAAAGTGAGGGATAACCGCCCGAAAGGGTGGCTAA
TACCGCATATGGTCTTCGGATTAAAGGATTTATCCGCTTTGGGAATGGCCTGCGTACGATTAGATAGT
TGGTAGGGTAAAGGCCTACCAAGTCGACGATCGTTAACTGGTCTGAGAGGATGATCAGTCAGACTGGA
ACTGAGACACGGTC
>Zotu732
GAACGCTAGCGGCAGGCTTAACACATGCAAGTCGAGGGGCAGCACGGTAGCAATACTGGTGGCGACCG

GCAAACGGGTGCGGAACACGTACAGAATCTACCTTGATCTGGGGCATAGCCCGGAGAAATCCGGATTA
ACTCCCCATAACATATAAGAGTGGCATCACTTTAATATTAAAAATTTATTGGATCGAGATGACTGTGC
GTCCCATTAGCTAGTTGGTAGGGTAACGGCCTACCAAGGCGACGATGGGTAGCTGGTGTGAGAGCACG
ACCAGCCACACGGGCACTGAGACACGGGC
>Zotu733
TAACGCTAGCGGCAGGCCTAATACATGCAAGTCGAACGGCAGCACGGGTAGCAATACCTGGTGGCGAG
TGGCGCACGGGTGCGTAACGCGTATGCAATCTACCTGTAACTGGGGAATAGCCTTCCGAAAGGAAGAT
TAACACCGCATAATATCACGAAGCGGCATCGCTTTGTGATCAAATCTCCGGAGGTTACAGATGAGCAT
GCGTGACATTAGCTAGTTGGTGAGGTAACGGCTCACCAAGGCGACGATGTCTAGGGGGTCTGAGAGGA
TGATCCCCCACACTGGTACTGAGACACGGAC
>Zotu734
GAACGCTGGCGGCGTGCTTAACACATGCAAGTCGAACGGTGAAAGAGGAGCTTGCTCCTCTGGATCAG
TGGCGAACGGGTGAGTAACACGTGAGTAACCTGCCCCAGACTCTGGGATAAGCACTGGAAACGGTGTC
TAATACCGGATACGAGCTTCAGCCGCATGGCTAGGAGCTGGAAAGAATTTCGGTCTGGGATGGACTCG
CGGCCTATCAGCTAGTTGGTGAGGTAATGGCTCACCAAGGCGACGACGGGTAGCCGGCCTGAGAGGGT
GACCGGCCACACTGGGACTGAGACACGGCC
>Zotu735
GAACGCCGGCGGTGTGCCTAATACATGCAAGTCGAACGCGTTGGCCCAATTGATTGACGGTGCTTGCA
CCTGATTGATTTTGGTCGCCAACGAGTGGCGGACGGGTGAGTAACACGTAGGTAACCTGCCCAGAAGC
GGGGGACAACATTTGGAAACAGATGCTAATACCGCATAACAACGTTGTTCGCATGAACAACGCTTAAA
AGATGGCTTCTCGCTATCACTTCTGGATGGACCTGCGGTGCATTAGCTTGTTGGTGGGGTAATGGCCT
ACCAAGGCGATGATGCATAGCCGAGTTGAGAGACTGATCGGCCACAATGGGACTGAGACACGGCC
>Zotu736
GAACGCTGGCGGCGTGCCTAATACATGCAAGTCGAGCGAACAGATGAGAAGCTTGCTTCTCTGATGTT
AGCGGCGGACGGGTGAGTAACACGTGGGTAACCTACCTATAAGACTGGGATAACTCCGGGAAACCGGG
GCTAATACCGGATAATATTTTGAACCGCATGGTTCAATAGTGAAAGACGGTTTCGGCTGTCACTTATA
GATGGACCCGCGCCGTATTAGCTAGTTGGTAAGGTAATGGCTTACCAAGGCGACGATACGTAGCCGAC
CTGAGAGGGTGATCGGCCACACTGGAACTGAGACACGGTC
>Zotu737
GAACGCTAGCTATAGGCTTAACACATGCAAGTCGAGGGGAAACGGCAAAGAGTGCTTGCACACTTTGG
ACGTCGACCGGCGCACGGGTGAGTAACGCGTATCCAACCTGCCCTTTACTTGGGGATAACCCGTTGAA
AGACGGACTAATACCCAATGATATTCATTGATGACATCTGATTTGAATTAAAGATTTATCGGTATAGG
ATGGGGATGCGTCTGATTAGCTTGTTGGTGAGGTAACGGCTCACCAAGGCAACGATCAGTAGGGGTTC
TGAGAGGAAGGTCCCCCACATTGGAACTGAGACACGGTC
>Zotu738
GAACGCTGGCGGCATGCCTAACACATGCAAGTCGAACGTGATCCAGGCTTCGGTCTGGTGAAAGTGGC
AGACGGGTGAGTAACGCGTGGGAACCTGCCCTGTGGTAGGGAATAACCGCGGGAAACTGCGGCTAATA
CCCTATGTGTCCTGAGGGACAAAGATTTATCGCCATAGGAGGGGCCCGCGTCGGATTAGGTAGTTGGT
GGGGTAACGGCCTACCAAGCCATCGATCCGTAGCTGGTCTGAGAGGATGATCAGCCACACTGGGACTG
AGATACGGCC
>Zotu739
GAACGCTGGCGGCGTGCTTAACACATGCAAGTCGAGCGGAAAGGCCCTTCGGGGTACTCGAGCGGCGA
ACGGGTGAGTAACACGTGAGTAACCTGCCCTGGACTTTGGGATAACCCTCGGAAACGGGGGCTAATAC
CGGATATGACTTTTCCTCGCATGGGGATTGGTGGAAAGTTTTTCGGTCTGGGATGGGCTCGCGGCCTA
TCAGCTTGTTGGTGGGGTAATGGCCTACCAAGGCGACGACGGGTAGCCGGCCTGAGAGGGCGACCGGC
CACACTGGGACTGAGACACGGCC
>Zotu740
GAACGCTGGCGGCGTGCCTAATACATGCAAGTAGAACGCTGAAGGAGGAGCTTGCTCTTCTGGATGAG
TTGCGAACGGGTGAGTAACGCGTAGGTAACCTGCCTGGTAGCGGGGGATAACTATTGGAAACGATAGC
TAATACCGCATAATAGCAGTTGTTGCATGACAGCTGTTTGAAAGGTGCAATTGCATCACTACCAGATG
GACCTGCGTTGTATTAGCTAGTTGGTGGGGTAACGGCTCACCAAGGCGACGATACATAGCCGACCTGA
GAGGGTGATCGGCCACACTGGGACTGAGACACGGCC
>Zotu741
GAACGCTAGCGGCAGGCTTAACACATGCAAGTCGAGGGGTATAAGGAGCTTGCTCCTTAGAGACCGGC

GCACGGGTGCGTAACGCGTATGCAACCTACCTTGAACAGGGGGATAGCCCAGAGAAATTTGGATTAAT
ACCCCATGGTATATTTGAGTGGCATCACTTGATTATTAAAGTTCCAACGGTTCAAGATGGGCATGCGT
CCCATTAGCTAGATGGTAAGGTAACGGCTTACCATGGCGACGATGGGTAGGGGTCCTGAGAGGGAGAT
CCCCCACACTGGTACTGAGACACGGAC
>Zotu742
GAACGCTGGCGGCAGGCCTAACACATGCAAGTCGAGCGAGGACTTCGGTTCTAGCGGCGGACGGGTGA
GTAACGCGTGGGAATGTGCCCTTCTCTACGGAATAGCCCTGGGAAACTGGGAGTAATACCGTATACGC
CCTATTGGGGAAAGATTTATCGGAGAAGGATCAGCCCGCGTTGGATTAGGTAGTTGGTGGGGTAATGG
CCTACCAAGCCGACGATCCATAGCTGGTTTGAGAGGATGATCAGCCACACTGGGACTGAGACACGGCC
>Zotu743
GAACGCTGGCGGCGTGCCTAACACATGCAAGTCGAGCGAGAGACTTCGGTCTCTAGCGGCGGACGGGT
GAGTAACACGTGGGTAACCTGCCTCGTAGAGGGGGATAGCCTTCCGAAAGGAAGATTAATACCGCATA
ACGTATATCTGGCGCATGCCAGATATATCAAAGGAGTAATCCGCTATGAGATGGACCCGCGGCGCATT
AGCTAGTTGGTGAGGTAACGGCTCACCAAGGCGACGATGCGTAGCCGACCTGAGAGGGTGATCGGCCA
CATTGGAACTGAGATACGGTC
>Zotu744
GAACGCTGACAGAATGCTTAACACATGCAAGTCTACTTGAACTTCGGTTTGGGTGGCGGACGGGTGAG
TAACGCGTAAAGAACTTGCCTCACAGTTAGGGACAACATTTGGAAACGAATGCTAATACCTGATATTA
TGATTTTAGGGCATCCTAAGATTATGAAAGCTATATGCGCTGTGAGAGAGCTTTGCGTCCCATTAGCT
AGTTGGAGAGGTAACGGCTCACCAAGGCGATGATGGGTAGCCGGCCTGAGAGGGTGAACGGCCACAAG
GGGACTGAGACACGGCC
>Zotu745
GAACGCTGGCGGCGTGCTTAACACATGCAAGTCGAGCGGAAAGGCTCCTTCGGGGGTACTCGAGCGGC
GAACGGGTGAGTAACACGTGAGTAATCTGCCCCTGGCTTTGGGATAGCCACCGGAAACGGTGATTAAT
ACCGGATACGACCACTTCTCGCATGGGATGGTGGTGGAAAGTTTTTCGGCCAGGGATGTGCTCGCGGC
CTATCAGCTTGATGGTGAGGTAATGGCTCACCATGGCTTCGACGGGTAGCCGGCCTGAGAGGGTGACC
GGCCACACTGGGACTGAGACACGGCC
>Zotu746
GAACGCTTGCGGCGTGCCTAAGAAATGCAAGTCGAACGAGAGCTTCGGCTCTAGTGGCGAACGGTCGC
GTAACACGTGGGCAACCTGCCCTGAAGCGGGGGACAACAGACCGAAAGGTCTGCTAATACCGCATATG
ATCCCTCTCGGCATCGAGAGTGCTCAAAGTCTTCGGACACTTCAGGATGGGCCCGCGGCCTATCAGCT
TGTTGGTGGGGTAACGGCTCACCAAGGCTACGACGGGTAGCTGGTCTGAGAGGACGACCAGCCGGATT
GGGACTGAGATACGGCC
>Zotu747
GAACGCTGGCGGCGTGCTTAACACATGCAAGTCGAACGGTAAGGCCCTTCGGGGTACACGAGTGGCGA
ACGGGTGAGTAACACGTGGGTAATCTGCCCTGCACTTCGGGATAAGCCTGGGAAACTGGGTCTAATAC
CGGATATTCAGCTTCTGCCGCATGGTGGTGGTTGGAAAGTTTTTCGGTGCAGGATGAGCCCGCGGCCT
ATCAGCTTGTTGGTGGGGTAATGGCCTACCAAGGCGACGACGGGTAGCCGGCCTGAGAGGGTGATCGG
CCACACTGGGACTGAGACACGGCC
>Zotu748
GAACGCTGGCGGCGCGCTTAACACATGCAAGTCGAACGGAGTCCTTCGGGGCTCAGTGGCGAACGGGT
GCGTAACACGTGAGCAACCTGCCTGGTCGTGGGGGATAACCAGTGGAAACGCTGGCTAATACCGCATG
AGCTCTTTGCTTCGATTGAGGCGTTGAGGAAAGCTCCTGCAAGGGAGTGCGACCAGAGGGGCTCGCGG
CGTATCAGCTAGTTGGAGGGGTAACGGCCCACCAAGGCGATGACGCGTAGCCGATCTGAGAGGATGAT
CGGCCACATTGGGACTGAGATACGGCC
>Zotu749
GAACGCTGGCGGCGTGCTTAACACATGCAAGTCGAACGGTGAAGGGACCAGCTTGCTGGTTCTGGATG
AGTGGCGAACGGGTGAGTAACACGTGAGTAACCTGCCCCCTTCTTCTGGATAACCGCATGAAAGTGTG
GCTAATACGGGATATTCTGGGTCTGTCGCATGACGGGCCCGGGAAAGATATTGTTTCGGTGGGGGATG
GGCTCGCGGCCTATCAGCTTGTTGGTGGGGTGATGGCCTACCAAGGCGGTGACGGGTAGCCGGCCTGA
GAGGGTGGACGGTCACACTGGGACTGAGACACGGCC
>Zotu750
GAACGCTGGCGGCGTGCTTAACACATGCAAGTCGAGCGGAAAGGCCCTTCGGGGTACTCGAGCGGCGA
ACGGGTGAGTAACACGTGGGCAACCTGCCCCTAGCTCTGGGATAACTCCGGGAAACCGGGGCTAATAC

CGGATACGACTTCCCCCGGCATCTGGGGAAGTGGAAAGTTCTTCGGCTAGGGATGGGCCCGCGGCCTA
TCAGCTTGTTGGTGGGGTAACGGCCCACCAAGGCGACGACGGGTAGCCGGCCTGAGAGGGCGACCGGC
CACACTGGGACTGAGACACGGCC
>Zotu751
GAACGCTGGCGGCGTGCCTAACACATGCAAGTCGAACGAGGTCCATCCGGTGGCAACACCGGTGAAGA
CCTAGTGGCGAACGGGTGAGTAACACGTGAGGAACCTGCCCCGAAGACCGGGATAACCCTCCGAAAGG
AGAGCTAATACCGGATACCCCCACCGGATCGCATGATCTGGTGAGGAAATGGATTCCGCTTCGGGAGG
GCCTCGCGGCCTATCAGCTAGTTGGTGGGGTAACGGCCCACCAAGGCTTCGACGGGTAGCTGGTCTGA
GAGGACGATCAGCCACACTGGGACTGAGACACGGCC
>Zotu752
GAACGCTGGCGGCGTGCTTAACACATGCAAGTCGAACGAGATCCAGCCGAGCCTTCGGGCAAAGCGAA
GATCTAGTGGCGAACGGGTGCGTAACACGTGAGAAACCTGCCCCGGACTTGGGAATAACAGTCGGAAA
CGGCTGCTAATACCGAATACCTTCGTAGCGTCGCATGGCGCATCGAAGAAAGCTTTTGCGGTTTGGGA
GGGTCTCGCGGCCTATCAGCTTGTTGGCGAGGTAACGGCTCACCAAGGCATCGACGGGTAGCTGGTCT
GAGAGGATGATCAGCCACACTGGGACTGAGACACGGCC
>Zotu753
GAACGCTGGCGGCGTGCTTAACACATGCAAGTCGAACGCTGAAGCCCGAGCTTGCTCGGGTGGATGAG
TGGCGAACGGGTGAGTAACACGTGAGTAACCTGCCCCGAACTCTGGGATAAGCGCTGGAAACGGCGTC
TAATACTGGATATGTCCTGTCACCGCATGGTGTGCAGGTGGAAAGATTTATCGGTTCGGGATGGACTC
GCGGCCTATCAGCTTGATGGTGAGGTAATGGCTCACCATGGCGACGACGGGTAGCCGGCCTGAGAGGG
TGACCGGCCACACTGGGACTGAGACACGGCC
>Zotu754
GAACGCTGGCGGCGTGCCTAATACATGCAAGTAGAACGCTGAAGGAGGAGCTTGCTCTTCTGGATGAG
TTGCGAACGGGTGAGTAACGCGTAGGTAACCTGCCTGGTAGCGGGGGATAACTATTGGAAACGATAGC
TAATACCGCATAAGAGTAGATGTTGCATGACATTTGCTTAAAAGGTGCAATTGCATCACTACCAGATG
GACCTGCGTTGTATTAGCTAGTTGGTGGGGTAACGGCTCACCAAGGCGACGATACATAGCCGACCTGA
GAGGGTGATCGGCCACACTGGGACTGAGACACGGCC
>Zotu755
GAACGCTAGCGGCAGGCTTAACACATGCAAGTCGAGGGGTAGAAGCAGCTTGCTGCTTTGAGACCGGC
GCACGGGTGCGTAACGCGTATGCAATCTGCCTTTCACAAAGGGATAGCCCAGAGAAATTTGGATTAAT
ACCTTATAGTATATTGATGTGGCATCACATTATTATTAAAGATTTATCGGTGAAAGATGAGCATGCGT
CCCATTAGCTAGTTGGTAAGGTAACGGCTTACCAAGGCAACGATGGGTAGGGGTCCTGAGAGGGAGAT
CCCCCACACTGGTACTGAGACACGGAC
>Zotu756
GAACGCTGGCGGCATGCCTAATACATGCAAGTCGAACGCTGAAGCACTCTGCTTGCAGAGTGCGGATG
AGTGGCGAACGGGTGAGTAACACGTGGGAAATCTGCCTAAGAGTGGGGGATAACATTCGGAAACGGAT
GCTAATACCGCATATATTCTTGAATCGCATGGTTCTGGAAAGAAAGACGGCTTTGCTGTCGCTTTTAG
ATGATCCCGCGGCGTATTAGTTAGTTGGTGGGGTAAAGGCCTACCAAGACCATGATACGTAGCCGACC
TGAGAGGGTGATCGGCCACACTGGGACTGAGACACGGCC
>Zotu757
GAACGCTGGCGGCGTGCCTAATACATGCAAGTAGAACGCTGAAGGAGGAGCTTGCTTCTCTGGATGAG
TTGCGAACGGGTGAGTAACGCGTAGGTAACCTGCCTGGTAGCGGGGGATAACTATTGGAAACGATAGC
TAATACCGCATAATAGTAGATGTTGCATGACATTTGCTTAAAAGGTGCAATTGCATCACTACCAGATG
GACCTGCGTTGTATTAGCTAGTTGGTGGGGTAACGGCTCACCAAGGCGACGATACATAGCCGACCTGA
GAGGGTGATCGGCCACACTGGGACTGAGACACGGCC
>Zotu758
GAACGTTGGCGGCGTGGATTAGGCATGCAAGTCGTACGCACCGCAAGGTGAGTGGCGCAAGGGTGAGT
AAGGCGAAGGTAACCAACCCCGAGATTGGGTATAGCCGTGGGAAACCGCGGGTAATCCCCAGCGATGT
TGTTGAGTGGCATCACTCGACAACCAAAGCTCCGGCGTCTTGGGACGGGCCTTCGTGGTATTAGGTTG
TTGGCGGGGTAATGGCCCACCAAGCCTCTGATGCCTACCGGGCGTGCGAGCGTGGCCCGGCACACTGG
GACTGAGACACTGCC
>Zotu759
GAACGCTGGCGGCGTGCTTAACACATGCAAGTCGAACGGTGAACCTGCTTCGGTGGGGGATCAGTGGC
GAACGGGTGAGTAACACGTGGGCAACCTGCCCCTGGCTCTGGGATAACTCCAAGAAATTGGGGCTAAT

ACCGGATATGACCGCTGACCGCATGGTCTGGTGGTGGAAAGATTTATCGGCTGGGGATGGGCCCGCGG
CCTATCAGCTTGTTGGTGGGGTAGTGGCCTACCAAGGCGACGACGGGTAGCCGGCCTGAGAGGGTGAC
CGGCCACACTGGGACTGAGACACGGCC
>Zotu760
GAACGCTGGCGGCGTGCTTAACACATGCAAGTCGAACGATGAAGCCCAGCTTGCTGGGTGGATTAGTG
GCGAACGGGTGAGTAACACGTGAGTAACCTGCCCTTAACTCTGGGATAAGCCTGGGAAACTAGGTCTA
ATACCGGATAGGAGCGCCTACCGCATGGTGGGTGTTGGAAAGATTTATCGGTTTTGGATGGACTCGCG
GCCTATCAGCTTGTTGGTGAGGTAATGGCTCACCAAGGCGACGACGGGTAGCCGGCCTGAGAGGGTGA
CCGGCCACACTGGGACTGAGACACGGCC
>Zotu761
GAACGCTGGTGGCGTGCTTAACACATGCAAGTCGAGCGAGGCGTCCGCTTTCGGGTGGATAGTCGAGC
GGCGAACGGGTGAGTAACACGTGAGTAATCTTCCCTGGGCTCGGGGATAACCTCGGGAAACTGAGGCT
AATACCGGATGTGCGTTGTTTGGGGCTTCCTGGATGACGGAAAGGTTCCATCTCGTGAGGGGTGGTTC
CGGCCTTGGATGAGCTCGCGGCCTATCAGCTTGTTGGTGCGGTGATGGCGTACCAAGGCGACGACGGG
TAGCCGGCCTGAGAGGGTGACCGGCCACACTGGGACTGAGATACGGCC
>Zotu762
GAACGCTGGCGGCGTGCTTAACACATGCAAGTCGAACGGTGAAAGAGGAGCTTGCTCCTCTGGATCAG
TGGCGAACGGGTGAGTAACACGTGAATAACCTGCCCCAGACTCTGGGATAAGCGTTGGAAACGACGTC
TAATACCGGATACGAGCTTCAGCCGCATGGCTAGGAGCTGGAAAGAATTTCGGTCTGGGATGGACTCG
CGGCCTATCAGGTAGTTGGTGAGGTAACGGCCCACCAAGCCTACGACGGGTAGCCGGCCTGAGAGGGT
GACCGGCCACACTGGGACTGAGACACGGCC
>Zotu763
GAACGCTGGCGGTATGCTTAACACATGCAAGTCGAACGGGGTACTTTCGGGTACCTAGTGGCGGACGG
GTGAGTAACGCGTGAGAACATGCCTTTAGATTGGGGACAACAGTTGGAAACGACTGCTAATACCGAAT
GTGCCGAGAGGTGAAAGATTAATTGTCTAAAGATTGGCTCGCGTCAGATTAGGTAGTTGGAGTGGTAA
CGGCACACCAAGCCTACGATCTGTACTTGGTCTGAGAGGATGACCAGGCACACTGGAACTGAGACACG
GTC
>Zotu764
TAACGCTGGCGGCGTGCATAACACATGCAAGTCGAACGATGAAACTTAGTAGACTTCTTCGGAAAGAC
ACTAAGCGGATTAGTGGCGAACGGGTGAGTAACGCGTGAGTAACCTGCCTTACACAAGGGGATAGCCT
TTGGAAACGAAGAATAATACCCTATAAAACCATAAAAGCATATGCAATTATGGTCAAAGTGATAGCGG
TGTAAGATGGACTTGCGTCTGATTAGCTAGTTGGTGAGATAAAAGCTCACCAAGGCAACGATCAGTAG
CCGGCTTGAGAGAGTGTACGGCCACATTGGGACTGAGACACGGCC
>Zotu765
GAACGCTGGCGGCGTGCCTAACACATGCAAGTCGAGCGAGGTCCATTCGGTAGCAATACCGATGAAGA
CCTAGCGGCGAACGGGTGAGTAACACGTGAGAAACCTGCCCCAAAGTCTGGGATAACCCGGGGAAACC
CGAGCTAATACCGGATACCTTCTTTTGATCGCATGGTCAGCTGAAGAAAGTTTTTTCGCTTTGGGAGG
GTCTCGCGGCCTATCAGTTTGTTGGTGGGGTAACGGCCTACCAAGACATCGACGGGTAGCTGGTCTGA
GAGGACGATCAGCCACACTGGGACTGAGACACGGCC
>Zotu768
GAACGCTGGCGGCGTGCCTAATACATGCAAGTCGAGCGGACCTTTTAAAGCTTGCTTTGAAAGGTTAG
CGGCGGACGGGTGAGTAACACGTGGGCAACCTGCCCTATAGTTTGGGATAACTCCGGGAAACCGGGGC
TAATACCGAATAACACAATCTATCTCCTGTTAGATTGTTGAAAGATGGTTTCGGCTATCGCTATAGGA
TGGGCCCGCGGCGCATTAGCTAGTTGGTAGGGTAACGGCCTACCAAGGCGACGATGCGTAGCCGACCT
GAGAGGGTGATCGGCCACACTGGGACTGAGACACGGCC
>Zotu769
GAACGCTGGCGGCGTGCCTAATACATGCAAGTCGAACGCATTTTCTTTCACCGTAGCTTGCTACACCG
AAAGAAAGTGAGTGGCGAACGGGTGAGTAACACGTGGGTAACCTGCCCATCAGCGGGGGATAACACTT
GGAAACAGGTGCTAATACCGCATAATTCCATTTACCGCATGGTAGATGGATGAAAGGCGCTTTTGCGT
CACTGATGGATGGACCCGCGGTGCATTAGCTAGTTGGTGGGGTAACGGCCTACCAAGGCTGCGATGCA
TAGCCGACCTGAGAGGGTGATCGGCCACACTGGGACTGAGACACGGCC
>Zotu770
GAACGCTGGCGGTGTGCTTCACACATGCAAGTCGAACGAGGTAGCAATACCTAGTGGCGGACGGGTGA
GTAACGCGTGGGAATCTGCCTTTAGATGGGGGATAACGGGCCGAAAGGTCCGCTAATACCGCATATGC

CGAGAGGTGAAAGGAGTAATCCGTCTAAAGATGAGCCCGCGTCCGATTAGCTAGTTGGTAGAGTAAGA
GCCTACCAAGGCGACGATCGGTAGCTGGTCTGAGAGGATGATCAGCCACAATGGGACTGAGACACGGC
C
>Zotu771
GAACGCTGGCGGCATGCCTTACACATGCAAGTCGAACGGCAGCACGGGTGCTTGCACCTGGTGGCGAG
TGGCGAACGGGTGAGTAATACATCGGAACGTACCTTATCGTGGGGGATAACGCAGCGAAAGCTGTGCT
AATACCGCATACGCCCTGAGGGGGAAAGCGGGGGATCGAAAGACCTCGCGCGATTAGAGCGGCCGATG
CCTGATTAGCTTGTTGGTGGGGTAAAAGCCCACCAAGGCGACGATCAGTAGCTGGTCTGAGAGGACGA
TCAGCCACACTGGGACTGAGACACGGCC
>Zotu772
GAACGCTGGCGGCGTGCCTAATACATGCAAGTCGAGCGAACAGATAAGGAGCTTGCTCCTTTGACGTT
AGCGGCGGACGGGTGAGTAACACGTGGATAACCTACCTATAAGACTGGGATAACTTCGGGAAACCGGA
GCTAATACCGGATAACATATTGAACCGCATGGTTCAATAGTGAAAGACGGCCTTGCTGTCACTTATAG
ATGGATCCGCGCCGTATTAGCTAGTTGGTAAGGTAACGGCTTACCAAGGCAACGATACGTAGCCGACC
TGAGAGGGTGATCGGCCACACTGGAACTGAGACACGGTC
>Zotu773
GAACGCTGGCGGCATGCTTAACACATGCAAGTCGAACGGCAGCATGACCTAGCTTGCTAGGTTGATGG
CGAGTGGCGGACGGGTGAGTAACGCGTAGGAATATGCCTTGAAGAGGGGGATAACTTGGGGAAACTCA
AGCTAATACCGCATAAGCTCTGAGGAGTGAAGCTGGGGACCGTAAGGCCTGGCGCTTTGAGATTAGCC
TGCGTCCGATTAGCTAGTTGGTGGGGTAAGGGCCTACCAAGGCGACGATCGGTAGCTGGTCTGAGAGG
ATGATCAGCCACACTGGGACTGAGACACGGCC
>Zotu774
GAACGCTAGCTATAGGCTTAACACATGCAAGTCGAGGGGCAGCGAATAGATAGCTTGCTATTTATGTC
GGCGACCGGCGCACGGGTGAGTAACGCGTATCCAACCTGCCCATAACTAAGGGATAACCCAGCGAAAG
TTGGACTAATACCTTATGTATTCGTTTGATCTCATGAGATTACGAATAAAGATTTATCGGTTATGGAT
GGGGATGCGTCTGATTAGCTTGTTGGCGGGGTAACGGCCCACCAAGGCAACGATCAGTAGGGGTTCTG
AGAGGAAGGTCCCCCACATTGGAACTGAGACACGGTC
>Zotu776
GAACGCTAGCGGGAGGCCTAACACATGCAAGCCGAGCGGTATTGTTTCTTCGGAAATGAGAGAGCGGC
GTACGGGTGCGGAACACGTGTGCAACCTGCCTTTATCTGGGGGATAGCCTTTCGAAAGGAAGATTAAT
ACTCCATAATATATTGATTGGCATCAATTAATATTGAAAGCTCCGGCGGATAGAGATGGGCACGCGCA
AGATTAGCTAGTTGGTGAGGTAACGGCTCACCAAGGCGATGATCTTTAGGGGGCCTGAGAGGGTGATC
CCCCACACTGGTACTGAGACACGGAC
>Zotu777
GAACGCTGGCGGCGTGCTTAACACATGCAAGTCGAACGATGAACCTCCCTCGGGAGGGGATTAGTGGC
GAACGGGTGAGTAACACGTGGGCAATCTGCCCTGCACTCTGGGATAACTCCGGGAAACCGGAGCTAAT
ACCGGATACGATCACCGCGGGCATCCGTGGTGGTGGAAAGCTCCGGCGGTGCAGGATGAGCCCGCGGC
CTATCAGCTTGTTGGTGGGGTGACGGCCCACCAAGGCGACGACGGGTAGCCGGCCTGAGAGGGCGACC
GGCCACACTGGGACTGAGACACGGCC
>Zotu778
GAACGCTGGCGGCGTGCCTAATACATGCAAGTCGAGCGAATCTGAGGGAGCTTGCTCCCAAAGATTAG
CGGCGGACGGGTGAGTAACACGTGGGTAACCTGCCTGTAAGATTGGGATAACTCCGGGAAACCGGAGC
TAATACCGGATAACATTTCGAACCGCATGGTTCGAAATTGAAAGATGGTTTCGGCTATCACTTACAGA
TGGACCCGCGGCGCATTAGCTAGTTGGTGAGGTAACGGCTCACCAAGGCAACGATGCGTAGCCGACCT
GAGAGGGTGATCGGCCACACTGGGACTGAGACACGGCC
>Zotu779
GAACGCTGGCGGCATGCTTAACACATGCAAGTCGAACGGCAGCATGACCTAGCTTGCTAGGTTGATGG
CGAGTGGCGGACGGGTGAGTAACGCGTAGGAATATGCCTTAGAGAGGGGGATAACTTGGGGAAACTCA
AGCTAATACCGCGTGATGTCTTAGGACTAAAGCTGGGGACCGCAAGGCCTGGCGCTTTAAGATTAGCC
TGCGTCCGATTAGCTAGTTGGTGGGGTAAGGGCTTACCAAGGCGACGATCGGTAGCTGGTCTGAGAGG
ATGATCAGCCACACTGGGACTGAGACACGGCC
>Zotu780
GAACGCTGGCGGCGTGCCTAATACATGCAAGTCGAGCGAATGGATTAAGAGCTTGCTCTTATGAAGTT
AGCGGCGGACGGGTGAGTAACACGTGGGTAACCTGCCCATAAGACTGGGATAACTCCGGGAAACCGGG

GCTAATACCGGATAACATTTTGAACCACATGGTTCGAAATTGAAAGGCGGCTTCGGCTGTCACTTATG
GATGGACCCGCGTCGCATTAGCTAGTTGGTGAGGTAACGGCTCACCAAGGCAACGATGCGTAGCCGAC
CTGAGAGGGTGATCGGCCACACTGGGACTGAGACACGGCC
>Zotu781
GAACGCTGGCGGCGTGCCTAATACATGCAAGTAGAACGCTGAAGCTTGGTGCTTGCACCGAGCGGATG
AGTTGCGAACGGGTGAGTAACGCGTAGGTAACCTGCCTGGTAGCGGGGGATAACTATTGGAAACGATA
GCTAATACCGCATAAGAGTAGATGTTGCATGACATTTGCTTAAAAGGTGCAATTGCATCACTACCAGA
TGGACCTGCGTTGTATTAGCTAGTTGGTGAGGTAACGGCTCACCAAGGCAACGATACATAGCCGACCT
GAGAGGGTGATCGGCCACACTGGGACTGAGACACGGCC
>Zotu782
GAACGCTGGCGGCAGGCTTAACACATGCAAGTCGAACGATGATTATCTAGCTTGCTAGATATGATTAG
TGGCGGACGGGTGAGTAACATTTAGGAATCTGCCTAGTAGTGGGGGATAGCTCGGGGAAACTCGAATT
AATACCGCATACGACCTACGGGTGAAAGGGGGCGCAAGCTCTTGCTATTAGATGAGCCTAAATCAGAT
TAGCTAGTTGGTGGGGTAAAGGCCCACCAAGGCGACGATCTGTAACTGGTCTGAGAGGATGATCAGTC
ACACCGGAACTGAGACACGGTC
>Zotu783
GAACGCTGGCGGCGTGCTTAACACATGCAAGTCGAGCGAGGCCCTCCTTCGGGGGGTGCCCTAGCGGC
GAACGGGTGAGTAACACGTGGGCAACCTGCCCTCAGCTCTGGGATAACTCCAAGAAATTGGTGCTAAT
ACCGGATGTGACCGCTGACCGCATGGTCTGGTGGTGGAAAGATTCATCGGCTGAGGATGGGCCCGCGG
CCTATCAGCTTGTTGGTGGGGTCACGGCCCACCAAGGCGACGACGGGTAGCCGGCCTGAGAGGGTGAC
CGGCCACACTGGGACTGAGACACGGCC
>Zotu784
GAACGCTAGCGACAGGCCTAACACATGCAAGTCGAGGGGCAGCGGGGATGAAGCTTGCTTCATCTGCC
GGCGACCGGCGCACGGGTGAGTAACACGTATGCAACCTGCCCGCCACAGGGGGATAAACGCTGGAAAC
GGCGCCTAATACCCCATACTATCGCTAAGCTGCATGGCTTTGCGATGAAAACTCCGGTGGTGGCGGAT
GGGCATGCGGCGCATTAGCTGGTTGGGGGGGTAACGGCCCACCAAGGCGACGATGCGTAGGGGTTCTG
AGAGGAAGGTCCCCCACACTGGTACTGAGACACGGAC
>Zotu785
GAACGCTGGCGGCGTGCTTAACACATGCAAGTCGAACGGGAACCTTTATTTCGGTAAAGGTGAGAGTG
GCAAACGGGTGAGTAACACGTAGGCAACCTGCCGACAGGATGGGGACAACATTCCGAAAGGAATGCTA
ATACCGAATGGAGTCCGGGGATGGCATCATCTCCGGATAAAAGATGGCCTCTGAATATGCTATCGCCT
GTCGATGGGCCTGCGTCTGATTAGCCAGTTGGCGGGGTAACGGCCCACCAAAGCGACGATCAGTAGCC
GGTCTGAGAGGATGAACGGCCACATTGGGACTGAGACACGGCC
>Zotu786
GAACGCTGACAGAATGCTTAACACATGCAAGTCAACTTGAACTTCGGTTTGGGTGGCGGACGGGTGAG
TAACGCGTAAAGAACTTGCCTCACAGCTAGGGACAACATTTGGAAACGAATGCTAATACCTGATATTA
TGATAATAGGGCATCCTATAATTATGAAAGCTATATGCGCTGTGAGAGAGCTTTGCGTCCCATTAGCT
AGTTGGAGAGGTAACGGCTCACCAAGGCGATGATGGGTAGCCGGCCTGAGAGGGTGATCGGCCACAAG
GGGACTGAGACACGGCC
>Zotu787
GAACGCTGGCGGCAGGCCTAACACATGCAAGTCGAACGGACCCTTCGGGGTTAGTGGCGGACGGGTGA
GTAACACGTGGGAACGTGCCTTTAGGTTCGGAATAGCTCCTGGAAACGGGTGGTAATGCCGAATGTGC
CCTTCGGGGGAAAGATTTATCGCCTTTAGAGCGGCCCGCGTCTGATTAGCTAGTTGGTGAGGTAATGG
CTCACCAAGGCGACGATCAGTAGCTGGTCTGAGAGGATGACCAGCCACATTGGGACTGAGACACGGCC
>Zotu788
GAACGCTTGCGGCGTGCCTAAGAAATGCAAGTCGAACGGAAAGGTCCTTCGGGACACTCGAGTGGCGA
ACGGTCGCGTAACACGTAAGCAACCTGCCTCAAAGTGGGGGACAACAGTCTGAAAAGACTGCTAATAC
CGCATGTGGTCAACTAAGGGCATCCTTGGTTGTCTAAAGGATTTATTCGCTTTGAGATGGGCTTGCGG
CCTATCAGGTAGTTGGTGGGGTAACGGCCTACCAAGCCTTCGACGGGTAGCTGGTCTGAGAGGACGAT
CAGCCGGATTGGGACTGAGATACGGCC
>Zotu789
GAACGCTAGCGGGAGGCCTAACACATGCAAGCCGAGCGGTATTTGTTCTTCGGAACAGAGAGAGCGGC
GCACGGGTGCGGAACACGTGTGCAACCTGCCTTTATCTGGGGGATAGCCTTTCGAAAGGAAGATTAAT
ACCCCATAATATATTGAGTGGCATCACTTGATATTGAAAACTCCGGTGGATAGAGATGGGCACGCGCA

AGATTAGATAGTTGGTGAGGTAACGGCTCACCAAGTCAATGATCTTTAGGGGGCCTGAGAGGGTGATC
CCCCACACTGGTACTGAGACACGGAC
>Zotu790
GAACGCTGGCGGCGTGCTTAACACATGCAAGTCGAACGGTGAAGCCAAGCTTGCTTGGTGGATCAGTG
GCGAACGGGTGAGTAACACGTGAGCAACCTGCCCTGGACTCTGGGATAAGCGCTGGAAACGGCGTCTA
ATACTGGATATGAGCTCCTATCGCATGGTGGGGGTTGGAAAGATTTTTCGGTCTGGGATGGGCTCGCG
GCCTATCAGCTTGTTGGTGAGGTAATGGCTCACCAAGGCGTCGACGGGTAGCCGGCCTGAGAGGGTGA
CCGGCCACACTGGGACTGAGACACGGCC
>Zotu791
GAACGCTGGCGGCGTGCTTAACACATGCAAGTCGAGCGGTAAGGCTCCTTCGGGAGTACACGAGCGGC
GAACGGGTGAGTAACACGTGAGTAATCTGCCCTTCACTTCGGGATAACCACCGGAAACGGTGGCTAAT
ACCGGATACGACCAGCCTCGGCATCGGGTGCTGGTGGAAAGTCTTTTCGGTGGAGGATGTGCTCGCGG
CCTATCAGCTTGTTGGTGGGGTAATGGCCTACCAAGGCTTCGACGGGTAGCCGGCCTGAGAGGGTGAC
CGGCCACACTGGGACTGAGACACGGCC
>Zotu792
GAACGCTGGCGGCGTGCTTAACACATGCAAGTCGAACGGAAAGGCCCTGCTTGCAGGGTGCTCGAGTG
GCGAACGGGTGAGTAACACGTGGGTGATCTGCCCCTTACTTTGGGATAAGCCTGGGAAACTGGGTCTA
ATACTGGATAGGACCATGCTGTAGGTGGTGTGGTGGAAAGATTTTTTCGGTAAGGGATGAGCTCGCGG
CCTATCAGCTTGTTGGTGGGGTAATGGCCTACCAAGGCGTCGACGGGTAGCCGGCCTGAGAGGGTGGA
CGGCCACATTGGGACTGAGATACGGCC
>Zotu793
GAACGCTGGCGGCGTGCTTAACACATGCAAGTCGAACGATGAAGCTTGGTGCTTGCACTGGGTGGATT
AGTGGCGAACGGGTGAGTAACACGTGAGTAACCTGCCCTTTACTTCGGGATAAGCTTGGGAAACTGGG
TCTAATACCGGATATTCTGCATGGTCGGATGGTTGTGTAGGAAAGATTTTTTGGTAAGGGATGGGCTC
GCGGCCTATCAGTTTGTTGGTGGGGTGATGGCCTACCAAGACGACGACGGGTAGCCGGCCTGAGAGGG
CGACCGGCCACACTGGGACTGAGACACGGCC
>Zotu794
GAACGCTGGCGGCGTGCTTAACACATGCAAGTCGAACGATGAACCGCTTTCGGGCGGGGATTAGTGGC
GAACGGGTGAGTAACACGTGGGCAATCTGCCCTGCACTCTGGGACAAGCCCTGGAAACGGGGTCTAAT
ACCGGATATGATTGTCCGCCGCATGGTGGATGGTGTAAAGCTCCGGCGGTGCAGGATGAGCCCGCGGC
CTATCAGCTTGTTGGTGAGGTAGTGGCTCACCAAGGCGACGACGGGTAGCCGGCCTGAGAGGGCGACC
GGCCACACTGGGACTGAGACACGGCC
>Zotu795
GAACGCTGGCGGCGTGCTTAAGACATGCAAGTCGAACGGCAGTCTTCGGACTGTAGTGGCGCACGGGT
GAGTAACGCGTAACTGACCTACCCCAAAGTCGCGGATAACGATTCGAAAGAATCGCTAATACGTGATG
TGCTGTCAGATTGTGTTCTGCCAGTAAAGATTGATTGCTTTGGGATGGGGTTGCGTTCCATCAGCTAG
TTGGTGGGGTAAAGGCCCACCAAGGCGACGACGGATAGCCGGCCTGAGAGGGTGGCCGGCCACAGGGG
CACTGAGACACGGGT
>Zotu796
GAACGCTGGCGGCGTGCTTAACACATGCAAGTCGAACGGGAAGAGATGAAGAGCTTGCTCTTTATCAA
ATCCAGTGGCAAACGGGTGAGTAACACGTAAACAACCTGCCTTCAGGATGGGGACAACAGACGGAAAC
GACTGCTAATACCGAATACGTTCCACGGGCCGCATGACCTGTGGAAGAAAGGGTAGCCTCTACCTGTA
AGCTATCGCCTGAAGAGGGGTTTGCGTCTGATTAGGCAGTTGGTGGGGTAACGGCTCACCAAACCAAC
GATCAGTAGCCGGTCTGAGAGGATGAACGGCCACACTGGAACTGAGACACGGTC
>Zotu797
GAACGCTAGCGGGAGGCCTAACACATGCAAGCCGAGCGGTATTTGTTCTTCGGGACAGAGAGAGCGGC
GCACGGGTGCGGAACACGTGTGCAACCTGCCTTTATCTGGGGGATAGCCTTTCGAAAGGAAGATTAAT
ACCCCATAATATATTGAGTGGCATCATTTGATATTGAAAACTCCGGTGGATAGAGATGGGCACGCGCA
AGATTAGATAGTTGGTGAGGTAACGGCTCACCAAGTCAATGATCTTTAGGGGGCCTGAGAGGGTGATC
CCCCACACTGGTACTGAGACACGGAC
>Zotu798
GAACGCTGGCGGCGTGCCTAACACATGCAAGTCGAGCGGAGTTGTTATGAGATTCATTAGATGATTAT
TTCAACTTAGCGGCGGATGGGTGAGTAACACGTGGGTAACCTGCCCTGCACTGGGGGATAACAGCTGG
AAACGGCTGTTAATACCGCATATGCACACACCGGCGCATGCCGGAGTGTGGAAAGATTCATCGGTGCA

GGATGGGCCCGCGCCGGATTAACTTGTTGGCAGGGTAATGGCCTACCAAGGTGATGATCCGTAGCCGG
CCTGAGAGGGCGACCGGCCACATTGGGACTGAGACACGGCC
>Zotu799
GAACGCTGGCGGCGTGCTTAACACATGCAAGTCGAGCGGAAAGGCTCCTTCGGGGGTACTCGAGCGGC
GAACGGGTGAGTAACACGTGAGTAATCTGCCCTTCACTTTGGGATAGCTCCCGGAAACGGGGATTAAT
ACCGGATATGACCATTCCAGGCATCTGGTGGTGGTGGAAAGTTTTTCGGTGGGGGATGTGCTCGCGGC
CTATCAGCTTGTTGGTGGGGTAATGGCCTACCAAGGCTTCGACGGGTAGCCGGCCTGAGAGGGTGACC
GGCCACACTGGGACTGAGACACGGCC
>Zotu800
GAACGCTGGCGGCATGCTTAACACATGCAAGTCGCACGGTCGGCGCAAGTCGGCAGTGGCGGACGGGT
GAGGAACGCGTAGGAACATGTCCTGAGGTGGGGGATAACGCTGGGAAACTGGCGCTAATACCGCATAT
GAGCTGAGGCTCAAAGCCGCGAGGCGCCTTGGGAGTGGCCTGCGTCCGATTAGGTAGTTGGTGGGGTA
ATGGCTTACCAAGCCTGCGATCGGTAGCTGGTCTGAGAGGACGACCAGCCACACTGGGACTGAGACAC
GGCC
>Zotu801
GAACGCTGGCGGCGTGCTTAACACATGCAAGTCGAACGATGAAAGAGGAGCTTGCTCCTCTGGATTAG
TGGCGAACGGGTGAGTAACACGTGAGCAATCTGCCCCTGACTCCGGAATAACTACGGGAAACTGTAGC
TAATACCGGATACGCGCCGAACAGGCATCTGTATCGGTGGGAAAGAATTTCGGTCAGGGATGAGCTCG
CGGCCTATCAGCTTGTTGGTGAGGTAACGGCTCACCAAGGCGACGACGGGTAGCCGGCCTGAGAGGGT
GACCGGCCACATTGGGACTGAGACACGGCC
>Zotu802
GAACGCTGGCGGCGTGCTTAACACATGCAAGTCGAACGCTGAAGCACCAGCTTGCTGGTGTGGATGAG
TGGCGAACGGGTGAGTAATACGTGAGTAACCTGCCCTTGACTCTGGGATAAGCCCGGGAAACTGGGTC
TAATACTGGATGCTACATGTCACCGCATGGTGGTGTGTGGAAAGGGTTTACTGGTCTTGGATGGGCTC
ACGGCCTATCAGCTTGTTGGTGAGGTAATGGCTCACCAAGGCGACGACGGGTAGCCGGCCTGAGAGGG
TGACCGGCAACACTGGGACTGAGACACGGCC
>Zotu803
GAACGCTGGCGGCGTGCTTAACACATGCAAGTCGAACGGAAAGGCCTCTGCTTGCAGAGGTACTCGAG
TGGCGAACGGGTGAGTAACACGTGGGTGATCTGCCTTGCACTTCGGGATAAGCCTGGGAAACTGGGTC
TAATACCGGATAGGACCATGCTTTAGTGTGTGTGGTGGAAAGTTTTTCGGTGCAAGATGAGCCCGCGG
CCTATCAGCTTGTTGGTGGGGTAATGGCCTACCAAGGCGACGACGGGTAGCCGGCCTGAGAGGGTGTA
CGGCCACATTGGGACTGAGACACGGCC
>Zotu805
GAACGCTGGCGGCATGCTTTACACATGCAAGTCGAACGGTAACAGGTCTTCGGATGCTGACGAGTGGC
GAACGGGTGAGTAACACATCGGAACGTGCCTAGTAGTGGGGGATAACTACTCGAAAGAGTAGCTAATA
CCGCATGAGATCTAAGGATGAAAGCAGGGGACCTTCGGGCCTTGCGCTACTAGAGCGGCTGATGGCAG
ATTAGGTAGTTGGTGGGATAAAAGCTTACCAAGCCGACGATCTGTAGCTGGTCTGAGAGGACGACCAG
CCACACTGGGACTGAGACACGGCC
>Zotu806
GAACGCTGGCGGCAGGCCTAACACATGCAAGTCGAACGGTAGCACAGAGAGCTTGCTCTCGGGTGACG
AGTGGCGGACGGGTGAGTAATGTCTGGGAAACTGCCTGATGGAGGGGGATAACTACTGGAAACGGTAG
CTAATACCGCATAACGTCGCAAGACCAAAGAGGGGGACCTTCGGGCCTCTTGCCATCAGATGTGCCCA
GATGGGATTAGCTAGTAGGTGGGGTAACGGCTCACCTAGGCGACGATCCCTAGCTGGTCTGAGAGGAT
GACCAGCCACACTGGAACTGAGACACGGTC
>Zotu807
GAATGCTGGCGGTGTGGATAGGGCATGCAAGTCAAGGGGACCGCAAGGTAACCGGCAGACGAGATAGT
AACGCGTAGGAACGTACCCAGAAGTCGGGCACAGCTCGCCGAAAGGCGGGGTAATTCCCGATGGTGTC
GCAAGACTAAAGATTTATCGCTTCTGGAGCGGCCTGCGTAATATCAGCTTGTTGGTGAGGTAATGGCT
CACCAAGGCTATGACGTTTAGGGGAGCTGAGAGGCTGACCCCCATCGATGGAATTGAGATACGGTC
>Zotu808
GAACGCTGGCGGCGTGCTTAACACATGCAAGTCGAACGGAAAGGCTCCTGCTTGCAGGGGTACTCGAG
TGGCGAACGGGTGAGTAACACGTGGGTGATCTGCCTTGTACTCTGGGATAAGCTTGGGAAACTGGGTC
TAATACCGGATAGTAGCCGCCTTTAGTGTGGTGGTTGGAAAGTTTTTTCGGTGCAAGATGAGCTCGCG
GCCTATCAGCTTGTTGGTGGGGTAATGGCCTACCAAGGCGTCGACGGGTAGCCGGCCTGAGAGGGTGT

ACGGCCACATTGGGACTGAGATACGGCC
>Zotu809
GAACGCTGGCGGCGTGCTTAACACATGCAAGTCGAACGAGATCCATTCAGTAGCAATACTGATGAAGA
TCTAGTGGCGAACGGGTGCGTAACACGTGAGGAACTTGTCCCGGTCTCTGGGATAACAGTTGGAAACG
ACTGCTAATACCGGATGACGTCGGAACTTCGCATGGAGTACTGACGAAAGGGTTACTGGATCGGGAGA
GCCTCGCGGCCTATCAGCTTGTTGGTGGGGTAACGGCCTACCAAGGCATCGACGGGTAGCTGGTCTGA
GAGGATGATCAGCCACACTGGGACTGAGACACGGCC
>Zotu810
TAACGCTGGCGGTATGCCTAATACATGCAAGTCGAACGCGCACTTCGGTGCGAGTGGCGGACGGGTGA
GTAACACGTTGGAACCTACCCCGAAGTGGGGAATAACCCATCGAAAGATGGGCTAATACCCCATAGTC
TTATGCTTGTCATAAGTAAAGTTTTTCGCTTCGGGAGGGGCCTGCGCAACATTAGCTAGTTGGTGAGA
TAAAAGCCCACCAAGGCGACGATGTTTAGCTGGATCGAGAGGTTGACCAGCCACACTGGGACTGAGAT
ACGGCC
>Zotu811
GAACGCTAGCGGCAGGCCTAACACATGCAAGTCGAGGGAGGAGCTTTCGGGCTTCGACCGGCGTACGG
GTGCGTAACGCGTATACAATCTGCCTTTCACATAGGGATAGCCCGAAGAAATTTGGATTAATACCTAA
TGGTATATAGGGATGGCATCATTTTTATATTAAAGCTGAGGCGGTGAAAGATGAGTATGCGTTCTATT
AGCTAGTTGGAGAGGTAACGGCTCCCCAAGGCGACGATAGATAGGGGTTCTGAGAGGGATATCCCCCA
CACTGGTACTGAGATACGGAC
>Zotu812
GAACGCTGGCGGCGTGCTTAACACATGCAAGTCGAGCGGAAAGGCCCTTCGGGGTACTCGAGCGGCGA
ACGGGTGAGTAACACGTGAGTAATCTGCCCTTGGCTTCGGGATAGCCACCGGAAACGGTGATTAATAC
CGGATATGACTACTTCCTGCATGGGATGGTAGTGGAAAGTTTTTCGGCCAGGGATGTGCTCGCGGCCT
ATCAGCTTGATGGTGAGGTAATGGCTCACCATGGCTTCGACGGGTAGCCGGCCTGAGAGGGTGACCGG
TCACACTGGGACTGAGACACGGCC
>Zotu813
GAACGCTGGCGGCATGCCTTACACATGCAAGTCGAACGGCAGCACGGAGCTTGCTCTGGTGGCGAGTG
GCGAACGGGTGAGTAATATATCGGAACGTACCCTGGAGTGGGGGATAACGTAGCGAAAGTTACGCTAA
TACCGCATACGATCTAAGGATGAAAGTGGGGGATCGCAAGACCTCATGCTCGTGGAGCGGCCGATATC
TGATTAGCTAGTTGGTAGGGTAAAAGCCTACCAAGGCATCGATCAGTAGCTGGTCTGAGAGGACGACC
AGCCACACTGGAACTGAGACACGGTC
>Zotu814
TAACGCTAGCGGCAGGCATAATACATGCAAGTTGAGCGAGATAAGTTGTAGCAATACGGCTTTGAAAG
CGGCGCACGGGTGCGTAACACGTATGCAACCTACCTTTTACTAAGGAATAGCCTCTCGAAAGAGAGAT
TAATACCTTATAATATGTTTTGAAGGCATCTTCAGAACCTTAAAGCTCCGGCGGTAAAAGATGGGCAT
GCGGTTGATTAGCTAGTTGGTGAGGTAATGGCTCACCAAGGCTTCGATCAATAGGGGGTCTGAGAGGA
TGTCCCCCCACACTGGTACTGAGACACGGAC
>Zotu815
AAACGCTGGCGGCGTGCCTAATGCATGCAAGTCGAACGGGGTCGCTTCGGCGGCTTACGTGGCGGACG
GGTGAGGAACACGTGGGCAATCTGCCGTCGGGTGGGGGGTAGCCGCTGGAAACAGCGGGTAATTCCGC
ATGTGCTTACCGATCTGTGGGGTTGGTAAGGAAAGCCTTCGGGCGCCCGACGAGGAGCCTGCGGCCGA
TTAGCTAGCTGGTGGGGTAACGGCCTACCAGGGCGATGATCGGTAGCTGGTCTGAGAGGACGGCCAGC
CACAGGGGAACTGAGACACGGTC
>Zotu816
GAACGCTGGCGGCGTGGATGAGGCATGCAAGTCGAAAGGAACCCCGCAAGGGGAAATCCTTGGCAGAC
GAGGTAGGAACACGTAGGTACGTACCCCAAAGTCACGGATAACCCGGAGAAATTCGGAATAATACGTG
ATAGTCTCGCGAGAGTAAAGATTTATCGCTTTGGGAACGGCCTGCGTTCTATCAGCTTGTTGGTAAGG
TAACGGCTTACCAAGGCTACGACGGATAGGGGAGGTGAGAGCCTGACCCCCACCGATGGAACTGCGAC
ACGGTC
>Zotu817
GAACGCTGGCGGCGTGCCTAATACATGCAAGTCGAGCGAAGTTTTTCTGGTGCTTGCACTAGAAAAAC
TTAGCGGCGAACGGGTGAGTAACACGTAAAGAACCTGCCTCATAGACTGGGACAACTATTGGAAACGA
TAGCTAATACCGGATAACAGCATTAACTGCATGGTTGATGTTTGAAAGTTGGTTTTGCTAACACTATG
AGATGGCTTTGCGGTGCATTAGCTAGTTGGTGGGGTAAAGGCCTACCAAGGCGACGATGCATAGCCGA

CCTGAGAGGGTGATCGGCCACACTGGGACTGAGACACGGCC
>Zotu818
GAACGCTGGCGGCGTGCCTAATACATGCAAGTCGAGCGAACCTTTAAAAGCTTGCTTTTAAAGGTTAG
CGGCGGACGGGTGAGTAACACGTGGGCAACCTACCTGTAAGACTGGGATAACTTCGGGAAACCGGAGC
TAATACCGGATAATTCTTTTCTACACATGTAGAAAAGCTGAAAGACGGTTTCGGCTGTCACTTACAGA
TGGGCCCGCGGCGCATTAGCTAGTTGGTGAGGTAATGGCTCACCAAGGCAACGATGCGTAGCCGACCT
GAGAGGGTGATCGGCCACACTGGGACTGAGACACGGCC
>Zotu819
GAACGCTGGCGGCGTGCCTAATACATGCAAGTCGAGCGAATGGATTAAGAGCTTGCTCTTATGAAGTT
AGCGGCGGACGGGTGAGTAACACGTGGGTAACCTGCCCATAAGACTGGGATAACTCCGGGAAACCGGG
GCTAATACCGGATAATATTTTGAACCGCATGGTTCGAAATTGAAAGGCGGCTTCGGCTGTCACTTATG
GATGGACCCGCGTCGCATTAGCTAGTTGGTGAGGTAACGGCTCACCAAGGCAACGATGCGTAGCCGAC
CTGAGAGGGTGATCGGCCACACTGGGACTGAGACACGGCC
>Zotu820
GAACGCTAGCGGCAGGCCTAATACATGCAAGTCGGACGGGATCCAGGAGCTTGCTTCTGGTGAGAGTG
GCGCACGGGTGCGTAACACGTATGTAACCTACCTTTGTCAGGGGGATAGCCTCTCGAAAGAGAGATTA
AGACCGCATAAAATCACAATGTGGCATCACAAAATGATCAAATATTTATAGGACGAAGATGGGCATGC
GGAACATTAGCTAGTTGGTAAGGTAACGGCTTACCAAGGCTACGATGTTTAGGGGATCTGAGAGGATG
ACCCCCCACACTGGTACTGAGACACGGAC
>Zotu821
GAACGCTGGCGGCAGGCCTAACACATGCAAGTCGAACGATGTAGCAATACATAGTGGCGCACGGGTGA
GGAACACGTGGGAATCTCCCTTCTAGTACGGGATAACACCGGGAAACTGGTGCTAATACCGTATGACC
CCTCCGGGGGAAAGATTTATCGCTAGAAGATGAGCCCGCGTAAGATTAGCTAGTTGGTGAGGTAATGG
CTCACCAAGGCTACGATCTTTAGCTGGTCTTAGAGGACGATCAGCCACACTGGAACTGAGACACGGTC
>Zotu822
GAACGCTGGCGGCGTGCCTAATACATGCAAGTCGAGCGAACTTGCGGGAGCTTGCTCCCAAAAGTTAG
CGGCGGACGGGTGAGTAACACGTGGGCAACCTGCCTATAAGACTGGGATAACTTCGGGAAACCGGAGC
TAATACCGGATAATTCTTTTCTACACATGTAGAAAAGCTAAAAGACGGTTTACGCTGTCACTTATAGA
TGGGCCCGCGGCGCATTAGCTAGTTGGTGAGGTAACGGCTCACCAAGGCCACGATGCGTAGCCGACCT
GAGAGGGTGATCGGCCACACTGGGACTGAGACACGGCC
>Zotu823
GAACGCTGGCGGCGTGCTTAACACATGCAAGTCGAACGAACTCGACCTTCGGGTCAATGAGTTAGTGG
CGAACGGGTGAGTAACACGTGAGAAACCTACCCTGGAGATGGGAATAACTCGGGGAAACCCGGGCTAA
TACTCGATACCTTCTCCCGGTCGCATGACCGGGAGAAGAAAGCTCAGGCGCTTTAGGATGGTCTCGCG
GCCTATCAGCTAGTTGGTGAGGTAACGGCTCACCAAGGCGACGACGGGTAGCTGGTCTGAGAGGACGA
TCAGCCACACTGGGACTGAGACACGGCC
>Zotu824
GAACGCTGGCGGCGTGCTTAACACATGCAAGTCGAACGGAAAGGCCCCCTTGTGGGGTACTCGAGTGG
CGAACGGGTGAGTAACACGTGAGTAACCTACCCTTGACTTTGGGATAACTTCAGGAAACTGGGGCTAA
TACCGGATAGGAGCTCCTGCTGCATGGTGGGGGTTGGAAAGTTTCGGCGGTTGGGGATGGACTCGCGG
CTTATCAGCTTGTTGGTGGGGTAGTGGCTCACCAAGGCTTTGACGGGTAGCCGGCCTGAGAGGGTGAC
CGGCCACATTGGGACTGAGATACGGCC
>Zotu825
GAACGCTGGCGGCGTGCCTAATACATGCAAGTCGAGCGGACAGATGGGAGCTTGCTCCCTGATGTTAG
CGGCGGACGGGTGAGTAACACGTGGGTAACCTGCCTGTAAGACTGGGATAACTCCGGGAAACCGGGGC
TAATACCGGATGGTTGTTTGAACCGCATGGTTCAGACATAAAAGGTGGCTTCGGCTACCACTTACAGA
TGGACCCGCGGCGCATTAGCTAGTTGGTGAGGTAACGGCTCACCAAGGCAACGATGCGTAGCCGACCT
GAGAGGGTGATCGGCCACACTGGGACTGAGACACGGCC
>Zotu826
GAACGCTGGCGGCATGCCTTACACATGCAAGTCGAACGGCAGCACGGGTGCTTGCACCTGGTGGCGAG
TGGCGAACGGGTGAGTAATACATCGGAACGTACCCGGAAGTGGGGGATAACGTAGCGAAAGTTACGCT
AATACCGCATATTCTGTGAGCAGGAAAGCAGGGGATCGCAAGACCTTGCGCTTTCGGAGCGGCCGATG
TCAGATTAGCTAGTTGGTGAGGTAAAGGCTCACCAAGGCGACGATCTGTAGCGGGTCTGAGAGGATGA
TCCGCCACACTGGAACTGAGACACGGTC

>Zotu827
GAACGCTGACAGAATGCTTAACACATGCAAGTCTTTGGCAAATCTGTGCTTGCACAGCCTAGCCAAGG
CGGACGGGTGAGTAACGCGTAAAGAACTTACCCTGCAGACAGGGATAACAGACGGAAACGACTGATAA
TACCTGATATAATTGCCAGCACGCATGTGCCCGGCAATGAAAAGTAATGCTGCAGGAGAGCTTTGCGT
CCTATTAGCTTGTTGGTGAGGTAACGGCTCACCAAGGCGATGATAGGTAGCCGGCCTGAGAGGGTGAA
CGGCCACAAGGGGACTGAGATACGGCC
>Zotu828
GAACGCTGGCGGCGTGCCTAATACATGCAAGTAGAACGCTGAAGAGAGGAGCTTGCTCTTCTTGGATG
AGTTGCGAACGGGTGAGTAACGCGTAGGTAACCTGCCTGGTAGCGGGGGATAACTATTGGAAACGATA
GCTAATACCGCATAAAATTGATTATTGCATGATAATTAATTGAAAGATGCAATTGCATCACTACCAGA
TGGACCTGCGTTGTATTAGCTAGTTGGTGAGGTAGCGGCTCACCAAGGCGACGATACATAGCCGACCT
GAGAGGGTGATCGGCCACACTGGGACTGAGACACGGCC
>Zotu829
GAACGCTGGCGGCAGGCCTAACACATGCAAGTCGAGCGAGATCTTCGGGTCTAGCGGCGGACGGGTGA
GTAACGCGTGGGAATATGCCTCTTGGTACGGAATAGCCTCGGGAAACTGGGAGTAATACCGTATGTGC
CCTTTGGGGGAAAGATTTATCGCCAAGAGATTAGCCCGCGTTGGATTAGGTAGTTGGTGGGGTAATGG
CCTACCAAGCCGACGATCCATAGCTGGTTTGAGAGGATGATCAGCCACACTGGGACTGAGACACGGCC
>Zotu830
GAACGCTGGCGGCGTGCTTAACACATGCAAGTCGAGCGGTAAGGCCTCTTCGGGGGTACACGAGCGGC
GAACGGGTGAGTAACACGTGGGTGACCTGCCCTCAGCTCTGGGATAAGCCTGGGAAACTGGGTCTAAT
ACCGGATATGACCTCTCATCGCATGGTGGGTGGTGGAAAGTTTTTTCGGCTGGGGATGGGCCCGCGGC
CTATCAGCTTGTTGGTGGGGTGATGGCCTACCAAGGCGACGACGGGTAGCCGGCCTGAGAGGGCGACC
GGCCACACTGGGACTGAGACACGGCC
>Zotu831
GAACGCTGGCGGCAGGCCTAACACATGCAAGTCGAACGAGCTGGGACCTTCGGGTCCCGATGCTAGTG
GCGCACGGGTGAGTAACGCGTGGGAACGTACCCTTTAGTACGGAATAACCCAGGGAAACTTGGGCTAA
TACCGTATGACTCCTTCGGGAGAAAGATTTATCGCTAAAGGATCGGCCCGCGTCTGATTAGGTAGTTG
GTAGGGTAATGGCCTACCAAGCCTACGATCAGTAGCTGGTCTGAGAGGATGATCAGCCACACTGGGAC
TGAGACACGGCC
>Zotu832
GAACGCTAGCGGCAGGCTTAATACATGCAAGTCGAACGGGTGTCAGCAATGGCATTAGTGGCGAACGG
GTGCGTAACGCGTACACAACATGCCTTCAACTGGGGGATAGCGCTGGGAAACTGGCAGTAACCCCCCA
TGTGGTCTTGGGTCGGCATCGACCTATGATTAAAATTACGGTGGTTGAAGATTGGTGTGCGTCCGATT
AGCTAGTTGGTAAGGTAACGGCTTACCAAGGCGACGATCGGTAGGGGGTGTGAGAGCATGACCCCCCA
CACGGGTACTGAGACACGGAC
>Zotu833
GAACGCTGGCGGCGTGCTTAACACATGCAAGTCGAACGATGAAGCCCAGCTTGCTGGGTGGATTAGTG
GCGAACGGGTGAGTAACACGTGAGCAACCTGCCCCTGACTCTGGGATAAGCGCTGGAAACGGCGTCTA
ATACTGGATATGTCCCGTCACCGCATGGTGTGCGGGTGGAAAGATTTTTCGGTTGGGGATGGGCTCGC
GGCCTATCAGCTTGTTGGTGAGGTAATGGCTCACCAAGGCGTCGACGGGTAGCCGGCCTGAGAGGGTG
ACCGGCCACACTGGGACTGAGACACGGCC
>Zotu834
GAACGCTGGCGGTGTGGATAAGCCATGCAAGTCGAACGGTCCGCAAGGACAGTGGCAGACGAGGTAGT
AACGCGCAGGGAGATCCCCCAAAGTCAGGGATAGCCAGCCGAAAGGTTGGGTAATACTTGATGGTCCC
GTAAGGGTAAAGATTTATCGCTTTGGGAGTCACCTGCGTAATATCAGCTAGTTGGTGAGGTAATGGCT
CACCAAGGCTACGACGTTTAGGGGAGCTGAGAGGCTGACCCCCACCGATGGGACTGCGATACGGCC
>Zotu835
GAACGCTAGCGGCAGGCTTAACACATGCAAGTCGAGGGGCAGCGCGGTAGCAATACTGGCGGCGACCG
GCAAACGGGTGCGGAACACGTACAGAACCTACCTTGATCTGGGGCATAGCCCGGAGAAATCCGGATTA
ACTCCCCATAATATACATGAGTGGCATCACTTGAGTATTAAAAATTTATTGGATCGAGATGGCTGTGC
GTCCCATTAGCTAGTTGGTAGGGTAACGGCCTACCAAGGCAACGATGGGTAGCTGGTGTGAGAGCACG
ACCAGCCACACGGGCACTGAGACACGGGC
>Zotu836
GAACGCTGGCGGCAGGCTTAACACATGCAAGTCGAACGGGTGTAGCAATACATCAGTGGCAGACGGGT

GAGTAACACGTGGGAACCTTCCTAGAGGTACGGAACAACGCAGGGAAACTTGCGCTAATACCGTATAC
GCTCTTGCGAGGAAAGATTTATCGCCTTTAGACGGGCCCGCGTAGGATTAGGTAGTTGGTGGGGTAAC
GGCCTACCAAGCCTACGATCCTTAGCTGATCTGAGAGGATGATCAGCCACACTGGAACTGAGACACGG
TC
>Zotu837
GAACGCTGGCGGCGTGCTTAACACATGCAAGTCGAACGGTGAACTCCGCTTGCGGGGGGATCAGTGGC
GAACGGGTGAGTAACACGTGGGCAACCTGCCCTCAGCTCTGGGATAACTCCAAGAAATTGGGGCTAAT
ACCGGATATGACTGCTGACCGCATGGTCTGGTGGTGGAAAGATTTATCGGCTGAGGATGGGCCCGCGG
CCTATCAGCTTGTTGGTGGGGTAGTGGCCTACCAAGGCGACGACGGGTAGCCGGCCTGAGAGGGTGAC
CGGCCACACTGGGACTGAGACACGGCC
>Zotu838
GAACGCTGGCGGCAGGCCTAACACATGCAAGTCGAACGGTAGCACAGAGAGCTTGCTCTTGGGTGACG
AGTGGCGGACGGGTGAGTAATGTCTGGGAAACTGCCCGATGGAGGGGGATAACTACTGGAAACGGTAG
CTAATACCGCATAACGTCTTCGGACCAAAGTGGGGGACCTTCGGGCCTCACACCATCGGATGTGCCCA
GATGGGATTAGCTAGTAGGTGGGGTAACGGCTCACCTAGGCGACGATCCCTAGCTGGTCTGAGAGGAT
GACCAGCCACACTGGAACTGAGACACGGTC
>Zotu839
TAACGCTGGCGGCATGCCTAACACATGCAAGTCGAATGTTGCCGCAAGGTAAACATGGCAGACGGCTG
AGTAACGCGTGGGGACTTACCCCGAAGTCGAGCATAACTCGCCGAAAGGCGAGATAATTCTCGATGTT
GTCGAAAGACTAAAGATTTATCGCTTCGGGAGAGACCTGCGTCCGATTAGCTTGTTGGTGAGGTAACG
GCTCACCAAGGCTTTGATCGGTAGGGGGCCTGAGAGGGTGACCCCCCAGACTGGGACTGAGACACGGC
C
>Zotu841
GAACGCTGGCGGCATGCTTAACACATGCAAGTCGGACGGGAAGTGGTGTTTCCAGTGGCGGACGGGTG
AGTAACGCGTAAGAACCTGCCCTTGGGAGGGGAACAACAGCTGGAAACGGCTGCTAATACCCCGTAGG
CTGAGGAGCAAAAGGAGGAATCCGCCTGAGGAGGGGCTCGCGTCTGATTAGCTAGTTGGTGAGGCAAT
AGCTTACCAAGGCGATGATCAGTAGCTGGTTCGAGAGGATGATCAGCCACACTGGGACTGAGACACGG
CC
>Zotu842
GAACGCTAGCGGCAGGCTTAATACATGCAAGTCGAGGGACGGTAGCAATACTGGACCGGCAAACGGGT
GCGGAACACGTACGCAACCTACCCCAAACAGGTGAATAGCCTTCTGAAAAGGAGATTAATACACCGTA
AAATATATGGATGGCATCATTTGTATATAATAGCTCAGGCGGTTTGGGATGGGCGTGCGCCTGATTAG
GTAGTTGGTAGGGTAACGGCCTACCAAGCCAGCGATCAGTAACTGGTGTGAGAGCACGACCAGTCACA
CGGGCACTGAGACACGGGC
>Zotu843
GAACGCTGGCGGCGTGCTTAACACATGCAAGTCGAACGATGAAGCCTTTCGGGGTGGATTAGTGGCGA
ACGGGTGAGTAACACGTGGGCAATCTGCCCTTCACTCTGGGACAAGCCCTGGAAACGGGGTCTAATAC
CGGATAACACTCTGTCCTGCATGGGACGGGGTTAAAAGCTCCGGCGGTGAAGGATGAGCCCGCGGCCT
ATCAGCTTGTTGGTGGGGTAATGGCCTACCAAGGCGACGACGGGTAGCCGGCCTGAGAGGGCGACCGG
CCACACTGGGACTGAGACACGGCC
>Zotu844
GAACGCTGGCGGCGTGCTTAACACATGCAAGTCGAACGATGACGACCGGGCTTGCCTGGTCTGATTAG
TGGCGAACGGGTGAGTAACACGTGAGTAACCTGCCCTTCACTCTGGGATAACCTCGGGAAATCGGGGC
TAATACCGGATATGAGTTTCCATCGCATGGTGGTTTCTGGAAAGTTTTTCGGTGAAGGATGGACTCGC
GGCCTATCAGTTTGTTGGTGAGGTAATGGCTCACCAAGGCGATGACGGGTAGCCGGCCTGAGAGGGCG
ACCGGCCACACTGGGACTGAGACACGGCC
>Zotu845
GAACGCTGGCGGCATGCCTAACACATGCAAGTCGAACGATGCTTTCGGGCATAGTGGCGCACGGGTGC
GTAACGCGTGGGAATCTGCCCTGGGGTACGGAATAACTCAGAGAAATTTGAGCTAATACCGTATGATG
ACGTAAGTCCAAAGATTTATCGCCCTGGGATGAGCCCGCGTAGGATTAGCTAGTTGGTGGGGTAAAGG
CCTACCAAGGCGACGATCCTTAGCTGGTCTGAGAGGATGATCAGCCACACTGGGACTGAGACACGGCC
>Zotu847
GAACGTTGGCGGCGTGCCTTAAGCATGCAAATCAAGGGGGTCCGATTTATCGGACTACCGGTGGACGG
GTGAGTAATGCGCGGGAATGTACCTCGAAGTGGGATATAGCCAGCCGAAAGGTTGGGTAATCTCCCAT

GTGAACGAAAGTTTAAAGCCGCAAGGCGCTTTGAGATCAGCCCGCGTCCTATCAGCTTGTTGGTGGGG
TAAAAGCCTACCAAGGCGACGACGGGTAGCTGGACTGAGAGGTCGGTCAGCCACAATGGGACTGCGAC
ACGGCC
>Zotu848
GAACGCTGGCGGCGTGCCTAACACATGCAAGTCGAGCGGTAACAGGCGTAGCAATACGTGCTGACGAG
CGGCGAACGGGTGAGCAATGCTTCGGAATCTGCCCAATGGTGGGGAATAACCAGTCGAAAGATTGGCT
AATACCGCATACGCCCTTCGGGGGAAAGCGGGGGTCGCAAGACCTCGCGCCGATGGATGAGCCGAAGT
CGGATTAGCTAGTTGGTAGGGTAACGGCCTACCAAGGCGACGATCCGTAGCTGGTCTGAGAGGACGAC
CAGCCACACTGGGACTGAGACACGGCC
>Zotu849
GAACGCTAGCTACAGGCTTAACACATGCAAGTCGAGGGGCAGCATGTCGGTTGCTTGCAACCGATGAT
GGCGACCGGCGCACGGGTGAGTAACGCGTATCCAACCTGCCTCTTACCAGGGAATAACCCGTCGAAAG
GCGGACTAATGCCCTATGCCCTCCGCGGCAGACATCTAATGCGGAGGAAAGATTCATCGGTAAGTGAT
GGGGATGCGTCTGATTAGCTTGTTGGCGGGGTAACGGCCCACCAAGGCAACGATCAGTAGGGGTTCTG
AGAGGAAGGTCCCCCACATTGGAACTGAGACACGGTC
>Zotu850
GGCAAGAATGTTGAAGAGCTAAGTACTGCTCACATGCACTGTCCTCCACAAAGGACGCTAACCGTGTG
TGTTGAGATTTCAGCACACATTAAAGAACCCCCAGGCGGGCAAAATTAAC
>Zotu851
GAACGCTGGCGGTGTGCTTCACACATGCAAGTCGAACGGGGTAGCAATACCTAGTGGCGGACGGGTGA
GTAACGCGTGGGAATCTGCCCAAGGGTGGGGGATAGCAGACCGAAAGGTCTATTAATACCGCATATGC
CGTGAGGTAAAAGGAGTAATCCGCCCCTGGATGAGCCTGCGTCCGATTAGCTAGTTGGTAGGGTAAAA
GCCTACCAAGGCTACGATCGGTAGCTGGTCTGAGAGGATGATCAGCCACAATGGGACTGAGACACGGC
C
>Zotu852
AAACGCTGGCGGCGTGCCTAACACATGCAAGTCGAGCGAGAAAGGTCTTCGGACTGAGTACAGCGGCG
GACGGGTGAGTAACGCGTAGGAATCTGCCCTTTAGTGAGGAATACCATCGAGAAATCGGTGTTAATAC
TGCATACGTTCCGAAAGGAAGAAAGGTGGCCTCTTTGCTACCGCTAAAGGATGAGCCTGCGTACTATT
AGCTAGTTGGTAGGGTAAAGGCCTACCAAGGCGATGATGGTTAGCCGGCCTGAGAGGGTGTACGGCCA
CATTGGAACTGAGACACGGTC
>Zotu853
GAACGCTGGCGGTATGCTTAACACATGCAAGTCGAACGGGGTTCTTCGGAACCTAGTGGCGGACAGGT
GAGTAACGCGTGAGAACATGCCTTTAGGACTGGGACAACAGATGGAAACGTCTGCTAATACCGGATGT
GCCCTTAGGGGTAAAAGATTTATTGTCTAAAGATTGGCTCGCGTCAGATTAGCTAGTAGGAGTGGTAA
CGGCACCCCTAGGCGACGATCTGTACTTGGTCTGAGAGGATGACCAGGCACACTGGAACTGAGACACG
GTC
>Zotu854
GAACGCTGGCGGCAGGCTTAACACATGCAAGTCGAGCGCCCCGCAAGGGGAGCGGCAGACGGGTGAGT
AACGCGTGGGAATCTACCTTTTGCTACGGAATAACGCAGGGAAACTTGTGCTAATACCGTATGTGCCC
TTTGGGGGAAAGATTTATCGGCAAAGGATGAGCCCGCGTTGGATTAGCTAGTTGGTGGGGTAAAGGCC
TACCAAGGCGACGATCCATAGCTGGTCTGAGAGGATGATCAGCCACATTGGGACTGAGACACGGCC
>Zotu855
GAACGCTGGCGGCGTGCTTAACACATGCAAGTCGAACGATGATGCCCAGCTTGCTGGGTTGATTAGTG
GCGAACGGGTGAGTAACACGTGAGTAACCTGCCCATCACTCTGGGATAACTCCGGGAAACCGGTGCTA
ATACCGGGTATGACTCCGATCCGCATGGGTACGGGGTGGAAAGATTTATCGGTGGTGGATGGACTCGC
GGCCTATCAGCTTGTTGGTGGGGTAATGGCCTACCAAGGCGACGACGGGTAGCCGGCCTGAGAGGGTG
ACCGGCCACACTGGGACTGAGACACGGCC
>Zotu856
GAACGCTGGCGGCATGCTTTACACATGCAAGTCGAACGGTAACAGGCCTTCGGGTGCTGACGAGTGGC
GAACGGGTGAGTAATGCATCGGAACGTACCCAGAGGTGGGGGATAACGCAGCGAAAGCTGTGCTAATA
CCGCATGTGATCTGAGGATGAAAGCGGGGGACCGAGCAGCAATGTTTGGCCTCGCGCCTTTGGAGCGG
CCGATGTCGGATTAGCTAGTTGGTGGGGTAAAGGCCTACCAAGGCGACGATCCGTAGCTGGTCTGAGA
GGACGACCAGCCACACTGGGACTGAGACACGGCC
>Zotu857

GAACGCTAGCGGCAGGCCTAATACATGCAAGTCGAGGGGTAGAGGTTCTTCGGAACCTTGAGACCGGC
GCACGGGTGCGTAACGCGTATGCAACCTACCTTTATCAGGGGGATAGCCCGAAGAAATTCGGATTAAT
ACCGCATAAAATCACAGTATAGCATTATACAATGATCAAACATTTATGGGATGAAGATGGGCATGCGT
GTCATTAGTTAGTTGGCGGGGTAACGGCCCACCAAGACGACGATGACTAGGGGATCTGAGAGGATGAC
CCCCCACACTGGTACTGAGACACGGAC
>Zotu858
GAACGCTGGCGGCGTGCCTAATACATGCAAGTAGAACGCTGAAGACTTTAGCTTGCTAAAGTTGGAAG
AGTTGCGAACGGGTGAGTAACGCGTAGGTAACCTGCCTACTAGCGGGGGATAACTATTGGAAACGATA
GCTAATACCGCATAACAGCATTTAACCCATGTTAGATGCTTGAAAGGAGCAATTGCTTCACTAGTAGA
TGGACCTGCGTTGTATTAGCTAGTTGGTGAGGTAACGGCTCACCAAGGCGACGATACATAGCCGACCT
GAGAGGGTGATCGGCCACACTGGGACTGAGACACGGCC
>Zotu859
GAACACTGGCGGCATGCCTAACACATGCAAGTCGAACGAGATCTTCGGGTCTAGTGGCGCACGGGTGC
GTAACGCGTGGGAATCTGCCCTTTGGTTCGGAATAACAGTTGGAAACGACTGCTAATACCGGATGATG
ACGAAAGTCCAAAGATTTATCGCCAGAAGATGAGCCCGCGTAGGATTAGCTAGTTGGTGTGGTAAAGG
CGCACCAAGGCGACGATCCTTAGCTGGTCTGAGAGGATGATCAGCCACACTGGGACTGAGACACGGCC
>Zotu860
GAACGCTGGAGGCACGCTTAAAACATGCAAGTCGAACGAGAAGCTTTGGTGCTTGCACCGAAGTGGGA
AGTGGCGAACGGGTGAGTAACACATGAGAAACCTGCCCCGAAGATTGGGATAAGCCTTGGAAACGAGG
TCTAATACCGAATACCTTCTAATTATCACATGGTAAATAGAAGAAAGCTCAGGCGCTTTGGGATGGTC
TCGTGGCCTATCAGCTTGTTGGTGAGGTAAAAGCTCACCAAGGCAACGACGGGTAGCTGGTCTGAGAG
GATGTCCAGCCACATTGGAACTGAGATACGGTC
>Zotu861
GAACGCTGGCGGCGTGCCTAACACATGCAAGTCGAGCGAACCCTTCGGGGTGAGCGGCGGACGGGTGA
GTAACGCGTGGGTAACCTGCCCTGTACACACGGATAACATACCGAAAGGTATGCTAATACGAGATAAA
ATGTAATGATCGCATGGTCGATACATCAAAGCTCCGGCGGTACAGGATGGACCCGCGTCTGATTAGCT
AGTTGGTGAGGTAATGGCTCACCAAGGCGACGATCAGTAGCCGACCTGAGAGGGTGATCGGCCACATT
GGAACTGAGACACGGTC
>Zotu862
GAACGCTAGCGGCAGGCCTAACACATGCAAGTCGAGGGGCAGCATAAAGATAGCTTGCTATCTTGGTG
GCGACCGGCGCACGGGTGCGTAACGCGTATGCAACCTACCTTATACTGGGGTATAGCCCGGAGAAATT
CGGATTAACCCCCCATAGTATTATCGGATGGCATCATTTGATAATTAAAGGTTACGGTATAAGATGGG
CATGCGTCCCATTAGCTAGATGGTGAGGTAACGGCTCACCATGGCGACGATGGGTAGGGGGCCTGAGA
GGGTGGTCCCCCACACTGGTACTGAGACACGGAC
>Zotu863
GAATGCTGGCGGCGTGCCTAACACATGCAAGTCGAGCGGTAAGGCCCCTTCGGGGGTACACGAGCGGC
GGACGGCTGAGTAACGCGTGGGAACATGCCCCAAAGTGAGGGATAAGCTCCAGAAATGGAGTCTAATA
CCGCATATGGTCTTCGGATTAAAGGATTTATCCGCTTTGGGAGTGGCCTGCGTACGATTAGATAGTTG
GTGAGGTAATGGCTTACCAAGTCTACGATCGTTAACTGGTCTGAGAGGATGATCAGTCAGACTGGAAC
TGAGACACGGTC
>Zotu864
GAACGCTGGCGGCGTGCTTAACACATGCAAGTCACGGGGGCCCGCAAGGGCAACCGGCGAACGGGTGC
GTAACACGTGAGCAATCTGCCGATTTCAGGGGGATAGCCGGCCCAACGGCCGGGTAATACCGCATACG
CTGGTGGAGGGGAGTCCCTCTATCAGGAAACCTCCGGGGGAAATCGAGGAGCTCGCGGCCTATCAGCT
AGTTGGCGGGGTAACGGCCCACCAAGGCGACGACGGGTAGCTGGTCTGAGAGGATGGCCAGCCACATT
GGGACTGAGAGACGGCC
>Zotu865
GAACGCTGGCGGCGTGCCTAATACATGCAAGTCGAGCGAATCGGAGGGAGCTTGCTCCCAAAGATTAG
CGGCGGACGGGTGAGTAACACGTGGGCAACCTGCCTGTAAGACTGGGATAACTTCGGGAAACCGGAGC
TAATACCGGATAATCCTTTTCCTCTCATGAGGAAAAGCTGAAAGACGGTTTCGGCTGTCACTTACAGA
TGGGCCCGCGGCGCATTAGCTAGTTGGTGAGGTAACGGCTCACCAAGGCAACGATGCGTAGCCGACCT
GAGAGGGTGATCGGCCACACTGGGACTGAGACACGGCC
>Zotu866
GAACGCTGGCGGTATGCTTTACACATGCAAGTCGTACGCAAGTTTACTTGAGTGGCGGACGGGTGAGT

AACACGTGAGAATTCACCTTTAGGTGGGGAATAACTTTTGGAAACGATAGCTAATGCCCCATAGACTC
GCAAGAGAAAAAAGAGTAATCTGCCTAAAGAGAAGCTCGCGGCTGATTAGCTAGTTGGTAAGGTAATG
GCTTACCAAGGCGACGATCAGTAGCTGGTTTGAGAGAACGATCAGCCACACTGGAACTGAGACACGGT
C
>Zotu867
GAACGCTGGCGGCGTGCCTAATACATGCAAGTCGAGCGAACCAATAAGAAGCTTGCTTTTTGTTGGTT
AGCGGCGGACGGGTGAGTAACACGTGGGTAACCTGCCTGTAAGACCGGGATAACTCCGGGAAACCGGT
GCTAATACCGGATAGATTATCTTTCCGCCTGGAGAGATAAGGAAAGATGGCTTTTGCCATCACTTACA
GATGGGCCCGCGGCGCATTAGCTAGTTGGTGAGGTAACGGCTCACCAAGGCGACGATGCGTAGCCGAC
CTGAGAGGGTGATCGGCCACACTGGGACTGAGACACGGCC
>Zotu868
GAACGCTGGCGGCGTGCTTAACACATGCAAGTCGAGCGGTAAGGCCCTTTCGGGGGTACACGAGCGGC
GAACGGGTGAGTAACACGTGAGTAATCTGCCCTCAGCTTTGGGATAAGCACTCGAAAGGGTGTCTAAT
ACCGGATATGACGATCCTCCGCATGGGGGGTTGTGGAAAGTTTTTTCGGCTGGGGATGTGCTCGCGGC
CTATCAGCTTGTTGGTGGGGTAATGGCCTACCAAGGCTTTGACGGGTAGCCGGCCTGAGAGGGTGACC
GGTCACACTGGGACTGAGACACGGCC
>Zotu869
GAACGCTAGCGGCAGGCCTAATACATGCAAGTCGAACGGTGTCTTCGGACATAGTGGCGCACGGGTGC
GTAACACGTATGCAACCTACCTTACATTGGGGGATAGCCTTTCGAAAGGGAGATTAATACCGCATAAG
ACAGTAACTAGGCATCTAGTAGCTGTTAAAGATTTATCGATGTAAGCTGGGCATGCGTCCAATTAGCT
AGTTGGTGAGGTAATGGCTCACCAAGGCAATGATTGGTAGGGGAACTGAGAGGTCAATCCCCCACACT
GGCACTGAGATACGGGC
>Zotu870
GAACGCTGGCGGCAGGCCTAACACATGCAAGTCGAGCGGCAGCGGAAAGTAGCTTGCTACTTTGCCGG
CGAGCGGCGGACGGGTGAGTAATGTCTGGGAAACTGCCTGATGGAGGGGGATAACTACTGGAAACGGT
AGCTAATACCGCATGACCTCGAAAGAGCAAAGTGGGGGACCTTCGGGCCTCACGCCATCGGATGTGCC
CAGATGGGATTAGCTAGTAGGTGAGGTAATGGCTCACCTAGGCGACGATCCCTAGCTGGTCTGAGAGG
ATGACCAGCCACACTGGAACTGAGACACGGTC
>Zotu871
GAACGCTGGCGGCGTGCCTAACACATGCAAGTGGAGCGACGAACCGGGCTTCGGCCCGGGGCAAAGCC
GCGAACGGGTGAGTAACACGTGGGTAACCTGCCCTGATGATTGGGACAACCCGAGGAAACTCGGGCTA
ATACCAAATGTGCCCCAAGACCTTAAGGTCTACGGGGAAAGGAAGCTTCGGCCTCCGCATCGGGATGG
GCCCGCGGCCCATTAGCTTGTTGGTGAGGTAATGGCTCACCAAGGCAACGATGGGTAGCTGGTCTGAG
AGGACGATCAGCCACACTGGGACTGAGACACGGCC
>Zotu872
GAACGCTAGCGGCAGGCTTAACACATGCAAGTCGAGGGGTATAGGTTTTCGGACCTAGAGACCGGCGC
ACGGGTGCGTAACGCGTATGCAATCTACCTTTCACAGAGGGATAGCCCAGAGAAATTTGGATTAATAC
CTCATAGTATAACGACTCGGCATCGAGATGTTATTAAAGTCACAACGGTGAAAGATGAGCATGCGTCC
CATTAGCTAGTTGGTAAGGTAACGGCTTACCAAGGCTACGATGGGTAGGGGTCCTGAGAGGGAGATCC
CCCACACTGGTACTGAGACACGGAC
>Zotu873
GAACGCTGGCGGCGCGCCTAACACATGCAAGTCGAGCGATAAGCCACCTTCGGGTGGTGGAAAGCGGC
GAACGGGTGAGTAACACGTGAGGAACCTGCCCCGGACTCTGGGATAACAGTGGGAAACCGCTGCTAAT
ACCGGATACACCATCTCTACGGCATCGCAGAAATGGGAAAGATTTATCGGTCTGGGATGGCCTCGCGG
CCTATCAGCTAGTTGGTGGGGTAACGGCCTACCAAGGCAACGACGGGTAGCTGGCGTGAGAGCGTGGC
CAGCCACACTGGGACTGAGATACGGCC
>Zotu874
GAACGCTGGCGGCGTGCCTAACACATGCAAGTCGAGCGATTCTCTTCGGAGAAGAGCGGCGGACGGGT
GAGTAACGCGTGGGTAACCTGCCCTGTACACACGGATAACATACCGAAAGGTATGCTAATACGGGATA
ACATATTTTGATGGCATCATCGAAATATCAAAGCTCCGGCGGTACAGGATGGACCCGCGTCTGATTAG
CTAGTTGGTGAGGTAATGGCTCACCAAGGCGACGATCAGTAGCCGACCTGAGAGGGTGATCGGCCACA
TTGGAACTGAGACACGGTC
>Zotu875
TAACGCTGGCGGCGTGCCTAACACATGCAAGTCGAACGGACGTAGCAATACGTTAGTGGCAGACGGGT

GAGTAACACGTAGATAACGTACCCCTCCGCTGGGGATAGCCACTGGAAACGGTGGGTAAAACCGAATA
TTCTTCAATATGGGCATCTATGTTGAAGGAAAACTTTGGTGTGGAGGGATCGGTTTGCGACCTATCAG
CTAGTTGGTGAGGTAATGGCTCACCAAGGCGACGACGGGTAACCGGTCTGAGAGGACGGTCGGTCACA
CTGGCACTGAGATACGGGC
>Zotu876
GAACGCTGGAAATGTGGATACCGGCATGCAAGTCAAGGGGCCGCAAGGCACCGGCGAACGGCTTAGTA
ACACGTGGATACATACCCAGAATTGGAGCACAGCTCGTCGAAAGACGGGGTAATTCTCCATAATACCG
CAAGGTTAAAGGCGCAAGCCGATTCTGGAATGGTCCGCGGTCTATCAGCTAGTTGGTGAGGTAATGGC
TCACCAAGGCTATGACGGATAGGGGGCGTGAGAGCGCGACCCCCAAGAATGGCACTGCGACACGGGC
>Zotu878
GAACACTAGCGGCGTGCCTGAGATATGCAAGTCGAATGTCTTTTTCTCCGCAAGGAGAGAAGGTAATG
GCGGACGGCTGAGTAACACGTGGGGACCTGCCCTAAAGTAGGGAATAGCTCACCGAAAGGTGGGGTAA
TACTCTATATCCAGTTTACTGGAAAGAGCTTAACTGCTCGCTTAAGGATGGACCTGCGTCCTATCAGA
TAGTTGGTGAGGTAAAGGCTTACCAAGTCTATGACGGGTAGCTGGCCTGAGAAGGTGGTCAGCCAGAG
GAGCACTGAGATACGGGC
>Zotu881
GAACGCTGGCGGCGTGCTTAACACATGCAAGTCGAACGGTGAACCTGCTTCGGTGGGGGATCAGTGGC
GAACGGGTGAGTAACACGTGGGCAACCTGCCCCCGGCTCTGGGATAACTCCAAGAAATTGGGGCTAAT
ACCGGATGTGACTGCTGACCGCATGGTCTGGTGGTGGAAAGATTTATCGGCTGGGGATGGGCCCGCGG
CCTATCAGCTTGTTGGTGGGGTGATGGCCTACCAAGGCGACGACGGGTAGCCGGCCTGAGAGGGTGAC
CGGCCACACTGGGACTGAGACACGGCC
>Zotu882
GAACGCTAGCGGTATGCTTAACACATGCAAGTCGTACGGAAGAGCAATCTTTAGTGGCGAACGGGTGA
GTAACGCGTGAGAATTTGCCTTTAGGAAGAGGATAACAATTGGAAACAGTTGCTAAAACTCTATATGC
CGTTTACGGTGAAAAAAGTAATTTGCCTAAAGAGAAGCTCGCGTCTGATTAGCTAGTTGGTGGGGTAA
AGGCCTACCAAGGCGACGATCAGTAGCTGGTCTGAGAGGACGATCAGCCACACTGGAACTGAGACACG
GTC
>Zotu883
GAACGCTGGCGGCGTGCTTAACACATGCAAGTCGAGCGGTAAGGCCCTTCGGGGTACACGAGCGGCGA
ACGGGTGAGTAACACGTGGGTGACCTGCCCTCCACTTCGGGATAAGCCTGGGAAACTGGGTCTAATAC
CGGATAGGACCAAGGGATGCATGTTCCTTGGTGGAAAGTTTTTCGGTGGGGGATGGGCCCGCGGTCT
ATCAGCTTGTTGGTGGGGTGATGGCCTACCAAGGCGACGACGGATAGCCGGCCTGAGAGGGTGATCGG
CCACACTGGGACTGAGACACGGCC
>Zotu884
GAACGCTGGCGGCGTGCCTAACACATGCAAGTCGAGCGAGATCCATCCAGTGGCAACACTGGTGAAGA
TCTAGCGGCGAACGGGTGAGTAACACGTGAGGAACCTGCCCCGAAGACCGGGATAACCCGAGGAAACT
CGAGCTAATACCGGATACCCCCATGAGATCGCATGGTCTTATGAGGAAATGTCTTTTCGCTTCGGGAG
GGCCTCGCGGCCTATCAGCTAGTTGGCGGGGTAACGGCCCACCAAGGCATCGACGGGTAGCTGGTCTG
AGAGGACGATCAGCCACACTGGGACTGAGACACGGCC
>Zotu885
GAACGCTGGCGGCGTGCTTAACACATGCAAGTCGAACGATGATGCCCAGCTTGCTGGGTGGATTAGTG
GCGAACGGGTGAGTAACACGTGAGTAACCTGCCCTTTCCTCTGGGATAACTCCGGGAAACCGGTGCTA
ATACTGGATATGACTCCAACCTGCATGGGTATGGGGTGGAAAGATTTATCGGGATGGGATGGACTCGC
GGCCTATCAGCTTGTTGGTGGGGTAATGGCCTACCAAGGCGACGACGGGTAGCCGGCCTGAGAGGGTG
ACCGGCCACACTGGGACTGAGACACGGCC
>Zotu886
GAACGCTAGCGGCAGGCTTAACACATGCAAGTCGAGGGGTATAGTAGCAATACTAGAGACCGGCGCAC
GGGTGCGTAACGCGTATGCAATCTACCTTTTACAGAGGGATAGCCCAGAGAAATTTGGATTAATACCT
CATAGTATTATAGAATGGCATCATTTTATAATTAAAGTCACAACGGTAAAAGATGAGCATGCGTCCCA
TTAGCTAGTTGGTAAGGTAACGGCTTACCAAGGCAACGATGGGTAGGGGTCCTGAGAGGGAGATCCCC
CACACTGGTACTGAGACACGGAC
>Zotu888
GAACGCTGGCGGCGTGCCTAATACATGCAAGTCGAGCGAAGTTTTTCTGGTGCTTGCACTAGAAAAAC
TTAGCGGCGAACGGGTGAGTAACACGTAAAGAACCTGCCTCATAGACGGGGACAACTATTGGAAACGA

TAGCTAATACCGGATAACAGCATAAATCGCATGATATATGTTTAAAAGTTGGTTTCGGCTAACACTAT
GAGATGGCTTTGCGGTGCATTAGCTAGTTGGTGGGGTAAAGGCCTACCAAGGCGACGATGCATAGCCG
ACCTGAGAGGGTGATCGGCCACACTGGGACTGAGACACGGCC
>Zotu889
GAACGCTGGCGGCGCGCCTAACACATGCAAGTCGAACGGAGCTGAGAGAAGCTTGCTTTTCTTAGCTT
AGTGGCGAACGGGTGAGTAACGCGTGAGTAACCTGCCCTGGAGTGGGGGACAACAGTTGGAAACGACT
GCTAATACCGCATAAGCCCACGATCCGGCATCGGATTGAGGGAAAAGGATTTATTCGCTTCAGGATGG
ACTCGCGTCCAATTAGCTAGTTGGTGAGGTAACGGCCCACCAAGGCGACGATTGGTAGCCGGACTGAG
AGGTTGAACGGCCACATTGGGACTGAGACACGGCC
>Zotu890
GAACGCTGGCGGCGTGCCTAAGACATGCAAGTCGAGTGGGTTTAGACCCACGGCGAACGGGTGAGTAA
TACCTAGGAACTTTCTTTAAGTGGAGCATAACCAGTCGAAAGATTGGATAATACTTCATGTGGCAGTA
ATGCTAAAGTCGCAAGACGCTTAAAGTGAGGCCTGGGCACCATCAGCTAGTTGGTAAGGTAATGGCTT
ACCAAGGCTATGACGGTTAGGGGTCCCGAGAGGGATAACCCCCACACTGGAACTGCGACACGGTC
>Zotu891
CAACGCTGGCGGCGTGCCTAATGCATGCAAGTCGAGCGGGGTGCATCTTCGGGTGGACTTACGCGGCG
GACGGGTGCGGAGCACGTGGGCAACCTGCCCTGGGGTGGGGGATAGCCGTGGGAAACCGCGGGTAATT
CCGCGTACGCTCGATTGCCGGAGGGGTGATCGAGGAAAGGCTCGTGAGAGCCGCCCTGGGAGGGGGCT
GCGGCTGATTAGCTGGTTGGTGGGGTAACGGCCTACCAAGGCGATGATCGGTAGCTGGTCTGAGAGGA
CGGCCAGCCACACGGGGACTGAGACACGGCC
>Zotu892
GAACGCTGGCGGCGTGCCTTAAGCATGCAAGTCGAACGGTCCTCGCAAGAGGGCAGTGGCGGACGGGT
GAGTAACGCGTGAGAATCTACCTTAAAGTGGGACATAGCTAGCCGAAAGGTTGGGTAATTTCCCATAG
TCCCCGATTTAATCGGGGTAAAGCCGCAAGGCGCTTTAAGATGAGCTCGCGTCCTATCAGCTTGTTGG
TGGGGTAAAAGCCTACCAAGGCGATGACGGGTAGCTGGTCTGAGAGGATGGTCAGCCACAATGGAACT
GCGACACGGTC
>Zotu893
GAACGCTAGCGGCAGGCTTAACACATGCAAGTCGAGGGGTAGAGTTCTTCGGAGCTTGAGACCGGCGC
ACGGGTGCGTAACGCGTATGCAATCTACCTTTTACAGAGGGATAGCCCAGAGAAATTTGGATTAATAC
CTCATAGTATAATGACTCGGCATCGAGATATTATTAAAGTCACAACGGTAAAAGATGAGCATGCGTCA
CATTAGCTAGTTGGTAAGGTAACGGCTTACCAAGGCAACGATGGGTAGGGGTCCTGAGAGGGAGATCC
CCCACACTGGTACTGAGACACGGAC
>Zotu894
GAACGCTAGCGGGAGGCCTAACACATGCAAGCTGAGCGGTATTTGTCTTTCGGGACAGAGAGAGCGGC
GTACGGGTGCGGAACACGTGTGCAACCTGCCTTTATCTGGGGGATAGCCTTTCGAAAGGGAGATTAAT
ACCCCATAATATAATGAGTGGCATCACTGATTATTGAAAACTCCGGTGGATAGAGATGGGCACGCGCA
AGATTAGATAGTTGGTGAGGTAACGGCTCACCAAGTCGATGATCTTTAGGGGGCCTGAGAGGGTGATC
CCCCACACTGGTACTGAGACACGGAC
>Zotu895
GAACGCTAGCGGCAGGCCTAATACATGCAAGTCGGACGGGATTTGGGAGCTTGCTCCCGATGAGAGTG
GCGCACGGGTGCGTAACACGTATGTAACCTACCCATATCAGGGGGATAGCCTCTCGAAAGAGAGATTA
AGACCGCATAACATAACGGAATGGCATCATTTTGTTATCAAATATTTATAGGATATGGATGGGCATGC
GGAACATTAGCTAGTTGGTGTGGTAACGGCATACCAAGGCTACGATGTTTAGGGGATCTGAGAGGATG
GCCCCCCACACTGGTACTGAGACACGGAC
>Zotu896
GAACGCTGGCGGCGTGCCTAACACATGCAAGTCGAGCGAGAGACTTCGGTCTCTAGCGGCGGACGGGT
GAGTAACACGTGGGTAACCTGCCTCGTAGAGGGGGATAGCCTTCCGAAAGGAAGATTAATACCGCATA
ACGTAGTTTAGTCGCATGACTAGATTATCAAAGGAGTAATCCGCTATGAGATGGACCCGCGGCGCATT
AGCTAGTTGGTGAGGTAACGGCTCACCAAGGCGACGATGCGTAGCCGACCTGAGAGGGTGATCGGCCA
CATTGGAACTGAGATACGGTC
>Zotu897
GAACGCTGGCGGCGTGCTTAACACATGCAAGTCGAACGGAAAGGCCCTGCTTGCAGGGTACTCGAGTG
GCGAACGGGTGAGTAACACGTGGGTGATCTGCCCTGCACTTTGGGATAAGCCTGGGAAACTGGGTCTA
ATACCGGATAGGACCACATCGTAGGGGTGTGGTGGAAAGATTTTTCGGTGCAGGATGAGCCCGCGGCC

TATCAGCTTGTTGGTGGGGTAATGGCCTACCAAGGCGGCGACGGGTAGCCGGCCTGAGAGGGTGTACG
GCCACATTGGGACTGAGACACGGCC
>Zotu898
GAACGCTGGCGGCGTGCTTAACACATGCAAGTCGAACGCTGAAGCACCAGCTTGCTGGTGTGGATGAG
TGGCGAACGGGTGAGTAATACGTGAGTAACCTGCCCTTGACTCTGGGATAAGCCCGGGAAACTGGGTC
TAATACTGGATGCTACATGTCACCGCATGGTGGTGTGTGGAAAGGGTTTACTGGTCTTGGATGGGCTC
ACGGCCTATCAGCTTGTTGGTGAGGTAATGGCTCACCAAGGCGACGACGGGTAGCCGGCCTGAGAGGG
TGACCGGCCACACTGGGACTGAGACACGGCC
>Zotu899
GAACGCTGGCGGCGTGCTTAACACATGCAAGTCGAACGATGAAGCCCAGCTTGCTGGGTGGATTAGTG
GCGAACGGGTGAGTAACACGTGAGTAACCTGCCCTTGACTCTGGGATAAGCACTGGAAACGGTGTCTA
ATACCGGATATTTCAGCTGGCCGCATGGTCTGGTTGTGGAAAGAATTTTGGTCAAGGATGGACTCGCG
GCCTATCAGGTTGTTGGTGAGGTAATGGCTCACCAAGCCTACGACGGGTAGCCGGCCTGAGAGGGTGA
CCGGCCACACTGGGACTGAGACACGGCC
>Zotu900
GAACGCTGGCGGCGTGCCTAATACATGCAAGTCGAGCGAAGTTTGATTGGTGCTTGCACCGATTAAAC
TTAGCGGCGAACGGGTGAGTAACACGTAAAGAACCTGCCTCATAGACTGGGACAACTACTGGAAACGG
TAGCTAATACCGGATAATATATTTTACTGCATGGTAAAATATTGAAAGTCGGTCATGCTGACACTATG
AGATGGCTTTGCGGTGCATTAGCTAGTTGGTAGGGTAAAGGCCTACCAAGGCGACGATGCATAGCCGA
CCTGAGAGGGTGATCGGCCACACTGGGACTGAGACACGGCC
>Zotu901
GAACGCTGGCGGCGTGCCTAACACATGCAAGTCGAACGAAGCTTTGAGGAGCTTGCTTTTTAAAGCTT
AGTGGCGGACGGGTGAGTAACGCGTGAGCAACCTGCCTCTCAGAGGGGGATAACGTTTTGAAAAGAAC
GCTAATACCGCATAATATATCGGAACCGCATGATTCTGATATCAAAGGAGCAATCCGCTGAGAGATGG
GCTCGCGTCCGATTAGTTAGTTGGTGAGGTAACGGCTCACCAAGACTACGATCGGTAGCCGGACTGAG
AGGTTGATCGGCCACATTGGGACTGAGACACGGCC
>Zotu902
GAACGCTGGCGGCGTGCCTAATACATGCAAGTGGAACGCATTGATATCACCGGAGCTTGCTCCACCGA
TATAAATGAGTCGCGAACGGGTGAGTAACGCGTAGGTAACCTGCCTGATAGCGGGGGATAACTATTGG
AAACGATAGCTAATACCGCATGAGAGTGTTTAACACATGTTAGAGACTTAAAAGATACAATTGTATCA
CTATCAGATGGACCTGCGTTGTATTAGCTAGTTGGTAGGGTAGCGGCCTACCAAGGCAACGATACATA
GCCGACCTGAGAGGGTGATCGGCCACACTGGGACTGAGACACGGCC
>Zotu903
GAACGCTGGCGGCGTGCCTAATACATGCAAGTCGAGCGAACAGATAAAGAGCTTGCTCCTTTGACGTT
AGCGGCGGACGGGTGAGTAACACGTGGGTAACCTACCTATAAGACTGGAATAACTCCGGGAAACCGGG
GCTAATGCCGGATAATACACAGAACCGCATGGTTCTGTGATGAAAGACGGTTTTGCTGTCACTTATAG
ATGGACCCGCGGCGTATTAGCTAGTTGGTAAGGTAACGGCTTACCAAGGCAACGATACGTAGCCGACC
TGAGAGGGTGATCGGCCACACTGGAACTGAGACACGGTC
>Zotu904
GAACGCTGGCGGCGTGCTTAACACATGCAAGTCACGGGGGCCCGCAAGGGTAACCGGCGAACGGGTGC
GTAACACGTGAGCAATCTGCCGTCCACTGGGGGATAGCCGGCCCAACGGCCGGGTAATACCGCATACG
CTTGCCGGGTGGCATCACCTGGCAAGGAAACCTCCGGGGGTGGACGAGGAGCTCGCGGCCTATCAGCT
AGTTGGTGGGGTCACGGCCCACCAAGGCGATGACGGGTAGCTGGTCTGAGAGGATGGCCAGCCACATT
GGGACTGAGATACGGCC
>Zotu906
GAACGCTGGCGGCGTGCCTAATACATGCAAGTCGAGCGAATGGATTAAGAGCTTGCTCTTATGAAGTT
AGCGGCGGACGGGTGAGTAACACGTGGGTAACCTGCCCATAAGACTGGGATAACTCCGGGAAACCGGG
GCTAATACCTGATAACATTTTGAACCGCATGGTTCGAAATTGAAAGGCGGCTTCGGCTGTCACTTATG
GATGGACCCGCGTCGCATTAGCTAGTTGGTGAGGTAACGGCTCACCAAGGCAACGATGCGTAGCCGAC
CTGAGAGGGTGATCGGCCACACTGGGACTGAGACACGGCC
>Zotu907
GAACGCTAGCTACAGGCTTAACACATGCAAGTCGAGGGGAAACGGCATTTGGTGCTTGCACCATTTGG
ACGTCGACCGGCGCACGGGTGAGTAACGCGTATCCAACCTGCCCATAAGTAGGGTATAACCCGCAGAA
ATGCGGACTAATCCCCTATGTTGTTCAAAGACGGCATCAGATTTGAACCAAAGGCTTGCCGCTTATGG

ATGGGGATGCGTCCGATTAGCTTGTTGGTGAGGTAACGGCTCACCAAGGCTTCGATCGGTAGGGGTTC
TGAGAGGAAGGCCCCCCACATAGGAACTGAGACACGGTC
>Zotu908
GAACGCTGGCTACAGGCTTAACACATGCAAGTCGAGGGGAAACGACGGGGAAGCTTGCTTCCCCGGGC
GTCGACCGGCGCACGGGTGAGTAACGCGTATCCAACCTGCCTCTGACTGAGGGATAACCCGTCGAAAG
TCGGCCTAATACCTCATGGCATCGTCTGCGGGCATCCAACGACGATTAAAGATTTCATCGGTCAGGGA
TGGGGATGCGTCTGATTAGCTTGTTGGCGGGGTAACGGCCCACCAAGGCGACGATCAGTAGGGGTTCT
GAGAGGAAGGTCCCCCACATTGGAACTGAGACACGGTC
>Zotu909
GAACGCTGGCGGCGTGCTTAACACATGCAAGTCGAACGCTGAAGCTCCAGCTTGCTGGGGTGGATGAG
TGGCGAACGGGTGAGTAATACGTGAGTAACCTGCCCTTGACTCTGGGATAAGCCTGGGAAACCGGGTC
TAATACTGGATACGACGTCCTACCGCATGGTGGGGCGTGGAAAGGGTTTTACTGGTTTTGGATGGGCT
CACGGCCTATCAGCTTGTTGGTGGGGTAATGGCCTACCAAGGCGACGACGGGTAGCCGGCCTGAGAGG
GTGACCGGCCACACTGGGACTGAGACACGGCC
>Zotu910
GAACGCTGGCGGCATGCCTTACACATGCAAGTCGAACGGCAGCACGGGAGCAATCCTGGTGGCGAGTG
GCGAACGGGTGAGTAATACATCGGAACGTGTCCTGGAGTGGGGGATAGCCCGGCGAAAGCCGGATTAA
TACCGCATACGCTCTATGGAGGAAAGCGGGGGATCTTCGGACCTCGCGCTCAAGGGGCGGCCGATGGC
GGATTAGCTAGTTGGTGGGGTAAAGGCCTACCAAGGCGACGATCCGTAGCTGGTCTGAGAGGACGACC
AGCCACACTGGGACTGAGACACGGCC
>Zotu911
GAACGCTGGCGGCATGCCTAATACATGCAAGTCGAACGCTGAAGCACTCTGCTTGCAGAGTGCGGATG
AGTGGCGAACGGGTGAGTAACACGTGGGAAATCTGCCTATAAGAGGGGGATAACATTCGGAAACGGAT
GCTAATACCGCATATATTCTTGGACCGCCTGGTCCTTGAAAGAAAGACGGCTTTGCTGTCGCTTATAG
ATGATCCCGCGGCGTATTAGTTAGTTGGTGGGGTAAAGGCCTACCAAGACAATGATACGTAGCCGACC
TGAGAGGGTGATCGGCCACACTGGGACTGAGACACGGCC
>Zotu912
GAACGCTGGCGGCGCGCTTAACACATGCAAGTCGAGCGAGAACCAGGCCTTCGGGCCTGGGGAAAGCG
GCGAACGGGTGAGTAACACGTGAGTAACCTGCCCTCGGCTCCGGGATAGCCCGTGGAAACGCGGATTA
ATACCGGATAGCCTTTTGAGCTCTCGGGCTCGTCAGAAAAGGTAGCTTCGGCCTCTGGCCGAGGAGGG
GCTCGCGGCGGATTAGCTTGTTGGCGGGGTAACGGCCCACCAAGGCGACGATCCGTAGCTGGTCTGAG
AGGACGATCAGCCACACTGGGACTGAGACACGGCC
>Zotu913
GAACGCTAGCGGCGAGCCTAACACATGCAAGTCGAGCGGGGAGCAATCCCAGCGGCGAACGGGTGAGT
AACACGTAGGTGACGTGTCCTTTGGTGGGGAACAACCGTGGGAAACCATGGCTAATTCCGCATATGTC
TTTCGGGATGAAAGCCTTTGGGCGCCAGAGGAGCGGCCTGCGGCCGATTATTTAGTCCTAGTTGGTAG
GGTAATGGCCTACCAAGGGTACGATCGGTAGCTGGTCTGAGAGGATGATCAGCCACACTGGAACTGAG
ACACGGTC
>Zotu914
GAACGCTGGCTGCGTGCTTAACACATGCAAGTCGAACGCTGAAGCCGACAGCTTGCTGTTGGTGGATG
AGTGGCGAACGGGTGAGTAACACGTGAGTAACCTGCCCCTGATTTCGGGATAAGCCTGGGAAACTGGG
TCTAATACCGGATATGACCAATCTTCGCATGAGGGTTGGTGGAAAGTTTTTCGATCGGGGATGGGCTC
GCGGCCTATCAGCTTGTTGGTGGGGTAATGGCCTACCAAGGCGACGACGGGTAGCCGGCCTGAGAGGG
CGACCGGCCACACTGGGACTGAGACACGGCC
>Zotu915
GAACGCTGGCGGCGTGCTTAACACATGCAAGTCGAACGGTGAACCCGCTTCGGTGGGGGATCAGTGGC
GAACGGGTGAGTAACACGTGGGCAACCTGCCCCCGGCTCTGGGATAACTCCAAGAAATTGGGGCTAAT
ACCGGATATGACCGCTGACCGCATGGTCTGGTGGTGGAAAGATTTATCGGCTGGGGATGGGCCCGCGG
CCTATCAGCTTGTTGGTGGGGTAGTGGCCTACCAAGGCGACGACGGGTAGCCGGCCTGAGAGGGTGAC
CGGCCACACTGGGACTGAGACACGGCC
>Zotu916
GAACGCTGGCGGCGTGCCTAATACATGCAAGTCGAGCGGCAGCGCGAATAGTTTACTATTTGGCGGCG
AGCGGCGGACGGCTGAGTAACGCGTAGGAATCTGCCCCAAAGTGAGGAATAACTCCGGGAAACCGGAG
CTAATACCGCATGTGGTCTTCGGATTAAAGCCTTCGGGCGCTTTGGGAGGAGCCTGCGTTCGATTAGC

TTGTTGGTGAGGTAATGGCTCACCAAGGCTACGATCGATAGCTGGTCTGAGAGGATGATCAGCCAGAC
TGGAACTGAGATACGGTC
>Zotu917
GAACGCTGGCGGCAGGCTTAACACATGCAAGTCGAGCGGGCCCTTCGGGGTCAGCGGCGGACGGGTTA
GTAACGCGTGGGAACGTGCCTCTTGCTCTGGAATAGCCCCGGGAAACTGGGGGTAATACCGGATGTGC
CCTGAGGGGGAAAGGTTTACCGGCAAGAGATCGGCCCGCGTCTGATTAGGTAGTTGGTGGGGTAACGG
CCCACCAAGCCGACGATCGGTAGCTGGTTTGAGAGGATGATCAGCAACACTGGGACTGAGACACGGCC
>Zotu918
GAACGCTGGCGGCGTGCTTAACACATGCAAGTCGAACGATGAAGCTCTAGCTTGCTGGGGTGGATTAG
TGGCGAACGGGTGAGTAACACGTGAGTAACCTGCCCTTTACTTCGGGATAAGCTTGGGAAACTGGGTC
TAATACCGGATATTCTGCGTGATCGGATGGTTGTGTAGGAAAGATTTTTTGGTAAGGGATGGGCTCGC
GGCCTATCAGTTTGTTGGTGGGGTGATGGCCTACCAAGACGACGACGGGTAGCCGGCCTGAGAGGGCG
ACCGGCCACACTGGGACTGAGACACGGCC
>Zotu919
GAATGTTGGCGGCGTGCTTGAGATATGCAAGTCGAACGATGTCCCTGCCGCAAGGTAGGGATAAAGTG
GCGGACGGCTGAGTAACACGTAGATATCTACCTCAAAGCGAATGATAGCTCATCGAAAGATGGGGTAA
CCATTCATGTGTCACATACCGCAAGGTATTTGATAAAGGGCATAACTGCTCACTTTAAGATGAGTCTG
CGGCCTATCAGGTAGTTGGTAAGGTAATGGCTTACCAAGCCTATGACGGGTAGCTGGCCTGAGAGGGT
GGTCAGCCAGAGGGGCACTGAGATACGGGC
>Zotu920
GAACGCTGGCGGCGTGCTTAACACATGCAAGTGGTGCGACGAACCAGAGCTTGCTCTGGGGCCAAGCC
GCGAACGGGTGAGTAACACGTGAGTAATCTGCCCCGATGACCGGGACAACCCGAGGAAACTCGGGCTA
ATACCGGATGTGGTGCACGGATTTAAGTCTGTGTACTAAAGGAAGCTTCGGCCTCCGCATTGGGAGGA
GCTCGCGGCCCATTAGCTTGTTGGTGGGGTAACGGCCTACCAAGGCAACGATGGGTAGCTGGTCTGAG
AGGACGATCAGCCACACTGGGACTGAGACACGGCC
>Zotu921
GAACGCTGGCGGCGTGCTTAACACATGCAAGTCGAACGGAAAGGCCCCTGCTTGCAGGGGTACTCGAG
TGGCGAACGGGTGAGTAACACGTGGGTGATCTGCCTTGCACTCTGGGATAAGCTTGGGAAACTGGGTC
TAATACCGGATAGGAACCGTCTTTAGTGTGGTGGTTGGAAAGTTTTTTTTCGGTGCAAGATGAGCTCG
CGGCCTATCAGCTTGTTGGTGGGGTAATGGCCTACCAAGGCGTCGACGGGTAGCCGGCCTGAGAGGGT
GTACGGCCACATTGGGACTGAGATACGGCC
>Zotu922
CAACGCTGGCGGCGTGCCTAACACATGCAAGTCGCACGAGAAAGGGGCTTCGGCCCTGAGTACAGTGG
CGCACGGGTGAGTAACACGTGACTTATCTACCCTCAAGTGGGGAATAACCTAGAGAAATCTGGGCTAA
TACCGCATAACACTTACGAGTCAAAGCAGCAATGCGCTTGGGGAGGAGGTCGCGGCCGATTAGCTAGT
TGGCGGGGTAATGGCCCACCAAGGCAATGATCGGTATCCGGCCTGAGAGGGCGCACGGACACACTGGA
ACTGAAACACGGTC
>Zotu923
GAACGCTGGCGGCGCGCCTAAAACATGCAAGTCGAACGAGAAGCCCGGGCTTGCCCGGGTGGACAGTG
GCGAACGGGTGAGTAACACGTGAGGAACCTGCCCCGAAGACCGGGACAACACCGGGAAACCGGTGCTA
ATACCGGATACCTTCGCCGGATCGCATGGTCTGGTGAAGAAATGGATTCCGCTTCGGGATGGCCTCGC
GGCCTATCAGCTTGTTGGTGAGGTAACGGCTCACCAAGGCAACGACGGGTAGCTGGTCTGAGAGGATG
TCCAGCCACACTGGGACTGAGACACGGCC
>Zotu924
GAACGCTGGCGGCGTGCCTAATACATGCAAGTCGAACGCTTTGTCTTTAACTGATCTGACGAGCTTGC
TCTGATTTGATTTTATCTGACAAAGAGTGGCGAACGGGTGAGTAACACGTGGGTAACCTACCTCTTAG
CAGGGGATAACATTTGGAAACAAGTGCTAATACCGTATAATACCAACAACCGCATGGTTGTTGGTTGA
AAGATGGTTCTGCTATCACTAAGAGATGGACCCGCGGTGCATTAGCTAGTTGGTAAGGTAACGGCTTA
CCAAGGCAATGATGCATAGCCGAGTTGAGAGACTGATCGGCCACAATGGGACTGAGACACGGCC
>Zotu925
GAACGCTGGCGGCACGCCTAATACATGCAAGTCGAGCGCGTGAAGCTTTCCGAAGCCTTCGGGCGGAC
GAAAGTGGATCGAGCGGCGGACGGGTGAGTAACACGTAGGCAACCTGCCTGTAAGACCGGGATAACTC
CGGGAAACCGGGGCTAATACCGGATAGGATCTCTTTTCGCATGAGAAGGGATTGAAAGGATGGCCGCA
AGGCTATCGCTTACAGATGGGCCTGCGGCGCATTAGCTGGTTGGTGAGGTAACGGCTCACCAAGGCGA

CGATGCGTAGCCGGCCTGAGAGGGTGACCGGCCACACTGGGACTGAGACACGGCC
>Zotu926
GAACGCTGGCGGCGTGCTTAACACATGCAAGTCGAGCGGAAAGGCCACTTCGGTGGTACTCGAGCGGC
GAACGGGTGAGTAACACGTGAGTAATCTGCCCCTGGCTTTGGGATAGCCACCGGAAACGGTGATTAAT
ACCGGATATGACATGGACTCGCATGGGTTTGTGTGGAAAGTTTTTCGGCCAGGGATGTGCTCGCGGCC
TATCAGCTTGATGGTGAGGTAATGGCTCACCATGGCTTCGACGGGTAGCCGGCCTGAGAGGGTGACCG
GTCACACTGGGACTGAGACACGGCC
>Zotu927
GAACGCTGGTAACGTGGATACCGGCATGCAAGTCAAGGGGCCGCAAGGCACCGGCGAACGGCTTAGTA
ACACGTAGATACATACCGTAAAGTGGGGCACAGCTCGTCGAAAGACGGGGTAATTCTCCATAGTTCTC
GCAAGAGTAAAGGCGCAAGCCGCTTTATGAATGGTCTGCGGTCTATCAGCTAGTTGGTGAGGTAATGG
CTCACCAAGGCTATGACGGATAGGGGGCGTGAGAGCGCGACCCCCAAGAATGGCACTGCGATACGGGC
>Zotu928
GAACGCTGGCGGCGTGCTTAACACATGCAAGTCGAACGGTGAACCAGAGCTTGCTCTGGGGATCAGTG
GCGAACGGGTGAGTAACACGTGGGCAACCTGCCCCAGACTCTGGGATAACCTCGGGAAACCGATGCTA
ATACCGGATACGACCACTTCGGACATCCGACGGTGGTGGAAAGTTTTTCGGTCTGGGATGGGCCCGCG
GCCTATCAGCTAGTTGGTGGGGTAATGGCCTACCAAGGCGACGACGGGTAGCCGGCCTGAGAGGGCGA
CCGGCCACACTGGAACTGAGACACGGTC
>Zotu929
GAACGCTGGCGGCGTGCTTAACACATGCAAGTCGAACGATGAAGCCCTTCGGGGTGGATTAGTGGCGA
ACGGGTGAGTAACACGTGGGCAATCTGCCCTGCACTCTGGGACAAGCCCTGGAAACGGGGTCTAATAC
CGGATGATATCCCCTCTCGCATGGGAGGGGGTTGAAAGCTCCGGCGGTGCAGGATGAGCCCGCGGCCT
ATCAGCTAGTTGGTGAGGTAGAAGCTCACCAAGGCGACGACGGGTAGCCGGCCTGAGAGGGCGACCGG
CCACACTGGGACTGAGACACGGCC
>Zotu930
GAACGCTGGCGGCGTGCCTAATACATGCAAGTCGAGCGGTAAGGCCACTTCGGTGGTACACGAGCGGC
GGACGGCTGAGTAACACGTAGGAACGTACCCCAAACTGAGGGATAAGCACCAGAAATGGTGTCTAATA
CCGCATGTGATCTTCGGATTAAAGCTTCGGCGGTTTGGGAACGGCCTGCGCATGATTAGCTTGTTGGT
GAGGTAAAAGCTCACCAAGGCGACGATCATTAGCTGGTCTGAGAGGATGATCAGCCAGACTGGGACTG
AGAACGGCC
>Zotu931
GAACGCTGGCGGCGTGCCTAATACATGCAAGTCGAGCGAATGGATTGAGAGCTTGCTCTCAAGAAGTT
AGCGGCGGACGGGTGAGTAACACGTGGGTAACCTGCCCATAAGACTGGGATAACTCCGGGAAACCGGG
GCTAATACCGGATAACACTTTGAACCGCATGGTTCGAAATTGAAAGGCGGCTTCGGCTGTCACTTATG
GATGGACCCGCGTCGCATTAGCTAGTTGGTGAGGTAACGGCTCACCAAGGCAACGATGCGTAGCCGAC
CTGAGAGGGTGATCGGCCACACTGGGACTGAGACACGGCC
>Zotu932
GAACGCTGGCGGCGTGCCTAATACATGCAAGTCGAGCGAATCTTTGGGAGCTTGCTCCTAAAGGTTAG
CGGCGGACGGGTGAGTAACACGTGGGCAACCTGCCTGTAAGACTGGGATAACTTCGGGAAACCGGAGC
TAATACCGGATAATCCTTTTCTTCTCATGAAGAAAAGCTGAAAGATGGCGCAAGCTATCACTTACAGA
TGGGCCCGCGGCGCATTAGCTAGTTGGTGAGGTAATGGCTCACCAAGGCGACGATGCGTAGCCGACCT
GAGAGGGTGATCGGCCACACTGGGACTGAGACACGGCC
>Zotu933
GAACGCTGGCGGCGTGCTTAACACATGCAAGTCGAACGGTGACGGAGAGCTTGCTCTCCTGATCAGTG
GCGAACGGGTGAGTAACACGTGGGCAACCTGCCCCTGACTTCGGGATAACGCTTCGAAAGAGGTGCTA
ATACCGGATACGAGCCTTCTAGGCATCTAGAGGGTTGGAAAGTTTTTCGGTCAGGGATGGGCCCGCGG
CCTATCAGCTTGTTGGTGAGGTAATAGCTCACCAAGGCGACGACGGGTAGCCGGCCTGAGAGGGCGAC
CGGCCACACTGGGACTGAGACACGGCC
>Zotu934
GAACGCTGGCGGCATGCCTAACACATGCAAGTCGAACGAGACCTTCGGGTCTAGTGGCGCACGGGTGC
GTAACGCGTGGGAACCTGCCTTTAGGTTCGGGATAACAGTTAGAAATGACTGCTAATACCGGATGATG
TCTTCGGACCAAAGATTTATCGCCTTTAGATGGGCCCGCGTTGGATTAGCTAGTTGGTGGGGTAAAGG
CCTACCAAGGCGACGATCCATAGCTGGTCTGAGAGGATGATCAGCCACACTGGGACTGAGACACGGCC
>Zotu935

GAACGCTGGCGGCAGGCCTAACACATGCAAGTCGAACGCCGTAGCAATACGGAGTGGCAGACGGGTGA
GTAACGCGTGGGAACATACCCTTCGGTTCGGAATAACTCAGGGAAACTTGAGCTAATACCGGATACGT
CCGCAAGGAGAAAGATTTATCGCCGAGGGATTGGCCCGCGTCTGATTAGCTTGTTGGTGAGGTAATGG
CTCACCAAGGCGACGATCAGTAGCTGGTCTGAGAGGATGACCAGCCACATTGGGACTGAGACACGGCC
>Zotu936
GAACGCTGGCGGCGTGCTTAACACATGCAAGTCGAACGGAAAGGCCAGTGCTTGCACTGGTACTCGAG
TGGCGAACGGGTGAGTAACACGTGGGTGATCTGCCCTGCACTTCGGGATAAGCCTGGGAAACTGGGTC
TAATACCGGATAGGACCACATTTTGGATGGTGTGGTGGAAAGTTTTTCGGTGTGGGATGAGCTCGCGG
CCTATCAGCTTGTTGGTGGGGTAATGGCCTACCAAGGCGTCGACGGGTAGCCGGCCTGAGAGGGTGTA
CGGCCACATTGGGACTGAGATACGGCC
>Zotu937
GAACGCTGGCGGCATGCTTAACACATGCAAGTCGCACGGGAACCGAGGGCTTGCTCTCGGGGAGAGTG
GCGCACGGGTGAGTAACACGTAGATAACGTTCCATTTGGTCTGGGATAACCTGCCGAAAGGCGGGCTA
ATACCGGATACGTTTCTTTGAGATTCGTCTTGGAGAAAGAAAGCAGGGCAACTTGCGCCAGTTGATCG
GTCTGCGTCCCATCAGCTAGTTGGTGAGGTAACGGCTCACCAAGGCGACGACGGGTAGCTGGTCTGAG
AGGATGATCAGCCACACTGGGACTGAGACACGGCC
>Zotu938
GAACGCTGGCGGCGTGCCTAATACATGCAAGTCGAGCGAACAGACGAGAGTGCTTGCACTCTCTGACG
TTAGCGGCGGACGGGTGAGTAACACGTGGGTAACCTACCTATAAGACTGGGATAACTTCGGGAAACCG
GAGCTAATACCGGATAATATTTAGCTTCGCATGAAGCAATAGTGAAAGACGGTTCTGCTGTCACTTAT
AGATGGACCCGCGGTGTATTAGCTAGTTGGTGAGGTAACGGCTCACCAAGGCAACGATACATAGCCGA
CCTGAGAGGGTGATCGGCCACACTGGGACTGAGACACGGCC
>Zotu939
TAACGCTAGCGGCAGGCCTAATACATGCAAGTCGAACGGTAACACGGGTAGCAATACCTGGTGACGAG
TGGCGCACGGGTGCGTAACGCGTATGTAATCTACCTCTAACTGGGGAATAGCCTTCCGAAAGGAAGAT
TAATACCGCATAATATAAAGGAGAGACATCTCCCCTTTATCAAAACTCAGGTGGTTAGAGATGAACAT
GCGTGACATTAGCTAGTTGGTAAGGTAACGGCTTACCAAGGCTACGATGTCTAGGGGGTCTGAGAGGA
TGATCCCCCACACTGGTACTGAGACACGGAC
>Zotu940
GAACGCTGGCGGCATGCTTAACACATGCAAGTCGGACGGGAAGTGGTGTCTCCAGTGGCGGACGGGTG
AGTAACGCGTAAGAACCTGCCCTTGGGAGGGGAACAACAACTGGAAACGGTTGCTAATACCCCGTAGG
CTGAGGAGCAAAAGGAGGAATCCGCCCGAGGAGGGGCTTGCGTCTGATTAGCTAGTTGGTGAGGTAAT
AGCTTACCAAGGCGATGATCAGTAGCTGGTCCGAGAGGATGATCAGCCACACTGGGACTGAGACACGG
CC
>Zotu941
GAACGCTGGCGGCGTGCCTAACACATGCAAGTCGAGCGGTAACAGGTGTAGCAATACATGCTGACGAG
CGGCGAACGGGTGAGTAATGCTTCGGAATCTACCTTATGGTGGGGAATAACTAGCCGAAAGGTTAGCT
AATACCGCATACGTCCTACGGGAGAAAGCAGGGGATCGCAAGACCTTGCGCCAAAAGATGAGCCGAAG
TCGGATTAGCTAGTTGGTAGGGTAACGGCCTACCAAGGCGACGATCCGTAGCTGGTCTGAGAGGACGA
CCAGCCACACTGGGACTGAGACACGGCC
>Zotu942
GAACGCTGGCGGCGTGCCTAACACATGCAAGTCGAACGGGGAGTCTATTTCGATAGGCTTCCAGTGGC
GAACGGGTGAGTAACACGTTGGTGACCTGCCCCGAAGAGGGGGACAACTCCTCGAAAGAGGAGCTAAT
ACCCCGGATGTCTGAGTGGTTAGAAGACTCAGACTAAAGTTTTTCGGAATGCTTTGGGAGGGGCCTGC
GGGCTATCAGCTAGTTGGTGAGGTAAGGGCTCACCAAGGCGAAGACGGCTAGGGGGCGTGAGAGCGTG
ACCCCCCACAAGGGAACTGAGAAACGGTC
>Zotu943
GAACGCTGGCGGCGTGCCTAATACATGCAAGTAGAACGCTGAAGAGAGGAGCTTGCTCTTCTTGGATG
AGTTGCGAACGGGTGAGTAACGCGTAGGTAACCTGCCTGGTAGCGGGGGATAACTATTGGAAACGATA
GCTAATACCGCATAATAGTAGATGTTGCATGACATTTGCTTAAAAGGTGCAAATGCATCACTACCAGA
TGGACCTGCGTTGTATTAGCTAGTTGGTGGGGTAACGGCTCACCAAGGCGACGATACATAGCCGACCT
GAGAGGGTGATCGGCCACACTGGGACTGAGACACGGCC
>Zotu944
GAACGCTAGCGGCAGGCCTTACACATGCAAGTCGAGGGGTAACAGGAGAGAGCTTGCTCTTTCGCTGA

CGACCGGCGCACGGGTGCGTAACACGTATGCAACCTACCTCTTACTGGGGAATAGCCCAGAGAAATTT
GGATTAACACCCCATAGTATTATTCTTCCGCATGGTTGAATGATTAAAGGTTACGGTAAGAGATGGGC
ATGCGCCCCATTAGCTAGTTGGTGAGGTAACGGCTCACCAAGGCTACGATGGGTAGGGGGTCTGAGAG
GACTATCCCCCACACTGGTACTGAGACACGGAC
>Zotu945
GAACGCTGGCGGAATGCTTTACACATGCAAGTCGAACGGCAGCACGGGTGCTTGCACCTGGTGGCGAG
TGGCGAACGGGTGAGTAACGCATCGGAACGTGCCTTTTAGTGGGGGATAACTATCCGAAAGGATAGCT
AATACCGCATACGCCCTGAGGGGGAAAGGGGGGGATCGCAAGACCTCTCGCTAAAAGAGCGGCCGATG
TCCGATTAGCTAGTTGGTGAGGTAAAGGCTCACCAAGGCGACGATCGGTAGCTGGTCTGAGAGGATGA
TCAGCCACACTGGAACTGAGACACGGTC
>Zotu946
GAACGCTGGCGGCGTGCTTAACACATGCAAGTCGAGCGGTAAGGCCTTTCGGGGTACACGAGCGGCGA
ACGGGTGAGTAACACGTGAGTAATCTGCCCTTCACTTCGGGATAAGCACCGGAAACGGTGTCTAATAC
CGGATATGAACCTCTTTCGCATGTTGGGGGTTGGAAAGTTCTGGCGGTGGAGGATGTGCTCGCGGCCT
ATCAGCTTGTTGGTGAGGTAATGGCTCACCAAGGCTTCGACGGGTAGCCGGCCTGAGAGGGTGACCGG
CCACACTGGGACTGAGACACGGCC
>Zotu947
GAACGCTGGCGGCATGCCTTACACATGCAAGTCGAACGGCAGCGGGCCCTTCGGGGTGCCGGCGAGTG
GCGAACGGGTGAGTAATGCATCGGAACGTACCCGGGAGAGGGGGATAACCGGCCGAAAGGCTGGCTAA
TACCGCATGAACTCGGAAGAGCAAAGCGGGGGACCTTCGGGCCTCGCGCTCTCGGAGCGGCCGATGTC
CGATTAGGCTAGTTGGTGGGGTAAAGGCCCACCAAGGCGACGATCGGTAGCGGGTCTGAGAGGACGAT
CCGCCACACTGGGACTGAGACACGGCC
>Zotu948
GAACGCTAGCGGCAGGCCTAATACATGCAAGTCGAACGGTGTCTTCGGACATAGTGGCGCACGGGTGC
GTAACACGTATGCAACCTACCTTACATTGGGGGATAGCCTTTCGAAAGGGAGATTAATACCGCATAAG
ACAGTAGCTGGGCATCCAGCAGCTGTTAAAGATTTATCGATGTAAGCTGGGCATGCGTCCAATTAGCT
AGTTGGTGAGGTAATGGCTCACCAAGGCAATGATTGGTAGGGGAACTGAGAGGTCAATCCCCCACACT
GGCACTGAGATACGGGC
>Zotu949
GAACGCTGGCGGCGTGCCTAACACATGCAAGTCGAACGGGACTATCAAGTGATGTTTTCGGACTGAAC
TTTTTAGTTTAGTGGCGGACGGGTGAGTAACGCGTGGACAACCTGCCTCTCACTGGGGGATAGCAGCT
GGAAACGGCTGGTAATACCGCATGCGTTCAATGTGCCGCATGACACATTGAAGAAAGATTTATCGGTG
AGAGATGGGTCCGCGTCTGATTAGCCAGTTGGCGGGGTAACGGCCCACCAAAGCGACGATCAGTAGCC
GGCCTGAGAGGGCGGACGGCCACATTGGGACTGAGACACGGCC
>Zotu950
GAACGCTAGCGGCAGGCTTAATACATGCAAGTCGAGGGGCAACAGGGGTAGCAATACCTGCTGGCGAC
CGGCAAACGGGTGCGGAACACGTACAGAACCTTCCTTTAAGCGGGGGATAGCCCATAGAAATGTGGAT
TAATACCCCATAGTAATGTTGACTGGCATCAGTTGATATTTAAAGATTTATCACTTAAAGATGGCTGT
GCGGCTGATTAGGTAGTTGGTCGGGGTAACGGCCCACCAAGCCAACGATCAGTAACTGGTGTGAGAGC
ACGACCAGTCACACGGGCACTGAGACACGGGC
>Zotu951
GAACGCTGGCGGCGTGCTTAACACATGCAAGTCGAACGGATCGGCTCTGACTTTTTTGTTGGGGTTTG
GTTAGTGGCGAACGGGTGAGTAATACGTGAGTAACCTGCCCTCTTCTCTGGGATAAGCCTTGGAAACG
GGGTCTAATACTGGATATTCTGCTTTCATCGCATGGTGGGGGTTGGAAAGGGTTTTCTGGTGGGGGAT
GGGCTCACGGCCTATCAGCTTGTTGGTGGGGTGATGGCCTACCAAGGCTTCGACGGGTAGCCGGCCTG
AGAGGGTGACCGGCCACATTGGGACTGAGATACGGCC
>Zotu952
GAACGCTGGCGGCAGGCCTAACACATGCAAGTCGAACGGAGTTATATTGTAGCTTGCTATAGTATAAC
TTAGTGGCAGACGGGTGAGTAATGTATAGGAATCTACCTAGTAGTACGGAATAATTGTTGGAAACGGC
AACTAATACCGTATACGCCCTACGGGGGAAAAATTTATTGCTATTAGATGAGCCTTATTAGATTAGCT
AGTTGGTGGAGTAATAGCCTACCAAGGCAATGATCTATAGCTGATCTGAGAGGATGATCAGCCACACT
GGAACTGAGATACGGTC
>Zotu953
GAACGCTAGCGGCAGGCTTAATACATGCAAGTCGAGGGGCAGCAGCTTTATAGCAATATAGAGGCTGG

CGACCGGCAAACGGGTGCGGAACACGTACACAACCTTCCTTGAATAGGGGGATAGCCCATAGAAATGT
GGATTAATACCCCGTAATATAGCGGTGTGGCATCACACTGTTATTATAGTTTCGACGATTCGAGATGG
GTGTGCGGCTGATTAGGTAGTTGGCGGGGTAAAGGCCCACCAAGCCTTCGATCAGTAGCTGATGTGAG
AGCATGATCAGCCACACGGGCACTGAGACACGGGC
>Zotu955
GAACGCTGGCGGCATGCCTAACACATGCAAGTCGAACGGCAGCACAGTAAAGATTTCGGTCTTTATGG
GTGGCGAGTGGCGGACGGGTGAGTAATGCGTAGGAATCTGTCTTGGAGCGTGGGATAACCCGAGGAAA
CTCGGGCTAATACCACATGATATCGAGAGATCAAAAGCAGGGGACCGAAAGGCCTTGCGCTCTGGGGT
GAGCCTACGTCGGATTAGCTAGTTGGTGGGGTAAAAGCCTACCAAGGCGACGATCCGTAGCTGGTCTG
AGAGGATGACCAGCCACACTGGGACTGAGACACGGCC
>Zotu956
GAACGCTAGCGGCAGGCCTAATACATGCAAGTCGAACGGTGATATTTATATCATAGTGGCGCACGGGT
GCGTAACACGTATGCAACCTACCTATTATTGGGGGATAGCCTTTGGAAACGGAGATTAATACCGCATA
ATACGATTGACTGGCATCAGTTGATTGTTAAAGCTTTGGCGATAATAGATGGGCATGCGTCCAATTAG
TTAGTTGGTTAGGTAACGGCTGACCAAGACGATGATTGGTAGGGGATCTGAGAGGATAATCCCCCACA
CTGGCACTGAGATACGGGC
>Zotu957
GAACGCTGGCGGCGTGCCTAACACATGCAAGTCGAACGAGGCCCAAGCAGTGGCAACACTGCGGAAGG
CCTAGTGGCGAACGGGTGAGTAACACGTGAGGAACCTGCCCCGAAGACCGGGATAACCCCCTGAAAAG
GGAGCTAATACCGGATGCCCCCACCGGACCGCATGGTCTGGTGAGGAAATGGATTCCGCTTCGAGAGG
GCCTCGCGGCCTATCAGCTTGTTGGTGAGGTAACGGCTCACCAAGGCATCGACGGGTAGCTGGTCTGA
GAGGACGATCAGCCACACTGGGACTGAGACACGGCC
>Zotu958
GAACGCTGGCGGCGTGCCTAACACATGCAAGTCGAGCGAACCCTTCGGGGTGAGCGGCGGACGGGTGA
GTAACGCGTGGGTAACCTGCCCTGTACACACGGATAACATACCGAAAGGTATGCTAATACGAGATAAT
ATGTAATGATCACATGGTCGATACATCAAAGCTCCGGCGGTACAGGATGGACCCGCGTCTGATTAGCT
AGTTGGTAAGGTAACGGCTTACCAAGGCGACGATCAGTAGCCGACCTGAGAGGGTGATCGGCCACATT
GGAACTGAGACACGGTC
>Zotu959
GAACGCTAGCGGCAGGCTTAACACATGCAAGTCGAGGGGTATAGTTCTTCGGAACTAGAGACCGGCGC
ACGGGTGCGTAACGCGTATGCAATCTACCTTTTACAGAGGGATAGCCCAGAGAAATTTGGATTAATAC
CTCATAGTATCTTCGATCCGCATGGATTGATTATTAAAGTCACAACGGTAAAAGATGAGCATGCGTCC
CATTAGCTTGTTGGTAAGGTAACGGCTTACCAAGGCAACGATGGGTAGGGGTCCTGAGAGGGAGATCC
CCCACACTGGTACTGAGACACGGAC
>Zotu960
GAACGCTGGCGGCGTGCCTAATACATGCAAGTAGAACGCTGAAGAGAGGAGCTTGCTCTTCTTGGATG
AGTTGCGAACGGGTGAGTAACGCGTAGGTAACCTGCCTGGTAGCGGGGGATAACTATTGGAAACGATA
GCTAATACCGCATAAAATGGATTATCGCATGATAATCGATTGAAAGGTGCAAATGCATCACTACCAGA
TGGACCTGCGTTGTATTAGCTAGTTGGTGGGGTAACGGCTCACCAAGGCAACGATACATAGCCGACCT
GAGAGGGTGATCGGCCACACTGGGACTGAGACACGGCC
>Zotu961
CAACGCTGGCGGCGCGCTTAACACATGCAAGTCGAACGAGAAAGGGTAGCAATACCTGAGTAAAGTGG
CGTACGGGTGAGTAACACGTGGATAATCTACCTCTTGGCGGGGAATACCTCTGGGAAACCGGAGACAA
TACCGCATAAGCTCGAGAGAGGAAACCCGCAAGGGGCCGAGAGAGGAGTCCGCGGCCGATTAGCTAGT
TGGTGAGGTAAAGGCTCACCAAGGCGAAGATCGGTAGCCGGCCTGAGAGGGCACACGGCCACACTGGC
ACTGAGACACGGGC
>Zotu962
GAACGTTGGCGGCGTGCCTTAAGCATGCAAATCAAAGGGGGCTCGCAAGAGTCAACCGGTGGACGGGT
GAGTAATACATGGGAATCTTCCTCGAAGTTGGACATAGCTAGCCGAAAGGTTAGGTAATTTCCAATGT
GACCGCAAGGTTAAAGCCGCAAGGCGCTTTGAGATGAGCCTGTGTCCTATCAGCTTGTTGGTGGGGTA
ATGGCCTACCAAGGCGACGACGGGTAGCTGGACTGAGAGGTCGGTCAGCCACAATGGGACTGCGACAC
GGCC
>Zotu963
GAACGCTGGCGGCAGGCCTAACACATGCAAGTCGAACGCTGTAGCAATACAGAGTGGCAGACGGGTGA

GTAACACGTGGGAATCTTCCTATCGGTACGGAATAGCTCAGGGAAACTTGGGGTAATACCGCATACGC
CCTTCGGGGGAAAGATTTATCGCCGATAGATGAGCCCGCGTCTGATTAGCTAGTTGGTGAGGTAATGG
CTCACCAAGGCGACGATCAGTAGCTGGTCTGAGAGGATGACCAGCCACACTGGGACTGAGACACGGCC
>Zotu964
GAACGCTGGCGGCGTGCCTAACACATGCAAGTCGAACGGAGTTACTTTAGTGAAGTTTTCGGATGGAT
CTGATTTAACTTAGTGGCGGACGGGTGAGTAATGCGTGAGCAACCTGCCCTTCAGAGGGGGACAACAG
TTGGAAACGACTGCTAATACCGCATAATGTATTCGGAAGGCATCTTCTGGATACCAAAGGAGAAATCC
GCTGATGGATGGGCTCACGTCTGATTAGCTAGTTGGTGAGGTAACGGCTCACCAAGGCTGCGATCAGT
AGCCGGACTGAGAGGTTGAACGGCCACATTGGGACTGAGATACGGCC
>Zotu965
GAACGCTGGCGGCGTGCCTAATACATGCAAGTCGAGCGGACTTAAAAAGCTTGCTTTTTAAGTTAGCG
GCGGACGGGTGAGTAACACGTGGGCAACCTGCCTGTAAGACTGGGATAACTTCGGGAAACCGGAGCTA
ATACCGGATAATCCTTTCCTACACATGTAGGGAAGTCTGAAAGACGGTTTACGCTGTCGCTTACAGAT
GGGCCCGCGGCGCATTAGCTGGTTGGTGAGGTAACGGCTCACCAAGGCGACGATGCGTAGCCGACCTG
AGAGGGTGATCGGCCACACTGGGACTGAGACACGGCC
>Zotu966
AAACGCTGGCGGCGTGCCTAAAACATGCAAGTCGAACGGGCAGTCTTCGGATTGTCAGTGGCAGACGG
CTGAGTAACGCGTGGGTGACGTACCTGTTGGTGGGGAATAACTGCCCGAAAGGGTGGCTAATTCCGCA
TACGTCCTTCAGTTCAGTTTTCTGAAGGAGAAAGCCGCAAGGCGCCGATGGAGCGGCCCGCGTTCGAT
TAGCTGGTTGGTGGGGTAACGGCCTACCAAGGCGACGATCGATAGCTGGTCTCAGAGGATGATCAGCC
AGACTGGGACTGAGACACGGCC
>Zotu968
GAACGCTAGCTACAGGCTTAACACATGCAAGTCGAGGGGAAACGGCATTAAGTGCTTGCACTTTTTGG
ACGTCGACCGGCGCACGGGTGAGTAACGCGTATCCAACCTTCCCATGACTAAGGGATAACCCGCCGAA
AGGCAGACTAATACCTTATGGTCTTCACTGACGGCATCAGATGTGAAGTAAAGATTTATCGGTTATGG
ATGGGGATGCGTCTGATTAGCTTGTTGGCGGGGTAACGGCCCACCAAGGCAACGATCAGTAGGGGTTC
TGAGAGGAAGGTCCCCCACATTGGAACTGAGACACGGTC
>Zotu969
GAACGCTGGCGGCAGGCCTAACACATGCAAGTCGAGCGGATGACGGGAGCTTGCTCCTTGATTCAGCG
GCGGACGGGTGAGTAATGCCTAGGAATCTGCCTGGTAGTGGGGGACAACGTTTCGAAAGGAACGCTAA
TACCGCATACGTCCTACGGGAGAAAGCAGGGGACCTTCGGGCCTTGCGCTATCAGATGAGCCTAGGTC
GGATTAGCTAGTTGGTGAGGTAATGGCTCACCAAGGCGACGATCCGTAACTGGTCTGAGAGGATGATC
AGTCACACTGGAACTGAGACACGGTC
>Zotu971
GAACGCTGGCGGCATGCCTTACACATGCAAGTCGAACGGCAGCGCGGACTTCGGTCTGGCGGCGAGTG
GCGAACGGGTGAGTAATACATCGGAACGTACCCTGTTGTGGGGGATAACTAGTCGAAAGATTAGCTAA
TACCGCATACGACCTGAGGGTGAAAGTGGGGGACCGCAAGGCCTCACGCAGCAGGAGCGGCCGATGTC
TGATTAGCTAGTTGGTGGGGTAAAGGCCCACCAAGGCGACGATCAGTAGCTGGTCTGAGAGGACGATC
AGCCACACTGGGACTGAGACACGGCC
>Zotu972
AAACGCTGGCGGTGTGCTTTATGCATGCAAGTCGAACGGGTTCCGCAAGGAATCAGTGGCAGACGGGT
GAGCAACACGTAAATGACCTACCCTAAAGTGGGGAACAACTCTTAGAAATGAGAGCTAATACCGCATG
TGATTCTGGTTTTTAATTATCAGAATTAAAGCCGCAAGGCGCTTTTGGAGGGGTTTGCGTCCGATTAG
ATAGTTGGTGAGGTAACGGCCCACCAAGTCGACGATCGGTAGCTGGTTTGAGAGGACGACCAGCCACA
CGGGGACTGAGACACGGCC
>Zotu973
GAACGCTGGCGGCGTGCCTAATACATGCAAGTCGAACGAGAGCGACCGGTGCTTGCACTGGTCAATCT
AGTGGCGAACGGGTGAGTAACACGTGGGTAACCTGCCCATCAGAGGGGGATAACATTCGGAAACGGAT
GCTAAAACCGCATAGGTCCTTTAACCGCATGGTTGAAGGAGGAAAAGAGGCGCAAGCTTCTGCTGATG
GATGGACCCGCGGTGCATTAGCTAGTTGGTGAGGTAACGGCTCACCAAGGCCGTGATGCATAGCCGAC
CTGAGAGGGTGATCGGCCACATTGGGACTGAGACACGGCC
>Zotu974
GAACGCTGGCGGCGTGCTTAACACATGCAAGTCGAACGATGACGACAGGGCTTGCCTTGTCTGATTAG
TGGCGAACGGGTGAGTAACACGTGAGTAACCTGCCCCCCTCTTCGGGATAACCTCGGGAAATCGGGGC

TAATACCGGATATGACTTCGCACCGCATGGTGTGTTGTGGAAAGTTTTTCGGTGGGGGATGGACTCGC
GGCCTATCAGTTTGTTGGTGAGGTAATGGCTCACCAAGGCGATGACGGGTAGCCGGCCTGAGAGGGCG
ACCGGCCACACTGGGACTGAGACACGGCC
>Zotu975
CAACGCTGGCGGCGTGCCTAATGCATGCAAGTCGAACGGGGTCATCTTCGGATGGCTTACGTGGCGGA
CGGGTGAGGAACACGTGGGTAACCTGCCCACTGGTGGGGGCTAGCCTTCGGAAACGGAGGGTAATTCC
GCATGAACTCGGATCCCGCGTGGGGGATGTGAGGAAAGCCGAGAGGCGCCAGTGGAGGGGCCCGCGTC
CGATTAGTTAGTTGGTGGGGTAACGGCTCACCAAGGCGATGATCGGTAGCTGGTTTGAGAGGATGATC
AGCCACACGGGGACTGAGACACGGCC
>Zotu976
GAACGCTGGCGGCGTGCTTAACACATGCAAGTCGAGCGAGGCCCATCTTTCGGGGTGGTGCCCTAGCG
GCGAACGGGTGAGTAACACGTGGGCAACCTGCCCTCAGCTCTGGGATAACTCCAAGAAATTGGTGCTA
ATACCGGATATGACTACTGGCCGCATGGTCTGGTGGTGGAAAGATTTATCGGCTGAGGATGGGCCCGC
GGCCTATCAGCTTGTTGGTGGGGTGATGGCCCACCAAGGCGACGACGGGTAGCCGGCCTGAGAGGGTG
ACCGGCCACACTGGGACTGAGACACGGCC
>Zotu977
GAACGCTGGCGGCGTGCCTAATACATGCAAGTCGAGCGGACCGAACGAGAGCTTGCTCTTGTTCGGTC
AGCGGCGGACGGGTGAGTAACACGTGGGCAACCTGCCCGCAAGACCGGGATAACTCCGGGAAACCGGA
GCTAATACCGGATAACACCAAAGACCGCATGGTCTTTGGTTGAAAGGCGGCTTCGGCTGCCACTTGCG
GATGGGCCCGCGGCGCATTAGCTAGTTGGTGAGGTAACGGCTCACCAAGGCGACGATGCGTAGCCGGC
CTGAGAGGGTGACCGGCCACACTGGGACTGAGACACGGCC
>Zotu978
GAACGCTGGCGGCGTGCTTAACACATGCAAGTCTTAGGGGATTTATTCTTTCGGGAATAAGTTCACTG
GCGCACGGGTGCGTAACGCGTAAGTAATCTGCCCTAGAGACTGGCACAACTCGTCGAAAGACGGGCTA
ATTCCAGATGGTACTGAAAGGTCAAAGGTTTCGACCGCTCTAGGATGAGCTTGCGTCTGATTAGCTAG
TTGGTGAGGTAATGGCTCACCAAGGCTACGATCAGTAGCCGGTCTGAGAGGATGATCGGCCACATTGG
AACTGAGACACGGTC
>Zotu979
GAACGCTAGCGATAGGCTTAACACATGCAAGTCGAGGGGCAGCATTATTTTAGCTTGCTAAGATAGAT
GGCGACCGGCGCACGGGTGCGTAACGCGTATGCAACCTGCCTGTAATTAGGGAATAACCCGGTGAAAG
TCGGACTAATACCCTATATTCTTCTTTCTCCGCATGGAGGAGGACGGAAAGATTTATTGATTACAGAT
GGGCATGCGTCCCATTAGCTAGTTGGTAAGGTAACGGCTTACCAAGGCAACGATGGGTAGGGGGACTG
AGAGGTTGACCCCCCACACTGGTACTGAGACACGGAC
>Zotu980
AAACGCTGGCGGCGTGCATGAGACATGCAAGTCGAACGAACGAGCTTGCTCGTTAGTGGCGAACGGCT
GAGTAACGCGTAGGTGACCTGCCCCGAAGTGGGGGATAACTGCCCGAAAGGGTGGCTAATACCGCATG
TGGCGACGTGTTCATTCACGTCATCAAAGCCGCGAGGCGCTTCGGGAGGGGCCTGCGTCCGATTAGCT
CGTTGGTGGGGTAACGGCTCACCAAGGCGACGATCGGTAGGTGGTCTGAGAGGACGATCACCCAGACT
GGGACTGAGACACGGCC
>Zotu981
GAACGCTGGCGGCGTGCTTAACACATGCAAGTCGAACGGTGACCCAGGGCTTGCCCTGGTGATCAGTG
GCGAACGGGTGAGTAACACGTGGGCAACCTGCCCCTGACTTCGGGATAACTCCTCGAAAGAGGAGCTA
ATACCGGATACGACCACTTCGGACATCCGATGGTGGTGGAAAGTTTTTCGGTCAGGGATGGGCCCGCG
GCCTATCAGCTAGTTGGTGGGGTAATGGCCTACCAAGGCGACGACGGGTAGCCGGCCTGAGAGGGCGA
CCGGCCACACTGGGACTGAGACACGGCC
>Zotu982
GAACGCTGGCGGCGTGCTTAACACATGCAAGTTGAACGGTGAAGCCCTTCGGGGTGGATCAGTGGCGA
ACGGGTGAGTAACACGTGAGCAACCTACCCTTGACTCTGGGATAACTCCGGGAAACCGGTGCTAATAC
CGGATACGAGCATCGCAGGCATCTGCTGGTGCTGGAAAGAATTTTGGTCAGGGATGGGCTCGCGGCCT
ATCAGCTTGTTGGTGAGGTAGTGGCTCACCAAGGCGACGACGGGTAGCCGGCCTGAGAGGGCGACCGG
CCACACTGGGACTGAGACACGGCC
>Zotu983
GAACGCTGGCGGCGTGCCTAATACATGCAAGTCGAACGCTTCCTACCGGTGCTTGCACCGGGAAAGAA
GAGTGGCGGACGGGTGAGTAACACGTGGGTAACCTACCTACAAGCGGGGGATAACATTCGGAAACGGA

TGCTAATACCGCATAGATCTTTTTGTCTCCTGACAGAAAGAAGAAAGACGGTTTCGGCTGTCACTTGT
AGATGGACCCGCGGCGCATTAGTTAGTTGGTGAGGTAATGGCTCACCAAGACGATGATGCGTAGCCGA
CCTGAGAGGGTGATCGGCCACACTGGGACTGAGACACGGCC
>Zotu984
GAACGCTGGCGGCGTGCTTAACACATGCAAGTCGAACGATGAAGCCCAGCTTGCTGGGTGGATTAGTG
GCGAACGGGTGAGTAACACGTGAGTAACCTGCCCTTAACTCTGGGATAAGCCTGGGAAACTGGGTCTA
ATACCGGATAGGAGCGCCTACCGCATGGTGGGTGTTGGAAAGATTTATCGGTTTTGGATGGACTCGCG
GCCTATCAGCTTGTTGGTGAGGTAATGGCTCACCAAGGCGACGACGGGTAGCCGGCCTGAGAGGGTGA
CCGGCCACACTGGGACTGAGACACGGCC
>Zotu985
GAACGCTGGCGGCGTGCTTAACACATGCAAGTCGAGCGGTAAGGCCCTTCGGGGTACACGAGCGGCGA
ACGGGTGAGTAACACGTGGGTGACCTGCCCTCCACTCTGGGATAAGCCTGGGAAACCGGGTCTAATAC
CGGATAGGACTCCTGGATGCATGTCCGGGGGTGGAAAGTTTTTCGGTGGGGGATGGGCCCGCGGTCT
ATCAGCTTGTTGGTGGGGTGATGGCCTACCAAGGCGACGACGGATAGCCGGCCTGAGAGGGTGATCGG
CCACACTGGGACTGAGACACGGCC
>Zotu986
CGCAAGCCTTGGGGAACTGTGCAGCTGGTTTGATCGGGAACGCGAGTACAGCCCAGGTAGCTCAGGCT
AGTGTCATGACCGACTGTCCAGCACGAGAACAGCTCAACGGCATCTTCTTCCTTCGCCGCCCCACAAT
ATACGCGCGTAACCGGCCGCGCACTTCAAATAGCATAAATTCCTATCACAATA
>Zotu987
GAACGCTGGCGGCGTGCTTAACACATGCAAGTCGAACGGTAAGGCTCCTTCGGGAGTACACGAGTGGC
GAACGGGTGAGTAACACGTGAGCAACCTGCCCTTGACTTTGGGATACCAGCTGGAAACAGCTGCTAAT
ACCGGATATGACCCCTTTACTCATGTTTTGGGGTGGAAAGCTCCGGCGGTCAGGGATGGGCTCGCGGC
CTATCAGCTTGTTGGTGAGGTAACGGCTCACCAAGGCTTCGACGGGTAGCCGGCCTGAGAGGGCGACC
GGCCACACTGGGACTGAGATACGGCC
>Zotu988
GAACGCTGGCGGCGTGCCTAATACATGCAAGTCGAGCGAACTGATTAGAAGCTTGCTTCTATGACGTT
AGCGGCGGACGGGTGAGTAACACGTGGGCAACCTGCCTGTAAGACTGGGATAACTTCGGGAAACCGAA
GCTAATACCGGATAGGATCTTCTCCTTCATGGGAGATGATTGAAAGATGGTTTCGGCTATCACTTACA
GATGGGCCCGCGGTGCATTAGCTAGTTGGTGAGGTAACGGCTCACCAAGGCAACGATGCATAGCCGAC
CTGAGAGGGTGATCGGCCACACTGGGACTGAGACACGGCC
>Zotu989
GAACGCTAGCGGCAGGCTTAATACATGCAAGTCGAGGGATGCCAGCAATGGTAGACCGGCAAACGGGT
GCGTAACACGTACACAACTTTCCGTCAAGTGGGGCATAGCCCGGGGAAACCCGGATTAATACCCCATA
AAATATAAGCTGCGGCATCGCAGTAATATTAAAGCTCCGGCGCTTGATGATGGGTGTGCGGCTGATTA
GGTAGTTGGCGGGGTAACGGCCCACCAAGCCTTCGATCAGTAGCTGATGTGAGAGCATGATCAGCCAC
ACGGGCACTGAGACACGGGC
>Zotu990
GAACGCTGGCGGCATGCCTAACACATGCAAGTCGAACGAACCCTTCGGGGTTAGTGGCGCACGGGTGC
GTAACGCGTGGGAATCTGCCCTTTGCTTCGGAATAACAGTTAGAAATGACTGCTAATACCGGATGATG
ACTTCGGTCCAAAGATTTATCGGCAAAGGATGAGCCCGCGTAGGATTAGGTAGTTGGTGGGGTAAAGG
CCTACCAAGCCGACGATCCTTAGCTGGTCTGAGAGGATGATCAGCCACACTGGGACTGAGACACGGCC
>Zotu991
GAACGCTGGCGGCGTGCCTAATACATGCAAGTCGAGCGAATGGATTAAGAGCTTGCTCTTATGAAGTT
AGCGGCGGACGGGTGAGTAACACGTGGGTAACCTGCCCATAAGACTGGGATAACTCCGGGAAACCGGG
GCTAATACCGGATAACATTTTGAACTGCATGGTTCGAAATTGAAAGGCGGCTTCGGCTGTCACTTATG
GATGGACCCGCGTCGCATTAGCTAGTTGGTGAGGTAACGGCTCACCAAGGCAACGATGCGTAGCCGAC
CTGAGAGGGTGATCGGCCACACTGGGACTGAGACACGGCC
>Zotu992
GAACGCTGGCGGCGTGCCTAATACATGCAAGTCGAGCGGATCTTGTCCTTCGGGATAAGGTTAGCGGC
GGACGGGTGAGTAACACGTAGGCAACCTGCCCTCGAGACTGGGATAACCTCCGGAAACGGATGCTAAT
ACCAGATACGCGATCTTCCCACCTGGGGAGATCGGGAAAGACGGAGCAATCTGTTACTCGGGGATGGG
CCTGCGGCGCATTAGCTAGTTGGTGAGGTAACGGCTCACCAAGGCGACGATGCGTAGCCGACCTGAGA
GGGTGATCGGCCACACTGGGACTGAGACACGGCC

>Zotu993
GAACGCTAGCGGCAGGCTTAACACATGCAAGTCGAGGGGTATATTTCTTCGGAGATAGAGACCGGCGC
ACGGGTGCGTAACGCGTATGCAATCTACCTTTTACAGAGGGATAGCCCAGAGAAATTTGGATTAATAC
CTCATAGTATAGCAGCTTCGCATGAAGCCACTATTAAAGTCACAACGGTAAAAGATGAGCATGCGTCC
CATTAGCTAGTTGGTAAGGTAACGGCTTACCAAGGCTACGATGGGTAGGGGTCCTGAGAGGGAGATCC
CCCACACTGGTACTGAGACACGGAC
>Zotu994
GAACGCTGGCGGCATGCCTTACACATGCAAGTCGAACGGTAACAGGTCTTTCGGGATGCTGACGAGTG
GCGAACGGGTGAGTAATACATCGGAACGTGCCTAGTAGTGGGGGATAACTACTCGAAAGAGTGGCTAA
TACCGCATGAGATCTATGGATGAAAGCAGGGGACCTTCGGGCCTTGTGCTACTAGAGCGGCCGATGGC
AGATTAGGTAGTTGGTGGGATAAAAGCTTACCAAGCCTACGATCTGTAGCTGGTCTGAGAGGACGATC
AGCCACACTGGGACTGAGACACGGCC
>Zotu995
GAACGCTGGCGGCAGGCCTAACACATGCAAGTCGAGCGGATGAGAGGAGCTTGCTCTTCGATTCAGCG
GCGGACGGGTGAGTAATGCCTAGGAATCTGCCTAGTAGTGGGGGACAACGTTTCGAAAGGAACGCTAA
TACCGCATACGTCCTACGGGAGAAAGTGGGGGATCTTCGGACCTCACGCTATTAGATGAGCCTAGGTC
GGATTAGCTAGTTGGTAGGGTAAAGGCCTACCAAGGCGACGATCCGTAACTGGTCTGAGAGGATGATC
AGTCACACTGGAACTGAGACACGGTC
>Zotu996
GAACGCTGGCGGCGTGCCTAATACATGCAAGTCGAGCGAACCTGCAGGAGCTTGCTCCTGTTGGTTAG
CGGCGGACGGGTGAGTAACACGTGGGCAACCTGCCTGTAAGACTGGGATAACACCGGGAAACCGGTGC
TAATACCGGATAATCCATTTCCTCTCATGAGGAAATGCTGAAAGTTGGTTTCGGCTGACACTTACAGA
TGGGCCCGCGGCGCATTAGCTAGTTGGTGAGGTAACGGCTCACCAAGGCGACGATGCGTAGCCGACCT
GAGAGGGTGATCGGCCACACTGGGACTGAGACACGGCC
>Zotu997
GAACGCTAGCGGGAGGCCTAACACATGCAAGCCGAGCGGTATTTGTTCTTCGGAACAGAGAGAGCGGC
GTACGGGTGCGGAACACGTGTGCAACCTGCCTTTATCAGGGAGATAGCCTTTCGAAAGGAAGATTAAT
ATCCCATAATATTTAGACTGGCATCAGTATAAATTGAAAGCTCCGGCGGATAGAGATGGGCACGCGCA
AGATTAGATAGTTGGCGGGGTAACGGCCCACCAAGTCAATGATCTTTAGGGGGCCTGAGAGGGTGATC
CCCCACACTGGTACTGAGACACGGAC
>Zotu998
GAACGCTAGCGATAGGCTTAACACATGCAAGTCGAGGGGCAGCACATGAGTGGTAACACGATGGTGGC
GACCGGCGCACGGGTGAGTAACACGTATGCAACCTGCCCATAACTGGGGGATAACCCGTCGAAAGGCG
GACTAATACCGCATAACAACATTGATCCGCATGGTGATGTGTTTAAAGATTTATCGGTTATGGATGGG
CATGCGTTCCATTAGATTGTTGGTAGGGTAACGGCCTACCAAGTCTGCGATGGATAGGGGTTCTGAGA
GGAAGGTCCCCCACACTGGTACTGAGACACGGAC
>Zotu999
TAACGCTGGCGGTATGCCTAACACATGCAAGTCGAACGGAATCTTATTTGTTCTACGCCTCAAACTAG
CAATAGTATGCGTCGTGGAACTGTAAGATTTAGTGGCGAACGGGTGAGTAACACGTTGGTACCTGCCC
TCTAGTGGGGAATAACTAGCCGAAAGGTTGGCTAATACCGCATGTGATCTTCGGATCAAAGCCTTCGG
GCGCTAGAGGAGGGGCCTGCGGATGATTAGCTAGTTGGTGAGGTAAAAGCTCACCAAGGCGACGATCA
TTAGCTGGATCGAGAGGTTGACCAGCCACACTGGGACTGAGATACGGCC
>Zotu1000
GAACGCTGGCGGCGTGCCTAACACATGCAAGTCGAGCGGGGGACTTCGGTCCCCAGCGGCGGACGGGT
GAGTAACACGTGGGTAACCTGCCTCATAGAGGGGGATAGCCTCCCGAAAGGGAGATTAATACCGCATA
ACACTGCGAAGTCACATGCCTTTGCAGTTAAAGGAGTAATCCGCTATGAGATGGGCCCGCGGCGCATT
AGCTAGTTGGTGAGGTAACGGCTCACCAAGGCCACGATGCGTAGCCGACCTGAGAGGGTGATCGGCCA
CATTGGAACTGAGACACGGTC
>Zotu1001
GAACGCTGGCGGCGTGCTTAACACATGCAAGTCGAACGGAAAGGCCCTTTCGGGGGTACTCGAGTGGC
GAACGGGTGAGTAACACGTGGGTGATCTGCCCTGCACTTCGGGATAAGCCTGGGAAACTGGGTCTAAT
ACCGGATATGATCATGCTCTTCATGGGGTGTGGTGGAAAGCTTTTGCGGTGTGGGATGGGCCCGCGGC
CTATCAGCTTGTTGGTGGGGTAATGGCCTACCAAGGCGACGACGGGTAGCCGGCCTGAGAGGGTGTCC
GGCCACACTGGGACTGAGATACGGCC

>Zotu1002
GAACGCTGGCGGCGTGCTTAACACATGCAAGTCGAACGGAAAGGCCCTGCTTGCAGGGTACTCGAGTG
GCGAACGGGTGAGTAACACGTGGGTGATCTGCCCTGCACTTCGGGATAAGCTTGGGAAACTGGGTCTA
ATACCGGATAGGAACCATCTTTAGTGTGGTGGTTGGAAAGTTTTTTCGGTGTAGGATGAGCTCGCGGC
CTATCAGCTTGTTGGTGGGGTAATGGCCTACCAAGGCGGCGACGGGTAGCCGGCCTGAGAGGGTGTAC
GGCCACATTGGGACTGAGATACGGCC
>Zotu1003
GAACGCTGGCGGCAGGCCTAACACATGCAAGTCGAGCGGATGAAGGGAGCTTGCTCCTGGATTCAGCG
GCGGACGGGTGAGTAATGCCTAGGAATCTGCCTGGTAGTGGGGGATAACGTCCGGAAACGGGCGCTAA
TACCGCATACGTCCTGAGGGAGAAAGTGGGGGATCTTCGGACCTCACGCTATCAGATGAGCCTAGGTC
GGATTAGCTAGTTGGTGGGGTAAAGGCCTACCAAGGCGACGATCCGTAACTGGTCTGAGAGGATGATC
AGTCACACTGGAACTGAGACACGGTC
>Zotu1004
GAACGCTGGCGGCGTGCCTAAGACATGCAAGTCGAGTGGGTTTAGACCCACGGCGAACGGGTGAGTAA
TACCTAGGAACTTTCTTTAAGTGGAGCATAACCAGTCGAAAGATTGGATAATACTTCATGTGACCGCA
AGGTTAAAGTCGCAAGACGCTTAAAGTGAGGCCTGGGCACCATCAGCTAGTTGGTAAGGTAATGGCTT
ACCAAGGCTATGACGGTTAGGGGTCCCGAGAGGGATAACCCCCACACTGGAACTGCGACACGGTC
>Zotu1005
GAACGCTGGCGGCGTGCTTAACACATGCAAGTCGAACGGAAAGGCTCCTGCTTGCAGGGGTACTCGAG
TGGCGAACGGGTGAGTAACACGTGGGTGATCTGCCTTGTACTCTGGGATAAGCTTGGGAAACTGGGTC
TAATACCGGATAGGAACTGCCTTTAGTGTGGTGGTTGGAAAGTTTTTTCGGTGCAAGATGAGCTCGCG
GCCTATCAGCTTGTTGGTGGGGTAATGGCCTACCAAGGCGTCGACGGGTAGCCGGCCTGAGAGGGTGT
ACGGCCACATTGGGACTGAGATACGGCC
>Zotu1006
GAACGCTGGCGGCGTGCTTAACACATGCAAGTCGAACGATGAAGCTTGGTGCTTGCACTGGGTGGATT
AGTGGCGAACGGGTGAGTAACACGTGAGTAACCTGCCCTTTACTTCGGGATAAGCTTGGGAAACTGGG
TCTAATACCGGATATTCTGCGTGATCGGATGGTTGTGTAGGAAAGATTTTTTGGTAAGGGATGGGCTC
GCGGCCTATCAGTTTGTTGGTGGGGTGATGGCCTACCAAGACGACGACGGGTAGCCGGCCTGAGAGGG
CGACCGGCCACACTGGGACTGAGACACGGCC
>Zotu1007
GAACGCTAGCGGTGTGCCTGAGATATGCAAGTCGAGCGACGTGTCTTTGTAGCTTGCTACAAAGATAA
CGAGCGGCGTCCGGCTGAGTAACGCGTAGGAATCTGCCCCCGACTGGGGAATAGCCAACCGAAAGGTT
GGGTAATACCCCATAGTCTCTTTCTTTATTGAGAGAGTAAAGGCCTTTACCGGCCGGTCAGGGATGAG
CCTGCGTCCTATCAGGTAGTTGGTGAGGTAACGGCTCACCAAGCCAATGACGGGTAGCTGGTCTCATG
AGGACGACCAGCCAGAGGGGGACTGAGACACGGCC
>Zotu1008
GAACGCTGGCGGCGTGCTTAACACATGCAAGTCGAACGATGATGCCCAGCTTGCTGGGCGGATTAGTG
GCGAACGGGTGAGTAATACGTGAGTAACCTGCCCTTGACTCTGGGATAAGCCTGGGAAACTGGGTCTA
ATACTGGATACTACCGTCCACCGCATGGTGGGTGGTGGAAAGGGTTTTACTGGTTTTGGATGGGCTCA
CGGCCTATCAGCTTGTTGGTGGGGTAATGGCTCACCAAGGCGACGACGGGTAGCCGGCCTGAGAGGGT
GACCGGCCACACTGGGACTGAGACACGGCC
>Zotu1009
GAACGCTGGCGGCGTGCTTAACACATGCAAGTCGAACGAAGAGCGATGGAAGCTTGCTTCTATCAATC
TTAGTGGCGAACGGGTGAGTAACGCGTAATCAACCTGCCCTTCAGAGGGGGACAACAGTTGGAAACGA
CTGCTAATACCGCATACGATCTAACCTCGGCATCGAGGATAGATGAAAGGTGGCCTCTATTTATAAGC
TATCACTGAAGGAGGGGATTGCGTCTGATTAGCTAGTTGGAGGGGTAATGGCCCACCAAGGCGATGAT
CAGTAGCCGGTCTGAGAGGATGAACGGCCACATTGGGACTGAGACACGGCC
>Zotu1010
AAACGCTGGCGGCGTGCTTAACACATGCAAGTCGAACGAAGTTTTTCTTTCGGGAGGAACTTAGTGGC
GGACGGGTGAGTAACGCGTGGGTAACCTGCCCTGTACAGGGGGACAACAGCTGGAAACGGCTGCTAAT
ACCGCATAAGCCCTTAGCACTGCATGGTGCATAGGGAAAAGGAGCAATCCGGTACAGGATGGACCCGC
GTCTGATTAGCCAGTTGGCAGGGTAACGGCCTACCAAAGCGACGATCAGTAGCCGATCTGAGAGGATG
TACGGCCACATTGGGACTGAGACACGGCC
>Zotu1011

GAACGTTGGCGGCGTGCCTAACACATGCAAGTCGAGCGAGAAAGGTCGAAAGGCTGAGTACAGCGGCG
AACGGGTGAGTAACGCGTGAGAATCTACCCTTTAGTGGAGAATACCCTGGAGAAATTCAGGTTAATAC
TGCATATTCTCGGAAACGAGTAAAGATGGCCTAATTAGCTGTCGCTAGAGGATGAGCTTGCGTACTAT
TATGCTAGTTGGTGGGGTAATGGCCTACCAAGGCGTTGATGGTTAGCCGGCCTGAGAGGGTGTACGGC
CACACTGGAACTTAGACACGGTC
>Zotu1012
GAACGCTGGCGGCGTGCTTAACACATGCAAGTCGAGCGGAAAGGCCTTTCGGGGTACTCGAGCGGCGA
ACGGGTGAGTAACACGTGAGTAATCTGCCCTTGGCTTTGGGATAACTACCGGAAACGGTGGCTAATAC
CGGATACGACACAGGTCTGCATGGGCTCTGTGTGGAAAGTTTTTTCGGCCAGGGATGTGCTCGCGGCC
TATCAGCTTGTTGGTGGGGTAATGGCCTACCAAGGCTTCGACGGGTAGCCGGCCTGAGAGGGTGACCG
GCCACACTGGGACTGAGACACGGCC
>Zotu1014
CTGACACTTATCAGTAGATTACATTTTAAGCCTACCAAATCCAAAATGCTATGTAAATGATATTCTTC
AGTTCCCAAGTTAACCTTGCACCTGGCAACCGTTGTCCCAAACAGCTTCCTCCTTCAGAAGGAGCTAT
TGATTAAAAGTCAGGATTGACAATATTTCTTCA
>Zotu1015
GAACGCTGGCGGCGTGCCTAATACATGCAAGTCGAGCGAAGTTTTTCTGGTGCTTGCACTAGAAAAAC
TTAGCGGCGAACGGGTGAGTAACACGTAAAGAACCTGCCTCATAGACTGGGACAACTATTGGAAACGA
TAGCTAATACCGGATAACAGCATTAACTGCATGGTTGATGTTTAAAAGTTGGTTTTGCTAACACTATG
AGATGGCTTTGCGGTGCATTAGCTAGTTGGTGGGGTAAAGGCCTACCAAGGCGACGATGCATAGCCGA
CCTGAGAGGGTGATCGGCCACACTGGGACTGAGACACGGCC
>Zotu1016
GAACGCTGGCGGCGTGCCTAATACATGCAAGTCGAGCGAATAGATTAAGAGCTTGCTCTTATGAAGTT
AGCGGCGGACGGGTGAGTAACACGTGGGTAACCTACCCATAAGACTGGGATAACTCCGGGAAACCGGG
GCTAATACCGGATAATATTTTGAACTGCATAGTTCGAAATTGAAAGGCGGCTTCGGCTGTCACTTATG
GATGGACCCGCGTCGCATTAGCTAGTTGGTGAGGTAACGGCTCACCAAGGCGACGATGCGTAGCCGAC
CTGAGAGGGTGATCGGCCACACTGGGACTGAGACACGGCC
>Zotu1017
GAACGCTGGCGGCGCGCTTAACACATGCAAGTCGAGCGATGAAATTTTGACAGATCCCTTCGGGGTGA
AGATAAAATGGATTAGCGGCGGACGGGTGAGTAACACGTGAGTAACCTGCCTTAGACATTGGGATAGC
CTCGGGAAACCGGGATTAATACCGAATAAAATCACAGATACACATGTATCCATGATCAAAACTCCGGT
GGTCTAAGATGGACTCGCGTCCCATTAGCTAGTTGGTGAGGTAACGGCCCACCAAGGCGACGATGGGT
AGCCGGCCTGAGAGGGTGAACGGCCACATTGGAACTGAGAGACGGTC
>Zotu1018
GAACGCTGGCGGCGTGCTTAACACATGCAAGTCGAACGCTGAAGCCCAGCTTGCTGGGTGGATGAGTG
GCGAACGGGTGAGTAACACGTGAGTAACCTGCCCCCTTCTTTGGGATAACGCCCGGAAACGGGTGCTA
ATACTGGATATTCACTGATCTTCGCATGGGGGTTGGTGGAAAGGTTTTTTCTGGTGGGGGATGGGCTC
GCGGCCTATCAGCTTGTTGGTGGGGTGATGGCCTACCAAGGCTTTGACGGGTAGCCGGCCTGAGAGGG
TGACCGGTCACATTGGGACTGAGATACGGCC
>Zotu1019
GAACGCTGGCGGCGTGCCTAATACATGCAAGTCGAACGCATTTTCTTTCACCGTAGCTTGCTACACCG
AAAGAAAATGAGTGGCGAACGGGTGAGTAACACGTGGGTAACTTGCCCATCAGCGGGGGATAACACTT
GGAAACAGGTGCTAATACCGCATAATTCCATTTACCGCATGGTAAATGGATGAAAGGCGCTTTTGCGT
CACTGATGGATGGACCCGCGGTGCATTAGCTAGTTGGTGGGGTAACGGCCTACCAAGGCTGCGATGCA
TAGCCGACCTGAGAGGGTGATCGGCCACACTGGGACTGAGACACGGCC
>Zotu1020
GAACGCTGGCGGCGTGCCTAATACATGCAAGTCGAGCGAATCGGAGGGAGCTTGCTCCCGAAGATTAG
CGGCGGACGGGTGAGTAACACGTGGGCAACCTGCCTGTAAGACTGGGATAACTTCGGGAAACCGGAGC
TAATACCGGATAATCCTTTTCCTCTCATGAGGAAAAGCTGAAAGACGGTTTCGGCTGTCACTTACAGA
TGGGCCCGCGGCGCATTAGCTAGTTGGTGAGGTAACGGCTCACCAAGGCAACGATGCGTAGCCGACCT
GAGAGGGTGATCGGCCACACTGGGACTGAGACACGGCC
>Zotu1021
GAACGCTGGCGGCGTGCTTAACACATGCAAGTCGAACGGAAAGGCCAGTGCTTGCACTGGTACTCGAG
TGGCGAACGGGTGAGTAACACGTGGGTGATCTGCCTTGCACTCTGGGATAAGCTTGGGAAACTGGGTC

TAATACCGGATAGGAACCGTCTTTAGTGTGGTGGTTGGAAAGTTTTTCGGTGCAAGATGAGCTCGCG
GCCTATCAGCTTGTTGGTGGGGTAATGGCCTACCAAGGCGTCGACGGGTAGCCGGCCTGAGAGGGTGT
ACGGCCACATTGGGACTGAGATACGGCC
>Zotu1023
GAACGCTGGCGGCGTGCTTAACACATGCAAGTCGAACGGTGAAGGTGGGAGCTTGCTCCCGCCGGATC
AGTGGCGAACGGGTGAGTAACACGTGAGCAATCTGCCCCGAACTCTGGGATAAGCACTGGAAACGGTG
TCTAATACTGGATACGACGCGAGAGGGCATCCTCATCGCGTGGAAAGAATTTCGGTTCGGGATGAGCT
CACGGCCTATCAGCTAGTTGGTGAGGTAATGGCTCACCAAGGCGACGACGGGTAGCCGGCCTGAGAGG
GTGACCGGCCACACTGGGACTGAGACACGGCC
>Zotu1024
GAACGCTGGCGGCGTGCTTAACACATGCAAGTCGAACGATGAAGCCCAGCTTGCTGGGTGGAAGAGTG
GCGAACGGGTGAGTAACACGTGAGTAACCTGCCCTGAACTCTGGGATAAGCACTGGAAACGGTGTCTA
ATACTGGATACGACCTATCACCGCATGGTGTGTGGGTGGAAAGATTTATCGGTTTTGGATGGACTCGC
GGCCTATCAGCTAGATGGTGAGGTAATGGCTCACCATGGCGACGACGGGTAGCCGGCCTGAGAGGGTG
ACCGGCCACACTGGGACTGAGACACGGCC
>Zotu1025
GAACGCTGGCGGCGTGCCTAATACATGCAAGTCGAGCGCGGGAAGCAGGCAGAACTCCTTCGGGAGGG
ACGCTTGTGGAACGAGCGGCGGACGGGTGAGTAACACGTGGGCAACCTACCTGTAAGACTGGGATAAC
TCCGGGAAACCGGGGCTAATACCGGATAGGCACCATAGGCGCATGCATGTGGTGTGAAAGGCGGGGCG
ACCTGCCGCTTACAGATGGGCCTGCGGCGCATTAGCTAGTTGGTGAGGTAACGGCTCACCAAGGCGAC
GATGCGTAGCCGACCTGAGAGGGTGACCGGCCACACTGGGACTGAGACACGGCC
>Zotu1026
GAACGCTGGCGGCGTGCCTAATACATGCAAGTCGAACGAACCGCGACTAGGTGCTTGCACTTGGTCAA
GGTGAGTGGCGAACGGGTGAGTAACACGTGGGTAACCTACCTCATAGTGGGGGATAACAGTCGGAAAC
GACTGCTAATACCGCATAGGACATGGAATCACATGGTTCAGTGAGGAAAGGTGGCGCAAGCTATCGCT
AAGAGATGGACCCGCGGTGCATTAGCTAGTTGGTAGGGTAACGGCCTACCAAGGCGATGATGCATAGC
CGACCTGAGAGGGTGATCGGCCACATTGGGACTGAGACACGGCC
>Zotu1027
GAACGCTGGCGGCATGCCTAATACATGCAAGTCGAACGGTCTTGATGGGCTTGCTCATCAAGGTAGTG
GCGGACGGGTGAGTAACGCGTGGGAACCTGCCTGGCAGTGGGGAATAACCGCGGGAAACTGCGGCTAA
TACCGCATACGCCCCCTGGACTTCGGTTTAGGGGGGAAAGATTTATCGCTGTCGGAGGGGCCCGCGTC
GGATTAGCTAGTTGGCGGGGTAACGGCCCACCAAGGCGACGATCCGTAGCTGGTCTGAGAGGATGATC
AGCCACACTGGGACTTAGACACGGCC
>Zotu1028
GAACGCTAGCGGCAGGCCTAATACATGCAAGTCGAACGGTTCGCAAGGACAGTGGCAAACGGGTGCGT
AACGCGTAAGCAACCTGCCTCATACTGGGGGATAGCCCGGCGAAAGCTGGGGTAAACCCGCACGGTCC
GATTGAGTCACCTGGCTTGATGGGTAAACATTTATGGGTATGAGAGGGGCTTGCGTCTGATTAGTTAG
TTGGCAGGGTAACGGCCTACCAAGACGATGATCAGTAGGGGTTCTGAGAGGATTGGCCCCCACATGGG
TACTGAGATACGGAC
>Zotu1029
GAACGCTGGCGGCAGGCCTAACACATGCAAGTCGAACGGTAACAGGAAGCAGCTTGCTGCTTCGCTGA
CGAGTGGCGGACGGGTGAGTAATGTCTGGGAAACTGCCTGATGGAGGGGGATAACTACTGGAAACGGT
AGCTAATACCGCATAACGTCGCAAGACCAAAGAGGGGGACCTTCGGGCCTCTTGCCATCGGATGTGCC
CAGATGGGATTAGCTTGTTGGTGGGGTAACGGCTCACCAAGGCGACGATCCCTAGCTGGTCTGAGAGG
ATGACCAGCCACACTGGAACTGAGACACGGTC
>Zotu1030
GAACGCTAGCGGCAGGCCTAATACATGCAAGTCGGACGGGATTGAGAAGCTTGCTTTTCATGAGAGTG
GCGCACGGGTGCGTAACACGTATGTAACCTACCTTCGTCAGGGGGATAGCCTCTCGAAAGAGAGATTA
AGACCGCATAACATTATAATGTGGCATCACAAAATAATCAAATATTTATAGGACGAAGATGGGCATGC
GGAACATTAGCTAGTTGGTAAGGTAACGGCTTACCAAGGCTACGATGTTTAGGGGATCTGAGAGGATG
GCCCCCCACACTGGTACTGAGACACGGAC
>Zotu1031
GAATGCTGGCGGCGTGCCTAACACATGCAAGTCGAGCGGCAGCGCGAGGAGTTTACTCCTTGGCGGCG
AGCGGCGGACGGCTGAGTAACGCGTGGGAACGTGCCCCAAAGTGAGGAATAACTGCCCGAAAGGGTGG

CTAATGCCGCATATGATCTTCGGATTAAAGGATTTATCCGCTTTGGGAGCGGCCCGCGTCGGATTAGG
TAGTTGGTGAGGTAATGGCTCACCAAGCCGACGATCCGTAGCTGGTCTGAGAGGATGATCAGCCAGAC
TGGAACTGAGACACGGTC
>Zotu1032
GAACGCTGGCGGCGTGCTTAACACATGCAAGTCGAACGATGATCTCCCGCTTGCGGGGGTGATTAGTG
GCGAACGGGTGAGTAATACGTGAGTAACCTGCCCTTGACTCTGGGATAAGCCTGGGAAACCGGGTCTA
ATACTGGATACTACTTCCTGCCGCATGGTGGGGGGTGGAAAGGGTTTTACTGGTTTTGGATGGGCTCA
CGGCCTATCAGCTTGTTGGTGGGGTAATGGCTCACCAAGGCGACGACGGGTAGCCGGCCTGAGAGGGT
GACCGGCCACACTGGGACTGAGACACGGCC
>Zotu1033
GAACGCTGGCGGTATGCTTAACACATGCAAGTCGAACGAATGAGATTTTAAGCTTGCTTAAGATCAAG
TTAGTGGCAAACGGGTGAGTAACACGTGGGAATCTACCCAGTAGTACGGAATAACGGTTAGAAATGAC
CGCTAATACCGTATAGTATCGAGAGATTAAAGATTTATCGCTACTGGATGAGCCCGCGTCAGATTAGG
TAGTTGGTGGGGTAAAAGCCTACCAAGCCCAAGATCTGTAGCTGGTCTGAGAGGATGACCAGCCACAC
TGGAACTGAGACACGGTC
>Zotu1034
GAACGCTGGCGGCGTGCCTAATACATGCAAGTCGAGCGAACAGATAAGGAGCTTGCTCCTTTGACGTT
AGCGGCGGACGGGTGAGTAACACGTGGGTAACCTACCTATAAGACTGGAATAACTCCGGGAAACCGGG
GCTAATGCCGGATAACATGTTGAACCGCATGGTTCTACAGTGAAAGGCGGCTTTGCTGTAACTTATAG
ATGGACCCGCGCCGTATTAGCTAGTTGGTGAGGTAACGGCTCACCAAGGCAACGATACGTAGCCGACC
TGAGAGGGTGATCGGCCACACTGGAACTGAGACACGGTC
>Zotu1035
GAACGCTGGCGGCAGGCCTAACACATGCAAGTCGGACGGTAGCACAGAGAGCTTGCTCTTGGGTGACG
AGTGGCGGACGGGTGAGTAATGTCTGGGGATCTGCCCGATAGAGGGGGATAACCACTGGAAACGGTGG
CTAATACCGCATAACGTCGCAAGACCAAAGAGGGGGACCTTCGGGCCTCTCACTATCGGATGAACCCA
GATGGGATTAGCTAGTAGGCGGGGTAATGGCCCACCTAGGCGACGATCCCTAGCTGGTCTGAGAGGAT
GACCAGCCACACTGGAACTGAGACACGGTC
>Zotu1036
TAACGCTGGCGGCGTGCATAACACATGCAAGTCGAACGATGAAACTTAGTAGACTTCTTCGGAAAGAC
ACTAAGCGGATTAGTGGCGAACGGGTGAGTAACGCGTGAGTAACCTGCCTTACACAAGGGGATAGCCT
TTGGAAACGAAGAATAATACCCTATAAAACCATAAAAGCACATGCTAGAATGGTCAAAGTGATAGCGG
TGTAAGATGGACTTGCGTCTGATTAGCTAGTTGGTGAGATAAAAGCTCACCAAGGCAACGATCAGTAG
CCGGCTTGAGAGAGTGTACGGCCACATTGGGACTGAGACACGGCC
>Zotu1037
GAACGCTAGCGGCAGGCCTAATACATGCAAGCCGGACGGCAAGTTGAATAGCAATATTTGACCTAGAG
TGGCGCAAGGGTGCGTAACACGTATGCAACCTACCCAGAACCGGGGCATAGCCCTCCGAAAGGAGGAT
TAACTTCCCATAGGAAGACGAATTGGCATCAATTTGTTTTTAAACTACGGGGTTCTGGATGGGCATGC
GTTTGATTAGCTAGTTGGCGGGGTAATGGCCCACCAAGGCGATGATCAATAGGGGACCTGAGAGGGTG
ATCCCCCACACTGGCACTGAGATACGGGC
>Zotu1038
GAATGCTGGCGGCGTGCTTAACACATGCAAGTCGAGCGGTAACAGGAATAATTTATTATTTGCTGACG
AGCGGCGGACGGCTGAGTAACGCGTAGGAACATGCCCCAAAGTGAGGAATAAGCTCCAGAAATGGAGT
CTAATGCCGCATATGATCTTCGGATTAAAGGATTTATCCGCTATGGGAGTGGCCTGCGTATGATTAGG
TAGTTGGTGAGGTAAAGGCTCACCAAGCCGACGATCATTAGCTGGTCTGAGAGGATGACCAGCCAGAC
TGGAACTGAGACACGGTC
>Zotu1039
GAACGCTGGCGGCGTGCTTAACACATGCAAGTCGAACGATGAAGCCAGCTTGCTGGTGGATTAGTGGC
GAACGGGTGAGTAACACGTGGGCAACCTGCCCTCAGCTCTGGGATAACTCCAAGAAATTGGTGCTAAT
ACCGGATATTCTTCTTTCACCGCATGGTGGTTGGAGGAAAGTTTTTCGGCTGGGGATGGGCCCGCGGC
CTATCAGCTTGTTGGTGGGGTGATGGCCTACCAAGGCGACGACGGGTAGCCGGCCTGAGAGGGTGACC
GGTCACACTGGGACTGAGACACGGCC
>Zotu1040
GAACGCTGGCGGCATGCCTAATACATGCAAGTCGAGCGAACAGATGAAGTGCTTGCACTTCTGACGTT
AGCGGCGAACGGGTGAGTAACACGTAAGGAATCTACCTATAAGCGGGGGATAACATTCGGAAACGGGT

GCTAATACCGCATAATATCTTCTTCCGCATGGAAGAAGATTGAAAGACGGCTCTGCTGTCACTTATAG
ATGACCTTGCGGTGCATTAGTTAGTTGGTGGGGTAACGGCCTACCAAGACGATGATGCATAGCCGACC
TGAGAGGGTGATCGGCCACATTGGGACTGAGACACGGCC
>Zotu1041
GAACGCTGGCGGCGTGCCTAATACATGCAAGTCGAGCGAACAGATAAGGAGCTTGCTCCTTTGACGTT
AGCGGCGGACGGGTGAGTAACACGTGGGTAACCTACCTATAAGACTGGGATAACTTCGGGAAACCGGA
GCTAATACCGGATAACATTTGGAACCGCATGGTTCTAAAGTGAAAGATGGTTTTGCTATCACTTATAG
ATGGACCCGCGCCGTATTAGCTAGTTGGTAAGGTAACGGCTTACCAAGGCAACGATACGTAGCCGACC
TGAGAGGGTGATCGGCCACACTGGAACTGAGACACGGTC
>Zotu1042
GAACGCTGGCGGCGTGCCTAATACATGCAAGTAGAACGCTGAAGGAGGAGCTTGCTCTTCTGGATGAG
TTGCGAACGGGTGAGTAACGCGTAGGTAACCTGCCTGGTAGCGGGGGATAACTATTGGAAACGATAGC
TAATACCGCATAAAAACAGTTGTTGCATGACAGCTGTTTGAAAGGTGCAATTGCATCACTACCAGATG
GACCTGCGTTGTATTAGCTAGTTGGTGGGGTAACGGCTCACCAAGGCGACGATACATAGCCGACCTGA
GAGGGTGATCGGCCACACTGGGACTGAGACACGGCC
>Zotu1043
GAACGCTGGCGGCGTGCTTAACACATGCAAGTCGAACGATGAACCACTTCGGTGGGGATTAGTGGCGA
ACGGGTGAGTAACACGTGGGCAATCTGCCCTTCACTCTGGGACAAGCCCTGGAAACGGGGTCTAATAC
CGGATACCACTACCGCAGGCATCTGTGGTGGTTGAAAGCTCCGGCGGTGAAGGATGAGCCCGCGGCCT
ATCAGCTTGTTGGTGAGGTAATGGCTCACCAAGGCGACGACGGGTAGCCGGCCTGAGAGGGCGACCGG
CCACACTGGGACTGAGACACGGCC
>Zotu1044
GAACGCTGGCGGCATGCCTTACACATGCAAGTCGAACGGCAGCACGGGAGCAATCCTGGTGGCGAGTG
GCGAACGGGTGAGTAAAGTATCGGAACGTGCCCAGTCGTGGGGGATAACGTAGCGAAAGTTACGCTAA
TACCGCATACGATCTATGGATGAAAGCGGGGGATCGCAAGACCTCGCGCGATTGGAGCGGCCGATATC
AGATTAGGTAGTTGGTGGGGTAAAGGCTCACCAAGCCGACGATCTGTAGCTGGTCTGAGAGGACGACC
AGCCACACTGGGACTGAGACACGGCC
>Zotu1045
GAACGTTAGCGGCGCGCCTAAGACATGCAAGTCGAGCGAGAAAGGGCTTCGGCCCCAGTAAAGCGGCG
CACGGGTGAGTAACACGTAGGTTATCAACCCCTGAGTGGTGGACAACCCTCCGAAAGGAGGGCTAATA
CAGCATAATGCCAGCAATGGCCAAAGCGGGCCTCTTCATGAAAGCTCGCGCTCGGGGATGAGCCTGCG
GCCCATCAGGTAGTTGGTTGGGGTAACGGCCCACCAAGCCTACGACGGGTAGCTGGTCTGAGAGGATG
AACAGCCACACTGGAACTGAGACACGGTC
>Zotu1046
GAACGCTAGCGGCAGGCCTAATACATGCAAGTCTAGGGGGCAGCAATGTCACCGGCGCACGGGTGCGT
AACACGTATGCAACCTACCTTCTACAGGGGTACAGCCTTTCGAAAGAGAGATTAATACCGCATGAGAC
CACACTTATTCATATAAGAGAGGTTAAAGATTTATCGGTAGAAGATGGGCATGCGTCTGATTAGTTAG
TTGGCGGGGTAATGGCTCACCAAGACGATGATCAGTAGGGGACCTGAGAGGGTAATCCCCCACACTGG
CACTGAGATACGGGC
>Zotu1047
GAACGCTGGCGGCATGCCTTACACATGCAAGTCGAACGGCAGCGCGGGAGCAATCCTGGCGGCGAGTG
GCGAACGGGTGAGTAATATATCGGAACGTGCCCATTAGTGGGGGATAGCCCGGCGAAAGCCGGATTAA
TACCGCATACGACCTGAGGGTGAAAGCGGGGGATCGCAAGACCTCGCGCTATTGGAGCGGCCGATATC
AGATTAGCTAGTTGGTGGGGTAAAGGCCTACCAAGGCAACGATCTGTAGTTGGTCTGAGAGGACGACC
AGCCACACTGGGACTGAGACACGGCC
>Zotu1048
GAACGCTGGCGGCGTGCTTAACACATGCAAGTCACGGGGGGTAGCAATACCAACCGGCGAACGGGTGC
GTAACACGTGAGCAATCTGCCGATCTCTGGGGGATAGCCGGCCCAACGGCCGGGTAATACCGCATACG
CTCATTGGGGGACATCCCTTGGTGAGGAAACCTCCGGGGGAGATCGAGGAGCTCGCGACCTATCAGCT
AGTTGGTGAGGTAATGGCTCACCAAGGCAACGACGGGTAGCTGGTCTGAGAGGATGGCCAGCCACATT
GGGACTGCGACACGGCC
>Zotu1049
GAACGCTGGCGGCGTGCTTAACACATGCAAGTGGAGCGACGAAGCGGACTTCGGTCCGTGGCAGAGCC
GCGAACGGGTGAGTAACACGTGGGTAACCCACCCCGATGACCGGGACAACCCGAGGAAACTCGGGCTA

ATACCGGATGTTCTCCTTTCTCATAAGGGAATCGAGCAAAGATAGCTTTTAGCCTTCGCATCGGGACG
GGCCCGCGGCCCATTAGCTTGTTGGTGAGGTAACGGCTCACCAAGGCGACGATGGGTAGCTGGTCTGA
GAGGACGATCAGCCACACTGGGACTGAGACACGGCC
>Zotu1050
GAACGCTGGCGGAATGCTTTACACATGCAAGTCGAGCGGCAGCGCGGGGCAACCTGGCGGCGAGCGGC
GAACGGGTGAGTAATACATCGGAACGTACCCAATCGTGGGGGATAACTATCCGAAAGGATAGCTAATA
CCGCATAAGTCCTGAGGGAGAAAGCAGGGGATCGCAAGACCTTGCGCGGATGGAGCGGCCGATGTCGG
ATTAGCTAGTTGGTGGGGTAAAGGCCTACCAAGGCGACGATCCGTAGCTGGTCTGAGAGGACGACCAG
CCACACTGGAACTGAGACACGGTC
>Zotu1051
GAACGCTAGCGGGAGGCCTAACACATGCAAGCCGAGCGGTATTTGTCTTTCGGGACAGAGAGAGCGGC
GTACGGGTGCGTAACACGTGTGCAACCTACCTTTATCAGGGGGATAGCCTTTCGAAAGGAAGATTAAT
ACCCCATAATATATTGAATGGCATCATTAAATATTGAAAACTCCGGTGGATAAAGATGGGCACGCGCA
AGATTAGATAGTTGGTGAGGTAACGGCTCACCAAGTCGATGATCTTTAGGGGTCCTGAGAGGGAGATC
CCCCACACTGGTACTGAGACACGGAC
>Zotu1052
TAACGCTAGCGGCAGGCCTAATACATGCAAGTCGAACGGCAGCACGGGTAGCAATACCTGGTGGCGAG
TGGCGCACGGGTGCGTAACGCGTATGCAATCTACCTATAACTGGGGAATAGCCTTCCGAAAGGAAGAT
TAATACCGCATAATATCACGAGACGGCATCGTTTTGTGATCAAATCTCCGGAGGTTATAGATGAGCAT
GCGTGACATTAGCTAGTTGGTGAGGTAACGGCTCACCAAGGCTACGATGTCTAGGGGGTCTGAGAGGA
TGATCCCCCACACTGGTACTGAGACACGGAC
>Zotu1053
GAACGCTGGCGGCGTGCTTAACACATGCAAGTCGAACGATGACCCAGAGCTTGCTCTGGTGATTAGTG
GAGAACGGGTGAGTAACACGTGAGTAACCTGCCCTTGACTCTGGGATAAGCACTGGAAACGGTGTCTA
ATACCGGATACGACAACCGACGGCATCGTCTGGTTGTGGAAAGAATTTTGGTCAAGGATGGACTCGCG
GCCTATCAGCTAGTTGGTGAGGTAATGGCCCACCAAGGCGACGACGGGTAGCCGGCCTGAGAGGGTGA
CCGGCCACACTGGGACTGAGACACGGCC
>Zotu1054
GAACGCTGGCGGCGTGCTTAACACATGCAAGTCGAACGGAAAGGCCCAGCTTGCTGGGTGCTCGAGTG
GCGAACGGGTGAGTAACACGTGGGTGATCTGCCTCTAACTTCGGGATAAGCTTGGGAAACTAGGTCTA
ATACCGGATAGGACAATCGTTTAGTGTCGGTTGTGGAAAGTTTTTTCGGTTAGGGATGAGCCCGCGGC
CTATCAGCTTGTTGGTGGGGTAATGGCCTACCAAGGCGTCGACGGGTAGCCGGCCTGAGAGGGTGGAC
GGCCACATTGGGACTGAGATACGGCC
>Zotu1056
GAACGCTGGCGGCAGGCTTAATACATGCAAGTCGAGCGGGCACTTCGGTGTCAGCGGCGCACGGGTGC
GTAACGCGTGGGAACATGCCTTTAGGTACGGAACAACCAAGGGAAACTTTGGCTAATACCGTATAATA
TCTCTGGATCAAAGATTTATCGCCTAAAGATTGGCCCGCGTTAGATTAGGTAGTTGGTGGGGTAATGG
CCTACCAAGCCGACGATCTATAGGGGGTCTGAGAGGATGATCCCCCACACTGGAACTGAGACACGGTC
>Zotu1057
GAACGCTGGCGGCATGCCTAACACATGCAAGTCGAACGAGACCTTCGGGTCTAGTGGCGCACGGGTGC
GTAACGCGTGGGAATCTGCCCTTGGGTTCGGGATAACTCGCCGAAAGGCGTGCTAATACCGGATGATG
ACGTAAGTCCAAAGATTTATCGCCCGAGGATGAGCCCGCGTTGGATTAGGTAGTTGGTGAGGTAAAGG
CTCACCAAGCCGACGATCCATAGCTGGTCTGAGAGGATGATCAGCCACACTGGGACTGAGACACGGCC
>Zotu1058
GAACGCTGGCGGCGTGCCTAATACATGCAAGTCGAGCGGATCTCTTCGGAGATCAGCGGCGGACGGGT
GAGTAACACGTGGGCAACCTGCCTATAAGACTGGGATAACTTCGGGAAACCGGAGCTAATACCGGATA
ACATCAAGGACCACATGGTCTTCGATTAAAAGATGGCGTCAAGCTATCACTTATAGATGGGCCCGCGT
CGCATTAGCTAGTTGGTGAGGTAACGGCTCACCAAGGCGACGATGCGTAGCCGACCTGAGAGGGTGAT
CGGCCACACTGGGACTGAGACACGGCC
>Zotu1059
GAACGCTGGCGGCACGCCTAATACATGCAAGTCGAGCGCGTGAAGCTTTCCGAAGCCTTCGGGCGGAC
GAAAGTGGATCGAGCGGCGGACGGGTGAGTAACACGTAGGCAACCTGCCTGTAAGACCGGGATAACTC
CGGGAAACCGGGGCTAATACCGGATAGGATCTCTTTTCGCATGGGAAGGGATTGAAAGGATGGCCGCA
AGGCTATCGCTTACAGATGGGCCTGCGGCGCATTAGCTGGTTGGTGAGGTAACGGCTCACCAAGGCGA

CGATGCGTAGCCGGCCTGAGAGGGTGACCGGCCACACTGGGACTGAGACACGGCC
>Zotu1060
GAACGCTGGCGGCGTGCCTAATACATGCAAGTCGAGCGAGCTTGCCTAGATGAATTTGGTGCTTGCAC
CAAATGAAACTAGATACAAGCGAGCGGCGGACGGGTGAGTAACACGTGGGTAACCTGCCCAAGAGACT
GGGATAACACCTGGAAACAGATGCTAATACCGGATAACAACACTAGACGCATGTCTAGAGTTTAAAAG
ATGGTTCTGCTATCACTCTTGGATGGACCTGCGGTGCATTAGCTAGTTGGTAAGGTAACGGCTTACCA
AGGCAATGATGCATAGCCGAGTTGAGAGACTGATCGGCCACATTGGGACTGAGACACGGCC
>Zotu1061
GAACGCTGGCGGCGTGCTTAACACATGCAAGTCGAACGGAAAGGCCCTTTCGGGGGTACTCGAGTGGC
GAACGGGTGAGTAACACGTGGGTGATCTGCCCTGCACTTCGGGATAAGCTTGGGAAACTGGGTCTAAT
ACCGGATAGGACTACGCGATGCATGTCGTGTGGTGGAAAGCTTTTGCGGTGTGGGATGGGCCCGCGGC
CTATCAGCTTGTTGGTGGGGTGATGGCCTACCAAGGCGACGACGGGTAGCCGGCCTGAGAGGGTGTCC
GGCCACACTGGGACTGAGATACGGCC
>Zotu1062
GAACGCTGGCGGCATGCCTAATACATGCAAGTCGAACGCTGAAGCACTCTGCTTGCAGAGTGCGGATG
AGTGGCGAACGGGTGAGTAACACGTGGGAAATCTGCCTAAAAGTGGGGGATAACATTCGGAAACGGAT
GCTAATACCGCATAGATTCTTGAACCGCATGGTTCTTGAAAGAAAGACGGCTTTGCTGTCGCTTTTAG
ATGATCCCGCGGCGTATTAGTTAGTTGGTGGGGTAAAGGCCTACCAAGACCATGATACGTAGCCGACC
TGAGAGGGTGATCGGCCACACTGGGACTGAGACACGGCC
>Zotu1064
GAACGCTAGCTACAGGCTTAACACATGCAAGTCGAGGGGCAGCATGGTCTTAGCTTGTTAAGGCCGAT
GGCGACCGGCGCACGGGTGAGTAACACGTATCCAACCTGCCGTCTACTCTTGGACAGCCTTCTGAAAG
GAAGATTAATACAAGATGGCATCATGAGTTCACATGTTCACATGATTAAAGGTATTCCGGTAGACGAT
GGGGATGCGTTCCATTAGATAGTAGGCGGGGTAACGGCCCACCTAGTCTTCGATGGATAGGGGTTCTG
AGAGGAAGGTCCCCCACATTGGAACTGAGACACGGTC
>Zotu1066
GAACGCTGGCGGCAGGCTTAACACATGCAAGTCGAGCGGGGTGATGTAGCTTGCTACCTGACCTAGCG
GCGGACGGGTGAGTAATGCTTAGGAATCTGCCTATTAGTGGGGGACAACATTCCGAAAGGAATGCTAA
TACCGCATACGCCCTACGGGGGAAAGCAGGGGATCTTCGGACCTTGCGCTAATAGATGAGCCTAAGTC
AGATTAGCTAGTTGGTGGGGTAAAGGCCTACCAAGGCGACGATCTGTAGCGGGTCTGAGAGGATGATC
CGCCACACTGGGACTGAGACACGGCC
>Zotu1067
GAACGCTAGCGGCAGGCTTAATACATGCAAGTCGAGGGGCAGCATGGGGTGTAGCAATACACTCTGAT
GGCGACCGGCAAACGGGTGCGGAACACGTACGCAACTTACCCTTAACTGGGGGATAGCCCGGGGAAAC
CCGGATTAATACCTCGTAACATAATTGGATGGCATCATTTGATTATTATAGCTTTATTGTGGTTAAGG
ATGGGCGTGCGGCTGATTAGGTAGTTGGCAGGGTAACGGCCTACCAAGCCATCGATCAGTAACTGGTG
TGAGAGCACGACCAGTCACACGGGCACTGAGACACGGGC
>Zotu1068
GAACGCTGGCGGCAGGCCTAACACATGCAAGTCGAACGGTAGCACAGAGGAGCTTGCTCCTTGGGTGA
CGAGTGGCGGACGGGTGAGTAATGTCTGGGAAACTGCCCGATGGAGGGGGATAACTACTGGAAACGGT
AGCTAATACCGCATAACGTCGCAAGACCAAAGAGGGGGACCTTCGGGCCTCTTGCCATCGGATGTGCC
CAGATGGGATTAGCTAGTAGGCGGGGTAACGGCTCACCTAGGCGACGATCCCTAGCTGGTCTGAGAGG
ATGACCAGCCACACTGGAACTGAGACACGGTC
>Zotu1069
AAACGCTGGCGGCGCACATAAGACATGCAAGTCGAACGAACTTAACCATTAGTTTACTATTGGAGCGG
TTAGTGGCGGACTGGTGAGTAACACGTAAGCAACCTGCCTATCAGAGGGGAACAACAGTTAGAAATGA
CTGCTAATACCGCATATGCCTTGAGTATCACATGGTACAAAAGGGAAAGGAGCAATCCGCTGATAGAT
GGGCTTGCGTCTGATTAGATAGTTGGTGAGGTAACGGCCCACCAAGTCGACGATCAGTAGCCGGACTG
AGAGGTTGAACGGCCACATTGGGACTGAGATACGGCC
>Zotu1070
GAACGCTGGCGGCGCGCCTAACACATGCAAGTCGAGCGATGAAAGTTGTTCAGATCCTTCGGGTGACG
AACTTCTGGATTAGCGGCGAACGGGTGAGTAACGCGTGAGGAACCTGCCTCTCACAACGGGATAGCCT
CGGGAAACCGGGATTAATACCGTATGACACTATCAGATGGCATCATCAGATAGTTAAAGTGCTAGCGG
TGAGAGATGGCCTCGCGTCTGATTAGTTAGTTGGTGGGGTAACGGCCTACCAAGACGACGATCAGTAA

CCGGCCTGAGAGGGTGAACGGTCACATTGGAACTGAGACACGGTC
>Zotu1071
CAACGCTGGCGGCGCGCTTAACACATGCAAGTCGAACGAGAAAGGGGAGCAATCCCTGAGTAAAGTGG
CGTACGGGTGAGTAACACGTGGGTGATCTGCCTGTTAGTGGGGAATACCCCCGGGAAACTGGGACAA
TACCGCATAATCCCGCAAGGGGAAAGTAGCAATACGCTGACAGAGGAGCCCGCGGCTGATTAGCTTGT
TGGTGAGGTAACGGCTCACCAAGGCTGCGATCGGTAGCCGGCCTGAGAGGGCACACGGCCACACTGGC
ACTGAAACACGGGC
>Zotu1072
GAACGCTAGCGGGATGCTTTACACATGCAAGTCGAACGGCAGCGCGAGAGAGCTTGCTCTCTTGGCGG
CGAGTGGCGGACGGGTGAGTAATATATCGGAACGTGCCCAGTAGCGGGGGATAACTACTCGAAAGAGT
GGCTAATACCGCATACGCCCTACGGGGGAAAGGGGGGGATCGCAAGACCTCTCACTATTGGAGCGGCC
GATATCGGATTAGCTAGTTGGTGGGGTAAAGGCTCACCAAGGCAACGATCCGTAGCTGGTTTGAGAGG
ACGACCAGCCACACTGGGACTGAGACACGGCC
>Zotu1074
GAATGCTGGCGGCGTGGATAAGTCATGCAAGTCAAGGGGGCCCGCAAGGGCAACCGGCAAACGAGGTA
GTAACACGTAGGTAGACCCCCCAGAGTCGGGCATAGCCGGTCGAAAGACCGGGTAATTCCCGATAGTC
CCGCAAGGGTAAAAATTTATTGCTCTGGGAGTTGCCTGCGTAGTATCAGCTAGTTGGTAGGGTAATGG
CCTACCAAGGCTACGACGCTTAGGGGAGCTGAGAGGCTGACCCCCACCGATGGAACTGCGACACGGTC
>Zotu1075
GAACGCTGGCGGCATGCCTTACACATGCAAGTCGAACGGCAGCACGGGAGCAATCCTGGTGGCGAGTG
GCGAACGGGTGAGTAATGTATCGGAACGTGCCCAGTCGTGGGGGATAACGTAGCGAAAGCTACGCTAA
TACCGCATACGATCTATGGATGAAAGCGGGGGACCGTAAGGCCTCGCGCGATTGGAGCGGCCGATATC
AGATTAGGTAGTTGGTGGGGTAAAGGCCCACCAAGCCGACGATCTGTAGCTGGTCTGAGAGGACGACC
AGCCACACTGGGACTGAGACACGGCC
>Zotu1076
GAACGCTGGCGGCGTGCTTAACACATGCAAGTCGAACGGAAAGGCTCCTGCTTGCAGGGGTACTCGAG
TGGCGAACGGGTGAGTAACACGTGGGTGATCTGCCTTGTACTTCGGGATAAGCCTGGGAAACTGGGTC
TAATACCGGATAGGACCAATCTTTAGTGTGGTTGGTGGAAAGTTTTGTCGGTACGAGATGAGCCCGCG
GCCTATCAGCTTGTTGGTGGGGTAATGGCCTACCAAGGCGACGACGGGTAGCCGGCCTGAGAGGGTGT
ACGGCCACATTGGGACTGAGACACGGCC
>Zotu1077
GAACGCTGGCGGTATGGATGAGGCATGCAAGTCGAATGCATTTAGTTGCATGGCAGACGAGGTAGTAA
CACGTAGGAACGTACCCAGAAGTCGGGCATAACCTGTCGAAAGGCAGAATAATTCTCGATAGTCTCGC
AAGAGTAAAGTTTTTCGCTTCTGGAGCGGCCTGCGCAATATCAGCTAGTTGGTGAGGTAATGGCTCAC
CAAGGCTATGACGTTTAGGGGAGCTGAGAGGCTGACCCCCACCGATGGGACTGAGACACGGCC
>Zotu1078
GAACGCTAGCTATATGCTTAACACATGCAAGTCGAACGTTGTTTTCGGGGAGCTGGGCAGAAGGAAAA
GAGGCTCCTAGCTAAAGTTGTCTCGCCCTGCTTCAAAACTACAGGGCGCGCGCTACGGCTTTGACCTA
ACGGCCTCCGTTTGCTGGAATCGGAATAGTTGAGAACAAAGTGGCGAACGGGTGCGTAACGCGTGGGA
ATCTGCCGAACAGTTCGGGCCAAATCCTGAAGAAAGCTCAAAAGCGCTGTTTGATGAGCCTGCGTAGT
ATTAGGTAGTTGGTCAGGTAAAGGCTGACCAAGCCAATGATGCTTAGCTGGTCTTTTCGGATGATCAG
CCACACTGGGACTGAGACACGGCC
>Zotu1080
GAACGCTGGCGGCGCGCTTAACACATGCAAGTCGAGCGAGAACCCTCCTTCGGGAGGGGGAAAGCGGC
GAACGGGTGAGTAACACGTGAGTAATCCACCCTCGGTACCGGGATAGCCCGGGGAAACCCGGATTAAT
ACCGGATGGCCCAGCAGCCCTGCGGGGCGGCTGGAAAAGGTAGCTTCGGCCTCCGACCGAGGACGAGC
TCGCGGCGGATTAGCTTGTTGGTGGGGTAACGGCCTACCAAGGCGACGATCCGTAGCTGGTCTGAGAG
GACGATCAGCCACACTGGGACTGAGACACGGCC
>Zotu1081
GAACGCTGGCGGCGTGCTTAACACATGCAAGTGGAGCGACGAACCAGGGCTTACCCTGGGGCAGAGCC
GCGAACGGGTGAGTAACACGTGGGTTACCTGCCTTGATGACCGGGACAACCCGAGGAAACTCGGGCTA
ATACCGGATGTGCCCGCAAGGGGAAAGGAAGCTTCGGCCTCCGCATCAAGATGGGCCCGCGGCCCATT
AGCTTGTTGGTGAGGTAACGGCTCACCAAGGCTTCGATGGGTAGCTGGTCTGAGAGGACGATCAGCCA
CACTGGGACTGAGACACGGCC

>Zotu1082
GAACGCTGGCGGCATGCCTAATACATGCAAGTCGAGCGAACAGACGAGGTGCTTGCACCTCTGACGTT
AGCGGCGGATGGGTGAGTAACACGTGGGCAACCTGCCCTGCAGATGGGGATAACTCCGGGAAACCGGG
GCTAATACCGAATAAACCATTGAACCTCCTGGTTCGATGTTGAAAGACGGTTTCGGCTGTCACTGCAG
GATGGGCCCGCGGCGCATTAGCTAGTTGGTGGGATAACGGCCTACCAAGGCAACGATGCGTAGCCGAC
CTGAGAGGGTGATCGGCCACACTGGGACTGAGACACGGCC
>Zotu1083
GAACGCTGGCGGCGTGCCTAATACATGCAAGTCGAGCGGAGTTGTGAGGGAGCTTGCTCCCGACCAAC
TTAGCGGCGGACGGGTGAGTAATACGTAGGCAACCTGCCCACAAGACCGGGATAACATTCGGAAACGA
ATGCTAATACCGGATACGCAGCATGGCCGCATGGCCGAGCTGAGAAAGACGGAGCAATCTGTTACTTG
TGGATGGGCCTACGGCGCATTAGCTAGTTGGTGGGGTAACGGCTCACCAAGGCGACGATGCGTAGCCG
ACCTGAGAGGGTGAACGGCCACACTGGGACTGAGACACGGCC
>Zotu1084
GAACGCTGGCGGCGTGCTTAACACATGCAAGTCGAACGGTGAAGGGACCAGCTTGCTGGTTCTGGATG
AGTGGCGAACGGGTGAGTAACACGTGAGTAACCTGCCCCCTTCTTCTGGATAACCGCATGAAAGTGTG
GCTAATACGGGATATTCTGCGCCTGCTGCATGGTGGGTGTGGGAAAGATATTGTTTCGGTGGGGGATG
GGCTCGCGGCCTATCAGCTTGTTGGTGGGGTGATGGCCTACCAAGGCGGTGACGGGTAGCCGGCCTGA
GAGGGTGGACGGTCACACTGGGACTGAGACACGGCC
>Zotu1085
GAACGCTAGCGGCAGGCTTAACACATGCAAGTCGAGGGGTAGAGTTCTTCGGAGCTTGAGACCGGCGC
ACGGGTGCGTAACGCGTATGCAATCTACCTTTTACAGAGGGATAGCCCAGAGAAATTTGGATTAATAC
CTCATAGTATAATTGAATGGCATCATTTAATTATTAAAGTCACAACGGTAAAAGATGAGCATGCGTCC
CATTAGCTAGTTGGTAAGGTAACGGCTTACCAAGGCTACGATGGGTAGGGGTCCTGAGAGGGAGATCC
CCCACACTGGTACTGAGACACGGAC
>Zotu1086
CAACGCTGGCGGCGTGCCTAATGCATGCAAGTTGAGCGGGGTCGTTCTTCGGAGGGGCTTACGCGGCG
GACGGGTGCGTAACACGTGGGGAACCTGCCCTGGGGTGGGGGATAGCCGAGGGAAACCTTGGGTAATT
CCGCATACGCTTGCGTACCGGAAGGGTACGTGAGGAAAGGGTCTTTCGGGACTCGCCTTGGGAGGGGC
CTGCGGCCGATTAGCTAGACGGTGGGGTAACGGCTCACCGTGGCGATGATCGGTAGCTGGTCTGAGAG
GACGGCCAGCCACACGGGGACTGAGAGACGGCC
>Zotu1087
GAACGCTGGCGGCGTGCTTAACACATGCAAGTCGAACGGAAAGGCCCTTCGGGGTGCTCGAGTGGCGA
ACGGGTGAGTAACACGTGGGTGATCTGCCCTGCACTTTGGGATAAGCCTGGGAAACTGGGTCTAATAC
CGGATAGGACCACGCGCTTCATGGTGTGTGGTGGAAAGCTTTTGCGGTGTGGGATGGGCCCGCGGCCT
ATCAGCTTGTTGGTGGGGTAATGGCCTACCAAGGCGTCGACGGGTAGCCGGCCTGAGAGGGTGTCCGG
CCACACTGGGACTGAGATACGGCC
>Zotu1088
GAACGCTGGCGGCATGCCTTACACATGCAAGTCGAACGGCAGCATAGGAGCTTGCTCCTGATGGCGAG
TGGCGAACGGGTGAGTAATATATCGGAACGTACCCAAGAGTGGGGGATAACGTAGCGAAAGTTACGCT
AATACCGCATACGATCTAAGGATGAAAGTGGGGGATCGCAAGACCTCATGCTCCTGGAGCGGCCGATA
TCTGATTAGCTAGTTGGTGAGGTAAAGGCTCACCAAGGCGACGATCAGTAGCTGGTCTGAGAGGACGA
CCAGCCACACTGGGACTGAGACACGGCC
>Zotu1089
GAACGCTGGAAATGTGGATACCGGCATGCAAGTCAAGGGGCCGCAAGGCACCGGCGAACGGCTTAGTA
ACACGTAGATACATACCGCAAAGTGGAGCATAGCTCGTCGAAAGACGGGGTAATTTTCCATAGTCCCG
CAAGGGTAAAGGCGCAAGCCGCTTTGTGAATGGTCTGCGGTCTATCAGCTAGTTGGTGAGGTAATGGC
TCACCAAGGCTATGACGGATAGGGGGCGTGAGAGCGCGACCCCCAAGAATGGCACTGCGACACGGGC
>Zotu1090
GAACGCTGGCGGCGTGCCTAATACATGCAAGTAGAACGCTGAAGGAGGAGCTTGCTCTTCTGGATGAG
TTGCGAACGGGTGAGTAACGCGTAGGTAACCTGCCTGGTAGCGGGGGATAACTATTGGAAACGATAGC
TAATACCGCATAATAGTAGATGTTGCATGACATTTGCTTGAAAGGTGCAATTGCATCACTACCAGATG
GACCTGCGTTGTATTAGCTAGTTGGTGGGGTAACGGCTCACCAAGGCGACGATACATAGCCGACCTGA
GAGGGTGATCGGCCACACTGGGACTGAGACACGGCC
>Zotu1091

GAACGCTGGCGGCATGCCTTACACATGCAAGTCGAACGGCAGCACGGACTTCGGTCTGGTGGCGAGTG
GCGAACGGGTGAGTAATACATCGGAACGTGCCCTGTTGTGGGGGATAACTAGTCGAAAGATTAGCTAA
TACCGCATACGACCTGAGGGTGAAAGCGGGGGACCGCAAGGCCTCGCGCAATAGGAGCGGCCGATGTC
TGATTAGCTAGTTGGTGGGGTAAAGGCCTACCAAGGCGACGATCAGTAGCTGGTCTGAGAGGACGATC
AGCCACACTGGGACTGAGACACGGCC
>Zotu1092
GAACGCTAGCGGCAGGCTTAACACATGCAAGTCGAGGGGTATAGGTTTTCGGACCTAGAGACCGGCGC
ACGGGTGCGTAACGCGTATGCAATCTACCTTTCACAGAGGGATAGCCCAGAGAAATTTGGATTAATAC
CTCATAGTATTATAGAGTGGCATCACTTTATAATTAAAGTCACAACGGTGAAAGATGAGCATGCGTCC
CATTAGCTAGTTGGTAAGGTAACGGCTTACCAAGGCAACGATGGGTAGGGGTCCTGAGAGGGAGATCC
CCCACACTGGTACTGAGACACGGAC
>Zotu1093
GAACGCTGGCGGCGTGCTTAACACATGCAAGTCGAACGAAGAGCGATGGAAGCTTGCTTCTATCAATC
TTAGTGGCGAACGGGTGAGTAACGCGTAATCAACCTGCCCTTCAGAGGGGGACAACAGTTGGAAACGA
CTGCTAATACCGCATACGATCTAACCTCGGCATCGAGGATAGATGAAAGGTGGCCTCTATTTATAAGC
TATCACTGAAGGAGGGGATTGCGTCTGATTAGCTAGTTGGAGGGGTAACGGCCCACCAAGGCGATGAT
CAGTAGCCGGTCTGAGAGGATGAACGGCCACATTGGGACTGAGACACGGCC
>Zotu1094
TAACGCTGGCGGCGTGCCTAACACATGCAAGTCGAGCGCCTCGCTTTGCGAGGAGCGGCGGATGGCTG
AGTAACACGTTGGTACCTACCCCGAACTCGGGAATAAGCGCTGGAAACGGCGTCTAATGCCCGATAGT
TCTCTTCGGAGTAAAGCTTTTGCGGTTCGGGAGGGGCCTGCGCCCGATTAGCTTGTTGGTGAGGTAAC
GGCTCACCAAGGCGATGATCGGTAGGTGGTCTGAGAGGATGATCACCCAGACTGGGACTGAGACACGG
CC
>Zotu1095
GAACGCTGGCGGCATGCCTAATACATGCAAGTCGAGCGAACAGACGAAGTGCTTGCACTTCTGACGTT
AGCGGCGAACGGGTGAGTAACACGTAAGGAATCTACCTATAAGCGGGGGATAACATTCGGAAACGGGT
GCTAATACCGCATAATATCTTCTTCCGCATGGAAGAAGATTGAAAGACGGCTCTGCTGTCACTTATAG
ATGACCTTGCGGTGCATTAGTTAGTTGGTGGGGTAATGGCCTACCAAGACGATGATGCATAGCCGACC
TGAGAGGGTGATCGGCCACATTGGGACTGAGACACGGCC
>Zotu1096
GAACGCTGGCGGCATGCCTCATACATGCAAGTCGAACGAAGGCTTCGGCCTTAGTGGCGCACGGGTGA
GTAGCGCGTGGGAACCTGCCCTGGAGTGGGGGATAACCGAGGGAAACTTCGGCTAATACCGCATGCGC
CCGGTAGCAATACCGGGGAAAGATTTATCGCTCTAGGAGGGGCCCGCGTCGGATTAGCTAGTTGGTGG
GGTAACGGCCTACCAAGGCAACGATCCGTAGCTGGTCTGAGAGGATGATCAGCCACACTGGGACTGAG
ACACGGCC
>Zotu1097
GAACGCTAGCGGCAGGCTTAACACATGCAAGTCGAGGGGCAGCACGATGTAGCAATACATGGGTGGCG
ACCGGCGCACGGGTGCGTAACACGTATGCAATCTTCCCTTCACTGGGGCATAGCCCGGAGAAATCCGG
ATTAATACCCCTTAATAGCTCGAGTGGCATCACTTGGGTTTGAAAGTTCCGACGGTGAAGGATGAGCA
TGCGCCCGATTAGCTAGTTGGTGAGGTAATGGCTCACCAAGGCTACGATCGGTAGGGGGCCTGAGAGG
GTTATCCCCCACACTGGTACTGAGACACGGAC
>Zotu1098
GAACGTTAGCGGCGCGCTTAACACATGCAAGTCGAGCGAGAAAGGGGCAACCCCGGTAAAGCGGCGCA
CGGGTGAGTAACACGTAGGTAATCTACCCCCAGGTGGTGGATAACCTTCCGAAAGGAAGGCTAATACA
GCATGGGACCACGACTCCGAAAGGAGTTGAGGTGAAAGTTGGCCTCTTCACGAAAGCCAACGCCAGGG
GATGAGCCTGCGGCCCATCAGCTAGTTGGTAGGGTAATGGCCTACCAAGGCAAAGACGGGTAGCTGGT
CTGAGAGGATGATCAGCCACACTGGAACTGAGACACGGTC
>Zotu1099
GAACGCTGGCGGCGTGCCTAAGACATGCAAGTCGAGTGGGTTTAGACCCACGGCGAACGGGTGAGTAA
TACCTAGGAACTTTCTTTAAGTAGAGGATAACTCCGCGAAAGCGGAGATAATACTCTATGTGATAGTA
ATATCAAAGTAGCAATACGCTTAAAGTGAGGCCTGGGCACCATCAGCTAGTTGGTAAGGTAACGGCTT
ACCAAGGCTATGACGGTTAGGGGTCCCGAGAGGGATAACCCCCACACTGGAACTGCGACACGGTC
>Zotu1100
GAACGCTGGCGGCGTGCTTAACACATGCAAGTCGAACGGAAAGGCCACAGCTTGCTGTGGTACTCGAG

TGGCGAACGGGTGAGTAACACGTGGGTGATCTGCCCTGTACTTCGGGATAAGCTTGGGAAACTGGGTC
TAATACCGGATAGGAATCACATGTGGGTGTGTGGTTGGAAAGCTTTTGCGGTATGGGATGAGCTCGCG
GCCTATCAGCTTGTTGGTGGGGTAATGGCCTACCAAGGCGTCGACGGGTAGCCGGCCTGAGAGGGTGT
ACGGCCACATTGGGACTGAGATACGGCC
>Zotu1101
GAACGCTGGCGGCGTGCTTAACACATGCAAGTCGAACGGTGAAGGGACTGGTTTTTGCTGGTTCTGGA
TGAGTGGCGAACGGGTGAGTAACACGTGAGTAACCTGCCCCCTTCTTCTGGATAACCGCATGAAAGTG
TGGCTAATACGGGATATTCTGCGCCTGCTGCATGGTGGGTGTGGGAAAGATATTGTTTCGGTGGGGGA
TGGGCTCGCGGCCTATCAGCTTGTTGGTGGGGTGATGGCCTACCAAGGCGGTGACGGGTAGCCGGCCT
GAGAGGGTGGACGGTCACACTGGGACTGAGACACGGCC
>Zotu1102
GAACGCTGGCGGTAGGCCTAACACATGCAAGTCGAACGGCAGCACAGAGGAGCTTGCTCCTTGGGTGG
CGAGTGGCGGACGGGTGAGGAATACATCGGAATCTACTTTTTCGTGGGGGATAACGTAGGGAAACTTA
CGCTAATACCGCATACGACCTACGGGTGAAAGCAGGGGATCTTCGGACCTTGCGCGATTGAATGAGCC
GATGTCGGATTAGCTAGTTGGCGGGGTAAAGGCCCACCAAGGCGACGATCCGTAGCTGGTCTGAGAGG
ATGATCAGCCACACTGGAACTGAGACACGGTC
>Zotu1103
GAACGCTAGCGGCAGGCTTAATACATGCAAGTCGAACGGTAACAGGTGTAGCAATACGCGCTGACGAG
TGGCAGACGGGTGAGGAACACGTACGCAATCTCCCTCTTTCTGGTGAATAGCCCTGAGAAATTAGGAT
TAATACACCATAGTATTACGAAACGGCATCGTTTTGTAATTAAAACTCCGGTGGAAAGAGATGAGCGT
GCGACTGATTAGCTAGTTGGTAAGGTAACGGCTTACCAAGGCAACGATCAGTAACTGGTGTGAGAGCA
CGACCAGTCACACGGGCACTGAGATACGGGC
>Zotu1104
GAACGCTGGCGGCAGGCTTAACACATGCAAGTCGAGCGGGGGAGGTTGCTTCGGTAACTGACCTAGCG
GCGGACGGGTGAGTAATACTTAGGAATCTGCCTATTAATGGGGGACAACATCTCGAAAGGGATGCTAA
TACCGCATACGCCCTACGGGGGAAAGCAGGGGATCACTTGTGACCTTGCGTTAATAGATGAGCCTAAG
TCGGATTAGCTAGTTGGTGGGGTAAAGGCCTACCAAGGCGACGATCTGTAGCGGGTCTGAGAGGATGA
TCCGCCACACTGGGACTGAGACACGGCC
>Zotu1105
GAACGCTGGCGGCATGCCTTACACATGCAAGTCGAACGGCAGCACGGGTGCTTGCACCTGGTGGCGAG
TGGCGAACGGGTGAGTAATACATCGGAACATGTCCTGTAGTGGGGGATAGCCCGGCGAAAGCCGGATT
AATACCGCATACGATCTACGGATGAAAGCGGGGGACCTTCGGGCCTCGCGCTATAGGGTTGGCCGATG
GCTGATTAGCTAGTTGGTGGGGTAAAGGCCTACCAAGGCGACGATCAGTAGCTGGTCTGAGAGGACGA
CCAGCCACACTGGGACTGAGACACGGCC
>Zotu1106
GAACGCTGGCGGCGTGCCTAATACATGCAAGTCGAGCGAACCAATAAGAAGCTTGCTTTTTGTTGGTT
AGCGGCGGACGGGTGAGTAACACGTGGGTAACCTGCCTGTAAGACCGGGATAACTCCGGGAAACCGGT
GCTAATACCGGATAGATTATCTTTCCGCCTGGAGAGATAAGGAAAGATGGCTATTGCCATCACTTACA
GATGGGCCCGCGGCGCATTAGCTAGTTGGTGAGGTAACGGCTCACCAAGGCGACGATGCGTAGCCGAC
CTGAGAGGGTGATCGGCCACACTGGGACTGAGACACGGCC
>Zotu1107
AAACGCTGGCGGCGTGCCTAATGCATGCAAGTCGAACGGGAGCCCCTTTGGGGGTCGACCGTGGCGGA
CGGGTGAGGAACACGTAGCTAACCTGCCCAGGTATGGGGGATATGCGCTGGAAACGGCGTGCAATACC
GCATACGTTCATCGGTCGGGAGACGGATGAGGAAAGCGCTTCGGCGTGTACCTGGAGGGGGCTGCGGC
CGATTAGCTAGTTGGAGTGGTAAGAGCACCCCAAGGCGATGATCGGTAGCTGGTCTGAGAGGACGATC
AGCCACACGGGGACTGAGACACGGCC
>Zotu1108
GAACGCTGGCGGCGTGCTTAACACATGCAAGTCGAACGGTGAACTCCGCTTGCGGGGGGATCAGTGGC
GAACGGGTGAGTAACACGTGGGCAACCTGCCCTCAGCTCTGGGATAACTCCAAGAAATTGGGGCTAAT
ACTGGATATGACTGCTGACCGCATGGTCTGGTGGTGGAAAGATTTATCGGCTGGGGATGGGCCCGCGG
CCTATCAGCTTGTTGGTGGGGTAGTGGCCTACCAAGGCGACGACGGGTAGCCGGCCTGAGAGGGTGAC
CGGCCACACTGGGACTGAGACACGGCC
>Zotu1109
GAACGCTGGCGGCGTGCCTAATACATGCAAGTAGAACGCACAGTTTATACCGTAGCTTGCTACACCAT

AGACTGTGAGTTGCGAACGGGTGAGTAACGCGTAGGTAACCTGCCTGGTAGCGGGGGATAACTATTGG
AAACGATAGCTAATACCGCATAATATTAATTATTGCATGATAATTAATTGAAAGGTGCAATTGCACCA
CTACCAGATGGACCTGCGTTGTATTAGCTAGTAGGTGAGGTAACGGCTCACCTAGGCGACGATACATA
GCCGACCTGAGAGGGTGATCGGCCACACTGGGACTGAGACACGGCC
>Zotu1111
GAACGCTGGCGGCGTGCTTAACACATGCAAGTCGAACGATGAACTCCAGCTTGCTGGGGGGATTAGTG
GCGAACGGGTGAGTAACACGTGAGTAACCTGCCCTGGACTCTGGGATAACCCCGAGAAATCGGAGCTA
ATACCGGATAGGACCTTTCCTCGCATGAGGGTTGGTGGAAAGTTTTTCGGTTTGGGATGGACTCGCGG
CCTATCAGCTTGTTGGTGAGGTAATGGCTCACCAAGGCGTCGACGGGTAGCCGGCCTGAGAGGGTGAC
CGGCCACACTGGGACTGAGACACGGCC
>Zotu1112
GAACGCTAGCGGCAGGCTTAACACATGCAAGTCGAGGGGCAGCAGGGACTTCGGTCCGCTGGCGACCG
GCAAACGGGTGCGGAACACGTACGCAACATACCTCTATCTGGGGCATAGCCCGGTGAAAACCGGATTA
ACTCCCCATAGTATTTTTTGGTGGCATCACCAGTTAATTAAAAATTCATTGGATAGAGATTGGCGTGC
GTCCTATTAGCTAGTTGGCGGGGTAAAGGCCCACCAAGGCGACGATAGGTAGCTGATGTGAGAGCATG
ATCAGCCACACGGGCACTGAGACACGGGC
>Zotu1113
GAACGCTGGCGGCATGCCTAACACATGCAAGTCGAACGAGACCTTCGGGTCTAGTGGCGCACGGGTGC
GTAACGCGTGGGAATCTGCCCTTGGGTTCGGAATAACAGTTGGAAACGACTGCTAATACCGGATGATG
ACGTAAGTCCAAAGATTTATCGCCCAGGGATGAGCCCGCGTAGGATTAGCTAGTTGGTGAGGTAAAGG
CTCACCAAGGCGACGATCCTTAGCTGGTCTGAGAGGATGATCAGCCACACTGGGACTGAGACACGGCC
>Zotu1114
GAACGCTGGCGGCAGGCTTAACACATGCAAGTCGAGCGCCCCGCAAGGGGAGCGGCAGACGGGTGAGT
AACGCGTGGGAATCTACCCTCTACTACGGAATAACGCAGGGAAACTTGTGCTAATACCGTATGTGTCC
TTTTTGGAGAAAGATTTATCGGTAGGGGATGAGCCCGCGTTGGATTAGCTAGTTGGTGGGGTAAAGGC
CTACCAAGGCGACGATCCATAGCTGGTCTGAGAGGATGATCAGCCACATTGGGACTGAGACACGGCC
>Zotu1115
GAACGCTAGCGGCAGGCCTAATACATGCAAGTCGGACGGGATTGGAGAGCTTGCTTTCCATGAGAGTG
GCGCACGGGTGCGTAACACGTATGTAACCTACCTTCTTCAGGGGGATAGCCTCTCGAAAGAGAGATTA
AGACCGCATAAAATCACAATGTGGCATCACAAAATGATCAAATATTTATAGGAAGAAGATGGGCATGC
GGAACATTAGCTAGTTGGTAAGGTAACGGCTTACCAAGGCTACGATGTTTAGGGGATCTGAGAGGATG
ACCCCCCACACTGGTACTGAGACACGGAC
>Zotu1116
GAACGCTGGCGGTAGGCCTAACACATGCAAGTCGAACGGCAGCACAGGAGAGCTTGCTCTCTGGGTGG
CGAGTGGCGGACGGGTGAGGAATACATCGGAATCTACTTTTTCGTGGGGGATAACGTAGGGAAACTTA
CGCTAATACCGCATACGACCTACGGGTGAAAGCAGGGGATCTTCGGACCTTGCGCGATTGAATGAGCC
GATGTCGGATTAGCTAGTTGGCGGGGTAAAGGCCCACCAAGGCGACGATCCGTAGCTGGTCTGAGAGG
ATGATCAGCCACACTGGAACTGAGACACGGTC
>Zotu1117
GAACGCTGGCGGCATGCTTTACACATGCAAGTCGAACGGCAGCGCGGGGCAACCTGGCGGCGAGTGGC
GAACGGGTGAGTAATATATCGGAACGTGCCCTAGAGTGGGGGATAACTAGTCGAAAGACTAGCTAATA
CCGCATACGATCTACGGATGAAAGTGGGGGATCGCAAGACCTCATGCTCCTGGAGCGGCCGATATCTG
ATTAGCTAGTTGGTGGGGTAAAAGCTCACCAAGGCGACGATCAGTAGCTGGTCTGAGAGGACGACCAG
CCACACTGGGACTGAGACACGGCC
>Zotu1118
GAACGCTAGCGGCAGGCCTAACACATGCAAGTCGAGCGGGATTCGGTGGCTTGCCACCGATGAGAGCG
GCGCACGGGTGCGTAACGCGTATACAACCTACCTTTTGCTACGGGATAGCCCAGAGAAATTTGGATTA
ACACCGTATGGTATAAGGACCCGGCATCGGGATCTTATTAAAGGCTACGGCAAAAGATGGGTATGCGT
CCTATTAGCTAGATGGTGCGGTAACGGCGCACCATGGCAACGATAGGTAGGGGCCCTGAGAGGGGGAT
CCCCCACACTGGTACTGAGACACGGAC
>Zotu1119
GAACGCTGGCGGCGTGCTTAACACATGCAAGTCGAGCGGTAAGGCCCTTCGGGGTACACGAGCGGCGA
ACGGGTGAGTAACACGTGAGCAATCTGCCCTTCACTTCGGGATAACCACCGGAAACGGTGGCTAATAC
CGGATATGACTGCACCAGGCATGTGGTGTGGTGGAAAGTTCTGGCGGTGGAGGATGAGCTCGCGGCCT

ATCAGCTTGTTGGTGGGGTAATGGCCTACCAAGGCGACGACGGGTAGCCGGCCTGAGAGGGTGACCGG
CCACACTGGGACTGAGACACGGCC
>Zotu1120
GAACGTTGGCGGCGTGGATTAGGCATGCAAGTCGTACGCACCGCAAGGTGAGTGGCGCAAGGGTGAGT
AAGGCGAAGGTAACCAACCCCGAGATTGGGTATAGCCGTGGGAAACCGCGGGTAATCCCCAGCGATGT
TGCTGAGTGGCATCACTCGACAACCAAAGCTCCGGCGTCTTGGGACGGGCCTTCGTGGTATTAGGTTG
TTGGCGGGGTAATGGCCCACCAAGCCTCTGATGCCTACCGGGCGTGCGAGCGTGGCCCGGCACACTGG
GACTGAGACACTGCC
>Zotu1121
GAACGCTGGCGGCGTGCTTAACACATGCAAGTCGAACGATGATCAGGAGCTTGCTCCTGTGATTAGTG
GCGAACGGGTGAGTAACACGTGAGTAACCTGCCCCTGACTCTGGGATAAGCGTTGGAAACGACGTCTA
ATACTGGATATGACAGCCGATCGCATGGTCTGGTTGTGGAAAGAATTTCGGTTGGGGATGGACTCGCG
GCCTATCAGGTAGTTGGTGAGGTAATGGCTCACCAAGCCGACGACGGGTAGCCGGCCTGAGAGGGTGA
CCGGCCACACTGGGACTGAGACACGGCC
>Zotu1122
GAACGCTGGCGGCGTGCTTAACACATGCAAGTCGAACGATGAAGCTCCAGCTTGCTGGGGTGGATTAG
TGGCGAACGGGTGAGTAACACGTGAGTAACCTGCCCTTCACTCTGGGATAAGCCTTGGAAACGAGGTC
TAATACTGGATATTCATACATGGTCGCATGGTTGTGTGTGGAAAGATTTTTTGGTGGGGGATGGACTC
GCGGCCTATCAGCTTGTTGGTGAGGTAGTGGCTCACCAAGGCTTTGACGGGTAGCCGGCCTGAGAGGG
TGACCGGCCACACTGGGACTGAGACACGGCC
>Zotu1123
GAACGCTGGCGGAATGCTTTACACATGCAAGTCGAACGGTAACAGGGACTTCGGTCCGCTGACGAGTG
GCGAACGGGTGAGTAATACATCGGAACGTACCCAGTTGTGGGGGATAACTACGCGAAAGCGTAGCTAA
TACCGCATACGACCTGAGGGTGAAAGCGGGGGACCGTAAGGCCTCGCGCAGTTGGAGCGGCCGATGTT
CGATTAGCTAGTTGGTGAGGTAAAGGCTCACCAAGGCGACGATCGATAGCTGGTCTGAGAGGATGATC
AGCCACACTGGGACTGAGACACGGCC
>Zotu1124
TAACGTTGGCAGTGTGCCTAACACATGCAAGTCGAGCGGCTCGCAAGAGCAGCGGCAGACGAGAGAGT
AATAACAAGGAACTTACCCCCGAGTGAAGGATAGCCATCCGAAAGGATGGGTAATCCTGCATAATATC
GAAAGATCAAAGCCTTCGGGCGCTTGGGGAGAGGCCTTGTCCCTATCAGCTAGTTGGTGAGGTAATGG
CTCACCAAGGCGATGACGGGTAGCTGGACTGAGAGGTTGACCAGCCTCGATGGGACTGAGACACGGCC
>Zotu1125
GAACGCTAGCGGCAGGCTTAACACATGCAAGTCGAGGGGTATAGTAGCAATACTAGAGACCGGCGCAC
GAGTGCGTAACGCGTATGCAATCTACCTTTTACAGAGGGATAGCCCAGAGAAATTTGGATTAATACCT
CATAGTATAGTGAGTTGGCATCAACACACTATTAAAGTCACAACGGTAAAAGATGAGCATGCGTCCCA
TTAGCTAGTTGGTAAGGTAACGGCTTACCAAGGCGACGATGGGTAGGGGTCCTGAGAGGGAGATCCCC
CACACTGGTACTGAGACACGGAC
>Zotu1126
GAACGCTGGCGGCATGCCTTACACATGCAAGTCGAACGGTAACAGGTCTTCGGACGCTGACGAGTGGC
GAACGGGTGAGTAATACATCGGAACGTGCCCGATCGTGGGGGATAACGAGGCGAAAGCTTTGCTAATA
CCGCATACGATCTACGGATGAAAGCGGGGGATCTTCGGACCTCGCGCGGACGGAGCGGCCGATGGCAG
ATTAGGTAGTTGGTGGGATAAAAGCTTACCAAGCCGACGATCTGTAGCTGGTCTGAGAGGATGATCAG
CCACACTGGGACTGAGACACGGCC
>Zotu1127
GAACGCTAGCGGCAGGCTTAATACATGCAAGTCGAGGGGCAACAGGGGTAGCAATACCTGCTGGCGAC
CGGCAAACGGGTGCGGAACACGTACAGAACCTTCCTTTAAGCGGGGGATAGCCCGGAGAAATCCGGAT
TAATACCCCATAGTAATATTGATTGGCATCAATTGATATTTAAAGATTTATCACTTAAAGATGGCTGT
GCGGCTGATTAGGTAGTTGGTTGAGGTAACGGCTCACCAAGCCAACGATCAGTAACTGGTGTGAGAGC
ACGACCAGTCACACGGGCACTGAGACACGGGC
>Zotu1128
GAACGCTGGCGGCGTGCTTAACACATGCAAGTCGAACGGTGAAGCGGGTGCTTGCACTCGTGGATCAG
TGGCGAACGGGTGAGTAACACGTGAGTAACCTGCCCATCACTCTGGGATAAGCGCTGGAAACGGCGTC
TAATACTGGATACGACCAACCCTTGCATGGGGTGTTGGTGGAAAGATTTATCGGTGGTGGATGGACTC
GCGGCCTATCAGCTTGTTGGTGGGGTAATGGCCTACCAAGGCGACGACGGGTAGCCGGCCTGAGAGGG

CGACCGGCCACACTGGGACTGAGACACGGCC
>Zotu1129
GAACGCTGGCGGCGTGCTTAACACATGCAAGTCGAACGCTGAAGCCTAGCTTGCTGGGTGGATGAGTG
GCGAACGGGTGAGTAACACGTGAGTAACCTGCCCTCTTCTTTGGGATAACGCCCGGAAATGGGTGCTA
ATACTGGATATTCACTGGTCTTCGCATGGGGGTTGGTGGAAAGGGTTTTTCTGGTGGGGGATGGGCTC
GCGGCCTATCAGCTTGTTGGTGGGGTGATGGCCTACCAAGGCTTTGACGGGTAGCCGGCCTGAGAGGG
TGACCGGTCACATTGGGACTGAGATACGGCC
>Zotu1130
GAACGCTAGCGGCAGGCTTAACACATGCAAGTCGAGGGGTAAGGTAGCAATATCTGAGACCGGCGCAC
GGGTGCGTAACGCGTATGCAATCTACCTTTTGCAGAGGGATAGCCCAGAGAAATTTGGATTAATACCT
CATAGTATATAGAGTTGGCATCAACACTATATTAAAGTCACAACGGCAAAAGATGAGCATGCGTCCCA
TTAGTTAGTTGGTAAGGTAACGGCTTACCAAGACTACGATGGGTAGGGGTCCTGAGAGGGAGATCCCC
CACACTGGTACTGAGACACGGAC
>Zotu1131
GAACGCTGGCGGCATGCCTAACACATGCAAGTCGAACGAGGGCTTCGGCCCTAGTGGCGCACGGGTGA
GTAACGCGTGGGAACCTGCCCTGTGGTACGGAATAACTCCGGGAAACTGGAGCTAATACCGTATGCGT
CCTGCGGGACAAAGATTTATCGCCATGGGATGGGCCCGCGTAGGATTAGCTTGTTGGTGGGGTAACGG
CCTACCAAGGCAACGATCCTTAGCTGGTCTGAGAGGATGATCAGCCACACTGGGACTGAGACACGGCC
>Zotu1132
GAACGCTGGCGGCATGCCTAATACATGCAAGTCGAACGCACGAAGTTGAAAAGCTTGCTTTTCGACCA
AGTGAGTGGCGGACGGGTGAGTAACACGTGGGTAACCTGCCCATAAGAGGGGGATAACATTCGGAAAC
GGATGCTAATACCGCATATTTCAAGTGACCGCATGGTCGCTTGATGAAAGGTGGCTTCGGCTACCACT
TATGGATGGACCCGCGGTGCATTAGTTAGTTGGTGAGGTAATGGCTCACCAAGACCATGATGCATAGC
CGACCTGAGAGGGTGATCGGCCACACTGGGACTGAGACACGGCC
>Zotu1134
GAACGCTGGCGGCGTGCTTAACACATGCAAGTGGAGCGACGAACCAAGGCTTGCCTTGGGGCAGAGCC
GCGAACGGGTGAGTAACACGTGGGTAACCCACCTCGATGACCGGGACAACCCGAGGAAACTCGGGCTA
ATACCGGATGAGATCCTCTTTCGTAAGGAAGATGATGAAAGATAGCTTCGGCCTTCGCATCGAGACGG
GCCCGCGGCCCATTAGCTTGTTGGTGGGGTAATGGCCTACCAAGGCTTCGATGGGTAGCTGGTCTGAG
AGGACGATCAGCCACACTGGGACTGAGACACGGCC
>Zotu1135
GAACGCTGGCGGCGTGCCTAATACATGCAAGTCGAGCGAACCACTTCGGTGGGAAGCGGCGAACGGGT
GAGTAACACGTAGGTAATCTGCCCATCAGACGGGGACAACGATTGGAAACGATCGCTAATACCGGATA
GGACGAAAGTTTAAAGATGCTCCTGGCATCACTGATGGATGAGCCTGCGGCGCATTAGCTAGTTGGTG
GGGTAAAGGCCTACCAAGGCGACGATGCGTAGCCGACCTGAGAGGGTGAACGGCCACACTGGGACTGA
GACACGGCC
>Zotu1136
GAACGCTAGCGGGAGGCTTAACACATGCAAGCCGAGGGGTATATAGTTTTCGGACTATAGAGACCGGC
GCACGGGTGCGTAACGCGTATGTAACTTGCCCTGATCAGTAGGATAGCCCTTCGAAAGGAGGATTAAT
ACTGCATAACACTAAGATTGGCATCAATTTTAGTTGAAAGATTTATCGGATCAGGATAGACATGCGTA
TGATTAGCTAGTTGGTAGAGTAACGGCCTACCAAGGCGATGATCATTAGGGGGCCTGAGAGGGTGAAC
CCCCACACTGGTACTGAGACACGGAC
>Zotu1137
GAACGCTGGCGGCGTGCTTAACACATGCAAGTCGAGCGGAAAGGCCTCTTCGGGGGTACTCGAGCGGC
GAACGGGTGAGTAACACGTGAGTAACCTGCCCCAGTCTTTGGGATAACCCTCGGAAACGGGGGCTAAT
ACCGGATATTACTGCCGGCCGCATGGTTGGTGGTGGAAAGTTTTTTCGGTCTGGGATGGGCTCGCGGC
CTATCAGCTTGTTGGTGGGGTGATGGCCTACCAAGGCGACGACGGGTAGCCGGCCTGAGAGGGCGACC
GGCCACACTGGGACTGAGACACGGCC
>Zotu1138
GAACGCTGGCGGCATGCTTTACACATGCAAGTCGAACGGTAACAGGCCTTCGGGTGCTGACGAGTGGC
GAACGGGTGAGTAATGCATCGGAACGTGCCCAGAGGTGGGGGATAACGCAGCGAAAGCTGTGCTAATA
CCGCATGTGATCTGAGGATGAAAGCGGGGGACCAAGCAGCAATGTTTGGCCTCGCGCCTCTGGAGCGG
CCGATGTCAGATTAGGTAGTTGGTGGGGTAAAGGCCTACCAAGCCGACGATCTGTAGCTGGTCTGAGA
GGACGACCAGCCACACTGGGACTGAGACACGGCC

>Zotu1139
GAACGCTGGCGGCGTGCCTAACACATTCAAGTCGAACGGAGTTAAGTTGAAAGCTTGCTTTTGACTTA
GCTTAGTGGCGGACGGGTGAGTAATGCATGAGCAACCTGCCTTTAAGAGGGGAATAACACAGAGAAAT
TTGTGCTAATACCGCATAGAACACTGAGAGGGCATCCTCATAGTGGGAAAGAAGCAATTCGCTTAAAG
ATGGGCTCATGTCTGATTAGATAGTTGGTGAGGTAACGGCTCACCAAGTCTGCGATCAGTAGCCGGAC
TGAGAGGTTGAACGGCCACATTGGAACTGAGATACGGTC
>Zotu1140
GAACGCTAGCGGCAGGCCTAATACATGCAAGTCGGACGGGATTGGGGCTTCGGCCCTATGAGAGTGGC
GCACGGGTGCGTAACACGTATGTAACCTACCTTTATCTGGGGGATAGCCTCTCGAAAGAGAGATTAAC
ACCGCATAAAACCACAATGCAGCATTGCATAATGGTCAAATATTTATAGGATAGAGATGGGCATGCGG
GACATTAGCTAGTTGGCGGGGTAACGGCCCACCAAGGCCACGATGTCTAGGGGATCTGAGAGGATGGC
CCCCCACACTGGTACTGAGACACGGAC
>Zotu1141
GAACGCTGGCGGCATGCCTAACACATGCAAGTCGAACGAGACCTTCGGGTCTAGTGGCGCACGGGTGC
GTAACGCGTGGGAATCTACCCTTGGGTTCGGAATAACAGTTAGAAATGACTGCTAATACCGGATGATG
ACGTAAGTCCAAAGATTTATCGCCCAGGGATGAGCCCGCGTAGGATTAGGTAGTTGGTGGGGTAAAGG
CTCACCAAGCCGACGATCCTTAGCTGGTCTGAGAGGATGATCAGCCACACTGGGACTGAGACACGGCC
>Zotu1142
GAACGCTGGCGGAATGCTTTACACATGCAAGTCGAGCGGCAGCGCGGGGGCAACCCTGGCGGCGAGCG
GCGAACGGGTGAGTAATACATCGGAACGTACCCTGTCGTGGGGGATAACTAACCGAAAGGTTGGCTAA
TACCGCATACGACCTGAGGGTGAAAGCGGGGGATCGCAAGACCTCGCGCGATAGGAGCGGCCGATGTC
GGATTAGCTAGTTGGTGAGGTAAAGGCTCACCAAGGCGACGATCCGTAGCGGGTCTGAGAGGACGGCC
CGCCACACTGGAACTGAGACACGGTC
>Zotu1143
GAACGCTGGCGGCATGCTTTACACATGCAAGTCGAACGGCAGCGAAGAGAAGCTTGCTTCTCTGTCGG
CGAGTGGCGAACGGGTGAGTATAGCATCGGAACGTGCCAAGTAGTGGGGGATAACCAAGCGAAAGTTT
GGCTAATACCGCGTAAGCTCTAAGGAGGAAAGTAGGGGACCGCAAGGCCTTACGCTATTTGATCGGCC
GATGTCGGATTAGCTAGTTGGTGAGGTAATGGCTCACCAAGGCGATGATCCGTAGCGGGTCTGAGAGG
ACGATCCGCCACACTGGGACTGAGACACGGCC
>Zotu1144
GAACGCTGGCGGCGTGCTTAACACATGCAAGTCGAGCGGTAAGGCCCTTCGGGGTACACGAGCGGCGA
ACGGGTGAGTAACACGTGAGTAATCTGCCCTTCACTTCGGGATAACCACCGGAAACGGTGGCTAATAC
CGGATATGACTTCTCTTCGCATGTTGAGAGGTGGAAAGTTCCGGCGGTGGAGGATGTGCTCGCGGCCT
ATCAGCTTGTTGGTGAGGTAATGGCTCACCAAGGCTTCGACGGGTAGCCGGCCTGAGAGGGTGACCGG
CCACACTGGGACTGAGACACGGCC
>Zotu1145
GAACGCTGGCGGCGTGCCTAATACATGCAAGTCGAACGCGCGGATCAGGAGCTTGCTCCTGTGACGCG
AGTGGCGGACGGGTGAGTAACACGTAGGCAACCTGCCCATCAGACCGGGATAACCACGGGAAACCGTG
GCTAATACCGGATAATCCTTTTCCACACAGGTGGAAAAGTTGAAAGGCGGTCTTTTGGCTGTCACTGA
TGGATGGGCCTGCGGCGCATTAGCTGGTTGGTGGGGTAACGGCCCACCAAGGCGACGATGCGTAGCCG
ACCTGAGAGGGTGATCGGCCACACTGGGACTGAGACACGGCC
>Zotu1146
GAACGCTAGCGGCAGGCCTAATACATGCAAGTCGAACGGTCTCTAGCAATAGGGATAGTGGCGCACGG
GTGCGTAACACGTAACTAACCTACCCACAACTGGGGGATAGCCCGCCGAAAGGCGGATTAATACCGCA
TAAGCTCTTTGGCTGGCATCAGCTGATTAGTAAAGATTTATTGGTTGTGGATGGGGTTGCGGGTCATT
AGCTAGTTGGTAAGGTAACGGCTTACCAAGGCGACGATGACTAGGGGAGCTGAGAGGCTGGTCCCCCA
CACGGGCACTGAGATACGGGC
>Zotu1147
GAACGCTGGCGGCGCGCTTAATACATGCAAGTCGAGCGAGAACCATGGCTTCGGCCATGGGGACAGCG
GCGAACGGGTGAGTAACACGTGGGTAATCAACCCTCGACACTGGGATAGCCCGGGGAAACCCGGATTA
ATACCGGATGTCCCATCTGGCCTTCGGGCCGGCTGGAAAAGGTAGCTTCGGCCTCCGGTCGGGGACGA
GCCCGCGGCGGATTAGCTTGTTGGTGGGGTAACGGCCCACCAAGGCGACGATCCGTAGCTGGTCTGAG
AGGACGATCAGCCACACTGGGACTGAGACACGGCC
>Zotu1148

GAACGCTGGCGTCGTGCTTAACACATGCAAGTCGAACGGATCCCAAGTGTCAGCAATGATGTGGAAGG
GTTAGTGGCGAACGGGTGAGTAACACGTGAGGAACCTGCCCCGGACTCAGGGATAACGGCTGGAAACG
GCTGCTAATACCTGATGCCGTCGTGTCATCGCAGGGTGATGTGACGAAAGAATCTCGGTTCGGGAGGG
TCTCGCGGCCTATCAGCTTGTTGGTGGGGTAATGGCCTACCAAGGCGTCGACGGGTAGCTGGTCTGAG
AGGATGATCAGCCACACTGGGACTGAGACACGGCC
>Zotu1150
GAACGCTGGCGGCGTGCCTAATACATGCAAGTCGAACGAACCGCGACTAGGTGCTTGCACTTGGTCAA
GGTGAGTGGCGAACGGGTGAGTAACACGTGGGTAACCTACCTCATAGTGGGGGATAACAGTCGGAAAC
GACTGCTAATACCGCATAGGACATGGAATCACATGATTCAGTGAGGAAAGGTGGCGCAAGCTATCGCT
AAGAGATGGACCCGCGGTGCATTAGCTAGTTGGTAGGGTAACGGCCTACCAAGGCGATGATGCATAGC
CGACCTGAGAGGGTGATCGGCCACATTGGGACTGAGACACGGCC
>Zotu1151
GAACGCTAGCGGGAGGCCTAACACATGCAAGCCGAGCGGGATTTGAGACTAGCTTGCTAGTCTTAATG
AGAGCGGCGTACGGGTGCGGAACACGTGTGCAACCTGCCTTTATCTGGGAGATAGCCTTTCGAAAGGA
AGATTAATATCCCATAATATAATGATTGGCATCAATTATTATTGAAAGCTCCGGCGGATAGAGATGGG
CACGCGCAAGATTAGATAGTTGGTGAGGTAACGGCTCACCAAGTCGATGATCTTTAGGGGGCCTGAGA
GGGTGATCCCCCACACTGGTACTGAGACACGGAC
>Zotu1153
GAACGCTGGCGACGCGTCTTAAGCATGCAAGTCGAACGGCAACACGGGGCAACCTGGTGGCGAGTGGC
GAACGGGTGAGTAACACGTAGGTGATCTGCCTTTGGGATGGGGATAGCCGTTAGAAATAGCGGGTAAT
ACCGAATGAAGTCATTCGACTATGGTTGGATGATGAAAGGGGCTAAGGCCTCGCCTGGAGATGAGCCT
GCGGCTGATTAGCTAGTTGGTGGGGTAAGAGCCTACCAAGGCGACGATCAGTAGCCGGCCTGAGAGGG
TGGCCGGCCACACTGGGACTGAGATACGGCC
>Zotu1154
GAACGCTAGCGGCAGGCCTAATACATGCAAGTCAAGGGGTAGCAATACACCGGCGGACGGGTGCGTAA
CGCGTACACAACGTACCTCAGAGCGGGGGATAGCATTGGGAAACTGGTGGTAAAACCCCATGGTGTTG
TTGAATGGCATCATTTAACAACTAAAGGTTACACTCTGAGATCGGTGTGCGTCTGATTAGCTAGTTGG
TAGGGTAACGGCCTACCAAGGCGACGATCAGTAGGGGGTGTGAGAGCATGACCCCCCACATGGGTACT
GAGACACGGAC
>Zotu1155
GAACGCTGGCGGCGTGCTTAACACATGCAAGTCGAACGATGAAGACCGGTGCTTGCACTGGTTGGATT
AGTGGCGAACGGGTGAGTAATACGTGAGTAACCTGCCTTTGACTCTGGGATAAGCCTGGGAAACTGGG
TCTAATACCGGATACGACACGAAACCGCATGGTTTGGTGTGGAAAGGGTTTGTACTGGTTTTAGATGG
GCTCACGGCCTATCAGCTTGTTGGTGAGGTAACGGCTCACCAAGGCGACGACGGGTAGCCGGCCTGAG
AGGGTGACCGGCCACACTGGGACTGAGACACGGCC
>Zotu1156
GAACGCTGGCGGCATGCCTAATACATGCAAGTCGAACGAGAGGAAGGAAAGCTTGCTTTCCTAAATCT
AGTGGCGAACGGGTGAGTAACACGTAGGTAACCTGCCCATGTGCCCGGGATAACTTCTGGAAACGGAT
GCTAAAACCGGATAGGTAGCAGACAAGCATTTGACTGCTATTAAAGTGGCTAAGGCCATGAACATGGA
TGGACCTGCGGTGCATTAGCTAGTTGGTGAGGTAACGGCCCACCAAGGCGACGATGCATAGCCGGCCT
GAGAGGGCGGACGGCCACATTGGGACTGAGACACGGCC
>Zotu1157
GAACGCTGGCGGCGTGCTTAACACATGCAAGTCGAACGATGAAGCCTAGCTTGCTAGGTGGATTAGTG
GCGAACGGGTGAGTAATACGTGAGTAACCTACCTTTAACTCTGGGATAAGCCCGGGAAACTGGGTCTA
ATACCGGATACGACCAATCTCCGCATGGGGTGTTGGTGGAAAGCGTTATGTAGTGGTTATAGATGGGC
TCACGGCCTATCAGCTTGTTGGTGAGGTAACGGCTCACCAAGGCGACGACGGGTAGCCGGCCTGAGAG
GGTGACCGGCCACACTGGGACTGAGACACGGCC
>Zotu1158
AAACGCTGGCGGCGTGCATAACACATGCAAGTCGAACGATGAAGCTTAGAAGATTTCTTCGGAATGAA
CTTAAGTGGATTAGTGGCGAACGGGTGAGTAACACGTGAGTAACCTGCCTTACACAAGGGGATAGCCT
CTGGAAACGGAGAATAATACCCTATAAAGTCTAATCAGCTCCTGCTGGAAAGACCAAAGATTTATCGG
TGTAAGATGGACTTGCGTCTGATTAGCTAGTTGGTGAGATAAAAGCTCACCAAGGCGACGATCAGTAG
CCGGCTTGAGAGAGTGTACGGCCACATTGGGACTGAGACACGGCC
>Zotu1159

GAACGCTAGCGGCAGGCCTAATACATGCAAGTCGAGGGGTATAGGGAGCTTGCTCCCTTGAGACCGGC
GCACGGGTGCGTAACGCGTATGCAACCTACCCAAACCTGGGGGATAGCCCGGAGAAATCCGGATTAAT
ACCGCATAAAATCACAGCAGGGCATCCTGCAATGATCAAACATTCATGGGGTTTGGATGGGCATGCGT
GCCATTAGTTAGTTGGCGGGGTAACGGCCCACCAAGACTTCGATGGCTAGGGGATCTGAGAGGATGGC
CCCCCACACTGGTACTGAGACACGGAC
>Zotu1160
GAACGCTGGCGGCGTGCTTAACACATGCAAGTCGAACGGAAAGGCCTGCTTTCCCTTGTGGATTGCGG
GTGCTCGAGTGGCGAACGGGTGAGTAACACGTGAGTGATCTGCCCCCAACTTGGGTATAAGCCTGGGA
AACTGGGTCTAATTCCCGATAGGACTGCAGGGTGGTGCCTGTGGTGGAAAACGTTTTCTAGTGGTTGG
GGATGAGCTCGCGGCCTATCAGCTTGTTGGTGGGGTAATGGCCTACCAAGGCGGCGACGGGTAGCCGG
CCTGAGAGGGTGGACGGCCACATTGGGACTGAGATACGGCC
>Zotu1161
GAACGCTGGCGGCGCGCCTAACACATGCAAGTCGAACGGAGCTTAGAGAGCTTGCTTTTTAAGCTTAG
TGGCGAACGGGTGAGTAACGCGTGAGTAACCTGCCCTAGAGTGGGGGACAACAGTTGGAAACGACTGC
TAATACCGCATAAGCCCACGGTACCGCATGGTACTGAGGGAAAAGGATTTATTCGCTTTAGGATGGAC
TCGCGTCCAATTAGCTAGTTGGTGAGGTAACGGCCCACCAAGGCGACGATTGGTAGCCGGACTGAGAG
GTTGAACGGCCACATTGGGACTGAGACACGGCC
>Zotu1162
GAACGCTAGCGGCAGGCTTAATACATGCAAGTCGAGGGGCAGCAGGGGTGTAGCAATACACTCGCTGG
CGACCGGCAAACGGGTGCGGAACACGTACGCAATCTACCCAAAACTGATGAATAGCCCTCCGAAAGGA
GGATTAATACATCGTAACATAATGAAGTGGCATCACTTTGTTATTATAGCTCCGGCGGTTTTGGATGA
GCGTGCGCCTGATTAGGTAGTTGGCGGGGTAACGGCCCACCAAGCCTACGATCAGTAACTGGTGTGAG
AGCACGACCAGTCACACGGGCACTGAGACACGGGC
>Zotu1163
GAACGCTGGCGGCGTGCTTAACACATGCAAGTGGTGCGACGAACCAGGGCTTGCCCTGGGGCCAAGCC
GCGAACGGGTGAGTAACACGTGAGTAACCTGCCCCGATGACCGGGACAACCCGAGGAAACTCGGGCTA
ATACCGGATGTGGTGCACGGATTTAAGTCTGTGTACTAAAGGAAGCTTCGGCCTCCGCATTGGGAGGG
GCTCGCGGCCCATTAGCTTGTTGGTGGGGTAACGGCCTACCAAGGCAACGATGGGTAGCTGGTCTGAG
AGGACGATCAGCCACACTGGGACTGAGACACGGCC
>Zotu1164
GAACGCTGGCGGCGTGCCTAATACATGCAAGTCGTGCGGACTTTTTAAAAGCTTGCTTTTAAAAGGTT
AGCGGCGGACGGGTGAGTAACACGTGGGCAACCTGCCTGTAAGATCGGGATAACGCCGGGAAACCGGG
GCTAATACCGGATATTTTTTTCCTCCGCATGGAGGAAAAAGGAAAGACGGCTTCGGCTGTCACTTACA
GATGGGCCCGCGGCGCATTAGCTAGTTGGTGGGGTAACGGCTCACCAAGGCCACGATGCGTAGCCGAC
CTGAGAGGGTGATCGGCCACATTGGGACTGAGACACGGCC
>Zotu1165
GAACGCTGGCGGCGTGCTTAACACATGCAAGTCGAGCGGGGTCCATCCAGTGGCAACACTGGGGAAGA
CCTAGCGGCGAACGGGTGAGTAACACGTGAGAAACCTGCCCCGAAGTCTGGGATAACCCGAGGAAACT
CGAGCTAATACCGGATACCTTCCACTGATCGCATGGTCGGTGGAAGAAAGAATTTCGCTTCGGGAGGG
TCTCGCGGCCTATCAGCTTGTTGGCGGGGTAACGGCCCACCAAGGCGTCGACGGGTAGCTGGTCTGAG
AGGACGATCAGCCACACTGGGACTGAGACACGGCC
>Zotu1166
GAACGCTGGCGGCGTGCTTAACACATGCAAGTCGCACGGGAAGCTGGGGGCAACCCCGGCCGAGAGTG
GCGAATGGGTGCGTAACACGTAGGCGACCTGCCCTCCAGCAGGGGATAACGCTCCGAAAGGAGCGCTA
ATACCGTATGCGGTGCACGATTCATTTTGTGCACTAAACCTGCGAGGTAACTCGTGGGGCTGGGGGAG
GGGCCTGCGGCCCATCAGCTAGTAGGTGAGGTAACGGCTCACCTAGGCGACGACGGGTAGGGGGCCTG
AGAGGGTGACCCCCCACACTGGCACTGACACACGGGC
>Zotu1168
GAATGCTGGCGGCGCGCCTAACACATGCAAGTCGAGCGGTAACAGGAGTAATTTATTACTTGCTGACG
AGCGGCGGACGGCTGAGTAACGCGTGGGAACATGCCCCAAAGTGAGGAATAACTACTCGAAAGAGTAG
CTAATGCCGCATATGATCTTCGGATTAAAGGATTTATCCGCTTTGGGAGTGGCCCGCGTACGATTAGG
TAGTTGGTGAGGTAATGGCTCACCAAGCCTACGATCGTTAACTGGTCTGAGAGGATGATCAGTCAGAC
TGGAACTGAGACACGGTC
>Zotu1169

GAACGCTGGCGGCGTGCCTAATACATGCAAGTTGAGCGCTGAAGGGAGGTACTTGTACCTTTTGGAAG
AGCAGCGAACGGGTGAGTAACGCGTGGGAATCTGCCTTTGAGCGGGGGACAACATTTGGAAACGAAT
GCTAATACCGCATAAAAGCTTTAAACATAAGTTAAAAGTTTGAAAGATGCAAAAGCATCACTCAAAGA
TGATCCCGCGTTGTATTAGCTAGTTGGTAGGGTAAAGGCCTACCAAGGCGATGATACATAGCCGACCT
GAGAGGGTGATCGGCCACATTGGGACTGAGACACGGCC
>Zotu1170
GAACGCTGGCGGCAGGCTTAACACATGCAAGTCGAGCGGAGAGAGGTAGCTTGCTACTGATCTTAGCG
GCGGACGGGTGAGTAATGCTTAGGAATCTGCCTATTAGTGGGGGACAACATTTCGAAAGGAATGCTAA
TACCGCATACGTCCTACGGGAGAAAGCAGGGGATCTTCGGACCTTGCGCTAATAGATGAGCCTAAGTC
GGATTAGCTAGTTGGTGGGGTAAAGGCCTACCAAGGCGACGATCTGTAGCGGGTCTGAGAGGATGATC
CGCCACACTGGGACTGAGACACGGCC
>Zotu1171
GAACGCTGGCGGCGTGCCTAATACATGCAAGTCGAGCGGACCGAATGGGAGCTTACTTTCGTTCGGTT
AGCGGCGGACGGGTGAGTAACACGTGGGCAACCTGCCCGTAAAACCGGGATAACTTCGGGAAACCGGA
GCTAATACCGGATAACACCGAAGATCGCATGATCTTTGGTTGAAAGGCGGCTTTGGCTGTCACTTACG
GATGGGCCCGCGGCGCATTAGCTAGTTGGTGAGGTAACGGCTCACCAAGGCGACGATGCGTAGCCGAC
CTGAGAGGGTGATCGGCCACACTGGGACTGAGACACGGCC
>Zotu1172
GAACGCTGGCGGCGTGCCTAATACATGCAAGTCGAGCGAACAGATAAGGAGCTTGCTCCTTTGACGTT
AGCGGCGGACGGGTGAGTAACACGTGGGTAACTTACCTATAAGACTGGAATAACTCCGGGAAACCGGG
GCTAATGCCGGATAACATTTAGAACCGCATGGTTCTAAAGTGAAAGATGGTTTTGCTATCACTTATAG
ATGGACCCGCGCCGTATTAGCTAGTTGGTAAGGTAACGGCTTACCAAGGCAACGATACGTAGCCGACC
TGAGAGGGTGATCGGCCACACTGGAACTGAGACACGGTC
>Zotu1173
GAACGCTGGCGGCGTGCCTAATACATGCAAGTCGAACGAACAGCGACTTGAACTTGTTCAAGTCAATG
TTAGTGGCGCACGGGTGAGTAACACGTGGAGAACCTACCCTTAAGCGGGGGATAACCATTGGAAACGA
TGACTAATACCGCATAGAATCACAGATCACATGATCAATGAAGGAAAGACGGCTTCGGCTGTCACTTA
AGGATGGCTCCGCGGTGCATTAGCTAGATGGTGGGGTAAAGGCCTACCATGGCAATGATGCATAGCCG
ACCTGAGAGGGTGATCGGCCACATTGGGACTGAGACACGGCC
>Zotu1174
GAACGCTGGCGGCGTGCCTAATACATGCAAGTCGAACGAACGGATAAAGAGCTTGCTCTTTTGAAGTT
AGTGGCGGACGGGTGAGTAACACGTGGGTAACCTACCTCACAGCTGGGGATAACATCGAGAAATCGAT
GCTAATACCGAATGTGCTGAACATCATAAGATGTTCAAGTGAAAGACGGTTTCGGCTGTCACTGTGAG
ATGGACCCGCGCTGGATTAGCTAGTTGGTAAGGTAATGGCTTACCAAGGCGACGATCCATAGCCGACC
TGAGAGGGTGATCGGCCACATTGGGACTGAGACACGGCC
>Zotu1175
GAACGCTGGCGGCGTGCCTAATACATGCAAGTCGAGCGGATCTTTAGAAGCTTGCTTCTAAAGATTAG
CGGCGGACGGGTGAGTAACACGTGGGCAACCTGCCTGTAAGACTGGGATAACTTCGGGAAACCGAGGC
TAATACCGGATAATCCTTTTTCTCACATGAGGGAAAGCTGAAAGACGGCGCAAGCTGTCACTTACAGA
TGGGCCCGCGGCGCATTAGCTAGTTGGTGAGGTAACGGCTCACCAAGGCGACGATGCGTAGCCGACCT
GAGAGGGTGATCGGCCACACTGGGACTGAGACACGGCC
>Zotu1176
GAACGCTGGCGGCGTGCTTAACACATGCAAGTCGAACGGAAAGGCCCTTCGGGGTACTCGAGTGGCGA
GCGGGTGAGTAACACGTGGGTGATCTGCCCTGCACTTTGGGATAAGCCTGGGAAACTGGGTCTAATAC
CGAATATGATCACGCTTCTCATGGGGTGTGGTGGAAAGCTTTTGCGGTGTGGGATGGGCCCGCGGCCT
ATCAGCTTGTTGGTGGGGTAATGGCCTACCAAGGCGACGACGGGTAGCCGGCCTGAGAGGGTGTCCGG
CCACACTGGGACTGAGATACGGCC
>Zotu1177
GAACGCTGGCGGCATGCCTAATACATGCAAGTCGAACGCGGCCATCCGGTGCTTGCACTGGTGAAGCC
GAGTGGCGAACGGGTGAGTAACACGTGAGAAACCTGCCCTGGAGTGGGGAATAACTCGAAGAAATTCG
AGCTAATACCGCATACCTTCTCTTCACCGCATGGTGAGTTGAAGAAAGATTTATCGCTCTAGGAGGGT
CTCGCGGCCTATCAGCTAGTTGGTGAGGTAATGGCTCACCAAGGCATCGACGGGTAGCTGGTCTGAGA
GGACGATCAGCCACACTGGGACTGAGACACGGCC
>Zotu1178

GAACGCTGGCGGCGTGCCTAATACATGCAAGTCGAACGGAGCAGCAATGCTTAGTGGCGACCGGGTGA
GTAACACGTAGTTGACCTGCCCCTCAGCGGGGGACAACAGTTCGAAAGGACTGCTAATACCGCATACA
TTGGTCGAGATTAGAAGTCGATCAAGAAACTTACGGGGTTGAGGGAGGGGACTGCGGCCCATCAGGTA
GTTGGTGAGGTAGAAGCTCACCAAGCCGACGACGGGTAGGGGGCCTGAGAGGGTGACCCCCCACACTG
GAACTGACATACGGTC
>Zotu1179
TAACGCTGGCGGCGTGCCTAATGCATGCAAGTCGAACGTGGTCCCTTCGGGGGCAAACGTGGCGGACG
GGTGAGGAACACGTGGGGAACCTGCCCATTGGTGGGGGGTAGCCCGCGGAAACGCGGGGTAATTCCGC
ATACGCTCGCTTGGGGGTAGCCCGAGTGAGGAAAGGGTCTTCGGACTCGCCGATGGAGGGGCCTGCGT
CCGATTAGCTAGTTGGTGGGGTAATGGCCTACCAAGGCGGTGATCGGTAGCTGGTCTGAGAGGACGGC
CAGCCACAGGGGGACTGAGACACGGCC
>Zotu1180
GAACGTTGGCGGCATGCCTAACACATGCAAGTCGAACGCGGACTTCGGTCCGAGTGGCGAACGGGTGC
GTAACACGTAGGTGACCTGCCCACCGGTGGGGGATAACCGCGAGAAATCGTGGCTAATACCGCATGCG
ATCCTGGTCTTTCGAAAGCCAGGAGGAAAGCTTCATGTGCCGGTGGAGGGGCCTGCGGCCTATCAGCT
AGTTGGTGAGGTAACGGCTCACCAAGGCGATGACGGGTAGCTGACCTGAGAGGGTGGTCAGCCACAGG
GGAACTGAGAGACGGTC
>Zotu1181
GAACGCTAGCGGCAGGCCTAATACATGCAAGTCAAGGGGTAGCAATACACCGGCGGACGGGTGCGTAA
CGCGTACACAACGTACCTCAGAGCGGGGGATAGTATCGGGAAACTGGTAGTAACACCCCATAGTGATA
TTGATTGGCATCAGTTAATATCTAAAGGTTACACTCTGAGATCGGTGTGCGTATGATTAGCTAGTTGG
TAGGGTAACGGCTTACCAAGGCGATGATCATTAGGGGGTGTGAGAGCATGACCCCCCACACGGGTACT
GAGACACGGAC
>Zotu1182
GAACGCTGGCGGCGTGCTTAACACATGCAAGTGGAGCGACGAACCAGGGCTTGCCCTGGGGCAAAGCC
GCGAACGGGTGAGTAACACGTGGGTAACCTGCCCCGACGACTGGGACAACCCGAGGAAACTCGGGCTA
ATACCGGATAATTTCACCACTCATAAGTGTGGTGATTAAAGGAGGCTTCGGCTTCCGCATCGGGAGGG
GCCCGCGGCCCATTAGCTTGTTGGTGAGGTAACGGCTCACCAAGGCGACGATGGGTAGCTGGTCTGAG
AGGACGATCAGCCACACTGGGACTGAGACACGGCC
>Zotu1183
GAACGCTGGCGGCGCGCTTAACACATGCAAGTCGAGCGGGAACCAGGCCTTCGGGCCTGGGGAAAGCG
GCGAACGGGTGAGTAACACGTGGGTAATCTGCCCTCGGCATCGGGATAGCCCGGGGAAACCCGGATTA
ATACCGGATAGCCTCCGAAGCTTTTGAGCATCGGAGAAAAGGTAGCTTCGGCTTCCGGCTGAGGATGA
GCCCGCGGCGGATTAGCTTGTTGGTGGGGTAAAGGCCCACCAAGGCGACGATCCGTAGCTGGTCTGAG
AGGACGATCAGCCACACTGGGACTGAGACACGGCC
>Zotu1184
GAACGCTGGCGGCGTGCCTAATACATGCAAGTAGAACGCTGAAGAGAGGAGCTTGCTCTTCTTGGATG
AGTTGCGAACGGGTGAGTAACGCGTAGGTAACCTGCCTGGTAGCGGGGGATAACTATTGGAAACGATA
GCTAATACCGCATGATATTAATTGTCGCATGATAATTAATTGAAAGATGCAATTGCATCACTACCAGA
TGGACCTGCGTTGTATTAGCTAGTTGGTGAGGTAACGGCTCACCAAGGCGACGATACATAGCCGACCT
GAGAGGGTGATCGGCCACACTGGGACTGAGACACGGCC
>Zotu1185
GAACGCTGGCGGCATGCCTTACACATGCAAGTCGAACGGCAGCACGGGGCAACCTGGTGGCGAGTGGC
GAACGGGTGAGTAATGCATCGGAACGTGCCATTGAGTGGGGGACAACCATCCGAAAGGATGGCTAATA
CCGCATATACTGTGCGCAGGAAAGCAGGGGATCGCAAGACCTTGCGCTCTTTGAGCGGCCGATGTCGG
ATTAGCTAGTTGGTGAGGTAAAGGCTCACCAAGGCGACGATCCGTAGCGGGTCTGAGAGGATGATCCG
CCACACTGGGACTGAGACACGGCC
>Zotu1186
GAACGCTGGCGGCGTGCTTAACACATGCAAGTCGAACGGGCCCGCTCCTTTTTTTGGGGGCGGGTTGA
GTGGCGAACGGGTGAGTATCACGTGAGCAACCTGCCCTCCTCTCCTGGATAACCGCTTGAAAGGGCGG
CTAATACGGGGTGCTCCGGTTGGTCCGCATGGGCTGGTCGGGAAAGATTCATCGTTGTGGTGTTTCGG
TGGGGGATGGGCTCGCGGCCTATCAGCTTGTTGGTGGGGTGATGGCCCACCAAGGCGGTGACGGGTAG
CCGGCCTGAGAGGGTGGGCGGCCACACTGGGACTGAGACACGGCC
>Zotu1187

GAACGCTGGCGGCGTGCCTAATACATGCAAGTCGAGCGAACCGGAGGGAGCTTGCTCCCAAAGGTTAG
CGGCGGACGGGTGAGTAATACGTAGGCAACCTGCCTGTAAGCTCGGGATAACTCACGGAAACGTGTGC
TAAAACCGGATAGGAACTTGTTGCACCTGCAATAAGTTGGAAAAACGGCGCAAGCTGTTACTTACAGA
GGGGCCTACGTCGCATTAGCTAGTTGGTGGGGTAACGGCTCACCAAGGCGACGATGCGTAGCCGACCT
GAGAGGGTGATCGGCCACACTGGGACTGAGACACGGCC
>Zotu1188
GAATGTTGGCGGCGTGCCTGAGATATGCAAGTCGAGCGAACTTGTTTCCGCAAGGGAACGAGTCAGCG
GCGGACGGCTGAGTAACACGTAGGTATCTCCCCCTATCTGGGGAATAGCTCCGCGAAAGCGGAGATAA
TACCCCATGATTCCGTCCTTTGGACGGACAAAGAGAGTAACTTTTCGGAAAGGGATGAACCTGCGGCT
TATCAGATAGTTGGTGGGGTAACGGCCTACCAAGTCTACGACGAGTAGCTGGCTTGAGAGAGTGGTCA
GCCAGAGGGGCACTGAGACACGGGC
>Zotu1189
GAACGCTGGCGGCGGGCTTAACACATGCAAGTCGAACGAACCCTTCGGGGTTAGTGGCGCACGGGTGA
GTAACGCGTAGGCAATCTGCCCCCAACTGAGGAATAACCCATCGAAAGGTGGGCTAATACCACATAAT
GTCGAACGGTCGCATGGCCGGTTGACCAAAGTTCAGGCGGTTGGGGATGAGCCTGCGTTGGATTAGCT
AGTTGGCGGGGTAACGGCCCACCAAGGCGACGATCCATAGCGGGTCTGAGAGGATGATCCGCCACACT
GGCACTGAGACACGGGC
>Zotu1190
GAACGCTGGCGGCGTGCTTAACACATGCAAGTCGAGCGGAAAGGCCACTTCGGTGGTACTCGAGCGGC
GAACGGGTGAGTAACACGTGAGTAATCTGCCCTTGGCTTTGGGATAGCCACCGGAAACGGTGATTAAT
ACCGGATACGACCACTCCTGGCATCAGGTGGTGGTGGAAAGTTTTTCGGCCAGGGATGTGCTCGCGGC
CTATCAGCTTGATGGTGAGGTAATGGCTCACCATGGCTTCGACGGGTAGCCGGCCTGAGAGGGTGACC
GGTCACACTGGGACTGAGACACGGCC
>Zotu1191
GAACGCTGGCGGCATGCCTAACACATGCAAGTCGAACGAACCCTTCGGGGTTAGTGGCGCACGGGTGC
GTAACGCGTGGGAATCTGCCCTTTGCTTCGGAATAACAGTTAGAAATGACTGCTAATACCGGATGATG
TCTTCGGACCAAAGATTTATCGGCAAAGGATGAGCCCGCGTAGGATTAGGTAGTTGGTGGGGTAAAGG
CCTACCAAGCCGACGATCCTTAGCTGGTCTGAGAGGATGATCAGCCACACTGGGACTGAGACACGGCC
>Zotu1192
GAACGCTAGCGGCAGGCTTAACACATGCAAGTCGAGGGGTAGAAGTTTTCGGACTTTGAGACCGGCGC
ACGGGTGCGTAACGCGTATGCAATCTACCTTTTACAGAGGGATAGCCCAGAGAAATTTGGATTAATAC
CTCATAGTGTTATAGAGTGGCATCACTTTATAACTAAAGTCACAACGGTAAAAGATGAGCATGCGTCC
CATTAGCTAGATGGTAAGGTAACGGCTTACCATGGCTACGATGGGTAGGGGTCCTGAGAGGGAGATCC
CCCACACTGGTACTGAGACACGGAC
>Zotu1193
GAACGCTGACAGAATGCTTAACACATGCAAGTCATTGGCGAAGCTGCGCTTGCGCAGCCTAGCCAAGG
CGGACGGGTGAGTAACGCGTAAGGAACTTGCCCTGCAGACAGGGATAACAGACGGAAACGACTGATAA
CACCTGGTATTGTCAGGCTGGCGCATGCCGGCCTGATGAAAGGAGACGCTGCAGGAGAGCCTTGCGTC
CTATTAGCTAGTTGGTGGGGTAACGGCCCACCAAGGCGATGATAGGTAGCCGGCCTGAGAGGGTGAAC
GGCCACAAGGGGACTGAGATACGGCC
>Zotu1194
GAACGCTAGCGGCAGGCTTAATACATGCAAGTCAAGGGGGTAGCAATACAACCGGCGCACGGGTGAGT
AACGCGTACACAACATACCATATACTGGAGTATAGCTTTGGGAAACTGAAGGTAATCCTCCATAACAT
TGCGAGAAGGCATCTAATTGCAATTAAAGTTTCGACGGTATATGATTGGTGTGCGTTTGATTAGCTAG
TTGGTAGGGTAACGGCCTACCAAGGCGACGATCAATAGCTGGCGTGAGAGCGCGACCAGCCACACTGG
AACTGAGACACGGTC
>Zotu1195
GAACGCTAGCGGCAGGCTTAACACATGCAAGTCGAGGGGTATGCTTCTTCGGGAGCAGAGACCGGCGC
ACGGGTGCGTAACGCGTATGCAATCTACCTTTTACAGAGGGATAGCCCAGAGAAATTTGGATTAATAC
CTCATAGCATAGCAGCTTCGCATGAAGCCACTATTAAAGTCACAACGGTAAAAGATGAGCATGCGTCC
CATTAGCTAGATGGTAAGGTAACGGCTTACCATGGCAACGATGGGTAGGGGTCCTGAGAGGGAGATCC
CCCACACTGGTACTGAGACACGGAC
>Zotu1196
GAACGCTTGCGGCGCGCCTAATACATGCAAGTCGAACGAGGTAGCAATACCTAGTGGCGCACGGGTGA

GTAACGCGTGAATAATCTGCCTACCAGTGGGGGATAACTAACCGAAAGGTTAGCTAATACCGCATGAG
GCCACAATGTCTGCGGACAAAGGGGTTAAAGATTTATCGCTGGTAGATGAGTTTGCGTGGGATTAGCT
AGTTGGTGGGGTAATGGCCTACCAAGGCAACGATCTCTAACTGGTCTGAGAGGATGGCCAGTCACACT
GGAACTGAGACACGGTC
>Zotu1197
GAACGCTGGCGGCATGCCTAACACATGCAAGTCGAACGAGACCTTCGGGTCTAGTGGCGCACGGGTGC
GTAACGCGTGGGAACCTGCCTTTAGGTTCGGAATAACTCTCTGAAAGGGGTGCTAATACCGAATAATG
TCTTCGGACCAAAGATTTATCGCCTTTAGATGGGCCCGCGTTGGATTAGCTAGTTGGTGGGGTAAAGG
CCTACCAAGGCGACGATCCATAGCTGGTCTTAGAGGATGATCAGCCACACTGGGACTGAGACACGGCC
>Zotu1198
GAACGCTGGCGGCGTGCTTAACACATGCAAGTGGAGCGACGAACCGGGGCTTGCCCCGGGGCAGAGCC
GCGAACGGGTGAGTAACACGTGGGTTACGTGCCCCGATGATTGGGACAACCCGAGGAAACTCGGGCTA
ATACCAAATGTGCTCGTGTCCGTGATGGACTCGAGGAAAGGAAGCTTCGGCCTCCGCATCGGGATCGG
CCCGCGGCCCATTAGCTAGTTGGTGGGGTAATGGCCTACCAAGGCGACGATGGGTAGCTGGTCTGAGA
GGACGATCAGCCACACTGGGACTGAGACACGGCC
>Zotu1199
GAACGCTGGCGGCATGCCTAATACATGCAAGTCGAACGCTGAAGCACTCTGCTTGCAGAGTGCGGATG
AGTGGCGAACGGGTGAGTAACACGTGGGAAATCTGCCTATAAGTGGGGGATAACATTCGGAAACGGAT
GCTAATACCGCATATATTCTTGGACCGCCTGGTCCTTGAAAGAAAGACGGCTTTGCTGTCGCTTATAG
ATGATCCCGCGGCGTATTAGTTAGTTGGTGGGGTAAAGGCCTACCAAGACAATGATACGTAGCCGACC
TGAGAGGGTGATCGGCCACACTGGGACTGAGACACGGCC
>Zotu1200
GAACGCTGGCGGCGTGCCTAATACATGCAAGTCGAGCGAATCGATGGGAGCTTGCTCCCTGAGATTAG
CGGCGGACGGGTGAGTAACACGTGGGTAACCTGCCTGTAAGATTGGGATAACTCCGGGAAACCGGAGC
TAATACCGGATAACATTTTGAACCGCATGGTTCAAAATTGAAAGATGGCTTCTAGCTATCACTTACAG
ATGGACCCGCGGCGCATTAGCTAGTTGGTGAGGTAATGGCTCACCAAGGCAACGATGCGTAGCCGACC
TGAGAGGGTGATCGGCCACACTGGGACTGAGACACGGCC
>Zotu1201
GAACGCTGGCGGCAGGCTTAACACATGCAAGTCGAACGATGACTCTCTAGCTTGCTAGAGATGATTAG
TGGCGGACGGGTGAGTAACATTTAGGAATCTACCTAGTAGTGGGGGATAGCTCGGGGAAACTCGAATT
AATACCGCATACGACCTACGGGTGAAAAGGGGCGCAAGCTCTTGCTATTAGATGAGCCTAAATCAGAT
TAGCTAGTTGGTGGGGTAAAGGCCCACCAAGGCGACGATCTGTAACTGGTCTGAGAGGATGATCAGTC
ACACCGGAACTGAGACACGGTC
>Zotu1202
GAACGCTAGCTACAGGCTTAACACATGCAAGTCGTGGGGCAGCGGATGCTTAGCTTGCTAAGTATGCC
GGCGACCGGCGCACGGGTGAGTAACGCGTACCGAACCTGCCCATCACACAGGGATAGGCTTGCGAAAG
CAAGATTAATACCTGATGGTCTCAATTTTATGCATGTATGATTGAGTAAAGCCTTCGGGCGGTGATGG
ATGGCGGTGCGTCCCATTAGGAAGTTGGCGGGGTAACGGCCCACCAATCCTTCGATGGGTAGGGGTTC
TGAGAGGAAGGTCCCCCACATTGGAACTGAGATACGGTC
>Zotu1203
GAACGCTGGCGGCGTGCTTAACACATGCAAGTGGAGCGACGAACCGGGGCTTGCCCCGGGGCAGAGCC
GCGAACGGGTGAGTAACACGTGGGTTACCTGCCCTGATGACCGGGACAACCCGTCGAAAGACGGGCTA
ATACCGGATGTGCCCGCAAGGGGAAAGGAGGCGTAAGCTTCCGCATCGGGATGGGCCCGCGGAGCATT
AGCTTGTTGGTGAGGTAAAAGCTCACCAAGGCTTCGATGCTTAGCTGGTCTGAGAGGACGATCAGCCA
CACTGGGACTGAGACACGGCC
>Zotu1204
GAACGCTGGCGGCGTGCTTAACACATGCAAGTCGAACGATGATGCCCAGCTTGCTGGGTGGATTAGTG
GCGAACGGGTGAGTAACACGTGAGTAACCTGCCCCTGACTCTGGGATAAGCGTTGGAAACGACGTCTA
ATACTGGATATGACTGCCGGCCGCATGGTCTGGTGGTGGAAAGATTTTTTGGTTGGGGATGGACTCGC
GGCCTATCAGCTTGTTGGTGAGGTAATGGCTCACCAAGGCGACGACGGGTAGCCGGCCTGAGAGGGTG
ACCGGCCACACTGGGACTGAGACACGGCC
>Zotu1205
GAACGCTGGCGGAATGCTTTACACATGCAAGTCGAACGGCAGCACGGACTTCGGTCTGGTGGCGAGTG
GCGAACGGGTGAGTAATATATCGGAACGTGCCCAGTCGTGGGGGATAACGTAGCGAAAGTTACGCTAA

TACCGCATACGATCTATGGATGAAAGCGGGGGATCGCAAGACCTCGCGCGATTGGAGCGGCCGATATC
AGATTAGGTAGTTGGTGGGGTAAAGGCTCACCAAGCCAACGATCTGTAGCTGGTCTGAGAGGACGACC
AGCCACACTGGAACTGAGACACGGTC
>Zotu1206
GAACGCTGGCGGCGTGCCTAACACATGCAAGTCGAGCGAGTGGATCTCCTTCGGGAGAGAAGCTAGCG
GCGGACGGGTGAGTAACACGTGGGCAACCTGCCTCGTAGAGGGGAATAGCCTCCCGAAAGGGAGATTA
ATACCGCATAAGATTGTAGCTTCGCATGAAGTAGCAATTAAAGGAGCAATCCGCTATGAGATGGGCCC
GCGGCGCATTAGCTAGTTGGTGAGGTAACGGCTCACCAAGGCGACGATGCGTAGCCGACCTGAGAGGG
TGATCGGCCACATTGGGACTGAGACACGGCC
>Zotu1207
GAACGCTGGCGGCAGGCCTAACACATGCAAGTCGAACGGACCCTTCGGGGTTAGTGGCGGACGGGTGA
GTAACACGTGGGAACGTGCCTTTAGGTTCGGAATAGCTCCTGGAAACGGGTGGTAATGCCGAATGTGC
CCTTCGGGGGAAAGATTTATCGCCTTTAGAGCGGCCCGCGTCTGATTAGCTAGTTGGTGAGGTAACGG
CTCACCAAGGCGACGATCAGTAGCTGGTCTGAGAGGATGACCAGCCACACTGGGACTGAGACACGGCC
>Zotu1208
GAACGCTGGCGGTATGCTTAACACATGCAAGTCGAACGGATGTTTTCGGACATTAGTGGCGGACGGGT
GAGTAACGCGTGAGAATCTAGCTTCAGGTTGGGGACAACAGTTGGAAACGACTGCTAATACCGAATGT
GCCGAAAGGTGAAAGATTTATTGCCTGAAGAAGAGCTCGCGTCTGATTAGCTAGTTGGTGGGGTAAGA
GCTTACCAAGGCGACGATCGGTAGCTGGTCTGAGAGGACGATCAGCCACACTGGGACTGAGACACGGC
C
>Zotu1209
GAACGCTGGCGGCAGGCTTAACACATGCAAGTCGAACGGTCTCTTCGGAGGCAGTGGCAGACGGGTGA
GTAACGCGTGGGAATCTACCTAGTTCTACGGAACAACAGAGGGAAACTTCTGCTAATACCGTATACGC
CCTACGGGGGAAAGATTTATCGGAATTAGATGAGCCCGCGTAAGATTAGCTAGTTGGTGGGGTAATGG
CCTACCAAGGCGACGATCTTTAGCTGGTCTGAGAGGATGATCAGCCACACTGGGACTGAGACACGGCC
>Zotu1210
GAACGCTAGCGGCAGACATAATACATGCAAGGCGAAGGGGCAGCAATGTCACTGTCGTACGGGTGCGT
AACGCGTATGCAACCTACCTATCACTGGGGGATAGCCCGGGGAAACCCGGATTAATACCGCATAAAAC
AGGGGCACCGCATGGTGATATTTGTTAAAGATTTATTGGTGATAGATGGGCATGCGTTCGATTAGCTA
GTTGGCGGGGTAACGGCCCACCAAGGCGACGATCGATAGGGGAGCTGAGAGGTTGGTCCCCCACACGG
GCACTGAGATACGGGC
>Zotu1212
GAACGCTGGCGGCAGGCCTAACACATGCAAGTCGAACGCTGTAGCAATACAGAGTGGCAGACGGGTGA
GTAACGCGTGGGAACATACCCTTTGGTTCGGAATAACTCAGGGAAACTTGAGCTAATACCGGATACGT
CCGCGAGGAGAAAGATTCATCGCCGAAGGATTGGCCCGCGTTCGATTAGCTAGTTGGTGAGGTAACGG
CTCACCAAGGCGACGATCGATAGCTGGTCTGAGAGGATGATCAGCCACATTGGGACTGAGACACGGCC
>Zotu1213
GAACGCTAGCGGCAGGCTTAACACATGCAAGTCGAGGGGTAGAGTTAGCTTGCTAACTTGAGACCGGC
GCACGGGTGCGTAACGCGTATGCAACCTACCTTTCACAGGGGGATAGCCCAGAGAAATTTGGATTAAT
ACCCCATGGTATATATGGATCGCATGGTTTATATATTAAAGTTCCAACGGTGAAAGATGGGCATGCGT
CCCATTAGCTAGATGGTAAGGTAACGGCTTACCATGGCGACGATGGGTAGGGGTCCTGAGAGGGAGAT
CCCCCACACTGGTACTGAGACACGGAC
>Zotu1214
GAACGCTGGCGGCGTGCCTAACACATGCAAGTCGAACGGGACTCGGGACTTCGGTTCTGAGTTTAGTG
GCGAACGGGTGAGTAACGCGTGAGGAACCTGCCTTTCAGTGGGGGACAACAGTTGGAAACGACTGCTA
ATACCGCATAATGTGTTTTGGGGGCATCCCCGAGACACCAAAGCTTTATGTGCTGAAAGATGGCCTCG
CGTCTGATTAGATAGTTGGTGAGGTAACGGCTCACCAAGTCTGCGATCAGTAGCCGGTCTGAGAGGAT
GAACGGCCACATTGGGACTGAGATACGGCC
>Zotu1215
GAACGCTGGCGGCGTGCTTAACACATGCAAGTGGAGCGACGAACCTGGCTTCGGCCAGGGGCAAAGCC
GCGAACGGGTGAGTAACACGTGGGTAACGTGCCCCAATGACTGGGACAACCCGGGGAAACCCGGGCTA
ATACCGGATGTGCTCCTTCCACATCAGTGGATTGAGTAAAGGTAGCTTCGGCTTCCGCATTGGGATCG
GCCCGCGGCGCATTAGCTTGTTGGTGAGGTAAAGGCTCACCAAGGCTTCGATGCGTAGCTGGTCTGAG
AGGACGATCAGCCACACTGGGACTGAGACACGGCC

>Zotu1216
GAACGCTAGCGGCAGGCTTAATACATGCAAGTCGAGGGGCAGCACGAATAGCAATATTTGGTGGCGAC
CGGCAAACGGGTGCGGAACACGTACGCAACCTTCCCATAACTGGTGAATAGCCTTCCGAAAGGGAGAT
TAATACACCGTAACATAAAGAGTAGGCATCTATTTTTTATTATAGCTTCGGCGGTTTTGGATGGGCGT
GCGCCTGATTAGGTAGTTGGCGGGGTAACGGCCCACCAAGCCTTCGATCAGTAACTGGTGTGAGAGCA
CGACCAGTCACACGGGCACTGAGACACGGGC
>Zotu1217
GAACGCTGGCGGCGTGCTTAATACATGCAAGTCGAGCGAGGTTACTGTTTTGGATCCCTTCGGGGTGA
AGAAGCTTTAACCTAGCGGCGGACGGGTGAGTAACGCGTGGGTAACCTACCCCAAAGTCCGGGATAAC
AGCGGGAAACCGCTGCTAATACCGGATGCCTTTACCTTGGGGCATCTTGAGGTAAAGAAAGGTAACGC
TTTGGGATGGGCCCGCGTCCCATTAGCTAGTTGGTGAGGTAACGGCTCACCAAGGCGACGATGGGTAG
CCGGCCTGAGAGGGTGGACGGCCACACTGGGACTGAGACACGGCC
>Zotu1218
GAACGCTGGCGGCGTGCTTAACACATGCAAGTCGAGCGGAAAGGCCCTTCGGGGTACTCGAGCGGCGA
ACGGGTGAGTAACACGTGAGTAACCTGCCCTAGGCTCTGGGATAACTGTCGGAAACGATGGCTAATAC
CGGATATTCATCTCCTGCCGCATGGTGGGGGTTGGAAAGTTTTTCGGCTTGGGATGGGCTCGCGGCCT
ATCAGCTTGTTGGTGGGGTGATGGCCTACCAAGGCTTCGACGGGTAGCCGGCCTGAGAGGGCGACCGG
CCACACTGGGACTGAGACACGGCC
>Zotu1219
GAACGCTGGCGGCAGGCTTAACACATGCAAGTCGAGCGGGCCCTTCGGGGTCAGCGGCAGACGGGTGA
GTAACACGTGGGAACGTACCCTTTGGTTCGGAATAACGCTGGGAAACTAGCGCTAATACCGGATACGC
CCTTTTGGGGAAAGGCTTGCTGCCGAAGGATCGGCCCGCGTCTGATTAGCTAGTTGGTGGGGTAATGG
CCTACCAAGGCGACGATCAGTAGCTGGTCTGAGAGGATGATCAGCCACACTGGGACTGAGACACGGCC
>Zotu1220
GAACGCTGGCGGCGTGCTTAACACATGCAAGTCGAACGGTGACGCTAGAGCTTGCTCTGGTTGATCAG
TGGCGAACGGGTGAGTAACACGTGAGTAACCTGCCCTTGACTCTGGGATAACTCCGGGAAACCGGGGC
TAATACCGGATACGAGACGCGATCGCATGGTCGGCGTCTGGAAAGTTTTTCGGTCAAGGATGGACTCG
CGGCCTATCAGCTTGTTGGTGAGGTAACGGCTCACCAAGGCGTCGACGGGTAGCCGGCCTGAGAGGGC
GACCGGCCACACTGGGACTGAGACACGGCC
>Zotu1221
TAACGCTGGCGGCGTGCATAACACATGCAAGTCGAACGATGAAACTTAGTAGACTTCTTCGGAAAGAC
ACTAAGCGGATTAGTGGCGAACGGGTGAGTAACGCGTGAGTAACCTGCCTTACACAAGGGGATAGCCT
TTGGAAACGAAGAATAATACCCTATAAAACCATAAAAGCACATGCTAGAATGGTCAAAGTGCTAGCGG
TGTAAGATGGACTTGCGTCTGATTAGCTAGTTGGTGAGATAAAAGCTCACCAAGGCAACGATCAGTAG
CCGGCTTGAGAGAGTGTACGGCCACATTGGGACTGAGACACGGCC
>Zotu1222
TAACGCTAGCGGCAGGCATAATACATGCAAGTTGAACGGGATTAATTGTAGCAATACGATTATGAGAG
TAGCGCACGGGTGCGTAACGCGTATGCAACCTGCCTTCATCTGGGGAATAGCCCCTCGAAAGAGGGAT
TAATACCGCATAAAATAGTGTATAGGCATCTATTTACTATTAAAACTATGGTGGATGAAGATGGGCAT
GCGTGACATTAGCTAGTTGGTAGGGTAATGGCCTACCAAGGCTACGATGTCTAGGGGGTCTGAGAGGA
TGTCCCCCCACACTGGTACTGAGATACGGAC
>Zotu1223
GAATGCTGGCGGCGTGCCTAACACATGCAAGTCGAGCGCGAAAAGGTTTCGGCCTTAGTAAAGCGGCG
AACGGGTGAGTAACGCGTGGAAATCTACCTTTTAGTGGAGAATACCCTAGAGAAATTTAGGTTAATAC
TGCATAAGTTCTGAAAAGAAGAAAGATGGCCTATTTGCTGTCGCTAGAAGATGAGTCTGCGTACTATT
AGTTAGTTGGTGGGGTAATGGCCTACCAAGACGATGATGGTTAGCCGGCCTGAGAGGGTGTACGGCCA
CACTGGAACTTAGACACGGTC
>Zotu1225
GAACGCTAGCGGCAGGCTTAATACATGCAAGTCGAGGGGCAGCGGAGTAGCAATACTGCCGGCGACCG
GCGCACGGGTGCGTAACACGTATACAATCTACCTTAAACAGGGGGATAGCCTTTCGAAAGAGAGATTA
ATACCCCGTAATAAATTGAATGGCATCATTTGATTTTGAAAGCTCCGGCGGTTTGAGATGAGTATGCG
TCTGATTAGCTAGTTGGTGAGGTAACGGCTCACCAAGGCGACGATCAGTAGGGGATCTGAGAGGACTA
ACCCCCACACTGGTACTGAGACACGGAC
>Zotu1226

GAACGCTGGCGGCGTGCTTAACACATGCAAGTCGAACGGTGAACCCGCTTCGGTGGGGATCAGTGGC
GAACGGGTGAGTAACACGTGGGCAACCTGCCCTCAGCTCTGGGATAACTCCAAGAAATTGGGGCTAAT
ACTGGATATGACCGCTGACCGCATGGTCTGGTGGTGGAAAGATTTATCGGCTGGGGATGGGCCCGCGG
CCTATCAGCTTGTTGGTGGGGTAGTGGCCTACCAAGGCGACGACGGGTAGCCGGCCTGAGAGGGTGAC
CGGCCACACTGGGACTGAGACACGGCC
>Zotu1227
GAACGCTAGCGGCAGGCTTAACACATGCAAGTCGAGGGGTATAGTAGCAATACTAGAGACCGGCGCAC
GGGTGCGTAACGCGTATGCAATCTACCTTTTACAGAGGGACAGCCCAGAGAAATTTGGATTAATACCT
CATAGTATTATACAATGGCATCATTATATAATTAAAGTCACAACGGTAAAAGATGAGCATGCGTCCCA
TTAGCTAGTTGGTAAGGTAACGGCTTACCAAGGCAACGATGGGTAGGGGTCCTGAGAGGGAGATCCCC
CACACTGGTACTGAGACACGGAC
>Zotu1228
GAACGCTGGCGGCGCGCCTAAAACATGCAAGTCGAACGAGAAGCCCGGGCTTGCCCGGGTGGACAGTG
GCGAACGGGTGAGTAACACGTGAGGAACCTGCCCCGAAGACCGGGACAACACCGGGAAACCGGTGCTA
ATACCGGATACCTTCACTGGATCGCATGGTCTGGTGAAGAAATGGATTCCGCTTCGGGATGGCCTCGC
GGCCTATCAGCTAGTTGGTGAGGTAACGGCTCACCAAGGCGACGACGGGTAGCTGGTCTGAGAGGATG
TCCAGCCACACTGGGACTGAGACACGGCC
>Zotu1229
GAACGCTAGCGGCAGGCCTAATACATGCAAGTCGAACGGATACCTTCGGGTATTAGTGGCGTACGGGT
GCGTAACGCGTAAACAATCTACCTCATACTGGGGGATAGCCCTGGGAAACCGGGAGTAAGCCCGCATG
GCATCGTTGGTCTTCATGGACTGATGATTAAAGATTTATTGGTATGAGATGAGTTTGCGTCTGATTAG
TTAGTTGGCGGGGTAACGGCCCACCAAGACGATGATCAGTAGGGGCTCTGAGAGGAGCGGCCCCCACA
CTGGCACTGAGACACGGGC
>Zotu1230
GAACGCTGGCGGCGTGCCTAATACATGCAAGTAGGACGCACAGCCTATACCGTAGCTTGCTACACCAT
AGGCTGTGAGTTGCGAACGGGTGAGTAACGCGTAGGTAACCTGCCTATTAGAGGGGGATAACTATTGG
AAACGATAGCTAATACCGCATAACAGTATGTAACACATGTTAGATGCTTGAAAGATGCAATTGCATCG
CTAGTAGATGGACCTGCGTTGTATTAGCTAGTAGGTAGGGTAATGGCCTACCTAGGCAACGATACATA
GCCGACCTGAGAGGGTGATCGGCCACACTGGGACTGAGACACGGCC
>Zotu1231
GAACGCTGGCGGCGTGCTTTAGGGATGCGAGTCGAGCGGTTTTTTAGGTGAAATGATGTTTCGCAAGA
AATTGAAGCGATCTTAAAAAATAGCGGCAGACGACTGAGTAACACGTAGACAACTTACCCATAACACA
GAGATAGCCACGCGAAAGCGTGGGTAATATCTGATAGCCCATATAGGTGAAATTCCGGTATGGTAAAG
GCGCAAGCCGGTTATGGAGGGGTCTGCGGCCCATCAGCTAGTTGGCAGGATAACAGCCTACCAAGGCG
AGGACGGGTAGGGGGCGTGAGAGCGCGACCCCCCAGGATGGCACTGAGATACGGGC
>Zotu1232
GAACGCTGGCGGCGTGCTTAACACATGCAAGTCGAACGGTGAAGCAGGAGCTTGCTCTTGTGGATCAG
TGGCGAACGGGTGAGTAACACGTGAGCAATCTGCCCCGAACTCTGGGATAAGCACTGGAAACGGTGTC
TAATACCGGATACGACGCACGAGGGCATCCTCTGTGCGCGGAAAGAATCTCGGTTCGGGATGAGCTCG
CGGCCTATCAGCTAGTTGGTGAGGTAATGGCTCACCAAGGCGACGACGGGTAGCCGGCCTGAGAGGGT
GACCGGCCACACTGGGACTGAGACACGGCC
>Zotu1233
GAACGCTGGCGGCGTGCCTAATACATGCAAGTCGAGCGGACCGAACGAGAGCTTGCTCTTGTTCGGTC
AGCGGCGGACGGGTGAGTAACACGTGGGCAACCTGCCCGCAAGACCGGGATAACTCCGGGAAACCGGA
GCTAATACCGGATAACACCAAAGACCGCATGGTCTTTGGTTGAAAGGCGGCTTCGGCTGTCACTTGCG
GATGGGCCCGCGGCGCATTAGCTAGTTGGTGAGGTAACGGCTCACCAAGGCGACGATGCGTAGCCGGC
CTGAGAGGGTGACCGGCCACACTGGGACTGAGACACGGCC
>Zotu1234
GAACGCTGGCGGCGTGCTTAACACATGCAAGTCGAACGATGAAGACCGGTGCTTGCACTGGTTGGATT
AGTGGCGAACGGGTGAGTAATACGTGAGTAACCTGCCTTTGACTCTGGGATAAGCCTGGGAAACTGGG
TCTAATACCGGATACGACACGAAACCGCATGATTTGGTGTGGAAAGGGTTTGTACTGGTTTTAGATGG
GCTCACGGCCTATCAGCTTGTTGGTGAGGTAACGGCTCACCAAGGCGACGACGGGTAGCCGGCCTGAG
AGGGTGACCGGCCACACTGGGACTGAGACACGGCC
>Zotu1235

GAACGCTAGCGGCAGGCTTAACACATGCAAGTCGAGGGGCAGCATGAGGAGTAGCAATACACCTTGAT
GGCGACCGGCGCAAGGGTGCGTAACGCGTGAGCAACATGCCCGTATCAGGGACATAACCGGTGGAAAC
GCCGCCTAATTTCCCATAACATCAGAGGTGACATCGTCTTTGGTTGAAAGATTCGTCGGATACGGATT
GGCTCGCGTGATATTAGCTGGTTGGTGAGGTAACGGCTCACCAAGGCTACGATATCTAGGGGTTCTGA
GAGGAAGGTCCCCCACATTGGAACTGAGACACGGTC
>Zotu1236
GAACGCTAGCGGCAGGCCTAATACATGCAAGTCGAACGGTAACATGAGTAGCAATACTTGATGACGAG
TGGCGCACGGGTGCGTAACGCGTATGCAATCTACCTTCAACCGGTGAATAGCCCGAAGAAATTCGGAT
TAATACACCATAGTATATTGAAGTGGCATCACTTTAATATTAAAACTCCGGTGGTTGAAGATGAGCAT
GCGTGACATTAGCTTGTTGGTGAGGTAACGGCTCACCAAGGCTACGATGTCTAGGGGATCTGAGAGGA
TTAACCCCCACACTGGTACTGAGACACGGAC
>Zotu1237
GAACGCTGGCGGCGTGCCTAATACATGCAAGTCGAGCGGATCTTGTCCTTCGGGATAAGGTTAGCGGC
GGACGGGTGAGTAACACGTAGGCAACCTGCCCTCGAGACTGGGATAACCTCCGGAAACGGATGCTAAT
ACCAGATACGCGATCTTCCCACCTGGGGAGATCGGGAAAGACGGAGCAATCTGTTACTCGGGGATGGG
CCTGCGGCGCATTAGCTAGTTGGTGAGGTAACGGCTCGCCAAGGCGACGATGCGTAGCCGACCTGAGA
GGGTGATCGGCCACACTGGGACTGAGACACGGCC
>Zotu1239
GAACGCTAGCGGGAGGCCTAACACATGCAAGCCGAGCGGTAGAGTTTCTTCGGAAACTTGAGAGCGGC
GCACGGGTGCGTAACACGTGTGCAACCTACCTTTATCTGGGGGATAGCCTTTCGAAAGGAAGATTAAT
ACCCCATAATATAATGATTGGCATCAATTATTATTGAAAACTCCGGTGGATAGAGATGGGCACGCGCA
AGATTAGATAGTTGGTGAGGTAACGGCTCACCAAGTCAATGATCTTTAGGGGGCCTGAGAGGGTGATC
CCCCACACTGGTACTGAGACACGGAC
>Zotu1241
GAACGCTGGCGGCGTGCTTAACACATGCAAGTCGAACGGTGACCTCGGAGCTTGCTCCGGGCGATCAG
TGGCGAACGGGTGAGTAACACGTGAGTAACCTGCCCCTGGCTCTGGGATAACTGTGGGAAACCGCAGC
TAATACCGGATACGACACAGACAGGCATCTGTACTGTGTGGAAAGTTTTTCGGCCAGGGATGGACTCG
CGGCCTATCAGCTTGTTGGTGGGGTAACGGCCCACCAAGGCGACGACGGGTAGCCGGCCTGAGAGGGC
GACCGGCCACACTGGGACTGAGACACGGCC
>Zotu1242
GAACGCTGGCGGCGTGCTTAACACATGCAAGTCGAACGATGATGAGGAGCTTGCTTCTCTGATTAGTG
GCGAACGGGTGAGTAACACGTGAGCAATCTGCCCTTGACTCTGGGATAAGCGTTGGAAACGACGTCTA
ATACCGGATACGACCCTTGGAGGCATCTCTGGGGGTGGAAAGAATTTTGGTCAAGGATGAGCTCGCGG
CCTATCAGCTAGTTGGTGAGGTAATGGCTCACCAAGGCGACGACGGGTAGCCGGCCTGAGAGGGTGAC
CGGCCACACTGGGACTGAGACACGGCC
>Zotu1243
GAACGCTGGCAGCGTGCTTAACACATGCAAGTCGAACGATGATGCCCAGCTTGCTGGGTTGATTAGTG
GCGAACGGGTGAGTAACACGTGAGTAACCTGCCCTTGACTTCGGGATAACTCCGGGAAACCGGGGCTA
ATACCGGATATGAGCCGTCCTCGCATGGGGGTGGTTGGAAAGTTTTTCGGTCAGGGATGGGCTCGCGG
CCTATCAGCTTGTTGGTGGGGTGATGGCCTACCAAGGCGACGACGGGTAGCCGGCCTGAGAGGGCGAC
CGGCCACACTGGGACTGAGACACGGCC
>Zotu1244
GAACGCTGGCGGCATGCTTAACACATGCAAGTCGCACGGTCGGGGGTAACTCCGGCAGTGGCGGACGG
GTGAGTAACGCGTAGGAATCTATCCTTGGGTGGGGGACAACCGTGGGAAACTACGGCTAATACCGCAT
GATACCTGTGGGTCAAAGGCGCAAGTCGCCTGAGGAGGAGCCTGCGTACGATTAGCTAGTTGGTGGGG
TAAAGGCCTACCAAGGCTACGATCGTTAGCTGGTCTGAGAGGATGATCAGCCACACTGGGACTGAGAC
ACGGCC
>Zotu1246
GAACGCTAGCGGCAGGCCTAATACATGCAAGTCGAACGGACAGTAGCAATACTGTTAGTGGCGCACGG
GTGCGTAACACGTAACTAACCTACCCACAACTGGGGGATAGCCCGCCGAAAGGCGGATTAATACCGCA
TAAGACTATAGAGTGGCATCACTTAGTAGTTAAAGATTTATTGGTTGTGGATGGGGTTGCGGGTCATT
AGCTAGTTGGTTGGGTAACGGCTGACCAAGGCGACGATGACTAGGGGAGCTGAGAGGCTGGTCCCCCA
CACGGGCACTGAGATACGGGC
>Zotu1247

GAACGCTGGCGGCGTGCCTAACACATGCAAGTCGAACGAGAAAGGGACTTCGGTCCTGAGTAAAGTGG
CGCACGGGTGAGTAACACGTAGGTAACCTACCTCTAAGATTAGGATAACCTGTCGAAAGATGAGCTAA
TACTGAATAAGACCACAGTTTCCTCGGAAGCAGGGGTCAAAGGTAGCCTCTATTTATAAGCTATCACT
TAGAGATGGGCCTGCGTACCATTAGCTAGTTGGTGAGGTAACGGCTCACCAAGGCAAAGATGGTTAGC
TGGTCTGAGAGGATGATCAGCCACACTGGAACTGAGACACGGTC
>Zotu1248
GAACGCTGGCGGCGTGCTTAACACATGCAAGTCGAACGGACCCTTCGGGGTTAGTGGCGGACGGGTGA
GTAACGCGTGAGGATCTGCCCTCAGGAGGGGAACAACAGTTGGAAACGACTGCTAATGCCCCATATGC
CGAGAGGTGAAAAGTTATTTCGCCTGAGGATGAACTCGCGTCTGATTAGCTAGTTGGTGGGGTAAGGG
CCTACCAAGGCGACGATCAGTAGCTGGTCTAAGAGGATGATCAGCCACACTGGGACTGAGACACGGCC
>Zotu1249
GAACGCTGGCGGCATGCCTTACACATGCAAGTCGAACGGTAACGCGGGGGCAACCCTGGCGACGAGTG
GCGAACGGGTGAGTAATACATCGGAACATATCCAGTAGCGGGGGATAACCTATCGAAAGATGGACTAA
TACCGCATACGATCTACGGATGAAAGCAGGGGACCGCAAGGCCTTGCACTATTGGAGTGGCCGATGTC
GGATTAGCTAGTTGGTGGGGTAAAAGCTTACCAAGGCGACGATCCGTAGCTGGTCTGAGAGGACGATC
AGCCACACTGGAACTGAGACACGGTC
>Zotu1250
GAACGCTGGTAACGTGGATACCGGCATGCAAGTCACGGGGGCCGCAAGGCAACCGGCGAACGACTTAG
TAACACGTGGGTACATACCCCAAACTGGAGCACAGCTCGTCGAAAGACGGGGTAATTCTCCATAGTCT
CGCAAGAGTAAACCCGCAAGGGGGTTTGGGAATGGCCTGCGGCCTATCAGATAGTTGGCGGTGTAATA
GACCACCAAGTCTATGACGGGTAGGGGGTGTGAGAGCACGACCCCCAAGAATGGCACTGCGACACGGG
C
>Zotu1252
GAACGCTGGCGGCGTGCCTAATACATGCAAGTCGAGCGGACCGGATCGGAGCTTGCTCTGATTTGGTC
AGCGGCGGACGGGTGAGTAACACGTGGGCAACCTGCCCGCAAGACCGGGATAACTCCGGGAAACCGGA
GCTAATACCGGATAACACCGAAGACCGCATGGTCTTTGGTTGAAAGGCGGCCTTTGGCTGTCACTTGC
GGATGGGCCCGCGGCGCATTAGCTAGTTGGTGAGGTAACGGCTCACCAAGGCGACGATGCGTAGCCGG
CCTGAGAGGGTGACCGGCCACACTGGGACTGAGACACGGCC
>Zotu1253
GAACGCTGGCGGCGTGCCTAACACATGCAAGTCAACGAGAAAGGGGTAGCAATATCCCGAGTAAAGTG
GCGCACGGGTGAGTAACGCGTAGGCAATTTGCCTTCAGGACGGGGATAATTCATCGAAAGGTGGACTA
ATACCCGATTATGCATCGGAATCGCCTGGTTCTGATGTTAAATGCCCGCAAGGGTGCCTGTTGATGAG
CCTGCGTCCCATTAGGTAGTTGGCGGGGTAATAGCCCACCAAGCCGACGATGGGTAGCTGGTCTGAGA
GGATGATCAGCCACACTGGAACTGAGACACGGTC
>Zotu1254
GAACGCTAGCGGGAGGCTTAATACATGCAAGTCGAGCGGTAATTTGTTTATAGCAATATAGATGAGCT
AGAGCGGCGCACGGGTGAGTAACGCGTACATGACCTACCCTATACTGGGGGATAGTCTTGGGAAACTG
AGAATAATCCCGCATGTGATTATTGGACTGCATGGTTTGATAATTAAAGCTACGGCGGTAAAGGATGG
ATGTGCGTCTGATTAGCTAGTTGGTAGGGTAACGGCCTACCAAGGCGATGATCAGTAGGGGGCGTGAG
AGCGTGAACCCCCACACGGGTACTGAGACACGGAC
>Zotu1255
GAACGCTGGCGGCGTGCTTAACACATGCAAGTCAAGGGGGCCCGCAAGGGCAACCGGCGAACGGGTGC
GTAACACGTGAGCAACCTGCCCTTGAGTGGGGGATAGCCGGCCCAACGGCCGGGTAATACCGCATACG
CTCGGTTGGGGACATCCCTGGCCGAGGAAACGCTTGCGGCTCAAGGAGGGGCTCGCGGCCTATCAGCT
TGTTGGTGGGGTAATGGCTCACCAAGGCGACGACGGGTAGCTGGTCTGAGAGGATGGCCAGCCACATT
GGGACTGAGAAACGGCC
>Zotu1256
GAACGCTAGCGGCAGGCCTAACACATGCAAGTCGAGGGGTAGAGGGTGCTTGCACCCTTGAGACCGGC
GCACGGGTGCGTAACGCGTATGCAACCTGCCTTCCACAGGGGAATAGCCCAGAGAAATTTGGATTAAT
GCCCCATGGCACAGCGAGACGGCATCGTCTTGCTGTTAAAGTAACAACGGTGGAAGATGGGCATGCGT
CCCATTAGCTAGTTGGTAGGGTAACGGCCTACCAAGGCTACGATGGGTAGGGGTCCTGAGAGGGAGAT
CCCCCACACTGGTACTGAGACACGGAC
>Zotu1257
GAACGCTAGCTACAGGCTTAACACATGCAAGTCGCAGGGTAACATGAGGAAAGCTTGCTTTCCTTGAT

GACGACTGGCGCACGGGTGAGTAACGCGTATCCAACCTTCCCATAACTACGGGATAACCCGTTGAAAG
ACGGCCTAATACCGTATGATATCGTTTGCTGACATCAAATAACGATTAAAGGTTTAGCGGTTATGGAT
GGGGATGCGTCTGATTAGCTTGTTGGCGGGGTAACGGCCCACCAAGGCGACGATCAGTAGGGGTTCTG
AGAGGAAGGTCCCCCACATTGGAACTGAGACACGGTC
>Zotu1258
GAACGCTGGCGGCGTGCCTAATACATGCAAGTCGTGCGGGTGAAGCCGACCACCCCTTCGGGGGCGGA
TGGCGGAACCAGCGGCGGACGGGTGAGTAACACGTGGGCAACCTGCCTGCAAGACCGGGATAACTGCG
GGAAACCGGAGCTAATACCGGATAAGAGGCTTTTCCGCATGGGGGAGCCTTAAAAGGCGGCGGAAGTC
GTCACTTGCAGATGGGCCCGCGGCGCATTAGCTAGTTGGTGAGGTAAGGGCTCACCAAGGCGACGATG
CGTAGCCGACCTGAGAGGGTGACCGGCCACACTGGGACTGAGACACGGCC
>Zotu1259
GAACGCTGGCGGCGTGCTTAACACATGCAAGTCGAACGGAAAGGCCCTTGCTTGCAGGGGTACTCGAG
TGGCGAACGGGTGAGTAACACGTGGGTGATCTGCCCTGCACTTCGGGATAAGCCTGGGAAACTGGGTC
TAATACCGGATAGGACGATGGTTTGGATGCCATTGTGGAAAGTTTTTTCGGTGTGGGATGAGCTCGCG
GCCTATCAGCTTGTTGGTGGGGTAATGGCCTACCAAGGCGTCGACGGGTAGCCGGCCTGAGAGGGTGT
ACGGCCACATTGGGACTGAGATACGGCC
>Zotu1260
GAACGCTGGCGGCGTGCTTAACACATGCAAGTCGAACGGAGGATTTATTTCGGTAGATCCTTAGTGGC
GAACGGGTGAGTAACGCGTGGGCAATCTGCCCTTCAGATGGGGACAACATCCCGAAAGGGGTGCTAAT
ACCGAATACGATCCTTCCACCGCATGGAGGGAGGATGAAAGATGGCCTCTATACATAAGCTATCGCTG
AAGGATGAGCCTGCGTCTGATTAGCTTGTTGGTGGGGTAACGGCCTACCAAGGCGATGATCAGTAGCC
GGTCTGAGAGGATGAACGGCCACATTGGGACTGAGACACGGCC
>Zotu1261
GAACGCTAGCGGCAGGCTTAATACATGCAAGTCGAGGGGCAGCGCAGTGTAGCAATACATGGGCGGCG
ACCGGCAAACGGGTGCGGAACACGTACGCAACCTACCCATAACTGATGAATAGCCCTCCGAAAGGAGG
ATTAATACATCGTAACTTAATGAAGTGGCATCACTTTATTAATATAGCTCCGGCGGTTTTGGATGGGC
GTGCGCCTGATTAGGTAGTTGGCGGGGTAATGGCCCACCAAGCCTGCGATCAGTAACTGGTGTGAGAG
CACGACCAGTCACACGGGCACTGAGACACGGGC
>Zotu1262
GAACGCTGGCGGCGCGCTTAACACATGCAAGTCGAGCGATGAAATCTTGACAGATCCCTTCGGGGTGA
AGATAAGATGGATTAGCGGCGGACGGGTGAGTAACACGTGAGTAACCTGCCTTAGACATTGGGATAGC
CTCGGGAAACCGGGATTAATACCAAATGAAATCATAGGTGCCCCTGCACTAATGATCAAAACTCCGGT
GGTCTAAGATGGACTCGCGTCCCATTAGCTAGTTGGTGAGGTAACGGCCCACCAAGGCGACGATGGGT
AGCCGGCCTGAGAGGGTGAACGGCCACATTGGAACTGAGAAACGGTC
>Zotu1263
GAACGCTGGCGGCGCGCCTAAAACATGCAAGTCGAACGAGAAGCCCGAGCTTGCTCGGGTGGAAAGTG
GCGAACGGGTGAGTAACACGTGAGGAACCTGCCCTGAAGACCGGGACAACATCGGGAAACCGTTGCTA
ATACCGGATACCTTCGATTGATCGCATGGTCGATCGAAGAAATGGATTCCGCTTCAGGATGGCCTCGC
GGCCTATCAGTTTGTTGGTGAGGTAACGGCTCACCAAGACGACGACGGGTAGCTGGTCTGAGAGGATG
TCCAGCCACACTGGGACTGAGACACGGCC
>Zotu1264
GAACGCTGGCGGTATGCTTAACACATGCAAGTCGAACGGAATCTTTAGGGATTTAGTGGCGGACGGGT
GAGTAACGCGTAAGAATCTACCTTCAGGTCGGGGACAACCACTGGAAACGGTGGCTAATACCGGATGT
GCCGCGAGGTGAAAGGCTTGCTGCCTGAAGAAGAGCTTGCGTCTGATTAGCTAGTTGGTGGGGTAAGA
GCTCACCAAGGCGACGATCAGTAGCTGGTCTGAGAGGATGATCAGCCACACTGGGACTGAGACACGGC
C
>Zotu1265
GAACGCTAGCGGGAGGCCTAATACATGCAAGTCGAACGGTAACAGGTAGCAATACGCTGACGAGTGGC
GCACGGGTGAGTAACACGTACACAATCTGCCTTATACTGGGGCATATCCTCGCGAAAGTGAGAGCAAT
TCCCCATAATATATTTGAATGGCATCATTTGAATATTAAAGCTCAGGCGGTATAAGATGAGTGTGCGT
CTGATTAGGTAGTTGGTGAGGTAATGGCTCACCAAGCCTGCGATCAGTAGCTGGTCTGAGAGGATGAT
CAGTCACACGGACACTGAGACACGGGT
>Zotu1266
GAACGCTAGCGGCAGGCCTAATACATGCAAGTCGGACGGGATTGGAGAGCTTGCTTTCCATGAGAGTG

GCGCACGGGTGCGTAACACGTATGTAACCTACCCTTGTCAGGGGGATAGCCTCTCGAAAGAGAGATTA
AGCCCGCATAACATGGAACTGTGGCATCACAGTTCCATCAAATATTTATAGGACAAGGATGGGCATGC
GGAACATTAGCTAGTTGGTAAGGTAACGGCTTACCAAGGCTACGATGTTTAGGGGATCTGAGAGGATG
ACCCCCCACACTGGTACTGAGACACGGAC
>Zotu1267
GAACGCTGGCGGCGTGCCTAATACATGCAAGTCGAGCGAATCAGATGGGAGCTTGCTCCCGTATGATT
AGCGGCGGACGGGTGAGTAACACGTGGGCAACCTGCCCGTAAGATTGGGATAACTCCGGGAAACCGGT
GCTAATACCGAATAGTTTCTGACACCTCATGGTATCAGAGGGAAAGTTGGCTTTGGCTAACACTTACG
GATGGGCCCGCGGCGCATTAGCTAGTTGGTGAGGTAACGGCTCACCAAGGCAACGATGCGTAGCCGAC
CTGAGAGGGTGATCGGCCACACTGGGACTGAGACACGGCC
>Zotu1269
GAACGTTAGCGGCACGCCTAACACATGCAAGTCGCACGAGAAAGGGGCAACCTGAGTAAAGTGGCGCA
CGGGTGCGTAACACGTAGATAACCTGCCCTTCGAAGGGGGATAACGTTCCGAAAGGAGCGCTAATACC
GCATACAGCAGCATTCGGTTCGCCGAGAGCTGGCAAAGGTAGGGACCGGCAACGGCCTATCGTCGAAG
GAGGGGTCTGCGGCCCATCAGCTAGTTGGGGAGGTAATGGCTCACCAAGGCAGAGACGGGTAGCTGGT
CTGAGAGGATGATCAGCCACACTGGTACTGGAACACGGAC
>Zotu1270
TAACGCTAGCGGCAGGCCTAATACATGCAAGTTGAACGGGATTTTGGGTAGCAATATCTGAATGAGAG
TAGCGCACGGGTGCGTAACGCGTATGCAACCTACCTTTGTCTGGGGGATAGCCTTCCGAAAGGAAGAT
TAATACCGCATAACGTATTGATGTAGCATTACATTAATACCAAACCTCCGGGGGACAAAGATGGGCAT
GCGTTCGATTAGCTAGTTGGTGAGGTAACGGCTCACCAAGGCTACGATCGATAGGGGGTCTGAGAGGA
TGATCCCCCACACTGGTACTGAGACACGGAC
>Zotu1271
GAACGCTGGCGGCGTGCTTAACACATGCAAGTCGAACGGAAAGGCCCTTCGGGGTACTCGAGTGGCGA
ACGGGTGAGTAACACGTGGGTGATCTGCCTCGTACTTTGGGATAAGCCTGGGAAACTGGGTCTAATAC
CGAATATGACCTTCCCTCGCATGGGGGTTGGTGGAAACTTTTTGGGTACGAGATGGGCCCGCGGCCTA
TCAGCTTGTTGGTGGGGTAATGGCCTACCAAGGCGACGACGGGTAGCCGGCCTGAGAGGGCGACCGGC
CACACTGGGACTGAGACACGGCC
>Zotu1272
GAACGCTAGCGGCAGGCCTAATACATGCAAGACGAACGGTAAGTTTGATAGCAATATTGAACCTAGAG
TGTCGTAAGGGTGCGTAACACGTATGCAATCTACCCGAAACTGGAGCATAGCTCCCCGAAAGGGGAAT
TAACACTCCATAAGATCATGGTGCGGCATCGCAGAGTGATTAAAACTCCGGTGGTTTCGGATGAGCAT
GCGTTTGATTAGTTAGTTGGTGAGGTAACGGCTCACCAAGACGATGATCAATAGGGGAACTGAGAGGT
TGATCCCCCACACTGGCACTGAGATACGGGC
>Zotu1273
GAATGCTGGCGGCGTGGATAAGGCATGCAAGTCAAGGGGGCCGCAAGGCAACCGGCAGACGAGGTAGT
AACACGTAGGTAGATACCCTGGAGTCGGGCATAGCCAGTCGAAAGACTGGGTAATTCCCGATGGTCTC
GCAAGAGTAAAGGTTTTTCGCTCCGGGAGTCGCCTGCGCAATATCAGCTAGTTGGTGAGGTAATGGCT
CACCAAGGCTACGACGTTTAGGGGAGCTGAGAGGCTGACCCCCACCGATGGGACTGAGATACGGCC
>Zotu1275
GAACGCTAGCGGCAGGCCTAACACATGCAAGTCGAGGGGTAGAGGGGAGCTTGCTCTCCTTGAGACCG
GCGGACGGGTGAGTAGCGCGTATGGAATCTGCCTTGTACAGGGGAATAGCCCAGGGAAACTTGGATTA
ATGCCCCATAGTGTTGAGGGGCGGCATTGTTCTTCAACTAAAGATTTATCGGTACAAGATGACCATGC
GTCCCATTAGCTTGTTGGTGTGGTAACGGCACACCAAGGCTACGATGGGTAGGGGCCCTGAGAGGGGG
ATCCCCCACACTGGTACTGAGACACGGAC
>Zotu1276
AAACGCTGGCGGCGCACATAAGACATGCAAGTCGAACGGAATTTAGTCGAATGTTTACATGAGACGAA
AATTTAGTGGCGGACGGGTGAGTAACACGTGAGCAACCTACCTTTAAGAGGGGGATAACATTGAGAAA
TCAGTGCTAATACCGCATAAAGCTATTTAACCGCATGATTAGATAGCCAAAGGAGAAATCCGCTTAAA
GATGGGCTCGCGGCTGATTAGTTAGTTGGTGGGGTAACGGCCTACCAAGACGATGATCAGTAGCCGAG
CTGAGAGGCACAACGGCCACATTGGGACTGAGACACGGCC
>Zotu1277
GAACGCTGGCGGCGCGCTTAACACATGCAAGTCGAACGGTGAACGGGGCTTCGGCCCCCGGATCAGTG
GCGAACGGGTGCGTAACACGTGGGCAACCTGCCCCAGACACTGGGATAACTCCGGGAAACCGGGGCTA

ATACCGGATACTCCGATCGGATCGCATGGTCTGATCGGGAAAGCTTTTGCGGTCTGGGATGGGCCCGC
GGCCTATCAGCTTGTTGGTGAGGTAACGGCTCACCAAGGCGACGACGGGTAGCTGGTCTGAGAGGACG
ACCAGCCACACTGGGACTGAGATACGGCC
>Zotu1278
GAACGCTGGCGGCGTGCTTAACACATGCAAGTCGAACGGAAAGGCCTTTTCGGAGGTACTCGAGTGGC
GAACGGGTGAGTAACACGTGGGTGATCTGCCCTGCACTCTGGGATAAGCCTGGGAAACTGGGTCTAAT
ACCGGATAGGACCGCACTTTAGTGTGTGTGGTGGAAAGTTTTTTCGGTGTAGGATGAGCCCGCGGCCT
ATCAGCTTGTTGGTGGGGTAATGGCCTACCAAGGCGGCGACGGGTAGCCGGACTGAGAGGTTGGTCGG
CCACATTGGGACTGAGATACGGCC
>Zotu1279
GAACGCTGGCGGCGTGCTTAACACATGCAAGTCGAACGGAAAGGCCCTGCTTGCAGGGTACTCGAGTG
GCGAACGGGTGAGTAACACGTGGGTGATCTGCCCTGTACTTTGGGATAAGCCTGGGAAACTGGGTCTA
ATACCGAATATGACCACTTCTTGGATGGGGTGGTGGAAAGCTTTTGCGGTATGGGATGAGCTCGCGGC
CTATCAGCTTGTTGGTGGGGTAATGGCCTACCAAGGCGTCGACGGGTAGCCGGCCTGAGAGGGTGTAC
GGCCACATTGGGACTGAGATACGGCC
>Zotu1280
GAACGCTGGCGGCAGGCCTAACACATGCAAGTCGAGCGGATGAGAGGAGCTTGCTCTTCGATTCAGCG
GCGGACGGGTGAGTAATACCTAGGAATCTGCCTGGTAGTGGGGGACAACGTTTCGAAAGGAACGCTAA
TACCGCATACGTCCTACGGGAGAAAGCAGGGGACCTTCGGGCCTTGCGCTATCAGATGAGCCTAGGTC
GGATTAGCTAGTTGGTGAGGTAATGGCTCACCAAGGCTACGATCCGTAACTGGTCTGAGAGGATGATC
AGTCACACTGGAACTGAGACACGGTC
>Zotu1281
GAACGCTAGCGGCAGGCCTAATACATGCAAGTCGAACGGGATTTGGGAGCTTGCTCCTGATGAGAGTG
GCGCACGGGTGCGTAACACGTATGTAACCTACCTTAATCTGGGGGATAGCCTCTCGAAAGAGAGATTA
ACACCGCATAACATTATCGGACAGCATTTTCTGATAATCAAATATTTATAGGATTAAGATGGGCATGC
GGAACATTAGCTAGTTGGTAAGGTAATGGCTTACCAAGGCTACGATGTTTAGGGGATCTGAGAGGATG
ACCCCCCACACTGGTACTGAGACACGGAC
>Zotu1282
GAACGCTGGCGGCAGGCCTAACACATGCAAGTCGAGCGGATGGGAAGAGCTTGCTCTTCGATTTAGCG
GCGGACGGGTGAGTAATACCTAGGAATCTGCCTGGTAGTGGGGGACAACGTTTCGAAAGGAACGCTAA
TACCGCATACGTCCTACGGGAGAAAGCAGGGGACCTTCGGGCCTTGCGCTATCAGATGAGCCTAGGTC
GGATTAGCTAGTTGGTGAGGTAATGGCTCACCAAGGCTACGATCCGTAACTGGTCTGAGAGGATGATC
AGTCACACTGGAACTGAGACACGGTC
>Zotu1283
GAACGCTAGCGGCAGGCTTAATACATGCAAGTCAAGGGGGTAGCAATACAACCGGCGCACGGGTGAGT
AACGCGTACACAACATGCCATATACTGGAGTATAGCTTTGGGAAACTGAAGGTAATCCTCCATAACAT
TTCGGATAGGCATCTATTTGAAATTAAAGTTTCGACGGTATATGATTGGTGTGCGTTTGATTAGCTAG
TTGGTAGGGTAACGGCCTACCAAGGCTACGATCAATAGCTGGCGTGAGAGCGCGACCAGCCACACTGG
AACTGAGACACGGTC
>Zotu1284
GAACGCTGGCGGCGTGCTTAACACATGCAAGTCGAACGGTAAGGCTCCTTCGGGAGTACACGAGTGGC
GAACGGGTGAGTAACACGTGAGCAACCTGCCCTTGACTTTGGGATACCAGCTGGAAACAGCTGCTAAT
ACCGGATATGACACCTCTTCGCATGTTGGGGTGTGGAAAGCTCCGGCGGTCAGGGATGGGCTCGCGGC
CTATCAGCTTGTTGGTGAGGTAATGGCTCACCAAGGCTTCGACGGGTAGCCGGCCTGAGAGGGCGACC
GGCCACACTGGGACTGAGATACGGCC
>Zotu1285
GAACGCTAGCGGCAGGCCTAATACATGCAAGTCGAACGATAGTCCCAGCTTGCTGGGGCAAAAGTGGC
GCACGGGTGCGTAACGCGTATGCAACCTACCTTAATCAGGGGGATAGCCCGAAGAAATTCGGATTAAC
ACCGCATAAAATCACAGCACAGCATTGTGCAATGATCAAATATTTATAGGATTAAGATGGGCATGCGT
GACATTAGTTAGTTGGCGGGGTAACGGCCCACCAAGACCACGATGTCTAGGGGATCTGAGAGGATGAC
CCCCCACACTGGTACTGAGACACGGAC
>Zotu1286
GAACGCTGGCGGCAGGCTTAACACATGCAAGTCGAGCGCCCCGAGTTTTAGCAATAAGACTTGGGGAG
CGGCAGACGGGTGAGTAACGCGTGGGAACGTACCCCCCGGTTCGGAATAACTCAGGGAAACTTGAGCT

AATACCGGATACGGCTGGAAGGCGAAAGATTTATCGCCGGGGGATCGGCCCGCGTCCGATTAGCTAGT
TGGTGTGGTAATGGCGCACCAAGGCGACGATCGGTAGCTGGTCTGAGAGGATGATCAGCCACATTGGG
ACTGAGACACGGCC
>Zotu1288
GAACGCTGGCGGCGTGCTTAACACATGCAAGTCGAACGGTGAAACAGGAGCTTGCTCCTGTGGATCAG
TGGCGAACGGGTGAGTAACACGTGAGTAACCTGCCCCAGACTCTGGGATAAGCACTGGAAACGGTGTC
TAATACCGGATACGAGCTTCAGCCGCATGGCTAGGAGCTGGAAAGAATTTCGGTCTGGGATGGACTCG
CGGCCTATCAGCTAGTTGGTGAGGTAACGGCCCACCAAGGCGACGACGGGTAGCCGGCCTGAGAGGGT
GACCGGCCACACTGGGACTGAGACACGGCC
>Zotu1289
GAACGCTGGCGGCGTGCTTAACACATGCAAGTCGAACGAAGCACTTTTACAGATTTCTTCGGAATGAA
GTTTTAGTGACTGAGTGGCGGACGGGTGAGTAACGCGTGGGTAACCTGCCTCACACAGGGGGATAACA
GTTGGAAACGGCTGCTAATACCGCATAAGCGCACAGTACCGCATGGTACAGTGTGAAAAACTCCGGTG
GTGTGAGATGGACCCGCGTCTGATTAGCTAGTTGGCAGGGCAACGGCCTACCAAGGCGACGATCAGTA
GCCGACCTGAGAGGGTGACCGGCCACATTGGGACTGAGACACGGCC
>Zotu1290
GAACGCTGGCGGCGTGCCTAATACATGCAAGTCGAGCGAACTTGCGGGAGCTTGCTCCCAAAAGTTAG
CGGCGGACGGGTGAGTAACACGTGGGCAACCTGCCTGTAAGACTGGGATAACTTCGGGAAACCGGAGC
TAATACCGGATAACTCTTATCAACTCATGTTGGTAAGCTAAAAGACGGTTTCGGCTGTCACTTACAGA
TGGGCCCGCGGCGCATTAGCTAGTTGGTGAGGTAACGGCTCACCAAGGCAACGATGCGTAGCCGACCT
GAGAGGGTGATCGGCCACACTGGGACTGAGACACGGCC
>Zotu1291
GAACGCTGGCGGCGTGCTTAACACATGCAAGTCGAACGGAAAGGCCCTTCGGGGTACTCGAGTGGCGA
ACGGGTGAGTAACACGTGGGTGATCTGCCTCGTACTCTGGGATAAGCCTGGGAAACTGGGTCTAATAC
CGGATAGGACCATTCCTCGCATGGGGGGTGGTGGAAAGTTTTTCGGTACGAGATGGGCCCGCGGCCTA
TCAGCTTGTTGGTGGGGTAATGGCCTACCAAGGCGACGACGGGTAGCCGGCCTGAGAGGGCGACCGGC
CACACTGGGACTGAGACACGGCC
>Zotu1292
GAACGCTGGCGGCGTGCCTAATACATGCAAGTCGAACGAATCTTCTTCGGAAGATGAGTGGCGAACGG
GTGAGTAATACATAGGTAACCTGCCCCTGTGCGGGGGATAACAGGAGGAAACTCCTGCTAATACCGCA
TAGCCATGAGCACCGCATGGAGCTCATGCCAAATATCCTTTCGGGGATAGCGCAGGGATGGACCTATG
GCGCATTAGCTAGTTGGCGGGGCAACGGCCCACCAAGGCAACGATGCGTAGCCGGCCTGAGAGGGCGG
ACGGCCACATTGGGACTGAGACACGGCC
>Zotu1293
GAACGCTGGCGGCGTGCTTAACACATGCAAGTCGAACGGAAAGGCCCTGCTTGCAGGGTACTCGAGTG
GCGAACGGGTGAGTAACACGTGGGTGATCTGCCCTGTACTTCGGGATAAGCTTGGGAAACTGGGTCTA
ATACCGGATAGGACCGGCCTTTAGTGTGGTTGGTGGAAAGCTTTTGCGGTATGGGATGAGCTCGCGGC
CTATCAGCTTGTTGGTGGGGTAATGGCCTACCAAGGCGTCGACGGGTAGCCGGCCTGAGAGGGTGTAC
GGCCACATTGGGACTGAGATACGGCC
>Zotu1294
GAACGCTGGCGGCGTGCTTAACACATGCAAGTCACGGGGGTCCCGCAAGGGATAACCGGCGAACGGGT
GCGTAACACGTGACCAACCTGCCCTCGTGTGGGGGATAGCCGGGCTAACGCCCGGGTAATACCGCATA
CGTTCCTCGAGCGGAGTCGCTTGGGGAGGAAAGCTCCGGCGCACGGGGAGGGGGTCGCGGCCTATCAG
CTCGTTGGCGGGGTAACGGCCCACCAAGGCGACGACGGGTAGCTGGTCTGAGAGGATGGCCAGCCACA
TTGGGACTGAGAGACGGCC
>Zotu1295
GAACGCTGGCGGCGTGCCTAATACATGCAAGTCGAACGAGAGCGACCGGTGCTTGCACTGGTCAATCT
AGTGGCGAACGGGTGAGTAACACGTGGGTAACCTGCCCATCAGAGGGGGATAACATTCGGAAACGGAT
GCTAAAACCGCATAGGTCTTCGAACCGCATGGTTTGAAGAGGAAAAGAGGCGCAAGCTTCTGCTGATG
GATGGACCCGCGGTGCATTAGCTAGTTGGTGAGGTAACGGCTCACCAAGGCCGTGATGCATAGCCGAC
CTGAGAGGGTGATCGGCCACATTGGGACTGAGACACGGCC
>Zotu1296
GAACGCTGGCGGCGTGCCTAACACATGCAAGTCGAGCGAGAGACTTCGGTCTCTAGCGGCGGACGGGT
GAGTAACACGTGGGTAACCTGCCTCAAACAGGGGGATAGCCTTCCGAAAGGAAGATTAATACCGCATA

ACGTAGTTTAGTCGCATGACTAGATTATCAAAGGAGTAATCCGGTTTGAGATGGACCCGCGGCGCATT
AGCTAGTTGGTGAGGTAACGGCTCACCAAGGCGACGATGCGTAGCCGACCTGAGAGGGTGATCGGCCA
CATTGGAACTGAGATACGGTC
>Zotu1297
GAACGCTGGCGGCGTGCTTAACACATGCAAGTCGAACGATGAACCAGGAGCTTGCTCTTGGGGATTAG
TGGCGAACGGGTGAGTAACACGTGAGTAACCTGCCCTTGACTCTGGGATAAGCGTTGGAAACGACGTC
TAATACCGGATACGAGCTTCCACCGCATGGTGAGTTGCTGGAAAGAATTTTGGTCAAGGATGGACTCG
CGGCCTATCAGCTTGTTAGTGAGGTAATGGCTCACCAAGGCGACGACGGGTAGCCGGCCTGAGAGGGT
GACCGGCCACACTGGGACTGAGACACGGCC
>Zotu1298
GAACGCTGGCGGCGTGTTTAACACATGCAAGTCGAGCGAGAAACTTCTCAGTTGATTCTTCGGATGAA
ACAGAGTTGTGGAAAGCGGCGGACGGGTGAGTAACGCGTAGGCAACCTGCCCCTTGCAGAGGGATAGC
CTCGGGAAACCGGGATTAAAACCTCATAATGCGGCACTGAGACATCTCGGAGCCGCCAAAGATTTATC
GGCAAGGGATGGGCCTGCGTCTGATTAGTTAGTTGGTGGGGTAACGGCCTACCAAGGCGACGATCAGT
AGCCGACCTGAGAGGGTGATCGGCCACATTGGAACTGAGACACGGTC
>Zotu1299
GAACGCTGGCGGCGCGCCTAACACATGCAAGTCGAACGGTTAAAGCACCTTTTAGGTGTGTATAAAGT
GGCGAACGGCTGAGTAACACGTGGGCAACCTGCCCTCCTCTTGGGGATAGCCTCGGGAAACCGAGGAT
AATACCCGATACTTCAATGTGGCCGCATGACGACATTGAGAAAGCTTTTGCGGAGAAGGATGGGCCCG
CGGCCTGTTAGCTTGTTGGTGGGGTAGAGGCCTACCAAGGCAATGATGGGTAGCTGGGTTGAGAGACC
GACCAGCCAGATTGGGACTGAGACACGGCC
>Zotu1300
GAACGCTGGTGGCGTGCTTAACACATGCAAGTCGAGCGAGGCGTCCGCTTTCGGGTGGATAGTCGAGC
GGCGAACGGGTGAGTAACACGTGAGTAATCTTCCCTGGGCTCGGGGATAACCTCGGGAAACTGAGGCT
AATACCGGATGTGCGTTGTTTGGGGCTTCCTGGATGACGGAAAGGTTGCGTGCCGTGAGGTGCGTTTC
CGGCCTTGGATGAGCTCGCGGCCTATCAGCTTGTTGGTGCGGTGATGGCGTACCAAGGCGACGACGGG
TAGCCGGCCTGAGAGGGTGACCGGCCACACTGGGACTGAGATACGGCC
>Zotu1301
GAACGCTGGCGGCGTGCCTAATACATGCAAGTCGAGCGAATCAGATGGGAGCTTGCTCCCGGATGATT
AGCGGCGGACGGGTGAGTAACACGTGGGCAACCTGCCCGTAAGATTGGGATAACTCCGGGAAACCGGT
GCTAATACCGAATAGTTTCTGACACCTCATGGTATCAGAGGGAAAGTTGGCTTCGGCTAACACTTACG
GATGGGCCCGCGGCGCATTAGCTAGTTGGTGAGGTAACGGCTCACCAAGGCAACGATGCGTAGCCGAC
CTGAGAGGGTGATCGGCCACACTGGGACTGAGACACGGCC
>Zotu1303
GAACGCTGGCGGCGTGCCTAACACATGCAAGTCGAGCGATTCTCTTCGGAGAATAGCGGCGGACGGGT
GAGTAACGCGTGGGTAACCTGCCCTGTACACACGGATAACATACCGAAAGGTATGCTAATACGGGATA
ATATATTTTGATGGCATCATCGAAATATCAAAGCTCCGGCGGTACAGGATGGACCCGCGTCTGATTAG
CTAGTTGGTGAGGTAATGGCTCACCAAGGCGACGATCAGTAGCCGACCTGAGAGGGTGATCGGCCACA
TTGGAACTGAGACACGGTC
>Zotu1304
GAACGCTGGCGGCGTGCCTAATACATGCAAGTCGAGCGAATCAATGGGAGCTTGCTCCCTGAGATTAG
CGGCGGACGGGTGAGTAACACGTGGGTAACCTGCCTGTAAGATTGGGATAACTCCGGGAAACCGGGGC
TAATACCGGATAATACTTTTAACCGCATGGTTAAAAGTTGAAAGATGGTTTCGGCTATCACTTACAGA
TGGACCCGCGGCGCATTAGCTAGTTGGTGAGGTAACGGCTCACCAAGGCAACGATGCGTAGCCGACCT
GAGAGGGTGATCGGCCACACTGGGACTGAGACACGGCC
>Zotu1305
GAACGCTGGCGGCGTGCTTAACACATGCAAGTCGAACGATGAAGCCTAGCTTGCTGGGTGGATTAGTG
GCGAACGGGTGAGTAACACGTGAGTAACCTGCCCTTGACTCTGGGATAAGCACTGGAAACGGTGTCTA
ATACCGGATATTTCAGCTGGCCGCATGGTCTGGTTGTGGAAAGAATTTTGGTCAAGGATGGACTCGCG
GCCTATCAGGTTGTTGGTGAGGTAATGGCTCACCAAGCCTACGACGGGTAGCCGGCCTGAGAGGGTGA
CCGGCCACACTGGGACTGAGACACGGCC
>Zotu1306
TAACGCTAGCGGCAGGCCTAATACATGCAAGTCGAACGGTAACAGGGTAGCAATACCGCTGACGAGTG
GCGCACGGGTGCGTAACGCGTATGCAATCTACCTTTAACAGGGGAATAGCTCAGAGAAATCTGAATTA

ATACCGCATAATATAGTGAGGAGGCATCTTCTTGCTATCAAACCTTCGGGGGTTAAAGATGAGCATGC
GTATCATTAGCTAGTTGGTGAGGTAACGGCTCACCAAGGCTACGATGATTAGGGGGTCTGAGAGGATG
ATCCCCCACACTGGTACTGAGACACGGAC
>Zotu1307
GAACGCTGGCGGCGTGCCTAATACATGCAAGTCGAACGAGAGCGACCGGTGCTTGCACTGGTTAATCT
AGTGGCGAACGGGTGAGTAACACGTGGGTAACCTGCCCATCAGAGGGGGATAACATCCGGAAACGGAT
GCTAAAACCGCATAGGTCTTCGAGCCGCATGGCTTGAAGAGGAAAAGAGGCGCAAGCTTCTGCTGATG
GATGGACCCGCGGTGCATTAGCTAGTTGGTGAGGTAACGGCTCACCAAGGCCGTGATGCATAGCCGAC
CTGAGAGGGTGATCGGCCACATTGGGACTGAGACACGGCC
>Zotu1308
GAACGCTGGCGGCGTGCCTAATACATGCAAGTCGAGCGGACCAAATCGGAGCTTGCTCTGGTTTGGTC
AGCGGCGGACGGGTGAGTAACACGTGGGCAACCTGCCCGCAAGACCGGGATAACTCCGGGAAACCGGA
GCTAATACCGGATAACACCGAAGACCGCATGGTCTTTGGTTGAAAGGCGGCCTTTGGCTGTCACTTGC
GGATGGGCCCGCGGCGCATTAGCTAGTTGGTGAGGTAACGGCTCACCAAGGCGACGATGCGTAGCCGG
CCTGAGAGGGTGACCGGCCACACTGGGGCTGAGACACGGCC
>Zotu1309
GAACGCTAGCGGCAGGCTTAACACATGCAAGTCGAGGGGTAACAGGGTCTTCGGACCGCTGACGACCG
GCAAACGGGTGCGGAACACGTACGCAACCTACCCTTTACTGGAGCATAGCCCGTAGAAATGCGGATTA
ACACTCCATAGTATTCAAGAGTGGCATCACTCAGGAATTAAAAATTTATTGGTAAAGGATGGGCGTGC
GTCCCATTAGCTAGTTGGTATGGGTAATGGCCTACCAAGGCGACGATGGGTAGCTGATGTGAGAGCAT
GATCAGCCACACGGGCACTGAGACACGGGC
>Zotu1310
GAACGCTGGCGGCATGCCTAACACATGCAAGTCGAACGAGATCTTCGGATCTAGTGGCGCACGGGTGC
GTAACGCGTGGGAATCTACCCTTGGGTTCGGAATAACAGCGAGAAATTGCTGCTAATACCGGATGATG
ACGTAAGTCCAAAGATTTATCGCCCAGGGATGAGCCCGCGTAGGATTAGGTAGTTGGTGTGGTAAAGG
CGCACCAAGCCTACGATCCTTAGCTGGTCTGAGAGGATGATCAGCCACACTGGGACTGAGACACGGCC
>Zotu1311
GAACGCTGGCGGCATGCCTAACACATGCAAGTCGAACGAGACCTTCGGGTCTAGTGGCGCACGGGTGC
GTAACGCGTGGGAATCTGCCCTTGGGTTCGGAATAACAGTTAGAAATGACTGCTAATACCGGATGATG
ACGAAAGTCCAAAGATTTATCGCCCAAGGATGAGCCCGCGTAGGATTAGCTAGTTGGTGGGGTAAAGG
CCTACCAAGGCGACGATCCTTAGCTGGTCTGAGAGGATGATCAGCCACACTGGGACTGAGACACGGCC
>Zotu1312
GAACGCTGGCGGCATGCCTAATACATGCAAGTCGAACGCTGAAGCACTCTGCTTGCAGAGTGCGGATG
AGTGGCGAACGGGTGAGTAACACGTGGGAAATCTGCCTAAGAGTGGGGGGATAACATTCGGAAACGGA
TGCTAATACCGCATAGATTCTTGGACCGCATGGTTCTGGAAAGAAAGACGGCTTTGCTGTCGCTTTTA
GATGATCCCGCGGCGTATTAGTTAGTTGGTGGGGTAACGGCCTACCAAGACCATGATACGTAGCCGAC
CTGAGAGGGTGATCGGCCACACTGGGACTGAGACACGGCC
>Zotu1313
GAACGCTAGCGGCAGGCTTAACACATGCAAGTCGAGGGGTAACAGGGTGCTTGCACCGCTGACGACCG
GCGCACGGGTGCGTAACGCGTATACAATCTACCTTATAGAGGGGGATAGCCCAGAGAAATTTGGATTA
ATACCCCATAGTACTTAGAGACAGCATTGTTTTTAGGTTAAACATTTATGGCTATAAGATGAGTATGC
GTCCTATTAGTTAGTTGGTAAGGTAACGGCTTACCAAGACCGCGATAGGTAGGGGTCCTGAGAGGGAG
ATCCCCCACACTGGTACTGAGACACGGAC
>Zotu1314
AAACGCTGGCGGCGCACATAAGACATGCAAGTCGAACGGAAGTCGTTATAATGATTTAGATTTTGATT
TGAGGATTTATCCAATTTGATGATGACGAAGATTTATAACAATGGCTTTAGTGGCGGACTGGTGAGTA
ACGCGTAAGCAATCTGCCTATTAGAGGGGAATAACAGTGAGAAATCATTGCTAATACCGCATATGCCA
TAATTATCGCATGATAGTAGTGGGAAAGGAGTAATCCGCTAATAGATGAGCTTGCGTCTGATTAGCTA
GTTGGTGGGGTAACGGCCTACCAAGGCGACGATCAGTAGCCGGACTGAGAGGTTGAACGGCCACATTG
GGACTGAGATACGGCC
>Zotu1315
GAACGCTAGCGGCAGGCCTAATACATGCAAGACGAACGGCAAGATTTTTAGCAATAAGAGTCCTAGAG
TGTCGTAAGGGTGCGTAACACGTATGCAATCTACCCTCAACAGGAGGATAGCTCCCCGAAAGGGGAAT
TAACACTCCATAAGATCATGGTTCGGCATCGGACAGTGATTAAAACTACGGTGGTTGAGGATGAGCAT

GCGCATGATTAGTTAGTTGGCGGGGTAATGGCCCACCAAGACGATGATCATTAGGGGAACTGAGAGGT
TGATCCCCCACACTGGCACTGAGATACGGGC
>Zotu1316
GAGCGCTGGCGGTGTGCCTAATACATGCAAGTCGAACGGACCTTTATTTTAGAAAATCCCAATGTGTT
TGGGGGTTTCCTTAAATAATGGTTAGTGGCGGACGGGTGAGTAACACGTTAGTACCTGCCCCAAAGTG
AGGAATAACCCGGCGAAAGTCGGGCTAATACCACATGTGACCGTTTACGGTTAAAGCCTTCGGGCGCT
TTGGGAGGGGCTTGCGTCTGATTAGCTAGTTGGTAGGGTAATGGCCTACCAAGGCTACGATCAGTAGC
TGGTTCGAGAGGATGACCAGCCACACTGGGACTGAGATACGGCC
>Zotu1317
GAACGCTGGCGGCGCGCTTAACACATGCAAGTCGAGCGATGAAATTTTGACAGATCCCTTCGGGGTGA
AGACAAAATGGATTAGCGGCGGACGGGTGAGTAACACGTGAGTAACCTGCCTTAGACATTGGGATAGC
CTCGGGAAACCGGGATTAATACCGAATAAAATCATAGGTGCACATGCACAAATGATCAAAACTCCGGT
GGTCTAAGATGGACTCGCGTCCCATTAGCTAGTTGGTAGGGTAACGGCCTACCAAGGCGACGATGGGT
AGCCGGCCTGAGAGGGTGAACGGCCACATTGGAACTGAGAAACGGTC
>Zotu1318
GAACGCTGGCGGCGTGCCTAATACATGCAAGTAGAACGCTGAAAGAGGAGCTTGCTTCTCTGGATGAG
TTGCGAACGGGTGAGTAACGCGTAGGTAACCTGCCTGGTAGCGGGGGATAACTATTGGAAACGATAGC
TAATACCGCATAATAGTAGATGTTGCATGACATTTGCTTAAAAGGTGCAATTGCATCACTACCAGATG
GACCTGCGTTGTATTAGCTAGTTGGTGGGGTAACGGCTCACCAAGGCGACGATACATAGCCGACCTGA
GAGGGTGATCGGCCACACTGGGACTGAGACACGGCC
>Zotu1319
GAACGCTGGCGGCGTGCCTAATACATGCAAGTCGAGCGAATCAGATGGGAGCTTGCTCCCGGATGATT
AGCGGCGGACGGGTGAGTAACACGTGGGCAACCTGCCCGTAAGATTGGGATAACTCCGGGAAACCGGT
GCTAATACCGAATAGTTTCTGACACCTCATGGTGTCAGAGGGAAAGTTGGCTTCGGCTAACACTTACG
GATGGGCCCGCGGCGCATTAGCTAGTTGGTGAGGTAACGGCTCACCAAGGCAACGATGCGTAGCCGAC
CTGAGAGGGTGATCGGCCACACTGGGACTGAGACACGGCC
>Zotu1320
GAACGCTGGCGGCGTGCCTAACACATGCAAGTCGAACGGTGATATATAGATGATTTCGGTCTGAAATA
TATCATAGTGGCGGACGGGTGAGTAACGCGTGGATAACCTGCCCCGTACTGGGGGATAGCAGCTGGAA
ACGGCTGGTAATACCGCATAAGCGCACGAGGGGGCCTCCCCTTGTGTGAAAAGATTCATCGGTACGGG
ATGGGTCCGCGTCTGATTAGCCAGTTGGCGGGGTAACGGCCCACCAAAGCGACGATCAGTAGCCGGCC
TGAGAGGGTGAACGGCCACATTGGGACTGAGACACGGCC
>Zotu1322
GCCGGCAATGTTGAAGAGGTAAGTACTGCTCACATGCACTGTCCTCCACGGAGGACGCTAACCATGTG
TGTCAAGATCTCAGCACACATTAAAGAACCCTCAGGCGGGCCAAAATTGAC
>Zotu1323
GAACGCTAGCTACAGGCTTAACACATGCAAGTCGAGGGGAAACGACATTGAAGCTTGCTTCGATGGGC
GTCGACCGGCGCACGGGTGAGTAACGCGTATCCAACCTGCCTCTGACTAAGGGATAACCCGGCGAAAG
TCGGACTAATACCTTATGAGGTTTTCTGCAGACATCTAATGAAAACGAAAGATTTATCGGTCAGTGAT
GGGGATGCGTCTGATTAGCTTGTTGGCGGGGTAATGGCCCACCAAGGCAACGATCAGTAGGGGTTCTG
AGAGGAAGGTCCCCCACATTGGAACTGAGACACGGTC
>Zotu1324
GAACGCTGGCGGCGTGCCTAATACATGCAAGTCGAGCGCGACTGATTACACCGTTCTTCGGAACACCG
TAATCATCGAGCGGCGGACGGGTGAGTAACACGTGGGCAACCTACCCTTAAGACTGGGATAACCGCGG
GAAACCGTGGCTAATACCGGATAATACTTTTATACAACGGTATAGAAGTTGAAAGGTGGATTTCTATC
ACTTAAGGATGGGCCTGCGGCGCATTAGCTGGTTGGTAAGGTAACGGCTTACCAAGGCGACGATGCGT
AGCCGACCTGAGAGGGTGATCGGCCACACTGGAACTGAGACACGGTC
>Zotu1325
GAACGCTGGCGGCGTGCTTAACACATGCAAGTCGAACGGTGAACTCCGCTTGCGGGGGGATCAGTGGC
GAACGGGTGAGTACACGTGGGCAACCTGCCCTCAGCTCTGGGATAACTCCAAGAAATTGGGGCTAATA
CCGGATATGACCGCTGACCGCATGGTCTGGTGGTGGAAAGATTTATCGGCTGGGGATGGGCCCGCGGC
CTATCAGCTTGTTGGTGGGGTAGTGGCCTACCAAGGCGACGACGGGTAGCCGGCCTGAGAGGGTGACC
GGCCACACTGGGACTGAGACACGGCC
>Zotu1326

GAACGCTAGCTACAGGCTTAACACATGCAAGTCGAGGGGAAACGGCATTAAGTGCTTGCACTTTTTGG
ACGTCGACCGGCGCACGGGTGAGTAACGCGTATCCAACCTGCCCCTTACCAGGGAATAACCCGTTGAA
AGACGGACTAATGCCCTATGCAGTCCTTTGACGGCATCAGATTAGGACTAAAGATTCATCGGTATGGG
ATGGGGATGCGTCTGATTAGCTTGTTGGCGGGGTAACGGCCCACCAAGGCATCGATCAGTAGGGGTTC
TGAGAGGAAGGTCCCCCACATAGGAACTGAGACACGGTC
>Zotu1327
GAACGCTGGCGGCAGGCCTAACACATGCAAGTCGAGCGGATGATGGGAGCTTGCTCCCGGATTCAGCG
GCGGACGGGTGAGTAATGCCTAGGAATCTGCCTGGTAGTGGGGGACAACGTTTCGAAAGGAACGCTAA
TACCGCATACGTCCTACGGGAGAAAGCAGGGGACCTTCGGGCCTTGCGCTATCAGATGAGCCTAGGTC
GGATTAGCTAGTTGGTGAGGTAATGGCTCACCAAGGCGACGATCCGTAACTGGTCTGAGAGGATGATC
AGTCACACTGGAACTGAGACACGGTC
>Zotu1328
GAACGCTGGCGGAATGCTTTACACATGCAAGTCGAACGGCAGCACGGGGGCAACCCTGGTGGCGAGTG
GCGAACGGGTGAGTAATACATCGGAACGTGCCCAGTCGTGGGGGATAACTACGCGAAAGCGTAGCTAA
TACCGCATACGATCTACGGATGAAAGCGGGGGACCGTAAGGCCTCGCGCGATTGGAGCGGCCGATGGC
AGATTAGGTAGTTGGTGGGGTAAAGGCCTACCAAGCCGACGATCTGTAGCTGGTCTGAGAGGACGACC
AGCCACACTGGGACTGAGACACGGCC
>Zotu1331
GAACGCTAGCGGCAGGCCTAATACATGCAAGTCTAAAGGGCAGCAATGTCCTTGGCGCACGGGTGCGT
AACACGTATGCAACCTACCCATAACTGGGGGATAACTTTTCGAAAGGGAGGATAATACCCCATAACAC
AATGATTCTGCATGGAAATATTGTTAAAGCTCCGGCGGTTATGGATGGGCATGCGAACGATTAGCTAG
TTGGTAGGGTAACGGCCTACCAAGGCTACGATCGTTAGGGGAACTGAGAGGTTGGTCCCCCACACTGG
CACTGAGATACGGGC
>Zotu1332
GAACGCTGGCGGCGTGCTTAACACATGCAAGTGGAGCGACGAAGCGGGCTTCGGCCCGTGGCAGAGCC
GCGAACGGGTGAGTAACACGTGGGTAACGTGCCCCAATGACTGGGACAACCCGGGGAAACCCGGGCTA
ATACCGGATGTGCTCCTTCCACATCAGTGGATTGAGTAAAGGTAGCTTCGGCTTCCGCATTGGGATCG
GCCCGCGGCGCATTAGCTTGTTGGTGAGGTAACGGCTCACCAAGGCTTCGATGCGTAGCTGGTCTGAG
AGGACGATCAGCCACACTGGGACTGAGACACGGCC
>Zotu1333
GAACGCTGGCGGTGTGCTTAACACATGCAAGTGGAGCGACGAACCAGGGCTTGCCCTGGGGCAGAGCC
GCGAACGGGTGAGTAACACGTGGGTAACCTACCCCGATGATTGGGACAACCCGAGGAAACTCGGGCTA
ATACCGAATGTGCTGTCAGTCCATGAGGACAGTCAGTAAAGGAAGCTTCGGCCTCCGCATCGGGAGGG
GCCCGCGGCCCATTAGTTAGTTGGTGGGGTAATGGCCTACCAAGACAGCGATGGGTAGCTGGTCTGAG
AGGACGATCAGCCACACTGGGACTGAGACACGGCC
>Zotu1334
GAACGCTAGCGGCAGGCTTAATACATGCAAGTCGAGGGGTAACATGGGTAGCAATACCTGATGACGAC
CGGCAAACGGGTGCGGAACACGTACGCAACCTTCCCATAACTGGTGAATAGCCCTCCGAAAGGAGGAT
TAATACATCGTAATATAATGGAGTGGCATCACTCTATTATCATAGGCTTCGGCCGGTTTTGGATGGGC
GTGCGCCTGATTAGGTAGTTGGCGGGGTAACGGCCCACCAAGCCTGCGATCAGTAACTGGTGTGAGAG
CACGACCAGTCACACGGGCACTGAGACACGGGC
>Zotu1335
GAACGCTGGCGGCAGGCTTAACACATGCAAGTCGAGCGGGGGAAGGTAGCTTGCTACCGGACCTAGCG
GCGGACGGGTGAGTAATGCTTAGGAATCTGCCTATTAGTGGGGGACAACATCTCGAAAGGGATGCTAA
TACCGCATACGTCCTACGGGAGAAAGCAGGGGATCTTCGGACCTTGCGCTAATAGATGAGCCTAAGTC
GGATTAGCTAGTTGGTGGGGTAAAGGCCTACCAAGGCGACGATCTGTAGCGGGTCTGAGAGGATGATC
CGCCACACTGGGACTGAGACACGGCC
>Zotu1337
GAACGCTTGCGACGTGCCTAAGAAATGCAAGTCGAACGGGGGAGCAATCCCTAGTGGCGAACGGTCGC
GTAACACGTGGGCAACCTGCCCTGAAGCGGGGGACAACAGTCCGAAAGGGCTGCTAATACCGCATGTG
AAGGGAGATGGCATCATCTCTTGTTCAAAGCTTTATGCACTTCAGGATGGGCCCGCGGCCTATCAGCT
AGTTGGTGGGGTAACGGCCTACCAAGGCTTCGACGGGTAGCTGGTCTGAGAGGACGGCCAGCCGGATT
GGGACTGAGATACGGCC
>Zotu1338

GAACGCTGGCGGCGCGCTTAACACATGCAAGTCGAGCGAGAACCTTGCCTTCGGGCTTGGGGACAGCG
GCGAACGGGTGAGTATCACGTGGGTAATCAACCCTCGACACTGGGATAGCCCGGGGAAACCCGGATTA
ATACCGGATACCCCATCTGGCCTTCGGGCCGGCTGGAAAAGGTAGCTTTGGCCTCCGGTCGAGGACGA
GCCCGCGGCGGATTAGCTTGTTGGCGGGGTAAAAGCCCACCAAGGCGACGATCCGTAGCTGGTCTGAG
AGGACGATCAGCCACACTGGGACTGAGACACGGCC
>Zotu1339
GAACGCTGGCGGCATGCCTAATACATGCAAGTTGAACGATGGTATTCTCTGCTTGCAGAGAATTGAAG
AGTAGCGAACGGGTGAGCAACACGTGGGGAACCTGCCCATGAGAGGGGGATAACATTCGGAAACGGAT
GCTAATACCGCATAATGCTTTGGACCGCCTGGTCCAGAGACGAAAGACGGCTTTTGCTGTCACTCATG
GATGGCCCCGCGCCGTATTAGCTAGTTGGTGAGGTAATGGCTCACCAAAGCTGTGATACGTAGCCGAC
CTGAGAGGGTGATCGGCCACACTGGGACTGAGACACGGCC
>Zotu1340
GAACGCTGGCGGCATGCCTAACACATGCAAGTCGAACGGGGGTAGCAATACCCCAGTGGCGGACGGGT
GAGGAATACGTAGGAATTTGCCCTTAAGTGGGGGATAGCCCGGGGAAACCCGGATTAATACCGCATGA
TCTCGAAAGAGCAAAGTGGGGGATCGCAAGACCTCACGCTTTTGGAGAAGCCTACGTCGGATTAGCTA
GTTGGTGGGGTAATGGCCCACCAAGGCAACGATCCGTAGCTGGTCTGAGAGGATGATCAGCCACACCG
GAACTGAGACACGGTC
>Zotu1341
GAATGCTGGCGGCGTGCCTAACACATGCAAGTCGAGCGGCAGCGCGAGTAGTTTACTACTTGGCGGCG
AGCGGCGGACGGCTGAGTAACGCGTGGGAACATGCCCCAAAGTGAGGAATAAGCTCCAGAAATGGAGT
CTAATGCCGCATATGATCTTCGGATTAAAGGATTTATCCGCTTTGGGAGTGGCCTGCGTACGATTAGA
TAGTTGGTGAGGTAATGGCTTACCAAGTCTACGATCGTTAGCTGGTCTGAGAGGATGACCAGCCAGAC
TGGAACTGAGACACGGTC
>Zotu1342
GAACGCTGGCGGCGTGCTTAACACATGCAAGTCGAACGGTGATGGAGAGCTTGCTCTCCGGATCAGTG
GCGAACGGGTGAGTAACACGTGAGCAATCTGCCCCTGACTCTGGGATAACTGCGGGAAACTGTAGCTA
ATACCGGATATGACGCGCGAAGGCATCTTCTGCGCGTGGAAAGAATTTCGGTCAGGGATGAGCTCGCG
GCCTATCAGCTTGTTGGTGAGGTAATGGCTCACCAAGGCGACGACGGGTAGCCGGCCTGAGAGGGTGA
CCGGCCACACTGGGACTGAGACACGGCC
>Zotu1343
GAACGCTAGCTACAGGCTTAACACATGCAAGTCGAGGGGCAGCATGGTCTTAGCTTGCTAAGACTGAT
GGCGACCGGCGCACGGGTGAGTAACACGTATCCAACCTGCCGTCTACTCTTGGACAGCCTTCTGAAAG
GAAGATTAATACAAGATGGCATCATGAGTCCGCATGTTCACATGATTAAAGGTATTCCGGTAGACGAT
GGGGATGCGTTCCATTAGATAGTAGGCGGGGTAACGGCCCACCTAGTCTTCGATGGATAGGGGTTCTG
AGAGGAAGGTCCCCCACATTGGAACTGAGACACGGTC
>Zotu1344
GAACGCTAGCGGCAGGCCTAATACATGCAAGTCGAACGATATTAAGGGGCTTGCTCCTTAAGAAAGTG
GCGCACGGGTGCGTAACGCGTATGCAACCTACCTTAATCAGGGGGATAGCCCGAAGAAATTCGGATTA
ACACCGCATAAAAACACAGGATAGCATTATCCAATGTTCAAATATTTATAGGATTAAGATGGGCATGC
GTGTCATTAGCTAGTTGGCGGGGTAACGGCCCACCAAGGCGACGATGACTAGGGGATCTGAGAGGATG
ACCCCCCACACTGGTACTGAGACACGGAC
>Zotu1345
GAACGCTGGCGGCGTGCTTAACACATGCAAGTCGAACGGTGAACGAGGGAGCTTGCTCCCTCCGGATC
AGTGGCGAACGGGTGAGTAACACGTGAGTAACCTGCCCTTGACTCTGGGATAAGCGTTGGAAACGACG
TCTAATACCGGATACGACCTTCGGAGGCATCTCCTGGGGGTGGAAAGAATTTCGGTCAAGGATGGACT
CGCGGCCTATCAGCTAGTTGGTGAGGTAATGGCTCACCAAGGCGACGACGGGTAGCCGGCCTGAGAGG
GTGACCGGCCACACTGGGACTGAGACACGGCC
>Zotu1346
GAACGCTGGCGGCGTGCTTAACACATGCAAGTCGAACGGAAAGGCCCTTCGGGGTACTCGAGTGGCGA
ACGGGTGAGTAACACGTGGGTGATCTGCCTCGTACTTTGGGATAAGCCTGGGAAACTGGGTCTAATAC
CGAATATGACCTCTGGATGCATGTCTGGTGGTGGAAAGTTTTTCGGTACGAGATGGGCCCGCGGCCTA
TCAGCTTGTTGGTGGGGTAATGGCCTACCAAGGCGACGACGGGTAGCCGGCCTGAGAGGGCGACCGGC
CACACTGGGACTGAGACACGGCC
>Zotu1347

GAACGCTGGCGGCAGGCTTAACACATGCAAGTCGAACGAGCTGGGTCCTTCGGGGCCCGATGCTAGTG
GCGCACGGGTGAGTAACACGTGGGAATATGCCCTTTAGTACGGAATAACCAAGGGAAACTTTGGCTAA
TACCGTATACGCCCTTCGGGGGAAAGATTTATCGCTAAAGGATTAGCCCGCGTCTGATTAGGTAGTTG
GTGAGGTAATGGCTCACCAAGCCGACGATCAGTAGCTGGTCTGAGAGGATGATCAGCCACACTGGGAC
TGAGACACGGCC
>Zotu1348
GAACGCTAGCTACAGGCTTAACACATGCAAGTCGCGGGGCAGCATGGGGGTTGCTTGCAACTCCCGAT
GGCGACCGGCGCACGGGTGAGTAACGCGTATCCAACCTGCCCTTCACCACGGGATAACCCGGCGAAAG
TCGGACTAATACCGTATGTTGTCCATTGACGGCATCCGATTTGGACGAAAGGCTTGGCGGTGAAGGAT
GGGGATGCGTCCGATTAGCCAGACGGCGGGGTAACGGCCCACCGTGGCTACGATCGGTAGGGGTTCTG
AGAGGAAGGTCCCCCACACTGGAACTGAGACACGGTC
>Zotu1349
GAACGCTGGCGGCGTGCTTAACACATGCAAGTCGAACGGTGAAGCACCTTCCTTTTGGTGGGTGTGGA
TGAGTGGCGAACGGGTGAGTAACACGTGAGTAACCTGCCCCCTTCTTCTGGATAACCGCATGAAAGTG
TGGCTAATACGGGATATTCTGCGCCTGCTGCATGGTGGGTGTGGGAAAGATGCGCCTTTGGGTGTTTT
TGGTGGGGGATGGGCTCGCGGCCTATCAGCTTGTTGGTGGGGTGATGGCCTACCAAGGCGGTGACGGG
TAGCCGGCCTGAGAGGGTGGACGGTCACACTGGGACTGAGACACGGCC
>Zotu1350
GAACGCTAGCGGCAGGCTTAACACATGCAAGTCGAGGGGTATAGTTCTTCGGAATTAGAGACCGGCGC
ACGGGTGCGTAACGCGTATGCAATCTACCTTTCACAGAGGGATAGCCCAGAGAAATTTGGATTAATAC
CTCATAGTATGCAGAGTTGGCATCAGCCCTGCATTAAAGTCACAACGGTGAAAGATGAGCATGCGTCC
CATTAGCTAGTTGGTAAGGTAACGGCTTACCAAGGCTACGATGGGTAGGGGTCCTGAGAGGGAGATCC
CCCACACTGGTACTGAGACACGGAC
>Zotu1351
GAACGCTAGCGGCGTGCTTAACACATGCAAGTGGAGCGACGAACCGGGGCTTGCCCCGGGGCAGAGCC
GCGAACGGGTGAGTAACACGTGGGTTACCTACCCCGATGATTGGGACAACCCGAGGAAACTCGGGCTA
ATACCAAATGTGCTCCTGGCCGTAATGGCCTTGAGGAAAGGAAGCTTCGGCCTCCGCATCGGGATGGG
CCCGCGGCCCATTAGCTAGTTGGTGGGGTAACGGCCTACCAAGGCTACGATGGGTAGCTGGTCTGAGA
GGACGATCAGCCACACTGGGACTGAGACACGGCC
>Zotu1353
GAACGCTGGCGGCGTGCTTAACACATGCAAGTCGAGCGGAAAGGCCACTTCGGTGGTACTCGAGCGGC
GAACGGGTGAGTAACACGTGAGTAATCTGCCCCTGGCTTTGGGATAGCCACCGGAAACGGTGATTAAT
ACCGGATATGACGACTTCTCGCATGAGTTGTTGTGGAAAGTTTTTCGGCCAGGGATGTGCTCGCGGCC
TATCAGCTTGATGGTGAGGTAATGGCTCACCATGGCTTCGACGGGTAGCCGGCCTGAGAGGGTGACCG
GCCACACTGGGACTGAGACACGGCC
>Zotu1354
GAACGCTAGCGGGAGGCCTAACACATGCAAGCCGAGCGGTATTGTTTCTTCGGAAATGAGAGAGCGGC
GTACGGGTGCGGAACACGTGTGCAACCTGCCTTTATCTGGGGGATAGCCTTTCGAAAGGAAGATTAAT
ACTCCATAACATATTGATTGGCATCAATTAATATTGAAAGCTCCGGCGGATAGAGATGGGCACGCGCA
AGATTAGCTAGTTGGTGAGGTAACGGCTCACCAAGGCGATGATCTTTAGGGGGCCTGAGAGGGTGATC
CCCCACACTGGTACTGAGACACGGAC
>Zotu1355
GAACGCTGGCGGCGTGCCTAAGACATGCAAGTCGAGTGGGTTTAGACCCACGGCGAACGGGTGAGTAA
TACCTAGGAACTTTACTTTAGTCGGGCATAACCAGTCGAAAGATTGGACAATTCCCGATATTATCGCA
AGATGAAAGCAGCAATGCGCTAAAGTTGAGGCCTGGGCACCATCAGCTAGTTGGTAAGGTAATGGCTT
ACCAAGGCTATGACGGTTAGGGGTCCCGAGAGGGATAACCCCCACACTGGAACTGCGACACGGTC
>Zotu1356
GAACGCTGGCGGCAGGCCTAACACATGCAAGTCGAGCGGATGAGAGGAGCTTGCTCCTGGATTCAGCG
GCGGACGGGTGAGTAATGCCTAGGAATCTGCCTGGTAGTGGGGGACAACGTTTCGAAAGGAACGCTAA
TACCGCATACGTCCTACGGGAGAAAGCAGGGGACCTTCGGGCCTTGCGCTATCAGATGAGCCTAGGTC
GGATTAGCTAGTTGGTGGGGTAAAGGCCTACCAAGGCGACGATCCGTAACTGGTCTGAGAGGATGATC
AGTCACACTGGAACTGAGACACGGTC
>Zotu1357
GAACGCTGGCGGCAGGCTTAACACATGCAAGTCGAGCGGGGAGATGTAGCTTGCTACATTTCCTAGCG

GCGGACGGGTGAGTAATGCTTAGGAATCTGCCTATTAGTGGGGGACAACATTCCGAAAGGAATGCTAA
TACCGCATACGCCCTACGGGGGAAAGCAGGGGATCTTCGGACCTTGCGCTAATAGATGAGCCTAAGTC
AGATTAGCTAGTTGGTGGGGTAAAGGCCTACCAAGGCGACGATCTGTAGCGGGTCTGAGAGGATGATC
CGCCACACTGGGACTGAGACACGGCC
>Zotu1358
GAACGCTGGCGGCGTGCCTAATACATGCAAGTCGAGCGGTAATCCCTCACCTTTTTCCATCAGCCATG
ATGCTTATACATCGTGCTAATTTGGTAGAAAAAGGTGAGGGAACGAGCGGCGGACGGCTGAGTAACGC
GTAGGAATCTACCCCAAACTGAGGGATAAGCACCGGAAACGGTGTCTAATACCGCATGTGCTCTAAGG
AGTAAAGCTTCGGCGGTTTGGGAAGAGCCTGCGTATGATTAGCTTGTTGGTGAGGTAATGGCTCACCA
AGGCTTCGATCATTAGCTGGTCTGAGAGGATGATCAGCCAGACTGGGACTGAGACACGGCC
>Zotu1360
GAACGCTGGCGGCGTGCCTAATACATGCAAGTAGAACGCTGAAGGAGGAGCTTGCTTCTCTGGATGAG
TTGCGAACGGGTGAGTAACGCGTAGGTAACCTGCCTGGTAGCGGGGGATAACTATTGGAAACGATAGC
TAATACCGCATAATAGTAGTTGTTGCATGACAACTGTTTGAAAGGTGCAATTGCATCACTACCAGATG
GACCTGCGTTGTATTAGCTAGTTGGTGGGGTAACGGCTCACCAAGGCGACGATACATAGCCGACCTGA
GAGGGTGATCGGCCACACTGGGACTGAGACACGGCC
>Zotu1361
GAACGCTGGCGGCGCGCCTAACACATGCAAGTCGAACGGAGCTGAGAGGAGCTTGCTTTTCTTAGCTT
AGTGGCGAACGGGTGAGTAACGCGTGAGTAACCTGCCCTGGAGTGGGGGACAACAGTTGGAAACGACT
GCTAATACCGCATAAGCCCACGGCCCGGCATCGGGCTGAGGGAAAAGGATTTATTCGCTTCAGGATGG
ACTCGCGTCCAATTAGCTAGTTGGTGAGGTAACGGCCCACCAAGGCGACGATTGGTAGCCGGACTGAG
AGGTTGAACGGCCACATTGGGACTGAGACACGGCC
>Zotu1362
GAACGCTAGCGGCAGGCTTAACACATGCAAGTCGAGGGGTATAGTAGCAATACTAGAGACCGGCGCAC
GGGTGCGTAACGCGTATGCAATCTACCTTTTGCAGGGGAATAGCCCAGAGAAATTTGGATTAATGCCC
CATAGTATAGTACAATCGCATGATTGAACTATTAAAGTTACAACGGCAAAAGATGAGCATGCGTCCCA
TTAGCTAGTTGGTAAGGTAACGGCTTACCAAGGCGACGATGGGTAGGGGTCCTGAGAGGGAGATCCCC
CACACTGGTACTGAGACACGGAC
>Zotu1363
GAACGCTGGCGGCAGGCCTAACACATGCAAGTCGAACGGCAGCACAGAAGAGCTTGCTCTTTGGGTGG
CGAGTGGCGGACGGGTGAGTAATGTCTGGGAAACTGCCCGATGGAGGGGGATAACTACTGGAAACGGT
AGCTAATACCGCATAACGTCGCAAGACCAAAGTGGGGGACCTTCGGGCCTCACACCATCGGATGTGCC
CAGATGGGATTAGCTAGTAGGTGGGGTAATGGCTCACCTAGGCGACGATCCCTAGCTGGTCTGAGAGG
ATGACCAGCCACACTGGAACTGAGACACGGTC
>Zotu1364
GAACGCTGGCGGCGTGCTTAACACATGCAAGTCGAACGATGATGCCCAGCTTGCTGGGTGGATTAGTG
GCGAACGGGTGAGTAACACGTGAGTAACCTGCCCCTGACTCTGGGATAAGCGTTGGAAACGACGTCTA
ATACTGGATACGACTGCCGGCCGCATGGTCTGGTGGTGGAAAGATTTTTTGGTTGGGGATGGACTCGC
GGCCTATCAGCTTGTTGGTGAGGTAATGGCTCACCAAGGCGACGACGGGTAGCCGGCCTGAGAGGGTG
ACCGGCCACACTGGGACTGAGACACGGCC
>Zotu1365
GAACGCTGGCGGCGTGCCTAATACATGCAAGTCGAACGCGACACTTTCGGGTGTCGAGTGGCGGACGG
GTGAGTAACACGTGGGTAACCTGCCCATCAGCCTGGGATAACCACCGGAAACGGTGGCTAATACCGGA
TATGTCTACGGATGAAAGTCGGATTTTCTGACACTGATGGATGGACCTGCGGCGCATTAGCTAGTTGG
TGAGGTAGTGGCTCACCAAGGCGACGATGCGTAGCCGACCTGAGAGGGTGATCGGCCACACTGGGACT
GAGACACGGCC
>Zotu1366
GAACGCTGGCGGCGTGCTTAACACATGCAAGTCGAACGATGATGCCCAGCTTGCTGGGTTGATTAGTG
GCGAACGGGTGAGTAACACGTGAGTAACCTGCCCTTGACTTCGGGATAACTCCGGGAAACCGGGGCTA
ATACCGGATATGAGCCGTCCTCGCATGGGGGTGGTTGGAAAGTTTTTCGGTCAGGGATGGGCTCGCGG
CCTATCAGCTTGTTGGTGGGGTGATGGCCTACCAAGGCGACGACGGGTAGCCGGCCTGAGAGGGCGAC
CGGCCACACTGGGACTGAGACACGGCC
>Zotu1367
GAACGCTAGCGGCAGGCCTAATACATGCAAGTCGAGGGGCAGCATAGTAGCAATACTGATGGCGACCG

GCGCACGGGTGCGTAACGCGTATGCAACCTACCTTTTACTGGGGGATAGCCCGGAGAAATTCGGATTA
ACACCGCATAACAAGTTGATATGGCATCATATTAATTTTAAATATTTATAGGTAAAAGATGGGCATGC
GTCCAATTAGCTAGTTGGTGAGGTAATGGCTCACCAAGGCGATGATTGGTAGGGGTTCTGAGAGGAAG
GTCCCCCACACTGGTACTGAGATACGGAC
>Zotu1368
CAACGCTGGCGGCGCGCTTAACACATGCAAGTCGAGCGAGAAAGTGGAGCAATCCATGAGTAAAGCGG
CGTACGGGTGAGTAACACGTGGGTAATCTACCTCTTAGTGGGGAATAACCCTGGGAAACCGGGGCTAA
TACCGCATAATCTCGAGAGAGGAAAGCAGCAATGCGCTGAGAGAGGAGCCCGCGGCCGATTAGCTAGT
TGGCAGGGTAAAAGCCTACCAAGGCATAGATCGGTAGCCGGCCTGAGAGGGCACACGGCCACACTGGC
ACTGAAACACGGGC
>Zotu1369
GAACGCTAGCGGCAGGCCTAATACATGCAAGTCTAAGGGGTAGCAATATCACTGGCGGACGGGTGCGT
AACGCGTATAGAACCTACCTATCACTGGGGGATAGCTTTTGGAAACAGGAGATAATACCGCATAAGAT
AGTAGCTTGGCATCAAGCAGCTATTAAAGATTTATTGGTGAGAGATGGCTATGCGTATGATTAGCTAG
TTGGTAAGGTAACGGCTTACCAAGGCTACGATCATTAGGGGTTCTGAGAGGATTGGCCCCCACACTGG
TACTGAGATACGGAC
>Zotu1370
GAGCGCTGGCGGCGTGCCTAATACATGCAAGTCGAGCGGCAGCGCGGTCTTCGGACTGGCGGCGAGCG
GCGGACGGCTGAGTAACACGTAGGAACCTACCCCAAAGTGAGGGATAACTGCTCGAAAGAGTAGCTAA
TACCGCATGTGATCCACGGATTAAAGCTTTATGCGCTTTGGGAAGGGCCTGCGTCCGATTAGCTAGTT
GGTGAGGTAATAGCTCACCAAGGCGATGATCGGTAGCTGGTCTGAGAGGATGATCAGCCAGACTGGGA
CTGAGACACGGCC
>Zotu1371
GAACGCTAGCTACAGGCTTAACACATGCAAGTCGAGGGGCAGCATGAACTTAGCTTGCTAAGTTTGAT
GGCGACCGGCGCACGGGTGAGTAACACGTATCCAACCTGCCGATGACTCGGGGATAGCCTTTCGAAAG
AAAGATTAATACCCGATGGCATAGTTCTTCCGCATGGTAGAACTATTAAAGGATTTCGGTCATCGATG
GGGATGCGTTCCATTAGGTTGTTGGCGGGGTAACGGCCCACCAAGCCTTCGATGGATAGGGGTTCTGA
GAGGAAGGTCCCCCACATTGGAACTGAGACACGGTC
>Zotu1372
GAACGCTGGCGGCGTGCCTAATACATGCAAGTCGAGCGGACCTCTTCGGAGGTCAGCGGCGGACGGGT
GAGTAACACGTGGGCAACCTGCCTATAAGACTGGGATAACTTCGGGAAACCGGAGCTAATACCGGATA
ATATCAGAGGCCACATGGTCTTCGATTAAAAGATGGCTTAGGCTATCACTTATAGATGGGCCCGCGTC
GCATTAGCTAGTTGGTGAGGTAACGGCTCACCAAGGCGACGATGCGTAGCCGACCTGAGAGGGTGATC
GGCCACACTGGGACTGAGACACGGCC
>Zotu1373
GAACGCTAGCGGCAGGCCTAATACATGCAAGTCGAACGCGATTGGGGAGCTTGCTCCTCATGAAAGTG
GCGCACGGGTGCGTAACGCGTATGCAACCTACCTTAATCAGGGGGATAGCCCGGAGAAATCCGGATTA
ACACCGCATAAAATCACAGTCTGGCATCAGATAATGATCAAATATTTATAGGATTAAGATGGGCATGC
GTGACATTAGTTAGTTGGTAAGGTAACGGCTTACCAAGACGACGATGTCTAGGGGATCTGAGAGGATG
ACCCCCCACACTGGTACTGAGACACGGAC
>Zotu1374
GAACGCTGGCGGCGTGCCTAATGCATGCAAGTCGCACGCGCCACCTTCGGGTGGTGAGTGGCGCACGG
CTGAGGATCACGTGGGAACATGCCTCCTTGTGGGGGACAACAGCGCGAAAGCGCTGCTAATCCCGCAT
ACGCTCGGCAACGAGGAAAGCGGTAACGCGCAGGGAGAGTGGCCCGCGGCCCATCAGGTCGTTGGTGG
GGTAATGGCCTACCAAGCCGATGACGGGTAGCTGGTCTGTGAGGACAGACAGCCAGACTGGGACTGAG
ACACGGCC
>Zotu1376
GAACGCTGGCGGCGTGCCTAACACATGCAAGTCGAGCGATTCTCTTCGGAGAAGAGCGGCGGACGGGT
GAGTAACGCGTGGGTAACCTGCCCTGTACACACGGATAACATACCGAAAGGTATGCTAATACGAGATA
ATATGCTTTTATCGCATGGTAGAAGTATCAAAGCTTTTGCGGTACAGGATGGACCCGCGTCTGATTAG
CTAGTTGGTAAGGTAACGGCTTACCAAGGCGACGATCAGTAGCCGACCTGAGAGGGTGATCGGCCACA
TTGGAACTGAGACACGGTC
>Zotu1377
GAACGCTGGCGGCGTGCCTAATACATGCAAGTCGAGCGAGTCCCTTCGGGGGCTAGCGGCGGACGGGT

GAGTAACACGTAGGCAACCTGCCTCTCAGACTGGAATAACGTAGGGAAACTTACGCTAATGCCAGATA
GGTTTCCGGACTGCATGGTCTGGAAAGGAAAGACGGCTCCGGCTGTCACTGAGAGATGGGCCTGCGGC
GCATTAGCTAGTTGGTGGGGTAACGGCCTACCAAGGCGACGATGCGTAGCCGACCTGAGAGGGTGACC
GGCCACACTGGGACTGAGACACGGCC
>Zotu1378
GAACGCTGGCGGCGTGCTTAACACATGCAAGTCGAACGGAAAGGCCCTTCGGGGTACTCGAGTGGCGA
ACGGGTGAGTAACACGTGGGTGATCTGCCCTGCACTTTGGGATAAGCCTGGGAAACTGGGTCTAATAC
CGAATATGATCATCGGCTTCATGGTCGGTGGTGGAAAGCTTTTGCGGTGTGGGATGGGCCCGCGGCCT
ATCAGCTTGTTGGTGGGGTAATGGCCTACCAAGGCGTCGACGGGTAGCCGGCCTGAGAGGGTGTCCGG
CCACACTGGGACTGAGATACGGCC
>Zotu1379
AAACGCTGGCGGCGTGCATGAGACATGCAAGTCGAACGAACGAGCTTGCTCGTTAGTGGCGGACGGCT
GAGTAACGCGTAGGTGACCTGCCCTGAAGTGGGGGATAACTGCCCGAAAGGGTGGCTAATACCGCATG
TGGCGACACGTTCATTTGTGTCATCAAAGCCGCGAGGCGCTTCGGGAGGGGCCTGCGTCCGATTAGCT
CGTTGGTGGGGTAATGGCTCACCAAGGCGACGATCGGTAGGTGGTCTGAGAGGACGATCACCCAGACT
GGGACTGAGACACGGCC
>Zotu1380
GAACGCTGGCGGCGTGCTTAACACATGCAAGTCGAACGGAAAGGCCCTTCGGGGTACTCGAGTGGCGA
ACGGGTGAGTAACACGTGGGTGATCTGCCTCGTACTCTGGGATAAGCCTGGGAAACTGGGTCTAATAC
TGGATAGGACCTTCTCTCGCATGGGGGTTGGTGGAAAGTTTTTCGGTACGAGATGGGCCCGCGGCCTA
TCAGCTTGTTGGTGGGGTAATGGCCTACCAAGGCGACGACGGGTAGCCGGCCTGAGAGGGCGACCGGC
CACACTGGGACTGAGACACGGCC
>Zotu1381
GAACGCTGGCGGCGTGCCTAATACATGCAAGTCGAGCGGTAAGGCCCCTTCGGGGGTACACGAGCGGC
GGACGGCTGAGTAACACGTAGGAACGTACCCCAAACTGAGGGATAAGCACCAGAAATGGTGTCTAATA
CCGCATGTGCTCTTCGGAGTAAAGCTACGGCGGTTTGGGAACGGCCTGCGCATGATTAGCTTGTTGGT
GAGGTAATAGCTCACCAAGGCGACGATCATTAGCTGGTCTGAGAGGATGACCAGCCAGACTGGGACTG
AGAACGGCC
>Zotu1382
GAACGCTAGCGGCAGGCTTAATACATGCAAGGCGAAGGGGCAGCAATGTCACTGTCGTACGGGTGCGT
AACGCGTATGCAACCTACCTATCACTGGGGGATAGCCCGGGGAAACCCGGATTAATACCGCATAACAC
AGGGGTCCCGCATGGGTACTATTTGTTAAAGATTTATTGGTGGTAGATGGGCATGCGTTCGATTAGCT
AGTTGGTATGGGTAACGGCCTACCAAGGCGACGATCGATAGGGGAGCTGAGAGGTTGATCCCCCACAC
GGGAACTGAGATACGGGC
>Zotu1383
GAACGCTGGCGGCGTGCCTAATACATGCAAGTCGAACGAACAGCGACTTGAACTTGTTCAAGTCAATG
TTAGTGGCGCACGGGTGAGTAACACGTGGAGAACCTACCCTTAAGCGGGGGATAACCATTGGAAACGA
TGACTAATACCGCATAGAATCACAGATCGCATGATCAATGAAGGAAAGACGGCTTCGGCTGTCACTTA
AGGATGGCTCCGCGGTGCATTAGCTAGATGGTGGGGTAAAAGCCTACCATGGCAATGATGCATAGCCG
ACCTGAGAGGGTGATCGGCCACATTGGGACTGAGACACGGCC
>Zotu1384
GAACGCTAGCGGCAGGCTTAACACATGCAAGTCGAGGGGCAGCACGATGTAGCAATACATTGGTGGCG
ACCGGCGCACGGGTGCGTAACACGTATGGAATCTGCCCTTTACTGGAGGATAGCCCGGAGAAATCCGG
ATTAATACTCCATAACAGTTCGAGTGGCATCACTTGGATTTGAAAGCTCCGGCGGTAAAGGATGACCA
TGCGCACGATTAGCTAGTAGGTGAGGTAACGGCTCACCTAGGCTACGATCGTTAGGGGGCCTGAGAGG
GTTATCCCCCACACTGGTACTGAGACACGGAC
>Zotu1385
GAACGCTGGCGGCGTGCTTAACACATGCAAGTCGAACGGAAAGGCCTCTGCTTGCAGGGGTGCTCGAG
TGGCGAACGGGTGAGTAACACGTGGGTGATCTGCCTTGTACTCTGGGATAAGCTTGGGAAACTGGGTC
TAATACCGGATAGGAACTGCCTTTAGTGTGGTGGTTGGAAAGTTTTTTCGGTGCAAGATGAGCTCGCG
GCCTATCAGCTTGTTGGTGGGGTAATGGCCTACCAAGGCGTCGACGGGTAGCCGGCCTGAGAGGGTGT
ACGGCCACATTGGGACTGAGATACGGCC
>Zotu1387
GAACGCTGGCGGCAGGCCTAACACATGCAAGTCGAGCGAGGTTTTCGGACCTAGCGGCGGACGGGTGA

GTAATGCGTAGGAAGCTGCCCGATAGAGGGGGATACCAGTTGGAAACGACTGTTAATACCGCATAATG
TCTACGGACCAAAGTGTGGGACCTTCGGGCCACATGCTATCGGATGCGCCTACGTGGGATTAGCTAGT
TGGTGGGGTAATGGCTCACCAAGGCGACGATCCCTAGCTGGTTTGAGAGGATGATCAGCCACACTGGG
ACTGAGACACGGCC
>Zotu1388
AAACGCTGGCGGCGTGCTTAACACATGCAAGTCGAACGAAGTTTTTCTTTCGGGAGAAACTTAGTGGC
GGACGGGTGAGTAACGCGTGGGTAACCTGCCCTGTACAGGGGGACAACAGCTGGAAACGGCTGCTAAT
ACCGCATAAGCCCTTAGCACTGCATGGTGCATAGGGAAAAGGAGCAATCCGGTACAGGATGGACCCGC
GTCTGATTAGCCAGTTGGCAGGGTAACGGCCTACCAAAGCGACGATCAGTAGCCGATCTGAGAGGATG
TACGGCCACATTGGGACTGAGACACGGCC
>Zotu1389
GAACGCTGGCGGCGTGCTTAACACATGCAAGTCGAACGATGAAGCCCAGCTTGCTGGGTGGATTAGTG
GCGAACGGGTGAGTAACACGTGAGTAACCTGCCCTTGACTCTGGGATAAGCCTGGGAAACTGGGTCTA
ATACTGGATATGACTTCTCACCGCATGGTGGGGGGTGGAAAGATTTATTGGTCTTGGATGGACTCGCG
GCCTATCAGCTTGTTGGTGAGGTAATGGCTCACCAAGGCGACGACGGGTAGCCGGCCTGAGAGGGTGA
CCGGCCACACTGGGACTGAGACACGGCC
>Zotu1390
GAACGCTAGCGGCAGGCTTAACACATGCAAGTCGAGGGGTATAGGTTTTCGGACCTAGAGACCGGCGC
ACGGGTGCGTAACGCGTATGCAATCTACCTTTTACAGCGGGATAGCCCAGAGAAATTTGGATTAATAC
CGCATAGTATAGCAGTTTCGCATGAGACCACTATTAAAGTCACAACGGTAAAAGATGAGCATGCGTCC
CATTAGTTAGTTGGTAAGGTAACGGCTTACCAAGACTACGATGGGTAGGGGTCCTGAGAGGGAGATCC
CCCACACTGGTACTGAGACACGGAC
>Zotu1391
GAATGCTGGCGGCGTGCCTAATACATGCAAGTCGAGCGGTAAGGCCCTTCGGGGTACACGAGCGGCGG
ACGGCTGAGTAACGCGTGGGAACATGCCCCAAAGTGAGGGATAAGCACCGGAAACGGTGTCTAATACC
GCATATGGTCTTCGGATTAAAGGATTTATCCGCTTTGGGAGTGGCCCGCGTACGATTAGTTTGTTGGT
GAGGTAATGGCTTACCAAGACTACGATCGTTAACTGGTCTGAGAGGATGATCAGTCAGACTGGAACTG
AGACACGGTC
>Zotu1392
GAACGCTGGCGGCAGGCTTAACACATGCAAGTCGAACGAAGTCTTCGGACTTAGTGGCGCACGGGTGA
GTAACACGTGGGAATATACCTCTTGGTGGGGAATAACATCGGGAAACTGATGCTAATACCGCATACGC
CCTTCGGGGGAAAGATTTATCGCCGAGAGATTAGCCCGCGTCCGATTAGCTAGTTGGTGAGGTAATGG
CTCACCAAGGCGACGATCGGTAGCTGGTCTGAGAGGATGATCAGCCACACTGGGACTGAGACACGGCC
>Zotu1393
GAACGCTGGCGGCATGCCTTACACATGCAAGTCGAACGGCAGCACGGGGGCAACCCTGGTGGCGAGTG
GCGAACGGGTGAGTAATATATCGGAACGTACCCAGTAGTGGGGGATAACTATCCGAAAGGATAGCTAA
TACCGCATACGATCTGTGGATGAAAGCAGGGGATCTTCGGACCTTGTGCTATTGGAATGGCCGATATC
TGATTAGCTAGTTGGTAGGGTAAAAGCCTACCAAGGCGACGATCAGTAGCTGGTCTGAGAGGACGACC
AGCCACACTGGAACTGAGACACGGTC
>Zotu1395
GAACGCTGGCGGCAGGCCTAACACATGCAAGTCGAACGGTAGCACAGAGGAGCTTGCTCCTTGGGTGA
CGAGTGGCGGACGGGTGAGTAATGTCTGGGAAACTGCCTGATGGAGGGGGATAACTACTGGAAACGGT
AGCTAATACCGCATAACGTCGCAAGACCAAAGAGGGGGACCTTCGGGCCTCTTGCCATCAGATGTGCC
CAGATGGGATTAGCTAGTAGGTGGGGTAATGGCTCACCTAGGCGACGATCCCTAGCTGGTCTGAGAGG
ATGACCAGCCACACTGGAACTGAGACACGGTC
>Zotu1396
GAACGCTGGCGGCAGGCTTAACACATGCAAGTCGAGCGCGCCCTTCGGGGTGAGCGGCGGACGGGTGA
GTAACGCGTGGGAACGTGCCCTTTGGTTCGGAATAGCCCCGGGAAACTGGGAGTAATGCCGAATAAGC
CCTTGGCAGCGATGTCTTGGGGAAAGATTTATCGCCAAAGGATCGGCCCGCGTCGGATTAGGTAGTTG
GTGGGGTAACGGCCTACCAAGCCGACGATCCGTAGCTGGTTTGAGAGGATGACCAGCCACACTGGGAC
TGAGACACGGCC
>Zotu1397
GAACGCTGGCGGCGTGCTTAACACATGCAAGTCGAGCGAGGAGCTTGCTCCTAGCGGCGAACGGGTGA
GTAACACGTGGGCAACCTGCCCCGAGCTCTGGGATAACTCCGGGAAACCGGAGCTAATACCGGATATG

ACCACGGAAGGCATCTTCTTGTGGTGGAAAGATTTATCGGCTCGGGATGGGCCCGCGGCCTATCAGCT
TGTTGGTGGGGTAACGGCCTACCAAGGCGACGACGGGTAGCCGGCCTGAGAGGGCGACCGGCCACACT
GGGACTGAGACACGGCC
>Zotu1398
GAACGCTAGCGGCAGGCCTAATACATGCAAGTCGAACGGGTCGCAAGGCCAGTGGCAAACGGGTGCGT
AACGCGTAAGCAACCTGCCTCATACTGGGGGATAGCCCGGCGAAAGCTGGGGTAAACCCGCACGGTCC
AGTTTTATCACCTGGTAAGATTGGTAAACATTTATGGGTATGAGAGGGGCTTGCGTCTGATTAGTTAG
TTGGCAGGGTAACGGCCTACCAAGACGATGATCAGTAGGGGTTCTGAGAGGATTGGCCCCCACATGGG
TACTGAGATACGGAC
>Zotu1399
GAACGCTGGCGGCGTGCTTAACACATGCAAGTCGAACGATGAAGCCCAGCTTGCTGGGTGGATTAGTG
GCGAACGGGTGAGTAACACGTGAGTAACCTGCCCTTGACTCTGGGATAAGCGTTGGAAACGACGTCTA
ATACCGGATACGAGCTTCCACCGCATGGTGAGTTGCTGGAAAGAATTTTGGTCAAGGATGGACTCGCG
GCCTATCAGCTTGTTGGTGAGGTAATGGCTCACCAAGGCGACGACGGGTAGCCGGCCTGAGAGGGTGA
CCGGCCACACTGGGACTGAGACACGGCC
>Zotu1400
GAACGCTGGCGGCGCGCTTAACACATGCAAGTCGAGCGAGAACCAGGGCTTCGGCCCTGGGGACAGCG
GCGAACGGGTGAGTAACACGTGGATAACCTGCCCTCGGCACCGGGATAGCCCGGGGAAACCCGGATTA
ATACCGGATAGCCACTCCGACTCTCGGGTCGGTCTGAAAAGGTAGCTTCGGCCTCTGGCCGAGGATGG
GTCCGCGGCGGATTAGCTTGTTGGTGGGGTAACGGCCTACCAAGGCTCCGATCCGTAGCTGGTCTGAG
AGGACGATCAGCCACACTGGGACTGAGACACGGCC
>Zotu1401
GAACGCTGGCGGCGTGCTTAACACATGCAAGTCGAACGGAAAGGCCCTTTCGGGGGTACTCGAGTGGC
GAACGGGTGAGTAACACGTGGGTGATCTGCCCTGCACTTTGGGATAAGCCTGGGAAACTGGGTCTAAT
ACCGGATATACCTCGCTGGTCGCATGGCCTGGTGGGGGAAAGCTTTTGCGGTGTGGGATGGGCCCGCG
GCCTATCAGCTTGTTGGTGAGGTTATGGCTTACCAAGGCGACGACGGGTAGCCGGCCTGAGAGGGTGA
CCGGCCACACTGGGACTGAGATACGGCC
>Zotu1402
GAACGCTGGCGGCGTGCCTAATACATGCAAGTCGAGCGGACAGATGGGAGCTTGCTCCCTGATGTTAG
CGGCGGACGGGTGAGTAACACGTGGGTAACCTGCCTGTAAGACTGGGATAACTCCGGGAAACCGGGGC
TAATACCGGATGCTTGTTTGAACCGCATGGTTCAGACATAAAAGGTGGCTTCGGCTACCACTTACAGA
TGGACCCGCGGCGCATTAGCTAGTTGGTGAGGTAACGGCTCACCAAGGCAACGATGCGTAGCCGACCT
GAGAGGGTGATCGGCCACACTGGGACTGAGACACGGCC
>Zotu1403
GAACGCTGGCGGCGTGCTTAACACATGCAAGTCGAACGATGAAGCTGGAGCTTGCTCCGGTGGATTAG
TGGCGAACGGGTGAGTAACACGTGAGCAATCTGCCCTTGACTCTGGGATAACTGCGGGAAACTGTAGC
TAATACCGGATACGACCCTTGGAGGCATCTCTGGGGGTGGAAAGAATTTCGGTCAAGGATGAGCTCGC
GGCCTATCAGCTAGTTGGTGAGGTAATGGCTCACCAAGGCGACGACGGGTAGCCGGCCTGAGAGGGTG
ACCGGCCACACTGGAACTGAGACACGGTC
>Zotu1404
GAACGCTGGCGGTATGCTTTACACATGCAAGTCGTACGAGAGTTTTTAACTCAAGTGGCGGACGGGTG
AGTAACACGTAAGAATTTGCCTTTAGGAGGGGGATAACAACTGGAAACGGTTGCTAATACCCCATATG
CTTTCGAGTAAAATGGATTTTCCGCCTAAAGAGAAGCTTGCGGCTGATTAGCTTGTTGGTGAGGTAAT
GGCTCACCAAGGCGACGATCAGTATCTGGTTTGAGAGGATGATCAGACACACTGGAACTGAGACACGG
TC
>Zotu1405
GAACGCTGGCGGCGTGCCTAATACATGCAAGTCGAGCGAACCAATAGGTGCTTGCGCCTGTTGGTTAG
CGGCGGACGGGTGAGTAACACGTGGGCAACCTGCCTGTAAGACTGGGATAACACCGGGAAACCGGTGC
TAATACCGGATAATCCTTTTCCTCTCATGAGGAAAAGCTGAAAGTCGGTGCAAGCTGACACTTACAGA
TGGGCCCGCGGCGCATTAGCTAGTTGGTGAGGTAACGGCTCACCAAGGCGACGATGCGTAGCCGACCT
GAGAGGGTGATCGGCCACACTGGGACTGAGACACGGCC
>Zotu1406
GAACGCTGGCGGCAGGCTTAACACATGCAAGTCGAACGATGAAACTCTAGCTTGCTAGAGATGATTAG
TGGCGGACGGGTGAGTAACATTTAGGAATCTGCCTAGTAGTGGGGGATAGCTCGGGGAAACTCGAATT

AATACCGCATACGACCTACGGGTGAAAGGGGGCGCAAGCTCTTGCTATTAGATGAGCCTAAATCAGAT
TAGCTAGTTGGTGGGGTAAAGGCCTACCAAGGCGACGATCTGTAACTGGTCTGAGAGGATGATCAGTC
ACACCGGAACTGAGACACGGTC
>Zotu1407
GAACGCTGGCGGCGTGCTTAACACATGCAAGTCGAACGATGAACCACTTCGGTGGGGATTAGTGGCGA
ACGGGTGAGTAACACGTGGGCAATCTGCCCTTCACTCTGGGACAAGCCCTGGAAACGGGGTCTAATAC
CGGATAACACTGCCACGGGCATCTGTGGTGGTTAAAAGCTCCGGCGGTGAAGGATGAGCCCGCGGCCT
ATCAGCTTGTTGGTGAGGTAATGGCTCACCAAGGCGACGACGGGTAGCCGGCCTGAGAGGGCGACCGG
CCACACTGGGACTGAGACACGGCC
>Zotu1408
GAACGCTGGCGGCGCGCTTAACACATGCAAGTCGAGCGAGAACCAGGGCTTCGGCCCTGGGGACAGCG
GCGAACGGGTGAGTAACACGTGGATAACCTGCCCTCGGCACCGGGATAGCCCGGGGAAACCCGGATTA
ATACCGGATAGCCTTTCCGACTCTCGAGTCGGTCCGAAAAGGTAGCTTCGGCCTCTGGCCGAGGATGG
GTCCGCGGCGGATTAGCTTGTTGGTGGGGTAACGGCCTACCAAGGCTCCGATCCGTAGCTGGTCTGAG
AGGACGATCAGCCACACTGGGACTGAGACACGGCC
>Zotu1409
TCGGGAAATCGGTGTTTAAGAAGAAACGCCACTGATTTCTTAAACCCGAAGCTTCCGTGTACAGGATC
CTCTTCTCCGCCCTTTTACAGCGCATCAAA
>Zotu1410
GAACGCTGGCGGCGTGCTTAACACATGCAAGTCGAACGGTAAGGCCCTTCGGGGTACACGAGTGGCGA
ACGGGTGAGTAACACGTGGGTAATCTGCCCTGCACTTCGGGATAAGCCTGGGAAACCGGGTCTAATAC
CGGATATTCAGTTCCTGCCGCATGGTGGGGGTTGGAAAGTTTTTCGGTGCAGGATGAGCCCGCGGCCT
ATCAGCTTGTTGGTGGGGTAATGGCCTACCAAGGCGACGACGGGTAGCCGGCCTGAGAGGGTGATCGG
CCACACTGGGACTGAGACACGGCC
>Zotu1411
GAACGCTGGCGGCGTGCTTAACACATGCAAGTCGAGCGGAAAGGCCACTTCGGTGGTACTCGAGCGGC
GAACGGGTGAGTAACACGTGAGTAATCTGCCCCTGGCTTTGGGATAGCCACCGGAAACGGTGATTAAT
ACCGGATATGACGACCTTTCGCATGATGGGTTGTGGAAAGTTTTTCGGCCAGGGATGTGCTCGCGGCC
TATCAGCTTGATGGTGAGGTAATGGCTCACCATGGCTTCGACGGGTAGCCGGCCTGAGAGGGTGACCG
GCCACACTGGGACTGAGACACGGCC
>Zotu1412
GAACGCTAGCGGCAGGCTTAACACATGCAAGTCGAGGGGCAGCGCGGTAGCAATACTGGCGGCGACCG
GCAAACGGGTGCGGAACACGTACAGAACCTACCTTTACCTGGGGCATAGCCCGGAGAAATCCGGATTA
ACTCCCCATAATATAATTGGATGGCATCATTTGGTTATTAAAAATTTATTGGGTAAAGATGGCTGTGC
GTCCCATTAGCTAGTTGGTAGGGTAACGGCCTACCAAGGCGACGATGGGTAGCTGGTGTGAGAGCACG
ACCAGCCACACGGGCACTGAGACACGGGC
>Zotu1413
GAACGCTAGCGGCAGGCCTAATACATGCAAGTCGAGGGGCAGCACGGTAGCAATACTGGTGGCGACCG
GCAAACGGGTGCGGAACACGTACGCAACCTTCCTTTAAGAGATGAATAGCCCGAAGAAATTCGGATTA
ATGCATCGTAAGATTGTGGGATGGCATCATCCAGCAATTAAAGATTTATTTCGCTTAAAGATGGGCGT
GCGCCTGATTAGGTAGTTGGTGAGGTAACGGCTCACCAAGCCGACGATCAGTAACTGGCGTGAGAGCG
CGACCAGTCACACGGGCACTGAGACACGGGC
>Zotu1414
GAACGCTGGCGGCGTGCTTAACACATGCAAGTCGAGCGGTAAGGCCCTTTCGGGGGTACACGAGCGGC
GAACGGGTGAGTAACACGTGAGTAATCTGCCCTTCACTTCGGGATAAGCCTCGGAAACGGGGTCTAAT
ACCGGATATGACCACTTCGGGCATCCGATGGTGGTGGAAAGTTTTTTCGGTGGGGGATGTGCTCGCGG
CCTATCAGCTTGTTGGTGGGGTAATGGCCTACCAAGGCTTCGACGGGTAGCCGGCCTGAGAGGGTGAC
CGGCCACACTGGGACTGAGACACGGCC
>Zotu1415
GAACGCTGGCGGCATGCCTAACACATGCAAGTCGAACGAGATCCTTCGGGGTCTAGTGGCGCACGGGT
GCGTAACGCGTGGGAATCTGCCCTTCGGTACGGAATAACTCAGGGAAACTTGAGCTAATACCGTATGA
TGTCTTCGGACCAAAGATTTATCGCCGAGGGATGAGCCCGCGTTGGATTAGCTAGTTGGTGGGGTAAA
GGCCTACCAAGGCGACGATCCATAGCTGGTCTGAGAGGATGATCAGCCACACTGGGACTGAGACACGG
CC

>Zotu1416
GAACGCTGGCGGCGTGCTTAACACATGCAAGTCGAACGAGGTCCATGAAGTAGCAATACTTCGGAAGA
CCTAGTGGCGAACGGGTGCGTAACACGTGAGGAACTTGTCCCGGTCTCTGGGATAACAGTTGGAAACG
ACTGCTAATACCGGATGACGTCGGAGCGTCGCATGGCGCGCTGACGAAAGGGTTACTGGATCGGGAGA
GCCTCGCGGCCTATCAGCTTGTTGGTGGGGTAACGGCCTACCAAGGCATCGACGGGTAGCTGGTCTGA
GAGGATGATCAGCCACACTGGGACTGAGACACGGCC
>Zotu1417
GAACGCTGGCGGCGTGCTTAACACATGCAAGTCGAACGGAAAGGCCTCTTCGGAGGTACTCGAGTGGC
GAACGGGTGAGTAACACGTGGGTAATCTGCCCTGCACTTCGGGATAAGCTTGGGAAACTGGGTCTAAT
ACCGGATATGACCTCGGGACGCATGTCCTCTGGTGGAAAGCTTTTGCGGTGTGGGATGGGCCCGCGGC
CTATCAGCTTGTTGGTGGGGTGATAGCCTACCAAGGCGACGACGGGTAGCCGGCCTGAGAGGGTGTCC
GGCCACACTGGGACTGAGATACGGCC
>Zotu1418
GAACGCTGGCGGCAGGCCTAACACATGCAAGTCGAGCGGATGAGTGGAGCTTGCTCCATGATTCAGCG
GCGGACGGGTGAGTAATGCCTAGGAATCTGCCTGGTAGTGGGGGACAACGTTTCGAAAGGAACGCTAA
TACCGCATACGTCCTACGGGAGAAAGCAGGGGACCTTCGGGCCTTGCGCTATCAGATGAGCCTAGGTC
GGATTAGCTAGTTGGTGGGGTAAAGGCCTACCAAGGCGACGATCCGTAACTGGTCTGAGAGGATGATC
AGTCACACTGGAACTGAGACACGGTC
>Zotu1419
GAACGCTGGCGGCGTGCCTAACACATGCAAGTCGAACGGGACTGTTTAGTGACGATTTCGGTCTGAAC
TTTACAGTTTAGTGGCGGACGGGTGAGTAACGCGTGGATAACCTGCCTTTCACAGGGGGATAGCAGCT
GGAAACGGCTAGTAATACCGCATACGCTCATGTAACCGCATGGTGATGTGAGGAAAGATTTATCGGTG
AAAGATGGATCCGCGTCTGATTAGCCAGTTGGCGGGGTAACGGCCCACCAAAGCGACGATCAGTAGCC
GGCCTGAGAGGGCGGACGGCCACATTGGGACTGAGACACGGCC
>Zotu1421
GAACGCTAGCGGCAGGCTTAATACATGCAAGTCGAGGGGCAGCGCGGGTAGCAATACCTGGCGGCGAC
CGGCAAACGGGTGCGGAACACGTACGCAACCTTCCTTCAAGTGGGGAATAGCCCAGGGAAACTTGGAT
TAATACCCCTTAATATTATTGAGTGGCATCACTTGATAATTAAAGCTCCGGCGCTTGAAGATGGGCGT
GCGCCTGATTAGGTAGTTGGCGGGGTAACAGCCCACCAAGCCTACGATCAGTAACTGGTGTGAGAGCA
CGACCAGTCACACGGGCACTGAGACACGGGC
>Zotu1422
GAACGCTAGCGGCATGCCTAACACATGCAAGTCGAACGAGATCTTCGGATCTAGTGGCGCACGGGTGC
GTAACGCGTGGGAATCTGCCTTGGGGTTCGGAATAACTCCCCGAAAGGGGTGCTAATACCGGATGATG
TCGAAAGACCAAAGATTTATCGCCCTGAGATGAGCCCGCGTAGGATTAGCTAGTTGGTGTGGTAAAGG
CGCACCAAGGCGACGATCCTTAGCTGGTCTGAGAGGATGATCAGCCACACTGGGACTGAGACACGGCC
>Zotu1424
GAACGCTGGCGGCGTGCTTAACACATGCAAGTCGAACGGTAAGGCCCTTCGGGGTACACGAGTGGCGA
ACGGGTGAGTAACACGTGAGTGACCTGCCCTTTACTTTGGGATAACCACCAGAAATGGTGGCTAATAC
CGAATATGACTCCTGCCTGCATGGGTGGGGGTGGAAAGCTCCGGCGGTAAGGGATGGACTCGCGGCCT
ATCAGCTTGTTGGTGAGGTAGTGGCTCACCAAGGCTTCGACGGGTAGCCGGCCTGAGAGGGTGACCGG
CCACATTGGGACTGAGATACGGCC
>Zotu1425
GAACGCTAGCGGCAGGCTTAACACATGCAAGTCGAGGGGTAGAAGGCTTCGGCCTTTGAGACCGGCGC
ACGGGTGCGTAACGCGTATGCAATCTACCTTTTACAGGGGGATAGCCCAGAGAAATTTGGATTAATAC
CCCATAGTATTATTGAATGGCATCATTTAATAATTAAAGTCACAACGGTAAAAGATGAGCATGCGTCC
CATTAGCTAGTTGGTATGGTAACGGCATACCAAGGCAATGATGGGTAGGGGTCCTGAGAGGGAGATCC
CCCACACTGGTACTGAGACACGGAC
>Zotu1426
GAACGCTAGCGGCAGGCCTAATACATGCAAGTCGAACGGGCTTTGGTTTTCGGATCAGGGCTAGTGGC
GCACGGGTGCGTAACGCGTACACAACATACCCTTTACTGGGGGATAGCGGTGGGAAACTGCCAGTAAT
ACCCCATGCCCTTACGAGGTGGCATTACCTTGTGAGGAAAGTTACGGCGGTAAAGGATTGGTGTGCGT
CCGATTAGCTAGTTGGCGGGGTAACGGCCCACCAAGGCGACGATCGGTAGGGGGTGTGAGAGCATGGC
CCCCCACACGGGTACTGAGACACGGAC
>Zotu1427

GAACGCTGGCGGCGTGCCTAACACATGCAAGTCGAGCGATCTTCTTCGGAAGAGAGCGGCGGACGGGT
GAGTAACGCGTGGGTAACCTGCCTCATACACATGGATAACATACCGAAAGGTATGCTAATACAAGATG
ATATAAGAGATTCGCATGGATTTCTTATCAAAGCTCCGGCGGTATGAGATGGACCCGCGTCTGATTAG
CTAGTTGGTAAGGTAACGGCTTACCAAGGCGACGATCAGTAGCCGACCTGAGAGGGTGATCGGCCACA
TTGGAACTGAGACACGGTC
>Zotu1428
GAACGCTAGCGGCAGGCCTAATACATGCAAGTCGAACGGTGTCTTCGGACATAGTGGCGCACGGGTGC
GTAACACGTATGCAACCTACCTTACATTGGGGGATAGCCTTTCGAAAGGGAGATTAATACCGCATAAG
ACAGTAGCTGGGCATCCAGCAGCTGTTAAAGATTTATCGATGTAAGCTGGGCATGCGTCCAATTAGCT
AGTTGGTGAGGTAACGGCTCACCAAGGCTATGATTGGTAGGGGAACTGAGAGGTCAATCCCCCACACT
GGCACTGAGATACGGGC
>Zotu1429
GAACGCTAGCGGCAGGCTTAATACATGCAAGTCGAGGGGCAGCGCAGTGTAGCAATACATGGGCGGCG
ACCGGCAAACGGGTGCGGAACACGTACGCAACTTACCCAAAACTGATGAATAGCCCTCCGAAAGGAGG
ATTAATACATCGTAACCTATTGAAGTGGCATCACTTTATTAGTATAGCTCCGGCGGTTTTGGATGGGC
GTGCGGCTGATTAGATAGTTGGCGGGGTAACGGCCCACCAAGTCTGCGATCAGTAACTGGTGTGAGAG
CACGACCAGTCACACGGGCACTGAGACACGGGC
>Zotu1430
TAACGCTGGCGGTATGCCTAATACATGCAAGTCGAACGCGCACTTCGGTGCGAGTGGCGGACGGGTGA
GTAACACGTTGGAACCTGCCCCGAAGTGGGGAATAACCCATCGAAAGATGGGCTAATACCCCATAGTC
TTATGCTTGTCATAAGTAAAGTTTTTCGCTTCGGGAGGGGCCTGCGCAACATTAGCTAGTTGGTGAGA
TAAAAGCCCACCAAGGCGACGATGTTTAGCTGGATCGAGAGGTTGACCAGCCACACTGGGACTGAGAT
ACGGCC
>Zotu1431
GAACGCTGGCGGCGCGCCTAACACATGCAAGTCGAGCGATGAACATTGAATGATCCCTTCGGGGTGAT
TTCGATCGGATTAGCGGCGAACGGGTGAGTAACGCGTGAGGAACCTGCCTCTTACAACGGGATAGCCT
CGGGAAACCGGGATTAATACCGTATAAGACTCCGACACCTCCTGGTGATGAAGTCAAAGCGTTAGCGG
TAAGAGATGGCCTCGCGTCTGATTAGTTAGTTGGCGGGGTAACGGCCCACCAAGACGACGATCAGTAA
CCGGCCTGAGAGGGTGAACGGTCACATTGGAACTGAGACACGGTC
>Zotu1433
GAACGCTGGCGGCGCGCCTAAAACATGCAAGTCGAACGAGAAACCCGGGCTTGCCCGGGTGGACAGTG
GCGAACGGGTGAGTAACACGTGAGGAACCTGCCCCGAAGACCGGGACAACACCGGGAAACCGGTGCTA
ATACCGGATGTCCTCACCGGATCGCATGATCTGGTGAGGAAATGGATTCCGCTTCGGGATGGCCTCGC
GGCCTATCAGCTAGTTGGTGGGGTAACGGCCCACCAAGGCGACGACGGGTAGCTGGTCTGAGAGGATG
TCCAGCCACACTGGGACTGAGACACGGCC
>Zotu1434
GAACGCTGGCGGCGCGCTTAACACATGCAAGTCGAGCGAGAACCGGGACTTCGGTCCTGGGGACAGCG
GCGAACGGGTGAGTAACACGTGGGTAATCTGCCCTCGATTCTGGGATAGCCCGGGGAAACCCGGATTA
ATACCGGATAGCCTTCCAAGCCCTCGGGCGAAGAAGAAAAGGTAGCTTCGGCCTCCGATCGAGGATGA
GCCCGCGGTGGATTAGCTTGTTGGCGGGGTAATGGCCCACCAAGGCGACGATCCATAGCTGGTCTGAG
AGGACGATCAGCCACACTGGGACTGAGACACGGCC
>Zotu1436
GAACGCTGGAAATGTGGATACCGGCATGCAAGTCAAGGGGCCGCAAGGCACCGGCGAACGGCTTAGTA
ACACGTGGATACATACCGCAAAGTGGGGCATAGCTCGTCGAAAGACGGGGTAATTTCCCATAGTCCCG
CAAGGGTAAAGGCGCAAGCCGCTTTGTGAATGGTCCGCGGCCTATCAGATAGTTGGCGAGGTAAAAGC
TCACCAAGTCTATGACGGGTAGGGGGCGTGAGAGCGCGACCCCCAAGAATGGCACTGCGACACGGGC
>Zotu1437
GAACGCTAGCGGCAGGCCTAATACATGCAAGTCGGACGGGATTGGAGAGCTTGCTTTCCATGAGAGTG
GCGCACGGGTGCGTAACACGTATGTAACCTACCTTCGTCAGGGGGATAGCCTCTCGAAAGAGAGATTA
AGACCGCATAAAATCACAATGTGGCATCACAAAATGATCAAATATTTATAGGACGAAGATGGGCATGC
GGAACATTAGCTAGTTGGTAAGGTAACGGCTTACCAAGGCTACGATGTTTAGGGGATCTGAGAGGATG
GCCCCCCACACTGGTACTGAGACACGGAC
>Zotu1438
GAACGCTGGCGGCAGGCTTAACACATGCAAGTCGAGCGCCCCGCAAGGGGAGCGGCAGACGGGTGAGT

AACACGTGGGAACGTACCTTTTGGTTCGGAACAACTCCGGGAAACTGGAGCTAATACCGGATAAGCCC
TAACGGGGAAAGATTTATCGCCAAAAGATCGGCCCGCGTCTGATTAGCTAGTTGGTGAGGTAACGGCT
CACCAAGGCGACGATCAGTAGCTGGTCTGAGAGGATGATCAGCCACACTGGGACTGAGACACGGCC
>Zotu1439
GAATGCTGGCGGCGTGCCTAATACATGCAAGTCGAGCGGTAAGGCCCTTCGGGGTACACGAGCGGCGG
ACGGCTGAGTAACGCGTGGGAACATGCCCCAAAGTGAGGGATAACCTCGAGAAATCGAGGCTAATACC
GCATATGGTCTTCGGATTAAAGGATTTATCCGCTTTGGGAGTGGCCCGCGTACGATTAGTTTGTTGGT
GAGGTAATGGCTCACCAAGACTACGATCGTTAACTGGTCTGAGAGGATGATCAGTCAGACTGGAACTG
AGACACGGTC
>Zotu1440
GAACGCTGGCGGCGTGCTTAACACATGCAAGTCGAACGAAGCACTTTATTTGATTTCCTTCGGGACTG
ATTATTTTGTGACTGAGTGGCGGACGGGTGAGTAACGCGTGGGTAACCTGCCTTGTACAGGGGGATAA
CAGTTGGAAACGGCTGCTAATACCGCATAAGCGCACAGCATCGCATGATGCAGTGTGAAAAACTCCGG
TGGTATAAGATGGACCCGCGTTGGATTAGCTAGTTGGTGAGGTAACGGCCCACCAAGGCGACGATCCA
TAGCCGACCTGAGAGGGTGACCGGCCACATTGGGACTGAGACACGGCC
>Zotu1441
GAACGCTGGCGGCGCGCCTAACACATGCAAGTCGAACGGAACTTGAGAGAGCTTGCTTTTTCAAGTTT
AGTGGCGAACGGGTGAGTAACGCGTGAGTAACCTGCCCTGGAGTGGGGGACAACAGTTGGAAACGACT
GCTAATACCGCATAAGCCCACGGTGCCGCATGGCACAGCGGGAAAAGGATTTATTCGCTTCAGGATGG
ACTCGCGTCCAATTAGCTAGTTGGTGAGGTAACGGCCCACCAAGGCGACGATTGGTAGCCGGACTGAG
AGGTTGAACGGCCACATTGGGACTGAGACACGGCC
>Zotu1442
GAACGCTAGCTACAGGCTTAACACATGCAAGTCGAGGGGCAGCATTGGAGTAGCAATACTTCAGATGG
TGACCGGCGCACGGGTGAGTAACACGTATCCAACCTGCCGCTTACTCGGGGATAGCCTTTCGAAAGAA
AGATTAATACCCGATGGTATAAAGAGTTCGCATGTTCTCTTTATTAAAGAATTTCGGTAAGCGATGGG
GATGCGTTCCATTAGATTGTTGGCGGGGTAACGGCCCACCAAGTCTACGATGGATAGGGGTTCTGAGA
GGAAGGTCCCCCACATTGGAACTGAGACACGGTC
>Zotu1443
GAACGCTGGCGGCGTGCCTAACACATGCAAGTCGAGCGATTTACTTCGGTAAAGAGCGGCGGACGGGT
GAGTAACGCGTGGGTAACCTGCCCTGTACACACGGATAACATACCGAAAGGTATGCTAATACGAGATA
ATATACTTTTATCGCATGGTAGAAGTATCAAAGCTCCGGCGGTACAGGATGGACCCGCGTCTGATTAG
CTAGTTGGTAAGGTAACGGCTTACCAAGGCGACGATCAGTAGCCGACCTGAGAGGGTGATCGGCCACA
TTGGAACTGAGACACGGTC
>Zotu1444
GAACGCTGGCGGCGTGCTTAACACATGCAAGTCGAGCGGAAAGGCCTCTTCGGGGGTACTCGAGCGGC
GAACGGGTGAGTAACACGTGAGTAATCTGCCCTTCACTTCGGGATAACCGCCGGAAACGGTGGCTAAT
ACCGGATATGACTGCGTCTCGCATGGGGTGTGGTGGAAAGCTTTTGTGGTGGAGGATGTGCTCGCGGC
CTATCAGCTTGTTGGTGGGGTAATGGCCTACCAAGGCTTCGACGGGTAGCCGGCCTGAGAGGGTGACC
GGTCACACTGGGACTGAGACACGGCC
>Zotu1445
TAACGCTGGCGGCATGCCTAATACATGCAAGTCGAACGAGAGTACTTGTACTCTAGTGGCGAACGGGT
GAGTAACACGTGGGCAACCTGTCCTAAAGACGAAGATAACAGTTGGAAACGACTGATAATACTGGATA
GTTTAACGAAACGCATGTTTTGTTAATTAAAGGTGGTAACACCGCTTTAGGAGGGGCCCGCGGTGCAT
TAGCTAGTTGGTGAGGTAACGGCCCACCAAGGCTACGATGCATAGCTGTGCTGAGAGGCAAAACAGCC
ACACTGGGACTGAGACACGGCC
>Zotu1446
GAACGCTGGCGGCGTGCTTAACACATGCAAGTCGAACGGTGAACCCGCTTCGGTGGGGGATCAGTGGC
GAACGGGTGAGTAACACGTGGGCAACCTGCCCTCAGCTCTGGGATAACTCCAAGAAATTGGGGCTAAT
ACCGGATATGACCGCTGACCGCATGGTCTGGTGGTGGAAAGATTTATCGGCTGGGGATGGGCCCGCGG
CCTATCAGCTTGTTGGTGGGGTAGTGGCCTACCAAGGCGACGACGGGTAGCCGGCCTGAGAGGGTGAC
CGGCCACACTGGGACTGAGACACGGCC
>Zotu1447
GAACGCTGGCGGCGTGCCTAATACATGCAAGTAGAACGCTGAAGGAGGAGCTTGCTTCTCCGGATGAG
TTGCGAACGGGTGAGTAACGCGTAGGTAACCTGCCTGGTAGCGGGGGATAACTATTGGAAACGATAGC

TAATACCGCATAAGAGTAGATGTTGCATGACATTTGCTTAAAAGGTGCAATTGCATCACTACCAGATG
GACCTGCGTTGTATTAGCTAGTTGGTGGGGTAACGGCTTACCAAGGCGACGATACATAGCCGACCTGA
GAGGGTGATCGGCCACACTGGGACTGAGACACGGCC
>Zotu1448
GAACGCTGGCGGCGTGCTTAACACATGCAAGTCGAGCGGAAAGGCCCCTTCGGGGGTACTCGAGCGGC
GAACGGGTGAGTAACACGTGAGTAATCTGCCCTCCACTTCGGGATAGCCCTCCGAAAGGGGGATTAAT
ACCGGATGGGCACCACGGACCGCATGGTGTGTGGTGGAAAGTTTTTCGGTGGGGGATGAGCTCGCGGC
CTATCAGCTTGTTGGTGAGGTGATGGCTCACCAAGGCTTCGACGGGTAGCCGGCCTGAGAGGGTGACC
GGCCACACTGGGACTGAGACACGGCC
>Zotu1449
GAACGCTGGCGGCGTGCTTAACACATGCAAGTCGAACGGTGATGGCCAGCTTGCTGGTCTGATCAGTG
GCGAACGGGTGAGTAACACGTGAGCAACCTGCCCTTCACTCTGGGATAAGCCCTGGAAACGGGGTCTA
ATACCGGATATGAGACGCACACGCATGTGTAGTGTCTGGAAAGATTTATCGGTGGGGGATGGGCTCGC
GGCCTATCAGCTTGTTGGTGGGGTAGTGGCCTACCAAGGCGACGACGGGTAGCCGGCCTGAGAGGGCG
ACCGGCCACACTGGGACTGAGACACGGCC
>Zotu1450
GAACGCTGGCGGCGTGCTTAACACATGCAAGTCGAGCGGAAAGGCCACTTCGGTGGTACTCGAGCGGC
GAACGGGTGAGTAACACGTGAGTAATCTGCCCTTGGCTTTGGGATAGCCACCGGAAACGGTGATTAAT
ACCGGATATGACTACTTCTCGCATGAGTTGTGGTGGAAAGTTTTTCGGCCAGGGATGTGCTCGCGGCC
TATCAGCTTGATGGTGAGGTAATGGCTCACCATGGCTTCGACGGGTAGCCGGCCTGAGAGGGTGACCG
GTCACACTGGGACTGAGACACGGCC
>Zotu1451
GAACGCTGGCGGCAGGCTTAACACATGCAAGTCGAGCGCCCCGCAAGGGGAGCGGCAGACGGGAGAGT
AACACGTGGGAACGTACCCTTCAGTACGGAACAACCAAGGGAAACTTTGGCTAATACCGTATACGTCC
GTAAGGAGAAAGATTTATCGCTGAAGGATCGGCCCGCGTCTGATTAGCTAGTTGGTGGGGTAATGGCC
CACCAAGGCGACGATCAGTAGCTGGTCTGAGAGGATGATCAGCCTCACTGGGACTGAGACACGGCC
>Zotu1453
GAACGCTGGCGGCGTGCTTAACACATGCAAGTCGAACGGAAAGGCCCTTCGGGGTACTCGAGTGGCGA
ACGGGTGAGTAACACGTGGGTGATCTGCCCTGCACTTTGGGATAAGCCTGGGAAACTGGGTCTAATAC
CGAATACACCTTCCTGGCTGCATGGTTGGGGGGGGAAAGCTTTTGCGGTGTGGGATGGGCCCGCGGCC
TATCAGCTTGTTGGTGAGGTTACGGCTCACCAAGGCGACGACGGGTAGCCGGCCTGAGAGGGTGACCG
GCCACACTGGGACTGAGATACGGCC
>Zotu1454
GAATGCTGGCGGCGTGGATAAGTCATGCAAGTCAAGGGGGCCCGCAAGGGCAACCGGCAGACGAGGTA
GTAACACGTAGGTAGATCCCCCGAAGTCGGGCATAGCCAGTCGAAAGACTGGGTAATTCCCGATAGTC
CCGCAAGGGTAAAGGTTTTTCGCTTCGGGAGTTGCCTGCGTAGTATCAGCTAGTTGGTAGGGTAATGG
CCTACCAAGGCTACGACGCTTAGGGGAGCTGAGAGGCTGACCCCCACCGATGGAACTGCGACACGGTC
>Zotu1455
GAACGCTGGCGGCAGGCTTAACACATGCAAGTCGAGCGGGTGTCCTTCGGGGCATCAGCGGCGGACGG
GTTAGTAACGCGTGGGAACGTGCCTTCTGCTCTGGAATAGCCCTGGGAAACTGGGGGTAATACCGGAT
GTGCCCTGAGGGGGAAAGGTTTATCTGGCAGGAGATCGGCCCGCGTCTGATTAGGTAGTTGGTGTGGT
AATGGCGCACCAAGCCGACGATCGGTAGCTGGTTTGAGAGGATGATCAGCAACACTGGGACTGAGACA
CGGCC
>Zotu1456
GAACGCTGGCGGCGTGCTTAACACATGCAAGTCGAACGGTGAAGGTGGAGCTTGCTCTGCTGGATCAG
TGGCGAACGGGTGAGTAACACGTGAGCAATCTGCCCCTGACTCTGGGATAACAGTTGGAAACAGCTGC
TAATACTGGATATGAACTGTGAAGGCATCTTCAGCAGTTGGAAAGAATTTCGGTCAGGGATGAGCTCG
CGGCCTATCAGCTTGTTGGTGAGGTAATGGCTCACCAAGGCGTCGACGGGTAGCCGGCCTGAGAGGGT
GACCGGCCACACTGGGACTGAGACACGGCC
>Zotu1457
GAACGCTGGCGGCGTGCTTAACACATGCAAGTCGAACGAGACCCTCGCTTCGGTGGGGTGTTCTAGTG
GCGAACGGGTGAGTAACACGTGGGCAACCTGCCCCTGGCTCTGGGATAACTCCAAGAAATTGGGGCTA
ATACCGGATATGACTGCTGACCGCATGGTCTGGTGGTGGAAAGATTTATCGGCTGGGGATGGGCCCGC
GGCCTATCAGCTTGTTGGTGGGGTAGTGGCCTACCAAGGCGACGACGGGTAGCCGGCCTGAGAGGGTG

ACCGGCCACACTGGGACTGAGACACGGCC
>Zotu1458
GAACGCTGGCGGCAGGCCTAACACATGCAAGTCGAGCGGATGAAGGGAGCTTGCTCCCTGATTTAGCG
GCGGACGGGTGAGTAATGCCTAGGAATCTGCCTGGTAGTGGGGGATAACGTTCCGAAAGGAACGCTAA
TACCGCATACGTCCTACGGGAGAAAGCAGGGGACCTTCGGGCCTTGCGCTATCAGATGAGCCTAGGTC
GGATTAGCTAGTTGGTGAGGTAATGGCTCACCAAGGCGACGATCCGTAACTGGTCTGAGAGGATGATC
AGTCACACTGGAACTGAGACACGGTC
>Zotu1460
GAACGCTGGCGGCATGCTTTACACATGCAAGTCGAACGGTAACAGGGAGCTTGCTCCGCTGACGAGTG
GCGAACGGGTGAGTAATATATCGGAACGTACCCAAGAGTGGGGGATAACGTAGCGAAAGTTACGCTAA
TACCGCATACGATCTATGGATGAAAGCAGGGGACCTTCGGGCCTTGTGCTCCTGGAGCGGCCGATATC
TGATTAGCTAGTTGGTGAGGTAAAGGCTCACCAAGGCGACGATCAGTAGCTGGTCTGAGAGGACGACC
AGCCACACTGGAACTGAGACACGGTC
>Zotu1461
GAACGCTGGCGGCAGGCCTAACACATGCAAGTCGAACGCGACCTTCGGGTCGAGTGGCGCACGGGTGA
GTAACGCGTGGGAACCTGTCCTGTGGTACGGAATAACATCGGGAAACTGGTGCTAATACCGTATATCC
CCTGAGGGGGAAAGATTTATCGCCATAGGAGGGGCCCGCGTAGGATTAGCTAGTTGGTGAGGTAACGG
CTCACCAAGGCGACGATCCTTAGCTGGTCTGAGAGGATGATCAGCCACACTGGGACTGAGACACGGCC
>Zotu1462
GAACGCTGGCGGCGTGCCTAATACATGCAAGTCGAGCGAATTTTTGGGAGCTTGCTCCCAAAGGTTAG
CGGCGGACGGGTGAGTAACACGTGGGCAACCTGCCTATAAGACTGGGATAACTTCGGGAAACCGGAGC
TAATACCGGATAATTCTTTTCCTCTCATGAGGAAAAGCTGAAAGACGGTTTACGCTGTCACTTATAGA
TGGGCCCGCGGCGCATTAGCTAGTTGGTGAGGTAACGGCTCACCAAGGCAACGATGCGTAGCCGACCT
GAGAGGGTGATCGGCCACACTGGGACTGAGACACGGCC
>Zotu1464
GAACGCTGGCGGCAGGCTTAACACATGCAAGTCGAACGGTCCTTCGGGATAGTGGCGGACGGGTGAGT
AACACGTGGGAACGTACCCTTAGGTTCGGAACAACACAGGGAAACTTGTGCTAATACCGGATAAGCCC
TTCGGGGGAAAGATTTATCGCCTTTGGAGCGGCCCGCGTCTGATTAGCTTGTTGGTGAGGTAATGGCT
CACCAAGGCTACGATCAGTAGCTGGTCTGAGAGGATGGCCAGCCACACTGGGACTGAGACACGGCC
>Zotu1465
GAACGCTGGCGGCGTGCTTAACACATGCAAGTCGAACGGGAAGAGATGAAGAGCTTGCTCTTTATCGA
ATCCAGTGGCAAACGGGTGAGTAACACGTAAACAACCTGCCTTCAGGATGGGGACAACAGACGGAAAC
GACTGCTAATACCGAATACGTTCCACGGGCCGCATGACCTGTGGAAGAAAGGGTAGCCTCTACCTGTA
AGCTATCGCCTGAAGAGGGGTTTGCGTCTGATTAGGCAGTTGGTGGGGTAACGGCCCACCAAACCAAC
GATCAGTAGCCGGTCTGAGAGGATGAACGGCCACACTGGAACTGAGACACGGTC
>Zotu1466
GAACGCTGGCGGCGTGCCTAATACATGCAAGTCGAGCGAACAGACGAGGAGCTTGCTCCTTTGATGTT
AGCGGCGGACGGGTGAGTAACACGTAGGTAACCTACCTATAAGACTGGGATAACTTCGGGAAACCGGA
GCTAATACCGGATAATATTTCGAACCGCATGGTTCGATAGTGAAAGATGGCTTTGCTATCACTTATAG
ATGGACCTGCGCCGTATTAGCTAGTTGGTAAGGTAACGGCTTACCAAGGCAACGATACGTAGCCGACC
TGAGAGGGTGATCGGCCACACTGGAACTGAGACACGGTC
>Zotu1467
GAACGCTGGCGGCGTGCCTAATACATGCAAGTAGAACGCTGAAGCTTGGTGCTTGCACCGAGCGGATG
AGTTGCGAACGGGTGAGTAACGCGTAGGTAACCTGCCTGGTAGCGGGGGATAACTATTGGAAACGATA
GCTAATACCGCATAAGAGTAGATGTCGCATGACATTTGCTTAAAAGGTGCAATTGCATCACTACCAGA
TGGACCTGCGTTGTATTAGCTAGTTGGTGAGGTAACGGCTCACCAAGGCAACGATACATAGCCGACCT
GAGAGGGTGATCGGCCACACTGGGACTGAGACACGGCC
>Zotu1468
GAACGCTGGCTGCGTGCTTAACACATGCAAGTCGAACGCTGAAGCCGACAGCTTGCTGTTGGTGGATG
AGTGGCGAACGGGTGAGTAACACGTGAGTAACCTGCCCCTGATTTCGGGATAAGCCTGGGAAACTGGG
TCTAATACCGGATACGACCTCCTGACGCATGTTGGGTGGTGGAAAGTTTTTCGATCGGGGATGGGCTC
GCGGCCTATCAGCTTGTTGGTGGGGTAATGGCCTACCAAGGCGACGACGGGTAGCCGGCCTGAGAGGG
CGACCGGCCACACTGGGACTGAGACACGGCC
>Zotu1469

GAACGCTGGCGGCGTGCCTAATACATGCAAGTCGAGCGGATTCATTCCTTCGGGGATGGGTTAGCGGC
GGACGGGTGAGTAACACGTAGGCAACCTGCCTATAAGCCTGGGATAACTTCCGGAAACGGATGCTAAT
ACCGGATACGTTGAATTCTCGCATGAGAATTCATGGAAAGACGGCGCAAGCTGTCACTTATGGATGGG
CCTGCGGCGCATTAGCTAGTTGGTGGGGTAACGGCTCACCAAGGCGACGATGCGTAGCCGACCTGAGA
GGGTGATCGGCCACACTGGGACTGAGACACGGCC
>Zotu1470
GAACGCTGGCGGCGTGCTTAACACATGCAAGTCGAACGAGATCCATTCAGTAGCAATACTGATGAAGA
TCTAGTGGCGAACGGGTGCGTAACACGTGAGGAACTTGTCCCGGTCTCTGGGATAACAGTTGGAAACG
ACTGCTAATACCGGATGACGTCGGAACTTCGCATGGAGTACTGACGAAAGGGTTACTGGATCGGGAGA
GCCTCGCGGCCTATCAGCTTGTTGGTGGGGTAATGGCCTACCAAGGCATCGACGGGTAGCTGGTCTGA
GAGGATGATCAGCCACACTGGGACTGAGACACGGCC
>Zotu1471
GAATGCTAGCGGTGTGCCTGAGATATGCAAGTCGAGCGCTTATTTTGCCGCAAGGTGAAGTGAGAGCG
GCGGACGGCTGAGTAATGTACAGGAATCTGCCCCTTAGTGGAACATAGCTCCGGGAAACCGGGGGTAA
TTTTCCATGGTTTCACCTTTTTGGTGAATAAAAGCCTTTACCGGTTGCTAAGGGATGAGCCTGTATCC
TATCAGGTAGTTGGTGGGGTAAAAGCCTACCAAGCCTATGACGGGTAGCTGATCTGGGAGGATGATCA
GCCAGAGGGGGACTGAGACACGGCC
>Zotu1472
GAACGCTAGCGGCAGGCTTAACACATGCAAGTCGAGGGGTATAGTTCTTCGGAACTAGAGACCGGCGC
ACGGGTGCGTAACGCGTATGCAATCTACCTTTTACAGCGGGATAGCCCAGAGAAATTTGGATTAATAC
CACATAGTATTATAAAATGGCATCATTTTATAATTAAAGTCACAACGGTAAAAGATGAGCATGCGTCC
CATTAGCTAGATGGTAAGGTAACGGCTTACCATGGCAACGATGGGTAGGGGTCCTGAGAGGGAGATCC
CCCACACTGGTACTGAGACACGGAC
>Zotu1473
GAACGCTGGCGGCGTGGATAAGGCATGCAAGTCGAAAGGGTTTAGGCCCTTGGCAAACGGGTTAGTAA
TGCATAGGAACTTTCCTTGAAGTCGTGAATAATCTGGAGAAATCCAGGATAATACACGATGTGCTCTA
CGGAGTAAAGATTTATCGCTTCAAGAGAGGCCTATGTCCTATCAGCTAGTTGGTAGTGTAATGGACTA
CCAAGGCTATGACGGGTAGGGGGTGTGAGAGCACGGCCCCCAACATTGGGACTGAGAACTGCC
>Zotu1474
GAACGCTGGCGGCGTGCTTAACACATGCAAGTCGAGCGATGAAGTTCCTTCGGGGACGGATTAGCGGC
GGACGGGTGAGTAACACGTGGGTAACCTGCCTCATAGAGGGGAATAGCCTTCCGAAAGGAAGATTAAT
ACCGCATAAGATTGTAGCTTCGCATGAAGTAGCAATTAAAGGAGTAATCCGCTATGAGATGGACCCGC
GTCGCATTAGCTAGTTGGTGAGGTAACGGCTCACCAAGGCGACGATGCGTAGCCGACCTGAGAGGGTG
ATCGGCCACATTGGGACTGAGACACGGCC
>Zotu1475
GAACGCTGGCGGCGTGCCTAACACATGCAAGTCGAACGGGGTCCATCTGGTGGCAACACCGGGGAAGA
CCTAGTGGCGAACGGGTGAGTAACACGTGAGAAACCCACCCCGATCTCTGGGATAACAGGTGGAAACA
CCTGCTAATACCGGATGCCTTCACCTCGTCGCATGGCGGGGTGAAGAAAGGGCTCTACCGGATCGGGA
CGGTCTCGCGGCCTATCAGCTTGTTGGTGGGGTAATGGCCCACCAAGGCTTCGACGGGTAGCTGGTCT
GAGAGGACGATCAGCCACACTGGGACTGAGACACGGCC
>Zotu1478
GAACGCTGGCGGCATGCTTTACACATGCAAGTCGAACGGTAACAGGGAGCTTGCTCCGCTGACGAGTG
GCGAACGGGTGAGTAATATATCGGAACGTACCCAAGAGTGGGGGATAACGTAGCGAAAGTTACGCTAA
TACCGCATACGATCCAAGGATGAAAGCAGGGGATCTTCGGACCTTGTGCTCCTGGAGCGGCCGATATC
TGATTAGCTAGTTGGTGAGGTAAAGGCTCACCAAGGCGACGATCAGTAGCTGGTCTGAGAGGACGACC
AGCCACACTGGAACTGAGACACGGTC
>Zotu1479
GAACGCTGGCGGCGTGCCTAATACATGCAAGTCGAGCGGACTGAATGAAAGCTTGCTTTCATTCGGTT
AGCGGCGGACGGGTGAGTAACACGTGGGCAACCTGCCCGTAAGACCGGGATAACTTCGGGAAACCGGA
GCTAATACCGGATAACACCGAAGATCGCATGATCTTTGGTTGAAAGGCGGTTTTGGCTGTCACTTACG
GATGGGCCCGCGGCGCATTAGTTAGTTGGTGAGGTAACGGCTCACCAAGGCGACGATGCGTAGCCGAC
CTGAGAGGGTGATCGGCCACACTGGGACTGAGACACGGCC
>Zotu1480
GAACGCTGGCGGCGTGCCTAAGACATGCAAGTCGAGTGGGTTTAGACCCACGGCGAACGGGTGAGTAA

TACCTAGGAACTTCCCTTTAGTTGAGCATAACCCATCGAAAGGTGGGATAATTCTCGATGTGACCGCA
AGGTTAAAGTCGCAAGACGCTAAAGAAAAGGCCTGGGCACCATCAGCTAGTTGGTAAGGTAAAAGCTT
ACCAAGGCTATGACGGTTAGGGGCCCCGAGAGGGGAAACCTCCACACTGGAACTGCGACACGGTC
>Zotu1481
GAACGCTAGCAATATGCTTTACACATGCAAGTCAAATGTGTCGTTTATATTAAACGCTACATGGCAAA
CGGGTGAGTAACGCGTAGGAATCTACCTAACAGTACGGAATAAGTTGCACAGGTATCCTGTGAAATCA
ATACCGTATATAAAAGATATATATCGCTGTTAGATGAGCCTGCGTAAGATTAGGTAGTTGGTAAGGTA
ATGGCTTACCAAGCCTTGGATCTTTAGCTGGTCTGAGAGGATGATCAGCCACACTAGGACTGAGACAC
GGCC
>Zotu1482
GAACGCTGGCGGCGTGCTTAACACATGCAAGTCGAACGGGATCCATCGCGAGCTTGCTCAAGAGAAGA
TCTAGTGGCGAACGGGTGCGTAACACGTGAGAAACCTGCCCCAGACTTGGGAATAACAGTCGGAAACG
ACTGCTAATACCTAATACCTTCGTAGCGTCGCATGGCGCATCGAAGAAAGCTTTATGCGGTTTGGGAG
GGTCTCGCGGCCTATCAGCTAGTTGGTGAGGTAACGGCTCACCAAGGCATCGACGGGTAGCTGGTCTG
AGAGGATGATCAGCCACACTGGGACTGAGACACGGCC
>Zotu1483
GAACGCTGGCGGCAGGCCTAACACATGCAAGTCGAGCGGTAGCACAGAGAGCTTGCTCTCGGGTGACG
AGCGGCGGACGGGTGAGTAATGTCTGGGAAACTGCCTGATGGAGGGGGATAACTACTGGAAACGGTAG
CTAATACCGCATAATGTCGCAAGACCAAAGTGGGGGACCTTCGGGCCTCATGCCATCAGATGTGCCCA
GATGGGATTAGCTAGTAGGTGGGGTAACGGCTCACCTAGGCGACGATCCCTAGCTGGTCTGAGAGGAT
GACCAGCCACACTGGAACTGAGACACGGTC
>Zotu1484
GAACGCTAGCGGCAGGCTTAACACATGCAAGTCGAGGGGTATAGTTCTTCGGAATTAGAGACCGGCGC
ACGGGTGCGTAACGCGTATGCAATCTACCTTTCACAGAGGGATAGCCCAGAGAAATTTGGATTAATAC
CTCATAGTATATAGCCCTGGCATCAGGATTATATTAAAGTCACAACGGTGAAAGATGAGCATGCGTCC
CATTAGCTAGTTGGTAAGGTAACGGCTTACCAAGGCTACGATGGGTAGGGGTCCTGAGAGGGAGATCC
CCCACACTGGTACTGAGACACGGAC
>Zotu1485
GAACGCTAGCGGCAGGCCTAACACATGCAAGTCGAGGGAGAGGTTACCTTCGGGTAACCAAAACCGGC
GCACGGGTGCGTAACGCGTATACAATCTGCCTTTCACATAGGGATAGCCCGAAGAAATTTGGATTAAT
ACCTAATGGTATATAAGAGCGGCATCGTTTTTATATTAAAGCTACGGTGGTGAAAGATGAGTATGCGT
TCTATTAGCTAGTTGGAGAGGTAACGGCTCCCCAAGGCGACGATAGATAGGGGTTCTGAGAGGGATGT
CCCCCACACTGGTACTGAGATACGGAC
>Zotu1486
GAACGCTAGCGGCAGGCTTAATACATGCAAGTCGTGGGGCAGCAGGACTGTAGCAATACAGTTGCTGG
CGACCGGCAAACGGGTGCGGAACACGTACGCAACCTACCCAAAACTGGGGGATAGCCCGGGGAAACCC
GGATTAATACCTCGTAACATAATGGTGTGGCATCACATTATTATTATAGCTCCGGCGGTTTTGGATGG
GCGTGCGCCTGATTAGGTAGTTGGCGGGGTAACGGCCCACCAAGCCTGCGATCAGTAACTGGTGTGAG
AGCACGACCAGTCACACGGGCACTGAGACACGGGC
>Zotu1487
GAACGCTAGCGGCAGGCCTAATACATGCAAGTCGAACGGTTGTATTTATACAATAGTGGCGCACGGGT
GCGTAACACGTATGAAACCTACCTTATAGACGGGGATAGCCTTTGGAAACGGAGATTAATACCCGATG
GTATATATTGATGGCATCATTAATATATTAAAGATTTATCGCTATAAGCTGGTCATGCGTCCAATTAG
TTAGTTGGCGGGGTAATGGCTCACCAAGACGATGATTGGTAGGGGAACTGAGAGGTTAATCCCCCACA
CTGGCACTGAGATACGGGC
>Zotu1488
GAACGTTGATGGCGTGCCTCATACATGCAAGTCGAGCGCCCCGCAAGGGGAGCGGCGGACGGGTGAGT
AACACGTAAAGAATCTGCCCTGAAGCGGGGAATAACTATCCCAACGGATAGCTAATACCGCATAATGA
TGGAGACGGCATCGTCTCTTCTCAAAAGGCTTCGGCCACTTCAGGATGACTTTGCGTCCCATTAGCTT
GTTGGCGGGGTAACGGCCCACCAAGGCTACGATGGGTATCCGGCCTGAGAGGGTGTACGGACACATTG
GGACTGAGATACGGCC
>Zotu1489
GAACGCTAGCGGCAGGCCTAATACATGCAAGTCGGACGGGATTGGAGAGCTTGCTTTCCATGAGAGTG
GCGCACGGGTGCGTAACACGTATGTAACCTACCCTTATCAGGGGGATAGCCTCTCGAAAGAGAGATTA

AGCCCGCATAATATGAAGACACAGCATTGTGATTTCATCAAATATTTATAGGATAAGGATGGGCATGC
GGAACATTAGCTAGTTGGTAAGGTAATGGCTTACCAAGGCTACGATGTTTAGGGGATCTGAGAGGATG
ACCCCCCACACTGGTACTGAGACACGGAC
>Zotu1490
GAACGCTAGCAGAACGCTTAACACATGCAAGTCGAACGAATATTAGTCTTCGGATTAATGTTAGTGGC
AAACGGGCGAGTAACGCGTATCTAACCTGCCCTTGAGTGAGGCACAACTATGGAGACATAGCTAATTC
CTCATACGCTCTAGAGAATGAAAATATCTAGAGGAAAGATTTATCGCTCAAGGAGGGGGATGCGTCCT
ATCAGGTAGTTGTTGAGGTAACGGCTCAACAAGCCTATGACGGGTAGCCGGTCTGAGAGGATGTCCGG
CCGCAATGGAACTGAGAACGGTC
>Zotu1491
GAACGCTGGCGGCGTGCCTAATACATGCAAGTCGAGCGAACCTTTGGGAGCTTGCTCCCAAAGGTTAG
CGGCGGACGGGTGAGTAACACGTGGGCAACCTGCCTATAAGACTGGGATAACTTCGGGAAACCGGAGC
TAATACCGGATAATTCTTTTCTACTCATGTAGAAAAGCTAAAAGACGGTTTACGCTGTCACTTATAGA
TGGGCCCGCGGCGCATTAGCTAGTTGGTGAGGTAACGGCTCACCAAGGCCACGATGCGTAGCCGACCT
GAGAGGGTGATCGGCCACACTGGGACTGAGACACGGCC
>Zotu1492
GAACGCTGGCGGCGTGCCTAATACATGCAAGTAGAACGCACAGTTTATACCGTAGCTTGCTACACCAT
AGACTGTGAGTTGCGAACGGGTGAGTAACGCGTAGGTAACCTGCCTGGTAGCGGGGGATAACTATTGG
AAACGATAGCTAATACCGCATAATATTAATTATTGCATGATAATTGATTGAAAGGTGCAAATGCATCA
CTACCAGATGGACCTGCGTTGTATTAGCTAGTAGTTGAGGTAACGGCTCACCTAGGCGACGATACATA
GCCGACCTGAGAGGGTGATCGGCCACACTGGGACTGAGACACGGCC
>Zotu1493
GAACGCTGGCGGCGTGCCTAATACATGAAAGTAGAACGCTGAAGACTTTAGCTTGCTAGAGTTGGAAG
AGTTGCGAACGGGTGAGTAACGCGTAGGTAACCTGCCTACTAGCGGGGGATAACTATTGGAAACGATA
GCTAATACCGCATAATAGTATTTAACACATGTTAGAGACTTGAAAGATGCAATTGCATCACTAGTAGA
TGGACCTGCGTTGTATTAGCTAGTTGGTGGGGTAACGGCCTACCAAGGCGACGATACATAGCCGACCT
GAGAGGGTGATCGGCCACACTGGGACTGAGACACGGCC
>Zotu1494
GAACGCTGGCGGCGTGCCTAATACATGCAAGTCGAGCGGTAAGGCCTCTTCGGAGGTACACGAGCGGC
GGACGGCTGAGTAACACGTAGGAACGTACCCCAAACTGAGGGATAAGCACCAGAAATGGTGTCTAATA
CCGCATGTGATCTTCGGATTAAAGCTTCGGCGGTTTGGGAACGGCCTGCGCATGATTAGCTTGTTGGT
GAGGTAAAAGCTCACCAAGGCGACGATCATTAGCTGGTCTGAGAGGATGATCAGCCAGACTGGGACTG
AGAACGGCC
>Zotu1495
GAACGCTGGCGGCGTGCTTAACACATGCAAGTCGAACGATGATGCCAGCTTGCTGGTGGATTAGTGGC
GAACGGGTGAGTAACACGTGAGCAACCTGCCCCTGACTTCGGGATAACCGCGGGAAACCGTGGCTAAT
ACCGGATATGACGCGCTATCGCATGGTGGTGTGTGGAAAGATTTATCGGTTGGGGATGGGCTCGCGGC
CTATCAGCTTGTTGGTGGGGTGATGGCCTACCAAGGCGACGACGGGTAGCCGGCCTGAGAGGGTGACC
GGCCACACTGGGACTGAGACACGGCC
>Zotu1496
GAACGCTGGCGGCGTGCTTAACACATGCAAGTCGAACGATGAAGCCCAGCTTGCTGGGTGGATTAGTG
GCGAACGGGTGAGTAACACGTGAGTAACCTGCCCTTGACTCTGGGATAAGCCTGGGAAACTGGGTCTA
ATACCGGATAGGAACGTCCACCGCATGGTGGGTGTTGGAAAGATTTATCGGTCATGGATGGACTCGCG
GCCTATCAGCTTGTTGGTGAGGTAATGGCTCACCAAGGCGACGACGGGTAGCCGGCCTGAGAGGGTGA
CCGGCCACACTGGGACTGAGACACGGCC
>Zotu1497
GAACGCTAGCGGCAGGCCTAATACATGCAAGTCGAGGGGCAGCACGGTGTAGCAATACACTGGTGGCG
ACCGGCGCACGGGTGCGTAACACGTATGTAATTTGCCCATAACTGGGGGATAGCCTTCCGAAAGGAAG
ATTAATACCCCATAACCCAGCGAAGTGGCATCACTTTTCTGGTAAACCTCCGGGGGTTATGGATAAGC
ATGCGTCCGATTAGCTAGTTGGCGAGGTAACGGCTCACCAAGGCAACGATCGGTAGGGGGTCTGAGAG
GACGATCCCCCACACGGGTACTGAGACACGGAC
>Zotu1498
CAACGCTGGCGGCGCGCTTAACACATGCAAGTCGCACGAGAAAGTGGAGCAATCCATGAGTACAGTGG
CGTACGGGTGAGTAACACGTGGATCATCTACCTCTTGGTGGGGAATAACCCTGGGAAACCGGGGCTAA

TACCGCATAAGCCTGCAAAGGGAAAGCAGCAATGCGCCGAGAGAGGAGTCCGCGGCCGATTAGCTGGT
TGGTAGGGTAACGGCCTACCAAGGCAATGATCGGTAGCCGGCCTGAGAGGGCACACGGCCACACTGGC
ACTGAAACACGGGC
>Zotu1499
GAACGCTGGCGGCGTGCTTAACACATGCAAGTCACGGGGGCCCGCAAGGGCAACCGGCGAACGGGTGC
GTAACACGTGAGCAATCTGCCGATCTCTGGGGGATAGCCGGCCCAACGGCCGGGTAATACCGCATACG
ATCACCGAGGGGATTCCCTTGGTGATGAAACCTCCGGGGGAGATCGAGGAGCTCGCGCCCTATCAGCT
AGTTGGCGGGGTAATGGCCCACCAAGGCTACGACGGGTAGCTGGTCTGAGAGGATGGCCAGCCACATT
GGGACTGCGACACGGCC
>Zotu1500
AAACGCTGGCGGCGTGCCTAATACATGCAAGTCGAACGAGGTAGCAATACCTAGTGGCGCACGGGTGA
GTAACGCGTGGATAATCTGCCTTTGAGTGGGGGATAACTAGTCGAAAGATTAGCTAATACCGCATAAG
ACCACAGGAACTGAGGTTCTAGGGGTTAAAGGTTTTTCGCTCATAGATGAGTCCGCGTGAGATTAGCT
AGTTGGTGAGGTAACGGCTCACCAAGGCGACGATCTCTAACTGGTCTGAGAGGATGATCAGTCACACT
GGAACTGAGACACGGTC
>Zotu1501
GAACGCTGGCGGCGTGCCTAAGACATGCAAGTCGAGTGGGTTTAGACCCACGGCGAACGGGTGAGTAA
TACCTAGGAACGTCCCTTAAGTTGAGGATATCCCATCGAAAGATGGTGCAATACTCAATGTGATCGCA
AGATCAAAGTCGCAAGACGCTTAAGGTGCGGCCTGGGCACCATCAGCTAGTTGGTAAGGTAATGGCTT
ACCAAGGCTATGACGGTTAGGGGCCCCGAGAGGGGAGACCCCCACACTGGAACTGCGACACGGTC
>Zotu1502
GAACGCTGGCGGCATGCTTTACACATGCAAGTCGAACGGCAGCGCGGGGCAACCTGGCGGCGAGTGGC
GAACGGGTGAGTAATATATCGGAACGTACCCAAGAGTGGGGGATAACGTAGCGAAAGTTACGCTAATA
CCGCATACGATCTCAGGATGAAAGCAGGGGACCGCAAGGCCTTGTGCTCCTGGAGCGGCCGATATCTG
ATTAGCTAGTTGGTGAGGTAAAGGCTCACCAAGGCGACGATCAGTAGCTGGTCTGAGAGGACGACCAG
CCACACTGGAACTGAGACACGGTC
>Zotu1503
GAACGCTAGCGGCAGGCCTAATACATGCAAGTCGGACGGGATTCCGTTGTAGCTTGCTATGACGGATG
AGAGTGGCGCACGGGTGCGTAACGCGTGAGCAACCTGCCCATGTCAGGGGGATGGCCCGTTGAAAGAC
GGATTAACACCGCATAATACATTATGCCCGCCTGGGTATTCTGTTAAATATTTATAGGACATGGATGG
GCTCGCGTGGCATTAGCTTGTTGGTAAGGTAACGGCTTACCAAGGCTATGATGCCTAGGGGCTCTGAG
AGGAGAATCCCCCACACTGGTACTGAGACACGGAC
>Zotu1504
GAACGCTGGCGGCGTGCTTAACACATGCAAGTCGAACGGGCCCGCCCCTGTTTTTGGGGGTGGGTTGA
GTGGCGAACGGGTGAGTATCACGTGAGTAACCTGCCCCCTGCTCCTGGATAACTGCTTGAAAGGGCGG
CTAATACGGGGTGTGCCGGCTGGTCCGCATGGGCCTGGTCGGGATAGTTTCATCTTCGGGTGTTTCGG
TGGGGGATGGGCTCGCGGCCTATCAGCTTGTTGGTGGGGTGATGGCCCACCAAGGCGGTGACGGGTAG
CCGGCCTGAGAGGGTGGGCGGCCACACTGGGACTGAGACACGGCC
>Zotu1505
GAACGCTGGCGGCAGGCTTAACACATGCAAGTCGAGCGGGCGTAGCAATACGTCAGCGGCAGACGGGT
GAGTAACGCGTGGGAACGTACCTTTTGGTTCGGAACAACACAGGGAAACTTGTGCTAATACCGGATAA
GCCCTTACGGGGAAAGATTTATCGCCGAAAGATCGGCCCGCGTCTGATTAGCTAGTTGGTGGGGTAAT
GGCCCACCAAGGCGACGATCAGTAGCTGGTCTGAGAGGATGATCAGCCACATTGGGACTGAGACACGG
CC
>Zotu1506
GAACGCTGGCGGCGTGCCTAATACATGCAAGTCGAGCGGTAAGGCTCTGCACAGTTTATTTAAGTGAT
GAGATTGGGAATGTTACGGCTGTAACCCCTTAAGAAGCGAAAGTTTCTCTCGTTCTTTACTACCTTGG
TAGTTGAAGATAGCTTGAATAAAGTGTGTAGAGTACACGAGCGGCGGACGGCTGAGTAACGCGTAGGA
ATGTACCCCAAAGTGAGGGATAAGCACCGGAAACGGTGTCTAATACCGCATGTGATCTTCGGATTAAA
GCTTTATGCGCTTTGGGAACAGCCTGCGTCTGATTAGCTTGTTGGTGAGGTAATAGCTCACCAAGGCT
TCGATCGGTAGCTGGTCTGAGAGGATGATCAGCCAGACTGGGACTGAGACACGGCC
>Zotu1507
GAACGCTGGCGGCGTGCTTAACACATGCAAGTCGAGCGGAGTTAATTGAAAGCTTGCTTTTGATTAAC
TTAGCGGCGGACGGGTGAGTAACGTGTGGGCAACCTGCCCTGTACTGTGGAATAATCACTTGAAAAGG

TGACTAATACCGCATGTGGTCCCTGGAAGGCATCTTCTGGGGAAGAAAGGATTTATTCGGTACAGGAT
GGGCCCGCATCTGATTAGCTAGTTGGTGAGATAACAGCCCACCAAGGCGACGATCAGTAGCCGACCTG
AGAGGGTGATCGGCCACATTGGGACTGAGACACGGCC
>Zotu1508
GAACGCTAGCGGCAGGCTTAACACATGCAAGTCGAGGGGTATAGGTTTTCGGACCTAGAGACCGGCGC
ACGGGTGCGTAACGCGTATGCAATCTACCTTTTACAGAGGGATAGCCCAGAGAAATTTGGATTAATAC
CTCATAGTATTATTGAATGGCATCATTTAATAATTAAAGTCACAACGGTAAAAGATGAGCATGCGTCC
CATTAGCTAGTTGGTAAGGTAACGGCTTACCAAGGCAACGATGGGTAGGGGTCCTGAGAGGGAGATCC
CCCACACTGGTACTGAGACACGGAC
>Zotu1509
GAACGCTGGCGGCAGGCCTAACACATGCAAGTCGAGCGCGCCCTTCGGGGTGAGCGGCGGACGGGTGA
GTAACGCGTGGGAACGTGCCCTTCTCTGCGGAATAGCCTCGGGAAACTGAGAGTAATACCGCATACGC
CCTTTGGGGGAAAGATTTATCGGAGAAGGATCGGCCCGCGTTGGATTAGGTAGTTGGTGGGGTAATGG
CCCACCAAGCCGACGATCCATAGCTGGTTTGAGAGGATGATCAGCCACACTGGGACTGAGACACGGCC
>Zotu1510
GAACGCTGGCGGCGTGCCTAACACATGCAAGTCGAACGGAAACAGGTCTAGTTTACTAGATGCTGTCG
AGTGGCGGACGGCTGAGTAACGCGTGGGAACGTGCCCCAAAGTGAGGGATAACTTCGAGAAATCGGAG
CTAATACCGCATATGATCTTCGGATTAAAGAATTTATTCGCTTTGGGAGCGGCCTGCGTCGGATTAGG
TTGTTGGTGAGGTAATGGCTCACCAAGCCGACGATCCGTAGCTGGTCTGAGAGGATGACCAGCCAGAC
TGGAACTGAGACACGGTC
>Zotu1511
GAACGCTGGCGGCGCGCTTCATACATGCAAGTCGAACGAAAAGCCCGGGCTTGCCCGGGTGGACAGTG
GCGAACGGGTGAGTAACACGTGAGCAACCTGCCCCGAAGACTGGGACAACACCGGGAAACCGGTGCTA
ATACCGGATGCCCTCACCACACCGCATGACGTGGTGAGGAAATGGATTCCGCTTCGGGATGGGCTCGC
GGTCTATCAGCTTGTTGGTGGGGTAACGGCCTACCAAGGCGACGACGGATAGCTGGTCTGAGAGGATG
TCCAGCCACACTGGGACTGAGACACGGCC
>Zotu1512
GAACGCTGGCGGCATGCCTAACACATGCAAGTCGGACGGGAAGTGGTGTTTCCAGTGGCGGACGGGTG
AGTAACGCGTAAGAACCTGCCCTTGGGAGGGGAACAACAACTGGAAACGGCTGCTAATACCCCGTAGG
CTGAGGAGCAAAAGGAGGAATCTGCCCGAGGAGGGGCTCGCGTCTGATTAGCTAGTTGGTGAGGCAAT
AGCTTACCAAGGCGATGATCAGTAGCTGGTCCGAGAGGATGATCAGCCACACTGGGACTGAGACACGG
CC
>Zotu1513
GAACGCTGGCGGCGTGCTTAACACATGCAAGTCGAACGGTGAACTCCGCTTGCGGGGGGATCAGTGGC
GAACGGGTGAGTAACACGTGGGCAACCTGCCCTCAGCTCTGGGATAACTCCAAGAAATTGGGGCTAAT
ACTGGATATGACCGCTGACCGCATGGTCTGGTGGTGGAAAGATTTATCGGCTGGGGATGGGCCCGCGG
CCTATCAGCTTGTTGGTGGGGTAGTGGCCTACCAAGGCGACGACGGGTAGCCGGCCTGAGAGGGTGAC
CGGCCACACTGGGACTGAGACACGGCC
>Zotu1514
GAACGCTGGCGGCGTGCCTAACACATGCAAGTCGAACGGAGATTTCATTTATGGAATCTTAGTGGCGA
ACGGGTGAGTAACGCGTAGACAACCTACCTTTTAGATGGGGACAACACTGCGAAAGTGGTGCTAATAC
CGAATGTTGCATTTTGGTTGCATAACCGAAATACTAAAGATGGCCTCTATTTATAAGCTATCGCTAAA
AGATGGGTCTGCGTCTGATTAGCTAGTTGGTGAGGTAATGGCTCACCAAGGCGACGATCAGTAGCCGG
TCTGAGAGGATGAACGGCCACACTGGGACTGAGACACGGCC
>Zotu1516
GAACGCTAGCGGCAGGCTTAACACATGCAAGTCGAGGGGTATAGTAGCAATACTAGAGACCGGCGCAC
GGGTGCGTAACGCGTATGCAATCTACCTTTTACAGCGGGATAGCCCAGAGAAATTTGGATTAATACCA
CATAGTATTATTGAGTGGCATCACTTAATAATTAAAGTTACAACGGTAAAAGATGAGCATGCGTCCCA
TTAGCTAGTTGGTAAGGTAACGGCTTACCAAGGCGACGATGGGTAGGGGTCCTGAGAGGGAGATCCCC
CACACTGGTACTGAGACACGGAC
>Zotu1517
CAACGCTGGCGGCGTGCCTAATGCATGCAAGTCGAACGGGGTCATCTTCGGATGGCTTACGTGGCGGA
CGGGTGAGGAGCACGTGGGCAACCTGCCCTTTGGTGGAGGATAACCGCTGGAAACAGTGGGTAATACT
GCATAACGCATTTTGACGAGTGGGTTGGAATGTAAAAGTCGCAAGACGCCGAAGGAGGGGCCTGCGTC

CGATTAGCTAGACGGTGGGGTAACGGCCTACCGTGGCGACGATCGGTAGCTGGTTTGAGAGGATGATC
AGCCACACGGGGACTGAGACACGGCC
>Zotu1518
GAACGCTGGCGGCGTGCTTAACACATGCAAGTCGAGCGGTAAGGCCCTTCGGGGTACACGAGCGGCGA
ACGGGTGAGTAACACGTGGGTGACCTGCCCTCCACTCTGGGATAAGCCTGGGAAACCGGGTCTAATAC
CGGATAGGACTCCTGGATGCATGTCTAGGGGTGGAAAGTTTTTTCGGTGGGGGATGGGCCCGCGGTCT
ATCAGCTTGTTGGTGGGGTGATGGCCTACCAAGGCGACGACGGATAGCCGGCCTGAGAGGGTGATCGG
CCACACTGGGACTGAGACACGGCC
>Zotu1519
GAACGCTGGCGGCGTGCCTAATACATGCAAGTCGAGCGGAAATTTTATTGGTGCTTGCACCTTTAAAA
TTTTAGCGGCGGACGGGTGAGTAACACGTGGGTAACCTACCTTATAGATTGGAATAACTCCGGGAAAC
CGGGGCTAATACCGAATAATACTTTTTAACACATGTTTGAAAGTTGAAAGACGGTTTCGGCTGTCACT
ATAAGATGGACCCGCGGCGCATTAGCTAGTTGGTGAGGTAACGGCTCACCAAGGCAACGATGCGTAGC
CGACCTGAGAGGGTGATCGGCCACACTGGGACTGAGACACGGCC
>Zotu1520
GAACGCTGGCGGCGTGCTTAACACATGCAAGTCGAACGATTAAAGCCCCTTCGGGGGTGTATAGAGTG
GCGAACGGGTGAGTAACACGTGGGCAATCTGCCCCTCACTTCGGGATAACTCAGGGAAACTTGAGCTA
ATACCGAATACTCCGAGCGGATCGCATGGTCTGCTCGGGAAAGCTCCGACGGTGAGGGATGAGCCCGC
GGCCCATTAGCTAGTTGGTGAGGTAATGGCCCACCAAGGCAACGATGGGTAGCCGCGCTGAGAGGCGG
ATCGGCCACACTGGGACTGAGACACGGCC
>Zotu1521
TAACGCTGGCGGCGTGCATAACACATGCAAGTCGAACGATGAAACTTAAAAGATTTCTTCGGAATGAA
CTTAAGTGGATTAGTGGCGAACGGGTGAGTAACGCGTGAGTAACCTGCCTTACACAAGGGGATAGCCT
CTGGAAACGGAGAATAATACCCTATGAAACTATCTTATCGCATGAAAAGATAGTCAAAGTGTTTTAGC
GGTGTAAGATGGACTTGCGTCTGATTAGCTAGTTGGTGAGATAACAGCCCACCAAGGCAACGATCAGT
AGCCGGCTTGAGAGAGTGTACGGCCACATTGGGACTGAGACACGGCC
>Zotu1522
GAACGCTGGCGGCGTGCCTAATACATGCAAGTCGAGCGAAGTTTTTCTGGTGCTTGCACTAGAAAAAC
TTAGCGGCGAACGGGTGAGTAACACGTAAAGAACCTGCCTCATAGACTGGGACAACTATTGGAAACGA
TAGCTAATACTGGATAACAGCATTAACTGCATGGTTGATGTTTAAAAGTTGGTTTTGCTAACACTATG
AGATGGCTTTGCGGTGCATTAGCTAGTTGGTGGGGTAAAGGCCTACCAAGGCGACGATGCATAGCCGA
CCTGAGAGGGTGATCGGCCACACTGGGACTGAGACACGGCC
>Zotu1523
GAACGCTGGCGGAATGCTTTACACATGCAAGTCGAACGGCAGCACGAGGGCAACCTTGGTGGCGAGTG
GCGAACGGGTGAGTAATATATCGGAACGTGCCCAGTCGTGGGGGATAACGTAGTGAAAATTACGCTAA
TACCGCATACGATCTATGGATGAAAGCGGGGGATCGCAAGACCTCGCGCGATTGGAGCGGCCGATATC
AGATTAGGTAGTTGGTGGGGTAAAGGCTCACCAAGCCAACGATCTGTAGCTGGTCTGAGAGGACGACC
AGCCACACTGGAACTGAGACACGGTC
>Zotu1524
GAACGCTGGCGGCGTGCTTAACACATGCAAGTCACGGGGGCCCGCAAGGGTAACCGGCGAACGGGTGC
GTAACACGTGAGCAATCTGCCGTCCACTGGGGGATAGCCGGCCCAACGGCCGGGTAATACCGCATACG
CTTGCCGGGTGGCATCACTTGGCAAGGAAACCTCCGGGGGTGGACGAGGAGCTCGCGGCCTATCAGCT
AGTTGGTGGGGTCACGGCCCACCAAGGCGATGACGGGTAGCTGGTCTGAGAGGATGGCCAGCCACATT
GGGACTGAGATACGGCC
>Zotu1525
GAACGCTAGCTACAGGCTTAACACATGCAAGTCGAGGGGAAACGATGAGAGAGCTTGCTCTCTTAGGC
GTCGACCGGCGCACGGGTGAGTAACGCGTATCCAACCTGCCCATTACAGGGGAATAACCTTGCGAAAG
TAAGACTAATGCCCCATGGTATTCTCAGCAGACATCTAATGAGGATTAAAGATTTATCGGTAATGGAT
GGGGATGCGTCTGATTAGCTTGTTGGCGGGGTAACGGCCCACCAAGGCAACGATCAGTAGGGGTTCTG
AGAGGAAGGTCCCCCACATTGGAACTGAGACACGGTC
>Zotu1526
GAATGCTGGCGGCGTGGATAAGGCATGCAAGTCAAGGGGACCGCAAGGTAACCGGCAGACGAGGTAGT
AACACGTACGAACGCACCCCAGAGTCTGGCATAGCTCGTCGAAAGACGGGGTAATTCCGGATGGTCCC
GAAAGGGTAAAGTTTTTTCGCTCTGGGAGCGGCGTGCGTAATATCAGCTTGTTGGTGAGGTAATGGCT

CACCAAGGCTACGACGTTTAGGGGCGCTGAGAGGCGGACCCCCACCGATGGGACTGAGACACGGCC
>Zotu1527
GAACGCTGGCGGCATGCCTAACACATGCAAGTCGAACGAAGGCTTCGGCCTTAGTGGCGCACGGGTGC
GTAACGCGTGGGAATCTGCCCTTGGGTCTGGGATAACTCAGAGAAATTTGAGCTAATACCGGATGATG
ACTTCGGTCCAAAGATTTATCGCCCGAGGATGAGCCCGCGTTGGATTAGCTAGTTGGTGGGGTAAAGG
CCTACCAAGGCGACGATCCATAGCTGGTCTGAGAGGATGATCAGCCACACTGGGACTGAGACACGGCC
>Zotu1528
GAACGCTGGCGGCAGGCCTAACACATGCAAGTCGAGCGGATGAGAGGGGCTTGCTCTTCGATTCAGCG
GCGGACGGGTGAGTAATGCCTAGGAATCTGCCTAGTAGTGGGGGACAACGTTTCGAAAGGAACGCTAA
TACCGCATACGTCCTACGGGAGAAAGTGGGGGATCTTCGGACCTCACGCTATTAGATGAGCCTAGGTC
GGATTAGCTAGTTGGTAGGGTAAAGGCCTACCAAGGCGACGATCCGTAACTGGTCTGAGAGGATGATC
AGTCACACTGGAACTGAGACACGGTC
>Zotu1529
GAACGCTGGCGGCATGCTTAACACATGCAAGTCGCACGGTCAGGTTTCGGCCTGGCAGTGGCGGACGG
GTGAGTAACGCGTAGGTATCTATCCATGGGTGGGGGATAACGGTTGGAAACGACTGCTAATACCGCAT
GATACCCGAGGGTCAAAGGCGCAAGTCGCCTGTGGAGGAGCCTGCGTACGATTAGCTAGTTGGTGGGG
TAAAGGCCTACCAAGGCGACGATCGTTAGCTGGTCTGAGAGGATGATCAGCCACACTGGGACTGAGAC
ACGGCC
>Zotu1530
GAACGCTAGCGGCAGGCTTAATACATGCAAGTCGAGGGGCAGCGCAGTGTAGCAATACATGGGCGGCG
ACCGGCAAACGGGTGCGGAACACGTACGCAACCTTCCTTCAAATGGGGGATAGCCCAGGGAAACTTGG
ATTAATACCCCGTAACATTATGAAATGGCATCATTTTGTAATTATAGCTACGGCGTTTGAAGATGGGC
GTGCGCCTGATTAGGTAGTTGGCGGGGTAACGGCCCACCAAGCCTTCGATCAGTAACTGGTGTGAGAG
CACGACCAGTCACACGGGCACTGAGACACGGGC
>Zotu1531
GAACGCTGGCGGCAGGCCTAACACATGCAAGTCGAGCGCCCCGCAAGGGGAGCGGCAGACGGGAGAGT
AACACGTGGGAACGTACCCTTCAGTTCGGAACAACCCAGGGAAACTTGGGCTAATACCGGATACGTCC
GTAAGGAGAAAGATTTATCGCTGAAGGATCGGCCCGCGTCTGATTAGCTAGTTGGTGAGGTAACGGCT
CACCAAGGCGACGATCAGTAGCTGGTCTGAGAGGATGATCAGCCTCACTGGGACTGAGACACGGCC
>Zotu1532
GAACGCTGGCGGCGTGCTTAACACATGCAAGTCGAACGATGACGGTGGTGCTTGCACCGCCTGATTAG
TGGCGAACGGGTGAGTAACACGTGAGTAACCTGCCCTCCACTTCGGGATAACCTCGGGAAATCGTGGC
TAATACCGGATATGAGCACTCATCGCATGGTGGGTGTTGGAAAGATTTATCGGTGGGGGATGGACTCG
CGGCCTATCAGTTTGTTGGTGAGGTGATGGCTCACCAAGACGATGACGGGTAGCCGGCCTGAGAGGGC
GACCGGCCACACTGGGACTGAGACACGGCC
>Zotu1533
GAACGCTGGCGGCGTGCTTAACACATGCAAGTCGAGCGGTAAGGCCCTTCGGGGTACACGAGCGGCGA
ACGGGTGAGTAACACGTGAGCAATCTGCCCTCCACATCGGGATAACCCCCGGAAACGGGAGCTAATAC
CGGATACGACCACTTCAGGCATCTGATGGTGGTGGAAAGTTCCGGCGGTGGAGGATGAGCTCGCGGCC
TATCAGCTTGTTGGTGAGGTAATGGCCCACCAAGGCTTCGACGGGTAGCCGGCCTGAGAGGGTGACCG
GCCACACTGGGACTGAGACACGGCC
>Zotu1534
GAACGCTGGCGGCGTGCCTAATACATGCAAGTCGAACGGTCTTTTCTATGGAAGCTTGCTTCCACTGA
GAAGATAGTGGCGAACGGGTGAGTAACACGTGGGTAACCTGCCCATAAGAGGGGGATAACATCCGGAA
ACGGGTGCTAATACCGCATAGTTTTCTTGATCGCATGATCGGGAAAGGAAAGACGGCCTTTGTGCTGT
CGCTTATGGATGGACCCGCGGCGTATTAGTTAGTTGGTGAGGTAACGGCTCACCAAGACGATGATACG
TAGCCGACCTGAGAGGGTGATCGTCCACATTGGGACTGAGACACGGCC
>Zotu1535
GAACGCTAGCGGCAGGCTTAATACATGCAAGTCGAGGGGCAGCACGGGTAGCAATACCTGGTGGCGAC
CGGCAAACGGGTGCGGAACACGTACGCAACCTTCCCAAAACTGGTGAATAGCCTTTCGAAAGAGAGAT
TAATACACCGTAAGATAACGAAGAGGCATCTCTTTGTTATTATAGTAGCAATACGGTTTTGGATGGGC
GTGCGCCTGATTAGGTAGTTGGCGGGGTAACGGCCCACCAAGCCTGCGATCAGTAACTGGTGTGAGAG
CACGACCAGTCACACGGGCACTGAGACACGGGC
>Zotu1536

GAACGCTGGCGGCGTGCCTAATACATGCAAGTCGAACGGTAACACCTCGGGTGACGAGTGGCGGACGG
CTGAGTAACGCGTAGGAATGTACCCCAAAGTGAGGGATAATCCACCGAAAGGTGGACTAATACCGCAT
ATTATCTTCGGATCAAAGCAGCAATGCGCTTTGGGAACAGCCTGCGTCTGATTAGCTAGTTGGTGAGG
TAATGGCTCACCAAGGCGACGATCGGTAGCTGGTCTGAGAGGATGATCAGCCAGACTGGGACTGAGAC
ACGGCC
>Zotu1537
GAACGCTGGCGGTATGCTTTACACATGCAAGTCGTACGAGAGTTTTTTACTCTAGTGGCGGACGGGTG
AGTAACACGTAAGAATTTGCCTTTAGGAGAGGGATAACAACTGGAAACGGTTGCTAATACCTCATATG
CTTTCGAGTGAAATGAATTTTTTTCGCCTAAAGAGAAGCTTGCGCCTGATTAGCTTGTTGGTAAGGTA
ATGGCTTACCAAGGCGACGATCAGTATCTGGTTTGAGAGGATGATCAGACACACTGGAACTGAGACAC
GGTC
>Zotu1538
GAACGCTAGCGGGAGGCCTAACACATGCAAGCCGAGCGGTATTGCTTCTTCGGAGGTGAGAGAGCGGC
GTACGGGTGCGGAACACGTGTGCAACCTGCCCTTATCAAGGGGATAGCCCAGAGAAATTTGGATTAAT
ACCCTATAACATATAGAGTGGCATCACTTTATATTAAAAACTACGGTGGATAAGGATGGGCACGCGCA
AGATTAGCTAGTTGGTGAGGTAACGGCTCACCAAGGCGACGATCTTTAGGGGGCCTGAGAGGGTGATC
CCCCACACTGGGACTGAGACACGGCC
>Zotu1539
GAACGCTGGCGGCGTGCTTAACACATGCAAGTCGAACGGTGAAGCGGGTGCTTGCACTCGTGGATCAG
TGGCGAACGGGTGAGTAACACGTGAGTAACCTGCCCTCCACTCTGGGATAAGCGCTGGAAACGGCGTC
TAATACTGGATACGACCGACCCTCGCATGGGGTGTTGGTGGAAAGATTTATCGGTGGTGGATGGACTC
GCGGCCTATCAGCTTGTTGGTGGGGTAATGGCCTACCAAGGCGACGACGGGTAGCCGGCCTGAGAGGG
CGACCGGCCACACTGGGACTGAGACACGGCC
>Zotu1540
GAACGCTAGCGGGAGGCCTAACACATGCAAGCTGAGCGGTAGAGTTCCTTCGGGGACTTGAGAGCGGC
GTACGGGTGCGGAACACGTGTGCAACCTGCCTTTATCAGGGGGATAGCCTTTCGAAAGGAAGATTAAT
ACCCCATAATATAATTGATGGCATCATTGATTATTGAAAACTCCGGTGGATAAAGATGGGCACGCGCA
AGATTAGATAGTTGGTGAGGTAACGGCTCACCAAGTCGATGATCTTTAGGGGGCCTGAGAGGGTGATC
CCCCACACTGGTACTGAGACACGGAC
>Zotu1541
GAACGCTGGCGGCGTGCTTAACACATGCAAGTGGAGCGACGAACCAGGCTTCGGCCTGGGGCAGAGCC
GCGAACGGGTGAGTAACACGTGGGTAACGTGCCCCGATGATCGGGACAACCCGGGGAAACCCGGGCTA
ATACCGAATGTGCTCCCCTCACACCAGTGAGGGGTGGAAAGGAAGCTTCGGCCTCCGCATCGGGATCG
GCCCGCGGCCCATTAGCTTGTTGGTGAGGTAACGGCTCACCAAGGCTGCGATGGGTAGCTGGTCTGAG
AGGACGATCAGCCACACTGGGACTGAGACACGGCC
>Zotu1543
GAACGCTAGCTACAGGCTTAACACATGCAAGTCGAGGGGCAGCATTGGAGTAGCAATACTTCAGATGG
CGACCGGCGCACGGGTGAGTAACACGTATCCAACCTGTCGCTTACTCGGGGATAGCCTTTCGAAAGAA
AGATTAATACCCGATGGCATAAAGAGTTCGCATGTTCTCTTTATTAAAGAATTTCGGTAAGCGATGGG
GATGCGTTCCATTAGATAGTTGGCGGGGTAACGGCCCACCAAGTCTACGATGGATAGGGGTTCTGAGA
GGAAGGTCCCCCACATTGGAACTGAGACACGGTC
>Zotu1544
GAACGCTAGCGGGAGGCCTAACACATGCAAGCCGAGCGGTATTGTTTCTTCGGAAATGAGAGAGCGGC
GTACGGGTGCGGAACACGTGTGCAACCTGCCTTTATCTGGGGGATAGCCTTTCGAAAGGAAGATTAAT
ACACCATAACATATTGAACGGCATCGTTTAATATTGAAAGCTCCGGCGGATAGAGATGGGCACGCGCA
AGATTAGCTAGTTGGTGAGGTAACGGCTCACCAAGGCGATGATCTTTAGGGGGCCTGAGAGGGTGATC
CCCCACACTGGTACTGAGACACGGAC
>Zotu1545
GAACGCTAGCGGGAGGCCTAACACATGCAAGCCGAGCGGTATTGTTTCTTCGGAAATGAGAGAACGGC
GTACGGGTGCGGAACACGTGTGCAACCTGCCTTTATCTGGGGGATAGCCTTTCGAAAGGAAGATTAAT
ACTCCATAACATATTGATTGGCATCAATTAATATTGAAAGCTCCGGCGGATAGAGATGGGCACGCGCA
AGATTAGCTAGTTGGTGAGGTAACGGCTCACCAAGGCGATGATCTTTAGGGGGCCTGAGAGGGTGATC
CCCCACACTGGTACTGAGACACGGAC
>Zotu1546

GAACGCTAGCGGCAGGCCTAATACATGCAAGACGAACGGTAACTTGAGTAGCAATATTTGAGCAAGAG
TGTCGTAAGGGTGCGTAACACGTATGCAATCTGCCCTCAACAGGAGGATAGCTCCCCGAAAGGGGAAT
TAACACTCCATAAGATCATGGTCTGGCATCAGACAGTGATTAAAACTACGGTGGTTGAGGATGAGCAT
GCGCATGATTAGTTAGTTGGCGGGGTAATGGCCCACCAAGACGATGATCATTAGGGGAACTGAGAGGT
TGATCCCCCACACTGGCACTGAGATACGGGC
>Zotu1547
GAACGCTGGCGGCGTGCCTAACACATGCAAGTCGAGCGAACCCTTCGGGGTGAGCGGCGGACGGGTGA
GTAACGCGTGGGTAACCTGCCCTGTACACACGGATAACATACCGAAAGGTATGCTAATACGAGATAAC
ATGTAATGATCGCATGGTCGATACATCAAAGCTCCGGCGGTACAGGATGGACCCGCGTCTGATTAGCT
AGTTGGTGAGGTAATGGCTCACCAAGGCGACGATCAGTAGCCGACCTGAGAGGGTGATCGGCCACATT
GGAACTGAGACACGGTC
>Zotu1548
GAACGCTGGCGGCGTGCCTAATACATGCAAGTCGAGCGAGGAGCTTTTAGCTCCTAGCGGCGAACGGG
TGAGTAACACGTGGGTAACCTGCCCGTAAGACTGGGATAACTTCGGGAAACCGGAGCTAATACTGGAT
AATCTTGATCTTCGCATGAAGGACAAGTAAAAGATTTATCACTTACGGATGGGCCCGCGGTGCATTAG
CTTGTTGGTGGGGTAATGGCCTACCAAGGCGACGATGCATAGCCGACCTGAGAGGGTGACCGGCCACA
CTGGGACTGAGACACGGCC
>Zotu1549
GAACGCTGGCGGCGTGCTTAACACATGCAAGTCGAACGATGAACCTGGAGCTTGCTCTAGGGAATTAG
TGGCGAACGGGTGAGTAACACGTGAGTAACCTGCCCTTGACTCTGGGATAACCTCCGGAAACGGAAGC
TAATACCGGATATGACGCACGGAGGCATCTCCTGTGCGTGGAAAGAACTTCGGTCAAGGATGGACTCG
CGGCCTATCAGGTAGTTGGTGAGGTAACGGCTCACCAAGCCTACGACGGGTAGCCGGCCTGAGAGGGT
GACCGGCCACACTGGGACTGAGACACGGCC
>Zotu1550
GAACGCTGGCGGCGTGCCTAATACATGCAAGTCGAGCGAAGTTTTTCTGGTGCTTGCACCGGAAAAAC
TTAGCGGCGAACGGGTGAGTAACACGTAAAGAACCTGCCTCATAGACTGGGACAACTATTGGAAACGA
TAGCTAATACCGGATAACAGCATTAACTGCATGGTTGATGTTTGAAAGTTGGTTTTGCTAACACTATG
AGATGGCTTTGCGGTGCATTAGCTAGTTGGTGGGGTAAAGGCCTACCAAGGCGACGATGCATAGCCGA
CCTGAGAGGGTGATCGGCCACACTGGGACTGAGACACGGCC
>Zotu1551
GAACGCTGGCGGCATGCCTAACACATGCAAGTCGAACGAAGGCTTCGGCCTTAGTGGCGCACGGGTGC
GTAACGCGTGGGAATCTGCCCTCAGGTTCGGAATAACAACGAGAAATTGCTGCTAATACCGGATGATA
TCGCGAGATCAAAGATTTATCGCCTGAGGATGAGCCCGCGTAGGATTAGCTAGTTGGTGTGGTAAAGG
CGCACCAAGGCGACGATCCTTAACTGGTCTGAGAGGATGATCAGCCACACTGGGACTGAGACACGGCC
>Zotu1552
GAACGCTGGCGGCGTGCCTAATACATGCAAGTCGAGCGGATCTCTTCGGAGATCAGCGGCGGACGGGT
GAGTAACACGTGGGCAACCTGCCTATAAGACTGGGATAACTTCGGGAAACCGGAGCTAATACCGGATA
ACATCAGAGGCCACATGGTCTTCGATTAAAAGATGGCTTTGGCTATCACTTATAGATGGGCCCGCGTC
GCATTAGCTAGTTGGTGAGGTAACGGCTCACCAAGGCGACGATGCGTAGCCGACCTGAGAGGGTGATC
GGCCACACTGGGACTGAGACACGGCC
>Zotu1553
CAACGCTGGCGGCGTGCATGAGACATGCAAGTCGAACGGACGACAAAGCTTGCTTTGTCGTTAGTGGC
GGACGGCTGAGTAACGCGTAGGTGACCTGCCTCGAAGTGGGGAATAACTGCCCGAAAGGGTGGCTAAT
ACCGCATGTGGTGGTGTATTCGATTACACCAGTAAAGCCGCAAGGCGCTTCGAGAGGGGCCTGCGTCC
GATTAGCTCGTTGGTGGGGTAACGGCTCACCAAGGCGACGATCGGTAGGTGGTCTGAGAGGACGATCA
CCCAGACTGGGACTGAGACACGGCC
>Zotu1554
GAACGCTGACAGAATGCTTAACACATGCAAGTCAACTTGAACTTCGGTTTGGGTGGCGGACGGGTGAG
TAACGCGTAAAGAACTTGCCTCACAGATAGGGACAACATTTGGAAACGAATGCTAATACCTGATATTA
TGATTATATGGCATCGTATAATTATGAAAGCTATATGCGCTGTGAGAGAGCTTTGCGTCCCATTAGCT
AGTTGGAGAGGTAACGGCTCACCAAGGCGATGATGGGTAGCCGGCCTGAGAGGGTGATCGGCCACAAG
GGGACTGAGACACGGCC
>Zotu1555
GAACGCTGGCGGCAGGCCTAACACATGCAAGTCGAACGCACCGCAAGGTGAGTGGCAGACGGGTGAGT

AACGCGTGGGAACCTTCCCTTTGGTTCAGGATAGCCCCGGGAAACTGGGATTAATACTGGATAAGATC
TACGGATGAAAGATTTATCGCCGAAGGATGGGCCCGCGTAAGATTAGCTAGTTGGTGAGGTAATGGCT
CACCAAGGCTTTGATCTTTAGCTGGTCTGAGAGGATGATCAGCCACACTGGAACTGAGACACGGTC
>Zotu1556
GAACGCTGGCGGCATGCTTTACACATGCAAGTCGAACGGTAACAGGGAGCTTGCTCCGCTGACGAGTG
GCGAACGGGTGAGTAATATATCGGAACGTGCCCAAGAGTGGGGGATAACGTAGCGAAAGTTACGCTAA
TACCGCATACGATCCAAGGATGAAAGCAGGGGACCTTCGGGCCTTGTGCTCCTGGAGCGGCCGATATC
TGATTAGCTAGTTGGTGAGGTAAAGGCTCACCAAGGCGACGATCAGTAGCTGGTCTGAGAGGACGACC
AGCCACACTGGAACTGAGACACGGTC
>Zotu1557
GAACGCTGGCGGCGTGCCTAAGACATGCAAGTCGAGTGGGTTTAGACCCACGGCGAACGGGTGAGTAA
TACCTAGGAACTTTCTTTAAGTGGAGGATAACCAGTCGAAAGATTGGATAATACTTCATGTGGCAGTA
ATGCTAAAGTCGCAAGACGCTTAAAGTGAGGCCTGGGCACCATCAGCTAGTTGGTAAGGTAATGGCTT
ACCAAGGCTATGACGGTTAGGGGTCCCGAGAGGGATAACCCCCACACTGGAACTGCGACACGGTC
>Zotu1558
GAACGCTGACAGAATGCTTAACACATGCAAGTCAACTTGAACTTCGGTTTGGGTGGCGGACGGGTGAG
TAACGCGTAAAGAACTTGCCTCACAGATAGGGACAACATTTGGAAACGAATGCTAATACCTGATATTA
TGATTTTAGGGCATCCTAGGATTATGAAAGCTATATGCGCTGTGAGAGAGCTTTGCGTCCCATTAGCT
AGTTGGAGAGGTAACGGCTCACCAAGGCGATGATGGGTAGCCGGCCTGAGAGGGTGATCGGCCACAAG
GGGACTGAGACACGGCC
>Zotu1559
GAACGCTGGCGGCGTGCTTAACACATGCAAGTCGAACGTGAACGGAGCAATCCTAGTAAAGTGGCGTA
CGGGTGAGTAACACGTAGGTAATCTACCTTAGAGTTCGGAATAACCTGCCGAAAGGCGGGCTAATACT
GGATAGAGCGACGGAATTCGTAAAGAATTTTTCGGGAAAGGGAGCTTGATAAGAGCCCTCGCTTTAAG
ATGAGCCTGCGTCTGATTAGTTAGTTGGTAAGGTAACGGCTTACCAAGACGATGATCAGTAGCTGGTC
TGAGAGGATGATCAGCCACACTGGAACTGAAACACGGTC
>Zotu1560
GAACGCTGGCGGCGTGCTTAACACATGCAAGTCGAACGGGATCCATCGCGAGCTTGCTCAAGAGAAGA
TCTAGTGGCGAACGGGTGCGTAACACGTGAGAAACCTGCCCCAGACTTGGGAATAACAGTCGGAAACG
ACTGCTAATACCGAATACCTTCGTAGCGTCGCATGGCGCATCGAAGAAAGCTTTATGCGGTTTGGGAG
GGTCTCGCGGCCTATCAGCTAGTTGGTGAGGTAACGGCTCACCAAGGCATCGACGGGTAGCTGGTCTG
AGAGGATGATCAGCCACACTGGGACTGAGACACGGCC
>Zotu1561
GAACGCTGACGGTGTGCTTTAGACATGCAAGTCGAACGAGAAAGCCCTTCGGGGTTAGTAAAGTGGCG
AACGGGTGAGTAACACGTGGGCAATCTGCCCCTCGCAGGGGGACAACCGGAGGAAACTCCGGCTAATA
CCCCGTACGCTTGTCGGACCGCATGGTACTGACAAGGAAAGGTAGCTTCGGCCATCCGGCGAGGGATG
AGCCCGCGGTACATTAGCTAGTTGGTGGGGTAACGGCCCACCAAGGCGACGATGTATAGCTGGTCTGA
GAGGATGATCAGCCACACTGGGACTGAGACACGGCC
>Zotu1562
GAACGCTGGCGGCACGCTTAACACATGCAAGTCGAACGAGAAAGTGGGTAACCATGAGTAAAGTGGCG
AACGGGTGCGTAACACGTGAGAATCTACCTTTTAGTGGAGGATACCCTGGAGAAATTTAGGTTAATCC
TGCATAAGCTCGAAAGAGGAAAGGCGGCTTAATTGCTGTCGCTAAAAGATGAGCTTGCGGCCTATTAG
CTAGTTGGTAAGGTAAAGGCTTACCAAGGCAAAGATGGGTATCCGGCCCGAGAGGGTGGTCGGACACA
CTGGGACTGAGATACGGCC
>Zotu1563
GAACGCTGGCGGCGTGCTTAACACATGCAAGTCGAACGGAAAGGCCCTTCGGGGTGCTCGAGTGGCGA
ACGGGTGAGTAACACGTGGGTGATCTGCCCTGCACTTTGGGATAAGCCTGGGAAACTGGGTCTAATAC
CGAATATGATCATGCCTCTCCTGGGGTGTGGTGGAAAGCTTTTGCGGTGTGGGATGGGCCCGCGGCCT
ATCAGCTTGTTGGTGGGGTAATGGCCTACCAAGGCGTCGACGGGTAGCCGGCCTGAGAGGGTGTCCGG
CCACACTGGGACTGAGATACGGCC
>Zotu1564
GAACGCTGGCGGCAGGCTTAACACATGCAAGTCGAGCGCACCTTCGGGTGAGCGGCAGACGGGTGAGT
AACGCGTGGGAATCTACCCATCTCTACGGAATAGCTCCGGGAAACTGGAATTAATACCGTATACGCCC
TTCGGGGGAAAGATTTATCGGAGATGGATGAGCCCGCGTTGGATTAGCTAGTTGGTGGGGTAAAGGCC

TACCAAGGCGACGATCCATAGCTGGTCTGAGAGGATGATCAGCCACATTGGGACTGAGACACGGCC
>Zotu1565
GAACGCTGGCGGCGTGCTTCACACATGCAAGTCGAGCGAGGCCCTTCCTTCGGGAAGGTGCCCTAGCG
GCGAACGGGTGAGTAACACGTGGGCAACCTGCCCCTGACTCTGGGATAAGCCCGGGAAACCGGGTCTA
ATACCGGATACGAACTCCACAGGCATCTGCGGAGTTGGAAAGATTTATCGGTCAGGGATGGGCCCGCG
GCCTATCAGCTTGTTGGTGGGGTAATGGCCCACCAAGGCGACGACGGGTAGCCGGCCTGAGAGGGCGA
CCGGCCACACTGGGACTGAGACACGGCC
>Zotu1566
GAACGCTGGCGGCGTGCTTAACACATGCAAGTCGAACGGTGACGCTGGAGCTTGCTCCAGCTAATCAG
TGGCGAACGGGTGAGTAACACGTGAGCAACCTGCCCTTGACTCTGGGATAACCCCGGGAAACCGGAGC
TAATACCGGATACGAACCGCACAGGCATCTGCGTGGTTGGAAAGTTTTTCGGTCAAGGATGGGCTCGC
GGCCTATCAGCTTGTTGGTGGGGTAACGGCCCACCAAGGCGACGACGGGTAGCCGGCCTGAGAGGGCG
ACCGGCCACACTGGGACTGAGACACGGCC
>Zotu1567
GAACGCTGGCGGCGTGCCTAATACATGCAAGTTGAGCGCTGAAGGTTGGTACTTGTACCAACTGGATG
AGCAGCGAACGGGTGAGTAACGCGTGGGGAATCTGCCTTTGAGCGGGGGACAACATTTGGAAACGAAT
GCTAATACCGCATAAAAACTTTAAACACAAGTTTTAAGTTTGAAAGATGCAATTGCATCACTCAAAGA
TGATCCCGCGTTGTATTAGCTAGTTGGTGAGGTAAAGGCTCACCAAGGCGATGATACATAGCCGACCT
GAGAGGGTGATCGGCCACATTGGGACTGAGACACGGCC
>Zotu1568
GAACGCTAGCGGCAGGCTTAATACATGCAAGTCGTGGGGCAGCAGCTTTGTAGCAATACAGAGGCTGG
CGACCGGCAAACGGGTGCGGAACACGTACACAACCTTCCTTTAAGTGGGGGATAGCCCAGGGAAACTT
GGATTAATACCCCGTAATACTATGAAGTGGCATCACTTTATATTTATAGCTCCGGCGCTTAATGATGG
GTGTGCGGCTGATTAGGTAGTTGGCGGGGTAACGGCCCACCAAGCCTGCGATCAGTAACTGGTGTGAG
AGCACGACCAGTCACACGGGCACTGAGACACGGGC
>Zotu1569
GAACGCTGGCGGCGTGCCTAATACATGCAAGTCGAGCGAATCTTTTGGGGGCTTGCTCCCAAAAGATT
AGCGGCGGACGGGTGAGTAACACGTGGGCAACCTGCCTATAAGACTGGGATAACTTCGGGAAACCGGA
GCTAATACCGGATATGTTCTTTTCTCGCATGAGAGAAGATGGAAAGACGGTTTCGGCTGTCACTTATA
GATGGGCCCGCGGCGCATTAGCTAGTTGGTGGGGTAATGGCTCACCAAGGCGACGATGCGTAGCCGAC
CTGAGAGGGTGATCGGCCACACTGGGACTGAGACACGGCC
>Zotu1570
GAATGCTGGCGGCGCGCCTAACACATGCAAGTCGAGCGGAAAGGCCCTTCGGGGTACTCGAGCGGCGG
ACGGCTGAGTAACGCGTGGGAACATGCCCCAAAGTGAGGAATAACTGCCCGAAAGGGTAGCTAATGCC
GCATATGATCTTCGGATTAAAGGATTTATCCGCTTTGGGAGTGGCCCGCGTACGATTAGGTAGTTGGT
GAGGTAAAGGCTCACCAAGCCCACGATCGTTAACTGGTCTGAGAGGATGACCAGTCAGACTGGAACTG
AGACACGGTC
>Zotu1572
GAACGCTAGCGGCAGGCTTAACACATGCAAGTCGAGGGGTATAGGTAGCTTGCTATCTAGAGACCGGC
GCACGGGTGCGTAACGCGTATGCAACCTACCTTTCACAAAGGGATAGCCCAGAGAAATTTGGATTAAT
ACCTTATAGTATATAAGAATCGCATGGTTTTTATATTAAAGTTCCAACGGTGAAAGATGGGCATGCGT
CCCATTAGCTAGATGGTAAGGTAACGGCTTACCATGGCAACGATGGGTAGGGGTCCTGAGAGGGAGAT
CCCCCACACTGGTACTGAGACACGGAC
>Zotu1573
GAACGCTAGCTACAGGCTTAACACATGCAAGTCGAGGGGAAACGACATCGAAAGCTTGCTTTTGATGG
GCGTCGACCGGCGCACGGGTGAGTAACGCGTATCCAACCTGCCCACCACTTGGGGATAACCTTGCGAA
AGTAAGACTAATACCCAATGATATCTCTAGAAGACATCTGAAAGAGATTAAAGATTTATCGGTGATGG
ATGGGGATGCGTCTGATTAGCTTGTTGGCGGGGTAACGGCCCACCAAGGCAACGATCAGTAGGGGTTC
TGAGAGGAAGGTCCCCCACATTGGAACTGAGACACGGTC
>Zotu1574
GAACGCTAGCGGCAGGCCTAATACATGCAAGTCGAACGGTCAGCAATGACAGTGGCGCACGGGTGCGT
AACGCGTATGCAACCTACCCATCAAAGGGGGATAGCCCGGGGAAACCCGGATTAATACCGCATAAGAT
AGTATTCTGGCATCAGAGAGCTATTAAACATTTATGGTTGATGGATGGGCATGCGTTAGATTAGCTAG
TTGGTGTAGTAACGGTGCACCAAGGCGATGATCTATAGGGGTTCTGAGAGGAAGATCCCCCACACTGG

TACTGAGATACGGAC
>Zotu1575
GAACGCTAGCGGCAGGCCTAATACATGCAAGTCGAACGATATTATCCAGCTTGCTGGATATGAAAGTG
GCGCACGGGTGCGTAACGCGTATGCAACCTACCTTAATCAGGGGGATAGCCCGAAGAAATTCGGATTA
AGACCGCATAAAAACACAGGATAGCATTATCCAATGTTCAAATATTTATAGGATTAAGATGGGCATGC
GTGACATTAGTTAGTTGGCGGGGTAACGGCCCACCAAGACCACGATGTCTAGGGGATCTGAGAGGATG
ACCCCCCACACTGGTACTGAGACACGGAC
>Zotu1576
GAACGCTGGCTGCGTGCTTAACACATGCAAGTCGAACGCTGAAGCCCCAGCTTGCTGGGGTGGATGAG
TGGCGAACGGGTGAGTAACACGTGAGTAACCTGCCCCTGACTTCGGGATAAGCCCGGGAAACTGGGTC
TAATACCGGATACGACTGCCGGACGCATGTCTGGTGGTGGAAAGTTTTTTCGGTTGGGGATGGGCTCG
CGGCCTATCAGTTTGTTGGTGAGGTAATGGCTCACCAAGACGACGACGGGTAGCCGGCCTGAGAGGGC
GACCGGCCACACTGGGACTGAGACACGGCC
>Zotu1577
GAACGCTGGCGGCACGTTTTAAGCATGCAAGTCGAACGGCAGTGGGGGTAACCCCATGAGAGTGGCGA
ACGGGTGAGTAACACGTAGGTGATCTGCCTTGAGGATGGGGATAGCTTGTGGAAACACAGGGTAATAC
CGAATAAGCTGGCGGGATTTTGGTCCTGTTAGTAAAGGTGCTACGGCACCGCCTTGAGAGGAGCCTGC
GGCTGATTAGCTAGTTGGTGGGGTAAAGGCCTACCAAGGCGACGATCAGTAGCCGGCCTGAGAGGGTG
TACGGCCACACTGGAACTGAGACACGGTC
>Zotu1578
GAACGCTGGCGGCGTGCCTAATACATGCAAGTCGAGCGGATTCATTCCTTCGGGAATGGGTTAGCGGC
GGACGGGTGAGTAACACGTAGGCAACCTGCCTATAAGCCTGGGATAACTTCCGGAAACGGATGCTAAT
ACCGGATACGTTGAATTCTCGCATGAGAATTCATGGAAAGACGGCGCAAGCTGTCACTTATGGATGGG
CCTGCGGCGCATTAGCTAGTTGGTGGGGTAACGGCTCACCAAGGCGACGATGCGTAGCCGACCTGAGA
GGGTGATCGGCCACACTGGGACTGAGACACGGCC
>Zotu1579
GAACGCTGGCGGTGTGCCTAATACATGCAAGTCGAGCGGTAAGGCTCCTTCGGGAGTACACGAGCGGC
GGACGGCTGAGTAACGCGTGGGAACGTACCCCAAAGTGAGGGATAAGCACCAGAAATGGTGTCTAATA
CCGCATATGCTCTAAGGAGTAAAGCTTCGGCGCTTTGGGAACGGCCTGCGTCTGATTAGCTTGTTGGT
GAGGTAAAAGCTCACCAAGGCGACGATCAGTAGCTGGTCTGAGAGGATGACCAGCCAGACTGGGACTG
AGAACGGCC
>Zotu1580
GAACGCTGGCGGCGTGCTTAACACATGCAAGTCGAACGGTAAGGCCCTTTCGGGGGTACACGAGTGGC
GAACGGGTGAGTAACACGTGGGTGATCTGCCCTGCACTTTGGGATAAGCCTGGGAAACTGGGTCTAAT
ACCGAATAGGACCATGCCATGCATGTGGTGTGGTGGAAAGCTTTTGCGGTGTGGGATGGGCCCGCGGC
CTATCAGCTTGTTGGTGAGGTTACGGCTCACCAAGGCGACGACGGGTAGCCGGCCTGAGAGGGTGACC
GGCCACACTGGGACTGAGATACGGCC
>Zotu1581
GAACGCTGGCGGCGTGCTTAACACATGCAAGTCGAACGGAATCTGATCTGTGGCAACACAGTGAAAGG
TTTAGTGGCGAACGGGTGAGTAACACGTGAGGAACCTGCCCCGAAGACCGGGATAACTCTCCGAAAGG
AGTGCTAATACCGGATACCCCCACCGAATCGCATGGTTTGGTGAGGAAATGGATTCCGCTTCGGGAGG
GCCTCGCGGCCTATCAGCTAGTTGGTGGGGTAATGGCCCACCAAGGCATCGACGGGTAGCTGGTCTGA
GAGGACGATCAGCCACACTGGGACTGAGACACGGCC
>Zotu1582
GAACGCTGGCGGCGCGCTTAACACATGCAAGTCGTACGGAGTTGTTCCTTCGGGAGCAGCTCAGTGGC
GAACGGGTGCGTAACACGTGAGCAACCTGCCTGCCGGTGGGGGATAACCCGGGGAAACCCGGGCTAAT
ACCGCATTCGCTCTTTCTCTCGATTGGGGGATTGAGGAAAGGTGCTGCAAGGCACTGCCGGCAGAGGG
GCTCGCGGCGTATCAGCTAGCTGGTGAGGTAACGGCTCACCAGGGCAACGACGCGTAGCCGATCTGAG
AGGATGATCGGCCACATTGGGACTGAGATACGGCC
>Zotu1584
GAACGCTTGCGGCGTGCCTAAGAAATGCAAGTCGAACGGGGAGATTTCGGTCTCCTAGTGGCGAACGG
TCGCGTAACACGTAAGCAACCTGCCCAGAAGTGGGGGACAACAGCCTGAAAGGGCTGCTAATACCGCA
TGTGAATGAGATTTGGCATCTTATCTTATTTAAAGCTTTATGCGCTTCTGGATGGGCTTGCGGCCTAT
CAGGTAGTTGGTGGGGTAACAGCCTACCAAGCCGACGACGGGTAGCTGGTCTGAGAGGACGATCAGCC

GGATTGGGACTGAGATACGGCC
>Zotu1585
GAACGCTGGCGGCATGCCTAACACATGCAAGTCGAACGAGGTTTCCGCTTCGGCGGTTGCCTAGTGGC
GCACGGGTGAGTAACGCGTGGGAACCTGCCCCGTGGTACGGAATAACTCCGGGAAACTGGAGCTAAGA
CCGTATGTGTCCCGAGGGACAAAGATTCATCGCCATGGGATGGGCCCGCGTAGGATTAGCTTGTTGGC
GGGGTAACGGCCCACCAAGGCGACGATCCTTAGCTGGTCTGAGAGGATGATCAGCCACACTGGGACTG
AGACACGGCC
>Zotu1586
GAACGCTGGCGGTATGCTTTACACATGCAAGTCATACGAGAATTTTTAATTCAAGTGGCGGACGGGTG
AGTAACACGTAAGAATTTACCTTTAGGAGGGGGATAACAACTGGAAACGGTTGCTAATACCCCATATG
CTTTCGAGTGAAATGGAGTTTTCCGCCTAGAGAGAAGCTTGCGGCTGATTAGCTTGTTGGTGAGGTAA
TGGCTTACCAAGGCGACGATCAGTATCTGGTTTGAGAGGACGATCAGACACACTGGAACTGAGACACG
GTC
>Zotu1587
GAACGCTAGCGGCAGGCATAATACATGCAAGGCGAAGGGGCAGCAATGTCACTGTCGTACGGGTGCGT
AACGCGTATGCAACTTATCTATCACTGGGGGATAGCCCGGGGAAACCCGGATTAATACCGCATAAAAC
AGGGGCACCGCATGGTGATATTTGTTAAAGATTTATTGGTGATAGATGGGCATGCGTTCGATTAGCTA
GTTGGCGGGGTAACGGCCCACCAAGGCGACGATCGATAGGGGAGCTGAGAGGTTGGTCTCCCACACGG
GCACTGAGATACGGGC
>Zotu1588
GAACGCTAGCGGCAGGCTTAATACATGCAAGTCGAGGGGCAGCACGAATAGCAATATTTGGTGGCGAC
CGGCGCACGGGTGCGTAACACGTATACAATCTACCTAAAACAGGGGGATAGCCTTTCGAAAGAGAGAT
TAATACCCCGTAATAAATTGAATGGCATCATTTGATTTTGAAAGCTCCGGCGGTTTTAGATGAGTATG
CGTCTGATTAGCTAGTTGGTGGTGTAATGGACCACCAAGGCTACGATCAGTAGGGGATCTGAGAGGAC
TACCCCCCACACTGGTACTGAGACACGGAC
>Zotu1589
GAACGCTGGCGGCGTGCTTAATACATGCAAGTCGAACGAGGCGTAAGCCTAGTGGCGAACGGGTGAGT
AACACGTGAGCAACCTGCCCCGAAGACCGGGATAACAGTGGGAAACCGCTGCTAATACCGGATACCCC
CATAGGGTCACATGGCCCAATGAGGAAATGGATTCCGCTTCGGGAGGGGCTCGCGGCCTATCAGCTTG
TTGGTGGGGTAACGGCCTACCAAGGCAACGACGGGTAGCTGGTCTGAGAGGACGATCAGCCACACTGG
GACTGAGACACGGCC
>Zotu1590
GAATGCTGGCGACGTGGATAAGGCATGCAAGTCAAGGGGGCCGCAAGGCAACCGGCAGACGAGGTAGT
AACACGTACGAACGCACCCCGAAGTCTGGTATAGCCGGCCGAAAGGCCGGGTAATCCCGGATGGTACC
GCAAGGTTAAAGATTTATCGCTTCGGGAGCGGCGTGCGCAATATCAGCTAGTTGGTAGGGTAATGGCC
TACCAAGGCTACGACGTTTAGGGGAGCTGAGAGGCTGACCCCCACCGATGGGACTGAGACACGGCC
>Zotu1591
AAACGCTGGCGGCGTGCATAACACATGCAAGTCGAACGATGAAGGTTAAAAGATTTCTTCGGAATGAA
CTTAATTGGATTAGTGGCGGACGGGTGAGTAACGCGTGAGTAACCTGCCTTGTACAAGGGGATAGCCG
TTGGAAACGACGAATAATACCCTATGACATCAAATAATCGCATGATAATTTGATCAAAGATTTATCGG
TATAAGATGGACTCGCGTCTGATTAGCTAGTTGGTAGAGTAAAAGCCTACCAAGGCAACGATCAGTAG
CCGGCTTGAGAGAGTGTACGGCCACATTGGGACTGAGACACGGCC
>Zotu1592
GAACGCTGGCGGCGTGCTTCACACATGCAAGTCGAACGAGGTCCAATCAGTCGCAAGACTGGTGAAGA
CCGAGTGGCGAACGGGTGCGTAACACGTGAGGAACCTGTCCCGGTCTCCGGAATAACAAATGGAAACA
CTTGCTAATACGGGATGACGTCGGAACGTCGCATGACGAACTGACGAAAGGGTTACCGGACCGGGAGG
GCCTCGCGGCCTATCAGCTCGTTGGTGGGGTAACGGCCCACCAAGGCTTCGACGGGTAGCTGGTCTGA
GAGGATGATCAGCCACACTGGGACTGAGACACGGCC
>Zotu1593
GAACGCTGGCGGCATGCCTAATACATGCAAGTCGAGCGGAATGATGGGAGCTTGCTCCCTGATTTCAG
CGGCGGACGGGTGAGTAACACGTGGGCAACCTGCCCTACAGATGGGGATAACTCCGGGAAACCGGGGC
TAATACCGAATAATCAGTTTGTCCGCATGGACAAACTCTGAAAGACGGTTTCGGCTGTCACTGTAGGA
TGGGCCCGCGGCGCATTAGCTAGTTGGTGGGGTAATGGCCTACCAAGGCGACGATGCGTAGCCGACCT
GAGAGGGTGATCGGCCACACTGGGACTGAGACACGGCC

>Zotu1595
GAACGCTGGCGGTATGCTTTACACATGCAAGTCGTACGAGAGTTTTTAACTCAAGTGGCGGACGGGTG
AGTAACACGTAAGAATTTGCCTTTAGGAGGGGGATAACAACTGGAAACGGTTGCTAATACCCCATATG
CTTTCGAGTGAAATGGATTTTTCCGCCTAAAGAGAAGCTTGCGGCTGATTAGCTTGTTGGTGAGGTAA
TGGCTTACCAAGGCGACGATCAGTATCTGGTTTGAGAGGACGATCAGACACACTGGAACTGAGAGACG
GTC
>Zotu1596
GAACGCTAGCGGCAGGCTTAATACATGCAAGTCGAACGGTATCAGCAATGGTATAGTGGCGCACGGGT
GAGTAACGCGTACATAACATGCCTTTATCTTTGGGATAGCCTTGGGAAACTGGGAGTAATACCGAATA
GTATTTTTGGATGGCATCATCTGGGAATTAAAGTTACGGCGGATAAAGATTGATGTGCGTAAGATTAG
GTAGTTGGTGAGGTAACGGCTCACCAAGCCAACGATCTTTAGGGGGCGTGAGAGCGTGACCCCCCACA
CGGGTACTGAGACACGGAC
>Zotu1597
GAACGCTGGCGGCATGCTTAACACATGCAAGTCGAACGGTAACGCGGGGGTAACCCTGGCGACGAGTG
GCGGACGGGTGAGTAATACTTAGGAATCTGCCCAGTAGTGGGGGACAACCCGGGGAAACCCGGGCTAA
TACCGCATACGCCCCATGGGGGAAAGCGGGGGACCGCAAGGCCTCGCGCTATTGGATGAGCCTAAGTC
CGATTAGCTAGTTGGTAGGGTAACGGCCTACCAAGGCGACGATCGGTAGCTGGTCTGAGAGGATGATC
AGCCACACTGGGACTGAGACACGGCC
>Zotu1598
GAACGCTGGCGGCGTGCTTAACACATGCAAGTCGAACGGAAAGGCCCTTCGGGGTACTCGAGTGGCGA
ACGGGTGAGTAACACGTGGGTGATCTGCCTCGTACTCTGGGATAAGCCTGGGAAACTGGGTCTAATAC
CGGATAGGACCATTCCTCGCATGGGGTGTGGTGGAAAGTTTTTCGGTACGAGATGGCCCGCGGCCTA
TCAGCTTGTTGGTGGGGTAATGGCCTACCAAGGCGACGACGGGTAGCCGGCCTGAGAGGGCGACCGGC
CACACTGGGACTGAGACACGGCC
>Zotu1599
GAACGCTAGCGGCAGGCCTAATACATGCAAGTCGAGGGGTAGCAATACCACTGGCGCACGGGTGCGTA
ACACGTTTGAAATCTGCCCTTTATTGGGGGATAGCCCGCCGAAAGGCGGATTAATACCGCATATGCCG
GTGGAGTGGCATCACTCAGCCGGGAAAGGCTTCGGCCGATAGAGGATGATCATGCGTCTGATTAGCTA
GTTGGCGGGGTAGAGGCCCACCAAGGCGACGATCAGTAGGGGACCTGAGAGGGCGATCCCCCACACGG
ACACTGAGATACGGGT
>Zotu1600
GAACGCTGGCGGCGTGCTTAACACATGCAAGTCGAACGATGATGTCCCGCTTGCGGGACGGATTAGTG
GCGAACGGGTGAGTAATACGTGAGTAACCTGCCCCTGACTCTGGGATAAGCCTGGGAAACCGGGTCTA
ATACTGGATACTACCTCGCACCGCATGGTGGGTGGTGGAAAGGGTTTTACTGGTTTTGGATGGGCTCA
CGGCCTATCAGCTTGTTGGTGGGGTAATGGCTCACCAAGGCGACGACGGGTAGCCGGCCTGAGAGGGT
GACCGGCCACACTGGGACTGAGACACGGCC
>Zotu1601
GAACGCTGGCGGCGTGCTTAACACATGCAAGTCGAACGGAAAGGCCCTGCTTGCAGGGTACTCGAGTG
GCGAACGGGTGAGTAACACGTGGGTGATCTGCCTCGTACTTCGGGATAAGCCTGGGAAACTGGGTCTA
ATACCGGATAGGACCTTCTTTTAGTGTGGAAGGTGGAAAGTTTTTCGGTACGAGATGAGCCCGCGGCC
TATCAGCTTGTTGGTGGGGTAATGGCCTACCAAGGCGACGACGGGTAGCCGGCCTGAGAGGGTGTACG
GCCACATTGGGACTGAGACACGGCC
>Zotu1603
GAACGCTGGCGGCGTGCTTAACACATGCAAGTCGAACGGTAAGGCCCTTCGGGGTACACGAGTGGCGA
ACGGGTGAGTAACGCGTGAGTAACCTGCCCCTGACTTTGGGATAACGGATGGAAACGTCTGCTAATAC
CGAATATTCAGTTCTCCGGGCATCCGGGGGGTTGGAAAGTTTTTTCGGTCAGGGATGGACTCGCGTCC
TATCAGCTTGTTGGTGGGGTAATGGCCTACCAAGGCGTCGACGGGTAGCCGGCCTGAGAGGGCGACCG
GCCACATTGGGACTGAGATACGGCC
>Zotu1604
GAACGCTGGCGGCAGGCTTAACACATGCAAGTCGAGCGCCCCGCAAGGGGAGCGGCAGACGGGTGAGT
AACGCGTGGGAATCTACCGTGCCCTACGGAATAGCTCCGGGAAACTGGAATTAATACCGTATACGCCC
TACGGGGGAAAGATTTATCGGGGTATGATGAGCCCGCGTTGGATTAGCTAGTTGGTGGGGTAAAGGCC
TACCAAGGCGACGATCCATAGCTGGTCTGAGAGGATGATCAGCCACATTGGGACTGAGACACGGCC
>Zotu1605

GAACGCTGGCGGCGTGCTTAACACATGCAAGTCGAGCGGTAAGGCCCTTCGGGGTACACGAGCGGCGA
ACGGGTGAGTAACACGTGGGTGACCTGCCCCCTACTCTGGGATAAGCCCGGGAAACTGGGTCTAATAC
CGGATATGACTACATGTCGCATGGTGTGTGGTGGAAAGTTTTTCGGTGGGGGATGGGCCCGCGGCCTA
TCAGCTTGTTGGTGGGGTGATGGCCTACCAAGGCGACGACGGGTAGCCGGCCTGAGAGGGCGACCGGC
CACACTGGGACTGAGACACGGCC
>Zotu1606
GCCGGCAATGTTGAAGAGGTAAGAACTGCTCACATGCACTGTCCTCCCCGGAGGACGCTAACCATGTG
TGTCGAGATTTCGGCACACGTTAAAGAACCCTCGGGTAGGCAAAATTAAC
>Zotu1607
GAACGCTAGCGGCAGGCCTAATACATGCAAGTCGAACGGGTCGCAAGACCAGTGGCAAACGGGTGCGT
AACGCGTAAGCAACCTGCCTCAAACTGGGGGATAGCCCGGCGAAAGCTGGGGTAAACCCGCACGGTTC
CCATTTATCACCTGGTAGTTGGGGTAAACATTTATGGGTTTGAGAGGGGCTTGCGTCTGATTAGTTAG
TTGGCAGGGTAACGGCCTACCAAGACGATGATCAGTAGGGGTTCTGAGAGGATTGGCCCCCACATGGG
TACTGAGATACGGAC
>Zotu1608
GAACGCTGGCGGCGTGCTTAACACATGCAAGTCAAAGGGTAGCAATATCCTTGGCGCACGGGTGAGTA
ACGCGTAGATTATCTGCCCCATACACAGACATAACTTTTCGAAAGAGACGATAATATCTGATGAGGTG
CTTCGGCATCAAAGTAGCAATACGGTATGGGATGAGTCTGCGTCTGATTAGCTAGTTGGCGAGGTAAT
GGCTCACCAAGGCGACGATCAGTAGCTGGTCTGAGAGGACGATCAGCCACACTGGAACTGAGACACGG
TC
>Zotu1609
GAACGTTGGCGGCGTGCCTTAAGCATGCAAATCAAGGGGGTCCCGCAAGGGACTACCGGTGGACGGGT
GAGTAATGCGTGGGAATCTACCTCGAAGTGGGATATAGCCAGCCGAAAGGTTGGGTAATCTCCCATGT
GATCGCAAGATCAAAGTCGCAAGACGCTTTGAGATGAGCCCACGTCCTATCAGCTAGTTGGTGGGGTA
ATGGCCTACCAAGGCAACGACGGGTAGCTGGACTGAGAGGTCGGTCAGCCACAATGGGACTGCGACAC
GGCC
>Zotu1610
GAACGCTGGCGGCGTGCCTAACACATGCAAGTCGAGCGATTCTCTTCGGAGAAGAGCGGCGGACGGGT
GAGTAACGCGTGGGTAACCTGCCCTGTACACACGGATAACATACCGAAAGGTATGCTAATACGAGATA
ACATACTTTTATCGCATGGTAGAAGTATCAAAGCTCCGGCGGTACAGGATGGACCCGCGTCTGATTAG
CTAGTTGGTAAGGTAACGGCTTACCAAGGCGACGATCAGTAGCCGACCTGAGAGGGTGATCGGCCACA
TTGGAACTGAGACACGGTC
>Zotu1611
GAACGCTGGCGGCGTGCCTAATACATGCAAGTCGTACGCATTGACGATGGAACTTGTTCCATCTGACG
TGAGTGGCGCACGGGTGAGTAACACGTGGGAAACCTACCCTTCAGCGGGGGATAACCGTCGGAAACGA
TGGCTAATACCGCATAGGTCTTTGAGTCACCTGGCTTAAAGAGGAAAGACGGCTTCGGCTGTCACTGA
AGGATGGTCCCGCGGTGCATTAGCTAGTTGGTAAGGTAATGGCTTACCAAGGCAATGATGCATAGCCG
ACCTGAGAGGGTGATCGGCCACATTGGGACTGAGACACGGCC
>Zotu1612
GAACGCTGGCGGCGTGCTTAACACATGCAAGTCGAACGATGAAGCTGGAGCTTGCTCTGGTGGATTAG
TGGCGAACGGGTGAGTAACACGTGAGCAACCTGCCCCTGACTCTGGGATAACAGCCGGAAACGGCTGC
TAATACCGGATATGCACCATGAACGCATGTTCTGTGGTGGGAAAGATTTTTCGGTTGGGGATGGGCTC
GCGGCCTATCAGCTTGTTGGTGAGGTAATGGCTCACCAAGGCGTCGACGGGTAGCCGGCCTGAGAGGG
TGACCGGCCACACTGGGACTGAGACACGGCC
>Zotu1613
GAACGCTAGCGGCAGGCTTAATACATGCAAGTCGAGGGGCAGCACAGGGTAGCAATACCTGGGTGGCG
ACCGGCAAACGGGTGCGGAACACGTACAGAACCTTCCTCTGAGTGTGGGATAGCCCGGAGAAATCCGG
ATTAATACCACATAGTAATATTAGAAGGCATCTTTTGATATTTAAAGATTTATCGCTTAGAGATGGCT
GTGCGGCTGATTAGGTAGTTGGTGCGGGTAACGGCCCACCAAGCCAGCGATCAGTAACTGGTGTGAGA
GCACGACCAGTCACACGGGCACTGAGACACGGGC
>Zotu1614
GAACGCTAGCGGCAGGCTTAATACATGCAAGTCGTGGGGCAGCAGGGGTAGCAATACTCGCTGGCGAC
CGGCAAACGGGTGCGGAACACGTACACAACCTTCCTTTAAGCGGGGAATAGCCCAGAGAAATTTGGAT
TAATACCCCATAAGATATAATATAGGCATCTATAATATATTAAAGCTTCGGCACTTGATGATGGGTGT

GCGTATGATTAGGTAGTTGGTAAGGTAACGGCTTACCAAGCCTACGATCATTAGCTGATGTGAGAGCA
TGATCAGCCACACGGGCACTGAGACACGGGC
>Zotu1615
GAACGCTGGCGGCGTGCCTAATACATGCAAGTAGAACGCTGAAGGAGGAGCTTGCTCTTCTAGATGAG
TTGCGAACGGGTGAGTAACGCGTAGGTAACCTGCCTGGTAGCGGGGGATAACTATTGGAAACGATAGC
TAATACCGCATAAGAGTAGATGTTGCATGACATTTGCTTAAAAGGTGCAATTGCATCACTACCAGATG
GACCTGCGTTGTATTAGCTAGTTGGTGGGGTAACGGCTCACCAAGGCGACGATACATAGCCGACCTGA
GAGGGTGATCGGCCACACTGGGACTGAGACACGGCC
>Zotu1616
GAACGCTGGCGGCGTGCCTAATACATGCAAGTCGAGCGGACTGAATGAAAGCTTGCTTTCATTCGGTT
AGCGGCGGACGGGTGAGTAACACGTGGGCAACCTGCCCGTAAGACCGGGATAACTTCGGGAAACCGGA
GCTAATACCGGATAACACCGAAGATCGCATGATCTTTGGTTGAAAGGCGGCTTTGGCTGTCACTTACG
GATGGGCCCGCGGCGCATTAGCTAGTTGGTGAGGTAACGGCTCACCAAGGCGACGATGCGTAGCCGAC
CTGAGAGGGTGATCGGCCACACTGGGACTGAGACACGGCC
>Zotu1617
GAACGCTGGCGGCATGCTTAACACATGCAAGTCGAACGGACACCTTCGGGTGTTAGTGGCGGACGGGT
GAGTAACGCGTGAGGAACCTGCCTCGCAGTGGGGAATAACGGTTGGAAACGACCGCTAATACCGCATA
ATGTATCGGGACCGCATGACTCTGATACCAAAGATTTATCGCTGTGAGATGGCCTCGCGTCTGATTAG
CTAGTTGGTGAGGTAACGGCCCACCAAGGCGACGATCAGTAGCCGGACTGAGAGGTTGACCGGCCACA
TTGGGACTGAGACACGGCC
>Zotu1618
GAACGCTGGCGGCGTGCCTAAGACATGCAAGTCGAGTGGGTTTAGACCCACGGCGAACGGGTGAGTAA
TACCTAGGAACTTTCTTTAAGTAGAGGATAACCAGCCGAAAGGTTGGATAATACTCCATGTGGCAGTA
ATGCTAAAGCAGCAATGCGCTTAAAGTGAGGCCTGGGCACCATCAGCTAGTTGGTAAGGTAATGGCTT
ACCAAGGCTTTGACGGTTAGGGGTCCCGAGAGGGATAACCCCCACACTGGAACTGCGACACGGTC
>Zotu1619
CAACGCTGGCGGCGCGCTTAACACATGCAAGTCGAACGAGAAAGGGAGCAATCCTGAGTAAAGTGGCG
TACGGGTGAGTAACACGTGGATCATCTGCCCTTTGGTGGGGAATAACTCCTGGAAACGGAGATAATA
CCGCATAAGTCCGAGAGGAGAACGCCGAAAGGCGCCGAAGGAGGAGTCCGCGGCTGATTAGCTAGTTG
GTAGGGTAATGGCCTACCAAGGCGACGATCGGTAGCCGGCCTGAGAGGGCACACGGCCACACTGGCAC
TGAGACACGGGC
>Zotu1620
GAACGCTGGCGGCGTGCTTAACACATGCAAGTCGAACGGTGATGGAGAGCTTGCTCTCCTGATCAGTG
GCGAACGGGTGAGTAACACGTGAGTAACCTGCCCTTGACTCTGGGATAAGCGTTGGAAACGACGTCTA
ATACCGGATAGGACTTCCACAGGCATCTGTGGGGGTGGAAAGATTTTTCGGTCATGGATGGACTCGCG
GCCTATCAGCTTGTTGGTGGGGTGATGGCCTACCAAGGCGACGACGGGTAGCCGGCCTGAGAGGGTGA
CCGGCCACACTGGGACTGAGACACGGCC
>Zotu1621
GAACGTTGATGGCGTGCCTCATACATGCAAGTCGAGCGCTGTAGCAATACGGAGCGGCGAACGGGTGA
GTAACACGTAAAGAATCTGCCCTGAAGCGGGGAATAACTATCCCAACGGATAGCTAATACCGCATAAC
GATGGAGGAGGCATCTCCTCTTCTCAAAAGGCTTCGGCCACTTCAGGATGACTTTGCGTCCCATTAGC
TTGTTGGCGGGGTAACGGCCCACCAAGGCTACGATGGGTATCCGGCCTGAGAGGGTGTACGGACACAT
TGGGACTGAGATACGGCC
>Zotu1622
GAACGCTGGCGACAGGCTTAACACATGCAAGTCGAACGCCCCACAAGGGAAGTGGCAGACGGGTGAGT
AACGCGTGGGAACATACCCTTTCCTGCGGAATAGCTCTGGGAAACTGGAATTAATACCGCATACGCCC
TACGGGGGAAAGATTTATCGGGGAAGGATTGGCCCGCGTTGGATTAGCTAGTTGGTGGGGTAAAGGCC
TACCAAGGCGACAATCCATAGCTGGTCTGAGAGGATGATCAGCCACATTGGGACTGAGACACGGCC
>Zotu1623
GAACGCTGGCGGCGTGCCTAACACATGCAAGTCTACGAGAAAAGGGTAGCAATATCCTGAGTAAAGTG
GCGCACGGGTGAGTAACGCGTAGGCAATCTGCCTTTAGGACGGGAACAACCCCGAGAAATCGAGGCTA
ATACCCGATAATGCAGCGGCACCGCATGGTGACAGTTGTTAAAAGCCCGCAAGGGCGCCTAGAGATGA
GCCTGCGCCCCATTAGGTAGTTGGTGAGGTAACGGCCCACCAAGCCTACGATGGGTAGCTGATCTGAG
AGGATGATCAGCCACACTGGAACTGAGACACGGTC

>Zotu1624
GAACGCTGGCGGCAGGCTTAACACATGCAAGTCGAACGCCCCGCAAGGGGAGTGGCAGACGGGTGAGT
AACGCGTGGGAATCTACCTAGTTCTACGGAACAACACAGGGAAACTTGTGCTAATACCGTATACGCCC
TACGGGGGAAAGATTTATCGGAATTAGATGAGCCCGCGTAAGATTAGCTAGTTGGTGGGGTAATGGCC
TACCAAGGCGACGATCTTTAGCTGGTCTGAGAGGATGATCAGCCACACTGGGACTGAGACACGGCC
>Zotu1625
GAACGCTAGCGGCAGGCCTAATACATGCAAGACGAACGGTAAGAATTGTAGCAATACGATTCCAAGAG
TGTCGTACGGGTGCGTAACACGTATGCAACCTACCTTGTACAGGAGTATAGCTCGGGGAAACTCGAAT
TAACCCTCCATAATATATTGATGTGGCATCACATTAATATTAAAACTACGGTGGTACAAGATGGGCAT
GCGTTTGATTAGCTAGTTGGCGGTGTAATGGACCACCAAGGCGATGATCAATAGGGGAACTGAGAGGT
TGATCCCCCACACTGGCACTGAGATACGGGC
>Zotu1626
GAACGCTAGCGGGAGGCCTAACACATGCAAGCCGAGCGGTAGAGTTCCTTCGGGAACTTGAGAGCGGC
GTACGGGTGCGTAACACGTGTGCAACCTGCCTTTATCTGGGAGATAGCCCTCCGAAAGGGGGATTAAT
ATTCCATAATATATCAGTTGGCATCAATTGATATTGAAAGCTCCGGCGGATAGAGATGGGCACGCGCA
AGATTAGATAGTTGGTGAGGTAACGGCTCACCAAGTCTGCGATCTTTAGGGGGCCTGAGAGGGTGATC
CCCCACACTGGTACTGAGACACGGAC
>Zotu1627
GAACGCTGGCGGCAGGCCTAACACATGCAAGTCGAGCGCCCCCGCAAGGGGGAGCGGCGGACGGGTGA
GTAACGCGTGGGAATTTTCCTCAAGGTACGGAACAACTCAGGGAAACTTGGGCTAATACCGTATGATG
CAGAGATGCCAAAGATTTATCGCCTTGGGATAAGCCCGCGTCAGATTAGGTAGTTGGTGGGGTAACGG
CCTACCAAGCCTGCGATCTGTAGCTGGTCTGAGAGGACGATCAGCCACACTGGGACTGAGACACGGCC
>Zotu1628
GAACGCTGGCGGCGTGCCTAATACATGCAAGTCGAGCGAATCAATGGGAGCTTGCTCCCTGAGATTAG
CGGCGGACGGGTGAGTAACACGTGGGTAACCTGCCTGTAAGACTGGGATAACTTCGGGAAACCGGAGC
TAATACCGGATAATTTCTTTTCTCGCATGAGAAGAGATGGAAAGATGGTTTTGCTATCACTTACAGAT
GGACCCGCGGCGCATTAGCTAGTTGGTGAGGTAACGGCTCACCAAGGCCACGATGCGTAGCCGACCTG
AGAGGGTGATCGGCCACACTGGGACTGAGACACGGCC
>Zotu1629
GAACGCTGGCGGCGTGCCTAATACATGCAAGTCGTGCGGGGGAGATCCTTCGGGATCGGACCCAGCGG
CGAACGGGTGAGTAACACGTGGGCAACCTGCCCGCAAGACGGGGATAACTCCGGGAAACCGGTGCTAA
TACCCGATAATCCCGGCTCCCGCATGGGAGCGGGGTGAAAGGCGGCTTCGGCTGTCACTTGCGGATGG
GCCCGCGGCGCATTAGCTGGTTGGTGAGGTAACGGCTCACCAAGGCGACGATGCGTAGCCGACCTGAG
AGGGTGATCGGCCACACTGGGACTGAGACACGGCC
>Zotu1630
CAACGCTGGCGGCGCGCCTAACACATGCAAGTCGAACGAGAAAGTGGAGCAATCCATGAGTACAGTGG
CGTACGGGTGAGTAACACGTGGGTAATCTACCTCTTAGTGGGGAATAACCAGGGGAAACTCTGGCTAA
TACCGCATAAGCCTGAGAAGGGAAAGCAGCAATGCGCTGAGAGAGGAGCCCGCGGCCGATTAGCTAGT
TGGTGGGGTAAGAGCCCACCAAGGCAGAGATCGGTAGCCGGCCTGAGAGGGCACACGGCCACACTGGC
ACTGAAACACGGGC
>Zotu1631
GAACGCTGGCGGCGTGCCTAATACATGCAAGTCGAGCGAGGTTCTTCGGAACCTAGCGGCGGACGGGT
GAGTAACACGTGGGTAACCTGCCTGTAAGACTGGGATAACTTCGGGAAACCGAAGCTAATACCGGATA
CTTTTGAGCATCGCATGATGCTTGATGGAAAGACGGCTTCGGCTGTCACTTACAGATGGACCCGCGTC
GCATTAGCTAGTTGGTGAGGTAACGGCTCACCAAGGCGACGATGCGTAGCCGACCTGAGAGGGTGATC
GGCCACACTGGGACTGAGACACGGCC
>Zotu1632
GAATGCTGGCGGCGTGGATGAGGCATGCAAGTCGAATGAGTTTATACTCATGGCAGACGAGGTAGGAA
CACGTGGGTACGTACCCAAGAGTCAGGGATAATCCGTCGAAAGGCGGGATAATACTTGATAGTCCCTT
CGGGGTAAAGATTTATCGCTCTTGGAACGGCCCGCGGGCTATCAGCTTGTTGGTAAGGTAACGGCTTA
CCAAGGCTACGACGGCTAGGGGAGGTGAGAGCCTGACCCCCACCGATGGAACTGCGACACGGTC
>Zotu1633
GAACGCTAGCGGCAGGCTTAACACATGCAAGTCGAGGGGTATTTATCTTCGGATAGAGAGACCGGCGC
ACGGGTGCGTAACGCGTATGCAATCTACCTTTTACAGCGGGATAGCCCAGAGAAATTTGGATTAATAC

CACATAGTATTATAAGATGGCATCATTTTATAATTAAAGTCACAACGGTAAAAGATGAGCATGCGTCC
CATTAGCTAGATGGTAAGGTAACGGCTTACCATGGCTACGATGGGTAGGGGTCCTGAGAGGGAGATCC
CCCACACTGGTACTGAGACACGGAC
>Zotu1634
GAACGCTAGCGGCAGGCCTAATACATGCAAGTCGAACGGACCTTGTTCCTTCGGGGACTTGGTTAGTG
GCGCACGGGTGCGTAACGCGTATGCAACCTACCTTCTATTGGGGGATAGCCCCGGGAAACCGGGATTA
ATACCGCATGAGATCACTTGAGGGCATCCGAAGGTGATTAAAGCCGAGGCGATAGAAGATGGGCATGC
GTGCCATTAGCTAGTTGGCGGGGTAAGGGCCCACCAAGGCGACGATGGCTAGGGGTTCTGAGAGGATG
GTCCCCCACACTGGTACTGAGACACGGAC
>Zotu1635
GAACGCTGGCGGCGTGCCTAACACATGCAAGTCGAGCGGAGATTGCAGCAATGTAATCTTAGCGGCGG
ACTGGTGAGTAACGCGTGGGCAACCTGCCTCACACAGGGGGATAACACAGGGAAACTTGTGCTAATAC
CGCATAACATCATGGAGGGACATCCCTTTATGATCAAAGGAGCAATCCGGTGTGAGATGGGCCCGCGT
CCGATTAGTTAGTTGGCGGTGTAACGGACCACCAAGACGACGATCGGTAGCCGAACTGAGAGGTTGAT
CGGCCACATTGGAACTGAGATACGGTC
>Zotu1636
TAACGTTGGCAGTGTGCCTAACACATGCAAGTCGCGCGATATAGCGGCAGACGAGAGAGTAATAACAA
GGAACTTACCCCCGAGTGAAGGATAGCCCACCGAAAGGTGGGGTAACCCTGCATAACATCGAAAGATC
AAAGCCGTAAGGCGCTTGGGGAGAGGCCTTGTTCCTATCAGCTAGTTGGTGAGGTAATGGCTCACCAA
GGCGATGACGGGTAGCTGGACTGAGAGGTTGACCAGCCTCGATGGGACTGAGACACGGCC
>Zotu1637
GAACGCTGGCGGCGTGCCTAATACATGCAAGTCGAGCGAATCTCTTCGGAGATTAGCGGCGGACGGGT
GAGTAACACGTGGGCAACCTGCCTATAAGACTGGGATAACTTCGGGAAACCGGAGCTAATACCGGATA
ACATCAAAGGCCACATGGTCTTCGATTAAAAGATGGCTTCGGCTATCACTTATAGATGGGCCCGCGTC
GCATTAGCTAGTTGGTGAGGTAACGGCTCACCAAGGCGACGATGCGTAGCCGACCTGAGAGGGTGATC
GGCCACACTGGGACTGAGACACGGCC
>Zotu1638
GAACGCTAGCGGCAGGCCTAATACATGCAAGTCGAACGGTTCGCAAGAATAGTGGCAAACGGGTGCGT
AACGCGTAAGCAACCTGCCTCAAACTGGGGGATAGCCCGGCGAAAGCTGGGGTAAACCCGCACGGTTC
CCATTTATCACCTGGTAGTTGGGGTAAACATTTATGGGTTTGAGAGGGGCTTGCGTCTGATTAGTTAG
TTGGCAGGGTAACGGCCTACCAAGACGATGATCAGTAGGGGTTCTGAGAGGATTGGCCCCCACATGGG
TACTGAGATACGGAC
>Zotu1639
GAACGCTGGCGGCGTGCCTAATACATGCAAGTAGAACGCTGAAGCTTGGTGATTGCACCGAGCGGATG
AGTTGCGAACGGGTGAGTAACGCGTAGGTAACCTGCCTCTTAGCGGGGGATAACTATTGGAAACGATA
GCTAATACCGCATAAAAGTCGACATTGCATGATGTTGACTTGAAAGGTGCAATTGCATCACTAAGAGA
TGGACCTGCGTTGTATTAGCTAGTTGGTGAGGTAACGGCTCACCAAGGCAACGATACATAGCCGACCT
GAGAGGGTGATCGGCCACACTGGGACTGAGACACGGCC
>Zotu1640
GAACGCTGGCGGCATGCCTTACACATGCAAGTCGAACGGCAGCACGGGAGCAATCCTGGTGGCGAGTG
GCGAACGGGTGAGTAAAGTATCGGAACGTGCCCAGTCGTGGGGGATAACGTAGCGAAAGCTACGCTAA
TACCGCATACGATCTATGGATGAAAGCGGGGGATCGCAAGACCTCGCGCGATTGGAGCGGCCGATATC
AGATTAGGTAGTTGGTGGGGTAAAGGCTCACCAAGCCAACGATCTGTAGCTGGTCTGAGAGGACGACC
AGCCACACTGGAACTGAGACACGGTC
>Zotu1641
GAACGCTGGCGGCATGCCTAACACATGCAAGTCGAACGAAGTGCCTTCGGGTGCTTAGTGGCGCACGG
GTGCGTAACGCGTGGGAATCTACCCCTTGGTTCGGAATAACAGTTGGAAACGACTGCTAATACCGGAT
GATGACGTAAGTCCAAAGATTTATCGCCGAGGGATGAGCCCGCGTAGGATTAGGTAGTTGGTGTGGTA
AAGGCGCACCAAGCCGACGATCCTTAGCTGGTCTGAGAGGATGATCAGCCACACTGGGACTGAGACAC
GGCC
>Zotu1642
GAACGCTGGCGGCGTGCCTTAGGCATGCAAGTCGAATGGGGCCGCAAGGCCCAATGGCAGACGGGTGA
GTAATGGTAAGGAATCTACCTTAAAGTGGAGGATAGCTCGTCGAAAGACGGGGTAATCCTGCATGTGG
TCGCAAGACTAAAGGGCTTAACTGCCCGCTTTAAGAGGAGCCTTACTCCTATCAGCTAGTTGGTGGGG

TAATGGCCTACCAAGGCTATGACGGGTAGCTGGTGTGAGAGCATGACCAGCCACAATGGCACTGAGAC
ACGGGC
>Zotu1643
GAACGCTGGCGGCATGCCTAACACATGCAAGTCGAACGAAGCCTTCGGGCTTAGTGGCGCACGGGTGC
GTAACGCGTGGGAATCTGCCCTTGGGTTCGGAATAACAGTTAGAAATGACTGCTAATACCGGATGATG
TCTTCGGACCAAAGATTTATCGCCCAGGGATGAGCCCGCGTAGGATTAGGTAGTTGGTGGGGTAAAGG
CCTACCAAGCCGACGATCCTTAGCTGGTCTGAGAGGATGATCAGCCACACTGGGACTGAGACACGGCC
>Zotu1644
GAACGCTGGCGGCGTGCTTAACACATGCAAGTCGAGCGGAAAGGCCCTTTCGGGGGTACTCGAGCGGC
GAACGGGTGAGTAACACGTGAGTAATCTGCCCTTCACTTGGGGATAACCTCCGGAAACGGTGGCTAAT
ACCCGATACGACCATGGATCGCATGATCTTGTGGTGGAAAGTTTTTCGGTGGGGGATGTGCTCGCGGC
CTATCAGCTTGTTGGTGGGGTAATGGCCTACCAAGGCTTCGACGGGTAGCCGGCCTGAGAGGGTGACC
GGCCACACTGGGACTGAGACACGGCC
>Zotu1645
GAACGCTAGCGGCAGGCTTAATACATGCAAGTCGAACGGGATTTAGGAGCTTGCTTCTAATGAGAGTG
GCGCACGGGTGAGTAACACGTACACAATCTACCTTGTACAGGGGGATAGCCTTGGGAAACTGAGAATA
ATACCCCATAGTTTTAATTTATCGCATGATAGATTAAATAAAGCTGAGGCGGTACAAGATGAGTGTGC
GGATGATTAGGTAGTTGGTAGGGTAACGGCCTACCAAGCCGACGATCAATAGCTGGCGTGAGAGCGCG
ACCAGCCACACTGGAACTGAGACACGGTC
>Zotu1646
GAACGCTGGCAGTGTGCCTTAGACATGCAAGTCGGACGGGGCGCTTCTCTTTGATCCTTTCGGGGTGA
TTTGGGTTGTTGCCTAGTGGCGTACGGGTGAGTAACACGTGGGTGACCTGCCCTCAAGTGGGGGATAA
CTCTGGGAAACTGGGGCTAATCCCGCATGTGCTGATCTCTTTTTGGGGGGTTAGTAAAGGAACTTGTT
CCGCTTGAGGATGGGCCCGCGGCCTATCAGCTTGTTGGTGTGGTAATGGCGCACCAAGGCTATGACAG
GTAGCCGGCCTGAGAGGGTGTTCGGCCACAGGGGCACTGAGATACGGGC
>Zotu1647
GAACGCTAGCGGCAGGCTTAACACATGCAAGTCGAGGGGCAGCATGAGTGTTAGCAATAACACTTGAT
GGCGACCGGCGCAAGGGTGCGTAACGCGTGAGCAACTTGCCCGTTTCAGGGGGATAACCGGTGGAAAC
GCCGACTAATACCCCATGGCATTGGTTTGAGGCATCTCTTACCAATTAAAGAACTTCGGAAACGGATA
GGCTCGCGTGACATTAGCTAGATGGCGGGGTAACGGCCCACCATGGCGACGATGTCTAGGGGTTCTGA
GAGGAAGGTCCCCCACACTGGAACTGAGACACGGTC
>Zotu1648
GAACGCTGGCGGCGTGCTTAACACATGCAAGTCGAACGGGAATTACTTTATTGAAACTTCGGTCGATA
TGATTTAATTCTAGTGGCGGACGGGTGAGTAACGCGTGGGTAACCTGCCTTATACAGGGGGATAACAG
TCAGAAATGACTGCTAATACCGCATAAGCGCACAGAGCTGCATGGCTCGGTGTGAAAAACTCCGGTGG
TATAAGATGGACCCGCGTTGGATTAGCTGGTTGGTGAGGTAACGGCCCACCAAGGCGACGATCCATAG
CCGGCCTGAGAGGGTGAACGGCCACATTGGGACTGAGACACGGCC
>Zotu1649
GAACGCTGGCGGCGCGCTTAACACATGCAAGTCGAGCGATGAAACTTTAACAGAACCCTTCGGGGCGA
AGATAAAGTGGATTAGCGGCGGACGGGTGAGTAACACGTGAGTAACCTGCCTTAGACATTGGGATAGC
CTCGGGAAACCGGGATTAATACCAAATAAAATCATAATGACACATGTCATAACGATCAAAACTCCGGT
GGTCTAAGATGGACTCGCGTCCCATTAGCTAGTTGGTAGAGTAAAAGCCTACCAAGGCGACGATGGGT
AGCCGGCCTGAGAGGGTGAACGGCCACATTGGAACTGAGAAACGGTC
>Zotu1650
GAACGCTGGCGGCAGGCTTAACACATGCAAGTCGAACGCTGTAGCAATACAGAGTGGCAGACGGGTGA
GTAACGCGTGGGAACGTGCCTTTCGGTTCGGGATAACTCAGGGAAACTTGAGCTAATACCGGATACGC
CCTTAGGGGGAAAGATTTATTGCCGAAAGATCGGCCCGCGTCCGATTAGCTAGTTGGTGAGGTAATGG
CTCACCAAGGCGACGATCGGTAGCTGGTCTGAGAGGATGATCAGCCACACTGGGACTGAGACACGGCC
>Zotu1651
GAACGCTAGCGGCAGGCTTAATACATGCAAGTCGAACGGTAACAGGTGTAGCAATACACGCTGACGAG
TGGCGCACGGGTGCGTAACGCGTATGCAACTTACCCTTAACTGGAGGATAGCTCGAAGAAATTCGAAT
TAATACTCCATAGTATTGTGATCCCGCATGGGATTGCTCTTAAAGCTCCGGCGGTTAAGGATGGGCAT
GCGTGACATTAGCTAGTTGGTGAGGTAAAAGCTCACCAAGGCAACGATGTCTAGGGGATCTGAGAGGA
TTATCCCCCACACTGGTACTGAGACACGGAC

>Zotu1652
GAACGCTGGCGGCGTGCCTAATACATGCAAGTCGAACGCATTTTCTTTCACCGTAGCTTGCTACACCG
AAAGAAAATGAGTGGCGAACGGGTGAGTAACACGTGGGTAACCTGCCCATCAGCGGGGGATAACACTT
GGAAACAGGTGCTAATACCGCATAATTCCATTTACTGCATGGTAGATGGATGAAAGGCGCTTTTGCGT
CACTGATGGATGGACCCGCGGTGCATTAGCTAGTTGGTGGGGTAACGGCCTACCAAGGCTGCGATGCA
TAGCCGACCTGAGAGGGTGATCGGCCACACTGGGACTGAGACACGGCC
>Zotu1653
GAACGCTGGCGGCGTGCTTAACACATGCAAGTCGAACGGTGAACCCGCTTCGGTGGGGATCAGTGGC
GAACGGGTGAGTAACACGTGGGCAACCTGCCCCCGGCTCTGGGATAACTCCAAGAAATTGGGGCTAAT
ACCGGATATGACCGCTGGCCGCATGGTCTGGTGGTGGAAAGATTTATCGGCTGGGGATGGGCCCGCGG
CCTATCAGCTTGTTGGTGGGGTAGTGGCCTACCAAGGCGACGACGGGTAGCCGGCCTGAGAGGGTGAC
CGGCCACACTGGGACTGAGACACGGCC
>Zotu1654
GAACGCTGGCGGCGTGCCTAATACATGCAAGTCGAACGCATTGTGGTTGAAATGATATGAAGAACTTG
TTCAGATTTGATTTTCAACATTGCAATGAGTGGCGAACGGGTGAGTAACACGTGGGAAACCTACCTCT
TAGCAGGGGATAACATTTGGAAACAAATGCTAATACCGTATAATAATTAAAACCGCATGGTTTTAGTT
TAAAAGATGGTCTTGCTATCACTAAGAGATGGTCCCGCGGTGTATTAGTTAGTTGGTGAGGTAATGGC
TCACCAAGACAATGATACATAGCCGAGTTGAGAGACTGACCGGCCACAATGGGACTGAGACACGGCC
>Zotu1655
GAACGCTGACAGAATGCTTAACACATGCAAGTCTTTGGCGAATCTGTGCTTGCACAGGCTAGCCAAGG
CGGACGGGTGAGTAACGCGTAAAGAACTTGCCCTGCAGACAGGGATAACAGACGGAAACGACTGATAA
CACCTGATACAATTGCCGGCACGCATGTGCCTGGCAATGAAAAGAGATGCTGCGGGAGAGCTTTGCGT
CCTATTAGCTTGTTGGTGAGGTAACGGCTCACCAAGGCGATGATAGGTAGCCGGCCTGAGAGGGTGAA
CGGCCACAAGGGGACTGAGATACGGCC
>Zotu1656
GAACGCTGGCGGCGTGCCTAATACATGCAAGTCGAGCGAATCTGAGGGAGCTTGCTCCCAAAGATTAG
CGGCGGACGGGTGAGTAACACGTGGGCAACCTGCCTGTAAGACCGGGATAACTTCGGGAAACCGGAGC
TAATACCGGATAATTCTTTCCCTCACATGAGGGAAAGCTGAAAGACGGTTTCGGCTGTCACTTACAGA
TGGACCCGCGGCGCATTAGCTAGTTGGTGAGGTAACGGCTCACCAAGGCGACGATGCGTAGCCGACCT
GAGAGGGTGATCGGCCACATTGGGACTGAGACACGGCC
>Zotu1657
GAACGCTGGCGGCGTGCTTAACACATGCAAGTCGAACGGTGACGGTGGAGCTTGCTCCACCTGATCAG
TGGCGAACGGGTGAGTAACACGTGAGTAACCTGCCCCTTACTTCGGGATAACCTCGGGAAATCGTGGC
TAATACCGGATATGAGACCTTGCTGCATGGCTGGGTCTGGAAAGATTTATCGGTGAGGGATGGACTCG
CGGCCTATCAGCTTGTTGGTGAGGTAATGGCTCACCAAGGCGACGACGGGTAGCCGGCCTGAGAGGGC
GACCGGCCACACTGGGACTGAGATACGGCC
>Zotu1658
GAACGCTAGCGGCAGGCTTAATACATGCAAGTCTAGGGGGCAGCAATGTCACCGGCGAACGGGTGCGT
AACACGTATGCAACCTACCCATATCAGGGGGATAGCCCGGGGAAACTCGGATTAATACCGCATAAGAT
GGTACTACGGCATCGTAGAGCTATTAAAGATTTATCGGATATGGATGGGCATGCGTTAGATTAGCTAG
TTGGGGAGGTAACGGCTCACCAAGGCTACGATCTATAGGGGCTCTGAGAGGAGTAACCCCCACACTGG
CACTGAGATACGGGC
>Zotu1659
GAACGCTGGCGGCGTGCCTAATACATGCAAGTCGAGCGGATCTCGTCAAGAGATCAGCGGCGGACGGG
TGAGTAACACGTGGGCAACCTGCCTATAAGACTGGGATAACTTCGGGAAACCGGAGCTAATACCGGAT
AACATCAAGGACCACATGGTCTTCGATTAAAAGATGGCGTCAAGCTATCACTTATAGATGGGCCCGCG
TCGCATTAGCTAGTTGGTGAGGTAACGGCTCACCAAGGCGACGATGCGTAGCCGACCTGAGAGGGTGA
TCGGCCACACTGGGACTGAGACACGGCC
>Zotu1660
GAACGCTAGCGGCAGGCCTAATACATGCAAGTCGGACGGGATTGAGATTAAAGCTTGCTTTGATTTCA
TGAGAGTGGCGCACGGGTGCGTAACGCGTGAGCAACCTACCCATATCAGGGGGATAGCCTCTCGAAAG
AGAGATTAACACCGCATAACATCATTAACCGGCATCGGATTATGATCAAATATTTATAGGATATGGAT
GGGCTCGCGTGACATTAGCTAGTTGGAGAGGTAACGGCTCACCAAGGCTACGATGTCTAGGGGCTCTG
AGAGGAGAATCCCCCACACTGGTACTGAGACACGGAC

>Zotu1661
GAACGCTGGCGGCGTGCCTAATACATGCAAGTCGAGCGCAGGAAATCGACGGAACCCTTCGGGGGGAA
GTCGACGGAATGAGCGGCGGACGGGTGAGTAACACGTAAAGAACCTGCCCTCAGGTCTGGGATAACCA
CGAGAAATCGGGGCTAATACCGGATGGGTCATCGGACCGCATGGTCCGAGGATGAAAGGCGCTTCGGC
GTCGCCTGGGGATGGCTTTGCGGTGCATTAGCTAGTTGGTGGGGTAATGGCCCACCAAGGCGACGATG
CATAGCCGACCTGAGAGGGTGATCGGCCACACTGGGACTGAGACACGGCC
>Zotu1662
GAACGCTGGCGGCATGCCTAACACATGCAAGTCGAACGAGACCTTCGGGTCTAGTGGCGCACGGGTGC
GTAACGCGTGGGAATCTGCCCTTGGGTTCGGAATAACAGTTGGAAACGACTGCTAATACCGGATGATG
ACGTAAGTCCAAAGATTTATCGCCCAGGGATGAGCCCGCGTAGGATTAGCTAGTTGGTGGGGTAAAGG
CCTACCAAGGCGACGATCCTTAGCTGGTCTGAGAGGATGATCAGCCACACTGGGACTGAGACACGGCC
>Zotu1663
GAACGCTAGCGGCAGGCCTAACACATGCAAGTCGAGGGGCAGCACAGTGTAGCAATACATGGGTGGCG
ACCGGCGCACGGGTGAGTAACGCGTATGCAACCTACCCTATACTGGGGGATAGCTCAGAGAAATCTGA
ATTAAACCCGCATAAAGCAGGGGTACCGCATGGTACTATTTGCTAAAACTCCGGTGGTATAGGATGGG
CATGCGTGTCATTAGCTAGTTGGTGAGGTAACGGCTCACCAAGGCGACGATGACTAGGGGGTCTGAGA
GGATGATCCCCCACACTGGTACTGAGACACGGAC
>Zotu1664
GAACGCTGGCGGCGTGCTTAACACATGCAAGTCGAACGGTGAAGCCCAGCTTGCTGGGTGGATCAGTG
GCGAACGGGTGAGTAACACGTGAGCAACCTGCCCCTGACTCTGGGATAAGCGCTGGAAACGGCGTCTA
ATACTGGATATGTGACGTGACCGCATGGTCTGCGTTTGGAAAGATTTTTCGGTTGGGGATGGGCTCGC
GGCCTATCAGCTTGTTGGTGAGGTAATGGCTCACCAAGGCGTCGACGGGTAGCCGGCCTGAGAGGGTG
ACCGGCCACACTGGGACTGAGACACGGCC
>Zotu1665
GAACGCTAGCGGCAGGCCTAATACATGCAAGTCGAACGCGATTATCCAGCTTGCTGGATATGAAAGTG
GCGCACGGGTGCGTAACGCGTGAGCAACCTACCTCTGTCAGGGGGATAGCCCGGCGAAAGTCGGATTA
ACACCGCATGACATTATTGATGTGGCATCACATTATAATCAAATATTTATAGGACAGAGATGGGCTCG
CGTGACATTAGCTAGTTGGAGAGGTAACGGCTCACCAAGGCAACGATGTCTAGGGGCTCTGAGAGGAG
AATCCCCCACACTGGTACTGAGACACGGAC
>Zotu1666
GAACGCTAGCGGGAGGCCTAACACATGCAAGCCGAGCGGTATTTGTTCTTCGGAACAGAGAGAGCGGC
GCACGGGTGCGGAACACGTGTGCAACCTGCCTTTATCTGGGGGATAGCCTTTCGAAAGGAAGATTAAT
ACCCCATAATATATTGAGTGGCATCATTTGATATTGAAAACTCCGGTGGATAGAGATGGGCACGCGCA
AGATTAGATAGTTGGTGAGGTAACGGCTCACCAAGTCAATGATCTTTAGGGGTCCTGAGAGGGAGATC
CCCCACACTGGTACTGAGACACGGAC
>Zotu1668
GAACGCTGGCGGTATGCCTAATACATGCAAGTCGAACGAGATCTTCGGATCTAGTGGCGCACGGGTGC
GTAACGCGTGGGAATCTGCCCTTGGGTTCGGAATAACGTCGGGAAACTGACGCTAATACCGGATGATG
ACGAAAGTCCAAAGATTTATCGCCCAGGGATGAGCCCGCGTAGGATTAGCTAGTTGGTGAGGTAAAGG
CTCACCAAGGCTACGATCCTTAGCTGGTCTGAGAGGATGATCAGCCACACTGGGACTGAGACACGGCC
>Zotu1669
GAACGCTGGCGGCGTGCTTAACACATGCAAGTCGAACGAAGAGCGATGGAAGCTTGCTTCTATCAATC
TTAGTGGCGAACGGGTGAGTAACGCGTAATCAACCTGCCCTTCAGAGGGGGACAACAGTTGGAAACGA
CTGCTAATACCGCATACGATCTAACCTCGGCATCGAGGAAAGATGAAAGGTGGCCTCTATGTATAAGC
TATCACTGAAGGAGGGGATTGCGTCTGATTAGCTAGTTGGAGGGGTAACGGCCCACCAAGGCGATGAT
CAGTAGCCGGTCTGAGAGGATGAACGGCCACATTGGGACTGAGACACGGCC
>Zotu1670
GAACGCTGGCGGCGTGCTTAACACATGCAAGTCGAACGGTGAACACGGAGCTTGCTCTGTGGGATCAG
TGGCGAACGGGTGAGTAACACGTGAGCAACCTGCCCCTGACTCTGGGATAAGCGCTGGAAACGGCGTC
TAATACTGGATACGAGTAGCGGCCGCATGGTCAGTTACTGGAAAGATTTATTGGTTGGGGATGGGCTC
GCGGCCTATCAGCTTGTTGGTGAGGTAATGGCTCACCAAGGCGTCGACGGGTAGCCGGCCTGAGAGGG
TGACCGGCCACACTGGGACTGAGACACGGCC
>Zotu1671
GAACGCTGGCGGTCTGCTTAACACATGCAAGTCGAGCGGAGGTTTTCGGACCTCAGCGGCGGACGGGT

GAGTAACGCGTGAGAACCTGCCCTCAGGTTGGGGACAACAGTTGGAAACGACTGCTAATACCCAATGT
GCTTTCGAGTGAAAGATTTATCGCCTGAGGATGGGCTCGCGTCCGATTAGGTAGTTGGAGTGGTAAGG
GCACCCCAAGCCATTGATCGGTAGCTGGTCTGAGAGGATGACCAGCCACACTGGGACTGAGACACGGC
C
>Zotu1672
TAACGCTGGCGGCATGCCTAATACATGCAAGTCGAACGAAAGTAGCAATCTTTAGTGGCGAACGGGTG
AGTAACACGTATCTAACCTACCATTTAGTGGGGAATAACTGTTGGAAACGGCAGCTAATACCGCATAT
GTTATTTATTTGCAATGATAAATAATGAAAGGAGCATTTGCTTCGCTAATTGATGGGGATGCGGCGCA
TTAACTAGTTGTGGTAAGGTAATGGCTTACCAAGGTAACGACGCGTAGCCGATCTGAGAGGATGAACG
GCCACATTGGGACTGAGACACGGCC
>Zotu1673
GAACGCTGGCGGCGTGCTTAACACATGCAAGTCGAACGATGATCCCCAGCTTGCTGGGGGGATTAGTG
GCGAACGGGTGAGTAACACGTGAGTAACCTGCCCCTGACTCTGGGATAAGCGCTGGAAACGGCGTCTA
ATACCGGATATGACAGCCGACGGCATCGTCTGGTTGTGGAAAGATTTTTCGGTTGGGGATGGACTCGC
GGCCTATCAGGTTGTTGGTGAGGTAATGGCTCACCAAGCCTACGACGGGTAGCCGGCCTGAGAGGGTG
ACCGGCCACACTGGGACTGAGACACGGCC
>Zotu1674
GAACGCTGGCGGCGCGCCTAATACATGCAAGTCGAGCGAGAAGACGTAGCAATACGTTTGTAAAGCGG
CGAACGGGTGAGGAATACATGGGTAACCTACCCTCGAGTGGGGAATAACTAGCCGAAAGGTTAGCTAA
TACCGCATACGACTCTTGGTCTGCGGATCGAGAGGGAAAGCGATACCGTGGGTATCGCGCTCATGGAT
GGGCTCATGTCCTATCAGCTTGTTGGTGAGGTAACGGCTCACCAAGGCTTCGACGGGTAGCTGGTCTG
AGAGGACGATCAGCCACACTGGCACTGCGACACGGGC
>Zotu1675
GAACGCTGGCGGCGTGCTTAACACATGCAAGTCGAACGGACCCTTCGGGGTTAGTGGCGGACGGGTGA
GTAACGCGTGAGGATCTGCCCTTAGGAGGGGAACAACAGTTGGAAACGACTGCTAATGCCCCATATGC
CGAGAGGTGAAAAGTTATATCGCCTGAGGATGAACTCGCGTCTGATTAGCTAGTTGGTAGTGTAATGG
ACTACCAAGGCGACGATCAGTAGCTGGTCTAAGAGGATGATCAGCCACACTGGGACTGAGACACGGCC
>Zotu1677
GAACGCTGGCGGCGTGCTTAACACATGCAAGTCGAACGGTGAACCCCTTCGGGGGGGATCAGTGGCGA
ACGGGTGAGTAACACGTGAGCAACCTGCCCCTGGCTCTGGGACAACCACTGGAAACGGTGGCTAATAC
CGGATACGACTCATCACCGCATGGTGTGTGGGTGGAAAGTTTTTCGGCTGGGGATGGGCTCGCGGCCT
ATCAGCTTGTTGGTGGGGTAATGGCCTACCAAGGCGACGACGGGTAGCCGGCCTGAGAGGGCGACCGG
CCACACTGGGACTGAGACACGGCC
>Zotu1678
GAACGCTGGCGGCGTGCCTAACACATGCAAGTCGAGCGAGAGACTTCGGTCTCTAGCGGCGGACGGGT
GAGTAACACGTGGGTAACCTGCCTCAAACAGGGGGATAGCCTTCCGAAAGGAAGATTAATACCGCATA
ACATAGTTTAGTCGCATGACTAGATTATCAAAGGAGTAATCCGGTTTGAGATGGACCCGCGGCGCATT
AGCTAGTTGGTGAGGTAACGGCTCACCAAGGCGACGATGCGTAGCCGACCTGAGAGGGTGATCGGCCA
CATTGGAACTGAGATACGGTC
>Zotu1679
GAACGCTAGCGGCAGGCTTAACACATGCAAGTCGAGGGGTATAGTAGCAATACTAGAGACCGGCGCAC
GGGTGCGTAACGCGTATGCAATCTACCTTTTACAGAGGGATAGCCCAGAGAAATTTGGATTAATACCT
CATAGTATTATTGATTGGCATCAATTGATAATTAAAGTTACAACGGTAAAAGATGAGCATGCGTCCCA
TTAGCTAGTTGGTAAGGTAACGGCTTACCAAGGCGACGATGGGTAGGGGTCCTGAGAGGGAGATCCCC
CACACTGGTACTGAGACACGGAC
>Zotu1680
GAACGCTGGCGGCATGCCTTACACATGCAAGTCGAACGGCAGCACGGGAGCAATCCTGGTGGCGAGTG
GCGAACGGGTGAGTAATACATCGGAACGTGCCCAATCGTGGGGGATAACGCAGCGAAAGCTGTGCTAA
TACCGCATACGATCTACGGATGAAAGCAGGGGACCGCAAGGCCTTGCGCGAATGGAGCGGCCGATGGC
AGATTAGGTAGTTGGTGGGGTAAAGGCTCACCAAGCCTGCGATCTGTAGCTGGTCTGAGAGGACGACC
AGCCACACTGGGACTGAGACACGGCC
>Zotu1681
GAACGCTGGCGGCGTGCTTAACACATGCAAGTCGAACGGTAAGGCGCTTTCGGGCGTACACGAGTGGC
GAACGGGTGAGTAACACGTGAGGAACGTGCCCTTGACTTTGGTATAGCACTTGGAAACAGGTGGTAAT

CCCGAATATTCAGTTTCCCGGGCATCCGGGTTGTTGGAAAGCTTTTTGCGGTCAGGGATCGTCTCGCG
GCCTATCAGCTTGTTGGTGGGGTAGTGGCCCACCAAGGCCTTGACGGGTAGCCGGCCTGAGAGGGCGA
CCGGCCACATTGGGACTGAGATACGGCC
>Zotu1682
GAACGCTGGCGGCGTGCTTAACACATGCAAGTCGAACGGTGAAGGAGAGCTTGCTCTCTGGATCAGTG
GCGAACGGGTGAGTAACACGTGAGCAATCTGCCCCTGACTCTGGGATAAGCGTTGGAAACGACGTCTA
ATACCGGATACGAGCTGCGAAGGCATCTTCAGCAGCTGGAAAGAACTTCGGTCAGGGATGAGCTCGCG
GCCTATCAGCTAGTTGGTGAGGTAATGGCTCACCAAGGCGTCGACGGGTAGCCGGCCTGAGAGGGTGA
CCGGCCACACTGGGACTGAGACACGGCC
>Zotu1683
GAACGCTGGCGGCGTGCCTAACACATGCAAGTCGAGCGAACCCTTCGGGGTGAGCGGCGGACGGGTGA
GTAACGCGTGGGTAACCTGCCCTATACACACGGATAACGTACCGAAAGGTACGCTAATACGAGATAAC
ATATTTTTATCGCATGGTAGAAATATCAAAGCTCCGGCGGTATAGGATGGACCCGCGTCTGATTAGCT
AGTTGGTAAGGTAATGGCTTACCAAGGCGACGATCAGTAGCCGACCTGAGAGGGTGATCGGCCACATT
GGAACTGAGACACGGTC
>Zotu1684
GAACGCTGGCGGCGTGCTTAACACATGCAAGTCGAGCGGAAAGGCCCACTTGTGGGTACTCGAGCGGC
GAACGGGTGAGTAACACGTGAGTAATCTGCCCTCCACTCTGGGATAACGTTCAGAAATGGGTGCTAAT
ACCGGATGTACACAGACTGGCGCATGTTGGTGTGTGGAAAGGTTTTTCTGGTGGGGGATGTGCTCGCG
GCCTATCAGCTTGTTGGTGGGGTAATGGCCTACCAAGGCTTTGACGGGTAGCCGGCCTGAGAGGGTGA
CCGGCCACACTGGGACTGAGACACGGCC
>Zotu1685
GAACGCTGGCGGCGTGCCTTAAGCATGCAAGTCGTACGAAGATATGAGATGTCGCAAGACAGATTGTT
GATTAGTGGCGGACGGGTGAGTATAAGCAGGGAACGTACCTTGAACTATCGGATAGCTCGTCGAAAGA
CGGGGTAATACGGTATGTGGACGCAAGTCTAAAGACCGCAAGGTCGGTTCTAGAGCGGCCCTGTTCCC
ATCAGCTTGTTGGTAGGGTAACGGCCTACCAAGGCTACGACGGGTAGGGGGTGTGAGAGCACGACCCC
CCACAATGGCACTGAGACACGGGC
>Zotu1686
GAACGCTGGCGGCGTGCTTAACACATGCAAGTCGAACGATGATGCCCAGCTTGCTGGGTGGATTAGTG
GCGAACGGGTGAGTAACACGTGAGTAATCTGCCCTTCACTTCGGGATAAGCCTCGGAAACGGGGTCTA
ATACTGGATATGACCTTCTCTCGCATGGGGGTTGGTGGAAAGTTTTTCGGTGGGGGATGTGCTCGCGG
CCTATCAGCTTGTTGGTGAGGTAATGGCTCACCAAGGCGACGACGGGTAGCCGGCCTGAGAGGGTGAT
CGGCCACACTGGGACTGAGACACGGCC
>Zotu1687
GAACGCTGGTGGCGTGCTTAACACATGCAAGTCGAGCGAGGCGTCCGCCTCCGGGCGGTCAGCCGAGC
GGCGAACGGGTGAGTAACACGTGAGCAATCTTCCCTAGGCTCGGGGATAACCTCGGGAAACTGAGGCT
AATACCGGATGAGCGTTTTTCGGGGCATCCCGGAAAACGAAAAGGTTTCGCACCGCGAGGTGCGTTTC
CGGCCTTGGATGAGCTCGCGGCCTATCAGCTTGTTGGTGTGGTAAATGCGCACCAAGGCGACGACGGG
TAGCCGGCCTGAGAGGGTGATCGGCCACACTGGGACTGAGACACGGCC
>Zotu1688
GAACGCTGGCGGCGCGCTTAACACATGCAAGTCGAGCGAGAACCTTGCCTTCGGGCTTGGGGACAGCG
GCGAACGGGTGAGTAACACGTGGGTAATCAACCCTCGACACTGGGATAGCCCGGGGAAACCCGGATTA
ATACCGGATACCCCATCTGGCCTTCGGGCCGGCTGGAAAAGGTAGCTTTGGCCTCCGGTCGAGGACGA
GCCCGCGGCGGATTAGCTTGTTGGTGGGGTAAAAGCCCACCAAGGCGACGATCCGTAGCTGGTCTGAG
AGGACGATCAGCCACACTGGGACTGAGACACGGCC
>Zotu1689
AAACGCTGGCGGCGTGCCTAAAACATGCAAGTCGAACGGGCACCTTCGGGTGTCAGTGGCAGACGGCT
GAGTAACGCGTGGGTGACGTACCTATGAGTGGGGAATAACTGCCCGAAAGGGTGGCTAATTCCGCATA
CGCCTGATCTTTCGGTAGGTCAGGGAAAGTCGCAAGACGCTCGTGGAGCGGCCCGCGTTCGATTAGCT
CGTTGGTGGGGTAATGGCCTACCAAGGCGACGATCGATAGCTGGTCTCAGAGGATGATCAGCCAGACT
GGGACTGAGACACGGCC
>Zotu1690
GAACGCTGGCGGCGTGCTTAACACATGCAAGTCGAACGGTGAACCTGCTTCGGTGGGGGATCAGTGGC
GAACGGGTGAGTAACACGTGGGCAACCTGCCCTCAGCTCTGGGATAACTCCAAGAAATTGGGGCTAAT

ACCGGATATGACCGCTGACCGCATGGTCTGGTGGTGGAAAGATTTATCGGCTGGGGATGGGCCCGCGG
CCTATCAGCTTGTTGGTGGGGTAGTGGCCTACCAAGGCGACGACGGGTAGCCGGCCTGAGAGGGTGAC
CGGCCACACTGGGACTGAGACACGGCC
>Zotu1691
GAACGCTGGCGGCGTGCCTTAAGCATGCAAGTCGTACGGCTCCGCAAGGAGCAGTGGCGGACGGTCGA
GTAACGCGTGGGGATCTACCTTGAAGTTGGACATAGCTCACCGAAAGGTGGGGTAATTTCCAATGTGA
ACGTAAGTTCAAAGCCGTAAGGCGCTTTAAGATGAACCCGCGTCCTATCAGCTAGTTGGTGAGGTAAT
GGCTCACCAAGGCATCGACGGGTAGCTGGTCTGAGAGGATGATCAGCCGGAATAGCACTGAGACACGG
GC
>Zotu1692
GAATGCTGGCGGCGTGGATGAGGCATGCAAGTCGAATGGGTTTAGGCCCATGGCAGACGAGGTAGGAA
CACGTAGGTACGTACCCCAAAGTCAGGGATAACCCGGAGAAATCCGGGATAATACTTGATAGTCTCTT
CGGAGTAAAGATTTATCGCTTTGGGAACGGCCTGCGGGCTATCAGCTTGTTGGTAAGGTAACGGCTTA
CCAAGGCTACGACGGCTAGGGGAGGTGAGAGCCTGACCCCCACCGATGGAACTGCGACACGGTC
>Zotu1693
GAACGCTGGCGGCAGGCTTAACACATGCAAGTCGAACGGGCTTCTTCGGAAGTCAGTGGCAGACGGGT
GAGTAACACGTGGGAACGTGCCCTTCGGTTCGGAATAACTCAGGGAAACTTGAGCTAATACCGGATAC
GCCCTTTTGGGGAAAGGTTGACTGCCGAAGGATCGGCCCGCGTCTGATTAGCTTGTTGGTGGGGTAAC
GGCCTACCAAGGCGACGATCAGTAGCTGGTCTGAGAGGATGATCAGCCACACTGGGACTGAGACACGG
CC
>Zotu1694
GAACGCTGGCGGCGTGCTTAACACATGCAAGTCGAACGGAAAGGCCCTTCGGGGTACTCGAGTGGCGA
ACGGGTGAGTAACACGTGGGTGATCTGCCTCGTACTTTGGGATAAGCCTGGGAAACTGGGTCTAATAC
CGAATATGACCTTGGGATGCATGTCTCTTGGTGGAAACTTTTTGGGTACGAGATGGGCCCGCGGCCTA
TCAGCTTGTTGGTGGGGTAATGGCCTACCAAGGCGACGACGGGTAGCCGGCCTGAGAGGGCGACCGGC
CACACTGGGACTGAGACACGGCC
>Zotu1695
GAACGCTGGCGGCGTGCTTTACACATGCAAGTCGAACGGCAGCATAGAATAGTAGCAATACTATTGGG
TGGCGAGTGGCGGACGGGTGAGTAATACATCGAGACGTGCCTAATAGATTGGGATAACCTGTCGAAAG
ATAGGCTAATACCGGATAATCTCTGAGGAGGAAAGCTGGGGACTTAGGAATAAGCCTGGCGCTAATAG
AGCGATCGATGGCCGATTAGTTAGTTGGTGGGGTAAAGGCTTACCAAGGCGATGATCGGTAGCTGTTC
TGAGAGGAAGATCAGCCACACTGGAACTGAGAGACGGTC
>Zotu1696
GAACGCTAGCGGCAGGCCTAATACATGCAAGTCGAACGGTAAGTGGAATAGCAATATTCTACCTAGAG
TGACGTACGGGTGCGTAACACGTATGCAACCTACCCGCAACTGGGGTATAGCTCGGTGAAAACCGGAT
TAACCCCCCATAAGACCACGCGAGGGCATCTGAGCGTGGTAAAAACTGAGGTGGTTGCGGATGGGCAT
GCGGATGATTAGCTAGTTGGCGGTGTAACGGACCACCAAGGCGATGATCATTAGGGGAACTGAGAGGT
TGATCCCCCACACTGGCACTGAGATACGGGC
>Zotu1697
GAACGCTGACAGAATGCTTAACACATGCAAGTCAACTTGAATTTGGGTTTTTAACTTAGATTTGGGTG
GCGGACGGGTGAGTAACGCGTAAAGAACTTGCCTCACAGCTAGGGACAACATTTAGAAATGAATGCTA
ATACCTGATATTATGATTTTAAGGCATCTTAGAATTATGAAAGCTATATGCACTGTGAGAGAGCTTTG
CGTCCCATTAGCTAGTTGGAGAGGTAACAGCTCACCAAGGCGATGATGGGTAGCCGGCCTGAGAGGGT
GAACGGCCACAAGGGGACTGAGACACGGCC
>Zotu1698
GAACGCTGGCGGCGTGCCTAATACATGCAAGTCGAGCGAATTTTTGGGAGCTTGCTCCCAAAAATTAG
CGGCGGACGGGTGAGTAACACGTGGGCAACCTGCCTATAAGACTGGGATAACTTCGGGAAACCGGAGC
TAATACCGGATAATTCTTTTCCTCTCATGAGGAAAAGCTGAAAGACGGTTTACGCTGTCACTTATAGA
TGGGCCCGCGGCGCATTAGCTAGTTGGTGAGGTAACGGCTCACCAAGGCAACGATGCGTAGCCGACCT
GAGAGGGTGATCGGCCACACTGGGACTGAGACACGGCC
>Zotu1699
GAACGCTGGCGGCGTGCTTAAGACATGCAAGTCGAACGATCGTCTTCGGACGGTAGTGGCGCACGGGT
GAGTAACGCGTAACTGACCTGCCCCCAAGTTCGGAATAACTTCCCGAAAGGGAAGCTAATACCGGATG
TGCTGTCCCCTCCTGTGGGGCCAGTAAAGGCTTGACCGCTTGGGGATGGGGTTGCGTTCCATCAGCTA

GATGGTGGGGTAAAGGCCTACCATGGCGACGACGGATAACCGGCCTGAGAGGGTGGCCGGTCACAGGG
GCACTGAGACACGGGT
>Zotu1700
GAACGCTGGCGGCGTGCCTAATACATGCAAGTCGAGCGAACAGATAAGGAGCTTGCTCCTTTGACGTT
AGCGGCGGACGGGTGAGTAACACGTGGGTAACCTACCTATAAGACTGGAATAACTCCGGGAAACCAGG
GCTAATGCCGGATAACATTTAGAACCGCATGGTTCTAAAGTGAAAGATGGTTTTGCTATCACTTATAG
ATGGACCCGCGCCGTATTAGCTAGTTGGTAAGGTAATGGCTTACCAAGGCAACGATACGTAGCCGACC
TGAGAGGGTGATCGGCCACACTGGAACTGAGACACGGTC
>Zotu1701
GAACGCTGGCGGCATGCCTTACACATGCAAGTCGAACGGTAACGCGGGGCAACCTGGCGACGAGTGGC
GAACGGGTGAGTAATGCATCGGAACGTGCCCAGTAGTGGGGGATAGCCCGGCGAAAGCCGGATTAATA
CCGCATACGACCTACGGGTGAAAGCGGGGGACCGCAAGGCCTCGCGCTATTGGAGCGGCCGATGTCAG
ATTAGGTAGTTGGTGGGGTAAAGGCCTACCAAGCCGACGATCTGTAGCTGGTCTGAGAGGACGACCAG
CCACACTGGGACTGAGACACGGCC
>Zotu1703
GAACGCTGGCGGCGTGCTTAACACATGCAAGTCGAACGGTGAAGGAGAGCTTGCTCTCTGGATCAGTG
GCGAACGGGTGAGTAACACGTGAGCAATCTGCCCTTGACTCTGGGATAAGCGCTGGAAACGGCGTCTA
ATACTGGATACGAGCTGCGAAGGCATCTTCAGCAGCTGGAAAGAACTTCGGTCAAGGATGAGCTCGCG
GCCTATCAGCTTGTTGGTGAGGTAATGGCTCACCAAGGCGTCGACGGGTAGCCGGCCTGAGAGGGTGA
CCGGCCACACTGGGACTGAGACACGGCC
>Zotu1704
GAACGCTGGCGGCGTGCTTAACACATGCAAGTGGAGCGACGAACCAGGGCTTGCCCTGGGGCAGAGCC
GCGAACGGGTGAGTAACACGTGGGTTACCTGCCTCGATGACCGGGACAACCCGAGGAAACTCGGGCTA
ATACCGGATGTGCCCGCAAGGGGAAAGGAAGCTTCGGCCTCCGCATCGAGATGGGCCCGCGGCCCATT
AGCTAGTTGGTGAGGTAACGGCTCACCAAGGCTCCGATGGGTAGCTGGTCTGAGAGGACGATCAGCCA
CACTGGGACTGAGACACGGCC
>Zotu1705
GAACGCTGGCGGCGTGCCTAATACATGCAAGTCGAGCGGTAAGGTTCTCACTCTGTATTTAACTAGTT
TGAATATAGTGTGAGAATACACGAGCGGCGGACGGCTGAGTAACACGTAGGAACGTACCCCAAAGTGA
GGGATAAGCATCGGAAACGATGTCTAATACCGCATGTGATCTTCGGATTAAAGCTTCGGCGCTTTGGG
AACGGCCTGCGCATGATTAGCTTGTTGGTGAGGTAATAGCTCACCAAGGCGATGATCATTAGCTGGTC
TGAGAGGATGACCAGCCAGACTGGGACTGAGAACGGCC
>Zotu1707
GAACGCTGGCGGCGTGCCTAATACATGCAAGTCGAACGCTTCTTTCCTCCCGAGTGCTTGCACTCAAT
TGGAAAGAGGAGTGGCGGACGGGTGAGTAACACGTGGGTAACCTACCCATCAGAGGGGGATAACACTT
GGAAACAGGTGCTAATACCGCATAACAGTTTATGCCGCATGGCATAAGAGTGAAAGGCGCTTTCGGGT
GTCGCTGATGGATGGACCCGCGGTGCATTAGCTAGTTGGTGAGGTAACGGCTCACCAAGGCCACGATG
CATAGCCGACCTGAGAGGGTGATCGGCCACACTGGGACTGAGACACGGCC
>Zotu1708
GAACGCTAGCGGCAGGCTTAACACATGCAAGTCGAGGGGCAGCACAGGTAGCAATACCGGGTGGCGAC
CGGCAAACGGGTGCGGAACACGTACTGAATCTACCTTTGTCTGGGGCATAGCCCGAAGAAATTCGGAT
TAACTCCCCATAATATAGATGAGTGGCATCACTCGACTATTAAAAATTTATTGGACAGAGATGACAGT
GCGTCCCATTAGCTAGTTGGTAGGGTAACGGCCTACCAAGGCGACGATGGGTAGCTGGTGTGAGAGCA
CGACCAGCCACACGGGCACTGAGACACGGGC
>Zotu1709
GAACGCTGGCGGCAGGCTTAACACATGCAAGTCGAACGCTCGTCTTCGGACGGGAGTGGCAGACGGGT
GAGTAACACGTGGGAACGTACCCTTTGGTTCGGAATAACGCAGGGAAACTTGCGCTAATACCGGATAC
GCCCTTTTGGGGAAAGGTTTACTGCCGAAGGATCGGCCCGCGTCTGATTAGCTAGTTGGTGGGGTAAC
GGCCTACCAAGGCGACGATCAGTAGCTGGTCTGAGAGGATGATCAGCCACACTGGGACTGAGACACGG
CC
>Zotu1710
GAACGCTAGCGACAGGCTTAACACATGCAAGTCGAGGGGCAGCGGAGGTAAAGGCTTGCCTTTACCTG
CCGGCGACCGGCGCACGGGTGAGTAACACGTATGCAACCTGGCCGTCACAGGAGGATAACCGGGAGAA
ATTCCGCCTAATACTCCATAACACCGGAGGGAGACATCTCCCACCGGTCAAAGGAATTTATTCCGGTG

ACGGATGGGCATGCGGCGCATTAGCTAGTCGGCGGGGTAACGGCCCACCGAGGCGACGATGCGTAGGG
GTTCTGAGAGGAAGGTCCCCCACATTGGAACTGAGACACGGTC
>Zotu1711
GAACGCTGGCGGCGTGCCTAATACATGCAAGTCGAGCGGACTGAATGGAAGCTTGCTTCCGTTCGGTT
AGCGGCGGACGGGTGAGTAACACGTGGGCAACCTGCCCGTAAGACCGGGATAACTTCGGGAAACCGGA
GCTAATACCGGATAACACCGAAGATCGCATGATCTTTGGTTGAAAGGCGGCTTTGGCTGTCACTTACG
GATGGGCCCGCGGCGCATTAGCTAGTTGGTGAGGTAACGGCTCACCAAGGCGACGATGCGTAGCCGAC
CTGAGAGGGTGATCGGCCACACTGGGACTGAGACACGGCC
>Zotu1712
GAACGCTGGCGGCGTGCTTAACACATGCAAGTCGAACGATGAAGCCCACTTGTGGGTGGATTAGTGGC
GAACGGGTGAGTAACACGTGAGTAACCTGCCCTTGACTCTGGGATAAGCCTGGGAAACTGGGTCTAAT
ACCGGATATGACTCCTCATCGCATGGTGGGGGGTGGAAAGCTTTTTGTGGTTTTGGATGGACTCGCGG
CCTATCAGCTTGTTGGTGAGGTAATGGCTTACCAAGGCGACGACGGGTAGCCGGCCTGAGAGGGTGAC
CGGCCACACTGGGACTGAGACACGGCC
>Zotu1713
GAACGCTAGCTACAGGCTTAACACATGCAAGTCGAGGGGAAACGGCATTAAGTGCTTGCACTTTTTGG
ACGTCGACCGGCGCACGGGTGAGTAACGCGTATCCAACCTTCCCATGACTAAGGGATAACCCGCCGAA
AGGCAGACTAATACCTTATGGCCTTCACTGACGGCATCAGATGTGAAGTAAAGATTTATCGGTTATGG
ATGGGGATGCGTCTGATTAGCTTGTTGGCGGGGTAACGGCCCACCAAGGCAACGATCAGTAGGGGTTC
TGAGAGGAAGGTCCCCCACATTGGAACTGAGACACGGTC
>Zotu1714
GAACGCTGGCGGCAGGCCTAACACATGCAAGTCGAGCGCGTGTAGCAATACACGAGCGGCGCACGGGT
GAGTAACGCGTGGGAATCTGCCCTACAGTGGGGAACAACCACGGGAAACTGTGGCTAATACCGCATAC
GTCCTTCGGGAGAAAGGCTTAGGCCGCTGTAGGAGGAGCCCGCGTCCGATTAGCTAGTTGGTGAGGTA
ATGGCTCACCAAGGCGACGATCGGTAACTGGTCTGAGAGGACGACCAGTCACACTGGGACTGAGACAC
GGCC
>Zotu1715
GAACGCTAGCGGCAGGCCTAACACATGCAAGTCGAGGGGCAGCACGAATTAGCTTGCTAATTTGGTGG
CGACCGGCGCACGGGTGCGTAACGCGTATGCAACCTACCTTATACAGGGGTATAGCCCGGAGAAATTC
GGATTAATCCCCCATAGTACTGAGACACCGCATGGTGATTCAGTTAAAGCTTCGGCGGTATAAGATGG
GCATGCGTCCTATTAGCTAGATGGTAAGGTAACGGCTTACCATGGCTACGATAGGTAGGGGGCCTGAG
AGGGTGGTCCCCCACACTGGTACTGAGACACGGAC
>Zotu1716
GAACGCTGGCGGCGTGCTTAACACATGCAAGTCGAACGGGATTTAGTAGACAGAAACCTCGGTGGAAG
ATTACTAATGAGAGTGGCGAACGGGTGAGTAACGCGTGAGCAACCTGCCTATGACAGTGGGATAGCCT
CGGGAAACCGGGATTAATACCGCATAAAATCGTAGAAACACATGTTTCAACGGTCAAAGATTTATCGG
TCATAGATGGGCTCGCGTCTGATTAGCTAGTTGGTGAGATAACAGCCCACCAAGGCGACGATCAGTAG
CCGGTCTGAGAGGATGAACGGCCACATTGGAACTGAGACACGGTC
>Zotu1717
GAATGTTGGCGGTGTGCCTGAGATATGCAAGTCGAATGCCTTGTGCTTTGCACAAGAAATTCCAATAA
CCAAATTACAAGAAACAAACAATTACCAATATCTAAATTTTCAATGTTCAAAACATTGGAGATTTGAA
TTTGTTTGTAATATTTGGAATTTTAAAGTTGGAGCTTCTCGAGCAGAGCACGAGTAATGGCGGACGGC
TGAGTAACACATAGGGACTTACCCTTTTTTGAAGAATAGCCCATCGAAAGGTGGGATAATACTTCATG
GTATGAATGTCGTAAGATATTTTTTAAAGAGATTAACTTCTCGAAAAAGGATGGACCTGTGGCTTATC
AGATAGTTGGTGGGGTAACGGCCTACCAAGTCTACGACGAGTAGCTGGCCTGAGAAGGTGGTCAGCCA
GAGGGGGACTGAGACACGGCC
>Zotu1718
GAACGCTGGCGGCGCGCTTAACACATGCAAGTCGAGCGAGAACCAGGACTTCGGTCCTGGGGACAGCG
GCGAACGGGTGAGTAACACGTGGGTAATCTGCCCTCGATTCTGGGATAGCCCGGGGAAACTCGGATTA
ATACCGGATAGCCTTTCAAGCTTTCGGGCGTGAAAGAAAAGGTAGCTTCGGCCTCCGATCGAGGATGA
GCCCGCGGTGGATTAGCTTGTTGGCGGGGTAATGGCCCACCAAGGCGACGATCCATAGCTGGTCTGAG
AGGACGATCAGCCACACTGGGACTGAGACACGGCC
>Zotu1719
TAACGCTAGCGGCAGGCCTAATACATGCAAGTCGAACGGGATTGATTGTAGCAATACGGTCATGAGAG

TGGCGCACGGGTGCGTAACACGTATGCAACCTACCTATAACTGGGGGATAGCCTCCCGAAAGGGAGAT
TAACACCGCATAACATTATGAAGTGGCATCATTTTATAATTAAATCTACGGAGGTTATAGATGGGCAT
GCGTCCGATTAGCTAGTTGGTGAGGTAACGGCTCACCAAGGCTCCGATCGGTAGGGGGTCTGAGAGGA
TGATCCCCCACACTGGTACTGAGACACGGAC
>Zotu1720
GAACGCTGGCGGCATGCCTAACACATGCAAGTCGAACGATGCTTTCGGGCATAGTGGCGCACGGGTGC
GTAACGCGTGGGAATCTGCCCTTGGGTCTGGGATAACAGTTGGAAACGACTGCTAATACCGGATGATA
TCGCGAGATCAAAGATTTATCGCCCGAGGATGAGCCCGCGTCAGATTAGCTAGTTGGTGGGGTAAAGG
CCTACCAAGGCGACGATCTGTAGCTGGTCTGAGAGGATGATCAGCCACACTGGGACTGAGACACGGCC
>Zotu1722
GAACGCTGGCGGCAGGCTTAACACATGCAAGTCGAACGGGTACCTTCGGGTGCTAGTGGCAGACGGGT
GAGTAACACGTGGGAACGTACCTTTTGGTTCGGAATAATACAGGGAAACTTGTACTAATACCGGATAC
GCCCTTCGGGGGAAAGATTTATCGCCGAAAGATCGGCCCGCGTCTGATTAGCTAGTTGGTGAGGTAAT
GGCTCACCAAGGCGACGATCAGTAGCTGGTCTGAGAGGATGATCAGCCACACTGGGACTGAGACACGG
CC
>Zotu1724
GAACGCTGGCGGCGTGCCTAATACATGCAAGTAGAACGCTGAAGGAGGAGCTTGCTTCTCCGGATGAG
TTGCGAACGGGTGAGTAACGCGTAGGTAACCTGCCTGGTAGTGGGGGATAACTATTGGAAACGATAGC
TAATACCGCATAACAGCAGTTGTTGCATGACAACTGTTTGAAAGGTGCAATTGCACCACTACCAGATG
GACCTGCGTTGTATTAGCTAGTTGGTGGGGTAACGGCTCACCAAGGCGACGATACATAGCCGACCTGA
GAGGGTGATCGGCCACACTGGGACTGAGACACGGCC
>Zotu1725
GAACGCTGGCGGCAGGCTTAACACATGCAAGTCGAGCGGGCGAGGTTGCTTCGGTAACTGAGCTAGCG
GCGGACGGGTGAGTAAAGCTTAGGAATCTGCCTATTAGTGGGGGACAACATTTCGAAAGGGATGCTAA
TACCGCATACGTCCTACGGGAGAAAGCAGGGGATCACTTGTGACCTTGCGCTAATAGATGAGCCTAAG
TCGGATTAGCTAGTTGGTGGGGTAAAGGCCTACCAAGGCGACGATCTGTAGCGGGTCTGAGAGGATGA
TCCGCCACACTGGGACTGAGACACGGCC
>Zotu1726
GAACGCTGGCGGCAGGCCTAACACATGCAAGTCGAGCGGTAGAGAGAAGCTTGCTTCTCTTGAGAGCG
GCGGACGGGTGAGTAATGCCTAGGAATCTGCCTGGTAGTGGGGGATAACGTTCGGAAACGGACGCTAA
TACCGCATACGTCCTACGGGAGAAAGCAGGGGACCTTCGGGCCTTGCGCTATCAGATGAGCCTAGGTC
GGATTAGCTAGTTGGTGAGGTAATGGCTCACCAAGGCTACGATCCGTAACTGGTCTGAGAGGATGATC
AGTCACACTGGAACTGAGACACGGTC
>Zotu1727
GAACGCTAGCGGCAGGCCTAATACATGCAAGTCGAGGGGTATGGGCAGCAATGCCCAGAGACCGGCAA
ACGGGTGCGGAACACGTACAGAACCTTCCCTCCAGCGGGGCATAGCCCTTGGAAACGAGGATTAATAC
CCCATGACATAACCGGGTGGCATCATCTGGTTATTAAAGGTTCACCACTGGAGGATGGCTGTGCGGCC
GATTAGGTAGTTGGCGGGGTAAAGGCCCACCAAGCCTTCGATCGGTAACTGGTGTGAGAGCACGACCA
GTCACACGGGCACTGAGACACGGGC
>Zotu1728
AAACGCTGGCGGCGTGCATGAGACATGCAAGTCGAACGAGTTCCTGGAGCTTGCTCCAGGCGCTAGTG
GCGGACGGCTGAGTAACGCGTGGGTGACCTACCCCGAAGTGGGGAATAACTGCCCGAAAGGGTGGCTA
ATACCGCATGTGATCGTTCTTTCAGTTGATCGATCAAAGCAGCAATGCGCTTCGGGAGGGGCCTGCGT
CCGATTAGCTCGTTGGTGGGGTAATGGCTCACCAAGGCGACGATCGGTAGGTGGTCTGAGAGGACGAT
CACCCAGACTGGGACTGAGACACGGCC
>Zotu1729
GAACGCTGGCGGTGTGCTTCATACATGCAAGTCGAACGAGGTAGCAATACCTAGTGGCGGACGGGTGA
GTAACGCGTGGGAATCTGCCTTAAACAGGGGGATAGCTGGCCGAAAGGCTAATTAATACCGCATATGC
ACACATCCCGACAGGGATATGTGTGAAAGGAGTAATCCAGTTTAAGATGAGCCCGCGTGCGATTAGCT
AGTTGGTGGGGTAAAGGCCCACCAAGGCGACGATCGCTAGGTGGTCTGAGAGGATGATCACCCACAAT
GGAACTGAGACACGGTC
>Zotu1730
GAACGCTGGCGGCGTGCTTAACACATGCAAGTCGAACGAAGAGCGATGGAAGCTTGCTTCTATCAATC
TTAGTGGCGAACGGGTGAGTAACGCGTAATCAACCTGCCCTTCAGAGGGGGACAACAGTTGGAAACGA

CTGCTAATACCGCATACGATCCAATCTCGGCATCGAGACTGGATGAAAGGTGGCCTCTATTTATAAGC
TATCACTGAAGGAGGGGATTGCGTCTGATTAGCTAGTTGGAGGGGTAACGGCCCACCAAGGCGATGAT
CAGTAGCCGGTCTGAGAGGATGAACGGCCACATTGGGACTGAGACACGGCC
>Zotu1731
GAACGCTAGCGGCAGGCTTAATACATGCAAGTCGAGGGGCAGCAGGATAGCAATATTGCTGGCGACCG
GCGCACGGGTGCGTAACACGTATACAATCTACCTTTAACAGGGGGATAGCCCGAAGAAATTCGGATTA
ATACCCCATGATAGTTTTCTTGGCATCAAGAGGATTTGAAAGCTCCGGCGGTTAGAGATGAGTATGCG
GTCTATTAGCTAGTAGGTGAGGTAACGGCTCACCTAGGCTACGATAGATAGGGGATCTGAGAGGACTA
CCCCCCACACTGGTACTGAGACACGGAC
>Zotu1732
GAACGCTGGCGGCACGCTTAACACATGCAAGTCGAACGAGAACTTCGGAGCTTGCTCCGAGGGGACAG
TGGCGAACGGGTGAGTAACACGTGAGGAACCTGCCCCGAAGACCGGGATAACACCGGGAAACCGGTGC
TAATACCGGATGCCCTCTCCGGATCGCATGATCTGGCGAGGAAATGGATTCCGCTTCGGGATGGCCTC
GCGGCCTATCAGCTTGTTGGTGGGGTAACGGCCTACCAAGGCTTCGACGGGTAGCTGGTCTGAGAGGA
TGTCCAGCCACACTGGGACTGAGACACGGCC
>Zotu1733
AAACGCTGGCGGCGTGCCTAACACATGCAAGTCGGACGCCCTATGCTTCGGTGTAGGGAGTGGCGGAC
GGGTGAGTAACACGTGGGATATCTACCCAGAGGTGAGGAATACCGGCGAGAAATCGCCGACAATACCG
CATACGCTCCGAGAGGAGCAAAGCTCGCAAGAGCGCCATTGGATGATCCTGCGGTCGATTAGCTAGTT
GGTGAGGTAATGGCTCACCAAGGCGACGATCGATAGCTGGTCTGAGAGGACGATCAGCCACACTGGGA
TTGAGAACGGCC
>Zotu1734
GAACGCTGGCGGCGTGCTTAACACATGCAAGTCGAACGATGAAGCCTAGCTTGCTGGGTGGATTAGTG
GCGAACGGGTGAGTAACACGTGAGCAACCTGCCCCTCACTCTGGGATAAGCGCTGGAAACGGCGTCTA
ATACTGGATACGAAACGCAACCGCATGGTTAGTGTTTGGAAAGATTTTTTGGTGGGGATGGGCTCGC
GGCCTATCAGCTTGTTGGTGAGGTAATGGCTCACCAAGGCGTCGACGGGTAGCCGGCCTGAGAGGGTG
ACCGGCCACACTGGGACTGAGACACGGCC
>Zotu1735
GAACGCTGGCGGCGTGCCTAACACATGCAAGTCGAACGAGGTCCATGCAGTGGCAACACTGCGGAAGA
CCTAGTGGCGAACGGGTGAGTAACACGTGAGGAACTTGCCCCGGAGACCGGGATAACCTTCCGAAAGG
ATGGCTAATACCGGATATCGTCACCGAGCCGCATGGCTTGGTGACGAAATGGATTCCGCTCCGGGAGA
GCCTCGCGGCCTATCAGCTAGTTGGTGGGGTAACGGCCCACCAAGGCATCGACGGGTAGCTGGTCTGA
GAGGACGATCAGCCACACTGGGACTGAGACACGGCC
>Zotu1736
GAACGCTGGCGGCAGGCTTAACACATGCAAGTCGAGCGGGGAAGAGTAGCTTGCTACTTTACCTAGCG
GCGGACGGGTGAGTAATGCTTAGGAATCTGCCTATTAGTGGGGGACAACATCTCGAAAGGGATGCTAA
TACCGCATACGTCCTACGGGAGAAAGCAGGGGACCTTCGGGCCTTGCGCTAATAGATGAGCCTAAGTC
GGATTAGCTAGTTGGTGGGGTAAAGGCCTACCAAGGCGACGATCTGTAGCGGGTCTGAGAGGATGATC
CGCCACACTGGGACTGAGACACGGCC
>Zotu1737
TAACGCTAGCGGCAGGCCTAATACATGCAAGTCGAACGGCAGCGGGTGTAGCAATACATGCCGGCGAG
TGGCGCACGGGTGCGTAACGCGTATGCAATCTACTTGTAACTGGGGAATAGCCTTCCGAAAGGAAGAT
TAATACCGCATAATATCATGAAGTGGCATCATTTTATGATCAAAACTCCGGTGGTTACAGATGAGCAT
GCGTGACATTAGCTAGTTGGTGAGGTAACGGCTCACCAAGGCTACGATGTCTAGGGGGTCTGAGAGGA
TGATCCCCCACACTGGTACTGAGACACGGAC
>Zotu1738
GAACGCTGGCGGCGTGCTTAACACATGCAAGTCGAACGGAAAGGCCTTGTGCTTGCACAAGGTACTCG
AGTGGCGAACGGGTGAGTAACACGTGGGTGATCTGCCCTGCACTGTGGGATAAGCCTGGGAAACTGGG
TCTAATACCATATAGGACCGCATCTTGGATGGTGTGGTGGAAAGCTTTTGCGGTGTGGGATGAGCCTG
CGGCCTATCAGCTTGTTGGTGGGGTAATGGCCTACCAAGGCGGCGACGGGTATCCGGCCTGAGAGGGT
GTACGGACACATTGGGACTGAGATACGGCC
>Zotu1739
GAACGCTGGCGGCGTGCCTAACACATGCAAGTCGAGCGAGATACTGTTGAACAGAGATTCGTCAACGG
GATACAGTGGAAAGCGGCGGACGGGTGAGTAACGCGTAGGCAACCTGCCCCTTACACAGGGATAGCCA

TTGGAAACGATGATTAAAACCTGATGACACCTGAGGATGACATCTTCCTGAGGTCAAAGATTTATCGG
TAAGGGATGGCCTGCGTCTGATTAGATAGTTGGTGAGGTAACGGCCCACCAAGTCAACGATCAGTAG
CCGACCTGAGAGGGTGATCGGCCACATTGGAACTGAGACACGGTC
>Zotu1741
GAACGCTGGCGGCGTGCTTAACACATGCAAGTCGAACGGAAAGGCCCTTTCGGGGGTACTCGAGTGGC
GAACGGGTGAGTAACACGTGGGTGATCTGCCCTGCACTTCGGGATAAGCTTGGGAAACTGGGTCTAAT
ACCGGATAGGACCATGGCATGCATGTGCTGTGGTGGAAAGCTTTTGCGGTGTGGGATGGGCCCGCGGC
CTATCAGCTTGTTGGTGGGGTGATGGCCTACCAAGGCGACGACGGGTAGCCGGCCTGAGAGGGTGTCC
GGCCACACTGGGACTGAGATACGGCC
>Zotu1742
CAACGCTGGCGGCGTGCCTAACACATGCAAGTCGAACGAGAAAGTGGAGCAATCCATGAGTAAAGTGG
CGCACGGGTGAGTAACACGTGACTAACCTACCCTTGAGTGGGGGATAACCTCGGGAAACCGGGGCTAA
TACCGCATAATACCGAGAGGTCAAAGGAGCAATTCGCTTAAGGAGGGGGTCGCGGCTGATTAGCTAGT
TGGCAGGGTAATGGCCTACCAAGGCAGTGATCAGTATCCGGCCTGAGAGGGCGCACGGACACACTGGA
ACTGAAACACGGTC
>Zotu1743
GAACGCTGGCGGCGCGCCTAAAACATGCAAGTCGAACGAGAAACCCGGGCTTGCCCGGGTGGACAGTG
GCGAACGGGTGAGTAACACGTGAGGAACCTGCCCCGAAGACCGGGACAACACCGGGAAACCGGTGCTA
ATACCGGATGTCCTCATCGGACCGCATGATCTGATGAGGAAATGGATTCCGCTTCGGGATGGCCTCGC
GGCCTATCAGCTAGTTGGTGGGGTAACGGCCCACCAAGGCGACGACGGGTAGCTGGTCTGAGAGGATG
TCCAGCCACACTGGGACTGAGACACGGCC
>Zotu1745
GAACGCTGGCGGCGTGCTTAACACATGCAAGTCGAACGCTGAAGCCGCCTTCGGGTGGTGGATGAGTG
GCGAACGGGTGAGTAACACGTGGGTAATCTGCCCTGTACTCTGGGATAAGCCTGGGAAACTGGGTCTA
ATACTGGATAGGACACATCGCTGCATGGTGGTGTGTGGAAAGCTTCGGCGGTACGGGATGAGCCCGCG
GCCTATCAGCTTGTTGGTGGGGTGAGGGCCTACCAAGGCGACGACGGGTAGCCGGCCTGAGAGGGTGA
CCGGCCACACTGGGACTGAGACACGGCC
>Zotu1746
CAACGCTGGCGGCGTGCATGAGACATGCAAGTCGAACGGACGACTCGGGCTTGCCTGAGTCGTTAGTG
GCGGACGGCTGAGTAACGCGTGGGTGACCTGCCCCGAAGTGGGGAATAACTGCCCGAAAGGGTGGCTA
ATACCGCATGTGACGGTGCGTTCGATCGCATCATCAAAGCAGCAATGCGCTTCGGGAGGGGCCTGCGT
TCGATTAGCTCGTTGGTGGGGTAACGGCTCACCAAGGCGACGATCGATAGGTGGTCTGAGAGGACGAT
CACCCAGACTGGGACTGAGACACGGCC
>Zotu1748
GAACGCTGGCGGCGTGCCTAATACATGCAAGTCGAGCGGATTGAAGGGAGCTTGCTCCCTGATATTAG
CGGCGGACGGGTGAGTAACACGTGGGCAACCTGCCCTGCAGATGGGGATAACTCCGGGAAACCGGGGC
TAATACCGAATAATCGGTTCTTCCGCATGGAAGAACTCTGAAAGACGGTTTCGGCTGTCACTGCAGGA
TGGGCCCGCGGCGCATTAGCTAGTTGGTGGGGTAACGGCCTACCAAGGCGACGATGCGTAGCCGACCT
GAGAGGGTGATCGGCCACACTGGGACTGAGACACGGCC
>Zotu1749
GAACGCTGGCGGCGTGCCTAACACATGCAAGTCGAACGGGGGTAGCAATACCCCAGTGGCGAACGGGT
GCGTAACACGTGAGGAACATTCCTTCCGGTGGGGGATAGCCGGCCTAACGGCCGGGTAATACCGCATA
CGCTGTTCGGGGTGCATACCCTGGATCGGAAAGCAGCAATGCGCCGGAAAGGTGCCTCGCGGCCTATC
AGCTAGTTGGTGAGGTAATGGCTCACCAAGGCGACGACGGGTAGCTGGTCTGAGAGGATGGCCAGCCA
CATTGGGACTGAGACACGGCC
>Zotu1751
GAACGCTAGCGGCAGGCTTAATACATGCAAGTCGTGGGGCAGCAGGTTTGTAGCAATACAAATGCTGG
CGACCGGCAAACGGGTGCGGAACACGTACAGAACCTTCCTTTAAGTGGGGAATAGCCCAGGGAAACTT
GGATTAATACCCCGTAATATAATGAAGTGGCATCACTTTATTATTATAGCAGCAATGCGCTTGAAGAT
GGCTGTGCGGCTGATTAGGTAGTTGGCGGGGTAATGGCCCACCAAGCCTACGATCAGTAACTGGTGTG
AGAGCACGACCAGTCACACGGGCACTGAGACACGGGC
>Zotu1752
GAACGCTAGCGGCAGGCTTAACACATGCAAGTCGAGGGGCAGCAGGTCTTCGGACGCTGGCGACCGGC
AAACGGGTGCGGAACACGTACGCAACATACCTTTATCTGGGGCATAGCCCGAAGAAATTCGGATTAAC

ACCCCATAAGATTATAGAGTGGCATCACTTAGTAATTAAAAATTTATTGGATAGAGATTGGCGTGCGT
CCTATTAGCTAGTTGGCGGGGTAAAGGCCCACCAAGGCGACGATAGGTAGCTGATGTGAGAGCATGAT
CAGCCACACGGGCACTGAGACACGGGC
>Zotu1753
GAACGCTGGCGGCAGGCCTAACACATGCAAGTCGAGCGGATGAGAGGAGCTTGCTCTTCGATTCAGCG
GCGGACGGGTGAGTAATGCCTAGGAATCTGCCTAGTAGTGGGGGACAACGTTTCGAAAGGAACGCTAA
TACCGCATACGTCCTACGGGAGAAAGTGGGGGATCTTCGGACTTTACGCTATTAGATGAGCCTAGGTC
GGATTAGCTAGTTGGTAGGGTAAAGGCCTACCAAGGCGACGATCCGTAACTGGTCTGAGAGGATGATC
AGTCACACTGGAACTGAGACACGGTC
>Zotu1755
GAACGCTGGTAACGTGGATACCGGCATGCAAGTCACGGGGGTCCCGTAAGGGACAACCGGCGAACGGC
TTAGTAACACGTAGATACATACCCCAAAGTGGAGAACAGCTCGTCGAAAGACGGGGTAATACTCCATA
GTCTCGCAAGAGTAAAGGCGCAAGCCGCTTTGGGAATGGTCTGCGGTCTATCAGCTAGTTGGCGAGGT
AAAGGCTCACCAAGGCTATGACGGATAGGGGGCGTGAGAGCGCGACCCCCAAGAATGGCACTGCGACA
CGGGC
>Zotu1756
CAACGCTGGCGGCGTGCCTAATGCATGCAAGTCGGACGTGGCCCTTTCGGGGGTGCGCGTGGCGGACG
GGTGAGGAACGCGTGGGAACCTGCCTGTTAGTGGGGGGTAGCCATCGGAAACGGTGGGTAATTCCGC
ATGAGCTCAGTTGCCGCGTGGGGTGATTGAGGAAAGCCTTCGGGCGCTGACAGATGGTCCTGCGGCCG
ATTAGCTCGTTGGTGGGGTAACGGCCTACCAAGGCGATGATCGGTAGCTGGTCTGAGAGGACGGCCAG
CCACATGGGGACTGAGACACGGCC
>Zotu1757
GAACGCTGGCGGCATGCCTAACACATGCAAGTCGAACGAGACCTTCGGGTCTAGTGGCGCACGGGTGC
GTAACGCGTGGGAACCTGCCTTTAGGTTCGGAATAACTCTCCGAAAGGGGTGCTAATACCGAATAATG
TCTTCGGACCAAAGATTTATCGCCTTTAGATGGGCCCGCGTTGGATTAGCTAGTTGGTGGGGTAAAGG
CCTACCAAGGCGACGATCCATAGCTGGTCTTAGAGGATGATCAGCCACACTGGGACTGAGACACGGCC
>Zotu1758
GAACGCTGGCGGCGTGCCTAATACATGCAAGTCGAACGCATTTTCTTTCACCGTAGCTTGCTACACCG
AAAGAAAATGAGTGGCGAACGGGTGAGTAACACGTGGGTAACCTGCCCATCAGCGGGGGATAACACTT
GGAAACAGGTGCTAATACCGCATAATTCCATTTACCGCATGGTAGATAGATGAAAGGCGCTTTTGCGT
CACTGATGGATGGACCCGCGGTGCATTAGCTAGTTGGTGGGGTAACGGCCTACCAAGGCTGCGATGCA
TAGCCGACCTGAGAGGGTGATCGGCCACACTGGGACTGAGACACGGCC
>Zotu1759
GAACGCTGGCGGCGCGCCTAACACATGCAAGTCGAGCGAGGTTCAAAGCTTCGGTTTTGAACCTAGCG
GCGGACGGGTGAGTAACGCGTGAGCAACCTGGCCTTCAGAGGGGGATACCGTCTGGAAACGGACGTTA
ATACCGCATAACATGTCGGGAGGGCATCTTCCTGATATCAAAGGAGAAATTCACTGAAGGATGGGCTC
GCGTCCGATTAGGTAGTTGGTGAGGTAACGGCTCACCAAGCCCGCGATCGGTAGCCGGACTGAGAGGT
TGGCCGGCCACATTGGGACTGAGACACGGCC
>Zotu1760
GAACGCTAGCGGCAGGCTTAATACATGCAAGTCGTGGGGCAGCGCAGTGTAGCAATACATGGGCGGCG
ACCGGCAAACGGGTGCGGAACACGTACGCAACCTTCCTTTAAGTGGAGAATAGCCCAGGGAAACTTGG
ATTAATACTCCGTAACATTACGAAGTGGCATCACTTTGTAATTATAGCAGCAATGCGCTTGAAGATGG
GCGTGCGCCTGATTAGGTAGTTGGCGGGGTAACGGCCCACCAAGCCTACGATCAGTAACTGGTGTGAG
AGCACGACCAGTCACACGGGCACTGAGACACGGGC
>Zotu1761
GAACGCTGGCGGCGTGCCTAATACATGCAAGTCGAGCGAAGAATTTAAGGTGCTTGCACCTAAAATTC
TTAGCGGCGAACGGGTGAGTAACACGTAAAGAACCTGCCTCATAGACTGGGACAACTACTGGAAACGG
TAGCTAATACCGGATAATATATTTTACTGCATGGTAAAATATTGAAAGTCGGTCATGCTGACACTATG
AGATGGCTTTGCGGTGCATTAGCTAGTTGGTAGGGTAAAGGCCTACCAAGGCGACGATGCATAGCCGA
CCTGAGAGGGTGATCGGCCACACTGGGACTGAGACACGGCC
>Zotu1762
GAACGCTGGCGGCGTGCTTAACACATGCAAGTCGAACGGAAAGGCCCTTTCGGGGGTACTCGAGTGGC
GAACGGGTGAGTAACACGTGGGTGATCTGCCCTGCACTTCGGGATAAGCTTGGGAAACTGGGTCTAAT
ACCGGATAGGATCACGCTCTTCATGGGGTGTGGTGGAAAGCTTTTGCGGTGTGGGATGGGCCCGCGGC

CTATCAGCTTGTTGGTGGGGTAATGGCCTACCAAGGCGACGACGGGTAGCCGGCCTGAGAGGGTGTCC
GGCCACACTGGGACTGAGATACGGCC
>Zotu1763
GAACGCTGGCGGCATGCCTTACACATGCAAGTCGAACGGCAGCACGGACTTCGGTCTGGTGGCGAGTG
GCGAACGGGTGAGTAATACATCGGAACGTGCCTGGTAGTGGGGGATAACTACTCGAAAGAGTGGCTAA
TACCGCATGAGATCTACGGATGAAAGCAGGGGATCGCAAGACCTTGTGCTACTAGAGCGGCCGATGGC
AGATTAGGTAGTTGGTGGGATAAAAGCTTACCAAGCCAACGATCTGTAGCTGGTCTGAGAGGACGATC
AGCCACACTGGGACTGAGACACGGCC
>Zotu1764
CAACGCTGGCGGCGTGCCTCAGACATGCAAGTCGTACGCGAAAGGCCCCTTCGGGGGCTGAGTAGAGT
GGCGTCCGGGTGAGTAACACGTGGGCAATCTGCCTCTGGGTGGGGAATAACCCGGGGAAACCCGGGCT
AATACCGCATACGGCGGCGCAGCCTGCGGGCCGCGTCGTGAAAGCGGGGGACCTCGGCAACGAGGCCT
CGCGCCCGGAGAGGAGCCTGCGGCGGATTAGCTAGTTGGTGAGGTAACGGCTCACCAAGGCGACGATC
CGTAGCCGGCCTGAGAGGGCGGTCGGCCACACTGGCACTGACACACGGGC
>Zotu1765
GAACGCTGGCGGCGTGCCTAATACATGCAAGTCGAGCGAACAGATAAGGAGCTTGCTCCTTTGACGTT
AGCGGCGGACGGGTGAGTAACACGTGGATAACCTACCTATAAGACTGGGATAACTTCGGGAAACCGGA
GCTAATACCGGATAACATATTGAACCGCATGGTTCAATAGTGAAAGACGGCCTTGCTGTGACTTATAG
ATGGATCCGCGCCGTATTAGCTAGTTGGTAAGGTAACGGCTTACCAAGGCAACGATACGTAGCCGACC
TGAGAGGGTGATCGGCCACACTGGAACTGAGACACGGTC
>Zotu1766
GAACGCTGGCGGCGTGCCTAATACATGCAAGTCGAGCGAACCAATAGGAGCTTGCTCCTGTTGGTTAG
CGGCGGACGGGTGAGTAACACGTGGGCAACCTGCCTGTAAGACTGGGATAACACCGGGAAACCGGTGC
TAATACCGGATAATCCTTTTCCTCTCATGAGGAAAAGCTGAAAGTCGGTGCAAGCTGACACTTACAGA
TGGGCCCGCGGCGCATTAGCTAGTTGGTGAGGTAACGGCTCACCAAGGCGACGATGCGTAGCCGACCT
GAGAGGGTGATCGGCCACACTGGGACTGAGACACGGCC
>Zotu1767
GAACGCTGGCGGCGTGCTTAACACATGCAAGTCGAACGGGCCTGCCTGGCTTTGGTTGGGTGGGTGAG
TGGCGAACGGGTGAGTAACACGTGAGTAACCTGCCCTCTTCTCTTGGATAACTGCTTGAAAGGGCGGC
TAATACGGGATGTTCTGGCTGCCTCGCATGGGGTGGTTTGGAAAGATTCGGCCTGGTTTTGGTTGTTT
TGGTGGGGGATGGGCTCGCGGCTTATCAGCTTGTTGGTGGGGTGATGGCCTACCAAGGCGGTGACGGG
TAGCCGGCCTGAGAGGGTGGACGGTCACACTGGGACTGAGACACGGCC
>Zotu1768
GAACGCTGGCGGCATGCCTAATACATGCAAGTCGAACGCTGAAGCACTCTGCTTGCAGAGTGCGGATG
AGTGGCGAACGGGTGAGCAACACGTGGGAAATCTGCCTATAAGTGGGGGATAACATTCGGAAACGGAT
GCTAATACCGCATATATTCTTGGACCGCCTGGTCCTTGAAAGAAAGACGGCTTTGCTGTCGCTTATAG
ATGATCCCGCGGCGTATTAGTTAGTTGGTGGGGTAAAGGCCTACCAAGACAATGATACGTAGCCGACC
TGAGAGGGTGATCGGCCACACTGGGACTGAGACACGGCC
>Zotu1769
GAACGCTGGCGGCAGGCTTAACACATGCAAGTCGAACGGGCCCTTCGGGGTCAGTGGCAGACGGGTGA
GTAACACGTGGGAACGTGCCCTTCGGTTCGGAACAACTCAGGGAAACTTGAGCTAATACCGGATACGC
CCTTCGGGGGAAAGATTTATCGCTGAAGGATCGGCCCGCGTCTGATTAGCTAGTTGGTGAGGTAATGG
CTCACCAAGGCGACGATCAGTAGCTGGTCTGAGAGGATGATCAGCCACACTGGGACTGAGACACGGCC
>Zotu1770
GAACGCTAGCGACAGGCTTAACACATGCAAGTCGAGGGGCAGCAATTTGTAGCAATACAAAGTTGGCG
ACCGGCGCACGGGTGAGTAACGCGTATGCAACCTACCTACAACAGAAGAATAATCCGGCGAAAGTCGG
TCTAATACTTCATAAAACAGGGACATCGCATGATGTTATTTGTTAAAGATTTATCGGTTGTAGATGGG
CATGCGTACCATTAGGTAGTTGGTGAGGTAACGGCCCACCAAGCCTACGATGGTTAGGGGTTCTGAGA
GGAAAGTCCCCCACACTGGTACTGAGACACGGAC
>Zotu1771
GAATGCTGGCGGCGTGGATAAGTCATGCAAGTCAAGGGGGCCCGCAAGGGCAACCGGCAGACGAGGTA
GTAACACGTAGGTAGATCCCCCGAAGTCGGGCATAGCCATCCGAAAGGATGGGTAATTCCCGATAGTC
CCGCAAGGGTAAAGGTTTTTCGCTTCGGGAGTTGCCTGCGTAGTATCAGCTAGTTGGTAGGGTAACGG
CCTACCAAGGCTACGACGCTTAGGGGAGCTGAGAGGCTGACCCCCACCGATGGAACTGCGACACGGTC

>Zotu1772
CAACGCTGGCGGCGCGCCTAACACATGCAAGTCGAACGAGAAAGTGGAGCAATCCATGAGTAAAGTGG
CGTACGGGTGAGTAACACGTGGATCATCTACCCTCTAGTGGGGAATACCGCTGGGAAACCGGCGACAA
TACCGCATAAGCTCGAGAGAGGAAAGCAGCAATGCGCTGGAGGAGGAGTCCGCGGTCGATTAGTTAGT
TGGCGGGGTAAAGGCCCACCAAGACTGTGATCGATAGCCGGCCTGAGAGGGCACACGGCCACACTGGC
ACTGAAACACGGGC
>Zotu1773
GAACGCTGGCGGCGTGCTTAACACATGCAAGTCGAGCGGAAAGGCCCACTTGTGGGTACTCGAGCGGC
GAACGGGTGAGTAACACGTGAGTAATCTGCCCTCCACTCTGGGATAACGTTCAGAAATGGGTGCTAAT
ACCGGATGTACACGCGCTGACGCATGTTGGTGTGTGGAAAGGTTTTTCTGGTGGGGGATGTGCTCGCG
GCCTATCAGCTTGTTGGTGGGGTAATGGCCTACCAAGGCTTTGACGGGTAGCCGGCCTGAGAGGGTGA
CCGGCCACACTGGGACTGAGACACGGCC
>Zotu1774
GAACGCTGGCGGCGTGCCTTAGGCATGCAAATCAAGGGGGGCTCGTAAGAGTCAACCGGTGGACGGGT
GAGTAACGCGTGAGAATCTACCTTAAAGTGGGGCATAGCTTCTCGAAAGAGAAGGTAATTTCCCATGT
GATCGCAAGATCAAAGCCGCAAGGCGCTTTAAGATGAGCTCGCGTCCTATCAGCTTGTTGGTGGGGTA
ATGGCCTACCAAGGCGACGACGGGTAGCTGGTCTGAGAGGATGGTCAGCCACAATGGAACTGCGACAC
GGTC
>Zotu1775
GAACGCTGGCGGCGGGCTTAACACATGCAAGTCGAACGAAGCCTTCGGGCTTAGTGGCGCACGGGTGC
GTAACGCGTAGGCAACCTACCCTCAGGCGGGGAATAACTACCGGAAACGGTAGCTAATACCGCATATT
GTCGGAGAGACACCTGTCTCACTGATGAAAGGCTTCGGCCACCTGAGGATGGGCCTGCGTTGGATTAG
GTAGTTGGTGAGGTAACGGCTCACCAAGCCGACGATCCATAGCTGGTCTGAGAGGATGATCAGCCACA
CTGGCACTGAGACACGGGC
>Zotu1776
GAACGCTGGCGGCGTGCTTAACACATGCAAGTCGAGCGATGAAGCACCTTCGGGTGTGAATTAGCGGC
GAACGGGTGAGGAACACGTGAGAAATCTGCCTTCAACTCTGGGATAACTCCGTGAAAACGGGGCTAAT
ACCGGATATGAAACCTTCAGGCATCTGAGGGTTTGGAAAGTTTTTCGGTTGAAGATGATCTCGCGGCC
TATCAGCTTGTTGGTGAGGTAATGGCTCACCAAGGCGACGACGGGTAGCCGGCCTGAGAGGGCGACCG
GCCACACTGGGACTGAGACACGGCC
>Zotu1777
GAACGCTGGCGGCGTGCTTAACACATGCAAGTCGAACGGTGACCTCGAGAGCTTGCTCTCGAGTGATC
AGTGGCGAACGGGTGAGTAACACGTGAGTAACCTGCCCCAGACTCTGGAATAACAATTGGAAACAGTT
GCTAATACCGGATACGACACGAACGGGCATCCGTATCGTGTGGAAAGTTTTTCGGTCTGGGATGGACT
CGCGGCCTATCAGCTTGTTGGTGAGGTAATGGCTCACCAAGGCGACGACGGGTAGCCGGCCTGAGAGG
GCGACCGGCCACACTGGGACTGAGACACGGCC
>Zotu1778
GAACGCTGGCGGCGTGCCTAATACATGCAAGTCGTACGCATTGATAATGGAACTTGTTCCATTTGACG
TGAGTGGCGCACGGGTGAGTAACACGTGGGAAACCTACCCTTCAGCGGGGGATAACCGTCGGAAACGA
TGGCTAATACCGCATAGGTCTTTGAGTCACCTGACTTAAAGAGGAAAGACGGCTTCGGCTGTCACTGA
AGGATGGTCCCGCGGTGCATTAGCTAGTTGGTAAGGTAATGGCTTACCAAGGCGATGATGCATAGCCG
ACCTGAGAGGGTGATCGGCCACATTGGGACTGAGACACGGCC
>Zotu1779
GAACGCTGGCGGCGTGCTTAACACATGCAAGTCGAACGAGATCCAACCAGTGGCAACACTGGGGAAGA
TCTAGTGGCGAACGGGTGAGTAACACGTGAGAAACCTACCCCGGACTCAGGAATAACAGTGGGAAACC
ACTGCTAATACCAGATACCTCCAACGAGCCTCCTGGCTCGATGAAGAAAGGGTTTACCGGTTCGGGAC
GGTCTCGCGGCCTATCAGCTTGTTGGTGGGGTAACGGCCCACCAAGGCGTCGACGGGTAGCTGGTCTG
AGAGGATGATCAGCCACACTGGGACTGAGACACGGCC
>Zotu1780
GAACGCTGGCGGCACGCCTAATGCATGCAAGTCGAACGACGCACCTTGGAGGAGTAATCCTCTGGGGT
GACGGAGTGGCGAACGGGTGCGTAACACGTGAGCAACCTGCCCCGAAGATCGGAATAGCCCAAGGAAA
CTTGGATTAATACCGAATACCCTCATCCTTCCGCATGGTCGGATGAGAAAAGGCCTTTGCGCTTCGGG
ATGGGCTCGCGGCCTATCAGCTAGTTGGTGAGGTAACGGCTCACCAAGGCAACGACGGGTAGCTGGTC
TGAGAGGACGATCAGCCACACTGGGACTGAGACACGGCC

>Zotu1781
GAACGCTGGCGGCAGGCTTAACACATGCAAGTCGAGCGCCCCGCAAGGGGAGCGGCAGACGGGTGAGT
AACGCGTGGGAACGTACCTTTTGGTTCGGAACAACTCCGGGAAACTGGAGCTAATACCGGATAAGCCC
TTCGGGGGAAAGATTTATCGCCGAAAGATCGGCCCGCGTCTGATTAGCTAGTTGGTGAGGTAACGGCT
CACCAAGGCGACGATCAGTAGCTGGTCTGAGAGGATGATCAGCCACACTGGGACTGAGACACGGCC
>Zotu1782
GAACGCTGGCGGCGTGCTTAACACATGCAAGTCGAACGATGACAGTGGTGCTTGCACCGCCTGATTAG
TGGCGAACGGGTGAGTAACACGTGAGTAACCTGCCCTCCACTTCGGGATAACCTCGGGAAATCGTGGC
TAATACCGGATACGAGCACTCATCGCATGGTGGGTGTTGGAAAGATTTATCGGTGGGGGATGGACTCG
CGGCCTATCAGTTTGTTGGTGAGGTGATGGCTCACCAAGACGATGACGGGTAGCCGGCCTGAGAGGGC
GACCGGCCACACTGGGACTGAGACACGGCC
>Zotu1783
GAACGCTGGCGGCGTGCTTAACACATGCAAGTCAGGGGGTGGGGCAACCCATCACCGGCGAACGGGTG
CGTAACACGTGAGCGACCTGCCTTCGAGTGGGGGATAGCCGGCCCAACGGCCGGGTAACACCGCATAC
GACACCGCCGGGGCATCCCGGGGGTGTGAAACCACTTGGGCTCGGAGAGGGGCTCGCGGCCTATCAGC
TAGTTGGCGAGGTAACGGCTCACCAAGGCGATGACGGGTAGCTGGTCTGAGAGGATGGCCAGCCACAT
TGGGACTGAGAGACGGCC
>Zotu1784
GAACGCTAGCGGCATGCTTTACACATGCAAGTCGAACGGTAACAGGTCTTCGGATGCTGACGAGTGGC
GAACGGGTGAGTAATACATCGGAACGTGCCTAGTAGTGGGGGATAACTACTCGAAAGAGTAGCTAATA
CCGCATGAGATCTACGGATGAAAGCAGGGGACCTTCGGGCCTTGTGCTACTAGAGCGGCTGATGGCAG
ATTAGGTAGTTGGTGGGGTAAAGGCTTACCAAGCCTGCGATCTGTAGCTGGTCTGAGAGGACGACCAG
CCACACTGGGACTGAGACACGGCC
>Zotu1785
GAACGCTGGCGGCATGCCTAACACATGCAAGTCGAACGAGACCTTCGGGTCTAGTGGCGCACGGGTGC
GTAACGCGTGGGAATCTGCCCTTTGCTTCGGAATAACAGTTAGAAATGACTGCTAATACCGGATGATG
TCTTCGGACCAAAGATTTATCGGCAAAGGATGAGCCCGCGTAGGATTAGGTAGTTGGTGGGGTAAAGG
CCTACCAAGCCGACGATCCTTAGCTGGTCTGAGAGGATGATCAGCCACACTGGGACTGAGACACGGCC
>Zotu1786
GAACGCTGGCGGCAGGCTTAACACATGCAAGTCGAACGGGCACCTTCGGGTGTCAGTGGCAGACGGGT
GAGTAACACGTGGGAACGTACCCTTCGGTTCGGAATAACTCAGGGAAACTTGAGCTAATACCGGATAC
GCCCTTTTGAGGAAAGGTTTACTGCCGAAGGATCGGCCCGCGTCTGATTAGCTTGTTGGTGGGGTAAC
GGCCTACCAAGGCGACGATCAGTAGCTGGTCTGAGAGAATGATCAGCCACACTGGGACTGAGACACGG
CC
>Zotu1787
GAACGCTGGCGGCGTGCTTAACACATGCAAGTCGAACGGATCAGGAGAGCTTGCTCTTCTGGTTAGTG
GCGAACGGGTGAGTAACACGTGAGCAATCTGCCCCTGACTCTGGGATAACTGCGGGAAACTGTAGCTA
ATACCGGATATGACGCGCGAAGGCATCTTCTGCGTGTGGAAAGAATTGTTGGTCAGGGATGAGCTCGC
GGCCTATCAGCTTGTTGGTGAGGTAATGGCTCACCAAGGCGACGACGGGTAGCCGGCCTGAGAGGGTG
ACCGGCCACACTGGGACTGAGACACGGCC
>Zotu1788
GAACGCTGGCGGCAGGCCTAACACATGCAAGTCGAGCGGCAGCGGGTCCTTCGGGATGCCGGCGAGCG
GCGGACGGGTGAGGAATGCGTCGGAATCTGCCTTTTTGTGGGGGATAACGTAGGGAAACTTACGCTAA
TACCGCATACGACCTACGGGTGAAAGTGGGGGACCTTCGGGCCTCACGCAGAAAGATGAGCCGACGCC
GGATTAGCTAGTTGGCGGGGTAAAGGCCCACCAAGGCGACGATCCGTAGCTGGTCTGAGAGGATGATC
AGCCACACTGGAACTGAGACACGGTC
>Zotu1789
GAACGCTGGCGGCGTGCTTAACACATGCAAGTCGAACGCTGAAGCTTGGTGCTTGCACTTAGGTGGGT
GAGTGGCGAACGGGTGAGTAATACGTGAGTAACCTGCCCTTGACTCTGGGATAAGCCTGGGAAACTGG
GTCTAATACTGGATATGACCTCCTGTCGCATGGTGGGGGGTGGAAAGGGTTTGTACTGGTTGTGGATG
GGCTCACGGCCTATCAGCTTGTTGGTGGGGTAATGGCCTACCAAGGCGACGACGGGTAGCCGGCCTGA
GAGGGTGACCGGCCACACTGGGACTGAGACACGGCC
>Zotu1790
GAACGCTGGCGGCGTGCTTAACACATGCAAGTCGAACGGAAAGGCCCTTTCGGGGGTACTCGAGTGGC

GAACGGGTGAGTAACACGTGGGTGATCTGCCCTGCACTTTGGGATAAGCCTGGGAAACTGGGTCTAAT
ACCGGATAGGACTACGCGATGCATGTCGTGTGGTGGAAAGCTTTTGCGGTGTGGGATGGGCCCGCGGC
CTATCAGCTTGTTGGTGGGGTGATGGCCTACCAAGGCGACGACGGGTAGCCGGCCTGAGAGGGTGACC
GGCCACACTGGGACTGAGATACGGCC
>Zotu1791
GAACGCTAGCGGCGTGCTTAACACATGCAAGTCGAACGCTGAAGCCCGCTTCGGTGGGTGGATGAGTG
GCGAACGGGTGAGTAACACGTGGGCAATCTACCCCGCACTCGGGGATAAGCCTTGGAAACGAGGTCTA
ATACCCGATAGGACATGCCATCGCATGGTGGTGTGTGGAAAGTGTGCAGCGGTGTGGGATGAGCCCGT
GGCCTATCAGCTTGTTGGTGGCGGTGATGGCGCACCAAGGCGACGACGGGTAGCCGGCCTGAGAGGGT
GACCGGCCACACTGGGACTGAGACACGGCC
>Zotu1792
GAACGCTGGCGGCGTGCTTAACACATGCAAGTGGAGCGACGAACCCGAGCTTGCTCGGGGGCAAAGCC
GCGAACGGGTGAGTAACACGTGGGTAATCTGCCCTGATGATTGGGACAACCCCGGGAAACCGGGGCTA
ATACCAAATGTGCTCTTTCCACATCAGTGGATCGAGGAAAGGAGGCTTCGGCTTCCGCATCGGGATGA
GCCCGCGGCCCATTAGCTTGTTGGTGGGGTAATGGCCTACCAAGGCTCCGATGGGTAGCTGGTCTGAG
AGGACGATCAGCCACACTGGGACTGAGACACGGCC
>Zotu1793
GAACGCTGGCGGCGTGCCTAATACATGCAAGTCGAGCGAACAGATGAGAAGCTTGCTTCTCTGATGTT
AGCGGCGGACGGGTGAGTAACACGTGGGTAACCTACCTATAAGACTGGGATAACTCCGGGAAACCGGG
GCTAATACCGGATAATATTTTGAACCGCATGGTTCAATAGTGAAAGACGGTTTCGGCTGTCACTTATA
GATGGACCCGCGCCGTATTAGCTAGTTGGTAAGGTAATGGCTTACCAAGGCAACGATACGTAGCCGAC
CTGAGAGGGTGATCGGCCACACTGGAACTGAGACACGGTC
>Zotu1794
GAACGCTGGCGGCGTGCTTAACACATGCAAGTCGAACGATGATGCTGGTGCTTGCACTGGTGGATTAG
TGGCGAACGGGTGAGTAACACGTGAGTAACCTGCCCCTGACTTCGGGATAAGCCCGGGAAACTGGGTC
TAATACCGGATATGACTTCCTGCTGCATGGCAGGGGGTGGAAAGATTTATCGGTTGGGGATGGACTCG
CGGCCTATCAGCTTGTTGGTGAGGTAATGGCTCACCAAGGCGACGACGGGTAGCCGGCCTGAGAGGGT
GACCGGCCACACTGGGACTGAGACACGGCC
>Zotu1796
GAACGCTGGCGGCGCGCCTAACACATGCAAGTCGAACGGACTTTACGGAAACAGAAGCTTCGGCAGAA
GAAGAAATAAAGTTAGTGGCGAACGGGTGAGTAACACGTGAGGAACCAACCCTTAAGAGGGGGATAAC
ACTTAGAAATAGGTGCTAATACCGCATAAGACCACACTCCGGCATCGGAGAGGGGTCAAAGGAGCAAT
CCGCTTAAGGACGGCCTCGCGTCCGATTAGGTAGTTGGCGGGGCAACGGCCCACCAAGCCGACGATCG
GTAGCCGGACTGAGAGGTTGAACGGCCACATTGGGACTGAGACACGGCC
>Zotu1797
GAACGCTGGCGGCGTGCTTAACACATGCAAGTCGAACGATGAAGCCAGCTTGCTGGTGGATTAGTGGC
GAACGGGTGAGTAACACGTGGGTGATCTGCCCCAAACTTTGGGATAAGCCTGGGAAACTGGGTCTAAT
ACCGAATATGACCACTGGATGCATGTCTGGTGGTGGAAAGCTCCGGCGGTTTGGGATGGGCCCGCGGC
CTATCAGCTTGTTGGTGGGGTAATGGCCTACCAAGGCGACGACGGGTAGCCGGCCTGAGAGGGCGACC
GGCCACACTGGGACTGAGACACGGCC
>Zotu1799
GAACGCTGGCGGCGTGCTTAACACATGCAAGTCGAGCGGAAAGGCCCACTTGTGGGTACTCGAGCGGC
GAACGGGTGAGTATCACGTGAGTAATCTGCCCCCCTCTTTTGGATAACCCTCGGAAACGGGGGCTAAT
ACGGAATATTCAGTGCTGCCTGCATGGGTGGTGTTGGAAAGATTTTTTGGTGGGGGATGAGCTCGCGG
CCTATCAGCTTGATGGTGGGGTAATGGCCTACCATGGCTTTGACGGGTAGCCGGCCTGAGAGGGTGTC
CGGCCACACTGGGACTGAGACACGGCC
>Zotu1800
GAACGCTGGCGGCGTGCCTAATACATGCAAGTCGAGCGAATCAATGGGAGCTTGCTCCCTGAGATTAG
CGGCGGACGGGTGAGTAACACGTGGGTAACCTGCCTGTAAGACTGGGATAACTTCGGGAAACCGGAGC
TAATACCGGATAATTTCTTTTCTCGCATGAGGAAAGATGGAAAGATGGTTCTGCTATCACTTACAGAT
GGACCCGCGGCGCATTAGCTAGTTGGTGAGGTAATGGCTCACCAAGGCGACGATGCGTAGCCGACCTG
AGAGGGTGATCGGCCACACTGGGACTGAGACACGGCC
>Zotu1801
AAACGCTGGCGGCGTGCTTAACACATGCAAGTCGAGCGAGAAAGGTCTTCGGACTGAGTAAAGCGGCA

AACGGGTGAGTAACACGTAGGAATCTGCCTTTCAGTGAGGAATACCCTCGAGAAATCGAGGTTAATAC
TGCATACGTCTAGAAATAGAAAAAGATGGCCTCTTTGCTGTCGCTGAAAGATGAGCCTGCGGACTATT
AGCTAGTTGGTAGGGTAATGGCCTACCAAGGCAATGATGGTTAGCCGACCTGAGAGGGTGTACGGCCA
CACTGGAACTTAGACACGGTC
>Zotu1802
GAACGCTGGCGGCGTGCCTAATACATGCAAGTCGAACGGTTTAGTTCCAAGAGGCTTGCCTTTTGGGA
CTAGACAGTGGCGGACGGGTGAGTAACACGTAGGCAACCTACCTGTCAAACCGGGATAACCTCGGGAA
ACCGAAGCTAAACCCGGATAAGCTCCTTTTTCGCATGAAGGGGGAGTGAAAAGGTGCGACCTGATGAC
AGATGGGCCTGCGGCGCATTAGCTAGTAGGTAGGGTAACGGCCTACCTAGGCGATGATGCGTAGCCGA
CCTGAGAGGGCGTACGGCCACACTGGGACTGAGATACGGCC
>Zotu1803
GAACGCTGGCGGCGTGCCTAATACATGCAAGTAGAACGCTGAAGGGGGAGCTTGCTTCTCCGGATGAG
TTGCGAACGGGTGAGTAACGCGTAGGTAACCTGCCTGGTAGCGGGGGATAACTATTGGAAACGATAGC
TAATACCGCATAAGAGTAGATGTTGCATGACATTTACTTAAAAGGTGCAATTGCATCACTACCAGATG
GACCTGCGTTGTATTAGCTAGTTGGTGGGGTAACGGCTCACCAAGGCGACGATACATAGCCGACCTGA
GAGGGTGATCGGCCACACTGGGACTGAGACACGGCC
>Zotu1804
GAACGCTGGCGGCGTGCCTAATACATGCAAGTCGAACGCTTCACTTCGGTGAAGAGTGGCGAACGGGT
GAGTAATACATAAGTAACCTGACATCTACAGGGGGATAACTGATGGAAACGTCAGCTAAGACCGCATA
GGTGTAGAGATCGCATGAACTCTATATGAAAAGTGCTACGGGACTGGTAGATGATGGACTTATGGCGC
ATTAGCTTGTTGGTAGGGTAACGGCCTACCAAGGCGACGATGCGTAGCCGACCTGAGAGGGTGACCGG
CCACACTGGGACTGAGACACGGCC
>Zotu1805
GAACGCTAGCGGCAGGCCTAATACATGCAAGTCGAACGGGATCCGTCGGAGAGCTTGCTCGAAGACGG
TGAGAGTGGCGCACGGGTGCGTAACGCGTGAGCAACCTACCTCTATCAGGGGGATAGCCTCTCGAAAG
AGAGATTAACACCGCATAACATATCTGACCGGCATCGGTTAGCTATTAAATATTTATAAGATAGAGAT
GGGCTCGCGTGACATTAGCTAGTTGGTAGGGTAACGGCTTACCAAGGCGACGATGTCTAGGGGCTCTG
AGAGGAGAATCCCCCACACTGGTACTGAGACACGGAC
>Zotu1806
GAACGCTAGCTACAGGCTTAACACATGCAAGTCGAGGGGAAACGACATTGAAGCTTGCTTCGATGGTC
GTCGACCGGCGCACGGGTGAGTAACGCGTATCCAACCTGCCTCTGACTAAGGGATAACCCGGCGAAAG
TCGGACTAATACCTTATGAGGTTTTCAGCAGACATCTAATGAAAACGAAAGATTTATCGGTCAGTGAT
GGGGATGCGTCTGATTAGCTTGTTGGCGGGGTAACGGCCCACCAAGGCAACGATCAGTAGGGGTTCTG
AGAGGAAGGTCCCCCACATTGGAACTGAGACACGGTC
>Zotu1807
GAACGCTGGCGGCGTGCCTAATACATGCAAGTCGAGCGGTAAGGCTCTGCACTTAGTATTTGATCGCA
AAGTCCTCGGACTTCTAAGCAGATTGATTCGACCCTAGGCTAGGGAACATGATACGAGCAGAGATCGA
ATAATGAGTGTAGAGTACACGAGCGGCGGACGGCTGAGTAACACGTAGGAACATACCCCAAAGTGAGG
GATAACTGCTCGAAAGAGTAGCTAATACCGCATGTGCTCTAAGGAGTAAAGCTTCGGCGCTTTGGGAA
TGGCCTGCGCATGATTAGCTTGTTGGTGAGGTAAAAGCTCACCAAGGCGACGATCATTAGCTGGTCTG
AGAGGATGATCAGCCAGACTGGGACTGAGACACGGCC
>Zotu1808
GAACGCTAGCGGCAGGCTTAACACATGCAAGTCGAGGGGCAGCGAGATGTAGCAATACATTGTCGGCG
ACCGGCGCACGGGTGCGTAACGCGTATGCAACCTACCATATACAGGAGGATAACCATTCGAAAGAATG
ACTAATACTCCATAGTATTATTTTGAGGCATCTTAGAATAATTAAAGAATTTCGGTATATGCTGGGCA
TGCGTGACATTAGCTAGTTGGAGAGGTAACGGCTCACCAAGGCTACGATGTCTAGGGGTTTTGAGAGG
AAGATCCCCCACACTGGTACTGAGACACGGAC
>Zotu1809
GAACGCTGGCGGCGTGCTTAACACATGCAAGTCGAACGTGAAATGTAGCAATACAGAGTAAAGTGGCG
TACGGGTGAGTAACACGTGGATAATCTACCAAAGTGCCCGGAATAACCGGCCGAAAGGCTGGCTAATA
CTGGATAGTTCAAGCCATCTGTAAGGGATGGATTGGGAAAGGGAGCGGGTAACCGCCTTCACATTTTG
ATGAGTCCGCGTCTGATTAGGTAGTTGGTGAGGTAATGGCTCACCAAGCCTGAGATCAGTAGCTGGTC
TGAGAGGATGATCAGCCACACTGGAACTGAAACACGGTC
>Zotu1810

GAACGCTGGCGGCAGGCTTAACACATGCAAGTCGAACGGATCCTTCGGGATTAGTGGCAGACGGGTGA
GTAACGCGTGGGAACGTACCCTTTGCTACGGAATAACTCAGGGAAACTTGAGCTAATACCGTATGTGC
CCTACGGGGGAAAGATTTATCGGCAAAGGATCGGCCCGCGTTGGATTAGCTAGTTGGTGGGGTAATGG
CCTACCAAGGCGACGATCCATAGCTGGTCTGAGAGGATGATCAGCCACACTGGGACTGAGACACGGCC
>Zotu1812
GAACGCTGGCGGCGTGCTTAACACATGCAAGTCGAGCGGAAAGGCCCTTCGGGGTACTCGAGCGGCGA
ACGGGTGAGTAACACGTGAGTAACCTGCCTCAGGCTTTGGGATAACCCTCGGAAACGGGGGCTAATAC
CGGATATGACCTGTCACCGCATGGTGGTGGGTGGAAAGTTTTTCGGCCTGGGATGGGCTCGCGGCCTA
TCAGCTTGTTGGTGGGGTGATGGCCTACCAAGGCGACGACGGGTAGCCGGCCTGAGAGGGCGACCGGC
CACACTGGGACTGAGACACGGCC
>Zotu1813
GAACGCTGGCGGCGTGCTTAACACATGCAAGTCGAGCGGAAAGGCCACTTCGGTGGTACTCGAGCGGC
GAACGGGTGAGTAACACGTGAGTAATCTGCCCCTGGCTTTGGGATAGCCACCGGAAACGGTGATTAAT
ACCGGATATGACATGGTCTCGCATGGGATTGTGTGGAAAGTTTTTCGGCCAGGGATGTGCTCGCGGCC
TATCAGCTTGATGGTGAGGTAATGGCTCACCATGGCTTCGACGGGTAGCCGGCCTGAGAGGGTGACCG
GTCACACTGGGACTGAGACACGGCC
>Zotu1814
GAACGCTGGCGGCGTGCTTAACACATGCAAGTCGAGCGACGCCTCATCTTCGGGTGGGTGACGGAGCG
GCGAACGGGTGAGTAACACGTGGGCAACCTGCCCCTAGCTTCGGGATAAGCCCGGGAAACCGGGTCTA
ATACCGGATATGAGACTGGATCGCATGGTCTGGTTTGGAAAGTTTTTCGGCTAGGGATGGGCCCGCGG
CCTATCAGCTTGTTGGTGGGGTAATGGCCCACCAAGGCGACGACGGGTAGCCGGCCTGAGAGGGCGAC
CGGCCACACTGGGACTGAGACACGGCC
>Zotu1816
GAACGCTAGCTGCAGGCTTAACACATGCAAGTCGAGGGGCAGCGGGAGTAGCAATACTTGCCGGCGAC
CGGCGGAAGGGTGCGTAACGCGTGAGCAACATTCCCGTATCAGGGGGATAACCGGTGGAAACGCCGAC
TAATACCCCATAAGAACATCTGCGGCATCAAAGGTGTTTGAAAGATTCGTCGGATGCGGATTGGCTCG
CGTGACATTAGCTAGTTGGTGAGGTAACGGCCCACCAAGGCGACGATGTCTAGGAGTTCTGAGAGGAA
GATCCCCCACATTGGAACTGAGACACGGTC
>Zotu1817
GAACGCTGGCGGCGTGCTTAACACATGCAAGTCGAGCGGAAAGGCCTCTTCGGGGGTACTCGAGCGGC
GAACGGGTGAGTAACACGTGAGTAATCTGCCTCTGGCTCTGGGATAGCCACCGGAAACGGTGATTAAT
ACCGGATATGACAGCTCCTCGCATGGGGTGGTTGTGGAAAGTTTTTTCGGCCAGGGATGTGCTCGCGG
CCTATCAGCTTGTTGGTGGGGTAATGGCCTACCAAGGCTTTGACGGGTAACCGGCCTGAGAGGGTGAC
CGGTCACACTGGGACTGAGACACGGCC
>Zotu1818
AAACGCTGGCGGCGTGCATGAGACATGCAAGTCGAACGGACGATTCGGGCTTGCTCGAATCGTTAGTG
GCGGACGGCTGAGTAACGCGTGGGTGACCTGCCCCGAAGTGGGGAATAACTGCCCGAAAGGGTGGCTA
ATACCGCATGTGGTCGCACGTTCAATCGTGCGAGTAAAGCCGCAAGGCGCTTCGGGAGGGGCCTGCGT
CCGATTAGCTCGTTGGTGGGGTAATGGCTCACCAAGGCGACGATCGGTAGGTGGTCTGAGAGGACGAT
CACCCAGACTGGGACTGAGACACGGCC
>Zotu1819
GAACGCTGGCGGCGTGCTTAACACATGCAAGTCGAACGATGACCCCGTGCTTGCACGGGTGATTAGTG
GCGAACGGGTGAGTAACACGTGAGTAACCTGCCCCTGACTCTGGGATAAGCCCGGGAAACTGGGTCTA
ATACTGGATATGACCTTTAACCGCATGGTTTTTGGTGGAAAGATTTATCGGTTGGGGATGGACTCGCG
GCCTATCAGCTTGTTGGTGAGGTAATGGCTCACCAAGGCGACGACGGGTAGCCGGCCTGAGAGGGTGA
CCGGCCACACTGGGACTGAGACACGGCC
>Zotu1820
GAACGCTGGCGGCAGGCCTAACACATGCAAGTCGAGCGCACCCTTCGGGGTGAGCGGCGGACGGGTGA
GTAACGCGTGGGAACGTGCCCTTCTCTGCGGAATAGCCTCGGGAAACTGAGAGTAATACCGCATACGC
CCTTTGGGGGAAAGATTTATCGGAGAAGGATCGGCCCGCGTTGGATTAGGTAGTTGGTGGGGTAATGG
CCCACCAAGCCGACGATCCATAGCTGGTTTGAGAGGATGATCAGCCACACTGGGACTGAGACACGGCC
>Zotu1821
GAACGCTGGCGGCATGCCTTACACATGCAAGTCGAACGGCAGCACGGGTGCTTGCACCTGGTGGCGAG
TGGCGAACGGGTGAGTAATACATCGGAACGTACCCGGAAGTGGGGGTAACGTAGCGAAAGTTACGCT

AATACCGCATATTCTGTGAGCAGGAAAGCAGGGGATCGCAAGACCTTGCGCTTTCGGAGCGGCCGATG
TCAGATTAGCTAGTTGGTGAGGTAAAGGCTCACCAAGGCGACGATCTGTAGCGGGTCTGAGAGGATGA
TCCGCCACACTGGAACTGAGACACGGTC
>Zotu1822
GAACGCTGGCGGAATGCTTTACACATGCAAGTCGAACGGCAGCACGGGGCAACCTGGTGGCGAGTGGC
GAACGGGTGAGTAATATATCGGAACGTGCCCAATCGTGGGGGATAACGTAGCGAAAGTTACGCTAATA
CCGCATACGATCTACGGATGAAAGCGGGGGATCGCAAGACCTCGCGCGAATGGAGCGGCCGATATCAG
ATTAGGTAGTTGGTGGGGTAAAGGCTCACCAAGCCAACGATCTGTAGCTGGTCTGAGAGGACGACCAG
CCACACTGGAACTGAGACACGGTC
>Zotu1823
GAACGCTGGCGGCAGGCTTAACACATGCAAGTCGAACGCCCCGCAAGGGGAGTGGCAGACGGGTGAGT
AACGCGTGGGAATCTACCTAGTTCTACGGAACAACAGAGGGAAACTTCTGCTAATACCGTATACGCCC
TAAGGGGGAAAGATTTATCGGAATTAGATGAGCCCGCGTAAGATTAGCTAGTTGGTGGGGTAATGGCC
TACCAAGGCGACGATCTTTAGCTGGTCTGAGAGGATGATCAGCCACACTGGGACTGAGACACGGCC
>Zotu1824
GAACGCTGACAGAATGCTTAACACATGCAAGTCGATGGGGAAGTGGTGCTTGCACCATGGTAACCATG
GCGGACGGGTGAGTAACGCGTAAGGAACTTGCCTGCGGGACTGGGATAACAGCAGGAAACTGCTGATA
ATACCGGATAAGGTCATTAAGCTGCATAGCTTAGTGATGAAAGGAGACGCCTGCGGAGAGCCTTGCGT
CCTATTAGCTAGTTGGAGGGGTAACGGCCCACCAAGGCGAAGATAGGTAGCCGGCCTGAGAGGGTGGA
CGGCCACAAGGGGACTGAGATACGGCC
>Zotu1825
GAACGCTGGCGGCGTGCCTAATACATGCAAGTCGAGCGAACAGATAAGGAGCTTGCTCCTTTGACGTT
AGCGGCGGACGGGTGAGTAACACGTGGGTAACCTACCTATAAGACTGGAATAACTCCGGGAAACCGGG
GCTAATGCCGGATAATACACAGAACCGCATGGTTCTGTGATGAAAGACGGTTTTGCTGTCACTTATAG
ATGGACCCGCGGCGTATTAGCTAGTTGGTAAGGTAACGGCTTACCAAGGCAACGATACGTAGCCGACC
TGAGAGGGTGATCGGCCACATTGGGACTGAGACACGGCC
>Zotu1826
CAACGCTGGCGGCGCGCTTAACACATGCAAGTCGAGCGAGAAAGGGGGCAACCCTGAGTAAAGCGGCG
TACGGGTGAGTAACACGTGGATTATCTACCTTTTAGTGGGGAATAACTTTGGGAAACCGAAGCTAATA
CCGCATAAGCCTGTAAAGGGAAAGCAGCAATGCGCTGGAAGAAGAGTCCGCGGCCGATTAGCTAGTTG
GTGAGGTAACGGCCCACCAAGGCGACGATCGGTAGCCGGCCTGAGAGGGCACACGGCCACACTGGCAC
TGAAACACGGGC
>Zotu1828
GAACGCTGGCGGCGTGCCTAACACATGCAAGTCGAGCGATGAAGCTTCTTCGGAAGTGGATTAGCGGC
GGACGGGTGAGTAACACGTGGGCAACCTGCCTCATAGAGAGGGATAGCCTTTCGAAAGGAAGATTAAT
ACCTCATAAGATTGTAGTTTCGCATGAAACGGCAATAAAAGGAGCAATCCGCTATGAGATGGGCCCGC
GTCGCATTAGCTAGTTGGTAAGGTAATGGCTTACCAAGGCGACGATGCGTAGCCGACCTGAGAGGGTG
ATCGGCCACATTGGGACTGAGACACGGCC
>Zotu1829
GAACGCTGGCGGCGTGCCTAATACATGCAAGTCGAGCGAACTGATTAAGAAGCTTGCTTCTTATGACG
TTAGCGGCGGACGGGTGAGTAACACGTGGGCAACCTGCCCTATAGTTGGGGATAACTCCGGGAAACCG
GGGCTAATACCGAATGATACATTTTAACTCCTGTTAGAATGTTGAAAGATGGTTTACGCTATCGCTAT
AGGATGGGCCCGCGGCGCATTAGCTAGTTGGTGAGGTAACGGCTCACCAAGGCGACGATGCGTAGCCG
ACCTGAGAGGGTGATCGGCCACACTGGGACTGAGACACGGCC
>Zotu1830
GAACGCTGGCGGCGTGCTTAACACATGCAAGTCGAACGCTGAAGCTTGGTGCTTGCACTGGGTGGATG
AGTGGCGAACGGGTGAGTAATACGTGAGTAACCTGCCCTTGACTCTGGGATAAGCCTGGGAAACTGGG
TCTAATACTGGATATGACATGCCACTGCATGGTGGTGTGTGGAAAGGGTTTTACTGGTTTTGGATGGG
CTCACGGCCTATCAGCTTGTTGGTGGGGTAATGGCCTACCAAGGCGACGACGGGTAGCCGGCCTGAGA
GGGTGACCGGCCACACTGGGACTGAGACACGGCC
>Zotu1831
GAACGCTGGCGGCGTGCTTAACACATGCAAGTCGAGCGGTAAGGCCCTTCGGGGTACACGAGCGGCGA
ACGGGTGAGTAACACGTGAGTAATCTGCCCTTCACTTCGGGATAACCACCGGAAACGGTGGCTAATAC
CGGATATGACTTCTCTTCGCATGTTGAGAGGTGGAAAGTTCTGGCGGTGGAGGATGTGCTCGCGGCCT

ATCAGCTTGTTGGTGAGGTAATGGCTCACCAAGGCTTCGACGGGTAGCCGGCCTGAGAGGGTGACCGG
CCACACTGGGACTGAGACACGGCC
>Zotu1832
GAACGCTAGCGGCAGGCTTAATACATGCAAGGCGAGGGGGCAGCAATGTCACCGTCGTACGGGTGCGT
AACGCGTATGCAACCTACCTTCAACTGGGGGATAGCCCGGGAAACCCGGATTAATACCGCATAATAC
AGGGGGCCCACATGGGTCTATTTGTTAAAGATTTATTGGTTGAAGATGGGCATGCGTTCGATTAGCTA
GTTGGTGTAGGTAACGGCTCACCAAGGCGACGATCGATAGGGGAGCTGAGAGGTTGATCCCCCACACG
GGCACTGAGATACGGGC
>Zotu1833
GAACGCTGGCGGCATGCTTAACACATGCAAGTCGCACGAGGGGGCAACCCCTAGTGGCGGACGGGTGA
GTAACGCGTAGGTATCTATCCTGAGGTGGGGGATAACAGCGGGAAACTGCTGCTAATACCGCATGACA
CCTGTGGGTCAAAGGCGCAAGTCGCCTTGGGAGGAGCCTGCGTCCGATTAGCTAGTTGGTGGGGTAAA
GGCCTACCAAGGCTTCGATCGGTAGCTGGTCTGAGAGGATGATCAGCCACACTGGGACTGAGACACGG
CC
>Zotu1834
GAACGCTGGCGGCGTGCCTAATACATGCAAGTAGAACGCTGAAGCTTGGTGCTTGCACCGAGCGGATG
AGTTGCGAACGGGTGAGTAACGCGTAGGTAACCTGCCTGGTAGCGGGGGATAACTATTGGAAACGATA
GCTAATACCGCATAACAGTAGATATTGCATGATATCTGCTTGAAAGGTGCAATTGCATCACTACCAGA
TGGACCTGCGTTGTATTAGCTAGTTGGTGAGGTAACGGCTCACCAAGGCGACGATACATAGCCGACCT
GAGAGGGTGATCGGCCACACTGGGACTGAGACACGGCC
>Zotu1835
GAACGCTGGCGGCAGGCTTAACACATGCAAGTCGAACGCCCCGCAAGGGGAGTGGCAGACGGGTGAGT
AACGCGTGGGAACGTACCTATCGGTTCGGAATAACCCAGGGAAACTTGGACTAATACCGGATACGCCC
TTTTGGGGGAAAGATTTATCGCCGATAGATCGGCCCGCGTCTGATTAGCTAGTTGGTGGGGTAATGGC
CTACCAAGGCGACGATCAGTAGCTGGTCTGAGAGGATGATCAGCCACACTGGGACTGAGACACGGCC
>Zotu1836
GAACGCTGGCGGCGTGCTTAACACATGCAAGTCGAACGGTGAACTCAGAGCTTGCTCTGAGGGATCAG
TGGCGAACGGGTGAGTAACACGTGAGTAACCTGCCCCAGACTCTGGAATAAGCGCTGGAAACGGCGTC
TAATACCGGATACGAGACAGAGAGGCATCTCTACTGTCTGGAAAGTTTTTCGGTCTGGGATGGACTCG
CGGCCTATCAGCTTGTTGGTGAGGTAACGGCTCACCAAGGCGACGACGGGTAGCCGGCCTGAGAGGGC
GACCGGCCACACTGGGACTGAGACACGGCC
>Zotu1837
GAACGCTAGCGGCAGGCCTAATACATGCAAGTCGAACGAGATTATCCAGCTTGCTGGATATGAAAGTG
GCGCACGGGTGCGTAACGCGTATGCAACCTACCTTTATCTGGGGGATAGCCCGGAGAAATCCGGATTA
ATACCGCATAAAATCACAGGATGGCATTATCCAATGATCAAACATTTATGGGATGAAGATGGGCATGC
GTGTCATTAGCTAGTTGGCGGGGTAACGGCCCACCAAGGCGACGATGACTAGGGGATCTGAGAGGATG
GCCCCCCACACTGGTACTGAGACACGGAC
>Zotu1839
GAACGCTGGCGGAATGCTTTACACATGCAAGTCGAACGGCAGCACGAGGGCAACCTTGGTGGCGAGTG
GCGAACGGGTGAGTAATGTATCGGAACGTGCCCAGTCGTGGGGGATAACGTAGCGAAAGCTACGCTAA
TACCGCATACGATCTATGGATGAAAGCGGGGGATCGCAAGACCTCGCGCGATTGGAGCGGCCGATATC
AGATTAGGTAGTTGGTGGGGTAAAGGCTCACCAAGCCAACGATCTGTAGCTGGTCTGAGAGGACGACC
AGCCACACTGGAACTGAGACACGGTC
>Zotu1840
GAACGCTGGCGGCGTGCCTAATACATGCAAGTCGAGCGAACAGATGAGGAGCTTGCTCCTCTGATGTT
AGCGGCGGACGGGTGAGTAACACGTGGGCAACCTGCCCTATAGTTTGGGATAACTTGCGGAAACGTGA
GCTAATACCGAATAATACAACGAAGCTCCTGCTTCGATGTTGAAAGATGGCTTTGCTATCGCTATAGG
ATGGGCCCGCGGCGCATTAGCTAGTAGGTGAGGTAACGGCTCACCTAGGCAACGATGCGTAGCCGACC
TGAGAGGGTGATCGGCCACACTGGGACTGAGACACGGCC
>Zotu1841
GAACGCTAGCGGCAGGCTTAACACATGCAAGTCGAACGGTAACAGATCTTCGGATGCTGACGAGTGGC
GCACGGGTGCGTAACGCGTATGCAACCTACCTCTAACTGGGGAATAGCCCGGGGAAACTCGGATTAAT
ACCCCATAACATTATGGAATCGCATGATTCTATAATTAAAACTCCGGTGGTTAGAGATGGGCATGCGT
CCTATTAGCTAGTTGGTGAGGTAACGGCTCACCAAGGCTACGATAGGTAGGGGGTCTGAGAGGATTAT

CCCCCACACTGGTACTGAGACACGGAC
>Zotu1842
GAACGCTGGCGGCATGCCTAATACATGCAAGTCGAACGCTGAAGTACTCTGCTTGCAGAGTGCGGATG
AGTGGCGAACGGGTGAGTAACACGTGGGGAATCTGCCTATAAGTGGGGGATAACATTCGGAAACGGAT
GCTAATACCGCATATATTCTTGGACCGCCTGATCCTTGAAAGAAAGACGGCTTTGCTGTCGCTTATAG
ATGATCCCGCGGCGTATTAGTTAGTTGGTGGGGTAAAGGCCTACCAAGACAATGATACGTAGCCGACC
TGAGAGGGTGATCGGCCACACTGGGACTGAGACACGGCC
>Zotu1843
GAACGCTAGCGGCAGGCTTAACACATGCAAGTCGAGGGGCAGCGCGGGTAGCAATACCTGGCGGCGAC
CGGCAAACGGGTGCGGAACACGTACTGAACCTACCTTGATCTGGGGCATAGCCCGATGAAAATCGGAT
TAACTCCCCATAATATAGATGAGTGGCATCACTTGACTATTAAAAATTTATTGGATCGAGATGGCAGT
GCGTCCCATTAGCTAGTTGGTAGGGTAACGGCCTACCAAGGCGACGATGGGTAGCTGGTGTGAGAGCA
CGACCAGCCACACGGGCACTGAGACACGGGC
>Zotu1844
GAACGCTGGCGGCGTGCTTAACACATGCAAGTGGAGCGACGAAGCGGACTTCGGTCCGTGGCAGAGCC
GCGAACGGGTGAGTAACACGTGGATAACCTACCCCAATGACCGGGACAACCCGAGGAAACTCGAGCTA
ATACCGGATGTTCTCTCTTCCTCTAAGGGAATTGAGCAAAGATAGCTTCGGCCTTCGCATTGGGACGG
GTCCGCGGCCCATTAGCTTGTTGGTGAGGTAACGGCTCACCAAGGCGACGATGGGTAGCTGGTCTGAG
AGGACGATCAGCCACACTGGGACTGAGACACGGCC
>Zotu1845
GAACGCTGGCGGCGTGCTTAACACATGCAAGTCGAACGATGATGCCCTGCTTGCAGGGTGGATTAGTG
GCGAACGGGTGAGTAACACGTGGGTGATCTGCCCTGCACTTCGGGATAAGCCTGGGAAACTGGGTCTA
ATACCGGATATGACCCTCCTTTAGTGTGGGGGGTGGAAAGCTTTTGCGGTGTGGGATGAGCCTGCAGC
CTATCAGCTTGTTGGTGGGGTAATGGCCTACCAAGGCGTCGACGGGTAGCCGGCCTGAGAGGGTGATC
GGCCACATTGGGACTGAGACACGGCC
>Zotu1846
GAACGCTGGCGGCGTGCCTAACACATGCAAGTCGAGCGAGAGACTTCGGTCTCTAGCGGCGGACGGGT
GAGTAACACGTGGGTAACCTGCCTCGTAGAGGGGGATAGCCTTCCGAAAGGAAGATTAATACCGCATA
ACATATATCTGGCGCATGCCAGATATATCAAAGGAGTAATCCGCTATGAGATGGACCCGCGGCGCATT
AGCTAGTTGGTGAGGTAACGGCTCACCAAGGCGACGATGCGTAGCCGACCTGAGAGGGTGATCGGCCA
CATTGGAACTGAGATACGGTC
>Zotu1847
GAACGCTAGCGGCAGGCTTAATACATGCAAGTCGAACGGATGTAGCAATACATTAGTGGCGCACGGGT
GAGTAACGCGTACATAACATGCCTTTAGCTTAGGAATAGCCTTGGGAAACTGGGAGTAATACCTAATA
GTATTTTTGGATGGCATCGTCTGGAAATTAAAGTTACGGCGGCTAAAGATTGATGTGCGTAAGATTAG
CTAGTTGGTGAGGTAACGGCTCACCAAGGCGACGATCTTTAGGGGGCGTGAGAGCGTGACCCCCCACA
CGGGTACTGAGACACGGAC
>Zotu1848
GAACGCTGGCGGCATGCCTAACACATGCAAGTCGAACGGAATCTTATATGTTCCTCGTTTCGGATTTT
TAATCCGCGTTGAGGAACAGTAAGATTTAGTGGCGGACGGGTGAGTAACACGTTGGTACCTACCCTCT
AGTGGGGAATAACTTCGAGAAATCGAGGCTAATACCGCATAATATCTACGGATTAAAGCATTTATGCG
CTAGAGGAGGGGCCTGCGTCTGATTAGCTAGTTGGTGAGGTAAAAGCTCACCAAGGCGACGATCAGTA
GCTGGATCGAGAGGTTGACCAGCCACACTGGGACTGAGATACGGCC
>Zotu1849
GAACGCTGGCGGCATGCCTTACACATGCAAGTCGAACGGCAGCACGGGAGCAATCCTGGTGGCGAGTG
GCGAACGGGTGAGTAATATATCGGAACGTGCCCAGTCGTGGGGGATAACGCAGCGAAAGCTGTGCTAA
TACCGCATACGATCTATGGATGAAAGCGGGGGACTCGTAAGGGCCTCGCGCGATTGGAGCGGCCGATA
TCAGATTAGCTAGTTGGTGGGGTAAAGGCCTACCAAGGCGACGATCTGTAGCTGGTCTGAGAGGACGA
CCAGCCACACTGGAACTGAGACACGGTC
>Zotu1850
GAACGCTAGCGGCAGGCCTAATACATGCAAGTCGAACGGGTTGAGGTAGCAATACTTCAATTAGTGGC
GCACGGGTGCGTAACGCGTATGCAACCTACCTTTTATTGGGGGATAGCCCGAGGAAACTCGGATTAAT
ACCGCATAACATCATTTGAGGGCATCTGAAGATGATTAAAGCTAAGGCGATAAAAGATGGGCATGCGT
GTCATTAGCTAGTTGGTTGAGGTAAAGGCTCACCAAGGCGACGATGACTAGGGGTTCTGAGAGGATGA

TCCCCCACACTGGTACTGAGATACGGAC
>Zotu1851
GAACGCTGGCGGCGCGCCTAACACATGCAAGTCGAGCGAGAACCAGGGCTTCGGCCCTGGGGACAGCG
GCGAACGGGTGAGTAACACGTGGATAACCTGCCCTCGGCACCGGAATAGCCCGGGGAAACCCGGATTA
ATGCCGGATAGCCCCACATCCCTGCGGGGAACGTGGGAAAAGGTAGCTTCGGCCTCTGGCCGAGGATG
GGTCCGCGGTGGATTAGCTTGTTGGCGGGGTAACGGCCCACCAAGGCGACGATCCATAGCTGGTCTGA
GAGGACGATCAGCCACACTGGGACTGAGACACGGCC
>Zotu1852
GAACGCTGGCGGCGTGCTTAACACATGCAAGTCGAGCGGTAAGGCTCCTTCGGGGGTACACGAGCGGC
GAACGGGTGAGTAACACGTGAGTAATCTGCCCTTGGCTTTGGGATAAGCACCGGAAACGGTGTCTAAT
ACCGGATATACACGCATTGTCGCATGGTGGTGTGTGGAAAGTTTTTCGGCCTTGGATGTGCTCGCGGC
CTATCAGCTTGATGGTGGGGTAATGGCCTACCATGGCTTCGACGGGTAGCCGGCCTGAGAGGGTGACC
GGTCACACTGGGACTGAGACACGGCC
>Zotu1853
GAACGCTGGCGGCGTGCTTAACACATGCAAGTGGAGCGACGAACCAGGGCTTGCCCTGGGGCAGAGCC
GCGAACGGGTGAGTAACACGTGGGTAACCTGCCCCGATCACTGGGACAACCCGAGGAAACTCGGGCTA
ATACCGGATGTGATCCCGTCACACCAGTGACGTGATGAAAGGTAGCTTCGGCTTCCGGATTGGGATGG
GCCCGCGGCCCATTAGCTTGTTGGTGGGGTAACGGCCTACCAAGGCGACGATGGGTAGCTGGTCTGAG
AGGACGATCAGCCACACTGGGACTGAGACACGGCC
>Zotu1854
GAACGCTGGCGGCGTGCTTAACACATGCAAGTCGAACGGAAAGGCCTCTGCTTGCAGAGGTACTCGAG
TGGCGAACGGGTGAGTAACACGTGGGTGATCTGCCTTGCACTTCGGGATAAGCCTGGGAAACTGGGTC
TAATACCGGATAGGACCATGCTTTAGTGTGTGTGGTGGAAAGTTTTTCGGTGCGAGATGAGCCCGCGG
CCTATCAGCTTGTTGGTGGGGTAATGGCCTACCAAGGCGACGACGGGTAGCCGGCCTGAGAGGGTGTA
CGGCCACATTGGGACTGAGACACGGCC
>Zotu1855
CAGGAAGAGGTCCCATAATGTCGATCACCATGCGCTTAAACGGCTCAGTAATGATCGGCACGAGCTGC
ATTGGCACCTTGCACCTGTCGTGCGGTTTTCCGGCCCGTTGGCAGCTGTCA
>Zotu1857
GAACGCTAGCGGCAGGCCTAATACATGCAAGTTGAACGGTTAGTAGCAATACTGATAGTGGCGCACGG
GTGCGTAACACGTAGCTAACCTGCCCAGAACTGGGGGATAGCCCGCCGAAAGGCGGATTAACACCGCA
TAATATCAGTACCTGGCATCAGGTGGTGATTAAATCTACGGAGGTTCTGGATGGGGCTGCGGGTCATT
AGCTAGTTGGTCAGGTAACGGCTTACCAAGGCGACGATGACTAGGGGAGCTGAGAGGCTGGTCCCCCA
CACGGGCACTGAGATACGGGC
>Zotu1858
GAACGCTGGCGGCGTGCCTAATACATGCAAGTCGAGCGAACAGATAAGGAGCTTGCTCCTTTGACGTT
AGCGGCGGACGGGTGAGTAACACGTGGGTAACCTACCTATAAGACTGGGATAACTTCGGGAAACCGGA
GCTAATACCGGATAATATTTCGAACCGCATGGTTCGATAGTGAAAGATGGTTTTGCTATCACTTATAG
ATGGACCCGCGCCGTATTAGCTAGTTGGTAAGGTAACGGCTTACCAAGGCGACGATACGTAGCCGACC
TGAGAGGGTGATCGGCCACACTGGAACTGAGACACGGTC
>Zotu1859
GAACGCTGGCGGCGTGCTTAACACATGCAAGTCGAACGGTGAAAGAGGAGCTTGCTCCTCTGGATCAG
TGGCGAACGGGTGAGTAACACGTGAGTAACCTGCCCCAGACTCTGGGATAAGCGTTGGAAACGACGTC
TAATACCGGATACGAGCTTCAGCCGCATGGCTAGGAGCTGGAAAGAATTTCGGTCTGGGATGGACTCG
CGGCCTATCAGGTAGTTGGTGAGGTAACGGCCCACCAAGCCTACGACGGGTAGCCGGCCTGAGAGGGT
GACCGGCCACACTGGGACTGAGACACGGCC
>Zotu1860
GAACGCTGGCGGCGTGCTTAACACATGCAAGTCGAACGATGAAACAATTCTTGATGTCTTCGGACTGA
TTGAGCTGTGGATTAGTGGCGGACGGGTGAGTAACGCGTGAGCAATCTGCCTTACACAAAGGGATAGC
CTCGGGAAACCGGGATTAATACCATATGATATCTACAAGCCGCATGGCCTATAGATTAAAGAATTTCG
GTGTAAGATGAGCTCGCGTCCCATTAATTAGTTGGTGAGGGTAACGGCCCACCAAGATAGCGATGGGT
AGCCGATCTAAGAGGATGACCGGCCACATTGGAACTGAGAAACGGTC
>Zotu1861
GAACGCTAGCGGGAGGCCTAACACATGCAAGCCGAGCGGTAGAAGATCTTCGGATCTTTGAGAGCGGC

GTACGGGTGCGGAACACGTGTGCAACCTGCCTTTATCAGGGGGATAGCCTTTCGAAAGGAAGATTAAT
ACCCCATAATATAATTGATGGCATCATTAATTATTGAAAACTCCGGTGGATAGAGATGGGCACGCGCA
AGATTAGATAGTTGGTAGGGTAACGGCCTACCAAGTCAGTGATCTTTAGGGGGCCTGAGAGGGTGATC
CCCCACACTGGTACTGAGACACGGAC
>Zotu1862
GAACGCTGGCGGCAGGCCTAACACATGCAAGTCGAGCGGTAGAGAGAAGCTTGCTTCTCTTGAGAGCG
GCGGACGGGTGAGTAATGCCTAGGAATCTGCCTGGTAGTGGGGGATAACGTTCGGAAACGGACGCTAA
TACCGCATACGTCCTACGGGAGAAAGCAGGGGACCTTCGGGCCTTGCGCTATCAGATGAGCCTAGGTC
GGATTAGCTAGTTGGTGGGGTAATGGCTCACCAAGGCGACGATCCGTAACTGGTCTGAGAGGATGATC
AGTCACACTGGAACTGAGACACGGTC
>Zotu1863
GAACGCTGGCGGCATGCTTTACACATGCAAGTCGAACGGCAACGAGGAGAAGCTTGCTTCTCTGTCGG
CGAGTGGCGAACGGGTGAGTATAGCATCGGAACGTGCCAAGTAGTGGGGGATAACCAAACGAAAGTTT
GGCTAATACCGCGTAAGCTCCAAGGAGGAAAGTAGGGGACCTAAATAAGGCCTTACGCTATTTGATCG
GCCGATGTCGGATTAGCTAGTTGGTGGGGTAATGGCTCACCAAGGCAATGATCCGTAGCGGGTCTGAG
AGGACGATCCGCCACACTGGGACTGAGACACGGCC
>Zotu1864
GAACGCTGGCGGCGTGCTTAACACATGCAAGTCGAACGGAAAGGCCTCTGCTTGCAGGGGTACTCGAG
TGGCGAACGGGTGAGTAACACGTGGGTGATCTGCCCTGCACTTCGGGATAAGCCTGGGAAACTGGGTC
TAATACCGGATAGGACCACATTTTGGATGGTGTGGTGGAAAGTTTTTCGGTGTGGGATGAGCTCGCGG
CCTATCAGCTTGTTGGTGGGGTAATGGCCTACCAAGGCGTCGACGGGTAGCCGGCCTGAGAGGGTGTA
CGGCCACATTGGGACTGAGATACGGCC
>Zotu1865
GAACGCTGGCGGCGTGCTTTAGGCATGCAAGTCGAACGGTTTCCGCAAGGAAGCAGTGGCAGACGGGT
GAGTAATACGTAGGAATCTACCCCTCGGTGGACCATAGCTCATCGAAAGATGGGGTAATAGTTCATGG
TCTCGTAAGAGTAAAGCGATTAACTTCGCGCCAAGGGAGGAGCCTGCGTCCTATCAGCTAGTTGGTGG
GGTAAAAGCCTACCAAGGCAATGACGGGTAGCTGGTCTGAGAGGATGGTCAGCCACAACTGCACTGAG
ACACGGGC
>Zotu1867
GAACGCTGGCGGCGTGCTTAACACATGCAAGTCGAACGGTAAGGCCCCTTCGGGGGTACACGAGTGGC
GAACGGGTGAGTAACACGTGGGTAATCTGCCCTGCACTTCGGGATAAGCCTGGGAAACTGGGTCTAAT
ACCGGATATGAACTCCTGCCGCATGGTGGGGGTTGGAAAGTTTTTCGGTGCAGGATGAGCCCGCGGCC
TATCAGCTTGTTGGTGGGGTAATGGCCTACCAAGGCGACGACGGGTAGCCGGCCTGAGAGGGTGATCG
GCCACACTGGGACTGAGACACGGCC
>Zotu1868
GAACGCTGGCGGCGTGCTTAACACATGCAAGTCGAGCGGAAAGGCCACTTCGGTGGTACTCGAGCGGC
GAACGGGTGAGTAACACGTGAGTAATCTGCCCCTGGCTTCGGGATAGCCACCGGAAACGGTGATTAAT
ACCGGATATGACATGGACTCGCATGGGTTTGTGTGGAAAGTTTTTCGGCCAGGGATGTGCTCGCGGCC
TATCAGCTTGATGGTGAGGTAATGGCTCACCATGGCTTCGACGGGTAGCCGGCCTGAGAGGGTGACCG
GCCACACTGGGACTGAGACACGGCC
>Zotu1869
GAACGCTAGCTACAGGCTTAACACATGCAAGTCGAGGGGCAGCATGAACTTAGCTTGCTAAGTTTGAT
GGCGACCGGCGCACGGGTGAGTAACACGTATCCAACCTGCCGATGACTCGGGGATAGCCTTTCGAAAG
AAAGATTAATACCCGATGGCATAGTTCTTCCGCATGGTGGAACTATTAAAGAATTTCGGTCATCGATG
GGGATGCGTTCCATTAGGTTGTTGGCGGGGTAACGGCCCACCAAGCCTTCGATGGATAGGGGTTCTGA
GAGGAAGGTCCCCCACATTGGAACTGAGACACGGTC
>Zotu1870
GAACGCTGGCGGCGCGCCTAATACATGCAAGTCGAGCGAGAACCGGGACTTCGGTCCCGGGGAGAGCG
GCGAACGGGTGCGTAACACGTGAGCAACCTGCCCCAAAGACCGGGACAACCCGGGGAAACCCGAGCTA
ATACCGGATGTCCTCATCGGATCGCATGGTCTGGTGAGGAAATGGATTCCGCTTTGGGATGGGCTCGC
GGTCTATCAGCTTGTTGGTGGGGTAACGGCTCACCAAGGCGACGACGGATAGCTGGTCTGAGAGGACG
ATCAGCCACACTGGGACTGAGACACGGCC
>Zotu1871
GAACGCTGGCGGCGTGCTTAACACATGCAAGTCGAGCGGAAAGGCCCTTCGGGGTACTCGAGCGGCGA

ACGGGTGAGTAACACGTGAGTAACCTGCCCTTGGCTCTGGGATAAGCCCGGGAAACTGGGTCTAATAC
CGGATATGACTCTCCACTGCATGGTGGAGGGTGGAAAGTTTTTCGGCTGGGGATGGACTCGCGGTCTA
TCAGCTTGTTGGTGGGGTAACGGCCTACCAAGGCGACGACGGATAGCCGGCCTGAGAGGGTGACCGGC
CACACTGGGACTGAGACACGGCC
>Zotu1872
GAACGCTGGCGGCGTGCCTAATACATGCAAGTCGAGCGGATCGAACGGGAGCTTGCTTCCGTTCGGTT
AGCGGCGGACGGGTGAGTAACACGTGGGCAACCTGCCCGTAAGGCCGGGATAACTTCGGGAAACCGGA
GCTAATACCGGATAACACCGAAGACCGCATGGTCTTCGGTTGAAAGGCGGCTTTGGCTGTCACTTACG
GATGGGCCCGCGGCGCATTAGCTAGTTGGTGAGGTAACGGCTCACCAAGGCGACGATGCGTAGCCGAC
CTGAGAGGGTGATCGGCCACACTGGGACTGAGACACGGCC
>Zotu1873
CAACGCTGGCGGCGCGCTTAACACATGCAAGTCGCACGAGAAAGTGGAGCAATCCATGAGTACAGTGG
CGTACGGGTGAGTAACACGTGGATCATCTACCTTCTAGTGGGGAATACCTCTGGGAAACCGGAGACAA
TACCGCATAAGCTCGTAAGAGGAAAGCAGTAATGCGCTGGAAGAGGAGTCCGCGGCTGATTAGCTAGT
TGGCGGGGTAATGGCCCACCAAGGCGAAGATCGGTAGCCGGCCTGAGAGGGCACACGGCCACACTGGC
ACTGAAACACGGGC
>Zotu1874
GAACGCTGGCGGCGTGCTTAACACATGCAAGTCGAACGGAAAGGCCCAGCTTGCTGGGTACTCGAGTG
GCGAACGGGTGAGTAACACGTGGGTGATCTGCCCCAAACTTCGGGATAAGCCTGGGAAACTGGGTCTA
ATACCGGATATGACCTGCTCCTGCATGGGGGTTGGTGGAAAGCTTTTGCGGTTTGGGATGGGCCCGCG
GCCTATCAGCTTGTTGGTGGGGTAATGGCCTACCAAGGCGACGACGGGTAGCCGACCTGAGAGGGTGA
TCGGCCACACTGGGACTGAGACACGGCC
>Zotu1875
GAACGCTAGCGGCAGGCTTAATACATGTAAGTCGAGGGGCAGCACGGTTAGCAATAACTGGTGGCGAC
CGGCGCACGGGTGCGTAACACGTATACAATCTACCTTAAACAGGGGGATAGCCTTTCGAAAGAGAGAT
TAATACCCCGTAATAAATTGAATGGCATCATTTGATTTTGAAAGCTCCGGCGGTTTGAGATGAGTATG
CGTCTGATTAGCTAGTTGGTGAGGTAACGGCTCACCAAGGCAACGATCAGTAGGGGATCTGAGAGGAC
TAACCCCCACACTGGTACTGAGACACGGAC
>Zotu1876
GAACGCTGGCGGCGTGCGTAACACATGCAAGTCGGACGGACGGAGTAATCCGTCAGTGGCGGACGGGT
GCGTAACACGTGGCTACCAACCCATCGCTGGGGGATACCCGGGGGAAACTCTGGTTAAGACCGCATAC
GCTCGAAAAGAGGAAAGCCCGCAAGGGCGGCGATGGCTGGGGCTGCGGCCGATTAGGTAGATGGTGGG
GTAAGGGCCTACCATGCCGACGATCGGTAGCTGGTCTGAGAGGATGGCCAGCCACACTGGGATTGAGA
ACGGCC
>Zotu1877
GAACGTTGGCGGCGTGCCTAACACATGCAAGTCGAGCGTGAAAGTCCTTCGGGATGAGTAAAGCGGCG
GACGGGTGAGTAACGCGTGAGAATCTACCTTTCAGTGAAGAATACCTTTGAGAAATTGAAGTTAATGC
TGCATACGTCTCGTAAGAGAGAAAGATGGCCTCTTTGCTGTCGCTGAAAGATGAGCTTGCGTTCTATT
AGCTAGTTGGTGGGGTAAAGGCCTACCAAGGCGATGATGGATAGCCGGCCTGAGAGGGCGAACGGCCA
CATTGGAACTTAGACACGGTC
>Zotu1878
GAACGCTAGCGGCAGGCTTAATACATGCAAGTCGAGGGGCAGCAGGGGTAGCAATACTCGCTGGCGAC
CGGCAAACGGGTGCGGAACACGTACAGAACCTTCCTTTAAGCGGGGGATAGCCCAGGGAAACCTGGAT
TAATACCCCATAGTATTGTTGGGTGGCATCACTTAATAATTAAAGATTTATCACTTAAAGATGGCTGT
GCGTATGATTAGGTAGTTGGCGGGGTAACGGCCCACCAAGCCTTCGATCATTAACTGGTGTGAGAGCA
CGACCAGTCACACGGGCACTGAGACACGGGC
>Zotu1879
GAACGCTGGCGGCGTGCTTAATACATGCAAGTCGAGCGGTAAGGCCCTTCGGGGTACACGAGCGGCGG
ACGGCTGAGTAACGCGTAGGAATGTACCCCAAAGTGAGGGATAAGCACCAGAAATGGTGTCTAATACC
GCATGTGATCTATGGATTAAAGCTTCGGCGCTTTGGGAACAGCCTGCGTATGATTAGCTTGTTGGTGA
GGTAAAGGCTCACCAAGGCGACGATCATTAGCTGGTCTGAGAGGATGACCAGCCAGACTGGGACTGAG
ACACGGCC
>Zotu1880
GAATGATGGCGGCGTGGATGAGGCATGCAAGTCGAATGAGTTTATACTCATGGCAGACGAGGTAGGAA

CACGTGGGTACGTACCCAAGAGTCAGGGATAATCCGTCGAAAGGCGGGATAATACTTGATAGTCCCTT
CGGGGTAAAGATTTATCGCTCTTGGAACGGCCCGCGGGCTATCAGCTTGTTGGTAAGGTAACGGCTTA
CCAAGGCTACGACGGCTAGGGGAGGTGAGAGCCTGACCCCCACCGATGGAACTGCGACACGGTC
>Zotu1882
GAACGCTGGCGGCGTGCTTAACACATGCAAGTCGAGCGGTAAGGCTCCTTCGGGAGTACACGAGCGGC
GAACGGGTGAGTAACACGTGAGTAATCTGCCCTTCACTTCGGGATAACCGCCGGAAACGGTGGCTAAT
ACCGGATACGACACAGTCCCGCATGGGATCTGAGTGGAAAGTTTTCTCGGTGGAGGATGTGCTCGCGG
CCTATCAGCTTGTTGGTGAGGTAACGGCTCACCAAGGCTTCGACGGGTAGCCGGCCTGAGAGGGTGAC
CGGCCACACTGGGACTGAGACACGGCC
>Zotu1883
GAACGCTGGCGGCAGGCTTAACACATGCAAGTCGAGCGGGGGAAGGTAGCTTGCTACTGGACCTAGCG
GCGGACGGGTGAGTAATGCTTAGGAATCTGCCTATTAGTGGGGGACAACATCTCGAAAGGGATGCTAA
TACCGCATACGTCCTACGGGAGAAAGCAGGGGATCTTCGGACCTTGCGCTAATAGATGAGCCTAAGTC
GGATTAGCTAGTTGGTGGGGTAAAGGCCTACCAAGGCGACGATCTGTAGCGGGTCTGAGAGGATGATC
CGCCACACTGGGACTGAGACACGGCC
>Zotu1884
AAACGCTGGCGGCGTGCATGAGACATGCAAGTCGAACGGACGAACGGGGCTTGCTCTGTTCGTTAGTG
GCGGACGGCTGAGTAACGCGTAGGTGACCTGTCCCGAAGTGGGGAATAACTGCCCGAAAGGGTGGCTA
ATACCGCATGTGGTGGTGTGTTCATTCACACCAGTAAAGCCGCAAGGCGCTTCGGGAGGGGCCTGCGT
CCGATTAGCTCGTTGGTGGGGTAACGGCTCACCAAGGCGACGATCGGTAGGTGGTCTGAGAGGACGAT
CACCCAGACTGGGACTGAGACACGGCC
>Zotu1885
GAACGCTGGCGGTAGGCCTAACACATGCAAGTCGAACGGCAGCACAGTAAGAGCTTGCTCTTACGGGT
GGCGAGTGGCGGACGGGTGAGGAATACATCGGAATCTACTTTTTCGTGGGGGATAACGTAGGGAAACT
TACGCTAATACCGCATACGACCTACGGGTGAAAGCAGGGGATCTTCGGACCTTGCGCGATTGAATGAG
CCGATGTCGGATTAGCTAGTTGGCGGGGTAAAGGCCCACCAAGGCGACGATCCGTAGCTGGTCTGAGA
GGATGATCAGCCACACTGGAACTGAGACACGGTC
>Zotu1887
GAACGCTGGCGGCGTGCTTAACACATGCAAGTCGAACGGTGAACCACCTTCGGGTGGGGATCAGTGGC
GAACGGGTGAGTAACACGTGACCAACCTGCCCCTTGCTCTGGGATAACCCCGGGAAACCGGAGCTAAT
ACCGGATACGAGACCGTGCGGCATCACGCGGTCTGGAAAGTTTATCGGCAAGGGATGGGGTCGCGGCC
TATCAGCTTGTTGGTGGGGTAACGGCCCACCAAGGCGACGACGGGTAGCCGGCCTGAGAGGGCGACCG
GCCACACTGGGACTGAGACACGGCC
>Zotu1888
GAACGCTGGCGGCGTGCCTAATACATGCAAGTAGAACGCTGAAGCTTGGTGCTTGCACCGAGCGGATG
AGTTGCGAACGGGTGAGTAACGCGTAGGTAACCTGCCTCTTAGCGGGGGATAACTATTGGAAACGATA
GCTAATACCGCATAAAAGTCGACATTGCATGATGTTGACTTGAAAGGTGCAATTGCATCACTAAGAGA
TGGACCTGCGTTGTATTAGCTAGTTGGTGAGGTAACGGCTCACCAAGGCGACGATACATAGCCGACCT
GAGAGGGTGATCGGCCACACTGGGACTGAGACACGGCC
>Zotu1889
GAACGCTGGCGGCGTGCTTAACACATGCAAGTCGAGCGGAAAGGCCACTTCGGTGGTACTCGAGCGGC
GAACGGGTGAGTAACACGTGAGTAATCTGCCCTTGGCTTTGGGATAGCCACCGGAAACGGTGATTAAT
ACCGGATACGACCACTTCTCGCATGGGATGGTGGTGGAAAGTTTTTCGGCCAGGGATGTGCTCGCGGC
CTATCAGCTTGATGGTGAGGTAATGGCTCACCATGGCTTCGACGGGTAGCCGGCCTGAGAGGGTGACC
GGTCACACTGGGACTGAGACACGGCC
>Zotu1890
GAACGCTGGCGGCGTGCTTAACACATGCAAGTCGAACGATGAAGCCCAGCTTGCTGGGTGGATTAGTG
GCGAACGGGTGAGTAACACGTGAGTAACCTGCCCTTGACTCTGGGATAAGCACTGGAAACGGTGTCTA
ATACCGGATATTTCAGCTGGCCGCATGGTCTGGTTGTGGAAAGAATTTTGGTCAAGGATGGACTCGCG
GCCTATCAGGTTGTTGGTGAGGTAATGGCTCACCAAGCCTACGACGGGTAGCCGGCCTGAGAGGGTGG
CCGGCCACACTGGGACTGAGACACGGCC
>Zotu1891
GAACGCTGGCGGCAGGCTTAACACATGCAAGTCGAACGCTGTAGCAATACAGAGTGGCAGACGGGTGA
GTAACACGTGGGAACGTACCCTTTGGTCTGGAATAACTCAGGGAAACTTGAGCTAATACCGGATACGC

CCTTATGGGGAAAGGTTTACTGCCGAAGGATCGGCCCGCGTCCGATTAGCTAGTTGGTGTTGGTAATG
GCTCACCAAGGCGACGATCGGTAGCTGGTCTGAGAGGATGGCCAGCCACATTGGGACTGAGACACGGC
C
>Zotu1892
GAACGCTGGCGGCATGCCTAACACATGCAAGTCGAACGAGACCTTCGGGTCTAGTGGCGCACGGGTGC
GTAACGCGTGGGAATCTGCCCTTCGGTTGGGAATAACACAGAGAAATTTGTGCTAATACCTTATAATG
TCTTCGGACCAAAGATTTATCGCCGAGGGATGAGCCCGCGTAGGATTAGCTAGTTGGTGGGGTAATGG
CCTACCAAGGCGACGATCCTTAGCTGGTCTGAGAGGATGATCAGCCACACTGGGACTGAGACACGGCC
>Zotu1893
GAACGCTGGCGGCGTGCTTAACACATGCAAGTCGAACGGTAAGGCCCCTTCGGGGGTACACGAGTGGC
GAACGGGTGAGTAACACGTGGGTAATCTGCCCTGCACTTCGGGATAAGCCTGGGAAACCGGGTCTAAT
ACCGGATATGAACTCCTGCCGCATGGTGGGGGTTGGAAAGTTTTTCGGTGCAGGATGAGCCCGCGGCC
TATCAGCTTGTTGGTGGGGTAATGGCCTACCAAGGCGACGACGGGTAGCCGGCCTGAGAGGGTGATCG
GCCACACTGGGACTGAGACACGGCC
>Zotu1894
GAACGCTAGCGGCAGGCTTAATACATGCAAGTCGAACGGTAAGTATGGTAGCAATATTATACGAGAGT
GACGTACGGGTGCGTAACACGTATGCAACCTACCTTGTACAGGAGGATAGCTCGGGGAAACTCGAATT
AACCCTCCATAAGATAGTGGTGTGGCATCACACAGCTATAAAAACTTTATTGTGGTACAAGATGGGCA
TGCGTCTGATTAGCTAGTTGGCGGGGTAATGGCCCACCAAGGCGATGATCAGTAGGGGACCTGAGAGG
GTGATCCCCCACACTGGCACTGAGATACGGGC
>Zotu1895
GAACGCTGGCGGCGTGCCTAATACATGCAAGTCGAGCGGTAAGGCCCCTTCGGGGGTACACGAGCGGC
GGACGGCTGAGTAACACGTAGGAACGTACCCCAAACTGAGGGATAAGCACCAGAAATGGTGTCTAATA
CCGCATGTGATCTTCGGATTAAAGCTTCGGCGGTTTGGGAACGGCCTGCGCATGATTAGCTTGTTGGT
GAGGTAAAAGCTCACCAAGGCGACGATCATTAGCTGGTCTGAGAGGATGATCAGCCAGACTGGAACTG
AGAACGGTC
>Zotu1896
GAACGCTGGCGGCGTGCTTAACACATGCAAGTCGAACGGAGACTTTTTCAGTGTAACCTTCGGGGAGC
AAGAGAAAGTCTTAGTGGCGGACGGGTGAGTAACGCGTGGGCAACCTGCCTCGCACCGTGGGATAACA
GGGGGAAGCTCCTGCTAATACCGCATAAAGTATATAGACGCATGTCAATATACAAAAAGGGATACCGG
TGAGAGATGGGCCCGCGTCCGATTAGATAGTTGGCAGGATAGCAGCCTACCAAGTCAACGATCGGTAG
CCGGCTTGAGAGAGTGGACGGCCACACTGGGACTGAGACACGGCC
>Zotu1897
GAACGCTGGCGGCGTGCTTAACACATGCAAGTCGAACGGTGACGGAGAGCTTGCTCTCCTGATCAGTG
GCGAACGGGTGAGTAACACGTGAGCAACCTACCCTTGACTCTGGGATAACAGTTGGAAACAGCTGCTA
ATACCGGATACGACCCTGGCCGGCATCGGCCGGGGTGGAAAGCTCCGACGGTCAAGGATGGGCTCGCG
GCCTATCAGCTTGTTGGTGAGGTAACGGCTCACCAAGGCGACGACGGGTAGCCGGCCTGAGAGGGCGA
CCGGCCACACTGGGACTGAGATACGGCC
>Zotu1898
CAACGCTGGCGGCGTGCCTCAGACATGCAAGTCGAACGATTAAGACTTCCTTCGGGAAGTGTATAAAG
TGGCGCACGGGTGAGTAACACGTAAGTAATCTGCCTTCGAGTGGGGAATACCTTCGGGAAACCGAAGA
TAATACCGCATAATGCAGCGGCACCGCAAGGTGACAGTTGTTAAAGGAGCAATCCGCTTGAAGAGGAG
CTTGCGGCAGATTAGCTAGTTGGTAAGGTAACGGCTTACCAAGGCTACGATCTGTATCCGGTCTAAGA
GGACGGTCGGACACACTGACACTGAATAACGGGT
>Zotu1899
GAACGCTGGCGGCGTGCTTAACACATGCAAGTCGAGCGGTAAGGCACTTCGGTGTACACGAGCGGCGA
ACGGGTGAGTAACACGTGAGTAATCTGCCCTTCACTTTGGGATAAGCCTCGGAAACGGGGTCTAATAC
CGGATACGACCACTTCGGGCATCTGATGGTGGTGGAAAGTTTTTTCGGTGGGGGATGTGCTCGCGGCC
TATCAGCTTGTTGGTGGGGTAATGGCCTACCAAGGCTTCGACGGGTAGCCGGCCTGAGAGGGTGACCG
GCCACACTGGGACTGAGACACGGCC
>Zotu1900
GAACGCTGGCGGCAGGCTTAACACATGCAAGTCGAACGCCCCGCAAGGGGAGTGGCAGACGGGTGAGT
AACGCGTGGGAATCTACCCAGATCTTCGGAACAACTGAGGGAAACTTCAGCTAATACCGGATACGCCC
TACGGGGGAAAGATTTATCGGATTTGGATGAGCCCGCGTTGGATTAGCTAGTTGGTGAGGTAACGGCT

CACCAAGGCGACGATCCATAGCTGGTCTGAGAGGATGATCAGCCACACTGGGACTGAGACACGGCC
>Zotu1901
GAATGCTGGCGGCGTGGATAAGGCATGCAAGTCGAACGGTCCGCAAGGACAGTGGCAGACGAGGTAGT
AACACGTAGGTACTCCCCCCAAAGTCTGGTATAGCTCGTCGAAAGACGGGGTAATCCCGGATGGTCCC
GAAAGGGTAAAGATTTATCGCTTTGGGAGAGGCCTGCGTAATATCAGCTTGTTGGTGAGGTAATGGCT
CACCAAGGCTACGACGTTTAGGGGCGCTGAGAGGCGGACCCCCACCGATGGGACTGAGACACGGCC
>Zotu1902
CAACGCTGGCGGCGCGCTTAACACATGCAAGTCGAACGGGAAAGTGGAGCAATCCATGAGTACAGTGG
CGTACGGGTGAGTAACACGTGGGTGATCTACCTTTTAGTGGGGAATAACCCTGGGAAACCGGGGCTAA
TACCGCATAAGCCCGAGAGGGGAAAGTCGCAAGACGCTGAAAGAGGAGCCCGCGGCCGATTAGCTAGT
TGGTAGGGTAAGAGCCTACCAAGGCAGAGATCGGTAGCCGGCCTGAGAGGGCACACGGCCACACTGGC
ACTGAAACACGGGC
>Zotu1903
GAACGCTGGCGGCGTGCTTAACACATGCAAGTCGAACGGTGAAGCGCCTTCCTTTGTGGTGGGTGTGG
ATGAGTGGCGAACGGGTGAGTAACACGTGAGTAACCTGCCCCCTTCTTCTGGATAACCGCATGAAAGT
GTGGCTAATACGGGATATTCTGGGTCTGTCGCATGGTGGGCCTGGGAAAGATTGCGCCTTTTGGGTGT
TTTTGGTGGGGGATGGGCTCGCGGCCTATCAGCTTGTTGGTGGGGTGATGGCCTACCAAGGCGGTGAC
GGGTAGCCGGCCTGAGAGGGTGGACGGTCACACTGGGACTGAGACACGGCC
>Zotu1904
GAACGCTGGCGGCGTGCTTAACACATGCAAGTCGAACGGTGAAGGAGAGCTTGCTCTCCGGATCAGTG
GCGAACGGGTGAGTAACACGTGAGCAACCTACCCTTGACTCTGGGATAACAGTTGGAAACAGCTGCTA
ATACCGGATATGACCCTGGCAGGCATCTGCCGGGGTGGAAAGCCTTGACGGTCAAGGATGGGCTCGCG
GCCTATCAGCTTGTTGGTGAGGTAACGGCTCACCAAGGCGTCGACGGGTAGCCGGCCTGAGAGGGCGA
CCGGCCACACTGGGACTGAGATACGGCC
>Zotu1906
GAACGCTGGCGGCGTGCCTAATACATGCAAGTGGAACGCATGATTGATACCGGAGCTTGCTCCACCAT
TAATCATGAGTCGCGAACGGGTGAGTAACGCGTAGGTAACCTACCTCATAGCGGGGGATAACTATTGG
AAACGATAGCTAATACCGCATAACAGTATTTATCGCATGGTAAATGCTTGAAAGGAGCAACTGCTTCA
CTATGAGATGGACCTGCGTTGTATTAGCTAGTTGGTGGGGTAACGGCTCACCAAGGCCTCGATACATA
GCCGACCTGAGAGGGTGATCGGCCACACTGGGACTGAGACACGGCC
>Zotu1907
GAACGCTGGCGGCGTGCTTAACACATGCAAGTCGAGCGGTAAGGCCCTTCGGGGTACACGAGCGGCGA
ACGGGTGAGTAACACGTGGGTGACCTGCCCTCAGCTCTGGGATAAGCCTGGGAAACTGGGTCTAATAC
CGGATACGACCACATGCTGCATGGCGTGTGGTGGAAAGTTTTTCGGCTGGGGATGGGCCCGCGGCCTA
TCAGCTTGTTGGTGGGGTGATGGCCTACCAAGGCGGTGACGGGTAGCCGGCCTGAGAGGGCGACCGGC
CACACTGGGACTGAGACACGGCC
>Zotu1908
GAACGCTTGCTGCGTGCCTGAGAAATGCAAGTCGAACGAGATTGAGCTTTCGGGTTCAGTCTAGTGGC
GAACGGTCGAGTAACACGTAAGCAACCTGCCCATCCGCGGGGGACAAGCCGTCGAAAGACGGGGTAAT
ACCGCATACGATCTACGGAGACATCTCCGGAGATGAAAGATTCATCACGGATGGAGGGGCTTGCGGCC
TATCAGCTAGTTGGTTGGGGTAACGGCTCACCAAGGCGACGACGGGTAGCGGGTCTGAGAGGACGACC
CGCCGGACTGGGACTGAGACACGGCC
>Zotu1909
GAACGCTGGCGGCGCGCTTAACACATGCAAGTCGAACGAGAAGGCGTAGCAATACGCTTGTACAGTGG
CGAACGGGTGAGGAATACGTGGGTAACCTACCCTCGAGTGGGGAATAACCAGCCGAAAGGTTGGCTAA
TGCCGCGTACGCTTCCAGGTCTGCGGATCGGGAAGGAAAGCCGTACAAAGTGTGCGGCGCTCAAGGAT
GGGCTCACGTCCTATCAGCTTGTTGGTGAGGTAACGGCTCACCAAGGCTTCGACGGGTAGCTGGTCTG
AGAGGACGATCAGCCACACTGGCACTGCGACACGGGC
>Zotu1910
GAACGCTGGCGGCGTGCTTAACACATGCAAGTCGAACGATGACGGTGGTGCTTGCATCACCTGATTAG
TGGCGAACGGGTGAGTAACACGTGAGTAACCTGCCCTTCACTTCGGGATAACCTCGGGAAATCGTGGC
TAATACCGGATATGAGCTCCTTCCGCATGGCGGGGGTTGGAAAGATTTATCGGTGAAGGATGGACTCG
CGGCCTATCAGTTTGTTGGTGAGGTAATGGCTCACCAAGGCGATGACGGGTAGCCGGCCTGAGAGGGC
GACCGGCCACACTGGGACTGAGACACGGCC

>Zotu1911
GAACGCTGGCGGCGTGCCTAATACATGCAAGTCGAACGAACCGCGACTGAGTGCTTGCACTTGGTCAA
GGTGAGTGGCGAACGGGTGAGTAACACGTGGGTAACCTACCTCATAGTGGGGGATAACAGTCGGAAAC
GACTGCTAATACCGCATAGGACATGGGATCACATGATTCTGTGAGGAAAGGTGGCGCAAGCTATCGCT
AAGAGATGGACCCGCGGTGCATTAGCTAGTTGGTAGGGTAACGGCCTACCAAGGCGATGATGCATAGC
CGACCTGAGAGGGTGATCGGCCACATTGGGACTGAGACACGGCC
>Zotu1912
GAACGCTGGCGGCGTGCCTAATACATGCAAGTCGAGCGGATTGATGGGAGCTTGCTCCCTGATATCAG
CGGCGGACGGGTGAGTAACACGTGGGCAACCTGCCTATAAGACTGGGATAACTTCGGGAAACCGGAGC
TAATACCGGATAATCCCTTTCCTCTCATGAGGAAAGGCTGAAAGATGGTTTACGCTATCACTTATAGA
TGGGCCCGCGGCGCATTAGCTAGTTGGTGAGGTAACGGCTCACCAAGGCAACGATGCGTAGCCGACCT
GAGAGGGTGATCGGCCACACTGGGACTGAGACACGGCC
>Zotu1913
GAATGCTGGCGGCGTGCCTAACACATGCAAGTCGAGCGGCAGCGCGAGTAGTTTACTACTTGGCGGCG
AGCGGCGGACGGCTGAGTAACGCGTAGGAACGTGCCCCAAAGTGAGGAATAACTGCCCGAAAGGGTGG
CTAATGCCGCATATGATCTTCGGATTAAAGTCTTCGGACGCTTTGGGAGCGGCCTGCGTTAGATTAGG
TAGTTGGTGAGGTAATGGCTCACCAAGCCGACGATCTATAGCTGGTCTGAGAGGATGATCAGCCAGAC
TGGGACTGAGACACGGCC
>Zotu1914
GAACGTTAGCGGCAGGCTTAACACATGCAAGTCGAGCGAGAACGGGACTTCGGTCCTTAGTAAAGCGG
CGGACGGGTGAGTAACACGTGGGTAATGTGCCCTTCAGCGGGGGATAACAGTCCGAAAGGATTGCTAA
TACCGCATACGTCAGCCGGGTCTTCGGATCTGGTTGGGAAAGGTGGCCTCTCCATGGAAGCTACCACT
GAAGGAGCAGCCCGCGGCCCATCAGCTAGTTGGCGGGGTAACGGCCCACCAAGGCAAAGACGGGTAGC
TGGTCTGAGAGGATGATCAGCCACACTGGAACTGAGACACGGTC
>Zotu1915
GAACGCTGGCGGCGTGCCTAATACATGCAAGTAGAACGCTGAAGGGAGAGCTTGCTCTTCTGGATGAG
TTGCGAACGGGTGAGTAACGCGTAGGTAACCTGCCTGGTAGCGGGGGATAACTATTGGAAACGATAGC
TAATACCGCATAATAGTAGATGTTGCATGACATTTGCTTGAAAGGTGCAATTGCATCACTACCAGATG
GACCTGCGTTGTATTAGCTAGTTGGTGGGGTAACGGCTCACCAAGGCGACGATACATAGCCGACCTGA
GAGGGTGATCGGCCACACTGGGACTGAGACACGGCC
>Zotu1916
GAACGCTGGCGGCGTGCCTAATACATGCAAGTCGAGCGGATCTTTAGAAGCTTGCTTCTAAAGATTAG
CGGCGGACGGGTGAGTAACACGTGGGCAACCTGCCTGTAAGACTGGGATAACTTCGGGAAACCGAGGC
TAATACCGGATAATCCTTTTCCTCACATGAGGGGAAGCTGAAAGACGGCGTAAGCTGTCACTTACAGA
TGGGCCCGCGGCGCATTAGCTAGTTGGTGAGGTAACGGCTCACCAAGGCAACGATGCGTAGCCGACCT
GAGAGGGTGATCGGCCACACTGGGACTGAGACACGGCC
>Zotu1917
GAACGCTGGCGGCGTGCTTAACACATGCAAGTCGAACGATGAAGCGTAGCTTGCTGCGTGGATTAGTG
GCGAACGGGTGAGTAACACGTGAGCAATCTGCCCTTGACTCTGGGATAAGCGCTGGAAACGGCGTCTA
ATACCGGATATGAGTAGTGATCGCATGATCGGCTACTGGAAAGATTTTTTGGTTGAGGATGAGCTCGC
GGCCTATCAGCTTGTTGGTGAGGTAATGGCTTACCAAGGCGTCGACGGGTAGCCGGCCTGAGAGGGTG
ACCGGCCACACTGGGACTGAGACACGGCC
>Zotu1918
GAACGCTAGCAGAACGCTTAACACATGCAAGTCGAACGAATGTATTGTCTTCGGATAATATGTTAGTG
GCAAACGGGCGAGTAACGCGTATCTAACCTGCCCTTGTGTGAGGCAAAACTATGGAGACATAGCTAAT
TCCTCATACGCTCTAAGAAATGAAAATACTTAGAGGAAAGATTTATCGCACAAGGAGGGGGATGCGTC
CTATCAGGTAGTTGGTGAGGTAACTGCTCACCAAGCCTATGACGGGTAGCCGGTCTGAGAGGATGTCC
GGCCGCAATGGAACTGAGACACGGTC
>Zotu1919
CAACGCTGGCGGCGCGCCTAACACATGCAAGTCGAACGAGAAAGCCGCAAGGCAAGTAAAGTGGCGTA
CGGGTGAGTAACACGTGGATTATCTACCTTCTGGTGGGGAATACCGTTGGGAAACCGACGACAATACC
GCATAAGCCGGAGACGGGAAAGCAGCAATGCGCCGGAAGAGGAGTCCGCGGTCGATTAGTTAGTTGGC
GGGGTAAAAGCCCACCAAGACTGTGATCGATAGCCGGCCTGAGAGGGCACACGGCCACACTGGCACTG
AAACACGGGC

>Zotu1920
GAACGCTAGCGGCAGGCCTAATACATGCAAGTCGGACGGGATTTAGGAGCTTGCTTCTAATGAGAGTG
GCGCACGGGTGCGTAACACGTATGTAACCTACCTTCTTCAGGGGGATAGCCTCTCGAAAGAGAGATTA
AGACCGCATAAAATCACAATGTGGCATCACAAAATGATCAAATATTTATAGGAAGAAGATGGGCATGC
GGAACATTAGCTAGTTGGTAAGGTAACGGCTTACCAAGGCTACGATGTTTAGGGGATCTGAGAGGATG
ACCCCCCACACTGGTACTGAGACACGGAC
>Zotu1921
GAACGCTGGAAATGTGGATACCGGCATGCAAGTCAAGGGGCCGCAAGGCACCGGCAAACGGCTTAGTA
ACACGTAGATACATACCCAAAAGTGGGGCATAGCTCGTCGAAAGACGGGGTAATTTCCCATAGTACCG
CAAGGTTAAAGACGCAAGTCGCTTTTGGAATGGTCTGCGGTCTATCAGCTAGTTGGTGAGGTAAAGGC
TCACCAAGGCTATGACGGATAGGGGGCGTGAGAGCGCGACCCCCAAGAATGGCACTGCGACACGGGC
>Zotu1922
GAATGCTGGCTGCGTGCTTAACACATGCAAGTCGAACGGTGAAGGAGGGCTTGCCCTCCGGATCAGTG
GCGAACGGGTGAGTAACACGTGGGCAACCTGCCCCTCACTCTGGGATAACACCGGGAAACCGGTGCTA
ATACCGGATACGACCAGGCGCGACATCTCGCCTGGTGGAAAGAACTTCGGTGAGGGATGGGCCCGCGG
CCTATCAGCTTGTTGGTGAGGTAACGGCTCACCAAGGCGACGACGGGTAGCCGGCCTGAGAGGGCGAC
CGGCCACACTGGGACTGAGACACGGCC
>Zotu1923
GAACGCTAGCGGCAGGCTTAATACATGCAAGGCGAAGGGGCAGCAATGTCACTGTCGTACGGGTGCGT
AACGCGTATGCAACCTACCTATCACTGGGGGATAGCCCGGGGAAACCCGGATTAATACCGCATAACAC
AGGGGTCCCGCATGGGTACTATTTGTTAAAGATTTATTGGTGGTAGATGGGCATGCGTTCGATTAGCT
AGTTGGTATGGGTAACGGCCTACCAAGGCGACGATCGATAGGGGAGCTGAGAGGTTGATCCCCCACAC
GGGCACTGAGATACGGGC
>Zotu1924
TAACGCTGACGGTGTGCCTAATACATGCAAGTCGAACGAGCAGTATGTTGGTGGCAACACCTTTATAC
TGCTAGTGGCGAACGGGTGAGTAACGCGTGGGTAATCTGCCTTAAAGACTGGGATAACTCCTCGAAAG
AGGTGCTAATACCGGATGGTCTATATTGATCTCATGATTTATATAGTAAAGGTAGCCGCAAGGCCTCT
GCTTTAAGATGAGCTCGCGTCCTATTAGCTAGTTGGTGGGGTAATGGCCTACCAAGGCAATGATGGGT
AGCCGGTCTGAGAGGATGGTCGGCCACACTGGGACTGAGATACGGCC
>Zotu1925
GAACGCTGGCGGCGTGCTTAACACATGCAAGTCGAACGATGAAGCCCAGCTTGCTGGGTGGATTAGTG
GCGAACGGGTGAGTAACACGTGAGTAACCTGCCCTTGACTCTGGGATAAGCGCTGGAAACGGCGTCTA
ATACCGGATACGACCTGCCCCGGCATCGGGTGCGGGTGGAAAGTTTTTCGGTCAAGGATGGACTCGCG
GCCTATCAGGTAGTTGGTGAGGTAATGGCTCACCAAGCCGACGACGGGTAGCCGGCCTAAGAGGGTGA
CCGGCCACACTGGGACTGAGACACGGCC
>Zotu1926
GAACGCTGGCGGCGTGCTTAACACATGCAAGTCGAACGAGGTCCACCGCGTAGCAATACAAGGGAAGA
CCTAGTGGCGAACGGGTGCGTAACACGTGAGGAACTTGTCCCGGTCTCTGGGATAACAGTTGGAAACG
ACTGCTAATACCGGATGACGTCGGAATCTCGCATGGGATACTGACGAAAGGGTTACTGGATCGGGAGA
GCCTCGCGGCCTATCAGCTTGTTGGTGAGGTAACGGCTCACCAAGGCATCGACGGGTAGCTGGTCTGA
GAGGATGATCAGCCACACTGGGACTGAGACACGGCC
>Zotu1927
GAACGCTGGCGGCATGCCTAACACATGCAAGTCGAACGATGCTTTCGGGCATAGTGGCGCACGGGTGC
GTAACGCGTGGGAATCTGCCCTTGGGTTCGGAATAACAGTTGGAAACGACTGCTAATACCGGATGATG
TCGATAAGACCAAAGATTTATCGCCTGAGGATGAGCCCGCGTTGGATTAGCTAGTTGGTGGGGTAAAG
GCCTACCAAGGCGACGATCCATAGCTGGTCTGAGAGGATGATCAGCCACACTGGGACTGAGACACGGC
C
>Zotu1928
GAACGCTGGTGGCGTGCCTAATACATGCAAGTCGAGCGAACGGACGAGAAGCTTGCTTCTCTGATGTT
AGCGGCGGACGGGTGAGTAACACGTGGATAACCTACCTATAAGACTGGGATAACTTCGGGAAACCGGA
GCTAATACCGGATAATATTTTGAACCGCATGGTTCAAAAGTGAAAGACGGTCTTGCTGTCACTTATAG
ATGGATCCGCGCTGCATTAGCTAGTTGGTAAGGTAACGGCTTACCAAGGCAACGATGCATAGCCGACC
TGAGAGGGTGATCGGCCACACTGGAACTGAGACACTGTC
>Zotu1929

GAACGCTGGCGGCGTGCCTAATACATGCAAGTCGAACGCACAGCGAAAGGTGCTTGCACCTTTCAAGT
GAGTGGCGAACGGGTGAGTAACACGTGGACAACCTGCCTCAAGGCTGGGGATAACATTTGGAAACAGA
TGCTAATACCGAATAAAACTTAGTGTCGCATGACACAAAGTTAAAAGGCGCTTCGGCGTCACCTAGAG
ATGGATCCGCGGTGCATTAGTTAGTTGGTGGGGTAAAGGCCTACCAAGACAATGATGCATAGCCGAGT
TGAGAGACTGATCGGCCACATTGGGACTGAGACACGGCC
>Zotu1930
GAACGCTGGCGGCGTGCCTAACACATGCAAGTCGAGCGCGAATAGGGGCAACCCTTAGTAGAGCGGCG
CACGGGTGCGTAACACGTGGATAATCTGCCTGGATGCTCGGGATAACCAGTCGAAAGATTGGCTAATA
CCGGATAAGCCCACGGTTTCTTCGGAGACTGAGGGAAAAGGTGGCCTCTGTATACAAGCTATCACAAC
CAGATGAGTCCGCGGCCCATCAGCTAGTTGGCGGGGTAATGGCCCACCAAGGCAACGACGGGTAGCTG
GTCTGAGAGGACGATCAGCCACACTGGAACTGAGACACGGTC
>Zotu1931
AAACGCTGGCGGCGTGCATAACACATGCAAGTCGAACGATGAAGATTAAAAGATTTCTTCGGAATGAA
CTTAATTGGATTAGTGGCGGACGGGTGAGTAACGCGTGAGTAACCTGCCTTGTACAAGGGGATAGCCG
TTGGAAACGACGAATAATACCCTATAACATGATATTATCGCATGAAAATATCATCAAAGATTTATCGG
TATAAGATGGACTCGCGTCTGATTAGCTAGTTGGTGGGGTAAAAGCCTACCAAGGCAACGATCAGTAG
CCGGCTTGAGAGAGTGTACGGCCACATTGGGACTGAGACACGGCC
>Zotu1932
GAACGCTGGCGGCGTGCTTAACACATGCAAGTCGAACGATGATCTCCCGCTTGCGGGGGTGATTAGTG
GCGAACGGGTGAGTAATACGTGAGTAACCTGCCCTTGACTCTGGGATAAGCCTGGGAAACCGGGTCTA
ATACTGGATACTACTTCCTGCCGCATGGTGGGGGGTGGAAAGGGTTTTACTGGTTTTGGATGGGCCCA
CGGCCTATCAGCTTGTTGGTGGGGTAATGGCTCACCAAGGCGACGACGGGTAGCCGGCCTGAGAGGGT
GACCGGCCACACTGGGACTGAGACACGGCC
>Zotu1933
GAACGCTGGCGGCGTGCTTAACACATGCAAGTCGAACGATGATCCTCAGCTTGCTGGGGGGATTAGTG
GCGAACGGGTGAGTAACACGTGAGTAACCTGCCCTTGACTCTGGGATAAGCCTGGGAAACTGGGTCTA
ATACCGGATATGACTTCTCATCGCATGGTGGGGGGTGGAAAGCTTTTTGTGGTTTTGGATGGACTCGC
GGCCTATCAGCTTGTTGGTGGGGTAATGGCCTACCAAGGCGACGACGGGTAGCCGGCCTGAGAGGGTG
ACCGGCCACACTGGGACTGAGACACGGCC
>Zotu1934
GAACGCTGGCGGCGTGCTTAACACATGCAAGTCGAACGATGAAGCCCAGCTTGCTGGGTGGATTAGTG
GCGAACGGGTGAGTAACACGTGAGTAACCTGCCCTTGACTCTGGGATAAGCGCTGGAAACGGCGTCTA
ATACCGGATACGACCTGCCCCGGCATCGGGTGCGGGTGGAAAGTTTTTCGGTCAAGGATGGACTCGCG
GCCTATCAGGTAGTTGGTGAGGTAATGGCTCACCAAGCCGACGACGGGTAGCCGGCCTGAGAGGGTGA
CCGGCCACACTGGGACTGAGACACGGCC
>Zotu1935
GAACGCTGGCGGCGTGCCTAATACATGCAAGTCGAGCGGACCGATGGGAGCTTGCTCCCTTAGGTCAG
CGGCGGACGGGTGAGTAACACGTGGGTAACCTGCCTGTAAGACTGGGATAACTCCGGGAAACCGGGGC
TAATACCGGATGCTTGATTGAACCGCATGGTTCAATCATAAAAGGTGGCTTTTAGCTACCACTTACAG
ATGGACCCGCGGCGCATTAGCTAGTTGGTGAGGTAACGGCTCACCAAGGCGACGATGCGTAGCCGACC
TGAGAGGGTGATCGGCCACACTGGGACTGAGACACGGCC
>Zotu1936
GAACGCTGGCGGCGTGCTTAACACATGCAAGTCGAACGGAAAGGCCAGTGCTTGCACTGGTGCTCGAG
TGGCGAACGGGTGAGTAACACGTGGGTGATCTGCCTTGCACTCTGGGATAAGCTTGGGAAACTGGGTC
TAATACCGGATAGGAACCGTCTTTAGTGTGGTGGTTGGAAAGTTTTTTCGGTGCAAGATGAGCTCGCG
GCCTATCAGCTTGTTGGTGGGGTAATGGCCTACCAAGGCGTCGACGGGTAGCCGGCCTGAGAGGGTGT
ACGGCCACATTGGGACTGAGATACGGCC
>Zotu1938
GAACGCTAGCGGGAGGCCTAACACATGCAAGCCGAGCGGTATTTATTCTTCGGAATAGAGAGAGCGGC
GTACGGGTGCGTAACACGTGTGCAACCTGCCTTTATCTGGGAGATAGCCCTCCGAAAGGGGGATTAAT
ATTCCATAATATATTAATTGGCATCAATTGATATTGAAAGCTCCGGCGGATAGAGATGGGCACGCGCA
AGATTAGATAGTTGGTGAGGTAACGGCTCACCAAGTCTGCGATCTTTAGGGGGCCTGAGAGGGTGATC
CCCCACACTGGTACTGAGACACGGAC
>Zotu1939

GGTGGTGTAGCTCCTGGAGGTGTCAATGAAGCCGTCGAAAGGCTCGGTGCTTGACCGAGCAGAGATGG
GCGAAGAGGTGGACAATCATCCGACATGGGGCTGTTACGCCTATCGCTGGAGAACGTCAGTCGTGGCC
AAAAATATTAGGAATATTTGATCATACT
>Zotu1940
GAATGCTGGCGACGTGGATAAGGCATGCAAGTCAAGGGGGCCCGCAAGGGCAACCGGCAGACGAGGTA
GTAACACGTACGAACGCACCCCAGAGTCTGGCATAGCTCGTCGAAAGACGGGGTAATTCCGGATGGTC
CCGCAAGGGTAAAGGTTTTTCGCTCTGGGAGCGGCGTGCGCAATATCAGCTAGTTGGTGAGGTAATGG
CTCACCAAGGCTACGACGTTTAGGGGAGCTGAGAGGCTGACCCCCACCGATGGGACTGAGACACGGCC
>Zotu1942
GAACGCTGGCGGCGTGCCTAATACATGCAAGTCGAGCGGGCCGAACGAGAGCTTGCTCTTGTTCGGTC
AGCGGCGGACGGGTGAGTAACACGTGGGCAACCTGCCCGCAAGACCGGGATAACTCCGGGAAACCGGA
GCTAATACCGGATAACACCAAAGACCGCATGGTCTTTGGTTGAAAGGCGGCTTCGGCTGCCACTTGCG
GATGGGCCCGCGGCGCATTAGCTAGTTGGTGAGGTAACGGCTCACCAAGGCGACGATGCGTAGCCGGC
CTGAGAGGGTGACCGGCCACACTGGGACTGAGACACGGCC
>Zotu1943
GAACGCTGGCGGCGTGGATTAGGCATGCAAGTCGAGCGACGGACAGTCTTCGGACTGTTACGGAGCGG
CGAACGGGTGAGTAACACGTGAACAACCTGCCCTCACATTCTGGATAATTCACCGAAAGGTGTTGTAA
TACAGGCGAGGATTCTTAAGAGGCATTTCTTGAGAAGGGAAGGCGCAAGCCGTGCGAGGAGGGGTTCG
CGGATTATCAGGTAGTTGGTGGGGTAACGGCCTACCAAGCCTACGACGATTAGCTGGTCTGAGAGGAT
GGTCAGCCACATTGGAACTGAGACACTGTC
>Zotu1944
GAACGCTGGCGGCGTGCCTAACACATGCAAGTTGAGCGACGAACCAGGGCTTGCCCTGGGGCAAAGCA
GCGAACGGGTGAGTAACACGTGGGTAACCTGCCCCGATGACCGGGACAACCCGAGGAAACTCGGGCTA
ATACCGGATGTGCCCTCCGGACACAAGTCCGGCGGGTAAAGGAAGCTTCGGCCTCCGTATCGGGAGGG
GCCCGCGGCCCATTAGCTAGTTGGTGAGGTAACGGCTCACCAAGGCCGCGATGGGTAGCTGGTCTGAG
AGGACGATCAGCCACACTGGGACTGAGACACGGCC
>Zotu1946
GAACGCTGGCGGCGTGCTTAACACATGCAAGTCGAACGGAAAGGCCCTAGCTTGCTGGGGTGCTCGAG
TGGCGAACGGGTGAGTAACACGTGGGTGATCTGCCCTGCACTTCGGGATAAGCCTGGGAAACTGGGTC
TAATACCGGATAGGACCACTTCTTGGAGGTTGTGGTGGAAAGTTTTTCGGTGTGGGATGAGCTCGCGG
CCTATCAGCTTGTTGGTGGGGTAATGGCCTACCAAGGCGTCGACGGGTAGCCGGCCTGAGAGGGTGTA
CGGCCACATTGGGACTGAGATACGGCC
>Zotu1947
GAACGCTGGCGGCATGCCTAACACATGCAAGTCGAACGAAGTCCTTCGGGGCTTAGTGGCGCACGGGT
GCGTAACGCGTGGGAATCTGCCCTTGGGTTCGGAATAACAGTTAGAAATGACTGCTAATACCGGATGA
TGTCGCAAGACCAAAGATTTATCGCCCAGGGATGAGCCCGCGTAGGATTAGGTAGTTGGTGGGGTAAA
GGCTCACCAAGCCGACGATCCTTAGCTGGTCTGAGAGGATGATCAGCCACACTGGGACTGAGACACGG
CC
>Zotu1948
GAACGCTGGCGGCATGCCTAACACATGCAAGTCGAACGAGATCTTCGGATCTAGTGGCGCACGGGTGC
GTAACGCGTGGGAATCTGCCCTTGGGTTCGGAATAACAGTGAGAAATTACTGCTAATACCGGATGATG
TCTTCGGACCAAAGATTTATCGCCCAGGGATGAGCCCGCGTAGGATTAGCTAGTTGGTGGGGTAATGG
CCTACCAAGGCGACGATCCTTAGCTGGTCTGAGAGGATGATCAGCCACACTGGGACTGAGACACGGCC
>Zotu1949
GAACGCTGGCGGCGTGCCTAATACATGCAAGTCGAGCGAGGGTCTTCGGACCCTAGCGGCGGACGGGT
GAGTAACACGTAGGCAACCTGCCTGTAAGACTGGGATAACATAGGGAAACTTATGCTAATACCAGATA
GCGTCTTCTCTTGCATGAGAGGAGACGGAAAGATGGCGCAAGCTATCACTTACAGATGGGCCTGCGGC
GCATTAGCTAGTTGGTGGGGTAATGGCCTACCAAGGCGACGATGCGTAGCCGACCTGAGAGGGTGACC
GGCCACACTGGGACTGAGACACGGCC
>Zotu1950
GAACGCTGGCGGCGTGCTTAACACATGCAAGTCGAACGAGATCCAGCCGACCTTCGGGGAAGCGAAGA
TCTAGTGGCGAACGGGTGCGTAACACGTGAGAAACCTGCCCCAGACTTGGGAATAACAGTCGGAAACG
TCTGCTAATACCGAATACCTTCGTAGCGTCGCATGGCGCATCGAAGAAAGCTTTTGCGGTTTGGGAGG
GTCTCGCGGCCTATCAGCTAGTTGGTGAGGTAACGGCTCACCAAGGCATCGACGGGTAGCTGGTCTGA

GAGGATGATCAGCCACACTGGGACTGAGACACGGCC
>Zotu1951
GAACGCTGGCGGCAGGCTTAACACATGCAAGTCGAACGCATCCTTCGGGGTGAGTGGCAGACGGGTGA
GTAACGCGTGGGAACGTGCCCTTCAGTTCGGAATAACCCAGGGAAACTTGGGCTAATACCGGATACGT
GCGAGAGCAGAAAGATTTATCGCTGAAGGATCGGCCCGCGTCTGATTAGCTAGTTGGTGAGGTAATGG
CTCACCAAGGCGACGATCAGTAGCTGGTCTGAGAGGATGATCAGCCACATTGGGACTGAGACACGGCC
>Zotu1952
GAACGCTGGCGGCGTGCTTTACACATGCAAGTCGAGCGGTAAGGCCCTTTCGGGGGTACACGAGCGGC
GAACGGGTGAGTAACACGTGAGTAATCTGCCCTTCACTTGGGGATAAGCACCGGAAACGGTGTCTAAT
ACCCGATACGACACAGTCCTGCATGGGATCTGTGTGGAAAGTTTTTTCGGTGGGGGATGTGCTCGCGG
CCTATCAGCTTGTTGGTGGGGTAATGGCCTACCAAGGCTTCGACGGGTAGCCGGCCTGAGAGGGTGAC
CGGCCACACTGGGACTGAGACATGGCC
>Zotu1953
GAACGCTGGCGGCGTGCTTAACACATGCAAGTCGAACGATGAACCAGGAGCTTGCTCCTGGGGATTAG
TGGCGAACGGGTGAGTAACACGTGAGCAATCTGCCCTTGACTCTGGGATAAGCGCTGGAAACGGCGTC
TAATACCGGATACGACGACCGGAGGCATCTCCTGGTCGTGGAAAGAACTTCGGTCAAGGATGAGCTCA
CGGCCTATCAGCTAGTTGGTGAGGTAACGGCTCACCAAGGCGACGACGGGTAGCCGGCCTGAGAGGGT
GACCGGCCACACTGGGACTGAGACACGGCC
>Zotu1954
GAACGCTGGCGGCGTGCTTAACACATGCAAGTCGAACGGTAAGGCTCCTTCGGGAGTACACGAGTGGC
GAACGGGTGAGTAACACGTGAGCAACCTGCCCTTGACTTTGGGATACCAGCTGGAAACAGCTGCTAAT
ACCGGATACGACCCCTTGACGCATGTCTTGGGGTGGAAAGCTCCGGCGGTCAGGGATGGGCTCGCGGC
CTATCAGCTTGTTGGTGAGGTAATGGCTCACCAAGGCTTCGACGGGTAGCCGGCCTGAGAGGGCGACC
GGCCACACTGGGACTGAGATACGGCC
>Zotu1955
GAACGCTGGCGGCATGCCTAACACATGCAAGTCGAACGGCAGCATAGTCTAGTTTACTAGATGGGTGG
CGAGTGGCGAACGGGTGAGTAATGCGTAGGAATATACCTTAAAGAGGGGGATAACCTGGGGAAACTCA
GGCTAATACCGCGTAATCTCCGAGGAGAAAAGCTGGGGACCTTCGGGCCTGGCGCTTTAAGAATAGCC
TGCGTCCGATTAGCTAGTTGGTGGGGTAAAGGCCTACCAAGGCGACGATCGGTAGCTGGTCTGAGAGG
ATGAACAGCCACACTGGGACTGAGACACGGCC
>Zotu1956
GAACGCTGGCGGCGTGCTTAACACATGCAAGTCGAGCGGAAAGGCCACTTCGGTGGTACTCGAGCGGC
GAACGGGTGAGTAACACGTGAGTAATCTGCCCTGGACTTTGGGATAGCCACCGGAAACGGTGATTAAT
ACCGGATATGACCGCGCATCGCATGGTGCGTGGTGGAAAGTTTTTCGGTTCAGGATGTGCTCGCGGCC
TATCAGCTTGATGGTGGGGTAATGGCCTACCATGGCTTCGACGGGTAGCCGGCCTGAGAGGGTGACCG
GCCACACTGGGACTGAGACACGGCC
>Zotu1957
GAACGCTGGCGGCGTGCTTAACACATGCAAGTCGAACGATGAACCACTTCGGTGGGGATTAGTGGCGA
ACGGGTGAGTAACACGTGGGCAATCTGCCCTGCACTCTGGGACAAGCCCTGGAAACGGGGTCTAATAC
CGGATATTGACCTTCACGGGCATCTGTGAGGTTCGAAAGCTCCGGCGGTGCAGGATGAGCCCGCGGCC
TATCAGCTTGTTGGTGAGGTAATGGCTCACCAAGGCGACGACGGGTAGCCGGCCTGAGAGGGCGACCG
GCCACACTGGGACTGAGACACGGCC
>Zotu1958
GAACGCTGGCGGCAGGCTTAACACATGCAAGTCGAACGCATCCTTCGGGGTGAGTGGCAGACGGGTGA
GTAACGCGTGGGAACGTGCCCTTCAGTTCGGGATAACCCAGGGAAACTTGGGCTAATACCGGATACGT
GCGAGAGCAGAAAGATTTATCGCTGAAGGATCGGCCCGCGTCTGATTAGCTAGTTGGTGAGGTAATGG
CTCACCAAGGCGACGATCAGTAGCTGGTCTGAGAGGATGATCAGCCACATTGGGACTGAGACACGGCC
>Zotu1959
GAACGCTGGCGGCAGGCCTAACACATGCAAGTCGAACGCCCCCTTCGGGGGGAGTGGCGCACGGGTGA
GTAACGCGTGGGAATCTACCCTTTGGTTCGGAATAACCCTGGGAAACTAGGGCTAACACCGGATAAGC
CCCGAGAGGGGGAAAGTTTACGCCGAAGGAGGAGCCCGCGTCCGATTAGCTAGTTGGTGAGGTAAGAG
CTCACCAAGGCTTCGATCGGTAGCTGGTCTGAGAGGACGATCAGCCACACTGGGACTGAGACACGGCC
>Zotu1960
GAACGCTGGCGGCGTGCTTAACACATGCAAGTCGAACGAGAAATGTTATTATGATCTCTTCGGAGTGA

TGATTTTATTTCGAGTGGCGGACGGGTGAGTAACGCGTGAGCAACCTGCCCTATACCGGGGGATACCA
GTTGGAAACGACTGTTAATACCGCATAAGCGCACGATAAGACATCTTGTTGTGTGAAAAACTCCGGTG
GTATAGGATGGGCTCGCGTATGATTAGCCAGTTGGCGGGGTAAAGGCCCACCAAAGCGACGATCATTA
GCCGACCTGAGAGGGCGACCGGCCACATTGGGACTGAGACACGGCC
>Zotu1961
GAACGCTGGCGGCGTGCTTAACACATGCAAGTCGAACGGAAAGGCCAGTGCTTGCACTGGTACTCGAG
TGGCGAACGGGTGAGTAACACGTGGGTGATCTGCCCCGCACTTCGGGATAAGCCTGGGAAACTGGGTC
TAATACCGGATAGGACCACTTCTTAGAGGTTGTGGTGGAAAGTTTTTTCGGTGTGGGATGAGCTCGCG
GCCTATCAGCTTGTTGGTGGGGTAATGGCCTACCAAGGCGTCGACGGGTAGCCGGCCTGAGAGGGTGT
ACGGCCACATTGGGACTGAGATACGGCC
>Zotu1962
GAACGCTGGCGGTATGCTTTACACATGCAAGTCGTACGGGAGTTTTTAACTCTAGTGGCGGACGGGTG
AGTAACACGTAAGAATTTGCCTTTAGGAGGGGGACAACAGCTGGAAACGGCTGCTAATACCCCATATG
CTTTCGAGTGAAATGGATTTCCGCCTAAAGAGAAGCTTGCGGCTGATTAGCTTGTTGGTAAGGTAATG
GCTTACCAAGGCGACGATCAGTATCTGGTTTGAGAGGATGATCAGACACACTGGAACTGAGACACGGT
C
>Zotu1963
GAACGCTAGCGGCAGGCCTAATACATGCAAGTCGGACGGGATAGGAGAGCTTGCTTTCCTTGAGAGTG
GCGCACGGGTGCGTAACACGTATGTAACCTACCTTGATCAGGGGGATAGCCTCTCGAAAGAGAGATTA
AGACCGCATAACATTGCAATATGGCATCATAAAGCAATCAAATATTTATAGGATCAAGATGGGCATGC
GGAACATTAGCTAGTTGGTAGGGTAACGGCCTACCAAGGCTACGATGTTTAGGGGATCTGAGAGGATG
GCCCCCCACACTGGTACTGAGACACGGAC
>Zotu1964
GAACGCTGGCGGCGTGCCTAACACATGCAAGTCGAGCGATTCTCTTCGGAGAAGAGCGGCGGACGGGT
GAGTAACGCGTGGGTAACCTGCCCTGTACACACGGATAACATATCGAAAGGTATGCTAATACGGGATA
ACATATTTTGATGGCATCATCGAAATATCAAAGCTCCGGCGGTACAGGATGGACCCGCGTCTGATTAG
CTAGTTGGTGAGGTAATGGCTCACCAAGGCGACGATCAGTAGCCGACCTGAGAGGGTGATCGGCCACA
TTGGAACTGAGACACGGTC
>Zotu1965
GAACGCTAGCGGCAGGCCTAATACATGCAAGTCAAAGGGGTAGCAATACCACTGGCGCACGGGTGCGT
AACGCGTATGCAACCTACCGTTCACTGGGGGATAACGTCGGGAAACCGGCGCTAATACCGCATAAAGC
AGGGGCTGGGCATCCAGATACTGCTAAAGTTCCGATGGTGAACGATGGGCATGCGTCTGATTAGCTAG
TTGGCGGGGTAATGGCCCACCAAGGCGACGATCAGTAGGGGATCTGAGAGGATAATCCCCCACACTGG
CACTGAGATACGGGC
>Zotu1966
GAACGCTGGCGGCGTGCTTAACACATGCAAGTCGAACGATGAAGCCCAGCTTGCTGGGTGGATTAGTG
GCGAACGGGTGAGTAACACGTGAGCAACGTGCCCATAACTCTGGGATAACCTCCGGAAACGGTGGCTA
ATACTGGATATCTAACACGATCGCATGGTCTGTGTTTGGAAAGATTTTTTGGTTATGGATCGGCTCAC
GGCCTATCAGCTTGTTGGTGAGGTAATGGCTCACCAAGGCGACGACGGGTAGCCGGCCTGAGAGGGTG
ACCGGCCACACTGGGACTGAGACACGGCC
>Zotu1967
GAACGCTGGCGGCGTGCCTAATACATGCAAGTAGAACGCTGAGGACAGGTGCTTGCACTAGTCTAAGG
AGTTGCGAACGGGTGAGTAACGCGTAGGTAACCTACCTGATAGCGGGGGATAACTATTGGAAACGATA
GCTAATACCGCATAAAAGTGCTTAACACATGTTAAGAACTTAAAAGGGGCAACTGCTCCACTATGAGA
TGGACCTGCGTTGTATTAGCTAGTTGGTGAGGTAAAGGCTCACCAAGGCGACGATACATAGCCGACCT
GAGAGGGTGATCGGCCACACTGGGACTGAGACACGGCC
>Zotu1968
GAACGCTGGCGGCAGGCTTAACACATGCAAGTCGAGCGGAAACGATAGTAGCTTGCTACTAGGCGTCG
AGCGGCGGACGGGTGAGTAATACTTAGGAATCTGCCCAGTAGTGGGGGATAGCTCGGGGAAACTCGAA
TTAATACCGCATACACCCTACGGGGAAAAGGGGGCGCTTGCGCTCTCGCTATTGGATGAGCCTAAGTC
GGATTAGCTAGTTGGTGGGGTAAAGGCCTACCAAGGCGACGATCTGTAGCTGGTCTGAGAGGATGATC
AGCCACACCGGAACTGAGACACGGTC
>Zotu1969
GAACGCTGGCGGCACGCCTAATACATGCAAGTCGAGCGCGTGAAGCTTTCCGAAGCCTTCGGGCGGAC

GAAAGTGGATCGAGCGGCGGACGGGTGAGTAACACGTAGGCAACCTGCCTGTAAGACCGGGATAACTC
CGGGAAACCGGGGCTAATACCGGATAGGATCTCTTTTCGCATGGGAAGGGATTGAAAGGATGGCCGCA
AGGCTATCGCTTACAGATGGGCCTGCGGCGCATTAGCTAGTTGGTGAGGTAACGGCTCACCAAGGCGA
CGATGCGTAGCCGGCCTGAGAGGGTGACCGGCCACACTGGGACTGAGACACGGCC
>Zotu1970
CAACGCTGGCGGCGTGCCTAATGCATGCAAGTCGCACGGGGTGGCCTTCGGGTCGCTTACGTGGCGGA
CGGGTGAGGAACACGTGGGCAACCTGCCCGGTGGTGGGGGCTAGCCCTCGGAAACGAGGGGTAATTCC
GCATGAGCTCATTTTCCGAGTGGGGAGGATGAGGAAAGCTCTTCGGAGCGCCATCGGAGGGGTCTGCG
TCCGATTAGTTAGCTGGTGGGGTAACGGCCTACCAGGGCGATGATCGGTAGCTGGTTTGAGAGGATGA
TCAGCCACACGGGGACTGAGACACGGCC
>Zotu1971
GAACGCTGGCGGCGTGCTTAACACATGCAAGTCGAACGATGAAGCCTAGCTTGCTAGGTGGATTAGTG
GCGAACGGGTGAGTAATACGTGAGTGACCTACCTTTGACTCTGGGATAAGCCTGGGAAACTGGGTCTA
ATACCGGATACGACCAATCTCCGCATGGGGTGTTGGTGGAAAGCGTTATGGAGTGGTTTTAGATGGGC
TCACGGCCTATCAGCTTGTTGGTGAGGTAATGGCTTACCAAGGCGACGACGGGTAGCCGGCCTGAGAG
GGTGACCGGCCACACTGGGACTGAGACACGGCC
>Zotu1972
GAACGCTGGCGGCGTGCTTAACACATGCAAGTCGAACGATGAAGCCTGGTGCTTGCACCGGGTGGATT
AGTGGCGAACGGGTGAGTATCACGTGAGTAACCTGCCCTTGACTCTGGGATAAGCCCGGGAAACTGGG
TCTAATACCGGATATGACCAGTCACCGCATGGTGTGCTGGTGGAAAGCTTTAGCGGTTTTGGATGGAC
TCGCGGCCTATCAGCTAGACGGTGAGGTAATGGCTCACCGTGGCGATGACGGGTAGCCGGCCTGAGAG
GGTGACCGGCCACACTGGGACTGAGACACGGCC
>Zotu1974
GAACGCTGGCGGCGTGCTTAACACATGCAAGTCGAACGATGATCTCCAGCTTGCTGGGGGGATTAGTG
GCGAACGGGTGAGTAACACGTGAGTAACCTGCCCTTGACTCTGGGATAAGCCTGGGAAACTGGGTCTA
ATACCGGATACGACCGCTCCACGCATGTGGTGGTGGTGGAAAGCTTTTGCGGTTTTGGATGGACTCGC
GGCCTATCAGCTTGTTGGTGGGGTAATGGCCTACCAAGGCGACGACGGGTAGCCGGCCTGAGAGGGTG
ACCGGCCACACTGGGACTGAGACACGGCC
>Zotu1975
GAATGCTGGCGGCGTGGATAAGTCATGCAAGTCAAGGGGGCCCGCAAGGGCAACCGGCAGACGAGGTA
GTAACACGTAGGTACGTCCCCCGAAGTCGGGCATAGCCATCCGAAAGGATGGGTAATTCCCGATAGTC
CCGCAAGGGTAAAGGTTTTTCGCTTCGGGAGCGGCCTGCGTAGTATCAGCTAGTTGGTAGGGTAACGG
CCTACCAAGGCTACGACGCTTAGGGGAGCTGAGAGGCTGACCCCCACCGATGGAACTGCGACACGGTC
>Zotu1977
GAACGCTGGCGGCGTGCCTAACACATGCAAGTCGAGCGAACCCTTCGGGGTGAGCGGCGGACGGGTGA
GTAACGCGTGGGTAACCTGCCCTGTACACACGGATAACATACTGAAAGGTATGCTAATACGAGATAAT
ATGTAATGATCGCATGATCGATACATCAAAGCTCCGGCGGTACAGGATGGACCCGCGTCTGATTAGCT
AGTTGGTGAGGTAATGGCTCACCAAGGCGACGATCAGTAGCCGACCTGAGAGGGTGATCGGCCACATT
GGAACTGAGACACGGTC
>Zotu1978
GAACGCTGGCGGCATGCCTAACACATGCAAGTCGAACGAAGGCTTCGGCCTTAGTGGCGCACGGGTGC
GTAACGCGTGGGAATCTGCCCTTGGGTTCGGAATAACAGCGAGAAATTGCTGCTAATACCGGATGAAG
ACGAAAGTCCAAAGATTTATCGCCTGAGGATGAGCCCGCGTTGGATTAGGTAGTTGGTGGGGTAAAGG
CCTACCAAGCCGACGATCCATAGCTGGTCTGAGAGGATGATCAGCCACACTGGGACTGAGACACGGCC
>Zotu1979
GAACGCTGGCGGCATGCCTTACACATGCAAGTCGAACGGTAACAGGTTAAGCTGACGAGTGGCGAACG
GGTGAGTAATATATCGGAACGTGCCCAATCGTGGGGGATAACGCAGCGAAAGTTGTGCTAATACCGCA
TAAGATCTAAGGATGAAAGCGGGGGATCGCAAGACCTCGCGCGAATGGAGCGGCCGATATCAGATTAG
CTAGTTGGTGGGGTAAAGGCCCACCAAGGCGACGATCTGTAGCTGGTCTGAGAGGACGACCAGCCACA
CTGGGACTGAGACACGGCC
>Zotu1980
GAACGCTGGCGGCAGGCCTAACACATGCAAGTCGAGCGGCAGCACGGGAGCTTGCTCCTGGTGGCGAG
CGGCGGACGGGTGAGTAATGCCTAGGAATCTGCCTGTTAGTGGGGGATAACTCGGGGAAACTCGAGCT
AATACCGCATACGTCCTACGGGAGAAAGTGGGGGACCTTCGGGCCTCACGCTAACAGATGAGCCTAGG

TCGGATTAGCTAGTTGGTGGGGTAAAGGCCTACCAAGGCGACGATCCGTAGCTGGTCTGAGAGGATGA
TCAGCCACACTGGAACTGAGACACGGTC
>Zotu1981
GAACGCTGGCGGCGTGCTTAACACATGCAAGTCGAACGGTGAAGCCCTTCGGGGTGGATCAGTGGCGA
ACGGGTGAGTAACACGTGAGCAATCTGCCCCCGACTCTGGGATAACTTCGGGAAACCGGAGCTAATAC
CGGATATGACACATGACCGCATGGTCTGTGTGTGGAAAGATTTATCGGTTGGGGATGAGCTCGCGGCC
TATCAGCTTGTTGGTGGGGTAAAGGCCTACCAAGGCGACGACGGGTAGCCGGCCTGAGAGGGCGACCG
GCCACACTGGGACTGAGACACGGCC
>Zotu1982
GAACGCTGGCGGCAGGCTTAACACATGCAAGTCGAGCGGGCCCTTCGGAGTCAGCGGCGGACGGGTGA
GTAACGCGTGGGAACGTGCCTTCTGGTCTGGAATAACCCTGGGAAACTAGGGCTAATACCGGATACGC
CCTTTTGGAGAAAGGTTTACTGCCGGAAGATCGGCCCGCGTCTGATTAGCTAGTTGGTGGGGTAACGG
CCTACCAAGGCGACGATCAGTAGCTGGTCTGAGAGGATGATCAGCCACACTGGGACTGAGACACGGCC
>Zotu1983
GAACGCTGGCGGCGTGCTTAACACATGCAAGTCGAACGGAAAGGCTCCTGCTTGCAGGGGTACTCGAG
TGGCGAACGGGTGAGTAACACGTGGGTGATCTGCCTTGTACTCTGGGATAAGCTTGGGAAACTGGGTC
TAATACCGGATAATAACTGCCTTTAGTGTGGTGGTTGGAAAGTTTTTTCGGTGCAAGATGAGCTCGCG
GCCTATCAGCTTGTTGGTGGGGTAATGGCCTACCAAGGCGTCGACGGGTAGCCGGCCTGAGAGGGTGT
ACGGCCACATTGGGACTGAGATACGGCC
>Zotu1984
GAACGCTGGTGGCGTGCCTAATACATGCAAGTCGAGCGAATCTGAGGGAGCTTGCTCCCAAAGATTAG
CGGCGGACGGGTGAGTAACACGTGGGCAACCTGCCTGTAAGACTGGGATAACTTCGGGAAACCGGAGC
TAATACCGGATAACATATGAGACCACATGGTCTTATATTAAAAGATGGCTTTTAGCTATCACTTACAG
ATGGGCCCGCGGCGCATTAGCTAGTTGGTGAGGTAACGGCTCACCAAGGCAACGATGCGTAGCCGACC
TGAGAGGGTGATCGGCCACACTGGGACTGAGACACGGCC
>Zotu1985
GAACGCTGGCGGCGTGCCTAACACATGCAAGTCGAACGGAGCAGATCCCTTCGGGGGTTTGCTTAGTG
GCGAACGGGTGAGTAACGCGTGAGGAACCTGCCTTTCAGTGGGGGACAACAGTTGGAAACGACTGCTA
ATACCGCATAATGCTTTTGAGCGGCATCGCTTGAAAGCCAAAGATTTATCGCTGAAAGATGGTCTCGC
GTCTGATTAGCTAGTTGGTGAGGTAACGGCTCACCAAGGCGGCGATCAGTAGCCGGTCTGAGAGGATG
AACGGCCACATTGGGACTGAGATACGGCC
>Zotu1986
GAACGCTGGCGGCGTGCTTAACACATGCAAGTCGAGCGGAAAGGCTCCCTTCGGGGGGTACTCGAGCG
GCGAACGGGTGAGTAACACGTGAGTGATCTGCCCCTCACTTCGGGATAACTGTCTGAAAGGGTGGCTA
ATACCGGATATTCTTTCATTGCTGCATGGTGGTGGAGGGAAAGTTTTTGCGGTGGGGGATGTGCTCGC
GGCCTATCAGCTTGTTGGTGGGGTAATGGCCTACCAAGGCTTTGACGGGTAGCCGGCCTGAGAGGGTG
ACCGGCCACACTGGGACTGAGACACGGCC
>Zotu1987
GAACGCTGGCGGCGTGCTTAACACATGCAAGTCGAACGGTAAGGCCCTTCGGGGTACACGAGTGGCGA
ACGGGTGAGTAACACGTGAGTAACCTGCCCCAGTCTCTGGGATAACCGCCGGAAACGGCGGCTAATAC
TGGATATTCAGCGTCTGCCGCATGGTGGGTGTTGGAAAGCTCCGGCGGATTGGGATGGGCTCGCGGCC
TATCAGCTTGTTGGTGAGGTAGTGGCTCACCAAGGCTTCGACGGGTAGCCGGCCTGAGAGGGCGACCG
GCCACATTGGGACTGAGATACGGCC
>Zotu1988
GAACGCTGGCGGCGTGCTTAACACATGCAAGTCGAACGATGATCAGGAGCTTGCTCCTGTGATTAGTG
GCGAACGGGTGAGTAACACGTGAGTAACCTGCCCCTGACTCTGGGATAAGCGTTGGAAACGACGTCTA
ATACTGGATATGATCACTGGCCGCATGGTCTGGTGGTGGAAAGATTTTTTGGTTGGGGATGGACTCGC
GGCCTATCAGCTTGTTGGTGAGGTAATGGCTCACCAAGGCGACGACGGGTAGCCGGCCTGAGAGGGTG
ACCGGCCACACTGGGACTGAGACACGGCC
>Zotu1990
GAACGCTAGCGGCAGGCTTAACACATGCAAGTCGAGGGGTAGGGTAGCAATACCTGAGACCGGCGCAC
GGGTGCGTAACGCGTATGCAATCTACCTTTTACAGAGGGATAGCCCAGAGAAATTTGGATTAATACCT
CATAGTATAGTACAATCGCATGATTGAACTATTAAAGTCACAACGGTAAAAGATGAGCATGCGTCCCA
TTAGCTAGTTGGTAAGGTAACGGCTTACCAAGGCGACGATGGGTAGGGGTCCTGAGAGGGAGATCCCC

CACACTGGTACTGAGACACGGAC
>Zotu1991
GAACGCTGGCGGTAGGCCTAACACATGCAAGTCGAACGGCAGCACAGGAGAGCTTGCTCTCTGGGTGG
CGAGTGGCGGACGGGTGAGGAATACATCGGAATCTACTCTGTCGTGGGGGATAACGTAGGGAAACTTA
CGCTAATACCGCATACGACCTACGGGTGAAAGCAGGGGATCTTCGGACCTTGCGCGATTGAATGAGCC
GATGTCGGATTAGCTAGTTGGCGGGGTAAAGGCCCACCAAGGCGACGATCCGTAGCTGGTCTGAGAGG
ATGATCAGCCACACTGGAACTGAGACACGGTC
>Zotu1992
GAACGCTGGCGGCAGGCTTAACACATGCAAGTCGAGCGGGGAAGAGTAGCTTGCTACTTAACCTAGCG
GCGGACGGGTGAGTAATGCTTAGGAATCTGCCTATTAGTGGGGGACAACATCTCGAAAGGGATGCTAA
TACCGCATACGTCCTACGGGAGAAAGCAGGGGACCTTCGGGCCTTGCGCTAATAGATGAGCCTAAGTC
GGATTAGCTAGTTGGTGGGGTAAAGGCCTACCAAGGCGACGATCTGTAGCGGGTCTGAGAGGATGATC
CGCCACACTGGGACTGAGACACGGCC
>Zotu1993
GAACGCTGGCGGCGTGCCTAACACATGCAAGTCGAGCGGTAACGCGGGAGCAATCCTGGCGACGAGCG
GCGAACGGGTGAGTAACGCTTCGGAATCTACCCAGTAGTGGGGAATAACCAACCGAAAGGTTGGCTAA
TGCCGCATACGACCTGAGGGTGAAAGGGGGGGATCGCAAGACCTCTCGCTATTGGATGAGCCGAAGTC
GGATTAGCTAGTTGGTAGGGTAACGGCCTACCAAGGCGACAATCCGTAGCTGGTCTGAGAGGACGACC
AGCCACACTGGGACTGAGACACGGCC
>Zotu1994
GAACGCTGGCGGCGTGCTTAACACATGCAAGTCGAGCGAAGCACTTGTTTGGAACCCTCCGGGAGGAA
GAACAATTGACTGAGCGGCGGACGGGTGAGTAACGCGTGGGGAACCTGCCTTATACCGGGGGATAACA
GCTGGAAACGGCTGCTAATACCGCATAAGCGCACGGCACCGCATGGTGCAGTGTGAAAAACTCCGGTG
GTATAAGATGGACCCGCGTTAGATTAGCTAGTTGGCGGGGTAACGGCCCACCAAGGCGACGATCTATA
GCCGGCCTGAGAGGGCGACCGGCCACATTGGGACTGAGACACGGCC
>Zotu1995
GAACGCTAGCGGGAGGCCTAACACATGCAAGCCGAGCGGTAGAAAGTAGCTTGCTACTTTTGAGAGCG
GCGTACGGGTGCGGAACACGTGTGCAACCTGCCTTTATCTGGGGAATAGCCTTTCGAAAGGAAGATTA
ATGCCCCATAACATATTGATTGGCATCAATTAATATTGAAAGCTCCGGCGGATAGAGATGGGCACGCG
CAAGATTAGCTAGTTGGTGAGGTAACGGCTCACCAAGGCGATGATCTTTAGGGGGCCTGAGAGGGTGA
TCCCCCACACTGGTACTGAGACACGGAC
>Zotu1996
GAACGCTGGCGGCAGGCCTAACACATGCAAGTCGAGCGGATGAAGGGAGCTTGCTCCCTGATTTAGCG
GCGGACGGGTGAGTAATGCCTAGGAATCTGCCTGGTAGTGGGGGACAACGTTTCGAAAGGAACGCTAA
TACCGCATACGTCCTACGGGAGAAAGTGGGGGATCTTCGGACCTCACGCTATCAGATGAGCCTAGGTC
GGATTAGCTAGTTGGTGAGGTAAAGGCTCACCAAGGCGACGATCCGTAACTGGTCTGAGAGGATGATC
AGTCACACTGGAACTGAGACACGGTC
>Zotu1997
GAACGCTGGCGGCATGCCTAACACATGCAAGTCGAACGAAGCCTTCGGGCTTAGTGGCGCACGGGTGC
GTAACGCGTGGGAATCTGCCCCTTGCTTCGGAATAACAGTTAGAAATGACTGCTAATACCGGATGATG
TCTTCGGACCAAAGATTTATCGGCAAGGGATGAGCCCGCGTAGGATTAGGTAGTTGGTGGGGTAAAGG
CCTACCAAGCCGACGATCCTTAGCTGGTCTGAGAGGATGATCAGCCACACTGGGACTGAGACACGGCC
>Zotu1998
GAACGCTGGCGGCGTGCTTAACACATGCAAGTCGAACGGGCCCGCCCTCTTTTTTGTGGGTGGGTTGA
GTGGCGAACGGGTGAGTATCACGTGAGTAACCTGCCCCCTGCTCCTGGATAACTGCTTGAAAGGGCGG
CTAATACGGGGTGTGCCGGCTGGTCCGCATGGGCCTGGTCGGGATAGTTTCATCTTCGGGTGTTTCGG
TGGGGGATGGGCTCGCGGCCTATCAGCTTGTTGGTGGGGTGATGGCCCACCAAGGCGGTGACGGGTAG
CCGGCCTGAGAGGGTGGGCGGCCACACTGGGACTGAGACACGGCC
>Zotu1999
GAACGCTGGCGGCGTGCTTAACACATGCAAGTCGAACGGAAAGGCTCCAGCTTGCTGGGGTGCTCGAG
TGGCGAACGGGTGAGTAACACGTAAGTGATCTGCCCTGCACTGGGGGATAAGCTTGGGAAACTGGGTC
TAATACCCTATAGGACCGCATCGTGGTTGGTGTGGTGGAAAGGTTTTTCTGGTGTGGGATGAGCTTGC
GGCCTATCAGCTTGTTGGTGGGGTAATGGCCTACCAAGGCGGCGACGGGTAGCCGGCCTGAGAGGGTG
TGCGGCCACATTGGGACTGAGATACGGCC

>Zotu2000
GAACGCTGGCGGCGTGCTTAACACATGCAAGTCGAGCGGTAAGGCACTTCGGTGTACACGAGCGGCGA
ACGGGTGAGTAACACGTGAGTAATCTGCCCTTCACTCTGGGATAAGCTTCGGAAACGGGGTCTAATAC
CGGATATGACCTGGCCCTGCATGGGGTTGGGTGGAAACTTTTATGGGTGGGGGATGTGCTCGCGGCCT
ATCAGCTTGTTGGTGGGGTAATGGCCTACCAAGGCTTCGACGGGTAGCCGGCCTGAGAGGGTGACCGG
CCACACTGGGACTGAGACACGGCC
>Zotu2001
GAACGCTGGCGGCGTGCTTAACACATGCAAGTCGAACGGAGAAGGAGGAGCTTGCTCCTCTGGATCAG
TGGCGAACGGGTGAGTAACACGTGAGTAACCTGCCCCAGACTCTGGGATAAGCGTTGGAAACGACGTC
TAATACCGGATACGAGCTTCAGCCGCATGGCTAGGAGCTGGAAAGAACTTCGGTCTGGGATGGACTCG
CGGCCTATCAGGTAGTTGGTGAGGTAACGGCCCACCAAGCCTACGACGGGTAGCCGGCCTGAGAGGGT
GACCGGCCACACTGGGACTGAGACACGGCC
>Zotu2002
GAACGCTGGCGGCAGGCTTAACACATGCAAGTCGAGCGCCCCGCAAGGGGAGCGGCAGACGGGTGAGT
AACGCGTGGGAATCTACCTTTTGCTACGGAATAACTCAGGGAAACTTGTGCTAATACCGTATGTGCCC
TTCGGGGGAAAGATTTATCGGCAAAGGATGAGCCCGCGTTGGATTAGCTAGTTGGTGGGGTAAAGGCC
CACCAAGGCGACGATCCATAGCTGGTCTGAGAGGATGATCAGCCACACTGGGACTGAGACACGGCC
>Zotu2003
GAACGCTGGCGGCATGCCTTACACATGCAAGTCGAACGGTAACAGGTCTTCGGATGCTGACGAGTGGC
GAACGGGTGAGTAATACATCGGAACGTGCCCGATCGTGGGGGATAACGAAGCGAAAGCTTTGCTAATA
CCGCATAAGATCTACGGATGAAAGCAGGGGACCGCAAGGCCTTGCGCGAACGGAGCGGCCGATGGCAG
ATTAGGTAGTTGGTGGGATAAAAGCTTACCAAGCCGACGATCTGTAGCTGGTCTGAGAGGACGACCAG
CCACACTGGGACTGAGACACGGCC
>Zotu2004
GAACGCTGGCGGCGTGCTTAACACATGCAAGTCGAACGATGAAGCGGTGCTTGCACCGTGGATTAGTG
GCGAACGGGTGAGTAATACGTGAGTGACCTGCCCTTGACTCTGGGATAAGCCTGGGAAACTGGGTCTA
ATACTGGATACGACCATCAGATGCATGTCTTGGTGGTGGAAAGGGTTTGTACTGGTTTTGGATGGGCT
CACGGCCTATCAGCTTGTTGGTGGGGTAATGGCTTACCAAGGCGACGACGGGTAGCCGGCCTGAGAGG
GTGACCGGCCACACTGGGACTGAGACACGGCC
>Zotu2005
GAACGCTGGCGGCGTGCCTAACACATGCAAGTCGAGCGGCAGCGCGGGAGCAATCCTGGCGGCGAGCG
GCGGACGGGTGAGCAATGCTTGGGAATCTGCCCATCAGTGGGGGACAACCCGGGGAAACTCGGGCTAA
TACCGCATACGTTCTGAGGAAGAAAGCAGGGGATCGCAAGACCTTGCGCTGATGGATGAGCCCAAGTC
GGATTAGCTAGTTGGTGAGGTAACGGCTCACCAAGGCGACGATCCGTAGCTGGTCTGAGAGGACGACC
AGCCACACCGGAACTGAGACACGGTC
>Zotu2006
GAACGCTGGCGGCAGGCTTAACACATGCAAGTCGAGCGGAGATGAGGTGCTTGCACCTTATCTTAGCG
GCGGACGGGTGAGTAATGCTTAGGAATCTGCCATTTAGTGGGGGACAACATTCCGAAAGGAATGCTAA
TACCGCATACGTCCTACGGGAGAAAGCAGGGGATCTTCGGACCTTGCGCTAAATGATGAGCCTAAGTC
GGATTAGCTAGTTGGTGGGGTAAAGGCCTACCAAGGCGACGATCTGTAGCGGGTCTGAGAGGATGATC
CGCCACACTGGGACTGAGACACGGCC
>Zotu2007
GAACGCTGGCGGCGTGCCTAATACATGCAAGTAGAACGCTGAAGGAGGAGCTTGCTCTTCTGGATGAG
TTGCGAACGGGTGAGTAACGCGTAGGTAACCTGCCTGGTAGCGGGGGATAACTATTGGAAACGATAGC
TAATACCGCATAATAGTAGATGTTGCATGATATTTACTTAAAAGGTGCAATTGCATCACTACCAGATG
GACCTGCGTTGTATTAGCTAGTTGGTGGGGTAACGGCTCACCAAGGCGACGATACATAGCCGACCTGA
GAGGGTGATCGGCCACACTGGGACTGAGACACGGCC
>Zotu2008
GAACGCTGGCGGCATGCCTAACACATGCAAGTCGAACGAGACCTTCGGGTCTAGTGGCGCACGGGTGC
GTAACGCGTGGGAATCTGCCCTTGGGTTCGGAATAACTCCTCGAAAGGGGTGCTAATACCGGATGATG
TCGTAAGACCAAAGATTTATCGCCCAAGGATGAGCCCGCGTAGGATTAGCTAGTTGGTGGGGTAAAGG
CCCACCAAGGCGACGATCCTTAGCTGGTCTGAGAGGATGATCAGCCACACTGGGACTGAGACACGGCC
>Zotu2009
TAACGCTGGCAGAGTGCATAACACATGCAAGTCGAGCGGAACTGTTTGAGATTACATCAAGGGCAGTT

TAGCGGCAGACGAGTGAGTAACACGTAAGCAATCTACCCTCGAATGGCGAATAACCAGTCGAAAGGCT
GGCTAATACGCCGTATTCTCCCCAGAAGGCATCTTAAGGGGAGGAGAGACCCGCAAGGGTCGTTCAAG
GAGGAGCTTGCGGCCCATCAGCTAGTTGGCGGTGTAACGGACCACCAAGGCTACGACGGGTACCCGGC
CTGAAAGGGCGGACGGGCACGATGGCACTGAGACACGGGC
>Zotu2010
GAACGCTGGCGGCGTGCTTAACACATGCAAGTCGAACGATGATCCGGTGCTTGCACCGGGGATTAGTG
GCGAACGGGTGAGTAACACGTGAGTAACCTGCCCTTGACTCTGGGATAAGCCTGGGAAACCGGGTCTA
ATACTGGATACGACCTTTCACCGCATGGTGGTTGGTGGAAAGATTTTTTGGTTTTGGATGGACTCGCG
GCCTATCAGCTTGTTGGTGAGGTAATGGCTCACCAAGGCGACGACGGGTAGCCGGCCTGAGAGGGTGA
CCGGCCACACTGGGACTGAGACACGGCC
>Zotu2012
GAACGCTGGCGGCAGGCTTAACACATGCAAGTCGAACGCGTGTAGCAATACACGAGTGGCGCACGGGT
GAGTAACGCGTGGATATCTGCCTTTTGGTTCGGAATAACCCCGGGAAACTGGGGCTAATACCGGATGG
TTCCTACGGGATAAAGATTTATCGCCAAAAGATGAGTCCGCGTCCGATTAGCTAGTTGGTGGGGTAAT
GGCCCACCAAGGCGACGATCGGTAGCTGGTCTGAGAGGACGATCAGCCACACTGGGACTGAGACACGG
CC
>Zotu2013
GAACGCTGGCGGCAGGCTTAACACATGCAAGTCGAGCGGGCGCTTTCGGGCGTCAGCGGCGGACGGGT
TAGTAACGCGTGGGAACGTGCCCTTTGCTCTGGAATAGCCCTGGGAAACTGGGGGTAATACCGGATGT
GCCCTGAGGGGGAAAGATTTATCGGCAAGGGATCGGCCCGCGTCTGATTAGGTAGTTGGTGAGGTAAC
GGCTCACCAAGCCGACGATCGGTAGCTGGTTTGAGAGGATGATCAGCAACACTGGGACTGAGACACGG
CC
>Zotu2015
GAACGCTGGCGGCGTGCCTAATACATGCAAGTCGAGCGGAAGTTGGAGAGCTTGCTCTTCAACTTTAG
CGGCGGACGGGTGAGTAACACGTGGGCAACCTGCCCTGCAGATCGGGATAACTCCGGGAAACCGGTGC
TAATACCGAATAGTTTTCGCCACCTCCTGGTGGCGTACGGAAAGACGGTTTCGGCTGTCACTGCAGGA
TGGGCCCGCGGCGCATTAGCTAGTTGGTGGGGTAACGGCCCACCAAGGCAACGATGCGTAGCCGACCT
GAGAGGGTGATCGGCCACACTGGGACTGAGACACGGCC
>Zotu2016
GAATGCTGGCGGTGTGGATAGGGCATGCAAGTCAAGGGGGCCGCAAGGCAACCGGCAGACGAGATAGT
AACGCGTAGGAACGTACCCAGAAGTCGGGCACAGCTCGCCGAAAGGCGGGGTAATTCCCGATGGTGTC
GCAAGACTAAAGATTTATCGCTTCTGGAGCGGCCTGCGTAGTATCAGCTAGTTGGTGAAGTAATGGTT
TACCAAGGCTATGACGCTTAGGGGAGCTGAGAGGCTGACCCCCATCGATGGAATTGAGATACGGTC
>Zotu2017
GAACGCTAGCGGCAGGCCTAATACATGCAAGTCGAACGGAGCAGCAATGCTTAGTGGCGCACGGGTGC
GTAACGCGTATGAAACCTGCCCTTCACTGGGGGATAACCTCTCGAAAGAGAGGCTAATACCGCATAAG
ATAGTAGAACGGCATCGTTCAGCTCTTAAAACTCCGGTGGTGAAGGATGGTCATGCGTCCAATTAGTT
AGTTGGTAGGGTAACGGCCTACCAAGACGATGATTGGTAGGGGGTCTGAGAGGATGATCCCCCACACT
GGTACTGAGATACGGAC
>Zotu2019
GAATGCTGGCGGCGTGCCTAATACATGCAAGTCGAGCGGTAACAGGGAGTTCCACCGTTTCCTTGACA
CACCTAGCGCTATGATCGTAGGTTGATGACTTCGAGGGAACAGTGGAATTCTGCTGACGAGCGGCGGA
CGGCTGAGTAACGCGTGGGAACGGACCCCAAACTGAGGGATAACTAGTCGAAAGATTAGCTAATACCG
CATGTGATCTTCGGATTAAAGCTTTCGAGCGGTTTGGGACTGGCCTGCGTACGATTAGATAGTTGGTA
GGGTAATGGCCTACCAAGTCGACGATCGTTAGATGGTTTGAGAAGATGATCATCCAGACTGGGACTGA
GACACGGCC
>Zotu2020
GAACGCTGGCGGCAGGCCTAACACATGCAAGTCGAGCGCCCCGCAAGGGGAGCGGCGGACGGGTGAGT
AACGCGTGGGAACGTGCCCTTTGCTTCGGAATAGCCCCGGGAAACTGGGAGTAATACCGAATGTGCCC
CCCATTTTGGAGATGGCGGGGGAAAGATTTATCGGCAAAGGATCGGCCCGCGTTGGATTAGGTAGTTG
GTGGGGTAACGGCCTACCAAGCCGACGATCCATAGCTGGTTTGAGAGGATGATCAGCCACACTGGGAC
TGAGACACGGCC
>Zotu2021
GAACGCTGGCGGCAGGCTTAACACATGCAAGTCGAGCGGGCATAGCAATATGTCAGCGGCAGACGGGT

GAGTAACGCGTGGGAACGTACCTTTTGGTTCGGAACAACACAGGGAAACTTGTGCTAATACCGGATAA
GCCCTTACGGGGAAAGATTTATCGCCGAAAGATCGGCCCGCGTCTGATTAGCTAGTTGGTGAAGTAAT
GGCTCACCAAGGCGACGATCAGTAGCTGGTCTGAGAGGATGATCAGCCACATTGGGACTGAGACACGG
CC
>Zotu2022
GAACGCTAGCGGCAGGCCTAATACATGCAAGTCGGACGGGATCCGGGGGTAGCTTGCTACCTTCGGTG
AGAGTGGCGCACGGGTGCGTAACGCGTGAGCAACCTACCCATATCAGGGGGATAGCCCGGAGAAATCC
GGATTAACACCGCATGCAACCAATGGACGGCATCGTTTTTTGGTTAAATATTTATAGGATATGGATGG
GCTCGCGTGACATTAGCTAGTTGGTGGGGTAACGGCCCACCAAGGCGACGATGTCTAGGGGCTCTGAG
AGGAGAATCCCCCACACTGGTACTGAGACACGGAC
>Zotu2023
AAACGCTGGCGGCGTGCTTAACACATGCAAGTCGAACGAAGTTTTTCATTCGTGGAAAACTTAGTGGC
GGACGGGTGAGTAACGCGTGGGTAACCTGCCCTGTACAGGGGGACAACAGCTGGAAACGGCTGCTAAT
ACCGCATAAGCCCTTAGCACTGCATGGTGCATAGGGAAAAGGAGTAATCCGGTACAGGATGGACCCGC
GTCTGATTAGCCAGTTGGCAGGGTAACGGCCTACCAAAGCAACGATCAGTAGCCGATCTGAGAGGATG
TACGGCCACATTGGGACTGAGACACGGCC
>Zotu2024
CAACGCTGGCGGCGTGCCTAATGCATGCAAGTCGAACGTGCGGATCTTCGGATCCGAGGCGTGGCGGA
CGGGTGAGGAACACGTGGGCAAGCTGCCCTGGGGTGGGGGGTAGCCCGCGGAAACGCGGGGTAATTCC
GCATGAGCTTCCGTGCTGAGTAGGGCATGGAAGGAAAGGGTCTTCGGACTCGCCCGAGGAGGCGCCTG
CGGCCGATTAGCTAGTTGGTGGGGTAACGGCCTACCAAGGCGGTGATCGGTAGCTGGTCTGAGAGGAC
GGCCAGCCACACGGGGACTGAGACACGGCC
>Zotu2025
GAACGCTGGCGGCGTGCCTAATACATGCAAGTAGAACGCACAGGATGCACCGTAGTTTACTACACCGT
ATTCTGTGAGTTGCGAACGGGTGAGTAACGCGTAGGTAACCTGCCTGGTAGCGGGGGATAACTATTGG
AAACGATAGCTAATACCGCATAAGAACATTTACTGCATGGTAGATGTTTAAAAGGTGCAAAAGCATCA
CTACCAGATGGACCTGCGTTGTATTAGCTAGTTGGTGAGGTAACGGCTCACCAAGGCAACGATACATA
GCCGACCTGAGAGGGTGATCGGCCACACTGGGACTGAGACACGGCC
>Zotu2027
GAACGCTGGCGGCGTGCTTAACACATGCAAGTCGAACGGAAAGGCCCTGCTTGCAGGGTACTCGAGTG
GCGAACGGGTGAGTAACACGTGGGTGATCTGCCCTGCACTTCGGGATAAGCCTGGGAAACTGGGTCTA
ATACCGGATAGGACCATCGTTTAGTGTCGGTGGTGGAAAGTTTTTCGGTGCAGGATGAGCTCGCGGCC
TATCAGCTTGTTGGTGGGGTAATGGCCTACCAAGGCGTCGACGGGTAGCCGGCCTGAGAGGGTGGACG
GCCACATTGGGACTGAGACACGGCC
>Zotu2028
GAACGCTGGCGGCGTGCTTAACACATGCAAGTCGAACGGAAAGGCCCTTCGGGGTACTCGAGTGGCGA
ACGGGTGAGTAACACGTGGGTGATCTGCCCTGCACTTTGGGATAAGCCTGGGAAACTGGGTCTAATAC
CGAATATGATCACTGGCTTCATGGTTGGTGGTGGAAAGCTTTTGCGGTGTGGGATGGGCCCGCGGCCT
ATCAGCTTGTTGGTGGGGTAATGGCCTACCAAGGCGTCGACGGGTAGCCGGCCTGAGAGGGTGTCCGG
CCACACTGGGACTGAGATACGGCC
>Zotu2029
GAACGCTGGCGGCGTGCCTAATACATGCAAGTCGAGCGGACTTGATAAAAGCTTGCTTTTATCAAGTT
AGCGGCGGACGGGTGAGTAACACGTGGGTAACCTGCCTATAAGACTGGGATAACTCCGGGAAACCGGT
GCTAATACCAGATAGTTTATCCTTTCGCATGAAAGGATAAGGAAAGACGGTTTCGGCTGTCACTTATG
GATGGACCCGCGGCGCATTAGCTAGTTGGTGAGGTAATGGCTCACCAAGGCAACGATGCGTAGCCGAC
CTGAGAGGGTGATCGGCCACACTGGGACTGAGACACGGCC
>Zotu2030
GAACGCTGGCGGTATGCTTAACACATGCAAGTCGTACGAAAATCTTTGATTTTAGTGGCGGACGGGTG
AGTAACACGTGAGAATCTGCCCTTAGGAAGAGGATAACAGCTGGAAACGGTTGCTAATACTCTATACG
CCGAGAGGTAAAAGGATCTATCCACCTTAGGAAGAGCTCGCGTCTGATTAGCTTGTTGGTAGGGTAAT
GGCCTACCAAGGCGACGATCAGTAGCTGGTTTGAGAGGACGACCAGCCACACTGGGACTGAGACACGG
CC
>Zotu2031
TAACGCTGGCGGCATGCCTAATACATGCAAGTCGAACGAAAGTAGCAATACTTTAGTGGCGAACGGGT

GAGTAACACGTATCTAACCTACCATTTAGTGGGGAATAACTGTTGGAAACGGCAGCTAATACCGCATA
TGTTATTTATTTGCAATGATAAATAATGAAAGGAGCATTTGCTTCGCTAATTGATGGGGATGCGGCGC
ATTAACTAGTTGGTAAGGTAATGGCTTACCAAGGTAACGATGCGTAGCCGATCTGAGAGGATGAACGG
CCACATTGGGACTGAGACACGGCC
>Zotu2032
GAACGCTGGCGGCATGCTTAACACATGCAAGTCGAACGAGATAAAACTCATGTTAAAGTGGGTTTTAT
CTAGTGGCGGACGGGTGAGTAACGCGTAAGAACCTACCCTTAGGAGGGGGACAACAATTGGAAACGGT
TGCTAATACCCCATATGCTGAGGAGTAAAACGGTAACAACCGCCTAGGGATGGGCTTGCGTCTGATTA
GCTTGTTGGTGGGGTAAATGCTTACCAAGGCAACGATCAGTAGTTGGTCTGAGAGGATGACCAGCCAC
ACTGGGACTGAGACACGGCC
>Zotu2034
GAACGCTGGCGGCGTGCTTAACACATGCAAGTCGAACGATGATCCAAAGCTTGCTTCGGGGATTAGTG
GCGAACGGGTGAGTAACACGTGAGTAACCTGCCCTTGACTCTGGGATAAGCGCTGGAAACGGCGTCTA
ATACCGGATACGACCTACCCCGGCATCGGGTGTGGGTGGAAAGTTTTTCGGTCAAGGATGGACTCGCG
GCCTATCAGCTTGTTGGTGAGGTAATGGCTCACCAAGGCGACGACGGGTAGCCGGCCTGAGAGGGTGA
CCGGCCACACTGGGACTGAGACACGGCC
>Zotu2035
GAACGCTGGTAACGTGGATACCGGCATGCAAGTCACGGGGGCCGCAAGGCAACCGGCGAACGGCTTAG
TAACACGTAGATACATACCGTAAAGAGGGGCATAGCTCGTTGAAAAACGGGGTAATTTCCCATAGTCC
CGCAAGGGTAAAGCAGCAATGCGCTTTATGAATGGTCTGCGGTCTATCAGCTTGTTGGTGAGGTAAAG
GCTTACCAAGGCTATGACGGATAGGGGGCGTGAGAGCGCGACCCCCAAGAATGGCACTGCGACACGGG
C
>Zotu2036
GAACGCTGGCGGCGTGCCTAATACATGCAAGTCGAACGCGAGCGATTGGAGCTTGCTCTAATCAATCG
AGTGGCGAACGGGTGAGTAACACGTGGGCAACCTGCCCATCAGCCTGGGATAACCACGGGAAACCGTG
GCTAATACCGGATAGACCGAGTGACAGAAGTCGCAACGGAGAAAGTCGGTTTTACTGACACTGATGGA
TGGGCCTGCGGCGCATTAGCTAGTTGGAGAGGTAGTGGCTCCCCAAGGCAACGATGCGTAGCCGACCT
GAGAGGGTGAACGGCCACACTGGAACTGAGACACGGTC
>Zotu2037
GAACGCTGGCGGCATGCTTTACACATGCAAGTCGAACGGTAACAGGGAGCTTGCTCCGCTGACGAGTG
GCGAACGGGTGAGTAATATATCGGAACGTGCCCAAGAGTGGGGGATAACGTAGCGAAAGTTACGCTAA
TACCGCATACGATCTAAGGATGAAAGCAGGGGACCTTCGGGCCTTGTGCTCCTGGAGCGGCCGATATC
TGATTAGCTAGTTGGTGAGGTAAAGGCTCACCAAGGCGACGATCAGTAGCTGGTCTGAGAGGACGACC
AGCCACACTGGAACTGAGACACGGTC
>Zotu2039
GAACGCTGGCGGCGTGCCTAATACATGCAAGTCGAGCGAACAGACGAGGAGCTTACTCCTCTGACGTT
AGCGGCGGACGGGTGAGTAACACGTGGATAACCTACCTATAAGACTGGGATAACTTCGGGAAACCGGA
GCTAATACCAGATAACATGTTGAACCGCATGGTTCAACAGTGAAAGACGGTCTTGCTGTCACTTATAG
ATGGATCCGCGTCGCATTAGCTAGTTGGTAAGGTAACGGCTTACCAAGGCAACGATGCGTAGCCGACC
TGAGAGGGTGATCGGCCACACTGGAACTGAGACACGGTC
>Zotu2040
GAACGCTGGCGGTATGCCTAACACATGCAAGTCGAACGGAACCTTCGGGTTTAGTGGCGGACGGGTGA
GTAACGCGTGAGAATCTGCCTCTAAGATGGGGACAACAGTCGGAAACGACTGCTAATACCCGATATGG
CGAAAGCTGAAAGATTTATCGCTTAGAGATGAGCTCGCGTTGGATTAGCTAGTTGGTAGTGTAATGGA
CTACCAAGGCGACGATCCATAGCTGGTCTGAGAGGATGAGCAGCCACACTGGGACTGAGACACGGCC
>Zotu2041
GAACGCTAGCGGCAGGCTTAACACATGCAAGTCGAGGGGTAACAGTCCAGCAATGGAGCTGACGACCG
GCAAACGGGTGCGGAACACGTACGCAACATACCTCTATCTGGGGCATAGCCTTCTGAAAAGGAGATTA
ACACCCCATAGTATCTTTGGTTGGCATCAATTGGAGATTAAAAATTCATTGGATAGAGATTGGCGTGC
GTCCTATTAGCTAGTTGGCGGGGTAAAGGCCCACCAAGGCGACGATAGGTAGCTGATGTGAGAGCATG
ATCAGCCACACGGGCACTGAGACACGGGC
>Zotu2042
AAACGTTGGCGGCGTGCTTAACACATGCAAGTCGAGCGAGAAAAGTTTTCGGACTTAGTACAGCGGCG
AACGGGTGAGTAACGCGTGAGAATCTGCCTTTTAGTGGAGAATACACTCGAGAAATCGAGATCAATAC

TGCATAAGTCCGGTAGCGGAGAAAGATGGCTTTAATGCTGTCGCTAGAAGATGAGCTTGCGTACTATT
AGCTAGTTGGTGGGGTAAAAGCCTACCAAGGCGATGATGGTTAGCCGGCCTGAGAGGGTGTGCGGCCA
CACTGGAACTTAGACACGGTC
>Zotu2043
TAACGCTGGCGGCGTGCATAACACATGCAAGTCGAACGATGAAACTTTTTTGATTTCTTCGGAATGAA
AAAAAGTGGATTAGTGGCGGACGGGTGAGTAACGCGTGAGTAACCTGCCTTAGAGAAGGGGATAGCCT
TTGGAAACGAAGAATAATACCCTATGAAACCATAACCTCGCATGAGGCCATGGTCAAAGATTTATCGC
TCTAAGATGGACTTGCGTCTGATTAGTTAGTTGGTGGGGTAACGGCCCACCAAGACAGCGATCAGTAG
CCGGCTTGAGAGAGTGTACGGCCACATTGGGACTGAGACACGGCC
>Zotu2044
GAACGCTAGCGGCAGGCTTAACACATGCAAGTCGAGGGGCAGCGGGGGTAGCAATACCTGCCGGCGAC
CGGCAAACGGGTGCGGAACACGTACGCAACATACCTCTTACTGGGGCATAGCCTTCTGAAAAGGAGAT
TAACACCCCATAGTATTATTAGCTGGCATCAGTTTGATAATTAAAAATTTATTGGTAAGAGATTGGCG
TGCGTCCCATTAGCTAGTTGGTAGGGTAACGGCCTACCAAGGCGACGATGGGTAGCTGGTGTGAGAGC
ACGACCAGCCACACGGGCACTGAGACACGGGC
>Zotu2045
GAACGCTGGCGGCATGCTTTACACATGCAAGTCGAACGGCAGCGCGGGGCAACCTGGCGGCGAGTGGC
GAACGGGTGAGTAATACATCGGAACGTACCCAGAAGTGGGGGATAACGTAGCGAAAGTTACGCTAATA
CCGCATACGTTCTACGGAAGAAAGTGGGGGACCTTCGGGCCTCATGCTTTTGGAGCGGCCGATGTCTG
ATTAGCTAGTTGGTGAGGTAAAGGCTCACCAAGGCGACGATCAGTAGCTGGTCTGAGAGGACGACCAG
CCACACTGGGACTGAGACACGGCC
>Zotu2046
GAACGCTGGCGGCGTGCCTAATACATGCAAGTCGAGCGAATCTTTAAAAGCTTGCTTTTGAAGATTAG
CGGCGGACGGGTGAGTAACACGTGGGCAACCTGCCTGTAAGACTGGGATAACTTCGGGAAACCGGAGC
TAATACCGGATGATCTTTTCTCTCGCATGAGGGAATTCTGAAAGACGGCGCAAGCTGTCACTTACAGA
TGGGCCCGCGGCGCATTAGCTAGTTGGTGAGGTAACGGCTCACCAAGGCGACGATGCGTAGCCGACCT
GAGAGGGTGATCGGCCACACTGGGACTGAGACACGGCC
>Zotu2047
GAACGCTGGCGGCGTGCTTAACACATGCAAGTCGAACGATGAACCAGGTGCTTGCACTTGGGGATTAG
TGGCGAACGGGTGAGTAACACGTGAGCAACCTGCCCCTCACTCTGGGATAAGCGCTGGAAACGGCGTC
TAATACTGGATACGAGTCGCAACCGCATGGTTAGTGACTGGAAAGATTTTTTGGTGGGGGATGGGCTC
GCGGCCTATCAGCTTGTTGGTGAGGTAATGGCTCACCAAGGCGTCGACGGGTAGCCGGCCTGAGAGGG
TGACCGGCCACACTGGGACTGAGACACGGCC
>Zotu2048
GAACGCTGGCGGCGTGCCTAATACATGCAAGTCGAGCGGACCACAAAGAAGCTTGCTTCTGAGTGGTT
AGCGGCGGACGGGTGAGTAACACGTGGGCAACCTGCCCGTAAGACCGGGATAACTTCGGGAAACCGGA
GCTAATACCGGATAACACCGAAGATCGCATGATCTTTGGTTGAAAGGCGGCTTTGGCTGTCACTTACG
GATGGGCCCGCGGCGCATTAGCTAGTTGGTGAGGTAACGGCTCACCAAGGCGACGATGCGTAGCCGAC
CTGAGAGGGTGATCGGCCACACTGGGACTGAGACACGGCC
>Zotu2049
GAACGCTGGCGGCAGGCCTAACACATGCAAGTCGAACGGTAGCACAGAGAGCTTGCTCTCGGGTGACG
AGTGGCGGACGGGTGAGTAATGTCTGGGAAACTGCCTGATGGAGGGGGATAACTACTGGAAACGGTAG
CTAATACCGCATAACGTCGCAAGACCAAAGAGGGGGACCTTCGGGCCTCTTGCCATCGGATGTGCCCA
GATGGGATTAGCTAGTAGGTGGGGTAACGGCTCACCTAGGCGACGATCCCTAGCTGGTCTGAGAGGAT
GACCAGCCACACTGGAACTGAGACACGGTC
>Zotu2050
GAACGCTGGCGGCGTGCCTAACACATGCAAGTCGAGCGATTCTCTTCGGAGAAGAGCGGCGGACGGGT
GAGTAACGCGTGGGTAACCTGCCCTGTACACACGGATAACATACCGAAAGGTATGCTAATACGGGATA
ACATATTTTGATGGCATCATCGAAATATCAAAGCTCCGGCGGTACAGGATGGACCCGCGTCTGATTAG
CTAGTTGGTGAGGTAATGGCTCACCAAGGCAACGATCAGTAGCCGACCTGAGAGGGTGATCGGCCACA
TTGGAACTGAGACACGGTC
>Zotu2051
GAACGCTGGCGGCGTGCCTTAAGCATGCAAGTCGGACGAAGATATGAGATGTCGCAAGACAGATTGTT
GATTAGTGGCGGACGGGTGAGTATAAGCAGGGAACGTACCTTGAACTATCGGATAGCTCGTCGAAAGA

CGGGGTAATACGGTATGTGGACGCAAGTCTAAAGACCGCAAGGTCGGTTCTAGAGCGGCCCTGTTCCC
ATCAGCTTGTTGGTAGGGTAACGGCCTACCAAGGCTACGACGGGTAGGGGGTGTGAGAGCACGACCCC
CCACAATGGCACTGAGACACGGGC
>Zotu2052
GAACGCTGGCGGCGTGCTTAACACATGCAAGTCGAACGCTGAAACCAGATGCTTGCATTTGGTGGATG
AGTGGCGAACGGGTGAGTAACACGTGGGTGATCTGCCCTACACTTTGGGATAAGCCTGGGAAACTGGG
TCTAATACCGAATATTCACACCACCGTAGGGGTGGTGTGGAAAGCTTTATGCGGTGTGGGATGAGCCT
GCGGCCTATCAGCTTGTTGGTGGGGTAATGGCCTACCAAGGCGTCGACGGGTAGCCGGCCTGAGAGGG
TGTACGGCCACATTGGGACTGAGACACGGCC
>Zotu2053
GAACGCTAGCGATAGGCTTAACACATGCAAGTCGAGGGGCAGCATGGTCTTAGCTTGCTAAGACTGAT
GGCGACCGGCGCACGGGTGCGTAACGCGTATGCAACTTGCCTCACAGAGGGGGATAACCCGTCGAAAG
ACGGACTAATACCGCGTACACTCCATCTTGGGCATCCAAGGTAGAGGAAATGAAATTCGCTGTGAGAT
AGGCATGCGTCCCATTAGCTAGTTGGTGAGGTAACGGCTCACCAAGGCGACGATGGGTAGGGGAACTG
AGAGGTTGAACCCCCACACTGGTACTGAGACACGGAC
>Zotu2054
GAACGCTGGCGGCGTGCCTAATACATGCAAGTCGAGCGGGCCGAATGGAAGCTTGCTTTCATTCGGTT
AGCGGCGGACGGGTGAGTAACACGTGGGTAACCTGCCCGTAAGACCGGGATAACTCCGGGAAACCGGG
GCTAATACCGGATAACACCGAAGACCGCATGGTCTTCGGTTGAAAGGTGGCTTCGGCTACCACTTACG
GATGGGCCCGCGGCGCATTAGCTAGTTGGTGAGGTAACGGCTCACCAAGGCGACGATGCGTAGCCGGC
CTGAGAGGGTGACCGGCCACACTGGGACTGAGACACGGCC
>Zotu2055
GAACGCTGGCGGCGTGCCTTAAGCATGCAAGTAGAACGAGTTCTATCTTGCCGCAAGGCAAGCTAAGA
ACTAGTTGCGAACGGCTGAGTAATGAATAGGAATCTTTCCTTTGGTGGAGAATACCTCATCGAAAGAT
GAGCTAATACTGCATAACATCTACGGATAAAAGCGAGTAACTTCGCGCCGATTGAGGAGCCTATTCCC
CATCAGGCAGTTGGTAGGGTAATGGCCTACCAAACCTACGACGGGTACCCGATCTGAGAGGATGGACG
GGCAGAATGGGACTGAGACACGGCC
>Zotu2057
TCTTTTGGTGGGGTCTCGTCTGTGTTTGAGTCACCTCTGGGGTGTGTGGAGTTTGGGGTTGGGAAAGG
CCAGTTCTCCCTCTTCCTCTGTCTACTCCC
>Zotu2058
GAACGCTGGCGGCGTGCTTAACACATGCAAGTCGAACGATGAAAGAGGAGCTTGCTCCTCTGGATTAG
TGGCGAACGGGTGAGTAACACGTGAGCAATCTGCCCCTGACTCCGGAATAACTACGGGAAACTGTAGC
TAATACCGGATACGCGCCGAACAGGCATCTGTATAGGTGGGAAAGAATTTCGGTCAGGGATGAGCTCG
CGGCCTATCAGCTTGTTGGTGAGGTAACGGCTCACCAAGGCGACGACGGGTAGCCGGCCTGAGAGGGT
GACCGGCCACATTGGGACTGAGACACGGCC
>Zotu2060
GAACGCTGGCGGCGTGCCTAATACATGCAAGTCGAGCGGTAAGGCCCCTTCGGGGGTACACGAGCGGC
GGACGGCTGAGTAACACGTAGGAACGTACCCCAAAGTGAGGCATAAGCACCAGAAATGGTGTCTAATA
CCGCATGTGATCTTCGGATTAAAGCTTCGGCGCTTTGGGAACGGCCTGCGCATGATTAGCTTGTTGGT
GAGGTAATAGCTCACCAAGGCGACGATCATTAGCTGGTCTGAGAGGATGACCAGCCAGACTGGGACTG
AGAACGGCC
>Zotu2061
GAACGCTGGCGGCATGCCTTACACATGCAAGTCGAACGGCAGCGCGGACTTCGGTCTGGCGGCGAGTG
GCGAACGGGTGAGTAATACATCGGAACGTACCCTGTTGTGGGGGATAACTAGTCGAAAGATTAGCTAA
TACCGCATACGACCTGAGGGTGAAAGTGGGGGACCGCAAGGCCTCACGCAGCAGGAGCGGCCGATGTC
TGATTAGCTAGTTGGTGGGGTAAGAGCCTACCAAGGCGACGATCAGTAGCTGGTCTGAGAGGACGATC
AGCCACACTGGGACTGAGACACGGCC
>Zotu2062
GAACGCTGGCGGCATGCCTAACACATGCAAGTCGAACGAAGGCTTCGGCCTTAGTGGCGCACGGGTGA
GTAACGCGTGGGAATCTGCCTATCAGTGGGGAATAACGACGGGAAACTGTCGCTAATACCGCATACGC
CCTTAGGGGGAAAGATTTATCGCTGATAGATGAGCCCGCGTACGATTAGCTAGTTGGTGAGGTAATGG
CTCACCAAGGCTACGATCGTTAGCTGGTCTGAGAGGATGATCAGCCACACTGGGACTGAGACACGGCC
>Zotu2063

GAACGCTGGCGGCAGGCCTAATACATGCAAGTCGAGCGGATGTAGCAATACATCAGCGGCGCACGGGA
GAATAACGCGTGGGAACCTTCCTTTTGGTTCGGAATAACACCGGGAAACTGGTGCTAATACCGAATAA
GCCCTTCGGGGGAAAGATTTATTGCCGAAAGATGGGCCCGCGTCTGATTAGCTAGTTGGTGAGGTAAC
GGCTCACCAAGGCTACGATCAGTAGCTGATCTGAGAGGATGATCAGCCTCACTGGGACTGAGACACGG
CC
>Zotu2064
GAACGCTGGCGGCGTGCCTAATGCATGCAAGTCGAGCGACGAATAGATGCGGGTAACCAAGTCTATTA
CGGAGCGGCGAACGGGTGAGTAACACGTGAGCAACCTGCCCCGAAGACCGGGATAGCCCAGGGAAACT
TGGATTAATACCGGATATCCTCTTTTGATCGCATGGTCGTTGGAGGAAATGCTTTTACGCTTCGGGAT
GGGCTCGCGGCCTATCAGCTTGTTGGTGGGGTAACGGCCTACCAAGGCGTCGACGGGTAGCTGGTCTG
AGAGGACGATCAGCCACACTGGGACTGAGACACGGCC
>Zotu2066
GCGATCCTGGAGACCCGGGATCGAATCCCACGTCGGGCTCCCGGTGCATGGAGCCTGCTGCTCCCTCT
GCCTGTGTCTCTGCCTCTCTCTGTGTGTCTTCCATGAATAAATGAAATATTTTTTTTAAATAAATAA
AGTTCCCAGGCCTTCACA
>Zotu2068
GAACGCTGGCGGCGTGCCTAACACATGCAAGTCGAGCGAACCCTTCGGGGTGAGCGGCGGACGGGTGA
GTAACGCGTGGGTAACCTGCCCTGTACACATGGATAACATACCGAAAGGTATGCTAATACGAGATAAT
ATGTAATGATCGCATGGTCGATACATCAAAGCTCCGGCGGTACAGGATGGACCCGCGTCTGATTAGCT
AGTTGGTAAGGTAACGGCTTACCAAGGCGACGATCAGTAGCCGACCTGAGAGGGTGATCGGCCACATT
GGAACTGAGACACGGTC
>Zotu2069
GAACGCTGGCGGCGTGCTTAACACATGCAAGTCGAGCGGTAAGGCCCTTCGGGGTACACGAGCGGCGA
ACGGGTGAGTAACACGTGGGTGATCTGCCCTGTACTTCGGGATAAGCCTGGGAAACTGGGTCTAATAC
CGGATATGACCTTACATCGCATGGTGTTTGGTGGAAAGATTTATCGGTACAGGATGGGCCCGCGGCCT
ATCAGCTTGTTGGTGGGGTAATGGCCTACCAAGGCGACGACGGGTAGCCGGCCTGAGAGGGCGACCGG
CCACACTGGGACTGAGACACGGCC
>Zotu2070
GAACGCTGGCGGCGTGCTTAACACATGCAAGTCGAACGATGATCCGGACCTTGCTTCGGGGATTAGTG
GCGAACGGGTGAGTAACACGTGAGTAACCTGCCCTTGACTCTGGGATAAGCGCTGGAAACGGCGTCTA
ATACCGGATACGACCTACCCCGGCATCGGGTGTGGGTGGAAAGTTTTTCGGTCAAGGATGGACTCGCG
GCCTATCAGCTTGTTGGTGAGGTAATGGCTCACCAAGGCGACGACGGGTAGCCGGCCTGAGAGGGTGA
CCGGCCACACTGGGACTGAGACACGGCC
>Zotu2071
GAACGCTGGCGGCAGGCTTAACACATGCAAGTCGAGCGGGGAAAGGTAGCTTGCTACCTGACCTAGCG
GCGGACGGGTGAGTAATGCTTAGGAATCTGCCTATTAGTGGGGGACAACATTCCGAAAGGAATGCTAA
TACCGCATACGCCCTACGGGGGAAAGCAGGGGATCTTCGGACCTTGCGCTAATAGATGAGCCTAAGTC
AGATTAGCTAGTTGGTGGGGTAAAGGCCTACCAAGGCGACGATCTGTAGCGGGTCTGAGAGGATGATC
CGCCACACTGGGACTGAGACACGGCC
>Zotu2072
TAACGCTGGCGGCGTGCATAACACATGCAAGTCGAACGATGAAGCTTAGATGATTTCTTCGGAATGAC
CTTAAGTGGATTAGTGGCGAACGGGTGAGTAACGCGTGAGTAACCTGCCTTACACAAGGGGATAGCCT
CTGGAAACGGAGAATAATACCCTATGACATTACAACCTCGCATGAGGAAGTAATCAAAGTGTTTTAGC
GGTGTAAGATGGACTTGCGTCTGATTAGCTAGTTGGTGAGATAACAGCCCACCAAGGCAACGATCAGT
AGCCGGCTTGAGAGAGTGTACGGCCACATTGGGACTGAGACACGGCC
>Zotu2073
GAACGCTGGCGGCAGGCTTAACACATGCAAGTCGAGCGGAGTGATGGTGCTTGCACTATCACTTAGCG
GCGGACGGGTGAGTAATGCTTAGGAATCTGCCATTTAGTGGGGGACAACATTCCGAAAGGAATGCTAA
TACCGCATACGTCCTACGGGAGAAAGCAGGGGATCTTCGGACCTTGCGCTAAATGATGAGCCTAAGTC
GGATTAGCTAGTTGGTGGGGTAAAGGCCTACCAAGGCGACGATCTGTAGCGGGTCTGAGAGGATGATC
CGCCACACTGGGACTGAGACACGGCC
>Zotu2074
GAACGCTAGCGGCAGGCTTAACACATGCAAGTCGAGGGGTAGAGTAGCAATACTTGAGACCGGCGCAC
GGGTGCGTAACGCGTATGCAACCTACCTTTTACAGGGGAATAGCCCAGAGAAATTTGGATTAATGCCC

CATGGTATATTTTAGTGGCATCACTAGATTATTAAAGTTCCAACGGTAAAAGATGGGCATGCGTCCTA
TTAGCTAGATGGTAAGGTAACGGCTTACCATGGCTACGATAGGTAGGGGTCCTGAGAGGGAGATCCCC
CACACTGGTACTGAGACACGGAC
>Zotu2076
GAACGCTAGCGGCAGGCCTAATACATGCAAGTCGGACGGGATCCATCGGAGAGCTTGCTCGAAGATGG
TGAGAGTGGCGCACGGGTGCGTAACGCGTGAGCAACCTACCTCTATCAGGGGGATAGCCTCTCGAAAG
AGAGATTAACACCGCATAACATCAACAGTTCGCATGTTCGGTTGATTAAATATTTATAGGATAGAGAT
GGGCTCGCGTGACATTAGCTAGTTGGTAGGGTAACGGCCTACCAAGGCGACGATGTCTAGGGGCTCTG
AGAGGAGAATCCCCCACACTGGTACTGAGACACGGAC
>Zotu2077
GAACGCTGGCGGCGTGCTTAACACATGCAAGTCGAACGATGAAGCCCTTCGGGGTGGATTAGTGGCGA
ACGGGTGAGTAACACGTGGGCAATCTGCCCTTCACTCTGGGACAAGCCCTGGAAACGGGGTCTAATAC
CGGATACAACCATCGGCCGCATGGTCTGGTGGTGGAAAGCTCCGGCGGTGAAGGATGAGCCCGCGGCC
TATCAGCTTGTTGGTGAGGTAGTGGCTCACCAAGGCGACGACGGGTAGCCGGCCTGAGAGGGCGACCG
GCCACACTGGGACTGAGACACGGCC
>Zotu2078
GAACGCTAGCGGCAGGCTTAACACATGCAAGTCGAGGGGTATAATTCTTCGGAATTAGAGACCGGCGC
ACGGGTGCGTAACGCGTATGCAATCTACCTTTTGCAGAGGGATAGCCCAGAGAAATTTGGATTAATAC
CTCATAGTATAGTACCTTGGCATCAAGGAACTATTAAAGTCACAACGGCAAAAGATGAGCATGCGTCC
CATTAGCTTGTTGGTAAGGTAACGGCTTACCAAGGCAACGATGGGTAGGGGTCCTGAGAGGGAGATCC
CCCACACTGGTACTGAGACACGGAC
>Zotu2079
GAACGCTAGCGGCAGGCCTAATACATGCAAGTCGGACGGGATTGGAGAGCTTGCTTTCCATGAGAGTG
GCGCACGGGTGCGTAACACGTATGTAACCTACCTTAATCTGGGGGATAGCCTCTCGAAAGAGAGATTA
ACACCGCATAACATGTTTGAATAGCATTATTTAAACATCAAATATTTATAGGATTAAGATGGGCATGC
GGAACATTAGCTAGTTGGTAAGGTAATGGCTTACCAAGGCTACGATGTTTAGGGGATCTGAGAGGATG
ACCCCCCACACTGGTACTGAGACACGGAC
>Zotu2081
GAACGCTAGCGATAGGCTTAACACATGCAAGTCGAGGGGCAGCATGGTCTTAGCTTGCTAAGACTGAT
GGCGACCGGCGCACGGGTGCGTAACGCGTATGCAACTTGCCTCACAGAGGGGGATAACCCGTCGAAAG
ACGGACTAATACCGCGTACACTCTATGTTGGGCATCCAATGTAGAGGAAATGAAATTCGCTGTGAGAT
AGGCATGCGTCCCATTAGCTAGTTGGTGAGGTAACGGCTCACCAAGGCGACGATGGGTAGGGGAACTG
AGAGGTTGAACCCCCACACTGGTACTGAGACACGGAC
>Zotu2082
GAACGCTGGCGGCGCGCTTAACACATGCAAGTCGAGCGAGAACCAAGGCTTCGGTCTTGGGGACAGCG
GCGAACGGGTGAGTAACACGTGGGTAATCAACCCTCGACACTGGGATAGCCCGGGGAAACCCGGATTA
ATACCGGATACGCCATCTGGCCTTCGGGCCGGCTGGAAAAGGTAGCTTTGGCCTCCGGTCGAGGACGA
GCCCGCGGCGGATTAGCTTGTTGGCGGGGTAAAAGCCCACCAAGGCGACGATCCGTAGCTGGTCTGAG
AGGACGATCAGCCACACTGGGACTGAGACACGGCC
>Zotu2083
GAACGCTGGCGGCATGCCTAACACATGCAAGTCGAACGAGGTTTCCGCTTCGGTGGGACCCTAGTGGC
GCACGGGTGAGTAACGCGTGGGAACCTGCCCTGTGGTACGGAATAACTCCGGGAAACTGGAGCTAATA
CCGTATGCGTCCTTTGGGACAAAGATTTATCGCCATGGGATGGGCCCGCGTAGGATTAGCTTGTTGGT
GGGGTAACGGCCTACCAAGGCAACGATCCTTAGCTGGTCTGAGAGGATGATCAGCCACACTGGGACTG
AGACACGGCC
>Zotu2084
GAACGCTGGCGGCGTGCTTAACACATGCAAGTCGAACGGTAAGGCCCTTCGGGGTACACGAGTGGCGA
ACGGGTGAGTAACACGTGGGTGATCTGCCCTGCACTTTGGGATAAGCCTGGGAAACTGGGTCTAATAC
CGAATAGGATCATGGCCTTCATGGGTTGTGGTGGAAAGCTTTTGCGGTGTGGGATGGGCCCGCGGCCT
ATCAGCTTGTTGGTGGGGTAATGGCCTACCAAGGCGACGACGGGTAGCCGGCCTGAGAGGGTGTCCGG
CCACACTGGGACTGAGATACGGCC
>Zotu2087
GAACGCCGGCGGTGTGCCTAATACATGCAAGTCGTACGCACTGGCCCAACTGATTGATGGTGCTTGCA
CCTGATTGACGATGGATCACCAGTGAGTGGCGGACGGGTGAGTAACACGTAGGTAACCTGCCCCGGAG

CGGGGGATAACATTTGGAAACAGATGCTAATACCGCATAACAACAAAAGCCGCATGGTTTTTGTTTGA
AAGATGGCTTTGGCTATCACTCTGGGATGGACCTGCGGTGCATTAGCTAGTTGGTAAGGTAACGGCTT
ACCAAGGCGATGATGCATAGCCGAGTTGAGAGACTGATCGGCCACAATGGAACTGAGACACGGTC
>Zotu2089
GAACGCTGGCGGTGTGCCTAATACATGCAAGTCGAACGAGATCTTCGGATCTAGTGGCGGACGGGTGA
GTAACACGTTGGTACCTACCCCTAAGTGGGGAATAACTGCCCGAAAGGGTGGCTAATACCGCATAATC
CCGCAAGGGTAAAGCAGTAATGCGCTTAGGGAGGGGCCTGCGTCTGATTAGCTAGTTGGTGAGGTAAT
TGCTCACCAAGGCGACGATCAGTAGCTGGATCGAGAGGTTGACCAGCCACACTGGGACTGAGATACGG
CC
>Zotu2090
GAACGCTAGCGGCAGGCTTAATACATGCAAGTCGAACGGTCTTAGCAATAAGACGAGTGGCGCACGGG
TGAGTAACGCGTACATAACATACCTTTAACTGGCGAATAGCCTTGGGAAACTGAGAGTAATACGCCAT
AACATTGTGAAACGGCATCGTTTTACAATTAAAGTTCCGGCGGTTAGAGATTGATGTGCGTTGGATTA
GGTAGTTGGCGAGGTAACGGCTCACCAAGCCGACGATCCATAGGGGGCGTGAGAGCGTGGTCCCCCAC
ACGGGTACTGAGACACGGAC
>Zotu2091
GAACGCTGGCGGCGTGCCTAATACATGCAAGTAGAACGCTGAAGCTTGGTGCTTGCACCGAGCGGATG
AGTTGCGAACGGGTGAGTAACGCGTAGGTAACCTGCCTCTTAGCGGGGGATAACTATTGGAAACGATA
GCTAATACCGCATAAAAGTCGATATCGCATGATGTTGACTTGAAAGGTGCAAATGCATCACTAAGAGA
TGGACCTGCGTTGTATTAGCTAGTTGGTGAGGTAACGGCTCACCAAGGCGACGATACATAGCCGACCT
GAGAGGGTGATCGGCCACACTGGGACTGAGACACGGCC
>Zotu2092
GAACGCTGGCGGCGTGCCTAAGACATGCAAGTCGAGTGGGTTTAGACCCACGGCAAACGGGTGAGTAA
TATCTAGGAACGTACCTCTAGTTGAGCATAACCTACCGAAAGGTAGAGCAATTCTCAATGTGACCGCA
AGGTTAAAGTCGCAAGACGCTAGAGAAGCGGCCTGGACACCATCAGCTTGTTGGTAAGGTAAAAGCTT
ACCAAGGCTATGACGGTTAGGGGCCCCGAGAGGGGAAACCCCCACACTGGAACTGAGACACGGTC
>Zotu2093
GAACGCTGGCGGCATGGATAGGGCATGCAAGTCGAGTGGGTTTAGACCCACGGCAAACGGGTTAGTAA
TGCGTAGGAATGTTCCTCAAAGTCGTGAATAATTTGTCGAAAGACAAGCTAATACACGATGTGCTCCA
CGGAGTAAAGGCTTGCCGCTTTGAGAGCAGCCTGCGTCCTATCAGCTAGTTGGTGGGGTAAAAGCCTA
CCAAGGCTTTGACGGGTAGGGGGTGTGAGAGCATGGCCCCCAACATTGGGACTGAGACACTGCC
>Zotu2094
GAACGCTAGCGACAGGCCTAACACATGCAAGTCGAGGGGCAGCACATGAGTAGCAATACGATGGTGGC
GACCGGCGCACGGGTGAGTAACACGTATGCAACCTACCTGTAAATGGGGAATAACCCGGAGAAATTCG
GACTAACACCGCATAACACTGTTTTTTCGCATGAAAGGACAGTTAAATATTTATAGTTTACAGATGGG
CATGCGTATGATTAGATAGTTGGTAGGGTAACGGCCTACCAAGTCTACGATCATTAGGGGTTCTGAGA
GGAAGGTCCCCCACACTGGTACTGAGACACGGAC
>Zotu2095
GAACGCTAGCGGGAGGCCTAACACATGCAAGCTGAGCGGTAGAGTTCCTTCGGGGACTTGAGAGCGGC
GTACGGGTGCGGAACACGTGTGCAACCTGCCTTTATCAGGGGGATAGCCTTTCGAAAGGAAGATTAAT
ACCCCATAATATAATTGATGGCATCATTGATTATTGAAAACTCCGGTGGATAAAGATGGGAACGCGCA
AGATTAGATAGTTGGTGAGGTAACGGCTCACCAAGTCGATGATCTTTAGGGGGCCTGAGAGGGTGATC
CCCCACACTGGTACTGAGACACGGAC
>Zotu2097
GAACGCTGGCGGCGTGCTTAACACATGCAAGTCGAACGGTGAAGGGGAGCTTGCTTCCTGGATCAGTG
GCGAACGGGTGAGTAACACGTGAGCAATCTGCCCCTGACTCTGGGATAAGCGCTGGAAACGGCGTCTA
ATACTGGATACGAGCTGCGATCGCATGGTCAGCAGTTGGAAAGAATTTCGGTCAGGGATGAGCTCGCG
GCCTATCAGCTTGTTGGTGAGGTAATGGCTCACCAAGGCGTCGACGGGTAGCCGGCCTGAGAGGGTGA
CCGGCCACACTGGGACTGAGACACGGCC
>Zotu2098
GAACGCTGGCGGCGTGGATAAGGCATGCAAGTCGCGTGGGTTTAGACCCACGGCAAACGGGTTAGTAA
TGCATAGGAACATTCCCAGAAGTCGTGAATAATCCGGAGAAATCCGGGACAATACACGATGTGCTCTA
CGGAGTAAAGATTTATCGCTTTTGGAGTGGCCTGTGTCCTATCAGCTAGTTGGTGAGGTAATGGCTCA
CCAAGGCTATGACGGGTAGGGGGCGTGAGAGCGTGGCCCCCAACATTGGGACTGAGACACTGCC

>Zotu2099
GAACGCTGGCGGCGTGCTTAACACATGCAAGTCGAACGGTGAAGCTCCAGCTTGCTGGAGTGGATCAG
TGGCGAACGGGTGAGTAACACGTGAGCAACCTGCCCCTGACTCTGGGATAACAGCCGGAAACGGCTGC
TAATACCGGATATGCACCATGAACGCATGTTCTGTGGTGGGAAAGATTTTTCGGTTGGGGATGGGCTC
GCGGCCTATCAGCTTGTTGGTGAGGTAATGGCTCACCAAGGCGTCGACGGGTAGCCGGCCTGAGGGGG
TGACCGGCCACACTGGGACTGAGACACGGCC
>Zotu2100
GAACGCTGGCGGCGTGCCTAAGACATGCAAGTCGAGCGGGGGACTGGGGAGCTTGCTCTCCGGTCTC
CTAGCGGCGGACGGGTGAGTAACACGTGGGCAACCTGCCCGGCAGTGGGGGATAACCCTGGGAAACTG
GGGCTAATACCGCATACGGTCCGCGTTCCGCATGGGGCGTGGAAGAAAGGCCGTCGTGAGGCGGTCGC
TGCCGGAGGGGCCCGCGGCCCATCAGCTGGTTGGTGGGGTAACGGCCCACCAAGGCGACGACGGGTAG
CCGGCCTGAGAGGGTGGCCGGCCACACTGGGACTGAGACACGGCC
>Zotu2101
GAACGCTGGCGGTATGCTTAACACATGCAAGTCGAACGGCAGCACGTTGCTTCTTTTCGGAGAAGTAA
TGGTGGCGAGTGGCGGACGGGTGAGTAACGCGTAGGAATCTACCCTAAAGTGGGGGATAACCCGGGGA
AACTCGGGCTAATACCGCATAATCTCTACGGAGCAAAGCTGGGGACCAGAGTTTACTTTGGCCTGGCG
CTATAGGATGAGCCTGCGTCGGATTAGCTAGTTGGTGGGGTAATGGCCTACCAAGGTAACGATCCGTA
GCTGGTCTGAGAGGATGATCAGCCACACTGGGACTGAGACACGGCC
>Zotu2102
GAACGTTGGCGGCGTGGATTAGGCATGCAAGTCGGACGGGCCGCAAGGCCAGTGGCGCAAGGGTGAGT
AAGGCGACGGTAACCAACCCCAGAGACGGGCATAGCCGGGGGAAACCCCGGGTAATTCCCGGCGACGT
GGCGACCCGGCATCGGGGTGCCACCAAAGATTCATCGCTCTGGGACGGGCCGTCGTGGTATTAGGTTG
TTGGCGGGGTGACGGCCCACCAAGCCTGCGATGCCTACCGGGCGTGCGAGCGTGGCCCGGCACACTGG
GACTGAGACACTGCC
>Zotu2103
GAACGCTAGCGGCAGGCCTAATACATGCAAGTCGAACGGAATATAGTTTTCGGATTATATTTAGTGGC
GCACGGGTGAGTAACACGTACATAACTTACCCTCAAGAGGGGGATAGCCTTGGGAAACTGAGCGTAAT
ACCCCATAGTATTATTGAACGGCATCGTTTGATAATTAAAGCTACGGCGCTTGAGGATAGATGTGCGT
CTGATTAGCTAGTTGGTAAGGTAACGGCTTACCAAGGCGACGATCAGTAGGGGGCGTGAGAGCGTGGC
CCCCCACACGGGTACTGAGACACGGAC
>Zotu2104
GAACGCTGGCGGCATGCCTAATACATGCAAGTCGAACGAGAGGAAGGAAAGCTTGCTTTTTTGAATCT
AGTGGCGAACGGGTGAGTAACACGTAGGTAACCTGCCCATGTGCCCGGGATAACTTCTGGAAACGGAT
GCTAAAACCGGATAGGTAGCAGACAAGCATTTGACTGCTATTAAAGTGGCTAAGGCCATGAACATGGA
TGGACCTGCGGTGCATTAGCTAGTTGGTGAGGTAACGGCCCACCAAGGCGACGATGCATAGCCGGCCT
GAGAGGGCGGACGGCCACATTGGGACTGAGACACGGCC
>Zotu2105
GAACGCTAGCGGCATGCTTAACACATGCAAGTCGAACGGACTAATTGGGGCTTGCTCCAATTAGTTAG
TGGCAGACGGGTGAGTAACACGTGGGAATCTACCCATCAGTACGGAATAACTTTTAGAAATAAAAGCT
AATACCGTATATTCTCTACGGAGGAAAGATTTATCGCTAATGGATGAGCCCGCGTCAGATTAGGTAGT
TGGTGAGGTAACGGCTCACCAAGCCGACGATCTGTAGCTGGTCTGAGAGGATGATCAGCCACACTGGG
ACTGAGACACGGCC
>Zotu2107
GAACGCTGGCGGCGCGCCTAAAACATGCAAGTCGAACGAGAAGCCCGGGCTTGCCCGGGTGGACAGTG
GCGAACGGGTGAGTAACACGTGAGGAACCTGCCCCGAAGACCGGGACAACATCGGGAAACCGTTGCTA
ATACCGGATACCTTCACCGGATCGCATGTTCTGGTGAAGAAATGGATTCCGCTTCGGGATGGCCTCGC
GGCCTATCAGCTAGTTGGTGAGGTAACGGCTCACCAAGGCGACGACGGGTAGCTGGTCTGAGAGGATG
TCCAGCCACACTGGGACTGAGACACGGCC
>Zotu2108
GAACGCTGGCGGCGTGCTTAACACATGCAAGTCGAACGGTGAACCTGCTTCGGTGGGGGATCAGTGGC
GAACGGGTGAGTAACACGTGGGCAACCTGCCCCCGGCTCTGGGATAACTCCAAGAAATTGGGGCTAAT
ACCGGATGTTCACGGCTGGCCGCATGGTCGGTGGTGGAAAGATTTATCGGCTGGGGATGGGCCCGCGG
CCTATCAGCTTGTTGGTGGGGTAGTGGCCTACCAAGGCGACGACGGGTAGCCGGCCTGAGAGGGTGAC
CGGCCACACTGGGACTGAGACACGGCC

>Zotu2109
GAACGCTGGCGGCGTGCTTAACACATGCAAGTCGAACGAGACCCTCGCTTCGGTGGGGTGTTCTAGTG
GCGAACGGGTGAGTAACACGTGGGCAACCTGCCCCCGGCTCTGGGATAACTCCAAGAAATTGGGGCTA
ATACCGGATATGACCGCTGACCGCATGGTCTGGTGGTGGAAAGATTTATCGGCTGGGGATGGGCCCGC
GGCCTATCAGCTTGTTGGTGGGGTAGTGGCCTACCAAGGCGACGACGGGTAGCCGGCCTGAGAGGGTG
ACCGGCCACACTGGGACTGAGACACGGCC
>Zotu2111
GAACGCTGGCGGAGTGCTTTAAGCATGCAAGTAGAACGGGTTTTGATAGCAATATTAAGATCAGTTGC
GAACGGCTGAGTAATAAGTAAGAATCTTTCCTTTGGTGGAGAATACCTCGTCGAAAGACGAGCTAATA
CTGCATAAGACCTACGGGTGAAAGCGCATAACTGCGCGCCGACTGAGGAGCTTACTTCCCATCAGGCA
GTTGGTAGGGTAATGGCCTACCAAACCTACGACGGGTACCCGATCTGAGAGGATGGACGGGCAGAATG
GGACTGAGACACGGCC
>Zotu2112
AAACGCTGGCGGCGTGCGTAACACATGCAAGTCGAACGAGAATGTACTGACGGACCCTTAGGGTGAAG
ATAGTACAGGACAGTGGCGGATGGGTGAGTAACGCGTAAGAAACCTGCCTTTCACACCGGGATAGCAG
CTGGAAACGGCTATTAATACCGGATGACACTTTTTCCCCGCATGAGGAAGAGGTTAAAGAATTTCGGT
GAAAGATGGTCTTGCGTCTGATTAGCTAGTTGGTGGGGTAACGGCCTACCAAGGCGACGATCAGTAGC
CGGCCTGAGAGGGTGTACGGCCACATTGGGACTGAGACACGGCC
>Zotu2113
GAACGCTGGCGGCAGGCTTAACACATGCAAGTCGAACGCACCTTCGGGTGAGTGGCAGACGGGTGAGT
AACACGTGGGAACATACCTCTTTGTTCGGAATAACGCATGGAAACGTGCGCTAATACCGGATACGCCC
TTATGGGGAAAGATTTATCGCAGAGAGATTGGCCCGCGTCTGATTAGCTAGTTGGTGAGGTAATGGCT
CACCAAGGCGACGATCAGTAGCTGGTCTGAGAGGATGATCAGCCACACTGGGACTGAGACACGGCC
>Zotu2114
GAACGCTGGCGGCGTGCTTAACACATGCAAGTCGAACGATGACGACCGGGCTTGCCTGGTCTGATTAG
TGGCGAACGGGTGAGTAACACGTGAGTAACCTGCCCCTCACTTCGGGATAACCTCGGGAAATCGAGGC
TAATACCGGATACGAGTTTCCGCCGCATGGTGGTTTCTGGAAAGTTTTTCGGTGAGGGATGGACTCGC
GGCCTATCAGTTTGTTGGTGAGGTAATGGCTCACCAAGGCGATGACGGGTAGCCGGCCTGAGAGGGCG
ACCGGCCACACTGGGACTGAGACACGGCC
>Zotu2115
GAACGCTGGCGGCGTGCTTAACACATGCAAGTCGAACGGAAAGGCCCTGCTTGCAGGGTACTCGAGTG
GCGAACGGGTGAGTAACACGTGGGTGATCTGCCCTGTACTTTGGGATAAGCCTGGGAAACTGGGTCTA
ATACCGAATATGACAATTGTTTGGATGCGATTGTGGAAAGCTTTTGCGGTATGGGATGAGCTCGCGGC
CTATCAGCTTGTTGGTGGGGTAATGGCCTACCAAGGCGTCGACGGGTAGCCGGCCTGAGAGGGTGTAC
GGCCACATTGGGACTGAGATACGGCC
>Zotu2117
GAACGCTGGCGGCGTGCTTAACACATGCAAGTCGAACGGATCACTCTTCGGGGTGGTTAGTGGCGAAC
GGGTGAGTAACACGTGAGCAACCTGCCCTTCACTCTGGGATAACTGTGGGAAACTTCAGCTAATACCG
GATATTACCTGTCACTGCATGGTGGTGGGTGGAAAGATTTTTTGGTGGGGGATGGGCTCGCGGCCTAT
CAGCTTGTTGGTGGGGTGATGGCCTACCAAGGCTTTGACGGGTAGCCGGCCTGAGAGGGCGACCGGCC
ACACTGGGACTGAGATACGGCC
>Zotu2118
GAACGCTGGCGGCGTGCTTAACACATGCAAGTCGAACGGAAAGGCCCCGCTCGCGGGGTACTCGAGTG
GCGAACGGGTGAGTAACACGTGGGTGATCTGCCCTGCACTTCGGGATAAGCCTGGGAAACTGGGTCTA
ATACCGGATAGGACCCTCCTTTAGTGTGGGGGGTGGAAAGTTTTTCGGTGCAGGATGAGCCCGCGGCC
TATCAGCTTGTTGGTGGGGTAATGGCCTACCAAGGCGTCGACGGGTAGCCGGCCTGAGAGGGTGGACG
GCCACATTGGGACTGAGATACGGCC
>Zotu2119
GAACGCTGGCGGCGTGCCTAATACATGCAAGTCGAGCGGACGAACTTTTGGTGCTTGCACCAAATTTT
CGTTAGCGGCGGACGGGTGAGTAACACGTGGGTAACCTGCCCTACAGACTGGGATAACTTCGGGAAAC
CGAGGCTAATACTGGATAATACTTTTTAACACATGTTAGAAAGTTGAAAGATGGTTTTGCTATCACTG
TAGGATGGACCCGCGGCGCATTAGCTAGTTGGTGAGGTAACGGCTCACCAAGGCAACGATGCGTAGCC
GACCTGAGAGGGTGATCGGCCACACTGGGACTGAGACACGGCC
>Zotu2120

AAACGCTGGCGGCGTGCTTTAAGCATGCAAGTCGCACGGATGCTCGCAAGAGTGTGAGTGGCAGACGG
CTGAGGAAGACGGAAGGAACCTGCCCTTCAGAGGGGGATAACTCCGCGAAAGCGGGGCTAAGACCGCG
TACGCTCGCCCGGGGGAGAGCCGGGTGAGGAAAACCGCGAGGTGCTGAAGGAGGGGCTTCCGGCCCAT
CAGGTAGTTGGGGGGGTAACGGCCCACCAAGCCGATGACGGGTCGCTGGTCTAAGAGGATGATCAGCC
AGACTGGGACTGAGACACGGCC
>Zotu2121
GAACGCTGGCGGCGTGCTTAACACATGCAAGTCGAACGATGATCAGGAGCTTGCTCCTGTGATTAGTG
GCGAACGGGTGAGTAACACGTGAGTAACCTGCCCCTGACTCTGGGATAAGCGTTGGAAACGACGTCTA
ATACTGGATATGATCACCGGCCGCATGGTCTGGTGGTGGAAAGATTTTTTGGTTGGGGATGGACTCGC
GGCCTATCAGCTTGTTGGTGAGGTAATGGCTCACCAAGGCGACGACGGGTAGCCGGCCTGAGAGGGTG
ACCGGCCACACTGGGACTGAGACACGGCC
>Zotu2122
GAACGCTGGCGGCGTGCTTAACACATGCAAGTCGAACGGAAAGGTCTCTTCGGAGATACTCGAGTGGC
GAACGGGTGAGTAACACGTGGGTGATCTGCCCTGCACTTCGGGATAAGCTTGGGAAACTGGGTCTAAT
ACCGGATATGATCATGCGCCTCATGGTGTGTGGTGGAAAGCTTTTGCGGTGTGGGATGGGCCCGCGGC
CTATCAGCTTGTTGGTGGGGTAATGGCCTACCAAGGCGACGACGGGTAGCCGGCCTGAGAGGGTGTCC
GGCCACACTGGGACTGAGATACGGCC
>Zotu2123
GAACGCTGGCGGCGTGCTTAACACATGCAAGTCGAACGATGATGCCCAGCTTGCTGGGTGGATTAGTG
GCGAACGGGTGAGTAACACGTGAGCAACCTGCCCCCGACTCTGGGATAAGCCCGGGAAACTGGGTCTA
ATACCGGATACGACCATTGGCCGCATGGCCTGGTGGTGGAAAGACTTGTTGGTTGGGGATGGGCTCGC
GGCCTATCAGCTTGTTGGTGGGGTAATGGCCCACCAAGGCGACGACGGGTAGCCGGCCTGAGAGGGTG
ACCGGCCACACTGGGACTGAGACACGGCC
>Zotu2124
GAACGCTGGCGGCGTGCTTAACACATGCAAGTCGAACGGTAAGGCTCCAGCTTGCTGGGGTACACGAG
TGGCGAACGGGTGAGTAACACGTGGGTGACCTGCCCTGCACTTCGGGATAAGCCTGGGAAACTGGGTC
TAATACCGGATAGGACCGCATCGTAGGTGGTGTGGTGGAAAGTTTTTCGGTGTGGGATGGGCCCGCG
GCCTATCAGCTTGTTGGTGGGGTAATGGCCTACCAAGGCGGCGACGGGTAGCCGGCCTGAGAGGGTGG
ACGGCCACATTGGGACTGAGACACGGCC
>Zotu2125
GAACGCTGGCGGCGTGCTTAACACATGCAAGTCGAACGGTGAAGGGGCCTGCTTTTGTGGGTCCTGGA
TGAGTGGCGAACGGGTGAGTAACACGTGAGTAACCTGCCCCCTTCTTCTGGATAACCGCATGAAAGTG
TGGCTAATACGGGATATTCTGGGTCTGTCGCATGGTGGGCCTGGGAAAGATTGCGCCTTTTTTGGTGT
TTTTGGTGGGGGATGGGCTCGCGGCCTATCAGCTTGTTGGTGGGGTGATGGCCTACCAAGGCGGTGAC
GGGTAGCCGGCCTGAGAGGGTGGACGGTCACACTGGGACTGAGACACGGCC
>Zotu2126
GAACGCTGGCGGCATGCCTAATACATGCAAGTCGAACGCCAAATCTTCCTGCTTGCAGGAAGAGGCGG
AGTGGCGAACGGGTGAGTAACACGTGAGGAACTTGCCCATAAGAGGGGGATAACACCCGGAAACGGGT
GCTAATACCGCATAGGTTTTCCCAGTGCATGCTGGGAAAATAAAAGGTGGCTCTGCTACCGCTTATGG
ATAGCCTCGCGGCGCATTAGCTAGTTGGTAAGGTAAAGGCCTACCAAGGCCATGATGCGTAGCCGACT
TGAGAGGGTGATCGGCCACACTGGAACTGAGACACGGTC
>Zotu2128
GAACGCTAGCGGCAGGCCTAATACATGCAAGTCGAACGAGATCAGAGACTTCGGTTTCTGGTTTAGTG
GCGCACGGGTGCGTAACGCGTATGCAACCTACCTTCCACAGGGGGATAGCCCACCGAAAGGTGGATTA
ATACCGCATGACACTATTTGAGGGCATCCGAAGATAGTCAAAGCTGAGGCGGTGGAAGATGGGCATGC
GTGCCATTAGCTAGTTGGTAGTGTAACGGACTACCAAGGCTTCGATGGCTAGGGGTTCTGAGAGGATG
GTCCCCCACACTGGTACTGAGACACGGAC
>Zotu2129
GAACGCTGGCGGCGTGCCTAATACATGCAAGTCGAGCGGACAGATGGGAGCTTGCTCCCTGATGTTAG
CGGCGGACGGGTGAGTAACACGTGGGTAACCTGCCTGTAAGACTGGGATAACTCCGGGAAACCGGGGC
TAATACCGGATGGTTGTTTGAACCGCATGGTTCAAACATAAAAGGTGGCTTCGGCTACCACTTACAGA
TGGACCCGCGGCGCATTAGCTAGTTGGTGAGGTAATGGCTCACCAAGGCAACGATGCGTAGCCGACCT
GAGAGGGTGATCGGCCACACTGGGACTGAGACACGGCC
>Zotu2130

GAACGCTGGCGGCATGCCTAATACATGCAAGTCGAGCGAATGAAGAAGAAGCTTGCTTCTTCTGATTT
AGCGGCGGACGGGTGAGTAACACGTGGGTAACCTACCCTATAGATTGGGATAACTCCGGGAAACCGGG
GCTAATACCGAATAATACCTTTCCTCTCATGAGGAGATGTTGAAAGATGGCTTCTCGCTATCACTATA
GGATGGGCCCGCGGCGCATTAGCTAGTTGGTGAGGTAACGGCTCACCAAGGCAACGATGCGTAGCCGA
CCTGAGAGGGTGATCGGCCACACTGGGACTGAGACACGGCC
>Zotu2131
GAACGCTGGCGGCATGCTTAACACATGCAAGTCGCACGGGCAGCAATGTCAGTGGCGGACGGGTGAGT
ATCGCGTAGGAATGTATCCTGAGGTGGGGGACAACCCTGGGAAACTGGGGCTAATACCGCATATGGAC
TGAGGTCCAAAGCCGTGAGGCGCCTTGGGAGCAGCCTGCGTCCGATTAGGTAGTTGGTGGGGTAAAGG
CCTACCAAGCCTGCGATCGGTAGCTGGTCTGAGAGGACGATCAGCCACACTGGGACTGAGACACGGCC
>Zotu2132
GAATGCTGGCGACATGGATAAGGCATGCAAGTCAAGGGGGGCCGCAAGGTCAACCGGCAGACGAGGTA
GTAACACGTAAGTACGTACCCCATAGTCTGGTATAGCTCGTCGAAAGACGGGGTAATCCCGGATGGTC
CCGCAAGGGTAAAGATTTATCGCTATGGGAGCGGCTTGCGTAATATCAGCTTGTTGGTGAGGTAATGG
CTCACCAAGGCTACGACGTTTAGGGGAGCTGAGAGGCTGACCCCCACCGATGGGACTGAGACACGGCC
>Zotu2133
GAATGCTAGCGGTGTGCCTGAGATATGCAAGTCGAGCGACCCCTAAGCCGCAAGGTTTAGGGGAGCGG
CGGACGGCTGAGTAACACGTAGGAACCTGCCCCTTTCTGGAGGATAGCTTACCGAAAGGTGAGGTAAT
ACTCCATAGTCCCCACCTTTGGTGGGGTAAAGATTTATCGGAAAGGGATGGGCCTGCGTCCTATCAGG
TAGTTGGTGGGGTAACGGCCTACCAAGCCAATGACGGGTAGCTAATCTGAGAGGATGGTTAGCCAGAG
GGGGACTGAGACACGGCC
>Zotu2134
GAACGCTGGCGGCGTGCCTAATACATGCAAGTCGAGCGAACAGAGAAGGAGCTTGCTCCTTCGACGTT
AGCGGCGGACGGGTGAGTAACACGTGGGCAACCTACCCTATAGTTTGGGATAACTCCGGGAAACCGGG
GCTAATACCGAATCATCTCTTTTACTTCATGGTAAAAGACTAAAAGACGGTTTCGGCTGTCGCTATAG
GATGGGCCCGCGGCGCATTAGCTAGTTGGTGAGGTAATGGCTCACCAAGGCGACGATGCGTAGCCGAC
CTGAGAGGGTGATCGGCCACACTGGGACTGAGACACGGCC
>Zotu2135
GAACGCTAGCGGCAGGCCTAACACATGCAAGTCGAGGGGCAGCATAAATAAAGCTTGCTTTATTGGTG
GCGACCGGCGCACGGGTGAGTAACGCGTATGCAACCTACCTTATACTGGGGAATAGCCCGAAGAAATT
CGGATTAATACCCCATAGTATTATCGAATGGCATTATTTGATGATTAAAGTTTCGGCGGTATAAGATG
GGCATGCGTCCTATTAGCTAGATGGTGAGGTAACGGCTCACCATGGCTACGATAGGTAGGGGGCCTGA
GAGGGTGGTCCCCCACACTGGTACTGAGACACGGAC
>Zotu2136
GAACGCTGGCGGCGTGCTTAACACATGCAAGTCGAGCGGAAAGGCCCTTCGGGGTACTCGAGCGGCGA
ACGGGTGAGTAACACGTGAGGAACCTGCCCTGGACTTTGGGATAACCCTCGGAAACGGGGGCTAATAC
CGGATACGACCTTCAACCGCATGGTTTGTTGGTGGAAAGTTTTTCGGTCTGGGATGGTCTCGCGGCCT
ATCAGCTTGTTGGTGGGGTAATGGCCTACCAAGGCGACGACGGGTAGCCGGCCTGAGAGGGCGACCGG
CCACACTGGGACTGAGACACGGCC
>Zotu2137
GAACGCTGGCGGCAGGCTTAACACATGCAAGTCGATCGCCCCGCAAGGGGAGCGGCAGACGGGTGAGT
AACACGTGGGAACGTACCCTTTGGTTCGGAACAACCCAGGGAAACTTGGGCTAATACCGGATAAGTCC
GTAAGGAGAAAGATTTATCGCCAAAGGATCGGCCCGCGTCTGATTAGCTAGTTGGTAGGGTAATGGCC
TACCAAGGCTACGATCAGTAGCTGGTCTGAGAGGATGATCAGCCACATTGGGACTGAGACACGGCC
>Zotu2138
GAACGCTGGCGGTATGCCTAAGACATGCAAGTCGCACGGTTTAGCAATAAACAGTGGCGGACGGGTGA
GTAACACGTGGGAGATCTGCCCCGTAGTAGAGGAAAACTGAGGGAAACTTCAGCTAATCCCCTATGTG
GATGCTGGTTCTGACCGGTATCTAAAGCTTTGGCGCTATGGGATGAGCCTGCGGCCCATCAGGTAGTT
GGCGGGGTAAAAGCCCACCAAGCCGACGACGGGTAGCTGGTCTGAGAGGACGACCAGCCACAGGGGTA
CTGAGACACGGAC
>Zotu2139
GGCAAGGTGGGAGGCTGCTGCACTCAAGTGCTCCCTCTGACTCCCAGAATCTGCAACTACACACAAGA
GCGGTTCCCTCTGAAGGAAAACCAAAACCCAGCTGA
>Zotu2140

GAACGCTGGCGGCAGGCCTAACACATGCAAGTCGAGCGGTAGCACGGGAGAGCTTGCTCTCTGGGTGA
CGAGCGGCGGACGGGTGAGTAATGTCTGGGAAACTGCCTGATGGAGGGGGATAACTACTGGAAACGGT
AGCTAATACCGCATGACCTCGCAAGAGCAAAGTGGGGGACCTTCGGGCCTCACGCCATCGGATGTGCC
CAGATGGGATTAGCTAGTAGGTGGGGTAATGGCTCACCTAGGCGACGATCCCTAGCTGGTCTGAGAGG
ATGACCAGCCACACTGGAACTGAGACACGGTC
>Zotu2141
GAACGCTGGCGGTGTGCTTCACACATGCAAGTCGAACGAGGTAGCAATACCTAGTGGCGGACGGGTGA
GTAACGCGTAGAAATCTGCCTTCAGGTGGGGGATAACAGTTCGAAAGAACTGCTAATACCGCATATGT
GTACAATTCGACAGTTTTGTACATGAAAGGAGCAATTTGCCTGGAGATGAGTCTGCGTCCGATTAGCT
AGTTGGTAGGGTAAAGGCCTACCAAGGCGACGATCGGTAGGTGGTCTGAGAGGATGATCACCCACAAT
GGGACTGAGACACGGCC
>Zotu2142
GAACGCTGGCGGCATGCTTTACACATGCAAGTCGAACGGCAGCGCGGACTTCGGTCTGGCGGCGAGTG
GCGAACGGGTGAGTAATACATCGGAACGTACCCAGAAGTGGGGGATAACGCAGCGAAAGTTGCGCTAA
TACCGCATACGTTCTACGGAAGAAAGTGGGGGATCGCAAGACCTCATGCTTTTGGAGCGGCCGATGTC
TGATTAGCTAGTTGGTAGGGTAAAGGCCTACCAAGGCGACGATCAGTAGCTGGTCTGAGAGGACGACC
AGCCACACTGGAACTGAGACACGGTC
>Zotu2143
GAACGCTGGCGGCGTGCTTAACACATGCAAGTCGAACGGAAAGGCCCTTCGGGGTACTCGAGTGGCGA
ACGGGTGAGTAACACGTGGGTGATCTGCCTCGTACTCTGGGATAAGCCTGGGAAACTGGGTCTAATAC
CGGATAGGACCTTCTCTCGCATGGGGGTTGGTGGAAAGTTTTTCGGTACGAGATGGGCCCGCGGCCTA
TCAGCTTGTTGGTGGGGTAATGGCCTACCAAGGCGACGACGGGTAGCCGGCCTGAGAGGGCGACCGGC
CACACTGGGACTGAGACACGGCC
>Zotu2144
GAACGCTGGCGGCGTGCTTAACACATGCAAGTCGAGCGGAAAGGCCCTTCGGGGTACTCGAGCGGCGA
ACGGGTGAGTAACACGTGAGCAACCTGCCCTTAGCTTTGGGATAACCCTCGGAAACGGGGGCTAATAC
CGAATGATCACTGCCGGGCGCATGTTTGGTGGTGGAAAGTTTTTCGGCTGGGGATGGGCTCGCGGCCT
ATCAGCTTGTTGGTGGGGTGATGGCCTACCAAGGCGATGACGGGTAGCCGGCCTGAGAGGGCGACCGG
CCACACTGGGACTGAGACACGGCC
>Zotu2145
GAACGCTGGCGGCATGCCTTACACATGCAAGTCGAACGGCAGCACGGGAGCAATCCTGGTGGCGAGTG
GCGAACGGGTGAGTAAAGTATCGGAACGTGCCCAGTCGTGGGGGATAACGTAGCGAAAGCTACGCTAA
TACCGCATACGATCTATGGATGAAAGCGGGGGATCGCAAGACCTCGCGCGATTGGAGCGGCCGATATC
AGATTAGGTAGTTGGTGGGGTAAAAGCCCACCAAGCCAACGATCTGTAGCTGGTCTGAGAGGACGACC
AGCCACACTGGGACTGAGACACGGCC
>Zotu2147
GAACGCTGGCGGCGTGCCTAATACATGCAAGTCGAGCGGACTGAATGGAAGCTTGCTTCCGTTCGGTT
AGCGGCGGACGGGTGAGTAACACGTGGGCAACCTGCCCGTAAGACCGGGATAACTTCGGGAAACCGGA
GCTAATACCGGATAACACCGAAGATCGCATGATCTTTGGTTGAAAGGCGGCTTTGGCTGTCACTTACG
GATGGGCCTGCGGCGCATTAGCTAGTTGGTGAGGTAACGGCTCACCAAGGCGACGATGCGTAGCCGAC
CTGAGAGGGTGATCGGCCACACTGGGACTGAGACACGGCC
>Zotu2149
GAACGCTGGCGGCGCGCTTAACACATGCAAGTCGTACGGGGGTCTTCGGACCTCAGTGGCGAACGGGT
GCGTAACACGTGAGCAACCTGCCTGGTCGTGGGGGATAACCAGTGGAAACGCTGGCTAATACCGCATG
AGCTCTTTCTGCCGATTGGTGGGATGAGGAAAGCTCCTGCAAGGGAGTGCGACCAGAGGGGCTCGCGG
CGTATCAGCTAGTTGGAGGGGTAACGGCCCACCAAGGCGATGACGCGTAGCCGATCTGAGAGGATGAT
CGGCCACATTGGGACTGAGATACGGCC
>Zotu2150
TAACGCTGGCGGCGTGCATAACACATGCAAGTCGAACGATGAAACTTAAATGATTTCTTCGGAATGAA
CTTAAGTGGATTAGTGGCGGACGGGTGAGTAACGCGTGAGTAACCTGCCTTACACAAGGGGATAGCCT
CTGGAAACGGAGAATAATACCCTATAAAACCATCATATCGCATGAAGTGATGGTCAAAGTGTTAGCGG
TGTAAGATGGACTTGCGTCTGATTAGCTAGTTGGTGAGAAAAAAGCCCACCAAGGCAACGATCAGTAG
CCGGCTTGAGAGAGTGTACGGCCACATTGGGACTGAGACACGGCC
>Zotu2153

GAACGCTGGCGGCATGCTTTACACATGCAAGTCGAACGGCAGCACGGGCTTCGGCCTGGTGGCGAGTG
GCGAACGGGTGAGTAATACATCGGAACGTACCCAGAAGTGGGGGATAACGTAGCGAAAGTTACGCTAA
TACCGCATACGTTCTACGGAAGAAAGTGGGGGATCGCAAGACCTCATGCTTTTGGAGCGGCCGATGTC
TGATTAGCTAGTTGGTGAGGTAAAGGCTCACCAAGGCGACGATCAGTAGCTGGTCTGAGAGGACGACC
AGCCACACTGGAACTGAGACACGGTC
>Zotu2154
GAACGCTAGCTACAGGCTTAACACATGCAAGTCGAGGGGCAGCATTGGAGTAGCAATACTTCAGATGG
CGACCGGCGCACGGGTGAGTAACACGTATCCAACCTGTCGCTTACTCGGGGATAGCCTTTCGAAAGAA
AGATTAATACCCGATGGCATAAGGAGTTCGCATGTTCTCTTTATTAAAGAATTTCGGTAAGCGATGGG
GATGCGTTCCATTAGATAGTTGGCGGGGTAACGGCCCACCAAGTCTACGATGGATAGGGGTTCTGAGA
GGAAGGTCCCCCACATTGGAACTGAGACACGGTC
>Zotu2155
GAACGCTGGCGGCATGCCTAACACATGCAAGTCGAACGCAGTAGCAATACTGAGTGGCGGACGGGTGA
GGAACACGTGGGAACATGCCGAATGGTGGGGGATAACTGCGGGAAACTGCAGCTAATACCGCATAAGC
CCTGAGGGGGAAAGATTTATCGCCATTCGATTGGCCCGCGGCAGATTAGGTAGTTGGTAGGGTAAAGG
CCTACCAAGCCGATGATCTGTAACTGGTCTGAGAGGATGACCAGTCACACTGGGACTGAGACACGGCC
>Zotu2156
CAACGTTGGCGGCGCGCCTAACACATGCAAGTCGAACGAGAAAGTGGAGCAATCCATGAGTAAAGTGG
CGTACGGGTGAGTAACACGTGGGTAATCTACCTCTTGATGGGGAATAACGCTGGGAAACTGGCGCTAA
TACCGCATAAGTTCGTAAGAAGAAAGTCGCAAGACGCCGAGAGAGGAGCCCGCGGCCGATTAGCTAGT
TGGTAGGGTAAAAGCCTACCAAGGCTATGATCGGTAGCCGGCCTGAGAGGGCACACGGCCACACTGGC
ACTGAAACACGGGC
>Zotu2157
GAACGCTGGCGGCAGGCTTAACACATGCAAGTCGAACGCCCCGCAAGGGGAGTGGCAGACGGGTGAGT
AACGCGTGGGAATCTACCTAGAACTACGGAACAACTGATGGAAACGTCAGCTAATACCGTATACGCCC
TACGGGGGAAAGATTTATCGGTTTTAGATGAGCCCGCGTAAGATTAGCTAGTTGGTGGGGTAATGGCC
TACCAAGGCGACGATCTTTAGCTGGTCTGAGAGGATGATCAGCCACACTGGGACTGAGACACGGCC
>Zotu2158
GAACGCTAGCGGCAGGCCTAATACATGCAAGTCGAACGGGCCGCAAGGTCAGTGGCGAACGGGTGCGT
AACGCGTAAGCAACCTGCCCACAACTGGGGGATAGCCTTGCGAAAGCGAGGGTAAACCCGCATGGTCC
TTTGGAGTCACCTGGCTTTTAAGGTAAACATTTATGGGTTGTGGAGGGGCTTGCGTCTGATTAGCTAG
TTGGTGGGGTAACGGCCCACCAAGGCGATGATCAGTAGGGGTTCTGAGAGGATTGGCCCCCACATGGG
TACTGAGACACGGAC
>Zotu2159
GAACGCTGGCGGCGTGCTTAACACATGCAAGTCGAGCGGTAAGGCCCTTCGGGGTACACGAGCGGCGA
ACGGGTGAGTAACACGTGAGCAATCTGCCCTTCTCTTTGGAATAACCATTGGAAACGATGGCTAATGC
CGAATATGAACCCCTTTCGCATGTTGGGGGTTGGAAAGCTCCGGCGGAGAAGGATGAGCTCGCGGCCT
ATCAGCTTGTTGGTGAGGTAATGGCTCACCAAGGCATTGACGGGTAGCCGGCCTGAGAGGGTGACCGG
CCACACTGGGACTGAGACACGGCC
>Zotu2160
GAACGCTGGCGGCGTGCCTAATACATGCAAGTCGAGCGGACTTGACAGAGCTTGCTCTGTTCAAGTTA
GCGGCGGACGGGTGAGTAACACGTGGGTAACCTGCCTGTAAGACTGGGATAACTCCGGGAAACCGGGG
CTAATACCGGATAATATCAAGAGCTGCATGGCTCTTGATTGAAAGGCGGCTTCGGCTGTCACTTACAG
ATGGACCCGCGGCGCATTAGCTAGTTGGTGAGGTAACGGCTCACCAAGGCGACGATGCGTAGCCGACC
TGAGAGGGTGATCGGCCACACTGGGACTGAGACACGGCC
>Zotu2161
GAACGCTGGCGGCATGCTTAACACATGCAAGTCGAACGAAAACAAAAAAATAAGTAATTATTTTTTTG
TTTTAGTGGCGGACGGGTGAGTAACGCGTAAGAATCTACCTTTAGGAGGGGGAAAACAACTGGAAACG
GTTGCTAACACCCCATATGCTGAGGAGTAAAATACATTGTGCCTAGAGATGAGCTTGCGTCTGATTAG
TTAGTTGGTAAGGTAATTGCTTCCCAAGACTATGATCAGTAGCTGGTCTGAGAGGATGATCAGCCACA
CTGGGACTGAGACACGGCC
>Zotu2162
GAACGCTGGCGGCGTGCCTAATACATGCAAGTCGAGCGAATGACGAGGAGCTTGCTCCTCTGATTTAG
CGGCGGACGGGTGAGTAACACGTGGGTAACCTGCCCTGTAGACTGGGATAACTTCGGGAAACCGGAGC

TAATACCGGATAATTCTTTTAACCTCATGGTTTTAAGCTGAAAGGCGCTTCGGCGTCACTACAGGATG
GACCCGCGGTGCATTAGCTAGTTGGTGAGGTAACGGCTCACCAAGGCAACGATGCATAGCCGACCTGA
GAGGGTGATCGGCCACATTGGGACTGAGACACGGCC
>Zotu2163
GAACGCTGGCGGCGTGCTTAACACATGCAAGTCGAACGGAAAGGCCTCTTGCTTGCAGGAGGTACTCG
AGTGGCGAACGGGTGAGTAACACGTGGGTGATCTGCCTTGCACTCTGGGATAAGCTTGGGAAACTGGG
TCTAATACCGGATAGGAACCGTCTTTAGTGTGGTGGTTGGAAAGTTTTTTCGGTGCAAGATGAGCTCG
CGGCCTATCAGCTTGTTGGTGGGGTAATGGCCTACCAAGGCGTCGACGGGTAGCCGGCCTGAGAGGGT
GTACGGCCACATTGGGACTGAGATACGGCC
>Zotu2164
GAACGCTAGCGGCAGGCTTAATACATGCAAGTCGAGGGGCAGCAGGGTGTAGCAATACATCGCTGGCG
ACCGGCAAACGGGTGCGGAACACGTACGCAACTTACCCATAACTGGTGAATAGCCCTCCGAAAGGAGG
ATTAATACACCGTAACCTTATGAAGTGGCATCACTTTATAAGTATAGCTCCGGCGGTTATGGATGGGC
GTGCGCCTGATTAGGTAGTTGGTGAGGGTAACGGCCCACCAAGCCTTCGATCAGTAACTGGTGTGAGA
GCACGACCAGTCACACGGGCACTGAGACACGGGC
>Zotu2165
AAACGCTGGCGGCGCACATAAGACATGCAAGTCGAACGGAATCGAGTCGAATGTTTACATGAGACGAA
GATTTAGTGGCGGACGGGTGAGTAACACGTGAGCAACCTACCTTTAAGAGGGGGATAACATTGAGAAA
TCAGTGCTAATACCGCATAAAGCAAGCGAATCGCATGATAAGCTTGCCAAAGGAGCAATCCGCTTAAA
GATGGGCTCGCGGCTGATTAGTTAGTTGGTGGGGTAAAGGCCTACCAAGACGATGATCAGTAGCCGAG
CTGAGAGGCACAACGGCCACATTGGGACTGAGACACGGCC
>Zotu2166
GAACGCTAGCGGGAGGCCTAACACATGCAAGCCGAGCGGTAGGGTTTCTTCGGAAACCTGAGAGCGGC
GCACGGGTGCGGAACACGTGTGCAACCTACCTTTATCAGGGGGATAGCCTTTCGAAAGGAAGATTAAT
ACCCCATAATATATTGAATGGCATCATTTGATATTGAAAACTCCGGTGGATAGAGATGGGCACGCGCA
AGATTAGATAGTTGGTGAGGTAACGGCTCACCAAGTCAATGATCTTTAGGGGGCCTGAGAGGGTGATC
CCCCACACTGGTACTGAGACACGGAC
>Zotu2167
GAACGCTGGCGGTGTGCTTCACACATGCAAGTCGAACGGGGTAGCAATACCCAGTGGCGGACGGGTGA
GTAACACGTGGGAATCTACCATAAGGTGGGGGATAGCAGACCGAAAGGTCTATTAATACCGCATATGT
AGGCATCTCGACAGAGGTGTTTATGAAAGGAGTAATCCGCCTTTTGATGAGCCCGCGTCCGATTAGCT
AGTTGGTAGGGTAAAAGCCTACCAAGGCGACGATCGGTAGGTGGTCTGAGAGGATGATCACCCACAAT
GGGACTGAGACACGGCC
>Zotu2168
GAACGCTAGCGGCAGGCCTAATACATGCAAGCCGAACGGCAAGCTTGGTAGCAATATCAAGCCTAGAG
TGGCGTAAGGGTGCGTAACACGTATGCAATCTGCCCGCAACAGGAGGATAGCTCCCCGAAAGGGGAAT
TAATACTCCATAAGATCACGAGAGGGCATCTGATTGTGATTAAACTACAAGGGGTTGCGGATGAGCAT
GCGGTTGATTAGCTAGTTGGTAAGGTAACGGCTTACCAAGGCGATGATCAATAGGGGAACTGAGAGGT
TTATCCCCCACACTGGCACTGAGATACGGGC
>Zotu2169
GAACGCTGGCGGCGTGCTTAACACATGCAAGTCGAACGAAGAGCGACGGAAGCTTGCTTCTATCAATC
TTAGTGGCGAACGGGTGAGTAACGCGTAATCAACCTGCCCTTCAGAGGGGGACAACAGTTGGAAACGA
CTGCTAATACCGCATACGATCTAATCTCGGCATCGAGGATGGATGAAAGGTGGCCTCTACATGTAAGC
TATCACTGAAGGAGGGGATTGCGTCTGATTAGCTAGTTGGAGGGGTAACGGCCCACCAAGGCGATGAT
CAGTAGCCGGTCTGAGAGGATGAACGGCCACATTGGGACTGAGACACGGCC
>Zotu2170
GAACGCTGGCGGCATGCCTAACACATGCAAGTCGAACGTGAAAGTACTTCGGTATGAGTAAAGTGGCG
CACGGGTGAGTAACACGTAGGTGACCTGCCTATTAATGGGGAATAACCAGAAGAAATTTTGGCTAATA
CCGCATACGAAGCACAAATCTTAGAGTAGTGCTTGAAAGGATGCCTCTGTATATGCATTCGTTGATAG
ATGGGCCTGCGGTACATTAGCTAGTTGGTGGGGTAAAGGCCTACCAAGGCAACGATGTATATCCGGTC
TGAGAGGATGATCGGACACACTGGAACTGAGACACGGTC
>Zotu2171
GAACGCTGGCGGCAGGCTTAACACATGCAAGTCGAGCGGGCCCTTCGGGGTCAGCGGCGGACGGGTTA
GTAACGCGTGGGAACGTGCCCTTTGCTCTGGAATAGCCCTGGGAAACTGGGGGTAATACCGGATGTGC

CCTGAGGGGGAAAGATTTATCGGCAAGGGATCGGCCCGCGTCTGATTAGGTAGTTGGTGGGGTAACGG
CTCACCAAGCCGACGATCGGTAGCTGGTTTGAGAGGATGATCAGCAACACTGGGACTGAGACACGGCC
>Zotu2172
GAACGCTAGCGGCAGGCCTAATACATGCAAGTCGAGGGGTAGTGGAAGCTTGCTTCCATGAGACCGGC
GCACGGGTGCGTAACGCGTATGCAACCTACCTTCATCTGGGGGATAGCCCGGAGAAATCCGGATTAAC
ACCGCATAAAATCACAGTACGGCATCGTACAATGATCAAATATTTATAGGATGGAGATGGGCATGCGT
GTCATTAGTTAGTTGGCGGGGTAACGGCCCACCAAGACCGCGATGACTAGGGGATCTGAGAGGATGGC
CCCCCACACTGGTACTGAGACACGGAC
>Zotu2173
GAACGCTGGCGGCGTGCCTAATACATGCAAGTCGAACGGGCTCTTCGGAGCTAGTGGCGGACGGGTGA
GTAACACGTTGGTACCTACCCCTAAGTGGGGAATAACTGCCCGAAAGGGTGGCTAATACCGCATAATC
CCGCAAGGGTAAAGCAGTAATGCGCTTAGGGAGGGGCCTGCGTCTGATTAGCTAGTTGGTAGTGTAAT
GGACTACCAAGGCGACGATCAGTAGCTGGATCGAGAGGTTGACCAGCCACACTGGGACTGAGATACGG
CC
>Zotu2174
GAACGCTGGCGGCGTGCCTAATACATGCAAGTCGAGCGGTAAGGCCCCTTCGGGGGTACACGAGCGGC
GGACGGCTGAGTAACACGTAGGAACGTACCCCAAACTGAGGGATAAGCACCAGAAATGGTGTCTAATA
CCGCATGTGGTCTTCGGATTAAAGCTTCGGCGGTTTGGGAACGGCCTGCGCATGATTAGCTTGTTGGT
GAGGTAATAGCTCACCAAGGCGACGATCATTAGCTGGTCTGAGAGGATGATCAGCCAGACTGGGACTG
AGAACGGCC
>Zotu2175
GAACGCTGGCGGCGTGCTTAACACATGCAAGTCGAACGGTGATGATGGGGCTTGCCCTGTCTGATCAG
TGGCGAACGGGTGAGTAACACGTGAGTAACCTGCCCCTCACTCTGGGATAACCTCGGGAAATCGTGGC
TAATACCGGATATGAGTTTCCACCGCATGGTGGTTTCTGGAAAGTTTTTCGGTGAGGGATGGACTCGC
GGCCTATCAGTTTGTTGGTGAGGTAATGGCTCACCAAGGCGATGACGGGTAGCCGGCCTGAGAGGGCG
ACCGGCCACACTGGGACTGAGACACGGCC
>Zotu2176
GAACGCTGGCGGCAGGCTTAACACATGCAAGTCGAGCGCGCCCTTCGGGGTGAGCGGCGAACGGGTGA
GTAACGCGTGGGAACGTGCCCCTTTCTTCGGAATAGCCTCGGGAAACTGGGAGTAATGCCGAATACGC
CCCTTACGGGGGAAAGATTTATCGGAGAGGGATCGGCCCGCGTTGGATTAGGTAGTTGGTGGGGTAAT
GGCCTACCAAGCCGACGATCCATAGCTGGTTTGAGAGGATGATCAGCCACACTGGGACTGAGACACGG
CC
>Zotu2177
GAACGCTGGCGGCGTGGCTAAGACATGCAAGTCGCACGGACCCGCAAGGGTTAGTGGCGAACGGGTGA
GTAATGAATCGCTAATGTACCCCGGACTCTGGGATAGCTAGCCGAAAGGCTAGGTAATACCAGATGAT
ATGCAGCTGTCGCATGACAGTTGCCTCAAAGGTCCGCCGGTCTGGGAGCAGGCGATTTCGTATCAGCT
TGTTGGTGAGGTAACGGCTCACCAAGGCGACGACGCGTAGCCGGACTGAGAGGTTGGCCGGCCACATT
GGGACTGAGACACTGCC
>Zotu2178
GAACGCTAGCGGCAGGCCTAATACATGCAAGTCGAACGGTTGTAGTAATACAATAGTGGCGCACGGGT
GCGTAACACGTATGCAACCTACCTTATAGCGGGGGATAGCCTTTGGAAACGGAGATTAATACCCCATA
ATATATTGACTCGGCATCGGGTTATTATTAAAGATTTATCGCTATAAGCTGGGCATGCGTCCAATTAG
TTAGTTGGCGGGGTAATGGCTCACCAAGACGATGATTGGTAGGGGAACTGAGAGGTTAATCCCCCACA
CTGGCACTGAGATACGGGC
>Zotu2179
GAATGCTGGCGGCGTGGATAAGTCATGCAAGTCAAGGGGACCGCAAGGTAACCGGCAAACGAGGTAGT
AACACGTAGGTACTCCCCCCGAAGTTTGGCATAGCTCGTCGAAAGACGGGGTAATTCCAAATAGTCCC
GCAAGGGTAAAGATTTATCGCTTCGGGAGAGGCCTGCGTAGTATCAGTTAGTTGGTAGGGTAACGGCC
TACCAAGGCTACGACGCTTAGGGGAGCTGAGAGGCTGACCCCCACCGATGGGACTGCGACACGGCC
>Zotu2180
GAACGCTGGCGGCATGCCTAACACATGCAAGTCGAACGAAGCCTTCGGGCTTAGTGGCGCACGGGTGC
GTAACGCGTGGGAATCTGCCCTTGGGTTCGGAATAACAGTTAGAAATGACTGCTAATACCGGATGATG
ACGTAAGTCCAAAGATTTATCGCCCAAGGATGAGCCCGCGTAGGATTAGCTTGTTGGTGAGGTAAAAG
CTCACCAAGGCGACGATCCTTAGCTGGTCTGAGAGGATGATCAGCCACACTGGGACTGAGACACGGCC

>Zotu2181
GAACGCTAGCGGCAGGCTTAACACATGCAAGTCGAGGGGTATAGTAGCAATACTAGAGACCGGCGCAC
GGGTGCGTAACGCGTATGCAATCTACCTTTTACAGAGGGATAGCCCAGAGAAATTTGGATTAATACCT
CATAGCATAGCAGCTTCGCATGAAGTCACTATTAAAGTTCCAACGGTAAAAGATGAGCATGCGTCCTA
TTAGCTAGATGGTAAGGTAACGGCTTACCATGGCTACGATAGGTAGGGGTCCTGAGAGGGAGATCCCC
CACACTGGTACTGAGACACGGAC
>Zotu2182
GAACGCTGGCGGCAGGCTTAACACATGCAAGTCGAGCGGGCGAGGTTGCTTCGGTAACTGAGCTAGCG
GCGGACGGGTGAGTAATGCTTAGGAATCTGCCTATTAGTGGGGGACAACATTCCGAAAGGAATGCTAA
TACCGCATACGCCCTACGGGGGAAAGCAGGGGATCTTCGGACCTTGCGCTAATAGATGAGCCTAAGTC
AGATTAGCTAGTTGGTGGGGTAAAGGCCTACCAAGGCGACGATCTGTAGCGGGTCTGAGAGGATGATC
CGCCACACTGGGACTGAGACACGGCC
>Zotu2183
GAACGCTGGCGGCGTGCCTAACACATGCAAGTCGAACGAGGTTGATCTGGTGGCAACACCAGAGACTA
CCTAGTGGCGAACGGGTGAGTAACACGTGAGGAACCTGCCCCGAAGACCGGGATAACCCTCCGAAAGG
AGAGCTAATACCGGATACCCCCATCGGATCGCATGATCTGATGAGGAAATGGATTCCGCTTCGGGAGG
GCCTCGCGGCCTATCAGCTTGTTGGCGGGGTAACGGCCCACCAAGGCATCGACGGGTAGCTGGTCTGA
GAGGACGATCAGCCACACTGGGACTGAGACACGGCC
>Zotu2184
GAACGCTGGCGGCGTGCCTAAGACATGCAAGTCGAGTGGGTTTAGACCCACGGCGAACGGGTGAGTAA
TACCTAGGAACTTTCTTTAAGTAGAGGATAACCAGCCGAAAGGTTGGATAATACTCTATGTGATAGTA
ATATCAAAGTCGCAAGACGCTTAAAGTGAGGCCTGGGCACCATCAGCTAGTTGGTAAGGTAATGGCTT
ACCAAGGCTATGACGGTTAGGGGTCCCGAGAGGGATAACCCCCACACTGGAACTGCGACACGGTC
>Zotu2185
GAATGCTGGCGACATGGATAAGTCATGCAAGTCAAGGGGGGCCGCAAGGTCAACCGGCAGACGAGGTA
GTAACACGTAGGTACGCACCCCAGAGTCTGGCATAGCTCGTCGAAAGACGGGGTAATTCCAGATGGTC
CCGCAAGGGTAAAGATTTATCGCTTTGGGAGCGGCCTGCGTAGTATCAGCTAGTTGGTAAGGTAATGG
CTTACCAAGGCTACGACGCTTAGGGGAGCTGAGAGGCTGACCCCCACCGATGGGACTGAGACACGGCC
>Zotu2186
GAACGCTGGCGGCGTGCTTAACACATGCAAGTCGAGCGGTAAGGCTCCTTCGGGAGTACACGAGCGGC
GAACGGGTGAGTAACACGTGAGTAACCTGCCCCATTCTTCGGAATAACCAGTGGAAACGCTGGCTAAT
GCCGAATACGACCATCGAAGGCATCTTCTGGTGGTGGAAAGCTCCGGCGGAATGGGATGGACTCGCGG
CCTATCAGCTTGTTGGTGAGGTAATGGCTCACCAAGGCGACGACGGGTAGCCGGCCTGAGAGGGTGAC
CGGCCACACTGGGACTGAGACACGGCC
>Zotu2187
GAACGCTGGCGGCATGCTTTACACATGCAAGTCGAACGGCAGCGCGGGGCAACCTGGCGGCGAGTGGC
GAACGGGTGAGTAAAATATCGGAACGTACCCAGAAGTGGGGGATAACGTAGCGAAAGTTACGCTAATA
CCGCATACGATCTACGGATGAAAGTGGGGGACCTTCGGGCCTCATGCTTTTGGAGCGGCCGATATCTG
ATTAGCTAGTTGGTGAGGTAAAGGCTCACCAAGGCGACGATCAGTAGCTGGTCTGAGAGGACGACCAG
CCACACTGGGACTGAGACACGGCC
>Zotu2188
GAACGCTGGCGGCGTGCCTAATACATGCAAGTCGAGCGAAGCAAACGCTTTTCTTCGGAAAAGCGTAA
GCTTAGCGGCGGACGGGTGAGTAACACGTGGGTAACCTGCCCATAAGCCTGGGATAACATTCGGAAAC
GAATGCTAATACCGGATATGCAACATCATCGCCTGGTGGTGTTGGGAAAGGCGGAGCAATCTGCCACT
TATGGATGGACCCGCGGTGCATTAGCTAGTTGGTGAGGTAAAGGCTCACCAAGGCGACGATGCATAGC
CGACCTGAGAGGGTGATCGGCCACACTGGGACTGAGACACGGCC
>Zotu2190
GAACGCTGGCGGCGTGCTTAACACATGCAAGTCGAGCGGAAAGGCCACTTCGGTGGTACTCGAGCGGC
GAACGGGTGAGTAACACGTGAGTAATCTGCCCTCCACTTCGGGATAGCCACCGGAAACGGTGATTAAT
ACCGGATATGACGCATGGTCGCATGGTCGTGTGTGGAAAGTTTTTTCGGTGGGGGATGTGCTCGCGGC
CTATCAGCTTGATGGTGAGGTAATGGCTCACCATGGCTACGACGGGTAACCGGCCTGAGAGGGTGACC
GGTCACACTGGGACTGAGACACGGCC
>Zotu2191
GAACGCTGGTGGCGTGCTTAACACATGCAAGTCGAGCGAGGCGTCCGCTTTCGGGTGGATAGTCGAGC

GGCGAACGGGTGAGTAACACGTGAGTAATCTTCCCTGGGCTCGGGGATAACCTTGGGAAACTGAGGCT
AATACCGGATGTGCGTTGTTTGGGGCTTCCTGGATGACGGAAAGGTTGCGTGCCGTGAGGTGCGTTTC
CGGCCTTGGATGAGCTCGCGGCCTATCAGCTTGTTGGTGCGGTGATGGCGTACCAAGGCGACGACGGG
TAGCCGGCCTGAGAGGGTGACCGGCCACACTGGGACTGAGATACGGCC
>Zotu2192
GAACGCTAGCGGCAGGCCTAATACATGCAAGTCGAACGGGTGTCCCTCGGGGCACCAGTGGCGCACGG
GTGCGTAACGCGTATGCAACCTACCCCCCACTGGGGAATAGCCTCCCGAAAGGGAGATTAATACCGCA
TAACACAGCTTGAGGGCATCTGAGGGCTGTTAAAGATTTATCGGTTGGGGATGGGCATGCGTGCCATT
AGCTAGTTGGTGGTGTAACGGACCACCAAGGCGACGATGGCTAGGGGTTCTGAGAGGACGGTCCCCCA
CACTGGCACTGAGATACGGGC
>Zotu2193
GAACGCTAGCGGCAGGCCTAATACATGCAAGCCGAACGGCAAGTGTTTTAGCAATAGAACACCTAGAG
TGGCGTAAGGGTGCGTAACACGTATGCAATCTGCCCTCAACAGGAGGATAGCTCCCCGAAAGGGGAAT
TAAGACTTCATAGGATCATAGAGTGGCATCACTTAGTGATTAAATTACAAGAGGTTGAGGATGAGCAT
GCGCATGATTAGTTAGTTGGCGGGGTAATGGCCCACCAAGACGATGATCATTAGGGGAACTGAGAGGT
TGATCCCCCACACTGGCACTGAGATACGGGC
>Zotu2194
GAACGTTGGCGGCGTGCCTAACACATGCAAGTCGAACGGAAATAGCAATATTTGAGTGGCGCACGGGT
GAGTAACACGTAGCTAATCTACCTCAAAGCGCGGGATAACGTGTCGAAAGACAGGCTAATACCGCATA
AGCTCACGGAAAGCAGAGGACTGTGAGAAAAGGCGCAAGTCACTTTGAGATGAGGCTGCGGCCCATCA
GCTAGTTGGTGCGGTAAAGGCGCACCAAGGCGACGACGGGTAGGGGACCTGAGAGGGTGACCCCCCAC
ACTGGGACTGAGACACGGCC
>Zotu2195
TAACGCTAGCGGCAGGCCTAATACATGCAAGTCGAACGGCAGCACGGGTAGCAATATCTGGTGGCGAG
TGGCGCACGGGTGCGTAACGCGTATGCAATCTACCTGTAACTGGGGAATAGCCTTCCGAAAGGAAGAT
TAATACCGCATAATATCACGAAACGGCATCGTTTTGTGATCAAATCTCCGGAGGTTACAGATGAGCAT
GCGTGACATTAGCTAGTTGGTGAGGTAACGGCTCACCAAGGCTACGATGTCTAGGGGGTCTGAGAGGA
TGATCCCCCACACTGGTACTGAGACACGGAC
>Zotu2196
GAACGCTGGCGGCGTGCTTAACACATGCAAGTCGAACGATGAACCTCCTTCGGGAGGGGATTAGTGGC
GAACGGGTGAGTAACACGTGGGCAATCTGCCCTGCACTCTGGGATAACTCCGGGAAACCGGAGCTAAT
ACCGGATACGACCACTGCGGGCATCCGTGGTGGTGGAAAGCTCCGGCGGTGCAGGATGAGCCCGCGGC
CTATCAGCTTGTTGGTGGGGTGATGGCCTACCAAGGCGACGACGGGTAGCCGGCCTGAGAGGGCGACC
GGCCACACTGGGACTGAGACACGGCC
>Zotu2197
GAACGCTGGCGGCGTGCCTAATACATGCAAGTCGAGCGGATGGATTCGGAGCTTGCTCCGATGAAGTC
AGCGGCGGACGGGTGAGTAACACGTGGGTAACCTGCCTGGCAGACTGGGATAACGCTTGGAAACGAGT
GCTAATACCGGATAACCTCTTGGATCGCATGGTTCGAGAGTAAAAGGAGCTTTTGCTTCACTGCCAGA
TGGACCCGCGGCGCATTAGCTAGTTGGTGAGGTAACGGCTCACCAAGGCGACGATGCGTAGCCGACCT
GAGAGGGTGACCGGCCACACTGGGACTGAGACACGGCC
>Zotu2198
GAACGCTAGCGGCAGGCTTAACACATGCAAGTCGAGGGGTAGTCTAGCAATAGATGAGACCGGCGCAC
GGGTGCGTAACGCGTATGCAATCTACCTTTTACAGAGGGATAGCCCAGAGAAATTTGGATTAATACCT
CATAGCATAGCAGCTTCGCATGAAGTCACTATTAAAGTTCCAACGGTAAAAGATGAGCATGCGTCCTA
TTAGCTAGATGGTAAGGTAACGGCTTACCATGGCTACGATAGGTAGGGGTCCTGAGAGGGAGATCCCC
CACACTGGTACTGAGACACGGAC
>Zotu2199
GAACGCTAGCGGCAGGCTTAACACATGCAAGTCGAGGGGTATAGTTCTTCGGAACTAGAGACCGGCGC
ACGGGTGCGTAACGCGTATGCAATCTACCTTTCACAGAGGGATAGCCCAGAGAAATTTGGATTAATAC
CTCATAGTATTATAGAATGGCATCATTTTATAATTAAAGTCACAACGGTGAAAGATGAGCATGCGTCC
CATTAGCTAGATGGTAAGGTAACGGCTTACCATGGCTACGATGGGTAGGGGTCCTGAGAGGGAGATCC
CCCACACTGGTACTGAGACACGGAC
>Zotu2200
GAACGCTGGCGGCAGGCTTAACACATGCAAGTCGAACGCCCCTTTCGGGGGGAGTGGCAGACGGGTGA

GTAACACGTGGGAACATACCCTTTGGTTCGGAACAACGTTTGGAAACGGACGCTAATACCGGATACGC
CCTTTGGGGGAAAGGCTTTGCTGCCGAAGGATTGGCCCGCGTTCGATTAGCTTGATGGCGGGGTAACG
GCCCACCATGGCGACGATCGATAGCTGGTCTGAGAGGATGGCCAGCCACATTGGGACTGAGACACGGC
C
>Zotu2202
GAACGCTGGCGGCGTGCCTAATACATGCAAGTCGAGCGGCAGCACGGTCTTCGGACTGGTGGCGAGCG
GCGGACGGCTGAGTAACACGTAGGAACGTACCCCAAAGTGAGGGATAAGCACCAGAAATGGTGTCTAA
TACCGCATATGCTCTACGGAGTAAAGCTTTTGCGCTTTGGGAGCGGCCTGCGTATGATTAGCTAGTTG
GTGAGGTAATAGCTCACCAAGGCGATGATCATTAGCTGGTCTGAGAGGATGATCAGCCAGACTGGGAC
TGAGACACGGCC
>Zotu2203
GAACGCTGGCGGCGTGCTTAACACATGCAAGTCGAGCGGAAAGGCCACTTCGGTGGTACTCGAGCGGC
GAACGGGTGAGTAACACGTGAGTAATCTGCCCCTGGCTTTGGGATAGCCACCGGAAACGGTGATTAAT
ACCGGATACGACCGCTCCTCGCATGAGGTGGTGGTGGAAAGTTTTTCGGCCAGGGATGTGCTCGCGGC
CTATCAGCTTGATGGTGAGGTAATGGCTCACCATGGCTTCGACGGGTAGCCGGCCTGAGAGGGTGACC
GGTCACACTGGGACTGAGACACGGCC
>Zotu2204
GAACGCTGGAAATGTGGATACCGGCATGCAAGTCAAGGGGCCGCAAGGCACCGGCGAACGGCTTAGTA
ACACGTAGATACATACCTTAAAGTGGAGCATAGCTCGTCGAAAGACGGGGTAATTTTCCATAGTCCCG
CAAGGGTAAAGGCGCAAGCCGCTTTAAGAATGGTCTGCGGTCTATCAGCTAGTTGGTGAGGTAATGGC
TCACCAAGGCTATGACGGATAGGGGGCGTGAGAGCGCGACCCCCAAGAATGGCACTGCGACACGGGC
>Zotu2206
AAACGCTGGCGGCGTGCCTAATGCATGCAAGTCGAACGGGATCACCCCGTTGCCAGTCCTTCGGGGCT
GGTGGGGTGAGGACCGTGGCGGACGGGTGAGGAACGCGTGGCCGACCTGCCCTGATCACGGGGATAAC
CCGGGGAAACCCGGGCGAATACCCGATGCCCTGGGGGATGGGGACATCCTTCAGGAAAGCGCTATGGA
GCGGATCAGGAGGGGGCTGCGCCCGATTAGCTAGCTGGTGGGGTAACGGCCTACCAGGGCGACGATCG
GTCGCTGGTCTGAGAGGATGATCAGCCACACGGGGACTGAGACACGGCC
>Zotu2207
GAACGTTGATGGCGTGCCTCATACATGCAAGTCGAGCGCGGTAGCAATACCGAGCGGCGAACGGGTGA
GTAACACGTAAAGAATCTGCCCTGAAGCGGGGAATAACTATCCCAACGGATAGCTAATACCGCATAAC
GATGGAGGAGGCATCTCCTCTTCTCAAAAGGCTTCGGCCACTTCAGGATGACTTTGCGTCCCATTAGC
TTGTTGGCGGGGTAACGGCCCACCAAGGCTACGATGGGTATCCGGCCTGAGAGGGTGTACGGACACAT
TGGGACTGAGATACGGCC
>Zotu2208
GAACGCTGGCGGCGTGCTTAACACATGCAAGTCGAACGATGAAGCTGCCTGCTTGCAGGTGGTGGATT
AGTGGCGAACGGGTGAGTATCACGTGAGTAACCTGCCCTTGACTCTGGGATAAGCCCGGGAAACTGGG
TCTAATACCGGATATGACCTCTCATCGCATGGTGGGGGGTGGAAAGTTTTTAACGGTCTTGGATGGGC
TCGCGGCCTATCAGCTTGACGGTGGGGTAGTGGCCTACCGTGGCTTTGACGGGTAGCCGGCCTGAGAG
GGTGACCGGCCACACTGGGACTGAGACACGGCC
>Zotu2209
GAACGCTGGCGGCGCGCCTAACACATGCAAGTCGAACGGCACCTGCCCTCGGGCAGAAGCGAGTGGCG
AACGGCTGAGTAACACGTGGAGAACCTGCCCCCTCCCCCGGGATAGCCGCCCGAAAGGACGGGTAATA
CCGGATACCCCGGGGTGCCGCATGGCACCCCGGCTAAAGCCCCGACGGGAGGGGATGGCTCCGCGGCC
CATCAGGTAGACGGCGGGGTGACGGCCCACCGTGCCGACAACGGGTAGCCGGGTTGAGAGACCGACCG
GCCAGATTGGGACTGAGACACGGCC
>Zotu2210
GAACGCTGGCGGCGTGCTTAATACATGCAAGTCGAACGAGGTTGATCCAGTGGAAACACTGGTGACTG
CCTAGTGGCGAACGGGTGAGTAACACGTGAGCAACCTGCCCCGAAGACCGGGATAACAGTGGGAAACC
GCTGCTAATACCGGATACCCCCACAGGGTCACATGGCCTGGTGAGGAAATGCCTGATTCTTTGGAGTC
ATACGCTTCGGGAGGGGCTCGCGGCCTATCAGCTTGTTGGTGGGGTAACGGCCTACCAAGGCAACGAC
GGGTAGCTGGTCTGAGAGGACGATCAGCCACACTGGGACTGAGACACGGCC
>Zotu2211
GAACGCTGGCGGCGTGCCTAACACATGCAAGTCGAGCGAGTGAATTTCCTTCGGGAGTGAAGCTAGCG
GCGGACGGGTGAGTAACACGTGGGCAACCTGCCTCATAGAGGGGAATAGCCTTTCGAAAGGAAGATTA

ATACCGCATAAGATTGTAGCTTCGCATGAAGTAGCAATTAAAGGAGCAATCCGCTATGAGATGGGCCC
GCGGCGCATTAGCTAGTTGGTGAGGTAACGGCTCACCAAGGCGACGATGCGTAGCCGACCTGAGAGGG
TGATCGGCCACATTGGGACTGAGACACGGCC
>Zotu2212
GAACGCTAGCGGCAGGCCTAATACATGCAAGTCGAACGGTAACATGAGTAGCAATACTTGATGACGAG
TGGCGCACGGGTGCGTAACGCGTATGCAATCTACCTTTAACTGGTGAATAGCCCGAAGAAATTCGGAT
TAATACACCATAGTATATTTGAATGGCATCATTTAAATATTAAAGTAACAACGGTTAGAGATGAGCAT
GCGTGACATTAGCTAGTTGGTGAGGTAATGGCTCACCAAGGCTACGATGTCTAGGGGATCTGAGAGGA
TTAACCCCCACACTGGTACTGAGACACGGAC
>Zotu2213
GAACGCTGGCGGCGTGCTTAACACATGCAAGTCGAACGAAGCACTTTAGACAGATTCTTCGGATGAAG
TCCTTAGTGACTGAGTGGCGGACGGGTGAGTAACGCGTGGGTAACCTGCCTCATACAGGGGGATAACA
GTTAGAAATGGCTGCTAATACCGCATAAGCGCACGGTACTGCATGGTACGGTGTGAAAAACTCCGGTG
GTATGAGATGGACCCGCGTTGGATTAGCTAGTTGGCAGGGTAACGGCCTACCAAGGCGACGATCCATA
GCCGGCCTGAGAGGGTGAACGGCCACATTGGGACTGAGACACGGCC
>Zotu2214
GAACGCTGGCGGCGTGCTTAACACATGCAAGTCGAACGAGGCGCTTGCGCCTAGTGGCGAACGGGTGA
GTAACACGTGAGCAACCTGCCCCTGGCTCTGGGATAGCCTCCGGAAACGGGGATTAATACCGGACATG
ACTGCTGCCGGCATCGGCGGCGGTGGAAAGTTTTTCGGCCAGGGAGGGGCTCGCGGCCTATCAGCTTG
TTGGGGGGGTAACGGCCCACCAAGGCGACGACGGGTAGCCGGCCTGAGAGGGCGACCGGCCACACTGG
GACTGAGACATGGCC
>Zotu2215
AAACGCTGGCGGCGTGCCTAATACATGCAAGTTGAGCGGTAAGGTTCTGCACCCACTATTTGACCAGA
TGAGTTTCCGCAAGGAAGCGCCATCTGCAGCGATAACTTCTGGCTGAAGTTATAGTCGAATGTTGAGT
GTAGAATACACGAGCGGCGGACGGCAGAGTAACGCGTAGGAACGTACCCCAAACAGAGGGATAAGCAC
CGGAAACGGTGTCTAATACCGCATAATATCTTCGGATTAAAGCTTCGGCGGTTTGGGAGCGGCCTGCG
TATGATTAGCTAGTTGGTGAGGTAATGGCTCACCAAGGCGACGATCATTAGCTGGTCTGAGAGGATGA
TCAGCCTGACTGGAACTGAGAACGGTC
>Zotu2216
GAACGCTAGCGGAAGGCCTAATACATGCAAGTCGAACGGAGATATTATTTCGGTAATATTTTAGTGGC
GCACGGGTGAGTAACGCGTACACAACCTACCTTATATTGGGGGATAGCCCTGGGAAACTGGGAGTAAT
ACCGCATAATATTATAAAGCGGCATCGTTTAATAATTAAAGTTACGGCGATATAAGATGGGTGTGCGT
CTGATTAGGTAGTTGGTAGGGTAATGGCCTACCAAGCCGACGATCAGTAGGGGGTGTGAGAGCATGGC
CCCCCACACGGGTACTGAGACACGGAC
>Zotu2218
GAACGCTGGCGGCGTGCTTAACACATGCAAGTCGAGCGGTAAGGCCCTTCGGGGTACACGAGCGGCGA
ACGGGTGAGTAACACGTGAGCAACCTGCCCCTGACTCCGGGATAAGCGGTGGAAACGCCGTCTAATAC
CGGATACGACCCGCCACCTCATGGTGGAGGGTGGAAAGTTTTATCGGTCAGGGATGGGCTCGCGGCCT
ATCAGCTTGTTGGTGGGGTAAAGGCCTACCAAGGCGATTACGGGTAGCCGGCCTGAGAGGGCGACCGG
CCACACTGGGACTGAGACACGGCC
>Zotu2219
GAATGCTGGCGGCGTGGATAAGTCATGCAAGTCAAGGGGGCCCGCAAGGGCAACCGGCAGACGAGGTA
GTAACACGTAGGTACGTCCCCCGAAGTCGGGCATAGCCAGTCGAAAGATTGGGTAATTCCCGATAGTC
CTGTAAAGGTAAAGTTTTTCGCTTCGGGAGCGGCCTGCGTAGTATCAGCTAGTTGGTAGGGTAACGGC
CTACCAAGGCTACGACGCTTAGGGGAGCTGAGAGGCTGACCCCCACCGATGGAACTGCGACACGGTC
>Zotu2220
GAACGCTGGCGGCGTGCTTAACACATGCAAGTCGAACGGAAAGGCCCCTTCGGGGGTACTCGAGTGGC
GAACGGGTGAGTAACACGTGGGTGATCTGCCCTGCACTTCGGGATAAGCTTGGGAAACTGGGTCTAAT
ACCGGATATACCTCATGGATCGCATGGTTTGTGGGGGAAAGCTTTTGCGGTGTGGGATGGGCCCGCGG
CCTATCAGCTTGTTGGTGGGGTGATGGCCTACCAAGGCGTCGACGGGTAGCCGGCCTGAGAGGGTGTC
CGGCCACACTGGGACTGAGATACGGCC
>Zotu2221
GAACGCTGGCGGCATGCCTAACACATGCAAGTCGAACGAGAGCTTTCGGGCTCTAGTGGCGCACGGGT
GCGTAACGCGTGGGAATCTGCCCTTTGGTTCGGGATAACTCAGGGAAACTTGAGCTAATACCGGATGA

TGTCGCGAGACCAAAGATTTATCGCCGGAGGATGAGCCCGCGTCGGATTAGCTAGTTGGTGGGGTAAA
GGCCTACCAAGGCGACGATCCGTAGCTGGTCTGAGAGGATGATCAGCCACACTGGGACTGAGACACGG
CC
>Zotu2222
GAACGCTGGCGGCATGCCTTACACATGCAAGTCGAACGGCAGCGCGGGGCAACCTGGCGGCGAGTGGC
GAACGGGTGAGTAATATATCGGAACGTACCCTGGAGTGGGGGATAACGTAGCGAAAGTTACGCTAATA
CCGCATACGATCTAAGGATGAAAGTGGGGGATCGCAAGACCTCATGCTCCTGGAGCGGCCGATATCTG
ATTAGCTAGTTGGTAGGGTAAAAGCCTACCAAGGCATCGATCAGTAGCTGGTCTGAGAGGACGACCAG
CCACACTGGAACTGAGACACGGTC
>Zotu2223
GAACGCTGGCGACATGGATAAGGCATGCAAGTCAAGGGGGCCCGCAAGGGCAACCGGCAGACGAGGTA
GTAACACGTAGGTACTCCCCCCGAAGTCCGGCATAGCTCGTCGAAAGACGGGGTAATTCCGGATAGTC
CCGCAAGGGTAAAGTTTTTCGCTTCGGGAGAGGCCTGCGTAGTATCAGCTTGTTGGTAAGGTAACGGC
TTACCAAGGCTACGACGCTTAGGGGAGCTGAGAGGCTGACCCCCACCGATGGGACTGAGACACGGCC
>Zotu2224
GAACGCTGGCGGCATGCCTAACACATGCAAGTCGAACGAGACCTTCGGGTCTAGTGGCGCACGGGTGC
GTAACGCGTGGGAATCTGCCCTTGGGTTCGGAATAACAGTGAGAAATTACTGCTAATACCGGATGATG
TCTTCGGACCAAAGATTTATCGCCCAAGGATGAGCCCGCGTAGGATTAGCTAGTTGGTGGGGTAATGG
CCTACCAAGGCGACGATCCTTAGCTGGTCTGAGAGGATGATCAGCCACACTGGGACTGAGACACGGCC
>Zotu2225
AAACGCTGGCGGCGTGCTTAACACATGCAAGTCGAGCGAGAAAGCTCTTTCGGGAGTGAGTAAAGCGG
CGGACGAGTTAGTAACACGTGAGAATCTACCCCTCAGTGAAGAATACCCTCGAGAAATCGAGGCTAAT
GCTGCATACGTCTCAACAGAGAGATCTGTTTAGAGAAAGGTGGCCTCTTTGCTGCCGCTGGGGGATGA
GCTTGCGCACTATTAGCTAGTTGGTGGGGTAAAGGCCTACCAAGGCTATGATGGTTAGCCGGCCTGAG
AGGGTGTACGGCAACGCTGGAACTTAGACACGGTC
>Zotu2226
GAACGCTGGAAACGTGGATACCGGCATGCAAGTCACGGGGACCGCAAGGTAACCGGCAAACGGCTTAG
TAACACGTGGGTACATACCCAGAAGTGGAGCACAGCTCGTCGAAAGACGGGGTAATTCTCCATAGTAT
CGCAAGATTAAACGCGCAAGCGGCTTCTGGAATGGCCTGCGGCCTATCAGCTTGTTGGTGAGGTAAAG
GCTCACCAAGGCTATGACGGGTAGGGGGCGTGAGAGCGCGACCCCCAAGAATGGCACTGCGACACGGG
C
>Zotu2228
GAACGCTAGCGGCAGGCCTAATACATGCAAGTCGAGGGGCAGCGGGAGTAGCAATACTTGCCGGCGAC
CGGCGCACGGGTGCGTAACACGTATACAATCTGCCCATGTCTGGGGGATAGCCCGGAGAAATCCGGAT
TAATACCCCATAATAAATGAAAGGGCATCCTTTTATTTTGAAAGCTCCGGCGGACATGGATGAGTATG
CGTCTGATTAGTTAGTTGGCGAGGTAACGGCTCACCAAGACCACGATCAGTAGGGGATCTGAGAGGAT
TAACCCCCACACGGATACTGAGACACGGAT
>Zotu2229
GAACGCTGGCGGCAGGCTTAACACATGCAAGTCGAACGAACGTAGCAATACGTTAGTGGCGCACGGGT
GAGTAACGCGTGGATATCTGCCTTTTGGTTCGGGATAACCTTGGGAAACTAGGGCTAATACCGAATGG
TTCCTACGGGATAAAGATTTATCGCCAAAAGATGAGTCCGCGTCCGATTAGCTAGTTGGCGGGGTAAT
GGCCCACCAAGGCGACGATCGGTAGCTGGTCTGAGAGGACGATCAGCCACACTGGGACTGAGACACGG
CC
>Zotu2231
GAACGCTGGCGGCAGGCTTAACACATGCAAGTCGAGCGGGGAGATGTAGCTTGCTACATTTCCTAGCG
GCGGACGGGTGAGTAATGCTTAGGAATCTGCCTATTAGTGGGGGACAACATTCCGAAAGGAATGCTAA
TACCGCATACGCCCTACGGGGGAAAGCAGGGGATCTTCGGACCTTGCGCTAATAGATGAGCCTAAGTC
AGATTAGCTAGTTGGTGGGGTAAAGGCCTACCAAGGCGACGATCTGTAACGGGTCTGAGAGGATGATC
CGCCACACTGGGACTGAGACACGGCC
>Zotu2232
GAACGCTGGCGGCGTGCCTAATACATGCAAGTCGAACGAATCTTGAAGTTAGCTTGCTAACTTCTTGG
TTAGTGGCGGACGGGTGAGTAACACGTAGGCAACCTGCCCTCAAGATCGGGATAACATTCGGAAACGG
ATGCTAAGACCGGATACACAATTTTCTCGCATGAGAGAATTGGGAAACACGGTGCAAGCTGTGGCTTG
GGGATGGGCCTGCGGCGCATTAGCTAGTTGGTGGGGTAACGGCTCACCAAGGCGACGATGCGTAGCCG

ACCTGAGAGGGTGAACGGCCACACTGGGACTGAGACACGGCC
>Zotu2233
GAACGCTGGCGGCGTGCTTAACACATGCAAGTCGAACGGTAAGGCCCTTCGGGGTACACGAGTGGCGA
ACGGGTGAGTAACACGTGAGTAACCTGCCCTTGACTCTGGGATAACAGTTGGAAACAGCTGCTAATAC
CGGATACGAACCCTTGTCGCATGATGTGGGTTGGAAAGCTCCGGCGGTCAGGGATGGACTCGCGGCCT
ATCAGCTTGTTGGTGAGGTAGTGGCTCACCAAGGCTTCGACGGGTAGCCGGCCTGAGAGGGCGACCGG
CCACATTGGGACTGAGATACGGCC
>Zotu2234
GAACGCTGGCGGCATGCCTAACACATGCAAGTCGAACGATAAGGCCCCTTCGGGGGTACATAAGTGGC
GAACGGGTGAGTAACACGTGGGTAACCTGCCCTTCACTGGGGGACAACCGAGGGAAACTTCGGCTAAT
ACCGCATGATGTGCACCGGCTCAAGTCGGTGTACCAAAACTCCGGTGGTGAAGGATGGGCCCGCGGCA
GATTAGCTTGTTGGTGGGGTAAAGGCCTACCAAGGCGACGATCTGTAGCTGGTCTGAGAGGACGATCA
GCCACACTGGGACTGAGACACGGCC
>Zotu2235
GAACGCTGGCGGCGTGCCTAATACATGCAAGTCGAACGAGAGCGACCGGTGCTTGCACTGGTCAATCT
AGTGGCGAACGGGTGAGTAACACGTGGGTAACCTGCCCATCAGAGGGGGATAACATTCGGAAACGGAT
GCTAAAACCGCATAGGTCTTCGAACCGCATGGTTTGAAGAGGAAAAGAGGCGCAAGCTTCTGCTGGTG
GATGGACCCGCGGTGCATTAGCTAGTTGGTGAGGTAACGGCTCACCAAGGCCGTGATGCATAGCCGAC
CTGAGAGGGTGATCGGCCACATTGGGACTGAGACACGGCC
>Zotu2237
GAACGCTGGCGGCGTGCCTAATACATGCAAGTAGAACGCTGAAGGAGGAGCTTGCTTCTCCGGATGAG
TTGCGAACGGGTGAGTAACGCGTAGGTAACCTGCCTGGTAGCGGGGGATAACTATTGGAAACGATAGC
TAATACCGCATAAGAGTAGATGTTGCATGACATTTACTTAAAAGGTGCAATTGCACCACTACCAGATG
GACCTGCGTTGTATTAGCTAGTTGGTGGGGTAACGGCTCACCAAGGCGACGATACATAGCCGACCTGA
GAGGGTGATCGGCCACACTGGGACTGAGACACGGCC
>Zotu2238
GAACGCTGGCGGCGTGCCTAATACATGCAAGTCGTGCGGACTTATCTTCGGATAAGTTAGCGGCGGAC
GGGTGAGTAACACGTGGGCAACCTGCCTGTAAGACTGGGATAACTCCGGGAAACCGGTGCTAATACCA
GATACAATCAAAGACTGCCTGGTTTTTGATAGAAAGGTCTTGTACCGCTTACAGATGGGCCCGCGGCG
CATTAGCTAGTTGGTGGGGTAATGGCTCACCAAGGCGACGATGCGTAGCCGACCTGAGAGGGTGATCG
GCCACACTGGGACTGAGACACGGCC
>Zotu2239
GAACGCTAGCTACAGGCTTAACACATGCAAGTCGAGGGGCAGCATGAATATTGGCTTGCCAATATTTG
ATGGCGACCGGCGCACGGGTGAGTAACACGTATCCAACCTTCCGGTTACTCGGGGATAGGCTTTCGAA
AGAAAGATTAATACCCGATGTTGTGTATCTTTCTCCTGATTGATACGCCAAAGGATTCCGGTAACCGA
TGGGGATGCGTTCCATTAGGCAGTTGGCGGGGTAACGGCCCACCAAACCTTCGATGGATAGGGGTTCT
GAGAGGAAGGTCCCCCACATTGGAACTGAGACACGGTC
>Zotu2240
GAACGCTGGCGGCATGCCTAACACATGCAAGTCGAACGAAGGCTTCGGCCTTAGTGGCGCACGGGTGC
GTAACGCGTGGGAATCTGCCCTTGGGTTCGGAATAACTCGCCGAAAGGCGTGCTAATACCGGATGATG
ACGTAAGTCCAAAGATTTATCGCCCAAGGATGAGCCCGCGTAGGATTAGCTAGTTGGTGAGGTAAAGG
CTCACCAAGGCGACGATCCTTAGCTGGTCTGAGAGGATGATCAGCCACACTGGGACTGAGACACGGCC
>Zotu2241
GAACGCTGGCGGCGTGCCTAATACATGCAAGTAGAACGCTGAAGCTTGGTGCTTGCACCGAGTGGATG
AGTTGCGAACGGGTGAGTAACGCGTAGGTAACCTGCCTCTTAGCGGGGGATAACTATTGGAAACGATA
GCTAATACCGCATAAAAGTCGATATCGCATGATGTTGACTTGAAAGGTGCAATTGCATCACTAAGAGA
TGGACCTGCGTTGTATTAGCTAGTTGGTGAGGTAACGGCTCACCAAGGCGACGATACATAGCCGACCT
GAGAGGGTGATCGGCCACACTGGGACTGAGACACGGCC
>Zotu2242
CAACGCTGGCGGCGTGCCTAACACATGCAAGTCGAACGAGAAAGTGGAGCAATCCATGAGTAAAGTGG
CGACCGGGTGAGTAACACGTGACTAACCTACCTTCGAGTGGGGGATAACCTCGGGAAACCGGGGCTAA
TACCGCATAACATCTTTGAAGTGGAAATTTCGGAGATCAAAGGAGCAATTCGCTGGAAGAGGGGGTCG
CGGCTGATTAGCTAGTTGGCGGGGTAACGGCCCACCAAGGCTTAGATCGGTAGCCGGCCTGAGAGGGC
GCACGGCCACACTGGAACTGAAACACGGTC

>Zotu2243
GAACGCTGGCGGCGTGCCTAATACATGCAAGTAGAACGCACAGGATACACCGTAGTTTACTACACCGT
ATTCTGTGAGTTGCGAACGGGTGAGTAACGCGTAGGTAACCTGCCTGGTAGCGGGGGATAACTATTGG
AAACGATAGCTAATACCGCATAAGAACATTTACTGCATGGTAGATGTTTAAAAGGTGCAAATGCATCA
CTACCAGATGGACCTGCGTTGTATTAGCTAGTAGGTGAGGTAACGGCTCACCTAGGCGACGATACATA
GCCGACCTGAGAGGGTGATCGGCCACACTGGGACTGAGACACGGCC
>Zotu2244
AAACGCTGGCGGCGTGCCTAACACATGCAAGTCGAACGAGAACGGTCCTTCGGGGCCTAGTACAGTGG
CGAACGGGTGAGTAATGTATAAGAATCAACCCTTTGGTGAAGGATACCCTTGAGAAATTGAGGACAAT
CCTGCATAAGCCTTTTTGGGAAAGATGGCAGGTTTCCTGCTGTCGCCAGAGGACGAGCTTATATGCTA
TCAGCTAGTTGGTGAGGTAATAGCTCACCAAGGCTATGACGGCTAGCCGGCCTGAGAGGGTGTACGGC
CACACTGGAACTTAGACACGGTC
>Zotu2245
TAACGTTGGCGGCGTGCATAACACATGCAAGTTGAGCGTGGCTCTAGGTTTACTTAGAGTCTAGCAGC
GGACGGCTGAGTAATGCACAGGAACATACCTCGCAGTGGGGAATAGCCCTTCGAAAGAAGGGGTAATA
CCGCATAGTCTCGCAAGAGTAAAGCCGCAAGGCGCTGTGAGAATGGCCTGTGTCCTATCAGGTAGTTG
GTGTGGGTAATGGCCTACCAAGCCTATGACGGGTAGCTGGTCTGAGAGGATGACCAGCCAGATCGGGA
CTGAGACACGGCC
>Zotu2246
GAACGCTGGCGGCGTGCTTAACACATGCAAGTCGAACGGAAAGGCCTTTCGGGGTACTCGAGTGGCGA
ACGGGTGAGTAACACGTGGGTGATCTGCCCTGCACTTTGGGATAAGCCTGGGAAACTGGGTCTAATAC
CGGATAGGACTACGCACTGCATGGTGTGTGGTGGAAAGCTTTTGCGGTGTGGGATGGGCCCGCGGCCT
ATCAGCTTGTTGGTGGGGTGATGGCCTACCAAGGCGACGACGGGTAGCCGGCCTGAGAGGGTGTCCGG
CCACACTGGGACTGAGATACGGCC
>Zotu2247
GAACGCTGGCGGCGTGCCTAATACATGCAAGTCGAGCGGAAGTTGGAGAGCTTGCTCTTCAACTTTAG
CGGCGGACGGGTGAGTAACACGTGGGCAACCTGCCCTGCAGATCGGGATAACTCCGGGAAACCGGTGC
TAATACCGAATAGTTTTCGCCACCTCCTGGTGGCGTACGGAAAGACGGTTTCGGCTGTCACTGCAGGA
TGGGCCCGCGGCGCATTAGCTAGTTGGTGGGGTAACGGCCCACCAAGGCGACGATGCGTAGCCGACCT
GAGAGGGTGATCGGCCACACTGGGACTGAGACACGGCC
>Zotu2248
GAACGCTGGCGGCGTGCTTAACACATGCAAGTCGAACGAGGTCCATTCCAGCTTGCTGGAGGAAGACC
TAGTGGCGAACGGGTGAGGAACACGTGAGAAACTTGCCCCGGAGAGGTGGACAACAGCGGGAAACCGC
TGCTAATACACCATGTTGAAACTGTGACCGCATGGTCATGTTTTGAAATGAATTTCGCTCCGGGAGAG
TCTCGCGGCCTATCAGCTTGTTGGTGAGGTAACGGCTCACCAAGGCTTCGACGGGTAGCTGGTCTGAG
AGGACGATCAGCCACATTGGGACTGAGACACGGCC
>Zotu2249
GAACGCTGGCGGCATGCTTAACACATGCAAGTCGCACGGGCAGTGGTAACATTGTCAGTGGCGGACGG
GTGAGTAACGCGTAGGAATCTGTCCTTGGGTGGGGGATAACAGTGGGAAACTACTGCTAATACCGCAT
GACAACTGAGGTTTAAAGGCGTGAGTCGCCTGAGGAGGAGCCTGCGTTCGATTAGCTAGTTGGTGGGG
TAAAGGCCTACCAAGGCTACGATCGATAGCTGGTCTGAGAGGATGATCAGCCACACTGGGACTGAGAC
ACGGCC
>Zotu2250
GAACGCTGGCGGCGTGCCTAATACATGCAAGTAGAACGCTGAAGGGAGAGCTTGCTCTTCTGGATGAG
TTGCGAACGGGTGAGTAACGCGTAGGTAACCTGCCTGGTAGCGGGGGATAACTATTGGAAACGATAGC
TAATACCGCATAACATTGACTATTGCATGATAGTCAATTGAAAGGTGCAATTGCACCACTACCAGATG
GACCTGCGTTGTATTAGCTAGTTGGTGGGGTAACGGCTCACCAAGGCGACGATACATAGCCGACCTGA
GAGGGTGATCGGCCACACTGGGACTGAGACACGGCC
>Zotu2251
AAATGCTGGCGGCGTGCCTAATACATGCAAGTCGAGCGGTAAGGCTTTCACAGATTATTTAAACGGAA
AAGTCCATTTATGGATACTATCCTTCAACGTTTCTTCACCCGGCTAGGTGATATATAAATGGTTGAAT
AATGTGTGAAAGTACACGAGCGGCGGACGGCTGAGTAACGCGTAGGAATGTACCCCAAAGTGAGGGAT
AAGCTCTGGAAACAGAGTCTAATACCGCATGTGATCTAAGGATTAAAGCCTTCGGGCGCTTTGGGAGC
AGCCTGCGTAAGATTAGTTTGTTGGTGAGGTAATGGCTCACCAAGACTACGATCTTTAGCTGGTCTGA

GAGGATGATCAGCCAGACTGGGACTGAGACACGGCC
>Zotu2252
GAACGCTGGCGGCATGCCTAATACATGCAAGTCGTGCGAATGCTTCAAGGAGCTTGCTCTTTGAAGTG
TTAGCGGCGGATGGGTGAGTAACACGTGGGCAACCTGCCCTACAGATGGGGATAACTCCGGGAAACCG
GGGCTAATACCGAATAAACAGTTTGTCCGCATGGACAAGCTCTAAAAGACGGTTTCGGCTGTCACTGT
AGGATGGGCCCGCGGCGCATTAGCTAGTTGGTGGGATAATGGCCTACCAAGGCAACGATGCGTAGCCG
ACCTGAGAGGGTGATCGGCCACACTGGGACTGAGACACGGCC
>Zotu2253
GAACGCTGGCGGCGCGCTTAACACATGCAAGTCGAGCGAGAACCAGGACTTCGGTCCTGGGGACAGCG
GCGAACGGGTGAGTAACACGTGGGTAATCTGCCCTCGATTCTGGGATAGCCCGGGGAAACCCGGATTA
ATACCGGATAGCCTTTCGAGCTTTCGGGCTTGGAAGAAAAGGTAGCTTCGGCCTCCGATCGAGGATGA
GCCCGCGGTGGATTAGCTTGTTGGCGGGGTAATGGCCCACCAAGGCGACGATCCATAGCTGGTCTGAG
AGGACGATCAGCCACACTGGGACTGAGACACGGCC
>Zotu2256
GAACGCTGGCGGCGTGCTTAACACATGCAAGTCGAGCGATGAAGCACCTTCGGGTGTGAATTAGCGGC
GAACGGGTGAGGAACACGTGAGAAATCTGCCTTCAACTCTGGGATAACTCCGTGAAAACGGGGCTAAT
ACCGGATATGAGATCTACAGGCATCTGTGGATCTGGAAAGTTTTTCGGTTGAAGATGATCTCGCGGCC
TATCAGCTTGTTGGTGAGGTAATGGCTCACCAAGGCGACGACGGGTAGCCGGCCTGAGAGGGCGACCG
GCCACACTGGGACTGAGACACGGCC
>Zotu2257
GAACGCTAGCGGCAGGCTTAACACATGCAAGTCGAGGGGTATAGTAGCAATACTAGAGACCGGCGCAC
GGGTGCGTAACGCGTATGCAATCTACCTTTTGCAGGGGAATAGCCCAGAGAAATTTGGATTAATGCCC
CATAGTATAGTGACTCGGCATCGAGATACTATTAAAGTTACAACGGCAAAAGATGAGCATGCGTCCCA
TTAGCTAGTTGGTAAGGTAACGGCTTACCAAGGCGACGATGGGTAGGGGTCCTGAGAGGGAGATCCCC
CACACTGGTACTGAGACACGGAC
>Zotu2258
GAACGCTGGCGGCGTGCTTAACACATGCAAGTCGAACGATGAAGCCCAGCTTGCTGGGTGGATTAGTG
GCGAACGGGTGAGTAACACGTGAGCAACCTGCCCCTGACTCTGGGATAACAGCCGGAAACGGTTGCTA
ATACCGGATATGCATCATGGCCGCATGGTCTGTGATGGGAAAGATTTTTCGGTTGGGGATGGGCTCGC
GGCCTATCAGCTTGTTGGTGAGGTAGTGGCTCACCAAGGCGTCGACGGGTAGCCGGCCTGAGAGGGTG
ACCGGCCACACTGGGACTGAGACACGGCC
>Zotu2260
GAACGCTGGCGGCGCGCTTAACACATGCAAGTCGAGGGAGAACCGGCTTTCGGGTCGGGGAGACCGGC
GAACGGGTGAGTAACACGTGAGGAACCTGCCCTGTAGATCGGAATAACCATGGGAAACTGTGGCTAAT
GCCGAATATGCTCTTCGCATCGCATGGTGTGGGGAGGAAATGGGTTTCTGCTACGGGATGGCCTCGCG
GCCTATCAGCTTGTTGGTGGGGTGATGGCCTACCAAGGCGACGACGGGTAGCTGGCGTGAGAGCGCGA
CCAGCCACACTGGGACTGAGACACGGCC
>Zotu2262
GAACGCTGGCGGCGTGCTTAACACATGCAAGTCGAGCGGAAAGGCCCTTCGGGGTACTCGAGCGGCGA
ACGGGTGAGTAACACGTGAGTAACCTGCCCCTAGCTCTGGGATAGCTCCGGGAAACCGGGATTAATAC
CAGATACGACCACTTTAGACATCTGTCGTGGTGGAAAGGATTCCGGCTAGGGATGGGCTCGCGGCCTA
TCAGCTTGTTGGTGGGGTAACGGCCTACCAAGGCGACGACGGGTAGCCGGCCTGAGAGGGCGACCGGC
CACACTGGGACTGAGACACGGCC
>Zotu2263
GAACGCTGGCGGCGTGCTTAACACATGCAAGTCGAACGATGAAGAAAAGAATTGAAGCTTCGGCAGAA
ATTCATTTCGGATTAGTGGCAGACGGGTGAGTAACGCGTGAGCAACCTACCTCTTACAAAGGGATAGC
CTCGGGAAACCGGGATTAATACCATATAACATTGCGACGAAGCATTTCGATGCAATCAAAGCTACGGC
GGTAAGAGATGGGCTCGCGTCCCATTAACCAGATGGTGAGGTAACGGCTCACCATAGTAACGATGGGT
AACCGGCCTGAGAGGGTGAACGGTCACACTGGAACTGAGACACGGTC
>Zotu2264
GAACGCTGGCGGCGTGCCTAATACATGCAAGTCGAGCGGACAGATGGGAGCTTGCTCCCTGATGTCAG
CGGCGGACGGGTGAGTAACACGTGGGTAACCTGCCTGTAAGACTGGGATAACTCCGGGAAACCGGGGC
TAATACCGGATGCTTGTTTGAACCGCATGGTTCAAACATAAAAGGTGGCTTCGGCTACCACTTACAGA
TGGACCCGCGGCGCATTAGCTAGTTGGTGAGGTAACGGCTCACCAAGGCAACGATGCGTAGCCGACCT

GAGAGGGTGATCGGCCACACTGGGACTGAGACACGGCC
>Zotu2265
GAACGCTGGCGGCGCGCTTAACACATGCAAGTCGAGCGAGAACCAGGACTTCGGTCCTGGGGACAGCG
GCGAACGGGTGAGTAACACGTGGGTAATCTGCCCTCGGTTCCGGGATAGCCCGGGGAAACTCGGATTA
ATACCGGATAGCCTCACGAACCTTCGGGCGAGTGAGAAAAGGTAGCTTTGGCCTCTGACCGAGGATGA
GCCCGCGGTGGATTAGCTTGTTGGCGGGGTAACGGCCCACCAAGGCGACGATCCATAGCTGGTCTGAG
AGGACGATCAGCCACACTGGGACTGAGACACGGCC
>Zotu2266
GAACGCTAGCGGCAGGCTTAATACATGCAAGTCGAACGGTCTTAGCAATAAGACGAGTGGCGCACGGG
TGAGTAACGCGTACATAACATACCTTTAACTGGCGAATAGCCTTGGGAAACTGAGAGTAATACGCCAT
AACATTGCGGTTCGGCATCGGACAGCAATTAAAGTTCCGGCGGTTAGAGATTGATGTGCGTTGGATTA
GGTAGTTGGTGAGGTAACGGCTCACCAAGCCAACGATCCATAGGGGGCGTGAGAGCGTGGTCCCCCAC
ACGGGTACTGAGACACGGAC
>Zotu2267
GAACGCTGGCAGCGTGCTTAACACATGCAAGTCGAACGGTGAAGCCCAGCTTGCTGGGTGGATCAGTG
GCGAACGGGTGAGTAACACGTGAGCAATCTGCCCCTGACTCTGGGATAAGCGCTGGAAACGGCGTCTA
ATACTGGATATGAACCGTGGAGGCATCTTCAACGGTTGGAAAGATTTTTTGGTCAGGGATGAGCTCGC
GGCCTATCAGCTTGTTGGTGAGGTAATGGCTCACCAAGGCGTCGACGGGTAGCCGGCCTGAGAGGGTG
ACCGGCCACACTGGGACTGAGACACGGCC
>Zotu2269
GAACGCTAGCTACAGGCTTAACACATGCAAGTCGAGGGGCAGCATGGTCTTAGCTTGCTAAGGCCGAT
GGCGACCGGCGCACGGGTGAGTAACACGTATCCAACCTGCCGTCTACTCTTGGACAGCCTTCTGAAAG
GAAGATTAATACAAGATGGCATCATGAGTTCACATGTTCACATGATTAAAGGTATTCCGGTAGACGAT
GGGGATGCGTTCCATTAGATAGTAGGCGGGGTAACGGCCCACCTAGTCTTCGATGGATAGGGGTTCTG
AGAGGAAGGTCCCCCACATTGGAACTGAGACACGGTC
>Zotu2270
GAACGCTGGCGGCGTGCTTAACACATGCAAGTCGAACGCTGAAGCCTAGCTTTTGTTGGGTGGATGAG
TGGCGAACGGGTGAGTAACACGTGAGTAACCTGCCCCCTTCTTTGGGATAACGCCCGGAAACGGGTGC
TAATACTGGATATTCACTGATCTTCGCATGGGGGTTGGTGGAAAGGTTTTTTCTGGTGGGGGATGGGC
TCGCGGCCTATCAGCTTGTTGGTGGGGTGATGGCCTACCAAGGCTTTGACGGGTAGCCGGCCTGAGAG
GGTGACCGGTCACATTGGGACTGAGATACGGCC
>Zotu2271
GAACGCTGGCGGCGTGCCTAATACATGCAAGTCGAGCGGACAGATGGGAGCTTGCTCCCTGAAGTTAG
CGGCGGACGGGTGAGTAACACGTGGGCAACCTGCCCTGCAGATGGGGATAACTCCGGGAAACCGGGGC
TAATACCGAATAATCAGTTTCTCCGCATGGAGAAACTCTGAAAGACGGTTTCGGCTGTCACTGCAGGA
TGGGCCCGCGGCGCATTAGCTAGTTGGTGGGATAACGGCCTACCAAGGCAACGATGCGTAGCCGACCT
GAGAGGGTGATCGGCCACACTGGGACTGAGACACGGCC
>Zotu2272
GAACGCTGGCGGCGTGCTTAACACATGCAAGTCGAACGATGAAGCCCAGCTTGCTGGGTGGAAGAGTG
GCGAACGGGTGAGTAACACGTGAGTAACCTGCCCTGAACTCTGGGATAAGCACTGGAAACGGTGTCTA
ATACTGGATACGACCTATCACCGCATGGTGTGTGGGTGGAAAGATTTATCGGTTTTGGATGGACTCGC
GGCCTATCAGCTAGATGGTGAGGTAATGGCTCACCATGGCGACGACGGGTAGCCGGCCTAAGAGGGTG
ACCGGCCACACTGGGACTGAGACACGGCC
>Zotu2273
GAACGCTGGCGGCATGCCTAACACATGCAAGTCGAACGGATGGTGGCAACACCATTAGTGGCGGACGG
GTGAGAAACGCGTGGGAACCTGCCTAGCTATGGGGGATAACCAGGGGAAACCTTGGCTAATACCGCAT
ACGCCCCGAGGGGGAAAGGCTTCGGCCGTAGTTAGAGGGGCCCGCGTCCGATTAGGTAGTTGGTGAGG
TAACGGCTCACCAAGCCGACGATCGGTAGCTGGTCTGAGAGGATGATCAGCCACACTGGGACTGAGAC
ACGGCC
>Zotu2274
GAACGCTGGCGGCGTGCTTAATACATGCAAGTCGAACGAGGTTGATCCAGTGGTAACACTGGTGACTA
CCTAGTGGCGAACGGGTGAGTAACACGTGAGCAACCTGCCCCGAAGACCGGGATAACAGTGGGAAACC
GCTGCTAATACCGGATACCCCCACCGGATCACATGGTCTGGTGAGGAAATGGATTCCGCTTCGGGAGG
GGCTCGCGGCCTATCAGCTTGTTGGTGGGGTAACGGCCTACCAAGGCTTCGACGGGTAGCTGGTCTGA

GAGGACGATCAGCCACACTGGGACTGAGACACGGCC
>Zotu2275
GAACGCTGGCGGCGTGCTTAATACATGCAAGTCAACTGAGCTTAAGGTAGCAATATCTTAAGTGCGGT
GGCGCACGGGTGAGTAACACGTAGGTAATCTGCCTTTAGGTCTGACATAATCCCGCGAAAGCGGGACT
AAAATCAGATGATGCAGCGGCTCGACATCGAGACAGTTGTTAAACCTTCGGGGCCTAAAGATGAGCCT
GCGTCTGATTAGGTAGTTGGCGGAGTAAAAGCCCACCAAGCCTGCGATCAGTAGCTGGTCTGAGAGGA
TGATCAGCCACACTGGAACTGAGACACGGTC
>Zotu2276
GAACGCTGGCGGCGTGGATAAGGCATGCAAGTCGAACGGAACTTATTTAGCTCGAGATGCGATTACAT
CAAGTCAAGAATTTTGTAAGTTTAGTGGCAGACGAGGTAGGAATACATATGTACATACCCCCGAGACG
GGCACAACACCGCGAAAGCGGTGCTAATTCCCGATATTGTGGTAACACGAAAGTCGCAAGACGCTCAG
GGATTGGCGTATGGGCCATCAGCTTGTTGGTAGGGTAACGGCCTACCAAGGCTATGACGGCTAGGAGG
CGTGAGAGCGTGACCTCCACCGACGGAACTGAGACACGGTC
>Zotu2277
GAACGCTAGCGGCAGGCTTAATACATGCAAGTCGAGGGGCAGCACAGTGTAGCAATACATGGGTGGCG
ACCGGCAAACGGGTGCGGAACACGTACACAACCTTCCTTCAAATGGGGAATAGCCCAGGGAAACTTGG
ATTAATACCCCGTAACATTATGAGGTGGCATCACTTTATAATTATAGCTCCGGCGTTTGAAGATGGGT
GTGCGGCTGATTAGGTAGTTGGCGGGGTAACGGCCCACCAAGCCTTCGATCAGTAACTGGTGTGAGAG
CACGACCAGTCACACGGGCACTGAGACACGGGC
>Zotu2278
TAACGCTGGCGGCGTGCATAACACATGCAAGTCGAACGATGAAACTTTTTTGATTTCTTCGGAATGAA
AAAAAGTGGATTAGTGGCGGACGGGTGAGTAACGCGTGAGTAACCTGCCTTAGAGAAGGGGATAGCCT
TTGGAAACGAAGAATAATACCCTATGAAACCATAACCTCGCATGAGGCCTTGGTCAAAGATTTATCGC
TCTAAGATGGACTTGCGTCTGATTAGTTAGTTGGTGGGGTAACGGCCTACCAAGACAGCGATCAGTAG
CCGGCTTGAGAGAGTGTACGGCCACATTGGGACTGAGACACGGCC
>Zotu2279
GAACGCTGGCGGCGTGCTTAACACATGCAAGTCGAACGGTGAAAGAGAGCTTGCTCTCTGGATCAGTG
GCGAACGGGTGAGTAACACGTGAGCAATCTGCCCCTGACTCTGGGATAAGCGCTGGAAACGGCGTCTA
ATACCGGATACGAGCTGCGAAGGCATCTTCGGCAGCTGGAAAGAATTTCGGTCAGGGATGAGCTCGCG
GCCTATCAGCTAGTTGGTGAGGTAATGGCTCACCAAGGCGTCGACGGGTAGCCGGCCTGAGAGGGTGA
CCGGCCACACTGGGACTGAGACACGGCC
>Zotu2280
GAACGCTGGCGGCGTGGATAAGGCATGCAAGTCGAGTGGGTTTAGACCCACGGCAGACGAGGTAGGAA
CACGTAGGTATGTACCCTCAAGTCGGAGATAACCCATCGAAAGGTGGAGTAATATTCGATAGTCCCTC
CGGGGTAAAGGCGCAAGCCGCTTGAGGAACAGCCTGCGGGCTATCAGCTAGTTGGTAGGGTAATGGCC
TACCAAGGCGATGACGGCTAGGGGAGCTGAGAGGCTGACCCCCACCGATGGAACTGAGACACGGTC
>Zotu2281
GAATGCTGGCGGCGTGGATAAGGCATGCAAGTCGAACGGTCCGCAAGGACAGTGGCAGACGAGGTAGT
AACGAGTAGGTACACCCCCCGAAGTCGGGCATAGCCAGCCGAAAGGTTGGGTAATTCCCGATGGTGTC
GCAAGACTAAAGATTTATCGCTTCGGGAGTGGCCTGCTTAATATCAGCTAGTTGGTGAGGTAATGGCT
CACCAAGGCTACGACGTTTAGGGGAGCTGAGAGGCTGACCCCCACCGATGGGACTGCGACACGGCC
>Zotu2283
GAACGCTGGCGGCGTGCTTAACACATGCAAGTCGAACGGATCGCATGTTTTCGGATGTGTGGTGAGTG
GCGAACGGGTGAGTAACACGTGAGTAACCTGCCCCGAACTCTGGGATAACGACTCGAAAGGGTTGCTA
ATACCGGATATTGAACTGTTGCCGTGTGGTGGTGGTTTGAAAGTTTTTCGGTTCGGGATGGGCTCGCG
GCCTATCAGCTTGTTGGTGGGGTGATGGCCTACCAAGGCGTCGACGGGTAGCCGGCCTGAGAGGGCGA
CCGGCCACACTGGGACTGAGATACGGCC
>Zotu2284
GAACGCTGGCGGCGTGCTTAACACATGCAAGTCGAACGGAAAGGCCCTTCGGGGTACTCGAGTGGCGA
ACGGGTGAGTAACACGTGGGTGATCTGCCCTGCACTTCGGGATAAGCTTGGGAAACTGGGTCTAATAC
CGGATATGATCATCGGCTTCATGGTCGGTGGTGGAAAGCTTTTGCGGTGTGGGATGGGCCCGCGGCCT
ATCAGCTTGTTGGTGGGGTAATGGCCTACCAAGGCGACGACGGGTAGCCGGCCTGAGAGGGTGTCCGG
CCACACTGGGACTGAGATACGGCC
>Zotu2286

GAACGCTGGCGGCAGGCTTAACACATGCAAGTCGAGCGCCCCGCAAGGGGAGCGGCAGACGGGTGAGT
AACGCGTGGGAATCTACCTTGAACTACGGGATAGCTCCGGGAAACTGGAATTAATACCGTATGTGCCC
TTTGGGGGAAAGATTTATCGGTTCAGGATGAGCCCGCGTCGGATTAGCTAGTTGGTGGGGTAAAGGCC
TACCAAGGCGACGATCCGTAGCTGGTCTAAGAGGATGATCAGCCACATTGGGACTGAGACACGGCC
>Zotu2288
GAACGCTAGCGGGAGGCTTAATACATGCAAGTCGAGCGGTAAGTTATTTATAGCAATATAGATGACCT
AGAGCGGCGCACGGGTGAGTAACGCGTACATAACCTACCCTTTACTGGGGGAAAGTCACGGGAAACTG
TGAATAATACCGCATAGTATTAATTAACTGCATGGTTGATTAATTAAAGATTTATTGGTAAGGGATGG
GTGTGCGTCTGATTAGCTAGTTGGTAGGGTAACGGCCTACCAAGGCGATGATCAGTAGGGGGCGTGAG
AGCGTGATCCCCCACACGGGTACTGAGACACGGAC
>Zotu2289
GAACGCTGGCGGCGTGCCTAATACATGCAAGTCGAGCGGATGACGGGGAGCTTGCTCCCCTGATTCAG
CGGCGGACGGGTGAGTAACACGTGGGCAACCTGCCTGTAAGATCGGGATAACTCCGGGAAACCGGGGC
TAATACCGGATGGTTCCTTCCTCCGCATGGAGGAAGGCGGAAAGACGGTTTCGGCTGTCACTTACAGA
TGGGCCCGCGGCGCATTAGCTAGTTGGTGGGGTAACGGCCCACCAAGGCAACGATGCGTAGCCGACCT
GAGAGGGTGATCGGCCACACTGGGACTGAGACACGGCC
>Zotu2290
GAACGCTGGCGGCAGGCTTAACACATGCAAGTCGAACGCTCGCAGCAATGCGGGAGTGGCAGACGGGT
GAGTAACGCGTGGGAACATACCCTTTGGTTCGGAATAACTGAGAGAAATTTCAGCTAATACCGGATGT
GCCCTTCGGGGGAAAGATTTATCGCCAAAGGATTGGCCCGCGTCTGATTAGCTAGTTGGTGAGGTAAC
GGCTCACCAAGGCGACGATCAGTAGCTGGTCTGAGAGGATGATCAGCCACACTGGGACTGAGACACGG
CC
>Zotu2291
GAACGCTGGCGGCATGCCTTACACATGCAAGTCGAACGGTAACAGGTCTTCGGACGCTGACGAGTGGC
GAACGGGTGAGTAATGCATCGGAACGTGCCCAGTCGTGGGGGATAACTGCTCGAAAGAGCAGCTAATA
CCGCATACGACCTGAGGGTGAAAGCGGGGGACCGTAAGGCCTCGCGCGATTGGAGCGGCCGATGTCAG
ATTAGCTAGTTGGTGGGGTAAAGGCCTACCAAGGCAACGATCTGTAGTTGGTCTGAGAGGACGACCAG
CCACACTGGGACTGAGACACGGCC
>Zotu2292
GAACGCTGGCGGCGTGCTTAACACATGCAAGTCGAACGGAAAGGCCGAAGCTTGCTTTGGTACTCGAG
TGGCGAACGGGTGAGTAACACGTGGGTGATCTGCCCTGCACTTTGGGATAAGCCTGGGAAACTGGGTC
TAATACCGGATAGGACCACGGTGTGGTGATCGTGGTGGAAAGTTTTTTCGGTGTGGGATGAGCTCGCG
GCCTATCAGCTTGTTGGTGGGGTAATGGCCTACCAAGGCGTCGACGGGTAGCCGGCCTGAGAGGGTGT
ACGGCCACATTGGGACTGAGATACGGCC
>Zotu2294
GAACGCTGGCGGCGTGCCTAATACATGCAAGTCGAGCGAGCAGAACCAGCAGATTTACTTCGGTAATG
ACGCTGGGGACGCGAGCGGCGGATGGGTGAGTAACACGTGGGGAACCTGCCCCATAGTCTGGGATACC
ACTTGGAAACAGGTGCTAATACCGGATAAGAAAGCAGATCGCATGATCAGCTTATAAAAGGCGGCGTA
AGCTGTCGCTATGGGATGGCCCCGCGGTGCATTAGCTAGTTGGTAAGGTAACGGCTTACCAAGGCGAT
GATGCATAGCCGAGTTGAGAGACTGATCGGCCACATTGGGACTGAGACACGGCC
>Zotu2295
GAACGCTAGCGGCAGGCTTAATACATGCAAGTCGTGGGACGATAGCAATATTGGACCGGCAAACGGGT
GCGTAACACGTACACAACTTTCCGTCAAGTGGGGGATAGCCTAAAGAAATTTGGATTAATACCCCATA
GAATAATGATGTGGCATCATATTATTATTAAAGCTCCGGCGCTTGATGATGGGTGTGCGGCTGATTAG
GTAGTTGGCGGGGTAACGGCCCACCAAGCCTTCGATCAGTAGCTGATGTGAGAGCATGATCAGCCACA
CGGGCACTGAGACACGGGC
>Zotu2296
CAACGCTGGCGGCGTGCCTAATGCATGCAAGTCGAGCGCGGGCCTCTTCGGGGGTGCTGCGCGGCGGA
CGGGTGCGTAACACGTGGGCAACCTGCCCTGGGGTGGGGGATAGCCGTGGGAAACCGCGGGTAATTCC
GCATACGGTGCTCTTTCGATCGGGAGGAGTACGAAAGACGCTTCGGCGTCGCCCTGGGAGGGGCCTGC
GGCCGATTAGCTAGACGGTGGGGTCACGGCCTACCGTGGCGATGATCGGTAGCTGGTCTGAGAGGACG
GCCAGCCACACGGGGACTGAGACACGGCC
>Zotu2297
GAACGCTGGCGGTATGCCTAACACATGCAAGTCGAACGGAATCTTCGGATTTAGTGGCGGACGGGTGA

GTAACGCGTGAGAATCTGCCTTCAGGATGGGGACAACAGTTGGAAACGACTGCTAATACCCGATATGC
AGCGATGTGAAAGATTTATCGCCTGGAGATGAGCTCGCGTTGGATTAGCTAGATGGTGTGGTAATGGC
GCACCATGGCGACGATCCATAGCTGGTCTGAGAGGATGAGCAGCCACACTGGGACTGAGACACGGCC
>Zotu2298
GAACGCTGGCGGCGTGCCTAATACATGCAAGTCGAGCGAATCAATGGGAGCTTGCTCCCTGAGATTAG
CGGCGGACGGGTGAGTAACACGTGGGTAACCTGCCTGTAAGACTGGGATAACTTCGGGAAACCGGAGC
TAATACCGGATAATTTCTTTTCTCGCATGAGAAGAGATGGAAAGATGGTTTCGGCTATCACTTACAGA
TGGACCCGCGGCGCATTAGCTAGTTGGTGAGGTAACGGCTCACCAAGGCCACGATGCGTAGCCGACCT
GAGAGGGTGATCGGCCACACTGGGACTGAGACACGGCC
>Zotu2299
GAACGCTGGCGGCGTGCTTAACACATGCAAGTCGAGCGAACCTTCGGGTTAGCGGCGAACGGGTGAGT
AACACGTGGGCAACCTGCCCCGAGCTCTGGGATAACCCCGGGAAACCGGGGCTAATACCGGATACGAC
ACCGACAGACATCTGTCGGTGTGGAAAGTTTTTCGGCTCGGGATGGGCCCGCGGCCTATCAGCTTGTT
GGTGGGGTAACGGCCTACCAAGGCGACGACGGGTAGCCGGCCTGAGAGGGCGACCGGCCACACTGGGA
CTGAGACACGGCC
>Zotu2300
GAACGCTGGCGGCGTGCTTAACACATGCAAGTCGAACGGGATTTAGTAAACAGAAGCTTCGGTGGAAG
ATTACTAATGAGAGTGGCGAACGGGTGAGTAACGCGTGAGCAACCTGCCTATGACAGTGGGATAGCCT
CGGGAAACCGGGATTAATACCGCATAAAATCGTAGAAACACATGTTTTAGCGGTCAAAGATTTATCGG
TCATAGATGGGCTCGCGTCTGATTAGCTAGTTGGTGAGATAACAGCCCACCAAGGCGACGATCAGTAG
CCGGTCTGAGAGGATGAACGGCCACATTGGAACTGAGACACGGTC
>Zotu2301
GAACGCTGGCGGCGTGCTTAAGACATGCAAGTCGAACGGGCTCTTCGGAGCCAGTGGCGCACGGGTGA
GTAACGCGTAACTGACCTACCCAGAAGTCCGGAATAACGGGTCGAAAGATCCGCTAATACTCGATGTG
CAGTGTCGCTGTGGCGACACTGTAAAGATTGATCGCTTGTGGATGGGGTTGCGTTTCATCAGCTAGTT
GGTGGGGTAAAGGCCTACCAAGGCGACGACGAATAGCCGGCCTGAGAGGGTGGCCGGCCACAGGGGCA
CTGAGACACGGGT
>Zotu2302
GAACGCTGGCGGCGTGCTTAACACATGCAAGTGGAGCGACGAACCGGGCTTCGGCCCGGGGCAGAGCC
GCGAACGGGTGAGTAACACGTGGGTCATCTGCCCAGTTGACAGGGACAACCCGGGGAAACCCGGGCTA
ATACCTGATGTGCCCTTCGGGGGAAAGGAAGCTTCGGCCTCCGCAACTGGATGAGCCCGCGGCCCATT
AGCTTGTTGGTGGGGTAACGGCCTACCAAGGCGACGATGGGTAGCTGGTCTGAGAGGACGATCAGCCA
CACTGGGACTGAGACACGGCC
>Zotu2303
GAACGCTGGCGGTATGCTTTACACATGCAAGTCATACGAGGATTTTTAATTCAAGTGGCGGACGGGTG
AGTAACACGTAAGAATTTACCTTTAGGAGGGGGATAACAACTGGAAACGGTTGCTAATACCCCATATG
CTTTCGAGTGAAATGGAGTTTTCCGCCTAGAGAGAAGCTTGCGGCTGATTAGCTTGTTGGTGAGGTAA
TGGCTTACCAAGGCGACGATCAGTATCTGGTTTGAGAGGACGATCAGACACACTGGAACTGATACACG
GTC
>Zotu2304
GAACGCTGGCGGCGTGCCTAACACATGCAAGTTGAGCGACGAACCAGGGCTTGCCCTGGGGCAAAGCA
GCGAACGGGTGAGTAACACGTGGGTAACCTGCCCCGATAACCGGGACAACCCGAGGAAACTCGGGCTA
ATACCGGATGTGCTCGTCAGACCCAAGTCTGACGCGTAAAGGAAGCTTCGGCCTCCGTATCGGGAGGG
GCCCGCGGCCCATTAGCTAGTTGGTGAGGTAACGGCTCACCAAGGCTGCGATGGGTAGCTGGTCTGAG
AGGACGATCAGCCACACTGGGACTGAGACACGGCC
>Zotu2305
GAACGCTGGCGGCGTGCTTAACACATGCAAGTCGAACGATGAAGCCCAGCTTGCTGGGTGGAAGAGTG
GCGAACGGGTGAGTAACACGTGAGTAACCTGCCCTGAACTCTGGGATAAGCACTGGAAACGGTGTCTA
ATACTGGATACGACCTATCACCGCATGGTGTGTGGGTGGAAAGATTTATCGGTTTTGGATGGACTCGC
GGCCTATCAGCTAGATGGTGAGGTAATGGCTCACCATGGCGACGACGGGTAGCCGGCCTGAGAGGGTG
ACCGGCCACACTGGGATTGAGACACGGCC
>Zotu2306
GAACGCTGGCGGCGTGCTTAACACATGCAAGTCGAGCGGTAAGGCTCCTTCGGGGGTACACGAGCGGC
GAACGGGTGAGTAACACGTGAGTAATCTGCCCTCCACTTCGGGATAAGCCTCGGAAACGGGGTCTAAT

ACCGGATACGACACAGTCTTGCATGAGATCTGTGTGGAAAGTTTTTCGGTGGGGGATGTGCTCGCGGC
CTATCAGCTTGTTGGTGGGGTAATGGCCTACCAAGGCTTCGACGGGTAGCCGGCCTGAGAGGGTGACC
GGCCACACTGGGACTGAGACACGGCC
>Zotu2307
GAACGCTAGCGGCAGGCTTAATACATGCAAGTCGAGGGGTAACAGGGGTAGCAATACCTGCTGACGAC
CGGCAAACGGGTGCGGAACACGTACGCAACCTCCCCATAACTGGTGAATAGCCCTCCGAAAGGAGGAT
TAATACACCGTAACATAATGAAATGGCATCATTTTATTATTATAGGCTTCGGTCGGTTTTGGATGGGC
GTGCGCCTGATTAGGTAGTTGGCGGGGTAACGGCCCACCAAGCCTGCGATCAGTAACTGGTGTGAGAG
CACGACCAGTCACACGGGCACTGAGACACGGGC
>Zotu2308
GAACGCTGGCGGCGTGCCTAATACATGCAAGTCGAACGAACCGACGGAGAACTTGTTCTCTTGACGTG
AGTGGCGGACGGGTGAGTAACACGTGGGAAACCTACCCTTCAGCGGGGGATAACGGTTCGAAAGAATC
GCTAATACCGCATAAAAGGATCAGTCGCCTGGCTGATTCGTAAAAGATGGCTCTGCTATCACTGAAGG
ATGGGCCCGCGGCGCATTAGCTAGTTGGTAAGGTAACGGCTTACCAAGGCGATGATGCGTAGCCGACC
TGAGAGGGTAATCGGCCACATTGGGACTGAGACACGGCC
>Zotu2309
GAACGCTGGCGGCGTGCCTAATACATGCAAGTCGAACGAAGTACAAGAAGCTTGCTTCTTGTAACTGA
GTGGCGAACGGGTGAGTAATACATAAGCAACCTGCCTTAATGCCTGGGATAACAGTTGGAAACGATTG
CTAATACCGGATAATTTCTTGATAGACATCTATTAAGAATAAAAGTTGGGATACAACACATTGAGATG
GGCTTATGGCGCATTAGTTAGTTGGTGAGGTAACGGCTCACCAAGACGATGATGCGTAGCCGACCTGA
GAGGGTGACCGGCCACACTGGGACTGAGACACGGCC
>Zotu2310
GAACGCTGGCGGTGTGCCTAATACATGCAAGTCGAGCGGTAAGGCCCCTTCGGGGGTACACGAGCGGC
GGACGGCTGAGTAACACGTAGGAACGTACCCCAAAGTGAGGGATAAGCACCAGAAATGGTGTCTAATA
CCGCATGTGCTCTAAGGAGTAAAGCTTTATGCGCTTTGGGAACGGCCTGCGCATGATTAGCTTGTTGG
TAAGGTAAAAGCTTACCAAGGCTACGATCATTAGCTGGTCTGAGAGGATGATCAGCCAGACTGGGACT
GAGACACGGCC
>Zotu2311
GAACGCTGGCGGCGTGCCTAATACATGCAAGTCGAGCGAAGTTTGATTGGTGCTTGCACCGATTAAAC
TTAGCGGCGAACGGGTGAGTAACACGTAAAGAACCTGCCTCATAGACTGGGACAACTACTGGAAACGG
TAGCTAATACCGGATAATATATTTTACTGCATGGTGAAATATTGAAAGTCGGTCATGCTGACACTATG
AGATGGCTTTGCGGTGCATTAGCTAGTTGGTAGGGTAAAGGCCTACCAAGGCGACGATGCATAGCCGA
CCTGAGAGGGTGATCGGCCACACTGGGACTGAGACACGGCC
>Zotu2312
TAACGCTGGCAGCGTGCATAACACATGCAAGTCGAGCGGAGTTGTTTGAGATTATATCAAAGACAATT
TAGCGGCAGACGAGTGAATAACACGTAAGGAATCTACCCCGGAGCGAGGAACAACCAGCCGAAAGGTT
GGCTAATACCTCGTACGACCCGAGAAAGGCATCTTTTTCGGTTGAAAGGCGTAAGCCGCTCTGGGAGG
ATCTTGCGGCCTATCAGCTAGTTGGCGGTGTAACGGACCACCAAGGCGACGACGGGTAGCCGGCCTGA
AAGGGCGATCGGCCACGGAGGCACTGAGACACGGGC
>Zotu2313
GAACGCTGGCGGCGTGCTTAACACATGCAAGTCGAGCGAAGCGGCTGAATTGATTCTTCGGATGATTT
TCAGTTGACTGAGCGGCGGACGGGTGAGTAACGCGTGGGTGACCTGCCCCATACCGGGTGATAACAGC
TGGAAACGGCTGCTAATACCGCATAAGCGCACAGAGCTGCATGGCTCCGTGTGAAAAACTCTGGTGGT
ATGGGATGGGCCCGCGTCTGATTAGGCAGTTGGCGGGGTAACGGCCCACCAAACCGACGATCAGTAGC
CGGCCTGAGAGGGCGACCGGCCACATTGGGACTGAGACACGGCC
>Zotu2314
GAACGCTGGCGGCGTGCCTAACACATGCAAGTCGAACGAGGCCCAAGCAGTGGCAACACTGCGGAAGG
CCTAGTGGCGAACGGGTGAGTAACACGTGAGGAACCTGCCCCGAAGACCGGGATAACCCCCTGAAAAG
GGAGCTAATACCGGATGCCCCCACCGGACCGCATGGTCTGGTGAGGAAATGGATTCCGCTTCGGGAGG
GCCTCGCGGCCTATCAGCTTGTTGGTGAGGTAACGGCTCACCAAGGCGTCGACGGGTAGCTGGTCTGA
GAGGACGATCAGCCACACTGGGACTGAGACACGGCC
>Zotu2315
GAACGCTGGCGGCATGCTTAACACATGCAAGTCGCACGGCCGGCAACGGCAGTGGCGGACGGGTGAGT
AACGCGTAGGTATCTATCTCCGGGTGGGGGACAACTGTGGGAAACTACAGCTAATACCGCATGATGCC

CGAGGGCCAAAGGCGCAAGTCGCCTGGAGAGGAGCCTGCGTACGATTAGCTTGTTGGCGGGGTAAAGG
CCCACCAAGGCGACGATCGTTAGCTGGTCTGAGAGGATGATCAGCCACACTGGGACTGAGACACGGCC
>Zotu2316
GAACGCTAGCGGCAGGCCTAATACATGCAAGTCGGACGGGATTTAGGAGCTTGCTTCTAATGAGAGTG
GCGCACGGGTGCGTAACACGTATGTAACCTACCTTGTTCAGGGGGATAGCCTCTCGAAAGAGAGATTA
AGACCGCATAAAATCACAATGTGGCATCACAAAATGATCAAATATTTATAGGAACAAGATGGGCATGC
GGAACATTAGCTAGTTGGTAAGGTAACGGCTTACCAAGGCTACGATGTTTAGGGGATCTGAGAGGATG
ACCCCCCACACTGGTACTGAGACACGGAC
>Zotu2317
GAACGCTGGCGGCGTGCCTAATACATGCAAGTCGAACGGTTTAGTTCCAAGAGGCAAGACTCTTGGAA
CTAAGCAGTGGCGGACGGGTGAGTAACACGTAGGCAACCTACCTGTCAAACCGGGATAACCTCGGGAA
ACCGAAGCTAAACCCGGATAAGCTCCTTTTTCGCATGAAGGGGGAGTGAAAAGGTGCGACCTGATGAC
AGATGGGCCTGCGGCGCATTAGCTAGTAGGTAGGGTAACGGCCTACCTAGGCGATGATGCGTAGCCGA
CCTGAGAGGGCGTACGGCCACACTGGGACTGAGATACGGCC
>Zotu2318
GAACGCTGGCGGCGTGCTTAACACATGCAAGTCGAACGGAAAGGCCTGCTTTCCCTTGTGGATTGCGG
GTGCTCGAGTGGCGAACGGGTGAGTAACACGTGAGTGATCTGCCCCCAACTTGGGTATAAGCCTGGGA
AACTGGGTCTAATTCCCGATAGGACTGCAGGGTGGTGCCTGTGGTGGAAAACGATTTTCTAGTGGTTG
GGGATGAGCTCGCGGCCTATCAGCTTGTTGGTGGGGTAATGGCCTACCAAGGCGGCGACGGGTAGCCG
GCCTGAGAGGGTGGACGGCCACATTGGGACTGAGATACGGCC
>Zotu2319
GAACGCTGGCGGCATGCCTAACACATGCAAGTCGAACGAGACCTTCGGGTCTAGTGGCGCACGGGTGC
GTAACGCGTGGGAATCTGCCCTTAGGTTCGGAATAACTCAGAGAAATTTGAGCTAATACCGGATAATG
ACGTAAGTCCAAAGATTTATCGCCTAGGGATGAGCCCGCGTAAGATTAGCTTGTTGGTGAGGTAAAGG
CTCACCAAGGCGACGATCTTTAGCTGGTCTGAGAGGATGATCAGCCACACTGGGACTGAGACACGGCC
>Zotu2320
GAACGCTAGCTACAGGCTTAACACATGCAAGTCGAGGGGAAACGGCATTGAGTGCTTGCACTCTTTGG
ACGTCGACCGGCGCACGGGTGAGTAACGCGTATCCAACCTTCCCATTACTGTGGGATAACCTGCCGAA
AGGCAGACTAATATCGCATAGTCTTCGATGACGGCATCAGATTTGAAGTAAAGATTTATCGGTAATGG
ATGGGGATGCGTCTGATTAGCTTGTTGGCGGGGTAACGGCCCACCAAGGCGACGATCAGTAGGGGTTC
TGAGAGGAAGGTCCCCCACATTGGAACTGAGACACGGTC
>Zotu2321
GAACGCTGGCGGCGTGCTTAACACATGCAAGTCGAACGGAAAGGCCGAAGCTTGCTTCGGTACTCGAG
TGGCGAACGGGTGAGTAACACGTGGGTGATCTGCCCTGCACTTTGGGATAAGCCTGGGAAACTGGGTC
TAATACCAGATAGGACCACGTCTTGGATGGTGTGGTGGAAAGTTTTTTCGGTGTGGGATGAGCTCGCG
GCCTATCAGCTTGTTGGTGGGGTAATGGCCTACCAAGGCGTCGACGGGTAGCCGGCCTGAGAGGGTGT
ACGGCCACATTGGGACTGAGATACGGCC
>Zotu2322
GAACGCTGGCGGCGTGCTTAACACATGCAAGTCGAACGGTGAAGCCAAGCTTGCTTGGTGGATCAGTG
GCGAACGGGTGAGTAACACGTGAGCAACCTGCCCTGGACTCTGGGATAAGCGCTGGAAACGGCGTCTA
ATACTGGATATGAGCCCCTATCGCATGGTGGGGGTTGGAAAGATTTTTTGGTCTGGGATGGGCTCGCG
GCCTATCAGCTTGTTGGTGAGGTAATGGCTCACCAAGGCGTCGACGGGTAGCCGGCCTGAGAGGGTGA
CCGGCCACACTGGGACTGAGACACGGCC
>Zotu2323
GAACGCTGGCGGCAGGCTTAACACATGCAAGTCGAGCGGTAGCACAAGGGAGCTTGCTCCCTGGGTGA
CGAGCGGCGGACGGGTGAGTAATGTCTGGGAAACTGCCTGATGGAGGGGGATAACTACTGGAAACGGT
AGCTAATACCGCATAACGTCGCAAGACCAAAGAGGGGGACCTTCGGGCCTCTTGCCATCAGATGTGCC
CAGATGGGATTAGCTAGTAGGTGGGGTAATGGCTCACCTAGGCGACGATCCCTAGCTGGTCTGAGAGG
ATGACCAGCCACACTGGAACTGAGACACGGTC
>Zotu2324
GAACGCTGGCGGCGTGCCTAATACATGCAAGTCGAGCGAATCAAAGGGAGCTTGCTCCCTGAGGTTAG
CGGCGGACGGGTGAGTAACACGTGGGCAACCTGCCTATAAGACTGGGATAACTTCGGGAAACCGGAGC
TAATACCGGATAATTCATATCTACTCATGTAGGTATGCTGAAAGACGGTTTACGCTGTCACTTATAGA
TGGGCCCGCGGCGCATTAGCTAGTTGGTGAGGTAACGGCTCACCAAGGCAACGATGCGTAGCCGACCT

GAGAGGGTGATCGGCCACACTGGGACTGAGACACGGCC
>Zotu2325
GAACGCTGGCGGCGTGCCTAATACATGCAAGTGGGACGCAAGAGAACACACTGTGCTTGCACACCGTG
TTTTCTTGAGTCGCGAACGGGTGAGTAACGCGTAGGTAACCTGCCTATTAGCGGGGGATAACTATTGG
AAACGATAGCTAATACCGCATAATATTAATTATTGCCTGATAATTGATTGAAAGATGCAAGCGCATCA
CTAGTAGATGGACCTGCGTTGTATTAGCTAGTTGGTAAGGTAAGAGCTTACCAAGGCGACGATACATA
GCCGACCTGAGAGGGTGATCGGCCACACTGGGACTGAGACACGGCC
>Zotu2326
GAACGCTGGCGGCGTGCTTAACACATGCAAGTCGAGCGATGAAGTTTCCTTCGGGAAACGGATTAGCG
GCGGACGGGTGAGTAACACGTGGGTAACCTGCCTCATAGAGTGGAATAGCCTTCCGAAAGGAAGATTA
ATACCGCATAACGTTGAAAGATGGCATCATCATTTAACCAAAGGAGTAATCCGCTATGAGATGGACCC
GCGGCGCATTAGCTAGTTGGTGGGGTAACGGCCTACCAAGGCGACGATGCGTAGCCGACCTGAGAGGG
TGATCGGCCACATTGGGACTGAGACACGGCC
>Zotu2327
GAACGCTGGCGGCGTGCCTAAGACATGCAAGTCGAGTGGGTTTAGACCCACGGCGAACGGGTGAGTAA
TACCTAGGAACTTTCTTTAAGTGGAGCATAACCAGTCGAAAGATTGGATAATTCCCCATACCATCGTA
AGATGAAAGTCGCAAGACGCTTAAAGTGAGGCCTGGGCACCATCAGCTAGTTGGTAAGGTAACGGCTT
ACCAAGGCTATGACGGTTAGGGGTCCCGAGAGGGATAACCCCCACACTGGAACTGCGACACGGTC
>Zotu2329
GAACGCTAGCGGGAGGCCTAACACATGCAAGCCGAGCGGTAGGGTTTCTTCGGAAACCTGAGAGCGGC
GCACGGGTGCGGAACACGTGTGCAACCTACCTTTATCAGGGGGATAGCCTTTCGAAAGGAAGATTAAT
ACCCCATAATATATTAGATGGCATCATTTGATATTGAAAACTCCGGTGGATAGAGATGGGCACGCGCA
AGATTAGATAGTTGGTGAGGTAACGGCTCACAAAGTCAGTGATCTTTAGGGGGCCTGAGAGGGTGATC
CCCCACACTGGTACTGAGACACGGAC
>Zotu2330
GAACGCTGGCGGCGTGCTTAACACATGCAAGTCGAACGGTGAAGGCGGAGCTTGCTCTGCTGGATCAG
TGGCGAACGGGTGAGTAACACGTGAGTAACCTGCCCTTGACTCTGGGATAAGCGTTGGAAACGACGTC
TAATACCGGATACGAGCCCCAACCGCATGGTTAGGGGTTGGAAAGAATTTCGGTCAAGGATGGACTCG
CGGCCTATCAGCTTGTTGGTGAGGTAATGGCTCACCAAGGCGACGACGGGTAGCCGGCCTGAGAGGGT
GACCGGCCACACTGGGACTGAGACACGGCC
>Zotu2331
CGCAAGCCTTGGGGAACTGTGCAGCTGGTTTGATCGGGAACGCGAGTACCGCCCAGGTAGCTCAGGCT
AGTGTCATGACCGACTGTCCAGCACGAGAACAGCTCAACGGCATCTTCTTCCTTCGCCGCCCCACAAT
ATACGCGCGTAATCGGCCGCGCACTTCAAATAGCATAAATTCCTATCACAATA
>Zotu2332
GAATGCTGGCGGCGTGCCTAACACATGCAAGTCGAGCGGTAACATGAATAATTTATTATTTGATGACG
AGCGGCGGACGGCTGAGTAACGCGTGGGAACATGCCCCAAAGTGAGGGATAAGCTCCAGAAATGGAGT
CTAATACCGCATATGATCTTCGGATTAAAGGATTTATCCGCTTTGGGAGTGGCCTGCGTAAGATTAGA
TTGTTGGTGAGGTAATGGCTCACCAAGTCTACGATCTTTAGCTGGTCTGAGAGGATGACCAGCCAGAC
TGGAACTGAGACACGGTC
>Zotu2333
GAACGCTGGCGGCGTGCTTAACACATGCAAGTCGAACGGTGAACACGGAGCTTGCTCTGTGGGATCAG
TGGCGAACGGGTGAGTAACACGTGAGCAACCTGCCCCTGACTCTGGGATAAGCGCTGGAAACGGCGTC
TAATACTGGATACGAACCACGACCGCATGGTCAGTGGTTGGAAAGAATTTCGGTTGGGGATGGGCTCG
CGGCCTATCAGCTTGTTGGTGAGGTAATGGCTCACCAAGGCGTCGACGGGTAGCCGGCCTGAGAGGGT
GACCGGCCACACTGGGACTGAGACACGGCC
>Zotu2334
GAACGCTGGCGGCGTGCCTAATACATGCAAGTCGAACGCATTTTCTTTCACCGTAGCTTGCTACACCG
AAAGAAAATGAGTGGCGAACGGGTGAGTAACACGTGGGTAACCTGCCCATCAGCGGGGGATAACACTT
GGAAACAGGTGCTAATACCGCATAATTCCATTTACCGCATGGTAGATGGATGAAAGGCGCTTTTGCGT
CACTGATGGATGGACCCGCGGTGCATTAGCTAGTTGGTGGGGTAACGGCCTACCAAGGCTGCGATGCA
TAGCCGACCTGAGAGGGTGATCGGCCACACTGGGACTGAGACACGGCC
>Zotu2335
GAACGCTGGCGGCATGCCTTACACATGCAAGTCGAACGGTAACAGGTTAAGCTGACGAGTGGCGAACG

GGTGAGTAATATATCGGAACGTGCCCAGTCGTGGGGGATAACTACTCGAAAGAGTAGCTAATACCGCA
TACGACCTGAGGGTGAAAGCGGGGGATCGCAAGACCTCGCGCGATTGGAGCGGCCGATATCAGATTAG
GTAGTTGGTGGGGTAAAGGCCTACCAAGCCGACGATCTGTAGCTGGTCTGAGAGGACGACCAGCCACA
CTGGGACTGAGACACGGCC
>Zotu2336
GAACGCTGGCGGCATGCCTAATACATGCAAGTCGAACGAGACCTTCGGGTCTAGTGGCGCACGGGTGC
GTAACGCGTGGGAATCTGCCCTTGGGTTCGGAATAACAGTTAGAAATGACTGCTAATACCGGATGATG
ACGTAAGTCCAAAGATTTATCGCCCAAGGATGAGCCCGCGTAGGATTAGCTAGTTGGTGAGGTAAAGG
CTCACCAAGGCTACGATCCTTAGCTGGTCTGAGAGGATGATCAGCCACACTGGGACTGAGACACGGCC
>Zotu2338
GAACGCTAGCAGAACGCTTAACACATGCAAGTCGAACGAATATATTGTCTTCGGATAATATGTTAGTG
GCAAACGGGCGAGTAACGCGTATCTAACCTGCCCTTGTGTGAGGCAAAACTATGGAGACATAGCTAAT
TCCTCATACGCTCTAAGAAATGAAAATACTTAGAGGAAAGATTTATCGCACAAGGAGGGGGATGCGTC
CTATCAGGTAGTTGGTGAGGTAACTGCTCACCAAGCCTATGACGGGTAGCCGGTCTGAGAGGATGTCC
GGCCGCAATGGAACTGAGACACGGTC
>Zotu2339
GAACGCTGGCGGCGTGCCTAACACATGCAAGTCGAGCGAGAGACTTCGGTCTCTAGCGGCGGACGGGT
GAGTAACACGTGGGTAACCTGCCTCAAACAGGGGGATAGCCTTCCGAAAGGAAGATTAATACCGCATA
ACGTATATCTGGCGCATGCCAGATATATCAAAGGAGTAATCCGGTTTGAGATGGACCCGCGGCGCATT
AGCTAGTTGGTGAGGTAACGGCTCACCAAGGCGACGATGCGTAGCCGACCTGAGAGGGTGATCGGCCA
CATTGGAACTGAGATACGGTC
>Zotu2340
GAACGCTAGCGGCAGGCCTAATACATGCAAGTCGAACGGTGATAGTAATATCATAGTGGCGCACGGGT
GCGTAACACGTATGCAACCTACCTTATACTGGGGGATAGCCTTTGGAAACGGAGATTAATACCCCATA
GTATATATTTATGGCATCATAGATATATTAAAGATTTATTGGTATAAGCTGGGCATGCGTCCAATTAG
TTAGTTGGTGAGGTAACGGCTCACCAAGACTTTGATTGGTAGGGGAACTGAGAGGTTAATCCCCCACA
CTGGCACTGAGATACGGGC
>Zotu2341
GAACGCTGGCGGCGTGCTTAACACATGCAAGTCGAGCGGTAAGGCCCTTCGGGGTACACGAGCGGCGA
ACGGGTGAGTAACACGTGGGTGACCTGCCCTCCACTTCGGGATAAGCCTGGGAAACTGGGTCTAATAC
CGGATAGGACCTCCTGGTGCATGCCGGGTGGTGGAAAGTTTTTCGGTGGGGGATGGCCCGCGGTCTA
TCAGCTTGTTGGTGGGGTGATGGCCTACCAAGGCGACGACGGATAGCCGGCCTGAGAGGGTGATCGGC
CACACTGGGACTGAGACACGGCC
>Zotu2342
GAACGCTGGCGGCAGGCCTAACACATGCAAGTCGAACGGATCCTTCGGGATTAGTGGCGGACGGGTGA
GGAACACGTGGGAACGTGCCTTTTGGTTCGGAACAACTCAGGGAAACTTGAGCTAATACCGGATAAGC
CCTTCGGGGGAAAGATTTATCGCCATTAGAGCGGCCCGCGTCTGATTAGCTAGTTGGTGGGGTAAAGG
CCCACCAAGGCTACGATCAGTAGCTGGTCTGAGAGGATGATCAGCCACATTGGGACTGAGACACGGCC
>Zotu2343
GAACGCTGGCGGCAGGCTTAACACATGCAAGTCGAACGATGACTAACTAGCTTGCTAGTTATGATTAG
TGGCGGACGGGTGAGTAACATTTAGGAATCTGCCTAGTAGTGGGGGATAGCTCGGGGAAACTCGAATT
AATACCGCATACGACCTACGGGTGAAAGGGGGCGCAAGCTCTTGCTATTAGATGAGCCTAAATCAGAT
TAGCTAGTTGGTGGGGTAAAGGCCCACCAAGGCGACGATCTGTAACTGGTCTGAGAGGATGATCAGTC
ACACCGGAACTGAGACACGGTC
>Zotu2344
GAACGCTGGCGGCGTGCCTAATACATGCAAGTCGAGCGGAGCGATCGTTTCCTTCGGGGAACGATTGG
CTTAGCGGCGGACGGGTGAGTAATACGTAGGTAACCTGCCCTTAAGACCGGGATAACTCACGGAAACG
TGGGCTAATACCGGATAGGCGATTTGCTCGCATGAGGGAATCGGGAAAGGCGGTTTCGATTTATCGGG
GCTGCCACTTATGGATGGACCTACGGCGCATTAGCTAGTTGGTGGGGTAACGGCTCACCAAGGCGACG
ATGCGTAGCCGACCTGAGAGGGTGAACGGCCACACTGGGACTGAGACACGGCC
>Zotu2345
GAACGCTAGCGGCAGGCCTAACACATGCAAGTCGAGGGGCAGCACGATTGAGCTTGCTTAATTGGTGG
CGACCGGCGCACGGGTGCGTAACGCGTATGCAACCTACCTCATACTGGGGTATAGCCCGAAGAAATTC
GGATTAACCCCCCATAGTATTATAGAATGGCATCATTTTATAATTAAAGGTTACGGTATGAGATGGGC

ATGCGTCCCATTAGCTAGATGGTGAGGTAACGGCTCACCATGGCAACGATGGGTAGGGGGCCTGAGAG
GGTGGTCCCCCACACTGGTACTGAGACACGGAC
>Zotu2347
GAACGCTGGCGGCATGCTTTACACATGCAAGTCGAACGGTAGCAGGGAGCTTGCTCCGCTGACGAGTG
GCGAACGGGTGAGTAATATATCGGAACGTGCCCAAGAGTGGGGGATAACGTAGCGAAAGTTACGCTAA
TACCGCATACGATCTATGGATGAAAGCAGGGGACCTTCGGGCCTTGTGCTCCTGGAGCGGCCGATATC
TGATTAGCTAGTTGGTGAGGTAAAGGCTCACCAAGGCGACGATCAGTAGCTGGTCTGAGAGGACGACC
AGCCACACTGGAACTGAGACACGGTC
>Zotu2348
GAACGCTGGCGGCGTGCCTAACACATGTAAGTCGAACGGGGCTGGGGGGGCAACTCTCCAGTTCAGTG
GCAGACGGGTGCGTAACACGTGAGAAACTTGTCCATCGGTGGGGGATAGCCGGCCCAACGGCCGGTTA
ATACCGCGTACGCTCGTTTGGGGGCATCCCTGAACGATGAAAGCATCGCAAGATGCGCCGGCGGAGAG
TCTCGCGGCCTATCAGCTAGTTGGCGGGGTAACGGCCCACTAAGGCGACGACGGGTAGCTGGTCTGAG
AGGATGGCCAGCCACATTGGGACTGAGACACGGCC
>Zotu2349
GAACGCTGGCGGCATGCTTTACACATGCAAGTCGAACGGCAGCACGGGCTTCGGTCTGGTGGCGAGTG
GCGAACGGGTGAGTAATACATCGGAACGTACCCAGAAGTGGGGGATAACGTAGCGAAAGTTACGCTAA
TACCGCATACGTTCTACGGAAGAAAGTGGGGGATCGCAAGACCTCATGCTTTTGGAGCGGCCGATGTC
TGATTAGCTAGTTGGTGAGGTAAAGGCTCACCAAGGCGACGATCAGTAGCTGGTCTGAGAGGACGACC
AGCCACACTGGAACTGAGACACGGTC
>Zotu2350
GAACGCTGGCGGCGTGCCTAAGACATGCAAGTCGAATGCTCCGCAAGGGGCATGGCGAACGGGTGAGT
AATACCTAGGAACTTTCTTTAAGTGGAGCATAACCAGTCGAAAGATTGGATAATACTCCATGTGGCAG
TAATGCTAAAGTCGCAAGACGCTTAAAGTGAGGCCTGGGCACCATCAGCTAGTTGGTAAGGTAATGGC
TTACCAAGGCTATGACGGTTAGGGGCCCCGAGAGGGGAAACCCCCACACTGGAACTGCGACACGGTC
>Zotu2351
GAACGCTAGCGGCAGGCTTAATACATGCAAGTCGAACGGATTCAGCAATGGATTAGTGGCGCACGGGT
GAGTAACGCGTACATAACATGCCTTTGACTGGAGAATAGCCCTTGGAAACGAGGAGTAATACTCCATG
GTATTATAGACTGGCATCGGTTTATAATTAAAGTTACGGCGGTCAAAGATTGATGTGCGTAAGATTAG
GTAGTTGGTGAGGTAACGGCTCACCAAGCCGACGATCTTTAGGGGGCGTGAGAGCGTGACCCCCCACA
CGGGTACTGAGACACGGAC
>Zotu2352
GAACGCTAGCGGGAGGCCTAACACATGCAAGCCGAGCGGTATTACTTCTTCGGAGGTGAGAGAGCGGC
GTACGGGTGCGGAACACGTGTGCAACCTGCCCTTATCAAGGGGATAGCCCAGAGAAATTTGGATTAAT
ACCCTATAACATATAGAGTGGCATCACTTTATATTAAAAACTACGGTGGATAAGGATGGGCACGCGCA
AGATTAGCTAGTTGGTGAGGTAACGGCTCACCAAGGCGACGATCTTTAGGGGGCCTGAGAGGGTGATC
CCCCACACTGGGACTGAGACACGGCC
>Zotu2354
GAACGCTAGCGATAGGCTTAACACATGCAAGTCGAGGGGCAGCATGGTCTTAGCTTGCTAAGACTGAT
GGCGACCGGCGCACGGGTGCGTAACGCGTATGCAACTTGCCTCACAGAGGGGGATAACCCGTCGAAAG
ACGGACTAATACCGCGTACACTCCAGTCTGGGCATCCAGGCTAGAGGAAATGAATTTCGCTGTGAGAT
AGGCATGCGTCCCATTAGCTAGTTGGTGAGGTAACGGCTCACCAAGGCAACGATGGGTAGGGGAACTG
AGAGGTTGAACCCCCACACTGGTACTGAGACACGGAC
>Zotu2355
GAACGCTGGCGGCGTGCCTAATACATGCAAGTCGAGCGAACTCTTTAAAAGCTTGCTTTTGAAGAGTT
AGCGGCGGACGGGTGAGTAACACGTGGGCAACCTGCCTATAAGACTGGGATAACTTCGGGAAACCGGA
GCTAATACCAGATAATTCTTTTCTACACATGTAGGAAAGCTGAAAGACGGTTTACGCTGTCACTTATA
GATGGGCCCGCGGCGCATTAGCTAGTTGGTGAGGTAACGGCTCACCAAGGCCACGATGCGTAGCCGAC
CTGAGAGGGTGATCGGCCACACTGGGACTGAGACACGGCC
>Zotu2357
GAACGCTGGCGGCAGGCCTAACACACGCAAGTCGAACGGTAGCACAGAGGAGCTTGCTCCTTGGGTGA
CGAGTGGCGGACGGGTGAGTAATGTCTGGGAAACTGCCTGATGGAGGGGGATAACTACTGGAAACGGT
AGCTAATACCGCATAACGTCGCAAGACCAAAGAGGGGGACCTTCGGGCCTCTTGCCATCAGATGTGCC
CAGATGGGATTAGCTAGTAGGTGGGGTAATGGCTCACCTAGGCGACGATCCCTAGCTGGTCTGAGAGG

ATGACCAGCCACACTGGAACTGAGACACGGTC
>Zotu2358
GAACGCTGGCGGCGTGCTTAACACATGCAAGTCGAACGATGAAGCCCAGCTTGCTGGGTGGATTAGTG
GCGAACGGGTGAGTAACACGTGAGCAACCTGCCCCTGACTCTGGGATAAGCGCTGGAAACGGCGTCTA
ATACTGGATACGAACAAGAATCGCATGGTTACTTGTTGGAAAGATTTTTTGGTTGGGGATGGGCTCGC
GGCCTATCAGCTTGTTGGTGAGGTAATGGCTCACCAAGGCGTCGACGGGTAGCCGGCCTGAGAGGGTG
ACCGGCCACACTGGGACTGAGACACGGCC
>Zotu2359
CAACGCTGGCGGCGTGCCTAACACATGCAAGTCGAACGAGAAAGTGGAGCAATCCATGAGTAAAGTGG
CGACCGGGTGAGTAACACGTGACTAACCTACCCTACAGTGGGGGATAACCTCGGGAAACCGGGGCTAA
TACCGCATAACATCGCAGGGTGAAATTCTTTGCGATCAAAGGAGCAATTCGCTGAAGGAGGGGGTCGC
GGCTGATTAGCTAGTTGGTGAGGTAACGGCTCACCAAGGCTTTGATCAGTAGCCGGCCTGAGAGGGCG
CACGGCCACACTGGAACTGAAACACGGTC
>Zotu2360
GAACGCTGGCGGCAGGCTTAACACATGCAAGTCGAGCGCCCCGCAAGGGGAGCGGCAGACGGGTGAGT
AACGCGTGGGAATCTACCTTTTGCTACGGAATAACTCAGGGAAACTTGTGCTAATACCGTATGTGCCC
TTCGGGGGAAAGATTTATCGGCAAAGGATGAGCCCGCGTTGGATTAGCTAGTTGGTGAGGTAAAGGCT
CACCAAGGCGACGATCCATAGCTGGTCTGAGAGGATGATCAGCCACACTGGGACTGAGACACGGCC
>Zotu2361
GAACGCTGGCGGCATGCCTTACACATGCAAGTCGAACGGCAGCACGGGAGCAATCCTGGTGGCGAGTG
GCGAACGGGTGAGTAATATATCGGAACGTGCCCAGTCGTGGGGGATAACGCAGCGAAAGCTGTGCTAA
TACCGCATACGATCTATGGATGAAAGCGGGGGACTCGCAAGGGCCTCGCGCGATTGGAGCGGCCGATA
TCAGATTAGCTAGTTGGTGGGGTAAAGGCCTACCAAGGCGACGATCTGTAGCTGGTCTGAGAGGACGA
CCAGCCACACTGGAACTGAGACACGGTC
>Zotu2364
GAACGCTGGCGGCAGGCTTAACACATGCAAGTCGAACGCATCCTTCGGGGTGAGTGGCAGACGGGTGA
GTAACGCGTGGGAACGTGCCCTTCAGTTCGGAATAACCCAGGGAAACTTGGGCTAATACCGGATACGT
GCGAGAGCAGAAAGATTTATCGCTGAAGGATCGGCCCGCGTCTGATTAGCTAGTTGGTAGGGTAATGG
CCTACCAAGGCGACGATCAGTAGCTGGTCTGAGAGGATGATCAGCCACATTGGGACTGAGACACGGCC
>Zotu2365
AAACGCAAGAGACGTGCTTAACACATGCAAATTAAATAGTTTTAACTAACTATAGTGAATTGGTGAGT
AATATATGAAAATTTAAATTTAGATTAGAAGTATTCTTAGTAACCTAGGAGTAATCTCTGATAAGTGC
ATAAGTGCTTTTAAAAGTTAATACTGTCTAAAGAAAAGTTCATATCTGATTAGCTAGTTAGTTAGGTA
AAAGCTTACTAAGGCGAAGATCAGTAGCTACCCTGAGAGGGGGATTAGCCACATTGGGATTGAAATAC
AGCC
>Zotu2366
GAACGCTGGCGGCGTGCCTAATACATGCAAGTCGAGCGGAGTTGATGAGGTGCTTGCACCTCTGATGC
TTAGCGGCGGACGGGTGAGTAACACGTAGGTAACCTGCCTGTAAGACTGGGATAACATTCGGAAACGA
ATGCTAATACCGGATACGCGATTTTCTCGCATGAGAGAACCGGGAAAGACGGAGCAATCTGTCACTTA
CGGATGGACCTGCGGCGCATTAGCTAGTTGGTGAGGTAACGGCTCACCAAGGCGACGATGCGTAGCCG
ACCTGAGAGGGTGATCGGCCACACTGGGACTGAGACACGGCC
>Zotu2367
GAACGCTAGCGGCAGGCTTAACACATGCAAGTCGAGGGGTATAGTTCTTCGGAACTAGAGACCGGCGC
ACGGGTGCGTAACGCGTATGCAATCTACCTTTTACAGAGGGATAGCCCAGAGAAATTTGGATTAATAC
CTCATAGTATATAGCCCTGGCATCAGGATTATATTAAAGTCACAACGGTAAAAGATGAGCATGCGTCC
CATTAGCTAGTTGGTAAGGTAACGGCTTACCAAGGCTACGATGGGTAGGGGTCCTGAGAGGGAGATCC
CCCACACTGGTACTGAGACACGGAC
>Zotu2368
GAACGCTGGCGGCAGGCCTCAAACATGCAAGTCTAGGGGGTGTAGCAATACATCACCGGCGGACGGGT
GCGTAACACGTGGGAATATACCTTTCGGTACGGAACAACTGCGGGAAACTGCAGCTAATACCGTATAC
GTCCTTGCGGAGAAAGATTTATCGCCGAAGGATTAGCCCGCGTTCGATTAGCTAGTTGGTGAGGTAAT
GGCTCACCAAGGCGACGATCGATAGCTGTTCTGAGAGGAAGATCAGCCACACTGGGACTGAGACACGG
CC
>Zotu2369

GAACGCTAGCGGCAGGCCTAATACATGCAAGTCGAACGGTGATAGCAATATCATAGTGGCGCACGGGT
GCGTAACACGTATGCAACCTACCTTATACTGAGGGATAGCCTTTCGAAAGGGAGATTAATACCTCATA
ACACATTGAACTGGCATCAGTTTATTGTTAAAGATTTATTGGTATAAGATGGGCATGCGTCCAATTAG
TTAGTTGGCGAGGTAACGGCTCACCAAGACGATGATTGGTAGGGGAACTGAGAGGTTAATCCCCCACA
CTGGCACTGAGATACGGGC
>Zotu2370
GAACGCTGGCGACATGCCTAATACATGCAAGTCGAACGCTGAAGCACTCTGCTTGCAGAGTGCGGATG
AGTGGCGAACGGGTGAGTAACACGTGGGGAATCTGCCTATAAGTGGGGGATAACATTCGGAAACGGAT
GCTAATACCGCATATATTCTTGGACCGCCTGGTCCTTGAAAGAAAGACGGCTTTGCTGTCGCTTATAG
ATGATCCCGCGGCGTATTAGTTAGTTGGTGGGGTAAAGGCCTACCAAGACAATGATACGTAGCCGACC
TGAGAGGGCGATCGGCCACACTGGGACTGAGACACGGCC
>Zotu2371
GAACGCTGGCGGCAGGCTTAACACATGCAAGTCGAGCGGGCCCTTCGGGGTCAGCGGCGGACGGGTTA
GTAACGCGTGGGAACGTGCCCTTTGCTCCGGAATAGCCCTGGGAAACCGGGGGTAATACCGGATGTGC
CCCGAGGGGGAAAGGTTTACCGGCAAGGGATCGGCCCGCGTCTGATTAGGTAGTTGGTGGGGTAATGG
CCCACCAAGCCGACGATCGGTAGCTGGTTTGAGAGGATGATCAGCAACACTGGGACTGAGACACGGCC
>Zotu2372
CAACGCTGGCGGCGTGCCTAATGCATGCAAGTCGGACGGGCTTGATCTTTGGATCGAGTCACGTGGCG
GACGGGTGCGGAGCACGTGGGCAAGCTGCCCCGGGGCGGGGGATAGCCGGCGGAAACGCCGGGCAATT
CCGCGTACGTTGGCCGGTCGGTGGGACTGGTCAGGAAAGGGTCTTGGGACTCACCCTGGGAGGCGCCT
GCGGCCGATTAGCTAGTTGGGGGGGTAACGGCTTCCCAAGGCGATGATCGGTAGCTGGTCTGAGAGGA
CGGCCAGCCACACGGGGACTGAGACACGGCC
>Zotu2373
GAACGCTGGCGGCGTGCTTAACACATGCAAGTCGAACGATGACGGTGGTGCTTGCACCGCCTGATTAG
TGGCGAACGGGTGAGTAACACGTGAGTAACCTGCCCCCCACTTCGGGATAACCTCGGGAAATCGTGGC
TAATACCGGATATGAGCACTCATCGCATGGTGGGTGTTGGAAAGATTTATCGGTGGGGGATGGACTCG
CGGCCTATCAGTTTGTTGGTGAGGTGATGGCTCACCAAGACGATGACGGGTAGCCGGCCTGAGAGGGC
GACCGGCCACACTGGGACTGAGACACGGCC
>Zotu2374
GAATGCTGGCTGTATGGATAAGGCATGCAAGTCGAACGGTCCGCAAGGACAGTGGCAGACGAGGTAGT
AACACGTAAGTACGTACCCTGTAGTCTGGCATAGCTCGTCGAAAGACGGGGTAATACCGGATGGTCCC
GTAAGGGTAAAGTTTTTCGCTACAGGAGCGGCTTGCGTAATATCAGCTTGTTGGTGAGGTAATGGCTC
ACCAAGGCTACGACGTTTAGGGGAGCTGAGAGGCTGACCCCCACCGATGGGACTGAGACACGGCC
>Zotu2375
GAACGCTGGCGGCGTGCTTAACACATGCAAGTCGAACGATGAACCGCTTTCGGGCGGGGATTAGTGGC
GAACGGGTGAGTAACACGTGGGCAATCTGCCCTGCACTCTGGGACAAGCCCTGGAAACGGGGTCTAAT
ACCGGATACTCACTGCCTTGGGCATCCTTGGTGGTGGAAAGCTCCGGCGGTGCAGGATGAGCCCGCGG
CCTATCAGCTAGTTGGTGAGGTAATGGCTCACCAAGGCGACGACGGGTAGCCGGCCTGAGAGGGCGAC
CGGCCACACTGGGACTGAGACACGGCC
>Zotu2376
GAACGCTGGCGGCAGGCTTAACACATGCAAGTCGAACGGTTGTAGCAATACAGCAGTGGCGCACGGGT
GAGTAACGCGTGGGAACGTACCTCTTAGTTCGGAATAACTATTGGAAACGATAGCTAATACCGGATAC
TCCCTTCGGGGGAAAGATTTATCGCTGAGAGATCGGCCCGCGTAAGATTAGGTAGTTGGTAGGGTAAT
GGCCTACCAAGCCTACGATCTTTAGCTGGTCTGAGAGGATGATCAGCCACACTGGAACTGAGACACGG
TC
>Zotu2377
GAACGCTGGCGGCGTGCCTAATACATGCAAGTCGAGCGGACAGAAGGGAGCTTGCTCCCGGAAGTCAG
CGGCGGACGGGTGAGTAACACGTGGGCAACCTGCCCTGCAGATTGGGATAACTCCGGGAAACCGGGGC
TAATACCGAATAACATTGGAAACCTCATGGTTTCCTTTTAAAAGGCGGCTTCGGCTGTCACTGCAGGA
TGGGCCCGCGGCGCATTAGCTAGTTGGTGAGGTAACGGCTCACCAAGGCGACGATGCGTAGCCGACCT
GAGAGGGTGATCGGCCACACTGGGACTGAGACACGGCC
>Zotu2378
GAACGCTGGCGGCGTGCTTAACACATGCAAGTCGAACGGTGAAGCCAAGCTTGCTTGGTGGATCAGTG
GCGAACGGGTGAGTAACACGTGAGCAACCTGCCCTGGACTCTGGGATAAGCGCTGGAAACGGCGTCTA

ATACTGGATATGAGACGTGATCGCATGGTCGTGTTTGGAAAGATTTTTCGGTCTGGGATGGGCTCGCG
GCCTATCAGCTTGTTGGTGAGGTAATGGCTCACCAAGGCGTCGACGGGTAGCCGGCCTGAGAGGGTGA
CCGGCCACACTGGGACTGAGACACGGCC
>Zotu2379
GAACGCTAGCGGGAGGCTTAACACATGCAAGCCGAGGGGTATATTTCTTTCGGGAGATAGAGACCGGC
GCACGGGTGCGTAACGCGTATGCAACTTGCCCTACTGAAAAGGATAGCCCTTCGAAAGGAGGATTAAT
ACTTTATAATAGAAATCATGGCATCATGAATTTTTGAAAGATTTATCGCAGTAGGATAGGCATGCGTA
AGATTAGTTAGTTGGTGAGGTAACGGCTCACCAAGACGATGATCTTTAGGGGGCCTGAGAGGGTGAAC
CCCCACACTGGTACTGAGACACGGAC
>Zotu2380
GAACGCTGGCGGCGTGCTTAACACATGCAAGTCGAACGGGAAGCTTTGACAAGTTTTACTTTGATGAG
TGGACAGTGGCGAACGGGTGAGTAACGCGTAGGTAACCAACCTTATGCTGGGGGATAGCCTTTGGAAA
CGAAGAGTAATACCGCATAAGACCACACTGCCGCATGGCAGAGGGGTAAAAGATTTATTGGCATGAGA
CGGACCTGCGTACCATTAGATAGTTGGTGGGGTAACGGCCTACCAAGTCGACGATGGTTAGCCGACCT
GAGAGGGTGATCGGCCACATTGGGACTGAGACACGGCC
>Zotu2381
GAACGCTAGCGGCAGGCTTAACACATGCAAGTCGAGGGGTATAGTTCTTCGGAATTAGAGACCGGCGC
ACGGGTGCGTAACGCGTATGCAATCTACCTTTCACAGAGGGATAGCCCAGAGAAATTTGGATTAATAC
CTCATAGTATAACGACTCGGCATCGAGATGTTATTAAAGTCACAACGGTGAAAGATGAGCATGCGTCC
CATTAGCTAGTTGGTAAGGTAACGGCTTACCAAGGCTACGATGGGTAGGGGTCCTGAGAGGGAGATCC
CCCACACTGGTACTGAGACACGGAC
>Zotu2384
GAACGCTGGCGGCGTGCTTAACACATGCAAGTCGAACGGTGAAGCAGGAGCTTGCTCCTGTGGATCAG
TGGCGAACGGGTGAGTAACACGTGAGCAATCTGCCCTGGACTCTGGGATAAGCGTTGGAAACGACGTC
TAATACCGGATACGAGACGCGAAGGCATCTTCAGCGTCTGGAAAGAACTTCGGTCCAGGATGAGCTCG
CGGCCTATCAGCTAGTTGGTGAGGTAATGGCTCACCAAGGCGACGACGGGTAGCCGGCCTGAGAGGGT
GACCGGCCACACTGGGACTGAGACACGGCC
>Zotu2385
GAACGCTGGCGGCGTGCTTAACACATGCAAGTCGAGCGGTAAGGCCCTTCGGGGTACACGAGCGGCGA
ACGGGTGAGTAACACGTGAGCAATCTGCCCTGCACTTTGGGATAACCACCGGAAACGGTGGCTAATAC
CGAATACGACCTCGCACTGCATGGTGTGTGGTGGAAAGTTCTGGCGGTGCAGGATGAGCTCGCGGCCT
ATCAGCTTGTTGGTGAGGTAATGGCTCACCAAGGCTTCGACGGGTAGCCGGCCTGAGAGGGTGACCGG
CCACACTGGGACTGAGACACGGCC
>Zotu2387
GAACGCTGGCGGTGTGCTTCACACATGCAAGTCGAACGAGGTAGCAATACCTAGTGGCGGACGGGTGA
GTAACGCGTGGGAATCTGCCTTTAGATGGGGGATAACGGGTCGAAAGACTCGCTAATACCGCATATGA
CGAAAGTTGAAAGGAGTAATCCGTCTAAAGATGAGCCCGCGTCCGATTAGCTAGTTGGTAGAGTAAAA
GCCTACCAAGGCGACGATCGGTAGCTGGTCTGAGAGGATGATCAGCCACAATGGGACTGAGACACGGC
C
>Zotu2388
GAACGCTGGCGGCGTGCCTAATACATGCAAGTCGAGCGGATCTTTAGAAGCTTGCTTCTAAAGATTAG
CGGCGGACGGGTGAGTAACACGTGGGCAACCTGCCTGTAAGACTGGGATAACTTCGGGAAACCGAGGC
TAATACCGGATAATCCTTTTCCTCACATGAGGGAAAGCTGAAAGACGGCGCAAGCTGTCACTTACAGA
TGGGCCCGCGGCGCATTAGCTAGTTGGTGAGGTAACGGCTCACCAAGGCAACGATGCGTAGCCGACCT
GAGAGGGTGATCGGCCACACTGGGACTGAGACACGGCC
>Zotu2389
GAACGCTGGCGGCGTGCCTAATACATGCAAGTCGAGCGAATGGAAGGGAGCTTGCTCCCTGAAGTTAG
CGGCGGACGGGTGAGTAACACGTGGGCAACCTGCCTGTAAGACTGGGATAACTTCGGGAAACCGGAGC
TAATACCGGATACGTTCTTTTCTCGCATGAGGAAAGATGGAAAGATGGTTTCGGCTATCACTTACAGA
TGGGCCCGCGGCGCATTAGCTAGTTGGTGGGGTAATGGCCTACCAAGGCAACGATGCGTAGCCGACCT
GAGAGGGTGATCGGCCACACTGGGACTGAGACACGGCC
>Zotu2392
TAACGCTAGCGGCAGGCCTAATACATGCAAGTCGAGGGGTAACAGGGTAGCAATACCGCTGACGACCG
GCGCACGGGTGAGTAACACGTATGTAACCTACCTATAACTGGGGAATAGCCTTCCGAAAGGAAGATTA

ACACCGCATAATATCACGAGACGGCATCGTTTTGTGATCAAACCTCCGGGGGTTATAGATGGACATGC
GTAACATTAGCTAGTTGGTGAGGTAACGGCTCACCAAGGCTACGATGTTTAGGGGGTCTGAGAGGATG
ATCCCCCACACTGGTACTGAGACACGGAC
>Zotu2393
GAACGCTGGCGGCAGGCTTAACACATGCAAGTCGAGCGGGCGTAGCAATACGTCAGCGGCAGACGGGT
GAGTAACACGTGGGAACGTACCTTTTGGTTCGGAACAACACAGGGAAACTTGTGCTAATACCGGATAA
GCCCTTACGGGGAAAGATTTATCGCCGAAAGATCGGCCCGCGTCTGATTAGCTAGTTGGTGAGGTAAT
GGCTCACCAAGGCGACGATCAGTAGCTGGTCTGAGAGGATGATCAGCCACATTGGGACTGAGACACGG
CC
>Zotu2395
GAACGCTAGCGGCAGGCCTAACACATGCAAGTCGAGGGGCATCAGAGGTAGCAATACTTGCTGGCGAC
CGGCAAACGGGTGCGTAACACTTACGCAATCTTCCCCTAACTGGAGGATAGCCTCTCGAAAGAGAGAT
TAATACTCCGTAACATTAAGATGTGGCATCACATTTTAATTATAGCTCCGGCGGTTAGGGATGAGCGT
GAGGCTGATTAGATAGTTGGTGAGGTAACGGCTCACCAAGTCAACGATCAGTAGCTGATGTGAGAGCA
TGATCAGCCACACGGGCACTGAGACACGGGC
>Zotu2396
GAACGCTGGCGGCAGGCCTAACACATGCAAGTCGAGCGGTAGCACAGGAGAGCTTGCTCTCTGGGTGA
CGAGCGGCGGACGGGTGAGTAATGTCTGGGAAACTGCCTGATGGAGGGGGATAACTACTGGAAACGGT
AGCTAATACCGCATAACGTCTTCGGACCAAAGTGGGGGACCTTCGGGCCTCACGCCATCAGATGTGCC
CAGATGGGATTAGCTAGTAGGTGGGGTAATGGCTCACCTAGGCGACGATCCCTAGCTGGTCTGAGAGG
ATGACCAGCCACACTGGAACTGAGACACGGTC
>Zotu2397
GAACGCTGGCGGCATGCCTAACACATGCAAGTCGAACGAAGCCTTCGGGCTTAGTGGCGCACGGGTGC
GTAACGCGTGGGAATCTGCCCCTCGGTTCGGAATAACAGTTAGAAATGACTGCTAATACCGGATGATG
ACGAAAGTCCAAAGATTTATCGCCGAGGGATGAGCCCGCGTAGGATTAGCTAGTTGGTGAGGTAATGG
CTCACCAAGGCGACGATCCTTAGCTGGTCTGAGAGGATGATCAGCCACACTGGGACTGAGACACGGCC
>Zotu2398
GAACGCTGGCAGTGTGCCTTAGACATGCAAGTCGAACGGATGGAGGAGTTTACTCCTCTATTAGTGGC
GAACGGGTGAGTAACACGTAGGTGACCTACCATGAAGCGTGGGATAACAGTGGGAAACTGCTGCTAAT
ACCGCATGTGCTCTAGACTCTTGTGACTAGAGTAAAGGATTAATTGCTTTGTGATGGGCCTGCGGCGC
ATCAGCTAGTTGGTAGGGTAAAGGCCTACCAAGGCAACGACGCGTAGCCGACCTGAGAGGGTGACCGG
CCACAGGGGCACTGAGACACGGGC
>Zotu2399
GAACGCTGGCGGCGTGCTTAACACATGCAAGTCGAACGAAGCGCTTTGTTTTGATTCCTTCGGGATGA
AGAACTTAGTGACTGAGTGGCGGACGGGTGAGTAACGCGTGGGTAACCTGCCTCATACAGGGGGATAA
CAGCTGGAAACGGTTGCTAATACCGCATAACCCGCTAGGGCCACATGGCCCGGACGGAAAAGATTTTA
TCGGTATGAGATGGACCCGCGTCTGATTAGCTAGTTGGAGGGGTAACGGCCCACCAAGGCGACGATCA
GTAGCCGACTTGAGAGAGTGATCGGCCACATTGGGACTGAGACACGGCC
>Zotu2400
GAACGCTAGCGGCAGGCCTAATACATGCAAGTCGGACGGGATTGAAGAGCTTGCTTTTCATGAGAGTG
GCGCACGGGTGCGTAACACGTATGTAACCTACCTTCTTCAGGGGGATAGCCTCTCGAAAGAGAGATTA
AGACCGCATAAAATCACAATGTGGCATCACAAAATGATCAAATATTTATAGGAAGAAGATGGGCATGC
GGAACATTAGCTAGTTGGTAAGGTAACGGCTTACCAAGGCTACGATGTTTAGGGGATCTGAGAGGATG
ACCCCCCACACTGGTACTGAGACACGGAC
>Zotu2402
TAACGCTGGCGGCGTGCCTAATACATGCAAGTCGAACGCGAAAGTCCTTCGGGATGAGTAGAGTGGCG
CACGGGTGAGTAACACGTGGGTAATCTGCCTTAAGATGGGGAATAACCCCTCGAAAGAGGGGCTAATA
CCGCATAACATCGCAGCTACTGTGGTAGCAGTGATTAAACGTGACCTCTATTTATAAGTTACGGTCTT
AAGATGAGCCTGCGCACTATTAGCTAGTTGGTGGGGTAATGGCCTACCAAGGCTACGATAGTTAGCTG
GTCTGAGAGGATGATCAGCCACACTGGAACTGAGACACGGTC
>Zotu2403
GAACGCTGGCGGCGTGCCTAATACATGCAAGTCGAGCGAACAGACGAGGAGCTTGCTCCTTTGACGTT
AGCGGTGGACGGGTGAGTAACACGTGGATAACCTACCTATAAGACTGGGATAACTTCGGGAAACCGGA
GCTAATACCGGATAACATGTTGAACCGCATGGTTCAACAGTGAAAGACGGTCTTGCTGTCACTTATAG

ATGGATCCGCGCCGCATTAGCTAGTTGGTAAGGTAACGGCTTACCAAGGCAACGATGCGTAGCCGACC
TGAGAGGGTGATCGGCCACACTGGAACTGAGATACGGTC
>Zotu2405
GAACGCTGGCGGCGTGCCTAACACATGCAAGTCGAGCGATCTTCTTCGGAAGAGAGCGGCGGACGGGT
GAGTAACGCGTGGGTAACCTGCCCTGTACACACGGATAACATACCGAAAGGTATGCTAATACGAGATA
ATATGCTTTTATCGCATGGTAGAAGTATCAAAGCTCTGGCGGTACAGGATGGACCCGCGTCTGATTAG
CTAGTTGGTAAGGTAACGGCTTACCAAGGCGACGATCAGTAGCCGACCTGAGAGGGTGATCGGCCACA
TTGGAACTGAGACACGGTC
>Zotu2406
CAACGCTGGCGGCGTGCCTCAGACATGCAAGTCGAACGATTAAGACTCCCTTCGGGGAGTGCATAAAG
TGGCGCACGGGTGAGTAACACGTAAGTAATCTACCCTCGAGTGGGGAATAACATCGGGAAACCGATGC
TAATACCGCATAATGCAGCGGCACCGCAAGGTGACAGTTGTTAAAGGAGCAATTCGCTTGAGGAGGAG
CTTGCGGCAGATTAGCTTGTTGGTAAGGTAACGGCTTACCAAGGCTACGATCTGTATCCGGCCTGAGA
GGGCGGTCGGACACACTGACACTGAATAACGGGT
>Zotu2407
GAATGTTGGCGGTGCGTCTAATACATGCAAGTCGAGCGAGGCTTCGAGCCGAGCGGCGAACGGCTGAG
TAACACGTTGGTAACTGCCCCATACTCAGGGATAACCATGAGAAATCATGGCTAATACCGGATGGTCC
TGCAAAGGTAAAGCTTTTGCGGTATGGGAGGTGCCTGCGGCCTATCAGTTTGTTGGTGAGGTAATGGC
TCACCAAGACTATGACGGGTAACTGGTCTGAGAGGATGATCAGTCAGAGGGAAACTGAGACACGGTT
>Zotu2408
GAACGCTGGCGGCGTGCTTAACACATGCAAGTGGAGCGACGAACCAGGGCTTGCCCTGGGGCAGAGCC
GCGAACGGGTGAGTAACACGTGGGTAACCTACCCCGAAGACTGGGACAACCCGAGGAAACTCGGGCTA
ATACCAGATGTGGTCCCTCTTCTTAAGGAGAGTGACTAAAGGGAGCTTCGGCCCCCGCTTTGGGAGGG
GCCCGCGGCGCATTAGCTTGTTGGTGAGGTAACGGCTCACCAAGGCTTCGATGCGTAGCTGGTCTGAG
AGGACGATCAGCCACACTGGGACTGAGACACGGCC
>Zotu2409
GAACGCTAGCTACAGGCTTAACACATGCAAGTCGAGGGGCAGCATGGTCTTAGCTTGCTAAGGCTGAT
GGCGACCGGCGCACGGGTGAGTAACACGTATCCAACCTGCCGTCTACTCTTGGCCAGCCTTCTGAAAG
GAAGATTAATCCAGGATGGGATCATGAGTTCACATGTCCGCATGATTAAAGGTATTTTCCGGTAGACG
ATGGGGATGCGTTCCATTAGATAGTAGGCGGGGTAACGGCCCACCTAGTCAACGATGGATAGGGGTTC
TGAGAGGAAGGTCCCCCACATTGGAACTGAGACACGGTC
>Zotu2410
GAACGCTGGCGGCGCGCCTAAAACATGCAAGTCGAGCGAGAACCAGGGCTTCGGCCCTGGGGAGAGCG
GCGAACGGGTGCGTAACACGTGAGCAACCTGCCCCGAAGACCGGGACAACACCGGGAAACCGGTGCTA
ATACCGGATGTCCTCACCGGATCGCATGGTCTGGTGAGGAAATGGATTCCGCTTCGGGATGGGCTCGC
GGTCTATCAGCTTGTTGGTGGGGTAACGGCCTACCAAGGCGACGACGGATAGCTGGTCTGAGAGGACG
ATCAGCCACACTGGGACTGAGACACGGCC
>Zotu2411
GAACGCTGGCGGCGCGCTTAACACATGCAAGTCGAGCGAGAACCAGGGCTTCGGCCCTGGGGACAGCG
GCGAACGGGTGAGTAACACGTGGGTAATCAACCCTCGACATCGGGATAGCCCGGGGAAACCCGGATTA
ATACCGAATGCCCCGGCCGGCTCTCGGGCTGGACGGAAAAGGTAGCTTCGGCCTCCGGTCGGGGACGA
GCCCGCGGCGGATTAGCTTGTTGGCGGGGTAACGGCCCACCAAGGCGACGATCCGTAGCTGGTCTGAG
AGGACGATCAGCCACACTGGGACTGAGACACGGCC
>Zotu2412
GAACGCTGGCGGCAGGCCTAACACATGCAAGTCGAACGGGTCCTTCGGGACTAGTGGCGGACGGGTGA
GTAACGCGTGGGAACATACCCAAAGGTACGGAATAGCCTCGGGAAACTGAGAGTAATACCGTATGTGC
CCTTCGGGGGAAAGATTTATCGCCTTTGGATTGGCCCGCGTTGGATTAGGTAGTTGGTGGGGTAATGG
CCTACCAAGCCGACGATCCATAGCTGGTTTGAGAGGATGATCAGCCACACTGGGACTGAGACACGGCC
>Zotu2413
GAACGCTGGCGGCGTGCTTAACACATGCAAGTCGAACGGTGATGTCAGAGCTTGCTCTGGCGGATCAG
TGGCGAACGGGTGAGTAACACGTGAGTAACCTGCCCCCGACTCTGGGATAACTGCTAGAAATGGTAGC
TAATACCGGATATGACGATTGGCCGCATGGTCTGGTCGTGGAAAGAATTTCGGTTGGGGATGGACTCG
CGGCCTATCAGGTTGTTGGTGAGGTAATGGCTCACCAAGCCTACGACGGGTAGCCGGCCTGAGAGGGT
GACCGGCCACACTGGGACTGAGACACGGCC

>Zotu2416
GAACGCTGGCGGCATGCTTTACACATGCAAGTCGAACGGCAGCGCGGGGCAACCTGGCGGCGAGTGGC
GAACGGGTGAGTAATACATCGGAACGTACCCAGAAGTGGGGGATAACGTAGCGAAAGTTACGCTAATA
CCGCATACGTTCTACGGAAGAAAGTGGGGGATCTTCGGACCTCATGCTTTTGGAGCGGCCGATGTCTG
ATTAGCTAGTTGGTGAGGTAAAGGCTCACCAAGGCGACGATCAGTAGCTGGTCTGAGAGGACGACCAG
CCACACTGGGACTGAGACACGGCC
>Zotu2417
GAACGCTGGCGGCGTGCCTAATACATGCAAGTGGGACGCAAGGGAACACACTGTGCTTGCACACCGTG
TTTTCTTGAGTCGCGAACGGGTGAGTAACGCGTAGGTAACCTGCCTATTAGCGGGGGATAACTATTGG
AAACGATAGCTAATACCGCATAATATTAATTATTGCATGATAATTGATTGAAAGATGCAAGCGCATCA
CTAGTAGATGGACCTGCGTTGTATTAGCTAGTTGGTAAGGTAAGAGCTTACCAAGGCGACGATACATA
GCCGACCTGAGAGGGTGATCGGCCACACTGGGACTGAGACACGGCC
>Zotu2418
GAACGCTGGCGGCAGGCCTAAAACATGCAAGTCGAACGCCGTAGCAATACGGAGTGGCAGACGGGTGA
GTAACACGTGGGAACGTACCCTTTGGTTCGGAACAACACAGGGAAACTTGTGCTAATACCGGATAAGC
CCTTACGGGGAAAGATTTATCGCCAGAGGATCGGCCCGCGTCTGATTAGCTAGTTGGTGGGGTAACGG
CCCACCAAGGCTACGATCAGTAGCTGGTCTGAGAGGATGATCAGCCACACTGGGACTGAGACACGGCC
>Zotu2419
GAACGCTGGCGGCGTGCTTAACACATGCAAGTCGAACGATGAAGGGGGAGCTTGCTCCTCCGGATTAG
TGGCGAACGGGTGAGTAATACGTGAGCAATGTGCCCTTGACTCTGGGATAAGCGCTGGAAACGGCGTC
TAATACCGGATACGACCATCGGAGGCATCTCCTGGTGGTGGAAAGAACTTCGGTCAAGGATCAGCTCA
CGGCCTATCAGCTTGTTGGTGAGGTAATGGCTCACCAAGGCGACGACGGGTAGCCGGCCTGAGAGGGT
GACCGGCCACACTGGGACTGAGACACGGCC
>Zotu2421
GAACGCTGGCGGCGTGCTTAACACATGCAAGTCGAACGGTGAAGCTCCAGCTTGCTGGAGTGGATCAG
CGGCGAACGGGTGAGTAACACGTGAGCAACCTGCCCCTGACTCTGGGATAACAGCCGGAAACGGCTGC
TAATACCGGATATGCACCATGAACGCATGTTCTGTGGTGGGAAAGATTTTTCGGTTGGGGATGGGCTC
GCGGCCTATCAGCTTGTTGGTGAGGTAATGGCTCACCAAGGCGTCGACGGGTAGCCGGCCTGAGAGGG
TGACCGGCCACACTGGGACTGAGACACGGCC
>Zotu2422
GAACGCTGGCGGCGTGCTTAACACATGCAAGTCGAACGGTGAAGGGACCGGTTTTTGCTGGTTCTGGA
TGAGTGGCGAACGGGTGAGTAACACGTGAGTAACCTGCCCCCTTCTTCTGGATAACCGCATGAAAGTG
TGGCTAATACGGGATATTCTGCGCCTGCTGCATGGTGGGTGTGGGAAAGATATTGTTTCGGTGGGGGA
TGGGCTCGCGGCCTATCAGCTTGTTGGTGGGGTGATGGCCTACCAAGGCGGTGACGGGTAGCCGGCCT
GAGAGGGTGGACGGTCACACTGGGACTGAGACACGGCC
>Zotu2423
GAACGCTGGCGGCAGGCTTAACACATGCAAGTCGAGCGGGGAAAGGTAGCTTGCTACCTGACCTAGCG
GCGGACGGGTGAGTAATGCTTAGGAATCTGCCTATTAGTGGGGGACAACATCTCGAAAGGGATGCTAA
TACCGCATACGTCCTACGGGAGAAAGCAGGGGACCTTCGGGCCTTGCGCTAATAGATGAGCCTAAGTC
GGATTAGCTAGTTGGTGGGGTAAAGGCCTACCAAGGCGACGATCTGTAGCGGGTCTGAGAGGATGATC
CGCCACACTGGGACTGAGACACGGCC
>Zotu2424
GAACGCTGGCGGCGTGCTTAACACATGCAAGTCGAACGATGAAGCCCAGCTTGCTGGGTGGATTAGTG
GCGAACGGGTGAGTAACACGTGAGTAACCTGCCCCTGACTCTGGGATAAGCACTGGAAACGGTGTCTA
ATACCGGATACGAACTTTGACCGCATGGTCAGGAGTTGGAAAGAATTTTGGTCTGGGATGGACTCGCG
GCCTATCAGCTTGTTGGTGAGGTAATGGCTCACCAAGGCGACGACGGGTAGCCGGCCTGAGAGGGTGA
CCGGCCACACTGGGACTGAGACACGGCC
>Zotu2425
GAACGCTGGCGGCGTGCCTAAGACATGCAAGTCGAGTGGGTTTAGACCCACGGCGAACGGGTGAGTAA
TACCTAGGAACGTTCTTTAAGTAGAGGATAATCAGCCGAAAGGTTGGATAATACTCTATGTGATAGCA
ATATCAAAGCAGCAATGCGCTTAAAGTGCGGCCTGGGCACCATCAGCTAGTTGGTAAGGTAACGGCTT
ACCAAGGCTATGACGGTTAGGGGTCCCGAGAGGGATAACCCCCACACTGGAACTGCGACACGGTC
>Zotu2426
GAACGCTAGCGGCAGGCTTAACACATGCAAGTCGAGGGGCAGCAGGGATTTCGGTCCGCTGGCGACCG

GCAAACGGGTGCGGAACACGTACGCAACATACCTCTATCTGGGGCATAGCCTTCTGAAAAGAAGATTA
ACACCCCATAAGATCATAGAATGGCATCATTTAGTGATTAAAAATTTATTGGATAGAGATTGGCGTGC
GTCCTATTAGCTAGTTGGTGCGGTAAAGGCCCACCAAGGCGACGATAGGTAGCTGGTGTGAGAGCAC
GACCAGCCACACGGGCACTGAGACACGGGC
>Zotu2427
GAACGCTGGCGGCGTGCTTAACACATGCAAGTCGAACGAAGCACTTTATTTGATTTCCTTCGGGACTG
ATTATTTTGTGACTGAGTGGCGGACGGGTGAGTAACGCGTGGGTAACCTGCCTTGTACAGGGGGATAA
CAGTTGGAAACGGCTGCTAATACCGCATAAGCGCACGGCATCGCATGATGCAGTGTGAAAAACTCCGG
TGGTATAAGATGGACCCGCGTTGGATTAGCTAGTTGGTGAGGTAACGGCCCACCAAGGCGACGATCCA
TAGCCGACCTGAGAGGGTGACCGGCCACATTGGGACTGAGACACGGCC
>Zotu2429
GAACGCTGGCGGCGTGCCTAACACATGCAAGTCGAACGAAGAGGCCCCTTCGGGGGTACTTCAGTGGC
GGACGGGTGAGTACAACATAGGTGACCTGCCCCAAAGCGGGGGATAACAGTCCGAAAGGATTGCTAAT
ACCCCATGTGGTCCTTGAGATTTGAGGCTCAATGACTAAAGGAGTAATCCGCTTTGGGAGGGGCCTGT
GGCCCATCAGCTTGTTGGTAGGGTAATGGCCTACCAAGGCTACGACGGGTAGGGGGCCTGAGAGGGTG
ACCCCCCACATTGGCACTGACACACGGGC
>Zotu2430
GAACGCTGGCGGCGTGCTTAACACATGCAAGTCGAACGGTGAAACAGGAGCTTGCTCCTGCGGATCAG
TGGCGAACGGGTGAGTAACACGTGAGAAATGTGCCCTGGACTCTGGGATAAGCGTTGGAAACGACGTC
TAATACCGGATACGAGCTGCGAAGGCATCTTCAGCAGCTGGAAAGAACTTCGGTCCAGGATCATCTCG
CGGCCTATCAGCTAGTTGGTGAGGTAACGGCTCACCAAGGCGACGACGGGTAGCCGGCCTGAGAGGGT
GACCGGCCACACTGGGACTGAGACACGGCC
>Zotu2431
GAACGCTAGCGGCAGGCCTAACACATGCAAGTCGAGGGAGGAGCTTTCGGGCTTCGACCGGCGTACGG
GTGCGTAACGCGTATACAATCTGCCTTTCACATAGGGATAGCCCGAAGAAATTTGGATTAATACCTAA
TGGTATATAGATGTGGCATCACATTTATATTAAAGCTGAGGCGGTGAAAGATGAGTATGCGTTCTATT
AGCTAGTTGGAGAGGTAACGGCTCCCCAAGGCTACGATAGATAGGGGTTCTGAGAGGGATATCCCCCA
CACTGGTACTGAGATACGGAC
>Zotu2432
GAACGCTGGCGGCAGGCCTAACACATGCAAGTCGAACGGATCCTTCGGGATTAGTGGCGGACGGGTGA
GTAACGCGTGGGAACATACCCTTTGGTACGGAATAGCCTCGGGAAACTGGGAGTAATACCGTATGTGC
CCTTCGGGGGAAAGATTTATCGCCAAAGGATTAGCCCGCGTTGGATTAGGTAGTTGGTGGGGTAATGG
CCTACCAAGCCGACGATCCATAGCTGGTTTGAGAGGATGATCAGCCACACTGGGACTGAGACACGGCC
>Zotu2433
GAACGCTGGCGGCGTGCTTAACACATGCAAGTCGAGCGGTAAGGCCTTTCGGGGTACACGAGCGGCGA
ACGGGTGAGTAACACGTGGGTGATCTGCCCTGCACTTCGGGATAAGCCTGGGAAACTGGGTCTAATAC
CGGATATGACCTCAGGTTGCATGACTTGGGGTGGAAAGATTTATCGGTGCAGGATGGGCCCGCGGCCT
ATCAGCTTGTTGGTGGGGTAATGGCCTACCAAGGCGACGACGGGTAGCCGACCTGAGAGGGTGACCGG
CCACACTGGGACTGAGACACGGCC
>Zotu2434
GAACGCTAGCGGCAGGCTTAATACATGCAAGGCGAAGGGTCAGCAATGGCACTGTCGTACGGGTGCGT
AACGCGTATGCAACCTGCCTATCACTGGGGGATAGCCCGGGGAAACCCGGATTAATACCGCATAACAC
AGGGGTCCCGCATGGGTACTATTTGTTAAAGATTTATCGGTGGTAGATGGGCATGCGTTCGATTAGCT
GGTTGGTGCGGGTAACGGCCCACCAAGGCGACGATCGATAGGGGAGCTGAGAGGTTGATCCCCCACAC
GGGCACTGAGATACGGGC
>Zotu2435
GAACGCTGGCGGCGTGCCTAATACATGCAAGTCGAGCGAATCTTTGGGAGCTTGCTCCTAAAGATTAG
CGGCGGACGGGTGAGTAACACGTGGGCAACCTGCCTATAAGACTGGGATAACTTCGGGAAACCGGAGC
TAATACCGGATAATTCTTTTCTACTCATGTAGGAAAGCTAAAAGACGGTTTACGCTGTCACTTATAGA
TGGGCCCGCGGCGCATTAGCTAGTTGGTGAGGTAACGGCTCACCAAGGCCACGATGCGTAGCCGACCT
GAGAGGGTGATCGGCCACACTGGGACTGAGACACGGCC
>Zotu2436
GAACGCTGGCGGCGTGCCTAATACATGCAAGTCGAGCGGACTGATGGGAGCTTGCTCCCTGATGTTAG
CGGCGGACGGGTGAGTAACACGTGGGCAACCTGCCCTATAGCTTGGGATAACTCCGGGAAACCGGGGC

TAATACCGAATAATACAATTTAGCTCCTGCTAGATTGTTGAAAGATGGTTTCGGCTATCGCTATAGGA
TGGGCCCGCGGCGCATTAGCTAGTTGGTAAGGTAACGGCTTACCAAGGCGACGATGCGTAGCCGACCT
GAGAGGGTGATCGGCCACACTGGGACTGAGACACGGCC
>Zotu2437
GAACGCTGGCGGCGTGCTTAACACATGCAAGTGGAGCGACGAAGCGGGCTTCGGCCCGTGGCAGAGCC
GCGAACGGGTGAGTAACACGTGGGTAACGTGCCCCAATGACTGGGACAACCCGGGGAAACCCGGGCTA
ATACCAGATGTGGTCTCTTCACATCAGTGAGGTGACTAAAGGTAGCTTCGGCTTCCGCATTGGGATCG
GCCCGCGGCGCATTAGCTAGTTGGTGAGGTAAAGGCTTACCAAGGCAACGATGCGTAGCTGGTCTGAG
AGGACGATCAGCCACACTGGGACTGAGACACGGCC
>Zotu2439
GAACGCTGGCGGCGTGCTTAACACATGCAAGTCGAACGATGAAGCCCAGCTTGCTGGGTGTATTAGTG
GCGAACGGGTGAGTAACACGTGAGTAACCTGCCCTTGACTCTGGGATAAGCCTGGGAAACTGGGTCTA
ATACCGGATAGGAACGTCCACCGCATGGTGGTTGTTGGAAAGATTTATCGGTCATGGATGGACTCGCG
GCCTATCAGCTTGTTGGTGAGGTAATGGCTCACCAAGGCGACGACGGGTAGCCGGCCTGAGAGGGTGA
CCGGCCACACTGGGACTGAGACACGGCC
>Zotu2440
GAACGCTTGCGATGTGTCTAAGGCATGCAAGTCGAATGCTAGTAGCAATACTGGCATGGCAAACGGGA
GAGTAACACATTGGTAACTTACCTCGAAGACGGGCATAACCCGGAGAAATCCGGAGTAATTCCCGATG
GTCGCGGGGGGACACAAGTCATTCCCACGTAAAGTTTTTTCGCTTCGAGAGAGACCTATGTTCTATCA
GCTAGTTGGTGAGGTAAAGGCTCACCAAGGCGACGACGGATAGCGGGTGTGAGAGCATGGCCCGCCTA
ACTGGGACTGAGAAACTGCC
>Zotu2441
GAACGCTGGCGGCGTGCTTAACACATGCAAGTCGAACGGAAAGGCCCTTTCGGGGGTACTCGAGTGGC
GAACGGGTGAGTAACACGTGGGTGATCTGCCCTGCACTTCGGGATAAGCTTGGGAAACTGGGTCTAAT
ACCGGATATGATCACGGACTTCATGGTTTGTGGTGGAAAGCTTTTGCGGTGTGGGATGGGCCCGCGGC
CTATCAGCTTGTTGGTGGGGTAATGGCCTACCAAGGCGACGACGGGTAGCCGGCCTGAGAGGGTGTCC
GGCCACACTGGGACTGAGATACGGCC
>Zotu2442
GAATGCTGGCGGCGTGGATAAGGCATGCAAGTCGAACGGTCCGCAAGGACAGTGGCAGACGAGGTAGT
AACACGTAGGTACTCCCCCCGAAGTCTGGTATAGCTCGTCGAAAGACGGGGTAATCCCGGATGGTCCC
GAAAGGGTAAAGATTTATCGCTTCGGGAGAGGCCTGCGTAATATCAGCTTGTTGGTAAGGTAATGGCT
TACCAAGGCTACGACGTTTAGGGGAGCTGAGAGGCTGACCCCCACCGATGGGACTGAGACACGGCC
>Zotu2443
GAACGCTAGCGGCAGGCTTAACACATGCAAGTCGAGGGGTATAGTAGCAATACTAGAGACCGGCGCAC
GGGTGCGTAACGCGTATGCAATCTACCTTTTACAGAGGGATAGCCCAGAGAAATTTGGATTAATACCT
CATAGTATATTTGAGTGGCATCACTTGATTATTAAAGTTCCAACGGTAAAAGATGAGCATGCGTCCCA
TTAGCTAGATGGTAAGGTAACGGCTTACCATGGCTACGATGGGTAGGGGTCCTGAGAGGGAGATCCCC
CACACTGGTACTGAGACACGGAC
>Zotu2445
GAACGCTGGCGGAGTGCCTTAAGCATGCAAGTAGAACGAGTTCTATTGTGCCGCAAGGCACGATTGAA
CTAGTTGCGAACGGCTGAGTAATCAATAGGAATCTTTCCTTTGGTGGGGAATACCTCGTCGAAAGACG
AGCTAATACCGCATAAAATCTACGGATAAAAGCGCATAACTGCGCGCCGATTGAGGAGCCTATTGCCC
ATCAGGTAGTTGGTAGGGTAATGGCCTACCAAGCCTACGACGGGTACCCGATCTGAGAAGATGACCGG
GCAGAATGGGACTGAGACACGGCC
>Zotu2446
GAACGCTGGCGGTATGCTTAACACATGCAAGTCGAACGGAACCTTTCGGGGTTTAGTGGCGGACGGGT
GAGTAACGCGTGAGAATCGGCCCTCAGGTTGGGGACAACCATTGGAAACGGTGGCTAATACCGAATAA
GCCGAAAGGTGAAAGATTAATCGCCTGGGGAAGAGCTCGCGTCTGATTAGCTAGTTGGTGGGGTAAAG
GCCTACCAAGGCACCGATCAGTAACTGGTCTGAGAGGATGGCCAGTCACACTGGGACTGAGACACGGC
C
>Zotu2447
GAACGCTGGCGGCGTGCCTAATACATGCAAGTCGAGCGGACCGAACGAGGGCTTGCTCTTGTTTGGTC
AGCGGCGGACGGGTGAGTAACACGTGGGCAACCTGCCCGCAAGACCGGGATAACTCCGGGAAACCGGA
GCTAATACCGGATAACACCGAAGACCGCATGGTCTTCGGTTGAAAGGCGGCCTTTGGGCTGTCACTTG

CGGATGGGCCCGCGGCGCATTAGCTAGTTGGTGAGGTAACGGCTCACCAAGGCGACGATGCGTAGCCG
GCCTGAGAGGGTGACCGGCCACACTGGGACTGAGACACGGCC
>Zotu2448
GAACGCTAGCGGCAGGCCTAACACATGCAAGTCGAGCGGGGGAGCAATCCCTAGCAGCGGACGGGTGA
GTAACGCGTGGGAACATACCCTTTGGTACGGAATAGCCCCGGGAAACTGGGAGTAATACCGTATGTGG
TCGCGAGACGAAAGATTTATCGCCAAAGGATTGGCCCGCGTTGGATTAGGTAGTTGGTGGGGTAAAGG
CCTACCAAGCCGACGATCCATAGCTGGTTTGAGAGGATGATCAGCCACACTGGGACTGAGACACGGCC
>Zotu2449
GAACGCTGACGGCGTGCTTAACACATGCAAGTCGAACGATGAAGCCCAGCTTGCTGGGTGGAAGAGTG
GCGAACGGGTGAGTAACACGTGAGTAACCTGCCCCGGACTCTGGGATAAGCGCTGGAAACGGCGTCTA
ATACTGGATATGACCCATACCGGCATCGGTTGTGGGTGGAAAGATTTATCGGTTCGGGATGGGCTCGC
GGCCTATCAGCTGGATGGTGAGGTAACGGCTCACCATGGCGACGACGGGTAGCCGGCCTGAGAGGGTG
ACCGGCCACACTGGGACTGAGACACGGCC
>Zotu2450
GAACGCTGGCGGCGTGCCTTAAGCATGCAAGTCGTACGGCTCCGCAAGGAGCAGTGGCGGACGGTCGA
GTAACGCGTGGGGATCTACCTTGAAGTTGGACATAGCTCGTCGAAAGGCGGGGTAATTTCCAATGTGA
ACGCAAGTTCAAAGCCGCAGGGCGCTTTAAGATGAACCCGCGTCCTATCAGCTAGTTGGTGAGGTAAA
GGCTCACCAAGGCATCGACGGGTAGCTGGTCTGAGAGGATGATCAGCCGGAATAGCACTGAGACACGG
GC
>Zotu2451
GAACGCTGGCGGCAGGCCTAACACATGCAAGTCGAACGCCGTAGCAATACGGAGTGGCAGACGGGTGA
GTAACGCGTGGGAACCTACCTTACGGTCGGGAATAGCTCAGGGAAACTTGGGGTAATACCGGATACGC
CCTTACGGGGAAAGATTTATCGCCGTTAGATGGGCCCGCGTAGGATTAGCTAGTTGGTGAGGTAATGG
CTCACCAAGGCGACGATCCTTAGCTGGTCTGAGAGGATGATCAGCCACACTGGGACTGAGACACGGCC
>Zotu2452
GAACGCTAGCGAGAGGCCTAACACATGCAAGTCGAGGGGTAACAGGGGAGCTTGCTCCCGCTGACGAC
CGGCGGATGGGTGAGTAACGCGTATGCAACCTGCCTTTAACTGTGGGATAGCTCCCTGAAAGGGGAAT
TAATACCGCATAAGACCACGTTGTTTCATGACAGAGTGGTAAAATATTTATAGGTTAAAGATGGGCAT
GCGTGACATTAGCTAGTTGGTGAGGTAACGGCTCACCAAAGCTACGATGTCTAGGGGTTCTGAGAGGA
AGGTCCCCCACACTGGTACTGAGACACGGAC
>Zotu2453
GAACGCTGGCGGCATGCCTAACACATGCAAGTCGAACGAGGGTAGCAATACTCTAGTGGCGGACGGGT
GAGGAATACGTAGGAATCTGCCCTTAAGTGGGGGATAACCCGGGGAAACCCGGATTAATACCGCATGA
TCTCGAAAGAGCAAAGCAGGGGATCGCAAGACCTTGTGCTTTTGGAGGAGCCTACGTCGGATTAGCTA
GTTGGTGAGGTAAAGGCTCACCAAGGCAACGATCCGTAACTGGTCTGAGAGGATGATCAGTCACACCG
GAACTGAGACACGGTC
>Zotu2454
GAACGCTGGCGGCGTGCTTAACACATGCAAGTCGAACGATGAAGCCTGGCTTGCTGGGTGGATTAGTG
GCGAACGGGTGAGTAACACGTGAGTAACCTGCCCTTGACTCTGGGATAAGCGCTGGAAACGGCGTCTA
ATACCGGATACGACCTGCCCCGGCATCGGGTGTGGGTGGAAAGTTTTTCGGTCAAGGATGGACTCGCG
GCCTATCAGCTTGTTGGTGAGGTAATGGCTCACCAAGGCGACGACGGGTAGCCGGCCTGAGAGGGTGA
CCGGCCACACTGGGACTGAGACACGGCC
>Zotu2455
GAACGCTGGCGGCGTGCCTAACACATGCAAGTCGAACGAGGTCCAACCAATGGCAACATTGGGGAATA
CCTAGTGGCGAACGGGTGAGTAACACGTGAGGAACCTGCCCCGAAGACCGGGATAACGCTCCGAAAGG
AACGCTAATACCGGATACCTTCACCGGACCGCATGATCTGGTGAAGAAATGGATTCCGCTTCGGGAGG
GTCTCGCGGCCTATCAGCTTGTTGGTGGGGTAATGGCCCACCAAGGCTTCGACGGGTAGCTGGTCTGA
GAGGACGATCAGCCACACTGGGACTGAGACACGGCC
>Zotu2456
GAACGTTGGCGGCGTGCCTAACACATGCAAGTCGAGGGGTAGCAATACCACTGGCGGACGGGTGAGTA
ACGCGTAGATTATCTGCCCCTATCACAGACATAACGTCGGGAAACTGACGCTAATAGCTGATGAGGCA
ATTTATTGTCAAAGAAGAGATTCGGATAGGGATGAGTCTGCGTCTGATTAGCTTGTTGGTAGTGTAAC
GGACTACCAAGGCGATGATCAGTAACTGGTCTGAGAGGACGATCAGTCACACTGGAACTGAGACACGG
TC

>Zotu2458
GAACGCTAGCGGCAGGCTTAACACATGCAAGTCGAGGGGTAGAAGGAGCTTGCTCCTTTGAGACCGGC
GCACGGGTGCGTAACGCGTATGCAATCTACCTTATACAGGGGAATAGCCCAGAGAAATTTGGATTAAT
GCCCCATGGTATTATAGAATGGCATCATTTTATAATTAAAGTTCCAACGGTATAAGATGAGCATGCGT
CCCATTAGTTAGTTGGTAAGGTAACGGCTTACCAAGACGATGATGGGTAGGGGTCCTGAGAGGGAGAT
CCTCCACACTGGTACTGAGACACGGAC
>Zotu2459
GAACGCTGGCGGCGTGCTTAACACATGCAAGTCGAGCGGAAAGGCCCTTCGGGGTACTCGAGCGGCGA
ACGGGTGAGTAACACGTGAGTAACCTGCCCTGGACTTTGGGATAACCCTCGGAAACGGGGGCTAATAC
CGGATACGACCTTTTGGTGCATGCCTTGGGGTGTAAAGATTTTTTGGTCTGGGATGGGCTCGCGGCCT
ATCAGCTTGTTGGTGGGGTGATGGCCTACCAAGGCGACGACGGGTAGCCGGCCTGAGAGGGCGACCGG
CCACACTGGGACTGAGACACGGCC
>Zotu2460
GAACGCTGGCGGCGTGCTTAACACATGCAAGTCGAACGATGATGCCCAGCTTGCTGGGTTGATTAGTG
GCGAACGGGTGAGTAATACGTGAGTAACCTGCCCTTGACTCTGGGATAAGCCTGGGAAACCGGGTCTA
ATACTGGATGCGACGGGCCATCGCATGGTGGTTCGTGGAAAGGGTTTCTGGTCTTGGATGGGCTCACG
GCCTATCAGCTGGTTGGTGGGGTAATGGCTCACCAAGGCGACGACGGGTAGCCGGCCTGAGAGGGTGA
CCGGCCACACTGGGACTGAGACACGGCC
>Zotu2461
GAACGCTGGCGGCAGGCCTAACACATGCAAGTCGAGCGAGACCTTCGGGTCTAGCGGCGGACGGGTGA
GTAACGCGTGGGAACGTACCCTTTTCTGCGGAATAGCCTCGGGAAACTGAGAGTAATACCGCATACGC
CCTTTTGGGGAAAGATTTATCGGAGAAGGATCGGCCCGCGTTGGATTAGGTAGTTGGTGGGGTAATGG
CCTACCAAGCCGACGATCCATAGCTGGTTTGAGAGGATGATCAGCCACACTGGGACTGAGACACGGCC
>Zotu2462
GAACGCTGGCGGCGTGCTTAACACATGCAAGTCGCACGGGACTGGGGGCAACTCCAGTTCAGTGGCGG
ACGGGTGCGTAACACGTAAGCAACATGTCCAAGGGTGGGGGATAGCCGGCCCAACGGCCGGGTAATAC
CGCATGCAGTCACCGCTGGCATCTACGGTGACGAAAGCCGGGGCAACCTGGCGCCCATGGAGTGGCTT
GCGGCCTATCAGCTAGTTGGTGGGGTAACGGCTCACCAAGGCGACGACGGGTAGCTGGTCTGAGAGGA
TGACCAGCCACATTGGGACTGAGACACGGCC
>Zotu2463
GAACGCTGGCGGTATGCCTAACACATGCAAGTCGAACGGAGTTCTTCGGAACTCAGTGGCGGACGGGT
GAGTAACGCGTGAGAATCTGCCTTCAGGATGGGGACAACAGTGAGAAATCGCTGCTAAAACCCAATGT
GCCGAGAGGTGAAATACTTGTAGCCTGAAGAGGAGCTCGCGTCCGATTAGTTAGTTGGTGGGGTAAAA
GCCTACCAAGGCAGCGATCGGTAGCTGGTCTGAGAGGATGAGCAGCCACACTGGGACTGAGACACGGC
C
>Zotu2464
GAACGCTGGCGGCATGCCTAACACATGCAAGTCGAACGAAGCCTTCGGGCTTAGTGGCGCACGGGTGC
GTAACGCGTGGGAATCTGCCCTTGGGTTCGGAATAACAGTTAGAAATGACTGCTAATACCGGATGATG
TCTTCGGACCAAAGATTTATCGCCCAAGGATGAGCCCGCGTCGGATTAGCTAGTTGGTGAGGTAAAGG
CTCACCAAGGCGACGATCCGTAGCTGGTCTGAGAGGATGATCAGCCACACTGGGACTGAGACACGGCC
>Zotu2465
GAACGCTGGCGGCATGCCTAACACATGCAAGTCGAACGAGATCTTCGGATCTAGTGGCGCACGGGTGC
GTAACGCGTGGGAATCTGCCCTTTGGTTCGGAATAACAGTTGGAAACGACTGCTAATACCGGATGATG
ACGTAAGTCCAAAGATTTATCGCCAGAGGATGAGCCCGCGTAGGATTAGCTAGTTGGTGTGGTAAGAG
CGCACCAAGGCGACGATCCTTAGCTGGTCTGAGAGGATGATCAGCCACACTGGGACTGAGACACGGCC
>Zotu2467
GAACGCTGGCGGCATGCCTAACACATGCAAGTCGAACGAGACCTTCGGGTCTAGTGGCGCACGGGTGC
GTAACGCGTGGGAACCTGCCCTTAGGTTCGGAATAACTCAGAGAAATTTGAGCTAATACCGGATAATG
TCTTCGGACCAAAGATTTATCGCCTTTGGATGGGCCCGCGTAGGATTAGCTTGTTGGTGGGGTAATGG
CCTACCAAGGCGACGATCCTTAGCTGGTCTGAGAGGATGATCAGCCACACTGGGACTGAGACACGGCC
>Zotu2468
GAATGCTGGCGGCGTGCCTAACACATGCAAGTCGAGCGGCAGCGCGGTCTTCGGACTGGCGGCGAGCG
GCGGACGGCTGAGTAACGCGTGGGAACGTACCCCAAAGTGAGGAATAACTGCCCGAAAGGGTAGCTAA
TGCCGCATATGGTCTTCGGATTAAAGGATTTATCCGCTTTGGGAACGGCCTGCGTACGATTAGATTGT

TGGTGAGGTAATGGCTCACCAAGTCGACGATCGTTAACTGGTCTGAGAGGATGATCAGTCAGACTGGA
ACTGAGACACGGTC
>Zotu2469
GAACGCTGGCGGCGTGCCTAATACATGCAAGTCGAACGCACTGACAGAGAACTTGTTCTCTTGACGTG
AGTGGCGAACGGGTGAGTAACACGTGGGAAACCTACCTTCTAGCGGGGGATAACAGTCGGAAACGACT
GCTAATACCGCATAGTGCTTTTCTTGGCATCAAGGAAAGAGGAAAGATGGTTCTGCTATCACTAGAAG
ATGGTCCCGCGGTGCATTAGCTAGTTGGTAAGGTAACGGCTTACCAAGGCGATGATGCATAGCCGACC
TGAGAGGGTAATCGGCCACATTGGGACTGAGACACGGCC
>Zotu2470
GAACGCTGGCGGCATGCCTAACACATGCAAGTCGAACGAAGGCTTCGGCCTTAGTGGTGCACGGGTGC
GTAACGCGTGGGAATCTGCCCCTTGGTTCGGAATAACAGTTAGAAATGACTGCTAATACCGGATGATG
ACGTAAGTCCAAAGATTTATCGCCGAGGGATGAGCCCGCGTAGGATTAGCTAGTTGGTGTGGTAAGAG
CGCACCAAGGCGACGATCCTTAGCTGGTCTGAGAGGATGATTAGCCATACTGGGACTGAGACACGGCC
>Zotu2471
GAACGCTGGCGGCGTGCCTAATACATGCAAGTCGAGCGGAGTTGATGAGGTGCTTGCACCTCTGATGC
TTAGCGGCGGACGGGTGAGTAACACGTAGGTAACCTGCCTGTAAGACTGGGATAACATTCGGAAACGA
ATGCTAATACCGGATACGCGATCCTCTCGCATGAGGGGATCGGGAAAGACGGAGCAATCTGTCGCTTA
CGGATGGACCTGCGGCGCATTAGCTAGTTGGTGAGGTAACGGCTCACCAAGGCGACGATGCGTAGCCG
ACCTGAGAGGGTGATCGGCCACACTGGGACTGAGACACGGCC
>Zotu2472
GAACGCTAGCGGGATGCTTTACACATGCAAGTCGAACGGCAGCGCGGACTTCGGTCTGGCGGCGAGTG
GCGAACGGGTGAGTAATGTATCGGAACGTGCCCAGTAGCGGGGGATAACTACGCGAAAGCGTAGCTAA
TACCGCATACGCCCTACGGGGGAAAGCAGGGGATCGTAAGACCTTGCACTATTGGAGCGGCCGATATC
GGATTAGCTAGTTGGTGAGGTAACGGCTCACCAAGGCGACGATCCGTAGCTGGTTTGAGAGGATGACC
AGCCACACTGGGACTGAGACACGGCC
>Zotu2474
GAACGCTGGCGGCGTGCTTAACACATGCAAGTCGAACGGTGAAGCCCCTTCGGGGGTGGATCAGTGGC
GAACGGGTGAGTAACACGTGGGCAACCTACCCTTGACTCTGGGATAACCCCGGGAAACCGGAGCTAAT
ACCGGATACGACGCGGATCGACATCCTTCCGCGTGGAAAGTTTTTCGGTCAGGGATGGGCCCGCGGCC
TATCAGCTTGTTGGTGGGGTAATGGCCTACCAAGGCGACGACGGGTAGCCGGCCTGAGAGGGCGACCG
GCCACACTGGGACTGAGACACGGCC
>Zotu2475
GAACGCTGGCGGTGTGCTTAATACATGCAAGTCGAACGAGTCGCAAGACTAGTGGCGAACGGGTGAGG
AACACGTAGGAATCTGCCCCAAACTGGGAAATAACTAGCCGAAAGGTTGGCTAATGTCCCATAATCCG
GCAACGGTAAAGTCGCAAGACGGTTTGGGAGGAGCCTGCGTCCTATCAGCTTGTTGGTGAGGTAATGG
CTCACCAAGGCTATGACGGGTAGCTGGTCTAAGAGGATGATCAGCCACAGTGGGACTGAGACACGGCC
>Zotu2476
GAACGCTGGCGGCGTGCCTAATACATGCAAGTCGAGCGGCAGCGCGAATAGTTTACTATTTGGCGGCG
AGCGGCGGACGGCTGAGTAACACGTAGGAACGTACCCCAAAGTGAGGAATAACTGCCCGAAAGGGTGG
CTAATGCCGCATGTGCTCTAAGGAGTAAAGCTTCGGCGCTTTGGGAGCGGCCTGCGTCTGATTAGTTT
GTTGGTGAGGTAATGGCTCACCAAGACTACGATCGGTAGCTGGTCTGAGAGGATGATCAGCCAGACTG
GGACTGAGACACGGCC
>Zotu2477
GAACGCTGGCGGCGTGCCTAATACATGCAAGTCGAGCGGACCAATAGAAAAGCTTGCTTTTCTTGAGG
TTAGCGGCGGACGGGTGAGTAACACGTGGGCAACCTACCTGTAAGACTGGGATAACTTACGGAAACGT
GAGCTAATACCGGATAGTTTCACTTCTCGCATGAGAAGTGAAGGAAAGATGGCTTTTAGCTATCACTT
ACAGATGGGCCCGCGGTGCATTAGCTAGTTGGTGGGGTAAAGGCCTACCAAGGCAACGATGCGTAGCC
GACCTGAGAGGGTGATCGGCCACACTGGGACTGAGACACGGCC
>Zotu2478
AAACGCTGGCGGCGTACCTAAGACATGCAAGTCGAACGGGACTTTCCGCTTCGGCGGGGAGTCGAGTG
GCGGACGGGTGAGTAACGCGTAGGAACCTGTCGCACAGTGGGGGATAACACGGGGAAACCGGTGCTAA
TACCGCATGTGGTCAGAGATGACGAAAGCAGCAATGCGCTGTGTGAGGGGCCTGCGTCCGATTAGCTG
GTTGGTGGGGTAAAGGCCTACCAAGGCGACGATCGGTAGCTGGTCTGAGAGGATGGTCAGCCACACTG
GGACTGAGATACGGCC

>Zotu2479
GAACGCTGGCGGCGTGCTTAACACATGCAAGTCGAACGGAAAGGCCCTGCTTGCAGGGTGCTCGAGTG
GCGAACGGGTGAGTAACACGTGGGTGATCTGCCCTGTACTTTGGGATAAGCCTGGGAAACTGGGTCTA
ATACCGAATATGACCACTTCTTGGATGGGGTGGTGGAAAGCTTTTGCGGTATGGGATGAGCTCGCGGC
CTATCAGCTTGTTGGTGGGGTAATGGCCTACCAAGGCGTCGACGGGTAGCCGGCCTGAGAGGGTGTAC
GGCCACATTGGGACTGAGATACGGCC
>Zotu2480
GAACGCTGGCGGCGTGCTTAACACATGCAAGTCGAACGATGAAGCCGGAGCTTGCTCCGGCGGATTAG
TGGCGAACGGGTGAGTAACACGTGAGTAACCTGCCCCAGACTCTGGGATAAGCACTGGAAACGGTGTC
TAATACCGGATACGAGCTTCAGCCGCATGGCTAGGAGCTGGAAAGAATTTCGGTCTGGGATGGACTCG
CGGCCTATCAGGTAGTTGGTGAGGTAAAGGCTCACCAAGCCGACGACGGGTAGCCGGCCTGAGAGGGT
GACCGGCCACACTGGGACTGAGACACGGCC
>Zotu2481
GAACGCTGGCGGCATGCTTTACACATGCAAGTCGGACGGCAGCGGGGAAGTGCTTGCACTTCTGCCGG
CGAGTGGCGAACGGGTGAGTAATGCATTGGAACGTACCGGGTAATGGGGGATAACTTGCCGAAAGGTA
AGCTAATACCGCATACGCTCTTAGGAGGAAAGCGGGGGATCGTTTGACCTCGCGTTATCCGAGCGGCC
AATGTCTGATTAGCTAGTTGGTGGGGTAAAAGCCTACCAAGGCAACGATCAGTAGCGGGTCTGAGAGG
ATGATCCGCCACACTGGGACTGAGACACGGCC
>Zotu2482
GAACGCTAGCGGCAGGCTTAACACATGCAAGTCGAGGGGTATAGTAGCAATACTAGAGACCGGCGCAC
GGGTGCGTAACGCGTATGCAATCTACCTTTCACAGAGGGATAGCCCAGAGAAATTTGGATTAATACCT
CATAGTATTATGATGTGGCATCACATTATAATTAAAGTCACAACGGTGAAAGATGAGCATGCGTCCCA
TTAGCTTGTTGGTAAGGTAACGGCTTACCAAGGCAACGATGGGTAGGGGTCCTGAGAGGGAGATCCCC
CACACTGGTACTGAGACACGGAC
>Zotu2483
GAACGCTGGCGGCGTGCTTAACACATGCAAGTCGAGCGGTAAGGCCCTTCGGGGTACACGAGCGGCGA
ACGGGTGAGTAACACGTGAGCAATCTGCCCTTCACTTTGGGATAACCCCCGGAAACGGGAGCTAATAC
CGAATATGACCCTGCCATGCATGTGGTGGGGTGGAAAGTTCTGGCGGTGGGGGATGAGCTCGCGGCCT
ATCAGCTTGTTGGTGGGGTAATGGCCTACCAAGGCTTCGACGGGTAGCCGGCCTGAGAGGGTGACCGG
CCACACTGGGACTGAGACACGGCC
>Zotu2484
GAACGCTGGCGGCATGCCTAACACATGCAAGTCGAACGAGACCTTCGGGTCTAGTGGCGCACGGGTGC
GTAACGCGTGGGAATCTGCCCTTGGGTCCGGAATAACAGTTAGAAATGACTGCTAATACCGGATGATG
TCTTCGGACCAAAGATTTATCGCCCAGGGATGAGCCCGCGTAGGATTAGCTTGTTGGTGAGGTAAAGG
CTCACCAAGGCGACGATCCTTAGCTGGTCTGAGAGGATGATCAGCCACACTGGGACTGAGACACGGCC
>Zotu2485
GAACGCTGGCGGCATGCCTAACACATGCAAGTCGAACGAGATCTTCGGATCTAGTGGCGCACGGGTGC
GTAACGCGTGGGAATCTGCCCTTGGGTTCGGAATAACTCAGAGAAATTTGAGCTAATACCGGATGATG
ACGTAAGTCCAAAGATTTATCGCCCAAGGATGAGCCCGCGTAAGATTAGCTAGTTGGTGGGGTAAAAG
CCTACCAAGGCGACGATCTTTAGCTGGTCTGAGAGGATGATCAGCCACACTGGGACTGAGACACGGCC
>Zotu2486
GAACGCTGGCGGCGTGCTTAACACATGCAAGTCGAACGAGAAGCGGGAGCTTGCTCCCGCAGACAGTG
GCGAACGGGTGAGTAACACGTGAGTAACCTGCCCTTGACTCTGGGACAACTCTGGGAAACTGGAGCTA
ATACCGGATACGAGACGCTCAGGCATCTGAAGCGTCTGGAAAGAATTTCGGTCAAGGATGGACTCGCG
GCCTATCAGCTTGTTGGCGGGGTAATGGCCCACCAAGGCGACGACGGGTAGCCGGCCTGAGAGGGCGA
CCGGCCACACTGGAACTGAGACACGGTC
>Zotu2487
TAACGCTAGCTATGTGCCTAAGGTATGCAAGTCGAACGGTAAAATTCAAGTAGCAATACTTGAATTTT
ATAGTGGCGAACGGCTGAGTAACGCGTAGGAATATCCCCTTAAGCCGAGAATAGCTCGCCGAAAGGCG
GGGTAAAGCTCGATAGTCCAGAAATGGTAAATCTACGGAGCTTAAGGATTAGCCTGCGTCCTATCAGG
TAGTTGGTGGGGTAAAAGCCTACCAAGCCTATGACGGGTAGCTGATCTGAGAGGATGGTCAGCCAGAC
TGGGACTGCGATACGGCC
>Zotu2488
GAACGCTGGCGGCGCGCCTAACACATGCAAGTCGAACGGAGCTTAGAGAGCTTGCTTTTTAAGCTTAG

TGGCGAACGGGTGAGTAACGCGTGAGTAACCTGCCCTAGAGTGGGGGACAACAGTTGGAAACGACTGC
TAATACCGCATAAGCCCACGGTACCGCATGATACTGAGGGAAAAGGATTTATTCGCTTTAGGATGGAC
TCGCGTCCAATTAGCTAGTTGGTGAGGTAACGGCTCACCAAGGCGACGATTGGTAGCCGGACTGAGAG
GTTGAACGGCCACATTGGGACTGAGACACGGCC
>Zotu2489
GAACGCTGGCTGTATGGATAAGGCATGCAAGTCGAACGGACCGCAAGGTCAGTGGCAGACGAGGTAGT
AACACGTAGGTACGTACTCCGAAGTCGGGCATAGCTCACCGAAAGGTGAGGTAATTCCCGATAGTCCC
GCAAGGGTAAAGATTTATCGCTTCGGAAGCGGCCTGCGTAGTATCAGCTAGTTGGTAGGGTAACGGCC
TACCAAGGCGACGACGCTTAGGGGAGCTGAGAGGCTGACCCCCACCGATGGGACTGAGACACGGCC
>Zotu2490
GAACGCTGGCGGCGTGCCTAATACATGCAAGTCGAGCGAACTTGCGGGAGCTTGCTCCCAAAAGTTAG
CGGCGGACGGGTGAGTAACACGTGGGCAACCTGCCTATAAGACTGGGATAACTTCGGGAAACCGGAGC
TAATACCAGATAATTCTTTTCTACACATGTAGAAAAGCTAAAAGACGGTTTACGCTGTCACTTATAGA
TGGGCCCGCGGCGCATTAGCTAGTTGGTGAGGTAACGGCTCACCAAGGCCACGATGCGTAGCCGACCT
GAGAGGGTGATCGGCCACACTGGGACTGAGACACGGCC
>Zotu2491
GAACGCTGGCGGCGTGCTTAACACATGCAAGTGGAGCGACGAACCGGGGCTTGCCCCGGGGCAGAGCC
GCGAACGGGTGAGTAACACGTGGGTAACCCACCTCGATGACCGGGACAACCCGAGGAAACTCGGGCTA
ATACCGGATGTGATCCTCTTTCGTAAGGAAGAGAATGAAAGATAGCTTCGGCCTTCGCATCGAGACGG
GCCCGCGGCCCATTAGCTTGTTGGTGGGGTAATGGCCTACCAAGGCTTCGATGGGTAGCTGGTCTGAG
AGGACGATCAGCCACACTGGGACTGAGACACGGCC
>Zotu2492
GAACGCTGGCGGCGTGCCTAACACATGCAAGTCGAACGGAACTGTTTTGAAAGATTTCTTCGGAATGA
ATTTGATTTAGTTTAGTGGCGGACGGGTGAGTAACGCGTGAGTAACCTGCCTTCAAGAGGGGGATAAC
ATTCTGAAAAGAATGCTAATACCGCATGACATATCGGAACCACATGGTTCTGGTATCAAAGATTTTAT
CGCTTGAAGATGGACTCGCGTCCGATTAGTTAGTTGGTGAGGTAACGGCTCACCAAGACCGCGATCGG
TAGCCGGACTGAGAGGTTGAACGGCCACATTGGGACTGAGACACGGCC
>Zotu2493
GAACGCTGGCGGCGTGCTTAACACATGCAAGTCGAACGGAAAGGCCCTTGCTTGCAAGGGTGCTCGAG
TGGCGAACGGGTGAGTAACACGTAGGTGATCTGCCTTTCACTTCGGGATAAGCTTGGGAAACTGGGTC
TAATACCGGATATTCACGGCACCGTGGTTGGTGTGGTGGAAAGCTTTTGCGGTGGGAGATGAGCTTGC
GGCCTATCAGCTTGTTGGTGGGGTAATGGCCTACCAAGGCGGCGACGGGTAGCCGGCCTGAGAGGGTG
TGCGGCCACATTGGGACTGAGATACGGCC
>Zotu2494
GAACGCTAGCGGCAGGCCTAATACATGCAAGTCGAGGGGCAGCGGGGGTAGCAATACCTGCCGGCGAC
CGGCAAACGGGTGCGGAACACGTACGCAACCTTCCTTTAAGCGGGGAATAGCCCAGAGAAATTTGGAT
TAATACCCCATAAGAATGTGGATTGGCATCAGTCAGCATTTAAAGATTTATCACTTAGAGATGGGCGT
GCGTCTGATTAGGTAGTTGGTGAGGTAACGGCTCACCAAGCCGACGATCAGTAACTGGCGTGAGAGCG
CGACCAGTCACACGGGCACTGAGACACGGGC
>Zotu2495
GAACGCTAGCGGCAGGCTTAACACATGCAAGTCGAGGGGTATAGTTCTTCGGAACTAGAGACCGGCGC
ACGGGTGCGTAACGCGTATGCAATCTACCTTTTACAGAGGGATAGCCCAGAGAAATTTGGATTAATAC
CTCATAGTATATAGACCTGGCATCAGGATTATATTAAAGTCACAACGGTAAAAGATGAGCATGCGTCC
CATTAGCTAGTTGGTAAGGTAACGGCTTACCAAGGCTACGATGGGTAGGGGTCCTGAGAGGGAGATCC
CCCACACTGGTACTGAGACACGGAC
>Zotu2496
GAACGCTGGCGGCATGCCTAACACATGCAAGTCGAACGAAGGCTTCGGCCTTAGTGGCGCACGGGTGC
GTAACGCGTGGGAATCTGCCCCTCGGTTCGGAATAACAGTTAGAAATGACTGCTAATACCGGATAATG
ACGAAAGTCCAAAGATTTATCGCCGAGGGATGAGCCCGCGTAGGATTAGGTAGTTGGTAGGGTAAAGG
CCTACCAAGCCGACGATCCTTAGCTGGTCTGAGAGGATGATCAGCCACACTGGGACTGAGACACGGCC
>Zotu2497
GAACGCTGGCGGCATGCCTAACACATGCAAGTCGAACGCACCGCAAGGTGAGTGGCGCACGGGTGAGT
AACGCGTGGGAACCTGCCCTATGGTACGGAATAACACCGGGAAACTGGTGCTAATACCGTATAAGCCC
CTTGGTCGTGGAGATTGAGGGGGAAAGATTTATTGCCATGGGAGGGGCCCGCGTCCGATTAGGTAGTT

GGTGGGGTAATGGCCTACCAAGCCTTCGATCGGTAGCTGGTCTGAGAGGATGATCAGCCACACTGGGA
CTGAGACACGGCC
>Zotu2498
CAACGCTGGCGGCGTGCCTAACACATGCAAGTCGAACGCGGTCACCCCGCAAGGGGTGACTGAGTGGC
AAACGGGTGAGTAACACGTGGGTGACCTGCCTCTGAGTGGGGGATAACGTTTCGAAAGGAACGCTAAT
ACCGCATGACATCCTGCCTTTGGACAGGCGGAGATCAAAGCCGGGGATCGCAAGACCTGGCGCTTGGA
GAGGGGCCCGCGTCCGATTAGCTAGTTGGTGAGGTAACGGCTCACCAAGGCGACGATCGGTATCCGGC
CTGAGAGGGCGGACGGACACACTGGGACTGAGACACGGCC
>Zotu2499
GAATGCTAGCGGTGTGCCTGAGATATGCAAGTCGAGCGATTATTTTGCCGCAAGGTGGAATAGTAGCG
GCGGACGGCTGAGTAATGTACAGGAATCTGCCCCTTAGTGGAACATAGCTCCGGGAAACCGGGGGTAA
TTTTCCATGGTTCCCGATTTTTTCGGGATAAAAGCCTTTACCGGTTGCTAAGGGATGAGCCTGTATCC
TATCAGGTAGTTGGTGGGGTAAAAGCCTACCAAGCCTATGACGGGTAGCTGATCTGGGAGGATGATCA
GCCAGAGGGGGACTGAGACACGGCC
>Zotu2500
GAACGCTGGCGGCGTGCCTAAGACATGCAAGTCGAGTGGGTTTAGACCCACGGCAAACGGGTGAGTAA
TACCTAGGAACTTTCTTTAAGTGGAGCATAACCAGCCGAAAGGTTGGATAATACTCCATACCATCGCA
AGATGAAAGTCGCAAGACGCTTAAAGTGAGGCCTGGGCACCATCAGCTAGTTGGTAAGGTAATGGCTT
ACCAAGGCTATGACGGTTAGGGGCCCCGAGAGGGGAGTCCCCCACACTGGAACTGCGACACGGTC
>Zotu2501
GAACGCTGGCGGCAGGCCTAACACATGCAAGTCGAACGGACCCTTCGGGGTTAGTGGCGGACGGGTGA
GTAACACGTGGGAACGTGCCTTTTGGTTCGGAACAACTCAGGGAAACTTGAGCTAATACCGGATGTGC
CCTTCGGGGGAAAGATTTATCGCCATTAGAGCGGCCCGCGTCTGATTAGCTTGTTGGTGAGGTAAAGG
CTCACCAAGGCGACGATCAGTAGCTGGTCTGAGAGGATGATCAGCCACATTGGGACTGAGACACGGCC
>Zotu2502
GAACGCTGGCGGCATGCCTTACACATGCAAGTCGAACGGCAGCACGGGGGCAACCCTGGTGGCGAGTG
GCGAACGGGTGAGTAACATATCGGAACGTGCCCTGGAGTGGGGGATAACTAGTCGAAAGACTAGCTAA
TACCGCATACGATCTACGGATGAAAGTGGGGGATCGCAAGACCTCATGCTCGTGGAGCGGCCGATATC
TGATTAGCTAGTTGGTGGGGTAAAGGCCTACCAAGGCATCGATCAGTAGCTGGTCTGAGAGGACGACC
AGCCACACTGGGACTGAGACACGGCC
>Zotu2503
GAACGCTGGCGGCGTGCTTAACACATGCAAGTCGAACGATGAAGCCTAGCTTGCTAGGTGGATTAGTG
GCGAACGGGTGAGTAATACGTGAGTGACCTACCTTTGACTCTGGGATAAGCCTGGGAAACTGGGTCTA
ATACCGGATACGACCAATCTCTGCATGGGGTGTTGGTGGAAAGCGTTATGGAGTGGTTTTAGATGGGC
TCACGGCCTATCAGCTTGTTGGTGAGGTAATGGCTTACCAAGGCGACGACGGGTAGCCGGCCTGAGAG
GGTGACCGGCCACACTGGGACTGAGACACGGCC
>Zotu2505
GAACGCTGGCGGTATGTCTAACACATGCAAGTCGAACGAGATCTTTTTTTTGTTTACAAGAAAAAGAT
TGCAGTGGCGGACGGGTGAGGAACACGTAAGAACTTACCTTTTGGCGGGGGATAACATCGGAAACTG
TTGCTAATACCCCATAATGCTGAGGAGCGAAAGGGAAACTGCCAAAAGAGAGGCTTGCGTCTGATTAG
CTAGTTGGTGGAGGTAAATGCTCCCCAAGGCGACGATCAGTAGCTGGTCTGAGAGGATGATCAGCCAC
ACTGGGACTGAGACACGGCC
>Zotu2506
GAACGCTGGCGGCATGCCTAACACATGCAAGTCGAACGATGCTTTCGGGCATAGTGGCGCACGGGTGC
GTAACGCGTGGGAATCTGCCCCTTGGTTCGGAATAACCGTTGGAAACGACGGCTAATACCGGATGACG
ACGTAAGTCCAAAGATTTATCGCCGAGGGATGAGCCCGCGTAGGATTAGCTAGTTGGTGTGGTAAAGG
CGCACCAAGGCGACGATCCTTAGCTGGTCTGAGAGGATGATCAGCCACACTGGGACTGAGACACGGCC
>Zotu2507
GAACGCTAGCGGGAGGCCTAACACATGCAAGCCGAGCGGTAGAAGTCCTTCGGGACTTTGAGAGCGGC
GTACGGGTGCGGAACACGTGTGCAACCTGCCTTTATCAGGGGGATAGCCTTTCGAAAGGAAGATTAAT
ACCCCATAATATAATGAACGGCATCGTTTATTATTGAAAACTCCGGTGGATAGAGATGGGCACGCGCA
AGATTAGATAGTTGGTAGGGTAACGGCCTACCAAGTCAGTGATCTTTAGGGGGCCTGAGAGGGTGATC
CCCCACACTGGTACTGAGACACGGAC
>Zotu2508

GAGCGCTGGCGGCGTGCCTAATACATGCAAGTCGAGCGGTAACAGGTCTAGTTTACTAGATGCTGACG
AGCGGCGGACGGCTGAGTAACACGTAGGAATGTACCCCAAAGTGAGGGATAACTGCTCGAAAGAGTAG
CTAATACCGCATGTGATCTAAGGATTAAAGCTTTATGCGCTTTGGGAACAGCCTGCGTTCGATTAGCT
AGTTGGTGAGGTAATTGCTCACCAAGGCGATGATCGATAGCTGGTCTGAGAGGATGATCAGCCAGACT
GGGACTGAGACACGGCC
>Zotu2509
GAACGCTAGCGGCATGCTTAACACATGCAAGTCGGACGGGAAGTGGTGTTTCCAGTGGCGGACGGGTG
AGTAACGCGTAAGAACCTGCCCTTGGGAGGGGAACAACAACTGGAAACGGTTGCTAATACCCCGTAGG
CTGAGGAGCAAAAGGAGGAATCCGCCCGAGGAGGGGCTCGCGTCTGATTAGCTAGTTGGTGAGGCAAT
AGCTTACCAAGGCGATGATCAGTAGCTGGTCCGAGAGGATGATCAGCCACACTGGGACTGAGACACGG
CC
>Zotu2510
GAACGCTGGCGGCGTGCCTAATACATGCAAGTCGAACGCATTTTCTTTCACCGTAGCTTGCTACACCG
AAAGAAAATGAGTGGCGAACGGGTGAGTAACACGTGGGTAACCTGCCCATCAGCGGGGGATAACACTT
GGAAACAGGTGCTAATACCGCATAATTCCATTTACCGCATGGTAAATGGATGAAAGGCGCTTTTGCGT
CACTGATGGATGGACCCGCGGTGCATTAGCTAGTTGGTGGGGTAACGGCCTACCAAGGCTGCGATGCA
TAGCCGACCTGAGAGGGTGATCGGCCACACTGGGACTGAGACACGGCC
>Zotu2511
GAACGCTGGCGGCGTGCCTAACACATGCAAGTCGAACGAGGTCCATGCAGTGGCAACACTGCGGAAGA
CCTAGTGGCGAACGGGTGAGTAACACGTGAGGAACTTGCCCCGGAGACCGAGATAACCTTCCGAAAGG
ATGGCTAATACCGGATATCGTCACCGAGCCGCATGGCTTGGTGACGAAATGGATTCCGCTCCGGGAGA
GCCTCGCGGCCTATCAGCTAGTTGGTGGGGTAACGGCCCACCAAGGCATCGACGGGTAGCTGGTCTGA
GAGGACGATCAGCCACACTGGGACTGAGACACGGCC
>Zotu2512
GAACGCTGGCGGCGTGCTTAACACATGCAAGTCGCGGGAGAAAGTCTTCGGATGAGTACACCGGCGCA
CGGGTGAGTAACACGTGGGTAATCTTCCTTCAGGCCTGGAATAACCCGGGGAAACTCGGACTAATACC
AGATAAGACCACGTCTCGAAAGAGATGTGGGAAAAGGGCGGGACCTCTTCGGAGGCCGTTCACCAGTC
GATGAGCCCGCGTCCCATCAGCTAGTTGGCGGGGTAACGGCCCACCAAGGCTAAGACGGGTAGCTGAT
CTGAGAGGATGATCAGCCACACTGGAACTGAGACACGGTC
>Zotu2513
GAACGCTGGCGGCGTGCCTAAGACATGCAAGTCGAGTGGGTTTAGACCCACGGCGAACGGGTGAGTAA
TACCTAGGAACTTTCTTTAAGTGGAGCATAACCCATAGAAATGTGGGATAATTCTCCATGTGCCCCGG
AGTTACATCCGGGGCAAAGTCGCAAGACGCTTAAAGTGAGGCCTGGGCACCATCAGCTAGTTGGTAAG
GTAATGGCTTACCAAGGCTATGACGGTTAGCGGTCCCGAGAGGGAGAACCGCCACACTGGAACTGCGA
CACGGTC
>Zotu2514
GAACGCTGGCGGCGTGCCTAATACATGCAAGTCGAGCGGAAACTTGGGAGCTTGCTCTCAAGTTTTAG
CGGCGGACGGGTGAGTAACACGTGGGCAACCTGCCCTGCAGATCGGGATAACTCCGGGAAACCGGTGC
TAATACCGAATAGTTTCCGGCCCCTCCTGGGGCGGGACGGAAAGACGGTTTCGGCTGTCACTGCAGGA
TGGGCCCGCGGCGCATTAGCTAGTTGGTGGGGTAACGGCCCACCAAGGCGACGATGCGTAGCCGACCT
GAGAGGGTGATCGGCCACACTGGGACTGAGACACGGCC
>Zotu2515
GAACGCTGGCGGCGTGCTTAACACATGCAAGTCGAGCGACAGCACTCTTCGGGGTGCATGGAGCGGCG
AACGGGTGAGTAACACGTGGGCAACCTGCCCTCAGCTCTGGGACAACTCCGGGAAACCGGGGCTAATA
CCGGATACGACCTTCCCTCGCATGGGGGTCGGTGGAAAGTTTTTCGGCTGAGGATGGGCCCGCGGCCT
ATCAGCTTGTTGGTGGGGTGATGGCCTACCAAGGCGACGACGGGTAGCCGGCCTGAGAGGGCGACCGG
CCACACTGGGACTGAGACACGGCC
>Zotu2516
GAACGTTAGCGGCGCGCCTAACACATGCAAGTCGCGCGAGAAAGGGCTTCGGCCCCAGTAAAGCGGCG
CACGGGTGAGTAACACGTAGGTCATCTTCCCCTGAGTGGTGGATAACTCTCCGAAAGGAGAGCTAATA
CAGCATAAGACCACGACCTCGCCGAGGTTGTGGCCAAAGCAGACCTCTTCACGAAAGTTTGCGCTTAG
GGATGAGCCTGCGGCCCATCAGCTAGTTGGTAGGGTAATGGCCTACCAAGGCTAAGACGGGTAGCTGG
TCTGAGAGGATGAACAGCCACACTGGAACTGAGACACGGTC
>Zotu2517

GAACGCTGGCGGCGTGCTTAACACATGCAAGTCGAACGGAAAGGCCCTTCGGGGTACTCGAGTGGCGA
ACGGGTGAGTAACACGTGGGTGATCTGCCCTGCACTTCGGGATAAGCCTGGGAAACTGGGTCTAATAC
CGGATATACCTCATGGATTGCATGGTTTGTGGGGGAAAGCTTTTGCGGTGTGGGATGGGCCCGCGGCC
TATCAGCTTGTTGGTGGGGTGATGGCCTACCAAGGCGTCGACGGGTAGCCGGCCTGAGAGGGTGTCCG
GCCACACTGGGACTGAGATACGGCC
>Zotu2518
GAACGCTAGCGGCAGGCCTAACACATGCAAGTCGAGGGGCAGCACGAATTAGCTTGCTAATTTGGTGG
CGACCGGCGCACGGGTGCGTAACGCGTATGCAACCTACCTTATACAGGAGTATAGCCCGGAGAAATTC
GGATTAATCCTCCATGGTACTGAGACACCGCATGGTGATTCAGTTAAAGCTTCGGCGGTATAAGATGG
GCATGCGTCCTATTAGCTAGATGGTAAGGTAACGGCTTACCATGGCTACGATAGGTAGGGGGCCTGAG
AGGGTGGTCCCCCACACTGGTACTGAGACACGGAC
>Zotu2519
GAACGCTGGCGGCATGCCTAACACATGCAAGTCGAACGAGACCTTCGGGTCTAGTGGCGCACGGGTGC
GTAACGCGTGGGAATCTGCCCTTGGGTTCGGAATAACAGTGAGAAATTACTGCTAATACCGGATGATG
TCTTCGGACCAAAGATTTATCGCCCAGGGATGAGCCCGCGTAGGATTAGCTAGTTGGTGGGGTAATGG
CCTACCAAGGCGACGATCCTTAGCTGGTCTGAGAGGATGATCAGCCACACTGGGACTGAGACACGGCC
>Zotu2520
AGCCGTTGGAAATCGCTGTATTCAACTCAGATACACGGGAGTTCAGGCCAAGGGCTCACACATGCTTT
TCCCGCAACCTCTCCCAGCCACTAACATTAGGATATTAGCA
>Zotu2521
GAACGCTGGCGGCGTGCTTAACACATGCAAGTCGAACGGAAAGGCCCTTCGGGGTACTCGAGTGGCGA
ACGGGTGAGTAACACGTGGGTGATCTGCCCTGCACTTTGGGATAAGCCTGGGAAACTGGGTCTAATAC
CGAATAGGACTACGCTCTTCATGGGGTGTGGTGGAAAGCTTTTGCGGTGTGGGATGGGCCCGCGGCCT
ATCAGCTTGTTGGTGGGGTAATGGCCTACCAAGGCGACGACGGGTAGCCGGCCTGAGAGGGTGACCGG
CCACACTGGGACTGAGATACGGCC
>Zotu2522
GAACGCTGGCGGCGTGCCTAATACATGCAAGTCGAGCGAATTCTTTAAAAGCTTGCTTTTGAAGAGTT
AGCGGCGGACGGGTGAGTAACACGTGGGCAACCTGCCTATAAGACTGGGATAACTTCGGGAAACCGGA
GCTAATACCGGATAATTCTTTTCTACACATGTAGGAAAGCTGAAAGACGGTTTACGCTGTCACTTATA
GATGGGCCCGCGGCGCATTAGCTAGTTGGTGAGGTAACGGCTCACCAAGGCCACGATGCGTAGCCGAC
CTGAGAGGGTGATCGGCCACACTGGGACTGAGACACGGCC
>Zotu2523
GAACGCTGGCGGCATGCCTAACACATGCAAGTCGAACGGCAGCACAGCAGTAGCAATACTGTGGGTGG
CGAGTGGCGGACGGGTGAGTAATGCATCGGGACCTACCTGAACGTGGGGGATAACGTAGGGAAACTTA
CGCTAATACCGCATACGTCCTACGGGAGAAAGCGGGGGACCTTCGGGCCTCGCGCGGTCAGACGGACC
GATGTTCGATTAGCTAGTTGGTAAGGTAACGGCTTACCAAGGCGACGATCGATAGCTGGTCTGAGAGG
ATGATCAGCCACACTGGAACTGAGACACGGTC
>Zotu2524
GAACGCTGGCGGCATGCCTAACACATGCAAGTCGAACGAGATCTTCGGATCTAGTGGCGCACGGGTGC
GTAACGCGTGGGAATCTGCCTTGGGGTTCGGAATAACTCCCCGAAAGGGGTGCTAATACCGGATGATG
TCGAGAGACCAAAGATTTATCGCCCTGAGATGAGCCCGCGTAGGATTAGCTAGTTGGTGTGGTAAAGG
CGCACCAAGGCGACGATCCTTAGCTGGTCTGAAAGGATGATCAGCCACACTGGGACTGAGACACGGCC
>Zotu2525
GAACGCTGGCGGCAGGCCTAAAACATGCAAGTCGAACGAACCTTCGGGTTAGTGGCGCACGGGTGAGT
AACGCGTGGGAATGTACCTCTGGGTGCGGAACAACGTCGGGAAACTGACGCTAATACCGCATAATGTC
TTCGGACCAAAGATTTATCGCCTAGAGATCAGCCCGCGTTAGATTAGCTAGTTGGTGAGGTAAGAGCT
CACCAAGGCTACGATCTATAGCTGGTCTGAGAGGATGATCAGCCACACTGGAACTGAGACACGGTC
>Zotu2526
GAACGCTAGCGGCAGGCCTAATACATGCAAGTCGGACGAGAATCAGGGAAGTAGCAATACGGAACTGA
GGACAGTGGCGCACGGGTGCGTAACGCGTATGCAACCTACCTTGATCAGGGGGATAGCCTCTCGAAAG
AGAGATTAACACCGCATAAGAACATAGAGTGGCATCACTCAGTGTTTAAATATTTATAGGATCAAGAT
GGGCATGCGTGACATTAGCTAGTTGGTGAGGTAACGGCTCACCAAGGCGACGATGTCTAGGGGATCTG
AGAGGATGACCCCCCACACTGGTACTGAGACACGGAC
>Zotu2528

GAACGCTAGCGGCAGGCCTAATACATGCAAGTCGAACGGTGGTAGCAATATCATAGTGGCAAACGGGT
GCGTAACGCGTAAGGAACCTGCCCCTGATTGGGGGATAGCCCGGCGAAAGCTGGGGTAAACCCGCATG
GTTCAGTTGGATGGCATCATTTGACTGATAAATATTTATAGATCAGGGAGGGTCTTGCGTCTGATTAG
TTAGTTGGTGGGGTAACGGCTCACCAAGACGATGATCAGTAGGGGCTCTGAGAGGAGCGGCCCCCACA
TGGACACTGAGACACGGGT
>Zotu2529
GAACGCTGGCGGCGTGGATGAGGCATGCAAGTCAAGGGGGCCCGCAAGGGCAACCGGCAGACGAGGTA
GGAACACGTAGGTACGCACCCCAAAGTCGGGCACAGCTCGTCGAAAGACGGGGTAATTCCCGATGTTG
TGGAAACACCAAAGAAGCAATTCGCTTTGGGAGCGGCCTGCGGGCTATCAGCTAGTTGGTACGGTAAC
GGCGTACCAAGGCTACGACGGCTAGGGGAGGTGAGAGCCTGACCCCCACCGATGGAACTGCGACACGG
TC
>Zotu2530
GAACGCTGGCGGCGTGCTTAACACATGCAAGTCGAACGAGGTCCAGCCGAGCTTTCGGGCAGAGCGAA
GACCTAGTGGCGAACGGGTGCGTAACACGTGAGGAACCTGCCCCGGACTTGGGAATAACAGTCGGAAA
CGGCTGCTAATACCGAATATCTCCGTAGCGTCGCATGGCGCATCGAAGAAAGCTTTTGCGGTCTGGGA
GGGCCTCGCGGCCTATCAGCTAGTTGGCGAGGTAACGGCTCACCAAGGCATCGACGGGTAGCTGGTCT
GAGAGGATGATCAGCCACACTGGGACTGAGACACGGCC
>Zotu2531
GAACGCTGGCGGCATGCCTAACACATGCAAGTCGAACGAGACCTTCGGGTCTAGTGGCGCACGGGTGC
GTAACGCGTGGGAATCTGCCCTTGGGTTCGGAATAACTCAGAGAAATTTGAGCTAATACCGGATGATG
ACGTAAGTCCAAAGATTTATCGCCCAAGGATGAGCCCGCGTAAGATTAGGTAGTTGGTGGGGTAAAGG
CCTACCAAGCCGACGATCTTTAGCTGGTCTGAGAGGATGATCAGCCACACTGGGACTGAGACACGGCC
>Zotu2532
GAACGCTGGCGGCGTGCTTAACACATGCAAGTCGAACGGTAAGGCCCCTTCGGGGGTACACGAGTGGC
GAACGGGTGAGTAACACGTGGGTAATCTGCCCTGCACTTCGGGATAAGCTTGGGAAACCGGGTCTAAT
ACCGGATATGAACTCCTGCCGCATGGTGGGGGTTGGAAAGTTTTTTGGTGCAGGATGAGCCCGCGGCC
TATCAGCTTGTTGGTGGGGTAATGGCTTACCAAGGCGACGACGGGTAGCCGGCCTGAGAGGGTGATCG
GCCACACTGGGACTGAGACACGGCC
>Zotu2533
TAACGCTGGCGGCGTGCATAACACATGCAAGTCGAACGATGAAACTTTACTGACTTCTTCGGAATGAT
TTAAAGCGGATTAGTGGCGAACGGGTGAGTAACGCGTGAGTAACCTGCCTTACACAAGGGGATAGCCT
CTGGAAACGGAGAATAATACCCTATGAAACCAGACAATCGCATGATAATCTGGTCAAAATGCTTAGTG
GTGTAAGATGGACTTGCGTCTGATTAGCTAGTTGGTGAGATAAAAGCTCACCAAGGCAACGATCAGTA
GCCGGCTTGAGAGAGTGTACGGCCACATTGGGACTGAGACACGGCC
>Zotu2534
GAACGCTAGCTACAGGCTTAACACATGCAAGTCGCAGGGTAACGTGAGGGAAGCTTGCTTCCCTTGAC
GACGACTGGCGCACGGGTGAGTAACGCGTATCCAACCTTCCCATGACCACGGGATAACCCGTTGAAAG
ACGGACTAATACCGTATGACGTCGTTTGCTGACATCAAATAACGATTAAAGGTTTGGCGGTGATGGAT
GGGGATGCGTCTGATTAGCTTGTTGGCGGGGTAACGGCCCACCAAGGCGACGATCAGTAGGGGTTCTG
AGAGGAAGGTCCCCCACATTGGAACTGAGACACGGTC
>Zotu2535
GAACGCTAGCGATAGGCTTAACACATGCAAGTCGAGGGGCAGCATGGTCTTAGCTTGCTAAGACTGAT
GGCGACCGGCGCACGGGTGCGTAACGCGTATGCAACTTGCCTCACAGAGGGGGATAACCCGTCGAAAG
ACGGACTAATACCGCGTACACTTGGGATAGGGCATCCTAATCCAAGGAAAAGAAATTCGCTGTGAGAT
AGGCATGCGTCCCATTAGCTAGTTGGTGAGGTAACGGCTCACCAAGGCAACGATGGGTAGGGGAACTG
AGAGGTTGAACCCCCACACTGGTACTGAGACACGGAC
>Zotu2536
GAACGCTGACAGAATGCTTAACACATGCAAGTCGATGGGGAAGCGGCGCTTGCGCCGTGGCAACCATG
GCGGACGGGTGAGTAACGCGTAAAGAACTTGCCGGATGGACCGGGATAACAGCTGGAAACGGCTGGCA
AGACCGGATACTGTGGGCTGGCCGCATGGCTGGCCCATGAAAAGGGATGCCGTCCGAGAGCTTTGCGT
CCTATTAGCTGGTTGGTAAGGTGACGGCTTACCAAGGCGATGATAGGTAGCCGGCCTGAGAGGGTGGA
CGGCCACAAGGGGACTGAGATACGGCC
>Zotu2537
GAACGCTAGCAATATGCTTAACACATGCAAGTTGAGCGTGAAAGAAATTTCGTCGCAGCACACGGGTG

AGTAATACGTGAGAATTTGCCTTTAGGAAGAGGATAGCAAATAGAAATGTTTGATAATACTCTATAGG
CCACGAAGCTAAAAAAGTAATTTGCCTAAAGAAAAGCTCACGTCTGATTAGCTAGTTGGTGGGGTAAA
AGCCTACCAAGGCGATGATCAGTAGCTGCTTTTAGTGAAGAGACAGCCACATTGGAACTGAGACACGG
TC
>Zotu2538
GAACGCTAGCGGCAGGCTTAATACATGCAAGTCGAGGGAGATTTTAGCTTCGGCTAAATGGAACCGGC
GCACGGGTGCGTAACGCGTACACAATCTGCCTAATACTGGAGTATAGCCTCGGGAAACTGGGAGTAAT
CCTCCATAACATTATCGGGTGGCATCACTTGATAATTAAAGCTCAGGCGGTATTAGATGAGTGTGCGT
ATGATTAGCTAGTTGGTAGGGTAACGGCCTACCAAGGCGACGATCATTAGCTGGCGTGAGAGCGCGAC
CAGCCACACTGGAACTGAGACACGGTC
>Zotu2539
GAACGCTGGCGGTATGCCTTACACATGCAAGTCGTACGAGAGTTTTTAACTCTAGTGGCGGACGGGTG
AGTAACACGTAAGAATTTACCTTTAGGAGGGGGATAACAACTGGAAACGGTTGCTAATACCCCATATG
CTTTCGAGTGAAATGGGTTTCCGCCTAAAGAAAAGCTTGCGGCTGATTAGCTTGTTGGTGAGGTAATG
GCTCACCAAGGCGACGATCAGTATCTGGTTTGAGAGGATGATCAGACACACTGGAACTGAGACACGGT
C
>Zotu2540
GAACGCTGGCGGCGTGCTTAACACATGCAAGTCGAACGATGATGCCCAGCTTGCTGGGCGGATTAGTG
GCGAACGGGTGAGTAACACGTGAGTAACCTGCCCCTGACTCTGGGATAAGCGTTGGAAACGACGTCTA
ATACTGGATATGATCGCCGACCGCATGGTCTGGTGGTGGAAAGATTTTTTGGTTGGGGATGGACTCGC
GGCCTATCAGCTTGTTGGTGAGGTAATGGCTCACCAAGGCGACGACGGGTAGCCGGCCTGAGAGGGTG
ACCGGCCACACTGGGACTGAGACACGGCC
>Zotu2541
GAACGCTAGCGGTATGCTTAACACATGCAAGTCGTACGGAAAAGCAATTTTTAGTGGCGAACGGGTGA
GTAACGCGTGAGAATTTGCCTTTAGGAAGAGGATAACAGTTGGAAACAATTGCTAAAACTCTATATGC
CGTTTACGGTGAAAAAAGTAATTTGCCTAAAGAGAAGCTCGCGTCTGATTAGCTAGTTGGTGGGGTAA
AGGCCTACCAAGGCGACGATCAGTAGCTGGTCTGAGAGGACGATCAGCCACACTGGAACTGAGACACG
GTC
>Zotu2542
GAACGCTGGCGGCAGGCTTAACACATGCAAGTCGAGCGCCCCGCAAGGGGAGCGGCAGACGGGTGAGT
AACGCGTGGGAATCTACCCATCTCTACGGAATAACGCATGGAAACGTGTGCTAATACCGTATACGTCC
TTTTGGAGAAAGATTTATCGGAGATGGATGAGCCCGCGTTGGATTAGCTAGTTGGTGGGGTAAAGGCC
TACCAAGGCGACGATCCATAGCTGGTCTGAGAGGATGATCAGCCACACTGGGACTGAGACACGGCC
>Zotu2544
GAGCGCTGGCGGTATGCCTAACACATGCAAGTCGTACGCCTCACTTCGGTGAGGAGTGGCGGACGGGT
GAGTAACACGTTAGTACCTGCCCCAAAGTGGGGAATAACTGCCCGAAAGGGTAGCTAATACCCCATAG
TCTCCGGTTTATCCGGAGTAAAGATTTATCGCTTTGGGAGGGGCTTGCGTTTGATTAGCTAGTTGGTG
AGGGTAATGGCCCACCAAGGCGACGATCAATAGCTGGATCGAGAGGTTGACCAGCCACACTGGGACTG
AGATACGGCC
>Zotu2545
GAACGCTGGCGGCGTGCCTAACACATGCAAGTCGAGCGAACCCTTCGGGGTGAGCGGCGGACGGGTGA
GTAACGCGTGGGTAACCTGCCCTGTACACACGGATAACATACCGAAAGGTATGCTAATACGAGATAAT
ATGTAATGATCGCATGATCGATACATCAAAGCTCCGGCGGTACAGGATGGACCCGCGTCTGATTAGCT
AGTTGGTGAGGTAATGGCTCACCAAGGCGACGATCAGTAGCCGACCTGAGAGGGTGATCGGCCACATT
GGAACTGAGACACGGTC
>Zotu2546
GAACGCTGGCGGCGTGCTTAACACATGCAAGTCGGGGGGCTCAGCAATGGGTCACCGGCGAACGGGTG
CGTAACACGTGAGCGACCTGCCTTCGAGAGGGGGATAGCCGGCCCAACGGCCGGGTAATACCGCATAC
GACCGCCGCGGGGCATCCCGGGGCGGTGAAACCTTTCGGGCTCGGAGAGGGGCTCGCGGCCTATCAGC
TAGTTGGCGAGGTAACGGCTCACCAAGGCGATGACGGGTAGCTGGTCTGAGAGGATGGCCAGCCACAT
TGGGACTGAGAGACGGCC
>Zotu2547
GAACGCTAGCGGCGTGCTTAACACATGCAAGTCGAACGCTGAAGCCCGCTTCGGTGGGTGGATGAGTG
GCGAACGGGTGAGTAACACGTGGGCAATCTACCCCGCACTCGGGGATAAGCCTTGGAAACGAGGTCTA

ATACCCGATAGGACATGCCATCGCATGGTGGTGTGTGGAAAGTGTGTAGCGGTGTGGGATGAGCCCGC
GGCCTATCAGCTTGTTGGTGGCGGTGATGGCGCACCAAGGCGACGACGGGTAGCCGGCCTGAGAGGGT
GACCGGCCACACTGGGACTGAGACACGGCC
>Zotu2549
GAACGCTGGCGGCGTGCTTAACACATGCAAGTCGAGCGAAGCACTTATCATTGACTCTTCGGAAGATT
TGATATTTGACTGAGCGGCGGACGGGTGAGTAACGCGTGGGTAACCTGCCTCATACAGGGGAATAACA
GTTAGAAATGGCTGCTAATGCCGCATAAGCGCACAGGACCGCATGGTCTGGTGTGAAAAACTGAGGTG
GTATGAGATGGACCCGCGTCTGATTAGGTAGTTGGTGGGGTAACGGCCTACCAAGCCGACGATCAGTA
GCCGGCCTGAGAGGGTGAACGGCCACATTGGGACTGAGACACGGCC
>Zotu2550
GAACGCTGGCGGCGTGCCTAATACATGCAAGTCGAGCGCCTTCACTGTGTGCTTCTCGGTAACGAGGA
ACATATTGTGAAGGAGCGGCGGACGGCTGAGTAACGCGTGGGAACATACCCCAAAGTGAGGGATAAGC
ACCGGAAACGGTGTCTAATACCGCATATGATCTTCGGATTAAAGCCTTCGGGCGCTTTGGGAGTGGCC
TGCGTAAGATTAGCTAGTTGGTGAGGTAAAGGCTCACCAAGGCGACGATCTTTAGTTGGTCTGAGAGG
ATGACCAGCCAGACTGGAACTGAGACACGGTC
>Zotu2551
GAACGCTGGCGGCATGCCTAATACATGCAAGTCGAGCGAACAGGCGAGGTGCTTGCACCTCTGACGTT
AGCGGCGGATGGGTGAGTAACACGTGGGCAACCTGCCCTGCAGATGGGGATAACTCCGGGAAACCGGG
GCTAATACCGAATAAACCATTGAACCTCCTGGTTCGATGTTGAAAGACGGTTTCGGCTGTCACTGCAG
GATGGGCCCGCGGCGCATTAGCTAGTTGGTGGGATAACGGCCTACCAAGGCAACGATGCGTAGCCGAC
CTGAGAGGGTGATCGGCCACACTGGGACTGAGACACGGCC
>Zotu2552
GAACGCTGGCGGCGTGCTTAACACATGCAAGTCGAGCGGAAAGGCCCCTTTGGGGGTACTCGAGCGGC
GAACGGGTGAGTATCACGTGAGTAATCTGCCCTTCACTTTTGGATAACCCTCGGAAACGGGGGCTAAT
ACGGAATATTCAGTGCTGCCTGCATGGGTGGTGTTGGAAAGATTTTTTGGTGGGGGATGAGCTCGCGG
CCTATCAGCTTGATGGTGGGGTAATGGCCTACCATGGCTTTGACGGGTAGCCGGCCTGAGAGGGTGTC
CGGCCACACTGGGACTGAGACACGGCC
>Zotu2553
GAACGCTGGCGGCGTGCCTAATACATGCAAGTCGAGCGAATCAAAGGGAGCTTGCTCCCTTTTGATTA
GCGGCGGACGGGTGAGTAACACGTGGGCAACCTGCCTATAAGACTGGGATAACTTCGGGAAACCGGAG
CTAATACCGGATAATTCATATCTACTCATGTAGATATGCTGAAAGACGGTTTACGCTGTCACTTATAG
ATGGGCCCGCGGCGCATTAGCTAGTTGGTGAGGTAACGGCTCACCAAGGCGACGATGCGTAGCCGACC
TGAGAGGGTGATCGGCCACACTGGGACTGAGACACGGCC
>Zotu2554
GAACGCTGGCGGCGTGCTTAACACATGCAAGTCGAACGGAAAGGCCCTTCGGGGTACTCGAGTGGCGA
ACGGGTGAGTAACACGTGGGTGATCTGCCTCGTACTTTGGGATAAGCCTGGGAAACTGGGTCTAATAC
CGAATATGACCTTGAGATGCATGTCTCTTGGTGGAAACTTTTTGGGTACGAGATGGGCCCGCGGCCTA
TCAGCTTGTTGGTGGGGTAATGGCCTACCAAGGCGACGACGGGTAGCCGGCCTGAGAGGGCGACCGGC
CACACTGGGACTGAGACACGGCC
>Zotu2556
GAACGCTGGCGGCGTGCTTAACACATGCAAGTCGAGCGGTAAGGCTCCTTCGGGAGTACACGAGCGGC
GAACGGGTGAGTAACACGTGAGCAATCTGCCCTTCTCATCGGAATAACCATTGGAAACGATGGCTAAT
GCCGAATATGACCTCGGATCACATGTTCTGAGGTGGAAAGCTCCGGCGGAGAAGGATGAGCTCGCGGC
CTATCAGCTTGTTGGTGAGGTAATGGCTCACCAAGGCATTGACGGGTAGCCGGCCTGAGAGGGTGACC
GGCCACACTGGGACTGAGACACGGCC
>Zotu2557
GAACGCTGGCGGCGTGCCTAATACATGCAAGTCGAGCGAACGGATAGGAGCTTGCTCCTTGAAGTTAG
CGGCGGATGGGTGAGTAACACGTGGGCAACCTGCCTGTAAGACTGGGATAACTTCGGGAAACCGGAGC
TAATACCGGATAATTTCTTTTTCCTCCTGGGGGAAGGTTGAAAGACGGCTTCGGCTGTCACTTACAGA
TGGGCCCGCGGCGCATTAGCTAGTTGGTGGGATAACGGCCTACCAAGGCGACGATGCGTAGCCGACCT
GAGAGGGTGATCGGCCACACTGGGACTGAGACACGGCC
>Zotu2558
GAACGCTGGCGGCGTGCTTAACACATGCAAGTCGAACGAAGAGCGATGGAAGCTTGCTTCTATCAATC
TTAGTGGCGAACGGGTGAGTAACGCGTAATCAACCTGCCCTTCAGAGGGGGACAACAGTTGGAAACGA

CTGCTAATACCGCATACGATCTAATCTCGGCATCGAGGATAGATGAAAGGTGGCCTCTATGTATAAGC
TATCACTGAAGGAGGGGATTGCGTCTGATTAGCTAGTTGGAGGGGTAACGGCCCACCAAGGCGATGAT
CAGTAGCCGGTCTGAGAGGATGAACGGCCACATTGGGACTGAGACACGGCC
>Zotu2559
GAACGCTAGCGGCAGGCCTAATACATGCAAGTCGAGGGGCAGCACGGGTAGCAATATCTGGTGGCGAC
CGGCGCACGGGTGCGTAACGCGTATGCAATCTACCTATTACTGGAGAATAGCCTTCCGAAAGGGAGAT
TAATGCTCCATAACATTATGATGTGGCATCACATTATAATTAAAGTTCCGGCGGTAATAGATGAGCAT
GCGTGACATTAGCTAGTTGGTGAGGTAATGGCTCACCAAGGCTACGATGTCTAGGGGCTCTGAGAGGA
TTGTCCCCCACACTGGTACTGAGACACGGAC
>Zotu2560
GAATGCTGGCGGTGTGCTTCACACATGCAAGTCGAACGGGGTAGCAATACCCAGTGGCGGACGGGTGA
GTAACGCGTGGGAATCTGCCTTAAGATGGGGGATAGCTAGCCGAAAGGCTAATTAATACCGCATATGT
ATATATCGCGACAGTTTTATATATAAAAGTAGTAATACGTCTTAAGATGAGCCCGCGTACGATTAGCT
AGTTGGTAGGGTAAAGGCCTACCAAGGCAACGATCGTTAGGTGGTCCGAGAGGATGATCACCCACAAT
GGAACTGAGACACGGTC
>Zotu2561
GAACGCTGGCGGCGTGCCTAATACATGCAAGTCGAGCGAACAGATAAGGAGCTTGCTCCTTTGACGTT
AGCGGCGGACGGGTGAGTAACACGTGGGTAACCTACCTATAAGACTGGGATAACTTCGGGAAACCGGA
GCTAATACCGGATAATATTTCGAACCGCATGGTTCGATAGTGAAAGATGGCCCTGCTATCACTTATAG
ATGGACCTGCGCCGTATTAGCTAGTTGGTAAGGTAACGGCTTACCAAGGCAACGATACGTAGCCGACC
TGAGAGGGTGATCGGCCACACTGGAACTGAGACACGGTC
>Zotu2562
GAACGCTGGCGGCGTGCCTAATACATGCAAGTAGAACGCTGAAGGAGGAGCTTGCTCTTCTGGATGAG
TTGCGAACGGGTGAGTAACGCGTAGGTAACCTGCCTGGTAGCGGGGGATAACTATTGGAAACGATAGC
TAATACCGCATAAGAGTAGATGTTGCATGACATTTACTTAAAAGGTGCAATTGCATCACTACCAGATG
GACCTGCGTTGTATTAGCTAGTTGGTGGGGTAACGGCTCACCAAGGCGACGATACATAGCCGACCTGA
GAGGGTGATCGGCCACACTGGGACTGAGACACGGCC
>Zotu2563
GAACGCTAGCGGGAGGCTTAACACATGCAAGTCGAGGGGCAGCACGAGGTAGCAATACTTTGGTGGCG
ACCGGCGCACGGGTGAGTAACGCGTATGCAACCTACCCTGTACAGGGGGATAGCCCGGAGAAATTCGG
ATTAATACCCCATAAAGATATCTGGAGGCATCTTTGGATATTTAAAGATTTATCGGTACGGGATGGGC
ATGCGTAACATTAGCTAGTTGGTAGGGTAACGGCCTACCAAGGCAACGATGTTTAGGGGTCCTGAGAG
GGTGATCCCCCACACTGGTACTGAGACACGGAC
>Zotu2564
GAACGCTGGCGGTGTGCCTTAAGCATGCAAGTTGAGCGGTCCCCGCAAGGGGACAGCAGCAGACGGGT
GAGTAACGCGTAGGAATCTACCTCGAAGTGGGGAATAGCCTTCCGAAAGGGAGGGTAATACCGCATAA
TCCCGCAAGGGCAAAGCAGCAATGCGCTTTGAGAGGAGCCTGCGTCCTATCAGTTAGTTGGTGGGGTA
ATAGCCTACCAAGACGATGACGGGTAGCCGATGTGAGAGCATGATCGGCCACAAGGTTACTGAGACAC
GGAA
>Zotu2566
GAACGCTGGCGGCATGCCTAACACATGCAAGTCGAACGATGCCTTCGGGCATAGTGGCGCACGGGTGC
GTAACGCGTGGGAATCTGCCCTTGGCTTCGGAATAACAGTTAGAAATGACTGCTAATACCGGATGATG
TCCCCCTGAGAGATCTTGGGGACCAAAGATTTATCGGCCAGGGATGAGCCCGCGTAGGATTAGGTAGT
TGGTGGGGTAAAGGCCTACCAAGCCGACGATCCTTAGCTGGTCTGAGAGGATGATCAGCCACACTGGG
ACTGAGACACGGCC
>Zotu2567
GAACGCTAGCGATAGGCTTAACACATGCAAGTCGAGGGGCAGCATGGTCTTAGCTTGCTAAGACTGAT
GGCGACCGGCGCACGGGTGCGTAACGCGTATGCAACTTGCCTCACAGAGGGGGATAACCCGTCGAAAG
ACGGACTAATACCGCGTACACTTACAGGAGGGCATCCTCCAGTAAGGAAATGAAATTCGCTGTGAGAT
AGGCATGCGTCCCATTAGCTAGTTGGTGAGGTAACGGCTCACCAAGGCGACGATGGGTAGGGGAACTG
AGAGGTTGAACCCCCACACTGGTACTGAGACACGGAC
>Zotu2569
GAACGCTGGCGGCGTGCCTAATACATGCAAGTCGAACGCACAGCGAAAGGTGCTTGCACCTTTCAAGT
GAGTGGCGAACGGGTGAGTAACACGTGGACAACCTGCCTCAAGGCTGGGGATAACATTTGGAAACAGA

TGCTAATACCGAATAAAACTTAGTATCGCATGATACCAGGTTAAAAGGCGCTACGGCGTCACCTAGAG
ATGGATCCGCGGTGCATTAGTTAGTTGGTGGGGTAAAGGCCTACCAAGACAATGATGCATAGCCGAGT
TGAGAGACTGATCGGCCACATTGGGACTGAGACACGGCC
>Zotu2570
GAACGCTGGCGGCAGGCCTAACACATGCAAGTCGAGCGAGACCTTCGGGTCTAGCGGCGGACGGGTGA
GTAACGCGTGGGAACGTGCCCTTTTCTACGGAATAGCCTCGGGAAACTGAGCGTAATACCGTATACGC
CCTTTGGGGGAAAGATTTATCGGAAAAGGATCGGCCCGCGTTGGATTAGGTAGTTGGTGGGGTAATGG
CCTACCAAGCCTACGATCCATAGCTGGTTTGAGAGGATGATCAGCCACACTGGGACTGAGACACGGCC
>Zotu2571
GAACGCTGGCGGCGTGCCTAATACATGCAAGTAGAACGCTGAAGGGGGAGCTTGCTTCTCCGGATGAG
TTGCGAACGGGTGAGTAACGCGTAGGTAACCTGCCTGGTAGCGGGGGATAACTATTGGAAACGGTAGC
TAATACCGCATAAGAGTAGATGTTGCATGACATTTACTTAAAAGGTGCAATTGCATCACTACCAGATG
GACCTGCGTTGTATTAGCTAGTTGGTGGGGTAACGGCTCACCAAGGCGACGATACATAGCCGACCTGA
GAGGGTGATCGGCCACACTGGGACTGAGACACGGCC
>Zotu2572
GAACGCTAGCGGCAGGCTTAATACATGCAAGTCGAGGGGCAGCACAGGTAGCAATACTGGGTGGCGAC
CGGCAAACGGGTGCGGAACACGTACGCAACCTTCCTTTAAGTGGGGGATAGCCCAGGGAAACTTGGAT
TAATACCCCTTAATATTATGAGATGGCATCATTTTATAATTAAAGCTCCGGCGCTTAAAGATGGGCGT
GCGCCTGATTAGGTAGTTGGCGGGGTAACAGCCCACCAAGCCTACGATCAGTAACTGGTGTGAGAGCA
CGACCAGTCACACGGGCACTGAGACACGGGC
>Zotu2573
GAACGCTGGCGGCGTGCTTAACACATGCAAGTCGAGCGGTAAGGCCCTTTCGGGGGTACACGAGCGGC
GAACGGGCGAGTAACACGTGAGTAATCTGCCCTTCACTTGGGGATAAGCCTCGGAAACGGGGTCTAAT
ACCTGATATGACGGCCGTCTGCATGGGCTGGTCGTGGAAAGTTTTTTCGGTGGGGGATGTGCTCGCGG
CCTATCAGCTTGTTGGTGGGGTAATGGCCTACCAAGGCTTCGGCGGGTAGCCGGCCTGAGAGGGTGAC
CGGCCACACTGGGACTGAGACACGGCC
>Zotu2574
GAACGCTGGCGGCATGCTTAACACATGCAAGTCGAACGGTTTATGAGTGCTTGCATTCATAGATAGTG
GCAGACGGGTGAGTAACGCGTGGGAACGTACCCCTTGGTTCAGAATAACTACTGGAAACGGTAGCTAA
TACTGGATACGTCCGAAAGGAGAAAGATTTATTGCCGAGGGAGCGGCCCGCGTTGGATTAGGTAGTTG
GTTAGGTAAAGGCTGACCAAGCCGATGATCCATAGCTGGTTTGAGAGGATGATCAGCCACACTGGGAC
TGAGACACGGCC
>Zotu2577
GAACGCTAGCGGCAGGCCTAATACATGCAAGTCGAGGGGTAGAGAGAGCTTGCTTTCTTGAGACCGGC
GCACGGGTGCGTAACGCGTATGCAACCTACCTTTATCTGGGGGATAGCCCGAAGAAATTCGGATTAAC
ACCGCATAAAATCACAGTATCGCATGGTACAATGATCAAATATTTATAGGATAAAGATGGGCATGCGT
GTCATTAGTTAGTTGGCGGGGTAACGGCCCACCAAGACCACGATGACTAGGGGATCTGAGAGGATGAC
CCCCCACACTGGTACTGAGACACGGAC
>Zotu2578
GAACGCTGGCGGCATGCCTAACACATGCAAGTCGAACGAGGGCCCCGCTTCGGTGGGACCCTAGTGGC
GCACGGGTGAGTAACGCGTGGGAACCTGCCCTGTGGTACGGAATAACTCCGGGAAACTGGAGCTAATA
CCGTATGTGTCTTGAGGGACAAAGATTTATCGCCATGGGATGGGCCCGCGTAGGATTAGCTTGTTGGT
GGGGTAACGGCCTACCAAGGCTACGATCCTTAGCTGGTCTGAGAGGATGATCAGCCACACTGGGACTG
AGACACGGCC
>Zotu2579
GAACGCTAGCTACAGGCTTAACACATGCAAGTCGAGGGGCATCAGTTTGGTTTGCTTGCAAACCAAAG
CTGGCGACCGGCGCACGGGTGAGTAACACGTATCCAACCTGCCTCATACTCGGGGATAGCCTTTCGAA
AGAAAGATTAATATCCGATAGCATATATTTCCCGCATGGGTTTTATATTAAAGAAATTCGGTATGAGA
TGGGGATGCGTTCCATTAGTTTGTTGGGGGGGTAACGGCCCACCAAGACTACGATGGATAGGGGTTCT
GAGAGGAAGGTCCCCCACATTGGAACTGAGACACGGTC
>Zotu2580
GAACGCTGGCGGCGTGCCTAACACATGCAAGTCGAGCGATGAAACTTCCTTCGGGGAGTGGATTAGCG
GCGGACGGGTGAGTAACACGTGGGTAACCTGCCTCAAAGAGGGGGATAGCCTCCCGAAAGGGAGATTA
ATACCGCATAACATTATTTTATGGCATCATAGAATAATCAAAGGAGCAATCCGCTTTGAGATGGACCC

GCGGCGCATTAGCTAGTTGGTGAGGTAAGGGCTCACCAAGGCGACGATGCGTAGCCGACCTGAGAGGG
TGATCGGCCACATTGGAACTGAGACACGGTC
>Zotu2581
GAACGCTAGCGGCAGGCCTAACACATGCAAGTCGAGGGGTAGATATCTTCGGGTATTGAGACCGGCGC
ACGGGTGCGTAACGCGTATGCAATCTACCTTTTACTAAGGGATAGCCCAGAGAAATTTGGATTAATAC
CTTATAGTATGTTTGAATGGCATCGTTTGAACATTAAAGATTTATTGGTAAAAGATGAGCATGCGTCC
CATTAGTTAGTTGGTAAGGTAACGGCTTACCAAGACGATGATGGGTAGGGGTCCTGAGAGGGAGATCC
CCCACACTGGTACTGAGACACGGAC
>Zotu2582
GAACGCTGGCGGCGTGCTTAACACATGCAAGTCGAACGGTAAGGCCCTTTCGGGGGTACACGAGTGGC
GAACGGGTGAGTAACACGTGGGTGATCTGCCCTGCACTTTGGGATAAGCCTGGGAAACTGGGTCTAAT
ACCGAATACTCCTTGCTGGTCGCATGGCCTGGTGGGGGAAAGCTTTTGCGGTGTGGGATGGGCCCGCG
GCCTATCAGCTTGTTGGTGGGGTGATGGCCTACCAAGGCGACGACGGGTAGCCGGCCTGAGAGGGTGT
CCGGCCACACTGGGACTGAGATACGGCC
>Zotu2585
GAACGCTGGCGGCAGGCTTAACACATGCAAGTCGAGCGCCCCGCAAGGGGAGCGGCAGACGGGTGAGT
AACACGTGGGAATCTACCCATCCCTACGGAACAACTCCGGGAAACTGGAGCTAATACCGTATACGCCC
TTCGGGGGAAAGATTTATCGGGGATGGATGAGCCCGCGTTGGATTAGCTAGTTGATGGGGTAAAGGCC
TACCAAGGCGACGATCCATAGCTGGTCTGAGAGGATGATCAGCCACATTGGGACTGAGACACGGCC
>Zotu2586
GAACGCTGGCGGCAGGCTTAACACATGCAAGTCGAGCGCCCCGCAAGGGGAGCGGCAGACGGGTGAGT
AACGCGTGGGAATCTACCCATCTCTACGGAATAACGCATGGAAACGTGTGCTAATACCGTATACGTCC
TTCGGGAGAAAGATTTATCGGAGATGGATGAGCCCGCGTTGGATTAGCTAGTTGGTGGGGTAAAGGCC
TACCAAGGCGACGATCCATAGCTGGTCTGAGAGGATGATCAGCCACACTGGGACTGAGACACGGCC
>Zotu2587
AAACGCTGGCGGCGTGCTTAACACATGCAAGTCGAACGGACTGATTTTGGTGCTTGCACCGAAAGAAA
GTTAGTGGCGGACGGGTGAGTAACACGTGAGTAACCTGCCTTTCAGAGGGGAACAACAGTTGGAAACG
ACTGCTAATACCGCATAACGTACAGGTGTCGCATGGCACATGTACCAAAGATTTATCGCTGAGGGATG
GGCTCGCGTCCGATTAGGCAGTTGGCGGGGTAAAGGCCCACCAAACCGACGATCGGTAGCCGGACTGA
GAGGTTGAACGGCCACATTGGGACTGAGACACGGCC
>Zotu2588
CAACGCTGGCGGCGTGCCTAACACATGCAAGTCGAACGAGAAAGGGACTTCGGTCCCGAGTAAAGTGG
CGCACGGGTGAGTAACACGTGACTAACCTACCCTGGAGTGGGGAATAACTTCGAGAAATCGAGGCTAA
TACCGCATAACACCTACGGGTCAAAGGAGCAATTCGCTTTGGGAGGGGGTCGCGGCAGATTAGCTAGT
TGGCGGGGTAATGGCCCACCAAGGCAGTGATCTGTATCCGGCCTGAGAGGGCGCACGGACACACTGGA
ACTGAAACACGGTC
>Zotu2589
CAACGCTGGCGGCGTGCCTCAGACATGCAAGTCGAACGATTAAACTTCCCTTCGGGGAAGATATAAAG
TGGCGCACGGGTGAGTAACACGTAGGTAATCTACCTTTGAGTGGGGAATAACAATCGGAAACGATTGC
TAATACCGCATAATGCAGCGGCATCTTATGATGACAGTTGTTAAAGGAGCAATCCGCTTAAAGAGGAG
CCTGCGGCAGATTAGCTTGTTGGTAAGGTAATGGCTTACCAAGGCTACGATCTGTAACCGACCTGAGA
GGGTGGTCGGTCACACTGACACTGAATAACGGGT
>Zotu2590
GAACGCTGGCGGCGTGCTTAACACATGCAAGTCGAACGGAAAGGCCCTGCTTGCAGGGTACTCGAGTG
GCGAACGGGTGAGTAACACGTGGGTGATCTGCCCTGTACTTTGGGATAAGCCTGGGAAACTGGGTCTA
ATACCGGATATGACAATTGTTTGGATGCGATTGTGGAAAGCTTTTGCGGTATGGGATGAGCTCGCGGC
CTATCAGCTTGTTGGTGGGGTAATGGCCTACCAAGGCGTCGACGGGTAGCCGGCCTGAGAGGGTGTAC
GGCCACATTGGGACTGAGATACGGCC
>Zotu2593
GAACGCTGGCGGCATGCCTAATACATGCAAGTCGAGCGAATGATGAAGAAGCTTGCTTCTTCTGATTT
AGCGGCGGACGGGTGAGTAACACGTGGGCAACCTACCCTATAGATTGGGATAACACAGGGAAACCTGT
GCTAATACCGAATAATACATTTTCTTTCATGAGGAGATGTTGAAAGATGGTTTCGGCTATCACTATAG
GATGGGCCCGCGGCGCATTAGCTAGTTGGTGAGGTAATGGCTCACCAAGGCAACGATGCGTAGCCGAC
CTGAGAGGGTGATCGGCCACACTGGGACTGAGACACGGCC

>Zotu2594
GAACGCTGGCGGCAGGCTTAACACATGCAAGTCGAACGCATCCTTCGGGGTGAGTGGCAGACGGGTGA
GTAACGCGTGGGAATCTACCTCTTGCTACGGGATAGCTCCGGGAAACTGGAATTAATACCGTATGTGC
CCTTCGGGGGAAAGATTTATCGGCAAGGGATGAGCCCGCGTTGGATTAGCTAGTTGGTGGGGTAAAGG
CCTACCAAGGCGACGATCCATAGCTGGTCTGAGAGGATGATCAGCCACATTGGGACTGAGACACGGCC
>Zotu2595
GAACGCTAGCGGCAGGCCTAATACATGCAAGTCGAACGGACCGCAAGGTTAATGGCGAACGGGTGCGT
AACGCGTAAACAACCTGCCCCTGTTTGGGGGATAGCCCGGCGAAAGCTGGGGTAAACCCGCGTAGTTT
CTAGTTATCGCATGGTGATTAGAATAGACATTTATGGAACAGGGAGGGGTTTGCGTCTGATTAGTTAG
TTGGCAGGGTAACGGCCTACCAAGGCGATGATCAGTAGGGGCTCTGAGAGGAGGGTCCCCCACATGGG
CACTGAGACACGGGC
>Zotu2596
GAACGCTAGCGGCAGGCTTAATACATGCAAGTCGAGGGGGCAGCAATGTCACCGGCGAACGGGTGCGT
AACGCGTATGCAACCTACCTACATCAGGGGGATAGCCCTAGGAAACTGGGATTAACACCGCATAACAA
TAGAGTTGGCATCAACTTTATTTGAAATATTTATAGGATGTAGATGGGCATGCGTAGGATTAGCTAGT
TGGTAAGGTAACGGCTTACCAAGGCTACGATCCTTAGGGGCTCTGAGAGGAGTATCCCCCACACTGGC
ACTGAGATACGGGC
>Zotu2597
GAACGCTGGCGGCATGCTTAACACATGCAAGTCGAACGGCAGCACGGCCTAGCTTGCTAGGTGGGTGG
CGAGTGGCGCACGGGTGAGTAACGCGTAGGAATATGCCTTAAAGAGGGGGATAACTTGGGGAAACTCA
AGCTAATACCGCATAAACTCCGAGGAGCGAAGCTGGGGACCTTAGGGCCTGGCGCTTTAAGAATAGCC
TGCGTCCGATTAGCTAGTTGGTGGGGTAAAGGCCTACCAAGGCGACGATCGGTAGCTGGTCTGAGAGG
ATGATCAGCCACACTGGGACTGAGACACGGCC
>Zotu2598
GAACGCTAGCGGCAGGCTTAACACATGCAAGTCGAGGGGTATAAGGAGCTTGCTCCTTAGAGACCGGC
GCACGGGTGCGTAACGCGTATGCAACCTACCTTGAACAGGGGGATAGCCCAGAGAAATTTGGATTAAT
ACCCCATGGTATATTTGAGTGGCATCACTTGATTATTAAAGTTCCAACGGTTCAAGATGGGCATGCGT
CCCATTAGCTAGATGGTAAGGTAACGGCTTACCATGGCGACGATGGGTAAGGGTCCTGAGAGGGAGAT
CCCCCACACTGGTACTGAGACACGGAC
>Zotu2600
GAACGCTGGCGGCGCGCCTAACACATGCAAGTCGAACGGTTAAAGCATCTTCGGATGTGTATAAAGTG
GCGAACGGCTGAGTAACACGTGGGCAACCTGCCCTTTGCACTGGGATAGCCTCGGGAAACCGAGGTTA
ATACCGGATACTCCATATTTGTCGCATGGCGAATATGGGAAAGCTCCGGCGGCAAAGGATGGGCCCGC
GGCCTGTTAGCTAGTTGGTGGGGTAGTGGCCTACCAAGGCAATGATGGGTAGCCGGGTTGAGAGACCG
ACCGGCCAGATTGGGACTGAGACACGGCC
>Zotu2601
GAACGCTGGCGGCATGCCTTACACATGCAAGTCGAACGGCAGCGCGGGCTTCGGCCTGGCGGCGAGTG
GCGAACGGGTGAGTAATACATCGGAACGTGCCCTGTTGTGGGGGATAACTAGTCGAAAGATTAGCTAA
TACCGCATACGACCTGAGGGTGAAAGCGGGGGACCGTAAGGCCTCGCGCAATAGGAGCGGCCGATGTC
TGATTAGCTAGTTGGTGGGGTAAAGGCCTACCAAGGCGACGATCAGTAGCTGGTCTGAGAGGACGATC
AGCCACACTGGGACTGAGACACGGCC
>Zotu2602
AAACGCTGGCGGCGTGCCTAAAACATGCAAGTCGAACGATCACTTCGGTGATAGTGGCGGACGGCTGA
GTAACGCGTAGGTAACCTGCCCCGGAGTGGGGAATAACTCCGCGAAAGCGGAGCTAATACCGCATACG
ATCACTGGTCTACGGGTTGGTGAGGAAAGTCGTAAGGCGCTCTGGGATGGGCCTGCGTCCGATTAGCT
TGTTGGTGGGGTAATGGCCTACCAAGGCTTCGATCGGTAGCTGGTCTGAGAGGATGATCAGCCAGACT
GGGACTGCGACACGGCC
>Zotu2603
GAACGCTAGCGGAACGCCTAACACATGCAAGTCGAACGGGTTTTATTTTGTAGCAATACATTATAAAT
CTAGTGGCAAACGAGCGAGTAACGCGTACTTAACCTGCCCTTGAGTTGAGAACAACTACGGAGACGTA
GCTAATTCTCAATATGCTCACGATGGTGAAATACCAATGTGAGGAAAGATTTATCGCTCAAGGAGGGG
GGTGCGTCCTATCAGGTTGTTGGTGGGGTAATGGCCTACCAAGCCTATGACGGGTAGCCGGTCTGAGA
GGATGTCCGGCCGCGATGGGACTGAGATACGGCC
>Zotu2604

GAACGCTAGCGGCAGGCCTAATACATGCAAGTCGAGGGGTAACAGGGTAGCAATACCGCTGACGACCG
GCGCACGGGTGCGTAACACGTATGCAATCTACCTCTAACAGAGGGATAGCCCGAAGAAATTCGGATTA
ATACCTCATAACATCACGGGAAGGCATCTTCCTGTGCTTAAAGATTTATCGGTTAGAGATGAGCATGC
GTCCGATTAGCTAGTTGGTGAGGTAATGGCTCACCAAGGCTACGATCGGTAGGGGGTCTGAGAGGATG
ATCCCCCACACTGGTACTGAGATACGGAC
>Zotu2605
GAATGCTGGCGGCGTGCCTAATACATGCAAGTCGTGCGGAAACACTTCGGGTGTCGAGCGGCGGACGG
CTGAGTAACGCATAGGAATGTACCCCAAAGTGAGGGATAACTCCGAGAAATCGGGGCTAATACCGCAT
ATGCTCTAAGGAGTAAAGCTTCGGCGCTTTGGGAACAGCCTATGTCCGATTAGCTAGTTGGTGAGGTA
AAGGCTTACCAAGGCGACGATCGGTAGCTGGTCTGAGAGGATGATCAGCCAGACTGGGACTGAGACAC
GGCC
>Zotu2606
GAACGCTGGCGGCGTGCTTAACACATGCAAGTCGAACGGAAAGGCCCTGCTTGCAGGGTACTCGAGTG
GCGAACGGGTGAGTAACACGTGGGTGATCTGCCCTGCACTTCGGGATAAGCTTGGGAAACTGGGTCTA
ATACCGGATAGGACCGCACTTTAGTGTGTGTGGTGGAAAGTTTTTTCGGTGTAGGATGAGCTCGCGGC
CTATCAGCTTGTTGGTGGGGTAATGGCCTACCAAGGCGGCGACGGGTAGCCGGCCTGAGAGGGTGTAC
GGCCACATTGGGACTGAGATACGGCC
>Zotu2608
GAACGCTGGCGGCATGCCTTACACATGCAAGTCGAACGGTAACAGGTCTTCGGATGCTGACGAGTGGC
GAACGGGTGAGTAATACATCGGAACGTGCCCGATCGTGGGGGATAACGGAGCGAAAGCTTTGCTAATA
CCGCATACGATCTACGGATGAAAGCAGGGGACCGCAAGGCCTTGCGCGGACGGAGCGGCCGATGGCAG
ATTAGGTAGTTGGTGGGATAAAAGCTTACCAAGCCGACGATCTGTAGCTGGTCTGAGAGGACGACCAG
CCACACTGGGACTGAGACACGGCC
>Zotu2609
GAACGCTGGCTGCGTGCTTAACACATGCAAGTCGAACGCTGAAGCCTGGTGCTTGCACCGGGTGGATG
AGTGGCGAACGGGTGAGTAACACGTGAGTAACCTGCCCCTGACTTCGGGATAAGCCCGGGAAACTGGG
TCTAATACCGGATACAACTGCCGGACGCATGTCTGGTGGTGGAAAGTTTTTTCGGTTGGGGATGGGCT
CGCGGCCTATCAGTTTGTTGGTGAGGTAATGGCTCACCAAGACGACGACGGGTAGCCGGCCTGAGAGG
GCGACCGGCCACACTGGGACTGAGACACGGCC
>Zotu2610
GAACGCTTGCGGCGCGCCTAATACATGCAAGTCGAACGAACCAGCAATGGTGAGTGGCGCACGGGTGA
GTAACGCGTGGATAATCTGCCCTCTACTGGGGAATAACTAACCGAAAGGTTAGCTAATACCGCATGAG
ACCACGATCCCTGAGGGGAACGAGGTTAAAGATTTATTGGTAGAGGATGAGTCTGCGTGGGATTAGCT
AGTTGGTGGGGTAATGGCCTACCAAGGCAACGATCTCTAACAGGTCTGAGAGGATGACCTGTCACACT
GGAACTGAGACACGGTC
>Zotu2611
GAACGCTTGCGGCGTACCTAAGAAATGCAAGTCGAGCGGGGGAGCAATCCCTAGCGGCGAACGGTCGC
GTAACACGTAGGCAACCTGCCCGAGAGACCGGGACAACACTTCGAAAGAGGTGCTAATACCGGATGTG
GCCGGCGGGAGACATCTCCTGCCGATTAAACCCCTTCGGGGGCTCTTGGAGGGGCCTGCGGCCTATCA
GCTAGATGGTGGGGTAACGGCCTACCATGGCGACGACGGGTAGCTGGTCTGAGAGGATGGTCAGCCGG
ATTGGGACTGAGATACGGCC
>Zotu2612
GAACGTTGGCGGCGCGTCTAATACATGCAAGTCGAGTGGGTTTAGGCCCACGGCGGACGGCTGAGTAA
CACGTTGGTAGCTGCCCCCCATTCAGGGATAACCTTTCGAAAGAAAGATTAATACCGGATAGTCCCGC
AAGGGTAAAGATTTATCGATGGGGGAGGTGCCTGCGTCCTATCAGCTAGTTGGTGAGGTAATGGCTCA
CCAAGGCTATGACGGGTAGGTGGTCTGAGAGGATGACCACCCAGAGGGAAACTGAGACACGGTT
>Zotu2613
GAACGCTAGCGGCAGGCCTAACACATGCAAGTCGAGGGGTATAGTAGCAATACTAGAGACCGGCGCAC
GGGTGCGTAACGCGTATGCAATCTACCTTTTACAGGGGAATAGCCCAGAGAAATTTGGATTAATGCCC
CATGGTATTATCGGATGGCATCATTTGATAATTAAAGTTCCAACGGTAAAAGATGAGCATGCGTCCTA
TTAGCTAGTTGGTAAGGTAACGGCTTACCAAGGCTACGATAGGTAGGGGTCCTGAGAGGGAGATCCCC
CACACTGGTACTGAGACACGGAC
>Zotu2616
GAACGCTGGCGGCGTGCTTAACACATGCAAGTCGAGCGGTAAGGCTCCTTCGGGAGTACACGAGCGGC

GAACGGGTGAGTAACACGTGAGCAATCTGCCCTTCTCATCGGAATAACCATTGGAAACGATGGCTAAT
GCCGAATATGACCTCGGATCACATGTTCTGAGGTGGAAAGCTCCGGCGGAGAAGGATGAGCTCGCGGC
CTATCAGCTTGTTGGTGAGGTAATGGCTCACCAAGGCGATGACGGGTAGCCGGCCTGAGAGGGTGACC
GGCCACACTGGGACTGAGACACGGCC
>Zotu2617
ATGATCCCAGAGTCCTGGGATTGAGTGCCACATTGGGATCCCTGCTCAGCAGGGAGTCTGCTTCTCCC
TCTGCTTCTCACCCCACTCGTGCTTTCTCTCTCACTCTCAAATAAATAAATAAGTAAAATCTTAAA
>Zotu2618
GAACGCTGGCGGCGTGCCTAATACATGCAAGTCGAGCGGACTTGATGAGAAGCTTGCTTCTCTGAGAG
TTAGCGGCGGACGGGTGAGTAACACGTAGGCAACCTGCCTGTAAGACTGGGATAACTACCGGAAACGG
TAGCTAATACCGGATAGGTGATTTTCTCGCATGAGGGAATCAAGAAAGACGGAGCAATCTGTCACTTA
CGGATGGGCCTGCGGCGCATTAGCTAGTTGGTGAGGTAATGGCTCACCAAGGCGACGATGCGTAGCCG
ACCTGAGAGGGTGAACGGCCACACTGGGACTGAGACACGGCC
>Zotu2619
GAACGCTGGCGGCGTGCCTAACACATGCAAGTCGAACGGGGTAGCAATACCCAGTGGCGAACGGGTGC
GTAACACGTGAGGAACATACCCTTCGGCGGGGGATAGCCGGCCCAACGGCCGGGTAATACCGCGTACG
ATCTTCGGGTGGCATCACCTGGGGATGAAAGCAGCAATGCGCCGATGGAGTGCCTCGCGGCCTATCAG
CTAGTTGGTGAGGTAATGGCTCACCAAGGCGACGACGGGTAGCTGGTCTGAGAGGATGATCAGCTACA
TTGGGACTGAGACACGGCC
>Zotu2620
GAACGCTGGCGGCGTGCTTAACACATGCAAGTGGAGCGACGAACCGGGCTTCGGCCCGGGGCAAAGCC
GCGAACGGGTGAGTAACACGTGGGTAACCTGCCCCGATGACCGGGACAACCCGAGGAAACTCGGGCTA
ATACCGGATGTGCTCCCCTCACACCAGTGAGGTGAGTAAAGGTAGCTTCGGCCTCCGCATCGGGATGG
GCCCGCGGCCCATTAGCTTGTTGGTGGGGTAACGGCCTACCAAGGCTGCGATGGGTAGCTGGTCTGAG
AGGACGATCAGCCACACTGGGACTGAGACACGGCC
>Zotu2622
GAACGCTGGCGGCGTGCCTAATACATGCAAGTCGAGCGGACTTGATGAGGAGCTTGCTCCTCTGAGAG
TTAGCGGCGGACGGGTGAGTAACACGTAGGCAACCTGCCCTCAAGACTGGGATAACTACCGGAAACGG
TAGCTAATACCGGATAATTCGTGCTGCTGCATGGCAGCGTGATGAAAGACGGAGCAATCTGTCACTTG
AGGATGGGCCTGCGGCGCATTAGCTAGTTGGTGGGGTAATGGCCTACCAAGGCGACGATGCGTAGCCG
ACCTGAGAGGGTGATCGGCCACACTGGGACTGAGACACGGCC
>Zotu2623
GAACGCTAGCGGCAGGCCTAATACATGCAAGTCGAACGAGATTGGGGAGCTTGCTCCCCATGAGAGTG
GCGTACGGGTGCGTAACGCGTGAGCAACCTACCGTTGTCAGGGGGATAGCCCGAAGAAATTCGGATTA
ATACCGCATAACATCATCGAACAGCATTGTTTGATGATCAAACATTTATGGGACAACAATGGGCTCGC
GTGACATTAGCTAGTTGGAGTGGTAACGGCACACCAAGGCGACGATGTCTAGGGGATCTGAGAGGATG
ACCCCCCACACTGGGACTGAGACACGGCC
>Zotu2624
GAACGCTGGCGGCGTGCTTAACACATGCAAGTCGAACGGAAAGGCCACAGCTTGCTGTGGTACTCGAG
TGGCGAACGGGTGAGTAACACGTGGGTGATCTGCCCTGTACTTCGGGATAAGCTTGGGAAACTGGGTC
TAATACCGGATAGGAATCACATGTGGGTGTGTGGTTGGAAAGCTTTTGCGGTATGGGATGAGCTCGCG
GCCTATCAGCTTGTTGGTGGGGTAATAGCCTACCAAGGCGTCGACGGGTAGCCGGCCTGAGAGGGTGT
ACGGCTACATTGGGACTGAGATACGGCC
>Zotu2625
GAACGCTGGCGGCAGGCCTAACACATGCAAGTCGAGCGAAGTCTTCGGACTTAGCGGCGGACGGGTGA
GTAACGCGTGGGAACATACCCTTTGGTACGGAATAGCCTCGGGAAACTGGGAGTAATACCGTATGTGC
CCTTCGGGGGAAAGATTTATCGCCAAAGGATTGGCCCGCGTTGGATTAGGTAGTTGGTGGGGTAATGG
CCTACCAAGCCGACGATCCATAGCTGGTTTGAGAGGATGATCAGCCACACTGGGACTGAGACACGGCC
>Zotu2626
GAACGCTGGCGGCGTGCTTAACACATGCAAGTCGAGCGGTAAGGCCCTTCGGGGTACACGAGCGGCGA
ACGGGTGAGTAACACGTGGGTGACCTGCCCCCTACTCTGGGATAAGCCTGGGAAACTGGGTCTAATAC
CGGATATGACTACATGTCGCATGGTGTGTGGTGGAAAGTTTTTCGGTGGGGGATGGGCCCGCGGCCTA
TCAGCTTGTTGGTGGGGTGATGGCCTACCAAGGCGACGACGGGTAGCCGGCCTGAGAGGGCGACCGGC
CACACTGGGACTGAGACACGGCC

>Zotu2627
GAACGCTAGCGGCAGGCTTAATACATGCAAGTCGAGGGGTAACATGGGTAGCAATACCTGATGACGAC
CGGCAAACGGGTGCGGAACATGTACGCAACCTTCCCAAAACTGGTGAATAGCCCTCCGAAAGGAGGAT
TAATACACCGTAACATAATGAAGAGGCATCTCTTTATTATTATAGCTCCGGCGGTTTTGGATGGGCGT
GCACCTGATTAGGTAGTTGGCGGGGTAACGGCCCACCAAGCCTACGATCAGTAACTGGTGTGAGAGCA
CGACCAGTCACACGGGCACTGAGACACGGGC
>Zotu2628
GAACGCTGGCGGCGTGCCTAATACATGCAAGTCGAGCGGGGATGCTTCCGGAAGCCCTTCGGGGCGGA
AGGCTGCATCCTAGCGGCGGACGGGTGAGTAACGCGTAGGGAACCTGCCCCCAACACCGGGATAACCC
GGGGAAACTCGGGCTAATACCGGATGCGGCCGACGAATCGCATGGTTTGTCGGTCAAAGGGCTTACCG
GTTGGGGATGGCCCTGCGTCCCATTAGCTAGTTGGTGAGGTAACGGCTCACCAAGGCGACGATGGGTA
GCCGGCCTGAGAGGGTGACCGGCCACACTGGCACTGAGACACGGGC
>Zotu2629
GAACGCTGGCGGCAGGCCTAACACATGCAAGTCGAGCGGATGAGAGGAGCTTGCTCCTTGATTCAGCG
GCGGACGGGTGAGTAATGCCTAGGAATCTGCCTATTAGTGGGGGACAACGTTTCGAAAGGAACGCTAA
TACCGCATACGTCCTACGGGAGAAAGCAGGGGACCTTCGGGCCTTGCGCTAATAGATGAGCCTAGGTC
GGATTAGCTAGTTGGTGAGGTAATGGCTCACCAAGGCGACGATCCGTAACTGGTCTGAGAGGATGATC
AGTCACACTGGAACTGAGACACGGTC
>Zotu2630
GAACGCTGGCGGCGTGCCTAATACATGCAAGTCGAGCGCAGGAAGCTGACGGAACTCTTCGGAGGGAA
GGCAGTGGAATGAGCGGCGGACGGGTGAGTAACACGTAAGGAACCTGCCTCAAGGATTGGGATAACTC
CGAGAAATCGGAGCTAATACCGGATAGTTCAACGGACCGCATGGTCCGCTGATGAAAGGCGCTTCGGC
GTCACCTTGAGATGGCCTTGCGGTGCATTAGCTAGTTGGTGGGGTAACGGCCCACCAAGGCGACGATG
CATAGCCGACCTGAGAGGGTGATCGGCCACACTGGGACTGAGACACGGCC
>Zotu2631
GAACGCTGGCGGCGTGCTTAACACATGCAAGTCGAACGGTGAAGCGCTTTCCTTTTTGGTTGGTGTGG
ATGAGTGGCGAACGGGTGAGTAACACGTGAGTAACCTGCCCCCTTCTTCTGGATAACCGCATGAAAGT
GTGGCTAATACGGGATATTCTGCGCCTGCTGCATGGTGGGTGTGGGAAAGATTGTGCCTTTGGGTGTT
TTCGGTGGGGGATGGGCTCGCGGCCTATCAGCTTGTTGGTGGGGTGATGGCCTACCAAGGCGGTGACG
GGTAGCCGGCCTGAGAGGGTGGACGGTCACACTGGGACTGAGACACGGCC
>Zotu2632
GAACGCTGGCGGCGTGCCTAAGACATGCAAGTCGAGTGGGTTTAGGCCCACGGCGAACGGGTGAGTAA
TACCTAGGAACGTATCTTTAGTTGAGGATATCCCGCCGAAAGGCGGAGCAATACTCAATGTCATCGTA
AGATGAAAGTCGCAAGACGCTAAAGAAGCGGCCTGGGCACCATCAGCTAGTTGGTAAGGTAAAGGCTT
ACCAAGGCTATGACGGTTAGGGGCCCCGAGAGGGGAGACCCCCACACTGGAACTGCGACACGGTC
>Zotu2633
GAACGCTGGCGGCAGGCTTAACACATGCAAGTCGAGCGGGCACTTCGGTGTCAGCGGCAGACGGGTGA
GTAACACGTGGGAACGTACCCTTCGGTTCGGAATAACGCTGGGAAACTAGCGCTAATACCGGATACGC
CCTTTTGGGGAAAGGTTTACTGCCGAAGGATCGGCCCGCGTCTGATTAGCTAGTTGGTGGGGTAACGG
CCTACCAAGGCGACGATCAGTAGCTGGTCTGAGAGGATGATCAGCCACACTGGGACTGAGACACGGCC
>Zotu2634
AAACGCTGGCGGCGTGCCTTATGCATGCAAGTCGAACGAGGCTAGGACTTCGGTCCGAAACCTAGTGG
CGGACGGGTGAGTAACACGTACGTGACCTGTCCGGAAGTGGGGAATAACCAGCCGAAAGGTTGGCTAA
TACCGCATACGGTCACCGCTTGGAAGCGGATGACCAAAGCCGAAAGGCGCTTCCGGAGGGGCGTGCGG
CGGATTAGCTAGTTGGTGTGGTAACGGCGCACCAAGGCGATGATCCGTAGCTGGTCTGAGAGGATGGT
CAGCCACACTGGGACTGAGACACGGCC
>Zotu2635
GAATGCTGGCGGCGTGCCTAATACATGCAAGTCGAGCGGTAACAGGCTTTCACTGTTTACTTCTCTCG
ATTCGGAAACGAAACGAGAATGAGGAGTAAAGAGTGAAAGTGCTGACGAGCGGCGGACGGCTGAGTAA
CGCGTGGGAACATGCCCCAAAGTGAGGGATAAGCATCGGAAACGGTGTCTAATACCGCATATGGTCTT
CGGATTAAAGGATTTATCCGCTTTGGGAGTGGCCCGCGTACGATTAGGTTGTTGGTGAGGTAATGGCT
CACCAAGCCTACGATCGTTAACTGGTCTGAGAGGATGATCAGTCAGACTGGAACTGAGACACGGTC
>Zotu2636
GAACGCTGGCGGCGTGCCTAATACATGCAAGTCGAGCGAACTGACGAGGAGCTTGCTCCTTTGACGTT

AGCGGCGGACGGGTGAGTAACACGTGGGTAACCTACCTATAAGACTGGAATAACTCCGGGAAACCGGG
GCTAATGCCGGATAACATATCGAACCGCATGGTTCGATAGTGAAAGACGGTCTTGCTGTCACTTATAG
ATGGACCCGCGCCGTATTAGCTAGTTGGTGAGGTAACGGCTCATCAAGGCGACGATACGTAGCCGACC
TGAGAGGGTGATCGGCCACACTGGAACTGAGACACGTTC
>Zotu2637
GAACGCTGGCGGCGTGCCTAACACATGCAAGTCGAGCGAGTGAATCTCCTTCGGGAGTGAAGCTAGCG
GCGGACGGGTGAGTAACACGTGGGCAACCTGCCTCATAGAGGGGAATAGCCTTTCGAAAGGAAGATTA
ATACCGCATAAGATTGTAGCTTCGCATGAAGTAGCAATTAAAGGAGCAATCCGCTATGAGATGGGCCC
GCGGCGCATTAGCTAGTTGGTGAGGTAACGGCTCACCAAGGCGACGATGCGTAGCCGACCTGAGAGGG
TGATCGGCCACATTGGGACTGAGACACGGCC
>Zotu2638
GAACGCTGGCGGCGTGCTTAACACATGCAAGTCGAGCGGAAAGGCCCCTTCGGGGGTACTCGAGCGGC
GAACGGGTGAGTAACACGTGGGTAATCTGCCCTAGACACAGGAATAACCAGTGGAAACGCTGGCTAAT
GCCTGATGATGTGCCGGCCTCACGGGGCTGGTACCAAAGGTAGCTTCGGCTTCCGGTCTTGGATGAGC
CCGCGGCCCATTAGCTAGTTGGCGGGGTAATGGCCCACCAAGGCTTCGATGGGTAGCTGGTCTGAGAG
GACGATCAGCCACACTGGGACTGAGACACGGCC
>Zotu2639
GAACGCTGGCGGCGTGTTTAACACATGCAAGTCGAGCGAGATGTTTCTCAGTAGATTCTTCGGATGAT
ACAGAGTTACGGAAAGCGGCGGACGGGTGAGTAACGCGTAGGCAACCTGCCCCTTGCAGAGGGATAGC
CTCGGGAAACCGGGATTAAAACCTCATAATGCGGCACTGAGACATCTCAGAGCCGCCAAAGATTTATC
GGCAAGGGATGGGCCTGCGTCTGATTAGTTAGTTGGTGGGGTAACGGCCTACCAAGGCGACGATCAGT
AGCCGACCTGAGAGGGTGATCGGCCACATTGGAACTGAGACACGGTC
>Zotu2640
GAACGCTGGCGGCGTGCTTAACACATGCAAGTCGAGCGAAGCACTCTGACTTCTTCGGAATGATGAGT
GACTGAGCGGCGGACGGGTGAGTAACGCGTGGGTAACCTGCCTCACACAGGGGGATAACAGTTAGAAA
TGACTGCTAATACCGCATAAGACCACAGCACCGCATGGTGCAGGGGTAAAAACTCCGGTGGTGTGAGA
TGGACCCGCGTCTGATTAGCTAGTTGGTGAGGTAACGGCTCACCAAGGCGACGATCAGTAGCCGACCT
GAGAGGGTGACCGGTCACATTGGGACTGAGACACGGCC
>Zotu2641
GAACGCTAGCGGCAGGCCTAATACATGCAAGTCGGACGGGATTGGAGAGCTTGCTTTCCATGAGAGTG
GCGCACGGGTGCGTAACACGTATGTAACCTACCTTCGTCAGGGGGATAGCCTCTCGAAAGAGAGATTA
AGACCGCATAACATTATCTGGTGGCATCATTAGGTAATCAAATATTTATAGGACGAAGATGGGCATGC
GGAACATTAGCTAGTTGGTAAGGTAACGGCTTACCAAGGCTACGATGTTTAGGGGATCTGAGAGGATG
ACCCCCCACACTGGTACTGAGACACGGAC
>Zotu2642
GAACGCTGGCGGCATGCCTAACACATGCAAGTCGAACGAAGCCTTCGGGCTTAGTGGCGCACGGGTGC
GTAACGCGTGGGAATCTGCCCCTCGGTTCGGAATAACAGTTAGAAATGACTGCTAATACCGGATAATG
ACGTAAGTCCAAAGATTTATCGCCGAGGGATGAGCCCGCGTAGGATTAGCTTGTTGGTGGGGTAATGG
CCCACCAAGGCGACGATCCTTAGCTGGTCTGAGAGGATGATCAGCCACACTGGGACTGAGACACGGCC
>Zotu2643
GAACGCTGGCGGCAGGCCTAACACATGCAAGTCGAGCGCTCCGCAAGGAGAGCGGCGCACGGGAGAGT
AACGCGTGGGAACCTTCCTTTTGGTTCGGAATAACACCGGGAAACTGGTGCTAATACCGGATAAGCCC
TTCGGGGGAAAGATTTATTGCCGAAAGATGGGCCCGCGTTGGATTAGCTAGTTGGTGAGGTAACGGCT
CACCAAGGCACCGATCCATAGCTGATCTGAGAGGATGATCAGCCTCACTGGGACTGAGACACGGCC
>Zotu2644
GAATGCTGGCGGCGCGCCTAACACATGCAAGTCGAGCGGTAACATGGACTTCGGTCTGATGACGAGCG
GCGGACGGCTGAGTAACGCGTGGGAACATGCCCCAAAGTGAGGAATAAGCTCCAGAAATGGAGTCTAA
TGCCGCATATGATCTTCGGATTAAAGGATTTATCCGCTTTGGGAGTGGCCTGCGTATGATTAGATAGT
TGGTGAGGTAATGGCTCACCAAGTCTACGATCATTAACTGGTCTGAGAGGATGATCAGTCAGACTGGA
ACTGAGACACGGTC
>Zotu2646
GAACGCTAGCGGCAGGCCTAATACATGCAAGTCGAGCGGAGAGTAGCAATACTTTTAGCGGCGGACGG
GTGAGTAACGCGTACACAACATGCCTTTGGTTGGGGGATAGCCAGTGGAAACGCTGAGTAATACCGCA
TAAGACTACGTTTCGGCATCGGAGAGTAGTTAAAGTTACGGCGACCAGAGATTGGTGTGCGTTCGATT

AGCTAGTTGGCGGGGTAACGGCCCACCAAGGCGACGATCGATAGGGGGTGTGAGAGCATGGCCCCCCA
CACGGGTACTGAGACACGGAC
>Zotu2647
GAACGCTAGCGGCAGGCTTAATACATGCAAGTCGAGGGGCAGCACGGGGGCAACCCTGGTGGCGACCG
GCAAACGGGTGCGGAACACGTACGCAACTTTCCCAAAACTGGTGAATAGCCTTCCGAAAGGGAGATTA
ATACACCGTAAGATATAGGAGTGGCATCACTTTTATATTATAGCTCCGGCGGTTTTGGATGGGCGTGC
GCCTGATTAGGTAGTTGGCGGGGTAACGGCCCACCAAGCCTGCGATCAGTAACTGGTGTGAGAGCACG
ACCAGTCACACGGGCACTGAGACACGGGC
>Zotu2648
GAACGCTGGCGGCGTGCCTAATACATGCAAGTCGAGCGAAAGTTTCAAAAGCTTGCTTTTGGAACTTT
AGCGGCGGACGGGTGAGTAACACGTGGGCAACCTGCCTGTAAGACTGGGATAACTCCGGGAAACCGGG
GCTAATACCGGATAATCCTTTTCCTTGCATAGGGAAAAGCTGAAAGACGGTTTCGGCTGTCACTTACA
GATGGGCCCGCGGCGCATTAGCTAGTTGGTGGGGTAACGGCCCACCAAGGCGACGATGCGTAGCCGAC
CTGAGAGGGTGATCGGCCACACTGGGACTGAGACACGGCC
>Zotu2649
GAACGCTGGCGGCGTGCCTAATACATGCAAGTCGAGCGGATCTTCGGGAGCTTGCTCCCGATGGTCAG
CGGCGGACGGGTGAGTAACACGTGGGCAACCTGCCTGTAAGACTGGGATAACTCCGGGAAACCGGGGC
TAATACCGGATAATTCATTCCCTCACATGAGGGAATGCTGAAAGACGGCTTTTGCTGTCACTTACAGA
TGGGCCCGCGGCGCATTAGCTAGTTGGTGAGGTAACGGCTCACCAAGGCCACGATGCGTAGCCGACCT
GAGAGGGTGATCGGCCACACTGGGACTGAGACACGGCC
>Zotu2650
TAACGCTGGCGGCGTGCATAACACATGCAAGTCGAACGATGAAACTTAAATGATCTCTTCGGAGTGAT
TCTAAGTGGATTAGTGGCGGACGGGTGAGTAACGCGTGAGTAACCTGCCTTAGAGAAGGGGATAGCCT
TTGGAAACGAAGAATAATACCCTATGATACCAACCCTACGCATGTAGAGTTGGTCAAAGATTTATCGC
TCTAAGATGGACTTGCGTCTGATTAGTTAGTTGGTGAGGTAACGGCCCACCAAGACAGCGATCAGTAG
CCGGCTTGAGAGAGTGTACGGCCACATTGGGACTGAGACACGGCC
>Zotu2651
GAACGCTGGCGGCGTGCTTAACACATGCAAGTCGAACGGACAGACAGAGAGCTTGCTCTCTTGAAGTT
AGTGGCGAACGGGTGAGTAACGCGTAATCAACCTGCCCTTCAGAGGGGGATAACAACGGGAAACCGTT
GCTAATACCGCGTACGATTCATAGACGGCATCGTTTGTGAATGAAAGGTGGCCTCTATTTATAAGCTA
TCGCTGAAGGAGGGGATTGCGTCTGATTAGCTAGTTGGAGGGGTAACGGCCCACCAAGGCAATGATCA
GTAGCCGGTCTGAGAGGATGAACGGCCACATTGGGACTGAGACACGGCC
>Zotu2652
GAACGCTGGCGGCGTGCTTAACACATGCAAGTCGAACGATGATCAGGAGCTTGCTCCTGTGATTAGTG
GCGAACGGGTGAGTAACACGTGAGTAACCTGCCCCTGACTCTGGGATAAGCGTTGGAAACGACGTCTA
ATACTGGATATGATCACTGGTCGCATGGCCTGGTGGTGGAAAGATTTTTTGGTTGGGGATGGACTCGC
GGCCTATCAGCTTGTTGGTGAGGTAATGGCTCACCAAGGCGACGACGGGTAGCCGGCCTGAGAGGGTG
ACCGGCCACACTGGGACTGAGACACGGCC
>Zotu2653
GAACGCTGGCGGCGTGCCTAATACATGCAAGTAGAACGCTGAAGAGAGGAGCTTGCTCTTCTTGAATG
AGTTGCGAACGGGTGAGTAACGCGTAGGTAACCTGCCTTGTAGCGGGGGATAACTATTGGAAACGATA
GCTAATACCGCATAACAATGGGTGACACATGTCATTTATTTGAAAGGGGCAATTGCTCCACTACAAGA
TGGACCTGCGTTGTATTAGCTAGTAGGTGAGGTAACGGCTCACCTAGGCGACGATACATAGCCGACCT
GAGAGGGTGATCGGCCACACTGGGACTGAGACACGGCC
>Zotu2654
GAACGCTGGCGGCGTGCTTAACACATGCAAGTCGAGCGGTAAGGCCTTTCGGGGTACACGAGCGGCGA
ACGGGTGAGTAACACGTGGGTGATCTGCCCTGCACTTCGGGATAAGCCTGGGAAACTGGGTCTAATAC
CGGATATGACTTCAGGTTGCATGACTTGGGGTGGAAAGATTTATCGGTGCAGGATGGGCCCGCGGCCT
ATCAGCTTGTTGGTGGGGTAATGGCCTACCAAGGCGACGACGGGTAGCCGACCTGAGAGGGTGACCGG
CCACACTGGGACTGAGACACGGCC
>Zotu2655
GAACGCTAGCGGGAGGCCTAACACATGCAAGCCGAGCGGTAGAGATCTTTCGGGATCTTGAGAGCGGC
GTACGGGTGCGGAACACGTGTGCAACCTGCCTTTATCTGGGGGATAGCCTTTCGAAAGGAAGATTAAT
ACCCCATAATATTTTGAATGGCATCATTTGAAATTGAAAACTCCGGTGGATAGAGATGGGCACGCGCA

AGATTAGATAGTTGGTGAGGTAACGGCTCACCAAGTCTGCGATCTTTAGGGGGCCTGAGAGGGTGATC
CCCCACACTGGTACTGAGACACGGAC
>Zotu2656
GAACGCTGGCGGCAGGCTTAACACATGCAAGTCGAACGATGACTCTCTAGCTTGCTAGAGATGATTAG
TGGCGGACGGGTGAGTAACATTTAGGAATCTACCTAGTAGTGGGGGATAGCTCGGGGAAACTCGAATT
AATACCGCATACGACCTACGGGTGAAAGGGGGCGCAAGCTCTTGCTATTAGATGAGCCTAAATCAGAT
TAGCTAGTTGGTGGGGTAAAGGCCCACCAAGGCGACGATCTGTAACTGGTCTGAGAGGATGATCAGTC
ACACCGGAACTGAGACACGGTC
>Zotu2659
GAACGCTGGCGGCGTGCCTAATACATGCAAGTCGAGCGGACTTTTTAAAAGCTTGCTTTTAAAAAGTT
AGCGGCGGACGGGTGAGTAACACGTGGGCAACCTGCCCTGTAGACTGGGATAACCTCGGGAAACCGAA
GCTAATACCGGATAATCAATTGAAGCTCCTGCTTCAATTGTAAAAGTTGGGATACTAACACTACAGGA
TGGGCCCGCGGCGCATTAGCTAGTTGGTAAGGTAATGGCTTACCAAGGCGACGATGCGTAGCCGACCT
GAGAGGGTGATCGGCCACACTGGGACTGAGACACGGCC
>Zotu2660
GAATGCTGGCGGCGTGGATAAGTCATGCAAGTCAAGGGGGCCGCAAGGCAACCGGCAGACGAGGTAGT
AACACGTAGGTAGATCCCCCGAAGTCGGGCATAGCCATCCGAAAGGATGGGTAATTCCCGATAGTCCC
GCAAGGGTAAAGGTTTTTCGCTTCGGGAGTTGCCTGCGTAGTATCAGCTAGTTGGTAGGGTAACGGCC
TACCAAGGCTACGACGCTTAGGGGAGCTGAGAGGCTGACCCCCACCGATGGAACTGCGACACGGTC
>Zotu2661
GAACGCTGGCAGAATGCTTAACACATGCAAGTCGAACGGATCTTGTCAATCTGATTTTGAAGCTTCGG
CCGATGATGAAGTGAGACGCTGATTAGTGGCAAACGGGCGAGTAACGCGTTTCTAATCTACCCCGGAG
ATGAGGACAACTACAGAGATGTAGCTAAACCTCGATACGCTCTACAAAATGAAAATATGTAGAGGAAA
TATTTATAGCTCTGGGACGAGGAAGCGTCCTATCAGGTAGTTGGCGAGATAATAGCTCACCAAGCCTA
TGACGGGTAGCCGGTCTGAGAGGATGTCCGGCCGCAATGGAACTGAGACACGGTC
>Zotu2662
GAACGCTAGCGGCAGGCCTAATACATGCAAGTCGAACGGGATGGAGTAGCAATACTCTATCTAGTGGC
GCACGGGTGCGTAACGCGTATGCAACCTACCTTTTATTGGGGGATAGCCCGAGGAAACTCGGATTAAT
ACCGCATGATACTATTTGAGGGCATCTTCATTATAGTTAAAGTTAAGGCGATAAAAGATGGGCATGCG
TGACATTAGCTAGTTGGTAGGGGTAACGGCCCACCAAGGCGACGATGTCTAGGGGTTCTGAGAGGATG
GTCCCCCACACTGGTACTGAGATACGGAC
>Zotu2663
GAACGCTGGCGGCGTGCCTAATACATGCAAGTCGAGCGGAGCCTTTCTTCTGAAACCTTCGGGTGGAA
GAAGAAAGTGCTCAGCGGCGGACGGGTGAGTAACACGTAGGCAACCTACCCATAAGATCGGGATAACC
CACGGAAACGTGAGCTAATACCGGATAAGCGGAGCCACTGCATAGTGGCTTCGGGAAAGGCGGAGCAA
TCTGTCACTTATGGATGGGCCTGCGGCGCATTAGCTAGTTGGTGGGGTAGAGGCCTACCAAGGCGACG
ATGCGTAGCCGACCTGAGAGGGTGACCGGCCACACTGGGACTGAGACACGGCC
>Zotu2664
GAACGCTGGTAACGTGGATACCGGCATGCAAGTCAAGGGGCCGCAAGGCACCGGCAAACGGCTTAGTA
ACACGTGGATACATACCCAAAAGTGGGGCACAGCTCGTCGAAAGACGGGGTAATTTCCCATAGTCTCG
CAAGAGTAAAGCAGCAATGCGCTTTTGGAATGGTCTGCGGTCTATCAGCTAGTTGGTGAGGTAATGGC
TCACCAAGGCTATGACGGATAGGGGGCGTGAGAGCGCGACCCCCAAGAATGGCACTGCGACACGGGC
>Zotu2665
GAACGCTGGCGGCATGCCTAATACATGCAAGTCGAGCGAATGATGAAGAAGCTTGCTTCTTCTGATTT
AGCGGCGGACGGGTGAGTAACACGTGGGCAACCTACCTTATAGCTTGGGATAACTTCGGGAAACCGGA
GCTAATACCGAATAATTCATTTTCTTTCATGAGAAAATGCTGAAAGATGGTTTCGGCTATCACTATAG
GATGGGCCCGCGGCGCATTAGCTAGTTGGTGAGGTAACGGCTCACCAAGGCGACGATGCGTAGCCGAC
CTGAGAGGGTGATCGGCCACACTGGGACTGAGACACGGCC
>Zotu2666
GAACGCTGGCGGCGTGCTTAACACATGCAAGTCGAGCGGAAAGGCTCCTTTCGGGGGGTACTCGAGCG
GCGAACGGGTGAGTAACACGTGAGTAATCTGCCCCTCACTCTGGGATAACTGCCTGAAAGGGTGGCTA
ATACCGGATGCGACCGTCCCCTGCATGGGGTGGTGGTGGAAAGGGTTTCTGGTGGGGGATGTGCTCGC
GGCCTATCAGCTTGTTGGTGGGGTAATGGCCTACCAAGGCTTTGACGGGTAGCCGGCCTGAGAGGGTG
ACCGGCCACACTGGGACTGAGACACGGCC

>Zotu2668
GAACGCTGGCGGCGTGCTTAACACATGCAAGTCGGATGTGCCGCAAGGTGCATGGCAGACGGGTGAGT
AACGCGTGGGGAACCTACCTCTTTGTGGGGAATAACGCTCCGAAAGGAGTGGTAATACCGCATGTGAT
CATCGACTGGAATGTTGGTGATGAAAGCTGGGGCAACCTAGCGCAAGGAGAGGGTCCTGCGTCTGATT
AGCTAGTTGGTGGGGTAACGGCCTACCAAGGCGACGATCAGTAGCCGGCCTGAGAGGGTGATCGGCCA
CACTGGAACTGAGACACGGTC
>Zotu2669
GAACGCTGGCGGCGTGCCTAATACATGCAAGTAGAACGCTGAAGAGAGGAGCTTGCTCTTCTTGGATG
AGTTGCGAACGGGTGAGTAACGCGTAGGTAACCTGCCTTGTAGCGGGGGATAACTATTGGAAACGATA
GCTAATACCGCATAACAATGGATGACCCATGTCATTTATTTGAAAGGGGCAAATGCTCCACTACAAGA
TGGACCTGCGTTGTATTAGCTAGTAGGTGAGGTAACGGCTCACCTAGGCGACGATACATAGCCGACCT
GAGAGGGTGATCGGCCACACTGGGACTGAGACACGGCC
>Zotu2670
GAACGCTAGCGATAGGCCTAACACATGCAAGTCGAGGGGCAGCACATGAAGTAGCAATACGGATGGTG
GCGACCGGCGCACGGGTGAGTAACGCGTATGCAACCTACCTACAACAGGGGAATAACTCGTTGAAAGA
CGGACTAATACCCCATAATACAGGGGTCCCGCATGGGAATATTTGTTAAAGATTTATCGGTTGTAGAT
GGGCATGCGTTCCATTAGCTAGTTGGTGAGGTAACGGCTCACCAAGGCAACGATGGATAGGGGAACTG
AGAGGTTTATCCCCCACACTGGTACTGAGACACGGAC
>Zotu2671
GAACGCTGGCGGCGTGCCTAATACATGCAAGTCGAGCGGACTTGATGGAGTGCTTGCACTCCTGAGAG
TTAGCGGCGGACGGGTGAGTAACACGTAGGTAACCTGCCCATAAGACCGGGATAACATTCGGAAACGA
ATGCTAATACCGGATATGCAATTCTTTCGCATGAGAGAGTTGGGAAAGACGGAGCAATCTGTCACTTA
TGGATGGACCTGCGGTGCATTAGCTAGTTGGTGAGGTAATGGCTCACCAAGGCGACGATGCGTAGCCG
ACCTGAGAGGGTGATCGGCCACACTGGGACTGAGACACGGCC
>Zotu2672
GAACGCTGGCGTCGTGCTTAACACATGCAAGTCGAACGGATCCCAAGTGTCAGCAATGATGTGGAAGG
GTTAGTGGCGAACGGGTGAGTAACACGTGAGGAACCTGCCCCGGACTCAGGGATAACGGCTGGAAACG
GCTGCTAATACCTGATGCCGTCGTGTCATCGCATGGTGATGTGACGAAAGAATCTCGGTTCGGGAGGG
TCTCGCGGCCTATCAGCTTGTTGGTGGGGTAATGGCCTACCAAGGCGTCGACGGGTAGCTGGTCTGAG
AGGATGATCAGCCACACTGGGACTGAGACACGGCC
>Zotu2673
GAACGCTGGCGGCGTGCTTAACACATGCAAGTCGAACGGAAAGGCCCTTCGGGGTACTCGAGTGGCGA
ACGGGTGAGTAACACGTGGGTGATCTGCCCTGCACTTCGGGATAAGCCTGGGAAACTGGGTCTAATAC
CGGATAGGACCACGGTCTGCATGGACTGTGGTGGAAAGCTTTTGCGGTGTGGGATGGGCCCGCGGCCT
ATCAGCTTGTTGGTGGGGTGATGGCCTACCAAGGCGACGACGGGTAGCCGGCCTGAGAGGGTGACCGG
CCACACTGGGACTGAGATACGGCC
>Zotu2674
GAACGCTGGCGGCAGGCCTAACACATGCAAGTCGAGCGGTAGCACAGGAGAGCTTGCTCTCCGGGTGA
CGAGCGGCGGACGGGTGAGTAATGTCTGGGAAACTGCCTGATGGAGGGGGATAACTACTGGAAACGGT
AGCTAATACCGCATAACGTCTTCGGACCAAAGTGGGGGACCTTCGGGCCTCACGCCATCAGATGTGCC
CAGATGGGATTAGCTAGTAGGTGGGGTAATGGCTCACCTAGGCGACGATCCCTAGCTGGTCTGAGAGG
ATGACCAGCCACACTGGAACTGAGACACGGTC
>Zotu2675
GAACGCTGGCGGCAGGCTTAACACATGCAAGTCGAACGCTTCATCTTCGGGTGGGGAGTGGCAGACGG
GTGAGTAACGCGTGGGAACGTACCCTTCGGTTCGGAATAACTCAGGGAAACTTGAGCTAATACCGGAT
AAGCCCTTTTTGGGGAAAGGTTGATCTGCCGAAGGATCGGCCCGCGTCCGATTAGCTAGTTGGTGTTG
GTAATGGCTCACCAAGGCGACGATCGGTAGCTGGTCTGAGAGGATGGCCAGCCACATTGGGACTGAGA
CACGGCC
>Zotu2676
GAACGCTGGCGGCATGCCTTACACATGCAAGTCGAACGGTAACGGGCCTTCGGGTGCCGACGAGTGGC
GAACGGGTGAGTAATACATCGGAACGTGCCCGATCGTGGGGGATAACGAGGCGAAAGCTTTGCTAATA
CCGCATACGATCTACGGATGAAAGCAGGGGACCGCAAGGCCTTGCGCGGACGGAGCGGCCGATGGCAG
ATTAGGTAGTTGGTGGGATAAAAGCTTACCAAGCCGACGATCTGTAGCTGGTCTGAGAGGATGATCAG
CCACACTGGGACTGAGACACGGCC

>Zotu2679
TAAAGTTGGCGGCGTGCCTAAGGTATGCAAGTCGCGCGGTTTTTATTGATTCGCAAGATGATTTAAGA
ATAGCGGCGAACGGCTGAGTAACGCGTGGGAATCTCCCTCGAAGTCAAGGATAGCCCACCGAAAGGTG
GAGTAATCCTGGATGTGAACTGTGGTTTAAAGCCGCAAGGCGCTTCGAGATGAGCCTGCGTCCTATCA
GGTTGTTGGCGGGGTAAATGCCCACCAAGCCTATGACGGGTAGCTGGTCTGAGAGGATGACCAGCCAG
AATGGGACTGAGACACGGCC
>Zotu2680
GAACGCTGGCGGCGTGCTTAACACATGCAAGTCGAGCGGAAAGGCCACTTCGGTGGTACTCGAGCGGC
GAACGGGTGAGTAACACGTGAGTAATCTGCCCCCACTTCGGGATAGCCACCGGAAACGGTGATTAAT
ACCGGATATGACACATGGTCGCATGGTTGTGTGTGGAAAGTTTTTCGGTGGGGGATGTGCTCGCGGC
CTATCAGCTTGATGGTGAGGTAATGGCTCACCATGGCTTCGACGGGTAACCGGCCTGAGAGGGTGACC
GGTCACACTGGGACTGAGACACGGCC
>Zotu2681
GAACGCTGGCGGCGTGCTTAACACATGCAAGTCGAGCGGAAAGGCTCCTTCGGGGGTACTCGAGCGGC
GAACGGGTGAGTAACACGTGAGTAATCTGCCCTGGACTTCGGGATAGCCCACCGAAAGGTGGATTAAT
ACCGGATATGACGCGTCCTCGCATGGGGGTGTGTGGAAAGTTTTTCGGTTCAGGATGTGCTCGCGGC
CTATCAGCTTGATGGTGAGGTAATGGCTCACCATGGCTTCGACGGGTAGCCGGCCTGAGAGGGTGACC
GGCCACACTGGGACTGAGACACGGCC
>Zotu2682
GAACGCTGGCGGCGTGCTTAACACATGCAAGTCGAGCGGTAAGGCCCTTCGGGGTACACGAGCGGCGA
ACGGGTGAGTAACACGTGGGTAATCTGCCTCATACTCTGGGATAAGCCTGGGAAACTGGGTCTAATAC
CGGATAGGACCATTGCGGGCATCCGTGGTGGTGGAAAGTTCCGGCGGTATGGGATGAGCCCGCGGCCT
ATCAGCTTGTTGGTGGGGTAGTGGCCTACCAAGGCGACGACGGGTAGCCGGCCTGAGAGGGTGACCGG
CCACACTGGGACTGAGACACGGCC
>Zotu2683
TGTCCGAGAACTGCTCGGGAGGAGGCGAGCCGACAGGGATGCGGCAAGAAACATGGCACGACGTACGG
GAAATGTGTCAAAGGCGTGGTGTATGAAATTACCCTTTCTTGTG
>Zotu2685
GAACGCTGGCGGCAGGCCTAACACATGCAAGTCGAGCGGTAACAGAGGAAAGCTTGCTTTCTTGCTGA
CGAGCGGCGGACGGGTGAGTAATGTATGGGGATCTGCCCGATAGAGGGGGATAACTACTGGAAACGGT
GGCTAATACCGCATGACGTCTACGGACCAAAGCAGGGGCTCTTCGGACCTTGCGCTATCGGATGAACC
CATATGGGATTAGCTAGTAGGTGAGGTAATGGCTCACCTAGGCGACGATCTCTAGCTGGTCTGAGAGG
ATGATCAGCCACACTGGGACTGAGACACGGCC
>Zotu2687
GAACGCTAGCGGCAGGCCTAATACATGCAAGTCGAACGGGATCCACTGGAGAGCTTGCTCGAAGGTGG
TGAGAGTGGCGAATGGGTGCGTAACGCGTGAGCAACTTACCCTTGTCAGGGGGATAGCCCGGCGAAAG
TCGGATTAACACCGCATAACATCATAGGATGGCATCATTTTATGATCAAATATTTATAGGACAAGGAT
GGGCTCGCGTGACATTAGCTAGTTGGTAAGGTAACGGCTTACCAAGGCTTCGATGTCTAGGGGCTCTG
AGAGGAGAATCCCCCACACTGGTACTGAGACACGGAC
>Zotu2688
GAACGCTGGCGGCGTGCTTAACACATGCAAGTCGAGCGGTAAGGCCCTTCGGGGTACACGAGCGGCGA
ACGGGTGAGTAACACGTGAGCAATCTGCCCTCCACATCGGGATAACCCCCGGAAACGGGAGCTAATAC
CGGATACGACCACTTCAGGCATCTGATGGTGGTGGAAAGTTCCGGCGGTGGAGGATGAGCTCGCGGCC
TATCAGCTTGTTGGTGGGGTAACGGCCCACCAAGGCTTCGACGGGTAGCCGGCCTGAGAGGGTGACCG
GCCACACTGGGACTGAGACACGGCC
>Zotu2689
GAACGCTGGCGGCGTGCTTAACACATGCAAGTCGAACGGTGAACCAGAGCTTGCTCTGGGGATCAGTG
GCGAACGGGTGAGTAACACGTGGGCAACCTGCCCCAGACTCTGGGATAACCTCGGGAAACCGATGCTA
ATACCGGATACGACCACTTCGGACATCCGACGGTGGTGGAAAGTTTTTCGGTCTGGGATGGGCCCGCG
GCCTATCAGCTAGTTGGTGAGGTAATGGCCTACCAAGGCGACGACGGGTAGCCGGCCTGAGAGGGCGA
CCGGCCACACTGGAACTGAGACACGGTC
>Zotu2690
GAACGCTGGCGGCGTGCCTAATACATGCAAGTCGAGCGGACCTTTAGGAGCTTGCTCCTGAAGGTTAG
CGGCGGACGGGTGAGTAACACGTGGGCAACCTGCCCTATAGTTGGGGATAACTCCGGGAAACCGGAGC

TAATACCGAATGATACAATTTAACTCCTGTTAGATTGTTGAAAGATGGTTTTGTGCTATCGCTATAGG
ATGGGCCCGCGGCGCATTAGCTAGTTGGTGAGGTAACGGCTCACCAAGGCGACGATGCGTAGCCGACC
TGAGAGGGTGATCGGCCACACTGGGACTGAGACACGGCC
>Zotu2691
GAACGCTGGCGGCGTGCTTAACACATGCAAGTCGAGCGGAAAGGCTCCTTCGGGGGTACTCGAGCGGC
GAACGGGTGAGTAACACGTGAGTAATCTGCCCCTGGCTTTGGGATAGCCACCGGAAACGGTGATTAAT
ACCGGATATGACCACTTCACGCATGTGATGGTGGTGGAAAGTTTTTCGGCCAGGGATGTGCTCGCGGC
CTATCAGCTTGATGGTGAGGTAATGGCTCACCATGGCTTCGACGGGTAGCCGGCCTGAGAGGGTGACC
GGTCACACTGGGACTGAGACACGGCC
>Zotu2692
GAACGCTAGCGGCAGGCCTAATACATGCAAGTCGGACGGGATTGAGAAGCTTGCTTTTCATGAGAGTG
GCGCACGGGTGCGTAACACGTATGTAACCTACCTTATCCAGGGGGATAGCCTCTCGAAAGAGAGATTA
ACACCGCATAACATTATTGAACAGCATTGTTTAATAATCAAATATTTATAGGGATAAGATGGGCATGC
GGGACATTAGCTAGTTGGCGGGGTAACGGCCCACCAAGGCGACGATGTCTAGGGGATCTGAGAGGATG
ACCCCCCACACTGGTACTGAGACACGGAC
>Zotu2693
GAACGCTAGCGGCAGGCTTAATACATGCAAGTCGAGGGGCAGCACGGGTAGTAATACCTGGTGACGAC
CGGCAAACGGGTGCGGAACACGTACGCAACCTTCCCATAACTGGTGAATAGCCCTCCGAAAGGAGGAT
TAATACACCGTAACATAATGAAGTGGCATCACTTTATTATTATAGGCAGCAATGTCGGTTTTGGATGG
GCGTGCGCCTGATTAGGTAGTTGGCGGGGTAACGGCCCACCAAGCCTGCGATCAGTAACTGGTGTGAG
AGCACGACCAGTCACACGGGCACTGAGACACGGGC
>Zotu2694
GAACGCTGGCGGCATGCCTTACACATGCAAGTCGAACGGCAGCACGGGAGCAATCCTGGTGGCGAGTG
GCGAACGGGTGAGTAATACATCGGAACGTGCCCAGTAGTGGGGGATAGCCCGGCGAAAGCCGGATTAA
TACCGCATGAGACCTGAGGGTGAAAGCGGGGGATCGCAAGACCTCGCGCTATTGGAGCGGCCGATGTC
AGATTAGCTAGTTGGTGGGGTAAAGGCCTACCAAGGCAACGATCTGTAGTTGGTCTGAGAGGACGACC
AGCCACACTGGGACTGAGACACGGCC
>Zotu2695
GAACGCTGGCGGCGTGCTTAACACATGCAAGTCGAACGCACCTTCGGGTGAGTGGCGGACGGGTGAGT
AACGCGTGGGAATCTGCCCTCAGGAGGGGGATAACGACTGGAAACGGTCGCTAATACCCCATATGCCG
AGAGGTGAAATGAATTTCGCCTGAGGATGAGCCCGCGTCTGATTAGCTAGTTGGTGGGGTAAGAGCTC
ACCAAGGCGACGATCAGTAGCTGGTCTGAGAGGATGATCAGCCACACTGGGACTGAGACACGGCC
>Zotu2696
GAACGCTGGCGGCGTGCTTAACACATGCAAGTCGAACGTGAAATGTAGCAATACAGAGTAAAGTGGCG
TACGGGTGAGTAACACGTGGGTAATCTACCAAAGAGTCTGGAATAACCGGCCGAAAGGCTGGCTAATA
CCAGATAGTTCAAGCGAGATATAATGTTTCGATTGGGAAAGGGAGCGGGCAACCGCCTTCACTTTTTG
ATGAGCCCGCGTCTGATTAGCTAGTTGGTGAGGTAATGGCTCACCAAGGCAGAGATCAGTAGCTGGTC
TGAGAGGATGATCAGCCACACTGGAACTGAAACACGGTC
>Zotu2697
GAACGCTGGCGGCGTGCTTAACACATGCAAGTCGAACGAAAAGACGGAAAGAGCTTGCTCTTTTCAGA
ATTGAGTGGCAAACGGGTGAGTAACACGTAAACAACCTGCCTTCAGGATGGGGACAACAGACGGAAAC
GACTGCTAATACCGAATAAGTTCCAAGGGCCGCATGGCCCATGGAAGAAAAGGTGGCCTCTACCTGTA
AGCTATCGCCTGAAGAGGGGTTTGCGTCTGATTAGCTGGTTGGAGGGGTAACGGCCCACCAAGGCGAC
GATCAGTAGCCGGTCTGAGAGGATGAACGGCCACACTGGAACTGAGACACGGTC
>Zotu2698
GAACGCTAGCGGCAGGCTTAATACATGCAAGTCGAACGGTCTTAGCAATAAGACGAGTGGCGCACGGG
TGAGTAACGCGTACATAACATACCTTTCACTGGCGAATAGCCTTGGGAAACTGAGAGTAATACGCCAT
AACATTGTGATTCGGCATCGGATTACAATTAAAGTTCCGGCGGTGAGAGATTGATGTGCGTTGGATTA
GGTAGTTGGTGGGGTAATGGCTCACCAAGCCCGCGATCCATAGGGGGCGTGAGAGCGTGGTCCCCCAC
ACGGGTACTGAGACACGGAC
>Zotu2699
GAACGCTGGCGGCATGCCTAACACATGCAAGTCGAACGAAGCCTTCGGGCTTAGTGGCGCACGGGTGC
GTAACGCGTGGGAATCTGCCCCTTGCTTCGGAATAACAGTTAGAAATGACTGCTAATACCGGATGAAG
TCTTCGGACCAAAGATTTATCGGCAAGGGATGAGCCCGCGTAGGATTAGGTAGTTGGTGGGGTAAAGG

CCTACCAAGCCGACGATCCTTAGCTGGTCTGAGAGGATGATCAGCCACACTGGGACTGAGACACGGCC
>Zotu2700
GAACGCTGGCGGCGTGCTTAACACATGCAAGTCGAGCGGTAAGGCCCTTTCGGGGTACACGAGCGGC
GAACGGGTGAGTAACACGTGAGTAATCTGCCCTTCACTTGGGGATAAGCCTCGGAAACGGGGTCTAAT
ACCTGATATGACGGCCGTCTGCATGGCTGGTCGTGGAAAGTTTTTTCGGTGGGGGATGTGCTCGCGG
CCTATCAGCTTGTTGGTGGGGTAATGGCCTACCAAGGCTTCGACGGGTAGCCGGCCTGAGAGGGTGAC
CGGCCACACTGGGACTGAGACACGGCC
>Zotu2701
GAACGCTGGCGGCATGCCTAACACATGCAAGTCGGACGGGAAGCATCCTCGTGGTGTTTCCAGTGGCG
GACGGGTGAGTAACGCGTAAGAACTTGCCCTTGGGAGGGGGACAACAATTGGAAACGGTTGCTAATAC
CCCGTAAGCTGAGGAGCAAAAGGGAGGGATCCGCCCAAGGATGGGCTTGCGTCTGATTAGCTGGTTGG
TGAGGCAATGGCTTACCAAGGCAACGATCAGTAGCTGGTCTGAGAGGATGATCAGCCACACTGGGACT
GAGACACGGCC
>Zotu2703
GAACGCTGGCGGCGTGCTTAACACATGCAAGTCGAACGGTGAACCTGCTTCGGTGGGGGATCAGTGGC
GAACGGGTGAGTAACACGTGGGCAACCTGCCCCCGGCTCTGGGATAACTCCAAGAAATTGGGGCTAAT
ACCGGATGTTCACGGCTGGCCGCATGGTCGGTGGTGGAAAGATTTATCGGCTGGGGATGGGCCCGCGG
CCTATCAGCTTGTTGGTTGGGTAGTGGCCTACCAAGGCGACGACGGGTAGCCGGCCTGAGAGGGTGAC
CGGCCACACTGGGACTGAGACACGGCC
>Zotu2705
GAACGCTGGCGGCGTGCCTAATACATGCAAGTCGAACGGTTTAGTTCCAAGAGGCTTGCCTGTTGGAA
CTAGACAGTGGCGGACGGGTGAGTAACACGTAGGCAACCTACCTGTCAAACCGGGATAACCTCGGGAA
ACCGAAGCTAAACCCGGATAAGCTCCTTTTTCGCATGAAGGGGGAGTGAAAAGGTGCGACCTGATGAC
AGATGGGCCTGCGGCGCATTAGCTAGTAGGTAGGGTAACGGCCTACCTAGGCGATGATGCGTAGCCGA
CCTGAGAGGGCGTACGGCCACACTGGGACTGAGATACGGCC
>Zotu2706
GAACGCTGGCGGCGTGCCTAATACATGCAAGTCGAGCGGACGTTTTTGAAGCTTGCTTCAAAAACGTT
AGCGGCGGACGGGTGAGTAACACGTGGGCAACCTACCTTATCGACTGGGATAACTCCGGGAAACCGGG
GCTAATACCGGATAATATCTAGCACCTCCTGGTGCCGGATTAAAAGAGGGCTTCTTGCTCTCGCGATG
AGATGGGCCCGCGGCGCATTAGCTAGTTGGAGAGGTAACGGCTCCCCAAGGCAACGATGCGTAGCCGA
CCTGAGAGGGTGATCGGCCACACTGGGACTGAGACACGGCC
>Zotu2708
GAACGCTGGCGGCGTGCCTAATACATGCAAGTCGAGCGGATCTTTAGAAGCTTGCTTCTAAAGATTAG
CGGCGGACGGGTGAGTAACACGTGGGCAACCTGCCTGTAAGACTGGGATAACTTCGGGAAACCGAGGC
TAATACCGGATAATCCTTTTTTTCACATGAGGGAAAGCTGAAAGACGGCGCAAGCTGTCACTTACAGA
TGGGCCCGCGGCGCATTAGCTAGTTGGTGAGGTAACGGCTCACCAAGGCAACGATGCGTAGCCGACCT
GAGAGGGTGATCGGCCACACTGGGACTGAGACACGGCC
>Zotu2709
GAACGCTGGCGGCGTGCTTAACACATGCAAGTCGAACGATGAAGCCCAGCTTGCTGGGTGGATTAGTG
GCGAACGGGTGAGTAACACGTGAGTAACCTGCCCACCACTCTGGGATAACTCCGGGAAACCGGTGCTA
ATACTGGATATGACTCCAACTCGCATGGGTATGGGGTGGAAAGATTTATCGGTGGTGGATGGACTCGC
GGCCTATCAGCTTGTTGGTGGGGTAATGGCCTACCAAGGCGACGACGGGTAGCCGGCCTGAGAGGGTG
ACCGGCCACACTGGGACTGAGACACGGCC
>Zotu2712
GAACGCTGGAAATGTGGATACCGGCATGCAAGTCAAGGGGCCGCAAGGCACCGGCGAACGGCTTAGTA
ACACGTAGATACATACCGCAAAGTGGGGCATAGCTCGTTGAAAAACGGGGTAATTTCCCATAGTTCCC
GCAAGGGTAAAGACGCAAGTCGCTTTGTGAATGGTCTGCGGTCTATCAGCTAGTTGGTGGGGTAAAAG
CCTACCAAGGCTATGACGGATAGGGGGCGTGAGAGCGCGACCCCCAAGAATGGCACTGCGACACGGGC
>Zotu2713
GAACGCTGGCGGCGTGCCTAATACATGCAAGTCGAGCGGATCCATTCCTTCGGGGATGGGTTAGCGGC
GGACGGGTGAGTAACACGTAGGCAACCTGCCTATAAGCCTGGGATAACTTCCGGAAACGGATGCTAAT
ACCGGATACGTTGAATTCTCGCATGAGAATTCATGGAAAGACGGCGCAAGCTGTCACTTATGGATGGG
CCTGCGGCGCATTAGCTAGTTGGTGGGGTAACGGCTCACCAAGGCGACGATGCGTAGCCGACCTGAGA
GGGTGATCGGCCACACTGGGACTGAGACACGGCC

>Zotu2714
CAACGCTGGCGGCGCGCCTAACACATGCAAGTCGAACGAGAAAGCCGCAAGGCAAGTAAAGTGGCGTA
CGGGTGAGTAACACGTGGATAATCTGCCTTTTAGTGGGGAATACCGTTGGGAAACCGACGACAATACC
GCATAATCCCGAGAGGGGAAAGCCGTAAGGCGCTGAAAGAGGAGTCCGCGGTCGATTAGGTAGTTGGC
GGGGTAAAAGCCCACCAAGCCTGTGATCGATAGCCGGCCTGAGAGGGCACACGGCCACACTGGCACTG
AAACACGGGC
>Zotu2716
GAACGCTGGCGGCAGGCCTAACACATGCAAGTCGAACGGTAGCACAGAGGAGCTTGCTCCTTGGGTGA
CGAGTGGCGGACGGGTGAGTAATGTCTGGGAAACTGCCCGATGGAGGGGGATAACTACTGGAAACGGT
AGCTAATACCGCATAACGTCGCAAGACCAAAGAGGGGGACCTTCGGGCCTCTTGCCATCAGATGTGCC
CAGATGGGATTAGCTAGTAGGTGGGGTAATGGCTCACCTAGGCGACGATCCCTAGCTGGTCTGAGAGG
ATGACCAGCCACACTGGAACTGAGACACGGTC
>Zotu2717
GAACGCTGGCGGCGTGCTTAACACATGCAAGTCGAACGGTGACGAGCAGCTTGCTGCTCTGATCAGTG
GCGAACGGGTGAGTAACACGTGAGCAACCTACCCTTGACTCCGGGATAACAGCTGGAAACAGCTGCTA
ATACCGGATACGACCTCGACCGACATCAGTTGAGGTGGAAAGTTCTGGCGGTCAAGGATGGGCTCGCG
GCCTATCAGCTAGTTGGTGAGGTAACGGCTCACCAAGGCTTCGACGGGTAGCCGGCCTGAGAGGGCGA
CCGGCCACACTGGGACTGAGATACGGCC
>Zotu2718
GAACGCTAGCGGCAGGCTTAATACATGCAAGTCGAACGGAATTAGTAGCAATACTGGTTTAGTGGCGC
ACGGGTGAGTAACGCGTACATAACATGCCTCGAAGTAAGGGATAGCCTTGGGAAACTGGGAGTAATAC
CTTATGTGATTGTTGGTTGGCATCAGCTGACAATTAAAGCTACGGTGCTTTGAGATTGATGTGCGTAA
GATTAGGTAGTTGGTAAGGTAACGGCTTACCAAGCCTACGATCTTTAGGGGGCGTGAGAGCGTGGACC
CCCACACGGGTACTGAGACACGGAC
>Zotu2719
GAACGCTGGCGGCGTGCCTAATACATGCAAGTCGTGCGGAAAGGCCTCTTCGGAGGTACTCGAGCGGC
GGACGGCTGAGTAACGCGTAGGAACGTACCCCAAACTGAGGGATAACTGCTCGAAAGAGTAGCTAATA
CCGCATGTGCTCTAAGGAGTAAAGCTTCGGCGGTTTGGGAACGGCCTGCGTAAGATTAGCTTGTTGGT
GAGGTAATGGCTCACCAAGGCGACGATCTTTAGCTGGTCTGAGAGGATGATCAGCCAGACTGGGACTG
AGACACGGCC
>Zotu2720
GAACGCTAGCGGCAGGCTTAATACATGCAAGTCGTGGGGCAGCAGGGGTGTAGCAATACATCTGCTGG
CGACCGGCAAACGGGTGCGTAACACGTACGCAACCTTCCCGTTACTGGTGAATAGCCCAGGGAAACTT
GGATTAATACACCATATTATATTGACTAGGCATCTGGTTGATATCAAAGCTTCGGCGGTAACGGATGG
GCGTGCGGTTGATTAGGTAGTTGGCGGGGTAACGGCCCACCAAGCCTACGATCAATAGCTGATGTGAG
AGCATGATCAGCCACACGGGCACTGAGACACGGGC
>Zotu2721
GAACGCTGGCGGCGCGCCTAATACATGCAAGTCGAGCGAGAAGACGTAGCAATACGTTTGTAAAGCGG
CGAACGGGTGAGGAATACATGGGTAACCTACCCTCGAGTGGGGAATAACTAGCCGAAAGGTTAGCTAA
TACCGCGTACGCTCCTTGGACTTCGGTTCAGGGAGGAAAGCGATACCGTGGGTATCGCGCTCTTGGAT
GGGCTCATGTCCTATCAGCTTGTTGGTGAGGTAACGGCTCACCAAGGCTTCGACGGGTAGCTGGTCTG
AGAGGACGATCAGCCACACTGGCACTGCGACACGGGC
>Zotu2722
TAACGCTGGCGGCGTGCATAACACATGCAAGTCGAACGATGAAACTTAAAAGATTTCTTCGGAATGAA
CTTAAGTGGATTAGTGGCGGACGGGTGAGTAACGCGTGAGTAACCTGCCTTACACAAGGGGATAGCCT
CTGGAAACGGAGAATAATACCCTATGATGTTTCTATCTCGCATGAGATATTAACCAAAGTGTTAGCGG
TGTAAGATGGACTTGCGTCTGATTAGCTAGTTGGTGAGAAAAAAAGCCCACCAAGGCAACGATCAGTA
GCCGGCTTGAGAGAGTGTACGGCCACATTGGGACTGAGACACGGCC
>Zotu2723
GAACGCTAGCGGCAGGCTTAATACATGCAAGTCGAGGGGCAGCACGGATAGCAATATTTGGTGGCGAC
CGGCGCACGGGTGCGTAACACGTATACAACCTACCTATAACAGGGGGATAGCCTTTCGAAAGAGAGAT
TAATACCCCGTAATACAAGACTCGGCATCGAGCTTTGTTGAAAGCTCCGGCGGTTATAGATGGGTATG
CGTTTGATTAGCTAGTTGGTGAGGTAACGGCTCACCAAGGCTACGATCAATAGGGGATCTGAGAGGAC
TAACCCCCACACTGGTACTGAGACACGGAC

>Zotu2724
GAACGCTAGCGGCAGGCCTAATACATGCAAGTCGAACGGCAGCAGGCTGTAGCAATACAGTGCTGGCG
AGTGGCGCACGGGTGCATAACACGTATGTAATTTGCCCATAACTGGGGGATAGCCTTCCGAAAGGAAG
ATTAATACCCCATAACCCAGCGAGGTGGCATCACCTTTCTGGTAAAACTCCGGTGGTTATGGATAAGC
ATGCGTCTGATTAGCTAGTTGGCGAGGTAACGGCTCACCAAGGCAACGATCAGTAGGGGGTCTGAGAG
GATGATCCCCCACACGGGTACTGAGACACGGAC
>Zotu2725
GAACGCTAGCGGGAGGCCTAACACATGCAAGCCGAGCGGTAGAGATCTTTCGGGATCTTGAGAGCGGC
GTACGGGTGCGGAACACGTGTGCAACCTGCCTTTATCTGGGGGATAGCCTTTCGAAAGGAAGATTAAT
ACCCCATAATATTTTGAATGGCATCATTTAAAATTGAAAACTCCGGTGGATAGAGATGGGCACGCGCA
AGATTAGATAGTTGGTGAGGTAACGGCTCACCAAGTCTGCGATCTTTAGGGGGCCTGAGAGGGTGATC
CCCCACACTGGTACTGAGACACGGAC
>Zotu2726
GAACGCTGGCGGTATGCTTAACACATGCAAGTCGAACGGAACCTTTCGGGGTTTAGTGGCGGACGGGT
GAGTAACGCGTGAGAATCGGCCCTCAGGTTGGGGACAACCATTGGAAACGGTGGCTAATACCGAATAA
GCCGAGAGGTGAAAGATTAATTGCCTGGGGAAGAGCTCGCGTCTGATTAGCTAGTTGGTGGGGTAAAG
GCCTACCAAGGCACCGATCAGTAACTGGTCTGAGAGGATGGCCAGTCACACTGGGACTGAGACACGGC
C
>Zotu2728
AAACGCTGGCGGCGTGCCTAATGCATGCAAGTCGAACGGAGTAGTCTTTCGGGACTACTGAGTGGCGG
ACGGGTGAGGAACACGTACGAGACCTGCCCTCGGATGGGGAATAAGCCCTCGAAAGGGGGTCTAATGC
CGCATACGGTCCCAAGCAGGAAGGTTTGGGAGGAAAGCTCGAGAGGGCGTCCGAGGAGGGGCGTGCGG
CCGATTAGCTAGTTGGTGGGGTAAAGGCCTACCAAGGCGACGATCGGTAGCTGGTCTGAGAGGATGGT
CAGCCACACTGGGACTGAGATACGGCC
>Zotu2729
GAACGCTGGCGGCGTGCCTAATACATGCAAGTCGAGCGGAGTTTCAAGAAGCTTGCTTTTTGAAACTT
AGCGGCGGACGGGTGAGTAACACGTGGGCAACCTGCCCCTTAGACTGGGATAACTCCGGGAAACCGGA
GCTAATACCGGATAATCCCTTTCTCCACCTGGAGAGAGGGTGAAAGATGGCTTCGGCTATCACTAGGG
GATGGGCCCGCGGCGCATTAGCTAGTTGGTAAGGTAACGGCTTACCAAGGCGACGATGCGTAGCCGAC
CTGAGAGGGTGATCGGCCACACTGGGACTGAGACACGGCC
>Zotu2730
GAACGCTAGCGACAGGCCTAACACATGCAAGTCGAGGGGCAGCACGAATAGCAATATTTGGTGGCGAC
CGGCGCACGGGTGCGTAACACGTATGCAATCTACCTGTAAGAGGAGGATAACCCGGAGAAATCCGGAC
TAATACTCCATAAGACTATAAAATCGCATGATTTATTAGTTAAACATTTATGGCTTACAGATGAGCAT
GCGCATGATTAGCTGGTTGGTGGAGTAACGGCCCACCAAGGCAACGATCATTAGGGGTTCTGAGAGGA
AGGTCCCCCACACTGGTACTGAGACACGGAC
>Zotu2732
GAACGCTGGCGGCGTGCTTAACACATGCAAGTCGAGCGAAGCAGTTAAGTTGATTTCTTCGGATTGAT
TCTTGACTGACTGAGCGGCGGACGGGTGAGTAACGCGTGGGTGACCTGCCCCATACCGGGGGATAACA
GCTGGAAACGGCTGCTAATACCGCATAAGCGCACAGAGCTGCATGGCTCCGTGTGAAAAACTCCGGTG
GTATGGGATGGGCCCGCGTCTGATTAGGCAGTTGGCGGGGTAACGGCCCACCAAACCGACGATCAGTA
GCCGGCCTGAGAGGGCGACCGGCCACATTGGGACTGAGACACGGCC
>Zotu2733
GAACGCTGGCGGCGTGCCTAACACATGCAAGTCGAACGGAGAATTTCAAAGAGCTTGCTCAGAGAAAT
TTTTAGTGGCGGACGGGTGAGTAACGCGTGGGTAACCTACCCATAAAGCCGGGACAACCCTTGGAAAC
GAGGGCTAATACCGGATAATCTTAGATCTTGGCATCAGGATTTAAGGAAAGGTGGCCTCTGAAAATGC
TACCGATTATGGATGGACCCGCGTCTCATTAGCTAGTTGGTGGGGTAAAGGCCTACCAAGGCGACGAT
CAGTAGCCGGCCTGAGAGGGTGAACGGCCACACTGGGACTGAGACACGGCC
>Zotu2734
GAACGCTGGCGGCGTGCCTAATACATGCAAGTCGAGCGGACTTGACAGAGCTTGCTCTGTTCAAGTCA
GCGGCGGACGGGTGAGTAACACGTGGGTAACCTGCCTGTAAGACTGGGATAACTCCGGGAAACCGGGG
CTAATACCGGATAACATCAAGAGCTGCATGGCTCTTGATTGAAAGGCGGCTTCGGCTGTCACTTACAG
ATGGACCCGCGGCGCATTAGCTAGTTGGTGAGGTAACGGCTCACCAAGGCGACGATGCGTAGCCGACC
TGAGAGGGTGATCGGCCACACTGGGACTGAGACACGGCC

>Zotu2735
GAACGCTGGCGGCAGGCTTAACACATGCAAGTCGAACGCCCCGCAAGGGGAGTGGCAGACGGGTGAGT
AACGCGTGGGAATCTACCCAGAACTACGGAACAACTGAGGGAAACTTCAGCTAATACCGTATACGCCC
TACGGGGGAAAGATTTATCGGTTTTGGATGAGCCCGCGTAAGATTAGCTAGTTGGTGGGGTAATGGCC
TACCAAGGCGACGATCTTTAGCTGGTCTGAGAGGATGATCAGCCACACTGGGACTGAGACACGGCC
>Zotu2736
GAATGCTGGCGGCGTGCCTAACACATGCAAGTCGAGCGGTAACAGGTCTAGTTTACTAGATGCTGACG
AGCGGCGGACGGCTGAGTAACGCGTGGGAACGTGCCCCAAAGTGAGGAATAACTGCCCGAAAGGGTAG
CTAATGCCGCATATGATCTTCGGATTAAAGTCTTCGGACGCTTTGGGAGCGGCCCGCGTTAGATTAGG
TAGTTGGTAGGGTAATGGCCTACCAAGCCGACGATCTATAGCTGGTCTGAGAGGATGATCAGCCAGAC
TGGGACTGAGACACGGCC
>Zotu2737
GAACGCTAGCGGCAGGCCTAACACATGCAAGTCGAGGGGTAGAAGTCTTCGGACTTTGAGACCGGCGC
ACGGGTGCGTAACGCGTATGCAATCTGCCTTACACTAAGGGATAGCCCAGAGAAATTTGGATTAATAC
CTTATAGTATATGACTGGGACATCCCTAACATATTAAAGTTCCAACGGTGTAAGATGAGCATGCGTCC
CATTAGCTAGTTGGTGTGGTAACGGCACACCAAGGCAATGATGGGTAGGGGTCCTGAGAGGGAGATCC
CCCACACTGGTACTGAGACACGGAC
>Zotu2738
GAACGCTGGCGGCGTGCCTAACACATGCAAGTCGAGCGATTCTCTTTTGAGAAGAGCGGCGGACGGGT
GAGTAACGCGTGGGTAACCTGCCCTGTACACACGGATAACATACCGAAAGGTATGCTAATACGGGATA
ATATATTTTGATGGCATCATCGAAATATCAAAGCTCCGGCGGTACAGGATGGACCCGCGTCTGATTAG
CTAGTTGGTGAGGTAATGGCTCACCAAGGCAACGATCAGTAGCCGACCTGAGAGGGTGATCGGCCACA
TTGGAACTGAGACACGGTC
>Zotu2739
GAACGCTGGCGGCGTGCTTAACACATGCAAGTCGAACGAAGCACTTTATTTGATTTCCTTCGGGACTG
ATTATTTGGTGACTGAGTGGCGGACGGGTGAGTAACGCGTGGGTAACCTGCCTTGTACAGGGGGATAA
CAGTTGGAAACGGCTGCTAATACCGCATAAGCGCACGGCATCGCATGATGCAGTGTGAAAAACTCCGG
TGGTATAAGATGGACCCGCGTTGGATTAGCTAGTTGGTGAGGTAACGGCCCACCAAGGCGACGATCCA
TAGCCGACCTGAGAGGGTGACCGGCCACATTGGGACTGAGACACGGCC
>Zotu2740
GAACGCTGGCGGCGTGCTTAACACATGCAAGTCGAACGAACCTTCGGGTTAGTGGCGAACGGGTGAGT
AACACGTGGGCAACCTGCCTCGAGCTCTGGGATAACTCCGGGAAACCGGGGCTAATACCGGATATGAC
CTGCTGAGACATCTTGGTGGGTGGAAAGTTTTTCGGCTCGAGATGGGCCCGCGGCCTATCAGCTTGTT
GGTGGGGTAATGGCCTACCAAGGCGACGACGGGTAGCCGGCCTGAGAGGGCGACCGGCCACACTGGGA
CTGAGACACGGCC
>Zotu2741
GAACGCTGGCGGCGTGCTTAACACATGCAAGTCGAACGAAAATACCAAGCTTGCTTGGTTGATTAGTG
GCGAACGGGTGAGTAACACGTGAGTAACCTGCCCCTTACTTTCCTGGATAACTGCTCGAAAGGGTAGC
TAATACGGGATATTCTGACCATCCTGCATGGGTTGGTTTGGAAAGGTTGTTTTTTCTGGTGAGGGATG
GGCTCGCGGCCTATCAGCTTGTTGGTGGGGTGATGGCCTACCAAGGCTTTGACGGGTAGCCGGCCTGA
GAGGGTGGACGGTCACACTGGGACTGAGATACGGCC
>Zotu2742
GAACGCTGGCGGCGTGCCTAACACATGCAAGTCGAGCGGCAGCGAGTTTTACACTAAGTTCTGGAAGA
TATTCGAGAATTTTGTGTAAAATGTCGGCGAGCGGCGGACGGCTGAGTAACGCGTGGGAACATACCCC
AAAGTGAGGGATAACTGCCCGAAAGGGTGGCTAATACCGCATATGATCTTCGGATTAAAGCATTTATG
CGCTTTGGGAATGGCCTGCGTCGGATTAGGTAGTTGGTGAGGTAAAGGCTCACCAAGCCGACGATCCG
TAACTGGTTTGAGAGGATGACCAGTCAGATTGGAACTGAGACACGGTC
>Zotu2744
TAACGCTAGCGGCAGGCCTAATACATGCAAGTCGAACGGTAACAGGATAGCAATATCGCTGACGAGTG
GCGCACGGGTGCGTAACGCGTATGCAATCTACCTTTAACAGGGGAATAGCTCAGAGAAATCTGAATTA
ATACCGCATAATATAGTGAGTGGGCATCCACTTACTATCAAACCTTCGGGGGTTAAAGATGAGCATGC
GTATCATTAGCTAGTTGGTGAGGTAACGGCTCACCAAGGCTACGATGATTAGGGGGTCTGAGAGGATG
ATCCCCCACACTGGTACTGAGACACGGAC
>Zotu2745

GAACGCTAGCAGAACGCCTAACACATGCAAGTCGAACGAACCAGCAATGGTTAGTGGCAAACGGGCGA
GTAACGCGTATCTAACCTACCCTTGAGTGAGGCACAACTACAGAGATGTAGCTAATTCCTCATACGTT
CTAGAGAATGAGAATATCTAGAAGAAAGATTTATCGCTCAGGGAGGGGGGTGCGTCCTATCAGGTAGT
TGGTGAGGTAATAGCTCACCAAGCCTATGACGGGTAGCCGGTCTGAGAGGATGTCCGGCCGCAATGGA
ACTGAGAACGGTC
>Zotu2746
GAACGCTGGCGGCGTGCTTAACACATGCAAGTCGAGCGGTAAGGCTCCTTCGGGAGTACACGAGCGGC
GAACGGGTGAGTAACACGTGAGTAATCTGCCCTTCACTTCGGGATAACCGCCGGAAACGGTGGCTAAT
ACCGAATACGACGCAGTCTCGCATGAGATCTGCGTGGAAAGTTTTCTCGGTGGAGGATGTGCTCGCGG
CCTATCAGCTTGTTGGTGAGGTAACGGCTCACCAAGGCTTCGACGGGTAGCCGGCCTGAGAGGGTGAC
CGGCCACACTGGGACTGAGACACGGCC
>Zotu2747
GAACGCTGGCGGCATGCTTAACACATGCAAGTCGAACGGTAGCAGGCCCTTCGGGGTGCTGACGAGTG
GCGGACGGGTGAGTAATGCGTAGGAATCTACCTAGTTGAGTGGGATAGCCTAGGGAAACTTAGGGTAA
TACCGCGTAAGCCCGAGAGGGGAAAGCGGGGGACCGAAAGGCCTTGCGCGATTAGATGAGCCTACGTC
GGATTAGCTTGTTGGTGGGGTAAAGGCCTACCAAGGCGATGATCCGTAGCTGGTCTGAGAGGACGACC
AGCCACACTGGGACTGAGACACGGCC
>Zotu2748
GAACGCTGGCGGCGTGCTTAACACATGCAAGTCGAGCGGTAAGGCTCCTTCGGGAGTACACGAGCGGC
GAACGGGTGAGTAACACGTGAGTAACCTGCCCCATTCATCGGAATAACCAGTGGAAACGCTGGCTAAT
GCCGAATACGACCACCGAAGGCATCTTCTGGTGGTGGAAAGCTCCGGCGGAATGGGATGGACTCGCGG
CCTATCAGCTTGTTGGTGAGGTAATGGCTCACCAAGGCGACGACGGGTAGCCGGCCTGAGAGGGTGAC
CGGCCACACTGGGACTGAGACACGGCC
>Zotu2749
TCGAAATCGCGTAGACCTGGAGTCCACCATTGCTTGGTCGCGGAAAATTTTGTCCCAAAAATCCAACT
TTTGGCTTTATTTTGTCTTCAATATCTTTTGTTCATATAG
>Zotu2750
GAACGCTGGCGGCGTGCCTAATACATGCAAGTAGAACGCTGAAGAGAGGAGCTTGCTCTTCTTGGATG
AGTTGCGAACGGGTGAGTAACGCGTAGGTAACCTGCCTGGTAGCGGGGGATAACTATTGGAAACGATA
GCTAATACCGCATAACAACAGTTGTTGCATGACAGCTGTTTAAAAGATGCAATTGCATCACTACCAGA
TGGACCTGCGTTGTATTAGCTAGTTGGTGAGGTAACGGCTCACCAAGGCGACGATACATAGCCGACCT
GAGAGGGTGATCGGCCACACTGGGACTGAGACACGGCC
>Zotu2751
GAACGCTGGCGGCAGGCCTAACACATGCAAGTCGAGCGCGAAAGTCTTCGGATGAGTAGAGCGGCGAA
CGGGTGAGTAACGCGTGGGAATCTGCCTAGTAGTGGGGGACAACGTTCCGAAAGGAACGCTAATACCG
CATACGCCCTACGGGGGAAAGGGGGGGATCTTCGGACCTCTCGCTATTAGATGAGCCCGCGTCGGATT
AGCTAGTTGGTAGGGTAAAGGCCTACCAAGGCTACGATCCGTAACTGGTCTGAGAGGATGATCAGTCA
CACTGGAACTGAGACACGGTC
>Zotu2752
GAACGCTGGCGGCGTGCTTAACACATGCAAGTCGAACGATGATCCCCAGCTTGCTGGGGGGATTAGTG
GCGAACGGGTGAGTAACACGTGAGTAACCTGCCCTTAACTCTGGGATAAGCCTGGGAAACTGGGTCTA
ATACCGGATATGACTCCTCATCGCATGGTGGGGGGTGGAAAGCTTTATTGTGGTTTTGGATGGACTCG
CGGCCTATCAGCTTGTTGGTGAGGTAATGGCTCACCAAGGCGACGACGGGTAGCCGGCCTGAGAGGGT
GACCGGCCACACTGGGACTGAGACACGGCC
>Zotu2753
GAACGCTGGCGGCGTGCTTAACACATGCAAGTCGAACGATGAAAGCGGGAGCTTGCTCCTGCTGGATT
AGTGGCGAACGGGTGAGTAACACGTGAGTAACCTGCCCTTGACTCTGGGATAAGCGTTGGAAACGACG
TCTAATACCGGATACGAGCTTCGGTCGCATGACCAGGAGCTGGAAAGAACTTCGGTCAAGGATGGACT
CGCGGCCTATCAGCTAGTTGGTGAGGTAACGGCTCACCAAGGCGACGACGGGTAGCCGGCCTGAGAGG
GTGACCGGCCACACTGGGACTGAGACACGGCC
>Zotu2754
CAACGCTGGCGGCGTGCCTAACACATGCAAGTCGAACGAGAAAGGGACTTCGGTCCTGAGTAAAGTGG
CGCACGGGTGAGTAACACGTGACTAACCTACCCTGAAGTGGGGAATAACTTCGAGAAATCGAGGCTAA
TACCGCATAATACCCACGGGTCAAAGGAGCAATTCGCTTCAGGAGGGGGTCGCGGCAGATTAGCTAGT

TGGCGGGGTAATGGCCCACCAAGGCAGTGATCTGTATCCGGCCTGAGAGGGCGCACGGACACACTGGA
ACTGAAACACGGTC
>Zotu2756
GAACGCTAGCGGGAGGCTTAACACATGCAAGCCGAGGGGTATAGTTCTTTCGGGAACTAGAGACCGGC
GCACGGGTGCGTAACGCGTATGCAACTTACCCTACTGAAAAGGATAGCCCTTCGAAAGGAGGATTAAT
ACTTTATAACAGATTTAATGGCATCATTAGATTTTGAAAGATTTATCGCAGTAGGATAGGCATGCGTA
AGATTAGTTAGTTGGTGAGGTAACGGCTCACCAAGACGATGATCTTTAGGGGGCCTGAGAGGGTGAAC
CCCCACACTGGTACTGAGACACGGAC
>Zotu2757
GAACGCTGGCGGCGCGCCTAACACATGCAAGTCGAACGGAGTCAAGAGGAGCTTGCTTTTCTTGACTT
AGTGGCGAACGGGTGAGTAACGCGTGAGTAACCTGCCCTGGAGTGGGGGACAACAGTTGGAAACGACT
GCTAATACCGCATAAGCCCACGGATCCGCATGGATCTGCGGGAAAAGGATTTATTCGCTTCAGGATGG
ACTCGCGTCCAATTAGCTAGTTGGTGAGGTAACGGCCCACCAAGGCGACGATTGGTAGCCGGACTGAG
AGGTTGAACGGCCACATTGGGACTGAGACACGGCC
>Zotu2758
GAACGCTGGCGGTGTGCTTCACACATGCAAGTCGAACGAGGTAGCAATACCTAGTGGCGGACGGGTGA
GTAACGCGTGGGAATCTGCCTTTAGATGGGGGATAACGAGCCGAAAGGTTCACTAATACCGCATATGC
CGCAAGGTGAAAGGAGTAATCCGTCTAAAGATGAGCCCGCGTCCAATTAGCTAGTTGGTGGGGTAAAG
GCCTACCAAGGCGACGATCGGTAGCTGGTCTGAGAAGATGATCAGCCACAATGGGACTGAGACACGGC
C
>Zotu2759
GAACGCTGGCGGCGTGCTTAACACATGCAAGTCGAACGATGAACCTCCTTCGGGAGGGGATTAGTGGC
GAACGGGTGAGTAACACGTGGGCAATCTGCCCTGCACTCTGGGATAACTCCGGGAAACCGGAGCTAAT
ACCGGATACGACTACTGCGGGCATCCGTGGTGGTGGAAAGCTCCGGCGGTGCAGGATGAGCCCGCGGC
CTATCAGCTTGTTGGTGGGGTGATGGCCTACCAAGGCGACGACGGGTAGCCGGCCTGAGAGGGCGACC
GGCCACACTGGGACTGAGACACGGCC
>Zotu2760
GAACGTTGGCGGCAGGCTTAACACATGCAAGTCGAGCGGAGAGAGGTAGCTTGCTACTGATCTTAGCG
GCGGACGGGTGAGTAATGCTTAGGAATCTGCCTATTAGTGGGGGACAACATTTCGAAAGGAATGCTAA
TACCGCATACGTCCTACGGGAGAAAGCAGGGGATCTTCGGACCTTGCGCTAATAGATGAGCCTAAGTC
GGATTAGCTAGTTGGTGGGGTAAAGGCCTACCAAGGCGACGATCTGTAGCGGGTCTGAGAGGATGATC
CGCCACACTGGGACTGAGACACGGCC
>Zotu2761
GAACGCTGGCGGCGTGCCTAAGACATGCAAGTCGAGGGGGTTTAGACCCCCGGCGAACGGGTGAGTAA
CACGTGGGAACTTCCATTAAGTTGAGGATAACTCTCCGAAAGGAGAGACAATACTCAATGTGATCGTA
AGATTAAAGCCGCAAGGCGCTTAATGCGAGGCCCGCGCTCCATCAGCTAGTTGGTAGGGTAAAGGCCT
ACCAAGGCTATGACGGATAGGGGTCCCGAGAGGGATAACCCCCACACTGGGACTGCGACACGGCC
>Zotu2762
GAACGCTAGCGGCAGGCCTAACACATGCAAGTCGAGGGGTAGAAGTCTTCGGACTTTGAGACCGGCGC
ACGGGTGCGTAACGCGTATGCAATCTACCTTGTACTGAGGGATAGCCCAGAGAAATTTGGATTAATAC
CTCATAGTATTTTCGGGTGACATCACCTGATTATTAAAGTTCCAACGGTACAAGATGAGCATGCGTCC
CATTAGCTAGTTGGTATGGTAACGGCATACCAAGGCTATGATGGGTAGGGGTCCTGAGAGGGAGATCC
CCCACACTGGTACTGAGACACGGAC
>Zotu2763
GAACGTTAGCGGCGCGCCTAACACATGCAAGTCGAGCGAGAAAGGGAGCAATCCCCGGTACAGCGGCG
CACGGGTGAGTACCACGTAGATTACCTTCCCTGAAGTGGTGGATAACCTGCCGAAAGGCGGGCTAATA
CAGCATGAGACCACGGGGTTTCGATCCTGGGGTTAAAGCGGGCCTCTGTTCACATGCTCGCGCTTTGG
GATGGGTCTGCGGCCCATCAGCTAGTTGGTAGGGTAACGGCCTACCAAGGCAGAGACGGGTAGCTGGT
CTGAGAGGATGATCAGCCACACTGGAACTGAGACACGGTC
>Zotu2764
GAACGCTGGCGGCGTGCCTAATACATGCAAGTCGAGCGCCCTTGACAGCGCATTTCTTTCCTTCGTTT
TAGACGGGGGGAATCAAGGAATGTAGTGTCAAGGGAGCGGCGGACGGCTGAGTAACGCGTAGGAACGT
ACCCCAAAGTGAGGGATAAGCACCGGAAACGGTGTCTAATACCGCATAATATCTCCGGATTAAAGCTT
CGGCGCTTTGGGAGCGGCCTGCGTATGATTAGCTTGTTGGTGAGGTAATGGCTCACCAAGGCGACGAT

CATTAGCTGGTCTGAGAGGATGACCAGCCAGACTGGAACTGAGACACGGTC
>Zotu2765
GAACGCTGGCGGCGTGCTTTACACATGCAAGTCGAGCGGCAGCATGACCTTCGGGTCGATGGCGAGCG
GCGAACGGGTGAGTAATACATCGGAACGTACCTTTTAGTGGGGGATAACGCATCGAAAGATGTGCTAA
TACCGCATAATCTCTGAGGAGGAAAGGCGGGGATCACGCAAGTGACCTGTCGCTGAAAGAGCGGCCGA
TGGTCGATTAGCTAGTTGGCGGGGTAAAAGCCCACCAAGGCGATGATCGATAGCGGGTCTGAGAGGAC
GATCCGTCACACTGGGACTGAGACACGGCC
>Zotu2766
GAACGCTGGCGGCGTGCCTAATACATGCAAGTCGAGCGAACGGACAGGAGTGCTTGCACTCCTTGAAG
TTAGCGGCGGACGGGTGAGTAACACGTGGGTAACCTACCTGTAAGACTGGGATAACTTCGGGAAACCG
GAGCTAATACCGGATAATATTTTCCACCTCATGGCGGAATAGTGAAAGACGGTTTCGGCTGTCACTTA
CAGATGGACCCGCGGCGCATTAGCTAGTTGGTGAGGTAACGGCTCACCAAGGCGACGATGCGTAGCCG
ACCTGAGAGGGTGATCGGCCACACTGGGACTGAGACACGGCC
>Zotu2767
GAACGCTGGCGGCGTGCCTAATACATGCAAGTCGAGCGGAGCTGATGAGGTGCTTGCACCTCTGATGC
TTAGCGGCGGACGGGTGAGTAACACGTAGGTAACCTGCCTGTAAGACTGGGATAACATTCGGAAACGA
ATGCTAATACCGGATACGCGATCCTCTCGCATGAGAGAATCGGGAAAGACGGAGCAATCTGTCACTTA
CGGATGGACCTGCGGCGCATTAGCTAGTTGGTGAGGTAACGGCTCACCAAGGCGACGATGCGTAGCCG
ACCTGAGAGGGTGATCGGCCACACTGGGACTGAGACACGGCC
>Zotu2768
GAACGCTGGCGGCGTGCTTAACACATGCAAGTCGAACGCTGAAGCCCAGCTTGCTGGGTGGATGAGTG
GCGAACGGGTGAGTAACACGTGAGTAACCTGCCCCCTTCTTTGGGATAACGCCCGGAAACGGGTGCTA
ATACTGGATATTCACTGATCTTCGCATGGGGGTTGGTGGAAAGGTTTGTTCTGGTGGGGGATGGGCTC
GCGGCCTATCAGCTTGTTGGTGGGGTGATGGCCTACCAAGGCTTTGACGGGTAGCCGGCCTGAGAGGG
TGACCGGTCACATTGGGACTGAGATACGGCC
>Zotu2769
GAACGCTGGCGGCGTGCTTAACACATGCAAGTCGAACGGTGAAGGAGAGCTTGCTCTTTGGATCAGTG
GCGAACGGGTGAGTAACACGTGAGTAACCTGCCCTTGACTCTGGGATAACTGCTAGAAATGGTAGCTA
ATACCGGATATGACGACCGATGGCATCATCTGGTTGTGGAAAGAATTTTGGTCAAGGATGGACTCGCG
GCCTATCAGGTAGTTGGTGAGGTAATGGCTCACCAAGCCTACGACGGGTAGCCGGCCTGAGAGGGTGA
CCGGCCACACTGGGACTGAGACACGGCC
>Zotu2770
GAACGCTAGCGGCAGGCCTAATACATGCAAGTCGGACGGGATTGAGGAGCTTGCTCTTCATGAGAGTG
GCGCACGGGTGCGTAACACGTATGTAACCTACCTTGTTCAGGGGGATAGCCTCTCGAAAGAGAGATTA
ACACCGCATAAAATCAATTTATGGCATCATAGAATGATCAAATATTTATAGGAACAAGATGGGCATGC
GGAACATTAGCTAGTTGGTAAGGTAACGGCTTACCAAGGCTACGATGTTTAGGGGATCTGAGAGGATG
ACCCCCCACACTGGTACTGAGACACGGAC
>Zotu2771
GAACGCTGGCGGCAGGCTTAACACATGCAAGTCGAACGCGTGTAGCAATACACGAGTGGCGCACGGGT
GAGTAACACGTGGATATCTGCCTTTTGGTTCGGAATAACTCCGGGAAACTGGAGCTAATACCGGATGG
TTCCTACGGGATAAAGATTTATCGCCAAAAGATGAGTCCGCGCACGATTAGCTAGTTGGTGAGGTAAT
GGCTCACCAAGGCGACGATCGTTAGCTGGTCTGAGAGGACGATCAGCCACACTGGGACTGAGACACGG
CC
>Zotu2772
GAACGCTGGCGGCGTGCCTAATACATGCAAGTCGAGCGGACGTTTTATTGGTGCTTGCACCGATTTAA
CGTTAGCGGCGGACGGGTGAGTAACACGTGGGCAACCTACCCTGTAGATTGGGATAACTCCGGGAAAC
CGGGGCTAATACCGAATAATACTTTTTAACTCATGTTAGAAAGTTGAAAGATGGTTTACGCTATCACT
ACAGGATGGGCCCGCGGCGCATTAGCTAGTTGGTGAGGTAATGGCTCACCAAGGCCACGATGCGTAGC
CGACCTGAGAGGGTGATCGGCCACACTGGGACTGAGACACGGCC
>Zotu2773
GAACGCTGGCGGCGTGCCTAACACATGCAAGTCGAACGAGGTCCATCCGGTAGCAATACTGGTGAAGA
TCGAGTGGCGAACGGGTGAGTAGCACGTGAGCAACCTGCCCCGAAGACCGGGACAACACCGGGAAACC
GGTGCTAATACCGGATACCCCCACCGAGTCGCATGGCCCGGTGAGGAAATGGATTCCGCTTCGGGAGG
GGCTCGCGGCCTATCAGCTTGTTGGTGAGGTAACGGCTCACCAAGGCGTCGACGGGTAGCTGGTCTGA

GAGGACGATCAGCCACACTGGGACTGAGACACGGCC
>Zotu2774
TAACGCTAGCGGCAGGCCTAATACATGCAAGTCGAACGGTAACATGGGTAGCAATATCTGATGACGAG
TGGCGCACGGGTGCGTAACACGTATGCAATCTACCTCTAACTGGGGAATAGCCTTCCGAAAGGGAGAT
TAAAACCGCATAATATCACGAGACGGCATCGCCTTGTGATTAAATCTACGGAGGTTAGAGATGAGCAT
GCGTCCGATTAGCTAGTTGGTAGAGTAACAGCCTACCAAGGCTACGATCGGTAGGGGGTCTGAGAGGA
TGATCCCCCACACTGGTACTGAGACACGGAC
>Zotu2775
GAACGCTAGCGGCAGGCTTAATACATGCAAGTCGAACGGTCTTAGCAATAAGACGAGTGGCGCACGGG
TGAGTAACGCGTACATAACATACCTTTAACTGGCGAATAGCCTTGGGAAACTGAGAGTAATACGCCAT
AACATTGCGGTTCGGCATCGGACTGCAATTAAAGTTCCGGCGGTTAGAGATTGATGTGCGTTGGATTA
GGTAGTTGGTGAGGTAACGGCTCACCAAGCCAGCGATCCATAGGGGGCGTGAGAGCGTGGTCCCCCAC
ACGGGTACTGAGACACGGAC
>Zotu2776
GAACGCTGGCGGCGTGCTTAACACATGCAAGTCGAACGGGAAATACTTTATTGAAACTTCGGTGGATT
TAATTTATTTCTAGTGGCGGACGGGTGAGTAACGCGTGGGTAACCTGCCTTATACTGGGGGATAACAG
CCAGAAATGACTGCTAATACCGCATAAGCGCACAGAACCGCATGGTTCGGTGTGAAAAACTCCGGTGG
TATAAGATGGACCCGCGTTGGATTAGCTAGTTGGCAGGGCAGTGGCCTACCAAGGCGACGATCCATAG
CCGGCCTGAGAGGGTGAACGGCCACATTGGGACTGAGACACGGCC
>Zotu2780
GAACGCTGGCGGCAGGCTTAACACATGCAAGTCGAACGGTTGTAGCAATACAGCAGTGGCGCACGGGT
GAGTAACGCGTGGGAACGTACCTCTTAGTTCGGAATAACTATTGGAAACGATAGCTAATACCGGATAC
TCCCTTCGGGGGAAAGATTTATCGCTGAGAGATCGGCCCGCGTAAGATTAGGTAGTTTGTAGGGTAAT
GGCCTACCAAGCCTACGATCTTTAGCTGGTCTGAGAGGATGATCAGCCACACTGGAACTGAGACACGG
TC
>Zotu2781
GAACGCTGGCGGCGTGCTTAACACATGCAAGTCGAACGGAAAGGCCCTGCTTGCAGGGTACTCGAGTG
GCGAACGGGTGAGTAACACGTGGGTGATCTGCCCTGCACTTCGGGATAAGCCTGGGAAACTGGGTCTA
ATACCGGATAGGACCATCGTTTAGTGTCGGTGGTGGAAAGTTTTTCGGTGCAGGATGAGCCCGCGGCC
TATCAGCTTGTTGGTGGGGTAATGGCCTACCAAGGCGTCGACGGGTAGCCGGCCTGAGAGGGTGGACG
GCCACATTGGGACTGAGACACGGCC
>Zotu2782
GAACGCTGGCGGCGTGCTTAAGACATGCAAGTCGAACGCAGTCTTCGGACTGAGTGGCGCACGGGTGA
GTAACACGTAACTGACCTACCCCCAAGTCGCGGATAACTGGCCGAAAGGTCAGCTAATACGTGATGTG
ATGTCAACTTTCTGGGTTGCCATTAAAGGTTTACTGCTTGGGGATGGGGTTGCGTTCCATCAGCTAGT
TGGCGGGGTAAAGGCCCACCAAGGCAACGACGGATAGCCGGCCTGAGAGGGTGGCCGGCCACAGGGGC
ACTGAGACACGGGT
>Zotu2783
GAACGCTGGCGGCGTGCCTAATACATGCAAGTCGAGCGAATCGATGGGAGCTTGCTCCCTGAGATTAG
CGGCGGACGGGTGAGTAACACGTGGGTAACCTGCCTGTAAGACTGGGATAACTTCGGGAAACCGGAGC
TAATACCGGATAGTTTCTTTTCTCGCATGAGAAGAGATGGAAAGATGGTTCTGCTATCACTTACAGAT
GGACCCGCGGCGCATTAGCTAGTTGGTGAGGTAACGGCTCACCAAGGCGACGATGCGTAGCCGACCTG
AGAGGGTGATCGGCCACACTGGGACTGAGACACGGCC
>Zotu2784
GAACGCTAGCGGGAGGCCTAACACATGCAAGTCGAACGGGATTGGGAGCTTGCTCCCATGAGAGTGGC
GCACGGGTGAGTAACGCGTATGCAACCTACCTTTAACTGAGGGATAGCCCATAGAAATGTGGATTAAT
ACCTCATAGTTCTGAGAGACCGCATGGTTTTTCAGATAAAGCTCCGGCGGTTAAAGATGGGCATGCGT
GACATTAGTTTGTTGGTGAGGTAACGGCTCACCAAGACTACGATGTCTAGGGGTTCTGAGAGGATGAT
CCCCCACACTGGTACTGAGACACGGAC
>Zotu2785
GAACGCTGGCGGCAGGCCTAACACATGCAAGTCGAACGGACTCTTCGGAGTTAGTGGCGGACGGGTGA
GTAACACGTGGGAACGTGCCTTTAGGTTCGGAATAAGCCCGGGAAACTGGGTCTAATACCGAATGTGC
CCTTCGGGGGAAAGATTTATCGCCTTTAGAGCGGCCCGCGTCTGATTAGCTAGTTGGTGGTGTAATGG
ACCACCAAGGCGACGATCAGTAGCTGGTCTGAGAGGATGACCAGCCACATTGGGACTGAGACACGGCC

>Zotu2787
GAACGCTGGCGGCATGCCTAACACATGCAAGTCGAACGAGATCTTCGGATCTAGTGGCGCACGGGTGC
GTAACGCGTAGGAATCTGCCTTGGGGTTCGGAATAACTCCCCGAAAGGGGTGCTAATACCGGATGATG
TCGAGAGACCAAAGATTTATCGCCCTGAGATGAGCCCGCGTAGGATTAGCTAGTTGGTGTGGTAAAGG
CGCACCAAGGCGACGATCCTTAGCTGGTCTGAGAGGATGATCAGCCACACTGGGACTGAGACACGGCC
>Zotu2788
GAACGCTAGCGGCAGGCCTAATACATGCAAGTCGAACGGTGATAGCAATATCATAGTGGCGCACGGGT
GCGTAACACGTATGCAACCTACCTTATACTGAGGGATAGCCTTTGGAAACGGAGATTAATACCTCATA
GGACAGTTGACTTGGCATCAAGTTACTGTTAAAGATTTATTGGTATAAGATGGGCATGCGTCCAATTA
GTTAGTTGGCGAGGTAACGGCTCACCAAGACGATGATTGGTAGGGGAACTGAGAGGTTAATCCCCCAC
ACTGGCACTGAGATACGGGC
>Zotu2790
GAACGCTAGCGGGAGGCCTAACACATGCAAGCCGAGCGGTATTTGTTCTTCGGAACAGAGAGAGCGGC
GTACGGGTGCGGAACACGTGTGCAACTTACCTTTATCAGGGGGATAGCCTTTCGAAAGGAAGATTAAT
ACCCCATAATATATTGAATGGCATCATTTGATATTGAAAACTCCGGTGGATAGAGATGGGCACGCGCA
AGATTAGATAGTTGGTGAGGTAACGGCTCACCAAGTCAATGATCTTTAGGGGGCCTGAGAGGGTGATC
CCCCACACTGGTACTGAGACACGGAC
>Zotu2791
GAACGCTGGCGGCGCGCTTAATACATGCAAGTCGAGCGAGAACCGGCCCTTCGGGGCCGGGGAAAGCG
GCGAACGGGTGAGTAACACGTGGGTAATCTGCCCTCGGCACCGGAATAGCCCGGGGAAACCCGGATTA
ATGCCGGATGGCCTCTCGAGCTCTTGGGCTCGTGAGAAAAGGTAGCTTCGGCCTCCGGCCGAGGAGGA
GCCCGCGGCGGATTAGCTTATTGGTGGGGTAACGGCCCACCAAGGCGACGATCCGTAGCTGGTCTGAG
AGGACGATCAGCCACACTGGGACTGAGACACGGCC
>Zotu2793
GAATGCTGGCTGCGTGCTTAACACATGCAAGTCGAACGGTGACCCAGGGCTTGCCCTGGTGATCAGTG
GCGAACGGGTGAGTAACACGTGGGCAACCTGCCCCTCACTCTGGGATAACTCCGGGAAACCGGGGCTA
ATACCGGATACGACCTCGTGCGACATCTCGCGAGGTGGAAAGAATTTCGGTGAGGGATGGGCCCGCGG
CCTATCAGCTAGTTGGTGAGGTAACGGCCCACCAAGGCGACGACGGGTAGCCGGCCTGAGAGGGCGAC
CGGCCACACTGGGACTGAGACACGGCC
>Zotu2794
GAACGCTGGCGGCGTGCCTAACACATGCAAGTCGAACGAGGTCCATCCTTCGGGAGAAGACCTAGTGG
CGAACGGGTGAGTAACACGTGAGGAACCTGCCCCGAAGACCGGGATAACCCTCCGAAAGGAGAGCTAA
TACCGGATACCCCCACCGAACCGCATGGTTTGGTGAGGAAATGGATTCCGCTTCGGGAGGGCCTCGCG
GCCTATCAGCTAGTTGGTGGGGTAACGGCCCACCAAGGCATCGACGGGTAGCTGGTCTGAGAGGACGA
TCAGCCACACTGGGACTGAGACACGGCC
>Zotu2795
GAACGCTGATGGCGTGCCTCATACATGCAAGTCGAGCGGGTAGAAATACCAGCGGCGGACGGGTGAGT
AACGCGTAAACAATCTGCCCGAAAATGGGGGATAACCATCCCAACGGATGGCTAATACCGCATAGGGT
TCTTGGGGGACATCCCTCGACAACGAAAGGCTTCGGCCGTTTTCGGATGAGTTTGCGTCCCATTAGTT
AGTTGGCGGGGTAACGGCCCACCAAGACCGCGATGGGTAGCCGGCCTGAGAGGGTGTACGGCCACATT
GGGACTGAGATACGGCC
>Zotu2798
GAACGCTAGCGGCAGGCCTAATACATGCAAGACGAACGGCAAGTTTGATAGCAATATTGAACCTAGAG
TGTCGTAAGGGTGCGTAACACGTATGCAATCTACCCGAAACTGGAGCATAGCTCCCCGAAAGGGGAAT
TAACACTCCATAAGATCATAGGATGGCATCATCTAGTGATTAAAACTGAGGTGGTTTCGGATGAGCAT
GCGTTTGATTAGTTAGTTGGTGAGGTAACGGCTCACCAAGACGATGATCAATAGGGGAACTGAGAGGT
TGATCCCCCACACTGGCACTGAGATACGGGC
>Zotu2799
GAACGCTGGCGGCGTGGATAAGGCATGCAAGTCAAGGGGGTTTCGCAGCAATGCGAAGCAACCGGCGA
ACGAGTTAGTAACACGTAGGTATCTACCCCAAAGTGGGACATAGCCCCGCGAAAGCGGGGGTAATTTC
CCATGGTCTCGCAAGAGTAAAGTCGCAAGACGCTTTGGGATGAGCCTGCGTCCTATCAGCTAGTTGGT
GAGGTAATGGCTCACCAAGGCAATGACGGGTAGGGGGTGTGAGAGCACGACCCCCAACGATGGGACTG
AGATACGGCC
>Zotu2800

GAACGCTAGCGGCAGGCTTAACACATGCAAGTCGAGGGGTATAGTTCTTCGGAATTAGAGACCGGCGC
ACGGGTGCGTAACGCGTATGCAATCTACCTTTCACAGAGGGATAGCCCAGAGAAATTTGGATTAATAC
CTCATAGTATAGCAACTTCGCATGAAGTCACTATTAAAGTCACAACGGTGAAAGATGAGCATGCGTCC
CATTAGCTAGTTGGTAAGGTAACGGCTTACCAAGGCTACGATGGGTAGGGGTCCTGAGAGGGAGATCC
CCCACACTGGTACTGAGACACGGAC
>Zotu2801
GAACGCTGGCGGCGTGCCTAATACATGCAAGTCGAACGGTTTAGTTCCAAGAGGCGTGCCTGTTGGGA
CTAGGCAGTGGCGGACGGGTGAGTAACACGTAGGCAACCTACCTGTCAAACCGGGATAACCTCGGGAA
ACCGAAGCTAAACCCGGATAAGCTCCTTTTTCGCATGAAGGGGGAGTGAAAAGGTGCGACCTGATGAC
AGATGGGCCTGCGGCGCATTAGCTAGTAGGTAGGGTAACGGCCTACCTAGGCGATGATGCGTAGCCGA
CCTGAGAGGGCGTACGGCCACACTGGGACTGAGATACGGCC
>Zotu2802
GAACGCTGGCGGCGTGCTTAACACATGCAAGTCGAGCGGAAAGGCCCTTCGGGGTACTCGAGCGGCGA
ACGGGTGAGTAACACGTGAGCAACCTGCCCTAGGCTTTGGGATAACCCTCGGAAACGGGGGCTAATAC
CGAATATTACTTGACCTCGCATGGGGTTGGGTGGAAAGTTTTTCGGCCTGGGATGGGCTCGCGGCCTA
TCAGCTTGTTGGTGGGGTGATGGCCTACCAAGGCGACGACGGGTAGCCGGCCTGAGAGGGCGACCGGC
CACACTGGGACTGAGACACGGCC
>Zotu2804
GAACGCTGGCGGCATGCCTTACACATGCAAGTCGAACGGTAGAGGAGCAATCCTCGAGAGTGGCGAAC
GGGTGAGTAATATATCGGAACGTGCCCAGTAGTGGGGGATAGCCCGGCGAAAGCCGGATTAATACCGC
ATGAGACCTGAGGGTGAAAGCGGGGGACTCGCAAGAGCCTCGCGCTACTGGAGCGGCCGATATCAGAT
TAGGTAGTTGGTGGGGTAAAGCCTACCAAGCCTGCGATCTGTAGCTGGTCTGAGAGGACGACCAGCC
ACACTGGGACTGAGACACGGCC
>Zotu2805
GAACGCTAGCGGCAGGCTTAACACATGCAAGTCGAGGGGTAGAAGTCTTCGGACTTTGAGACCGGCGC
ACGGGTGCGTAACGCGTATGCAATCTACCTTTTACAGAGGGATAGCCCAGAGAAATTTGGATTAATAC
CTCATAGTATATAGCCCTGGCATCAGGATTATATTAAAGTCACAACGGTAAAAGATGAGCATGCGTCC
CATTAGCTAGTTGGTAAGGTAACGGCTTACCAAGGCTACGATGGGTAGGGGTCCTGAGAGGGAGATCC
CCCACACTGGTACTGAGACACGGAC
>Zotu2806
GAACGCTGGCGGCGTGCCTAATACATGCAAGTCGAGCGGACAGACAGGAGTGCTTGCACTCCTTGAAG
TCAGCGGCGGACGGGTGAGTAACACGTGGGTAACCTACCTATAAGACTGGGATAACTTCGGGAAACCG
GAGCTAATACCGGATAATATGTTTCACCTCATGGTGAAACAGTGAAAGACGGTTCTGCTGTCACTTAT
AGATGGACCCGCGGCGCATTAGCTAGTTGGTGAGGTAACGGCTCACCAAGGCGACGATGCGTAGCCGA
CCTGAGAGGGTGATCGGCCACACTGGGACTGAGACACGGCC
>Zotu2807
GAACGCTGGCGGCGTGCCTAATACATGCAAGTAGAACGCTGAAGGAGGAGCTTGCTCTTCTGGATGAG
TTGCGAACGGGTGAGTAACGCGTAGGTAACCTGCCTGGTAGCGGGGGATAACTATTGGAAACGATAGC
TAATACCGCATAATAGCAGTTGTTGCATGACAACTGTTTGAAAGGTGCAATTGCACCACTACCAGATG
GACCTGCGTTGTATTAGCTAGTTGGTGGGGTAACGGCTCACCAAGGCGACGATACATAGCCGACCTGA
GAGGGTGATCGGCCACACTGGGACTGAGACACGGCC
>Zotu2808
GAACGCTGGCGGCGTGCCTAATACATGCAAGTGGAACGCATTGATTTCACCGGAGCTTGCTCCACCGA
AATTAGTGAGTCGCGAACGGGTGAGTAACGCGTAGGTAACCTACCTCTTAGCGGGGGATAACTATTGG
AAACGATAGCTAATACCGCATAACAATTGGATACACATGTACCCTATTTAAAAGGAGCAACTGCTTCA
CTAAGAGATGGACCTGCGTTGTATTAGCTAGTTGGTAGGGTAACGGCCTACCAAGGCATCGATACATA
GCCGACCTGAGAGGGTGATCGGCCACACTGGGACTGAGACACGGCC
>Zotu2809
GCCGTGTCTCAGTCCCAGTGTGGCTGATCATCCTCTCAGACCAGCTACGGATCGTCGCCTTGGTAGGC
AATTACCCCACCAACAAGCTAATCCGACATAGGCTCATCTTATAGCGCGAGGTCAGAAGATCCCCCGC
TTTACTCCATAGAGATCATGCGGTATTAGCCCGAGTTTCCCCGGGTTGTCCCCCTCTATAAGGTAGAT
TCCTACGTATTACTCACCCGTCCGCCACTCGCCGCCAGGGAGCATAAACTCCCCGCTCTGCCGTTCGA
CTTGCATGTGTTAAGCATGCCGCTAGCGTTCA
>Zotu2810

GAACGCTGGCGGCGTGCCTAATACATGCAAGTCGAGCGGTAAGGCCTCTTCGGAGGTACACGAGCGGC
GGACGGCTGAGTAACACGTAGGAACGTACCCCAAAGTGAGGGATAAGCACCAGAAATGGTGTCTAATA
CCGCATGTGATCTTCGGATTAAAGCTCCGGCGCTTTGGGAACGGCCTGCGCATGATTAGCTTGTTGGT
GAGGTAATAGCTCACCAAGGCGACGATCATTAGCTGGTCTGAGAGGATGACCAGCCAGACTGGGACTG
AGAACGGCC
>Zotu2811
GAACGCTAGCTACAGGCTTAACACATGCAAGTCGAGGGGAAACGACATCGAAAGCTTGCTTTTGGTGG
GCGTCGACCGGCGCACGGGTGAGTAACGCGTATCCAACCTGCCCACCACTTGGGGATAACCTTGCGAA
AGTAAGACTAATACCCAATGATATCCCACGAAGGCATCCGAATGGGATTAAAGATTTATCGGTGATGG
ATGGGGATGCGTCTGATTAGCTTGTTGGCGGGGTAACGGCCCACCAAGGCTACGATCAGTAGGGGTTC
TGAGAGGAAGGTCCCCCACATTGGAACTGAGACACGGTC
>Zotu2812
GAACGCTAGCGGCAGGCTTAACACATGCAAGTCGAGGGGTAGGGTAGCAATACCTGAGACCGGCGCAC
GGGTGCGTAACGCGTATGCAATCTACCTTATACAGAGGGATAGCCCAGAGAAATTTGGATTAATACCT
CATGGTATTTTCGAATGGCATCATTTGATTATTAAAGTTCCAACGGTATAAGATGAGCATGCGTCCCA
TTAGCTAGATGGTAAGGTAACGGCTTACCATGGCTACGATGGGTAGGGGTCCTGAGAGGGAGATCCCC
CACACTGGTACTGAGACACGGAC
>Zotu2813
GAACGCTGGCGGCGTGCTTAACACATGCAAGTCGAGCGGAAAGGCCCACTTGTGGGTACTCGAGCGGC
GAACGGGTGAGTAACACGTGAGTAATCTGCCCTGTGCTCTGGGATAGCCACCGGAAACGGTGATTAAT
ACCGGATATGACGCGCTCCTGCATGGGGGTGTGTGGAAAGTTTTTTCGGCATGGGATGTGCTCGCGGC
CTATCAGCTTGTTGGTGAGGTAATGGCTTACCAAGGCTTTGACGGGTAACCGGCCTGAGAGGGTGACC
GGTCACACTGGGACTGAGACACGGCC
>Zotu2814
GAACGCTGGCGGCGTGCCTAACACATGCAAGTCGAACGTGAAAATCTTCGGATGAGTAAAGTGGCGTA
CGGGTGAGTAACACGTGGGTAATCTACCTTAAAGTTTGGAATAACCTGCCGAAAGGCGGGCTAATACC
AGATAGAGCGAGAAAGACGTGAAGTCTTTTTCGGGAAAGGGAGCTCGCAAGAGCCCTTGCTTTAAGAT
GAGCCTGCGCTCCATTAGCTAGTTGGTAGGGTAAAAGCCTACCAAGGCGATGATGGATAGCTGATTTA
AGAGGATGATCAGCCACACTGGAACTGAAACACGGTC
>Zotu2816
GAACGCTGGCGGCAGGCCTAACACATGCAAGTCGAACGCCCCGCAAGGGGAGTGGCAGACGGGTGAGT
AACACGTGGGAACCTTCCCTATAGTACGGAATAGCCCAGGGAAACTTGGAGTAATACCGTATACGCCC
GAAAGGGGAAAGATTTATCGCTATAGGATGGGCCCGCGTAGGATTAGCTAGTTGGTGAGGTAATGGCT
CACCAAGGCGACGATCCTTAGCTGGTTTGAGAGAACGACCAGCCACACTGGGACTGAGACACGGCC
>Zotu2817
GAACGCTGGCGGCGTGCCTAATACATGCAAGTCGAGCGAATCAATAGGTGCTTGCACCTATTGATTAG
CGGCGGACGGGTGAGTAACACGTGGGCAACCTGCCTGTAAGACTGGGATAACACCGGGAAACCGGTGC
TAATACCGGATAATCCTTTTCCTCTCATGAGGAAAAGCTGAAAGTCGGTTTCGGCTGACACTTACAGA
TGGGCCCGCGGCGCATTAGCTAGTTGGTGAGGTAACGGCTCACCAAGGCGACGATGCGTAGCCGACCT
GAGAGGGTGATCGGCCACACTGGGACTGAGACACGGCC
>Zotu2818
GAACGCTGGCGGCGTGCTTAACACATGCAAGTCGAACGGAAAGGCCCTTCGGGGTACTCGAGTGGCGA
ACGGGTGAGTAACACGTGGGTGATCTGCCCTGCACTTTGGGATAAGCCTGGGAAACTGGGTCTAATAC
CGAATAGGACCGCGCTCTTCATGGGGTGTGGTGGAAAGCTTTTGCGGTGTGGGATGGGCCCGCGGCCT
ATCAGCTTGTTGGTGGGGTAATGGCCTACCAAGGCGACGACGGGTAGCCGGCCTGAGAGGGTGACCGG
CCACACTGGGACTGAGATACGGCC
>Zotu2820
GAACGCTGGCGGCGTGCCTAATACATGCAAGTCGAGCGGAGTCATTCCTTCGGGGATGGCTTAGCGGC
GGACGGGTGAGTAACACGTAGGCAACCTGCCTGCAAGATCGGGATAACATTCGGAAACGAATGCTAAT
ACCGGATACACAATTTCCTCGCATGGGGAGATTGGGAAAGACGGCGCAAGCTGTCACTTGCAGATGGG
CCTGCGGTGCATTAGCTAGTTGGTGGGGTAACGGCTCACCAAGGCGACGATGCATAGCCGACCTGAGA
GGGTGATCGGCCACACTGGGACTGAGACACGGCC
>Zotu2821
GAACGCTGGCGGCAGGCCTAACACATGCAAGTCGAACGGACCTTTCGGGGTTAGTGGCGGACGGGTGA

GTAACACGTGGGAACGTGCCTTTAGGTTCGGAATAGCTCCTGGAAACGGGTGGTAATGCCGAATGTGC
CCTTCGGGGGAAAGATTTATCGCCTTTAGAGCGGCCCGCGTCTGATTAGCTAGTTGGTGAGGTAATGG
CTCACCAAGGCGACGATCAGTAGCTGGTCTGAGAGGATGACCAGCCACATTGGGACTGAGACACGGCC
>Zotu2822
GAACGCTGGCGGCGTGCTTAACACATGCAAGTGGAGCGACGAACCAGGGCTTGCCCTGGGGCAGAGCC
GCGAACGGGTGAGTAACACGTGGGTAACCTACCCCGAAGACTGGGACAACCCGAGGAAACTCGGGCTA
ATACCAGATGTGGTCTCTCTTCATAAGTAGAGCGACTAAAGGGAGCTTCGGCCCCCGCTTTGGGAGGG
GCCCGCGGCCCATTAGCTTGTTGGTGAGGTAACGGCTCACCAAGGCTTCGATGGGTAGCTGGTCTGAG
AGGACGATCAGCCACACTGGGACTGAGACACGGCC
>Zotu2823
GAACGCTAGCGGCAGGCCTAATACATGCAAGTCGAGGGGTAGTGTAGCAATACATGAGACCGGCGCAC
GGGTGCGTAACGCGTATGGAACTTGTCCGATCCTCCGGGATAGCCCCCGGAAACGGGGATTAATACCG
GATGGTCCAATGGAACGGCATCGTTTTATTGGTAAAGCTCAGGCGGGATCGGGTGGCCATGCGTTCGA
TTAGCTAGTTGGCGGGGTAACGGCCCACCAAGGCGACGATCGATAGGGGTCCTGAGAGGGAGATCCCC
CACACTGGTACTGAGACACGGGC
>Zotu2824
GAACGCTGGCGGCGTGCCTAATACATGCAAGTCGAGCGAACAGATAAGGAGCTTGCTCCTTTGACGTT
AGCGGCGGACGGGTGAGTAACATGTGGGTAACCTACCTATAAGACTGGAATAACTCCGGGAAACCGGG
GCTAATGCCGGATAACATATAGAACCGCATGGTTCTATAGTGAAAGATGGTTTTGCTATCACTTATAG
ATGGACCCGCGCCGTATTAGCTAGTTGGTAAGGTAATGGCTTACCAAGGCGACGATACGTAGCCGACC
TGAGAGGGTGATCGGCCACACTGGAACTGAGACACGGTC
>Zotu2825
GAACGCTGGCGGCGTGCCTAATACATGCAAGTCGAGCGGACCTCTTCGGAGGTCAGCGGCGGACGGGT
GAGTAACACGTGGGCAACCTGCCTATAAGACTGGGATAACTTCGGGAAACCGGAGCTAATACCGGATA
ATATCAGAGACCACATGGTCTTCGATTAAAAGATGGCTTCGGCTATCACTTATAGATGGGCCCGCGTC
GCATTAGCTAGTTGGTGAGGTAACGGCTCACCAAGGCGACGATGCGTAGCCGACCTGAGAGGGTGATC
GGCCACACTGGGACTGAGACACGGCC
>Zotu2826
GAACGCTGGCGGCGTGCTTAACACATGCAAGTCGAACGGAAAGGCCCTTCGGGGTACTCGAGTGGCGA
ACGGGTGAGTAACACGTGGGTGATCTGCCCTGCACTTTGGGATAAGCCTGGGAAACTGGGTCTAATAC
CGAATAGGACCACGCGCTTCATGGTGTGTGGTGGAAAGCTTTTGCGGTGTGGGATGGGCCCGCGGCCT
ATCAGCTTGTTGGTGGGGTAATGGCCTACCAAGGCGACGACGGGTAGCCGGCCTGAGAGGGTGACCGG
CCACACTGGGACTGAGATACGGCC
>Zotu2827
GAACGCTGGCGGCGTGCCTAATACATGCAAGTCGAGCGGAATTATGGGAGCTTGCTCCCGTAATTTAG
CGGCGGACGGGTGAGTAACACGTGGGCAACCTGCCTATAAGACTGGGATAACTTCGGGAAACCGGAGC
TAATACCGGATAATTCTTTTCCTCTCATGAGGAAAAGCTGAAAGACGGTTTACGCTGTCACTTATAGA
TGGGCCCGCGGCGCATTAGCTAGTTGGTGAGGTAACGGCTCACCAAGGCAACGATGCGTAGCCGACCT
GAGAGGGTGATCGGCCACACTGGGACTGAGACACGGCC
>Zotu2829
GAACGCTGGCGGCGTGCCTAATACATGCAAGTAGAACGCTGAAGCTTGGTGCTTGCACCGAGCGGATG
AGTTGCGAACGGGTGAGTAACGCGTAGGTAACCTGCCTCTTAGCGGGGGATAACTATTGGAAACGATA
GCTAATACCGCATAAAAGACGATATCGCATGATATTGATTTGAAAGGTGCAATTGCATCACTAAGAGA
TGGACCTGCGTTGTATTAGCTAGTTGGTGAGGTAACGGCTCACCAAGGCGACGATACATAGCCGACCT
GAGAGGGTGATCGGCCACACTGGGACTGAGACACGGCC
>Zotu2830
GAACGCTGGCGGCGTGCTTAACACATGCAAGTCGAACGATGAACCCCGCTTGCGGGGGGATTAGTGGC
GAACGGGTGAGTAACACGTGAGTAACCTGCCCTTAACTCTGGGATAAGCCTTGGAAACGGGGTCTAAT
ACTGGATATTGACTTTTCCTCGCATGGGGATTGGTTGAAAGATTTATTGGTTTTGGATGGACTCGCGG
CCTATCAGCTTGTTGGTGAGGTAATGGCTCACCAAGGCGACGACGGGTAGCCGGCCTGAGAGGGTGAC
CGGCCACACTGGGACTGAGACACGGCC
>Zotu2831
GAACGCTGGCGGCATGCCTTACACATGCAAGTCGAACGGTAACAGGTCTTCGGACGCTGACGAGTGGC
GAACGGGTGAGTAACACATCGGAACGTGCCCGATCGTGGGGGATAACGGAGCGAAAGCTTTGCTAATA

CCGCATACGATCTACGGATGAAAGCAGGGGACCGCAAGGCCTTGCGCGGACGGAGCGGCCGATGGCAG
ATTAGGTAGTTGGTGGGATAAAAGCTTACCAAGCCGACGATCTGTAGCTGGTCTGAGAGGATGATCAG
CCACACTGGGACTGAGACACGGCC
>Zotu2832
GAACGCTGGCGGCGTGCCTAATACATGCAAGTCGAGCGAACTTGCGGGAGCTTGCTCCTGTAAGTTAG
CGGCGGACGGGTGAGTAACACGTGGGCAACCTGCCTATAAGACTGGGATAACTTCGGGAAACCGGAGC
TAATACCGGATAATTCTTTTCTACACATGTAGGAAAGCTGAAAGACGGTTTACGCTGTCACTTATAGA
TGGGCCCGCGGCGCATTAGCTAGTTGGTGAGGTAACGGCTCACCAAGGCCACGATGCGTAGCCGACCT
GAGAGGGTGATCGGCCACACTGGGACTGAGACACGGCC
>Zotu2834
GAACGCTGGCGGCAGGCTTAACACATGCAAGTCGAGCGGGGGAGATTGCTTCGGTGATTGACCTAGCG
GCGGACGGGTGAGTAATACTTAGGAATCTGCCTATTAATGGGGGACAACATCTCGAAAGGGATGCTAA
TACCGCATACGCCCTACGGGGGAAAGCAGGGGATCACTTGTGACCTTGCGTTAATAGATGAGCCTAAG
TCGGATTAGCTAGTTGGTGGGGTAAAGGCCTACCAAGGCGACGATCTGTAGCGGGTCTGAGAGGATGA
TCCGCCACACTGGGACTGAGACACGGCC
>Zotu2835
CAACGCTGGCGGCGTGCCTAATGCATGCAAGTCGAACGGGGTGTCCTTCGGGGCACTTACGTGGCGGA
CGGGTGAGGAACACGTGGGTAACCTGCCCACTGGTGGGGGCTAGCCTTCGGAAACGGAGGGTAATTCC
GCATGATCTCGCAGCCCGCGTGGGGGGTGTGAGGAAAGCCTTCGGGCGCCAGTGGAGGGGCCCGCGCC
CGATTAGCTTGTTGGTGGGGTAACGGCTTACCAAGGCGATGATCGGTAGCTGGTTTGAGAGGATGATC
AGCCACACGGGGACTGAGACACGGCC
>Zotu2836
GAATGCTGGCGGTGTGGATAGGGCATGCAAGTCAAGGGGACTCAGTTAGTAATAATTGGGTAACCGGC
AGACGAGATAGTAACGCGTAGGAACGTACCCAGAAGTCGGGCACAGCTCGCCGAAAGGCGGGGTAATT
CCCGATGGTGACGAAAGTCTAAAGATTTATCGCTTCTGGAGCGGCCTGCGTAATATCAGCTTGTTGGT
GAGGTAATGGCTCACCAAGGCTATGACGTTTAGGGGAGCTGAGAGGCTGACCCCCATCGATGGAATTG
AGATACGGTC
>Zotu2837
GAACGCTGGCGGCGTGCTTAACACATGCAAGTCGAACGGAAAGGCCCTTCGGGGTACTCGAGTGGCGA
ACGGGTGAGTAACACGTGGGTGATCTGCCCTGCACTTTGGGATAAGCCTGGGAAACTGGGTCTAATAC
CGAATACACCTCGCTGGCCGCATGGTCTGGTGGGGGAAAGCTTTTGCGGTGTGGGATGGGCCCGCGGC
CTATCAGCTTGTTGGTGGGGTGATGGCCTACCAAGGCGACGACGGGTAGCCGGCCTGAGAGGGTGACC
GGCCACACTGGGACTGAGATACGGCC
>Zotu2838
AAACGCTGGCGGCGTGCCTAATACATGCAAGTCGATCGCTGGAATTCCAGCTTGCTGGAATGAAGGAG
AGGCGAACGGGTGAGTAATACATAAGCAACCTGCCCACGAAGACTGGGATAATTCCTGGAAACGGGGA
CTAATACCGGATAGGTAGTGTGATCGCATGATCGCACTATTAAAGGTTCAAGACACTGGTGGATGGGC
TTATGGCGCATTAGCTGGTTGGTGAGGTAACGGCCCACCAAGGCGATGATGCGTAGCCGGCCTGAGAG
GGTGAACGGCCACATTGGGACTGAGACACGGCC
>Zotu2839
GAACGCTGGCGGCAGGCCTAACACATGCAAGTCGAGCGGATGAATGGAGCTTGCTCCATGATTCAGCG
GCGGACGGGTGAGTAATGCCTAGGAATCTGCCTATTAGTGGGGGACAACGTTTCGAAAGGAACGCTAA
TACCGCATACGTCCTACGGGAGAAAGCAGGGGACCTTCGGGCCTTGCGCTAATAGATGAGCCTAGGTC
GGATTAGCTAGTTGGTGAGGTAATGGCTCACCAAGGCGACGATCCGTAACTGGTCTGAGAGGATGATC
AGTCACACTGGAACTGAGACATGGTC
>Zotu2840
GAACGCTGGCGGCATGCCTTACACATGCAAGTCGAACGCGAAAGGGGGGGCAACTCCCCAAGTAGAGT
GGCGAACGGGTGAGTAATACATCGGAACGTGTCCAGTAGCGGGGGATAACCTCTCGAAAGAGAGGCTA
ATACCGCATACGTCCTACGGGAGAAAGAGGGGGATCGCAAGACCTCTCACTATTGGAGCGGCCGATGT
CGGATTAGCTAGTTGGTGGGGTAAAAGCCCACCAAGGCGACGATCCGTAGCTGGTCTGAGAGGACGAC
CAGCCACACTGGAACTGAGACACGGTC
>Zotu2841
GAACGCTGGCGGCGTGCTTAACACATGCAAGTCGAACGATGACGACAGGGCTTGCCTTGTCTGATTAG
TGGCGAACGGGTGAGTAACACGTGAGTAACCTGCCCCCTCTTCGGGATAACCTCGGGAAATCGGGGC

TAATACCGGATATGACTTCGCACCGCATGGTGTGTTGTGGAAAGTTTTTCGGTGGGGGATGGACTCGC
GGCCTATCAGTTTGTTGGTGAGGTAATGGCTCACCAAGGCGATGACGGGTAGCCGGCCTGAGAGGGCG
ACCGGCCACACTGGGACTGAGGCACGGCC
>Zotu2842
GAACGTTAGCGGCAGGCCTAACACATGCAAGTCGAGCGAGAATAGGGCTTCGGCTCTTAGTAAAGCGG
CGGACGGGTGCGTAACACGTAGATAATCTGCCCTCTAGCGGGGAACAACAGTCCGAAAGGATTGCTAA
TGCCGCATGTCACTGCGGGGACTCCGGTCCCTGTTGGAAAAGGGGCCCGCAAGGGTCTCACTAGAGGA
TGAGTCTGCGGCCCATCAGCTTGTTGGCGGGGTAATGGCCCACCAAGGCTAAGACGGGTAGCTGGTCT
GAGAGGATAACCAGCCACACTGGGACTGAGACACGGCC
>Zotu2843
GAACGCTGGCGGCGTGCCTTAAGCATGCAAGTAGAACGAGTTCTAACTTGCCGCAAGGCAAGCTTAGA
ACTAGTTGCGAACGGCTGAGTAATAAGTAAGAATCTTTCCTTTGGTGGAGAATACCTCGTCGAAAGAC
GAGCTAATACTGCATAAAATCTACGGATAAAAGCAATTAACTTTGCGCCGATTGATGAGCTTACTTCC
CATCAGGCAGTTGGTAGGGTAATGGCCTACCAAACCTACGACGGGTACCCGATCTGAGAGGATGAACG
GGCAGAATGGGACTGAGACACGGCC
>Zotu2845
GAACGCTGGCGGCAGGCCTAACACATGCAAGTCGAGCGGTAGAGAGGAGCTTGCTCCTCTTGAGAGCG
GCGGACGGGTGAGTAATGCCTAGGAATCTGCCTGGTAGTGGGGGATAACGTTCGGAAACGGACGCTAA
TACCGCATACGTCCTACGGGAGAAAGCAGGGGACCTTCGGGCCTTGCGCTATCAGATGAGCCTAGGTC
GGATTAGCTAGTTGGTGAGGTAATGGCTCACCAAGGCGACGATCCGTAACTGGTCTGAGAGGATGATC
AGTCACACTGGAACTGAGACACGGTC
>Zotu2847
GAACGCTGGCGGCGTGCCTAATACATGCAAGTCGAACGAGTTCTCGTTGATGATCGGTGCTTGCACCG
AGATTCAACATGGAACGAGTGGCGGACGGGTGAGTAACACGTGGGTAACCTGCCCTTAAGTGGGGGAT
AACATTTGGAAACAGATGCTAATACCGCATAGATCCAAGAACCGCATGGTTCTTGGCTGAAAGATGGC
GTAAGCTATCGCTTTTGGATGGACCCGCGGCGTATTAGCTAGTTGGTGAGGTAATGGCTCACCAAGGC
GATGATACGTAGCCGAACTGAGAGGTTGATCGGCCACATTGGGACTGAGACACGGCC
>Zotu2848
GAATGCTGGCGGCGTGGATGAGGCATGCAAGTCGAATGGGTTTAGACCCATGGCAGACGAGGTAGGAA
CACGTAGGTACGTACCGGAGAGTCAGGGATAACCTGGAGAAATCCAGAATAATACTTGATGGTCCCGA
GAGGGTAAAGATTTATCGCTCTCTGAACGGCCTGCGTTCTATCAGCTTGTTGGTAAGGTAACGGCTTA
CCAAGGCTACGACGGATAGGGGAGGTGAGAGCCTGACCCCCACCGATGGAACTGCGACACGGTC
>Zotu2849
GAACGCTGGCGGCGTGCTTAACACATGCAAGTCGAACGATGAAGCCTCTTCGGGGGTGGATTAGTGGC
GAACGGGTGAGTAACACGTGAGTAACCTGCCCTTCACTTCAGGATAAGCCTTGGAAACGGGGTCTAAT
ACTGGATATGACTCAAGCTCGCATGGGTTTGGGTGGAAAGATTTTTTGGTGGGGGATGGGCTCGCGGC
CTATCAGCTTGTTGGTGAGGTAGTGGCTTACCAAGGCGACGACGGGTAGCCGGCCTGAGAGGGTGACC
GGCCACACTGGGACTGAGACACGGCC
>Zotu2850
GAACGCTGGCGGCGTGCTTAACACATGCAAGTCGAACGATGAAGCCCAGCTTGCTGGGTGGAAGAGTG
GCGAACGGGTGAGTAACACGTGAGTAACCTGCCCCGGACTCTGGGATAAGCGCTGGAAACGGCGTCTA
ATACTGGATATGACCCATACCGGCATCGGTTGTGGGTGGAAAGATTTATCGGTTCGGGATGGGCTCGC
GGCCTATCAGCTGGATGGTGAGGTAACGGCTCACCATGGCGACGACGGGTAGCCGGCCTGAGAGGGTG
ACCGGCCACACTGGGACTGAGACACGGCC
>Zotu2851
GAACGCTGGCGGCGTGCCTAATACATGCAAGTCGAGCGAATCAATAGGAGCTTGCTCCTGTTGGTTAG
CGGCGGACGGGTGAGTAACACGTGGGCAACCTGCCTGTAAGACTGGGATAACACCGGGAAACCGGTGC
TAATACCAGATAATCCTTTTTCTCACATGAGGAAAAGCTGAAAGACGGTTTCGGCTGTCACTTACAGA
TGGGCCCGCGGCGCATTAGCTAGTTGGTGAGGTAACGGCTCACCAAGGCGACGATGCGTAGCCGACCT
GAGAGGGTGATCGGCCACACTGGGACTGAGACACGGCC
>Zotu2852
GAACGCTGGCGGTATGCTTAACACATGCAAGTCGAACGGAGCTTTTCGGAGCTTAGTGGCGGACGGGT
GAGTAACGCGTGAGAATCTGCCTTTTGGACCGGGACAACTGCGGGAAACGGCAGCTAAGACCGGATGT
GCCTTCGGGTGAAATATTCATAGCCAAAAGAGGAGCTCGCGACCGATTAGCTAGTTGGTGGGGTAAGA

GCCTACCAAGGCTGCGATCGGTAGCCGGTTTGAGAGGACGACCGGCCACACTGGGACTGAGACACGGC
C
>Zotu2853
GAACGCTGGCGGCGTGCTTAACACATGCAAGTGGTGCGACGAACCAGGGCTTGCCCTGGGGCTAAGCC
GCGAACGGGTGAGTAACACGTGGGTAATCTGCCCCGATGACCGGGACAACCCGAGGAAACTCGGGCTA
ATACCGGATGTGGTGCACACACATAAGTGTGTTCATCAAAGGTAGCTTCGGCCTCCGCATCGGGAGGA
GCTCGCGGCCCATTAGCTTGTTGGTGAGGTAACGGCTCACCAAGGCGACGATGGGTAGCTGGTCTGAG
AGGACGATCAGCCACACTGGGACTGAGACACGGCC
>Zotu2854
GAACGCTAGCGGCAGGCCTAATACATGCAAGTCGAACGGTCAGTAGCAATACTGATAGTGGCGCACGG
GTGCGTAACACGTAACTAACCTACCTATAACTGGGGGATAGCCCGCCGAAAGGCGGATTAATACCGCA
TAAGCTAGTTAAACGGCATCGTTTGATTAGTAAAGATTTATTGGTTATAGATGGGGTTGCGGGTCATT
AGCTAGTTGGTAAGGTAACGGCTTACCAAGGCGACGATGACTAGGGGAGCTGAGAGGCTGGTCCCCCA
CACGGGCACTGAGATACGGGC
>Zotu2855
GAACGCTAGCGGCAGGCCTAATACATGCAAGTCAAGGGGTAGCAATACACCGGCGGACGGGTGCGTAA
CGCGTACACAACATACCTTTAAGCGGGGGATAGCATTGGGAAACTGGTGGTAATACCCCATAACATTG
CGAGGAGGCATTTTTTTGCAATTAAAGTTTCGGCGCTTAGAGATTGGTGTGCGTCTGATTAGCTAGTT
GGCGGGGTAACGGCCCACCAAGGCGACGATCAGTAGGGGGTGTGAGAGCATGACCCCCCACATGGGTA
CTGAGACACGGAC
>Zotu2857
GAACGCTGGCGGTATGCCTTAAGCATGCAAGTCGAGCGCATTGGAAGTCGCAAGATTTTCAGTGAGCG
GCGGACGGGTGAGTAATAGTAAGGAACGTACCCCGAAGTGGAGGATAGCTCGCCGAAAGGCGGGGTAA
GCCTGCATAGTATCGTAAGATTAAAGAGCGTAACTGCTCGCTTTGGGAGCGGCCTTATTCCTATCAGC
TTGTTGGTGAGGTAATAGCTCACCAAGGCTATGACGGGTAGGGGGCGTGAGAGCGTGACCCCCCACAA
TGGCACTGAGACACGGGC
>Zotu2858
GAACGCTGGCGGCGTGCTTAACACATGCAAGTCGAGCGGTAAGGCTCCTTCGGGAGTACACGAGCGGC
GAACGGGTGAGTAACACGTGAGCAATCTGCCCTTCTCATCGGAATAACCATTGGAAACGATGGCTAAT
GCCGAATATGACCTCGGAACACATGTTCTGAGGTGGAAAGCTCCGGCGGAGAAGGATGAGCTCGCGGC
CTATCAGCTTGTTGGTGAGGTAATAGCTCACCAAGGCGATGACGGGTAGCCGGCCTGAGAGGGTGACC
GGCCACACTGGGACTGAGACACGGCC
>Zotu2859
GAACGCTGGCGGCGTGCTTAACACATGCAAGTCGAACGGAAAGGCCCTTCGGGGTACTCGAGTGGCGA
ACGGGTGAGTAACACGTGGGTGATCTGCCCTGCACTTCGGGATAAGCTTGGGAAACTGGGTCTAATAC
CGGATAGGATCATGGCTCTCATGGGTTGTGGTGGAAAGCTTTTGCGGTGTGGGATGGGCCCGCGGCCT
ATCAGCTTGTTGGTGGGGTAATGGCCTACCAAGGCGTCGACGGGTAGCCGGCCTGAGAGGGTGTCCGG
CCACACTGGGACTGAGATACGGCC
>Zotu2860
GAACGCTAGCGGGAGGCCTAACACATGCAAGCCGAGCGGTATTTCTTCTTCGGGAGAGAGAGAGCGGC
GTACGGGTGCGGAACACGTGTGCAACCTGCCTTTATCTAGAGGATAGCCTTTCGAAAGGAAGATTAAT
ACTCTATAATATATTAATTGGCATCAATTGATATTGAAAACTCCGGTGGATAGAGATGGGCACGCGCA
AGATTAGCTAGTTGGTGAGGTAACGGCTCACCAAGGCAATGATCTTTAGGGGGCCTGAGAGGGTGATC
CCCCACACTGGTACTGAGACACGGAC
>Zotu2861
GAACGCTGGCGGCATGCCTTACACATGCAAGTCGAACGGTAACGCGGGGCAACCTGGCGACGAGTGGC
GAACGGGTGAGTAATACATCGGAACGTGCCCAGTAGTGGGGGATAGCCCGGCGAAAGCCGGATTAATA
CCGCATACGACCTACGGGTGAAAGCGGGGGATCGCAAGACCTCGCGCTACTGGAGCGGCCGATGTCAG
ATTAGGTAGTTGGTGGGGTAAAGGCCTACCAAGCCGACGATCTGTAGCTGGTCTGAGAGGACGACCAG
CCACACTGGGACTGAGACACGGCC
>Zotu2862
GAACGCTGGCGGCGTGCCTAATACATGCAAGTCGAGCGAATCAATAGGAGCTTGCTCCTGTTGATTAG
CGGCGGACGGGTGAGTAACACGTGGGCAACCCGCCTGTAAGACTGGGATAACACCGGGAAACCGGTGC
TAATACCGGATAATCTTTTCCCTCACATGAGGGAAAACTGAAAGACGGTTTCGGCTGTCACTTACAGA

TGGGCCCGCGGCGCATTAGCTAGTTGGTGAGGTAACGGCTCACCAAGGCGACGATGCGTAGCCGACCT
GAGAGGGTGATCGGCCACACTGGGACTGAGACACGGCC
>Zotu2863
GAACGCTGGCGGCGTGCTTAACACATGCAAGTCGAGCGGAAAGGCCCTTTGGGGTACTCGAGCGGCGA
ACGGGTGAGTAACACGTGGGTAACCTGCCCCTTGCTCTGGGATAACTCCGGGAAACCGGTGCTAATAC
TGGATATTCATGCTTGCAGGCATCTGTGGGTGTGGAAAGTTTTTTCGGCGAGGGATGGACCCGCGGCC
TATCAGCTTGTTGGTGGGGTGATGGCCTACCAAGGCTTTGACGGGTAGCCGGCCTGAGAGGGCGACCG
GCCACACTGGGACTGAGACACGGCC
>Zotu2864
GAACGCTGATGGCGTGCCTCATACATGCAAGTCGAGCGGGTAGCAATACCAGCGGCGGACGGGTGAGT
AACGCGTGAACAATCTGCCTAGAAGTGGGGGATAACCATTCCAACGGATGGCTAATACCGCATAGGGT
AGCGAGAGGGCATCTTTTTGCTACGAAAGGCTTCGGCCGCTTTTGGATGAGTTCGCGTCCCATTAGCT
AGTTGGCGGGGTAATGGCCCACCAAGGCGACGATGGGTAGCCGGCCTGAGAGGGTGTCCGGCCACATT
GGGACTGAGATACGGCC
>Zotu2866
GAACGCTAGCGGCAGGCCTAATACATGCAAGTCGAGGGGCAGCACGATCTTCGGATTGGTGGCGACCG
GCGCACGGGTGCGTAACGCGTATGCAACCTACCTATTACTAAGGGATAGCCCGGGGAAACTCGGATTA
ATACCTTATAGCATTACAAGATGGCATCTGAAAGTAATTAAAGATTTATCGGTAATAGATGGGCATGC
GTATCATTAGTTAGTTGGTGAGGTAATGGCTCACCAAGACCGCGATGATTAGGGGGCCTGAGAGGGTG
ATCCCCCACACTGGTACTGAGATACGGAC
>Zotu2867
GAACGCTGGCGGCATGCTTAACACATGCAAGTCGAACGAGACCTTCGGGTCTAGTGGCGCACGGGTGC
GTAACGCGTGGGAATCTGCCCTTAGGTACGGAATAACTCAGAGAAATTTGTGCTAATACCGTATGATG
TCGAAAGACCAAAGATTTATCGCCTAAGGATGAGCCCGCGTAAGATTAGCTTGTTGGTGAGGTAAAAG
CTCACCAAGGCGACGATCTTTAGCTGGTCTGAGAGGATGATCAGCCACACTGGGACTGAGACACGGCC
>Zotu2868
GAACGCTGGCGGCGTGCCTAACACATGCAAGTCGAACGAGAAAGGGGCTTCGGCTCCGAGTAAAGTGG
CGCACGGGTGAGTAACACGTAGGTAACCTGCCTCTAAGATTGGGATAACCTGTCGAAAGACGAGCTAA
TACCAAATAAGACCACGGTCTCCTCGGGGACAGGGGTCAAAGGTAGCCTCTATTTATAAGCTATCGCT
GAGAGATGGGCCTGCGTACCATTAGCTAGTTGGTGAGGTAACGGCTCACCAAGGCAAAGATGGTTAGC
TGGTCTGAGAGGATGATCAGCCACACTGGAACTGAGACACGGTC
>Zotu2869
GAACGCTGGCGGCGTGCCTAACACATGCAAGTCGAACGGAGGTAGCAATACCTTAGTGGCGCACGGGT
GAGTAATGCATGGGTATCTGCCTTTTGGAGGGGGATAACTACGGGAAACTGTAGCTAAGACCGCGTAA
GCAGCAGCGATGCTGGAAATGCCGGGACCGCAAGGCCGGCAGCCAAGAGATGAGCCCATGTTCCATTA
GCTAGTTGGCGGGGTAACAGCCCACCAAGGCGAAGATGGATAGCTGGTCTGAGAGGACGATCAGCCAC
ACTGGGACTGAGACACGGCC
>Zotu2871
GAACGCTGGCGGCGTGCCTAACACATGCAAGTCGAGCGATTCTCTTCGGAGAAGAGCGGCGGACGGGT
GAGTAACGCGTGGGTAACTTGCCCTGTACACACGGATAACATACCGAAAGGTATGCTAATACGAGATA
ATATACTTTTATCGCATGGTAGAAGTATCAAAGCTCCGGCGGTACAGGATGGACCCGCGTCTGATTAG
CTAGTTGGTAAGGTAACGGCTTACCAAGGCGACGATCAGTAGCCGACCTGAGAGGGTGATCGGCCACA
TTGGAACTGAGACACGGTC
>Zotu2872
GAACGCTGGCGGCGTGCCTAACACATGCAAGTGGAGCGACGAACCGGGCTTCGGCCCGGGGCAAAGCC
GCGAACGGGTGAGTAACACGTGGGTAACCTGCCCCGATGACCGGGACAACCCGAGGAAACTCGGGCTA
ATACCAGATGTGCCCTGCGATCGCAAGATCGTTGGGGAAAGGAAGCTTCGGCCTCCGCATCGGGATGG
GCCCGCGGCGCATTAGCTTGTTGGTGAGGTAACGGCTCACCAAGGCGACGATGCGTAGCTGGTCTGAG
AGGACGATCAGCCACACTGGGACTGAGACACGGCC
>Zotu2873
GAACGCTAGCGGCAGGCTTAACACATGCAAGTCGAGGGGTAGAAGCAGCTTGCTGCTTTGAGACCGGC
GCACGGGTGCGTAACGCGTATACAATCTACCTTTTACAGAGGGATAGCCCAGAGAAATTTGGATTAAT
ACCTCATAGTATTTTCGAATCGCATGATTTGATTATTAAAGTTCCAACGGTAAAAGATGAGTATGCGT
CCCATTAGCTAGATGGTAAGGTAACGGCTTACCATGGCAACGATGGGTAGGGGTTCTGAGAGGGAGAT

CCCCCACACTGGTACTGAGACACGGAC
>Zotu2874
GAACGCTGGCGGCAGGCTTAACACATGCAAGTCGAACGGGTGTAGCAATACATCAGTGGCAGACGGGT
GAGTAACACGTGGGAACCTTCCTAGGGGTACGGAACAACTCAGGGAAACTTGAGCTAATACCGTATAC
GTCCGTAAGGAGAAAGTTTTAACGCCCCTAGACGGGCCCGCGTAGGATTAGGTAGTTGGTGAGGTAAC
GGCTCACCAAGCCTACGATCCTTAGCTGATCTGAGAGGATGATCAGCCACACTGGGACTGAGACACGG
CC
>Zotu2878
GAACGCTGGCGGCGTGCCTAACACATGCAAGTCGAGCGGGGTGAGTTCTTCGGAACGATCCTAGCGGC
GGACGGGTGAGTAACACGTGGGTAACCTGCCTCAAAGTGGGGGATAGCCTTTCGAAAGGAAGATTAAT
ACCGCATAACCCATAGCTTCCGCATGGAAGCAATGGTAAAGATTTATCGCTTTGAGATGGACCCGCGG
CGCATTAGCTAGTTGGTGAGGTAACGGCTCACCAAGGCCACGATGCGTAGCCGACCTGAGAGGGTGAT
CGGCCACATTGGAACTGAGACACGGTC
>Zotu2879
GAACGCTGGCGGCGTGCTTAACACATGCAAGTCGAACGGGGTGTTTATTTCGGTAAACACCAAGTGGC
GAACGGGTGAGTAACGCGTAAGCAATCTACCTTCAAGATGGGGACAACACTTCGAAAGGGGTGCTAAT
ACCGAATGAATGTAAGAGTATCGCATGAGACACTTACTAAAGGAGGCCTCTGAATATGCTTCCGCTTG
AAGATGAGCTTGCGTCTGATTAGCTAGTTGGTGAGGGTAAAGGCCTACCAAGGCGACGATCAGTAGCC
GGTCTGAGAGGATGAACGGCCACATTGGGACTGAGACACGGCC
>Zotu2880
TAACGCTGGCGGTATGCCTAACACATGCAAGTCGAACGGAATGCTATTTAAGAAAATCTCACACTAGT
GAGGGATTTTCACGAGTAGTATTTAGTGGCGGACGGGTGAGTAACACGTTGGAACCTGCCCCAAAGTG
GGGAATAACTGCCCGAAAGGGTAGCTAATACCCCATAGTATCCAGTTTATCTGGATTAAAGATTTATC
GCTTTGGGAGGGGCCTGCGTCTGATTAGCTAGTTGGTGAGGTAAAAGCTCACCAAGGCGACGATCAGT
AGCTGGTTCGAGAGGATGACCAGCCACACTGGGACTGAGATACGGCC
>Zotu2882
GAACGCTAGCGGCAGGCCTAATACATGCAAGTCGAACGGTGGTAGTAATACCATAGTGGCGCACGGGT
GCGTAACGCGTAACCAACCTACCCTTGACTGGGGGATAGCCCGCCGAAAGGCGGATTAATACCGCATA
AACCAGCAGAACGGCATCGTTTAATTGGTAAAGATTTATTGGTCAAGGATGGGGTTGCGTAGCATTAG
CTAGATGGCGGGGTAACGGCCCACCATGGCGACGATGCTTAGGGGACCTGAGAGGGTGATCCCCCACA
CTGGCACTGAGATACGGGC
>Zotu2883
GAACGCTGGCGGCGTGCCTAATACATGCAAGTCGAGCGAACAGATAAGGAGCTTGCTCCTTTGACGTT
AGCGGCGGACGGGTGAGTAACACGTGGGTAACCTACCTATAAGACTGGAATAACTCCGGGAAACCGGG
GCTAATGCCGGATAACATATAGAACCGCATGGTTCTATAGTGAAAGATGGTTTTGCTATCACTTATAG
ATGGACCCGCGCCGTATTAGCTAGTTGGTAAGGTAACGGCTTACCAAGGCGACGATACGTAGCCGACC
TGAGAGGGTGATCGGCCACACTGGAACTGAGACACGGTC
>Zotu2884
GAACGCTGGCGGCGTGCTTAACACATGCAAGTCGAACGATGAAGGGGAGCTTGCTTCCTGGATTAGTG
GCGAACGGGTGAGTAACACGTGAGTAACCTGCCCTTGACTCTGGGATAAGCGTTGGAAACGACGTCTA
ATACCGGATATGACCTGTGACGGCATCGTCTGCAGGTGGAAAGAATTTTGGTCAAGGATGGACTCGCG
GCCTATCAGGTAGTTGGTGAGGTAACGGCTCACCAAGCCGACGACGGGTAGCCGGCCTGAGAGGGTGA
CCGGCCACACTGGGACTGAGACACGGCC
>Zotu2885
GAACGCTGGCGGCGTGCTTAACACATGCAAGTGGAGCGACGAACCGGGGCTTGCCCCGGGGCAGAGCC
GCGAACGGGTGAGTAACACGTGGGTAACCCACCTCGATGACCGGGACAACCCGAGGAAACTCGGGCTA
ATACCGGATGTGATCCTCTTCCGCAAGGAAGTTGATGAAAGATAGCTTCGGCCTTCGCATCGAGACGG
GCCCGCGGCCCATTAGCTTGTTGGTGGGGTAACGGCCTACCAAGGCTTCGATGGGTAGCTGGTCTGAG
AGGACGATCAGCCACACTGGGACTGAGACACGGCC
>Zotu2886
AAACGCTGGCGGCGTGCTTAACACATGCAAGTCGAACGAAGTTTTTCTTTCGGGAGGAACTTAGTGGC
GGACGGGTGAGTAACGCGTGGGTAACCTGCCCTGTACAGGGGGACAACAGCTGGAAACGGCTGCTAAT
ACCGCATAAGCCCTTAGCACTGCATGGTGCATAGGGAAAAGGAGTAATCCGGTACAGGATGGACCCGC
GTCTGATTAGCCAGTTGGCAGGGTAACGGCCTACCAAAGCGACGATCAGTAGCCGATCTGAGAGGATG

TACGGCCACATTGGGACTGAGACACGGCC
>Zotu2888
GAACGCTGGCGGCAGGCTTAACACATGCAAGTCGAACGGGCACTTCGGTGCTAGTGGCAGACGGGTGA
GTAACACGTGGGAACGTACCTTTCGGTTCGGAATAATTCAGGGAAACTTGGACTAATACCGGATACGC
CCTTCGGGGGAAAGATTTATCGCCGATAGATCGGCCCGCGTCTGATTAGCTAGTTGGTGAGGTAATGG
CTCACCAAGGCGACGATCAGTAGCTGGTCTGAGAGGATGGCCAGCCACACTGGGACTGAGACACGGCC
>Zotu2889
GAACGCTGGCGGCAGGCCTAACACATGCAAGTCGAGCGGACCTTTCGGGGTCAGCGGCGGACGGGTGA
GTAACGCGTGGGAATATGCCCTTCTCTACGGAATAGTCCTGGGAAACTGGGGGTAATACCGTATACGC
CCTTTGGGGGAAAGATTTATCGGAGAAGGATTAGCCCGCGTTGGATTAGGTAGTTGGTGGGGTAATGG
CCTACCAAGCCGACGATCCATAGCTGGTTTGAGAGGATGATCAGCCACACTGGGACTGAGACACGGCC
>Zotu2890
GAACGCTGGCGGCATGCTTAACACATGCAAGTTGAACGCAGAATAAACCTTCGGGTTTATTGCTGAGT
AGCAAACGGGTGAGTAACGCGTGGGAATCTGCCTTTTTGTGGGGGATAACTGAGGGAAACTTCAGCTA
ATACCGCATACGCTCTACGGAGGAAAGACTTCGGTCGCAAAAAGAGGAGCCCGCGTCAGATTAGGTAG
TTGGTAGGGTAAAGGCCTACCAAGCCAATGATCTGTAGCTGGTCTGAGAGGATGATCAGCCACACTGG
GACTGAGACACGGCC
>Zotu2891
GAACGCTGGCGGCGTGCTTAACACATGCAAGTCGAGCGAGCCCCACCTTTCGGGGTGGTGTGCTAGCG
GCGAACGGGTGAGTAACACGTGGGCAACCTGCCCCCGGCTCTGGGATAACTCCAAGAAATTGGGGCTA
ATACCGGATATGACTGCTGACCGCATGGTCTGGTGGTGGAAAGATTTATCGGCTGGGGATGGGCCCGC
GGCCTATCAGCTTGTTGGTGGGGTAGTGGCCTACCAAGGCGACGACGGGTAGCCGGCCTGAGAGGGTG
ACCGGCCACACTGGGACTGAGACACGGCC
>Zotu2892
GAACGCTGGCGGCGTGCCTAATACATGCAAGTCGAGCGAACTGATGAGAAGCTTGCTTCTCTGACGTT
AGCGGCGGACGGGTGAGTAACACGTGGGCAACCTGCCCTATAGTTGGGGATAACTCCGGGAAACCGGG
GCTAATACCGAATGATACATTTTAACTCCTGTTAGAATGTTGAAAGATGGTTTACGCTATCGCTATAG
GATGGGCCCGCGGCGCATTAGCTAGTTGGTGAGGTAACGGCTCACCAAGGCGACGATGCGTAGCCGAC
CTGAGAGGGTGATCGGCCACACTGGGACTGAGACACGGCC
>Zotu2893
CAACGCTGGCGGCGTGCCTAACACATGCAAGTCGAACGAGAAAGGGACTTCGGTCCTGAGTATAGTGG
CGCACGGGTGAGTAACACGTGAATGATCTACCTCCGAGTGGGGAATAACCAAGAGAAATCTTGGCTAA
TACCGCATAACACTTACGAGTCAAAGCAGCAATGCGCTTGGAGAGGAGTTCGCGGCAGATTAGTTAGT
TGGCGGGGTAATGGCCCACCAAGACGATGATCTGTATCCGGCCTGAGAGGGCGCACGGACACACTGGA
ACTGAAACACGGTC
>Zotu2894
GAACGCTAGCGGCAGGCCTAATACATGCAAGTCGAACGGTCAGCAATGACAGTGGCGCATGGGTGCGT
AACGCGTATGCAACCTACCCATCAAAGGGGGATAGCCCGGGGAAACCCGGATTAATACCGCATAAGAT
AGTATTCTGGCATCAGAGAGCTATTAAACATTTATGGTTGATGGATGGGCATGCGTTAGATTAGCTAG
TTGGTGTAGTAACGGTGCACCAAGGCGATGATCTATAGGGGTTCTGAGAGGAAGATCCCCCACACTGG
TACTGAGATACGGAC
>Zotu2895
GAACGCTGGCGGCGTGCCTAATACATGCAAGTAGAACGCTGATGTTTGGTGTTTACACTAGACTGATG
AGTTGCGAACGGGTGAGTAACGCGTAGGTAACCTGCCTCATAGCGGGGGATAACTATTGGAAACGATA
GCTAATACCGCATAAGAGTAATTAACACATGTTAGTTATTTAAAAGGAGCAATTGCTTCACTGTGAGA
TGGACCTGCGTTGTATTAGCTAGTTGGTGAGGTAAAGGCTCACCAAGGCGACGATACATAGCCGACCT
GAGAGGGTGATCGGCCACACTGGGACTGAGACACGGCC
>Zotu2896
GAACGCTAGCGGCAGGCCTAATACATGCAAGTCGAACGGTTGTAGCAATACAATAGTGGCGCACGGGT
GCGTAACACGTATGCAACCTACCTTATAGAGGGGGATAGCCTTTGGAAACGGAGATTAATACCCCATA
ACACATTGATACGGCATCGTATTATTGTTAAAGATTTATCGCTATAAGCTGGGCATGCGTCCAATTAG
TTAGTTGGTGAGGTAATGGCTCACCAAGACTTTGATTGGTAGGGGAACTGAGAGGTTAATCCCCCACA
CTGGCACTGAGATACGGGC
>Zotu2897

GAACGCTAGCGGCAGGCCTAACACATGCAAGTCGAGGGGCAGCGCGGGGTAGCAATACCCTGGCGGCG
ACCGGCGCACGGGTGCGTAACGCGTATGCAACCTACCTCCGACAGGGGGATAAGCCAGGGAAACCTGG
TCTAATATCCCATATGGTCGCGGACCGCATGGTCCTGCGGCGAAAGCCCTGGTGGTCGGAGATGGGC
ATGCGTGGCATTAGATAGTTGGCGGGGCAACGGCCCACCAAGTCGACGATGCCTAGGGGAGCTGAGAG
GCTTATCCCCCACACTGGTACTGAGACACGGAC
>Zotu2898
CAACGCTGGCGGCGTGCCTAACACATGCAAGTCGAACGAGAAAGGGCTTTCGGGCCTGAGTAAAGTGG
CGCACGGGTGAGTAACACGTGACTAACCTACCCTTGAGCGGGGGATAACCTAGGGAAACCTGGGCTAA
TACCGCATAACACCTACGGGTCAAAGCGAGCAATCGCACTTAAGGAGGGGGTCGCGGCAGATTAGCTA
GTTGGTGAGGTAATGGCTCACCAAGGCGATGATCTGTATCCGGCCTGAGAGGGCGCACGGACACACTG
GAACTGAAACACGGTC
>Zotu2899
GAACGCTGGCTACAGGCTTAACACATGCAAGTCGAGGGGAAACGGCATTGAGTGCTTGCACTGAATGG
ACGTCGACCGGCGCACGGGTGAGTAACGCGTATCCAACCTTCCCGTTACTGCGGGATAACCTGCCGAA
AGGCAGACTAATACCGCATGTTCTTCGATGACGGCATCAGATTCGAAGCAAAGATTCTTCGGTAACGG
AGGGGGATGCGTCTGATTAGCTAGTTGGCGGGGCGACGGCCCACCAAGGCGACGATCAGTAGGGGTTC
TGAGAGGAAGGTCCCCCACATTGGAACTGAGACACGGTC
>Zotu2900
GAACGCTGGCGGCGTGCATAACACATTCAAGTCGAACGGTGATGAGGACTTCGGTCCTCTGATCAGTG
GCGGACGGGTGAGTAACGCGTGAGCAACCTGCCCTTGTGTGGGGGATAACGTCCGGAAACAGACGCTA
ATACCGCATAACGTATCGAGACCGCATGGTCATGATACCAAAGATTTATTGCGCAAGGATGGGCTCGC
GTTGGATTAGATAGTTGGTGGGGTAACGGCCTACCAAGTCAACGATCCATAGCCGGACTGAGAGGTTG
AACGGCCACATTGGAACTGAGAAACGGTC
>Zotu2901
GAACGCTGGCGGCAGGCTTAACACATGCAAGTCGAGCGAGATCTTCGGGTCTAGCGGCGGACGGGTGA
GTAACGCGTGGGAACGTGCCCTTCTCTACGGAATAGCCCCGGGAAACTGGGAGTAATACCGTATACGC
CCTTTGGGGGAAAGATTTATCGGAGAAGGATCGGCCCGCGTTGGATTAGGTAGTTGGTGGGGTAATGG
CCCACCAAGCCGACGATCCATAGCTGGTTTGAGAGGATGATCAGCCACACTGGGACTGAGACACGGCC
>Zotu2902
GAACGCTAGCGGCAGGCCTAATACATGCAAGTCGGACGGGATCCATCGGAGAGCTTGCTCGAGGATGG
TGAGAGTGGCGCACGGGTGCGTAACGCGTGAGCAACCTACCTCTATCAGGGGGATAGCCTCTCGAAAG
AGAGATTAACACCGCATAACATCAACAGTTCGCATGTTCGGTTGATTAAATATTTATAGGATAGAGAT
GGGCTCGCGTGACATTAGCTAGTTGGTAGGGTAACGGCTTACCAAGGCGACGATGTCTAGGGGCTCTG
AGAGGAGAATCCCCCACACTGGTACTGAGACACGGAC
>Zotu2903
GAACGCTGGCGGCGTGCTTAACACATGCAAGTCGAGCGGAAAGGCCTCTTCGGGGGTACTCGAGCGGC
GAACGGGTGAGTAACACGTGAGTAATCTGCCCTTGGCTTCGGGATAGCCACCGGAAACGGTGATTAAT
ACCGGATATGACCACTCCAGGCATCTGGTGGTGGTGGAAAGTTTTTCGGCCAGGGATGTGCTCGCGGC
CTATCAGCTTGATGGTGAGGTAATGGCTCACCATGGCTTCGACGGGTAGCCGGCCTGAGAGGGTGACC
GGTCACACTGGGACTGAGACACGGCC
>Zotu2904
GAACGCTGGCGGCATGCCTAACACATGCAAGTCGAACGAGATCTTCGGATCTAGTGGCGCACGGGTGC
GTAACGCGTGGGAATCTGCCCTTGGGTTCGGAATAACAGTGAGAAATTACTGCTAATACCGGATGATG
TCTTCGGACCAAAGATTTATCGCCCAAGGATGAGCCCGCGTAGGATTAGGTAGTTGGTGGGGTAATGG
CCTACCAAGCCGACGATCCTTAGCTGGTCTGAGAGGATGATCAGCCACACTGGGACTGAGACACGGCC
>Zotu2905
CAACGCTGGCGGCGTGCCTAATGCATGCAAGTCGAACGGGGGTTCCTTCGGGGACCTTACGTGGCGGA
CGGGTGAGGAACACGTGGCCAATCTGCCCACTGGCAGGGGGTAGCCGGCGGAAACGCCGGGTAATTCC
GTATACGCTCAGGGTCCGAGTGGGGATGCTGAGGAAAGGGTCTTCGGACTCGCCGGTGGAGGAGGCTG
CGGCCGATTAGCTAGTTGGTGGGGTAACGGCCTACCAAGGCGATGATCGGTAGCTGGTCTGAGAGGAT
GATCAGCCACACGGGGACTGAGACACGGCC
>Zotu2906
GAACGCTGGCGGCAGGCCTCAAACATGCAAGTCGAGCGCCCAGCAATGGGAGCGGCGGACGGGTGCGT
AACACGTGGGAATGTACCTATTGGTTCGGAACAACGCAGGGAAACTTGCGCTAATACCGAATGTGCCC

TTACGGGGAAAGATTCATCGCCAATAGATCAGCCCGCGTCCGATTAGCTAGTTGGTGAGGTAAAGGCT
CACCAAGGCGACGATCGGTAGCTGTTCTGAGAGGAAGATCAGCCACACTGGGACTGAGACACGGCC
>Zotu2907
GAATGCTGGCGGCGTGGATGAGTCATGCAAGTCGAACGGCCCGCAAGGGCAGTGGCAGACGAGGTAGT
AACACGTAGGTAGATACCCCAGAGTCGGGCATAGCCAGCCGAAAGGTTGGGTAATTCCCGATAGTCTT
GTGAAAGTAAAGGAGCAATCCGCTCTGGGAGTCGCCTGCGTAATATCAGCTAGTTGGTGAGGTAATGG
CTCACCAAGGCTACGACGTTTAGGGGAGCTGAGAGGCTGACCCCCACCGATGGGACTGAGATACGGCC
>Zotu2908
GAACGCTGGCGGCGTGCCTAACACATGCAAGTCGAGCGAACCCTTCGGGGTGAGCGGCGGACGGGTGA
GTAACGCGTGGGTAACCTGCCCTGTACACACGGATAACATACCGAAAGGTATGCTAATACGAGATAAT
ATATTTTAATCGCATGGTTAAGATATCAAAGCTCCGGCGGTACAGGATGGACCCGCGTCTGATTAGCT
AGTTGGTGAGGTAATGGCTCACCAAGGCGACGATCAGTAGCCGACCTGAGAGGGTGATCGGCCACACT
GGAACTGAGACACGGTC
>Zotu2909
GAACGCTAGCTACAGGCTTAACACATGCAAGTCGAGGGGCAGCATGGTCTTAGCTTGCTAAGGCCGAT
GGCGACCGGCGCACGGGTGAGTAACACGTATCCAACCTGCCGTCTACTCTTGGACAGCCTTCTGAAAG
GAAGATTAATACAAGATGGCATCATGAGTCCGCATGTTCACATGATTAAAGGTATTCCGGTAGACGAT
GGGGATGCGTTCCATTAGATAGTAGGCGGGGTAACGGCCCACCTAGTCTTCGATGGATAGGGGTTCTG
AGAGGAAGGTCCCCCACATTGGAACTGAGACACGGTC
>Zotu2913
GAACGCTGGTGGTATGCTTAACACATGCAAGTCGAACGGGCTTGGAGGGGCAACTCTTTAAGTCAGTG
GCAAACGGGTGAGGAATATATAGGAATCTCCCCAACAGTAAGGAATAACATTGGGAAACCAATGCTAA
TGCCATATATTACCGATTTATCGGGAAAGATTTATCGCTGTTGGATGAGCCTTTATCGGATTAGCTAG
TTGGTAGGGTAATGGCCTACCAAGGCAATGATCCGTAGCTGGTCTGAGAGGATGATCAGCCACATTGG
GACTGCGACACGGCC
>Zotu2914
CAACGCTGGCGGCGCGCTTAACACATGCAAGTCGAGCGAGAAAGGGGAGCAATCCCTGAGTAAAGCGG
CGTACGGGTGAGTAACACGTGGGTAATCTACCTTTTAGTGGGGAATAACCCTGGGAAACCGGGGCTAA
TACCGCATAATCCTGTGAAGGGAAAGCAGTAATGCGCTGAAAGAGGAGCCCGCGGCCGATTAGCTAGT
TGGTGAGGTAAAGGCTCACCAAGGCGGTGATCGGTAGCCGGCCTGAGAGGGCACACGGCCACACTGGC
ACTGAAACACGGGC
>Zotu2915
GAACGCTGGCGGCAGGCTTAACACATGCAAGTCGAACGGGCTTCTTCGGAAGTCAGTGGCAGACGGGT
GAGTAACACGTGGGAACGTGCCCTTCGGTTCGGAATAACTCAGGGAAACTTGAGCTAATACCGGATAC
GCCCTTTTGGGGAAAGGTTTACTGCCGAAGGATCGGCCCGCGTCTGATTAGCTTGTTGGTGGGGTAAC
GGCCTACCAAGGCGACGATCAGTAGCTGGTCTGAGAGGATGATCAGCCACACTGGGACTGAGACACGG
CC
>Zotu2916
GAACGCTAGCGGCAGGCTTAATACATGCAAGGCGAGGGGGCAGCAATGTCACCGTCGTACGGGTGCGT
AACGCGTATGCAACCTACCTTCAACTGGGGGATAGCCCGGGGAAACCCGGATTAATACCGCATGACAC
ATTTGGATCACATGGTTTGGATTGTTAAAGATTTATTGGTTGAAGATGGGCATGCGTTCGATTAGCTA
GTTGGCAGGGTAACGGCCTACCAAGGCGACGATCGATAGGGGAGCTGAGAGGTTGATCCCCCACACGG
GCACTGAGATACGGGC
>Zotu2917
GAACGCTGGCGGCAGGCTTAACACATGCAAGTCGAGCGGGCACTTCGGTGTCAGCGGCAGACGGGTGA
GTAACACGTGGGAACGTACCCTTCGGTTCGGAATAACGCTGGGAAACTAGCGCTAATACCGGATACGC
CCTTATGGGGAAAGGTTTACTGCCGAAGGATCGGCCCGCGTCTGATTAGCTAGTTGGTGGGGTAATGG
CCTACCAAGGCGACGATCAGTAGCTGGTCTGAGAGGATGATCAGCCACACTGGGACTGAGACACGGCC
>Zotu2919
GAACGCTGGCGGCAGGCTTAACACATGCAAGTCGAGCGGGGTGATGTAGCTTGCTACATTACCTAGCG
GCGGACGGGTGAGTAATGCTTAGGAATCTGCCTATTAGTGGGGGACAACATTCCGAAAGGAATGCTAA
TACCGCATACGCCCTACGGGGGAAAGCAGGGGATCTTCGGACCTTGCGCTAATAGATGAGCCTAAGTC
AGATTAGCTAGTTGGTGGGGTAAAGGCCTACCAAGGCGACGATCTGTAGCGGGTCTGAGAGGATGATC
CGCCACACTGGGACTGAGACACGGCC

>Zotu2920
GAACGCTGGCGGTATGCTTAACACATGCAAGTCGAACGGGGTGCTTCGGCACCTAGTGGCGGACGGGT
GAGTAACGCGTGAGAATCTGACTCGAGGACGGGGACAACCACTGGAAACGGTGGCTAAGACCCGATAT
GCCGAGAGGTGAAAATAATTTAGCCTTGAGAGGAGCTCGCGTCTGATTAGCTAGTTGGTGGGGTAAGA
GCCTACCAAGGCGACGATCAGTAGCTGGTCTGAGAGGATGATCAGCCACACTGGGACTGAGACACGGC
C
>Zotu2921
GAACGCTGGCGGCAGGCCTAACACATGCAAGTCGAACGGTAGCACAGAGGAGCTTGCTCCTTGGGTGA
CGAGTGGCGGACGGGTGAGTAATGTCTGGGAAACTGCCTGATGGAGGGGGATAACTACTGGAAACGGT
AGCTAATACCGCATAACGTCGCAAGACCAAAGAGGGGGACCTTCGGGCCTCTTGCCATCAGATGTGCC
CAGATGGGATTAGCTAGTAGGTGGGGTAACGGCTCACCTAGGCGACGATCCCTAGCTGGTCTGAGAGG
ATGACCAGCCACACTGGAACTGAGACACGGTC
>Zotu2922
GAACGCTGGCGGCACGCTTAAAACATGCAAGTCGAACGTGAAACCCGGGCTTGCCTGGGTGGAAAGTG
GCGAACGGGTGCGTAACACGTGAGCAACCTGCCCCGAAGACTGGGATAACACCGGGAAACCGGTGCTA
ATACCGGATGCCTTCACCACGTCGCATGGCGAGGTGAAGAAATGGATTCCGCTTCGGGATGGGCTCGC
GGCCTATCAGCTTGTTGGTGGGGTAACGGCCTACCAAGGCAACGACGGGTAGCTGGTCTGAGAGGATG
TCCAGCCACACTGGGACTGAGACACGGCC
>Zotu2923
GAACGCTGGCGAGATGCTTTACACATGCAAGTCGTACGAGAGTTTTTAACTCAAGTGGCGGACGGGTG
AGTAACACGTAAGAATTTGCCTTTAGGAGGGGGACAACAATTGGAAACGATTGCTAATACCCCATATG
CTTTCGAGTGAAATGGATTTTTCCGCCTAAAGAGAAGCTTGCGGCTGATTAGCTTGTTGGTAAGGTAA
TGGCTTACCAAGGCGACGATCAGTATCTGGTTTGAGAGGACGATCAGACACACTGGAACTGAGACACG
GTC
>Zotu2926
GAACGCTGGCGGCGCGCTTAACACATGCAAGTCGAGCGATGAAATCTTGACAGATCCCTTCGGGGTGA
AGATAAGATGGATTAGCGGCGGACGGGTGAGTAACACGTGAGTAACCTGCCTTAGACATTGGGATAGC
CTCGGGAAACCGGGATTAATACCAAATGAAATCATAGGTGCCCATGCACTAATGATCAAAACTCCGGT
GGTCTAAGATGGACTCGCGTCCCATTAGCTAGTTGGTGAGGTAACGGCCCACCAAGGCGACGATGGGT
AGCCGGCCTGAGAGGGTGAACGGCCACATTGGAACTGAGAAACGGTC
>Zotu2927
GAACGCTGGCGGCGTGCTTAACACATGCAAGTCGAACGATGACCCCGTGCTTGCACGGGTGATTAGTG
GCGAACGGGTGAGTAACACGTGAGTAACCTGCCCCTGACTCTGGGATAAGCCCGGGAAACTGGGTCTA
ATACTGGATATGACCTTTGACCGCATGGTTTTTGGTGGAAAGATTTATCGGTTGGGGATGGACTCGCG
GCCTATCAGCTTGTTGGTGAGGTAATGGCTCACCAAGGCGACGACGGGTAGCCGGCCTGAGAGGGTGA
CCGGCCACACTGGGACTGAGACACGGCC
>Zotu2928
GAACGCTGGCGGCGTGCTTAACACATGCAAGTCGAGGGGGCGGGAGGGGCAACCCTGTCGTCACCGGC
GAACGGGTGCGTAACACGTGAGCGACCTACCTTTCTGCGGGGGATAGCCGGCCCAACGGCCGGGTAAT
ACCGCATACGCTCTTCTTCGGGCATCCGGGGGGGAGGAAACTTTCGAGGCAGAGAGAGGGGCTCGCGG
CCTATCAGCTAGTTGGCGGGGTAACGGCCCACCAAGGCAGTGACGGGTAGCTGGTCTGAGAGGATGGC
CAGCCACATTGGGACTGAGAGACGGCC
>Zotu2929
GAATGCTGGCGGCGTGCCTAATACATGCAAGTCGTACGGAAAGGCCCTTCGGGGTACTCGAGTGGCGG
ACGGCTGAGTAACGCGTGGGAACGTGCCCCAAAGTGAGGGATAACGCACCGAAAGGTGTGCTAATACC
GCATATGATCTTCGGATTAAAGCTTCGGCGCTTTGGGAGCGGCCCGCGTACGATTAGATAGTTGGTGA
GGTAATGGCTCACCAAGTCGACGATCGTTAGCTGGTCTGAGAGGATGATCAGCCAGACTGGAACTGAG
ACACGGTC
>Zotu2930
GAACGCTAGCGGTATGCTTAACACATGCAAGTCGTACGGAAAAGCAATTTTTAGTGGCGAACGGGTGA
GTAACGCGTGAGAATTTGCCTTTAGGAAGAGGATAACAATTGGAAACAGTTGCTAAAACTCTATATGC
CGTTTACGGTGAAAAAAGTAATTTGCCTAAAGAGAAGCTCGCGTCTGATTAGCTAATTGGTGGGATAA
AGGCCTACCAAGGCGACGATCAGTAGCTGGTCTGAGGACGATCAGCCACACTGGAACTGAGACACG
GTC

>Zotu2931
GAACGCTGGCGGCGTGCTTAACACATGCAAGTCGAACGATGAAGCTGTGCTTGCACAGTGGATTAGTG
GCGAACGGGTGAGTAACACGTGAGTAACCTGCCCTTCACTTTGGGATAACTCCGAGAAATCGGGGCTA
ATACTGGATATGACTACTGGCCGCATGGTTGGTGGTGGAAAGTTTTTCGGTGAGGGATGGGCTCGCGG
CCTATCAGTTTGTTGGTGAGGTAGTGGCTCACCAAGGCGATGACGGGTAGCCGGCCTGAGAGGGCGAC
CGGCCACACTGGGACTGAGACACGGCC
>Zotu2932
GAACGCTGGCGGCGTGCCTAACACATGCAAGTCGAACGGAGTCAATTGAGGAAGTTTTTGGATGGAAT
CAGCTGACTTAGTGGCGGACGGGTGAGTAACGCGTGGGAAACCTGCCCTGTACTGGGGGATAACACTT
AGAAATAGGTGCTAAAACCGCATAAGCGCACGGGATCGCATGGTCTTGTGTGAAAAACTCCGGTGGTA
CAGGATGGTCCCGCGTCTGATTAGCCAGTTGGCAGGGTAACGGCCTACCAAAGCGACGATCAGTAGCC
GGCCTGAGAGGGTGGACGGCCACATTGGGACTGAGACACGGCC
>Zotu2934
GAACGCTGGCGGCGTGCCTAATACATGCAAGTCGAGCGAATGACGAAGAAGCTTGCTTCTTCTGATTT
AGCGGCGGACGGGTGAGTAACACGTGGGCAACCTACCCTATAGCTTGGGATAACTCCGGGAAACCGGG
GCTAATACCGAATAATACATTTCTTCACATGTTGAAATGTTGAAAGATGGTTTCGGCTATTACTATAG
GATGGGCCCGCGGCGCATTAGCTAGTAGGTAAGGTAACGGCTTACCTAGGCGACGATGCGTAGCCGAC
CTGAGAGGGTGATCGGCCACACTGGGACTGAGACACGGCC
>Zotu2935
GAACGCTGGCGGCGTGCCTAATACATGCAAGTCGAGCGAACCAATGGGAGCTTGCTCCCTGAGGTTAG
CGGCGGACGGGTGAGTAACACGTGGGTAACCTGCCTGTAAGATTGGGATAACTCCGGGAAACCGGAGC
TAATACCGGATAACATTTCGAACCGCATGGTTCGAAATTGAAAGATGGCTTCGGCTATCACTTACAGA
TGGACCCGCGGCGCATTAGCTAGTTGGTGAGGTAACGGCTCACCAAGGCAACGATGCGTAGCCGACCT
GAGAGGGTGATCGGCCACACTGGGACTGAGACACGGCC
>Zotu2936
GAACGCTGGCGGCGTGCCTAATACATGCAAGTCGAGCGCGGGAAGCAAACTGAAACCTTCGGGTGGAC
GTTTGTGGAACGAGCGGCGGATGGGTGAGTAACACGTGGGTAACCTGCCTGTAAGCTTGGGATAACTT
GCGGAAACGTGAGCTAATACCAAATAATGCTTATTACCTCCTGGTAACAAGCTGAAAGGCGGCATTTA
TATGCTGTCACTTACAGATGGACCCGTGGCGCATTAGCTAGTTGGTGGGATAAAAGCCTACCAAGGCG
ACGATGCGTAGCCGACCTGAGAGGGTGATCGGCCACACTGGGACTGAGACACGGCC
>Zotu2937
GAACGCTAGCGGCAGGCTTAACACATGCAAGTCGAGGGGTAGAAGGAGCTTGCTCCTTTGAGACCGGC
GCACGGGTGCGTAACGCGTATGCAATCTACCTTTTACAGAGGGATAGCCCAGAGAAATTTGGATTAAT
ACCTCATAGTATTATGAAATGGCATCATTTTATAATTAAAGTTCCAACGGTAAAAGATGAGCATGCGT
CCCATTAGCTAGTTGGTGTGGTAACGGCACACCAAGGCAACGATGGGTAGGGGTCCTGAGAGGGAGAT
CCCCCACACTGGTACTGAGACACGGAC
>Zotu2938
GAACGCTGGCGGCGTGCCTAATACATGCAAGTCGAGCGAACAAATTAAAAGCTTGCTTTTGATTTGTT
AGCGGCGGACGGGTGAGTAACACGTGGGCAACCTACCTATAAGACTGGGATAACTTGCGGAAACGTGA
GCTAATACCAGATAGTTTTTCTTCCTGCATGGGAGGGAAAGGAAAGATGGTTTCGGCTATCACTTATA
GATGGGCCCGCGGCGCATTAGCTAGTTGGTGAGGTAATGGCTCACCAAGGCAACGATGCGTAGCCGAC
CTGAGAGGGTGATCGGCCACACTGGGACTGAGACACGGCC
>Zotu2940
GAACGCTGGCGGCGTGCCTAATACATGCAAGTAGAACGCTGAAGCTTGGTGCTTGCACCGAGCGGATG
AGTTGCGAACGGGTGAGTAACGCGTAGGTAACCTGCCTGGTAGCGGGGGATAACTATTGGAAACGATA
GCTAATACCGCATGACAACAGTTATTGCATGATAACTGTTTAAAAGGGGCAACTGCTCCACTACCAGA
TGGACCTGCGTTGTATTAGCTAGTTGGTGAGGTAACGGCTCACCAAGGCGACGATACATAGCCGACCT
GAGAGGGTGATCGGCCACACTGGGACTGAGACACGGCC
>Zotu2941
GAACGCTGGCGGCATGCCTTACACATGCAAGTCGAACGGCAGCGCGGGGCAACCTGGCGGCGAGTGGC
GAACGGGTGAGTAATATATCGGAACGTACCCTGGAGTGGGGGATAACGTAGCGAAAGTTACGCTAATA
CCGCATACGATCTAAGGATGAAAGTGGGGGCTTCGCAAGAACCTCATGCTCCTGGAGCGGCCGATATC
TGATTAGCTAGTTGGTGAGGTAAAGGCTCACCAAGGCTTCGATCAGTAGCTGGTCTGAGAGGACGACC
AGCCACACTGGAACTGAGACACGGTC

>Zotu2942
GAACGCTGGCGGCGTGCCTAATACATGCAAGTCGAGCGGACAGACGAGGTGCTTGCACCTCTGAAGTC
AGCGGCGGACGGGTGAGTAACACGTGGGTAACCTACCTGTAAGACTGGGATAACTTCGGGAAACCGGA
GCTAATACCGGATAATATTTTCCACCTCATGGTGGAATAGTGAAAGACGGTTTTGCTGTCACTTACAG
ATGGACCCGCGGCGCATTAGCTAGTTGGTGAGGTAACGGCTCACCAAGGCGACGATGCGTAGCCGACC
TGAGAGGGTGATCGGCCACACTGGGACTGAGACACGGCC
>Zotu2943
GAACGCTAGCGGGAGGCTTAACACATGCAAGTCGAGGGGCAGCACGAGGTAGCAATACTTTGGTGGCG
ACCGGCGCACGGGTGAGTAACGCGTATGTAACCTACCTTGTACAGGGAGATAGCCCGGAGAAATTCGG
ATTAATACCCCATAATGATATTTAGAGGCATCTTTGAATATTTAAAGTTTCGACGGTACGAGATGGGC
ATGCGTGACATTAGCTAGTTGGTAAGGTAACGGCTTACCAAGGCTTCGATGTCTAGGGGTCCTGAGAG
GGTGATCCCCCACACTGGTACTGAGACACGGAC
>Zotu2945
GAACGCTGGCGGCGTGCCTAACACATGCAAGTCGAGCGGTAACAGGCGTAGCAATACGTGCTGACGAG
CGGCGAACGGGTGAGCAATGCTTCGGAATCTGCCCAATGATGGGGAATAACCAGTCGAAAGATTGGCT
AATACCGCATACGCCCTTCGGGGGAAAGCAGGGGATCGCAAGACCTTGCGTCGATGGATGAGCCGAAG
TCGGATTAGCTAGTTGGTAGGGTAACGGCCTACCAAGGCGACGATCCGTAGCTGGTCTGAGAGGACGA
CCAGCCACACTGGGACTGAGACACGGCC
>Zotu2946
AAACGCTGGCGGCGCACATAAGACATGCAAGTCGAACGAACTTAACCATTAGTTTACTATTGGAGCGG
TTAGTGGCGGACTGGTGAGTAACACGTAAGCAACCTGCCTATCAGAGGGGAACAACAGTTAGAAATGA
CTGCTAATACCGCATATGCCTTGAGTATCACATGGTACAAAAGGGAAAGGAGCAATCCGCTGATAGAT
GGGCTTGCGTCTGATTAGATAGTTGGTGAGGTAACGGCCCACCAAGTCGACGATCAGTAGCCGGACTG
AGGGGTTGAACGGCCACATTGGGACTGAGATACGGCC
>Zotu2947
GAACGCTGGCGGCATGCCTAACACATGCAAGTCGAACGGGCACCTTCGGGTGCTAGTGGCGCACGGGT
GCGTAACGCGTGGGAATCTGCCCTTGGGTTCGGAATAACTCAGCGAAAGCTGTGCTAATACCGGATGA
TGACGTAAGTCCAAAGATTTATCGCCCGAGGATGAGCCCGCGTTGGATTAGCTAGTTGGTGGGGTAAA
GGCCTACCAAGGCGACGATCCATAGCTGGTCTGAGAGGATGATCAGCCACACTGGGACTGAGACACGG
CC
>Zotu2949
GAACGCTGGCGGCAGGCTTAACACATGCAAGTCGAACGGTAACATAAAGAAGCTTGCTTCTTTGATGA
CGAGTGGCGGACGGGTGAGTAATGCTTGGGAATCTAGCTTATGGAGGGGGATAACTACGGGAAACTGT
AGCTAATACCGCGTAATATCGAAAGATTAAAGTGTGGGACCTTTCGGGCCACATGCCATAGGATGAGC
CCAAGTGGGATTAGGTAGTTGGTGAGGTAAAGGCTCACCAAGCCGACGATCTCTAGCTGGTCTGAGAG
GATGACCAGCCACACTGGGACTGAGACACGGCC
>Zotu2950
GAACGCTGGCGGCGTGCCTAATACATGCAAGTCGAGCGAATTTTTGGGAGCTTGCTCCCGAAAATTAG
CGGCGGACGGGTGAGTAACACGTGGGCAACCTGCCTATAAGACTGGGATAACTTCGGGAAACCGGAGC
TAATACCGGATAATTCTTTTCCTCTCATGAGGAAAAGCTGAAAGACGGTTTCGGCTGTCACTTATAGA
TGGGCCCGCGGCGCATTAGCTAGTTGGTGAGGTAACGGCTCACCAAGGCAACGATGCGTAGCCGACCT
GAGAGGGTGATCGGCCACACTGGGACTGAGACACGGCC
>Zotu2952
GAACGCTGGCGGCAGGCTTAACACATGCAAGTCGAACGCCCCGCAAGGGGAGTGGCAGACGGGTGAGT
AACGCGTGGGAATCTACCTAGTTCTACGGAACAACAGTTGGAAACGACTGCTAATACCGTATACGCCC
TATGGGGGAAAGATTTATCGGAATTAGATGAGCCCGCGTAAGATTAGCTAGTTGGTGGGGTAATGGCC
TACCAAGGCGACGATCTTTAGCTGGTCTGAGAGGATGATCAGCCACACTGGGACTGAGACACGGCC
>Zotu2953
CAACGCTGGCGGCGTGCCTAACACATGCAAGTCGAACGAGAAAGTGGGGGCAACCCTGCGAGTACAGT
GGCAGACGGGTGAGTAACACGTGAACAACCTACCCTTGAGTGGGGAATAACACCGCGAAAGCGGTGCT
AATACCGCATAACATCTTTGGGTCGCATGACCTGGGGATCAAAAGCGCAAGTTGCTTGAGGAGGGGTT
CGCGGCTGATTAGCTAGTTGGTGAGGTAACGGCCCACCAAGGCAACGATCAGTAGCCGGCCTGAGAGG
GCGAACGGCCACACTGGAACTGAGACACGGTC
>Zotu2954

GAACGCTGGCGGCGTGCTTAACACATGCAAGTCGAACGGTAAGGCTCCAGCTTGCTGGGGTACACGAG
TGGCGAACGGGTGAGTAACACGTGGGTGACCTGCCCTGCACTTCGGGATAAGCCTGGGAAACTGGGTC
TAATACCGGATAGGACCGCACCGTGAGGGTGTGGTGGAAAGTTTTTTCGGTGTGGGATGGGCCCGCGG
CCTATCAGCTTGTTGGTGGGGTAATGGCCTACCAAGGCGGCGACGGGTAGCCGGCCTGAGAGGGTGGA
CGGCCACATTGGGACTGAGACACGGCC
>Zotu2956
GAACGCTGGCGGCGTGCCTAACACATGCAAGTCGCGCGAGAAAGTCCTTCGGGATGAGTAGAGCGGCG
CACGGGTGAGTAATACATAGGAACGTACCTTTCGGTGGGGGATAACCACGGGAAACTGTGGCTAATAC
CGCATAAAGACGTGAGTCTAAAGCAGGCCTCTATTTATAAGCTTGTGCCGGTAGAGCGGCCTATGTCC
CATTAGCTAGTTGGTAGGGTAATGGCCTACCAAGGCGACGATGGGTAGCTGGTCTGAGAGGATGATCA
GCCACACTGGGACTGAGACACGGCC
>Zotu2957
GAACGCTGGCGGCGTGCCTAATACATGCAAGTCGAGCGGACTTGATGGAGAGCTTGCTCTCCTGATGG
TTAGCGGCGGACGGGTGAGTAACACGTAGGCAACCTGCCTGCAAGACCGGGATAACCCACGGAAACGT
GAGCTAATACCGGATATCTCATTTCCTCTCCTGAGGGGATGATGAAAGACGGAGCAATCTGTCGCTTG
CGGATGGGCCTGCGGCGCATTAGCTAGTTGGTGAGGTAACGGCTCACCAAGGCGACGATGCGTAGCCG
ACCTGAGAGGGTGAACGGCCACACTGGGACTGAGACACGGCC
>Zotu2959
GAACGCTGGCGGCGGGCCTCATACATGCAAGTCGAGCGCGAAAGTCCGCAAGGATGAGTAAAGCGGCG
CACGGGTGAGTAACACGTGGGTAATCTACCTTTTGGATTGGAATAACCTGTCGAAAGACGGGCTAATG
CCGAGTAAGCTTTCTTGAACGTGAAGTTCGAGCAAGGAAAGATGGCGCAAGCTATCGCCGAAAGATGA
GCCTGCGTCCCATTAGTTAGTTGGTGGGGTAATGGCTTACCAAGACAATGATGGGTACCTGGTTTGAG
AGAATGATCAGGCACACTGGAACTGAAACACGGTC
>Zotu2960
GAACGCTGGCGGCGTGCTTAACACATGCAAGTCGAGCGATGAAGTTTCCTTCGGGAAACGGATTAGCG
GCGGACGGGTGAGTAACACGTGGGTAACCTGCCTCATAGAGTGGAATAGCCTTCCGAAAGGAAGATTA
ATACCGCATAACGTTGAAAGATGGCATCATCATTCAACCAAAGGAGCAATCCGCTATGAGATGGACCC
GCGGCGCATTAGCTAGTTGGTGGGGTAACGGCCTACCAAGGCGACGATGCGTAGCCGACCTGAGAGGG
TGATCGGCCACATTGGGACTGAGACACGGCC
>Zotu2963
GAACGCTTGCGGCAGGCTTAATACATGCAAGTCGGACGGTAACAGGGTCTTCGGACTGCTGACGAGTG
GCGGATGGGTGAGTAACGCGTATGCAACCTTCCTCACACCGAGAAAAAGTCGACTGAAAAGTTGAATA
AAGTCTCGTGGTGTGCATTTCTCGCATGAGATGTGTATGATAGAAGCAATTTGGTGTGAGATGGGCAT
GCGTATCATTAGCTAGTTGGTAGGGTAACGGCTTACCAAGGCGACGATGATTAGGGGGCCTGAGAGGG
TGGTCCCCCACACTGGGACTGAGACACGGCC
>Zotu2964
GAACGCTGGCGGCATGCCTTACACATGCAAGTCGAACGGCAGCACGGGTGCTTGCACCTGGTGGCGAG
TGGCGAACGGGTGAGTAATACATCGGAACGTACCTTATCGTGGGGGATAACGCAGCGAAAGCTGTGCT
AATACCGCATACGCCCTGAGGGGGAAAGCGGGGGATCGCAAGACCTCGCGCGATTAGAGCGGCCGATG
CCTGATTAGCTAGTTGGTGGGGTAAAAGCCCACCAAGGCGACGATCAGTAGCTGGTCTGAGAGGACGA
TCAGCCACACTGGGACTGAGACACGGCC
>Zotu2965
GAACGCTGGCGGCGCGCCTAACACATGCAAGTCGAGCGCTCACCTTCGGGTGGGAGCGGCGGACGGGT
GAGTAACGCGTGGGAACCTGCCCTGGAGTTCGGGACAACCGCGGGAAACTGCGGCTGACACCGGATAC
GCCCCTTTGGGGGGAAAGTTTACGCTCTGGGATGGGCCCGCGTCCGATTAGGTAGTTGGTGGGGTAAT
GGCCTACCAAGCCAATGATCGGTAGCCGGTCTGAGAGGACGATCGGCCACACTGGGACTGAGACACGG
CC
>Zotu2966
GAACGCTGGCGGCGTGCCTAATACATGCAAGTCGAGCGGAGTTATTCCTTCGGGGATAACTTAGCGGC
GGACGGGTGAGTAACACGTAGGCAACCTGCCTGTAAGACTGGGATAACTACCGGAAACGGTAGCTAAT
ACCGGATATGCAAAAGAGTCGCATGACACTTTTGAGAAAGACGGAGTAATCTGTCACTTACAGATGGG
CCTGCGGCGCATTAGCTAGTTGGTGAGGTAATGGCTCACCAAGGCGACGATGCGTAGCCGACCTGAGA
GGGTGAACGGCCACACTGGGACTGAGACACGGCC
>Zotu2967

GAACGCTGGCGGCAGGCCTAACACATGCAAGTCGAACGGATCCTTCGGGATTAGTGGCGGACGGGTGA
GTAACACGTGGGAACGTGCCCTTTGGTTCGGAACAACTCAGGGAAACTTGAGCTAATACCGGATGTGC
CCTTCGGGGGAAAGATTTATCGCCATTGGAGCGGCCCGCGTCTGATTAGCTAGTTGGTGAGGTAAAGG
CTCACCAAGGCGACGATCAGTAGCTGGTCTGAGAGGATGATCAGCCACATTGGGACTGAGACACGGCC
>Zotu2968
GAACGCTAGCGGGAGGCTTAATACATGCAAGTCGAACGGCAGCAGGTCCTTCGGGACGCTGGCGAGTG
GCGAACGGGTGAGTAACGCGTACACGATCTGCCCATAAGTCTGGGATAGTCACTGGAAACGGTGAATA
ATACCGGATGTGGATAAATTGCTTCATGGTAATTTATTTAAAGGCTGGTCTGCTTATGGATGAGTGTG
CGTCCTATTAGTTAGTTGGTGAGGTAACGGCTCACCAAGACGATGATAGGTAGGGGGCGTGAGAGCGT
GGACCCCCACACGGGTACTGAGACACGGAC
>Zotu2969
GAACGCTGGCGGCGTGCTTAACACATGCAAGTCGAACGGTGAAGGAGAGCTTGCTCTTTGGATCAGTG
GCGAACGGGTGAGTAACACGTGAGCAATCTGCCCTTGACTCTGGGATAAGCGTTGGAAACGACGTCTA
ATACCGGATACGAGCTGTGACCGCATGGTCAACAGTTGGAAAGAACTTCGGTCAAGGATGAGCTCGCG
GCCTATCAGCTTGTTGGTGAGGTAATGGCTCACCAAGGCGTCGACGGGTAGCCGGCCTGAGAGGGTGA
CCGGCCACACTGGGACTGAGACACGGCC
>Zotu2970
GAATGCTGGCGGCGTGCCTAACACATGCAAGTCGAGCGGTAACATGAATAATTTATTATTTGATGACG
AGCGGCGGACGGCTGAGTAACGCGTGGGAACATGCCCCAAAGTGAGGGATAAGCTCCAGAAATGGAGT
CTAATACCGCATATGATCTTCGGATTAAAGGATTTATCCGCTTTGGGAGTGGCCTGCGTACGATTAGG
TAGTTGGTGAGGTAATGGCTCACCAAGCCTACGATCGTTAGCTGGTCTGAGAGGATGACCAGCCAGAC
TGGAACTGAGACACGGTC
>Zotu2971
GAACGTTAGCGGCGCGCTTAACACATGCAAGTCGAACGAGAAAGGGCTTCGGCCCCGGTAAAGTGGCG
CACGGGTGAGTAACACGTGGGTAAACTTCCCTTGAGCGGGGGATAACATTCCGAAAGGAGTGCTAATA
CCGCATAAGACCACGGAGTCGAAAGATTCTGAGGTAAAAGCAGGCCTCTATACATAAGCTTGCACTCG
AGGATGTGCCCGCGGCCCATCAGGTAGTTGGTAGGGTAATGGCCTACCAAGCCAAAGACGGGTAGCTG
GTCTGAGAGGATGATCAGCCACACTGGAACTGAGACACGGTC
>Zotu2972
GAACGCTGGCGGCGTGCTTAACACATGCAAGTCAAGGGGGCCCGCAAGGGCAACCGGCGAACGGGTGC
GTAACACGTGAGCAAGCTTCCGATTTGTGGGGGATAGCCGGCCCAACGGCCGGGTAATACCGCATACG
TTCACCGGACGGCATCGTCTGGTGAGGAAACCTCCGGGGCAAATCGAGGCGCTCGCGGCCTATCAGCT
AGTTGGCGGGGTAATGGCCCACCAAGGCTACGACGGGTAGCTGGTCTGAGAGGATGGCCAGCCACATT
GGGACTGCGACACGGCC
>Zotu2973
GAACGTTAGCGGCGCGCCTAATACATGCAAGTCGTGCGAGAAAGGGCTTCGGCCCCGGTACAGCGGCG
CACGGGTGAGTAACACGTAGGTAATCTTCCCTCGAATGGTGGATAACCCTCCGAAAGGAGAGCTAATA
CAGCATAAGACCACGACCTCGTTGAGGTTGCGGCCAAAGCAGGCCTCTACTTGTAAGCTTGCGTTTGA
GGATGAGCCTGCGGCCCATCAGCTAGTTGGTAGGGTAATGGCCTACCAAGGCTAAGACGGGTAGCTGG
TCTGAGAGGATGAACAGCCACACTGGAACTGAGACACGGTC
>Zotu2974
GAACGCTGGCGGCAGGCTTAACACATGCAAGTCGAGCGCCCGCAAGGGAGCGGCAGACGGGTGAGTAA
CGCGTGGGAATCTACCCAGTTCTACGGAACAACTGAGGGAAACTTCAGCTAATACCGTATACGCCCTA
CGGGGGAAAGATTTATCGGAATTGGATGAGCCCGCGTTGGATTAGCTAGTTGGTGAGGTAACGGCTCA
CCAAGGCGACGATCCATAGCTGGTCTGAGAGGATGATCAGCCACACTGGGACTGAGACACGGCC
>Zotu2975
GAACGCTGGCGGCGTGCCTAATACATGCAAGTCGAGCGAACCAATAAGAAGCTTGCTTTTTGTTGGTT
AGCGGCGGACGGGTGAGTAACACGTGGGTAACCTGCCTGTAAGACCGGGATAACTCCGGGAAACCGGT
GCTAATACCGGATAGATTATCTTTCCGCCTGGAGAGATAAGGAAAGATGGCTTTCGCCATCACTTACA
GATGGGCCCGCGGCGCATTAGCTAGTTGGTGAGGTAACGGCTCACCAAGGCGACGATGCGTAGCCGAC
CTGAGAGGGTGATCGGCCACACTGGGACTGAGACACGGCC
>Zotu2977
GAACGCTGGCGGCGTGCTTAACACATGCAAGTCGAACGGTGAACCTGCTTCGGTGGGGGATCAGTGGC
GAACGGGTGAGTAACACGTGGGCAACCTGCCCTCAGCTCTGGGATAACTCCAAGAAATTGGGGCTAAT

ACCGGATATGACTGCTGACCGCATGGTCTGGTGGTGGAAAGATTTATCGGCTGGGGATGGGCCCGCGG
CCTATCAGCTTGTTGGTGGGGTAGTGGCCTACCAAGGCGACGACGGGTAGCCGGCCTGAGAGGGTGAC
CGGCCACACTGGGACTGAGACACGGCC
>Zotu2978
GAACGTTGGCGGCGTGTCTAAGGCATGCAAGTCGAGGGACAGATCTAAGGGTAGCAATATTTTTAGGT
ACTGACCGGCAGACGGGTGCGTAACACATTGGTAACCTGCCTTCGAGACGGGGATAGCTTTCCGAAAG
GAAAATTAATACCCGATGGTCATGAGGGGTCGTATGACATTCTCATGTAAATGATTTATCGCTCGAAG
AGGGGCCTATGGCCTATCAGCTAGTTGGTGAGGTAATGGCTCACCAAGGCTATGACGGGTAGGGGATG
TGAGAGCATGATCCCCCTCACTGGGACTGAGACACTGCC
>Zotu2979
GAACGCTGGCGGCGTGCCTAATACATGCAAGTCGAGCGGACCTGAATGGAGCTTGCTCCAGACAGGTT
AGCGGCGGACGGGTGAGTAACACGTAGGCAACCTGCCCGTAAGACCGGGATAACGTAGGGAAACTTAC
GCTAATACCGGATAGGGCACTCTCTCGCATGAGAGGGTGCGGAAAGGTGGCGCAAGCTACCACTTGCG
GATGGGCCTGCGGCGCATTAGCTAGTTGGTGGGGTAACGGCCTACCAAGGCGACGATGCGTAGCCGAC
CTGAGAGGGTGACCGGCCACACTGGGACTGAGACACGGCC
>Zotu2980
GAACGCTGGCGGCGTGCTTAACACATGCAAGTCGAACGGAAAGGCCTCTTGCTTGCAGGAGGTACTCG
AGTGGCGAACGGGTGAGTAACACGTGGGTGATCTGCCTTGCACTCTGGGATAAGCTTGGGAAACTGGG
TCTAATACCGGATATGAACTGCCTTTAGTGTGGTGGTTGGAAAGTTTTTTCGGTGCAAGATGAGCTCG
CGGCCTATCAGCTTGTTGGTGGGGTAATGGCCTACCAAGGCGTCGACGGGTAGCCGGCCTGAGAGGGT
GTACGGCCACATTGGGACTGAGATACGGCC
>Zotu2981
GAACGCTGGCGGCGCGCTTAACACATGCAAGTCGAGCGAGAACCAAGCCTTCGGGTTTGGGGACAGCG
GCGAACGGGTGAGTAACACGTGGGTAATCAACCCTCGACACTGGGATAGCCCGGGGAAACTCGGATTA
ATACCGGATACCCCATCTGGCCTTCGGGCCGGCTGGAAAAGGTAGCTTTGGCCTCCGGTCGAGGACGA
GCCCGCGGCGGATTAGCTTGTTGGTGGGGTAAAAGCCCACCAAGGCGACGATCCGTAGCTGGTCTGAG
AGGACGATCAGCCACACTGGGACTGAGACACGGCC
>Zotu2982
GAACGCTGGCGGCGTGCTTAACACATGCAAGTCGAACGAGAACGGATTATAGCTTGCTATAATTGTCA
GCTAAGTGGCGCACGGGTGAGTAATGTATAGGTAATATGCCTCTTACTAAGGGATAACAATTGGAAAC
GATTGCTAATACCTTATACTCCTTGTTAACTTAAGTTAATAAGGGAAAGATTTATTGGTAAGAGATTA
GCCTGTATTGTATCAGTTAGTTGGTGGGGTAATGGCCTACCAAGACTATGACACATAACTGGTTTGAG
AGGATGATCAGTCACACTGGAACTGAGACACGGTC
>Zotu2983
GAACGCTAGCGGCAGGCCTAATACATGCAAGACGAACGGTAAGATTGATAGCAATATTGATTCTAGAG
TGTCGTAAGGGTGCGTAACACGTATGCAATCTGCCCTCAACAGGAGAATAGCTCCCCGAAAGGGGAAT
TAACACTCCGTAAGATTGTGGTTTGGCATCAAACAGCAATTAAAACTGAGGTGGTTGAGGATGAGCAT
GCGCATGATTAGTTAGTTGGCGGGGTAACGGCCCACCAAGACGATGATCATTAGGGGAACTGAGAGGT
TGATCCCCCACACTGGCACTGAGATACGGGC
>Zotu2984
GAACGCTGGCGGCATGCCTAACACATGCAAGTCGAACGAGACCTTCGGGTCTAGTGGCGCACGGGTGC
GTAACGCGTGGGAATCTGCCCTTGGGTGCGGAATAACTTTCCGAAAGGAATGCTAATACCGCATAATG
TCGTAAGACCAAAGATTTATCGCCCAAGGATGAGCCCGCGTAAGATTAGCTAGTTGGTGGGGTAAAAG
CCTACCAAGGCGACGATCTTTAGCTGGTCTGAGAGGATGATCAGCCACACTGGGACTGAGACACGGCC
>Zotu2985
GAACGCTGGCGGCGTGCCTAATACATGCAAGTAGAACGCTGAAGGGAGAGCTTGCTCTTCTGGATGAG
TTGCGAACGGGTGAGTAACGCGTAGGTAACCTGCCTGGTAGCGGGGGATAACTATTGGAAACGATAGC
TAATACCGCATAACAGTAAATGTTGCATGACATTTGCTTAAAAGGTGCAATTGCATCACTACCAGATG
GACCTGCGTTGTATTAGCTAGTTGGTGGGGTAACGGCTCACCAAGGCGACGATACATAGCCGACCTGA
GAGGGTGATCGGCCACACTGGGACTGAGACACGGCC
>Zotu2986
GAACGCTGGCGGCGTGCTTAACACATGCAAGTCGAACGGTGAAAGAGAGCTTGCTCTCTGGATCAGTG
GCGAACGGGTGAGTAACACGTGAGCAATCTGCCCTTGACTCTGGGATAAGCGTTGGAAACGACGTCTA
ATACCGGATACGAGCTGTGAAGGCATCTTCAACAGCTGGAAAGAACTTCGGTCAAGGATGAGCTCGCG

GCCTATCAGCTAGTTGGTGAGGTAATGGCTCACCAAGGCGTCGACGGGTAGCCGGCCTGAGAGGGTGA
CCGGCCACACTGGGACTGAGACACGGCC
>Zotu2987
GAACGCTGGCGGCGTGCCTAACACATGCAAGTCGAACGAAGCACTGAAAACGGAGATTCGTCGAAGTT
TTCTTTGACTTAGTGGCGGACGGGTGAGTAATGCGTGAGCAACCTGCCTTTCAGAGGGGGACAACAGT
TGGAAACGACTGCTAATACCGCATAATGTATTTTGGGGGCATCCCTGAGATACCAAAGGAGCAATCCG
CTGAAAGATGGGCTCACGTCTGATTAGCTAGTTGGTGAGGTAACGGCTCACCAAGGCTGCGATCAGTA
GCCGGACTGAGAGGTTGAACGGCCACATTGGGACTGAGATACGGCC
>Zotu2988
GAACGCTAGCGGCAGGCCTAATACATGCAAGTCGAGGGGTTAGCAATAACACCGGCGCACGGGTGCGT
AACACGTATGCAACCTACCTTATACAGGGGGATAGCCTTTGGAAACGGAGATTAATACCCCATAGTAT
TAAATGATGGCATCATTATTTAATTAAAGATTTATTGGTATAAGATGGGCATGCGTCCAATTAGTTAG
TTGGTTAGGTAACGGCTGACCAAGACGATGATTGGTAGGGGAACTGAGAGGTTAATCCCCCACACTGG
CACTGAGATACGGGC
>Zotu2989
GAACGCTGGCGGCGTGCCTTAAGCATGCAAGTCAAGGGGGGCTCGCAAGAGTCAACCGGCGGACGGGT
GAGTAATGCGTAGGAATCTTCCTCGAAGTTGGACATAGCTAGCCGAAAGGTTGGGTAATTTCCAATGT
GACCGTAAGGTCAAAGCAGCAATGCGCTTTGAGATGAGCCTGCGTCCTATCAGCTAGTTGGTGGGGTA
AAAGCCTACCAAGGCGACGACGGGTAGCTGGTCTGAGAGGATGGTCAGCCACAATGGGACTGCGACAC
GGCC
>Zotu2990
GAACGCTAGCGGCAGGCTTAATACATGCAAGTCGAGGGGCAGCACAGGTAGCAATACTGGGTGGCGAC
CGGCAAACGGGTGCGGAACACGTACGCAACCTTCCTTTAAGTGGGGGATAGCCCAGGGAAACTTGGAT
TAATACCCCTTAACATTATGAGATGGCATCATTTTATAATTAAAGCTCCGGCGCTTAAAGATGGGCGT
GCGCCTGATTAGGTAGTTGGCGGGGTAACAGCCCACCAAGCCTACGATCAGTAACTGGTGTGAGAGCA
CGACCAGTCACACGGGCACTGAGACACGGGC
>Zotu2991
GAACGCTGGCGGCGTGCCTAATACATGCAAGTCGAGCGGACTTTGCCTTCGGGTAAAGTTAGCGGCGG
ACGGGTGAGTAACACGTGGGTAACCTGCCCATAAGACTGGGATAACATTCGGAAACGAATGCTAATAC
CGGATACGCGAATCGGTCGCATGATCGGATCGGGAAAGGCGGAGCAATCTGCCGCTTATGGATGGACC
CGCGGCGCATTAGCTAGTTGGTGGGGTAACGGCTCACCAAGGCGACGATGCGTAGCCGACCTGAGAGG
GTGATCGGCCACACTGGGACTGAGACACGGCC
>Zotu2992
GAACGCTGGCGGCGTGCCTAACACATGCAAGTCGAACGGAGAGATACGAGAGCTTGCTCTTGTAAATC
TTAGTGGCAAACGGGTGAGTAACGCGTAAACAACCTGCCCAACGGATGGGGACAACAGACGGAAACGA
CTGCTAATACCCAATAGACTCCGGTTTCGCATGAAACCGACAGGAAAGGTGGCCTCTCGAAGAAGCTA
CCGCCGCAGGAGGGGTTTGCGTTTGATTAGTTAGTTGGTGGGGTAACGGCCTACCAAGACGATGATCA
ATAGCCGGTCTGAGAGGATGAACGGCCACACTGGAACTGAGACACGGTC
>Zotu2994
GAACGCTAGCTACAGGCTTAACACATGCAAGTCGAGGGGCAGCGGGAGAAAAGCTTGCTTTTCTTGCC
GGCGACCGGCGCACGGGTGCGTAACGCGTATCGAATCTGCCCGTTACTCGGGGATAGCCTTGCGAAAG
TAAGATTAATACCCGATGTTGTTGCATTGCCGCATGACGATGTAACCAAAGATTAATCGGTAACGGAT
GACGATGCGTCTGATTAGGTAGTAGGCGGGGTAACGGCCCACCTAGCCATCGATCAGTAGGGGTTCTG
AGAGGAAGGTCCCCCACATTGGAACTGAGACACGGTC
>Zotu2995
GAACGCTGGCGGCGTGCCTAATACATGCAAGTCGAGCGAACGGAGGAGAGCTTGCTCTCCGAAGTTAG
CGGCGGACGGGTGAGTAACACGTGGGCAACCTGCCTATAAGACTGGGATAACTTCGGGAAACCGGAGC
TAATACCGGATAGATTCTTTTCTCTCATGAGGAAAGAAGGAAAGATGGTTTCGGCTATCACTTATAGA
TGGGCCCGCGGCGCATTAGCTAGTTGGTAAGGTAACGGCTTACCAAGGCGACGATGCGTAGCCGACCT
GAGAGGGTGATCGGCCACACTGGGACTGAGACACGGCC
>Zotu2996
GAACGCTGGCGGCGTGCCTAACACATGCAAGTGGAGCGACGAACCAGGCTTCGGCCTGGGGCAAAGCC
GCGAACGGGTGAGTAACACGTGGGTTACCTACCCCGAAGACCGGGATAACCCGGGGAAACTCGGGCTA
ATACCGGATGTGCTCCGTCTGCTGCGGCAGGCGGTGGAAAGGAAGCTTCGGCCTCCGCTTCGGGATGG

GCCCGCGCCCCATTAGCTAGTTGGTGAGGTAACGGCTCACCAAGGCGACGATGGGTAGCTGGTCTGAG
AGGATGGCCAGCCACACTGGGACTGAGACACGGCC
>Zotu2997
GAACGCTGGCGGCAGGCTTAACACATGCAAGTCGAGCGCCCTCTTCGGAGGGAGCGGCGGACGGGTGA
GTAACACGTGGGAACGTGCCCTTCGGTTCGGAATAACTCAGGGAAACTTGAGCTAACACCGGATACGC
CCTTCGGGGGAAAGGTTACGCCGATGGAGCGGCCCGCGTTGGATTAGCTAGTTGGTGAGGTAAAGGCT
CACCAAGGCTACGATCCATAGCTGGTCTGAGAGGATGATCAGCCACACTGGGACTGAGACACGGCC
>Zotu2998
GAACGCTGGCGGCGTGCCTAACACATGCAAGTCGAGCGATGAAGTTTCCTTCGGGGAACGGATTAGCG
GCGGACGGGTGAGTAACACGTGGGTAACCTGCCTTGTAGAGGGGGATAGCCTTCCGAAAGGAAGATTA
ATACCGCATAAGATTGTAGTTTCGCATGAAACAGCAATTAAAGGAGCAATCCGCTACAAGATGGACCC
GCGGCGCATTAGCTAGTTGGTGAGGTAACGGCTCACCAAGGCGACGATGCGTAGCCGACCTGAGAGGG
TGATCGGCCACATTGGGACTGAGACACGGCC
>Zotu2999
GAACGCTAGCGGCAGGCTTAACACATGCAAGTCGAGGGGCAGCGCGGGTAGCAATACCTGGCGGCGAC
CGGCAAACGGGTGCGGAACACGTACAGAACCTACCTTTACCTGGGGCATAGCCCGGAGAAATCCGGAT
TAACTCCCCATAACATAAGAGAGTGGCATCACTCATTTATTAAAAATTTATTGGGTAAAGATGGCTGT
GCGTCCCATTAGCTAGTTGGTAGGGTAACGGCCTACCAAGGCGACGATGGGTAGCTGGTGTGAGAGCA
CGACCAGCCACACGGGCACTGAGACACGGGC
>Zotu3000
GAACGCTGGCGGCGTGCCTAATACATGCAAGTCGAACGAACCGCGACTGAGTGCTTGCACTTGGTCAA
GGTGAGTGGCGAACGGGTGAGTAACACGTGGGTAACCTACCTCATAGTGGGGGATAACAGTCGGAAAC
GACTGCTAATACCGCATAGGACATGGAATCACATGATTCCGTGAGGAAAGGTGGCGCAAGCTATCGCT
AAGAGATGGACCCGCGGTGCATTAGCTAGTTGGTAGGGTAAAGGCCTACCAAGGCGATGATGCATAGC
CGACCTGAGAGGGTGATCGGCCACATTGGGACTGAGACACGGCC
>Zotu3001
GAACGCTAGCGGCAGGCCTAACACATGCAAGTCGAGGGGTATAAGGAGCTTGCTCCTTAGAGACCGGC
GCACGGGTGCGTAACGCGTATGCAACCTACCTTGAACAGGGGGATAGCCCAGAGAAATTTGGATTAAT
ACCCCATGGTATATTTGAGTGGCATCACTTGATTATTAAAGTTCCAACGGTTCAAGATGGGCATGCGT
CCCATTAGCTAGATGGTAAGGTAACGGCTTACCATGGCGACGATGGGTAGGGGTCCTGAGAGGGAGAT
CCCCCACACTGGTACTGAGACACGGAC
>Zotu3002
GAACGCTGGCGGCGTGCTTAACACATGCAAGTCGAGCGGTAAGGCCCTTCGGGGTACACGAGCGGCGA
ACGGGTGAGTAACACGTGGGTGACCTGCCCTCCACTCTGGGATAAGCCTGGGAAACTGGGTCTAATAC
CGGATATGACCACATGTCGCATGGTGTGTGGTGGAAAGCTTTACGTGGTGGGGGATGGACCCGCGGCC
TATCAGCTTGTTGGTGGGGTAATGGCCTACCAAGGCGACGACGGGTAGCCGGCCTGAGAGGGCGACCG
GCCACACTGGGACTGAGACACGGCC
>Zotu3003
GAACGCTGGCGGCGTGCCTAATACATGCAAGTAGAACGCACAGTTTATACCGTAGCTTGCTACACCAT
AGACTGTGAGTTGCGAACGGGTGAGTAACGCGTAGGTAACCTGCCTGGTAGCGGGGGATAACTATTGG
AAACGATAGCTAATACCGCATAATATTAATTATTGCATGATAATTGATTGAAAGGTGCAAATGCATCA
CTACCAGATGGACCTGCGTTGTATTAGCTAGTAGGTGAGGTAACGGCTCACCTAGGCGACGATACATA
GCCGACCTGAGAGGGTGATCGGCCACACTGGGACTGAGACACGGCC
>Zotu3004
GAACGCTGGCGGCGTGCCTAACACATGCAAGTGGAGCGACGGACCAGGGCTTGCCCTGGGGCCAAGCC
GCGAACGGGTGAGTAACACGTGGGTAACCTGCCCCGATGACCGGGACAACCCGAGGAAACTCGGGCTA
ATACCGGATGTGCTCGCCGCACTCAAGTGTGGTGAGTAAAGGAAGCTTCGGCCTCCGCTTCGGGAGGG
GCCCGTGGCCCATTAGCTTGTTGGTGAGGTAACGGCTCACCAAGGCAACGATGGGTAGCTGGTCTGAG
AGGACGATCAGCCACACTGGGACTGAGACACGGCC
>Zotu3005
GAACGCTGGCGGCGTGCCTAATACATGCAAGTCGAGCGGACGACTTTTTGGTGCTTGCACCAGAATTT
CGTTAGCGGCGGACGGGTGAGTAACACGTGGGTAACCTACCTTGTAGACTGGGATAACTTCGGGAAAC
CGAGGCTAATACCGGATAATACTTTTAAACACATGTTTGAAAGTTGAAAGATGGTCTTGCTATCACTA
CAAGATGGACCCGCGGCGCATTAGCTAGTTGGTGGGGTAATGGCTCACCAAGGCAACGATGCGTAGCC

GACCTGAGAGGGTGATCGGCCACACTGGGACTGAGACACGGCC
>Zotu3006
GAACGCTGGCGGCGTGCTTAACACATGCAAGTCGAGCGGAAAGGCCACTTCGGTGGTACTCGAGCGGC
GAACGGGTGAGTAACACGTGAGTAATCTGCCCTTGGCTTTGGGATAGCCACCGGAAACGGTGATTAAT
ACCGAATATGCACTACTTCTCGCATGGGTTGTGGTGGAAAGTTTTTCGGCCAGGGATGTGCTCGCGGC
CTATCAGCTTGATGGTGAGGTAATGGCTCACCATGGCTTCGACGGGTAACCGGCCTGAGAGGGTGACC
GGTCACACTGGGACTGAGACACGGCC
>Zotu3007
GAACGCTGGCGGCATGCTTTACACATGCAAGTCGAACGGCAGCGCGGGGCAACCTGGCGGCGAGTGGC
GAACGGGTGAGTAATACATCGGAACGTACCCAGAAGTGGGGGATAACGTAGCGAAAGTTACGCTAATA
CCGCATACGTTCTACGGAAGAAAGTGGGGGATCGCAAGACCTCATGCTTTTGGAGCGGCCGATGTCTG
ATTAGCTAGTTGGTGAGGTAAAGGCTCACCAAGGCGACGATCAGTAGCTGGTCTGAGAGGACGACCAG
CCACACTGGGACTGAGACACGGCC
>Zotu3008
GAACGCTGGCGGCGTGCTTAACACATGCAAGTCGAACGATGATCAGGAGCTTGCTTCTGTGATTAGTG
GCGAACGGGTGAGTAACACGTGAGTAACCTGCCCCTGACTCTGGGATAAGCGTTGGAAACGACGTCTA
ATACTGGATAGGACTACTGGCCGCATGGTCTGGTGGTGGAAAGATTTTTTGGTTGGGGATGGACTCGC
GGCCTATCAGCTTGTTGGTGAGGTAATGGCTCACCAAGGCGACGACGGGTAGCCGGCCTGAGAGGGTG
ACCGGCCACACTGGGACTGAGACACGGCC
>Zotu3009
GAACGCTGGCGGTGTGCTTAACACATGCAAGTGGAGCGACGAACCAGGGCTTGCCCTGGGGCAGAGCC
GCGAACGGGTGAGTAACACGTGGGTAACCTACCCTGATGACTGGGACAACCCGAGGAAACTCGGGCTA
ATACCGGATGTGCTCTCTCCTCACGAGAGGAGCGAGTAAAGGAAGCTTCGGCCTCCGCATCAGGAGGG
GCCCGCGGCCCATTAGTTTGTTGGTGGGGTAACGGCCTACCAAGACTGCGATGGGTAGCTGGTCTGAG
AGGACGATCAGCCACACTGGGACTGAGACACGGCC
>Zotu3010
GAACGCTGGCGGCGTGCCTAACACATGCAAGTCGAACGGAATGAGGGGGCTTGCTCCTTCATTTAGTG
GCGCACGGGTGAGTAACGCGTAGATAATCTGCCTGATGATCTGGGATAACACTTCGAAAGAGGTGCTA
ATACCGGATAAGCCCACGGAGTCTTTGGATTCTGCGGGAAAAGGGGGGGACCTTCGGGCCTTCTGTCA
TCAGATGAGTCTGCGTACCATTAGCTAGTTGGTAGGGTAATGGCCTACCAAGGCTACGATGGTTAGCT
GGTCTGAGAGGATGATCAGCCACACTGGAACTGAGACACGGTC
>Zotu3011
GAACGCTGGCGGCGTGCTTAACACATGCAAGTGGTGCGACGAACCAGGGCTTGCCCTGGGGCCAAGCC
GCGAACGGGTGAGTAACACGTGAGTAACCTGCCCCGATGACCGGGACAACCCGAGGAAACTCGGGCTA
ATACCGGATGTGGCGCACAGGCGTAAGCCTGTTCGCTAAAGGAAGCTTCGGCCTCCGCATTGGGAGGG
GCTCGCGGCCCATTAGCTTGTTGGTGGGGTTATGGCCTACCAAGGCGACGATGGGTAGCTGGTCTGAG
AGGACGATCAGCCACACTGGGACTGAGACACGGCC
>Zotu3012
GAACGCTGGCGGCGTGCCTAACACATGCAAGTTGAGCGACGAACCGGGGCTTGCCCCGGGGCAAAGCA
GCGAACGGGTGAGTAACACGTGGGGAACCTGCCCCGAAGACCGGGACAACCCGAGGAAACTCGGGCTA
ATACCGGATATGTCCAACGAGCATGAGCTCATTGGGCAAAGGAAGCTTCGGCCTCCGCTTCGGGAGGG
TCCCGCGGCCCATTAGCTAGTCGGTGAGGTAACGGCTCACCGAGGCAACGATGGGTAGCTGGTCTGAG
AGGACGATCAGCCACACTGGGACTGAGACACGGCC
>Zotu3014
GAACGCTGGCGGTGTGCTTAATACATGCAAGTCGAGCGGTAAGGCCTCTTCGGAGGTACACGAGCGGC
GGACGGCTGAGTAACGCGTAGGAACGTACCCCAAAGTGAGGGATAAGCACCAGAAATGGTGTCTAATA
CCGCATGTGATCTTCGGATTAAAGCTTTATGCGCTTTGGGAACGGCCTGCGTATGATTAGCTTGTTGG
TGAGGTAATGGCTCACCAAGGCAATGATCATTAGCTGGTCTGAGAGGATGACCAGCCAGACTGGGACT
GAGACACGGCC
>Zotu3015
GAACGCTGGCGGCGTGCTTAATACATGCAAGTCGAACGAGGTCCTTCGGGACCTAGTGGCGAACGGGT
GAGTAACACGTGAGCAACCTGCCCCGAAGACCGGGATAACAGTGGGAAACCGCTGCTAATACCGGATA
CCCCCACCGGATCACATGGTCTGGTGAGGAAATGCCTGATTCTTCTAGATTCAGACGCTTCGGGAGGG
GCTCGCGGCCTATCAGCTTGTTGGTGGGGTAACGGCCTACCAAGGCTTCGACGGGTAGCTGGTCTGAG

AGGACGATCAGCCACACTGGGACTGAGACACGGCC
>Zotu3016
GAACGCTGGCGGCATGCCTTACACATGCAAGTCGAACGGCAGCGCGGGGGCAACCCTGGCGGCGAGTG
GCGAACGGGTGAGTAATACTTCGGAACGTGCCCAGTAGCGGGGGATAACGCAGCGAAAGTTGTGCTAA
TACCGCATACGCTCTACGGAGGAAAGAGGGGGATCGCAAGACCTCTCACTATTGGAGCGGCCGAAGCC
TGATTAGCTAGTTGGTGGGGTAAAAGCCCACCAAGGCGACGATCAGTAGCTGGTTTGAGAGAACGACC
AGCCACACTGGGACTGAGACACGGCC
>Zotu3017
GAACGCTGGCGGCGTGCTTAACACATGCAAGTCGAACGGATCCCATGGCTTAGCAATAAGTCGGAAGG
GTTAGTGGCGAACGGGTGAGTAACACGTGAGGAACCTGCCCCGGACTCAGGGATAACGGCTGGAAACG
GCTGCTAATACCTGATGCCGTCGTATCATCGCATGGTGGTGTGACGAAAGAATCTCGGTTCGGGAGGG
TCTCGCGGCCTATCAGCTTGTTGGTGGGGTAATGGCCTACCAAGGCGTCGACGGGTAGCTGGTCTGAG
AGGATGATCAGCCACACTGGGACTGAGACACGGCC
>Zotu3018
GAACGCTGGCGGCGTGCTTAACACATGCAAGTCGAGCGGTAAGGCTCCTTCGGGAGTACACGAGCGGC
GAACGGGTGAGTAACACGTGAGTAATCTGCCCTTCACTTCGGGATAACCGCCGGAAACGGTGGCTAAT
ACCGGATACGACGCAGTCTCGCATGAGATCTGAGTGGAAAGTTTTTCGGTGGAGGATGTGCTCGCGG
CCTATCAGCTTGTTGGTGAGGTAACGGCTCACCAAGGCTTCGACGGGTAGCCGGCCTGAGAGGGTGAC
CGGCCACACTGGGACTGAGACACGGCC
>Zotu3019
GAACGCTGGCGGTGTGCTTAATACATGCAAGTCGAGCGGTAAGGCCCCTTCGGGGGTACACGAGCGGC
GGACGGCTGAGTAACGCGTGGGAACGTACCCCAAAGTGAGGGATAAGCACCAGAAATGGTGTCTAATA
CCGCATGTGCTCTACGGAGTAAAGCTTCGGCGCTTTGGGAACGGCCCGCGTCCGATTAGCTAGTTGGT
GAGGTAATAGCTTACCAAGGCTACGATCGGTAGCTGGTCTGAGAGGATGACCAGCCAGACTGGGACTG
AGAACGGCC
>Zotu3020
GAACGCTGGCGGCGTGCCTAAGACATGCAAGTCGAGTGGGTTTAGGCCCACGGCGAACGGGTGAGTAA
TACCTAGGAACGTTCTTTAAGTTGAGGACATCCCATCGAAAGATGGTGCAATACTCAATGTGGTAGCA
ATACTAAAGTCGCAAGACGCTTAAAGTGCGGCCTGGGCACCATCAGCTAGTTGGTAGGGTAAAGGCCT
ACCAAGGCTATGACGGTTAGGGGCCCCGAGAGGGGAAACCCCCACACTGGGACTGCGACACGGCC
>Zotu3021
GAACGCTGGCGGCGTGCCTAACACATGCAAGTCGTACGGAAAGGCCCTTTTAGGGTACTCGAGTGGCG
GACGGCTGAGTAACGCGTGGGAACGTGCCCCAAAGTGAGGAATAACTGCCCGAAAGGGTAGCTAATGC
CGCATATGGTCTTCGGATTAAAGTATTTATACGCTTTGGGAGCGGCCCGCGTCGGATTAGGTTGTTGG
TGAGGTAAAGGCTCACCAAGCCGACGATCCGTAGCTGGTCTGAGAGGATGATCAGCCAGACTGGAACT
GAGACACGGTC
>Zotu3022
GAACGCTGGTGGCGTGCTTAACACATGCAAGTCGAGCGAGGCGTCCGCTTTCGGGTGGATAGTCGAGC
GGCGAACGGGTGAGTAACACGTGAGTAATCTTCCCTGGGCTCTGGGATAACCTCGGGAAACTGAGGGT
AATACCGGATGTGCGTTTTCTGGCGCCTGCTGGGAGACGGAAAGGTTGCGTGCCGTGAGGTGCGTTTC
CGGCCTTGGATGAGCTCGCGGCCTATCAGCTTGTTGGTGCGGTGATGGCGTACCAAGGCGACGACGGG
TAGCCGGCCTGAGAGGGTGACCGGCCACACTGGGACTGAGATACGGCC
>Zotu3023
CCCACACGCTATTGGATGACTTTTCTGACGACCTTTACACCACATTTCAGCATAATCAGTTCGCGTGT
GCGCTCTTCTTGAATGTGGCGAAAGGTTTCGATTCTCTTAGTCATAAAGTTCTATTAAGTGAGCTTTA
TTATTACGGTTTCCGCGGCCCTTTTTTCTCATTGCTCAAGAGCTTCTTAACTGATAGGTTCCAGCAGG
TGTCCATAGGCGGTGCAGTAAGTTCCAAAATACATTTGAAGGCTGGGGTGCCTCTGGGTTCAGTGCTA
TCTACACTTTTGTTTAATCTATATGTCAATGACCTCTCCCTTTCGACATCTACTTGTATTGTAAATCA
ATATGCGGATGATACTCTACTGTTATCGCGGCATGTGAATAACGGTGACGCGTT
>Zotu3024
GAACGCTGACGGCGTGCTTAACACATGCAAGTCGAACGGGATCCATTAGCGCTTTTGTGTTTTTGGTG
AGAGTGGCGAACGGGTGAGTAACACGTGAGTGACCTGCCCTTTTCTTTGGGATAAGCCTGGGAAACTG
GGTCTAATACTGGATGTTCCGGCTTCCTCGCATGGGGTTGTTGGGAAAGGTTTTTTCTGGATTGGGAT
GGGCTCGCGGCTTATCAGCTTGTTGGTGGGGTGATGGCTTACCAAGGCTTTGACGGGTAGCCGGCCTG

AGAGGGTGGTCGGTCACACTGGGACTGAGATACGGCC
>Zotu3025
GAACGCTGGCGGCGTGCCTAATACATGCAAGTCGAGCGGAGCACTTCGGTGCTTAGCGGCGGACGGGT
GAGTAACACGTAGGCAACCTGCCTGTAAGACCGGGATAACTACCGGAAACGGTAGCTAAGACCGGATA
ACTGGTTTCTCCGCATGGAGGGATCATGAAACACGGTGCAAGCTGTGGCTTACGGATGGGCCTGCGGC
GCATTAGCTAGTTGGTGGGGTAACGGCCTACCAAGGCGACGATGCGTAGCCGACCTGAGAGGGTGAAC
GGCCACACTGGGACTGAGACACGGCC
>Zotu3026
GAACGCTGGCGGCAAGCTTAACACATACAAGTCGAGCGCCCCGCAAGGGGAGCGGCAGACGGGTGAGT
AACGCGTGGGAACGTACCATTTGCTACGGAATAACTCAGGGAAACTTGTGCTAATACCGTATGTGCCC
GAAAGGGGAAAGATTTATCGGCAAATGATCGGCCCACGTTGGATTAGCTAGTTGGTGGGGTAAAGGCC
TACCAAGGCGACGATCCATAGCTGGTCTGAGAGGATGATCAGCCACACTGGGACTGAGACACGGCC
>Zotu3027
GAACGCTGGCGGCGTGCTTAACACATGCAAGTCGAACGAGATTCAGTCAGTAGCAATACTGACGAAAA
TCTAGTGGCGAACGGGTGCGTAACACGTGAGGAACTTGTCCCGGTCTCTGGGATAACAGTTGGAAACG
ACTGCTAATACCGGATGACGTCGGAACTTCGCATGGAGTACTGACGAAAGGGTTACTGGATCGGGAGA
GCCTCGCGGCCTATCAGCTTGTTGGTGGGGTAACGGCCTACCAAGGCATCGACGGGTAGCTGGTCTGA
GAGGATGATCAGCCACACTGGGACTGAGACACGGCC
>Zotu3028
AAACGCTGGCGGCGTGCTTAATACATGCAAGTCGAACGGTCCTCCTTCGGGAGGATAGTGGCGGACGG
GTGAGTAACACGTAGATAACCTGCCCTGCTGAGGGGAATAACATCTCGAAAGAGATGCTAATACCGCA
TAAGCTCTCATTTGCGAGAATGAGAGGAAACCTTTTCTTTCGAGAAAGGGGCTGCAGGAGGGGTCTGC
GGCGGATTAGCTAGTTGGTGAGGTAAAGGCTCACCAAGGCGACGATCCGTAGCTGGTCTGAGAGGACG
GCCAGCCACACTGGGACTGAGACACGGCC
>Zotu3029
GAACGCTGGCGGCACGTTTTAAGCATGCAAGTCGAACGGCAGTGGGGGCTTGCTCCCATGAAAGTGGC
GAACGGGTGAGTAACACGTAGGTGATCTGCCTTGAGGATGGGGATAGCCTGTGGAAACACAGGGTAAT
ACCGAATGAGCTGACGACACTTTGGTGATGTTAGTAAAGGTGCTACGGCACCGCCTTAAGAGGAGCCT
GCGGCTGATTAGCTAGTTGGTGGGGTAAAGGCCTACCAAGGCGACGATCAGTAGCCGGCCTGAGAGGG
TGTACGGCCACACTGGAACTGAGACACGGTC
>Zotu3030
GGTCAGGAGGGCAGACGAAGGATTTATAGCTGAGGAGTTTCGGGGAGTCCTACACTAATTATTTGTAG
TATTGAGGTTTTAAGAGGTAATTG
>Zotu3032
TAATGCTGGTGGTATGCATAACACATGCAAGTTGAACGAAGAGGGCTTGCCCTCTTAGTGGCGAACGG
GCGAGTAACGCATATTTAACTTACTTACGCGAGGAGAATAGCAATCCGAAAGGGTTATTAATATTTCA
TAGGTTTAAGACTTAAATTAAAGGGGGCGTTCCTTCGGGAGCCCCGCGCGTAAATAGGAATATGTCCT
ATTAGGTTGTTGGTAGGGTAAATGCCTACCAAGCCGTTGATGGGTAGCTGGGCTGAGAGGCCGACCAG
CCGCAATGGGATTGAAATATGGCC
>Zotu3033
GAACGCTGGCGGCGCGCTTAACACATGCAAGTCGAGCGAGGCCCCGGGCTTGCCCGGGTGTCCTAGCG
GCGAACGGGTGAGTAACACGTGAGGAACCTGCCCCGAAGACCGGGATAACACCGGGAAACCGGTGCTA
ATACCGGATGCCCTCATCAGACCGCATGATCTGGTGAGGAAATGGATTCCGCTTCGGGATGGTCTCGC
GGCCTATCAGCTTGTTGGTGGGGTAACGGCCTACCAAGGCAACGACGGGTAGCTGGTCTGAGAGGATG
TCCAGCCACACTGGGACTGAGACACGGCC
>Zotu3034
GAACGCTGGCGGCAGGCCTAACACATGCAAGTCGAGCGCCCCGCAAGGGGAGCGGCAGACGGGAGAGT
AACACGTGGGAACATGCCCTTCAGTACGGAACAACCAAGGGAAACTTTGGCTAATACCGTATACGTCC
GTAAGGAGAAAGATTTATCGCTGAAGGATTGGCCCGCGTCTGATTAGCTAGTTGGTGAGGTAATGGCT
CACCAAGGCGACGATCAGTAGCTGGTCTGAGAGGATGATCAGCCTCACTGGGACTGAGACACGGCC
>Zotu3036
GAACGCTGGCGGCGTGCTTAACACATGCAAGTCGAACGGAAAGGCCCTAGCTTGCTAGGGTGCTCGAG
TGGCGAACGGGTGAGTAACACGTGGGTGATCTGCCTTGTACTCTGGGATAAGCTTGGGAAACTGGGTC
TAATACCGGATAGGAACTGCCTTTAGTGTGGTGGTTGGAAAGTTTTTCGGTGCAAGATGAGCTCGCG

GCCTATCAGCTTGTTGGTGGGGTAATGGCCTACCAAGGCGTCGACGGGTAGCCGGCCTGAGAGGGTGT
ACGGCCACATTGGGACTGAGATACGGCC
>Zotu3037
GAACGCTGGCGGCGCGCCTAACACATGCAAGTCGAACGAGCGAGAGAGAGCTTGCTTTCTCGAGCGAG
TGGCGAACGGGTGAGTAACGCGTGAGGAACCTGCCTCAAAGAGGGGGACAACAGTTGGAAACGACTGC
TAATACCGCATAAGCCCACGACCCGGCATCGGGAAGAGGGAAAAGGAGCAATCCGCTTTGAGATGGCC
TCGCGTCCGATTAGCTAGTTGGTGAGGTAACGGCCCACCAAGGCGACGATCGGTAGCCGGACTGAGAG
GTTGAACGGCCACATTGGGACTGAGACACGGCC
>Zotu3038
GAACGCTGGCGGCATGCCTTACACATGCAAGTCGAACGGCAGCACGGGGCAACCTGGTGGCGAGTGGC
GAACGGGTGAGTAATATATCGGAACGTACCCTGGAGTGGGGGATAACGTAGCGAAAGTTACGCTAATA
CCGCATACGATCTAAGGATGAAAGTGGGGGCTTCGCAAGAACCTCATGCTCCTGGAGCGGCCGATATC
TGATTAGCTAGTTGGTAGGGTAAAAGCCTACCAAGGCTTCGATCAGTAGCTGGTCTGAGAGGACGACC
AGCCACACTGGAACTGAGACACGGTC
>Zotu3039
GAACGCTGGCGGCGCGCCTAACACATGCAAGTCGAACGAGAGATGAGGAGCTTGCTCTTCAAATCGAG
TGGTGAACGGGTGAGTAACGCGTGAGGAACCTGCCTCAAAGAGGGGGACAACAGTTGGAAACGACTGC
TAATACCGCATAAGCCCACGGCTCGGCATCGAGCAGAGGGAAAAGGAGCAATCCGCTTTGAGATGGCC
TCGCGTCCGATTAGCTAGTTGGTGAGGTAACGGCCCACCAAGGCGACGATCGGTAGCCGGACTGAGAG
GTTGAACGGCCACATTGGGACTGAGACACGGCC
>Zotu3040
GAACGCTGGCGGCATGCCTAACACATGCAAGTCGAACGAGGGCTTCGGCCCTAGTGGCGCACGGGTGA
GTAACGCGTGGGAACCTGCCCTGTGGTACGGAATAACTCCGGGAAACTGGAGCTAATACCGTATGCGT
CCTGCGGGACAAAGATTTATCGCCATGGGATGGGCCCGCGTAGGATTAGCTTGTTGGTGGGGTAACGG
CCTACCAAGGCTTCGATCCTTAGCTGGTCTGAGAGGATGATCAGCCACACTGGGACTGAGACACGGCC
>Zotu3041
GAACGCTGGCGGCGTGCTTAACACATGCAAGTCGAACGATGAACCTGTGCTTGCACGGGGGATTAGTG
GCGAACGGGTGAGTAACACGTGAGTAACCTGCCCTTGACTTCGGGATAAGCCTGGGAAACTGGGTCTA
ATACTGGATACGACCTCTCATCGCATGGTGTGGGGGTGGAAAGTTTTTGCGGTTTTGGATGGACTCGC
GGCCTATCAGCTTGTTGGTGAGGTAATGGCTCACCAAGGCGACGACGGGTAGCCGGCCTGAGAGGGTG
ACCGGCCACACTGGGACTGAGACACGGCC
>Zotu3042
GAACGCTAGCGGCAGGCTTAACACATGCAAGTCGAGGGGTATAGTTCTTCGGAGCTAGAGACCGGCGC
ACGGGTGCGTAACGCGTATGCAATCTACCTTTTACAGAGGGATAGCCCAGAGAAATTTGGATTAATAC
CTCATAGTATTATGAAATGGCATCATTTTATAATTAAAGTCACAACGTTAAAAGATGAGCATGCGTCC
CATTAGCTTGTTGGTAAGGTAACGGCTTACCAAGGCTACGATGGGTAGGGGTCCTGAGAGGGAGATCC
CCCACACTGGTACTGAGACACGGAC
>Zotu3043
GAACGCTGGCGGCGTGCTTAACACATGCAAGTCGAGCGGTAAGGCTCCTTCGGGAGTACACGAGCGGC
GAACGGGTGAGTAACACGTGAGTAATCTGCCCCTCACTTCGGGATAAGCCTCGGAAACGAGGTCTAAT
ACCGGATACGACCACTTCTTGCATGAGATGGTGGTGGAAAGTTTTTCGGTGGGGGATGTGCTCGCGGC
CTATCAGCTTGTTGGTGGGGTAATGGCCTACCAAGGCTTCGACGGGTAGCCGGCCTGAGAGGGTGACC
GGCCACACTGGGACTGAGACACGGCC
>Zotu3044
GAACGCTGGCGGCGTGCCTAATACATGCAAGTCGAGCGAACCTGCAGGAGCTTGCTCCTGTTGGTTAG
CGGCGGACGGGTGAGTAACACGTGGGCAACCTGCCTGTAAGACTGGGATAACACCGGGAAACCGGTGC
TAATACCGGATAATCCTTTTCCTCTCATGAGGAAAAGCTGAAAGTCGGTTTCGGCTGACACTTACAGA
TGGGCCCGCGGCGCATTAGCTAGTTGGTGAGGTAACGGCTCACCAAGGCGACGATGCGTAGCCGACCT
GAGAGGGTGATCGGCCACACTGGGACTGAGACACGGCC
>Zotu3045
GAACGCTGGCGGCATGCCTTACACATGCAAGTCGAACGCGAAAGCGGAGCAATCCGCAAGTAGAGTGG
CGAACGGGTGAGTAATACATCGGAACATTTCCAGTAGCGGGGGATAACCTGTCGAAAGATAGGCTAAT
ACCGCATACGCCCCACGGGGGAAAGCGGAGGACCGCAAGGCTTCGCACTATTGGAGTGGCCGATGTCG
GATTAGCTAGTTGGCGGGGTAAAAGCCCACCAAGGCTACGATCCGTAGCTGGTCTGAGAGGACGATCA

GCCACACTGGAACTGAGACACGGTC
>Zotu3046
GAACGCTAGCGGCAGGCTTAACACATGCAAGTCGAGGGGTATACTTCTTCGGAAGTAGAGACCGGCGC
ACGGGTGCGTAACGCGTATGCAATCTACCTTTCACAAAGGAATAGCCCAGAGAAATTTGGATTAATGC
CTTATAGTATAACGACTTCGCATGGAGATGTTATTAAAGATTTATCGGTGAAAGATGAGCATGCGTCC
CATTAGCTAGATGGTAAGGTAACGGCTTACCATGGCTACGATGGGTAGGGGTCCTGAGAGGGAGATCC
CCCACACTGGTACTGAGACACGGAC
>Zotu3047
GAACGCTGGCGGCGTGCTTAACACATGCAAGTCGAGCGGTAAGGCCCTTCGGGGTACACGAGCGGCGA
ACGGGTGAGTAACACGTGGGTGACCTGCCCTCAGCTCTGGGATAAGCCTGGGAAACCGGGTCTAATAC
CGGATACGACCATTGGACGCATGTCTGGTGGTGGAAAGTTTTTCGGCTGGGGATGGCCCGCGGCCTA
TCAGCTTGTTGGTGGGGTGATGGCCTACCAAGGCGGTGACGGGTAGCCGGCCTGAGAGGGCGACCGGC
CACACTGGGACTGAGACACGGCC
>Zotu3048
GAACGCTGGCGGCGTGCTTAACACATGCAAGTCGAACGATGAAGCCCAGCTTGCTGGGTGGATTAGTG
GCGAACGGGTGAGTAACACGTGAGTAACCTGCCCACCACTCTGGGATAACTCCGGGAAACCGGTGCTA
ATACTGGATATGACTCCAACTCGCATGGGTATGGGGTGGAAAGATTTATTGGTGGTGGATGGACTCGC
GGCCTATCAGCTTGTTGGTGGGGTAATGGCCTACCAAGGCGACGACGGGTAGCCGGCCTGAGAGGGTG
ACCGGCCACACTGGGACTGAGACACGGCC
>Zotu3049
GAACGCTGGCGGCAGGCCTAACACATGCAAGTTGAACGATGTAGCAATACAGAGTAGCGCACGGGTGC
GTAACGCGTGGGAATATGCCCTTCACTACGGAACAACGTTTGGAAACGAACGCTAATACCGTATAATG
ACTTCGGTCCAAAGATTTATCGGTGAAGGATTAGCCCGCGTCTGATTAGCTAGTTGGTGAGGTAATGG
CTCACCAAGGCGACGATCAGTAGCTGGTCTGAGAGGATGATCAGCCACACTGGGACTGAGACACGGCC
>Zotu3050
GAACGCTGGCGGCGTGCCTAACACATGCAAGTCGAGCGAGTGGAGTTCTTCGGAACAAAGCTAGCGGC
GGACGGGTGAGTAACACGTGGGCAACCTGCCTCATAGAGGGGAATAGCCTCCCGAAAGGGAGATTAAT
ACCGCATAAGATTGTAGCTTCGCATGAAGTAGCAATTAAAGGAGCAATCCGCTATGAGATGGGCCCGC
GGCGCATTAGCTAGTTGGTGAGGTAACGGCTCACCAAGGCGACGATGCGTAGCCGACCTGAGAGGGTG
ATCGGCCACATTGGGACTGAGACACGGCC
>Zotu3051
GAACGCTGGCGGCAGGCCTAACACATGCAAGTCGAACGGCAGCACAGCAGTAGCAATACTGTGGGTGG
CGAGTGGCGGACGGGTGAGGAATACATCGGAATCTGCCTAGTCGTGGGGGATAACGTAGGGAAACTTA
CGCTAATACCGCATACGACCTACGGGTGAAAGCGGGGGATCGCAAGACCTCGCGCGATTAGATGAGCC
GATGTCCGATTAGCTAGTTGGCGGGGTAATGGCCCACCAAGGCGACGATCGGTAGCTGGTCTGAGAGG
ATGATCAGCCACACTGGAACTGAGACACGGTC
>Zotu3052
GAACGCTGGCGGCGCGCTTAACACATGCAAGTCGAACGGAGTCCTTCGGGACTCAGTGGCGAACGGGT
GCGTAACACGTGAGCAACCTGCCTGGTCGTGGGGGATAACCAGTGGAAACGCTGGCTAATACCGCATG
AGCTCTTTGCTTCGATTGAGGCGTTGAGGAAAGCTCCTGCAAGGGAGTGCGACCAGAGGGGCTCGCGG
CGTATCAGCTAGTTGGAGGGGTAACGGCCCACCAAGGCGATGACGCGTAGCCGATCTGAGAGGATGAT
CGGCCACATTGGGACTGAGATACGGCC
>Zotu3053
GAACGCTGGCGGCGTGCCTAATACATGCAAGTAGAACGCTGAAGGAATAGCTTGCTATTCTGGAAGAG
TTGCGAACGGGTGAGTAACGCGTAGGTAACCTACCTCATAGCGGGGGATAACTATTGGAAACGATAGC
TAATACCGCATAAGAATACTTATTGCATGATAAGTATTTAAAAGGAACAACAGTTTCACTGTGAGATG
GACCTGCGTTGTATTAGCTAGTTGGTAAGGTAACGGCTTACCAAGGCGACGATACATAGCCGACCTGA
GAGGGTGATCGGCCACACTGGGACTGAGACACGGCC
>Zotu3054
GAACGCTGGCGGCGTGCCTAACACATGCAAGTCGAGCGGAGTTTAGCGGGCGGCGACTTCGGTCAAAG
CCTGTTAAACTTAGCGGCGGACGGGTGAGTAACACGTAGGCAACCTGCCCATGACACGGGGATAGCTT
CGGGAAACCGGAGGTAATACCCGATACCTTTACTTATCGCATGGTAAGTAAAGAAAGCGACTTAGCGG
TCATGGATGGGCCTGCGTCCCATTAGCTAGTTGGTGAGATAACAGCCCACCAAGGCGACGATGGGTAG
CCGACCTGAGAGGGTGACCGGCCACACTGGAACTTAGACACGGTC

>Zotu3055
GAACGCTGGCGGCGTGCCTAATACATGCAAGTCGAGCGAACAGATGAGGAGCTTGCTCCTCTGATGTT
AGCGGCGGACGGGTGAGTAACACGTGGGCAACCTGCCCTATAGTTTGGGATAACTCGCGGAAACGCGA
GCTAATACCGAATAATACATTGAAGCTCCTGCTTTAATGTTGAAAGATGGTTTCGGCTATCGCTATAG
GATGGGCCCGCGGCGCATTAGCTAGTAGGTGAGGTAACGGCTCACCTAGGCAACGATGCGTAGCCGAC
CTGAGAGGGTGATCGGCCACACTGGGACTGAGACACGGCC
>Zotu3056
GAACGCTGGCGGCGTGCCTAATACATGCAAGTCGAGCGGATCTGAAGGAGTGCTTGCACTCCTGAAGG
TTAGCGGCGGACGGGTGAGTAACACGTAGGTAACCTGCCTACAAGACTGGGATAACATTCGGAAACGA
ATGCTAATACCGGATACGCGATTTCCTCGCATGGGGAGATCGGGAAAGACGGAGCAATCTGTCACTTG
TAGATGGACCTGCGGCGCATTAGCTAGTTGGTGGGGTAACGGCTCACCAAGGCGACGATGCGTAGCCG
ACCTGAGAGGGTGATCGGCCACACTGGGACTGAGACACGGCC
>Zotu3058
GAACGCTGGCGGCGTGCTTAACACATGCAAGTCGAGCGGAAAGGCCCTTTCGGGGGTACTCGAGCGGC
GAACGGGTGAGTAACACGTGAGCAACCTGCCCCAGGCTTTGGGATAACCCTCGGAAACGGGGGCTAAT
ACCGGATAGGACCTGTCATCGCATGGTGATGGGTGGAAAGTTTTTCGGCTTGGGATGGGCTCGCGGCC
TATCAGCTTGTTGGTGGGGTGATGGCCTACCAAGGCTTCGACGGGTAGCCGGCCTGAGAGGGCGACCG
GCCACACTGGGACTGAGACACGGCC
>Zotu3060
GAACGCTAGCGGCAGGCTTAATACATGCAAGTCGAGGGGCAGCACAGTGTAGCAATACATGGGTGGCG
ACCGGCAAACGGGTGCGGAACACGTACAGAACCTTCCTTTAAGTGGTGAATAGCCCAGAGAAATTTGG
ATTAATACACCGTAACATTATGAAGTGGCATCACTTTTTAATTATAGCTCCGGCGCTTGAAGATGGCT
GTGCGGCTGATTAGGTAGTTGGCGGGGTAACGGCCCACCAAGCCTTCGATCAGTAACTGGTGTGAGAG
CACGACCAGTCACACGGGCACTGAGACACGGGC
>Zotu3061
GAACGCTGGCGGCATGCCTAACACATGCAAGTCGAACGAAGCCTTCGGGCTTAGTGGCGCACGGGTGC
GTAACGCGTGGGAATCTGCCCTTCGGTTCGGAACAACAGTTAGAAATGACTGCTAATACCGGATAATG
ACGTAAGTCCAAAGATTTATCGCCGAAGGATGAGCCCGCGTAGGATTAGCTAGTTGGTAAGGTAAAAG
CTTACCAAGGCGACGATCCTTAGCTGGTCTGAGAGGATGATCAGCCACACTGGGACTGAGACACGGCC
>Zotu3063
GAACGCTGGCGGCGTGCTTAACACATGCAAGTCGAACGGTGAAGTCAGAGCTTGCTCTGGCGGATCAG
TGGCGAACGGGTGAGTAACACGTGAGTAACCTGCCCCCGACTCTGGGATAACTGCTAGAAATGGTAGC
TAATACCGGATATGACGACTGGCCGCATGGTCTGGTCGTGGAAAGAATTTCGGTTGGGGATGGACTCG
CGGCCTATCAGGTTGTTGGTGAGGTAATGGCTCACCAAGCCTACGACGGGTAGCCGGCCTGAGAGGGT
GACCGGCCACACTGGGACTGAGACACGGCC
>Zotu3064
GAACGCTGGCGGCGTGCTTAACACATGCAAGTCGAACGGTGATGATCAAGCTTGCTTGGTCGGATCAG
TGGCGAACGGGTGAGTAACACGTGAGCAACCTGCCCCAGACTCTGGGATAACTTCGGGAAACCGGAGC
TAATACCGGATATGACACTCGCACGCATGTGCTGGGTGTGGAAAGTTTTTCGGTCTGGGATGGGCTCG
CGGCCTATCAGCTTGTTGGTGAGGTAGTGGCTCACCAAGGCGACGACGGGTAGCCGGCCTGAGAGGGC
GACCGGCCACACTGGGACTGAGACACGGCC
>Zotu3065
GAACGCTGGCGGCAGGCTTAACACATGCAAGTCGAGCGGGCACTTCGGTGTCAGCGGCAGACGGGTGA
GTAACACGTGGGAACGTACCCTTTGGTTCGGAATAACGCTGGGAAACTAGCGCTAATACCGGATACGC
CCTTTTGGGGAAAGGCTTGCTGCCGAAGGATCGGCCCGCGTCTGATTAGCTAGTTGGTGGGGTAATGG
CCTACCAAGGCGACGATCAGTAGCTGGTCTGAGAGGATGATCAGCCACACTGGGACTGAGACACGGCC
>Zotu3066
GAACGCTGGCGGCAGGCTTAACACATGCAAGTCGAACGCCCCGCAAGGGGAGTGGCAGACGGGTGAGT
AACGCGTGGGAATCTACCTAGTTCTACGGAACAACAGTTGGAAACGACTGCTAATACCGTATACGCCC
TACGGGGGAAAGATTTATCGGAATTAGATGAGCCCGCGTAAGATTAGCTAGTTGGTGGGGTAATGGCC
TACCAAGGCGACGATCTTTAGCTGGTCTGAGAGGATGATCAGCCACACTGGGACTGAGACACGGCC
>Zotu3067
GAACGCTGGCGGCGTGCCTAATACATGCAAGTCGAACGAGAGCGACCGGTGCTTGCACTGGTCAATCT
AGTGGCGAACGGGTGAGTAACACGTGGGTAACCTGCCCATCAGAGGGGGATAACATTCGGAAACGGAT

GCTAAAACCGCATAGGTTCTTTAGTCGCATGACTGAAGAAGGAAAAGAGGCTTCGGCTTCTGCTGATG
GATGGACCCGCGGTGCATTAGCTAGTTGGTGAGGTAACGGCTCACCAAGGCGATGATGCATAGCCGAC
CTGAGAGGGTGATCGGCCACATTGGGACTGAGACACGGCC
>Zotu3068
GAACGCTGGCGGCAGGCTTAACACATGCAAGTCGAGCGGGGAAAGGTAGCTTGCTACCGGACCTAGCG
GCGGACGGGTGAGTAATGCTTAGGAATCTGCCTATTAGTGGGGGACAACATCTCGAAAGGGATGCTAA
TACCGCATACGTCCTACGGGAGAAAGCAGGGGATCTTCGGACCTTGCGCTAATAGATGAGCCTAAGTC
GGATTAGCTAGTTGGTGGGGTAAAGGCCTACCAAGGCGACGATCTGTAGCGGGTCTGAGAGGATGATC
CGCCACACTGGGACTGAGACACGGCC
>Zotu3069
GAACGCTGGCGGCGTGCTTAACACATGCAAGTCGAACGGTGAAGCTCCAAGCTTGCTTGGGGTGGATC
AGTGGCGAACGGGTGAGTAACACGTGAGCAACCTGCCCCGGACTCTGGAATAAGCGCTGGAAACGGCG
TCTAATACTGGATATGACATCAAGCCGCATGGTTATGGTGTGGAAAGTTTTTCGGTCCGGGATGGGCT
CGCGGCCTATCAGCTTGTTGGTGAGGTAATGGCTCACCAAGGCGACGACGGGTAGCCGGCCTGAGAGG
GCGACCGGCCACACTGGGACTGAGACACGGCC
>Zotu3070
GAACGCTGGCGGCGTGCTTTACACATGCAAGTCGAACGGCAGCATAGATAGTAGCAATACTATTGGGT
GGCGAGTGGCGGACGGGTGAGTAATACATCGAGACGTGCCTAATAGATTGGGATAACCTGTCGAAAGA
TAGGCTAATACCGGATAGTCTCTGAGGAGGAAAGCTGGGGACTTAGGAATAAGCCTGGCGCTAATAGA
GCGATCGATGGCCGATTAGTTAGTTGGTGGGGTAAAGGCTTACCAAGGCGATGATCGGTAGCTGTTCT
GAGAGGAAGATCAGCCACACTGGAACTGAGAGACGGTC
>Zotu3072
GAACGCTGGCGGCATGCTTAACACATGCAAGTCGCACGGGGGGATTTCGGTCCTTCAGTGGCGGACGG
GTGAGTATCGCGTAGGGACCTATCGTTGGGTGGGGGATAACCTTGGGAAACTGGGGCTAATACCGCAT
GACGCCTTGGGGCCAAAGCTATGGCGCCTGACGAGGGGCCTGCGTCCGATTAGGTAGTTGGTTGGGTA
ATGGCTGACCAAGCCTGCGATCGGTAGCTGGTCTGAGAGGATGATCAGCCACACTGGGACTGAGACAC
GGCC
>Zotu3073
GAACGCTGGCGGTATGCCTAACACATGCAAGTCGAACGGTCTCTTCGGAGATAGTGGCGGACGGGTGA
GTAACGCGTGAGAATCTGCCTTCAGGATGGGGACAACAGAGGGAAACTTCTGCTAAAACCCAATGTGC
CGAAAGGTGAAATATTTATAGCCTGAAGAGGAGCTCGCGTCCGATTAGTTAGTTGGTGGGGTAAAGGC
CTACCAAGGCAGCGATCGGTAGCTGGTCTGAGAGGATGAGCAGCCACACTGGGACTGAGACACGGCC
>Zotu3074
GAACGCTGGCGGCGTGCTTAACACATGCAAGTCGAACGATGAAGCAGAGCTTGCTCTGTGGATTAGTG
GCGAACGGGTGAGTAACACGTGAGTAACCTGCCCCAGACTCTGGGATAAGCACTGGAAACGGTGTCTA
ATACCGGATACGAGCTTCAGCCGCATGGCTAGGAGCTGGAAAGAATTTCGGTCTGGGATGGACTCGCG
GCCTATCAGCTAGTTGGTGAGGTAATGGCCCACCAAGGCGACGACGGGTAGCCGGCCTGAGAGGGTGA
CCGGCCACACTGGGACTGAGACACGGCC
>Zotu3075
GAACGCTGGCGGCATGCCTAACACATGCAAGTCGAACGAGACCTTCGGGTCTAGTGGCGCACGGGTGC
GTAACGCGTGGGAACCTGCCTTTAGGTTCGGAATAACTCCTCGAAAGGGGTGCTAATACCGAATAATG
TCTTCGGACCAAAGATTTATCGCCTTTAGATGGGCCCGCGTTGGATTAGCTAGTTGGTGGGGTAAAGG
CTCACCAAGGCGACGATCCATAGCTGGTCTTAGAGGATGATCAGCCACACTGGGACTGAGACACGGCC
>Zotu3076
GAACGCTGGCGGCGTGCCTAATACATGCAAGTCGAGCGAACAGATAAGGAGCTTGCTCCTTTGACGTT
AGCGGCGGACGGGTGAGTAACACGTGGATAACCTACCTATAAGACCGGGATAACTTCGGGAAACCGGA
GCTAATACCGGATAACATATTGAACCGCATGGTTCAATAGTGAAAGACGGCCTTGCTGTCACTTATAG
ATGGATCCGCGCCGTATTAGCTAGTTGGTAAGGTAACGGCTTACCAAGGCAACGATACGTAGCCGACC
TGAGAGGGTGATCGGCCACACTGGAACTGAGACACGGTC
>Zotu3077
GAACGCTGGCGGCGTGCCTAATACATGCAAGTCGAGCGAGAAAGTCTTCGGATGAGTAAAGCGGCGGA
CGGGTGAGTAACACGTGGGTAATCAACCTTCTAGTGGGGAATAACGTTTCGAAAGGAGCGCTAATACC
GCATGTGAATACGAGTCGTTGGATTTGTATTCAAAGGGGGGTCGTAAGATCTCTCGCTAGAAGACGAG
CCTGCGTCCCATTAGCTAGTTGGTGAGGTAAAGGCTCACCAAGGCATCGATGGGTAGCTGGTCTGAGA

GGACGACCAGCCACACTGGAACTGACACACGGTC
>Zotu3078
GAACGCTGGCGGCGTGCCTAAGACATGCAAGTCGAGTGGGTTTAGACCCACGGCGAACGGGTGAGTAA
TACCTAGGAACTTTTCTTTAGTCGGGCATATCCCACCGAAAGGTGGAGCAATTCCCGATGTGATCCGC
CTTTGGCGGATTAAAGTCGCAAGACGCTAAAGGTGAGGCCTGGGCACCATCAGCTAGTTGGTAAGGTA
ACGGCTTACCAAGGCTATGACGGTTAGGGGCCCCGAGAGGGGAGACCCCCACACTGGGACTGCGACAC
GGCC
>Zotu3079
GAACGCTGGCGGCGTGCTTAACACATGCAAGTCGAACGGTGAAGCAAGAGCTTGCTCTTGTGGATCAG
TGGCGAACGGGTGAGTAACACGTGAGTAACCTGCCCCAGACTCTGGGATAACCTCGGGAAACCGTGGC
TAATACCGGATACGAAACGAGATCGCATGATCATCGTTTGGAAAGAATTTCGGTCTGGGATGGACTCG
CGGCCTATCAGCTTGTTGGTGAGGTAATGGCTCACCAAGGCGACGACGGGTAGCCGGCCTGAGAGGGC
GACCGGCCACACTGGAACTGAGACACGGTC
>Zotu3080
GAACGCTGGCGGCATGCCTTACACATGCAAGTCGAACGGTAACGCGGGGCAACCTGGCGACGAGTGGC
GAACGGGTGAGTAATATATCGGAACGTGCCCAGTAGTGGGGGATAGCCCGGCGAAAGCCGGATTAATA
CCGCATGAGACCTGAGGGTGAAAGCGGGGGACTCGCAAGAGCCTCGCGCTATTGGAGCGGCCGATATC
AGATTAGCTAGTTGGTGGGGTAAAGGCCTACCAAGGCGACGATCTGTAGCTGGTCTGAGAGGACGACC
AGCCACACTGGGACTGAGACACGGCC
>Zotu3081
GAACGCTGGCGGCGTGCCTAACACATGCAAGTCGAGCGGGATCCAACCAGTGGCAACACTGGGGAAGA
TCTAGCGGCGAACGGGTGAGTAACACGTGAGAAACTTGCCCCGGAGTTCGGGATAACCCGAGGAAACT
CGAGCTAATACCGAATGCCCTCTTCTGATCGCATGGTCGGATGAGGAAAGATTTATCGCTCCGGGAGA
GTCTCGCGGCCTATCAGCTAGTTGGCGGGGTAACGGCCCACCAAGGCATCGACGGGTAGCTGGTCTGA
GAGGACGATCAGCCACACTGGGACTGAGACACGGCC
>Zotu3082
GAACGCTGGCGGCGTGCCTAATACATGCAAGTCGAGCGAACTGAATTGGAGCTTGCTCCAATGACGTT
AGCGGCGGACGGGTGAGTAACACGTGGGTAACCTGCCCTACAGACTGGGATAACTTCGGGAAACCGGA
GCTAATACCGGATAATACTTTTTAACACATGTTAGAAAGTTGAAAGATGGTTCTGCTATCACTGTAGG
ATGGACCCGCGGCGCATTAGCTAGTTGGTGAGGTAACGGCTCACCAAGGCAACGATGCGTAGCCGACC
TGAGAGGGTGATCGGCCACACTGGGACTGAGACACGGCC
>Zotu3083
GAACGCTGGCGGCGTGCTTAACACATGCAAGTCGAACGGTGATCCTGGTGCTTGCACCGGGTGATCAG
TGGCGAACGGGTGAGTAACACGTGAGCAACCTGCCCCTGACTCTGGGATAACCCCGAGAAATCGGAGC
TAATACCGGATACGAGACGCACAGGCATCTGTAGCGTCTGGAAAGAATTTTGGTCAGGGATGGGCTCG
CGGCCTATCAGCTTGTTGGTGGGGTAACGGCCCACCAAGGCGACGACGGGTAGCCGGCCTGAGAGGGC
GACCGGCCACACTGGGACTGAGACACGGCC
>Zotu3085
GAACGCTAGCGGCAGGCCTAATACATGCAAGTCGGACGGGATAGGAGAGCTTGCTTTCCTTGAGAGTG
GCGCACGGGTGCGTAACACGTATGTAACCTACCCTTGTCAGGGGGATAGCCTCTCGAAAGAGAGATTA
AGACCGCATAACATTATGATATAGCATTATATTATAATCAAATATTTATAGGACAAGGATGGGCATGC
GGAACATTAGCTAGTTGGTGTGGTAACGGCATACCAAGGCTACGATGTTTAGGGGATCTGAGAGGATG
ACCCCCCACACTGGTACTGAGACACGGAC
>Zotu3086
GAACGCTGGCGGCATGCCTAACACATGCAAGTCGAACGAAGGCTTCGGCCTTAGTGGCGCACGGGTGC
GTAACGCGTGGGAATCTGCCCTTGGGTTCGGAATAACAGTTAGAAATGACTGCTAATACCGGATGATG
ACGAAAGTCCAAAGATTTATCGCCCAGGGATGAGCCCGCGTAGGATTAGGTAGTTGGTGGGGTAAAGG
CTCACCAAGCCGACGATCCTTAGCTGGTCTGAGAGGATGATCAGCCACACTGGGACTGAGACACGGCC
>Zotu3087
GAACGCTGGCGGCGTGCCTAATACATGCAAGTCGAGCGGAGTTGAATAAGTGCTTGCACTAATTCAAC
TTAGCGGCGAACGGGTGAGTAACACGTAAAGAACCTGCCTCATAGACTGGGACAACTACTGGAAACGG
TAGCTAATACCGGATAACATATTTTTCTGCATGGAAGAATATTGAAAGTCGGTCATGCTGACACTATG
AGATGGCTTTGCGGTGCATTAGCTAGTTGGTAGGGTAAAGGCCTACCAAGGCGACGATGCATAGCCGA
CCTGAGAGGGTGATCGGCCACACTGGGACTGAGACACGGCC

>Zotu3088
GAACGCTGGCGGCAGGCTTAACACATGCAAGTCGAACGATGTAGCAATACATAGTGGCGCACGGGTGA
GTAACGCGTGGGAACATGCCCTTCAGTTCAGAATAACAGCGGGAAACTGCTGCTAATACTGGATATTC
CCTCCGGGGGAAAGATTTATCGCTGAAGGATTGGCCCGCGTAAGATTAGGTAGTTGGTGGGGTAATGG
CCTACCAAGCCTACGATCTTTAGCTGGTCTGAGAGGATGATCAGCCACACTGGAACTGAGACACGGTC
>Zotu3089
GAACGTTGGCGGCGTGCCTTAAGCATGCAAATCAAGGGGGTCCCGCAAGGGACTACCGGTGGACGGGT
GAGTAATGCGTGGGAATCTACCTGGAAGTTGGACATAGCTTGCCGAAAGGCGAGGTAATTTCCAATGT
GACCGCAAGGTCAAAGTCGCAAGACGCTTTTAGATGAGCCCACGTCCTATCAGCTAGTTGGTGGGGTA
ATGGCCTACCAAGGCAACGACGGGTAGCTGGACTGAGAGGTCGGTCAGCCACAATGGGACTGCGACAC
GGCC
>Zotu3090
GAACGCTGGCGGCGTGCCTAACACATGCAAGTGGAGCGACGAACCCGGACTTGTCCGGGGGCAAAGCC
GCGAACGGGTGAGTAACACGTGGATTATCTGCCCCAATGACCGGGACAACTCGAGGAAACTCGAGCTA
ATACCGGATGTGCTCTTCGGAGGAAAGGAAGCTTCGGCCTCCGCATTGGGATGAGTCCGCGGCGCATT
AGCTAGTTGGTGAGGTAAAGGCTCACCAAGGCGACGATGCGTAGCTGGTCTGAGAGGACGATCAGCCA
CACTGGAACTGAGACACGGTC
>Zotu3091
GAACGCTGGCGGCGTGCTTAACACATGCAAGTCGAACGGTGAACTCGCTTCGGTGGGGGATCAGTGGC
GAACGGGTGAGTAACACGTGGGCAACCTGCCCCCGGCTCTGGGATAACTCCAAGAAATTGGGGCTAAT
ACCGGATATGACCGCTGACCGCATGGTCTGGTGGTGGAAAGATTTATCGGCTGGGGATGGGCCCGCGG
CCTATCAGCTTGTTGGTGGGGTAGTGGCCTACCAAGGCGACGACGGGTAGCCGGCCTGAGAGGGTGAC
CGGCCACACTGGGACTGAGACACGGCC
>Zotu3092
GAACGCTGGCGGCGTGCTTAACACATGCAAGTCGAACGGTGAAGCTCCAGCTTGCTGGAGTGGATCAG
TGGCGAACGGGTGAGTAACACGTGAGCAACCTGCCCCTGACTCTGGAATAAGCGCTGGAAACGGCGTC
TAATACTGGATACGAGAAGGGTCCGCATGGGCACTTTCTGGAAAGTTTTTCGGTTGGGGATGGGCTCG
CGGCCTATCAGCTTGTTGGTGAGGTAATGGCTCACCAAGGCGACGACGGGTAGCCGGCCTGAGAGGGC
GACCGGCCACACTGGGACTGAGACACGGCC
>Zotu3094
GAACGCTGGCGGCACGCCTAACACATGCAAGTCGAACGGAGAAAATAGCTTCGGTTGTTTTCTTAGTG
GCGGACGGGTGAGTAACACGTGAGGAACCTGCCTTTGAGAGTGGGATAGCTTCTGGAAACGGATGGTA
ATACCGCATAACATAGCGAAACCGCATGATTTTGCTATCAAAGTATTTCGCTCAGAGATGGCCTCGCG
TCTGATTAGATAGTTGGTGAGGTAACGGCTCACCAAGTCGACGATCAGTAGCCGGACTGAGAGGTTGA
ACGGCCACATTGGGACTGAGACACGGCC
>Zotu3095
GAACGCTGGCGGCGTGCCTAATACATGCAAGTCGAGCGAATCTTGAGGTGCTTGCACCTCTTGGTTAG
CGGCGGACGGGTGAGTAACACGTGGGCAACCTGCCTGTAAGACTGGGATAACACCGGGAAACCGGTGC
TAATACCGGATAATCCTTTTCCTCTCATGAGGAAAAGCTGAAAGTCGGTTTACGCTGACACTTACAGA
TGGGCCCGCGGCGCATTAGCTAGTTGGTGAGGTAACGGCTCACCAAGGCGACGATGCGTAGCCGACCT
GAGAGGGTGATCGGCCACACTGGGACTGAGACACGGCC
>Zotu3096
GAACGCTGGCGGCGTGCTTAACACATGCAAGTCGAACGGTGAAGGGATCAGCTTGCTGGTTCTGGATG
AGTGGCGAACGGGTGAGTAACACGTGAGTAACCTGCCCCCTTCTTCTGGATAACCGCATGAAAGTGTG
GCTAATACGGGATATTCTGGGTCTGTCGCATGACGGGTCCGGGAAAGATGCGCCTTTGGGTGTTTTTG
GTGGGGGATGGGCTCGCGGCCTATCAGCTTGTTGGTGGGGTGATGGCCTACCAAGGCGGTGACGGGTA
GCCGGCCTGAGAGGGTGGACGGTCACACTGGGACTGAGACACGGCC
>Zotu3097
GAACGCTGGCGGCAGGCCTAACACATGCAAGTCGAGCGGTCGTAGCAATACGGCAGCGGCAGACGGGA
GAGTAACACGTGGGAACGCGCCCTTCGGTTCGGAATAACTCAGGGAAACTTGGGCTAATACCGGATAC
GCCCTTACGGGGAAAGATTTATTGCCGAAGGAACGGCCCGCGTCCGATTAGCTTGTTGGTGAGGTAAT
GGCTCACCAAGGCGACGATCGGTAGCTGGTCTGAGAGGATGACCAGCCTCACTGGGACTGAGACACGG
CC
>Zotu3098

GAACGCTGGCGGCGTGCTTAACACATGCAAGTCGAGCGAAGAGCGATGGAAGCTTGCTTCTATCAATC
TTAGTGGCGAACGGGTGAGTAACGCGTAATCAACCTGCCCTTCAGAGGGGGACAACAGTTGGAAACGA
CTGCTAATACCGCATACGATTTAATCTCGGCATCGAGGATAGATGAAAGGTGGCCTCTACATGTAAGC
TATCACTGAAGGAGGGGATTGCGTCTGATTAGCTAGTTGGAGGGGTAACGGCCCACCAAGGCGATGAT
CAGTAGCCGGTCTGAGAGGATGAACGGCCACATTGGGACTGAGACACGGCC
>Zotu3099
GAACGCTGGCGGCATGCCTAACACATGCAAGTCGAACGAAACCTTCGGGTTTAGTGGCGCACGGGTGC
GTAACGCGTGGGAATCTGCCCCTTGGTTCGGAATAACAGTTGGAAACGACTGCTAATACCGGATGACG
ACGTAAGTCCAAAGATTTATCGCCGAGGGATGAGCCCGCGTAGGATTAGGTAGTTGGTGTGGTAAAGG
CGCACCAAGCCGACGATCCTTAGCTGGTCTGAGAGGATGATCAGCCACACTGGGACTGAGACACGGCC
>Zotu3100
GAACGCTGGCGGCGTGCTTAACACATGCAAGTGGAGCGACGAACCAGGGCTTGCCCTGGGGCAGAGCC
GCGAACGGGTGAGTAACACGTGGGTAACCTACCCCGATGATTGGGACAACCCGAGGAAACTCGGGCTA
ATACCAAATGTGCTCGAAGTCCATGAGGACTCCGAGTAAAGATAGCTTCGGCCTTCGCATCGGGACGG
GCCCGCGGCCCATTAGCTTGTTGGTGGGGTAACGGCCTACCAAGGCGACGATGGGTAGCTGGTCTGAG
AGGACGATCAGCCACACTGGGACTGAGACACGGCC
>Zotu3101
GAACGCTAGCGGCAGGCTTAATACATGCAAGTCGAGGGGCAGCAGGAATAGCAATATTTGCTGGCGAC
CGGCGCACGGGTGCGTAACACGTATACAATCTGCCTTTAACAGGGGGATAGCCCGAAGAAATTCGGAT
TAATACCCCATAACAGTGATCTTGGCATCAAGGACACTTAAAAGCTCCGGCGGTTAAAGATGAGTATG
CGTCTGATTAGCTTGTTGGTGAGGTAACGGCTCACCAAGGCGACGATCAGTAGGGGATCTGAGAGGAT
TAACCCCCACACGGATACTGAGACACGGAT
>Zotu3102
GAACGCTAGCTACAGGCTTAACACATGCAAGTCGAGGGGCAGCATGGTCTTAGCTTGCTAAGGCTGAT
GGCGACCGGCGCACGGGTGAGTAACACGTATCCAACCTGCCGTCTACTCTTGGCCAGCCTTCTGAAAG
GAAGATTAATCCAGGATGGCATCATGAGTTCACATGTCCGCATGATTAAAGGTATTTTCCGGTAGACG
ATGGGGATGCGTTCCATTAGATAGTAGGCGGGGTAACGGCCCACCTAGTCAACGATGGATAGGGGTTC
TGAGAGGAAGGTCCCCCACATTGGAACTGAGACACGGTC
>Zotu3103
GAACGCTGGCGGCGTGCTTAACACATGCAAGTCGAGCGGAAAGGCCCTTCGGGGTACTCGAGCGGCGA
ACGGGTGAGTAACACGTGGGCAACCTGCCCTCAGCTCTGGGATAACTCCGGGAAACCGGGGCTAATAC
TGGATACGACAGATCTCCGCATGGGGTGTCTGTGGAAAGATTCATCGGCTGAGGATGGGCCCGCGGCC
TATCAGCTTGTTGGTGGGGTGATGGCCTACCAAGGCGACGACGGGTAGCCGGCCTGAGAGGGCGACCG
GCCACACTGGGACTGAGACACGGCC
>Zotu3105
GAACGCTGGCGGCGTGCTTAACACATGCAAGTCGAACGGGATCCCAGGAGCTTGCTCCTGGGTGAGAG
TGGCGAACGGGTGAGTAATGCGTGACCGACCTGCCCCATGCACCGGAATAGCTCCTGGAAACGGGTGG
TAATGCCGGATGCTCCATCACACTGCATGGTGTGTTGGGAAAGCCTTTGCGGCATGGGATGGGGTCGC
GTCCTATCAGCTTGATGGCGGGGTAACGGCCCACCATGGCTTCGACGGGTAGCCGGCCTGAGAGGGCG
ACCGGCCACATTGGGACTGAGATACGGCC
>Zotu3106
GAACGCTGGCGGCGTGCTTAACACATGCAAGTCGAACGTGAAATATAGCAATACAGAGTAAAGTGGCG
TACGGGTGAGTAACACGTGGGTAATCTACCTTAGAGTTTGGAATAACCGGTCGAAAGGCTGGCTAATA
CTGAATAGATCAAGTGCACTGTAGAGGTGCGATTGGGAAAGGGAGCCGGTAACGGCCTTCGCTTTAAG
ATGAGCCCGCGTCTGATTAGCTAGTTGGTGAGGTAATGGCTCACCAAGGCATTGATCAGTAGCTGGTC
TGAGAGGATGATCAGCCACACTGGAACTGAAACACGGTC
>Zotu3107
GAACGCTGGCGGCGTGCCTAACACATGCAAGTCGAGCGATTTACTTCGGTAAAGAGCGGCGGACGGGT
GAGTAACGCGTGGGTAACCTGCCCTGTACACACGGATAACATACCGAAAGGTATGCTAATACGAGATA
ATATGTTTTTATCGCATGGTAGAAGTATCAAAGCTTTTGCGGTACAGGATGGACCCGCGTCTGATTAG
CTAGTTGGTAAGGTAACGGCTTACCAAGGCGACGATCAGTAGCCGACCTGAGAGGGTGATCGGCCACA
TTGGAACTGAGACACGGTC
>Zotu3108
GAACGCTGGCGGCGTGCTTAACACATGCAAGTCGAGCGGTAAGGCCTTTCGGGGTACACGAGCGGCGA

ACGGGTGAGTAACACGTGGGTGATCTGCCCTGCACTCTGGGATAAGCTTGGGAAACTGGGTCTAATAC
CGGATATGACCACATGCCGCATGGTGTGTGGTGGAAAGATTTATCGGTGCAGGATGGGCCCGCGGCCT
ATCAGCTTGTTGGTGGGGTAATGGCCTACCAAGGCGACGACGGGTAGCCGACCTGAGAGGGTGACCGG
CCACACTGGGACTGAGACACGGCC
>Zotu3109
GAACGCTGGCGGCGTGCTTAACACATGCAAGTCGAACGCTGAAGCTTGGTGCTTGCACTGGGTGGATG
AGTGGCGAACGGGTGAGTAATACGTGAGTAACCTGCCCTTGACTCTGGGATAAGCCCGGGAAACTGGG
TCTAATACTGGATGCTACATGTCACCGCATGGTGGTGTGTGGAAAGGGTTTACTGGTCTTGGATGGGC
TCACGGCCTATCAGCTTGTTGGTGAGGTAATGGCTCACCAAGGCGACGACGGGTAGCCGGCCTGAGAG
GGTGACCGGCCACACTGGGACTGAGACACGGCC
>Zotu3110
GAACGCTAGCGGCAGGCCTAACACATGCAAGTCGAGGGGTATAGTAGCAATACTAGAGACCGGCGCAC
GGGTGCGTAACGCGTATGCAATCTACCTTTTACAGGGGAATAGCCCAGAGAAATTTGGATTAATGCCC
CATGGTATATATGAATGGCATCATTTATATATTAAAGTTCCAACGGTAAAAGATGAGCATGCGTCCCA
TTAGTTAGTTGGTAAGGTAACGGCTTACCAAGACGATGATGGGTAGGGGTCCTGAGAGGGAGATCCCC
CACACTGGTACTGAGACACGGAC
>Zotu3111
GAACGCTGGCGGCATGCCTAACACATGCAAGTCGAACGAGGGCTTCGGCCCTAGTGGCGCACGGGTGA
GTAACGCGTGGGAACCTGCCCCGTGGTACGGAATAACTCCGGGAAACTGGAGCTAATACCGTATGTGT
CCCGAGGGACAAAGATTCATCGCCATGGGATGGGCCCGCGTAGGATTAGCTTGTTGGCGGGGTAACGG
CCCACCAAGGCGACGATCCTTAGCTGGTCTGAGAGGATGATCAGCCACACTGGGACTGAGACACGGCC
>Zotu3113
GAACGCTGGCGGCGTGCTTAACACATGCAAGTGGAGCGACGAACCAGGGCTTGCCCTGGGGCAGAGCC
GCGAACGGGTGAGTAACACGTGGGTAACCTACCTTGATGACCGGGACAACCCGAGGAAACTCGGGCTA
ATACCGGATGTGCTCCTTCCACATCAGTGGATTGAGGAAAGATAGCTTCGGCCTTCGCATCAAGATGG
GCCCGCGGCGCATTAGCTTGTTGGTGGGGTAACGGCCTACCAAGGCTTCGATGCGTAGCTGGTCTGAG
AGGACGATCAGCCACACTGGGACTGAGACACGGCC
>Zotu3114
ACCGTGTCTCAGTTCCAGTGTGGCTGATCATCCTCTCAGACCAACTAAAGATCGTTGCCTTGGTGAGC
CATTACCTCACCAACTAGCTAATCCTGCATGGGCCCATCTTGCAGCAGTAAACCTTTCACCTCTCGGC
TATATGCGACATTAGCAATTGCTTCCAACTGTTATTTCGCACTGCAAGGTAGATTCCCATGTATTACT
CACCCGTCTGCCACTAGCAGAACCCTGTATTGCTACAGCTCTTGCTCGTTCGACTTGCATGTGTTAAG
CACGCCGCCAGCGTTCA
>Zotu3115
GAACGCTGGCGGCGTGCTTAACACATGCAAGTCGAGCGGTAAGGCCCTTCGGGGTACACGAGCGGCGA
ACGGGTGAGTAACACGTGGGTGACCTGCCCTCCACTTCGGGATAAGCCCGGGAAACTGGGTCTAATAC
CGGATATGACCTTGAAGCGCATGTTTCTTGGTGGAAAGTTTTTTCGGTGGGGGATGGGCCCGCGGTCT
ATCAGCTTGTTGGTGGGGTGATGGCCTACCAAGGCGACGACGGATAGCCGGCCTGAGAGGGTGATCGG
CCACACTGGGACTGAGACACGGCC
>Zotu3116
GAACGCTGGCGGCAGGCTTAACACATGCAAGTCGAACGCACCTTTCGGGGTGAGTGGCAGACGGGTGA
GTAACGCGTGGGAACGTGCCCTTCAGTTCGGAATAACCCAGGGAAACTTGGGCTAATACCGGATACGT
GCGAGAGCAGAAAGATTTATCGCTGAAGGATCGGCCCGCGTCTGATTAGCTAGTTGGTAGGGTAATGG
CCTACCAAGGCGACGATCAGTAGCTGGTCTGAGAGGATGATCAGCCACATTGGGACTGAGACACGGCC
>Zotu3117
GAACGCTGGCGGCGTGCTTAACACATGCAAGTCGAACGGAAAGGCCAGTGCTTGCACTGGTACTCGAG
TGGCGAACGGGTGAGTAACACGTGGGTGATCTGCCTTGTACTCTGGGATAAGCTTGGGAAACTGGGTC
TAATACCGGATAGGAACTGCCTTTAGTGTGGTGGTTGGAAAGTTTTTTCGGTGCAAGATGAGCTCGCG
GCCTATCAGCTTGTTGGTGGGGTAATGGCCTACCAAGGCGTCGACGGGTAGCCGGCCTGAGAGGGTGT
ACGGCCACATTGGGACTGAGATACGGCC
>Zotu3118
GAACGCTGGCGGCGTGCTTAACACATGCAAGTCGAGCGGTAAGGCCCTTCGGGGTACACGAGCGGCGA
ACGGGTGAGTAACACGTGAGCAACCTGCCCCTGACACCGGGATAAGCGGCGGAAACGCTGTCTAATAC
CGGATACGACCTTCCGGCGCCTGCCGGGGGGTGGAAAGTCTTGTCGGTCGGGGATGGGCTCGCGGCCT

ATCAGCTTGTTGGTGGGGTAACGGCCTACCAAGGCGATTACGGGTAGCCGGCCTGAGAGGGCGACCGG
CCACACTGGGACTGAGACACGGCC
>Zotu3119
GAACGCTGGCGGCGTGCTTAACACATGCAAGTCGAACGGTGAAGGTCAGCTTGCTGACCGGATCAGTG
GCGAACGGGTGAGTAACACGTGAGCAACCTGCCCTCCACTCTGGGATAAGCCTTGGAAACGAGGTCTA
ATACTGGATACGAGACGCCCGGGCATCTGGAGCGTCTGGAAAGATTTATCGGTGGGGGATGGGCTCGC
GGCCTATCAGCTTGTTGGTGGGGTGATGGCCTACCAAGGCGACGACGGGTAGCCGGCCTGAGAGGGCG
ACCGGCCACACTGGGACTGAGACACGGCC
>Zotu3120
GAACGCTGGCGGCGTGCCTAATACATGCAAGTCGAGCGGCAGCGCGGTCTTCGGACTGGCGGCGAGCG
GCGGACGGCTGAGTAACGTGTAGGAATGTACCCCAAAGTGAGGGATAAGCACCAGAAATGGTGTCTAA
TACCGCATGTGCTCTAAGGAGTAAAGCTTTAGCGCTTTGGGAACAGCCTGCGTCCGATTAGCTAGTTG
GTGAGGTAATGGCTTACCAAGGCGATGATCGGTAGCTGGTCTGAGAGGATGATCAGCCAGACTGGGAC
TGAGAACGGCC
>Zotu3122
GAACGCTGGCGGCGTGCTTAACACATGCAAGTCGAGGGGGCGGGAGGGGCAACCCTGTCGTCACCGGC
GAACGGGTGCGTAACACGTGAGCGACCTACCTTTTTGCGGGGGATAGCCGGCCCAACGGCCGGGTAAT
ACCGCATACGCTCTTCTTCGGGCATCCGGGGGGGAGGAAACTTTCGAGGCAGAGAGAGGGGCTCGCGG
CCTATCAGCTAGTTGGCGGGGTAACGGCCCACCAAGGCAGTGACGGGTAGCTGGTCTGAGAGGATGGC
CAGCCACATTGGGACTGAGAGACGGCC
>Zotu3123
GAACGCTGGCGGCATGCCTAATACATGCAAGTCGCGCGAGAAAGTCCTTCGGGATGAGTAGAGCGGCG
CACGGGTGAGTAATGCATAGGAACGTACCTTCTGATGGGGAATAACCACGGGAAACTGTGGCTAATAC
CGCATAATGACGCGAGTCTAAATCCGGCCTCTATTTATAAGCTGGAGTCAGTAGAGCGGCCTATGTCC
CATTAGCTAGTTGGTAGGGTAATGGCCTACCAAGGCGACGATGGGTAGCTGGTCTGAGAGGATGATCA
GCCACACTGGGACTGAGACACGGCC
>Zotu3124
GAACGCTGGCGGCGTGCTTAACACATGCAAGTCGAACGATGAAGCTGGAGCTTGCTCCGGTGGATTAG
TGGCGAACGGGTGAGTAACACGTGAGCAATCTGCCCTTGACTCTGGGATAACTGCGGGAAACTGTAGC
TAATACCGGATACGAGGCTTGGAGGCATCTCCAGGCCTGGAAAGAATTTCGGTCAAGGATGAGCTCGC
GGCCTATCAGCTAGTTGGTGAGGTAATGGCTCACCAAGGCGACGACGGGTAGCCGGCCTGAGAGGGTG
ACCGGCCACACTGGGACTGAGACACGGCC
>Zotu3125
GAACGCTGGCGGCGTGCCTAATACATGCAAGTCGAGCGAACCGAATGAAAGCTTGCTTTCATTCGGTT
AGCGGCGGACGGGTGAGTAACACGTGGGCAACCTGCCCGTAAGACCGGGATAACTTCGGGAAACCGGA
GCTAATACCGGATAACACCGAAGATCGCATGATCTTTGGTTGAAAGGCGGCTTTGGCTGTCACTTACG
GATGGGCCCGCGGCGCATTAGCTAGTTGGTGAGGTAACGGCTCACCAAGGCGACGATGCGTAGCCGAC
CTGAGAGGGTGATCGGCCACACTGGGACTGAGACACGGCC
>Zotu3126
GAACGCTGGCGGCGTGCCTAATACATGCAAGTCGAGCGGTAAGGCCACTTCGGTGGTACACGAGCGGC
GGACGGCTGAGTAACGCGTAGGAACGTACCCCAAACTGAGGGATAACTGCTCGAAAGAGTAGCTAATA
CCGCATGTGCTCTACGGAGTAAAGCTTCGGCGGTTTGGGAACGGCCTGCGTATGATTAGCTTGTTGGT
GAGGTAATGGCTCACCAAGGCGACGATCATTAGCTGGTCTGAGAGGATGATCAGCCAGACTGGGACTG
AGACACGGCC
>Zotu3127
GAACGCTGGCGGCGTGCTTAACACATGCAAGTCGAGCGGAAAGGCCACTTCGGTGGTACTCGAGCGGC
GAACGGGTGAGTAACACGTGAGTAATCTGCCCTTGGCTTCGGGATAGCCACCGGAAACGGTGATTAAT
ACCGGATATGACGCGGGCCCGCATGGGTTTGTGTGGAAAGTTTTTCGGCCAGGGATGTGCTCGCGGCC
TATCAGCTTGATGGTGAGGTAATGGCTCACCATGGCTTCGACGGGTAGCCGGCCTGAGAGGGTGACCG
GTCACACTGGGACTGAGACACGGCC
>Zotu3129
GAACGCTGGCGGCGTGCATAACACATTCAAGTCGAACGGTGAAGCACCTTCGGGTGTGGATCAGTGGC
GGACGGGTGAGTAACGCGTGAGCAATCTGCCCTGCACTGAGGGATAACGACCGGAAACAGTCGCTAAT
ACCTCATGATGTTTTCTTACCGCATGGTAGGACTACCAAAGATTTATTGGTGCAGGATGAGCTCGCGT

CCGATTAGATAGTTGGCGGGGCAACGGCCCACCAAGTCTGCGATCGGTAGCCGGACTGAGAGGTTGAA
CGGCCACATTGGAACTGAGAAACGGTC
>Zotu3130
GAACGCTGGCGGCGTGCTTAACACATGCAAGTCGAACGATGATCTCCCGCTTGCGGGGGTGATTAGTG
GCGAACGGGTGAGTAATACGTGAGTAACCTGCCCTTGACTCTGGGATAAGCCTGGGAAACCGGGTCTA
ATACTGGATACTACCTTTCACCGCATGGTGGGGGGTGGAAAGGGTTTTACTGGTTTTGGATGGGCTCA
CGGCCTATCAGCTTGTTGGTGGGGTAATGGCTCACCAAGGCGACGACGGGTAGCCGGCCTGAGAGGGT
GACCGGCCACACTGGGACTGAGACACGGCC
>Zotu3131
GAACGCTGGCGGCGTGCTTAACACATGCAAGTCGAACGGTGACCTCGAGAGCTTGCTCTTGGGTGATC
AGTGGCGAACGGGTGAGTAACACGTGAGTAACCTGCCCCAGACTCTGGAATAACCCCGGGAAACCGGA
GCTAATACCGGATACGAGACGGAGCCGCATGGCTACCGTTTGGAAAGTTCTTCGGTCTGGGATGGACT
CGCGGCCTATCAGCTAGTTGGTGAGGTAACGGCTCACCAAGGCGACGACGGGTAGCCGGCCTGAGAGG
GCGACCGGCCACACTGGGACTGAGACACGGCC
>Zotu3132
GAACGCTGGCGGCGTGCTTAACACATGCAAGTCGAACGGAAAGGCCTCAGCTTGCTGGGGTACTCGAG
TGGCGAACGGGTGAGTAACACGTGGGTGATCTGCCTTGCACTTCGGGATAAGCTTGGGAAACTGGGTC
TAATACCGGATAGGACAGCTGTTTAGTGTCAGTTGTGGAAAGTTTTTTCGGTGCAAGATGAGCTCGCG
GCCTATCAGCTTGTTGGTGGGGTAATGGCCTACCAAGGCGTCGACGGGTAGCCGGCCTGAGAGGGTGG
ACGGCCACATTGGGACTGAGATACGGCC
>Zotu3133
GAACGCTGGCGGCAGGCTTAACACATGCAAGTCGAGCGCCCCGCAAGGGGAGCGGCAGACGGGTGAGT
AACGCGTGGGAATCTACCCTTTACTACGGAATAACGCATGGAAACGTGTGCTAATACCGTATGTGTCC
TTCGGGAGAAAGATTTATCGGTAAAGGATGAGCCCGCGTTGGATTAGCTAGTTGGTGGGGTAAAGGCC
TACCAAGGCGACGATACATAGCTGGTCTGAGAGGATGATCAGCCACATTGGGACTGAGACACGGCC
>Zotu3134
GAACGCTGGCGGCATGCCTAACACATGCAAGTCGAACGAGACCTTCGGGTCTAGTGGCGCACGGGTGC
GTAACGCGTGGGAATCTGCCCTTGGGTTCGGAATAACTCGCCGAAAGGCGTGCTAATACCGGATGATG
TCGCAAGACCAAAGATTTATCGCCCAAGGATGAGCCCGCGTAAGATTAGGTAGTTGGTGAGGTAAAGG
CTCACCAAGCCGACGATCTTTAGCTGGTCTGAGAGGATGATCAGCCACACTGGGACTGAGACACGGCC
>Zotu3135
GAACGCTGGCGGCAGGCTTAACACATGCAAGTCGAGCGCCCCGCAAGGGGAGCGGCAGACGGGTGAGT
AACGCGTGGGAATCTACCTAACTCTACGGAATAACCCAGGGAAACTTGGACTAATACCGTATACGCCC
CTCGGGGGAAAGATTTATCGGAGTTGGATGAGCCCGCGTTGGATTAGCTAGTTGGTGGGGTAATGGCC
TACCAAGGCGACGATCCATAGCTGGTCTGAGAGGATGATCAGCCACACTGGGACTGAGACACGGCC
>Zotu3137
GAACGCTGGCGGCGTGCTTAACACATGCAAGTCGAACGGTAAGGCCCCTTCGGGGGTACACGAGTGGC
GAACGGGTGAGTAACACGTGAGTAACCTGCCCTTGACTCTGGGATAACAGCTGGAAACAGCTGCTAAT
ACCGGATATCAGCCCCGATGGCATCTTCGAGGCTTGAAAGCTCCGGCGGTCATGGATGGACTCGCGGC
CTATCAGCTTGTTGGTGAGGTAACGGCTCACCAAGGCGACGACGGGTAGCCGGCCTGAGAGGGCGACC
GGCCACATTGGGACTGAGATACGGCC
>Zotu3138
GAACGCTGGCGGCGGGTTTAACACATGCAAGTCGAGGGACGAAGCCTTTCTTCGGAGAGGTGGAGACC
GGCGAACGGGTGAGTAACACGTGAGGAACCTACCCGGTACTTCGGGATAACCGCGGGAAACCGTGGCT
AATACCGGATATTCCATGTCTGGCGCATGTCAGGTTTGGGAAAGTTCAGGCGGTATCGGACGGCCTCG
CGGCGTATCAGCTTGTTGGTGGGGTAATGGCTTACCAAGGCGTCGACGCGTAGCTGGAGTGAGAGCTC
GATCAGCCACACTGGGACTGAGACACGGCC
>Zotu3139
GAACGCTGGCGGCAGGCTTAACACATGCAAGTCGAACGGTCCTTCGGGATAGTGGCGGACGGGTGAGT
AACACGTGGGAACGTACCCTTAGGTTCGGAACAACACAGGGAAACTTGTGCTAATACCGGATGAGCCC
TTCGGGGGAAAGATTTATCGCCTTTGGAGCGGCCCGCGTCTGATTAGCTAGTTGGTGAGGTAATGGCT
CACCAAGGCGACGATCAGTAGCTGGTCTGAGAGGATGATCAGCCACATTGGGACTGAGACACGGCC
>Zotu3140
GAACGCTAGCGGCAGGCCTAATACATGCAAGTCGAACGGTCGTAGTGATACGATAGTGGCGCACGGGT

GCGTAACACGTATGCAACCTACCTTATAGAGGGGGATAGCCTTTGGAAACGGAGATTAATACCCCATA
ATACATTTGATAGGCATCTATTTAATGTTAAAGATTTATCGCTATAAGCTGGGCATGCGTCCAATTAG
TTAGTTGGCGGGGTAATGGCTCACCAAGACTTTGATTGGTAGGGGAACTGAGAGGTTAATCCCCCACA
CTGGCACTGAGATACGGGC
>Zotu3141
GAACGCTGGCGGCGCGCCTAACACATGCAAGTCGAACGAGAGAGAGGGAGCTTGCTTCCTCAATCGAG
TGGCGAACGGGTGAGTAACGCGTGAGGAACCTGCCTCAAAGAGGGGGACAACAGTTGGAAACGACTGC
TAATACCGCATAAGCCCACGACCCGGCATCGGGTTGAGGGAAAAGGAGCAATCCGCTTTGAGATGGCC
TCGCGTCCGATTAGCTAGTTGGTGAGGTAATGGCCCACCAAGGCGACGATCGGTAGCCGGACTGAGAG
GTTGAACGGCCACATTGGGACTGAGACACGGCC
>Zotu3142
GAACGCTGGCGGCGTGCTTAACACATGCAAGTCGAACGAAGCATTCGCAGCTGAGGCCCTTCGGGGTT
GACGCAGCGAATGACTGAGTGGCGGACGGGTGAGTAACACGTGAGTAACCTGCCTTTCACCGTGGAAT
AACCATTGGAAACGATGGCTAATACCGCATAATGCTACATGACGGCATCGTCGTGTATCCAAAGATTT
TATCAGTGAAAGATGGACTCGCGCCCGATTAGATAGTTGGTGAGGTAACGGCTCACCAAGTCTGCGAT
CGGTAGCCGAACTGAGAGGTTGATCGGCCACATTGGGACTGAGACACGGCC
>Zotu3143
GAACGCTGGCGGCGTGCCTAATACATGCAAGTTGAGCGATGAAGATTGGTGCTTGCACCAATTTGAAG
AGCAGCGAACGGGTGAGTAACGCGTGGGGAATCTGCCTTTGAGCGGGGGACAACATTTGGAAACGAAT
GCTAATACCGCATAATAACTTTAAACATAAGTTTTAAGTTTGAAAGATGCAATTGCATCACTCAAAGA
TGATCCCGCGTTGTATTAGCTAGTTGGTGAGGTAAAGGCTCACCAAGGCGATGATACATAGCCGACCT
GAGAGGGTGATCGGCCACATTGGGACTGAGACACGGCC
>Zotu3144
GAACGCTGGCGGCATGCCTAATACATGCAAGTCGAACGAAGTTATGATGGAGCTTGCTCCTGATTAGC
TTAGTGGCGGACGGGTGAGTAACACGTGGGCAACCTGCCTACAAGTTGGGGATAACTAGCGGAAACGT
TAGCTAATACCGAATGGTCAAGGATGAGGCATCTTATCCTTGGGAAAGACGGAGCAATCTGTCACTTG
TAGATGGGCCCGCGGTGCATTAGCTAGTTGGTGAGGTAACGGCTCACCAAGGCGACGATGCATAGCCG
ACCTGAGAGGGTGAACGGCCACACTGGGACTGAGACACGGCC
>Zotu3145
GAACGCTGGCGGCAGGCTTAACACATGCAAGTCGAACGCCCCGCAAGGGGAGTGGCAGACGGGTGAGT
AACGCGTGGGAATCTACCCATCTCTACGGAATAACGCATGGAAACGTGTGCTAATACCGTATACGCCC
TTTTGGGGAAAGATTTATCGGAGATGGATGAGCCCGCGTTGGATTAGCTAGTTGGTGGGGTAAAGGCC
TACCAAGGCGACGATCCATAGCTGGTCTGAGAGGATGATCAGCCACATTGGGACTGAGACACGGCC
>Zotu3146
GAACGCTGGCGGCAGGCTTAACACATGCAAGTCGAACGCCCCGCAAGGGGAGTGGCAGACGGGTGAGT
AACGCGTGGGAATCTACCGTGCCCTGCGGAATAGCTCCGGGAAACTGGAATTAATACCGCATACGCCC
TACGGGGGAAAGATTTATCGGGGTATGATGAGCCCGCGTTGGATTAGCTAGTTGGTGGGGTAAAGGCC
TACCAAGGCGACGATCCATAGCTGGTCTGAGAGGATGATCAGCCACATTGGGACTGAGACACGGCC
>Zotu3147
GAACGCTGGCGGCGTGCCTAATACATGCAAGTCGAGCGAATCTGAGGGAGCTTGCTCCCAAAGATTAG
CGGCGGACGGGTGAGTAACACGTGGGCAACCTGCCCCTAAGATTGGGATAACTCCGGGAAACCGGTGC
TAATACCGAATAGTTTCTTTCCTCTCATGAGGAAAGAAGGAAAGTTGGCTTCGGCTAACACTTAGGGA
TGGGCCCGCGGCGCATTAGCTAGTTGGTGAGGTAACGGCTCACCAAGGCAACGATGCGTAGCCGACCT
GAGAGGGTGATCGGCCACACTGGGACTGAGACACGGCC
>Zotu3148
GAACGCTGGCGGCGTGCCTAACACATGCAAGTCGAACGGATGCTTTCGGGCATTAGTGGCGCACGGGT
GAGTAACACGTGGTTGACCTACCTCAAAGTAAGGGATAACGTCTCGAAAGGGATGCTAATACCTTATA
AGCTCCCGGCATTTAGAAAGTTGGGAGAAAAGTTCCGGCGCTTTGAGAGGGGACTGCGAGCCATCAGC
TAGTTGGTGAGGTAATAGCTCACCAAGGCGACGACGGCTAGGAGGCCTGAGAGGGTGACCTCCCACAC
TGGAACTGAGACACGGTC
>Zotu3150
GAACGCTGGCGGCAGGCTTAACACATGCAAGTCGAACGCCCTCTTCGGAGGGAGTGGCAGACGGGTGA
GTAACGCGTGGGAACGTACCTTTCGGTTCGGAATAACTCGGGGAAACTCGTGCTAATACCGGATACGC
CCGCAAGGGGAAAGATTTATCGCCGAAAGATCGGCCCGCGTCCGATTAGCTAGTTGGTGGGGTAATGG

CCTACCAAGGCGACGATCGGTAGCTGGTCTGAGAGGATGATCAGCCACACTGGGACTGAGACACGGCC
>Zotu3151
GAACGCTGGCGGCGTGCTTAACACATGCAAGTCGAACGATGATCCCCAGCTTGCTGGGGGGATTAGTG
GCGAACGGGTGAGTAACACGTGAGTAACCTGCCCTTAACTCTGGGATAAGCCTGGGAAACTGGGTCTA
ATACCGGATATGACTGATCATCGCATGGTGGTTGGTGGAAAGCTTTTTGTGGTTTTGGATGGACTCGC
GGCCTATCAGCTTGTTGGTGAGGTAATGGCTTACCAAGGCGACGACGGGTAGCCGGCCTGAGAGGGTG
ACCGGCCACACTGGGACTGAGACATGGCC
>Zotu3152
GAACGCTGGCGGCGTGCCTAAGACATGCAAGTCGAGTGGGTTTAGACCCACGGCGAACGGGTGAGTAA
TACCTAGGAACGTCCCTTAAGTTGAGGATATCCCATCGAAAGATGGTGCAATACTCAATGTGATCGCA
AGATCAAAGTCGCAAGACGCTTAAGGCGCGGCCTGGGCACCATCAGCTAGTTGGTAGGGTAAAAGCCT
ACCAAGGCTATGACGGTTAGGGGTCCCGAGAGGGATAACCCCCACACTGGAACTGCGACACGGTC
>Zotu3153
GAACGCTAGCGGCAGGCTTAACACATGCAAGTCGAGGGGTAGAGTTCTTCGGAACTTGAGACCGGCGC
ACGGGTGCGTAACGCGTATGCAATCTACCTTTCACAGAGGGATAGCCCAGAGAAATTTGGATTAATAC
CTCATAGTATAACGACCTGGCATCAGGATGTTATTAAAGTCACAACGGTGAAAGATGAGCATGCGTCC
CATTAGCTAGTTGGTAAGGTAACGGCTTACCAAGGCTACGATGGGTAGGGGTCCTGAGAGGGAGATCC
CCCACACTGGTACTGAGACACGGAC
>Zotu3154
GAACGCTGGCGGCGTGCCTAATACATGCAAGTAGAACGCTGAAGCTTGGTGCTTGCACCGAGTGGATG
AGTTGCGAACGGGTGAGTAACGCGTAGGTAACCTGCCTCTTAGCGGGGGATAACTATTAGAAACGATA
GCTAATACCGCATAAAAGTCGATATCGCATGATGTTGACTTGAAAGGTGCAATTGCATCACTAAGAGA
TGGACCTGCGTTGTATTAGCTAGTTGGTGAGGTAACGGCTCACCAAGGCGACGATACATAGCCGACCT
GAGAGGGTGATCGGCCACACTGGGACTGAGACACGGCC
>Zotu3155
CAACGCTGGCGGCGCGCTTAACACATGCAAGTCGCACGAGAAAGCCCGCAAGGGTGAGTAAAGTGGCG
CACGGGTGAGTAACACGTGGATAATCTACCTCTTAGTGGGGAATAACTTTGGGAAACCGAAGCTAATA
CCGCATAAGCTCGGAAGAGGAAAGCAGCAATGCGCTGAGAGAGGAGTCCGCGGCCGATTAGCTAGTTG
GTAGGGTAAAGGCCTACCAAGGCAATGATCGGTAGCCGGCCTGAGAGGGCACACGGCCACACTGGCAC
TGAAACACGGGC
>Zotu3156
TAACGCTGGCGGCGTGCCTAACACATGCAAGTCGAACGATTCTTTGATTTATAGCAATATTTATTAAA
GAGGAGTGGCGAACGGGTGAGTAACACGTAGATAATCTGCCTTTCAGACGGGGACAACTCAGGGAAAC
TTGTGCTAATACCCGATGAGACCACAAGAAGGGATTTTTGTGGCCAAAAGCGGGCAGCAATGCTCGTG
CTGAAAGATGAGTCTGCGTCCTATCAGCTAGTTGGTGGGGTAAATGCCTACCAAGGCTAAGACGGGTA
GCCGGCCTGAGAGGGTGAACGGCCACACTGGGACTGAGACACGGCC
>Zotu3157
GAACGCTGGCGGCGTGCCTAATACATGCAAGTCGAACGGTCTTTTCTATGGAAGCTTGCTTCCACTGA
GAAGATAGTGGCGGACGGGTGAGTAACACGTGGGTAACCTGCCCATAAGAGGGGGATAACATCCGGAA
ACGGGTGCTAATACCGCATAGTTTTCTTGATCGCATGATCGAGAAAGGAAAGACGGCCTTTGTGCTGT
CGCTTATGGATGGACCCGCGGCGTATTAGTTAGTTGGTGAGGTAATGGCTCACCAAGACGATGATACG
TAGCCGACCTGAGAGGGTGATCGGCCACATTGGGACTGAGACACGGCC
>Zotu3158
GAACGCTGGCGGCAGGCCTAACACATGCAAGTCGAGCGGATGACGGGAGCTTGCTCCTTGATTCAGCG
GCGGACGGGTGAGTAATACCTAGGAATCTGCCTGGTAGTGGGGGACAACGTTTCGAAAGGAACGCTAA
TACCGCATACGTCCTACGGGAGAAAGCAGGGGACCTTCGGGCCTTGCGCTATCAGATGAGCCTAGGTC
GAATTAGCTAGTTGGTGAGGTAATGGCTCACCAAGGCTACGATCCGTAACTGGTCTGAGAGGATGATC
AGTCACACTGGAACTGAGACACGGTC
>Zotu3159
GAACGCTGGCGGCGTGCCTAATACATGCAAGTCGAGCGGACTTGATGGAGAGCTTGCTCTCCTGATGG
TTAGCGGCGGACGGGTGAGTAACACGTAGGCAACCTGCCTGCAAGACCGGGATAACCCACGGAAACGT
GAGCTAATACCGGATATCTCATTTCCTCTCCTGAGGGGATGATGAAAGACGGAGCAATCTGTCACTTG
CGGATGGGCCTGCGGCGCATTAGCTAGTTGGTGAGGTAACGGCTCACCAAGGCGACGATGCGTAGCCG
ACCTGAGAGGGTGAACGGCCACACTGGGACTGAGACACGGCC

>Zotu3160
GAACGCTGGCGGAATGCTTTACACATGCAAGTCGAACGATGAACCCTAGCTTGCTAGGGGAATTAGTG
GCGAACGGGTGAGTAATATATCGGAACGTATCCAATAATGGGGGATAACGCAGCGAAAGTTGCGCTAA
TACCGCATACGCCCTGAGGGGGAAAGCTAGGGATCTTCGGACCTGGCGTTAATGGAGCGGCCGATATC
GGATTAGCTAGTTGGTGGGGTAAAGGCCCACCAAGGCGACGATCCGTAGCTGGTCTGAGAGGACGACC
AGCCACACTGGAACTGAGACACGGTC
>Zotu3161
GAACGCTGGCGGCGTGCCTAATACATGCAAGTCGAGCGGACCGACGGGAGCTTGCTCCCTTAGGTCAG
CGGCGGACGGGTGAGTAACACGTGGGTAACCTGCCTGTAAGACTGGGATAACTCCGGGAAACCGGGGC
TAATACCGGATGCTTGATTGAACCGCATGGTTCCAATCATAAAAGGTGGCTTTTAGCTACCACTTACA
GATGGACCCGCGGCGCATTAGCTAGTTGGTGAGGTAACGGCTCACCAAGGCGACGATGCGTAGCCGAC
CTGAGAGGGTGATCGGCCACACTGGGACTGAGACACGGCC
>Zotu3162
GAACGCTGGCGGCGTGCTTAACACATGCAAGTCGAACGGTAAGGCCCTAGCTTGCTGGGGTACACGAG
TGGCGAACGGGTGAGTAACACGTGGGTGACCTGCCCTGCACTTCGGGATAAGCCTGGGAAACTGGGTC
TAATACCGGATAGGACCGCATCGTAGGTGGTGTGGTGGAAAGTTTTTTCGGTGTGGGATGGGCCCGCG
GCCTATCAGCTTGTTGGTGGGGTAATGGCCTACCAAGGCGGCGACGGGTAGCCGGCCTGAGAGGGTGG
ACGGCCACATTGGGACTGAGACACGGCC
>Zotu3163
GAACGCTGGCGGCATGCCTAATACATGCAAGTCGAACGCTGAAGCACTCTGCTTGCAGAGTGCGGATG
AGTGGCGAACGGGTGAGTAACACGTGGGAAATCTGCCTAAGAGTGGGGGATAACATTCGGAAACGGAT
GCTAATACCGCATATATTCTTGAACCGCATGGTTCTGGAAAGAAAGACGGCTTTGCTGTCGCTTTTAG
ATGATCCCGCGGCGTATTAGTTAGTTGGTGGGGTAAAGGCCTACCAAGACCATGATACGTAGCCGACC
TGAGAGGGTGATCGGCCACACTGGGACTGAGACACGGCC
>Zotu3164
GAACGCTGGCGGCAGGCTTAACACATGCAAGTCGAGCGCGTAGCAATACGAGCGGCAGACGGATGAGT
AACGCGTGGGAACCTACCCTTTTCTACGGAATAACGCATGGAAACGTGTGCTAATACCGTATACGCCC
TTTGGGGGAAAGATTTATCGGAGAAGGATGGGCCCGCGTTGGATTAGCTAGTTGGTGGGGTAAAGGCC
TACCAAGGCGACGATCCATAGCTGGTCTGAGAGGATGATCAGCCACATTGGGACTGAGACACGGCC
>Zotu3165
GAACGCTGGCGGCAGGCTTAACACATGCAAGTCGAACGCCCGAGCAATCGGGAGTGGCAGACGGGTGA
GTAACACGTGGGAACATACCCTTCGGTTCGGAACAACGTTTGGAAACGAACGCTAATACCGGATACGG
CTCGAGGGCGAAAGATTCATCGCCGAAGGATTGGCCCGCGTTCGATTAGCTAGATGGCGGGGTAACGG
CCCACCATGGCGACGATCGATAGCTGGTCTGAGAGGATGGCCAGCCACATTGGGACTGAGACACGGCC
>Zotu3166
GAACGCTGGCGGCGTGCCTAATACATGCAAGTCGAGCGAGTCTGCCTTGAAGATCGGAGTGCTTGCAC
TCTGTGAAACAAGATACAGGCTAGCGGCGGACGGGTGAGTAACACGTGGGTAACCTGCCCAAGAGATC
GGGATAACACCTGGAAACAGATGCTAATACCGGATAACAACAGATGATGCCTATCAACTGTTTAAAAG
ATGGTTCTGCTATCACTCTTGGATGGACCTGCGGTGCATTAGCTAGTTGGTAGGGTAACGGCCTACCA
AGGCGATGATGCATAGCCGAGTTGAGAGACTGATCGGCCACATTGGGACTGAGACACGGCC
>Zotu3167
GAACGCTGGCGGCAGGCCTAACACATGCAAGTCGAGCGGCAGCGGGTCCTTCGGGATGCCGGCGAGCG
GCGGACGGGTGAGGAATGCGTCGGAATCTGCCTTTTTGTGGGGGATAACGTAGGGAAACTTACGCTAA
TACCGCATACGACCTACGGGTGAAAGTGGGGGACCGCAAGGCCTCACGCAGAAAGATGAGCCGACGCC
GGATTAGCTAGTTGGCGGGGTAAAGGCCCACCAAGGCGACGATCCGTAGCTGGTCTGAGAGGATGATC
AGCCACACTGGAACTGAGACACGGTC
>Zotu3168
GAACGCTGGCGGTGTGCTTCACACATGCAAGTCGAACGGGGTAGCAATACCCAGTGGCGGACGGGTGA
GTAACGCGTAGGAATCTGCCTTTAGGTGGGGGATAACAGTTCGAAAGAACTGCTAATACCGCATATGT
GTACGATACGACAGTTTTGTACATGAAAGGAGCAATTTGCCTGAAGATGAGCCTGCGTCCGATTAGCT
AGTTGGTAGGGTAAAAGCCTACCAAGGCGACGATCGGTAGGTGGTCTGAGAGGATGATCACCCACAAT
GGGACTGAGACACGGCC
>Zotu3169
GAATGCTGGCGGCGTGGATAAGTCATGCAAGTCAAGGGGGCCCGCAAGGGCAACCGGCAAACGAGGTA

GTAACACGTAGGTAGACCCCCCGAAGTCGGGCATAGCCCTCCGAAAGGAGGGGTAATTCCCGATAGTC
CCGCAAGGGTAAAGTTTTTCGCTTCGGGAGTCGCCTGCGTAGTATCAGCTAGTTGGTAGGGTAACGGC
CTACCAAGGCTACGACGCTTAGGGGAGCTGAGAGGCTGACCCCCACCGATGGGACTGCGACACGGCC
>Zotu3170
GAACGCTGGCGGCAGGCCTAACACATGCAAGTCGAGCGGATGACGGGAGCTTGCTCCTTGATTCAGCG
GCGGACGGGTGAGTAATGCCTAGGAATCTGCCTGGTAGTGGGGGACAACGTTTCGAAAGGAACGCTAA
TACCGCATACGTCCTACGGGAGAAAGCAGGGGACCTTCGGGCCTTGCGCTATCAGATGAGCCTAGGTC
GGATTAGCTTGTTGGTGAGGTAATGGCTCACCAAGGCGACGATCCGTAACTGGTCTGAGAGGATGATC
AGTCACACTGGAACTGAGACACGGTC
>Zotu3171
GAACGCTGGCGGCGTGCTTAACACATGCAAGTCGAACGATGAACCCCAGCTTGCTGGGGGGATTAGTG
GCGAACGGGTGAGTAACACGTGAGTAACCTGCCCCTGACTCTGGGATAAGCGCTGGAAACGGCGTCTA
ATACCGGATATGACAACCGATGGCATCGTCTGGTTGTGGAAAGAATTTCGGTTGGGGATGGACTCGCG
GCCTATCAGGTTGTTGGTGAGGTAATGGCTCACCAAGCCTACGACGGGTAGCCGGCCTGAGAGGGTGA
CCGGCCACACTGGGACTGAGACACGGCC
>Zotu3173
GAACGCTGGCGGCGTGGATAAGGCATGCAAGTCGAACGGTCCGCAAGGACAGTGGCAGACGAGGTAGT
AACGCGTAGGTAGATCCCCCGGAGTCGGGCATAGCCAGCCGAAAGGTTGGGTAATTCCCGATAATCCC
GCAAGGGCAAAGATTTTTTGCTCCGGGAGTTGCCTGCGCCGTATCAGCTAGTTGGTAGGGTAACGGCC
TACCAAGGCTACGACGCGTAGGGGAGCTGAGAGGCTGACCCCCACCGATGGGACTGCGACACGGCC
>Zotu3174
GAACGCTGGCGGCATGCCTAACACATGCAAGTCGAACGAAGCCTTCGGGCTTAGTGGCGCACGGGTGC
GTAACGCGTGGGAACCTGCCTTTAGGTTCGGAATAACAGTTGGAAACGACTGCTAATACCGGATGATG
TCTTCGGACCAAAGATTTATCGCCTTTAGATGGGCCCGCGTTGGATTAGCTAGTTGGTGGGGTAAAGG
CCTACCAAGGCGACGATCCATAGCTGGTCTGAGAGGATGATCAGCCACACTGGGACTGAGACACGGCC
>Zotu3175
GAACGCTGGCGGCGTGCTTAACACATGCAAGTCGAGCGGTAAGGCCCTTCGGGGTACACGAGCGGCGA
ACGGGTGAGTAACACGTGAGTAACCTGCCCCTGACTCTGGGATAAGCCCGGGAAACCGGGTCTAATAC
CGGATATGACTCCTGCTCGCATGGGTGGGGGTGGAAAGGTTGGTTTTCCTTCCGGTCGGGGATGGGCT
CGCGGCCTATCAGCTTGTTGGTGGGGTGACGGCCTACCAAGGCGATGACGGGTAGCCGGCCTGAGAGG
GCGACCGGCCACACTGGGACTGAGACACGGCC
>Zotu3176
GAACGCTGGCGGCATGCCTAACACATGCAAGTCGAACGAGACCTTCGGGTCTAGTGGCGCACGGGTGC
GTAACGCGTGGGAATCTGCCCCTTGGTTCGGGATAACAGTTGGAAACGACTGCTAATACCGGATGATG
ACGAAAGTCCAAAGATTTATCGCCGAGGGATGAGCCCGCGTAGGATTAGGTAGTTGGTGGGGTAAAGG
CCTACCAAGCCGACGATCCTTAGCTGGTCTGAGAGGATGATCAGCCACACTGGGACTGAGACACGGCC
>Zotu3177
GAACGCTGGCGGCATGCTTAACACATGCAAGTCGAACGATGCTTTCGGGCATAGTGGCGCACGGGTGA
GTAACGCGTGGGAACCTGCCTCTCGGGTCGGAATAACTGCGGGAAACTGCAGCTAATACCGGATACGT
CGAGCTGCTGTGGTGGTGGCTTGGGAAAGATTTATCGCCGAGAGAGGGGCCCGCGTCCGATTAGGTAG
TTGGTGAGGTAACGGCTCACCAAGCTTATGATCGGTAGCTGGTCTGAGAGGATGATCAGCCACACTGG
GACTGAGACACGGCC
>Zotu3179
GAACGCTGGCGGCGTGGATAAGGCATGCAAGTCGAGGGCCGATTTATCGGCCCGGCAAACGGGTTAGT
AATACATAGGAACATTCCCAAAAGTCGTGAATAATCCGGAGAAATCCGGAATAATACACGATGTGCTC
TACGGAGTAAAGATTTATCGCTTTTGGAGTGGCCTGTGTCCTATCAGCTTGTTGGTGAGGTAATGGCT
CACCAAGGCTATGACGGGTAGGGGGCGTGAGAGCGTGGCCCCCAACATTGGGACTGAGACACTGCC
>Zotu3180
GAACGCTGGCGGAATGCTTTACACATGCAAGTCGAGCGGCAGCGCGGGGGCAACCCTGGCGGCGAGCG
GCGAACGGGTGAGTAATGTATCGGAACGTACCCAGTCGTGGGGGATAACTATCCGAAAGGATAGCTAA
TACCGCATACGACCTGAGGGTGAAAGCGGGGGACCGAAAGGCCTCGCGCGATTGGAGCGGCCGATATC
GGATTAGCTAGTTGGTGAGGTAAAGGCTCACCAAGGCGACGATCCGTAGCTGGTCTGAGAGGACGACC
AGCCACACTGGAACTGAGACACGGTC
>Zotu3181

GAACGCTAGCGGCGTGCTTAACACATGCAAGTCGAACGATGAAGCCCAGCTTGCTGGGTGGATTAGTG
GCGAACGGGTGAGTAACACGTGAGTAACCTGCCCTTCACTCTGGGATAAGCCTTGGAAACGAGGTCTA
ATACTGGATATGACCTGTCACCGCATGGTGGTGGGTGGAAAGTTTTTCGGTGGGGGATGGACTCGCGG
CCTATCAGCTTGTTGGTGAGGTAGTGGCTCACCAAGGCGACGACGGGTAGCCGGCCTGAGAGGGTGAT
CGGCCACACTGGGACTGAGACACGGCC
>Zotu3182
GAACGCTGGCGGCGTGCCTAATACATGCAAGTCGAACGAGGTGCTTTTGTAGCAATACGAGAGTATCC
TAGTGGCGAACGGGTGAGTAACGCGTGGGTGACCTGCCCCAAAGTGTGGGATAACAGTCCGAAAGGAT
TGCTAATACCTCATGTGGTTGTTGGGTTTAGAAACTGACAACTAAAGGAGTAATCCGCTTTGGGAGGG
ACCTGCGTCCCATCAGCTAGTTGGTGAGGTAATGGCTCACCAAGGCTACGACGGGTAGGGGACCTGAG
AGGGTGGCCCCCCACAATGGAACTGAAACACGGTC
>Zotu3183
GAACGCTGGCGGCGTGCTTAACACATGCAAGTCGAGCGGTAAGGCCCTTCGGGGTACACGAGCGGCGA
ACGGGTGAGTAACACGTGAGCAACCTGCCCCTGACTCTGGGATAAGCGGTGGAAACGCCGTCTAATAC
CGGATATGACCTGCCACCTCATGGTGGCGGGTGGAAAGTTTTTCGGTCAGGGATGGCTCGCGGCCTA
TCAGCTTGTTGGTGGGGTAACGGCCTACCAAGGCGATTACGGGTAGCCGGCCTGAGAGGGCGACCGGC
CACACTGGGACTGAGACACGGCC
>Zotu3184
GAACGCTGGCGGCGTGCTTAACACATGCAAGTCGAGCGGTAAGGCCCTTCGGGGTACACGAGCAGCGA
ACGGGTGAGTAACACGTGGGTGATCTGCCCTGTACTTCGGGATAAGCCTGGGAAACTGGGTCTAATAC
CGGATATGACCTTACATCGCATGGTGTTTGGTGGAAAGATTTATCGGTACAGGATGGGCCCGCGGCCT
ATCAGCTTGTTGGTGGGGTAATGGCCTACCAAGGCGACGACGGGTAGCCGGCCTGAGAGGGCGACCGG
CCACACTGGGACTGAGACACGGCC
>Zotu3186
GAACGCTAGCGGCAGGCCTAATACATGCAAGTCGGACGGGATTGGAGAGCTTGCTTTCCATGAGAGTG
GCGCACGGGTGCGTAACACGTATGTAACCTACCCTTATCAGGGGGATAGCCTCTCGAAAGAGAGATTA
AGCCCGCATAATATGAAGACACAGCATTGTGATTTCATCAAATATTTATAGGATAAGGATGGGCATGC
GGAACATTAGCTAGTTGGTAAGGTAATGGCTTACCAAGGCTACGATGTTTAGGAGATCTGAGAGGATG
ACCCCCCACACTGGTACTGAGACACGGAC
>Zotu3188
GAACGCTGGCGGTCTGCTTAACACATGCAAGTCGAACGGAGTAGAAATACTTAGTGGCGGACGGGTGA
GTAACGCGTGAGAATCTGCCTTCAGGACGGAGACAACAGTTGGAAACGACTGCTAAGCCCCGATGAAC
CGAAAGGGAAAATATTTATAGCCTGAAGATGAGCTCGCGTCCGATTAGCTAGTTGGCGGAGTAAAAGC
CCACCAAGGCGACGATCGGTAGCTGGTCTGAGAGGACGATCAGCCACACTGGGACTGAGACACGGCC
>Zotu3189
GAACGCTGGCGGCGTGCTTAACACATGCAAGTCGAACGGTGAAGGGACTGGCTTTTGCTGGTTCTGGA
TGAGTGGCGAACGGGTGAGTAACACGTGAGTAACCTGCCCCCTTCTTCTGGATAACCGCATGAAAGTG
TGGCTAATACGGGATATTCTGCGCCTGCTGCATGGTGGGTGTGGGAAAGATATTGTTTCGGTGGGGGA
TGGGCTCGCGGCCTATCAGCTTGTTGGTGGGGTGATGGCCTACCAAGGCGGTGACGGGTAGCCGGCCT
GAGAGGGTGGACGGTCACACTGGGACTGAGACACGGCC
>Zotu3190
GAACGCTGGCGGCGTGCCTAACACATGCAAGTCGAACGATCAGATGTCTTCGGACATTTGGGAGTGGC
GCACGGGTGAGTAATGCATAGGAACATACCTAAAGGCGGGGAATAACTGCGGGAAACTTCAGCTAATA
CCCCATGAAGTCGCAAGACCAAAGGAGGCCTCTACTTGTAAGCTTTCACCTTTAGAATGGCCTATGTC
CCATTAGCTAGTTGGTAGGGTAAAGGCCTACCAAGGCGACGATGGGTAGCTGGTCTGAGAGGACGATC
AGCCACACTGGAACTGAGACACGGTC
>Zotu3191
GAACGCTGGCGGCATGCTTTACACATGCAAGTCGAACGGCAGCACGGACTTCGGTCTGGTGGCGAGTG
GCGAACGGGTGAGTAATACATCGGAACGTGCCTAGTAGTGGGGGATAACTACTCGAAAGAGTAGCTAA
TACCGCATGATATCTACGGATGAAAGCAGGGGACCTTCGGGCCTTGTGCTACTAGAGCGGCTGATGGC
AGATTAGGTAGTTGGTGGGGTAAAGGCTTACCAAGCCCGTGATCTGTAGCTGGTCTGAGAGGACGACC
AGCCACACTGGGACTGAGACACGGCC
>Zotu3192
GAACGCTGGCGGCGCGCTTAACACATGCAAGTCGAGCGAGAACCGGGGCTTCGGCTCCGGGGAAAGCG

GCGAACGGGTGAGTAACACGTGGGTAACCTGCCCTCGGCACCGGGATAGCCCGGGGAAACCCGGATTA
ATACCGGACGGCCTCCGGAGCCTTCGGGCGCCGGAGAGAAGGTGGCTTCGGCTTCCGGCCGAGGAGGG
GCCTGCGGCGGATTAGCTTGTTGGCGGGGTAACGGCCCACCAAGGCGACGATCCGTAGCTGGTCTGAG
AGGACGATCAGCCACACTGGGACTGAGACACGGCC
>Zotu3193
GAACGCTGGCGGCGTGCCTAACACATGCAAGTCAACTTGAAGGAAAGAGCAATCTGACCTGATGGTGG
CGCAAGGGTGCGTAACGCGTAAGTAACTTGCCCCAAGGTAAGGAATAACGTCGAGAAATCGGCGCTAA
TACCTCATAATGCAGCGGATCCGCATGGATATGTTGTTAAAGGTTTACCGCCTTGGGATAGGCTTGCG
TCTGATTAGCTAGTTGGTGTGGTAACGGCGCACCAAGGCTACGATCAGTAACTAGTCTGAGAGGATGA
TCAGTCACACTGGAACTGAGACACGGTC
>Zotu3194
GAACGCTAGCGGCAGGCCTAATACATGCAAGTCGAACGCGATTAGGGAGCTTGCTCCCTATGAAAGTG
GCGCACGGGTGCGTAACGCGTATGCAACCTACCTTAATCAGGGGGATAGCCCGGAGAAATCCGGATTA
ACACCGCATAAAATCACACTTCGGCATCGAGGAATGATCAAATATTTATAGGATTAAGATGGGCATGC
GTGACATTAGTTAGTTGGTGAGGTAACGGCTCACCAAGGCGACGATGTCTAGGGGATCTGAGAGGATG
ACCCCCCACACTGGTACTGAGACACGGAC
>Zotu3195
GAACGCTGGCGGCATGCTTTACACATGCAAGTCGAACGCGAAAGGGGGCAACCCTGAGTAGAGTGGCG
CACGGGTGAGTAATACATCGGAACGTGTCCTGTAGTGGGGGATAACGCAGCGAAAGCTGTGCTAATAC
CGCATACGTCCTACGGGAGAAAGTGGGGGACCGCAAGGCCTCACGCTATAGGAGCGGCCGATGTCGGA
TTAGCTAGTTGGTGGGGTAAAGGCTCACCAAGGCGACGATCCGTAGCTGGTCTGAGAGGATGATCAGC
CACACTGGGACTGAGACACGGCC
>Zotu3196
GAACGCTGGCGGCGTGCTTAACACATGCAAGTCGAGCGGAAAGGCCCTTCGGGGTACTCGAGCGGCGA
ACGGGTGAGTAACACGTGAGTAATCTGCCCTTGGCTTCGGGATAGCCACCGGAAACGGTGATTAATAC
CGGATATGACTACTTCCTGCATGGGGTGGTAGTGGAAAGTTTTTCGGCCAGGGATGTGCTCGCGGCCT
ATCAGCTTGATGGTGAGGTAATGGCTCACCATGGCTTCGACGGGTAGCCGGCCTGAGAGGGTGACCGG
TCACACTGGGACTGAGACACGGCC
>Zotu3197
GAACGCTGGCGGCGTGCTTAACACATGCAAGTGGAGCGACGAAGCGGGCTTCGGCCCGTGGCAGAGCC
GCGAACGGGTGAGTAACACGTGGGTAACGTGCCCCAATGACTGGGACAACCCGGGGAAACCCGGGCTA
ATACCGGATGTGCTCCTTCCACATCAGTGGATTGAGTAAAGGTAGCTTCGGCTTCCGCATTGGGATCG
GCCCGCGGCGCATTAGCTTGTTGGTGAGGTAACGGCTTACCAAGGCAACGATGCGTAGCTGGTCTGAG
AGGACGATCAGCCACACTGGGACTGAGACACGGCC
>Zotu3198
GAACGCTAGCGGCAGGCCTAATACATGCAAGTCGAACGAGATTAGTAGCAATACTATGAAAGTGGCGC
ACGGGTGCGTAACACGTATGCAATCTACCTTTAATTGGAGGATAGCCTCGAGAAATCGGGATTAATAC
TCCATAACATTATGAAATGGCATCATTTTATAATTAAACCTCCGGGGATTAAAGATGAGCATGCGCAA
CATTAGCTAGTTGGTAAGGTAATGGCTTACCAAGGCTACGATGTTTAGGGGGTCTGAGAGGATGATCC
CCCACACTGGTACTGAGACACGGAC
>Zotu3199
GAACGCTGGCGGCAGGCTTAACACATGCAAGTCGAACGCCCTCGCAAGGGGGAGTGGCAGACGGGTGA
GTAACACGTGGGAACCTACCCTTCGGTTCGGAACAACTCGGGGAAACCCGAGCTAATACCGGATGAGC
GCGAGAGCGGAAAGATTGATCGCCGAAGGATGGGCCCGCGTCCGATTAGCTAGTTGGTGAGGTAACGG
CTCACCAAGGCGACGATCGGTAGCTGGTCTGAGAGGATGGCCAGCCACATTGGGACTGAGACACGGCC
>Zotu3202
GAACGCTGGCGGCGTGCTTAACACATGCAAGTCGAACGGAAAGGCCCTTCGGGGTACTCGAGTGGCGA
ACGGGTGAGTAACACGTGGGTGATCTGCCCTGCACTTTGGGATAAGCCTGGGAAACTGGGTCTAATAC
CGAATACACCTCATTGACTGCATGGTTGGTGGGGGAAAGCTTTTGCGGTGTGGGATGGGCCCGCGGCC
TATCAGCTTGTTGGTGAGGTTACGGCTCACCAAGGCGACGACGGGTAGCCGGCCTGAGAGGGTGACCG
GCCACACTGGGACTGAGATACGGCC
>Zotu3203
GAACGCTGGCGGCGTGCCTAATACATGCAAGTCGAGCGGATGACGGGGAGCTTGCTCCCCTGATTCAG
CGGCGGACGGGTGAGTAACACGTGGGCAACCTGCCTGTAAGATCGGGATAACTCCGGGAAACCGGGGC

TAATACCGGATGGTTCCTTCCTCCGCATGGAGGAAGGCGGAAAGACGGTTTCGGCTGTCACTTACAGA
TGGGCCCGCGGCGCATTAGCTAGTTGGTGGGGTAATGGCCCACCAAGGCAACGATGCGTAGCCGACCT
GAGAGGGTGATCGGCCACACTGGGACTGAGACACGGCC
>Zotu3204
GAACGCTAGCGGCAGGCCTAATACATGCAAGTCGGACGGGATAGAAGAGCTTGCTTTTCTTGAGAGTG
GCGCACGGGTGCGTAACACGTATGTAACCTACCTTATCCAGGGGGATAGCCTCTCGAAAGAGAGATTA
AGACCGCATAATATTATTGAATAGCATTATTTAATAATCAAATATTTATAGGGATAAGATGGGCATGC
GGGACATTAGCTAGTTGGCGGGGTAACGGCCCACCAAGGCGACGATGTCTAGGGGATCTGAGAGGATG
ACCCCCCACACTGGTACTGAGACACGGAC
>Zotu3205
GAACGCTGGCGGCAGGCCTAACACATGCAAGTCGAGCGGATGACGGGAGCTTGCTCCCGGATTCAGCG
GCGGACGGGTGAGTAATGCCTAGGAATCTGCCTGGTAGTGGGGGACAACGTTTCGAAAGGAACGCTAA
TACCGCATACGTCCTACGGGAGAAAGCAGGGGACCTTCGGGCCTTGCGCTATCAGATGAGCCTAGGTC
GGATTAGCTGGTTGGTGAGGTAACGGCTCACCAAGGCGACGATCCGTAACTGGTCTGAGAGGATGATC
AGTCACACTGGAACTGAGACACGGTC
>Zotu3206
CAACGCTGGCGGCGCGCTTAACACATGCAAGTCGCACGAGAAAGGGAGCAATCCTGAGTAAAGTGGCG
TACGGGTGAGTAACACGTGGATCATCTACCTTTAGTGGGGAATACCCCCGGGAAACTGGGGACAATA
CCGCATAAGCTCGTGAGAGGAAAGCCGAAAGGCGCTGAAAGAGGAGTCCGCGGCTGATTAGCTAGTTG
GTGAGGTAACGGCCCACCAAGGCGAAGATCGGTAGCCGGCCTGAGAGGGCACACGGCCACACTGGCAC
TGAAATACGGGC
>Zotu3208
GAACGCTGGCGGCGTGCTTAACACATGCAAGTCGAACGGTGAACAAGGAGCTTGCTTCTTGGGATCAG
TGGCGAACGGGTGAGTAACACGTGAGTAACCTGCCCTTGACTCTGGGATAAGCGTTGGAAACGACGTC
TAATACCGGATATGACCTCCGACGGCATCGTCTGGGGGTGGAAAGAATTTTGGTTGAGGATGGACTCG
CGGCCTATCAGCTTGTTGGTGAGGTAATGGCTCACCAAGGCGACGACGGGTAGCCGGCCTGAGAGGGT
GACCGGCCACACTGGGACTGAGACACGGCC
>Zotu3209
CAACGCTAGCGGCGTGCCTAACACATGCAAGTCGAACGCGAAAGGGACTTCGGTTCTTAGTAGAGTGG
CGTACGGGTGAGTAACGCGTGGGTCATCTGCCCTCGGGTGGGGAATAACTAACCGAAAGGTTAGCTAA
TACCGCATAACACAGTGATGTCAAAGCAGCAATGTGCCTGAGGAGGAGCCTGCGTCTGATTAGGTAGT
TGGTGGGGTAACGGCCTACCAAGCCTGCGATCAGTAGCTGGTCTGAGAGGACGGCCAGCCACAATGGC
ACTGAAACACGGGC
>Zotu3210
GAACGCTGGCGGCATGCCTAACACATGCAAGTCGAACGAAGCCTTCGGGTTTAGTGGCGCACGGGTGC
GTAACGCGTGGGAATCTGCCCCTTGGTTCGGAATAACAGTTGGAAACGACTGCTAATACCGGATGATG
ACGTAAGTCCAAAGATTTATCGCCGAGGGATGAGCCCGCGTAGGATTAGGTAGTTGGTGTGGTAAAGG
CGCACCAAGCCGACGATCCTTAGCTGGTCTGAGAGGATGATCAGCCACACTGGGACTGAGACACGGCC
>Zotu3211
GAACGCTGGCGGCGTGCTTAACACATGCAAGTCGAACGCTGAAGCCCAGCTTGCTGGGTGGATGAGTG
GCGAACGGGTGAGTAACACGTGAGCAACCTGCCCTTCACTCTGGGATAACTCCGGGAAACCGGGGCTA
ATACCGGATGCGAGCACTGCACGCATGTGCGGGTGCTGGAAAGTTTTTCGGTGAGGGATGGGCTCGCG
GCCTATCAGCTTGTTGGTGAGGTAGTGGCTCACCAAGGCGACGACGGGTAGCCGGCCTGAGAGGGCGA
CCGGCCACACTGGGACTGAGACACGGCC
>Zotu3212
GAACGCTGGCGGCATGCCTAACACATGCAAGTCGAACGAGATCTTCGGGTCTAGTGGCGCACGGGTGC
GTAACGCGTGGGAATCTGCCCTTGGGTTCGGAATAACTTCGGGAAACTGAAGCTAATACCGGATGATG
ACGTAAGTCCAAAGATTTATCGCCCAAGGATGAGCCCGCGTAGGATTAGCTAGTTGGTGAGGTAAAGG
CTCACCAAGGCGACGATCCTTAGCTGGTCTGAGAGGATGATCAGCCACACTGGGACTGAGACACGGCC
>Zotu3213
GAACGCTGGCGGCGTGCCTAATACATGCAAGTCGAGCGAACAGATAAGGAGCTTGCTCCTTTGACGTT
AGCGGCGGACGGGTGAGTAACACGTGGGTAACCTACCTATAAGACTGGAATAACTCCGGGAAACCGGG
GCTAATGCCGGATAATATTTAGAACCGTATGGTTCTAAAGTGAAAGATGGTTTTACTATCACTTATAG
ATGGACCCGCGCCGTATTAGCTAGTTGGTGGGGTAATGGCTTACCAAGGCAACGATACGTAGCCGACC

TGAGAGGGTGATCGGCCACACTGGAACTGAGACACGGTC
>Zotu3214
GAACGCTGGCGGCGTGCCTAATACATGCAAGTCGAGCGAATCTTTGGGAGCTTGCTCCCAAAAGTTAG
CGGCGGACGGGTGAGTAACACGTGGGCAACCTGCCTATAAGACTGGGATAACTTCGGGAAACCGGAGC
TAATACCGGATAATTCTTTTCTACACATGTAGGAAAGCTAAAAGACGGTTTACGCTGTCACTTATAGA
TGGGCCCGCGGCGCATTAGCTAGTTGGTGAGGTAACGGCTCACCAAGGCCACGATGCGTAGCCGACCT
GAGAGGGTGATCGGCCACACTGGGACTGAGACACGGCC
>Zotu3216
GAACGCTGGCGGCGTGCCTAATACATGCAAGTCGAGCGGACTTTTAAAAGCTTGCTTTTAAAAGTTAG
CGGCGGACGGGTGAGTAACACGTGGGTAACCTGCCTGTAAGACTGGGATAACTCCGGGAAACCGGGGC
TAATACCGGATAACTTTTTTCCTCGCATGAGGAAAAATTGAAAGATGGCTTCGGCTATCACTTACAGA
TGGACCCGCGGCGCATTAGCTAGTTGGTGAGGTAACGGCTCACCAAGGCAACGATGCGTAGCCGACCT
GAGAGGGTGATCGGCCACACTGGGACTGAGACACGGCC
>Zotu3217
GAACGCTGGCGGCATGCCTTACACATGCAAGTCGGACGGGAAGTGGTGTTTCCAGTGGCGGACGGGTG
AGTAACGCGTAAGAACCTACCCTTGGGAGGGGAACAACAGCTGGAAACGGCTGCTAATACCCCGTAGG
CTGAGGAGCAAAAGGAGGAATCCGCCCGAGGAGGGGCTCGCGTCTGATTAGCTAGTTGGTGAGGCAAT
AGCTTACCAAGGCGATGATCAGTAGCTGGTCCGAGAGGATGATCAGCCACACTGGGACTGAGACACGG
CC
>Zotu3219
GAACGCTGGCGGCGTGCTTAACACATGCAAGTCGAACGGTGAAGCCCAGCTTGCTGGGTGGATCAGTG
GCGAACGGGTGAGTAACACGTGAGCAATCTGCCCCTGACTCTGGGATAAGCGCTGGAAACGGCGTCTA
ATACTGGATACGAGCCGTGAAGGCATCTTCAACGGTTGGAAAGATTTTTCGGTCAGGGATGAGCTCGC
GGCCTATCAGCTTGTTGGTGAGGTAATGGCTCACCAAGGCGTCGACGGGTAGCCGGCCTGAGAGGGTG
ACCGGCCACACTGGAACTGAGACACGGCC
>Zotu3220
GAACGCTGGCGGCGTGCCTAATACATGCAAGTCGTGCGAACTGATTAGAAGCTTGCTTCTATGACGTT
AGCGGCGGACGGGTGAGTAACACGTGGGCAACCTGCCTGTAAGACTGGGATAACTTCGGGAAACCGAA
GCTAATACCGGATAGGATCTTCTCCTTCATGGGAGATGATTGAAAGATGGTTTCGGCTATCACTTACA
GATGGGCCCGCGGTGCATTAGCTAGTTGGTGAGGTAACGGCTCACCAAGGCAACGATGCATAGCCGAC
CTGAGAGGGTGATCGGCCACACTGGGACTGAGACACGGCC
>Zotu3221
GAACGCTGGCGGCGTGCCTAATACATGCAAGTCGAGCGGATCTTTAGAAGCTTGCTTCTAAAGATTAG
CGGCGGACGGGTGAGTAACACGTGGGCAACCTGCCTGTAAGACTGGGATAACTTCGGGAAACCGAGGC
TAATACCGGATAATCCTTTTCCTCACATGAGGGAAAGCTGAAAGACGGCGTAAGCTGTCACTTACAGA
TGGGCCCGCGGCGCATTAGCTAGTTGGTGAGGTAACGGCTCACCAAGGCAACGATGCGTAGCCGACCT
GAGAGGGTGATCGGCCACACTGGGACTGAGACACGGCC
>Zotu3222
GAACGCTGGCGGCGTGCTTAACACATGCAAGTCGAACGCTGAAGACCTGGCTTTTGTTGGGTTGGATG
AGTGGCGAACGGGTGAGTAACACGTGAGTAACCTGCCCTCTTCTTTGGGATAACGCCCGGAAACGGGT
GCTAATACTGGATATTCACTGATCTTCGCATGGGGGTTGGTGGAAAGGTTTGTTCTGGTGGGGGATGG
GCTCGCGGCCTATCAGCTTGTTGGTGGGGTGATGGCCTACCAAGGCTTTGACGGGTAGCCGGCCTGAG
AGGGTGACCGGTCACATTGGGACTGAGATACGGCC
>Zotu3223
GAACGCTGGCGGCGTGCCTAATACATGCAAGTCGAGCGGTAAGGCTTCCACAGTTTGCTGGATGGAAT
TAGTAATAATTCACTACATCGAGCAAAGTGTGGAAGTACACGAGCGGCGGACGGCTGAGTAACGCGTA
GGAATGTACCCCAAAGTGAGGGATAAGCACCGGAAACGGTGTCTAATACCGCATGTGCTCTTCGGAGT
AAAGCTTTATGCGCTTTGGGAACGGCCTGCGTCTGATTAGCTTGTTGGTGAGGTAAAAGCTCACCAAG
GCGACGATCGGTAGCTGGTCTGAGAGGATGATCAGCCAGACTGGGACTGAGACACGGCC
>Zotu3225
GAACGCTAGCGGCAGGCCTAATACATGCAAGTCGGACGGGATCCGTCGGAGAGCTTGCTCGAAGACGG
TGAGAGTGGCGCACGGGTGCGTAACGCGTGAGCAACCTACCTCTATCAGGGGGATAGCCTCTCGAAAG
AGAGATTAACACCGCATAATATCAACAGTTCGCATGTTCGGTTGATTAAATATTTATAGGATAGAGAT
GGGCTCGCGTGACATTAGCTAGTTGGTAGGGTAACGGCTTACCAAGGCGACGATGTCTAGGGGCTCTG

AGAGGAGAATCCCCCACACTGGTACTGAGACACGGAC
>Zotu3226
GAACGCTGGCGGCGTGCCTAAGACATGCAAGTCGAACGCGTGTAGCAATACACGAGTGGCGAACGGGT
GAGTAACGCGTGGGAACTTTCTACAAGTTTGGAACAACCTTCCGAAAGGAGGGCTAATACCAAATGTG
GCCGTAAGGCTAAAGGAGCAATCCGCTTGTGGTGAGGCCCGCGTCCCATCAGCTAGTTGGTAGGGTAA
AGGCCTACCAAGGCTATGACGGGTAGGGGGACCGAGAGGTTTTACCCCCACACTGGGACTGCGACACG
GCC
>Zotu3227
GAACGCTGGCGGTATGCTTAACACATGCAAGTCGAACGGAGTACTTCGGTACTTAGTGGCGGACGGGT
GAGTAACTCGTGAGAAACTGCCCCTTGGACTGGGACAACAGCTGGAAACGGCTGCTAACACCGGATGT
GCCGAAAGGTGAAATATTTATAGCCAGGGGATGTGCTCGCGTCTGATTAGCTAGTAGGTGAGGTAAAG
GCTTACCTAGGCGACGATCAGTAGCTGGTCTGAGAGGATGATCAGCCACACTGGGACTGAGACACGGC
C
>Zotu3228
GAACGCTAGCGGCAGGCCTAACACATGCAAGTCGAGGGGTAGAAATAGCTTGCTATTTTGAGACCGGC
GCACGGGTGAGTAACGCGTATGTAATCTACCTTGTACAGAGGGATAGCCCAGAGAAATTTGGATTAAT
ACCTCATAGTATAATAGAATGGCCTCATTTTATTATTAAAGTTCCAACGGTACAAGATGAACATGCGT
CCCATTAGTTAGTTGGTAAGGTAACGGCTTACCAAGACTATGATGGGTAGGGGTCCTGAGAGGGAGAT
CCCCCACACTGGTACTGAGACACGGAC
>Zotu3229
GAACGCTAGCTACAGGCTTAACACATGCAAGTCGCAGGGTAACGTGAGGGAAGCTTGCTTCCCTTGAC
GACGACTGGCGCACGGGTGAGTAACGCGTATCCAACCTTCCCATGACCACGGGATAACCCGTTGAAAG
ACGGACTAATACCGTATGACGTCGTTTGCTGACATCAAATAACGATTAAAGGTTTAGCGGTGATGGAT
GGGGATGCGTCTGATTAGCTTGTTGGCGGGGTAACGGCCCACCAAGGCTACGATCAGTAGGGGTTCTG
AGAGGAAGGTCCCCCACATTGGAACTGAGACACGGTC
>Zotu3230
GAACGCTGGCGGCGTGCCTAATACATGCAAGTCGAGCGGAAGTTGGAGAGCTTGCTCTTCAACTTTAG
CGGCGGACGGGTGAGTAACACGTGGGCAACCTGCCCTGCAGATCGGGATAACTCCGGGAAACCGGTGC
TAATACCGAATAGTTTTCGCCACCTCCTGGTGGCGTACGGAAAGACGGTTTCGGCTGTCACTGCAGGA
TGGGCCCACGGCGCATTAGCTAGTTGGTGGGGTAACGGCCCACCAAGGCAACGATGCGTAGCCGACCT
GAGAGGGTGATCGGCCACACTGGGACTGAGACACGGCC
>Zotu3231
GAACGCTGGCGGCGTGCTTAACACATGCAAGTCGAACGGAAAGGTCTCTTCGGAGATACTCGAGTGGC
GAACGGGTGAGTAACACGTGGGTAATCTGCCCTGCACTTCGGGATAAGCCTGGGAAACTGGGTCTAAT
ACCGAATAGGACCACTTGGCGCATGCCTTGTGGTGGAAAGCTTTTGCGGTGTGGGATGGGCCCGCGGC
CTATCAGCTTGTTGGTGGGGTGATGGCCTACCAAGGCGACGACGGGTAGCCGGCCTGAGAGGGTGTCC
GGCCACACTGGGACTGAGATACGGCC
>Zotu3232
GAACGCTAGCGGCAGGCCTAATACATGCAAGTCTAGGGGGCAGCAATGTCACCGGCGCACGGGTGCGT
AACACGTATGCAACCTACCTTCTACAGGGGTACAGCCTTCCGAAAGGGAGATTAATACCGCATGAGAC
CACACCTATTCATATAGGAGGGGTTAAAGATTTATCGGTAGAAGATGGGCATGCGTCTGATTAGTTAG
TTGGCGGGGTAACGGCTCACCAAGACGATGATCAGTAGGGGATCTGAGAGGACTATCCCCCACACTGG
CACTGAGATACGGGC
>Zotu3233
GAACGCTGGCGGCGTGCCTAATACATGCAAGTAGAACGCTGAAGACTTTAGCTTGCTAAAGTTGGAAG
AGTTGCGAACGGGTGAGTAACGCGTAGGTAACCTGCCTACTAGCGGGGGATAACTATTGGAAACGATA
GCTAATACCGCATAACAGCATTTAACACATGTTAGATGCTTGAAAGGAGCAATTGCTTCACTAGTAGA
TGGACCTGCGTTGTATTAGCTAGTTGGTGAGGTAACGGCTCACCAAGGCGACGATACATAGCCGACCT
GAGAGGGTGATCGGCCACACTGGGACTGAGACACGGCC
>Zotu3234
GAACGCTAGCGGCAGGCTTAACACATGCAAGTCGAGGGGTAGGGTAGCAATACCTGAGACCGGCGCAC
GGGTGCGTAACGCGTATGCAATCTACCTTTTACAGAGGGATAGCCCAGAGAAATTTGGATTAATACCT
CATAGCATAGCAGCTTCGCATGAAGTCACTATTAAAGTTCCAACGGTAAAAGATGAGCATGCGTCCTA
TTAGCTAGATGGTAAGGTAACGGCTTACCATGGCTACGATAGGTAGGGGTCCTGAGAGGGAGATCCCC

CACACTGGTACTGAGACACGGAC
>Zotu3235
GAACGCTGGCGGTATGCCTAACACATGCAAGTCGTACGAGAGTTTTTAACTCTAGTGGCGGACGGGTG
AGTAACACGTGAGAATTTACCTTTAGGAGGGGGATAACAATTGGAAACGAATGCTAATACCCCATATG
CTTTCGAGTGAAATGGATTTATAATTATCTGCCTAAAGAAAAGCTCGCGGCTGATTAGCTTGTTGGTA
AGGTAAGAGCTTACCAAGGCGACGATCAGTATCTGGTTTGAGAGGACGATCAGACACACTGGAACTGA
GACACGGTC
>Zotu3236
GAACGCTAGCGACAGGCTTAACACATGCAAGTCGAGGGGCAGCATAAAAGTAGCAATACTTTGGTGGC
GACCGGCGCACGGGTGAGTAACGCGTATGCAACCTACCTATCAGAGGGGAATAACCCGGCGAAAGTCG
GACTAATACCGCATAAAACAGGGGCACCGCATGGTGCTATTTGTTAAAAATTTATTGCTGATAGATGG
GCATGCGTTCCATTAGGTAGTTGGAGAGGTAACGGCTCACCAAGCCTACGATGGATAGGGGAACTGAG
AGGTTGGTCCCCCACACTGGTACTGAGACACGGAC
>Zotu3237
GAACGCTGGCGGCAGGCTTAACACATGCAAGTCGAACGCTCCGCAAGGAGAGTGGCAGACGGGTGAGT
AACGCGTGGGAATCTACCCATCTCTACGGAATAACGCAGGGAAACTTGTGCTAATACCGTATACGCCC
TTCGGGGGAAAGATTTATCGGAGATGGATGAGCCCGCGTTGGATTAGCTAGTTGGTGGGGTAAAGGCC
TACCAAGGCGACGATCCATAGCTGGTCTGAGAGGATGATCAGCCACATTGGGACTGAGACACGGCC
>Zotu3238
GAACGCTGGCGGCAGGCTTAACACATGCAAGTCGAACGATGACTCTCTAGCTTGCTAGAGAAGATTAG
TGGCGGACGGGTGAGTAACATTTAGGAATCTGCCTAGTAGTGGGGGATAGCTCGGGGAAACTCGAATT
AATACCGCATACGACCTACGGGTGAAAGGGGGCGCAAGCTCTTGCTATTAGATGAGCCTAAATCAGAT
TAGCTAGTTGGTGGGGTAAAGGCCCACCAAGGCGACGATCTGTAACTGGTCTGAGAGGATGATCAGTC
ACACCGGAACTGAGACACGGTC
>Zotu3239
TAACGCTGGCGGCGTGCATAACACATGCAAGTCGAACGATGAAACTTTACTGACTTCTTCGGAATGAT
TTAAAGCGGATTAGTGGCGAACGGGTGAGTAACGCGTGAGTAACCTGCCTTACACAAGGGGATAGCCT
CTGGAAACGGAGAATAATACCCTATGAAATTACATTCTCGCATGAGGAAATAATCAAAATGTTTAGTG
GTGTAAGATGGACTTGCGTCTGATTAGCTAGTTGGTGAGATAAAAGCTCACCAAGGCAACGATCAGTA
GCCGGCTTGAGAGAGTGTACGGCCACATTGGGACTGAGACACGGCC
>Zotu3241
GAACGCTGGCGGCGTGCCTAATACATGCAAGTCGAGCGGTAAGGTTCTGCACTTAGTATTTGACCCAG
TAAGTCCCTCAGAGGATACTGCTTGGAAAATCGAGCTCTAGGCTAGAGTAAGATGTCGAATGTTGAGT
GTAGAATACACGAGCGGCGGACGGCTGAGTAACGCGTAGGAATGTACCCCAAAGTGAGGGATAAGCAC
CGGAAACGGTGTCTAATACCGCATGTGATCTTCGGATTAAAGCTTTATGCGCTTTGGGAGCGGCCTGC
GTAAGATTAGCTTGTTGGTGAGGTAAAGGCTCACCAAGGCGACGATCTTTAGCTGGTCTGAGAGGATG
ACCAGCCAGACTGGGACTGAGACACGGCC
>Zotu3243
GAACGCTGGCGGCGCGCTTAACACATGCAAGTCGAGCGATGAAATTTTAACAGATCCCTTCGGGGTGA
AGATAAAATGGATTAGCGGCGGACGGGTGAGTAACACGTGAGTAACCTGCCTTAGACATTGGGATAGC
CTCGGGAAACCGGGATTAATACCGAATAAAATCACAGATACACATGTATCCATGATCAAAACTCCGGT
GGTCTAAGATGGACTCGCGTCCCATTAGCTAGTTGGTGAGGTAACGGCCCACCAAGGCGACGATGGGT
AGCCGGCCTGAGAGGGTGAACGGCCACATTGGAACTGAGAAACGGTC
>Zotu3244
GAACGCTGGCGGCGCGCTTAACACATGCAAGTCGAGCGAGAACCATTCCTTCGGGAATGGGGACAGCG
GCGAACGGGTGAGTAACACGTGGGTAATCTGCCCTCGATTCTGGGATAGCCCGGGGAAACCCGGATTA
ATACCGGATAGCCTCTCGAGCTTTCGAGCTCGTGAGAAAAGGTAGCTTCGGCCTCCGATCGAGGATGA
GCCCGCGGCGGATTAGCTTGTTGGTGGGGTAATGGCCCACCAAGGCGACGATCCGTAGCTGGTCTGAG
AGGACGATCAGCCACACTGGGACTGAGACACGGCC
>Zotu3245
GAACGCTGGCGGCGTGCCTAATACATGCAAGTCGAGCGGAGTTACAGGAGCTTGCTCCTGTAACTCAG
CGGCGGACGGGTGAGTAACACGTGGGCAACCTGCCTATAAGACTGGGATAACTTCGGGAAACCGGAGC
TAATACCGGATAATTCTTTTCCTCTCATGAGGAAAAGCTGAAAGACGGTTTACGCTGTCACTTATAGA
TGGGCCCGCGGCGCATTAGCTAGTTGGTGAGGTAACGGCTCACCAAGGCAACGATGCGTAGCCGACCT

GAGAGGGTGATCGGCCACACTGGGACTGAGACACGGCC
>Zotu3246
GAACGCTAGCGGGAGGCTTAACACATGCAAGCCGAGGGGTATTCAGTTGCTTGCAACTGGAGAGACCG
GCGTACGGGTGAGTAACGCGTATGTAACTTGCCTCATTCAACGGGATAGCCCGGGGAAACCCGGATTA
ATATCGTATAACAGCAATTCGGGCATCCGGAATGTTTGAAAGATTTATCGGAATGAGATAGACATGCG
TATGATTAGTTAGTTGGCGGGGTAACGGCCCACCAAGACGATGATCATTAGGGGTCCTGAGAGGGTGA
TCCCCCACACTGGTACTGAGACACGGAC
>Zotu3247
GAACGCTGGCGGCGTGCTTAACACATGCAAGTCGAACGGTGAAGCGCTTTCCTTTTTGGTTGGTGTGG
ATGAGTGGCGAACGGGTGAGTAACACGTGAGTAACCTGCCCCTTCTTCTGGATAACCGCATGAAAGT
GTGGCTAATACGGGATATTCTGTGCCTGCTGCATGGTGGGTGTGGGAAAGATTGTGCCTTTGGGTGTT
TTCGGTGGGGGATGGGCTCGCGGCCTATCAGCTTGTTGGTGGGGTGATGGCCTACCAAGGCGGTGACG
GGTAGCCGGCCTGAGAGGGTGGACGGTCACACTGGGACTGAGACACGGCC
>Zotu3248
GAACGCTGGCGGCGTGCCTAATACATGCAAGTCGAGCGCGAAGATCAGGAGCTTGCTCCTGAGATTCG
AGCGGCGGACGGGTGAGTAACACGTAGGCAACCTACCCTTGAGATTGGGATAACTACCGGAAACGGTA
GCTAATACCGGATACGACATTCCTGCATAAGTAAGAATGTTAAAAGGCGGATTTATCTGCCGCTCATG
GATGGGCCTGCGGTGCATTAGCTAGTTGGTGAGATAGTAGCTCACCAAGGCGACGATGCATAGCCGAC
CTGAGAGGGTGATCGGCCACACTGGGACTGAGACACGGCC
>Zotu3249
GAACGCTGGCGGCGTGCTTAACACATGCAAGTCGAGCGGTAAGGCCCTTCGGGGTACACGAGCGGCGA
ACGGGTGAGTAACACGTGGGTGACCTGCCCTCAGCTCTGGGATAAGCCCGGGAAACTGGGTCTAATAC
CGGATAGGACTGCATGCTTCATGGTGTGTGGTGGAAAGTTTTTCGGCTGGGGATGGGCCCGCGGCCTA
TCAGCTTGTTGGTGGGGTGATGGCTCACCAAGGCGACGACGGGTAGCCGGCCTGAGAGGGCGACCGGC
CACACTGGGACTGAGACACGGCC
>Zotu3250
GAACGCTGGCGGCGTGCTTAACACATGCAAGTCGAGCGGTAAGGCCTTTCGGGGTACACGAGCGGCGA
ACGGGTGAGTAACACGTGGGTGACCTGCCCTCCACTCTGGGATAAGCCTGGGAAACTGGGTCTAATAC
TGGATATGACTTCTGGGTGCATGCCTGGTTGTGGAAAGATTTATCGGTGGGGGATGGGCCCGCGGTCT
ATCAGCTTGTTGGTGGGGTGATGGCCTACCAAGGCGACGACGGATAGCCGGCCTGAGAGGGTGATCGG
CCACACTGGGACTGAGACACGGCC
>Zotu3252
GAACGCTGGCGGCGTGCCTAACACATGCAAGTGGAGCGACGAACCAGACTTCGGTCTGGGGCCAAGCC
GCGAACGGGTGAGTAACACGTGGGTAACCTGCCCCAATGATCGGGACAACCCGGGGAAACCCGGGCTA
ATACCGAATGTGGTCGCAGTCCGTGAGGACGGTGACGAAAGGAAGCTTCGGCCTCCGCATTGGGATGG
GCCCGCGGCCCATTAGCTTGTTGGTGGGGTAATGGCCTACCAAGGCATCGATGGGTAGCTGGTCTGAG
AGGACGATCAGCCACACTGGGACTGAGACACGGCC
>Zotu3253
GAACGCTGGCGGTATGCTTAACACATGCAAGTCGTACGAGAGTTTTTAACTCTAGTGGCGGACGGGTG
AGTAACACGTGAGAATCTACCCCAAGGAGGGGAATAACAGCTGGAAACGGTTGCTAATACCCCATATG
CCGAAAGGTAAAAGGATGTATCCACCTTAGGAAGAGCTCGCGTCTGATTAGCTAGTTGGTGAGGTAAT
AGCTCACCAAGGCCATGATCAGTAGCTGGTTTGAGAGGACGATCAGCCACACTGGGACTGAGACACGG
CC
>Zotu3254
GAACGCTGGCGGCGTGCCTAATACATGCAAGTCGAGCGAACTTTTGGGAGCTTGCTCCTAAAGGTTAG
CGGCGGACGGGTGAGTAACACGTGGGCAACCTGCCTATAAGACTGGGATAACTTCGGGAAACCGGAGC
TAATACCGGATAATTCTTTTCTACACATGTAGAAAAGCTAAAAGACGGTTTACGCTGTCACTTATAGA
TGGGCCCGCGGCGCATTAGCTAGTTGGCGAGGTAACGGCTCACCAAGGCCACGATGCGTAGCCGACCT
GAGAGGGTGATCGGCCACACTGGGACTGAGACACGGCC
>Zotu3255
GAATGCTGGCGGCGTGGATAAGGCATGCAAGTCAAGGGGGCCCGCAAGGGCAACCGGCAAACGAGGTA
ATAACACGTGGGTACGTACCTGGAAGTCAGGCATAGCTCATCGAAAGGTGAGGTAATTCCTGATAGTC
TCTTCGGAGTAAAGTTTTTTCGCTTCCAGAGCGGCCTGCGGGATATCAGCTAGTTGGTAGGGTAATGG
CCTACCAAGGCGATGACGTCTAGGGGAGCTGAGAGGCTGACCCCCACCGATGGAACTGAGACACGGTC

>Zotu3256
GAACGCTGGCGGCATGCCTAACACATGCAAGTCGAACGAGACCTTCGGGTCTAGTGGCGCACGGGTGC
GTAACGCGTGGGAATCTGCCCTTGGGTTCGGAATAACTCAGAGAAATTTGAGCTAATACCGGATGATG
TCGCAAGACCAAAGATTTATTGCCCAAGGATGAGCCCGCGTAGGATTAGCTAGTTGGTGAGGTAAAGG
CTCACCAAGGCGACGATCCTTAGCTGGTCTGAGAGGATGATCAGCCACACTGGGACTGAGACACGGCC
>Zotu3257
GAACGCTAGCGATAGGCCTAACACATGCAAGTCGAGGGGCAGCATAGAGGTAGTAATACTTTGGTGGC
GACCGGCGCACGGGTGAGTAACACGTATGCAACCTGCCTTCAACACGGGGATAACCCGATGAAAGTCG
GACTAATATTCGATAACACAGGGGCACCGCATGGTGATATTTGTTAAAGATTAATTGGTTGAAGATGG
GCATGCGCATGATTAGTTAGTTGGTAGGGTAACGGCCTACCAAGGCAACGATCATTAGGGGTTCTGAG
AGGAAGATCCCCCACACTGGTACTGAGACACGGAC
>Zotu3258
GAACGCTGGCGGCATGCCTAACACATGCAAGTCGAACGAAGTGCCTTCGGGTGCTTAGTGGCGCACGG
GTGCGTAACGCGTGGGAATCTGCCCCTTGGTTCGGAATAACAGTTGGAAACGACTGCTAATACCGGAT
GACGACGTAAGTCCAAAGATTTATCGCCGAGGGATGAGCCCGCGTAGGATTAGGTAGTTGGTGTGGTA
AAGGCGCACCAAGCCGACGATCCTTAGCTGGTCTGAGAGGATGATCAGCCACACTGGGACTGAGACAC
GGCC
>Zotu3259
GAACGCTAGCGGCAGGCCTAATACATGCAAGTCGAACGGTTGTATTTATACAATAGTGGCGCACGGGT
GCGTAACACGTATGCAACCTACCTTATAGACGGGGATAGCCTTTGGAAACGGAGATTAATACCCGATA
GTACTGTTGACTGGCATCAGTTAATATTTAAAGATTTATCGCTATAAGCTGGGCATGCGTCCAATTAG
TTAGTTGGTGAGGTAACGGCTCACCAAGACGATGATTGGTAGGGGAACTGAGAGGTTAATCCCCCACA
CTGGCACTAAGATACGGGC
>Zotu3260
GAACGCTGGCGGCGTGCTTAACACATGCAAGTCGAGCGGTAAGGCCCTTCGGGGTACACGAGCGGCGA
ACGGGTGAGTAACACGTGAGCAATCTGCCCTCCACATCGGGATACCCCCCGGAAACGGGAGCTAATAC
CGGATACGACCACTTCAGGCATCTGATGGTGGTGGAAAGTTCCGGCGGTGGAGGATGAGCTCGCGGCC
TATCAGCTTGTTGGTGGGGTAATGGCCCACCAAGGCTTCGACGGGTAGCCGGCCTGAGAGGGTGACCG
GCCACACTGGGACTGAGACACGGCC
>Zotu3261
GAATGCTGGCGATGTGGATAAGTCATGCAAGTCAAGGGGGCCCGCAAGGGCAACCGGCAGACGAGGTA
GTAACACGTAGGTACGTCCCCTTTAGTCGGGCATAGCCAGTCGAAAGATTGGGTAATTCCCGATGGTC
CCGCAAGGGTAAAGGTTTTCCGCTAGAGGAGCGGCCTGCGTAGTATCAGCTAGTTGGTAGGGTAATGG
CCTACCAAGGCTACGACGCTTAGGGGAGCTGAGAGGCTGACCCCCACCGATGGGACTGCGACACGGCC
>Zotu3262
CAACGCTGGCGGCGTGCCTCAGACATGCAAGTCGAACGATTAAACTTCCCTTCGGGGAAGATATAAAG
TGGCGCACGGGTGAGTAACACGTAAGTAATCTACCTTCGAGTGGGGAATAACAGTCGGAAACGACTGC
TAATACCGCATAATGCAGCGGCATCGCAAGATGACAGTTGTTAAAGGAGCAATCCGCTTGAAGAGGAG
CTTGCGGCAGATTAGCTAGTTGGTAAGGTAATGGCTTACCAAGGCTACGATCTGTATCCGGTCTAAGA
GGACGGTCGGACACACTGACACTGAACAACGGGT
>Zotu3263
GAACGCTGGCGGCGTGCCTAACACATGCAAGTCGAGCGAGAAAGTAGCAATACGAGTAAAGCGGCGAA
CGGGTGAGTAACACGTAGGTAATCTACCTTTTAGTGGGGAATACCGTTTCGAAAGGAACGTTAATACC
GCATGTGGATCCTTTCCGATGGGAGAGTATCTAAAGGGGGGTGTAGCAATACATCTCTCGCTAATCGA
TGAGCCTGCGTCCCATTAGTTAGTTGGTAAGGTAATGGCTTACCAAGACTTCGATGGGTAGCTGGTCT
GAGAGGACGACCAGCCACACTGGAACTGACACACGGTC
>Zotu3264
GAACGCTGGCGGCGTGCCTAATACATGCAAGTCGAGCGGACGAATTTTCGGTGCTTGCACCGAATTTT
CGTTAGCGGCGGACGGGTGAGTAACACGTGGGTAACCTACCCTACAGACTGGGATAACTTCGGGAAAC
CGAGGCTAATACCGGATAATACTTTTTAACACATGTTAGAAAGTTGAAAGATGGTTTTGCTATCACTG
TAGGATGGACCCGCGGCGCATTAGCTAGTTGGTGAGGTAACGGCTCACCAAGGCAACGATGCGTAGCC
GACCTGAGAGGGTGATCGGCCACACTGGGACTGAGACACGGCC
>Zotu3265
GAACGCTGGAAATGTGGATACCGGCATGCAAGTCAAGGGGCCGCAAGGCACCGGCGAACGGCTTAGTA

ACACGTGGATACATACCGCAAAGCGGAGCACAGCTCGTCGAAAGACGGGGTAATTCTCCATGATAACG
CAAGTTTAAAGGCGCAAGCCACTTTGTGAATGGTCCGCGGTCTATCAGCTAGTTGGTGAGGTAATGGC
TCACCAAGGCTATGACGGATAGGGGGCGTGAGAGCGCGACCCCCAAGAATGGCACTGCGACACGGGC
>Zotu3266
GAACGTTGGCGGCGTGCCTAACACATGCAAGTCGAACGGAAATAGCAATATTTGAGTGGCGCACGGGT
GAGTAACACGTAGCTAATCTACCTCAAAGCGCGGGATAACGTGTCGAAAGACAGGCTAATACCCCATA
AGCTCACGGAAAGCAGAGGACTGTGAGAAAAGGCGCAAGTCACTTTGAGATGAGGCTGCGGCCCATCA
GCTAGTTGGTGCGGTAAAGGCGCACCAAGGCGACGACGGGTAGGGGACCTGAGAGGGTGACCCCCCAC
ACTGGGACTGAGACACGGCC
>Zotu3267
GAACGCTGGCGGCGTGCCTAACACATGCAAGTCGAGCGAAGCACTCTGCTTCAATTTCTTCGGATTGA
GAAGTAGTTTGACTGAGCGGCGGACGGGTGAGTAACGCGTGGGTAATCTGCCTCACACAGGGGGATAA
CAGTTAGAAATGACTGCTAATACCGCATAAGGCCACAGCACCGCATGGTGCAGGGGTAAAAACTCCGG
TGGTGTGAGATGAGCCCGCGTCTGATTAGCCAGTTGGCGGGGTAACGGCCCACCAAAGCGACGATCAG
TAGCCGGCCTGAGAGGGTGATCGGCCACATTGGGACTGAGACACGGCC
>Zotu3269
GAACGCTGGCGGCGTGCCTAATACATGCAAGTCGAGCGAACAGATGAGGAGCTTGCTCCTTTGACGTT
AGCGGCGGACGGGTGAGTAACACGTGGGTAACCTACCTATAAGACTGGAATAACTCCGGGAAACCGGG
GCTAATGCCGGATAATACACAGAACCGCATGGTTCTGTGATGAAAGACGGTTTTGCTGTCACTTATAG
ATGGACCCGCGGCGTATTAGCTAGTTGGTAAGGTAACGGCTTACCAAGGCAACGATACGTAGCCGACC
TGAGAGGGTGATCGGCCACATTGGGACTGAGACACGGCC
>Zotu3270
GAACGCTGGCGGTATGCTTTACACATGCAAGTCGTACGAGAGTTTTGAACTCAAGTGGCGGACGGGTG
AGTAACACGTAAGAATTTGCCTTTAGGAGGGGGACAACAACTGGAAACGGTTGCTAATACCCCATATG
CTTTCGAGTGAAACGGTTTTTCCGCCTAAAGAGAAGCTTGCGGCTGATTAGCTTGTTGGTAAGGTAAT
GGCTTACCAAGGCGACGATCAGTATCTGGTTTGAGAGGACGATCAGACACACTGGAACTGAGACACGG
TC
>Zotu3272
GAACGCTGGCGGCAGGCTTAACACATGCAAGTCGAACGGGCACTTTCGGGTGTCAGTGGCAGACGGGT
GAGTAACACGTGGGAACGTGCCCTTCGGTTCGGAACAACTCAGGGAAACTTGAGCTAATACCGGATAC
GCCCTTACGGGGAAAGGTTTACTGCCGAAGGATCGGCCCGCGTCTGATTAGCTAGTTGGTGAGGTAAT
GGCTCACCAAGGCGACGATCAGTAGCTGGTCTGAGAGGATGATCAGCCACACTGGGACTGAGACACGG
CC
>Zotu3273
GAACGCTGGCGGCGTGCTTAACACATGCAAGTCGAGCGGTAAGGCCCTTCGGGGTACACGAGCGGCGA
ACGGGTGAGTAACACGTGGGTGATCTGCCCTGCACTTCGGGATAAGCCTGGGAAACTGGGTCTAATAC
CGGATATGACCTCCGATCGCATGGTTGGTGGTGGAAAGATTTATCGGTGCAGGATGGGCCCGCGGCCT
ATCAGCTTGTTGGTGGGGTAATGGCCTACCAAGGCGACGACGGGTAGCCGACCTGAGAGGGTGACCGG
CCACACTGGGACTGAGACACGGCC
>Zotu3274
GAACGCTGGCGGCGCGCCTCATACATGCAAGTCGAACGTGAAGCCCGGAGCTTGCTCTGGGTGGAAAG
TGGCGAACGGGTGAGGAATACGTGAGGAACCTGCCCCGAAGACCGGGATAACTCCGGGAAACCGGTGC
TAATACCGGATGCCCTCATCGGAACTCATGTTCCGATGAGGAAATGGATTCCGCTTCGGGATGGCCTC
GCGGCCTATCAGCTTGTTGGTGGGGTAACGGCCCACCAAGGCTTCGACGGGTAGCTGGTCTGAGAGGA
TGTCCAGCCACACTGGGACTGAGACACGGCC
>Zotu3275
GAACGCTGGCGGCGTGCTTAACACATGCAAGTCGAGCGGTAAGGCACCTTCGGGTGTACACGAGCGGC
GAACGGGTGAGTAACACGTGAGCAATCTGCCCTTCTCATCGGAATAAGCGCGGGAAACTGCGTCTAAT
GCCGAATATGACCTCCTTTCGCATGATCGGAGGTGGAAAGCTCCGGCGGAGAAGGATGAGCTCGCGGC
CTATCAGCTAGTTGGTGAGGTAACGGCCCACCAAGGCGACGACGGGTAGCCGGCCTGAGAGGGTGACC
GGCCACACTGGGACTGAGACACGGCC
>Zotu3277
GAACGCTGGCGGCAGGCTTAACACATGCAAGTCGAGCGGGCCCTTCGGGGTCAGCGGCGGACGGGTTA
GTAACGCGTGGGAACGTGCCCTTTGCTCTGGAATAGCCCTGGGAAACCGGGGGTAATACCGGATGTGC

CCTGAGGGGGAAAGGTTTACCGGCAAGGGATCGGCCCGCGTCTGATTAGGTAGTTGGTGGGGTAACGG
CCCACCAAGCCGACGATCGGTAGCTGGTTTGAGAGGATGATCAGCAACACTGGGACTGAGACACGGCC
>Zotu3278
GAACGCTGGCGGCATGCCTAACACATGCAAGTCGAACGAAGGCTTCGGTCTTAGTGGCGCACGGGTGC
GTAACGCGTGGGAATCTGTCCCTCGGTTCGGAATAACAGTTAGAAATGACTGCTAATACCGGATGATG
ACGTAAGTCCAAAGATTTATCGCCGAGGGATGAGCCCGCGTAGGATTAGCTAGTTGGTGTGGTAAGAG
CGCACCAAGGCGACGATCCTTAGCTGGTCTGAGAGGATGATCAGCCACACTGGGACTGAGACACGGCC
>Zotu3279
GAACGCTGGCGGCGTGCCTAATACATGCAAGTAGAACGCTGAAGGAGGAGCTTGCTTCTCTGGATGAG
TTGCGAACGGGTGAGTAACGCGTAGGTAACCTGCCTGGTAGCGGGGGATAACTATTGGAAACGATAGC
TAATACAGCATAAGAGTAGATGTTGCATGACATTTGCTTAAAAGGTGCAATTGCATCACTACCAGATG
GACCTGCGTTGTATTAGCTAGTTGGTGAGGTAACGGCTCACCAAGGCGACGATACATAGCCGACCTGA
GAGGGTGATCGGCCACACTGAGACTGAGACACGGCC
>Zotu3281
GAACGCTAGCGGCAGGCCTAATACATGCAAGTCGAACGGTAACATGAGTAGCAATACTTGATGACGAG
TGGCGCACGGGTGCGTAACGCGTATGCAATCTACCTTCAACTGGTGAATAGCCCGAAGAAATTCGGAT
TAATACACCATAGTATTGTTTGTTGGCATCAACAGACAATTAAAACTCCGGTGGTTGAAGATGAGCAT
GCGTGACATTAGCTAGTTGGTGAGGTAATGGCTCACCAAGGCTACGATGTCTAGGGGATCTGAGAGGA
TTAACCCCCACACTGGTACTGAGACACGGAC
>Zotu3282
GAACGCTGGCGGCGTGCTTAACACATGCAAGTCTACGAGAATAAGGTAGCAATATCTTTAGTAAAGTG
GCGCACGGGTGCGTAACACGTAAGCAATCTACCTTTGGGTCTGGAATAACTTCGAGAAATCGAGGCTA
ATACCGGATAATGCAGCGGCACCGCATGGTGATGTTGTTAAAGTTTAAGTAATGCCTATAGATGAGCT
TGCGCCTGATTAGCTAGTTGGTAGGGTAAAGGCCTACCAAGGCTACGATCAGTAGCTGGTCTGAGAGG
ATGATCAGCCACACTGGAACTGAGACACGGTC
>Zotu3283
GAACGCTGGCGGCGTGCTTAACACATGCAAGTCGAACGATGATGCTCAAGCTTGCTTGGGTTGATTAG
TGGCGAACGGGTGAGTAACACGTGGGTGATCTGCCCTGCACTTCGGGATAAGCCTGGGAAACTGGGTC
TAATACCGGATATGACCCTCCTTTAGTGTGGGGGGTGGAAAGCTTTTGCGGTGTGGGATGAGCCTGCG
GCCTATCAGCTTGTTGGTGGGGTAATGGCCTACCAAGGCGTCGACGGGTAGCCGGCCTGAGAGGGTGA
TCGGCCACATTGGGACTGAGACACGGCC
>Zotu3284
CAACGCTGGCGGCGTGCCTCAGACATGCAAGTCGAACGGTTAAGACTCCCTTCGGGGAGTGTATAAAG
TGGCGCACGGGTGAGTAACACGTGGGTAATCTACCTCCGGGTGGGGGATAACCCGCCGAAAGGTGGGC
TAATACCGCATAATGCAGCGGCACCGAATGGTGACGGTTGTTAAAGGAGCAATCCGCCTGGAGAGGAG
CCCGCGGCCGATTAGCTAGTTGGTAGGGTAATGGCCTACCAAGGCGACGATCGGTAGCCGGCCTGAGA
GGGCGGTCGGCCACACTGGCACTGAAACACGGGC
>Zotu3285
GAACGCTGGCGGCGTGCTTAACACATGCAAGTCGAGCGAGGCCCTCCTTCGGGGGGTGCCCTAGCGGC
GAACGGGTGAGTAACACGTGGGCAACCTGCCCTCAGCTCTGGGATAACTCCAAGAAATTGGTGCTAAT
ACCGGATGTGACCGCTGACCGCATGGTCTGGTGGTGGAAAGATTCATCGGCTGAGGATGGGCCCGCGG
CCTATCAGCTTGTTGGTGGGGTGATGGCCTACCAAGGCGACGACGGGTAGCCGGCCTGAGAGGGTGAC
CGGCCACACTGGGACTGAGACACGGCC
>Zotu3286
GAATGCTGGCGGCGTGCCTAACACATGCAAGTCGAACGGTAACAGAGATAATTTATTATTTGCTGACG
AGTGGCGGACGGCTGAGTAATGCGTGGGAACATGCCCCAAAGTGAGGGATAAGCTCCAGAAATGGAGT
CTAATACCGCATATGATCTTCGGATTAAAGGATTTATCCGCTTTGGGAGTGGCCTGCGTACGATTAGA
TAGTTGGTGAGGTAATGGCTTACCAAGTCTACGATCGTTAGCTGGTCTGAGAGGATGACCAGCCAGAC
TGGAACTGAGACACGGTC
>Zotu3287
GAATGCTGGCGGCGTGCTTAACACATGCAAGTCGAGCGGTAAGGCCCTTCGGGGTACACGAGCGGCGG
ACGGCTGAGTAACGCGTGGGAACATGCCCCAAAGTGAGGAATAACTGCCCGAAAGGGTGGCTAATGCC
GCATATGATCTTCGGATTAAAGGATTTATCCGCTTTGGGAGTGGCCTGCGTACGATTAGGTAGTTGGT
GAGGTAATGGCTCACCAAGCCGACGATCGTTAGCTGGTCTGAGAGGATGACCAGCCAGACTGGAACTG

AGACACGGTC
>Zotu3288
GAACGCTGGTGGCGTGCTTAACACATGCAAGTCGAACGGTGAAGCTCCTGCTTGCAGGGGTGGATCAG
TGGCGAACGGGTGAGTAACACGTGAGCAACCTGCCCTCCACTTCGGGATAAGCCTTGGAAACGGGGTC
TAATACCGGATATGAGCACTCACTGCATGGTGGGTGTTGGAAAGTTTTTCGGTGGGGGATGGGCTCGC
GGCCTATCAGCTTGTTGGTGGGGTAATGGCCTACCAAGGCGTCGACGGGTAGCCGGCCTGAGAGGGCG
ACCGGCCACACTGGGACTGAGACACGGCC
>Zotu3289
GAACGCTGGCGGCGTGCTTAACACATGCAAGTCGAACGCTGAAGCTCCCAGCTTGCTGGGGGTGGATG
AGTGGCGAACGGGTGAGTATCACGTGAGGAACCTGCCCTGAACTTCGGGATAAGCCTGGGAAACTGGG
TCTAATACCGGATAGGACATTCTACCGCATGGTGGGGTGTGGAAAGTTTTAACGGTTTGGGATGGTCT
CGCGGCCTATCAGCTTGTTGGTGGGGTAATGGCCTACCAAGGCGATGACGGGTAGCCGGCCTGAGAGG
GTGACCGGCCACACTGGGACTGAGACACGGCC
>Zotu3290
GAACGCTGGCGGCGTGCTTAACACATGCAAGTCGAACGGTGACCTCGAGAGCTTGCTCTTGGGTGATC
AGTGGCGAACGGGTGAGTAACACGTGAGTAACCTGCCCCAGACTCTGGAATAACCCCGGGAAACCGGA
GCTAATACCGGATACGAAGTGGAGCCGCATGGCTACCACTTGGAAAGTTTTTCGGTCTGGGATGGACT
CGCGGCCTATCAGCTAGTTGGTGAGGTAACGGCTCACCAAGGCGACGACGGGTAGCCGGCCTGAGAGG
GCGACCGGCCACACTGGGACTGAGACACGGCC
>Zotu3291
TAACGCTGGCGGCGTGCATAACACATGCAAGTCGAACGATGAAACTTAAATGATTTCTTCGGAATGAA
CTTAAGTGAATTAGTGGCGAACGGGTGAGTAACGCGTGAGTAACCTGCCTTACACAAGGGGATAGCCT
CTGGAAACGGAGAATAATACCCTATGACATTAATACTTCGCATGAAGCATTAATCAAAGTGTTAGCGG
TGTAAGATGGACTTGCGTCTGATTAGCTAGTTGGTGAGATAACAGCCCACCAAGGCAACGATCAGTAG
CCGGCTTGAGAGAGTGTACGGCCACATTGGGACTGAGACACGGCC
>Zotu3292
GAACGCTGGCGGCAGGCTTAACACATGCAAGTCGAACGCCCCGCAAGGGGAGTGGCAGACGGGTGAGT
AACACGTGGGAACCTTCCCTATAGTACGGAATAGCCCAGGGAAACTTGGAGTAATACCGTATACGCCC
GAAAGGGGAAAGAATTTCGCTATAGGATGGGCCCGCGTAGGATTAGCTAGTTGGTGAGGTAATGGCTC
ACCAAGGCGACGATCCTTAGCTGGTTTGAGAGAACGACCAGCCACACTGGGACTGAGACACGGCC
>Zotu3293
GAACGCTGGCGGCAGGCTTAACACATGCAAGTCGAGCGGGCGTAGCAATACGTCAGCGGCAGACGGGT
GAGTAACGCGTGGGAACGTACCCTTTGGTTCGGAACAACACAGGGAAACTTGTGCTAATACCGGATAA
GCCCTTACGGGGAAAGATTTATCGCCGAAGGATCGGCCCGCGTCTGATTAGCTAGTTGGTGAGGTAAT
GGCTCACCAAGGCGACGATCAGTAGCTGGTCTGAGAGGATGATCAGCCACACTGGGACTGAGACACGG
CC
>Zotu3294
GAACGCTGGCGGCGTGCTTTACACATGCAAGTCGAACGGCAGCATAGATAGTAGCAATACTATTGGGT
GGCGAGTGGCGGACGGGTGAGTAATACATCGAGACGTGCCTAATAGATTGGGATAACCTGTCGAAAGA
TAGGCTAATACCGGATAATCTCTGAGGAGGAAAGCTGGGGACTTAGGAATAGGCCTGGCGCTAATAGA
GCGATCGATGGCCGATTAGTTAGTTGGTGGGGTAAAGGCTTACCAAGGCGATGATCGGTAGCTGTTCT
GAGAGGAAGATCAGCCACACTGGAACTGAGAGACGGTC
>Zotu3295
GAACGCTGGCGGCGTGCTTAACACATGCAAGTCGAACGGTGAAGCGCCTTCCTTTGTGGTGGGTGTGG
ATGAGTGGCGAACGGGTGAGTAACACGTGAGTAACCTGCCCCCTTCTTCTGGATAACCGCATGAAAGT
GTGGCTAATACGGGATATTCTGTGCCTGCTGCATGGTGGGTGTGGGAAAGATTGCGCCTTTGGGTGTT
TTTGGTGGGGGATGGGCTCGCGGCCTATCAGCTTGTTGGTGGGGTGATGGCCTACCAAGGCGGTGACG
GGTAGCCGGCCTGAGAGGGTGGACGGTCACACTGGGACTGAGACACGGCC
>Zotu3296
GAACGCTGGCGGCATGCCTTACACATGCAAGTCGAACGGTAACGGGTTAAGCCGACGAGTGGCGAACG
GGTGAGTAATATATCGGAACGTGCCCAGTCGTGGGGGATAACGTAGAGAAATTTACGCCAATACCGCA
TACGATCTAAGGATGAAAGCGGGGGATCGCAAGACCTCGCGCGATTGGAGCGGCTGATATCAGATTAG
GTTGTTGGTGAGGTAAAAGCTCACCAAGCCGACGATCTGTAGCTGGTTTGAGAGAACGACCAGCCACA
CTGGGACTGAGACACGGCC

>Zotu3298
GAACGCTAGCGGCAGGCCTAATACATGCAAGTCGAGGGGTATAGGAGGCTTGCTTCCTAGAGACCGGC
GCACGGGTGCGTAACGCGTATGCAACCTACCCTTTTCTGGGGGATAGCCCGAAGAAATTCGGATTAAT
ACCGCATAAGACCACACGATGGCATCATCGAGAGGTCAAACATTTATGGGAGAAGGATGGGCATGCGT
GCCATTAGCTAGTTGGCGGGGTAACGGCCCACCAAGGCGACGATGGCTAGGGGATCTGAGAGGATGAC
CCCCCACACTGGTACTGAGACACGGAC
>Zotu3299
GAACGCTAGCGGCAGGCTTAATACATGCAAGTCGAGGGGCAGCACGAATAGCAATATTTGGTGGCGAC
CGGCAAACGGGTGCGGAACACGTACGCAACCTTCCCATAACTGGTGAATAGCCTTCCGAAAGGGAGAT
TAATACACCGTAACATAGTTGAGTGGCATCACTTGACTATTATAGCTTCGGCGGTTTTGGATGGGCGT
GCGCCTGATTAGGTAGTTGGCGGGGTAACGGCCCACCAAGCCTCCGATCAGTAACTGGTGTGAGAGCA
CGACCAGTCACACGGGCACTGAGACACGGGC
>Zotu3300
GAACGCTGGCGGCGCGCCTAATACATGCAAGTCGAACGGGCACACTTCGGTGTGTCAGTGGCGGACGG
GTGAGTAACACGTGAGTGACCTGCCTCCTAGTACGGAATAACTCTGGGAAACCAGAGCTAATACCGCA
TGTGCTTAGTGTTTCATGATGCTAAGTAAAGATTTATTCGCTAGGAGAGGGGCTTGCGGCGGATTAGC
TAGTCGGTGGGGTAACGGCCTACCGAGGCGACGATCCGTAGCTGGTCTGAGAGGACGACCAGCCACAC
TGGGACTGAGACACGGCC
>Zotu3301
GAACGCTGGCGGCAGGCCTAACACATGCAAGTCGAACGAGCTGAGACCTTCGGGTCTCGATGCTAGTG
GCGCACGGGTGAGTAACGCGTGGGAATATGCCCTTCAGTACGGAATAACCAAGGGAAACTTTGGCTAA
TACCGTATACGCCCTCCGGGGGAAAGATTTATCGCTGAAGGATTAGCCCGCGTCTGATTAGGTAGTTG
GTGAGGTAACGGCTCACCAAGCCGACGATCAGTAGCTGGTCTGAGAGGATGATCAGCCACACTGGGAC
TGAGACACGGCC
>Zotu3302
GAACGCTAGCGGCAGGCCTAATACATGCAAGCCGAACGGCAAGTCTGATAGCAATATTGGACCTAGAG
TGGCGCAAGGGTGCGTAACACGTATGCAATCTGCCCACGACAGGAGTATAGCTCCCCGAAAGGGGAAT
TAACCCTCCATAACACCGCGCGAGGGCATCCGAGAGCGGTTAAACCAAAAGGGGTCGTGGATGAGCAT
GCGGTTGATTAGCTAGTTGGTAGGGTAACGGCCTACCAAGGCGATGATCAATAGGGGTTCTGAGAGGA
AGGTCCCCCACACTGGCACTGAGATACGGGC
>Zotu3303
GAACGCTGGCGGCATGCTTAACACATGCAAGTCGAACGGGCCCTTCGGGGTTAGTGGCGGACGGGTGA
GTAACGCGTGGGAACCTGCCCTGGGGTGGGGGATAACCGCTGGAAACGGCGGCTAATACCGCATGTGC
CTTGAGGGGCAAAGGTCTTTATGGCCGCCTTGGGAGGGGCCCGCGTCTGATTAGCTTGTTGGTGGGGT
AATGGCCTACCAAGGCTTCGATCGGTAGCTGGTCTGAGAGGATGATCAGCCACACTGGGACTGAGACA
CGGCC
>Zotu3304
GAACGCTGGTGGCGTGCTTAACACATGCAAGTCGAGCGAGGCGTCCGCCTCCGGGCGGTCAGCCGAGC
GGCGAACGGGTGAGTAACACGTGAGCAATCTTCCCTCGGCTCGGGGATAACCTCGGGAAACTGAGGCT
AATACCGGATGAACGTTTTTCGGGGCATCCCGGAAAACGAAAAGGTTTCGCACCGCGAGGTGCGTTTC
CGGCCGGGGATGAGCTCGCGGCCTATCAGTTTGTTGGTGTGGTAAATGCGCACCAAGACGACGACGGG
TAGCCGGCCTGAGAGGGTGATCGGCCACACTGGGACTGAGACACGGCC
>Zotu3306
GAACGCTTGCGGCGTGCCTAAGAAATGCAAGTCGAGCGAACCGAGGGTGCTTGCACCCAAAGGTTAGC
GGCGAACGGTCGCGTAACACGTAAGCAACCTGCCCTAAAATGGGGGATAGCCCACCGAAAGGTGGATT
AATACCGCATGTGGATGTCTCTTCGCATGTAGAGACATCTAAAGCAGCAATGCGTTTTAGGATGGGCT
TGCGGCCTATCAGCTAGTTGGCGAGGTAACGGCTCACCAAGGCGACGACGGGTAGCTGGTCTGAGAGG
ATGGTCAGCCGGACTGGGACTGAGATACGGCC
>Zotu3307
GAACGCTGGCGGCGTGCTTAACACATGCAAGTCGAACGATGAACCTCCTTCGGGAGGGGATTAGTGGC
GAACGGGTGAGTAACACGTGGGCAATCTGCCCTGCACTCTGGGACAAGCCCTGGAAACGGGGTCTAAT
ACCGGATACTGATCATCTTGGGCATCCTTGGTGATCGAAAGCTCCGGCGGTGCAGGATGAGCCCGCGG
CCTATCAGCTTGTTGGTGAGGTAATGGCTCACCAAGGCGACGACGGGTAGCCGGCCTGAGAGGGCGAC
CGGCCACACTGGGACTGAGACACGGCC

>Zotu3308
GAACGCTGGCGGCGTGCCTAATACATGCAAGTCGAGCGAATCAATGGGAGCTTGCTCCCTGAGATTAG
CGGCGGACGGGTGAGTAACACGTGGGTAACCTGCCTGTAAGATTGGGATAACTCCGGGAAACCGGAGC
TAATACCGGATAACATTTTGAACCGCATGGTTCAAAATTAAAAGGTGGCTTTTGCTACCACTTACAGA
TGGACCCGCGGCGCATTAGCTAGTTGGTGAGGTAACGGCTCACCAAGGCAACGATGCGTAGCCGACCT
GAGAGGGTGATCGGCCACACTGGGACTGAGACACGGCC
>Zotu3309
TAACGCTGGCGGCGTGCATAACACATGCAAGTCGAACGATGAAACTTAAATGATTTCTTCGGAATGAA
CTTAAGTGGATTAGTGGCGGACGGGTGAGTAACGCGTGAGTAACCTGCCTTACACAAGGGGATAGCCT
CTGGAAACGGAGAATAATACCCTATGATGTTTCTATCTCGCATGAGATATTAACCAAAGTGTTAGCGG
TGTAAGATGGACTTGCGTCTGATTAGCTAGTTGGTGAGAAAAAAGCCCACCAAGGCAACGATCAGTAG
CCGGCTTGAGAGAGTGTACGGCCACATTGGGACTGAGACACGGCC
>Zotu3312
CAACGCTGGCGGCGCGCTTAACACATGCAAGTCGCACGAGAAAGCCGGGGCAACCTGGTGAGTACAGT
GGCGTACGGGTGAGTAACACGTGGATCATCTACCTCTTGGTGGGGAATAACCCTGGGAAACCGGGGCT
AATACCGCATAAGCCTGCAAAGGGAAAGCAGCAATGCGCCGAGAGAGGAGTCCGCGGCCGATTAGCTA
GTTGGTAGGGTAAAAGCCTACCAAGGCAATGATCGGTAGCCGGCCTGAGAGGGCACACGGCCACACTG
GCACTGAAACACGGGC
>Zotu3314
GAACGCTGGCGGCATGCCTTACACATGCAAGTCGAACGGCAGCATGACGTGTAGCAATACACGTTGAT
GGCGAGTGGCGAACGGGTGAGTAATATATCGGAACATGTCCTGTAGCGGGGGATAACCTATCGAAAGA
TGGACTAATACCGCATACGCTCTACGGAGGAAAGCGGGGGTCGCAAGACCTCGCACTATAGGAGTGGC
CGATATCGGATTAGCTAGTTGGCGGGGTAAAAGCCCACCAAGGCGACGATCCGTAGCTGGTCTGAGAG
GATGATCAGCCACACTGGAACTGAGACACGGTC
>Zotu3315
GAACGCTGGCGGCGTGCTTAACACATGCAAGTCGAACGGAGTCTAAAAATTACCACTGAGATCTTAGT
CAGTGGGCGAGCAGCGTCATGGACACGAAGTGGCCATCACGCGTTAAAAAAGTGCCAACATAGGGCTG
AGGCCTGAGTGGTGATTTTTAGACTGAGTGGCAAACGGGTGAGTAACACGTAGGCAACCTGCCGACAG
GATGGGGACAACATTCCGAAAGGAATGCTAATACCGAATGGAGTCCGGGGATGGCATCATCCCTGGAT
AAAAGATGGCCTCTGAATATGCTATCGCCTGTCGATGGGCCTGCGTCTGATTAGCCAGTTGGCGGGGT
AACGGCCCACCAAAGCGACGATCAGTAGCCGGTCTGAGAGGATGAACGGCCACATTGGGACTGAGACA
CGGCC
>Zotu3318
GAACGCTGGCGGTGTGCTTAACACATGCAAGTGGAGCGACGAACCAGGGCTTGCCCTGGGGCAGAGCC
GCGAACGGGTGAGTAACACGTGGGTAACCTACCCCGATGACTGGGACAACCCGAGGAAACTCGGGCTA
ATACCGGATGTGCTCGGAGATCACGAGATTCTCGAGTAAAGGAAGCTTCGGCCTCCGCATTGGGAGGG
GCCCGCGGCCCATTAGCTTGTTGGTGGGGTAACGGCCTACCAAGGCTGCGATGGGTAGCTGGTCTGAG
AGGACGATCAGCCACACTGGGACTGAGACACGGCC
>Zotu3320
AAAGAGGCTAACTCGGGCCAGGCTTCCTGGTTTTTTGCCTGGAAGGAGAGGAGGTGCATGTTTTGAAG
GCCTTGTACACGTTCCCATCTCAAAGGCCAGGAGAATACCATGGGCCAGACTCTCTGTG
>Zotu3321
GAACGCTGGCGGCGTGCTTAACACATGCAAGTCGAGCGAGGCCCTCTCTTCGGGGGGGTGCCCTAGCG
GCGAACGGGTGAGTAACACGTGGGCAACCTGCCCTCAGCTCTGGGATAACTCCAAGAAATTGGTGCTA
ATACCGGATGTGACCGCTGACCGCATGGTCTGGTGGTGGAAAGATTCATCGGCTGGGGATGGGCCCGC
GGCCTATCAGCTTGTTGGTGGGGTCACGGCCCACCAAGGCGACGACGGGTAGCCGGCCTGAGAGGGTG
ACCGGCCACACTGGGACTGAGACACGGCC
>Zotu3323
GAACGCTGGCGGCGTGCTTAACACATGCAAGTCGAGCGGAAAGGCTCCTTCGGGGGTACTCGAGCGGC
GAACGGGTGAGTAACACGTGAGTAATCTGCCCTTGGCTTCGGGATAGCCACCGGAAACGGTGATTAAT
ACCGGATATGACCACTTCTCGCATGGGATGGTGGTGGAAAGTTTTTCGGCCAGGGATGTGCTCGCGGC
CTATCAGCTTGATGGTGAGGTAATGGCTCACCATGGCTTCGACGGGTAGCCGGCCTGAGAGGGTGACC
GGTCACACTGGGACTGAGACACGGCC
>Zotu3324

GAACGCTGGCGGCGCGCCTAAAACATGCAAGTCGAACGAGAAACCCGGGCTTGCCCGGGTGGACAGTG
GCGAACGGGTGAGTAACACGTGAGGAACCTGCCCCGAAGACCGGGACAACACCGGGAAACCGGTGATA
ATACCGGATGTCCTCACCGGATCGCATGATCTGGTGAGGAAATGGATTCCGCTTCGGGATGGCCTCGC
GGCCTATCAGCTAGTTGGTGGGGTAACGGCCCACCAAGGCGACGACGGGTAGCTGGTCTGAGAGGATG
TCCAGCCACACTGGGACTGAGACACGGCC
>Zotu3325
GAACGCTAGCGGCAGGCCTAACACATGCAAGTCGAGGGAGGAGCTTTCGGGCTTCGACCGGCGTACGG
GTGCGTAACGCGTATACAATCTGCCTTTCACATAGGGATAGCCCGAAGAAATTTGGATTAATACCTAA
TGGTATATAGTTGTGGCATCACATTTATATTAAAGCTGAGGCGGTGAAAGATGAGTATGCGTTCTATT
AGCTAGTTGGAGAGGTAACGGCTCCCCAAGGCTACGATAGATAGGGGTTCTGAGAGGGATATCCCCCA
CACTGGTACTGAGATACGGAC
>Zotu3327
GAACGCTGGCGGCGTGCCTAATACATGCAAGTCGAACGCTTTTTCCAATCATCGTAGCTTGCTACACC
GATTGGAAATTGAGTGGCGAACGGGTGAGTAACACGTGGGTAACCTGCCCAAAAGAGGGGGATAACAC
TTGGAAACAGGTGCTAATACCGCATAACCATGATGACCGCATGGTCATTATGTAAAAGATGGTTTCGG
CTATCACTTTTGGATGGACCCGCGGCGTATTAACTTGTTGGTGGGGTAACGGCCTACCAAGGTAATGA
TACGTAGCCGAACTGAGAGGTTGATCGGCCACATTGGGACTGAGACACGGCC
>Zotu3328
GAACGCTGGCGGCGTGCCTAACACATGCAAGTCGAGCGAACCCCTTCGGGGGAGAGCGGCGGACGGGT
GAGTAACGCGTGGGTAACCTACCCTGTACACACGGATAACATACCGAAAGGTTTACTAATACGTGATG
ACATATCAGACAGGCATCTGTTTGATATCAAAGGTCAGCTGGTACAGGATGGACCCGCGTCTGATTAG
CTAGTTGGTGAGATAAAAGCTCACCAAGGCGACGATCAGTAGCCGACCTGAGAGGGTGATCGGCCACA
TTGGAACTGAGACACGGTC
>Zotu3330
GAACGCTGGCGGCGTGCCTAATACATGCAAGTCGAACGAAGTTATGAGAGAGCTTGCTCTCGATTAGC
TTAGTGGCGGACGGGTGAGTAACACGTAGGCAACCTGCCCCTAAGATCGGGATAACATTCGGAAACGA
ATGCTAAGACCGGATAAACAATCTACCCGCATGGGTGGATTGGGAAACACGGAGCAATCTGTGGCTTA
GGGATGGGCCTGCGGCGCATTAGCTAGTTGGTGGGGTAACGGCTCACCAAGGCGACGATGCGTAGCCG
ACCTGAGAGGGTGAACGGCCACACTGGGACTGAGACACGGCC
>Zotu3331
GAACGCTAGCGGCAGGCTTAATACATGCAAGTCGTGGGGCAGCAGGGTGTAGCAATACATCGCTGGCG
ACCGGCAAACGGGTGCGGAACACGTACACAACCTTCCTTTAAGTGGGGAATAGCCCAGGGAAACTTGG
ATTAATACCCCGTAACATATAGATGAGGCATCTCAATTATATTATAGCTACGGCGCTTGAAGATGGGT
GTGCGGCTGATTAGGTAGTTGGCGGGGTAATGGCCCACCAAGCCTACGATCAGTAACTGGTGTGAGAG
CACGACCAGTCACACGGGCACTGAGACACGGGC
>Zotu3335
GAACGCTAGCGGCAGGCCTAATACATGCAAGTCGAACGGGCTTGGGTTCTTCGGGACTCGAGTCAGTG
GCGCACGGGTGCGTAACGCGTATGCAACCTACCTTCCACAGGGGGATAGCCCCGGGAAACCGGGATTA
ATACCGCATGACACCATTTTGTGGCATCACAGAATGGTCAAAGTTTCGGCGGTGGAAGATGGGCATGC
GTGCCATTAGCTAGTTGGCGGGGTAGAGGCCCACCAAGGCTACGATGGCTAGGGGTTCTGAGAGGATG
GTCCCCCACACTGGTACTGAGACACGGAC
>Zotu3336
GAACGCTGGCGGTGTGCTTTAGGTATGCAAGTCGAGCGAATAAGTAGCAATACTTGTTAGCGGCGGAC
GGGTGAGTAACATACAAGAATCTACCCCCGAGTGGAGAATAACTGACCGAAAGGTTGGCTAATGTTCC
ATGGTAACTACGGTTTAAAGCGATTAACTTCGCGCTTGGGGAGGAGCTTGTATCCTATCAGCTAGTTG
GCGGGGTAAATGCCCACCAAGGCTATGACGGGTATCTGGACTGAGAGGTCGGCCAGACACAGGGGCAC
TGAGACACGGGC
>Zotu3337
GAACGCTGGCGGCGTGCTTAACACATGCAAGTCGAACGAGGCCCTCTCTTCGGAGGGGTGTTCTAGTG
GCGAACGGGTGAGTAACACGTGGGCAACCTGCCCCCGGCTCTGGGATAACTCCAAGAAATTGGGGCTA
ATACCGGATATGACCGCTGACCGCATGGTCTGGTGGTGGAAAGTTTTTCGGCTGGGGATGGCCCGCG
GCCTATCAGCTTGTTGGTGGGGTAGTGGCCTACCAAGGCGACGACGGGTAGCCGGCCTGAGAGGGTGA
CCGGCCACACTGGGACTGAGACACGGCC
>Zotu3338

GAACGCTAGCGGCAGGCCTAACACATGCAAGTCGAGGGGTAGAGGGGAGCTTGCTCTCCTTGAGACCG
GCGGACGGGTGAGTAGCGCGTATGGAATCTGCCTTGTACAGGGGAATAGCCCAGGGAAACTTGGATTA
ATGCCCCATAGTGTTGAGGGGCGGCATTGTTCTTCAACCAAAGATTTATCGGTACAAGATGACCATGC
GTCCCATTAGCTTGTTGGTGTGGTAACGGCACACCAAGGCTACGATGGGTAGGGGCCCTGAGAGGGGG
ATCCCCCACACTGGTACTGAGACACGGAC
>Zotu3339
GAACGCTGGCGGCGTGCTTAACACATGCAAGTGGTGCGACGAACCAGGGCTTGCCCTGGGGCAAAGCC
GCGAACGGGTGAGTAACACGTGAGTAACCTGCCCCGATGACCGGGACAACCCGAGGAAACTCGGGCTA
ATACCGGATTTGGCGCACAGATTTAAGTTTGTGCGCTAAAGGAAGCTTCGGCCTCCGCATTGGGAGGG
GCTCGCGGCCCATTAGCTTGTTGGTGGGGTAACGGCCTACCAAGGCAACGATGGGTAGCTGGTCTGAG
AGGACGATCAGCCACACTGGGACTGAGACACGGCC
>Zotu3340
GAACGCTAGCGGCAGGCTTAACACATGCAAGTCGAGGGGCAGCACGATGTAGCAATACATTGGTGGCG
ACCGGCGCACGGGTGCGTAACACGTATGGAACTTACCCTTTACTGGGAGATAGCCCGGAGAAATCCGG
ATTAATATCCCATAATAAACGAGGTGGCATCACTTTGTTTTGAAAGCTCCGGCGGTAAAGGATGGCCA
TGCGCACGATTAGCTAGTAGGTGAGGTAACGGCTCACCTAGGCTACGATCGTTAGGGGGCCTGAGAGG
GTTATCCCCCACACTGGTACTGAGACACGGAC
>Zotu3341
GGACGCTGGCGGCGTGCTTAACACATGCAAGTCGAACGATGATCCCCAGCTTGCTGGGGGGATTAGTG
GCGAACGGGTGAGTAACACGTGAGTAACCTGCCCCTGACTCTGGGATAAGCGCTGGAAACGGCGTCTA
ATACCGGATATGACAGCCGACGGCATCGTCTGGTTGTGGAAAGATTTTTCGGTTGGGGATGGACTCGC
GGCCTATCAGGTTGTTGGTGAGGTAATGGCTCACCAAGCCTACGACGGGTAGCCGGCCTGAGAGGGTG
ACCGGCCACACTGGGACTGAGACACGGCC
>Zotu3342
GAACGCTGGCGGCGTGCTTAACACATGCAAGTCGAACGGGATCCATCGCGAGCTTGCTCAAGAGAAGA
TCTAGTGGCGAAAGGGTGCGTAACACGTGAGAAACCTGCCCCAGACTTGGGAATAACAGTCGGAAACG
ACTGCTAATACCGAATACCTTCGTAGCGTCGCATGGCGCATCGAAGAAAGCTTTATGCGGTTTGGGAG
GGTCTCGCGGCCTATCAGCTAGTTGGTGAGGTAACGGCTCACCAAGGCATCGACGGGTAGCTGGTCTG
AGAGGATGATCAGCCACACTGGGACTGAGACACGGCC
>Zotu3345
GAACGCTGGCGGCGTGCTTAACACATGCAAGTCGAGCGGAAAGGCCCTTCGGGGTACTCGAGCGGCGA
ACGGGTGAGTAACACGTGAGTAATCTGCCCTTCACTCTGGGATAGCCACCGGAAACGGTGATTAATAC
CGGATATGACACGTCCCTGCATGGGGTGCGTGTGGAAACTTTTTTGGGTGGGGGATGTGCTCGCGGCC
TATCAGCTTGTTGGTGGGGTAATGGCCTACCAAGGCTTCGACGGGTAGCCGGCCTGAGAGGGTGACCG
GCCACACTGGGACTGAGACACGGCC
>Zotu3346
GAACGCTGGCGGCGTGCTTAACACATGCAAGTCGAACGGTGAAGGAGGAGCTTGCTCTTCTGGATCAG
TGGCGAACGGGTGAGTAACACGTGAGCAATCTGCCCCTGACTCTGGGATAACAGTTGGAAACAGCTGC
TAATACCGGATACGAGACGTGACCGCATGGTCAACGTTTGGAAAGAACTTCGGTCAGGGATGAGCTCA
CGGCCTATCAGCTTGTTGGTGAGGTAATGGCTCACCAAGGCGACGACGGGTAGCCGGCCTGAGAGGGT
GACCGGCCACACTGGGACTGAGACACGGCC
>Zotu3347
GAACGCTGGCGGCGTGCTTAACACATGCAAGTCGAACGGAAAGGCCTGCTTTCCCTTGTGGATTGCGG
GTACTCGAGTGGCGAACGGGTGAGTAACACGTGAGTGATCTGCCCCCAACTTGGGTATAAGCCTGGGA
AACTGGGTCTAATTCCCGATAGGACTGCAGGGTGGTGCCTGTGGTGGAAAACGATTTTTTAGTGGTTG
GGGATGAGCTCGCGGCCTATCAGCTTGTTGGTGGGGTAATGGCCTACCAAGGCGGCGACGGGTAGCCG
GCCTGAGAGGGTGGACGGCCACATTGGGACTGAGATACGGCC
>Zotu3348
GAACGTTGGCGGCGTGCCTTAAGCATGCAAATCGAGGGGGTCCGATTTATCGGACTACCGGTGGACGG
GTGAGTAATGCGCGGGAATGTACCTCGAAGTGGGATATAGCCAGCCGAAAGGTTGGGTAATCTCCCAT
GTGAACGAAAGTTTAAAGCCGCAAGGCGCTTTGAGATCAGCCCGCGTCCTATCAGCTTGTTGGTGGGG
TAAAAGCCTACCAAGGCGACGACGGGTAGCTGGACTGAGAGGTCGGTCAGCCACAATGGGACTGCGAC
ACGGCC
>Zotu3349

GAACGCTGGCGGCGTGCCTAAGACATGCAAGTCGAACGGGTTTAGACCCGTGGCGAACGGGTGAGTAA
CGCGTGGGTACTTCCATTAAGTTTGGAATAACCTTTCGAAAGAGAGGACAATACCAAATGTGGTCGCA
AGACTAAAGCCGCAAGGCGCTTAATGCGAGGCCCGCGTCCCATCAGCTAGTTGGTAGGGTAAAGGCCT
ACCAAGGCTATGACGGGTAGCGGAACCGAGAGGTTGATCCGCCACACTGGGACTGCGACACGGCC
>Zotu3350
GAACGCTGGCGGCGTGTCTAAGGTATGCAAGTCGAACGAATTTATTGCCGTCGCAAGACGGCGATTGA
TTAGTGGCAAACGGGTGCGTAACACATTGGTAACCTGCCTCCATGACGGGCACAACCTGCCGAAAGGC
GGGCTAATTCCCGATAGTCTCTTCCCGTGAAAATCGGGTTGAGTAAAGTACAAAATACACATGGAGAG
GGGCCTATGGCCTATCAGCTAGTTGGTGAGGTAATGGCTCACCAAGGCTATGACGGGTAGCGGGCGTG
AGAGCGCGACCCGCCTCAGTGGGACTGAGACACTGCC
>Zotu3354
GAACGCTGGCGGCGTGCCTAATACATGCAAGTAGAACGCTGAAGGAATAGCTTGCTATTCTGGAAGAG
TTGCGAACGGGTGAGTAACGCGTAGGTAACCTACCTCATAGCGGGGGATAACTATTGGAAACGATAGC
TAATACCGCATAAGAATACTTATTGCATGATAAGTGTTTAAAAGGAACAACTGTTTCACTGTGAGATG
GACCTGCGTTGTATTAGCTAGTTGGTAAGGTAACGGCTTACCAAGGCGACGATACATAGCCGACCTGA
GAGGGTGATCGGCCACACTGGGACTGAGACACGGCC
>Zotu3356
GAACGCTGGCGGCGTGCTTAACACATGCAAGTGGAGCGACGAACCGGGCTTCGGCCCGGGGCAAAGCC
GCGAACGGGTGAGTAACACGTGGGTCATCTGCCCAGTTGACAGGGACAACCCGGGGAAACCCGGGCTA
ATACCTGATGTGCCCCTCGGGGGAAAGGAAGCTTCGGCCTCCGCAACTGGATGAGCCCGCGGCCCATT
AGCTTGTTGGTGGGGTAATGGCCTACCAAGGCTGCGATGGGTAGCTGGTCTGAGAGGACGATCAGCCA
CACTGGGACTGAGACACGGCC
>Zotu3357
GAACGCTAGCGGCAGGCCTAATACATGCAAGACGAACGGCAAGAATTGTAGCAATACGATTCCTAGAG
TGTCGCACGGGTGCGTAACACGTATGCAACCTACCTTGTACAGGAGTATAGCTCGGGGAAACTCGAAT
TAACCCTCCATAATATGATTGAATGGCATCATTTAATTATTAAAACTTCGGTGGTACAAGATGGGCAT
GCGTTTGATTAGCTAGTTGGCGGGGTAATGGCCCACCAAGGCGATGATCAATAGGGGAACTGAGAGGT
TGATCCCCCACACTGGCACTGAGATACGGGC
>Zotu3358
GAACGCTGGCGGCGTGCCTAATACATGCAAGTCGAGCGAATGATGAAAGAAGCTTGCTTCTTTCTGAT
TTAGCGGCGGACGGGTGAGTAACACGTGGGCAACCTGCCCTTTAGTTTGGGATAACTTCGGGAAACCG
GAGCTAATACCGAATAATACTCTTTCCTTCATGGGAGAAAGTTGAAAGATGGTTTCGGCTATCGCTAA
AGGATGGGCCCGCGGTGCATTAGCTAGTTGGTGAGGTAACGGCTCACCAAGGCAACGATGCATAGCCG
ACCTGAGAGGGTGATCGGCCACACTGGGACTGAGACACGGCC
>Zotu3359
GAACGCTGGCGGCGTGCTTAACACATGCAAGTCGAGCGGTAAGGCCCTTTCGGGGGTACACGAGCGGC
GAACGGGTGAGTAACACGTGAGTAATCTGCCCTTCACTTCGGGATAACCACCGGAAACGGTGGCTAAT
ACCGGATATGACCATTCCCGGCATCGGGTGGTGGTGGAAAGTTCTGGCGGTGGAGGATGAGCTCGCGG
CCTATCAGCTTGTTGGTGGGGTAGTGGCCTACCAAGGCGACGACGGGTAGCCGGCCTGAGAGGGTGAC
CGGCCACACTGGGACTGAGACACGGCC
>Zotu3360
GAACGCTAGCGGCAGGCTTAATACATGCAAGGCGAAGGGGCAGCAATGTCACTGTCGTACGGGTGCGT
AACGCGTATGCAACCTACCTATCACTGGGGGATAGCCCGGGGAAACCCGGATTAATACCGCATAACAC
AGGGGTCCCGCATGGGTACTATTTGTTAAAGATTTATCGGTGGTAGATGGGCATGCGTTCGATTAGCT
AGTTGGTTGAGGTAACGGCTCACCAAGGCGACGATCGATAGGGGAGCTGAGAGGTTGATCCCCCACAC
GGGCACTGAGATACGGGC
>Zotu3361
GAACGCTGGCGGCAGGCCTAACACATGCAAGTCGAACGGATTCTTCGGGATTAGTGGCGGACGGGTGA
GTAACACGTGGGAACGTGCCTTTTGGTTCGGAACAACTCAGGGAAACTTGAGCTAATACCGGATAAGC
CTTTCGAGGGAAAGATTTATCGCCATTAGAGCGGCCCGCGTAGGATTAGCTAGTTGGTGGGGTAAAGG
CCCACCAAGGCTACGATCCTTAGCTGGTCTGAGAGGATGATCAGCCACATTGGGACTGAGACACGGCC
>Zotu3362
GAACGCTGGCGGCGTGCTTAACACATGCAAGTCGAGCGGAAAGGCCCTTCGGGGTACTCGAGCGGCGA
ACGGGTGAGTAACACGTGAGTAACCTGCCCCCGACTTTGGGATAAGCCTGGGAAACCGGGTCTAATAC

CGAATACGACCGGCCTTGGCATCGGGTGCCGGTGGAAAGTTTTTTCGGTTGGGGATGGGCTCGCGGCC
TATCAGCTTGTTGGTGGGGTAGTGGCCTACCAAGGCGACGACGGGTAGCCGGCCTGAGAGGGCGACCG
GCCACACTGGGACTGAGACACGGCC
>Zotu3364
GAACGCTGGCGGCGTGCTTCACACATGCAAGTCGAGCGAGGCCCTTCCTTCGGGAAGGAGCCCTAGCG
GCGAACGGGTGAGTAACACGTGGGCAACCTGCCCTCAGCTCTGGGATAAGCCCGGGAAACCGGGTCTA
ATACCGGATATGACTCCGACAGGCATCTTGCGGAGTGGAAAGATTCATCGGCTGAGGATGGGCCCGCG
GCCTATCAGCTTGTTGGTGGGGTAACGGCCTACCAAGGCGACGACGGGTAGCCGGCCTGAGAGGGCGA
CCGGCCACACTGGGACTGAGACACGGCC
>Zotu3366
GAACGCTGGCGGCAGGCCTAACACATGCAAGTCGAGCGGATGACGGGAGCTTGCTCCTTGATTCAGCG
GCGGACGGGTGAGTAATGCCTAGGAATCTGCCTGGTAGTGGGGGACAACGTTTCGAAAGGAACGCTAA
TACCGCATACGTCCTACGGGAGAAAGCAGGGGACCTTCGGGCCTTGCGCTATCAGATGAGCCTAGGTC
GGATTAGCTAGTTGGTGGGGTAATGGCTCACCAAGGCGACGATCCGTAACTGGTCTGAGAGGATGATC
AGTCACACTGGAACTGAGACACGGTC
>Zotu3367
GAACGCTGGCGGCAGGCTTAACACATGCAAGTCGAACGATGAAACTCTAGCTTGCTAGAGATGATTAG
TGGCGGACGGGTGAGTAACATTTAGGAATCTGCCTAGTAGTGGGGGATAGCTCGGGGAAACTCGAATT
AATACCGCATACGACCTACGGGTGAAAGGGGGCGCAAGCTCTTGCTATTAGATGAGCCTAAATCAGAT
TAGCTAGTTGGTGGGGTAAAGGCCCACCAAGGCGACGATCTGTAACTGGTCTGAGAGGATGATCAGTC
ACACCGGAACTGAGACACGGTC
>Zotu3368
GAACGCTGGCGGCGCGCCTAACACATGCAAGTCGAGCGAGGTTCAAAGCTTCGGTTTTGAACCTAGCG
GCGGACGGGTGAGTAACGCGTGAGCAACCTGGCCTTCAGAGGGGGATACCGTCTGGAAACGGACGTTA
ATACCGCATAACATGTCGGGAGGGCATCTTCCTGATATCAAAGGAGAAATTCACTGAAGGATGGGCTC
GCGTCCGATTAGGTAGTTGGTGAGGTAACGGCTCACCAAGCCCGCGATCGGTAGCCGGACTGAGAGGT
TGGCCGGCCACATTGGGACTGAGATACGGCC
>Zotu3369
CAACGCTGGCGGCGTGCCTAATGCATGCAAGTCGAACGGGGTGTCCTTCGGGGCACTTACGTGGCGGA
CGGGTGAGGAACACGTGGGTAACCTGCCCACTGGTGGGGGCTAGCCTTCGGAAACGGAGGGTAATTCC
GCATGATCTCGCAGCCCGCGTGGGGGTTGTGAGGAAAGCCTTCGGGCGCCAGTGGAGGGGCCCGCGCC
CGATTAGCTTGTTGGTGGGGTAACGGCTTACCAAGGCGATGATCGGTAGCTGGTTTGAGAGGATGATC
AGCCACACGGGGACTGAGACACGGCC
>Zotu3370
GAACGCTGGCGGCATGCCTAACACATGCAAGTCGAACGATGCTTTCGGGCATAGTGGCGCACGGGTGC
GTAACGCGTGGGAATCTGCCCTTTGGTCTGGGATAACTGTTGGAAACGACAGCTAATACCGGATGATG
ACGTAAGTCCAAAGATTTATCGCCAGAGGATGAGCCCGCGTCGGATTAGCTAGTTGGTGGGGTAAAGG
CCTACCAAGGCGACGATCCGTAGCTGGTCTGAGAGGATGATCAGCCACACTGGGACTGAGACACGGCC
>Zotu3371
GAACGCTGGCGGCGTGCTTAACACATGCAAGTCGAACGGAAAGGCCCTGCTTGCAGGGTACTCGAGTG
GCGAACGGGTGAGTAACACGTGGGTGATCTGCCCTGCACTTCGGGATAAGCCTGGGAAACTGGGTCTA
ATACCGGATAGGAACCATCTTTAGTGTGGTGGTTGGAAAGTTTTTCGGTGTAGGATGAGCTCGCGGC
CTATCAGCTTGTTGGTGGGGTAATGGCCTACCAAGGCGGCGACGGGTAGCCGGCCTGAGAGGGTGTAC
GGCCACATTGGGACTGAGATACGGCC
>Zotu3372
GAACGCTGGCGGCAGGCTTAACACATGCAAGTCGAACGCCCCGCAAGGGGAGTGGCAGACGGGTGAGT
AACGCGTGGGAACATACCCATCACTACGGAATAACTCAGGGAAACTTGTGCTAATACCGTATACGACC
GAGAGGTGAAAGATTTATCGGTGATGGATTGGCCCGCGTTGGATTAGCTAGTTGGTGGGGTAAAGGCC
TACCAAGGCGACGATCCATAGCTGGTCTGAGAGGATGATCAGCCACATTGGGACTGAGACACGGCC
>Zotu3373
GAACGCTGGCGGCGTGCTTAACACATGCAAGTCGAACGGTGAAGATCCGGCTTTGGTCGGGTTGGATC
AGTGGCGAACGGGTGAGTAACACGTGAGTAACCTGCCCTTGACTCTGGGATAACTCCGGGAAACCGGT
GCTAATACTGGATATGACGCTGCCCTGCCTGGGGTGGTGTGGAAAGATTTATCGGTTGGGGATGGACT
CGCGGCCTATCAGCTTGTTGGTGAGGTAGTGGCTTACCAAGGCGACGACGGGTAGCCGGCCTGAGAGG

GTGATCGGCCACACTGGGACTGAGACACGGCC
>Zotu3374
GAACGCTGGCGGCGTGCTTAACACATGCAAGTCGAACGTGAAGGTGGAGCAATCCACCTGAAAGTGGC
GAACGGGTGAGTAACACGTGGGTGACCTGCCTCGGAGTGGGGGATAACCATGGGAAACTGTGGCTAAT
ACCGCATGGGCTTGTTAGACTTGGCGTCTAACGAGTAAAGCTTTAGTGCTTCGAGAGGGGCCTGCGTC
CGATTAGGTCGTTGGTGAGGTAATGGCTCACCAAGCCGATGATCGGTAGCTGGTCTGAGAGGACGATC
AGCCACACGGGAACTGAGAAACGGTC
>Zotu3375
GAACGCTGGCGGCGTGCCTAATACATGCAAGTAGAACGCTGAAGCTTGGTGCTTGCACCGAGCGGATG
AGTTGCGAACGGGTGAGTAACGCGTAGGTAACCTGCCTCTTAGCGGGGGATAACTATTGGAAACGATA
GCTAATACCGCATAAAAGTCGATATCGCATGATATTGATTTGAAAGGTGCAAATGCATCACTAAGAGA
TGGACCTGCGTTGTATTAGCTAGTTGGTGAGGTAACGGCTCACCAAGGCGACGATACATAGCCGACCT
GAGAGGGTGATCGGCCACACTGGGACTGAGACACGGCC
>Zotu3376
GAATGCTGGCGGCGCGCCTAACACATGCAAGTCGAGCGGTAACATGGACTTCGGTCTGATGACGAGCG
GCGGACGGCTGAGTAACGCGTGGGAACATGCCCCAAAGTGAGGAATAAGCTCCAGAAATGGAGTCTAA
TGCCGCATATGATCTTCGGATTAAAGGATTTATCCGCTTTGGGAGTGGCCTGCGTATGATTAGATAGT
TGGTAAGGTAATGGCTTACCAAGTCTACGATCATTAACTGGTCTGAGAGGATGATCAGTCAGACTGGA
ACTGAGACACGGTC
>Zotu3377
GAACGCTGGAAATGTGGATACCGGCATGCAAGTCAAGGGGCCGCAAGGCACCGGCGAACGGCTTAGTA
ACACGTAGATACATACCCAAAAGTGGGGCATAGCTCGTCGAAAGACGGGGTAATTTCCCATGTGCTCG
CAAGAGTAAAGACGCAAGTCGCTTTTGGAATGGTCTGCGGTCTATCAGCTAGTTGGCGGGGTAAAAGC
CCACCAAGGCTATGACGGATAGGGGGCGTGAGAGCGCGACCCCCAAGAATGGCACTGCGACACGGGC
>Zotu3378
GAACGCTGGCGGCGTGCTTAACACATGCAAGTCGAACGGTGACGGAGAGCTTGCTCTCCTGATCAGTG
GCGAACGGGTGAGTAACACGTGGGCAACCTGCCCCAGACTTCGGGATAACGCTTCGAAAGAGGTGCTA
ATACCGAATACGAACGCCGTGGGCATCCACGGTGTTGGAAAGTTTTTCGGTCTGGGATGGGCCCGCGG
CCTATCAGCTTGTTGGTGAGGTAATGGCTCACCAAGGCGACGACGGGTAGCCGGCCTGAGAGGGCGAC
CGGCCACACTGGGACTGAGACACGGCC
>Zotu3379
GAACGCTGGCGGCATGCCTAACACATGCAAGTCGAACGAAGGCTTCGGCCTTAGTGGCGCACGGGTGC
GTAACGCGTGGGAATCTGCCCCTTGGTTCGGAATAACAGTTGGAAACGACTGCTAATACCGGATGATG
ACGTTAAGTCCAAAGATTTATCGCCGAGGGATGAGCCCGCGTAGGATTAGCTAGTTGGTGTGGTAAGA
GCGCACCAAGGCGACGATCCTTAGCTGGTCTGAGAGGATGATCAGCCACACTGGGACTGAGACACGGC
C
>Zotu3380
GAACGCTGGCGGCGTGCTTAACACATGCAAGTCGAGCGGAAAGGCCCCTTTGGGGGTACTCGAGCGGC
GAACGGGTGAGTATCACGTGAGTAATCTGCCCCTCTCTTTTGGATAACCCTCGGAAACGGGGGCTAAT
ACGGAATATTCAGGGCTGCCTGCATGGGTGGTTTTGGAAAGATTTTTTGGTGGGGGATGAGCTCGCGG
CCTATCAGCTTGATGGTGGGGTAATGGCCTACCATGGCTTTGACGGGTAGCCGGCCTGAGAGGGTGTC
CGGCCACACTGGGACTGAGACACGGCC
>Zotu3381
GAACGCTGGCGGCGTGCTTAACACATGCAAGTCGAGCGAGGCCCTCCTTCGGGGGGTGCCCTAGCGGC
GAACGGGTGAGTAACACGTGGGCAACCTGCCCTCAGCTCTGGGATAACTCCAAGAAATTGGTGCTAAT
ACCGGATGTGACCGCTGACCGCATGGTCTGGTGGTGGAAAGATTCATCGGCTGAGGATGGGCCCGCGG
CCTATCAGCTTGTTGGTGGGGTAACAGCCTACCAAGGCGACGACGGGTAGCCGGCCTGAGAGGGTGAC
CGGCCACACTGGGACTGAGACACGGCC
>Zotu3382
GAACGCTGGCGGCATGCTTAACACATGCAAGTCGGACGGGAAGTGGTGTTTCCAGTGGCGGACGGGTG
AGTAACGCGTAAGAACCTGCCCTTGGGAGGGGAACAACAGCTGGAAACGGCTGCTAATACCCCGTAGG
CTGAGGAGCAAAAGGAGGAATCCGCCTGAGGAGGGGCTCGCGTCTGATTAGCTAGTTGGTGAGGCAAT
AGCTTACCAAGGCGATGATCAGTAACTGGTTCGAGAGGATGATCAGCCACACTGGGACTGAGACACGG
CC

>Zotu3383
GAACACTGGCGGCAGGCCTAACACATGCAAGTCGAGCGGATGACGGGAGCTTGCTCCTTGATTCAGCG
GCGGACGGGTGAGTAATGCCTAGGAATCTGCCTGGTAGTGGGGGACAACGTTTCGAAAGGAACGCTAA
TACCGCATACGTCCTACGGGAGAAAGCAGGGGACCTTCGGGCCTTGCGCTATCAGATGAGCCTAGGTC
GGATTAGCTAGTTGGTGAGGTAATGGCTCACCAAGGCGACGATCCGTAACTGGTCTGAGAGGATGATC
AGTCACACTGGAACTGAGACACGGTC
>Zotu3384
GAACGCTGGCGGCGTGCCTAACACATGCAAGTCGAGCGAAAAGTGTAGCAATACACTTTTAGCGGCGG
ACGGGTGAGTAACGCGTGGGTAACCTGCCTTAGGCTGGGGGATAACACTGGGAAACTAGTGCTAATAC
CGCATAACGCAGAACTGGGGCATCCCAGGACTGCCAAAGGAGTAATCCGGCTTTAGATGGACCCGCGT
CCGATTAGCTAGTAGGTGAGGTAATGGCTCACCTAGGCGACGATCGGTAGCCGAACTGAGAGGTTGAT
CGGCCACATTGGGACTGAGACACGGCC
>Zotu3385
GAACGCTAGCGGCAGGCTTAACACATGCAAGTCGAGGGGTATATTTCTTCGGAGATAGAGACCGGCGC
ACGGGTGCGTAACGCGTATGCAATCTACCTTTTACAGAGGGATAGCCCAGAGAAATTTGGATTAATAC
CTCATAGTATTATGAAATGGCATCATTTTATAATTAAAGTCACAACGGTAAAAGATGAGCATGCGTCC
CATTAGCTAGTTGGTAAGGTAACGGCTTACCAAGGCTACGATGGGTAGGGGTCCTGAGAGGGAGATCC
CCCACACTGGTACTGAGACACGGAC
>Zotu3386
GAACGCTGGCGGTATGCCTAACACATGCAAGTCGAACGAGGTAGCAATACCTAGTGGCGGACGGGTGA
GGAATACGTAGGGATCTGCCTTTAAGTGGGGGATAACCCGGGGAAACCCGGATTAATACCGCATGATC
TCGCAAGAGCAAAGAGGGGGACCGCAAGGCCTCTCGCTTTTAGAGGAACCTACGTCGGATTAGCTAGT
TGGTGAGGTAATGGCTCACCAAGGCGACGATCCGTAACTGGTCTGAGAGGATGATCAGTCACACCGGA
ACTGAGACACGGTC
>Zotu3387
GAACGCTAGCGGCAGGCCTAATACATGCAAGTCGAACGGGGTAGCAATACCCAGTGGCGCACGGGTGC
GTAACGCGTATGCAACCTACCTTTTGCTGGGGGATAGCCCGGAGAAATCCGGATTAATACCGCATAAC
ACCTTTTGATGGCATCTGAGGAAGGTTAAAGATTTATCGGCAAAAGATGGGCATGCGTGCCATTAGCT
AGTTGGTGGGGTAACAGCCCACCAAGGCGACGATGGCTAGGGGGTCTGAGAGGATGGTCCCCCACACT
GGCACTGAGATACGGGC
>Zotu3389
GAATGCTGGCGGCGTGGATAAGGCATGCAAGTCAAGGGGGCCGCAAGGCAACCGGCAGACGAGGTAGT
AACACGTAGGTAGATACTCCAGAGTCGGGCATAGCTAGTCGAAAGATTAGGTAATTCCCGATGGTCCC
GAAAGGGTAAAGGTTTTTCGCTCTGGAAGTCGCCTGCGCAGTATCAGCTAGTTGGTAGGGTAATGGCC
TACCAAGGCTACGACGCTTAGGGGAGCTGAGAGGCTGACCCCCACCGATGGGACTGAGAGACGGCC
>Zotu3390
GAACGCTGGCGGCGCGCTTAACACATGCAAGTCGAACGGAGGGCTTCGGCCCTCAGTGGCGAACGGGT
GCGTAACACGTGAGCAACCTGCCCCTGTGCGGGGGATAACCGGGGGAAACCCCGGCTAATACCGCATG
AGACCACGACCTCCGTGGAGGTCGTGCTTAAAGGTCCTTTCGGGGGCTGCGCAGGGAGGGGCTCGCGG
CGTATCAGCTAGCTGGTGAGGTAACGGCTCACCAGGGCAACGACGCGTAGGGGGTCTGAGAGGATGGC
CCCCCACATTGGGACTGAGATACGGCC
>Zotu3391
GAACGCTGGCGGCAGGCTTAACACATGCAAGTCGAGCGGGGAAAGGTAGCTTGCTACCTAACCTAGCG
GCGGACGGGTGAGTAATGCTTAGGAATCTGCCTATTAGTGGGGGACAACATTCCGAAAGGAATGCTAA
TACCGCATACGCCCTACGGGGGAAAGCAGGGGATCTTCGGACCTTGCGCTAATAGATGAGCCTAAGTC
AGATTAGCTAGTTGGTGGGGTAAAGGCCTACCAAGGCGACGATCTGTAGCGGGTCTGAGAGGATGATC
CGCCACACTGGGACTGAGACACGGCC
>Zotu3392
GAACGCTGACAGAATGCTTAACACATGCAAGTCTACTTGAATTTGGGTTTTTAACTTAGATTTGGGTG
GCGGACGGGTGAGTAACGCGTAAAGAACTTGCCTCACAGCTAGGGACAACATTTAGAAATGAATGCTA
ATACCTAATATTATGATAATAGGGCATCCTATAATTATGAAAGCTATATGCGCTGTGAGAGAGCTTTG
CGTCCCATTAGCTAGTTGGAGAGGTAACGGCTCACCAAGGCGATGATGGGTAGCCGGCCTGAGAGGGT
GATCGGCCACAAGGGGACTGAGACACGGCC
>Zotu3393

GAACGCTGGCGGCGTGCCTAATACATGCAAGTCGAGCGGTAAGGCCCCTTCGGGGTACACGAGCGGC
GGACGGCTGAGTAACACGTAGGAACGTACCCCAAACTGAGGGATAAGCACCAGAAATGGTGTCTAATA
CCGCATGTGGTCTTCGGATTAAAGCTTCGGCGGTTTGGGAACGGCCTGCGCATGATTAGCTTGTTGGT
GAGGTAATGGCTCACCAAGGCGACGATCATTAGCTGGTCTGAGAGGATGATCAGCCAGACTGGGACTG
AGAACGGCC
>Zotu3394
GAACGCTGGCGGCATGCCTAACACATGCAAGTCGAACGAGATCTTCGGGTCTAGTGGCGCACGGGTGC
GTAACGCGTGGGAATCTGCCCTTTGGTTCGAAATAACAGTTGGAAACGACTGCTAATACCGGATGATG
ACGAAAGTCCAAAGATTTATCGCCAGAAGATGAGCCCGCGTAGGATTAGCTAGTTGGTGTGGTAAAGG
CGCACCAAGGCGACGATCCTTAGCTGGTCTGAGAGGATGATCAGCCACACTGGGACTGAGACACGGCC
>Zotu3395
GAACGCTGGCGGCGTGCTTAACACATGCAAGTCGAGCGGTAAGGCCTCTTCGGGGGTACACGAGCGGC
GAACGGGTGAGTAACACGTGAGTAATCTGCCCTCCACTTCGGGATAAGCCTCGGAAACGGGGTCTAAT
ACCGGATATGACTCGCTCCGGCATCGGGGTGGGTGGAAAGTTTTTTCGGTGGGGGATGTGCTCGCGGC
CTATCAGCTTGTTGGTGGGGTAATGGCCTACCAAGGCTTCGACGGGTAGCCGGCCTGAGAGGGTGACC
GGCCACACTGGGACTGAGACACGGCC
>Zotu3396
GAACGCTGGCGGCATGCCTTACACATGCAAGTCGAACGGTAGAGGGGGCAACCCCTTGAGAGTGGCGA
ACGGGTGAGTAATGCATCGGAACGTGCCCAGTCGTGGGGGATAACTATCCGAAAGGATGGCTAATACC
GCATGAGATCTGTGGATGAAAGCGGGGGACCTGAAAGGGCCTCGCGCGATTGGAGCGGCCGATGTCAG
ATTAGCTAGTTGGTGGGGTAAAGGCCCACCAAGGCGACGATCTGTAACTGGTTTGAGAGAACGACCAG
TCACACTGGGACTGAGACACGGCC
>Zotu3397
GAACGCTGGCGGCGTGCTTAACACATGCAAGTCGAACGGAAAGGCCTCTGCTTGCAGAGGTACTCGAG
TGGCGAACGGGTGAGTAACACGTGGGTGATCTGCCCTGCACTTCGGGATAAGCCTGGGAAACTGGGTC
TAATACCGGATAGGACCACATTTTGGATGGTGTGGTGGAAAGTTTTTCGGTGTGGGATGAGCTCGCGG
CCTATCAGCTTGTTGGTGGGGTAATGGCCTACCAAGGCGTCGACGGGTAGCCGGCCTGAGAGGGTGTA
CGGCCACATTGGGACTGAGATACGGCC
>Zotu3398
GAACGCTGGCGGCGCACCTAAGACATGCAAGTCGAACGGGGGCTTCGGCCCCAGTGGCGGACGGGTGA
GTAACACGTAGGGGACCTGCCCCGTGGCGGGGGATAACACCGGGAAACCGGTGCTAAGACCGCATACG
TCCGATGGCATAGCGGCCATCGGGGAAATGCGCGAGCAGCCACGGGAGGGCCTTGCGGCGGATTAGGT
AGTTGGTGGGGTAGAAGCCTACCAAGCCGACGATCCGTAGCTGGTCTGAGAGGACGACCAGCCACACC
GGGACTGAGACACGGCC
>Zotu3399
GAACGCTAGCGGCAGGCCTAACACATGCAAGTCGAGGGGTAGAAGGCTTCGGCCTTTGAGACCGGCGC
ACGGGTGCGTAACGCGTATGCAATCTGCCTTACACTAAGGGATAGCCCAGAGAAATTTGGATTAATAC
CTTATAGTATATTTTTGTGGCATCACAAGATTATTAAAGATTTATTGGTGTAAGATGAGCATGCGTCC
CATTAGCTAGTTGGTATGGTAACGGCATACCAAGGCTATGATTGGTAGGGGTCCTGAGAGGGAGATCC
CCCACACTGGTACTGAGACACGGAC
>Zotu3400
GAACGCTGGCGGCAGGCCTAACACATGCAAGTCGAGCGGCAGCGGGAAGGTAGCTTGCTACTTTTGCC
GGCGAGCGGCGGACGGGTGAGTAATGCCTGGGAAATTGCCCAGTCGAGGGGGATAACAGTTGGAAACG
ACTGCTAATACCGCATACGCCCTACGGGGGAAAGCAGGGGACCTTCGGGCCTTGCGCGATTGGATATG
CCCAGGTGGGATTAGCTAGTTGGTGAGGTAATGGCTCACCAAGGCGACGATCCCTAGCTGGTCTGAGA
GGATGATCAGCCACACTGGAACTGAGACACGGTC
>Zotu3401
GAACGCTGGCGGCGTGCCTAACACATGCAAGTGGAGCGACGAACCGGGGCTTGCCCCGGGGCAGAGCC
GCGAACGGGTGAGTAACACGTGGGTTACCTGCCTCGATGACTGGGACAACCCGTCGAAAGACGGGCTA
ATACCGGATGTGCCCCTTGGGGGAAAGGAAGCTTTGGCCTCCGCATCGGGATGGGCCCGCGGCGCATT
AGCTTGTTGGTGCGGTAATGGCGTACCAAGGCGACGATGCGTAGCTGGTCTGAGAGGACGATCAGCCA
CACTGGGACTGAGACACGGCC
>Zotu3402
GAACGCTGGCGGCGTGCTTAACACATGCAAGTCGAACGGTGAAGGAAGAGCTTGCTCTTTTGGATCAG

TGGCGAACGGGTGAGTAACACGTGAGTAACCTGCCCTTGACTCTGGGATAAGCGTTGGAAACGACGTC
TAATACCGGATACGAGCTTCAGCCGCATGGCTAGGAGTTGGAAAGAATTTTGGTCAAGGATGGACTCG
CGGCCTATCAGCTAGTTGGTGAGGTAATGGCCCACCAAGGCGACGACGGGTAGCCGGCCTGAGAGGGT
GACCGGCCACACTGGGACTGAGACACGGCC
>Zotu3404
GAACGCTGGCGGCGTGCCTAACACATGCAAGTGGAGCGACGACCCGGACTTCGGTCCGGTGCAAAGCC
GCGAACGGGTGAGTAACACGTGGGTTACCTGCCCCGATGACAGGGACAACCTCGGGAAACCGAGGCTA
ATACCTGATGAGCCCGCAAGGGGAAAGGAAGCTTCGGCCTCCGCATCGGGATGGGCCCGCGGCGCATT
AGCTTGTTGGTGGGGTAAAGGCCTACCAAGGCGACGATGCGTAGCTGGTCTGAGAGGACGATCAGCCA
CACTGGGACTGAGACACGGCC
>Zotu3405
GAACGCTGGCGGCGCGCTTAACACATGCAAGTCGTACGGAGTTGTTCCTTCGGGAGCGGCTCAGTGGC
GAACGGGTGCGTAACACGTGAGCAACCTGCCTGCCGGTGGGGGATAACCCGGGGAAACCCGGGCTAAT
ACCGCATTCGCTCTTTCTCTCGATTGGGGGATTGAGGAAAGGTGCTGCAAGGCACTGCCGGCAGAGGG
GCTCGCGGCGTATCAGCTGGATGGTGGGGTAACGGCCTACCATGGCGACGACGCGTAGCCGATCTGAG
AGGATGATCGGCCACATTGGGACTGAGATACGGCC
>Zotu3406
GAACGCTAGCTACAGGCTTAACACATGCAAGTCGAGGGGCAGCATGAACTTAGCTTGCTAAGTTTGAT
GGCGACCGGCGCACGGGTGAGTAACACGTATCCAACCTGCCGATGACTCGGGGATAGCCTTTCGAAAG
AAAGATTAATACCCGATGGCATAGTTCTTCCGCATGGTAGAACTATTAAAGAATTTCGGTCATCGATG
GGGATGCGTTCCATTAGGTTGTTGGCGGGGTAACGGCCCACCAAGCCTTCGATGGATAGGGGTTCTGA
GAGGAAGGTCCCCCACATTGGAACTGAGACACGGTC
>Zotu3407
TAACGCTGGCAGAGTGCATAACACATGCAAGTCGAGCGAGACTGGTCGCAAGATCAGTCTAGCGGCAG
ACGAGTGAGTAACACGTAAACAATCTACCCTTGAATGGGGGATAACGGTTCGAAAGGATCGCTAATAC
CCCGTACTCTCCCCGGAAGGCATCTTCTGGGGAGGAGAGATCCGCAAGGATCGTTCTTGGAGGAGTTT
GCGGCCCATCAGCTAGTTGGCGGTGTAATAGACCACCAAGGCTACGACGGGTACCCGGCCTGAAAGGG
CGGACGGGCACGATGGCACTGAGACACGGGC
>Zotu3408
GAACGCTGGCGGCGTGCTTAACACATGCAAGTCGAGCGGTAAGGCCCTTTCGGGGGTACACGAGCGGC
GAACGGGTGAGTAACACGTGAGTAATCTGCCCTTCACTCTGGGATAAGCACTCGAAAGGGTGTCTAAT
ACCGGATACGACACAGACTGGCATCGGTTCTGTGTGGAAACTTTTGTGGGTGGGGGATGTGCTCGCGG
CCTATCAGCTTGTTGGTGGGGTAATGGCCTACCAAGGCTTTGACGGGTAGCCGGCCTGAGAGGGTGAC
CGGCCACACTGGGACTGAGACACGGCC
>Zotu3409
GAACGCTGGCGGCGTGCTTAACACATGCAAGTCGAACGATGAAGCCGGAGCTTGCTCTGGTGAATTAG
TGGCGAACGGGTGAGTAACACGTGAGCAATCTGCCCTTGACTCTGGGATAACTGCGGGAAACTGTAGC
TAATACCGGATACGACGTACGGAGGCATCTCCTGTACGTGGAAAGAATTTTGGTCAAGGATGAGCTCG
CGGCCTATCAGCTAGTTGGTGAGGTAATGGCTCACCAAGGCGACGACGGGTAGCCGGCCTGAGAGGGT
GACCGGCCACACTGGAACTGAGACACGGTC
>Zotu3410
GAACGCTGGCGGCGTGCTTAACACATGCAAGTCGAGCGGTAAGGCTCCTTCGGGGGTACACGAGCGGC
GAACGGGTGAGTAACACGTGAGTAATCTGCCCTTCACTTCGGGATAACCATCGGAAACGGTGGCTAAT
ACCGGATACGACGTACTCCTGCATGGGATGTGCGTGGAAAGTTTTTCGGTGGGGGATGTGCTCGCGG
CCTATCAGCTTGTTGGTGAGGTAATGGCTCACCAAGGCTTCGACGGGTAGCCGGCCTGAGAGGGTGAC
CGGCCACACTGGGACTGAGACACGGCC
>Zotu3411
GAACGCTGGCGGCGTGCTTAACACATGCAAGTCGAGCGGTAAGGCCCTTCGGGGTACACGAGCGGCGA
ACGGGTGAGTAACACGTGGGTGACCTGCCCTCAGCTTCGGGATAAGCCTGGGAAACCGGGTCTAATAC
CGGATACGACACATGGGCGCATGCTCGTGTGTGGAAAGTTTTTTCGGCTGGGGATGGGCCCGCGGCCT
ATCAGCTTGTTGGTGGGGTGATGGCCTACCAAGGCGGTGACGGGTAGCCGGCCTGAGAGGGCGACCGG
CCACACTGGGACTGAGACACGGCC
>Zotu3412
GAACGCTGGCGGCGTGCCTTATACATGCAAGTCACACGGGTGTAGCAATACACCAGTGGCGAACGGCT

GAGTAACACGTGGGAACTTCCTTTAAGTTCGGAATAACCCGCCGAAAGGCGGACTAATACCGGATGTG
CCCGAAAGGGTAAAGCCGCAAGGCGCTTAAAGCGAGGCCCGCGCACCATCAGGTAGTTGGTAGGGTAA
TGGCCTACCAAGCCTATGACGGTTAGAGGGCCCGAGAGGGTGTACCTCCAGACGGGAACTGCGACACG
GTC
>Zotu3413
GAACGCTGGCGGCGTGCTTAACACATGCAAGTCGAACGGTGATCTTGAGAGCTTGCTCTTGGGTGATC
AGTGGCGAACGGGTGAGTAACACGTGAGGAACCTGCCCCAGACTCTGGAATAACCCCGGGAAACCGGA
GCTAATACCGGATATGACATCAGACCGCATGGTCATGGTGTGGAAAGTTTTTCGGTCTGGGATGGCCT
CGCGGCCTATCAGCTTGTTGGTAAGGTAGTGGCTCACCAAGGCGACGACGGGTAGCCGGCCTGAGAGG
GCGACCGGCCACACTGGGACTGAGACACGGCC
>Zotu3414
GAACGCTAGCGATAGGCTTAACACATGCAAGTCGAGGGGCAGCATTATTTTAGCTTGCTAAGATAGAT
GGCGACCGGCGCACGGGTGCGTAACGCGTATGCAACCTGCCTGTAATTAGGGAATAACCCGGTGAAAG
TCGGACTAATACCCTATACTCTTCTTTCTCCGCATGGAGGAGGACGGAAAGATTTATTGATTACAGAT
GGGCATGCGTCCCATTAGCTAGTTGGTAAGGTAACGGCTTACCAAGGCAACGATGGGTAGGGGGACTG
AGAGGTTGACCCCCCACACTGGTACTGAGACACGGAC
>Zotu3415
GAACGCTGGCGGCATGCCTAACACATGCAAGTCAAGGGGTAGCAATACCACTGGCGCACGGGTGAGTA
ACACGTAGGTTATCTGCCCCCATCACAGACATAACCTCGGGAAACCGAGGCTAATATCTGATGAGGTC
GTGAGATCAAAGCTTCGGCGGATGGGGATGAGCCTGCGGCTGATTAGCTAGTTGGTGAGGTAATGGCT
CACCAAGGCGACGATCAGTAGCTGGTCTGAGAGGACGAACAGCCACACTGGAACTGAGACACGGTC
>Zotu3416
GAACGCTGGCGGCGTGCTTAAGACATGCAAGTCGAACGATCGTCTTCGGACGGTAGTGGCGCACGGGT
GAGTAACACGTAACTGACCTGCCCCCAAGTTCCGAATAACCCTCCGAAAGGAGGGCTAATACGGAATG
TGCTGTCACCTCCTGTGGTGCCAGTAAAGATTGATCGCTTGGGGATGGGGTTGCGTTCCATCAGCTTG
TTGGTGGGGTAAAGGCCCACCAAGGCAACGACGGATAACCGGCCTGAGAGGGTGGCCGGTCACAGGGG
CACTGAGACACGGGT
>Zotu3417
GAACGCTGGCGGAATGCTTTACACATGCAAGTCGAACGGCAGCACGAGAGCTTGCTCTTGGTGGCGAG
TGGCGAACGGGTGAGTAATATATCGGAACGTATCCAATAATGGGGGATAACTAATCGAAAGGTTGGCT
AATACCGCATACGCCCTACGGGGGAAAGCAGGGGATCTTCGGACCTTGCGTTAATGGAGCGGCTGATA
TCTGATTAGCTAGTAGGTGAGGTAAAGGCTCACCTAGGCTTCGATCAGTAGCTGGTCTGAGAGGACGA
CCAGCCACACTGGAACTGAGACACGGTC
>Zotu3418
GAACGCTGGCGGCGTGCTTAACACATGCAAGTCGAGCGATTTACTTCGGTAAAGAGCGGCGGACGGGT
GAGTAACGCGTGGGTAACCTGCCCTGTACACACGGATAACATACCGAAAGGTATGCTAATACGAGATA
ATATGCTTTTATCGCATGGTAGAAGTATCAAAGCTTTTGCGGTACAGGATGGACCCGCGTCTGATTAG
CTAGTTGGTAAGGTAACGGCTTACCAAGGCGACGATCAGTAGCCGACCTGAGAGGGTGATCGGCCACA
TTGGAACTGAGACACGGTC
>Zotu3419
GAACGCTGGCGGCGTGCTTAACACATGCAAGTCGAACGAGAGCGTAGAGAAAGCTTGCTTTTTCTACA
ATCTAGTGGCAAACGGGTGAGTAACGCGTAAACAACCTGCCCTACGGATGGGGACAACAGCTGGAAAC
GGCTGCTAATACCGAATACGTTCCGAAAGTCGCATGACTATCGGAAGAAAGGATGGCCTCTATTTATA
AGCTATCGCCGGAGGAGGGGTTTGCGTCTGATTAGCTAGTTGGAGGGGTAACGGCCCACCAAGGCGAC
GATCAGTAGCCGGTCTGAGAGGATGAACGGCCACATTGGAACTGAGACACGGTC
>Zotu3420
GAACGCTGGCGGTGTGCTTAACACATGCAAGTCGAACGGGCAAGAGCTGTAGCAATACAGAATTCTGC
TAGTGGCAAACGGGTGAGTAATACATGGGAATCTACCTTGCAGTGCGAAATAACAGTTGGAAGCAATT
GCTAATGTCGCATATAGCCGAGAGGTGAAAGATTTATCGCTGCAAGACGAGCCCATGCAGGATTAGCT
AGTTGGTGGGGTAACGGCCTACCAAGGCGACGATACGTAGCCGACCTGAGAGGGTGATCGGCCACACT
GGAACTGAGACACGGTC
>Zotu3421
GAACGCTAGCTACAGGCTTAACACATGCAAGTCGAGGGGAAACGACATCGGAAGCTTGCTTTTGATGG
GCGTCGACCGGCGCACGGGTGAGTAACGCGTATCCAACCTGCCCACCACTTGGGGATAACCTTGCGAA

AGTAAGACTAATACCCAATGATATCTCTAGAAGACATCTGAAAGAGATTAAAGATTTATCGGTGATGG
ATGGGGATGCGTCTGATTAGCTTGTTGGCGGGGTAACGGCCCACCAAGGCAACGATCAGTAGGGGTTC
TGAGAGGAAGGTCCCCCACATTGGAACTGAGACACGGTC
>Zotu3422
TAACGCTGGCGGCGTGCCTAATACATGCAAGTCGAACGGATAGTAATATCAGTGGCGAACGGGTGAGT
AATACGTGGGTAATCTACCTTCTCATGGGGAATAACTCTCCGAAAGGAGAGCTAATACCGCATAAGAC
CACGGGTGCTGCGGCACCTGAGGTTAAAGGCGACCTCTACTTGTAAGTTGTCGTGAGAAGATGAGCTT
GCGTCTGATTAGCTAGTTGGTGAGGTAAAAGCTTACCAAGGCTCTGATCAGTAGCTGGTCTGAGAGGA
TGGTCAGCCACACTGGAACTGAGACACGGTC
>Zotu3423
GAACGCTGGCGGCGTGCCTAACACATGCAAGTCGAGCGAGTGAATCTCCTTCGGGGGTGAAGCTAGCG
GCGGACGGGTGAGTAACACGTGGGCAACCTGCCTCATAGAGGGGAATAGCCTTTCGAAAGGAAGATTA
ATACCGCATAAGATTGTAGCTTCGCATGAAGTAGCAATTAAAGGAGCAATCCGCTATGAGATGGGCCC
GCGGCGCATTAGCTAGTTGGTGAGGTAACGGCTCACCAAGGCGACGATGCGTAGCCGACCTGAGAGGG
TGATCGGCCACATTGGGACTGAGACACGGCC
>Zotu3424
GAACGCTGGCGGCGTGCTTAACACATGCAAGTCGAACGGGATTTAGTAGACAGAAGCTTCGGTGGAAG
ATTACTAATGAGAGTGGCGAACGGGTGAGTAACGCGTGAGCAACCTGCCTATGACAGTGGGATAGCCT
CGGGAAACCGGGATTAATACCGCATAAAATCGTAGAAACACATGTTTTAACGGTCAAAGATTTATCGG
TCATAGATGGGCTCGCGTCTGATTAGCTAGTTGGTGAGATAACAGCCCACCAAGGCGACGATCAGTAG
CCGGTCTGAGAGGATGAACGGCCACATTGGAACTGAGACACGGTC
>Zotu3425
AAACGCTGGCGGCGTGCCTAATACATGCAAGTCGATCGCTGGAGCTCAAGCTTGCTTGAGTGAAGGAG
AGGCGAACGGGTGAGTAATACATAAGCAACCTGCCCACGAAGACTGGGATAATCTCTGGAAACGGGGA
CTAATACCGGATAGGTGACAAGATCGCATGATCATGTCATTAAAGATTCAAAGCACTGGTGGATGGGC
TTATGGCGCATTAGCTGGTTGGTGAGGTAACGGCCCACCAAGGCGATGATGCGTAGCCGGCCTGAGAG
GGTGAACGGCCACATTGGGACTGAGACACGGCC
>Zotu3426
GAACGCTGGCGGCGTGCTTAACACATGCAAGTCGAACGGTGAAGCAGAGCTTGCTCTGTGGATCAGTG
GCGAACGGGTGAGTAACACGTGAGCAACCTGCCCCTGACTCTGGGATAAGCGCTGGAAACGGCGTCTA
ATACTGGATATGAGCTGCGATCGCATGGTCAGCAGTTGGAAAGATTTTTCGGTCTGGGATGGGCTCAC
GGCCTATCAGCTTGTTGGTGAGGTAATGGCTCACCAAGGCGTCGACGGGTAGCCGGCCTGAGAGGGTG
ACCGGCCACACTGGGACTGAGACACGGCC
>Zotu3427
GAACGCTGGCGGCATGCCTAACACATGCAAGTCGAACGAAGCCTTCGGGCTTAGTGGCGCACGGGTGC
GTAACACGTGGGAATCTGCCCTTCGGTTCGGAATAACAGTGGGAAACTACTGCTAATACCGGATAATG
ACGTAAGTCCAAAGATTTATCGCCGAAGGATGAGCCCGCGTAGGATTAGGTAGTTGGTGGGGTAATGG
CCTACCAAGCCGACGATCCTTAGCTGGTCTGAGAGGATGATCAGCCACACTGGGACTGAGACACGGCC
>Zotu3428
CAACGCTGGCGGCGTGCCTAACACATGCAAGTCGAACGAGAAAGTGGAGCAATCCATGAGTAAAGTGG
CGACCGGGTGAGTAACACGTGACTAACCTACCTTCGAGTGGGGGATAACCTCGGGAAACCGAGGCTAA
TACCGCATAATATCGCAAGATCAAAGCAGCAATGCGCTGGAAGAGGGGGTCGCGGCTGATTAGCTAGT
TGGCGGGGTAACGGCCCACCAAGGCGAAGATCGGTAGCCGGCCTGAGAGGGCGCACGGCCACACTGGA
ACTGAAACACGGTC
>Zotu3429
GAACGCTGGCGGCGTGCCTAATACATGCAAGTCGAGCGAGGTTCTTCGGAACCTAGCGGCGGACGGGT
GAGTAACACGTGGGTAACCTGCCTGTAAGACCGGGATAACTTCGGGAAACCGAAGCTAATACCGGATA
CTTTCGAGCATCGCATGATGCTTGATGGAAAGACGGCTTCGGCTGTCACTTACAGATGGACCCGCGTC
GCATTAGCTAGTTGGTGAGGTAACGGCTCACCAAGGCGACGATGCGTAGCCGACCTGAGAGGGTGATC
GGCCACACTGGGACTGAGACACGGCC
>Zotu3430
GAACGCTGGCGGCATGCCTAACACATGCAAGTCGAACGAAGCCTTCGGGCTTAGTGGCGCACGGGTGC
GTAACGCGTGGGAATCTGCCCTTCGGTTCGGAACAACAGTTAGAAATGACTGCTAATACCGGATGATG
ACGTAAGTCCAAAGATTTATCGCCGAAGGATGAGCCCGCGTAGGATTAGCTTGTTGGTGAGGTAAAAG

CTCACCAAGGCGACGATCCTTAGCTGGTCTGAGAGGATGATCAGCCACACTGGGACTGAGACACGGCC
>Zotu3431
GAACGCTGGCGGCATGCTTTACACATGCAAGTCGAACGGCAGCGAAGAGAAGCTTGCTTCTCTGTCGG
CGAGTGGCGAACGGGTGAGTATAGCATCGGAACGTGCCAAGTAGTGGGGGATAACCAAACGAAAGTTT
GGCTAATACCGCATAAGCTCTAAGGAGGAAAGTAGGGGACCGTAAGGCCTTACGCTATTTGATCGGCC
GATGTCGGATTAGCTAGTTGGTGAGGTAATGGCTCACCAAGGCGATGATCCGTAGCGGGTCTGAGAGG
ACGATCCGCCACACTGGGACTGAGACACGGCC
>Zotu3432
GAACGTTGGCGGCGTGCCTAACACATGCAAGTCGAGCGTGAAAGTCTTCGGATGAGTAAAGCGGCGGA
CGGGTGAGTAACGCGTAGATAATCTACCTTTTAGTGGGGAATAACTAAGGGAAACTTTAGCTAATACC
GCATGTGGATCCGGATCTTTTGATCTGTATCTAAAGGGGGCTTCGGCTCTCGCTAATTGATGAGTCTG
CGTCCCATTAGCTAGTTGGTAGGGTAATGGCCTACCAAGGCGACGATGGGTAGCTGGTTTGAGAGAAC
GACCAGCCACACTGGAACTGACACACGGTC
>Zotu3433
GAACGCTGGCGGCGTGCTTAACACATGCAAGTCGAACGATGACGGAGGAGCTTGCTCCTCCTGATTAG
TGGCGAACGGGTGAGTAACACGTGAGCAATGTGCCCTTGACTCTGGGATAAGCGCTGGAAACGGCGTC
TAATACCGGATACGACCACCGGAGGCATCTCCTGGTGGTGGAAAGAACTTCGGTCAAGGATCAGCTCA
CGGCCTATCAGCTAGTTGGTGAGGTAATGGCTCACCAAGGCGACGACGGGTAGCCGGCCTGAGAGGGT
GACCGGCCACACTGGGACTGAGACACGGCC
>Zotu3435
GAACGCTGGCGGCGTGCTTAACACATGCAAGTCGAACGAAGAGCGATGGAAGCTTGCTTCTATCAATC
TTAGTGGCGAACGGGTGAGTAACGCGTAATCAACCTGCCCTTCAGAGGGGGACAACAGTTGGAAACGA
CTGCTAATACCGCATACGATCTAACCTCGGCATCGAGGATGGATGAAAGGTGGCCTCTATTTATAAGC
TATCACTGAAGGAGGGGATTGCGTCTGATTAGCTAGTTGGAGGGGTAACGGCCCACCAAGGCGATGAT
CAGTAGCCGGTCTGAGAGGATGAACGGCCACATTGGGACTGAGACACGGCC
>Zotu3437
GAACGCTGGCGGCGTGCCTAATACATGCAAGTCGAGCGGTAAGGCTCTGCACTTAGTATTTGATCGTA
AAGTCCTCGGACTTCTAAGCAGATTGATTCGACCCTAGGCTAGGGAACATGATACGAGTAGAGATCGA
ATAATGAGTGTAGAGTACACGAGCGGCGGACGGCTGAGTAACACGTAGGAACATACCCCAAAGTGAGG
GATAACTGCTCGAAAGAGTAGCTAATACCGCATGTGCTCTAAGGAGTAAAGCTTCGGCGCTTTGGGAA
TGGCCTGCGCATGATTAGCTTGTTGGTGAGGTAAAAGCTCACCAAGGCGACGATCATTAGCTGGTCTG
AGAGGATGATCAGCCAGACTGGGACTGAGACACGGCC
>Zotu3438
GAACGCTGGCGGCGTGCTTAACACATGCAAGTCGAGCGAGAGCCGGCTGAGGGGTAACCCTCGGCCGG
GGACAGCGGCGAACGGGTGCGTAACACGTGGGCAACCTGCCCCTGACTCTGGGATAACCCCGGGAAAC
CGGAGCTAATACCGGATACTCTCGGCCCTCCTCATGGTGGGCCGAGGAAAGATCTGTCGGTCAGGGAT
GGGCCCGCGGCCTATCAGCTTGTTGGTGGGGTAACGGCCTACCAAGGCGACGACGGGTAGCTGGTCTG
AGAGGACGACCAGCCACACTGGAACTGAGATACGGTC
>Zotu3440
AAACGCTGGCGGCGTGCTTAACACATGCAAGTCGAACGGTAAAGCCCTTCGGGGTACACGAGTGGCGA
ACGGGTGAGTAACGCGTGAGTAACCTGCCCTTCACTCTGGGATAACAGTTGGAAACGGCTGCTAATAC
CGGATATTCAGCTCCTGTCGCATGGTGGGGGTTGGAAAGTTTTGGTGGTGGAGGATGGGCTCGCGTCC
TATCAGCTTGTTGGTGGGGTGATGGCCTACCAAGGCGTCGACGGGTAGCCGGCCTGAGAGGGCGACCG
GCCACATTGGGACTGAGATACGGCC
>Zotu3441
GAACGCTAGCGGCAGGCTTAATACATGCAAGGCGAGGGGGCAGCAATGTCACCGTCGTACGGGTGCGT
AACGCGTATGTAACCTACCTTTTACTGGGGGATAGCCCGGGGAAACTCGGATTAATACCGCATAAGAC
ATTTGGATCACCTGATTTGATTTGTTAAAGATTTATTGGTAAGAGATGGGCATGCGTTCGATTAGCTA
GTTGGCAGGGTAACGGCCTACCAAGGCGACGATCGATAGGGGAGCTGAGAGGTTGATCCCCCACACGG
GCACTGAGATACGGGC
>Zotu3442
GAACGCTGGCGGCGTGCTTAACACATGCAAGTCGAGCGGAAAGGCCCACTTGTGGGTACTCGAGCGGC
GAACGGGTGAGTAACACGTGAGTAATCTGCCCTCCACTCTGGGATAACGTTCAGAAATGGGTGCTAAT
ACCGGATGTACACGTGCTGACGCATGTTGGTGTGTGGAAAGGTTTTCTGGTGGGGGATGTGCTCGCGG

CCTATCAGCTTGTTGGTGGGGTAATGGCCTACCAAGGCTTTGACGGGTAGCCGGCCTGAGAGGGTGAC
CGGCCACACTGGGACTGAGACACGGCC
>Zotu3444
AAACGCTGGCGGCGTGCTTAACACATGCAAGTCGAACGAAGTTTTTCTTTCGGGAGGAACTTAGTGGC
GGACGGGTGAGTAACGCGTGGGTAACCTGCCCCGTACAGGGGGACAACAGCTGGAAACGGCTGCTAAT
ACCGCATAAGCCCTTAGCACTGCATGGTGCATAGGGAAAAGGAGCAATCCGGTACAGGATGGACCCGC
GTCTGATTAGCCAGTTGGCAGGGTAACGGCCTACCAAAGCGACGATCAGTAGCCGATCTGAGAGGATG
TACGGCCACATTGGGACTGAGACACGGCC
>Zotu3446
GAACGCTGGCGGCGTGCCTAACACATGCAAGTCGAACGGGGTAGAGAGCTTCGGTTTTCTACTTAGTG
GCGAACGGGTGAGTAACGCGTGAGAAACCTGCCTTTCAGTGGGGGACAACAGTTGGAAACGACTGCTA
ATACCGCATAATGTTATTTGGGGGCATCCCCGAATAACCAAAGCTTTATGTGCTGAAAGATGGTCTCG
CGTCTGATTAGCTAGATGGCGGGGTAACGGCCCACCATGGCGACGATCAGTAGCCGGTCTGAGAGGAT
GAACGGCCACATTGGGACTGAGATACGGCC
>Zotu3447
GAACGCTAGCGGCAGGCCTAACACATGCAAGTCGAGGGAGAAGCCTTTCGGGGCAGAAACCGGCGCAC
GGGTGCGTAACGCGTATGCAACCTACCTTTTACAGGGGGATAGCCCGAAGAAATTTGGATTAATACCC
CATAATATTATTGTACGACATCGTTTGATAATTAAAGCTGAGGCGGTAGAAGATGGCATGCGTCCTA
TTAGCTAGTTGGAGTGGTAACGGCACCCCAAGGCTACGATAGGTAGGGGTCCTGAGAGGGAGATCCCC
CACACTGGTACTGAGACAGGGAC
>Zotu3448
GAACGCTGGCGGCGTGCTTAACACATGCAAGTCGAGCGGAAAGGCCCTTCGGGGTACTCGAGCGGCGA
ACGGGTGAGTAACACGTGAGTAATCTGCCCTTGGCTTCGGGATAGCCACCGGAAACGGTGATTAATAC
CGGATACGACAACTTCTCGCATGGGATGGTTGTGGAAAGTTTTTCGGCCAGGGATGTGCTCGCGGCCT
ATCAGCTTGATGGTGAGGTAATGGCTCACCATGGCTTCGACGGGTAGCCGGCCTGAGAGGGTGACCGG
TCACACTGGGACTGAGACACGGCC
>Zotu3449
GAACGCTGGCGGCGTGCTTAACACATGCAAGTCGAGCGGAAAGGCCCTTCGGGGTACTCGAGCGGCGA
ACGGGTGAGTAACACGTGAGTAACCTGCCCTGGACTTTGGGATAACCCTCGGAAACGGGGGCTAATAC
CGGATACGACCCACTTCTGCATGGTGGTGGGTGTAAAGATTTTTTGGTCTGGGATGGGCTCGCGGCCT
ATCAGCTTGTTGGTGGGGTGATGGCCTACCAAGGCGACGACGGGTAGCCGGCCTGAGAGGGCGACCGG
CCACACTGGGACTGAGACACGGCC
>Zotu3450
GAACGCTGGCGGCAGGCTTAACACATGCAAGTCGAACGCCCCGCAAGGGGAGTGGCAGACGGGTGAGT
AACACGTGGGAACATACCCTTCGGTTCGGAACAACGTTTGGAAACGAACGCTAATACCGGATACGGCT
TACGAGCGAAAGATTGATCGCCGAAGGATTGGCCCGCGTTCGATTAGCTTGATGGTGAGGTAACGGCT
CACCATGGCGACGATCGATAGCTGGTCTGAGAGGATGGCCAGCCACATTGGGACTGAGACACGGCC
>Zotu3451
GAACGCTGGCGGCGTGCTTAACACATGCAAGTCGAACGGTGAAGCCAAGCTTGCTTGGTGGATCAGTG
GCGAACGGGTGAGTAACACGTGAGCAACCTGCCCTGAACTCTGGGATAAGCGCTGGAAACGGCGTCTA
ATACTGGATATGAGCTGCGGAGGCATCTTCGTGGTTGGAAAGATTTTTCGGTTTGGGATGGGCTCGCG
GCCTATCAGCTTGTTGGTGAGGTAATGGCTCACCAAGGCGTCGACGGGTAGCCGGCCTGAGAGGGTGA
CCGGCCACACTGGGACTGAGACACGGCC
>Zotu3452
GAACGCTGGCGGCGTGCCTAACACATGCAAGTCGAGCGAACCCTTCGGGGTGAGCGGCGGACGGGTGA
GTAACGCGTGGGTAACCTGCCCTGTACACACGGATAACATACCGAAAGGTATGCTAATACGAGATAAT
ATGTAATGATCGCATGGTCAATACATCAAAGCTCCGGCGGTACAGGATGGACCCGCGTCTGATTAGCT
AGTTGGTAAGGTAACGGCTTACCAAGGCGACGATCAGTAGCCGACCTGAGAGGGTGATCGGCCACATT
GGAACTGAGACACGGTC
>Zotu3453
GAACGCTAGCGGGAGGCTTAACACATGCAAGTCGAGCGGGATTCCCTTCGGGGATGAGAGCGGCGCAC
GGGTGCGTAACGCGTATGCAACCTGCCTTATACAGGGGGATAGCCCATGGAAACGTGGATTAATATCC
CATAGGATTACAAATTCGCATGGAAAAGTAATTAAAACTACGGTGGTATAAGATGGCATGCGTCTGA
TTAGGTAGTTGGTAGGGTAACGGCCTACCAAGCCGACGATCAGTAGGGGTTCTGAGAGGATTACCCCC

CACACTGGTACTGAGACACGGAC
>Zotu3455
GAACGCTAGCGGCAGGCCTAATACATGCAAGTCGAGGGGTAGTGTAGCAATACATGAGACCGGCGCAC
GGGTGCGTAACGCGTATGAAAGGTGTCCAAATCTCTGGGATAGCCCTCGGAAACGGGGATTAATACTG
GATGGTCTATATGTGTGGCATCACACTTATAGTAAAGCTCAGGCGGATTTGGGTCCTCATGCGTACGA
TTAGCTAGTTGGCGGGGTAATGGCCCATCAAGGCGACGATCGTTAGGGGTCCTGAGAGGGAGATCCCC
CACACTGGTACTGAGACACGGGC
>Zotu3459
GAACGCTGGCGGCGTGCCTAATACATGCAAGTCGAGCGAATCAATAGGAGCTTGCTCCTGTTGATTAG
CGGCGGACGGGTGAGTAACACGTGGGCAACCTGCCTGTAAGACTGGGATAACTTCGGGAAACCGGAGC
TAATACCGGATAATCCTTTTCTACTCATGTAGAAAAGCTGAAAGACGGTTTCGGCTGTCACTTACAGA
TGGGCCCGCGGCGCATTAGCTAGTTGGTGAGGTAACGGCTCACCAAGGCGACGATGCGTAGCCGACCT
GAGAGGGTGATCGGCCACACTGGGACTGAGACACGGCC
>Zotu3460
GAACGCTGGCGGCGTGCTTAACACATGCAAGTCGAACGGAAAGGCCTCTAGCTTGCTAGAGGTACTCG
AGTGGCGAACGGGTGAGTAACACGTGGGTGATCTGCCCTGCACTGTGGGATAAGCCTGGGAAACTGGG
TCTAATACCATATAGGACCGCATCTTGGATGGTGTGGTGGAAAGCTTTTGCGGTGTGGGATGAGCCTG
CGGCCTATCAGCTTGTTGGTGGGGTAATGGCCTACCAAGGCGGCGACGGGTATCCGGCCTGAGAGGGT
GTACGGACACATTGGGACTGAGATACGGCC
>Zotu3461
GAACGCTGGCGGCAGGCCTAACACATGCAAGTCGAGCGGATGAAAGGAGCTTGCTCCTGGATTCAGCG
GCGGACGGGTGAGTAATGCCTAGGAATCTGCCTGGTAGTGGGGGACAACGTTTCGAAAGGAACGCTAA
TACCGCATACGTCCTACGGGAGAAAGCAGGGGACCTTCGGGCCTTGCGCTATCAGATGAGCCTAGGTC
GGATTAGCTAGTTGGTGGGGTAAAGGCCTACCAAGGCGACGATCCGTAACTGGTCTGAGAGGATGATC
AGTCACACTGGAACTGAGACACGGTC
>Zotu3465
GAACGCTGGCGGCGTGCTTAACACATGCAAGTCGAACGAGACCCCTTCTTCGGGAGGGTGTTCTAGTG
GCGAACGGGTGAGTAACACGTGGGCAACCTGCCCCTGGCTCTGGGATAACTCCAAGAAATTGGGGCTA
ATACCGGATATGACCGCTGACCGCATGGTCTGGTGGTGGAAAGATTTATCGGCTGGGGATGGGCCCGC
GGCCTATCAGCTTGTTGGTGGGGTAGTGGCCTACCAAGGCGACGACGGGTAGCCGGCCTGAGAGGGTG
ACCGGCCACACTGGGACTGAGACACGGCC
>Zotu3466
GAACACTGGCGACGTGCTTAACACATGCAAGTCGAACGGAAAGGCCCTGCTTGCAGGGTACTCGAGTG
GCGAACGGGTGAGTAACACGTGGGTGATCTGCCCTGCACTTCGGGATAAGCCTGGGAAACTGGGTCTA
ATACCGGATAGTACCATCGTTTAGTGTCGGTGGTGGAAAGTTTTTCGGTGCAGGATGAGCCCGCGGCC
TATCAGCTTGTTGGTGGGGTAATGGCCTACCAAGGCGTCGACGGGTAGCCGGCCTGAGAGGGTGGACG
GCCACATTGGGACTGAGACACGGCC
>Zotu3467
GAACGCTGGCGGTGTGCTTCACACATGCAAGTCGAACGGGGTAGCAATACCCAGTGGCGGACGGGTGA
GTAACGCGTAGAAATCTGCCTTTAGGTGGGGGATAACAGTCCGAAAGGACTGCTAATACCGCATATGT
GTGTAATTCGACAGTTTTACACATGAAAGAATTTCGCCTGAAGATGAGTCTGCGTCCGATTAGCTAGT
TGGTAGGGTAAAGGCCTACCAAGGCGACGATCGGTAGGTGGTCTGAGAGGATGATCACCCACAATGGG
ACTGAGACACGGCC
>Zotu3468
GAACGCTGGCGGCGCGCTTAACACATGCAAGTCGTACGGGGGTCTTCGGACCTCAGTGGCGAACGGGT
GCGTAACACGTGAGCAACCTGCCTGGTCGTGGGGGATAACCAGTGGAAACGCTGGCTAATACCGCATG
AGCCCTTTCTGCCGATTGGTGGGATGAGGAAAGCTCCTGCAAGGGAGTGCGACCAGAGGGGCTCGCGG
CGTATCAGCTAGTTGGAGGGGTAACGGCCCACCAAGGCGATGACGCGTAGCCGATCTGAGAGGATGAT
CGGCCACATTGGGACTGAGATACGGCC
>Zotu3469
GAACGCTGGCGGCAGGCTTAACACATGCAAGTCGAGCGCCCCGCAAGGGGAGCGGCAGACGGGTGAGT
AACGCGTGGGAATCTACCCAGCTCTACGGAATAACCCAGGGAAACTTGGACTAATACCGTATACGTCC
TCCGGGAGAAAGATTTATCGGAGTTGGATGAGCCCGCGTTGGATTAGCTAGTTGGTGGGGTAATGGCC
TACCAAGGCGACGATCCATAGCTGGTCTGAGAGGATGATCAGCCACACTGGGACTGAGACACGGCC

>Zotu3470
GAACGTTGGTAACGTGGATACCGGCATGCAAGTCAAGGGGCCCGCAAGGGCACCGGCGAACGGCTTAG
TAACACGTGGATACATACCGTAAAGTGGGGCATAGCTCGTCGAAAGACGGGGTAATTTCCCATGTGAA
CGCAAGTTTAAACACGCAAGTGGCTTTACGAATGGTCTGCGGTCTATCAGCTAGTTGGTGGGGTAATG
GCCTACCAAGGCTATGACGGATAGGGGGCGTGAGAGCGCGACCCCCAAGAATGGAACTGCGACACGGT
C
>Zotu3471
GAACGCTGGCGGCAGGCCTAACACATGCAAGTCGAGCGCGAAAGGCCTTCGGGCTGAGTAGAGCGGCG
GACGGGTGAGTAACGCGTAGGAATCTACCTGGTAGTGGGGGACAACTTGGGGAAACTCAAGCTAATAC
CGCATACGCCCTAAGGGGGAAAGCGGGGGACCTTCGGGCCTCGTGCTATCGGATGAGCCTGCGTAGGA
TTAGCTAGTTGGTGAGGTAAAGGCTCACCAAGGCGACGATCCTTAGCTGGTCTGAGAGGATGATCAGC
CACACTGGGACTGAGACACGGCC
>Zotu3473
AAACGCTGGCGGCGTGCATGAGACATGCAAGTCGAACGAACGAGCTTGCTCGTTAGTGGCGGACGGCT
GAGTAACGCGTAGGTGACCTGCCCTGAAGTGGGGGATAACTGCCCGAAAGGGTGGCTAATACCGCATG
TGGCGACACGTTCATTCGTGTCATCAAAGCCGCGAGGCGCTTCGGGAGGGGCCTGCGTCCGATTAGCT
CGTTGGTGGGGTAATGGCTCACCAAGGCGACGATCGGTAGGTGGTCTGAGAGGACGATCACCCAGACT
GGGACTGAGACACGGCC
>Zotu3475
GAACGCTGGCGGCGCGCTTAACACATGCAAGTCGAGCGATGAAATTTCGACAGATCCCTTCGGGGTGA
AGATGAAATGGATTAGCGGCGGACGGGTGAGTAACACGTGAGTAACCTGCCTTAGACATTGGGATAGC
CTCGGGAAACCGGGATTAATACCAAATGAAATCACATATGCACATGCATAAATGATCAAAGCTCCGGC
GGTCTAAGATGGACTCGCGTCCCATTAGCTAGTTGGTGAGGTAACGGCCCACCAAGGCGACGATGGGT
AGCCGGCCTGAGAGGGTGAACGGCCACATTGGAACTGAGAAACGGTC
>Zotu3476
GAACGCTGGCGGCGTGCCTAATACATGCAAGTCGAGCGGACCGGATGGAAGCTTGCTTTCGTTCGGTT
AGCGGCGGACGGGTGAGTAACACGTGGGCAACCTGCCCGTAAGACCGGGATAACTTCGGGAAACCGGA
GCTAATACCGGATAACACTGAAGACCGCATGGTCTTTGGTTGAAAGGCGGCTTTAGCTGTCACTTACG
GATGGGCCCGCGGCGCATTAGCTAGTTGGTGAGGTAACGGCTCACCAAGGCGACGATGCGTAGCCGAC
CTGAGAGGGTGATCGGCCACACTGGGACTGAGACACGGCC
>Zotu3477
GAACGCTGGCGGCGTGCCTAATACATGCAAGTCGAGCGAACTTGCGGGAGCTTGCTCCCAAAAGTTAG
CGGCGGACGGGTGAGTAACACGTGGGCAACCTGCCTATAAGACTGGGATAACTTCGGGAAACCGGAGC
TAATACCGGATAATTCTTTTCTACACATGTAGAAAAGCTAAAAGACGGTTTGCGCTGTCACTTATAGA
TGGGCCCGCGGCGCATTAGCTAGTTGGTGAGGTAACGGCTCACCAAGGCCACGATGCGTAGCCGACCT
GAGAGGGTGATCGGCCACACTGGGACTGAGACACGGCC
>Zotu3480
GAACGCTGGCGGCGTGCTTAACACATGCAAGTCGAACGATGAAGCCCTGCTTGCAGGGTGGATTAGTG
GCGAACGGGTGAGTAACACGTGGGTGATCTGCCCTGCACTTCGGGATAAGCCTGGGAAACTGGGTCTA
ATACCGGATATGACCCTCCTTTAGTGTGGGGGGTGGAAAGCTTTTGCGGTGTGGGATGAGCCTGCGGC
CTATCAGCTTGTTGGTGGGGTAATGGCCTACCAAGGCGTCGACGGGTAGCCGGCCTGAGAGGGTGATC
GGCCACATTGGGACTGAGACACGGCC
>Zotu3483
GAACGCTGGCGGCGTGCCTAATACATGCAAGTCGAGCGGACTTTTCGGGAGCTTGCTTCCGGAAAGTC
AGCGGCGAACGGGTGAGTAACACGTGGGTAACCTGCCTGCAAGACCGGGATAACTTCGGGAAACCGGA
GCTAATACCGGATGATCTCCTTTTCCGCATGGAGAAGGACTGAAAGGACGGCGCAAGCTGTCGCTTGC
AGATGGGCCCGCGGCGCATTAGCCAGTTGGTGAGGTAACGGCTCACCAAGGCGACGATGCGTAGCCGA
CCTGAGAGGGTGATCGGCCACACTGGGACTGAGACACGGCC
>Zotu3484
GAACGCTAGCGGGATGCCTTACACATGCAAGTCGAACGGCAGCACGGACTTCGGTCTGGTGGCGAGTG
GCGAACGGGTGAGTAATGTATCGGAACGTGCCCAGTAGCGGGGGATAACTACGCGAAAGCGTAGCTAA
TACCGCATACGCCCTTAGGGGGAAAGCAGGGGATCGCAAGACCTTGCACTATTGGAGCGGCCGATATC
GGATTAGCTAGTTGGTGAGGTAATGGCTCACCAAGGCCGCGATCCGTAGCTGGTTTGAGAGGACGACC
AGCCACACTGGGACTGAGACACGGCC

>Zotu3485
GAACGCTGGCGGCAGGCTTAACACATGCAAGTCGAGCGGGCCCTTCGGGGTCAGCGGCGGACGGGTGA
GTAACGCGTGGGAACGTGCCCTTCGGTTCGGAATAGCCCCGGGAAACTGGGATTAACGCCGGATGCGC
CCTCTGGGTCACACTGGGGGGGAAAGGTTTGCGCCGAGGGATCGGCCCGCGTCCGATTAGGTAGTTGG
TGGGGTAACGGCTCACCAAGCCGACGATCGGTAGCTGGTTTGAGAGGATGATCAGCCACACTGGGACT
GAGACACGGCC
>Zotu3486
GAACGCTGGCGGCGTGCTTAACACATGCAAGTCGAACGATGAACCTCCTTCGGGAGGGGATTAGTGGC
GAACGGGTGAGTAACACGTGGGCAATCTGCCCTGCACTCTGGGATAACTCCGGGAAACCGGAGCTAAT
ACCGGATACGACCACTGCGGGCATCCGTGGTGGTGGAAAGCTCCGGCGGTGCAGGATGAGCCCGCGGC
CTATCAGCTTGTTGGTGGGGTGATGGCCTACCAAGGCGACGACGGGTAGCCGGCCTGAGAGGGCGACC
GGCCACACTGGGACTGAGGCACGGCC
>Zotu3490
GAACGCTGGCGGCGTGCTTAACACATGCAAGTCGAACGGTGAAGGAGAGCTTGCTCTCTGGATCAGTG
GCGAACGGGTGAGTAACACGTGAGCAATCTGCCCCTGACTCTGGGATAAGCGCTGGAAACGGCGTCTA
ATACCGGATACGAGCTGCGAAGGCATCTTCAGCAGCTGGAAAGAACTTCGGTCAGGGATGAGCTCGCG
GCCTATCAGCTAGTTGGTGAGGTAAAGGCTCACCAAGGCGTCGACGGGTAGCCGGCCTGAGAGGGTGA
CCGGCCACACTGGGACTGAGACACGGCC
>Zotu3491
TCGCATTTCGATGGAGGCGAAATGGTAGAGGCCCGTGCACTGTGCGATGTCAGTGCATATTAAAGAAC
ACCAGATGGTCGAAACTTCCGGAGCCTCCCACTACGGCGTGCTCCATAACCATATCCTGATATTATTA
GTATATTCTCAAACGTACAGTCAGTGTCAAAGCTTCAAAGACCTCCGGACACAGGAAAAAAACTGAAA
GTT
>Zotu3492
GAACGCTGGCGGCGTGCTTAACACATGCAAGTCGAGCGGAAAGGCCCTTCGGGGTACTCGAGCGGCGA
ACGGGTGAGTAACACGTGGGCAACCTGCCCTCAGCTCTGGGATAACTCCGGGAAACCGGGGCTAATAC
CGGATATGACCTTCACCGGCATCGGTGGTGGTGGAAAGTTCTTCGGCTGAGGATGGGCCCGCGGCCTA
TCAGCTTGTTGGTGGGGTGATGGCCTACCAAGGCGACGACGGGTAGCCGGCCTGAGAGGGCGACCGGC
CACACTGGGACTGAGACACGGCC
>Zotu3494
GAACGCTGGCGGCGTGCCTAATACATGCAAGTCGAGCGAGAAAGTCCTTCGGGATGAGTACAGCGGCG
CACGGGTGAGTAACACGTAGGTACCTGCCCTTTAGTGGGGAATAACACCGGGAAACCGGTGCTAATAC
CGCATATGTAGGAGACTACAAAGGGGGCGTAGCAATACGCTCTCGCTAGAGGAGGGACCTGCGCGACA
TTAGGTAGTTGGTGGGGTAATGGCCCACCAAGCCGACGATGTCTATCCGGTCTGAGAGGATGATCGGA
CACACTGGAACTGAGACACGGTC
>Zotu3495
GAACGCTGGCGGCGTGCCTAACACATGCAAGTCGAGCGATTTACTTCGGTAAAGAGCGGCGGACGGGT
GAGTAACGCGTGGGTAACCTGCCCTGTACACACGGATAACATACCGAAAGGTATGCTAATACGAGATA
ATATGCTTTTATCGCATGGTAGAAGTATCAAAGCTTTTGCGGTACAGGATGGACCCGCGTCTGATTAG
CTAGTTGGTAAGGGAACGGCTTACCAAGGCGACGATCAGTAGCCGACCTGAGAGGGTGATCGGCCACA
TTGGAACTGAGACACGGTC
>Zotu3496
GAACGCTGGCGGCATGCCTAACACATGCAAGTCGAACGGTAGCATGAACTAGCTTGCTAGTTTGATGA
CGAGTGGCGAACGGGTGAGTAACGCGTAGGAATACATCCTAGAGAGGGGGACAACCTGGGGAAACTCA
GGCTAATACCGCATGACATCTGAGGATTAAAGGCTGGGGACCTTCGGGCCTGGCGCTTTAGGGGTAGC
CTGCGTACGATTAGCTAGTTGGTGGGGTAAGGGCCTACCAAGGCGACGATCGTTAGCTGGTCTGAGAG
GACGAACAGCCACACTGGGACTGAGACACGGCC
>Zotu3497
GAACGCTAGCGGCAGGCTTAACACATGCAAGTCGAGGGGCAGCACGATGTAGCAATACATTGGTGGCG
ACCGGCGCACGGGTGCGTAACACGTATGGAATCTGCCCTTTACTGGAGGATAGCCCGGGGAAATCCGG
ATTAATACTCCATAACAGTTCGAGTGGCATCACTTGGATTTGAAAGCTCCGGCGGTAAAGGATGACCA
TGCGCACGATTAGCTAGTAGGTGAGGTAACGGCTCACCTAGGCTACGATCGTTAGGGGGCCTGAGAGG
GTTATCCCCCACACTGGTACTGAGACACGGAC
>Zotu3498

GAACGCTGGCGGCGTGCCTAATACATGCAAGTCGAGCGGTAAGGCCCCTTCGGGGGTACACGAGAGGC
GGACGGCTGAGTAACACGTAGGAACGTACCCCAAACTGAGGGATAAGCACCAGAAATGGTGTCTAATA
CCGCATGTGCTCTTCGGAGTAAAGCTACGGCGGTTTGGGAACGGCCTGCGCATGATTAGCTTGTTGGT
GAGGTAATAGCTCACCAAGGCGACGATCATTAGCTGGTCTGAGAGGATGACCAGCCAGACTGGGACTG
AGAACGGCC
>Zotu3499
GAACGCTGGCGGCGTGCCTAACACATGCAAGTCGAACGTGAAATCGAAAGAAAGTAAAGTGGCGTACG
GGTGAGTAACACGTAGGTAATCTACCTAATAGTTTGGAATAACCTGCCGAAAGGCGGGCTAATACTGG
ATAGAGCGAAGGACTCGTGAGGAGTTTTTCGGGAAAGGGAGGTCGTAAAGATCCCTCGCTATTGGATG
AGCCTGCGTCTCATTAGCTAGTTGGTAGGGTAAAGGCCTACCAAGGCTTTGATGGGTAGCTGGTCTGA
GAGGATGATCAGCCACACTGGAACTGAAACACGGTC
>Zotu3500
GAACGCTGGCGGCGTGCTTAACACATGCAAGTCGAACGATGAAGCCCAGCTTGCTGGGTGGATTAGTG
GCGAACGGGTGAGTAACACGTGAGCAACGTGCCCATAACTCTGGGATAACCTCCGGAAACGGTGGCTA
ATACTGGATATCTAACATGATCGCATGGTCTGTGTTTGGAAAGATTTTTTGGTTATGGATCGGCTCAC
GGCCTATCAGCTTGTTGGTGAGGTAATGGCTCACCAAGGCGACGACGGGTAGCCGGCCTGAGAGGGTG
ACCGGCCACACTGGGACTGAGACACGGCC
>Zotu3502
GAACGCTGGCGGTATGCCTTATACATGCAAGTCGTACGAGAGTTTTTAACTCTAGTGGCGGACGGGTG
AGTAACACGTAAGAATTTGCCTTTAGGAGGGGGACAACAACTGGAAACGGTTGCTAATACCCCATATG
CTTTCGAGTGAAATGGATTTCCGCCTAAAGAGAAGCTTGCGGCTGATTAGCTTGTTGGTAGGGTAATG
GCCTACCAAGGCGACGATCAGTATCTGGTTTGAGAGGACGATCAGACACACTGGAACTGAGACACGGT
C
>Zotu3503
GAACGCTAGCGGCAGGCTTAATACATGCAAGTCGAGGGGCAGCACGGGTAGCAATACCTGGTGGCGAC
CGGCAAACGGGTGCGGAACACGTACGCAACCTTCCCATAACTGGTGAATAGCCCTCCGAAAGGAGGAT
TAATACACCGTAACATAATGAAGTGGCATCACTTTATTATTATAGGCAGCAATGTCGGTTTTGGATGG
GCGTGCGCCTGATTAGGTAGTTGGCGGGGTAACGGCCCACCAAGCCTGCGATCAGTAACTGGTGTGAG
AGCACGACCAGTCACACGGGCACTGAGACACGGGC
>Zotu3504
GAATGCTGGCGATGTGGATAAGTCATGCAAGTCGAACGGTCCGCAAGGACAGTGGCAGACGAGGTAGT
AACACGTAGGTACACCCCCCGAAGTCGGGCATAGCCAGCCGAAAGGTTGGGTAATTCCCGATAGTCGC
GCAAGCGTAAAGATTTATCGCTTCGGGAGTGGCCTGCGTAATATCAGCTAGTTGGTAAGGTAACGGCT
TACCAAGGCTACGACGTTTAGGGGAGCTGAGAGGCTGACCCCCACCGATGGGACTGCGATACGGCC
>Zotu3505
CAACGCTGGCGGCGTGCCTAACACATGCAAGTCGAACGATTAAAGCCCCTTCGGGGGTGTATAGAGTG
GCGTACGGGTGAGTAACACGTGGGTAATCTACCTTTGGGTGGGGAATAACTCAGGGAAACTTGAGCTA
ATACCGCATAATGCAGCGGCTCGACATCGAGACAGTTGTTAAAGATTTATCGCCTGAAGAGGAGCCTG
CGCCTGATTAGCTAGTTGGTGGGGTAACGGCCCACCAAGGCTTCGATCAGTAGCTGGTCTGAGAGGAC
GGCCAGCCACAATGGCACTGAAACACGGGC
>Zotu3506
GAACGCTGGCGGCGTGCCTAATACATGCAAGTCGAACGCATTTTCTTTCACCGTAGCTTGCTACACCG
AAAGAAAATGAGTGGCGAACGGGTGAGTAACACGTGGGTAACCTGCCCATCAGCGGGGGATAACACTT
GGAAACAGGTGCTAATACCGCATAATTCCATTTACCGCATGGTAGATGGGTGAAAGGCGCTTTTGCGT
CACTGATGGATGGACCCGCGGTGCATTAGCTAGTTGGTGGGGTAACGGCCTACCAAGGCTGCGATGCA
TAGCCGACCTGAGAGGGTGATCGGCCACACTGGGACTGAGACACGGCC
>Zotu3507
GAACGCTGGCGGCGTGCCTAATACATGCAAGTCGAGCGAAGAATTTAAGGTGCTTGCACCTAAAATTC
TTAGCGGCGAACGGGTGAGTAACACGTAAAGAACCTGCCTCATAGACTGGGACAACTACTGGAAACGG
TAGCTAATACCGGATAATATATTTTACTGCATGGTGAAATATTGAAAGTCGGTCATGCTGACACTATG
AGATGGCTTTGCGGTGCATTAGCTAGTTGGTAGGGTAAAGGCCTACCAAGGCGACGATGCATAGCCGA
CCTGAGAGGGTGATCGGCCACACTGGGACTGAGACACGGCC
>Zotu3508
GAACGCTGGCGGCGTGCTTAACACATGCAAGTCGAGCGAGAAAGCTCCTTCGGGAGTGAGTACAGCGG

CGAACGGGTGAGTAACACGTGGGCAACCTGCCCTAGGGAGCGGGACAACCCGGGGAAACTCGGGCTAA
TACCGCATGAATCGTCGTGCCCCAAGGCGCGGCGGGAAAAGGTAGCTTCGGCCTCCGCCCTAGGATGG
GCCCGCGCCGCATTAGCTTGTTGGTGGGGTAACGGCTCACCAAGGCGACGATGCGTAGCTGGTCTGAG
AGGACGATCAGCCACACTGGGACTGAGACACGGCC
>Zotu3509
GAACGCTGGCGGCGTGCTTAACACATGCAAGTCGAACGGTGAAGCCAAGCTTGCTTGGTGGATCAGTG
GCGAACGGGTGAGTAACACGTGAGCAACCTGCCCTGGACTCTGGGATAAGCGCTGGAAACGGCGTCTA
ATACTGGATATGAGCTCTCATCGCATGGTGGGGGTTGGAAAGATTTTTCGGTCTGGGATGGGCTCGCG
GCCTATCAGCTTGTTGGTGAGGTAATGGCTCACCAAGGCGTCGACGGGTAGCCGGCCTGAGAGGGTGA
CCGGCCACACTGGGACTGAGACACGGCC
>Zotu3510
GAACGCTGGCGGCGTGCTTAACACATGCAAGTCACGGGGGCCCGCAAGGGCAACCGGCGAACGGGTGC
GTAACACGTGAACAACCTGCCCTCGTGTGGGGGATAGCCATGCTAACGCGTGGGTAATACCGCATACG
TTCCCTTGGAGGAGTCTCTGGGGGAGGAAAGCTCCGGCGCACGGGGAGGGGTTCGCGGCCTATCAGCT
AGTTGGCGGGGTAATGGCCCACCAAGGCGACGACGGGTAGCTGGTCTGAGAGGATGGCCAGCCACATT
GGGACTGAGAGACGGCC
>Zotu3512
GAACGCTGGCGGCATGCCTAATACATGCAAGTCGAACGCTGAAGCACTCTGCTTGCAGAGTGCGGATG
AGTGGCGAACGGGTGAGTAACACGTGGGGAATCTGCCTAGGAGTGAGGGACAACATTCGGAAACGGAT
GCTAATACCTTATATCTTAATGGACCGCATGGTCCATTAATGAAAGACGGCTTCGGCTGTCGCTCTTA
GATGATCCCGCGGCGTATTAGTTAGTTGGTGAGGTAATGGCTCACCAAGACCATGATACGTAGCCGAC
CTGAGAGGGTGATCGGCCACACTGGGACTGAGACACGGCC
>Zotu3513
GAACGCTGGCGGCGTGCTTAACACATGCAAGTCGAACGGTGAAGGGGCCTGCTTTTGTGGGTCCTGGA
TGAGTGGCGAACGGGTGAGTAACACGTGAGTAACCTGCCCCCTTCTTCTGGATAACCGCATGAAAGTG
TGGCTAATACGGGATATTCTGTGCCTGCTGCATGGTGGGTGTGGGAAAGATTGCGCTTTTGGGTGTTT
TTGGTGGGGGATGGGCTCGCGGCCTATCAGCTTGTTGGTGGGGTGATGGCCTACCAAGGCGGTGACGG
GTAGCCGGCCTGAGAGGGTGGACGGTCACACTGGGACTGAGACACGGCC
>Zotu3514
TAACGCTGGCGGCGTGCCTAACACATGCAAGTCGAACGGGGCCCTGGTTCGCCAGGGTTCAGTGGCAG
ACGGGTGAGTAACACGTAGGTAATGTACCTTCCCGCTGGGGATACCTGCCCGAAAGGGCAGTTAATAC
CGAATGTGCCAGGCCTGGGGCATCCCGGGGTTGGCAAACCGGCAACGGGCGGGAGGATCAGCCTGCGG
ACCATCAGCTAGTTGGTGGGGTAACGGCCTACCAAGGCGACGACGGTTAGCCGGTCTGAGAGGACGAT
CGGCCACACTGGGACTGAGACACGGCC
>Zotu3516
GAACGCTGGCGGCGTGCTTAACACATGCAAGTCGAACGGAAAGGCTCCTGCTTTTGTGGGGGTGCTCG
AGTGGCGAACGGGTGAGTAACACGTGGGTGATCTGCCCTCAACTTCGGGATAAGCTTGGGAAACTGGG
TCTAATACCGGATAGGACCATGCAGTGGTGTGTGTGGTGGAAAGATGTTTTTTGGTTGGGGATGAGCT
CGCGGCCTATCAGCTTGTTGGTGGGGTAATGGCCTACCAAGGCGTCGACGGGTAGCCGGCCTGAGAGG
GTGGACGGCCACATTGGGACTGAGATACGGCC
>Zotu3517
GAACGCTGGCGGCGTGCTTAACACATGCAAGTCGAACGGTGATCTTGGGAGCTTGCTCCTGGGTGATC
AGTGGCGAACGGGTGAGTAACACGTGAGTAACCTGCCCCAGACTCTGGAATAACTTCGGGAAACCGAT
GCTAATACCGGATATGACACAGAATCGCATGGTTTCTGTGTGGAAAGTTTTTCGGTTTGGGATGGACT
CGCGGCCTATCAGCTTGTTGGTGAGGTAATGGCTCACCAAGGCGACGACGGGTAGCCGGCCTGAGAGG
GCGATCGGCCACACTGGGACTGAGACACGGCC
>Zotu3518
GAACGCTGGCGGCATGCCTTACACATGCAAGTCGAACGGCAGCACGGGAGCAATCCTGGTGGCGAGTG
GCGAACGGGTGAGTAAAGCATCGGAACGTGCCCAGTCGTGGGGGATAACGTAGCGAAAGCTACGCTAA
TACCGCATACGATCTGTGGATGAAAGCGGGGGACCGTAAGGCCTCGCGCGATTGGAGCGGCCGATGTC
AGATTAGGTAGTTGGTGGGGTAAAGGCTCACCAAGCCGACGATCTGTAGCTGGTCTGAGAGGACGACC
AGCCACACTGGGACTGAGACACGGCC
>Zotu3519
GAACGCTGGCGGCGTGCTTAACACATGCAAGTCGAGCGAGGCTTCTCCTTCGGGAGGATGTCCTAGCG

GCGAACGGGTGAGTAACACGTGGGCAACCTGCCCCTAGCACTGGGATAACCCCGGGAAACCGGGGCTA
ATACCGGATACGACCTCGAAAGGCATCCTTCGAGGTGGAAAAGGTTTACTGGCTAGGGATGGGCCCGC
GGCCTATCAGCTTGTTGGTGGGGTAACGGCCTACCAAGGCGACGACGGGTAGCCGGCCTGAGAGGGCG
ACCGGCCACACTGGGACTGAGACACGGCC
>Zotu3520
GAACGCTGGCGGCGTGCTTAACACATGCAAGTCGAGCGAACCCTTCGGGGTGAGCGGCGGACGGGTGA
GTAACGCGTGGGTAACCTGCCCTGTACACACGGATAACATACCGAAAGGTATGCTAATACGAGATAAT
ATGTAATGATCGCATGATCGATACATCAAAGCTCCGGCGGTACAGGATGGACCCGCGTCTGATTAGCT
AGTTGGTGAGGTAATGGCTCACCAAGGCGACGATCAGTAGCCGACCTGAGAGGGTGATCGGCCACATT
GGAACTGAGACACGGTC
>Zotu3521
GAACGCTAGCGGCAGGCCTCATACATGCAAGTCGAGGGGCAGCGGGACATTTCGGTGTTGCTGGCGAC
CGGCGAACGGGTGCGTAACACGTATGCAATCTACCTCGTACTGGGGAATAGCTCTCGGAAACGAGAAT
TAATACCCCATATACTCGTAACAGCACATGCTGATACGAGAAAAGCTCCGGCGGTACGAGATGAGCAT
GCGTCCCATTAGCTAGTTGGTAAGGTAACGGCTTACCAAGGCAACGATGGGTAGGGGTTCTGAGAGGA
AGATCCCCCACACTGGCACTGAGATACGGGC
>Zotu3522
GAACGCTGGCGGCATGCCTAACACATGCAAGTCGAACGAGACCTTCGGGTCTAGTGGCGCACGGGTGC
GTAACGCGTGGGAACCTGCCCTTAGGTTCGGAATAACTTCCCGAAAGGGATGCTAATACCGGATAATG
TCTTCGGACCAAAGATTTATCGCCTTTGGATGGGCCCGCGTAGGATTAGCTTGTTGGTGGGGTAATGG
CCTACCAAGGCGACGATCCTTAGCTGGTCTGAGAGGATGATCAGCCACACTGGGACTGAGACACGGCC
>Zotu3523
GAACGCTGGCGGCGTGCCTAACACATTCAAGTCGAACGGTGACGGAGACTTCGGTCTCCTGATCAGTG
GCGGACGGGTGAGTAACGCGTGAAGAACCTGCCTGAGATTGGGGAATAACATCGGGAAACCGGTGCTA
ATACCGCATAACATGTTGATGCCGCATGACATTAACATCAAAGATTTATCGATCCCAGATGGCTTCGC
GTCCGATTAGACAGTTGGCGGGGTAACGGCCCACCAAATCTACGATCGGTAACCGGACTGAGAGGTTG
AACGGTCACATTGGAACTGAGAAACGGTC
>Zotu3525
GAACGCTGGCGGCGTGCTTAACACATGCAAGTCGAGCGAGAAAGCCCCTTCGGGGGTGAGTACAGCGG
CGAACGGGTGAGTAACACGTGGGTAACCTGCCTTGAGGAGCGGGACAACCCGAGGAAACTCGGGCTAA
TACCGCATGACTCGTCGCGTCCCAAGGCGCGACGGCAAATGGTAGCTTCGGCCTCCGCCTCAAGATGG
GCCCGCGCCGCATTAGCTTGTTGGTGGGGTAATGGCTCACCAAGGCGACGATGCGTAGCTGGTCTGAG
AGGACGATCAGCCACACTGGGACTGAGACACGGCC
>Zotu3526
GAACGCTGGCGGCGTGCCTAATACATGCAAGTCGAGCGGAGTTGATGAGGTGCTTGCACCTCTGATAC
TTAGCGGCGGACGGGTGAGTAACACGTAGGTAACCTGCCTGTAAGACTGGGATAACATTCGGAAACGG
ATGCTAATACCGGATACGCGGATTGATCGCATGATCAAACTGGGAAAGATGGAGCAATCTATCACTTA
CAGATGGACCTGCGGCGCATTAGCTAGTTGGTGAGGTAACGGCTCACCAAGGCGACGATGCGTAGCCG
ACCTGAGAGGGTGATCGGCCACACTGGGACTGAGACACGGCC
>Zotu3528
GAACGCTGGCGGCGTGCTTAACACATGCAAGTCGAACGGTGAAGCAGAGCTTGCTCTGTGGATCAGTG
GCGAACGGGTGAGTAACACGTGAGCAATCTGCCCCTGACTCTGGGATAAGCGCTGGAAACGGCGTCTA
ATACCGGATACGAGACGTGAAGGCATCTTCAACGTCTGGAAAGAACTTCGGTCAGGGATGAGCTCGCG
GCCTATCAGCTAGTTGGTGAGGTAATGGCTCACCAAGGCGTCGACGGGTAGCCGGCCTGAGAGGGTGA
CCGGCCACACTGGGACTGAGACACGGCC
>Zotu3529
GAACGCTGGCGGCGTGCTTAACACATGCAAGTCGAACGATGAAGCCCAGCTTGCTGGGTGGATTAGTG
GCGAACGGGTGAGTAACACGTGAGTAACCTGCCCTTGACTCTGGGATAAGCACTGGAAACGGTGTCTA
ATACCGGATATATCAGCTGGCCGCATGGTCTGGTTGTGGAAAGAATTTTGGTCAAGGATGGACTCGCG
GCCTATCAGGTAGTTGGTGAGGTAATGGCTCACCAAGCCTACGACGGGTAGCCGGCCTGAGAGGGTGA
CCGGCCACACTGGGACTGAGACACGGCC
>Zotu3530
GAACGCTAGCGGCAGGCTTAATACATGCAAGTCGAGGGGCAGCACGAATAGCAATATTTGGTGGCGAC
CGGCAAACGGGTGCGGAACACGTACGCAACCTTCCCATAACTGGTGAATAGCCTTCCGAAAGGGAGAT

TAATACACCGTAACATAGTTGAGTGGCATCACTTGACTATTATAGCTTCGGCGGTTTTGGATGGGCGT
GCGCCTGATTAGGTAGTTGGCGGGGTAACGGCCCACCAAGCCTTCGATCAGTAACTGGTGTGAGAGCA
CGACCAGTCACACGGGCACTGAGACACGGGC
>Zotu3531
GAACGCTGGCGGCGTGCTTAACACATGCAAGTCGAGCGGTAAGGCCCTTCGGGGTACACGAGCGGCGA
ACGGGTGAGTAACACGTGGGTGACCTGCCCTCCACTTCGGGATAAGCCTGGGAAACTGGGTCTAATAC
CGGATAGGACCATTCCGCGCATGTGGGGTGGTGGAAAGTTTTTTCGGTGGGGGATGGGCCCGCGGTCT
ATCAGCTTGTTGGTGGGGTGATGGCCTACCAAGGCGACGACGGATAGCCGGCCTGAGAGGGTGATCGG
CCACACTGGGACTGAGACACGGCC
>Zotu3533
GAACGCTGGCGGCGTGCTTAACACATGCAAGTCGAACGATGAAGCCCTTCGGGGTGGATTAGTGGCGA
ACGGGTGAGTAACACGTGGGCAATCTGCCCTTCACTCTGGGACAAGCCCTGGAAACGGGGTCTAATAC
CGGATAACACTCTGTCCCGCATGGGGCGGGGTTGAAAGCTCCGGCGGTGAAGGATGAGCCCGCGGCCT
ATCAGCTTGTTGGTGGGGTAATGGCCTACCAAGGCGACGACGGGTAGCCGGCCTGAGAGGGCGACCGG
CCACACTGGGACTGAGACACGGCC
>Zotu3534
GAACGCTGGCGGCGTGCTTAACACATGCAAGTCGAACGTGAAAGTAGCAATACGAGTAAAGTGGCGTA
CGGGTGAGTAACACGTGGGTAATCTACCTTCAAGCCTGGAATAACGTGCCGAAAGGCGCGCTAATACT
GGATAGATCAAGCTTGTTGTAATGACAAGATTGGAAAAGGGAGCCGGCAATGGCCCTCACTTTTAGAT
GAGCCCGCGTCCCATTAGCTAGTTGGTAGGGTAAAGGCCTACCAAGGCGATGATGGGTAGCTGGTCTG
AGAGGATGATCAGCCACACTGGAACTGAAACACGGTC
>Zotu3535
GAACGCTGGCGGCATGCCTTACACATGCAAGTCGAACGGCAGCATAGGAGCTTGCTCCTGATGGCGAG
TGGCGAACGGGTGAGTAATATATCGGAACGTACCCAAGAGTGGGGGATAACGTAGCGAAAGTTACGCT
AATACCGCATACGATCTAAGGATGAAAGTGGGGGATCGCAAGACCTCATGCTCCTGGAGCGGCCGATA
TCTGATTAGCTAGTTGGTGGGGTAAAAGCTCACCAAGGCGACGATCAGTAGCTGGTCTGAGAGGACGA
CCAGCCACACTGGGACTGAGACACGGCC
>Zotu3537
GAACGCTGGCGGCATGCCTAACACATGCAAGTCGAACGAAGGCTTCGGCCTTAGTGGCGCACGGGTGC
GTAACGCGTGGGAATCTGCCCTTGGGTTCGGAATAACAGTGGGAAACTACTGCTAATACCGGATAATG
ACGTAAGTCCAAAGATTTATCGCCCAAGGATGAGCCCGCGTAGGATTAGCTAGTTGGTGGGGTAAAGG
CCTACCAAGGCGACGATCCTTAGCTGGTCTGAGAGGATGATCAGCCACACTGGGACTGAGACACGGCC
>Zotu3539
GAACGCTGGCGGCGTGCCTAATACATGCAAGTCGAGCGGACTGATGGGAGCTTGCTCCCTGATGTTAG
CGGCGGACGGGTGAGTAACACGTGGGCAACCTGCCCTATAGCTTGGGATAACTCCGGGAAACCGGGGC
TAATACCGGATAATACAATCTAGCTCCTGCTAGATTGTTGAAAGATGGTTTCGGCTATCGCTATAGGA
TGGGCCCGCGGCGCATTAGCTAGTTGGTAGGGTAATGGCCTACCAAGGCGACGATGCGTAGCCGACCT
GAGAGGGTGATCGGCCACACTGGGACTGAGACACGGCC
>Zotu3540
GAATGCTGGCGGCGTGGATAAGTCATGCAAGTCAAGGGGGCCCGCAAGGGCAACCGGCAGACGAGGTA
GTAACACGTAGGTACGTCCCCCGAAGTCGGGCATAGCCGGTCGAAAGACCGGGTAATTCCCGATAGTC
CCGCAAGGGTAAAGGTTTTTCGCTTCGGGAGCGGCCTGCGTAGTATCAGCTAGTTGGTAGGGTAATGG
CCTACCAAGGCTACGACGCTTAGGGGGGCTGAGAGGCCGATCCCCACCGATGGAACTGCGACACGGTC
>Zotu3541
GAACGCTAGCGGCAGGCCTAATACATGCAAGTCGAACGGTAACAGGGTAGCAATACCGCTGACGAGTG
GCGCACGGGTGCGTAACGCGTATGCAATCTACCTTCAACTAGTGAATAGCTCGAAGAAATTCGGATTA
ATACACCATAGAATTATTGATTGGCATCAATTAATAATTAAAGCTCAGGCGGTTGAAGATGAGCATGC
GTGACATTAGCTAGTTGGTGAGGTAACGGCTCACCAAGGCTACGATGTCTAGGGGATCTGAGAGGATT
AACCCCCACACTGGTACTGAGACACGGAC
>Zotu3542
GAACGCTGGCGGCATGCCTAACACATGCAAGTCGAACGAGACCTTCGGGTCTAGTGGCGCACGGGTGC
GTAACGCGTGGGAATCTGCCCTTAGGTACGGAATAACTCAGGGAAACTTGAGCTAATACCGTATAATG
ACTCCGGTCCAAAGATTTATCGCCTAGGGATGAGCCCGCGTAGGATTAGCTAGTTGGTGAGGTAAAGG
CTCACCAAGGCGACGATCCTTAGCTGGTCTGAGAGGATGATCAGCCACACTGGGACTGAGACACGGCC

>Zotu3543
GAACGCTGGCGGCGTGCTTAACACATGCAAGTCGAACGTGAGGGTGTAGCAATATGCCTGAAAGTGGC
GAACGGGTGAGTAACACGTAGGTAACCTGCCTTGAAGCGGGGGATAACGGCTGGAAACGGCTGCTAAT
ACCGCATATTGTGAGTGGAGTGGGAACTTCACTTGTGAAAATTTTGGTGCTTCAAGAGGGGCCTGCGT
CCGATTAGCTAGTTGGTGGGGTAAGAGCCTACCAAGGCGATGATCGGTAGCTGGTCTGAGAGGACGAT
CAGCCACACGGGAACTGAGAAACGGTC
>Zotu3544
GAACGCTGGTGGCGTGCCTAATACATGCAAGTCGAGCGAATCTTTGGGAGCTTGCTCCCAAAGGTTAG
CGGCGGACGGGTGAGTAACACGTGGGCAACCTGCCTATAAGACTGGGATAACTTCGGGAAACCGGAGC
TAATACCGGATAATTCTTTTCTACACATGTAGAAAAGCTAAAAGACGGTTTACGCTGTCACTTATAGA
TGGGCCCGCGGCGCATTAGCTAGTTGGTGAGGTAACGGCTCACCAAGGCCACGATGCGTAGCCGACCT
GAGAGGGTGATCGGCCACACTGGGACTGAGACACGGCC
>Zotu3545
GAACGCTGGCGGCATGCCTTACACATGCAAGTCGAACGGCAGCGCGGGAGCAATCCTGGCGGCGAGTG
GCGAACGGGTGAGTAATATATCGGAACGTGCCCATTAGTGGGGGATAGCCCGGCGAAAGCCGGATTAA
TACCGCATACGACCTGAGGGTGAAAGCGGGGGATCGCAAGACCTCGCGCTATTGGAGCGGCCGATATC
AGATTAGCTAGTTGGTGGGGTAAAAGCCTACCAAGGCAACGATCTGTAGTTGGTCTGAGAGGACGACC
AGCCACACTGGGACTGAGACACGGCC
>Zotu3546
GAACGCTGGCGGCGTGCCTAATACATGCAAGTCGAGCGAATCTTTGGGAGCTTGCTCCCAAAGATTAG
CGGCGGACGGGTGAGTAACACGTGGGCAACCTGCCTATAAGACTGGGATAACTTCGGGAAACCGGAGC
TAATACCGGATAATTCTTTTCTACTCATGTAGAAAAGCTAAAAGACGGTTTACGCTGTCACTTATAGA
TGGGCCCGCGGCGCATTAGCTAGTTGGTGAGGTAACGGCTCACCAAGGCCACGATGCGTAGCCGACCT
GAGAGGGTGATCGGCCACACTGGGACTGAGACACGGCC
>Zotu3547
AAACGCTAGCGGCAGGCCTAATACATGCAAGTCGAACGGGGTCCTTCGGGGCCCAGTGGCGCACGGGT
GCGTAACGCGTATGCAACCTACCTTGAACTGGGGGATAGCCCGGAGAAATCCGGATTAATACCGCATA
AGACCACGTGAGAACATCCGAGTGTGGTTAAAGATTCATCGGTTCAAGATGGGCATGCGTGCCATTAG
CTAGTTAGTGGTGTAACGGACCACCAAGGCGACGATGGCTAGGGGTTCTGAGAGGACGGTCCCCCACA
CTGGCACTGAGATACGGCC
>Zotu3548
GAACGCTGGCGGCAGGCTTAACACATGCAAGTCGAGCGGGGAAAAGTAGCTTGCTACTGGACCTAGCG
GCGGACGGGTGAGTAATGCTTAGGAATCTGCCTATTAGTGGGGGACAACATCTCGAAAGGGATGCTAA
TACCGCATACGTCCTACGGGAGAAAGCAGGGGACCTTCGGGCCTTGCGCTAATAGATGAGCCTAAGTC
GGATTAGCTAGTTGGTGGGGTAAAGGCCTACCAAGGCGACGATCTGTAGCGGGTCTGAGAGGATGATC
CGCCACACTGGGACTGAGACACGGCC
>Zotu3550
GAACGCTAGCTACAGGCTTAACACATGCAAGTCGAGGGGTAACATGACGGTGGCTTGCCATCGTTGAT
GACGACCGGCGCACGGGTGAGTAACGCGTATCCAACCTGGCCCACACTGGGGAATAGTCCCTCGAAAG
AGAGCTTAATGCCCCATGTTGCAGCATTGTCACATGTTGATGCTGCCAAAGGCTTGCCGGTATGGGAT
GGGGATGCGTCCGATTAGCTAGTTGGCGGGGTAACGGCCCACCAAGGCGACGATCGGTAGGGGTTCTG
AGAGGAAGGTCCCCCACATTGGTACTGAGACACGGAC
>Zotu3551
GAACGCTGGCGGCGTGCCTAATACATGCAAGTCGAGCGGACTTTCAAGAAGCTTGCTTTTTGAAAGTT
AGCGGCGGATGGGTGAGTAACACGTGGGCAACCTACCTGCAAGACGGGGATAACTCCGGGAAACCGGG
GCTAATACCGGATAATATCTTCCTTCGCATGAAGGAAGGTTGAAAGGCGGCGCAAACTGCCGCTTGCA
GATGGGCCCGCGGCGCATTAGCTAGTTGGTGAGGTAACGGCTCACCAAGGCGACGATGCGTAGCCGAC
CTGAGAGGGTGATCGGCCACACTGGGACTGAGACACGGCC
>Zotu3552
GAACGCTGGCGGCATGCCTAACACATGCAAGTCGAACGAAGGCTTCGGCCTTAGTGGCGCACGGGTGC
GTAACGCGTGGGAATCTGCCCTTGGGTTCGGAATAACTCCCCGAAAGGGGTGCTAATACCGGATGATG
TCGCAAGACCAAAGATTTATCGCCCAAGGATGAGCCCGCGTTGGATTAGCTAGTTGGTGAGGTAAAAG
CTCACCAAGGCGACGATCCATAGCTGGTCTGAGAGGATGATCAGCCACACTGGGACTGAGACACGGCC
>Zotu3555

GAACGCTGGCGGCAGGCCTAACACATGCAAGTCGAACGGACCCTTCGGGGTTAGTGGCGGACGGGTGA
GTAACACGTGGGAACGTGCCTTTAGGTTCGGAATAGCTCCTGGAAACGGGTGGTAATGCCGAATGTGC
CCTTCGGGGGAAAGATTTATCGCCTTTAGAGCGGCCCGCGTCTGATTAGCTTGTTGGTGAGGTAATGG
CTCACCAAGGCTACGATCAGTAGCTGGTCTGAGAGGATGACCAGCCACATTGGGACTGAGACACGGCC
>Zotu3556
GAACGCTGGCGGCATGCTTAACACATGCAAGTCGCACGCCCAGCAATGGGAGTGGCGGACGGGTGAGT
AACGCGTAGGAACGTATCCAGAGGTGGGGGACAACCCCGGGAAACTGGGGCTAATACCGCATATGGGC
TGAGGCCCAAAGCAGTGATGCGCCTTTGGAGCGGCCTGCGTCCGATTAGGTTGTTGGTGGGGTAATGG
CCTACCAAGCCTGCGATCGGTAGCTGGTCTGAGAGGATGATCAGCCACACTGGGACTGAGACACGGCC
>Zotu3557
GAACGCTAGCGGCAGGCCTAATACATGCAAGTCGAACGGTAACATGAGTAGCAATACTTGATGACGAG
TGGCGCACGGGTGCGTAACGCGTATGCAATCTACCTTCAACCGGTGAATAGCCCGAAGAAATTCGGAT
TAATACACCATAATATATAGAAGTGGCATCACTTTTATATTAAAACTCCGGTGGTTGAAGATGAGCAT
GCGTGACATTAGCTAGTTGGTGAGGTAATGGCTCACCAAGGCTACGATGTCTAGGGGATCTGAGAGGA
TTAACCCCCACACTGGTACTGAGACACGGAC
>Zotu3558
GAACGCTGGCGGCGTGCTTAACACATGCAAGTCGAGCGGTAAGGCTCCTTCGGGAGTACACGAGCGGC
GAACGGGTGAGTAACACGTGAGCAATCTGCCCTTCACACTGGGATAACCTTCGGAAACGAGGACTAAT
ACCGGATACGACCACTTCAGACATCTGATGGTGGTGGAAAGCTCCGGCGGTGGAGGATGAGCTCGCGG
CCTATCAGCTTGTTGGTGAGGTAACGGCTCACCAAGGCTTCGACGGGTAGCCGGCCTGAGAGGGTGAC
CGGCCACACTGGGACTGAGACACGGCC
>Zotu3559
GAACGCTGGCGGCGTGCCTAACACATGCAAGTCGAGGGAGAAAGCCGCAAGGCGAGTAAACCGGCGAA
CGGGTGCGTAACACGTGGACAACCTACCTTCTCGACGGGGATAACTGGCCGAAAGGCTTGCTAATACC
CGATAAGCCCACGGAGTCTTAGGACTCAGAGGGAAAAGGCGGCCTCTGTAAACAAGCTGTCACGAGAA
GATGGGTCCGCGGCCCATCAGCTAGTTGGCGGGGTAACGGCCCACCAAGGCAACGACGGGTAGCTGGT
CTGAGAGGATGATCAGCCACACTGGAACTGAGACACGGTC
>Zotu3560
GAACGCTGGCGGCAGGCTTAACACATGCAAGTCGAACGCCCCGCAAGGGGAGTGGCAGACGGGTGAGT
AACGCGTGGGAATCTACCCAGTTCTACGGAACAACTGAGGGAAACTTCAGCTAATACCGTATACGCCC
TACGGGGGAAAGATTTATCGGAATTGGATGAGCTCGCGTTGGATTAGCTAGTTGGTGGGGTAATGGCC
TACCAAGGCGACGATCCATAGCTGGTCTGAGAGGATGATCAGCCACACTGGGACTGAGACACGGCC
>Zotu3561
GAACGCTGGCGGCGTGCTTAACACATGCAAGTCGAACGATGAAGCCCTTCGGGGTGGATTAGTGGCGA
ACGGGTGAGTAACACGTGGGCAATCTGCCCTTCACTCTGGGACAAGCCCTGGAAACGGGGTCTAATAC
CGGATGATACTTCTCCTCGCATGGGGGGAGGTTGAAAGCTCCGGCGGTGAAGGATGAGCCCGCGGCCT
ATCAGCTTGTTGGTGAGGTAGTGGCTCACCAAGGCGACGACGGGTAGCCGGCCTGAGAGGGCGACCGG
CCACACTGGGACTGAGACACGGCC
>Zotu3562
GAACGCTAGCTACAGGCTTAACACATGCAAGTCGAGGGGAAACGGCATTTGGTGCTTGCACCATTTGG
ATGTCGACCGGCGCACGGGTGAGTAACGCGTATCCAACCTGCCCATAAGTAGGGTATAACCCGCAGAA
ATGCGGACTAATCCCCTATGTTGTTCAAAGACGGCATCAGATTTGAACCAAAGGCTTGCCGCTTATGG
ATGGGGATGCGTCCGATTAGCTTGTTGGTGAGGTAACGGCTCACCAAGGCTTCGATCGGTAGGGGTTC
TGAGAGGAAGGCCCCCCACATAGGAACTGAGACACGGTC
>Zotu3564
GAACGCTAGCGGCAGGCCTAATACATGCAAGTCGAACGGTTCGCAAGGACAGTGGCAAACGGGTGCGT
AACGCGTAAGCAACCTGCCTCATACTGGGGGATAGCCCGGCGAAAGCTGGGGTAAACCCGCACGGTTC
CATTGTTTCACCTGGGGTGATGGATAAACATTTATGGGTATGAGAGGGGCTTGCGTCTGATTAGCTAG
TTGGGGGGGTAACGGCCCACCAAGGCGATGATCAGTAGGGGTTCTGAGAGGATTGGCCCCCACATGGG
TACTGAGATACGGAC
>Zotu3565
GAACGCTGGCGGCATGCCTAACACATGCAAGTCGAACGAGAGGACCCCGCAAGGGGTTAGTAAAGTGG
CGCACGGGTGAGTAACACGTGGGTAATCTGCCCTTTAGTTTGGGATAACCCTTCGAAAGAAGGGCTAA
TACCGGAGAGACCACAGGGACATCAGTCCTAGGGGTTGAAGATGGGGACCGCAAGGCCTATCGCTAAA

GGATGAGCCCGCATACTATCAGCTAGTTGGTGAGGTAATGGCTCACCAAGGCTATGACGGTTAGCTGG
TCTGAGAGGATGATCAGCCACACTGGAACTGAGACACGGTC
>Zotu3567
GAACGCTAGCGGGAGGCTTAACACATGCAAGCCGAGGGGTATAGTTCTTTCGGGGACTAGAGACCGGC
GCACGGGTGCGTAACGCGTATGCAACTTGCCCTACTGAAAAGGATAGCCCTTCGAAAGGAGGATTAAT
ACTTTATAACAGATTTAATGGCATCATTAGATTTTGAAAGATTTATCGCAGTAGGATAGGCATGCGTA
AGATTAGTTAGTTGGTGAGGTAACGGCTCACCAAGACGATGATCTTTAGGGGGCCTGAGAGGGTGAAC
CCCCACACTGGTACTGAGACACGGAC
>Zotu3568
GAACGCTGGCGGCGTGCTTAACACATGCAAGTCGAACGGAAAGGCCCTTCGGGGTACTCGAGTGGCGA
ACGGGTGAGTAACACGTGGGTGATCTGCCCTGCACTTCGGGATAAGCCTGGGAAACTGGGTCTAATAC
CGGATAGGACTACGGCCTGCATGGGTTGTGGTGGAAAGCTTTTGCGGTGTGGGATGGGCCCGCGGCCT
ATCAGCTTGTTGGTGGGGTGATGGCCTACCAAGGCGACGACGGGTAGCCGGCCTGAGAGGGTGACCGG
CCACACTGGGACTGAGATACGGCC
>Zotu3569
GAACGCTGGCGGCGTGCCTAATACATGCAAGTCGAGCGGCAGCGCAGGGTGCTTGCACCCTGGCGGCG
AGCGGCGGACGGCTGAGTAACGCGTAGGAACGTACCCCAAAGTGAGGAATAACTGCCCGAAAGGGTGG
CTAATGCCGCATGTGCTCTTCGGAGTAAAGCTTCGGCGCTTTGGGAACGGCCTGCGTCCGATTAGCTA
GTTGGTAAGGTAATGGCTTACCAAGGCTACGATCGGTAGCTGGTCTGAGAGGATGATCAGCCAGACTG
GGACTGAGAACGGCC
>Zotu3570
GAACGCTGGCGGCATGCCTAATACATGCAAGTCGAGCGAATGATGAAGAAGCTTGCTTCTTCTGATTT
AGCGGCGGACGGGTGAGTAACACGTGGGTAACCTACCTTACAGATTGGGATAACTTCGGGAAACCGGA
GCTAATACCGAATAACACATTTCTTCTCAGGAAGAGATGTTGAAAGACGGTTTCGGCTGTCACTGTAA
GATGGGCCCGCGGCGCATTAGCTAGTTGGTGAGGTAACGGCTCACCAAGGCAACGATGCGTAGCCGAC
CTGAGAGGGTGATCGGCCACACTGGGACTGAGACACGGCC
>Zotu3571
GAACGCTGGCGGCATGCCTAACACATGCAAGTCGAACGAGACCTTCGGGTCTAGTGGCGCACGGGTGC
GTAACGCGTGGGAACCTGCCCTTAGGTTCGGAATAACTCCCCGAAAGGGGTGCTAATACCGGATAATG
TCTTCGGACCAAGGATTTATCGCCTTTGGATGGGCCCGCGTTGGATTAGCTAGTTGGTGAGGTAAAAG
CTCACCAAGGCGACGATCCATAGCTGGTCTGAGAGGATGATCAGCATCACTGGGACTGAGACACGGCC
>Zotu3572
GAACGCTGACAGAATGCTTAACACATGCAAGTCAACTTGAATTTGGGTTTTTAACTTAGATTTGGGTG
GCGGACGGGTGAGTAACGCGTAAAGAACTTGCCTCACAGCTAGGGACAACATTTAGAAATGAATGCTA
ATACCTGATATTATGATTTTAAGGCATCTTAGAATTATGAAAGCTATAAGCACTGTGAGAGAGCTTTG
CGTCCCATTAGCTAGTTGGAGAGGTAACAGCTCACCAAGGCGATGATGGGTAGCCGGCCTGAGAGGGT
GAACGGCCACAAGGGGACTGAGACACGGCC
>Zotu3575
GAACGCTGGCGGCGCGCCTAAAACATGCAAGTCGAACGAGAAGCCCGGGCTTGCCCGGGTGGACAGTG
GCGAACGGGTGAGTAACACGTGAGGAACCTGCCCCGAAGACCGGGACAACACCGGGAAACCGGTGCTA
ATACCGGATACCTTCATCGGACCGCATGATCTGATGAAGAAATGGATTCCGCTTCGGGATGGCCTCGC
GGCCTATCAGCTTGTTGGTGGGGTAATGGCCCACCAAGGCAACGACGGGTAGCTGGTCTGAGAGGATG
TCCAGCCACACTGGGACTGAGACACGGCC
>Zotu3576
GAACGCTGGCGGCGTGCTTAACACATGCAAGTCGAGCGGAAAGGCCCTTCGGGGTACTCGAGCGGCGA
ACGGGTGAGTAACACGTGAGTAACCTGCCCTGGACTTTGGGGTAACCCTCGGAAACGGGGGCTAATAC
CGGATATGACTTTTCCTCGCATGGGGATTGGTGGAAAGTTTTTCGGTCTGGGATGGGCTCGCGGCCTA
TCAGCTTGTTGGTGGGGTAATGGCCTACCAAGGCGACGACGGGTAGCCGGCCTGAGAGGGCGACCGGC
CACACTGGGACTGAGACACGGCC
>Zotu3577
GAACGCTGGCGGCGTGCTTAACACATGCAAGTCGAACGATGAAGCTGGAGCTTGCTCCGGTGGATTAG
TGGCGAACGGGTGAGTAACACGTGAGCAATCTGCCCTTGACTCTGGGATAACTGCGGGAAACTGTAGC
TAATACCGGATACGAAGCCTTGGGGCATCCCAAGGTTTGGAAAGAATTTCGGTCAAGGATGAGCTCGC
GGCCTATCAGCTAGTTGGTGAGGTAATGGCTCACCAAGGCGACGACGGGTAGCCGGCCTGAGAGGGTG

ACCGGCCACACTGGAACTGAGACACGGTC
>Zotu3578
GAATGCTGGCGGTGTGGATAGGGCATGCAAGTCAAGGGGACCGCAAGGTAACCGGCAGACGAGATAGT
AACGCGTAGGAACGTACCCAGAAGTCGGGCACAGCTCGCCGAAAGGCGGGGTAATTCCCGATGGTGAC
GAAAGTCTAAAGATTTATCGCTTCTGGAGCGGCCTGCGTAATATCAGCTAGTTGGTGAGGTAATGGCT
CACCAAGGCTATGACGTTTAGGGGAGCTGAGAGGCTGACCCCCATCGATGGAATTGAGATACGGTC
>Zotu3579
GAACGCTAGCGGCAGGCCTAATACATGCAAGACGAACGGCAAGATTTTTAGCAATAAGAGTCCTAGAG
TGTCGTAAGGGTGCGTAACACGTATGCAATCTACCCTCAACAGGAGGATAGCTCCCCGAAAGGGGAAT
TAACACTCCATAAGATCATGGTCCGGCATCGGACAGTGATTAAAACTACGGTGGTTGAGGATGAGCAT
GCGCATGATTAGTTAGTTGGCGGGGTAATGGCCCACCAAGACGATGATCATTAGGGGAACTGAGAGGT
TGATCCCCCACACTGGCACTGAGATACGGGC
>Zotu3580
GAACGCTGGCGGCAGGCTTAACACATGCAAGTCGAGCGCACTCATTTAGAGTGAGCGGCAGACGGGTG
AGTAACGCGTGGGAATCTACCCTTTTCTACGGAATAACACAGAGAAATTTGTGCTAATACCGTATACG
TCCTACTGGAGAAAGATTTATCGGAGAAGGATGAGCCCGCGTTGGATTAGCTAGTTGGTGAGGTAAAG
GCTCACCAAGGCGACGATCCATAGCTGGTCTGAGAGGATGATCAGCCACACTGGGACTGAGACACGGC
C
>Zotu3581
GAACGCTAGCGGCAGGCTTAACACATGCAAGTCGAGGGGTAGGGTAGCAATACCTGAGACCGGCGCAC
GGGTGCGTAACGCGTATGCAATCTACCTTTTACAGAGGGATAGCCCAGAGAAATTTGGATTAATACCT
CATAGTATAGTACAATCGCATGATTGAACTATTAAAGTCACAACGGTAAAAGATGAGCATGCGTCCCA
TTAGCTAGTTGGTAAGGTAACGGCTTACCAAGGCGACGATGGGTAGGGGTCCTGAGAGGGAGATCCCC
CACACTGGTACTGAGGCACGGAC
>Zotu3582
GAACGCTAGCGGCAGGCTTAACACATGCAAGTCGAGGGGTATATCATTTCGGTGATAGAGACCGGCGC
ACGGGTGCGTAACGCGTATGCAATCTACCTTTTACAGGGGAATAGCCCAGAGAAATTTGGATTAATGC
CCCATAGTATTATGAAATGGCATCATTTTATAATTAAAGTTCCAACGGTAAAAGATGAGCATGCGTCC
CATTAGTTAGTTGGTAAGGTAACGGCTTACCAAGACGATGATGGGTAGGGGTCCTGAGAGGGAGATCC
CCCACACTGGTACTGAGACACGGAC
>Zotu3583
AAACGCTGGCGGCGTGCGTAACACATGCAAGTCGAACGAGAATGTACTGACGGATCCTTAGGGTGAAG
ATAGTACAGGACAGTGGCGGATGGGTGAGTAACGCGTAAGAAACCTGCCTTTCACACCGGGATAGCAG
CTGGAAACGGCTATTAATACCGGATGACACTTTTTCCCCGCATGAGGAAGAGGTTAAAGAATTTCGGT
GAAAGATGGTCTTGCGTCTGATTAGCTAGTTGGTGGGGTAACGGCCTACCAAGGCGACGATCAGTAGC
CGGCCTGAGAGGGTGTACGGCCACATTGGGACTGAGACACGGCC
>Zotu3585
GAATGCTGGCGGTGTGGATAGGGCATGCAAGTCAAGGGGACTCAGTTAGTAATAATTGGGTAACCGGC
AGACGAGATAGTAACGCGTAGGAACGTACCCAGAAGTCGGGCACAGCTCGCCGAAAGGCGGGGTAATT
CCCGATGGTGTCGCAAGACTAAAGATTTATCGCTTCTGGAGCGGCCTGCGTAATATCAGCTTGTTGGT
GAGGTAATGGCTCACCAAGGCTATGACGTTTAGGGGAGCTGAGAGGCTGACCCCCATCGATGGAATTG
AGATACGGTC
>Zotu3586
GAACGCTGGCGGCGTGCTTAACACATGCAAGTCGAACGATGATGCCCTGCTTGCAGGGTGGATTAGTG
GCGAACGGGTGAGTAACACGTGGGTGATCTGCCTCGTACTTCGGGATAAGCCTGGGAAACTGGGTCTA
ATACCGGATAGGACCTTCCTTTAGTGTGGAAGGTGGAAAGTTTTTTCGGTACGAGATGAGCCCGCGGC
CTATCAGCTTGTTGGTGGGGTAATGGCCTACCAAGGCGACGACGGGTAGCCGGCCTGAGAGGGTGTAC
GGCCACATTGGGACTGAGACACGGCC
>Zotu3587
GAACGCTGGCGGCGTGCCTAATACATGCAAGTCGAGCGAATCTTTGGGAGCTTGCTCCCGAAGGTTAG
CGGCGGACGGGTGAGTAACACGTGGGCAACCTGCCTGTAAGACTGGGATAACACCGGGAAACCGGTGC
TAATACCGGATAATTCTTTTCCTCTCATGAGGAAAAGCTGAAAGTCGGTTTCGGCTGACACTTACAGA
TGGGCCCGCGGCGCATTAGCTAGTTGGTGAGGTAACGGCTCACCAAGGCGACGATGCGTAGCCGACCT
GAGAGGGTGATCGGCCACACTGGGACTGAGACACGGCC

>Zotu3588
GAACGCTAGCGGCAGGCTTAATACATGCAAGGCGAGGGGGCAGCAATGTCACCGTCGTACGGGTGCGT
AACGCGTATGCAACCTACCTATCACTGGGGGATAGCCCGGGGAAACCCGGATTAATACCGCATAACAC
AGGGGTCCCGCATGGGTACTATTTGTTAAAGATTTATCGGTGGTAGATGGGCATGCGTTCGATTAGCT
AGTTGGTGCAGGTAACGGCTCACCAAGGCGACGATCGATAGGGGAGCTGAGAGGTTGATCCCCCACAC
GGGCACTGAGATACGGGC
>Zotu3589
GAACGCTAGCGGCAGGCTTAATACATGCAAGGCGAGGGGGCAGCAATGTCACCGTCGTACGGGTGCGT
AACGCGTATGCAACCTACCTATCACTGGGGGATAGCCCGGGGAAACCCGGATTAATACCGCATAACAC
AGGGGCTCCACATGGAGATATTTGTTAAAGATTTATCGGTGGTAGATGGGCATGCGTTCGATTAGCTA
GTTGGCAGGGTAACGGCCTACCAAGGCGACGATCGATAGGGGAGCTGAGAGGTTGATCCCCCACACGG
GCACTGAGATACGGGC
>Zotu3590
GAACGCTGGCGGCATGCTTAACACATGCAAGTCGAACGGGAAGTGGTGTTTCCAGTGGTGAACGGGTG
AGTAACGCGTAAGAACCTGCCCTTGGGAGGGGAACAACAACTGGAAATGGTTGCTAATACCCTATAGG
CTGAGGAGCAAAAGGAGAAATCCGCCCAAGGAGGGGCTCGCGTCTGATTAGCTAGTTGGTGAGGTAAT
AGCTTACCAAGGCGATGATCAGTAGCTGGTCCGAGAGGATGATCAGCCACAATGGGACTGAGACACGG
CC
>Zotu3591
GAACGCTGGCGGCGTGCTTAACACATGCAAGTCGAACGAGAACGGATTATAGCTTGCTATAATTGTCA
GCTAAGTGGCGCACGGGTGAGTAATGTATAGGTAATATGCCTCTTACTAAGGGATAACAATTGGAAAC
GATTGCTAATACCTTATACTCCTTATTAACCTAAGTTAATAAGGGAAAGATTTATTGGTAAGAGATTA
GCCTGTATTGTATCAGTTAGTTGGTGGGGTAATGGCCTACCAAGACTATGACACATAACTGGTTTGAG
AGGATGATCAGTCACACTGGAACTGAGACACGGTC
>Zotu3592
TAACGCTAGCAGGATGCCTAACACATGCAAGTCGAACGAGGTAGCAATACCTAGTGGCAAACGGGTGA
GTAATACATGCTCAATCTACCCATAAGAGGGAGATAACAGCTCGAAAGAGTTGCTAATATCCCATAAC
ATGTAGAAACATAAGTTTTTACATCTAAAGTTGCGTATGCAACACTTTTGGATGAGGGTGTGTCTTAT
CAGATAGTTGGTGAGGTAACGGCCCACCAAGTCGATGACGAGTAGCTGTGCTGAGAGGCAGAACAGCC
ACAATGGGACTGAGACACGGTC
>Zotu3593
GAACGCTGGCGGTATGCCTTATACATGCAAGTCGAACGGGAGTTTTTAACTCTAGTGGCGGACGGGTG
AGTAACACGTAAGAATTTGCCTTTAGGAGGGGGACAACAACTGGAAACGGTTGCTAATACCCCATATG
CTTTCGAGTAAAATGGATTTCCGCCTAAAGAGAAGCTTGCGGCTGATTAGCTTGTTGGTAGGGTAATG
GCCTACCAAGGCGACGATCAGTATCTGGTTTGAGAGGACGATCAGACACACTGGAACTGAGACACGGT
C
>Zotu3594
GAACGCTAGCGGCAGGCTTAATACATGCAAGTCGAGGGGCAGCATGAGTGTAGCAATACATTTGATGG
CGACCGGCAAACGGGTGCGGAACACGTACAGAACCTTCCTTCGAGCGGGGAATAGCCCAGAGAAATTT
GGATTAATACCCCATAGTATATTGAAGAGGCATCTCTTTAATATTAAAGATTTATCACTTGAAGATGG
CTGTGCGGCTGATTAGATAGTTGGCGGGGTAATGGCCCACCAAGTCTACGATCAGTAACTGGTGTGAG
AGCACGACCAGTCACACGGGCACTGAGACACGGGC
>Zotu3596
GAATGCTGGCGGCGTGTCTAAGGCATGCAAGTCGAATGTCTAGTTTACTAGGCATGGCAAACGGGAGA
GTAACACATTGATAACCTACCCTGAAGACGGGGATAACCTTGCGAAAGCGAGAATAATACCCGATAGT
ATGAAACGGCATGAGCTGTTTCATTAAAGCTACGACGCTTCAGGAGGGGTCTATGGCCTATCAGCTTG
TTGGTGAGGTAATGGCTCACCAAGGCAACGACGGGTATCGGGCGCGAGAGCGTGACCCGACTCACTGG
GACTGAGAAACTACC
>Zotu3598
GAACGCTGGCGGCGTGCCTAATACATGCAAGTCGAGCGGTAAGGCTCTCACTCTGTACTTTAGTTTCT
AGAGTATGGTGTGAGAGTACACGAGCGGCGGACGGCTGAGTAACGCGTAGGAACGTACCCCAAAGTGA
GGGATAGCCCACCGAAAGGTGGATTAATACCGCATGTGCTCTTCGGAGTAAAGCTTTATGCGCTTTGG
GAACGGCCTGCGTATGATTAGTTTGTTGGTGAGGTAATGGCTCACCAAGACTACGATCATTAGCTGGT
CTGAGAGGATGATCAGCCAGACTGGGACTGAGACACGGCC

>Zotu3599
GAACGCTAGCGGCAGGCCTAATACATGCAAGTCGAACGGTAAGTTGAATAGCAATATTTGACCTAGAG
TGACGTACGGGTGCGTAACACGTATGCAACCTACCCTTTACAGGAGGATAGCTCGGGGAAACTCGAAT
TAACCCTCCATGAGATCATAGAGTAGCATTACTCAGTGATTAAAACTCAGGTGGTAAAGGATGGGCAT
GCGTCTGATTAGTTAGTTGGCGGGGTAACGGCCCACCAAGACGATGATCAGTAGGGGAACTGAGAGGT
TGATCCCCCACACTGGCACTGAGATACGGTC
>Zotu3600
GAACGCTGGCGGCGTGCTTAACACATGCAAGTCGAACGGAAAGGCCTGTGCTTGCACGGGTACTCGAG
TGGCGAACGGGTGAGTAACACGTGGGTGATCTGCCCTTCACTTCGGGATAAGCCTGGGAAACTGGGTC
TAATACTGGATAGGACCATCGTTTAGTGTCGGTGGTGGAAAGTTTTTCGGTGGAGGATGAGCTCGCG
GCCTATCAGCTTGTTGGTGGGGTAATGGCCTACCAAGGCGACGACGGGTAGCCGGCCTGAGAGGGTGG
ACGGCCACATTGGGACTGAGACACGGCC
>Zotu3601
GAATGCTGGCGGTGTGGATAGGGCATGCAAGTCAAGGGGACCGCAAGGTAACCGGCAGACGAGATAGT
AACGCGTAGGAACGTACCCAGAAGTCGGGCACAGCTCGCCGAAAGGCGGGGTAATTCCCGATATTGTC
GCGAGATGAAAGATTTATCGCTTCTGGAGCGGCCTGCGTAATATCAGCTAGTTGGTGAGGTAATGGCT
CACCAAGGCTATGACGTTTAGGGGAGCTGAGAGGCTGACCCCCATCGATGGAATTGAGATACGGTC
>Zotu3602
GGTGGTGCTCCCCGCAGCTCACCTGGCAGCCCCAGGGCACCTGCTGTGCCTCCCTGGTCTCTTCGTCC
CACCGCCCTTGCCAGCTCCGCATTGGGTGGCACAT
>Zotu3603
GAACGCTGGCGGCGTGCTTAAGACATGCAAGTCGAACGCAGAGTGAGTAGCAATACTGGCTCTGAGTG
GCGAATGGGTGCGTAACACGTAGGTGACCTGCCCTGAAGTGGGGAATAACAGTCCGAAAGGATTGCTA
ATACCGCATACGTTCTTGGGCGTTAGAGAACCAAGCACAAAGGCTGGCGTAAGCCAGTCGCTTCAGGA
GGGGCCTGCGGCCCATCAGTTAGTTGGTAGGGTAACGGCCTACCAAGACAACGACGGGTAGGGGACCT
GAGAGGGTGGCCCCCCACACTGGCACTGACACACGGGC
>Zotu3604
GAACGCTGGCGGCGTGCCTAATACATGCAAGTCGAGCGAATCTGAGGGAGCTTGCTCCCAAAGATTAG
CGGCGGACGGGTGAGTAACACGTGGGCAACCTGCCTGTAAGACTGGGATAACTTCGGGAAACCGGAGC
TAATACCGGATAACATATGAGACCACATGGTCTTATATTAAAAGATGGCTTTTAGCTATCACTTACAG
ATGGGCCCGCGGCGCATTAGCTAGTTGGTGAGGTAACGGCTCACCAAGGCAACGATGCGTAGCCGACC
TGAGAGGGTGATCGGCCACACTGGGACTGAGACACGGCC
>Zotu3605
GAACGCTGGCGGCGTGCCTAATACATGCAAGTCGAGCGAACCAATAGAAGCTTGCTTCTATTGGTTAG
CGGCGGACGGGTGAGTAACACGTGGGCAACCTACCTGTAAGACTGGGATAACTTCGGGAAACCGAAGC
TAATACCGGATAACTCTTCTTTACACATGTAGAGAAGCTAAAAGACGGTTTCGGCTGTCACTTACAGA
TGGGCCCGCGGCGCATTAGCTAGTTGGTGAGGTAACGGCTCACCAAGGCAACGATGCGTAGCCGACCT
GAGAGGGTGATCGGCCACACTGGGACTGAGACACGGCC
>Zotu3607
GAACGCTGGCGGCGTGCCTAATACATGCAAGTCGAGCGGATCTTGTCCTTCGGGATAAGGTTAGCGGC
GGACGGGTGAGTAACACGTGGGTAACCTGCCCATAAGACTGGGATAACATTCGGAAACGAATGCTAAT
ACCGGATACGCGAAGCGGTCGCATGATCGAATCGGGAAAGGCGGAGCAATCTGCCACTTATGGATGGA
CCCGCGGCGCATTAGCTAGTTGGTGGGGTAACGGCTCACCAAGGCGACGATGCGTAGCCGACCTGAGA
GGGTGATCGGCCACACTGGGACTGAGACACGGCC
>Zotu3608
GAACGCTGGCGGCGTGCTTAACACATGCAAGTCGAGCGAGAAAGGCTCTTCGGAGCTGAGTACAGCGG
CGAACGGGTGAGTAACACGTGGGCAACCTGCCCCAAGGAGCGGGACAACCCGGGGAAACTCGGGCTAA
TACCGCATGAATCGCCGGCCCTCAAGGGTCTGCGGGAAAAGGTAGCTTCGGCCTCCGCCTTGGGATGG
GCCCGCGGCGCATTAGCTTGTTGGTGAGGTAACGGCTCACCAAGGCGACGATGCGTAGCTGGTCTGAG
AGGACGATCAGCCACACTGGGACTGAGACACGGCC
>Zotu3609
GAACGCTGGCGGCAGGCTTAACACATGCAAGTCGAACGGTAACATAAAGAAGCTTGCTTCTTTGATGA
CGAGTGGCGGACGGGTGAGTAATGCTTGGGAATCTAGCTTATGGAGGGGGATAACTACGGGAAACTGT
AGCTAATACCGCGTAATATCGGAAGATTAAAGTGTGGGACCTTCGGGCCACATGCCATAGGATGAGCC

CAAGTGGGATTAGGTAGTTGGTGAGGTAAAGGCTCACCAAGCCGACGATCTCTAGCTGGTCTGAGAGG
ATGACCAGCCACACTGGGACTGAGACACGGCC
>Zotu3610
GAACGCTAGCGGCAGGCCTAACACATGCAAGTCGAGGGGTATTGGTTTTCGGACCAAGAGACCGGCGC
ACGGGTGCGTAACGCGTATGCAATCTACCTTTTACAGGGGAATAGCCCAGAGAAATTTGGATTAATGC
CCCATAGTATATATCATTCGCATGTTTGATATATTAAAGTTCCAACGGTAAAAGATGAGCATGCGTCC
CATTAGTTAGTTGGTAAGGTAACGGCTTACCAAGACGATGATGGGTAGGGGTCCTGAGAGGGAGATCC
CCCACACTGGTACTGAGACACGGAC
>Zotu3611
GAACGCTGGCGGCGTGCCTAATACATGCAAGTAGAACGCTGAAGAGAGGGGCTTGCTCTTCTTGGATG
AGTTGCGAACGGGTGAGTAACGCGTAGGTAACCTGCCTGGTAGCGGGGGATAACTATTGGAAACGATA
GCTAATACCGCATAAAATGGATTATCGCATGATAATCCATTGAAAGGTGCAAATGCATCACTACCAGA
TGGACCTGCGTTGTATTAGCTAGTTGGTGGGGTAACGGCTCACCAAGGCGACGATACATAGCCGACCT
GAGAGGGTGATCGGCCACACTGGGACTGAGACACGGCC
>Zotu3612
GAACGCTGGCGGCGTGCTTAACACATGCAAGTCGAACGATGAAGCCCTTCGGGGTGGATTAGTGGCGA
ACGGGTGAGTAACACGTGGGCAATCTGCCCTTCACTCTGGGACAAGCCCTGGAAACGGGGTCTAATAC
CGGATAACACTCTGTCCCGCATGGGACGGGGTTAAAAGCTCCGGCGGTGAAGGATGAGCCCGCGGCCT
ATCAGCTTGTTGGTGGGGTGATGGCCTACCAAGGCGACGACGGGTAGCCGGCCTGAGAGGGCGACCGG
CCACACTGGGACTGAGACACGGCC
>Zotu3613
GAACGCTGGCGGCGTGCTTAACACATGCAAGTCGAACGGGCCGTTTTCGGACGGTCAGTGGCAGACGG
GTGCGTAACACGTGGGTGACCTGCCCTCAAGTGGGGAACAACGCGACTAACGTCGCGCTAATACCGCA
TAAGTTCGCTTCTTCGGTGGGGCGAGTAAAGCAGCAATGCGCTTGGGGATGGGCCTGCGGCGGATTAG
GTAGTTGGTGGGGTAACGGCCTACCAAGCCGATGATCCGTAGGGGGCGTGAGAGCGTGACCCCCCACA
TTGGCACTGAGACACGGGC
>Zotu3614
GAACGCTGGCGGCGCGCCTAACACATGCAAGTCGAGGGAGAACCACATCTTCGGATGTGGGGAGACCG
GCGAACGGGTGAGTAACACGTGAGGAACCTGCCCTGTAGATCGGAATAACCGCGGGAAACTGCGGCTA
ATGCCGAATGATCTCGCCGCTCCGCATGGAGCAGCGAGGAAATGGTCTGCCGCTACAGGATGGCCTCG
CGGCCTATCAGCTTGTTGGTGAGGTAACGGCTCACCAAGGCGACGACGGGTAGCTGGCGTGAGAGCGC
GACCAGCCACACTGGGACTGAGACACGGCC
>Zotu3617
GAACGCTGGCGGCGTGCTTAACACATGCAAGTCGAGCGGTAAGGCCCTTCGGGGTACACGAGCGGCGA
ACGGGTGAGTAACACGTGAGCAATCTGCCCTTCACATCGGGATAACCCCCGGAAACGGGAGCTAATAC
CGGATACGACCACTTCAGGCATCTGATGGTGGTGGAAAGTTCCGGCGGTGGAGGATGAGCTCGCGGCC
TATCAGCTTGTTGGTGAGGTAATGGCTCACCAAGGCTTCGACGGGTAGCCGGCCTGAGAGGGTGACCG
GCCACACTGGGACTGAGACACGGCC
>Zotu3618
GAACGCTGGCGGTGTGCTTAACACATGCAAGTCGAACGGCAAGAGCTGTAGCAATACAGAATTCTGC
TAGTGGCAAACGGGTGAGTAATACATGGGAATCTACCTTGCAGTGCGAAATAACAGTTGGAAGCAATT
GCTAATGTCGCATATAGCCGAGAGGTGAAAGATTTATCGCTGCAAGACGAGCCCATGCAGGATTAGCT
AGTTGGTGGGGTAATGGCTCACCAAGGCGACGATACGTAGCCGACCTGAGAGGGTGATCGGCCACACT
GGAACTGAGACACGGTC
>Zotu3619
GAACGCTAGCGGCAGGCCTAACACATGCAAGTCGAGGGAGGAGCCTTCGGGCTTCGACCGGCGTACGG
GTGCGTAACGCGTATACAATCTGCCTTTCACATAGGGATAGCCCGAAGAAATTTGGATTAATACCTAA
TGGTATATAGGAGTGGCATCACATTTATATTAAAGCTGAGGCGGTGAAAGATGAGTATGCGTTCTATT
AGCTAGTTGGAGAGGTAACGGCTCCCCAAGGCTACGATAGATAGGGGTTCTGAGAGGGATATCCCCCA
CACTGGTACTGAGATACGGAC
>Zotu3621
GAACGCTGGCGGCGTGCTTAACACATGCAAGTCGAGCGGTAAGGCCCTTCGGGGTACACGAGCGGCGA
ACGGGTGAGTAACACGTGAGCAATCTGCCCTCCACATCGGGATACCCCCCGGAAACGGGAGCTAATAC
CGGATACGACCACTTCAGGCATCTGATGGTGGTGGAAAGTTCCGGCGGTGGAGGATGAGCTCGCGGCC

TATCAGCTTGTTGGTGGGGTAACGGCCCACCAAGGCTTCGACGGGTAGCCGGCCTGAGAGGGTGACCG
GCCACACTGGGACTGAGACACGGCC
>Zotu3623
GAACGCTGGCGGCGTGCTTAACACATGCAAGTCGAACGATGATGCCCAGCTTGCTGGGTGGATTAGTG
GCGAACGGGTGAGTAACACGTGAGTAACCTGCCCCTGACTCTGGGATAAGCGTTGGAAACGACGTCTA
ATACTGGATACGACTACCGGCCGCATGGTCTGGTGGTGGAAAGATTTTTTGGTTGGGGATGGACTCGC
GGCCTATCAGCTTGTTGGTGAGGTAATGGCTCACCAAGGCGACGACGGGTAGCCGGCCTGAGAGGGTG
ACCGGCCACACTGGGACTGAGACACGGCC
>Zotu3624
GAACGCTAGCGGCAAGCTTAATACATGCAAGTCGAGGGGCAGCATGAGTGTAGCAATACATTTGATGG
CGACCGGCAAACGGGTGCGGAACACGTACAGAACCTTCCTTCGAGCGGGGAATAGCCCAGAGAAATTT
GGATTAATACCCCATAGTATTACGGAGTGGCATCACTTTGTAATTAAAGATTTATCACTTGAAGATGG
CTGTGCGGCTGATTAGATAGTTGGCGGGGTAACGGCCCACCAAGTCTGCGATCAGTAACTGGTGTGAG
AGCACGACCAGTCACACGGGCACTGAGACACGGGC
>Zotu3625
GAACGCTAGCTACAGGCTTAACACATGCAAGTCGAGGGGAAACGACATCGAAAGCTTGCTTTTGATGG
GCGTCGACCGGCGCACGGGTGAGTAACGCGTATCCAACCTGCCCACCACTTGGGGATAACCTTGCGAA
AGTAAGACTAATACCCAATGACGTCTCTAGAAGACATCTGAAAGAGATTAAAGATTTATCGGTGATGG
ATGGGGATGCGTCTGATTAGCTTGTTGGCGGGGTAACGGCCCACCAAGGCAACGATCAGTAGGGGTTC
TGAGAGGAAGGTCCCCCACATTGGAACTGAGACACGGTC
>Zotu3626
GAACGCTGGCGGCATGCTTTACACATGCAAGTCGAACGGCAGCGCGGGGCAACCTGGCGGCGAGTGGC
GAACGGGTGAGTAATATATCGGAACGTACCCAGAAGTGGGGGATAACGTAGCGAAAGTTACGCTAATA
CCGCATACGATCTACGGATGAAAGTGGGGGATCGCAAGACCTCATGCTTTTGGAGCGGCCGATATCTG
ATTAGCTAGTTGGTGAGGTAAAGGCTCACCAAGGCGACGATCAGTAGCTGGTCTGAGAGGACGACCAG
CCACACTGGGACTGAGACACGGCC
>Zotu3627
GAACGCTGGCGGCAGGCTTAACACATGCAAGTCGAGCGCCTCGCAAGAGGAGCGGCAGACGGGTGAGT
AACGCGTGGGAATCTACCCATCTCTACGGAACAACTCCGGGAAACTGGAGCTAATACCGTATACGTCC
TTCGGGAGAAAGATTTATCGGAGATGGATGAGCCCGCGTTGGATTAGCTAGTTGGTGGGGTAATGGCC
TACCAAGGCGACGATCCATAGCTGGTCTGAGAGGATGATCAGCCACATTGGGACTGAGACACGGCC
>Zotu3628
GAACGCTGGCGGCGTGCCTAATACATGCAAGTCGAGCGAATCTGAGGGAGCTTGCTCCCAAAGGTTAG
CGGCGGACGGGTGAGTAACACGTGGGCAACCTGCCTGTAAGACTGGGATAACTTCGGGAAACCGGAGC
TAATACCGGATAATTCTTTTCTTCACATGAAGGAAAGCTGAAAGATGGTTTCGGCTATCACTTACAGA
TGGGCCCGCGGCGCATTAGCTAGTTGGTGAGGTAACGGCTCACCAAGGCAACGATGCGTAGCCGACCT
GAGAGGGTGATCGGCCACACTGGGACTGAGACACGGCC
>Zotu3629
GAACGCTGGCGGCATGCCTAACACATGCAAGTCGAACGAGACCTTCGGGTCTAGTGGCGCACGGGTGC
GTAACGCGTGGGAATCTGCCCTTAGGTTCGGAATAACAGTTAGAAATGACTGCTAATACCGGATAATG
ACGTAAGTCCAAAGATTTATCGCCTAGGGATGAGCCCGCGTAAGATTAGCTTGTTGGTGAGGTAAAGG
CTCACCAAGGCGACGATCTTTAGCTGGTCTGAGAGGATGATCAGCCACACTGGGACTGAGACACGGCC
>Zotu3630
GAACGCTGGCGGCGTGCCTAATACATGCAAGTCGAGCGAATGACGAGAGAAGCTTGCTTCTCTCTGAT
TTAGCGGCGGACGGGTGAGTAACACGTGGGTAACCTACCTTATAGATTGGGATAACTCCGGGAAACCG
GGGCTAATACCGAATAATACTTTTAAACACATGTTTAAAAGTTGAAAGACGGTCTTGCTGTCACTATA
AGATGGACCCGCGGCGCATTAGCTAGTTGGTGAGGTAACGGCTCACCAAGGCAACGATGCGTAGCCGA
CCTGAGAGGGTGATCGGCCACACTGGGACTGAGACACGGCC
>Zotu3631
GAACGCTGGCGGCATGCCTAACACATGCAAGTCGAACGAGACCTTCGGGTCTAGTGGCGCACGGGTGC
GTAACGCGTGGGAATCTACCCTTGGGTTCGGAATAACAGCTAGAAATGGCTGCTAATACCGGATGATG
TCTTCGGACCAAAGATTTATCGCCCAGGGATGAGCCCGCGTAGGATTAGCTTGTTGGTGAGGTAAAAG
CTCACCAAGGCGACGATCCTTAGCTGGTCTGAGAGGATGATCAGCCACACTGAGACTGAGACACGGCC
>Zotu3632

GAACGCTGGCGGCATGCTTTACACATGCAAGTCAAACGGCAGCGCGGGGCAACCTGGCGGCGAGTGGC
GAACGGGTGAGTAATATATCGGAACATACCCAAGAGTGGGGGATAACGTAGCGAAAGTTACGCTAATA
CCGCATACGATCTAAGGATGAAAGTGGGGGATCGCAAGACCTCATGCTCCTGGAGCGGCCGATATCTG
ATTAGCTAGTTGGTGAGGTAAAGGCTCACCAAGGCGACAATCAGTAGCTGGTCTGAGAGGACGACCAG
CCACACTGGGACTGAGACACGGCC
>Zotu3633
GAACGCTGGCGGCGTGCCTAATACATGCAAGTCGAACGCTTCACTTCGGTGAAGAGTGGCGAACGGGT
GAGTAATACATAAGTAACCTGGCATCTACAGGGGGATAACTGATGGAAACGTCAGCTAAGACCGCATA
GGTGTAGAGATCGCATGAACTCTATATGAAAAGTGCTACGGGACTGGTAGATGATGGACTTATGGCGC
ATTAGCTAGTTGGTAGGGTAACGGCCTACCAAGGCGACGATGCGTAGCCGACCTGAGAGGGTGACCGG
CCACACTGGGACTGAGACACGGCC
>Zotu3634
GAACGCTGGCGGCGTGCCTAATACATGCAAGTCGAGCGAATCAAAGGGAGCTTGCTCCCTGAGATTAG
CGGCGGACGGGTGAGTAACACGTGGGCAACCTGCCTATAAGACTGGGATAACTTCGGGAAACCGGAGC
TAATACCGGATAATTCATATCTACTCATGTAGGTATGCTGAAAGACGGTTTACGCTGTCACTTATAGA
TGGGCCCGCGGCGCATTAGCTAGTTGGTGAGGTAACGGCTCACCAAGGCAACGATGCGTAGCCGACCT
GAGAGGGTGATCGGCCACACTGGGACTGAGACACGGCC
>Zotu3635
GAACGCTGGCGGAATGCTTTACACATGCAAGTCGAGCGGCAGCACGGGGGCAACCCTGGTGGCGAGCG
GCGAACGGGTGAGTAATACATCGGAACATGCCCAGTCGTGGGGGATAACACTTCGAAAGAAGTGCTAA
TACCGCATACGTCCTGAGGGAGAAAGCGGGGGATCGCAAGACCTCGCGCGATTGGAGTGGCCGATGTC
AGATTAGCTAGTTGGTGGGGTAAAGGCCTACCAAGGCAACGATCTGTAGCTGGTCTGAGAGGATGATC
AGCCACACTGGGACTGAGACACGGCC
>Zotu3636
TAACGCTGGCGGCGTGCATAACACATGCAAGTCGAACGATGAAACTTAGTAGACTTCTTCGGAATGAC
ACTAAGCGAATTAGTGGCGAACGGGTGAGTAACGCGTGAGTAACCTGCCTTACACAAGGGGATAGCCT
TTGGAAACGAAGAATAATACCCTATAAAACCATAAAAGCACATGCTAGAATGGTCAAAGTGCTAGCGG
TGTAAGATGGACTTGCGTCTGATTAGCTAGTTGGTGAGATAAAAGCTCACCAAGGCAACGATCAGTAG
CCGGCTTGAGAGAGTGTACGGCCACATTGGGACTGAGACACGGCC
>Zotu3637
GAACGCTTGCGACATGCCTAAGAAATGCAAGTCGAGCGGGGGTACGGCTTCGGTTGTACCTTTAGCGG
CGAACGGTCGCGTAACACGTAGGCAACCTGCCTGCAAGACCGGGACAACAGTTCGAAAGGACTGCTAA
TACCGGATGTGTTCACGGGGAGGCATCTTCCTGTGAATAAATCCCTTACGGGGCTTGTGGAGGGGCCT
GCGGCCTATCAGCTAGATGGTGGGGTAACAGCCTACCATGGCGACGACGGGTAGCTGGTCTGAGAGGA
TGGTCAGCCGGATTGGGACTGAGACACGGCC
>Zotu3639
GAACGCTAGCGGCAGGCTTAATACATGCAAGGCGAGGGGGCAGCAATGTCACCGTCGTACGGGTGCGT
AACGCGTATGCAACCTACCTTCAACTGGGGGATAGCCCGGGGAAACCCGGATTAATACCGCATAATAC
ATTGATATAGCATTATATTAATTGTTAAAGATTTATTGGTTGAAGATGGGCATGCGTTCGATTAGCTA
GTTGGTATGGGTAACGGCCTACCAAGGCAACGATCGATAGGGGAGCTGAGAGGTTGATCCCCCACACG
GGCACTGAGATACGGGC
>Zotu3640
GAACGCTAGCGAGAGGCCTAACACATGCAAGTCGAGGGGTAACAGGGGAGCTTGCTCCCGCTGACGAC
TGGCGGATGGGTGAGTAACGCGTATGCAACCTGCCTTTAACTGTGGGATAGCTCCCTGAAAGGGGAAT
TAATACCGCATAAGACCACGTTGTTTCATGACAGAGTGGTAAAATATTTATAGGTTAAAGATGGGCAT
GCGTGACATTAGCTAGTTGGTGAGGTAACGGCTCACCAAAGCTACGATGTCTAGGGGTTCTGAGAGGA
AGGTCCCCCACACTGGTACTGAGACACGGAC
>Zotu3641
GAACGCTAGCTACAGGCTTAACACATGCAAGTCGAGGGGAAACGACATCGAAAGCTTGCTTTTGATGG
GCGTCGACCGGCGCACGGGTGAGTAACGCGTATCCAACCTGCCCATCACTTGGGGATAACCTTGCGAA
AGTAAGACTAATACCCAATGATATCTCTAGAAGACATCTGAAAGAGATTAAAGATTTATCGGTGATGG
ATGGGGATGCGTCTGATTAGCTTGTTGGCGGGGTAACGGCCCACCAAGGCGACGATCAGTAGGGGTTC
TGAGAGGAAGGTCCCCCACATTGGAACTGAGACACGGTC
>Zotu3642

GAACGCTGGCGGCGTGGATTAGGCATGCAAGTCGAACGAGAAAGGGTTCGCAAGAACCCGAGTAGAGT
GGCGAAAGGGTGAGTAGTACCTAGGTAACCTGCCCGGAAGACGGGGACAACGTCGTGAAAACGACGCT
AATACCCGATATGTCGGCGAGATCGCATGGTATTGCCGGCAAACGGTAGCCTCTATTTATAAGCTTCC
GCTTTCGGAGGGGCCTCGGGCCTATCAGCTAGTTGGCGGGGTAACGGCCCACCAAGGCAGGGACGGGT
AGCCGGTCTGAGAGGATGGTCGGCCACACTGGGACTGAGACACTGCC
>Zotu3644
GAACGCTGGCGGCACGCTTAACACATGCAAGTCGAACGAAGTTTTGGAAGTTTACTTTTAAAACTTAG
TGGCGGACGGGTGAGTAACACGTGAGCAATCTGCCTTTGAGTGGGGAATACCGGTTGGAAACGACTGT
TAATACCGCATAAAATACTTAAGCCGCATGACTTTAGTATCAAAGATTTATCGCTTGAAGATGAGCTC
GCGTCTGATTAGCTAGTTGGTGAGGTAACGGCTCACCAAGGCGACGATCAGTAGCCGGACTGAGAGGT
TGAACGGCCACATTGGGACTGAGACACGGCC
>Zotu3645
GAACGCTGGCGGCGTGCCTTAAGCATGCAAGTCGTACGGCTCCGCAAGGAGCAGTGGCGGACGGTCGA
GTAACGCGTGGGGATCTACCTTGAAGTTGGACATAGCTCGTCGAAAGGCGGGGTAATTTCCAATGTGA
ACGCAAGTTCAAAGCCGCAAGGCGCTTTAAGATGAACCCGCGTCCTATCAGCTAGTTGGTGAGGTAAA
GGCTCACCAAGGCATCGACGGGTAGCTGGTCTGAGAGGATGATCAGCCGGAATAGCACTGAGACACGG
GC
>Zotu3646
GAACGCTGGCGGCGTGCTTAACACATGCAAGTCGAACGATGATCCGGAGCTTGCTTCGGGGATTAGTG
GCGAACGGGTGAGTAACACGTGAGTAACCTGCCCTTGACTCTGGGATAAGCGCTGGAAACGGCGTCTA
ATACCGGATACGACCTACCCCGGCATCGGGTGTGGGTGGAAAGTTTTTCGGTCAAGGATGGACTCGCG
GCCTATCAGCTTGTTGGTGAGGTAATGGCTCACCAAGGCGACGACGGGTAGCCGGCCTGAGAGGGTGA
CCGGCCACACTGGGACTGAGACACGGCC
>Zotu3647
GAACGCTAGCGGCAGGCCTAATACATGCAAGTCGGACGGGATCCAGGAGCTTGCTTCTGGTGAGAGTG
GCGCACGGGTGCGTAACACGTATGTAACCTACCCTTGTCAGGGGGATAGCCTCTCGAAAGAGAGATTA
AGACCGCATAACATTATGATGTGGCATCACAAAATAATCAAATATTTATAGGACAAGGATGGGCATGC
GGAACATTAGCTAGTTGGTAAGGTAACGGCTTACCAAGGCTACGATGTTTAGGGGATCTGAGAGGATG
ACCCCCCACACTGGTACTGAGACACGGAC
>Zotu3650
GAACGCTGGCGGCATGCCTAACACATGCAAGTCGAACGAGATCTTCGGATCTAGTGGCGCACGGGTGC
GTAACGCGTGGGAATCTGCCTTGGGGTTCGGAATAACTCCCCGAAAGGGGTGCTAATACCGGATGATG
TCGAGAGACCAAAGATTTATCGCCCTGAGATGAGCCCGCGTAGGATTAGCTAGTTGGTGTGGTAAAGG
CGCATCAAGGCGACGATCCTTAGCTGGTCTGAGAGGATGATCAGCCACACTGGGACTGAGACACGGCC
>Zotu3651
GAACGCTGGTAACGTGGATACCGGCATGCAAGTCACGGGGGCCGCAAGGCAACCGGCGAACGGCTTAG
TAACACGTAGATACATACCGCAAAGTGGAGCATAGCTCGTCGAAAGACGGGGTAATTCTCCATAGTCC
CGCAAGGGTAAAGCAGCAATGCGCTTTGTGAATGGTCTGCGGCCTATCAGATAGTTGGCGAGGTAAAA
GCTCACCAAGTCTATGACGGGTAGGGGGCGTGAGAGCGCGACCCCCAAGAATGGCACTGCGACACGGG
C
>Zotu3652
GAACGCTGGCGGCGTGCTTAACACATGCAAGTCGAGCGGAAAGGCCCTTTCGGGGGTACTCGAGCGGC
GAACGGGTGAGTAACACGTGAGTAATCTGCCCTTCACTTGGGGATAACCACCGGAAACGGTGGCTAAT
ACCCGATACGACGTAGTCTCGCATGAGATCTGCGTGGAAAGTTTTTTCGGTGGGGGATGTGCTCGCGG
CCTATCAGCTTGTTGGTGGGGTAATGGCCTACCAAGGCTTCGACGGGTAGCCGGCCTGAGAGGGTGAC
CGGCCACACTGGGACTGAGACACGGCC
>Zotu3653
GAACGCTGGCGGCATGCCTAATACATGCAAGTCGAGCGAATGATGAAGAAGCTTGCTTCTTCTGATTT
AGCGGCGGACGGGTGAGTAACACGTGGGTAACCTACCTTACAGATTGGGATAACTTCGGGAAACCGGA
GCTAATACCGAATAATACATTTCTTTTCATGAAGAGATATTGAAAGACGGTTTCGGCTGTCACTGTAA
GATGGGCCCGCGGCGCATTAGCTAGTTGGTGAGGTAACGGCTCACCAAGGCAACGATGCGTAGCCGAC
CTGAGAGGGTGATCGGCCACACTGGGACTGAGACACGGCC
>Zotu3654
GAACGCTGGCGGCGTGCCTAACACATGCAAGTCGAACGAGGTCCATGCAGTGGCAACACTGCGGAAGA

CCTAGTGGCGAACGGGTGAGTAACACGTGAGGAACCTGCCCCGAAGACCGGGATAACCCTCCGAAAGG
AGAGCTAATACCGGATACCCCCATCTGATCGCATGATCGGATGAGGAAATGGATTCCGCTTCGGGAGG
GCCTCGCGGCCTATCAGCTAGTTGGTGGGGTAACGGCCCACCAAGGCATCGACGGGTAGCTGGTCTGA
GAGGACGATCAGCCACACTGGGACTGAGACACGGCC
>Zotu3655
GAACGCTGGCGGCATGCTTAACACATGCAAGTCGCACGGATGGGTTTCGGCCTGTCAGTGGCGGACGG
GTGAGTAACGCGTAGGGATTTATCCATGGGTGGGGGATAACCGCGGGAAACTGCGGCTAATACCGCAT
GAAGCCTGAGGGCCAAAGGCGCGAGTCGCCTGTGGAGGAGCCTGCGTTCGATTAGCTTGTTGGTGGGG
TAAAGGCCTACCAAGGCGATGATCGATAGCTGGTCTGAGAGGATGATCAGCCACACTGGGACTGAGAC
ACGGCC
>Zotu3656
GAACGCTGGCGGCGTGCTTAACACATGCAAGTCGAACGGGGACCATGAAGTGGCAACACTTCGGAAGT
CCGAGTGGCGAACGGGTGAGTAACACGTGAGGAACTTGTCCCGGTCTCTGGGATAACAGTTGGAAACG
ACTGCTAATACCGGATGACGTCGGAGCACCGCATGGTGCACTGACGAAAGGGTTACTGGATCGGGAGA
GCCTCGCGACCTATCAGCTAGTTGGTGGGGTAACGGCCTACCAAGGCATCGACGGGTAGCTGGTCTGA
GAGGATGATCAGCCACACTGGGACTGAGACACGGCC
>Zotu3657
GAACGCTGGCGGCGTGCTTAACACATGCAAGTCGAACGTGAAAGTAGCAATATGAGTAAAGTGGCGTA
CGGGTGAGTAACACGTGGGTAATCTACCAAAGAGTCTGGAATAACCGGCCGAAAGGCTGGCTAATACT
GGATAGTTCAAGCGCACTGTAGAGGTGTGATTGGGAAAGGGAGCTGGCAACAGCCTTCGCTTTTTGAT
GAGCCCGCGTCTGATTAGCTAGTTGGTAAGGTAATGGCTTACCAAGGCGACGATCAGTAGCTGGTCTG
AGAGGATGATCAGCCACACTGGAACTGAAACACGGTC
>Zotu3658
GAACGCTGGCGGCGTGCTTAATACATGCAAGTCAACTGAACTGTGAGTAGCAATATTTGCAGGGAGGT
GGCGCACGGGTGCGTAACACGTAGGTAATCTGCCTTCAGGTCTGACATAACCCACCGAAAGGTGAGCT
AATATCAGATGATGCAGCGGCTTGACATCAAGACAGTTGTTAAAGATTTATCGCCTGAAGATGAGCCT
GCGCCTGATTAGGTAGTTGGCGGGGTAATGGCCCACCAAGCCTTCGATCAGTAGCTGGTCTGAGAGGA
TGATCAGCCACACTGGAACTGAGACACGGTC
>Zotu3663
GAACGCTAGCGGCAGGCTTAACACATGCAAGTCGAGGGGCAGCGCGGAGTAGCAATACTTTGGCGGCG
ACCGGCGGAAGGGTGCGTAACGCGTGAGCAACATGCCCGTCTTTGGGAGATAACCGATGGAAACGTCG
CCTAATATCCCATAACAACTGAGGAGGCATCTTTTCAGTTTGAAAGTTTCGATGAAGACGGATTGGTT
CGCGTGACATTAGCTAGTTGGAGTGGTAACGGCACACCAAGGCGACGATGTCTAGGGGTTCTGAGAGG
AAGGTCCCCCACACTGGAACTGAGACACGGTC
>Zotu3665
GAACGCTGGCGGCGTACTTAACACATGCAAGTCGAACGGGGAGTGCATCGGTAGCAATACTGATGGGC
TTCTAGTGGCGCACGGGTGAGTAACACGTGAGTGACCTGCCCTGAGGTCCGGGATACCCCTTCGAAAG
GAGGGCTAATACCGGGTGCGATTCCCTGGCTGCGAGGCTGGGGAAGGAAAGGCCGTGGCAACACGGTC
GCCGCAGGAGGGGCTCGCGGCCTATCAGCTAGTTGGCGGGGTAACGGCCCACCAAGGCGATGACGGGT
AGCTGGTCTGAGAGGACGACCAGCCACACGGGGACTGAGACACGGCC
>Zotu3666
GAACGCTAGCGGCAGGCTTAACACATGCAAGTCGAGGGGTATGTTTCTTCGGAAGCAGAGACCGGCGC
ACGGGTGCGTAACGCGTATGCAATCTACCTTTTACAGAGGGATAGCCCAGAGAAATTTGGATTAATAC
CTCATAGTATTATGAAATGGCATCATTTTATAATTAAAGTCACAACGGTAAAAGATGAGCATGCGTCC
CATTAGCTAGTTGGTAAGGTAACGGCTTACCAAGGCTACGATGGGTAGGGGTCCTGAGAGGGAGATCC
CCCACACTGGTACTGAGACACGGAC
>Zotu3667
GAACGCTGGCGGCGTGCCTAATACATGCAAGTCGAGCGAATGAATGGGAGCTTGCTCCCTGACATTAG
CGGCGGACGGGTGAGTAACACGTGGGCAACCTGCCCTGCAGATGGGGATAACTCCGGGAAACCGGGGC
TAATACCGAATAATCAATTCCTCCGCATGGAGGAATTCTGAAAGACGGCTTCGGCTGTCACTGCAGGA
TGGGCCCGCGGCGCATTAGCTAGTTGGTGGGGTAACGGCCTACCAAGGCGACGATGCGTAGCCGACCT
GAGAGGGTGATCGGCCACACTGGGACTGAGACACGGCC
>Zotu3668
GAACGCTGGCGGCATGCCTAATACATGCAAGTCGAGCGGAACACTTGGAGCTTGCTCCAAGCGTTTAG

CGGCGGACGGGTGAGTAACACGTGGGCAACCTGCCCTGCAGATCGGGATAACTCCGGGAAACCGGTGC
TAATACCGAATAGTTTTCGGCCCCTCCTGGGGCCGTACGGAAAGACGGCATCTCGCTGTCACTGCAGG
ATGGGCCCGCGGCGCATTAGCTAGTTGGTGGGGTAACGGCCCACCAAGGCGACGATGCGTAGCCGACC
TGAGAGGGTGATCGGCCACACTGGGACTGAGACACGGCC
>Zotu3669
GAACGCTGGCGGCAGGCCTAACACATGCAAGTCGAACGCCGTAGCAATACGGAGTGGCAGACGGGTGA
GTAACGCGTGGGAACCTACCTTACGGTCGGGAATAGCTCAGGGAAACTTGGGGTAATACCGGATAAGC
CCTTACGGGGAAAGATTTATCGCCGTTAGATGGGCCCGCGTAGGATTAGCTAGTTGGTGAGGTAATGG
CTCACCAAGGCGACGATCCTTAGCTGGTCTGAGAGGATGATCAGCCACACTGGGACTGAGACACGGCC
>Zotu3671
GAACGCTGGCGGCGTGCCTAATACATGCAAGTCGAGCGGACAGATGGGAGCTTGCTCCCTGATGTTAG
CGGCGGACGGGTGAGTAACACGTGGGTAACCTGCCTGTAAGACTGGGATAACTCCGGGAAACCGGGGC
TAATACCGGATGGTTGTTTGAACCGCATGGTTCAAACATAAAAGGTGGCTTCGGCTACCACTTACAGA
TGGACCCGCGGCGCATTAGCTAGTTGGTGAGGTAACGGCTCACCAAGGCAACGATGCGTAGCCGACCT
GAGAGGGTGATCGGCCACACTGGGACTGAGACACGGCC
>Zotu3673
GAACGCTGGCGGCAGGCTTAACACATGCAAGTCGAGCGGGCACTTCGGTGTCAGCGGCAGACGGGTGA
GTAACACGTGGGAACGTACCCTTCGGTTCGGAATAACTCTGGGAAACTAGAGCTAATACCGGATACGT
CCGAGAGGAGAAAGGTTTACTGCCGAAGGATCGGCCCGCGTCTGATTAGCTAGTTGGTGGGGTAATGG
CCTACCAAGGCGACGATCAGTAGCTGGTCTGAGAGGATGATCAGCCACATTGGGACTGAGACACGGCC
>Zotu3674
GAACGCTGGCGGCGTGCTTAACACATGCAAGTCGAGCGGTAAGGCCCTTCGGGGTACACGAGCGGCGA
ACGGGTGAGTAACACGTGAGCAATCTGCCCTTCACTCTGGGATAACCCCCGGAAACGGGAGCTAATAC
CGGATATGACCACTACACGCATGTGTTGGTGGTGGAAAGTTCTGGCGGTGGAGGATGAGCTCGCGGCC
TATCAGCTTGTTGGTGGGGTAATGGCCCACCAAGGCTTCGACGGGTAGCCGGCCTGAGAGGGTGACCG
GCCACACTGGGACTGAGACACGGCC
>Zotu3675
GAACGCTGGCGGCGTGCTTAACACATGCAAGTCACGGGGGCCCAGTAGCAATACTGGGTAACCGGCGA
ACGGGTGCGTAACACGTGAGCAATCTGCCGATTTCTGGGGGATAGCCGGCCCAACGGCCGGGTAATAC
CGCATAATCTCATTGGGGGACATCCCCTGATGAGCAAACCTCCGGGGGAAATCGAGGAGCTCGCGCCC
TATCAGCTTGTTGGCGGGGTAATGGCCCACCAAGGCTATGACGGGTAGCTGGTCTGAGAGGATGGCCA
GCCACATTGGGACTGCGACACGGCC
>Zotu3676
GAACGCTGGCGGCAGGCCTAACACATGCAAGTCGAACGCCCTAGCAATAGGGAGTGGCAGACGGGTGA
GTAACGCGTGGGAACGTACCCTTCGGTTCGGAATAAGCCGGGGAAACCTGGTCTAATACCGGATACGT
GCGAGAGCAGAAAGATTTATCGCCGAAGGATCGGCCCGCGTCCGATTAGCTAGTTGGTGAGGTAATGG
CTCACCAAGGCGACGATCGGTAGCTGGTCTGAGAGGATGATCAGCCACACTGGGACTGAGACACGGCC
>Zotu3678
GAACGCTGGCGGCGCGCCTAACACATGCAAGTCGAACGGTTAAAGCCCCTTCGGGGGTGCATAAAGTG
GCGAACGGCTGAGTAACACGTGGGCAACCTGCCCCTCGCACCGGGACAGCCTCGGGAAACCGTGGTTA
ATACCGGATACGCCCCCCTGAGGACATCCTCTCGGGGGGAAAGCCCCGACGGCGAGGGATGGGCCCGC
GGCCTGTTAGCTTGTTGGTGGGGTAACGGCCCACCAAGGCGATTATGGGTAGCTGGGTTGAGAGACCG
ACCAGCCAGATTGGGACTGAGACACGGCC
>Zotu3679
GCCGTGTCTCAGTCCCAGTGTGGCTGATCGTCCTCTCAGACCAGCTACGGATCGTCGCCTTGGTAGGC
CATTACCCTACCAACTAGCTAATCCGACGCAGGCTCATCTCATAGCACGAGGTCCGAAGATCCCCCGC
TTTGCTTTGAAGAGATTATGCGGTATTAGCCCGAGTTTCCCCGGGTTATCCCCCACTAACTATTATAA
TTAAGGTAGATTCCTACGTGTTACTCACCCGTCCGCCACTCGCCGTCAGGGAGCAAGCTCCCCCCGCT
GCCGTTCGACTTGCATGTGTTAGGCATGCCGCTAGCGTTCA
>Zotu3680
GAACGCTAGCGGCAGGCCTAACACATGCAAGTCGAGGGGTAGAGGAAGCTTGCTTCCTTGAGACCGGC
GCACGGGTGCGTAACGCGTATGCAATCTACCTTATACAGAGGGATAGCCCAGAGAAATTTGGATTAAT
ACCTCATAGTATTATGAAATCGCATGGTTTTATAATTAAAGTTCCAACGGTATAAGATGAGCATGCGT
CCCATTAGTTAGTTGGTAAGGTAACGGCTTACCAAGACAATGATGGGTAGGGGTCCTGAGAGGGAGAT

CCCCCACACTGGTACTGAGACACGGAC
>Zotu3681
GAACGCTGGCGGCAGGCCTAACACATGCAAGTCGAGCGGATGACGGGAGCTTGCTCCTTGATTCAGCG
GCGGACGGGTGAGTAATGCCTAGGAATCTGCCTGGTAGTGGGGGACAACGTTCCGAAAGGAGCGCTAA
TACCGCATACGTCCTACGGGAGAAAGTGGGGGATCTTCGGACCTCACGCTATCAGATGAGCCTAGGTC
GGATTAGCTAGTTGGTGAGGTAAAGGCTCACCAAGGCGACGATCCGTAACTGGTCTGAGAGGATGATC
AGTCACACTGGAACTGAGACACGGTC
>Zotu3682
GAACGCTGGCGGCAGGCTTAACACATGCAAGTCGAACGATGACTAACTAGCTTGCTAGATATGATTAG
TGGCGGACGGGTGAGTAATATTTAGGAATCTGCCTAGTAGTGGGGGATAGCTCGGGGAAACTCGAATT
AATACCGCATACGACCTACGGGTGAAAGGGGGCGCAAGCTCTTGCTATTAGATGAGCCTAAATCAGAT
TAGCTAGTTGGTGGGGTAAAGGCCCACCAAGGCGACGATCTGTAACTGGTCTGAGAGGATGATCAGTC
ACACCGGAACTGAGACACGGTC
>Zotu3683
GAACGCTAGCTACAGGCCTAACACATGCAAGTCGTGGGGCAGCGGGTATGCTTGTTTTCAAGTATATG
CCGGCGACCGGCGCACGGGTGAGTAACGCGTATCCAACCTGCCCATCACAAGGGAATAACCCGTCGAA
AGTCGGCCTAATGCCCTATGTATTCCGATGAAGTCCTCTGAATCGGAATAAAGCATTTGCGGTGATGG
ATGGGGATGCGTCTGATTAGCTTGTCGGCGGGGTAACGGCCCACCGAGGCGACGATCAGTAGGGGTTC
TGAGAGGAAGGTCCCCCACATTGGAACTGAGACACGGTC
>Zotu3684
GAACGCTGGCGGCGTGCCTAATACATGCAAGTCGAGCGAACCAATAGGAGCTTGCTCCTGTTAGTTAG
CGGCGGACGGGTGAGTAACACGTGGGCAACCTGCCTGTAAGACTGGGATAACACCGGGAAACCGGTGC
TAATACCGGATAATCCTTTTCCTCTCATGAGGAAAAGCTGAAAGTCGGTGCAAGCTGACACTTACAGA
TGGGCCCGCGGCGCATTAGCTAGTTGGTGAGGTAACGGCTCACCAAGGCGACGATGCGTAGCCGACCT
GAGAGGGTGATCGGCCACACTGGGACTGAGACACGGCC
>Zotu3685
GAACGCTGGCGGCGTGCTTAACACATGCAAGTCGAGCGGAAAGGCCTCCTTCGGGGGGTACTCGAGCG
GCGAACGGGTGAGTAACACGTGAGTAATCTGCCCTTCACTTCGGGATAGCTCTCGGAAACGGGGATTA
ATACCGGATATGACTGCGCTACGCATGTGGTGTGGTGGAAAGTTTTTTTCGGTGGGGGATGTGCTCGC
GGCCTATCAGCTTGTTGGTGGGGTAATGGCCTACCAAGGCTTCGACGGGTAGCCGGCCTGAGAGGGTG
ACCGGCCACACTGGGACTGAGACACGGCC
>Zotu3686
AAACGCTGGCGGCGCACATAAGACATGCAAGTCGAACGGACTTAACTCATTCTTTTAGTTTGAGAGCG
GTTAGTGGCGGACTGGTGAGTAACACGTAAGCAACCTGCCTATCAGAGGGGAATAACAGTGAGAAATC
ACTGCTAATACCGCATAAGCTAGTAGTATCGCATGATACAGCTAGAAAAGGAGCAATCCGCTGATAGA
TGGGCTTGCGTCTGATTAGCTAGTTGGTGGGGCAACGGCCTACCAAGGCGACGATCAGTAGCCGAACT
GAGAGGTTGAACGGCCACATTGGGACTGAGATACGGCC
>Zotu3687
GAACGCTGGCGGCGTGCTTAACACATGCAAGTCGAACGGTGAAAGAGAGCTTGCTCTCTGGATCAGTG
GCGAACGGGTGAGTAACACGTGAGCAATCTGCCCCTGACTCTGGGATAACAGTTGGAAACAGCTGCTA
ATACCGGATACGAGACGTGAAGGCATCTTCAACGTCTGGAAAGAACTTCGGTCAGGGATGAGCTCGCG
GCCTATCAGCTAGTTGGTGAGGTAATGGCTCACCAAGGCGTCGACGGGTAGCCGGCCTGAGAGGGTGA
CCGGCCACACTGGGACTGAGACACGGCC
>Zotu3688
GAACGCTAGCGGCAGGCCTAATACATGCAAGTCGAACGTGATTGTTGTAGCAATATGACATGAAAGTG
GCGCACGGGTGCGTAACACGTATGCAATCTACCTTTAATAGGAGAATAGCTTCGAGAAATCGGAATTA
ATGCTCCATAACATTACTAAATGGCATCATTTGGTAATCAAACCTCCGGGGATTAAAGATGAGCATGC
GCAATATTAGCTAGTTGGTAAGGTAATGGCTTACCAAGGCTACGATATTTAGGGGGTCTGAGAGGATG
ATCCCCCACACTGGTACTGAGACACGGAC
>Zotu3689
GAACGCTGGCGGCGTGCCTAATACATGCAAGTCGAGCGAATTGATGGGAGCTTGCTCCCTGATATTAG
CGGCGGACGGGTGAGTAACACGTGGGCAACCTGCCCTGCAGATGGGGATAACTCCGGGAAACCGGGGC
TAATACCGAATAATCGGTTCTTCCGCATGGAAGAACTCTGAAAGACGGTTTCGGCTGTCACTGCAGGA
TGGGCCCGCGGCGCATTAGCTAGTTGGTGGGGTAATGGCCTACCAAGGCGACGATGCGTAGCCGACCT

GAGAGGGTGATCGGCCACACTGGGACTGAGACACGGCC
>Zotu3690
GAACGCTAGCGGCAGGCTTAACACATGCAAGTCGAGGGGCAGCGGGAGTAGCAATACTCGCCGGCGAC
CGGCGGAAGGGTGCGTAACGCGTGAGCAACATTCCCGTATCAGGGAGATAACCGATGGAAACGTCGCC
TAATATCCCATAACAGGTCATATCGCATGTCATGACCTTGAAAGCTACGGTGGATACGGATTGGCTCG
CGTGACATTAGCTGGTTGGAGTGGTAACGGCACACCAAGGCTACGATGTCTAGGGGTTCTGAGAGGAA
GGTCCCCCACACTGGAACTGAGACACGGTC
>Zotu3691
GAACGCTGGCGGCAGGCTTAACACATGCAAGTCGAACGAACCCTTCGGGGTTAGTGGCGGACGGGTGA
GTAACACGTGGGAACGTGCCCTTAGGTTCGGAACAACACAGGGAAACTTGTGCTAATACCGGATGTGC
CCCTCACGGGGGAAAGATTTATCGCCTTTGGAGCGGCCCGCGTCTGATTAGCTTGTTGGTGAGGTAAA
GGCTCACCAAGGCTACGATCAGTAGCTGGTCTGAGAGGATGGCCAGCCACATTGGGACTGAGACACGG
CC
>Zotu3694
GAACGCTGGCGGCGTGCCTAAGACATGCAAGTCGAGTGGTTGTCGTAAGACAACCACGGCAAACGGGT
GAGTAATACCTAGGAACTTTCTTTAAGTGGAGCATAACCAGTCGAAAGATTGGATAATTCTCCATGTG
ACCGCAAGGTCAAAGTCGCAAGACGCTTAAAGTGAGGCCTGGGCACCATCAGCTAGTTGGTAAGGTAA
TGGCTTACCAAGGCTATGACGGTTAGGGGCCCTCAGAGGGGAGTCCCCCACACTGGAACTGCGACACG
GTC
>Zotu3695
GAACGCTAGCGGCAGGCCTAATACATGCAAGTCGAACGGACCTTGTTCCTTCGGGGACTTGGTTAGTG
GCGCACGGGTGCGTAACGCGTATGCAACCTACCTTCTATTGGGGGATAGCCCCGGGAAACCGGGATTA
ATACCGCATGAGATCACTTGAGGGCATCCGAAGGTGATTAAAGCTGAGGCGATAGAAGATGGGCATGC
GTGCCATTAGCTAGTTGGCGGGGTAAGGGCCCACCAAGGCGACGATGGCTAGGGGTTCTGAGAGGATG
GTCCCCCACACTGGTACTGAGACACGGAC
>Zotu3698
GAACGCTAGCGGCAGGCCTAATACATGCAAGTCGAACGGGTCGCAAGACCAGTGGCAAACGGGTGCGT
AACGCGTAAGCAACCTGCCTCATACTGGGGGATAGCCCGGCGAAAGCTGGGGTAAACCCGCACGGTTC
CCATTTATCACCTGGTAGTTGGGGTAAACATTTATGGGTATGAGAGGGGCTTGCGTCTGATTAGTTAG
TTGGCAGGGTAACGGCCTACCAAGACGATGATCAGTAGGGGTTCTGAGAGGATTGGCCCCCACATGGG
TACTGAGATACGGAC
>Zotu3699
GAATGCTGGCGGTGTGGATAGGGCATGCAAGTCAAGGGGACCCGCAAGGGTAACCGGCAGACGAGATA
GTAACGCGTAGGAACGTACCCAGAAGTCGGGCACAGCTCGCCGAAAGGCGGGGTAATTCCCGATGGTG
ACGAAAGTCTAAAGATTTATCGCTTCTGGAGCGGCCTGCGTAATATCAGCTTGTTGGTGAGGTAATGG
CTCACCAAGGCTATGACGTTTAGGGGAGCTGAGAGGCTGACCCCCATCGATGGAATTGAGATACGGTC
>Zotu3700
GAACGCTGGCGGCGTGCTTAACACATGCAAGTCGAGCGGAAAGGCCACTTCGGTGGTACTCGAGCGGC
GAACGGGTGAGTAACACGTGAGTAATCTGCCCCTGGCTTTGGGATAGCCACCGGAAACGGTGATTAAT
ACCGGATATGACCATTCTTCGCATGAGGTGTGGTGGAAAGTTTTTCGGCCAGGGATGTGCTCGCGGCC
TATCAGCTTGATGGTGAGGTAATGGCTCACCATGGCTTCGACGGGTAGCCGGCCTGAGAGGGTGACCG
GTCACACTGGGACTGAGACACGGCC
>Zotu3702
GAACGTTGGCGGCGTGCCTTAAGCATGCAAATCAAGGGGGTCCCGCAAGGGACTACCGGTGGACGGGT
GAGTAATGCGTGGGAATGTACCTCGAAGTTGGATATAGCTAGCCGAAAGGTTGGGTAATCTCCAATGT
GAACGAAAGTTTAAAGCCGCAAGGCGCTTTGAGATCAGCCCGCGTCCTATCAGCTTGTTGGTGGGGTA
ATGGCCTACCAAGGCGACGACGGGTAGCTGGACTGAGAGGTCGGTCAGCCACAATGGGACTGCGACAC
GGCC
>Zotu3703
TAACGCTGGCGGCGTGCATAACACATGCAAGTCGAACGATGAAACTTAAATGATTTCTTCGGAATGAA
CTTAAGCGGATTAGTGGCGGACGGGTGAGTAACGCGTGAGTAACCTGCCTTACACAAGGGGATAGCCT
CTGGAAACGGAGAATAATACCCTATAAAACCATCATATCGCATGAAGTGATGGTCAAAGTGTTAGCGG
TGTAAGATGGACTTGCGTCTGATTAGCTAGTTGGTGAGAAAAAAGCCCACCAAGGCAACGATCAGTAG
CCGGCTTGAGAGAGTGTACGGCCACATTGGGACTGAGACACGGCC

>Zotu3704
GAACGCTGGCGGCAGGCTTAACACATGCAAGTCGAGCGGATGAAAGTAGCTTGCTACTGAATTCAGCG
GCGGACGGGTGAGTAATGCTTAGGAATCTGCCCATTAATGGGGGACAACAGTTGGAAACGACTGCTAA
TACCGCATACGCCCTACGGGGGAAAGCAGGGGATCTTCGGACCTTGCGTTAATGGATGAGCCTAAGTC
GGATTAGCTAGTTGGTGGGGTAAAGGCCTACCAAGGCGACGATCTGTAGCGGGTCTGAGAGGATGATC
CGCCACACTGGGACTGAGACACGGCC
>Zotu3705
GAATGCTGGCGGTATGGATAAGGCATGCAAGTCGAACGGCCCGCAAGGGCAGTGGCAGACGAGGTAGT
AACACGTAGGAACGTACACGGAAGTCAGGGATAGCCAGTCGAAAGGTTGGGTAATACTTGATGGTCTC
GCGAGAGTAAAGATTTATCGCTTCCGTAGCGGCCTGCGCAATATCAGCTTGTTGGTGAGGTAACGGCT
CACCAAGGCTACGACGTTTAGGGGCGCTGAGAGGCGGACCCCCACCGATGGGACTGAGATACGGCC
>Zotu3706
GAACGCTAGCGGCAGGCTTAATACATGCAAGTCGAGGGGCAGCAAGGGTAGCAATACCTGTTGGCGAC
CGGCAAACGGGTGCGGAACACGTACAGAACCTTCCTTTAAGCGGGGGATAGCCCATAGAAATGTGGAT
TAATACCCCATAGCAATATTTGATGGCATCATTAGATATTTAAAGATTTATCACTTAAAGATGGCTGT
GCGGCTGATTAGGTAGTTGGTGTGGGTAACGGCCCACCAAGCCAGCGATCAGTAACTGGTGTGAGAGC
ACGACCAGTCACACGGGCACTGAGACACGGGC
>Zotu3707
GAACGCTGGCGGCGTGGATGAGGCATGCAAGTCGAATGGGTTTAGACCCATGGCAGACGAGGTAGGAA
CACGTAGGTACATACCCTAAAGTCACGGATAATCTGCCGAAAGGCAGGATAATACGTGATAGTCCCTT
CGGGGTAAAGATTTATCGCTTTAGGAATGGCCTGCGTTCTATCAGCTTGTTGGTAAGGTAACGGCTTA
CCAAGGCTATGACGGATAGGGGAGGTGAGAGCCTGACCCCCACCGATGGAACTGCGACACGGTC
>Zotu3709
GAACGCTGGCGGCGCGCTTAACACATGCAAGTCGTACGGAGTTGTCCCTTCGGGGGCGGCTCAGTGGC
GAACGGGTGCGTAACACGTGAGCAACCTGCCTGCCGGTGGGGGATAACCCGGGGAAACCCGGGCTAAT
ACCGCATTCGCTCTTTCTCTCGATTGGGAGATTGAGGAAAGGTGCTGCAAGGCACTGCCGGCAGAGGG
GCTCGCGGCGTATCAGCTAGCTGGTGGGGTAACGGCCTACCAGGGCAACGACGCGTAGCCGATCTGAG
AGGATGATCGGCCACATTGGGACTGAGATACGGCC
>Zotu3710
GAACGCTGGCGGTATGCTTTACACATGCAAGTCGTACGGGAGTTTTTAACTCAAGTGGCGGACGGGTG
AGTAACACGTAAGAATTTGCCTTTAGGAGGGGGATAACAACTGGAAACGGTTGCTAATACCCCATATG
CTTTTGAGTGAAATGGAATTTCCGCCTAAAGAGAAGCTTGCGGCTGATTAGCTTGTTGGTAAGGTAAT
GGCTTACCAAGGCGACGATCAGTATCTGGTTTGAGAGGACGATCAGACACACTGGAACTGAGACACGG
TC
>Zotu3711
GAACGCTGGCGGCATGCTTAACACATGCAAGTCGAACGGAATTAAGTTTTACATTTTGAATCCTCAAA
ATGCGAGCTTAATTTAGTGGCGGACGGGTGAGTAACGCGTAAGAACCTACCTTTAGGAGGGGGACAAC
AGCTGGAAACGGCTGCTAATACCCCATATGCTGAGGAGTAAAATGGTGTAACAACCGCCTAAAGATGG
GCTTGCGTCTGATTAGCTTGTTGGTAAGGTAAAGGCTTACCAAGGCAACGATCAGTAGCTGGTCTGAG
AGGATGATCAGCCACACTGGGACTGAGACACGGCC
>Zotu3712
GAACGCTGGCGGCAGGCTTAACACATGCAAGTCGAGCGGTAGCACAAGGGAGCTTGCTCCCTGGGTGA
CGAGCGGCGGACGGGTGAGTAATGTCTGGGAAACTGCCTGATGGAGGGGGATAACTACTGGAAACGGT
AGCTAATACCGCATAACGTCGCAAGACCAAAGAGGGGGACCTTCGGGCCTCTTGCCATCGGATGTGCC
CAGATGGGATTAGCTAGTAGGTGGGGTAACGGCTCACCTAGGCGACGATCCCTAGCTGGTCTGAGAGG
ATGACCAGCCACACTGGAACTGAGACACGGTC
>Zotu3715
GAACGCTGGCGGCATGCTTTACACATGCAAGTCGAACGGCAGCGCGGGGCAACCTGGCGGCGAGTGGC
GAACGGGTGAGTAATACATCGGAACGTACCCAGAAGTGGGGGATAACGTAGCGAAAGTTACGCTAATA
CCGCATACGTTCTACGGAAGAAAGCGGGGGATCGCAAGACCTCGTGCTTTTGGAGCGGCCGATGTCTG
ATTAGCTAGTTGGTGAGGTAAAGGCTCACCAAGGCGACGATCAGTAGCTGGTCTGAGAGGACGACCAG
CCACACTGGGACTGAGACACGGCC
>Zotu3716
GAACGCTAGCGGCAGGCCTAATACATGCAAGTCGAACGGTGTAGCAATACATAGTGGCGCACGGGTGC

GTAACACGTATGCAACCTACCTTATACTTAGGGATAGCCTTTGGAAACGGAGATTAATACCTAATAGC
ACATTAGACTGGCATCGGTTATATGTTAAAGATTTATTGGTATAAGATGGGCATGCGTCCAATTAGTT
AGTTGGTGAGGTAACGGCTCACCAAGACGATGATTGGTAGGGGAACTGAGAGGTTAATCCCCCACACT
GGCACTGAGATACGGGC
>Zotu3717
GAACGCTGGCGGCAGGCTTAACACATGCAAGTCGAGCGGGGCCTTTCGGGGCTCAGCGGCGGACGGGT
TAGTAACGCGTGGGAACGTGCCTTTTGCTTCGGAATAGCCCTGGGAAACTGGGGGTAATACCGGATGT
GCCCTGAGGGGGAAAGATTTATCGGCAAGAGATCGGCCCGCGTCTGATTAGGTAGTTGGTGGGGTAAT
GGCCCACCAAGCCGACGATCGGTAGCTGGTTTGAGAGGATGATCAGCAACACTGGGACTGAGACACGG
CC
>Zotu3718
GAACGCTGGCGGTATGCTTAACACATGCAAGTCGTACGAAGATCTTTGATCTTAGTGGCGGACGGGTG
AGTAACACGTGAGAATCTGCCCTTAGGAAGAGGATAACAGCTGGAAACGGTTGCTAATACTCTATACG
CCGAGAGGTAAAAGGATCTATCCACCTTAGGAAGAGCTCGCGTCTGATTAGCTTGTTGGTAAGGTAAT
GGCTTACCAAGGCGACGATCAGTAGCTGGTTTGAGAGGACGACCAGCCACACTGGGACTGAGACACGG
CC
>Zotu3719
GAATGCTGGCGGCGTGGATAAGCCATGCAAGTCGAACGGTCCGCAAGGACAGTGGCAGACGAGGTAGT
AAGGCATAGGGAGATCCCCCAGAGTCAGGGATAGCCAGTCGAAAGATTGGGTAATACTTGATGGTCCG
GTGACGGTAAAGATTTATCGCTCTGGGAGTCACCTATGTAATATCAGCTAGTTGGTGAGGTAATGGCT
CACCAAGGCTACGACGTTTAGGGGAGCTGAGAGGCTGACCCCCACCGATGGGACTGCGATACGGCC
>Zotu3720
GAACGCTGGCGGCGTGCCTAATACATGCAAGTCGAGCGCGTTTCGAAGGTGCTTGCACCTTCAATGAA
ACGAGCGGCGGACGGGTGAGTAACACGTAGGTAACCTACCTTTATGACTGGGATAACCACGGGAAACC
GGGGCTAATACCGGATAAGATTTAGCTTCGCATGAAGCAGAAGTGAAAGGCAGTTTCGGCTGTCACAT
AGAGATGGACCTGCGGCGCATTAGCTAGTTGGAGAGGTAAGAGCTCCCCAAGGCGACGATGCGTAGCC
GACCTGAGAGGGTGATCGGCCACACTGGGACTGAGACACGGCC
>Zotu3721
GAACGCTAGCGGGAGGCTTAACACATGCAAGTCGAGGGGCATCACGAGGTAACAATACTTTGGTGGCA
ACCGGCGCACGGGTGAGTAACGCGTATGCAACCTACCCTGTACAGGGGGATAGCCCGGAGAAATTCGG
ATTAATACCCCATAAAGATATTTGAAGGCATCTTTAGATATTTAAAGTTTCGACGGTACGGGATGGGC
ATGCGTAACATTAGCTAGTTGGTAGGGTAACGGCCTACCAAGGCAACGATGTTTAGGGGTCCTGAGAG
GGTGATCCCCCACACTGGTACTGAGACACGGAC
>Zotu3722
GAACGCTGGCGGCGTGCCTAATACATGCAAGTCGAACGAGAGCGACCGGTGCTTGCACTGGTCAATCT
AGTGGCGAACGGGTGAGTAACACGTGGGTAACCTGCCCATCAGAGGGGGATAACATTCGGAAACGGAT
GCTAAAACCGCATAGGTTCTACAGTCGCATGACTGAAGAAGGAAAAGAGGCTTCGGCTTCTGCTGATG
GATGGACCCGCGGTGCATTAGCTAGTTGGTGAGGTAACGGCTCACCAAGGCCGTGATGCATAGCCGAC
CTGAGAGGGTGATCGGCCACATTGGGACTGAGACACGGCC
>Zotu3723
GAACGCTGGCGGCGTGCTTAACACATGCAAGTCGAACGGTGAAGCCCAGCTTGCTGGGTGGATCAGTG
GCGAACGGGTGAGTAACACGTGAGCAACCTGCCCCTGACTCTGGGATAAGCGCTGGAAACGGCGTCTA
ATACTGGATACGAGTAGCGACCGCATGGTCAGTTACTGGAAAGATTTATTGGTTGGGGATGGGCTCGC
GGCCTATCAGCTTGTTGGTGAGGTAATGGCTCACCAAGGCGTCGACGGGTAGCCGGCCTGAGAGGGTG
ACCGGCCACACTGGGACTGAGACACGGCC
>Zotu3724
GAACGCTGGCGGCAGGCTTAACACATGCAAGTCGAGCGGAGATGAGGTGCTTGCACCTTATCTTAGCG
GCGGACGGGTGAGTAATGCTTAGGAATCTGCCTATTAGTGGGGGACAACATTTCGAAAGGAATGCTAA
TACCGCATACGCCCTACGGGGGAAAGCAGGGGATCTTCGGACCTTGCGCTAATAGATGAGCCTAAGTC
GGATTAGCTAGTTGGTGGGGTAAAGGCCTACCAAGGCGACGATCTGTAGCGGGTCTGAGAGGATGATC
CGCCACACTGGGACTGAGACACGGCC
>Zotu3725
GAACGCTGGCGGCATGCTTAACACATGCAAGTCGGACAGGAAGTGGTGTTTCCAATGGCGGACGGGTG
AGTAACGCGTAAGAACCTGCCCTTGGGAGGGGAACAACAGCTAGAAACGGCTACTAATACCCCGTAGG

CTGAGGAGCAAAAGGAGATATCCGCCTGAGGAGGGGCTCGCGTCTGATTAGCTAGTTGGTGAGGCAAT
AGCTTACCAAGGCGATGATCAGTAGCTGGTTCGAGAGGATGATCAGCCACACTGGGACTGAGACACGG
CC
>Zotu3726
GAACGCTGGCGGCGCGCTTAACACATGCAAGTCGAGCGAGAACCGGTTCTTCGGAACCGGGGAAAGCG
GCGAACGGGTGAGTAACACGTGAGTAATCCACCCTCGGCACCGGGATAGCCCGGGGAAACTCGGATTA
ATACCGGATGGCCCAGCAGCTCTTCGGGGCGGTTGGAAAAGGTAGCTTAGGCCTCTGGCCGAGGACGA
GCTCGCGGCGGATTAGCTTGTTGGTGGGGTAATGGCCTACCAAGGCGACGATCCGTAGCTGGTCTGAG
AGGACGATCAGCCACACTGGGACTGAGACACGGCC
>Zotu3727
GAACGCTGGCGGCGTGCCTAATACATGCAAGTCGAGCGAATCAATGGGAGCTTGCTCCCTGAGATTAG
CGGCGGACGGGTGAGTAACACGTGGGTAACCTGCCTGTAAGACTGGGATAACTTCGGGAAACCGGAGC
TAATACCGGATAATTTCTTTTCTCGCATGAGGGAAGATGGAAAGATGGTTCTGCTATCACTTACAGAT
GGACCCGCGGCGCATTAGCTAGTTGGTGAGGTAATGGCTCACCAAGGCAACGATGCGTAGCCGACCTG
AGAGGGTGATCGGCCACACTGGGACTGAGACACGGCC
>Zotu3728
GAACGCTGGCGGCGTGCCTAATACATGCAAGTCGAGCGAATCTGAGGGAGCTTGCTCCCAAAGGTTAG
CGGCGGACGGGTGAGTAACACGTGGGCAACCTGCCTGTAAGACTGGGATAACTTCGGGAAACCGGAGC
TAATACCGGATAATTCTTACCAACACATGTTGGTAAGCTAAAAGACGGCGCAAGCTGTTACTTACAGA
TGGGCCCGCGGCGCATTAGCTAGTTGGTAAGGTAATGGCTTACCAAGGCAACGATGCGTAGCCGACCT
GAGAGGGTGATCGGCCACACTGGGACTGAGACACGGCC
>Zotu3729
GAACGCTGGCGGCGTGCCTAATACATGCAAGTCGTGCGAGGCTCTTCGGAGTCGAGCGGCGGACGGGT
GAGTAACACGTGGGCAACCTGCCCGACAGATCGGGATAACGCTTGGAAACGAGTGCTAATACCGGATA
CGCTTTTGAGGCGCATGCCTCGAAAGGGAAAGTTGCTTTTGCAGCACTGTCGGATGGGCCCGCGGCGC
ATTAGCTAGTTGGTGAGGTAACGGCTCACCAAGGCGACGATGCGTAGCCGACCTGAGAGGGTGACCGG
CCACACTGGGACTGAGACACGGCC
>Zotu3730
GAATGCTGGCGGTATGGATAAGGCATGCAAGTCGAACGGCCCGCAAGGGTAGTGGCAGACGAGGTAGT
AACACGTACGAACGTACCCTGTAGTCTGGCATAGCTCGTCGAAAGACGGGGTAATTCCGGATGGTCCC
GAAAGGGTAAAGTTTTTCGCTACAGGAACGGCGTGCGTAGTATCAGCTTGTTGGTAAGGTAATGGCTT
ACCAAGGCTACGACGCTTAGGGGAGCTGAGAGGCTGACCCCCACCGATGGGACTGAGACACGGCC
>Zotu3731
GAACGCTGGCGGCGTGCCTAATACATGCAAGTCGAGCGGTAAGGCTTCCACAGTTTGTATCTCCCTTA
TAACAAAGTTATGAGATACGGAGGTGCAAAGCGTGGAAGTACACGAGCGGCGGACGGCTGAGTAACGC
GTAGGAACGTACCCCAGAGTGAGGGATAAGCACCGGAAACGGTGTCTAATACCGCATACTACCTTCGG
GTCAAAGCTTTAGCGCTTTGGGAACGGCCTGCGTATGATTAGCTTGTTGGTGAGGTAATGGCTTACCA
AGGCGACGATCATTAGCTGGTCTGAGAGGATGATCAGCCAGACTGGGACTGAGACACGGCC
>Zotu3732
GAACGCTGGCGGCAGGCTTAACACATGCAAGTCGAACGCTCCGCAAGGAGAGTGGCAGACGGGTGAGT
AACACGTGGGAACGTACCCTTTACTACGGAACAACTCCGGGAAACTGGAGCTAATACCGTATGTGCCC
TTCGGGGGAAATATTTATCGGTAAAGGATCGGCCCGCGTTGGATTAGCTAGTTGGTGGGGTAAAGGCC
TACCAAGGCGACGATCCATAGCTGGTCTGAGGGGATGATCAGCCACATTGGGACTGAGACACGGCC
>Zotu3733
GAACGCTGGCGGCAGGCTTAACACATGCAAGTCGAGCGGAAACGATGGGAGCTTGCTCCCAGGCGTCG
AGCGGCGGACGGGTGAGTAATACTTAGGAATCTGCCCAGTAGTGGGGGATAGCTCGGGGAAACTCGAA
TTAATACCGCATACACCCTACGGGGAAAGGGGGCGCTTGCGCTCTCGCTATTGGATGAGCCTAAGTC
GGATTAGCTAGTTGGTGGGGTAAAGGCCTACCAAGGCGACGATCTGTAGCTGGTCTGAGAGGATGATC
AGCCACACCGGGACTGAGACACGGCC
>Zotu3735
GAACGCTAGCGGCAGGCCTAATACATGCAAGTCGGACGGGATCCATCGGAGAGCTTGCTCGAAGACGG
TGAGAGTGGCGCACGGGTGCGTAACGCGTGAGCAACCTACCTCTATCAGGGGGATAGCCTCTCGAAAG
AGAGATTAACACCGCATAACATATCTGACCGGCATCGGTTAGCTATTAAATATTTATAGGATAGAGAT
GGGCTCGCGTGACATTAGCTAGTTGGTAGGGTAACGGCTTACCAAGGCGACGATGTCTAGGGGCTCTG

AGAGGAGAATCCCCCACACTGGTACTGAGACACGGAC
>Zotu3736
GAACGCTGGCGGCAGGCCTAACACATGCAAGTCGAACGGATCCTTCGGGATTAGTGGCGGACGGGTGA
GGAACACGTGGGAACGTGCCTTTTGGTTCGGAACAACTCAGGGAAACTTGAGCTAATACCGGATAAGC
CCTTCGGGGGAAAGATTTATCGCCATTAGAGCGGCCCGCGTCTGATTAGCTAGTTGGTGAGGTAAAGG
CTCACCAAGGCGACGATCAGTAGCTGGTCTGAGAGGATGATCAGCCACATTGGGACTGAGACACGGCC
>Zotu3737
GAACGCTGGCGGCGTGCCTAATACATGCAAGTCGAGCGAATCTTGAGGTGCTTGCACCTCTTGGTTAG
CGGCGGACGGGTGAGTAACACGTGGGCAACCTGCCTGTAAGACTGGGATAACACCGGGAAACCGGTGC
TAATACCGGATAATACTTTTCCTCTCATGAGGAAAAGTTGAAAGTCGGTTTACGCTGACACTTACAGA
TGGGCCCGCGGCGCATTAGCTAGTTGGTGAGGTAACGGCTCACCAAGGCGACGATGCGTAGCCGACCT
GAGAGGGTGATCGGCCACACTGGGACTGAGACACGGCC
>Zotu3738
GAACGCTGGCGGCGTGCCTAATACATGCAAGTCGAGCGAATCTTCAGGAGCTTGCTCCTGAAGATTAG
CGGCGGACGGGTGAGTAACACGTGGGCAACCTGCCTGTAAGACTGGGATAACTTCGGGAAACCGAGGC
TAATACCGGATAATCCTTTTCCTCACATGAGGGGAAGCTGAAAGACGGCGCAAGCTGTCACTTACAGA
TGGGCCCGCGGCGCATTAGCTAGTTGGTGAGGTAACGGCTCACCAAGGCGACGATGCGTAGCCGACCT
GAGAGGGTGATCGGCCACACTGGGACTGAGACACGGCC
>Zotu3740
GAACGCTGGCGGCATGCCTTACACATGCAAGTAGAACGGCAGCGCGGACTTCGGTCTGGCGGCGAGTG
GCGAACGGGTGAGTAATACATCGGAACGTACCCTGTTGTGGGGGATAACTAGTCGAAAGATTAGCTAA
TACCACATACGACCTGAGGGTGAAAGCGGGGGACCGTAAGGCCTCGCGCAGCAGGAGCGGCCGATGTC
TGATTAGCTAGTTGGTGGGGTAAAAGCCCACCAAGGCGACGATCAGTAGCTGGTCTGAGAGGACGATC
AGCCACACTGGGACTGAGACACGACC
>Zotu3741
GAATGCTGGCGGCGTGGATAAGTCATGCAAGTAGAACGCCCCGCAAGGGGAGTTGCGAACGAGGTAGT
AACACGTAGGTACGTCCCCCGAAGTCGGGCATAGCCAGTCGAAAGACTGGGTAATTCCCGATAGTCCC
GAAAGGGTAAAGGTTTTTCGCTTCGGGAGCGGCCTGCGTAGTATCAGCTAGTTGGTAGGGTAATGGCC
TACCAAGGCTACGACGCTTAGGGGGGCTGAGAGGCCGATCCCCACCGATGGAACTGCGACACGGTC
>Zotu3742
GAACGCTGGCGGCAGGCCTAACACATGCAAGTCGAACGGCAGCACAGGGGAGCTTGCTCCCTGGGTGG
CGAGTGGCGGACGGGTGAGGAATACATCGGAATCTGCCCTGTCGTGGGGGATAACGTAGGGAAACTTA
CGCTAATACCGCATACGACCTTCGGGTGAAAGAGGGGGATCGCAAGACCTCTCGCGATTGGATGAGCC
GATGTCGGATTAGCTAGTTGGCGGGGTAAAGGCCCACCAAGGCGACGATCCGTAGCTGGTCTGAGAGG
ATGATCAGCCACACTGGAACTGAGACACGGTC
>Zotu3743
GAACGCTGGCGGCATGCCTAACACATGCAAGTCGAACGAGACCTTCGGGTCTAGTGGCGCACGGGTGC
GTAACGCGTGGGAATCTGCCCTTGGGTTCGGAATAACTCGCCGAAAGGCGTGCTAATACCGGATGATG
TCGTAAGACCAAAGATTTATCGCCCAGGGATGAGCCCGCGTAAGATTAGCTAGTTGGTGAGGTAAAGG
CTCACCAAGGCGACGATCTTTAGCTGGTCTGAGAGGATGATCAGCCACACTGGGACTGAGACACGGCC
>Zotu3744
GAACGCTGGCGGCGTGCTTAACACATGCAAGTCGAACGGACTGCTTGGGGGCTTGCTCCTGGGTGGTG
AGTGGCGAACGGGTGAGTAACACGTGAGTAACCTGCCCTCTTCTTCGGGATAACGGTCGGAAACGGCT
GCTAATACTGGATATTCACTTCCTGTCGCATGGTGGGGGGTGGAAAGGTTTTTTCTGGTGGGGGATGG
GCTCGCGGCCTATCAGCTTGTTGGTGGGGTGATGGCCTACCAAGGCTTTGACGGGTAACCGGCCTGAG
AGGGTGACCGGTCACATTGGGACTGAGATACGGCC
>Zotu3745
GAACGCTGGCGGCGTGCTTAACACATGCAAGTGGAGCGACGAACCAGGCTTCGGCCTGGGGCAGAGCC
GCGAACGGGTGAGTAACACGTGGGTAACGTGCCCCGATGATCGGGACAACCCGGGGAAACCCGGGCTA
ATACCGAATGTGCTCCCCTCACACCAGTGAGGGGTGGAAAGGAAGCTTCGGCCTCCGCATCGGGATCG
GCCCGCGGCCCATTAGCTTGTTGGTGAGGTAACGGCTCACCAAGGCTGCGATGGGTAGCTGGTCTGAG
AGGACGATCAGCCACACTGGGACTGAGACACGGTC
>Zotu3746
AAACGCTGGCGGCGTGCCTAATACATGCAAGTCAAACGGGGAAAGGCTTCGGCCGAGTACTAGTGGCG

CACGGGTGAGTAACACGTGGGTAATCTGCCTGGAAGTGGGGGATAACCAGTCGAAAGATTGGTTAATA
CCGCATAAGACCACAGAGTCTAAGGACTCAGGGGTCAAAGGTTTTTCGCTTCCAGATGAGCCTGCGCG
GGATTAGCTAGTTGGTGAGGTAACGGCTCACCAAGGCTATGATCTCTAGCTGGTCTGAGAGGATGATC
AGCCACACTGGAACTGAGACACGGTC
>Zotu3747
GAACGCTGGCGGTATGCTTAACACATGCAAGTCGAACGGAACCTTTCGGGGTTTAGTGGCGGACGGGT
GAGTAACGCGTGAGAATCGGCCCTCAGGTTGGGGACGACCATTGGAAACGGTGGCTAATACCGAATAA
GCCGAGAGGTGAAAGATTAATTGCCTGGGGAAGAGCTCGCGTCTGATTAGCTAGTTGGTGGGGTAAAG
GCCTACCAAGGCACCGATCAGTAACTGGTCTGAGAGGATGGCCAGTCACACTGGGACTGAGACACGGC
C
>Zotu3748
GAACGCTAGCGACAGGCTTAACACATGCAAGTCGAGGGGCAGCGGGGGAGTGGCTTGCCACTCCCGCC
GGCGACCGGCGCACGGGTGAGTAACACGTATGCAACCTGCCCTTGACAGGGGAATAAGCGGCGGAAAC
GCCGTCTAATTCCGCGTACACCGCGGGAGGGGCATCCCTCCGGCGGAAAAGGAGGCGACTCCGGTCAA
GGATGGGCATGCGGCGCATTAGCTAGACGGCGGGGTAACGGCCCACCGTGGCGACGATGCGTAGGGGT
TCTGAGAGGAAGGTCCCCCACATTGGAACTGAGACACGGTC
>Zotu3749
GAACGCTGGCGGCATGCCTAACACATGCAAGTCGAACGAAGGCTTCGGCCTTAGTGGCGCACGGGTGC
GTAACGCGTGGGAATCTACCCTTGGGTTCGGAATAACAGCTAGAAATGGCTGCTAATACCGGATGATG
ACGTAAGTCCAAAGATTTATCGCCCAAGGACGAGCCCGCGTAGGATTAGCTAGTTGGTGAGGTAAAGG
CTCACCAAGGCGACGATCCTTAGCTGGTCTGAGAGGATGATCAGCCACACTGGGACTGAGACACGGCC
>Zotu3750
GAACGCTAGCGGCAGGCCTAACACATGCAAGTCGAGGGGCAGCGCGAGGTAGCAATACCTTGGCGGCG
ACCGGCGGACGGGTGCGTAACGCGTATGGAACCAACCCGTCACAGGGGGACAAGCCAGGGAAACTTGG
TCTAATACCCCATATTACTGTTTGACCGCATGATTAGACAGTGAAAGCCTACGGGCGGTGACGGACGG
GCATGCGTGACATTAGCTAGTTGGTAAGGCAACGGCTTACCAAGGCGACGATGTCTAGGGGAGCTGAG
AGGCTAATCCCCCACACTGGTACTGAGACACGGAC
>Zotu3751
GAACGCTGGCGGCAGGCTTAACACATGCAAGTCGAACGCATCCTTCGGGGTGAGTGGCAGACGGGTGA
GTAACGCGTGGGAACGTGCCCTTCAGTTCGGGATAACCCAGGGAAACTTGGGCTAATACCGGATACGT
GCGAGAGCAGAAAGATTTATCGCTGAAGGATCGGCCCGCGTCTGATTAGCTGGTTGGTGAGGTAATGG
CTCACCAAGGCGACGATCAGTAGCTGGTCTGAGAGGATGATCAGCCACATTGGGACTGAGACACGGCC
>Zotu3752
AAACGCTGACAGAATGCTTAACACATGCAAGTCTATGAGGAAGTTTAGCTTGCTAAATGGACTCATGG
CGGACGGGTGAGTAACGCGTAAAGAACTTGCCCTTTAGACTGGGATAACAGAGGGAAACTTCTGATAA
TACTGGATAAGTTAGTATATCGCATGATATGCAAATGAAAGCTACGGCACTAAAGGAGAGCTTTGCGT
CCTATTAGCTAGTTGGTAAGGTAAGAGCTTACCAAGGCGATGATAGGTAGCCGGCCTGAGAGGGTGGA
CGGCCACAAGGGGACTGAGATACGGCC
>Zotu3753
GAACGCTGGCGGCGTGCTTAACACATGCAAGTCGAGCGATGAAGCTTCTTCGGAAGTGGATTAGCGGC
GGACGGGTGAGTAACACGTGGGTAACCTGCCTCATAGAGGGGAATAGCCTTTCGAAAGGAAGATTAAT
ACCGCATAAGATTGTAATATCGCATGATATAGCAATTAAAGGAGCAATCCGCTATGAGATGGACCCGC
GTCGCATTAGCTAGTTGGTGAGGTAACGGCTCACCAAGGCGACGATGCGTAGCCGACCTGAGAGGGTG
ATCGGCCACATTGGGACTGAGACACGGCC
>Zotu3754
GAACGCTGGCGGCAGGCTTAACACATGCAAGTCGAACGCCCCGCAAGGGGAGTGGCAGACGGGTGAGT
AACGCGTGGGAATCTACCCTGATCTGCGGGATAGCTCCGGGAAACTGGAATTAATACCGCATGTGCCC
TGAGGGGCAAAGGTTTACCGGATTGGGATGAGCCCGCGTCCGATTAGCTAGTTGGTGGGGTAAAGGCC
TACCAAGGCGACGATCGGTAGCTGGTCTAAGAGGATGATCAGCCACATTGGGACTGAGACACGGCC
>Zotu3755
GAACGCTGGCGGCATGCCTAACACATGCAAGTCGAACGGAGATTAGATTGAAGCTTGCGATTTCTAAT
CTTAGTGGCGCACGGGTGAGTAACGCGTGGATAACCTGCCTGGTAGACCGGGATAACAGCTGGAAACG
GCTGCTAATACCGGATACGCTCGAGGAACTGCATGGTTTCTTGAGGAAAGATTTATTACTATCAGATG
GATCCGCGTCCCATTAGCTAGTTGGTGGTGTAACGGACCACCAAGGCGACGATGGGTAGCCGGCCTGA

GAGGGCGACCGGCCACACTGGGACTGAGACACGGCC
>Zotu3756
GAACGCTAGCGGCAGGCCTAATACATGCAAGTCGAACGGTAACATGAATAGCAATATTTGATGACGAG
TGGCGCACGGGTGCGTAACGCGTATGCAATCTACCTTCAACTGGTGAATAGCCCGAAGAAATTCGGAT
TAACACACCATAGTATAATAGAATGGCATCATTTTATTATTAAAACTCCGGTGGTTGAAGATGAGCAT
GCGTGACATTAGCTAGTTGGTGAGGTAATGGCTCACCAAGGCTACGATGTCTAGGGGATCTGAGAGGA
TTAACCCCCACACTGGTACTGAGACACGGAC
>Zotu3757
GAACGCTGGCGGCGTGCTTAACACATGCAAGTCGAACGAGGTCCATGGAGCTTGCTCCGGAAGATCTA
GTGGCGAACGGGTGCGTAACACGTGAGAAACCTGCCCCGATCTCTGGGATAACAGTTGGAAACGACTG
CTAATACCGGATGCCGTCACACCGCTTCATGGCGGAGTGCCGAAAGATTTATCGGATTGGGAGGGTCT
CGCGGCCTATCAGCTAGTTGGAGAGGTAACGGCTCACCAAGGCATCGACGGGTAGCTGGTCTGAGAGG
ATGATCAGCCACACTGGGACTGAGACACGGCC
>Zotu3758
GAACGCTGGCGGCGTGCCTAATACATGCAAGTCGAGCGGACAGATGGGAGCTTGCTCCCTGATGTTAG
CGGCGGACGGGTGAGTAACACGTGGGTAACCTGCCTGTAAGACTGGGATAACTCCGGGAAACCGGGGC
TAATACCGGATGGTTGTTTGAACCGCATGGTTCAGACATAAAAGGTGGCTTCGGCTATCACTTACAGA
TGGACCCGCGGCGCATTAGCTAGTTGGTGAGGTAACGGCTCACCAAGGCGACGATGCGTAGCCGACCT
GAGAGGGTGATCGGCCACACTGGGACTGAGACACGGCC
>Zotu3759
GAACGCTGGCGGCGTGCCTAACACATGCAAGTCGAACGGGGTCCATGGAGTGCTTGCACTCTTGAAGA
CCTAGTGGCGAACGGGTGAGTAACACGTGAGAAACCTGCCCCAGACTCTGGGATAACTCCGGGAAACC
GGAGCTAATACCGGATACTCTCATTTGATCGCATGGTCGTTTGAGGAAAGTTTTTCGGTCTGGGAGGG
TCTCGCGACCTATCAGCTAGTTGGTGAGGTAACGGCTCACCAAGGCTACGACGGGTAGCTGGTTTGAG
AGAACGATCAGCCACACTGGAACTGAGACACGGTC
>Zotu3760
GAACGCTAGCGGCAGGCTTAATACATGCAAGTCGAACGGATGTAGCAATACATTAGTGGCGCACGGGT
GAGTAACGCGTACATAACATGCCTTTAGCTTAGGAATAGCCTTGGGAAACTGGGAGTAATACCTAATA
GTATTTTTGGATGGCATCATCTGGAAATTAAAGTTACGGCGGCTAAAGATTGATGTGCGTAAGATTAG
CTAGTTGGCGAGGTAACGGCTCACCAAGGCGACGATCTTTAGGGGGCGTGAGAGCGTGACCCCCCACA
CGGGTACTGAGACACGGAC
>Zotu3761
CAACGCTGGCGGCGCGCTTAACACATGCAAGTCGAACGAGAAAGGGTAGCAATACCTGAGTAAAGTGG
CGTACGGGTGAGTAACACGTGGATCATCTACCCTCTTGTGGGGAATACCGATGGGAAACCGTCGACAA
TACCGCATAAGCTCGAGAGAGGAAAGCCGAAAGGCGCAGGAGGAGGAGTCCGCGGTCGATTAGTTAGT
TGGCAGGGTAATGGCCTACCAAGACTGTGATCGATAGCCGGCCTGAGAGGGCTCACGGCCACACTGGC
ACTGAAACACGGGC
>Zotu3763
GAACGCTGGCGGCGTGCTTAACACATGCAAGTCGAGCGAGGTTCAACTGGTGGCAACACCAGGGAAGA
CCGAGCGGCGAACGGGTGAGTAACACGTGAGCAACCTGTCCCGAAGACCGGGATAACACCGGGAAACC
GGTGCTAATACCGGATGTCCCCACCGGACCACATGGTTCGGTGAGGAAATGGATTCCGCTTCGGGAGG
GGCTCGCGGGCTATCAGCTTGTTGGTGGGGTAACGGCCTACCAAGGCAACGACGGCTAGCTGGTCTGA
GAGGACGACCAGCCACACTGGGACTGAGACACGGCC
>Zotu3764
GAACGCTGGCGGCGTGCTTAACACATGCAAGTCGAACGGAAAGGCCCAGCTTGCTGGGTACTCGAGTG
GCGAACGGGTGAGTAACACGTGGGTGATCTGCCCTGCACTCTGGGATAAGCCTGGGAAACTGGGTCTA
ATACCGGATATGACCAACTGTCGCATGGTGGTTGGTGGAAAGCTTTTGCGGTGTGGGATGGGCCCGCG
GCCTATCAGCTTGTTGGTGGGGTAATGGCCTACCAAGGCGACGACGGGTAGCCGACCTGAGAGGGTGA
TCGGCCACACTGGGACTGAGACACGGCC
>Zotu3765
GAACGCTAGCTACAGGCTTAACACATGCAAGTCGAGGGGCAGCATGGTCTTAGCTTGCTAAGACTGAT
GGCGACCGGCGCACGGGTGAGTAACACGTATCCAACCTGCCGTCTACTCTTGGACAGCCTTCTGAAAG
GAAGATTAATACAAGATGGCATCATGAGTCCACATGTTCACATGATTAAAGGTATTCCGGTAGACGAT
GGGGATGCGTTCCATTAGATAGTAGGCGGGGTAACGGCCCACCTAGTCTTCGATGGATAGGGGTTCTG

AGAGGAAGGTCCCCCACATTGGAACTGAGACACGGTC
>Zotu3766
GAACGCTAGCGGCAGGCTTAACACATGCAAGTCGAGGGGTATAGTAGCAATACTAGAGACCGGCGCAC
GGGTGCGTAACGCGTATGCAATCTACCTTTTACAGAGGGATAGCCCAGAGAAATTTGGATTAATACCT
CATAGTATAGTACAATCGCATGATTGAATTATTAAAGTCACAACGGTAAAAGATGAGCATGCGTCCCA
TTAGCTAGTTGGTAAGGTAACGGCTTACCAAGGCGACGATGGGTAGGGGTCCTGAGAGGGAGATCCCC
CACACTGGTACTGAGACACGGAC
>Zotu3767
GAACGCTGGCGGCGTGCTTAACACATGCAAGTCGAACGGAAAGGCCCTTCGGGGTACTCGAGTGGCGA
ACGGGTGAGTAACACGTGGGTGATCTGCCCTGCACTCTGGGATAAGCCTGGGAAACTGGGTCTAATAC
CGGATAGGACCGCATGCTTCATGGTGTGTGGTGCAAAGCTTTTGCGGTGTGGGATGAGCCCGCGGCCT
ATCAGCTTGTTGGTGGGGTAATGGCCTACCAAGGCGACGACGGGTAGCCGGCCTGAGAGGGTGACCGG
CCACACTGGGACTGAGATACGGCC
>Zotu3768
GAACGCTGGCGGCGTGCCTAATACATGCAAGTCGAGCGAACTCTTCAAAAGCTTGCTTTTGAAGAGTT
AGCGGCGGACGGGTGAGTAACACGTGGGCAACCTGCCTATAAGACTGGGATAACTTCGGGAAACCGGA
GCTAATACCGGATAATTCTTTTCTACTCATGTAGAAAAGCTGAAAGACGGTTTACGCTGTCACTTATA
GATGGGCCCGCGGCGCATTAGCTAGTTGGTGAGGTAACGGCTCACCAAGGCCACGATGCGTAGCCGAC
CTGAGAGGGTGATCGGCCACACTGGGACTGAGACACGGCC
>Zotu3769
GAACGCTGGCGGCAGGCTTAACACATGCAAGTCGAACGGGCACCTTCGGGTGTCAGTGGCAGACGGGT
GAGTAACACGTGGGAACGTGCCCTTCGGTTCGGAATAACTCAGGGAAACTTGAGCTAATACCGGATAC
GCCCTTATGGGGAAAGGTTTACTGCCGAAGGATCGGCCCGCGTCTGATTAGCTTGTTGGTGGGGTAAC
GGCCTACCAAGGCGACGATCAGTAGCTGGTCTGAGAGGATGATCAGCCACACTGGGACTGAGACACGG
CC
>Zotu3771
GAACGCTTGCGGCGTGCCTAAGAAATGCAAGTCGAACGGACAGTCCAGCAATGGTCTGTTAGTGGCGA
ACGGTCGCGTAACACGTAAGCAACCTGCCTCGAAGTGGGGGACAACAGTCCGAAAGGTCTGCTAATAC
CGCATGTGGCCACAGTTTCGCATGATTCTGTGTCTAAAGGATTTATTCGCTTCGAGATGGGCTTGCGG
CCTATCAGGTAGTTGGTGGGGTAATGGCCTACCAAGCCGACGACGGGTAGCTGGTCTGAGAGGACGAT
CAGCCGGATTGGGACTGAGATACGGCC
>Zotu3772
AAACGCTGGCGGTGTGCCTGAGATATGCAAGTCGAGCGGGAATCATTGAAGTCGCAAGATGGATATGA
TTTCAGCGGCGGACGGCTGAGTAACACGTAAGAATCTCCCCCAAAGTCTCACATAGCTCACCGAAAGG
TGGGGTAATTTGAGATAGTCTCCCCTCGCAAGAGGGGTGTAAAGCCGCAAGGCGCTTCGGGATGAGCT
TGCGTCCTATCAGGTAGTTGGTGGGGTAATGGCCCACCAAGCCTACGACGGGTAGCTGGCCTCATGAG
GGTGGTCAGCCAGAAGGGGACTGAGATACGGCC
>Zotu3773
CAACGCTGGCGGCGTGCCTAACACATGCAAGTCGCACGAGAAAGTGGAGCAATCCATGAGTAAAGTGG
CGCACGGGTGAGTAACACGTGACTAACCTACCCTCGAGTGGGGAATAACCTAGAGAAATCTGGGCTAA
TACCGCATAACACTTACGAGTCAAAGCAGCAATGCGCTTGGGGAGGGGGTCGCGGCCGATTAGTTAGT
TGGCAGGGTAATGGCCTACCAAGACGATGATCGGTATCCGGCCTGAGAGGGCGCACGGACACACTGGA
ACTGAAACACGGTC
>Zotu3774
GAACGCTGGCGGCGGGCTTAACACATGCAAGTCGAACGCGAAAGCCCCTTCGGGGGTGAGTAGAGTGG
CGCACGGGTGAGTATCGCGTAGACAACCTGCCCTTTGCTGGGGAATAACCACGGGAAACCGTGGCTAA
TACCGCATATTGTCTTAACCAGGCATCTGGATTAGATGAAAGCCTTCGGGCGGCAAAGGATGGGTCTG
CGTCGGATTAGCTAGTAGGTAGGGTAAAAGCCTACCTAGGCGACGATCCGTAGCTGGTCTGAGAGGAT
GATCAGCCACACTGGCACTGAGACACGGGC
>Zotu3775
GAACGCTGGCGGCGTGCTTAACACATGCAAGTCGAACGGTGAAAGCGGGAGCTTGCTCCTGCTGGATC
AGTGGCGAACGGGTGAGTAACACGTGAGTAACCTGCCCTTGACTCTGGGATAAGCGTTGGAAACGACG
TCTAATACCGGATACGAGCTTCGGAGGCATCTCCAGGAGCTGGAAAGAACTTCGGTCAAGGATGGACT
CGCGGCCTATCAGCTAGTTGGTGAGGTAACGGCTCACCAAGGCGACGACGGGTAGCCGGCCTGAGAGG

GTGACCGGCCACACTGGGACTGAGACACGGCC
>Zotu3777
GAACGCTGGCGGCGTGCTTAACACATGCAAGTCGAACGATGAAGCCAGCTTGCTGGTGGATTAGTGGC
GAACGGGTGAGTAACACGTGAGTAACCTGCCCTTGACTCTGGGATAAGCCTGGGAAACCGGGTCTAAT
ACTGGATACGACCTTCCCACGCATGTGGTGTTGGTGGAAAGCTTTTGTGGTTTTGGATGGACTCGCGG
CCTATCAGCTTGTTGGTGGGGTAATGGCCTACCAAGGCGACGACGGGTAGCCGGCCTGAGAGGGTGAC
CGGCCACACTGGGACTGAGACACGGCC
>Zotu3778
GAACGCTGGCGGCAGGCTTAACACATGCAAGTCGAACGCACCTTTACGGGTGAGTGGCAGACGGGTGA
GTAACGCGTGGGAACGTGCCCTTCAGTTCGGGATAACCCAGGGAAACTTGGGCTAATACCGGATACGT
GCGAGAGCAGAAAGATTTATCGCTGAAGGATCGGCCCGCGTCTGATTAGCTAGTTGGTGAGGTAATGG
CTCACCAAGGCGACGATCAGTAGCTGGTCTGAGAGGATGATCAGCCACATTGGGACTGAGACACGGCC
>Zotu3779
GAACGCTGGCGGCATGCTTAACACATGCAAGTCGAACGGTAACAGGTCTAGCTTGCTAGATGCTGACG
AGTGGCGGACGGGTGAGTAACGCGTAGGAATCTACCCCGCGGTGGGGGATAACACGAGGAAACTCGTG
CTAATACCGCATACGCTCTACGGAGGAAAATTTTTTTGCCGTGGGATGAGCCTGCGTTAGATTAGCTT
GTTGGTAGGGTAATGGCCTACCAAGGCGATGATCTATAGCTGTTCTGAGAGGAAGATCAGCCACACTG
GGACTGAGACACGGCC
>Zotu3780
GAACGCTGGCGGCATGCTTTACACATGCAAGTCGAACGGCAGCGCGGGGCAACCTGGCGGCGAGTGGC
GAACGGGTGAGTAATATATCGGAACGTACCCAGAAGTGGGGGATAACGTAGCGAAAGTTACGCTAATA
CCGCATACGATCTACGGATGAAAGTGGGGGACCTTCGGGCCTCATGCTTGTGGAGCGGCCGATATCTG
ATTAGCTAGTTGGTGAGGTAATGGCTCACCAAGGCGACGATCAGTAGCTGGTCTGAGAGGACGACCAG
CCACACTGGGACTGAGACACGGCC
>Zotu3781
GAACGCTGGCGGCGTGCCTAATACATGCAAGTCGAGCGGAAGTTGAAGAGCTTGCTCTTCAACTTTAG
CGGCGGACGGGTGAGTAACACGTGGGCAACCTGCCCTGCAGATCGGGATAACTCCGGGAAACCGGTGC
TAATACCGAATAGTTTTGGGTCCCTCCTGGGACCCAACGGAAAGACGGTTTCGGCTGTCACTGCAGGA
TGGGCCCGCGGCGCATTAGCTAGTTGGTGGGGTAACGGCCCACCAAGGCCACGATGCGTAGCCGACCT
GAGAGGGTGATCGGCCACACTGGGACTGAGACACGGCC
>Zotu3782
GAACGCTGGTGGCATGCTTTACACATGCAAGTCGAACGGCAGCATGGGCTTCGGCCTGATGGCGAGTG
GCGAACGGGTGAGTAATACATCGGAACGTGCCTAGTAGTGGGGGATAACTACTCGAAAGAGTAGCTAA
TACCGCATGAGATCTAAGGATGAAAGCAGGGGACCTTCGGGCCTTGTGCTACTAGAGCGGCCGATGGC
AGATTAGGTAGTTGGTGGGATAAAAGCTTACCAAGCCGACGATCTGTAGCTGGTCTGAGAGGACGACC
AGCCACACTGGGACTGAGACACGGCC
>Zotu3783
GAACGCTAGCGGCAGGCTTAATACATGCAAGTCGAGGGGTAACAGGGGTAGCAATACCTGCTGACGAC
CGGCAAACGGGTGCGGAACACGTACGCAACCTTCCCATAACTGGTGAATAGCCCTCCGAAAGGAGGAT
TAATACACCGTAACATAATGAAGTGGCATCACTTTATTATTATAGGCTTCGGCCGGTTTTGGATGGGC
GTGCGCCTGATTAGGTAGTTGGCGGGGTAACGGCCCACCAAGCCTGCGATCAGTAACTGGTGTGAGAG
CACGACCAGTCACACGGGCACTGAGACACGGGC
>Zotu3784
GAACGCTGGCGGCATGCCTAACACATGCAAGTCGAACGGCAGCACAGTAAAGATTTCGGTCTTTATGG
GTGGCGAGTGGCGGACGGGTGAGTAACGCGTAGGAATCTGTCCTGGAGCGTGGGATAACCCGAGGAAA
CTCGGGCTAATACCGCATGATATCGAGAGATCAAAAGCAGGGGACCGAAAGGCCTTGCGCTCTGGGGT
GAGCCTACGTCGGATTAGCTAGTTGGTGGGGTAAAAGCCTACCAAGGCGACGATCCGTAGCTGGTCTG
AGAGGATGACCAGCCACACTGGGACTGAGACACGGCC
>Zotu3785
GAACGCTGGCGGCGTGGATAAGTCATGCAAGTCAAGGGGACCGCAAGGTAACCGGCAAACGAGGTAGT
AACACGTAGGTACTCCCCCCGAAGTTTGGCATAGCTCGTCGAAAGACGGGGTAATTCCAAATAGTCCC
GCAAGGGTAAAGATTTATCGCTTCGGGAGAGGCCTGCGTAGTATCAGTTAGTTGGTAGGGTAACGGCC
TACCAAGGCTACGACGCTTAGGGGAGCTGAGAGGCTGACCCCCACCGATGGGACTGCGACACGGCC
>Zotu3786

GAACGCTGGCGGCGTGCCTAATACATGCAAGTCGAGCGAATTACTGGGAGCTTGCTCCTGGTAGTTAG
CGGCGGACGGGTGAGTAACACGTGGGCAACCTGCCTGTAAGACTGGGATAACACCGGGAAACCGGTGC
TAATACCGGATAATCCTTTTCCTCGCGTGGGGGAAAGCTGAAAGTCGGTTTCGGCTGGCACTTACAGA
TGGGCCCGCGGCGCATTAGCTAGTTGGTGAGGTAACGGCTCACCAAGGCGACGATGCGTAGCCGACCT
GAGAGGGTGATCGGCCACACTGGGACTGAGACACGGCC
>Zotu3787
GAATGCTGGCGGCGTGCCTAATACATGCAAGTCGAGCGGTAACAGGGCTTCACCGTTTCCTTGACACA
CCTAGCGCTTTATCGTAGGTTGGTTAGATTCGAGGGAACAGTGAAGCTGCTGACGAGCGGCGGACGGC
TGAGTAACGCGTGGGAACGGACCCCAAACTGAGGGATAACGCACCGAAAGGTGTGCTAATACCGCATG
TGATCTTCGGATTAAAGTCTTCGGACGGTTTGGGACTGGCCTGCGTACGATTAGATAGTTGGTAGGGT
AATGGCCTACCAAGTCGACGATCGTTAGATGGTTTGAGAAGATGATCATCCAGACTGGGACTGAGACA
CGGCC
>Zotu3788
GAACGCTGGCGGCGTGCTTCATACATGCAAGTCGAACGGACACCATCCAGTAGCAATACTGGTGAAGT
GTTAGTGGCGAACGGGTGCGTAACACGTGAGAAACTCACCCCGGTCTCTGGGATAACAGATGGAAACA
ACTGCTAATACCGGATACCTTCCCGAAGTCGCATGGCTATGGGAAGAAAGATTTATCGGACTGGGAGA
GTCTCGCGGCCTATCAGCTAGTTGGTGGGGTAAAGGCCTACCAAGGCATCGACGGGTAGCTGGTCTGA
GAGGATGATCAGCCACACTGGGACTGAGACACGGCC
>Zotu3789
GAACGCTGGCGGCGTGCCTAACACATGCAAGTCGAGCGATCTTCTTCGGAAGAGAGCGGCGGACGGGT
GAGTAACGCGTGGGTAACCTGCCCTGTACACACGGATAACATACCGAAAGGTATGCTAATACGAGATA
ACATACTTTTATCGCATGGTAGAAGTATCAAAGCTCCGGCGGTACAGGATGGACCCGCGTCTGATTAG
CTAGTTGGTAAGGTAACGGCTTACCAAGGCGACGATCAGTAGCCGACCTGAGAGGGTGATCGGCCACA
TTGGAACTGAGACACGGTC
>Zotu3790
GAACGCTGGCGGCATGCCTTACACATGCAAGTCGAACGGCAGCACGGGAGCAATCCTGGTGGCGAGTG
GCGAACGGGTGAGTAAAGTATCGGAACGTGCCCAGTCGTGGGGGATAACGTAGCGAAAGTTACGCTAA
TACCGCATACGATCTACGGATGAAAGCGGGGGATCGCAAGACCTCGCGCGATTGGAGCGGCTGATATC
AGATTAGGTAGTTGGTGGGGTAAAGGCTCACCAAGCCGACGATCTGTAGCTGGTCTGAGAGGACGACC
AGCCACACTGGGACTGAGACACGGCC
>Zotu3792
GAATGCTGGCGGCGTGCCTAATACATGCAAGTCGAGCGGTAACAGGGGGTCTCACCGTTTCCTTGACA
CACCTAGCGCTATGATCGTAGGTTGTTGACTTCGAGGGAACAGTGAGATCCTGCTGACGAGCGGCGGA
CGGCTGAGTAACGCGTGGGAACGGACCCCAAACTGAGGGATAACGCACCGAAAGGTGTGCTAATACCG
CATGTGATCTTCGGATTAAAGGATTTATCCGGTTTGGGACTGGCCTGCGTACGATTAGATAGTTGGTA
GGGTAACGGCCTACCAAGTCGACGATCGTTAGATGGTTTGAGAAGATGATCATCCAGACTGGGACTGA
GACACGGCC
>Zotu3793
GAACGCTAGCGGCAGGCCTAATACATGCAAGACGAACGGCAAGATTGATAGCAATATTGATCCTAGAG
TGTCGCACGGGTGCGTAACACGTATGCAACCTACCTTGTACAGGAGTATAGCTCGGGGAAACTCGAAT
TAACCCTCCATAATATGTCGATGTGGCATCACATTGATATTAAAACTTCGGTGGTACAAGATGGGCAT
GCGTTTGATTAGCTAGTTGGCGGGGTAATGGCCCACCAAGGCGATGATCAATAGGGGAACTGAGAGGT
TGATCCCCCACACTGGCACTGAGATACGGGC
>Zotu3794
GAACGCTAGCGGGAGGCCTAACACATGCAAGCCGGGCGGGATCCCTTCGGGGTGAGAGCGGCGTACGG
GTGAGGAGCACGTGTGCAACCTGCCCCTGTCTGGGGGATAGCCCTCCGAAAGGAGGATTAACACCCCA
TAGGATGTTCGCCGGCATCGGCGGTCATTGAAAGGTTACGGACGGGGATGGGCACGCGCAGGATTAGG
TAGTTGGCAGGGTAACGGCCTACCAAGCCTTTGATCCTTAGGGGGCCTGAGAGGGTGATCCCCCACAC
TGGTACTGAGACACGGAC
>Zotu3795
GAACGCTGGCGGCGTGCCTTAAGCATGCAAGTAGAACGAGTTCTATCTTGCCGCAAGGCAAGCTTAGA
ATGAGTTGCGAACGGCTGAGTAATGAATAAGAATCTTTCCTTTAGTGGGGAATACCTCATCGAAAGAT
GAGCTAATACCGCATAACATCTACGGATAAAAGCGATTAACTTCGCGCTGATTGATGAGCTTATTCCC
TATCAGGTAGTTGGTGGGGTAATGGCCTACCAAGCCTACGACGGGTACCCGATCTGAGAGGATGGACG

GGCAGAATGGGACTGAGACACGGCC
>Zotu3796
GAACGCTGGCGGCATGCTTTACACATGCAAGTCGAACGGCAGCGCGGGGCAACCTGGCGGCGAGTGGC
GAACGGGTGAGTAATATATCGGAACGTACCCAAGAGTGGGGGATAACGTAGCGAAAGTTACGCTAATA
CCGCATACGATCCAAGGATGAAAGTGGGGGATCTTCGGACCTCATGCTCCTGGAGCGGCCGATATCTG
ATTAGCTAGTTGGTGAGGTAAAGGCTCACCAAGGCGACGATCAGTAGCTGGTCTGAGAGGACGACCAG
CCACACTGGGACTGAGACACGGCC
>Zotu3797
GAACGCTAGCGGCAGGCCTAACACATGCAAGTCGAGGGGTATATGTCTTCGGATATAGAGACCGGCGC
ACGGGTGCGTAACGCGTATGCAATCTACCTTTTACAGGGGAATAGCCCAGAGAAATTTGGATTAATGC
CCCATAGTATATAAGAATCGCATGGTTTTTATATTAAAGTTCCAACGGTAAAAGATGAGCATGCGTCC
CATTAGTTAGTTGGTAAGGTAACGGCTTACCAAGACAATGATGGGTAGGGGTCCTGAGAGGGAGATCC
CCCACACTGGTACTGAGACACGGAC
>Zotu3798
GAATGCTGGCGACGTGGATAAGGCATGCAAGTCAAGGGGGCCGCAAGGCAACCGGCAGACGAGGTAGT
AACACGTACGAACGCACCCCGAAGTCTGGAATAGCTCGTCGAAAGACGGGGTAATACCGGATGGTACC
GCAAGGTTAAAGATTTATCGCTTCGGGAGCGGCGTGCGCAATATCAGCTAGTTGGTGAGGTAATGGCT
CACCAAGGCTACGACGTTTAGGGGAACTGAGAGGTTGACCCCCACCGATGGGACTGAGACACGGCC
>Zotu3799
GAACGCTGGCGGCGTGCCTAACACATGCAAGTCGAGCGGAGAGGTTGCGGAGATTCAATCGATGATCT
GCTTCTTCTTAGCGGCGGATGGGTGAGTAACACGTGGGTAACCTGCCCTGCACTGGGGAATAACAGTT
GGAAACGACTGTTAATACCGCATATGCGCACGGGGTCGCATGACCCCGGGCGGAAAGATTTATCGGTG
CAGGATGGGCCCGCGTCGGATTAACTGGTTGGTGAGGTAATGGCCCACCAAGGTGATGATCCGTAGCC
GACCTGAGAGGGTGACCGGCCACATTGGGACTGAGACACGGCC
>Zotu3800
GAACGCTAGCGGGAGGCCTAACACATGCAAGCCGAGCGGTATTTGTCTTTCGGGACGAAGAGAGCGGC
GTACGGGTGCGGAACACGTGTGCAACCTGCCTTTATCAGGGGGATAGCCTTTCGAAAGGAAGATTAAT
ACCCCATAATATATTAATTGGCATCAATTGATATTGAAAACTACGGTGGATAGAGATGGGCACGCGCA
AGATTAGATAGTTGGTAGGGTAACGGCCTACCAAGTCAGTGATCTTTAGGGGGCCTGAGAGGGTGATC
CCCCACACTGGTACTGAGACACGGAC
>Zotu3801
GAACGCTGGCGGCGTGCCTAATACATGCAAGTCGAGCGGACCTTTTAAAACTTGCTTTGAAAGGTTAG
CGGCGGACGGGTGAGTAACACGTGGGCAACCTGCACTATAGTTTGGGATAACTCCGGGAAACCGGGGC
TAATACCGAATAACACAATCTATCTCCTGTTAGATTGTTGAAAGATGGTTTCGGCTATCGCTATAGGA
TGGGCCCGCGGCGCATTAGCTAGTTGGTAGGGTAACGGCCTACCAAGGCGACGATGCGTAGCCGACCT
GAGAGGGTGATCGGCCACACTGGGACTGAGACACGGCC
>Zotu3802
GAACGCTGGCGGCAGGCTTAACACATGCAAGTCGAGCGGTAACAGGAGAAGCTTGCTTCTCGCTGACG
AGCGGCGGACGGGTGAGTAATACTTAGGAATCTACCTAGTAGTGGGGGATAGCTCGGGGAAACTCGAA
TTAATACCGCATACGACCTACGGGAGAAAGGGGGCAACTTGTTGCTCTCGCTATTAGATGAGCCTAAG
TCGGATTAGCTAGATGGTGGGGTAAAGGCCTACCATGGCGACGATCTGTAGCTGGTCTGAGAGGATGA
TCAGCCACACCGGGACTGAGACACGGCC
>Zotu3804
GAACGCTGGCGGCAGGCCTAACACATGCAAGTCGAACGATAAGCCACCTTCGGGTGGTGGACAGTGGC
AGACGGGTGAGTAACGCGTGGGAACGTGCCTTTCGGTTCGGGATAACCCAGGGAAACTTGGGCTAATA
CCGGATACGCCCTTCGGGGGAAAGATTCATTGCCGAAAGATCGGCCCGCGTCGGATTAGCTTGTTGGT
GGGGTAATGGCCTACCAAGGCGACGATCCGTAGCTGGTCTGAGAGGATGACCAGCCACACTGGGACTG
AGACACGGCC
>Zotu3805
GAACGCTAGCGGCAGGCCTAATACATGCAAGTCAAACGGCTAGCAATAGCAGTGGCGCACGGGTGAGT
AACGCGTACACAACGTACCTTATAGAGGGGTATAGCATCGGGAAACTGGTGGTAATCCCCCATAGTAT
TATTGAATGGCATCATTTAATAATTAAACTTTTTGGCTATGAGATCGGTGTGCGTCTGATTAGCTAGT
TGGTAGGGTAACGGCTTACCAAGGCTACGATCAGTAGGGGGCGTGAGAGCGTGGTCCCCCACACGGGT
ACTGAGACACGGAC

>Zotu3806
GAACGCTGGCGGCGTGCTTAACACATGCAAGTCGAGCGGTAAGGCCCTTCGGGGTACACGAGCGGCGA
ACGGGTGAGTAACACGTGGGTGACCTGCCCTCCACTTCGGGATAAGCCTGGGAAACCGGGTCTAATAC
CGGATATGACTTCTGGGTGCATGCCTGGTTGTGGAAAGTTTTTTCGGTGGGGGATGGGCCCGCGGTCT
ATCAGCTTGTTGGTGGGGTGATGGCCTACCAAGGCGACGACGGATAGCCGGCCTGAGAGGGTGATCGG
CCACACTGGGACTGAGACACGGCC
>Zotu3807
GAACGCTGGCGGTATGCCTAATACATGCAAGTCGAGCGGTAAGGCCCCTTCGGGGGTACACGAGCGGC
GGACGGCAGAGTAACGCGTAGGAACATACCCAAAGTGAGGAATAACTGCTCGAAAGAGTAGCTAATA
CCGCATGTGATCTTCGGATTAAAGCCTTAGGGCGCTTTGGGAATGGCCTGCGTTCGATTAGCTAGTTG
GTGAGGTAAATGCTCACCAAGGCGACGATCGATAGCTGGTCTGAGAGGATGATCAGCCTGACTGGAAC
TGAGACACGGTC
>Zotu3809
GAACGCTGGCGGAATGCTTAACACATGCAAGTCGCACGGGGGCTTTCGGGCTCTAGTGGCGGACGGGT
GAGTAACGCGTAGGAACGTGTCTTGGGGTGGGGGATAACGCTGGGAAACTGGCGCTAATACCGCATAT
CTGCTGTGGCAGAAAGCCTTCGGGTGCCTTGAGAGCGGCCTGCGTCCGATTAGCTTGTTGGTGGGGTA
ATGGCTTACCAAGGCGACGATCGGTAGCTGGTCTGAGAGGACGATCAGCCACACTGGGACTGAGACAC
GGCC
>Zotu3810
GAACGCTGGCGGCGTGCCTAATACATGCAAGTCGAGCGGTAAGGTTCTCACTCTGTATTTAACGAGTT
TGAATATGGTGTGAGAATACACGAGCGGCGGACGGCTGAGTAACACGTAGGAACGTACCCCAAAGTGA
GGGATAAGCACCAGAAATGGTGTCTAATACCGCATGTGCTCTTCGGAGGAAAGCTTCGGCGCTTTGGG
AACGGCCTGCGCATGATTAGCTTGTTGGTGAGGTAATGGCTCACCAAGGCGATGATCATTAGCTGGTC
TGAGAGGATGATCAGCCAGACTGGGACTGAGAACGGCC
>Zotu3812
GAACGCTAGCGGCAGGCCTAACACATGCAAGTCGAGGGGTATAGGTAGCAATACTTAGAGACCGGCGC
ACGGGTGAGTAACGCGTATGCAATCTGCCCCATATCGGGGGATAGCTCGTCGAAAGGCGAATTAATAC
CCCATAATACTACGAGAAGGCATCTTTTTGTATTTAAAACTCCGGTGATATGGGATGAGCATGCGTGA
CATTAGCTAGTTGGTGAGGTAACGGCTCACCAAGGCTACGATGTCTAAGGGGTCTGAGAGGATGATCC
CTCACACTGGTACTGAGACACGGAC
>Zotu3815
TAACGCTGGCGGCGTGCTTAACACATGCAAGTCGAACGAGGGCCTTTCTCCCGCAAGGGAGGGGGGTT
CCTAGTGGCGAACGGGTGAGTAACACGTGGGCAACCTGTCCTTTCGTGGGGGATAACACCGGGAAACC
GGTGCTAATACCGCGTACGGTCGCCGAAGGCATCTTCGGTGAAGAAAGCAGCAATGCGCGAAAGGGTG
GGCCCGCGGCCTATCAGCTAGTCGGTGGGGTAACGGCCTACCGAGGCTTTGACGGGTAGCTGGTCTGA
GAGGACGACCAGCCACACGGGGACTGAGACACGGCC
>Zotu3816
GAACGCTGGCGGCGTGCTTAACACATGCAAGTCGAACGCTGAAGCCTGGTGCTTGCACCGGGTGGATG
AGTGGCGAACGGGTGAGTAATACGTGAGTGACCTGCCTTTGACTCTGGGATAAGCCTGGGAAACTGGG
TCTAATACCGGATGCGACTACTGCCCGCATGGGCTGGTGGTGGAAAGGGTTATGTACTGGTCTTAGAT
GGGCTCACGGCCTATCAGCTGGTTGGTGGGGTAATGGCCTACCAAAGCGACGACGGGTAGCCGGCCTG
AGAGGGTGACCGGCCACACTGGGACTGAGACACGGCC
>Zotu3818
GAACGCTGGCGGCGTGCCTAATACATGCAAGTCGAGCGGAGCACTTCGGTGCTTAGCGGCGGACGGGT
GAGTAACACGTAGGCAACCTGCCTATAAGACTGGGATAACTATCGGAAACGATAGCTAAGACCGGATA
ACTGGTTTTCTCGCATGAGAGAATCATGAAACACGGAGCAATCTGTGGCTTATAGATGGGCCTGCGGC
GCATTAGCTAGTTGGTGAGGTAACGGCTCACCAAGGCGACGATGCGTAGCCGACCTGAGAGGGTGAAC
GGCCACACTGGGACTGAGACACGGCC
>Zotu3819
GAACGCTGGCGGCGTGCCTAACACATGCAAGTCGAGCGATTCTCTTCGGAGAAGAGCGGCGGACGGGT
GAGTAACGCGTGGGTAACCTGCCCTGTACACACGGATAACATACCGAAAGGTATGCTAATACGGGATA
ATATATTTTGATGGCATCATCGAAATATCAAAGCTCCGGCGGTACAGGATGGACCCGCGTCTGATTAG
CTAGTTGGTGAGGTAATGGCTCACCAAGGCGACGATCAGTAGCCGACCTGAGAGGGTGATCGGCCACA
TTGGAACTGAGACACGGTC

>Zotu3820
GAATGCTGGCGGCGCGCCTAACACATGCAAGTCGAGCGGTAACAGGTCTAGTTTACTAGATGCTGACG
AGCGGCGGACGGCTGAGTAACGCGTGGGAACATGCCCCAAAGTGAGGAATAAGCTCCAGAAATGGAGT
CTAATGCCGCATATGGTCTTCGGATTAAAGGATTTATCCGCTTTGGGAGTGGCCTGCGTATGATTAGA
TAGTTGGTGAGGTAATGGCTCACCAAGTCTACGATCATTAACTGGTCTGAGAGGATGATCAGTCAGAC
TGGAACTGAGACACGGTC
>Zotu3821
GAACGCTGGCGGCGTGCCTAATACATGCAAGTCGAGCGGTAACACTTCGGGTGACGAGCGGCGGACGG
CAGAGTAACGCGTAGGAACGTACCCCAAACTGAGGGATAAGCCCGGGAAACCGGGTCTAATACCGCAT
ATTATTTTCGAATCAAAGCCTTCGGGCGGTTTGGGAGCGGCCTGCGTATGATTAGCTTGTTGGTGAGG
TAATAGCTCACCAAGGCGACGATCATTAGCTGGTCTGAGAGGATGATCAGCCTGACTGGAACTGAGAA
CGGTC
>Zotu3822
GAACGCTGGCGGCGTGCCTAACACATGCAAGTCGCGGGAGAAAGTCTTCGGATGAGTAAACCGGCGCA
CGGGTGAGTAACACGTGGGTAATCTTCCTTCAGGCCTGGAATAACCCGGGGAAACTCGGGCTAATACC
GGATAAGACCACGCAACGAGAGTTGTGTGAGAAAAGATGAGGACCTCTTCGGAGGCTTTTCACCAGAA
GATGAGCCCGCGGCCCATCAGCTAGTTGGCGGGGTAACGGCCCACCAAGGCTAAGACGGGTAGCTGGT
CTGAGAGGACGACCAGCCACACTGGAACTGAGACACGGTC
>Zotu3823
GAACGCTGGCGGCATGGTTAAAGCATGCAAGTCGAACGGACCCGCAAGGGTCAGTGGCGGAAGGGTGA
GTAATATGATCGAACGTGCCCTGAGGTGAGGGATAGCCATGGGAAACTGTGGGTAATACTTCATGATC
TCTACGGAGCAAAGGTTTACCGCCTTGGGATCGGCGATCATCCTATCAGCTAGTTGGTGAGGTAAAGG
CTCACCAAGGCGATGACGGGTAGCAGGTGTGAGAGCACGGCCTGCCACATCGAAACTGAGACACTGTT
>Zotu3824
GAACGCTGGCGGCGTGCTTAACACATGCAAGTCGAGCGAAGCAGTTAAGTTGATTTCTTCGGATTGAT
TCTTGACTGACTGAGCGGCGGACGGGTGAGTAACGCGTGGGTGACCTGCCCCATACCGGGGGATAACA
GCTGGAAACGGCTGCTAATACCGCATAAGCGCACAGAGCTGCATGGCTCGGTGTGAAAAACTCCGGTG
GTATGGGATGGGCCCGCGTCTGATTAGGCAGTTGGCGGGGTAACGGCCCACCAAACCGACGATCAGTA
GCCGGCCTGAGAGGGCGACCGGCCACATTGGGACTGAGACACGGCC
>Zotu3826
GAACGCTGGCGGCAGGCTTAACACATGCAAGTCGAGCGGAGATGAGGTGCTTGCACCTTATCTTAGCG
GCGGACGGGTGAGTAATGCTTAGGAATCTGCCTATTAGTGGGGGACAACATTCCGAAAGGAATGCTAA
TACCGCATACGTCCTACGGGAGAAAGCAGGGGATCTTCGGACCTTGCGCTAATAGATGAGCCTAAGTC
GGATTAGCTAGTTAGTGAGGTAAAGGCCTACCAAGGCGACGATCTGTAGCGGGTCTGAGAGGATGATC
CGCCACACTGGGACTGAGACACGGCC
>Zotu3827
GAACGCTGGCGGCGTGCTTAACACATGCAAGTCGAACGATGAAGCCAGCTTGCTGGTGGATTAGTGGC
GAACGGGTGAGTAACACGTGGGTGATCTGCCCCAAACTTTGGGATAAGCCTGGGAAACTGGGTCTAAT
ACCGAATATTACCTTCTCCTGCATGGGGGTTGGTGGAAAGCTCCGGCGGTTTGGGATGGGCCCGCGGC
CTATCAGCTTGTTGGTGGGGTAATGGCCTACCAAGGCGACGACGGGTAGCCGGCCTGAGAGGGCGACC
GGCCACACTGGGACTGAGACACGGCC
>Zotu3828
GAACGCTGGCGGCGTGCTTAACACATGCAAGTCGAGTGCACAGTCTGTAGCAATACAGATGGGTGCAC
GGCGCACGGGTGCGTAACACGTGGGTACCTGCCCTTTGATGGGGAATAACCCCGCGAAAGCGGGGCTA
ATTCCGCATAAGATCCGCAAGGATGAAAGCCGAAAGGCGTCGAAGGAGGGGCCTGCGCCCGATTAGCT
AGTTGGTGAGGTAACGGCTCACCAAGGCAATGATCGGTAGCTGGTCTGAGAGGATGATCAGCCACACT
GGAACTGAGACACGGTC
>Zotu3829
GAACGCTGGTGGCGTGCTTAACACATGCAAGTCGAACGGTGAAGCCCAGCTTGCTGGGTGGATCAGTG
GCGAACGGGTGAGTAACACGTGAGCAACCTGCCCTCCACTTCGGGATAAGCCTTGGAAACGAGGTCTA
ATACCGGATACGAGCACTTACCGCATGGTGGGTGTTGGAAAGTTTTTCGGTGGGGGATGGGCTCGCGG
CCTATCAGCTTGTTGGTGGGGTAATGGCCTACCAAGGCGTCGACGGGTAGCCGGCCTGAGAGGGCGAC
CGGCCACACTGGGACTGAGACACGGCC
>Zotu3830

GAACGCTAGCTACAGGCTTAACACATGCAAGTCGAGGGGCAGCATGGTCTTAGCTTGCTAAGGCCGAT
GGCGACCGGCGCACGGGTGAGTAACACGTATCCAACCTGCCGTCTACTCTTGGACAGCCTTCTGAAAG
GAAGATTAATACAAGATGGCATCATGAGTCCGCATGTTCACATGATTAAAGGTATTCCGGTAGACGAT
GGGGATGCGTTCCATTAGATAGTAGGCGGGGTAACGGCCCACCTAGTCTTCGATGGATAGGGGTCCTG
AGAGGAAGGTCCCCCACATTGGAACTGAGACACGGTC
>Zotu3831
AAACGCTGGCGGCGTACCTAAAACATGCAAGTCGAACGGCTCACTTCGGTGGGTAGTGGCGAACGGCT
GAGTAACACGTGGGTGACCTGCCTTCGAACGGGGGATAACTGCCCGAAAGGGTGGCTAATACCGCAGA
TGTCGGTTCTTCGTTGAGCTGACTAAACCGTTTAGGGTTCGAAGAGGGGCCTGCGTCCGATTAGCTAG
CTGGTGGGGTAAAGGCCTACCAGGGCGACGATCGGTAGCTGGTCTGAGAGGATGATCAGCCAGACTGG
GACTGCGAGACGGCC
>Zotu3832
GAACGCTGGCGGCAGGCTTAACACATGCAAGTCGAACGCCCCGCAAGGGGAGTGGCAGACGGGTGAGT
AACACGTGGGAACATACCCTTCGGTTCGGAACAACGCCGGGAAACTGGCGCTAATACCGGATACGGCC
TGTGGGCGAAAGATTCATCGCCGAAGGATTGGCCCGCGTTCGATTAGCTTGTTGGTGAGGTAACGGCT
CACCAAGGCGACGATCGATAGCTGGTCTGAGAGGATGGCCAGCCACATTGGGACTGAGACACGGCC
>Zotu3833
GAACGCTGGCGGCATGCCTAACACATGCAAGTCGAACGAGACCTTCGGGTCTAGTGGCGCACGGGTGC
GTAACGCGTGGGAATCTGCCCTTGGGTTCGGGATAACAGTTGGAAACGACTGCTAATACCGGATGATG
ACTTCGGTCCAAAGATTTATCGCCCAGGGATGAGCCCGCGTAGGATTAGCTAGTTGGTGGGGTAAAAG
CTCACCAAGGCGACGATCCTTAGCTGGTCTGAGAGGATGATCAGCCACACTGGGACTGAGACACGGCC
>Zotu3834
GAACGCTGGCGGAATGCTTTACACATGCAAGTCGAACGGCAGCACGGACTTCGGTCTGGTGGCGAGTG
GCGAACGGGTGAGTAATATATCGGAACGTGCCCAGTCGTGGGGGATAACGTAGCGAAAGTTACGCTAA
TACCGCATACGATCTATGGATGAAAGCGGGGGATCGCAAGACCTCGCGCGATTGGAGCGGCCGATATC
AGATTAGGTAGTTGGTGGGGTAAAGGCCTACCAAGCCGACGATCTGTAGCTGGTCTGAGAGGACGACC
AGCCACACTGGGACTGAGACACGGCC
>Zotu3835
GAACGCTAGCTATATGCTTAACACATGCAAGTCGAACGTTGTTTTCGGGGAGCTGGGCAGAAGGAAAA
GAGGCTCCTAGCTAAAGGTAGCTTGTCTCGCCCAGGAGGTGGGAACAGTTGAGAACAAAGTGGCGAAC
GGGTGCGTAACGCGTGGGAATCTGCCGAACAGTTCGGGCCAAATCCTGAAGAAAGCTAAAAAGCGCTG
TTTGATGAGCCTGCGTAGTATTAGGTAGTTGGTCAGGTAAAGGCTGACCAAGCCAATGATGCTTAGCT
GGTCTTTTCGGATGATCAGCCACACTGGGACTGAGACACGGCC
>Zotu3836
GAACGCTGGCGGCAGGCCTAACACATGCAAGTCGAGCGAGACCTTCGGGTCTAGCGGCGGACGGGTGA
GTAACGCGTGGGAACGTGCCCTTTTCTGCGGAATAGCCTCGGGAAACTGAGCGTAATACCGCATACGC
CCTTTGGGGGAAAGATTTATCGGAAAAGGATCGGCCCGCGTTGGATTAGGTAGTTGGTGGGGTAACGG
CCTACCAAGCCGACGATCCATAGCTGGTTTGAGAGGATGATCAGCCACACTGGGACTGAGACACGGCC
>Zotu3837
GAACGTTGATGGCGTGCCTCATACATGCAAGTCGAGCGCCCCGCAAGGGGAGCGGCGGACGGGTGAGT
AACACGTAAAGAATCTGCCCTGAAGCGGGGAATAACTATCCCAACGGATAGCTAATACCGCATAATGA
TGGAGGAGGCATCTCCTTTTCTCAAAAGGCTTCGGCCACTTCAGGATGACTTTGCGTCCCATTAGCTT
GTTGGCGGTGTAACGGACCACCAAGGCTACGATGGGTATCCGGCCTGAGAGGGTGTACGGACACATTG
GGACTGAGATACGGCC
>Zotu3838
GAACGCTGGCGGCATGCCTAACACATGCAAGTCGAACGAGACCTTCGGGTCTAGTGGCGCACGGGTGC
GTAACGCGTGGGAATCTGCCCTTGGGTTCGGAATAACTCAGAGAAATTTGAGCTAATACCGGATGATG
ACGTAAGTCCAAAGATTTATCGCCCAAGGATGAGCCCGCGTAGGATTAGCTAGTTGGTGAGGTAAAGG
CTCACCAAGGCGACGATCCTTAGCTGGTCTGAGAGGATGATCAGCCACACTGGGACTGAGACACGGCC
>Zotu3839
GAACGCTGGCGGCGTGCTTAACACATGCAAGTCGAGCGGAAAGGCCACTTCGGTGGTACTCGAGCGGC
GAACGGGTGAGTAACACGTGAGTAATCTGCCCTTGGCTTTGGGATAGCCACCGGAAACGGTGATTAAT
ACCGGATACGACAACTACTCGCATGGGTTGGTTGTGGAAAGTTTTTCGGCCAGGGATGTGCTCGCGGC
CTATCAGCTTGATGGTGAGGTAATGGCTCACCATGGCTTCGACGGGTAGCCGGCCTGAGAGGGTGACC

GGTCACACTGGGACTGAGACACGGCC
>Zotu3840
TAACGCTGGCGGTATGCCTTATACATGCAAGTCGAGCGGAAAGGCCCTTCGGGGTACTCGAGCGGCGG
ACGGGTGAGTAACACGTAGGAATCTGCCCTAAAGCGGGGGACAACCAGGGGAAACTTTGGCTAATACC
CCATAGGTCTAACGATTAAAGAAGCAATTCGCTTTAGGAGGAGCCTGCGGCTGATTAGCTAGTTGGTA
GAGTAATAGCCTACCAAGGCTTCGATCAGTAGCTGGTCTGAGAAGACGACCAGCCACAATGGAACTGA
GACACGGTC
>Zotu3841
GAACGCTGGCGGCGTGCCTAACACATGCAAGTCGAGCGAGTGGAGTTCTTCGGAACAAAGCTAGCGGC
GGACGGGTGAGTAACACGTGGGCAACCTGCCTCATAGAGGGGAATAGCCTCCCGAAAGGGAGATTAAT
ACCGCATAAGATTGTAGCTTCGCATGAAGTAGCAATTAAAGGAGCAATCCGCTATGAGATGGGCCCGC
GGCGCATTAGCTAGTTGGTGAGGTAGCGGCTCACCAAGGCGACGATGCGTAGCCGACCTGAGAGGGTG
ATCGGCCACATTGGGACTGAGACACGGCC
>Zotu3842
GAACGCTGGCGGCGTGCTTAACACATGCAAGTCGAGCGGAAAGGCCCTTCGGGGTACTCGAGCGGCGA
ACGGGTGAGTAACACGTGGGCAATCTGCCCCTCGCTCTGGGATAACTCCGGGAAACCGGGGCTAATAC
CGGATATGACCCTTTGAGGCATCTCTTTGGGTGGAAAGTTTTTCGGCGAGGGATGAGCCCGCGGCCTA
TCAGCTTGTTGGTGGGGTAATGGCCTACCAAGGCAGTGACGGGTAGCCGGCCTGAGAGGGCGACCGGC
CACACTGGGACTGAGACACGGCC
>Zotu3844
GAACGCTAGCGGCAGGCCTAATACATGCAAGCTGAACGGCAAGAGTTGTAGCAATACGATTCCTAGAG
TAGCGTAAGGGTGTGTAACACGTATGCAATCTACCCACAACAGGAGGATAGCTCCCCGAAAGGGGAAT
TAAGACTCCATAATATCATATGAGGGCATCTGAGTATGATTAAAGTACAAGCGGTTGTGGATGAGCAT
GCGTCTGATTAGTTAGTTGGCGGGGTAACGGCCCACCAAGACGATGATCAGTAGGGGAACTGAGAGGT
TGGTCCCCCACACTGGCACTGAGATACGGGC
>Zotu3845
GAACGCTAGCGGCAGGCTTAATACATGCAAGTCGAGGGGCAGCGGGGGTAGCAATACCTGCCGGCGAC
CGGCAAACGGGTGCGGAACACGTACAGAACCTTCCTTTAAGCGGGGGATAGCCCATAGAAATGTGGAT
TAATACCCCATAGTATTATTGGTGTGGCATCACACTATAATTAAAGATTTATCACTTAAAGATGGCTG
TGCGGCTGATTAGGTAGTTGGTGCGGGTAACGGCCCACCAAGCCTTCGATCAGTAACTGGTGTGAGAG
CACGACCAGTCACACGGGCACTGAGACACGGGC
>Zotu3846
AAACGCTGGCGGCGTGCCTAACACATGCAAGTCGAACGCCCTATGCTTCGGTGTAGGGAGTGGCGGAC
GGGTGAGTAACACGTGGGATATCTACCCAGAGGTGAGGAATACCGGCGAGAAATCGCCGACAATACCG
CATGCGCTTCGCAAGAAGCAAAGCCTGGCAACAGGCGCCACTGGACGATCCTGCGGTCGATTAGCTAG
TTGGCGGGGTCAAAGCCCACCAAGGCGACGATCGATAGCTGGTCTGAGAGGACGATCAGCCACACTGG
GATTGAGAACGGCC
>Zotu3847
GAACGCTGGCGGCGTGCCTAATACATGCAAGTCGAGCGAATCAATGGGAGCTTGCTCCCTGAGATTAG
CGGCGGACGGGTGAGTAACACGTGGGTAACCTGCCTGTAAGATTGGGATAACTCCGGGAAACCGGGGC
TAATACCGGATAATACTTTTAACCACATGGTTAAAAGTTGAAAGATGGTTTCGGCTATCACTTACAGA
TGGACCCGCGGCGCATTAGCTAGTTGGTGAGGTAACGGCTCACCAAGGCAACGATGCGTAGCCGACCT
GAGAGGGTGATCGGCCACACTGGGACTGAGACACGGCC
>Zotu3848
GAACGCTGGCGGCGTGCTTAACACATGCAAGTCGCGTGCGCAGTTCGCGTAGCAATACGTGGATGGGC
GCACGGCGCACGGGTGCGTAACACGTGGGCAATCTGCCCTCCGATGGGGAATAACCCCGCGAAAGCGG
GGCTAATACCGCATAATATTCCGAGGACTTCGGTTCTTGGATTCAAAGCAGTGATGCGTCGGAGGAGG
AGCCCGCGCCCGATTAGCTAGTTGGTAGGGTAACGGCCTACCAAGGCGATGATCGGTAGCTGGTCTGA
GAGGATGATCAGCCACACTGGAACTGAGACACGGTC
>Zotu3849
GAACGCTAGCGGCAGGCTTAACACATGCAAGTCGAGGGGTATAGTAGCAATACTAGAGACCGGCGCAC
GGGTGCGTAACGCGTATGCAATCTACCTTTTACAGAGGGATAGCCCAGAGAAATTTGGATTAATACCT
CATAGTATATCGTTTGGGCATCCTAACGATATTAAAGTTTCAACGGTAAAAGATGAGCATGCGTCCTA
TTAGCTAGATGGTAAGGTAACGGCTTACCATGGCTACGATAGGTAGGGGTCCTGAGAGGGAGATCCCC

CACACTGGTACTGAGACACGGAC
>Zotu3850
GAACGCTGGCGGCGTGCCTAACACATGCAAGTCGAGCGATGAAACCCTTCGGGGTGGATTAGCGGCGG
ACGGGTGAGTAACACGTGGGTAACCTGCCTCAAAGAGGGGAATAGCCTCCCGAAAGGGAGATTAATAC
CGCATAATATGTTTAGATCGCATGATCTTAACATCAAAGGAATTTCTTCGGAAATTTCACTTTGAGAT
GGACCCGCGGCGCATTAGCTAGTTGGTGAGGTAAAGGCCCACCAAGGCAACGATGCGTAGCCGACCTG
AGAGGGTGATCGGCCACATTGGAACTGAGACACGGTC
>Zotu3851
GAACGCTAGCGGCAGGCCTAATACATGCAAGTCGGACGGGATTGCAGTATAGCTTGCTATACTGCATG
AGAGTGGCGCACGGGTGCGTAACGCGTGAGCAACCTGCCCATGTCAGGGGGATAGCCCGTTGAAAGAC
GGATTAATACCGCATAACACATAGAGACCACCTGGTTTCTATGTCAAATATTTATAGGACATGGATGG
GCTCGCGTGGCATTAGTTAGTTGGTAAGGTAACGGCTTACCAAGACGATGATGCCTAGGGGCTCTGAG
AGGAGTGACCCCCACACTGGTACTGAGACACGGAC
>Zotu3852
GAACGCTGGCGGCGTGCCTAATACATGCAAGTCGAGCGGAACACTTGGAGCTTGCTCCAAGCGTTTAG
CGGCGGACGGGTGAGTAACACGTGGGCAACCTGCCCTGCAGATCGGGATAACTCCGGGAAACCGGTGC
TAATACCGAATAGTTTGAAGCCTCTCCTGAGGCTTCACGGAAAGACGGTTTCGGCTGTCACTGCAGGA
TGGGCCCGCGGCGCATTAGCTAGTTGGTGGGGTAACGGCCCACCAAGGCCACGATGCGTAGCCGACCT
GAGAGGGTGACCGGCCACACTGGGACTGAGACACGGCC
>Zotu3853
GAACGCTGGCGGCAGGCCTAACACATGCAAGTCGAGCGCCCCGCAAGGGGAGCGGCGGACGGGTGAGT
AACACGTGGGAATTTTCCTCAAGGTACGGAACAACTCAGGGAAACTTGGGCTAATACCGTATGATGCA
GAGATGCCAAAGATTTATCGCCTTGGGATAAGCCCGCGTCAGATTAGGTAGTTGGTGGGGTAATGGCC
TACCAAGCCTGTGATCTGTAGCTGGTCTGAGAGGACGATCAGCCACATTGGGACTGAGACACGGCC
>Zotu3854
GAACGCTGGCGGAATGCTTTACACATGCAAGTCGAGCGGCAGCGCGGGGCAACCTGGCGGCGAGCGGC
GAACGGGTGAGTAATACATCGGAACATATCCATTTGTGGGGGATAGCCCGGCGAAAGCCGGATTAATA
CCGCATGAGCTCGGGAGAGGAAAGCCGGGGACCGCAAGGCCTGGCGCGAATGGGTTGGCCGATGTCCG
ATTAGCTTGTTGGTGAGGTAAAGGCTTACCAAGGCGATGATCGGTAGCTGGTCTGAGAGGATGATCAG
CCACACTGGGACTGAGACACGGCC
>Zotu3856
GAACGCTAGCGGTGCGCCTAACACATGCAAGTCGAGTGCCCCGCCTCTGGTGGGGCACGGCAAACGGG
TGAGTAACACGTTGGTAACTTACCCCGGAGTGAGGAATAACCCGCCGAAAGGCGGGCTAATGCCGCAT
AAGAAAGGGGGGACGAAAGTCATTCCCTTTTAAAGTCGTAAGACGCTTCGGGAGAGGCCTGCGTAGTA
TCAGCTAGTTGGTAGGGTAACGGCCTACCAAGGCTATGACGCTTAGTTGGTCTGAGAGGACGACCAGC
CACAATGGGACTGAGACACGGCC
>Zotu3857
GAACGCTGGCGGCGTGCCTAACACATGCAAGTCGAACGGAGATTTCAGCAATGAAATCTTAGTGGCGA
ACGGGTGAGTAACGCGTAGACAACCTACCTTTTAGATGGGGACAACACTGCGAAAGTGGTGCTAATAC
CGAATGTTGCATTTTGATTGCATAATCGAAATACTAAAGATGGCCTCTATTTATAAGCTATCGCTAAA
AGATGGGTCTGCGTCTGATTAGCTAGTTGGTGAGGTAATGGCTCACCAAGGCGACGATCAGTAGCCGG
TCTGAGAGGATGAACGGCCACACTGGGACTGAGACACGGCC
>Zotu3858
GAACGCTGGCGGCGTGCTTAACACATGCAAGTCGAGCGGTAAGGCTCCTTCGGGAGTACACGAGCGGC
GAACGGGTGAGTAACACGTGAGTAATCTGCCCTCCACTTCGGGATAAGCGCCGGAAACGGCGTCTAAT
ACCGAATACGACGCACTCTCGCATGAGATGTGCGTGGAAAGTTCTATCGGTGGAGGATGTGCTCGCGG
CCTATCAGCTTGTTGGTGAGGTAACGGCTCACCAAGGCTTCGACGGGTAGCCGGCCTGAGAGGGTGAC
CGGCCACACTGGGACTGAGACACGGCC
>Zotu3859
GAACGCTGGCGGCGTGCCTAATACATGCAAGTCGAGCGCCCCTTTAAGAGGTGACAATTTCGATTGTC
GGTTCCTATGGGGAGCGGCGGACGGCTGAGTAACGCGTGGGAACCTGCCCCAAAGTGAGGGATAACGC
ACCGAAAGGTGTGCTAATACCGCATATGATCTTCGGATTAAAGCTTTATGCGCTTTGGGAGGGGCCTG
CGTCCTATTAGATAGTTGGTGAGGTAAAGGCTCACCAAGTCAACGATGGGTAGCTGGTCTGAGAGGAT
GACCAGCCAGACTGGAACTGAGACACGGTC

>Zotu3860
GAACGCTGGCGGCATGCCTAACACATGCAAGTCGAACGAAGCCTTCGGGCTTAGTGGCGCACGGGTGC
GTAACGCGTGGGAATCTGCCCTTGGGTTCGGAATAACGTCGGGAAACTGACGCTAATACCGGATAATA
TCGCGAGATCAAAGATTTATCGCCTGAGGATGAGCCCGCGTAGGATTAGCTAGTTGGTGGGGTAAAGG
CCTACCAAGGCGACGATCCTTAGCTGGTCTGAGAGGATGATCAGCCACACTGGGACTGAGACACGGCC
>Zotu3861
GAACGCTGGCGGCGTGCTTAACACATGCAAGTGGAGCGACGAACCGGGGCTTGCCCCGGGGCAGAGCC
GCGAACGGGTGAGTAACACGTGGGTTACCTGCCCCGTTGACAGGGACAACCCGGGGAAACCCGGGCTA
ATACCTGATGTGCCCCTTGGGGGAAAGGAAGCTTCGGCCTCCGCAACGGGATGGGCCCGCGGCGCATT
AGCTTGTTGGTGGGGTAACGGCCTACCAAGGCGACGATGCGTAGCTGGTCTGAGAGGACGATCAGCCA
CACTGGGACTGAGACACGGCC
>Zotu3862
GAACGCTGGCGGCGTGCCTAACACATGCAAGTCGAGCGATCTCTTCGGAGAGAGCGGCGGACGGGTGA
GTAACGCGTGGGTAACCTGCCCTGTACACACGGATAACATACCGAAAGGTATACTAATACGGGATAAC
ATACGAAAGTCGCATGGCTTTTGTATCAAAGCTCCGGCGGTACAGGATGGACCCGCGTCTGATTAGCT
AGTTGGTAAGGTAATGGCTTACCAAGGCAACGATCAGTAGCCGACCTGAGAGGGTGATCGGCCACACT
GGAACTGAGACACGGTC
>Zotu3863
GAACGCTGGCGGCATGCCTAACACATGCAAGTCGAACGAGACCTTCGGGTCTAGTGGCGCACGGGTGC
GTAACGCGTGGGAATCTGCCCTTGGGTTCGGAATAACTTCTGGAAACGGAAGCTAATACCGGATGATG
ACGTAAGTCCAAAGATTTATCGCCTGAGGATGAGCCCGCGTGAGATTAGGTAGTTGGTGGGGTAAAGG
CCTACCAAGCCGACGATCTCTAGCTGGTCTGAGAGGATGATCAGCCACACTGGGACTGAGACACGGCC
>Zotu3864
GAACGCTGGCGGCGTGCTTAACACATGCAAGTCGAACGGTGAAGGACGGAGCTTGCTCTGTCTGGATC
AGTGGCGAACGGGTGAGTAACACGTGAGTAACCTGCCCCAGACTCTGGAATAAGCGCTGGAAACGGCG
TCTAATACCGGATACGAGACGGAGAGGCATCTCTACCGTCTGGAAAGTTTTTCGGTCTGGGATGGACT
CGCGGCCTATCAGCTTGTTGGTGAGGTAATGGCTCACCAAGGCAACGACGGGTAGCCGGCCTGAGAGG
GCGACCGGCCACACTGGGACTGAGACACGGCC
>Zotu3865
TAACGTTGGCAGCGTGCCTAATACATGCAAGTCGAGCGGCCCGCAAGGGCAGCGGCAGACGAGAGAGT
AATAGTAAGGAACTTACCCCCGAGTGAAGGATAGCCATCCGAAAGGATGGGTAATCCTGCATAATATC
GAAAGATCAAAGCCGCAAGGCGCTTGGGGAGAGGCCTTACACCTATCAGCTTGTTGGCGAGGTAAATG
CTCACCAAGGCGATGACGGGTAGCTGGTCTGAGAGGATGACCAGCCTCGATGGGACTGAGACACGGCC
>Zotu3868
GAACGCTGGCGGCGTGCTTAACACATGCAAGTCGAACGAAGCACTCTATTTGATTTTCTTCGGAAATG
AAGATTTTGTGACTGAGTGGCGGACGGGTGAGTAACGCGTGGGTAACCTGCCTCATACAGGGGGATAA
CAGTTGGAAACGACTGCTAATACCGCATAAGCGCACAGGATTGCATGATCCGGTGTGAAAAACTCCGG
TGGTATGAGATGGACCCGCGTCTGATTAGCCAGTTGGCAGGGTAACGGCCTACCAAAGCGACGATCAG
TAGCCGACCTGAGAGGGTGACCGGCCACATTGGGACTGAGACACGGCC
>Zotu3869
GAACGCTGGCGGCGTGCCTAATACATGCAAGTCGAGCGAAGTTTTTCTGGTGCTTGCACTAGAAAAAC
TTAGCGGCAAACGGGTGAGTAACACGTAAAGAACCTGCCTCATAGACTGGGACAACTATTGGAAACGA
TAGCTAATACCGGATAACAGCATTAACTGCATGGTTGATGTTTAAAAGTTGGTTTTGCTAACACTATG
AGATGGCTTTGCGGTGCATTAGCTAGTTGGTGGGGTAAAGGCCTACCAAGGCGACGATGCATAGCCGA
CCTGAGAGGGTGATCGGCCACACTGGGACTGAGACACGGCC
>Zotu3870
AAACGCTGGCGGCGTGCTTAATACATGCAAGTCGAGCGGCAGCATGGTCTTCGGACTGATGGCGAGCG
GCGGACGGCTGAGTAACGCGTAGGAACGTACCCCAAAGTGAGGAATAACTGCCCGAAAGGGTAGCTAA
TGCCGCATGTGGTCTTCGGATTAAAGCTTTATGCGCTTTGGGAACGGCCTGCGTACGATTAGCTTGTT
GGTGAGGTAATGGCTCACCAAGGCTGTGATCGTTAGCTGGTCTGAGAGGATGACCAGCCAGACTGGGA
CTGAGACACGGCC
>Zotu3871
GAACGCTGGCGGCGTGCTTAACACATGCAAGTCGAACGCTGAAGCTTGGTGCTTGCACTGGGTGGATG
AGTGGCGAACGGGTGAGTAATACGTGAGTAACCTGCCCTTGACTCTGGGATAAGCCTGGGAAACTGGG

TCTAATACTGGATACGACATGTCACTGCATGGTGGTGTGTGGAAAGGGTTTTACTGGTTTTGGATGGG
CTCACGGCCTATCAGCTTGTTGGTGGGGTAATGGCCTACCAAGGCGACGACGGGTAGCCGGCCTGAGA
GGGTGACCGGCCACACTGGGACTGAGACACGGCC
>Zotu3872
GAACGCTAGCGATAGGCTTAACACATGCAAGTCGAGGGGTAACGTGTTGGAAGCTTGCTTCCGATGAC
GACGACCGGCGGATGGGTGCGTAACGCGTATGCAACTTGCCTCACAGCGGAGAATAACCCGGAGAAAT
CCGGACTAATGCTCCATACACTCTTAGGGACCCATGTCCCTGAGAGGAAAGATTTATCGCTGTGAGAT
AGGCATGCGTCCTATTAGGTAGTTGGTGAGGTAACGGCTCACCAAGCCGACGATAGGTAGGGGTGCTG
AGAGGCAGATCCCCCACATTGGGACTGAGACACGGCC
>Zotu3876
GAACGCTAGCGGCAGGCCTAATACATGCAAGTCGAGGGGTAAATAATGTAGCAATACTTTATGAGACC
GGCGCACGGGTGCGTAACACGTATGCAACCTACCTTTTACTGGGGCATAGCTCCGAGAAATCGGAATT
AATACCCCATATTTTTTGTGATTGGCATCAATTGCAAAACAAAGCTCAGGCGGTAAAAGATGGGCATG
CGGAACATTAGCTAGTTGGTGAGGTAACGGCTCACCAAGGCTACGATGTTTAGGGGGTCTGAGAGGAT
GATCCCCCACACTGGTACTGAGACACGGAC
>Zotu3878
GAACGCTGGCGGCGTGCCTAACACATGCAAGTCGAACGGAACTGTTTTGAAAGATTTCTTCGGAATGA
ATTTGATTTAGTTTAGTGGCGGACGGGTGAGTAACGCGTGAGTAACCTGCCTTCAAGAGGGGGATAAC
ATTCTGAAAAGAATGCTAATACCGCATGACATATCGGAACCACATGGTTTTGATATCAAAGATTTTAT
CGCTTGAAGATGGACTCGCGTCCGATTAGTTAGTTGGTGAGGTAACGGCTCACCAAGACCGCGATCGG
TAGCCGGACTGAGAGGTTGAACGGCCACATTGGGACTGAGACACGGCC
>Zotu3879
GAACGCTGGCGGCGTGCTTAACACATGCAAGTCGAACGGAAAGGCCTCAGCTTGCTGGGGTGCTCGAG
TGGCGAACGGGTGAGTAACACGTAGGTGATCTGCCTTTCACTTCGGGATAAGCTTGGGAAACTGGGTC
TAATACCGGATATTCACGGCACCGTGGTTGGTGTGGTGGAAAGCTTTTGCGGTGGGAGATGAGCTTGC
GGCCTATCAGCTTGTTGGTGGGGTAATGGCCTACCAAGGCGGCGACGGGTAGCCGGCCTGAGAGGGTG
TGCGGCCACATTGGGACTGAGATACGGCC
>Zotu3880
GAACGCTAGCGGCAGGCCTAATACATGCAAGTCGAGGGGTAGTGTAGCAATACATGAGACCGGCGCAC
GGGTGCGTAACGCGTATGAAAGGTGTCCAAATCTCTGGGATAGCCCTCGGAAACGGGGATTAATACCG
GATGGTCCGTAGTAGTGGCATCACTTATACGGTAAAGCTCAGGCGGATTTGGGTCCTCATGCGTACGA
TTAGCTAGTTGGCGAGGTAACGGCTCACCAAGGCGACGATCGTTAGGGGTCCTGAGAGGGAGATCCCC
CACACTGGTACTGAGACACGGGC
>Zotu3881
GAATGCTGGCGGCGTGGATAAGTCATGCAAGTCAAGGGGGCCCGCAAGGGCAACCGGCAGACGAGGTA
GTAACACGTAGGTACGTCCCCCGAAGTCGGGCATAGCTATCCGAAAGGATGGGTAATTCCCGATAGTC
CCGCAAGGGTAAAGGTTTTTCGCTTCGGGAGCGGCCTGCGTAGTATCAGCTAGTTGGTAAGGTAACGG
CTTACCAAGGCTACGACGCTTAGGGGAGCTGAGAGGCTGACCCCCACCGATGGAACTGCGACACGGTC
>Zotu3882
GAACGCTAGCGGCAGGCCTAATACATGCAAGTCGGACGGGATTGGAGAGCTTGCTTTCCATGAGAGTG
GCGCACGGGTGCGTAACACGTATGTAACCTACCTTCGTCAGGGGGATAGCCTCTCGAAAGAGAGATTA
AGACCGCATAACATTATTGGATAGCATTATTTAATAATCAAATATTTATAGGACGAAGATGGGCATGC
GGAACATTAGCTAGTTGGTAAGGTAACGGCTTACCAAGGCTACGATGTTTAGGGGATCTGAGAGGATG
ACCCCCCACACTGGTACTGAGACACGGAC
>Zotu3883
GAACGCTGGCGGCGTGCCTAATACATGCAAGTCGAGCGAATTTTCAGGAGCTTGCTCCTGAAGGTTAG
CGGCGGACGGGTGAGTAACACGTGGGCAACCTGCCTGTAAGACTGGGATAACATCGGGAAACCGATGC
TAATACTGGATAATCCTTTTCCTCTCATGAGGAAAAGTTGAAAGACGGTTTCGGCTGTCACTTACAGA
TGGGCCCGCGGCGCATTAGCTAGTTGGTGAGGTAACGGCTCACCAAGGCGACGATGCGTAGCCGACCT
GAGAGGGTGATCGGCCACACTGGGACTGAGACACGGCC
>Zotu3884
GAACGCTGGCGGCGTGCCTAATACATGCAAGTCGAACGAAGGTACTTGTACCTTAGTGGCGAACGGGT
GAGTAACACGTAGGTAACCTGCCCTCAAGACGAGGATAACAGTTGGAAACGACTGCTAATACTCGATA
GGTAATCACTAGGCATCTAGAGATTATTAAAGATCCGTTTGGATTACTTGAGGAGGGACCTGCGCCGC

ATTAGTTAGTTGGTAAGGTAATGGCTTACCAAGACCATGATGCGTAGCCGGACTGAGAGGTTGAACGG
CCACACTGGGACTGAGACACGGCC
>Zotu3885
GAACGCTGGCGGCGTGCCTAATACATGCAAGTTGAGCGATGAAGATTGGTGCTTGCACCAATTTGAAG
AGCAGCGAACGGGTGAGTAACGCTTGGGGAATCTGCCTTTGAGCGGGGGACAACATTTGGAAACGAAT
GCTAATACCGCATAATAACTTTAAACATAAGTTTTAAGTTTGAAAGATGCAATTGCATCACTCAAAGA
TGATCCCGCGTTGTATTAGCTAGTTGGTGAGGTAAAGGCTCACCAAGGCGATGATACATAGCCGACCT
GAGAGGGTGATCGGCCACATTGGGACTGAGACACGGCC
>Zotu3886
GAACGCTAGCGGCAGGCCTGATACATGCAAGTCGGACGGGATTGGAGGGCTTGCTTTCCATGAGAGTG
GCGCACGGGTGCGTAACACGTATGTAACCTACCCATATCAGGGGGATAGCCTCTCGAAAGAGAGATTA
AGCCCGCATAACATCACGGGGCAGCATTGTACTGTGATCAAATATTTATAGGATATGGATGGGCATGC
GGAACATTAGCTAGTTGGCGGGGTAACGGCCCACCAAGGCGACGATGTTTAGGGGATCTGAGAGGATG
GCCCCCCACACTGGTACTGAGACACGGAC
>Zotu3887
GAACGCTGGCGGCATGCCTAACACATGCAAGTCGAACGAGACCTTCGGGTCTAGTGGCGCACGGGTGC
GTAACGCGTGGGAACCTGCCCTTAGGTTCGGAATAACTCCTCGAAAGGGGTGCTAATACCGGATAATG
TCTTCGGACCAAAGATTTATCGCCTTTGGATGGGCCCGCGTTGGATTAGCTAGTTGGTGGGGTAAAGG
CCTACCAAGGCGACGATCCATAGCTGGTCTTAGAGGATGATCAGCCACACTGGGACTGAGACACGGCC
>Zotu3888
TAACGCTGGCGGCGTGCCTAATACATGCAAGTCGAACGGAACACTATTTTTGAGAACCTCGCTCCTTT
GCGGGATTGTCATCAGTAGTGTTTAGTGGCGGACGGGTGAGTAACACGTTGGTACCTGCCCTTTAGTG
GGGAATAACTCGGCGAAAGTCGGGCTAATACCGCATGTGATCCTTCGGGATTAAAGCCTTCGGGCGCT
AAAGGAGGGGCCTGCGTCTGATTAGCTTGTTGGTAAGGTAATGGCTTACCAAGGCGATGATCAGTAGC
TGGTTCGAGAGGATGATCAGCCACACTGGAACTGAGATACGGTC
>Zotu3889
GAACGTTGGCGGCGTGGATTAGGCATGCAAGTCGAACGAATCCCAGTGGGGGTAACTCTGCTGGGGGA
AGTGGCGAAAGGGGCAGTAAGGCGTGGGTAACCTACCTCCGGGACGGGGATAGCCATTCTAACGAGTG
GGTAATACCCGGCGATTCGACGGCGGGGCATCTCGCTGTCGGGAAATGAGTTTCGCCTGGAGAGGGGC
TCACGTGGTATTAGCTTGTTGGCAGGGTAACGGCCTACCAAGGCAAAGATGCCTAGCGGGTGTGAGAG
CACGACCCGCGCCACTGGCACTGAGACACTGGC
>Zotu3890
GAACGCTGGCGGCGTGCTTAACACATGCAAGTCGAACGATGAACCACCTTCGGGTGGGGATTAGTGGC
GAACGGGTGAGTAACACGTGGGCAATCTGCCCTGCACTCTGGGACAAGCCCTGGAAACGGGGTCTAAT
ACCGGATACTGACCTGCCAAGGCATCTTGGCGGGTCGAAAGCTCCGGCGGTGCAGGATGAGCCCGCGG
CCTATCAGCTTGTTGGTGAGGTAATGGCTCACCAAGGCGACGACGGGTAGCCGGCCTGAGAGGGCGAC
CGGCCACACTGGGACTGAGACACGGCC
>Zotu3891
GAACGCTGGCGGCATGCTTAACACATGCAAGTCGAACGGGATCTTCGGATCTAGTGGCGCACGGGTGA
GTAGCGCGTGGGAACCTGCCCTTTTGTTCGGAACAACCGCGGGAAACTGCGGCTAATACCGGATACGC
CTGTAGGCTTTGGCGGCTTACGGGGAAAGATTTATCGCTGAAGGAGGGGCCCGCGTCGGATTAGGTAG
TTGGTGGGGTAACGGCCTACCAAGCCTTCGATCCGTAGCTGGTCTGAGAGGATGATCAGCCACACTGG
GACTGAGACACGGCC
>Zotu3893
GAACGCTGGCGGCGTGCTTAACACATGCAAGTCGAGCGATGAAGTTTCCTTCGGGAAACGGATTAGCG
GCGGACGGGTGAGTAACACGTGGGTAACCTGCCTCATAGAGTGGGATAGCCTTCCGAAAGGAAGATTA
ATACCGCATAATGTTGAAAGATGGCATCATCATTTAACCAAAGGAGCAATCCGCTATGAGATGGACCC
GCGGCGCATTAGCTAGTTGGTGGGGTAACGGCCTACCAAGGCGACGATGCGTAGCCGACCTGAGAGGG
TGATCGGCCACATTGGGACTGAGACACGGCC
>Zotu3894
GAACGCTGGCGGCATGCTTAACACATGCAAGTCGAACGAGCTAATGGGTAACTATTAGTTTAGTGGCG
GACGGGTGCGTAATGCGTAAGAACCTACCTTTTGGAGGGGGATAACAGCGGGAAACTGTTGCTAATAC
CCCATAATGCTGAGGAGTTAAAGCAGCGAAAGCTGGCCAAAAGAGGGGCTTGCGTCTGATTAGCTAGT
TGGTGGGGGTAAAAGCCTCCCAAGGCCACGATCAGTAGCTGGTCTGAGAGGATGATCAGCCACACTGG

GACTGAGACACGGCC
>Zotu3897
GAACGCTGGCGGCAGGCCTAACACATGCAAGTCGAGCGGCAGCGGACCTTCGGGTGCCGGCGAGCGGC
GGACGGGTGAGTAAGGCATCGGAATCTGCCCTGTTGTGGGGGATAACGTAGGGAAACTTACGCTAATA
CCGCATACGACCTACGGGTGAAAGCGGGGGACCTTCGGGCCTCGCGCAGCAGGATGAGCCGATGTCCG
ATTAGCTAGTTGGTGGGGTAAAGGCCCACCAAGGCGACGATCGGTAGCCGGTCTGAGAGGATGATCGG
CCACACTGGGACTGAGACACGGCC
>Zotu3898
GAACGCTGGCGGCGTGCTTAACACATGCAAGTCGAACGGATCACACCCTTCGGGGTGTGGTGAGTGGC
GAACGGGTGAGTAACACGTGAGCAACCTGCCCCCCTCTCTGGGATAACTCCGGGAAACCGGGGCTAAT
ACCGGATACGACAGGTCACCGCATGGTGGTGTGTGGAAAGGGTTTCTGGTGGGGGATGGGCTCGCGGC
CTATCAGCTTGTTGGTGGGGTGATGGCCTACCAAGGCGACGACGGGTAGCCGGCCTGAGAGGGTGACC
GGCCACACTGGGACTGAGACACGGCC
>Zotu3900
GAACGCTAGCGGCAGGCTTAATACATGCAAGTCGAACGGCAAGGATCTTCGGATCCCTAGAGTGGCGC
ACGGGTGAGTAACACGTACACAACATACCCTATATTGGAGGATAGCCTTGGGAAACTGGGGGTAATAT
TCCATAGTATTTATTGGAGGCATCTCCTGATAAATTAAAGAGCAATCGATATAGGATTGGTGTGCGTC
CCATTAGCTAGTTGGTAGGGTAACGGCCTACCAAGGCTACGATGGGTAGCTGGCGTGAGAGCGCGACC
AGCCACACTGGAACTGAGACACGGTC
>Zotu3901
GAACGCTGGCGGCAGGCTTAACACATGCAAGTCGAGCGAGGTTTTCGGACCTAGCGGCGGACGGGTGA
GTAACGCGTGGGAACGTGCCCTTTGCTACGGAATAGCCCCGGGAAACTGGGAGTAATACCGTATGTGC
CCCTTGGCCTTGGCGGTCATGGGGGAAAGATTTATCGGCAAAGGATCGGCCCGCGTTGGATTAGGTAG
TTGGTGGGGTAATGGCCCACCAAGCCGACGATCCATAGCTGGTTTGAGAGGATGATCAGCCACACTGG
GACTGAGACACGGCC
>Zotu3902
CAACGCTGGCGGCGTGCCTCAGACATGCAAGTCGAACGATTAAGACTTCCTTCGGGAAGTGTATAAAG
TGGCGCACGGGTGAGTAACACGTAAGTAATCTACCTTCGAGTGGGGAATAACCCGGGGAAACCCGAGC
TAATACCGCATAACGCAGCGGCATCGCAAGATGACAGTTGTTAAAGGAATTTATTTCGCTTGAAGAGG
AGCTTGCGGCAGATTAGCTAGTTGGTAAGGTAACGGCTTACCAAGGCTACGATCTGTATCCGGTCTAA
GAGGACGGTCGGACACACTGACACTGAATAACGGGT
>Zotu3903
GAACGCTGGCGGCGTGCTTAACACATGCAAGTCGAACGCTGAAGCCTGGTGCTTGCACCGGGTGGATG
AGTGGCGAACGGGTGAGTAATACGTGAGTGACCTGCCTTTGACTCTGGGATAAGCCTGGGAAACTGGG
TCTAATACCGGATGCGACTACTGCCCGCATGGGCTGGTGGTGGAAAGGGTTATGTACTGGTCTTAGAT
GGGCTCACGGCCTATCAGCTGGTTGGTGGGGTAATGGCCTACCAAGGCGACGACGGGTAGCCGGCCTG
AGAGGGTGACCGGCCACACTGGGACTGAGACACGGCC
>Zotu3904
GAACGCTGGCGGCAGGCCTAATACATGCAAGTCGAACGCTGTAGCAATACAGAGTGGCGCACGGGTGA
GTAATGCATGGGAACATACCCCTTAGTACGGAACAACACCGGGAAACTGGTGCTAATACCGTATAATG
TCTACGGACCAAAGATTTATCGCTGAGGGATTGGCCCATGTTGGATTAGCTTGTTGGCGAGGTAATGG
CTCACCAAGGCGACGATCCATAGCTGGTCTGAGAGGATGATCAGCCACACTGGGACTGAGACACGGCC
>Zotu3906
CAACGCTGGCGGCGTGCCTAACACATGCAAGTCGAACGAGAAAGGGCTTTCGGGCCTGAGTAAAGTGG
CGCACGGGTGAGTAACACGTGACTAACCTACCCTTGAGCGGGGGATAACCTAGGGAAACCTGGGCTAA
TACCGCATAACACCTACGGGTCAAAGCGAGCAATCGCGCTTAAGGAGGGGGTCGCGGCAGATTAGCTA
GTTGGTGAGGTAATGGCTCACCAAGGCGATGATCTGTATCCGGCCTGAGAGGGCGCACGGACACACTG
GAACTGAAACACGGTC
>Zotu3907
GAACGCTGGCGGCGTGCTTAACACATGCAAGTCGAACGGTGATCTCAGAGCTTGCTCTGGGTGATCAG
TGGCGAACGGGTGAGTAACACGTGAGTAACCTGCCCTTGACTCTGGGATAACTCCGGGAAACCGGGGC
TAATACTGGATACGAGATGCGACCGCATGGTCTGTGTCTGGAAAGTTTTTCGGTCAAGGATGGACTCG
CGGCCTATCAGCTTGTTGGTGGGGTAACGGCCCACCAAGGCGTCGACGGGTAGCCGGCCTGAGAGGGC
GACCGGCCACACTGGGACTGAGACACGGCC

>Zotu3908
GAACGCTGGCGGCGTGCCTAAGACATGCAAGTCGAGTGGGTTTAGACCCACGGCGAACGGGTGAGTAA
TACCTAGGAATGTCCTTTAAGTTGAGCATAACTAGTCGAAAGATTAGACAATTCTCAATGTGGTCGTA
AGACTAAAGTCGCAAGACGCTTAAAGCGCAGCCTGGGCACCATCAGCTAGTTGGTAAGGTAATGGCTT
ACCAAGGCTATGACGGTTAGGGGCCCCGAGAGGGGAAACCCCCACACTGGAACTGCGACACGGTC
>Zotu3909
AAACGCTGGCGGCGTGCCTAACACATGCAAGTCGAACGCACTCTCGCGAGAGAGTGAGTGGCGGACGG
GTGAGTAACACGTGGGTACCTACCGTGAGGTGAGGAATACCACCGAGAAATCGGTGACAAGACCGCAT
ATGCTCGCAAGAGCAAAGTTTCTTTTCGGAGAGACGCCACACGATGGGCCTGCGCCTGATTAGCTGGT
TGGTGAGGTAATGGCTCACCAAGGCGACGATCAGTAGCTGGTCTGAGAGGACGATCAGCCACACTGGG
ATTGAGAACGGCC
>Zotu3910
GAACGCTGGCGGCGTGCCTAATACATGCAAGTCGTGCGGCAGCATGATTCTCACTATTTGCTTCTCTC
TTTGTTGTTTGACAGAGAACCGGAGGAGTAAGTTGTGAGAGTTGATGGCGAGCGGCGGACGGCTGAGT
AACGCGTGGGAACGTGCCCCAAAGTGAGGAATAAGCTCCAGAAATGGAGTCTAATGCCGCATATGGTC
TTCGGATTAAAGCTTCGGCGCTTTGGGAGCGGCCTGCGTTTGATTAGATTGTTGGTGAGGTAAAGGCT
CACCAAGTCGACGATCAATAGCTGGTCTGAGAGGATGATCAGCCAGACTGGAACTGAGACACGGTC
>Zotu3911
GAACGCTGGCGGCGTGCTTAACACATGCAAGTCGAACGGTGAAAGAGAGCTTGCTCTCTGGATCAGTG
GCGAACGGGTGAGTAACACGTGAGCAATCTGCCCCTGACTCTGGGATAAGCGTTGGAAACGACGTCTA
ATACCGGATACGAGCTGCGAAGGCATCTTCAGCAGCTGGAAAGAACTTCGGTCAGGGATGAGCTCGCG
GCCTATCAGCTAGTTGGTGAGGTAATGGCTCACCAAGGCGTCGACGGGTAGCCGGCCTGAGAGGGTGA
CCGGCCACACTGGGACTGAGACACGGCC
>Zotu3912
GAACGCTGGCGGCATGGATAAGGCATGCAAGTCGAGTGGGTTTAGACCCACTGCAAACGGGTTAGTAA
TGCGTAGGAATGTTCCTCAAAGTCGTGAATAATTTGTCGAAAGACAAGCTAATACACGATGTGCTCCA
CGGAGTAAAGGCTTGCCGCTTTGAGAGCAGCCTGCGTCCTATCAGCTAGTTGGTGGGGTAAAAGCCTA
CCAAGGCTTTGACGGGTAGGGGGTGTGAGAGCATGGCCCCCAACATTGGGACTGAGACACTGCC
>Zotu3913
GAACGCTGGCGGCAGGCTTAACACATGCAAGTCGAACGGGCGTAGCAATACGTCAGTGGCAGACGGGT
GAGTAACACGTGGGAACGTACCTTTTGGTTCGGAACAACACAGGGAAACTTGTGCTAATACCGGATAA
GCCCTTACGGGGAAAGATTTATCGCCGAAAGATCGGCCCGCGTCTGATTAGCTAGTTGGTAGGGTAAC
GGCCTACCAAGGCGACGATCAGTAGCTGGTCTGAGAGGATGATCAGCCACATTGGGACTGAGACACGG
CC
>Zotu3914
GAACGCTGGCGGCGTGCTTAACACATGCAAGTCGAACGAGAGGATGAGGAAGCTTGCTTCTTCAGAAT
CGAGTGGCAAACGGGTGAGTAACGCGTAAACAACCTGCCCCCGGGATGGGGACAACAGCTGGAAACGG
CTGCTAATACCGAATACGATCATACTGCCGCATGGCAGCATGATGAAAGGATGGCCTCTTAAAAAAGC
TATCGCACGGGGAGGGGTTTGCGTCTGATTAGCTGGTTGGAGGGGTAACGGCCCACCAAGGCGATGAT
CAGTAGCCGGTCTGAGAGGACGAACGGCCACATTGGAACTGAGACACGGTC
>Zotu3915
GAACGCTGGCGGCGTGCCTAATACATGCAAGTCGAGCGGACCGGATTGGGGCTTGCCTTGATTCGGTC
AGCGGCGGACGGGTGAGTAACACGTGGGCAACCTGCCCGCAAGACCGGGATAACTCCGGGAAACCGGA
GCTAATACCGGATAACACCGAAGACCGCATGGTCTTCGGTTGAAAGGCGGCCTTTGGGCTGTCACTTG
CGGATGGGCCCGCGGCGCATTAGCTAGTTGGTGAGGTAACGGCTCACCAAGGCGACGATGCGTAGCCG
GCCTGAGAGGGTGACCGGCCACACTGGGACTGAGACACGGCC
>Zotu3916
GAACGCTGGCGACATGCTTGACACATGCAAGTCGAACGAGAACTTGTAAGCAACTTGTTGTTTACTTG
TTCTAGTGGCGGACGGGTGAGTAACGCGTAAGAATCTGCCTCGGGGCGAGGGATACCAACTGGAAACG
GTTGCTAATACCTCATAAGCTGAGAAGTAAAAGGTGTACTACACCACCAAGAGAGGAGCTTGCGTCTG
ATTAGTTAGTTGGTAAGGTAATGGCTTACCAAGGCTATGATCAGTAGCTGGTCTGAGAGGACAATCAG
CCACACTGGGACTGAGACACGGCC
>Zotu3917
GAACGCTGGCGGCATGCCTAACACATGCAAGTCGAACGAGACCTTCGGGTCTAGTGGCGCACGGGTGC

GTAACGCGTGGGAATCTGCCCTTGGGTTCGGAATAACTCGCCGAAAGGCGTGCTAATACCGGATGATG
TCGTAAGACCAAAGATTTATCGCCCAGGGATGAGCCCGCGTAAGATTAGGTAGTTGGTGAGGTAAAGG
CTCACCAAGCCGACGATCTTTAGCTGGTCTGAGAGGATGATCAGCCACACTGGGACTGAGACACGGCC
>Zotu3918
GAACGCTGGTGGCGTGCTTAACACATGCAAGTCGAGCGAGGCGTCCGCTTTCGGGTGGATAGTCGAGC
GGCGAACGGGTGAGTAACACGTGAGTAATCTTCCCTGGGCTCGGGGATAACCTCGGGAAACTGAGGGT
AATACCGGATGTGCGTTTTCTGGCGCCTGCTGGGAGACAGAAAGGTTGCGTGCCGTGAGGTGCGTTTC
CGGCCTTGGATGAGCTCGCGGCCTATCAGCTTGTTGGTGCGGTGATGGCGTACCAAGGCGACAACGGG
TAGCCGGCCTGAGAGGGTGACCGGCCACACTGGGACTGAGATACGGCC
>Zotu3919
GAACGCTGGCGGCATGCTTTACACATGCAAGTCGAACGGCAGCGCGGGGCAACCTGGCGGCGAGTGGC
GAACGGGTGAGTAATATATCGGAACGTACCCAAGAGTGGGGGATAACGTAGCGAAAGTTACGCTAATA
CCGCATACGATCTAAGGATGAAAGCAGGGGACCGCAAGGCCTTGTGCTCCTGGAGCGGCCGATATCTG
ATTAGCTAGTTGGTGAGGTAAAGGCTCACCAAGGCGACGATCAGTAGCTGGTCTGAGAGGACGACCAG
CCACACTGGGACTGAGACACGGCC
>Zotu3922
GAACGCTGGCGGCGTGCCTAACACATGCAAGTCGAACGGAGATTTCATTTATGAAATCTTAGTGGCGA
ACGGGTGAGTAACGCGTAGATAACCTGCCTTTTAGATGGGGACAACACTGGGAAACTAGTGCTAATAC
CGAATGTTGTATTTTGATTGCATGATTAAGATACTAAAGATGGCCTCTATTTATAAGCTATCACTAAA
AGATGGGTCTGCGTCTGATTAGCTAGTTGGTGAGGTAATGGCTCACCAAGGCGACGATCAGTAGCCGG
TCTGAGAGGATGAACGGCCACACTGGGACTGAGACACGGCC
>Zotu3923
GAACGCTGGCGGCGTGCCTAATACATGCAAGTCGAACGAAACTTCTTTATCACCGAGTGCTTGCACTC
ACCGATAAAGAGTTGAGTGGCGAACGGGTGAGTAACACGTGGGCAACCTGCCCAAAAGAGGGGGATAA
CACTTGGAAACAGGTGCTAATACCGCATAACCATAGTTACCGCATGGTAACTATGTAAAAGGTGGCTA
TGCTACCGCTTTTGGATGGGCCCGCGGCGCATTAGCTAGTTGGTGGGGTAAAGGCTTACCAAGGCAAT
GATGCGTAGCCGAACTGAGAGGTTGATCGGCCACATTGGGACTGAGACACGGCC
>Zotu3925
GAACGCTGGCGGCAGGCTTAACACATGCAAGTCGAGCGGTAGCACAAGGGAGCTTGCTCCCTGGGTGA
CGAGCGGCGGACGGGTGAGTAATGTCTGGGAAACTGCCTGATGGAGGGGGATAACTACTGGAAACGGT
AGCTAATACCGCATAACGTCGCAAGACCAAAGAGGGGGACCTTCGGGCCTCTTGCCATCAGATGTGCC
CAGATGGGATTAGCTAGTAGGTGGGGTAACGGCTCACCTAGGCGACGATCCCTAGCTGGTCTGAGAGG
ATGACCAGCCACACTGGAACTGAGACACGGTC
>Zotu3926
GAACGCTAGCGGCAGGCTTAACACATGCAAGTCGAGGGGCAGCGCGGTAGCAATACTGGCGGCGACCG
GCAAACGGGTGCGGAACACGTACAGAACCTACCTTGATCTGGGGCATAGCCCGGAGAAATCCGGATTA
ACTCCCCATAATATACACGAGTGGCATCACTTGAGTATTAAAAATTTATTGGATCGAGATGGCTGTGC
GTCCCATTAGCTAGTTGGTAGGGTAACGGCCTACCAAGGCAACGATGGGTAGCTGGTGTGAGAGCACG
ACCAGCCACACGGGCACTGAGACACGGGC
>Zotu3927
GAACGCTGGCGGCAGGCCTAACACATGCAAGTCGAGCGCGAAAAGTTTTCGGACTTAGTAAAGCGGCG
GACGGGTGAGTAACGCGTGGGAATCTGCCCAGTAGTGGGGGACAACAGCCGGAAACGGCTGCTAATAC
CGCATACGCCCTACGGGGGAAAGGGGGGGATCTTCGGACCTCTCGCTATTGGATGAGCCCGCGTCGGA
TTAGCTAGTTGGTAGGGTAAAGGCCTACCAAGGCTACGATCCGTAACTGGTCTGAGAGGATGATCAGT
CACACTGGAACTGAGACACGGTC
>Zotu3930
GAACGCTGGCGGCGTGCTTAACACATGCAAGTCGAACGATGAAGCCGGAGCTTGCTCTGGTGGATTAG
TGGCGAACGGGTGAGTAACACGTGAGTAACCTACCCTTGACTCTGGGATAAGCCCGGGAAACTGGGTC
TAATACCGGATATGAGTCATCACCGCATGGTGGTGGTTTGAAAGATTTATCGGTCTTGGATGGACTCG
CGGCCTATCAGCTTGTTGGTGAGGTAATGGCTCACCAAGGCGACGACGGGTAGCCGGCCTGAGAGGGT
GACCGGCCACACTGGGACTGAGACACGGCC
>Zotu3931
GAACGCTAGCGGCAGGCCTAATACATGCAAGTCGAACGGTGTAGCAATACACAGTGGCGCACGGGTGC
GTAACACGTATGCAACCTACCTATCATTGGGGGATAGCCTTTGGAAACGGAGATTAATACCGCATAAG

ACAGTAGCATGGCATCATGTAGCTGTTAAAGCTTTGGCGATGATAGATGGGCATGCGTCCAATTAGTT
AGTTGGTGAGGTAACGGCTCACCAAGACGATGATTGGTAGGGGATCTGAGAGGATAATCCCCCACACT
GGCACTGAGATACGGGC
>Zotu3932
GAACGCTGGCGGCAGGCCTAACACATGCAAGTCGAACGCTGTAGCAATACAGAGTGGCAGACGGGTGA
GTAACACGTGGGAACATGCCCTTCGGTTCGGAATAACTCAGGGAAACTTGAGCTAATACCGGATACGT
CCGCAAGGAGAAAGATTTATCGCCGAAGGATTGGCCCGCGTCTGATTAGCTTGTTGGTGAGGTAATGG
CTCACCAAGGCGACGATCAGTAGCTGGTCTGAGAGGATGATCAGCCACATTGGGACTGAGACACGGCC
>Zotu3933
GAACGCTGGCGGCGTGCCTAACACATGCAAGTCGAACGGAAACAGGCTTTCACTGTTTGCTTCTCGCT
GATCGAAGTAATTCGATAGAATATCGAGGAGTGAAGAGTGAAAGTGCTGTCGAGTGGCGGACGGCTGA
GTAACGCGTGGGAACGTGCCCCAAAGTGAGGGATAACTTCGAGAAATCGGAGCTAATACCGCATATGA
TCTTCGGATTAAAGTATTTATACGCTTTGGGAGCGGCCCGCGTCGGATTAGGTTGTTGGTGAGGTAAT
GGCTCACCAAGCCGACGATCCGTAGCTGGTCTGAGAGGATGACCAGCCAGACTGGAACTGAGACACGG
TC
>Zotu3935
GAACGCTGGCGGCATGCCTTACACATGCAAGTCGAACGGTAACGCGGGGCAACCTGGCGACGAGTGGC
GAACGGGTGAGTAATATATCGGAACGTGCCCAGTAGTGGGGGATAGCCCGGCGAAAGCCGGATTAATA
CCGCATGAGACCTGAGGGTGAAAGCGGGGGATCGCAAGACCTCGCGCTATTGGAGCGGCCGATATCAG
ATTAGCTAGTTGGTGGGGTAAAGGCCTACCAAGGCAACGATCTGTAGTTGGTCTGAGAGGACGACCAG
CCACACTGGGACTGAGACACGGCC
>Zotu3936
GAACGCTAGCGGCAGGCTTAACACATGCAAGTCGAGGGGTATAGTAGCAATACTAGAGACCGGCGCAC
GGGTGCGTAACGCGTATGCAATCTACCTTTTACAGCGGGATAGCCCAGAGAAATTTGGATTAATACCT
CATAGTATAATGAGTTGGCATCAACACATTATTAAAGTCACAACGGTAAAAGATGAGCATGCGTCCCA
TTAGCTAGTTGGTAAGGTAACGGCTTACCAAGGCAACGATGGGTAGGGGTCCTGAGAGGGAGATCCCC
CACACTGGTACTGAGACACGGAC
>Zotu3937
GAACGCTGGCGGCGTGCTTAACACATGCAAGTCGAGCGGTAAGGCCCTTCGGGGTACACGAGCGGCGA
ACGGGTGAGTAACACGTGGGTGACCTGCCCTCCACTTCGGGATAAGCCTGGGAAACTGGGTCTAATAC
CGGATAGGACCATTCCATGCATGTGGTTTGGTGGAAAGTTTTTTCGGTGGGGGATGGGCCCGCGGTCT
ATCAGCTTGTTGGTGGGGTGATGGCCTACCAAGGCGACGACGGATAGCCGGCCTGAGAGGGTGATCGG
CCACACTGGGACTGAGACACGGCC
>Zotu3938
GAACGCTGGCGGCGTGCTTTACACATGCAAGTCGAGCGAGGCGTGTAGCAATACATAGTCGAGCGGCG
AACGGGTGAGTAACGCGTGGATAACCTGCCCTTCAGTGGGGAACAACCGGGGGAAACTCCGGCTGATA
CCGCATACGATCGAGAGATGAAAGGGGCCCGCAAGGGTCTCGCTGATGGAGGGGTCCGCGTCCGATTA
GCTAGTTGGCAGGGTAATGGCCTACCAAGGCGACGATCGGTAGCCGGCCTGAGAGGGTGATCGGCCAC
ACTGGAACTGAGACACGGTC
>Zotu3939
GAACGCTGGCGGCAGGCTTAACACATGCAAGTCGAACGCCCGAGCAATCGGGAGTGGCAGACGGGTGA
GTAACACGTGGGAACATACCCTTCGGTTCGGAACAACGTTTGGAAACGAACGCTAATACCGGATACGG
CCTCTGGGCGAAAGATTCATTGCCGAAGGATTGGCCCGCGTTCGATTAGCTTGTTGGTGAGGTAACGG
CTCACCAAGGCGACGATCGATAGCTGGTCTGAGAGGATGGCCAGCCACATTGGGACTGAGACACGGCC
>Zotu3941
GAACGCTGGCGGCAGGCTTAACACATGCAAGTCGAGCGGGGTCCTTCGGGGCTCAGCGGCGGACGGGT
TAGTAACGCGTGGGAACGTGCCCTTCACTGCGGAATAGCATTGGGAAACCGGTGGTAATACCGCATAC
GCCCTTAGGGGGAAAGATTTATCGGTGAGGGATCGGCCCGCGTCTGATTAGGTAGTTGGTGAGGTAAT
GGCTCACCAAGCCTACGATCGGTAGCTGGTTTGAGAGGATGATCAGCAACACTGGGACTGAGACACGG
CC
>Zotu3942
GAACGCTGGCAGTGTGCCTAAGACATGCAAGTCGAACGGGAGTCTTCGGACTCTAGTGGCGAACGGGT
GAGTAACACGTGGGCGACCTACCCCCAAGTACGGCATAACCCCGGGAAACCGGAGCTAATTCCGTATG
TGCTTACGTCTCATTTGACGTACGTAAAGATTTATCGCTTGGGGATGGGCCCGCGGCGCATCAGCTAG

TTGGTGGGGTAACGGCCTACCAAGGCGACGACGCGTAGCCGACCTGAGAGGGTGACCGGCCACAGGGG
CACTGAGACACGGGC
>Zotu3943
GAACGCTGGCGGCGTGCTTAACACATGCAAGTCGAACGGTGAACCGCCTTCGGGTGGGGATCAGTGGC
GAACGGGTGAGTAACACGTGAGCAACCTGCCCTTAGCTCTGGGATAACTCCGGGAAACCGGGGCTAAT
ACCGGATATGAGATCGGACGGCATCGTCTGGTCTGTAAAGTTTTTCGGCTAGGGATGGGCTCGCGGCC
TATCAGCTTGTTGGTGGGGTGATGGCCTACCAAGGCGACGACGGGTAGCCGGCCTGAGAGGGCGACCG
GCCACATTGGGACTGAGACACGGCC
>Zotu3944
GAACGCTGGCGGTATGCTTAACACATGCAAGTCGAACGGATATAGCAATATATTAGTGGCGAACGGGT
GAGTAATGCAAAGGAATCTACCTAGCAGTAAGGGATAACTTTGGGAAACCAAAGCTAATACCATATAT
TACCGCCTGATTAACTTCAGGGGGGAAAGATTTATCGCTGTTAGATGAGCCTTTGTCGGATTAGCTAG
TTGGTAGGGTAATGGCCTACCAAGGCTATGATCCGTAGCTGGTCTGAGAGGATGATCAGCCACACTGG
AACTGAGACACGGTC
>Zotu3945
GAACGCTGGCGGCGTGCTTAATACATGCAAGTCGAGCGGTAACACTTCGGGTGACGAGCGGCGGACGG
CTGAGTAACGCGTAGGAATGTACCCCAAAGTGAGGGATAAGCACCAGAAATGGTGTCTAATACCGCAT
GTGATCTATGGATTAAAGCTTCGGCGCTTTGGGAACAGCCTGCGTATGATTAGCTTGTTGGTGAGGTA
AAGGCTCACCAAGGCGACGATCATTAGCTGGTCTGAGAGGATGACCAGCCAGACTGGGACTGAGACAC
GGCC
>Zotu3946
GAACGCTGGCGGCGTGCCTAATACATGCAAGTCGAGCGGACGAATTTTTGGTGCTTGCACCAGACTTT
CGTTAGCGGCGGACGGGTGAGTAACACGTGGGTAACCTACCCTATAGACTGGGATAACTTCGGGAAAC
CGAGGCTAATACCGGATAATACTTGAAACCACATGGTTTCTTGTTAAAAGATGGTTCTGCTATCACTA
TAGGATGGACCCGCGGCGCATTAGCTAGTTGGTGAGGTAACGGCTCACCAAGGCGACGATGCGTAGCC
GACCTGAGAGGGTGATCGGCCACACTGGGACTGAGACACGGCC
>Zotu3947
GAATGCTGGCGGCGTGGATAAGTCATGCAAGTCAAGGGGGCCCGCAAGGGCAACCGGCAGACGAGGTA
GTAACACGTAGGTACGTCCCCCGAAGTCGGGCATAGCCATCCGAAAGGATGGGTAATTCCCGATAGTC
CTGTAAAGGTAAAGGTTTTTCGCTTCGGGAGCGGCCTGCGTAGTATCAGCTAGTTGGTAAGGTAATGG
CTTACCAAGGCTACGACGCTTAGGGGAGCTGAGAGGCTGACCCCCACCGATGGAACTGCGACACGGTC
>Zotu3948
GAACGCTGGCGGCATGCCTTACACATGCAAGTCGAACGGTAACAGGCCGCAAGGTGCTGACGAGTGGC
GAACGGGTGAGTAATGCATCGGAACGTGCCCAGTAGTGGGGGATAGCCCGGCGAAAGCCGGATTAATA
CCGCATACGACCTGAGGGTGAAAGGGGGGGATCGCAAGACCTCTCGCTATTGGAGCGGCCGATGTCAG
ATTAGCTAGTTGGTGGGGTAAAGGCCTACCAAGGCAACGATCTGTAGTTGGTCTGAGAGGACGACCAG
CCACACTGGGACTGAGACACGGCC
>Zotu3949
GAACGCTGGCGGCGTGCCTAACACATGCAAGTTGAGCGACGAACCAGGGCTTGCCCTGGGGCAAAGCA
GCGAACGGGTGAGTAACACGTGGGTAACCTGCCCCGATAACCGGGACAACCCGAGGAAACTCGGGCTA
ATACCGGATGTGCTCGCCGGACTTCGGTCCGGCGCGTAAAGGAAGCTTCGGCCTCCGTATCGGGAGGG
GCCCGCGGCCCATTAGTTAGTTGGTGAGGTAACGGCTCACCAAGACCGCGATGGGTAGCTGGTCTGAG
AGGACGATCAGCCACACTGGGACTGAGACACGGCC
>Zotu3950
GAACGCTGGCGGCGTGCCTAATACATGCAAGTCGAGCGAGCAGAACCAGCAGATTTACTTCGGTAATG
ACGCTGGGGACGCGAGCGGCGGATGGGTGAGTAACACGTGGGGAACCTGCCCCATAGTCTGGGATACC
ACTTGGAAACAGGTGCTAATACCGGATAAGAAAGCAGATCGCATGATCAGCTTATAAAAGGCGGCGTA
AGCTGTCGCTATGGGATGGCCCCGCGGTGCATTAGCTAGTTGGTAAGGTAACGGCTTACCAAGGCAAT
GATGCATAGCCGAGTTGAGAGACTGATCGGCCACATTGGGACTGAGACACGGCC
>Zotu3951
GAATGCTGGCTGTATGGATAAGGCATGCAAGTCGAACGGACCGCAAGGTCAGTGGCAGACGAGGTAGT
AACACGTAGGTACGTACTCCAAAGTCTGGTATAGCTCGTCGAAAGACGGGGTAATCCCGGATGGTCCC
GCAAGGGTAAAGATTTATCGCTTTGGAAGCGGCCTGCGTAGTATCAGCTAGTTGGTAAGGTAATGGCT
TACCAAGGCTACGACGCTTAGGGGAGCTGAGAGGCTGACCCCCACCGATGGGACTGAGACACGGCC

>Zotu3952
GAACGCTGGCGGCGTGCCTAAGACATGCAAGTCGAGTGGGTTTAGGCCCACGGCAAACGGGTGAGTAA
TATCTAGGAACGTACCACTAGTTGAGCATAACCTACCGAAAGGTAGAGCAATTCTCAATGTGACCGCA
AGGTTAAAGTCGCAAGACGCTAGTGAAGCGGCCTGGACACCATCAGCTTGTTGGTAAGGTAAAAGCTT
ACCAAGGCTATGACGGTTAGGGGCCCCGAGAGGGGAAACCCCCACACTGGAACTGAGACACGGTC
>Zotu3953
GAACGCTGGCGGCGTGCTTAACACATGCAAGTCAGGGGGCTCCGCAAGGGGTCACCGGCGAACGGGTG
CGTAACACGTGAGCGACCTGCCCTCGAGTGGGGGATAGCCGGCCCAACGGCCGGGTAATACCGCATAC
GTCTCCGGCTTGGACGCCGAGAGAGAAACCCGCAAGGGGTTCGAGGAGGGGCTCGCGGCCTATCAGCT
AGTTGGTGTGGTAACGGCGCACCAAGGCGATGACGGGTAGCTGGTCTGAGAGGATGGCCAGCCACATT
GGGACTGAGAGACGGCC
>Zotu3954
GAACGCTGGCGGCATGCCTAATACATGCAAGTCGAACGATGATATCACTGCTTGCAGTGATTGATTAG
TGGCGAACGGGTGAGTAACACGTGAGGAACTTGCCCATGAGCGGGGGACAACATTCGGAAACGGATGC
TAATACCCCATAGGTGGATTGGTCGCATGACTAATTCATTAAAGGTGGCTCTGCTACCACTCATGGAT
AGCCTCGCGGCGTATTAGCTAGTTGGTAAGGTAATGGCTTACCAAGGCAGTGATACGTAGCCGACTTG
AGAGGGTGATCGGCCACACTGGGACTGAGACACGGCC
>Zotu3955
GAACGCTGGCGGCGTGCCTAATACATGCAAGTCGAGCGAATCTTTGGGAGCTTGCTCCCAAAGATGAG
CGGCGGACGGGTGAGTAACACGTGGGCAACCTGCCTGTAAGACTGGGATAACTTCGGGAAACCGGAGC
TAATACCGGATCATTCTTTTTCTCACATGAGGGAAAGCTGAAAGACGGCGTAAGCTGTCACTTACAGA
TGGGCCCGCGGCGCATTAGCTAGTTGGTGAGGTAACGGCTCACCAAGGCGACGATGCGTAGCCGACCT
GAGAGGGTGATCGGCCACATTGGGACTGAGACACGGCC
>Zotu3956
GAACGCTAGCTACAGGCTTAACACATGCAAGTCGAGGGGCAGCATGACGATAGCTTGCTATTGTTGAT
GGCGACCGGCGCACGGGTGAGTAACGCGTATCCAACCTGCCCTTGTCCATCGGATAACCCGTCGAAAG
GCGGCCTAACACGATATGCAGTTCTCAGCAGGCATCTAACGAGAACGAAATGTGAAGGAGAAGGATGG
GGATGCGTCTGATTAGCTTGTTGGCGGGGTAACGGCCCACCAAGGCGACGATCAGTAGGGGTTCTGAG
AGGAAGGTCCCCCACATTGGAACTGAGACACGGTC
>Zotu3957
GAACGCTAGCGGCAGGCCTAATACATGCAAGTCGAGGGGCAGCACGGTAGCAATACTGGTGGCGACCG
GCGCACGGGTGCGTAACACGTATACAATCTGCCTTTAACAGGGGGATAGCCTTTCGAAAGAGAGATTA
ATACCCCATAATACTTGAAGTAGCATTACTTTAAGTTAAAAGCTCCGGCGGTTAAAGATGAGTATGCG
TCTGATTAGCTAGTTGGTGAGGTAACGGCTCACCAAGGCGACGATCAGTAGGGGATCTGAGAGGACTA
CCCCCCACACGGATACTGAGACACGGAT
>Zotu3958
GAACGCTGGCGGCGTGCCTAATACATGCAAGTCGAGCGAATGGATGGGAGCTTGCTCCCGGAAATTAG
CGGCGGACGGGTGAGTAACACGTGGGCAACCTGCCTGTAAGACTGGGATAACTTCGGGAAACCGGAGC
TAATACCGGATAACTTCTTTTCTCGCATGGGAAAAGGTTGAAAGACGGCTTCGGCTGTCACTTACAGA
TGGGCCCGCGGCGCATTAGCTAGTTGGTGAGGTAACGGCTCACCAAGGCAACGATGCGTAGCCGACCT
GAGAGGGTGATCGGCCACACTGGGACTGAGACACGGCC
>Zotu3959
GAACGCTGGCGGCATGCCTAACACATGCAAGTCGAACGAAGGCTTCGGCCTTAGTGGCGCACGGGTGC
GTAACGCGTGGGAATCTGCCCCTCGGTTCGGAATAACAGCGAGAAATTGCTGCTAATACCGGATAATG
ACGTAAGTCCAAAGATTTATCGCCGAGGGATGAGCCCGCGTAGGATTAGCTAGTTGGTGTGGTAAAGG
CGCACCAAGGCGACGATCCTTAGCTGGTCTGAGAGGATGATCAGCCACACTGGGACTGAGACACGGCC
>Zotu3960
GAACGCTGGCGGCAGGCTTAACACATGCAAGTCGAGCGGGCATCCTTGTGGTGTCAGCGGCAGACGGG
TGAGTAACGCGTGGGAACGTGCCCTTCGGTTCGGAATAACTCAGGGAAACTTGAGCTAATACCGGATA
CGCCCTTTTGGGGAAAGGTTTACTGCCGAAGGATCGGCCCGCGTCTGATTAGCTAGTTGGTGAGGTTA
TGGCTCACCAAGGCGACGATCAGTAGCTGGTCTGAGAGGATGATCAGCCACACTGGGACTGAGACACG
GCC
>Zotu3961
GAACGCTAGCTACAGGCTTAACACATGCAAGTCGAGGGGCAGCATGTCGGTTGCTTGCAACCGACGAT

GGCGACCGGCGCACGGGTGAGTAACGCGTATCCAACCTACCCTTGTCCATCGGATAACCCGTCGAAAG
GCGGCCTAACACGATATGCGGTTCTCAGCAGGCATCTAACGAGAACGAAATGTGAAGGAGAAGGATGG
GGATGCGTCTGATTAGCTTGTTGGCGGGGTAACGGCCCACCAAGGCGACGATCAGTAGGGGTTCTGAG
AGGAAGGTCCCCCACATTGGAACTGAGACACGGTC
>Zotu3962
GAACGCTGGCGGCGCGCTTAACACATGCAAGTCGAGCGAGAACCAGGGCTTCGGCCCTGGGGACAGCG
GCGAACGGGTGAGTAACACGTGGATAACCTGTCCTCGGCACCGGGATAGCCCGGGGAAACCCGGATTA
ATACCGGATAGCCTTTCCGACTCTCGAGTCGGTCCGAAAAGGTAGCTTCGGCCTCTGGCCGAGGATGG
GTCCGCGGCGGATTAGCTTGTTGGTGGGGTAACGGCCTACCAAGGCTCCGATCCGTAGCTGGTCTGAG
AGGACGATCAGCCACACTGGGACTGAGACACGGCC
>Zotu3963
GAACGCTGGCGGCGTGCTTCATACATGCAAGTCGAACGAGAAGTTACCTTCGGGTGATGGAAAGTGGC
GGACGGGTGAGTAATATGTAGAGAATCTGCCCTTGAGAGGGGGACAACAGAGGGAAACTTCTGCTAAT
ACCCCATATGAGCGTAGCTGAAATGCTATTCTTGAAAACTCCGGTGCTCAAGGATGAGTCTGCATCTG
ATTAGCTAGTTGGTGGTGTAATGGACTACCAAGGCGACGATCAGTAGCTGGTTTGAGAGGATGATCAG
CCACAATGGGACTGAGACACGGCC
>Zotu3965
GAACGCTGGCGGCGTGCTTAACACATGCAAGTCGAACGGTGAAAGAGGAGCTTGCTCCTCTGGATCAG
TGGCGAACGGGTGAGTAACACGTGAGCAATCTGCCCCGAACTCTGGGATAAGCGCTGGAAACGGCGTC
TAATACCGGATACGACGCGGGAGGGCATCCTCTCCGCGTGGAAAGAATTTCGGTTCGGGATGAGCTCG
CGGCCTATCAGCTAGTTGGTGAGGTAATGGCTCACCAAGGCGACGACGGGTAGCCGGCCTGAGAGGGT
GACCGGCCACACTGGGACTGAGACACGGCC
>Zotu3967
GAACGCTAGCGGCAGGCTTAACACATGCAAGTCGAGGGGTATAGTTCTTCGGAATTAGAGACCGGCGC
ACGGGTGCGTAACGCGTATGCAATCTACCTTTCACAGAGGGATAGCCCAGAGAAATTTGGATTAATAC
CTCATAGTATATAGAGTTGGCATCAACATTATATTAAAGTCACAACGGTGAAAGATGAGCATGCGTCC
CATTAGCTAGTTGGTAAGGTAACGGCTTACCAAGGCTACGATGGGTAGGGGTCCTGAGAGGGAGATCC
CCCACACTGGTACTGAGACACGGAC
>Zotu3968
GAACGCTAGCGACAGGCCTAACACATGCAAGTCGAGGGGCAGCGCGGGTAGCAATACCTGGCGGCGAC
CGGCGCACGGGTGCGTAACAGGTGTGCAATCTGTCCATAATCAGGGAATAGCCCAGCGAAAGTTGGAT
TAAAGCTCTATGTGTCATTGGTTCGCATGGACTGATGATGAAACGCGAGGATTATGGGTGAGCACGCT
TCTGATTAGATAGTTGGTGAGGTAACGGCCCACCAAGTCGACGATCAGTAGGGGTTCTGAGAGGAAGG
TCCCCCACATTGGAACTGAGACACGGTC
>Zotu3969
GAACGCTGGCGGCAGGCTTAACACATGCAAGTCGAACGCCCCGCAAGGGGAGTGGCAGACGGGTGAGT
AACGCGTGGGAATCTACCCAGTTCTACGGAACAACTGAGGGAAACTTCAGCTAATACCGTATACGCCC
TACGGGGGAAAGATTTATCGGAATTGGATGAGCCCGCGTTGGATTAGCTAGTTGGTGGGGTAATGGCC
TACCAAGGCGACGATCCATAGCTGGTCTGAGAGGATGATCAGCCACACTGGGACTGAGACACGGCC
>Zotu3972
GAACGCTGGCGGCGTGCCTAATACATGCAAGTCGAGCGGATCTCGAGGAGCTTGCTCCTTGAGATTAG
CGGCGGACGGGTGAGTAACACGTGGGCAACCTGCCTGTAAGACTGGGATAACACCGGGAAACCGGTGC
TAATACCGGATAATTCTTCCCCTCACATGAGGGAAAGCTGAAAGTCGGTTTCGGCTGACACTTACAGA
TGGGCCCGCGGCGCATTAGCTAGTTGGTGAGGTAACGGCTCACCAAGGCGACGATGCGTAGCCGACCT
GAGAGGGTGATCGGCCACACTGGGACTGAGACACGGCC
>Zotu3973
GAACGCTAGCGGCAGGCCTAATACATGCAAGTCGAACGAGCTACAGGACTTCGGTTCTGTGGTTAGTG
GCGCACGGGTGCGTAACGCGTATGCAACTTACCTTCCACAGGGGGATAGCCCACCGAAAGGTGGATTA
ATACCGCATAACATCATTTGAGGGCATCCGAAGATGATCAAAGCTGCGGCGGTGGAAGATGGGCATGC
GTGCCATTAGCTAGTTGGTAGGGTAACGGCCTACCAAGGCGACGATGGCTAGGGGTTCTGAGAGGATG
GTCCCCCACACTGGTACTGAGACACGGAC
>Zotu3974
GAACGCTGGCGGCAGGCTTAACACATGCAAGTCGAACGGGCATAGCAATATGTCAGTGGCAGACGGGT
GAGTAACGCGTGGGAACGTACCTTTTGGTTCGGAACAACACAGGGAAACTTGTGCTAATACCGGATAA

GCCCTTACGGGGAAAGATTTATCGCCGAAAGATCGGCCCGCGTCTGATTAGCTAGTTGGTAGGGTAAT
GGCCTACCAAGGCGACGATCAGTAGCTGGTCTGAGAGGATGATCAGCCACATTGGGACTGAGACACGG
CC
>Zotu3976
GAACGCTGGCGGCGTGCTTAACACATGCAAGTCGAACGGAAAGGCTCTCTTCGGAGAGTACTCGAGTG
GCGAACGGGTGAGTAACACGTGGGTAACCTACCTTTGACTGGGGGACAACCGGGGGAAACTCCGGCTA
ATACCGCATAACGTGCGACGTCATAAGGCGACGTACCAAAGATAGCTTCGGCTTTCGGTTAAAGATGG
GCCCGCGGCGGATTAGCTTGTTGGTGGGGTAACGGCCTACCAAGGCTACGATCCGTAGCTGGTCTGAG
AGGACGATCAGCCACACTGGAACTGAGACACGGTC
>Zotu3978
GAACGCTAGCGGCAGGCTTAACACATGCAAGTCGAGGGGTATGGTTCTTCGGAACCAGAGACCGGCGC
ACGGGTGCGTAACGCGTATGCAATCTACCTTTCACAGAGGGATAGCCCAGAGAAATTTGGATTAATAC
CTCATAGTATATTAGTCTCGCATGAGATTATTATTAAAGATTTATCGGTGAAAGATGAGCATGCGTCC
CATTAGTTAGTTGGTAAGGTAACGGCTTACCAAGACTACGATGGGTAGGGGTCCTGAGAGGGAGATCC
CCCACACTGGTACTGAGACACGGAC
>Zotu3979
GAACGCTGGCGGCGTGCCTAACACATGCAAGTCGTGCGGGAATACGGGAGCAATCCTGTTAGTACAGC
GGCGCACGGGTGAGTAACACGTAGGTAACCTGCCTTGGAGCCTGGGATAACCTGTCGAAAGATGGGCT
AATACCAGATAAGACCACGAGAACCTCGGTTCTTGGGGTAAAAGATGGCCTCTGTTTACAAGCTATCA
CTCGGAGATGGGCCTGCGTGCCATTAGCTAGTTGGTGAGGTAACGGCTCACCAAGGCAGAGATGGCTA
GCTGGTCTGAGAGGATGGCCAGCCACACTGCGACTGAGACACGGCC
>Zotu3980
AAACGCTGGCGGCGTGCCTAATACATGCAAGTCGAGCGGTAAGGCTTTCACAGTTTATATCTCTCCCA
TTTACTTGGGACCAAGAGGTGTAAAGCGTGAAAGTACACGAGCGGCGGACGGCTGAGTAACACGTAGG
AATCTACCCCAAACTGAGGGATAACTGCTCGAAAGAGTAGCTAATACCGCATACCTTCTTCGGAAGAA
AGCTTCGGCGGTTTGGGAAGAGCCTGCGCAAGATTAGCTAGTTGGTGAGGTAATAGCTCACCAAGGCG
ACGATCTTTAGCTGGTCTGAGAGGATAATCAGCCAGACTGGGACTGAGACACGGCC
>Zotu3981
GAACGCTGGCGGCGTGCTTAACACATGCAAGTCGAGCGGAAAGGCCTCTTCGGGGGTACTCGAGCGGC
GAACGGGTGAGTAACACGTGAGTAATCTGCCCTTCACACATGGATAACCACCGGAAACGGTGGCTAAT
ACATGATACGACACGTCTTCGCATGAGGTGCGTGTGGAAAGTTTTTCGGTGGAGGATGTGCTCGCGG
CCTATCAGCTTGTTGGTGGGGTAATGGCCTACCAAGGCTTCGACGGGTAGCCGGCCTGAGAGGGTGAC
CGGCCACACTGGGACTGAGACACGGCC
>Zotu3982
GAACGCTAGCGGCAGGCCTAACACATGCAAGTCGAGGGGTAAGGAGAGCTTGCTCTCTCTAGACCGGC
GTACGGGTGCGTAACACGTGTACAATCTACCTTTTACTAAGGAATAGCCCGAAGAAATTTGGATTAAT
ACCTTATAGTATTATTTTATGGCATCATTAAATAATTAAAGCTACGGTGGTAAAAGATGAGTACGCGT
CCCATTAGCTAGTTGGAGTGGTAACGGCACACCAAGGCTACGATGGGTAGGGGTTCTGAGAGGGATGT
CCCCCACACTGGTACTGAGACACGGAC
>Zotu3983
GAACGCTGGCGGCATGCTTTACACATGCAAGTCGAACGGCAGCGCGGGGCAACCTGGCGGCGAGTGGC
GAACGGGTGAGTAATATATCGGAACGTACCCAAGAGTGGGGGATAACGTAGCGAAAATTACGCTAATA
CCGCATAAGATCTAAGGATGAAAGCGGGGGATCGCAAGACCTCGTGCTCCTGGAGCGGCCGATATCTG
ATTAGCTAGTTGGTGAGGTAAAGGCTCACCAAGGCGACGATCAGTAGCTGGTCTGAGAGGACGACCAG
CCACACTGGAACTGAGACACGGTC
>Zotu3984
GAACGCTAGCGGCAGGCTTAACACATGCAAGTCGAGGGGTATAGTAGCAATACTAGAGACCGGCGCAC
GGGTGCGTAACGCGTATGCAACCTACCTTTTACAGGGGAATAGCCCAGAGAAATTTGGATTAATGCCC
CATGGTATAATTGAATGGCATCATTTAATTATTAAAGTTCCAACGGTAAAAGATGGCATGCGTCCTA
TTAGCTAGATGGTAAGGTAACGGCTTACCATGGCTACGATAGGTAGGGGTCCTGAGAGGGAGATCCCC
CACACTGGTACTGAGACACGGAC
>Zotu3985
GAACGCTGGCGGCGTGCTTAACACATGCAAGTCGAGCGGAAAGGCCCTTTCGGGGGTACTCGAGCGGC
GAACGGGTGAGTAACACGTGAGTAATCTGCCCTTCACTTGGGGATAACCTCCGGAAACGGTGGCTAAT

ACCCGATACGACCATGGATCGCATGATCTTGTGGTGGAAAGTTTTTTCGGTGGGGGATGTGCTCGCGG
CCTATCAGCTTGTTGGTGGGGTAATGGCCTACCAAGGCTTCGACGGGTAGCCGGCCTGAGAGGGTGAC
CGGCCACACTGGGACTGAAACACGGCC
>Zotu3988
GAACGCTGGCGGCGTGCTTAACACATGCAAGTCACGGGGGCCCGCAAGGGCAACCGGCGAACGGGTGC
GTAACACGTGAACAACCTGCCCTCGTGTGGGGGATAGCCGGGCTAACGCCCGGGTAATACCGCATACG
GTCCTTTGGGGGAGTCCCTGGGGGAAGAAAGCTCCGGCGCACGGGGAGGGGTTCGCGGCCTATCAGCT
AGTTGGCGAGGTAACGGCTCACCAAGGCGACGACGGGTAGCTGGTCTGAGAGGATGGCCAGCCACATT
GGGACTGAGAAACGGCC
>Zotu3989
GAACGCTGGCGGCGTGCCTAATACATGCAAGTCGAGCGGAACTTTGATTGGTGCTTGCACCGATTAAA
GTTTAGCGGCGGACGGGTGAGTAACACGTGGGTAACCTGCCCTACAGACTGGGATAACTTCGGGAAAC
CGAGGCTAATACCGGATAATACTTTTTAACACATGTTAGAAAGTTGAAAGATGGTTTCGGCTATCACT
GTAGGATGGACCCGCGGCGCATTAGCTAGTTGGTGAGGTAACGGCTCACCAAGGCAACGATGCGTAGC
CGACCTGAGAGGGTGATCGGCCACACTGGGACTGAGACACGGCC
>Zotu3990
GAACGCTGGCGGCGTGCCTAATACATGCAAGTCGAGCGAACCTGAGGGAGCTTGCTCCCAAAGGTTAG
CGGCGGACGGGTGAGTAACACGTGGGCAACCTGCCTGTAAGACTGGGATAACTTCGGGAAACCGGAGC
TAATACCGGATAATTCTTTTCTTCACATGAGGAAAAGCTGAAAGACGGCGCAAGCTGTCACTTACAGA
TGGGCCCGCGGCGCATTAGCTAGTTGGTGAGGTAACGGCTCACCAAGGCGACGATGCGTAGCCGACCT
GAGAGGGTGATCGGCCACATTGGGACTGAGACACGGCC
>Zotu3991
GAACGCTAGCGGCAGGCTTAACACATGCAAGTCGAGGGGTATAGTTCTTCGGAGCTAGAGACCGGCGC
ACGGGTGCGTAACGCGTATGCAATCTACCTTTTACAGAGGGATAGCCCAGAGAAATTTGGATTAATAC
CTCATAGCATAGCAGCTTCGCATGAAGTCACTATTAAAGTCACAACGGTAAAAGATGAGCATGCGTCC
CATTAGCTAGTTGGTAAGGTAACGGCTTACCAAGGCTACGATGGGTAGGGGTCCTGAGAGGGAGATCC
CCCACACTGGTACTGAGACACGGAC
>Zotu3992
GAACGCTGGCGGCGTGCTTAACACATGCAAGTCGAGCGGTAAGGCCCTTCGGGGTACACGAGCGGCGA
ACGGGTGAGTAACACGTGGGTGACCTGCCCTCAGCTCCGGGATAAGCCTGGGAAACCGGGTCTAATAC
CGGATACGACCATTGGATGCATGTCCGGTGGTGGAAAGTTTTTCGGCTGGGGATGGCCCGCGGCCTA
TCAGCTTGTTGGTGGGGTGATGGCCTACCAAGGCGGTGACGGGTAGCCGGCCTGAGAGGGCGACCGGC
CACACTGGGACTGAGACACGGCC
>Zotu3995
GAACGCTGGCGGCGTGCCTAATACATGCAAGTCGAGCGGACAGATGGGAGCTTGCTCCCTGAAGTTAG
CGGCGGACGGGTGAGTAACACGTGGGCAACCTGCCCTGCAGATGGGGATAACTCCGGGAAACCGGGGC
TAATACCGAATAATCAGTTTCTTCGCATGGAGAAATTCTGAAAGACGGTTTCGGCTGTCACTGCAGGA
TGGGCCCGCGGCGCATTAGCTAGTTGGTGGGATAACGGCCTACCAAGGCAACGATGCGTAGCCGACCT
GAGAGGGTGATCGGCCACACTGGGACTGAGACACGGCC
>Zotu3996
GAACGCTGGCGGCGCGCTTAACACATGCAAGTCGAGCGATCGGCCGACCCTTCGGGGACGGTACGGAG
CGGCGAACGGGTGAGTAACACGTGGGTAATCTGCCCTCGGCACTGGGATAGCCCGGGGAAACCCGGAT
TAATACCGGATGGTCTCCGGAGCCTGCGGGCGCCGGAGAAAAGGAAGCTTCGGCCTCCGACCGAGGAT
GAGCCCGCGGCGGATTAGCTTGTTGGTGGGGTAAAGGCCCACCAAGGCGACGATCCGTAGCTGGTGTG
AGAGCATGACCAGCCACACTGGGACTGAGACACGGCC
>Zotu3997
GAACGCTGGCGGCAGGCTTAATACATGCAAGTCGAGGGGCAGCAGCTTTATAGCAATATAGAGGCTGG
CGACCGGCAAACGGGTGCGGAACACGTACACAACCTTCCTTGAATAGGGGGATAGCCCATAGAAATGT
GGATTAATACCCCGTAATATAGCGGTGTGGCATCACACTGTTATTATAGTTTCGACGATTCGAGATGG
GTGTGCGGCTGATTAGGTAGTTGGCGGGGTAAAGGCCCACCAAGCCTTCGATCAGTAGCTGATGTGAG
AGCATGATCAGCCACACGGGCACTGAGACACGGGC
>Zotu3998
GAACGCTGGCGGCGTGCCTAATACATGCAAGTCGAGCGGACCTGAATGGAGCTTGCTCCAGACAGGTT
AGCGGCGGACGGGTGAGTAACACGTAGGCAACCTGCCCGCAAGACCGGGATAACGTAGGGAAACTTAC

GCTAATACCGGATAGGGCACTCTATCGCATGAGAGAGTGCGGAAAGGTGGCGCAAGCTACCACTTGCG
GATGGGCCTGCGGCGCATTAGCTAGTTGGTGGGGTAACGGCCTACCAAGGCGACGATGCGTAGCCGAC
CTGAGAGGGTGACCGGCCACACTGGGACTGAGACACGGCC
>Zotu3999
GAACGCTGGCGGCGTGCCTAACACATGCAAGTCGAACGAGGTCCATGCAGTGGCAACACTGCGGAAGA
CCTAGTGGCGAACGGGTGAGTAACACGTGAGGAACCTGCCCCGAAGACCGGGATAACTCTCCGAAAGG
AGCGCTAATACCGGATACCCCCACCTAACCGCATGGTCTGGTGAGGAAATGGATTCCGCTTCGGGAGG
GCCTCGCGGCCTATCAGCTAGTTGGTGGGGTAACGGCCCACCAAGGCGTCGACGGGTAGCTGGTCTGA
GAGGACGATCAGCCACACTGGGACTGAGACACGGCC
>Zotu4000
GAACGCTAGCGGCAGGCCTAATACATGCAAGTCGAACGATAGTGAAGGGCTTGCTCTTCACGAAAAGT
GGCGCACGGGTGCGTAACGCGTATGCAACCTACCTTAATCAGGGGGATAGCCCGAAGAAATTCGGATT
AACACCGCATAAAATCACAGTATAGCATTATACAATGATCAAATATTTATAGGATTAAGATGGGCATG
CGTGACATTAGTTAGTTGGCGAGGTAACGGCTCACCAAGACCACGATGTCTAGGGGATCTGAGAGGAT
GACCCCCCACACTGGTACTGAGACACGGAC
>Zotu4002
GAACGCTAGCGGCAGGCTTAATACATGCAAGTCGAGGGGCAGCACGGGTAGCAATACCTGGTGGCGAC
CGGCAAACGGGTGCGGAACACGTACGCAACCTTCCCATAACTGGTGAATAGCCTTTCGAAAGAGAGAT
TAATACACCGTAATATAACGAAGAGGCATCTCTTTGTTATCATAGGCTTCGGCCGGTTTTGGATGGGC
GTGCGCCTGATTAGGTAGTTGGCGGGGTAACGGCCCACCAAGCCTGCGATCAGTAACTGGTGTGAGAG
CACGACCAGTCACACGGGCACTGAGACACGGGC
>Zotu4003
GAACGCTGGCGGCAGGCTTAACACATGCAAGTCGAGCGAGCCCTTCGGGGTCAGCGGCGGACGGGTGA
GTAACGCGTGGGAACGTGCCTTCTGGTCTGGAATAACCCTGGGAAACTAGGACTAATACCGGATACGC
CCTTTTGGGGAAAGGTTTACTGCCGGAAGATCGGCCCGCGTCTGATTAGCTAGTTGGTGGGGTAACGG
CCTACCAAGGCGACGATCAGTAGCTGGTCTGAGAGGATGATCAGCCACACTGGGACTGAGACACGGCC
>Zotu4004
CAACGCTGGCGGCGTGCCTAACACATGCAAGTCGCACGAGAAAGGGACTTCGGTCCTGAGTAAAGTGG
CGCACGGGTGAGTAACACGTGACTAATCTACCCTCGAGTGGGGAATAACCTAGGGAAACCTGGGCTAA
TACCGCATAACACTTACGAGTCAAAGCAGCAATGCGCTTGGGGAGGAGGTCGCGGCAGATTAGCTAGT
TGGCGGGGTAATGGCCCACCAAGGCGATGATCTGTATCCGGCCTGAGAGGGCGCACGGACACACTGGA
ACTGAAACACGGTC
>Zotu4005
GAACGCTGGCGGCGTGGATTAGGCATGCAAGTCGAGCGATCTCGCAAGAGATAGCGGCAAACGGGTGA
GGAACACGTAAATAATCTTCCCCCTATCTGGACATAGCCCTATGAAAATAGGAGTAATATCCAATGAC
AACATTTAGCAGCATTGCTTTATGTTTAAAGACAGGGACCGCAAGGCCTGTCGGTAAGGGATGAGTTT
GCGTAGCATCAGCTAGTTGGTAGGGTAATGGCCTACCAAGGCTATGACGCTTAGCTGGTCTGAGAGGA
TGGTCAGCCACACTGGGACTGAGACACTGCC
>Zotu4006
AAACGCTGGCGGCGTGCATAAAACATGCAAGTCGAACGGACTTCAGTCCGCAAGGACTGAAGTGAGTG
GCGAACGGCTGAGTAACACGTAGGTGACCTGCCCCGAAGTTTGGGATAACAACTCGAAAGAGTTGCTA
ATACCGGATGTGACCACCGATTCATTTCGGTGCTTAAAGTCGCAAGACGCTTTGGGAGGGGCCTGCGG
CGGATTAGCTAGTTGGTGTGGTAACGGCATACCAAGGCTTCGATCCGTAGCTGGTCTGAGAGGATGGC
CAGCCAGACTGGGACTGAGACACGGCC
>Zotu4007
GAACGCTAGCGGCAGGCCTAATACATGCAAGTCGAGGGGTAGAGGTTCTTCGGAACCTTGAGACCGGC
GCACGGGTGCGTAACGCGTATGCAACCTACCTTTATCAGGGGGATAGCCCGAAGAAATTCGGATTAAT
ACCGCATAAAATCACAGTATAGCATTATACAATGATCAAACATTTATGGGATGAAGATGGGCATGCGT
GTCACTAGTTAGTTGGCGGGGTAACGGCCCACCAAGACGACGATGACTAGGGGATCTGAGAGGATGAC
CCCCCACACTGGTACTGAGACACGGAC
>Zotu4008
GAACGCTGGCGGCGTGCCTAACACATGCAAGTCGAGCGATTCTCTTCGGAGAAGAGCGGCGGACGGGT
GAGTAACGCGTGGGTAACCTGCCCTGTACACACGGATAACATACCGAAAGGTATGCTAATACGAGATA
ATATACTTTTATCGCATGATAGAAGTATCAAAGCTCCGGCGGTACAGGATGGACCCGCGTCTGATTAG

CTAGTTGGTAAGGTAACGGCTTACCAAGGCGACGATCAGTAGCCGACCTGAGAGGGTGATCGGCCACA
TTGGAACTGAGACACGGTC
>Zotu4009
GAACGCTGGCGGCGTGCTTAACACATGCAAGTCGAACGGTGATGCAGAGCTTGCTCTGCGGATCAGTG
GCGAACGGGTGAGTAACACGTGAGTAACCTGCCCTTGACTCTGGGATAAGCGTTGGAAACGACGTCTA
ATACCGGATATGACCCGTGGAGGCATCTCCTGCGGGTGGAAAGATTTTTCGGTTGAGGATGGACTCGC
GGCCTATCAGCTTGTTGGTGAGGTAATGGCTCACCAAGGCGACGACGGGTAGCCGGCCTGAGAGGGTG
ACCGGCCACACTGGGACTGAGACACGGCC
>Zotu4010
AAACGCTGGCGGCGTGCATAACACATGCAAGTCGAACGATGAAACTTAAATGATTTCTTCGGAATGAA
ATTAAGTGGATTAGTGGCGAACGGGTGAGTAACACGTGAGTAACCTGCCTTACACAAGGGGATAGCCT
TTGGAAACGAAGAATAATACCCTATAAAGTCAAAATATCGCATGATAAAGTGACCAAAGATTTATCGG
TGTAAGATGGACTTGCGTCTGATTAGCTAGTTGGTGAGATAAAAGCCCACCAAGGCGACGATCAGTAG
CCGGCTTGAGAGAGTGTACGGCCACATTGGGACTGAGACACGGCC
>Zotu4011
AAACGCTGGCGGCGTGCATAACACATGCAAGTCGAACGATGAACCTTAATTGATTTCTTCGGAATGAT
TTTAAGCGGATTAGTGGCGAACGGGTGAGTAACACGTGAGTAACCTGCCTTACACAAGGGGATAGCCT
TTGGAAACGAAGAATAATACCCTATAAAGTCAAAATATCGCATGATAAAGTGACCAAAGATTTATCGG
TGTAAGATGGACTTGCGTCTGATTAGCTAGTTGGTGAGATAAAAGCCCACCAAGGCGACGATCAGTAG
CCGGCTTGAGAGAGTGTACGGCCACATTGGGACTGAGACACGGCC
>Zotu4012
GAACGCTGGCGGCGTGCCTAATACATGCAAGTCGAGCGAATTTTTGGGAGCTTGCTCCCGAAGGTTAG
CGGCGGACGGGTGAGTAACACGTGGGCAACCTGCCTATAAGACTGGGATAACTTCGGGAAACCGGAGC
TAATACCGGATAATTCTTTTCTACACATGTAGAAAAGCTAAAAGACGGTTTACGCTGTCACTTATAGA
TGGGCCCGCGGCGCATTAGCTAGTTGGTGAGGTAACGGCTCACCAAGGCCACGATGCGTAGCCGACCT
GAGAGGGTGATCGGCCACACTGGGACTGAGACACGGCC
>Zotu4013
GAACGCTGGCGGCAGGCTTAACACATGCAAGTCGAGCGGGCGTAGCAATACGTCAGCGGCAGACGGGT
GAGTAACACGTGGGAACGTACCTTTTGGTTCGGAACAACACAGGGAAACTTGTGCTAATACCGGATAA
GCCCTTACGGGGAAAGATTTATCGCCGAAAGATCGGCCCGCGTCTGATTAGCTAGTTGGTGAGGTAAT
GGCTCACCAAGGCGACGATCAGTAGCTGGTCTGAGAGGATGATCAGCCACACTGGGACTGAGACACGG
CC
>Zotu4015
GAACGCTGGCGGCATGCCTAACACATGCAAGTCGAACGAGACCTTCGGGTCTAGTGGCGCACGGGTGC
GTAACGCGTGGGAATCTGCCCTTGGGTACGGAATAACTCTCCGAAAGGAGTGCTAATACCGTATGATG
TCGCAAGACCAAAGATTTATCGCCCAGGGATGAGCCCGCGTAGGATTAGCTAGTTGGTGAGGTAAAGG
CTCACCAAGGCGACGATCCTTAGCTGGTCTGAGAGGATGATCAGCCACACTGGGACTGAGACACGGCC
>Zotu4016
GAACGCTGGCGGTGTGTCTAAGGCATGCAAGTCGAACGAATCGCTTTTTTGTAGCAATACTTGAAAGT
GGTTAGTGGCAAACGGGTGAGTAACACATTGGTAACCTGCCTTCAAGACGGGCACAACATTGCGAAAG
CGATGCTAATTCCCGATGGTCTCTTTGGTGAAAGTCCAAAAGAGTAAACGTCCAAAAGACGCTTGAAG
AGGGGCCTATGGCCTATCAGCTTGTTGGTGAGGTAATGGCTCACCAAGGCGACGACGGGTAGCAGGCG
TGAGAGCGCGACCTGCCTCAGTGGGACTGCGATACTGCC
>Zotu4019
GAACGCTGGCGGCGTGCCTAATACATGCAAGTAGAACGCTGAAGGAGGAGCTTGCTCTTCTGGATGAG
TTGCGAACGGGTGAGTAACGCGTAGGTAACCTGCCTGGTAGCGGGGGATAACTATTGGAAACGATAGC
TAATACCGCATAATAGTAGATGTTGCATGACATTTGCTTGAAAGGTGCAATTGCATCACTACCAGATG
GACCTGCGTTGTACTAGCTAGTTGGTGGGGTAACGGCTCACCAAGGCGACGATACATAGCCGACCTGA
GAGGGTGATCGGCCACACTGGGACTGAGACACGGCC
>Zotu4020
GAACGCTAGCTACAGGCTTAACACATGCAAGTCGAGGGGAAACGACATCGAAAGCTTGCTTTTGATGG
GCGTCGACCGGCGCACGGGTGAGTAACGCGTATCCAACCTGCCCATCACTTGGGGATAACCTTGCGAA
AGTAAGACTAATACCCAATGATATCTCTAGAAGACATCTGAAAGGGATTAAAGATTTATCGGTGATGG
ATGGGGATGCGTCTGATTAGCTTGTTGGCGGGGTAACGGCCCACCAAGGCGACGATCAGTAGGGGTTC

TGAGAGGAAGGTCCCCCACATTGGAACTGAGACACGGTC
>Zotu4021
GAACGCTGGCGGCACGCTTAACACATGCAAGTCGAACGGAGAATTTTGGTTTACTGGAATTCTTAGTG
GCGGACGGGTGAGTAACACGTGAGCAACCTGCCTCTGAGAGAGGAATAGCTTCTGGAAACGGATGGTA
ATACCTCATAACATACATTTATCGCATGGTAGATGTATCAAAGATTATATCACTCAGAGATGGGCTCG
CGTCTGATTAGATAGTTGGTGAGGTAACGGCTCACCAAGTCGACGATCAGTAGCCGGACTGAGAGGTT
GAACGGCCACATTGGGACTGAGACACGGCC
>Zotu4022
AAACGCTGGCGGCGTGCTTAACACATGCAAGTCGAACGAAGTTTTTCATTCGTGGAAAACTTAGTGGC
GGACGGGTGAGTAACGCGTGGGTAACCTGCCCTGTACAGGGGGACAACAGCTGGAAACGGCTGCTAAT
ACCGCATAAGCCCTTAGCACTGCATGGTGCATAGGGAAAAGGAGCAATCCGGTACAGGATGGACCCGC
GTCTGATTAGCCAGTTGGCAGGGTAACGGCCTACCAAAGCGACGATCAGTAGCCGATCTGAGAGGATG
TACGGCCACATTGGGACTGAGACACGGCC
>Zotu4023
GAACGCTGGCGGCAGGCTTAACACATGCAAGTCGAGCGCCGTAGCAATACGGAGCGGCAGACGGGTGA
GTAACGCGTGGGAACATGCCCTTTGGTTCGGAATAACTCAGGGAAACTTGAGCTAATACCGGATAAGC
CCTTTTGGGGAAAGATTTATCGCCGAAGGATTGGCCCGCGTCCGATTAGCTAGTTGGTGTGGTAATGG
CGCACCAAGGCGACGATCGGTAGCTGGTCTGAGAGGATGATCAGCCACACTGGGACTGAGACACGGCC
>Zotu4024
GAACGCTGGCGGCGTGCTTAACACATGCAAGTCGAGCGGTAAGGCACTTCGGTGTACACGAGCGGCGA
ACGGGTGAGTAACACGTGAGTAATCTGCCCTTCACTCTGGGATAAGCACTAGAAATGGTGTCTAATAC
CGGATACGACCAATCCCTGCATGGGGTGTTGGTGGAAACTTTTGTGGGTGGGGGATGTGCTCGCGGCC
TATCAGCTTGTTGGTGGGGTAATGGCCTACCAAGGCTTCGACGGGTAGCCGGCCTGAGAGGGTGACCG
GCCACACTGGGACTGAGACACGGCC
>Zotu4025
GAACGCTGGCGGCGTGCCTAATACATGCAAGTCGAACGCATTTTCTTTCACCGTAGCTTGCTACACCG
AAAGAAAATGAGTGGCGAACGGGTGAGTAACACGTGGGTAACCTGCCCATCAGCGAGGGATAACACTT
GGAAACAGGTGCTAATACCGCATAATTCCATTTACCGCATGGTAGATGGATGAAAGGCGCTTTTGCGT
CACTGATGGATGGACCCGCGGTGCATTAGCTAGTTGGTGGGGTAACGGCCTACCAAGGCTGCGATGCA
TAGCCGACCTGAGAGGGTGATCGGCCACACTGGGACTGAGACACGGCC
>Zotu4026
GAATGCTGGCGGCATGCCTTACACATGCAAGTCGAACGGCAGCGCGGACTTCGGTCTGGCGGCCAGTG
GCGAACGGGTGAGTAATACATCGGAACGTACCCTGTTGTGGGGGATAACTAGTCGAAAGATTAGCTAA
TACTGCATATGACCTGAGGGTGAAAGCGGGGGACCGTAAGGCCTCGCGCAGCAGGAGCGGCCGATGTC
TGATTAGCTAGTTGGTGGGGTAAAGGCCCACCAAGGCGACGATCAGTAGCTGGTCTGAGAGGACGATC
AGCCACACTGGGACTGAGACACGGCC
>Zotu4027
GAACGCTGGCGGCGTGCTTAACACATGCAAGTCGAACGGTGAACCCGCTTCGGTGGGGGATCAGTGGC
GAACGGGTGAGTAACACGTGGGCAACCTGCCCTCAGCTCTGGGATAACTCCAAGAAATTGGGGCTAAT
ACCGGATATGACTGCTGACCGCATGGTCTGGTGGTGGAAAGATTTATCGGCTGGGGATGGGCCCGCGG
CCTATCAGCTTGTTGGTGGGGTAGTGACCTACCAAGGCGACGACGGGTAGCCGGCCTGAGAGGGTGAC
CGGCCACACTGGGACTGAGACACGGCC
>Zotu4028
GAACGCTGGCGGCGTGCCTAATACATGCAAGTCGAACGGTGCAGCAATGTACAGTGGCGACCGGGTGA
GTAACACGTAGTTGACCTACCCTTCCGCGGGGGACAACAGTTCGAAAGGACTGCTAATACCGCATACA
TTGGCAGAGTTTAGAAGTCTGTCAAGAAACTTCAAGGGCGGAAGGAGGGGGCTGCGGCCCATCAGGTA
GTTGGTGAGGTAGTGGCTCACCAAGCCGACGACGGGTAGGGGGCCTGAGAGGGTGACCCCCCACACTG
GAACAGACATACGGTC
>Zotu4029
GAACGCTGGCGGCAGGCCTAACACATGCAAGTCGAGCGGGGTTTTCGGACCTAGCGGCGGACGGGTGA
GTAATGCGTAGGAAGCTGCCCGATAGAGGGGGATACCAGTTGGAAACGACTGTTAATACCGCATAATG
TCTACGGACCAAAGTGTGGGACCTTCGGGCCACATGCTATCGGATGCGCCTACGTGGGATTAGCTAGT
TGGTGAGGTAATGGCTCACCAAGGCGACGATCTCTAGCTGGTTTGAGAGGATGATCAGCCACACTGGA
ACTGAGACACGGTC

>Zotu4030
GAACGCTGGCGGCGTGCTTAACACATGCAAGTCGAGCGAGGCCCGTTCCTTCGGGAACGGTGCCCTAG
CGGCGAACGGGTGAGTAACACGTGGGCAACCTGCCCCTAGCACTGGGATAACTCCGGGAAACCGGGGC
TAATACCGGATACGACCTCTGAGGGCATCCTCGGAGGTGGAAAGGATTCTGGCTAGGGATGGGCCCGC
GGCCTATCAGCTTGTTGGTGGGGTAATGGCCTACCAAGGCGACGACGGGTAGCCGGCCTGAGAGGGCG
ACCGGCCACACTGGGACTGAGACACGGCC
>Zotu4031
GAACGCTGGCGGCGTGCTTAACACATGCAAGTCGAACGGTGAAGGGGCCTGCTTTTGTGGGTCCTGGA
TGAGTGGCGAACGGGTGAGTAACACGTGAGTAACCTGCCCCCTTCTTCTGGATAACCGCATGAAAGTG
TGGCTAATACGGGATATTCTGGGTCTGTCGCATGGTGGGCCTGGGAAAGATTGCGCTTTTTGGTGTTT
TTGGTGGGGGATGGGCTCGCGGCCTATCAGCTTGTTGGTGGGGTGATGGCCTACCAAGGCGGTGACGG
GTAGCCGGCCTGAGAGGGTGGACGGTCACACTGGGACTGAGACACGGCC
>Zotu4032
GAATGCTGGCGGTGTGCCTTAAGCATGCAAGTCGAGCGGCTCGCAAGAGCAGCGGCAGACGGGTGAGT
AACACGTAGGAATCTACCTCAAAGTGGGGAATAGCCAGCCGAAAGGTTGGGTAATACCGCATGTGATC
GAAAGATTAAAGCAGCAATGCGCTTTGAGAGGAGCCTGCGTCCTATCAGCTAGTTGGTGAGGTAATAG
CTTACCAAGGCAATGACGGGTAGCCGATGTGAGAGCATGATCGGCCACAAGGTCACTGAGACACGGGA
>Zotu4035
GAACGCTAGCGGCAGGCTTAACACATGCAAGTCGAGGGGTAGGGTAGCAATACCTGAGACCGGCGCAC
GGGTGCGTAACGCGTATGCAATCTACCTTTTACAGAGGGATAGCCCAGAGAAATTTGGATTAATACCT
CATAGCATAGCAGCTTCGCATGAAGTCACTATTAAAGTTCCAACGGTAAAAGATGAGCATGCGTCCTA
TTAGCTAGATGTTAAGGTAACGGCTTACCATGGCTACGATAGGTAGGGGTCCTGAGAGGGAGATCCCC
CACACTGGTACTGAGACACGGAC
>Zotu4037
GAACGCTGGCGGCATGCCTAACACATGCAAGTCGAACGAGACCTTCGGGTCTAGTGGCGCACGGGTGC
GTAACGCGTGGGAATCTGCCCCTTGGTTCGGAATAACAGTTAGAAATGACTGCTAATACCGGATGATG
ACTTCGGTCCAAAGATTTATCGCCAAGGGATGAGCCCGCGTAGGATTAGCTAGTTGGTGGGGTAAAGG
CCTACCAAGGCGACGATCCTTAGCTGGTCTGAGAGGATGATCAGCCACACTGGGACTGAGACACGGCC
>Zotu4038
GAACGCTGGCGGCATGCCTTACACATGCAAGTCGAACGCGAAAGCAGGGGCAACCCTGTTAGTAGAGT
GGCGAACGGGTGAGTAATACATCGGAACGTGCCCAGTTGTGGGGGATAACCAGTCGAAAGACTGGCTA
ATACCGCATGAGACCTGAGGGTGAAAGCAGGGGATCCGCAAGGACCTTGCGCGACTGGAGCGGCCGAT
GTCGGATTAGCTAGTTGGTGAGGTAAAAGCTCACCAAGGCGACGATCCGTAGCTGGTCTGAGAGGACG
ATCAGCCACACTGGAACTGAGACACGGTC
>Zotu4039
GAACGCTGGCGGCGTGCCTAATACATGCAAGTCGAGCGAACCTTTGGGAGCTTGCTCCCAAAGGTTAG
CGGCGGACGGGTGAGTAACACGTGGGCAACCTGCCTATAAGACTGGGATAACTTCGGGAAACCGGAGC
TAATACCGGATAACTCATATCTACTCATGTAGATATGCTAAAAGACGGTTTCGGCTGTCACTTATAGA
TGGGCCCGCGGCGCATTAGCTAGTTGGTGAGGTAACGGCTCACCAAGGCGACGATGCGTAGCCGACCT
GAGAGGGTGATCGGCCACACTGGGACTGAGACACGGCC
>Zotu4042
GAACGCTGGCAGCAGGCTTAACACATGCAAGTCGAGCGGAGAGAGGTAGCTTGCTACTGATCTTAGCG
GCGGACGGGTGAGTAATGCTTAGGAATCTGCCTATTAGTGGGGGACAACATTTCGAAAGGAATGCTAA
TACCGCATACGTCCTACGGGAGAAAGCAGGGGATCTTCGGACCTTGCGCTAATAGATGAGCCTAAGTC
GGATTAGCTAGTTGGTGGGGTAAAGGCCTACCAAGGCGACGATCTGTAGCGGGTCTGAGAGGATGATC
CGCCACACTGGGACTGAGACACGGCC
>Zotu4043
GAACGCTAGCGGCAGGCTTAATACATGCAAGTCGAGGGGCAGCATGGTGTAGCAATACACTGATGGCG
ACCGGCAAACGGGTGCGGAACACGTACGCAACCTTCCTTCAACTGAGGAATAGCCCAGGGAAACTTGG
ATTAATACCTCGTAACATTATGAAGTGGCATCACTTTATAATTATAGCTCAGGCGGTTGAAGATGGGC
GTGCGACTGATTAGGTAGTTGGCGGAGTAACAGCCCACCAAGCCTGCGATCAGTAACTGGTGTGAGAG
CACGACCAGTCACACGGGCACTGAGACACGGGC
>Zotu4045
GAACGCTGGCGGCGTGCCTAATACATGCAAGTCGAGCGAATCTGAGGGAGCTTGCTCCCAAAGGTTAG

CGGCGGACGGGTGAGTAACACGTGGGCAACCTGCCTATAAGACTGGGATAACTTCGGGAAACCGGAGC
TAATACCGGATAATTCATATCTACTCATGTAGATATGCCAAAAGACGGTTTACGCTGTCACTTATAGA
TGGGCCCGCGGCGCATTAGCTAGTTGGTGAGGTAACGGCTCACCAAGGCAACGATGCGTAGCCGACCT
GAGAGGGTGATCGGCCACACTGGGACTGAGACACGGCC
>Zotu4046
GAACGCTGGCGGCGTGCCTAATACATGCAAGTCGAGCGAATCCGAGGGAGCTTGCTCCCAAAGATTAG
CGGCGGACGGGTGAGTAACACGTGGGCAACCTGCCTGTAAGACTGGGATAACTTCGGGAAACCGGAGC
TAATACCGGACATTTCTTTTCTTCACATGAAGGAAAGTTGAAAGACGGCGCAAGCTGTCACTTACAGA
TGGGCCCGCGGCGCATTAGCTAGTTGGTGAGGTAACGGCTCACCAAGGCGACGATGCGTAGCCGACCT
GAGAGGGTGATCGGCCACATTGGGACTGAGACACGGCC
>Zotu4047
GAACGCTGGCGGCGTGCCTAATACATGCAAGTCGAACGAATCTCGAAGTTAGCTTGCTAACTTCTTGG
TTAGTGGCGGACGGGTGAGTAACACGTAGGCAACCTGCCCTCAAGATCGGGATAACATTCGGAAACGA
ATGCTAAGACCGGATACACAATTTTCTCGCATGAGAGAATTGGGAAACACGGTGCAAGCTGTGGCTTG
GGGATGGGCCTGCGGCGCATTAGCTAGTTGGTGGGGTAACGGCTCACCAAGGCGACGATGCGTAGCCG
ACCTGAGAGGGTGAACGGCCACACTGGGACTGAGACACGGCC
>Zotu4049
GAACGCTGGCGGCATGCCTAACACATGCAAGTCGAACGGCAGCACAGCAGTAGCAATACTGTGGGTGG
CGAGTGGCGAACGGGTGAGGAATGCGTCGGAATCTGCCCAGTTGTGGGGGATAACTAGCCGAAAGGTT
AGCTAATACCGCATACGACCGAGAGGTGAAAGCGGGGGACCGTAAGGCCTCGCGCGATTGGATGAGCC
GACGCCGGATTAGCTTGTTGGTGGGGTAAAGGCCTACCAAGGCGACGATCCGTAGCTGGTCTGAGAGG
ATGATCAGCCACACTGGAACTGAGACACGGTC
>Zotu4050
GAACGCTGGCGGCGTGCCTAATACATGCAAGTCGAGCGAATCTTTGGGAGCTTGCTCCCAAAGGTTAG
CGGCGGACGGGTGAGTAACACGTGGGCAACCTGCCTGTAAGACTGGGATAACACCGGGAAACCGGTGC
TAATACCGGATAATTCTTTTCCTCTCATGAGGAAAAGCTGAAAGTCGGTTTCGGCTGACACTTACAGA
TGGGCCCGCGGCGCATTAGCTAGTTGGTGAGGTAACGGCTCACCAAGGCGACGATGCGTAGCCGACCT
GAGAGGGTGATCGGCCACACTGGGACTGAGACACGGCC
>Zotu4051
TAACGCTAGCGGCAGGCCTAACACATGCAAGTCGAGGGGCAACAGTTGTAGCAATACAAGCTGGCGAC
CGGCGCACGGGTGCGTAACGCGTATGCAACCTACCTTTTACAGGGGGATAGTCTGAAGAAATTTAGAA
TAATACCCCATAGTATTATGTATTGGCATCAATATATAATTAAAGATTTATCGGTAAAAGATGGGCAT
GCGTATCATTAGCTAGTTGGAGAGGTAACGGCTCACCAAGGCTATGATGATTAGGGGTTCTGAGAGGA
TTTACCCCCACACTGGTACTGAGACAGGGAC
>Zotu4053
GAACGCTGGCGGCGCGTCTTAAGCATGCAAGTCGAACGGCAAGAAGAAGCTTGCTTCTTCCTAGAGTG
GCGGACTGGTGAGTAACGCGTGGATGACGTACCCTCGTGACGGGGATAGCCGGTAGAAATACCGGATA
ATACCGGATAAGGTCTCACGGAATAAAGGCGTGAGAGGAAAGCAGCGTATGCTGCGCAGGAGGAACGG
TTCGCGGCCTATCAGCTAGTTGGCAGGGTAAAGGCCTACCAAGGCGACGACGGGTATCCGGCCTGAGA
GGGTGGACGGACACATTGGGACTGAGATACGGCC
>Zotu4054
GAACGCTGGCGGTGTGCCTAATACATGCAAGTCGAGCGGTAACACGTCATGGTGACGAGCGGCGGACG
GCTGAGTAACGCGTAGGAACATACCCCAAACTGAGGGATAACTGCCCGAAAGGGTAGCTAATACCGCA
TGTGATCTTCGGATTAAAGCTTCGGCGGTTTGGGAATGGCCTGCGTTCGATTAGGTAGTTGGTGAGGT
AAAAGCTCACCAAGCCGACGATCGATAGCTGGTCTGAGAGGATGATCAGCCAGACTGGGACTGAGACA
CGGCC
>Zotu4055
GAACGCTGGCGGCATGCCTAACACATGCAAGTCGAACGGAGAGCATTCGCTGAAGTTTTCGGATGGAC
GTGGATGTATCTTAGTGGCGGACGGGCGAGTAACGCGTGAGCAACCTGCCCCTCAGTGGGGAACAACA
GTTGGAAACGACTGCTAATACCGCATAACGTATAGTTGCCGCATGGCGACGATACCAAAGGATTTATT
CGCTGAGGGATGGGCTCGCGTCCGATTAGATAGTTGGTGAGGTAACGGCCCACCAAGTCGACGATCGG
TAGCCGAACTGAGAGGTTGATCGGCCGCATTGGGACTGAGACACGGCC
>Zotu4058
GAACGCTGGCGGCAGGCTTAACACATGCAAGTCGAGCGGGCGATGTTGCTTCGGTAACTGAGCTAGCG

GCGGACGGGTGAGTAAAGCTTAGGAATCTGCCTATTAGTGGGGGACAACATTCCGAAAGGGATGCTAA
TACCGCATACGTCCTACGGGAGAAAGCAGGGGATCACTTGTGACCTTGCGCTAATAGATGAGCCTAAG
TCGGATTAGCTAGTTGGTGGGGTAAAGGCCTACCAAGGCGACGATCTGTAGCGGGTCTGAGAGGATGA
TCCGCCACACTGGGACTGAGACACGGCC
>Zotu4059
GAACGCTGGCGGCATGCTTTACACATGCAAGTCGAACGGTAACAGGGAGCTTGCTCCGCTGACGAGTG
GCGAACGGGTGAGTAATATATCGGAACGTACCCAAGAGTGGGGGATAACGTAGCGAAAGTTACGCTAA
TACCGCATACGATCCAAGGATGAAAGCAGGGGACCGCAAGGCCTTGTGCTCCTGGAGCGGCCGATATC
TGATTAGCTAGTTGGTAGGGTAAAGGCCTACCAAGGCATCGATCAGTAGCTGGTCTGAGAGGACGACC
AGCCACACTGGAACTGAGACACGGTC
>Zotu4060
GAACGCTGGCGGCGTGCTTAACACATGCAAGTCGAACGATGACCTCCAGCTTGCTGGGGTGATTAGTG
GCGAACGGGTGAGTAACACGTGAGTAACCTGCCCTTGACTCTGGGATAAGCACTGGAAACGGTGTCTA
ATACCGGATACGACAACCGACGGCATCGTCTGGTTGTGGAAAGAATTTTGGTCAAGGATGGACTCGCG
GCCTATCAGCTAGTTGGTGAGGTAATGGCCCACCAAGGCGACGACGGGTAGCCGGCCTGAGAGGGTGA
CCGGCCACACTGGGACTGAGACACGGCC
>Zotu4061
GAATGCTGGCGACGTGGATAAGGCATGCAAGTCAAGGGGGCCGCAAGGCAACCGGCAGACGAGGTAGT
AACACGTACGAACGCACCCCGAAGTCTGGAATAGCTCACCGAAAGGTGAGGTAATACCGGATGGTACC
GCAAGGTTAAAGATTTATCGCTTCGGGAGCGGCGTGCGCAATATCAGCTTGTTGGTGAGGTAACGGCT
CACCAAGGCTACGACGTTTAGGGGAACTGAGAGGTTGACCCCCACCGATGGGACTGAGACACGGCC
>Zotu4062
GAACGCTGGCGGCGTGCTTAACACATGCAAGTCGAGCGGAAAGGCCCTTTCGGGGGTACTCGAGCGGC
GAACGGGTGAGTAACACGTGAGTAATCTGCCTCTGGCTTCGGGATAGCCACCGGAAACGGTGATTAAT
ACCGGATATGACACATGGTCGCATGGTTGTGTGTGGAAAGTTTTTTCGGCCAGGGATGTGCTCGCGGC
CTATCAGCTTGATGGTGAGGTAATGGCTCACCATGGCTTCGACGGGTAGCCGGCCTGAGAGGGTGACC
GGCCACACTGGGACTGAGACACGGCC
>Zotu4064
GAACGCTGGCGGCGTGCTTAACACATGCAAGTCGAGCGGTAAGGCCCTTCGGGGTACACGAGCGGCGA
ACGGGTGAGTAACACGTGGGTGACCTGCCCTCCACTTCGGGATAAGCCTGGGAAACTGGGTCTAATAC
CGGATAGGACCTCTGGGTGCATGCCTGGTGGTGGAAAGTTTTTTCGGTGGGGGATGGGCCCGCGGTCT
ATCAGCTTGTTGGTGGGGTGATGGCCTACCAAGGCGACGACGGATAGCCGGCCTGAGAGGGTGATCGG
CCACACTGGGACTGAGACACGGCC
>Zotu4065
GAACGCTGGCGGCGTGCCTAATACATGCAAGTCGAGCGGATCTTTGGGAGCTTGCTCCCAAAGGTTAG
CGGCGGACGGGTGAGTAACACGTGGGCAACCTGCCTGTAAGACTGGGATAACTTCGGGAAACCGGAGC
TAATACCGGATAACTTCTTTTCTCGCATGAGGAGAGGTTGAAAGATGGCTTCGGCTATCACTTACAGA
TGGGCCCGCGGCGCATTAGCTAGTTGGTGAGGTAACGGCTCACCAAGGCAACGATGCGTAGCCGACCT
GAGAGGGTGATCGGCCACACTGGGACTGAGACACGGCC
>Zotu4066
GAACGCTGGCGGCGTGCCTAATACATGCAAGTAGGACGCACAGTTTATACCGTAGCTTGCTACACCAT
AGACTGTGAGTTGCGAACGGGTGAGTAACGCGTAGGTAACCTGCCTATTAGAGGGGGATAACTATTGG
AAACGATAGCTAATACCGCATAACAGTATGTAACACATGTTAGATGCTTGAAAGATGCAATTGCATCG
CTAGTAGATGGACCTGCGTTGTATTAGCTAGTAGGTAGGGTAAAGGCCTACCTAGGCAACGATACATA
GCCGACCTGAGAGGGTGATCGGCCACACTGGGACTGAGACACGGCC
>Zotu4067
GAACGCTGGCGGCGTGCTTAACACATGCAAGTCGAACGGTGACGACGGGGCTTGCCCTGTCTGATCAG
TGGCGAACGGGTGAGTAACACGTGAGTAACCTGCCCTTGACTCTGGGATAACCGCGGGAAACGGCGGC
TAATACCGGATATGAGACGTACAGGCATCTGTTGCGTCTGGAAAGATTTATCGGTCAAGGATGGGCTC
GCGGCCTATCAGCTTGTTGGTGGGGTGATGGCCTACCAAGGCGACGACGGGTAGCCGGCCTGAGAGGG
CGACCGGCCACACTGGGACTGAGACACGGCC
>Zotu4068
AAACGCTGGCGGCGTGCCTAATACATGCAAGTCGAACGGGGAAAGCTTTCGGGTGAGTACTAGTGGCG
CACGGGTGAGTAACACGTGGGTAATTTTCCTTAGAGTGGGGGATAACTAGTCGAAAGATTAGCTAATA

CCCCATAAGACCACAGGATCTGCGGATCTAGGGGTCAAAGTTTTTCGCTCTAAGATAAGCCCGCGCAA
GATTAGCTAGTTGGTGAGGTAACGGCTCACCAAGGCGACGATCTTTAGCTGGTCTGAGAGGATGATCA
GCCACACTGGAACTGAGACACGGTC
>Zotu4069
GAATGCTGGCGGTATGCTTAACACATGCAAGTCGAACGGAACCTTTCGGGGTTTAGTGGCGGACGGGT
GAGTAACGCGTGAGAATCGGCCCTCAGGTTGGGGACAACCATTGGAAACGGTGGCTAATACCGAATAA
GCCGAAAGGTGAAAGATTAATCGCCTGGGGAAGAGCTCGCGTCTGATTAGCTAGTTGGTGGGGTAAAG
GCCTACCAAGGCACCGATCAGTAACTGGTCTGAGAGGATGGCCAGTCACACTGGGACTGAGACACGGC
C
>Zotu4070
GAACGCTGGCGGCGTGCCTAACACATGCAAGTCAACTTGAAGGAAAGAGCAATCTGACCTGATGGTGG
CGCAAGGGTGCGTAACGAGTAAGTAACTTGCCCCAAGGTAAGGAATAACGTCGAGAAATCGGCGCTAA
TACCTCATAATGCAGCGGATCCGCATGGATATGTTGTTAAAGGTTTACCGCCTTGGGATAGGCTTGCG
TCTGATTAGCTAGTTGGTGTGGTAACGGCGCACCAAGGCTACGATCAGTAACTAGTCTGAGAGGATGA
TCAGTCACACTGGAACTGAGACACGGTC
>Zotu4071
CAACGCTGGCGGCGTGCCTAACACATGCAAGTCGAACGAGAAAGGGACTTCGGTCCTGAGTAAAGTGG
CGCACGGGTGAGTAACACGTGACTAACCTACCCTTGAGTGGGGAATAACTTCGAGAAATCGAGGCTAA
TACCGCATAACACCTACGGGTCAAAGGAGCAATTCGCTTAAGGAGGGGGTCGCGGCAGATTAGCTAGT
TGGCGGGGTAATGGCCCACCAAGGCAGTGATCTGTATCCGGCCTGAGAGGGCGCACGGACACACTGGA
ACTGAAACACGGTC
>Zotu4072
GAACGCTGGCGGCGTGCCTAACACATGCAAGTCGAACGGAGCTGCTTTGATGAAGTTTTCGGATGGAT
TTAAAACAGCTTAGTGGCGGACGGGTGAGTAACGCGTGGGTAACCTGCCTCACACTGGGGGATAACAG
TTAGAAATAGCTGCTAATACCGCATAAGCGCACGGTTCCGCATGGAACAGTGTGAAAAACTCCGGTGG
TGTGAGATGGACCCGCGTCTGATTAGCCAGTTGGCGGGGTAACGGCCCACCAAAGCGACGATCAGTAG
CCGGCCTGAGAGGGTGAACGGCCACATTGGGACTGAGACACGGCC
>Zotu4073
GAACGCTGGCGGCGTGCTTAACACATGCAAGTCGAACGGTGACCTCGAGAGCTTGCTCTTGGGTGATC
AGTGGCGAACGGGTGAGTAACACGTGAGTAACCTGCCCCAGACTCTGGAATAACCCCGGGAAACCGGA
GCTAATACCGGATACGAGACGGAGCCGCATGGCTACCGTCTGGAAAGTTTTTCGGTCTGGGATGGACT
CGCGGCCTATCAGTTAGTTGGTGAGGTAACGGCTCACCAAGACGACGACGGGTAGCCGGCCTGAGAGG
GCGACCGGCCACACTGGGACTGAGACACGGCC
>Zotu4074
GAACGCTGGCGGCATGCCTAAGACATGCAAGTCGAACGAGATGGCCCAGTGAAGGTTGAGTGCTTGCA
CGAGACTGAATCTGGATTCCCATCTAGTGGCAGACGGGTGAGTAACACGTGGGTGACCTACCTTTTCG
TTGGGGATAACAGTTGGAAACGATTGCTAATACCGAATAAAATGTAAGAGTCAATTTTACATTGAAAG
GAGCCTTTAAAGCTTCGCGATTAGATGGGCCTGCGGCGTATTAACTAGTTGGTGAGGTAATGGCTTAC
CAAGGTGACGATGCGTAGCCGAACTGAGAGGTTGATCGGCCACATTGGGACTGAGACACGGCC
>Zotu4075
GAACGCTGGCGGCAGGCTTAACACATGCAAGTCGAGCGGGCACTTTCGGGTGTCAGCGGCGGACGGGT
TAGTAACGCGTGGGAACGTGCCCTTTGCTCTGGAATAGCCCTGGGAAACTGGGGGTAATACCGGATGT
GCCCTGAGGGGGAAAGATTTATCGGCAAGGGATCGGCCCGCGTCTGATTAGGTAGTTGGTGAGGTAAC
GGCTCACCAAGCCGACGATCGGTAGCTGGTTTGAGAGGATGATCAGCAACACTGGGACTGAGACACGG
CC
>Zotu4076
GAACGCTGGCGGCGTGCCTAATACATGCAAGTCGAGCGAACCCTTCGGGGTTAGCGGCGGACGGGTGA
GTAACACGTGGGCAACCTACCTTGTAGATGGGGATAACATCGAGAAATCGGTGCTAATACCGAATAAT
ACTTTTCTTCTCATGAAGGAAAGTTAAAAGTTGGCTTTAGCTAACACTACGAGATGGGCCCGCGGCGC
ATTAGCTAGTTGGTGAGGTAACGGCTCACCAAGGCGACGATGCGTAGCCGACCTGAGAGGGTGATCGG
CCACACTGGGACTGAGACACGGCC
>Zotu4077
GAACGCTGGCGGCGTGCTTAACACATGCAAGTCGAGCGGTAAGGCCCTTTCGGGGGTACACGAGCGGC
GAACGGGTGAGTAACACGTGAGTAATCTGCCCTTCACTTCGGGATAACCGCCGGAAACGGTGGCTAAT

ACCGGATACGACGCACTCCTGCATGGGATGTGTGTGGAAAGTTTTTTCGGTGGGGGATGTGCTCGCGG
CCTATCAGCTTGTTGGTGAGGTAACGGCTCACCAAGGCTTCGACGGGTAGCCGGCCTGAGAGGGTGAC
CGGCCACACTGGGACTGAGACACGGCC
>Zotu4079
GAACGCTGGCGGCGTGCCTTAAGCATGCAAGTCGAACGGTCCTCGCAAGAGGGCAGTGGCGGACGGGT
GAGTAACGCGTGAGAATCTACCTTAAAGTGGGACATAGCTAGCCGAAAGGTTGGGTAATTTCCCATAG
TCCCCGATTTAATCGGGGTAAAGCCGCAAGGCGCTTTAAGATGAGCTCGCGTCCTATCAGCTTGTTGG
TGGGGTAAAAGCCTACCAAGGCGATGACGGGTAGCTGGTCTGAGAGGATTGTCAGCCACAATGGAACT
GCGACACGGTC
>Zotu4080
GAACGCTGGCGGCATGCCTTACACATGCAAGTCGAACGGCAGCACGGGAGCAATCCTGGTGGCGAGTG
GCGAACGGGTGAGTAAAGTATCGGAACGTGCCCAGTCGTGGGGGATAACGTAGCGAAAGCTACGCTAA
TACCGCATACGATCTATGGATGAAAGCGGGGGACCGTAAGGCCTCGCGCGATTGGAGCGGCCGATATC
AGATTAGGTAGTTGGTGGGGTAAAGGCCCACCAAGCCGACGATCTGTAGCTGGTCTGAGAGGACGACC
AGCCACACTGGGACTGAGACACGGCC
>Zotu4081
GAACGCTGGCGGCGTGCCTAAGACATGCAAGTCGAGTGGGTTTAGACCCACGGCGAACGGGTGAGTAA
TACCTAGGAACTTTCCTTAAGTGGAGCATAACCAGCCGAAAGGTTGGATAATACTCCATGTGGCCGTA
AGGCCAAAGTAGCAATACGCTTAAGTTGAGGCCTGGGCACCATCAGCTAGTTGGTAGGGTAAAGGCCT
ACCAAGGCTATGACGGTTAGGGGGCCCGAGAGGGTGAACCCCCACACTGGGACTGCGACACGGCC
>Zotu4082
GAACGCTGGCGGCAGGCCTAACACATGCAAGTCGAGCGAACCCTTCGGGGTTAGCGGCGGACGGGTGA
GTAACGCGTGGGAACGTGCCCTTTGCTACGGAATAGCCCCGGGAAACTGGGAGTAATACCGTATACGC
CCTTAGGGGGAAAGATTTATCGGCAAGGGATCGGCCCGCGTTGGATTAGGTAGTTGGTGGGGTAATGG
CCTACCAAGCCGACGATCCATAGCTGGTTTGAGAGGATGATCAGCCACACTGGGACTGAGACACGGCC
>Zotu4083
GAACGCTGGCGGCGTGCTTAACACATGCAAGTCGAGCGGAAAGGCCACCTCGGTGGTACTCGAGCGGC
GAACGGGTGAGTAACACGTGAGTAATCTGCCCCTGGCTTTGGGATAGCCACCGGAAACGGTGATTAAT
ACCGGATATGACATGGACTCGCATGGGTTTGTGTGGAAAGTTTTTCGGCCAGGGATGTGCTCGCGGCC
TATCAGCTTGATGGTGAGGTAATGGCTCACCATGGCTTCGACGGGTAGCCGGCCTGAGAGGGTGACCG
GTCACACTGGGACTGAGACACGGCC
>Zotu4084
AAACGCTGGCGGCGTGCTTAACACATGCAAGTCGAACGTGAAAGTTTCTTCGGAAATGAGTAAAGTGG
CGAACGGGTGAGTAACGCGTGAGAATCTGCCCTTTAGTGAAGGATACCATCGAGAAATCGATGTTAAT
ACTGCATATCTTTGCAAAAAGAAAGGTGGCCTATAAGCTGCCGCTAAAGGATGAGCTTGCGTGCTATT
AGCTAGTTGGTGGGGTAATGGCCTACCAAGGCGATGATGGCTAGCCGGCCTGAGAGGGTGTACGGCCA
CATTGGAACTGAGAAACGGTC
>Zotu4085
GAACGCTGGCGGCATGCCTAACACATGCAAGTCGAACGAAGGTCCTGGTTCGCTGGGGCTTTAGTGGC
GCACGGGTGAGTAACGCGTGGGAACCTGCCCTTTTGTTCGGAATAACGTTGGGAAACTGACGCTAATA
CCGGATACGCCCTACGGGGGAAAGATTTATCGCAGAAGGAGGGGCCCGCGTCAGATTAGCTAGTTGGT
GGGGTAATGGCCTACCAAGGCGATGATCTGTAGCTGGTCTGAGAGGATGATCAGCCACACTGGGACTG
AGACACGGCC
>Zotu4086
GAACGCTGACGGCGTGCTTAACACATGCAAGTCGAACGGGATCCATTAGCGCTTTTGTGTTTTTGGTG
AGAGTGGCGAACGGGTGAGTAACACGTGAGTAACCTGCCCTTTTCTTTGGGATAAGCCTGGGAAACTG
GGTCTAATACTGGATGTTCCGGCTTCCTCGCATGGGGTTGTTGGGAAAGGTTTTTTCTGGATTGGGAT
GGGCTCGCGGCTTATCAGCTTGTTGGTGGGGTGATGGCTTACCAAGGCTTTGACGGGTAGCCGGCCTG
AGAGGGTGGTCGGTCACACTGGGACTGAGATACGGCC
>Zotu4088
GAACGCTGGCGGCGTGCCTAACACATGCAAGTCGAGCGCGTCTGATTTGATGCTTGCATTAATGAAAG
ATGAGCGGCGGACGGGTGAGTAACGCGTGGGTAACCTGCCCTATACACATGGATAACATACTGAAAAG
TTTACTAATACATGATAATATATATTTACGGCATCGTAGATATATCAAAGTGTTAGCGGTATAGGATG
GACCCGCGTCTGATTAGCTAGTTGGTGAGATAACTGCCCACCAAGGCGACGATCAGTAGCCGACCTGA

GAGGGTGATCGGCCACATTGGAACTGAGACACGGTC
>Zotu4089
GAACGCTGGCGGCACGCCTAACACATGCAAGTCGAGCGGTAGCAGGATTAGTAGCAATACTAATCGCT
GACGAGCGGCGGACGGGTGAGTAATACGTAGGAACCTGCCCTTAAGCGGGGGATAACTAAGGGAAACT
TTAGCTAATACCGCATACACTCGAGAGAGGAAAGCTGCCGTAAGGTGGCACTTGAGGAGGGGCCTGCG
TCCGATTAGCTAGTTGGTAGGGTAAGAGCCTACCAAGGCAATGATCGGTAACTGGTCTGAGAGGACGA
CCAGTCACACTGGGACTGAGACACGGCC
>Zotu4090
GAACGCTGGTAACGTGGATACTGGCATGCAAGTCACGGGGGTACCGAGCCGCAAGGCGAGGTGCAACC
GGCGAACGGCTTAGTAACACGTGGATACATACCGTAAAGCGGAGTACAGCTCGTCGAAAGACGGGGTA
ATCCTCCATAGTCCCCGAGGCCGCAAGGCCGAGGGGTAAAGGCGCAAGCCGCTTTATGAATGGTCCGC
GGCCTATCAGCTTGTTGGTGAGGTAAAAGCTCACCAAGGCGATGACGGGTAGGGGGCGTGAGAGCGCG
ACCCCCAAGAATGGCACTGCGACACGGGC
>Zotu4091
GAACGCTGGCGGCGTGCTTAACACATGCAAGTCGAACGGTGACAGGGAGCTTGCTCCCTCGATCAGTG
GCGAACGGGTGAGTAACACGTGAGTAACCTGCCCCAGACTCTGGGATAACGTCGAGAAATTGGCGCTA
ATACCGGATACGAGACGAGACCGCATGGTCATTGTCTGGAAAGAATTTCGGTCTGGGATGGACTCGCG
GCCTATCAGCTTGTTGGTGAGGTAATGGCTCACCAAGGCGACGACGGGTAGCCGGCCTGAGAGGGCGA
CCGGCCACACTGGAACTGAGACACGGTC
>Zotu4092
GAACGCTGGCGGCATGCCTAACACATGCAAGTCGAACGAGACCTTTCGGGGTCTAGTGGCGCACGGGT
GCGTAACGCGTGGGAATCTGCCCTTGGGTTCGGAATAACAGTTGGAAACGACTGCTAATACCGGATGA
TGACGTAAGTCCAAAGATTTATCGCCCAGGGATGAGCCCGCGTAGGATTAGCTAGTTGGTGAGGTAAA
GGCTCACCAAGGCGACGATCCTTAGCTGGTCTGAGAGGATGATCAGCCACACTGGGACTGAGACACGG
CC
>Zotu4093
GAACGCTGGCGGCGTGCTTAACACATGCAAGTCGAACGATTAAACCGGCTTCGGTCGGATATAGAGTG
GCGAACGGGTGAGTAACACGTGACCAACCTACCCCTTACACTGGGACAACCACTGGAAACGGTGGCTA
ATACCGGATACTCCGAGTATCTCGCATGAGGTATTCGGGAAAGCCTTCACGGTAAGGGATGGGGTCGC
GGCCCATTAGGTAGACGGCGGGGTAACGGCCCACCGTGCCTGCGATGGGTAGCCGGGCTGAGAGGCTG
ATCGGCCACATTGGGACTGAGATACGGCC
>Zotu4094
GAACGCTGGCGGCGTGCCTAATACATGCAAGTCGAGCGGATTTTTGGGAGCTTGCTCCCAAAAATTAG
CGGCGGACGGGTGAGTAACACGTGGGCAACCTGCCTATAAGACTGGGATAACTTCGGGAAACCGGAGC
TAATACCGGATAACTCATATCTACTCATGTAGATATGCTGAAAGACGGTTTCGGCTGTCACTTATAGA
TGGGCCCGCGGCGCATTAGCTAGTTGGTGAGGTAACGGCTCACCAAGGCAACGATGCGTAGCCGACCT
GAGAGGGTGATCGGCCACACTGGGACTGAGACACGGCC
>Zotu4095
GAACGTTAGCGGCGCGCCTAACACATGCAAGTCGCACGAGAAAGGGCTTCGGCCCCGGTAAAGTGGCG
CACGGGTGAGTAACACGTGGGTAATCTTCCTCCGAGCGGGGGATAACATTCCGAAAGGAGTGCTAATA
CCGCATAAGACCACGGTGTCGAAAGACGCTGAGGTAAAAGCTGGCCAATTCATGAAAGCTAGCACTTG
GAGATGAGCCCGCGGCCCATCAGGCAGTTGGCAGGGTAATGGCCTACCAAACCGAAGACGGGTAGCTG
GTCTGAGAGGATGATCAGCCACACTGGAACTGAGACACGGTC
>Zotu4096
GAACGCTAGCGGCAGGCTTAATACATGCAAGTCGTACGGGATCCGAGGTAGCAATACTTTGGTGAGAG
TGGCGCACGGGTGCGTAACACGTATGCAACCTACCTTTAACCGGGACACAACCTCGAGAAATTGAGGC
TAATATCCCATAACACTGCGAGATGGCATCATTTTGCAGTTAAAACTCCGGTGGTTATTGATGGGCAT
GCGCGACATTAGCTAGTTGGTGAGGTAACGGCCCACCAAGGCTACGATGTCTAGGGGTTCTGAGAGGA
TTATCCCCCACACTGGTACTGAGACACGGAC
>Zotu4097
GAACGCTGGCGGCGTGCTTAACACATGCAAGTCGAACGGTGAACACGGAGCTTGCTCTGTGGGATCAG
TGGCGAACGGGTGAGTAACACGTGAGCAACCTGCCCCTGACTCTGGGATAAGCGCTGGAAACGGCGTC
TAATACTGGATACGAGTAGCGACCGCATGGTCAGTTACTGGAAAGATTTATTGGTTGGGGATGGGCTC
GCGGCCTATCAGCTTGTTGGTGAGGTAATGGCTCACCAAGGCGTCGACGGGTAGCCGGCCTGAGAGGG

TGACCGGCCACACTGGGACTGAGACACGGCC
>Zotu4098
GAACGCTGGCGGCGTGCCTTAAGCATGCAAGTAGAACGAGTTTCTATCTTGCCGCAAGGCAAGCTAGG
AACTAGTTGCGAACGGCTGAGTAATGAATAAGAATCTTTCCTTTGGTGGAGAATACCTCGTCGAAAGA
CGAGCTAATACTGCATAACATCTACGGATAAAAGGACTTAACTGTCCGCCGATTGAGGAGCTTATTCC
CCATCAGGTAGTTGGTAGGGTAATGGCCTACCAAGCCTACGACGGGTACCCGATCTGAGAGGATGGAC
GGGCAGAATGGGACTGAGACACGGCC
>Zotu4099
GAACGCTGGCGGCATGCCTTACACATGCAAGTCGAACGGCAGCACGGGGGCAACCCTGGTGGCGAGTG
GCGAACGGGTGAGTAATACATCGGAACGTGTCCTGTAGTGGGGGATAGCCCGGCGAAAGCCGGATTAA
TACCGCATACGATCTACGGAAGAAAGCGGGGGATCCTTCGGGACCTCGCGCTATAGGGGCGGCCGATG
GCAGATTAGCTAGTTGGTGGGGTAAAGGCCTACCAAGGCGACGATCTGTAGCTGGTCTGAGAGGACGA
CCAGCCACACTGGGACTGAGACACGGCC
>Zotu4101
AAACGCTGGCGGTGTACATAAGACATGCAAGTCGAACGGGAGAAGACAGGAAGATTTATCTACTTGTT
GGACACCGTGGCGGACGGGTGAGTAACACGTAAGCAACCTGCCTATAAGAGGGGAATAACAGTGAGAA
ATTACTGATAATACCGCATAAGCTGGAGGTATCGCATGATACATTCAGAAAAGATTTATCGCTTATAG
ATGGGCTTGCGTCTGATTAGCTAGTTGGTGAGATAACAGCTCACCAAGGCGACGATCAGTAGCCGGAT
TGAGAGGTTGAACGGCCACATTGGGACTGAGACACGGCC
>Zotu4102
GAACGCTGGCGGCGTGCTTAACACATGCAAGTCGAACGGTAAGGCCCCTTCGGGGGTACACGAGTGGC
GAACGGGTGAGTAACACGTGAGCAACCTACCCTTGACTCTGGAATAACGGTTGGAAACAGCCGCTAAT
ACCGGATACGACCTTGAGCGTCATCGCTCGAGGTGGAAAGCTCCGGCGGTCAGGGATGGGCTCGCGGC
CTATCAGCTAGTTGGTGAGGTAACGGCTCACCAAGGCTTCGACGGGTAGCCGGCCTGAGAGGGCGACC
GGCCACACTGGGACTGAGATACGGCC
>Zotu4103
GAACGCTGGCGGCATGCCTTACACATGCAAGTCGAACGCGAAAGTCCTTCGGGATGAGTAGAGTGGCA
AACGGGTGAGTAACGCGTGGGAACATACCTTCAAGTACGGGATAGCTCCGGGAAACTGGAATTAATAC
CGTATATTCCCTATGGGGGAAAGATTTATCGCTTGAAGATTGGCCCGCGTCAGATTAGCTAGTTGGTG
GGGTAACAGCCTACCAAGGCAACGATCTGTAGCTGGTCTGAGAGGACGATCAGCCACATTGGGACTGA
GACACGGCC
>Zotu4104
GAACGCTGGCGGCGTGCCTTAAGCATGCAAGTCAAGGGGGGCTCGCAAGAGTCAACCGGCGGACGGGT
GAGTAACGCGTGAGAATCTACCTTAAAGTGGGACATAGCCCTCCGAAAGGAGGGGTAATTTCCCATGT
GATCCGGCTTTGCCGGATTAAAGCCGCAAGGCGCTTTAAGATGAGCTCGCGTCCTATCAGCTAGTTGG
TGGGGTAAAAGCCTACCAAGGCGACGACGGGTAGCTGGACTGAGAGGTTGGTCAGCCACAATGGCACT
GCGACACGGGC
>Zotu4105
GAACGCTGGCGGCGTGGATGAGGCATGCAAGTCGAATGGGTTTAGACCCATGGCAGACGAGGTAGGAA
CACGTAGGTACATACCCCAAAGTCAGGGATAATCTGTCGAAAGGCAGGATAATACTTGATGGTCTCGA
AAGAGTAAAGATTTATCGCTTTGGGAATGGCCTGCGTTCTATCAGCTTGATGGTGAGGTAACGGCTCA
CCATGGCTACGACGGATAGGGGAGGTGAGAGCCTGACCCCCACCGATGGAACTGCGACACGGTC
>Zotu4106
GAACGCTGGCGGCGTGCTTAACACATGCAAGTCGAACGCTGAAGCACCAGCTTGCTGGTGTGGATGAG
TGGCGAACGGGTGAGTAATACGTGAGTAACCTGCCCTTGACTCTGGGATAAGCCTGGGAAACTGGGTC
TAATACTGGATATGACCTCCTGTCGCATGGTGGGGGGTGGAAAGGGTTTGTACTGGTTGTGGATGGGC
TCACGGCCTATCAGCTTGTTGGTGGGGTAATGGCCTACCAAGGCGACGACGGGTAGCCGGCCTGAGAG
GGTGACCGGCCACACTGGGACTGAGACACGGCC
>Zotu4107
GAACGCTGGCGGCATGCCTAACACATGCAAGTCGAACGATGCTTTCGGGCATAGTGGCGCACGGGTGC
GTAACGCGTGGGAATCTGCCCTTGGGTCTGGGATAACAGCGGGAAACTGCTGCTAATACCGGATGATG
ACGTTAAGTCCAAAGATTTATCGCCCAGGGATGAGCCCGCGTAGGATTAGCTAGTTGGTGGGGTAAAG
GCCTACCAAGGCGACGATCCTTAGCTGGTCTGAGAGGATGATCAGCCACACTGGGACTGAGACACGGC
C

>Zotu4108
GAACGCTGGCGGCGTGCTTAACACATGCAAGTCGAACGATGAAGCCCAGCTTGCTGGGTGGATTAGTG
GCGAACGGGTGAGTAACACGTGAGTAACCTGCCCCCGACTCTGGGATAAGCCCGGGAAACTGGGTCTA
ATACCGGATATGACTTCGCACCGCATGGTGTGGGGTGGAAAGATTTATCGGTGGGGGATGGACTCGCG
GCCTATCAGCTTGTTGGTGAGGTAATGGCTCACCAAGGCGACGACGGGTAGCCGGCCTGAGAGGGTGA
CCGGCCACACTGGGACTGAGACACGGCC
>Zotu4109
GAACGCTAGCGGCAGGCCTAATACATGCAAGTCGGACGGGATTTGGGAGCTTGCTCCTGATGAGAGTG
GCGCACGGGTGCGTAACACGTATGTAACCTACCTTAATCTGGGGGATAGCCTCTCGAAAGAGAGATTA
ACACCGCATAACATTATCGGATAGCATTTTTGATAATCAAATATTTATAGGATTAAGATGGGCATGC
GGAACATTAGCTAGTTGGTAAGGTAATGGCTTACCAAGGCTACGATGTTTAGGGGATCTGAGAGGATG
ACCCCCCACACTGGTACTGAGACACGGAC
>Zotu4110
GAACGCTGGCGGCAGGCTTAACACATGCAAGTCGAACGCCCCGCAAGGGGAGTGGCAGACGGGTGAGT
AACACGTGGGAATGTGCCCTTTGGTTCGGAATAACTTGGGGAAACTCAAGCTAATACCGGATACGCCC
TTTGGGGGAAAGGTTTACTGCCGAAGGAGCAGCCCGCGTCCGATTAGCTAGTTGGTGAGGTAATGGCT
CACCAAGGCGACGATCGGTAGCTGGTCTGAGAGGATGGCCAGCCACATTGGGACTGAGACACGGCC
>Zotu4111
GAACGCTGGCGGCGTGCTTAACACATGCAAGTCGAACGAAGAGCGACGGAAGCTTGCTTCTATCAATC
TTAGTGGCGAACGGGTGAGTAACGCGTAATCAACCTGCCCTTCAGAGGGGGACAACAGTTGGAAACGA
CTGCTAATACCGCATACGATCTAATCTCGGCATCGAGGATGGATGAAAGATGGCCTCTACATGTAAGC
TATCACTGAAGGAGGGGATTGCGTCTGATTAGCTAGTTGGAGGGGTAACGGCCCACCAAGGCGATGAT
CAGTAGCCGGTCTGAGAGGATGAACGGCCACATTGGGACTGAGACACGGCC
>Zotu4112
GAACGCTGGCGGCGTGCCTAATACATGCAAGTCGAGCGAATCAAAGGGAGCTTGCTCCCGGAGATTAG
CGGCGGACGGGTGAGTAACACGTGGGCAACCTGCCTATAAGACTGGGATAACTTCGGGAAACCGGAGC
TAATACCAGATAATAATTGGAACTGCATAGTTCCAATTTGAAAGATGGCCTCGGCTATCACTTATAGA
TGGGCCCGCGGCGCATTAGCTAGTTGGTAAGGTAATGGCTTACCAAGGCAACGATGCGTAGCCGACCT
GAGAGGGTGATCGGCCACACTGGGACTGAGACACGGCC
>Zotu4113
GAACGCTGGCGGCGTGCCTAATACATGCAAGTCGAGCGGACTTTGCCTTCGGGACAAGATTAGCGGCG
GACGGGTGAGTAACACGTGGGTAACCTGCCCATAAGACTGGGATAACATTCGGAAACGAATGCTAATA
CCGGATACGCGAAACGGTCGCATGATCGAATCGGGAAAGGCGGAGCAATCTGCCACTTATGGATGGAC
CCGCGGCGCATTAGCTAGTTGGTGGGGTAACGGCTCACCAAGGCGACGATGCGTAGCCGACCTGAGAG
GGTGATCGGCCACACTGGGACTGAGACACGGCC
>Zotu4114
GAACGCTGGCGGCGTGCTTAACACATGCAAGTCGAGCGGAAAGGCTCCTTCGGGGGTACTCGAGCGGC
GAACGGGTGAGTAACACGTGAGTAATCTGCCCTGGACTTCGGGATAGCCACCGGAAACGGTGATTAAT
ACCGGATATGACATGGCCTCGCATGGGGTGGTGTGGAAAGTTTTTCGGTTCAGGATGTGCTCGCGGC
CTATCAGCTTGATGGTGAGGTAATGGCTCACCATGGCTTCGACGGGTAGCCGGCCTGAGAGGGTGACC
GGTCACACTGGGACTGAGACACGGCC
>Zotu4115
GAACGCTGGCGGCGTGCCTAACACATGCAAGTCGAACGGAACTTCTTTAAAGGATTTCTTCGGAATGA
ATTTGATTAAGTTTAGTGGCGGACGGGTGAGTAACGCGTGAGTAACCTGCCTCTAAGAGGGGAATAAC
ATTCTGAAAAGAATGCTAATACCGCATAATATATATTTATCGCATGGTAGATATATCAAAGATTTATC
GCTTAGAGATGGACTCGCGTCCGATTAGTTAGTTGGTGAGGTAACGGCTCACCAAGACCGCGATCGGT
AGCCGGACTGAGAGGTTGAACGGCCACATTGGGACTGAGACACGGCC
>Zotu4116
GAACGCTGGCGGCGTGCTTAACACATGCAAGTCGAACGAGGTCCATGCAGTCGCAAGACTGCGGACGA
CCTAGTGGCGAACGGGTGCGTAACACGTGAGGAACTTGTCCCGGTCTCTGGAATAACAGGTGGAAACA
CCTGCTAATACCGGATGCCGTCGGATCATCGCATGGTGGTCTGACGAAAGGGTTACCGGATCGGGAGA
GCCTCGCGGCCTATCAGCTTGTTGGTGAGGTAATGGCTCACCAAGGCATCGACGGGTAGCTGGTCTGA
GAGGATGATCAGCCACACTGGGACTGAGACACGGCC
>Zotu4117

GAACGCTGGCGGCATGCCTAACACATGCAAGTCGAACGATCTCTTCGGAGATAGTGGCGCACGGGTGC
GTAACGCGTGGGAATCTGCCCTTGGGTTCGGAATAACTTCTGGAAACGGAAGCTAATACCGGATGATG
ACGTAAGTCCAAAGATTTATCGCCTGAGGATGAGCCCGCGTGAGATTAGGTAGTTGGTGGGGTAAAGG
CCTACCAAGCCGACGATCTCTAGCTGGTCTGAGAGGATGATCAGCCACACTGGGACTGAGACACGGCC
>Zotu4118
GAACGCTGGCGGCATGCCTAACACATGCAAGTCGAACGAGATCTTCGGATCTAGTGGCGCACGGGTGC
GTAACGCGTGGGAATCTGCCCTTGGGTTCGGAATAACAGTGGGAAACTACTGCTAATACCGGATAATG
ACGTAAGTCCAAAGATTTATCGCCCTGGGATGAGCCCGCGTAGGATTAGCTAGTTGGTGGGGTAAAGG
CCTACCAAGGCGACGATCCTTAGCTGGTCTGAGAGGATGATCAGCCACACTGGGACTGAGACACGGCC
>Zotu4119
GAACGCTGGCGGCAGGCATAATACATGCAAGGCGAAGGGGCAGCAATGTCACTGTCGTACGGGTGCGT
AACGCGTATGCAACTTATCTATCACTGGGGGATAGCCCGGGGAAACCCGGATTAATACCGCATAAAAC
AGGGGCACCGCATGGTGATATTTGTTAAAGATTTATTGGTGATAGATGGGCATGCGTTCGATTAGCTA
GTTGGCGGGGTAACGGCCCACCAAGGCGACGATCGATAGGGGAGCTGAGAGGTTGGTCTCCCACACGG
GCACTGAGATACGGGC
>Zotu4120
GAACGCTGGCGGCGTGCCTAAGACATGCAAGTCGAGTGGGTTTAGACCCACGGCGAACGGGTGAGTAA
TACCTAGGAACTTTCTTTAAGTGGAGCATAACCAGTCGAAAGATTGGGCAATTCTTCATGTGGCAGTA
ATGCTAAAGTCGCAAGACGCTTAAAGTGAGGCCTGGGCACCATCAGCTAGTTGGTAAGGTAATGGCTT
ACCAAGGCTATGACGGTTAGGGGTCCCGAGAGGGATAACCCCCACACTGGAACTGCGACACGGTC
>Zotu4123
GAACGCTAGCGGCAGGCCTAACACATGCAAGTCGAGGGGTAGTATAGCAATATATGAGACCGGCGCAC
GGGTGCGTAACGCGTATGCAATCTACCTTTTACAGAGGGATAGCCCAGAGAAATTTGGATTAATACCT
CATAGTATATTTGAGTGGCATCACTTGATTATTAAAGTTCCAACGGTAAAAGATGAGCATGCGTCCTA
TTAGCTAGTTGGTGTGGTAACGGCACACCAAGGCTACGATAGGTAGGGGTCCTGAGAGGGAGATCCCC
CACACTGGTACTGAGACACGGAC
>Zotu4124
GAATGCTGGCGGTGTGGATAAGTCATGCAAGTCGAACGGTCCGCAAGGACAGTGGCAGACGAGGTAGT
AACACGTAGGTACACCCCCCGAAGTCGGGCATAGCCAGCCGAAAGGTTGGGTAATTCCCGATAGTCGC
GCAAGCGTAAAGATTTATCGCTTCGGGAGTGGCCTGCGTAATATCAGCTAGTTGGTAAGGTAACGGCT
TACCAAGGCTACGACGTTTAGGGGAGCTGAGAGGCTGACCCCCACCGATGGGACTGCGATACGGCC
>Zotu4128
AAACGCTGGCGGCGTGCCTAACGCATGCAAGTCGAACGAGGCCTTCGGGCCTAGTGGCGGACGGGTGA
GTAACACGTAGGAACCTGCCCGGAAGTGGGGGACAACTCCACGAAAGCGGGGCTAATACCGCATACGG
TCTACGGACCAAAGCCTTCGGGCGCTTTCGGAGGGGCCTGCGGCGGATTAGCTAGTTGGTGGGGTAAC
GGCCTACCAAGGCGACGATCCGTAGCTGGTCTGAGAGGATGATCAGCCACACTGGGACTGAGACACGG
CC
>Zotu4132
GAATGCTGGCGGCGTGCCTAACACATGCAAGTCGAGCGGCAGCGAGTTTTACACTGAATTCTGGAAGC
TTCTGGGTAATATAGAAGATCTATTCAAGAATTTTGTGTAAAATGTCGGCGAGCGGCGGACGGCTGAG
TAACGCGTAGGAATTTGCCCCAAAGTGAGGAATAACTGCCCGAAAGGGTAGCTAATGCCGCATATGGT
CTTCGGATTAAAGCCTTCGGGCGCTTTGGGAGAAGCCTGCGTTGGATTAGGTAGTTGGTAGGGTAATG
GCCTACCAAGCCGACGATCCATAGCTGGTCTGAGAGGATGATCAGCCAGACTGGAACTGAGACACGGT
C
>Zotu4133
GAACGCTGGCGGCGTGCTTAACACATGCAAGTCGAACGGTGAAAGAGGAGCTTGCTCCTCTGGATCAG
TGGCGAACGGGTGAGTAACACGTGAGCAATCTGCCCTGGACTCTGGGATAAGCGTTGGAAACGACGTC
TAATACCGGATACGCGACGCGAAGGCATCTTCAGCGTCGGGAAAGAACTTCGGTCCAGGATGAGCTCG
CGGCCTATCAGCTAGTTGGTGAGGTAACGGCCCACCAAGGCGACGACGGGTAGCCGGCCTGAGAGGGT
GACCGGCCACACTGGGACTGAGACACGGCC
>Zotu4134
GAATGCTGGCGATGTGGATAAGTCATGCAAGTCAAGGGGGCCGCAAGGCAACCGGCAGACGAGGTAGT
AACACGTACGAACGCACCCCGAAGTCTGGTATAGCTCACCGAAAGGTGAGGTAATCCCGGATGGTACC
GCAAGGTTAAAGATTTATCGCTTCGGGAGCGGCGTGCGCAATATCAGCTAGTTGGTAGGGTAATGGCC

TACCAAGGCTACGACGTTTAGGGGAGCTGAGAGGCTGACCCCCACCGATGGGACTGAGACACGGCC
>Zotu4135
GAACGCTGGCGGCATGCTTAACACATGCAAGTCGAACGAACCTATGGGGCTTGCTTCATAGGTTAGTG
GCAGACGGGTGAGTAACACGTGGAAACGTACCCTAAAGTTCGGAATAACTATTGGAAACGATAGCTAA
TACCGAATGTGGCCGAGAGGCTAAAGGTTTACTGCTTTAGGAGCGGTCCGCGCTAGATTAGCTAGTTG
GTGGGGTAATGGCCTACCAAGGCGATGATCTATAGCTGGTCTGAGAGGATGACCAGCCACACTGGGAC
TGAGACACGGCC
>Zotu4136
GAACACTGGCGGCAGGCTTAACACATGCAAGTCGAGCGGGGTGATGTAGCTTGCTACCTGACCTAGCG
GCGGACGGGTGAGTAATGCTTAGGAATCTGCCTATTAGTGGGGGACAACATTCCGAAAGGAATGCTAA
TACCGCATACGCCCTACGGGGGAAAGCAGGGGATCTTCGGACCTTGCGCTAATAGATGAGCCTAAGTC
AGATTAGCTAGTTGGTGGGGTAAAGGCCTACCAAGGCGACGATCTGTAGCGGGTCTGAGAGGATGATC
CGCCACACTGGGACTGAGACACGGCC
>Zotu4138
GAACGCTGGCGGCAGGCCTCAAACATGCAAGTCTAGGGGGGTAGCAATACCCACCGGCGGACGGGTGC
GTAACGCGTGGGAACATACCTTTCGGTACGGAACAACTGCGGGAAACTGCAGCTAATACCGTATACGT
CCTTCGGGAGAAAGATTTATCGCCGAAAGATTGGCCCGCGTTCGATTAGCTAGTTGGTGGGGTAACGG
CCCACCAAGGCGACGATCGATAGCTGTTCTGAGAGGAAGATCAGCCACACTGGGACTGAGACACGGCC
>Zotu4139
GAACGCTGGCGGCATGCTTTACACATGCAAGTCGAACGGTAACAGGGAGCTTGCTCCGCTGACGAGTG
GCGAACGGGTGAGTAATATATCGGAACGTACCCAAGAGTGGGGGATAACGTAGCGAAAGTTACGCTAA
TACCGCATACGATCTAAGGATGAAAGCAGGGGATCTTCGGACCTTGTGCTCCTGGAGCGGCCGATATC
TGATTAGCTAGTTGGTGAGGTAAAGGCTCACCAAGGCGACGATCAGTAGCTGGTCTGAGAGGACGACC
AGCCACACTGGAACTGAGACACGGTC
>Zotu4141
GAACGCTGGCGGCAGGCTTAACACATGCAAGTCGAACGCCCCGCAAGGGGAGTGGCGCACGGGTGAGT
AACGCGTGGGAATATGCCCTTCACTACGGAACAACATCGGGAAACTGATGCTAATACCGTATAATGAC
TCCGGTCCAAAGATTTATCGGTGAAGGATTAGCCCGCGTAAGATTAGGTAGTTGGTGAGGTAACGGCT
CACCAAGCCGACGATCTTTAGCTGGTCTGAGAGGATGATCAGCCACACTGGGACTGAGACACGGCC
>Zotu4143
GAACGCTGGCGGCGCGCCTAATACATGCAAGTCGAACGAAAACCTCGGACTTCGGTCTGGGGGGACAG
TGGCGAACGGGTGCGTAACACGTGAGCAACCTGCCTCGGAGACCGGGACAACGCGGGGAAACCCGTGC
TAATACCGGATACCCTCCTCGGATCGCATGGTCTGATGAGGAAATGGATTCCGCTCCGAGATGGGCTC
GCGGCCTATCAGCTTGTTGGTGAGGTAACGGCTCACCAAGGCTTCGACGGGTAGCTGGTCTGAGAGGA
CGATCAGCCACACTGGGACTGAGACACGGCC
>Zotu4144
AAACGCTGGCGGCGTGCTTAACACATGCAAGTCGAACGAAGTTTTTCTTTCGGGAGGAACTTAGCGGC
GGACGGGTGAGTAACGCGTGGGTAACCTGCCCTGTACAGGGGGACAACAGCTGGAAACGGCTGCTAAT
ACCGCATAAGCCCTTAGCACTGCATGGTGCATAGGGAAAAGGAGCAATCCGGTACAGGATGGACCCGC
GTCTGATTAGCCAGTTGGCAGGGTAACGGCCTACCAAAGCGACGATCAGTAGCCGATCTGAGAGGATG
TACGGCCACATTGGGACTGAGACACGGCC
>Zotu4146
GAACGCTGGCGGCGTGCCTAATACATGCAAGTCGAGCGGAGTTACAGGAGCTTGCTCCTGTAACTTAG
CGGCGGACGGGTGAGTAACACGTGGGCAACCTGCCTATAAGACTGGGATAACTTCGGGAAACCGGAGC
TAATACCGGATAATTCTTTTCCTCTCATGAGGAAAAGCTGAAAGACGGTTTCGGCTGTCACTTATAGA
TGGGCCCGCGGCGCATTAGCTAGTTGGTGAGGTAACGGCTCATCAAGGCAACGATGCGTAGCCGACCT
GAGAGGGTGAACGGCCACACTGGGACTGAGACACGGCC
>Zotu4147
GAACGCTGGCGGCATGCTTTACACATGCAAGTCGAACGGCAGCGCGGGGCAACCTGGCGGCGAGTGGC
GAACGGGTGAGTAATATATCGGAACGTACCCAGAAGTGGGGGATAACGTAGCGAAAGTTACGCTAATA
CCGCATACGATCTACGGATGAAAGTGGGGGATCGCAAGACCTCATGCTTTTGGAGCGGCCGATATCTG
ATTAGCTAGTTGGTAGGGTAAAGGCCTACCAAGGCGACGATCAGTAGCTGGTCTGAGAGGACGACCAG
CCACACTGGGACTGAGACACGGCC
>Zotu4149

GAACGCTGGCGGCGTGCCTAACACATGCAAGTCGGATGTGCCCGCAAGGGTGCATGGCAGACGGGTGA
GTAACGCGTGGGAAACCTACCTCTTTGTGGGGAATAACGCTCCGAAAGGAGTGGTAATACCGCATGAG
ACCATGAGCTGGGACGCTCATGGTGAAACCTGGGGACCGCAAGGCCTGGGGCAAGGAGAGGGTCCTGC
GTCTGATTAGCTAGTTGGTGGGGTAACGGCCTACCAAGGCAACGATCAGTAGCCGGCCTGAGAGGGTG
ATCGGCCACACTGGAACTGAGACACGGTC
>Zotu4150
GAACGCTGGCGGCGTGCCTAATACATGCAAGTCGAGCGGATGATGAAGAAGCTTGCTTCTTCTGATTT
AGCGGCGGACGGGTGAGTAACACGTGGGCAACCTGCCCTGTAGATTGGGATAACTCCGGGAAACCGGG
GCTAATACCGAATAATCCATTCCCTCACATGAGAGAATGTTAAAAGACGGTCTCGGCTGTCACTACAG
GATGGGCCCGCGGCGCATTAGCTAGTTGGTGAGGTAACGGCTCACCAAGGCGACGATGCGTAGCCGAC
CTGAGAGGGTGATCGGCCACACTGGGACTGAGACACGGCC
>Zotu4151
GAACGCTGGCGGCGTGCCTAATACATGCAAGTCGAGCGGACTGAATGGAAGTTTGCTTTCGTTCGGTT
AGCGGCGGACGGGTGAGTAACACGTGGGCAACCTGCCCGTAAGACCGGGATAACTTCGGGAAACCGGA
GCTAATACCGGATAACACCGAAGATCGCATGGTCTTTGGTTGAAAGGCGGCTTTGGCTGTCACTTACG
GATGGGCCCGCGGCGCATTAGCTAGTTGGTGAGGTAACGGCTCACCAAGGCGACGATGCGTAGCCGAC
CTGAGAGGGTGATCGGCCACACTGGGACTGAGACACGGCC
>Zotu4154
GAACGCTGGCGGCAGGCCTAACACATGCAAGTCGAACGGTAACAGGAAGCAGCTTGCTGCTTTGCTGA
CGAGTGGCGGACGGGTGAGTAATGTCTGGGGATCTGCCTGATGGAGGGGGATAACTACTGGAAACGGT
AGCTAATACCGCATAACGTCGCAAGACCAAAGAGGGGGACCTTCGGGCCTCTTGCCATCAGATGAACC
CAGATGGGATTAGCTAGTAGGTGGGGTAACGGCTCACCTAGGCGACGATCCCTAGCTGGTCTGAGAGG
ATGACCAGCCACACTGGAACTGAGACACGGTC
>Zotu4155
GAACGCTGGCGGCGTGCTTAACACATGCAAGTGGAGCGACGAACCAGGGCTTGCCCTGGGGCAGAGCC
GCGAACGGGTGAGTAACACGTGGGTAACCTGCCCCGATCACTGGGACAACCCGAGGAAACTCGGGCTA
ATACCGGATGTGATCCCGTCACACCAGTGACGTGATGAAAGGTAGCTTCGGCTTCCGGATTGGGATGG
GCCCGCGGCCCATTAGCTCGTTGGTGGGGTAACGGCCTACCAAGGCGACGATGGGTAGCTGGTCTGAG
AGGACGATCAGCCACACTGGGACTGAGACACGGCC
>Zotu4156
GAACGCTGGCGGCGTGCTTAACACATGCAAGTCGAACGATGACGGTGGTGCTTGCATCACCTGATTAG
TGGCGAACGGGTGAGTAACACGTGAGTAACCTGCCCTTCACTTCGGGATAACCTCGGGAAATCGTGGC
TAATACCGGATATGAGCTCTTGCCGCATGGTGGGGGTTGGAAAGATTTATCGGTGAAGGATGGACTCG
CGGCCTATCAGTTTGTTGGTGAGGTAATGGCTCACCAAGGCGATGACGGGTAGCCGGCCTGAGAGGGC
GACCGGCCACACTGGGACTGAGACACGGCC
>Zotu4157
GATCGCTGGCGGCGTGCTTAACACATGCAAGTCGAGCGAATTCTAGCAGCGTATCGCTAGAGTTAGCG
GCAGACGGGTGAGTAATACTTGGGCAATCTGCCCTCGAGAGGGAAACAACCGGCCGAAAGGCTGGCTA
ATGTCCCATAAGACCACAGGGATGCATATTCCAGGGGTCAAAGCTCCGCAAGGGGCGCTTGAGGAGGA
GCCCGAGTCCTATCATTGGTAGTTGGTGAGGTAACGGCTCACCAAGCCTCTGACGGGTAGCCGGCCTG
AAAGGGTGACCGGCCACACTGGCACTGAGACACGGGC
>Zotu4158
GAACGCTGGCGGCAGGCTTAACACATGCAAGTCGAACGGGCTTCTTCGGAAGTCAGTGGCAGACGGGT
GAGTAACACGTGGGAACGTGCCCTTCGGTTCGGAATAACTCAGGGAAACTTGAGCTAATACCGGATAC
GCCCTTATGGGGAAAGGTTTACTGCCGAAGGATCGGCCCGCGTCTGATTAGCTTGTTGGTGGGGTAAC
GGCCTACCAAGGCGACGATCAGTAGCTGGTCTGAGAGGATGATCAGCCACACTGGGACTGAGACACGG
CC
>Zotu4162
GAATGCTGGCGGCGCGCCTAACACATGCAAGTCGAGCGGTAACATGTTTCTCACTGTCTTCTTCTGTT
TTTACAGGAGGGAATTGTGAGAAATGATGACGAGCGGCGGACGGCTGAGTAACGCGTGGGAACATGCC
CCAAAGTGAGGAATAACTGCCCGAAAGGGTGGCTAATGCCGCATATGGTCTTCGGATTAAAGGATTTA
TCCGCTTTGGGAGTGGCCTGCGTATGATTAGATAGTTGGTGAGGTAATGGCTCACCAAGTCTACGATC
ATTAACTGGTCTGAGAGGATGATCAGTCAGACTGGAACTGAGACACGGTC
>Zotu4163

GAACGCTGGCGGCATGCCTAACACATGCAAGTCGAACGCAGTAGCAATACTGAGTGGCGGACGGGTGA
GGAACACGTGGGAACATGCCGAATGGTGGGGGATAACTGCGGGAAACTGCAGCTAATACCGCATAAGC
CCTGAGGGGGAAAGATTTATCGCCATTCGATTGGCCCGCGGCAGATTAGGTAGTTGGTGGGGTAAAGG
CCTACCAAGCCGATGATCTGTAACTGGTCTGAGAGGATGACCAGTCACACTGGGACTGAGACACGGCC
>Zotu4164
GAACGCTGGCGGCGTGCCTTAAGCATGCAAGTCGTATGATCGCAAGATAATGGCGGACGGGTGAGTAA
CGCGTGAGAATCTGCCTTGAAGTGGGGCATAGCCCTCCGAAAGGAGGGTAATACCGCATGTGATAGC
AATATCAAAGCAGCAATGCGCTTTAAGATGAGCTCGCGTAAGATTAGCTAGTTGGTGGGGTAAAGGCC
TACCAAGGCGACGATCTTTAGCTGGTCTGAGAGGATGGTCAGCCACAATGGAACTGAGAACGGTC
>Zotu4166
GAACGCTGGCGGCGTGCTTAACACATGCAAGTCGAGCGGTAAGGCCCTTCGGGGTACACGAGCGGCGA
ACGGGTGAGTAACACGTGAGCAACCTGCCCCTGACTCCGGGATAAGCGGTGGAAACGCCGTCTAATAC
CGGATATGACCTGTCACCTCATGGTGGCGGGTGGAAAGTTTTATCGGTCAGGGATGGGCTCGCGGCCT
ATCAGCTTGTTGGTGGGGTAACGGCCTACCAAGGCGATTACGGGTAGCCGGCCTGAGAGGGCGACCGG
CCACACTGGGACTGAGACACGGCC
>Zotu4167
GAACGCTAGCGGCAGGCCTAACACATGCAAGTCGAGGGGTAGTATAGCAATATATGAGACCGGCGCAC
GGGTGCGTAACGCGTATGCAATCTACCTTTTACAGAGGGATAGCCCAGAGAAATTTGGATTAATACCT
CATAGCATTGCAGAATGGCATCATTCAGCAATTAAAGTCACAACGGTAAAAGATGAGCATGCGTCCTA
TTAGCTAGTTGGTAAGGTAACGGCTTACCAAGGCTACGATAGGTAGGGGTCCTGAGAGGGAGATCCCC
CACACTGGTACTGAGACACGGAC
>Zotu4168
GAACGCTTGCGGCGTGCCTGAGAAATGCAAGTCGAACGAGATTGAGCTTTCGGGTTCAGTCTAGTGGC
GAACGGTCGAGTAACACGTAAGCAACCTGCCCATCCGCGGGGGACAAGCCGTCGAAAGACGGGGTAAT
ACCGCATACGATCTACGGAGACATCTCCGGAGATGAAAGATTCATCACGGATGGAGGGGCTTGCGGCC
TATCAGCTAGTTGGTTGGGGTAACGGCTCACCAAGGCGACGACGGGTAGCGGGTCTGAGAGGACGACC
CGCCGGACTGGGACTGAGACACGGCC
>Zotu4169
TAACGCTGGCGGCGTGCATAACACATGCAAGTCGAACGATGAAACTCTAAAGATTTCTTCGGAATGAA
TTAGAATGGATTAGTGGCGAACGGGTGAGTAACACGTGAGTAACCTGCCTTAGAGAAGGGGATAGCCT
TTGGAAACGAAGAATAATACCCTATGAAATGATAATATCGCATGATAGTATCATCAAAGATTTATCGC
TCTAAGATGGACTTGCGCCTGATTAGCTAGTTGGTGGAGTAACAGCCCACCAAGGCAACGATCAGTAG
CCGGCTTGAGAGAGTGTACGGCCACATTGGGACTGAGACACGGCC
>Zotu4170
GAACGCTGGCGGCATGCCTAACACATGCAAGTCGAACGAACCCTTCGGGGTTAGTGGCGCACGGGTGC
GTAACGCGTGGGGATCTGCCCTTTGCTTCGGAATAACAGTTAGAAATGACTGCTAATACCGGATGATG
TCGAAAGACCAAAGATTTATCGGCAAAGGATGAACCCGCGTAGGATTAGCTTGTTGGTGGGGTAAAGG
CCTACCAAGGCGACGATCCTTAGCTGGTCTGAGAGGATGATCAGCCACACTGGGACTGAGACACGGCC
>Zotu4172
GAACGCTGGCGGCATGCCTAACACATGCAAGTCGAACGAGACCTTCGGGTCTAGTGGCGCACGGGTGC
GTAACGCGTGGGAATCTGCCCTTGGGTTCGGAATAACTCCTCGAAAGGGGTGCTAATACCGGATGATG
TCGCAAGACCAAAGATTTATCGCCCAAGGATGAGCCCGCGTAGGATTAGCTTGTTGGTGAGGTAAAAG
CTCACCAAGGCGACGATCCTTAGCTGGTCTGAGAGGATGATCAGCCACACTGGGACTGAGACACGGCC
>Zotu4174
GAACGCTGGCGGCGTGCTTAACACATGCAAGTCGAACGGTGAAGCCCAGCTTGCTGGGTGGATCAGTG
GCGAACGGGTGAGTAACACGTGAGCAATCTGCCCCTGACTCTGGGATAAGCGCTGGAAACGGCGTCTA
ATACTGGATACGAACCGTGGAGGCATCTTCAACGGTTGGAAAGATTTTTTGGTCAGGGATGAGCTCGC
GGCCTATCAGCTTGTTGGTGAGGTAATGGCTCACCAAGGCGTCGACGGGTAGCCGGCCTGAGAGGGTG
ACCGGTCACACTGGGACTGAGACACGGCC
>Zotu4177
GAACGCTGGCGGCGTGCTTAACACATGCAAGTCGAGTGCGCGTCTGTAGCAATACAGATGGCGCACGG
CGCACGGGTGCGTAACACGTGGGCAATCTGCCCTTCGATGGGGAATATCTTCGGGAAACCGAAGCGAA
TTCCGCATAACATTCCTTGGACTTCGGTTTGAGGACTCAAAGCCGTAAGGCGTCGAAGGAGGAGCCCG
CGCACGATTAGCTAGTTGGTAGGGTAACGGCCTACCAAGGCTTTGATCGTTAGCTGGTCTGAGAGGAT

GGCCAGCCACACTGGAACTGAGACACGGTC
>Zotu4178
AAACGCTGGCGGCGTGCCTAACACATGCAAGTCGAACGCCCTTAGCAATAAGGGAGTGGCGGACGGGT
GAGGAACACGTGGGTGACCTACCTCGAAGTGAGGAATACCGGCGAGAAATCGCCGACAATACCGCATA
CGCTTCGCAAGAAGCAAAGTCGCAAGACGCTTTGAGATGGGCCCGCGGCCGATTAGCTAGTTGGTGGG
GTAATGGCCTACCAAGGCGACGATCGGTAGCTGGTCTGAGAGGACGATCAGCCACACTGGGATTGAGA
ACGGCC
>Zotu4181
GAACGCTGGCGGCGTGCTTAACACATGCAAGTCGAACGATGAAGCTCCAGCTTGCTGGGGTGGATTAG
TGGCGAACGGGTGAGTAACACGTGAGTAACCTGCCCTTGACTCTGGGATAAGCCTTGGAAACGGGGTC
TAATACCGGATAGGAGCATTCACCGCATGGTGGGTGTTGGAAAGAATTTCGGTCTTGGATGGACTCGC
GGCCCATCAGCTTGTTGGTGAGGTAATGGCTCACCAAGGCGACGACGGGTAGCCGGCCTGAGAGGGTG
ACCGGCCACACTGGGACTGAGACACGGCC
>Zotu4182
GAATGCTGGCGGCGTGGATAAGGCATGCAAGTCGAACGGTCCGCAAGGACAGTGGCAGACGAGGTAGT
AACACGTACGAACGCACCCCATAGTCTGGCATAGCTCGTCGAAAGACGGGGTAATTCCGGATGGTCCC
TACCAAGGGTAAAGTTTTTCGCTATGGGAGCGGCGTGCGTAATATCAGCTTGTTGGTAAGGTAATGG
CTTACCAAGGCTACGACGTTTAGGGGAGCTGAGAGGCTGACCCCCACCGATGGGACTGAGACACGGCC
>Zotu4184
GAACGCTGGCGGCGTGCCTAACACATGCAAGTCGAGCGAGGCCCGCGCCTCCGGGCGCGGAGCCGAGC
GGCGAACGGGTGAGTAACACGTGAGCAACCTGCCCCCGGCATCGGGATAACCCCGGGAAACCGGGGCT
AATACCGGATACGACCGCGGAGGGCATCCTCGCGCGGTGGAAAGGTCCTCTCCCGTGAGGGAGGGTTC
CGGCCGGGGATGGGCTCGCGGCCTATCAGCTCGTTGGTGGGGTGACGGCCCACCAAGGCGACGACGGG
TAGCCGGCCTGAGAGGGCGACCGGCCACACTGGGACTGAGACACGGCC
>Zotu4185
GAACGCTAGCGGCAGGCCTAATACATGCAAGTCGAACGGGATCCGTCGGAGAGCTTGCTCGAAGACGG
TGAGAGTGGCGCACGGGTGCGTAACGCGTGAGCAACCTACCTCTATCAGGGGGATAGCCTCTCGAAAG
AGAGATTAACACCGCATAACATATCTGACCGGCATCGGTTAGCTATTAAATATTTATAGGATAGAGAT
GGGCTCGCGTGACATTAGCTAGTTGGTAGGGTAACGGCTTACCAAGGCGACGATGTCTAGGGGCTCTG
AGAGGAGAATCCCCCACACTGGTACTGAGACACGGAC
>Zotu4186
GAACGCTGGCGGCGTGCTTAACACATGCAAGTCGAACGGACAGACAGAGAGCTTGCTCTCTTGAAGTT
AGTGGCGAACGGGTGAGTAACGCGTAATCAACCTGCCCTTCAGAGGGGGATAACAACGGGAAACCGTT
GCTAATACCGCGTACGATTCATGAACGGCATCGTTTATGAATGAAAGGTGGCCTCTATGTATAAGCTA
TCGCTGAAGGAGGGGATTGCGTCTGATTAGCTAGTTGGAGGGGTAACGGCCCACCAAGGCAATGATCA
GTAGCCGGTCTGAGAGGATGAACGGCCACATTGGGACTGAGACACGGCC
>Zotu4187
GAACGCTGGCGGCGTGCCTAATACATGCAAGTCGAGCGAATCAATAGGAGCTTGCTCCTGTTGGTTAG
CGGCGGACGGGTGAGTAACACGTGGGCAACCTGCCTGTAAGACTGGGATAACACCGGGAAACCGGTGC
TAATACCGGATAATCCTTTTCCTCTCATGAGGAAAAGCTGAAAGTCGGTGCAAGCTGACACTTACAGA
TGGGCCCGCGGCGCATTAGCTAGTTGGTGAGGTAACGGCTCACCAAGGCGACGATGCGTAGCCGACCT
GAGAGGGTGATCGGCCACACTGGGACTGAGACACGGCC
>Zotu4189
GAACGCTGGCGGCGTGCTTAACACATGCAAGTCGAACGAGAATCTACGGATCGAGGATTCGTCCAAGT
GAAGTAGAGGACAGTGGCGGACGGGTGAGTAACGCGTGAGGAACCTGCCTTTCAGAGGGGGACAACAG
TTGGAAACGACTGCTAATACCGCATGACACATTGGGGTCGCATGGCCTTGATGTCAAAGATTTATCGC
TGAAAGATGGCCTCGCGTCTGATTAGCTAGTTGGTGAGGTAACGGCCCACCAAGGCGACGATCAGTAG
CCGGACTGAGAGGTTGACCGGCCACATTGGGACTGAGATACGGCC
>Zotu4190
GAACGCTAGCGGCAGGCCTAATACATGCAAGTCGGACGGGATTGGAGAGCTTGCTTTCCATGAGAGTG
GCGCACGGGTGCGTAACACGTATGTAACCTACCTTCACCAGGGGGATAGCCTCTCGAAAGAGAGATTA
AGACCGCATAAAATTGTTTAATGGCATCGTTGAACAATCAAATATTTATAGGGTGAAGATGGGCATGC
GGAACATTAGCTAGTTGGTAAGGTAACGGCTTACCAAGGCTACGATGTTTAGGGGATCTGAGAGGATG
ACCCCCCACACTGGTACTGAGACACGGAC

>Zotu4191
GAACGCTGGCGGCATGCCTAACACATGCAAGTCGAACGAAGTCCTTCGGGGCTTAGTGGCGCACGGGT
GCGTAACGCGTGGGAATCTGCCCTTGGGTTCGGAATAACAGTTAGAAATGACTGCTAATACCGGATGA
TGTCGCAAGACCAAAGATTTATCGCCCAGGGATGAGCCCGCGTAGGATTAGGTAGTTGGTGGGGTAAA
GGCCTACCAAGCCGACGATCCTTAGCTGGTCTGAGAGGATGATCAGCCACACTGGGACTGAGACACGG
CC
>Zotu4193
GAATGCTGGCGGCGTGCCTAACACATGCAAGTCGAGCGGTAACAGGAGTAATTTATTACTTGCTGACG
AGCGGCGGACGGCTGAGTAACGCGTGGGAACGTGCCCCAAAGTGAGGAATAAGCTCCAGAAATGGAGT
CTAATGCCGCATATGATCTTCGGATTAAAGGATTTATCCGCTTTGGGAGCGGCCCGCGTTAGATTAGG
TAGTTGGTAGGGTAATGGCCTACCAAGCCGACGATCTATAGCTGGTCTGAGAGGATGATCAGCCAGAC
TGGGACTGAGACACGGCC
>Zotu4194
GAACGCTGGCGGCGTGCCTAATACATGCAAGTCGAGCGAATGTCAGGGAGCTTGCTCCCTGACATTAG
CGGCGGACGGGTGAGTAACACGTGGGCAACCTGCCCTGCAGATGGGGATAACTCCGGGAAACCGGGGC
TAATACCGAATAATCAGTTCCTCCGCATGGAGGAACTCTGAAAGACGGTTTCGGCTGTCACTGCAGGA
TGGGCCCGCGGCGCATTAGCTAGTTGGTGGGGTAATGGCCTACCAAGGCGACGATGCGTAGCCGACCT
GAGAGGGTGATCGGCCACACTGGGACTGAGACACGGCC
>Zotu4195
GAACGCTGGCGGCGTGCCTAATACATGCAAGTCGAGCGGACCGAATAGAAGCTTGCTCCTGTTCGGTT
AGCGGCGGACGGGTGAGTAACACGTGGGCAACCTGCCCGTAAGACCGGGATAACTTCGGGAAACCGGA
GCTAATACCGGATAACACCGAAGACCGCATGGTCTTCGGTTGAAAGGCGGCTTTGGCTGTCACTTACG
GATGGGCCCGCGGCGCATTAGCTAGTTGGTGAGGTAACGGCTCACCAAGGCGACGATGCGTAGCCGAC
CTGAGAGGGTGATCGGCCACACTGGGACTGAGACACGGCC
>Zotu4196
CAACGCTGGCGGCGTGCCTAACACATGCAAGTCGAACGAGAAAGCAGGAGCAATCCTGTGAGTACAGT
GGCGACCGGGTGAGTAACACGTGACTAACCTGCCCTTGAGCGTGGAATAACTCCGGGAAACCGGAGCT
AATACCGCATAACACCGTAAGGTCAAAGGAGCAATCCACTCATGGAGGGGGTCGCGGCTGATTAGCTA
GTTGGTAGGGTAATGGCCTACCAAGGCAGTGATCAGTATCCGGCCTGAGAGGGCGCACGGACACACTG
GAACTGAAACACGGTC
>Zotu4197
GAACGCTGGCGGCGTGCTTAACACATGCAAGTCGAACGATGACGGTGGTGCTTGCACCACCTGATTAG
TGGCGAACGGGTGAGTAACACGTGAGTAACCTGCCCCCCACTTCGGGATAACCTCGGGAAATCGTGGC
TAATACCGGATACGAGCACTCATCGCATGGTGGGTGCTGGAAAGATTTATCGGTGGGGGATGGACTCG
CGGCCTATCAGTTTGTTGGTGAGGTGATGGCTCACCAAGACGATGACGGGTAGCCGGCCTGAGAGGGC
GACCGGCCACACTGGGACTGAGACACGGCC
>Zotu4198
GAACGCTGGCGGCGTGCTTAACACATGCAAGTCGAACGATGAAGCCCAGCTTACTGGGTGGAAGAGTG
GCGAACGGGTGAGTAACACGTGAGTAACCTGCCCTGAACTCTGGGATAAGCACTGGAAACGGTGTCTA
ATACTGAATACGACCTATCACCGCATAGTGTGTGGGTGGAAAGATTTATCGGTTTTGGATAGACTCGC
GGCCTATCAGCTAGATGGTGAGGTAATGGCTCACCATGGCGACGACGGGTAGCCGGCCTGAGAGGGTG
ACCGGCCACACTGGGACTGAGACACGGCC
>Zotu4199
GAACGCTAGCGATAGGCCTAACACATGCAAGTCGAGGGGCAGCACATGACGTAGCAATACAGATGGTG
GCGACCGGCGCACGGGTGAGTAACACGTATGCAACCTACCTTCAACAGGAGAATAACCCGTCGAAAGA
CGGACTAATACTCCATAACACAGGGATCCCGCATGGGAATATTTGTTAAAGATTTATCGGTTGAAGAT
GGGCATGCGCTCCATTAGCTAGTTGGTGAGGTAACGGCTCACCAAGGCAACGATGGATAGGGGAACTG
AGAGGTTTATCCCCCACACTGGTACTGAGACACGGAC
>Zotu4200
GAACGCTGGCGGCGTGCTTAACACATGCAAGTCGAACGGAAAGGCCCTTCGGGGTACTCGAGTGGCGA
GCGGGTGAGTAACACGTGGGTGATCTGCCTCGTACTTTGGGATAAGCCTGGGAAACTGGGTCTAATAC
CGAATATGACCTTCCCTCGCATGGGGGTTGGTGGAAACTTTTTGGGTACGAGATGGGCCCGCGGCCTA
TCAGCTTGTTGGTGGGGTAATGGCCTACCAAGGCGACGACGGGTAGCCGGCCTGAGAGGGCGACCGGC
CACACTGGGACTGAGACACGGCC

>Zotu4201
GAACGCTGGCGGCGTGCTTAACACATGCAAGTCGAACGGTGAGGGAGAGCTTGCTCTCTGGATCAGTG
GCGAACGGGTGAGTAACACGTGGGCAATCTGCCCCTGACTCTGGGATAAGCGCTGGAAACGGCGTCTA
ATACCGGATACGAGCTGCGAAGGCATCTTCAGCAGCTGGAAAGAACTTCGGTCAGGGATGAGCTCGCG
GCCTATCAGCTAGTTGGTGAGGTAACGGCTCACCAAGGCGTCGACGGGTAGCCGGCCTGAGAGGGTGA
CCGGCCACACTGGGACTGAGACACGGCC
>Zotu4202
GAACGCTGGCGGCAGGCTTAACACATGCAAGTCGAGCGGGGGAGATTGCTTCGGTAATTGACCTAGCG
GCGGACGGGTGAGTAATACTTAGGAATCTGCCTATTAATGGGGGACAACATCTCGAAAGGGATGCTAA
TACCGCATACGCCCTACGGGGGAAAGCAGGGGATCACTTGTGACCTTGCGTTAATAGATGAGCCTAAG
TCGGATTAGCTAGTTGGTGGGGTAAAGGCCTACCAAGGCGACGATCTGTAGCGGGTCTGAGAGGATGA
TCCGCCACACTGGGACTGAGACACGGCC
>Zotu4203
GAACGCTGGCGGCGTGCTTAACACATGCAAGTCGAACGGTGAAGGACGGAGCTTGCTCCGACCGGATC
AGTGGCGAACGGGTGAGTAACACGTGAGTAACCTGCCCCAGACTCTGGAATAACCCCGGGAAACCGGA
GCTAATACCGGATACGAAGTAGAGCTGCATGGCTACTACTTGGAAAGTTTTTCGGTCTGGGATGGACT
CGCGGCCTATCAGCTAGTTGGTGAGGTAAAGGCTCACCAAGGCGACGACGGGTAGCCGGCCTGAGAGG
GCGACCGGCCACACTGGGACTGAGACACGGCC
>Zotu4204
GAACGCTGGCGGCGCGCTTAACACATGCAAGTCGAGCGAGAACCAGACCTTCGGGTCTGGGGACAGCG
GCGAACGGGTGAGTAACACGTGGGTAATCAACCCTCGGTACTGGGATAGCCCGGGGAAACCCGGATTA
ATACCGGATGGCCCTTTGGATCTTCGGATCTGACGGAAAAGGTAGCTTCGGCCTCCGACCGAGGACGA
GCCCGCGGCGGATTAGCTTGTTGGTGGGGTAACGGCTCACCAAGGCGACGATCCGTAGCTGGTCTGAG
AGGACGATCAGCCACACTGGGACTGAGACACGGCC
>Zotu4205
GAACGCTGGCGGCAGGCTTAACACATGCAAGTCGAGCGCCCCGCAAGGGGAGCGGCAGACGGGTGAGT
AACGCGTGGGAATCTACCCAATACTACGGAATAGCTCCGGGAAACTGGAATTAATACCGTATGTGCCC
GACAGGGGAAAGATTTATCGGTATTGGATGAGCCCGCGTTGGATTAGCTAGTTGGTGGGGTAATGGCC
TACCAAGGCGACGATCCATAGCTGGTCTGAGAGGATGATCAGCCACACTGGGACTGAGACACGGCC
>Zotu4206
GAACGCTAGCGGCATGCTTAACACATGCAAGTCGAACGGTAACAGGCCTAGCTTGCTAGGCGCTGACG
AGTGGCGGACGGGTGAGTAACGCGTAGGAATCTACCTCGTAGTGGGGAATAACTTGGGGAAACCCAAG
CTAATACCGCATAAGCCCGCAAGGGGAAAGCAGGGGACCGAAAGGCCTTGTGCTATGAGATGAGCCTG
CGTCGGATTAGCTAGTTGGTAGGGTAAAGGCCTACCAAGGCGAAGATCCGTAGCTGGTCTGAGAGGAC
GACCAGCCACACTGGGACTGAGACACGGCC
>Zotu4207
GAACGCTGGCGGCAGGCTTAACACATGCAAGTCGAGCGGACCTTTCGGGGTCAGCGGCGGACGGGTGA
GTAACGCGTGGGAACGTGCCTTCTGGTTCGGAATAACCCTGGGAAACTAGGGCTAATACCGGATACGC
CCTTATGGGGAAAGGTTTACTGCCGGAAGATCGGCCCGCGTCTGATTAGCTAGTTGGTGGGGTAACGG
CCTACCAAGGCGACGATCAGTAGCTGGTCTGAGAGGATGATCAGCCACACTGGGACTGAGACACGGCC
>Zotu4208
GAACGCTGGCGGCGTGCTTAACACATGCAAGTCGAACGGTGAAGCAGAGCTTGCTCTGTGGATCAGTG
GCGAACGGGTGAGTAACACGTGAGCAACCTGCCCTGGACTCTGGGATAAGCGCTGGAAACGGCGTCTA
ATACTGGATATGAGCCTCTTCCGCATGGTGGGGGTTGGAAAGATTTTTAGGTCTGGGATGGGCTCGCG
GCCTATCAGCTTGTTGGTGAGGTAATGGCTCACCAAGGCGTCGACGGGTAGCCGGCCTGAGAGGGTGA
CCGGCCACACTGGGACTGAGACACGGCC
>Zotu4209
GAACGCTAGCGGCAGGCTTAATACATGCAAGTCGAGGGGGCAGCAATGTCACCGGCGAACGGGTGCGT
AACGCGTATGCAACCTACCTACATCAGGGGGATAGCCCTAGGAAACTGGGATTAACACCGCATAATAT
TATTAATGGCATCATTGGTAATTGAAATATTTATAGGATGTAGATGGGCATGCGTAGGATTAGCTAGT
TGGTAAGGTAACGGCTTACCAAGGCTACGATCCTTAGGGGCTCTGAGAGGAGTATCCCCCACACTGGC
ACTGAGATACGGGC
>Zotu4210
GAACGCTGGCGGCATGCCTAACACATGCAAGTCGAACGGTAGCAGGCCCTTCGGGGTGCTGACGAGTG

GCGGACGGGTGAGTAATGCGTAGGGATCTGCCTCATTGAGTGGGATAGCCTAGGGAAACTTAGGGTAA
TACCGCGTAAGCCTGAAAGGGGAAAGCCGGGGACTGTAATAGGCCTGGCGCGATGAGATGAACCTACG
TCGGATTAGCTTGTTGGTGAGGTAAAGGCTCACCAAGGCGATGATCCGTAGCTGGTCTGAGAGGACGA
CCAGCCACACTGGGACTGAGACACGGCC
>Zotu4212
GAACGCTGGCGGCGTGCCTAATACATGCAAGTCGAGCGAATTTTTTGGAGCTTGCTCCTAAAAATGAG
CGGCGGACGGGTGAGTAACACGTGGGCAACCTGCCTGTAAGACTGGGATAACTTCGGGAAACCGGAGC
TAATACCGGATAATTCTTTCTCTCACATGAGGGAAAGCTGAAAGACGGTTTCGGCTGTCACTTACAGA
TGGACCCGCGGCGCATTAGCTAGTTGGTGAGGTAACGGCTCACCAAGGCGACGATGCGTAGCCGACCT
GAGAGGGTGATCGGCCACATTGGGACTGAGACACGGCC
>Zotu4213
GAACGCTGGCGGCGTGCCTAATACATGCAAGTAGAACGCTGAAGCTTGGTGCTTGCACCGAGCGGATG
AGTTGCGAACGGGTGAGTAACGCGTAGGTAACCTGCCTGGTAGCGGGGGATAACTATTGGAAACGATA
GCTAATACCGCATAACAGTCGACATTGCATGATGTTGATTTGAAAGGGGCAATTGCTCCACTACCAGA
TGGACCTGCGTTGTATTAGCTAGTTGGTGAGGTAACGGCTCACCAAGGCGACGATACATAGCCGACCT
GAGAGGGTGATCGGCCACACTGGGACTGAGACACGGCC
>Zotu4215
GAACGCTAGCGGCAGGCTTAATACGTGCAAGGCGAGGGGGCAGCAATGTCACCGTCGTACGGGTGCGT
AACGCGTATGCAACCTACCTATCACTGGGGGATAGCCCGGGGAAACCCGGATTAATACCGCATAACAC
AGGGGTCCCGCATGGGTACTATTTGTTAAAGATTTATCGGTGGTAGATGGGCATGCGTTCGATTAGCT
AGTTGGTGCAGGTAACGGCTCACCAAGGCGACGATCGATAGGGGAGCTGAGAGGTTGATCCCCCACAC
GGGCACTGAGATACGGGC
>Zotu4216
GAACGCTGGCGGCGTGCTTAACACATGCAAGTCGAACGGAAAGGCCCTTCGGGGTACTCGAGTGGCGA
ACGGGTGAGTAACACGTGGGTGATCTGCCCTGCACTTTGGGATAAGCCTGGGAAACTGGGTCTAATAC
CGAATACACCCTTCTGGCTGCATGGTCTGGGAGGGGAAAGCTTTTGCGGTGTGGGATGGGCCCGCGGC
CTATCAGCTTGTTGGTGAGGTTATGGCTTACCAAGGCGACGACGGGTAGCCGGCCTGAGAGGGTGACC
GGCCACACTGGGACTGAGATACGGCC
>Zotu4217
GAACGCTGGCGACGTGCCTAATGCATGCAAGTCGAACGCGGGGTGCTTGCACCGCCGCGTGGCGCACG
GCTGAGGAACACGTGGGTATCAACCTCGGAGTGGGGGATACCTCGTCGAAAGACGAGACAATCCCGCA
TACGACCTGCGCAAGCAGATGAAAGCCCTCGGACGCTCCGGGACGAGCCTGCGGCCCATCAGGTAGTT
GGTGAGGTAATGGCTCACCAAGCCTCTGACGGGTCCCTGGTCTGAGAGGACGACCAGGCAGACTGGGA
CTGAGACACGGCC
>Zotu4218
GAACGCTGGCGGCGTGTCTAAGGCATGCAAGTCGAACGGGCCCTCGGAGTTAGCAATAATTTTGAGGG
CTAGTGGCAAACGGGTGAGTAACACATTGGTAACCTGCCCCGAAGTTCGGAATAACGGTCCGAAAGGA
CCGCTAATACCGGATGTGATCCCCTGTCATATGACGGGTGAGGAAAGCCCAAAAGGCGCTTCGGGAGG
GACCTATGGTCTATCAGCTAGTTGGTGCGGGTAATGGCCCACCAAGGCTACGACGGATAGCAGGCGTG
AGAGCGCGACCTGCCTCAGTGGAACTGAGACACTGTC
>Zotu4219
GAACGCTGGCAGTGTGCCTAAGACATGCAAGTCGAACGGGAGGCTTCGGCCTCTAGTGGCGAACGGGT
GAGTAACACGTGGGCGACCTACCTCCAAGTACGGCATAACCCCGGGAAACCGGAGCTAATTCCGTATG
TGCCCGCATCTTATGTGATGCGAGTAAAGATTTATCGCTTGGAGATGGGCCCGCGGCGCATCAGCTAG
TTGGTAGGGTAACGGCCTACCAAGGCGACGACGCGTAGCCGACCTGAGAGGGTGACCGGCCACAGGGG
CACTGAGACACGGGC
>Zotu4220
GAACGCTAGCGGCAGGCCTAATACATGCAAGCCGAACGGCAAGATTTTCAGCAATGAGAATCCTAGAG
TGGCGTAAGGGTGCGTAACACGTATGCAATCTACCCTCAACAGGAGGATAGCTCCCCGAAATGGGAAT
TAAGACTCCATAAGATCACACGAAGGCATCTGAGAGTGATTAAAGTACAAGCGGTTGAGGATGAGCAT
GCGTCTGATTAGTTAGTTGGCGGGGTAACGGCCCACCAAGACGATGATCAGTAGGGGAACTGAGAGGT
TGATCCCCCACACTGGCACTGAGATACGGGC
>Zotu4221
GAACGCTTGCGACGTGCCTAAGAAATGCAAGTCGAACGGGGGAGCAATCCCTAGTGGCGAACGGTCGC

GTAACACGTAGGCAACCTGCCCTGAAGCGGGGGACAACAGCTTGAAAGAGCTGCTAATACCGCATGTG
TCTATTTTTCGGCATCGAGGAATAAACAAAGCTTAATGCACTTCAGGATGGGCCTGCGGCCTATCAGC
TAGTTGGTGAGGTAACGGCTCACCAAGGCTTCGAAGGGTAGCTGGTCTGAGAGGACGATCAGCCGGAT
TGGGACTGAGATACGGCC
>Zotu4222
GAACGCTGGCGGTGCGCCTAAAACATGCAAGTCGAGTGGGTTTAGACCCACGGCGAACGGCTGCGTAA
CACGTGGATACTTGCCTCGTACTCAGGGATACCTCTCCGAAAGGAGAGCTAATACCGGATGGTCCCGA
AAGGGTAAAGATTTATCGGTACGAGAGAGGTCTGCGGCCTATCAGATAGTTGGCAGGGTAATGGCCTA
CCAAGTCGATGACGGGTAGCTGGATTGAGAGATCGACCAGCCAGAATGGAACTGAGACACGGTC
>Zotu4223
GAACGCTGGCGGCGCGCCTAATACATGCAAGTCGAGCGAGAAGACGTAGCAATACGTTTGTAAAGCGG
CGAACGGGTGAGGAATACATGGGTAACCTACCCTCGAGTGGGGAATAACTAGCCGAAAGGTTAGCTAA
TACCGCGTACGCTTCTTGGACTTCGGTTCAGGGAGGAAAGCGCTACCGTGGGTAGCGCGCTCTTGGAT
GGGCTCATGTCCTATCAGCTTGTTGGTGAGGTAACGGCTCACCAAGGCTTCGACGGGTAGCTGGTCTG
AGAGGACGATCAGCCACACTGGCACTGCGACACGGGC
>Zotu4224
AAACGCTGGCGGCGTGCATGAGACATGCAAGTCGAACGAACGAGCTTGCTCGTTAGTGGCGAACGGCT
GAGTAACGCGTAGGTGACCTGCCCCGAAGTGGGGGATAACTGCCCGAAAGGGTGGCTAATACCGCATG
TGGCGACGCTTTCATTGGCGTCATCAAAGCCGCGAGGCGCTTCGGGAGGGGCCTGCGTCCGATTAGCT
CGTTGGTGGGGTAATGGCTCACCAAGGCGACGATCGGTAGGTGGTCTGAGAGGACGATCACCCAGACT
GGGACTGAGACACGGCC
>Zotu4227
GAATGCTGGCTGCGTGCTTAACACATGCAAGTCGAACGGTGAAGGAGGGCTTGCCCTCCGGATCAGTG
GCGAACGGGTGAGTAACACGTGGGCAACCTGCCCCTCACTCTGGGATAACGCCGGGAAACCGGCGCTA
ATACCGGATACGACCAGAAGCGACATCTCTTCTGGTGGAAAGAATTTCGGTGAGGGATGGGCCCGCGG
CCTATCAGCTAGTTGGTGGGGTAATGGCCCACCAAGGCGACGACGGGTAGCCGGCCTGAGAGGGCGAC
CGGCCACACTGGGACTGAGACACGGCC
>Zotu4228
GAACGCTGGCGGCGTGCTTAACACATGCAAGTCGAGCGGTAAGGCTCCTTCGGGAGTACACGAGCGGC
GAACGGGTGAGTAACACGTGAGTAATCTGCCCTCCACTTTGGGATAAGCCTCGGAAACGAGGTCTAAT
ACCGAATACGACCACTTCCTGCATGGGATGGTGGTGGAAAGTTTTTTCGGTGGGGGATGTGCTCGCGG
CCTATCAGCTTGTTGGTGGGGTAATGGCCTACCAAGGCTTCGACGGGTAGCCGGCCTGAGAGGGTGAC
CGGCCACACTGGGACTGAGACACGGCC
>Zotu4230
GAACGCTAGCGGCAGGCCTAACACATGCAAGTCGAACGGTCTCAGCAGTGGGATAGTGGCGCACGGGT
GAGTAACGCGTATGCAACCTGCCTTGTACAGGGGGATAGCTTGGGGAAACCCAGGGTAACACCGCGTA
AGCTCTCGTTTCGGCATCGAGGTGAGAGGGAATCTACGGAGGTACAAGATGGGCATGCGTTCCATTAG
CTAGTTGGTGGGGTAACGGCCCACCAAGGCGACGATGGATAGGAGGTCTAAAAGGATGGTCTCCCACA
CTGGTACTGAGACACGGAC
>Zotu4231
GAACGCTGGCGGCATGCCTAACACATGCAAGTCGAACGAGACCTTCGGGTCTAGTGGCGCACGGGTGC
GTAACGCGTGGGAATCTGCCCTTGGGTACGGAATAACTCAGAGAAATTTGAGCTAATACCGTATAATG
ACTTCGGTCCAAAGATTTATCGCCCAAGGATGAGCCCGCGTAAGATTAGCTAGTTGGTGGGGTAAAGG
CCTACCAAGGCGACGATCTTTAGCTGGTCTGAGAGGATGATCAGCCACACTGGGACTGAGACACGGCC
>Zotu4232
GAACGCTGGCGGCGTGCCTAATACATGCAAGTCGAGCGAACAGATAAGGAGCTTGCTCCTTTGACGTT
AGCGGCGGACGGGTGAGTAACACGTGGGTAACCTACCTATAAGACTGGGATAACTTCGGGAAACCGGA
GCTAATACCGGATAATATTTTGAACCGCATGGTTCGATAGTGAAAGATGGCCCTGCTATCACTTATAG
ATGGACCTGCGCCGTATTAGCTAGTTGGTAAGGTAACGGCTTACCAAGGCAACGATACGTAGCCGACC
TGAGAGGGTGATCGGCCACACTGGAACTGAGACACGGTC
>Zotu4233
GAACGCTGGCGGCGTGCTTAACACATGCAAGTCGAACGGAAAGGCCCTTCGGGGTACTCGAGTGGCGA
ACGGGTGAGTAACACGTGGGTGATCTGCCCTGCACTTCGGGATAAGCCTGGGAAACTGGGTCTAATAC
CGGATATACCTCATGGACTGCATGGTTCGTGGGGGAAAGCTTTTGCGGTGTGGGATGGGCCCGCGGCC

TATCAGCTTGTTGGTGGGGTGATGGCCTACCAAGGCGTCGACGGGTAGCCGGCCTGAGAGGGTGTCCG
GCCACACTGGGACTGAGATACGGCC
>Zotu4235
GAACGCTGGCGGCGTGCTTAACACATGCAAGTGGAGCGACGAACCAGGGCTTGCCCTGGGGCAGAGCC
GCGAACGGGTGAGTAACACGTGGGTAACCTACCCCGATGACCGGGACAACCCGAGGAAACTCGAGCTA
ATACCGGATGTTCTCGCTTCCTATAAGGGAAACGAGCAAAGATAGCTTCGGCCTTCGCATCGGGACGG
GCCCGCGGCCCATTAGCTTGTTGGTGGGGTAACGGCCTACCAAGGCGACGATGGGTAGCTGGTCTGAG
AGGACGATCAGCCACACTGGGACTGAGACACGGCC
>Zotu4236
GAACGCTGGCGGCGTGCTTAACACATGCAAGTGGAGCGACGAACCAGGGCTTGCCCTGGGGCAGAGCC
GCGAACGGGTGAGTAACACGTGGGTAACCTGCCCAGATGACCGGGACAACCCGAGGAAACTCGGGCTA
ATACCGGATGTGCTCCTTCCACATCAGTGGATTGAGGAAAGGTAGCTTCGGCTTCCGCATCTGGATGG
GCCCGCGGCGCATTAGCTTGTTGGTGAGGTAACGGCTCACCAAGGCGACGATGCGTAGCTGGTCTGAG
AGGACGATCAGCCACACTGGGACTGAGACACGGCC
>Zotu4237
GAACGCTGGCGGCAGGCTTAACACATGCAAGTCGAGCGGTAGCACAGGGGAGCTTGCTCCCTGGGTGA
CGAGCGGCGGACGGGTGAGTAATGTCTGGGAAACTGCCTGATGGAGGGGGATAACTACTGGAAACGGT
AGCTAATACCGCATAACGTCGCAAGACCAAAGAGGGGGACCTTCGGGCCTCTTGCCATCAGATGTGCC
CAGATGGGATTAGCTAGTAGGTGGGGTAATGGCTCACCTAGGCGACGATCCCTAGCTGGTCTGAGAGG
ATGACCAGCCACACTGGAACTGAGACACGGTC
>Zotu4238
GAACGCTGGCGGCGTGCCTAATACATGCAAGTCGAGCGAACTTGAGGGAGCTTGCTCCCAAAAGTTAG
CGGCGGACGGGTGAGTAACACGTGGGCAACCTGCCTATAAGACTGGGATAACTTCGGGAAACCGGAGC
TAATACCGGATAATTCATATCTACTCATGTAGATATGCTGAAAGACGGTTTACGCTGTCACTTATAGA
TGGGCCCGCGGCGCATTAGCTAGTTGGTGAGGTAACGGCTCACCAAGGCAACGATGCGTAGCCGACCT
GAGAGGGTGATCGGCCACACTGGGACTGAGACACGGCC
>Zotu4239
GAACGCTAGCTATATGCTTAACACATGCAAGTCGAACGTTGTTTTCGGGGAGCTGGGCAGAAGGAAAA
GAGGCTCCTAGCTAAAGGTAGCTTGTCTCGCCCAGGAGGTGAGAAGAGTTGAGAACAAAGTGGCGAAC
GGGTGCGTAACGCGTGGGAATCTGCCGAACAGTTCGGGCCAAATCCTGAAGAAAGCTAAAAAGCGCTG
TTTGATGAGCCTGCGTAGTATTAGGTAGTTGGTCAGGTAAAGGCTGACCAAGCCAATGATGCTTAGCT
GGTCTTTTCGGATGATCAGCCACACTGGGACTGAGACACGGCC
>Zotu4240
GAACGCTGGCGGCATGCTTTACACATGCAAGTCGAACGGTAACAGGTCTTCGGATGCTGACGAGTGGC
GAACGGGTGAGTAATGCATCGGAACGTGCCCAGTCGTGGGGGATAACGCAGCGAAAGCTGTGCTAATA
ACGCATACGATCTATGGATGAAAGCGGGGGATCGCAAGACCTCGCGCGATTGGAGCGGCCGATGTCAG
ATTAGCTAGTTGGTGGGGTAAAGGCCCACCAAGGCGACGATCTGTAGCTGGTCTGAGAGGACGACCAG
CCACACTGGGACTGAGACACGGCC
>Zotu4241
GAACGCTGGCGGCATGCCTAACACATGCAAGTCGAACGAAGCCTTCGGGCTTAGTGGCGCACGGGTGC
GTAACGCGTGGGAATCTGCCTTGGGGTTCGGAATAACTCAGAGAAATTTGAGCTAATACCGGATGATG
TCTACGGACCAAAGATTTATCGCCCTGAGATGAGCCCGCGTGAGATTAGCTAGTTGGTGGGGTAAAGG
CCCACCAAGGCGACGATCTCTAGCTGGTCTGAGAGGATGATCAGCCACACTGGGACTGAGACACGGCC
>Zotu4243
GAACGCTGGCGGCGTGCTTAACACATGCAAGTCGAACGGTGAAGCCCAGCTTGCTGGGCGGATCAGTG
GCGAACGGGTGAGTAACACGTGAGCAACCTGCCCTCCACTTCGGGATAAGCCTTGGAAACGGGGTCTA
ATACCGGATATGAGCGCTCACCGCATGGTGGGTGTTGGAAAGTTTTTCGGTGGGGGATGGGCTCGCGG
CCTATCAGCTTGTTGGTGGGGTGATGGCCTACCAAGGCGTCGACGGGTAGCCGGCCTGAGAGGGCGAC
CGGCCACACTGGGACTGAGACACGGCC
>Zotu4245
GAACGCTAGCGGCAGGCCTAATACATGCAAGTCAAGGGGTTCAGCAATGGACACCGGCGGACGGGTGC
GTAACGCGTACACAACGTGCCCAAGGGCGGGGGAAAGCGGCGGGAAACTGCCGGTAATACCCCATGGT
GTTGAAGGGTGGCATCACTTTTCAACTAAAGTACAAGCGCCCTTGGATCGGTGTGCGTCCGATTAGCT
AGTTGGCGGGGTAACGGCCCACCAAGGCGTCGATCGGTAGGGGGTGTGAGAGCATGGCCCCCCACACG

GGTACTGAGACACGGAC
>Zotu4247
GAACGCTGGCGGCGTGCCTAATACATGCAAGTCGTGCGGAACCTTCGGGTTCAGCGGCGGACGGGTGA
GTAACACGTGGGCAACCTGCCCGACAGATCGGGATAACGCTTGGAAACGAGTGCTAATACCGGATACG
CTTTTGAGGCGCATGCCTCGAAAGGGAAAGTTGCTTTTGCAGCACTGTCGGATGGGCCCGCGGCGCAT
TAGCTAGTTGGTGAGGTAACGGCTCACCAAGGCGACGATGCGTAGCCGACCTGAGAGGGTGACCGGCC
ACACTGGGACTGAGACACGGCC
>Zotu4249
GAACGCTGGCGGCGTGCTTAACACATGCAAGTCGAGCGGTAAGGCCCTTTCGGGGGTACACGAGCGGC
GAACGGGTGAGTAACACGTGAGTAATCTGCCCTCAGCTTTGGGATAAGCACTCGAAAGGGTGTCTAAT
ACCGGATATGACGATCCTCCGCATGGGGGGTTGTGGAAAGTTTTTTCGGCTGGGGATGTGCTCGCGGC
CTATCAGCTTGTTGGTGGGGTAATGGCCTACCAAGGCTTTGACGGGTAGCCGGCCTGAGAGGGTGATT
AGCCACATTGGGACTGAGATACGGCC
>Zotu4250
GAACGCTGGCGGCAGGCTTAACACATGCAAGTCGAACGCCCCGCAAGGGGAGTGGCAGACGGGTGAGT
AACGCGTGGGAATCTACCCTTTACTACGGAATAACGCATGGAAACGTGTGCTAATACCGTATGTGTCC
TTCGGGAGAAAGATTTATCGGTAAAGGATGAGCCCGCGTTGGATTAGCTAGTTGGTGGGGTAAAGGCC
TACCAAGGCGACGATCCATAGCTGGTCTGAGAGGATGATCAGCCACATTGGGACTGAGACACGGCC
>Zotu4251
GAACGCTGGCGGCATGCCTAACACATGCAAGTCGAACGAGACCTTCGGGTCTAGTGGCGCACGGGTGC
GTAACGCGTGGGAATCTACCCTTGGGTTCGGAATAACAGCTAGAAATGGCTGCTAATACCGGATGATG
TCTTCGGACCAAAGATTTATCGCCCAGGGATGAGCCCGCGTAGGATTAGCTTGTTGGTGAGGTAAAAG
CTCACCAAGGCGACGATCCTTAGCTGGTCTGAGAGGATGATCAGCCACACTGGGACTGAGACACGGCC
>Zotu4252
AAATGCTGGCGGCGTGCCTAATACATGCAAGTCGTGCGGAAACACTTCGGGTGTCGAGCGGCGGACGG
CTGAGTAACGCATAGGAATGTACCCCAAAGTGAGGGATAACTCCGAGAAATCGGGGCTAATACCGCAT
ATGCTCTAAGGAGTAAAGCTTCGGCGCTTTGGGAACAGCCTATGTCCGATTAGCTAGTTGGTGAGGTA
AAGGCTTACCAAGGCGACGATCGGTAGCTGGTCTGAGAGGATGATCAGCCAGACTGGGACTGAGACAC
GGCC
>Zotu4253
GAACGCTGGCGGCGTGCTTAACACATGCAAGTCGAGCGGTAAGGCCCTTCGGGGTACACGAGCGGCGA
ACGGGTGAGTAACACGTGGGTGACCTGCCCTCAGCTCTGGGATAAGCCTGGGAAACTGGGTCTAATAC
CGGATATGACCATGGGATGCATGTCCTGTGGTGGAAAGTTTTTCGGCTGGGGATGGGCCCGCGGCCTA
TCAGCTTGTTGGTGGGGTGATGGCCTACCAAGGCGGTGACGGGTAGCCGGCCTGAGAGGGCGACCGGC
CACACTGGGACTGAGACACGGCC
>Zotu4254
GAACGCTGGCGGCATGCCTAACACATGCAAGTCGAACGATGCTTTCGGGCATAGTGGCGCACGGGTGC
GTAACGCGTGGGAATCTGCCCTTGGGTCTGGGATAACAGTTGGAAACGACTGCTAATACCGGATGATA
TCGCGAGATCAAAGATTTATCGCCCGAGGATGAGCCCGCGTAGGATTAGCTAGTTGGTGGGGTAAAGG
CCTACCAAGGCGACGATCCTTAGCTGGTCTGAGAGGATGATCAGCCACACTGGGACTGAGACACGGCC
>Zotu4256
GAACGCTGGCGGCAGGCCTAATACATGCAAGTCGAGCGGATGTAGCAATACATCAGCGGCGCACGGGA
GAATAACACGTGGGAACCTACCTTTTGGTTCGGAATAACACCGGGAAACTGGTGCTAATACCGGATAA
GCCCTTCGGGGGAAAGATTTATTGCCGAAAGATGGGCCCGCGTTGGATTAGCTAGTTGGTGAGGTAAC
GGCTCACCAAGGCAACGATCCATAGCTGGTCTGAGAGGATGATCAGCCTCACTGGGACTGAGACACGG
CC
>Zotu4257
GAACGCTGGCGGCGTGCTTAACACATGCAAGTCGAACGGAAAGGCCCCCACCATTTTTGGTGGGGGTG
CTCGAGTGGCGAACGGGTGAGTAACACGTGGGTGATCTGCCCTGTACTTCGGGATAAGCTTGGGAAAC
TGGGTCTAATACCGGATAGGACCATGGTTTAGTGTCTCATGGTGGAAAGATTATTGTTTTGGTGCAGG
ATGAGCTCGCGGCCTATCAGCTTGTTGGTGGGGTAATGGCCTACCAAGGCGTCGACGGGTAGCCGGCC
TGAGAGGGTGGACGGCCACATTGGGACTGAGATACGGCC
>Zotu4258
GAACGCTGGCGGCGTGCTTAACACATGCAAGTCGAGCGGTAAGGCCCTTTCGGGGGTACACGAGCGGC

GAACGGGTGAGTAACACGTGAGTAATCTGCCCTTCACTTCGGGATAACCATCGGAAACGATGGCTAAT
ACCGGATATGACACCTTCTTGCATGAGATGGGTGTGGAAAGTTTTTCGGTGGAGGATGTGCTCGCGGC
CTATCAGCTTGTTGGTGAGGTAATGGCTCACCAAGGCTTCGACGGGTAGCCGGCCTGAGAGGGTGACC
GGCCACACTGGGACTGAGACACGGCC
>Zotu4260
GAACGCTGGTAACGTGGATACCGGCATGCAAGTCAAGGGGGCCCGCAAGGGCAACCGGCGAACGGCAT
AGTAACACGTGGGTACATACCCTAAAGTGGAGGACAGCTCGTCGAAAGACGGGGTAATACTCCATAGT
CTCGCAAGAGTAAAGTCGCAAGACGCTTTAGGAATGGCCTGCGGCCTATCAGGTAGTTGGCGGTGTAA
TAGACCACCAAGCCTATGACGGGTAGGGGGCGTGAGAGCGCGACCCCCATGAATGGCACTGCGACACG
GGC
>Zotu4261
GAACGCTGGAAATGTGGATACCGGCATGCAAGTCAAGGGGCCGCAAGGCACCGGCGAACGGCTTAGTA
ACACGTAGATACATACCGCAAAGTGGGGCATAGCTCGTCGAAAGACGGGGTAATTTCCCATAGTCCCG
CAAGGGTAAACACGCAAGTGGCTTTGTGAATGGTCTGCGGTCTATCAGCTAGTTGGTGAGGTAATGGC
TCACCAAGGCTATGACGGATAGGGGGCGTGAGAGCGCGACCCCCAAGAATGGCACTGCGACACGGGC
>Zotu4262
GAACGCTGGCGGCGTGCCTAATACATGCAAGTCGAGCGAACTCTTTAAAAGCTTGCTTTTGAAGAGTT
AGCGGCGGACGGGTGAGTAACACGTGGGCAACCTGCCTATAAGACTGGGATAACTTCGGGAAACCGGA
GCTAATACCGGATAATTCTTTTCTACACATGTAGGAAAGCTGAAAGACGGTTTACGCTGTCACTTATA
GATGGGCCCGCGGCGCATTAGCTAGTTGGTGAGGTAACGGCTCACCAAGGCTACGATGCGTAGCCGAC
CTGAGAGGGTGATCGGCCACACTGGGACTGAGACACGGCC
>Zotu4263
GAACGCTGGCGGCACGTTTTAAGCATGCAAGTCGAACGGCAGTGGGGGCTTGCTCCCATGAGAGTGGC
GAACGGGTGAGTAACACGTAGGTGATCTGCCTTGAGGATGGGGATAGCTTGTGGAAACACAGGGTAAT
ACCGAATAAGCTGGCGGTACTTTGGTACTGTTAGTAAAGGTGCTACGGCACCGCCTTGAGAGGAGCCT
GCGGCTGATTAGCTAGTTGGTGGGGTAAAGGCCTACCAAGGCGACGATCAGTAGCCGGCCTGAGAGGG
TGTACGGCCACACTGGAACTGAGACACGGTC
>Zotu4265
GAACGCTGGCGGCGTGCATAACACATTCAAGTCGAACGGAGATACATTGATTAATCCTTCGGGAAGCG
TTGATGTATTTTAGTGGCGGACGGGTGAGTAACGCGTGAGCAACCTGCCCTTCATTGAGGGATAACGT
CTGGAAACGGACGCTAATACCTCATGACACATAGTTACCGCATGGTAGGTATGTTAAAGGAATCTGAT
GAAGGATGGGCTCGCGTATGATTAGACAGTTGGCGGGGTAACGGCCCACCAAATCAACGATCATTAGC
CGAACTGAGAGGTTGAACGGCCACATTGGAACTGAGAAACGGTC
>Zotu4266
GAACGCTGGCGGCATGCCTAACACATGCAAGTCGAACGAGACCTTCGGGTCTAGTGGCGCACGGGTGC
GTAACGCGTGGGAATCTGCCCTTGGGTCCGGAATAACAGTTAGAAATGACTGCTAATACCGGATGATG
ACGTAAGTCCAAAGATTTATCGCCCAGGGATGAGCCCGCGTAAGATTAGCTAGTTGGTGGGGTAAAGG
CCTACCAAGGCAACGATCTTTAGCTGGTCTGAGAGGATGATCAGCCACACTGGGACTGAGACACGGCC
>Zotu4267
GAACGCTGGCGGCATGCCTAACACATGCAAGTCGAACGAGGCCTTCGGGTCTAGTGGCGCACGGGTGC
GTAACGCGTGGGAATCTGCCCCTTGGTTCGGAATAACAGCGAGAAATTGCTGCTAATACCGGATAATG
ACGTAAGTCCAAAGATTTATCGCCAAGGGATGAGCCCGCGTAGGATTAGCTAGTTGGTGTGGTAAAGG
CGCACCAAGGCGACGATCCTTAGCTGGTCTGAGAGGATGATCAGCCACACTGGGACTGAGACACGGCC
>Zotu4268
GAACGCTGGCGGCGTGCCTAATACATGCAAGTCGAGCGCAGGAAGCAGACAGAATCCTTTGGGAGGAC
GTTTGTGGAATGAGCGGCGGACGGGTGAGTAACACGTGGGTAACCTGCCTGTAAGATTGGGATAACTC
CGGGAAACCGGTGCTAATACCGAATAAAGTTCTTTACCGCCTGGTAAAGAAATGAAAGATGGTTTCGG
CTATCACTTACAGATGGACCTGCGGCGCATTAGCTAGTTGGTGGGGTAAAGGCCTACCAAGGCAACGA
TGCGTAGCCGACCTGAGAGGGTGATCGGCCACACTGGGACTGAGACACGGCC
>Zotu4269
GAACGCTGGCGGCAGGCTTAACACATGCAAGTCGAACGCCCCGCAAGGGGAGTGGCAGACGGGTGAGT
AACGCGTGGGAATCTACCCAGATCTACGGAACAACAGAGGGAAACTTCTGCTAATACCGTATACGCCC
TACGGGGGAAAGATTTATCGGATTTGGATGAGCCCGCGTAAGATTAGCTAGTTGGTGAGGTAATGGCT
CACCAAGGCGACGATCTTTAGCTGGTCTGAGAGGATGATCAGCCACACTGGGACTGAGACACGGCC

>Zotu4271
GAACGCTGGCGGCGTGCCTAATACATGCAAGTCTAAGGGGTGCAGATCCTTCGGGGTCTGTATCACTG
GCGCACGGGTGAGTAACGCGTAAGTCATCTGCCCCTGGGTCTGGCATAACGTCGGGAAACCGGCGCTA
ATTCCAGATGATACCGCAAGGTCAAAGAATTTCGCCCAGGGATGAGCTTGCGTCTGATTAGCTAGTTG
GCGGGGTAATGGCCCACCAAGGCGACGATCAGTAGCCGGTCTGAGAGGATGATCGGCCACATTGGGAC
TGAGACACGGCC
>Zotu4273
GAACGCTGGCGGCATGCCTAACACATGCAAGTCGAACGAGACCTTCGGGTCTAGTGGCGCACGGGTGC
GTAACGCGTGGGAATCTGCCCTTCGGTTGGGAATAACTCAGGGAAACTTGAGCTAATACCTTATAATG
TCTTCGGACCAAAGATTTATCGCCGAGGGATGAGCCCGCGTAGGATTAGCTAGTTGGTGGGGTAATGG
CCTACCAAGGCGACGATCCTTAGCTGGTCTGAGAGGATGATCAGCCACACTGGGACTGAGACACGGCC
>Zotu4274
GAACGCTGGCGGCGTGCTTAAGACATGCAAGTCGAACGCAGTCTTCGGACTGAGTGGCGCACGGGTGA
GTAACACGTAACTGACCTACCCCCAAGTCGCGAATAACCAGCCGAAAGGATGGCTAATACGTGATGTG
ATGATCCGCTGTGGCGGATCATTAAAGACTTGATCGCTTGGGGATGGGGTTGCGTTCCATCAGCTAGT
TGGTAAGGTAAGGGCTTACCAAGGCAACGACGGATAGCCGGCCTGAGAGGGTGGCCGGCCACAGGGGC
ACTGAGACACGGGT
>Zotu4275
GAACGCTGGCGGCAGGCTTAACACATGCAAGTCGAGCGGGCCCTTCGGGGTCAGCGGCGGACGGGTTA
GTAACGCGTGGGAACGTGCCCTTTGCTTCGGAATAGCCCTGGGAAACTGGGGGTAATACCGGATGTGC
CCTGAGGGGGAAAGATTTATCGGCAAGGGATCGGCCCGCGTCTGATTAGGTAGTTGGTGCGGTAACGG
CGCACCAAGCCGACGATCGGTAGCTGGTTTGAGAGGATGATCAGCAACACTGGGACTGAGACACGGCC
>Zotu4276
GAACGCTGGCGGCGTGCCTAACACATGCAAGTCGAGCGAGAGCCGGCCGAGGGGTAACCCTCGACCGG
GGACAGCGGCGAACGGGTGCGTAACACGTGGGCAACCTGCCCCTGACTCCGGGATAACCCCGGGAAAC
CGGAGCTAATACCGGATACTCTCGGCCAGCCTCATGGCGGGCCGAGGAAAGATCCGTCGGTCAGGGAT
GGGCCCGCGGCCTATCAGCTTGTTGGCGGGGTGACGGCCCACCAAGGCGACGACGGGTAGCTGGTCTG
AGAGGACGACCAGCCACACTGGAACTGAGATACGGTC
>Zotu4277
GAACGCTGGCGGCGTGCTTAACACATGCAAGTCGAACGGAAAGGCCTGTTTTCCCTTGTGGATTGCGG
GTACTCGAGTGGCGAACGGGTGAGTAACACGTGAGTGATCTGCCCCCAACTTGGGTATAAGCCTGGGA
AACTGGGTCTAATTCCCGATAGGACTGCAGGGTGGTGCCTGTGGTGGAAAACGATTTTTTAGTGGTTG
GGGATGAGCTCGCGGCCTATCAGCTTGTTGGTGGGGTAATGGCCTACCAAGGCGGCGACGGGTAGCCG
GCCTGAGAGGGTGGACGGCCACATTGGGACTGAGATACGGCC
>Zotu4278
TAACGCTGGCGGCGTGCATAACACATGCAAGTCGAACGATGAAACTCATTAGATTTCTTCGGAATGAA
AATGAATGGATTAGTGGCGAACGGGTGAGTAACGCGTGAGTAACCTGCCTTACACAAGGGGATAGCCT
CTGGAAACGGAGAATAATACCCTATGAAATTACATTCTCGCATGAGGAAATAATCAAAATGTTTAGTG
GTGTAAGATGGACTTGCGTCTGATTAGCTAGTTGGTGAGATAAAAGCTCACCAAGGCAACGATCAGTA
GCCGGCTTGAGAGAGTGTACGGCCACATTGGGACTGAGACACGGCC
>Zotu4279
GAACGCTGGCGGCGTGCTTAACACATGCAAGTCACGGGGGCCCGCAAGGGCAACCGGCGAACGGGTGC
GTAACACGTGAGCGACCTGCCGAGGTGTGGGGGATAGCCGGGCCAACGCCCGGGTAATACCGCATACG
ATCACCGGGGGGCATCCCCTGGTGATGAAACCTCCGGGGCACTTCGAGGGGCTCGCGGCTTATCAGCT
AGTTGGCGGGGTAACGGCCCACCAAGGCGACGACGAGTAGCTGGTCTGAGAGGATGGCCAGCCACATT
GGGACTGAGAGACGGCC
>Zotu4280
GAACGCTGGCGGCAGGCTTAACACATGCAAGTCGAACGCATCCTTCGGGATGAGTGGCAGACGGGTGA
GTAACGCGTGGGAATCTACCCATCTCTACGGAATAGCTCTGGGAAACTGGGATTAATACCGTATACGC
CCTTTTGGGGAAAGATTTATCGGAGATGGATGAGCCCGCGTTGGATTAGCTAGTTGGTGGGGTAAAGG
CCTACCAAGGCGACGATCCATAGCTGGTCTGAGAGGATGATCAGCCACATTGGGACTGAGACACGGCC
>Zotu4281
GAACGCTGGCGGCAGGCCTAACACATGCAAGTCGAGCGGCAGCGGGAAAGTAGCTTGCTACTTTTGCC
GGCGAGCGGCGGACGGGTGAGTAATGCCTGGGAAATTGCCCAGTCGAGGGGGATAACAGTTGGAAACG

ACTGCTAATACCGCATACGCCCTACGGGGGAAAGCAGGGGACCTTCGGGCCTTGCGCGATTGGATATG
CCCAGGTGGGATTAGCTAGTTGGTGAGGTAATGGCTCACCAAGGCGACGATCCCTAGCTGGTCTGAGA
GGATGATCAGCCACACTGGAACTGAGACACGGTC
>Zotu4282
GAACGCTGGCGGCGTGCTTAATACATGCAAGTCGAGCGGTAAGGCCCCTTCGGGGGTACACGAGCGGC
GGACGGCTGAGTAACGCGTGGGAACGTACCCCAAAGTGAGGGATAAGCACCAGAAATGGTGTCTAATA
CCGCATGTGCTCTAAGGAGTAAAGCTCCGGCGCTTTGGGAACGGCCTGCGTCCGATTAGCTTGTTGGT
GAGGTAATGGCTTACCAAGGCTACGATCGGTAGCTGGTCTGAGAGGATGACCAGCCAGACTGGGACTG
AGAACGGCC
>Zotu4283
GAACGCTGGCGGCGTGCCTAAGACATGCAAGTCGAGTGGGTTTAGACCCACGGTAAACGGGTGAGTAA
TACCTAGGAACTTTCTTTAAGTGGAGCATAACCAGCCGAAAGGTTGGATAATACTCCATACCATCGCA
AGATGAAAGTCGCAAGACGCTTAAAGTGAGGCCTGGGCACCATCAGCTAGTTGGTAAGGTAATGGCTT
ACCAAGGCTATGACGGTTAGGGGCCCCGAGAGGGGAGTCCCCCACACTGGAACTGCGACACGGTC
>Zotu4284
GAACGCTGGCGGCGTGCTTAACACATGCAAGTCGAACGGTGAAGCCATTTCGGTGGTGGATCAGTGGC
GAACGGGTGAGTAACACGTGAGCAATCTGCCCTTGACTCTGGGATAACTTCGGGAAACCGGAGCTAAT
ACCGGATATGACGCATGGAGGCATCTCCTGTGCGTGGAAAGATTTATCGGTTGGGGATGAGCTCGCGG
CCTATCAGCTTGTTGGTGGGGTAATGGCCTACCAAGGCGACGACGGGTAGCCGGCCTGAGAGGGCGAC
CGGCCACACTGGGACTGAGACACGGCC
>Zotu4285
GAACGCTAGCGGCAGGCCTAATACATGCAAGTCGGACGGGATCCGGCGGTAGCTTGCTACCTTCGGTG
AGAGTGGCGCACGGGTGCGTAACGCGTGAGCAACCTACCCATATCAGGGGGATAGCCCGGAGAAATCC
GGATTAACACCGCATGCAACCAATGGACGGCATCGTTTTTTGGTTAAATATTTATAGGATATGGATGG
GCTCGCGTGACATTAGCTAGTTGGTGGGGTAACGGCCCACCAAGGCGACGATGTCTAGGGGCTCTGAG
AGGAGAATCCCCCACACTGGTACTGAGACACGGAC
>Zotu4286
GAACGCTGGCGGCAGGCTTAACACATGCAAGTCGAACGGTTGTAGCAATACAGCAGTGGCGCACGGGT
GAGTAACGCGTGGGAACATACCTTAGAGTTCGGAATAACAGCGGGAAACTGCTGCTAATACCGGATAT
TCTCTCCGGAGGAAAGATTTATCGCTCTAAGATTGGCCCGCGTCAGATTAGCTAGTTGGTGGGGTAAT
GGCCTACCAAGGCTCCGATCTGTAGCTGGTCTGAGAGGACGACCAGCCACACTGGAACTGAGACACGG
TC
>Zotu4287
GAACGCTGGCGGCGTGCCTAATACATGCAAGTCGAGCGAATGAATGGGAGCTTGCTCCCTGACATTAG
CGGCGGACGGGTGAGTAACACGTGGGCAACCTGCCCTGCAGATGGGGATAACTCCGGGAAACCGGGGC
TAATACCGAATAATCAGTTTGTCCGCATGGACAAACTCTGAAAGACGGTTTCGGCTGTCACTGCAGGA
TGGGCCCGCGGCGCATTAGCTAGTTGGTGGGGTAATGGCCTACCAAGGCAACGATGCGTAGCCGACCT
GAGAGGGTGATCGGCCACACTGGGACTGAGACACGGCC
>Zotu4288
GAACGCTGGCGGCGTGCCTAATACATGCAAGTCGAACGCATTTTCTTTCACCGTAGCTTGCTACACCG
AAAGAAAATGAGTGGCGAACGGGTGAGTAACACGTGGGCAACCTGCCCATCAGCGGGGGATAACACTT
GGAAACAGGTGCTAATACCGCATAATTCCATTTACCGCATGGTAGAGGGATGAAAGGCGCTTTTGCGT
CACTGATGGATGGACCCGCGGTGCATTAGCTAGTTGGTGGGGTAACGGCCTACCAAGGCTGCGATGCA
TAGCCGACCTGAGAGGGTGATCGGCCACACTGGGACTGAGACACGGCC
>Zotu4289
GAACGCTGGCGGCGTGCCTAACACATGCAAGTCAAGGATGTAGCAATACATACTGGCGCACGGGTGCG
GAACACGTAGGTAATCTGCCCTCTGGGGGGGAACAACCATCCGAAAGGATGGCTAAATCTCCGTAACG
TTGTAGCAAGGCATCTTGCAGCAACCAAGCTTTCCAGGGGATGAGCCTGCGTCTGATTAGCTAGTTGG
TGAGGTAACGGCTCACCAAGGCTACGATCAGTAGCTGGTCTGAGAGGATGATCAGCCACATTGGGACT
GAGATACGGCC
>Zotu4290
GAACGCTGGCGGCGTGCCTAATACATGCAAGTCGAGCGAACTGACGAGGAGCTTGCTCCTTTGACGTT
AGCGGCGGACGGGTGAGTAACACGTGGGTAACCTACCTATAAGACTGGAATAACTCCGGGAAACCGGG
GCTAATGCCGGATAACATGTTGAACCGCATGGTTCAACAGTGAAAGACGGTCTTGCTGTCACTTATAG

ATGGACCCGCGCCGCATTAGCTAGTTGGTGGGGTAATGGCCTACCAAGGCAACGATGCGTAGCCGACC
TGAGAGGGTGATCGGCCACACTGGAACTGAGACACGGTC
>Zotu4291
GAACGCTGGCGGCGTGCTTAACACATGCAAGTCGAACGATTGAAGCTGGAGCTTGCTCCGGCCGATTT
AGTGGCGGACGGGTGAGTAACGCGTGGGTAACCTGCCTCATACAGGGGGATAGCAGTTGGAAACGACT
GGTAAAACCGCATAAGCGCACGGTGTCGCATGACACAGTGTGAAAACACTCCGGTGGTATGAGATGGA
CCCGCGTCTGATTAGCTAGTTGGTGAGGTAACGGCCCACCAAGGCGACGATCAGTAGCCGGCCTGAGA
GGGTGAACGGCCACATTGGGACTGAGACACGGCC
>Zotu4292
CAACGCTGGCGGCGTGCCTAACACATGCAAGTCGAACGTGAAAGGGGCTTCGGCCCCGAGTAAAGTGG
CAAACGGGTGAGTAACGCGTGGGTGATCTGCCTCTGAGTGGGGGATAACCTTCCGAAAGGAGGGCTAA
TACCGCATGACATCCTGTTCTTGGATGAACGGTGATCGAAGCTGGGGACCGCAAGGCCTGGCGCTTGG
AGAGGAGCCCGCGTCCGATTAGCTAGTTGGTGGGGTAATGGCCTACCAAGGCGACGATCGGTAGCCGG
CCTGAGAGGGCGGACGGCCACACTGGGACTGAGACACGGCC
>Zotu4293
GAACGCTGGCGGCGTGGATAAGACATGCAAGTCGAACGGGATCATTGAGGTAGCAATATTTCGATGGT
CTAGTGGCGAACGGGTGCGTAACACGTGGATACATTCCGGGAAGCGGGGGATAGCCCAGGGAAACTTG
GATTAATACCGCATGTGGTCGAGAGACTAAAGGCAGGGACCGCAAGGCCTGCCACTTTCCGATTGGTC
CGCGGCCTATCAGCTAGTTGGCGGGGTAAAAGCCCACCAAGGCTACGACGGGTAGCTGGTCTTAGAGG
ACGACCAGCCACACTGGAACTGAGACACGGTC
>Zotu4294
GAACGCTGGCGGCGTGCCTAACACATGCAAGTCGAACGCGAAAGGGAGCAATCCCCGGTAGCGTGGCA
GACGGGTGAGTAACACGTGGGTAACCTACCCGGAGGTGGGGGATAACCCTTCGAAAGGAGGGCTAATA
CCGCATGACACCGCGGGGACCTCGGTCCTTGCGATCAAAGCTGGCCTCTGTTTACAAGCTAGCGCCAC
CGGATGGGCCCGCGCCCCATTAGCTAGTTGGTGAGGTAACGGCTCACCAAGGCGACGATGGGTAGCCG
GCCTGAGAGGGCGATCGGCCACACTGGAACTGCGACACGGTC
>Zotu4295
GAACGCTGGCGGCAGGCTTAACACATGCAAGTCGAGCGGACCTTTCGGGGTCAGCGGCGGACGGGTGA
GTAACGCGTGGGAACGTGCCTTCTGGTTCGGAATAACCCTGGGAAACTAGGGCTAATACTGGATACGC
CCTTATGGGGAAAGGTTTACTGCCGGAAGATCGGCCCGCGTCTGATTAGCTAGTTGGTGGGGTAACGG
CCTACCAAGGCGACGATCAGTAGCTGGTCTGAGAGGATGATCAGCCACACTGGGACTGAGACACGGCC
>Zotu4296
GAACGCTGGTAACGTGGATACCGGCATGCAAGTCGAGCGGCCAGCAATGGCAGCGGCGAACGGCTTAG
TAACACGTGGGTACCTACCCTAAACTGGAGGACAGCTCGTCGAAAGACGGGGTAATACTCCATAGTCT
CGCAAGAGTAAAGTCGCAAGACGGTTTAGGAAGGGCCTGCGGCCTATCAGATAGTTGGCAGGGTAACG
GCCTACCAAGTCTATGACGGGTAGGGGGCGTGAGAGCGCGACCCCCAAGAATGGCACTGCGACACGGG
C
>Zotu4298
GAACGCTGGCGGAATGCTTTACACATGCAAGTCGAACGGCAGCACGGGGGCAACCCTGGTGGCGAGTG
GCGAACGGGTGAGTAATACATCGGAACGTGCCCAGTCGTGGGGGATAACGTAGCGAAAGCTACGCTAA
TACCGCATACGAACTGAGGTTGAAAGCGGGGGATCGCAAGACCTCGCGCGATTGGAGCGGCCGATGGC
AGATTAGGTAGTTGGTGGGGTAAAGGCCTACCAAGCCGACGATCTGTAGCTGGTCTGAGAGGACGACC
AGCCACACTGGGACTGAGACACGGCC
>Zotu4299
GAACGCTAGCGGCAGGCTTAATACATGCAAGTCGAGGGGCAGCAGGTCTGTAGCAATACAGATGCTGG
CGACCGGCAAACGGGTGCGGAACACGTACGCAATCTACCCATAACTGATGAATAGCCCTCCGAAAGGA
GGATTAATACATCGTAACCTATTTGAGTGGCATCACTTGATTAGTATAGCTCCGGCGGTTTTGGATGA
GCGTGCGGCTGATTAGGTAGTTGGTTGAGGTAACGGCTCACCAAGCCTGCGATCAGTAACTGGTGTGA
GAGCACGACCAGTCACACGGGCACTGAGACACGGGC
>Zotu4300
GAACGCTGGCGGCATGCCTAACACATGCAAGTCGAACGAAGCCTTCGGGCTTAGTGGCGCACGGGTGC
GTAACGCGTGGGAATCTGCCCTTGGGTTCGGAATAACAGTTAGAAATGACTGCTAATACCGGATGATG
TCTTCGGACCAAAGATTTATCGCCCAGGGATGAGCCCGCGTAGGATTAGGTAGTTGGTGGGGTAATGG
CCTACCAAGCCGACGATCCTTAGCTGGTCTGAGAGGATGATCAGCCACACTGGGACTGAGACACGGCC

>Zotu4301
GAACGCTGGCGGCGTGCCTAATACATGCAAGTAGAACGCTGAAGCTTGGTGCTTGCACCGAGCGGATG
AGTTGCGAACGGGTGAGTAACGCGTAGGTAACCTGCCTGGTAGCGGGGGATAACTATTGGAAACGATA
GCTAATACCGCATGACAACAGTTATTGCATGATAACTGTTTAAAAGGGGCAATTGCTCCACTACCAGA
TGGACCTGCGTTGTATTAGCTAGTTGGTGAGGTAACGGCTCACCAAGGCGACGATACATAGCCGACCT
GAGAGGGTGATCGGCCACACTGGGACTGAGACACGGCC
>Zotu4302
GAACGCTGGCGGCGTGCCTAATACATGCAAGTCGAGCGGAACATTTATTGGTGCTTGCACCGATAAAT
GTTTAGCGGCGGACGGGTGAGTAACACGTGGGCAACCTGCCCTGTAGATTGGGATAACTCCGGGAAAC
CGGGGCTAATACCGAATAATACTTTTTAACTCATGTTAGAAAGTTGAAAGATGGTTCTGCTATCACTA
CAGGATGGGCCCGCGGCGCATTAGCTAGTTGGTGAGGTAACGGCTCACCAAGGCGACGATGCGTAGCC
GACCTGAGAGGGTGATCGGCCACACTGGGACTGAGACACGGCC
>Zotu4303
GAACGCTGGCGGCAGGCTTAACACATGCAAGTCGAGCGGGCCCTTCGGGGTCAGCGGCGGACGGGTTA
GTAACGCGTGGGAACGTGCCCTTTGCTTCGGAATAGCCCTGGGAAACTGGGGGTAATACCGGATGTGC
CCTGAGGGGGAAAGATTTATCGGCAAGGGATCGGCCCGCGTCTGATTAGGTAGTTGGTGTGGTAACGG
CGCACCAAGCCGACGATCGGTAGCTGGTTTGAGAGGATGATCAGCAACACTGGGACTGAGACACGGCC
>Zotu4304
GAACGCTGGCGGCAGGCTTAACACATGCAAGTCGAGCGGAGATGAGGTGCTTGCACCTTATCTTAGCG
GCGGACGGGTGAGTAATGCTTAGGAATCTGCCTATTAGTGGGGGACAACATTCCGAAAGGAATGCTAA
TACCGCATACGTCCTACGGGAGAAAGCAGGGAATCTTCAGACCTTGCGCTAATAGATGAGCCTAAGTC
GGATTAGCTAGTTGGTGGGGTAAAGGCCTACCAAGGCGACGATCTGTAGCGGGTCTGAGAGGATGATT
CGCCACACTGGGACTGAGACACGGCC
>Zotu4305
GAACGCTAGCGGCAGGCTTAATACATGCAAGTCGTGGGGCAGCAGGGTTGTAGCAATACAATCGCTGG
CGACCGGCAAACGGGTGCGTAACACGTACGCAACCTTCCCGTTACTGTGGAATAGCCCAGGGAAACTT
GGATTAATACCACATATTATATTGATGAGGCATCTCGTTGATATCAAAGCTTCGGCGGTAACGGATGG
GCGTGCGGTTGATTAGGTAGTTGGCGGGGTAACGGCCCACCAAGCCTACGATCAATAGCTGATGTGAG
AGCATGATCAGCCACACGGGCACTGAGACACGGGC
>Zotu4306
GAACGCTGGCGGCGTGCCTAATACATGCAAGTCGAGCGGACAGATGGGAGCTTGCTCCCTGATGTTAG
CGGCGGACGGGTGAGTAACACGTGGGTAACCTGCCTGTAAGACTGGGATAACTCCGGGAAACCGGGGC
TAATACCGGATGCTTGATTGAACCGCATGGTTCAATTATAAAAGGTGGCTTTTAGCTACCACTTACAG
ATGGACCCGCGGCGCATTAGCTAGTTGGTGAGGTAACGGCTCACCAAGGCGACGATGCGTAGCCGACC
TGAGAGGGTGATCGGCCACACTGGGACTGAGACACGGCC
>Zotu4307
GAACGCTGGCGGCATGCCTAACACATGCAAGTCGAACGAGACCTTCGGGTCTAGTGGCGCACGGGTGC
GTAACGCGTGGGAATCTGCCCCTTGGTTCGGAATAACAGCGAGAAATTGCTGCTAATACCGGATAATG
ACGTAAGTCCAAAGATTTATCGCCAAGGGATGAGCCCGCGTAGGATTAGCTAGTTGGTGTGGTAAAGG
CGCACCAAGGCGACGATCCTTAGCTGGTCTGAGAGGATGATCAGCCACACTGGGACTGAGACACGGCC
>Zotu4308
GAACGCTGGCGGCAGGCTTAACACATGCAAGTCGAGCGCCGTAGCAATACGGAGCGGCAGACGGGTGA
GTAACACGTGGGAACGTACCTTTTGGTTCGGAACAACTGAGGGAAACTTCAGCTAATACCGGATAAGC
CCTTACGGGGAAAGATTTATCGCCGAAAGATCGGCCCGCGTCTGATTAGCTAGTTGGTGAGGTAACGG
CTCACCAAGGCGACGATCAGTAGCTGGTCTGAGAGGATGATCAGCCACACTGGGACTGAGACACGGCC
>Zotu4310
GAACGCTGGCGGCGTGCTTAACACATGCAAGTCGAGCGGTAAGGCCTCTTCGGGGGTACACGAGCGGC
GAACGGGTGAGTAACACGTGAGTGATCTGCCCTTCACTCTGGGATAAGCCCGGGAAACTGGGTCTAAT
ACCGGATGTTCACGCGTCCGTGCATGCGGGTGTGTGGAAAGCCTTTTTGGCGGTGGGGGATGAGCTCG
CGGCCTATCAGCTTGTTGGTGGGGTGATGGCCTACCAAGGCTTTGACGGGTAGCCGGCCTGAGAGGGT
GACCGGCCACACTGGGACTGAGACACGGCC
>Zotu4311
GAACGCTGGCGGCATGCCTAACACATGCAAGTCGAACGAGACCTTCGGGTCTAGTGGCGCACGGGTGC
GTAACGCGTGGGAATCTGCCCTTAGGTTCGGGATAACTCAGGGAAACTTGAGCTAATACCGGATGACG

ACTTCGGTCCAAAGATTTATCGCCTAAGGATGAGCCCGCGTAGGATTAGCTTGTTGGTGAGGTAAAGG
CTCACCAAGGCGACGATCCTTAGCTGGTCTGAGAGGATGATCAGCCACACTGGGACTGAGACACGGCC
>Zotu4312
GAACGCTGGCGGCGTGCCTAATACATGCAAGTCGAACGCTTCACTTCGGTGAAGAGTGGCGAACGGGT
GAGTAATACATAAGTAACCTGGCATCTACAGGGGGATAACTGATGGAAACGTCAGCTAAGACCGCATA
GGTGTAGAGATCGCATGAACTCTATATGAAAAGTGCTACGGGACTGGTAGATGATGGACTTATGGCGC
ATTAGCTGGTTGGTAGGGTAACGGCCTACCAAGGCGACGATGCGTAGCCGACCTGAGAGGGTGACCGG
CCACACTGGGACTGAGACACGGCC
>Zotu4313
GAACGCTGGCGGCGTGCTTAACACATGCAAGTCGAACGGAAAGGCCCTGCTTGCAGGGTACTCGAGTG
GCGAACGGGTGAGTAACACGTGGGTGATCTGCCCTGTACTTTGGGATAAGCCTGGGAAACTGGGTCTA
ATACCGGATAGGACCACATCGTAGGGGTGTGGTGGAAAGATTTTTCGGTGCAGGATGAGCCCGCGGCC
TATCAGCTTGTTGGTGGGGTAATGGCCTACCAAGGCGTCGACGGGTAGCCGGCCTGAGAGGGTGTACG
GCCACATTGGGACTGAGACACGGCC
>Zotu4314
GAACGCTGGCGGCAGGCCTAACACATGCAAGTCGAACGCCCCGCAAGGGGAGTGGCAGACGGGTGAGT
AACACGTGGGAACGTACCCTTCGGTTCGGAATAACACAGAGAAATTTGTGCTAATACCGGATACGTCC
GAGAGGAGAAAGATTTATCGCCGAAGGATCGGCCCGCGTCTGATTAGCTTGTTGGTGAGGTAACGGCT
CACCAAGGCGACGATCAGTAGCTGGTCTGAGAGGATGATCAGCCACACTGGGACTGAGACACGGCC
>Zotu4315
GAACGCTGGCGGCGTGCATAACACATGCAAGTCGAACGATGAAACTTAGTAGACTTCTTCGGAAAGAC
ACTAAGCGGATTAGTGGCGAACGGGTGAGTAACGCGTGAGTAACCTGCCTTACACAAGGGGATAGCCT
TTGGAAACGAAGAATAATACCCTATAAAACCATAAAAGCACATGCTAGAATGGTCAAAGTGATAGCGG
TGTAAGATGGACTTGCGTCTGATTAGCTAGTTGGTGAGATAAAAGCTCACCAAGGCAACGATCAGTAG
CCGGCTTGAGAGAGTGTACGGCCACATTGGGACTGAGACACGGCC
>Zotu4317
GAACGCTGGCGGCGTGCCTAATACATGCAAGTCGAGCGGACTATCGGGAGCTTGCTCCTGATAGTTAG
CGGCGGACGGGTGAGTAACACGTGGGCAACCTGCCCTGCAGATGGGGATAACTCCGGGAAACCGGGGC
TAATACCGAATAATCAATACCTCCGCATGGAGGTATTCTAAAAGACGGTTTCGGCTGTCACTGCAGGA
TGGGCCCGCGGCGCATTAGCTTGTTGGTGGGGTAATGGCCTACCAAGGCGACGATGCGTAGCCGACCT
GAGAGGGTGATCGGCCACACTGGGACTGAGACACGGCC
>Zotu4318
GAACGCTGGCGGCGTGCCTAATACATGCAAGTCGAACGCATTTTCTTTCACCGTAGCTTGCTACACCG
AAAGAAAATGAGTGGCGAACGGGTGAGTAACACGTGGGTAACCTGCCCATCAGCGGGGGATAACACTT
GGAAACAGGTGCTAATACCGCATAATTCCATTTACTGCATGGTAGATGGATGAAAGGCGCTTTTGCGT
CACTGATGGATGGACCCGCGGTGCATTAGCTAGTTGGTGGGGTAACGGCCTACCAAGGCTGCGATGCA
TAGCCGACCTGAGAGGGTGATCGGCCACACTGGGACTGAGACACGACC
>Zotu4319
GAACGCTAGCGGCAGGCTTAATACATGCAAGTCGAACGGCAAGGACAATTTCGGTTGTCCCTAGAGTG
GCGCACGGGTGAGTAACGCGTACACAACTTGCCCTGTACTGGGGAATATCCTTGGGAAACTAAGGTTA
ATACCCCATAGTATTCCGAAATGGCATCGTTTTGGAATTAAAGCTTCGGCGGTACAGGATGGGTGTGC
GTATGATTAGGTAGTTGGTAGGGTAACGGCCTACCAAGCCGACGATCATTAACTGGCGTGAGAGCGCG
ACCAGTCACACTGGAACTGAGACACGGTC
>Zotu4320
GAACGCTGGCGGCGTGCCTAACACATGCAAGTCGAACGAGGTCCATCCTTCGGGAGAAGACCTAGTGG
CGAACGGGTGAGTAACACGTGAGGAACCTGCCCCGAAGACCGGGATAACTCTCCGAAAGGATAGCTAA
TACCGGATACCCCCACCGAGCCGCATGGTTTGGTGAGGAAATGGATTCCGCTTCGGGAGGGCCTCGCG
GCCTATCAGCTAGTTGGTGGGGTAACGGCCCACCAAGGCATCGACGGGTAGCTGGTCTGAGAGGACGA
TCAGCCACACTGGGACTGAGACACGGCC
>Zotu4322
GAACGCTGGCGGCAGGCCTAACACATGCAAGTCGAACGGTAGCACAGAGGAGCTTGCTCCTTGGGTGA
CGAGTGGCGGACGGGTGAGGAATACATCGGAATCTACCCTGTCGTGGGGGATAACGTAGGGAAATTTA
CGCTAATACCGCATACGAACTGAGGTTGAAAGCGGCGGACCGCAAGGCGTCGCGCGATTGGATGAGCC
GATGTCCGATTAGCTAGTTGGCGGGGTAATGGCCCACCAAGGCGACGATCGGTAGCTGGTCTGAGAGG

ATGATCAGCCACACTGGAACTGAGACACGGTC
>Zotu4325
GAACGCTAGCGGCAGGCCTAATACATGCAAGTCGAGCGGGTGTTAGCAATAACATTAGCGGCGGACGG
GTGAGTAACGCGTACACAACATGCCTTTGGTTGGGGGATAGCCAGTGGAAACGCTGAGTAATACCGCA
TAAGACTCCGGCACGGCATCGTGCAGAAGTTAAAGTTACGGCGACCAGAGATTGGTGTGCGTTCGATT
AGCTAGTTGGCGGGGTAACGGCCCACCAAGGCGACGATCGATAGGGGGTGTGAGAGCATGGCCCCCCA
CACGGGTACTGAGACACGGAC
>Zotu4326
GAACGCTGGCGGCGTGCTTAACACATGCAAGTCGAACGGTGACGGAGAGCTTGCTCTCCTGATCAGTG
GCGAACGGGTGAGTAACACGTGGGCAACCTGCCCCAGACTTCGGGATAACGCTTCGAAAGAGGTGCTA
ATACCGAATACGAACTCCCAAGGCATCTTGGGAGTTGGAAAGTTTTTCGGTCTGGGATGGGCCCGCGG
CCTATCAGCTTGTTGGTGAGGTAACGGCTCACCAAGGCGACGACGGGTAGCCGGCCTGAGAGGGCGAC
CGGCCACACTGGGACTGAGACACGGCC
>Zotu4327
GAACGCTGGCGGCGTGCTTAACACATGCAAGTCGAACGGGATCCAAAGGCTTTTTGTTTTTGGTGAGA
GTGGCGAACGGGTGAGTAACACGTGAGTAACCTGCCCCCTTCTTTTGGATAACCGCATGAAAGTGTGG
CTAATACAGGATATTCCAGGCTGGTCGCATGATTGGTTTGGGAAATGGTTTGGTCTGGTGGGGGATGG
GCTCGCGGCCTATCAGCTTGTTGGTGGGGTGATGGCCTACCAAGGCGGTGACGGGTAGCCGGCCTGAG
AGGGTGGGCGGTCACACTGGGACTGAGATACGGCC
>Zotu4328
GAACGCTGGCGGCGTGCTTAACACATGCAAGTCGAACGGTGAAAGAGGAGCTTGCTCCTCTGGATCAG
TGGCGAACGGGTGAGTAACACGTGAGTAACCTGCCCTTGACTCTGGGATAAGCGTTGGAAACGACGTC
TAATACCGGATATGAACCGCGAAGGCATCTTCTGCGGTTGGAAAGAATTTTGGTCAAGGATGGACTCG
CGGCCTATCAGCTAGTTGGTGAGGTAACGGCCCACCAAGGCGACGACGGGTAGCCGGCCTGAGAGGGT
GACCGGCCACACTGGGACTGAGACACGGCC
>Zotu4329
GAACGCTGGCGGCAGGCCTAACACATGCAAGTCGAACGGATCCTTCGGGATTAGTGGCAGACGGGTGA
GTAACACGTGGGAACGTGCCCTTTGGTTCGGAACAACTCAGGGAAACTTGAGCTAATACCGGATGTGC
CCTTCGGGGGAAAGATTTATCGCCATTGGAGCGGCCCGCGTCTGATTAGGTAGTTGGTGGGGTAAAGG
CCCACCAAGCCTACGATCAGTAGCTGGTCTGAGAGGATGATCAGCCACACTGGGACTGAGACACGGCC
>Zotu4330
GAACGCTGGCGGTATGCCTAACACATGCAAGTCGTACGAGAGTTTTTAACTCTAGTGGCGGACGGGTG
AGTAACACGTGAGAATTTGCCTTTAGGAGGGGGATAACAATTGGAAACGAATGCTAATACCCCATATG
CTTTTGAGTGAAATGGATTTATCCGCCTAAAGAGAAGCTCGCGGCTGATTAGCTTGTTGGTAAGGTAA
GAGCTTACCAAGGCGACGATCAGTATCTGGTTTGAGAGGACGATCAGACACACTGGAACTGAGACACG
GTC
>Zotu4331
GAACGCTGGCGGCGTGCCTAACACATGCAAGTCGAACGGGTGTAGCAATACATCAGTGGCGGACGGGT
GAGTATAGCATGGGTATCTGCCTTGTGGAGGGGAATAGCTGCGGGAAGCTGCAGGTAAGTCCGCGTAA
GCAAAGTGTACGAGAGTGCATTTTGGAAAGTATCGGGAACAGTAATGTCCGGTAGCCATGAGATGAGC
CCATGTCCCATTAGCTAGTTGGTAGGGTAAAGGCCTACCAAGGTAAAGATGGTTAGCTGGTCTGAGAG
GACGACCAGCCACACTGGAACTGAGATACGGTC
>Zotu4333
TAACGCTAGCGGCAGGCCTAATACATGCAAGTCGAACGGGATTTTGGGTAGCAATATCTGAATGAGAG
TGGCGCACGGGTGCGTAACGCGTATGCAACCTACCTTTATCTGGGGGATAGCCTTCCGAAAGGAAGAT
TAATACCGCATAAAGTATTGATGAGGCATTTCATTAATACCAAACCTCCGGGGGATAAAGATGGGCAT
GCGTCCGATTAGCTAGTTGGTGAGGTAACGGCTCACCAAGGCATCGATCGGTAGGGGGTCTGAGAGGA
TGATCCCCCACACTGGTACTGAGACACGGAC
>Zotu4334
GAACGCTGGCGGCGTGCCTAACACATGCAAGTCGAGCGGTAGCAGGCTTTCACTGTTTGCTTCTCTCT
CGTTGGTAATCAATGAGATCAAAGAGGAGTGAAGAGTGAAAGTGCTGACGAGCGGCGGACGGCTGAGT
AACACGTGGGAACGTGCCCCAAAGTGAGGGATAAGCTCCAGAAATGGAGTCTAATACCGCATATGGTC
TTCGGATTAAAGTATTTATACGCTTTGGGAGCGGCCTGCGTACGATTAGATAGTTGGTGAGGTAAAGG
CTCACCAAGTCTACGATCGTTAACTGGTCTGAGAGGATGATCAGTCAGACTGGAACTGAGACACGGTC

>Zotu4335
GAACGCTGGCGGCATGCCTAACACATGCAAGTCGAACGAAGGCTTCGGCCTTAGTGGCGCACGGGTGC
GTAACGCGTGGGAATCTGCCTTGGGGTTCGGAATAACTCCCCGAAAGGGGTGCTAATACCGGATGATG
TCGAAAGACCAAAGATTTATCGCCCTGAGATGAGCCCGCGTAAGATTAGCTAGTTGGTGGGGTAAAGG
CCTACCAAGGCGACGATCTTTAGCTGGTCTGAGAGGATGATCAGCCACACTGGGACTGAGACACGGCC
>Zotu4336
GAACGCTGGCGGCATGCCTAACACATGCAAGTCGAACGGACTAATTGGGGCTTGCTCCAATTAGTTAG
TGGCAGACGGGTGAGTAACATGTGGGAATCTACCCATCAGTACGGAATAACTTTTAGAAATAAAAGCT
AATACCGTATATTCTCTACGGAGGAAAGATTTATCGCTGATGGATGAGCCCGCGTCAGATTAGGTAGT
TGGTGAGGTAATGGCTCACCAAGCCGACGATCTGTAGCTGGTCTGAGAGGATGACCAGCCACACTGGG
ACTGAGACACGTCC
>Zotu4337
GAACGCTGGCGGCGTGCTTAACACATGCAAGTCGAGCGGTAAGGCCCTTCGGGGTACACGAGCGGCGA
ACGGGTGAGTAACACGTGAGCAATCTGCCCTTCACTCTGGGATAGCCTCGGGAAACTGAGATTAATAC
CGGATACGACCACTTCAGGCATCTGATGGTGGTGGAAAGTTCCGGCGGTGAAGGATGAGCTCGCGGCC
TATCAGCTTGTTGGCGGGGTAATGGCCCACCAAGGCTTCGACGGGTAGCCGGCCTGAGAGGGTGACCG
GCCACACTGGGACTGAGACACGGCC
>Zotu4338
GAACGCTGGCGGCGTGCCTAATACATGCAAGTCGAGCGAACCTGCAGGAGCTTGCTCCTGTTGGTTAG
CGGCGGACGGGTGAGTAACACGTGGGCAACCTGCCTGTAAGACTGGGATAACACCGGGAAACCGGTGC
TAATACCGGATAATCCTTTTCCTCTCATGAGGAAAAGCTGAAAGTCGGTGCAAGCTGACACTTACAGA
TGGGCCCGCGGCGCATTAGCTAGTTGGTGAGGTAACGGCTCACCAAGGCGACGATGCGTAGCCGACCT
GAGAGGGTGATCGGCCACACTGGGACTGAGACACGGCC
>Zotu4339
GAACGCTGGCGGCGTGCCTAATACATGCAAGTCGAGCGAACCTCAAGAAGCTTGCTTCTTGAGGTTAG
CGGCGGACGGGTGAGTAACACGTGGGCAACCTGCCTATAAGACTGGGATAACTTCGGGAAACCGGAGC
TAATACCGGATAATTCTTCTTTACACATGTAGAGAAGCTGAAAGGCGGCTTCGGCTGTCACTTATAGA
TGGGCCCGCGGCGCATTAGCTAGTTGGTGAGGTAACGGCTCACCAAGGCAACGATGCGTAGCCGACCT
GAGAGGGTGATCGGCCACACTGGGACTGAGACACGGCC
>Zotu4340
GAACGCTGGCGGCGTGCCTAATACATGCAAGTCGTGCGGACTTATCTTCGGATAAGTTAGCGGCGGAC
GGGTGAGTAACACGTGGGCAACCTGCCTGTAAGACTGGGATAACTCCGGGAAACCGGTGCTAATACTA
GATACAATCAAAGACTGCCTGGTTTTTGATGGAAAGGTCTTGTACCGCTTACAGATGGGCCCGCGGCG
CATTAGCTAGTTGGTGGGGTAATGGCTCACCAAGGCGACGATGCGTAGCCGACCTGAGAGGGTGATCG
GCCACACTGGGACTGAGACACGGCC
>Zotu4341
CAACGCTGGCGGCGTGCCTAATGCATGCAAGTCGAGCGGGGTGCCTTTCGGGGCACTTACGCGGCGGA
CGGGTGCGGAACACGTGGGCAACCTGCCCCGAGGTGGGGGATAGCCGGTGGAAACACCGGGTAATTCC
GCATGAGGTCACTGGCTGGAGGGCTGGTGAGGAAAGGGTCTTTGACCCGCCTTGGGAGGGGCCTGCGG
CCGATTAGCTAGACGGTGGGGTAACGGCCTACCGTGGCGATGATCGGTAGCTGGTCTGAGAGGACGGC
CAGCCACACGGGGACTGAGACACGGCC
>Zotu4342
GAACGCTAGCGGCAGGCCTAATACATGCAAGTTGAGCGAGAATCTATCTTCGGGTAGAGGAAAGCGGC
GCACGGGTGAGTAACGCGTACGAAATCTGGCCTAAGGTGAGGGATACCTCAGGGAAACTTGAGACAAT
ACCACATGTGATTGCGACTCGGCATCGGGATGCAATGAAAGCAGTGATGCGCCTTAGGATGATCGTGC
GTACGATTAGATAGTTGGTAAGGTAACGGCTTACCAAGTCTACGATCGTTAGGGGGCGTGAGAGCGTG
GCCCCCCACACGGGTACTGAGACACGGAC
>Zotu4343
GAACGCTAGCGGCAGGCTTAACACATGCAAGTCGAGGGGTATAAGGAGCTTGCTCCTTAGAGACCGGC
GCACGGGTGCGTAACGCGTATGCAACCTACCTTGAACAGGGGAATAGCCCAGAGAAATTTGGATTAAT
GCCCCATGGTATTCTGAGATGGCATCATCTTATTATTAAAGATTCATCGGTTCAAGATGGGCATGCGT
CCCATTAGCTTGTTGGTGAGGTAACGGCTCACCAAGGCGATGATGGGTAGGGGTCCTGAGAGGGAGAT
CCCCCACACTGGTACTGAGACACGGAC
>Zotu4344

GAACGCTAGCGGCATGCCTAACACATGCAAGTCGAACGGCAGCATGTTACTGGGTAACCAGTAATGAT
GGCGAGTGGCGGACGGGTGAGTAACGCGTAGGAATCTGCCTCGTAGTGGGGGATAACCCGGGGAAACT
CGGGCTAATACCGCATATTTTCTTCGGAACAAAGGAAGCCTCTATTTATAAGCTTTCGCTATGAGATG
AGCCTGCGTTGGATTAGCTAGTTGGTGGGGTAAAGGCCTACCAAGGCAACGATCCATAGCTGGTCTGA
GAGGACGATCAGCCACACTGGGACTGAGACACGGCC
>Zotu4346
GAACGCTGGCGGCATGCTTAACACATGCAAGTCGCACGAACCTTTCGGGGTTAGTGGCGGACGGGTGA
GTAACGCGTAGGGATCTGTCCATGGGTGGGGGATAACACTGGGAAACTGGTGCTAATACCGCATGACA
CCTGAGGGTCAAAGGCGAGAGTCGCCTGTGGAGGAACCTGCGTTCGATTAGCTAGTTGGTGGGGTAAC
TGCCTACCAAGGCGATGATCGATAGCTGGTCTGAGAGGATGATCAGCCACACTGGGACTGAGACACGG
CC
>Zotu4347
GAACGCTGGCGGCGTGCCTAATACATGCAAGTCGAACGAACCGCGACTGAGTGCTTGCACTTGGTCAA
GGTGAGTGGCGAACGGGTGAGTAACACGTGGGTAACCTACCTCATAGTGGGGGATAACAGTCGGAAAC
GACTGCTAATACCGCATAGGACATGGAATCACATGATTCCGTGAGGAAAGGTGGCGCAAGCTATCGCT
AAGAGATGGACCCGCGGTGCATTAGCTAGTTGGTAGGGTAACGGCCTACCAAGGCGATGATGCATAGC
CGACCTGAGAGGGTGATCGGCCACATTGGGACTGAGACACGGCC
>Zotu4350
GAACGCTGGCGGCAGGCTTAACACATGCAAGTCGAGCGGGGAGATGTAGCTTGCTACATTTCCTAGCG
GCGGACGGGTGAGTAATGCTTAGGAATCTGCCTATTAGTGGGGGACAACATTCCGAAAGGAATGCTAA
TACCGCATACGCCCTACGAGGGAAAGCAGGGGATCTTCGGACCTTGCGCTAATAGATGAGCCTAAGTC
AGATTAGCTAGTTGGTGGGGTAAAGGCCTACCAAGGCGACGATCTGTAGCGGGTCTGAGAGGATGATC
CGCCACACTGGGACTGAGACACGGCC
>Zotu4351
GAACGCTGGCGGCATGCCTTACACATGCAAGTCGAACGCGAAAGAGGGGCAACCCTTGAGTAGAGTGG
CGAACGGGTGAGTAACGCATCGGAACGTATCCAGTAGTGGGGGATAACCCATCGAAAGATGGGCTAAT
ACCGCATACGCCCTGAGGGGGAAAGTGGGGGATCCGCAAGGACCTCACGTTATTGGAGCGGCCGATGT
CCGATTAGCTAGTTGGCGGGGTAAGAGCCCACCAAGGCGACGATCGGTAGCTGGTCTGAGAGGATGAT
CAGCCACACTGGGACTGAGACACGGCC
>Zotu4352
GAACGCTAGCGGCAGGCTTAACACATGCAAGTCGAGGGGCATCGGGGATAGCAATATCCGCCGGCGAC
CGGCGAAAGGGTGCGTAACACGTGAGCAACATTCCCGTATCTGGGAGATAACCGATGGAAACGTCGAC
TAACATCCCATAACAACCATGAAGGCATCTTCGAGGTTTGAAAACTCCGGTGGATACGGATTGGCTCG
CGTCCCATTAGCTAGTTGGCGTGGTAACGGCACACCAAGGCGACGATGGGTAGGGGTTCTGAGAGGAA
GGTCCCCCACATTGGAACTGAGACACGGTC
>Zotu4353
GAACGCTGGCGGCATGCTTTACACATGCAAGTCGAACGGCAGCATGGGCTTCGGCCTGATGGCGAGTG
GCGAACGGGTGAGTAATACATCGGAACGTGCCTAGTAGTGGGGGATAACTACTCGAAAGAGTAGCTAA
TACCGCATGAGATCTAAGGATGAAAGCAGGGGACCTTCGGGCCTTGTGCTACTAGAGCGGCCGATGGC
AGATTAGGTAGTTGGTGGGATAAAAGCTTACCAAGCCGACGATCTGTAGCTGGTCTGAGAGGACGACC
AGCCACACTGGGACTGAGACACGGCC
>Zotu4354
GAACGCTAGCGGCAGGCCTAATACATGCAAGTCGAACGGTGGCAGCAATGCCATAGTGGCGCACGGGT
GCGTAACGCGTAACCAACCTACCCAGAACTGGGGGATAGCCCGCCGAAAGGCGGATTAATACCGCATA
AGCTCTGCTTCCGGCATCGGAAGCTTAGTAAAGATTTATTGGTTTTGGATGGGGTTGCGTGCCATTAG
CTAGTTGGTGGGGTAACGGCCCACCAAGGCGACGATGGCTAGGGGAGCTGAGAGGCTGGTCCCCCACA
CGGGCACTGAGATACGGGC
>Zotu4355
GAACGCTGGCGGCAGGCTTAACACATGCAAGTCGAACGATGAAACTCTAGCTTGCTAGAGAAGATTAG
TGGCGGACGGGTGAGTAACATTTAGGAATCTGCCTAGTAGTGGGGGATAGCTCGGGGAAACTCGAATT
AATACCGCATACGACCTACGGGTGAAAGGGGGCGCAAGCTCTTGCTATTAGATGAGCCTAAATCAGAT
TAGCTAGTTGGTGGGGTAAAGGCCCACCAAGGCGACGATCTGTAACTGGTCTGAGAGGATGATCAGTC
ACACCGGAACTGAGACACGGTC
>Zotu4357

GAACGCTAGCGGCAGGCTTAATACATGCAAGTCGAACGGTTACAGCAATGTGACGAGTGGCGCACGGG
TGAGTAACGCGTACATAACATACCTTTAACTGGCGAATAGCCTTGGGAAACTGAGAGTAATACGCCAT
AACATTGTGATTCGGCATCGGATTACAATTAAAGTTCCGGCGGTTAGAGATTGATGTGCGTTGGATTA
GGTAGTTGGTGAGGTAACGGCTCACCAAGCCAGCGATCCATAGGGGGCGTGAGAGCGTGGTCCCCCAC
ACGGGTACTGAGACACGGAC
>Zotu4359
GAACGCTGGCGGCGTGCCTAATACATGCAAGTCGAGCGAATCTTTGGGAGCTTGCTCCTAAAGGTTAG
CGGCGGACGGGTGAGTAACACGTGGGCAACCTGCCTATAAGACTGGGATAACTTCGGGAAACCGGAGC
TAATACCGGATAATTCTTTTCTACACATGTAGAAAAGCTAAAAGACGGTTTACGCTGTCACTTATAGA
TGGGCCCGCGGCGCATTAGCTAGTTGGTGAGGTAACGGCTCACCAAGGCCACGATGCGTAGCCGACCT
GAGAGGGTGATCGGCCACACTGGGACTGAGACACGGCC
>Zotu4360
GAACGCTAGCGGCAGGCTTAACACATGCAAGTCGAGGGGTATAGTTCTTCGGAGCTAGAGACCGGCGC
ACGGGTGCGTAACGCGTATGCAATCTACCTTTTACAGAGGGATAGCCCAGAGAAATTTGGATTAATAC
CTCATAGTATAGTACAATCGCATGATTGAACTATTAAAGTCACAACGGTAAAAGATGAGCATGCGTCC
CATTAGCTTGTTGGTAAGGTAACGGCTTACCAAGGCTACGATGGGTAGGGGTCCTGAGAGGGAGATCC
CCCACACTGGTACTGAGACACGGAC
>Zotu4363
GAACGCTAGCTACAGGCTTAACACATGCAAGTCGTGCGGTGCGCTTTCGGGCGCACAGCGGCAAACGG
GTGAGTAACACGTATCCAACCTGGCCCTTACTTGGGGATAGCCCCTCGAAAGAGAGATTAATACCCGA
TGTTCTGGAAGTCCCACATGCCACTTCCAGCAAAGCTCCGGCGGTAAGGGATGGGGATGCGCTCCATT
AGGTAGTAGGCGGGGTAACGGCCCACCTAGCCGATGATGGATAGGGGTTCTGAGAGGAAGGTCCCCCA
CATTGGAACTGCGACACGGTC
>Zotu4365
GAACGCTGGCGGCGTGCTTAACACATGCAAGTCGAGCGAAGCACTTCCTTCGGGGAGTGACTGAGCGG
CGGACGGGTGAGTAACGCGTGGGTAACCTGCCTCATACAGGGGGATAACAGTTAGAAATGACTGCTAA
TACCGCATAAGCGCACAGAGTCGCATGACTCAGTGTGAAAAACTCCGGTGGTATGAGATGGACCCGCG
TTGGATTAGGCAGTTGGCGGGGTAACGGCCCACCAAACCGACGATCCATAGCCGGCCTGAGAGGGTGA
ACGGCCACATTGGGACTGAGACACGGCC
>Zotu4366
GAACGCTGGCGGCGTGCCTAATACATGCAAGTCGAGCGGATCTCTTCGGAGATCAGCGGCGGACGGGT
GAGTAACACGTGGGCAACCTGCCTATAAGACTGGGATAACTTCGGGAAACCGGAGCTAATACCGGATA
ACATCAAGGGCCACATGGTCTTCGATTAAAAGATGGCGTCAAGCTATCACTTATAGATGGGCCCGCGT
CGCATTAGCTAGTTGGTGAGGTAACGGCTCACCAAGGCGACGATGCGTAGCCGACCTGAGAGGGTGAT
CGGCCACACTGGGACTGAGACACGGCC
>Zotu4367
GAACGCTGGCGGCATGCCTAACACATGCAAGTCGAACGAAGGCTTCGGCCTTAGTGGCGCACGGGTGC
GTAACGCGTGGGAATCTGCCCCTTGGTTCGGAATAACAGTTGGAAACGACTGCTAATACCGGATGACG
ACGTAAGTCCAAAGATTTATCGCCGAGGGATGAGCCCGCGTAGGATTAGCTAGTTGGTGTGGTAAGAG
CGCACCAAGGCGACGATCCTTAGCTGGTCTGAGAGGATGATCAGCCACACTGGGACTGAGACACGGCC
>Zotu4368
GAACGCTGGCGGCATGCCTTACACATGCAAGTCGAACGGCAGCGCGGGGCAACCTGGCGGCGAGTGGC
GAACGGGTGAGTAATATATCGGAACGTGCCCTAGAGTGGGGGATAACTAGTCGAAAGACTAGCTAATA
CCGCATACGATCTACGGATGAAAGTGGGGGATCGCAAGACCTCATGCTCCTGGAGCGGCCGATATCTG
ATTAGCTAGTTGGTGGGGTAAAAGCTCACCAAGGCGACGATCAGTAGCTGGTCTGAGAGGACGACCAG
CCACACTGGGACTGAGACACGGCC
>Zotu4370
GAACGCTGGCGGCGTGCCTAATACATGCAAGTCGAGCGAATCAAAGGGAGCTTGCTCCCTTTTGGTTA
GCGGCGGACGGGTGAGTAACACGTGGGCAACCTGCCTATAAGACTGGGATAACTTCGGGAAACCGGAG
CTAATACCGGATAATTCATATCTACTCATGTAGGTATGCTGAAAGACGGTTTCGGCTGTCACTTATAG
ATGGGCCCGCGGCGCATTAGCTAGTTGGTGAGGTAACGGCTCACCAAGGCAACGATGCGTAGCCGACC
TGAGAGGGTGATCGGCCACACTGGGACTGAGACACGGCC
>Zotu4371
GAACGCTGGCGGCGCGCCTAACACATGCAAGTCGAGCGATGAAGAGAAGACAGAAGCCCTCGGGCAGA

CGTCGTCTTGGATTAGCGGCGGACGGGTGAGTAACAGGTGAGTAACCTACCTTTGACACAGGGATAGC
CTCGGGAAACCGGGATTAATACCGGATGACATTACACTTTCGCATGAAAGAGTAATCAAAGAACTTCG
GTCAAAGATGGACTCGCCGCTGATTAGCTAGTTGGTGAGATAACAGCCCACCAAGGCAACGATCAGTA
GCCGGCCTGAGAGGGTGAACGGCCACATTGGAACTGAGACACGGTC
>Zotu4374
GAACGCTAGCGGCAGGCCTAATACATGCAAGTCGAACGGGATTGGGGAGCTTGCTCCCCATGAGAGTG
GCGCACGGGTGCGTAACACGTATGCAACCTACCTTTACCAGGGGGATAGCCTCTCGAAAGAGAGATTA
ACACCGCATAACATTGAGCTATGGCATCATAGTTTAATCAAATATTTATAGGGTAAAGATGGGCATGC
GGAACATTAGCTAGTTGGTGAGGTAACGGCTCACCAAGGCGACGATGTTTAGGGGATCTGAGAGGATG
ACCCCCCACACTGGTACTGAGACACGGAC
>Zotu4375
GAACGCTGGCGGCGTGCTTAACACATGCAAGTCGAACGATGATGCCCAGCTTGCTGGGCGGATTAGTG
GCGAACGGGTGAGTAATACGTGAGTAACCTGCCCTTGACTCTGGGATAAGCCTGGGAAACCGGGTCTA
ATACTGGATACTACCGTCCACCGCATGGTGGGTGGTGGAAAGGGTTTTACTGGTTTTGGATGGGCTCA
CGGCCTATCAGCTTGTTGGTGGGGTAATGGCTCACCAAGGCGACGACGGGTAGCCGGCCTGAGAGGGT
GACCGGCCACACTGGGACTGAGACACGGCC
>Zotu4376
GAACGCTAGCGACAGGCTTAACACATGCAAGTCGAGGGGCAGCATTTTATTAGCTTGCTAATAAAGAT
GGCGACCGGCGCACGGGTGAGTAACGCGTATGCAACCTACCTATCAGAGGGGGATAGCCCATTGAAAG
ATGGATTAATACCGCATAAAGCAGGGGTCCCGCATGGGAATATTTGCTAAAGATTTATTGCTGATAGA
TGGGCATGCGTTCCATTAGTTAGTTGGTAAGGTAACGGCTTACCAAGACATCGATGGATAGGGGTTCT
GAGAGGAAGGTCCCCCACACTGGTACTGAGACACGGAC
>Zotu4377
GAACGCTGGCGGCGTGCCTAATACATGCAAGTCGAGCGAATGAAGAGAGAAGCTTGCTTCTCTCGGAT
TTAGCGGCGGACGGGTGAGTAACACGTGGGTAACCTACCTTATAGTTTGGGATAACTCCGGGAAACCG
GGGCTAATACCGAATAATACTTTTAAACGCATGTTTGAAAGTTGAAAGACGGTCTCGGCTGTCACTAT
AAGATGGACCCGCGGCGCATTAGCTAGTTGGTGAGGTAATGGCTCACCAAGGCCACGATGCGTAGCCG
ACCTGAGAGGGTGATCGGCCACACTGGGACTGAGACACGGCC
>Zotu4378
GAACGCTGGCGGCGTGCTTAACACATGCAAGTCGAGCGGAAAGGCCACTTCGGTGGTACTCGAGCGGC
GAACGGGTGAGTAACACGTGAGTAATCTGCCCCCCACTTTGGGATAGCCACCGGAAACGGTGATTAAT
ACCGGATATGACCCTTGACCGCATGGTTGTGGGTGGAAAGTTTTTCGGTGGGGGATGTGCTCGCGGCC
TATCAGCTTGATGGTGGGGTAATGGCCTACCATGGCTTCGACGGGTAGCCGGCCTGAGAGGGTGACCG
GCCACACTGGGACTGAGACACGGCC
>Zotu4379
GAACGCTGGCGGCATGCCTTACACATGCAAGTCGAACGGCAGCGCAGGAGCAATCCTGGCGGCGAGTG
GCGAACGGGTAAGTAATACATCGGAACGTGCCCAATCGTGGGGGATAACGCAGCGAAAGCTGTGCTAA
TACCGCATACGATCTACAGATGAAAGCAGGGGATCGCAAGACCTTGCGCGAATGGAGCGGCCGATGGC
AGATTAGGTAGTTGGTGAGGTAAAGGCTCACCAAGCCTTCGATCTGTAGCTGGTCTGAGAGGACGACC
AGCCACACTGGGACTGAGACACGGCC
>Zotu4382
GAACGCTGGCGGCGTGCCTAATACATGCAAGTCGAGCGCGTGTAGCAATACACGGGCGGCGGACGGGT
GAGTAACGCGTGGGCAACCTGCCCTCTAGCCTGGGATAACAGCGCGAAAGTGCTGCTAAAACCGGATA
TGCTCACAAGGAGGCATCTCCTGGTGAGGAAAGTTTACACTAGAGGATGGGCCCGCGTCCCATTAGCT
AGTTGGTGAGGTAACGGCCCACCAAGGCGACGATGGGTAGCCGACCTGAGAGGGTGATCGGCCACACT
GGGACTGAGACACGGCC
>Zotu4384
GAACGCTATCGGTATGCTTAACACATGCAAGTCGAACGGCAGCACAGTAAAGATTTCGGTCTTTACGG
GTGGCGAGTGGCGGACGGGTGAGTAAAGCGTAGGAATCTGTCCTGGAGAGTGGGATAACCCGAGGAAA
CTCGGGCTAATACCGCATGATATCGAGAGATCAAAGCAGGGGACCCTTCGGGGCCTTGCGCTCTAGGA
TGAGCCTACGTCGGATTAGCTAGTTGGTGGGGTAAAGGCCTACCAAGGCGACGATCCGTAGCTGGTCT
GAGAGGATGACCAGCCACACTGGGACTGAGACACGTCC
>Zotu4385
GAACGCTGGCGGCGTGCTTAACACATGCAAGTCGAGCGGAAAGGCCCTTCGGGGTACTCGAGCGGCGA

ACGGGTGAGTAACACGTGAGTAACCTGCCCTTGGCTCTGGGATAAGCCCGGGAAACTGGGTCTAATAC
CGGATATGACTCTCCACCGCATGGTGGAGGGTGGAAAGTTTTTCGGCTGGGGATGGACTCGCGGTCTA
TCAGCTTGTTGGTGGGGTAACGGCCTACCAAGGCGACGACGGATAGCCGGCCTGAGAGGGTGACCGGC
CACACTGGGACTGAGACACGGCC
>Zotu4386
GAACGCTAGCTGCAGGCTTAACACATGCAAGTCGAGGGGCAGCGGGAGTAGCAATACTTGCCGGCGAC
CGGCGGAAGGGTGCGTAACGCGTGAGCAACATTCCCGTATCAGGGGGATAACCGGTGGAAACGCCGAC
TAATACCCCATAAGAACATCTGCGGCATCAAAGGTGTTTGAAAGATTCGTCGGATGCGGATTGGCTCG
CGTGACATTAGCTAGTTGGTGAGGTAACGGCCCACCAAGGCGACGATGTCTAGGGGTTCTGAGAGGAA
GATCCCCCACATTGGAACTGAGACACGGTC
>Zotu4387
GAACGCTAGCGGCAGGCCTAATACATGCAAGTCGAGGGGCAGCACGGGTAGCAATACTTGGTGGCGAC
CGGCGCACGGGTGCGTAACGCGTATGCAACTTACCCATTACTGGGGGATAGCCCGGGGAAACTCGGAT
TAATACCCCATAGTATCTTAATGCCGCATGGTATAAAGATTAAAGATTTATTGGTAATGGATGGGCAT
GCGTGTCATTAGTTAGTTGGTGAGGTAATGGCTCACCAAGACTTCGATGACTAGGGGGCCTGAGAGGG
TGATCCCCCACACTGGTACTGAGATACGGAC
>Zotu4388
GAACGCTGGCGGCGTGGATGAGGCATGCAAGTCGAATGGGCTTAGACCCATGGCAGACGAGGTAGGAA
CACGTAGGTACATACCCTAAAGTCACGGATAACCCGGCGAAAGTCGGAATAATACGTGATAGTCCCTT
CGGGGTAAAGATTTATCGCTTTAGGAATGGCCTGCGTTCTATCAGCTTGTTGGTAAGGTAACGGCTTA
CCAAGGCTACGACGGATAGGGGAGGTGAGAGCCTGACCCCCACCGATGGAACTGCGACACGGTC
>Zotu4389
GAACGCTGGCGGCGTGCTTAACACATGCAAGTCGAACGGTGATCCCAAGAGCTTGCTTTTGGGTGAGC
AGTGGCGAACGGGTGAGTAACACGTGAGTAACCTGCCCCTGACTCTGGAATAAGCGCTGGAAACGGCG
TCTAATACCGGATATGACACCAATCCGCATGGGTATGGTGTGGAAAGTTTTTCGGTTGGGGATGGACT
CGCGGCCTATCAGCTTGTTGGTGAGGTAATGGCTCACCAAGGCGATGACGGGTAGCCGGCCTGAGAGG
GCGATCGGCCACACTGGGACTGAGACACGGCC
>Zotu4390
GAACGCTGGCGGCGTGCCTAATACATGCAAGTCGAGCGGACTTTAAAAGCTTGCTTTTAAAGTTAGCG
GCGGACGGGTGAGTAACACGTGGGCAACCTGCCTGTAAGACTGGGATAACTTCGGGAAACCGGAGCTA
ATACCGGATAATCCTTTTCCTCTCATGAGGAAAAGCTGAAAGACGGTTTACGCTGTCACTTACAGATG
GGCCCGCGGCGCATTAGCTAGTTGGTGAGGTAACGGCTCACCAAGGCGACGATGCGTAGCCGACCTGA
GAGGGTGATCGGCCACACTGGGACTGAGACACGGCC
>Zotu4391
GAACGCTAGCGGGAGGCCTAACACATGCAAGCCGAGCGGTATTTGTTCTTCGGAACAGAGAGAGCGGC
GTACGGGTGCGGAACACGTGTGCAACCTGCCTTTATCAGGGGGATAGCCTTTCGAAAGGAAGATTAAT
ACCCCATAATATATTAAGTGGCATCACTTGATATTGAAAACTCCGGTGGATAGAGATGGGCACGCGCA
AGATTAGATAGTTGGTAGGGTAACGGCCTACCAAGTCAGTGATCTTTAGGGGGCCTGAGAGGGTGATC
CCCCACACTGGTACTGAGACACGGAC
>Zotu4392
GAACGCTGGCGGCGTGCTTAACACATGCAAGTCGAGCGAAGCAGTTAAGAAGATTCTTCGGATGATTC
TTAACTGACTGAGCGGCGGACGGGTGAGTAACGCGTGGGTGACCTGCCCCATACCGGGGGATAACAGC
TGGAAACGGCTGCTAATACCGCATAAGCGCACAGAGCTGCATGGCTCGGTGTGAAAAACTCCGGTGGT
ATGGGATGGGCCCGCGTCTGATTAGGCAGTTGGCGGGGTAACGGCCCACCAAACCGACGATCAGTAGC
CGGCCTGAGAGGGCGACCGGCCACATTGGGACTGAGACACGGCC
>Zotu4393
GAACGCTGGCGGCGTGCCTAATACATGCAAGTCGAGCGGTAACAGACCTTCGGGTGCTGACGAGCGGC
GGACGGGTGAGTAACGCGTGGGAATCTACCCCAGACTGAGGGATAACTCCGAGAAATCGGGGCTAATA
CCGCATACGGTCTTCGGATTAAAGCTTCGGCGGTTTGGGAAGAGCCCGCGTAAGATTAGCTAGTTGGT
AAGGTAATGGCTTACCAAGGCAACGATCTTTAACTGGTTTGAGAGGATGATCAGTCACACTGGAACTG
AGACACGGTC
>Zotu4394
GAACGCTGGCGGCGTGCTTAACACATGCAAGTCGAACGGAAAGGCCTCTGCTTGCAGAGGTACTCGAG
TGGCGAACGGGTGAGTAACACGTGGGTGATCTGCCCTGCACTTCGGGATAAGCCTGGGAAACTGGGTC

TAATACCGGATAGGACGATGGTTTGGATGCCATTGTGGAAAGTTTTTCGGTGTGGGATGAGCTCGCGG
CCTATCAGCTTGTTGGTGGGGTAATGGCCTACCAAGGCGTCGACGGGTAGCCGGCCTGAGAGGGTGTA
CGGCCACATTGGGACTGAGATACGGCC
>Zotu4395
GAACGCTGGCGGCGTGCCTAATACATGCAAGTCGAGCGGACTTTTAAAAGCTTGCTTTTAAAAGTTAG
CGGCGGACGGGTGAGTAACACGTGGGTAACCTGCCTGTAAGACTGGGATAACTCCGGGAAACCGGGGC
TAATACCGGATAACTTTTTTCCTCGCATGAGGAAAAATTGAAAGATGGCTCCGGCTATCACTTACAGA
TGGACCCGCGGCGCATTAGCTAGTTGGTGAGGTAACGGCTCACCAAGGCAACGATGCGTAGCCGACCT
GAGAGGGTGATCGGCCACACTGGGACTGAGACACGGCC
>Zotu4396
GAACGCTGGCGGCGTGCTTAACACATGCAAGTCGAACGGTGAACCCTCTTCGGAGGGGGATCAGTGGC
GAACGGGTGAGTAACACGTGGGCAACCTGCCCTCAGCTCTGGGATAACTCCGGGAAACCGGGGCTAAT
ACCGGATACGACTTCCTGTGGCATCTCGGGAAGTGGAAAGTTTTTCGGCTGAGGATGGGCCCGCGGCC
TATCAGCTTGTTGGTGGGGTAATGGCCCACCAAGGCGACGACGGGTAGCCGGCCTGAGAGGGCGATCG
GCCACACTGGGACTGAGACACGGCC
>Zotu4397
TAACGTTGGCGGCGTGCGTAATACATGCAAGTCACACGGGATTGGGGCAACTCAATTCAGTGGCGAAC
GGGTGAGTAACACGTGGATGATCTACCCTTAAGATCGGGATAACTAGCCGAAAGGTTAGCTAATACCG
GGTATGACCACGGTCTGTATCGTCAGATCGAGGTAAGAGCTGGGGACCGCAAGGCCTAGCGCTTAAGG
ATGAGTCCGCGGCTGATTAGCTAGTTGGCGGGGTAATGGCCCACCAAGGCGACGATCAGTAGCCGGCC
TGAGAGGGTGACCGGCCACACTGGGACTGAGACACGGCC
>Zotu4398
GAACGCTGGCGGCGTGCTTAACACATGCAAGTCGAACGGGAAACCTTTTATTGAAGCTTCGGCAGATT
TAGATTGTTTCTAGTGGCGGACGGGTGAGTAACGCGTGGGTAACCTGCCTTGTACAGGGGGATAACAG
TCAGAAATGACTGCTAATACCGCATAAGCGCACAGGACCGCATGGTCCGGTGTGAAAAACTCCGGTGG
TATAAGATGGACCCGCGTTGGATTAGCTAGTTGGTGAGGTAACGGCCCACCAAGGCGACGATCCATAG
CCGGCCTGAGAGGGTGAACGGCCACATTGGGACTGAGACACGGCC
>Zotu4399
GAACGCTGGCGGCGTGCTTAACACATGCAAGTCGAACGATGAAAGCGGGAGCTTGCTCCTGCTGGATT
AGTGGCGAACGGGTGAGTAACACGTGAGTAACCTGCCCTTGACTCTGGGATAAGCGTTGGAAACGACG
TCTAATACCGGATACGACCCTCGGAGGCATCTCCTGGGGGTGGAAAGAATTTCGGTCAAGGATGGACT
CGCGGCCTATCAGCTAGTTGGTGAGGTAATAGCTCACCAAGGCGACGACGGGTAGCCGGCCTGAGAGG
GTGACCGGCCACACTGGGACTGAGACACGGCC
>Zotu4400
GAACGCTGGAAATGTGGATACCGGCATGCAAGTCAAGGGGCCGCAAGGCACCGGCAAACGGCTTAGTA
ACACGTAGATACATACCGCAAAGTGGGGCATAGCTCGTCGAAAGACGGGGTAATTTCCCATAGTCCCG
CAAGGGTAAAGGCGCAAGCCGCTTTGTGAATGGTCTGCGGTCTATCAGCTAGTTGGTGAGGTAAAGGC
TTACCAAGGCTATGACGGATAGGGGGCGTGAGAGCGCGACCCCCAAGAATGGCACTGCGACACGGGC
>Zotu4401
GAACGCTAGCGGCAGGCTTAATACATGCAAGTCGAGGGGCAGCATGGGTGTAGCAATACATCTGATGG
CGACCGGCAAACGGGTGCGGAACACGTACAGAACCTTCCTTCGAGCGGGGAATAGCCCAGAGAAATTT
GGATTAATACCCCATAGTATTACGGAGTGGCATCACTTTGTAATTAAAGATTTATCACTTGAAGATGG
CTGTGCGGCTGATTAGGTAGTTGGCGGGGTAACGGCCCACCAAGCCTACGATCAGTAACTGGTGTGAG
AGCACGACCAGTCACACGGGCACTGAGACACGGGC
>Zotu4402
GAGCGCTGGCGGCGTGCTTAACACATGCAAGTCGAGCGGAAAGGCCCTTCGGGGTACTCGAGCGGCGA
ACGGGTGAGTAACACGTGAGTAATCTGCCCTTGGCTTCGGGATAGCCACCGGAAACGGTGATTAATAC
CGGATACGACAACTTCTCGCATGGGATGGTTGTGGAAAGTTTTTCGGCCAGGGATGTGCTCGCGGCCT
ATCAGCTTGATGGTGAGGTAATGGCTCACCATGGCTTCGACGGGTAGCCGGCCTGAGAGGGTGACCGG
TCACACTGGGACTGAGACACGGCC
>Zotu4403
GAACGCTGGCGGCGTGCTTAACACATGCAAGTCGAACGATGAACTGGAGCTTGCTCCAGGGATTAGTG
GCGAACGGGTGAGTAACACGTGAGTAACCTGCCCTTGACTCTGGGATAAGCGCTAGAAATGGCGTCTA
ATACCGGATACGAGCTTCAGCCGCATGGCTGGGAGTTGGAAAGAATTTTGGTCAAGGATGGACTCGCG

GCCTATCAGCTAGTTGGTGAGGTAATGGCTCACCAAGGCGACGACGGGTAGCCGGCCTGAGAGGGTGA
CCGGCCACACTGGGACTGAGACACGGCC
>Zotu4404
GAACGCTGGCGGCAGGCTTAACACATGCAAGTCGAACGATGATTTCCCAGCTTGCTGGGAATGATTAG
TGGCGGACGGGTGAGTAACATTTAGGAATCTGCCTAGTAGTGGGGGATAGCTCGGGGAAACTCGAATT
AATACCGCATACGACCTACGGGTGAAAGGGGGCGCAAGCTCTTGCTATTAGATGAGCCTAAATCAGAT
TAGCTAGTTGGTGGGGTAAAGGCCCACCAAGGCGACGATCTGTAACTGGTCTGAGAGGATGATCAGTC
ACACCGGAACTGAGACACGGTC
>Zotu4405
GAACGCTAGCGGCAGGCTTAATACATGCAAGTCAAGGGGGTAGCAATACAACCGGCGCACGGGTGAGT
AACGCGTACACAACATACCATATACTGGAGTATAGCTTTGGGAAACTGAAGGTAATCCTCCATAACAT
TGCGAAAGGGCATCTGATTGCAATTAAAGTTTCGACGGTATATGATTGGTGTGCGTTTGATTAGCTAG
TTGGTAGGGTAACGGCCTACCAAGGCGACGATCGATAGCTGGCGTGAGAGCGCGACCAGCCACACTGG
AACTGAGACACGGTC
>Zotu4406
GAACGCTGGCGGTATGCCTAACACATGCAAGTCGAACGAAGCCTTCGGGCTTAGTGGCGCACGGGTGC
GTAACGCGTGGGAATCTGCCCTTGGGTTCGGAATAACAGTTAGAAATGACTGCTAATACCGGATGATG
ACGTAAGTCCAAAGATTTATCGCCCAAGGATGAGCCCGCGTAGGATTAGCTTGTTGGTGAGGTAAAAG
CTCACCAAGGCGACGATCCTTAGCTGGTCTGAGAGGATGATCAGCCACACTGGGACTGAGACACGGCC
>Zotu4407
GAACGCTGGCGGCGTGCTTAACACATGCAAGTCGAACGGTGATCTTGAGAGCTTGCTCTTGGGTGATC
AGTGGCGAACGGGTGAGTAACACGTGAGGAACTTGCCCCAGACTCTGGAATAACCCCGGGAAACCGGA
GCTAATACCGGATATGACACCAGATCGCATGGTCATGGTGTGGAAAGTTTTTCGGTCTGGGATGGCCT
CGCGGCCTATCAGCTTGTTGGTGAGGTAGTGGCTCACCAAGGCGACGACGGGTAGCCGGCCTGAGAGG
GCGACCGGCCACACTTGGACTGAGACACGGCC
>Zotu4408
GAACGCTGGCGGCATGCCTAACACATGCAAGTCGAACGAGATCTTCGGATCTAGTGGCGCACGGGTGC
GTAACGCGTGGGAATCTGCCCTTGGGTTCGGAATAACTCAGAGAAATTTGAGCTAATACCGGATGATG
ACGTAAGTCCAAAGATTTATCGCCCAAGGATGAGCCCGCGTAGGATTAGCTAGTTGGTGAGGTAAAGG
CTCACCAAGGCGACGATCCTTAGCTGGTCTGAGAGGATGATCAGCCACACTGGGACTGAGACACGGCC
>Zotu4410
GAACGCTGGCGGCGTGCTTAACACATGCAAGTCGAACGATGAAGCTCCTGCTTGCAGGGGTGGATTAG
TGGCGAACGGGTGAGTATCACGTGAGTAACCTGCCCTTGACTCTGGGATAAGCCTGGGAAACTGGGTC
TAATACCGGATAGTACTTCGCACCGCATGGTGTGGGGTGGAAAGATTTTATCGGTCTTGGATGGGCTC
GCGGCCTATCAGCTTGACGGTGGGGTAGTGGCTCACCGTGGCTTTGACGGGTAGCCGGCCTGAGAGGG
TGACCGGCCACACTGGGACTGAGACACGGCC
>Zotu4411
GAACGCTGGCGGCGTGCCTAATACATGCAAGTCGAGCGGTAAGGCCTCTTCGGAGGTACACGAGCGGC
GGACGGCTGAGTAACGCGTAGGAACGTACCCCAAACTGAGGGATAAGCACCAGAAATGGTGTCTAATA
CCGCATGTGCTCTAAGGAGTAAAGCTTCGGCGGTTTGGGAACGGCCTGCGTACGATTAGATTGTTGGT
GAGGTAATGGCTCACCAAGTCGACGATCGTTAGCTGGTCTGAGAGGATGATCAGCCAGACTGGGACTG
AGAACGGCC
>Zotu4412
AAACGCTGGCGGCGTGCTTAACACATGCAAGTCGAACGGAAAGGCCCTTTCGGGGGTACTCGAGTGGC
GAACGGGTGAGTAACACGTGGGTGATCTGCCCTGCACTTCGGGATAAGCTTGGGAAACTGGGTCTAAT
ACCGGATAGGACTACGCACTGCATGGTGTGTGGTGGAAAGCTTTTGCGGTGTGGGATGGGCCCGCGGC
CTATCAGCTTGTTGGTGGGATGATGGCCTACCAAGGCGACGACGGGTAGCCGGCCTGAGAGGGTGTCC
GGCCACACTGGGACTGAGATACGGCC
>Zotu4413
GAACGCTAGCGGCAGGCTTAATACATGCAAGTCGAACGGTAACATGAGTAGCAATACTTGATGACGAG
TGGCGCACGGGTGCGTAACGCGTATGCAATCTACCTTTAACTGGTGAATAGCCCGAAGAAATTCGGAT
TAACACACCATAGTATTATGAAGTGGCATCATTTTATAATTAAAACTCCGGTGGTTAAAGATGAGCAT
GCGTGACATTAGCTAGTTGGTGAGGTAATGGCTCACCAAGGCTACGATGTCTAGGGGATCTGAGAGGA
TTAACCCCCACACTGGTACTGAGACACGGAC

>Zotu4414
GAACGCTAGCGGCAGGCCTAATACATGCAAGTCGAACGGTCAGTAGCAATACTGATAGTGGCGCACGG
GTGCGTAACACGTAACTAACCTACCCACAACTGGGGGATAGCCCGCCGAAAGGCGGATTAATACCGCA
TAAGCTCTTGACCCGGCATCGGGTTATTAGTAAAGATTTATTGGTTGTGGATGGGGTTGCGGGTCATT
AGCTAGTTGGTTGGGTAACGGCTGACCAAGGCGACGATGACTAGGGGAGCTGAGAGGCTGGTCCCCCA
CACGGGCACTGAGATACGGGC
>Zotu4415
GAACGCTGGCGGCATGCCTAACACATGCAAGTCGAACGAGACCTTCGGGTCTAGTGGCGCACGGGTGC
GTAACGCGTGGGAATCTGCCCTTGGGTTCGGAATAACAGTTAGAAATGACTGCTAATACCGGATGATG
ACGTAAGTCCAAAGATTTATCGCCCAAGGATGAGCCCGCGTAGGATTAGCTAGTTGGTGAGGTAAAGG
CTCACCAAGGCGACGATCCTTAGCTGGTCTGAGAGGATGATCAGCCACACTGGGACTGAGACACGGCC
>Zotu4417
GAACGCTGGCGGCATGCCTAACACATGCAAGTCGAACGAGATCCTTCGGGGTCTAGTGGCGCACGGGT
GCGTAACGCGTGGGAATCTGCCCTTGGGTTCGGAATAACACAGAGAAATTTGTGCTAATACCGGATGA
TGTCTTCGGACCAAAGATTTATCGCCCAAGGATGAGCCCGCGTAGGATTAGGTAGTTGGTGGGGTAAT
GGCCTACCAAGCCGACGATCCTTAGCTGGTCTGAGAGGATGATCAGCCACACTGGGACTGAGACACGG
CC
>Zotu4418
GAACGCTGGCGGTATGCTTAACACATGCAAGTCGAACGGGATGCTTCGGCATCTAGTGGCGGACGGGT
GAGTAACGCGTGAGAATCTGGCTTTAGGTTCGGGACAACAGTGGGAAACTGCTGCTAATACCGAATGT
GCAGGAATGTGAAAGATTAATTGCCTGAAGATGAGCTCGCGTCTGATTAGCTAGTTGGTGAGGTAAAG
GCTCACCAAGGCGACGATCAGTAACTGGTCTGAGAGGATGGCCAGTCACACTGGGACTGAGACACGGC
C
>Zotu4419
GAACGCTAGCGGCAGGCCTAATACATGCAAGTCGAACGGCTCGCAAGAGCAGTGGCAAACGGGTGCGT
AACGCGTAAGCAACCTGCCTCAAACTGGGGGATAGCCCGGCGAAAGCTGGGGTAAACCCGCACGGTCC
CCTTGAGTCACCTGGCTTATTGGGTAAACATTTATGGGTTTGAGAGGGGCTTGCGTCTGATTAGTTAG
TTGGCAGGGTAACGGCCTACCAAGACGATGATCAGTAGGGGTTCTGAGAGGATTGGCCCCCACATGGG
TACTGAGATACGGAC
>Zotu4420
GAACGCTGGCGGCGTGCCTAATACATGCAAGTCGATCGAGTAGCAATACTAGAGGCGCACGGGAGAAT
AACACGTAGGTAATCTACCTTTGAGTGGGGAATAACCAGTCGAAAGATTGGCTAATACCGCATAAGAC
CACGAGATCTGCGGATCATGAGGTCAAAGGAGCAATTCGCTTGAAGATGAGCCTGCGTCCGATTAGCT
AGTTGGTAAGGTAATGGCTTACCAAGGCGAGGATCGGTAGCTGGTCTGAGAGGATGATCAGCCTCACT
GGAACTGAGACACGGTC
>Zotu4421
GAACGCTGGCGGCGTGCTTAACACATGCAAGTCGAACGATGACGGTGGTGCTTGCACCACCTGATTAG
TGGCGAACGGGTGAGTAACACGTGAGTAACCTGCCCCTCACTTCGGGATAACCTCGGGAAATCGTGGC
TAATACCGGATATGAACTCCTGCCGCATGGTGGGTGTTGGAAAGATTTATCGGTGAGGGATGGACTCG
CGGCCTATCAGTTTGTTGGTGAGGTAATGGCTCACCAAGACGATGACGGGTAGCCGGCCTGAGAGGGC
GACCGGCCACACTGGGACTGAGACACGGCC
>Zotu4423
GAATGCTGGCGGCGTGGATAAGTCATGCAAGTCAAGGGGGCCCGCAAGGGCAACCGGCAGACGAGGTA
GTAACACGTAGGTACGTCCCCCGAAGTCGGGCATAGCCAGTCGAAAGACTGGGTAATTCCCGATAGTC
CCGCAAGGGTAAAGGTTTTTCGCTTCGGGAGCGGCCTGCGTAGTATCAGCTAGTTGGTAGGGTAATGG
CCTACCAAGGCTACGACGCTTAGGGGAGCTGAGAGGCTGACCCCCACCGATGGAACTGCGACACGGTC
>Zotu4424
GAACGCTGGCGGCAGGCCTAACACATGCAAGTCGAACGCCCCGCAAGGGGAGTGGCGGACGGGTGAGT
AACACGTGGGAATTTTCCTCAAGGTACGGAACAACTCAGGGAAACTTGGGCTAATACCGTATGATGCA
GAGATGCCAAAGATTTATCGCCTTGGGATAAGCCCGCGTCAGATTAGGTAGTTGGTGGGGTAATGGCC
TACCAAGCCTGTGATCTGTAGCTGGTCTGAGAGGACGATCAGCCACATTGGGACTGAGACACGGCC
>Zotu4425
GAACGCTGGCGGCGTGCCTAATACATGCAAGTCGAGCGAACTTGCGGGAGCTTGCTCCCAAAAGTTAG
CGGCGGACGGGTGAGTAACACGTGGGCAACCTGCCTGTAAGACTGGGATAACTTCGGGAAACCGGAGC

TAATACCGGATAATTCTTTTCTGCTCATGCAGGAAAGCTGAAAGATGGTTTCGGCTATCACTTACAGA
TGGGCCCGCGGCGCATTAGCTAGTTGGTGAGGTAACGGCTCACCAAGGCAACGATGCGTAGCCGACCT
GAGAGGGTGATCGGCCACACTGGGACTGAGACACGGCC
>Zotu4426
ACCGTGTCTCAGTTCCAGTGTGGCTGATCATCCTCTCAGACCAACTAAAGATCGTCGCCTTGGTGAGC
CATTACCCCACCAACTAGCTAATCCTGCATGGGCTCGTCTTGCAGCGATAAATCTTTCACCTCTCGGC
TATATGCGACATTAGCAATTGCTTCCAACTGTTATTTCGCACTGCAAGGTAGATTCCCATGTATTACT
CACCCGTCTGCCACTAGCAGAATTCTGTATTGCTACAGCTCTTGCCCGTTCGACTTGCATGTGTTAAG
CACACCGCCAGCGTTCA
>Zotu4427
GAACGCTGGCGGTGTGCTTAACACATGCAAGTGGAGCGACGAACCAGAGCTTGCTCTGGGGCAGAGCC
GCGAACGGGTGAGTAACACGTGGGTAACGTACCCCGATAACCGGGACAACCCGAGGAAACTCGGGCTA
ATACCGGATGTGCTCCTCCCTCATGAGAGGGTTGAGTAAAGGAAGCTTCGGCCTCCGTATCGGGAGCG
GCCCGCGGGCTATTAGTTTGTTGGTGGGGTAACGGCCTACCAAGACTGCGATAGCTAGCTGGTCTGAG
AGGACGATCAGCCACACTGGGACTGAGACACGGCC
>Zotu4428
GAACGCTGGCGGCAGGCTTAACACATGCAAGTCGAACGCTCCGCAAGGAGAGTGGCAGACGGGTGAGT
AACGCGTGGGAACGTACCTTTCAGTTCGGAATAACTCAGGGAAACTTGTGCTAATACCGGATACGCCC
GCAAGGGGAAAGATTTATCGCTGAAAGATCGGCCCGCGTTGGATTAGCTAGTTGGTGGGGTAAAGGCC
TACCAAGGCGACGATCCATAGCTGGTCTGAGAGGATGATCAGCCACACTGGGACTGAGACACGGCC
>Zotu4429
GAACGCTGGCGGCATGCCTAACACATGCAAGTCGAACGAGACCCCGACTTCGGTTGGACGTCTAGTGG
CGCACGGGTGCGTAACGCGTGGGAATCTGCCCTTCGGTTCGGAACAACAGTTAGAAATGACTGCTAAT
ACCGGATAATGACGTAAGTCCAAAGATTTATCGCCGAAGGATGAGCCCGCGTAGGATTAGCTAGTTGG
TAAGGTAAAAGCTTACCAAGGCGACGATCCTTAGCTGGTCTGAGAGGATGATCAGCCACACTGGGACT
GAGACACGGCC
>Zotu4430
CAACGCTGGCGGCGTGCCTAACACATGCAAGTCGAACGAGAAAGGGTGAGTGTAGCAATACATTTGCC
TGAGTAAAGTGGCGACCGGGTGAGTAACACGTGGCCAACCTACCCTCGAACGGGGGATAACCTGGGGA
AACTCGGGCTAATACCGCATAACGTCCCGGCGACATAAGCCGCTGGGACCAAAGGCCCGCAAGGGTCA
CTTGAGGAGGGGGCTGCGGCCGATTAGCTAGTTGGTGAGGTAACGGCCCACCAAGGCAATGATCGGTA
GCCGGCCTGAGAGGGCGCACGGTCACACTGGCACTGAAACACGGGC
>Zotu4431
GAACGCTGGCGGCGTGCCTAATACATGCAAGTCGAGCGAATCTTTGGGAGCTTGCTCCCGAAGGTTAG
CGGCGGACGGGTGAGTAACACGTGGGCAACCTGCCTATAAGACTGGGATAACTTCGGGAAACCGGAGC
TAATACCGGATAATTCTTTTCTACACATGTAGAAAAGCTAAAAGACGGTTTACGCTGTCACTTATAGA
TGGGCCCGCGGCGCATTAGCTAGTTGGTGAGGTAACGGCTCACCAAGGCCACGATGCGTAGCCGACCT
GAGAGGGTGATCGGCCACACTGGGACTGAGACACGGCC
>Zotu4432
GAACGCTGGCGGCGTGCCTAATACATGCAAGTCGAACGAGAAAGTGGCTTCGGCCATGAGTACAGTGG
CAGACGGGTGAGTAATACATAGGTAACCTTCCCTCAAGATGGGGACAACCTTCCGAAAGGAGGGCTAA
TACCGAATGAGACCACAGTGACATATGTCACAGGGGTTAAAGACGGCCTCTATTTATAAGCTTTCGCT
TGAGGATGGGCTTATGCACCATTAGCTAGTTGGTAGGGTAACGGCCTACCAAGGCTGCGATGGTTAGC
TGATCTGAGAGGTTGATCAGCCACACTGGAACTGAGACACGGTC
>Zotu4433
GAACGCTGGCGGCGTGCCTAATACATGCAAGTCGAGCGAACTGACTTGAGAAGCTTGCTTCTCAATGA
CGTTAGCGGCGGACGGGTGAGTAACACGTGGGTAACCTACCCTATAGACTGGGATAACTTCGGGAAAC
CGGAGCTAATACCGGATAATTGTTTTCTTCGCATGAAGAAAATTGGAAAGACGGTTTCGGCTGTCACT
ATAGGATGGACCCGCGGCGCATTAGCTAGTTGGTGAGGTAACGGCTCACCAAGGCGACGATGCGTAGC
CGACCTGAGAGGGTGATCGGCCACACTGGGACTGAGACACGGCC
>Zotu4434
GAACGCTGGCGGCGTGCCTAACACATGCAAGTCGAACGGGTGTAGCAATACATCAGTGGCGGACGGGT
GAGTATAGCATGGGTATCTGCCTTGTGGAGGGGAATAGCTGCGGGAAGCTGCAGGTAAGTCCGCGTAA
GCAAAGTGTACGAGAGTGCATTTTGGAAAGTATCGGGAACAGTAATGTCCGGTAGCCATGAGATGAGC

CCATGTCCCATTAGCTAGTTGGTAGGGTAAAGGCCTACCAAGGTAAAGATGGGTAGCTGGTCTGAGAG
GACGACCAGCCACACTGGAACTGAGATACGGTC
>Zotu4435
GAACGCTGGCGGCGTGCCTAATACATGCAAGTCGAACGAAGTTGTTACTTGTAACAACTTAGTGGCGA
ACGGGTGAGTAACACGTAGGTAACCTGCCCCTTAGTTGGGAATAACCATTGGAAACGATGGCTAATAC
CGAATAAGTAATTGAGTGGCATCACTTAATTATAAAAGAACCTGAAAGGGTTCGCTAAGGGAGGGACC
TGCGTCGCATTAGTTAGTTGGTAAGGTAACGGCTTACCAAGACAGTGATGCGTAGCCGGACTGAGAGG
TTGAACGGCCACACTGGGACTGAGACACGGCC
>Zotu4436
GAACGCTAGCGGCAGGCCTAATACATGCAAGTCGAGGGGCAGCACGGGTAGTAATACTTGGTGGCGAC
CGGCGCACGGGTGCGTAACGCGTATGCAACCTACCTTATACAGGGGGATAGCCCAGAGAAATTTGGAT
TAATACCCCATAGTATCATTGAGAGGCATCTTGATATGATTAAAGATTTATTGGTATAAGATGGGCAT
GCGTCTGATTAGTTAGTTGGTGAGGTAACGGCTCACCAAGACATCGATCAGTAGGGGTTCTGAGAGGA
TGATCCCCCACACTGGCACTGAGATACGGGC
>Zotu4437
GAACGCTGGCGGCGTGCCTAATACATGCAAGTCGAACGAACCAACGCTAGAACTTGTTCTAGCAGCGG
TTAGTGGCGCACGGGTGAGTAACACGTGGGGAACCTGCCCTTTAGTGGGGGATAACAGTCGGAAACGA
CTGCTAATACCGCATAGACATTTTAACCGCCTGGTTAGAATGGGAAAGGTGCTACGGCATCATTAAAG
GATGGCCCCGCGGTGCATTAGTTAGTTGGTAAGGTAATGGCTTACCAAGACGATGATGCATAGCCGAC
CTGAGAGGGTGATCGGCCACATTGGGACTGAGACACGGCC
>Zotu4438
GAACGCTAGCGGCAGGCCTAATACATGCAAGTCGAGCGGATTGTAGCAATACAGTTAGCGGCGCACGG
GTGAGTAACGCGTACACAATCTACCTTCAACTGGGGAATAGCCTCGGGAAACTGGGAGTAATACCCCA
TAGTATAACAGAGCGGCATCGCTTTGTTATTAAAGTTCCGGCGGTTGAAGATGGGTGTGCGTCTGATT
AGATTGTTGGTGAGGTAACGGCTCACCAAGTCGATGATCAGTAAGGGGCGTGAGAGCGTGGCCCCTCA
CATGGACACTGAGACACGGGT
>Zotu4439
GAACGCTGGCGGCATGCCTAACACATGCAAGTCGAACGAAGCCTTCGGGCTTAGTGGCGCACGGGTGC
GTAACACGTGGGAATCTGCCCTCTGGTACGGAATAACTCAGGGAAACTTGAGCTAATACCGTATGATG
TCTTCGGACCAAAGATTTATCGCCAGAGGATGAGCCCGCGTCGGATTAGCTAGTTGGTGGGGTAAAGG
CCTACCAAGGCGACGATCCGTAGCTGGTCTGAGAGGATGATCAGCCACACTGGGACTGAGACACGGCC
>Zotu4440
GAACGCTGGCGGCGCGCCTAACACATGCAAGTCGAACGAGCGAGAGAGAGCTTGCTTTCTCGAGCGAG
TGGCGAACGGGTGAGTAACGCGTGAGGAACCTGCCTCAAAGAGGGGGACAACAGTTGGAAACGACTGC
TAATACCGCATAAGCCCACGGCTCGGCATCGAGCAGAGGGAAAAGGAGCAATCCGCTTTGAGATGGCC
TCGCGTCCGATTAGCTAGTTGGTGAGGTAACGGCCCACCAAGGCGACGATCGGTAGTCGGACTGAGAG
GTTGAACGGCCACATTGGGACTGAGACACGGCC
>Zotu4442
GAACGCTGGCGGCGTGCCTAAGACATGCAAGTCGAGGGGGTTTAGACCCCCGGCGAACGGGTGAGTAA
CACGTGGGAACTTTCATTAAGTTGAGGATAACCTCTCGAAAGGGAGGACAATACTCAATGTGGCCGCA
AGGCTAAAGATTTATCGCTTAATGTGAGGCCCGCGTCCCATCAGCTAGTTGGTAGGGTAAAAGCCTAC
CAAGGCTACGACGGGTAGCGGAACCGAGAGGTTGATCCGCCACACTGGGACTGCGACACGGCC
>Zotu4443
GAACGCTGGAAATGTGGATACTGGCATGCAAGTCAAGGGGACCGCAAGGTCACCGGCGAACGGCTTAG
TAACACGTAGATACATACCGCAAAGTGGGGCATAGCTCGTTGAAAAACGGGGTAATTTCCCATAGTCT
CGCAAGAGTAAACACGCAAGTGGCTTTGTGAATGGTCTGCGGTCTATCAGCTAGTTGGTGAGGTAAAG
GCTCACCAAGGCTATGACGGATAGGGGGCGTGAGAGCGCGACCCCCAAGAATGGCACTGCGACACGGG
C
>Zotu4444
GAACGCTGGCGGCAGGCTTAACACATGCAAGTCGAACGCACCGCAAGGTGAGTGGCAGACGGGTGAGT
AACACGTGGGAACGTGCCCTTCGGTTCGGAATAACTCAGGGAAACTTGAGCTAATACCGGATACGCCC
TTTTGGGGAAAGGTTTACTGCCGAAGGATCGGCCCGCGTCTGATTAGCTAGTTGGTGAGGTAATGGCT
CACCAAGGCGACGATCAGTAGCTGGTCTGAGAGGATGATCAGCCACACTGGGACTGAGACACGGCC
>Zotu4445

GAACGCTGGCGGCATGCCTTACACATGCAAGTCGAACGGCAGCACGGGGGCAACCCTGGTGGCGAGTG
GCGAACGGGTGAGTAATACATCGGAACGTGTCCTGTAGTGGGGGATAGCCCGGCGAAAGCCGGATTAA
TACCGCATACGACCTAAGGGAGAAAGCGGGGGATCTTCGGACCTCGCGCTATAGGGGCGGCCGATGGC
AGATTAGCTAGTTGGTGGGGTAAAGGCCTACCAAGGCGACGATCTGTAGCTGGTCTGAGAGGACGACC
AGCCACACTGGGACTGAGACACGGCC
>Zotu4446
GAACGCTGGCGGCGTGCCTAATACATGCAAGTCGAGCGGACGGATGGGAGCTTGCTCCCTGAAGTTAG
CGGCGGACGGGTGAGTAACACGTGGGCAACCTGCCCTGCAGATGGGGATAACTCCGGGAAACCGGGGC
TAATACCGAATAATCAGTTTCTCCGCATGGAGAAACTCTGAAAGACGGTTTCGGCTGTCACTGCAGGA
TGGGCCCGCGGCGCATTAGCTAGTTGGTGGGATAACGGCCTACCAAGGCAACGATGCGTAGCCGACCT
GAGAGGGTGATCGGCCACACTGGGACTGAGACACGGCC
>Zotu4447
GAACGCTGGCGGCGTGCCTAATACATGCAAGTCGAGCGGACGTTTTATTGGTGCTTGCACCGATTTAA
CGTTAGCGGCGGACGGGTGAGTAACACGTGGGCAACCTGCCCTGTAGATTGGGATAACTCCGGGAAAC
CGGGGCTAATACCGAATAATACTTTTTAACTCATGTTAGAAAGTTGAAAGATGGTTCTGCTATCACTA
CAGGATGGGCCCGCGGCGCATTAGCTAGTTGGTGAGGTAACGGCTCACCAAGGCGACGATGCGTAGCC
GACCTGAGAGGGTGATCGGCCACACTGGGACTGAGACACGGCC
>Zotu4448
GAACGCTGGCGGCGTGCTTAACACATGCAAGTCGAACGGAAAGGCCCACCACCTTGGTGGTGGGTACT
CGAGTGGCGAACGGGTGAGTAACACGTGGGTGATCTGCCCCGCACTGTGGGATAAGCCTGGGAAACTG
GGTCTAATACCACATACGACCACACTGTAGACAGTGTGGTGGAAAGCCTTGTGCGGTGTGGGATGAGC
CCGCGGCCTATCAGCTTGTTGGTGGGGTAATGGCCTACCAAGGCGGCGACGGGTAGCCGGCCTGAGAG
GGTGGACGGCCACATTGGGACTGAGACACGGCC
>Zotu4449
GAACGCTGGCGGCGTGCTTAACACATGCAAGTCGAACGGTGATCCTCTTTCGGGGGGTGATCAGTGGC
GAACGGGTGAGTAACACGTGAGCAACCTGCCCCTGGCTCTGGGACAACCACTGGAAACGGTGGCTAAT
ACCGGATACGACCTACCTCGGCATCGGGTGTGGGTGGAAAGTTTTTCGGCTGGGGATGGGCTCGCGGC
CTATCAGCTTGTTGGTGGGGTAATGGCCCACCAAGGCGACGACGGGTAGCCGGCCTGAGAGGGCGACC
GGCCACACTGGGACTGAGACACGGCC
>Zotu4452
GAACGCTGGCGGCGTGCCTAATACATGCAAGTCGAGCGGACCTGAATGGAGCTTGCTCCAGACAGGTT
AGCGGCGGACGGGTGAGTAACACGTAGGCAACCTGCCCGCAAGACCGGGATAACGTAGGGAAACTTAC
GCTAATACCGGATAGGGCACTCTCTCGCATGAGAGAGTGCGGAAAGGTGGCGCAAGCTACCACTTGCG
GATGGGCCTGCGGCGCATTAGCTAGTTGGTGGGGTAACGGCCTACCAAGGCGACGATGCGTAGCCGAC
CTGAGAGGGTGACCGGCCACACTGGGACTGAGACACGGCC
>Zotu4453
GAACGCTGGCGGCGCGCTTAACACATGCAAGTCGAGCGAGAACCTGGCTTCGGCCAGCGGAAAGCGGC
GAACGGGTGAGTAACACGTGGGCAACCCACCCTTGGTATCCGGATAGCCCGGGGAAACCCGGATTAAT
ACGGAATGGCCCAACAGCTCTTCGGGGCGGTTGGAAAAGGTAGCTTCGGCCTCCGACCAAGGACGGGC
CCGCGGCGGATTAGCTTGTTGGTGGGGTAACGGCCCACCAAGGCGACGATCCGTAGCTGGTCTGAGAG
GACGATCAGCCACACTGGGACTGAGACACGGCC
>Zotu4454
GAACGCTGGCGGCGTGCCTAATACATGCAAGTCGAGCGGACCTCTTCGGAGGTCAGCGGCGGACGGGT
GAGGAACACGTGGGTAATCTGCCTTTCAGACCGGAATAACGCCCGGAAACGGGCGCTAATGCCGGATA
CGCCCGCGAGGAGGCATCTTCTTGCGGGGAAAGGCCCAACTGGGCCGCTGAGAGAGGAGCCCGCGGCG
CATTAGCTGGTTGGCGGGGTAACGGCCCACCAAGGCGACGATGCGTAGCCGACCTGAGAGGGTGACCG
GCCACACTGGGACTGAGACACGGCC
>Zotu4456
GAACGCTGGCGGCGCGCTTAACACATGCAAGTCGAGCGAGAACCAGGGCTTCGGCCCTGGGGACAGCG
GCGAACGGGTGAGTAACACGTGAGTAATCTGCCCTCGGCACCGGGATAGCCCGGGGAAACCCGGATTA
ATACCGGATAGCCTCCGAAGCCTTCGGGCGTCGGAGCAAAGGTAGCTTCGGCCTCTGGCCGAGGATGA
GCTAGCGGCGGATTAGCTTGTTGGTGGGGTAATGGCCCACCAAGGCGACGATCCGTAGCTGGTCTGAG
AGGACGATCAGCCACACTGGGACTGAGACACGGCC
>Zotu4458

GAACGCTGGCGGCGTGCTTAACACATGCAAGTCGAGCGGTAAGGCCCTTCGGGGTACACGAGCGGCGA
ACGGGTGAGTAACACGTGGGTGATCTGCCTCGTACTTCGGGATAAGCCTGGGAAACTGGGTCTAATAC
CGGATATGACCACGGACTGCATGGTTTGTGGTGGAAAGATTTATCGGTGCGAGATGGGCCCGCGGCCT
ATCAGCTTGTTGGTGGGGTAATGGCCTACCAAGGCGACGACGGGTAGCCGACCTGAGAGGGTGACCGG
CCACACTGGGACTGAGACACGGCC
>Zotu4459
GAACGCTAGCGATGTGCTTTAAGCATGCAAGTGGACGAGTTGTAGCAATACAACTAGTCGCGAACGG
CTGAGTATAAGATAGGAATCTACCTCTTGGTGGGGAATACCTCGTCGAAAGACGAGCTAATACCGCAT
AAGAATCTAATTGAAAGCGATTAACTTCGCGCCGAGAGAGGAGCCTATCTCCCATCAGCTTGTTGGTA
GGGTAATGGCCTACCAAGGCTACGACGGGTAGGGGGCGTGAGAGCGTGACCCCCCAGAACAGGACTGA
GACACGGCC
>Zotu4460
GAACGCTAGCGGCAGGCTTAACACATGCAAGTCGAGGGGTATAGTAGCAATACTAGAGACCGGCGCAC
GGGTGCGTAACGCGTATGCAACCTACCTTTTACAGGGGAATAGCCCAGAGAAATTTGGATTAATGCCC
CATGGTATATTTTAGTGGCATCACTAGATTATTAAAGTTCCAACGGTAAAAGATGGGCATGCGTCCTA
TTAGCTAGATGGTAAGGTAACGGCTTACCATGGCTACGATAGGTAGGGGTCCTGAGAGGGAGATCCCC
CACACTGGTACTGAGACACGGAC
>Zotu4461
CAACGCTGGCGGCGTGCCTAACACATGCAAGTCGAACGAGAAAGTGGAGCAATCCATGAGTAAAGTGG
CGACCGGGTGAGTAACACGTGACTAACCTACCCTAGAGTGGGGGATAACCTCGGGAAACCGGGGCTAA
TACCGCATAACATCACGAGGGGAAGATCCTTGTGATCAAAGGAGCAATTCGCTCAAGGAGGGGGTCGC
GGCTGATTAGCTAGTTGGTGGGGTAACGGCCCACCAAGGCAAAGATCAGTAGCCGGCCTGAGAGGGCG
CACGGCCACACTGGAACTGAAACACGGTC
>Zotu4463
GAACGCTGGCGGCGTGCTTAACACATGCAAGTCGAACGGTGAAGCGCCTTCCTTTTTGGGGGGTGTGG
ATGAGTGGCGAACGGGTGAGTAACACGTGAGTAACCTGCCCCCTTCTTCTGGATAACCGCATGAAAGT
GTGGCTAATACGGGATATTCTGGGTCTGTCGCATGATGGGTCTGGGAAAGATATTGTTTCGGTGGGGG
ATGGGCTCGCGGCCTATCAGCTTGTTGGTGGGGTGATGGCCTACCAAGGCGGTGACGGGTAGCCGGCC
TGAGAGGGTGGACGGTCACACTGGGACTGAGACACGGCC
>Zotu4464
GAACGCTGGCGGCATGCCTTACACATGCAAGTCGAACGGCAGCACGGGAGCTTGCTCCTGGTGGCGAG
TGGCGAACGGGTGAGTAATGCATCGGAACGTGCCCTGAAGTGGGGGATAACGTAGCGAAAGTTACGCT
AATACCGCATATTCTGTGAGCAGGAAAGCAGGGGATCTTCGGACCTTGCGCTTTTGGAGCGGCCGATG
TCGGATTAGCTAGTTGGTGGGGTAAAGGCTCACCAAGGCAACGATCCGTAGCGGGTCTGAGAGGATGA
TCCGCCACACTGGAACTGAGACACGGTC
>Zotu4465
GAATGCTGGCAGCGTGGATGAGGCATGCAAGTCGAACGAGACTAGAGGGCAACTTTTAGTTTAGTGGC
GAAAGGGTTAGTAATACATGAATAACTTACCCTTTACCTGGGGATAACGACTGGAAACAGGCGCTAAT
ACCGAATGAGGTACCGATAAGGAGTTATTGGCTATCAAAGTGGGGGACCTTGTAAAGAGGCCTTGCGG
TAAAGGATAGGTTCATGGGATATCAGCTAGTTGGTGTGGTAAAGGCGCACCAAGGCTAAGACGTCTAG
GCGGATTGAGAGATTGACCGCCAACATTGGGACTGAGACACTGCC
>Zotu4466
GAACGCTGGCGGCATGCCTAACACATGCAAGTCGAACGAAGTCCTTCGGGGCTTAGTGGCGCACGGGT
GCGTAACGCGTGGGAATCTGCCCTTAGGTTCGGAATAACAGTGGGAAACTACTGCTAATACCGGATGA
TGACGTAAGTCCAAAGATTTATCGCCTAGGGATGAGCCCGCGTAAGATTAGCTAGTTGGTGGGGTAAA
GGCCTACCAAGGCTACGATCTTTAGCTGGTCTGAGAGGATGATCAGCCACACTGGGACTGAGACACGG
CC
>Zotu4468
GAACGCTGGCGGTATGCTTAACACATGCAAGTCGAACGGAGTACTTCGGTACTTAGTGGCGGACGGGT
GAGTAACGCGTGAGAAACTGCCCCTTGGACTGGGACAACAGCTGGAAACGGCTGCTAACACCGGATGT
GCCGAAAGGTGAAATATTTATAGCCAGGGGATGTGCTCGCGTCTGATTAGCTAGTAGGTGAGGTAAAG
GCTTACCTAGGCGACGATCAGTAGCTGGTCTGAGAGGATGATCAGCCACACTGGGACTGAGACACGGC
C
>Zotu4471

GAACGCTAGCGACAGGCTTAACACATGCAAGTCGAGGGGCAGCGGGGGATAAGCTTGCTTTTCCTGCC
GGCGACCGGCGCACGGGTGAGTAACACGTATGCAACCTGCCCCTGTCAGAGGGATAATCCGGAGAAAT
CCGGTCTAATATCGCATAAAGCGTTTGCGGGGCATCCTGCGAGCGCCAAAGGAGTAATCCGGACAGGG
ATGGGCATGCGGCGCATTAGCTAGTTGGGGGGGTAACGGCCCACCAAGGCGACGATGCGTAGGGGTTC
TGAGAGGAAGGTCCCCCACATTGGTACTGAGACACGGAC
>Zotu4472
GAACGCTAGCTACAGGCTTAACACATGCAAGTCGAGGGGCAGCATGGGGCAGCAATGTCCCGATGGCG
ACCGGCGAATGGGTGAGTAACACGTATCCAACCTGCCCCGTGCTCAGGGATAGCCCTTAGAAATGAGG
ATTAATACCTGATGCAGTGCGCTGACCGCATGGGATGTTCACTAAAGATTCATCGGCACGGGATGGGG
ATGCGTCTGATTAGCTTGTTGGCGGGGTAACGGCCCACCAAGGCTTCGATCAGTAGGGGTTCTGAGAG
GAAGGTCCCCCACATTGGTACTGAGACACGGAC
>Zotu4473
GAACGCTGGCGGCGTGCCTAATACATGCAAGTCGAGCGCTTGCACAAGAAAGCTTGCTTTCTTGTTGC
TTGAGCGGCGGACGGGTGAGTAACACGTGGGCAACCTGCCTATAAGACTGGGATAACTTCGGAAAACC
GGAGCTAATACCGGATAATGCTTTTGAACACATGTTTGAAAGCTGAAAGATGGTTTCGGCTATCACTT
ATAGATGGGCCCGCGGCGCATTAGCTAGTTGGTGAGGTAACGGCTCACCAAGGCGACGATGCGTAGCC
GACCTGAGAGGGTGATCGGCCACACTGGGACTGAGACACGGCC
>Zotu4474
GAACGCTAGCGGGATGCCTTACACATGCAAGTCGAACGGCAGCGGGAAAGTAGCTTGCTACTTTTGCC
GGCGAGTGGCGAACGGGTGAGTAATGTATCGGAACGTGCCCAGTAGCGGGGGATAACTACGCGAAAGC
GTGGCTAATACCGCATACGCCCTACGGGGGAAAGGGGGGGATCTTAGGACCTCTCACTATTGGAGCGG
CCGATATCGGATTAGCTAGTTGGTGGGGTAAAGGCCTACCAAGGCGACGATCCGTAGCTGGTTTGAGA
GGACGACCAGCCACACTGGGACTGAGACACGGCC
>Zotu4476
GAACGCTGGCGGCGTGCCTAACACATGCAAGTCGAACGAGGCCCAAGCAGTGGCAACACTGCGGAAGG
CCTAGTGACGAACGGGTGAGTAACACGTGAGGAACCTGCCCCGAAGACCGGGATAACCCCCTGAAAAG
GGAGCTAATACCGGATGCCCCCACCGGACCGCATGGTCTGGTGAGGAAATGGATTCCGCTTCGGGAGG
GCCTCGCGGCCTATCAGCTTGTTGGTGAGGTAACGGCTCACCAAGGCGTCGACGGGTAGCTGGTCTGA
GAGGACGATCAGCCACACTGGGACTGAGACACGGCC
>Zotu4478
GAACGCTGGCGGCGTGCTTAACACATGCAAGTCGAACGGTGAAGCCCAGCTTGCTGGGTGGATCAGTG
GCGAACGGGTGAGTAACACGTGAGCAACCTGCCCCTGACTCTGGGATAAGCGCTGGAAACGGCGTCTA
ATACTGGATACGAACCACAGTCGCATGGCTCGTGGTTGGAAAGATTTTTTGGTTGGGGATGGGCTCGC
GGCCTATCAGCTTGTTGGTGAGGTAATGGCTCACCAAGGCGTCGACGGGTAGCCGGCCTGAGAGGGTG
ACCGGCCACACTGGGACTGAGACACGGCC
>Zotu4479
GAACGCTGGCGGCGTGCCTAATACATGCAAGTGGAACGCATGATTGATACCGGAGCTTGCTCCACCAT
TAATCATGAGTCGCGAACGGGTGAGTAACGCGTAGGTAACCTGCCTGATAGTGGGGGATAACTATTGG
AAACGATAGCTAATACCGCATAAGAGTCGTTAACACATGTTAATGATTTAAAAGGAGCAATTGCTTCA
CTATCAGATGGACCTGCGTTGTATTAGCTAGTTGGTGAGGTAACGGCTCACCAAGGCCTCGATACATA
GCCGACCTGAGAGGGTGATCGGCCACACTGGGACTGAGACACGGCC
>Zotu4480
GAACGCTGGCGGCGTGCTTAACACATGCAAGTCGAACGGAAAGGCCTGTACTTGTACAGGTACTCGAG
TGGCGAACGGGTGAGTAACACGTGGGTGATCTGCCCTGCACTATGGGATAAGCTTGGGAAACTGGGTC
TAATACCATATAGGACCACTTCTTGGATGTTGTGGTGGAAAGCTTTTACGGTGTGGGATGAGCCTGCG
GCCTATCAGCTTGTTGGTGGGGTAATGGCCTACCAAGGCGGCGACGGGTATCCGGCCTGAGAGGGTGT
ACGGACACATTGGGACTGAGATACGGCC
>Zotu4481
GAACGCTGGCGGCGTGCTTAACACATGCAAGTCGAGCGGAAAGGCCCTTCGGGGTACTCGAGCGGCGA
ACGGGTGAGTAACACGTGAGTAACCTGCCCCTGACTCTGGGATAAGCCTGGGAAACTGGGTCTAATAC
CGGATACGACCATTTCTCGCATGGGATGGTGGTGGAAAGTCTTTTCGGTTGGGGATGGGCTCGCGGCC
TATCAGCTTGTTGGTGGGGTGATGGCCTACCAAGGCGACGACGGGTAGCCGGCCTGAGAGGGCGACCG
GCCACACTGGGACTGAGACACGGCC
>Zotu4482

GAACGCTGGCGGCAGGCTTAACACATGCAAGTCGAGCGCCCCGCAAGGGGAGCGGCAGACGGGTGAGT
AACGCGTGGGAATCTACCTTTTGCTACGGAATAACGCATGGAAACGTGTGCTAATACCGTATGTGCCC
TTCGGGGGAAAGATTTATCGGCAAAGGATGAGCCCGCGTTGGATTAGCTAATTGGTGGGGTAAAGGCC
TACCAAGGCGACGATCCATAGCTGGTCTGAGAGGATGATCAGCCACATTGGGACTGAGACACGGCC
>Zotu4484
GAACGCTGGCGGCGTGCCTAATACATGCAAGTCGAGCGAACCAACAGGAGCTTGCTCCTGTTGGTTAG
CGGCGGACGGGTGAGTAACACGTGGGCAACCTGCCTGTAAGACTGGGATAACACCGGGAAACCGGTGC
TAATACCGGATAATCCTTTTCCTCTCATGAGGAAAAGCTGAAAGTCGGTTTCGGCTGACACTTACAGA
TGGGCCCGCGGCGCATTAGCTAGTTGGTGAGGTAACGGCTCACCAAGGCGACGATGCGTAGCCGACCT
GAGAGGGTGATCGGCCACACTGGGACTGAGACACGGCC
>Zotu4485
GAACGCTGGCGGCGTGCTTAACACATGCAAGTCGAACGGAGCTATTTATTTCGGTAAATCGCTTAGTG
GCAAACGGGTGAGTAACGCGTAGGCAACCTGCCCTCAGGATGGGGACAACGGCTCGAAAGAGCCGCTA
ATACCGAATGAGGTAACTTTTTCACATGGAAGAGATACTAAAGATGGGTCAAACCATCGCCTGAGGAT
GGGCCTGCGTCTGATTAGCCAGTTGGTGAGGTAACGGCTCACCAAAGCGACGATCAGTAGCCGGTCTG
AGAGGATGAACGGCCACAATGGGACTGAGACACGGCC
>Zotu4486
GAACGCTGGCGGTATGCTTAACACATGCAAGTCGAACGAGTAGCTACTTCGGTAGCGAGAGTGGCGGA
CGGGTGAGTAACGCGTAGGAATCTGCCTGTTAGTGGGGGACAACGTGGGGAAACGCACGCTAATACCG
CATACGCCCTACGGGGGAAAGGAGGGGACCTTCGGGCCTTTCGCTAACAGATGAGCCTGCGTGGGATT
AGCTAGTTGGTAGGGTAAAGGCCTACCAAGGCGACGATCCCTAGCTGGTCTGAGAGGACGATCAGCCA
CACTGGGACTGAGACACGGCC
>Zotu4487
GAACGCTGGCGGCGTGCTTAACACATGCAAGTCGAACGATGATGGTGGTGCTTGCACCACCTGATTAG
TGGCGAACGGGTGAGTAACACGTGAGTAACCTGCCCCTCACTTCGGGATAACCTCGGGAAATCGTGGC
TAATACCGGATATGAACTTCTACCGCATGGTGGTCGTTGGAAAGATTTATCGGTGAGGGATGGACTCG
CGGCCTATCAGTTTGTTGGTGAGGTAATGGCTCACCAAGACGATGACGGGTAGCCGGCCTGAGAGGGC
GACCGGCCACACTGGGACTGAGACACGGCC
>Zotu4488
GAACGCTGGCGGCGTGCTTAACACATGCAAGTCGAACGGTGAAACAGGAGCTTGCTCCTGCGGATCAG
TGGCGAACGGGTGAGTAACACGTGAGTAACCTGCCCTTGACTCTGGGATAAGCGTTGGAAACGACGTC
TAATACCGGATACGAGCTTCAGTCGCATGGCTAGGAGCTGGAAAGAACTTCGGTCAAGGATGGACTCG
CGGCCTATCAGCTAGTTGGTGAGGTAACGGCCCACCAAGGCGACGACGGGTAGCCGGCCTGAGAGGGT
GACCGGCCACACTGGGACTGAGACACGGCC
>Zotu4489
GAACGCTGGCGGCGTGCTTAACACATGCAAGTCGAGCGAGGCCCTTCCTTCGGGAGGGTGTCCTAGCG
GCGAACGGGTGAGTAACACGTGGGCAACCTGCCCTCAGCTCTGGGATAACTCCAAGAAATTGGGGCTA
ATACCGGATATGACTGCTGACCGCATGGTCTGGTGGTGGAAATATTTATCGGCTGGGGATGGGCCCGC
GGCCTATCAGCTTGTTGGTGGGGTAGTGGCCTACCAAGGCGACGACGGGTAGCCGGCCTGAGAGGGTG
ACCGGCCACACTGGGACTGAGACACGGCC
>Zotu4490
GAACGCTGGCGGCGTGCTTAACACATGCAAGTCACGGGGGCCCGCAAGGGCAACCGGCGAACGGGTGC
GTAACACGTGAGCAATTTGCCGATTTCTGGGGGATAGCCGGCCCAACGGCCGGGTAATACCGCATACG
CTCAGTGGGGGGAGTCCCCTGCTGAGGAAACCTCCGGGGGAAATCGAGGAGCTCGCGGCCTATCAGCT
AGTTGGCGGGGTAACGGCCCACCAAGGCGACGACGGGTAGCTGGTCTGAGAGGATGGCCAGCCACATT
GGGACTGAGAGACGGCC
>Zotu4491
GAACGCTGGCGGCGTGCCTAAGACATGCAAGTCGAGCGGGGGGACTGGGGAGCTTGCTCTCCGGTCTC
CTAGCGGCGGACGGGTGAGTAACACGTGGGCAACCTGCCCGGCAGTGGGGGATAACCCTGGGAAACTG
GGGCTAATACCGCATACGGTCCGCGTTCCGCATGGGGCGTGAAAGAAAGGCCGTCGTGAGGCGGTCGC
TGCCGGAGGGGCCCGCGGCCCATCAGCTGGTTGGTGGGGTAACGGCCCACCAAGGCGACGACGGGTAG
CCGGCCTGAGAGGGTGGCCGGCCACACTGGGACTGAGACACGGCC
>Zotu4492
GAACGCTGGCGGCGTGCTTAACACATGCAAGTCGAACGGAAAGGCCCTTCGGGGTACTCGAGTGGCGA

ACGGGTGAGTAACACGTGGGTGATCTGCCCTGCACTTCGGGATAAGCCTGGGAAACTGGGTCTAATAC
CGGATAGGACTACGCGATGCATGTCGTGTGGTGGAAAGCTTTTGCGGTGTGGGATGGGCCCGCGGCCT
ATCAGCTTGTTGGTGGGGTGATGGCCTACCAAGGCGACGACGGGTAGCCGGCCTGAGAGGGTGTCCGG
CCACACTGGGACTGAGATACGGCC
>Zotu4495
GAACGCTGGTGGCGTGCTTAACACATGCAAGTCGAGCGAGGCGTCCGCCTCCGGGCGGTCAGCCGAGC
GGCGAACGGGTGAGTAACACGTGAGCAATCTTCCCTCGGCTCGGGGATAACCTCGGGAAACTGAGGCT
AATACCGGATGCACGTTTTTCGGGGCATCCCGGAAAACGAAAAGGTTTCGCACCGCGAGGTGCGTTTC
CGGCCGGGGATGAGCTCGCGGCCTATCAGTTTGTTGGTGTGGTAAAAGCGCACCAAGGCGACGACGGG
TAGCCGGCCTGAGAGGGTGATCGGCCACACTGGGACTGAGACACGGCC
>Zotu4496
GAACGCTGGCGGCGTGCTTAACACATGCAAGTCGAGTGCGCGTCTGTAGCAATACAGATGGCGCACGG
CGCACGGGTGCGTAACACGTGGGTAATCTGCCCTTCGAAGGGGAATAACTCGCCGAAAGGCGAGCTAA
TTCCGCATAACATTCCGAGAACCTTGGTTTTTGGATTCAAAGGCGTAAGTCGTCGGAGGAGGAGCCCG
CGCACGATTAGCTAGTTGGTAGGGTAACGGCCTACCAAGGCTATGATCGTTAGCTGGTCTGAGAGGAT
GGCCAGCCACACTGGAACTGAGACACGGTC
>Zotu4498
GAACGCTGGCGGCGTGCTTAACACATGCAAGTCGAACGGTAAGGCCCCTTCGGGGGTACACGAGTGGC
GAACGGGTGAGTAACACGTGAGCAACCTGCCCTTGACTCTGGAATAACAGCTGGAAACAGCTGCTAAT
ACCGGATATGACCTCAAACGACATCGTCTGAGGTGGAAAGCTCCGGCGGTCAAGGATGGGCTCGCGGC
CTATCAGCTAGTTGGTGAGGTAACGGCTCACCAAGGCTTCGACGGGTAGCCGGCCTGAGAGGGCGACC
GGCCACACTGGGACTGAGATACGGCC
>Zotu4499
GAACGCTGGCGGCGTGCCTAATACATGCAAGTCGAGCGAATCTTTGGGAGCTTGCTCCCAAAGATTAG
CGGCGGACGGGTGAGTAACACGTGGGCAACCTGCCTATAAGACTGGGATAACTTCGGGAAACCGGAGC
TAATACCGGATAATTCATATCTACTCATGTAGATATGCTGAAAGACGGTTTACGCTGTCACTTATAGA
TGGGCCCGCGGCGCATTAGCTAGTTGGTGAGGTAACGGCTCACCAAGGCAACGATGCGTAGCCGACCT
GAGAGGGTGATCGGCCACACTGGGACTGAGACACGGCC
>Zotu4500
GAACGCTAGCGGAAGGCCTAATACATGCAAGTCGAACGGTGTAGCAATACATAGTGGCGGACGGGTGC
GTAACACGTATGCAACCTACCCAAGGGTGTGGAATAACAATTTGAAAAGATTGCTAATACCGCATAAT
GCAGCGGACCCGCATGGGTATGTTGTTAAAGGCGAAAGTCGCCTTTGGATGGGCGTGCGTCCGATTAG
GTAGTTGGTAGGGTAACGGCCTACCAAGCCGACGATCGGTAGCTGGTCTGAGAGGATGATCAGCCACA
CTGGCACTGAGACACGGGC
>Zotu4503
GAACGCTAGCGGCAAGCTTAATACATGCAAGTCGAGGGGCAGCGCAGTGTAGCAATACATGGGCGGCG
ACCGGCAAACGGGTGCGGAACACGTACAGAACCTTCCTTCGAGCGGGGAATAGCCCAGAGAAATTTGG
ATTAATACCCCATAGCATATTGAAGTGGCATCACTTTAATATTAAAGATTTATCACTTGAAGATGGCT
GTGCGGCTGATTAGATAGTTGGCGGGGTAACGGCCCACCAAGTCTGCGATCAGTAACTGGTGTGAGAG
CACGACCAGTCACACGGGCACTGAGACACGGGC
>Zotu4504
CAACGCTGGCGGCGTGCCTAACACATGCAAGTCGAACGAGAAAGGGACTTCGGTCCTGAGTAAAGTGG
CGCACGGGTGAGTAACACGTGACTAACCTACCTTTGAGCGGGGGATAACCTAGGGAAACCTGGGCTAA
TACCGCATAATACCTACGGGTCAAAGCGAGCAATCGCACTTAAAGAGGGGGTCGCGGCAGATTAGTTA
GTTGGTGAGGTAATGGCTCACCAAGACGATGATCTGTATCCGGCCTGAGAGGGCGCACGGACACACTG
GAACTGAAACACGGTC
>Zotu4505
GAACGCTGGCGGCGTGCCTAATACATGCAAGTTGAACGATGATTTCTGGTACTTGTATCAGAATGAAG
AGTAGCGAACGGGTGAGTAACGCGTGGGTAACCTACCTCTTAGCGGGGGACAACTATTGGAAACGATA
GCTAATACCGCATAACAATGCTTAACACATGTTAAGGATTTGAAAGTACCAATTGGTACACTAGGAGA
TGGACCCGCGTTGTATTAGCTAGTTGGTAGTGTAATGGACTACCAAGGCGATGATACATAGCCGACCT
GAGAGGGTGATCGGCCACATTGGGACTGAGACACGGCC
>Zotu4506
GAACGCTAGCGGCAGGCTTAACACATGCAAGTCGAGGGGTATAGTAGCAATACTAGAGACCGGTGCAC

GGGTGCGTAACGCGTATGCAATCTACCTTTTGCAGGGGAATAGCCCAGAGAAATTTGGATTAATGCCC
CATAGTATATAGAGTTGGCATCAACATTATATTAAAGTCACAACGGCAAAAGATGAGCATGCGTCCCA
TTAGCTAGTTGGTAAGGTAACGGCTTACCAAGGCGACGATGGGTAGGGGTCCTGAGAGGGAGATCCCC
CACACTGGTACTGAGACACGGAC
>Zotu4507
GAACGCTGGCGGCGTGCCTAACACATGCAAGTCGAGCGGAGTTTAGCGGGCGGCGACTTCGGTCAAAG
CCTGTTAAACTTAGCGGCGGACGGGTGAGTAACACGTAGGCAACCTGCCCATGACACGGGGATAGCTT
CGGGAAGCCGGAGGTAATACCCGATACCTTTACTTATCGCATGGTAAGTAAAGAAAGCGACTTAGCGG
TCATGGATGGGCCTGCGTCCCATTAGCTAGTTGGTGAGATAACAGCCCACCAAGGCGACGATGGGTAG
CCGACCTGAGAGGGTGACCGGCCACACTGGAACTTAGACACGGTC
>Zotu4508
GAACGCTGGCGGCGCGCCTAATACATGCAAGTCGAACGGGCACACTTCGGTGTGTCAGTGGCGGACGG
GTGAGTAACACGTGAGTGACCTGGCTCCTAGTACGGAATAACTCTGGGAAACCAGAGCTAATACCGCA
TGTGCTTAGTGTTTCATGATGCTAAGTAAAGATTTATTCGCTAGGAGAGGGGCTTGCGGCGGATTAGC
TAGTCGGTGGGGTAACGGCCTACCGAGGCGACGATCCGTAGCTGGTCTGAGAGGACGACCAGCCACAC
TGGGACTGAGACACGGCC
>Zotu4509
GAACGCTGGCGGCGTGCTTAACACATGCAAGTCGAACGGAAAGGCCCTTCGGGGTACTCGAGTGGCGA
ACGGGTGAGTAACACGTGGGTGATCTGCCCTGCACTTCGGGATAAGCTTGGGAAACTGGGTCTAATAC
CGGATATGATCATGCTCTTCATGGGGTGTGGTGGAAAGCTTTTGCGGTGTGGGATGGGCCCGCGGCCT
ATCAGCTTGTTGGTGGGGTAATGGCCTACCAAGGCGACGACGGGTAGCCGGCCTGAGAGGGTGTCCGG
CCACACTGGGACTGAGATACGGCC
>Zotu4510
GAACGCTGGCGGCGTGCTTAACACATGCAAGTCGAACGATGATGCTGAGCTTGCTTAGCGGATTAGTG
GCGAACGGGTGAGTAACACGTGAGTAACCTGCCCTTGACTCTGGGATAAGCGTTGGAAACGACGTCTA
ATACCGGATATGACCTTCAGCCGCATGGTTTGTTGGTGGAAAGATTTTTTGGTCAAGGATGGACTCGC
GGCCTATCAGGTTGTTGGTGAGGTAATGGCTCACCAAGCCTACGACGGGTAGCCGGCCTGAGAGGGTG
ACCGGCCACACTGGGACTGAGACACGGCC
>Zotu4511
GAACGCTGGCGGCATGCCTTACACATGCAAGTCGAACGCGAAAGGGGGCAACCCTGAGTAGAGTGGCG
AACGGGTGAGTAACACATCGGAACATACCCAGTAGTGGGGGATAACCAGTCGAAAGACTGGCTAATAC
CGCATACGACCTACGGGTGAAAGCGGGGGACCGCAAGGCCTCGCGCCATTGGATTGGCCGATGTCCGA
TTAGCTAGTTGGCGGGGTAAAAGCCCACCAAGGCGACGATCGGTAGCTGGTCTGAGAGGATGATCAGC
CACACTGGGACTGAGACACGGCC
>Zotu4512
GAACGCTGGCGGCAGGCTTAACACATGCAAGTCGAGCGCCCCGCAAGGGGAGCGGCAGACGGGTGAGT
AACACGTGGGAACGTACCCTTTGGTTCGGAACAACCCAGGGAAACTTGGGCTAATACCGGATAAGTCC
GCGAGGAGAAAGATTTATCGCCGAAGGATCGGCCCGCGTCTGATTAGCTAGTTGGTGTGGTAACGGCG
CACCAAGGCGACGATCAGTAGCTGGTCTGAGAGGATGATCAGCCACACTGGGACTGAGACACGGCC
>Zotu4514
GAACGCTGGCGGCGTGCCTAATACATGCAAGTCGAGCGAATTGATGGGAGCTTGCTCCCTGATGTTAG
CGGCGGACGGGTGAGTAACACGTGGGCAACCTGCCCTGCAGATGGGGATAACTCCGGGAAACCGGGGC
TAATACCGAATAATCGGTTCTTCCGCATGGAAGAACTCTGAAAGACGGTTTCGGCTGTCACTGCAGGA
TGGGCCCGCGGCGCATTAGCTAGTTGGTGGGGTAATGGCCTACCAAGGCGACGATGCGTAGCCGACCT
GAGAGGGTGATCGGCCACACTGGGACTGAGACACGGCC
>Zotu4515
GAACGCTGGCGGCGTGCCTAATACATGCAAGTCGAGCGAATGGATGGGAGCTTGCTCCCGGAAATTAG
CGGCGGACGGGTGAGTAACACGTGGGCAACCTGCCTGTAAGACTGGGATAACTTCGGGAAACCGGAGC
TAATACCGGATAACTTCTTTTCTCACATGGGAAAAGGTTGAAAGACGGCTTCGGCTGTCACTTACAGA
TGGGCCCGCGGCGCATTAGCTAGTTGGTGAGGTAACGGCTCACCAAGGCAACGATGCGTAGCCGACCT
GAGAGGGTGATCGGCCACACTGGGACTGAGACACGGCC
>Zotu4516
GAATGCTGGCGGTATGGATAAGGCATGCAAGTCAAGGGGGGCCGCAAGGTCAACCGGCAGACGAGGTA
GTAACACGTACGAACGCGCCCTGTAGTCTGGCATAGCTCGTCGAAAGACGGGGTAATTCCGGATGGTC

CCGAAAGGGTAAAGATTTATCGCTACAGGAGCGGCGTGCGTAGTATCAGCTAGTTGGTAAGGTAATGG
CTTACCAAGGCTACGACGCTTAGGGGGGGTGAGAGCCCGATCCCCACCGATGGGACTGAGACACGGCC
>Zotu4518
GAACGCTGGCGGCGCGCTTAACACATGCAAGTCGAGCGATGAAATCTTGACAGATCCCTTCGGGGTGA
AGATAAGATGGATTAGCGGCGGACGGGTGAGTAACACGTGAGTAACCTGCCTTAGACATTGGGATAGC
CTCGGGAAACCGGGATTAATACCAAATGAAATCATAGGTGCCCCTGCACTAATGATCAAAACTCCAGT
GGTCTAAGATGGACTCGCGTCCCATTAGCTAGTTGGTGAGGTAACGGCCCACCAAGGCGACGATGGGT
AGCCGGCCTGAGAGGGTGAACGGCCACATTGGAACTGAGAAACGGTC
>Zotu4519
GAACGCTGGCGGCGTGCCTAACACATGCAAGTCGAACGGTCCTTCGGGACAGTGGCGCACGGGGGAGG
AACACGTGGCTGACCTGCCCTCAAGTGCGGGATAACTTCGGGAAACCGAAGCTAATACCGCGTAAGCT
CACGGTCGTTAGGGGGCTGTGAGAAAAGGCTTCGGTCGCTTGAGGAGGGGGCTGCGGCGCATCAGGTA
GATGGTGGGGTAACGGCCTACCATGCCGACGACGCGTCGGGGGTCTGAGAGGATGATCCCCCACACTG
GGACTGAGACACGGCC
>Zotu4520
GAACGCTGGCGGCGTGCTTAACACATGCAAGTCGAACGGAAAGGCCCTTCGGGGTGCTCGAGTGGCGA
ACGGGTGAGTAACACGTGGGTGATCTGCCCTGCACTTTGGGATAAGCCTGGGAAACTGGGTCTAATAC
CGAATACACCTCTTCAGCTGCATGGTTGTTGGGGGAAAGCTTTTGCGGTGTGGGATGGGCCCGCGGCC
TATCAGCTTGTTGGTGGGGTGATGGCCTACCAAGGCGACGACGGGTAGCCGGCCTGAGAGGGTGTCCG
GCCACACTGGGACTGAGATACGGCC
>Zotu4521
GAACGCTGGCGGCGCGCTTAACACATGCAAGTCGAACGGTTAAGGCCCCCTCGGGGGCGAATAGAGTG
GCGAACGGCTGAGTAACACGTGGGCAACCTGCCCCCCGCACCGGGATAGCCTCGGGAAACCGTGGGTA
ATACCGGATACTCCCCCACCGGCGCATGCCGGAGGGGGGAAAGCGTACACGGCGGGGGATGGGCCCGC
GGCCTGTTAGCTCGTTGGCGGGGTAACGGCCCACCAAGGCGATTATGGGTAGCCGGGTTGAGAGACCG
ACCGGCCAGATTGGGACTGAGACACGGCC
>Zotu4523
GAACGCTAGCGGCGTGACTAACACATGCAAGTCGAACGTGAGAGGGTACTTGTATCCTTGAAAGTGGC
GGACGGGTGAGTAAGACGTAGGTGACCTGCCTCGGAGTGGGGGATAACCACTGGAAACGGTGGCTAAT
ACCGCATACGTTGGTGAAGATGGGAATCTTTATCAGGAAAGCTTAAGTGCTTTGAGAGGGGCCTACGG
CCGATTAGCTAGTTGGTGAGGTAAAGGCTCACCAAGGCGATGATCGGTAGCTGGTCTGAGAGGACGAT
CAGCCACACGGGAACTGAGACACGGTC
>Zotu4524
GAACGCTGGCGGCATGCTTAACACATGCAAGTCGAACGGTTATATGGGGCTTGCTCCATATGATAGTG
GCAGACGGGTGAGGAACGCGTGGGAACGTACCCCAAGGTTCGGAATAACAGTTGGAAACGACTGCTAA
TACCGGATAAGTCCGAGAGGAGAAAGGTTTACCGCCTTGGGAGCGGCCCGCGTTAGATTAGGTAGTTG
GTAGGGTAAAGGCCTACCAAGCCGATGATCTATAGCTGGTTTGAGAGGATGATCAGCCACACTGGGAC
TGAGACACGGCC
>Zotu4525
GAACGCTGGCGGCAGGCTTAACACATGCAAGTCGAGCGCCCCGCAAGGGGAGCGGCAGACGGGTGAGT
AACGCGTGGGAATCTACCCATCCCTACGGAACAACTCCGGGAAACTGGAGCTAATACCGTATACGCCC
TTCGGGGGAAAGATTTATCGGGGATGGATGAGCCCGCGTTGGATTAGCTAGTTGGTGGGGTAATGGCC
TACCAAGGCGACGATCCATAGCTGGTCTGAGAGGATGATCAGCCACACTGGGACTGAGACACGGCC
>Zotu4526
GAACGCTGGCGGCGTGCCTAACACATGCAAGTCGAACGAGATCCATGGAGTGCTTGCACTCCGGAAGA
TCTAGTGGCGAACGGGTGAGTAACACGTGAGAAACCTGCCCCAGACTCTGGGATAACCTCGGGAAACC
GAAGCTAATACCGGATATTCTCCTTTGGTCGCATGGCCAGTTGAGGAAAGTTTTTCGGTCTGGGAGGG
TCTCGCGGCCTATCAGCTAGTTGGTGGGGTAACGGCCCACCAAGGCGACGACGGGTAGCTGGTTTGAG
AGAACGATCAGCCACACTGGAACTGAGACACGGTC
>Zotu4527
GAACGCTGGCGGCGTGGATTAGGCATGCAAGTCGAACGCGAAAGGGGAGCAATCCCTGAGTAGAGTGG
CGAACGGGTGAGTAACACGTGAGAAACCTGCCTTCATCAGGGGGATAACACTCCCAACGGGGTGCTAA
TACCGCATGAGACCACGGGATCGCATGGTCCTGAGGTCAAAGGTGGCCTCTGCTTGCAAGCTACCGGA
TGAAGATGGTCTCGCGTCCCATTAGCTAGTTGGTGGGGTAATGGCCTACCAAGGCGACGATGGGTAGC

TGGTCTGAGAGGATGGTCAGCCACACTGGGACTGAGATACGGCC
>Zotu4528
GAACGCTGGCGGCGTGCTTAACACATGCAAGTCGAACGGGATCCATCAGGCTTTGCTTGGTGGTGAGA
GTGGCGAACGGGTGAGTAATGCGTGACCGACCTGCCCCATACACCGGAATAGCTCCTGGAAACGGGTG
GTAATGCCGGATGCTCCAGTTGATCGCATGGTCTTCTGGGAAAGCTTTCGCGGTATGGGATGGGGTCG
CGTCCTATCAGCTTGACGGCGGGGTAACGGCCCACCGTGGCTTCGACGGGTAGCCGGCCTGAGAGGGC
GACCGGCCACATTGGGACTGAGATACGGCC
>Zotu4531
GAGCGCTGGCGGCATGCCTAATACATGCAAGTCGAACGGAACACTATTTTTGAGAATCCCGATATCCT
CGGGGCGTCTCATAAATCGTGTTTAGTGGCGGACGGGTGAGTAACACGTTGGTACCTGCCCTCTAGTG
GGGAATAACTGGCCGAAAGGTCGGCTAATACCGCATGTGGTCTACGGATCAAAGCCCTTCGGGGCGCT
AGAGGAGGGGCCTGCGTCTGATTAGCTAGTTGGTAGGGTAAAGGCCTACCAAGGCGACGATCAGTAGC
TGGTTCGAGAGGATGACCAGCCACACTGGGACTGAGATACGGCC
>Zotu4532
GAACGCTGGCGGCGTGGATAAGGCATGCAAGTCGAGAGGGTTAGACCCTCGGCAAACGGGTTAGTAAT
GCATAGGAATTTACCCTGAAGTCGTGGATAATCCGGAGAAATCCGGAATAATACACGATATGTTCTAC
GGAATAAAGATTTATCGCTTCAGGAGAAGCCTATGTCCTATCAGCTTGTTGGTGGTGTAAAGGACCAC
CAAGGCTATGACGGGTAGGGGGTGTGAGAGCACGGCCCCCAACATTGGAACTGCGACACTGTC
>Zotu4535
GAACGCTGGCGGCAGGCCTAACACATGCAAGTCGAACGGGCGTCTTCGGACGTCAGTGGCGGACGGGT
GAGTAACGCGTGGGAACATGCCCTTTGGTTCGGAACAACTCGGGGAAACTTGAGCTAATACCGGATAA
GCCCTTTTGGGGAAAGATTTATCGCCGAAGGATTGGCCCGCGTCTGATTAGCTAGTTGGTGTGGTAAC
GGCGCACCAAGGCGACGATCAGTAGCTGGTCTGAGAGGATGATCAGCCACATTGGGACTGAGACACGG
CC
>Zotu4537
GAACGCTAGCGGCAGGCCTAATACATGCAAGTCGAGGGGCAGCGGGGTAGCAATACTGCCGGCGACCG
GCGCACGGGTGCGTAACACGTATGCAACCTACCTTAAACAGGGGGATAGCCCGGGGAAACTCGGATTA
ATACCCCATAACACGGTGATACCACATGGTAATGTCGTTAAAGATTTATCGGTTTAAGCTGGGCATGC
GTTCGATTAGCTAGTTGGTAAGGTAATGGCTTACCAAGGCTACGATCGATAGGGGGTCTGAGAGGATG
ATCCCCCACACTGGTACTGAGATACGGAC
>Zotu4538
GAACGCTGGCGGCGTGCTTAACACATGCAAGTCGAACGGTGATGCAGAGCTTGCTCTGCGGATCAGTG
GCGAACGGGTGAGTAACACGTGAGTAACCTGCCCTTGACTCTGGGATAAGCGTTGGAAACGACGTCTA
ATACCGGATACGACCCGTGGAGGCATCTCCTGCGGGTGGAAAGAATTTTGGTTGAGGATGGACTCGCG
GCCTATCAGCTTGTTGGTGAGGTAACGGCTCACCAAGGCGACGACGGGTAGCCGGCCTGAGAGGGTGA
CCGGCCACACTGGGACTGAGACACGGCC
>Zotu4539
GAACGCTGGCGGCAGGCTTAACACATGCAAGTCGAGCGGGGAAAGGTAGCTTGCTACCTGACCTAGCG
GCGGACGGGTGAGTAATGCTTAGGAATCTGCCTATTAGTGGGGGACAACATTCCGAAAGGAATGCTAA
TACCGCATACGCCCTACGGGGGAAAGCAGGGGATCTTCGGACCTTGCGCTAATAGATGAGCCTAAGTC
AGATTAGCTAGTTGGTGGGGTAAAGGCCTACCAAGGCGACGATCTGTAGCGGGTCTGAGAGGATGATC
CGCCACACTGGGACTGAGACACAGCC
>Zotu4541
GAACGCTGGAAATGTGGATACCGGCATGCAAGTCAAGGGGCCGCAAGGCACCGGCGAACGGCTTAGTA
ACACGTAGATACATACCCAAAAGTGGGGCATAGCTCGTCGAAAGACGGGGTAATTTCCCATAGCACCG
CAAGGTTAAACACGCAAGTGGCTTTTGGAATGGTCTGCGGTCTATCAGCTAGTTGGTGAGGTAAAGGC
TCACCAAGGCTATGACGGATAGGGGGCGTGAGAGCGCGACCCCCAAGAATGGCACTGCGACACGGGC
>Zotu4543
GAACGCTAGCGGCGTGCTTAACACATGCAAGTGGAGCGACGAACCAGGGCTTGCCCTGGGGCAGAGCC
GCGAACGGGTGAGTAACACGTGGGTAACCTACCCCGATGATTGGGACAACCCGGGGAAACCCGGGCTA
ATACCAAATGTGGTCTCTCTTCATAAGTAGAGCGACTAAAGGAAGCTTCGGCCTCCGCATTGGGAGGG
GCCCGCGGCCCATTAGCTTGTTGGTGAGGTAACGGCTCACCAAGGCTTCGATGGGTAGCTGGTCTGAG
AGGACGATCAGCCACACTGGGACTGAGACACGGCC
>Zotu4544

TTTCAGCGAAACCTGAGAAAAGCGCTCTTCCCCTCCATCAGAAAATGCCTGGAGTGCGCCAACAGGCC
TGGATTCGTGTGCTTACTGTGCGCGCAACGGAGTT
>Zotu4545
GAACGCTAGCGGCAGGCTTAATACATGCAAGTCGAGGGGCAGCGCGGGTAGCAATACCTGGCGGCGAC
CGGCAAACGGGTGCGGAACACGTACGCAACCTTCCCAAAACTGGTGAATAGCCCTCCGAAAGGAGGAT
TAATACACCGTACCATAACGAAGAGGCATCTCTTTGTTATTATAGTAGCAATACGGTTTTGGATGGGC
GTGCGCCTGATTAGGTAGTTGGCGGGGTAACGGCCCACCAAGCCTGCGATCAGTAACTGGTGTGAGAG
CACGACCAGTCACACGGGCACTGAGACACGGGC
>Zotu4546
GAACGCTGGCGGCAGGCCTAACACATGCAAGTCGAGCGCGCCCTTCGGGGTGAGCGGCGGACGGGTGA
GTAACGCGTGGGAACGTGCCCTTTGGTTCGGAATAGTCCTGGGAAACTGGGGGTAATACCGGATACGC
CCTTTTTGGGGAAAGATTTATCGCCGAAGGATCGGCCCGCGTTGGATTAGGTAGTTGGTGGGGTAATG
GCCTACCAAGCCGACGATCCATAGCTGGTTTGAGAGGATGATCAGCCACACTGGGACTGAGACACGGC
C
>Zotu4547
GAACGCTGGCGGCGTGCCTTAGGCATGCAAGTCGAACGGTTTCCGCAAGGAAGCAGTGGCAGACGGGT
GAGTAACGCGTAGAAATCTACCCCTAGGAGGACCATAGCTCATCGAAAGATGGGGTAATAGTCCATGG
TCTCGCAAGAGTAAAGCAATTAACTTTGCGCCAAGGGAGGAGTCTGCGTCCTATCAGCTTGTTGGTGG
GGTAATGGCCTACCAAGGCAATGACGGGTAGCTGGACTGAGAGGTCGATCAGCCACAACTGCACTTAG
ACACGGGC
>Zotu4548
GAACGCTGGCGGCGTGCCTAACACATGCAAGTCGAGCGGAGAGATTGTCGTATTTATATGACATGATC
TTAGCGGCGGACGGGTGAGTAACACGTGGGTAACCTGCCTCAAAGTGGGGGATAGCCTTTCGAAAGGA
AGATTAATACCGCATAAGATTGTAGCATCACATGATGCAGCAAGAAAAGGAGCAATCCGCTTTGAGAT
GGACCCGCGGCGCATTAGCTAGTTGGTGAGGTAACGGCTCACCAAGGCCACGATGCGTAGCCGACCTG
AGAGGGTGATCGGCCACATTGGAACTGAGATACGGTC
>Zotu4549
GAATGCTGGCGGCGTGGATAAGTCATGCAAGTCAAGGGGGCCTGTAAGGGCAACCGGCAGACGAGGTA
GTAACACGTAGGTACGTCCCCCGAAGTCGGGCATAGCCATCCGAAAGGATGGGTAATTCCCGATAGTC
CCGCAAGGGTAAAGGTTTTTCGCTTCGGGAGCGGCCTGCGTAGTATCAGCTAGTTGGTAGGGTAATGG
CCTACCAAGGCTACGACGCTTAGGGGAGCTGAGAGGCTGACCCCCACCGATGGAACTGCGACACGGTC
>Zotu4550
CAACGCTGGCGGCGCGCCTAACACATGCAAGTCGAACGAGAAAGCCGGAGCAATCTGGTGAGTAAAGT
GGCGTACGGGTGAGTAACACGTGGGTGATTTACCCTTTAGTGGGGGATAACCCGGGGAAACCTGGGCT
AATACCGCATAAGTCCGAGAGGAGAAAGTCGCAAGACGCTGAAGGAGAAGCCCGCGGCCGATTAGCTA
GTTGGTGGGGTAATGGCCTACCAAGGCAACGATCGGTAGCCGGCCTGAGAGGGCACACGGCCACACTG
GCACTGAAACACGGGC
>Zotu4551
GAACGCTGGCGGCAGGCTTAACACATGCAAGTCGAGCGGGCTCTTTCGGGGGTCAGCGGCGGACGGGT
TAGTAACGCGTGGGAACGTGCCTTTTGCTTCGGAATAGCCCTGGGAAACTGGGGGTAATACCGGATGT
GCCCTGAGGGGGAAAGATTTATCGGCAAGAGATCGGCCCGCGTCTGATTAGGTAGTTGGTGTGGTAAC
GGCGCACCAAGCCGACGATCGGTAGCTGGTTTGAGAGGATGATCAGCAACACTGGGACTGAGACACGG
CC
>Zotu4552
GAACGCTGGCGGCAGGCTTAACACATGCAAGTCGAGCGGGCCCTTCGGGGTCAGCGGCAGACGGGTGA
GTAACGCGTGGGAACGTACCATTTGCTACGGAATAACTCAGGGAAACTTGTGCTAATACCGTATGAGC
CCCCCTTTAAAATTTCAGGAATCATAAAATGCCCTGGCATTTTATGGGGGGGAAAGATTTATCGGCAA
ATGATCGGCCCGCATTGGATTAGCTAGTTGGTGGGGTAAAGGCCTACCAAGGCGACGATCCATAGCTG
GTCTGAGAGGATGATCAGCCACACTGGGACTGAGACACGGCC
>Zotu4554
GAACGCTGGCGGCGTGCCTAATACATGCAAGTCGAGCGGACTTTTAAAAGCTTGCTTTTAAAAGTTAG
CGGCGGACGGGTGAGTAACACGTGGGCAACCTGCCTGTAAGACTGGGATAACTTCGGGAAACCGGAGC
TAATACCGGATAATGTTTTTTCTCTCATGAGGAGAAACTGAAAGATGGTTTACGCTATCACTTACAGA
TGGGCCCGCGGCGCATTAGCTAGTTGGTGAGGTAACGGCTCACCAAGGCGACGATGCGTAGCCGACCT

GAGAGGGTGATCGGCCACACTGGGACTGAGACACGGCC
>Zotu4555
GAACGCTGGCGGCATGCTTAACACATGCAAGTCGAACGAGACTTGGTAACAAGTTGCAGTGGCGGACG
GGTGCGTAACGCGTAAGAATCTACCTTTCGGAGGGGGATAACATCGGGAAACTGATGCTAATACCCCA
TAATGCTGAGGAGCTAAAGGTGAAAATCGCCGAAAGAGGAGCTTGCGTCTGATTAGCTAGTTGGTGGG
GGTAAAAGCCTCCCAAGGCCACGATCAGTAGCTGGTCTAAGAGGATGATCAGCCACACTGGGACTGAG
ACACGGCC
>Zotu4556
CAACGCTGGCGGCGTGCCTAACACATGCAAGTCGAACGCGGTTGCCCCGCAAGGGGCAACTGAGTGGC
GAACGGGTGAGTAACGCGTGGGTGATCTACCTTCGAGTGGGGGATAACGTCCCGAAAGGGACGCTAAT
ACCGCATGACATCCTGCCTTTGAACAGGCGGAGATCAAAGCCGGGGATCGCAAGACCTGGCGCTTGTT
GAGGAGCCCGCGTCCGATTAGCTAGTTGGTGGGGTAATGGCCCACCAAGGCGACGATCGGTAGCCGGC
CTGAGAGGGCGGACGGCCACACTGGGACTGAGACACGGCC
>Zotu4557
GAACGCTAGCGGCAGGCTTAATACATGCAAGTCGAGGGGCAGCGGGGGTAGCAATATCTGCCGGCGAC
CGGCAAACGGGTGCGGAACACGTACGCAACCTTCCTTTAAGCGGGGAATAGCCCAGAGAAATTTGGAT
TAATACCCCATAACATAATGAAGAAGCATTTCTTTATTATTAAAGATTTATCACTTAGAGATGGGCGT
GCGGCTGATTAGGTAGTTGGCGGAGTAACAGCCCACCAAGCCTGCGATCAGTAGCTGATGTGAGAGCA
TGATCAGCCACACGGGCACTGAGACACGGGC
>Zotu4558
GAACGCTGGCGGCATGCCTAACACATGCAAGTCGAACGAGACCTTCGGGTCTAGTGGCGCACGGGTGC
GTAACGCGTGGGAATCTGCCCTTTGCTTCGGAATAACTCAGGGAAACTTGAGCTAATACCGGATAATG
TCGCAAGACCAAAGATTTATCGGCAAAGGATGAGCCCGCGTAGGATTAGCTTGTTGGTGGGGTAATGG
CCCACCAAGGCGACGATCCTTAGCTGGTCTGAGAGGATGATCAGCCACACTGGGACTGAGACACGGCC
>Zotu4559
GAACGCTGGCGGCGTGCCTTAAGCATGCAAGTAGAACGAGTTTCTATCTTGCCGCAAGGCAAGCTTAG
AAACTAGTTGCGAACGGCTGAGTAATCAATAGGAATCTTTCCTTTAGTGGGGAATACCTCATCGAAAG
ATGAGCTAATACCGCATAACATCTCCGGATAAAAGCGATTAACTTCGCGCTGATTGAGGAGCCTATTG
CCTATCAGGTAGTTGGTGGGGTAAAAGCCTACCAAGCCTACGACGGGTACCCGATCTGAGAGGATGGA
CGGGCAGAATGGGACTGAGACACGGCC
>Zotu4560
GAACGCTAGCGGCAGGCTTAACACATGCAAGTCGAGGGGTATAGTAGCAATACTAGAGACCGGCGCAC
GGGTGCGTAACGCGTATGCAATCTACCTTTTGCAGGGGAATAGCCCAGAGAAATTTGGATTAATGCCC
CATAGTATAGTACAATCGCATGATTGAACTATTAAAGTCACAACGGCAAAAGATGAGCATGCGTCCCA
TTAGCTAGTTGGTAAGGTAACGGCTTACCAAGGCGACGATGGGTAGGGGTCCTGAGAGGGAGATCCCC
CACACTGGTACTGAGACACGGAC
>Zotu4562
GAACGCTGGCGGCATGCCTAACACATGCAAGTCGAACGAGACCTTCGGGTCTAGTGGCGCACGGGTGC
GTAACGCGTGGGAATCTGCCCTTGGGTTCGGAATAACAGTTAGAAATGACTGCTAATACCGGATGATG
ACGTAAGTCCAAAGATTTATCGCCCAGGGATGAGCCCGCGTAAGATTAGCTAGTTGGTAGGGTAAAGG
CCTACCAAGGCTACGATCTTTAGCTGGTCTGAGAGGATGATCAGCCACACTGGGACTGAGACACGGCC
>Zotu4563
GAACGCTGGCGGCATGCCTAACACATGCAAGTCGAACGAAGCCTTCGGGCTTAGTGGCGCACGGGTGC
GTAACGCGTGGGAATCTGCCCTTCGGTTCGGAACAACAGTTAGAAATGACTGCTAATACCGGATGATG
ACGTAAGTCCAAAGATTTATCGCCGAAGGATGAGCCCGCGTAGGATTAGCTAGTTGGTAAGGTAAAAG
CTTACCAAGGCGACGATCCTTAGCTGGTCTGAGAGGATGATCAGCCACACTGGGACTGAGACACGGCC
>Zotu4564
GAACGCTGGCGGCATGCCTAACACATGCAAGTCGAACGAACTCTTCGGAGTTAGTGGCGCACGGGTGC
GTAACGCGTGGGAATCTGCCCTTGGGTTCGGAATAACAGTTAGAAATGACTGCTAATACCGGATGATG
TCTTCGGACCAAAGATTTATCGCCCAGGGATGAGCCCGCGTAGGATTAGGTAGTTGGTGGGGTAAAGG
CCTACCAAGCCGACGATCCTTAGCTGGTCTGAGAGGATGATCAGCCACACTGGGACTGAGACACGGCC
>Zotu4565
GAACGCTGGCGGCGTGCTTAACACATGCAAGTCGAACGGAAAGGCCCGCTTGCGGGTACTCGAGTGGC
GAACGGGTGAGTAACACGTGGGCAACCTGCCCTCAGCTTTGGGATAACTCCCGGAAACGGGTGCTAAT

ACCGAATATTGACACGATGTCACATGGTGTTGTGTTGAAAGTTTTTCGGCTGGGGATGGGCCCGCGGC
CTATCAGCTTGTTGGTGGGGTGATGGCCTACCAAGGCGACGACGGGTAGCCGGCCTGAGAGGGCGATC
GGCCACACTGGAACTGAGACACGGTC
>Zotu4566
GAACGCTGGCGGCATGCCTAACACATGCAAGTCGAACGAGATCTTCGAGTCTAGTGGCGCACGGGTGC
GTAACGCGTGGGAATCTGCCCTTTGGTTCGGAATAACAGTTGGAAACGACTGCTAATACCGGATGATG
ACGAAAGTCCAAAGATTTATCGCCAGAGGATGAGCCCGCGTAGGATTAGCTAGTTGGTGTGGTAAAGG
CGCACCAAGGCGACGATCCTTAGCTGGTCTGAGAGGATGATCAGCCACACTGGGACTGAGACACGGCC
>Zotu4567
GAACGCTGGCGGCATGCTTTACACATGCAAGTCGAACGGCAGCGAAGAGAAGCTTGCTTCTCTGTCGG
CGAGTGGCGAACGGGTGAGTATAGCATCGGAACGTGCCAAGTAGTGGGGGATAACCAAGCGAAAGTTT
GGCTAATACCGCGTAAGCTCTAAGGAGGAAAGTAGGGGACCGCAAGGCCTTACGCTATTTGATCGGCC
GATGTCGGATTAGCTAGTTGGTGAGGTAATGGCTCACCAAGGCGATGATCCGTAGCGGGTCTGAGAGG
ACGATCAGCCACACTGGGACTGAGACACGGCC
>Zotu4568
GAACGCTGGCGGCGTGCCTTAAGCATGCAAGTCGAACGGACCGCAAGGTCAGTGGCGGACGGGTGAGT
AACGCGTGGGAATCTGCCTTAAAGTTGGACATAGCTCATCGAAAGATGGGGTAATTTCCAATGTGGAT
GTAAATCTAAAGTCGCAAGACGCTTTAAGATGAGCCCGCGTCCTATCAGCTAGTTGGTGGGGTAATGG
CCTACCAAGGCGACGACGGGTAGCTGGTCTGAGAGGATGGTCAGCCACAATAGCATTGAGATACGGGC
>Zotu4570
GAACGCTGGCGGCGCGCCTAACACATGCAAGTCGAGCGATGAAGAGAAGACAGAAGCCCTCGGGCAGA
CGTCATCTTGGATTAGCGGCGGACGGGTGAGTAACAGGTGAGTAACCTACCTTTGACACAGGGATAGC
CTCGGGAAACCGGGATTAATACCGGATGACATTACACTTTCGCATGAAAGAGTAATCAAAGAGATTCG
GTCAAAGATGGACTCGCCGCTGATTAGCTAGTTGGTGAGATAACAGCCCACCAAGGCAACGATCAGTA
GCCGGCCTGAGAGGGTGAACGGCCACATTGGAACTGAGACACGGTC
>Zotu4571
GAACGCTGGCGGCGGGCCTCATACATGCAAGTCGAGCGCGAAAGTCCGCAAGGATGAGTAAAGCGGCG
CACGGGTGAGTAACACGTGGATAATCTACCCTTTGGATTGGAATAACCTGTCGAAAGACGGGCTAATG
CCGAGTAAGCCCTCGAGAGCGTGAAGCTCTTGCGGGGAAAGATGGCGCAAGCTATCGCCGATGGATGA
GTCTGCGTCCCATTAGTTAGTTGGTGAGGTAACGGCTTACCAAGACAATGATGGGTACCTGGTCTGAG
AGGATGACCAGGCACACTGGAACTGAAACACGGTC
>Zotu4572
GAACGCTGGCGGCGTGCCTAACACATGCAAGTCGAGCGAACCCTTCTGGGTGAGCGGCGGACGGGTGA
GTAACGCGTGGGTAACCTGCCCTGTACACACGGATAACATACCGAAAGGTATGCTAATACGAGATAAT
ATGTAATGATCGCATGGTCAATACATCAAAGCTCCGGCGGTACAGGATGGACCCGCGTCTGATTAGCT
AGTTGGTGAGGTAATGGCTCACCAAGGCGACGATCAGTAGCCGACCTGAGAGGGTGATCGGCCACATT
GGAACTGAGACACGGTC
>Zotu4573
GAACGCTGGAAATGTGGATACTGGCATGCAAGTCAAGGGGCCGCAAGGCACCGGCGAACGGCTTAGTA
ACACGTAGATACATACCGTAAAGTGGGGCATAGCTCGTCGAAAGACGGGGTAATTTCCCATGTGAACG
CAAGTTTAAAGGCGCAAGCCGCTTTATGAATGGTCTGCGGTCTATCAGCTAGTTGGTGAGGTAAAGGC
TTACCAAGGCTATGACGGATAGGGGGCGTGAGAGCGCGACCCCCAAGAATGGCACTGCGACACGGGC
>Zotu4575
GAACGCTGGCGGCGTGCCTAATACATGCAAGTCGAGCGAATTTTTGGGAGCTTGCTCCCAAAAGTTAG
CGGCGGACGGGTGAGTAACACGTGGGTAACCTGCCTATAAGACTGGGATAACTTCGGGAAACCGGAGC
TAATACCGGATAATTCTTTTCCTCTCATGAGGAAAAGCTGAAAGACGGTTTCGGCTGTCACTTATAGA
TGGGCCCGCGGCGCATTAGCTAGTTGGTGAGGTAACGGCTCACCAAGGCAACGATGCGTAGCCGACCT
GAGAGGGTGATCGGCCACACTGGGACTGAGACACGGCC
>Zotu4577
GAATGCTGGCGGCGTGCCTAACACATGCAAGTCGAGCGGCAGCGCGAGTAGTTTACTACTTGGCGGCG
AGCGGCGGACGGCTGAGTAACGCGTGGGAACGTGCCCCAAAGTGAGGAATAACTGCCCGAAAGGGTAG
CTAATGCCGCATATGGTCTTCGGATTAAAGTATTTATACGCTTTGGGAGCGGCCCGCGTCGGATTAGG
TTGTTGGTGAGGTAATGGCTCACCAAGCCGACGATCCGTAGCTGGTCTGAGAGGATGATCAGCCAGAC
TGGAACTGAGACACGGTC

>Zotu4578
AAACGCTGGCGGCGTGCTTAACACATGCAAGTCGAGCGAGAACGTTTCTTCGGAAACTAGTACAGCGG
CGGACGGGTGAGTAACACGTGAGAATTTACCCTTTAGTGAAGAATACTCCTGAGAAATCAGGCTTAAT
ACTGCATAAGTCCCTACGGGGAGAAAGGTGGCCTCTTTGCTGCCGCTAAAGGATAAGCTTGCGGGCTA
TTAGCTAGTTGGTGAGGTAAAGGCTCACCAAGGCGATGATGGCTAGCCGACCTGAGAGGGTGTACGGC
CACACTGGAACTTAGACACGGTC
>Zotu4580
GAACGCTGGCGGCATGCCTTACACATGCAAGTCGAACGGCAGCGCGGGGCAACCTGGCGGCGAGTGGC
GAACGGGTGAGTAATATATCGGAACGTACCCTGGAGTGGGGGATAACGTAGCGAAAGTTACGCTAATA
CCGCATACGATCTAAGGATGAAAGTGGGGGATGCGTAAGCACCTCATGCTCCTGGAGCGGCCGATATC
TGATTAGCTAGTTGGTGGGGTAAAGGCCCACCAAGGCATCGATCAGTAGCTGGTCTGAGAGGACGACC
AGCCACACTGGAACTGAGACACGGTC
>Zotu4581
GAACGCTGGCGGCAGGCCTAACACATGCAAGTCGAGCGGCAGCACGGGTACTTGTACCTGGTGGCGAG
CGGCGGACGGGTGAGTAATGCCTAGGAATCTGCCTGGTAGTGGGGGATAACGCTCGGAAACGGACGCT
AATACCGCATACGTCCTACGGGAGAAAGCAGGGGACCTTCGGGCCTTGCGCTATCAGATGAGCCTAGG
TCGGATTAGCTAGTTGGTGAGGTAATGGCTCACCAAGGCTACGATCCGTAACTGGTCTGAGAGGATGA
TCAGTCACACTGGAACTGAGACACGGTC
>Zotu4582
GAACGCTGGCGGCGTGCCTAACACATGCAAGTCGAACGGTCCATTTCGGTGGATAGTGGCGCACGGGT
GAGTAACACGTGGTTGACCTACCTCAGAGTAAGGGATAACGTCTCGAAAGGGATGCTAATACCTTATA
AGCTTCCGGTATTTAGAAAACTGGGAGAAAAGTTCCGGCGCTTTGAGAGGGGACTGCGAGCCATCAGC
TAGTTGGTGTGGTAATGGCACACCAAGGCGACGACGGCTAGGGGGCCTGAGAGGGTGACCCCCCACAC
TGGAACTGAGACACGGTC
>Zotu4584
GAACGCTGGCGGCGTGCTTAACACATGCAAGTCGAACGGTAAGGCCCTTTCGGGGGTACACGAGTGGC
GAACGGGTGAGTAACACGTGGGTGATCTGCCCTGCACTTTGGGATAAGCCTGGGAAACTGGGTCTAAT
ACCGAATAGGACTCCGGACTGCATGGTCTGGGGTGGAAAGCTTTTGCGGTGTGGGATGGGCCCGCGGC
CTATCAGCTTGTTGGTGGGGTGATGGCCTACCAAGGCGACGACGGGTAGCCGGCCTGAGAGGGTGTCC
GGCCACACTGGGACTGAGATACGGCC
>Zotu4585
GAACGCTGGCGGCGTGCTTAACACATGCAAGTCGAACGGTGACGGAGAGCTTGCTCTCCTGATCAGTG
GCGAACGGGTGAGTAACACGTGGGCAACCTGCCCCTGACTTCGGGATAACGCTTCGAAAGAGGTGCTA
ATACCGAATACGAACTCCCTTGGCATCTTGGGAGTTGGAAAGTTTTTCGGTCAGGGATGGGCCCGCGG
CCTATCAGCTTGTTGGTGAGGTAATGGCTCACCAAGGCGACGACGGGTAGCCGGCCTGAGAGGGCGAC
CGGCCACACTGGGACTGAGACACGGCC
>Zotu4586
GAACGCTGGCGGCAGGCCTAACACATGCAAGTCGAGCGGACCTTTCGGGGTCAGCGGCGGACGGGTGA
GTAACGCGTGGGAATATGCCCTTCTCTACGGAATAGTCCTGGGAAACTGGGGGTAATACCGTATACGC
CCTTTGGGGGAAAGATTTATCGGGGAAGGATTAGCCCGCGTTGGATTAGGTAGTTGGTGGGGTAATGG
CCTACCAAGCCGACGATCCATAGCTGGTTTGAGAGGATGATCAGCCACACTGGGACTGAGACACGGCC
>Zotu4587
GAACGCTGGCGGCATGCCTAACACATGCAAGTCGAACGAGACCTTCGGGTCTAGTGGCGCACGGGTGC
GTAACGCGTGGGAATCTGCCCCTTGATTCGGAATAACTCAGAGAAATTTGAGCTAATACCGGATGATG
ACGTAAGTCCAAAGATTTATCGCCAAGGGATGAGCCCGCGTAGGATTAGCTAGTTGGTGAGGTAAAGG
CTCACCAAGGCGACGATCCTTAGCTGGTCTGAGAGGATGATCAGCCACACTGGGACTGAGACACGGCC
>Zotu4588
GAACGCTGGCGGCGTGCTTAACACATGCAAGTCGAACGGTAAGGCCCCTTCGGGGGTACACGAGTGGC
GAACGGGTGAGTAACACGTGGGTAATCTGCCCTGCACTTCGGGATAAGCCTGGGAAACTGGGTCTAAT
ACCGGATATGAGCTCTCACCGCATGGTGGGGGTTGGAAAGTTTTTCGGTGCAGGATGAGCCCGCGGCC
TATCAGCTTGTTGGTGGGGTAATGGCCTACCAAGGCGACGACGGGTAGCCGGCCTGAGAGGGTGATCG
GCCACACTGGGACTGAGACACGGCC
>Zotu4589
GAACGCTGGCGGCGTGCTTAACACATGCAAGTCGAGCGGAAAGGCCCTTTCGGGGGTACTCGAGCGGC

GAACGGGTGAGTAACACGTGAGTAATCTGCCCCTGGCTTTGGGATAGCCACCGGAAACGGTGATTAAT
ACCGGATATGACCACTTTTCGCATGGATGGTGGTGGAAAGTTTTTCGGCCAGGGATGTGCTCGCGGCC
TATCAGCTTGATGGTGAGGTAATGGCTCACCATGGCTTCGACGGGTAGCCGGCCTGAGAGGGTGACCG
GCCACACTGGGACTGAGACACGGCC
>Zotu4591
GAACGCTGGCGGCGTGCCTAATACATGCAAGTCGAGCGATCGAGCTTTCCTTCGGGAAAGCTCGGTAG
CGGCGGACGGGTGAGTAACACGTAGGCAACCTGCCCATAAGACCGGGATAACCCACGGAAACGTGAGC
TAATACCGGATACACAGTTTCTTCGCATGGAGGGACTGGGAAAGGCGGAGCAATCTGCCACTTACGGA
TGGGCCTGCGGCGCATTAGCTAGTTGGTGAGGTAACGGCTCACCAAGGCGACGATGCGTAGCCGACCT
GAGAGGGTGAACGGCCACACTGGGACTGAGACACGGCC
>Zotu4592
GAACGCTGGCGGCGTGCTTAACACATGCAAGTCGAACGAGGTCCATCCGGTAGCAATACCGGAGAAGA
CCTAGTGGCGAACGGGTGAGTAACACGTGAGGAACCTGCCCCGAAGACCGGGATAACAGCGGGAAACC
GCTGCTAATACCGGATACCCCCATCGGACCACATGGCCTGATGAGGAAATGGATTCCGCTTCGGGAGG
GCCTCGCGGCCTATCAGCTTGTTGGTGAGGTGACGGCTCACCAAGGCGACGACGGGTAGCTGGTCTGA
GAGGACGATCAGCCACACTGGGACTGAGACACGGCC
>Zotu4595
GAACGCTGGCGGCGTGCTTAACACATGCAAGTCGAGCGGAAAGGCCACTTCGGTGGTACTCGAGCGGC
GAACGGGTGAGTAACACGTGAGTAATCTGCCCCTGGCTTTGGGATAGCCACCGGAAACGGTGATTAAT
ACCGGATACGACCACTTTGCGCATGCGATGGTGGTGGAAAGTTTTTCGGCCAGGGATGTGCTCGCGGC
CTATCAGCTTGATGGTGAGGTAATGGCTCACCATGGCTTCGACGGGTAGCCGGCCTGAGAGGGTGACC
GGTCACACTGGGACTGAGACACGGCC
>Zotu4596
GAACGCTGGCGGCAGGCTTAACACATGCAAGTCGAACGCCCCGCAAGGGGAGTGGCAGACGGGTGAGT
AACACGTGGGAACATACCCTTCGGTTCGGAACAACGCCGGGAAACTGGCGCTAATACCGGATACGGCT
TGAGGGCGAAAGATTCATCGCCGAAGGATTGGCCCGCGTTCGATTAGCTTGTTGGTGAGGTAACGGCT
CACCAAGGCGACGATCGATAGCTGGTCTGAGAGGATGGCCAGCCACATTGGGACTGAGACACGGCC
>Zotu4597
GAATGCTGGCGATGTGGATGAGTCATGCAAGTCAAGGGGGCCCGCAAGGGCAACCGGCAAACGAGGTA
GTAACACGTAGGTAGATCCCCCGGAGTCGGGCATAGCTTGCCGAAAGGCAAGGTAATTCCCGATGGTC
TCGCAAGAGTAAAGTTTTTCGCTCCGGGAGTCGCCTGCGCAGTATCAGCTAGTTGGTAGGGTAACGGC
CTACCAAGGCTACGACGCTTAGGGGAGGTGAGAGCCTGACCCCCACCGATGGGACTGCGACACGGCC
>Zotu4598
GAACGCTGGCGGCAGGCTTAACACATGCAAGTCGAGCGGGGAAAGGTAGCTTGCTACCTAACCTAGCG
GCGGACGGGTGAGTAATGCTTAGGAATCTGCCTATTAGTGGGGGACAACATTCCGAAAGGAATGCTAA
TACCGCATACGCCCTACGGGGAAAAGCAGGGGATCTTCGGACCTTGCGCTAATAGATGAGCCTAAGTC
AGATTAGCTAGTTGGTGGGGTAAAGGCCTACCAAGGCGACGATCTGTAGCGGGTCTGAGAGGATGATC
CGCCACACTGGGACTGAGACACGGCC
>Zotu4599
GAACGCTGGCGGCGTGCCTAATACATGCAAGTCGAGCGAACTTTTGGGAGCTTGCTCCCAAAAGTTAG
CGGCGGACGGGTGAGTAACACGTGGGCAACCTGCCTATAAGACTGGGATAACTTCGGGAAACCGGAGC
TAATACCGGATAATTCCTTTTCTCTCATGAGGAAAGGCTGAAAGACGGTTTCGGCTGTCACTTATAGA
TGGGCCCGCGGCGCATTAGCTAGTTGGTGAGGTAACGGCTCACCAAGGCAACGATGCGTAGCCGACCT
GAGAGGGTGATCGGCCACACTGGGACTGAGACACGGCC
>Zotu4600
GAACGCTGGCGGCGTGCCTAATACATGCAAGTCGAACGGTAAGGCTTTCACAGTTTGCTGGTAGTACG
TAAGTACTCGAGCAAAGCGTGAAAGTACACGAGTGGCGGACGGCAGAGTAACGCGTAGGAATGTACCC
CAAAGTGAGGGATAACTGCTCGAAAGAGTAGCTAATACCGCATGTGGTCTTAGGATTAAAGCTTCGGC
GCTTTGGGAACAGCCTGCGTAAGATTAGCTAGTTGGTAGTGTAATGGACTACCAAGGCGACGATCTTT
AGCTGGTCTGAGAGGATGATCAGCCTGACTGGAACTGAGACACGGTC
>Zotu4601
GAACGCTGGCGGCGTGCTTAACACATGCAAGTCGAACGGAAAGGCCCTTCGGGGTACTCGAGTGGCGA
ACGGGTGAGTAACACGTGGGTGATCTGCCCTGCACTTTGGGATAAGCCTGGGAAACTGGGTCTAATAC
CGAATATGATCATCGGCTTCATGGTCGGTGGTGGAAAGCTTTTGCGGTGTGGGATGGGCCCGCGGCCT

ATCAGCTTGTTGGTGGGGTAATGGCCTACCAAGGCGACGACGGGTAGCCGGCCTGAGAGGGTGACCGG
CCACACTGGGACTGAGATACGGCC
>Zotu4602
GAACGCTAGCGGCAGGCTTAACACATGCAAGTCGTGGGGCAGCGCGGGTAGCAATACTTGGCGGCGAC
CGGCAAACGGGTGCGGAACACGTACGCAACCTCCCTCAAAGCGGGGAATAGCCTCTCGAAAGAGAGAT
TAATACCCCATAGTATCGTCAGAAGGCATCTGAAGACGATTAAAGCTCCGGCACTTTGAGATGGGCGT
GCGGCTGATTAGGTAGTTGGTAGGGTAACGGCCTACCAAGCCGACGATCAGTAGCTGATGTGAGAGCA
TGATCAGCCACACGGGCACTGAGACACGGGC
>Zotu4603
GAACGCTGGTGGTATGCTTAACACATGCAAGTCGAACGGGCATAGCAATATGTCAGTGGCAAACGGGT
GAGGAATATATAGGAATCTACCTAGCAGTAAGGAATAACCTTGGGAAATCAAGGCTAATGCCATATAT
TACCGATTTATCGGGAAAGATTTATCGCTGTTAGATGAGCCTGTATCAGATTAGCTAGTTGGTGGGGT
AATGGCCTACCAAGGCAATGATCTGTAGCTGGTCTGAGAGGATGATCAGCCACACTGGAACTGCGACA
CGGTC
>Zotu4604
GAACGCTGGCGGCGTGCTTAACACATGCAAGTCGAACGGTGATGATCAAGCTTGCTTGGTCTGATCAG
TGGCGAACGGGTGAGTAACACGTGAGTAACCTGCCCTTCACTCTGGGATAAGCCTTGGAAACGAGGTC
TAATACCGGATATGAGACGTACAGGCATCTGTTGCGTCTGGAAAGATTTATCGGTGAAGGATGGGCTC
GCGGCCTATCAGCTTGTTGGTGGGGTGATGGCCTACCAAGGCGACGACGGGTAGCCGGCCTGAGAGGG
CGACCGGCCACACTGGGACTGAGACACGGCC
>Zotu4605
GAACGCTGGCGGCGTGCTTAACACATGCAAGTCGAACGGTGAAAGAGGAGCTTGCTCCTCTGGATCAG
TGGCGAACGGGTGAGTAACACGTGAGTAACCTGCCCCAGACTCTGGGATAAGCGTTGGAAACGACGTC
TAATACCGGATACGAGCTTCAGCCGCATGGCTAGGAGCTGGAAAGAATTTCGGTCTGGGATGGACTCG
CGGCCTATCAGGTAGTTGGTGAGGTAAAGGCCCACCAAGCCTACGACGGGTAGCCGGCCTGAGAGGGT
GACCGGCCACACTGGGACTGAGACACGGCC
>Zotu4606
GAACGCTGGCGGCGTGCTTAACACATGCAAGTCGAACGGTAAGGCCCTTTCGGGGGTACACGAGTGGC
GAACGGGTGAGTAACACGTGGGTAATCTGCCCTGCACTTCGGGATAAGCCTGGGAAACTGGGTCTAAT
ACCGGATATTCAGCTCCTGCCGCATGGTGGGGGTTGGAAAGTTTTTCGGTGCAGGATGAGCCCGCGGC
CTATCAGCTTGTTGGTGGGGTAATGGCCTACCAAGGCGACGACGGGTAGCCGGCCTGAGAGGGTGATC
GGCCACACTGGGACTGAGACACGGCC
>Zotu4608
GAACGCTGGCGGCAGGCTTAACACATGCAAGTCGAGCGCCCCGCAAGGGGAGCGGCAGACGGGTGAGT
AACGCGTGGGAATCTACCCATCTCTACGGAACAACTCCGGGAAACTGGAGCTAATACCGTATACGCCC
TTCGGGGGAAAGATTTATCGGAGATGGATGAGCCCGCGTTGGATTAGGTAGTTGGTGGGGTAATGGCC
TACCAAGCCGACGATCCATAGCTGGTCTGAGAGGATGATCAGCCACATTGGGACTGAGACACGGCC
>Zotu4609
GAACGCTGGCTGTATGGATGAGTCATGCAAGTCGAACGGTCCGCAAGGACAGTGGCAGACGAGGTAGT
AACACGTACGTACGTACTCTGTAGTCTGGAATAGCTCACCGAAAGGTGAGGTAATACCGGATGGTCCC
TAAGGGGTAAAGTTTTTCGCTACAGAAGCGGCGTGCGTAGTATCAGCTAGTTGGTAAGGTAATGGCTT
ACCAAGGCTACGACGCTTAGGGGAGCTGAGAGGCTGACCCCCACCGATGGGACTGAGACACGGCC
>Zotu4610
GAACGCTAGCGGGAGGCCTAATACATGCAAGTCGAACGGTAACAGGGGTAGCAATACCTGCTGACGAG
TGGCGTACGGGTGCGTAACACGTACACAACCTGCCTTATACTGGAGCATACCCCCAAGAAATTGGGAC
TAATTCTCCATAATATATTTGAAAGGCATCTTTTGAATATTAAAACTCCGGTGGTATAAGATGGGTGT
GCGTCCGATTAGATAGTTGGTGAGGTAACGGCTCACCAAGTCAACGATCGGTAGCTGGTCTGAGAGGA
CGATCAGCCACACGGGCACTGAGACACGGGC
>Zotu4611
GAACGCTGGCGGCGTGCCTAATACATGCAAGTCGAACGCTTCTTTCCTCCCGAGTGCTTGCGCTCAAT
TGGAAAAAGGAGTGGCGGACGGGTGAGTAACACGTGGGTAACCTACCCATCAGAGGGGGATAACACTT
GGAAACAGGTGCTAATACCGCATAACAGTTTATGCCGCATGGCATAAGAGTGAAAGGCGCTTTCGGGT
GTCGCTGATGGATGGACCCGCGGTGCATTAGCTAGTTGGTGAGGTAATGGCTCACCAAGGCCACGATG
CATAGCCGACCTGAGAGGGTGATCGGCCACACTGGGACTGAGACACGGCC

>Zotu4612
GAACGCTGGCGGCAGGCCTAACACATGCAAGTCGAACGAGCTGGGACCTTCGGGTCCCGATGCTAGTG
GCGCACGGGTGAGTAACACGTGGGAATCTACCCAATAGTACGGAATAACTGAGGGAAACTTTGGCTAA
TACCGTATACGCCCTTCGGGGGAAAGATTTATCGCTATTGGATGAGCCCGCGTCTGATTAGGTAGTTG
GTGAGGTAACGGCTCACCAAGCCGACGATCAGTAGCTGGTCTGAGAGGATGATCAGCCACACTGGGAC
TGAGACACGGCC
>Zotu4613
GAACGCTGGCGGCGTGCTTAACACATGCAAGTCGAACGATGAAGCCCAGCTTGCTGGGTGGATTAGTG
GCGAACGGGTGAGTAACACGTGAGTAACCTGCCCTTCACTCTGGGATAACTCCGGGAAACCGGTGCTA
ATACTGGATATGACTCCAACCTGCATGGGTATGGGGTGGAAAGATTTATCGGTGGAGGATGGACTCGC
GGCCTATCAGCTTGTTGGTGGGGTAATGGCCTACCAAGGCGACGACGGGTAGCCGGCCTGAGAGGGTG
ACCGGCCACACTGGGACTGAGACACGGCC
>Zotu4614
GCCGTGTCTCAGTCCCAGTGTGGCTGGTCATCCTCTCAGACCAGCTACGGATCGTCGCCTTGGTAGGC
TTTTACCCTACCAACTAGCTAATCCGATGCAGGCTCACCCTAGAGCGCAAGGCCTCGAAAGGTCCCCT
GCCTTTGATTTTTCAATATCATGTGGTATTAGCTCTCGTTTCCGAAAGTTATCCCCCTCTCTAGGACA
GATTCCTACCTATTACTCACCCGTCCGCCACTTGCCATCATTCCTCACTCAAATCACTTTCCGTATTT
CTTTTCCAAAAAGAAATTAAAGTGATTTGAGTGAGGAACATGCTGCCGTTCGACTTGCATGTGTTAAG
CATACCGCCAGCGTTCA
>Zotu4615
GAACGCTGGCGGCATGCCTAATACATGCAAGTCGAGCGGAGTTGATAGGAAGCTTGCTTCCTTGATAC
TTAGCGGCGGACGGGTGAGTAACACGTAGGCAACCTGCCCTCAAGTTTGGGACAACTACCGGAAACGG
TAGCTAATACCGAATAGTTGTTTTCTTCGCCTGAAGAGAACTGGAAAGACGGAGCAATCTGTCACTTG
GGGATGGGCCTGCGGCGCATTAGCTAGTTGGTGGGGTAACGGCTCACCAAGGCGACGATGCGTAGCCG
ACCTGAGAGGGTGATCGGCCACACTGGGACTGAGACACGGCC
>Zotu4616
GAACGCTGGCGGTGTGCCTAACACATGCAAGTCGAACGAAGCAGCAATGCTTAGTGGCGAACGGGTGA
GTAATGCACAGGAATTTGCCCCGAACTAGGAAATACCCTGCCGAAAGGTTGGCTAATGTCCTATGGTC
TCGCAAGAGTAAAGATTTATCGGTTCGGGAGAAGCCTGTGTCCTATCAGCTAGTTGGTGGGGTAAAAG
CCTACCAAGGCGTTGACGGGTAGCTGGTCTGAGAGGATGGTCAGCCACAATGGGACTGAGACACGGCC
>Zotu4617
GAACGCTAGCGGCAGGCCTAATACATGCAAGTCGAACGGTAAATATTCAGCAATGGATATGAGAGTGA
CGTACGGGTGCGTAACACGTATGCAACCTACCCTTTACAGGCGGATAGCTCGGGGAAACTCGAATTAA
CCCGTCATAAGATCATAGAGTGGCATCACTTAGTGATTAAAATTACGGTGGTAAAGGATGGGCATGCG
TCTGATTAGTTAGTTGGCGGGGTAACGGCCCACCAAGACGATGATCAGTAGGGGAACTGAGAGGTTGA
TCCCCCACACTGGCACTGAGATACGGGC
>Zotu4618
GAACGCTAGCGGCAGGCCTAATACATGCAAGTCGAACGATATTAAGGAGCTTGCTCCTTAAGAAAGTG
GCGCACGGGTGCGTAACGCGTATGCAACCTACCTTAATCAGGGGGATAGCCCGGAGAAATCCGGATTA
ACACCGCATAAAAACACAGGATAGCATTATCCAATGTTCAAATATTTATAGGATTAAGATGGGCATGC
GTGTCATTAGCTAGTTGGCGGGGTAACGGCCCACCAAGGCGACGATGACTAGGGGATCTGAGAGGATG
ACCCCCCACACTGGTACTGAGACACGGAC
>Zotu4619
GAACGCTGGCGGCGTGCCTAATACATGCAAGTCGAGCGGAGTTGATGAGGTGCTTGCACCTCTGATGC
TTAGCGGCGGACGGGTGAGTAACACGTAGGTAACCTGCCTGTAAGACTGGGATAACATTCGGAAACGA
ATGCTAATACCGGATACGCGATTCTCTCGCATGGGAGAATCGGGAAAGACGGAGCAATCTGTCACTTA
CGGATGGACCTGCGGCGCATTAGCTAGTTGGTGAGGTAACGGCTCACCAAGGCGACGATGCGTAGCCG
ACCTGAGAGGGTGATCGGCCACACTGGGACTGAGACACGGCC
>Zotu4620
GAACGCTAGCGGCAGGCCTAATACATGCAAGTCGAGGGGTAGAGGTTGCTTGCAACCTTGAGACCGGC
GCACGGGTGCGTAACGCGTATGCAACCTACCTTCATCTGGGGGATAGCCCGGAGAAATCCGGATTAAT
ACCGCATAAAATCACAGGATGGCATTATCCAATGATCAAACATTTATGGGATGGAGATGGGCATGCGT
GTCATTAGCTAGTTGGCGGGGTAACGGCCCACCAAGGCGACGATGACTAGGGGATCTGAGAGGATGGC
CCCCCACACTGGTACTGAGACACGGAC

>Zotu4621
GAACGCTGGCTGCGTGCTTAACACATGCAAGTCGAACGATGACGGTGGTGCTTGCATCACCTGATTAG
TGGCGAACGGGTGAGTAACACGTGAGTAACCTGCCCTTCACTTCGGGATAACCTCGGGAAATCGTGGC
TAATACCGGATATGAGCTCCTTCCGCATGGCGGGGGTTGGAAAGATTTATCGGTGAAGGATGGACTCG
CGGCCTATCAGTTTGTTGGTGAGGTAATGGCTCACCAAGGCGATGACGGGTAGCCGGCCTGAGAGGGC
GACCGGCCACACTGGGACTGAGACACGGCC
>Zotu4623
GAACGCTAGCGGCAGGCTTAATACATGCAAGTCGAACGGGATTTAAGATAGCAATATTTTGATGAGAG
TGGCGCACGGGTGCGTAACACGTATGCAACCTACCTTTAACTGGTGAATAGCCCCGGGAAATCGGGAT
TAATACACCATAACATTACGGAATGGCATCATTCTGCAATTAAAGATATTCGGTTAGAGATGGGCATG
CGGGACATTAGCTAGTTGGTGAGGTAACGGCTCACCAAGGCAACGATGTCTAGGGGTCCTGAGAGGGT
TTTCCCCCACACTGGTACTGAGACACGGAC
>Zotu4624
GAACGCTGGCGGCGTGCCTAATACATGCAAGTCGAGCGAGTCCCTTCGGGGGCTAGCGGCAGACGGGT
GAGTAACACGTAGGCAACCTGCCCGTAAGATCGGGATAACATAGGGAAACTTATGCTAATACCGAATA
GAGTCTTTTCTCGCATGAGGAGAGACGGAAAGATGGCGCAAGCTATCACTTACGGATGGGCCTGCGGC
GCATTAGCTAGTTGGTGGGGTAACGGCCTACCAAGGCGACGATGCGTAGCCGACCTGAGAGGGTGACC
GGCCACACTGGGACTGAGACACGGCC
>Zotu4625
GAACGCTGGCGGCGTGCTTAACACATGCAAGTCGAACGCTGAAGCTTGGTGCTTGCACTGGGTGGATG
AGTGGCGAACGGGTGAGTAATACGTGAGTAACCTGCCCTTGACTCTGGGATAAGCCTGGGAAACTGGG
TCTAATACTGGATGCTACATGTCACCGCATGGTGGTGTGTGGAAAGGGTTTACTGGTCTTGGATGGGC
TCACGGCCTATCAGCTTGTTGGTGAGGTAATGGCTCACCAAGGCGACGACGGGTAGCCGGCCTGAGAG
GGTGACCGGCCACACTGGGACTGAGACACGGCC
>Zotu4626
GAACGCTGGCGGCAGGCCTAACACATGCAAGTCGAGCGCCGTAGCAATACGGAGCGGCAGACGGGTGA
GTAACGCGTGGGAACGTGCCCTTCAGTTCGGAATAACCCAGGGAAACTTGGGCTAATACCGGATACGT
CCGTAAGGAGAAAGATTTATCGCTGAAGGATCGGCCCGCGTAAGATTAGCTAGTTGGTGAGGTAACGG
CTCACCAAGGCTATGATCTTTAGCTGTTCTGAGAGGAAGACCAGCCACACTGGGACTGAGACACGGCC
>Zotu4627
GAACGCTGGCGGCGTGCTTAACACATGCAAGTCGAGCGAGGCTCCTCCTTCGGGAGGATGTCCTAGCG
GCGAACGGGTGAGTAACACGTGGGCAACCTGCCCCTAGCTCTGGGATAACCCCGGGAAACCGGGGCTA
ATACCGGATATGACTTCAGAAGGCATCTTCCGAAGTGGAAAAGGTTTACTGGCTAGGGATGGGCCCGC
GGCCTATCAGCTTGTTGGTGGGGTAATGGCCTACCAAGGCGACGACGGGTAGCCGGCCTGAGAGGGCG
ACCGGCCACACTGGGACTGAGACACGGCC
>Zotu4628
GAACGCTGGCGGCGTGCCTAATACATGCAAGTCGAGCGAATCGATGGGAGCTTGCTCCCTGAGATTAG
CGGCGGACGGGTGAGTAACACGTGGGCAACCTGCCTATAAGACTGGGATAACTTCGGGAAACCGGAGC
TAATACCGGATACGTTCTTTTCTCGCATGAGAGAAGATGGAAAGACGGTTTACGCTGTCACTTATAGA
TGGGCCCGCGGCGCATTAGCTAGTTGGTGAGGTAATGGCTCACCAAGGCGACGATGCGTAGCCGACCT
GAGAGGGTGATCGGCCACACTGGGACTGAGACACGGCC
>Zotu4629
GAACGCTGGCGGCGTGCCTAATACATGCAAGTCGAGCGAACTTGCGGGAGCTTGCTCCTGTAAGTTAG
CGGCGGACGGGTGAGTAACACGTGGGCAACCTGCCTATAAGACTGGGATAACTTCGGGAAACCGGAGC
TAATACCGGATAATTCTTTTCTACTCATGTAGGAAAGCTGAAAGACGGTTTACGCTGTCACTTATAGA
TGGGCCCGCGGCGCATTAGCTAGTTGGTGAGGTAACGGCTCACCAAGGCCACGATGCGTAGCCGACCT
GAGAGGGTGATCGGCCACACTGGGACTGAGACACGGCC
>Zotu4630
GAACGCTGGCGGCGTGCTTAACACATGCAAGTCGAACGGAAAGGCCCTTCGGGGTACTCGAGTGGCGA
ACGGGTGAGTAACACGTGGGTGATCTGCCCTGCACTTCGGGATAAGCTTGGGAAACTGGGTCTAATAC
CGGATATGACTACTGGCTTCATGGTTGGTGGTGGAAAGCTTTTGCGGTGTGGGATGGGCCCGCGGCCT
ATCAGCTTGTTGGTGGGGTAATGGCCTACCAAGGCGACGACGGGTAGCCGGCCTGAGAGGGTGTCCGG
CCACACTGGGACTGAGATACGGCC
>Zotu4631

GAATGCTGGCGATGTGGATGAGTCATGCAAGTCAAGGGGGCCCGCAAGGGCAACCGGCAGACGAGGTA
GTAACGCGTAGGTAGATCCCCCGAAGTCGGGCATAGCCGGCCGAAAGGCCGGGTAATTCCCGATAGTC
CCGCAAGGGTAAAGGTTTTTCGCTTCGGGAGTCGCCTGCGCAGTATCAGCTAGTTGGTAAGGTAACGG
CTTACCAAGGCTACGACGCTTAGGGGAGCTGAGAGGCTGACCCCCACCGATGGGACTGCGACACGGCC
>Zotu4632
AAACGCTGGCGGCGTGCATAAAACATGCAAGTCGAACGGACTTCAGTCCGCAAGGACTGAAGTGAGTG
GCGAACGGCTGAGTAACACGTAGGTGACCTGCCCCGAAGTTTGGGATAACAACTCGAAAGAGTTGCTA
ATACCGGATGTGACCACCGATTCATTTCGGTGCTTAAAGTCGTAAGACGCTTTGGGAGGGGCCTGCGG
CGGATTAGCTAGTTGGTGTGGTAACGGCATACCAAGGCTTCGATCCGTAGCTGGTCTGAGAGGATGGC
CAGCCAGACTGGGACTGAGACACGGCC
>Zotu4634
GAACGCTAGCGGCAGGCTTAACACATGCAAGTCGAGGGGTAACAGGGTAGCAATACTGCTGACGACCG
GCAAACGGGTGCGGAACACGTACGCAACATACCCGCTACTGGGGCATAGCCTTCTGAAAAGGAGATTA
ACACCCCATAATATCGATTGGTGGCATCACTAGACGATTAAAAATTTATTGGTAGCGGATTGGCGTGC
GTCCTATTAGGTAGTTGGTGTGGGTAACGGCCTACCAAGCCGACGATAGGTAGCTGGTGTGAGAGCAC
GACCAGCCACACGGGAACTGAGACACGGTC
>Zotu4635
GAACGCTGGCGACAGGCTTAACACATGCAAGTCGAACGATGACTAACTAGCTTGCTAGATATGATTAG
TGGCGGACGGGTGAGTAACATTTAGGAATCTGCCTAGTAGTGGGGGATAGCTCGGGGAAACTCGAATT
AATACCGCATACGACCTACGGGTGAAAGGGGGCGCAAGCTCTTGCTATTAGATAAGCCTAAATCAGAT
TAGCTAGTTGGTGGGGTAAAGGCCCACCAAGGCGACGATCTGTAACTGGTCTGAGAGGATGATCAGTC
ACACCGGAACTGAGACACGGTC
>Zotu4636
GAACGCTGGCGGCAGGCCTAACACATGCAAGTCGAACGGCAGCACAGAAGAGCTTGCTCTTTGGGTGG
CGAGTGGCGGACGGGTGAGTAATGTCTGGGAAACTGCCCGATGGAGGGGGATAACTACTGGAAACGGT
AGCTAATACCGCATAACGTCGCAAGACCAAAGTGGGGGACCTTCGGGCCTCACACCATCGGATGTGCC
CAGATGGGATTAGCTAGTAGGTGGGGTAACGGCTCACCTAGGCGACGATCCCTAGCTGGTCTGAGAGG
ATGACCAGCCACACTGGAACTGAGACACGGTC
>Zotu4637
GAACGCTAGCGGCAGGCCTAATACATGCAAGTCGAACGGTAACATGAGTAGCAATACTTGATGACGAG
TGGCGCACGGGTGCGTAACGCGTATGCAATCTACCTTTAACCGGTGAATAGCCCGAAGAAATTCGGAT
TAATACACCATAGTATGTTTGAGTGGCATCACTTAAACATTAAAACTCCGGTGGTTAAAGATGAGCAT
GCGTGACATTAGCTAGTTGGTGAGGTAATGGCTCACCAAGGCTACGATGTCTAGGGGATCTGAGAGGA
TTAACCCCCACACTGGTACTGAGACACGGAC
>Zotu4638
GAACGCTGGCGGCAGGCTTAACACATGCAAGTCGAGCGCGTAGCAATACGAGCGGCAGACGGGTGAGT
AACGCGTGGGAATCTACCCATCTCTACGGAACAACTCCGGGAAACTGGAGCTAATACCGTATACGTCC
TTTTGGAGAAAGATTTATCGGAGATGGATGAGCCCGCGTTGGATTAGCTAGTTGGTGGGGTAATGGCC
TACCAAGGCGACGATCCATAGCTGGTCTGAGAGGATGATCAGCCACACTGGGACTGAGACACGGCC
>Zotu4639
GAACGCTGGCGGTATGCTTTACACATGCAAGTCGAACGGGGGTTTTTAACTCTAGTGGCGGACGGGTG
AGTAACACGTAAGAATTTGCCTTTAGGAGGGGGACAACAGCTGGAAACGGCTGCTAATACCCCATATG
CTTTCGAGTGAAATGGATTTCCGCCTGAAGAGAAGCTTGCGGCTGATTAGCTTGTTGGTGAGGTAATG
GCTCACCAAGGCGACGATCAGTATCTGGTTTGAGAGGACGATCAGACACACTGGAACTGAGACACGGT
C
>Zotu4640
GAACGCTGGCGGCGTGCTTAACACATGCAAGTCGAACGGTGAAGCCAAGCTTGCTTGGTGGATCAGTG
GCGAACGGGTGAGTAACACGTGAGCAACCTGCCCTGGACTCTGGGATAAGCGCTGGAAACGGCGTCTA
ATACTGGATATGAGCCTCTTCCGCATGGTGGGGGTTGGAAAGATTTTTTGGTCTGGGATGGGCTCGCG
GCCTATCAGCTTGTTGGTGAGGTAATGGCTCACCAAGGCGTCGACGGGTAGCCGGCCTGAGAGGGTGA
CCGGCCACACTGGGACTGAGACACGGCC
>Zotu4642
GAACGCTAGCGGCAGGCCTAATACATGCAAGTCGAGGGGTAACAGGAATAGCAATATTTGCTGACGAC
CGGCGCACGGGTGCGTAACACGTATGTAATCTGCCCATCACTGGAGGATAGCCCGGAGAAATCCGGAT

TAATACTCCATAATAAAAGAGAAAGCATTTTCTTTTTTTGAAAGCTCCGGCGGTGATGGATGAACATG
CGACTGATTAGTTTGTTGGTGAGGTAACGGCTCACCAAGACTTCGATCAGTAGGGGTACTGAGAGGTT
AAACCCCCACACTGGTACTGAGACACGGAC
>Zotu4643
GAACGCTAGCGGGAGGCCTAACACATGCAAGCCGAGCGGTATTTGTTCTTCGGAACAGAGAGAGCGGC
GTACGGGTGCGTAACACGTGTGCAACCTACCTTTATCTGGGAGATAGCCTTTCGAAAGGAAGATTAAT
ATCCCATAATATATTAGATGGCATCATTTGATATTGAAAGCTCCGGCGGATAGAGATGGGCACGCGCA
AGATTAGATAGTTGGTGAGGTAACGGCTCACCAAGTCAATGATCTTTAGGGGGCCTGAGAGGGTGATC
CCCCACACTGGTACTGAGACACGGAC
>Zotu4644
AAACGCTGGCGGCATGCATGAGACATGCAAGTCGAACGAGTTCCTGGAGCTTGCTCCAGGCGCTAGTG
GCGGACGGCTGAGTAACGCGTGGGTGACCTACCCCGAAGTGGGGAATAACTGCCCGAAAGGGTGGCTA
ATACCGCATGTGATCGTTCTTTCAGTTGATCGATCAAAGCAGCAATGCGCTTCGGGAGGGGCCTGCGT
CCGATTAGCTCGTTGGTGGGGTAATGGCTCACCAAGGCGACGATCGGTAGGTGGTCTGAGAGGACGAT
CACCCAGACTGGGACTGAGACACGGCC
>Zotu4645
GAACGCTGGCGGCGTGCCTAATACATGCAAGTCGAGCGAATTTTTGGGAGCTTGCTCCTAAAAGTTAG
CGGCGGACGGGTGAGTAACACGTGGGCAACCTGCCTATAAGACTGGGATAACTTCGGGAAACCGGAGC
TAATACCGGATAATTCTTTTCTACACATGTAGAAAAGCTGAAAGACGGTTTACGCTGTCACTTATAGA
TGGGCCCGCGGCGCATTAGCTAGTTGGTGAGGTAACGGCTCACCAAGGCGACGATGCGTAGCCGACCT
GAGAGGGTGATCGGCCACACTGGGACTGAGACACGGCC
>Zotu4646
GAACGCTGGCGGCGTGCTTAACACATGCAAGTCGAACGGTGACCTTGGAGGCTTGCCTTCAAGTGATC
AGTGGCGAACGGGTGAGTAACACGTGAGCAACCTGCCCCAGACTCTGGGATAACTTCGGGAAACCGGA
GCTAATACCGGATATGACACGGAGATGCATGTCTACCGTGTGGAAAGTTTTTCGGTCTGGGATGGGCT
CGCGGCCTATCAGCTTGTTGGTGAGGTAATGGCTCACCAAGGCGACGACGGGTAGCCGGCCTGAGAGG
GCGACCGGCCACACTGGGACTGAGACACGGCC
>Zotu4648
GAACGCTGGCGGCGTGCTTAACACATGCAAGTCGAGCGGTAAGGCCTTTCGGGGTACACGAGCGGCGA
ACGGGTGAGTAACACGTGGGTGATCTGCCCTGCACTCTGGGATAAGCCTGGGAAACTGGGTCTAATAC
TGGATATGACCGTGGGATGCATGTTTCATGGTGGAAAGCTTTTGTGGTGCAGGATGGGCCCGCGGCCT
ATCAGCTTGTTGGTGGGGTAATGGCCTACCAAGGCGACGACGGGTAGCCGACCTGAGAGGGTGACCGG
CCACACTGGGACTGAGACACGGCC
>Zotu4649
GAACGCTGGCGGCGTGCTTAACACATGCAAGTCGAGCGGTAAGGCCCTTCGGGGTACACGAGCGGCGA
ACGGGTGAGTAACACGTGGGTGACCTGCCCTCAGCTCCGGGATAAGCCTGGGAAACCGGGTCTAATAC
CGGATAGGACCATTGGATGCATGTCCGGTGGTGGAAAGTTTTTCGGCTGGGGATGGGCCCGCGGCCTA
TCAGCTTGTTGGTGGGGTGATGGCCTACCAAGGCGGTGACGGGTAGCCGGCCTGAGAGGGCGACCGGC
CACACTGGGACTGAGACACGGCC
>Zotu4651
GAACGCTGGCGGCGTGCCTAACACATGCAAGTCGAACGAGGTAGCAATACCGAGTGGCGGACGGGTGA
GTAATACATAGGCAACCTGCCCAACAGTGGGGGATACCCAGTCGAAAGATTGGCTAATACCGCATACG
ATCCCTGGTTGGAATACCTGGGATGAAAGATTTATCGCTGTTGGATGGGCCTGTGGTGGATTAGCTAG
TTGGTGGGGTAACGGCCTACCAAGGCGACGATCCATAGCTGGTCTGAGAGGATGATCAGCCACACTGG
GACTGAGACACGGCC
>Zotu4652
GAACGCTGGCGGCAGGCTTAACACATGCAAGTCGAACGCCCCGCAAGGGGAGTGGCAGACGGGTGAGT
AACACGTGGGAACGTACCTTTCGGTTCGGAATAACTTGGGGAAACTCAAGCTAATACCGGATACGCCC
TTACCGGGAAAGGTTTACCGCCGAAAGATCGGCCCGCGTCTGATTAGCTAGTTGGTGAGGTAATGGCT
CACCAAGGCGACGATCAGTAGCTGGTCTGAGAGGATGATCAGCCACATTGGGACTGAGACACGGCC
>Zotu4653
GAACGCTGGCGGCGTGCTTAACACATGCAAGTCGAACGATGAAGCCCAGCTTGCTGGGTGGAAGAGTG
GCGAACGGGTGAGTAACACGTGAGTAACCTGCCCCGGACTCTGGGATAAGCGCTGGAAACGGCGTCTA
ATACTGGATATGACCCATACCGGCATCGGTTGTGGGTGGAAAGATTTATCGGTTCGGGATGGGCTCGC

GGCCTATCAGCTGGATGATGAGGTAACGGCTCACCATGGCGACGACGGGTAGCCGGCCTGAGAGAGTG
ACCGGCCACACTGGGACTGAGACACGGCC
>Zotu4654
GAACGCTGGCGGTATGCCTAACACATGCAAGTCGTACGAGAGTTTTTAACTCAAGTGGCGGACGGGTG
AGTAACACGTGAGAATTTACCTTTAGGAGGGGACAACAATTGGAAACGATTGCTAATACCCCATATG
CTCTAGAGTGAAACGGATTTTTCCGCCTAAAGAAAAGCTCGCGGCTGATTAGCTTGTTGGTAAGGTAA
TGGCTTACCAAGGCGACGATCAGTATCTGGTTTGAGAGGACGATCAGACACACTGGAACTGAGACACG
GTC
>Zotu4655
GAACGCTGGCGGCGTGCTTAACACATGCAAGTCAAGGGGGCCCGCAAGGGCAACCGGCGAACGGGTGC
GTAACACGTGAGCGACCTGCCCGCTTGTGGGGGATAGCCGGCCCAACGGCCGGGTAATACCGCATACG
CTTTCCCTGGGGCATCTTGGGGATCGGAAAGATTTATCGCAGGCGGAGGGGCTCGCGGCCTATCAGCT
AGTTGGCGAGGTAACGGCTCACCAAGGCGACGACGGGTAGCTGGTCTGAGAGGATGGCCAGCCACATT
GGGACTGAGAGACGGCC
>Zotu4657
GAACGCTGGCGGCATGCCTTACACATGCAAGTCGAACGGCAGCACGGGAGCTTGCTCCTGGTGGCGAG
TGGCGAACGGGTGAGTAATATATCGGAACGTACCCTAGAGTGGGGGATAACGCAGCGAAAGTTGCGCT
AATACCGCATACGATCTACGGATGAAAGTGGGGGATCGCAAGACCTCATGCTCATGGAGCGGCCGATA
TCTGATTAGCTAGTTGGTGGGGTAAAGGCCTACCAAGGCATCGATCAGTAGCTGGTCTGAGAGGACGA
CCAGCCACACTGGAACTGAGACACGGTC
>Zotu4658
GAACATTGGCGGTAGGCTTAACACATGCAAGTCGAGCGAGGCGTAATAGTCGAGCGGCGAACGGGTGA
GTAATACATGGGAATCTACCTCGAAGTGGGGGATAACGTTCAGAAATGGACGCTAATACCGCATAATC
CGGAAACGGGAAAGCGCGCTGCGCTTTGAGATGAGCCCATGGCTGATTAGCTAGTTGGTAGGGTAAAG
GCTTACCAAGGCGACGATCAGTAGCTGGTCTGAGAGGATGATCAGCCACACTGGAACTGAGACACGGT
C
>Zotu4659
GAACGCTGGCGGCGTGCCTAATACATGCAAGTCGAGCGGATCTCGAGGAGCTTGCTCCTTGAGATTAG
CGGCGGACGGGTGAGTAACACGTGGGCAACCTGCCTGTAAGACTGGGATAACACCGGGAAACCGGTGC
TAATACCGAATAATTCTTTCTCTCTCATGAGGGAAAGCTGAAAGTCGGTTTCGGCTGACACTTACAGA
TGGGCCCGCGGCGCATTAGCTAGTTGGTGAGGTAACGGCTCACCAAGGCGACGATGCGTAGCCGACCT
GAGAGGGTGATCGGCCACACTGGGACTGAGACACGGCC
>Zotu4660
GAACGCTGGCGGCGTGCCTAATACATGCAAGTCGTGCGGGGTCTTGAAGGGCTTGCCCTTTAAGACCC
AGCGGCGAACGGGTGAGTAACACGTGGGTAACCTGCCCGCAAGACCGGGATAACTCCGGGAAACCGGG
GCTAATACCGGATAAGACTCCGGATCGCATGATCCGAAGTGGAAAGGCGGCGTCAAGCTGCCACTTGC
GGATGGGCCCGCGGCGCATTAGCTGGTTGGTGAGGTAACGGCTCACCAAGGCGACGATGCGTAGCCGA
CCTGAGAGGGTGATCGGCCACACTGGGACTGAGACACGGCC
>Zotu4661
GAACGCTGGCGGCATGCCTAACACATGCAAGTCGAACGAGACCTTCGGGTCTAGTGGCGCACGGGTGC
GTAACGCGTGGGAATCTGCCCTTGGGTTCGGAATAACAGTTAGAAATGACTGCTAATACCGGATGATG
ACTTCGGTCCAAAGATTTATCGCCCAGGGATGAGCCCGCGTAGGATTAGCTTGTTGGTGAGGTAAAAG
CTCACCAAGGCGACGATCCTTAGCTGGTCTGAGAGGATGATCAGCCACACTGGGACTGAGACACGGCC
>Zotu4662
GAACGCTGGCGGCGTGCCTAACACATGCAAGTCGAGCGAAGTTTTTAAAGTGAATCCCTTCGGGGTGA
AACTTTAATTATCTTAGCGGCGGACGGGTGAGTAACGCGTGAGAAACCTGCCTTTCACAAAGGGATAG
CCTCGGGAAACTGGGATTAATACCTTATGATACTTTTACTTCACATGAAGAAAAAGTTAAAGCGTAAA
GCGGTGAAAGATGGTCTCGCGTCCCATTAGCTAGTTGGTAAGGTAATAGCTTACCAAGGCGACGATGG
GTAGCCGGCCTGAGAGGGTGAACGGCCACACTGGAACTGAGACACGGTC
>Zotu4666
GAACGCTGGCGGCGTGCCTAATACATGCAAGTCGAGCGGTAAGGCTTCCACAGTTTGCTTCTTACGCT
CCTGAGACAGGTCGTCGATTGCGTAGCAATCGACATCAAAAACGAAGGAGTGATGGCTGGTCGTATGA
CCAGCCAAAGAAGGAGCAAAGTGTGGAAGTACACGAGCGGCGGACGGCAGAGTAACGCGTAGGAACGT
ACCCCAAACTGAGGGATAAGCCCGGGAAACCGGGTCTAATACCGCATATTATCTTCGGATCAAAGCTT

TAAGCGGTTTGGGAGCGGCCTGCGTATGATTAGCTTGTTGGTGAGGTAATGGCTCACCAAGGCGACGA
TCATTAGCTGGTCTGAGAGGATGATCAGCCTGACTGGGACTGAGACACGGCC
>Zotu4667
GAACGCTGGCGGCATGCCTAACACATGCAAGTCGAACGAGACCTTCGGGTCTAGTGGCGCACGGGTGC
GTAACGCGTGGGAATCTACCCTTGGGTTCGGAATAACAGTTGGAAACGACTGCTAATACCGGATGATG
ACGTAAGTCCAAAGATTTATCGCCCAAGGATGAGCCCGCGTAGGATTAGCTTGTTGGTGAGGTAAAAG
CTCACCAAGGCGACGATCCTTAGCTGGTCTGAGAGGATGATCAGCCACACTGGGACTGAGACACGGCC
>Zotu4668
GAACGCTAGCGGCAGGCCTAATACATGCAAGTCGAGGGGTAGAGTAAGCTTGCTTACTTGAGACCGGC
GCACGGGTGCGTAACGCGTATGCAACCTACCTTTATCTGGGGGATAGCCCGGAGAAATCCGGATTAAT
ACCGCATAAAATCACAGTACTGCATAGTACAATGATCAAACATTTATGGGATGAAGATGGGCATGCGT
GTCATTAGCTAGTTGGCGGGGTAATGGCCCACCAAGGCGACGATGACTAGGGGATCTGAGAGGATGAC
CCCCCACACTGGTACTGAGACACGGAC
>Zotu4669
GAACGCTGGCGGCGTGCCTAACACATGCAAGTCGAGCGATGAAGTTTCCTTCGGGAAACGGATTAGCG
GCGGACGGGTGAGTAACACGTGGGTAACCTGCCTCATAGAGGGGAATAGCCTCCCGAAAGGGAGATTA
ATACCGCATAACATTGCAGTTTCGCATGAAACAGCAATTAAAGGAGCAATCCGCTATGAGATGGACCC
GCGGCGCATTAGCTAGTTGGTAAGGTAATGGCTTACCAAGGCGACGATGCGTAGCCGACCTGAGAGGG
TGATCGGCCACATTGGGACTGAGACACGGCC
>Zotu4670
GAACGCTAGCGGGCGGCTTAACACATGCAAGTCGAGCGGGGGGTTTCGGCCCTCAGCGGCGGACGGGT
GAGTAACACGTAGGAATCTGCCTTTTGGTGGGGGATAGCCGGGGGAAACCCCGATTAATACCGCATAT
GGCCCATGGGTCAAAGCCGTAAGGCGCCAGAAGATGAGCCTGCGTCCGATTATTTAGTCCTTGTTGGT
GGGGTAACGGCCTACCAAGGGTACGATCGGTAGCTGGTCTGAGAGGACGATCAGCCACACTGGAACTG
AGACACGGTC
>Zotu4671
GAACGCTGGCGGCATGCTTTACACATGCAAGTCGAACGGCAGCGCGGGGCAACCTGGCGGCGAGTGGC
GAACGGGTGAGTAATATATCGGAACGTACCCAGAAGTGGGGGATAACGTAGCGAAAGTTACGCTAATA
CCGCATACGATCTACGGATGAAAGTGGGGGACCGCAAGGCCTCATGCTTTTGGAGCGGCCGATATCTG
ATTAGCTAGTTGGTGAGGTAAAGGCTCACCAAGGCGACGATCAGTAGCTGGTCTGAGAGGACGACCAG
CCACACTGGGACTGAGACACGGCC
>Zotu4672
GAACGCTGGCGGCATGCCTTACACATGCAAGTCGAACGGCAGCACGGGGGCAACCCTGGTGGCGAGTG
GCGAACGGGTGAGTAATATATCGGAACATGCCCTGAAGTGGGGGATAACGTAGCGAAAGTTACGCTAA
TACCGCATATTCTGTGAGCAGGAAAGCAGGGGATCGCAAGACCTTGCGCTTTAGGAGTGGCCGATATC
GGATTAGCTAGTTGGTGAGGTAAAGGCTCACCAAGGCGACGATCCGTAGCGGGTCTGAGAGGATGATC
CGCCACACTGGGACTGAGACACGGCC
>Zotu4674
GAACGCTAGCGGCAGGCCTAATACATGCAAGTCGAGGGGTAGTGTAGCAATACATGAGACCGGCGCAC
GGGTGCGTAACGCGTATGAAAGGTGTCCAAATCTCTGGGATAGCCCTCGGAAACGGGGATTAATACTG
GATGGTCTATATGTGTGGCATCACACTTATAGTAAAGCTCAGGCGGATTTGGGTCCTCATGCGTACGA
TTAGCTAGTTGGCGGGGTAATGGCCCACCAAGGCGACGATCGTTAGGGGTCCTGAGAGGGAGATCCCC
CACACTGGTACTGAGACACGGGC
>Zotu4675
GAACGCTAGCGGCAGGCCTAATACATGCAAGTCGGACGGGATAGAAGAGCTTGCTTTTCTTGAGAGTG
GCGCACGGGTGCGTAACACGTATGTAACCTACCTTGATCAGGGGGATAGCCTCTCGAAAGAGAGATTA
AGACCGCATAACATTATGATATGGCATCGTATTATAATCAAATATTTATAGGATCAAGATGGGCATGC
GGAACATTAGCTAGTTGGTAAGGTAACGGCTTACCAAGGCTACGATGTTTAGGGGATCTGAGAGGATG
ACCCCCCACACTGGTACTGAGACACGGAC
>Zotu4677
GAACGCTAGCGGCAGGCTTAACACATGCAAGTCGAGGGGTATAGTAGCAATACTAGAGACCGGCGCAC
GGGTGCGTAACGCGTATGCAATCTACCTTTTACAGAGGGATAGCCCAGAGAAATTTGGATTAATACCT
CATAGTATGTGAGAATGGCATCATTTTCACATTAAAGTCACAACGGTAAAAGATGAGCATGCGTCCCA
TTAGCTAGTTGGTAAGGTAACGGCTTACCAAGGCTACGATGGGTAGGGGTCCTGAGAGGGAGATCCCC

CACACTGGTACTGAGACACGGAC
>Zotu4678
GAACGCTGGCGGCAGGCCTAACACATGCAAGTCGAACGGATGTAGCAATACATTAGTGGCGCACGGGA
GAGTAACGCGTGGGAATATACCTTTTGGTTTGGGATAACACCGGGAAACTGGTGCTAATACCGGATAA
GCCCTTCGGGGGAAAGATTTATCGCCGAAAGATTAGCCCGCGTAGGATTAGCTAGTTGGTGAGGTAAA
GGCTCACCAAGGCTACGATCCTTAGCTGGTCTAAGAGGATGATCAGCCTCACTGGGACTGAGACACGG
CC
>Zotu4679
GAACGCTGGCGGCGTGCTTAACACATGCAAGTCGAGCGGAAAGGCCCTTCGGGGTACTCGAGCGGCGA
ACGGGTGAGTAACACGTGGGCAACCTGCCCCTAGCTCTGGGATAACTCCGGGAAACCGGGGCTAATAC
CGGATACGACTCCTCCTGGCATCTGGAGGAGTGGAAAGTTCTTCGGCTAGGGATGGGCCCGCGGCCTA
TCAGCTTGTTGGTGGGGTAACGGCCCACCAAGGCGACGACGGGTAGCCGGCCTGAGAGGGCGACCGGC
CACACTGGGACTGAGACACGGCC
>Zotu4680
GAACGCTGGCGGCGCGCTTAACACATGCAAGTCGAGCGAGAACCAGTCCTTCGGGGCTGGGGACAGCG
GCGAACGGGTGAGTAACACGTGGGTAATCTGCCCTCGGCACCGGAATAGCCCGGGGAAACCCGGATTA
ATGCCGGATGGCCTCGCCGGCCTTCGGGCTGGCGAGAAAAGGTAGCTTCGGCCTCCGGCCGAGGATGA
GCCCGCGGCCGATTAGCTTGTTGGTGGGGTAACGGCTCACCAAGGCGACGATCGGTAGCTGGTCTGAG
AGGACGATCAGCCACACTGGGACTGAGACACGGCC
>Zotu4682
GAACGCTGGCGGCATGCCTTACACATGCAAGTCGAACGGCAGCACGGGTGCTTGCACCTGGTGGCGAG
TGGCGAACGGGTGAGTAATACATCGGAACATGTCCTGTAGTGGGGGATAGCCCGGCGAAAGCCGGATT
AATACCGCATACGATCTACGGATGAAAGCGGGGGACCTTCGGGCCTCGCGCTATAGGGTTGGCCGATG
GCTGATTAGCTAGTTGGTGGGGTAAAGGCCCACCAAGGCGACGATCAGTAGCTGGTCTGAGAGGACGA
CCAGCCACACTGGGACTGAGACACGGCC
>Zotu4683
GAACGCTAGCGGCAGGCCTAATACATGCAAGTCGAACGAGATTGAAGAGCTTGCTTTTCATGAAAGTG
GCGCACGGGTGCGTAACGCGTATGCAACCTACCTTCTTCAGGGGGATAGCCTCTCGAAAGAGAGATTA
ACACCGCATAATATTATTGGATGGCATCATTTAATAATCAAATATTTATAGGAAGAAGATGGGCATGC
GTGACATTAGTTAGTTGGCGGGGTAATGGCCCACCAAGACCACGATGTCTAGGGGATCTGAGAGGATG
ACCCCCCACACTGGTACTGAGACACGGAC
>Zotu4685
GAACGCTGGCGGCGTGCCTAATACATGCAAGTAGAACGCTGAAGCTTGGTGCTTGCGCCGAGCGGATG
AGTTGCGAACGGGTGAGTAACGCGTAGGTAACCTGCCTGGTAGCGGGGGATAACTATTGGAAACGATA
GCTAATACCGCATAACAGTAGATATTGCATGATATCTGCTTGAAAGGTGCAATTGCATCACTACCAGA
TGGACCTGCGTTGTATTAGCTAGTTGGTGAGGTAACGGCTCACCAAGGCGACGATACATAGCCGACCT
GAGAGGGTGATCGGCCACACTGGGACTGAGACACGGCC
>Zotu4687
GAACGCTGGCGGCGTGCTTAACACATGCAAGTCGAACGATGAAGCCCAGCTTGCTGGGTGGATTAGTG
GCGAACGGGTGAGTAACACGTGAGTAACCTGCCCTTCACTCTGGGATAAGCCTTGGAAACGAGGTCTA
ATACTGGATATGACCTGTCACCGCATGGTGGTGGGTGGAAAGTTTTTCGGTGGGGGATGGACTCGCGG
CCTATCAGCTTGTTGGTGAGGTAGTGGCTCACCAAGGCGACGACGGGTAGCCGGCCTGAGAGGGTGAT
CGGCCACACTGGGACTGAGACACGGCC
>Zotu4689
GAACGCTGGCGGCGTGCTTAACACATGCAAGTCGAACGGTGAAGGAGGAGCTTGCTCTTCTGGATCAG
TGGCGAACGGGTGAGTAACACGTGAGTAACCTGCCCTTGACTCTGGGATAAGTGTTGGAAACGACGTC
TAATACCGGATACGAACCTCAACCGCATGGTTAGGGGTTGGAAAGATTTTTTGGTCAAGGATGGACTC
GCGGCCTATCAGCTTGTTGGTGAGGTAATGGCTCACCAAGGCGACGACGGGTAGCCGGCCTGAGAGGG
TGACCGGCCACACTGGGACTGAGACACGGCC
>Zotu4691
GAACGCTGGCGGTATGGATAAGGCATGCAAGTCGAGCGCCCCGCAAGGGGAGCGGCAGACGATGTAGT
AACACGTAGGTACATCCCCCAAAGTCAGGAATAGCTAGTCGAAAGATTAGGTAATACTTGATATGCTG
CAAAGTGAAAGATTTATCGCTTTGGGAGTGGCCTGCGTCGTATCAGCTAGTTGGTGAGGTAATGGCTC
ACCAAGGCTATGACGCGTAGGGGAGCTGAGAGGCTGACCCCCACAGATGAGACTGAGACACGGCT

>Zotu4693
GAACGCTGGCGGCGTGCTTAACACATGCAAGTCGAACGAAGAGCGATGGAAGCTTGCTTCTATCAATC
TTAGTGGCGAACGGGTAAGTAACGCGTAATCAACCTGCCCTTCAGAGGGGGACAACAGTTGGAAACGA
CTGCTAATACCGCATACGATCTAACCTCGGCATCGAGGAAAGATGAAAGGTGGCCTCTATGTATAAGC
TATCACTGAAGGAGGGGATTGCGTCTGATTAGCTAGTTGGAGGGGTAACGGCCCACCAAGGCGATGAT
CAGTAGCCGGTCTGAGAGGATGAACGGCCACATTGGGACTGAGACACGGCC
>Zotu4694
GAACGCTGGCGGCGTGCCTAATACATGCAAGTCGAACGCAACACCTTCGGGTGTTGAGTGGCGGACGG
GTGAGTAACACGTGGGTAACCTGCCCATCAGCCTGGGATAACCACCGGAAACGGTGGCTAATACCGGA
TATGTCTACGGATGAAAGTCGGATTATCTGACGCTGATGGATGGACCTGCGGCGCATTAGCTAGTTGG
TGAGGTAGAGGCTCACCAAGGCGACGATGCGTAGCCGACCTGAGAGGGTGATCGGCCACACTGGGACT
GAGACACGGCC
>Zotu4696
GAACGCTGGCGGCGTGCTTAACACATGCAAGTCGAACGGAAAGGCTCCTGCTTGCAGGGGTACTCGAG
TGGCGAACGGGTGAGTAACACGTGGGTGATCTGCCCTGCACTTCGGGATAAGCCTGGGAAACTGGGTC
TAATACCGGATAGGACCATCGTTTAGTGTCGGTGGTGGAAAGTTTTTCGGTGTGGGATGAGCTCGCGG
CCTATCAGCTTGTTGGTGGGGTAATGGCCTACCAAGGCGTCGACGGGTAGCCGGCCTGAGAGGGTGTA
CGGCCACATTGGGACTGAGATACGGCC
>Zotu4697
GAACGCTGGCGGCGTGCTTAACACATGCAAGTCGAGGGAACCTGGGGGGCAACCTTCAGGTGACCGGC
GAACGGGTGCGTAACACGTGAGCGACCTACCGTTTTGCGGGGGATAGCCGGCCCAACGGCCGGGTAAT
ACCGCATACGCTCATCGGGCGGCATCGTCTGGTGAGGAAACCTCAGGGGCGGAACGAGGGGCTCGCGG
CCTATCAGCTAGTTGGTGAGGTAACGGCTCACCAAGGCAACGACGGGTAGCTGGTCTGAGAGGATGGC
CAGCCACATTGGGACTGCGACACGGCC
>Zotu4698
GAACGCTGGCGGCAGGCTTAACACATGCAAGTCGAGCGGGGAAAAGTAGCTTGCTACTTTACCTAGCG
GCGGACGGGTGAGTAATGCTTAGGAATCTGCCTATTAGTGGGGGACAACATTTCGAAAGGAATGCTAA
TACCGCATACGTCCTACGGGAGAAAGCAGGGGACCTTCGGGCCTTGCGCTAATAGATGAGCCTAAGTC
GGATTAGCTAGTTGGTGGGGTAAAGGCCTACCAAGGCGACGATCTGTAGCGGGTCTGAGAGGATGATC
CGCCACACTGGGACTGAGACACGGCC
>Zotu4700
GAACGCTAGCGGCAGGCCTAATACATGCAAGTCGAGGGGCAGCGCAGTGTAGCAATACATGGGCGGCG
ACCGGCAAACGGGTGCGGAACACGTACGCAACCTTCCTTCAAGCGGGGAATAGCCCAGAGAAATTTGG
ATTAATACCCCATAGCACTGTCCGGTGGCATCATTGGACAGTTAAAGATTTATCACTTGAAGATAGGC
GTGCGTCTGATTAGGTAGTTGGTGAGGTAACGGCTCACCAAGCCGACGATCAGTAACTGGCGTGAGAG
CGCGACCAGTCACACGGGCACTGAGACACGGGC
>Zotu4701
GAACGCTGGCGGCGCGCCTAACACATGCAAGTCGAACGAGAGATGAGGAGCTTGCTCTTCAAATCGAG
TGGCGAACGGGTGAGTAACGCGTGAGGAACCTGCCTCAAAGAGGGGGACAACAGTTGGAAACGACTGC
TAATACCGCATAAGCCCACGGCTCGGCATCGAGCAGAGGGAAAAGGAGCAATCCGCTTTGAGATGGCC
TCGCGTCCGATTAGCTAGTTGGTGAGGTAATGGCCCACCAAGGCGACGATCGGTATCCGGACTGAGAG
GTTGAACGGCCACATTGGGACTGAGACACGGCC
>Zotu4703
GAACGCTAGCGGCAGGCTTAACACATGCAAGTCGAGGGGTATAGTAGCAATACTAGAGACCGGCGCAC
GGGTGCGTAACGCGTATGCAATCTACCTTTAGCAGGGGCATAGCCCAGAGAAATTTGGATTAATTCCC
CATAGTATTATAGTATGGCATCATATTATAATTAAAGTAACAACGGCTAAAGATGAGCATGCGTCCCA
TTAGCTAGTTGGTATGGTAACGGCATACCAAGGCAATGATGGGTAGGGGTCCTGAGAGGGAGATCCCC
CACACTGGTACTGAGACACGGAC
>Zotu4704
TAACGCTAGCGGCAGGCCTAATACATGCAAGTCGAACGGCAGCACGGGTAGCAATATCTGGTGGCGAG
TGGCGCACGGGTGCGTAACGCGTATGCAATCTACCTGTAACTGGGGAATAGCCTTCCGAAAGGAAGAT
TAATACCGCATAATATCACGAAACGGCATCGTTTTGTGATCAAATCTCCGGAGGTTACAGATGAGCAT
GCGTGACATTAGCTAGTTGGTGAGGTAACGGCCCACCAAGGCTACGATGTCTAGGGGGTCTGAGAGGA
TGATCCCCCACACTGGTACTGAGACACGGAC

>Zotu4705
GAACGCTGGCGGCGTGCTTAACACATGCAAGTCGAACGGTGACCTCGGAGCTTGCTCCGGGTGATCAG
TGGCGAACGGGTGAGTAACACGTGAGTAACCTGCCCCAGACTCTGGGATAACCCCGGGAAACCGGAGC
TAATACCGGATACGACGCGAACAGGCATCTGTATCGCGTGGAAAGTTCTTCGGTCTGGGATGGACTCG
CGGCCTATCAGCTTGTTGGTGAGGTAACGGCTCACCAAGGCGACGACGGGTAGCCGGCCTGAGAGGGC
GACCGGCCACACTGGGACTGAGACACGGCC
>Zotu4706
GAACGCTGGCGGCATGCCTTACACATGCAAGTCGAACGGCAGCACGGGTGCTTGCACCTGGTGGCGAG
TGGCGAACGGGTGAGTAATATATCGGAACATACCTTATAGTGGGGGATAACTATCCGAAAGGATAGCT
AATACCGCATACGCTCTACGGAGGAAAGCGGGGGATCGTAAGACCTCGTGCTATAAGAGTGGCCGATA
TCTGATTAGCTAGTTGGTGAGGTAAAGGCTCACCAAGGCGACGATCAGTAGCTGGTCTGAGAGGACGA
CCAGCCACACTGGAACTGAGACACGGTC
>Zotu4707
GAACGCTGGCGGCGTGCTTCATACATGCAAGTCGAACGAGAAGCTGACTTCGGTCAGTGGAAAGTGGC
GGACGGGTGAGTAATATGTAGGAAATCTGCCTTAGAGAGGGGGACAACAGAGGGAAACTTCTGCTAAT
ACCCCATATGAGCGTACCTGAAATGGTATTCTTGAAAACTCCGGTGCTCTAAGATGAGCCTGCATCTG
ATTAGCTTGTTGGTGGGGTAATGGCCTACCAAGGCGACGATCAGTAGCTGGTTTGAGAGGATGATCAG
CCACAATGGGACTGAGACACGGCC
>Zotu4709
AAACGCTGGCGGCATGCCTAACACATGCAAGTCGAACGGACTAATTGGGGCTTGCTCCAATTAGTTAG
TGGCAGACGGGTGAGTAACATGTGGGAATCTACCCATCAGTACGGAATAACTTTTAGAAATAAAAGCT
AATACCGTATATTCTCTACGGAGGAAAGATTTATCGCTGATGGATGAGCCCGCGTCAGATTAGGTAGT
TGGTGAGGTAATGGCTCACCAAGCCGACGATCTGTAGCTGGTCTGAGAGGATGACCAGCCACACTGGG
ACTGAGACACGTCC
>Zotu4710
GAACGCTAGCGGGAGGCCTAACACATGCAAGCCGAGCGGTAGGGTTTCTTCGGAAACCTGAGAGCGGC
GCACGGGTGCGGAACACGTGTGCAACCTACCTTTATCAGGGGGATAGCCTTTCGAAAGGAAGATTAAT
ACCCCATAATATATTAGATGGCATCATTTGATATTGAAAACTCCGGTGGATAGAGATGGGCACGCGCA
AGATTAGATAGTTGGTGAGGTAACGGCTCACCAAGTCCATGATCTTTAGGGGGCCTGAGAGGGTGATC
CCCCACACTGGTACTGAGACACGGAC
>Zotu4711
GAACGCTGGCGGCGTGCCTAATACATGCAAGTCGAGCGAATCTTTGGGAGCTTGCTCCCAAAGATTAG
CGGCGGACGGGTGAGTAACACGTGGGTAACCTGCCTGTAAGACTGGGATAACTTCGGGAAACCGGAGC
TAATACCGGATAACATATGAGACCGCATGGTCTTATATTAGAAGATGGCTTTTAGCTATCACTTACAG
ATGGACCCGCGGCGCATTAGCTAGTTGGTGAGGTAATGGCTCACCAAGGCGACGATGCGTAGCCGACC
TGAGAGGGTGATCGGCCACACTGGGACTGAGACACGGCC
>Zotu4713
GAACGCTGGCGGCATGCTTTACACATGCAAGTCGGACGGCAGCACAGAGAAGCTTGCTTCTTGGGTGG
CGAGTGGCGAACGGGTGAGTAACATATCGGAACGTACCGGGCAGTGGGGGATAACTAATCGAAAGATT
AGCTAATACCGCATATATTCTGAGGAAGAAAGCAGGGGACCATTTGGCCTTGCGCTGTTTGAGCGGCC
GATATCTGATTAGCTGGTTGGTGGGGTAAAGGCCTACCAAGGCGACGATCAGTAGCGGGTCTGAGAGG
ATGATCCGCCACACTGGGACTGAGACACGGCC
>Zotu4715
GAACGCTAGCGGGATGCTTTACACATGCAAGTCGAACGGCAGCGCGAGAGAGCTTGCTCTCTTGGCGG
CGAGTGGCGGACGGGTGAGTAATATATCGGAACGTGCCCAGTAGCGGGGGATAACTACTCGAAAGAGT
GGCTAATACCGCATACGCCCTACGGGGGAAAGGGGGGGATCGCAAGACCTCTCACTATTGGAGCGGCC
GATATCGGATTAGCTAGTTGGTGAGGTAATGGCTCACCAAGGCAACGATCTTTAGTTGGTCTGAGAGG
ATGATCAGCCACACTGGAACTGAGACACGGTC
>Zotu4719
GAACGCTGGCGGCGTGCTTAACACATGCAAGTCGAACGATGACGGAGAGCTTGCTCTTCTGATTAGTG
GCGAACGGGTGAGTAACACGTGAGTAACCTGCCCTTGACTCTGGGATAAGCACTGGAAACGGTGTCTA
ATACCGGATACGAGCTTCAGCCGCATGGCTAGGAGCTGGAAAGAATTTTGGTCAAGGATGGACTCGCG
GCCTATCAGCTAGTTGGTGAGGTAACGGCCCACCAAGGCGACGACGGGTAGCCGGCCTGAGAGGGTGA
CCGGCCACACTGGGACTGAGACACGGCC

>Zotu4720
GAACGCTGGCGGCGTGCTTAACACATGCAAGTCGAGCGGTAAGGCACCTTCGGGTGTACACGAGCGGC
GAACGGGTGAGTAACACGTGAGTAATCTGCCCTTCACTTCGGGATAACCGCCGGAAACGGTGGCTAAT
ACCGGATACGACGCACTCTCGCATGGGATGTGCGTGGAAAGTCTTATCGGTGGAGGATGTGCTCGCGG
CCTATCAGCTTGTTGGTGAGGTAACGGCTCACCAAGGCTTCGACGGGTAGCCGGCCTGAGAGGGTGAC
CGGCCACACTGGGACTGAGACACGGCC
>Zotu4721
GAACGCTAGCGGCAGGCCTAATACATGCAAGTCGAACGGGCTGGTAGCAATACCAGTTAGTGGCGAAC
GGGTGCGTAACACGTACACAACATGCCTTCAACTGGGGGATAGCGGTGGGAAACTGCCAGTAATACCC
CATGTGATTGCGGAATGGCATCATTTTGCAATTAAAACCTTGGTGGTTGAAGATTGGTGTGCGTCCGA
TTAGCTAGTTGGTAGGGTAACGGCCTACCAAGGCGACGATCGGTAGGGGGTGTGAGAGCATGGCCCCC
CACACGGGTACTGAGACACGGAC
>Zotu4722
GAACGCTGGCGGCGTGCTTAACACATGCAAGTCGAACGGTGAAGCCCAGCTTGCTGGGTGGATCAGTG
GCGAACGGGTGAGTAACACGTGAGCAATCTGCCCCTGACTCTGGGATAAGCGCTGGAAACGGCGTCTA
ATACTGGATACGAACAGCGACCGCATGGTCAGTTGTTGGAAAGATTTTTCGGTCAGGGATGAGCTCGC
GGCCTATCAGCTTGTTGGTGAGGTAATGGCTCACCAAGGCGTCGACGGGTAGCCGGCCTGAGAGGGTG
ACCGGCCACACTGGGACTGAGACACGGCC
>Zotu4724
GAACGCTGGCGGCGTGCTTAACACATGCAAGTCGAACGGTGAAGCAGGAGCTTGCTCTTGTGGATCAG
TGGCGAACGGGTGAGTAACACGTGAGCAACCTGCCCCTGACTCTGGGATAAGCGCTGGAAACGGCGTC
TAATACTGGATATGTGACGTGACCGCATGGTCTGCGTTTGGAAAGATTTTTCGGTTGGGGATGGGCTC
GCGGCCTATCAGCTTGTTGGTGAGGTAATGGCTCACCAAGGCGTCGACGGGTAGCCGGCCTGAGAGGG
TGACCGGCCACACTGGGACTGAGACACGGCC
>Zotu4725
GAACGCTGGCGGCATGCCTTACACATGCAAGTCGAACGGCAGCGCGGGCTTCGGCCTGGCGGCGAGTG
GCGAACGGGTGAGTAACATATCGGAACGTGCCCTGGAGTGGGGGATAACTAGTCGAAAGATTAGCTAA
TACCGCATACGATCTACGGAGGAAAGTGGGGGACCGCAAGGCCTCACGCTCGTGGAGCGGCCGATATC
TGATTAGCTAGTTGGTGGGGTAAAGGCCTACCAAGGCATCGATCAGTAGCTGGTCTGAGAGGACGACC
AGCCACACTGGGACTGAGACACGGCC
>Zotu4729
GAACGCTAGCGGCAGGCTTAATACATGCAAGTCGAGGGGCAGCACGGGTAGCAATACCTGGTGGCGAC
CGGCAAACGGGTGCGGAACACGTACGCAACTTTCCTCTGAGCGGGGAATAGCCCGGGGAAACCCGGAT
TAATACCCCATAATATAATGGAGAAGCATTTCTTTATTATTAAAGATTTATCACTCAGAGATGGGCGT
GCGCCTGATTAGGTAGTTGGCGGGGTAACGGCCCACCAAGCCTGCGATCAGTAGCTGATGTGAGAGCA
TGATCAGCCACACGGGCACTGAGACACGGGC
>Zotu4730
CAACGCTGACGGCGTGCCTAACACATGCAAGTCGAACGTGAAAGGGGCTTCGGCCCTGAGTAAAGTGG
CAAACGGGTGAGTAACGCGTGGGTGATCTGCCTCTGAGTGGGGGATAACCTTCCGAAAGGAGGGCTAA
TACCGCATGACATCCTGTTCTTGGATGAACGGTGATCGAAGCTGGGGACCGCAAGGCCTGGCGCTTGG
AGAGGAGCCCGCGTCCGATTAGCTAGTTGGTGGGGTAATGGCCTACCAAGGCGACGATCGGTAGCCGG
CCTGAGAGGGCGGACGGCCACACTGGGACTGAGACACGGCC
>Zotu4731
GAACGCTGGCGGCGTGCTTAACACATGCAAGTCGAACGCTGAAGCCCAGCTTGCTGGGTGGATGAGTG
GCGAACGGGTGAGTAACACGTGAGTAACCTGCCCTTCACTCTGGGATAACTCCGGGAAACCGGTGCTA
ATACTGGATAGGACTCCTGCCTGCATGGGTGGGGGTGGAAAGATTTATCGGTGGAGGATGGACTCGCG
GCCTATCAGCTTGTTGGTGGGGTAATGGCCTACCAAGGCGACGACGGGTAGCCGGCCTGAGAGGGTGA
CCGGCCACACTGGGACTGAGACACGGCC
>Zotu4732
GAACGCTGGCGGCGTGCCTAACACATGCAAGTCGAACGAGATGCGTATCGGAAAGCTTCGGCCGGAAG
ATACGTTATCTAGTGGCGGACGGGTGAGTAACACGTGGGTAACCTGCCTTATGGAGGGGGATAACAGA
GAGAAATCACTGCTAATACCGCATAAGCACACAGTACCGCATGGTACAGGGTGAAAAGATTTATCGCC
ATAAGATGGACCCGCGTCTGATTAGCCAGTTGGCAGGGTAAAGCCTACCAAAGCAACGATCAGTAGC
CGATCTGAGAGGATGACCGGCCACATTGGGACTGAGACACGGCC

>Zotu4733
GAACGCTAGCGGCAGGCTTAACACATGCAAGTCGAGGGGTATAGTAGCAATACTAGAGACCGGCGCAC
GGGTGCGTAACGCGTATGCAACCTACCTTTCACAGAGGGATAGCCCAGAGAAATTTGGATTAATACCT
CATAGTATATATGAATCGCATGGTTTATATATTAAAGTTCCAACGGTGAAAGATGGGCATGCGTCCCA
TTAGCTAGATGGTAAGGTAACGGCTTACCATGGCAACGATGGGTAGGGGTCCTGAGAGGGAGATCCCC
CACACTGGTACTGAGACACGGAC
>Zotu4734
GAACGCTGGCGGCGTGCCTAATACATGCAAGTCGAGCGAACAGATGAGGAGCTTGCTCCTCTGATGTT
AGCGGCGGACGGGTGAGTAACACGTGGGCAACCTGCCCTATAGTTTGGGATAACTTGCGGAAACGTGA
GCTAATACCGGATAATACAACGAAGCTCCTGCTTCGTTGTTGAAAGATGGCTTTGCTATCGCTATAGG
ATGGGCCCGCGGCGCATTAGCTAGTAGGTGAGGTAACGGCTCACCTAGGCAACGATGCGTAGCCGACC
TGAGAGGGTGATCGGCCACACTGGGACTGAGACACGGCC
>Zotu4736
GAACGCTGGCGGCACGTTTTAAGCATGCAAGTCGAACGGCAGTGGGGGCAACCCCATGAGAGTGGCGA
ACGGGTGAGTAACACGTAGGTGATCTGCCTTGAGGATGGGGATAGCTTGTGGAAACACAGGGTAATAC
CGAATAAGCTGACGCGGCTTTGGCTGAGTTAGTAAAGGTGCTACGGCACCGCCTTGAGAGGAGCCTGC
GGCTGATTAGCTAGTTGGTGGGGTAAAGGCCTACCAAGGCGACGATCAGTAGCCGGCCTGAGAGGGTG
TACGGCCACACTGGAACTGAGACACGGTC
>Zotu4737
GAACGCTGGCGGCGTGCCTAACACATGCAAGTCAAGGGTTTGATTTATCAGACACTGGCATCCGGGTG
AGTAACGCGTAGGTCATCTGCCCCTATCACAGACATAACAGTTCTAACGGACTGCTAATATCTGATGA
GGCGAGCAATCGTCAAAGCTTAGGCGGATAGGGATGAGCCTGCGTCTGATTAGGTAGTTGGTGAGGTA
ATGGCTCACCAAGCCTTCGATCAGTAGCTGGTCTGAGAGGACGAACAGCCACACTGGAACTGAGACAC
GGTC
>Zotu4738
GAACGCTGGCGGCGTGCTTAACACATGCAAGTCGAACGGAAAGGCCTTGTGCTTGCACAAGGTACTCG
AGTGGCGAACGGGTGAGTAACACGTGGGTGATCTGCCCTGCACTGTGGGATAAGCCTGGGAAACTGGG
TCTAATACCATATAGGACCGCACTTTGGATGGTGTGGTGGAAAGCTTTTGCGGTGTGGGATGAGCCTG
CGGCCTATCAGCTTGTTGGTGGGGTAATGGCCTACCAAGGCGGCGACGGGTATCCGGCCTGAGAGGGT
GTACGGACACATTGGGACTGAGATACGGCC
>Zotu4740
GAACGCTAGCGGCAGGCTTAACACATGCAAGTCGAGGGGTATAGTTAGCTTGCTAATTAGAGACCGGC
GCACGGGTGCGTAACGCGTATGCAATCTACCTTTCACAGAGGGATAGCCCAGAGAAATTTGGATTAAT
ACCTCATAGTATTATAGAATGGCATCATTTTATAATTAAAGTCACAACGGTGAAAGATGAGCATGCGT
CCCATTAGCTATTTGGTATGGTAACGGCATACCAAGGCTACGATGGGTAGGGGTCCTGAGAGGGAGAT
CCCCCACACTGGTACTGAGACACGGAC
>Zotu4741
GAACGCTGGCGGCGTGCTTAACACATGCAAGTGGAGCGACGAACCGGGGCTTGCCCCGGGGCAGAGCC
GCGAACGGGTGAGTAACACGTGGGTAACCCACCTTGATGACTGGGACAACCCGAGGAAACTCGGGCTA
ATACCGGATGTGATCCTCTTTCGTAAGGAAGATGATGAAAGATAGCTTCGGCTTTCGCATCAAGACGG
GCCCGCGGCCCATTAGCTTGTTGGTGGGGTAATGGCCTACCAAGGCTTCGATGGGTAGCTGGTCTGAG
AGGACGATCAGCCACACTGGGACTGAGACACGGCC
>Zotu4743
GAACGCTGGCGGCGTGCTTAACACATGCAAGTCGAACGATGAAGCCCAGCTTGCTGGGTGAAAGAGTG
GCGAACGGGTGAGTAACACGTGAGTAACCTGCCCTGAACTCTGGGATAAGCACTGGAAACGGTGTCTA
ATACTGGATACGACCTATCACCGCATGGTGTGTGGGTGGAAAGATTTATCGGTTTTGGATGGACTCGC
GGCCTATCAGCTAGATGGTGAGGTAATGGCTCACCATGGCGACGACGGGTAGCCGGCCTGAGAGGGTA
ACCGGCCACACTGGGACTGAGACACAGCC
>Zotu4744
GAACGCTGGCGGCGTGCCTAATACATGCAAGTCGAGCGGATCTTTAGAAGCTTGCTTCTAAAGATTAG
CGGCGGACGGGTGAGTAACACGTGGGCAACCTGCCTGTAAGACTGGGATAACTTCGGGAAACCGAGGC
TAATACCGGATAATCCTTTTCCTCACATGAGGGAAAGCTGAAAGACGGTGCAAGCTGTCACTTACAGA
TGGGCCCGCGGCGCATTAGCTAGTTGGTGAGGTAACGGCTCACCAAGGCGACGATGCGTAGCCGACCT
GAGAGGGTGATCGGCCACACTGGGACTGAGACACGGCC

>Zotu4745
GAACGCTGGCGGCAGGCCTAACACATGCAAGTCGAACGGGTGTCTTCGGGCACTAGTGGCGGACGGGT
GAGTAACGCGTGGGAACATACCCCTTTCTTCGGAATAGCCTCGGGAAACTGGGAGTAATACCGGATAC
GCCCTTCGGGGGAAAGATTTATCGGTAAGGGATTGGCCCGCGTTGGATTAGGTAGTTGGTGGGGTAAT
GGCCTACCAAGCCGACGATCCATAGCTGGTTTGAGAGGATGATCAGCCACACTGGGACTGAGACACGG
CC
>Zotu4746
GAACGCTGGCGGTATGCTTAACACATGCAAGTCGAACGGACTCAGGGAGCTTGCTCCCAAAAGTTAGT
GGCAGACGGGTGAGTAACGCGTGGGAATCTGCCTATCAGTAGGGAATAACATTTGGAAACAAATGCTA
ATACCCTATACGCTCTTCGGAGGAAAGATTTATCGCTGATAGATGAGCCCGCGTCAGATTAGGTAGTT
GGTAGGGTAATGGCCTACCAAGCCGACGATCTGTAGCTGGTCTGAGAGGATGATCAGCCACACTGGGA
CTGAGACACGGCC
>Zotu4748
GAACGCTGGCGGCGTGCCTAATACATGCAAGTCGAGCGAATCACTGGGAGCTTGCTCCCGGTGGTTAG
CGGCGGACGGGTGAGTAACACGTGGGCAACCTGCCTGTAAGACTGGGATAACTTCGGGAAACCGGAGC
TAATACCGGATAATTTCTTCCCTCGCATGAGGGAAGGTTGAAAGTCGGTTTCGGCTGACACTTACAGA
TGGGCCCGCGGCGCATTAGCTAGTTGGTGAGGTAACGGCTCACCAAGGCGACGATGCGTAGCCGACCT
GAGAGGGTGATCGGCCACACTGGGACTGAGACACGGCC
>Zotu4749
GAACGCTGGCGGCATGCTTTACACATGCAAGTCGAACGGCAGCGCGGGGCAACCTGGCGGCGAGTGGC
GAACGGGTGAGTAATACATCGGAACGTACCCAGAAGTGGGGGATAACGTAGCGAAAGTTACGCTAATA
CCGCATACGTTCTACGGAAGAAAGTGGGGGACCTTCGGACCTCATGCTTTTGGAGCGGCCGATGTCTG
ATTAGCTAGTTGGTGAGGTAAAGGCTCACCAAGGCGACGATCAGTAGCTGGTCTGAGAGGACGACCAG
CCACACTGGGACTGAGACACGGCC
>Zotu4750
GAACGCTGGCGGCATGCCTAACACATGCAAGTCGAACGAGACCTTCGGGTCTAGTGGCGCACGGGTGC
GTAACGCGTGGGAACCTGCCCTTAGGTTCGGAATAACTCAGAGAAATTTGAGCTAATACCGGATAATG
TCTTCGGACCAAAGATTTATCGCCTTTGGATGGGCCCGCGTTGGATTAGCTAGTTGGTGGGGTAATGG
CCTACCAAGGCGACGATCCATAGCTGGTCTGAGAGGATGATCAGCCACACTGGGACTGAGACACGGCC
>Zotu4752
GAACGCTAGCGGCAGGCTTAACACATGCAAGTCGAGGGGTATAGTTCTTCGGAATTAGAGACCGGCGC
ACGGGTGCGTAACGCGTATGCAATCTACCTTTCACAGAGGGATAGCCCAGAGAAATTTGGATTAATAC
CTCATAGTATTACGAGATGGCATCATTTTGTAATTAAAGTCACAACGGTGAAAGATGAGCATGCGTCC
CATTAGCTTGTTGGTAAGGTAACGGCTTACCAAGGCTACGATGGGTAGGGGTCCTGAGAGGGAGATCC
CCCACACTGGTACTGAGACACGGAC
>Zotu4754
GAACGCTAGCGGCAGGCCTAATACATGCAAGTCGAGCGGATTGTAGCAATACAGTTAGCGGCGCACGG
GTGAGTAACGCGTACACAATCTACCTTCAACAGGGGAATAGCCTCGGGAAACTGGGAGTAATACCCCA
TAGCATCTTTGGGTCGCATGACCTGATTATTAAAGTTACGGCGGTTGAAGATGAGTGTGCGTCTGATT
AGATTGTTGGTGAGGTAACGGCTCACCAAGTCGATGATCAGTAAGGGGCGTGAGAGCGTGGCCCCTCA
CATGGACACTGAGACACGGGT
>Zotu4755
GAACGCTGGCGGCAGGCTTAACACATGCAAGTCGAGCGGAGAGAGGTAGCTTGCTACTGATCTTAGCG
GCGGACGGGTGAGTAATGCTTAGGAATCTGCCTATTAGTGGGGGACAACATTTCGAAAGGAATGTTAA
TACCGCATACGTCCTACGGGAGAAAGCAGGGGATCTTCGGACCTTGCGCTAATAGATGAGCCTAAGTC
GGATTAGCTAGTTGGTGGGGTAAAGGCCTACCAAGGCGACGATCTGTAGCGGGTCTGAGAGGATGATC
CGCCACACTGGGACTGAGACACGGCC
>Zotu4756
GAACGCTGGCGGCATGCCTAACACATGCAAGTCGAACGAGACCTTCGGGTCTAGTGGCGCACGGGTGC
GTAACGCGTGGGAATCTGCCCTTGGGTTCGGAACAACAGTTGGAAACGACTGCTAATACCGGATGATG
ACTTCGGTCCAAAGATTTATCGCCCAAGGATGAGCCCGCGTAGGATTAGCTTGTTGGTGAGGTAAAAG
CTCACCAAGGCGACGATCCTTAGCTGGTCTGAGAGGATGATCAGCCACACTGGGACTGAGACACGGCC
>Zotu4757
GAACGCTGGCGGTAGGCCTAACACATGCAAGTCGAACGGCAGCACAGTAAGAGCTTGCTCTTACGGGT

GGCGAGTGGCGGACGGGTGAGGAATACATCGGAATCTACTTTTTCGTGGGGGATAACGTAGGGAAACT
TACGCTAATACCGCATACGACCTACGGGTGAAAGCAGGGGATCTTCGGGCCTTGCGCGATTGAATGAG
CCGATGTCGGATTAGCTAGTTGGCGGGGTAAAGGCCCACCAAGGCGACGATCCGTAGCTGGTCTGAGA
GGATGATCAGCCACACTGGAACTGAGACACGGTC
>Zotu4759
GAACGCTGGCGGCAGGCTTAACACATGCAAGTCGAACGCCCCGCAAGGGGAGTGGCAGACGGGTGAGT
AACGCGTGGGAATCTACCCAGATCTTCGGAACAACTGAGGGAAACTTCAGCTAATACCGGATACGCCC
TACGGGGGAAAGATTTATCGGTTCTGGATGAGCCCGCGTTGGATTAGCTAGTTGGTGGGGTAATGGCC
TACCAAGGCGACGATCCATAGCTGGTCTGAGAGGATGATCAGCCACACTGGGACTGAGACACGGCC
>Zotu4760
GAACGCTAGCGGCAGGCCTAATACATGCAAGTCAAGGGGTCAGCAATGACACCGGCGGACGGGTGCGT
AACGCGTACACAACATGCCTTCGAGCGGGGGATAGCGGCGGGAAACTGCCGGTAATACCCCATAACAC
TGCGTTTTGGCATCAGGATGCAGTTAAAGTTCCGGCGCTCGGAGATTGGTGTGCGTCTGATTAGCTAG
TTGGCGGGGTAACGGCCCACCAAGGCGACGATCAGTAGGGGGTGTGAGAGCATGGCCCCCCACATGGG
TACTGAGACACGGAC
>Zotu4761
GAACGCTGGCGGCATGCCTAACACATGCAAGTCGAACGAAGGCCTTCGGGCTTTAGTGGCGCACGGGT
GCGTAACGCGTGGGAATCTGCCCTTAGGTTCGGAATAACAGCTGGAAACGGCTGCTAATACCGGATGA
AGACGAAAGTCCAAAGATTTATCGCCTGAGGATGAGCCCGCGTTGGATTAGGTAGTTGGTGGGGTAAA
GGCCTACCAAGCCGACGATCCATAGCTGGTCTGAGAGGATGATCAGCCACACTGGGACTGAGACACGG
CC
>Zotu4762
GAACGCTGGCGGCGCGCCTAAAACATGCAAGTCGAACGAGAAGCCCGGGCTTGCCCGGGTGGACAGTG
GCGAACGGGTGAGTAACACGTGAGGAACCTGCCCCGAAGACCGGGACAACATCGGGAAACCGTTGCTA
ATACCGGATGCCCTCGAAGAATCGCATGGTTCATCGAGGAAATGGATTCCGCTTCGGGATGGCCTCGC
GGCCTATCAGCTTGTTGGTGAGGTAATGGCTCACCAAGGCAACGACGGGTAGCTGGTCTGAGAGGATG
TCCAGCCACACTGGGACTGAGACACGGCC
>Zotu4763
GAACGCTGGCGGCGTGCTTAACACATGCAAGTCGAACGAACCTTCGGGTTAGTGGCGGACGGGTGAGT
AACGCGTGGGAATCTGCCCTCAGGAGGGGGATAACGGCTGGAAACGGCCGCTAATACCCCATATGCCG
AGAGGTGAAACGAATTTCGCCTGAGGATGAGCCCGCGTCTGATTAGCTAGTTGGTGGGGTAAGAGCCT
ACCAAGGCGACGATCAGTAGCTGGTCTGAGAGGATGATCAGCCACACTGGGACTGAGACACGGCC
>Zotu4764
GAACGCTGGCGGCGTGCTTAACACATGCAAGTCGAGCGGTAAGGCCTTTCGGGGTACACGAGCGGCGA
ACGGGTGAGTAACACGTGGGTGATCTGCCCTGCACTCTGGGATAAGCTTGGGAAACTGGGTCTAATAC
CGGATATGACCGCATGCCGCATGGTGTGTGGTGGAAAGATTTATCGGTGCAGGATGGGCCCGCGGCCT
ATCAGCTTGTTGGTGGGGTAATGGCCTACCAAGGCGACGACGGGTAGCCGACCTGAGAGGGTGACCGG
CCACACTGGGACTGAGACACGGCC
>Zotu4765
GAACGCTGGCGGCGTGCCTAATACATGCAAGTCGAGCGAACCACTTCGGTGGTGAGCGGCGAACGGGT
GAGTAACACGTAGGTGATCTGCCCATCAGACGGGGACAACGATTGGAAACGATCGCTAATACCGGATA
GGACGAAAGTTTAAAGGTGCTTCTGGCACCGCTGATGGATGAGCCTGCGGCGCATTAGCTAGTTGGTA
GGGTAAAGGCCTACCAAGGCGACGATGCGTAGCCGACCTGAGAGGGTGAACGGCCACACTGGGACTGA
GACACGGCC
>Zotu4766
GAACGCTGGCGGCATGCCTAACACATGCAAGTCGAACGATGGCTTCGGCCATAGTGGCGCACGGGTGA
GTAACGCGTGGGAACCTGCCTTTTGGTTTGGGATAACTCCGGGAAACTGGAGCTAATACCGGATGAGC
CCGAGCAATCGGGGAAAGATTTATCGCCAAAAGAGGGGCCCGCGTCCGATTAGGTAGTTGGTGGGGTA
ACGGCCTACCAAGCCTACGATCGGTAGCTGGTCTGAGAGGATGATCAGCCACACTGGGACTGAGACAC
GGCC
>Zotu4767
GAACGCTGGCGGCGTGCCTAATACATGCAAGTTGAGCGGCAGCGAGTTTTGCACTTATCCTTCCGACC
CATACGTGGGTGGAAGGATGTGTGCAGAATGTCGGCGAGCGGCGGACGGCAGAGTAACGCGTAGGAAT
GTACCCCAAAGTGAGGGATAACTGCTCGAAAGAGTAGCTAATACCGCATGTGATCTTCGGATTAAAGC

TTTATGCGCTTTGGGAACAGCCTGCGTAAGATTAGCTAGTTGGTGAGGTAATGGCTCACCAAGGCGAC
GATCTTTAGCTGGTCTGAGAGGATGATCAGCCTGACTGGAACTGAGACACGGTC
>Zotu4768
GAACGCTGGCGGCGTGCCTAATACATGCAAGTCGAACGGTAAGGCTTTCACAGTTTGCTGGTAGTGGT
AAAACACTCGAGCAAAGCGTGAAAGTACACGAGTGGCGGACGGCAGAGTAACGCGTAGGAATGTACCC
CAAAGTGAGGGATAACTGCTCGAAAGAGTAGCTAATACCGCATGTGGTCTTCGGATTAAAGCTTCGGC
GCTTTGGGAACAGCCTGCGTAAGATTAGCTAGTTGGTAGTGTAATGGACTACCAAGGCGACGATCTTT
AGCTGGTCTGAGAGGATGATCAGCCTGACTGGAACTGAGACACGGTC
>Zotu4769
GAACGCTGGCGGCAGGCTTAACACATGCAAGTCGAACGCCCCGCAAGGGGAGTGGCAGACGGGTGAGT
AACGCGTGGGAATCTACCTAGAACTACGGAACAACAGTTGGAAACGACTGCTAATACCGTATACGCCC
TACGGGGGAAAGATTTATCGGTTTTAGATGAGCCCGCGTAAGATTAGCTAGTTGGTGGGGTAATGGCC
TACCAAGGCGACGATCTTTAGCTGGTCTGAGAGGATGATCAGCCACACTGGGACTGAGACACGGCC
>Zotu4770
GAACGCTGGCGGCGTGCCTAACACATGCAAGTCGAACGAGTCGCAAGACTAGTGGCGAACGGGTGAGT
AACACGTAGGAATTTGCCCCAGAGCGGAGGATACCTAGCCGAAAGGTTGGTTAATACTCCATAGTCCC
GCAAGGGTAAAGCAGCAATGCGCTTTGGGAGAAGCCTGCGTCCTATCAGCTAGTTGGTGAGGTAATGG
CTTACCAAGGCTATGACGGGTAGGGGGCGTGAGAGCGTGACCCCCCACAATGGGACTGAGACACGGCC
>Zotu4771
GAACGCTGGCGGCGTGCTTAACACATGCAAGTCGAGCGACGCTCCTCCTTCGGGAGGATGACGGAGCG
GCGAACGGGTGAGTAACACGTGGGCAACCTGCCCCTAGCTCTGGGATAAGCCCGGGAAACCGGGTCTA
ATACCGGATATGAGTTCGGAAGGCATCTTCTGACCTGGAAAGTTTTTCGGCTAGGGATGGGCCCGCGG
CCTATCAGCTTGTTGGTGAGGTAACGGCCCACCAAGGCGACGACGGGTAGCCGGCCTGAGAGGGCGAC
CGGCCACACTGGGACTGAGACACGGCC
>Zotu4772
GAACGCTGGCGAGATGCCTTACACATGCAAGTCGTACGGGAATTTTTAATTCAAGTGGCGGACGGGTG
AGTAACACGTAAGAATTTGCCTTTAGGAGGGGGACAACAACTGGAAACGGTTGCTAATACCCCATATG
CTTTCGAGTGAAATGGATTTTTCCGCCTAAAGAGAAGCTTGCGGCTGATTAGCTTGTTGGTGAGGTAA
TGGCTCACCAAGGCGACGATCAGTATCTGGTTTGAGAGGATGATCAGACACACTGGAACTGAGACACG
GTC
>Zotu4773
GAACGCTGGCGGCGTGCTTAACACATGCAAGTGGAGCGACGAACCGGGGCTTGCCCCGGGGCAGAGCC
GCGAACGGGTGAGTAACACGTGGGTAACCCACCTCGATGACCGGGACAACCCGAGGAAACTCGGGCTA
ATACCGGATGTGATCCTCTTTCGTAAGGAAGATGATGAAAGATAGCTTCGGCCTTCGCATCGAGACGG
GCCCGCGGCCCATTAGCTTGTTGGTGGGGTAATGGCCTACCAAGGCTTCGATGGGTAGCTGGTCTGAG
AGGACGATCAGCCACACTGGGACTGAGACACGGCC
>Zotu4774
GAACGCTGGCGGCATGCCTTACACATGCAAGTCGAACGGCAGCACGGGAGCTTGCTCCTGGTGGCGAG
TGGCGAACGGGTGAGTAAAGCATCGGAACGTACCGTGAAGTGGGGGATAACGTAGCGAAAGTTACGCT
AATACCGCATATTCTGTGAGCAGGAAAGCAGGGGATCGCAAGACCTTGTGCTTTACGAGCGGCCGATG
TCGGATTAGCTAGTTGGTGAGGTAAAGGCTCACCAAGGCGACGATCCGTAGCGGGTCTGAGAGGATGA
TCCGCCACACTGGGACTGAGACACGGCC
>Zotu4775
GAACGCTGGCGGCGTGCCTAATACATGCAAGTCGAGCGGACAGATGGGAGCTTGCTCCCTGATGTTAG
CGGCGGACGGGTGAGTAACACGTGGGTAACCTGCCTGTAAGACTGGGATAACTCCGGGAAACCGGGGC
TAATACCGGATGCTTGTTTGAACCGCATGGTTCAAACATAAAAGGTGGCTTCGGCTACCACTTACAGA
TGGACCCGCGGCGCATTAGCTAGTTGGTGAGGTAATGGCTCACCAAGGCAACGATGCGTAGCCGACCT
GAGAGGGTGATCGGCCACACTGGGACTGAGACACGGCC
>Zotu4776
GAACGCTGGCGGCGTGCTTAACACATGCAAGTCGAACGATGAACCTCACTTGTGGGGGGATTAGTGGC
GAACGGGTGAGTAACACGTGAGTAACCTGCCCTTGACTCTGGGATAAGCCTGGGAAACCGGGTCTAAT
ACTGGATACGACCTTCCCGCGCATGCGGTGTTGGTGGAAAGCTTTTGCGGTTTTGGATGGACTCGCGG
CCTATCAGCTTGTTGGTGGGGTAATGGCCTACCAAGGCGACGACGGGTAGCCGGCCTGAGAGGGTGAC
CGGCCACACTGGGACTGAGACACGGCC

>Zotu4778
GAACGCTGGCGGCGTGCCTAATACATGCAAGTCGAGCGGACTTGTGGGAGCTTGCTCCCGCAAGTTAG
CGGCGGACGGGTGAGTAACACGTGGGCAACCTGCCTGTAAGACTGGGATAACTTCGGGAAACCGGAGC
TAATACCGGATAACTCCTTTCCTCACCTGGGGAAAGGTTGAAAGATGGCTTCGGCTATCACTTACAGA
TGGGCCCGCGGCGCATTAGCTAGTTGGTGAGGTAACGGCTCACCAAGGCGACGATGCGTAGCCGACCT
GAGAGGGTGATCGGCCACACTGGGACTGAGACACGGCC
>Zotu4780
GAACGCTGGCGGCGTGCTTAACACATGCAAGTCGAACGGTGAAGGGGAGCTTGCTCCCCGGATCAGTG
GCGAACGGGTGAGTAACACGTGGGCAACCTGCCCCAGACTTCGGGATAACGCTTCGAAAGAGGTGCTA
ATACCGGATACGAACCTCCCTGGCATCTGGGAGGTTGGAAAGTTTTTCGGTCTGGGATGGGCCCGCGG
CCTATCAGCTTGTTGGTGAGGTAACGGCTCACCAAGGCGACGACGGGTAGCCGGCCTGAGAGGGCGAC
CGGCCACACTGGGACTGAGACACGGCC
>Zotu4781
GAACGCTAGCGGGAGGCTCAACACATGCAAGCCGAGGGGTATATAATTGCTTGCAATTATAGAGACCG
GCGGATGGGTGAGTAACGCGTATGTAACTTACCCTATTCATTGGGATAGCCCGAGGAAACTCGGATTA
ATACCAGATAACACTAAGGTCGGCATTGACTTTAGTTGAAAGATTTATCGGAATAGGATAGACATGCG
TCAGATTAGCTAGTTGGTGAGATAGAGGCTCACCAAGGCAATGATCTGTAGGGGGCCTGAGAGGGTGA
TCCCCCACACTGGTACTGAGACACGGAC
>Zotu4786
GAACGCTGGCGGTATGCTTAACACATGCAAGTCGAACGGACTCTTCGGAGTTAGTGGCGGACGGGTGA
GTAACGCGTGAGAATCTGCCTTTAGGTCGGGGACAACAGTTGGAAACGACTGCTAATACCGGATGTGC
AGCAATGTGAAAGATTTATTGCCTAAAGATGAGCTCGCGTCTGATTAGCTAGTAGGTGTGGTAATGGC
GCACCTAGGCGACGATCAGTAACTGGTCTGAGAGGATGACCAGTCACACTGGGACTGAGACACGGCC
>Zotu4788
GAACGCTAGCGGCAGGCCTAACACATGCAAGTCGAGCGGGATTCGGTGGCTTGCCACCGATGAGAGCG
GCGCACGGGTGCGTAACGCGTATACAGCCTACCTTTTGCTACGGGATAGCCCAGAGAAATTTGGATTA
ACACCGTATGGTATAAGGACCCGGCATCGGGATCTTATTAAAGGCTACGGCAAAAGATGGGTATGCGT
CCTATTAGCTAGATGGTGCGGTAACGGCGCACCATGGCAACGATAGGTAGGGGCCCTGAGAGGGGGAT
CCCCCACACTGGTACTGAGACACGGAC
>Zotu4789
GAACGCTGGCGGCGTGCTTAACACATGCAAGTCGAACGGTGAAAGAGGAGCTTGCTCCTCTGGATCAG
TGGCGAACGGGTGAGTAACACGTGAGTAACCTGCCCCAGACTCTGGGATAAGCACTGGAAACGGTGTC
TAATACCGGATACGAACTTCAGCCGCATGGCTAGGAGTTGGAAAGAATTTCGGTCTGGGATGGACTCG
CGGCCTATCAGCTAGTTGGTGAGGTAATGGCCCACCAAGGCGACGACGGGTAGCCGGCCTGAGAGGGT
GACCGGCCACACTGGGACTGAGACACGGCC
>Zotu4790
CAACGCTGGCGGCGCGCTTAACACATGCAAGTCGAGCGAGAAAGGGGGCAACCCTGAGTAAAGCGGCG
TACGGGTGAGTAACACGTGGATTATCTACCTTTTAGTGGGGAATAACTTTGGGAAACCGAAGCTAATA
CCGCATAAGCCTGTAAAGGGAAAGCAGCAATGCGCTGGAAGAAGAGTCCGCGGCCGATTAGCTAGTTG
GTGAGGTAACGGCCCACCAAGGCGATGATCGGTAGCCGGCCTGAGAGGGCACACGGCCACACTGGCAC
TGAAACACGGGC
>Zotu4791
GAACGCTAGCGGTGCGCCTAACACATGCAAGTCGAGCGGGAGTATTTTGTAGCTTGCTACAAAATATG
AGAGCGGCGAACGGGTGAGTAACACGTTGGAATCTGCCCCTAAGTCAGGGATAGCCCAGGGAAACCTG
GATTAATACCGGATAGTCTCTTTGGAGTAAAAATTTATTGCTTAGGGAGGAGCCTGCGTACTATCAGC
TAGTTGGTGAGGTAATAGCTTACCAAGGCAATGACGGTTAACTGGTCTGAGAGGATGATCAGTCACAA
TGGAACTGAGACACGGTC
>Zotu4792
GAACGCTGACAGAATGCTTAACACATGCAAGTCTACTTGAAGTAACTTCGGTTATGGATGGTGGCGGA
CGGGTGAGTAACGCGTAAATAACTTGCCCCCTAGTCTGGGACAACTGTTGGAAACGACAGCTAATACT
AGATATTATGAGACCTTGGCATCAAGGATTTATGAAAGCTATATGCGCTAGGGGAGAGATTTGCGTCC
TATTAGCTAGTTGGTGAGGTAATGGCTCACCAAGGCGACGATAGGTAGCCGGCCTGAGAGGGTGAACG
GCCACAAGGGGACTGAGACACGGCC
>Zotu4793

GAACGCTGGCGGCATGCCTAATACATGCAAGTCGAGCGGAATGATGAGAGCTTGCTCTCTGATTTTAG
CGGCGGACGGGTGAGTAACACGTGGGCAACCTGCCCTACAGATGGGGATAACTCCGGGAAACCGGGGC
TAATACCGAATAATCAGTTTGTCCGCATGGACAAACTCTGAAAGACGGTTTCGGCTGTCACTGTAGGA
TGGGCCCGCGGCGCATTAGCTAGTTGGTGGGGTAATGGCCTACCAAGGCGACGATGCGTAGCCGACCT
GAGAGGGTGATCGGCCACACTGGGACTGAGACACGGCC
>Zotu4795
GAACGCTGGCGGCGTGCCTAATACATGCAAGTCGAGCGGACTTGACAGAGCTTGCTCTGTTCAAGTTA
GCGGCGGACGGGTGAGTAACACGTGGGCAACCTGCCCTGCAGATTGGGATAACTCCGGGAAACCGGGG
CTAATACCGAATAATATCGAAGATTTCATGATCTTCTTTTGAAAGATGGCTTTAGCTGTCACTGCAGG
ATGGGCCCGCGGCGCATTAGCTAGTTGGTGAGGTAACGGCTCACCAAGGCGACGATGCGTAGCCGACC
TGAGAGGGTGATCGGCCACACTGGGACTGAGACACGGCC
>Zotu4796
GAACGCTGGCGGCGTGCCTAATACATGCAAGTCGAGCGCCCCTTTAAGAGGTGACAAAGAGATTTGTC
GGTTCCTATGGGGAGCGGCGGACGGCTGAGTAACGCGTGGGAACGTGCCCCAAAGTGAGGGATAAGCA
CCGGAAACGGTGTCTAATACCGCATATGATCTTCGGATTAAAGCCTTGTGCGCTTTGGGAGCGGCCTG
CGTCCTATTAGATAGTTGGTGAGGTAAAGGCTCACCAAGTCAACGATGGGTAGCTGGTCTGAGAGGAT
GACCAGCCAGACTGGAACTGAGACACGGTC
>Zotu4797
GAACGCTGGCGGCGTGCTTAACACATGCAAGTCGAGCGGAAAGGCCCCTTCGGGGGTACTCGAGCGGC
GAACGGGTGAGTAACACGTGAGTAACCTGCCCCAGGCTCTGGGATAACCCTCGGAAACGGGGGCTAAT
ACCGGATATGACCTGCTGCCGCATGGTGGTGGGTGGAAAGGTGTTTTTCCGGCCTGGGATGGGCTCGC
GGCCTATCAGCTTGTTGGTGGGGTAATGGCTCACCAAGGCGATGACGGGTAGCCGGCCTGAGAGGGCG
ACCGGCCACACTGGGACTGAGACACGGCC
>Zotu4800
GAACGCTGGCGGCATGCTTAACACATGCAAGTCGAACGGTAACAGGCCCGCAAGGGTGCTGACGAGTG
GCGGACGGGTGAGTAAAGCGTAGGAATCTATCTAGTGGAGTGGGATAACCTAGGGAAACTTAGGGTAA
TACCGCATAAGTTCTAAGGAATAAAGCAGGGGACCTTCGGGCCTTGCGCTATTAGCTGAGCCTACGTC
GGATTAGCTAGTTGGTGGGGTAAAGGCCTACCAAGGCGATGATCCGTAGCTGGTTTGAGAGGACGACC
AGCCACACTGGGACTGAGACACGGCC
>Zotu4801
AAACGCTGGCGGCGTGCTTAACACATGCAAGTCGAACGATGAAGCCCAGCTTGCTGGGTGGATTAGTG
GCGAACGGGTGAGTAATACGTGAGTAACCTGCCTTTAACTCTGGGATAAGCCTTGGAAACGGGGTCTA
ATACCGGATACGACCAGTTCCCGCATGGGATGCTGGTGGAAAGGGATTTTGTACTGGTTTTAGATGGG
CTCACGGCCTATCAGCTTGTTGGTGAGGTAACGGCTCACCAAGGCGACGACGGGTAGCCGGCCTGAGA
GGGTGACCGGCCACACTGGGACTGAGACACGGCC
>Zotu4802
CAACGCTGGCGGCGTGCCTCAGACATGCAAGTCGAACGATTAAACTTTCCTTCGGGGAAGATATAGAG
TGGCGCACGGGTGAGTAACACGTAGGTAATCTACCTCTGGGTGGGGAATAACTTGTGGAAACGCAAGC
TAATACCGCATAATGCAGCGGCACCGAATGGTGACAGTTGTTAAAGCTTTATGTGCCTGGAGAGGAGC
CTGCGGCGGATTAGGTAGTTGGTAAGGTAACGGCTTACCAAGCCTGCGATCCGTATCCGGTCTAAGAG
GACGGTCGGACACACTGACACTGAATAACGGGT
>Zotu4804
GAACGCTGGCGGCGTGCTTAACACATGCAAGTGGAGCGACGAACCAGGGCTTGCCCTGGGGCAAAGCC
GCGAACGGGTGAGTAACACGTGGGTAACCTGCCTCGATGATCGGGACAACCCGGGGAAACCCGAGCTA
ATACCGAATGTGTCCCGTCCACATCAGTGGACGGTGTAAAGGAAGCTTCGGCCTCCGCATCGAGATGG
GCCCGCGGCCCATTAGCTTGTTGGTGGGGTAACGGCCTACCAAGGCTCCGATGGGTAGCTGGTCTGAG
AGGACGATCAGCCACACTGGGACTGAGACACGGCC
>Zotu4805
GAACGCTGGCGGCATGCCTTACACATGCAAGTCGAACGGTAACAGGTCTTCGGATGCTGACGAGTGGC
GAACGGGTGAGTAATACATCGGAACGTGCCCAATCGTGGGGGATAACACATCGAAAGATGTGCTAATA
CCGCATAAGATCTACGGATGAAAGCGGGGGACTCGCAAGAGCCTCGCGCGAATGGAGCGGCCGATGGC
AGATTAGGTAGTTGGTGTGGTAAAAGCGCACCAAGCCGACGATCTGTAGCTGGTCTGAGAGGACGACC
AGCCACACTGGGACTGAGACACGGCC
>Zotu4806

GAACGCTGGCGGCGTGCTTAACACATGCAAGTCGAACGGAAAGGCCCCGCTCGCGGGGTACTCGAGTG
GCGAACGGGTGAGTAACACGTGGGTGATCTGCCCTGCACTTTGGGATAAGCCTGGGAAACTGGGTCTA
ATACCGGATAGGACCCTCCTTTAGTGTGGGGGGTGGAAAGTTTTTCGGTGCAGGATGAGCCCGCGGCC
TATCAGCTTGTTGGTGGGGTAATGGCCTACCAAGGCGTCGACGGGTAGCCGGCCTGAGAGGGTGGACG
GCCACATTGGGACTGAGATACGGCC
>Zotu4808
CAACGCTGGCGGCGTGCCTCAGACATGCAAGTCGAACGCGAAAGTTTCCTTCGGGAGACGAGTAGAGT
GGCGCACGGGTGAGTAACACGTAGGTAATTTGCCTTCGGGTGGGGAACAACTCAGGGAAACTTGAGCT
AATACCGCATAATGCAGCGGCATTTAATTATGACAGTTGTTAAAGCGGAGGATCGAAAGACTTCGCGC
CTGAAGAGAAGCCTGCGGCAGATTAGCTAGTTGGTAAGGTAATGGCTTACCAAGGCGACGATCTGTAA
CCGGTCTAAGAGGACGGTCGGTCACACTGACACTGAATAACGGGT
>Zotu4810
GAACGCTGGCGGCGTGCTTAACACATGCAAGTCGAACGGAGTTTATGTAGAATGAGTCTTCGGACAAA
TGAAATATAAACTTAGTGGCGGACGGGTGAGTAACGTGTGGGTAACCTACCCTATACAGGAGGATAAC
AGTAAGAAATTATTGCTAAAACTGCATAAGATTACGAGTTCGCATGAACAAGTGATAAAAACTTTAGT
GGTATAGGATGGGCCCGCATCTGATTAGGTAGTTGGTGGAGTAAAAGCCTACCAAGCCGACGATCAGT
AGCCGATCTGAGAGGATGACCGGCCACATTGGGACTGAGACACGGCC
>Zotu4811
GAACGCTGGCGGCGTGCCTAATACATGCAAGTCGAGCGAATCTTCAGGAGCTTGCTCCTGAAGATTAG
CGGCGGACGGGTGAGTAACACGTGGGCAACCTGCCTGTAAGACTGGGATAACTTCGGGAAACCGAGGC
TAATACCGGATAATCCTTTTTCTCACATGAGGGAAAGCTGAAAGACGGCGCAAGCTGTCACTTACAGA
TGGGCCCGCGGCGCATTAGCTAGTTGGTGAGGTAACGGCTCACCAAGGCAACGATGCGTAGCCGACCT
GAGAGGGTGATCGGCCACACTGGGACTGAGACACGGCC
>Zotu4812
GAACGCTGGCGGCGCGCTTAACACATGCAAGTCGAGCGAGAACCGAAGCCTTCGGGCCTCGGGGAAAG
CGGCGAACGGGTGAGTAACACGTGGGTAACCCACCCTTGGTACCGGGATAGCCCGGGGAAACTCGGAT
TAATACCGGATGGCCCAACAGCTCTTCGGGGCGGTTGGAAAAGGTAGCTTCGGCCTCCGACCAAGGAC
GGGCCCGCGGCGGATTAGCTTGTTGGTGGGGTAACGGCCTACCAAGGCGACGATCCGTAGCTGGTCTG
AGAGGACGATCAGCCACACTGGGACTGAGACACGGCC
>Zotu4813
GAACGCTAGCGGCAGGCTTAACACATGCAAGTCGAGGGGTAGTAGGAGCTTGCTTCTATGAGACCGGC
GCACGGGTGCGTAACGCGTATGCAATCTGCCTCTTACTGGGGGATAGCCCGGAGAAATCCGGATTAAT
ACCCCATGGTATTTTGGAATGGCATCATTCTATTATTAAAGATTTATCGGTAAGAGATGAGCATGCGT
CCCATTAGTTAGTTGGTGTGGTAACGGCACACCAAGACGATGATGGGTAGGGGTCCTGAGAGGGAGAT
CCCCCACACTGGTACTGAGACACGGAC
>Zotu4814
GAACGCTGGCGGCGTGCTTAACACATGCAAGTCGAACGGTGAAGGAGAGCTTGCTTTCTGGATCAGTG
GCGAACGGGTGAGTAACACGTGAGCAATCTGCCCCTGACTCTGGGATAACAGTTGGAAACAGCTGCTA
ATACCGGATACGAGACGTGACCGCATGGTCAACGTTTGGAAAGAACTTCGGTCAGGGATGAGCTCGCG
GCCTATCAGCTTGTTGGTGAGGTAATGGCTCACCAAGGCGTCGACGGGTAGCCGGCCTGAGAGGGTGA
CCGGCCACACTGGGACTGAGACACGGCC
>Zotu4815
GAACGCTAGCGGCAGGCTTAACACATGCAAGTCGAGGGGTATAGTTCTTCGGAACTAGAGACCGGCGC
ACGGGTGCGTAACGCGTATGCAATCTACCTTTCACAAAGGGATAGCCCAGAGAAATTTGGATTAATAC
CTTATAGTATAGCGACTTCGCATGGAGCTGCTATTAAAGATTTATCGGTGAAAGATGAGCATGCGTCC
CATTAGCTAGTTGGTAAGGTAACGGCTTACCAAGGCTACGATGGGTAGGGGTCCTGAGAGGGAGATCC
CCCACACTGGTACTGAGACACGGAC
>Zotu4816
GAACGCTGGCGGCGTGCTTAACACATGCAAGTCGAGCGGTAAGGCCCTTCGGGGTACACGAGCGGCGA
ACGGGTGAGTAACACGTGAGCAATCTGCCCTCCACATCGGGATAACCCCCGGAAACGGGAGCTAATAC
CGGATACGACCACTTCAGGCATCTGATGGTGGTGGAAAGTTCCGGCGGTGGAGGATGAGCTCGCGGCC
TATCAGCTTGTTGGTGGGGTAATGGCCCACCAAGGCTTCGACGGGTAGCCGGCCTGAGAGGGTGACCG
GCCACACTGGGACTGAGACACGGCC
>Zotu4818

AAACGCTGGCGGCGTGCCTAATACATGCAAGTCGAACGGGGAAAGTCTTCGGATGAGTACTAGTGGCG
AACGGGAGAGGAACACGTGGGTAATCTACCTTTAGTTGAAGAATAACTAGTCGAAAGATTAGCTAATA
CTTCGTAAGACCACAGTATCTAATGATACAGGGGTTATAGGAGCAATCCGACTTTAGATGAGCCCGCG
TCCGATTAGCTAGTTGGTGAGGTAATGGCTCACCAAGGCTGTGATTGGTAGCTGGTCTGAGAGGATGA
CCAGCCTCACTGGAACTGACACACGGTC
>Zotu4819
GAACGCTGGCGGCGTGCCTAATACATGCAAGTCGAGCGGTAAGGCTCTGCACAGGTTATTCGATCGGG
ATTTATCCTACGCTTTCTCCGGCTAGAGAAGGTAAAAGATCGAGTGATGCGTGTAGAGTACACGAGCG
GCGGACGGCTGAGTAACACGTAGGAACGTACCCCAAACTGAGGGATAAGCACCAGAAATGGTGTCTAA
TACCGCATGTGATCTTCGGATTAAAGCTTCGGCGGTTTGGGAACGGCCTGCGCATGATTAGCTTGTTG
GTGAGGTAAAAGCTCACCAAGGCGACGATCATTAGCTGGTCTGAGAGGATGATCAGCCAGACTGGGAC
TGAGACACGGCC
>Zotu4820
GAACGCTGGCGGCGTGCCTAAAACATGCAAGTCGAACGAGAAAGTGGGCAACCATGAGTATAGTGGCG
CACGGGTGAGTAACACGTGGATAACCTACCTTAAGAACTGGGATAACAAGCCGAAAGGTTTGCTAATA
CCGGATGAAGTGTCGGAGGCATTGGGGCCTCTGCCATGAAAACGGGCGCAAGCTACGGTCTTGAGAGG
GGTCTGCGTCCCATTAGCTAGTTGGTGAGGTAAAAGCTTATCCAAGGCGAAGATGGGTAGCTGGTCTG
AGAGGACGACCAGCCACACTGGAACTGAAACACGGTC
>Zotu4821
GAACGCTGGCGGCGTGCTTAACACATGCAAGTCGAACGGTAAGGCTCTTCGGAGTACACGAGTGGCGA
ACGGGTGAGTAACACGTGAGCAACCTGCCCTTGACTCTGGGATACCAGTTGGAAACAACTGCTAATAC
CGGATATCACCTCTAGAGGCATCTCCTGAGGTTGAAAGCTCCGGCGGTCAAGGATGGGCTCGCGGCCT
ATCAGCTAGTTGGTGAGGTAACGGCTCACCAAGGCTTCGACGGGTAGCCGGCCTGAGAGGGCGACCGG
CCACACTGGGACTGAGATACGGCC
>Zotu4823
GAACGCTGGCGGCGTGCCTAATACATGCAAGTCGAGCGGACGTTTTATTGGTGCTTGCACCGATTTAA
CATTAGCGGCGGACGGGTGAGTAACACGTGGGCAACCTACCCTGTAGATTGGGATAACTCCGGGAAAC
CGGGGCTAATACCGAATAATACTTTTTAACTCATGTTAGAAAGTTGAAAGATGGTTTACGCTATCACT
ACAGGATGGGCCCGCGGCGCATTAGCTAGTTGGTGAGGTAATGGCTCACCAAGGCCACGATGCGTAGC
CGACCTGAGAGGGTGATCGGCCACACTGGGACTGAGACACGGCC
>Zotu4824
GAACGCTGGCGGCGTGCCTAATACATGCAAGTCGAGCGAACAGATGAGGAGCTTGCTCCTCTGACGTT
AGCGGCGGATGGGTGAGTAACACGTGGGCAACCTGCCCTGCAGATGGGGATAACTCCGGGAAACCGGG
GCTAATACCGAATAATCAGTTCCTTCGCATGAAGGAACTCTGAAAGACGGCTTCGGCTGTCACTGCAG
GATGGGCCCGCGGCGCATTAGCTAGTTGGTAAGGTAATGGCTTACCAAGGCGACGATGCGTAGCCGAC
CTGAGAGGGTGATCGGCCACACTGGGACTGAGACACGGCC
>Zotu4825
GAACGCTGGCGGCGTGCCTAACACATGCAAGTCGAACGGGGTTAAGTGAAATTTTCGGATGGAGCTTA
ACTTAGTGGCGGACGGGTGAGTAACGCGTGGATAACCTGCCTCACACTGGGGGATAGCAGCTGGAAAC
GGCTGGTAATACCGCATAAGACCACAGCACCGCATGGTGCAGGGGTAAAAGATTTATCGGTGTGAGAT
GGATCCGCGTCTGATTAGCCAGTTGGCGGGGTAACGGCCCACCAAAGCGACGATCAGTAGCCGGCCTG
AGAGGGTGAACGGCCACATTGGGACTGAGACACGGCC
>Zotu4826
GAACGCTGGCGGCGTGCCTAATACATGCAAGTCGAACGGTCTTTTCTATGGAAGCTTGCTTCCGCTGA
GAAGATAGTGGCGAACGGGTGAGTAACACGTGGGTAACCTGCCCATAAGAGGGGGATAACATCCGGAA
ACGGGTGCTAATACCGCATAGTTTTCTTGATCGCATGATTGAGAAAGGAAAGACGGCCTTTGTGCTGT
CGCTTATGGATGGACCCGCGGCGTATTAGTTAGTTGGTGAGTTAACGGCTCACCAAGACGATGATACG
TAGCCGACCTGAGAGGGTGATCGGCCACATTGGGACTGAGACACGGCC
>Zotu4828
GAACGCTGGCGGCGTGCCTAACACATGCAAGTCGAACGAAGCCTTCGGGCTTAGTGGCGGACGGGTGA
GTAACGCGTTGGTACCTGCCCTCTAGTGGGGAATAACTGGCCGAAAGGTCAGCTAATACCGCATGTGA
CAGTAATGTCAAAGCCGTAAGGCGCTAGAGGAGGGGCCTGCGTCCGATTAGCTAGTTGGCGGGGTAAA
TGCCCACCAAGGCGACGATCGGTAGCTGGATCGAGAGGTTGACCAGCCACACTGGGACTGAGATACGG
CC

>Zotu4829
GAACGCTGGCGGCGTGCCTAATACATGCAAGTCGAGCGAATCTCGAGGTGCTTGCACCTCTTGGTTAG
CGGCGGACGGGTGAGTAACACGTGGGCAACCTGCCTGTAAGACTGGGATAACACCGGGAAACCGGTGC
TAATACCGGATAATTCTTTTCCTCTCATGAGGAAAAACTGAAAGTCGGCTTACGCTGACACTTACAGA
TGGGCCCGCGGCGCATTAGCTAGTTGGTGAGGTAACGGCTCACCAAGGCGACGATGCGTAGCCGACCT
GAGAGGGTGATCGGCCACACTGGGACTGAGACACGGCC
>Zotu4830
GAACGCTGGCGGCGTGCTTAACACATGCAAGTCGAGCGGAAAGGCCCTTCGGGGTACTCGAGCGGCGA
ACGGGTGAGTAACACGTGAGTAATCTGCCCTTGGCTTTGGGATAACTACCGGAAACGGTGGCTAATAC
CGGATACGACCAACCTCTGCATGGGGTGTTGGTGGAAAGTTTTTCGGCCAGGGATGTGCTCGCGGCC
TATCAGCTTGTTGGTGGGGTAATGGCCTACCAAGGCTTCGACGGGTAGCCGGCCTGAGAGGGTGACCG
GCCACACTGGGACTGAGACACGGCC
>Zotu4831
GAACGCTGGCGGCGTGCTTAACACATGCAAGTCGAGCGGAAAGGCCACTTCGGTGGTACTCGAGCGGC
GAACGGGTGAGTAACACGTGAGTAATCTGCCCCCCACTTTGGGATAGCCACCGGAAACGGTGATTAAT
ACCGGATATGACCCTTGGCCGCATGGTTGTGGGTGGAAAGTTTTTCGGTGGGGGATGTGCTCGCGGCC
TATCAGCTTGATGGTGGGGTAATGGCCTACCATGGCTTCGACGGGTAGCCGGCCTGAGAGGGTGACCG
GCCACACTGGGACTGAGACACGGCC
>Zotu4833
GAACGCTGGCGGCGTGCCTTAAGCATGCAAGTCGAACGATTTCAGTAGCAATATTGTTTTAGTGGCAG
ACGGGTGAGTAATATATAGGAATCTACCTCTACGTGGGGGATAGCTCGCCGAAAGGCGGGGTAATACC
GCATATGGCCTACGGGTCAAAGTCCTTTACCGGACGCGAAGAGAGGAGCCTGTATCCTATCAGCTAGT
TGGTGAGGTAATGGCTCACCAAGGCTACGACGGGTAGGGGGTGTGAGAGCACGACCCCCCACAAGGAC
ACTGAGACACGGGT
>Zotu4834
GAACGCTGGCGGCGTGCTTAACACATGCAAGTCGAGCGGAAAGGCCACTTCGGTGGTACTCGAGCGGC
GAACGGGTGAGTAACACGTGAGTAATCTGCCCTTCACTTGGGGATAACTACCGGAAACGGTGGCTAAT
ACCCGATACGACCATGGATCGCATGATCTTGTGGTGGAAAGTTTTTTCGGTGGAGGATGTGCTCGCGG
CCTATCAGCTTGTTGGTGGGGTAATGGCCTACCAAGGCTTCGACGGGTAGCCGGCCTGAGAGGGTGAC
CGGCCACACTGGGACTGAGACACGGCC
>Zotu4835
GAACGCTGGCGGCGTGCCTAAGACATGCAAGTCGAGTGGGTTTAGACCCACGGCGAACGGGTGAGTAA
TACCTAGGAACTTTCTTTAAGTGGAGCATAACCCATAGAAATGTGGGATAATTCTCCATGTGCCCCGG
AGTTACATCCGGGGCAAAGTCGCAAGACGCTTAAAGTGAGGCCTGGGCACCATCAGCTAGTTGGTAAG
GTAATGGCTTACCAAGGCTATGACGGTTAGCGGCCCCGAGAGGGAGAACCGCCACACTGGAACTGCGA
CACGGTC
>Zotu4836
GAACGCTGGCGGCGTGCTTAACACATGCAAGTCGAACGGTGACGGAGAGCTTGCTCTCCTGATCAGTG
GCGAACGGGTGAGTAACACGTGGGCAACCTGCCCCTGACTTCGGGATAACGCTTCGAAAGAGGTGCTA
ATACCGAATACGAACTTCCTTGGCATCTGGGAAGTTGGAAAGTTTTTCGGTCAGGGATGGGCCCGCGG
CCTATCAGCTTGTTGGTGAGGTAATGGCTCACCAAGGCGACGACGGGTAGCCGGCCTGAGAGGGCGAC
CGGCCACACTGGGACTGAGACACGGCC
>Zotu4837
GAACGCTGGCGGCGTGCTTAACACATGCAAGTCGAACGGAAAGGCCTCTTGCTTGCAAGGGGTACTCG
AGTGGCGAACGGGTGAGTAACACGTGGGTGATCTGCCTTGCACTCTGGGATAAGCTTGGGAAACTGGG
TCTAATACCGGATATGAACTGCCTTTAGTGTGGTGGTTGGAAAGTTTTTTCGGTGCAAGATGAGCTCG
CGGCCTATCAGCTTGTTGGTGGGGTAATGGCCTACCAAGGCGTCGACGGGTAGCCGGCCTGAGAGGGT
GTACGGCCACATTGGGACTGAGATACGGCC
>Zotu4838
GAACGCTGGCGGCAGGCTTAACACATGCAAGTCGAGCGGGGATAGGGTGCTTGCACCTGATTCCTAGC
GGCGGACGGGTGAGTAATGCTTAGGAATCTGCCTATTAGTGGGGGACAACGTTCCGAAAGGGACGCTA
ATACCGCATACGTCCTACGGGAGAAAGCAGGGGATCTTCGGACCTTGCGCTAATAGATGAGCCTAAGT
CGGATTAGCTAGTTGGTGGGGTAAAGGCCTACCAAGGCGACGATCTGTAGCGGGTCTGAGAGGATGAT
CCGCCACACTGGGACTGAGACACGGCC

>Zotu4839
GAACGCTGGCGGCGTGCTTAACACATGCAAGTCGAGCGGTAAGGCCCTTTGGGGTACACGAGCGGCGA
ACGGGTGAGTAACACGTGGGTGACCTGCCCTCCACTTCGGGATAAGCCTGGGAAACTGGGTCTAATAC
CGGATAGGACCATTGGATGCATGTCTGGTGGTGGAAAGTTTTGTCGGTGGGGGATGGGCCCGCGGTCT
ATCAGCTTGTTGGTGGGGTGATGGCCTACCAAGGCGACGACGGGTAGCCGGCCTGAGAGGGTGATCGG
CCACACTGGGACTGAGACACGGCC
>Zotu4840
GAACGCTGGCGGCGTGCTTAACACATGCAAGTCGAACGGTAAGGCCCTTCGGGGTACACGAGTGGCGA
ACGGGTGAGTAACACGTGGGTAATCTGCCCTGCACTTCGGGATAAGCCTGGGAAACTGGGTCTAATAC
CGGATATTCAGCTCCTGCCGCATGGTGGGGGTTGGAAAGTTTTTCGGTGCAGGATGAGCCCGCGGCCT
ATCAGCTTGTTGGTGGGGTAATGGCCCACCAAGGCGACGACGGGTAGCCGGCCTGAGAGGGTGATCGG
CCACACTGGGACTGAGACACGGCC
>Zotu4841
TAACGCTGGCGGTATGCCTAACACATGCAAGTCGAACGGAATACTCGAAAAGAGAATCTCACACTAGT
GAGAGGTTCTCATACCGAGTATTTAGTGGCGGACGGGTGAGTAACACGTTGGAACCTGCCCCGAAGTG
GGGAATAACTGCTCGAAAGAGTAGCTAATACCCCATAGTCTCCGGTTTATCCGGAGTAAAGATTTATC
GCTTCGGGAGGGGCCTGCGTCTGATTAGCTAGTTGGTGAGGTAAAAGCTCACCAAGGCGACGATCAGT
AGCTGGTTCGAGAGGATGACCAGCCACACTGGGACTGAGATACGGCC
>Zotu4843
GAACGCTGGCTGCGTGCTTAACACATGCAAGTCGAACGCTGAAGCCCTGGTGCTTGCACCGGGGTGGA
TGAGTGGCGAACGGGTGAGTAACACGTGAGTAACCTGCCCCCGACTTCGGGATAAGCCCGGGAAACTG
GGTCTAATACCGGATATGACCATGACTCGCATGAGTGATGGTGGAAAGTTTTTCGGTTGGGGATGGG
CTCGCGGCCTATCAGTTTGTTGGTGAGGTAATGGCTCACCAAGACGACGACGGGTAGCCGGCCTGAGA
GGGCGACCGGCCACACTGGGACTGAGACACGGCC
>Zotu4844
GAACGCTGGCGGCGTGCCTAATACATGCAAGTCGAGCGAACTTGCGGGAGCTTGCTCCCAAAAGTTAG
CGGCGGACGGGTGAGTAACACGTGGGCAACCTGCCTATAAGACTGGGATAACTTCGGGAAACCGGAGC
TAATACCGGATAATTCTTTTCTACTCATGTAGAAAAGCTGAAAGACGGTTTACGCTGTCACTTATAGA
TGGGCCCGCGGCGCATTAGCTAGTTGGTGAGGTAACGGCTCACCAAGGCCACGATGCGTAGCCGACCT
GAGAGGGTGATCGGCCACACTGGGACTGAGACACGGCC
>Zotu4845
GAACGCTGGCGGCGTGCTTAACACATGCAAGTCGAGCGGTAAGGCCCTAGCTTGCTGGGGTACACGAG
CGGCGAACGGGTGAGTAACACGTGAGTAATCTGCCCTTCACTCTGGGATAAGCACTCGAAAGGGTGTC
TAATACCGGATATGACTGCTTCCTGCATGGGATGGTGGTGGAAACTTTTGTGGGTGGGGGATGTGCTC
GCGGCCTATCAGCTTGTTGGTGGGGTAATGGCCTACCAAGGCTTCGACGGGTAGCCGGCCTGAGAGGG
TGACCGGTCACACTGGGACTGAGACACGGCC
>Zotu4846
GAACGCTGGCGGCACGTTTTAAGCATGCAAGTCGAACGGCAGTGGGGGGCAACCTCCATGAGAGTGGC
GAACGGGTGAGTAACACGTAGGTGATCTGCCCTGGGGATGGGGATAGCCTGTGGAAACACAGGGTAAT
ACCGAATAAGCTGACGGGATTTTGATTCTGTTAGTAAAGGTGCTAAGGCACCGTCCTGGGAGGAGCCT
GCGGCTGATTAGCTAGTTGGTGGGGTAAAGGCCTACCAAGGCGACGATCAGTAGCCGGCCTGAGAGGG
TGTACGGCCACACTGGAACTGAGACACGGTC
>Zotu4847
GAACGCTGGCGGCGTGCTTAACACATGCAAGTCGAACGTGAAATGGGGCAACCCAAAGTAAAGTGGCG
TACGGGTGAATAACGCGTAGGTAATCTACCCCAAAATCTGGAATAACCGGCCGAAAGGCTGGCTAATA
CTAGATAGAGCGATGGAATCCGTAATTGGGTTTTTCGGGAAAGGGAGCTCGCAAGAGCCTTCGTTTTG
GGATGAGCCTGCGTCTCATTAGCTAGTTGGCGAGGTAACGGCTCACCAAGGCATTGATGAGTAGCTGG
TCTGAGAGGATGATCAGCCACACTGGAACTGAAACACGGTC
>Zotu4848
GAACGCTGGCGGCGTGCCTAATACATGCAAGTCGAGCGAATCTTTAGAAGCTTGCTTCTAAAGATTAG
CGGCGGACGGGTGAGTAACACGTGGGCAACCTGCCTGTAAGACTGGGATAACTTCGGGAAACCGAGGC
TAATACCGGATAATCCTTTTCCTCACATGAGGAAAAGCTGAAAGACGGCGCAAGCTGTCACTTATAGA
TGGGCCCGCGGCGCATTAGCTAGTTGGTGAGGTAACGGCTTACCAAGGCGACGATGCGTAGCCGACCT
GAGAGGGTGATCGGCCACACTGGGACTGAGACACGGCC

>Zotu4852
GAACGCTGGCGGCGTGCTTAACACATGCAAGTCGAACGATGAAGCCCAGCTTGCTGGGTGGAAGAGTG
GCGAACGGGTGAGTAACACGTGAATAACCTGCCCCGGACTCTGGGATAAGCGCTGGAAACGGCGTCTA
ATACTGGATATGACCCATACCGGCATCGGTTGTGGGTGGAAAGATTTATCGGTTCGGGATGGGCTCGC
GGCCTATCAGCTGGATGGTGAGGTAACGGCTCACCATGGCGACAACGGGTAGCCGGCCTGAGAGGGTG
ACCGGCCACACTGGGACTGAGACACGGCC
>Zotu4855
GAACACTGGCGGCAGGCCTAACACATGCAAGTCGAGCGGATGAGTGGAGCTTGCTCCATGATTCAGCG
GCGGACGGGTGAGTAATGCCTAGGAATCTGCCTGGTAGTGGGGGACAACGTTTCGAAAGGAACGCTAA
TACCGCATACGTCCTACGGGAGAAAGCAGGGGACCTTCGGGCCTTGCGCTATCAGATGAGCCTAGGTC
GGATTAGCTAGTTGGTGGGGTAAAGGCCTACCAAGGCGACGATCCGTAACTGGTCTGAGAGGATGATC
AGTCACACTGGAACTGAGACACGGTC
>Zotu4856
GAACGCTGGCGGCGTGCCTAATACATGCAAGTCGTGCGGGTGAAGCCGACCACCCCTTCGGGGGCGGA
TGGCGGAACCAGCGGCGGACGGGTGAGTAACACGTGGGCAACCTGCCTGCAAGACCGGGATAACTGCG
GGAAACCGGAGCTAATACCGGATAGGAGGCTTTTCCGCATGGGGGAGCCTTAAAAGGCGGCGGAAGTC
GTCACTTGCAGATGGGCCCGCGGCGCATTAGCTAGTTGGTGAGGTAAGGGCTCACCAAGGCGACGATG
CGTAGCCGACCTGAGAGGGTGACCGGCCACACTGGGACTGAGACACGGCC
>Zotu4857
GAACGCTAGCGGCAGGCCTAATACATGCAAGTCGAACGGTGGTAGCGATACCATAGTGGCGCACGGGT
GCGTAACGCGTAACCAACCTGCCCTTGACTGGGGGATAGCCCGCCGAAAGGCGGATTAATACCGCATA
AGACTCGTTCCTGGCATCAGGGGTTAGTTAAAGATTTATTGGTCAAGGATGGGGTTGCGTGACATTAG
CTGGTTGGCGGGGTAACGGCCCACCAAGGCGACGATGTCTAGGGGAGCTGAGAGGCTGGTCCCCCACA
CGGGCACTGAGATACGGGC
>Zotu4858
GAACGCTGGCGGCATGCTTAACACATGCAAGTCGCACGGGCAGCAATGTCAGTGGCGGACGGGTGAGT
AACGCGTAGGAACGTGTCCAGAGGTGGGGGACAACCCCGGGAAACTGGGGCTAATACCGCATAGGGGC
TGAGGCCCAAAGCCGAAAGGCGCCTTTGGAGCGGCCTGCGTCCGATTAGGTAGTTGGTGGGGTAAAGG
CCTACCAAGCCTGCGATCGGTAGCTGGTCTGAGAGGACGACCAGCCACACTGGGACTGAGACACGGCC
>Zotu4859
GAACGCTGGCGGCGTGCTTAACACATGCAAGTCGAGCGAAGCAGTTAAGTTGATTTCTTCGGATTGAT
TCTTAATTGACTGAGCGGCGGACGGGTGAGTAACGCGTGGGTGACCTGCCCCATACCGGGGGATAACA
GCTGGAAACGGCTGCTAATACCGCATAAGCGCACAGAGCTGCATGGCTCAGTGTGAAAAACTCCGGTG
GTATGGGATGGGCCCGCGTCTGATTAGGCAGTTGGCGGGGTAACGGCCCACCAAACCGACGATCAGTA
GCCGGCCTGAGAGGGCGACCGGCCACATTGGGACTGAGACACGGCC
>Zotu4860
GAACGCTGGCGGCGTGCCTAATACATGCAAGTCGAGCGCGAGCGATTGGAGCTTGCTCCAATCAATCG
AGCGGCGGACGGGTGAGTAACACGTAGGCAACCTACCCATCAGACTGGGATAACCACGGGAAACCGTG
GCTAATACCGGATGAACCTTTGAGACACAAGTCTTAAAGTTGAAAGGCGCTTTTGGTGCCGTTGATGG
ATGGGCCTGCGGCGCATTAGCTAGTTGGTGAGATAGTGGCTCACCAAGGCGACGATGCGTAGCCGACC
TGAGAGGGTGATCGGCCACACTGGGACTGAGACACGGCC
>Zotu4861
GAACGCTGGCGGCGTGCTTAACACATGCAAGTCGAACGAGAACGGATTATAGCTTGCTATAATTGTCA
GCTAAGTGGCGCACGGGTGAGTAATATATAGGTAATGTGCCCTAGAGAAGAGGATAACAGTTGGAAAC
GACTGCTAAGACTCTATATGCCTTTAAGGCAGAAGTCTGCAAGGGAAATATTTATAGCTCTAGGATCA
GCCTGTACAGTATCAGTTAGTTGGTGAGGTAATGGCTCACCAAGACAATGACGCTTAACTGGTTTGAG
AGGATGATCAGTCACACTGGAACTGAGACACGGTC
>Zotu4862
GAACGCTAGCGGCAGGCCTAATACATGCAAGTCGAACGAAACAGCAATGTTTAGTGGCGCACGGGTGC
GTAACGCGTATGTAACCTACCTTTTACTGGGGGATAACTTCTCGAAAGAGAAGCTAATACCGCATAAT
ACATTTGGTTCTCATGAACTGATTGTTAAAGATTTATTGGTAAAAGATGGGCATGCGTCCAATTAGCT
AGTTGGTAGTGTAACGGACTACCAAGGCTACGATTGGTAGGGGGTCTGAGAGGATGGTCCCCCACACT
GGTACTGAGATACGGAC
>Zotu4863

TAACGCTGGCGGCGTGCCTAACACATGCAAGTCGAACGGTTCTAAGCGCGAGCTTAGGATAGTGGCGA
ACGGGTGAGTAAAAGGTAGGAATCTACCCCTTGGACGGAGATAGCTACTGGAAACGGTAGGTAAACTT
CGATAAGCCCGAGAGGGGAAAGTGGTGAGACAGCCGAGGGATGAGCCTACTCTCCATCAGGTAGTTGG
TGAGATAAAAGCTTACCAAGCCGATGACGGATAACCGGTGTGAGAGCATGAACGGTCACAAGGGCACT
GAGAGACGGGC
>Zotu4864
GAACGCTGGCGGCATGCCTTACACATGCAAGTCGAACGGCAGCACGGGAGCAATCCTGGTGGCGAGTG
GCGAACGGGTGAGTAATATATCGGAACGTGCCCAGTCGTGGGGGATAACGCAGCGAAAGCTGTGCTAA
TACCGCATACGATCTCTGGATGAAAGCGGGGGACTCGCAAGAGCCTCGCGCGATTGGAGCGGCCGATA
TCAGATTAGCTAGTTGGTGGGGTAAAGGCCCACCAAGGCGACGATCTGTAGCTGGTCTGAGAGGACGA
CCAGCCACACTGGAACTGAGACACGGTC
>Zotu4865
GAACGCTAGCGGCAGGCCTAACACATGCAAGTCGAGGGGTAGTATAGCAATATATGAGACCGGCGCAC
GGGTGCGTAACGCGTATGCAATCTACCTTTTACAGAGGGATAGCCCAGAGAAATTTGGATTAATACCT
CATAGTGTATTTGAGTGGCATCACTTGATTACTAAAGTCACAACGGTAAAAGATGAGCATGCGTCCCA
TTAGCTTGTTGGTAAGGTAACGGCTTACCAAGGCTACGATGGGTAGGGGTCCTGAGAGGGAGATCCCC
CACACTGATACTGAGACACGGAC
>Zotu4866
GAACGCTGGCGGCGTGCTTAACACATGCAAGTCGAACGGAAAGGCCCAGCTTGCTGGGTACTCGAGTG
GCGAACGGGTGAGTAACACGTGGGTGATCTGCCCTGGACTCTGGGATAAGCCTGGGAAACTGGGTCTA
ATACCGGATATGACCTTACATCGCATGGTGTTTGGTGGAAAGCTTTTGCGGTTCAGGATGGGCCCGCG
GCCTATCAGCTTGTTGGTGGGGTAATGGCCTACCAAGGCGACGACGGGTAGCCGACCTGAGAGGGTGA
TCGGCCACACTGGGACTGAGACACGGCC
>Zotu4867
GAACGCTGGCGGCGTGCTTAACACATGCAAGTCGAACGGTAAGGCCCCTTCGGGGGTACACGAGTGGC
GAACGGGTGAGTAACACGTGAGCAACCTGCCCTTGACACTGGAATAACAGTTGGAAACAGCTGCTAAT
GCCGGATATGACCTTCGGTGACATCACCTGGAGGTGGAAAGTGCTTAGCGGTCAAGGATGGGCTCGCG
GCCTATCAGCTAGTTGGTGAGGTAACGGCTCACCAAGGCTTCGACGGGTAGCCGGCCTGAGAGGGCGA
CCGGCCACACTGGGACTGAGATACGGCC
>Zotu4869
GAACGCTGGCGGCGTGCCTAATACATGCAAGTCGAGCGGACCTTTTAAAAGCTTGCTTTTGAAAGGTT
AGCGGCGGACGGGTGAGTAACACGTGGGCAACCTGCCCTATAGTTTGGGATAACTCCGGGAAACCGGG
GCTAATACCGGATAATACAATTTAACTCCTGTTAGATTGTTGAAAGATGGTTTCGGCTATCGCTATAG
GATGGGCCCGCGGCGCATTAGCTAGTTGGTAAGGTAACGGCTTACCAAGGCGACGATGCGTAGCCGAC
CTGAGAGGGTGATCGGCCACACTGGGACTGAGACACGGCC
>Zotu4870
GAACGCTAGCGGCAGGCCTAATACATGCAAGTCGAACGAGATTAGTAGCAATACTATGAAAGTGGCGC
ACGGGTGCGTAACACGTATGCAATCTACCTTTAATTGGAGCATAGCCTCGAGAAATCGGGATTAATAC
TCCATAACATTATGAAATGGCATCATTTTATAATTAAACCTCCGGGGATTAAAGATGAGCATGCGCAA
CATTAGCTAGTTGGTAAGGTAATGGCTTACCAAGGCTACGATGTTTAGGGGGTCTGAGAGGATGATCC
CCCACACTGGTACTGAGACACGGAC
>Zotu4871
GAACGCTGGCGGCATGCCTTACACATGCAAGTCGAACGGCAGCACGGGTGCTTGCACCTGGTGGCGAG
TGGCGAACGGGTGAGTAATGCATCGGAACGTACCCGGAAGTGGGGGATAACGTAGCGAAAGTTACGCT
AATACCGCATATTCTGTGAGCAGGAAAGAGGGGGCTCGTAAGACCTCTCGCTTTCGGAGCGGCCGATG
TCGGATTAGCTAGTTGGTGAGGTAAAGGCTCACCAAGGCGACGATCCGTAGCGGGTCTGAGAGGATGA
TCCGCCACACTGGGACTGAGACACGGCC
>Zotu4872
CAACGCTGGCGGCGTGCCTCAGACATGCAAGTCGAACGATTAAGACTCCCTTCGGGGAGTGTATAAAG
TGGCGTACGGGTGAGTAACACGTAAGTAATCTACCTTCGAGTGGGGAATAACAGTCGGAAACGGCTGC
TAATACCGCATAATGCAGCGGCATCGAAAGATGACAGTTGTTAAAGGAGCAATTCGCTTGAAGAGGAG
CTTGCGGCAGATTAGCTAGTTGGTAAGGTAATGGCTTACCAAGGCTACGATCTGTATCCGGTCTAAGA
GGACGGTCGGACACACTGACACTGAATAACGGGT
>Zotu4874

CAACGCTGGCGGCGTGCCTAACACATGCAAGTCGAACGCGAAAGCCCCGCAAGGGGTAAGTAGAGTGG
CAAACGGGTGAGTAACACGTGGGTGATCTGCCTCAGAGTGGGGGATAACGACCCGAAAGGGTCGCTAA
TACCGCATAACATCCTGCCTTTGGATAGGTGGAGATCAAAGCCGGGGACCGCAAGGCCTGGCGCTTGG
AGAGGAGCCCGCGGCCGATTAGCTAGTTGGTGAGGTAACGGCTCACCAAGGCGACGATCGGTATCCGG
CCTGAGAGGGCGGACGGACACACTGGGACTGAGACACGGCC
>Zotu4875
GAATGCTGGCGGCGTGGATAAGGCATGCAAGTCGAGTGGGTTTAGACCCACGGCAGACGAGGTAGTAA
CACGTAGGAACGTACCCCAAAGTCCGGGATATTCCATCGAAAGATGGCGCAATACCGGATGGTCTCTA
AGGAGTAAAGATTTATCGCTTTGGGAGCGGCCTGCGCAGTATCAGCTAGTTGGTAGGGTAATGGCCTA
CCAAGGCTATGACGCTTAGGGGAGCTGAGAGGCTGACCCCCACCGATGGAACTGAGATACGGTC
>Zotu4876
GAACGCTGGCGGCGTGCCTAATACATGCAAGTCGAGCGGAGTTACAGGAGCTTGCTCCTGTAACTTAG
CGGCGGACGGGTGAGTAACACGTGGGCAACCTGCCTATAAGACTGGGATAACTTCGGGAAACCGGAGC
TAATACCGGATAATTCTTTTCCTCTCATGAGGAAAAGATGAAAGACGGTTTCGGCTGTCACTTATAGA
TGGGCCCGCGGCGCATTAGCTAGTTGGTGAGGTAACGGCTCACCAAGGCAACGATGCGTAGCCGACCT
GAGAGGGTGATCGGCCACACTGGGACTGAGACACGGCC
>Zotu4877
GAATGCTGTCGGCGTGGATAAGGCATGCAAGTCAAGGGGGCCGCAAGGCAACCGGCAGACGAGGTAGT
AACACGTAGGTAGATACCCTGGAGTCGGGCATAGCCAGTCGAAAGACTGGGTAATTCCCGATGGTCTC
GCAAGAGTAAAGGTTTTTCGCTCCGGGAGTCGCCTGCGCAATATCAGCTAGTTGGTGAGGTAATGGCT
CACCAAGGCTACGACGTTTAGGGGAGCTGAGAGGCTGACCCCCACCGATGGGACTGAGATACGGCC
>Zotu4878
GAACGCTGGCGGCGTGCTTAACACATGCAAGTCGAACGGAGATAATAGTAATATTATCTTAGTGGCGG
ACGGGTGAGTAACGCGTGGGCAACTTGCCTCGGACTGGGGGATAACACAGGGAAACTTGTGCTAATAC
CGCATAATATGATTGAAGGACATCCTTCGATCATCAAAGGAGCAATCCGGTTTGAGATGGGCCCGTGT
CCGATTAGCTAGTTGGTAGTGTAATGGACTACCAAGGCGACGATCGGTAGCCGAACTGAGAGGTTGAT
CGGCCACATTGGGACTGAGACACGGCC
>Zotu4879
GAACGCTAGCGGGAGGCCTAACACATGCAAGCCGAGCGGTATTTCTTCTTCGGAAGAGAGAGAGCGGC
GTACGGGTGCGTAACACGTGTGCAACCTACCTTTATCTGGGGGATAGCCTTTCGAAAGGAAGATTAAT
ACCCCATAATATATTGAATGGCATCATTTAATATTGAAAACTCCGGTGGATAGAGATGGGCACGCGCA
AGACTAGATAGTTGGTGAGGTAACGGCTCACCAAGTCTGTGATCTTTAGGGGTCCTGAGAGGGAGATC
CCCCACACTGGTACTGAGACACGGAC
>Zotu4880
GAACGCTGGCGGCGCGCTTAACACATGCAAGTCGAACGGTTAAAGCCCCTTCGGGGGTGCATACAGTG
GCGAACGGCTGAGTAACACGTGGGCAACCTGCCCCCCGCACCGGGATAGCCTCGGGAAACCGTGGGTA
ATACCGGATACTCCGCAGCCCCCGCATGGGGGAGGCGGGAAAGCGTACACGGCGGGGGATGGGCCCGC
GGCCTGTTAGCTCGTTGGCGGGGTAACGGCCCACCAAGGCGATTATGGGTAGCCGGGTTGAGAGACCG
ACCGGCCAGATTGGGACTGAGACACGGCC
>Zotu4881
GAACGCTGGCGGCAGGCTTAACACATGCAAGTCGAGCGGGGAGATGTAGCTTGCTACATTTCCTAGCG
GCGGACGGGTGAGTAATGCTTAGGAATCTGCCTATTAGTGGGGGACAACATTCCGAAAGGAATGCTAA
TACCGCATACGCCCTACGGGAGAAAGCAGGGGATCTTCGGACCTTGCGCTAATAGATGAGCCTAAGTC
AGATTAGCTAGTTGGTGGGGTAAAGGCCTACCAAGGCGACGATCTGTAGCGGGTCTGAGAGGATGATC
CGCCACACTGGGACTGAGACACGGCC
>Zotu4882
GAACGCTGGCGGCGCGCCTAACACATGCAAGTCGAACGGAGTCAAGAGGAGCTTGCTTTTCTTGACTT
AGTGGCGAACGGGTGAGTAACGCGTGAGTAACCTGCCCTGGAGTGGGGGACAACAGTTGGAAACGACT
GCTAATACCGCATAAGCACACGGCCCGGCATCGGGCTGCGGGAAAAGGATTTATTCGCTTCAGGATGG
ACTCGCGTCCAATTAGCTAGTTGGTGAGGTAACGGCCCACCAAGGCGACGATTGGTAGCCGGACTGAG
AGGTTGAACGGCCACATTGGGACTGAGACACGGCC
>Zotu4883
GAACGCTAGCGGCAGGCCTAACACATGCAAGTCGAGGGGTATCGGGGGCTTGCCCCCGAGAGACCGGC
GCACGGGTGAGCAACGCGTATAGAACCTACCCGTTACAGGGGGATAGCCCAGAGAAATTTGGATCAAT

ACCCCGTGGGGCCCTTGACCGGCATCGGTCTTGGGCTAAAGAACCTCGGTAACGGATGGCTATGCGTC
CCATTAGCTAGTTGGTAGGGTAACGGCCTACCAAGGCCGCGATGGGTAGGGGCCCTGAGAGGGGGATC
CCCCACACTGGTACTGAGACACGGAC
>Zotu4884
GAACGCTGGCGGCGTGCTTAACACATGCAAGTCGAACGGTGAAGGACGGAGCTTGCTCCGACCGGATC
AGTGGCGAACGGGTGAGTAACACGTGAGTAACCTGCCCCAGACTCTGGAATAACCCCGGGAAACCGGA
GCTAATACCGGATACGAAGTGGAGCCGCATGGCTACTACTTGGAAAGTTTTTCGGTCTGGGATGGACT
CGCGGCCTATCAGCTAGTTGGTGAGGTAACGGCTCACCAAGGCGACGACGGGTAGCCGGCCTGAGAGG
GCGACCGGCCACACTGGGACTGAGACACGGCC
>Zotu4885
GAACGCTGGCGGCGTGCCTAATACATGCAAGTCGAACGGTCTTTTCTATGGAAGCTTGCTTCCATAGA
AAAGATAGTGGCGAACGGGTGAGTAACACGTGGGTAACCTGCCCATAAGAGGGGGATAACATCCGGAA
ACGGGTGCTAATACCGCATAGTTTTCTTGATCGCATGATCGGGAAAGGAAAGACGGCCTTTGTGCTGT
CGCTTATGGATGGACCCGCGGCGTATTAGTTAGTTGGTGAGGTAACGGCTCACCAAGACGATGATACG
TAGCCGACCTGAGAGGGTGATCGGCCACATTGGGACTGAGACACGGCC
>Zotu4886
GAACGCTGGCGGCGCGCTTAACACATGCAAGTCGAGCGAGAACCGATCCTTCGGGATTGGGGAAAGCG
GCGAACGGGTGAGTAACACGTGAGTAATCCACCCTCGGCACCGGGATAGCCCGGGGAAACTCGGATTA
ATACCGGATGGCCCAGCAGCTCTTCGGGGCGGCTGGAAAAGGTAGCTTAGGCCTCCGGCCGAGGACGA
GCTCGCGGCGGATTAGCTTGTTGGTGGGGTAACGGCCTACCAAGGCGACGATCCGTAGCTGGTCTGAG
AGGACGATCAGCCACACTGGGACTGAGACACGGCC
>Zotu4887
GAACGCTGGCGGCGTGCCTAATACATGCAAGTCGAGCGAATCAAAGGGAGCTTGCTCCCTTTGATTAG
CGGCGGACGGGTGAGTAACACGTGGGCAACCTGCCTATAAGACTGGGATAACTTCGGGAAACCGGAGC
TAATACCGGATAATTCATATCTACTCATGTAGATATGCTGAAAGACGGTTCACGCTGTCACTTATAGA
TGGGCCCGCGGCGCATTAGCTAGTTGGTGAGGTAACGGCTCACCAAGGCGACGATGCGTAGCCGACCT
GAGAGGGTGATCGGCCACACTGGGACTGAGACACGGCC
>Zotu4890
GAACGCTAGCGGAAGGCCTAATACATGCAAGTCGAGCGGTAGAATAACTTCGGTTGTTTGAGAGCGGC
GCACGGGTGAGTAACGCGTACATGACCTACCTTTAAGAATTGGATAGCCCTGGGAAACTGGATTAAT
ACAGTATGTGATATGTGTTATAGAGGATACATATTAAAGCTTCGGCGCTTAGAGATGGATGTGCGTCT
GATTAGCTAGTTGGTAGGGTAAAGGCCTACCAAGGCGACGATCAGTAGGGGGCGTGAGAGCGTGATCC
CCCACACGGGTACTGAGACACGGAC
>Zotu4891
GAACGCTGGCGGCGTGCCTAATACATGCAAGTCGAGCGAACCGTAAAGGAGCTTGCTCCTTTACGGTT
AGCGGCGGACGGGTGAGTAACACGTGGGTAACCTGCCCTCAAAACTGGGATAACATCGAGAAATCGGT
GCTAATACCGGATAATCAATAAGACCGCATGGTCTTATTATAAAAGGTGGCTTCGGCTACTATTTGAG
GATGGGCCCGCGGCGCATTAGCTAGTTGGTAAGGTAACAGCTTACCAAGGCGACGATGCGTAGCCGAC
CTGAGAGGGTGATCGGCCACATTGGGACTGAGACACGGCC
>Zotu4892
GAACGCTAGCGGGAGGCTTAATACATGCAAGTCGAACGGTAAATGGTGTAGCAATACACTATGAGAGT
GGCGAACGGGTGAGGAATACATACGCAACTTACCTCTGACTGGGGGATAGCCCTTCGAAAGGAGGATT
AATACCCCATAATATCCTGGGAAGGCATCTTCCTGGGATTAAAACTCCGGTGGTCAGAGATGGGCGTG
TGCCCCATTAGCTAGTTGGTAAGGTAACGGCTTACCAAGGCAACGATGGGTAGCTGGCGTGAGAGCGC
GACCAGCCACACGGGCACTGAGACACGGGC
>Zotu4893
GAACGCTAGCGGCATGCCTAACACATGCAAGTCGAACGGCAGCGAAGAGAAGCTTGCTTCTCTGTCGG
CGAGTGGCGAACGGGTGAGTATAGCATCGGAACGTGCCAAGTAGTGGGGGATAACCAAGCGAAAGTTT
GGCTAATACCGCGTAAGCTCTAAGGAGGAAAGTAGGGGACCGCAAGGCCTTACGCTATTTGATCGGCC
GATGTCGGATTAGCTAGTTGGTGAGGTAATGGCTCACCAAGGCGATGATCCGTAGCGGGTCTGAGAGG
ACGATCCGCCACACTGGGACTGAGACACGGCC
>Zotu4894
GAACGCTGGCGGCGTGCCTAATACATGCAAGTCGAGCGAACGGACAAGGAGCTTGCTCCTTTGAAGTT
AGCGGCGGACGGGTGAGTAACACGTGGGTAACCTACCTATAAGACTGGAATAACTCCGGGAAACCGGG

GCTAATGCCGGATAATATGTTGAACCGCATGGTTCAACAGTGAAAGACGGTCTTGCTGTCACTTATAG
ATGGACCCGCGCCGTATTAGCTAGTTGGTGGGGTAACGGCCTACCAAGGCAACGATACGTAGCCGACC
TGAGAGGGTGATCGGCCACACTGGAACTGAGACACGGTC
>Zotu4895
CAACGCTGGCGGCGTGCCTAACACATGCAAGTCGAACGAGAAAATTCCGGGTGTAGCAATACATCCGG
GGTGAGTAAAGTGGCGTACGGGTGAGTAACACGTAGCCAACCTACCCTCAAGTGGGGAATAACCCGGG
GAAACCTGGGCTAATGCCGCATAACATCCCGGTTCCACAAGAGATTGGGATCAAAGGCCCGCAAGGGT
CGCTGGAGGAGGGGGCTGCGGCCGATTAGCTAGTTGGTGAGGTAACGGCTCACCAAGGCGATGATCGG
TAGCCGGCCTGAGAGGGCGCACGGTCACACTGGCACTGAAACACGGGC
>Zotu4896
GAACGCTGGCGGCGTGCTTAACACATGCAAGTCGAACGGAAAGGCCCTTCGGGGTACTCGAGTGGCGA
ACGGGTGAGTAACACGTGGGTGATCTGCCCTGCACTTCGGGATAAGCCTGGGAAACTGGGTCTAATAC
CGGATATGACCTTGGGATGCATGTCCTTTGGTGGAAAGCTTTTGCGGTGTGGGATGGGCCCGCGGCCT
ATCAGCTTGTTGGTGGGGTTAAGGCCTACCAAGGCGACGACGGGTAGCCGGCCTGAGAGGGTGACCGG
CCACACTGGGACTGAGATACGGCC
>Zotu4897
GAACGCTAGCGGCAGGCTTAATACATGCAAGTCGAGGGGTAACAGGGGTAGCAATACCTGCTGACGAC
CGGCAAACGGGTGCGGAACACGTACGCAACCTCCCCATAACTGGTGAATAGCCCTCCGAAAGGAGGAT
TAATACACCGTAACATAATGAAATGGCATCATTTTATTATTATAGGCTTCGGCCGGTTTTGGATGGGC
GTGCGCCTGATTAGGTAGTTGGCGGGGTAACGGCCCACCAAGCCTGCGATCAGTAACTGGTGTGAGAG
CACGACCAGTCACACGGGCACTGAGACACGGGC
>Zotu4898
TAACGCTGGCGGCGTGCCTAACACATGCAAGTCGAACGGTGCAGCAATGCACAGTGGCAGACGGGTGA
GTAACGCGTAGATAACGTGCCCTCTTGCGGGGGATACCAGTTCGAAAGGACTGTTAATACCGCATAAC
ATCTCCGAGGGGCATCCTTTGGAGAGCAAACTGATTCAGGGCGAGAGGAGCGGTCTGCGTACTATCAG
GTAGTTGGTGAGGTAATGGCTCACCAAGCCTACGACGGTTAACTGGTCTGAGAGGATGATCAGTCACA
CTGGAACTGAGACACGGTC
>Zotu4899
GAACGCTGGCGGCAGGCTTAACACATGCAAGTCGAGCGCCCCGCAAGGGGAGCGGCAGACGGGTGAGT
AACGCGTGGGAATCTACCCAGTTCTACGGAATAACACAGGGAAACTTGTGCTAATACCGTATACGCCC
TACGGGGGAAAGATTTATCGGAATTGGATGAGCCCGCGTTGGATTAGCTAGTTGGTGGGGTAATGGCC
TACCAAGGCGACGATCCATAGCTGGTCTGAGAGGATGATCAGCCACACTGGGACTGAGACACGGCC
>Zotu4900
GAACGCTGGCGGCATGCTTAACACATGCAAGTCGAACGGCAGCATGGTTTAGTTTACTAGACTGATGG
CGAGTGGCGGACGGGTGAGTAACGCGTAGGAATATGCCTTGAAGAGGGGGATAACTTGGGGAAACTCA
AGCTAATACCGCATGAGATCTTAGGATGAAAGCTGGGGACCGTAAGGCCTGGCGCTTTAAGAATAGCC
TGCGTCCGATTAGCTAGTTGGTGGGGTAATGGCCTACCAAGGCGACGATCGGTAGCTGGTCTGAGAGG
ATGGCCAGCCACACTGGGACTGAGACACGGCC
>Zotu4901
GAACGCTGGCGGCGTGCCTAATACATGCAAGTCGAGCGGACCTGTATGGAGCTTGCTCCAGACAGGTT
AGCGGCGGACGGGTGAGTAACACGTAGGCAACCTGCCCGCAAGACCGGGATAACGTAGGGAAACTTAC
GCTAATACCGGATAGGGCACTCTCTCGCATGAGAGGGTGCGGAAAGGTGGCGCAAGCTACCACTTGCG
GATGGGCCTGCGGCGCATTAGCTAGTTGGTGGGGTAACGGCCTACCAAGGCGACGATGCGTAGCCGAC
CTGAGAGGGTGACCGGCCACACTGGGACTGAGACACGGCC
>Zotu4903
CAACGCTGGCGGCGTGCCTAACACATGCAAGTCGAACGAGAAAGTCCCGCAAGGGATGAGTAAAGTGG
CGACCGGGTGAGTAACACGTGACTAACCTACCCTAGAGTGGGGAATAACCTCGGGAAACCGGGGCTAA
TACCGCATAACATCGCAGAGTGAAAGTCTTTGCGATCAAAGGAGTAATTCGCTCAAGGAGGGGGTCGC
GGCTGATTAGTTAGTTGGCGGGGTAACGGCCCACCAAGACTTAGATCAGTAGCCGGCCTGAGAGGGCG
CACGGCCACACTGGAACTGAAACACGGTC
>Zotu4905
GAACGCTGGCGGAATGCTTTACACATGCAAGTCGAGCGGCAGCGCGGGGGCAACCCTGGCGGCGAGCG
GCGAACGGGTGAGTAATACATCGGAACGTACCCTGTCGTGGGGGATAACTAACCGAAAGGTTGGCTAA
TACCGCATACGACCTGAGGGTGAAAGCGGGGGATCGCAAGACCTCGCGCGATTGGAGCGGCCGATATC

AGATTAGGTAGTTGGTGGGGTAAAGGCCTACCAAGCCGACGATCTGTAGCTGGTCTGAGAGGACGACC
AGCCACACTGGGACTGAGACACGGCC
>Zotu4906
GAACGCTAGCGGCAGGCTTAACACATGCAAGTCGAGGGGTAAGGTAGCAATATCTGAGACCGGCGCAC
GGGTGCGTAACGCGTATGCAATCTACCTTTTACAGAGGGATAGCCCAGAGAAATTTGGATTAATACCT
CATAGCATAGCAGCTTCGCATGAAGTCACTATTAAAGTTCCAACGGTAAAAGATGAGCATGCGTCCTA
TTAGCTAGATGGTAAGGTAACGGCTTACCATGGCTACGATAGGTAGGGGTCCTGAGAGGGAGATCCCC
CACACTGGTACTGAGACACGGAC
>Zotu4907
GAACGCTGGCGGTATGCTTAACACATGCAAGTCGTACAAAAATCTTTGATTTTAGTGGCGGACGGGTG
AGTAACACGTGAGAATCTGCCCTTAGGAAGAGGATAACAGCTGGAAACGGTTGCTAATACTCTATACG
CCGAGAGGTAAAAGGATCTATCCACCTTAGGAAGAGCTCGCGTCTGATTAGCTTGTTGGTAGGGTAAT
GGCCTACCAAGGCGACGATCAGTAGCTGGTTTGAGAGGACGACCAGCCACACTGGGACTGAGACACGG
CC
>Zotu4909
GAACGCTAGCGGCGGGCCTAATACATGCAAGTCGAACGGATTAAGTCTTTCGGGATTTAGTTAGTGGC
GCACGGGTGAGTAACGCGTACACAACGTGGCTCTCAGAGGGGGATAGCCTTGGGAAACTGAGCGTAAT
ACCCCATAGTATTATCGGAAGGCATCTTTTGATAATTAAACCTACGGGGCTGAGAGATCGGTGTGCGT
TAGATTAGGTAGTTGGTGGGGTAACGGCTCACCAAGCCTGCGATCTATAAGGGGCGTGAGAGCGTGAT
CCCTCACACGGGTACTGAGACACGGAC
>Zotu4911
GAACGCTGGCGGCGTGCTTAACACATGCAAGTCGAACGAGGTCCATGCAGTCGCAAGACTGCGGAAGA
CCGAGTGGCGAACGGGTGCGTAACACGTGAGGAACTTGTCCCGGTCTCTGGAATAACAAGTGGAAACA
CTTGCTAATACCGGATGCCGTCGGGCCATCGCATGGTGGTCTGACGAAAGGGTTACCGGATCGGGAGA
GCCTCGCGGCCTATCAGCTTGTTGGTGAGGTAACGGCTCACCAAGGCATCGACGGGTAGCTGGTCTGA
GAGGATGATCAGCCACACTGGGACTGAGACACGGCC
>Zotu4912
GAACGCTGGCGGCATGCCTTACACATGCAAGTCGAACGGTAACGCGGGGCAACCTGGCGACGAGTGGC
GAACGGGTGAGTAATGTATCGGAACGTGCCCAGTTGTGGGGGATAACTGCTCGAAAGAGCAGCTAATA
CCGCATACGACCTGAGGGTGAAAGCGGGGGATCGCAAGACCTCGCGCAATTGGAGCGGCCGATATCAG
ATTAGGTAGTTGGTGGGGTAAAGGCTCACCAAGCCAACGATCTGTAGCTGGTCTGAGAGGACGACCAG
CCACACTGGGACTGAGACACGGCC
>Zotu4913
GAACGCTGGCGGTATGCCTTACACATGCAAGTCGAACGGTAGAGGAGCAATCCTCGAGAGTGGCGAAC
GGGTGAGTAATACATCGGAACGTGCCCAGTCGTGGGGGATAACGTAGCGAAAGCTACGCTAATACCGC
ATACGACCCGAGGGTGAAAGCGGGGGACTCGCAAGAGCCTCGCGCGATTGGAGCGGCCGATGGCAGAT
TAGGTAGTTGGTGGGGTAAAGGCCCACCAAGCCTGCGATCTGTAGCTGGTCTGAGAGGACGACCAGCC
ACACTGGGACTGAGACACGGCC
>Zotu4914
GAACGCTGGCGGCAGGCCTAACACATGCAAGTCGAGCGGATGAAGGGAGCTTGCTCCCTGATTTAGCG
GCGGACGGGTGAGTAATGCCTAGGAATCTGCTTGGTAGTGGGGGATAACGTTCCGAAAGGAACGCTAA
TACCGCATACGTCCTACGGGAGAAAGCAGGGGACCTTCGGGCCTTGCGCTATCAGATGAGCCTAGGTC
GGATTAGCTAGTTGGTGAGGTAATGGCTCACCAAGGCGACGATCCGTAACTGGTCTGAGAGGATGATC
AGTCACACTGGAACTGAGACACGGTC
>Zotu4916
GAACGCTGGCGGCAGGCTTAACACATGCAAGTCGAACGGATCCTTCGGGATTAGTGGCAGACGGGTGA
GTAACGCGTGGGAACGTACCCTTTGCTACGGAATAACTCAGGGAAACTTGAGCTAATACCGTATGTGC
CCTACGGGGGAAAGATTTATCGGCAAAGGATCGGCCCGCGTTGGATTAGCTAGTTGGTGGGGTAATGG
CCTACCAAGGCGACGATCCATAGCTGGTCTGAGAGGATGATCAGTCACACTGGGACTGAGACACGGCC
>Zotu4917
GAACGCTGGCGGCGTGCTTAACACATGCAAGTCGAACGGGGTCCATCCTTCGGGAGAAGACCTAGTGG
CGAACGGGTGAGTAACACGTGAGCAACCTGTCTCAAAGTCTGGGATAAGCCAGGGAAACTTGGTCTAA
TACCGGATGTCCTCATCGGATCCTATGTTCTGATGAGGAAAGATTTATCGCTTTGAGAGGGGCTCGCG
GCCTATCAGCTTGTTGGTGGGGTAATGGCCTACCAAGGCGTCGACGGGTAGCTGGTCTGAGAGGATGA

TCAGCCACACTGGAACTGAGACACGGTC
>Zotu4918
GAACGTTGGCGGCGTGGATTAGGCATGCAAGTCGAACGAATCCCTGGGGGCTTGCTCTCAGGGGGAAG
TGGCGAAAGGGGCAGTAATGCGTGGGTAACCTACCTCGGGGACCGGGATAGCCGTCCTAACGGACGGG
TAATACCGGGCGATGTAGCGAGGAGGCATCTTCTTGCTACGAAATGAATTTCGCCTCGAGAGGGGCCC
ACGTGGTATTAGCTTGTTGGTGAGGTAACGGCTCACCAAGGCCAAGATGCCTAGCGGGTGTGAGAGCA
CGACCCGCGCCACTGGCACTGCGACACTGGC
>Zotu4919
GAACGCTGGCGGCATGCCTAACACATGCAAGTCGAACGAGATCTTCGGATCTAGTGGCGCACGGGTGC
GTAACGCGTGGGAATCTGCCCTTGGGTTCGGAATAACAGTGAGAAATTACTGCTAATACCGGATGAAG
TCTTCGGACCAAAGATTTATCGCCCAGGGATGAGCCCGCGTAGGATTAGGTAGTTGGTGGGGTAATGG
CCTACCAAGCCGACGATCCTTAGCTGGTCTGAGAGGATGATCAGCCACACTGGGACTGAGACACGGCC
>Zotu4920
GAACGCTGGCGGCGTGCTTAACACATGCAAGTCGAACGATGAAGCCCGGTGCTTGCACCGGGTGGATT
AGTGGCGAACGGGTGAGTATCACGTGAGTAACCTGCCCTTGACTCTGGGATAAGCCTGGGAAACTGGG
TCTAATACCGGATACGACCAGTCACCGCATGGTGTGCTGGTGGAAAGCTTTAGCGGTTTTGGATGGAC
TCGCGGCCTATCAGCTAGACGGTGAGGTAATGGCTCACCGTGGCGATGACGGGTAGCCGGCCTGAGAG
GGTGACCGGCCACACTGGGACTGAGACACGGCC
>Zotu4922
GAACGCTGGCGGCATGCCTTACACATGCAAGTCGAACGGCAGCACGGGAGCAATCCTGGTGGCGAGTG
GCGAACGGGTGAGTAATATATCGGAACGTGCCCAGTCGTGGGGGATAACGCAGCGAAAGCTGTGCTAA
TACCGCATACGATCTCTGGATGAAAGCGGGGGACCTGCAAGGGCCTCGCGCGATTGGAGCGGCCGATA
TCAGATTAGCTAGTTGGTGGGGTAAAAGCCCACCAAGGCGACGATCTGTAGCTGGTCTGAGAGGACGA
CCAGCCACACTGGAACTGAGACACGGTC
>Zotu4923
GAACGCTAGCTACAGGCTTAACACATGCAAGTCGAGGGGCAGCATGAACTTAGCTTGCTAAGTTTGAT
GGCGACCGGCGCACGGGTGCGTAACGCGTATCCAACTTTCCCGTTACTCAGGTATAGCCTTGCGAAAG
TAAGATTAATCCCTGATGATATGTTGTTGTTGCATGACTTTGACATTAAAGATTTATCGGTAACGGAT
AGGGATGCGTTCCATTAGGTAGTTGGTGAGGTAACGGCTCACCAAGCCCGCGATGGATAGGGGTTCTG
AGAGGAAGGTCCCCCACACTGGAACTGAGACACGGTC
>Zotu4924
GAACGCTGGCGGCAGGCTTAACACATGCAAGTCGAACGGGCCCTTCGGGGTCAGTGGCAGACGGGTGA
GTAACACGTGGGAACGTGCCCTTCAGTTCGGAATAACCCAGGGAAACTTGGGCTAATACCGGATACGC
CCGCAAGGGGAAAGATTTTATCGCTGAAGGATCGGCCCGCGTCTGATTAGCTAGTTGGTGAGGTAAAG
GCTCACCAAGGCGACGATCAGTAGCTGGTCTGAGAGGATGATCAGCCACACTGGGACTGAGACACGGC
C
>Zotu4925
GAACGCTGGCGGCGCGCTTAACACATGCAAGTCGAGCGAGAACCGGTCCTTCGGGACCGGGGAAAGCG
GCGAACGGGTGAGTAACACGTGGGTAATCAACCCCTGGCACTGGGATAGCCCGAGGAAACTCGGATTA
ATACCGGATGGCCCAGCAGTCTTTCGGGACGGCTGGAAAAGGTAGCTTCGGCCTCCGGCCGGGGACGA
GCCCGCGGCGGATTAGCTTGTTGGTGGGGTAATGGCCTACCAAGGCGACGATCCGTAGCTGGTCTGAG
AGGACGATCAGCCACACTGGGACTGAGACACGGCC
>Zotu4926
GAACGCTGGCGGCATGCTTTACACATGCAAGTCGAACGGCAGCGAGGAGGAGCTTGCTTCTCTGTCGG
CGAGTGGCGAACGGGTGAGTATAGCATCGGAACGTGCCAAGTAGTGGGGGATAACCAAGCGAAAGTTT
GGCTAATACCGCATAAGCTCTAAGGAGGAAAGTAGGGGACCGTAAGGCCTTACGCTATTTGATCGGCC
GATGTCGGATTAGCTAGTTGGTGAGGTAATGGCTCACCAAGGCGATGATCCGTAGCGGGTCTGAGAGG
ACGATCCGCCACACTGGGACTGAGACACGGCC
>Zotu4927
GAACGCTGGCGGCGCGCCTAACACATGCAAGTCGAACGGCACCTATCTTCGGATAGAAGCGAGTGGCG
AACGGCTGAGTAACACGTGGAGAACCTGCCCCCTCCCCCGGGATAGCCGCCCGAAAGGACGGGTAATA
CCGGATACCCCGGGGTGCCGCATGGCACCCCGGCTAAAGCCCCGACGGGAGGGGATGGCTCCGCGGCC
CATCAGGTAGACGGCGGGGTGACGGCCCACCGTGCCGACAACGGGTAGCCGGGTTGAGAGACCGACCG
GCCAGATTGGGACTGAGACACGGCC

>Zotu4928
GAACGCTGGCGGCGTGCTTAACACATGCAAGTCGAGCGAATTTTGACAGCGTATCGTCAGAGTTAGCG
GCAGACGGGTGAGTAATACTTGGGCAATCTGCCCTTGAGAGGGAAACAACCTGCCGAAAGGTGGGCTA
ATGTCCCGTAAGACCACAAGATGGCATCATCTAGGGGTCAAAGCTCCGCAAGGGGCGCTCGAGGAGGA
GCCTAAGTTCTATCATGGTAGTTGGCGGGGTAACGGCCCACCAAGCCTTTGACGGATAGCCGGCCTGA
AAGGGTGATCGGCCACACTGGCACTGAGACACGGGC
>Zotu4931
GAACGCTGGCGGCGTGCCTAATACATGCAAGTAGAACGCTGAAGGAGGAGCTTGCTTCTCTGGATGAG
TTGCGAACGGGTGAGTAACGCGTAGGTAACCTGCCTGGTAGCGGGGGATAACTATTGGAAACGATAGC
TAATACCGCATAAAATTGATTATTGCATGATAGTCAATTAAAAGGTGCAATTGCATCACTACCAGATG
GACCTGCGTTGTATTAGCTAGTTGGTGAGGTAACGGCTCACCAAGGCGACGATACATAGCCGACCTGA
GAGGGTGATCGGCCACACTGGGACTGAGACACGGCC
>Zotu4933
GAACGCTGGCGGCATTCCTTACACATGCAAGTCGAACGGTAACAGGTTAAGCTGACGAGTGGCGAACG
GGTGAGTAATATATCGGAACGTGCCCAGTCGTGGGGGATAACGTAGCGAAAGCTACGCTAATACCGCA
TACGATCTATGGATGAAAGCGGGGGATCGCAAGACCTCGCGCGATTGGAGCGGCCGATATCAGATTAG
GTAGTTGGTGGGGTAAAGGCCCACCAAGCCGACGATCTGTAGCTGGTCTGAGAGGACGACCAGCCACA
CTGGGACTGAGACACGGCC
>Zotu4934
GAACGCTGGCGGCGTGCTTAACACATGCAAGTCGAACGGTGAACTCCGCTTGCGGGGGGATCAGTGGC
GAACGGGTGAGTACACGTGGGCAACCTGCCCTCAGCTCTGGGATAACTCCAAGAAATTGGGGCTAATA
CCGGATATGACCGCTGACCGCATGGTCTGGTGGTGGAAAGATTTATCGGCTGGGGATGGGCCCGCGGC
CTATCAGCTTGTTGGTGGGGTAGTGGCCTACCAAGGCGACGACGGGTAGCCGGCCTGAGATGGTGACC
GGCCACACTGGGACTGAGACACGGCC
>Zotu4936
CAACGCTGGCGGCGTGCCTAACACATGCAAGTCGAACGAGAAAGGGCTTTCGGGCCTGAGTAAAGTGG
CGCACGGGTGAGTAACACGTGACTAACCTACCTTTGAGCGGGGGATAACCTAGGGAAACCTGGGCTAA
TACCGCATAATACCTACGGGTCAAAGCGAGCAATCGCACTTAAAGAGGGGGTCGCGGCAGATTAGCTA
GTTGGTGAGGTAATGGCTCACCAAGGCGATGATCTGTATCCGGCCTGAGAGGGCGCACGGACACACTG
GAACTGAAACACGGTC
>Zotu4937
GAACGCTGGCGGTATGCTTTACACATGCAAGTCGTACGAGAGTTTTTAACTCAAGTGGCGGACGGGTG
AGTAACACGTAAGAATTTGCCTTTAGGAGGGGGATAACAACTGGAAACGGTTGCTAATACCCCATATG
CTTTCGAGTGAAACGGATTTTCCGCCTAAAGAGAAGCTTGCGGCTGATTAGCTTGTTGGTGAGGTAAT
GGCTCACCAAGGCGACGATCAGTATCTGGTTTGAGAGGATGATCAGACACACTGGAACTGAGACACGG
TC
>Zotu4938
GAACGCTGGCGGCGTGCCTAACACATGCAAGTCGAGGGGCTGAGTAGCAATACTTAGTCACTGGCGCA
CGGGTGAGTAACGCGTAGATTATCTGCCCTTAACACTGACATAACGTTGAGAAATTGACGCTAATATC
AGATAATACCGCAAGGTCAAAGAGCAATCGGTTAAGGATGAGTCTGCGTTAGATTAGCTAGTTGGCGG
GGTAATGGCCCACCAAGGCAACGATCTATAGCTGGTCTGAGAGGACGATCAGCCACACTGGAACTGAG
ACACGGTC
>Zotu4939
GAACGCTGGCGGCGTGCCTAATACATGCAAGTCGAACGAAGAGTATTTGTAGTAATACAGATGCTTCT
TAGTGGCGAACGGGTGAGTAACGCGTTGGTGACCTGCCCCAAAGTGTGGGATAACAGTCCGAAAGGAT
TGCTAATACCTCATGTGGTCTTCGGAATTAGAAACCGATTTCTAAAGGAGTAATCCGCTTTGGGAGGG
GCCTGCGTCCCATCAGCTAGTTGGTGAGGTAACGGCTCACCAAGGCTTCGACGGGTAAGGGGCCTGAG
AGGGTGGCCCCTCACAATGGAACTGAAACACGGTC
>Zotu4940
GAACGCTGGCGGCATGCTTTACACATGCAAGTCGAACGGCAGCGCGGGCGGGGGTAACCTCGCCTGGC
GGCGAGTGGCGAACGGGTGAGTAATATATCGGAACGTACCCAGAAGTGGGGGATAACGTAGCGAAAGT
TACGCTAATACCGCATACGATCTACGGATGAAAGTGGGGGACCTTCGGGCCTCATGCTTGTGGAGCGG
CCGATATCTGATTAGCTAGTTGGTGAGGTAAAGGCTCACCAAGGCGACGATCAGTAGCTGGTCTGAGA
GGACGACCAGCCACACTGGAACTGAGACACGGTC

>Zotu4941
GAACGCTGGCGGCGTGCTTAACAAATGCAAGTCGAACGAGGTAAGAAGCTTCGGTTTTTACCGAGTG
GCGGACTGGTGAGTAACACGTGAGTAACCAACCTTATACAGGGGGATACCACAAAGAAATTTGTGCTA
ATACCGCATAATACAAGAAAGCCACATGGCAGAGAAGTTAAAGGAGAAATCCGGTATAAGACGGACTC
GCGCATCATTAGCTAGTTGGTGAGGTAACGGCCCACCAAGGCAACGATGATTAGCCGGCCTGAGAGGG
TGTACGGCCACATTGGGACTGAGATACGGCC
>Zotu4942
GAACGCTAGCGGCAGGCTTAACACATGCAAGTCGAGGGGTATGGTTCTTCGGAACCAGAGACCGGCGC
ACGGGTGCGTAACGCGTATGCAATCTACCTTTTACAGAGGGATAGCCCAGAGAAATTTGGATTAATAC
CTCATAGTATATAGACCTGGCATCAGGATTATATTAAAGTCACAACGGTAAAAGATGAGCATGCGTCC
CATTAGCTAGTTGGTAAGGTAACGGCTTACCAAGGCTACGATGGGTAGGGGTCCTGAGAGGGAGATCC
CCCACACTGGTACTGAGACACGGAC
>Zotu4943
GAACACTGGCGGCATGCCTAACACATGCAAGTCGAACGATTGCCTTCGGGTGATAGTGGCGCACGGGT
GCGTAACACGTGGGAATCTGCCCTTAGGTTCGGAATAACAGCTGGAAACGGCTGCTAATACCGGATGA
TATCGCGAGATCAAAGATTTATCGCCTGAGGATGAGCCCGCGTTGGATTAGGTAGTTGGTAGGGTAAA
GGCCTACCAAGCCGACGATCCATAGCTGGTCTGAAAGGATGATCAGCCACACTGGGACTGAGACACGG
CC
>Zotu4944
GAACGCTGGCGGCGTGCCTAATACATGCAAGTCGAGCGGTAAGGCCCCTTCGGGGGTACACGAGCGGC
GGACGGCTGAGTAACGCGTAGGAACGTACCCCAAAGTGAGGGATAAGCATTGGAAACAGTGTCTAATA
CCGCATGTGATCCACGGATTAAAGCTTCGGCGCTTTGGGAACGGCCTGCGTAAGATTAGCTTGTTGGT
GAGGTAATGGCTCACCAAGGCGACGATCTTTAGCTGGTCTGAGAGGATGATCAGCCAGACTGGGACTG
AGACACGGCC
>Zotu4945
GAACGCTGGCGGCGTGCTTAACACATGCAAGTCGAACGGTGAAAGAGGAGCTTGCTCCTCTGGATCAG
TGGCGAACGGGTGAGTAACACGTGAGAAATGTGCCCTGGACTCTGGGATAAGCGTTGGAAACGACGTC
TAATACCGGATACGCGACGCGAAGGCATCTTCAGCGTCGGGAAAGAACTTCGGTCCAGGATCATCTCG
CGGCCTATCAGCTAGTTGGTGAGGTAACGGCCCACCAAGGCGACGACGGGTAGCCGGCCTGAGAGGGT
GACCGGCCACACTGGGACTGAGACACGGCC
>Zotu4946
GAACGCTGGCGGCGTGCCTAATACATGCAAGTCGAGCGGACGAAAATTTGGTGCTTGCACCAAATTTT
CGTTAGCGGCGGACGGGTGAGTAACACGTGGGTAACCTGCCCTACAGACTGGGATAACTTCGGGAAAC
CGAGGCTAATACCGGATAATACTTTTTAACACATGTTAGAAAGTTGAAAGATGGTTTTGCTATCACTG
TAGGATGGACCCGCGGCGCATTAGCTAGTTGGTGAGGTAACGGCTCACCAAGGCAACGATGCGTAGCC
GACCTGAGAGGGTGATCGGCCACACTGGGACTGAGACACGGCC
>Zotu4947
CAACGCTGGCGGCGCGCCTAACACATGCAAGTCGCACGAGAAAGGGAGCAATCCTGAGTAAAGTGGCG
TACGGGTGAGTAACACGTGGGAATCTACCTCTTGGCGGGGAATAACCCTGGGAAACCGGGGCTAATAC
CGCATAAGCCCGTGAGGGGAAAGCAGCAATGCACCGAGAGAGGAGCCCGCGGCCGATTAGCTAGTTGG
TGAGGTAATGGCTCACCAAGGCGAAGATCGGTAGCCGGCCTGAGAGGGCACACGGCCACACTGGCACT
GAAACACGGGC
>Zotu4948
GAACGCTGGCGGCGTGCTTAACACATGCAAGTCGAACGATGAAGCCCAGCTTGCTGGGTGGATTAGTG
GCGAACGGGTGAGTAACACGTGAGTAACCTGCCCTTGACTCTGGGATAAGCGCTGGAAACGGCGTCTA
ATACCGGATATGACAACCGACGGCATCGTCTGGTTGTGGAAAGAATTTTGGTCAAGGATGGACTCGCG
GCCTATCAGCTTGTTGGTGAGGTAATGGCTCACCAAGGCGACGACGGGTAGCCGGCCTGAGAGGGTGA
CCGGCCACACTGGGACTGAGACACGGCC
>Zotu4951
GAACGCTGGCGGCAGGCCTAACACATGCAAGTCGAGCGGTAGAGAGTGCTTGCACTCTTGAGAGCGGC
GGACGGGTGAGTAATGCCTAGGAATCTGCCTGGTGGTGGGGGATAACGTTCGGAAACGGACGCTAATA
CCGCATACGTCCTACGGGAGAAAGCGGGGGATCTTCGGACCTCGCGCCATCAGATGAGCCTAGGTCGG
ATTAGCTAGTTGGTGGGGTAATGGCTCACCAAGGCGACGATCCGTAACTGGTCTGAGAGGATGATCAG
TCACACTGGAACTGAGACACGGTC

>Zotu4952
GAACGCTGGCGGCATGCCTAACACATGCAAGTCGAACGAGACCTTCGGGTCTAGTGGCGCACGGGTGC
GTAACGCGTGGGAATCTGCCCTTGGGTTCGGAATAACAGTTAGAAATGACTGCTAATACCGGATGATG
TCTTCGGACCAAAGATTTATCGCCCAGGGATGAGCCCGCGTAGGATTAGCTAGTTGGTGGGGTAAAGG
CCTACCAAGGCGACGATCCTTAGCTGGTCTGAGAGGATGATCAGCCACACTGGGACTGAGACACGGCC
>Zotu4953
GAACGCTAGCGGCAGGCTTAACACATGCAAGTCGAGGGGTAGGGCAGCAATGCTTGAGACCGGCGCAC
GGGTGCGTAACGCGTATGCAATCTACCTTTTGCAGAGGGATAGCCCAGAGAAATTTGGATTAATACCT
CATAGTAATATGACTTCGCATGGAGATATATTTAAAGTAACAACGGCAAAAGATGAGCATGCGTATCA
TTAGCTAGATGGTAAGGTAACGGCTTACCATGGCTACGATGATTAGGGGTCCTGAGAGGGAGATCCCC
CACACTGGTACTGAGACACGGAC
>Zotu4954
GAACGCTGGCGGCGTGCTTAACACATGCAAGTCGAACGAGGCGCTTGCGCCTAGTGGCGAACGTGTGA
GTAACACGTGAGCAACCTGCCCCTGGCTCTGGGATAGCCTCCGGAAACGGGGATTAATACCGGACATG
ACTGCTGCCGGCATCGGCGGCGGTGGAAAGTTTTTCGGCCAGGGAGGGGCTCGCGGCCTATCAGCTTG
TTGGGGGGGTAACGGCCCACCAAGGCGACGACGGGTAGCCGGCCTGAGAGGGCGACCGGCCACACTGG
GACTGAGACATGGCC
>Zotu4956
GAACGCTGGCGGCGTGCTTAACACATGCAAGTCGAGCGGTAAGGCCCTTCGGGGTACACGAGCGGCGA
ACGGGTGAGTAACACGTGAGCAACCTGCCCCTGACTCCGGGATAAGCGGTGGAAACGCCGTCTAATAC
CGGATATGACACGCCACCTCATGGTGGTGTGTGGAAAGTTTTTCGGTCAGGGATGGGCTCGCGGCCTA
TCAGCTTGTTGGTGGGGTAACGGCCTACCAAGGCGATTACGGGTAGCCGGCCTGAGAGGGCGACCGGC
CACACTGGGACTGAGACACGGCC
>Zotu4957
GAACGCTGGCGGCGTGCCTAATACATGCAAGTCGAGCGAACTTGCGGGAGCTTGCTCCCAAAAGTTAG
CGGCGGACGGGTGAGTAACACGTGGGCAACCTGCCTATAAGACTGGGATAACTTCGGGAAACCGGAGC
TAATACCGGATAATTCATATCTACTCATGTAGATATGCTGAAAGACGGTTTACGCTGTCACTTATAGA
TGGGCCCGCGGCGCATTAGCTAGTTGGTGAGGTAACGGCTCACCAAGGCGACGATGCGTAGCCGACCT
GAGAGGGTGATCGGCCACACTGGGACTGAGACACGGCC
>Zotu4958
GAACGCTGGCGGCGTGCTTAACACATGCAAGTCGAACGGTGACCTCGAGAGCTTGCTCTTGGGTGATC
AGTGGCGAACGGGTGAGTAACACGTGAGTAACCTGCCCCAGACTCTGGAATAACCCCGGGAAACCGGA
GCTAATACCGGATACGAGACGGAGCCGCATGGCTACCGTTTGGAAAGTTTTTCGGTCTGGGATGGACT
CGCGGCCTATCAGCTAGTTGGTGAGGTAACGGCTCACCAAGGCGACGACGGGTAGCCGGCCTGAGAGG
GCGACCGGCCACACTGGGACTGAGACACGGCC
>Zotu4959
GAACGCTGGCGGCGTGCTTAACACATGCAAGTCGAGCGGTAAGGCCCTTTCGGGGGTACACGAGCGGC
GAACGGGTGAGTAACACGTGAGTAATCTGCCCTTCACTTCGGGATAACCGCCGGAAACGGCGGCTAAT
ACCGGATACGACCGAGTTTTGCATGAAATCTTGGTGGAAAGTTTTTTCGGTGGAGGATGTGCTCGCGG
CCTATCAGCTTGTTGGTGAGGTAATGGCTCACCAAGGCTTCGACGGGTAGCCGGCCTGAGAGGGTGAC
CGGCCACACTGGGACTGAGACACGGCC
>Zotu4960
GAACGCTGGCGGCGTGCCTAACACATGCAAGTCGAACGGAAGTATACGAAGTCTTCGGACGGAATATA
CTTTAGTGGCGGACGGGTGAGTAACGCGTGGGTAACCTGCCTCATACAGGGGGATAACAGCTGGAAAC
GGCTGCTAAAACCGCATAAGCATACACTGCCGCATGGCAGAGTGTGAAAATACTTGTAGGTATGAGAT
GGGCCCGCGTCTGATTAGCTAGTTGGTAGGGTAACGGCCTACCAAGGCGACGATCAGTAGCCGGCTTG
AGAGAGTGACCGGCCACATTGGGACTGAGACACGGCC
>Zotu4961
GAGCGCTGGCGGCGTGCCTAATACATGCAAGTCGAGCGGAAAGGCCTCTTCGGAGGTACTCGAGCGGC
GGACGGCTGAGTAACGCGTAGGAATGTACCCCAAAGTGAGGGATAACTGCTCGAAAGAGTAGCTAATA
CCGCATGTGATCCACGGATTAAAGCTTTATGCGCTTTGGGAACAGCCTGCGTCCGATTAGCTAGTTGG
TGAGGTAAAAGCTTACCAAGGCTATGATCGGTAGCTGGTCTGAGAGGATGATCAGCCAGACTGGGACT
GAGACACGGCC
>Zotu4962

GAACGCTGGCGGCATGCCTAACACATGCAAGTCGAACGAGACCTTCGGGTCTAGTGGCGCACGGGTGC
GTAACGCGTGGGAATCTGCCCTTGGGTTCGGGATAACAGTTGGAAACGACTGCTAATACCGGATGATG
ACTTCGGTCCAAAGATTTATCGCCCAGGGATGAGCCCGCGTAGGATTAGCTAGTTGGTGAGGTAAAGG
CTCACCAAGGCGACGATCCTTAGCTGGTCTGAGAGGATGATCAGCCACACTGGGACTGAGACACGGCC
>Zotu4963
GAACGCTGGCGGCGTGCTTAACACATGCAAGTCGAGCGAGGCCCTTCCTTCGGTAAGGTGCCCTAGCG
GCGAACGGGTGAGTAACACGTGGGCAACCTGCCCCTAGCACTGGGATAACTCCGGGAAACCGGGGCTA
ATACCGGATATGACCTCCGAGGGCATCCTCGTTGGTGTAAAGGATTCTGGCTAGGGATGGGCCCGCGG
CCTATCAGCTTGTTGGTGGGGTAATGGCCTACCAAGGCGACGACGGGTAGCCGGCCTGAGAGGGCGAC
CGGCCACACTGGGACTGAGACACGGCC
>Zotu4964
GAACGCTGGCGGCGTGCTTAACACATGCAAGTCGAACGATGATGCCCAGCTTGCTGGGTGGATTAGTG
GCGAACGGGTGAGTAACACGTGAGTAACCTGCCCCTGACTCTGGGATAAGCGTTGGAAACGACGTCTA
ATACTGGATATGACTACCGGCCGCATGGTCTGGTGGTGGAAAGATTTCTTGGTTGGGGATGGACTCGC
GGCCTATCAGCTTGTTGGTGAGGTAATGGCTCACCAAGGCGACGACGGGTAGCCGGCCTGAGAGGGTG
ACCGGCCACACTGGGACTGAGACACGGCC
>Zotu4965
GAACGCTGGCGGCGTGCTTAACACATGCAAGTCGAACGAACCTTCGGGTTAGTGGCGGACGGGTGAGT
AACGCGTGAGAATCTGCCCTCAGGAGGGGGATAACGGCTGGAAACGGCCGCTAATACCCCATATGCCG
AGAGGTGAAATGAATTTCGCCTGAGGATGAGCTCGCGTCTGATTAGCTAGTTGGTGTGGTAATGGCTC
ACCAAGGCTTCGATCAGTAGCTGGTCTGAGAGGATGATCAGCCACACTGGGACTGAGACACGGCC
>Zotu4966
GAACGCTGGCGGCGTGCCTAACACATGCAAGTCGAACGGAATATTTCGGATGAGGCCTTCGGGCGGAT
GAAGAGATATTTAGTGGCGGACGGGTGAGTAACGCGTGGGTAACCTGCCCTGTACCGGGGGATAACAG
TTGGAAACGACTGCTAATACCGCATAAGCACACGGTGCCGCATGGCACAGGGTGAAAAACTCCGGTGG
TACAGGATGGACCCGCGTCTGATTAGCTTGTTGGTGAGGTAACGGCCCACCAAGGCGACGATCAGTAG
CCGACTTGAGAGAGTGATCGGCCACATTGGGACTGAGACACGGCC
>Zotu4967
GAACGCTAGCGGCAGGCCTAATACATGCAAGTCGAACGGTAAGCGCCCCAGCGATGGGGTGCGAGAGT
GGCGCACGGGTGCGTAACGCGTATGCAACCAGCCCTCTGCCGGGGGATAGCCCGGAGAAATCCGGGGT
AACACCCCATGACGGGCCGCGGGGGCATCCCCGCGGCCTGAAAACCACGGTGGCGGGGGACGGGCATG
CGTCCCATTAGGTAGTCGGCGGGGTGACGGCCCACCGAGCCCACGATGGGTAGGGGATCTGAGAGGAC
GGTCCCCCACACTGGTACTGAGACACGGAC
>Zotu4969
GAACGCTGGCGGCGTGCCTAATACATGCAAGTCGAACGATGGCGATTTTCTCCTTACGGAGAAAACGC
CGTAGTGGCGGACGGGTGAGTAACACGTGGGCAACCTGCCTGTAAGCTGGGGATAACTAGCGGAAACG
TTAGCTAATACCGAATGGTCAAGAGGAAGGCATCTTCCACTTGGGAAAGATGGAGCAATCTATCGCTT
ACAGATGGGCCCGCGTCGCATTAGCTAGTAGGTGAGGTAACGGCTCACCTAGGCGACGATGCGTAGCC
GACCTGAGAGGGTGAACGGCCACACTGGGACTGAGACACGGCC
>Zotu4970
GAACGCTGGCGGCGTGCCTAATACATGCAAGTCGAGCGAATGACGAGAAGCTTGCTTCTCTGATTTAG
CGGCGGACGGGTGAGTAACACGTGGGCAACCTGCCCTGTAGACTGGGATAACTTCGGGAAACCGGAGC
TAATACCGGATAATCCATTTCACCTCATGGTGAAATGTTGAAAGGCGCTTCGGCGTCACTACAGGATG
GGCCCGCGGTGCATTAGCTAGTTGGTGGGGTAACGGCCTACCAAGGCAACGATGCATAGCCGACCTGA
GAGGGTGATCGGCCACATTGGGACTGAGACACGGCC
>Zotu4971
GAACGCTGGCGGCGTGCTTAACACATGCAAGTCGAGCGAGGCTCCCTCTTCGGAGGGATGTCCTAGCG
GCGAACGGGTGAGTAACACGTGGGCAACCTGCCCCTAGCACTGGGATAACCCCGGGAAACCGGGGCTA
ATACCGGATATGACCTCGATGGGCATCCGTCGAGGTGGAAAAGGTTACTGGCTAGGGATGGGCCCGCG
GCCTATCAGCTTGTTGGTGGGGTAACGGCCTACCAAGGCGACGACGGGTAGCCGGCCTGAGAGGGCGA
CCGGCCACACTGGGACTGAGACACGGCC
>Zotu4972
GAACGCTAGCGGCAGGCCTAATACATGCAAGTCGAGGGGTAGCATAGTAGCAATACTGATGGCGACCG
GCGCACGGGTGCGTAACGCGTATGCAACCTACCTTTTACTGGGGGATAGCCCGGAGAAATTCGGATTA

ACACCGCATAACAAGTTGATATGGCATCATATTAATTTTAAATATTTATAGGTAAAAGATGGGCATGC
GTCCAATTAGCTAGTTGGTGAGGTAATGGCTCACCAAGGCGATGATTGGTAGGGGTTCTGAGAGGAAG
GTCCCCCACACTGGTACTGAGATACGGAC
>Zotu4973
GAACGCTGGCGGCGTGCCTAAGACATGCAAGTCGAGTGGGTTTAGACCCACGGCGAACGGGTGAGTAA
TACCTAGGAACGTACCTTTAGTCGAGCATACGCAGCCGAAAGGTTGTTTAATTCTCGATGTGACCGCA
AGGTTAAAGTCGTAAGACGCTAAAGAATCGGCCTGGACACCATCAGCTAGTTGGTAGGGTAAAAGCCT
ACCAAGGCGACGACGGTTAGGGGTCCCGAGAGGGATAACCCCCACACTGGGACTGCGACACGGCC
>Zotu4974
GAACGCTGGCGGCGTGCCTAATACATGCAAGTCGAGCGCCTCTACTAAGTTTACGAAGTAATTCGTAG
GTTTGGTTGAGGAGCGGCGGACGGCAGAGTAACGCGTAGGAACGTACCCCAAACTGAGGGATAAGCAC
CGGAAACGGTGTCTAATACCGCATAATATCTTCGGATTAAAGCTACGGCGGTTTGGGAGCGGCCTGCG
TCTGATTAGTTAGTTGGTGAGGTAATGGCTTACCAAGACAATGATCGGTAGCTGGTCTGAGAGGATGA
TCAGCCTGACTGGAACTGAGAACGGTC
>Zotu4975
GAACGCTGGCGGCATGCCTTACACATGCAAGTCGAACGGCAGCATAGGAGCTTGCTCCTGATGGCGAG
TGGCGAACGGGTGAGTAATACATCGGAACGTACCCTGTTGTGGGGGATAACTAGTCGAAAGACTAGCT
AATACCGCATACGATCTACGGATGAAAGTGGGGGATCGCAAGACCTCATGCTCCTGGAGCGGCCGATA
TCTGATTAGCTAGTTGGTGGGGTAAAAGCTCACCAAGGCGACGATCAGTAGCTGGTCTGAGAGGACGA
CCAGCCACACTGGGACTGAGACACGGCC
>Zotu4978
GAACGCTGGCGGCATGCCTAACACATGCAAGTCGAACGAAGGCCCGATTTCGGTTGGGTCCTTAGTGG
CGCACGGGTGCGTAACGCGTGGGAATCTGCCCCTTGGTTCGGAATAACAGTTAGAAATGACTGCTAAT
ACCGGATGATGACGTAAGTCCAAAGATTTATCGCCGAGGGATGAGCCCGCGTAGGATTAGCTAGTTGG
TGTGGTAAGAGCGCACCAAGGCGACGATCCTTAGCTGGTCTGAGAGGATGATCAGCCACACTGGGACT
GAGACACGGCC
>Zotu4980
GAACGCTAGCGGCAGGCTTAACACATGCAAGTCGAGGGGTATAGTTCTTCGGAACTAGAGACCGGCGC
ACGGGTGCGTAACGCGTATGCAATCTACCTTTCACAGAGGGATAGCCCAGAGAAATTTGGATTAATAC
CTCATAGTATGCAGAGTTGGCATCAACGCTGCATTAAAGTCACAACGGTGAAAGATGAGCATGCGTCC
CATTAGCTAGTTGGTAAGGTAACGGCTTACCAAGGCTACGATGGGTAGGGGTCCTGAGAGGGAGATCC
CCCACACTGGTACTGAGACACGGAC
>Zotu4982
GAACGCTGGCGGCGTGCCTAATACATGCAAGTCGAGCGAATGATGAGAGAAGCTTGCTTCTCTCTGAT
TTAGCGGCGGACGGGTGAGTAACACGTGGGTAACCTGCCCTACAGACTGGGATAACTTCGGGAAACCG
AGGCTAATACCGGATAATACTTTTGATCACATGGTCGGAAGTTGAAAGATGGTTTCGGCTATCACTGT
AGGATGGACCCGCGCTGCATTAGCTAGTTGGTGAGGTAACGGCTCACCAAGGCCACGATGCATAGCCG
ACCTGAGAGGGTGATCGGCCACACTGGGACTGAGACACGGCC
>Zotu4984
GAACGCTAGCGGCAGGCTTAACACATGCAAGTCGAGGGGTAGTAGGAGCTTGCTTCTATGAGACCGGC
GCACGGGTGCGTAACGCGTATGCAATCTGCCTCTCACTGGGGGATAGCCCGGAGAAATCCGGATTAAT
ACCCCATGGTATTTTGGAATGGCATCATTCTATTATTAAAGATTTATCGGTGAGAGATGAGCATGCGT
CCCATTAGTTAGTTGGTGTGGTAACGGCACACCAAGACGATGATGGGTAGGGGTCCTGAGAGGGAGAT
CCCCCACACTGGTACTGAGACACGGAC
>Zotu4985
GAACGCTAGCGGCAGGCTTAACACATGCAAGTCGAGGGGTATAGTTCTTCGGAGCTAGAGACCGGCGC
ACGGGTGCGTAACGCGTATGCAATCTACCTTTTACAGAGGGATAGCCCAGAGAAATTTGGATTAATAC
CTCATAGTATTATGAAATGGCATCATTTTATAATTAAAGTCACAACGGTAAAAGATGAGCATGCGTCC
CATTAGCTTGTTGGTAAGGTAACGGCTTACCAAGGCTACGATGGGTAGGGGTCCTGAGAGGGAGATCC
CCCACACTGGTACTGAGACACGGAC
>Zotu4986
GAACGCTGGCGGCGTGCCTAATACATGCAAGTCGAGCGAATCTTTGGGAGCTTGCTCCTAAAGGTTAG
CGGCGGACGGGTGAGTAACACGTGGGCAACCTGCCTATAAGACTGGGATAACTTCGGGAAACCGGAAC
TAATACCGGATAATTCTTTTCTACACATGTAGAAAAGCTAAAAGACGGTTTACGCTGTCACTTATAGA

TGGGCCCGCGGCGCATTAGCTAGTTGGTGAGGTAACGGCTCACCAAGGCCACGATGCGTAGCCGACCT
GAGAGGGTGATCGGCCACACTGGGACTGAGACACGGCC
>Zotu4987
GAACGCTGGCGGCGTGCCTAATACATGCAAGTCGAGCGAAGTTTTTCTGGTGCTTGCACTAGAAAAAC
TTAGCGGCGAACGGGTGAGTAACACGTAAAGAACCTGCCTCATAGACTGGGACAACTATTGGAAACGA
TAGCTAATACCGGATAACAGTATTTCTCGCATGGGAGATATTTAAAAGTTGGTTATGCTAACACTATG
AGATGGCTTTGCGGTGCATTAGCTAGTTGGTGGGGTAAAGGCCCACCAAGGCGACGATGCATAGCCGA
CCTGAGAGGGTGATCGGCCACACTGGGACTGAGACACGGCC
>Zotu4988
GAACGCTGGCGGCAGGCTTAACACATGCAAGTCGAGCGGGCATCCTTGTGGTGTCAGCGGCAGACGGG
TGAGTAACGCGTGGGAACGTGCCCTTCGGTTCGGAATAACTCAGGGAAACTTGAGCTAATACCGGATA
CGTGCGAGAGCAGAAAGGTTTATCTGCCGAAGGATCGGCCCGCGTCTGATTAGCTAGTTGGTGAGGTT
ACGGCTCACCAAGGCGACGATCAGTAGCTGGTCTGAGAGGATGATCAGCCACACTGGGACTGAGACAC
GGCC
>Zotu4989
CAACGCTGGCGGCGCGCTTAACACATGCAAGTCGCACGAGAAAGGGAGCAATCCTGAGTATAGTGGCG
CACGGGTGAGTAACACGTGGATGATCTACCTCCTTGTGGGGAATAACCCTGGGAAACCGGGGCTAATA
CCGCATACGTTCGAGAGAAGAAAGCCGAAAGGCGCAGGGAGAGGAGTCCGCGGTCGATTAGCTAGTTG
GCAGGGTAATGGCCTACCAAGGCGACGATCGATAGCCGGCCTGAGAGGGCACACGGCCACACTGGCAC
TGAAACACGGGC
>Zotu4991
CAACGCTGGCGGCGTGCCTAACACATGCAAGTCGCACGAGAAAGGGGCTTCGGCCCTGAGTAAAGTGG
CGCACGGGTGAGTAACACGTGAATCATCTACCTTCGAGTGGGGAATAACCTAGAGAAATCTGGGCTAA
TACCGCATAACACTTACGAGTCAAAGCAGCAATGCGCTTGAAGAGGAGTTCGCGGCAGATTAGTTAGT
TGGTGGGGTAATGGCCTACCAAGACGATGATCTGTATCCGGCCTGAGAGGGCGCACGGACACACTGGA
ACTGAAACACGGTC
>Zotu4992
GAACGCTAGCGGCAGGCTTAACACATGCAAGTCGAGGGGCAGCACGATGTAGCAATACATGGGTGGCG
ACCGGCGCACGGGTGCGTAACACGTATGGAACTTGCCCTTTACTGGGGGATAGCCCGGAGAAATCCGG
ATTAATACCCCATAACAAGCGGAGTGGCATCACTCTGCTTTGAAAGCTCCGGCGGTAAAGGATGGCCA
TGCGCCCGATTAGCTAGTTGGTGAGGTAACGGCTCACCAAGGCTATGATCGGTAGGGGGCCTGAGAGG
GTTATCCCCCACACTGGTACTGAGACACGGAC
>Zotu4993
GAACGCTGGCGGCGTGCCTAATACATGCAAGTCGAGCGAATCTTTGGGAGCTTGCTCCCAAAGATTAG
CGGCGGACGGGTGAGTAACACGTGGGCAACCTGCCTATAAGACTGGGATAACTTCGGGAAACCGGAGC
TAATACCGGATAATTCTTTTCTACTCATGTAGGAAAGCTAAAAGACGGTTTACGCTGTCACTTATAGA
TGGGCCCGCGGCGCATTAGCTAGTTGGTGAGGTAACGGCTCACCAAGGCCACGATGCGTAGCCGACCT
GAGAGGGTGATCGGCCACACTGGGACTGAGACACGGCC
>Zotu4994
GAACGCTGGCGGCGTGCTTAACACATGCAAGTCGAACGAAGCACTTTACCGGATTTCTTCGGGATGAA
AGTTTTGTGACTGAGTGGCGGACGGGTGAGTAACGCGTGGGTAACCTGCCTCATACAGGGGGATAACA
GTTAGAAATGACTGCTAATACCGCATAAGACCACAGGATTGCATGATCCGGTGGTAAAAACTCCGGTG
GTATGAGATGGACCCGCGTCTGATTAGGTAGTTGGTGGGGTAACGGCTCACCAAGCCGACGATCAGTA
GCCGACCTGAGAGGGTGACCGGCCACATTGGGACTGAGACACGGCC
>Zotu4995
GAACGCTGGCGGCAGGCCTAACACATGCAAGTCGAACGGACCCTTCGGGGTTAGTGGCGGACGGGTGA
GTAACACGTGGGAACGTGCCTTTAGGTTCGGAATAGCTCCTGGAAACGGGTGGTAATGCCGAATGTGC
CCTTCGGGGGAAAGATTTATCGCCTTTAGAGCGGCCCGCGTCTGATTAGCTAGTTGGTGAGGTAAAGG
CTCACCAAGGCTACGATCAGTAGCTGGTCTGAGAGGATGACCAGCCACATTGGGACTGAGACACGGCC
>Zotu4997
GAACGCTGGCGGCGTGCCTAAGACATGCAAGTCGAGTGGGTTTAGACCCACGGCGAACGGGTGAGTAA
TACCTAGGAACTTTCTTTAAGTGGAGCATAACCTATAGAAATGTAGGATAATTCTCCATGTGGCAGTA
ATGCTAAAGTCGCAAGACGCTTAAAGTGAGGCCTGGGCACCATCAGCTAGTTGGTAAGGTAACGGCTT
ACCAAGGCTATGACGGTTAGGGGTCCCGAGAGGGATAACCCCCACACTGGAACTGCGACACGGTC

>Zotu4998
GAACGCTAGCGGCAGGCTTAATACATGCAAGTCGTACGGGATCCGAGGTAGCAATACTTTGGTGAGAG
TGGCGCACGGGTGCGTAACACGTATGCAACCTACCTTTAACCGGGACACAACCTCGAGAAATTGTGGC
TAATATCCCATAACACTGCGAAGTGGCATCACTTTGCAGTTAAAACTCCGGTGGTTATTGATGGGCAT
GCGCGACATTAGCTAGTTGGTGAGGTAACGGCCCACCAAGGCTACGATGTCTAGGGGTTCTGAGAGGA
TTATCCCCCACACTGGTACTGAGACACGGAC
>Zotu4999
GAACGCTGGCGGCAGGCTTAACACATGCAAGTCGAGCGCCCCGCAAGGGGAGCGGCAGACGGGTGAGT
AACGCGTGGGAATCTACCTATCTCTACGGAATAACCCAGGGAAACTTGGACTAATACCGTATACGCCC
TTCGGGGGAAAGATTTATCGGAGATGGATGAGCCCGCGTTGGATTAGCTAGTTGGTGGGGTAAAGGCC
TACCAAGGCGACGATCCATAGCTGGTCTGAGAGGATGATCAGCCACATTGGGACTGAGACACGGCC
>Zotu5002
GAACGCTGGCGGCGTGCTTAACACATGCAAGTCGAACGGTGACGAGCAGCTTGCTGCTCTGATCAGTG
GCGAACGGGTGAGTAACACGTGAGCAACCTACCCTTGACTCTGGGATAACAGCTGGAAACAGCTGCTA
ATACCGGATACGACCTCGACCGACATCTGTTGAGGTGGAAAGTTCTGGCGGTCAAGGATGGGCTCGCG
GCCTATCAGCTAGTTGGTGAGGTAACGGCTCACCAAGGCTTCGACGGGTAGCCGGCCTGAGAGGGCGA
CCGGCCACACTGGGACTGAGATACGGCC
>Zotu5003
GAACGCTGGCGGCGTGCTTAACACATGCAAGTCGAGCGGTAAGGCCCCTTCGGGGGTACACGAGCGGC
GAACGGGTGAGTAACACGTGAGCAACCTACCCATCACATCGGGATAACTCCGGGAAACCGGAGCTAAT
ACCGAATACGACCACTTCAGGCATCTGATGGTGGTGGAAAGTTCCGGCGGTGATGGATGGGCTCGCGG
CCTATCAGCTTGTTGGTGGGGTAACGGCCCACCAAGGCTTCGACGGGTAGCCGGCCTGAGAGGGTGAC
CGGCCACACTGGGACTGAGACACGGCC
>Zotu5004
GAACGCTAGCGGCAAGCTTAATACATGCAAGTCGAGGGGCAGCGCAGTGTAGCAATACATGGGCGGCG
ACCGGCAAACGGGTGCGGAACACGTACAGAACCTTCCTTCGAGCGGGGAATAGCCCAGAGAAATTTGG
ATTAATACCCCATAGTATATTGGAGTGGCATCACTTTAATATTAAAGATTTATCACTTGAAGATGGCT
GTGCGGCTGATTAGATAGTTGGCGGGGTAACGGCCCACCAAGTCTGCGATCAGTAACTGGTGTGAGAG
CACGACCAGTCACACGGGCACTGAGACACGGGC
>Zotu5005
GAACGCTAGCGGCAGGCCTAATACATGCAAGTCGAACGAGACCAGAGACTTCGGTTTCTGGTTTAGTG
GCGCACGGGTGCGTAACGCGTATGCAACCTACCTTCCACAGGGGGATAGCCCACCGAAAGGTGGATTA
ATACCGCATAAGACCACAGCCTGGCATCAGGCAGTGCTCAAAGATTTATCGGTGGAAGATGGGCATGC
GTGCCATTAGCTAGATGGTGTGGGTAACGGCCCACCATGGCGACGATGGCTAGGGGTTCTGAGAGGAT
GGTCCCCCACACTGGTACTGAGACACGGAC
>Zotu5006
GAACGCTGGCGGCAGGCTTAACACATGCAAGTCGAACGGGCACTTCGGTGCTAGTGGCAGACGGGTGA
GTAACGCGTGGGAACGTGCCTTTCGGTTCGGAATAATCCAGGGAAACTTGGACTAATACCGGATACGC
CCTTCGGGGGAAAGATTTATCGCCGAAAGATCGGCCCGCGTCTGATTAGCTAGTTGGTGAGGTAAAGG
CTCACCAAGGCGACGATCAGTAGCTGGTCTGAGAGGATGATCAGCCACATTGGGACTGAGACACGGCC
>Zotu5007
GAACGCTGGCGGCAGGCCTAACACATGCAAGTCGAACGAGGTCTTCGGACCTAGTGGCGGACGGGTGA
GTAACACGTGGGAACGTGCCCTTAGGTACGGAATAACTCAGGGAAACTTGAGCTAATACCGTATGTGC
CCTTCGGGGGAAAGATTTATCGCCTTTGGAGCGGCCCGCGTCTGATTAGCTTGTTGGTGAGGTAATGG
CTCACCAAGGCGACGATCAGTAGCTGGTCTGAGAGGATGACCAGCCACATTGGGACTGAGACACGGCC
>Zotu5008
GAACGCTGGCGGCATGCCTAAAACATGCAAGTCGAGCGGTAAGGCCCCTTCGGGGGTACACGAGCGGC
GGACGGGTGAGTAACGCGTGGGAATCTACCCCAAACTGAGGGATAAGCATTGGAAACGATGTCTAATA
CCGCATATGGTCTTCGGATTAAAGCTTCGGCGGTTTGGGAAGAGCCCGCGTAAGATTAGCTAGTTGGT
GAGGTAAAGGCTCACCAAGGCGACGATCTTTAACTGGTTTGAGAGAATGATCAGTCACACTGGAACTG
AGACACGGTC
>Zotu5009
GAACGCTGGCGGCGTGCCTAAGACATGCAAGTCGAGTGGGTTTAGACCCACGGCGAACGGGTGAGTAA
TACCTAGGAACTTTACTTTAGTCGGGCATAACCAGTCGAAAGATTGGACAATTCCCGATATTATCGCA

AGATGAAAGCAGCAATGCGCTAAAGTTGAGGCCTGGGCACCATCAGCTAGTTGGTAAGGTAATGGCTT
ACCAAGGCTATGACGGTTAGGGGTCCCGAGAGGGATAACCCCCACACTGGAACTGCGACGCGGTC
>Zotu5011
GAACGCTGGCGGCGTGCTTAACACATGCAAGTCGAACGGTGATGATGGAGCTTGCTCTGTCGGATCAG
TGGCGAACGGGTGAGTAACACGTGAGTAACCTGCCCTCAACTCTGGGATAACTTCCCGAAAGGGAAGC
TAATACTGGATATGAACTGCACGGGCATCTGTGTGGTTGTAAAGATTTTTTGGTTGAGGATGGACTCG
CGGCCTATCAGCTTGTTGGTGGGGTAATGGCCTACCAAGGCGACGACGGGTAGCCGGCCTGAGAGGGC
GACCGGCCACACTGGGACTGAGACACGGCC
>Zotu5012
GAACGCTAGCGGCAGGCCTAATACATGCAAGTCGAACGGGATTGCAGTGTAGCTTGCTATACTGCATG
AGAGTGGCGTACGGGTGCGTAACGCGTGAGCAACCTGCCCATATCTGGGGGATAGCCCGGCGAAAGTC
GGATTAACACCGCATAACAATGGATTACCGCATGGTTTTCCATTTAAATATTTATAGGATATGGATGG
GCTCGCGTGACATTAGCTAGTTGGTAAGGTAACGGCTTACCAAGGCGACGATGTCTAGGGGCTCTGAG
AGGAGAATCCCCCACACTGGTACTGAGACACGGAC
>Zotu5013
GAACGCTAGCGGGAGGCCTAACACATGCAAGCCGAGCGGTAGAAGTCCTTCGGGACTTTGAGAGCGGC
GTACGGGTGCGGAACACGTGTGCAACCTGCCTTTATCAGGGGGATAGCCTTTCGAAAGGAAGATTAAT
ACCCCATAATATAATGAACGGCATCGCTTATTATTGAAAACTCCGGTGGATAGAGATGGGCACGCGCA
AGATTAGATAGTTGGTAGGGTAACGGCCTACCAAGTCAGTGATCTTTAGGGGGCCTGAGAGGGTGATC
CCCCACACTGGTACTGAGACACGGAC
>Zotu5014
GAACGCTGGCGGCGCGCTTAACACATGCAAGTCGAGCGAGAACCTTTCCTTCGGGATTGGGGACAGCG
GCGAACGGGTGAGTAACACGTGGGTAATCTGCCCTTGACATCGGGATAGCCCGGGGAAACCCGGATTA
ATACCGAATAGCCTCCGGAGCCTTCGGGCGCCGGAGAAAAGGTAGCTTCGGCCTCTGGTCAAGGATGA
GCCCGCGGCGGATTAGCTTGTTGGCGGGGTAATGGCCCACCAAGGCGACGATCCGTAGCTGGTCTGAG
AGGACGATCAGCCACACTGGGACTGAGACACGGCC
>Zotu5015
GAACGCTGGCGGCGTGCTTAACACATGCAAGTCGAACGATGAAGCTCCAGCTTGCTGGGGTGGAAGAG
TGGCGAACGGGTGAGTAACACGTGAGTAACCTGCCCTGAACTCTGGGATAAGCGCTGGAAACGGTGTC
TAATACTGGATACGACCCATGGGGGCATCTTCTGTGGGTGGAAAGATTTATCGGTTTGGGATGGGCTC
GCGGCCTATCAGCTGGATGGTGGGGTAACGGCTCACCATGGCGACGACGGGTAGCCGGCCTGAGAGGG
TGACCGGCCACACTGGGACTGAGACACGGCC
>Zotu5018
GAACGCTGGCGGCAGGCTTAACACATGCAAGTCGAACGATGATTCTCTAGCTTGCTAGAGATGATTAG
TGGCGAACGGGTGAGTAACATTTAGGAATCTACCTTTTAGTGGGGGATAGCTCGGGGAAACTCGAATT
AATACCGCATACGACCTACGGGTGAAAGGGGGCGCAAGCTCTTGCTAGGAGATGAGCCTAAATCAGAT
TAGCTAGTTGGTAGGGTAAAGGCCTACCAAGGCGACGATCTGTAACTGGTCTGAGAGGATGATCAGTC
ACACCGGAACTGAGACACGGTC
>Zotu5019
GAACGCTGGCGGCGTGCCTAACACATGCAAGTCGAACGTGAACGGGGCAACCCTAGTAAAGTGGCGTA
CGGGTGAGTAACGCGTAGGTAATCTACCCCAGAGTTTGGAATAACCGGCCGAAAGGCTGGCTAATACT
GGATAGAGCGACGGAATCCGTAATTGGGTTTTTCGGGAAAGGGAGCTTGATAAGAGCCCTCGCTTTGG
GATGAGCCTGCGTCTCATTAGCTAGTTGGTGGGGTAATGGCCCACCAAGGCTTTGATGGGTAGCTGGT
CTGAGAGGATGATCAGCCACACTGGAACTGAAACACGGTC
>Zotu5021
GAACGCTAGCGGGATGCTTTACACATGCAAGTCGAACGGCAGCGCGAGAGAGCTTGCTCTCTTGGCGG
CGAGTGGCGGACGGGTGAGTAATATATCGGAACGTGCCCAGTAGCGGGGGATAACTACTCGAAAGAGT
GGCTAATACCGCATACGCCCTACGGGGGAAAGGGGGGGATCGCAAGACCTCTCACTATTGGAGCGGCC
GATATCGGATTAGCTAGTTGGTGGGGTAAAGGCTCACCAAGGCAACGATCTTTAGTTGGTCTGAGAGG
ATGATCAGCCACACTGGAACTGAGACACGGTC
>Zotu5022
CAACGCTGGCGGCGTGCCCAACACATGCAAGTCGAACGAGAAAGGGCTTTCGGGCCTGAGTAAAGTGG
CGCACGGGTGAGTAACACGTGACTAACCTACCCTTGAGCGGGGGATAACCTAGGGAAACCTGGGCTAA
TACCGCATAACACCTACGGGTCAAAGCGAGCAATCGCACTTAAGGAGGGGGTCGCGGCAGATTAGCTA

GTTGGTGAGGTAATGGCTCACCAAGGCGATGATCTGTATCCGGCCTGAGAGGGCGCACGGACACACTG
GAACTGAAACACGGTC
>Zotu5023
CAACGCTGGCGGCGTGCCTAACACATGCAAGTCGAACGCGAAAGTCCCGCAAGGGATCAGTAGAGTGG
CAAACGGGTGAGTAACACGTGGGCGACCTACCTTCGAGTGGGGGATAACCTTCCGAAAGGAGGGCTAA
TACCGCATGACATCCCATGTTTGGATACATGGACATCAAAGCCGGGGATCGCAAGACCTGGCGCTTGA
AGAGGGGCCTGCGCCCGATTAGCTAGTTGGTGGGGTCATGGCTCACCAAGGCTTCGATCGGTATCCGG
CCTGAGAGGGCGGACGGACACACTGGAACTGAGACACGGTC
>Zotu5025
GAACGCTGGCGGCGTGCCTAATACATGCAAGTCGAGCGAATTAATAGGAGCTTGCTCCTGTTAGTTAG
CGGCGGACGGGTGAGTAACACGTGGGCAACCTGCCTGTAAGACTGGGATAACACCGGGAAACCGGTGC
TAATACCGGATAATCCTTTTCCTCTCATGAGGAAAAGCTGAAAGTCGGTTTCGGCTGACACTTACAGA
TGGGCCCGCGGCGCATTAGCTAGTTGGTGAGGTAACGGCTCACCAAGGCGACGATGCGTAGCCGACCT
GAGAGGGTGATCGGCCACACTGGGACTGAGACACGGCC
>Zotu5026
GAACGCCGGCGGTGTGCCTAATACATGCAAGTCGAACGCGTTGGCCCAACTGATTGATTGTGCTTGCA
CAGGATTGACGTTGGTTTCCCAACGAGTGGCGGACGGGTGAGTAACACGTAGGCAACCTGCCCCGAAG
CGGGGGACAACATTTGGAAACAAGTGCTAATACCGCATAACAGCTTTGACCGCCTGGTCAAAGCTTAA
AAGATGGTTTCGGCTATCACTTCGGGATGGGCCTGCGGCGCATTAGCTTGTTGGTGGGGTAACGGCCT
ACCAAGGCGATGATGCGTAGCCGAGTTGAGAGACTGATCGGCCACAATGGGACTGAGACACGGCC
>Zotu5027
GAACGCTGGCGGCGCGCTTAACACATGCAAGTCGAGCGAGAACCAAGCCTTCGGGCTTGGGGACAGCG
GCGAACGGGTGAGTAACACGTGGGTAATCAACCCTCGACACTGGGATAGCCCGGGGAAACCCGGATTA
ATACCGGATACTCCATCTGGCTTTCGGGCCGGCTGGAAAAGGTAGCTTTGGCCTCCGGTCGAGGACGA
GCCCGCGGCGGATTAGCTTGTTGGTGGGGTAAAGGCCCACCAAGGCGACGATCCGTAGCTGGTCTGAG
AGGACGATCAGCCACACTGGGACTGAGACACGGCC
>Zotu5028
GAACGCTAGCGGCAGGCCTAATACATGCAAGTCGAACGCGATTGCGGTGCTTGCACCGCATGAGAGTG
GCGTACGGGTGCGTAACGCGTGAGCAACCTACCGTTGTCTGGGGGATAGCCCGGAGAAATCCGGATTA
ATACCGCATAATATTAGAGAGCAGCATTGCTTTCTGATCAAACATTTATGGGACAGCGATGGGCTCGC
GTGACATTAGCTAGTTGGAGTGGTAACGGCACACCAAGGCGACGATGTCTAGGGGATCTGAGAGGATG
ACCCCCCACACTGGGACTGAGACACGGCC
>Zotu5029
GAACGCTGGCGGCGTGCTTAACACATGCAAGTCGAGCGGTAAGGCTCCTTCGGGAGTACACGAGCGGC
GAACGGGTGAGTAACACGTGAGCAATCTGCCCTTCACTTTGGGATAACTTCCGGAAACGGAAGCTAAT
ACCGAATACGACCACTTCAGGCATCTGATGGTGGTGGAAAGTTCCGGCGGTGAAGGATGAGCTCGCGG
CCTATCAGCTTGTTGGTGAGGTAATGGCTCACCAAGGCTTCGACGGGTAGCCGGCCTGAGAGGGTGAC
CGGCCACACTGGGACTGAGACACGGCC
>Zotu5030
GAACGCTGGCGGCGCGCCTAATACATGCAAGTCGAGCGAGAAGACGTAGCAATACGTTTGTAAAGCGG
CGAACGGGTGAGGAATACATGGGTAACCTACCCTCGAGTGGGGAATAACTAGCCGAAAGGTTAGCTAA
TACCGCATACGATTCCCGGACTGCGGTTCGGGAAGGAAAGCGATACCGTGGGTATCGCGCTCTTGGAT
GGGCTCATGTCCTATCAGCTTGTTGGTGAGGTAATGGCTCACCAAGGCTTCGACGGGTAGCTGGTCTG
AGAGGACGATCAGCCACACTGGCACTGCGACACGGGC
>Zotu5032
GAACGCTGGCGGCAGGCTTAACACATGCAAGTCGAGCGCCCCGCAAGGGGAGCGGCAGACGGGTGAGT
AACGCGTGGGAATCTACCTAGCTCTACGGAATAGCTCCGGGAAACTGGAATTAATACCGTATACGTCC
TTCGGGAGAAAGATTTATCGGAGTTGGATGAGCCCGCGTTGGATTAGCTAGTTGGTGGGGTAATGGCC
TACCAAGGCGACGATCCATAGCTGGTCTGAGAGGATGATCAGCCACACTGGGACTGAGACACGGCC
>Zotu5033
GAACGCTAGCGGCAGGCTTAACACATGCAAGTCGAGGGGTATAGTTCTTCGGAATTAGAGACCGGCGC
ACGGGTGCGTAACGCGTATGCAATCTACCTTTCACAGAGGGATAGCCCAGAGAAATTTGGATTAATAC
CTCATAGTATAACGACTCGGCATCGAGATGTTATTAAAGTCACAACGGTGAAAGATGAGCATGCGTCC
CATTAGCTAGTTGGTAAGGTAACGGCTTACCAAGGCTATGATGGGTAGGGGTCCTGAGAGGGAGATCC

CCCACACTGGTACTGAGACACGGAC
>Zotu5034
GAACGCTGGCGGCGTGCTTCACACATGCAAGTCGAGCGAGGCCCTTTCTTCGGGAAGGTGCCCTAGCG
GCGAACGGGTGAGTAACACGTGGGCAACCTGCCCCTGACTCTGGGATAAGCCCGGGAAACCGGGTCTA
ATACCGGATACGAACTCCACAGGCATCTGCGGAGTTGGAAAGATTTATCGGTCAGGGATGGCCCGCG
GCCTATCAGCTTGTTGGTGGGGTAATGGCCCACCAAGGCGACGACGGGTAGCCGGCCTGAGAGGGCGA
CCGGCCACACTGGGACTGAGACACGGCC
>Zotu5035
GAACGTTGGCGGCGTGCCTAACACATGCAAGTCGAGCGAGAGAGTCCTTCGGGATGAGTAAAGCGGCG
AACGGGTGAGTAATATATAAGAATCTGCCTTTTAGTGAAGAATACCTTTGGGAAACTAAAGTTAATAC
TGCATACGTCCCTTCGGGGAGAAAGATGGCCTCTTTGCTGTCGCTAAAAGATGAGCTTATACTCTATT
AGTTAGTTGGTGGGGTAAAGGCCTACCAAGACTATGATGGATAGCCGGCCTGAGAGGGTGTACGGCCA
CATTGGAACTTAGATACGGTC
>Zotu5037
GAACGCTAGCGGCGGGCTTAATACATGCAAGTCAAGGGGTAGCAATACACCGGCGTACGGGTGCGTAA
CGCGTATGGAACCTGCCCTTATCTGGGGGATAACTCGGGGAAACTCGAGCTAATACCGCATAAGATAG
TAGAATGGCATCATTCAGCTATTAAAGATTTATTGGATAAGGATGGCCATGCGTAAGATTAGATAGTT
GGCGAGGTAACGGCTCACCAAGTCTACGATCTTTAGGGGCTCTGAGAGGAGAATCCCCCACACTGGCA
CTGAGATACGGGC
>Zotu5040
GAACGCTGGCGGCATGCTTAACACATGCAAGTCGAACGAGACCTTCGGGTCTAGTGGCGCACGGGTGC
GTAACGCGTGGGAATCTACCCTTTGCTACGGAATAACTCAGAGAAATTTGTGCTAATACCGTATAATG
TCGCAAGACCAAAGATTTATCGGCAGAGGACGAGCCCGCGTAAGATTAGCTTGTTGGTGAGGTAAAGG
CTCACCAAGGCGACGATCTTTAGCTGGTCTGAGAGGATGATCAGCCACACTGGGACTGAGACACGGCC
>Zotu5041
GAACGCTGGCGGCGCGCCTAATACATGCAAGTCGAGCGAGAAGACGTAGCAATACGTTTGTAAAGCGG
CGAACGGGTGAGGAATACATGGGTAACCTACCCTAGAGTGGGGAATAACTAGCCGAAAGGTTAGCTAA
TACCGCGTACGCTTCTTGGACTTCGGTTCAGGGAGGAAAGCGCTACCGAGGGTAGCGCGCTCTTGGAT
GGGCTCATGTCCTATCAGCTTGTTGGTGAGGTAATGGCTCACCAAGGCTTCGACGGGTAGCTGGTCTG
AGAGGACGATCAGCCACACTGGCACTGCGACACGGGC
>Zotu5043
GAACGCTGGCGGCGTGCTTAACACATGCAAGTCGAGCGGTAAGGCTCTTCGGAGTACACGAGCGGCGA
ACGGGTGAGTAACACGTGAGCAACCTGCCCATGACACTGGGATAACGATTGGAAACAGTCGCTAATAC
CGGATACGACCTTGGTGGACATCTACACGAGGTGGAAAGCTCCGGCGGTCATGGATGGGCTCGCGGCC
TATCAGCTTGTTGGTGAGGTAACGGCTCACCAAGGCGTCGACGGGTAGCCGGCCTGAGAGGGCGACCG
GCCACACTGGGACTGAGATACGGCC
>Zotu5044
GAACGCTGGCGGCATGCCTAACACATGCAAGTCGAACGAAGGCTTCGGCCTTAGTGGCGCACGGGTGC
GTAACGCGTGGGAATCTGCCCTGGGGTACGGAATAACTCAGAGAAATTTGAGCTAATACCGTATGATA
TCGCGAGATCAAAGATTTATCGCCCTGGGATGAGCCCGCGTTGGATTAGCTAGTTGGTGGGGTAAAGG
CTTACCAAGGCGACGATCCATAGCTGGTCTGAGAGGATGATCAGCCACACTGGGACTGAGACACGGCC
>Zotu5046
GAACGCTGGCGGCATGCCTAACACATGCAAGTCGAACGAGACCTTCGGGTCTAGTGGCGCACGGGTGC
GTAACGCGTGGGAACCTGCCTTTAGGTTCGGAATAACTCCCCGAAAGGGGTGCTAATACCGGATAATG
TCTTCGGACCAAAGATTTATCGCCTTTAGATGGGCCCGCGTTGGATTAGCTAGTTGGTGGGGTAAAGG
CCTACCAAGGCGACGATCCATAGCTGGTCTGAGAGGATGATCAGCCACACTGGGACTGAGACACGGCC
>Zotu5047
GAACGTTAGCGGCGCGCTTAACACATGCAAGTCGCACGAGAAAGGGCTTCGGCCCCGGTAAAGTGGCG
CACGGGTGAGTAACACGTGGATAACCTTCCCTCGGGCGGGGGATAACACTCCGAAAGGAGTGCTAATA
CCGCATAAGACCACGTCCTCGAAAGAGGATGAGGTAAAAGCCGGCCTCTATATATAAGCTGGTACCTG
AGGATGGGTCCACGGCCCATCAGGCAGTTGGCGGGGTAATGGCCCACCAAACCTAAGACGGGTAGCTG
GTCTGAGAGGATGATCAGCCACACTGGAACTGAGACACGGTC
>Zotu5048
GAACGCTGGCGGCGTGCTTAACACATGCAAGTCGAACGGTGATGGAGGAGCTTGCTCTTCTTGATCAG

TGGCGAACGGGTGAGTAACACGTGAGTAACCTGCCCTTGACTCTGGGATAACTCCGGGAAACCGGGGC
TAATACTGGATACGAAGCGCCTCGGCATCGGGTGCGCTTGGAAAGTTTTTCGGTCAAGGATGGACTCG
CGGCCTATCAGCTTGTTGGCGGGGTAACGGCCCACCAAGGCGTCGACGGGTAGCCGGCCTGAGAGGGC
GACCGGCCACACTGGGACTGAGACACGGCC
>Zotu5049
GAACGCTAGCGGCAGGCCTAATACATGCAAGTCGAACGGGTCGCAAGACCAGTGGCAAACGGGTGCGT
AACGCGTAAGCAACCTGCCTCATACTGGGGGATAGCCCGGCGAAAGCTGGGGTAAACCCGCACGGTCC
AGTGGTGTCACCTGGCATTTCTGGTAAACATTTATGGGTATGAGAGGGGCTTGCGTCTGATTAGCTAG
TTGGGGGGGTAACGGCCCACCAAGGCGATGATCAGTAGGGGTTCTGAGAGGATTGGCCCCCACATGGG
TACTGAGATACGGAC
>Zotu5050
GAACGCTGGCGGCATGCCTAACACATGCAAGTCGAACGAGACCTTCGGGTCTAGTGGCGCACGGGTGC
GTAACGCGTGGGAATCTGCCCTTGGGTTCGGAATAACAGTTAGAAATGACTGCTAATACCGGATGATG
TCTTCGGACCAAAGATTTATCGCCCAAGGATGAGCCCGCGTAGGATTAGGTAGTTGGTGAGGTAAAGG
CTCACCAAGCCGACGATCCTTAGCTGGTCTGAGAGGATGATCAGCCACACTGGGACTGAGACACGGCC
>Zotu5051
GAACGCTGGCGGCGTGGATGAGGCATGCAAGTCGAATGGGTTTAGGCCCATGGCAGACGAGGTAGGAA
CACGTAGGTACACACCCCGAAGTCACGAATAACCGGCCGAAAGGTCGGATAATACGTGATGGTCTCGC
AAGAGTAAAGATTTATCGCTTCGGGAATGGCCTGCGGGCTATCAGCTAGTTGGTAAGGTAACGGCTTA
CCAAGGCTACGACGGCTAGGGGAGCTGAGAGGCTGACCCCCACCGATGGAACTGCGACACGGTC
>Zotu5052
CCCGTGTCTCAGTGCCCGTGTGGCTGATCATGCTCTCACATCAGCTACTGATCGTAGACTTGGTGAGC
CGTTACCTCACCAACTATCTAATCAGCCGCACACCCATCATCAAGTGCCGAAGCTTTAATATTAATAA
GATGCCTTATCAAATATATTATGGGGTATTAATCCAAATTTCTCTGGGCTATTCCCCGCTTGACGGAA
GGTTGTGTACGTGTTCCGCACCCGTTTGCCGGTCGCCATCAAGTATTGCTACTCATGCTGCCCCACGA
CTTGCATGTATTAAGCCTGCCGCTAGCGTTCA
>Zotu5054
GAACGCTAGCGGGAGGCCTAATACATGCAAGTCGAGCGGTAGTATCCTTCGGGATATGAGAGCGGCGC
ACGGGTGAGTAACGCGTACATGACCTACCTTTAAGAACGGGATAGCCCTGGGAAACTGGGATTAATAC
CGTATGTGATAATAGATATAAGGATTTATTATTAAAGCTGAGGCGCTTAGAGATGGATGTGCGTCTGA
TTAGCTAGTTGGTGAGGTAACGGCTCACCAAGGCGACGATCAGTAGGGGGCGTGAGAGCGTGGTACTG
AGACACGGAC
>Zotu5055
GAATGCTGGCGGCGTGGATAAGTCATGCAAGTCAAGGGGGCCCGCAAGGGCAACCGGCAGACGAGGTA
GTAACACGTAGGTACGTCCCCCGAAGTCGGGCATAGCCAGCCTAAAGGTTGGGTAATTCCCGATAGTC
CCGCAAGGGTAAAGGTTTTTCGCTTCGGGAGCGGCCTGCGTAGTATCAGCTAGTTGGTAGGGTAACGG
CCTACCAAGGCTACGACGCTTAGGGGAGCTGAGAGGCTGACCCCCACCGATGGAACTGCGACACGGTC
>Zotu5056
GAACGCTGGCGGCAGGCCTAACACATGCAAGTCGAGCGGTAGCACAAGAGAGCTTGCTCTCTGGGTGA
CGAGCGGCGGACGGGTGAGTAATGTCTGGGAAACTGCCTGATGGAGGGGGATAACTACTGGAAACGGT
AGCTAATACCGCATAACGTCTTCGGACCAAAGTGGGGGACCTTCGGGCCTCACGCCATCAGATGTGCC
CAGATGGGATTAGCTAGTAGGTGGGGTAATGGCTCACCTAGGCGACGATCCCTAGCTGGTCTGAGAGG
ATGACCAGCCACACTGGAACTGAGACACGGTC
>Zotu5057
GAACGCTGGCGGCAGGCCTAACACATGCAAGTCGAGCGGATGACAGGAGCTTGCTCCTGAATTCAGCG
GCGGACGGGTGAGTAATGCCTAGGAATCTGCCTGGTAGTGGGGGACAACGTCTCGAAAGGGACGCTAA
TACCGCATACGTCCTACGGGAGAAAGTGGGGGATCTTCGGACCTCACGCTATCAGATGAGCCTAGGTC
GGATTAGCTAGTTGGTGAGGTAATGGCTCACCAAGGCGACGATCCGTAACTGGTCTGAGAGGATGATC
AGTCACACTGGAACTGAGACACGGTC
>Zotu5060
GAACGCTGGCGGCGTGCCTAATACATGCAAGTCGAGCGAATCAAAGGGAGCTTGCTCTCTGAGGTTAG
CGGCGGACGGGTGAGTAACACGTGGGCAACCTGCCTATAAGACTGGGATAACTTCGGGAAACCGGAGC
TAATACCGGATAATTCATATCTACTCATGTAGATATGCTGAAAGACGGTTTCGACTGTCACTTATAGA
TGGGCCCGCGGCGCATTAGCTAGTTGGTGAGGTAACGGCTCACCAAGGCAACGATGCGTAGCCGACCT

GAGAGGGTGATCGGCCACACTGGGACTGAGACACGGCC
>Zotu5061
GAACGCTAGCGGCAGGCTTAACACATGCAAGTCGAGGGGTATAGTAGCAATACTAGAGACCGGCGCAC
GGGTGCGTAACGCGTATGCAATCTACCTTTTACAGAGGGATAGCCCAGAGAAATTTGGATTAATACCT
CATAGTATAATGACTCGGCATCGAGATGTTATTAAAGTTACAACGGTAAAAGATGAGCATGCGTCCCA
TTAGCTAGTTGGTGTGGTAACGGCACACCAAGGCAACGATGGGTAGGGGTCCTGAGAGGGAGATCCCC
CACACTGGTACTGAGACACGGAC
>Zotu5063
GAACGCTGGCGGCGTGCCTAATACATGCAAGTCGAGCGAACAGACGAGAGTGCTTGCACTCTCTGACG
TTAGCGGCGGACGGGTGAGTAACACGTGGGTAACCTACCTATAAGACTGGGATAACTTCGGGAAACCG
GAGCTAATACCGGATAATATTTTCCACCACATGGTGGAATAGTGAAAGACGGTTCTGCTGTCACTTAT
AGATGGACCCGCGGTGTATTAGCTAGTTGGTGAGGTAACGGCTCACCAAGGCGACGATACATAGCCGA
CCTGAGAGGGTGATCGGCCACACTGGGACTGAGACACGGCC
>Zotu5064
GAACGCTGGCGGCGTGCCTAATACATGCAAGTCGAGCGAACTTTTGAGAGCTTGCTCTCAAAAGTTAG
CGGCGGACGGGTGAGTAACACGTGGGCAACCTACCTATAAGACTGGGATAACTTACGGAAACGTGAGC
TAATACCGGATAATTCTTTCTAACACATGTTAGGAAGTTGAAAGATGGCTTCTCGCTATCACTTATAG
ATGGGCCCGCGGCGCATTAGCTAGTTGGTGGGGTAATGGCCTACCAAGGCAACGATGCGTAGCCGACC
TGAGAGGGTGATCGGCCACACTGGGACTGAGACACGGCC
>Zotu5067
GAATGCTGGCGGCGTGGATAAGGCATGCAAGTCAAGGGGGCTCTTCGGAGCAACCGGCAGACGAGGTA
GTAACACGTACGAACGCACCCCATAGTCTGGTATAGCTCGTCGAAAGACGGGGTAATCCCGGATGGTC
CCGCAAGGGTAAAGATTTTTCGCTATGGGAGCGGCGTGCGTAATATCAGCTTGTTGGTGAGGTAATGG
CTCACCAAGGCTACGACGTTTAGGGGCGCTGAGAGGCGGACCCCCACCGAGGGAACTGAGACACGGTC
>Zotu5068
GAACGCTAGCGGCAGGCCTAACACATGCAAGTCGAGGGGTAGAGCTACTTTAGTAGTTTGAGACCGGC
GCACGGGTGCGTAACGCGTATGCAATCTACCTTATACAGAGGGATAGCCCAGAGAAATTTGGATTAAT
ACCTCATAGTATATTTGAGTGGCATCATTTAGATATTAAAGATTTATCGGTATAAGATGAGCATGCGT
CCCATTAGTTAGTTGGTGTGGTAACGGCATACCAAGACGATGATGGGTAGGGGTCCTGAGAGGGAGAT
CCCCCACACTGGTACTGAGACACGGAC
>Zotu5069
GAACGCTGGCGGCGTGCTTAACACATGCAAGTCGAACGGGGTCCTGGTTGGTGGCAACACCTTCCAGT
ACCTAGTGGCGAACGGGTGAGGAACACGTGAGCAACCCACCCCGAACTCCGGGATAACAGCGGGAAAC
CGCTGCTAATACCGGATACCTTCCTGCAGTCGCATGGCTGTACGAAGAAAGGGTTACTGGTTCGGGAC
GGGCTCGCGGCCTATCAGCTTGTTGGTGGGGTAACGGCCTACCAAGGCTTCGACGGGTAGCTGGTCTG
AGAGGATGATCAGCCACACTGGGACTGAGACACGGCC
>Zotu5070
GAACGCTAGCGGCAGGCCTAATACATGCAAGTCGAGGGGCATCAGGCTGTAGCAATACAGTGCTGGCG
ACCGGCAAACGGGTGCGAAACACGTACATAACTTTCCGATAACTGGGGCATAACATTAAGAAATTGGT
GCTAATTCCCCATAGCATTGTTGAGAGGCATCTCTTGGCAATTAAAGCTCAGGCGGTTATTGATAGAT
GTGCGGCTGATTATGGTAGTTGGTGAGGTAACGGCTCACCAAGCCTACGATCAGTAACTGGTGTGAGA
GCACGACCAGTCACACGGGCACTGAGACACGGGC
>Zotu5071
GAACGCTGGCGGCGTGCCTAATACATGCAAGTCGAGCGGATGACGAAAGAAGCTTGCTTCTTTCTGAT
TTAGCGGCGGACGGGTGAGTAACACGTGGGCAACCTGCCCTTTAGTTTGGGATAACTTCGGGAAACCG
GAGCTAATACCGAATAATACTTTTAGCTTCATGGCTTTAAGTTGAAAGACGGCATCTCGCTGTCGCTA
AAGGATGGGCCCGCGGTGCATTAGCTAGTTGGTGAGGTAACGGCTCACCAAGGCGACGATGCATAGCC
GACCTGAGAGGGTGATCGGCCACACTGGGACTGAGACACGGCC
>Zotu5072
GAACGCTGGCGGCGTGCCTAACACATGCAAGTCGAGCGATTTACTTCGGTAAAGAGCGGCGGACGGGT
GAGTAACGCGTGGGTAACCTGCCTCATACACATGGATAACATACCGAAAGGTATGCTAATACAAGATA
ATATAAGAGATTCGCATGGATTTCTTATCAAAGCTCCGGCGGTATGAGATGGACCCGCGTCTGATTAG
CTAGTTGGTAAGGTAACGGCTTACCAAGGCGACGATCAGTAGCCGACCTGAGAGGGTGATCGGCCACA
TTGGAACTGAGACACGGTC

>Zotu5073
GAACGCTAGCGGCAGGCTTAATACATGCAAGTCGTACGGGATCCGAGGTAGCAATACTTTGGTGAGAG
TGGCGCACGGGTGCGTAACACGTATGCAACCTACCTTTAACCGGGACACAACCTTGAGAAATTGAGGC
TAATATCCCATAACACTGCGAGATGGCATCATTTTGCAGTTAAAACTCCGGTGGTTATTGATGGGCAT
GCGCGACATTAGCTAGTTGGTGAGGTAACGGCCCACCAAGGCTACGATGTCTAGGGGTTCTGAGAGGA
TTATCCCCCACACTGGTACTGAGACACGGAC
>Zotu5074
GAACGCTGGCGGCATGCCTAACACATGCAAGTCGAACGGCAGCATGAATGGGGGTAACCCTATTTGAT
GGCGAGTGGCGGACGGGTGAGTAATATGTAGGAATCTACCTAGAGGAGGGGGATAACTTGGGGAAACC
CAAGCTAATACCACATAAGCCCTACGGGGGAAAGCAGGGGACCGCAAGGCCTTGCGCCTTATGATGAG
CCTACATCGGATTAGCTAGTTGGTGGGGTAAAGGCCTACCAAGGCGACGATCCGTAGCTGGTCTGAGA
GGATGATCAGCCACACTGGGACTGAGACACGGCC
>Zotu5075
GAACGCTGGCGGCATGCCTAACACATGCAAGTCGAACGAGACCTTCGGGTCTAGTGGCGCACGGGTGC
GTAACGCGTGGGAATCTGCCCTTCGGTTCGGAACAACAGTTAGAAATGACTGCTAATACCGGATGATG
ACGTAAGTCCAAAGATTTATCGCCGAAGGATGAGCCCGCGTAGGATTAGCTAGTTGGTAAGGTAAAAG
CTTACCAAGGCGACGATCCTTAGCTGGTCTGAGAGGATGATCAGCCACACTGGGACTGAGACACGGCC
>Zotu5077
GAACGCTGGCGGCAGGCTTAACACATGCAAGTCGAGCGGGCACTTCGGTGTCAGCGGCAGACGGGTGA
GTAACGCGTGGGAACGTGCCCTTCGGTTCGGAATAACTCAGGGAAACTTGAGCTAATACCGGATACGC
CCTTTTGGGGAAAGGTTTACTGCCGAAGGATCGGCCCGCGTCTGATTAGCTAGTTGGTGAGGTTACGG
CTCACCAAGGCGACGATCAGTAGCTGGTCTGAGAGGATGATCAGCCACACTGGGACTGAGACACGGCC
>Zotu5080
GAACGCTAGCGGCAGGCTTAACACATGCAAGTCGAGGGGTATAGTAGCAATACTAGAGACCGGCGCAC
GGGTGCGTAACGCGTATGCAATCTACCTTTTACAGAGGGATAGCCCAGAGAAATTTGGATTAATACCT
CATAGCATAGCAACTTCGCATGAAGTCACTATTAAAGTTCCAACGGTAAAAGATGAGCATGCGTCCTA
TTAGCTAGATGGTAAGGTAACGGCTTACCATGGCTACGATAGGTAGGGGTCCTGAGAGGGAGATCCCC
CACACTGGTACTGAGACACGGAC
>Zotu5081
CAACGCTGGCGGCGCGCCTAACACATGCAAGTCGAACGAGAAAGCAGGAGCAATCTTGTGAGTAAAGT
GGCGTACGGGTGAGTAACACGTGGATTATCTACCTCTTTGTGGGGGATAACCCTGGGAAACCGGGGCT
AATACCGCATAAGTCCGAGAGGAGAAAGCAGCAATGCGCAGAGAGAGGAGTCCGCGGCCGATTAGCTA
GTTGGTAGGGTAAAAGCCTACCAAGGCGACGATCGGTAGCCGGCCTGAGAGGGCACACGGCCACACTG
GCACTGAAACACGGGC
>Zotu5082
CAACGCTGGCGGCGTGCCTAACACATGCAAGTCGCACGAGAAAGTGGAGCAATCCATGAGTAAAGTGG
CGCACGGGTGAGTAACACGTGAATTATCTACCTCCGAGTGGGGAATAACTGAGAGAAATCTTAGCTAA
TACCGCATAACACTTACGAGTCAAAGCAGCAATGCGCTTGGAGAGGAGTTCGCGGCCGATTAGTTAGT
TGGCGGGGTAATGGCCCACCAAGACGATGATCGGTATCCGGCCTGAGAGGGCGCACGGACACACTGGA
ACTGAAACACGGTC
>Zotu5084
GAACGCTGGCGGCGTGCTTAACACATGCAAGTCGAACGAGGTCCATGAAGTAGCAATACTTCGGAAGA
CCTAGTGGCGAACGGGTGCGTAACACGTGAGGAACTTGTCCCGGTCTCTGGGATAACAGTTGGAAACG
ACTGCTAATACCGGATGACGTCGGAGCGTCGCATGGCGCGCTGACGAAAGGGTTACTGGATCGGGAGA
GCCTCGCGGCCTATCAGCTTGTTGGTGGGGTAACGGCCTACCAAGGCATCGACGGGTAGCTGGTCTGA
GAGGATGAGCAGCCACACTGGGACTGAGACACGGCC
>Zotu5085
GAACGCTAGCGGCAGGCCTAACACATGCAAGTCGAGGGGTAACAGGGTAGCAATATCGCTGACGACCG
GCGCACGGGTGAGTAACGCGTATGCAACCTTCCTTTAACTGGGGGATAGCCCGGAGAAATTCGGATTA
ATACCCCATAAGATCATAGAAAGACATCTTTTAGTGATTAAAGCTCCGGCGGTTAAAGATGGGCATGC
GTGCCATTAGCTAGTTGGTGAGGTAACGGCTCACCAAGGCTACGATGGTTAGGGGGTCTGAGAGGATG
GTCCCCCACACTGGTACTGAGACACGGAC
>Zotu5086
GAACGCTGGCGGCGTGCTTAACACATGCAAGTCGAACGGTGAAGGGACCAGCTTGCTGGTTCTGGATG

AGTGGCGAACGGGTGAGTAACACGTGAGTAACCTGCCCCCTTCTTCTGGATAACCGCATGAAAGTGTG
GCTAATACGGGATATTCTGGGTCTGTCGCATGATGGGCCCGGGAAAGATGCGCCTTTGGGTGTTTTTG
GTGGGGGATGGGCTCGCGGCCTATCAGCTTGTTGGTGGGGTGATGGCCTACCAAGGCGGTGACGGGTA
GCCGGCCTGAGAGGGTGGACGGTCACACTGGGACTGAGACACGGCC
>Zotu5087
GAACGCTGGCGGCGTGCTTAATACATGCAAGTCGAACGGACAATATTAGATAGCTTGCTATTTGATAT
TGTTAGTGGCGAACGGGTGAGTAATACATAGGTAATCTGCCCTGAAGAGCGGGATAGCTGTTGGAAAC
GACAATTAATACCGCATAGGTAATCGGGAGGCATCTTCTGATTATTAAAGATCCGTTTGGATCACTAC
AGGATGAGCTTATGGTGTATTAGCTAGTTGGCAGGGTAAAGGCCTACCAAGGCGATGATACATAGCCG
GCTTGAGAGGGTGAACGGCCACATTGGGACTGAGACACGGCC
>Zotu5088
GAACGCTAGCGGCAGGCTTAATACATGCAAGGCGAGGGGGCAGCAATGTCACCGTCGTACGGGTGCGT
AACGCGTATGCAACCTACCTATCACTGGGGGATAGCCCGGGGAAACCCGGATTAATACCGCATAATAC
ATATGGGTCACCTGATCTGTTTTGTTAAAGATTTATTGGTGATAGATGGGCATGCGTTCGATTAGCTA
GTTGGCAGGGTAACGGCCTACCAAGGCAACGATCGATAGGGGAGCTGAGAGGTTGATCCCCCACACGG
GCACTGAGATACGGGC
>Zotu5089
GAACGCTAGCGACAGGCCTAACACATGCAAGTCGAGGGGCAGCACATGAGTAGCAATACGATGGTGGC
GACCGGCGCACGGGTGAGTAACACGTATGCAACCTACCTGTAAGAGGGGAATAACCCGGAGAAATTCG
GACTAATACCGCATAATACCTTGAAGCTGCATGGCTTTGAGGTTAAACATTTATGGCTTACAGATGGG
CATGCGTATGATTAGCTAGTTGGTGAGGTAACGGCTCACCAAGGCAACGATCATTAGGGGTTCTGAGA
GGAAGGTCCCCCACACTGGTACTGAGACACGGAC
>Zotu5090
GAACGCTGGCGGCAGGCTTAACACATGCAAGTCGAGCGGGCCCTTCGGGGTCAGCGGCAGACGGGTGA
GTAACGCGTGGGAACGTGCCCTTCGGTTCGGAATAACTCAGGGAAACTTGAGCTAATACCGGATACGT
GCTTAGGCAGAAAGGTTTATCTGCCGAAGGATCGGCCCGCGTCTGATTAGCTAGTTGGTGAGGTTATG
GCTCACCAAGGCGACGATCAGTAGCTGGTCTGAGAGGATGATCAGCCACACTGGGACTGAGACACGGC
C
>Zotu5091
GAACGCTGGCGGCGCGCCTAACACATGCAAGTCGAACGGAGTCAAGAGGAGCTTACTTTTCTTGACTT
AGTGGCGAACGGGTGAGTAACGCGTGAGTAACCTGCCCTGGAGTGGGGGACAACAGTTGGAAACGACT
GCTAATACCGCATAAGCCCACGGATTCGCATGGATCTGCGGGAAAAGGATTTATTTGCTTCAGGATGG
ACTCGCGTCCAATTAGCTAGTTGGTGAGGTAACGGCCCACCAAGGCGACGATTGGTAGCCGGACTGAG
AGGTTGAACGGCCACATTGGGACTGAGACACGGCC
>Zotu5092
GAACGCTAGCGGCAGGCTTAATACATGCAAGTCGTACGGGATCCGAGGTAGCAATACTTTGGTGAGAG
TGGCGCACGGGTGCGTAACACGTATGCAACCTACCTTTAACCGGGACACAACCTCGAGAAATTGAGGC
TAATATCCCATAACACTGCGAAATGGCATCATTTTGCAGTTAAAACTCCGGTGGTTATAGATGGGCAT
GCGCGACATTAGCTAGTTGGTGAGGTAACGGCCCACCAAGGCTACGATGTCTAGGGGTTCTGAGAGGA
TTATCCCCCACACTGGTACTGAGACACGGAC
>Zotu5093
GAACGCTGGCGGCGTGCCTAAGACATGCAAGTCGTACGTGAAAGTAGCAATATGAGTAAAGTGGCGCA
CGGGTGAGTAATAACTAGGTAATGCGCCCATTCGCGGGGAATAACCAGGCGAAAGCTTGGCTAATGCC
GCATGAGTCGGGAGGTCTCCGGATCTTTTTGACAAAGCCGTAAGGCGCGAATGGAGTGGCCTAGTCCC
TATCAGCTAGTTGGTGAGGTAACGGCTCACCAAGGCTTCGACGGGTAGCTGGTTTGAGAGGACGATCA
GCCACACTGGTACTGGAACACGGAC
>Zotu5095
GAACGCTGGCGGCAGGCCTAACACATGCAAGTCGAGCGGTAGCACAGGGAGCTTGCTCCTGGGTGACG
AGCGGCGGACGGGTGAGTAATGTCTGGGAAACTGCCTGATGGAGGGGGATAACTACTGGAAACGGTAG
CTAATACCGCATAACGTCTACGGACCAAAGTGGGGGACCTTCGGGCCTCATGCCATCAGATGTGCCCA
GATGGGATTAGCTAGTAGGTGGGGTAATGGCTCACCTAGGCGACGATCCCTAGCTGGTCTGAGAGGAT
GACCAGCCACACTGGAACTGAGACACGGTC
>Zotu5096
GAACGCTAGCGGCAGGCCTAATACATGCAAGTCGAGGGGCAGCAGGATAGCAATATCGCTGGCGACCG

GCGCACGGGTGCGTAACACGTATACAATCTGCCTTTAACAGGGGGATAGCCTTTCGAAAGAGAGATTA
ATACCCCATAACACATGAAGTAGCATTACTTTGTGTTAAAAGCTCCGGCGGTTAAAGATGAGTATGCG
TCTGATTAGCTAGTTGGTGAGGTAACGGCTCACCAAGGCGACGATCAGTAGGGGATCTGAGAGGACTA
CCCCCCACACGGATACTGAGACACGGAT
>Zotu5098
GAACGCTGGCGGCGTGCTTAACACATGCAAGTCGAACGGAAAGGCCCTTCGGGGTACTCGAGTGGCGA
ACGGGTGAGTAACACGTGGGTGATCTGCCCTGCACTTTGGGATAAGCCTGGGAAACTGGGTCTAATAC
CGGATAGGACCACGCGCTTCATGGTGTGTGGTGGAAAGCTTTTGCGGTGTGGGATGGGCCCGCGGCCT
ATCAGCTTGTTGGTGGGGTAATGGCCTACCAAGGCGACGACGGGTAGCCGGCCTGAGAGGGTGACCGG
CCACACTGGGACTGAGATACGGCC
>Zotu5099
GAACGCTGGCGGCGTGCCTAATACATGCAAGTCGAACGAGAGCGACCGGTGCTTGCACTGGTCAATCT
AGTGGCGAACGGGTGAGTAACACGTGGGTAACCTGCCCATCAGAGGGGGATAACATTCGGAAACGGAT
GCTAAAACCGCATAGGTCTTTGAACCGCATGGTTTGAAGAGGAAAAGAGGCGCAAGCTTCTGCTGATG
GATGGACCCGCGGTGCATTAGCTAGTTGGTGAGGTAACGGCTCACCAAGGCCGTGATGCATAGCCGAC
CTGAGAGGGTGATCGGCCACATTGGGACTGAGACACGGCC
>Zotu5101
GAACGCTGGCGGCGTGCTTAACACATGCAAGTCGAGCGGAAAGGCTCCTTCGGGGGTACTCGAGCGGC
GAACGGGTGAGTAACACGTGAGTAATCTGCCCTCCACTTCGGGATAGCCACCGGAAACGGTGATTAAT
ACCGGATATGACTACTGCTCGCATGAGTTGGTGGTGGAAAGTTTTTCGGTGGGGGATGTGCTCGCGGC
CTATCAGCTTGTTGGTGAGGTAATGGCTCACCAAGGCTTCGACGGGTAGCCGGCCTGAGAGGGTGACC
GGTCACACTGGGACTGAGACACGGCC
>Zotu5103
GAACGCTGACAGAATGCTTAACACATGCAAGTCTACTTGAACTTCGGTTTGGGTGGCGGACGGGTGAG
TAACGCGTAAAGAACTTGCCTCACAGTTTGGGACAACATTTGGAAACGAATGCTAATACCGAATATTA
TGATCGAATGGCATCATTTGATTATGAAAGCTATATGCGCTGTGAGAGAGCTTTGCGTCCCATTAGCT
AGTTGGAGAGGTAACGGCTCACCAAGGCGATGATGGGTAGCCGGCCTGAGAGGGTGAACGGCCACAAG
GGGACTGAGACACGGCC
>Zotu5104
GAACGATGGCGAGATGCCTTACACATGCAAGTCGTACGGGAATTTTTAATTCAAGTGGCGGACGGGTG
AGTAACACGTAAGAATTTGCCTTTAGGAGGGGGACAACAACTGGAAACGGTTGCTAATACCCCATATG
CTTTCGAGTGAAATGGATTTTTCCGCCTAAAGAGAAGCTTGCGGCTGATTAGCTTGTTGGTGAGGTAA
TGGCTCACCAAGGCGACGATCAGTATCTGGTTTGAGAGGATGATCAGACACACTGGAACTGAGACACG
GTC
>Zotu5105
GAACGCTGGCGGCGTGCCTAACACATGCAAGTCGAGCGGAGAAGAGCATTTATGTTCTTTTCAGCGGC
GGACGGGTGAGTAACACGTGGGTAACCTGCCTCGAAATGGGGGATAGCCTTCCGAAAGGAAGATTAAT
ACCGCATAACCTCGGATCTCCGCATGGAGGACCGAGAAAAGGAGAAATCCGTTTCGAGATGGACCCGC
GGCGCATTAGCTAGTTGGTGAGGTAACGGCCCACCAAGGCGACGATGCGTAGCCGACCTGAGAGGGTG
ATCGGCCACATTGGAACTGAGACACGGTC
>Zotu5106
GAACGCTGGCGGCATGCCTTACACATGCAAGTCGAACGGCAGCATGACGTGTAGCAATACACGTTGAT
GGCGAGTGGCGAACGGGTGAGTAATATATCGGAACGTGCCCTGTAGCGGGGGATAACCTATCGAAAGA
TGGACTAATACCGCATACGCTCTACGGAGGAAAGCGGGGGTCGCAAGACCTCGCACTATAGGAGCGGC
CGATATCGGATTAGCTAGTTGGCGGGGTAAAAGCCCACCAAGGCGACGATCCGTAGCTGGTCTGAGAG
GATGATCAGCCACACTGGAACTGAGACACGGTC
>Zotu5107
GAACGCTGGCGGCATGCCTAACACATGCAAGTCGAACGAGATCTTCGGGTCTAGTGGCGCACGGGTGC
GTAACGCGTGGGAATCTGCCCTTTGGTTCGGAATAACAGTTGGAAACGACTGCTAATACCGGATGATG
ACGAAAGTCCAAAGATTTATCGCCAGAGGATGAGCCCGCGTAGGATTAGCTAGTTGGTGTGGTAAAGG
CGCACCAAGGCGACGATCCTTAGCTGGTCTGAGAGGATGATCAGCCACACTGGGACTGAGACACGGCC
>Zotu5108
TAACGCTAGCGGCAGGCCTAATACATGCAAGTCGAACGGTAACATGGGTAGCAATATCTGATGACGAG
TGGCGCACGGGTGCGTAACGCGTATGTAATCTACCTCTAACTGGGGGATAGCCTTCCGAAAGGAAGAT

TAATACCGCATAATATATTAAAGAGGCATCTCTTTGTTATCAAAACTCTGGTGGTTAGAGATGAACAT
GCGTGACATTAGCTAGTTGGTGAAGTAATAGCTCACCAAGGCTACGATGTCTAGGGGGTCTGAGAGGA
TGATCCCCCACACTGGTACTGAGACACGGAC
>Zotu5110
GAACGCTAGCGGCAGGCCTAATACATGCAAGTCGGACGGGATAGGGGAGCTTGCTTTCCTTGAGAGTG
GCGCACGGGTGCGTAACACGTATGTAACCTACCTTGACCTGGGGGATAGCCTCTCGAAAGAGAGATTA
AGACCGCATAACATCATTTGGCAGCATTGTCGGATGATCAAATATTTATAGGGACAAGATGGGCATGC
GGGACATTAGCTAGTTGGCGGGGTAACGGCCCACCAAGGCGACGATGTCTAGGGGATCTGAGAGGATG
GCCCCCCACACTGGTACTGAGACACGGAC
>Zotu5111
GAACGCTGGCGGCATGCCTAACACATGCAAGTCGAACGGAGATTATTTCGGTAATCTTAGTGGCGAAC
GGGTGAGTAACGCGTAGGCAACCTGCCCTTTAGTTGGGGACAACATCCCGAAAGGGGTGCTAATACCG
AATGTGATCGCTTTCTCGCATGAGAGAGCGATGAAAGATGGCCTCTATTTATAAGCTATCGCTAAAGG
ATGGGCCTGCGTCTGATTAGCTAGTTGGTGGGGTAACGGCCTACCAAGGCGATGATCAGTAGCCGGTC
TGAGAGGATGAACGGCCACATTGGGACTGAGACACGGCC
>Zotu5112
GAACGCTGGCGGCGTGCCTAACACATGCAAGTCGAACGGAGATTACGGACGGAAGTTTTCGGATGGAA
GACTGTAATTCTTAGTGGCGGACGGGTGAGTAACGCGTGGGCAACCTGCCCTATACAGGGGGATAGCA
GCTGGAAATGGCTGGTAAGACCGCATAAGTCGGTTAAATCGCATGATTTGATCGGGAAATGAGCAATC
AGGTATAGGATGGGCCCGCGTCCGATTAGCCAGTTGGCAGGGTAAAAGCCTACCAAAGCGACGATCGG
TAGCCGGACTGAGAGGTCGGACGGCCACATTGGGACTGAGACACGGCC
>Zotu5113
GAACGCTGGCGGCGTGCATAACACATGCAAGTCGAACGATGAAACTTTTTTGATTTCTTCGGAATGAA
AAAAAGTGGATTAGTGGCGGACGGGTGAGTAACGCGTGAGTAACCTGCCTTAGAGAAGGGGATAGCCT
TTGGAAACGAAGAATAATACCCTATGAAACCATAACCTCGCATGAGGCCATGGTCAAAGATTTATCGC
TCTAAGATGGACTTGCGTCTGATTAGTTAGTTGGTGGGGTAACGGCCCACCAAGACAGCGATCAGTAG
CCGGCTTGAGAGAGTGTACGGCCACATTGGGACTGAGACACGGCC
>Zotu5114
GAACGCTGGCGGCGTGCCTAATACATGCAAGTCGAGCGAATCTTTAGGAGCTTGCTCCTGAAGGTTAG
CGGCGGACGGGTGAGTAACACGTGGGCAACCTGCCTGTAAGACTGGGATAACTTCGGGAAACCGGAGC
TAATACCGGATAATCCTTTTCCTCTCATGAGGGAAAGCTGAAAGACGGCGCAAGCTGTCACTTACAGA
TGGGCCCGCGGCGCATTAGCTAGTTGGTGAGGTAACGGCTCACCAAGGCGACGATGCGTAGCCGACCT
GAGAGGGTGATCGGCCACACTGGGACTGAGACACGGCC
>Zotu5115
GAACGCTGGCGGCGTGCCTAATACATGCAAGTCGAGCGAACTTGCGGGAGCTTGCTCCCAAAAGTTAG
CGGCGGACGGGTGAGTAACACGTGGGCAACCTGCCTATAAGACTGGGATAACTTCGGGAAACCGGAGC
TAATACCGGATAATTCTTTTCTACACATGTAGGAAAATTAAAAGACGGTTTACGCTGTCACTTATAGA
TGGGCCCGCGGCGCATTAGCTAGTTGGTGAGGTAACGGCTCACCAAGGCCACGATGCGTAGCCGACCT
GAGAGGGTGATCGGCCACACTGGGACTGAGACACGGCC
>Zotu5116
TAACGCTAGCGGCAGGCCTAATACATGCAAGTCGAGGGGCAGCGGGGTAGCAATACTGCCGGCGACCG
GCGCACGGGTGCGTAACACGTATGCAACCTACCTTTCATTGGGGAATAGCTCTGCGAAAGTAGAATTA
ATACCGCATAATATTGTTGTTCGGCATCGTTTGACAATTAAAACTGAGGTGATGAAAGATGGGCATGC
GTCTGATTAGCTAGTTGGCGAGGTAACGGCTCACCAAGGCGACGATCAGTAGGGGGTCTGAGAGGATG
ATCCCCCACACCGGTACTGAGACACGGAC
>Zotu5118
GAACGCTGGCGGCATGCCTTACACATGCAAGTCGAGCGGCAGCGCGGGGGCAACCCTGGCGGCGAGCG
GCGAACGGGTGAGTAATACATCGGAACGTGTCCAGTTGTGGGGGATAACCAGTCGAAAGACTGGCTAA
TACCGCATACGATCTACGGATGAAAGCGGGGGATCGCAAGACCTCGCGCTATTGGAGCGGCCGATGTC
CGATTAGCTAGTTGGCGGGGTAACGGCCCACCAAGGCGACGATCGGTAGCTGGTCTGAGAGGATGATC
AGCCACACTGGAACTGAGACACGGTC
>Zotu5119
GAACGCTAGCGGCAGGCTTAATACATGCAAGTCGTGGGGCAGCAGCTTTGTAGCAATACAGAGGCTGG
CGACCGGCAAACGGGTGCGGAACACGTACGCAACCTTCCTCTAACTGGAAGATAGCCCACCGAAAGGT

GGATTAATACTCCATAATATATTGAAGTGGCATCACTTTAATATTAAAGCTCCGGCGGTAAGAGATGG
GCGTGCGGCTGATTAGGTAGTTGGCGGGGTAACGGCCCACCAAGCCTACGATCAGTAGCTGATGTGAG
AGCATGATCAGCCACACGGGCACTGAGACACGGGC
>Zotu5122
GAACGCTGGCGGCGTGCCTAATACATGCAAGTCGAGCGAATCTTTGGGAGCTTGCTCCCGAAGATTAG
CGGCGGACGGGTGAGTAACACGTGGGCAACCTGCCTATAAGACTGGGATAACTTCGGGAAACCGGAGC
TAATACCGGATAATTCTTTTTCTCTCATGAGGAAAAGCTGAAAGACGGTTTACGCTGTCACTTATAGA
TGGGCCCGCGGCGCATTAGCTAGTTGGTGAGGTAACGGCTCACCAAGGCAACGATGCGTAGCCGACCT
GAGAGGGTGATCGGCCACACTGGGACTGAGACACGGCC
>Zotu5123
GAACGCTGGCGGCGTGCCTTAAGCATGCAAGTCGAACGGTAGTAATACAGTGGCGGACGGGTGAGTAA
CGCGTGGGAATCTGCCTCAAAGTGGAGTATAGCTTGCCGAAAGGTGAGGTAATCCTCCATGTGACCGC
AAGGTTAAAGTCGCAAGACGCTTTGAGATGAGCCCGCGTAAGATTAGCTAGTTGGTGGGGTAAAGGCC
TACCAAGGCAACGATCTTTAGCTGGTCTGAGAGGATGATCAGCCACAATGGAACTGAGACACGGTC
>Zotu5124
GAACGCTGGCGGCAGGCCTAACACATGCAAGTCGAGCGGATGATGGGAGCTTGCTCCCGGATTCAACG
GCGGACGGGTGAGTAATGCCTAGGAATCTGCCTAGTAGTGGGGGACAACGTTTCGAAAGGAACGCTAA
TACCGCATACGTCCTACGAGAGAAAGCAGGGGACCTTCGGGCCTTGCACTATCAGATGAGCCTAGGTC
GGATTAGCTAGTTGGTGGGGTAAAGGCCTACCAAGGCGACGATCCGTAACTGGTCTGAGAGGATGATC
AGTCACACTGGAACTGAGACACGGTC
>Zotu5125
GAACGCTGGCGGCGTGCTTAACACATGCAAGTCGAACGAAGCGCTTGGATGGAATTCTTCGGAAGGAA
GCTCAAGTGACTGAGTGGCGGACGGGTGAGTAACGCGTGGGTAACCTGCCTCATACAGGGGGATAACA
GTTAGAAATGACTGCTAATACCGCATAAGCACACGTGATCGCATGATCGAGTGTGAAAAACTCCGGTG
GTATGAGATGGACCCGCGTCTGATTAGCTAGTTGGTGGGGTAACGGCCCACCAAGGCGACGATCAGTA
GCCGGCCTGAGAGGGTGAACGGCCACATTGGGACTGAGACACGGCC
>Zotu5126
GAACGCTGGCGGCGTGCTTAACACATGCAAGTCGAACGATGAAGACCGGTGCTTGCACTGGTTGGATT
AGTGGCGAACGGGTGAGTAATACGTGAGTAACCTGCCTTTGACTCTGGGATAAGCCTTGGAAACGAGG
TCTAATACCGGATATGACACAGTTTCGCATGAAATCTGTGTGGAAAGGGTTTGTACTGGTTTTAGATG
GGCTCACGGCCTATCAGCTTGTTGGTGAGGTAATGGCTCACCAAGGCGACGACGGGTAGCCGGCCTGA
GAGGGTGACCGGCCACACTGGGACTGAGACACGGCC
>Zotu5127
GAACGCTGGCGGCGTGCCTAATACATGCAAGTCGAGCGCAGGAAATCGACGGAACCCTTCGGGGGGAA
GTCGACGGAATGAGCGGCGGACGGGTGAGTAACACGTAAAGAACCTGCCCTCAGGTCTGGGATAACCA
CGAGAAATCGGGGCTAATACCGGATGGGTCATCGGACCGCATGGTCCGAGGATGAAAGGCGCTTCGGC
GTCGCCTGGGGATGGCTTTGCGGTGCATTAGCTAGTTGGTGGGGTAATGGCCCACCAAGGCGACGATG
CATAGCCGACCTGAGAGGGTGATCGGCCACACTGGGACTGAGACACGGAC
>Zotu5128
GAACGCTAGCGGCAGGCTTAATACATGCAAGTCGTGGGGCAGCACAGAGTAGCAATACTTGGGTGGCG
ACCGGCAAACGGGTGCGGAACACGTACGCAACCTTCCTTCAGGCGGGGAATAGCCCAGAGAAATTTGG
ATTAATACCCCATAAGATTTTGAGATGGCATCATCTTATTATTAAAGATTTATCACCTGAAGATGGGC
GTGCGGCTGATTAGGTAGTTGGCGGGGTAACGGCCCACCAAGCCTACGATCAGTAGCTGATGTGAGAG
CATGATCAGCCACACGGGCACTGAGACACGGGC
>Zotu5129
GAACGCTAGCGGTGTGCTTTAAGCATGCAAGTAGAGCGAGTTCCGCAAGGGACGAGCTGCGAACGGCT
GAGTATAAAATAGGTATCTACCTCTTGGTGGTGAATACCCATCCGAAAGGATGGCTAATACAGCATAA
GATTCTAAATGAAAGCGTGTAACTACGCGCCGAGAGAGGAGCCTATTTCCCATCAGCTTGTTGGTAGG
GTAATGGCCTACCAAGGCTACGACGGGTAGGGGGCGTGAGAGCGTGACCCCCCAGAACGGGACTGAGA
CACGGCC
>Zotu5130
CAACGCTGGCGGCGTGCCTAACACATGCAAGTCGAGCGAGAGGAGGTAGCAATACCTTAGTAAAGCGG
CGCACGGGTGAGTAACACGTGGATAACCTGCCTCGAAGTGGGGAATAACCCCGGGAAACCGGAGCTAA
TACCGCATATTCTGACGAGATCTGCGGATTTTGTCAGGAAAGGAGCAATTCGCTTTGAGAGGGGTCCG

CGGCTGATTAGCTAGTTGGTGAGGTAATGGCTCACCAAGGCCACGATCAGTAGCCGGTCTGAGAGGAC
GGTCGGCCACACTGGAACTGAGACACGGTC
>Zotu5131
GAACGCTGGCGGCAGGCTTAACACATGCAAGTCGAGCGCCCCGCAAGGGGAGCGGCAGACGGGTGAGT
AACGCGTGGGAACATACCCTCCGGTTCGGAATAACTCAGGGAAACTTGAGCTAATACCGGATACGGTC
GAGAGGCGAAAGATTTATCGCCGGAGGATTGGCCCGCGTCCGATTAGCTAGTTGGTGTGGTAATGGCG
CACCAAGGCGACGATCGGTAGCTGGTCTGAGAGGATGATCAGCCACATTGGGACTGAGACACGGCC
>Zotu5133
GAACGCTGGCGGCATGCCTAACACATGCAAGTCGAACGAGATCTTCGGATCTAGTGGCGCACGGGTGC
GTAACGCGTGGGAATCTGCCCTGGGGTACGGAATAACTCAGAGAAATTTGAGCTAATACCGTATGATG
ACGTAAGTCCAAAGATTTATCGCCCTGGGATGAGCCCGCGTAGGATTAGCTAGTTGGTGGGGTAAAGG
CCTACCAAGGCGACGATCCTTAGCTGGTCTGAGAGGATGATCAGCCACACTGGGACTGAGACACGGCC
>Zotu5134
GAACGCTGGCGGCGTGCTTAACACATGCAAGTGGAGCGACGAACCGGGGCTTGCCCCGGGGCAGAGCC
GCGAACGGGTGAGTAACACGTGGGTAACCTGCCTTGATGATTGGGACAACCCGGGGAAACTCGGGCTA
ATACCAAATGTGCTCTCTCCCTCCAAGGGGATTGAGGAAAGGAAGCTTCGGCCTCCGCATCAAGATGG
GCCCGCGGCCCATTAGCTTGTTGGTGAGGTAACGGCTCACCAAGGCTTCGATGGGTAGCTGGTCTGAG
AGGACGATCAGCCACACTGGGACTGAGACACGGCC
>Zotu5135
GAACGCTGGCGGCAGGCTTAACACATGCAAGTCGAGCGGACCTTTCGGGGTCAGCGGCGGACGGGTGA
GTAACGCGTGGGAACGTGCCTTCCGGTTCGGAATAACCCTGGGAAACTAGGGCTAATACCGGATACGC
CCTTATGGGGAAAGGTTTACTGCCGGAAGATCGGCCCGCGTCTGATTAGCTAGTTGGTGGGGTAACGG
CCTACCAAGGCGACGATCAGTAGCTGGTCTGAGAGGATGATCAGCCACACTGGGACTGAGACACGGCC
>Zotu5136
GAACGCTGGCGGCATGCTTTACACATGCAAGTCGAACGGCAGCACGGGCTTCGGCCTGGTGGCGAGTG
GCGAACGGGTGAGTAATATATCGGAACGTACCCAAGAGTGGGGGATAACGTAGCGAAAGTTACGCTAA
TACCGCATACGATCTCAGGATGAAAGCAGGGGACCGCAAGGCCTTGTGCTCCTGGAGCGGCCGATATC
TGATTAGCTAGTTGGTAGGGTAAAGGCCTACCAAGGCGACGATCAGTAGCTGGTCTGAGAGGACGACC
AGCCACACTGGAACTGAGACACGGTC
>Zotu5137
GAACGCTGGCGGCGTGCCTAACACATGCAAGTCGAGCGATCTTCTTCGGAAGAGAGCGGCGGACGGGT
GAGTAACGCGTGGGTAACCTGCCCTGTACACACGGATAACATACCGAAAGGTATGCTAATACGAGATA
ATATGCTTTTATCGCATGGTAGAAGTATCAAAGCTTTTGCGGTACAGGATGGACCCGCGTCTGATTAG
CTAGTTGGTGAGGTAACGGCTCACCAAGGCGACGATCAGTAGCCGACCTGAGAGGGTGATCGGCCACA
TTGGAACTGAGACACGGTC
>Zotu5138
GAACGCTGGCGGCGTGCCTAATACATGCAAGTCGAGCGAATCTTTGGGAGCTTGCTCCCGAAGATTAG
CGGCGGACGGGTGAGTAACACGTGGGCAACCTGCCTATAAGACTGGGATAACTTCGGGAAACCGGAGC
TAATACCGGATAATTCTTTTCCTCTCATGAGGAAAAGCTGAAAGACGGTTTACGCTGTCACTTATAGA
TGGGCCCGCGGCGCATTAGCTAGTTGGTGAGGTAACGGCTCACCAAGGCAACGATGCGTAGCCGACCT
GAGAGGGTGAACGGCCACACTGGGACTGAGACACGGCC
>Zotu5139
GAACGCTGGCGGCAGGCCTAACACATGCAAGTCGAGCGCCCCGCAAGGGGAGCGGCAGACGGGAGAGT
AACACGTGGGAACGTACCCTTCAGTTCGGAACAACCCAGGGAAACTTGGGCTAATACCGAATACGTCC
GTAAGGAGAAAGATTTATCGCTGAAGGATCGGCCCGCGTCTGATTAGCTAGTTGGTGAGGTAACGGCT
CACCAAGGCGACGATCAGTAGCTGGTCTGAGAGGATGATCAGCCTCACTGGGACTGAGACACGGCC
>Zotu5140
GAACGCTAGCGGCAGGCTTAATACATGCAAGTCGAACGGTCTTAGCAATAAGACGAGTGGCACACGGG
TGAGTAACGCGTACATAACATACCTTTAACTGGCGAATAGCCTTGGGAAACTGAGAGTAATACGCCAT
AACATTGCGGTTCGGCATCGGACTGCAATTAAAGTTCCGGCGGTTAGAGATTGATGTGCGTTGGATTA
GGTAGTTGGTGAGGTAACGGCTCACCAAGCCAGCGATCCATAGGGGGCGTGAGAGCGTGGTCCCCCAC
ACGGGTACTGAGACACGGAC
>Zotu5141
GAATGCTGGCGGCGTGGATGAGTCATGCAAGCCGAACGGCCCGCAAGGGCAGTGGCAGACGAGGTAGT

AACACGTAGGTAGATACCCCAGAGTCGGGCATAGCCAGCCGAAAGGTTGGGTAATTCCCGATAGTCTT
GTGAAAGTAAAGGAGCAATCCGCTCTGGGAGTCGCCTGCGTAATATCAGCTAGTTGGTGAGGTAATGG
CTCACCAAGGCTACGACGTTTAGGGGAGCTGAGAGGCTGACCCCCACCGATGGGACTGAGATACGGCC
>Zotu5142
GAACGCTAGCGGCGTGCCTAACACATGCAAGTCGAACGTGAGAGGGTACTTGTATCCTTGAAAGTGGC
GGACGGGTGAGTAAGACGTAGGTGACCTGCCTCGGAGTGGGGGATAACCACTGGAAACGGTGGCTAAT
ACCGCATACGTTGGTGAAGATGGGAATCTTTATCAGGAAAGCTTAAGTGCTTTGAGAGGGGCCTACGG
CCGATTAGCTAGTTGGTGAGGTAAAGGCTCACCAAGGCGATGATCGGTAGCTGGTCTGAGAGGACGAT
CAGCCACACGGGAACTGAGACACGGTC
>Zotu5143
GAACGCTAGCGGCAGGCCTAATACATGCAAGTCGAGGGGCAGCACGGGTAGCAATACCTGGTGACGAC
CGGCAAACGGGTGCGGAACACGTACGCAACCTTCCCATAACTGGTGAATAGCCCTCCGAAAGGAGGAT
TAATACACCGTAACATAATGAAGTGGCATCACTTTATTATTATAGGCAGCAATGTCGGTTTTGGATGG
GCGTGCGCCTGATTAGGTAGTTGGCGGGGTAACGGCCCACCAAGCCTGCGATCAGTAACTGGTGTGAG
AGCACGACCAGTCACACGGGCACTGAGACACGGGC
>Zotu5144
GAACGCTGGCGGCATGCCTAACACATGCAAGTCGAACGAGGGTAGCAATACCTTAGTGGCGGACGGGT
GAGGAATACATAGGAATTTGCCCTTAAGTGGGGGATAGCCCGGGGAAACCCGGATTAATACCGCATGA
ACTCGTGAGAGCAAAGTCGGGGACCTTCGGGCCTGGCGCTTTTGGAGAAGCCTATGTCGGATTAGCTA
GTTGGTGAGGTAATGGCTCACCAAGGCTGCGATCCGTAACTGGTCTGAGAGGATGATCAGTCACACCG
GAACTGAGACACGGTC
>Zotu5145
GAACGCTGGCGGCGTGCCTAATACATGCAAGTCGAACGAGAAAGTGTAGCAATACATTAGTAAAGTGG
CAGACGGGTGAGTAATACGTAGATAATCTTCCTCCAAGATGGGGATAACCTTCCGAAAGGAGGGCTAA
TACCGAATGAGACCACACTGACTATGGTCAGAGCGGTCAAAGGTGACCTCTATTTATAAGTTATCGCT
TGGAGATGAGTTTATGCACCATCAGGTAGTTGGTAAGGTAATGGCTTACCAAGCCTTTGACGGTTAGC
TGATCTGAGAGGTTGATCAGCCACACTGGAACTGAGACACGGTC
>Zotu5146
GAACGCTGGCGGCGTGCCTAATACATGCAAGTCGAGCGAATCAATGGGAGCTTGCTCCCTGAGATTAG
CGGCGGACGGGTGAGTAACACGTGGATAACCTGCCTGTAAGATTGGGATAACTCCGGGAAACCGGAGC
TAATACCGGATAACATTTTGAACTGCATGGTTCAAAATTGAAAGGTGGCTTTTGCTACCACTTACAGA
TGGATCCGCGGCGCATTAGCTAGTTGGTGAGGTAACGGCTCACCAAGGCAACGATGCGTAGCCGACCT
GAGAGGGTGATCGGCCACACTGGGACTGAGACACGGCC
>Zotu5147
GAACGCTGGCGGCAGGCTTAACACATGCAAGTCGAGCGGGCCCTTTCGGGGGTCAGCGGCAGACGGGT
GAGTAACACGTGGGAACGTACCCTTTGGTTCGGAATAACGCTGGGAAACTAGCGCTAATACCGGATAC
GCCCTTGGCCTTTGGCCCTGGGGAAAGGCTTGTCTGCCGAAGGATCGGCCCGCGTCTGATTAGCTAGT
TGGTGGGGTAATGGCCTACCAAGGCGACGATCAGTAGCTGGTCTGAGAGGATGATCAGCCACACTGGG
ACTGAGACACGGCC
>Zotu5148
GAACGCTGGCGGCAGGCTTAACACATGCAAGTCGAGCGGAGATGAGATGCTTGCACCTTATCTTAGCG
GCGGACGGGTGAGTAATGCTTAGGAATCTGCCTATTAGTGGGGGACAACATTCCGAAAGGAATGCTAA
TACCGCATACGTCCTACGGGAGAAAGCAGGGGATCTTCGGACCTTACGCTAATAGATGAGCCTAAGTC
GGATTAGCTAGTTGGTGGGGTAAAGGCCTACCAAGGCGACGATCTGTAGCGGGTCTGAGAGGATGATC
CGCCACACTGGGACTGAGACACGGCC
>Zotu5149
GAACGCTGGCGGCATGCTTAACACATGCAAGTTGAACGCAGAATAGGCCTTCGGGTCTATTGCTGAGT
AGCAAACGGGTGAGTAACGCGTGGGAATCTACCTTTATGTGGGGGATAACTGAGGGAAACTTCAGCTA
ATACCGCATACGCTCTACGGAGGAAAAGCTTCACTTTTCAGTAATGAGTAATTGTATTGCTCATTACT
GAGTAGTGAAGTTGCATAAAGAGGAGCCCGCGTCAGATTAGGTAGTTGGTGGGGTAAAGGCCTACCAA
GCCAATGATCTGTAGCTGGTCTGAGAGGATGATCAGCCACACTGGGACTGAGACACGGCC
>Zotu5151
GAACGCTGGCGGCATGCCTTACACATGCAAGTCGAACGGTAACGGGTTAAGCCGACGAGTGGCGAACG
GGTGAGTAATATATCGGAACGTGCCCAGTCGTGGGGGATAACGTAGAGAAATTTACGCTAATACCGCA

TACGATCTAAGGATGAAAGCGGGGGATCGCAAGACCTCGCGCGATTGGAGCGGCTGATATCAGATTAG
GTTGTTGGTGAGGTAAAAGCTCACCAAGCCGACGATCTGTAGCTGGTTTGAGAGAACGACCAGCCACA
CTGGGACTGAGACACGGCC
>Zotu5152
GAACGCTGGCGGCATGCTTAACACATGCAAGTCGCACGGGCACTTTCGGGTGTTAGTGGCGGACGGGT
GAGTATCGCGTAGGGATTTATCGTTGGGTGGGGGATAACCTTGGGAAACTGGGGCTAATACCGCATGA
CGCCTGGGGGCCAAAGCTACGGCGCCTGACGAGGAGCCTGCGTCCGATTAGGTAGTTGGTTGGGTAAT
GGCTGACCAAGCCTGCGATCGGTAGCTGGTCTGAGAGGATGATCAGCCACACTGGGACTGAGACACGG
CC
>Zotu5153
GAACGTTAGCGGCAGGCCTAACACATGCAAGTCGCACGAGAATTGGGCTTCGGCCCATAGTAAAGTGG
CGGACGGGTGAGTAACACGTGGATAATCTGCCCTTCGGCGGGGGACAACAGTCCGAAAGGATTGCTAA
TACCGCATTCGTCAACGAGGTCTTCGGACCTTGTTGGGAAAGATGGCCTCTTCATGAAAGCTATCACC
GAAGGATGAGTCCGCGTCCCATCAGCTAGTTGGCGGGGTAATGGCCCACCAAGGCTAAGACGGGTAGC
TGGTCTGAGAGGATGATCAGCCACACTGGAACTGAGACACGGTC
>Zotu5154
GAACGCTGGCGGCGTGCTTAACACATGCAAGTCGAACGATGAAGCTTCCTGCTTGCAGGAAGTGGATT
AGTGGCGAACGGGTGAGTATCACGTGAGCAACCTGCCCTTGACTCTGGGATAAGCCTGGGAAACTGGG
TCTAATACCGGATATAACCTCTTACCGCATGGTGAGTGGTGGAAAGCTTTTGCGGTCTTGGATGGGCT
CGCGGCCTATCAGCTTGACGGTGAGGTAGTGGCTCACCGTGGCGATGACGGGTAGCCGGCCTGAGAGG
GTGACCGGCCACACTGGGACTGAGACACGGCC
>Zotu5155
GAACGCTGGCGGCGTGCCTAATACATGCAAGTCGAGCGAACGGACAGGAGTGCTTGCACTCCTTGAAG
TTAGCGGCGGACGGGTGAGTAACACGTGGGTAACCTACCTGTAAGACTGGGATAACTTCGGGAAACCG
GAGCTAATACCGGATAATATTTTCCACCTCATGGTGGAATAGTGAAAGACGGTTTCGGCTGTCACTTA
CAGATGGACCCGCGGCGCATTAGCTAGTTGGTGAGGTAACGGCTCACCAAGGCGACGATGCGTAGCCG
ACCTGAGAGGGTGATCGGCCACACTGGGACTGAGACACGGCC
>Zotu5156
GAACGCTGGCGGCGTGCTTAACACATGCAAGTCGAGCGGTAAGGCTCCTTCGGGGGTACACGAGCGGC
GAACGGGTGAGTAACACGTGAGTAATCTGCCCATGGCTCTGGGATAAGCACTCGAAAGGGTGTCTAAT
ACCGGATATGACAATCCTCCGCATGGGGTGGTTGTGGAAAGTTTTTTCGGCTATGGATGTGCTCGCGG
CCTATCAGCTTGTTGGTGGGGTAATGGCCTACCAAGGCTTTGACGGGTAGCCGGCCTGAGAGGGTGAC
CGGTCACACTGGGACTGAGACACGGCC
>Zotu5157
GAACGCTGGCGGCGCGCCTAACACATGCAAGTCGAACGGACGATGAAAAGCTTGCTTTTCAAAGTTAG
TGGCGGACGGGTGAGTAACGCGTGGGTAACCTGCCTCATACAGGGGAATAGCAGCTGGAAACGGCTGA
TAAAACCGCATAAGCGCACAGTGCCGCATGGCACAGTGTGAAAAACTCCGGTGGTATGAGATGGACCC
GCGTCTGATTAGCTGGTTGGCGGGGTAACGGCCCACCAAGGCGACGATCAGTAGCCGGCCTGAGAGGG
TGATCGGCCACATTGGGACTGAGACACGGCC
>Zotu5158
GAACGCTGGCGGCGTGCCTAACACATGCAAGTCGAACGAAGCGATTTGGAGGAAGTTTTCGGATGGAA
TCTGAATTGACTGAGTGGCGGACGGGTGAGTAACGCGTGGGTAACCTGCCTCACACTGGGGGACAACA
GCTGGAAACGGCTGCTAATACCGCATAAGCGCACAGCTTCGCATGAAGCAGTGTGAAAAACTCCGGTG
GTGTGAGATGGACCCGCGTCTGATTAGCTAGTTGGTAAGGTAACGGCTTACCAAGGCGACGATCAGTA
GCCGACCTGAGAGGGTGACCGGCCACATTGGGACTGAGACACGGCC
>Zotu5161
GAACGCTGGCGGCAGGCTTAACACATGCAAGTCGAACGCCCCGCAAGGGGAGTGGCAGACGGGTGAGG
AACACGTGGGAACGTACCCTTCGGTTCGGAACAACCCTGGGAAACTAGGGCTAATACCGGATACGCGC
GAGAGCGGAAAGGTTTACCGCCGAAGGATCGGCCCGCGTCTGATTAGCTAGTTGGTGAGGTAACGGCT
CACCAAGGCGACGATCAGTAGCTGGTCTGAGAGGATGATCAGCCACATTGGGACTGAGACACGGCC
>Zotu5162
GAACGCTGGCGGCGTGCCTAATACATGCAAGTCGAGCGAAGTGGAGGAAGCTTGCTTCCAAAGCTTAG
CGGCGAACGGGTGAGTAACACGTGGGCAACCTGCCTGACAGACTGGGATAACTTGGGGAAACTCAAGC
TAATACCGGATAGTATCGCAAACTGCATGGTTTGCGATGGAAAGTCTGAACGACACTGTCAGATGGGC

CCGCGGCGCATTAGCTAGTTGGTGGGGTAATGGCCTACCAAGGCGACGATGCGTAGCCGACCTGAGAG
GGTGACCGGCCACACTGGGACTGAGACACGGCC
>Zotu5163
GAACGCTGGCGGCAGGCTTAACACATGCAAGTCGAGCGCCTCGCAAGAGGAGCGGCAGACGGGTGAGT
AACGCGTGGGGATGTGCCTGGTGGTACGGGACAACTCAGGGAAACTTGAGCTAATACCGCATAAGCCC
TTAGGGGGAAAGATTTATCGCCACCAGATCAACCCGCGTTGGATTAGCTAGTTGGTGAGGTAACGGCT
CACCAAGGCGACGATCCATAGCTGGTCTGAGAGGATGATCAGCCACACTGGGACTGAGACACGGCC
>Zotu5165
GAACGCTGGAAATGTGGATACCGGCATGCAAGTCAAGGGGCCGCAAGGCACCGGCGAACGGCTTAGTA
ACACGTAGATACATACCGTGAAGTGGGGCACAGCTCGTCGAAAGACGGGGTAATTTCCCATAGTCTCG
CAAGAGTAAACTCGCAAGAGGCTTCATGAATGGTCTGCGGTCTATCAGCTAGTTGGTGAGGTAATGGC
TCACCAAGGCTATGACGGATAGGGGGCGTGAGAGCGCGACCCCCAAGAATGGCACTGCGACACGGGC
>Zotu5168
TAACGCTGGCGGCGTGCCTAACACATGCAAGTCGAACGGACCTTTATTTTTGAGACTCCCCATTTCCT
TGGGGCGCCCCATAAATAATGGTTAGTGGCGGACGGGTGAGTAACACGTTGGTACCTGCCCTCTAGTG
GGGGATAATCCGCCGAAAGGCGGACTAATACCGCATGTGATCTACGGATCAAAGCAGCAATGCGCTAG
AGGAGGGGCCTGCGGCTGATTAGCTTGTTGGTAGGGTAATGGCCTACCAAGGCGACGATCAGTAGCTG
GATCGAGAGGTTGACCAGCCACACTGGGACTGAGATACGGCC
>Zotu5169
GAACGCTGGCGGCGTGCTTAACACATGCAAGTCGAACGGAAAGGCCTCTTCGGAGGTACTCGAGTGGC
GAACGGGTGAGTAACACGTGGGTGATCTGCCCTGCACTCTGGGATAAGCCTGGGAAACTGGGTCTAAT
ACCGGATAGGACCATGCTTTAGTGTGTGTGGTGGAAAGTTTTTTCGGTGTAGGATGAGCCCGCGGCCT
ATCAGCTTGTTGGTGGGGTAATGGCCTACCAAGGCGGCGACGGGTAGCCGGACTGAGAGGTTGGTCGG
CCACATTGGGACTGAGATACGGCC
>Zotu5170
GAACGCTGGCGGCGCGCCTAAAACATGCAAGTCGAGCGAGAAGCAGGACTTCGGTCTTGTGGAGAGCG
GCGAACGGGTGCGTAACACGTGAGCAACCTGCCCCGAAGACCGGGACAACTCGAGGAAACTCGGGCTA
ATACCGGATGTCCTCACCGGATCGCATGGTCTGGTGAAGAAATGGATTCCGCTTTGGGATGGGCTCGC
GGTCTATCAGCTTGTTGGTGGGGTAACGGCCTACCAAGGCGACGACGGATAGCTGGTCTGAGAGGACG
ATCAGCCACACTGGGACTGAGACACGGCC
>Zotu5171
GAACGCTGGCGGCGTGCCTAATACATGCAAGTCGAGCGAGTCCCTTCGGGGGCTAGCGGCGGACGGGT
GAGTAACACGTAGGCAACCTGCCCGTAAGATCGGGATAACATAGGGAAACTTATGCTAATACCGAATA
GAGTCTTTTCTCGCATGAGGAGAGACGGAAAGATGGCGCAAGCTATCACTTACGGATGGGCCTGCGGC
GCATTAGCTAGTTGGTGGGGTAACGGCCTACCAAGGCGACGATGCGTAGCCGACCTGAGAGGGTGACC
GGCCACACTGGGACTGAGACACGGCC
>Zotu5172
GAACGCTGGCGGCGTGCTTAACACATGCAAGTCGAACGGTGAAAGAGGAGCTTGCTCCTCTGGATCAG
TGGCGAACGGGTGAGTAACACGTGAGTAACCTGCCCCAGACTCTGGGATAAGCACTGGAAACGGTGTC
TAATACCGGATACGAGCTTCAGCCGCATGGCTAGGAGCTGGAAAGAATTTCGGTCTGGGATGGACTCG
CGGCCTATCAGCTAGTTGGTGAGGTAACGGCCCACCAAGGCGACGACGGGTAGCCGGCCTGAGAGGGT
GACCGGCCACACTGGGACTGAGACACGGCC
>Zotu5173
GAACGCTAGCGGGAGGCCTAATACATGCAAGTCGAGCGTCCAGCAATGGAAGCGGCAGACGGGTGCGT
AACGCGTATGAAACTTGCCCTACACAGGAGAATAGCCTTGGGAAACTGAGAGTAATACTCCATAGCAT
TATAGAGTCGCATGATTTTATAATTAAAGCTCCGGCGGTGTAGGATAGTCGTGCGTCCGATTAGCTAG
TTGGCGGGGTAACGGCCCACCAAGGCGACGATCGGTAGCTGGTCTGAGAGGATGATCAGCCACACGGG
CACTGAGACACGGGC
>Zotu5174
GAACGCTGGCGGCGTGCTTAACACATGCAAGTCGAACGGGAAATACTTTATTGAAACTTCGGTGGATT
TAATTTATTTCTAGTGGCGGACGGGTGAGTAACGCGTGGGTAACCTGCCTTATACTGGGGGATAACAG
CCAGAAATGACTGCTAATACCGCATAAGCGCACAGAACCGCATGGTTCGGTGTGAAAAACTCCGGTGG
TATAAGATGGACCCGCGTTGGATTAGCTAGTTGGCAGGGCAGCGGCCTACCAAGGCGACGATCCATAG
CCGGCCTGAGAGGGTGAACGGCCACATTGGGACTGAGACACGGCC

>Zotu5176
GAACGCTGGCGGCGCGCCTAACACATGCAAGTCGAACGAGAGATGAGGAGCTTGCTCTTCAGATCGAG
TGGCGAACGGGTGAGTAACGCGTGAGGAACCTGCCTCAAAGAGGGGGACAACAGTTGGAAACGACTGC
TAATACCGCATAAGCCCACAGCTCGGCATCGAGCAGAGGGAAAAGGAGTGATCCGCTTTGAGATGGCC
TCGCGTCCGATTAGCTGGTTGGTGAGGTAACGGCCCACCAAGGCGACGATCGGTAGCCGGACTGAGAG
GTTGAACGGCCACATTGGGACTGAGACACGGCC
>Zotu5177
GAACGCTAGCGGCAGGCTTAATACATGCAAGTCGAGGGGCAGCGCGGGTAGCAATACCTGGCGGCGAC
CGGCAAACGGGTGCGGAACACGTACGCAACCTTCCCATAACAGGTGAATAGCCTTCCGAAAGGGAGAT
TAATACACCGTAACATAACGAAGTGGCATCACTTTGTTATTATAGGCTTCGGCCGGTTTTGGATGGGC
GTGCGGCTGATTAGGTAGTTGGCGGGGTAACGGCCCACCAAGCCTGCGATCAGTAACTGGTGTGAGAG
CACGACCAGTCACACGGGCACTGAGACACGGGC
>Zotu5178
GAACGCTGGCGGCATGCCTTACACATGCAAGTCGAACGGCAGCACGGGGGCAACCCTGGTGGCGAGTG
GCGAACGGGTGAGTAATACATCGGAACGTGCCCTGAAGTGGGGGATAACGTAGCGAAAGTTACGCTAA
TACCGCATATTCTGTGAGCAGGAAAGCAGGGGATCGCAAGACCTTGCGCTTTAGGAGCGGCCGATGTC
GGATTAGCTAGTTGGTGAGGTAAAGGCTCACCAAGGCGACGATCCGTAGCGGGTCTGAGAGGATGATC
CGCCACACTGGGACTGAGACACGGCC
>Zotu5179
GAACGCTAGCGGCAGGCCTAATACATGCAAGTCGAATGGTAACATGAGTAGCAATACTTGATGACGAG
TGGCGCACGGGTGCGTAACACGTATGCAATCTACCTTTAACCGGGGGATAGCTCCGAGAAATCGGAAT
TAATACCCCATAACACCTTGAAGTAGCATTACTTTAAGGTTAAAACTTCGGTGGTTAAAGATGGGCAT
GCGCGACATTAGCTAGTTGGTGAGGTAATGGCTCACCAAGGCTACGATGTCTAGGGGATCTGAGAGGA
TTAACCCCCACACTGGTACTGAGACACGGAC
>Zotu5180
GAACGCTGGCGGCAGGTCTTAGGCATGCAAGTCGAACGGGCGTAGCAATACGTCAGTGGCGCACGGGT
GAGTAACGCGTGGGAATATGCCCATTACTGGGGGATAACGTTTGGAAACGAACGCTAATACCGCATAA
GCCGGAAACGGGAAAGATTTATCGGTAATGGATTAGCCCGCGTTGGATTAGCTTGTTGGTGGGGTAAC
GGCCTACCAAGGCGATGATCCATAGCTGGTCTGAGAGGACGATCAGCCACACTGGAACTGAGACACGG
TC
>Zotu5182
GAACGCTGGCGGCGTGCCTAACACATGCAAGTCGAGCGATCGTCTTCGGAAGAGAGCGGCGGACGGGT
GAGTAACGCGTGGGTAACCTGCCTCATACACATGGATAACATACCGAAAGGTATGCTAATACAAGATG
ATATAAGAGATTCGCATGGATTTCTTATCAAAGCTTTTGCGGTATGAGATGGACCCGCGTCTGATTAG
CTGGTTGGTAAGGTAATGGCTTACCAAGGCGACGATCAGTAGCCGACCTGAGAGGGTGATCGGCCACA
TTGGAACTGAGACACGGTC
>Zotu5183
AGACATTTGTTCTGCGCGCAGACGCATCGTCTACCAGTGTGGGAGCCGTGCTCATGCGGAATTATGAC
GGTGTTCTCCATCCAGTCGCCTACGCCAGCAGGAA
>Zotu5184
GAACGCTGGCGGCGTGCTTAACACATGCAAGTCGAACGGTAAGGCCCCTTCGGGGGTACACGAGTGGC
GAACGGGTGAGTAACACGTGAGCAACCTGCCCTTGACTCTGGGATAACAGTTGGAAACAGCTGCTAAT
ACCGGGTGTGACCACTTCCTGCATGGGATGGTGGTGGAAAGTTTTTCGGTCAAGGATGGGCTCGCGGC
CTATCAGCTTGTTGGTGAGGTAATGGCTCACCAAGGCTTCGACGGGTAGCCGGCCTGAGAGGGCGACC
GGCCACACTGGGACTGAGATACGGCC
>Zotu5185
GAACGCTAGCGGCAGGCTTAACACATGCAAGTCGAGGGGTATAGTAGCAATACTAGAGACCGGCGCAC
GGGTGCGTAACGCGTATGCAATCTACCTTTTACAGAGGGATAGCCCAGAGAAATTTGGATTAATACCT
TATAGTATATATGAATGGCATCATTTATATATTAAAGTTCCAACGGTAAAAGATGAGCATGCGTCCCA
TTAGCTAGATGGTAAGGTAACGGCTTACCATGGCTACGATGGGTAGGGGTCCTGAGAGGGAGATCCCC
CACACTGGTACTGAGACACGGAC
>Zotu5186
GAACGCTGGCGGCATGCTTAACACATGCAAGTCGAACGGTAACGCGGGGGCAACCCTGGCGACGAGTG
GCGGACGGGTGAGTAATACGTAGGAATCTGCCCATTAGTGGGGGACAACCCGGCGAAAGCCGGGCTAA

TACCGCATACGCTCTACGGAGGAAAACGGGGGATCGTAAGACCTCGTGCTAATGGATGAGCCTACGTC
GGATTAGCTAGTTGGTAGGGTAACGGCCTACCAAGGCGACGATCCGTAGCTGGTCTGAGAGGACGATC
AGCCACACTGGGACTGAGACACGGCC
>Zotu5187
GAACGCTGGCGGCATGCCTAACACATGCAAGTCGAACGAAGGCTTCGGCCTTAGTGGCGCACGGGTGC
GTAACGCGTGGGAATCTGCCCCTTGGTTCGGAATAACAGTTGGAAACGACTGCTAATACCGGATGACG
ACGTAAGTCCAAAGATTTATCGCCGAGGGATGAGCCCGCGTAGGATTAGCTAGTTGGTGTGGTAAAGG
CGCACCAAGGCGACGATCCTTAGCTGGTCTGAGAGGATGATCAGCCACACTGGGACTGAGACACGGCC
>Zotu5188
GAACGCTGGCGGCGTGCCTAATACATGCAAGTAGGACGCACAGTTTATACCGTAGCTTGCTACACCAT
AGACTGTGAGTTGCGAACGGGTGAGTAACGCGTAGGTAACCTGCCTATTAGAGGGGGATAACTATTGG
AAACGATAGCTAATACCGCATAACAGTATGTAACACATGTTAGATGCTTGAAAGATGCAATTGCATCG
CTAGTAGATGGACCTGCGTTGTATTAGCTAGTAGGTAGGGTAATGGCCTACCTAGGCGACGATACATA
GCCGACCTGAGAGGGTGATCGGCCACACTGGGACTGAGACACGGCC
>Zotu5189
GAACGCTGGCGGCAGGCCTAACACATGCAAGTCGAGCGCGCCTTCGGGTGAGCGGCGGACGGGTGAGT
AACACGTGGGAATATGCCTTTCGGTGCGGAATAGTCTTGGGAAACTGAGGGTAATACCGCATACGCCC
CCTGGGGGAAAGATTTATCGCCGAAAGATTAGCCCGCGTTGGATTAGGTAGTTGGTGGGGTAATGGCC
TACCAAGCCGACGATCCATAGCTGGTTTGAGAGGATGATCAGCCACACTGGGACTGAGACACGGCC
>Zotu5190
GAACGCTGGCGGCGTGCCTAATACATGCAAGTCGAGCGGATCTCGAGGAGCTTGCTCCTTGAGATTAG
CGGCGGACGGGTGAGTAACACGTGGGCAACCTGCCTGTAAGACTGGGATAACACCGGGAAACCGGTGC
TAATACCGGATAATTCTTTCTCTCACATGAGGGAAAGCTGAAAGTCGGCTTCGGCTGACACTTACAGA
TGGGCCCGCGGCGCATTAGCTAGTTGGTGAGGTAACGGCTCACCAAGGCGACGATGCGTAGCCGACCT
GAGAGGGTGATCGGCCACACTGGGACTGAGACACGGCC
>Zotu5191
GAACGCTGGCGGCATGCCTTACACATGCAAGTCGAACGGCAGCACGGGAGCAATCCTGGTGGCGAGTG
GCGAACGGGTGAGTAAAGTATCGGAACGTGCCCAGTCGTGGGGGATAACGTAGCGAAAGTTACGCTAA
TACCGCATACGATCTATGGATGAAAGCGGGGGATCGCAAGACCTCGCGCGATTGGAGCGGCCGATATC
AGATTAGGTAGTTGGTGGGGTAAAGGCTCACCAAGCCAACGATCTGTAGCTGGTCTGAGAGGACGACC
AGCCACACTGGAACTGAGACACGGTC
>Zotu5192
GAACGCTGGAAATGTGGATACCGGCATGCAAGTCAAGGGGCCGCAAGGCACCGGCGAACGGCTTAGTA
ACACGTAGATACATACCGTAAAGTGGGGCATAGCTCGTCGAAAGACGGGGTAATTTCCCATGGTCCCG
CAAGGGTAAAGGCGCAAGCCGCTTTATGAATGGTCTGCGGTCTATCAGCTAGTTGGTGAGGTAATGGC
TCACCAAGGCTATGACGGATAGGGGGCGTGAGAGCGCGACCCCCAAGAATGGCACTGCGACACGGGC
>Zotu5194
GAACGTTGGCGGCGTGGATTAGGCATGCAAGTCGTACGGGCCGCAAGGCCAGTGGCGCAAGGGTGAGT
AAGGCGACGGTAACCAACCCCAGGACTGGGTATAGCCGCGGGAAACTGCGGGTAATCCCCAGCGACGT
CGCGGGGGGGCATCCCCCCGCGACCAAAGCTCCGGCGTCGTGGGACGGGCCGTCGTGGTATTAGGCTG
TTGGCGGGGTAACGGCCCACCAAGCCGGCGATGCCTACCGGGCGTGCGAGCGTGGCCCGGCACACTGG
GACTGAGACACTGCC
>Zotu5196
GAACGCTGGCGGCGTGCTTAACACATGCAAGTCGAACGGAAAGGCCTTGTGCTTGCACAAGGTACTCG
AGTGGCGAACGGGTGAGTAACACGTGGGTGATCTGCCCTGCACTGTGGGATAAGCCCGGGAAACTGGG
TCTAATACCATATAGGACCACTTCTTGGATGTTGTGGTGGAAAGCTTTTGCGGTGTGGGATGAGCCTG
CGGCCTATCAGCTTGTTGGTGGGGTAATGGCCTACCAAGGCGGCGACGGGTATCCGGCCTGAGAGGGT
GTACGGACACATTGGGACTGAGATACGGCC
>Zotu5197
GAACGCTGGCGGCGTGCCTAATACATGCAAGTCGAGCGAATGATGGAGAAGCTTGCTTCTCCTGATTT
AGCGGCGGACGGGTGAGTAACACGTGGGTAACCTACCCTATAGATTGGGATAACTCCGGGAAACCGGG
GCTAATACCGAATAATACATTCTTCTTCATGGGAGAGTGTTAAAAGATGGCTTCTCGCTATCACTATA
GGATGGGCCCGCGGCGCATTAGCTAGTTGGTGAGGTAACGGCTCACCAAGGCAACGATGCGTAGCCGA
CCTGAGAGGGTGATCGGCCACACTGGGACTGAGACACGGCC

>Zotu5198
GAACGCCGGCGGTGTGCCTAATACATGCAAGTCGAACGCGTTGGCCCAATTGATTGATGGTGCTTGCA
CCTGATTGATTTTGGTCGCCAACGAGTGGCGGACGGGTGAGTAACACGTAGGTAACCTGCCCAGAAGC
GGGGGACAACATTTGGAAACAGATGCTAATACCGCATAACAGCGTTGTTCGCATGAACAACGCTTAAA
AGATGGCTTCTCGCTATCACTTCTGGATGGACCTGCGGTGCATTAGCTTGTTGGTGGGGTAACGGCCT
ACCAAGGCGATGATGCATAGCCGAGTTGAGAGACTGATCGGCCACAATGGGACTGAGACACGGCC
>Zotu5199
GAACGCTGGCGGCGTGCCTAACACATGCAAGTCGAGCGCGAACGGCTCTTTCGGGAGTCTAGTAGAGC
GGCGCACGGGTGAGTAACACGTAGACAACCTGCCTTCCAGCCTGGGATAACACCGCGAAAGTGGTGCT
AATACCGGATAACAATGGGGCCCGCATGGGCCTTATTTGAAAGGCTTGCTACTGGGAGAGGGGTCTGC
GCGACATTAGCTAGTTGGTGAGGTAACGGCTCACCAAGGCTACGATGTCTAGCGGGTCTGAGAGGATG
ATCCGCCACACTGGAACTGAGACACGGTC
>Zotu5200
GAACGCTGGCGGCGCGCCTAACGCATGCAAGTCGAACGAGGCTGACAAGGTGCTTGCACCTTCGATGC
CTAGTGGCGAACGGGTGAGGAACACGTGAGAAACCTGCCCCAAACACGGGAATAACCCGAGGAAACTC
TGGCTAATGCCCGATACCCTCCGCAGTTCGCATGAACAGCGAAGGAAAGATCAGTCGGTTTGGGAGGG
TCTCGCGTCCTATCAGCTAGTTGGTGAGGTAACGGCTCACCAAGGCGACGACGGGTAGCTGGTTTGAG
AGAACGATCAGCCACACTGGAACTGAGACACGGTC
>Zotu5201
GAACGCTGGCGGCGTGCCTAACACATGCAAGTCGAACGGAGATTTCAGTAATGAAATCTTAGTGGCGA
ACGGGTGAGTAACGCGTAGACAACCTACCTTCTAGATGGGGACAACACTGCGAAAGTGGTGCTAATAC
CGAATGTTGTATTAAGGGTGCATATCCTAAATACTAAAGGTGGCCTCTATTTATAAGCTACCACTAGA
AGATGGGTCTGCGTCTGATTAGCTAGTTGGTGAGGTAATGGCTCACCAAGGCGACGATCAGTAGCCGG
TCTGAGAGGATGAACGGCCACACTGGGACTGAGACACGGCC
>Zotu5202
GAACGCTAGCGGCAGGCCTAACACATGCAAGTCGAGGGGCAGCGGGTTTTAGCAATAAGATGCCGGCG
ACCGGCGCACGGGTGCGTAACGCGTATACAATTTGCCTTATACTGGGAGATAGCCCGGAGAAATTCGG
ATTAATATCCTATGATAATGTTGAATCGCATGGTTTGACATTTAAAGCTACGGCGGTATAAGATGAGT
ATGCGTCTGATTAGCTAGTTGGTGAGGTAATGGCTCACCAAGGCGACGATCAGTAGGGGGCCTGAGAG
GGTTATCCCCCACACTGGTACTGAGACACGGAC
>Zotu5203
GAACGCTGGCGGCGTGCTTAACACATGCAAGTCGAACGGTAAGGCCCCTTCGGGGGTACACGAGTGGC
GAACGGGTGAGTAACACGTGAGCAACCTACCCTTGACTCTGGAATAACAGCTGGAAACAGCTGCTAAT
ACCGGATATGACCTTCTGCGTCATCGCGGAAGGTGGAAAGCTCCGGCGGTCAAGGATGGGCTCGCGGC
CTATCAGCTAGTTGGTGAGGTAACGGCTCACCAAGGCTTCGACGGGTAGCCGGCCTGAGAGGGCGACC
GGCCACACTGGGACTGAGATACGGCC
>Zotu5204
GAACGCTGGCGGCGTGCCTAATACATGCAAGTCGAGCGAACAGATAAAGAGCTTGCTCCTTTGACGTT
AGCGGCGGACGGGTGAGTAACACGTGGGTAACCTACCTATAAGACTGGAATAACTCCGGGAAACCGGG
GCTAATGCCGGATAATACACAGAACCGCATGGTTCTGTGATGAAAGACGGTTTTGCTGTCACTTATAG
ATGGACCCGCGGCGTATTAGCTAGTTGGTAAGGTAACGGCTTACCAAGGCAACGATACGTAGCCGACC
TGAGAGGGTGATCGGCCACATTGGGACTGAGACACGGCC
>Zotu5205
GAACGCTGGCGGCAGGCCTAACACATGCAAGTCGAGCGCCCAGCAATGGGAGCGGCAGACGGGAGAGT
AACACGTGGGAACGTACCCTTCAGTTCGGAACAACCCAGGGAAACTTGGGCTAATACCGAATACGTCC
GTAAGGAGAAAGATTTATCGCTGAAGGATCGGCCCGCGTCTGATTAGCTAGTTGGTGAGGTAACGGCT
CACCAAGGCGACGATCAGTAGCTGGTCTGAGAGGATGATCAGCCTCACTGGGACTGAGACACGGCC
>Zotu5206
GAACGCTGGCGGCGTGCATAACACATTCAAGTCGAACGGTGCGAGGGAGCAATCCCTCAATCAGTGGC
GGACGGGTGAGTAACGCGTGAGCAACCTGCCCTTATGTGTGGGATAACGTTTGGAAACGGACGCTAAT
ACCGCATAATGCATCTGAATCGCATGTTTCGGATGCCAAAGATTTATTGCATAAGGATGGGCTCGCGT
CTGATTAGATAGTTGGTGGGGTAACGGCCTACCAAGTCTGCGATCAGTAGCCGGACTGAGAGGTTGAA
CGGCCACATTGGAACTGAGAAACGGTC
>Zotu5207

GCCAAGGACAGAGAGGGAACGGCCGGAGGAGTGGACACCAGCGTGGCCGAGTGGGAATGACGTGGCTA
GCCTTGCTGGGAGGCTGGGGGGAGGAGGG
>Zotu5209
GAACGCTGGCGGCGTGCTTCACACATGCAAGTCGAACGAGGTCCAATCAGTCGCAAGACTGGTGAAGA
CCGAGTGGCGAACGGGTGCGTAACACGTGAGGAACCTGTCCCGGTCTCCGGAATAACAAGTGGAAACA
CTTGCTAATACGGGATGACGTCGGAACGTCGCATGACGAACTGACGAAAGGGTTACCGGACCGGGAGG
GCCTCGCGGCCTATCAGCTCGTTGGTGGGGTAACGGCCCACCAAGGCTTCGACGGGTAGCTGGTCTGA
GAGGATGATCAGCCACACTGGGACTGAGACACGGCC
>Zotu5210
GAACGTTGATGGCGTGCCTCATACATGCAAGTCGAGCGCCCCGCAAGGGGAGCGGCGGACGGGTGAGT
AACACGTAAAGAATCTGCCCTGAAGCGGGGAATAACTATCCCAACGGATAGCTAATACCGCATAACGA
TGAAGGTGGCATCACCTTTTCTCAAAAGGCTTCGGCCACTTCAGGATGACTTTGCGTCCCATTAGCTT
GTTGGCGGTGTAACGGACCACCAAGGCTACGATGGGTATCCGGCCTGAGAGGGTGTACGGACACATTG
GGACTGAGATACGGCC
>Zotu5211
GAACGCTGGCGGCAGGCTTAACACATGCAAGTCGAGCGGATACTTCGGTATCAGCGGCAGACGGGTGA
GTAACGCGTGGGGATCTGCCTAGTGGTACGGAACAACACAGGGAAACTTGTGCTAATACCGTATAAGT
CCTTCGGGAGAAAGATTTATCGCCATTAGATGAACCCGCGTCTGATTAGCTAGTTGGTGAGGTAATGG
CTCACCAAGGCGACGATCAGTAGCTGGTCTGAGAGGATGATCAGCCACACTGGGACTGAGACACGGCC
>Zotu5212
GAACGCTGGCGGCAGGCCTAATACATGCAAGTCGAACGGCAGCACAGCAGAGCTTGCTCTGTGGGTGG
CGAGTGGCGGACGGGTGAGGAATACATGGGAATCTGCCCAGTCGTGGGGGATAACGTAGGGAAACTTA
CGCTAATACCGCATGCGCCCTTCGGGGGAAAGCCGGGGACCTTCGGGCCTGGCGCGATTGGATGAGCC
CATGTCGGATTAGCTAGTTGGTGGGGTAAAGGCCCACCAAGGCGACGATCCGTAGCTGGTCTGAGAGG
ATGATCAGCCACACTGGAACTGAGACACGGTC
>Zotu5213
GAACGCTGGCGGCGTGGCTAAGACATGCAAGTCGCACGGTGTAGCAATACACAGTGGCGAACGGGAGA
GTAATGAATCGCTAATGTACCCCGGACTCTGGGATAGCTATCCGAAAGGGTAGGTAATACCAGATGAG
ACCACGGCAGGGCATCCTGCTGTGCTCAAAGGTCCGCCGGTCTGGGAGCAGGCGATTTCGTATCAGCT
AGTTGGTGAGGTAACGGCTCACCAAGGCGACGACGCGTAGCCGGACTGAGAGGTTGGCCGGCCTCATT
GGGACTGAGACACTGCC
>Zotu5214
GAACGCTGGCGGCGCGCTTAAAACATGCAAGTCGAACGAAAAGCCCGGGCTTGCCCGGGTGGAAAGTG
GCGAACGGGTGCGTAACACGTGAGCAACCTGCCCCGAAGACTGGGATAACACCGGGAAACCGGTGCTA
ATACCGGATGCCTTCATCATGTCGCATGGCGAGATGAAGAAATGGATTCCGCTTCGGGATGGGCTCGC
GGCCTATCAGCTTGTTGGTGGGGTAATGGCCTACCAAGGCAACGACGGGTAGCTGGTCTGAGAGGATG
TCCAGCCACACTGGGACTGAGACACGGCC
>Zotu5215
GAACGCTGGCGGCGTGCTTAACACATGCAAGTCGAACGGAAAGGCCCGCTTGCGGGTACTCGAGTGGC
GAACGGGTGAGTAACACGTGGGCAACCTGCCCCCGGCTTTGGGATAACACAAAGAAATTTGTGCTAAT
ACCGAATATTGACAACTTGAGGCATCTTTTGTTGTTGAAAGTTTTTTCGGCTGGGGATGGGCCCGCGG
CCTATCAGCTTGTTGGTGGGGTAATGGCCTACCAAGGCGACGACGGGTAGCCGGCCTGAGAGGGCGAC
CGGCCACACTGGAACTGAGACACGGTC
>Zotu5216
GAACGCTGGCGGCGTGCTTAACACATGCAAGTCGAGCGGTAAGGCCCTTCGGGGTACACGAGCGGCGA
ACGGGTGAGTAACACGTGAGCAATCTGCCCTTCACTCTGGGATAACTCTCGGAAACGAGAGCTAATAC
CGGATACGACCACTTCAGACATCTGACGGTGGTGGAAAGTTCTGGCGGTGAAGGATGAGCTCGCGGCC
TATCAGCTTGTTGGTGAGGTAATGGCTCACCAAGGCTTCGACGGGTAGCCGGCCTGAGAGGGTGACCG
GCCACACTGGGACTGAGACACGGCC
>Zotu5218
GAACGCTGGCGGCGTGCTTAACACATGCAAGTCGAACGATGAAGCTCCAGCTTGCTGGAGTGGATTAG
TGGCGAACGGGTGAGTAACACGTGAGTAACCTGCCCTTGACTCTGGGATAAGCCTTGGAAACGAGGTC
TAATACCGGATAGGAGCACTCATCGCATGGTGGGTGTTGGAAAGAATTTCGGTCATGGATGGACTCGC
GGCCTATCAGCTTGTTGGTGAGGTAATGGCTCACCAAGGCGACGACGGGTAGCCGGCCTGAGAGGGTG

ACCGGCCACACTGGGACTGAGACACGGCC
>Zotu5220
GAACGCTGGCGGCATGCTTAACACATGCAAGTTGAACGGCAGCATGACTTAGCTTGCTAAGTTGATGG
CGAGTAGCGAACGGGTGAGTAACGCGTAGGAATATGCCTTAAAGAGGGGGACAACTTGGGGAAACTCA
AGCTAATACCGCATAATGTCTGAGGACGAAAGCTGGGGACCTTATGGCCTGGCGCTTTAAGATTAGCC
TGCGTCCGATTAGCTAGTTGGTAGGGTAAAGGCCTACCAAGGCGACGATCGGTAGCTGGTCTGAGAGG
ATGACCAGCCACACTGGAACTGAGACACGGTC
>Zotu5221
CAACGCTGGCGGCGTGCCTAATGCATGCAAGTCGTGCGGGCTGCCTTCGGGCAGTCACGCGGCGGACG
GGTGCGGAACACGTGGGCAACCTGCCCTGGGGCGGGGGATATTCCCCGGAAACGGGGAGTAATACCGC
ATACGCTCGATCCTCCGTGGGAGGGTTGAGGAAAGGCGAAAGCCACCCCTGGAGGGGCCTGCGGCCGA
TTAGCTAGACGGCGGGGTAACGGCCCACCGTGGCTGTGATCGGTAGCTGGTCTGAGAGGACGGCCAGC
CACACGGGGACTGAGACACGGCC
>Zotu5222
AAACGCTGGCGGCGTGCATGAGACATGCAAGTCGAACGAACGAGCTTGCTCGTTAGTGGCGGACGGCT
GAGTAACGCGTAGGTGACCTGCCCCGAAGTGGGGGATAACTGCCCGAAAGGGTGGCTAATACCGCATG
TGGCGATGTGTTCATTCACATCATCAAAGCCGCGAGGCGCTTCGGGAGGGGCCTGCGTCCGATTAGCT
CGTTGGTGGGGTAACGGCTCACCAAGGCGACGATCGGTAGGTGGTCTGAGAGGACGATCACCCAGACT
GGGACTGAGACACGGCC
>Zotu5223
GAACGCTGGCGGCAGGCTTAACACATGCAAGTCGAACGGGCACCTTCGGGTGTCAGTGGCAGACGGGT
GAGTAACACGTGGGAACGTACCCTTCGGTTCGGAATAACTCAGGGAAACTTGAGCTAATACCGGATAC
GCCCTTACGGGGAAAGGTTTACTGCCGAAGGATCGGCCCGCGTCTGATTAGCTAGTTGGTGGGGTAAT
GGCCTACCAAGGCGACGATCAGTAGCTGGTCTGAGAGGATGATCAGCCACACTGGGACTGAGACACGG
CC
>Zotu5224
GAACGCTGGCGGCGTGCCTAATACATGCAAGTCGAGCGAACAGATAAGGAGCTTGCTCCTTTGACGTT
AGCGGCGGACGGGTGAGTAACACGTGGGTAACCTACCTATAAGACTGGAATAACTCCGGGAAAACGGG
GCTAATGCCGGATAACATGTTGAACCGCATGGTTCTACAGAGAAAGGCGGCTTTGCTGTCACTTATAG
ATGGACCCGCGCCGTATTAGCTAGTTGGTGAGGTAATGGCTCACCAAGGCAACGATACGTAGCCGACT
TGAGAGGGTGATCGGCCACACTGGAACTGAGACACGGTC
>Zotu5225
GAACGCTGGCGGCGTGCCTAATACATGCAAGTCGAGCGAACGGACAGGAGTGCTTGCACTCCTTGAAG
TTAGCGGCGGACGGGTGAGTAACACGTGGGTAACCTACCTTTAAGACTGGGATAACTTCGGGAAACCG
GAGCTAATACCGGATAATATTTTCCACCTCATGGTGGAATAGTGAAAGACGGTTTCGGCTGTCACTTA
CAGATGGACCCGCGGCGCATTAGCTAGTTGGTGAGGTAACGGCTCACCAAGGCGACGATGCGTAGCCG
ACCTGAGAGGGTGATCGGCCACACTGGGACTGAGACACGGCC
>Zotu5226
GAACGCTAGCGGCAAGCTTAACACATGCAAGTCGAACGCATACCTTCGGGTATGAGTGGCGAACGGGT
GAGTAACACGTAGGTTACGTACCCTCTGGTGGGGAATAACTTGGGGAAACCTAAGCTAATACCGCATG
TGTCCTTCGGGATGAAAGCCTTCGGGCGCCAGAGGAGCGGCCTGCGGCCCATTATTTAATCCTTGTTG
GTGAGGTAACGGCTCACCAAGGGTACGATGGGTAGCTGGTCTGAGAGGATGATCAGCCACACTGGAAC
TGAGACACGGTC
>Zotu5229
TAACGCTAGCGGCAGGCCTAATACATGCAAGTCGAGGGGTAACAGGGTAGCAATACCGCTGACGACCG
GCGCACGGGTGAGTAACGCGTATGTAACCTACCTATAACTGGGGAATAGCCTTCCGAAAGGAAGATTA
ACACCGCATAATATCACGAGACGGCATCGTTTTGTGATCAAACCTCCGGGGGTTATAGATGGACATGC
GTAACATTAGCTAGTTGGTGAGGTAACGGCTCACCAAGGCGACGATGTTTAGGGGGTCTGAGAGGATG
ATCCCCCACACTGGTACTGAGACACGGAC
>Zotu5230
GAACGCTGGCGGCGTGCCTAATACATGCAAGTCGAGCGAACAGACGGGGAGCTTGCTCCCCCGACGTT
AGCGGCGGACGGGTGAGTAACACGTGGGTAACCTACCTATAAGACTGGAATAACTCCGGGAAACCGGG
GCTAATGCCGGATAACATGTTGGACCGCATGGTCCTACAGTGAAAGACGGTCTTGCTGTCACTTATAG
ATGGACCCGCGCCGTATTAGCTAGTTGGTAAGGTAACGGCTTACCAAGGCAACGATACGTAGCCGACC

TGAGAGGGTGATCGGCCACACTGGAACTGAGACACGGTC
>Zotu5231
GAACGCTGGCGGCGTGCTTAACACATGCAAGTCGAACGAGGATGTTGGCTTCGGCTGACATTCGAGTG
GCGGACGGGTGAGTAACGCGTGAGCAATCTGTCTCAGAGAGGGGGATAACAACTGGAAACAGTTGCTA
ATACCGCATAAGACCACGAAGGGGCATCCCTTTGAGGTAAAAGCAGCAATGCGCTTTGAGGTGAGCTC
GCGTCCCATTAGATAGTTGGTGAGGTAACGGCCCACCAAGTCAGCGATGGGTAGCCGACCTGAGAGGG
TGACCGGCCACATTGGGACTGAGACACGGCC
>Zotu5232
GAACGCTGGCGGAGTGCCTAATACATGCAAGTCGAGCGGCAGCGCAGAGTAGTTTACTACTCGGGCGG
CGAGCGGCGGACGGCTGAGTAACGCGTGGGAACGTGCCCTAAAGTGAGGAATAAGCTCCAGAAATGGA
GTCTAATGCCGCATATGCTCTTCGGAGTAAAGCTTTCGAGCGCTTTGGGAGCGGCCCGCGTCCGATTA
GATAGTTGGTGAGGTAAAGGCTCACCAAGTCTACGATCGGTAACTGGTCTGAGAGGATGATCAGTCAG
ACTGGAACTGAGACACGGTC
>Zotu5233
GAACGCTAGCGGCAGGCTTAATACATGCAAGTCAAGGGGGTAGCAATACAACCGGCGCACGGGTGAGT
AACGCGTACACAACATACCATATACTGGGGTATAGCTTTGGGAAACTGAAGGTAATCCCCCATAACAT
TGCGAGAGGGCATCTGATTGCAATTAAAGTTTCGACGGTATATGATTGGTGTGCGTTTGATTAGCTAG
TTGGTAGGGTAACGGCCTACCAAGGCGACGATCAATAGCTGGCGTGAGAGCGCGACCAGCCACACTGG
AACTGAGACACGGTC
>Zotu5235
GAACGCTGGCGGCGCGCTTAACACATGCAAGTCGAGCGATGAAATTTTGACAGATCCCTTCGGGGTGA
AGATAAAATGGATTAGCGGCGGACGGGTGAGTAACACGTGAGTAACCTGCCTTAGACATTGGGATAGC
CTCGGGAAACCGGGATTAATACCGAATAAAATCATAGGTGCACATGCACTAATGATCAAAACTCCGGT
GGTCTAAGATGGACTCGCGTCCCATTAGCTAGTTGGTAGGGTAACGGCCTACCAAGGCGACGATGGGT
AGCCGGCCTGAGAGGGTGAACGGCCACATTGGAACTGAGAAACGGTC
>Zotu5236
GAACGCTAGCGGCAGGCTTAATACATGCAAGTCGAACGGCAAGGATTCTTCGGAATCCCTAGAGTGGC
GCACGGGTGAGTAACACGTACACAACATGCCCTATATTGGGAGATAGCATCGGGAAACTGGTGGTAAT
ACCCCATAGTGTTTATGATAGGCATCTATTTTAAACTAAAGATTTATTGATATAGGATTGGTGTGCGT
CCCATTAGCTAGTTGGTAGGGTAACGGCCTACCAAGGCGACGATGGGTAGCTGGCGTGAGAGCGCGAC
CAGCCACACTGGAACTGAGACACGGTC
>Zotu5238
GAACGCTGGCGGCAGGCCTAACACATGCAAGTCGAGCGGACCCTTCGGGGTTAGCGGCGGACGGGTGA
GTAACGCGTGGGAATATGCCCTTCTCTACGGAATAGTCCTGGGAAACTGGGGGTAATACCGTATACGC
CTTTTGGGGGAAAGATTTATCGGGGAAGGATTAGCCCGCGTTGGATTAGGTAGTTGGTGGGGTAATGG
CTCACCAAGGCTACGATCCGTAGCTGGTCTGAGAGGACGACCAGCCACACCGGAACTGAGACACGGTC
>Zotu5239
GAACGCTGGTAACGTGGATACCGGCATGCAAGTCACGGGGGCCGCAAGGCAACCGGCGAACGGCTTAG
TAACACGTGGGTACATACCTTAAACTGGAGCATAGCTCGTCGAAAGACGGGGTAATTCTCCATAGTCC
CGCAAGGGTAAAGCAGCAATGCGGTTTAAGAATGGCCTGCGGCCTATCAGATAGTTGGCGAGGTAATA
GCTCACCAAGTCTATGACGGGTAGGGGGCGTGAGAGCGCGACCCCCAAGAATGGCACTGCGACACGGG
C
>Zotu5240
GAACGCTGGCGGCATGCTTAACACATGCAAGTCGAACGGCAGCACAAACTAGTTTACTAGTTGGGTGG
CGAGTGGCGAACGGGTGAGTAACGCGTAGGAACATGCCTTAAAGAGTGGGACAACCTGGGGAAACTCA
GGCTAATACCGCATAACCTCCGAGGAGAGAAGCTGGGGACCTTAGGGCCTGGCGCTTTAAGATTGGCC
TGCGTCTGATTAGCTAGTTGGTGGGGTAAGGGCCTACCAAGGCGACGATCAGTAGCTGGTCTGAGAGG
ATGACCAGCCACACTGGAACTGAGACACGGTC
>Zotu5241
GAACGCTGGCGGCGTGCCTAATACATGCAAGTCGAACGCATTTTCTTTCACCGTAGCTTGCTACACCG
AAAGAAAGTGAGTGGCGAACGGGTGAGTAACACGTGGGTAACCTGCCCATCAGCGGGGGATAGCACTT
GGAGACAGGTGCTAATACCGCATAATTCCATTTACCGCATGGTAGATGGATGAAAGGCGCTTTTGCGT
CACTGATGGATGGACCCGCGGTGCATTAGCTAGTTGGTGGGGTAACGGCCTACCAAGGCTGCGATGCA
TAGCCGACCTGAGAGGGTGATCGGCCACACTGGGACTGAGACACGGCC

>Zotu5242
GAACGCTGGCGGCGTGCCTAATACATGCAAGTCGAGCGGATCTTTTCCTTCGGGGAAAGGTTAGCGGC
GGACGGGTGAGTAACACGTGGGTAACCTGCCCATAAGACTGGGATAACATTCGGAAACGAATGCTAAT
ACCGGATACGCGAATTGGTCGCATGGCCGAATCGGGAAAGGCGGAGTAATCTGCCACTTATGGATGGA
CCCGCGGCGCATTAGCTAGTTGGTGAGGTAACGGCTCACCAAGGCGACGATGCGTAGCCGACCTGAGA
GGGTGATCGGCCACACTGGGACTGAGACACGGCC
>Zotu5244
CAACGCTGGCGGCGTGCCTAACACATGCAAGTCGCACGAGAAAGTGGAGCAATCCATGAGTAAAGTGG
CGTACGGGTGAGTAACACGTGACTAACCTACCCTGGAGTGGGGAATAACCTCGGGAAACCGGGGCTAA
TACCGCATAACACCTACGGGTCAAAGCCGAAAGGCGCTTTGGGAGGGGGTCGCGGCTGATTAGCTAGT
TGGTAGGGTAATGGCCTACCAAGGCGACGATCAGTATCCGGCCTGAGAGGGCGCACGGACACACTGGA
ACTGAAACACGGTC
>Zotu5245
GAACGCTGGCGGCGTGCCTAATACATGCAAGTCGAGCGGACGTTTTATTGGTGCTTGCACCGATAAAA
CGTTAGCGGCGGACGGGTGAGTAACACGTGGGCAACCTGCCCTGTAGATTGGGATAACTCCGGGAAAC
CGGGGCTAATACCGAATAATACTTTTTAACTCATGTTAGAAAGTTGAAAGATGGTTTACGCTATCACT
ACAGGATGGGCCCGCGGCGCATTAGCTAGTTGGTGAGGTAACGGCTCACCAAGGCGACGATGCGTAGC
CGACCTGAGAGGGTGATCGGCCACACTGGGACTGAGACACGGCC
>Zotu5246
GAACGCTAGCGGCAGGCCTAATACATGCAAGTCGAGGGGTATTGTAGCAATACGAGAGACCGGCGCAC
GGGTGCGTAACGCGTATGAAAGGTGTCCAAATCACTAGGATAGCCCTCGGAAACGGGGATTAATACTG
GATGGTCTGTAGGGGTGGCATCATCTTTACGGTAAAGCTCAGGCGGATTTGGGTCCTCATGCGTACGA
TTAGCTAGTTGGCGAGGTAACGGCTCCCCAAGGCGACGATCGTTAGGGGTCCTGAGAGGGAGATCCCC
CACACTGGTACTGAGACACGGGC
>Zotu5247
GAACGCTGGCGGCAGGCCTAACACATGCAAGTCGAACGCACCGCAAGGTGAGTGGCAGACGGGTGAGT
AACACGTGGGAACATGCCCTTCGGTTCGGAATAACTCAGGGAAACTTGAGCTAATACCGGATACGTCC
GCGAGGAGAAAGATTCATCGCCGAAGGATTGGCCCGCGTCTGATTAGCTAGTTGGTGAGGTAACGGCT
CACCAAGGCGACGATCAGTAGCTGGTCTGAGAGGATGATCAGCCACATTGGGACTGAGACACGGCC
>Zotu5248
GAACGCTGGCGGCGTGCTTAACACATGCAAGTCGAGTGCGCGCCCGTAGCAATACGGGTGGCGCACGG
CGCACGGGTGCGTAACACGTGGGCAATCTGCCCTCTGATGGGGAATAACCCGCCGAAAGGCGGGCTAA
TACCGCATAACATTCCGAGAACTCTGGTTTTTGGATTCAAAGGCGTAAGTCGTCAGAGGAGGAGCCCG
CGCACGATTAGCTAGTTGGCAGGGTAACGGCCTACCAAGGCTTTGATCGTTAGCTGGTCTGAGAGGAT
GGCCAGCCACACTGGAACTGAGACACGGTC
>Zotu5250
AAACGCTGGCGGCGTGCCTAATGCATGCAAGTCGAACGGGAGTGCTTTCGGGCATCGACCGTGGCGGA
CGGGTGAGGAACACGTAGCTAACCTGCCCAGGTATGGGGGATATGCGCTGGAAACGGCGTGCAATACC
GCATACGTTCGGGTCACGGGAGTGACTTGAGGAAAGCCCGTGAGGGTGTACCTGGAGGGGGCTGCGTC
CGATTAGCTAGTTGGTGTGGTAAGAGCGCACCAAGGCGATGATCGGTAGCTGGTCTGAGAGGACGATC
AGCCACACGGGGACTGAGACACGGCC
>Zotu5251
GAACGCTGGCGGCGTGCCTAATACATGCAAGTCGAACGAGAAAGTGTAGCAATACATTAGTAAAGTGG
CAGACGGGTGAGTAATACATAGGTAACCTCCCCTCGAGATGGGAATAACCTTCCGAAAGGAGGGCTAA
TACCAAATGAGACCACGGGAACTAAGGTTCCTGAGGTTAAAGATGACCTCTATTTATAAGTTATCGCT
TGAGGATGGGCTTATGCACCATCAGCTAGTTGGTAAGGTAACGGCTTACCAAGGCTTTGACGGTTAGC
TGATCTGAGATGTTGATCAGCCACACTGGAACTGAGACACGGTC
>Zotu5252
GAACGCTGGCGGCACGCTTAACACATGCAAGTCGAACGTGAAGCTCGGAGCTTGCTCCGGGTGGAAAG
TGGCGAACGGGTGAGTAACACGTGAGGAACCTGCCCCGAAGACTGGGATAACGCCGGGAAACCGACGC
TAATACTGGATGCCTTCTCCGGATCGCATGATCTGGCGAAGAAATGGATTCCGCTTCGGGATGGCCTC
GCGGCCTATCAGCTTGTTGGTGGGGTAATGGCCTACCAAGGCAACGACGGGTAGCTGGTCTGAGAGGA
TGTCCAGCCACACTGGGACTGAGACACGGCC
>Zotu5253

GAACGCTGGCGGCGTGCCTAATACATGCAAGTCGAGCGAATTTTTGGGAGCTTGCTCCTAAAAGTTAG
CGGCGGACGGGTGAGTAACACGTGGGCAACCTACCTATAAGACTGGGATAACTTCGGGAAACCGGAGC
TAATACCGGATAATTCATATTTACTCATGTAGATATGCTGAAAGACGGTTTACGCTGTCACTTATAGA
TGGGCCCGCGGCGCATTAGCTAGTTGGTGAGGTAACGGCTCACCAAGGCGACGATGCGTAGCCGACCT
GAGAGGGTGATCGGCCACACTGGGACTGAGACACGGCC
>Zotu5254
GAACGCTGGCGGCATGCCTAACACATGCAAGTCGAACGAGACCTTCGGGTCTAGTGGCGCACGGGTGC
GTAACGCGTGGGAATCTGCCCTTCGGTTCGGAATAACTCAGGGAAACTTGAGCTAATACCGAATGATG
TCTTCGGACCAAAGATTTATCGCCGAAGGATGAGCCCGCGTCGGATTAGCTAGTTGGTGGGGTAAAGG
CCCACCAAGGCGACGATCCGTAGCTGGTCTGAGAGGATGATCAGCCACACTGGGACTGAGACACGGCC
>Zotu5256
GAACGCTGGCGGCATGCCTAACACATGCAAGTCGAACGAAGTCTTCGGACTTAGTGGCGCACGGGTGA
GTAACGCGTGGGAACCTGCCCTTCGGTACGGAATAACGTCGGGAAACTGACGCTAATACCGTATATGT
CTCTGGATGAAAGATTTATCGCCGGAGGAGGGGCCCGCGTCTGATTAGCTAGTTGGTGAGGTAATGGC
TCACCAAGGCGACGATCAGTAGCTGGTCTGAGAGGATGATCAGCCACACTGGGACTGAGACACGGCC
>Zotu5259
GAACGCTGGCGGCATGCCTTACACATGCAAGTCGAACGGCAGCATAGGAGCTTGCTCCTGATGGCGAG
TGGCGAACGGGTGAGTAATATATCGGAACGTACCCAAGAGTGGGGGATAACGTAGCGAAAGTTACGCT
AATACCGCATACGATCTACGGATGAAAGTGGGGGATCGCAAGACCTCATGCTCCTGGAGCGGCCGATA
TCTGATTAGCTAGTTGGTGGGGTAAAAGCTCACCAAGGCGACGATCAGTAGCTGGTCTGAGAGGACGA
CCAGCCACACTGGGACTGAGACACGGCC
>Zotu5261
GAACGCTGGCGGCGTGCCTAATACATGCAAGTCGAGCGGACCTCTTCGGAGGTCAGCGGCGGACGGGT
GAGGAACACGTGGGTAATCTGCCTTTCAGACCGGAATAACGCCCGGAAACGGGCGCTAATGCCGGATA
CGCCCGCGAGGAGGCATCTTCTTGCGGGGAAAGGCCCGATTGGGCCGCTGAGAGAGGAGCCCGCGGCG
CATTAGCTGGTTGGCGGGGTAACGGCCCACCAAGGCGATGATGCGTAGCCGACCTGAGAGGGTGACCG
GCCACACTGGGACTGAGACACGGCC
>Zotu5263
GAACGCTGGCGGCGTGGATAAGGCATGCAAGTCGAGTGGGTTTAGGCCCACGGCAAACGGGTTAGTAA
TGCATAGGAATTTTCCCTGAAGTCGTGAATAATCTGGAGAAATCCAGGATAATACACGATGTGCTCTA
CGGAGTAAAGATTTATCGCTTCAGGAGAAGCCTATGTCCTATCAGCTAGTTGGTAGTGTAATGGACTA
CCAAGGCTATGACGGGTAGGGGGTGTGAGAGCACGGCCCCCAACATTGGGACTGAGAACTGCC
>Zotu5264
GAACGCTAGCGGCAGGCCTAATACATGCAAGTCGAACGATAGAAGAGGGCTTGCTCTCTTCAAAAGTG
GCGCACGGGTGCGTAACGCGTATGCAACCTACCTTAATCAGGGGGATAGCCCGAAGAAATTCGGATTA
ACACCGCATAAAATCACAGTACAGCATTGTACAATGATCAAATATTTATAGGATTAAGATGGGCATGC
GTGACATTAGTTAGTTGGCGGGGTAACGGCCCACCAAGACCACGATGTCTAGGGGATCTGAGAGGATG
ACCCCCCACACTGGTACTGAGACACGGAC
>Zotu5265
GAACGCTGGCGGCGTGCTTAACACATGCAAGTCGAACGAAGCACTTTTTATAAAGTTTTCGGATGGAG
TAAAAAGTGACTGAGTGGCGGACGGGTGAGTAACGCGTGGGTAACCTGCCTTATACAGGGGGATAACA
GTTGGAAACGACTGCTAATACCGCATAAGCGCACAATGCTGCATGGCATCGTGTGAAAAGATTTATCG
GTATAAGATGGACCCGCGTCTGATTAGCTGGTTGGTGAGGTAACGGCCCACCAAGGCGACGATCAGTA
GCCGGCTTGAGAGAGTGAACGGCCACATTGGGACTGAGACACGGCC
>Zotu5266
GAACGCTGGCGGCATGCCTAACACATGCAAGTCGAACGAGACCTTCGGGTCTAGTGGCGCACGGGTGC
GTAACGCGTGGGAATCTGCCCCTCGGTTCGGAATAACTCAGGGAAACTTGAGCTAATACCGGATGATG
ACTTCGGTCCAAAGATTTATCGCCGAGGGATGAGCCCGCGTAGGATTAGCTAGTTGGTGGGGTAATGG
CCCACCAAGGCGACGATCCTTAGCTGGTCTGAGAGGATGATCAGCCACACTGGGACTGAGACACGGCC
>Zotu5268
GAACGCTGGCGGCGTGCTTAACACATGCAAGTCGAGCGGTAAGGCCCCTTCGGGGGTACACGAGCGGC
GAACGGGTGAGTAACACGTGAGTAATCTGCCCTTCACTTCGGGATAACCGCCGGAAACGGTGGCTAAT
ACCGGATACGACGCACTCTCGCATGAGATGTGCGTGGAAAGTCTTCTCGGTGAAGGATGTGCTCGCGG
CCTATCAGCTTGTTGGTGGGGTAACGGCCTACCAAGGCTTCGACGGGTAGCCGGCCTGAGAGGGTGAC

CGGCCACACTGGGACTGAGACACGGCC
>Zotu5269
GAACGCTGGCGGCATGCCTAACACATGCAAGTCGAACGAAGGCTTCGGCCTTAGTGGCGCACGGGTGC
GTAACGCGTGGGAATCTGCCCCTCGGTTCGGAATAACAGTTAGAAATGACTGCTAATACCGGATAATG
ACGAAAGTCCAAAGATTTATCGCCGAGGGATGAGCCCGCGTAGGATTAGGTAGTTGGTAGGGTAAAGG
CCTACCAAGCCGACGATCCTTAGCTGGTCTGAGAGGATGATCAGCCACACTGGGGCTGAGACACGGCC
>Zotu5270
GAACGCTGGCGGCGTGCCTAACACATGCAAGTCGAACGAGGTCCATGAGAGCTTGCTCTCGGACGACC
TAGTGGCGAACGGGTGAGTAACACGTGAGAAACCTGCCTCGGAGTGGGGAATAACTCGGGGAAACTCG
AGCTAATACCGCATACCTTCAGATGATCGCATGGTCGACTGAAGAAAGAATTTCGCTCTGAGAGGGTC
TCGCGGCCTATCAGCTAGTTGGTGGGGTAACGGCCTACCAAGGCAACGACGGGTAGCTGGATTGAGAG
ATCGATCAGCCACACTGGAACTGAGACACGGTC
>Zotu5271
GAACGCTGGCGGCAGGCCTAACACATGCAAGTCGAGCGAGGTTTTCGGACCTAGCGGCGGACGGGTGA
GTAATGCGTAGGAAGCTGCCCGATAGAGGGGGATACCAGTTGGAAACGACTGTTAATACCGCATAATG
TCTACGGACCAAAGTGTGGGACCTTCGGGCCACATGCTATCGGATGCGCCTACGTGGGATTAGCTAGT
TGGTGGGGTAATGGCTCACCAAGGCGACGATCCCTAGCTGGTTTGAGAGGATGATCAGCCACACTGGA
ACTGAGACACGGTC
>Zotu5272
GAACGCTGGCGGCGTGCCTAATACATGCAAGTCGAGCGAACTTGCGGGAGCTTGCTCCCAAAAGTTAG
CGGCGGACGGGTGAGTAACACGTGGGCAACCTACCTATAAGACTGGGATAACTTCGGGAAACCGGAGC
TAATACCGGATAATTCTTTTCTACACATGTGGAAAAGCTAAAAGACGGTTTACGCTGTCACTTATAGA
TGGGCCCGCGGCGCATTAGCTAGTTGGTGAGGTAACGGCTCACCAAGGCCACGATGCGTAGCCGACCT
GAGAGGGTGATCGGCCACACTGGGACTGAGACACGGCC
>Zotu5273
GAACGCTGGCGGCGTGCCTTAAGCATGCAAGTCGAACGGCTCCGCAAGGAGCAGTGGCGGACGGTCGA
GTAACGCGTGGGGATCTGCCTTAAAGTTGGACATAGCTCGTCGAAAGACGGGGTAATTTCCAATGTGA
ATGTAAGTTCAAAGCCGCAAGGCGCTTTAAGATGAACCCGCGTCCTATCAGCTAGTTGGTGAGGTAAT
GGCTCACCAAGGCGTCGACGGGTAGCTGGTCTGAGAGGATGATCAGCCGGAATAGCACTGAGACACGG
GC
>Zotu5274
GAACGCTGGCGGCGTGCCTAACACATGCAAGTCGAACGAGAAAGCCGCAAGGTGAGTAAAGTGGCGGA
CGGGTGAGTAACGCGTAGGTAATCTACCTTTTAGTGGGGAATAGCCATTGGAAACGATGGATAATACC
GCATAAGATTTATGGTTGCAAAACTGTAAAAGAAAGGGGGGTCGCAAGATCTTTCGCTAGAAGATGAG
CCTGCGTCCCATTAGCTAGTTGGTGGGGTAAGAGCCTACCAAGGCAACGATGGGTAGCTGGTCTGAGA
GGATGATCAGCCACACTGGAACTGACACACGGTC
>Zotu5276
GAACGCTGGCGGCATGCCTAATACATGCAAGTCGAACGCCAAATCTTCCTGCTTGCAGGAAGAGGCGG
AGTGGCGAACGGGTGAGTAACACGTGAGGAACTTGCCCATAAGAGGGGGATAACACCCGGAAACGGGT
GCTAATACCGCATAAGTTTTCCCAGTGCATGCTGGGAAAATGGAAGGTGGCTCTGCTACCGCTTATGG
ATAGCCTCGCGGCGCATTAGCTAGTTGGTAGGGTAAAGGCCTACCAAGGCCATGATGCGTAGCCGACT
TGAGAGGGTGATCGGCCACACTGGAACTGAGACACGGTC
>Zotu5277
GAACGCTGGCGGCGTGCTTAACACATGCAAGTCGAACGCTGAACTCCGCTTGCGGAGGGATGAGTGGC
GAACGGGTGAGTAACACGTGGGCAACCTGCCCCCGGCTCTGGGATAACTCCAAGAAATTGGGGCTAAT
ACCGGATGGTCACCGGCTACCGCATGGTGGTGGGTGGAAAGGATTTCCGGCTGGGGATGGGCCCGCGG
CCTATCAGCTTGTTGGTGGGGTAGTGGCCTACCAAGGCGACGACGGGTAGCCGGCCTGAGAGGGTGAC
CGGCCACACTGGGACTGAGACACGGCC
>Zotu5278
GAACGCTAGCGGCAGGCTTAATACATGCAAGACGAACGGTAAGTAGTGTAGCAATACATTGCCTAGAG
TGTCGTACGGGTGCGTAACACGTATGCAACCTACCTATAACAGGAGTATAGCTCGGGGAAACTCGAAT
TAACCCTCCATAAGATCGTGAAGTGGCATCACTTAGCGCTTAAAACTGAGGTGGTTATAGATGGGCAT
GCGTCTGATTAGCTAGTTGGCGGGGTAATGGCCCACCAAGGCGATGATCAGTAGGGGAACTGAGAGGT
TGATCCCCCACACTGGCACTGAGATACGGGC

>Zotu5279
GAACGCTGGCGGCGTGCTTAACACATGCAAGTCGAACGGTGAAAGAGGGAGCTTGCTCCTTCTGGATC
AGTGGCGAACGGGTGAGTAACACGTGAGTAACCTGCCCTTGACTCTGGGATAAGCGTTGGAAACGACG
TCTAATACCGGATACGACCTTCGGAGGCATCTCCTGAAGGTGGAAAGAATTTCGGTCAAGGATGGACT
CGCGGCCTATCAGCTAGTTGGTGAGGTAATGGCCCACCAAGGCGACGACGGGTAGCCGGCCTGAGAGG
GTGACCGGCCACACTGGGACTGAGACACGGCC
>Zotu5280
GAACGCTGGCGGCGTGCTTAACACATGCAAGTCGAGCGGTAAGGCCCCTTCGGGGGTACACGAGCGGC
GAACGGGTGAGTAACACGTGAGCAATCTGCCCATCACATCGGGATAACTCCGGGAAACCGAAGCTAAT
ACCGAATACGACCACTTCAGGCATCTGACGGTGGTGGAAAGTTCCGGCGGTGATGGATGAGCTCGCGG
CCTATCAGCTTGTTGGTGGGGTAACGGCCCACCAAGGCTTCGACGGGTAGCCGGCCTGAGAGGGTGAC
CGGCCACACTGGGACTGAGACACGGCC
>Zotu5281
CAACGCTGGCGGCGCGCTTAACACATGCAAGTCGAACGAGAAAGGGGAGCAATCCCTGAGTAAAGTGG
CGTACGGGTGAGTAACACGTGGGTAATCTACCTTTTGGTGGGGAATAACGCTGGGAAACCGGCGCTAA
CACCGCATAAGCTCGAAAGAGGAAAAGAGAAATCTGCCGAAAGAGGAGCCCGCGGCTGATTAGCTAGT
TGGTGGGGTAAAGGCTCACCAAGGCTTTGATCGGTAGCCGGCCTGAGAGGGCACACGGCCACACTGGC
ACTGAAACACGGGC
>Zotu5283
GAACGCTGGCGGCAGGCCTAACACATGCAAGTCGAGCGCCCCGCAAGGGGAGCGGCAGACGGGTGAGA
AACGCGTGGGAACATACCTTTTAGTACGGAATAGCCTAGGGAAACTTAGATTAATACCGTATACGCCC
TATGGGGGAAAGATTTATCGCTAAAAGATTGGCCCGCGTAAGATTAGCTAGTTGGTGAGGTAATGGCT
CACCAAGGCTACGATCTTTAGCTGGTTTGAGAGAATGATCAGCCACACTGGAACTGAGACACGGTC
>Zotu5285
GAACGCTAGCGGCAGGCCTAATACATGCAAGTCGAGGGGCAGCGGGAATAGCAATATTTGCCGGCGAC
CGGCGCACGGGTGCGTAACACGTATACAATCTGCCTATAACAGGGGGATAGCCCGGAGAAATCCGGAT
TAACCCCCCATAACAATAGAAGAAGCATTTCTTTTATTTGAAAACTACGGTGGTTATAGATGAGTATG
CGTATGATTAGCTAGTTGGTAGTGTAATGGACTACCAAGGCGACGATCATTAGGGGTACTGAGAGGTT
AAACCCCCACACGGATACTGAGACACGGAT
>Zotu5286
GAACGCTGGCAGCGTGCCTTAGGCATGCAAGTCGAACGGTAGTAATACAGTGGCAGACGGTCGAGTAA
CGCGTGGGAATCTACCTTAAAGTGGGGGATAGCTCTCCGAAAGGAGAGGTAATACCGCATGTGATCCG
GCGCAAGTCGGATCAAAGTCGCAAGACGCTTTAAGATGAGCCTGCGTCCTATCAGCTTGTTGGTGGGG
TAAAAGCCTACCAAGGCGACGACGGGTAGCTGGTCTGAGAGGATGGTCAGCCGGAAAGGGACTGAGAC
ACGGCC
>Zotu5289
CAACGCTGGCGGCGTGCCTCAGACATGCAAGTCGAACGATTAAAGCTCTCTTCGGAGAGTGCATAGAG
TGGCGCACGGGTGAGTAACACGTAAGTAATCTACCCTCGAGTGGGGAATAACGTCCGGAAACGGACGC
TAATACCGCATAATGCAGCGGCACCGCAAGGTGACAGTTGTTAAAGATTTATCGCTTGAGGAGGAGCT
TGCGGCAGATTAGCTAGTTGGTAAGGTAATGGCTTACCAAGGCTACGATCTGTAACCGGTCTAAGAGG
ACGGTCGGTCACACTGACACTGAATAACGGGT
>Zotu5290
GAACGCTGGCGGCGTGCTTAACACATGCAAGTCGAACGGAAAGGCCCCAGCTTGCTGGGGTACTCGAG
TGGCGAACGGGTGAGTAACACGTGGGTGATCTGCCCTGCACTCTGGGATAAGCCTGGGAAACTGGGTC
TAATACCGGATAGGACCGTGCTTTAGTGTGTGCGGTGGAAAGTTTTTCGGTGCAGGATGAGCCCGCGG
CCTATCAGCTTGTTGGTGGGGTAATGGCCTACCAAGGCGGCGACGGGTAGCCGGCCTGAGAGGGTGTA
CGGCCACATTGGGACTGAGACACGGCC
>Zotu5291
GAACGCTGGCGGCATGCCTTACACATGCAAGTCGAACGGTAACAGGTTAAGCTGACGAGTGGCGAACG
GGTGAGTAATATATCGGAACGTGCCCAGTTGTGGGGGATAACTACTCGAAAGAGTGGCTAATACCGCA
TACGACCTGAGGGTGAAAGCGGGGGATCGCAAGACCTCGCGCAATTGGAGCGGCCGATATCAGATTAG
GTAGTTGGTGGGGTAAAGGCTCACCAAGCCAACGATCTGTAGCTGGTCTGAGAGGACGACCAGCCACA
CTGGGACTGAGACACGGCC
>Zotu5292

GAACGCTGGCGGCGTGCTTAACACATGCAAGTCGAACGATGAACCCCGCTTGCGGGGGGATTAGTGGC
GAACGGGTGAGTAACACGTGAGTAACCTGCCCTTAACTCTGGGATAAGCCTTGGAAACGGGGTCTAAT
ACTGGATATTGACTTTTCCTCGCATGGGGTTTGGTTGAAAGATTTATTGGTTTTGGATGGACTCGCGG
CCTATCAGCTTGTTGGTGAGGTAATGGCTCACCAAGGCGACGACGGGTAGCCGGCCTGAGAGGGTGAC
CGGCCACACTGGGACTGAGACACGGCC
>Zotu5294
GAACGCTGGCGGCGTGCTTAACACATGCAAGTCGAACGGTGAACCCTTCGGGGGGATCAGTGGCGAAC
GGGTGAGTAACACGTGAGCAACCTGCCCTTGGCTCTGGGACAACCACTGGAAACGGTGGCTAATACCG
GATACGACCCATGCAGGCATCTGTTGTGGGTGGAAAGATTTTTCGGCTGGGGATGGGCTCGCGGCCTA
TCAGCTTGTTGGTGGGGTAATGGCCTACCAAGGCGACGACAGGTAGCCGGCCTGAGAGGGCGACCGGC
CACACTGGGACTGAGACACGGCC
>Zotu5295
GAACGTTGGTAACGTGGATACCGGCATGCAAGTCAAGGGGCCGCAAGGCACCGGCGAACGGCTTAGTA
ACACGTGGATACATACCGCAAAGTGGGGCACAGCTCGTCGAAAGACGGGGTAATTTCCCATGATCTCG
CAAGAGTAAAGGCGCAAGCCGCTTTGTGAATGGTCCGCGGCCTATCAGCTTGTTGGCGGGGTAAAAGC
CCACCAAGGCGATGACGGGTAGGGGGCGTGAGAGCGCGACCCCCAAGAATGGCACTGCGACACGGGC
>Zotu5296
CAACGCTGGCGGCGTGCCTAACACATGCAAGTCGAACGAGAAAGTGGAGCAATCCATGAGTAAAGTGG
CGACCGGGTGAGTAACACGTGACTAACCTACCCTAGAGTGGGGAATAACCTCGGGAAACCGGGGCTAA
TACCGCATAACATCGCGAGGTGTAAATTCCTTGTGATCAAAGGAGCAATTCGCTCAAGGAGGGGGTCG
CGGCTGATTAGCTAGTTGGTGAGGTAACGGCCCACCAAGGCTAAGATCAGTATCCGGCCTGAGAGGGC
GCACGGACACACTGGAACTGAAACACGGTC
>Zotu5298
GAACGCTAGCGGCAGGCTTAACACATGCAAGTCGAGGGGTATGGCAGCAATGCCAGAGACCGGCGCAC
GGGTGCGTAACGCGTATGCAACCTACCTTTCACAGGGGGATAGCCCAGAGAAATTTGGATTAATACCC
CATGGCATATATGGACCGCATGGTCCTTATATTAAAGTCACAACGGTGAAAGATGGGCATGCGTCCCA
TTAGCTTGTTGGTAAGGTAACGGCTTACCAAGGCAACGATGGGTAGGGGTCCTGAGAGGGAGATCCCC
CACACTGGTACTGAGACACGGAC
>Zotu5300
GAACGCTGGCGGCATGCCTAACACATGCAAGTCGAACGAGATTATTGACTTCGGTTGATAGTCTAGTG
GCGCACGGGTGCGTAACGCGTGGGAATCTGCCCTTAGGTTCGGAATAACTCAGAGAAATTTGAGCTAA
TACCGGATAATGACGTAAGTCCAAAGATTTATCGCCTAGGGATGAGCCCGCGTAAGATTAGCTAGTTG
GTGGGGTAAAGGCCTACCAAGGCGACGATCTTTAGCTGGTCTGAGAGGATGATCAGCCACACTGGGAC
TGAGACACGGCC
>Zotu5301
GAATGCTGGCGACGTGGATAAGGCATGCAAGTCAAGGGGGCCGCAAGGCAACCGGCAGACGAGGTAGT
AACACGTACGAACGCACCCCGAAGTCTGGCATAGCTCACCGAAAGGTGAGGTAATTCCGGATGGTACC
GCAAGGTTAAAGATTTATCGCTTCGGGAGCGGCGTGCGCAATATCAGCTAGTTGGTGAGGTAATGGCT
CACCAAGGCTACGACGTTTAGGGGAACTGAGAGGTTGACCCCCACCGATGGGACTGAGACACGGCC
>Zotu5302
GAACGCTGGCGGCGTGCTTAACACATGCAAGTCGAACGAGACAATTAAAAGCTTGCTTTTAAACGTCG
AGTGGCAAACGGGTGAGTAACGCGTAGACAACCTGCCGCAAAGATGGGGACAACAGTTCGAAAGGACT
GCTAATACCGAATGTTCTGGAAGTTCCGCATGGAAACTCCAGCAAAGATGGCCTCTACTTGTAAGCGA
TCGCTTTGCGATGGGTCTGCGTCTGATTAGCTAGTTGGTGGGGCAAGGGCCTACCAAGGCGACGATCA
GTAGCCGGTCTGAGAGGATGAACGGCCACATTGGGACTGAGACACGGCC
>Zotu5303
GAACGCTGGCGGCGTGCCTAATACATGCAAGTCGAGCGCGTGTAGCAATACACGAGCGGCGGACGGGT
GAGTAACGCGTGGGCAACCTGCCCTCTAGCCTGGGATAACAGCGCGAAAGTGCTGCTAAAACCGGATA
TGCTCACAAGGAGGCATCTCCTGGTGAGGAAAGTTTACACTAGAGGATGGGCCCGCGTCCCATTAGCT
AGTTGGTGAGGTAACGGCCCACCAAGGCGACGATGGGTAGCCGACCTGAGAGGGTGATCGGCCACACT
GGGACTGAGACACGGCC
>Zotu5304
GAACGCTGGCGGCATGCCTAACACATGCAAGTCGAACGAGACCTTCGGGTCTAGTGGCGCACGGGTGC
GTAACGCGTGGGAATCTGCCCTTGGGTTCGGAATAACAGTTAGAAATGACTGCTAATACCGGATGATG

TCTTCGGACCAAAGATTTATCGCCCAAGGATGAGCCCGCGTAGGATTAGCTAGTTGGTGAGGTAAAGG
CTCACCAAGGCGACGATCCTTAGCTGGTCTGAGAGGATGATCAGCCACACTGGGACTGAGACACGGCC
>Zotu5305
GAACGCTGGCGGCGTGCCTAACACATGCAAGTGGAGCGACGAACCGGGGCTTGCCCCGGGGCAGAGCC
GCGAACGGGTGAGTAACACGTGGGTTACCTGCCTCGATGACTGGGACAACCCGTCGAAAGACGGGCTA
ATACCGGATGTGCCCTTTGGGGGAAAGGAAGCTTTGGCCTCCGCATCGGGATGGGCCCGCGGCGCATT
AGCTTGTTGGTGCGGTAATGGCGTACCAAGGCGACGATGCGTAGCTGGTCTGAGAGGACGATCAGCCA
CACTGGGACTGAGACACGGCC
>Zotu5306
AAACGCTGGCGGCGTGCTTAACACATGCAAGTCGAACGAAGTTTTTCTTTCGGGAGAAACTTAGTGGC
GGACGGGTGAGTAACGCGTGGGTAACCTGCCCTGTACAGGGGGACAACAGCTGGAAACGGCTGCTAAT
ACCGCATAAGCCCTTAGCACTGCATGGTGCATAGGGAAAAGGAGTAATCCGGTACAGGATGGACCCGC
GTCTGATTAGCCAGTTGGCAGGGTAACGGCCTACCAAAGCGACGATCAGTAGCCGATCTGAGAGGATG
TACGGCCACATTGGGACTGAGACACGGCC
>Zotu5307
AAACGCTGGCGGCGTGCATAACACATGCAAGTCGAACGATGAAGATTAAATGATTTCTTCGGAATGAA
CTTAATTGGATTAGTGGCGGACGGGTGAGTAACGCGTGAGTAACCTGCCTTGTACAAGGGGATAGCCG
TTGGAAACGACGAATAATACCCTATGACATCAAATAATCGCATGATAATTTGATCAAAGATTTATCGG
TATAAGATGGACTCGCGTCTGATTAGCTAGTTGGCGGGGTAACAGCCCACCAAGGCAACGATCAGTAG
CCGGCTTGAGAGAGTGTACGGCCACATTGGGACTGAGACACGGCC
>Zotu5308
GAACGCTGGCGGCATGCCTTACACATGCAAGTCGAACGGCAGCACGGAGCTTGCTCTGGTGGCGAGTG
GCGAACGGGTGAGTAATATATCGGAACGTACCCAAGAGTGGGGGATAACGTAGCGAAAGTTACGCTAA
TACCGCATACGATCTAAGGATGAAAGTGGGGGATCGCAAGACCTCATGCTCCTGGAGCGGCCGATATC
TGATTAGCTAGTTGGTGAGGTAAAGGCTCACCAAGGCATCGATCAGTAGCTGGTCTGAGAGGACGACC
AGCCACACTGGAACTGAGACACGGTC
>Zotu5310
GAACGCTGGCGGCGTGCTTAACACATGCAAGTCGAACGGTAAGGCCCCTTCGGGGGTACACGAGTGGC
GAACGGGTGAGTAACACGTGAGCAACCTGCCCTTGACTCTGGAATAACAGTTGGAAACAGCTGCTAAT
ACTGGATATTCATCGTCTCCTTCCTGGGGGGTGGTGGAAAGCTCCGGCGGTCAGGGATGGGCTCGCGG
CCTATCAGCTTGTTGGTGAGGTAATGGCTCACCAAGGCTTCGACGGGTAGCCGGCCTGAGAGGGCGAC
CGGCCACACTGGGACTGAGATACGGCC
>Zotu5312
GAACGCTGGCGGCATGCCTAACACATGCAAGTCGAACGAAGCCTTCGGGTTTAGTGGCGCACGGGTGC
GTAACGCGTGGGAATCTGCCCTTTGGTTCGGAATAACAGTTGGAAACGACTGCTAATACCGGATGATG
ACGAAAGTCCAAAGATTTATCGCCAGAGGATGAGCCCGCGTAGGATTAGCTAGTTGGTGTGGTAAAGG
CGCACCAAGGCGACGATCCTTAGCTGGTCTGAGAGGATGATCAGCCACACTGGGACTGAGACACGGCC
>Zotu5314
GAATGCTGGCGGCGTGGATAAGTCATGCAAGTCGAACGGCCCGCAAGGGCAGTGGCAAACGAGGTAGT
AACACGTAGGTACGTCCCCCCGAGTCGGGCATAGCTAGCCGAAAGGTTAGGTAATTCCCGATGGTCCC
GCAAGGGTAAAGTTTTTCGCTCGGGGAGCGGCCTACGTAGTATCAGCTAGTTGGTAGGGTAACGGCCT
ACCAAGGCTACGACGCTTAGGGGCGCTGAGAGGCGGACCCCCACCGATCGGACTGAGACACGGCC
>Zotu5315
GAACGCTAGCGGCAGGCCTAATACATGCAAGTCGGACGGGATTTTGGAGCTTGCTTCAAATGAGAGTG
GCGCACGGGTGCGTAACACGTATGTAACCTACCTTGTTCAGGGGGATAGCCTCTCGAAAGAGAGATTA
AGACCGCATAAAATCACAATGTGGCATCACAAAATGATCAAATATTTATAGGAACAAGATGGGCATGC
GGAACATTAGCTAGTTGGTAAGGTAACGGCTTACCAAGGCTACGATGTTTAGGGGATCTGAGAGGATG
ACCCCCCACACTGGTACTGAGACACGGAC
>Zotu5316
GAACGCTGGCGGCGTGCTTAACACATGCAAGTCGAACGATGAAGCCTAGCTTGCTAGGTGGATTAGTG
GCGAACGGGTGAGTAACACGTGAGCAACCTGCCCCGAACTTTGGGATAAGCGCTGGAAACGGCGTCTA
ATACCGAATACGCACCATGACGGCATCGTCAATGGTGGGAAAGATTTTTCGGTTCGGGAGGGGCTCGC
GGCCTATCAGCTTGTTGGTGAGGTAATGGCTCACCAAGGCGTCGACGGGTAGCCGGCCTGAGAGGGTG
ACCGGCCACACTGGGACTGAGACACGGCC

>Zotu5317
GAACGCTGGCGGCGTGCTTAACACATGCAAGTCGAACGGTGAAGGACGGAGCTTGCTCCGACCGGATC
AGTGGCGAACGGGTGAGTAACACGTGAGTAACCTGCCCCAGACTCTGGAATAACCCCGGGAAACCGGA
GCTAATACCGGATACGAAGTGGAGCCGCATGGCTACCACTTGGAAAGTTTTTCGGTCTGGGATGGACT
CGCGGCCTATCAGCTAGTTGGTGAGGTAACGGCTCACCAAGGCGACGACGGGTAGCCGGCCTGAGAGG
GCGACCGGCCACACTGGGACTGAGACACGGCC
>Zotu5319
GAACGCTGGCGGCATGCCTTACACATGCAAGTCGAACGGCAGCACGGGGTAACCTGGTGGCGAGTGGC
GAACGGGTGAGTAATATATCGGAACGTACCCTGGAGTGGGGGATAACGTAGCGAAAGTTACGCTAATA
CCGCATACGATCTACGGATGAAAGTGGGGGATTCGCAAGAACCTCATGCTCGTGGAGCGGCCGATATC
TGATTAGCTAGTTGGTAGGGTAAAAGCCTACCAAGGCATCGATCAGTAGCTGGTCTGAGAGGACGACC
AGCCACACTGGAACTGAGACACGGTC
>Zotu5320
GAACGCTGGCGGCATGCCTTACACATGCAAGTCGAACGGCAGCATAGGAGCTTGCTCCTGATGGCGAG
TGGCGAATGGGTGAGTAATATATCGGAACGTGCCCTAGAGTGGGGGATAACTAGTCGAAAGACTAGCT
AATACCGCATACGATCTACGGATGAAAGCGGGGGACCGTAAGGCCTCGCGCAGCAGGAGCGGCCGATG
TCTGATTAGCTAGTTGGTGGGGTAAAGGCCCACCAAGGCGACGATCAGTAGCTGGTCTGAGAGGACGA
TCAGCCACACTGGGACTGAGACACGGCC
>Zotu5321
GAACGCTAGCGGCAGGCTTAATACATGCAAGTCGAGGGGCAGCACAGATAGCAATATTGGGTGGCGAC
CGGCAAACGGGTGCGGAACACGTACACAACCTTCCTTTCGGTGGGGGATAGCCTTTGGAAACGAAGAT
TAATACCCCATAAAACATATTTACCGCATGGTATGATATGTTAAAGATTTATTGCCGGAAGATGGGTG
TGCGTATGATTAGGTAGTTGGTAGGGTAATGGCCTACCAAGCCGACGATCATTAACTGGTGTGAGAGC
ACGACCAGTCACACGGGCACTGAGACACGGGC
>Zotu5322
GAACGCTAGCGGCAGGCTTAACACATGCAAGTCGAGGGGTATAGTTCTTCGGGACTAGAGACCGGCGC
ACGGGTGCGTAACGCGTATGCAATCTACCTTTTACAGAGGGATAGCCCAGAGAAATTTGGATTAATAC
CTCATAGTATAACGACTCGGCATCGAGATGTTATTAAAGTCACAACGGTAAAAGATGAGCATGCGTCC
CATTAGCTAGTTGGTAAGGTAACGGCTTACCAAGGCTACGATGGGTAGGGGTCCTGAGAGGGAGATCC
CCCACACTGGTACTGAGACACGGAC
>Zotu5323
GAACGCTGGCGGCGTGCCTAATACATGCAAGTCGAACGAAGTTATGAGAGAGCTTGCTCTCGATTAGC
TTAGTGGCGGACGGGTGAGTAACACGTAGGCAACCTGCCCCTAAGATCGGGATAACATTCGGAAACGA
ATGCTAAGACCGGATAAACAATCTGCCCGCATGGGCGGATTGGGAAACACGGAGCAATCTGTGGCTTA
GGGATGGGCCTGCGGCGCATTAGCTAGTTGGTGGGGTAACGGCTCACCAAGGCGACGATGCGTAGCCG
ACCTGAGAGGGTGAACGGCCACACTGGGACTGAGACACGGCC
>Zotu5324
GAACGCTGGCGGCGTGCTTAACACATGCAAGTCGAACGATGAAGCCAGCTTGCTGGTGGATTAGTGGC
GAACGGGTGAGTAACACGTGGGTGATCTGCCCCAAACTTTGGGATAAGCCTGGGAAACTGGGTCTAAT
ACCGAATATGACTGATACTCGCATGGGTGTTGGTGGAAAGCTCCGGCGGTTTGGGATGGGCCCGCGGC
CTATCAGCTTGTTGGTGGGGTAATGGCCTACCAAGGCGACGACGGGTAGCCGGCCTGAGAGGGCGACC
GGCCACACTGGGACTGAGACACGGCC
>Zotu5325
CAACGCTGGCGGCGTGCCTAACACATGCAAGTCGCACGAGAAAGGGGCTTCGGCCCTGAGTACAGTGG
CGCACGGGTGAGTAACACGTGAATTATCTACCTCCGAGTGGGGAATAACTGAGAGAAATCTTAGCTAA
TACCGCATAACACTTACGAGTCAAAGCAGCAATGCGCTTGGAGAGGAGTTCGCGGCAGATTAGTTAGT
TGGCGGGGTAATGGCCCACCAAGACGATGATCTGTATCCGGCCTGAGAGGGCGCACGGACACACTGGA
ACTGAAACACGGTC
>Zotu5327
GAACGCTGGCGGCGTGGATTAGGCATGCAAGTCGAACGCGAAAGAGGGGGCAACTCCTCGAGTAGAGT
GGCGAACGGGTGAGTAACGCGTGAAGAACCTGCCTTCAACAGGGGGATAACACTCCCAACGGGGTGCT
AATACCGCATGAGACCACGGATCTGCATGGATCCGAGGTCAAAGGTGGCCTCTGCTTGCAAGCTACCG
GTTGAAGATGGCTTCGCGTCCCATTAGCTAGTTGGCGGTGTAAAAGACCACCAAGGCGACGATGGGTA
GCTGGTCTGAGAGGATGGTCAGCCACACTGGGACTGAGATACGGCC

>Zotu5329
AAACGCTGGCGGCGCACATAAGACATGCAAGTCGAACGGAAGTCGTTGTAATGAAATAGAAACGGACA
GAGAACTTGTTCGAAGAAAGTGGAAGTAGACTTACAACAATGGCTTTAGTGGCGGACTGGTGAGTAAC
GCGTAAGGAACCTGCCTTCTAGAGGGGAATAACAATGAGAAATCATTGCTAATACCGCATATGCCGAG
AGAATCACATGGTACAATCGGGAAAAGAGTAATCTGCTAGAAGATGGCCTTGCGTCTGATTAGATAGT
TGGTGGGGTAACGGCCTACCAAGTCGACGATCAGTAGCCGGACTGAGAGGTTGAACGGCCACATTGGG
ACTGAGATACGGCC
>Zotu5330
GAACGCTGGCGGCGTGCCTAATACATGCAAGTCGAGCGAATCTTTGGGAGCTTGCTCCCAAAGATTAG
CGGCGGACGGGTGAGTAACACGTGGGCAACCTGCCTATAAGACTGGGATAACTTCGGGAAACCGGAGC
TAATACCGGATAATTCATATCTACTCATGTAGATATGCTGAAAGACGGTTTACGCTGTCACTTATAGA
TGGGCCCGCGGCGCATTAGCTAGTTGGTGAGGTAACGGCTCACCAAGGCAGCGATGCGTAGCCGACCT
GAGAGGGTGATCGGCCACACTGGGACTGAGACACGGCC
>Zotu5331
GAACGCTGGCGGCAGGCCTAACACATGCAAGTCGAACGGATGTAGCAATACATTAGTGGCGCACGGGA
GAGTAACGCGTGGGAACCTACCTTTTGGTTTGGGATAACATCGGGAAACTGGTGCTAATACCGGATAA
GCCCTTCGGGGGAAAGATTTATCGCCGAAAGATGGGCCCTCGTTGGATTAGCTAGTTGGTGAGGTAAC
GGCTCACCAAGGCTACGATCCATAGCTGGTCTGAGAGGATGATCAGCCTCACTGGGACTGAGACACGG
CC
>Zotu5332
GAACGCTGGCGGCGTGCCTAATACATGCAAGTCGAGCGAATCTTTGGGAGCTTGCTCCCAATGGTTAG
CGGCGGACGGGTGAGTAACACGTGGGCAACCTGCCTGTAAGACTGGGATAACTTCGGGAAACCGGAGC
TAATACCGGATAATCCTTTTCCTCACATGAGGAAAAGCTGAAAGTCGGTTTCGGCTGACACTTACAGA
TGGGCCCGCGGCGCATTAGCTAGTTGGTGAGGTAACGGCTCACCAAGGCGACGATGCGTAGCCGACCT
GAGAGGGTGATCGGCCACACTGGGACTGAGACACGGCC
>Zotu5333
GAACGCTGGCGGCGTGCCTAAGACATGCAAGTCGAGTGGGTTTAGACCCACGGCGAACGGGTGAGTAA
TACCTAGGAACTTTCTTTAAGTGGAGCATAACCAGTCGAAAGATTGGATAATACTCCATGCCATCGCA
AGATGAAAGTCGCAAGACGCTTAAAGTGAGGCCTGGGCACCATCAGCTAGTTGGTAAGGTAATGGCTT
ACCAAGGCTATGACGGTTAGGGGTCCCGAGAGGGAGAACCCCCACACTGGAACTGCGACACGGTC
>Zotu5334
GAACGCTGGCGGCGTGCTTAACACATGCAAGTCGAACGAGGTCCATGGAGCTTGCTCCGGAAGATCTA
GTGGCGAACGGGTGCGTAACACGTGAGAAACCTGCCCCGATCTCTGGGATAACAGTTGGAAACGACTG
CTAATACCGGATGCCGTCACACCGCTTCATGGCGGAGTGCCGAAAGATTTATCGGATTGGGAGGGTCT
CGCGGCCTATCAGCTAGTTGGAGAGGTAACTGCTCACCAAGGCATCGACGGGTAGCTGGTCTGAGAGG
ATGATCAGCCACACTGGGACTGAGACACGGCC
>Zotu5335
GAACGCTGGCGGCAGGCTTAACACATGCAAGTCGAACGCATCCTTCGGGGTGAGTGGCAGACGGGTGA
GTAACGCGTGGGAACGTGCCCTTCAGTTCGGGATAACCCAGGGAAACTTGGGCTAATACCGGATACGT
GCGAGAGCAGAAAGATTTATCGCTGAAGGATCGGCCCGCGTCTGATTAGCTAGTTGGTGAGGTAATGG
CTCACCAAGGCGACGATCAGTAGCTGGTCTGAGAGGATGATCAGCCACACTGGGACTGAGACACGGCC
>Zotu5337
GAACGCTAGCGGCAGGCTTAACACATGCAAGTCGAGGGGTATAGTAGCAATACTAGAGACCGGCGCAC
GGGTGCGTAACGCGTATGCAACCTACCTTTTACAGGGGAATAGCCCAGAGAAATTTGGATTAATGCCC
CATGGTATAATCGGATGGCATCATTTGATTATTAAAGTTCCAACGGTAAAAGATGGGCATGCGTCCTA
TTAGCTAGATGGTAAGGTAACGGCTTACCATGGCTACGATAGGTAGGGGTCCTGAGAGGGAGATCCCC
CACACTGGTACTGAGACACGGAC
>Zotu5338
GAACGCTGGCGGTATGCTTAACACATGCAAGTCGAACGGTGTCTTCGGACACAGTGGCGGACGGGTGA
GTAACGCGTGAGAATCTGGCTTTAGGTCTGGGACAACCACTGGAAACGGTGGCTAATACCGGATGTGC
CGAGAGGTGAAAGGTTAACTGCCTGAAGATGAGCTCGCGTCTGATTAGCTAGTTGGTGGTGTAATGGA
CTACCAAGGCGACGATCAGTAGCTGGTCTGAGAGGACGATCAGCCACACTGGGACTGAGACACGGCC
>Zotu5339
GAACGCTAGCGATAGGCTTAACACATGCAAGTCGAGGGGCAGCAGGGACTTCGGTCTGCTGGCGACCG

GCGCACGGGTGCGTAACGCGTATGCAACTTGCCTTATAGAGGGGGATAACCCGTTGAAAGACGGACTA
ATACCGCGTATTCTTATTTAGTGGCATCATTGGATAAGGAAAGATTTATTGCTATAAGATAGGCATGC
GTCCCATTAGCTAGTTGGTGAGGTAACGGCTCACCAAGGCGACGATGGGTAGGGGAACTGAGAGGTTG
AACCCCCACACTGGTACTGAGACACGGAC
>Zotu5340
GAACGCTGGCGGCATGCCTAACACATGCAAGTCGAGCGGAGTTTGATGGAGTGCTTGCACAAAGTCAA
ACTTAGCGGCGGACGGGTGAGTAACGCGTGAGTAATCTGCCTTTTACAGGGGAATAACAACGGGAAAC
CGTTGCTAATACCGCATAACATACTTGGGTGGCATCACTTGAGTATCAAAGGAGCAATCCGGTAGAAG
ATGAGCTCGCGTCCGATTAGCTAGTTGGTGAGGTAAAGGCTCACCAAGGCAACGATCGGTAGCCGAAC
TGAGAGGTTGATCGGCCACATTGGGACTGAGACACGGCC
>Zotu5341
GCCGTGTCTCAGTCCCAGTGTGGCTGATCGTCCTCTCAGACCAGCTACGGATCGTCGCCTTGGTAGGC
CATTACCCTACCAACTAGCTAATCCGACGCAGGCTCATCTCATAGCACGAGGTCCGAAGATCCCCCGC
TTTGCTTTGAAGAGATTATGCGGTATTAGCCCGAGTTTCCCCGGGTTATCCCCCACTAACTATTATAA
TTAAGGTAGATTCCTACGTGTTACTCCCCCGTCCGCCACTCGCCGTCAGGGAGCAAGCTCCCCCCGCT
GCCGTTCGACTTGCATGTGTTAGGCATGCCGCTAGCGTTCA
>Zotu5342
GAACGCTGGCGGCGTGCCTAACACATGCAAGTCGAGCGGAGATCTTGGAAGTAGCAATACGGAAGAGA
TTTTAGCGGCGGACGGGTGAGTAACACGTGAGGAACCTACCTTCAACTGGGGAATAACACCGTGAAAA
CGGTGCTAATACCGCATAACGTTGCACCATGGCATCATGGAGCAACCAAAGGAGCAATCCGGTTAAAG
ATGGCCTCGCGTCCGATTAGTTAGTTGGTGGGGTAATGGCCTACCAAGACAGCGATCGGTAGCCGGAC
TGAGAGGTCGATCGGCCACATTGGAACTGAGAAACGGTC
>Zotu5343
GAACGTTGGCGGTGCGCTTAACACATGCAAGTCGAGCGAGAAAGATCTTCGGATTGAGTACAGCGGCA
GACGGGTGAGTAACGCGTGGGAATCTACCTTCTAGTGAGGAATACCTCTGAGAAATCGGAGTTAATAC
TGCATAAGTCCCTTCGGGGAGAAAGATGGCCTCTTTGCTGTCGCTAGAAGACGAGCCTGCGTACTATT
AGCTAGTTGGTGGGGTAAAGGCCTACCAAGGCAATGATGGTTAACCGGCCTGAGAGGGTGATCGGTCA
CATTGGAACTGAGATACGGTC
>Zotu5344
GAACGCTGGCGGCGTGCTTAACACATGCAAGTCGAGCGGTAAGGCCCTTCGGGGTACACGAGCGGCGA
ACGGGTGAGTAACACGTGAGCAATCTGCCCATCACTTCGGGATAACTCCGGGAAACCGGAGCTAATAC
CGGATATGACCACTTCAGGCATCTGATGGTGGTGGAAAGTTCTGGCGGTGATGGATGAGCTCGCGGCC
TATCAGCTTGTTGGTGGGGTAACGGCCCACCAAGGCTTCGACGGGTAGCCGGCCTGAGAGGGTGACCG
GCCACACTGGGACTGAGACACGGCC
>Zotu5345
GAACGCTGGCGGCGTGCTTAAGACATGCAAGTCGAACGGCCTTCTTCGGAAGGCAGTGGCGCACGGGT
GAGTAACGCGTAACTGACCTACCCTCAAGTCTGGAATAACGGGTCGAAAGATCCGCTAATACTGGATG
TGCAGTGTCGCTGTGGCGACACTGTAAAGATTGATCGCTTGAGGATGGGGTTGCGTTTCATCAGCTGG
TTGGTGGGGTAAAGGCCTACCAAGGCGACGACGAATAGCCGGCCTGAGAGGGTGGCCGGCCACAGGGG
CACTGAGACACGGGT
>Zotu5346
GAACGCTGGCGGCGTGCTTAACACATGCAAGTCGAGCGGAAAGGCCCTTCGGGGCACTCGAGCGGCGA
ACGGGTGAGTAACACGTGGGCAACCTGCCCTCAGCTCTGGGATAACTCCGGGAAACCGGGGCTAATAC
CGGATACGACTTCTGCCTGCATGGGTGGGAGTGGAAAGTTCTTCGGCTGAGGATGGGCCCGCGGCCTA
TCAGCTTGTTGGTGGGGTGATGGCCTACCAAGGCGACGACGGGTAGCCGGCCTGAGAGGGCGACCGGC
CACACTGGGACTGAGACACGGCC
>Zotu5347
GAACGCTGATGGCGTGCCTCATACATGCAAGTCGAGCGCTGTAGCAATACGGAGCGGCGGACGGGTGA
GTAACACGTAAACAATCTGCCCTCAAGTGGGGAATAACTATCCCAACGGATAGCTAATACCGCATAAG
GATGGAGGTGGCATCACCTCTTCTCGAAAGGCTTCGGCCGCTTAAGGATGAGTTTGCGTCCCATTAGC
TTGTTGGCGGGGTAACGGCCCACCAAGGCTACGATGGGTATCCGGCCTGAGAGGGTGTACGGACACAT
TGGGACTGAGATACGGCC
>Zotu5349
GAACGCTGGCGGCGTGCTTAACACATGCAAGTGGAGCGACGAAGCGGACTTCGGTCCGTGGCAGAGCC

GCGAACGGGTGAGTAACACGTGGGTAACCCACCCCGATGACCGGGACAACCCGAGGAAACTCGGGCTA
ATACCGGATGTTCTCTTCTCCTTCAAGGGAGTTGAGCAAAGATAGCTTCGGCCTTCGCATCGGGACGG
GCCCGCGGCCCATTAGCTTGTTGGTGGGGTAACGGCCTACCAAGGCGACGATGGGTAGCTGGTCTGAG
AGGACGATCAGCCACACTGGGACTGAGACACGGCC
>Zotu5351
GAACGCTAGCGGCAGGCCTAATACATGCAAGTCGGACGGGATTGGAGAGCTTGCTTTCCATGAGAGTG
GCGCACGGGTGCGTAACACGTATGTAACCTACCTTAATCTGGGGGATAGCCTCTCGAAAGAGGGATTA
ACACCGCATAACATGTTTGAATAGCATTATTTAAACATCAAATATTTATAGGATTAAGATGGGCATGC
GGAACATTAGCTAGTTGGTAAGGTAATGGCTTACCAAGGCTACGATGTTTAGGGGATCTGAGAGGATG
ACCCCCCACACTGGTACTGAGACACGGAC
>Zotu5353
GAACGCTGGCGGCGTGCTTAACACATGCAAGTCGAACGGAAAGGCCCTTTCGGGGGTACTCGAGTGGC
GAACGGGTGAATAACACGTGGGTAATCTGCCCTGCACTTCGGGATAAGCTTGGGGAACTGGGTCTAAT
ACCGGATAGGACTACGCACTGCATGGTGTGTGGTGGAAAGCTTTTGCGGTGTGGGATGGGCCCGCGGC
CTATCAGCTTGTTGGTGGGGTGATGGCCTACCAAGGCGACGACGGGTAGCCGGCCTGAGAGGGTGTCC
GGCCACACTGGGACTGAAATACGGCC
>Zotu5354
CGTCAGGTCCCAGTGCGCTTGGTTAATTACACCTTAGAAGACCTTTCCCTGCCGCGAAAAAGGGTAGT
AGCGCAATTCATCCCGGCGAACATTACAGAAAGCGGACAAACGGAGAGTATCGGACTAACAGGA
>Zotu5355
GAACGCTGGCGGCGTGCTTAACACATGCAAGTCGAGCGGTAAGGCCCTTCGGGGTACACGAGCGGCGA
ACGGGTGAGTAACACGTGAGCAATCTGCCCTTCACTTTGGGATAACTCCCGGAAACGGGTGCTAATAC
CGGATACGACCACATTCCGCATGGTGTGTGGTGGAAAGTTCTGGCGGTGGAGGATGAGCTCGCGGCCT
ATCAGCTTGTTGGTGGGGTAATGGCCTACCAAGGCTTCGACGGGTAGCCGGCCTGAGAGGGTGACCGG
CCACACTGGGACTGAGACACGGCC
>Zotu5356
GAACGCTGGCGGCGTGCCTAATACATGCAAGTCGAGCGGATCTTTAGGAGCTTGCTCCTAAAGATTAG
CGGCGGACGGGTGAGTAACACGTGGGCAACCTACCTGTAAGACTGGGATAACTTCGGGAAACCGGAGC
TAATACCGGATAATTTCTTTCTCCACATGGAGAAAGGTTGAAAGACGGCTCACGCTGTCACTTACAGA
TGGGCCCGCGGCGCATTAGCTAGTTGGTGAGGTAACGGCTCACCAAGGCAACGATGCGTAGCCGACCT
GAGAGGGTGATCGGCCACACTGGGACTGAGACACGGCC
>Zotu5357
GAACGCTGGCGGCAGGCCTAACACATGCAAGTCGAGCGGTAACAGGGGGTGCTTGCACTCTGCTGACG
AGCGGCGGACGGGTGAGCAATACTTGGGTATCTGCCCAATAGTGGGGGACAACACGGGGAAACTCGTG
CTAATACCGCATACGCCCTACGGGGGAAAGCAGGCTTCGGCTTGCGCTATCGGATGAGCCCAAGTCGG
ATTAGCTAGTTGGTGAGATAAAAGCTCACCAAGGCGACGATCCGTAACTGGTCTGAGAGGATGATCAG
TCACACCGGAACTGAGACACGGTC
>Zotu5358
GAACGCTGGCGGCGTGCTTAACACATGCAAGTCGAACGCTGAAGCCTGGTGCTTGCACTGGGTGGATG
AGTGGCGAACGGGTGAGTAACACGTGAGTAACCTGCCCTTGACTTCGGGATAAGCCCGGGAAACTGGG
TCTAATACTGGATATGACTCCTTACCGCATGGTGGGGGGTGGAAAGATTTATCGGTTGGGGATGGACT
CGCGGCCTATCAGTTTGTTGGTGGGGTAATGGCCTACCAAGACGACGACGGGTAGCCGGCCTGAGAGG
GTGACCGGCCACACTGGGACTGAGACACGGCC
>Zotu5359
GAACGCTGGCGGCGTGCCTAACACATGCAAGTCGAGGGAGAAAGCCGCAAGGTGAGTAAACCGGCGAA
CGGGTGCGTAACACGTGGATAATCTACCTCTGTGACTGGGACAACTGGCCGAAAGGCTTGCTAATACC
AGATAAGCCCACGGGGACTTCGGTTCCTGAGGGAAAAGGCGGCCTCTGTATACAAGCTGTCACATGGA
GATGAGTCCGCGGCCCATCAGCTAGTTGGCGGGGTAAAAGCCCACCAAGGCAACGACGGGTAGCTGGT
CTGAGAGGATGATCAGCCACACTGGAACTGAGACACGGTC
>Zotu5360
GAACGCTGGCGGCACGCTTAACACATGCAAGTCGAACGGAGAATTTTGATTTATTGAAATTCTTAGTG
GCGGACGGGTGAGTAACACGTGAGCAACCTGCCTCTGAGAGAGGAATAATTTCTGGAAACGGATTGTA
ATACCTCATAACATACAGTTATCGCATGGTGATTGTATCAAAGATTTATCACTCAGAGATGGGCTCGC
GTCTGATTAGATAGTTGGTGAGGTAACGGCTCACCAAGTCGACGATCAGTAGCCGGACTGAGAGGTTG

AACGGCCACATTGGGACTGAGACACGGCC
>Zotu5361
CAACGCTGGCGGCGTGCCTCAGACATGCAAGTCGAACGCGAAAGTTTCCTTCGGGAGATGAGTAGAGT
GGCGCACGGGTGAGTAACACGTAGGTAATCTACCTTTGGATGGGGAATAACAATCGGAAACGATTGCT
AATACCGCATAATGCAGCGGCACCTAATGGTGACAGTTGTTAAAGCGGGGGATCGAAAGACCTCGCGT
CTGAAGAGGAGCCTGCGGCGGATTAGCTAGTTGGTAAGGTAATGGCTTACCAAGGCTACGATCCGTAA
CCGGCCTGAGAGGGCGGTCGGTCACACTGACACTGACATACGGGT
>Zotu5362
GAATGCTGGCGGCGTGCCTAACACATGCAAGTCGAGCGGCAGCGCGAATAGTTTACTATTTGGCGGCG
AGCGGCGGACGGCTGAGTAACGCGTGGGAATTTGCCCCAAAGTGAGGAATAACTGCCCGAAAGGGTAG
CTAATACCGCATATGGTCTTCGGATTAAAGCTTTCGAGCGCTTTGGGAGAAGCCCGCGTCGGATTAGA
TAGTTGGTGAGGTAATGGCTCACCAAGTCGACGATCCGTAGCTGGTCTGAGAGGATGATCAGCCAGAC
TGGAACTGAGACACGGTC
>Zotu5363
CAACGCTGGCGGCGCGCTTAACACATGCAAGTCGAACGAGAAAGGGGAGCAATCCCTGAGTACAGTGG
CGTACGGGTGAGTAACACGTGGATGATCTACCTCTTAGTGGGGGATACCTCTGGGAAACCGGAGACAA
TACCGCATAAGCTCGAGAGAGGAAAGCAGCAATGCGCTGAGAGAGGAGTCCGCGGCCGATTAGCTAGT
TGGTAGGGTAAAGGCCTACCAAGGCAAAGATCGGTAGCCGGCCTGAGAGGGCACACGGCCACACTGGC
ACTGAAACACGGGC
>Zotu5364
GAATGCTGGCGGCGCGCCTAACACATGCAAGTCGAGCGGTAACAGGGGTAATTTATTACCTGCTGACG
AGCGGCGGACGGCTGAGTAACGCGTGGGAACGTACCCCAAAGTGAGGAATAACTGCCCGAAAGGGTGG
CTAATGCCGCATATGATCTTCGGATTAAAGGATTTATCCGCTTTGGGAACGGCCTGCGTAAGATTAGA
TAGTTGGTGAGGTAAAGGCTCACCAAGTCAACGATCTTTAACTGGTCTGAGAGGATGACCAGTCAGAC
TGGAACTGAGACACGGTC
>Zotu5365
GAACGCTGGCGGCATGCCTTACACATGCAAGTCGAACGGCAGCACGGGAGCAATCCTGGTGGCGAGTG
GCGAACGGGTGAGTAATATATCGGAACGTACCCAGTAGTGGGGGATAGCCCGGCGAAAGCCGGATTAA
TACCGCATACGACCTGTGGGTGAAAGCGGGGGATCGCAAGACCTCGCGCTATTGGAGCGGCCGATATC
AGATTAGCTAGTTGGCGGGGTAAAAGCCCACCAAGGCGACGATCTGTAGCTGGTCTGAGAGGACGACC
AGCCACACTGGGACTGAGACACGGCC
>Zotu5366
GAACGCTGGCGGCGTGCCTAATACATGCAAGTCGAGCGGACAGACGGGAGCTTGCTCCCTGAAGTCAG
CGGCGGACGGGTGAGTAACACGTGGGCAACCTGCCTGTAAGACTGGGATAACTCCGGGAAACCGGGGC
TAATACCGGATAATTCTTTTCTCTTCATGGAGAAAAGCTGAAAGACGGTCTCGGCTGTCACTTACAGA
TGGGCCCGCGGCGCATTAGCTAGTTGGTGAGGTAACGGCTCACCAAGGCGACGATGCGTAGCCGACCT
GAGAGGGTGATCGGCCACACTGGGACTGAGACACGGCC
>Zotu5367
GAACGCTGGCGGCAGGCTTAACACATGCAAGTCGAACGCCCCGCAAGGGGAGTGGCAGACGGGTGAGT
AACGCGTGGGAATCTACCTTTTGCTACGGAATAACTCAGGGAAACTTGTGCTAATACCGTATGTGCCC
TTCGGGGGAAAGATTTATCGGCAAAGGATGAGCCCGCGTTGGATTAGCTAGTTGGTGGGGTAAAGGCC
TACCAAGGCGACGATCCATAGCTGGTCTGAGAGGATGATCAGCCACATTGGGACTGAGACACGGCC
>Zotu5368
TAACGCTGGCGGTATGCCTAACACATGCAAGTCGAACGAGGCTTCGAGCCGAGTGGCGGACGGGTGAG
TAACACGTTGGAACCTACCCCGAAGTGGGGGATAACTGGCCGAAAGGTCGGCTAATACCCCATGGTAT
CCGGTTTATCCGGATTAAAGCTTTTGCGCTTCGGGAGGGGCCTACGTAGCATTAGTTTGTTGGTGAGG
TAAAAGCTCACCAAGACTACGATGCTTAGCTGGATCGAGAGGTTGACCAGCCACACTGGGACTGAGAT
ACGGCC
>Zotu5369
GAACGCTGGCGGCAGGCCTAACACATGCAAGTCGAGCGAGGACTTCGGTCCTAGCGGCGGACGGGTGA
GTAACGCGTGGGAACGTGCCCTTTGCTTCGGAATAGCCCTGGGAAACTGGGAGTAATACCGAATGTGC
CCTTAGGGGGAAAGATTTATCGGCAAAGGATCGGCCCGCGTTGGATTAGGTAGTTGGTGGGGTAACGG
CCTACCAAGCCGACGATCCATAGCTGGTTTGAGAGGATGATCAGCCACACTGGGACTGAGACACGGCC
>Zotu5370

GAACGTTGGCGGCATGCCTAACACATGCAAGTCGAACGCGGTCTTCGGACCGAGTGGCGAACGGGTGC
GTAACACGTGGGTGACCTACCTGGAAGAGGGGGATAACCGCCGGAAACGGTGGCTAATACCGCATGAC
CTAACGAGCGCTTGAATCTCGTTAGTAAAACTCCGGTGCTTCCAGCGGGGCCCGCGGCCTATCAGGTA
GTTGGTGTGGTAACGGCACACCAAGCCAATGACGGGTAGCTGACCTGAGAGGGTGGTCAGCCACAGGG
GAACTGAGACACGGTC
>Zotu5371
GAACGTTAGCGGCGCGCTTAATACATGCAAGTCGTGCGAGAAAGGGCTTCGGCCCCGGTACAGCGGCG
CACGGGTGAGTAACACGTAGATCATCTCCCCTCGAGTGGTGGATAACCCTCCGAAAGGAGGGCTAATA
CAGCATGAGACCACGACCTCGCTGAGGTTGCGGCCAAAGTAGGCCTCTACTTGTAAGCTTACGCTTGA
GGATGAGTCTGCGGCCCATCAGCTAGTTGGTAAGGTAACGGCTTACCAAGGCGAAGACGGGTAGCTGG
TCTGAGAGGATGAACAGCCACACTGGAACTGAGACACGGTC
>Zotu5372
GAACGCTGGCGGCGTGCTTAACACATGCAAGTCGAACGGTAAGGCCCTTTCGGGGGTACACGAGTGGC
GAACGGGTGAGTAACACGTGAGTAACCTACCCTCAACTCTGGGATAACGGCTGGAAACGGTTGCTAAT
ACTGGATATGAGCTGGGTCTGCATGGGTCTGGTTGGAAAGATTTTTTGGTTGGGGATGGGCTCGCGGC
CTATCAGCTTGTTGGTGAGGTAGTGGCTCACCAAGGCTTCGACGGGTAGCCGGCCTGAGAGGGCGACC
GGCCACATTGGGACTGAGATACGGCC
>Zotu5373
AGTAGATTAAGTGTGATTACTACCGTCAAATATAGCGAGCGTCTCAGGGTCTGGCAACGTTCGTTTTT
CTCCGTCATGGCGGAACCCATGCGGTTAGAGGTTTCGATGTATGGGCCCTATGGCGAGCTTTTCCGCT
AGAGTCAGTATATTAACCCTATGGACACGACGATGCACGCGGCGACAGCAGACGACGATACACCACCG
ACAAGCCGAGACACTAAGGTGCTTCGCCCAAAATAAACAGGTGGTGTACACACTTGTGTGCATGACGC
ATCAAGAGG
>Zotu5374
GAACGCTGGCGGCGTGCTTAACACATGCAAGTCGAGCGAGGCCCCTTCCTTCGGGAAGGGAGCCCTAG
CGGCGAACGGGTGAGTAACACGTGGGCAATCTGCCCCTAGCACTGGGATAACTCCGGGAAACCGGGGC
TAATACCGGATATGACCTTCGACGGCATCGTCTGAAGGTGGAAAGGATTCCGGCTAGGGATGAGCCCG
CGGCCTATCAGCTTGTTGGTGGGGTAATGGCCTACCAAGGCGACGACGGGTAGCCGGCCTGAGAGGGC
GACCGGCCACACTGGGACTGAGACACGGCC
>Zotu5376
GAACGCTGGCGGCAGGCCTAACACATGCAAGTCGAGCGGGGGTAGCAATACCCTAGCGGCGAACGGGT
GAGGAATGCTTGGGAATCTGCCTGGTAGTGGGGGATAACGTTCCGAAAGGAACGCTAATACCGCATAC
GTCCTACGGGAGAAAGGGGGGGATCTTCGGACCTCTCGCTATCAGATGAGCCCAAGCGGGATTAGCTA
GTTGGTGAGGTAAAGGCTCACCAAGGCGACGATCCCTAGCTGGTCTGAGAGGATGATCAGCCACACTG
GAACTGAGACACGGTC
>Zotu5378
GAACGCTGGCGGCGTGCCTAACACATGCAAGTCGAACGTGAATGGGAGCAATCCCTAGTAAAGTGGCG
TACGGGTGAGTAACACGTAGGTAATCTACCTGTGAGTCTGGAATAACCTGCCGAAAGGCGGGCTAATA
CTGGATAGAGCGAGGGACTTATAAAGAGTTTCTCGGGAAAGGGAGCCCGCAAGGGCCTTTGCTTACAG
ATGAGCCTGCGTCCCATCAGCTAGTTGGTAGGGTAACGGCCTACCAAGGCTATGACGGGTAGCTGGTC
TGAGAGGATGATCAGCCACACTGGAACTGAAACACGGTC
>Zotu5379
GAACGCTAGCGGCAGGCTTAACACATGCAAGTCGAGGGGCAGCGGGAGTAGCAATACTTGCCGGCGAC
CGGCGGAAGGGTGCGTAACGCGTGAGCAACATTCCCGTATCAGGGGGATAACCGGTGGAAACGCCGAC
TAATACCCCATAAGAACATCTGCGGCATCAAAGGTGTTTGAAAGATTCGTCGGATGCGGATTGGCTCG
CGTGACATTAGCTAGTTGGTGAGGTAACGGCCCACCAAGGCGACGATGTCTAGGGGTTCTGAGAGGAA
GATCCCCCACATTGGAACTGAGACACGGTC
>Zotu5380
GAACGCTGGCGGCGTGCCTTAAGCATGCAAGTCGAATGATCCGCCGCAAGGCGGATAATGGCGGACGG
GTGAGTAACGCGTGGGAATCTGCCTTAAAGTTGGACATAGCTCATCGAAAGATGGGGTAATTTCCAAT
GTGCTCGCAAGAGTAAAGTCGCAAGACGCTTTAAGATGAGCCCGCGTCCTATCAGCTAGTTGGTGGGG
TAATGGCCTACCAAGGCAACGACGGGTAGCTGGTCTGAGAGGATGGTCAGCCACAATGGGATTGAGAA
CGGCC
>Zotu5381

GAACGCTAGCGGGAGGCTTAACACATGCAAGCCGAGGGGTATATAATTGCTTGCAATTATAGAGACCG
GCGGATGGGTGAGTAACGCGTATGTAACTTACCCTATTCATTGGGATAGCCCGAGGAAACTCGGATTA
ATACCAGATAACACTAAGGTCGGCATTGACTTTAGTTGAAAGATTTATCGGAATAGGATAGACATGCG
TCAGATTAGCTAGTTGGTGAGATAGAGGCTCACCAAGGCAATGATCTGTAGGGGGCCTGAGAGGGTGA
TCCCCCACACTGGTACTGAGACACGGAC
>Zotu5382
GAACGCTGGCGGCGTGCCTAACACATGCAAGTCGAGCGAGAAATAAAGGAATGAAGCTTCGGTAGATT
TTCTTTATGGAAAGCGGCGGACGGGTGAGTAACGCGTAGGCAACCTGCCCTGTACAGGGGGATAGCCA
CCCGAAAAGGTGATTAATACCCCATAACGCAAGAGATACACATGTATCACTTGTCAAAGATTTATCGG
TATAGGATGGGCCTGCGTCTGATTAGCTAGTTGGTAGGGTAACGGCTTACCAAGGCAACGATCAGTAG
CCGACCTGAGAGGGTGATCGGCCACATTGGAACTGAGACACGGTC
>Zotu5383
GAACGCTGGCGGCGTGCTTAACACATGCAAGTCGAACGATGAAGGGGGAGCTTGCTTCCCTGGATTAG
TGGCGAACGGGTGAGTAACACGTGAGCAATCTGCCCTTGACTCTGGGATAAGCGTTGGAAACGACGTC
TAATACCGGATATGAGCTGTGAAGGCATCTTCAACAGCTGGAAAGAACTTCGGTCAAGGATGAGCTCG
CGGCCTATCAGGTAGTTGGTGAGGTAATGGCTCACCAAGCCTACGACGGGTAGCCGGCCTGAGAGGGT
GACCGGCCACACTGGGACTGAGACACGGCC
>Zotu5384
GAACGCTGGCGGTATGCTTAACACATGCAAGTCGAACGAGAAGCTTTTGATGGATCTTTCGGGAGAAA
TGAGAAGCGGAAAGTGGCGAACGGGTGAGTAACGCGTGGGTAACCTACCTCATGGAAAGGGATAGCCC
CGGGAAACTGGGAATAAAACCTTATAGATATTGTAATCGCATGGATACAGTAAGAAAACTCCGGTGCC
ATGAGATGGACCCGCGTCCCATTAGCTAGTTGGTGAGATAACAGCCCACCAAGGCGACGATGGGTAAC
CGGTCTGAGAGGGCGAACGGTCACACTGGAACTGAGACACGGTC
>Zotu5385
GAACGCTGGCGGCGTGCTTAACACATGCAAGTCGAACGGGATCCATCCAGTGGCAACACTGGTGAAGA
TCTAGTGGCGAACGGGTGAGTAACACGTGAGAAACCTGTCCCGGTCTCTGGAATAACAGTGGGAAACC
ACTGCTAATACCGGATACCTTCACTGCGTCGCATGGCGCGGTGAAGAAAGGCTTGCCGGATTGGGAGG
GTCTCGCGGCCTATCAGCTAGTTGGTGGGGTAACGGCCTACCAAGGCGTCGACGGGTAGCTGGTCTGA
GAGGACGATCAGCCACACTGGGACTGAGACACGGCC
>Zotu5386
GAACGCTAGCGGCAGGCTTAATACATGCAAGTCGTGGGGCAGCGCAGTGTAGCAATACATGGGCGGCG
ACCGGCAAACGGGTGCGGAACACGTACGCAATCTACCCAGAACTGGGGCATAGCCCGGGGAAACCCGG
ATTAATACCCCGTAACATAATTGAAGTGGCATCACTTTATTATAAAAGCTCCGGCGGTTTTGGATGAG
CGTGCGGCTGATTAGGTAGTTGGTGAGGGTAACGGCCCACCAAGCCTTCGATCAGTAACTGGTGTGAG
AGCACGACCAGTCACACGGGCACTGAGACACGGGC
>Zotu5387
GAACGCTGGCGGCGTGCTTAACGCATGCAAGTCGAACGGTGAACCTGCTTCGGTGGGGGATCAGTGGC
GAACGGGTGAGTAACACGTGGGCAACCTGCCCTCAGCTCTGGGATAACTCCAAGAAATTGGGGCTAAT
ACCGGATATGACCGCTGACCGCATGGTCTGGTGGTGGAAAGATTTATCGGCTGGGGATGGGCCCGCGG
CCTATCAGCTTGTTGGTGGGGTAGTGGCCTACCAAGGCGACGACGGGTAGCCGGCCTGAGAGGGTGAC
CGGCCACACTGGGACTGAGACACGGCC
>Zotu5389
GAACGCTGGCGGCGTGCTTCAGACATGCAAGTCGTACGAGAAAGTGACTTCGGTCATAAGTATAGTGG
CGCACGGGTGAGTAATACTTGGGTAATGTACCTTATCGCTGGGGATAACTAGACGAAAGTTTAGCTAA
TACCGAATACATCGCCTTTTACTACGGTAGAAGTCGAGAAAGCCTCACGGCGCGGTAAGATCAGCCCA
AGTCCCATTAGCTAGTTGGAGGGGTAATGGCCCACCAAGGCTTCGATGGGTAGCTGGTCTGAGAGGAT
GATCAGCCACACTGGAACTGGAATACGGTC
>Zotu5390
GAACGCTAGCGGGAGGCTTAATACATGCAAGTCGTACGGTAACAGGTGTAGCAATACATGCTGACGAG
TGGCGCACGGGTGAGGAACACGTACACAATCTGCCTTTGACTGGGGGATAGCCTTGGGAAACTAAGAT
TAATACCCCATAATATTACGATGTGGCATCACATTGTAATTAAACCTCCGGGGGTCAAAGATGAGTGT
GCGTCTGATTAGGTAGTTGGTAGGGTAACGGCCTACCAAGCCAACGATCAGTAGCTGGCGTGAGAGCG
CGACCAGCCACACGGGAACTGAGACACGGTC
>Zotu5391

GAACGCTGGCGGCATGCCTTACACATGCAAGTCGAACGGCAGCACGGGAGCAATCCTGGTGGCGAGTG
GCGAACGGGTGAGTAAAGTATCGGAACGTGCCCAGTCGTGGGGGATAACGTAGCGAAAGCTACGCTAA
TACCGCATACGATCTATGGATGAAAGCGGGGGATCGCAAGACCTCGCGCGATAGGAGCGGCCGATGTC
GGATTAGCTAGTTGGTGAGGTAAAGGCTCACCAAGGCGACGATCCGTAGCGGGTCTGAGAGGACGGCC
CGCCACACTGGAACTGAGACACGGTC
>Zotu5392
GAACGCTGGCGGCATGCCTAACACATGCAAGTCGAACGAAGGCTTCGGCCTTAGTGGCGCACGGGTGC
GTAACGCGTGGGAATCTGCCCTCAGGTTCGGAATAACAGCGAGAAATTGCTGCTAATACCAAATGATA
TCGCGAGATCAAAGATTTATCGCCTGAGGATGAGCCCACGTAGGATTAGCTAGTTGGTGTGGTAAAGG
CGCACCAAGGCGACGATCCTTAGCTGGTCTGAGAGGATGATCAGCCACACTGGGACTGAGACACGGCC
>Zotu5393
GAACGCTGGCGGCATGCTTTACACATGCAAGTCGAACGGCAGCGCGGGGCAACCTGGCGGCGAGTGGC
GAACGGGTGAGTAATATATCGGAACGTACCCAAGAGTGGGGGATAACGTAGCGAAAGTTACGCTAATA
CCGCATACGATCTACGGATGAAAGTGGGGGATCGCAAGACCTCATGCTCCTGGAGCGGCCGATATCTG
ATTAGCTAGTTGGTAGGGTAATGGCCTACCAAGGCGACGATCAGTAGCTGGTCTGAGAGGACGACCAG
CCACACTGGAACTGAGACACGGTC
>Zotu5394
GAACGCTGGCGGCATGCCTAACACATGCAAGTCGAACGAAGGCTTCGGCCTTAGTGGCGCACGGGTGC
GTAACGCGTGGGAATCTGCCCCTTGGTTCGGAATAACAGCGAGAAATTGCTGCTAATACCGGATAATG
ACGTAAGTCCAAAGATTTATCGCCTAGGGATGAGCCCGCGTAGGATTAGCTAGTTGGTGTGGTAAAGG
CGCACCAAGGCGACGATCCTTAGCTGGTCTGAGAGGATGATCAGCCACACTGGGACTGAGACACGGCC
>Zotu5395
GAACGCTAGCGACAGGCCTAACACATGCAAGTCGAGGGGCAACAGGAAGTAGCAATACTTTGCTGGCG
ACCGGCGCACGGGTGAGTAACGCGTATGCAACCTGCCTACTACAGGGGGATAACCCGTCGAAAGACGG
CCTAATACCGCATAATACAGGTTTACTGCATGGTAAATTTGTTAAAGAACTTCGGTAGTAGATGGGCA
TGCGTTCCATTAGCTAGTTGGTGAGGTAACGGCTCACCAAGGCAACGATGGATAGGGGAACTGAGAGG
TCGATCCCCCACACTGGAACTGAGACACGGAC
>Zotu5396
GAACGCTGGCGGCGCGCCTAACACATGCAAGTCGAACGAGCGAGAGAGAGCTTGCTTTCTCGAGCGAG
TGGCGAACGGGTGAGTAACGCGTGAGGAACCTGCCTCAAAGAGGGGGACAACAGTTGGAAACGACTGC
TAATACCGCATAAGCCCACGGCTCGGCATCGAGCAGAGGGAAAAGGAGCAATCCGCTTTGAGATGGCC
TCGCGTCCGATTAGCTAGTTGGTGAGGTAACGGCCCACCAAGGCGACGATCGGTAGCCGGACTGAGAG
GTTGAACGGCCACATTGGGACTGAGACACGGCC
>Zotu5397
GAACGCTAGCGATAGGCCTAACACATGCAAGTCGAGGGGCAGCACATGAAGTAGCAATACGGATGGTG
GCGACCGGCGCACGGGTGAGTAACGCGTATGCAATCTACCTACAACAAGGGAATAACTCGTTGAAAGA
CGGACTAATACCCTATAAAACAGGGGTCCCGCATGGGAATATTTGTTAAAGATTTATCGGTTGTAGAT
GAGCATGCGTTCCATTAGCTTGTTGGTGAGGTAACGGCCCACCAAGGCAACGATGGATAGGGGAACTG
AGAGGTTTATCCCCCACACTGGTACTGAGACACGGAC
>Zotu5398
GAACGCTGGCGGCGTGCTTAACACATGCAAGTCAGGGGGCCCGCAAGGGTCACCGGCGAACGGGTGCG
TAACACGTGAGCGACCTGCCCTGGTGTGGGGGATAGCCGGCCCAACGGCCGGGTAATACCGCATACGT
CACTTGGCCTGCATGCGCCGAGTGAGAAACTTTCGAGGCACCAGGAGGGGCTCGCGGCCTATCAGCTA
GTTGGTGGGGTAACGGCCCACCAAGGCGATGACGGGTAGCTGGTCTGAGAGGATGGCCAGCCACATTG
GGACTGAGAAACGGCC
>Zotu5399
CAACGCTGGCGGCGTGCCTAACACATGCAAGTCGAACGCGAAAGTGGAGCAATCCATGAGTAGAGTGG
CAAACGGGTGAGTAACGCGTGGGTGACCTACCTCCGAGTGGGGGATAACGTCTCGAAAGGGACGCTAA
TACCGCATAACATCCCGTCTTTTAAGAGATGGCGATCAAAGCCTGGGACCGCAAGGCCAGGCGCTTGA
AGAGGGGCCCGCGTCTGATTAGCTAGTTGGTGGGGTAATGGCTCACCAAGGCAACGATCAGTATCCGG
CCTGAGAGGGCGGACGGACACACTGGGACTGAGACACGGCC
>Zotu5400
GAACGCTAGCGGCAGGCCTAATACATGCAAGTCGAGGGGCAGCATAGTAGCAATACTGATGGCGACCG
GCGCACGGGTGCGTAACGCGTATGCAACCTACCTTTTACTGGGGGATAGCCCGGAGAAATTCGGATTA

ACACCGCATAATAAGTTGAAGTGGCATCACTTTAATTTTAAATATTTATAGGTAAGAGATGGGCATGC
GTCCAATTAGCTAGTTGGTGAGGTAATGGCTCACCAAGGCGATGATTGGTAGGGGTTCTGAGAGGAAG
GTCCCCCACACTGGTACTGAGATACGGAC
>Zotu5401
GAACGCTAGCGGCAGGCTTAATACATGCAAGTCGTGGGGCAGCGCAGTGTAGCAATACATGGGCGGCG
ACCGGCAAACGGGTGCGGAACACGTACACAACCTTCCTTCAAATGGGGAATAGCCCAGGGAAACTTGG
ATTAATACCCCGTAATATTATGGAGTGGCATCACTTTATAATTATAGCTCCGGCGTTTGAAGATGGGT
GTGCGGCTGATTAGGTAGTTGGCGGGGTAACGGCCCACCAAGCCTTCGATCAGTAACTGGTGTGAGAG
CACGACCAGTCACACGGGCACTGAGACACGGGC
>Zotu5402
GAACGTTGGCGGCGTGGATTAGGCATGCAAGTCGAACGAGAACTCCTGGGCAACTGGGAGGGAAAGTG
GCGAAAGGGGCAGTAAGGCGTGGGTAACCTACCCTGGGGATGAGGATAGCCGTTCTAACGGACGGGTA
ATACTCAGCGACGTAGCGAGATCGCATGGTTTTGCTACCAAATGAATTTCGCCCTGGGAGGGGCTCAC
GTGGTATTAGCTAGTTGGTGGGGTAACGGCCTACCAAGGCGAAGATGCCTAGCGGGTGTGAGAGCACG
ACCCGCGCCACTGGCACTGAGACACTGGC
>Zotu5403
GAACGCTGGCGGCGCGCTTAACACATGCAAGTCGAGCGAGAACCATGGCTTCGGCTATGGGGACAGCG
GCGAACGGGTGAGTAACACGTGGGTAATCAACCCTCGACACTGGGATAGCCCGGGGAAACTCGGATTA
ATACCGGATACCCCATCTGGCCTTCGGGCCGGCTGGAAAAGGTAGCTTTGGCCTCCGGTCGAGGACGA
GCCCGCGGCGGATTAGCTTGTTGGTGGGGTAAAAGCCCACCAAGGCGACGATCCGTAGCTGGTCTGAG
AGGACGATCAGCCACACTGGGACTGAGACACGGCC
>Zotu5404
GAACGCTGGCGGCATGCCTTACACATGCAAGTCGAACGGCAGCGCGGGGCAACCTGGCGGCGAGTGGC
GAACGGGTGAGTAAAATATCGGAACATACCCTAGAGTGGGGGATAACGTAGCGAAAGTTACGCTAATA
CCGCATACGCACTAAGGTGGAAAGTGGGGGATCGCAAGACCTCATGCTCATGGAGTGGCCGATATCTG
ATTAGCTAGTTGGTAGGGTAAAAGCCTACCAAGGCGACGATCAGTAGCTGGTTTGAGAGAACGACCAG
CCACACTGGAACTGAGACACGGTC
>Zotu5406
GAACGCTGGCGGCGTGCTTAACACATGCAAGTCGAGCGGTAAGGCCCTTCGGGGTACACGAGCGGCGA
ACGGGTGAGTAACACGTGGGTGATCTGCCCTGTACTTCGGGATAAGCCTGGGAAACTGGGTCTAATAC
CGGATATGACTCCAGGATGCATGTCTTGGGGTGGAAAGATTTATCGGTACAGGATGGGCCCGCGGCCT
ATCAGCTTGTTGGTGGGGTAATGGCCTACCAAGGCGACGACGGGTAGCCGACCTGAGAGGGTGACCGG
CCACACTGGGACTGAGACACGGCC
>Zotu5407
GAATGCTAGCGGTGTGCCTGAGATATGCAAGTCGAGCGACATGTTCCCGCAAGGGAACGTGAGCGGCG
GACGGCTGAGTAATACATAGGAATCTGCCCCTTTCTGGAGGATAGCTCGCCGAAAGGTGAGGTAATAC
TTCATGGTCCCTTCTTTTTGAAGGGTAAAGATTTATCGGAAAGGGATGAGCCTATGTCCTATCAGGTA
GTTGGTAGAGTAAAAGCCTACCAAGCCTATGACGGGTAGCTAATCTGGGAGGATGATTAGCCAGAGGG
GGACTGAGACACGGCC
>Zotu5408
GAATGCTGGCGGCGTGCCTAACACATGCAAGTCGAGCGGTAACAGGAGTAGTTTACTACTTGCTGACG
AGCGGCGGACGGCTGAGTAACGCGTGGGAACATGCCCCAAAGTGAGGGATAAGCTCCAGAAATGGAGT
CTAATACCGCATATTATCTTCGGATTAAAGGATTTATCCGCTTTGGGAGTGGCCTGCGTACGATTAGA
TAGTTGGTGAGGTAATGGCTCACCAAGTCTACGATCGTTAACTGGTCTGAGAGGATGATCAGTCAGAC
TGGAACTGAGACACGGTC
>Zotu5409
GAACGCTGGCGGCGTGCCTAATACATGCAAGTCGAGCGAATTTTTGGGAGCTTGCTCCCAAAAATTAG
CGGCGGACGGGTGAGTAACACGTGGGCAACCTGCCTGTAAGACTGGGATAACTTCGGGAAACCGGAGC
TAATACCAGATAATTCTTATCAACTCATGTTGATAAGCTGAAAGACGGTTTCGGCTGTCACTTACAGA
TGGGCCCGCGGCGCATTAGCTAGTTGGTGAGGTAACGGCTCACCAAGGCAACGATGCGTAGCCGACCT
GAGAGGGTGATCGGCCACACTGGGACTGAGACACGGCC
>Zotu5410
GAACGCTAGCGACAGGCCTAATACATGCAAGTCGAACGGTGTCTTCGGACATAGTGGCGCACGGGTGC
GTAACACGTATGCAACCTACCTTACATTGGGGGATAGCCTTTCGAAAGGGAGATTAATACCGCATAAG

ACAGTAGCTGGGCATCCAGCAGCTGTTAAAGATTTATCGATGTAAGCTGGGCATGCGTCCAATTAGCT
AGTTGGTGAGGTAATGGCTCACCAAGGCTATGATTGGTAGGGGAACTGAGAGGTCAATCCCCCACACT
GGCACTGAGATACGGGC
>Zotu5411
GAACGCTGGCGGCAGGCCTAACACATGCAAGTCGAACGGCAGCACAGGAGAGCTTGCTCTCTGGGTGG
CGAGTGGCGGACGGGTGAGGAATACATCGGAATCTACCTTGTCGTGGGGGATAACGTAGGGAAACTTA
CGCTAATACCGCATACGACCTACGGGTGAAAGCAGGGGACCTTCGGGCCTTGCGCGATTGGATGAGCC
GATGTCGGATTAGCTAGTTGGCGGGGTAAAGGCCCACCAAGGCGACGATCCGTAGCTGGTCTGAGAGG
ATGATCAGCCACACTGGAACTGAGACACGGTC
>Zotu5412
GAACGCTGGCGGCGTGCCTAACACATGCAAGTCGAGCGATCTTCTTCGGAAGAAAGCGGCGGACGGGT
GAGTAACGCGTGGGTAACCTGCCCTGTACACACGGATAACATACCGAAAGGTATGCTAATACGAGATA
ATATGCTTTTATCGCATGGTAGAAGTATCAAAGCTCCGGCGGTACAGGATGGACCCGCGTCTGATTAG
CTAGTTGGTAAGGTAACGGCTTACCAAGGCGACGATCAGTAGCCGACCTGAGAGGGTGATCGGCCACA
TTGGAACTGAGACACGGTC
>Zotu5413
GAACGCTGGCGGCATGCTTTACACATGCAAGTCGAACGGTAACAGGTCTTCGGATGCTGACGAGTGGC
GAACGGGTGAGTAATACATCGGAACGTGCCTAGTAGTGGGGGATAACTACTCGAAAGAGTAGCTAATA
CTGCATGAGATCTACGGATGAAAGCAGGGGACCTTCGGGCCTTGTGCTACTAGAGCGGCTGATGGCAG
ATTAGGTAGTTGGTGGGGTAAAAGCTTACCAAGCCTGCGATCTGTAGCTGGTCTGAGAGGACGACCAG
CCACACTGGGACTGAGACACGGCC
>Zotu5414
GAACGCTGGCGGCGTGCCTAATACATGCAAGTCGAACGAAGGTACTTGTACCTTAGTGGCGAACGGGT
GAGTAACACGTAGGTAACCTGCCCTCAAGACGAGGATAACAGTTGGAAACGACTGCGAATACTCGATA
GGTAATCACTAGGCATCTAGAGATTATTAAAGATCCGTTTGGATTACTTGAGGAGGGACCTGCGCCGC
ATTAGTTAGTTGGTAAGGTAATGGCTTACCAAGACCATGATGCGTAGCCGGACTGAGAGGTTGAACGG
CCACACTGGGACTGAGACACGGCC
>Zotu5416
GAACGCTAGCGGCAGGCTTAATACATGCAAGTCGAGGGGCAGCACGGGTAGCAATACCTGGTGACGAC
CGGCAAACGGGTGCGGAACACGTACGCAACCTTCCCATAACTGGTGAATAGCCCTCCGAAAGGAGGAT
TAATACACCGTAACATAATGAAGTGGCATCACTTTATTATTATAGGCAGCAATGTCGGTTTTGGATGG
GCGTGCGCCTGATTAGGTAGTTGGCGGGGTAACGGCCCACCAAGCCTGCGATCAGTAACTGGTGTGAG
AGCACGACCAGTCACACGGGCACTGAGACACGGGC
>Zotu5417
GAACGCTGGCGGCGTGCTTAACACATGCAAGTCGAGCGACAGCACTCTTCGGGGTGCATGGAGCGGCG
AACGGGTGAGTAACACGTGGGCAACCTGCCCTCAGCTCTGGGACAACTCCGGGAAACCGGGGCTAATA
CCGGATACGACCTTCCCTCGCATGGGGGTCGGTGGAAAGTTTTTCGGCTGAGGATGGGCCCGCGGCCT
ATCAGATTGTTGGTGGGGTGATGGCCTACCAAGGCGACGACGGGTAGCCGGCCTGAGAGGGCGACCGG
CCACACTGGGACTGAGACACGGCC
>Zotu5418
GAACGCTGGCGGCATGCTTTACACATGCAAGTCGAACGGCAGCGGGGAGGAGCTTGCTTCTCTGCCGG
CGAGTGGCGAACGGGTGAGTAATACATCGGAACGTGTCGAGAAGAGGGGGATAACCACCCGAAAGGGT
GGCTAATACCGCATAAATTCTGAGGAAGAAAGCTGGGGACCTAGGAATAGGCCTAGCGCTTTTTGAGC
GGCCGATGTCGGATTAGCTAGTTGGTGAGGTAAAGGCTCACCAAGGCGAAGATCTGTAGCGGGTCTGA
GAGGATGATCCGCCACACTGGGACTGAGACACGGCC
>Zotu5419
GAACGCTGGCGGCACGCTTAACACATGCAAGTCGAACGGAGATATGAAGATTTATTGGATTATCTTAG
TGGCGGACGGGTGAGTAACACGTGAGCAACCTGCCTCTGAGAGAGGGATAATTTCTGGAAACGGATTG
TAATACCTCATAATGTACAGTTTTCGCATGGAGATTGTACCAAAGATTTATCACTCAGAGATGGGCTC
GCGTCTGATTAGCTGGATGGTGAGGTAACGGCTCACCATGGCGACGATCAGTAGCCGGACTGAGAGGT
TGAACGGCCACATTGGGACTGAGACACGGCC
>Zotu5420
GAACGCTAGCGGCAGGCTTAACACATGCAAGTCGAGGGGTATAGTAGCAATACTAGAGACCGGCGCAC
GGGTGCGTAACGCGTATGCAATCTACCTTTTGCAGGGGAATAGCCCAGAGAAATTTGGATTAATGCCC

CATAGTATATAGAGTTGGCATCAACATTATATTAAAGTCACAACGGCAAAAGATGAGCATGCGTCCCA
TTAGCTAGTTGGTAAGGTAACGGCTTACCAAGGCGACGATGGGTAGGGGTCCTGAGAGGGAGATCCCC
CACACTGGTACTGAGACACGGAC
>Zotu5421
GAACGCTAGCGGCAGGCCTAATACATGCAAGTCGGACGGGATAGAGGAGCTTGCTTTTCTTGAGAGTG
GCGCACGGGTGCGTAACACGTATGTAACCTACCTTGATCAGGGGGATAGCCTCTCGAAAGAGAGATTA
AGACCGCATAACATTATGAGATAGCATTATTTTATAATCAAATATTTATAGGATCAAGATGGGCATGC
GGAACATTAGCTAGTTGGTAAGGTAACGGCTTACCAAGGCTACGATGTTTAGGGGATCTGAGAGGATG
ACCCCCCACACTGGTACTGAGACACGGAC
>Zotu5422
GAACGCTGGCGGCGTGCCTAATACATGCAAGTCGAGCGGTAAGGCCTCTTCGGAGGTACACGAGCGGC
GGACGGCTGAGTAACGCGTAGGAACGTACCCCAAAGTGAGGGATAAGCACCGGAAACGGTGTCTAATA
CCGCATGTGATCTAAGGATTAAAGCTTCGGCGCTTTGGGAACGGCCTGCGTATGATTAGCTTGTTGGT
GAGGTAAAAGCTCACCAAGGCGACGATCATTAGCTGGTCTGAGAGGATGATCAGCCAGACTGGGACTG
AGACACGGCC
>Zotu5423
GAACGCTGGCGGCATGCCTAACACATGCAAGTCGAACGGACATCTTCGGATGTTAGTGGCGCACGGGT
GCGTAACGCGTGGGAATCTACCCCTGGGTTCGGAATAACGGTTGGAAACGACTGCTAATACCGGATGA
TGACGTAAGTCCAAAGATTTATCGCCCAGGGATGAGCCCGCGTAGGATTAGGTAGTTGGTGTGGTAAA
GGCGCACCAAGCCGACGATCCTTAGCTGGTCTGAGAGGATGATCAGCCACACTGGGACTGAGACACGG
CC
>Zotu5425
GAACGCTAGCGGGAGGTCTAACACATGCAAGTCGAACGGGATTGGGAGCTTGCTCCCATGAGAGTGGC
GCACGGGTGAGTAACGCGTATGCAACCTACCTTTAACTGAGGGATAGCCCATAGAAATGTGGATTAAT
ACCTCATAGTTCTGAGAGACCGCATGGTTTTTCAGATAAAGCTCCGGCGGTTAAAGATGGGCATGCGT
GACATTAGTTTGTTGGTGAGGTAACGGCTCACCAAGACTACGATGTCTAGGGGTTCTGAGAGGATGAT
CCCCCACACTGGTACTGAGACACGGAC
>Zotu5426
GAACGCTGGCGGCATGGATAAGGCATGCAAGTCGAGTGGGTTTAGACCCACGGCAAACGGGTTAGTAA
TGCGTAGGAATGTCCCTCAAAGTCGTGAATAATCCGTCGAAAGGCGGAAGAATACACGATGTGCTCCA
CGGAGTAAAGATTTATCGCTTTGAGAGCAGCCTGCGTCCTATCAGCTAGTTGGTGGGGTAAAAGCCTA
CCAAGGCTTTGACGGGTAGGGGGTGTGAGAGCATGGCCCCCAACATTGGGACTGAGACACTGCC
>Zotu5428
GAACGCTAGCGGCAGGCCTAACACATGCAAGTCGAGGGGTAGTATAGCAATATATGAGACCGGCGCAC
GGGTGCGTAACGCGTATGCAACCTACCTTTTACAGGGGAATAGCCCAGAGAAATTTGGATTAATGCCC
CATGGTATATTTTAGTGGCATCACTGGATTATTAAAGTTCCAACGGTAAAAGATGGGCATGCGTCCTA
TTAGCTAGATGGTAAGGTAACGGCTTACCATGGCTACGATAGGTAGGGGTCCTGAGAGGGAGATCCCC
CACACTGGTACTGAGACACGGAC
>Zotu5429
GAACGCTGGCGGCGTGCCTAAGACATGCAAGTCGAGTGGGTTTAGACCCACGGCGAACGGGTGAGTAA
TACCTAGGAACTTTCTCTAAGTGGAGCATAACCAGTCGAAAGATTGGATAATACTTCATGTGACCGCA
AGGTTAAAGTCGCAAGACGCTTAAAGTGAGGCCTGGGCACCATCAGCTAGTTGGTAAGGTAATGGCTT
ACCAAGGCTATGACAGTTAGGGGTCCCGAGAGGGATAACCCCCACACTGGAACTGCGACACGGTC
>Zotu5430
GAACGCTAGCGGTATGCTTAACACATGCAAGTCGAACGAAAAAATTTGAAAGAATTAAAGAAATTTAA
AAAATCTATTTTTTAGTGGCGAACGGGTGAGTAATGCGTGAGAATTTGCCTTTAGGAGGAGGATAACT
ATTAGAAACAATAGCTAATACTCCGTAAAGTCTTATTATGTAAGATGAAAGAAGAAATTCGCCTAAAG
AGAAGCTCACGTCTGATTAGCTAGTTGGTAAGGTAAAGGCTTACCAAGGCGACGATCAGTAGCTGGTT
TGAGAGGATGATCAGCCACATTGGAACTGAGACACGGTC
>Zotu5431
GAACGCTGGCGGCAGGCCTAACACATGCAAGTCGAGCGCCCCGCAAGGGGAGCGGCAGACGGGAGAGT
AACACGTGGGAACGTACCCTTCAGTACGGAACAACCAAGGGAAACTTTGGCTAATACCGTATACGTCC
GTAAGGAGAAAGATTTATCGCTGAAGGATCGGCCCGCGTCTGATTAGCTAGTTGGTGAGGTAACGGCT
CACCAAGGCGACGATCAGTAGCTGGTCTGAGAGGATGATCAGCCTCATTGGGACTGAGACACGGCC

>Zotu5432
GAACGCTGGCGGCGTGCTTAACACATGCAAGTCGAACGGTGATGGCCAGCTTGCTGGTCTGATCAGTG
GCGAACGGGTGAGTAACACGTGAGCAACCTGCCCTTCACTCTGGGATAAGCCCTGGAAACGGGGTCTA
ATACCGGATATGAGACGCACACGCATGTGTAGTGTCTGGAAAGATTTATCGGTGGGGGATGGGCTCGC
GGCCTATCAGCTTGTTGGTGGGGTAGTGGCCTACCAAGGCGACGACGGGTAGCCGGCCTGAGAGGGCG
ACCGGCCACACTGGGACTGAGACACGGTC
>Zotu5433
GAACGCTAGCGGCAGGCTTAACACATGCAAGTCGAGGGGTATGGTTCTTCGGAACCAGAGACCGGCGC
ACGGGTGCGTAACGCGTATGCAATCTACCTTTTACAGAGGGATAGCCCAGAGAAATTTGGATTAATAC
CTCATAGTATATAGAGTTGGCATCAACACTATATTAAAGTTCCAACGGTAAAAGATGAGCATGCGTCC
CATTAGCTAGATGGTAAGGTAACGGCTTACCATGGCAACGATGGGTAGGGGTCCTGAGAGGGAGATCC
CCCACACTGGTACTGAGACACGGAC
>Zotu5434
GAACGCTGGCGGCGTGCCTAACACATGCAAGTCGAGCGAGAAAGTCCTTCGGGATGAGTACAGCGGCA
GACGAGTGAGTAACACGTGAGAATCTACCCTTGAATGGAGAATACCCTCGAGAAATCGAGGTTAATAC
TGCATACGTCCGATACGCGAGAGCGTGATGGAGAAAGATGGCTTTATTGCTGTCGTTCGAGGAGGAGC
TTGCGGCCTATCAGCTAGTTGGTGAGGTAATGGCTCACCAAGGCGATGACGGGTAGCTGACCTGAGAG
GGTGACCAGCCACGTTGGAACTGAGACACGGTC
>Zotu5435
GAACGCTGGCGGCATGCCTAACACATGCAAGTCGAACGAGACCTTCGGGTCTAGTGGCGCACGGGTGC
GTAACGCGTGGGAACCTGCCCTTAGGTTCGGAATAACTCAGAGAAATTTGAGCTAATACCGGATAATG
TCTTCGGACCAAAGATTTATCGCCTTTGGATGGGCCCGCGTAGGATTAGCTAGTTGGTGGGGTAATGG
CCTACCAAGGCGACGATCCTTAGCTGGTCTGAGAGGATGATCAGCCACACTGGGACTGAGACACGGCC
>Zotu5436
GAACGCTGGCGGCGTGCCTAATACATGCAAGTCGAGCGAACAGAAAAGGAGCTTGCTCCTTTGAAGTT
AGCGGCGGACGGGTGAGTAACACGTGGGCAACCTACCCTATAGTTTGGGATAACTCCGGGAAACCGGG
GCTAATACCGAATAATCTCTTTTGCTTCATGGTAAAAGACTGAAAGACGGTTTCGGCTGTCGCTATAG
GATGGGCCCGCGGCGCATTAGCTAGTTGGTGAGGTAACGGCTCACCAAGGCGACGATGCGTAGCCGAC
CTGAGAGGGTGATCGGCCACACTGGGACTGAGACACGGCC
>Zotu5437
GAACGCTGGCGGCGTGCCTAACACATGCAAGTCGAACGGGATCCATGAGAGCTTGCTCTCGGAAGATC
TAGTGGCGAACGGGTGAGTAACACGTGAGAAACCTGCCTCGGAGTGGGGAATAACTCGGGGAAACTCG
AGCTAATACCGCATACCTTCAGATGATCGCATGGTCGACTGAAGAAAGAATTTCGCTCTGAGAGGGTC
TCGCGGCCTATCAGCTAGTTGGTGGGGTAACGGCCTACCAAGGCAACGACGGGTAGCTGGATTGAGAG
ATCGATCAGCCACACTGGAACTGAGACACGGTC
>Zotu5438
GAACGCTGGCGGCGTGCCTAAGACATGCAAGTCGAGGGGGTTTAGACCCCCGGCGAACGGGTGAGTAA
CACGTGGGAACTTTCATTAAGTTGAGGATAACCCCTCGAAAGGGAGGACAATACTCAATGTGGCCGCA
AGGCTAAAGATTTATCGCTTAATGTGAGGCCCGCGTCCCATCAGCTAGTTGGTAGGGTAAAAGCCTAC
CAAGGCTACGACGGGTAGCGGAACCGAGAGGTTGATCCGCCACACTGGGACTGCGACACGGCC
>Zotu5440
GAACGCTGGCGGCAGGCCTAATACATGCAAGTCGAACGGCAGCACAGCAGAGCTTGCTCTGTGGGTGG
CGAGTGGCGGACGGGTGAGGAATACATGGGAATCTGCCCAGTCGTGGGGGATAACGTAGGGAAACTTA
CGCTAATACCGCATGCGCCCTACGGGGGAAAGCAGGGGATCTTCGGACCTTGTGCGATTGGATGAGCC
CATGTTCGATTAGCTAGTTGGCGGGGTAACGGCCCACCAAGGCGACGATCGATAGCTGGTCTGAGAGG
ATGATCAGCCACACTGGAACTGAGACACGGTC
>Zotu5441
GAACGCTAGCGGCAGGCTTAATACATGCAAGTCGAGGGGCAGCACGGGGGCAACCCTGGTGGCGACCG
GCAAACGGGTGCGGAACACGTACGCAACTTTCCCAAAACTGGTGAATAGCCTTCCGAAAGGGAGATTA
ATACACCGTAAGATATAGGAGTGGCATCACTTTTATATTATAGCTCCGGCGGTTTTGGATGGGCGTGC
GCCTGATTAGGTAGTTGGCGGGGTAGCGGCCCACCAAGCCTGCGATCAGTAACTGGTGTGAGAGCACG
ACCAGTCACACGGGCACTGAGACACGGGC
>Zotu5442
GAACGCTGGCGGCACGTTTTAAGCATGCAAGTCGAACGGCAGTGGGGGCAACCCCATGAGAGTGGCGA

ACGGGTGAGTAACACGTAGGTGATCTGCCTTGAGGATGGGGATAGCTTGTGGAAACACAGGGTAATAC
CGAATGAGCTGACGCGGCTATGGCTGAGTTAGTAAAGGTGCTACGGCACCGCCTTGAGAGGAGCCTGC
GGCTGATTAGCTAGTTGGTGGGGTAAAGGCCTACCAAGGCGACGATCAGTAGCCGGCCTGAGAGGGTG
TACGGCCACACTGGGACTGAGACACGGCC
>Zotu5443
GAACGCTAGCGGCGGGCTTAATACATGCAAGTCGAGCGGTAACAGGTGTAGCAATACATGCTGACGAG
CGGCAGACGGGTGAGGAACACGTACGCAACCTCCCTCCAACTGGGGCACAACTCCGTGAAAACGGGGC
TAATTCCCCATAACATTGCGAAAGGGCATCTGATTGCAATTAAAGATTCGTCGGTTGGAGATGGGCGT
GCGACTGATTAGCCAGTTGGTAGGGTAACGGCCTACCAAAGCAACGATCAGTAGCTGGTGTGAGAGCA
CGACCAGCCACACGGGCACTGAGATACGGGC
>Zotu5444
GAACGCTGGCGGCGTGCTTAACACATGCAAGTCGAACGGGCCTTGTCCTCTTTTTTTTTGGGGGTGGG
GTTAGTGGCGAACGGGTGAGTAACACGTGAGTAACCTGCCCCTCACTTCTGGATAACCGCTTGAAAGG
GTGGCTAATACGGGGTGTTCTGGCCTGCTCGCATGGGTGGGTTTGGAAAGATTCGACCGGTTTTGGTT
GTTTTGGTGGGGGATGGGCTCGCGGCCTATCAGCTTGTTGGTGGGGTGATGGCCTGCCAAGGCTTTGA
CGGGTAGCCGGCCTGAGAGGGTGGGCGGTCACACTGGGACTGAGACACGGCC
>Zotu5445
GAACGCTGGCGGCGTGCTTAACACATGCAAGTCGAACGAAGCGCTTAGATCTCTTCGGAGTGATGAGT
GACTGAGTGGCGGACGGGTGAGTAACGCGTGGGTAACCTGCCTCGTACAGGGGGACAACAGTTGGAAA
CGACTGCTAATACCGCATAAGCGCACGGTATCGCATGATACAGTGTGAAAAGCTGAGGCGGTACGAGA
TGGACCCGCGTTGGATTAGCTAGTTGGTGGGGTAACGGCCTACCAAGGCGACGATCCATAGCCGACCT
GAGAGGGTGACCGGCCACATTGGGACTGAGACACGGCC
>Zotu5447
GAACGCTAGCGGCAGGCTTAACACATGCAAGTCGAGGGGTATAGTAGCAATACTAGAGACCGGCAAAC
GGGTGCGGAACACGTACGCAATCTACCCTTACCCGGGGCATAGCCCGAAGAAATTCGGATTAACACCC
CATAAGATATTAGAGAGGCATCTCTTAGATATTAAAAATTTATTGGGTAAGGATGAGCGTGCGTCCCA
TTAGCTAGTTGGTAGGGTAACGGCCTACCAAGGCGACGATGGGTAGCTGGTGTGAGAGCACGACCAGC
CACACGGGCACTGAGACACGGGC
>Zotu5448
GAACGCTGGCGGCATGCTTTACACATGCAAGTCGAACGGCAGCGCGGGGCAACCTGGCGGCGAGTGGC
GAACGGGTGAGTAATATATCGGAACGTACCCAGAAGTGGGGGATAACGTAGCGAAAGTTACGCTAATA
CCGCATACGATCTACGGATGAAAGTGGGGGACCTTCGGGCCTCATGCTTTTGGAGCGGCCGATATCTG
ATTAGCTAGTTGGTGAGGTAAAGGCTCACCAAGGCGACGATCAGTAGCTGGTCTGAGAGGACGACCAG
CCACACTGGGACTGAGACACGGCC
>Zotu5449
GAACGCTGGCGGCGTGGATAAGACATGCAAGTCGAACGGGACCGGACGGTAGCAATATTGAACGGTCG
AGTGGCGAACGGGTGCGTAACACGTGGGTCACATTCCGGGAAGTGAGGAATAGCCCAGGGAAACTTGG
ATTAATACCTCATGTGGTCGAAAGACTAAAGGCGCAAGCCGCTTCCTGATTGGCCCGCGGCCTATCAG
CTAGTTGGTGGGGTAATGGCTCACCAAGGCTACGACGGGTAGCTGGTCTAAGAGGACGACCAGCCACA
CTGGAACTGAGACACGGTC
>Zotu5450
GCCGTGTCTCAGTCCCAGTGTGGCTGGTCGTCCTCTCAGACCAGCTACTGATCGTCGCCTTGGTGAGC
TTTTACCCCACCAACTAGCTAATCAGATATCGGCCGCTCCAGGAGCATGAGGTCTTGCGATCCCCCAC
TTTCATCCGTAGATCGTATGCGGTATTAGCTAGTCTTTCGACTAGTTATCCCCCACTCTAGGGCACGT
TCCGATATATTACTCACCCGTTCGCCACTCGCCATCAGGAGCAAGCTCCTATGCTGCCGTTCGACTTG
CATGTGTAAGGCATGCCGCCAGCGTTCA
>Zotu5451
GAACGCTGGCGGCAGGCCTAACACATGCAAGTCGAACGCTCGTAGCAATACGGGAGTGGCGCACGGGT
GAGTAACGCGTGGGCACCTGCCCGGTGGTCCGGAATAACCGCGGGAAACTGCGGCTAATACCGAATAC
GCCCTTAGGGGGAAAGATTTATCGCCATCGGATGGGCCCGCGTAGGATTAGCTAGTTGGTGAGGTAAC
GGCTCACCAAGGCAACGATCCTTAGCTGGTCTGAGAGGATGATCAGCCACACTGGGACTGAGACACGG
CC
>Zotu5452
GAACGCTGGCGGCGTGCCTAATACATGCAAGTCGAGCGGTAAGGCTACTTCGGTAGTACACGAGCGGC

GGACGGCTGAGTAACACGTAGGAACATACCCCAAACTGAGGGATAACTGCTCGAAAGAGTAGCTAATA
CCGCATGTGCTCTTCGGAGTAAAGCTACGGCGGTTTGGGAATGGCCTGCGTAAGATTAGCTAGTTGGT
GAGGTAATAGCTCACCAAGGCGACGATCTTTAGCTGGTCTGAGAGGATGATCAGCCAGACTGGGACTG
AGAACGGCC
>Zotu5453
GAACGCTGGCGGCAGGCCTAACACATGCAAGTCGAGCGGTAACAGGGGGTGCTTGCACTCTGCTGACG
AGCGGCGGACGGGTGAGTAATACTTGGGAATCTGCCCGATAGTGGGGGACAACACGGGGAAACTCGTG
CTAATACCGCATACGCCCTACGGGGGAAAGCAGGCTTAGGCTTGCGCTATCGGATGAGCCCAAGTCGG
ATTAGCTAGTTGGTAGGGTAAAGGCCTACCAAGGCGACGATCCGTAACTGGTCTGAGAGGATGATCAG
TCACACCGGAACTGAGACACGGTC
>Zotu5454
GAACGCTGGCGGCGTGCCTAACACATGCAAGTCGAGCGGCAGCGCGGGAGCAATCCTGGCGGCGAGCG
GCGGACGGGTGAGCAATGCTTGGGAATCTGCCCATCAGTGGGGGACAACCCGGGGAAACTCGAGCTAA
TACCGCATACGTTCTTTGGAAGAAAGCAGGGGACCGCAAGGCCTTGCGCTGATGGATGAGCCCAAGTC
GGATTAGCTAGTTGGTGAGGTAACGGCTCACCAAGGCGACGATCCGTAGCTGGTCTGAGAGGACGACC
AGCCACACCGGAACTGAGACACGGTC
>Zotu5457
GAACGTTGGCGGCGTGCCTTAGGCATGCAAATCAAGGGGATCCGCCGCAAGGCGGGTAACCGGTGGAC
GGGTGAGTAATGCACAGGAATCTACCTAGAAGTGGGATATAGCTAGCCGAAAGGTTGGGTAATCTCCC
ATGTGAACGTAAGTTTAAAGCCGCAAGGCGCTTTTAGATGAGCCTGTGTCCTATCAGCTTGTTGGTGG
GGTAATGGCCTACCAAGGCGACGACGGGTATCTGGACTGAGAGGTCGGCCAGACACAATGGGACTGCG
ACACGGCC
>Zotu5458
GAACGCTGGCGGCGTGCCTAATACATGCAAGTCGAGCGAATCTTTGGGAGCTTGCTCCCAAAGATTAG
CGGCGGACGGGTGAGTAACACGTGGGCAACCTGCCTATAAGACTGGGATAACTTCGGGAAACCGGAGC
TAATACCGGATAATTCATATCTACTCATGTAGGTATGCTGAAAGACGGTTTACGCTGTCACTTATAGA
TGGGCCCGCGGCGCATTAGCTAGTTGGTGAGGTAACGGCTCACCAAGGCAACGATGCGTAGCCGACCT
GAGAGGGTGATCGGCCACACTGGGACTGAGACACGGCC
>Zotu5459
GAACGCTGGCGGCGTGCTTAACACATGCAAGTCGAACGGAAAGGCCCTTCGGGGTACTCGAGTGGCGA
ACGGGTGAGTAACACGTGGGTGATCTGCCTCGTACTCTGGGATAAGCCTGGGAAACTGGGTCTAATAC
TGGATAGGACCACTGGTCGCATGGTTGGTGGTGGAAAGTTTTTCGGTACGAGATGGGCCCGCGGCCTA
TCAGCTTGTTGGTGGGGTAATGGCCTACCAAGGCGACGACGGGTAGCCGGCCTGAGAGGGCGACCGGC
CACACTGGGACTGAGACACGGCC
>Zotu5460
GAACGCTGGCGGCATGCTTAACACATGCAAGTCTCACGGGCAGCAATGTCAGTGGCGGACGGGTGAGT
AACGCGTAGGTATCTATCCTGAGGTGGGGGACAACCGTGGGAAACTACGGCTAATACCGCATAATGCC
TGAGGGCCAAAGGCGCGAGTCGCCACAGGAGGAGCCTGCGTACGATTAGCTAGTTGGTGGGGTAAAGG
CCTACCAAGGCGACGATCGTTAGCTGGTCTGAGAGGATGATCAGCCACACTGGGACTGAGACACGGCC
>Zotu5461
GAATGCTGGCGGTGTGGATAGGGCATGCAAGTCAAGGGGACTCAGTTAGTAATAATTGGGTAACCGGC
AGACGAGATAGTAACGCGTAGGAACGTACCCAGAAGTCGGGCACAGCTCGCCGAAAGGCGGGGTAATT
CCCGATGGTGACGAAAGTCTAAAGATTTATCGCTTCTGGAGCGGCCTGCGTAATATCAGCTTGTTGGT
GAGGTAATGGCTCACCAGGGCTATGACGTTTAGGGGAGCTGAGAGGCTGACCCCCATCGATGGAATTG
AGATACGGTC
>Zotu5462
GAACGCTGGCGGAATGCTTTACACATGCAAGTCGAACGGCAGCACGAGGGCAACCTTGGTGGCGAGTG
GCGAACGGGTGAGTAATATATCGGAACGTGCCCAGTCGTGGGGGATAACGTAGTGAAAATTACGCTAA
TACCGCATACGATCTATGGATGAAAGCGGGGGATCGCAAGACCTCGCGCGATTGGAGCGGCCGATATC
AGATTAGGTAGTTGGTGGGGTAAAGGCCTACCAAGCCGACGATCTGTAGCTGGTCTGAGAGGACGACC
AGCCACACTGGGACTGAGACACGGCC
>Zotu5463
GAACGCTAGCGGCAGGCCTAATACATGCAAGTCGGACGGGATTGAGGAGCTTGCTTCTCATGAGAGTG
GCGCACGGGTGCGTAACACGTATGTAACCTACCTTCGTCAGGGGGATAGCCTCTCGAAAGAGAGATTA

AGACCGCATAACATTGAGACACAGCATTGTGATTCAATCAAATATTTATAGGACGAAGATGGGCATGC
GGAACATTAGCTAGTTGGTAAGGTAACGGCTTACCAAGGCTACGATGTTTAGGGGATCTGAGAGGATG
ACCCCCCACACTGGTACTGAGACACGGAC
>Zotu5464
GAACGCTGGCGGCGCGCCTAACACATGCAAGTCGAGCGATGAAAGTCGTTCTGATCCTTCGGGTGACG
AACTTCTGGATTAGCGGCGAACGGGTGAGTAACGCGTGAGGAACCTGCCTCTTACAACGGGATAGCCT
CGGGAAACCGGGATTAATACCGTATAAGACTCCGACACCTCCTGGTGATGAAGTCAAAGTGCTAGCGG
TAAGAGATGGCCTCGCGTCTGATTAGTTAGTTGGTGGGGTAACGGCCTACCAAGACGACGATCAGTAA
CCGGCCTGAGAGGGTGAACGGTCACATTGGAACTGAGACACGGTC
>Zotu5465
GAACGCTGGCGGCATGCCTAACACATGCAAGTCGAACGAGACCTTCGGGTCTAGTGGAGCACGGGTGC
GTAACGCGTGGGAATCTGCCCTTCGGTTGGGAATAACTCAGGGAAACTTGAGCTAATACCTTATAATG
TCTTCGGACCAAAGATTTATCGCCGAGGGATGAGCCCGCGTAGGATTAGCTAGTTGGTGGGGTAATGG
CCTACCAAGGCAACGATCCTTAGCTGGTCTGAGAGGATGATCAGCCACACTGGGACTGAGACACGGCC
>Zotu5466
GAACGCTGGCGGCGTGCCTAATACATGCAAGTCGAGCGAGGTTGTTTGGAATGCTTGCATTTCAAACA
ACCTAGCGGCGGACGGGTGAGTAACACGTGGGCAACCTGCCCTGTAGACTGGGATAACTTCGGGAAAC
CGGAGCTAATACCGGATAACTCTTTTAGCCTCATGGCTTTAAGCTAAAAGGCGCTTCGGCGTCACTAC
AGGATGGGCCCGCGGTGCATTAGCTAGTTGGTAGGGTAACGGCCTACCAAGGCAACGATGCATAGCCG
ACCTGAGAGGGTGATCGGCCACATTGGGACTGAGACACGGCC
>Zotu5468
GAACGCTGGCGGCGTGCTTAACACATGCAAGTCGAGCGGTAAGGCTCCTTCGGGAGTACACGAGCGGC
GAACGGGTGAGTAACACGTGAGCAATCTGCCCTTCTCATCGGAATAACCATTGGAAACGATGGCTAAT
GCCGAATATGACCTCGGATCACATGTTCTGAGGTGGAAAGCTCCGGCGGAGAAGGATGAGCTCGCGGC
CTATCAGCTTGTTGGTGAGGTAATGGCTCACCAAGGCAATGACGGGTAGCCGGCCTGAGAGGGTGACC
GGCCACACTGGGACTGAGACACGGCC
>Zotu5469
GAACGCTGGCGGCATGCCTAACACATGCAAGTCGAACGAGGGTAGCAATACCCTAGTGGCGGACGGGT
GAGGAATACGTAGGAATTTGCCCTTAAGTGGGGGATAGCCCGGGGAAACCCGGATTAATACCGCATGA
TCTCGAAAGAGCAAAGTGGGGGATCGCAAGACCTCACGCTTTTGGAGAAGCCTACGTCGGATTAGCTA
GTTGGTGAGGTAATGGCTCACCAAGGCGACGATCCGTAACTGGTCTGAGAGGATGATCAGTCACACCG
GAACTGAGACACGGTC
>Zotu5470
GAACGCTGGCGGCGTGCCTAATACATGCAAGTCGAGCGGTGAAGCACCTTCGGGTGTGGATCAGCGGC
GAACGGGTGAGTAACACGTGGGCAACCTGCCCTCAAGCCTGGGATAACTCCGGGAAACCGGGGCTAAC
ACCAGATAATCTTCTTGGACGCATGTGTGAGAAGTAAAAGTTTACACTTGAGGATGGGCCCGCGGTGC
ATTAGCTAGTTGGTGGGGTAACGGCCTACCAAGGCGACGATGCATAGCCGACCTGAGAGGGTGACCGG
CCACACTGGGACTGAGACACGGCC
>Zotu5471
GAACGCTAGCGGCAGGCTTAACACATGCAAGTCGAGGGGTATTTATCTTCGGATAGAGAGACCGGCGC
ACGGGTGCGTAACGCGTATGCAATCTACCTTTTACAGCGGGATAGCCCAGAGAAATTTGGATTAATAC
CACATAGTATTATAAGATGGCATCATTTTATAATTAAAGTCACAACGGTAAAAGATGAGCATGCGTCC
CATTAGCTAGATGGTAAGGTAACGGCTTACCATGGCAACGATGGGTAGGGGTCCTGAGAGGGAGATCC
CCCACACTGGTACTGAGACACGGAC
>Zotu5473
GAACGCTGGCGGCGTGCCTAACACATGCAAGTCGAGCGGGGATGAGGGAGCTTGCTTCCGAATCATAG
CGGCGGACGGGTGAGTAACGCGTGAGCAACCTGCCTCTTACAGGGGGATAACGCATCGAAAGATGTGC
TAATACCGCGTAAGACCACAGTACCGCATGGTACAGGGGTCAAAGGAGCAATCCGGTAAGAGATGGGC
TCGCGTCCGATTAGCTAGTTGGTGAGATAAAAGCCCACCAAGGCGACGATCGGTAGCCGACCTGAGAG
GGTGATCGGCCACATTGGAACTGAGAAACGGTC
>Zotu5474
GAACGCTGGCGGCGTGCCTAATACATGCAAGTCGAACGCATTTTCTTTCACCGTAGCTTGCTACACCG
AAAGAAAATGAGTGGCGAACGGGTGAGTAACACGTGGGTAACCTGCCCATCAGCGGGGGATAACACTT
GGAAACAGGTGCTAATACCGCATAATTCCATTTACCGCATGGTAAATGGATGAAAGGCGCTTTTGCGT

CACTGATGGATGGACCCGCGGTGCATTAGCTAGTTGGTGGGGTAACGGCCTACCAAGGCTGCGATACA
TAGCCGACCTGAGAGGGTGATCGGCCACACTGGGACTGAGACACGGCC
>Zotu5475
AAACGCTGGCGGCGTGCATAACACATGCAAGTCGAACGATGAAGATTAAATGATTTCTTCGGAATGAA
CTTAATTGGATTAGTGGCGGACGGGTGAGTAACGCGTGAGTAACCTGCCTTGTACAAGGGGATAGCCG
TTGGAAACGACGAATAATACCCTATGACATCAAATAATCGCATGATAATTTGATCAAAGATTTATCGG
TATAAGATGGACTCGCGTCTGATTAGCTAGTTGGTAGGGTAAAAGCCTACCAAGGCGACGATCAGTAG
CCGGCTTGAGAGAGTGTACGGCCACATTGGGACTGAGACACGGCC
>Zotu5476
GAATGCTAGCGGTGTGCCTGAGATATGCAAGTCGAGCGACCCTTTGACCGCAAGGTTAAAGGGAGCGG
CGGACGGCTGAGTAACACGTGGGAATCTGCCCCTTTCTAGAGAATAGCTTGCCGAAAGGTGAGGTAAT
GCTCTATAGTCTAAGCCTAGCTTAGTAAAGATTTATCGGGAAGGGATGAGCCTGCGGCCTATCAGGTA
GTTGGTGGGGTAACGGCCTACCAAGCCAGTGACGGGTAGCTAATCTGGGAGGATGGTTAGCCAGAGGG
GGACTGAGACACGGCC
>Zotu5477
GAACGCTGGCGGCGTGCCTAATACATGCAAGTCGAGCGGAAACCAAGGAGCTTGCTCCTCGGTTTTAG
CGGCGGACGGGTGAGTAACACGTGGGCAACCTGCCCTGCAGATCGGGATAACTCCGGGAAACCGGTGC
TAATACCGAATAGGTTCTGGTCCCTCCTGGGACCGGACGGAAAGATGGTTTCGGCTATCGCTGCAGGA
TGGGCCCGCGGCGCATTAGCTAGTTGGTGGGGTAACGGCCCACCAAGGCGACGATGCGTAGCCGACCT
GAGAGGGTGATCGGCCACACTGGGACTGAGACACGGCC
>Zotu5478
GAACGCTAGCGACAGGCCTAACACATGCAAGTCGAGGGGTAACAGGGAGCTTGCTCCGCTGACGACCG
GCGCACGGGTGAGTAACGCGTATGCAACCTGCCTATTACAGGGGGATAACCCGGAGAAATTCGGATTA
ATACCGCATAACACTATTGAGTGGCATCATTTAATAGTTAAAGATTTATTGGTAATAGATGGGCATGC
GTTCCATTAGTTAGTTGGTGAGGTAACGGCTCACCAAGACGTCGATGGATAGGGGTTCTGAGAGGAAG
GTCCCCCACACTGGTACTGAGACACGGAC
>Zotu5479
GAACGCTGGCGGCGTGCTTAACACATGCAAGTGGAGCGACGAACCGGGGCTTGCCCCGGGGCAAAGCC
GCGAACGGGTGAGTAACACGTGGGTAACCTACCCAATTGACAGGGACAACCCGAGGAAACTCGAGCTA
ATACCTGATGTGTCCAGGTGGCATCAGCCATCTGGGGAAAGGAAGCTTCGGCCTCCGCAATTGGATGG
GCCCGCGGCCCATTAGCTTGTTGGTGAGGTAACGGCTCACCAAGGCTTCGATGGGTAGCTGGTCTGAG
AGGACGATCAGCCACACTGGGACTGAGACACGGCC
>Zotu5482
GAACGCTGGCGGCGTGCCTAATACATGCAAGTCGAGCGGTAAGGCCACTTCGGTGGTACACGAGCGGC
GGACGGCTGAGTAACGCGTAGGAACATACCCCAAAGTGAGGGATAACTGCTCGAAAGAGTAGCTAATA
CCGCATATGGTCTAAGGATTAAAGCTTCGGCGCTTTGGGAATGGCCTGCGTATGATTAGTTTGTTGGT
GAGGTAATGGCTCACCAAGACTACGATCATTAGCTGGTCTGAGAGGATGATCAGCCAGACTGGGACTG
AGACACGGCC
>Zotu5484
CAACGCTGGCGGCGTGCCTAACACATGCAAGTCGCACGAGAAAGGGGCTTCGGCCCTGAGTAAAGTGG
CGCACGGGTGAGTAACACGTGAATAATCTACCTTCGAGTGGGGAATAACTGAGGGAAACCTTAGCTAA
TACCGCATAACACTTACGAGTCAAAGCAGCAATGCGCTTGAAGAGGAGTTCGCGGCAGATTAGTTAGT
TGGTGAGGTAATGGCTCACCAAGACGATGATCTGTATCCGGCCTGAGAGGGCGCACGGACACACTGGA
ACTGAAACACGGTC
>Zotu5485
GAACGCTGGCGGCGTGCCTAACACATGCAAGTCGAGCGCGATTTATGAAGACGAAACTTCGGTCTAGG
AATCATAATGAAAGCGGCGGACGGGTGAGTAACGCGTAGGCAACCTGCCCCAAACACAGGGATAGCCT
CGGGAAACCGGGATTAAAACCTGATAATGCCAAAGAGTCACATGGCTCGTTGGCCAAAGATTTATCGG
TTTGGGATGGGCCTGCGTCTGATTAGTTTGTTGGTGGGGTAACGGCCTACCAAGACTACGATCAGTAG
CCGACCTGAGAGGGTGATCGGCCACATTGGAACTGAGACACGGTC
>Zotu5486
GAACGCTGGCGGCGTGCTTAACACATGCAAGTCGAACGGTGAAGCAGAGCTTGCTCTGCGGATCAGTG
GCGAACGGGTGAGTAACACGTGAGTAACCTGCCCTTGACTCTGGGATAAGCGTTGGAAACGACGTCTA
ATACCGGATACGACCCGTGGAGGCATCTCCTACGGGTGGAAAGAATTTTGGTTGAGGATGGACTCGCG

GCCTATCAGCTTGTTGGTGAGGTAATGGCTCACCAAGGCGACGACGGGTAGCCGGCCTGAGAGGGTGA
CCGGCCACACTGGGACTGAGACACGGCC
>Zotu5487
GAACGCTGGCGGCGTGCCTAAGACATGCAAGTCGAGCGGGGCAGGTTTATACCTGTCCAGCGGCGGAC
GGGTGAGTAACGCGTGGGTGACCTACCCGGAAGAGGCGGACAACCTGGGGAAACCCAGGCTAATCCGC
CATGTGGTCCTGTCCTGTGGGGCAGGACTAAAGGGTGAATAGCCCGCTTCCGGATGGGCCCGCGTCCC
ATCAGCTAGTTGGTGGGGTAAAGGCCCACCAAGGCGACGACGGGTAGCCGGTCTGAGAGGATGGCCGG
CCACAGGGGCACTGAGACACGGGC
>Zotu5488
GAACGCTAGCGGTATGCTTAACACATGCAAGTCGAACGAAAAAGTAATTTTTAGTGGCGAACGGGTGA
GTAACGCGTGAGAATTCGCCTCTAAGAGAAGGATAACGTTTAGAAATAAATGCTAAAACTTTATAATC
AAAAAAGTAATTTGCTTAGAGAGAAGCTCGCGTCTGATTAGCTAGTTGGTAGGGTAAAGGCCTACCAA
GGCGATGATCAGTAACTGGTCTGAGAGGATGATCAGTCACATTGGAACTGAGAAACGGTC
>Zotu5489
GAACGCTGGCGGCATGCTTAACACATGCAAGTCGAACGAGCTAATGGGTAACTATTAGTATAGTGGCG
GACGGGTGCGTAACGCGTAAGAACCTACCTTTTGGAGGGGGATAACATCGGGAAACCTTTGGTAATAC
CCCATAATGCTGAGGAGTTAAAGCAGCGAGAGCTGGCCAAAAGAGGGGCTTGCGTCTGATTAGCTAGT
TGGTGGGGGTAAAAGCCTCCCAAGGCCACGATCAGTAGCTGGTCTGAGAGGATGATCAGCCACACTGG
GACTGAGACACGGCC
>Zotu5491
GAACGCTAGCGGCAGGCCTAACACATGCAAGTCGAGGGGTAGAACACTTCGGTGTTTGAGACCGGCGC
ACGGGTGCGTAACGCGTATGCAATCTACCTTGTACAGGGGGATAGCCCGAAGAAATTCGGATTAATAT
CCCATGGTATTTTAGAGTGGCATCACTTTATTATTAAAGATTTATCGGTACAAGATGAGCATGCGTCC
CATTAGTTAGTTGGTGTGGTAACGGCACACCAAGACGATGATGGGTAGGGGTCCTGAGAGGGAGATCC
CCCACACTGGTACTGAGACACGGAC
>Zotu5494
GAACGCTGGCGGCAGGCCTAACACATGCAAGTCGAGCGGCAGCACGGGTACTTGTACCTGGTGGCGAG
CGGCGGACGGGTGAGTAATGCCTAGGAATCTGCCTGGTAGTGGGGGATAACGCTCGGAAACGGACGCT
AATACCGCATACGTCCTACGGGAGAAAGCAGGGGACCTTCGGGCCTTGCGCTATCAGATGAGCCTAGG
TCGGATTAGCTAGTTGGTGAGGTAATGGCTCACCAAGGCGACGATCCGTAACTGGTCTGAGAGGATGA
TCAGTCACACTGGAACTGAGACACGGTC
>Zotu5495
GAACGCTGGCGGCAGGCTTAACACATGCAAGTCGAGCGGGCGAGGTTGCTTCGGTAACTGAGCTAGCG
GCGGACGGGTGAGTAAAGCTTAGGAATCTGCCTATTAGTGGGGGACAACATTTCGAAAGGGATGCTAA
TACCGCATACGTCCTACGGGAGAAAGCAGGGGACCTTCGGGCCTTGCGCTAATAGATGAGCCTAAGTC
GGATTAGCTAGTTGGTGGGGTAAAGGCCTACCAAGGCGACGATCTGTAGCGGGTCTGAGAGGATGATC
CGCCACACTGGGACTGAGACACGGCC
>Zotu5497
GAACGCTGGCGGCAGGCCTAACACATGCAAGTCGAGCGCGCCCTTCGGGGTGAGCGGCGGACGGGTGA
GTAACGCGTGGGAACGTGCCCTTCTCTACGGAATAGCCCCGGGAAACTGGGAGTAATGCCGTATACGC
CTTTCGAGGGAAAGATTTATCGGAGAAGGATCGGCCCGCGTCTGATTAGGTAGTTGGTGGGGTAATGG
CCTACCAAGCCGACGATCAGTAGCTGGTTTGAGAGGATGATCAGCCACACTGGGACTGAGACACGGCC
>Zotu5498
GAACGCTGGCGGCGTGCTTAACACATGCAAGTCGAGCGGAAAGGCCACTTCGGTGGTACTCGAGCGGC
GAACGGGTGAGTAACACGTGAGTAATCTGCCCCTGGCTTTGGGATAGCCACCGGAAACGGTGATTAAT
ACCGGATACGACAACTTCTCGCATGGGATGGTTGTGGAAAGTTTTTCGGCCAGGGATGTGCTCGCGGC
CTATCAGCTTGATGGTGAGGTAATGGCTCACCATGGCTTCGACGGGTAGCCGGCCTGAGAGGGTGACC
GGCCACACTGGGACTGAGACACGGCC
>Zotu5499
GAACGCTTGCGGCGTGCCTAAGAAATGCAAGTCGAGCGGGGGAGCAATCCCTAGCGGCGAACGGTCGC
GTAACACGTAGGCAACCTGCCCGAAAGACCGGGACAACACTTCGAAAGGGGTGCTAATACCGGATGTG
CCCGCTTCTTCACCTGGGGAGGCGAGTAAATCCTCGCAAGGGGGCTTTTGGAGGGGCCTGCGGCCTAT
CAGCTAGATGGTGAGGTAACGGCCCACCATGGCGACGACGGGTAGCTGGTCTGAGAGGATGGTCAGCT
GGACTGGGACTGAGATACGGCC

>Zotu5502
GAACGCTGGCGGCGTGCCTAATACATGCAAGTCGAGCGAATCAAAGGGAGCTTGCTCCCTTTTGATTA
GCGGCGGACGGGTGAGTAACACGTGGGCAACCTGCCTATAAGACTGGGATAACTTCGGGAAACCGGAG
CTAATACCGGATAATTCTTTTCTACACATGTAGGAAAGCTGAAAGACGGTTTACGCTGTCACTTATAG
ATGGGCCCGCGGCGCATTAGCTAGTTGGTGAGGTAACGGCTCACCAAGGCGACGATGCGTAGCCGACC
TGAGAGGGTGATCGGCCACACTGGGACTGAGACACGGCC
>Zotu5503
GAACGCTGGCGGCATGCCTAATACATGCAAGTCGAACGAGCTACTTCGGTAGCTAGTGGCGAACGGGT
GAGTAACACGTAGATAACCTGCCCATACCCGGGGGATACGCTTTGGAAACGAAGTCTAAAACCCCATA
GGAAGATTTAAGGCATCTTAAATTTTTGAAATAAGCTTTGGCTTAGGGGATGGATGGATCTGCGGTGC
ATTAGCTAGTTGGTGAGGTAACAGCTCACCAAGGCGATGATGCATAGCCGGCCTGAGAGGGCGATCGG
CCACACTGGGACTGAGACACGGCC
>Zotu5504
GAACGCTGGCGGCGTGCTTAACACATGCAAGTCGAACGATGATCAGGAGCTTGCTCCTGTGATTAGTG
GCGAACGGGTGAGTAACACGTGAGTAACCTGCCCCTGACTCTGGGATAAGCGTTGGAAACGACGTCTA
ATACTGGATACGACTACTGGCCGCATGGTCTGGTGGTGGAAAGATTTTTTGGTTGGGGATGGACTCGC
GGCCTATCAGCTTGTTGGTGAGGTAATGGCTCACCAAGGCGACGACGGGTAGCCGGCCTGAGAGGGTG
ACCGGCCACACTGGGACTGAGACACGGCC
>Zotu5505
GAACGCTGGCGGCGTGCCTAATACATGCAAGTCGAACGCACGGAGGAAGAACTTGTTCTTCCAAAGTG
AGTGGCGCACGGGTGAGTAACACGTGGGAAACCTACCCTTCAGCGGGGGATAACCGTCGGAAACGATG
ACTAATACCGCATAGACGACAGAGTCGCATGACTCAATCGGGAAAGACGGCTTCGGCTGTCACTGAAG
GATGGTCCCGCGCTGCATTAGCTAGTTGGTAAGGTAACGGCTTACCAAGGCGATGATGCATAGCCGAC
CTGAGAGGGTGATCGGCCACATTGGGACTGAGACACGGCC
>Zotu5507
GAACATTGGCGGTAGGCTTAACACATGCAAGTCGAGCGAGGCGTAATAGTCGAGCGGCGAACGGGTGA
GTAATACATGGAAATCTACCCTGAAGTGGGGGATAACGTTCAGAAATGAACGCTAATACCGCATAATC
CATATTGGGAAAGCGTTGCAGCGCTTTAGGATGAGTCCATGGCTGATTAGCTTGTTGGTGAGGTAATG
GCTCACCAAGGCGACGATCAGTAGCTGGTCTGAGAGGATGACCAGCCACACTGGAACTGAGACACGGT
C
>Zotu5508
GAACGCTAGCGGCAGGCCTAATACATGCAAGTCGAACGGTCCCCAGCAATGGGGATAGTGGCGCACGG
GTGCGTAACGCGTATGAAACCTGCCCTTCACTGGGGGATAACCTCTCGAAAGAGAGGCTAATACCGCA
TAAGATAGTAGAACGGCATCGTTCAGCTCTTAAAACTCCGGTGGTGAAGGATGGTCATGCGTCCAATT
AGTTAGTTGGTAGGGTAACGGCCTACCAAGACGATGATTGGTAGGGGGTCTGAGAGGATGATCCCCCA
CACTGGTACTGAGATACGGAC
>Zotu5509
GAACGTTGATGGCGTGCCTCATACATGCAAGTCGAGCGGGTAGCAATACCAGCGGCGGACGGGTGAGT
AACACGTAAGGAATCTGCCCTGAAGCGGGGAATAACTATCCCAACGGATAGCTAATACCGCATAATGA
TGGAGACGGCATCGTCTCTTCTCAAAAGGCTTCGGCCACTTCAGGATGACCTTGCGGCCCATTAGCTT
GTTGGCGGGGTAACGGCCCACCAAGGCTACGATGGGTATCCGGCCTGAGAGGGTGTACGGACACATTG
GGACTGAGATACGGCC
>Zotu5510
GAACGCTGGCGGCGTGCTTAACACATGCAAGTCGAACGGTGAACTCAGCTTGCGGGGGGATCAGTGGC
GAACGGGTGAGTAACACGTGGGCAACCTGCCCTCAGCTCTGGGATAACTCCAAGAAATTGGGGCTAAT
ACTGGATATGACCGCTGACCGCATGGTCTGGTGGTGGAAAGATTTATCGGCTGGGGATGGGCCCGCGG
CCTATCAGCTTGTTGGTGGGGTAGTGGCCTACCAAGGCGACGACGGGTAGCCGGCCTGAGAGGGTGAC
CGGCCACACTGGGACTGAGACACGGCC
>Zotu5512
GAACGCTGGCGGCGTGCTTAACACATGCAAGTCGAACGGAAAGGCCTTGCGCTTGCACAAGGTACTCG
AGTGGCGAACGGGTGAGTAACACGTGGGTGATCTGCCCTGCACTGTGGGATAAGCCTGGGAAACTGGG
TCTAATACCATATAGGACCGCATCTTGGATGGTGTGGTGGAAAGCTTTTGCGGTGTGGGATGAGCCTG
CGGCCTATCAGCTTGTTGGTGGGGTAATGGCCTACCAAGGCGGCGACGGGTATCCGGCCTGAGAGGGT
GTACGGACACATTGGGACTGAGATACGGCC

>Zotu5514
GAACGCTGGCGGCGCGCCTAAAACATGCAAGTCGAACGAGAAGCCTGGGCTTGCCCGGGTGGACAGTG
GCGAACGGGTGAGTAACACGTGAGGAACCTGCCCCGAAGACCGGGACAACACCGGGAAACCGGTGCTA
ATACCGGATACCTTCACCGAATCGCATGGTCTGGTGAAGAAATGGATTCCGCTTCGGGATGGCCTCGC
GGCCTATCAGCTTGTTGGTGAGGTAATGGCTCACCAAGGCGACGACGGGTAGCTGGTCTGAGAGGATG
TCCAGCCACACTGGGACTGAGACACGGCC
>Zotu5515
GAACGCTGGCGGCATGCTTAACACATGCAAGTCGAGCGGTAAGGCTCCTTCGGGAGTACACGAGCGGC
GAACGGGTGAGTAACACGTGAGCAATCTGCCCTTCACTTTGGGATAACCCCGGGAAACTGGGGCTAAT
ACCGAATACGACCACCGATGGCATCTTTGGTGGTGGAAAGTTCCGGCGGTGAAGGATGAGCTCGCGGC
CTATCAGCTTGTTGGTGAGGTAATGGCTCACCAAGGCAACGACGGGTAGCCGGCCTGAGAGGGTGACC
GGCCACACTGGGACTGAGACACGGCC
>Zotu5517
GAACGCTGGCGGCATGCCTTACACATGCAAGTCGAACGGCAGCACGGGTGCTTGCACCTGGTGGCGAG
TGGCGAACGGGTGAGTAACGCATCGGAACGTGCCTTTTAGTGGGGGATAACTATCCGAAAGGATAGCT
AATACCGCATACGCCCTGAGGGGGAAAGGGGGGGATCGCAAGACCTCTCGCTAAAAGAGCGGCCGATG
TCCGATTAGCTAGTTGGTGAGGTAAAGGCTCACCAAGGCGACGATCGGTAGCTGGTCTGAGAGGATGA
TCAGCCACACTGGAACTGAGACACGGTC
>Zotu5518
GAACGCTGGCGGCATGCCTAATACATGCAAGTCGAACGAGATCTTCGGATCTAGTGGCGCACGGGTGC
GTAACGCGTGGGAATCTGCCCTTGGGTTCGGAATAACTTCTGGAAACGGAAGCTAATACCGGATGATG
ACGTAAGTCCAAAGATTTATCGCCCAAGGATGAGCCCGCGTAGGATTAGCTAGTTGGTGGGGTAAAGG
CCCACCAAGGCGACGATCCTTAGCTGGTCTGAGAGGATGATCAGCCACACTGGGACTGAGACACGGCC
>Zotu5519
GAACGCTGGCGGCGTGCCTAATACATGCAAGTCGAGCGAATCTTTGGGAGCTTGCTCCCTATAGATTA
GCGGCGGACGGGTGAGTAACACGTGGGTAACCTGCCTGTAAGACTGGGATAACTTCGGGAAACCGGAG
CTAATACCGGATAGTTTCTTTTCTCGCATGAGAAGAGATGGAAAGATGGTTCTGCTATCACTTACAGA
TGGACCCGCGGCGCATTAGCTAGTTGGTGAGGTAACGGCTCACCAAGGCGACGATGCGTAGCCGACCT
GAGAGGGTGATCGGCCACACTGGGACTGAGACACGGCC
>Zotu5520
GAACGCTGGCAGCGTGCTTAACACATGCAAGTCGAACGGTGAAGCCCCGCTTGCGGGGTGGATCAGTG
GCGAACGGGTGAGTAACACGTGAGCAATCTGCCCCTGACTCTGGGATAAGCGCTGGAAACGGCGTCTA
ATACTGGATACGAACCGTGGAGGCATCTTCAACGGTTGGAAAGATTTTTTGGTCAGGGATGAGCTCGC
GGCCTATCAGCTTGTTGGTGAGGTAATGGCTCACCAAGGCGTCGACGGGTAGCCGGCCTGAGAGGGTG
ACCGGCCACACTGGGACTGAGACACGGCC
>Zotu5521
GAACGCTGGCGGCATGCCTAACACATGCAAGTCGAACGAAGGCTTCGGCCTTAGTGGCGCACGGGTGC
GTAACGCGTGGGAATCTGCCCTTAGGTTCGGAATAACAGCTGGAAACGGCTGCTAATACCGGATGAAG
ACGAAAGTCCAAAGATTTATCGCCTGAGGATGAGCCCGCGTTGGATTAGGTAGTTGGTGGGGTAAAGG
CCTACCAAGCCGACGATCCATAGCTGGTCTGAGAGGATGATCAGCCACACTGGGACTGAGACACGGCC
>Zotu5522
GAACGCTGGCGGCAGGCTTAACACATGCAAGTCGAGCGGGCCCTTCGGGGTCAGCGGCAGACGGGTGA
GTAACGCGTGGGAACGTGCCCTTCGGTTCGGAATAACCCTGGGAAACTAGGGCTAATACCGGATACGC
CCCTTGGGGGAAAGGTTTACCGCCGAAGGATCGGCCCGCGTCTGATTAGCTTGTTGGTGAGGTAACGG
CTCACCAAGGCGACGATCAGTAGCTGGTCTGAGAGGATGATCAGCCACACTGGGACTGAGACACGGCC
>Zotu5523
GATCATTGGCGGCAGGCTTAACACATGCAAGTCGAGCGAGGCTAAGAGCCGAGCGGCGAACGGGTGAG
TAATACTTGGGAATCTACCCTGGAGTGGGGGATAACAACCAGAAATGGTTGCTAATACCGCATAATCC
ATATTGGGAAAGCGCTTAGCGCTCTAGGATGAGCCCAAGTCTGATTAGCTAGTTGGGGGGGTAATGGC
CCACCAAGGCGACGATCAGTAGCTGGTTTGAGAGGATGACCAGCCACACTGGAACTGAGACACGGTC
>Zotu5524
GAACGCTGGCGGCGTGCTTAAGACATGCAAGTCGAACGGCTGGAAGCTTGCTTCCAGCAGTGGCGCAC
GGGTGAGTAACACGTAACTGACCTGCCCCAAAGTTCCGAATAACTCTCCGAAAGGAGAGCTAACACGG
GATGTGCAGTCTGGTCCTGTCCAGCCTGTAAAGATTTATCGCTTTGGGATGGGGTTGCGTTCCATCAG

CTTGTTGGTGAGGTAAAGGCTCACCAAGGCGACGACGGATAGCCGGCCTGAGAGGGTGGCCGGCCACA
GGGGCACTGAGACACGGGC
>Zotu5525
GAACGCTGGCGGCATGCCTAACACATGCAAGTCGAACGATGCCTTCGGGCATAGTGGCGCACGGGTGC
GTAACGCGTGGGAATCTGCCCTTTGGTTCGGGATAACTGTTGGAAACGACAGCTAATACCGGATGATG
ACGTAAGTCCAAAGATTTATCGCCAGAGGATGAGCCCGCGTCGGATTAGCTAGTTGGTGGGGTAAAGG
CCTACCAAGGCGACGATCCGTAGCTGGTCTGAGAGGATGATCAGCCACACTGGGACTGAGACACGGCC
>Zotu5526
GAACGCTGGCTGCGTGCTTAACACATGCAAGTCGAACGCTGAAGCCCGCAGCTTGCTGTGGGTGGATG
AGTGGCGAACGGGTGAGTAACACGTGAGTAACCTGCCCCCGATTTCGGGATAAGCCCGGGAAACTGGG
TCTAATACCGGATATGACCTCCAGCCGCATGGTTGGTGGTGGAAAGTTTTTCGATCGGGGATGGGCTC
GCGGCCTATCAGCTTGTTGGTGGGGTAATGGCCTACCAAGGCGACGACGGGTAGCCGGCCTGAGAGGG
CGACCGGCCACACTGGGACTGAGACACGGCC
>Zotu5528
GAACGCTGGCGGCAGGCTTAACACATGCAAGTCGAGCGGGCCCTTCGGGGTCAGCGGCGGACGGGTGA
GTAACGCGTGGGAACGTGCCCTCTTCTACGGAATAGCCCAGGGAAACTTGGATTAATGCCGTATACGC
CCGAAAGGGGAAAGATTTATCGGAGGAGGATCGGCCCGCGTTGGATTAGGTAGTTGGTGGGGTAATGG
CCTACCAAGCCGACGATCCATAGCTGGTTTGAGAGGATGATCAGCCACACTGGGACTGAGACACGGCC
>Zotu5529
GAACGCTAGCGGCAGGCTTAATACATGCAAGTCGAGGGGTAACATGGATAGCAATACCTGATGACGAC
CGGCAAACGGGTGCGGAACACGTACGCAACCTTCCCATAACTGGTGAATAGCCTTTCGAAAGAGAGAT
TAATACACCGTAACATAACGAGTAGGCATCTACTTGTTATTATAGGCTTCGGCCGGTTTTGGATGGGC
GTGCGCCTGATTAGGTAGTTGGCGGGGTAACGGCCCACCAAGCCTGCGATCAGTAACTGGTGTGAGAG
CACGACCAGTCACACGGGCACTGAGACACGGGC
>Zotu5531
GAACGCTGGCGGCGTGCTTAACACATGCAAGTCGAACGGTAAGGCCCTTCGGGGTACACGAGTGGCGA
ACGGGTGAGTAACACGTGGGTAATCTGCCCTGCACTTCGGGATAAGCCTGGGAAACTGGGTCTAATAC
CGGATATTCAGTTCCTGCCGCATGGTGGGGGTTGGAAAGTTTTTCGGTGCAGGATGAGCCCGCGGCCT
ATCAGCTTGTTGGTGGGGTAATGGCCTACCAAGGCGACGACGGGTAGCCGGCCTGAGAGGGTGATCGG
CCACACTGGGACTGAGACACGGCC
>Zotu5533
GAACGCTGGCGGCGTGCCTAATACATGCAAGTCGAACGGTTTAGTTCCAAGAGGCAAGACTCTTGGGA
CTAGGCAGTGGCGGACGGGTGAGTAACACGTAGGCAACCTACCTGTCAAACCGGGATAACCTCGGGAA
ACCGAAGCTAAACCCGGATAAGCTCCTTTTTCGCATGAAGGGGGAGTGAAAAGGTGCGACCTGATGAC
AGATGGGCCTGCGGCGCATTAGCTAGTAGGTAGGGTAACGGCCTACCTAGGCGATGATGCGTAGCCGA
CCTGAGAGGGCGTACGGCCACACTGGGACTGAGATACGGCC
>Zotu5534
GAACGCTGGCGGCAGGCTTAACACATGCAAGTCGAGCGGGCCCTTCGGGGTCAGCGGCAGACGGGTGA
GTAACACGTGGGAACGTGCCCTTCGGTTCGGAATAACTCAGGGAAACTTGAGCTAATACCGGATACGC
CCTTTTGGGGAAAGGTTTACTGCCGAAGGATCGGCCCGCGTCTGATTAGCTAGTTGGTGGGGTAATGG
CCCACCAAGGCGACGATCAGTAGCTGGTCTGAGAGGATGATCAGCCACACTGGGACTGAGACACGGCC
>Zotu5535
TAACGCTGGCGGCATGCCTTATGCATGCAAGTAAAACGGGTGAGTAGCAATACTCACAAGTTGCGAAC
GGCTGAGTAATGGATAGGAATCTCCCCGATGGCGTGGAATACCTCATCGAAAGATGAGCTAATACCGC
ATAACCCCGCAAGGGAAAAGCGCTTAACTGCGCACCGACGGATGAGCCTATCTCCCATCAGGCTGTTG
GTAGGGTAATGGCCTACCAAACCTATGACGGGTACCCGCCCTGAGAGGGGAAACGGGCAGAATGGGAC
TGAGACACGGCC
>Zotu5536
GAACGCTGGCGGCAGGCTTAACACATGCAAGTCGAGCGGGGAAGGTTGCTTCGGTAACTGACCTAGCG
GCGGACGGGTGAGTAATGCTTAGGAATCTGCCTATTAGTGGGGGACAACATTCCGAAAGGAATGCTAA
TACCGCATACGTCCTACGGGAGAAAGCAGGGGATCATTTGACCTTGCGCTAATAGATGAGCCTAAGTC
GGATTAGCTAGTTGGTGGGGTAAAGGCCTACCAAGGCGACGATCTGTAGCGGGTCTGAGAGGATGATC
CGCCACACTGGGACTGAGACACGGCC
>Zotu5538

GAACGCTGGCGGCAGGCTTAACACATGCAAGTCGAGCGGGGAAAAGTAGCTTGCTACTTTACCTAGCG
GCGGACGGGTGAGTAATGCTTAGGAATCTGCCTATTAGTGGGGGACAACATCTCGAAAGGGATGCTAA
TACCGCATACGTCCTACGGGAGAAAGCAGGGGACCTTCGGGCCTTGCGCTAATAGATGAGCCTAAGTC
GGATTAGCTAGTTGGTGGGGTAAAGGCCTACCAAGGCGACGATCTGTAGCGGGTCTGAGAGGATGATC
CGCCACACTGGGACTGAGACACGGCC
>Zotu5539
TAACGCTGGCGGCGTGCCTAACACATGCAAGTCGAACGGACCTTTATTTTTGAGACGCCCCATTTCCT
TGGAGCGCCCCATAAATAATGGTTAGTGGCGAACGGGTGAGTAACACGTTGGTACCTGCCCTCTAGTG
GGGGATAACTGCCCGAAAGGGTAGCTAATACCGCATGTGATCTACGGATCAAAGCAGCAATGCGCTAG
AGGAGGGGCCTGCGGCTGATTAGCTTGTTGGTAGGGTAATGGCCTACCAAGGCGACGATCAGTAGCTG
GATCGAGAGGTTGACCAGCCACACTGGGACTGAGATACGGCC
>Zotu5540
GAACGCTGGCGGCATGCCTAACACATGCAAGTCGAACGGAGCCGCAAGGCTCAGTGGCGAACGGGTGC
GTAACACGTGAGGAACATGGCAATCGGCGGGGGATAGCCGGCCCAACGGCCGGGTAATACCGCGTACG
ACCTTCGGGTGGCATCATCTGGAGGTGAAAGCAGCAATGCGCCGATTGGGTGCCTCGCGGCCTATCAG
CTAGTTGGTGAGGTAATGGCTCACCAAGGCTACGACGGGTAGCTGGTCCGAGAGGATGACCAGCCACA
TTGGGACTGAGACACGGCC
>Zotu5541
GAACGCTGGCGGCGTGCTTAACACATGCAAGTCGAACGATGACGGTGGTGCTTGCACTGCCTGATTAG
TGGCGAACGGGTGAGTAACACGTGAGTAACCTGCCCTTCACTTCGGGATAACCTCGGGAAATCGTGGC
TAATACCGTATATGAACTCCTGCCGCATGGTGGGGGTTGGAAAGATTTATCGGTGAAGGATGGACTCG
CGGCCTATCAGTTTGTTGGTGAGGTAATGGCTCACCAAGACGATGACGGGTAGCCGGCCTGAGAGGGC
GACCGGCCACACTGGGACTGAGACACGGCC
>Zotu5542
GAACGCTGGCGGCGTGCTTAACACATGCAAGTCGAACGATGAACCCGGAGCTTGCTCCGGTGGATTAG
TGGCGAACGGGTGAGTAACACGTGAGTAACCTGCCCTTGACTCTGGGATAACTGCTAGAAATGGTAGC
TAATACCGGATACGACCCTCGGAGGCATCTCCAGGGGGTGGAAAGAATTTCGGTCAAGGATGGACTCG
CGGCCTATCAGCTAGTTGGTGAGGTAAAGGCTCACCAAGGCGACGACGGGTAGCCGGCCTGAGAGGGT
GACCGGCCACACTGGGACTGAGACACGGCC
>Zotu5543
GAACGCTAGCGGGATGCCTTACACATGCAAGTCGAACGGCAGCACGGACTTCGGTCTGGTGGCGAGTG
GCGAACGGGTGAGTAATGTATCGGAACGTGCCTAGTAGCGGGGGATAACTACGCGAAAGCGTAGCTAA
TACCGCATACGCCCTACGGGGGAAAGCGGGGGATCGTAAGACCTCGCACTATTAGAGCGGCCGATATC
GGATTAGCTAGTTGGTGAGGTAAAGGCTCACCAAGGCGACGATCCGTAGCTGGTTTGAGAGGACGACC
AGCCACACTGGGACTGAGACACGGCC
>Zotu5544
GAACGCTGGCGGTGCGCCTAAAACATGCAAGTCGAGTGGGTTTAGACCCACGGCAAACGGCTGAGGAA
CACATGGATACCTGCCCCGAACTCAGGGATAGCCCGCCGAAAGGCGGAGTAATACCGGATGGTCCCGC
AAGGGTAAAGATTTATCGGTTCGGGAGGGGTCTGTGTCCTATCAGGTAGTTGGTAAGGTAATGGCTTA
CCAAGCCTATGACGGGTAACTGGTCTGAGAGGACGGCCAGTCAGAATGGAACTGAGACACGGTC
>Zotu5545
GAACGCTGGCGGCGTGCTTAATACATGCAAGTCGAACGAGGTCCTTCGGGACCTAGTGGCGAACGGGT
GAGTAACACGTGAGCAACCTGCCCCGAAGACCGGGATAACAGTGGGAAACCGCTGCTAATACCGGATA
CCCCCACCGGATCACATGGTCTGGTGAGGAAATGCTGACTTTCGAGTCATACGCTTCGGGAGGGGCTC
GCGGCCTATCAGCTTGTTGGTGGGGTAACGGCCTACCAAGGCAACGACGGGTAGCTGGTCTGAGAGGA
CGATCAGCCACACTGGGACTGAGACACGGCC
>Zotu5547
GAACGCTGGCGGCGTGCTTAACACATGCAAGTCGAACGATGATGTCCCAGCTTGCTGGGGTGGATTAG
TGGCGAACGGGTGAGTAACACGTGGGTGATCTGCCCTGCACTTCGGGATAAGCCTGGGAAACTGGGTC
TAATACCGGATATGACCCTCCTTTAGTGTGGGGGGTGGAAAGCTTTAGCGGTGTGGGATGAGCCTGCG
GCCTATCAGCTTGTTGGTGGGGTAATGGCCTACCAAGGCGTCGACGGGTAGCCGGCCTGAGAGGGTGA
TCGGCCACATTGGGACTGAGACACGGCC
>Zotu5548
GAATGCTGGCGGCGTGCTTAACACATGCAAGTCGAGCGGTAAGGCCCCTTCGGGGGTACACGAGCGGC

GGACGGCTGAGTAACGCGTAGGAACATGCCCCAAAGTGAGGAATAAGCTCCAGAAATGGAGTCTAATG
CCGCATATGATCTTCGGATTAAAGGATTTATCCGCTATGGGAGTGGCCTGCGTATGATTAGGTAGTTG
GTGAGGTAAAGGCTCACCAAGCCGACGATCATTAGCTGGTCTGAGAGGATGACCAGCCAGACTGGAAC
TGAGACACGGTC
>Zotu5549
GAATGCTGGCGGCGTGGATAAGGCATGCAAGTCGAGCGGGTTTAGACCCGCGGCAGACGAGGTAGTAA
CACGTAGGAACATACCCCGAAGTCTGGCATATTCCGTCGAAAGACGGTGCAATTCCAGATGGTCTCTA
AGGAGTAAAGAAGCAATTCGCTTCGGGAGTGGCCTGCGCAATATCAGCTAGTTGGTGAGGTAATGGCT
CACCAAGGCTATGACGTTTAGGGGAGCTGAGAGGCTGACCCCCACCGATGGGACTGAGATACGGCC
>Zotu5550
GAACGTTGGCGGCGTGCCTAACACATGCAAGTCGAACGGAAATAGCAATATTTGAGTGGCGCACGGGT
GAGTAACACGTAGCTAATCTACCTCAAAGCGCGGGATAACGTGTCGAAAGACAGGCTAATACCGCATA
AGCTCGCGACCTGTAGAGGGTTGCGAGAAAAGGCGAAAGTCACTTTGAGATGAGGCTGCGGCCCATCA
GCTAGTTGGGGTGGTAAAAGCACACCAAGGCAACGACGGGTAGGGGACCTGAGAGGGTGACCCCCCAC
ACTGGGACTGAGACACGGCC
>Zotu5551
GAACGCTGGCGGCGTGCCTAACACATGCAAGTCGAGCGGAGACATTGGTTTCGGCCAATGTTTTAGCG
GCGGACGGGTGAGTAACGCGTGAGCAATCTGTCCTCTACAGGGGGATACCAGTTGGAAACAACTGCTA
ATACCGCATAAGACCACAGAGTCACATGGCTTAGGGGTCAAAGGAGTAATTCGGTAGGGGGTGAGCTC
GCGTCTGATTAGATAGTTGGTGAGGTAACGGCCCACCAAGTCAACGATCAGTAGCCGACCTGAGAGGG
TGATCGGCCACATTGGAACTGAGACACGGTC
>Zotu5552
GAACGCTAGCGGCAGGCCTAATACATGCAAGCTGAACGGCAAGTTGAATAGCAATATTTGACCTAGAG
TAGCGTAAGGGTGCGTAACACGTATGCAATCTGCCCGCAACAGGAGGATAGCTTGATGAAAGTTGAGA
TAAGACTCCATAAGATCACACGAGGGCATCTGAGGGTGATTAAACTACAAGGGGTTGCGGATGAGCAT
GCGTCTGATTAGTTAGTTGGCGGGGTAACGGCCCACCAAGACGATGATCAGTAGGGGAACTGAGAGGT
TGGTCCCCCACACTGGCACTGAGATACGGGC
>Zotu5553
GAACGCTGGCGGCATGCCTAACACATGCAAGTCGAACGAAGGCTTCGGCCTTAGTGGCGCACGGGTGC
GTAACGCGTGGGAATCTGCCCTGGGGTACGGAATAACTCAGAGAAATTTGAGCTAATACCGTATGATA
TCGCGAGATCAAAGATTTATCGCCCTGGGATGAGCCCGCGTTGGATTAGCTAGTTGGTGGGGTAAAGG
CCTACCAAGGCGACGATCCATAGCTGGTCTGAGAGGATGATCAGCCACACTGGGACTGAGACACGGCC
>Zotu5554
GAACGCTGGCGGCGCGCCTAAAACATGCAAGTCGAACGAGAAACTCGGGCTTGCTCGGGTGGACAGTG
GCGAACGGGTGAGTAACACGTGAGGAACCTGCCCCGAAGACCGGGACAACACCGGGAAACCGGTGCTA
ATACCGGATGTCCTCATCGGACCGCATGATCTGATGAGGAAATGGATTCCGCTTCGGGATGGCCTCGC
GGCCTATCAGCTAGTTGGTGGGGTAACGGCCCACCAAGGCGACGACGGGTAGCTGGTCTGAGAGGATG
TCCAGCCACACTGGGACTGAGACACGGCC
>Zotu5557
GAACGCTGGCGGCATGCTTTACACATGCAAGTCGAACGGCAGCGGGGGCTTCGGCCTGCCGGCGAGTG
GCGAACGGGTGAGTAATGCATCGGAACGTGCCCAGTCATGGGGGATAACTACGCGAAAGCGTAGCTAA
TACCGCATACGCCCTGAGGGGGAAAGTGGGGGATCGCAAGACCTCACGTGATTGGAGCGGCCGATGTC
AGATTAGCTAGTTGGTGGGGTAAAAGCCTACCAAGGCGACGATCTGTAGCGGGTCTGAGAGGATGATC
CGCCACACTGGGACTGAGACACGGCC
>Zotu5558
GATCGCTGGCGGCGTGCTTAACACATGCAAGTCGAGCGGATTTCTGCAGTGTATCGCAGGAATTAGCG
GCAGACGGGTGAGTAATACTTGGGCAATCTGCCCTTGAGTGGGAAACAACCGGCCGAAAGGCTGGCTA
ATGTCCCATAAGACCACAGGATGGCATCATCCAGGGGTCAAAGCTCCGTAAGGGGCGCTTGAGGAGGA
GCCTGAGTCCTATCATGGTAGTTGGTGAGGTAACGGCTCACCAAGCCTTTGACGGGTAGCCGGCCTGA
AAGGGTGATCGGCCACACTGGCACTGAGACACGGGC
>Zotu5559
GAACGCTGGCGGCGTGCTTAACACATGCAAGTCGAACGGAATCCAACCGGTGGCAACACTGGTGAAGA
TTCAGTGGCGAACGGGTGCGGAACACGTGAGCAACCTGCCCCAAAGACCGGGATAACACCGGGAAACT
GGTGCTAATACCGGATACCCCCACGAGTTCGCATGGGCTTGTGAGGAAAGGGTTTCCGCTTTGGGAGG

GGCTCGCGGCCTATCAGCTAGTTGGTGAGGTAACGGCTCACCAAGGCGACGACGGGTAGCTGGTCTGA
GAGGACGATCAGCCACACTGGGACTGAGACACGGCC
>Zotu5561
CAACGCTGGCGGCGTGCCTAACACATGCAAGTCGAACGCGAAAGTGGAGCAATCCATGAGTAGAGTGG
CAAACGGGTGAGTAACACGTGGGTGACCTGCCTTCGAGTGGGGGATAACGTCCCGAAAGGGACGCTAA
TACCGCATGACATCCTGCCTTTGAACAGGCGGAGATCAAAGCCGGGGATCGCAAGACCTGGCGCTTGA
AGAGGGGCCCGCGTCCGATTAGCTAGTTGGTGAGGTAATGGCTCACCAAGGCAACGATCGGTAGCCGG
CCTGAGAGGGCGGACGGCCACACTGGGACTGAGACACGGCC
>Zotu5563
GAACGCTGGCGGCATGCTTAACACATGCAAGTCGCACGAGGGGGCAACCTCTAGTGGCGGACGGGTGA
GTAACGCGTAGGTATCTATCCTGGGGTGGGGGATAACAGCGGGAAACTGCTGCTAATACCGCATGACA
CCTGAGGGTCAAAGGCGCGAGTCGCCTTGGGAGGAGCCTGCGTCCGATTAGCTAGTTGGTGGGGTAAG
AGCCTACCAAGGCGATGATCGGTAGCTGGTCTGAGAGGATGATCAGCCACATTGGGACTGAGACACGG
CC
>Zotu5564
GAACGCTAGCGGCAGGCTTAATACATGCAAGGCGAGGGGGCAGCAATGTCACCGTCGTACGGGTGCGT
AACGCGTATGCAACCTACCTTCAACTGGGGGATAGCCCGGGGAAACCCGGATTAATACCGCATAATAC
ATTGGTGTAGCATTACATTATTTGTTAAAGATTTATTGGTTGAAGATGGGCATGCGTTCGATTAGCTA
GTTGGCAGGGTAACGGCCTACCAAGGCGACGATCGATAGGGGAGCTGAGAGGTTGATCCCCCACACGG
GCACTGAGATACGGGC
>Zotu5565
GAACGCTGGCGGCGTGCCTAAGACATGCAAGTCGAGTGGGTTTAGACCCACGGCGAACGGGTGAGTAA
TACCTAGGAACGTTCTTTAGGTGGAGCATAACCAGCCGAAAGGTTGGATAATTCTCCATGTAGTCGAA
AGACAAAAGTCGCAAGACGCCTAAAGTGCGGCCTGGGCACCATCAGCTAGTTGGTAGGGTAAAAGCCT
ACCAAGGCTATGACGGTTAGGGGCCCCGAGAGGGGAGACCCCCACACTGGGACTGCGACACGGCC
>Zotu5566
GAACGCTGGCGGCGTGCTTAACACATGCAAGTCGAACGAATAACCCTTCTTCGGAAGGTTATAGAGTG
GCGAACGGGTGAGTAACACGTGACCAACCTGCCTCTTAGATTGGGATAACTCAGAGAAATTTGAGCTA
ATACCGAATACTCCGATATCTTCGCATGGAGATGTCGGGAAAGCTTTTGCGCTAAGAGATGGGGTCGC
GGCCTATTAGATAGTTGGTGGGGTAACGGCCTACCAAGTCAACGATAGGTAGCCGGGTTGAGAGACTG
ATCGGCCACATTGGGACTGAGATACGGCC
>Zotu5568
GAACGCTGGCGGCGTGCTTAACACATGCAAGTCGAGCGGTAAGGCCCTTCGGGGTACACGAGCGGCGA
ACGGGTGAGTAACACGTGGGTGACCTGCCCTCCACTTCGGGATAAGCCTGGGAAACTGGGTCTAATAC
CGGATAGGACCATTGGATGCATGTCTGGTGGTGGAAAGTTTTGTCGGTGGGGGATGGGCCCGCGGTCT
ATCAGCTTGTTGGTGGGGTGATGGCCTACCAAGGCGACGACGGATAGCCGGCCTGAGAGGGTGATCGG
CCACACTGGGACTGAGACACGGCC
>Zotu5569
GAACGCTGGCGGCATGCTTTACACATGCAAGTCGAACGGCAGCGCGGGGCAACCTGGCGGCGAGTGGC
GAACGGGTGAGTAATATATCGGAACGTACCCAGAAGTGGGGGATAACGTAGCGAAAGTTACGCTAATA
CCGCATACGATCTACGGATGAAAGTGGGGGACCTTCGGGCCTCATGCTTGTGGAGCGGCCGATATCTG
ATTAGCTAGTTGGTGGGGTAAAGGCTCACCAAGGCAACGATCAGTAGCTGGTCTGAGAGGACGACCAG
CCACACTGGGACTGAGACACGGCC
>Zotu5572
GAACGCTGGCGGCGTGCCTAACACATGCAAGTCGAACGCGAAAGGGAGCAATCCCCTGTAGCGTGGCA
GACGGGTGAGTAACACGTGGGTAACCTACCCGGAGGTGGGGGATAACCCTTCGAAAGGAGGGCTAATA
CCGCATGACACCGTGGGGACCTCGGTCCTTGCGATCAAAGCTGGCCTCTGTTTACAAGCTAGCGCCAC
CGGATGGGCCCGCGCCCCATTAGCTAGTTGGTGAGGTAACGGCTCACCAAGGCGACGATGGGTAGCCG
GCCTGAGAGGGCGATCGGCCACACTGGAACTGCGACACGGTC
>Zotu5575
GAACGCTGGCGGCGTGCTTAACACATGCAAGTCGAACGGTGATCTCGGTGCTTGCACCGGGTGATCAG
TGGCGAACGGGTGAGTAACACGTGAGCAACCTGCCCCTGACTCTGGGATAACCCCGAGAAATCGGAGC
TAATACCGGATACGAGACGCACGGGCATCCGTAGCGTCTGGAAAGAATTTTGGTCAGGGATGGGCTCG
CGGCCTATCAGCTTGTTGGTGGGGTAACGGCCCACCAAGGCGACGACGGGTAGCCGGCCTGAGAGGGC

GACCGGCCACACTGGGACTGAGACACGGCC
>Zotu5581
GAGCGCTGGCGGCGTGCTTAACACATGCAAGTCGAACGGTGATCCCAAGAGCTTGCTTTTGGGTGAGC
AGTGGCGAACGGGTGAGTAACACGTGAGTAACCTGCCCCTGACTCTGGAATAAGCGCTGGAAACGGCG
TCTAATACCGGATATGACACCAATCCGCATGGGTATGGTGTGGAAAGTTTTTCGGTTGGGGATGGACT
CGCGGCCTATCAGCTTGTTGGTGAGGTAATGGCTCACCAAGGCGATGACGGGTAGCCGGCCTGAGAGG
GCGATCGGCCACACTGGGACTGAGACACGGCC
>Zotu5583
GAACGCTGGCGGCAGGCTTAACACATGCAAGTCGAACGCTCACTTTCGGGTGGGAGTGGCAGACGGGT
GAGTAACACGTGGGAACATACCCTTCGGTTCGGAACAACGCCGGGAAACTGGCGCTAATACCGGATAC
GGCCTTGGGGCGAAAGATTGATCGCCGAAGGATTGGCCCGCGTTCGATTAGCTTGTTGGTGAGGTAAC
GGCTCACCAAGGCGACGATCGATAGCTGGTCTGAGAGGATGGCCAGCCACATTGGGACTGAGACACGG
CC
>Zotu5584
GAACGCTAGCGGCAGGCCTAATACATGCAAGTCGAACGGTAACATGAGTAGCAATACTTGATGACGAG
TGGCGCACGGGTGCGTAACGCGTATGCAATCTACCTTTAACAGGTGAATAGCCCGAAGAAATTCGGAT
TAAAACACCATAGTATTGTTTGTTGGCATCAACAGACAATTAAAACTCCGGTGGTTAAAGATGAGCAT
GCGTGACATTAGCTAGTTGGTGAGGTAATGGCTCACCAAGGCAACGATGTCTAGGGGATCTGAGAGGA
TTAACCCCCACACTGGTACTGAGACACGGAC
>Zotu5586
GAACGCTGGCGGCATGCCTAACACATGCAAGTCGAACGAAGCCTTCGGGCTTAGTGGCGCACGGGTGC
GTAACACGTGGGAATCTGCCCTTAGGTACGGAATAACAGTTAGAAATGACTGCTAATACCGTATGATG
CCGCAAGGCCAAAGATTTATCGCCTAGGGATGAGCCCGCGTCGGATTAGCTAGTTGGTGAGGTAAAGG
CTCACCAAGGCGACGATCCGTAGCTGGTCTGAGAGGATGATCAGCCACACTGGGACTGAGACACGGCC
>Zotu5588
GAACGCTAGCGGCAGGCCTAATACATGCAAGGCGAACGGCAAGTTTGATAGCAATATTGAACCTAGAG
TGTCGTAAGGGTGCGTAACACGTATGCAATCTGCCCTGTACAGGAGTATAGCTCCCCGAAAGGGGAAT
TAACACTGCATAAGATTAAGGAGTGGCATCACTTTTTAATTAAAACTGAGGTGGTACAGGATGAGCAT
GCGCATGATTAGTTAGTTGGCGGGGTAACGGCCCACCAAGACGATGATCATTAGGGGAACTGAGAGGT
TGATCCCCCACACTGGCACTGAGATACGGGC
>Zotu5590
GAACGCTGGCGGCATGCCTTACACATGCAAGTCGAACGGCAGCATAGGAGCTTGCTCCTGATGGCGAG
TGGCGAACGGGTGAGTAATATATCGGAACGTGCCCTAGAGTGGGGGATAACTAGTCGAAAGATTAGCT
AATACCGCATACGACCTGAGGGTGAAAGCGGGGGACCGTAAGGCCTCGCGCAGCAGGAGCGGCCGATG
TCTGATTAGCTAGTTGGTGGGGTAAAAGCTCACCAAGGCGACGATCAGTAGCTGGTCTGAGAGGACGA
CCAGCCACACTGGGACTGAGACACGGCC
>Zotu5591
GAACGCTGGCGGCAGGCTTAACACATGCAAGTCGAACGGGCACTTCGGTGTCAGTGGCAGACGGGTGA
GTAACACGTGGGAACGTGCCCTTCGGTTCGGAACAACTCAGGGAAACTTGAGCTAATACCGGATACGC
CCTTACGGGGAAAGGTTTACTGCCGAAGGATCGGCCCGCGTCTGATTAGCTAGTTGGTGAGGTAATGG
CTCACCAAGGCGACGATCAGTAGCTGGTCTGAGAGGATGATCAGCCACACTGGGACTGAGACACGGCC
>Zotu5592
CGTCAGGTCCCAGTGCGCTTGGTTAATTACACCTTAGAAGACCTTTCCCTGCCGCAAAAAAGGGTAGT
AGCGCAATTCATCCCGGCGAACATTACAGAAAGCGGGCAAAGGGAGAGTATCGGACTAACAGGA
>Zotu5593
GAACGCTGGCGGCAGGCTTAACACATGCAAGTCGAGCGGGCACTTCGGTGTCAGCGGCAGACGGGTGA
GTAACACGTGGGAACGTACCCTTTGGTTCGGAACAACTCTGGGAAACTGGAGCTAATACCGGATACGC
CCTTTTGGGGAAAGGCTTGCTGCCGAAGGATCGGCCCGCGTCTGATTAGCTAGTTGGTGGGGTAATGG
CCTACCAAGGCGACGATCAGTAGCTGGTCTGAGAGGATGATCAGCCACACTGGGACTGAGACACGGCC
>Zotu5594
GAACGCTGGCGGCAGGCCTAACACATGCAAGTCGAACGAACTCTTCGGAGTTAGTGGCGGACGGGTGA
GTAACACGTGGGAACGTGCCTTTTGGTTCGGAATAACTCAGGGAAACTTGTGCTAATACCGAATGTGC
CCTTCGGGGGAAAGATTTATCGCCATTAGAGCGGCCCGCGTCTGATTAGCTAGTTGGTGAGGTAAAGG
CTCACCAAGGCTACGATCAGTAGCTGGTCTGAGAGGATGACCAGCCACATTGGGACTGAGACACGGCC

>Zotu5595
GAACGCTAGCGGTTTGCCTAACACATGCAAGTTGCACGGCGCCATGCCTCTCGAGGAATGGCGGAGTG
GCGGACGGGTGAGGAACGCGTAAGGACCTGCCCCTGGGAGGGGGATAACAGCTGGAAACGGCTGCTAA
TACCCCATATCCTGAGGAGGGAAAGGAGAGATCCGCCCAGGGAGGGGCTTGCGTCTGATTAGCTAGTT
GGGGGGGTAATGGCCTCCCAAGGCAACGATCAGTTGCTGGTCTGAGAGGATGATCAGCCACACTGGGA
CTGAGACACGGCC
>Zotu5596
GAACGCTGGCGGCGTGCCTAATACATGCAAGTCGAGCGAATCAAAGGGAGCTTGCTCCCAATGATTAG
CGGCGGACGGGTGAGTAACACGTGGGCAACCTGCCCGTAAGATTGGGATAACTCCGGGAAACCGGAGC
TAATACCGAATAGGTTCTTTCTTTGCATAAAGGAAGAAGGAAAGTTGGCCTCGGCTAACACTTACGGA
TGGGCCCGCGGCGCATTAGCTAGTTGGTGAGGTAACGGCTCACCAAGGCAACGATGCGTAGCCGACCT
GAGAGGGTGATCGGCCACACTGGGACTGAGACACGGCC
>Zotu5598
GAACGCTGGCGGCGTGCTTAACACATGCAAGTCGAACGGTGAAACAGGAGCTTGCTCCTGCGGATCAG
TGGCGAACGGGTGAGTAACACGTGAGGAATGTGCCCTGGACTCTGGGATAAGCGTTGGAAACGACGTC
TAATACCGGATACGAGCTGCGAAGGCATCTTCAGCAGCTGGAAAGAACTTCGGTCCAGGATCATCTCG
CGGCCTATCAGCTAGTTGGTGAGGTAACGGCTCACCAAGGCGACGACGGGTAGCCGGCCTGAGAGGGT
GACCGGCCACACTGGGACTGAGACACGGCC
>Zotu5599
GAACGCTGGCGGCGTGCCTAATACATGCAAGTCGAACGGGCTGCCATAAGGCAGCCAGTGGCGAACGG
GTGAGGAGCACATGGGGAACCTGCCCGTCAGAGGGGGACAACAGCTGGAAACGGCTGCTAAGACCGCA
TAGGCTCAAGGAGGGCATCCTCGTTGAGTTAAAGTGTCCTTTCGGGGACAGCTGACGGATGGACCTGT
GGCGCATTAGCCAGTTGGCGGGGTAAAGGCCCACCAAGGCGACGATGCGTAGCCGGCCTGAGAGGGCG
GACGGCCACACTGGGACTGAGACACGGCC
>Zotu5600
GAACGCTGGCGGCGTGCCTAACACATGCAAGTCGAACGAGAAAGTTCCTTCGGGAGCGAGTACAGTGG
CGCACGGGTGAGTAACACGTAGGTGACCTTCCCTTTTGAGTGGGACAACTAGAAGAAATTTTAGCTAA
TACCACATACGCACAACCGACATGAATGAGGTTGTGGAAAGAATGCCTCTACTTGTAAGCATTTACTT
AAGGATGGGCCTGCGGGACATTAGCTAGTTGGTGGGGTAAAGGCCTACCAAGGCAACGATGTCTAGCC
GGATTGAGAGATTGATCGGCCACACTGGAACTGAGACACGGTC
>Zotu5601
GAACGCTGGCGGCGTGCTTAACACATGCAAGTCGAACGCTGAAGCCCAGCTTGCTGGGTGGATGAGTG
GCGAACGGGTGAGTAACACGTGAGTAACCTGCCCTTCACTCTGGGATAACTCCGGGAAACCGGTGCTA
ATACTGGATAGGACTCCTGCCTGCATGGGTGGGGGTGGAAAGATTTATCGGTGATGGATGGACTCGCG
GCCTATCAGCTTGTTGGTGGGGTAATGGCCTACCAAGGCGACGACGGGTAGCCGGCCTGAGAGGGTGA
CCGGCCACACTGGGACTGAGACACGGCC
>Zotu5602
GAACGCTGGCGGCGTGCTTAACACATGCAAGTCGAACGGAAAGGCCCCTTTGGGGGTACTCGAGTGGC
GAACGGGTGAGTAACACGTGAGTAACCTGCCCTTGACTTCGGGATAACTTCAGGAAACTGGGGCTAAT
ACCGGATAGGAATCCTTGCTGCATGGTGGGGGTTGGAAAGCTTCGGCGGTTTTGGATGGACTCGCGGC
TTATCAGCTTGTTGGTGGGGTAGTGGCTTACCAAGGCTTTGACGGGTAGCCGGCCTGAGAGGGCGACC
GGCCACATTGGGACTGAGATACGGCC
>Zotu5604
GAACGCTAGCGGTGCGCCTAACACATGCAAGTCGAGCGAGAAATTGAGATAGCTTGCTATCAAGATGG
AAAGCGGCGAACGGGTGAGTAACACGTTGGTATCTGCCCTCTGGTCAGGGATAGCCATTCGAAAGGAT
GATTAATACCGGATAGTCTCGAAAGAGTAAAGATTTATTGCCAGAGGAGGAACCTGCGGCCTATCAGC
TAGTTGGTGAGGTAGAAGCTTACCAAGGCGATGACGGGTAGCTGATCTGAGAGGATGATCAGCCACAA
TGGAACTGAGACACGGTC
>Zotu5605
GAACGCTTGCGGCGTGCCTAAGAAATGCAAGTCGAACGGACAGTGGCTTCGGTTACTGTTAGTGGCGA
ACGGTCGCGTAACACGTAAGAAACCTGCCTTGAAGCGGGGGACAACAGTCCGAAAGGGCTGCTAATAC
CGCATGTGGCCAGTGAGGGGCATCCCTTACTGTCTAAAGATTTATCACTTCAAGATGGTCTTGCGGCC
TATCAGCTAGTTGGTGAGGTAACGGCTTACCAAGGCGACGACGGGTAGCTGGTCTGAGAGGACGATCA
GCCGGATTGGGACTGAGATACGGCC

>Zotu5607
GAATGCTGGCTGTATGGATAAGGCATGCAAGTCGAACGGACCGCAAGGTCAGTGGCAGACGAGGTAGT
AACACGTAAGTACGTACCCTGTAGTCTGGAATAGCTCGTCGAAAGGCGGGGTAATACCGGATGGTGTA
GCAATACTAAAGATTTATCGCTACAGGAGCGGCTTGCGTAATATCAGCTTGTTGGTGAGGTAATGGCT
CACCAAGGCTACGACGTTTAGGGGAGCTGAGAGGCTGACCCCCACCGATGGGACTGAGACACGGCC
>Zotu5608
GAACGCTGGCGGCAGGCTTAACACATGCAAGTCGAGCGGGCGATGTTGCTTCGGTAACTGAGCTAGCG
GCGGACGGGTGAGTAAAGCTTAGGAATCTGCCTATTAGTGGGGGACAACATTTCGAAAGGGATACTAA
TACCGCATACGTCCTACGGGAAAAAGCAGGGGACCTTCGGGCCTTGTGCTAATAGATGAGCCTAAGTC
GGATTAGCTAGTTGGTGGGGTAAAGGCCTACCAAGGCGACGATCTGTAGCGGGTCTGAGAGGATGATC
CGCCACACTGGGACTGAGACACGGCC
>Zotu5610
GAACGCTGGCGGCAGGCTTAACACATGCAAGTCGAACGGCTGTAGCAATACAGCAGTGGCAGACGGGT
GAGTAACGCGTGGGAACGTACCTTTCAGTTCGGAATAACTCAGGGAAACTTGTGCTAATACCGGATAC
GCCCTTAAGGGGAAAGATTTATCGCTGAAAGATCGGCCCGCGTTGGATTAGCTAGTTGGTGGGGTAAT
GGCCCACCAAGGCGACGATCCATAGCTGGTCTGAGAGGATGACCAGCCACACTGGGACTGAGACACGG
CC
>Zotu5611
GAACGTTGGCGGCGTGCCTAATACATGCAAGTCGAACGCGGTCTCCTAATTGAAAAACCGTGCTTGCA
CGGGTTGGATTTTAGATCGGACCGAGTGGCGAACTGGTGAGTAACACGTGGGTAACCTGCCCAGAAGT
AGGGGATAACACCTGGAAACAGATGCTAATACCGTATAACAACTAAAACCACATGGTTTTAGTTTGAA
AGCTGGCCTTTGCGCTAGTGCTTTTGGATGGACCCGCGGCGTATTAGCTAGTTGGTGAGATAATAGCT
CACCAAGGCGATGATACGTAGCAGACCTGAGAGGGTAATCTGCCACAATGGGACTGAGACACGGCC
>Zotu5612
GAACGCTGGCGGCGTGCTTAACACATGCAAGTCGAACGGTAAGGCCTTTCGGGGTCATACGAGTGGCG
AACGGGTGAGTAACACGTGAGCAACCTGCCCTTGACTTCGGAATACCAGCTGGAAACAGCTGCTAATA
CCGGATATGACCCTGGTTCTCTTGGACTGGGGTGGAAAGCTCCGGCGGTCAGGGATGGGCTCGCGGCC
TATCAGCTTGTTGGTGAGGTAATGGCTCACCAAGGCTTCGACGGGTAGCCGGCCTGAGAGGGCGACCG
GCCACACTGGGACTGAGATACGGCC
>Zotu5613
GAATGCTGGCGGCGTGGATAAGTCATGCAAGTCAAGGGGGCCCGCAAGGGCAACCGGCAGACGAGGTA
GTAACACGTAGGTACGTCCCCCGAAGTCGGGCATAGCCAATCGAAAGGTTGGGTAATTCCCGATAGTC
CCGCAAGGGTAAAGGTTTTTCGCTTCGGGAGCGGCCTGCGTAGTATCAGCTAGTTGGTAGGGTAATGG
CCTACCAAGGCGACGACGCTTAGGGGAGCTGAGAGGCTGACCCCCACCGATGGAACTGCGACACGGTC
>Zotu5616
GAACGCTGGCGGCAGGCTTAACACATGCAAGTCGAGCGGAGCGAGGGTGCTTGCACCTTAGCTTAGCG
GCGGACGGGTGAGTAATGCTTAGGAATCTGCCTATTAGTGGGGGACAACATTCCGAAAGGAATGCTAA
TACCGCATACGCCCTACGGGGGAAAGCAGGGGATCTTCGGACCTTGCGCTAATAGATGAGCCTAAGTC
GGATTAGCTAGTTGGTGGGGTAAAGGCCTACCAAGGCGACGATCTGTAGCGGGTCTGAGAGGATGATC
CGCCACACTGGGACTGAGACACGGCC
>Zotu5617
GAACGCTGGCGGCGTGCTTAACACATGCAAGTCGAACGATGAAGCCCAGCTTGCTGGGTGGAAGAGTG
GCGAACGGGTGAGTAACACGTGAGTAACCTGCCCCGGACTCTGGGATAAGCGCTGGAAACGGCGTCTA
ATACTGGATATGACCCATACCGGCATCGGTTGTGGGTGGAAAGATTTATCGGTTCGGGATGGGCTCGC
GGCCTATCAGCTGGATGGTGAGGTAACGGCTCACCATGGCAACAACGGGTAGCCGGCCTGAGAGGGTG
ACCGGCCACACTGGGACTGAGACACGGCC
>Zotu5618
TAACGTTGGCAGTGTGCCTAACACATGCAAGTCGAGCGATATAGCGGCAGACGAGAGAGTAATAACAA
GGAACTTACCCCCAAGTGAAGGATAGCCACCCGAAAGGGTGGGTAATCCTGCATAACATCGAAAGATC
AAAGCCTTCGGGCGCTTGGGGAGAGGCCTTGTCCCTATCAGCTAGTTGGTGAGGTAATGGCTTACCAA
GGCGATGACGGGTAGCTGGCCTGAGAGGGTGACCAGCCTCGATGGGACTGAGACACGGCC
>Zotu5620
GAACGCTGGCGGCGTGCTTAACACATGCAAGTCGAACGGTGAAGGGACCGGCTTTTGCTGGTTCTGGA
TGAGTGGCGAACGGGTGAGTAACACGTGAGTAACCTGCCCCCTTCTTCTGGATAACCGCATGAAAGTG

TGGCTAATACGGGATATTCTGGGTCTGTCGCATGGCGGGCCTGGGAAAGATGCGCCTTTGGGTGTTTT
TGGTGGGGGATGGGCTCGCGGCCTATCAGCTTGTTGGTGGGGTGATGGCCTACCAAGGCGGTGACGGG
TAGCCGGCCTGAGAGGGTGGACGGTCACACTGGGACTGAGACACGGCA
>Zotu5621
GAACGCTGGCGGCGTGCCTAACACATGCAAGTCGAGCGAGAAATCCATGATCGAAACTTCGGTTGATT
GATTGGACGGAAAGCGGCGGACGGGTGAGTAACGCGTAGGCAACCTGCCTCTGGCTGAGGGACAGCCT
CGGGAAACCGGGATTAATACCTCATAACACGAAGGGAAGACATCTTTTTTTCGTCAAAGATTTATCGG
CTGGAGATGGGCCTGCGTCTGATTAGTTAGTTGGTGGGGTAACGGCCTACCAAGGCGACGATCAGTAG
CCGACCTGAGAGGGTGATCGGCCACATTGGAACTGAGACACGGTC
>Zotu5622
GAACGCTGGCGGCGTGCTTAACACATGCAAGTCGAACGGATCCTCGGCTTCGGTTGGGGGTTAGTGGC
GAACGGGTGAGTAACACGTGAGCAACCTGCCCTTCACTCTGGGATAACTCCGGGAAACCGGGGCTAAT
ACCGGATAGGACTCCTCGTCGCATGGTGGGGGGTGGAAAGGTGCCCTGTTGGGGGTTTCTGGTGGGGG
ATGGGCTCGCGGCCTATCAGCTTGTTGGTGGGGTGATGGCCTACCAAGGCGACGACGGGTAGCCGGCC
TGAGAGGGTGACCGGCCACACTGGGACTGAGACACGGCC
>Zotu5624
GAATGCTGGCGGCGTGCCTAACACATGCAAGTCGAGCGGCAGCGCGAGTAGTTTACTACTTGGCGGCG
AGCGGCGGACGGCTGAGTAACGCGTGGGAACGTGCCCCAAAGTGAGGAATAACTGCCCGAAAGGGTGG
CTAATACCGCATATGGTCTTCGGATTAAAGTCTTCGGACGCTTTGGGAGCGGCCTGCGTCGGATTAGG
TTGTTGGTGAGGTAATGGCTCACCAAGCCGACGATCCGTAGCTGGTCTGAGAGGATGATCAGCCAGAC
TGGGACTGAGACACGGCC
>Zotu5626
GAACGCTGGCGGTATGCCTAATACATGCAAGTCGTACGCGATCACTTTTTTGCTCGCAGCAGTGGTTA
TTTATAACCACCTTTGCGAGCAGAATTATGGTGATTGAGTGGCGGACGGGTGAGTAACACGTTGGTAC
CTGCCCCAAAGTGGGGAATAATTCACCGAAAGGTGGACTAATACCCCATAGTCTCCGGTTTATCCGGA
GTAAAGCTTTAGCGCTTTGGGAGGGGCCTGCGTCCGATTAGCTAGTTGGTAGAGTAACGGTTTACCAA
GGCTACGATCGGTAGCTGGATCGAGAGGTTGACCAGCCACACTGGGACTGAGATACGGCC
>Zotu5628
GAACGCTGGCGGCGTGCCTAACACATGCAAGTCGAGCGATGAACCCTTCGGGGGGATTAGCGGCGGAC
GGGTGAGTAACACGTGGGTAACCTGCCTCAAAGAGGGGGATAGCCTCCCGAAAGGGAGATTAATACCG
CATAACATTGAGCTATCGCATGGTAGTTTAATCAAAGGAGTAATCCGCTTTGAGATGGACCCGCGGCG
CATTAGCTAGTTGGTGAGGTAACGGCTCACCAAGGCGACGATGCGTAGCCGACCTGAGAGGGTGATCG
GCCACATTGGAACTGAGACACGGTC
>Zotu5629
GAACGCTGGCGGCGTGCTTAAGGTATGCAAGTCGAACGAGAATTTTGCAGTAGCAATATTGTAAAATT
CTAGTGGCAAACGGGTGAGTAATGCGTAGATAATCTACCCTTAACATTGAAATAACTTTGCGAAAGTA
AGGCTAATGTCATGTGTGGTGTATAAGTGAAATTCTTATATATTAAAGGAGCAATCCGGTTAAGGATG
AGTCTGCGTCCTATCAGCTAGTTGGTGAGGTAAATGCTCACCAAGGCGACGACGGGTAGCTGGTCTGA
GAGGATGATCAGCCACATTGGAACTGCGACACGGTC
>Zotu5630
GAACGCTGGCGGCGTGCTTAACACATGCAAGTCGAACGAGAATTCTTCAAATAGAAGCTTCGGTGGAA
ACGAGAAGAAGGAAAGTGGCGAACGGGTGAGTAACGCGTGAGCAACCTACCTTATACAAAGGGATAGC
CTCGGGAAACCGGGATTAATACCATATGAGACCCCACTAACGAGTGTTAGAGAGGTTAAAGAATTTCG
GTATAAGATGGGCTCGCGTCTGATTAGCTAGTTGGTAGGGTAAAGGCCTACCAAGGCGACGATCAGTA
ACCGGCTTGAGAGAGTGAACGGTCACATTGGAACTGAGACACGGTC
>Zotu5631
GAACGCTGGCGGCATGCCTTACACATGCAAGTCGAACGGCAGCACGGGAGCAATCCTGGTGGCGAGTG
GCGAACGGGTGAGTAATATATCGGAACGTGCCCAGTCGTGGGGGATAACGTAGCGAAAGCTACGCTAA
TACCGCATACGATCTATGGATGAAAGCGGGGGATCGCAAGACCTCGCGCGATTGGAGCGGCCGATATC
AGATTAGCTAGTTGGTGGGGTAAAGGCCCACCAAGGCGACGATCTGTAGCTGGTCTGAGAGGACGACC
AGCCACACTGGGACTGAGACACGGCC
>Zotu5632
GAACGCTAGCGGCGTGCTTTAAGCATGCAAGTAGAACGAATCTAGTAGCAATACTAGATAAGTTGCGA
ACGGCTGAGTAATCAATAGGAATCTTTCCTTTAGTGGGGAATACCTCGTCGAAAGACGAGCTAATACC

GCATAATACCTCCGGGTGAAAGCGATTAACTTCGCGCTGATTGAGGAGCCTATTGCCCATCAGGTAGT
TGGTAGGGTAAAAGCCTACCAAGCCTACGACGGGTACCCGATCTGAGAGGATGGACGGGCAGAATGGG
ACTGAGACACGGCC
>Zotu5635
GAACGCTGGCGGCGTGCCTAATACATGCAAGTCGAGCGGACTTGATGGAGAGCTTGCTCTCCTGATAG
TTAGCGGCGGACGGGTGAGTAACACGTAGGCAACCTGCCTGCAAGACCGGGATAACCCACGGAAACGT
GAGCTAATACCGGATATCTCATTTCCTCTCCTGAGGAGATGATGAAAGACGGAGCAATCTGTCACTTG
CGGATGGGCCTGCGGCGCATTAGCTAGTTGGTGAGGTAACGGCTCACCAAGGCGACGATGCTTAGCCG
ACCTGAGAGGGTGAACGGCCACACTGGGACTGAGACACGGCC
>Zotu5637
GAACGCTGGCGGCGTGCTTAACACATGCAAGTCGAACGGAAAGGCCCTTTCGGGGGTACTCGAGTGGC
GAACGGGTGAGTAACACGTGGGTGATCTGCCCTGCACTTCGGGATAAGCTTGGGAAACTGGGTCTAAT
ACCGGATATACCTCATGGATCGCATGGTTTGTGGGGGAAAGCTTTTGCGGTGTGGGATGGGCCCGCGG
CCTATCAGCTTGTTGGTGGGGTGATGGCCTACCAAGGCGTCGACGGGTAGCCGGCCTGAGAGGGTGTC
CGGCCACACTGGGACTGAGATACGGCC
>Zotu5638
GAACGCTAGCTACAGGCTTAACACATGCAAGTCGTGGGGCAGCGGATACTTAGCTTGCTAAGTATGCC
GGCGACCGGCGCACGGGTGAGTAACGCGTACCGAACCTGCCCATCACACAGGGATAGGCTTGCGAAAG
CAAGATTAATACCTGATGGTCTCAGTTGTATGCATGTATAATTGAGTAAAGCCTTCGGGCGGTGATGG
ATGGCGGTGCGTCCCATTAGGAAGTTGGCGGGGTAACGGCCCACCAATCCTTCGATGGGTAGGGGTTC
TGAGAGGAAGGTCCCCCACATTGGAACTGAGATACGGTC
>Zotu5639
TAACGCTGGCGGCGCGCATAACACATGCAAGTCGAGCGAGGGCTTACTTCTCGCAAGAGAGGTGAGTT
CCTAGCGGCGAACGGGTGAGTAACACGTGGGCAACCTTTCCTATCGCGGGGGATAACACCGGGAAACC
GATGCTAATACCGCATGTCGTCACTGGTCGCATGGCTGGTGAAGAAAGCAGCAATGCACGATGGGGTG
GGCCCGCGGCCTATCAGCTAGTCGGTGGGGTAACGGCCTACCGAGGCGATGACGGGTAGCTGGTCTGA
GAGGACGGCCAGCCACACGGGGACTGAGACACGGCC
>Zotu5640
GAACGCTGGAAATGTGGATACCGGCATGCAAGTCAAGGGGCCGCAAGGCACCGGCGAACGGCTTAGTA
ACACGTAGATACATACCTTAAAGTGGGGCATAGCTCGTCGAAAGACGGGGTAATTTCCCATAGTCTCG
CAAGAGTAAAGGCGCAAGCCGCTTTAAGAATGGTCTGCGGTCTATCAGCTAGTTGGTGAGGTAATGGC
TCACCAAGGCTATGACGGATAGGGGGCGTGAGAGCGCGACCCCCAAGAATGGCACTGCGACACGGGC
>Zotu5641
GAACGCTGGCGGCAGGCTTAACACATGCAAGTCGAGCGCCCCGCAAGGGGAGTGGCAGACGGGTGAGT
AACGCGTGGGAATCTACCTTTTGCTACGGAATAACTCAGGGAAACTTGTGCTAATACCGTATGTGCCC
TTTGGGGGAAAGATTTATCGGCAAAGGATGAGCCCGCGTTGGATTAGCTAGTTGGTGAGGTAAAGGCT
CACCAAGGCGACGATCCATAGCTGGTCTGAGAGGATGATCAGCTACACTGGGACTGAGACACGGCC
>Zotu5642
GAACGCTGGCGGCGTGCTTAACACATGCAAGTCGAGCGGAAAGGCCCTTTCGGGGGTACTCGAGCGGC
GAACGGGTGAGTAACACGTGAGTAATCTGCCCTTGGCTTTGGGATAACCACCGGAAACGGTGGCTAAT
ACCGGATACGACCAATCTCTGCATGGAGTGTTGGTGGAAAGTTTTTCGGCCAGGGATGTGCTCGCGG
CCTATCAGCTTGTTGGTGGGGTAATGGCCTACCAAGGCTTCGACGGGTAGCCGGCCTGAGAGGGTGAC
CGGCCACACTGGGACTGAGACACGGCC
>Zotu5643
GAACGCTGGCGGCATGCCTAACACATGCAAGTCGAACGAAGGCTTCGGCCTTAGTGGCGCACGGGTGC
GTAACGCGTGGGAATCTACCCTTGGGTTCGGAATAACAGTGAGAAATTACTGCTAATACCGGATGATG
TCGCAAGACCAAAGATTTATCGCCCAGGGATGAGCCCGCGTAGGATTAGCTTGTTGGTGAGGTAAAAG
CTCACCAAGGCGACGATCCTTAGCTGGTCTGAGAGGATGATCAGCCACACTGGGACTGAGACACGGCC
>Zotu5645
GAACGCTGGCGGCGCGCTTAACACATGCAAGTCGAGCGAGAACCAGGCCTTCGGGCCTGGGGACAGCG
GCGAACGGGTGAGTAACACGTGGGTAATCAACCCTCGGTACTGGGATAGCCCGGGGAAACCCGGATTA
ATACCGGATGGCCCTTCGGATCTTCGGATCTGACGGAAAAGGTAGCTTCGGCCTCCGACCGAGGACGA
GCCCGCGGCGGATTAGCTTGTTGGTGGGGTAACGGCTCACCAAGGCGACGATCCGTAGCTGGTCTGAG
AGGACGATCAGCCACACTGGGACTGAGACACGGCC

>Zotu5646
GAACGCTAGCGGCAGGCCTAATACATGCAAGTCGAACGGGCGGTAGCAATACCGCTAGTGGCGCACGG
GTGCGTAACGCGTAACCAACTTACCCACTACTGGGGGATAGCCCGCCGAAAGGCGGATTAATACCGCA
TATACCAGCAGCCTGGCATCAGGCAATTGGGAAAGATTTATTGGTAGTGGATGGGGTTGCGTAGCATT
AGCTAGATGGCGGGGTAACGGCCCACCATGGCGACGATGCTTAGGGGACCTGAGAGGGTGATCCCCCA
CACTGGCACTGAGATACGGGC
>Zotu5647
GAACGCTGGCGGCATGCTTAACACATGCAAGTCGAACGAGCTAATGGGTAACTATTAGTATAGTGGCG
GACGGGTGAGTAACGCGTAAGAACCTACCTTTTGGAGGGGGATAACAGCGGGAAACTGTTGCTAATAC
CCCATAATGCTGAGGAGTTAAAGCAGTGAAAACTGGCCAAAAGAGGGGCTTGCGTCTGATTAGCTAGT
TGGTGGGGTAAAAGCCTACCAAGGCCACGATCAGTAGTCGGTCTGAGAGGACTAACGGCCACACTGGG
ACTGAGACACGGCC
>Zotu5648
GAACGCTGGCGGCGTGCATAACACATTCAAGTCGAACGGAGTTAAATTTGTTAATCCTTCGGGAAGCG
CAGAAATAACTTAGTGGCGGACGGGTGAGTAACGCGTGAGCAACCTGCCTCTGCATGGGGGATAACGT
TTGGAAACGGACGCTAATACCTCATAACACAAGGACACCGCATGATGACCTTGTCAAAGATTTATTGT
GCAGAGATGGGCTCGCGTCTGATTAGGTAGTTGGTGAGGTAACGGCCCACCAAGCCGACGATCAGTAG
CCGGACTGAGAGGTTGAACGGCCACATTGGAACTGAGATACGGTC
>Zotu5649
GAACGCTAGCGGCAGGCCTAATACATGCAAGTCGAACGGGATTCCGGTTTAGCTTGCTAAGCTGGATG
AGAGTGGCGTACGGGTGCGTAACGCGTGAGCAACCTGCCCATGTCAGGGGTATAGCCCGTTGAAAGAC
GGATTAACACCGCATAATACATTTGAATCGCATGGTTTTGATGTTAAATATTTATAGGACATGGATGG
GCTCGCGTGGCATTAGCTAGTTGGTATGGTAACGGCATACCAAGGCTATGATGCCTAGGGGCTCTGAG
AGGAGAATCCCCCACACTGGTACTGAGACACGGAC
>Zotu5652
GAACGCTGGCGGCATGCCTAATACATGCAAGTCGAACGATCACTTCGGTGATAGTGGCGCACGGGTGC
GTAACGCGTGGGAATCTGCCCTTGGGTTCGGAATAACTTCTGGAAACGGAAGCTAATACCGGATGATG
ACGTAAGTCCAAAGATTTATCGCCCAAGGATGAGCCCGCGTAGGATTAGCTAGTTGGTGGGGTAAAGG
CCCACCAAGGCGACGATCCTTAGCTGGTCTGAGAGGATGATCAGCCACACTGGGACTGAGACACGGCC
>Zotu5653
GAACGCTGGCGGCATGCTTAACACATGCAAGTCGAACGAATGATTTCTTCTACTTGTAGAAGAAATTA
ATGAGTGGCGGACGGGTGAGTAACACGTAAGAACCTACCTTTTGGAGAGGGACAACCACTGGAAACGG
TGGCTAAAACCTCATAAAAAGCTGAGAAGTGAAAAGATGAAAAATCGCCAAAAGATGGGCTTGCGGCT
GATTAGCTTGTTGGTGAGGTAATGGCTTACCAAGGCAATGATCAGTAGCTGGTCTGAGAGGATGATCA
GCCACACTGGGACTGAGACACGGCC
>Zotu5654
GAACGCTGGCGGCATGCTTTACACATGCAAGTCGAACGGCAGCGCGGGCTTCGGCCTGGCGGCGAGTG
GCGAACGGGTGAGTAATACATCGGAACGTGCCCAGTAGTGGGGGATAGCTCGGCGAAAGCCGGATTAA
TACCGCATACGACCTGAGGGTGAAAGCGGGGGATCTTCGGACCTCGCGCTATTGGAGCGGCCGATGGC
AGATTAGCTAGTTGGTGGGGTAAAGGCCTACCAAGGCGACGATCTGTAGCTGGTCTGAGAGGACGACC
AGCCACACTGGGACTGAGACACGGCC
>Zotu5655
CAACGCTGGCGGCGTGCCTAACACATGCAAGTCGAACGAGAAAGTGGAGCAATCCATGAGTAAAGTGG
CGACCGGGTGAGTAACACGTGACTAACCTACCTCCGAGTGGGGAATAACTCCGGGAAACCGGGGCTAA
TACCGCATAACATCGCAAGATCAAAGCAGCAATGCGCTTGGAGAGGGGGTCGCGGCTGATTAGCTAGT
TGGCGGGGTAACGGCCCACCAAGGCGAAGATCGGTATCCGGCCTGAGAGGGCGCACGGACACACTGGA
ACTGAAACACGGTC
>Zotu5656
GAACGCTGGCGGCAGGCTTAACACATGCAAGTCGAGCGGGCATAGCAATATGTCAGCGGCAGACGGGT
GAGTAACGCGTGGGAACGTACCTTTTGGTTCGGAACAACACAGGGAAACTTGTGCTAATACCGGATAA
GCCCTTACGGGGAAAGATTTATCGCCGAAAGATCGGCCCGCGTCTGATTAGCTAGTTGGTGGGGTAAC
GGCCCACCAAGGCGACGATCAGTAGCTGGTCTGAGAGGATGATCAGCCACATTGGGACTGAGACACGG
CC
>Zotu5658

AAACGCTGGCGGCGTGCCTAACACATGCAAGTCGAACGCCCTTAGCAATAAGGGAGTGGCGGACGGGT
GAGGAACACGTGGGTGACCTACCTCGAAGTGAGGAATACCGGCGAGAAATCGCCGACAATACCGCATA
CGCTTCGCAAGAAGCAAAGTCGCAAGACGCTTTGAGATGGGCCCGCGGCCGATTAGCTGGTTGGTGGG
GTAATGGCCTACCAAGGCGACGATCGGTAGCTGGTCTGAGAGGACGATCAGCCACACTGGGATTGAGA
ACGGCC
>Zotu5659
GAACGCTAGCGGCAGGCCTAATACATGCAAGTCGAACGGTAACAGGCCCAGCAATGGGTGCTGACGAG
TGGCGCACGGGTGCGTAACACGTATGCAATCTACCTTTAACTGGAGCATAGCCCCGAGAAATCGGGAT
TAATTCTCCATAGTATTATGAAGTGGCATCACTTTATAATTAAAGTCACAACGGTTAAAGATGAGCAT
GCGCGACATTAGCTAGTTGGTGAGGTAACGGCTCACCAAGGCTACGATGTCTAGGGGTTCTGAGAGGA
TTAACCCCCACACTGGTACTGAGACACGGAC
>Zotu5661
GAACGCTAGCGACAGGCTTAACACATGCAAGTCGAGGGGTAACAGGGGAGCTTGCTTCCGCTGACGAC
CGGCGCACGGGTGAGTAACGCGTATGCAACCTACCTATAAGAGGGGAATAACCCGGAGAAATTCGGAC
TAATACCGCATAATAACAAATTAAGGCATCTTGATTTGTTTAAATATTTATAGCTTATAGATGGGCAT
GCGTCTGATTAGTTTGTTGGTGAGGTAACGGCTCACCAAGACTACGATCAGTAGGGGTTCTGAGAGGA
AGGTCCCCCACACTGGTACTGAGACACGGAC
>Zotu5663
GAACGCTGGCGGCGTGCATAACACATGCAAGTCAAGGGGGCCAGCAATGGCAACCGGCGAACGGGTGC
GTAACACGTGAGCAATCTGCCTCTATGTGGGGCATAGCCGGCCTAACGGCCGGGTAATTCCGCATACG
ATCTTTTGGGGACATCCCCGATTGATCAAACGCTTCGGCGGCATAGAGAGGAGCTCGCGGCCTATCAG
CTAGTTGGTGAGGGTAACGGCCCACCAAGGCGACGACGGGTAGCTGGTCTGAGAGGATGGCCAGCCAC
ATTGGGACTGAGATACGGCC
>Zotu5664
GAACGCTGGTAACGTGGATACCGGCATGCAAGTCACGGGGGCCGCAAGGCAACCGGCGAACGGCTTAG
TAACACGTAGATATATACCTTAAAGTGGAGCATAGCTCGTTGAAAAGCGGGGTAATTCTCCATAGTCC
CGCAAGGGTAAAGGCGCAAGCCGCTTTAAGAATAGTCTGCGGTCTATCAGCTAGTTGGTGAGGTAATG
GCTCACCAAGGCTATGACGGATAGGGGGCGTGAGAGCGCGACCCCCAAGAATGGCACTGCGACACGGG
C
>Zotu5665
GAACGCTGGCGGCGTGCCTAATACATGCAAGTCGAGCGGATCTTTGGGAGCTTGCTCCCAAAGATTAG
CGGCGGACGGGTGAGTAACACGTGGGCAACCTGCCTGTAAGACTGGGATAACTTCGGGAAACCGGAGC
TAATACCGGATAACTTCTTTTCTCGCATGAGGAGAGGTTGAAAGATGGTTTCGGCTATCACTTACAGA
TGGGCCCGCGGCGCATTAGCTAGTTGGTGAGGTAACGGCTCACCAAGGCGACGATGCGTAGCCGACCT
GAGAGGGTGATCGGCCACACTGGGACTGAGACACGGCC
>Zotu5667
GAACGCTGGCGGCGTGCTTAACACATGCAAGTCGAACGCTGAAGCCCAGCTTGCTGGGTGGATGAGTG
GCGAACGGGTGAGTAACACGTGGGTAATCTGCCCCGTACTCCGGGATAAGCCCGGGAAACTGGGTCTA
ATACCGGATAGGACACGCTATCGCATGGTGGTGTGTGGAAAGCTCCGGCGGTACGGGATGAGCCCGCG
GCCTATCAGCTTGTTGGTGGGGTGATGGCCTACCAAGGCGACGACGGGTAGCCGGCCTGAGAGGGTGA
CCGGCCACACTGGGACTGAGACACGGCC
>Zotu5668
GAACGCTGGCGGCGTGCTTAACACATGCAAGTCGAGCGGTAAGGCCCTTCGGGGTACACGAGCGGCGA
ACGGGTGAGTAACACGTGGGTGATCTGCCCTGTACTTCGGGATAAGCCTGGGAAACTGGGTCTAATAC
CGGATATGACCTTACATCGCATGGTGTTTGGTGGAAAGATTTATCGGTACAGGATGGGCCCGCGGCCT
ATCAGCTTGTTGGTGGGGTAATGGCCTACCAAGGCGACGACGGGTAGCCTGCCTGAGAGGGCGACCGG
CCACACTGGGACTGAGACACGGCC
>Zotu5669
GAATGCTGGCGGCGTGCCTAATACATGCAAGTCGAGCGGTAACAGGGCTTCACCGTTTCCTTGACACA
CCTGACCATTGTGTAGGTTGTTTGATTTCGAGGGAACAGTGAAGCTGCTGACGAGCGGCGGACGGCTG
AGTAACGCGTGGGAACATACCCCAAACTGAGGGATAACTAGTCGAAAGATTAGCTAATACCGCATGTG
ATCTTCGGATTAAAGCTTTCGGGCGGTTTGGGAATGGCCTGCGTACGATTAGATAGTTGGTGAGGTAA
TGGCTCACCAAGTCGACGATCGTTAGATGGTTTGAGAGGATGATCATCCAGACTGGGACTGAGACACG
GCC

>Zotu5671
GAACGCTGGCGGCATGCCTTACACATGCAAGTCGAACGGCAGCACGGGAGCAATCCTGGTGGCGAGTG
GCGAACGGGTGAGTAATATATCGGAACGTGCCCAGTCGTGGGGGATAACGTAGCGAAAGCTACGCTAA
TACCGCATACGATCTATGGATGAAAGCGGGGGATCGCAAGACCTCGCGCGATTGGAGCGGCCGATATC
AGATTAGCTAGTTGGTGGGGTAAAAGCTCACCAAGGCGACGATCTGTAGCTGGTCTGAGAGGACGACC
AACCACACTGGAACTGAGACACGGTC
>Zotu5672
GAACGCTGGCGGTATGCCTAACACATGCAAGTCGTACGGAAAAGAAATTTTTAGTGGCGAACGGGTGA
GTAACGCGTGAGAATTTGCTTCTAGGTAGAGGATAACCAATAGAAATATTGGCTAAAACTCTATAATT
CTATTAAAAAAATAGCTAAAAAAAGAAATTTGCCTAGAAAAAAGCTCGCGTCTGATTAGCTAGTTGGT
AAGGGAAAGGCTTACCAAGGCGACGATCAGTAGCTGATTTGAGAGGATGATCAGCCACACTGGAACTG
AGACACGGTC
>Zotu5673
GAACGCTGGCGGCGTGCCTAATACATGCAAGTCGAGCGAATTGATGGGAGCTTGCTCCCTGATATTAG
CGGCGGACGGGTGAGTAACACGTGGGCAACCTGCCCTGCAGATGGGGATAACTCCGGGAAACCGGGGC
TAATACCGAATAATCAGTTCCTCCGCATGGAGGAACTCTGAAAGACGGTTTCGGCTGTCACTGCAGGA
TGGGCCCGCGGCGCATTAGCTAGTTGGTGGGGTAATGGCCTACCAAGGCGACGATGCGTAGCCGACCT
GAGAGGGTGATCGGCCACACTGGGACTGAGACACGGCC
>Zotu5675
GAACGCTAGCGATAGGCTTAACACATGCAAGTCGAGGGGCAGCATAGAGTCAGCTTGCTGATTTGGAT
GGCGACCGGCGGATGGGTGCGTAACGCGTATGCAACTTACCTGTCAGAGGGGGATAACCCGGAGAAAT
TCGGACTAATACCGCATATACTTGAGATGCTGCATGGTATTTCAAGGAAATATTTATAGCTGACAGAT
AGGCATGTGTTCCATTAGCTAGTAGGTGAGGTAACGGCTCACCTAGGCGACGATGGATAGGGGAACTG
AGAGGTTAAACCCCCACACTGGTACTGAGACACGGAC
>Zotu5676
GAACGCTGGCGGCATGCTTTACACATGCAAGTCGAACGGCAGCACGGACTTCGGTCTGGTGGCGAGTG
GCGAACGGGTGAGTAATACATCGGAACGTGCCCAGTTGTGGGGGATAACTACTCGAAAGAGTAGCTAA
TACCGCATGAGAACTGAGGTTGAAAGCAGGGGATCGCAAGACCTTGCGCAACTGGAGCGGCCGATGGC
AGATTAGGTAGTTGGTGGGATAAAAGCTTACCAAGCCGACGATCTGTAGCTGGTCTGAGAGGACGACC
AGCCACACTGGGACTGAGACACGGCC
>Zotu5677
GAACGCTGGCGGCGTGCCTAATACATGCAAGTCGAGCGGATTCATTCCTTCGGGGATGGGTTAGCGGC
GGACGGGTGAGTAACACGTAGGCAACCTGCCTATAAGCCTGGGATAACTTCCGGAAACGGATGCTAAT
ACCGGATACGTTGAATTCTCGCATGAGGATTCATGGAAAGACGGCGCAAGCTGTCACTTATGGATGGG
CCTGCGGCGCATTAGCTAGTTGGTGGGGTAACGGCTCACCAAGGCGACGATGCGTAGCCGACCTGAGA
GGGTGATCGGCCACACTGGGACTGAGACACGGCC
>Zotu5678
GAACGCTGGCGGCGTGCCTAATACATGCAAGTCGAGCGGGAGAATCATCCTCCGGGATGAGGATCCAG
CGGCGAACGGGTGAGTAACACGTGGGCAACCTGCCCGCAAGACCGGGATAACTCCGGGAAACCGGGGC
TAATACCGGATAGGCCTTTTCCCTGCATGGGGAAAAGGGGAAAGGCGGCTTCGGCTGTCACTTGCGGA
TGGGCCCGCGGCGCATTAGCTGGTTGGTGAGGTAACGGCTCACCAAGGCGACGATGCGTAGCCGACCT
GAGAGGGTGATCGGCCACACTGGGACTGAGACACGGCC
>Zotu5679
GAACGCTGGCGGCATGCTTAACACATGCAAGTCGAACGGACACCTTCGGGTGTTAGTGGCGGACGGGT
GAGTAACGCGTGAGGAACCTGCCTCGCAGTGGGGAATAACGGTTGGAAACGACCGCTAATACCGCATA
ATGTATCGGGACCGCATGATTCTGATACCAAAGATTTATCGCTGTGAGATGGCCTCGCGTCTGATTAG
CTAGTTGGTGAGGTAACGGCCCACCAAGGCGACGATCAGTAGCCGGACTGAGAGGTTGACCGGCCACA
TTGGGACTGAGACACGGCC
>Zotu5680
GAACGCTGGCGGAATGCTTTACACATGCAAGTCGAACGGCAGCACGGGAGCTTGCTCCTGGTGGCGAG
TGGCGAACGGGTGAGTAACATATCGGAACGTATCCAATAATGGGGGATAACTAATCGAAAGGTTGGCT
AATACCGCATACGCCCTGAGGGGGAAAGCTGGGGATCTTCGGACCTAGCGTTAATGGAGCGGCCGATA
TCTGATTAGCTAGTTGGTGGGGTAAAGGCCCACCAAGGCGACGATCAGTAGCTGGTCTGAGAGGACGA
CCAGCCACACTGGAACTGAGACACGGTC

>Zotu5682
GAACGCTGGCGGCGTGCCTAATACATGCAAGTCGAGCGGATTGAAGGGAGCTTGCTCCCTGATATCAG
CGGCGGACGGGTGAGTAACACGTGGGCAACCTGCCCTGCAGATGGGGATAACTCCGGGAAACCGGGGC
TAATACCGAATAATCAGTTCCTCCGCATGGAGGAACTCTGAAAGACGGTTTCGGCTGTCACTGCAGGA
TGGGCCCGCGGCGCATTAGCTAGTTGGTGGGATAACGGCCTACCAAGGCGACGATGCGTAGCCGACCT
GAGAGGGTGATCGGCCACACTGGGACTGAGACACGGCC
>Zotu5683
GAACGCTGGCGGCGTGCCTTAAGCATGCAAGTCGAACGAATCGTAAGATTAGTGGCGGACGGTCGAGT
AACGCGTAGGGATCTGCCTTAAAGTTGGACATAGCTCATCGAAAGGTGGGGTAATTTCCAATGTGAAT
GCAAATTTAAAGCCGCAAGGCGCTTTAAGATGAACCTGCGTCCTATCAGCTAGTTGGTGAGGTAATGG
CTCACCAAGGCATCGACGGGTAGCTGGTCTGAGAGGACGACCAGCCGGAATAGCACTGAGACACGGGC
>Zotu5684
GAACGCTGGCGGCGTGCCTAATACATGCAAGTCGAGCGACGAAAGGAGAAGGGGTAACCCTTCTCCCT
GAAAGCGGCGAACGGGTGAGTAACACGTGGGTAACGTGCCCTGGACTCTGGGATAACAACGGGAAACC
GTTGCTAATACCGGATGACCCTGCACCACCGCATGGTGTGAGCAGGCAAAGTTTTTCGGTCCAGGATC
GGCCCGCGGTGTATCAGCTTGTTGGTGGGGTAACGGCCTACCAAGGCTTCGACGCATAGCTGGCGTGA
GAGCGCGACCAGCCACACTGGGACTGAGACACGGCC
>Zotu5685
GAACGCTGGCGGCAGGCCTAACACATGCAAGTCGAACGCCCCGCAAGGGGAGTGGCAGACGGGTGAGT
AACACGTGGGAACCTTCCCAACGGTACGGAATAGCCCAGGGAAACTTGGAGTAATACCGTATACGCCC
CTAGGGGGAAAGATTTATCGCCGTTGGATGGGCCCGCGTCTGATTAGCTAGTTGGTGAGGTAACGGCT
CACCAAGGCGACGATCAGTAGCTGGTCTGAGAGGATGATCAGCCACACTGGGACTGAGACACGGCC
>Zotu5686
GAACGCTGGCGGCGTGCTTAACACATGCAAGTCGAACGGAAAGGCCCTTCGGGGTGCTCGAGTGGCGA
ACGGGTGAGTAACACGTGGGTGATCTGCCCTGCACTTTGGGATAAGCCTGGGAAACTGGGTCTAATAC
CGAATATGACCACGCATTTCATGGTGTGTGGTGGAAAGCTTTTGCGGTGTGGGATGGGCCCGCGGCCT
ATCAGCTTGTTGGTGGGGTAATGGCCTACCAAGGCGACGACGGGTAGCCGGCCTGAGAGGGTGACCGG
CCACACTGGGACTGAGATACGGCC
>Zotu5687
GAACGCTGGCGGCAGGCTTAACACATGCAAGTCGAACGCCCCGCAAGGGGAGTGGCAGACGGGTGAGT
AACGCGTGGGAATCTACCCAGTTCTACGGAACAACTGAGGGAAACTTCAGCTAATACCGTATACGCCC
TACGGGGGAAAGATTTATCGGAATTGGATGAGCCCGCGTTGGATTAGCTAGTTGGTGAGGTAACGGCT
CACCAAGGCGACGATCCATAGCTGGTCTGAGAGGATGATCAGCCACACTGGGACTGAGACACGGCC
>Zotu5688
GAACGCTGGCGGCAGGCTTAACACATGCAAGTCGAGCGCCCCGCAAGGGGAGCGGCAGACGGGTGAGT
AACGCGTGGGAACGTACCATTTGCTACGGAATAACTCAGGGAAACTTGTGCTAATACCGTATGTGCCC
GAAAGGGGAAAGATTTATCGGCAAATGATCGGCCCGCGTTGGATTAGCTAGTTGGTGGGGTAAAGGCC
TACCAAGGCGACGATCCATAGCTGGTCTGAGAGGATGATCAGCCACACTGGGACTGAGACACGGCC
>Zotu5689
GAACGCTGGCGGCGTGCTTAAGACATGCAAGTCGAACGCAGTCTTCGGACTGAGTGGCGCACGGGTGA
GTAACACGTAACTGACCTGCCCCAAAGTCGCGGATAACTGGGTGAAAGTCCAGCTAATACGTGATGTG
CTGTCAGATTTTGTTCTGCCAGTAAAGGTTTACTGCTTTGGGATGGGGTTGCGTTCCATCAGCTAGTT
GGCGGGGTAAAGGCCCACCAAGGCGACGACGGATAGCCGGCCTGAGAGGGTGGCCGGCCACAGGGGCA
CTGAGACACGGGT
>Zotu5690
GAACGTTGGCGGCGTGGATTAGGCATGCAAGTCGTACGGGCCGCAAGGCCAGTGGCGCAAGGGTGAGT
TAGGCGATGGCAACCAACCCCGCGAATGGGCATAGCCACGGGAAACTGTGGGTAATTCCCAGCGATGT
TCGAGATGGGCATCCGTCTTGAACCAAAGGTGCGATTCCGTCGCGGGACGGGCCATCGTGATATTAGA
TTGTTGGCGGGGTAACGGCCCACCAAGTCTATGATGTCTACCGGGCGTGTGAGCGTGGCCCGGCACAC
TGGGACTGAGACACTGCC
>Zotu5692
GAACGCTGGCGGCGTGCTTAACACATGCAAGTCGAACGAGGTCCACGGTGTAGCAATACACCTGAAGA
CCTAGTGGCGAACGGGTGCGTAACACGTGAGGAACTTGTCCCGGTCTCTGGGATAACAGTTGGAAACG
ACTGCTAATACCGGATGACGTCGGAGCATCGCATGGTGCGCTGACGAAAGGGTTACTGGATCGGGAGA

GCCTCGCGGCCTATCAGCTTGTTGGTGGGGTAACGGCCCACCAAGGCATCGACGGGTAGCTGGTCTGA
GAGGATGATCAGCCACACTGGGACTGAGACACGGCC
>Zotu5694
GAACGCTAGCGGCAGGCCTAATACATGCAAGTCGAGGGGTAGGGTCGGAGTAATCTGACCTGAGACCG
GCGCACGGGTGCGTAACACGTATGCAACCTACCTTTTACTGGGGTATAGCTCAGAGAAATCTGAATTA
ATCCCCCATAATGTTATTGAATGGCATCATTTAATAACTAAAGCTGAGGCGGTAAAAGATGGGCATGC
GTTGTATTAGCTAGTTGGTGAGGTAATGGCTCACCAAGGCTACGATACATAGGGGTTCTGAGAGGATG
ATCCCCCACACTGGTACTGAGACACGGAC
>Zotu5696
GAACGCTGGCGGCGTGCTTAACACATGCAAGTCGAACGGTGACGGAGAGCTTGCTCTCCTGATCAGTG
GCGAACGGGTGAGTAACACGTGGGCAACCTGCCCCTGACACTGGGATAACTCCGGGAAACCGGGGCTA
ATACCGGATATTACGCGAGATGGCATCTTCTCGCGTGGAAAGTTTTTCGGTCAGGGATGGGCCCGCGG
CCTATCAGCTTGTTGGTGAGGTAACGGCCCACCAAGGCGACGACGGGTAGCCGGCCTGAGAGGGCGAC
CGGCCACACTGGGACTGAGACACGGCC
>Zotu5698
GAACGCTAGCGGCAGGCTTAACACATGCAAGTCGAGGGGTATAGTTCTTCGGAGCTAGAGACCGGCGC
ACGGGTGCGTAACGCGTATGCAATCTACCTTTTACAGAGGGATAGCCCAGAGAAATTTGGATTAATAC
CTCATAGCATTATGATATGGCATCGTATTATAATTAAAGTTCCAACGGTAAAAGATGAGCATGCGTCC
CATTAGCTAGTTGGTGTGGTAACGGCACACCAAGGCTACGATGGGTAGGGGTCCTGAGAGGGAGATCC
CCCACACTGGTACTGAGACACGGAC
>Zotu5699
GAACGCTGGCGGCATGCCTAACACATGCAAGTCGAACGAGACCTTCGGGTCTAGTGGCGCACGGGTGC
GTAACGCGTGGGAATCTACCCTTAGGTACGGAATAACTCTTCGAAAGGAGTGCTAATACCGTATGATG
TCGCAAGACCAAAGATTTATCGCCTAAGGATGAGCCCGCGTTGGATTAGCTTGTTGGTGAGGTAAAAG
CTCACCAAGGCGACGATCCATAGCTGGTCTGAGAGGATGATCAGCCACACTGGGACTGAGACACGGCC
>Zotu5700
GAACGCTGGCGGCAGGCTTAACACATGCAAGTCGAACGCTCGCAGCAATGCGGGAGTGGCAGACGGGT
GAGTAACGCGTGGGAACATACCCTTTGGTTCGGAATAACTGAGAGAAATTTCAGCTAATACCGGATGT
GCCCTTCGGGGGAAAGATTTATCGCCAAAGGATTGGCCCGCGTCTGATTAGCTAGTTGGTGAGGTAAC
GGCTCACCAAGGCGACGATCAGTAGCTGGTCTGAGAGGATGATCAGCTACACTGGGACTGAGACACGG
CC
>Zotu5701
GAACGCTGGCGGCGCGCTTAACACATGCAAGTCGTACGGGATCATGGTTTCGGCCCTGGTTCAGTGGC
GAACGGGTGCGTAACACGTGAGCAACCTGCCTGCCGGTGGGGGATAACCCGGGGAAACCCGGGCTAAT
ACCGCATTCGCTTCACGTCTCGAGTGGGACATGAAGGAAAGGTGCTGCAAGGCACTGCCGGCAGAGGG
GCTCGCGGCGTATCAGCTAGCTGGTGGGGTAACGGCCCACCAGGGCAACGACGCGTAGCCGATCTGAG
AGGATGATCGGCCACATTGGGACTGAGATACGGCC
>Zotu5702
CAACGCTGGCGGCGCGCTTAACACATGCAAGTCGCACGAGAAAGGGAGCAATCCTGAGTATAGTGGCG
CACGGGTGAGTAACACGTGGATGATCTACCTCCTTGTGGGGAATAACCCTGGGAAACCGGGGCTAATA
CCGCATACGTCCGAGAGGAGAAAGCCGAAAGGCGCAGGGAGAGGAGTCCGCGGTCGATTAGCTAGTTG
GCAGGGTAATGGCCTACCAAGGCGACGATCGATAGCCGGCCTGAGAGGGCACACGGCCACACTGGCAC
TGAAACACGGGC
>Zotu5703
GAACGCTGGCGGCGTGCTTAACACATGCAAGTCGAGCGAAGCAATTTAAGTGAAGTTTTCGGATGGAT
CTTAAATTGACTGAGCGGCGGACGGGTGAGTAACGCGTGGATAACCTGCCTCACACAGGGGGATAACA
GTTAGAAATGACTGCTAATACCGCATAAGCGCACGGTACCGCATGGTACAGTGTGAAAAACTCCGGTG
GTGTGAGATGGATCCGCGTCTGATTAGGTAGTTGGTGAGGTAACGGCCCACCAAGCCGACGATCAGTA
GCCGACCTGAGAGGGTGACCGGCCACATTGGGACTGAGACACGGCC
>Zotu5704
GAACGCTGGCGGCGCGCCTAACACATGCAAGTCGAGCGGGGACATCTGGAGAGTAATCAAGAGATGTT
CTAGCGGCGGACGGGTGAGTAACGCGTGAGTGACCTGCCTTACACAGGGGGATACCGTACCGAAAGGT
ACACTAATACCGCATGAGACCACAGCAGCGCATGCTGCAGAGGTCAAAGGAGCAATCCGGTGTAAGAT
GGACTCGCGTCCGATTAGATAGTTGGCGGGGTAACGGCCCACCAAGTCAGTGATCGGTAGCCGACCTG

AGAGGGTGATCGGCCACATTGGAACTGAGAGACGGTC
>Zotu5705
GAACGCTGGCGGCGTGCTTAAGACATGCAAGTCGAACGCAGTCTTCGGACTGAGTGGCGCACGGGTGA
GTAACACGTAACTGACCTACCCCCAAGTCGCGAATAACCAGCCGAAAGGATGGCTAATACGTGATGTG
ATGATCCGCTGTGGCGGATCATTAAAGACTTGATCGCTTGGGGATGGGGTTGCGTTCCATCAGCTAGT
TGGTAAGGTAAAGGCTTACCAAGGCAACGACGGATAGCCGGCCTGAGAGGGTGGCCGGCCACAGGGGC
ACTGAGACACGGGT
>Zotu5706
GAACGCTGGCGGCAGGCCTAACACATGCAAGTCGAGCGAGATCTTCGGATCTAGCGGCGGACGGGTGA
GTAACGCGTGGGAACGTACCTCTTGCTACGGAATAGCCTCGGGAAACTGGGAGTAATACCGTATGTGC
CCTACGGGGGAAAGATTTATCGGCAAGAGATCGGCCCGCGTTGGATTAGGTAGTTGGTGAGGTAACGG
CTCACCAAGCCGACGATCCATAGCTGGTTTGAGAGGATGATCAGCCACACTGGGACTGAGACACGGCC
>Zotu5707
GAACGCTGGCGGCGTGCCTAATACATGCAAGTCGAGCGAACAGATAAGGAGCTTGCTCCTTTGACGTT
AGCGGCGGACGGGTGAGTAACACGTGGATAACCTACCTATAAGACCGGGATAACTTCGGGAAACCGGA
GCTAATACCGGATAACATATTGAACCGCATGGTTCAATAGTGAAAGACGGCCTTGCTGTGACTTATAG
ATGGATCCGCGCCGTATTAGCTAGTTGGTAAGGTAACGGCTTACCAAGGCAACGATACGTAGCCGACC
TGAGAGGGTGATCGGCCACACTGGAACTGAGACACGGTC
>Zotu5709
AAACGCTGGCGGCGCACATAAGACATGCAAGTCGAACGGAAGTCGTTATGAAATGAATTGAAGATTTA
TCTAATTGAAGTTTCATAACAATGGCTTTAGTGGCGGACTGGTGAGTAACACGTAAGCAATCTACCTA
TTAGAGGGGAATAACAATGAGAAATCATTGCTAATACCGCATAAGCTTGTAGCATCGCATGATGCAGC
AAGAAAAGGAGTAATCCGCTAATAGATGAGCTTGCGGCTGATTAGCTAGTTGGTAAGGTAACGGCTTA
CCAAGGCAACGATCAGTAGCCGGGCTGAGAGGCTCTGCGGCCACATTGGGACTGAGATACGGCC
>Zotu5713
GAACGCTGGCGGCGTGCCTAAGACATGCAAGTCGAGTGGGTTTAGACCCACGGCGAACGGGTGAGTAA
TACCTAGGAACTTTCTTTAAGTGGAGCATAACCCATAGAAATGTGGGATAATACCCCATGTGACCGCA
AGGTCAAAGTCGCAAGACGCTTAAAGTGAGGCCTGGGCACCATCAGCTAGTTGGTAAGGTAATGGCTT
ACCAAGGCTATGACGGTTAGGGGTCCCGAGAGGGATAACCCCCACACTGGAACTGCGACACGGTC
>Zotu5716
GAACGCTGGCGGCAGGCTTAACACATGCAAGTCGAACGCATCCTTCGGGGTGAGTGGCAGACGGGTGA
GTAACGCGTGGGAACATGCCCTTCAGTTTGGAATAACCCAGGGAAACTTGGGCTAATACCGGATACGC
CCTTAGGGGGAAAGATTTATCGCTGAAGGAGTGGCCCGCGTCTGATTAGCTAGTTGGTGAGGTAATGG
CTCACCAAGGCGACGATCAGTAGCTGGTCTGAGAGGATGATCAGCCACACTGGGACTGAGACACGGCC
>Zotu5717
GAACGCTGGCGGCGTGCTTAACACATGCAAGTCGAGCGGTAAGGCCTCTTCGGGGGTACACGAGCGGC
GAACGGGTGAGTAACACGTGAGTAATCTGCCCTTCACTTTGGGATAAGCTTCGGAAACGGGGTCTAAT
ACCGAATACGACACAGATCTGCATGGATTCTGTGTGGAAACTTTTGTGGGTGGGGGATGTGCTCGCGG
CCTATCAGCTTGTTGGTGGGGTAATGGCCTACCAAGGCTTTGACGGGTAGCCGGCCTGAGAGGGTGAC
CGGCCACACTGGGACTGAGACACGGCC
>Zotu5718
GAACGCTGGCGGCGTGCCTAATACATGCAAGTCGAGCGCGAAGATCAGGAGCTTGCTCCTGAGATTTG
AGCGGCGGACGGGTGAGTAACACGTAGGCAACCTACCCTTGAGATTGGGATAACTACCGGAAACGGTA
GCTAATACCGGATACGACTTTTTCACATAAGTGGAACTGTTAAAAGGCGGATTTATCTGCCGCTCATG
GATGGGCCTGCGGTGCATTAGCTAGTTGGTGAGATAGTAGCTCACCAAGGCGACGATGCATAGCCGAC
CTGAGAGGGTGATCGGCCACACTGGGACTGAGACACGGCC
>Zotu5719
GAACGCTGGCGGCGTGCCTAATACATGCAAGTCGAGCGGTAAGGCTCTGCACCCATTATTTGACCGGA
TAAGTCTGTTTTACAGAAACTGTCCGAGAGGTTGTTCTCTAGGCTAGAGGACAAGCGTCGAATACTGA
GTGTAGAGTACACGAGCGGCGGACGGCTGAGTAACGCGTAGGAATGTACCCCGAAGTGAGGGATAAGC
ACCAGAAATGGTGTCTAATACCGCATGTGCTCTTTCGAGAGGAAAGCTTTAGCGCTTTGGGAGCAGCC
TGCGTATGATTAGTTAGTTGGTGAGGTAATGGCTTACCAAGACGATGATCATTAGCTGGTCTGAGAGG
ATGATCAGCCAGACTGGGACTGAGAACGGCC
>Zotu5720

GAACGCTAGCTACAGGCTTAACACATGCAAGTCGCAGGGTAACGTGAGGGAAGCTTGCTTCCCTTGAC
GACGACTGGCGCACGGGTGAGTAACGCGTATCCAACCTTCCCATGACCACGGGATAACCCGTTGAAAG
ACGGACTAATACCGTATGACGTCGTTTGCTGACATCAAATAACGATTAAAGGTTTAGCGGTGATGGAT
GGGGATGCGTCTGATTAGCTTGTTGGCGGGGTAACGGCCCACCAAGGCGACGATCAGTAGGGGTTCTG
AGAGGAAGGTCCCCCACATTGGAACTGAGACACGGTC
>Zotu5722
GAACGCTAGCGGCATGCCTAACACATGCAAGTCGAACGGCAGCATAGGAGCTTGCTCCTGATGGCGAG
TGGCGAACGGGTGAGTAATATATCGGAACGTGCCCTAGAGTGGGGGATAACTAGTCGAAAGACTAGCT
AATACCGCATACGATCTACGGATGAAAGTGGGGGATCGCAAGACCTCATGCTCCTGGAGCGGCCGATA
TCTGATTAGCTAGTTGGTAGGGTAATGGCCTACCAAGGCGACGATCCGTAGCTGGTCTGAGAGGACGA
TCAGCCACACTGGGACTGAGACACGGCC
>Zotu5724
GAACGCTGGCGGCGTGCCTAATACATGCAAGTCGAGCGAATCTTTGGGAGCTTGCTCCCAAAGGTTAG
CGGCGGACGGGTGAGTAACACGTGGGCAACCTGCCTATAAGACTGGGATAACTTCGGGAAACCGGAGC
TAATACCGGATAATTCTTTCCTATACATGTAGGAAAGCTAAAAGACGGTTTACGCTGTCACTTATAGA
TGGGCCCGCGGCGCATTAGCTAGTTGGTGAGGTAACGGCTCACCAAGGCCACGATGCGTAGCCGACCT
GAGAGGGTGATCGGCCACACTGGGACTGAGACACGGCC
>Zotu5726
GAACGCTGGCGGCGTGCCTAATACATGCAAGTCGAGCGAATCAAAGGGAGCTTGCTCCCTGAGATTAG
CGGCGGACGGGTGAGTAACACGTGGGCAACCTGCCTATAAGACTGGGATAACTTCGGGAAACCGGAGC
TAATACCGGATAATTCTTATCAACTCATGTTGGTAAGCTGAAAGACGGTTTACGCTGTCACTTATAGA
TGGGCCCGCGGCGCATTAGCTAGTTGGTGAGGTAACGGCTCACCAAGGCGACGATGCGTAGCCGACCT
GAGAGGGTGATCGGCCACACTGGGACTGAGACACGGCC
>Zotu5727
GAACGCTGGCGGCATGCTTTACACATGCAAGTCGAACGGCAGCGCGGGGCAACCTGGCGACGAGTGGC
GAACGGGTGAGTAATACATCGGAACGTACCCAGAAGTGGGGGATAACGTAGCGAAAGTTACGCTAATA
CCGCATACGTTCTACGGAAGAAAGCGGGGGATCGCAAGACCTCGTGCTTTTGGAGCGGCCGATGTCTG
ATTAGCTAGTTGGTGAGGTAAAGGCTCACCAAGGCGACGATCAGTAGCTGGTCTGAGAGGACGACCAG
CCACACTGGGACTGAGACACGGCC
>Zotu5729
GAACGCTGGCGGCGTGCTTAACACATGCAAGTCGAACGATGAAGCCCTTCGGGGTGGATTAGTGGCGA
ACGGGTGAGTAACACGTGGGCAATCTGCCCTGCACTCTGGGACAAGCCCTGGAAACGGGGTCTAATAC
CGGATATGACCTTTCCCCTCCTGGGGTTTGGTGGAAAGCTCCGGCGGTGCAGGATGAGCCCGCGGCCT
ATCAGCTTGTTGGTGGGGTAATGGCCTACCAAGGCGACGACGGGTAGCCGGCCTGAGAGGGCGACCGG
CCACACTGGGACTGAGACACGGCC
>Zotu5730
GAACGCTGGCGGCAGGCCTAACACATGCAAGTCGAGCGGATGAAGGGAGCTTGCTCCCTGATTTAGCG
GCGGACGGGTGAGTAATGCCTAGGAATCTGCCTGGTAGTGGGGGATAACGTCCGGAAACGGGCGCTAA
TACCGCATACGTCCTACGGGAGAAAGCAGGGGACCTTCGGGCCTTGCGCTATCAGATGAGCCTAGGTC
GGATTAGCTAGTTGGTGAGGTAATGGCTCACCAAGGCGACGATCCGTAACTGGTCTGAGAGGATGATC
AGTCACACTGGAACTGAGACACGGTC
>Zotu5732
GAACGCTGGCGGCGTGCTTAACACATGCAAGTCAAGGGGGCCCGCAAGGGTAACCGGCGAACGGGTGC
GTAACACGTGAGCAATCTGCCTAGATGTGGGGGATAGCCGGCCCAACGGCCGGGTAATACCGCATACG
CTCTCTGGTCGGCATCGACTGGAGAGGAAACATTCGTGGCATCTAGAGGAGCTCGCGGCCTATCAGCT
AGTTGGTGGGGTAACGGCCTACCAAGGCTTTGACGGGTAGCTGGTCTGAGAGGATGGCCAGCCACATT
GGGACTGAGATACGGCC
>Zotu5733
GAACGCTGGCGGCGTGCCTAATACATGCAAGTCGAGCGAACCAAAAAGGAGCTTGCTCCTTTACGGTT
AGCGGCGGACGGGTGAGTAACACGTGGGTAACCTGCCCTCAAAACTGGGATAACATCGAGAAATCGGT
GCTAATACCGGATAATCAATAAGACCGCATGGTCTTATTATAAAAGGTGGCTTCGGCTACTATTTGAG
GATGGGCCCGCGGCGCATTAGCTAGTTGGTAAGGTAACAGCTTACCAAGGCAACGATGCGTAGCCGAC
CTGAGAGGGTGATCGGCCACATTGGGACTGAGACACGGCC
>Zotu5734

GAACGCTAGCTGCAGGCTTAACACATGCAAGTCGAGGGGCAGCGGGAGTAGCAATACTTGCCGGCGAC
CGGCGGAAGGGTGCGTAACGCGTGAGCAACATTCCCGTATCAGGGGGATAACCGGTGGAAACGCCGAC
TAATACCCCATAAGAACATCTGCGGCACCAAAGGTGTTTGAAAGATTCGTCGGATGCGGATTGGCTCG
CGTGACATTAGCTAGTTGGTGAGGTAACGGCCCACCAAGGCGACGATGTCTAGGGGTTCTGAGAGGAA
GATCCCCCACATTGGAACTGAGACACGGTC
>Zotu5735
GAATGCTGGCGGCGTGGATAAGGCATGCAAGTCGAGTGGGTTTAGACCCACGGCAGACGAGGTAGTAA
CACGTAGGAACGTACCCCAAAGTCGGGAATATTCCATCGAAAGATGGCACAATACCCGATGGTCTCTA
AGGAGTAAAGATTTATCGCTTTGGGAGCGGCCTGCGCAATATCAGCTAGTTGGTGAGGTAATGGCTCA
CCAAGGCTATGACGTTTAGGGGAGCTGAGAGGCTGACCCCCACCGATGGAACTGAGAAACGGTC
>Zotu5736
GAACGCTGGCGGTGTGCCTAATACATGCAAGTCGAACGGACCTTCGGGTTAGTGGCGAACGGGTGAGT
AACACGTTGGTACCTGCCCTCTAGTGGGGAATAATTCGCCGAAAGGCGGACTAATACCGCATATTGCC
GCAAGGTGAAAGCAGTAATGCGCTAGAGGAGGGGCCTGCGGCCCATTAGCTTGTTGGTAGTGTAAAAG
ACTACCAAGGCTACGATGGGTAGCTGGATCGAGAGGTTGACCAGCCACACTGGGACTGAGATACGGCC
>Zotu5737
GAACGCTGGCGGCGTGCTTAACACATGCAAGTCGAACGGAAAGGCCCTTTCGGGGGTACTCGAGTGGC
GAACGGGTGAGTAACACGTGGGTGATCTGCCCTGCACTTTGGGATAAGCCTGGGAAACTGGGTCTAAT
ACCGGATATACCCTGCTGGTCGCATGGCCTGGTGGGGGAAAGCTTTTGCGGTGTGGGATGGGCCCGCG
GCCTATCAGCTTGTTGGTGGGGTGATGGCCTACCAAGGCGACGACGGGTAGCCGGCCTGAGAGGGTGT
CCGGCCACACTGGGACTGAGATACGGCC
>Zotu5738
GAACGCTGGCGGCGTGCTTAACACATGCAAGTCGAACGGTGAACCAGGAGCTTGCTCTTGGGGATCAG
TGGCGAACGGGTGAGTAACACGTGAGCAATCTGCCCCTGACTCTGGGATAAGCGCTGGAAACGGCGTC
TAATACTGGATACGAGCCGTGAAGGCATCTTCAACGGTTGGAAAGATTTTTCGGTCAGGGATGAGCTC
GCGGCCTATCAGCTTGTTGGTGAGGTAATGGCTCACCAAGGCGTCGACGGGTAGCCGGCCTGAGAGGG
TGACCGGCCACACTGGGACTGAGACACGGCC
>Zotu5739
GAACGCTGGCGGTATGCTTAACACATGCAAGTCGAACGAATGAGATTTTAAGCTTGCTTAAAATCGAG
TTAGTGGCAAACGGGTGAGTAACACGTGGGAATCTACCCAGTAGTACAGAATAACAGTTAGAAATGAC
TGCTAATACTGTATAGTATCGAGAGATTAAAGATTTATCGCTACTGGATGAGCCCGCGTCAGATTAGG
TAGTTGGTGGGGTAATGGCCTACCAAGCCAAAGATCTGTAGCTGGTCTGAGAGGATGATCAGCCACAC
TGGAACTGAGACATGGTC
>Zotu5740
GAACGCTAGCGGCAGGCCTAACACATGCAAGTCGAGGGGTAGAGGAAGCTTGCTTCCTTGAGACCGGC
GCACGGGTGCGTAACGCGTATGCAATCTACCTTGTACAGAGGGATAGCCCAGAGAAATTTGGATTAAT
ACCTCATGGTATTATTGAATGGCATCATTTGATAATTAAAGTTCCAACGGTACAAGATGAGCATGCGT
CCTATTAGCTAGATGGTAAGGTAACGGCTTACCATGGCTACGATAGGTAGGGGTCCTGAGAGGGAGAT
CCCCCACACTGGTACTGAGACACGGAC
>Zotu5741
TAACGCTGGCGGCGTGCATAACACATGCAAGTCGAACGATGAAGCTTAAATGATTTCTTCGGAATGAA
CTTAAGTGAATTAGTGGCGAACGGGTGAGTAACGCGTGAGTAACCTGCCTTACACAAGGGGATAGCCT
CTGGAAACGGAGAATAATACCCTATGACATTACAGCCTCGCATGAAGTAGTAATCAAAGTGTTAGCGG
TGTAAGATGGACTTGCGTCTGATTAGCTAGTTGGTGAGATAACAGCCCACCAAGGCAACGATCAGTAG
CCGGCTTGAGAGAGTGTACGGCCACATTGGGACTGAGACACGGCC
>Zotu5742
GAACGCTGGCGGCGTGCCTAATACATGCAAGTCGAGCGGTAACAGGTCTAGTTTACTAGATGCTGACG
AGCGGCGGACGGCTGAGTAACACGTAGGAACGTACCCCAAAGTGAGGGATAACTGCTCGAAAGAGTAG
CTAATACCGCATGTGATCCACGGATTAAAGCTTTATGCGCTTTGGGAACGGCCTGCGTTCGATTAGCT
AGATGGTAGTGTAATGGACTACCATGGCGACGATCGATAGCTGGTCTGAGAGGATGATCAGCCAGACT
GGGACTGAGACACGGCC
>Zotu5743
GAACGCTGGCGGCAGGCCTAACACATGCAAGTCGAACGGCAGCGGGTCCTTCGGGATGCCGGCGAGTG
GCGGACGGGTGAGGAATGCGTCGGAATCTGCCTCTTTGTGGGGGATAACGTAGGGAAACTTACGCTAA

TACCGCATACGACCTACGGGTGAAAGCCGGGGACCTTCGGGCCTGGCGCAGAGAGATGAGCCGACGCC
GGATTAGCTAGTTGGCGGGGTAAAGGCCCACCAAGGCGACGATCCGTAGCTGGTCTGAGAGGATGATC
AGCCACACTGGAACTGAGACACGGTC
>Zotu5744
GAACGCTGGCGGCATGCCTAACACATGCAAGTCGAACGAGATCTTTCGGGGTCTAGTGGCGCACGGGT
GCGTAACGCGTGGGAATCTGCCTTGGGGTTCGGAATAACGTCGGGAAACTGACGCTAATACCGGATGA
TGACGTAAGTCCAAAGATTTATCGCCCTGAGATGAGCCCGCGTCCGATTAGCTAGTTGGTGGGGTAAA
GGCCTACCAAGGCGACGATCGGTAGCTGGTCTGAGAGGATGATCAGCCACACTGGGACTGAGACACGG
CC
>Zotu5745
GAACGCTGGCGGCAGGCTTAACACATGCAAGTCGAACGCCCCGCAAGGGGAGTGGCAGACGGGTGAGT
AACGCGTGGGAATCTACCCAGCTCTACGGAACAACTCCGGGAAACTGGAGCTAATACCGTATACGTCC
GATAGGAGAAAGATTTATCGGAGTTGGATGAGCCCGCGTTGGATTAGCTAGTTGGTGGGGTAATGGCC
CACCAAGGCGACGATCCATAGCTGGTCTGAGAGGATGATCAGCCACACTGGGACTGAGACACGGCC
>Zotu5746
GAACGCTGGCGGCGTGCCTAAAACATGCAAGTCGTGCGGTAACAGGCCCTTCGGGGTGCTGACGAGCG
GCGGACGGCTGAGTAACGCGTGGGAATCTGCCCCAAAGTGAGGGATAACTGCCCGAAAGGGTGGCTAA
TACCGCATATGGTCTTCGGATTAAAGGAGTAATCCGCTTTGGGAAGAGCCCGCGTAAGATTAGATAGT
TGGTGAGGTAATGGCTCACCAAGTCGACGATCTTTAACTGGTTTGAGAGGATGATCAGTCAGACTGGA
ACTGAGACACGGTC
>Zotu5747
GAACGCTGGCGGCGTGCCTAATACATGCAAGTAGAACGCTGAAGGGGGAGCTTGCTTCTCCGGATGAG
TTGCGGACGGGTGAGTAACGCGTAGGTAACCTGCCTGGTAGCGGGGGATAACTATTGGAAACGATAGC
TAATACCGCATAAGAGTAGATGTTGCATGACATTTACTTAAAAGGTGCAATTGCATCACTACCAGATG
GACCTGCGTTGTATTAGCTAGTTGGTGGGGTAACGGCTCACCAAGGCGACGATACATAGCCGACCTGA
GAGGGTGATCGGCCACACTGGGACTGAGACACGGCC
>Zotu5748
GAACGCTGGCGGCGTGCCTAATACATGCAAGTCGAACGGTCTTTTCTATGGAAGCTTGCTTCCGCTGA
GAAGATAGTGGCGGACGGGTGAGTAACACGTGGGTAACCTGCCCATAAGAGGGGGATAACATCCGGAA
ACGGGTGCTAATACCGCATAGTTTTTCTGATCGCATGATCGGGAAAGGAAAGACGGCCTTTGTGCTGT
CGCTTATGGATGGACCCGCGGCGTATTAGTTAGTTGGTGAGGTAATGGCTCACCAAGACGATGATACG
TAGCCGACCTGAGAGGGTGATCGGCCACATTGGGACTGAGACACGGCC
>Zotu5749
GAACGCTGGCGGCGTGCTTAACACATGCAAGTCGAACGATGACGGTGGTGCTTGCATCACCTGATTAG
TGGCGAACGGGTGAGTAACACGTGAGTAACCTGCCCTTCACTTCGGGATAACCTCGGGAAATCGTGGC
TAATACCGGATATGAGCTCCTTCCGCATGGCGGGGGTTGGAAAGATTTATCGGTGAGGGATGGACTCG
CGGCCTATCAGTTTGTTGGTGAGGTAATGGCTCACCAAGGCGATGACGGGTAGCCGGCCTGAGAGGGC
GACCGGCCACACTGGGACTGAGACACGGCC
>Zotu5751
GAACGCTGGCGGCGTGCTTAACACATGCAAGTCGAACGGTGAAGGGACTGGTTTTGCTGGTTCTGGAT
GAGTGGCGAACGGGTGAGTAACACGTGAGTAACCTGCCCCCTTCTTCTGGATAACCGCATGAAAGTGT
GGCTAATACGGGATATTCTGGGTCTGTCGCATGGTGGGCCTGGGAAAGATTGCGCTTTTTTGGTGTTT
TTGGTGGGGGATGGGCTCGCGGCCTATCAGCTTGTTGGTGGGGTGATGGCCTACCAAGGCGGTGACGG
GTAGCCGGCCTGAGAGGGTGGACGGTCACACTGGGACTGAGACACGGCC
>Zotu5752
GAACGCTGGCGGCGTGCCTAACACATGCAAGTCGAACGAGGTATTTTGATTGAAGTTTTCGGACAGAT
TTCAAGATACCGAGTGGCGGACGGGTGAGTAACGCGTGGGTAACCTGCCTCATACAGGGGGATAACGG
TTAGAAATGACTGCTAATACCGCATAAGCGCACAGTACCGCATGGTACGGTGTGAAAAACTCCGGTGG
TATGAGATGGACCCGCGTCTGATTAGCTAGTTGGTGGGGTAACGGCCCACCAAGGCGACGATCAGTAG
CCGACCTGAGAGGGTGACCGGCCACATTGGGACTGAGACACGGCC
>Zotu5753
GAACGCTGGCGGCGTGCTTAACACATGCAAGTCGAACGGATCACTTTTACTTCGGTTTTGGTGGTTAG
TGGCGAACGGGTGAGTAACACGTGAGTAACCTGCCCTTCACTGTGGGATAACGAGAAGAAATTCTTGC
TAATACCGCATATGACGCGTCTCCGCATGGGGGTGTGTGGAAAGTTTTTTCGGTGGGGGATGGACTCG

CGGCCTATCAGCTTGTTGGTGGGGTAATGGCTCACCAAGGCTTTGACGGGTAGCCGGCCTGAGAGGGC
GACCGGCCACACTGGGACTGAGATACGGCC
>Zotu5754
TAACGCTGGCGGCGTGCATAACACATGCAAGTCGAACGATGAAATTTATTAGATTCTTCGGATGAAAA
TAAACGGATTAGTGGCGGACGGGTGAGTAACGCGTGAGTAACCTGCCTTATACAAGGGGATAGCCGTT
GGAAACGACGAATAATACCCTATAATATAAACTCCTCGCATGAGGAGCTTATCAAAGATTTATCGGTA
TAAGATGGACTCGCGTCTGATTAGCTAGTTGGTGAGATAAAAGCTCACCAAGGCGACGATCAGTAGCC
GGCTTGAGAGAGTGTACGGCCACATTGGGACTGAGACACGGCC
>Zotu5755
GAACGCTGGCGGCAGGCTTAACACATGCAAGTCGAACGATGATTACCTAGCTTGCTAGGTATGATTAG
TGGCGAACGGGTGAGTAATGCTTAGGAATCTGCCTGATAGTGGGGGACAACTTGGGGAAACCCAAGCT
AATACCGCATACGTCCTACGGGAGAAAGGGGGCTTTTAGCTCTCGCTATCAGATGAGCCTAAGTCGGA
TTAGCTAGTTGGTGGGGTAAAGGCCTACCAAGGCGACGATCTGTAGCTGGTCTTAGAGGATGATCAGC
CACACTGGGACTGAGACACGGCC
>Zotu5756
GAACGCTGGCGGCGTGCTTAACACATGCAAGTCGAACGATGATCAAGGCTTCGGTTTTGTGATTAGTG
GCGAACGGGTGAGTAACACGTGAGCAACCTGCCCTTCACTTCGGGATAACTACCGGAAACGGTGGCTA
ATACCGGATATTCATTTCTCCATGCCTGTGGGGTTGTGGAAAGTTTTTTCGGTGGGGGATGGGCTCGC
GGCCTATCAGCTTGTTGGTGGGGTAATGGCCTACCAAGGCGTCGACGGGTAGCCGGCCTGAGAGGGCG
ACCGGCCACACTGGGACTGAGATACGGCC
>Zotu5760
GAACGCTAGCGGCAGGCTTAACACATGCAAGTCGAGGGGCAGCGCGGGTAGCAATACCTGGCGGCGAC
CGGCAAACGGGTGCGGAACACGTACAGAACCTACCTTTGTCTGGGGCATAGCCCGGAGAAATCCGGAT
TAACTCCCCATAATATAATTGAGTGGCATCACTTGACTATTAAAAATTTATTGGACAGAGATGGCTGT
GCGTCCCATTAGCTAGTTGGTAGGGTAACGGCCTACCAAGGCAACGATGGGTAGCTGGTGTGAGAGCA
CGACCAGCCACACGGGCACTGAGACACGGGC
>Zotu5763
GAACGCTGGCGGCGTGCTTCACACATGCAAGTCGAGCGAGGCCCTTCCTTCGGGAAGGTGCCCTAGCG
GCGAACGGGTGAGTAACACGTGGGCAACCTGCCCTCAGCTCTGGGATAAGCCCGGGAAACCGGGTCTA
ATACCGGATATGACTCCGACAGGCATCTTGCGGAGTGGAAAGATTCATCGGCTGAGGATGGGCCCGCG
GCCTATCAGCTTGTTGGTGGGGTAACGGCCTACCAAGGCGACGACGGGTAGCCGGCCTGAGAGGGCGA
CCGGCCACACTGGGACTGAGACACGGCC
>Zotu5764
GAACGCTGGCGGCATGCCTAACACATGCAAGTCGAACGAAACCTTCGGGTTTAGTGGCGCACGGGTGC
GTAACGCGTGGGAATCTGCCTCTCGGTTCGGAATAACAGTTAGAAATGACTGCTAATACCGGATGATG
ACGAAAGTCCAAAGATTTATCGCCGAGAGATGAGCCCGCGTAGGATTAGCTAGTTGGTGGGGTAAAGG
CCTACCAAGGCGACGATCCTTAGCTGGTCTGAGAGGATGATCAGCCACACTGGGACTGAGACACGGCC
>Zotu5765
GAACGCTGGTAACGTGGATACCGGCATGCAAGTCACGGGGGCCGCAAGGCAACCGGCGAACGGCTTAG
TAACACGTAGATATATACCTTAAAGTGGAGCATAGCTCGTTGAAAAACGGGGTAATTCTCCATAGTCC
CGCAAGGGTAAAGGCGCAAGCCGCTTTAAGAATAGTCTGCGGTCTATCAGCTAGTTGGTGAGGTAATG
GCTCACCAAGGCTATGACGGATAGGGGGCGTGAGAGCGCGACCCCCAAGAATGGCACTGCGACACGGG
C
>Zotu5766
GAACGCTGGCGGCAGGCCTAACACATGCAAGTCGAACGCCCCGCAAGGGGAGTGGCAGACGGGTGAGT
AACACGTGGGAACCTTCCCTTTGGTACGGAATAACCCACCGAAAGGTGGATTAATGCCGCATAAGCCC
GTGAGGGGAAAGATTTATCGCCAAAGGATGGGCCCGCGTAGGATTAGCTAGTTGGTGAGGTAATGGCT
CACCAAGGCGACGATCCTTAGCTGGTCTGAGAGGACGACCAGCCACACTGGGACTGAGACACGGCC
>Zotu5767
GAACGCTGGCGGCGTGCTTAACACATGCAAGTCGAACGATGACCCGGAGCTTGCTTCGGTGATTAGTG
GCGAACGGGTGAGTAACACGTGAGTAACCTGCCCTTGACTCTGGGATAAGCACTGGAAACGGTGTCTA
ATACCGGATATGAACCTCAGCCGCATGGCTAGGGGTTGGAAAGAATTTCGGTCAAGGATGGACTCGCG
GCCTATCAGCTAGTTGGTGAGGTAATGGCCCACCAAGGCGACGACGGGTAGCCGGCCTGAGAGGGTGA
CCGGCCACACTGGGACTGAGACACGGCC

>Zotu5771
GAACGCTGGCGGCAGGCTTAACACATGCAAGTCGAGCGGGGAAAGGTAGCTTGCTACCTAACCTAGCG
GCGGACGGGTGAGTAATGCTTAGGAATCTGCCTATTAGTGGGGGACAACATCTCGAAAGGGATGCTAA
TACCGCATACGTCCTACGGGAGAAAGCAGGGGACCTTCGGGCCTTGCGCTAATAGATGAGCCTAAGTC
GGATTAGCTAGTTGGTGGGGTAAAGGCCTACCAAGGCGACGATCTGTAGCGGGTCTGAGAGGATGATC
CGCCACACTGGGACTGAGACACGGCC
>Zotu5772
GAACGCTGGCGGCATGCCTTACACATGCAAGTCGAACGGCAGCACGGGGCAACCTGGTGGCGAGTGGC
GAACGGGTGAGTAATATATCGGAACGTACCCTGGAGTGGGGGATAACGTAGCGAAAGTTACGCTAATA
CCGCATACGATCTAAGGATGAAAGTGGGGGATCGCAAGACCTCATGCTCCTGGAGCGGCCGATATCTG
ATTAGCTAGTTGGTAGGGTAAAAGCCTACCAAGGCTTCGATCAGTAGCTGGTCTGAGAGGACGACCAG
CCACACTGGAACTGAGACACGGTC
>Zotu5773
GAACGCTGGCGGCGTGCTTAACACATGCAAGTCGAACGGGAATTACTTTATTGAAACTTCGGTCGATT
TAATTTAATTCTAGTGGCGGACGGGTGAGTAACGCGTGGGTAACCTGCCTTATACAGGGGGATAACAG
TCAGAAATGGCTGCTAATACCGCATAAGCGCACAGAGCTGCATGGCTCAGTGTGAAAAACTCCGGTGG
TATAAGATGGACCCGCGTTGGATTAGCTAGTTGGTGGGGTAACGGCCCACCAAGGCGACGATCCATAG
CCGGCCTGAGAGGGTGAACGGCCACATTGGGACTGAGACACGGCC
>Zotu5775
GAACGCTGGCGGCGTGCTTAACACATGCAAGTGGTGCGACGAACCAGGGCTTGCCCTGGGGCAAAGCC
GCGAACGGGTGAGTAACACGTGAGTAATCTGCCCCGATGACCGGGACAACCCGAGGAAACTCGGGCTA
ATACCGGATGATGAGCCAAGGCGTAAGCCTTTTCTCTAAAGGAAGCTTCGGCCTCCGCATCGGGAGGA
GCTCGCGGCCCATTAGCTTGTTGGTGAGGTAACGGCTCACCAAGGCGACGATGGGTAGCTGGTCTGAG
AGGACGATCAGCCACACTGGAACTGAGACACGGTC
>Zotu5777
GAACGCTGGCGGCAGGCCTAACACATGCAAGTCGAACGGTAACACAGAGAGCTTGCTCTCGGGTGACG
AGTGGCGGACGGGTGAGTAATGTCTGGGAAACTGCCTGATGGAGGGGGATAACTACTGGAAACGGTAG
CTAATACCGCATAACGTCGCAAGACCAAAGAGGGGGACCTTCGGGCCTCTTGCCATCGGATGTGCCCA
GATGGGATTAGCTAGTAGGTGGGGTAACGGCTCACCTAGGCGACGATCCCTAGCTGGTCTGAGAGGAT
GACCAGCCACACTGGAACTGAGACACGGTC
>Zotu5780
GAACGCTGGCGGCGTGCTTAACACATGCAAGTCGAACGGTGACCTTGGAGCTTGCTCCGAGCGATCAG
TGGCGAACGGGTGAGTAACACGTGAGTAACCTGCCCCAGACTCTGGGATAACCTCGGGAAACCGGGGC
TAATACCGGATATGAGACGCTTCTGCATGGAATGCGTCTGGAAAGATTTATCGGTCTGGGATGGACTC
GCGGCCTATCAGCTTGTTGGTGAGGTAATGGCTCACCAAGGCGACGACGGGTAGCCGGCCTGAGAGGG
CGACCGGCCACACTGGAACTGAGACACGGTC
>Zotu5781
GAATGGTGGCAGCGTGGATGAGTCATGCAAGTCAAGGGGGCCGCAAGGCAACCGGCAAACGAGGTAGT
AACACGTAGGTAGATCCCCCCGAGTCGGGCATAGCTTGCCGAAAGGCAAGGTAATTCCCGATGGTCCC
GCAAGGGTAAAGTTTTTCGCTCGGGGAGTCGCCTGCGTAGTATCAGCTAGTTGGTAGGGTAACGGCCT
ACCAAGGCTACGACGCTTAGGGGGGGTGAGAGCCCGATCCCCACCGATGGGACTGCGACACGGCC
>Zotu5782
CAACGCTGGCGGCGCGCTTAACACATGCAAGTCGAACGAGAAAGCCCGCAAGGGTGAGTAAAGTGGCG
TACGGGTGAGTAACACGTGGGTAATCTACCTTTTGGTGGGGAATAACCCTGGGAAACCGGGGCTAATA
CCGCATAAGCTCGAAAGAGGAAAGGAGCAATTCGCCGAAAGAGGAGCCCGCGGCTGATTAGCTAGTTG
GTGAGGTAAAGGCTCACCAAGGCTATGATCGGTAGCCGGCCTGAGAGGGCACACGGCCACACTGGCAC
TGAAACACGGGC
>Zotu5783
GAACGCTGGCGGCGTGCTTAACACATGCAAGCCGAACGATGAAGCCCAGCTTGCTGGGTGGATTAGTG
GCGAACGGGTGAGTAACACGTGAGTAACCTGCCCTTGACTCTGGGATAAGCACTGGAAACGGTGTCTA
ATACCGGATATATCAGCTGGCCGCATGGTCTGGTTGTGGAAAGAATTTTGGTCAAGGATGGACTCGCG
GCCTATCAGGTAGTTGGTGAGGTAATGGCTCACCAAGCCTACGACGGGTAGCCGGCCTGAGAGGGTGA
CCGGCCACACTGGGACTGAGACACGGCC
>Zotu5785

GAACGCTGGCGGCGTGCCTAACACATGCAAGTCGAGCGATTTACTTCGGTAAAGAGCGGCGGACGGGT
GAGTAACGCGTGGGTAACCTGCCTCATACACATGGATAACATACCGAAAGGTATGCTAATACAAGATG
ATATAAGAGATTCGCATGGATTTCTTATCAAAGCTTTTGCGGTATGAGATGGACCCGCGTCTGATTAG
CTAGTTGGTAAGGTAACGGCTTACCAAGGCGACGATCAGTAGCCGACCTGAGAGGGTGATCGGCCACA
TTGGAACTGAGACACGGTC
>Zotu5786
GAACGCTGGCGGCGTGCTTAACACATGCAAGTCGAACGGACTCCATTCAGTAGCAATACTGAGGAAGA
GTTAGTGGCGAACGGGTGAGTAACACGTGAGAAACCTATCCTGGTCTCTGGGATAACAGTCGGAAACA
ACTGCTAATACCGGATGCCGTCGGAGCGTCGCATGGCGCGCTGACGAAAGGGTTACTGGATCAGGAGG
GTCTCGCGGCCTATCAGCTAGTTGGTGGGGTAAAGGCCTACCAAGGCATCGACGGGTAGCTGGTCTGA
GAGGATGATCAGCCACACTGGGACTGAGACACGGCC
>Zotu5787
GAACGCTGGCGGCATGCCTAACACATGCAAGTCGAACGAGACCTTCGGGTCTAGTGGCGCACGGGCGC
GTAACGCGTGGGAACCTGCCTTTAGGTTCGGAATAACTCCTCGAAAGGGGTGCTAATACCGAATAATG
TCTTCGGACCAAAGATTTATCGCCTTTAGATGGGCCCGCGTTGGATTAGCTAGTTGGTGGGGTAAAGG
CTCACCAAGGCGACGATCCATAGCTGGTCTTAGAGGATGATCAGCCACACTGGGACTGAGACACGGCC
>Zotu5789
TAACGCTGGCGGCGTGCATGAGACATGCAAGTCGAACGGACGATCAGGGCTTGCTCTGATCGTTAGTG
GCGGACGGCTGAGTAACGCGTAGGTGACCTGCCCCGAAGTGGGGGATAACTGCCCGAAAGGGTGGCTA
ATACCGCATGTGGTGGTTCGTTCAATCGAACCATCAAAGCAGTGATGCGCTTCGGGAGGGGCCTGCGT
CCGATTAGCTCGTTGGTGGGGTAACGGCTCACCAAGGCGACGATCGGTAGGTGGTCTGAGAGGACGAT
CACCCAGACTGGGACTGAGACACGGCC
>Zotu5790
GAACGCTGGAAATGTGGATACTGGCATGCAAGTCAAGGGGCCGCAAGGCACCGGCGAACTGCTTAGTA
ACACGTAGATACATACCGTAAAGTGGAACATAGCTCGTCGAAAGACGGGGTAATTTTCCATGTGAACG
CAAGTTTAAAGGCGCAAGCCGCTTTACGAATGGTCTGCGGTCTATCAGCTAGTTGGTGAGGTAAAGGC
TCACCAAGGCTATGACGGATAGGGGGCGTGAGAGCGCGACCCCCAAGAATGGCACTGCGACACGGGC
>Zotu5791
GAACGCTGGCGGAGTGCCTTAAGCATGCAAGTGGGACGGGCAGTAGCAATACTGTCAGTCGCGAACGG
CTGAGTAATGAATAGGAATCTTTCCTTTGGTGGAGAATACCTCATCGAAAGATGAGCTAATACTGCAT
AATTTCATTCATATGAAAATATGAATGAGGAAAGCGCATAACTGCGCGCCGATTGAGGAGCCTATTCC
CCATCAGGCAGTTGGTAGGGTAATGGCCTACCAAACCTACGACGGGTAACCGATCTGAGAGGATGACC
GGTCAGAATGGGACTGAGACACGGCC
>Zotu5792
GAACGCTGGCGGCGTGCTTAACACATGCAAGTCGAACGAGGACATTGACTTCGGTTGGTGTTCGAGTG
GCGGACGGGTGAGTAACGCGTGAGGAATCTGTCTCACAGAGGGGGATACCAACTGGAAACAGTTGCTA
ATACCGCATAAGACCACGGTACCGCATGGTAGTGAGGTCAAAGGAGAGATCCGCTGTGAGGTGAGCTC
GCGTCTGATTAGCTGGTTGGTGAGGTAACGGCCCACCAAGGCGACGATCAGTAGCCGACCTGAGAGGG
TGATCGGCCACATTGGGACTGAGACACGGCC
>Zotu5793
GAACGCTGGCGGCGTGCCTAATACATGCAAGTCGAGCGGAGATTTGAAAAGCTTGCTTTTCAAATCTT
AGCGGCGGACGGGTGAGTAACACGTGGGCAACCTGCCCTTGAGACGGGGATAACTCCGGGAAACCGGA
GCTAATACCGGATAACACATATCTTCGCATGAGGATATGTTAGAAGGTGGCTTTTAGCTACCACTCAA
GGATGGGCCCGCGGCGCATTAGCTAGTTGGTGAGGTAACGGCTCACCAAGGCGACGATGCGTAGCCGA
CCTGAGAGGGTGATCGGCCACACTGGGACTGAGACACGGCC
>Zotu5795
GAACGCTGGCGGCGCGCCTAACACATGCAAGTCGAACGGAGTTATTTTCTTCACTCCGAGCTTTTTGC
CGGTTAGCCGGAGCGGCAATCGGTTTTGCCCAGTATAAAAAGCAAAACCAATTTAGGCTCATAAAGTA
AGACTAACACAGAGGGCAGAGAGTTGGGAGTGAAGAAAATAACTTAGTGGCGAACGGGTGAGTAACGC
GTGAGTAACCTGCCCTGGAGTGGGGGACAACAGTTGGAAACGACTGCTAATACCGCATAAGCCCACGG
CCCGGCATCGGGCTGCGGGAAAAGGATTTATTCGCTTCAGGATGGACTCGCGTCCAATTAGCTAGTTG
GTGAGGTAACGGCCCACCAAGGCGACGATTGGTAGCCGGACTGAGAGGTTGAACGGCCACATTGGGAC
TGAGACACGGCC
>Zotu5796

GAACGCTGGCGGCGTGCCTAACACATGCAAGTCGAGCGAGATCCATCCAGTGGCAACACTGGTGAAGA
TCTAGCGGCGAACGGGTGAGTAACACGTGAGAAACCTGCCCCGGAGACCGGGATAACCCGAGGAAACT
CGGGCTAATACCGGATACCCCCACCGAACCGCATGATTTGGTGAGGAAATGCTCCGACGCTCCGGGAG
GGTCTCGCGGCCTATCAGCTAGTTGGTGAGGTAACGGCTCACCAAGGCTAAGACGGGTAGCTGGTCTG
AGAGGACGATCAGCCACACTGGGACTGAGACACGGCC
>Zotu5797
GAACGCTGGCGGCGGGGTTTAGGCATGCAAGTCGCGGGGGAAAGTCGCAAGACGAGTATACCGGCGTA
AGGGTGAGTAATGGCTCGTCAACGTACCTCTATGACGCGGATAACTGGCCGAAAGGCCTCGTAAGACG
CGATGTTGTGATTAGGTCTCCGGACTTGATCATGAAAGGGCTGGGATCCTTTGGGACCAGTCGCCTAG
AGAGCGGACGAGCTCCGATTAGCTAGATGGCGGGGTAACGGCCCACCATGGCGATGATCGGTAGCCGG
CCTGAGAGGGTGTCCGGCCACACTGGGACTGAGACACTGCC
>Zotu5798
GAACGCTGGCGGCGCGTCTTAAGCATGCAAGTCGAACGGCAACACGGGGCAACCTGGTGGCGAGTGGC
GAACGGGTGAGTAACACGTAGGTGATCTGCCTTTGGGCTGGGGATAGCCATTAGAAATAGTGGGTAAT
ACCGAATGAAGTCACGGAGCTTTGGCACTGTGATGAAAGGGGCTACGGCCTCGCCTGGAGATGAGCCT
GCGTCTGATTAGCTAGTTGGTGGGGTAAGAGCCTACCAAGGCGACGATCAGTAGCCGGCCTGAGAGGG
TGGTCGGCCACACTGGGACTGAGATACGGCC
>Zotu5799
GAACGCTGGCGGCGTGCTTAACACATGCAAGTCGAACGAAGCACAGAGGGACGATTCTTCGGAAGAGA
TCTTCTGTGACTTAGTGGCGGACGGGTGAGTAACGCGTGGGTAACCTGCCTCACACAGGGGGATAACA
GTTGGAAACGACCGCTAATACCGCATGAGCCAACGGGACCGCATGGTCTAGTTGGAAAAGATTTATCG
GTGTGAGATGGACCCGCGTCTGATTAGGCAGTTGGCAGGGTAACGGCCTACCAAACCGACGATCAGTA
GCCGGCCTGAGAGGGTGAACGGCCACATTGGGACTGAGACACGGCC
>Zotu5800
GAACGCTAGCGGCAGGCTTAACACATGCAAGTCGAGGGGTAGTCTAGCAATAGATGAGACCGGCGCAC
GGGTGCGTAACGCGTATGCAATCTACCTTTTACAGAGGGATAGCCCAGAGAAATTTGGATTAATACCT
CATAGTATAGTACAATCGCATGATTGAACTATTAAAGTTACAACGGTAAAAGATGAGCATGCGTCCCA
TTAGCTAGTTGGTAAGGTAACGGCTTACCAAGGCGACGATGGGTAGGGGTCCTGAGAGGGAGATCCCC
CACACTGGTACTGAGACACGGAC
>Zotu5801
GAACGCTAGCGGCAGGCCTAATACATGCAAGTCGAACGGTGGTAGCAATACCATAGTGGCGCACGGGT
GCGTAACACGTAACTAACCTACCTATAACTGGGGGATAGCCCGCCGAAAGGCGGATTAATACCGCATA
AGCTCTTTGGCTGGCATCAGCTGATTAGGAAAGATTTATTGGTTATAGATGGGGTTGCGGGTCATTAG
CTAGTTGGGTGGGTAACGGCTGCCCAAGGCGACGATGACTAGGGGAGCTGAGAGGCTGGTCCCCCACA
CGGGCACTGAGATACGGGC
>Zotu5802
GAACGCTGGCGGCAGGCTTAACACATGCAAGTCGAGCGGGGAAAGGTAGCTTGCTACCTGACCTAGCG
GCGGACGGGTGAGTAATGCTTAGGAATCTGCCTATTAGTGGGGGACAACATTCCGAAAGGAATGCTAA
TACCGCATACGTCCTACGGGAGAAAGCAGGGGATCTTCGGACCTTGCGCTAATAGATGAGCCTAAGTC
AGATTAGCTAGTTGGTGGGGTAAAGGCCTACCAAGGCGACGATCTGTAGCGGGTCTGAGAGGATGATC
CGCCACACTGGGACTGAGACACGGCC
>Zotu5804
GAACGCTGGCGGCAGGCCTAACACATGCAAGTCGAACGAACTCTTCGGAGTTAGTGGCGGACGGGTGA
GTAACACGTGGGAACGTGCCTTTAGGTTCGGAATAACTCAGGGAAACTTGTGCTAATACCGAATGTGC
CCTTCGGGGGAAAGATTTATCGCCTTTAGAGCGGCCCGCGTCTGATTAGCTAGTTGGTTGGGGTAATG
GCCCACCAAGGCTACGATCAGTAGCTGGTCTGAGAGGATGACCAGCCACATTGGGACTGAGACACGGC
C
>Zotu5805
GAACGCTGGCGGCATGCCTTACACATGCAAGTCGAACGGTAACAGGGAGCTTGCTCCGCTGACGAGTG
GCGAACGGGTGAGTAATATATCGGAACGTACCTTTGAGTGGGGGATAACTAGTCGAAAGATTAGCTAA
TACCGCATACGATCTACGGATGAAAGCGGGGGATCGCAAGACCTCGTGCTCATAGAGCGGCCGATATC
TGATTAGCTAGTTGGTGAGGTAAAGGCTCACCAAGGCATCGATCAGTAGCTGGTCTGAGAGGACGACC
AGCCACACTGGGACTGAGACACGGCC
>Zotu5806

GAACGCTGGCGGCATGCCTAACACATGCAAGTCGAACGGTGCCTTCGGGCACAGTGGCGCACGGGTGC
GTAACGCGTGGGAATCTGCCCTTGGGTTCGGAATAACAGTGGGAAACTACTGCTAATACCGGATGATG
ACGTAAGTCCAAAGATTTATCGCCCGAGGATGAGCCCGCGTAGGATTAGCTAGTTGGTGGGGTAATGG
CTTACCAAGGCGACGATCCTTAGCTGGTCTGAGAGGATGATCAGCCACACTGGGACTGAGACACGGCC
>Zotu5807
GAACGCTGGCGGCGTGCCTAATACATGCAAGTCGAGCGGATCTTTGGGAGCTTGCTCCCAAAGATTAG
CGGCGGACGGGTGAGTAACACGTGGGCAACCTGCATATGAGACTGGGATAACTCCGGGAAACCGGGGC
TAATACCGGATAATTCTTTTCTACACATGTAGAAAAGCTGAAAGATGGTTTCGGCTATCACTCATAGA
TGGGCCCGCGGCGCATTAGCTAGTTGGTGAGGTAACGGCTCACCAAGGCGACGATGCGTAGCCGACCT
GAGAGGGTGATCGGCCACACTGGGACTGAGACACGGCC
>Zotu5808
GAACGCTGGAAATGTGGATACCGGCATGCAAGTCAAGGGGCCGCAAGGCACCGGCGAACGGCTTAGTA
ACACGTAGATACATACCGTAAAGTGGGGCATAGCTCGTCGAAAGACGGGGTAATTTCCCATAGTCCCG
CAAGGGTAAAGCAGCAATGCGCTTTATGAATGGTCTGCGGTCTATCAGCTAGTTGGTGAGGTAATGGC
TCACCAAGGCTATGACGGATAGGGGGCGTGAGAGCGCGACCCCCAAGAATGGCACTGCGACACGGGC
>Zotu5809
GAATGGTGGCGATGTGGATGAGTCATGCAAGTCAAGGGGGCCTTCGGGCAACCGGCAGACGAGGTAGT
AACACGTAGGTAGATCCCCCGGAGTCGGGCATAGCCATTCGAAAGGATGGGTAATTCCCGATGGTCTC
GTGAGAGTAAAGCCGTTGTATTTATATAACTGGTCGCTCCGGGAGTCGCCTGCGTAGTATCAGCTAGT
TGGTAGGGTAATGGCCTACCAAGGCTACGACGCTTAGGGGAGCTGAGAGGCTGACCCCCACCGATGGG
ACTGAGACACGGCC
>Zotu5810
GAACGCTAGCGGGAGGCTTAATACATGCAAGTCGAACGGCAGCAGGTCCTTCGGGATGCTGGCGAGTG
GCGAACGGGTGAGTAACGCGTACACGATCTGCCCATAAGTCTGGGATAGTCACTGGAAACGGTGAATA
ATACCGGATGTGGATAAATTGCTTCATGGTAATTTATTTAAAGGCTGGTCTGCTTATGGATGAGTGTG
CGTCCTATTAGTTAGTTGGTGAGGTAACGGCTCACCAAGACGATGATAGGTAGGGGGCGTGAGAGCGT
GGACCCCCACACGGGTACTGAGACACGGAC
>Zotu5811
GAACGCTGGCGGCGTGCTTAACACATGCAAGTCGAACGGTGATCCTGGTGCTTGCACCGGGTGATCAG
TGGCGAACGGGTGAGTAACACGTGAGCAACCTGCCCCCGACTCTGGGATAACTTCGGGAAATCGGAGC
TAATACCGGATACGAGACGCACGGGCATCCGTAGCGTCTGGAAAGAATTTCGGTCGGGGATGGGCTCG
CGGCCTATCAGCTTGTTGGTGGGGTAACGGCCTACCAAGGCGACGACGGGTAGCCGGCCTGAGAGGGC
GACCGGCCACACTGGGACTGAGACACGGCC
>Zotu5813
GAACGCTGGCGGCATGCCTAATACATGCAAGTCGAACGCTTCTCGAGGTGCTTGCACCTCACTAGAAG
AGTGGCGGACGGGTGAGTAACACGTGGGAAACCTGCCTGTAAGTATGGAATAACCTTCGGAAACGGAT
GCTAATACCATATAGGTTTCACTTCCTCATGGAAGTGAAAGGAAAGACGGCTCTGCTGTCGCTTACAG
ATGGTCCCGCGGCGCATTAGCTAGTTGGTGAGATAAAAGCTCACCAAGGCGATGATGCGTAGCCGACC
TGAGAGGGTAACCGGCCACACTGGGACTGAGACACGGCC
>Zotu5815
GAACGCTGGCGGCGTGCCTAACACATGCAAGTCGAACGGAGTCAAGAAGCTTGCTTTTTGACTTAGTG
GCGGACGGGTGAGTAACGCGTGAGTAACCTGCCTCTGAGAGGGGAATAACGTTCTGAAAAGAACGCTA
ATACCGCATGACGTATCGAAGCCGCATGACTTTGATACCAAAGATTTTATCGCTCGGAGATGGACTCG
CGTCCGATTAGTTAGTTGGTGAGGTAACGGCTCACCAAGACCGCGATCGGTAGCCGGACTGAGAGGTT
GATCGGCCACATTGGGACTGAGACACGGCC
>Zotu5816
GAACGCTGGCGGCGTGCCTAATACATGCAAGTCGAGCGAATGATGAGGAGCTTGCTCCTCTGATTTAG
CGGCGGACGGGTGAGTAACACGTGGGTAACCTGCCCTGTAGACTGGGATAACTTCGGGAAACCGGAGC
TAATACCGGATAATCCTTTTAACCACATGGTTTTAAGTTGAAAGGCGCTTCGGCGTCACTACAGGATG
GACCCGCGGTGCATTAGCTAGTTGGTGAGGTAACGGCTCACCAAGGCAACGATGCATAGCCGACCTGA
GAGGGTGATCGGCCACATTGGGACTGAGACACGGCC
>Zotu5818
GAACGCTGGCGGCGTGCTTAACACATGCAAGTCGAACGGTAAGGCCCTAGCTTGCTGGGGTACACGAG
TGGCGAACGGGTGAGTAACACGTGGGTGACCTGCCCTGCACTTCGGGATAAGCCTGGGAAACTGGGTC

TAATACCGGATAGGACCGCATCGTAGGTGGTGTGGTGGAAAGTTTTTCGGTGTGGGATGGGCCCGCG
GCCTATCAGCTTGTTAGTGGGGTAATGGCCTACCAAGGCGGCGACGGGTAGCCGGCCTGAGAGGGTGG
ACGGCCACATTGGGACTGAGACACGGCC
>Zotu5820
GAACGCTGGCGGCATGCCTAACACATGCAAGTCGAACGAGACCTTCGGGTCTAGTGGCGCACGGGTGC
GTAACGCGTGGGAACCTGCCCCTTGGTTCGGAATAACACAGAGAAATTTGTGCTAATACCGGATGATG
TCTTCGGACCAAAGATTTATCGCCAAGGGATGGGCCCGCGTAGGATTAGCTTGTTGGTGGGGTAATGG
CCTACCAAGGCGACGATCCTTAGCTGGTCTGAGAGGATGATCAGCCACACTGGGACTGAGACACGGCC
>Zotu5822
GAACGCTGGCGGTATGCTTTACACATGCAAGTCGAACGGAGTTGGAGCTTGCTCCAATTTAGTGGCGA
ACGGGTGAGTAATATATCGGAACATATCCGGAAGTGGGGGATAACGTAGCGAAAGTTACGCTAATACC
GCATATGCCCTGAGGGGGAAAGGGGGGGATCGCAAGACCTCTCGCTTTCGGAGTGGCCGATACCGGAT
TAGCTAGATGGTGAGGTAAAGGCTCACCATGGCGACGATCCGTAGCTGGTCTGAGAGGACGACCAGCC
ACACTGGAACTGAGACACGGTC
>Zotu5824
GAACGCTGGCGGCGTGCCTAAGACATGCAAGTCGAGGGGGTTTAGACCCCCGGCGAACGGGTGAGTAA
CGCGTGGGTACTTTCGTTAAGTTCGGGATAACTCTCCGAAAGGAGAGACAATACCGGATGTGGCCGCA
AGGCTAAAGATTTATCGCTTAACGTGAGGCCCGCGTCCCATCAGCTAGTTGGTAAGGTAATGGCTTAC
CAAGGCTATGACGGGTAGCGGAAGCGAGAGCTTGATCCGCCACACTGGGACTGCGACACGGCC
>Zotu5825
GAACGCTGGCGGCGCGCCTAACACATGCAAGTCGAACGGCACCCCTCTCCGGAGGGAAGCGAGTGGCG
AACGGCTGAGTAACACGTGGAGAACCTGCCCCCTCCCCCGGGATAGCCGCCCGAAAGGACGGGTAATA
CCGGATACCCCCGGGCGCCGCATGGCGCCCGGGCTAAAGCCCCGACGGGAGGGGATGGCTCCGCGGCC
CATCAGGTAGACGGCGGGGTGACGGCCCACCGTGCCGACAACGGGTAGCCGGGTTGAGAGACCGACCG
GCCAGATTGGGACTGAGACACGGCC
>Zotu5826
GAACGCTGGCGGCAGGCCTCAAACATGCAAGTCTAGGGGGTGTAGCAATACATCACCGGCGGACGGGT
GCGTAACGCGTGGGAATGTACCTATTGGTTCGGAACAACTGAGGGAAACTTCAGCTAATACCGAATGT
GCCCGTAAGGGGAAAGATTTATCGCCAATGGATCAGCCCGCGTAGGATTAGCTAGTTGGTGAGGTAAT
GGCTCACCAAGGCTCCGATCCTTAGCTGTTCTGAGAGGAAGATCAGCCACACTGGGACTGAGACACGG
CC
>Zotu5827
GAACGCTGGCGGCGCGCCTAATACATGCAAGTCGAGCGAGAAGGTGTAGCAATACACTTGTAAAGCGG
CGAACGGGTGAGGAACACATGGGTAACCTACCATCGAGTGGGGAATAACCAGCCGAAAGGTTGGCTAA
TACCGCGTACGCCTGCGGGTCTTCGGATTCGCAGGGAAAGCCACACTGAGAGTGTGGCGCTCATTGAT
GGGCTCATGTCCTATCAGCTTGTTGGTAGGGTAACGGCCTACCAAGGCTTTGACGGGTAGCTGGTCTG
AGAGGACGATCAGCCACACTGGCACTGCGACACGGGC
>Zotu5828
GAACGCTGGCGGCGTGCCTAAGACATGCAAGTCGAGTGGGTTTAGACCCACGGCGAACGGGTGAGTAA
TACCTAGGTACTTTCTTTAAGTGGAGCATAACCAGTCGAAAGATTGGATAATACTTCATGTGCCCCGG
AGTTAAATCCGGGGTAAAGTCGCAAGACGCTTAAAGTGAGGCCTGGGCACCATCAGCTAGTTGGTAAG
GTAATGGCTTACCAAGGCTATGACGGTTAGGGGTCACGAGAGGGATAACCCCCACACTGGAACTGCGA
CACGGTC
>Zotu5829
GAACGCTGGCGGCAGGCTTAACACATGCAAGTCGAGCGGGGAAAAGTAGCTTGCTACTTGACCTAGCG
GCGGACGGGTGAGTAATGCTTAGGAATCTGCCTATTAGTGGGGGACAACATCTCGAAAGGGATGCTAA
TACCGCATACGTCCTACGGGAGAAAGCAGGGGACCTTCGGGCCTTGCGCTAATAGATGAGCCTAAGTC
GGATTAGCTAGTTGGTGGGGTAAAGGCCTACCAAGGCGACGATCTGTAGCGGGTCTGAGAGGATGATC
CGCCACACTGGGACTGAGACACGGCC
>Zotu5830
GAACGCTGGCGGCGTGCCTAACACATGCAAGTCGGATGTGCCGCAAGGTGCATGGCAGACGGGTGAGT
AACGCGTAGGGAATCTACCTTTTAGTGGGGAATAACGCTCCGAAAGGAGTGGTAATACCGCATGAGAC
CGCGAGCTGGGACGCTTGTGGTGAAACCTGGGGACCGCAAGGCCTGGGGCTAGAAGAGGATCCTGCGT
CTGATTAGCTAGTTGGCGGGGTAATGGCCCACCAAGGCGACGATCAGTAGCCGGCCTGAGAGGGTGAT

CGGCCACACTGGAACTGAGACACGGTC
>Zotu5831
GAACGCTGGCGGCGTGCCTAACACATGCAAGTCGAGCGGGAAGTCCTTAAACGAAACTTCGGTCGAGT
GAGAGGATGGATAGCGGCGGACGGGTGAGTAACGCGTAGGTAAGCTGCCCCACGCACAGGGATAGCCT
CGGGAAACTGGGATTAATACCTGATAATGCGGAGAGATCACATGGTCACTCCGCCAAAGATTTATCGG
CATGGGATGCACCTGCGTCTGATTAGTTAGTTGGTGAGGTAACGGCCCACCAAGACAGCGATCAGTAG
CCGACCTGAGAGGGTAATCGGCCACATTGGAACTGAGACACGGTC
>Zotu5832
GAACGCTGGCGGCGCGCTTAACACATGCAAGTCGAGCGAGAACCTTTCCTTCGGGATTGGGGACAGCG
GCGAACGGGTGAGTAACACGTGGGTAATCTGCCCTCGACATCGGGATAGCCCGGGAAACCCGGATTA
ATACCGGATAGCCTTACTGGTTTTCGGGCTGGTAAGAAAAGGTAGCTTTGGCCTCCGGTCGAGGAGGA
GCCCGCGGCGGATTAGCTTGTTGGCGGGGTAAAAGCCCACCAAGGCGACGATCCGTAGCTGGTCTGAG
AGGACGATCAGCCACACTGGGACTGAGACACGGCC
>Zotu5833
GAACGCTAGCTACAGGCTTAACACATGCAAGTCGAGGGGCATCGCGGCGTAGCAATACGCTGGCGGCG
ACCGGCGCACGGGTGAGTAACGCGTATCCGACCTGGCCTTCACTCTGGGATAGCCCCTCGAAAGAGGG
ATTAATACCGGATATGTGGCAGAGTTACATGGCACTGCCACGAAAGATTTATCGGTGAAGGATGGGGA
TGCGTCCCATTAGCTTGTTGGCGGGGTAACGGCCCACCAAGGCGACGATGGGTAGGGGTACTGAGAGG
TAGGTCCCCCACATTGGAACTGAGACACGGTC
>Zotu5834
GAACGCTGGCGGCAGGCTTAACACATGCAAGTCGAGCGGGCATCCTTGTGGTGTCAGCGGCAGACGGG
TGAGTAACGCGTGGGAACGTGCCCTTCGGTTCGGAATAACTCAGGGAAACTTGAGCTAATACCGGATA
CGCCCTTATGGGGAAAGGTTTATCTGCCGAAGGATCGGCCCGCGTCTGATTAGCTGGTTGGTGAGGTT
ATGGCTCACCAAGGCGACGATCAGTAGTTGGTCTGAGAGGATGATCAGCCACACTGGGACTGAGACAC
GGCC
>Zotu5835
GAACGCTGGCGGCGTGCTTAACACATGCAAGTCGAACGGGAAACATTTTATTGAAGCTTCGGCAGATT
TAGCTTGTTTCTAGTGGCGGACGGGTGAGTAACGCGTGGGTAACCTGCCTTATACTGGGGGATAACAG
CCAGAAATGACTGCTAATACCGCATAAGCGCACAGGACCGCATGGTTCGGTGTGAAAAACTCCGGTGG
TATAAGATGGACCCGCGTTGGATTAGCTAGTTGGCAGGGCAGCGGCCTACCAAGGCGACGATCCATAG
CCGGCCTGAGAGGGTGAACGGCCACATTGGGACTGAGACACGGCC
>Zotu5836
GAACGCTGGCGGCAGGCTTAACACATGCAAGTCGAACGCCCCGCAAGGGGAGTGGCAGACGGGTGAGT
AACGCGTGGGAACATACCCAACTCTACGGAATAACTCAGGGAAACTTGTGCTAATACCGTATACGCCC
TTTGGGGGAAAGATTTATCGGAGTTGGATTGGCCCGCGTTGGATTAGCTAGTTGGTGGGGTAAAGGCC
TACCAAGGCGACGATCCATAGCTGGTCTGAGAGGATGATCAGCCACATTGGGACTGAGACACGGCC
>Zotu5837
GAACGCTGGCGGCGTGCTTAACACATGCAAGTCGAACGGAAAGGCCCCTTTTTGGGGTACTCGAGTGG
CGAACGGGTGAGTAACACGTGGGTGATCTGCCCTCAACTTCGGGATAAGCTTGGGAAACTGGGTCTAA
TACCGGATAGGACTCCTCTTTAGTGTGAGGGGTGGAAAGTTTTTTCGGTTGGGGATGAGCTCGCGGCC
TATCAGCTTGTTGGTGGGGTAATGGCCTACCAAGGCGTCGACGGGTAGCCGGCCTGAGAGGGTGTACG
GCCACATTGGGACTGAGATACGGCC
>Zotu5838
GAACGCTGGCGGCAGGCCTAACACATGCAGGTCGAGCGCGCCCTTCGGGGTGAGCGGCGGACGGGTGA
GTAACGCGTGGGAACGTGCCCTTTGGTTCGGAATAGTCCTGGGAAACTGGGGGTAATACCGGATACGC
CCTTTTTGGGGAAAGATTTATCGCCGAAGGATCGGCCCGCGTTGGATTAGGTAGTTGGTGGGGTAATG
GCCTACCAAGCCGACGATCCATAGCTGGTTTGAGAGGATGATCAGCCACACTGGGACTGAGACACGGC
C
>Zotu5839
GAACGCTGGCGGCATGCCTTACACATGCAAGTCGAACGGCAGCACGGGAGCAATCCTGGTGGCGAGTG
GCGAACGGGTGAGTAATACATCGGAACGTGTCCTGTCGTGGGGGATAGCCCGGCGAAAGCCGGATTAA
TACCGCATACGACCTACGGGTGAAAGCGGGGGATCGCAAGACCTCGCGCGATTGGAGCGGCCGATGTC
AGATTAGCTTGTTGGTGGGGTAAAAGCCTACCAAGGCAACGATCTGTAGCTGGTCTGAGAGGACGACC
AGCCACACTGGGACTGAGACACGGCC

>Zotu5840
GAACGCTGGCGGCACGCTTAACACATGCAAGTCGAACGAGAAACTCGGAGCTTGCTCTGGGTGGACAG
TGGCGAACGGGTGAGGAACACGTGAGGAACCTGCCCTGGAGACCGGGACAACATCGGGAAACCGTTGC
TAATACCGGATGCCTTCATCGGACCGCATGATCTGATGAGGAAATGGATTCCGCTCCAGGATGGCCTC
GCGGCCTATCAGCTTGTTGGTGGGGTAACGGCCCACCAAGGCGTCGACGGGTAGCTGGTCTGAGAGGA
TGTCCAGCCACACTGGGACTGAGACACGGCC
>Zotu5841
GAACGCTGGCGGTATGCTTAACACATGCAAGTCGAACGGACTTAGGGAGCTTGCTCCCGAAAGTTAGT
GGCAAACGGGTGAGTAACGCGTGGGAATCTCCCCATCAGTACGGAACAACATTTGGAAACAAATGCTA
ATACCGTATATTCTCGAGAGAGGAAAGATTTATCGCTGATGGATGAGCCCGCGTCGGATTAGGTAGTT
GGTGAGGTAATGGCTCACCAAGCCGACGATCCGTAGCTGGTCTGAGAGGATGATCAGCCACACTGGGA
CTGAGACACGGCC
>Zotu5842
GAACGCTGGCGGCGTGCCTAACACATGCAAGTCGAGCGAACCCTTCGGGGTGAGCGGCGGACGGGTGA
GTAACGCGTGGGTAACCTGCCCTGTACACACGGATAACATACCGAAAGGTATGCTAATACGAGATAAT
ATGTTTTAATCGCATGGTTAAGATATCAAAGCTCCGGCGGTACAGGATGGACCCGCGTCTGATTAGCT
AGTTGGTGAGGTAATGGCTCACCAAGGCGACGATCAGTAGCCGACCTGAGAGGGTGATCGGCCACACT
GGAACTGAGACACGGTC
>Zotu5843
GAACGCTGACAGAATGCTTAACACATGCAAGTCTACTTGAATTTGGGTTTTTAACTTAGATTTGGGT
GGCGGACGGGTGAGTAACGCGTAAAGAACTTGCCTCACAGCTAGGGACAACATTTGGAAACGAATGCT
AATACCTGATATTATGATTATAAGGCATCTTAGAATTATGAAAGCTATATGCGCTGTGAGAGAGCTTT
GCGTCCCATTAGCTAGTTGGAGAGGTAACGGCTCACCAAGGCGATGATGGGTAGCCGGCCTGAGAGGG
TGAACGGCCACAAGGGGACTGAGACACGGCC
>Zotu5844
GAACGCTGGCGGCGTGCTTAACACATGCAAGTCGAGCGGTAAGGCCCTTTCGGGGGTACACGAGCGGC
GAACGGGTGAGTAACACGTGAGTAATCTGCCCTCCACTTTGGGATAAGCCTCGGAAACGGGGTCTAAT
ACCGGATATGAGCATCCCACGCATGTGGGGTGTTGGAAAGTTTTTCGGTGGGGGATGTGCTCGCGGCC
TATCAGCTTGTTGGTGGGGTAATGGCCTACCAAGGCTTCGACGGGTAGCCGGCCTGAGAGGGTGACCG
GCCACACTGGGACTGAGACACGGCC
>Zotu5845
GAACGCTGGCGGCGTGCCTAATACATGCAAGTCGAGCGAATCTTTGGGAGTTTGCTCCTAAAGGTTAG
CGGCGGACGGGTGAGTAACACGTGGGCAACCTGCCTATAAGACTGGGATAACTTCGGGAAACCGGAGC
TAATACCGGATAATTCTTTTCTACACATGTAGAAAAGCTAAAAGACGGTTTACGCTGTCACTTATAGA
TGGGCCCGCGGCGCATTAGCTAGTTGGTGAGGTAACGGCTCACCAAGGCCACGATGCGTAGCCGACCT
GAGAGGGTGATCGGCCACACTGGGACTGAGACACGGCC
>Zotu5847
GAACGCTGGCGGCATGCTTAACACATGCAAGTCGTACGAATGAGTTTTGGCTTGCCAAAACTTAATGA
GTGGCGGACGGGTGAGTAACACGTAAGAACCTACCTTTTGGAGAGGGACAACCATTGGAAACGATGGC
TAATACCTCATAATACTGAGAAGTTAAATGATGAATAATCGCCAAGAGATGGGCTTGCGGCTGATTAG
CTAGTTGGTGAGGTAATAGCTTACCAAGGCGATGATCAGTAGCTGGTCTGAGAGGATGATCAGCCACA
CTGGGACTGAGACACGGCC
>Zotu5848
GAACGCTGGCGGCGTGCTTAACACATGCAAGTCGAACGAACCTTCGGGTTAGTGGCGAACGGGTGAGT
AACACGTGGGCAACCTGCCTCGAGCTCTGGGATAACTCCGGGAAACCGGGGCTAATACCGGATATGAC
CTACTGAGACATCTTGGTGGGTGGAAAGTTTTTCGGCTCGAGATGGGCCCGCGGCCTATCAGCTTGTT
GGTGGGGTAACGGCCTACCAAGGCGACGACGGGTAGCCGGCCTGAGAGGGCGACCGGCCACACTGGGA
CTGAGACACGGCC
>Zotu5849
GAACGCTGGCGGCGTGCCTAAAACATGCAAGTCGAACGAGGGAGCAATCCCTAGTGGCGAACGGCGGA
GTAAGACATGAACAACGTGCCCTAAGGTCCGGGACAAACGCAGAAATGCCATAAATACCGGATGTGGC
CCCGATTCGGCATCGAGTTGGGATTAAACGGATTTTCGCCTTAGGAGCGGTTCATGGCCTATCAGGTA
GTTGGTGGGGTAACGGCCCACCAAGCCGACGACAGGTAGCGGGTCTGAGAGGATGATCCGCCCGAGTG
GGACTGAGACACGGCC

>Zotu5853
GAACGCTGGCGGCGTGCCTAATACATGCAAGTCGAGCGAACGGACAGGAGTGCTTGCACTCCTTGATG
TTAGCGGCGGACGGGTGAGTAACACGTGGGTAACCTACCTATAAGACTGGGATAACTTCGGGAAACCG
GAGCTAATACCGGATAATATGTTTCACCTCATGGTGAAACAGTGAAAGACGGTTCTGCTGTCACTTAT
AGATGGACCCGCGGTGTATTAGCTAGTTGGTGAGGTAACGGCTCACCAAGGCGACGATACATAGCCGA
CCTGAGAGGGTGATCGGCCACACTGGGACTGAGACACGGCC
>Zotu5854
GAATGCTGGCGGCGTGGATGAGTCATGCAAGTCGAACGGCCCGCAAGGGCAGTGGCAGACGAGGTAGT
AACACGTAGGTAGATACCCCAGAGTCGGGCATAGCCAGCCGAAAGGTTGGGTAATTCCCGATAGTCTT
GTGAAAGTAAAGGAGCAATCCGCTCTGGGAGTCGCCTGCGTAATATCAGCTAGTTGGTGAGGTAATGG
CTCACCGAGGCTACGACGTTTAGGGGAGCTGAGAGGCTGACCCCCACCGATGGGACTGAGATACGGCC
>Zotu5855
GAGCGCTGGCGGCGTGCTTAACACATGCAAGTCGAGCGGAAAGGCCCTTCGGGGTACTCGAGCGGCGA
ACGGGTGAGTAACACGTGAGTAATCTGCCCTTGGCTTCGGGATAGCCACCGGAAACGGTGATTAATAC
CGGATACGACAACTTCTCGCATGGGATGGTTGTGGAAAGTTTTTCGGCCAGGGATGTGCTCGCGGCCT
ATCAGCTTGATGGTGAGGTAATGGCTCACCATGGCTTCGACGGGTAGCCGGCCTGAGAGGGTGACCGG
TCACACTGGGACTGGGACACGGCC
>Zotu5856
GAACGCTGGCGGCAGGCTTAACACATGCAAGTCGAGCGGGGAAGGGTAGCTTGCTACCTAACCTAGCG
GCGGACGGATGAGTAATGCTTAGGAATCTGCCTATTAGTGGGGGACAACATTCCGAAAGGAATGCTAA
TACCGCATACGCCCTACGGGGGAAAGCAAGGGATCTTCGGACCTTGCGCTAATAGATGAGCCTAAGTC
AGATTAGCTAGTTGGTGGGGTAAAGACCTACCAAGGCGACGATCTGTAGCGGGTCTGAGAGGATGATC
CGCCACACTGGGACTGAGACACGGCC
>Zotu5857
GAACGCTGGCGGCATGCCTAATACATGCAAGTCGAACGATCACTTCGGTGGTAGTGGCGCACGGGTGC
GTAACGCGTGGGAATCTGCCCTTGGGTTCGGAATAACTTCTGGAAACGGAAGCTAATACCGGATGATG
ACGTAAGTCCAAAGATTTATCGCCCAGGGATGAGCCCGCGTAGGATTAGCTAGTTGGTGGGGTAAAGG
CTTACCAAGGCGACGATCCTTAGCTGGTCTGAGAGGATGATCAGCCACACTGGGACTGAGACACGGCC
>Zotu5858
GCGATCCTGGAGACCCGGGATCGAATCCCACGTCGGGCTCCCGGTGCATGGAGCCTGCTGCTCCCTCT
GCCTGTGTCTCTGCCTCTCTCTGTGTGTCTTCCATGAATAAATGAAATATTTTTTTTAAGTAAATAA
AGTTCCCAGGCCTTCACA
>Zotu5859
GAACGCTGGCGGCATGCTTTACACATACAAGTCGAACGGTAACAGGTCTTCGGATGCTGACGAGTGGC
GAACGGGTGAGTAATACATCGGAACGTGCCTAGTAGTGGGGGATAACTACTCGAAAGAGTAGCTAATA
CCGCATGAGATCTACGGATGAAAGCAGGGGACCTTCGGGCCTTGTACTACTAGAGCGGCTGATGGCAG
ATTAGGTAGTTGGTGGGGTAAAGGCTTACCAAGCCTGCGATCTGTAGCTGGTCTGAGAGGACAACCAG
CCACACTGGGACTGAGACACGGCC
>Zotu5860
GAACGCTATCGGCAGGCCTAATACATGCAAGTCGGACGGGATAGGAGAGCTTGCTTTCCTTGAGAGTG
GCGCACGGGTGCGTAACACGTATGTAACCTACCTTGATCAGGGGGATAGCCTCTCGAAAGAGAGATTA
AGACCGCATAACATTGCAATATGGCATCATAAAGCAATCAAATATTTATAGGATCAAGATGGGCATGC
GGAACATTAGCTAGTTGGTAGGGTAACGGCCTACCAAGGCTACGATGTTTAGGGGATCTGAGAGGATG
GCCCCCCACACTGGTACTGAGACACGGAC
>Zotu5861
GAACGCTGGCGGCAGGCCTAACACATGCAAGTCGAACGGTAACACAGAGAGCTTGCTCTCGGGTGACG
AGTGGCGGACGGGTGAGTAATGTCTGGGAAACTGCCTGATGGAGGGGGATAACTACTGGAAACGGTAG
CTAATACCGCATAACGTCGCAAGACCAAAGAGGGGGACCTTCGGGCCTCTTGCCATCAGATGTGCCCA
GATGGGATTAGCTAGTAGGTGGGGTAACGGCTCACCTAGGTGACGATCCCTAGCTGGTCTGAGAGGAT
GACCAGCCACACTGGAACTGAGACACGGTC
>Zotu5862
GAACGCTGGCGGCAGGCTTAACACATGCAAGTCGAACGGTCTCTTCGGAGGCAGTGGCAGACGGGTGA
GTAATGCATGGGAATCTACCGTTCTCTACGGAATAACTCAGGGAAACTTGTGCTAATACCGTATACGC
CCTTTTGGGGAAAGATTTATCGGAGAATGATGAGCCCATGTTGGATTAGCTAGTTGGTAGGGTAAAGG

CCTACCAAGGCGACGATCCATAGCTGGTCTGAGAGGATGATCAGCCACACTGGGACTGAGACACGGCC
>Zotu5865
GAACGCTGGCGGCAGGCTTAACACATGCAAGTCGAGCGGGTGTAGCAATACATCAGCGGCAGACGGGT
GAGTAACGCGTGGGAACGTACCTTTTGGTTCGGAACAACACAGGGAAACTTGTGCTAATACCGGATAA
GCCCTTACGGGGAAAGATTTATCGCCGAAAGATCGGCCCGCGTCTGATTAGCTAGTTGGTGAGGTAAT
GGCTCACCAAGGCGACGATCAGTAGCTGGTCTGAGAGGATGATCAGCCACATTGGGACTGAGACACGG
CC
>Zotu5866
GAACGCTGGCGGCGTGCTTAACACATGCAAGTGGAGCGACGAACCAGGGCTTGCCCTGGGGCAGAGCC
GCGAACGGGTGAGTAACACGTGGGCAACCTACCCCGAAGACTGGGACAACCCGAGGAAACTCGGGCTA
ATACCAGATGTGGTCCCTCTTCTTAAGGAGAGTGACTAAAGGGAGCTTCGGCCCCCGCTTTGGGAGGG
GCCCGCGGCGCATTAGCTTGTTGGTGAGGTAACGGCTCACCAAGGCTTCGATGCGTAGCTGGTCTGAG
AGGACGATCAGCCACACTGGGACTGAGACACGGCC
>Zotu5867
GAACGCTGGCGGCATGCTTAACACATGCAAGTCGAACGCAGCATCAGGGCTTGCTCTGATGGCTGAGT
GGCAAACGGGTGAGTAACACGTGGGAACTTACCCTTTAGACTGGGACAACCACTGGAAACGGTGGCTA
ATACCGGATACGCCCTGAGGGGGAAAGCAGCAATGCGCTAAGGGAGAGGCCCGCGGCAGATTAGGTAG
TTGGTGGGGTAAAGGCCTACCAAGCCTGTGATCTGTAGCTGGTTTGAGAGGATGATCAGCCACACTGG
GACTGAGACACGGCC
>Zotu5868
GAACGCTGGCGGCAGGCTTAACACATGCAAGTCGAGCGCCGTAGCAATACGGAGCGGCAGACGGGTGC
GTAACGCGTGGGAACATACCCTTTGGTTCGGAATAACTCAGGGAAACTTGAGCTAATACCGGATAAGT
CCGAGAGGAGAAAGATTTATCGCCGAAGGATTGGCCCGCGTCTGATTAGCTAGTTGGTGAGGTAACGG
CCCACCAAGGCTACGATCAGTAGCTGGTCTGAGAGGATGATCAGCCACACTGGGACTGAGACACGGCC
>Zotu5869
GAACGCTGGCGGTGTGCTTAATACATGCAAGTCGAGCGGTAAGGCCTCTTCGGAGGTACACGAGCGGC
GGACGGCTGAGTAACGCGTAGGAATCTACCCCGAACTGAGGAATAAGCACCAGAAATGGTGTCTAATG
CCGCATATGCTCTTAGGAGTAAAGCTTCGGCGGTTTGGGAAGAGCCTGCGTTCGATTAGCTTGTTGGT
AAGGTAACGGCTTACCAAGGCGACGATCGATAGCTGGTCTGAGAGGATGACCAGCCAGACTGGGACTG
AGAACGGCC
>Zotu5871
GAACGCTGGCGGCATGCCTTACACATGCAAGTCGAACGGCAGCACGGGAGCAATCCTGGTGGCGAGTG
GCGAACGGGTGAGTAATACATCGGAACGTGCCCAGTAGTGGGGGATAGCCCGGCGAAAGCCGGATTAA
TACCGCATGAGACCTGAGGGTGAAAGCGGGGGATCGCAAGACCTCGCGCTATTGGAGCGGCCGATGTC
AGATTAGCTAGTTGGTGGGGTAAAAGCCTACCAAGGCAACGATCTGTAGTTGGTCTGAGAGGACGACC
AGCCACACTGGGACTGAGACACGGCC
>Zotu5872
GAACGCTGGCGGCGTGCTTAACACATGCAAGTCGAACGGTGAAGCAGAGCTTGCTCTGTGGATCAGTG
GCGAACGGGTGAGTAACACGTGAGCAACCTGCCCCTGACTCTGGGATAAGCGCTGGAAACGGCGTCTA
ATACTGGATATGAGCTGCGACCGCATGGTCAGCAGTTGGAAAGATTTTTCGGTCTGGGATGGGCTCAC
GGCCTATCAGCTTGTTGGTGAGGTAATGGCTCACCAAGGCGTCGACGGGTAGCCGGCCTGAGAGGGTG
ACCGGCCACACTGGGACTGAGACACGGCC
>Zotu5873
AAATGCTGGCGGCGTGCCTAATACATGCAAGTCGAGCGGTAAGGCTTTCACAGATTATTTGAACGGAT
CAGTCCCGCACTTTTTCCTGAATTTCCGGCAGTCTGCAATCAGATTGTCGAAAACCAAGAAAATGTGC
GGGATACTATCCTTCAACGTTTAACTCCCGGCTAGGAGAAAAATGGTTGAATAATGTGTGAAAGTACA
CGAGCGGCGAACGGCTGAGTAACGCGTAGGAATGTACCCCAAAGTGAGGGATAAGCACCGGAAACGGT
GTCTAATACCGCATGTGGTCTAAGGACTAAAGACTTCGGTCGCTTTGGGAGCAGCCTGCGTAAGATTA
GTTTGTTGGTGAGGTAATGGCTCACCAAGACTACGATCTTTAGCTGGTCTGAGAGGATGATCAGCCAG
ACTGGAACTGAGACACGGTC
>Zotu5874
GAATGCTGGCGGCGCGCCTAACACATGCAAGTCGAGCGGTAACATGTTTCTCACTGTCTTCTTCTTTT
TATAAAAGGAGGGAATTGTGAGAAATGATGACGAGCGGCGGACGGCTGAGTAACGCGTGGGAACATGC
CCCAAAGTGAGGGATAAGCTCCAGAAATGGAGTCTAATACCGCATATGATCTTAGGATTAAAGGATTT

ATCCGCTTTGGGAGTGGCCTGCGTACGATTAGATAGTTGGTGAGGTAAAGGCTCACCAAGTCTACGAT
CGTTAACTGTTCTGAGAGGATGATCAGTCAGACTGGAACTGAGACACGGTC
>Zotu5878
GAACGCTGGCGGCGTGCTTAACACATGCAAGTCGAGCGGTAAGGCCCCTTCGGGGGTACACGAGCGGC
GAACGGGTGAGTAACACGTGAGCAATCTGCCCTTCACACGGGGATAACCACCGGAAACGGTGGCTAAT
ACCCGATACGACCACTTCAGGCATCTGATGGTGGTGGAAAGTTCCGGCGGTGGAGGATGAGCTCGCGG
CCTATCAGCTTGTTGGTGGGGTAATGGCCTACCAAGGCAACGACGGGTAGCCGGCCTGAGAGGGTGAC
CGGCCACACTGGGACTGAGACACGGCC
>Zotu5879
GAACGCTGGCGGTGTGCCTAATACATGCAAGTCGAGCGGTAAGGCCCCTTCGGGGGTACACGAGCGGC
GGACGGCTGAGTAACACGTAGGAACGTACCCCAAACTGAGGGATAAGCACCAGAAATGGTGTCTAATA
CCGCATGTGCTCTAAGGAGTAAAGCTTCGGCGGTTTGGGAGCGGCCTGCGTCCGATTAGCTAGTTGGT
GAGGTAATAGCTTACCAAGGCGACGATCGGTAGCTGGTCTGAGAGGATGATCAGCCAGACTGGGACTG
AGAACGGCC
>Zotu5880
GAACGCTGGCGGCGTGCCTAATACATGCAAGTCGAGCGGATTGATGGGAGCTTGCTCCCTGATATTAG
CGGCGGACGGGTGAGTAACACGTGGGCAACCTGCCCTGCAGATGGGGATAACTCCGGGAAACCGGGGC
TAATACCGAATAATCAGTTCCTCCGCATGGAGGAACTCTGAAAGACGGTTTCGGCTGTCACTGCAGGA
TGGGCCCGCGGCGCATTAGCTAGTTGGTGGGGTAATGGCCTACCAAGGCGACGATGCGTAGCCGACCT
GAGAGGGTGATCGGCCACACTGGGACTGAGACACGGCC
>Zotu5882
GAACGCTGGCGGCGTGCCTAATACATGCAAGTCGAGCGAAGTTGAGGGAGCTTGCTCCTGAGACTTAG
CGGCGAACGGGTGAGTAACACGTGGGCAACCTGCCCGATAGACCGGGATAACTTGGGGAAACCCAAGC
TAATACCGGATAGGATCATTGACCGCCTGGTTGATGATGGAAAGTCTGAACGACACTATCGGATGGGC
CCGCGGCGCATTAGCTAGTTGGTGGGGTAACGGCCTACCAAGGCGACGATGCGTAGCCGACCTGAGAG
GGTGACCGGCCACACTGGGACTGAGACACGGCC
>Zotu5883
GAACGCTGGCGGCAGGCTTAACACATGCAAGTCGAACGGACCCTTCGGGGTTAGTGGCGGACGGGTGA
GTAACACGTGGGAACGTGCCTTTAGGTTCGGAACAACACAGGGAAACTTGTGCTAATACCGGATGTGC
CCTTCGGGGGAAAGATTTATCGCCTTTAGAGCGGCCCGCGTCTGATTAGCTAGTTGGTGGGGTAAAGG
CCTACCAAGGCGACGATCAGTAGCTGGTCTGAGAGGATGGCCAGCCACATTGGGACTGAGACACGGCC
>Zotu5885
GAACGCTGGCGGCATGCCTTACACATGCAAGTCGAACGGCAGCGCGGGGCAACCTGGCGGCGAGTGGC
GAACGGGTGAGTAATATATCGGAACGTACCCTGGAGTGGGGGATAACGTAGCGAAAGTTACGCTAATA
CCGCATACGATCTAAGGATGAAAGCAGGGGACCGCAAGGCCTTGTGCTCCTGGAGCGGCCGATATCTG
ATTAGCTAGTTGGTGGGGTAAAGGCCCACCAAGGCATCGATCAGTAGCTGGTCTGAGAGGACGACCAG
CCACACTGGAACTGAGACACGGTC
>Zotu5887
GAATGCTGGCGGCGTGCCTAATACATGCAAGTCGAGCGGAAAGGCCCTTCGGGGTACTCGAGCGGCGG
ACGGCTGAGTAACGCGTGGGAACGTACCCCAAAGTGAGGAATAACCGCCCGAAAGGGTGGCTAATGCC
GCATATGATCTTCGGATTAAAGGATTTATCCGCTTTGGGAACGGCCTGCGTATGATTAGATTGTTGGT
GAGGTAAAGGCTCACCAAGTCTACGATCATTAACTGGTCTGAGAGGATGATCAGTCAGACTGGAACTG
AGACACGGTC
>Zotu5889
GAACGCTGGCGGCAGGCTTAACACATGCAAGTCGAACGCCCCGCAAGGGGAGTGGCAGACGGGTGAGT
AACGCGTGGGAATCTACCTAGTTCTGCGGAACAACAGAGGGAAACTTCTGCTAATACCGCATACGCCC
TACGGGGGAAAGATTTATCGGAATTAGATGAGCCCGCGTAAGATTAGCTAGTTGGTGGGGTAATGGCC
TACCAAGGCGACGATCTTTAGCTGGTCTGAGAGGATGATCAGCCACACTGGGACTGAGACACGGCC
>Zotu5890
GAACGCTGGCGGCGTGCTTAACACATGCAAGTCGAACGATGAACTGGAGCTTGCTCCAGGGATTAGTG
GCGAACGGGTGAGTAACACGTGAGTAACCTGCCCTTGACTCTGGGATAAGCGCTGGAAACGGCGTCTA
ATACCGGATACGAGCTTCAGCCGCATGGCTAGGAGTTGGAAAGAATTTCGGTCAAGGATGGACTCGCG
GCCTATCAGCTAGTTGGTGAGGTAATGGCTCACCAAGGCGACGACGGGTAGCCGGCCTGAGAGGGTGA
CCGGCCACACTGGGACTGAGACACGGCC

>Zotu5891
GAACGCTGGCGGCATGCCTAACACATGCAAGTCGAACGAGACCTTCGGGTCTAGTGGCGCACGGGTGC
GTAACGCGTGGGAATCTGCCCTTGGGTTCGGAATAACAGTTAGAAATGACTGCTAATACCGGATAATG
ACGTAAGTCCAAAGATTTATCGCCCAAGGATGAGCCCGCGTAGGATTAGCTAGTTGGTGGGGTAAAGG
CCCACCAAGGCGACGATCCTTAGCTGGTCTGAGAGGATGATCAGCCACACTGGGACTGAGACACGGCC
>Zotu5893
GAACGCTGGCGGCGTGCTTAACACATGCAAGTCGAACGGTGAAGCCCTTCGGGGTGGATCAGTGGCGA
ACGGGTGAGTAACACGTGAGTAATCTGCCCTTGACTCTGGGATAACTTCGGGAAACCGGAGCTAATAC
CGGATATTCACATTCTGTCGCATGGTGGGGTGTGGAAAGGATTCTGGTTGAGGATGAGCTCGCGGCCT
ATCAGCTTGTTGGTGGGGTAATGGCCTACCAAGGCGACGACGGGTAGCCGGCCTGAGAGGGCGACCGG
CCACACTGGGACTGAGACACGGCC
>Zotu5894
GAACGCTAGCGGCAGGCCTAATACATGCAAGTCGAGGGGCAGCAGGGTAGCAATATCGCTGGCGACCG
GCGCACGGGTGCGTAACACGTATACAATCTGCCTTTAACAGGGGGATAGCCTTTCGAAAGAGAGATTA
ATACCCCATAACACATGAAATAGCATTATTTTATGTTAAAAGCTCCGGCGGTTAGAGATGAGTATGCG
TCTGATTAGCTAGTAGGTGAGGTAACGGCTCACCTAGGCGACGATCAGTAGGGGATCTGAGAGGATTA
ACCCCCACACGGATACTGAGACACGGAT
>Zotu5895
GAACGCTGGAAATGTGGATACCGGCATGCAAGTCAAGGGGCCGCAAGGCACCGGCGAACGGCTTAGTA
ACACGTAGATACATACCGCAAAGTGGGGCACAGCTCGTCGAAAGACGGGGTAATTTCCCATAGTCCCG
CAAGGGTAAAGGCGCAAGCCGCTTTGTGAATGGTCTGCGGTCTATCAGCTAGTTGGCGGGGTAAAAGC
CCACCAAGGCTATGACGGATAGGGGGCGTGAGAGCGCGACCCCCAAGAATGGCACTGCGACACGGGC
>Zotu5897
TAACGCTAGCGGCAGGCCTAATACATGCAAGTCGAACGGGATTGATTGTAGCAATATGATCATGAGAG
TGGCGCACGGGTGCGTAACACGTATGCAACCTACCTATAACTGGGGGATAGCCTCCCGAAAGGGAGAT
TAACACCGCATAACATTATGAAGTGGCATCACTTTATAATTAAATCTACGGAGGTTATAGATGGGCAT
GCGTCCGATTAGCTAGTTGGTGAGGTAACGGCTCACCAAGGCTCCGATCGGTAGGGGGTCTGAGAGGA
TGATCCCCCACACTGGTACTGAGACACGGAC
>Zotu5898
GAACGCTGGCGGCGTGCTTAACACATGCAAGTCGAACGGATCACTCTTTGGGGTGGTTAGTGGCGAAC
GGGTGAGTAACACGTGAGCAACCTGCCCTTCACTCTGGGATAACTGTGGGAAACTTCAGCTAATACCG
GATATGACCTGCCACTGCATGGTGGTGGGTGGAAAGATTTTTTGGTGGGGGATGGGCTCGCGGCCTAT
CAGCTTGTTGGTGGGGTGATGGCCTACCAAGGCTTTGACGGGTAGCCGGCCTGAGAGGGCGACCGGCC
ACACTGGGACTGAGATACGGCC
>Zotu5899
GAACGCTGGCGGCGCGCTTAACACATGCAAGTCGAACGGGTGTCCTTCGGGGCATCAGTGGCGGACGG
GTGCGTAACACGTAAGCGACCTGCCTTCAGGTGGGGGATAACCCCGGGAAACCGGGGCTAATACCGCA
TGAGCTCTTCGGGCTGCATGGTCTGAAGAGGAAAGCTTCTGTAAGGGAGCGCTTGAAGAGGGGCTTGC
GGCGTATCAGCTAGTTGGCGAGGTAACGGCTCACCAAGGCGACGACGCGTAGGGGGTCTGAGAGGACG
GCCCCCCACATTGGGACTGAGATACGGCC
>Zotu5900
GAACGCTGGCGGCATGCTTAACACATGCAAGTCGCACGGGCATTTTCGGATGTCAGTGGCGGACGGGT
GAGTAACGCGTAGGAACATGTCCAGGGGTGGGGGATAACGCTGGGAAACTGGCGCTAATACCGCATGT
GAGCTGAGGCTCAAAGCCGCGAGGCGCCTTTGGAGTGGCCTGCGTCCGATTAGCTTGTTGGTGGGGTA
ATGGCTTACCAAGGCGACGATCGGTAGCTGGTCTGAGAGGACGACCAGCCACACTGGGACTGAGACAC
GGCC
>Zotu5901
GAACGCTGGCGGCAGGCTTAACACATGCAAGTCGAGCGGGCATCCTTGTGGTGTCAGCGGCAGACGGG
TGAGTAACGCGTGGGAACGTGCCCTTCGGTTCGGAATAACCCAGGGAAACTTGAGCTAATACCGGATA
CGTGCGAGAGCAGAAAGGTTTATCTGCCGAAGGATCGGCCCGCGTCTGATTAGCTAGTTGGTGAGGTT
ACGGCTCACCAAGGCGACGATCAGTAGCTGGTCTGAGAGGATGATCAGCCACACTGGGACTGAGACAC
GGCC
>Zotu5902
AAACGCTGGCGGCGTGCTTCAGGCATGCAAGTCGAACGAGTGTCCACCTTCGGGTGGTCTACTAGTGG

CGGACGGGTGAGTAACGCGTTAGCAACCTGCCCAAAAGTGGGGAATAACTAACCGAAAGGTTAGCTAA
TACCGCATGTGATAGCTGGTTCTTGGACCGGTTATGAAAGGCTTCGGTCGCTTTTGGAGGGGCTTGCG
TCAGATTAGCTTGTTGGTGGGGTAATGGCCTACCAAGGCGACGATCTGTAGCTGGTCTGAGAGGACGA
TCAGCCACACGGGGACTGAGATACGGCC
>Zotu5903
GAACGCTGGCGGCGTGCCTAATACATGCAAGTCGAGCGGACAGATGGGAGCTTGCTCCCTGATGTCAG
CGGCGGACGGGTGAGTAACACGTGGGCAACCTGCCTGTAAGACTGGGATAACTCCGGGAAACCGGGGC
TAATACCGGATAATTCTTTTCTCTTCATGGAGAAAAGCTGAAAGACGGTTTCGGCTGTCACTTACAGA
TGGGCCCGCGGCGCATTAGCTAGTTGGTGAGGTAACGGCTCACCAAGGCGACGATGCGTAGCCGACCT
GAGAGGGTGATCGGCCACACTGGGACTGAGACACGGCC
>Zotu5904
GAACGCTGGCGGCACGCCTAACACATGCAAGTCGAACGAAGCATAGACCTCGGTCTATGACTGAGTGG
CAGACGGGTGAGTAACACGTGGGAATCTGCCTTTTAGTGGGGAATAACTTCGGGAAACTGGAGCTAAT
ACCGCATACGTCCTTAGGGAGAAAGACTTCGGTCGCTAAAAGAGGAGCCCGCGTTGGATTAGGTAGTT
GGTGGGGTAAAAGCCTACCAAGCCTACGATCCATAGCTGGTCTAAGAGGATGATCAGCCACACTGGGA
CTGAGACACGGCC
>Zotu5905
GAACGCTAGCGGCAGGCTTAACACATGCAAGTCGAGGGGTAGAAGGAGCTTGCTTCTTTGAGACCGGC
GCACGGGTGCGTAACGCGTATGCAATCTACCTTATACAGGGGAATAGCCCAGAGAAATTTGGATTAAT
GCCCCATGGTATTATAGAATGGCATCATTTTATAATTAAAGTTCCAACGGTATAAGATGAGCATGCGT
CCCATTAGTTAGTTGGTAAGGTAACGGCTTACCAAGACGATGATGGGTAGGGGTCCTGAGAGGGAGAT
CCCCCACACTGGTACTGAGACACGGAC
>Zotu5907
GAACGCTGGCGGCGTGCCTAATACATGCAAGTCGAGCGAATCTGAGGGAGCTTGCTCCCAAAGATTAG
CGGCGGACGGGTGAGTAACACGTGGGCAACCTGCCTGTAAGACTGGGATAACTTCGGGAAACCGGAGC
TAATACCGGATAATTCTTATCAACTCATGTTGGTAAGCTAAAAGACGGCGCAAGCTGTCACTTACAGA
TGGGCCCGCGGCGCATTAGCTAGTTGGTGAGGTAACGGCTCACCAAGGCAACGATGCGTAGCCGACCT
GAGAGGGTGATCGGCCACACTGGGACTGAGACACGGCC
>Zotu5908
GAACGCTGGCGGCGTGCTTAACACATGCAAGTCGAACGGTGATGGGGAGCTTGCTCCTCTGATCAGTG
GCGAACGGGTGAGTAACACGTGAGTAACCTGCCCTCGACTCTGGGATAAGCGTTGGAAACGACGTCTA
ATACCGGATACGACCTCGGATGGCATCTTCCGGGGTGGAAAGAGTTTCGGTCGAGGATGGACTCGCGG
CCTATCAGCTTGTTGGTGAGGTAGTGGCTCACCAAGGCGACGACGGGTAGCCGGCCTGAGAGGGTGAC
CGGCCACACTGGGACTGAGACACGGCC
>Zotu5909
GAACGCTAGCTACAGGCTTAACACATGCAAGTCGAGGGGTCTGCTTCGGCAGACGACCGGCGCACGGG
TGAGTAACGCGTATCCAACCTGCCCTTCTCATTGGGATAGCCTTTCGAAAGAGAGATTAATACCGAAT
AACATAGTCAAGTTGCCTGATTTAACTATGAAAGCTCCGGCGGAGAAGGATGGGGATGCGTTCCATTA
GATAGTAGGCGGGGTAACGGCCCACCTAGTCAACGATGGATAGGGGTTCTGAGAGGAAGGTCCCCCAC
ATTGGAACTGAGACACGGTC
>Zotu5910
GAACGCTGGCGGCAGGCTTAACACATGCAAGTCGAGCGGTAGCACAAGGGAGCTTGCTCCCTGGGTGA
CGAGCGGCGGACGGGTGAGTAATGTCTGGGAAACTGCCCGATGGAGGGGGATAACTACTGGAAACGGT
AGCTAATACCGCATAACGTCGCAAGACCAAAGAGGGGGACCTTCGGGCCTCTTGCCATCGGATGTGCC
CAGATGGGATTAGCTAGTAGGTGGGGTAACGGCTCACCTAGGCGACGATCCCTAGCTGGTCTGAGAGG
ATGACCAGCCACACTGGAACTGAGACACGGTC
>Zotu5911
GAACGCTGGCGGCGTGCCTAATACATGCAAGTCGAGCGAATCTTTGGGAGCTTGCTCCCATTGGTTAG
CGGCGGACGGGTGAGTAACACGTGGGCAACCTGCCTGTAAGACTGGGATAACTTCGGGAAACCGGAGC
TAATACCGGATAATCCATTTCCTCTCATGGGGAGATGCTGAAAGACGGTTTCGGCTGTCACTTACAGA
TGGGCCCGCGGCGCATTAGCTAGTTGGTGAGGTAACGGCTCACCAAGGCGACGATGCGTAGCCGACCT
GAGAGGGTGATCGGCCACACTGGGACTGAGACACGGCC
>Zotu5913
GAACGCTAGCGGGAGGCCTAACACATGCAAGCCGAGCGGTATTGTTTCTTCGGAAATGAGAGAGCGGC

GTACGGGTGCGGAACACGTGTGCAACCTGCCTTTATCTAGAGGATAGCCTTTCGAAAGGAAGATTAAT
ACTCTATAATATATTAATTGGCATCAATTGATATTGAAAACTCCGGTGGATAGAGATGGGCACGCGCA
AGATTAGCTAGTTGGTGAGGTAACGGCTCACCAAGGCAATGATCTTTAGGGGGCCTGAGAGGGTGATC
CCCCACACTGGTACTGAGACACGGAC
>Zotu5914
GAACGCTAGCGGGAGGCCTAACACATGCAAGCTGAGCGGTATAGTTTCTTCGGAAATGAGAGAGCGGC
GTACGGGTGCGGAACACGTGTGCAACCTGCCTTTATCTAGGGGATAGCCTTTCGAAAGGAAGATTAAT
ACTCCATAATATATTGAACGGCATCGTTTAATATTGAAAGCTCCGGCGGATAGAGATGGGCACGCGCA
AGATTAGCTAGTTGGTGAGGTAACGGCTCACCAAGGCGATGATCTTTAGGGGGCCTGAGAGGGCGATC
CCCCACACTGGTACTAAGACACGGAC
>Zotu5915
CAACGCTGGCGGCGTGCCTCAGACATGCAAGTCGAACGATTAAAGCTCTCTTCGGAGAGTGCATAGAG
TGGCGCACGGGTGAGTAACACGTAAGTAATCTACCTTCGAGTGGGGAATAACGTCCGGAAACGGACGC
TAATACCGCATAATGCAGCGGCATCGTAAGATGACAGTTGTTAAAGATTTATCGCTTGAAGAGGAGCT
TGCGGCAGATTAGCTAGTTGGTAAGGTAATGGCTTACCAAGGCTACGATCTGTAACCGGTCTTAGAGG
ACGGTCGGTCACACTGACACTGAATAACGGGT
>Zotu5916
GAACGCTGGCGGCGTGCCTAACACATGCAAGTCGAACGGGGGTAGCAATACCCCAGTGGCGAACGGGT
GCGTAACACGTGAGGAACATTCCTTCCGGTGGGGGATAGCCGGCCTAACGGCCGGGTAATACCGCATA
CGCTGTTCGGGGTGCATACCCCGGATCGGAAAGCAGCAATGCGCCGGAAAGGTGCCTCGCGGCCTATC
AGCTAGTTGGTGAGGTAATGGCTCACCAAGGCGACGACGGGTAGCTGGTCTGAGAGGATGGCCAGCCA
CATTGGGACTGAGACACGGCC
>Zotu5917
GAACGCTGGCGGCAGGCCTAACACATGCAAGTCGAGCGGATGACGGGAGCTTGCTCCTTGATTCAGCG
GCGGACGGGTGAGTAATACCTAGGAATCTGCCTGGTAGTGGGGGACAACGTTTCGAAAGGAACGCTAA
TACCGCATACGTCCTACGGGAGAAAGCAGGGGACCTTCGGGCCTTGCGCTATCAGATGAGCCTAGGTC
GAATTAGCTAGTTGGTGAGGTAATGGCTCACCAAGGCTACGATCCGTAACTGGTCTGAGAGGATGGTC
AGTCACACTGGAACTGAGACACGGTC
>Zotu5921
GAACGCTGGCGGCAGGCTTAACACATGCAAGTCGAGCGCCCCGCAAGGGGAGCGGCAGACGGGTGAGT
AACGCGTGGGAATCTACCTTTTGCTACGGAATAACTCAGGGAAACTTGTGCTAATACCGTATGTGCCC
TTTGGGGGAAAGATTTATCGGCAAAGGATGAGCCCGCGTTGGATTAGGTAGTTGGTGGGGTAATGGCC
TACCAAGCCGACGATCCATAGCTGGTCTGAGAGGATGATCAGCCACATTGGGACTGAGACACGGCC
>Zotu5922
GAACGCTGGCGGCGTGCTTAACACATGCAAGTCGAACGATGACGGGGAGCTTGCTCCTCTGATTAGTG
GCGAACGGGTGAGTAACACGTGAGTAACCTGCCCTTGACTCTGGGATAAGCACTGGAAACGGTGTCTA
ATACCGGATACGAGCTTCAGCCGCATGGCTAGGAGCTGGAAAGAATTTCGGTCAAGGATGGACTCGCG
GCCTATCAGCTAGTTGGTGAGGTAACGGCCCACCAAGGCGACGACGGGTAGCCGGCCTGAGAGGGTGA
CCGGCCACACTGGGACTGAGACACGGCC
>Zotu5923
GAACGCTGGCGGCAGGCCTAATACATGCAAGTCGAGCGGTCCCTTCGGGGGCAGCGGCGGACGGGTGA
GTAACGCGTGGGAACGTGCCCTTTGGTACGGAACAACTGAGGGAAACTTCAGCTAATACCGTATGTGC
CCTTTGGGGGAAAGATTTATCGCCATTGGAGCGGCCCGCGTTAGATTAGCTAGTTGGTAGGGTAATGG
CCTACCAAGGCGACGATCTATAGCTGGTCTGAGAGGATGATCAGCCACACTGGGACTGAGACACGGCC
>Zotu5924
GAACGCTGGCGGCGTGCCTAATACATGCAAGTCGAGCGAACGAGGAAGAAACTTGTTTCTTCCTTTGT
TAGCGGCGGATGGGTGAGTAACACGTGGGCAACCTGCCCTGTAGTTGGGGATAACTCCGGGAAACCGG
GGCTAATACCGAATAATCAGTTCCTTCACATGAAGGAACTCTGAAAGACGGCTATGCTGTCACTACAG
GATGGGCCCGCGGCGCATTAGCTAGTTGGTGAGGTAACGGCTTACCAAGGCGACGATGCGTAGCCGAC
CTGAGAGGGTGATCGGCCACACTGGGACTGAGACACGGCC
>Zotu5926
GAACGCTGGCGGCATGCCTAACACATGCAAGTCGAACGAGATCCTTCGGGGTCTAGTGGCGCACGGGT
GCGTAACGCGTGGGAATCTGCCCTTAGGTTCGGAATAACAGTTAGAAATGACTGCTAATACCGGATGA
TGACGTTAAGTCCAAAGATTTATCGCCTGAGGATGAGCCCGCGTAGGATTAGCTAGTTGGTGTGGTAA

AGGCGCACCAAGGCGACGATCCTTAGCTGGTCTGAGAGGATGATCAGCCACACTGGGACTGAGACACG
GCC
>Zotu5927
GAACGCTGGCGGCGTGCTTAACACATGCAAGTCGAACGGAAAGGCCCTTCGGGGTGCTCGAGTGGCGA
ACGGGTGAGTAACACGTGGGTGATCTGCCCTGCACTTTGGGATAAGCCTGGGAAACTGGGTCTAATAC
CGAATAGGATCACTGGCTTCATGGTCGGTGGTGGAAAGCTTTTGCGGTGTGGGATGGGCCCGCGGCCT
ATCAGCTTGTTGGTGGGGTAATGGCCTACCAAGGCGACGACGGGTAGCCGGCCTGAGAGGGTGACCGG
CCACACTGGGACTGAGATACGGCC
>Zotu5929
GAACGCTGGCGGCAGGCTTAACACATGCAAGTCGAGCGAAGGCTTCGGCCTTAGCGGCGGACGGGTGA
GTAACGCGTGGGAACGTGCCCTTCACTACGGAATAGGCCCGGGAAACTGGGTTTAATACCGTATACGC
CCTTTGGGGGAAAGATTTATCGGTGAAGGATCGGCCCGCGTTGGATTAGGTAGTTGGTGGGGTAATGG
CCTACCAAGCCGACGATCCATAGCTGGTTTGAGAGGATGATCAGCCACACTGGGACTGAGACACGGCC
>Zotu5930
GAACGCTAGCGGCAGGCCTAATACATGCAAGTCGAGGGGTAGAGGGAGCTTGCTTCCTTGAGACCGGC
GCACGGGTGCGTAACGCGTATGCAACCTACCTTTATCTGGGGGATAGCCCGGAGAAATCCGGATTAAT
ACCGCATAAAATCACAGAATAGCATTATTTAATGATCAAACATTTATGGGATGAAGATGGGCATGCGT
GTCATTAGCTAGTTGGCGGGGTAACGGCCCACCAAGGCGACGATGACTAGGGGATCTGAGAGGATGGC
CCCCCACACTGGTACTGAGACACGGAC
>Zotu5931
GAACGCTGGCGGAATGCTTTACACATGCAAGTCGAACGGCAGCACGGACTTCGGTCTGGTGGCGAGTG
GCGAACGGGTGAGTAATATATCGGAACGTGCCCAGTCGTGGGGGATAACGTAGCGAAAGCTACGCTAA
TACCGCATACGATCTATGGATGAAAGCGGGGGATCGCAAGACCTCGCGCGATTGGAGCGGCCGATATC
AGATTAGGTAGTTGGTGGGGTAAAGGCCTACCAAGCCGACGATCTGTAGCTGGTCTGAGAGGACGACC
AGCCACACTGGGACTGAGACACGGCC
>Zotu5932
GAACGCTAGCGGCAGGCTTAATACATGCAAGTCGAACGGTAACATGAGTAGCAATACTTGATGACGAG
TGGCGCACGGGTGCGTAACGCGTATGCAACTTACCTTCAACTGGAAGATAGCCCCTCGAAAGAGGGAT
TAATATTCCATAATATTATGATTTGGCATCAAATTATAATTAAAGATCAGTCGGTTGAAGATAGGCAT
GCGTGACATTAGCTAGTTGGTGAGGTAATGGCTCACCAAGGCTACGATGTCTAGGGGATCTGAGAGGA
CTAACCCCCACACTGGTACTGAGACACGGAC
>Zotu5933
GAACGCTGGCGGCATGCCTAACACATGCAAGTCGAACGAAGCCTTCGGGCTTAGTGGCGCACGGGTGC
GTAACGCGTGGGAATCTGCCCTTGGGTTCGGAATAACAGTTAGAAATGACTGCTAATACCGGATGATG
TCTTCGGACCAAAGATTTATCGCCCAAGGATGAGCCCGCGTAGGATTAGCTTGTTGGTGAGGTAAAGG
CTCACCAAGGCGACGATCCTTAGCTGGTCTGAGAGGATGATCAGCCACACTGGGACTGAGACACGGCC
>Zotu5934
GAACGCTGGCGGCGTGCCTAATACATGCAAGTCGAGCGCGGGAAGCAGACTGAATCCTTCGGGAGGAC
GTTTGTGGAACGAGCGGCGGACGGGTGAGTAACACGTGGGTAACCTGCCTACAAGATCGGGATAACAC
CGGGAAACCGGTGCTAATACCGGATAATACTTTTTGTCTCCTGACAGAAAGATGAAAGACGGTTTCGG
CTGTCACTTGTAGATGGACCCGCGGCGCATTAGCTAGTTGGTGGGGTAATGGCTCACCAAGGCAACGA
TGCGTAGCCGACCTGAGAGGGTGATCGGCCACACTGGGACTGAGACACGGCC
>Zotu5935
GAACGCTGGCGGCGTGCCTAATACATGCAAGTCGAACGGTTTAGTTCCAAGAGGCTTGCCTTTTGGAA
CTAAGCAGTGGCGGACGGGTGAGTAACACGTAGGCAACCTACCTGTCAAACCGGGATAACCTCGGGAA
ACCGAAGCTAAACCCGGATAAGCTCCTTTTTCGCATGAAGGGGGAGTGAAAAGGTGCGACCTGATGAC
AGATGGGCCTGCGGCGCATTAGCTAGTAGGTAGGGTAACGGCCTACCTAGGCGATGATGCGTAGCCGA
CCTGAGAGGGCGTACGGCCACACTGGGACTGAGATACGGCC
>Zotu5936
GAACGCTGGCGGCAGGCTTAACACATGCAAGTCGAACGCCCCGCAAGGGGAGTGGCAGACGGGTGAGT
AACGCGTGGGAATCTACCCAATTCTTCGGAACAACACATGGAAACGTGTGCTAATACCGAATGAGCCC
TTCGGGGGAAAGATTTATCGGAATTGGATGAGCCCGCGTTGGATTAGCTAGTTGGTGGGGTAAAGGCC
TACCAAGGCGACGATCCATAGCTGGTCTGAGAGGATGATCAGCCACACTGGGACTGAGACACGGCC
>Zotu5937

GAACGCTGGCGGCGTGCTTAACACATGCAAGTCGAACGGTGAAGGAGGAGCTTGCTCTTCTGGATCAG
TGGCGAACGGGTGAGTAACACGTGAGCAATCTGCCCTTGACTCTGGGATAACAGTTGGAAACAGCTGC
TAATACCGGATACGAGACGTGACCGCATGGTCAACGTCTGGAAAGAACTTCGGTCAAGGATGAGCTCA
CGGCCTATCAGCTAGTTGGTGAGGTAATGGCTCACCAAGGCGACGACGGGTAGCCGGCCTGAGAGGGT
GACCGGCCACACTGGGACTGAGACACGGCC
>Zotu5939
GAACGCTGGCGGCGTGCTTAACACATGCAAGTCGAACGGAAAGGCCCTTCGGGGTACTCGAGTGGCGA
ACGGGTGAGTAACACGTGGGTGATCTGCCCTGCACTTTGGGATAAGCCTGGGAAACTGGGTCTAATAC
CGAATATGATCATCGGCTTCATGGTCGGTGGTGGAAAGCTTTTGCGGTGTGGGATGGGCCCGCGGCCT
ATCAGCTTGTTGGTGGGGTGATGGCCTACCAAGGCGACGACGGGTAGCCGGCCTGAGAGGGTGTCCGG
CCACACTGGGACTGAGATACGGCC
>Zotu5940
GAACGCTGGCGGCAGGCTTAACACATGCAAGTCGAGCGGGCACTTTCGGGTGTCAGCGGCGGACGGGT
GAGTAACGCGTGGGAACGTGCCCTCTGGTTCGGAATAACTCAGGGAAACTTGAGCTAATACCGGATAC
GCCCTTTTGGGGAAAGGCTTGCTGCCGGAGGATCGGCCCGCGTCTGATTAGCTAGTTGGTGAGGTAAC
GGCTCACCAAGGCGACGATCAGTAGCTGGTCTGAGAGGATGATCAGCCACACTGGGACTGAGACACGG
CC
>Zotu5942
GAACGCTGGCGGCAGGCTTAACACATGCAAGTCGAGCGGGCCCTTCGGGGTCAGCGGCAGACGGGTGA
GTAACGCGTGGGAACGTGCCCTTCGGTTCGGAATAATTCAGGGAAACTTGAGCTAATACCGGATACGC
CCTTCTGGGGAAAGGTTTACTGCCGAAGGATCGGCCCGCGTCTGATTAGCTGGTTGGTGAGGTTACGG
CTCACCAAGGCGACGATCAGTAGCTGGTCTGAGAGGATGATCAGCCACACTGGGACTGAGACACGGCC
>Zotu5943
GAACGCTGGCGGCGTGCCTAATACATGCAAGTCGAGCGGTAAGGCTCTCACTCTGTGCTTAAAGTTTT
GAGCATGGTGTGAGAGTACACGAGCGGCGGACGGCTGAGTAATACGTAGGAACGTACCCCAAAGTGAG
GGATAAGCATCGGAAACGGTGTCTAATACCGCATATTATCTTCGGATCAAAGCTTCGGCGCTTTGGGA
ACGGCCTACGTCCGATTAGGTTGTTGGTGAGGTAACGGCTCACCAAGCCGACGATCGGTAGCTGGTCT
GAGAGGATGATCAGCCAGACTGGGACTGAGACACGGCC
>Zotu5945
GAACGCTGGCGGCGTGCTTAACACATGCAAGTCGAGCGGTAAGGCTCCTTCGGGAGTACACGAGCGGC
GAACGGGTGAGTAACACGTGAGCAATCTGCCCTTCACTTTGGGATAACCTCCGGAAACGGTGGCTAAT
ACCGAATATGACCACTTCACGCATGTGATGGTGGTGGAAAGTTCTGGCGGTGGAGGATGAGCTCGCGG
CCTATCAGCTTGTTGGTGAGGTAATGGCTCACCAAGGCTTCGACGGGTAGCCGGCCTGAGAGGGTGAC
CGGCCACACTGGGACTGAGACACGGCC
>Zotu5946
GAACGCTGGCGGCATGCTTAACACATGCAAGTCGAACGATGGCTTCGGCCATAGTGGCGCACGGGTGA
GTAACGCGTGGGAATCTACCCAGCAGTGGGGAATAACCGAGGGAAACTTCGGCTAATACCGCATACGC
CCTTTGGGGGAAAGATTTATTGCTGTTGGATGAGCCCGCGTCCGATTAGGTAGTTGGTGAGGTAACGG
CTCACCAAGCCTTCGATCGGTAGCTGGTCTGAGAGGATGATCAGCCACACTGGGACTGAGACACGGCC
>Zotu5947
GAACGCTAGCTACAGGCTTAACACATGCAAGTCGAGGGGAAACGACATCGAAAGCTTGCTTTTGATGG
GCGTCGACCGGCGCACGGGTGAGTAACGCGTATCCAACCTGCCCATCACTTGGGGATAACCTTGCGAA
AGTAAGACTAATACCCAATGATATCTCTAGAAGACATCTGAAAGGGATTAAAGATTTATCGGTGATGG
ATGGGGATGCGTCTGATTAGCTTGTTGGCGTGGTAACGGCCCACCAAGGCGACGATCAGTAGGGGTTC
TGAGAGGAAGGTCCCCCACATTGGAACTGAGACACGGTC
>Zotu5949
GAACGCTAGCGGCAGGCCTAATACATGCAAGTCGAACGGTAACATGAGTAGCAATACTTGATGACGAG
TGGCGCACGGGTGCGTAACGCGTATGCAATCTACCTTTAACAGGTGAATAGCCCGAAGAAATTCGGAT
TAAAACACCATAGTATAATGAATGGGCATCTATTTATTATTAAAACTCCGGTGGTTAAAGATGAGCAT
GCGTGACATTAGCTAGTTGGTGAGGTAATGGCTCACCAAGGCAACGATGTCTAGGGGATCTGAGAGGA
TTAACCCCCACACTGGTACTGAGACACGGAC
>Zotu5950
GAACGCTGGCGGCAGGCCTAACACATGCAAGTCGAACGCTCGTAGCAATACGGGAGTGGCAGACGGGT
GAGTAACGCGTGGGAACCTTCCCAGTGGTACGGAATAGCTCAGGGAAACTTGAGGTAATACCGTATAC

GCCCCTAGGGGGAAAGATTTATCGCCATTGGATGGGCCCGCGTCGGATTAGCTAGTTGGTGAGGTAAC
GGCTCACCAAGGCGACGATCCGTAGCTGGTTTGAGAGAATGACCAGCCACACTGGGACTGAGACACGG
CC
>Zotu5951
GAACGCTAGCGGCAGGCATAATACGTGCAAGGCGAGGGGGTAGCAATACCACCGTCGTACGGGTGCGT
AACACGTATGCAACCTGCCCTTCACTGGGGGATAGCCTGGGAAACTCGGATTAATACCGCATAAGAC
AGTAGACTGGCATCAGTTAGCTGTTAAAGATTTATTGGTGAAGGATGGCATGCGTCTGATTAGCTAG
TTGGCGGGGTAAAGGCCCACCAAGGCTACGATCAGTAGGGGTTCTGAGAGGATTGGCCCCCACACTGG
CACTGAGATACGGGC
>Zotu5952
GAACGCTGGCGGCGTGCCTAATACATGCAAGTCGAGCGGTAAGGCTCCTACAGTTTATTTGATCATAA
TGACTTCGGTCATTGCAGCAAACCACCTGCGACCCCGGCTAGGGAAGTAGTGGCGAGGGAAGATCGAA
TGAAGTGTTGGAGTACACGAGCGGCGGACGGCTGAGTAACACGTAGGAACGTACCCCAAAGTGAGGCA
TAAGCACCAGAAATGGTGTCTAATACCGCATGTGCTCTTCGGAGTAAAGCTACGGCGCTTTGGGAACG
GCCTACGCATGATTAGCTTGTTGGTGAGGTAAAAGCTCACCAAGGCGACGATCATTAGCTGGTCTGAG
AGGATGATCAGCCAGACTGGGACTGAGAACGGCC
>Zotu5953
GAACGTTAGCGGCGCGCCTAATACATGCAAGTCGTGCGAGAAAGGGCTTCGGCCCCGGTACAGCGGCG
CACGGGTGAGTAACACGTAGGTAATCTTCCCTTGAATGGTGGATAACCCTCCGAAAGGAGAGCTAATA
CAGCATAAGACCACGGCCTCGTTGAGGTTGCGGCCAAAGCAGGCCTCTACTTGTAAGCTTGCGTTTGA
GGATGAGCCTGCGGCCCATCAGCTAGTTGGTAGGGTAATGGCCTACCAAGGCTAAGACGGGTAGCTGG
TCTGAGAGGATGAACAGCCACACTGGAACTGAGACACGGTC
>Zotu5954
GAACGCTGGCGGCATGCCTAACACATGCAAGTCGAACGAGACCTTCGGGTCTAGTGGCGCACGGGTGC
GTAACGCGTGGGCATCTACCCTTGGGTTCGGAATAACTCAGAGAAATTTGAGCTAATACCGGATGATG
TCGTAAGACCAAAGATTTATCGCCCAGGGATGAGCCCGCGTAAGATTAGCTAGTTGGTGGGGTAAAGG
CCTACCAAGGCGACGATCTTTAGCTGGTCTGAGAGGATGATCAGCCACACTGGGACTGAGACACGGCC
>Zotu5955
GAATGCTGGCGGCGTGGATAAGGCATGCAAGTCGAGTGGGTTTAGACCCACGGCAGACGAGGTAGTAA
CACGTAGGAACATACCCCGAAGTCTGGCATATTCCATCGAAAGATGGCGCAATTCCAGATGGTCTCTA
AGGAGTAAAGAAGCAATTCGCTTCGGGAGTGGCCTGCGCAGTATCAGCTAGTTGGTAGGGTAATGGCC
TACCAAGGCTATGACGCTTAGGGGAGCTGAGAGGCTGACCCCCACCGATGGGACTGAGATACGGCC
>Zotu5956
GAATGCTGGCGATGTAGATGAGTCATGCAAGTCAAGGGGGCCCGCAAGGGCAACCGGCAGACGAGGTA
GTAACGCGTAGGTAGACCCCCCGAAGTCGGGCATAGCCAGCCGAAAGGCTGGGTAATTCCCGATAGTC
CCGCAAGGGTAAAGGTTTTTCGCTTCGGGAGTCGCCTGCGCAGTATCAGCTAGTTGGTAAGGTAACGG
CTTACCAAGGCTACGACGCTTAGGGGAGCTGAGAGGCTGACCCCCACCGATGGGACTGCGACACGGCC
>Zotu5957
GAACGCTGGCGGCGTGCTTAACACATGCAAGTCGAACGATGAAGCCAGCTTGCTGGTGGATTAGTGGC
GAACGGGTGAGTAACACGTGGGTGATCTGCCCTGTACTTTGGGATAAGCCTGGGAAACTGGGTCTAAT
ACCGAATATGACCACTTAACGCATGTTTTGTGGTGGAAAGCTCCGGCGGTATGGGATGGGCCCGCGGC
CTATCAGCTTGTTGGTGGGGTAATGGCCTACCAAGGCGACGACGGGTAGCCGGCCTGAGAGGGCGACC
GGCCACACTGGGACTGAGACACGGCC
>Zotu5958
GAACGCTGGCGGCGTGCTTAACACATGCAAGTCGAGCGAGGCCCTGCTCTTCGGAGCAGGTGCCCTAG
CGGCGAACGGGTGAGTAACACGTGGGCAATCTGCCCCTAGCACTGGGATAACCCCGGGAAACCGGGGC
TAATACCGGATACGACCTCTGAGGGCATCCTCCGGAGGTAAAAAGGATTCTGGCTAGGGATGAGCCCG
CGGCCTATCAGCTTGTTGGTGGGGTAATGGCCTACCAAGGCGACGACGGGTAGCCGGCCTGAGAGGGC
GACCGGCCACACTGGGACTGAGACACGGCC
>Zotu5959
GAACGCTGGCGGCATGCTTAATACATGCAAGTCGAACGAGACCTTCGGGTCTAGTGGCGCACGGGTGC
GTAACGCGTGGGAATCTACCCTTTGCTACGGAATAACTCAGAGAAATTTGTGCTAATACCGTATAATG
TCGCAAGACCAAAGATTTATCGGCAGAGGACGAGCCCGCGTAAGATTAGCTTGTTGGTGAGGTAAAGG
CTCACCAAGGCGACGATCTTTAGCTGGTCTGAGAGGATGATCAGCCACACTGGGACTGAGACACGGCC

>Zotu5961
AAACGCTGGCGGCGTGCCTAAGACATGCAAGTCGGACGGGGAAGCTTTCGGGCTTCCGAGTGGCGGAC
GGGTGAGGAGCGCGTGGAGAATCTACCCTGGGGTGGGGGATACCCGCCAGAAATGGCGGTTAAGACCG
CATACGACCACCCAGCGCCGGCTGGGTGGGGAAAGCCGCAAGGTGCCCTGGGCGGAGTCCGCGTCGGA
TTAGCTAGTTGGTGGGGTAACGGCCCACCAAGGCCGCGATCCGTAGCTGGTCTGAGAGGACGGCCAGC
CACACTGGGACTGAGACACGGCC
>Zotu5965
GCCGTATCTCAGTCCCAATGTGGCCGGTCACCCTCTCAGGCCGGCTACCCGTCAAAGCCTTGGTAAGC
CACTACCCCACCAACAAGCTGATAAGCCGCGAGTCCATCCCCAACCGCCGAAACTTTCCAACCCCCAC
CATGCAGCAGGAGCTCCTATCCGGTATTAGCCCCAGTTTCCTGAAGTTATCCCAAAGTCAAGGGCAGG
TTACTCACGTGTTACTCACCCGTTCGCCACTCGAGCACCCCACAAAAGCAGGGCCTTTCCGTTCGACT
TGCATGTGTTAAGCACGCCGCCAGCGTTCG
>Zotu5966
GAACGCTGGCGGCGTGCCTAAAACATGCAAGTCGAACGGGGGAGCAATCCCTAGTGGCGAACGGCGGA
GTAATACATGAGGAACGTGCCCTGAAGACTGGGATAGCCGTTGGAAACGACGAGTAATACCAGATGTG
GCGGACTTTTGGCATCAAGAGTCCACTAAACGGATTTTCGCTTCAGGAGTGCCTCATGGCCTATCAGG
TAGTTGGTGAGGTAATGGCTCACCAAGCCGACGACGGGTAGCGGGTCTCAGAGGATGATCCGCTCGAG
TGGGACTGAGACACGGCC
>Zotu5967
GAACGCTGGCGGCGTGCCTAATACATGCAAGTCGTGCGGACTTATCTTCGGATAAGTTAGCGGCGGAC
GGGTGAGTAACACGTGGGCAACCTGCCTGTAAGACTGGGATAACTCCGGGAAACCGGTGCTAATACCA
GATACAATCAAAGACTGCCTGGTTTTTGATGGAAAGGTCTTGTACCGCTTACAGATGGGCCCGCGGCG
CATTAGCTAGTTGGTGGGGTAATGGCTCACCAAGGCGACGATGCGTAGCCGACCTGAGAGGGTGATCG
GCCACACTGGGACTGAGACACGGCC
>Zotu5969
GAACGCTAGCGGCATGCCTAACACATGCAAGTCGAACGGGGAGTCTATTTCGATAGGCTTCCAGTGGC
GAACGGGTGAGTAACACGTTGGTGACCTGCCCCGAAGAGGGGGACAACTCCTCGAAAGAGGAGCTAAT
ACCCCGGATGTCTGAGTGGTTAGAAGACTCAGACTAAAGTTTTTCGGAATGCTTTGGGAGGGGCCTGC
GGGCTATCAGCTAGTTGGTGAGGTAAGGGCTCACCAAGGCGAAGACGGCTAGGGGGCGTGAGAGCGTG
ACCCCCCACAAGGGAACTGAGAAACGGTC
>Zotu5970
GAACGCTGGCGGCATGCCTAACACATGCAAGTCGAACGAGACCTTCGGGTCTAGTGGCGCACGGGTGC
GTAACGCGTGGGAATCTGCCCTTAGGTTCGGAATAACAGTTAGAAATGACTGCTAATACCGGATAATG
ACGTAAGTCCAAAGATTTATCGCCTAGGGATGAGCCCGCGTAGGATTAGCTAGTTGGTGGGGTAAAAG
CCTACCAAGGCGACGATCCTTAGCTGGTCTGAGAGGATGATCAGCCACACTGGGACTGAGACACGGCC
>Zotu5971
GAACGCTGGCGGCGTGCTTAACACATGCAAGTCGAGCGGTAAGGCCCTTCGGGGTACACGAGCGGCGA
ACGGGTGAGTAACACGTGGGTGACCTGCCCCCAACTCTGGGATAAGCCCGGGAAACTGGGTCTAATAC
CGGATATGACTTCACACTGCATGGTGTGTTGTGGAAAGTTTTTCGGTTGGGGATGGGCCCGCGGCCTA
TCAGCTTGTTGGTGGGGTGATGGCCTACCAAGGCGACGACGGGTAGCCGGCCTGAGAGGGCGACCGGC
CACACTGGGACTGAGACACGGCC
>Zotu5972
GAACGCTGGCGGCGTGCCTAACACATGCAAGTCGAGCGATTTACTTCGGTAAAGAGCGGCGGACGGGT
GAGTAACGCGTGGGTAACCTGCCCTGTACACACGGATAACATACCGAAAGGTATGCTAATACGAGATA
ATATGCTTTTATCGCATGGTAGAAGTATCAAAGCTTTTGCGGTACAGGATGGACCCGCGTCTGATTAG
CTAGTTGGTGAGGTAACGGCTCACCAAGGCGACGATCAGTAGCCGACCTGAGAGGGTGATCGGCCACA
TTGGAACTGAGACACGGTC
>Zotu5973
GAACGCTGGCGGCGTGCCTAACACATGCAAGTCGAACGGAATGAGGGGCTTGCTCCTCATTTAGTGGC
GCACGGGTGAGTAACGCGTAGATAATCTGCCTGATGATCTGGGATAACACTTCGAAAGGGGTGCTAAT
ACCGGATAAGCCCACGATGGCCTTGGTCGTTGCGGGAAAAGGGGGGGACCTTCGGGCCTTCTGTCATC
AGATGAGTCTGCGTACCATTAGCTAGTTGGTAGGGTAAAGGCCTACCAAGGCTACGATGGTTAGCTGG
TCTGAGAGGATGATCAGCCACACTGGAACTGAGACACGGTC
>Zotu5974

GAACGCTGGCGGCGTGCCTAATACATGCAAGTCGAGCGAATCAAAGGGAGCTTGCTCCCTTTTGGTTA
GCGGCGGACGGGTGAGTAACACGTGGGCAACCTGCCTATAAGACTGGGATAACTTCGGGAAACCGGAG
CTAATACCGGATCATTCATATCTACTCATGTGGATATGCTGAAAGACGGTTTACGCTGTCACTTATAG
ATGGGCCCGCGGCGCATTAGCTAGTTGGTGAGGTAACGGCTCACCAAGGCGACGATGCGTAGCCGACC
TGAGAGGGTGATCGGCCACACTGGGACTGAGACACGGCC
>Zotu5975
GAACGCTAGCGGCAGGCCTAATACATGCAAGTCGAACGATAGTGAGAAGCTTGCTTCTCACGAAAGTG
GCGCACGGGTGCGTAACGCGTATGCAACCTACCTTAATCAGGGGGATAGCCCGAAGAAATTCGGATTA
ACACCGCATAAAATCACAGTATAGCATTATACAATGATCAAATATTTATAGGATTAAGATGGGCATGC
GTGACATTAGTTAGTTGGCGGGGTAACGGCCCACCAAGACCACGATGTCTAGGGGATCTGAGAGGATG
ACCCCCCACACTGGTACTGAGACACGGAC
>Zotu5977
GAACGCTAGCGGCAGGCCTAATACATGCAAGTCGAGGGGTATTGTAGCAATACGAGAGACCGGCGCAC
GGGTGCGTAACGCGTATGAAAGGTGTCCAAATCACTGGGATAGCCCTCGGAAACGGGGATTAATACCG
GATGGTCTCTTTATGTGGCATCACATTAAGAGTAAAGCTCAGGCGGATTTGGGTCCTCATGCGTACGA
TTAGCTAGTTGGCGGGGTAATGGCCCACCAAGGCGACGATCGTTAGGGGTCCTGAGAGGGAGATCCCC
CACACTGGTACTGAGACACGGGC
>Zotu5978
GAACGCTGGCGGCGTGCCTAATACATGCAAGTCGTGCGGCAGCATGCCCTTCGGGGTGATGGCGAGCG
GCGGACGGCTGAGTAACGCGTAGGAACCTACCCCAAAGTGAGGGATAACCTCGAGAAATCGAGGCTAA
TACCGCATGTGATCTTCGGATTAAAGCTTCGGCGCTTTGGGAAGGGCCTGCGTTCGATTAGCTAGTTG
GTGAGGTAATTGCTCACCAAGGCTACGATCGATAGCTGGTCTGAGAGGATGACCAGCCAGACTGGAAC
TGAGACACGGTC
>Zotu5979
GAACGCTAGCGGGAGGCCTAATACATGCAAGTCGAGCGGTAACTTTCTTCGGGAAGCTAGAGCGGCGC
ACGGGTGAGTAACGCGTACATGACCTACCTTTAAGACTGTGATAGCCCTGGGAAACTGGGATTAATAA
CGGATGTGATAATAGGTATAAGGATTTATTATTAAAGATGAGTCGCTTAGAGATGGGTGTGCGTCTGA
TTAGCTAGTTGGTAGGGTAACGGCCTACCAAGGCGACGATCAGTAGGGGGCGTGAGAGCGTGACCCCC
CACACGGGTACTGAGACACGGAC
>Zotu5981
GAACGCTGGCGGCGTGCATAACACATTCAAGTCGAACGAACTGATAAGACAGCTTGCTGTTTTTGAAA
GTTAGTGGCGGACGGGTGAGTAACGCGTGAGCAACCTGCCTGACGGCGAGGAATAACACCCGGAAACA
GGTGCTAATACCTCATGACCCATAAAGCCCGCATGGGCATTATGGCAAAGATTTATCGCCGATAGATG
GGCTCGCGTCTGATTAGATAGTTGGTGAGGTAACGGCTCACCAAGTCGACGATCAGTAGCCGGACTGA
GAGGTTGAACGGCCACATTGGAACTGAGATACGGTC
>Zotu5982
GAACGCTGGCGGCAGGCTTAACACATGCAAGTCGAGCGGGCATCCTTGTGGTGTCAGCGGCAGACGGG
TGAGTAACGCGTGGGAACGTGCCCTTCGGTTCGGAATAACTCAGGGAAACTTGAGCTAATACCGGATA
CGCCCTTATGGGGAAAGGTTTACTGCCGAAGGATCGGCCCGCGTCTGATTAGCTGGTTGGTGAGGTTA
CGGCTCACCAAGGCGACGATCAGTAGTTGGTCTGAGAGGATGATCAGCCACACTGGGACTGAGACACG
GCC
>Zotu5985
GAACGCTGGTAACGTGGATACCGGCATGCAAGTCAAGGGGGCCCGCAAGGGCAACCGGCGAACGGCTT
AGTAACACGTGGGTACATACCCCAAAGTGGAGGACAGCTCGTCGAAAGACGGGGTAATACTCCATAGT
CCTGCAAAGGTAAAGTCGCAAGACGCTTTGGGAATGGCCCGCGGCCTATCAGCTTGTTGGTGAGGTAA
AAGCTCACCAAGGCGATGACGGGTAGGGGGTGTGAGAGCACGACCCCCAAGAATTGCACTGCGACACG
GGC
>Zotu5987
GAACGCTGGCGGCATGCCTAACACATGCAAGTCGAACGAAGGCTTCGGCCTTAGTGGCGCACGGGTGC
GTAACGCGTGGGAATCTGCCCTTGGGTTCGGAATAACAGTTAGAAATGACTGCTAATACCGGATGATG
ACGTAAGTCCAAAGATTTATCGCCCAGGGATGAGCCCGCGTAGGATTAGCTAGTTGGTGAGGTAAAGG
CTCACCAAGGCGACGATCCTTAGCTGGTCTGAGAGGATGATCAGCCACACTGGGACTGAGACACGGCC
>Zotu5988
TAGCGCTAGCGATGTGCCTAAGGTATGCAAGTTGAACGGTCTAGATAGCAATATCTAAGACAGTGGCA

GACGGCTGAGTAACACGTAGGAATATTCCCTTAAGTCAAGCATAGCTCACCGAAAGGTGGGGTAATTC
TTGATGGTCCCTTCGGGGTAAAGATTTTTCGCTTAAGGATTAGCCTGCGTCCTATCAGATTGTTGGTG
AGGTAAAAGCTCACAAAGTCTATGACGGGTAGCTGGCCTGAGAGGGTGGTCAGCCAGACTGGGACTGC
GATACGGCC
>Zotu5989
GAACGCTAGCGGCAGGCCTAACACATGCAAGTCGAGGGGCAGCATAAAACAAGCTTGCTTGTTTGGTG
GCGACCGGCGCACGGGTGCGTAACGCGTATGCAACCTACCCTGTACTGAGGGATAGCCCGGAGAAATT
CGGATTAACACCTCATAGGATCTTAGATCGGCATCGATTAGAGATTAAAGGTTACGGTACAGGATGGG
CATGCGTCCCATTAGCTAGATGGTAAGGTAACGGCTTACCATGGCAACGATGGGTAGGGGGCCTGAGA
GGGTGGTCCCCCACACTGGTACTGAGACACGGAC
>Zotu5990
GAACGCTGGCGGCGTGCCTAACACATGCAAGTCGAACGCGAACGGTGCTTTCGGGTGCCTAGTAGAGT
GGCGCACGGGTGAGTAACACGTAGACAACCTGCCTTCGAGCCCGGAATAACCTTTCGAAAGAAGGGCT
AATACCGGATAACATCCTGGGTTGCATGACTTGGGATTGAAAGGCTTGCTACTCGAAGAGGGGTCTGC
GGGACATTAGCTAGTTGGTGGGGTAACGGCCTACCAAGGCGACGATGTCTAGCGGGTCTGAGAGGATG
ATCCGCCACACTGGAACTGAGACACGGTC
>Zotu5991
GAACGCTGGCGGCATGCCTTACACATGCAAGTCGAACGGCAGCGCGGGAACAATCCTGGCGGCGAGTG
GCGAACGGGTGAGTAATACATCAGAACGTGCCCAATCGTGGGGGATAACGCAGCGAAAGCTGTGCTAA
TACCGCATACGATCTACGGATGAAAGCAGGGGATCACAAGACCTTGCGCGAATGGAGCGGCCGATGGC
AGATTAGGTAGTTGGTGAGGTAAAGGCTCACCAAGCCTTCGACCTGTAGCTGGTCTGAGAGGACGACC
AGCCACACTGGGACTGAGACACGGCC
>Zotu5992
GAACGCTGGCGGCGTGCCTAACACATGCAAGTTGAGCGACGAACCGGGGCTTGCCCCGGGGCAAAGCA
GCGAACGGGTGAGTAACACGTGGGTAACGTGCCCCGACGACCGGGACAACCCGAGGAAACTCGGGCTA
ATACCGGATGTGCCCTCCGGCCTCAAGGCCGGCGGGTAAAGGAAGCTTCGGCCTCCGCGTTGGGAGCG
GCCCGCGGCCCATTAGCTAGTTGGTGAGGTAAAGGCTCACCAAGGCAACGATGGGTAGCTGGTCTGAG
AGGACGATCAGCCACACTGGGACTGAGACACGGCC
>Zotu5993
GAACGCTGGCGGCAGGCTTAACACATGCAAGTCGAGCGCCCCGCAAGGGGAGCGGCAGACGGGTGAGT
AACGCGTGGGAATCTACCGTACCCTACGGAATAACGCATGGAAACGTGTGCTAATACCGTATACGCCC
TACGGGGGAAAGATTTATCGGGGTATGATGAGCCCGCGTTGGATTAGCTAGTTGGTGGGGTAAAGGCC
TACCAAGGCGACGATCCATAGCTGGTCTGAGAGGATGATCAGCCACATTGGGACTGAGACACGGCC
>Zotu5995
GAACGCTGGCGGCGTGCTTAACACATGCAAGTCGAACGGTGAAGGGACTGGTTTTTGCTGGTTCTGGA
TGAGTGGCGAACGGGTGAGTAACACGTGAGTAACCTGCCCCCTTCTTCTGGATAACCGCATGAAAGTG
TGGCTAATACGGGATATTCTGCGCCTGCTGCATGGTGGGTGTGGGAAAGATGCGCCTTTGGGTGTTTT
TGGTGGGGGATGGGCTCGCGGCCTATCAGCTTGTTGGTGGGGTGATGGCCTACCAAGGCGGTGACGGG
TAGCCGGCCTGAGAGGGTGGACGGTCACACTGGGACTGAGACACGGCC
>Zotu5996
GAACGCTGGCGGCGTGCCTAATACATGCAAGTCGAGCGGACCTGAATGGAGCTTGCTCCAGACAGGTT
AGCGGCGGACGGGTGAGTAACACGTAGGCAACCTGCCCGCAAGACCGGGATAACGTAGGGAAACTTAC
GCTAATACCGGATAGGGCACTCTCTCGCATGAGAGGGTGCGGAAAGGTGGCGCAAGCTACCACTTGCG
GATGGGCCTGCGGCGCATTAGCTAGTTGGTGGGGTAACGGCCTACCAAGGCGACGATGCGTAGCCGAC
CTGAGAGGGTGACCGGCCACACTGGGACTGAGACACGGCC
>Zotu5997
GAACGCTGGCGGCATGCTTAACACATGCAAGTCGAACGGTAACAGGCTCGCAAGGGCGCTGACGAGTG
GCGGACGGGTGAGTAGAGCGTAGGGATCTGCCTAGATGATTGGGATAGCCTAGGGAAACTTAGGGTAA
TACCGGATACTCCTTAAGAGGGAAAGCTGGGGACTTTTAATAGAGCCTGGCGCATTTAGATGAACCTA
CGTCGGATTAGCTAGTTGGTAAGGTAAAGGCTTACCAAGGCGATGATCCGTAGCTGGTCTGAGAGGAC
GGCCAGCCACACTGGGACTGAGACACGGCC
>Zotu5998
GAACGCTGGCGGCATGCCTAACACATGCAAGTCGAACGAAGGCTTCGGCCTTAGTGGCGCACGGGTGA
GTAACACGTGGGAACCTGCCTTTCGGTTCGGAATAACGTTTGGAAACGAACGCTAACACCGGATACGC

CCTTCGGGGGAAAGTTTACGCCGAGAGAGGGGCCCGCGTCCGATTAGGTAGTTGGTGAGGTAACGGCT
CACCAAGCCGACGATCGGTAGCTGGTCTGAGAGGATGATCAGCCACACTGGGACTGAGACACGGCC
>Zotu5999
GAACGCTGGCGGCGCGCTTAACACATGCAAGTCGAACGGTTAAGGCCCCCTCGGGGGCGAATAGAGTG
GCGAACGGCTGAGTAACACGTGGGCAACCTGCCCCCCGCACCGGGATAGCCTCGGGAAACCGTGGGTA
ATACCGGATACTCCCCCACCGGCGCATGCCGGAGGGGGGAAAGCGTACACGGCGGGGGATGGGCCCGC
GGCCTGTTAGCTCGTTGGCGGGGCAACGGCCCACCAAGGCGATTATGGGTAGCCGGGTTGAGAGACCG
ACCGGCCAGATTGGGACTGAGACACGGCC
>Zotu6000
GAACGCTGGCGGCGTGCTTAACACATGCAAGTCGAGCGGAAAGGCCACTTCGGTGGTACTCGAGCGGC
GAACGGGTGAGTAACACGTGAGTAATCTGCCCTCCACTTTGGGATAGCCACCGGAAACGGTGATTAAT
ACCGGATATGACCTTTGCCTGCATGGGTTTTGGTGGAAAGTTTTTCGGTGGGGGATGTGCTCGCGGCC
TATCAGCTTGATGGTGGGGTAATGGCCTACCATGGCTTCGACGGGTAGCCGGCCTGAGAGGGTGACCG
GCCACACTGGGACTGAGACACGGCC
>Zotu6001
GAACGCTGGCGGCATGCCTAACACATGCAAGTCGAACGAGACCTTCGGGTCTAGTGGCGCACGGGTGC
GTAACGCGTGGGAATCTGCCCTTGGGTTCGGAATAACAGTTAGAAATGACTGCTAATACCGGATGATG
ACGTAAGTCCAAAGATTTATCGCCCAGGGATGAGCCCGCGTAAGATTAGCTAGTTGGTGGGGTAAAGG
CCTACCAAGGCGACGATCTTTAGCTGGTCTGAGAGGATGATCAGCCACACTGGGACTGAGACACGGCC
>Zotu6002
GAACGCTGGCGGCGTGCTTAACACATGCAAGTCAAGCGGTAAGGCCCTTTCGGGGGTACACGAGCGGC
GAACGGGTGAGTAACACGTGAGTAATCTGCCCTTCACTTGGAAATAAGCCTCGGAAACGGGGTCTAAT
ACCTGATATGACGGCCGTCTGCATGGGCTGGTCGTGGAAAGTTTTTTCGGTGGGGGATGTGCTCGCGG
CCTATCAGCTTGTTGGTGGGGTAATGGCCTACCAAGGCTTCGACGGGTAGCCGGCCTGAGAGGGTGAC
CGGCCACACTGGGACTGAGACACGGCC
>Zotu6003
GAACGCTGGCGGTGTGCTTCACACATGCAAGTCGAACGGGGTAGCAATACCCAGTGGCGGACGGGTGA
GTAACGCGTAGGAATCTGCCTATAGATGGGGGATAGCAGACCGAAAGGTCTATTAATACCGCATATGT
ATACACTCAGACATTTGTGTATATGAAAGGAGTAATCCGTCTGTAGATGAGCCTGCGTCCGATTAGCT
AGTTGGTAGGGTAAAAGCCTACCAAGGCGACGATCGGTAGGTGGTCTGAGAGGATGATCACCCACAAT
GGGACTGAGACACGGCC
>Zotu6004
GAACGCTGGCGGCGTGCCTAATACATGCAAGTCGAGCGGTAAGGTTCTGCACTCAGTATTTGACCAGA
TAAGTTTCGAAAGAAATACTGTCTGACGCAAGGTGACTCCCCGGCGGGAGGATCCGAGTCGAATCATG
AGTGTAGAATACACGAGCGGCGGACGGCTGAGTAACACGTAGGAACGTACCCCAAACTGAGGGATAAG
CACCAGAAATGGTGTCTAATACCGCATGTGATCTTCGGATTAAAGCTTCGGCGGTTTGGGAACGGCCT
GCGCATGATTAGCTTGTTGGTGAGGTAATGGCTCACCAAGGCGACGATCATTAGCTGGTCTGAGAGGA
TGATCAGCCAGACTGGAACTGAGAACGGTC
>Zotu6005
GAACGCTGGCGGCGTGCCTAATACATGCAAGTCGAGCGGACAGATGGGAGCTTGCTCCCTGATGTTAG
CGGCGGACGGGTGAGTAACACGTGGGTAACCTGCCTGTAAGACTGGGATAACTCCGGGAAACCGGGGC
TAATACCGGATGCTTGATTGAACCGCATGGTTCAATTATAAAAGGTGGCTTTTAGCTACCACTTACAG
ATGGACCCGCGGCGCATTAGCTAGTTGGTGAGGTAACGGCTCACCAAGGCAACGATGCGTAGCCGACC
TGAGAGGGTGATCGGCCACACTGGGACTGAGACACGGCC
>Zotu6007
GAACGCTGGCGGCAGGCCTAACACATGCAAGTCGAACGGTAGCGGGAAGAAGCTTGCTTCTTTGCCGA
CGAGTGGCGGACGGGTGAGTAATGTCTGGGGATCTGCCCGATGGAGGGGGATAACCACTGGAAACGGT
GGCTAATACCGCATAACGTCGCAAGACCAAAGTGGGGGACCTTCGGGCCTCACACCATCGGATGAACC
CAGATGGGATTAGCTAGTAGGTGGGGTAACGGCTCACCTAGGCGACGATCCCTAGCTGGTCTGAGAGG
ATGACCAGCCACACTGGAACTGAGACACGGTC
>Zotu6008
GAACGCTGGCGGCATGCCTAATACATGCAAGTCGAGCGAATGATGAAGAAGCTTGCTTCTTCTGATTT
AGCGGCGGACGGGTGAGTAACACGTGGGTAACCTACCTTACAGATTGGGATAACTTCGGGAAACCGGA
GCTAATACCGAATAATATATTTCTTTTCATGAAGAAATATTGAAAGACGGTTTCGGCTGTCGCTGTAA

GATGGGCCCGCGGCGCATTAGCTAGTTGGTGAGGTAACGGCTCACCAAGGCAACGATGCGTAGCCGAC
CTGAGAGGGTGATCGGCCACACTGGGACTGAGACACGGCC
>Zotu6009
GAACGCTGGCGGCGTGCCTAATACATGCAAGTCGAGCGAATGGATGAAGAAAGCTTGCTTTCTTCTGA
TGTTAGCGGCGGACGGGTGAGTAACACGTGGGCAACCTGCCCTTTAGATTGGGATAACTTCGGGAAAC
CGGAGCTAATACCAAATAATACTTTCGAATTCATGTTTGGAAGTTGAAAGACGGTTTCGGCTGTCACT
AAAGGATGGGCCCGCGGTGCATTAGCTAGTTGGTGAGGTAACGGCTCACCAAGGCAACGATGCATAGC
CGACCTGAGAGGGTGATCGGCCACACTGGGACTGAGACACGGCC
>Zotu6010
GAATGCTGGCGGCGTGCTTAACACATGCAAGTCGAGCGGTAACGGGTCCTTCGGGATGCCGACGAGCG
GCGGACGGCTGAGTAACGCGTGGGAACATGCCCCAAAGTGAGGAATAAGCACCGGAAACGGTGTCTAA
TGCCGCATATGGTCTTCGGATTAAAGGATTTATCCGCTTTGGGAGTGGCCCGCGTTCGATTAGGTAGT
TGGTGAGGTAAAGGCTCACCAAGCCTACGATCGATAACTGGTCTGAGAGGATGATCAGTCAGACTGGA
ACTGAGATACGGTC
>Zotu6011
GAACGCTGGCGGCGTGCCTAATACATGCAAGTAGAACGCTGAAGGGAGAGCTTGCTCTTCTGGATGAG
TTGCGAACGGGTGAGTAACGCGTAGGTAACCTGCCTGGTAGCGGGGGATAACTATTGGAAACGATAGC
TAATACCGCATAATAGTAGATGTTGCATGACATTTGCTTGAAAGGTGCAATTGCATCACTACCAGATG
GACCTGCGTTGTATTAGCTAGTTGGTGGGGTAACGGCTCACCAAGGCGACGATACATAGCCGACCTGA
GAGGGTGATCGGCCACACTGGGACTGAGACACGGTC
>Zotu6017
GAATGCTGGCGGCGTGGATAAGCCATGCAAGTCGAACGGTCCGCAAGGACAGTGGCAGACGAGGTAGT
AAGGCGCAGGGAGATCCCCCGGAGTCAGGGATAGCCAGCCGAAAGGTTGGGTAATACTTGATGGTCCA
GCGATGGTAAAGATTTATCGCTCCGGGAGTCACCTGCGTAATATCAGCTAGTTGGTGAGGTAATGGCT
CACCAAGGCTACGACGTTTAGGGGAGCTGAGAGGCTGACCCCCACCGATGGGACTGCGATACGGCC
>Zotu6018
GAACGCTGGCGGCAGGCCTAACACATGCAAGTCGAGCGAAGTCTTCGGACTTAGCGGCGGACGGGTGA
GTAACGCGTGGGAACATACCCAAAGGTACGGAATAGCCTCGGGAAACTGGGAGTAATACCGTATGTGC
CCTTCGGGGGAAAGATTTATCGCCTTTGGATTGGCCCGCGTTGGATTAGGTAGTTGGTGGGGTAATGG
CCTACCAAGCCGACGATCCATAGCTGGTTTGAGAGGATGATCAGCCACACTGGGACTGAGACACGGCC
>Zotu6019
GAACGCTGGCGGCGTGCTTAACACATGCAAGTCGAACGAGGATTTTTACTTCGGTGAAGATCCGAGTG
GCAAACGGGTGAGTAACGCGTAGACAACCTGCCTCTCTGATGGGGACAACAGTTCGAAAGGACTGCTA
ATACCGAATGAAGTGGAAGCTCCACATGGAGCCTCCACCAAAGACAGCCTACTAACAGTGCTGTCGCG
GAGAGATGGGTCTGCGTCTGATTAGCTAGTTGGAGGGGTAACGGCCCACCAAGGCAACGATCAGTAGC
CGGTCTGAGAGGATGGACGGCCACATTGGAACTGAGACACGGTC
>Zotu6020
GAACGCTGGCGGCATGCCTAATACATGCAAGTCGTGCGGACAGATGGAAGGCTTGCCTTCCTGAAGTC
AGCGGCGGACGGGTGAGTAACACGTGGGCAACCTGCCCGCAAGACCGGGATAACTGCGGGAAACCACA
GCTAATACCGGATACGACCTCTTCTCGCATGAGCGGAGGTGGAAAGGCGGCGTCAAGCTGTCACTTGC
GGATGGGCCCGCGGCGCATTAGCTAGTTGGTAAGGTAGCGGCTTACCAAGGCGACGATGCGTAGCCGA
CCTGAGAGGGTGACCGGCCACACTGGGACTGAGACACGGCC
>Zotu6022
GAACGCTGGCGGCAGGCCTAACACATGCAAGTCGAACGCTGTAGCAATACAGAGTGGCAGACGGGTGA
GTAACACGTGGGAACATACCCTTCGGTTCGGAATAACTCAGGGAAACTTGAGCTAATACCGGATACGT
TCGCGAGAAGAAAGATTTATCGCCGAAGGATTGGCCCGCGTCTGATTAGCTAGTTGGTGAGGTAACGG
CTCACCAAGGCGACGATCAGTAGCTGGTCTGAGAGGATGATCAGCCACATTGGGACTGAGACACGGCC
>Zotu6024
GAACGCTGGCGGCAGGCTTAACACATGCAAGTCGAACGCTCCGCAAGGAGAGTGGCAGACGGGTGAGT
AACGCGTGGGAATCTACCCATCTCTACGGAATAACGCATGGAAACGTGTGCTAATACCGTATACGCCC
TTAGGGGGAAAGATTTATCGGAGATGGATGAGCCCGCGTTGGATTAGCTAGTTGGTGGGGTAAAGGCC
TACCAAGGCGACGATCCATAGCTGGTCTGAGAGGATGATCAGCCACATTGGGACTGAGACACGGCC
>Zotu6025
GAACGCTAGCGGCAGGCCTAATACATGCAAGTCGGACGGGATTGAGAAGCTTGCTTTTCATGAGAGTG

GCGCACGGGTGCGTAACACGTATGTAACCTACCTTTATCAGGGGGATAGCCTCTCGAAAGAGAGATTA
AGCCCGCATAACATGGAGATGCAGCATTGCATTTCCATCAAATATTTATAGGATAAAGATGGGCATGC
GGAACATTAGCTAGTTGGTAAGGTAATGGCTTACCAAGGCTACGATGTTTAGGGGATCTGAGAGGATG
ACCCCCCACACTGGTACTGAGACACGGAC
>Zotu6026
GAACGCTGGCGGCGTGCTTAACACATGCAAGTCGAACGATGAAGCCCTTCGGGGTGGATTAGTGGCGA
ACGGGTGAGTAACACGTGGGCAATCTGCCCTTCACTCTGGGACAAGCCCTGGAAACGGGGTCTAATAC
CGGATAACACTCCTGCTCTCATGGGCAGGGGTTAAAAGCTCCGGCGGTGAAGGATGAGCCCGCGGCCT
ATCAGCTTGTTGGTGAGGTAATGGCTCACCAAGGCGACGACGGGTAGCCGGCCTGAGAGGGCGACCGG
CCACACTGGGACTGAGACACGGCC
>Zotu6027
GAACGCTGGCGGCGTGCCTAATACATGCAAGTCGAGCGAATGAATGGGAGCTTGCTCCCTGACATTAG
CGGCGGACGGGTGAGTAACACGTGGGCAACCTGCCCTGCAGATGGGGATAACTCCGGGAAACCGGGGC
TAATACCGAATAATCAGTTCCTCCGCATGGAGGAACTCTGAAAGACGGTTTCGGCTGTCACTGCAGGA
TGGGCCCGCGGCGCATTAGCTGGTTGGTGGGGTAATGGCCTACCAAGGCAACGATGCGTAGCCGACCT
GAGAGGGTGATCGGCCACACTGGGACTGAGACACGGCC
>Zotu6028
GAACGCTGGCGGCGTGCTTAACACATGCAAGTCGAACGATGATGCCTAGCTTGCTAGGTGGATTAGTG
GCGAACGGGTGAGTAATACGTGAGTAACCTGCCCCCTTCTTTGGGATAAGCCTTGGAAACGGGGTCTA
ATACCGGATATTCCTGGTCTGCCGCATGGTGGGTTGGGGAAAGTTTTTTCGGTGGGGGATGGGCTCGC
GGCCTATCAGCTTGTTGGTGGGGTGATGGCCTACCAAGGCGTCGACGGGTAGCCGGCCTGAGAGGGTG
ACCGGCCACACTGGGACTGAGATACGGCC
>Zotu6029
GAACGCTGGCGTCGTGCCTAATACATGCAAGTAGAACGCTGAAGGAGGAGCTTGCTTCTCCGGATGAG
TTGCGAACGGGTGAGTAACGCGTAGGTAACCTGCCTGGTAGCGGGGGATAACTATTGGAAACGATAGC
TAATACCGCATAAGAGTAGATATTGCATGATATCTGCTTGAAAGGTGCAATTGCATCACTACCAGATG
GACCTGCGTTGTATTAGCTAGTTGGTGAGGTAACGGCTCACCAAGGCGACGATACATAGCCGACCTGA
GAGGGTGATCGGCCACACTGGGACTGAGACACGGCC
>Zotu6030
CAACGCTGGCGGCGCGCCTAACACATGCAAGTCGAACGAGAAAGCCGCAAGGTGAGTAAAGTGGCGTA
CGGGTGAGTAACACGTGGATAATCTGCCCTTTAGTGGGGAATACCGCTGGGAAACCGGCGACAATACC
GCATAAGCTCGAGAGAGGAAAGCAGCAATGCGCTGAAGGAGGAGTCCGCGGTCGATTAGTTAGTTGGC
GGGGTAAAGGCCCACCAAGACTGTGATCGATAGCCGGCCTGAGAGGGCACACGGCCACACTGGCACTG
AAACACGGGC
>Zotu6031
GAACGTTGGCGGCGTGGATTAGGCATGCAAGTCGTACGGGCCGCAAGGCCAGTGGCGCAAGGGTGAGT
AAGGCGACGGTAACCAACCCCAGGACTGGGTATAGCCGCGGGAAACTGCGGGTAATCCCCAGCGACGT
CGCGGGGGGGCATCCCCCCGCGACCAAAGCTCCGGCGTCGTGGGACGGGCCGTCGTGGTATTAGGTTG
TTGGCGGGGTAACGGCCCACCAAGCCGGCGATGCCTACCGGGCGTGCGAGCGTGGCCCGGCACACTGG
GACTGAGACACGGCC
>Zotu6034
GAACGCTGGCGGCAGGCTTAACACATGCAAGTCGAACGGTAACATGAAGAAGCTTGCTTCTTTGATGA
CGAGTGGCGGACGGGTGAGTAATGCTTGGGAATCTAGCTTATGGAGGGGGATAACTACGGGAAACTGT
AGCTAATACCGCGTAGTATCGAGAGATGAAAGTGTGGGACCTTCGGGCCACATGCCATAGGATGAGCC
CAAGTGGGATTAGGTAGTTGGTGAGGTAAAGGCTCACCAAGCCGACGATCTCTAGCTGGTCTGAGAGG
ATGACCAGCCACACTGGGACTGAGACACGGCC
>Zotu6035
GAACGCTGGCGGCATGCCTAAGACATGCAAGTCGAACGAGACGACCTTTGAAAGACTGAGTGCTTGCA
CAAAGTTGGAAGAAGATCATCGTCTAGTGGCAGACGGGTGAGTAACACGTGGGTAACCTACCTTTAAG
CTGGGGATAACAGTTGGAAACGACTGATAATACCGAATAAGATATAGTAATATATTGAAAGAAGCCTT
TAAAGCTTCACTTAAAAATGGGCCTGCGGCGTATTAACTAGTTGGTGGGGTAATGGCTCACCAAGGTA
ACGATGCGTAGCCGAACTGAGAGGTTGAACGGCCACACTGGGACTGAGACACGGCC
>Zotu6036
CAACGCTGGCGGCGTGCCTAACACATGCAAGTAGCACGAGAAAGGGGCTTCGGTCCTGAGTAAAGTGG

CGCACGGGTGAGTAACACGTGACTAACCTACCTCCGAGTGGGGAATAACCTAGAGAAATCTGGGCTAA
TACCGCATAAAACTTACGAGTCAAAGCAGCAATGCGCTTGGAGAGGGGGTCGCGGTCGATTAGCTAGT
TGGCGGGGTAATGGCCCACCAAGGCGATGATCGATATCCGGCCTGAGAGGGCGCACGGACACACTGGA
ACTGAAACACGGTC
>Zotu6038
GAACGCTGGCGGCGTGCTTAAGACATGCAAGTCGGACGCATCCCCCTCGGGGGATGAGTGGCGCACGG
GTGAGTAACGCGTAACTGACCTACCCTCAAGTCCTGACCAACTCCTCGAAAGAGGAGCTAATGTGGGA
TGTGCTGCCTCACCGTGGTGAGGCAGTAAAGGCTATGACCGCTTGAGGATGGGGTTGCGTTTCATCAG
CTAGATGGTGGGGTAAAGGCCTACCATGGCGACGACGAATAGCCGGCCTGAGAGGGTGGCCGGCCACA
GGGGCACTGAGACACGGGC
>Zotu6039
GAACGCTGGCGGCGTGCCTAAGACATGCAAGTCGAATGCTCCGCAAGGGGCATGGCGAACGGGTGAGT
AATACCTAGGAACGTATCTTTAGTCGAGGATATCCCATCGAAAGATGGTGCAATACTCGATGTGGCCG
CAAGGCTAAAGTCGCAAGACGCTAAAGAAGCGGCCTGGGCACCATCAGCTAGTTGGTAAGGTAATGGC
TTACCAAGGCTATGACGGTTAGGGGCCCCGAGAGGGGGAACCCCCACACTGGAACTGCGACACGGTC
>Zotu6040
GAACGCTGGCGGCGTGCCTAATACATGCAAGTCGAGCGGATCTTTAGAAGCTTGCTTCTAAAGATTAG
CGGCGGACGGGTGAGTAACACGTGGGCAACCTGCCTGTAAGACTGGGATAACTTCGGGAAACCGAGGC
TAATACCGGATAATCCTTTTCCTCACATGAGGGAAAGCTGAAAGACGGCCTTAAAAGCTGTCACTTAC
AGATGGGCCCGCGGCGCATTAGCTAGTTGGTGAGGTAACGGCTCACCAAGGCAACGATGCGTAGCCGA
CCTGAGAGGGTGATCGGCCACACTGGGACTGAGACACGGCC
>Zotu6042
GAACGCTGGCGGCATGCCTAATACATGCAAGTCGAACGCTGAAGCACTCTGCTTGCAGAGTGCGGATG
AGTGGCGAACGGGTGAGTAACACGTGGGAAATCTGCCTAAAAGTGGGGGATAACATTCGGAAACGGAT
GCTAATACCGCATAGATTCTTGAACCGCATGGTTCTTGAAAGAAAGACGGCTTTGCTGTCGCTTTTAG
ATGATCCCGCGGCGTATTAGTTAGTTGGTGGGGTAATGGCTTACCAAGACCATGATACGTAGCCGACC
TGAGAGGGTGATCGGCCACACTGGGACTGAGACACGGCC
>Zotu6043
GAACGCTGGCGGCAGGCCTAACACATGCAAGTCGAGCGGATGAGAGGAGCTTGCTCCTGGATTCAGCG
GCGGACGGGTGAGTAATGCCTAGGAATCTGCCTGGTAGTGGGGGATAACGTCCGGAAACGGGCGCTAA
TACCGCATACGTCCTGAGGGAGAAAGTGGGGGATCTTCGGACCTCACGCTATCAGATGAGCCTAGGTC
GGATTAGCTAGTTGGTGGGGTAAAGGCCTACCAAGGCGACGATCCGTAACTGGTCTGAGAGGATGATC
AGTCACACTGGAACTGAGACACGGTC
>Zotu6044
GAACGCTGGCGGCAGGCCTAACACATGCAAGTCGAACGGAGTTATATTGTAGCTTGCTATGATGTGAC
TTAGTGGCAGACGGGTGAGTAATGTATAGGAATCTACCTAGTAGTACGGAATAATTGTTGGAAACGAC
AACTAATACCGTATACGCCCTACGGGGGAAAAATTTATTGCTATTAGATGAGCCTATATTAGATTAGC
TTGTTGGTGGGGTAATAGCCTACCAAGGCAATGATCTATAGCTGATCTGAGAGGATGATCAGCCACAC
TGGAACTGAGATACGGTC
>Zotu6045
GAACGCTGGCGGCGTGCTTAACACATGCAAGTCGAACGATGAAGCCCAGCTTGCTGGGCGGATTAGTG
GCGAACGGGTGAGTAACACGTGAGTAACCTGCCCTTGACTCTGGGATAAGCCCGGGAAACTGGGTCTA
ATACCGGATAGGAGCGCCTGTCGCATGACGGGTGTTGGAAAGATTTTTCGGTCATGGATGGACTCGCG
GCCTATCAGCTTGTTGGTGAGGTAATGGCTCACCAAGGCGACGACGGGTAGCCGGCCTGAGAGGGTGA
CCGGCCACACTGGGACTGAGACACGGCC
>Zotu6047
TAACGCTGGCGGCGTGCATGAGACATGCAAGTCGAACGGACACATGGGGCTTGCTCCATGTGTTAGTG
GCGGACGGCTGAGTAACGCGTAGGTGACCTGCCCCGAAGTGGGGGATAACTGCCCGAAAGGGTGGCTA
ATACCGCATGTGGAGGTTCGTTCATTCGAACCTTCAAAGCAGCAATGCGCTTCGGGAGGGGCCTGCGT
CCGATTAGCTCGTTGGTGGGGTAACGGCTCACCAAGGCGACGATCGGTAGGTGGTCTGAGAGGACGAT
CACCCAGACTGGGACTGAGACACGGCC
>Zotu6048
GAACGCTGGCGGCGTGCTTAACACATGCAAGTCGAGCGGAAAGGCCACTTCGGTGGTACTCGAGCGGC
GAACGGGTGAGTAACACGTGAGTAATCTGCCCCTGGCTTTGGGATAGCCACCGGAAACTGTGATTAAT

ACCGGATACGACAACTTCTCGCATGGGATGGTTGTGGAAAGTTTTTCGGCCAGGGATGTGCTCGCGGC
CTATCAGCTTGATGGTGAGGTAACGGCTCACCATGGCTTCGACGGGTAGCCGGCCTGAGAGGGTGACC
GGCCACACTGGGACTGAGACACGGCC
>Zotu6049
GAACGCTGGCGGCAGGCCTAAAACATGCAAGTCGAACGCTGTAGCAATACAGAGTGGCGCACGGGTGA
GTAACATGTGGGAATATGCCTTTTGGTACGGAACAACTTTTGGAAACGAAAGCTAATACCGTATGGTC
CCTTCGGGGTAAAGATTTATCGCCAAAAGATTAGCCCGCACTAGATTAGCTAGTTGGTAAGGTAATGG
CTTACCAAGGCGACGATCTATAGCTGGTCTGAGAGGACGATCAGCCACACTGGGACTGAGACACGGCC
>Zotu6050
GAACGCTGGCGGCGTGCTTAACACATGCAAGTCGAGCGGAAAGGCCCTTCGGGGTACTCGAGCGGCGA
ACGGGTGAGTAACACGTGAGCAACCTGCCCTTGGCTCTGGGATAGCCTCCGGAAACGGGGATTAATAC
CGGACATGCACTTCCCGCGGCATCGCGGGGGGTGGAAAGTTTTTCGGCCGGGGAGGGGCTCGCGGCC
TATCAGCTTGTTGGGGGGGTAACGGCCCACCAAGGCGACGACGGGTAGCCGGCCTGAGAGGGCGACCG
GCCACACTGGGACTGAGACACGGCC
>Zotu6051
GAACGCTAGCGGCAGGCCTAACACATGCAAGTCGAGGGGCAGCACAGTGTAGCAATACATGGGTGGCG
ACCGGCGCACGGGTGAGTAACGCGTATGCAACCTACCCTATACTGGGGGATAGCTCAGAGAAATCTGA
ATTAAACCCGCATAAAGCAGGGGTACCGCATGGTACTATTTGCTAAAACTCCGGTGGTATAGGATGGG
CATGCGTGTCATTAGCTAGTTGGTGAGGTAACGGCTCACCAAGGCGACGATAACTAGGGGGTCTGAGA
GGATGATCCCCCACACTGGTACTGAGACACGGAC
>Zotu6053
GAACGCTGGCGGCGTGCCTAACACATGCAAGTCGAACGGAGTAATTACTTCGGTGATTACTTAGTGGC
GAACGGGTGAGTAACGCGTGAGAAACCTGCCTTTCAGTGGGGGACAACAGTTGGAAACGACTGCTAAT
ACCGCATAATGTTATTTGGGGGCATCCCTGAATAACCAAAGCTTTATGTGCTGAAAGATGGTCTCGCG
TCTGATTAGCTAGTTGGTGAGGTAACGGCTCACCAAGGCGACGATCAGTAGCCGGTCTGAGAGGATGA
ACGGCCACATTGGGACTGAGATACGGCC
>Zotu6054
GAACGCTAGCGATAGGCTTAACACATGCAAGTCGAGGGGCAGCATAGAGTCAGCTTGCTGATTTGGAT
GGCGACCGGCGGATGGGTGCGTAACGCGTATGCAACTTACCTGTCAGAGGGGGATAACCCGGAGAAAT
TCGGACTAATACCGCATATACTTGAGATGCTGCATGGTATTTCAAGGAAATATTTATAGCTGACAGAT
AGGCATGCGTTCCATTAGCTAGTAGGTGAGGTAACGGCTCACCTAGGCGACGATGGATAGGGGAACTG
AGAGGTTAAACCCCCACACTGGTACTGAGACACGGAC
>Zotu6055
GAACGCTGGCGGCGTGCTTAACACATGCAAGTCGAACGGAAAGGCCCTTCGGGGTACTCGAGTGGCGA
ACGGGTGAGTAACACGTGGGTGATCTGCCCTGCACTTCGGGATAAGCCTGGGAAACTGGGTCTAATAC
CGGATAGGACCACGCGCTTCATGGTGTGTGGTGGAAAGCTTTTGCGGTGTGGGATGGGCCCGCGGCCT
ATCAGCTTGTTGGTGGGGTAATGGCCTACCAAGGCGTCGACGGGTAGCCGGCCTGAGAGGGTGTCCGG
CCACACTGGGACTGAGATACGGCC
>Zotu6056
TAACGCTGGCGGCGTGCCTAACACATGCAAGTCGAACGAAGACATGAATTTTGAATCTTGAAGCTTGA
AGCTGGAATTCCAGACTCAAGACTCCAGATTCCAGGTTCGGTTTTAGTGGCGGACGGGTGAGTAACAC
GTTGGTACATACCCTCAAGTGGGGAATAACTGGCCGAAAGGTCGGCTAATACCGCATAATATGCGCCT
TTGGCGCATTAAAGTCCGCCTCGGCGGACGCTTGAGGAATGGCCTGCGTCCGATTAGCTAGTTGGCGG
GGTAAAGGCCCACCAAGGCGACGATCGGTAGCTGGATCGAGAGGTTGACCAGCCACACTGGGACTGAG
ATACGGCC
>Zotu6058
GAACGCTGGCGGCGTGCTTAACACATGCAAGTCGAACGGATCACTCTTTGGGGTGGTTAGTGGCGAAC
GGGTGAGTAACACGTGAGCAACCTGCCCTTCACTCTGGGATAACTGTGGGAAACTTCAGCTAATACCG
GATATGACCTGTCACTGCATGGTGGTGGGTGGAAAGATTTTTTGGTGGGGGATGGGCTCGCGGCCTAT
CAGCTTGTTGGTGGGGTGATGGCCTACCAAGGCTTTGACGGGTAGCCGGCCTGAGAGGGCGACCGGCC
ACACTGGGACTGAGATACGGCC
>Zotu6059
GAACGCTGGCGGCAGGCCTAACACATGCAAGTCGAGCGGATGAAGGGAGCTTGCTCCCTGATTCAGCG
GCGGACGGGTGAGTAATGCCTAGGAATCTGCCTGGTAGTGGGGGACAACGTCTCGAAAGGGACGCTAA

TACCGCATACGTCCTACGGGAGAAAGCAGGGGACCTTCGGGCCTTGCGCTATCAGATGAGCCTAGGTC
GGATTAGCTAGTTGGTGAGGTAATGGCTCACCAAGGCGACGATCCGTAACTGGTCTGAGAGGATGATC
AGTCACACTGGAACTGAGACACGGTC
>Zotu6060
GAACGCTGGCGGCGTGCCTAATACATGCAAGTCGAGCGAATCTTTGGGAGCTTGCTCCCAAAGATTAG
CGGCGGACGGGTGAGTAACACGTGGGCAACCTGCCTGTAAGACTGGGATAACTTCGGGAAACCGGAGC
TAATACCGGATAATTCTTTTCTACTCATGTAGGAAAGCTGAAAGACGGTTTCGGCTGTCACTTACAGA
TGGGCCCGCGGCGCATTAGCTAGTTGGTGAGGTAACGGCTCACCAAGGCGACGATGCGTAGCCGACCT
GAGAGGGTGATCGGCCACACTGGGACTGAGACACGGCC
>Zotu6061
GAACGCTAGCGGCAGGCCTAACACATGCAAGTCGAGGGGCAGCATAAATAAAGCTTGCTTTATTGGTG
GCGACCGGCGCACGGGTGCGTAACGCGTATGCAACCTACCCCGTACTGAGGGATAGCCCGGAGAAATT
CGGATTAAGACCTCATGGTATTGTTTTATCGCATGGTAAGACAATTAAAGGTTACGGTACGGGATGGG
CATGCGTCCTATTAGCTAGATGGTGAGGTAACGGCTCACCATGGCTACGATAGGTAGGGGGCCTGAGA
GGGTGGTCCCCCACACTGGTACTGAGACACGGAC
>Zotu6062
GAACGTTAGCGGCGCGCCTAACACATGCAAGTCGCACGAGAAAGGGCTTCGGCCCCGGTACAGTGGCG
CACGGGTGAGTAACACGTAGATCATCTCCCCTCGAGTGGTGGATAACTCTCCGAAAGGAGAGCTAATA
CAGCATGAGACCACGGCTTCGTTGAAGCTGCGGCCAAAGCAGGCCTCTACACGTAAGCTTGCGCTTGA
GGATGAGTCTGCGGCCCATCAGCTAGTTGGTAGGGTAATGGCCTACCAAGGCTAAGACGGGTAGCTGG
TCTGAGAGGATGAACAGCCACACTGGAACTGAGACACGGTC
>Zotu6063
GAACGCTGGCGGCGCGCCTAAAACATGCAAGTCGAGCGAGAACCAGGGCTTCGGCCCTGGGGAGAGCG
GCGAACGGGTGCGTAACACGTGAGCAACCTGCCCCGAAGACCGGGACAACACCGGGAAACCGGTGCTA
ATACCGGATGTCCTCGCCGGATCGCATGGTCTGGTGAGGAAATGGATTCCGCTTCGGGATGGGCTCGC
GGTCTATCAGCTTGTTGGTGGGGTAACGGCCTACCAAGGCGACGACGGATAGCTGGTCTGAGAGGACG
ATCAGCCACACTGGGACTGAGACACGGCC
>Zotu6064
GAACGCTGGCGGCGTGCCTAATACATGCAAGTCGAGCGGACTTAAAAGGAGCTTGCTCCTATTAAGTT
AGCGGCGGACGGGTGAGTAACACGTGGGCAACCTGCCCTATAGTTTGGGATAACTCCGGGAAACCGGG
GCTAATACCGAATGATACATTTTAACTCCTGTTAGAATGTTGAAAGATGGTTCTGCTATCGCTATAGG
ATGGGCCCGCGGCGCATTAGCTAGTAGGTGAGGTAACGGCTCACCTAGGCGACGATGCGTAGCCGACC
TGAGAGGGTGATCGGCCACACTGGGACTGAGACACGGCC
>Zotu6065
GAACGCTGGCGGCGTGCTTAACACATGCAAGTCGAACGGTGAAAGAGAGCTTGCTCTCTGGATCAGTG
GCGAACGGGTGAGTAACACGTGAGCAATCTGCCCCTGACTCTGGGATAAGCGCTGGAAACGGCGTCTA
ATACCGGATACGAGCTGCGAAGGCATCTTCAGCAGCTGGAAAGAACTTCGGTCAGGGATGAGCTCGCG
GCCTATCAGCTAGTTGGTGAGGTAATGGCTCACCAAGGCGTCGACGGGTAGCCGGCCTGAGAGGGTGA
CCGGCCACACTGGGACTGAGACACGGCC
>Zotu6068
GAACGCTGGCGGCGTGCCTAATACATGCAAGTCGAGCGGACTAGAGGGAGCTTGCTCCTGATGGTCAG
CGGCGGACGGGTGAGTAACACGTGGGTAACCTGCCTGACAGATCGGGATAACGCTTGGAAACGAGTGC
TAATACCGGATAGTCTTTTGAACCGCATGGTTCGAAAGGGAAAGAAGCTATCGCTTCACTGTCAGATG
GACCCGCGGCGCATTAGCTAGTTGGTGAGGTAACGGCTCACCAAGGCGACGATGCGTAGCCGACCTGA
GAGGGTGACCGGCCACACTGGGACTGAGACACGGCC
>Zotu6069
GAACGCTAGCTACAGGCTTAACACATGCAAGTCGAGGGGCAGCATTTTAGTTTGCTTGCAAACTAAAG
ATGGCGACCGGCGCACGGGTGAGTAACACGTATCCAACCTGCCGATAACTCGGGGATAGCCTTTCGAA
AGAAAGATTAATATCCGATGGTATATTTTCTCCGCATGGTGAGAATATTAAAGAATTTCGGTTATCGA
TGGGGATGCGTTCCATTAGTTTGTTGGCGGGGTAACGGCCCACCAAGACCACGATGGATAGGGGTTCT
GAGAGGAAGGTCCCCCACATTGGAACTGAGACACGGTC
>Zotu6070
CAACGCTGGCGGCGTGCCTAACACATGCAAGTCGAACGCGGTTACCTCGCAAGGGGTGACTGAGTGGC
GAACGGGTGAGTAACACGTGGGTGACCTACCTTCGAGTGGGGGATAACGTCCCGAAAGGGACGCTAAT

ACCGCATAACATCCTGCCTTTGAATAGGCGGTGATCAAAGCCGGGGATCGTAAGACCTGGCGCTTGAT
GAGGGGCTCGCGTCTGATTAGCTAGTTGGTGGGGTAATGGCTCACCAAGGCGACGATCAGTATCCGGC
CTGAGAGGGCGGACGGACACACTGGGACTGAGACACGGCC
>Zotu6071
GAACGCTGGCGGCGTGCTTAACACATGCAAGTCGAACGGAAAGGCCCTTCGGGGTACTCGAGTGGCGA
ACGGGTGAGTAACACGTGGGTGATCTGCCCTGCACTTTGGGATAAGCCTGGGAAACTGGGTCTAATAC
CGAATAGGACCACGGCATTCATGTGCTGTGGTGGAAAGCTTTTGCGGTGTGGGATGGGCCCGCGGCCT
ATCAGCTTGTTGGTGGGGTAATGGCCTACCAAGGCGACGACGGGTAGCCGGCCTGAGAGGGTGACCGG
CCACACTGGGACTGAGATACGGCC
>Zotu6072
CAACGCTGGCGGCGTGCCTAACACATGCAAGTCGAGCGCGAAAGGGGAGCAATCCCCAAGTAGAGCGG
CGAACGGGTGAGTAACGCGTGGGTAATCTGCCTCCGAGTGGGGAACAACACCGGGAAACTGGTGCTAA
TACCGCATAACATCGTCGGGTTTTCGGATCTGACGATCAAAGCCGGGGACCGCAAGGCCTGGCGCTTG
GAGAGGAGCCCGCGTCCGATTAGCTAGTTGGTGGGGTAATGGCCCACCAAGGCGACGATCGGTAGCCG
GCCTGAGAGGGCGGACGGCCACACTGGGACTGAGACACGGCC
>Zotu6073
GAACGCTAGCGGCAGGCCTAATACATGCAAGTCGAGGGGCAGCGATGTAGCAATACAGTCGGCGACCG
GCGCACGGGTGCGTAACACGTATGCAACCTGCCTTCAACTGGGGGATAGCCCGGGGAAACTCGGATTA
ATACCCCATAACATGGTGAAACAGCATTGTTTTATCATTAAAGCTCCGGCGGTTGAAGATGGGCATGC
GTTCGATTAGCTAGTTGGTGTAGGTAACGGCTCACCAAGGCTACGATCGATAGGGGGTCTGAGAGGAT
GATCCCCCACACTGGTACTGAGATACGGAC
>Zotu6074
GAACGCTGGCGGCATGCTTTATACATGCAAGTCGAACGGCAGCGCGGACTTCGGTCTGGCGGCGAGTG
GCGAACGGGTGAGTAATACATCGGAACGTACCCAGAAGTGGGGGATAACGCAGCGAAAGTTGCGCTAA
TACCGCATACGTTCTACGGAAGAAAGTGGGGGATCGCAAGACCTCATGCTTTTGGAGCGGCCGATGTC
TGATTAGCTAGTTGGTAGGGTAAAGGCCTACCAAGGCGACGATCAGTAGCTGGTCTGAGAGGACGACC
AGCCACACTGGAACTGAGACACGGTC
>Zotu6075
GAACGCTGGCGGCGTGCCTAATACATGCAAGTCGAGCGAACTTTTGAGAGCTTGCTCTCAAAAGTTAG
CGGCGGACGGGTGAGTAACACGTGGGCAACCTACCTATAAGACTGGGATAACTTACGGAAACGTGAGC
TAATACCGGATAATTCTTTCTAACACATGTTAGGAAGTTGAAAGATGGCTTCTCGCTATCACTTATAG
ATGGGCCCGCGGCGCATTAGCTAGTTGGTGGGGTAATGGCCTACTAAGGCAACGATGCGTAGCCGACC
TGAGAGGGTGATCGGCCACACTGGGACTGAGACACGGCC
>Zotu6076
GAATGCTGGCTGTATGGATAAGGCATGCAAGTCGAACGGTCCGCAAGGACAGTGGCAGACGAGGTAGT
AACACGTAAGTACGTACCCTGTAGTCGGGCATAGCTTGTCGAAAGGCAAGGTAATTCCCGATAGTCCC
GCAAGGGTAAAGATTTATCGCTACAGGAGCGGCTTGCGTAATATCAGCTTGTTGGTGAGGTAATGGCT
CACCAAGGCTACGACGTTTAGGGGAGCTGAGAGGCTGACCCCCACCGATGGGACTGAGACACGGCC
>Zotu6080
GAATGCTGGCGGCGTGGATGAGGCATGCAAGTCGAATGGGTTTAGACCCATGGCAGACGAGGTAGTAA
CACGTAGGTACGTACCCCAAAGTCGGGCATAATCCGCCGAAAGGCGGGATAATTCCCGATGGTCCCGC
AAGGGTAAAGATTTATCGCTTTGGGAACGGCCTGCGACGTATCAGCTAGTTGGTGAGGTAATGGCTCA
CCAAGGCGACGACGCGTAGGGGAGCTGAGAGGCTGACCCCCACCGATGGCACTGCGACACGGGC
>Zotu6082
GAACGCTGGCGGCAGGCTTAACACATGCAAGTCGAACGCACCGCAAGGTGAGTGGCAGACGGGTGAGT
AACACGTGGGAACGTACCCTTTTGTTCGGAATAACCCTGGGAAACTAGGGCTAATACCGGATACGCCC
TAACGGGGAAAGGTTTACTGCAGGAGGATCGGCCCGCGTCCGATTAGCTAGTTGGTTGGGGTAATGGC
TCACCAAGGCGACGATCGGTAGCTGGTCTGAGAGGATGGCCAGCCACATTGGGACTGAGACACGGCC
>Zotu6084
GAACGCTGGCGGCGTGCTTAACACATGCAAGTCGAGTGCGCAGTCGTTTGTAGCAATACAAATGATGG
GCGCACGGCGCACGGGTGCGTAACACGTGGGTAATTTGCCCTCGAGTGGGGAATAACCCGCCGAAAGG
CGGGCTAATACCGCATAACACTCCCCGGACTACGGTCTAGGGATTCAAAGCCCGCAAGGGCGCTTGAG
GAGAAACCCGCGGCCGATTAGCTAGTTGGCGAGGTAACGGCTCACCAAGGCAATGATCGGTAGCTGGT
CTGAGAGGATGGCCGGCCACACTGGAACTGAGATACGGTC

>Zotu6086
GAACGCTAGCGGCAGGCTTAACACATGCAAGTCGAGGGGTATTCTAGCAATAGGAGAGACCGGCGCAC
GGGTGCGTAACGCGTATGCAATCTACCTTTCACAGAGGGATAGCCCAGAGAAATTTGGATTAATACCT
CATAGTATAACGAGTTCGCATGGATTTGTTATTAAAGTCACAACGGTGAAAGATGAGCATGCGTCCCA
TTAGCTAGTTGGTAAGGTAACGGCTTACCAAGGCAACGATGGGTAGGGGTCCTGAGAGGGAGATCCCC
CACACTGGTACTGAGACACGGAC
>Zotu6087
GAACGCTGGCGGCGTGCCTAATACATGCAAGTCGAGCGAATTGATGAGAAGCTTGCTTCTCTGAAGTT
AGCGGCGGACGGGTGAGTAACACGTGGGCAACCTGCCCTATAGTTTGGGATAACTCCGGGAAACCGGG
GCTAATACCGAATAATACATTTTAACTCCTGTTAGAATGTTGAAAGATGGTTTACGCTATCGCTATAG
GATGGGCCCGCGGCGCATTAGCTAGTAGGTGAGGTAACGGCTCACCTAGGCGACGATGCGTAGCCGAC
CTGAGAGGGTGATCGGCCACACTGGGACTGAGACACGGCC
>Zotu6088
GAACGCTGGCGGCGTGCTTAACACATGCAAGTCGAGCGGAAAGGCCCTTCGGGGTACTCGAGCGGCGA
ACGGGTGAGTAACACGTGAGTAACCTGCCCCAGACTTTGGGATAACCCCCGGAAACGAGGGCTAATAC
CGAATATTACTTGGTCCCGCATGGGATTTGGTGGAAAGTTTTTCGGTCTGGGATGGACTCGCGGCCTA
TCAGCTTGTTGGTGGGGTAATGGCCTACCAAGGCGAGGACGGGTAGCCGGCCTGAGAGGGCGACCGGC
CACACTGGGACTGAGACACGGCC
>Zotu6089
GAACGCTAGCGGCAGGCCTAACACATGCAAGTCGAGGGGTAGAAGGAGCTTGCTCCTTTGAGACCGGC
GCACGGGTGCGTAACGCGTATGCAACCTACCTTTCACAGGGGAATAGCCCAGAGAAATTTGGATTAAT
GCCCCATGGCACAGCGAGACGGCATCGTCTTGCTGTTAAAGTAACAACGGTGAAAGATGGGCATGCGT
CCCATTAGCTAGTTGGTAAGGTAACGGCTTACCAAGGCTATGATGGGTAGGGGTCCTGAGAGGGAGAT
CCCCCACACTGGTACTGAGACACGGAC
>Zotu6090
GAACGCTGACGGCAGGCTTAACACATGCAAGTCGAGCGGGCCCTTCGGGGTCAGCGGCAGACGGGTGA
GTAACGCGTGGGAACGTACCATTTGCTACGGAATAACTCAGGGAAACTTGTGCTAATACCGTATGAGC
CCCCCTTTAAAATTTCAGGAATCATAAAATGCCCTGGCATTTTATGGGGGGGAAAGATTTATCGGCAA
ATGATCGGCCCGCGTTGGATTAGCTAGTTGGTGGGGTAAAGGCCTACCAAGGCGACGATCCATAGCTG
GTCTGAGAGGATGATCAGCCACACTGGGACTGAGACACGGCC
>Zotu6091
GAACGCTAGCGGCAGGCTTAATACATGCAAGGCGAGGGGGCAGCAATGTCACCGTCGTACGGGTGCGT
AACGCGTATGCAACCTACCTATCACTGGGGGATAGCCCGGGAAACCCGGATTAATACCGCATAATAC
ATATGGTCCACCTGGATTGTTTTGTTAAAGATTTATTGGTGATAGATGGGCATGCGTTCGATTAGCTA
GTTGGTATGGGTAACGGCCTACCAAGGCAACGATCGATAGGGGAGCTGAGAGGTTGATCCCCCACACG
GGCACTGAGATACGGGC
>Zotu6092
GAACGCTGGCGGCGTGCTTAAGACATGCAAGTCGAACGGTTTCTTCGGAAACAGTGGCGCACGGGTGA
GTAACACGTGACTGACCTACCCCGAAGTTCTGAATAACCTGGCGAAAGTCGGGCTAATACAGGATGCG
CAGTATCGCTGTGGCGATACTGCAAAGATTTATCGCTTTGGGATGGGGTTGCGTTCCATCAGCTAGTT
GGTAGGGTAAAGGCCTACCAAGGCGACGACGGATAGCCGGCCTGAGAGGGTGGCCGGCCACAGGGGCA
CTGAGACACGGGC
>Zotu6093
GAACGCTGGCGGCGTGCCTAACACATGCAAGTCGAGCGGGAAAGTGGGGGCAACTCCACGAGTACAGC
GGCGCACGGGTGAGTAACACGTAGGTAACCTACCTACGAGACCGGGATAACCTGCCGAAAGGCGGGCT
AATACCGGATAAGACCACGGGAATCGCGGTTCCTGGGGTAAAAGGTGGCCTCTCCTTGGAAGCTACTA
CTCGAAGATGGGCCTGCGCGCCATTAGCTAGTTGGCGGGGTAATGGCCCACCAAGGCAAAGATGGCTA
GCTGGTCTGAGAGGATGGCCAGCCACACTGGGACTGAGACACGGCC
>Zotu6094
GAACGCTGGCGGCGTGCCTAATACATGCAAGTCGAGCGGTAAGGCCCCTTCGGGGGTACACGAGCGGC
GGACGGCTGAGTAACACGTAGGAACGTACCCCAAAGTGAGGGATAATCTCGAGAAATCGAGACTAATA
CCGCATGTGATCTACGGATTAAAGCGTTTACGCGCTTTGGGAACGGCCTGCGTCCGATTAGCTAGTTG
GTGAGGTAATAGCTCACCAAGGCGACGATCGGTAGCTGGTCTGAGAGGATGATCAGCCAGACTGGGAC
TGAGACACGGCC

>Zotu6095
CAACGCTGGCGGCGTGCCTCAGACATGCAAGTCGAACGATTAAACTTTCCTTCGGGGAAGATATAAAG
TGGCGCACGGGTGAGTAACACGTAAGTAATCTACCTTCGAGTGGGGAATAACGTTCGGAAACGAACGC
TAATACCGCATAATGCAGCGGCATCGCGAGATGACAGTTGTTAAAGATTTATCGCTTGAAGAGGAGCT
TGCGGCAGATTAGCTAGTTGGTAAGGTAACGGCTTACCAAGGCGACGATCTGTATCCGGTCTAAGAGG
ACGGTCGGACACACTGACACTGAATAACGGGT
>Zotu6096
TAACGCTGGCGGCGTGCATGAGACATGCAAGTCGAACGGACGATCAGGGCTTGCTCTGATCGTTAGTG
GCGGACGGCTGAGTAACGCGTAGGTGACCTGCCCCGAAGTGGGGGATAACTGCCCGAAAGGGTGGCTA
ATACCGCATGTGGAGGCTCGTTCATTCGAGCCTTCAAAGCAGCAATGCGCTTCGGGAGGGGCCTGCGT
CCGATTAGCTCGTTGGTGGGGTAACGGCTCACCAAGGCGACGATCGGTAGGTGGTCTGAGAGGACGAT
CACCCAGACTGGGACTGAGACACGGCC
>Zotu6097
GAACGCTGGCGGCGTGCCTAATACATGCAAGTCGAGCGGAGTTACAGGAGCTTGCTCCTGTAACTTAG
CGGCGGACGGGTGAGTAACACGTGGGCAACCTGCCTATAAGACTGGGATAACTTCGGGAAACCGGAGC
TAATACCGGATAATTCTTTTCCTCTCATGGGGAAAAGCTGAAAGACGGTTTACGCTGTCACTTATAGA
TGGGCCCGCGGCGCATTAGCTAGTTGGTGAGGTAACGGCTCACCAAGGCAACGATGCGTAGCCGACCT
GAGAGGGTGATCGGCCACACTGGGACTGAGACACGGCC
>Zotu6098
GAACGCTAGCGGGAGGCCTAACACATGCAAGTCGAGCGGGAATCTCTTTCGGGAGATGAGAGCGGCGC
ACGGGTGAGTAACGCGTATGCAACCTGCCCCACACAGGGGGATAGCCCGGAGAAATCCGGATTAATAC
CCCATGAGATTATGAAGTCGCATGATTTTATGATCAAAGTTCCGGCGGTTTGGGATGGGCATGCGTGA
CATTAGCTTGTTGGTGAGGTAACGGCTCACCAAGGCTGCGATGTCTAGGGGTTCTGAGAGGATGATCC
CCCACACTGGTACTGAGACACGGAC
>Zotu6099
GAACGCTAGCGGCAGGCCTAATACATGCAAGCCGAACGGCAAGATTTTCAGCAATGAGAATCCTAGAG
TGGCGTAAGGGTGCGTAACACGTATGCAATCTACCCTCAACAGGAGGATAGCTCCCCGAAAGGGGAAT
TAAGACTCCATAAGATCACACGAAGGCATCTGAGAGTGATTAAAGTACAAGCGGTTGAGGATGAGCAT
GCGTCTGATTAGTTAGTTGGCGGGGTAACGGCCCACCAAGACGATGATCAGTAGGGGAACTGAGAGGT
TGATCCCCCACACTGGCACTGAGATACGGGC
>Zotu6100
GAACGCTGGTAACGTGGATACCGGCATGCAAGTCACGGGGGCCGCAAGGCAACCGGCGAACGGCTTAG
TAACACGTAGATACATACCCCAAAGTGGAGCATAGCTCGTCGAAAGACGGGGTAATTCTCCATAGCAC
CGCAAGGTTAAAGGCGCAAGCCGCTTTGGGAATGGTCTGCGGTCTATCAGCTTGTTGGCGAGGTAATA
GCTCACCAAGGCTATGACGGATAGGGGGCGTGAGAGCGCGACCCCCAAGAATGGCACTGCGACACGGG
C
>Zotu6101
GAACGCTGGCGGCGTGCCTAATACATGCAAGTCGTGCGGTAACAGGTTCTCACTATTTGCTTCTCTCT
CAATCAGTAAAACGATCGGGACTCAGAGGAGTAAGTTGTGAGAATGCTGACGAGCGGCGGACGGCTGA
GTAACGCGTAGGAACGTGCCCCAAAGTGAGGGATAAGCTCCAGAAATGGAGTCTAATACCGCATATGG
TCTTCGGATTAAAGCTTCGGCGCTTTGGGAGCGGCCTGCGTTTGATTAGATAGTTGGTGAGGTAAAGG
CTCACCAAGTCGACGATCAATAGCTGGTCTGAGAGGATGATCAGCCAGACTGGAACTGAGACACGGTC
>Zotu6102
GAACGCTGGCGGAATGCTTTACACATGCAAGTCGAACGGCAGCACGGGGCAACCTGGTGGCGAGTGGC
GAACGGGTGAGTAATATATCGGAACGTGCCCAATCGTGGGGGATAACGTAGCGAAAGTTACGCTAATA
CCGCATACGATCTACGGATGAAAGCGGGGGATCGCAAGACCTCGCGCGAATGGAGCGGCCGATATCAG
ATTAGGTAGTTGGTGGGGTAAAGGCTCACCAAGCCAACGATCTGTAGCTGGTTTGAGAGGACGACCAG
CCACACTGGAACTGAGACACGGTC
>Zotu6103
GAACGCTGGCGGCATGCTTAACACATGCAAGTCGAACGCGGTGTCCTGGTTCGCCGGGGCACTGAGTG
GCGCACGGGTGAGTAGCGCGTGGGAACCTGCCCAGCAGTGGGGGATAACCGAGGGAAACCTCGGCTAA
TACCGCATGCGCCGGAGGTAACTCCGGGAAAGATTTATCGCTGTTGGAGGGGCCCGCGTCGGATTAGC
TAGTTGGTGGGGTAACGGCCCACCAAGGCGATGATCCGTAGCTGGTCTGAGAGGATGATCAGCCACAC
TGGGACTGAGACACGGCC

>Zotu6104
GAACGCTGGCGGCATGCCTAACACATGCAAGTCGAACGAAGGCTTCGGCCTTAGTGGCGCACGGGTGC
GTAACGCGTGGGAATCTGCCCTCAGGTACGGAATAACTCAGGGAAACTTGAGCTAATACCGTATAATG
ACTTCGGTCCAAAGATTTATCGCCTGAGGATGAGCCCGCGTCGGATTAGCTAGTTGGTGAGGTAAAGG
CTCACCAAGGCGACGATCCGTAGCTGGTCTGAGAGGATGATCAGCCACACTGGGACTGAGACACGGCC
>Zotu6105
GAACGCTGGCGGCGTGCCTAATACATGCAAGTCGAGCGAACAAGGAAGAAACTTGTTTCTTCCTTTGT
TAGCGGCGGATGGGTGAGTAACACGTGGGCAACCTGCCCTGCAGTTGGGGATAACTCCGGGAAACCGG
GGCTAATACCGAATAATCAGTTCCTTCGCATGAAGGAACTCTGAAAGACGGCTATGCTGTCACTGCAG
GATGGGCCCGCGGCGCATTAGCTAGTTGGTGGGGTAACGGCTTACCAAGGCGACGATGCGTAGCCGAC
CTGAGAGGGTGATCGGCCACACTGGGACTGAGACACGGCC
>Zotu6106
TAACGCTGGCGGCGTGCATAACACATGCAAGTCGAACGATGAAACTCATTAGACTTCTTCGGAATGAC
AATGAATGGATTAGTGGCGAACGGGTGAGTAACGCGTGAGTAACCTGCCTTACACAAGGGGATAGCCT
CTGGAAACGGAGAATAATACCCTATAACATTACAACTTCGCATGAAGTAGTAATCAAAGTGTTAGCGG
TGTAAGATGGACTTGCGTCTGATTAGCTAGTTGGTGAGATAACAGCCCACCAAGGCAACGATCAGTAG
CCGGCTTGAGAGAGTGTACGGCCACATTGGGACTGAGACACGGCC
>Zotu6107
GAACGCTGGCGGCATGCCTAACACATGCAAGTCGAACGGCATCACGTACTAGTTTACTAGTATGGTGG
CGAGTGGCGCACGGGTGAGTAACGCGTAGGAATATGCCTTAAAGAGGGGGACAACCTGGGGAAACTCA
GGCTAATACCGCATACGCTCTAAGGAGGAAAGCTGGGGACCTTCGGGCCTGGCGCTTTAAGAATAGCC
TGCGTACGATTAGCTAGTTGGTGGGGTAAAGGCCTACCAAGGCGACGATCGTTAGCTGGTCTGAGAGG
ATGGCCAGCCACACTGGGACTGAGACACGGCC
>Zotu6109
GAACGCTGGCGGCGTGCTTAACACATGCAAGTCGAACGGAAAGGCCCCTTCGGGGGTACTCGAGTGGC
GAACGGGTGAGTAACACGTGGGTGATCTGCCCTGCACTTCGGGATAAGCTTGGGAAACTGGGTCTAAT
ACCGGATATACCTCATGGATCGCATGGTTTGTGGGGGAAAGCTTTTGCGGTGTGGGATGGGCCCGCGG
CCTATCAGCTTGTTGGTGGGGTGATGGCCTACCAAGGCGACGACGGGTAGCCGGCCTGAGAGGGTGTC
CGGCCACACTGGGACTGAGATACGGCC
>Zotu6110
GAACGCTAGCGGCAGGCTTAACACATGCAAGTCGAGGGGTAGAGTAGCAATACTTGAGACCGGCGCAC
GGGTGCGTAACGCGTATGCAACCTACCTTTTACAGGGGAATAGCCCAGAGAAATTTGGATTAATGCCC
CATGGTATATTTTAGTGGCATCACTGGATTATTAAAGTTCCAACGGTAAAAGATGGGCATGCGTCCTA
TTAGCTAGATGGTAAGGTAACGGCTTACCATGGCTACGATAGGTAGGGGTCCTGAGAGGGAGATCCCC
CACACTGGTACTGAGACACGGAC
>Zotu6112
GAACGCTGGCGGCATGCCTAACACATGCAAGTCGAACGAGACCTTCGGGTCTAGTGGCGCACGGGTGC
GTAACGCGTGGGAATCTGCCCTTGGGTTCGGGATAACAGTTGGAAACGACTGCTAATACCGGATGATG
ACTTCGGTCCAAAGATTTATCGCCCAGGGATGAGCCCGCGTCGGATTAGCTAGTTGGTGAGGTAAAGG
CTCACCAAGGCGACGATCCGTAGCTGGTCTGAGAGGATGATCAGCCACACTGGGACTGAGACACGGCC
>Zotu6113
GAACGCTGGCGGCAGGCTTAACACATGCAAGTCGAACGGTAACATAAAGAAGCTTGCTTCTTTGATGA
CGAGTGGCGGACGGGTGAGTAATGCTTGGGAATCTGGCTTATGGAGGGGGATAACTACGGGAAACTGT
AGCTAATACCGCGTAGAATCGGGAGATGAAAGTGTGGGACCTTCGGGCCACATGCCATAGGATGAGCC
CAAGTGGGATTAGGTAGTTGGTGAGGTAAAGGCTCACCAAGCCGACGATCTCTAGCTGGTCTGAGAGG
ATGACCAGCCACACTGGGACTGAGACACGGCC
>Zotu6114
GAACGCTGGCGGCAGGCCTAACACATGCAAGTCGAGCGCGTGTAGCAATACACGAGCGGCGCACGGGT
GAGTAACGCGTGGGAATCTGCCCTACAGTGGGGAACAACCACGGGAAACTGTGGCTAATACCGCATAC
GTCCTTCGGGAGAAAGGCTTAGGCCGCTGTAGGAGGAGCCCGCGTCCGATTAGCTAGTTGGTGAGGTA
ACGGCTCACCAAGGCGACGATCGGTAACTGGTCTGAGAGGACGACCAGTCACACTGGGACTGAGACAC
GGCC
>Zotu6115
GAACGCTAGCGGCAGGCTTAATACATGCAAGTCGTGGGGCAGCATGGTGTAGCAATACACTGATGGCG

ACCGGCAAACGGGTGCGGAACACGTACGCAACCTTCCTTTAACTGGAGAATAGCCCGGGGAAACCCGG
ATTAATACTCCGTAACATTATGAAGTGGCATCACTTTATAATTATAGCTCCGGCGGTTGAAGATGGGC
GTGCGTCTGATTAGGTAGTTGGCGGGGTAACGGCCCACCAAGCCTACGATCAGTAACTGGTGTGAGAG
CACGACCAGTCACACGGGCACTGAGACACGGGC
>Zotu6116
GAACGCTGGCGGCATGCTTAACACATGCAAGTCGAACGAGCTAATGGGTAACTATTAGTGCAGTGGCG
GACGGGTGCGTAACGCGTAAGAACCTACCTTTTGGAGGGGGATAACAGCGGGAAACTGTTGCTAATAC
CCCATAATGCTGAGGAGTTAAAACAGTTTTTACTGGCCAAAAGAGGGGCTTGCGTCTGATTAGCTTGT
TGGTGGGGTAAAAGCCTCCCAAGGCCACGATCAGTAGCTGGTCTGAGAGGATGATCAGCCACACTGG
GACTGAGACACGGCC
>Zotu6117
GAACGCTGGCGGCAGGCTTAACACATGCAAGTCGAGCGGGCCTCTTCGGGGGTCAGCGGCGGACGGGT
TAGTAACGCGTGGGAACGTGCCTTCTGCTTCGGAATAGCCCTGGGAAACCGGGGGTAATACCGGATGT
GCCCTGAGGGGGAAAGATTTATCGGCAGGAGATCGGCCCGCGTTGGATTAGGTAGTTGGTGGGGTAAC
GGCCCACCAAGCCGACGATCCATAGCTGGTTTGAGAGGATGATCAGCAACACTGGGACTGAGACACGG
CC
>Zotu6118
GAACGCTGGCGGCGTGCTTAACACATGCAAGTCGAACGGAAAGGCCCCTTCGGGGGTACTCGAGTGGC
GAACGGGTGAGTAACACGTGGGCAACCTGCCCCTAGCTCTGGGATAACACTTGGAAACAGGTGCTAAT
ACCGGATAACAACCCTTCACGGCATCGCGATGGGTTCAAAGTTTTTCGGCTAGGGATGGGCCCGCGGC
CTATCAGCTTGTTGGTGGGGTAATGGCCTACCAAGGCGACGACGGGTAGCCGGCCTGAGAGGGCGACC
GGCCACACTGGGACTGAGACACGGCC
>Zotu6119
CAACGCTGGCGGCGTGCCTAACACATGCAAGTCGCACGAGAAAGTGGGGGCAACTCCGTGAGTAAAGT
GGCGCACGGGTGAGTAACACGTGACTAACCTACCTTCGAATGGGGAATAACCTCGGGAAACCGGGACT
AATACCGCATAATATCCGCAGTTGAGATGCTACGGATCAAACGCGCAAGCGGTTTGAAGAGGGGGTCG
CGGCTGATTAGCTAGTTGGTGAGGTAATGGCCCACCAAGGCAATGATCAGTATCCGGCCTGAGAGGGC
GCACGGACACACTGGAACTGAAACACGGTC
>Zotu6120
GAACGCTAGCGGCAGGCCTAACACATGCAAGTCGAGGGGTAGAAGTCTTCGGACTTTGAGACCGGCGC
ACGGGTGCGTAACGCGTATGCAATCTACCTTGTACAGAGGGATAGCCCAGAGAAATTTGGATTAATAC
CTCATAGTATTATTGAGTGGCATCACTTGATTATTAAAGTTCCAACGGTACAAGATGAGCATGCGTCC
CATTAGCTAGTTGGTATGGTAACGGCATACCAAGGCAATGATGGGTAGGGGTCCTGAGAGGGAGATCC
CCCACACTGGTACTGAGACACGGAC
>Zotu6121
GAACGCTGGCGGCAGGCTTAACACATGCAAGTCGAACGGTAACATAAAGAAGCTTGCTTCTTTGATGA
CGAGTGGCGGACGGGTGAGTAATGCTTGGGAATCTAGCTTATGGAGGGGGATAACTACGGGAAACTGT
AGCTAATACCGCGTAGAATCGGGAGATGAAAGTGTGGGACCTTCGGGCCACATGCCATAGGATGAGCC
CAAGTGGGATTAGGTAGTTGGTGAGGTAAAGGCTCACCAAGCCGACGATCTCTAGCTGGTCTGAGAGG
ATGACCAGCCACACTGGGACTGAGACACGGCC
>Zotu6124
GAACGCTGGCGGCGTGCCTAATACATGCAAGTCGAGCGGACCTTTGGGAGCTTGCTCCCAAAGGTTAG
CGGCGGACGGGTGAGTAACACGTGGGCAACCTGCCTGTAAGACTGGGATAACTTCGGGAAACCGGAGC
TAATACCGGATAACTTCTTTTCTCGCATGAGGAGAGGTTGAAAGATGGCTTCGGCTATCACTTACAGA
TGGGCCCGCGGCGCATTAGCTAGTTGGTGAGGTAACGGCTCACCAAGGCAACGATGCGTAGCCGACCT
GAGAGGGTGATCGGCCACACTGGGACTGAGACACGGCC
>Zotu6125
AAACGTTGGCGGTGCGTCTTAGACATGCAAGTCGAGCGTGAAAGCCCGCAAGGGTGAGTAAAGCGGCG
AACGGGTGAGTAATACTTGGGTAACGTACCTCCTAGATCGGGATAACGCGCCGAAAGGCGTGCTAATA
CCGGATGTGACCACGGTTCCTACGGGAGCAGGGGTAAAAGATGGCGCAAGCTATCACTGGGAGATCGG
CTCAAGCACCATTAGCTAGTTGGCGGGGTAACGGCCCACCAAGGCTACGATGGTTAGCCGGCCTGAGA
GGGTGATCGGCCACACTGGGACTGAGATACGGCC
>Zotu6126
GAACGTTGGCGGCGTGCCTTAGGCATGCAAATCAAGGGGATCCGCCGCAAGGCGGGTAACCGGTGGAC

GGGTGAGTAATGCACAGGAATCTACCTAGAAGTGGGATATAGCTAGCCGAAAGGTTGGGTAATCTCCC
ATGTGAACGTAAGTTTAAAGCCGCAAGGCGCTTTTAGATGAGCCTGTGTCCTATCAGCTTGTTGGTGG
GGTAATGGCCCACCAAGGCGACGACGGGTATCTGGACTGAGAGGTCGGCCAGACACAATGGGACTGCG
ACACGGCC
>Zotu6127
TAACGCTGGCGGCGTGCCTAACACATGCAAGTCGAACGGCCCAGCACCTTTTGCAGCATCCAGTGTCG
CAGCGATATTTCGTTGCGCTTTGGTTTATGATGGTACAAAAGGTGCTGGGTAGTGGCGAACGGGTGAG
TAACACGTTGGTACCTGCCCTCTAGTGGGGAATAATTCGCCGAAAGGCGGACTAATACCGCATGTGAT
CTACGGATCAAAGCAGCAATGCGCTAGAGGAGGGGCCTGCGGCTGATTAGCTTGTTGGTAGGGTAATG
GCCTACCAAGGCGACGATCAGTAGCTGGATCGAGAGGTTGACCAGCCACACTGGGACTGAGATACGGC
C
>Zotu6128
GAACGCTGGCGGCGTGCTTAACACATGCAAGTCGAACGATGAAGCCGGAGCTTGCTCCGGCGGATTAG
TGGCGGACGGGTGAGTAACACGTGAGTAACCTGCCCCAGACTCTGGGATAAGCACTGGAAACGGTGTC
TAATACCGGATACGAGCTTCAGCCGCATGGCTAGGAGCTGGAAAGAATTTCGGTCTGGGATGGACTCG
CGGCCTATCAGGTAGTTGGTGAGGTAAAGGCTCACCAAGCCGACGACGGGTAGCCGGCCTGAGAGGGT
GACCGGCCACACTGGGACTGAGACACGGCC
>Zotu6129
GAACGCTAGCGGCAGGCCTAATACATGCAAGTCGAACGGTAACAGGGTAGCAATACCGCTGACGAGTG
GCGCACGGGTGCGTAACGCGTATGCAATCTACCTTTAACTAGTGAATAGCTCGAAGAAATTCGAATTA
ATACACCATAGTATTATTAATTGGCATCAATTGATAATTAAAGCTCCGGCGGTTAAAGATGAGCATGC
GTGACATTAGCTAGTTGGTGAGGTAACGGCTCACCAAGGCTACGATGTCTAGGGGATCTGAGAGGATT
AACCCCCACACTGGTACTGAGACACGGAC
>Zotu6130
GAACGCTGGCTGCATGCTTGACACATGCAAGTTGAACGAAGACAGGATTTTACTTGTAAAACTCTGTA
CTTAGTAGCGGACGGGTGAGTAACGCGTAAGAATCTACCTTTAGGAACAGAATAACAGCTGGAAACGG
CTGCTAATACTGTATATGCTGAGAAGTTAAATGGGTATCCGCCTAAAGATGAGCTTGCGTCTGATTAG
CTAGTTGGGGGGGTAAAGGCTTCCCAAGGCGACGATCAGTAACTGGTCTGAGAGGATGATCAGTCACA
CTGGGACTGAGACACGGCC
>Zotu6131
GAACGCTGGCGGCAGGCTTAACACATGCAAGTCGAGCGCCCCGCAAGGGGAGCGGCAGACGGGTGAGT
AACGCGTGGGAACGTACCTTTTGGTTCGGAACAACTCCGGGAAACTGGAGCTAATACCGGATAAGCCC
TAACGGGGAAAGATTTATCGCCGAAAGATCGGCCCGCGTCTGATTAGCTAGTTGGTGAGGTAACGGCT
CACCAAGGCGACGATCAGTAGCTGGTCTGAGAGGATGATCAGCCACACTGGGACTGAGACACGGCC
>Zotu6132
GAACGCTGGCGGCGTGCTTAACACATGCAAGTCGAACGAAGAGCGATGGAAGCTTGCTTCTATCAATC
TTAGTGGCGAACGGGTGAGTAACGCGTAATCAACCTGCCCTTCAGAGGGGGACAACAGTTGGAAACGA
CTGCTAATACCGCATACGATCTGACCTCGGCATCGAGGATGGATGAAAGGTGGCCTCTATTTATAAGC
TATCACTGAAGGAGGGGATTGCGTCTGATTAGCTAGTTGGAGGGGTAACGACCCACCAAGGCGATGAT
CAGTAGCCGGTCTGAGAGGATGAACGGCCACATTGGGACTGAGACACGGCC
>Zotu6133
GAACGCTGGCGGCGTGCTTAACACATGCAAGTCGAACGAAGCACTTTTACAGATTTCTTCGGAATGAA
GTTTTAGTGACTGAGTGGCGGACGGGTGAGTAACGCGTGGGTAACCTGCCTCACACAGGGGGATAACA
GTTGGAAACGGCTGCTAATACCGCATAAGCGCACAGTACCGCATGGTACAGTGTGAAAAACTCTGGTG
GTGTGAGATGGACCCGCGTCTGATTAGCTAGTTGGCAGGGTAACGGCCTACCAAGGCGACGATCAGTA
GCCGACCTGAGAGGGTGACCGGCCACATTGGGACTGAGACACGGCC
>Zotu6134
GAACGCTGGCGGCAGGCTTAACACATGCAAGTCGAGCGGTAGCACAGGGGAGCTTGCTCCCTGGGTGA
CGAGCGGCGGACGGGTGAGTAATGTCTGGGAAACTGCCTGATGGAGGGGGATAACTACTGGAAACGGT
AGCTAATACCGCATAACGTCGCAAGACCAAAGAGGGGGACCTTCGGGCCTCTTGCCATCGGATGTGCC
CAGATGGGATTAGCTAGTAGGTGGGGTAACGGCTCACCTAGGCGACGATCCCTAGCTGGTCTGAGAGG
ATGACCAGCCACACTGGAACTGAGACACGGTC
>Zotu6135
GAACGCTGGCGGCGCGCCTAACACATGCAAGTCGAGCGAGAACCAGGGCTTGCCCTGGGGACAGCGGC

GAACGGGTGCGTAACACGTGAGCAACCTGCCCCAAAGACCGGGACAACCCGAGGAAACTCGGACTAAT
ACCGGATGCCCTCACCGGGTCGCATGGTCTGGTGAGGAAATGGATTCCGCTTTGGGATGGGCTCGCGG
TCTATCAGCTTGTTGGTGGGGTAACGGCCTACCAAGGCGACGACGGATAGCTGGTCTGAGAGGACGAT
CAGCCACACTGGGACTGAGACACGGCC
>Zotu6136
GAACGCTGGCGGCATGCCTAACACATGCAAGTCGAACGAGACCTTCGGGTCTAGTGGCGCACGGGTGC
GTAACGCGTGGGAACCTGCCCTTAGGTTCGGAATAACTCCTCGAAAGGGGTGCTAATACCGGATAATG
TCTTCGGACCAAAGATTTATCGCCTTTGGATGGGCCCGCGTTGGATTAGCTTGTTGGTGGGGTAAAGG
CCTACCAAGGCGACGATCCATAGCTGGTCTTAGAGGATGATCAGCCACACTGGGACTGAGACACGGCC
>Zotu6137
GAACGCTGGCGGCGTGCTTAACACATGCAAGTCGAACGATGACGGTGGTGCTTGCATCACCTGATTAG
TGGCGAACGGGTGAGTAACACGTGAGTAACCTGCCCTTCACTTCGGGATAACCTCGGGAAATCGTGGC
TAATACCGGATATGAGCTCCTTCCGCATGGCGGGGGTTGGAAAGATTTATCGGCGAAGGATGGACTCG
CGGCCTATCAGTTTGTTGGTGAGGTAATGGCTCACCAAGGCGATGACGGGTAGCCGGCCTGAGAGGGC
GACCGGCCACACTGGGACTGAGACACGGCC
>Zotu6138
GAACGCTGGCGGCGTGCCTAATACATGCAAGTCGAGCGAACGGACGAGAGTGCTTGCACTCTCTGATG
TTAGCGGCGGACGGGTGAGTAACACGTGGGTAACCTACCTATAAGACTGGGATAACTTCGGGAAACCG
GAGCTAATACCGGATAATATTTAGCTTCGCATGAAGCAATAGTGAAAGACGGTTCTGCTGTCACTTAT
AGATGGACCCGCGGTGTATTAGCTAGTTGGTGAGGTAACGGCTCACCAAGGCAACGATACATAGCCGA
CCTGAGAGGGTGATCGGCCACACTGGGACTGAGACACGGCC
>Zotu6139
GAACACTGGCGGCATGCCTAACACATGCAAGTCGAACGAAATCTTCGGATCTAGTGGCGCACGGGTGC
GTAACGCGTGGGAATCTGCCTTGGGGTTCGGAATAACTCCCCGAAAGGGGTGCTAATACCGGATGATG
TCGAAAGACCAAAGATTTATCGCCCTGAGATAAGCCCGCGTAGGATTAGCTAGTTGGTGTGGTAAAGG
CGCACCAAGGCGACGATCCTTAGCTGGTCTGAGAGGATGATCAGCCACACTGGGACTGAGACACGGCC
>Zotu6140
GAACGCTAGCGATAGGCTTAACACATGCAAGTCGAGGGGCAGCAGGGACTTCGGTCTGCTGGCGACCG
GCGCACGGGTGCGTAACGCGTATGCAACTTGCCTTACAGAGGGGGATAACCCGTTGAAAGACGGACTA
ATACCGCGTACACTCGTTTATGGGCATCTATAGACGAGGAAAGAGATTCGCTGTAAGATAGGCATGCG
TCCCATTAGCTAGTTGGTGAGGTAACGGCTCACCAAGGCAACGATGGGTAGGGGAACTGAGAGGTTGA
ACCCCCACACTGGTACTGAGACACGGAC
>Zotu6141
GAACGCTGGCGGCGTGCCTAATACATGCAAGTCGAACGAGAAAAGGGCTTCGGCTCCTAGTAAAGTGG
CAGACGGGTGAGTAATACATAGGTAACCTACCTCCAAGATGGGGATAACCTTCCGAAAGGAGGGCTAA
TACCGAATGAGACCACAGTCACATATGTGACCGAGGTCAAAGGTGGCCTCTACTTGTAAGCTATCGCT
TGGAGATGGGCTTATGCACCATCAGCTAGTTGGCGAGGTAACGGCTCACCAAGGCTTTGACGGTTAGC
TGATCTGAGAGGTTGATCAGCCACACTGGAACTGAGACACGGTC
>Zotu6142
GAACGCTGGCGGCGTGCTTAATACATGCAAGTCGAACGTGATTGTAGCGATACATGAAAGTGGCAAAC
GGGTGAGTAACACGTAGGAACCTTCCTTGAAGTGGGGGATAACCATTGGAAACGGTGGCTAATACCGC
ATAGTTTCACTCAAATGGGAATAGAGTGAATAAAGATTTATTGCTTTAAGAGGGGCCTGCGGTCGATT
AGCTAGTTGGTGAGGTAAAGGCTCACCAAGGCAGTGATCGATAGCTGGTCTGAGAGGATGATCAGCCA
CACGGGAACTGAGAAACGGTC
>Zotu6143
GAACGCTGGCGGCATGCCTTACACATGCAAGTCGAACGGTAACAGGTTAAGCTGACGAGTGGCGAACG
GGTGAGTAATATATCGGAACGTGCCCAGTCGTGGGGGATAACGTAGCGAAAGCTACGCTAATACCGCA
TACGATCTATGGATGAAAGCGGGGGATCGCAAGACCTCGCGCGATTGGAGCGGCCGATATCAGATTAG
GTAGTTGGTGGGGTAAAGGCCCACCAAGCCGACGATCTGTAGCTGGTCTGAGAGGACGACCAGCCACA
CTGGGACTGAGACACGGCC
>Zotu6144
GAACGCTGGCGGCGCGCTTAACACATGCAAGTCGAGCGAGAACCGGAGCTTCGGCTCCGGGGACAGCG
GCGAACGGGTGAGTAACACGTGGGTAATCTACCCTCGGTTCTGGGATAGCCCGGGGAAACTCGGATTA
ATACCGGATAGCCTCACCGGCTCTTGGGCTGGTGAGAAAAGGTAGCTTCGGCCTCCGACTGAGGATGA

GCCCGCGGCGGATTAGCTTGTTGGCGGGGTAACGGCCCACCAAGGCGACGATCCGTAGCTGGTCTGAG
AGGACGATCAGCCACACTGGGACTGAGACACGGCC
>Zotu6145
GAACGCTGGCGGCAGGCCTAAAACATGCAAGTCGAACGAACCTTCGGGTTAGTGGCGCACGGGTGCGT
AACGCGTGGGAATATGCCTCTTGGTGCGGAACAACGTCTGGAAACGGACGCTAATACCGCATAATATC
TACGGATCAAAGATTTATCGCCAAGAGATTAGCCCGCGTCTGATTAGCTAGTTGGTGAGGTAATGGCT
CACCAAGGTGACGATCAGTAGCTGGTCTGAGAGGATGATCAGCCACACTGGAACTGAGACACGGTC
>Zotu6147
GAACGCTGGCGGCATGCTTTACACATGCAAGTCGAACGGCAGCACGGGCTTCGGCCTGGTGGCGAGTG
GCGAACGGGTGAGTAATACATCGGAACGTACCCAGAAGTGGGGGATAACGCAGCGAAAGTTGCGCTAA
TACCGCATACGTTCTACGGAAGAAAGTGGGGGATCGCAAGACCTCATGCTTTTGGAGCGGCCGATGTC
TGATTAGCTAGTTGGTAGGGTAAAGGCCTACCAAGGCGACGATCAGTAGCTGGTCTGAGAGGACGACC
AGCCACACTGGAACTGAGACACGGTC
>Zotu6148
GAACGCTGGCGGCAGGCTTAACACATGCAAGTCGAGCGGGCATCCTTGTGGTGTCAGCGGCAGACGGG
TGAGTAACGCGTGGGAACGTGCCCTTCGGTTCGGAATAACTCAGGGAAACTTGAGCTAATACCGGATA
CGCCCTTTTGGGGAAAGGCTTGACTGCCGAAGGATCGGCCCGCGTCTGATTAGCTAGTTGGTGAGGTT
ACGGCTCACCAAGGCGACGATCAGTAGCTAGTCTGAGAGGATGATCAGCCACACTGGGACTGAGACAC
GGCC
>Zotu6149
GAACGCTGGCGGCATGCCTTACACATGCAAGTCGAACGCGAAAGTCCTTCGGGGCGAGTAGAGTGGCA
AACGGGTGAGTAACGCGTGGGAACCTTCCTTACAGTATGGAATAGCTCCGGGAAACTGGAATTAATAC
CGTATATTCCCTTCGGGGGAAAGATTTATCGCTGTAAGATGGGCCCGCGTCAGATTAGCTAGTTGGTG
GGGTAATAGCCTACCAAGGCAACGATCTGTAGCTGGTCTGAGAGGACGATCAGCCACACTGGGACTGA
GACACGGCC
>Zotu6152
GAACGCTGGCGGCATGCTTTACACATGCAAGTCGGACGGCAGCACGGGTGCTTGCACCTGGTGGCGAG
TGGCGAACGGGTGAGTAATGCATTGGAACGTACCGAGTAGTGGGGGATAACTGTCCGAAAGGATAGCT
AATACCGCATTCGACCTACGGGTGAAAGCAGGGGACCTTAGGGCCTTGCGCTATTTGAGCGGCCAATG
TCTGATTAGCTAGTTGGTGGGGTAAAGGCCTACCAAGGCGACGATCAGTAGCGGGTCTGAGAGGATGA
TCCGCCACACTGGGACTGAGACACGGCC
>Zotu6153
GAACGCTGGCGGCGTGCTTCATACATGCAAGTCGAACGATAAGGTCTCTTCGGAGATACATAAGTGGC
GGACGGGTGAGTAATATGTAGGAAATCTGCCCTAGAGAAGGGGACAACAGAGGGAAACTTCTGCTAAT
ACCCTATATGAGCGTAGTTGAAATACTATTCTTGAAATGAATTTCGCTCTAGGATGAGCCTGCATCTG
ATTAGTTTGTTGGCGGTGTAATGGACCACCAAGGCGACGATCAGTAGCTGGTTTGAGAGGATGATCAG
CCACAATGGGACTGAGACACGGCC
>Zotu6154
GAACGCTGGCGGCGTGCCTAATGCATGCAAGTCGCACGCAGTGCCTTCGGGCAGTGAGTGGCGCACGG
CTGAGTAGCACGTGGGAACATGCCCTCCAGTGGGGGATAACACCGCGAAAGCGGTGCTAAGCCCGCAT
ACGCTCGGGTGACCGAGGAAAGCCGTAAGGCGCTGGTGGAGTGGCCCGCGGCCCATCAGGTAGTTGGG
GGGGTAACGGCCTCCCAAGCCGATGACGGGTAGCTGGTCTGTGAGGACAACCAGCCAGACTGGGACTG
AGACACGGCC
>Zotu6155
GAACGCTGGCGGCGTGCCTAATACATGCAAGTCGAGCGAATCATTAGGAGCTTGCTCCTAATGATTAG
CGGCGGACGGGTGAGTAACACGTGGGCAACCTGCCTATAAGACTGGGATAACTTCGGGAAACCGGAGC
TAATACCGGATAATCCTTTTCCTCTCATGAGGAAAAGCTGAAAGACGGTTTTTAGCTGTCACTTATAG
ATGGGCCCGCGGCGCATTAGCTAGTTGGTGAGGTAATGGCTCACCAAGGCAACGATGCGTAGCCGACC
TGAGAGGGTGATCGGCCACACTGGGACTGAGACACGGCC
>Zotu6156
GAACGCTGGCGGCGTGCTTAACACATGCAAGTCGAGCGGTAAGGCCCTTCGGGGTACACGAGCGGCGA
ACGGGTGAGTAACACGTGAGCAACCTGCCCCTGACTCCGGGATAAGCGGTGGAAACGCCGTCTAATAC
CGGATATGACACACCACCTCATGGTGGTGTGTGGAAAGTTTTTCGGTCAGGGATGGCTCGCGGCCTA
TCAGCTTGTTGGTGGGGTAACGGCCTACCAAGGCGATTACGGGTAGCCGGCCTGAGAGGGCGACCGGC

CACACTGGGACTGAGACACGGCC
>Zotu6160
GAACGCTGGCGGCAGGCCTAATACATGCAAGTCGAACGCCCCGCAAGGGGAGTGGCGCACGGGTGAGT
AACGCGTGGGAATGTGCCCTTAGGTGCGGAACAACGTTTGGAAACGAACGCTAATACCGCATAATATC
TCCGGATCAAAGATTTATCGCCTAGGGATCAGCCCGCGTTGGATTAGCTAGTTGGTGGGGTAACGGCC
TACCAAGGCGACGATCCATAGCTGGTCTGAGAGGATGATCAGCCACACTGGGACTGAGACACGGCC
>Zotu6161
GAACGCTGGCGGCATGCCTAACACATGCAAGTCGAACGAGACCTTCGGGTCTAGTGGCGCACGGGTGC
GTAACGCGTGGGAATCTGCCCTTGGGTTCGGAATAACTCAGAGAAATTTGAGCTAATACCGGATGATG
ACGTAAGTCCAAAGATTTATCGCCCAGGGATGAGCCCGCGTAAGATTAGGTAGTTGGTGGGGTAAAGG
CCTACCAAGCCGACGATCTTTAGCTGGTCTGAGAGGATGATCAGCCACACTGGGACTGAGACACGGCC
>Zotu6163
GAACGCTGGCGGCATGCCTAACACATGCAAGTCGAACGAGACCTTCGGGTCTAGTGGCGCACGGGTGC
GTAACGCGTGGGAATCTGCCCTTGGGTTCGGAATAACTCAGAGAAATTTGAGCTAATACCGGATGATG
TCGAAAGACCAAAGATTTATCGCCTGAGGATGAGCCCGCGTTGGATTAGCTAGTTGGTGGGGTAAAGG
CCTACCAAGGCGACGATCCATAGCTGGTCTGAGAGGATGATCAGCCACACTGGGACTGAGACACGGCC
>Zotu6166
GAACGCTGGTAACGTGGATACCGGCATGCAAGTCACGGGGGCCGCAAGGCAACCGGCGAACGGCTTAG
TAACACGTGGGTACATACCCCAAAGCGGAGCACAGCTCGTCGAAAGACGGGGTAATTCTCCATAATAT
CGCAAGATTAAAGCAGCAATGCGCTTTGGGAATGGCCTGCGGCCTATCAGATAGTTGGCGAGGTAAAG
GCTCACCAAGTCTATGACGGGTAGGGGGCGTGAGAGCGCGACCCCCAAGAATGGCACTGCGACACGGG
C
>Zotu6167
GAACGCTGGCGGCGTGCTTAACACATGCAAGTCGAACGATGAAGCCGGAGCTTGCTCCGGTGGATTAG
TGGCGAACGGGTGAGTAACACGTGAGCAATCTGCCCTTGACTCTGGGATAACTGCGGGAAACTGTAGC
TAATACCGGATACGACACCTTGGGGCATCCCAGGGTGTGGAAAGAATTTCGGTCAAGGATGAGCTCGC
GGCCTATCAGCTAGTTGGTGAGGTAATGGCTCACCAAGGCGACGACGGGTAGCCGGCCTGAGAGGGTG
ACCGGCCACACTGGAACTGAGACACGGTC
>Zotu6169
GAACGCTGGCGGCATGCCTAACACATGCAAGTCGAACGGGTTTCGGCAATTCATCGTCGAGACTAGTG
GCAAACGGGTGAGTAATACGTGGACAATCTTCCTTTAAGAGGGGGTGAACCCCGGGAAACCGGGGCTA
ATACCGCGTGAGACCACGTCCTCGCATGAGGATGAGGTTAAAGCTCCGCAAGGGGCGCTTAAAGAGGA
GTCCACGGCCTATCAGCTAGTTGGCGGGGTAACGGCCCACCAAGGCAACGACGGGTAGCCGATCTGAA
AGGACGACCGGCCACAATGGCACTGAGACACGGGC
>Zotu6170
GAACGCTGGCGGCGTGCCTAATACATGCAAGTCGTGCGGACTTATCTTCGGATAAGTTAGCGGCGGAC
GGGTGAGTAACACGTGGGCAACCTGCCTGTAAGACTGGGATAACTCCGGGAAACCGGTGCTAATACCA
GATACAATCAAAGACTGCCTGGTTTTTGATAGAAAGGTCTTGTACCGCTTACAGATGGGCCCGCGGCG
CATTAGCTAGTTGGTGGGGTAACGGCTCACCAAGGCGACGATGCGTAGCCGACCTGAGAGGGTGATCG
GCCACACTGGGACTGAGACACGGCC
>Zotu6171
GAACGCTGGCGAGATGCTTTACACATGCAAGTCGTACGAGAGTTTCAAACTCAAGTGGCGGACGGGTG
AGTAACACGTAAGAATTTGCCTTTAGGAGGGGGACAACAACTGGAAACGGTTGCTAATACCCCATATG
CTTTCGAGTGAAACGGATTTTTCCGCCTAAAGAGAAGCTTGCGGCTGATTAGCTTGTTGGTGAGGTAA
TGGCTCACCAAGGCGACGATCAGTATCTGGTTTGAGAGGACGATCAGACACACTGGAACTGAGACACG
GTC
>Zotu6172
GAACGCTGGCGGCAGGCCTAACACATGCAAGTCGAGCGGCCGTAGCAATACGGCAGCGGCAGACGGGA
GAGTAACACGTGGGAACGCGCCCTTTGGTTCGGAATAACTCAGGGAAACTTGAGCTAATACCGGATAA
GCCCTTACGGGGAAAGATTTATCGCCAAAGGAACGGCCCGCGTCCGATTAGCTAGTTGGTGAGGTAAC
GGCTCACCAAGGCGACGATCGGTAGCTGGTCTGAGAGGATGACCAGCCTCACTGGGACTGAGACACGG
CC
>Zotu6174
GAACGCTGGCGGCATGCCTTACACATGCAAGTCGAACGGCAGCACGGGAGCAATCCTGGTGGCGAGTG

GCGAACGGGTGAGTAATACATCGGAACGTGTCCCGTAGTGGGGGATAGCCCGGCGAAAGCCGGATTAA
TACCGCATACGACCTACGGGTGAAAGCGGGGGACCGCAAGGCCTCGCGCTACAGGAGCGGCCGATGTC
AGATTAGCTAGTTGGTGGGGTAAAGGCCTACCAAGGCGACGATCTGTAGCTGGTCTGAGAGGACGACC
AGCCACACTGGGACTGAGACACGGCC
>Zotu6175
GAACGCTAGCGGCGGGCCTAATACATGCAAGTCGAGGGGCATCGGGAGTAGCAATACTTGCCGGCGAC
CGGCGCACGGGTGCGTAACGCGTATGCAATCTACCTCTGACATGGGGATAGCCCGAAGAAATTCGGAT
TAATACCCAATGGTATCTTAATATGACCTCATATAAAGATTAAAGATTTATCGGTCAGAGATGAGCAT
GCGTCTGATTAGCTAGTTGGTGATGTAATTGCTCACCAAGGCAATGATCAGTAGGGGTTCTGAGAGGA
TTATCCCCCACACCGGTACTGAGACAGGGAC
>Zotu6178
GAACGCTGGCGGCAGGCCTAACACATGCAAGTCGAACGGACCCTTCGGGGTTAGTGGCGGACGGGTGA
GTAACACGTGGGAACGTGCCTTTAGGTTCGGAATAGCTCCTGGAAACGGGTGGTAATGCCGAATGTGC
CCTTCGGGGGAAAGATTTATCGCCTTTAGAGCGGCCCGCGTCTGATTAGCTAGTTGGTAAGGTAAAAG
CTTACCAAGGCGACGATCAGTAGCTGGTCTGAGAGGATGACCAGCCACATTGGGACTGAGACACGGCC
>Zotu6179
GAACGCTGGCGGCGTGCTTAACACATGCAAGTCGAACGGAAAGGCCTCTGCTTGCAGGGGTGCTCGAG
TGGCGAACGGGTGAGTAACACGTGGGTGATCTGCCCTGCACTTTGGGATAAGCCTGGGAAACTGGGTC
TAATACCAGATAGGACCACGTCTTGGATGGTGTGGTGGAAAGTTTTTTCGGTGTGGGATGAGCTCGCG
GCCTATCAGCTTGTTGGTGGGGTAATGGCCTACCAAGGCGTCGACGGGTAGCCGGCCTGAGAGGGTGT
ACGGCCACATTGGGACTGAGATACGGCC
>Zotu6181
GAACGCTGGCGGCGTGCCTAACACATGCAAGTCGAACGCGAAAGTCCTTCGGGATGAGTAAAGTGGCG
CACGGGTGAGTAACACGTAGGTGACCTGCCTTTTAGAGGGGAATAACCAGAAGAAATTTTGGCTAATG
CCGCATACGAAGCACAAACTTAAATATAGTGCTTGAAAGGATGCCTCTGTATATGCATCCACTAAAAG
ATGGGCCTGCGGAACATTAGCTAGTTGGTGGGGTAATGGCCTACCAAGGCAACGATGTTTATCCGGTC
TGAGAGGATGATCGGACACACTGGAACTGAGACACGGTC
>Zotu6182
GAACGCTGGCGGCGTGCTTAACACATGCAAGTCGAACGGTGAACCAGGGCTTGCCTTGGGGATCAGTG
GCGAACGGGTGAGTAACACGTGGGCAACCTGCCCCAGACTCTGGGATAACTCCGGGAAACCGGGGCTA
ATACCGGATACGAATCGAGTTGGCATCTTCTCGATTGGAAAGTTTTTCGGTCTGGGATGGGCCCGCGG
CCTATCAGCTTGTTGGTGGGGTAATGGCCTACCAAGGCGACGACGGGTAGCCGGCCTGAGAGGGCGAA
CGGCCACACTGGGACTGAGACACGGCC
>Zotu6183
GAACGCTGGCGGCATGCTTAACACATGCAAGTCGAACGGCAGCACGACCTAGCTTGCTAGGTGGGTGG
CGAGTGGCGCACGGGTGAGTAACGCGTAGGAATATGCCTTAAAGAGGGGGATAACTTGGGGAAACTCA
AGCTAATACCGCATAAACTCCGAGGAGCGAAGCTGGGGACCTTAGGGCCTGGCGCTTTAAGAATAGCC
TGCGTCCGATTAGCTAGTTGGTGGGGTAAAGGCCTACCAAGGCGACGATCGGTAGCTGGTCTGAGAGG
ATGATCAGCCACACTGGGACTGAGACACGGCC
>Zotu6184
GAACGCTGGCGGCGTGCCTAATACATGCAAGTCGAACGAAGGACGCTGACGGAATTCTTCGGAAGGAA
GTCGGCATTCCTGAGTGGCGGACGGGTGAGTAACACGTAGGCAACCTGCCTGTAAGTCGGGGATAACT
TCGGGAAACCGGAGCTAATACCGGATAAGGCAAACCGATCGCATGGTTGGTTTGGTAAAGGAGGTACG
CTTCCGCTTACAGATGGGCCTGCGGCGCATTAGCTTGTTGGTGAGGTAACGGCTCACCAAGGCGACGA
TGCGTAGCCGACCTGAGAGGGTGACCGGCCACACTGGGACTGAGACACGGCC
>Zotu6186
GAACGCTGGCGGCATGCCTAACACATGCAAGTCGAACGAAGCCTTCGGGCTTAGTGGCGCACGGGTGC
GTAACGCGTGGGAATCTGCCCTTGGGTACGGAACAACAGTTAGAAATGACTGCTAATACCGTATGATG
TCTTCGGACCAAAGATTTATCGCCCTGGGATGAGCCCGCGTTGGATTAGGTAGTTGGTGGGGTAAAGG
CCTACCAAGCCGACGATCCATAGCTGGTCTGAGAGGATGATCAGCCACACTGGGACTGAGACACGGCC
>Zotu6188
GAACGCTGGCGGCGTGCCTAATACATGCAAGTCGAGCGAATCAATAGGAGCTTGCTCCTGTTGGTTAG
CGGCGGACGGGTGAGTAACACGTGGGCAACCTGCCTGTAAGACTGGGATAACACCGGGAAACCGGTGC
TAATACCGGATAATCCTTTTTCTCTCATGAGGAAGAGCTGAAAGTCGGTTTCTGCTGACACTTACAGA

TGGGCCCGCGGCGCATTAGCTAGTTGGTGAGGTAACGGCTCACCAAGGCGACGATGCGTAGCCGACCT
GAGAGGGTGATCGGCCACACTGGGACTGAGACACGGCC
>Zotu6191
GAACGCTGGCGGCAGGCTTAACACATGCAAGTCGAGCGGTAGCACAAGGGAGCTTGCTCCCCGGGTGA
CGAGCGGCGGACGGGTGAGTAATGTCTGGGAAACTGCCTGATGGAGGGGGATAACTACTGGAAACGGT
AGCTAATACCGCATAACGTCGCAAGACCAAAGAGGGGGACCTTCGGGCCTCTTGCCATCAGATGTGCC
CAGATGGGATTAGCTAGTAGGTGGGGTAATGGCTCACCTAGGCGACGATCCCTAGCTGGTCTGAGAGG
ATGACCAGCCACACTGGAACTGAGACACGGTC
>Zotu6192
GAACGCTGGCGGCGTGCTTAACACATGCAAGTCGAACGGAAAGGCCCTTTCGGGGGTACTCGAGTGGC
GAACGGGTGAGTAACACGTGGGTGATCTGCCCTGCACTTCGGGATAAGCTTGGGAAACTGGGTCTAAT
ACCGGATAGGATCATGGCTCTCATGGGTTGTGGTGGAAAGCTTTTGCGGTGTGGGATGGGCCCGCGGC
CTATCAGCTTGTTGGTGGGGTAATGGCCTACCAAGGCGTCGACGGGTAGCCGGCCTGAGAGGGTGTCC
GGCCACACTGGGACTGAGATACGGCC
>Zotu6193
GAACGCTGGCGGTGTGGATAGGGCATGCAAGTCAAGGGGACTCAGTTAGTAATAATTGGGTAACCGGC
AGACGAGATAGTAACGCGTAGGAACGTACCCAGAAGTCGGGCACAGCTCGCCGAAAGGCGGGGTAATT
CCCGATGGTGACGAAAGTCTAAAGATTTATCGCTTCTGGAGCGGCCTGCGTAATATCAGCTTGTTGGT
GAGGTAATGGCTCACCAAGGCTATGACGTTTAGGGGAGCTGAGAGGCTGACCCCCATCGATGGAATTG
AGATACGGTC
>Zotu6194
GAACGCTGGCGGTATGCCTAATACATGCAAGTCGTGCGGAAAGGCCCTTCGGGGTACTCGAGCGGCGG
ACGGCAGAGTAACGCGTAGGAACGTACCCCAAAGTGAGGAATAACTGCTCGAAAGAGTAGCTAATGCC
GCATGTGGTCTTCGGACTAAAGCCTTCGGGCGCTTTGGGAACGGCCTGCGTTCGATTAGCTAGTTGGT
GAGGTAAATGCTCACCAAGGCGATGATCGATAGCTGGTCTGAGAGGATGATCAGCCTGACTGGAACTG
AGACACGGTC
>Zotu6195
GAACGCTGGCGGCGTGCTTAACACATGCAAGTCGAGCGAAGCACTTTGGAAAGATTCTTCGGATGATT
TCCTTTGTGACTGAGCGGCGGACGGGTGAGTAACGCGTGGGTAACCTGCCTCATACAGGGGGATAACA
GTTAGAAATGACTGCTAATACCGCATAAGACCACGGTACCGCATGGTACAGTGGTAAAAACTCCGGTG
GTATGAGATGGACCCGCGTCTGATTAGGTAGTTGGTGGGGTAACGGCCTACCAAGCCGACGATCAGTA
GCCGACCTGAGAGGGTGACCGGCCACATTGGGACTGAGACACGGCC
>Zotu6198
GAACGCTGGCGGCGTGCTTAACACATGCAAGTCGAACGGAAAGGCCTCTAGCTTGCTAGAGGTACTCG
AGTGGCGAACGGGTGAGTAACACGTGGGTGATCTGCCCTGCACTGTGGGATAAGCCTGGGAAACTGGG
TCTAATACCATATAGGACCGCACTTTGGATGGTGTGGTGGAAAGCTTTTGCGGTGTGGGATGAGCCTG
CGGCCTATCAGCTTGTTGGTGGGGTAATGGCCTACCAAGGCGGCGACGGGTATCCGGCCTGAGAGGGT
GTACGGACACATTGGGACTGAGATACGGCC
>Zotu6200
GAACGCTGGCGGCGTGCCTAATACATGCAAGTTGAGCGGACAGATGGGAGCTTGCTCCCTGATGTTAG
CGGCGGACGGGTGAGTAACACGTGGGTAACCTGCCTGTAAGACTGGGATAACTCCGGGAAACCGGGGC
TAATACCGGATGCTTGTTTGAACCGCATGGTTCAGACATAAAAGGTGGCTTCGGCTACCACTTACAGA
TGGACCCGCGGCGCATTAGCTAGTTGGTGAGGTAACGGCTCACCAAGGCGACGATGCGTAGCCGACCT
GAGAGGGTGATCGGCCACACTGGGACTGAGACACGGCC
>Zotu6202
GAACGCTGGCGGCGTGCTTAACACATGCAAGTCGAGCGGTAAGGCTCCTTCGGGAGTACACGAGCGGC
GAACGGGTGAGTAACACGTGAGCAATCTGCCCTTCACTTTGGGATAACCCCGGGAAACCGGGGCTAAT
ACCGAATACGACCACCGATGGCATCGTCTGGTGGTGGAAAGTTCCGGCGGTGAAGGATGAGCTCGCGG
CCTATCAGCTTGTTGGTGGGGTAACGGCCCACCAAGGCATTGACGGGTAGCCGGCCTGAGAGGGTGAC
CGGCCACACTGGGACTGAGACACGGCC
>Zotu6203
CAACGCTGGCGGCGTGCCTAACACATGCAAGTCGAACGCGAAAGTCCCGCAAGGGATTAGTAGAGTGG
CAAACGGGTGAGTAACACGTGGGTGACCTGCCTTCGAGCGGGGGATAACGTCCCGAAAGGGACGCTAA
TACCGCATAACATCCTGCCTTTTAAGAGGCGGTGATCAAAGCCGGGGATTCGCAAGAACCTGGCACTT

GAAGAGGGGCCCGCGCCTGATTAGCTAGTTGGTGGGGTAATGGCCTACCAAGGCAACGATCAGTATCC
GGCCTGAGAGGGCGGACGGACACACTGGGACTGAGACACGGCC
>Zotu6204
GAACGCTGACAGAATGCTTAACACATGCAAGTCGACTTGAATTTGGGTTTTTAACTTAGGTTTGGGTG
GCGGACGGGTGAGTAATGCGTAAAGAACTTGCCTCACAGCTAGGGACAACATTTGGAAACGAATGCTA
ATACCTAATATTATGATAATAGGGCATCTTATAATTATGAAAGCTATAAGCGCTGTGAGAGAGCTTTG
CGTCCCATTAGCTAGTTGGAGAGGTAACAGCTCACCAAGGCGATGATGGGTAGCCGGCCTGAGAGGGT
GATCGGCCACAAGGGGACTGAGACACGGCC
>Zotu6205
GAACGCTGGCGGCGTGCCTAACACATGCAAGTGGAGCGACGAACCCGGACTTGTCCGGGGGCAAAGCC
GCGAACGGGTGAGTAACACGTGGATTATCTGCCCCAATGACCGGGACAACTCGAAGAAATTCGAGCTA
ATACCGGATGTGCTCTTCGGAGGAAAGGAAGCTTCGGCCTCCGCATTGGGATGAGTCCGCGGCGCATT
AGCTAGTTGGTGGGGTAAAGGCCTACCAAGGCGACGATGCGTAGCTGGTCTGAGAGGACGATCAGCCA
CACTGGAACTGAGACACGGTC
>Zotu6206
GAACGCTGGCGGCAGGCTTAACACATGCAAGTCGAACGCTCCGCAAGGAGAGTGGCAGACGGGTGAGT
AACGCGTGGGAACGTACCTTTCAGTTCGGAATAACTCAGGGAAACTTGTGCTAATACCGGATACGCCC
GTAAGGGGAAAGATTTATCGCTGAAAGATCGGCCCGCGTTGGATTAGCTAGTTGGTGGGGTAAAGGCC
TACCAAGGCGACGATCCATAGCTGGTCTGAGAGGATGATCAGCCACACTGGGACTGAGACACGGCC
>Zotu6208
GAACGCTAGCTACAGGCTTAACACATGCAAGTCGAGGGGAAACGATATTGGAAGCTTGCTTCCGATAG
GCGTCGACCGGCGCACGGGTGAGTAACGCGTATCCAACCTGCCCACCACTTGGGGATAACCTTGCGAA
AGTAAGACTAATACCCAATGATATCTCTAGAAGACATCTGAAAGAGATTAAAGATTTATCGGTGATGG
ATGGGGATGCGTCTGATTAGCTTGTTGGCGGGGTAACGGCCCACCAAGGCGACGATCAGTAGGGGTTC
TGAGAGGAAGGTCCCCCACATTGGAACTGAGACACGGTC
>Zotu6209
GAACGCTGGCGGCGTGCCTAAGACATGCAAGTCGAGTGGGTTTAGACCCACGGCAAACGGGTGAGTAA
TACCTAGGAACATACCTTTAGTTGAGCATAACCTACCGAAAGGTAGGACAATTCTCAATGTGGCAGCA
ATGCTAAAGTCGCAAGACGCTAAAGAAGTGGCCTGGGCACCATCAGCTAGTTGGTAAGGTAAAGGCTT
ACCAAGGCTATGACGGTTAGGGGCCCCGAGAGGGGAAACCCCCACACTGGAACTGAGACACGGTC
>Zotu6210
GAACGCTGGCGGCGTGCCTAATACATGCAAGTCGAGCGAATCTTTAGGAGCTTGCTCCTCAAAGGTTA
GCGGAGGACGGGTGAGTAACACGTGGGCAACCTGCCTGTAAGACTGGGATAACTTCGGGAAACCGGAG
CTAATACCGGATAATCCTTTTCCTCTCATGAGGAAAAGCTGAAAGTCGGTTTACGCTGACACTTACAG
ATGGGCCCGCGGCGCATTAGCTAGTTGGTGAGGTAATGGCTCACCAAGGCGACGATGCGTAGCCGACC
TGAGAGGGTGATCGGCCACACTGGGACTGAGACACGGCC
>Zotu6213
GAACGCTGGCGGCGTGCCTAAGACATGCGAGTCGAGTGGGTTTAGACCCACGGCAAACGGGTGAGTAA
TACCTAGGAACTTTCTTTAAGTGGAGCATAACCAGCCGAAAGGTTGGATAATACTCCATACCATCGCA
AGATGAAAGTCGCAAGACGCTTAAAGTGAGGCCTGGGCACCATCAGCTAGTTGGTAAGGTAATGGCTT
ACCAAGGCTATGACGGTTAGGGGCCCCGAGAGGGGAGTCCCCCACACTGGAACTGCGACACGGTC
>Zotu6214
GAACGCTGGCGGCGTGCCTAATACATGCAAGTCGAGCGGAGTTGATGAGGTGCTTGCACCTCTGATGC
TTAGCGGCGGACGGGTGAGTAACACGTAGGTAACCTGCCTGTAAGACTGGGATAACATTCGGAAACGA
ATGCTAATACCGGATACGCGATTTTCTCGCATGAGAGAACCGGGAAAGACGGAGCAATCTGTCACTTA
CGGATGGACCTGCGGCGCATTAGCTAGTTGGTGAGGTAACGGCTCACCAAGGCGACGATGCGTAGCCG
ACCTGAGAGTGTGATCGGCCACACTGGGACTGAGACACGGCC
>Zotu6215
GAACGCTGGCGGCGTGCTTAACACATGCAAGTCGAACGATGAAGCGCCCAGCTTGCTGGGTGTGGATT
AGTGGCGAACGGGTGAGTATCACGTGAGTAACCTGCCCTTGACTCTGGGATAAGCCTGGGAAACTGGG
TCTAATACCGGATACAACTGCCTACCGCATGGTGGGTGGTGGAAAGCTTTTGCGGTTTTGGATGGACT
CGCGGCCTATCAGCTTGACGGTGAGGTAATGGCTCACGGTGGCTTTGACGGGTAGCCGGCCTGAGAGG
GTGACCGGCCACACTGGGACTGAGACACGGCC
>Zotu6216

GAACATTGGCGGTAGGCTTAACACATGCAAGTCGAGCGAGGCGTAATAGTCGAGCGGCGAACGGGTGA
GTAATACATGGAAATCTACCCTGAAGTGGGGGATAACGTTCAGAAATGGACGCTAATACCGCATAATC
CATATTGGGAAAGCGTTGCAGCGCTTTAGGATGAGTCCATGGCTGATTAGCTTGTTGGTGAGGTAATG
GCTCACCAAGGCGACGATCAGTAGCTGGTCTGAGAGGATGATCAGCCACACTGGAACTGAGACACGGT
C
>Zotu6217
GAACGCTGGCGGCGTGCTTAACACATGCAAGTCGAGCGGAAAGGCCCCTTTGGGGGTACTCGAGCGGC
GAACGGGTGAGTAACACGTGAGTAATCTGCCCTTCACTTTGGGATAACCCCCGGAAACGGGAGCTAAT
ACCGGATACGACCACTCCGGGCATCTGGTGGTGGTGGAAACTTTTTGGGTGGGGGATGTGCTCGCGGC
CTATCAGCTGGTTGGTGGGGTAATGGCCTACCAAGGCTTTGACGGGTAGCCGGCCTGAGAGGGTGACC
GGCCACACTGGGACTGAGACACGGCC
>Zotu6218
GAACGCTAGCGGCGTGCTTAACACATGCAAGTCGAGCGGTAAGGCCCTTCGGGGTACACGAGCGGCGA
ACGGGTGAGTAACACGTGGGTGACCTGCCCCCTACTCTGGGATAAGCCTGGGAAACTGGGTCTAATAC
CGGATATGACTACATGTCGCATGGTGTGTGGTGGAAAGTTTTTCGGTGGGGGATGGCCCGCGGCCTA
TCAGCTTGTTGGTGGGGTGATGGCCTACCAAGGCGACGACGGGTAGCCGGCCTGAGAGGGCGACCGGC
CACACTGGGACTGAGACACGGCC
>Zotu6220
GAACGCTGGCGGCGTGCTTAACACATGCAAGTCACGGGGGCCCGCAAGGGCAACCGGCGAACGGGTGC
GTAACACGTGAGCAATCTGCCGATCTCTGGGGGATAGCCGGCCCAACGGCCGGGTAATACCGCATACG
TTCTCGAGGGGGAGTCCCCTTGAGAAGAAACCTCCGGGGGAGATCGAGGAGCTCGCGACCTATCAGCT
AGTTGGCGGGGTAACGGCCCACCAAGGCATCGACGGGTAGCTGGTCTGAGAGGATGGCCAGCCACATT
GGGACTGCGACACGGCC
>Zotu6221
GAACGCTGGCGGCGCGCTTAACACATGCAAGTCGTACGAGGGTCTTCGGACCTCAGTGGCGAACGGGT
GCGTAACACGTGAGCAACCTGCCTGGTCGTGGGGGATAACCAGTGGAAACGCTGGCTAATACCGCATG
AGCTCTTTCTGCCGATTGGTGGGATGAGGAAAGCTCCTGCAAGGGAGTGCGACCAGAGGGGCTCGCGG
CGTATCAGCTAGTTGGAGGGGTAACGGCCCACCAAGGCGATGACGCGTAGCCGATCTGAGAGGATGAT
CGGCCACATTGGGACTGAGATACGGCC
>Zotu6222
GAACGCTGGCGGCGTGCCTAATACATGCAAGTCGAGCGGACAGATAAGGAGCTTGCTCCTTTGACGTT
AGCGGCGGACGGGTGAGTAACACGTGGGCAACCTGCCCCTTAGACTGGGATAACTCCGGGAAACCGGA
GCTAATACCGGATAATCCCTTTCTCCACCTGGAGAGAGGGTGAAAGATGGCTTCGGCTATCACTAAGG
GATGGGCCCGCGGCGCATTAGCTAGTTGGTAAGGTAACGGCTTACCAAGGCAACGATGCGTAGCCGAC
CTGAGAGGGTGATCGGCCACACTGGGACTGAGACACGGCC
>Zotu6223
GAACGCTGGCGGCGTGCTTAACACATGCAAGTCGAACGATGAAGCACCAGCTTGCTGGTGTGGATTAG
TGGCGAACGGGTGAGTAACACGTGAGTAACCTGCCCCCTACTCTGGGATAAGCCTGGGAAACTGGGTC
TAATACTGGATATGACCAATCACTGCATGGTGTGTTGGTGGAAAGATTTTTTGGTGGGGGATGGACTC
GCGGCCTATCAGCTTGTTGGTGAGGTAATGGCCTACCAAGGCGACGACGGGTAGCCGGCCTGAGAGGG
CGACCGGCCACACTGGGACTGAGACACGGCC
>Zotu6224
GAACGCTGGCGGCATGCTTAACACATGCAAGTCGAACGGCAGCATAGCTTGGTTTACCAAGTGGATGG
CGAGTGGCGAACGGGTGAGTAACGCGTAGGAATATACCTTAAAGAGGGGGATAACTTGGGGAAACTCA
AGCTAATACCGCGTAATATCTGAGGATAAAAGCTGGGGACCTTCGGGCCTGGCGCTTTAAGAATAGCC
TGCGTCCGATTAGCTAGTTGGTGGGGTAAAGGCCTACCAAGGCGACGATCGGTAGCTGGTCTGAGAGG
ATGATCAGCCACACTGGGACTGAGACACGGCC
>Zotu6225
GAACGCTGGCGGCGTGCTTAACACATGCAAGTCGAACGGTGAAGCCCCGCTTGCGGGGTGGATCAGTG
GCGAACGGGTGAGTAACACGTGAGCAACCTGCCCCTGACTCTGGGATAAGCGCTGGAAACGGCGTCTA
ATACTGGATACGAGACGTGATCGCATGGTCAACGTTTGGAAAGATTTTTCGGTTGGGGATGGGCTCGC
GGCCTATCAGCTTGTTGGTGAGGTAATGGCTCACCAAGGCGTCGACGGGTAGCCGGCCTGAGAGGGTG
ACCGGCCACACTGGGACTGAGACACGGCC
>Zotu6226

GAACGCTGGCGGAATGCTTTACACATGCAAGTCGAACGGCAGCACGGACTTCGGTCTGGTGGCGAGTG
GCGAACGGGTGAGTAATATATCGGAACGTGTCCAATAATGGGGGATAACTAATCGAAAGGTTGGCTAA
TACCGCATACGCCCTACGGGGGAAAGCAGGGGATCTTCGGACCTTGCGTTAATGGAGCGGCCGATATC
TGATTAGCTAGTTGGTGGGGTAAAGGCCCACCAAGGCGACGATCAGTAGCTGGTCTGAGAGGACGACC
AGCCACACTGGAACTGAGACACGGTC
>Zotu6227
GAACGCTGGCGGCGTGCCTAATACATGCAAGTCGAACGCACTGACGGAGAACTTGTTCTCTTGGCGTG
AGTGGCGCACGGGTGAGTAACACGTGGGAAACCTACCCTTCAGCGGGGGATAACCATCGGAAACGATG
ACTAATACCGCATAGACGACAGAACCGCCTGGTTCAATCGGGAAAGACGGCTTCGGCTGTCACTGAAG
GATGGTCCCGCGCTGCATTAGCTAGTTGGTAAGGTAACGGCTTACCAAGGCGATGATGCATAGCCGAC
CTGAGAGGGTGATCGGCCACATTGGGACTGAGACACGGCC
>Zotu6228
GAACGCTGGCGGCATGCTTAACACATGCAAGTCGAACGAGACCTTCGGGTCTAGTGGCGCACAGGTGC
GTAACGCGTGGGAATCTGCCCTTAGGTACGGAATAACTCAGAGAAATTTGTGCTAATACCGTATGATG
TCGAAAGACCAAAGATTTATCGCCTAAGGATGAGCCCGCGTAAGATTAGCTTGTTGGTGAGGTAAAAG
CTCACCAAGGCGACGATCTTTAGCTGGTCTGAGAGGATGATCAGCCACACTGGGACTGAGACACGGCC
>Zotu6229
GAACGCTGGCGGCAGGCCTAACACATGCAAGTCGAACGCTCGTAGCAATACGAGAGTGGCAGACGGGT
GAGTAACGCGTGGGAACCTTCCCAGTGGTACGGAATAGCTCAGGGAAACTTGAGGTAATACCGTATAC
GCCCCTAGGGGGAAAGATTTATCGCCATTGGATGGGCCCGCGTCGGATTAGCTAGTTGGTGAGGTAAC
GGCTCACCAAGGCGACGATCCGTAGCTGGTTTGAGAGAATGACCAGCCACACTGGGACTGAGACACGG
CC
>Zotu6230
TAACGCTGGCGGTGTGCCTAATACATGCAAGTCGAACGGACCTTTATTTTTGAGAACTCCCTTATAGG
ATGTCTCACAAATAATGGTTAGTGGCGGACGGGTGAGTAACACGTTGGTACCTGCCCTCTAGTGGGGA
ATAACTGGCCGAAAGGTCGGCTAATACCGCATGTGATCGCAAGATCAAAGCAGCAATGCGCTAGAGGA
GGGGCCTGCGTCTGATTAGCTAGTTGGTAGTGTAATGGACTACCAAGGCGACGATCAGTAGCTGGTTC
GAGAGGATGACCAGCCACACTGGGACTGAGATACGGCC
>Zotu6233
GAACGCTGGCGGCGTGCCTAACACATGCAAGTCGAGCGATGAAACCCTTCGGGGTGGATTAGCGGCGG
ACGGGTGAGTAACACGTGGGTAACCTGCCTCAAAGAGGGGAATAGCCTCCCGAAAGGGAGATTAATAC
CGCATAATATGTTTTGATCGCATGATCGAGATATCAAAGGAATCTTCTTCGGAAGTTTCCACTTTGAG
ATGGACCCGCGGCGCATTAGCTAGTTGGTGAGGTAACGGCTCACCAAGGCAACGATGCGTAGCCGACC
TGAGAGGGTGATCGGCCACATTGGAACTGAGACACGGTC
>Zotu6234
GAACGCTGGCGGTGTGCTTAACACATGCAAGTCGAACGGGCAAGAGCTGTAGCAATACAGAATTCTGC
TAGTGGCAAACGGGTGAGTAATACATGGGAATCTACCTTGCAGTGCGAAATAACAGTTGGAAGCAATT
GCTAATGTCGCATATAGCCGAGAGGTGAAAGATTTATCGCTGCAAGACGAGCCCATGCAGGATTAGCT
AGTTGGTAAGGTAACGGCTTACCAAGGCAACGATACGTAGCCGACCTGAGAGGGTGATCGGCCACACT
GGAACTGAGACACGGTC
>Zotu6235
GAACGCTGGCGGCATGCCTAACACATGCAAGTCGAACGAGATCTTCGGGTCTAGTGGCGCACGGGTGC
GTAACGCGTGGGAATCTGCCCTTTGGTTCAGAATAACAGTTGGAAACGACTGCTAATACCGGATGATG
ACGAAAGTCCAAAGATTTATCGCCAGAGGATGAGCCCGCGTAGGATTAGCTAGTTGGTGTGGTAAAGG
CGCACCAAGGCGACGATCCTTAGCTGGTCTGAGAGGATGATCAGCCACACTGGGACTGAGACACGGCC
>Zotu6236
GAACGCTAGCGGCAGGCTTAATACATGCAAGTCGAACGGTAAGTGGTGTAGCAATACATCACCAAGAG
TGACGTACGGGTGCGTAACACGTATGCAACCTACCTATAACAGGAGTATAGCTCGGGGAAACTCGAAT
TAACCCTCCATAATATGATTGAATGGCATCATTTGATTATTAAAACTGAGGTGGTTATAGATGGGCAT
GCGTCTGATTAGCTAGTTGGCGGGGTAATGGCCCACCAAGGCGATGATCAGTAGGGGAACTGAGAGGT
TGATCCCCCACACTGGCACTGAGATACGGGC
>Zotu6238
GAACGCTAGCGGCAAGCTTAATACATGCAAGTCGTGGGGCAGCATGAAGTAGCAATACTTTGATGGCG
ACCGGCAAACGGGTGCGGAACACGTACACAACCTTCCGATAAGTGGGGAATAGCCCAGAGAAATTTGG

ATTAATACCCCGTAACATTGCGATGTGGCATCACATTGCAATTATAGCTTCGGCGCTTATTGATGGGT
GTGCGTATGATTAGATAGTTGGCGGGGTAACGGCCCACCAAGTCTACGATCATTAGCTGATGTGAGAG
CATGATCAGCCACACGGGCACTGAGACACGGGC
>Zotu6239
GAACGCTGGCGGCGTGCCTAATACATGCAAGTCGAGCGGATCTTTTAAAAGCTTGCTTTTAAAAGATT
AGCGGCGGACGGGTGAGTAACACGTGGGCAACCTGCCTGTAAGACTGGGATAACTCCGGGAAACCGGG
GCTAATACCGGATAATTCTTGTCCCCGCGTGGGGGCAAGCTGAAAGACGGCTTATGCTGTCACTTACA
GATGGGCCCGCGGCGCATTAGCTAGTTGGTGAGGTAATGGCTCACCAAGGCAACGATGCGTAGCCGAC
CTGAGAGGGTGATCGGCCACACTGGGACTGAGACACGGCC
>Zotu6240
GAACGCTAGCTACAGGCTTAACACATGCAAGTCGAGGGGCAGCATGAAGTCAGCTTGCTGACTTTGAT
GGCGACCGGCGCACGGGTGCGTAACGCGTATCAAACCTGCCGCATACTCGGGGATAGCCTTGCGAAAG
TAAGATTAATACCCGATGTTATTGTTTTCCCGCATGGGTAGATAATCAAAGATTTATCGGTATGCGAT
GGTGATGCGTCTGATTAGGTAGTAGGCGGGGTAACGGCCCACCTAGCCAACGATCAGTAGGGGTTCTG
AGAGGAAGGTCCCCCACACTGGAACTGAGACACGGTC
>Zotu6241
GAACGCTAGCGACAGGCCTAACACATGCAAGTCGAGGGGCAGCGAGTTTGTAGCAATACGAATGTCGG
CGACCGGCGCACGGGTGCGTAACAGGTGTGCAATCTGTCCATAGTCGGGGAATAGCCCAGCGAAAGTT
GGATTAATGCTCCATGTGTCATGATAGGGCATCCTATTATGATGAAACGAGAGGACTATGGGTGAGCA
CGCTTCTGATTAGATAGTTGGTGAGGTAACGGCTCACCAAGTCGACGATCAGTAGGGGTTCTGAGAGG
AAGGTCCCCCACATTGGAACTGAGACACGGTC
>Zotu6243
GAACGCTGGCGGTCTGCTTAACACATGCAAGTCGAACGGAGCCTTTCGGGGCTTAGTGGCGGACGGGT
GAGTAACGCGTGAGAATCTGCCCATAGGATAGGGACAACAGTTGGAAACGACTGCTAATACCTGATGT
GCCGAGAGGTGAAACTTTTATGGCCTGTGGATGAGCTCGCGTCTGATTAGCTAGTTGGTAGGGTAAAG
GCTTACCAAGGCGACGATCAGTAGCTGGTCTGAGAGGATGATTAGCCACACTGGAACTGAGACACGGT
C
>Zotu6244
GAACGCTGGCGGCATGCCTAACACATGCAAGTCGAACGAAGGCTTCGGCCTTAGTGGCGCACGGGTGC
GTAACGCGTGGGAATCTGCCCCTCGGTTCGGAATAACAGAGAGAAATTTCTGCTAATACCGGATAATG
ACGTAAGTCCAAAGATTTATCGCCGAGGGATGAGCCCGCGTAGGATTAGCTAGTTGGTGTGGTAAAGG
CGCACCAAGGCGACGATCCTTAGCTGGTCTGAGAGGATGATCAGCCACACTGGGACTGAGACACGGCC
>Zotu6245
GAACGCTGGCGGCGTGCTTAACACATGCAAGTCGAACGAGAAATGTTATTATGATCTCTTCGGAGTGA
TGATTTTATTTCGAGTGGCGGACGGGTGAGTAACGCGTGAGCAACCTGCCCTATACCGGGGGATACCA
GTTGGAAACGACTGTTAATGCCGCATAAGCGCACGATAAGACATCTTGTTGTGTGAAAAACTCCGGTG
GTATAGGATGGGCTCGCGTATGATTAGCCAGTTGGCGGGGTAAAGGCCCACCAAAGCGACGATCATTA
GCCGACCTGAGAGGGCGACCGGCCACATTGGGACTGAGACACGGCC
>Zotu6246
GAACGCTGGCGGCGTGCTTAACACATGCAAGTCGAGGGTAGCAATACACTGGCGCAAGGGTGCGTAAC
GCGTAGATTATCTGCCCCATACACTAACATAACGCTTGGAAACGGGTGCTAATATTAGATGAGGTGCT
TCGGCATCAAAGTTTCGACGGTATGGGATGAGTCTGCGTCTGATTAGCTAGTTGGTAGTGTAATGGAC
TACCAAGGCGACGATCAGTAGCTGGTCTGAGAGGACGATCAGCCACACTGGAACTGAGACACGGTC
>Zotu6247
GAACGCTGGCGGCGTGCCTAACACATGCAAGTCGAACGAGAAAGAGCTTCGGCTCAAGTACAGTGGCG
AACGGGTGAGTAATACGTGGGAATCAACCCTTTAGTGAAGGACATCCTCGAGAAATCGAGAGCAATCC
TGCATAAGTCCCTTCGGGGAGAAAGATGGCCTCTTTGCTGTCGCTAAAGGACGAGCCTGCGCGCTATC
AGCTAGTTGGTAGGGTAATGGCCTACCAAGGCTTTGACGGCTAGCCGGCCTGAGAGGGTGTACGGCCA
CACTGGAACTTAGACACGGTC
>Zotu6248
GAACGCTGGCGGCATGCCTAACACATGCAAGTCGAACGAGGGCTTCGGCCCTAGTGGCGCACGGGTGA
GTAACGCGTGGGAACCTGCCCTGTGGTACGGAATAACTCCGGGAAACTGGAGCTAATACCGTATGTGT
CCTGTGGGACAAAGATTTATCGCCATGGGATGGGCCCGCGTAGGATTAGCTTGTTGGTGGGGTAACGG
CCTACCAAGGCAACGATCCTTAGCTGGTCTGAGAGGATGATCAGCCACACTGGGACTGAGACACGGCC

>Zotu6249
GAACGCTGGCGGCGTGCTTAACACATGCAAGTCGAGCGGAAAGGCCCTTCGGGGTACTCGAGCGGCGA
ACGGGTGAGTAACACGTGGGTAACCTGCCCCTTGCTCTGGGATAACTCCGGGAAACCGGGGCTAATAC
CGGATATGACCATCTTAGGCATCTTTGGTGGTGGAAAGTTTTTCGGCGAGGGATGGACCCGCGGCCTA
TCAGCTTGTTGGTGGGGTGATGGCCTACCAAGGCAGTGACGGGTAGCCGGCCTGAGAGGGCGACCGGC
CACACTGGGACTGAGACACGGCC
>Zotu6251
GAACGCTAGCGGCAGGCCTAACACATGCAAGTCGAGGGGTAACAGGGTAGCAATATCGCTGACGACCG
GCGCACGGGTGAGTAACGCGTATGCAACCTTCCTTTAACTGGGGGATAGCCCGGAGAAATTCGGATTA
ATACCCCATAACATCATAGAAAGGCATCTTTCAGTGATTAAAGCTCCGGCGGTTAAAGATGGGCATGC
GTGCCATTAGCTAGTTGGTGAGGTAACGGCTCACCAAGGCTACGATGGTTAGGGGGTCTGAGAGGATG
ATCCCCCACACTGGTACTGAGACACGGAC
>Zotu6252
GAACGCTGGCGGCGTGCCTAACACATGCAAGTCGAACGGCTATTTCGGTAGCAGTGGCGCACGGGTGA
GTAACACGTAGCTGACCTGCCTTCGAGTGCAGGATAACGTCCCGAAAGGGACGCTAAGACTGCATAAG
CTCCCGGTCTCTAGAGGACTGAGAGAAAAGATTGATCGCTCGAAGAGGGGGCTGCGTCCCATCAGGTA
GTTGGTAGGGTAACGGCCTACCAAGCCAACGACGGGTAGGGGGTCTGAGAGGATGATCCCCCACACTG
GGACTGAGACACGGCC
>Zotu6253
GAACGCTGGCGGCATGCTTTACACATGCAAGTCGAACGGCAGCGAAGAGAAGCTTGCTTCTCTGTCGG
CGAGTGGCGAACGGGTGAGTATAGCATCGGAACGTGCCAAGTAGTGGGGGATAACCAAGCGAAAGTTT
GGCTAATACCGCGTAAGCTCTAAGGAGGAAAGTAGGGGACCGCAAGGCCTTACGCTATTTGATCGGCC
GATGTCGGATTAGCTAGTTGGTAGGGTAATGGCCTACCAAGGCGACGATCCGTAGCTGGTCTGAGAGG
ACGATCAGCCACACTGGGACTGAGACACGGCC
>Zotu6254
GAACGCTAGCGGCAGGCCTAATACATGCAAGTCGAGGGGCAGCAGGGATAGCAATATTCGCTGGCGAC
CGGCGCACGGGTGCGTAACACGTATACAATCTGCCTTTAACAGGGGGATAGCCCGGAGAAATCCGGAT
TAATACCCCATAACACAAGAAACGGCATCGTTTTTTGTTAAAAGCTCCGGCGGTTAGAGATGAGTATG
CGTCTGATTAGCTAGTTGGTGAGGTAACGGCTCACCAAGGCAATGATCAGTAGGGGATCTGAGAGGAT
TAACCCCCACACGGATACTGAGACACGGAT
>Zotu6255
GAACGCTGGCGGCGTGCCTAATACATGCAAGTCGAGCGGACCACAAAGAAGCTTGCTTCTGAGTGGTT
AGCGGCGGACGGGTGAGTAACACGTGGGCAACCTGCCCGTAAGACCGGGATAACTTCGGGAAACCGGA
GCTAATACCGGATAACACTGAAGATCGCATGATCTTTGGTTGAAAGGCGGCTTTGGCTGTCACTTACG
GATGGGCCCGCGGCGCATTAGCTAGTTGGTGAGGTAACGGCTCACCAAGGCGACGATGCGTAGCCGAC
CTGAGAGGGTGATCGGCCACACTGGGACTGAGACACGGCC
>Zotu6256
GAACGCTAGCGGGAGGCCTAACACATGCAAGCCGAGCGGTAGAGATTCTTCGGAATCTTGAGAGCGGC
GCACGGGTGCGGAACACGTGTGCAACCTGCCTTTATCTGGGGGATAGCCTTTCGAAAGGAAGATTAAT
ACCCCATAATATATTGAATGGCATCATTTGATATTGAAAACTCCGGTGGATAGAGATGGGCACGCGCA
GGATTAGATAGTTGGTGAGGTAACGGCTCACCAAGTCAACGATCCTTAGGGGGCCTGAGAGGGTGATC
CCCCACACTGGTACTGAGACACGGAC
>Zotu6257
GAACGCTGGCGGCAGGCCTAAAACATGCAAGTCGAACGCTCTAGCAATAGAGAGTGGCGCACGGGTGC
GTAACATGTGGGAATATGCCTTTTGGTACGGAACAACGTTTGGAAACGAACGCTAATACCGTATGGTC
CCTTCGGGGTAAAGATTTATCGCCAAAAGATTAGCCCGCACTGGATTAGCTAGTTGGTGAGGTAATGG
CTCACCAAGGCGACGATCCATAGCTGGTCTGAGAGGACGATCAGCCACACTGGGACTGAGACACGGCC
>Zotu6258
GAACGCTAGCGGCAGGCCTAATACATGCAAGTCGAACGGGCGATAGCAATATCGCTAGTGGCGCACGG
GTGCGTAACGCGTAACCAACCTGCCCTGAACTGGGGGATAGCCCGCCGAAAGGCGGATTAATACCGCA
TAAACCAACAGTGTGGCATCACATAATTGGTAAAGATTTATTGGTTCAGGATGGGGTTGCGTAGCATT
AGCTAGTTGGCGGGGTAATAGCCCACCAAGGCGACGATGCTTAGGGGACCTGAGAGGGTGATCCCCCA
CACTGGCACTGAGATACGGGC
>Zotu6259

GAACGCTGGCGGCGTGCTTAACACATGCAAGTCGAGCGGTAAGGCCCTTCGGGGTACACGAGCGGCGA
ACGGGTGAGTAACACGTGGGTGACCTGCCCTCCACTCTGGGATAAGCCTGGGAAACTGGGTCTAATAC
CGGATATGACCTCTGGGTGCATGCCTGGTGGTGGAAAGTTTTGTCGGTGGGGGATGGGCCCGCGGTCT
ATCAGCTTGTTGGTGGGGTGATGGCCTACCAAGGCGACGACGGATAGCCGGCCTGAGAGGGTGATCGG
CCACACTGGGACTGAGACACGGCC
>Zotu6262
CAACGCTGGCGGCGTGCCTAACACATGCAAGTCGAACGCGAAAGTGGAGCAATCCATGAGTAGAGTGG
CGAACGGGTGAGTAACACGTGGGTGACCTACCTTCGAATGGGGGATAACGTCCCGAAAGGGACGCTAA
TACCGCATAACATCCTGTCCTTGAACGGATGGAGATCAAAGCCGGGGATCGCAAGACCTGGCGTTTGA
AGAGGGGCCCGCGTCCGATTAGCTAGTTGGTGAGGTAATGGCTCACCAAGGCGACGATCGGTAACCGG
CCTGAGAGGGCGGACGGTCACACTGGGACTGAGACACGGCC
>Zotu6263
GAACGCTGGCGGCGTGCTTAACACATGCAAGTCGAACGATGATGCCCAGCTTGCTGGGTGGATTAGTG
GCGAACGGGTGAGTAACACGTGAGTAACCTGCCCACCACTCTGGGATAACTCCGGGAAACCGGTGCTA
ATACTGGATATGACTCCAACTCGCATGGGTATGGGGTGGAAAGATTTATTGGTGGTGGATGGACTCGC
GGCCTATCAGCTTGTTGGTGGGGTAATGGCCTACCAAGGCGACGACGGGTAGCCGGCCTGAGAGGGTG
ACCGGCCACACTGGGACTGAGACACGGCC
>Zotu6264
GAACGCTGGCGGCGTGCCTAACACATGCAAGTCGGATGTGCCGCAAGGTGCATGGCAGACGGGTGAGT
AACGCGTGGGCAATCTACCTATCAGTGGGGAATAACGATCCGAAAGGATTGGTAATACCGCATGTGAC
CATCGGCTGGGATGCTGGTGGTGAAACCCGGGGACCGCAAGGCCTGGGGCTGATAGAGGAGCCTGCGT
CTGATTAGCTAGTTGGCGGGGTAACGGCCCACCAAGGCGACGATCAGTAGCCGGCCTGAGAGGGTGAT
CGGCCACACTGGAACTGAGACACGGTC
>Zotu6265
GAACGCTGGCGGCGTGCCTAACACATGCAAGTCGAACGGAACTGTTTTGAAAGATTTCTTCGGAATGA
ATTTAATTTAGTTTAGTGGCGGACGGGTGAGTAACGCGTGAGTAACCTGCCTTCAAGAGGGGGATAAC
ATTCTGAAAAGAATGCTAATACCGCATGACATATCGGAACCACATGGTTTTGATATCAAAGATTTTAT
CGCTTGAAGATGGACTCGCGTCCGATTAGTTAGTTGGTGAGGTAACGGCTCACCAAGACCGCGATCGG
TAGCCGGACTGAGAGGTTGAACGGCCACATTGGGACTGAGACACGGCC
>Zotu6266
GAACGCTGGCGGCGTGCTTAACACATGCAAGTCGAACGATGATGCCTAGCTTGCTAGGTGGATTAGTG
GCGAACGGGTGAGTAATACGTGAGTAACCTGCCCCCTTCTTTGGGATAAGCCTTGGAAACGGGGTCTA
ATACCGGATATTCCTGGTCTGCCGCATGGTGGGTTGGGGAAAGTTTTTTCGGTGGGGGATGGGCTCGC
GGCCTATCAGCTTGTTGGCGGGGTGATGGCCTACCAAGGCGTCGACGGGTAGCCGGCCTGAGAGGGTG
ACCGGCCACACTGGGACTGAGATACGGCC
>Zotu6269
GAACGCTGGCGGCAGGCTTAACACATGCAAGTCGAACGCCCCGCAAGGGGAGTGGCAGACGGGTGAGT
AACACGTGGGAACGTACCCTTTACTACGGAATAACGCATGGAAACGTGTGCTAATACCGTATGTGCCC
TTCGGGGGAAAGATTTATCGGTAAAGGATCGGCCCGCGTTGGATTAGCTAGTTGGTGGGGTAAAGGCC
TACCAAGGCGACGATCCATAGCTGGTCTGAGAGGATGATCAGCCACATTGGGACTGAGACACGGCC
>Zotu6270
GAACGCTGGCGGCGTGCCTAAGACATGCAAGTCGAGTGGGTTTAGACCCACGGCGAACGGGTGAGTAA
TACCTAGGAATGTATCTTTAGTTGAGGATATCCCATCGAAAGATGGTGCAATACTCAATGTGATCGCA
AGATTAAAGTCGCAAGACGCTAAAGAAGCAGCCTGGGCACCATCAGCTAGTTGGTAAGGTAATGGCTT
ACCAAGGCTATGACGGTTAGGGGTCCCGAGAGGGAGAACCCCCACACTGGAACTGCGACACGGTC
>Zotu6271
GAACGCTGGCGGCGTGCCTAACACATGCAAGTCGAGCGATCTTCTTCGGAAGAGAGCGGCGGACGGGT
GAGTAACGCGTGGGTAACCTGCCCTGTACACACGGATAACATACCGAAAGGTATGCTAATACGAGATA
ATATGCTTTTATCGCATGGTAGAAGTATCAAAGCTCCGGCGGTACAGGATGGACCCGCGTCTGATTAG
CTAGTTGGTGAGGTAACGGCTCACCAAGGCGACGATCAGTAGCCGACCTGAGAGGGTGATCGGCCACA
TTGGAACTGAGACACGGTC
>Zotu6273
GAACGCTAGCGGCAGGCCTAATACATGCAAGTCGAACGGTAACAGGAGTAGCAATACTTGCTGACGAG
TGGCGCACGGGTGCGTAACGCGTATGCAATCTACCTTTAACAGGTGAATAGCCCGAAGAAATTCGGAT

TAAAACACCATAGTATAATGAATAGGCATCTATTTATTATTAAAACTCCGGTGGTTAAAGATGAGCAT
GCGTGACATTAGCTAGTTGGTGAGGTAATGGCTCACCAAGGCAACGATGTCTAGGGGATCTGAGAGGA
TTAACCCCCACACTGGTACTGAGACACGGAC
>Zotu6275
TAACGCTGGCGGCGCGCCTAATACATGCAAGTCGAACGATGTAGCAATACATAGTGGCGCACGGGAGA
GTAACACGTGGATAATCTCCCCTCGAGAGAAGAATAACTAGTCGAAAGATTGGCTAATACTTCATAAG
ACCACAGAGACTAATGTCTCAGAGGTAAAAGTCGTAAGACGCTTGAGGATGAGTCTGCGTCCCATTAG
CTAGTTGGTGAGGTAATGGCTCACCAAGGCGACGATGGGTAGCTGGTCTGAGAGGATGATCAGCCTCA
CTGGAACTGACACACGGTC
>Zotu6276
GAACGCTAGCGGCAGGCTTAATACATGCAAGGCGAGGGGGCAGCAATGTCACCGTCGTACGGGTGCGT
AACGCGTATGCAACCTACCTTCAACTGGGGGATAGCCCGGGGAAACCCGGATTAATACCGCATAATAC
ATTGGTGTAGCATTACATTATTTGTTAAAGATTTATTGGTTGAAGATGGGCATGCGTTCGATTAGCTA
GTTGGTGTAGGTAACGGCTCACCAAGGCGACGATCGATAGGGGAGCTGAGAGGTTGATCCCCCACACG
GGCACTGAGATACGGGC
>Zotu6277
GAACGCTAGCGGCAGGCTTAACACATGCAAGTCGAGGGGTATAGTAGCAATACTAGAGACCGGCGCAC
GGGTGCGTAACGCGTATGCAATCTACCTTTTGCAGGGGAATAGCCCAGAGAAATTTGGATTAATGCCC
CATAGTATAATGCCTCGGCATCGAGATATTATTAAAGTCACAACGGCAAAAGATGAGCATGCGTCCCA
TTAGCTAGTTGGTAAGGTAACGGCTTACCAAGGCGACGATGGGTAGGGGTCCTGAGAGGGAGATCCCC
CACACTGGTACTGAGACACGGAC
>Zotu6278
GAACGCTGGCGGCGTGCCTAACACATGCAAGTCGAGCGGGGTCCATCCAGTAGCAATACTGGGGAAGA
CCTAGCGGCGAACGGGTGAGTAACACGTGAGAAACCTGCCCCGAAGTCTGGGATAACCCGAGGAAACT
CGAGCTAATACCGGATGCCCTCTTCTGGTCGCATGGCCGGAAAAGGAAAGATTCATCGCTTCGGGAGG
GTCTCGCGGCCTATCAGCTAGTTGGTGGGGTAACGGCCCACCAAGGCGTCGACGGGTAGCTGGTCTGA
GAGGACGATCAGCCACACTGGGACTGAGACACGGCC
>Zotu6281
GAACGCTGGCGGCGTGCTTAACACATGCAAGTCGAGCGAAGCGCAGAAACGGATTTCTTCGGATTGAA
GGTTCTGTGACTGAGCGGCGGACGGGTGAGTAACGCGTGGGGAACCTGCCCTGTACCGGGGGATAACA
GACAGAAATGTCTGCTAATACCGCATAAGCGCACGAGAAGGCATCTTCTTGTGTGAAAAACTCCGGTG
GTACAGGATGGACCCGCGTTGGATTAGCCAGTTGGCGGGGTAACGGCCCACCAAAGCGACGATCCATA
GCCGGCCTGAGAGGGCGACCGGCCACATTGGGACTGAGACACGGCC
>Zotu6282
CAACGCTGGCGGCGTGCCTCAGACATGCAAGTCGAACGATTAAGACTTTCTTCGGAGAGTGAATAAAG
TGGCGCACGGGTGAGTAACACGTAGGTAATCTACCTTTGAGTGGGGAATAACAATCGGAAACGATTGC
TAATACCGCATAATGCAGCGGCATCGCAAGATGACAGTTGTTAAAGGAGCAATTCGCTTAAAGAGGAG
CCTGCGGCAGATTAGCTAGTTGGTAAGGTAATGGCTTACCAAGGCGACGATCTGTAACCGACCTGAGA
GGGTGGTCGGTCACACTGACACTGAATAACGGGT
>Zotu6284
GAACGCTAGCGGCAGGCCTAATACATGCAAGTCAAACGGCTAGCAATAGCAGTGGCGCACGGGTGAGT
AACGCGTACACAACGTACCTTATAGAGGGGTATAGCATCTGGAAACTGGTGGTAATCCCCCATAGTAT
TATTGAATGGCATCATTTAATAATTAAACTTTTTGGCTATGAGATCGGTGTGCGTCTGATTAGCTAGT
TGGTAGGGTAACGGCTTACCAAGGCTACGATCAGTAGGGGGCGTGAGAGCGTGGTCCCCCACACGGGT
ACTGAGACACGGAC
>Zotu6285
GAACGCTGGCGGCGTGCTTAACACATGCAAGTCGAACGGAAAGGCCCCCAGCTTGCTGGGGGTACTCG
AGTGGCGAACGGGTGAGTAACACGTGGGTGATCTGCCCTGCACTTCGGGATAAGCCTGGGAAACTGGG
TCTAATACCGGATAGGAGCCATCTTTGGTGTGATGGTTGGAAAGTTTTTTCGGTGTAGGATGAGCCTG
CGGCCTATCAGCTTGTTGGTGGGGTAATGGCCTACCAAGGCGTCGACGGGTAGCCGGCCTGAGAGGGT
GTACGGCCACATTGGGACTGAGATACGGCC
>Zotu6286
GAACGCTGGCGGCGTGCCTAACACATGCAAGTCGAGCGGGGTCCATCTAGTAGCAATACTGGGGAAGA
CCTAGCGGCGAACGGGTGAGTAACACGTGAGAAACCTGCCCCGAAGTCTGGGATAACCCGAGGAAACT

CGAGCTAATACCGGATGCCCTTTTCTGGTCGCATGGCCGGAAACGGAAAGATTTATCGCTTCGGGAGG
GTCTCGCGGCCTATCAGCTAGTTGGTGGGGTAACGGCCCACCAAGGCGTCGACGGGTAGCTGGTCTGA
GAGGACGATCAGCCACACTGGGACTGAGACACGGCC
>Zotu6287
CAACGCTGGCGGCGCGCTTAACACATGCAAGTCGCACGAGAAAGGGGAGCAATCCCTGAGTACAGTGG
CGTACGGGTGAGTAACACGTGGATCATCTACCTTCTAGTGGGGAATAACCCTGGGAAACCGGGGCTAA
TACCGCATAAGCTCGAGAGAGGAAAGCAGCAATGCGCTGGAAGAGGAGTCCGCGGCCGATTAGCTAGT
TGGCAGGGTAAAAGCCTACCAAGGCATCGATCGGTAGCCGGCCTGAGAGGGCACACGGCCACACTGGC
ACTGAAACACGGGC
>Zotu6288
GAACGCTGGCGGCAGGCTTAACACATGCAAGTCGAGCGGGCATTTTCGGATGTCAGCGGCAGACGGGT
GAGTAACACGTGGGAACGTACCCTTCGGTTCGGAACAACTCTGGGAAACTGGAGCTAATACCGGATAC
GCCCTTTTGGGGAAAGGTTTACTGCCGAAGGATCGGCCCGCGTCTGATTAGCTAGTTGGTGGGGTAAC
GGCCTACCAAGGCGACGATCAGTAGCTGGTCTGAGAGGATGATCAGCCACACTGGGACTGAGACACGG
CC
>Zotu6289
GAACGCTGGCGGCGTGCCTAACACATGCAGGTCGAGCGAGTGAATCTCCTTCGGGAGTGAAGCTAGCG
GCGGACGGGTGAGTAACACGTGGGCAACCTGCCTCATAGAGGGGAATAGCCTTTCGAAAGGAAGATTA
ATACCGCATAAGATTGTAGCTTCGCATGAAGTAGCAATTAAAGGAGCAATCCGCTATGAGATGGGCCC
GCGGCGCATTAGCTAGTTGGTGAGGTAACGGCTCACCAAGGCGACGATGCGTAGCCGACCTGAGAGGG
TGATCGGCCACATTGGGACTGAGACACGGCC
>Zotu6291
GAACGCTGGCGGAGTGCCTAACACATGCAAGTCGAGCGGCAGCACGAGTAGTTTACTACTTGGTGGCG
AGCGGCGGACGGCTGAGTAACGCGTGGGAACGTGCCCCAAAGTGAGGAATAACTGCCCGAAAGGGTAG
CTAATGCCGCATATGATCTTCGGATTAAAGTATTTATTACGCTTTGGGAGCGGCCCGCGTCCGATTAG
GTAGTTGGTGAGGTAAAGGCTCACCAAGCCCACGATCGGTAGCTGGTCTGAGAGGATGACCAGCCAGA
CTGGAACTGAGACACGGTC
>Zotu6293
CAACGCTGGCGGCGCGCTTAACACATGCAAGTCGCACGAGAAAGGGTAGCAATATCTGAGTAAAGTGG
CGTACGGGTGAGTAACACGTGGATCATCTACCTTTTGGCGGGGAATACCTCCTGGAAACGGGAGACAA
TACCGCATAAGCTCGAAAGAGGAAAGCAGCAATGCACCGAAAGAGGAGTCCGCGGTCGATTAGCTAGA
TGGTGAGGTAATGGCTCACCATGGCGAAGATCGATAGCCGGCCTGAGAGGGCTCACGGCCACACTGGC
ACTGAAACACGGGC
>Zotu6295
GAACGCTGGCGGCATGCCTAAGACATGCAAGTCGAACGAGGTGGCCCAAAGAAGATTGAGTGCTTGCA
CAAAATTGGACTTGGATTACCACCTAGTGGCGGACGGGTGAGTAACACGTAGGTGACCTACCTTTGAG
TTTGGGATAACTATTAGAAATGATAGCTAATACCGAATTAAATGCAGATTCGTTTCTGTATTAAAAGG
AGCCTTTAAAGCTTCGCTTAAAGATGGGCCTGCGGCGTATTAGCTAGTTGGTGAGGTAATGGCTTACC
AAGGCGACGATGCGTAGCCGAACTGAGAGGTTGATCGGCCACATTGGGACTGAGACACGGCC
>Zotu6296
GAACGCTGGCGGCGCGCTTAACACATGCAAGTCGAGCGAGAACCAGGCCTTCGGGCCTGGGGACAGCG
GCGAACGGGTGAGTAACACGTGGGTAATCAACCCTCGATACTGGGATAGCCCGGGGAAACCCGGATTA
ATACCGGATGGCCCTTTGGATCCTCGGATCTGAAGGAAAAGGTAGCTTCGGCCTCCGATCGAGGACGA
GCCCGCGGCGGATTAGCTTGTTGGTGAGGTAACGGCCCACCAAGGCGACGATCCGTAGCTGGTCTGAG
AGGACGATCAGCCACACTGGGACTGAGACACGGCC
>Zotu6297
GAACGCTAGCGATAGGCTTAACACATGCAAGTCGAGGGGTAACGTGTTGGAAGCTTGCTTCCGATGAC
GACGACCGGCGGATGGGTGCGTAACGCGTATGCAACTTGCCTCACAGTGGAGAATAACCCGGAGAAAT
CCGGACTAATGTTCCATACACTCTTAAGTACGCCTGTACATGAGAGGAAAGATTTATCGCTGTGAGAT
AGGCATGCGTCCTATTAGGTAGTTGGTGAGGTAACGGCTCACCAAGCCGACGATAGGTAGGGGTGCTG
AGAGGCAGATCCCCCACATTGGGACTGAGACACGGCC
>Zotu6298
CAACGCTGGCGGCGTGCCTAACACATGCAAGTCGAACGCGAAAGTGGAGCAATCCATGAGTAGAGTGG
CAAACGGGTGAGTAACACGTGGGTGACCTGCCTTCGAGCGGGGGATAACGTCCCGAAAGGGACGCTAA

TACCGCATAACATCCTGCCTTTTAAGAGGTGGAGATCAAAGCCGGGGATCGCAAGACCTGGCACTTGA
AGAGGGGCCCGCGCCTGATTAGCTAGTTGGTGGGGTAATGGCCTACCAAGGCAACGATCAGTATCCGG
CCTGAGAGGGCGGACGGACACACTGGGACTGAGACACGGCC
>Zotu6299
GAACGCTGGCGGCGTGCCTAATACATGCAAGTCGAGCGAATCAATAGGGAGCTTGCTCCCTTGAGATT
AGCGGCGGACGGGTGAGTAACACGTGGGTAACCTGCCTGTAAGACTGGGATAACTCCGGGAAACCGGG
GCTAATACCGAATAACTTCTTTCTCCACATGGAGAAAGATTGAAAGATGGCTTCGGCTATCACTTACA
GATGGACCCGCGGCGCATTAGCTAGTTGGTGAGGTAACGGCTCACCAAGGCAACGATGCGTAGCCGAC
CTGAGAGGGTGATCGGCCACACTGGGACTGAGACACGGCC
>Zotu6300
GAACGCTGGCGGCGTGCTTAACACATGCAAGTCGAGCGAAGCAGTTAAGAAGATTCTTCGGATGAAAC
TTGATTGACTGAGCGGCGGACGGGTGAGTAACGCGTGGGTGACCTGCCCCATACCGGGGGATAACAGC
TGGAAACGGCTGCTAATACCGCATAAGCGCACAGAGCTGCATGGCTCAGTGTGAAAAACTCCGGTGGT
ATGGGATGGGCCCGCGTCTGATTAGGCAGTTGGCGGGGTAACGGCCCACCAAACCGACGATCAGTAGC
CGGCCTGAGAGGGCGACCGGCCACATTGGGACTGAGACACGGCC
>Zotu6301
GAACGCTGGCGGCGTGCTTAACACATGCAAGTCGAACGAAGCACTTTGGACAGATTCTTCGGATGAAG
TCCTTAGTGACTGAGTGGCGGACGGGTGAGTAACGCGTGGGTAACCTGCCTCATACAGGGGGATAACA
GTTAGAAATGGCTGCTAATACCGCATAAGCGCACGGTACTGCATGGTACAGTGTGAAAAACTCCGGTG
GTATGAGATGGACCCGCGTTGGATTAGCTAGTTGGGAGGGTAACGGCCTACCAAGGCGACGATCCATA
GCCGGCCTGAGAGGGTGGACGGCCACATTGGGACTGAGACACGGCC
>Zotu6302
GAACGCTGGCGGCATGCCTAACACATGCAAGTCGAACGAGACCTTCGGGTCTAGTGGCGCACGGGTGC
GTAACGCGTGGGAATCTGCCCTTGGGTTCGGAATAACTCAGAGAAATTTGAGCTAATACCGGATGATG
ACGTAAGTCCAAAGATTTATCGCCCAGGGATGAGCCCGCGTAAGATTAGCTAGTTGGTGGGGTAAAGG
CCTACCAAGGCGACGATCTTTAGCTGGTCTGAGAGGATGATCAGCCACACTGGGACTGAGACACGGCC
>Zotu6303
GAACGCTGGCGGCGTGCCTAATACATGCAAGTCGAGCGGTAAGGCCTCTTCGGAGGTACACGAGCGGC
GGACGGCTGAGTAACGCGTAGGAACGTACCCCAAAGTGAGGGATAAGCACCGGAAACGGTGTCTAATA
CCGCATGTGCTCTAAGGAGTAAAGCTTCGGCGCTTTGGGAACGGCCTGCGTATGATTAGCTTGTTGGT
GAGGTAAAAGCTCACCAAGGCGACGATCATTAGCTGGTCTGAGAGGATGATCAGCCAGACTGGGACTG
AGACACGGCC
>Zotu6304
GAACGCTGGCGGCGTGCTTAACACATGCAAGTCGAACGATGAACCCGCTTGCGGGGGATTAGTGGCGA
ACGGGTGAGTAACACGTGAGTAACCTGCCCTTAACTCTGGGATAAGCCTTGGAAACGGGGTCTAATAC
TGGATATTGACTTTACCTCGCATGGGGTTTGTTGAAAGATTTATTGGTTTTGGATGGACTCGCGGCC
TATCAGCTTGTTGGTGAGGTAATGGCTCACCAAGGCGACGACGGGTAGCCGGCCTGAGAGGGTGACCG
GCCACACTGGGACTGAGACACGGCC
>Zotu6307
GAACGCTGGCGGCGTGCCTAACACATGCAAGTCGAACGGGATCCATGGAGTGCTTGCACTCCGGAAGA
TCTAGTGGCGAACGGGTGAGTAACACGTGAGAAACCTGCCCTAGACTCTGGGATAACCTCGGGAAACC
GAAGCTAATACCGGATATTCTCCTTTGGTCGCATGGCCAGTTGAGGAAAGTTTTTCGGTCTGGGAGGG
TCTCGCGGCCTATCAGCTAGTTGGTGGGGTAACGGCCCACCAAGGCTACGACGGGTAGCTGGTTTGAG
AGAACGATCAGCCACACTGGAACTGAGACACGGTC
>Zotu6309
GAACGCTGGCAGTGTGCCTGAGATATGCAAGTCGAGCGAAGTTCCGACCGCAAGGTTGGAATTCAGCG
GCAGACGGCTGAGTAACACGTGGGAATCTGCCCCGAAATAAAGGATAGCTCCTCGAAAGAGGGGGTAA
TACTTTATACTCTCCAACGCAAGTTGGAGCAAAGCGCTTTACCGCGCGTTTCGGGATGAGCCTGCGTC
CTATCAGGTAGTTGGTGGGGTAAAAGCCTACCAAGCCTACGACGGGTAGCTGGACCGAGAGGTTGGTC
AGCCAGAAGAGCACTGAGACACGGGC
>Zotu6310
GAACGCTGGCGGCGTGCTTAACACATGCAAGTCGAACGGAAAGGCCTCTTGCTTGCAGGAGGTACTCG
AGTGGTGAACGGGTGAGTAACACGTGGGTGATCTGCCTTGCACTCTGGGATAAGCTTGGGAAACTGGG
TCTAATACCGGATATGAACTGCCTTTAGTGTGGTGGTTGGAAAGTTTTTTCGGTGCAAGATGAGCTCG

CGGCCTATCAGCTTGTTGGTGGGGTAATGGCCTACCAAGGCGTCGACGGGTAGCCGGCCTGAGAGGGT
GTACGGCCACATTGGGACTGAGATACGGCC
>Zotu6311
GAACGCTGGCGGCGTGCCTAACACATGCAAGTCGAACGGAGATTCTAGCAATAGAATCTTAGTGGCGA
ACGGGTGAGTAACGCGTAGACAACCTACCTTCGAGATGGGGACAACACTGCGAAAGTGGTGCTAATAC
CGAATGTTGTATTTTGATTGCATAATTGAAATACTAAAGATGGCCTCTATTTATAAGCTATCGCTAGA
AGATGGGTCTGCGTCTGATTAGCTAGTTGGTGAGGTAATGGCTCACCAAGGCGACGATCAGTAGCCGG
TCTGAGAGGATGGACGGCCACACTGGGACTGAGACACGGCC
>Zotu6312
GAACGCTGGCGGCGTGCCTAAGACATGCAAGTCGAGTGGGTTTAGACCCACGGCGAACGGGTGAGTAA
TACCTAGGAACTTTCTTTAAGTGGAGCATAACTAGTCGAAAGATTAGATAATTCTCCATGTGGCAGCA
ATGCTAAAGTCGCAAGACGCTTAAAGTGAGGCCTGGGCACCATCAGCTAGTTGGTAAGGTAATGGCTT
ACCAAGGCTATGACGGTTAGGGGTCCCGAGAGGGATAACCCCCACACTGGAACTGCGACACGGTC
>Zotu6314
GAACGCTGGCGGCGTGCTTAACACATGCAAGTCGAACGGTGACGGAGAGCTTGCTCTCCTGATCAGTG
GCGAACGGGTGAGTAACACGTGGGCAACCTGCCCCTGACACTGGGATAACTCCGGGAAACCGGGGCTA
ATACCGGATATTACGCGAGATGGCATCTTCTCGCGCGGAAAGTTTTTCGGTCAGGGATGGGCCCGCGG
CCTATCAGCTTGTTGGTGAGGTAACGGCCCACCAAGGCGACGACGGGTAGCCGGCCTGAGAGGGCGAC
CGGCCACACTGGGACTGAGACACGGCC
>Zotu6315
GAACGCTAGCGGCGTGCCTAAGGTATGCAAGTCGAGCGGGTCCGGAGAGCTTGCTTTCCGGGCTAGCG
GCAAACGGTTGAGTAACACGTAGACAACCTGCCTCCGAGTGGGGGATAACAGCTCGAAAGGGCTGCTA
ATACCGCATGTGGCCGGGAGGGGCATCTCTTCCAGGCTAAACGATGGCGCAAGCCTTCGGCTCGGAGA
TGGGTCTGCGGCCTATCAGCTTGTTGGTGGGGTAACGGCCTACCAAGGCGACGACGGGTAGCTGGTCT
GAGAGGATGACCAGCCAGACGGGGACTGAGACACGGCC
>Zotu6316
GAACGCTAGCGGCGTGCCTAACACATGCAAGTCGAGCGATCTTCTTCGGAAGAGAGCGGCGGACGGGT
GAGTAACGCGTGGGTAACCTGCCCTGTACACACGGATAACATACCGAAAGGTATGCTAATACGAGATA
ATATGCTTTTATCGCATGGTAGAAGTATCAAAGCTCTGGCGGTACAGGATGGACCCGCGTCTGATTAG
CTAGTTGGTAAGGTAACGGCTTACCAAGGCGACGATCAGTAGCCAACCTGAGAGGGTGATCGGCCACA
TTGGAACTGAGACACGGTC
>Zotu6318
GAACGCTGGCGGCGTGCTTAACACATGCAAGTCGAACGATGATGCCCGTGCTTGCACGGGTGGATTAG
TGGCGAACGGGTGAGTAACACGTGAGTAACCTGCCCTTCACTCTGGGATAAGCCTGGGAAACTGGGTC
TAATACTGGATATGACCAATTGTCGCATGGTGGTTGGTGGAAAGATTTTTTGGTGGGGGATGGACTCG
CGGACTATCAGCTTGTTGGTGGGGTAGTGGCCTACCAAGGCTTTGACGGTTAGCCGGCCTGAGAGGGC
GACCGGCCACACTGGGACTGAGACACGGCC
>Zotu6319
GAACGCTGGCGGCGTGCTTAACACATGCAAGTGGAGCGACGAACCCGGACTTGTCCGGGGGCAGAGCC
GCGAACGGGTGAGTAACACGTGGGTAACCCACCTCGATGACCGGGACAACCCGAGGAAACTCGGGCTA
ATACCGGATGTGATGGTTCTCTATAAGGAGAACTAGGAAAGATAGCTTCGGCCTTCGCATCGAGACGG
GCCCGCGGCCCATTAGCTTGTTGGTGGGGTAATGGCCTACCAAGGCTTCGATGGGTAGCTGGTCTGAG
AGGACGATCAGCCACACTGGGACTGAGACACGGCC
>Zotu6321
GAACGCTGGCGGCATGCTTAACACATGCAAGTCGAACGAAACCAAGGCTTCGGCCTGAAGTTTAGTGG
CGCACGGGTGCGTAACGCGTGGGAATCTGCCCTTAGGTACGGAATAACTCAGAGAAATTTGTGCTAAT
ACCGTATGATGTCGAAAGACCAAAGATTTATCGCCTAAGGATGAGCCCGCGTAAGATTAGCTAGTTGG
TGAGGTAAAAGCTCACCAAGGCGACGATCTTTAGCTGGTCTGAGAGGATGATCAGCCACACTGGGACT
GAGACACGGCC
>Zotu6322
GAACGCTGGCGGCGTGCCTAATACATGCAAGTCGAGCGGACCTCTTTTGAGGTCAGCGGCGGACGGGT
GAGGAACACGTGGGTAATCTGCCTTTCAGACCGGAATAACGCCCGGAAACGGGCGCTAATGCCGGATA
CGCCCGCGAGGAGGCATCTTCTTGCGGGGAAAGGCCCGATTGGGCCGCTGAGAGAGGAGCCCGCGGCG
CATTAGCTAGTTGGCGGGGTAACGGCCCACCAAGGCGACGATGCGTAGCCGACCTGAGAGGGTGACCG

GCCACACTGGGACTGAGACACGGCC
>Zotu6323
GAACGCTGGCGGCATGCCTTACACATGCAAGTCGAACGGTAACGCGGGGGCAACCCTGGCGACGAGTG
GCGAACGGGTGAGTAATACATCGGAACATATCCAGTAGCGGGGGATAACCTATCGAAAGATGGACTAA
TACCGCATACGATCTACGGATGAAAGCAGGGGACCGCAAGGCCTTGCACTATTGGAGTGGCCGATGTC
GGATTAGCTAGTTGGTGGGGTAAAAGCTTACCAAGGCGACGATCCGTAGCTGGTCTGAGAGGACGATC
AGCCACACTGGGACTGAGACACGGCC
>Zotu6324
GAACGCTGGCGGCGTGCTTAACACATGCAAGTCGAACGGTAAGGCCCTTCGGGGTACACGAGTGGCGA
ACGGGTGAGTAACACGTGGGTAATCTGCCCTGCACATCGGGATAAGCCTGGGAAACTGGGTCTAATAC
CGAATAGGACCACAGGACACATGTCCTGTGGTGGAAAGCTTTTGCGGTGTGGGATGGGCCCGCGGCCT
ATCAGCTTGTTGGTGGGGTGATGGCCTACCAAGGCGACGACGGGTAGCCGGCCTGAGAGGGTGTCCGG
CCACACTGGGACTGAGATACGGCC
>Zotu6327
GAACGCTGGCGGCGTGCTTAATACATGCAAGTCGAGCGAGGTTACTGGAGTGGAGCCCTTCGGGGTGA
AGCTGCTTTAACCTAGCGGCGGACGGGTGAGTAACGCGTGGGTAACCTACCCCAAAGCCCGGGATAAC
AGCGGGAAACCGCTGCTAATACCGGATGACTTTACCTTGGGGCATCTTGAGGTAAAGAAAGGTTAACG
CTTTGGGATGGGCCCGCGTCCCATTAGCTAGTTGGTGAGGTAACGGCTCACCAAGGCGACGATGGGTA
GCCGGCCTGAGAGGGTGGACGGCCACACTGGGACTGAGACACGGCC
>Zotu6328
GAACGCTGGCGGCATGCCTAACACATGCAAGTCGAACGAGACCTTCGGGTCTAGTGGCGCACGGGTGC
GTAACGCGTGGGGATCTGCCCTTTGCTTCGGAATAACAGTTAGAAATGACTGCTAATACCGGATGATG
TCGTAAGACCAAAGATTTATCGGCAAAGGATGAACCCGCGTAGGATTAGCTAGTTGGTAGGGTAAAGG
CCTACCAAGGCGACGATCCTTAGCTGGTCTGAGAGGATGATCAGCCACACTGGGACTGAGACACGGCC
>Zotu6329
GAACGCTGGCGGCGTGCCTAACACATGCAAGTCGAGCGGAGCTTTACTCAACACTGAGTATTCATGTT
AAATGAGTGCTGAGTGTTGAGTAAAGTTTAGCGGCGGACGGGTGAGTAACGCGTGGGCAACCTGCCTC
GTACTGGGGGATAACACTGGGAAACTAGTGCTAATACCGCATAACAAAGCATTACCGCATGGTAGAGC
TTTCAAAGGAGCAATCCGGTATGAGATGGGCCCGCGTCCGATTAGCTAGTTGGTGGGGTAAAGGCCTA
CCAAGGCGACGATCGGTAGCCGAACTGAGAGGTTGATCGGCCACATTGGGACTGAGACACGGCC
>Zotu6330
GAACGCTAGCGGCAGGCTTAATACATGCAAGTCGAGGGGCAGCATGAGTGTAGCAATACATTTGATGG
CGACCGGCAAACGGGTGCGGAACACGTACAGAACCTCCCTTTAAGTGGTGAATAGCCCAGAGAAATTT
GGATTAATACACCGTAACATATAGAAGTGGCATCACTTTTATATTAAAGCTCCGGCGCTTAAAGATGG
CTGTGCGGCTGATTAGGTAGTTGGCGGGGTAACGGCCCACCAAGCCTGCGATCAGTAACTGGTGTGAG
AGCACGACCAGTCACACGGGCACTGAGACACGGGC
>Zotu6332
GAACGCTGGCGGCGTGTTTAACACATGCAAGTCGAGCGAGAAGCTTTCCAGTCGAATCTTCGGATGAA
ACAGGATTGCGGAAAGCGGCGGACGGGTGAGTAACGCGTAGGCAACCTGCCCCCTGCAGAGGGATAGC
CTCGGGAAACCGGGATTAAAACCTCATAATGCGCGAGTGAGACATCTCACATGCGCCAAAGATTTATC
GGCAGGGGATGGGCCTGCGTCTGATTAGTTAGTTGGTGGGGTAACGGCCTACCAAGGCGACGATCAGT
AGCCGACCTGAGAGGGTGATCGGCCACATTGGAACTGAGACACGGTC
>Zotu6335
GAACGCTGGCGGCATGCCTAACACATGCAAGTCGAACGAAGGCTTCGGCCTTAGTGGCGCACGGGTGC
GTAACGCGTGGGAATCTGCCCTTGGGTTCGGAATAACAGTTGGAAACGACTGCTAATACCGGATGATG
TCGCGAGACCAAAGATTTATCGCCCAGGGATGAGCCCGCGTCGGATTAGGTAGTTGGTGGGGTAAAGG
CCTACCAAGCCGACGATCCGTAGCTGGTCTGAGAGGATGATCAGCCACACTGGGACTGAGACACGGCC
>Zotu6336
GAACGCTGGCGGCGTGCTTAACACATGCAAGTCGAGCGGTAAGGCCCTTTCGGGGGTACACGAGCGGC
GAACGGGTGAGTAACACGTGAGTAATCTGCCCTTCACTTCGGGATAAGCTTCGGAAACGGGGTCTAAT
ACCGGATATGACACAGTTTCGCATGAGATCTGTGTGGAAAGTTTTTTCGGTGGGGGATGTGCTCGCGG
CCTATCAGCTTGTTGGTGGGGTAATGGCCTACCAAGGCTTCGACGGGTAACCGGCCTGAGAGGGTGAC
CGGTCACACTGGGACTGAGACACGGCC
>Zotu6338

GAACGCTGGCGGCACGCTTAACACATGCAAGTCGAACGAATTGGATCACTTCGGTGATTTAGTTAGTG
GCGGACGGGTGAGTAACACGTGGGAACCTGCCTTGTAGAGGGGGATAACTGCTGGAAACGGCAGCTAA
TACCGCATACGCCCTGAGGGGGAAAGATTTATCGCTACAAGATGGTCCCGCGTCGGATTAGGTAGTTG
GTAGGGTAACGGCCTACCAAGCCGATGATCCGTAGCTGGTCTGAGAGGATGGCCAGCCACACTGGGAC
TGAGACACGGCC
>Zotu6339
GAACGCTGGCGGCATGCTTAACACATGCAAGTCGAACGAGCTAATGGGTAACTATTAGTGTAGTGGCG
GACGGGTGCGTAACGCGTAAGAACCTACCTTTTGGAGGGGGATAACATCGGGAAACTGTTGGTAATAC
CCCATAATGCTGAGGAGTTAAAGCAGCGAGAGCTGGCCAAAAGAGGGGCTTGCGTCTGATTAGCTAGT
TGGTGGGGGTAAAAGCCTCCCAAGGCCACGATCAGTAGCTGGTTTGAGAGGATGATCAGCCACACTGG
GACTGAGACACGGCC
>Zotu6340
GAACGCTGGCGGCATGCCTAATACATGCAAGTCGAACGCTGAAGCACTCTGCTTGCAGAGTGCGGATG
AGTGGCGAACGGGTGAGTAACACGTGGGAAATCTGCCTAAGAGTGGGGGATAACATTCGGAAACGGAT
GCTAATACCGCATATATTCTTGAACCGCATGGTTCTGGAAAGAAAGACGGCTTTGCTGTCGCTTTTAG
ATGATCCCGCGGCGTATTAGTTAGTTGGTGGGGTAACGGCCTACCAAGACCATGATACGTAGCCGACC
TGAGAGGGTGATCGGCCACACTGGGACTGAGACACGGCC
>Zotu6341
GAACGCTGGCGGCGTGCTTAACACATGCAAGTCGAACGCTGAGGCTCCCAGCTTGCTGGGGGTGGATG
AGTGGCGAACGGGTGAGTATCACGTGAGGAACCTGCCCTGAACTTCGGGATAAGCCTGGGAAACTGGG
TCTAATACCGGATAGGACATTCTACCGCATGGTGGGGTGTGGAAAGTTTTAACGGTTTGGGATGGTCT
CGCGGCCTATCAGCTTGTTGGTGGGGTAATGGCCTACCAAGGCGATGACGGGTAGCCGGCCTGAGAGG
GTGACCGGCCACACTGGGACTGAGACACGGCC
>Zotu6344
GAACGCTAGCGGCAGGCCTAACACATGCAAGTCGAGGGGCAGCACGAATTAGCTTGCTAATTTGGTGG
CGACCGGCGCACGGGTGCGTAACGCGTATGCAACCTACCTTATACAGGAGTATAGCCCGGAGAAATTC
GGATTAATCCTCCATGGTACTGAGACACCGCATGGTGCTTCAGTTAAAGCTTCGGCGGTATAAGATGG
GCATGCGTCCTATTAGCTAGATGGTAAGGTAACGGCTTACCATGGCTACGATAGGTAGGGGGCCTGAG
AGGGTGGTCCCCCACACTGGTACTGAGACACGGAC
>Zotu6347
TAACGCTAGCGGCAGGCCTAATACATGCAAGTCGAACGGCAGCACGGGTAGCAATACCTGGTGGCGAG
TGGCGCACGGGTGCGTAACGCGTATGCAATCTACCTGTAACTGGGGAATAGCCTTCCGAAAGGAAGAT
TAATACCGCATAATATCACGAGACGGCATCGCCTTGTGATCAAATCTCCGGAGGTTACAGATGAGCAT
GCGTGACATTAGCTAGTTGGTGAGGTAACGGCTCACCAAGGCTACGATGTCTAGGGGGTCTGAGAGGA
TGATCCCCCACACTGGTACTGAGACACGGAC
>Zotu6348
GAACGCTAGCGGCAGGCTTAATACATGCAAGGCGAGGGGGCAGCAATGTCACCGTCGTACGGGTGCGT
AACGCGTATGCAACCTACCTTCAACTGGGGGATAGCCCGGGGAAACCCGGATTAATACCGCATAATAC
ATTGATATAGCATTATACTAATTGTTAAAGATTTATTGGTTGAAGATGGGCATGCGTTCGATTAGCTA
GTTGGTATGGGTAACGGCCTACCAAGGCAACGATCGATAGGGGAGCTGAGAGGTTGATCCCCCACACG
GGCACTGAGATACGGGC
>Zotu6350
GAACGCTGGCGGCGTGCCTAATACATGCAAGTCGAGCGCCCCTTTAAGAGGTGATAATCGAAAGATTA
TCGGTTCCTATGGGGAGCGGCGGACGGCTGAGTAACGCGTGGGAACGTGCCCCAAAGTGAGGGATAAG
CACCGGAAACGGTGTCTAATACCGCATATGATCTTCGGATTAAAGCTTTATGCGCTTTGGGAGCGGCC
TGCGTCCTATTAGATAGTTGGTGAGGTAAAGGCTCACCAAGTCAACGATGGGTAGCTGGTCTGAGAGG
ATGACCAGCCAGACTGGAACTGAGACACGGTC
>Zotu6351
GAACGCTAGCGGCAGGCTTAATACATGCAAGTCGTACGGGATCCGAGGTAGCAATACTTTGGTGAGAG
TGGCGCACGGGTGCGTAACACGTATGCAACCTACCTTTAACCGGGACACAACCTCGAGAAATTGAGGC
TAATATCCCATAACACTGCGAAATGGCATCATTTTGCAGTTAAAACTCCGGTGGTTATAGATGGGCAT
GAGCGACATTAGCTAGTTGGTGAGGTAACGGCCCACCAAGGCTACGATGTCTAGGGGTTCTGAGAGGA
TTATCCCCCACACTGGTACTGAGACACGGAC
>Zotu6353

GAACGCTGGCGGCAGGCTTAACACATGCAAGTCGAGCGGTAGCACAGGGGAGCTTGCTCCCTGGGTGA
CGAGCGGCGGACGGGTGAGTAATGTCTGGGAAACTGCCTGATGGAGGGGGATAACTACTGGAAACGGT
AGCTAATACCGCATAACGTCGCAAGACCAAAGAGGGGGACCTTCGGGCCTCTTGCCATCAGATGTGCC
CAGATGGGATTAGCTAGTAGGTGGGGTAACGGCTCACCTAGGCGACGATCCCTAGCTGGTCTGAGAGG
ATGACCAGCCACACTGGAACTGAGACACGGTC
>Zotu6354
GAATGCTGGCGGCGTGGATAAGTCATGCAAGTCAAGGGGGCCCGCAAGGGCAACCGGCAGACGAGGTA
GTAACACGTAGGTACGTCCCCCGAAGTCGGGCATAGCCAGCCGAAAGGTTGGGTAATTCCCGATAGTC
CCGCAAGGGTAAAGGTTTTTCGCTTCGGGAGCGGCCTGCGTAGTATCAGCTAGTTGGTAGGGTAACGG
CCTACCAAGGCTACGACGCTTAGGGGAGCTGAGAGGCTGACCCCCACCGATGGAACTGCGACACGGTC
>Zotu6355
GAACGCTGGCGGCATGCCTAACACATGCAAGTCGAACGGGATGGAGCCTTCGGGCTCCGGCGAGTGGC
GAACGGGTGAGTAACACGTGGGTAACCTGCCCTTCACTGGGGGACAACCGAGGGAAACTTCGGCTAAT
ACCGCATGATGTGCGTTGTCTCAAGGCGACGTACCAAAACTCCGGTGGTGAAGGATGGGCCCGCGGCG
GATTAGCTAGTTGGTGGGGTAATGGCCTACCAAGGCGACGATCCGTAGCTGGTCTGAGAGGACGATCA
GCCACACTGGGACTGAGACACGGCC
>Zotu6356
GAACGCTGGCGGCATGCCTAACACATGCAAGTCGTACGAGAAGATCCCGCAAGGGATTTGTAAAGTGG
CGCACGGGTGAGTAACACGTGGGTAATCTGCCCTTTAGCTTGGGATAACCCTTCGAAAGAAGGGCTAA
TACCGAAGAGACCACAAGGACATCAGTCCAAGGGGTTAAAGATTGGGACCGCAAGGCCAATCACTGAA
GGATGAGCCCGCGCACTATCAGCTAGTTGGTGAGGTAATGGCTCACCAAGGCTATGACGGTTAGCTGG
TCTGAGAGGATGATCAGCCACACTGGAACTGAGACACGGTC
>Zotu6357
GAACGCTGGCGGCGCACTTAACACATGCAAGTCGAGCGAGAACCAGGGCTTCGGCCCTGGGGACAGCG
GCGAACGGGTGAGTAACACGTGGATAACCTGCCCTCGGCACCGGGATAGCCCGGGGAAACCCGGATTA
ATACCGGATAGCCATTCCGACTCTCGGGTCGGTCTGAAAAGGTAGCTTCGGCCTCTGGCCGAGGATGG
GTCCGCGGCGGATTAGCTTGTTGGTGGGGTAACGGCCTACCAAGGCACCGATCCGTAGCTGGTCTGAG
AGGACGATCAGCCACACTGGGACTGAGACACGGCC
>Zotu6360
GAACGCTAGCGGCAGGCCTAATACATGCAAGTCGAACGGGATTGGGGCAGTAGCTTGCTACTGCCGCA
TGAGAGTGGCGCACGGGTGCGTAACGCGTGAGCAACCTGCCCATGTCAGGGGTATAGCCCGTTGAAAG
ACGGATTAACACCGCATGAAGATGGTTTCCGGCATCGGGGACTGTCCAAATATTTATAGGACATGGAT
GGGCTCGCGTGACATTAGCTAGTTGGTGCGGTAACGGCGCACCAAGGCGACGATGTCTAGGGGCTCTG
AGAGGAGAATCCCCCACACTGGTACTGAGACACGGAC
>Zotu6361
GAACGCTGGCGGCATGCCTAGCACATGCAAGTTGAACGAGATCTTCGGGTCTAGTGGCGCACGGGTGC
GTAACGCGTGGGAATCTGCCCTTTGGTTCGGAATAACAGTTGGAAACGACTGCTAATACCGGATGATG
ACGAAAGTCCAAAGATTTATCGCCAGAGGATGAGCCCGCGTAGGATTAGCTAGTTGGTGTGGTAAAGG
CGCACCAAGGCGACGATCCTTAGCTGGTCTGAGAGGATGATCAGCCACACTGGGACTGAGACACGGCC
>Zotu6362
GAACGTTGGCGGCATGCCTAACACATGCAAGTCGAACGCGGACTTCGGTCCGAGTGGCGAACGGGTGC
GTAACACGTAGGTGACCTGCCCATCGGTGGGGGATAACCGCGAGAAATCGTGGCTAATACCGCATGAG
ATCGTGCTGTTTTGAAAGGCACGAGGAAAGCTTGATGCGCCGGTGGAGGGGCCTGCGGCCTATCAGCT
AGTTGGTGAGGTAACGGCTCACCAAGGCGATGACGGGTAGCTGACCTGAGAGGGTGGTCAGCCACAGG
GGAACTGAGAGACGGTC
>Zotu6363
GAACGCTATCGGTATGCTTAACACATGCAAGTTGAACGGATTATTTAGGGCTTGCTCTAAATAATTAG
TAGCAAACGGGTGAGTAACACGTGGGAATCTACCCATCAGTACGGAATAACATTTAGAAATAAATGCT
AATACCGTATATTCTCTTAGGAGGAAAGATTTATCGCTGATGGATGAGCCCGCGTCAGATTAGGTAGT
TGGTAGGGTAATGGCCTACCAAGCCAACGATCTGTAGCTGGTCTGAGAGGATGATCAGCCACACTGGG
ACTGAGACACGGCC
>Zotu6364
GAACGCTGGCGGCGTGTTTAACACATGCAAGTCGAGCGAGAAACCTATGATGGATTCTTCGGATGACA
GATTAGGCGGAAAGCGGCGGACGGGTGAGTAACGCGTAGGCAACCTGCCCCTTGCAGAGGGATAGCCT

CGGGAAACCGGGATTAAAACCTCATAATGCCACTTGATGACATCTTCGGGTGGCCAAAGATTTATCGG
CAAGGGATGGGCCTGCGTCTGATTAGTTAGTTGGTGGGGTAACGGCCTACCAAGGCAGCGATCAGTAG
CCGACCTGAGAGGGTGATCGGCCACATTGGAACTGAGACACGGTC
>Zotu6365
GAACGCTAGCGGCAGGCCTAATACATGCAAGTCAAGGGGTAGCAATACACCGGCGGACGGGTGCGTAA
CGCGTACATAACATGCCCTTAGGTGGGGGATAGTATTGGGAAACTGATAGTAATACCCCATAGTATTG
ATTTATGGCATCATGAGTCAATTAAAGGTTACGCCTAAGGATTGATGTGCGTATGATTAGCTAGTTGG
TGGGGTAACGGCTCACCAAGGCGACGATCATTAGGGGGTGTGAGAGCATGACCCCCCACACGGGTACT
GAGACACGGAC
>Zotu6367
GAACGCTGGCGGCGTGCTTAACACATGCAAGTCGAACGGAAAGGCCACAGCTTGCTGTGGTACTCGAG
TGGCGAACGGGTGAGTAACACGTGGGTGATCTGCCCTGTACTTCGGGATAAGCTTGGGAAACTGGGTC
TAATACCGGATATGACCACTTTTTGGATGGGGTGGTGGAAAGCTTTTGCGGTATGGGATGAGCTCGCG
GCCTATCAGCTTGTTGGTGGGGTAATGGCCTACCAAGGCGTCGACGGGTAGCCGGCCTGAGAGGGTGT
ACGGCCACATTGGGACTGAGATACGGCC
>Zotu6369
GAACGTTAGCGGCGCGCCTAACACATGCAAGTCGAGCGAGAAAGGGCTTCGGCCCCGGTAAAGCGGCG
CACGGGTGAGTAACACGTAGGTAATCTGCCCCTGAGCGGGGGATAACCTTCCGAAAGGAGGGCTAATA
CCGCATAAGACCACGCCTGCGAGAGCAGGAGGGGTCAAAGCCGGCCTCTATACATAAGCTGGCACTTG
GGGATGAGCCTGCGGCCCATCAGCTAGTTGGTGGTGTAACGGACCACCAAGGCGAAGACGGGTAGCTG
GTCTGAGAGGATGATCAGCCACACTGGAACTGAGACACGGTC
>Zotu6371
CAACGCTGGCGGCGCGCCTAACACATGCAAGTCGAACGAGAAAGGGGAGCAATCCCTGAGTAAAGTGG
CGTACGGGTGAGTAACACGTGGATCATCTGCCTTTTAGTGGGGAATACCGCTGGGAAACCGGTGACAA
TACCGCATAATCTCGAGAGAGGAAAGCCGCAAGGCGCTGAAAGAGGAGTCCGCGGTCGATTAGCTAGT
TGGTAAGGTAATGGCTTACCAAGGCTGTGATCGATAGCCGGCCTGAGAGGGCACACGGCCACACTGGC
ACTGAAACACGGGC
>Zotu6372
CAACGCTGGCGGCGCGCTTAACACATGCAAGTCGCACGAGAAAGGGAGCAATCCTAAGTAAAGTGGCG
TACGGGTGAGTAACACGTGGATCATCTACCTTTTGGCGGGGAATACCTCCTGGAAACGGGAGACAATA
CCGCATAAGCTCGAAAGAGGAAAGCAGCAATGCACCGAAAGAGGAGTCCGCGGTCGATTAGCTAGATG
GTGAGGTAATGGCTCACCATGGCGAAGATCGATAGCCGGCCTGAGAGGGCTCACGGCCACACTGGCAC
TGAAACACGGGC
>Zotu6374
GAACGCTGGCGGCGTGCTTAACACATGCAAGTCGAGCGAACCTTCGGGTTAGCGGCGAACGGGTGAGT
AACACGTGGGCAACCTGCCCCGAGCTCTGGGATAACCCCGGGAAACCGGGGCTAATACCGGATACGAG
GCCGACAGACATCTGTCGGTCTGGAAAGTTTTTCGGCTCGGGATGGGCCCGCGGCCTATCAGCTTGTT
GGTGGGGTAACGGCCTACCAAGGCGACGACGGGTAGCCGGCCTGAGAGGGCGACCGGCCACACTGGGA
CTGAGACACGGCC
>Zotu6375
GAACGCTGGCGGCGTGCCTAATACATGCAAGTCGAGCGGACTTGAGGGAGCTTGCTCCTGATGGTCAG
CGGCGGACGGGTGAGTAACACGTGGGTAACCTGCCTGGCAGATCGGGATAACGCTTGGAAACGAGTGC
TAATACCGGATAGTCTTTTGAACCGCATGGTTCGAAAGGGAAAGAAGCTATCGCTTCACTGTCAGATG
GACCCGCGGCGCATTAGCTAGTTGGTGAGGTAACGGCTCACCAAGGCGACGATGCGTAGCCGACCTGA
GAGGGTGACCGGCCACACTGGGACTGAGACACGGCC
>Zotu6376
GAACGCTGGTGGCGTGTTTATACATGCAAGTCGAGCGAGGACGGTAGCAATACCGAGCCGAGCGGCG
AACGGGTGAGTAACGCGTAAGCAATCTGCCCCACAATCGGGAACAACCGTGCCAACGCGCGGCTAATG
CCGGATATTGTAGTCTACTGCATAGTAGGCCAACGAAAGATTTATTGTTGTGGGATGAGCTTGCGTCC
GATTAGCTAGTTGGCGGGGTAATAGCCCACCAAGGCGACGATCGGTAGCCGGCCTGAGAGGGTGATCG
GCCACATTGGGACTGAGATACGGCC
>Zotu6377
GAACGCTGGCGGCATGCCTAATACATGCAAGTCGAACGGAGGGTGCTTGCACCCTTAGTGGCAAACGG
GTGAGTAACGCGTGGGAATCTACCCTTTGGTGGGGGACAACTGGAGGAAACTTCAGCTAATACCGCAT

ATTCTTGGCAACAAGGAAAGATTTATCGCCAAAGGATGAGCCCGCGTCAGATTAGGTAGTTGGTAGGG
TAATGGCCTACCAAGCCTGTGATCTGTAGCTGGTCTGAGAGGATGATCAGCCACACTGGGATTGAGAT
ACGGCC
>Zotu6378
GAACGCTGGCGGCGTGGTTAAGGAATGCAAGTCGAACGGTAAGCAGGTAATTTATTATTTGCTTATAG
TGGCAAACGGGTTAGTAACGCGTAGGTAACTTACCTTGAAGCCCCGAATAATTCGCCGAAAGGCGAAC
TAATACGGGATGGTAATGAGGGGGCGAAAGCCATTCTCATTTAAAGATTCATCACTTCAAGAGAGGCC
TGCGTCCTATCAGCTAGTTGGCGAGGTAATTGCTCACCAAGGCTATGACGGGTAGGGGGACTGAGAGG
TTGGCCCCCAACATCGGAACTGAGACACTGTC
>Zotu6379
CAACGCTGGCGGCGTGCCTAACACATGCAAGTCGAACGCGAAAGTGGAGCAATCCATGAGTAGAGTGG
CAAACGGGTGAGTAACGCGTGGGCGACCTACCTTCGAGTGGGGGATAACATTCCGAAAGGAGTGCTAA
TACCGCATGACATCCCGTGTTTGGATACACGGAGATCAAAGCCGGGGATCGTAAGACCTGGCGCTTGA
AGAGGGGCCTGCGTCCGATTAGCTAGTTGGTGAGGTCACGGCTCACCAAGGCTCCGATCGGTATCCGG
CCTGAGAGGGCGGACGGACACACTGGAACTGAGACACGGTC
>Zotu6380
GAACGCTGGCGGCATGCCTAACACATGCAAGTCGAACGAAGGCCCGATTTCGGTTGGGTCCTTAGTGG
CGCACGGGTGCGTAACGCGTGGGAATCTGCCCCTCGGTTCGGAATAACAGTTAGAAATGACTGCTAAT
ACCGGATGATGACGTAAGTCCAAAGATTTATCGCCGAGGGATGAGCCCGCGTAGGATTAGCTAGTTGG
TGTGGTAAGAGCGCACCAAGGCGACGATCCTTAGCTGGTCTGAGAGGATGATCAGCCACACTGGGACT
GAGACACGGCC
>Zotu6383
GAACGCTGGCGGCATGCCTAACACATGCAAGTCGAACGAGACCTTCGGGTCTAGTGGCGCACGGGTGC
GTAACGCGTGGGAACCTGCCCTTAGGTTCGGAATAACTCCTCGAAAGGGGTGCTAATACCGGATGATG
TCTTCGGACCAAAGATTTATCGCCTATGGATGGGCCCGCGTTGGATTAGCTAGTTGGTAGGGTAAAGG
CCTACCAAGGCGACGATCCATAGCTGGTCTTAGAGGATGATCAGCCACACTGGGACTGAGACACGGCC
>Zotu6384
GAACGCTGGCGGCGTGCCTAATACATGCAAGTCGAGCGGACCTCTTCGGAGGTCAGCGGCGGACGGGT
GAGTAACACGTGGGCAACCTGCCTATAAGACTGGGATAACTTCGGGAAACCGGAGCTAATACCGGATA
ACATCAAGGACCACATGGTCTTCGATTAAAAGATGGCGTCAAGCTATCACTTATAGATGGGCCCGCGT
CGCATTAGCTAGTTGGTGAGGTAACGGCTCACCAAGGCGACGATGCGTAGCCGACCTGAGAGGGTGAT
CGGCCACACTGGGACTGAGACACGGCC
>Zotu6385
GAACGCTGGCGGCAGGCCTAACACATGCAAGTCGAGCGCCCCGCAAGGGGAGCGGCAGACGGGTGAGT
AACGCGTGGGTATCTACCTAGTGGTACGGAATAGCCCAGGGAAACTTGGAGTAATACCGTATACGCCC
GAAAGGGGAAAGATTTATCGCCATTAGATGAGCCCGCGTTGGATTAGCTAGTTGGTGGGGTAATGGCC
CACCAAGGCGACGATCCATAGCTGGTCTGAGAGGATGATCAGCCACACTGGGACTGAGACACGGCC
>Zotu6386
GAACGCTGGCGGCGTGCCTAATACATGCAAGTCGAGCGAATCAATGGGAGCTTGCTCCCATTGATTAG
CGGCGGACGGGTGAGTAACACGTGGGTAACCTGCCTGTAAGACTGGGATAACTTCGGGAAACCGGAGC
TAATACCGGATAGTTTCTTTTCTCGCATGAGAGAAGATGGAAAGATGGTTCTGCTATCACTTACAGAT
GGACCCGCGGCGCATTAGCTAGTTGGTGAGGTAACGGCTCACCAAGGCCACGATGCGTAGCCGACCTG
AGAGGGTGATCGGCCACACTGGGACTGAGACACGGCC
>Zotu6387
GAACGCTGGCGGCGTGCCTAATACATGCAAGTCGTGCGGTAACAGGTCTAGTTTACTAGATGCTGACG
AGCGGCGGACGGCTGAGTAACGCGTAGGAACGTGCCCCAAAGTGAGGGATAAGCTCCAGAAATGGAGT
CTAATACCGCATATGGTCTTCGGATTAAAGCTTCGGCGCTTTGGGAGCGGCCTGCGTTTGATTAGATA
GTTGGTGAGGTAAAGGCTCACCAAGTCTACGATCAATAGCTGGTCTGAGAGGATGATCAGCCAGACTG
GAACTGAGACACGGTC
>Zotu6389
GAACGTTGGCGGCGTGGATTAGGCATGCAAGTCGAACGCCCCGCAAGGGGAGTGGCGCAAGGGTGAGT
AAGGCGATGGTAACCAACCCCGAGGTCGGGCATAGCCGAGGGAAACCTCGGGTAATTCCCGGCGATGC
AGGAGACTGGCATCAGTTTCCTGTCAAAGGCTTCGGCCGCCGCGGGACGGGCCGTCGTGGTATTAGGT
AGTTGGTGGGGTGACGGCTCACCAAGCCTGCGATGCCTACCGGGTGTGAGAGCATGGCCCGGCACACT

GGGACTGAGACACTGCC
>Zotu6390
GAACGCTGGCGGCAGGCCTAACACATGCAAGTCGAACGGATGTAGCAATACATTAGTGGCGCACGGGA
GAGTAACGCGTGGGAACCTACCTTTTGGTTTGGGATAACATCGGGAAACTGGTGCTAATACCGGATAA
GCCCTTCGGGGGAAAGATTTATCGCCGAAAGATGGGCCCGCGTTGGATTAGCTAGTTGGTGAGGTAAC
GGCTCACCAAGGCTACGATCCATAGCTGGTCTGAGAGGATGATCAGCCTCACTGGGACTGAGACACGG
CC
>Zotu6391
GAACGCTAGCGGGAGGCCTAATACATGCAAGTCGAGCGGTAACTTTCTTCGGGAAGCTAGAGCGGCGC
ACGGGTGAGTAACGCGTACATGACCTACCTTTAAGACCGGGATAGCCCTGGGAAACTGGGATTAATAC
CGGATGTGATAATAGATATAAAGATTTATTATTAAAGTTCCGGCGCTTAGAGATGGGTGTGCGTCTGA
TTAGCTAGTTGGTAGGGTAACGGCCTACCAAGGCGACGATCAGTAGGGGGCGTGAGAGCGTGGCCCCC
CACACGGGTACTGAGACACGGAC
>Zotu6392
GAACGCTGGCGGCAGGCCTAAAACATGCAAGTCGAGCGCTCCAGCAATGGAGAGCGGCGCACGGGTGC
GTAACGCGTGGGAATGTGCCTCTGGGTGCGGAACAACACCGGGAAACTGGTGCTAATACCGCATAATG
ACTTCGGTCCAAAGATTTATCGCCCAGAGATCAGCCCGCGTCGGATTAGGTAGTTGGTAGGGTAATGG
CCTACCAAGCCGACGATCCGTAGCTGGTCTTAGAGGATGATCAGCCACACTGGAACTGAGACACGGTC
>Zotu6393
GAACGCTGGCGGCATGCCTAACACATGCAAGTCGAACGGCAGCACAGTAAAGATTTCGGTCTTTATGG
GTGGCGAGTGGCGGACGGGTGAGTAATACGTAGGAATCTGTCCTGGAGAGGGGGATAACCCGAGGAAA
CTCGGGCTAATACCGCATGATATTGAGAAATCAAAAGCAGGGGACCTGAAAGGGCCTTGCGCTCTGGG
TTGAGCCTACGTCGGATTAGCTAGTTGGTGGGGTAAAGGCCTACCAAGGCAACGATTCGTAGTTGGTC
TGAGAGGATGACCAGCCACACTGGGACTGAGACACGGCC
>Zotu6394
GAACGCTGGCGGCGTGCCTAACACATGCAAGTCGAGCGAACCCTTCGGGGTGAGCGGCGGACGGGTGA
GTAACGCGTGGGTAACCTGCCCTATACACACGGATAACGTACCGAAAGGTACGCTAATACGAGATGAT
ATATTTTTATCGCATGGTAGAAATATCAAAGCTCCGGCGGTATAGGATGGACCCGCGTCTGATTAGCT
AGTTGGTAAGGTAATGGCTTACCAAGGCGACGATCAGTAGCCGACCTGAGAGGGTGATCGGCCACATT
GGAACTGAGACACGGTC
>Zotu6395
GAACGCTGGCGGCATGCCTAATACATGCAAGTCGAACGCTGAAGCACTCTGCTTGCAGAGTGCGGATG
AGTGGCGAACGGGTGAGTAACACGTGGGAAATCTGCCTAAGAGTGGGGGGATAACATTCGGAAACGGA
TGCTAATACCGCATAGATTCTTGGACCGCATGGTTCTGGAAAGAAAGACGGCTTTGCTGTCGCTTTTA
GATGATCCCGCGGCGTATTAGTTAGTTGGTGGGGTAATGGCTTACCAAGACCATGATACGTAGCCGAC
CTGAGAGGGTGATCGGCCACACTGGGACTGAGACACGGCC
>Zotu6396
GAACGCTGGCGGCGTGCTTAACACATGCAAGTCGAACGGTAAGGCCCTTCGGGGTACACGAGTGGCGA
ACGGGTGAGTAACACGTGGGTGATCTGCCCTGCACTTTGGGATAAGCCTGGGAAACTGGGTCTAATAC
CGAATATGACTGCATCCTGCATGGGGTGTGGTGGAAAGCTTTTGCGGTGTGGGATGGGCCCGCGGCCT
ATCAGCTTGTTGGTGGGGTGATGGCCTACCAAGGCGACGACGGGTAGCCGGCCTGAGAGGGTGACCGG
CCACACTGGGACTGAGATACGGCC
>Zotu6398
GAACGCTGGCAGCGTGCCTAACACATGCAAGTCGAACGGAACTTCTTTAAAGGATTTCTTCGGAATGA
ATTTGATTAAGTTTAGTGGCGGACGGGTGAGTAACGCGTGAGTAACCTGCCTCTAAGAGGGGAATAAC
ATTCTGAAAAGAATGCTAATACCGCATAATATATATTTATCGCATGGTAGATATATCAAAGATTTATC
GCTTAGAGATGGACTCGCGTCCGATTAGTTAGTTGGTGAGGTAACGGCTCACCAAGACCGCGATCGGT
AGCCGGACTGAGAGGTTGAACGGCCACATTGGGACTGAGACACGGCC
>Zotu6399
GAACGCTGGCGGCAGGCTTAACACATGCAAGTCGAGCGAGATCTTCGGGTCTAGCGGCGGACGGGTGA
GTAACGCGTGGGAACGTGCCCTTTGCTACGGAATAGTCCTGGGAAACTGGGGGTAATACCGTATACGC
CCTTTTGGGGAAAGATTTATCGGCGAAGGATCGGCCCGCGTTGGATTAGGTAGTTGGTGGGGTAATGG
CCTACCAAGCCGACGATCCATAGCTGGTTTGAGAGGATGATCAGCCACACTGGGACTGAGACACGGCC
>Zotu6400

GAACGCTGGCGGCATGCTTAACACATGCAAGTCGAACGGCAGCACGGTGTGTAGCGATACACATGGGT
GGCGAGTGGCGGACGGGTGAGTAATGCGTAGGAATCTGCCTTAAAGAGTGGGACAACCCGAGGAAACT
CGGGCTAATACCGCATGAAGTCGGAAGACTAAAGGCTGGGGTAACCTGGCGCTTTGAGAGGAGCCTGC
GTCTGATTAGCTAGTTGGTGGGGTAAGAGCTTACCAAGGCGATGATCGGTAGCTGGTCTGAGAGGATG
ATCAGCCACACTGGGACTGAGACACGGCC
>Zotu6401
GAACGCTGGCGGCGTGCCTAATACATGCAAGTCGAGCGAATCAATTGGGAGCTTGCTCCCTTTTGGTT
AGCGGCGGACGGGTGAGTAACACGTGGGTAACCTGCCTGTAAGACTGGGATAACTCCGGGAAACCGGG
GCTAATACCGGATAAGTTGTTCCTCCGCATGGAGGAACATTGAAAGATGGCTTCGGCTATCACTTACA
GATGGACCCGCGGCGCATTAGCTAGTTGGTGAGGTAACGGCTCACCAAGGCAACGATGCGTAGCCGAC
CTGAGAGGGTGATCGGCCACACTGGGACTGAGACACGGCC
>Zotu6403
GAACGCTGGCGGTATGCCTAACACATGCAAGTCGAACGAGATCTTCGGATCTAGTGGCGCACGGGTGC
GTAACGCGTGGGAATCTGCCTTGGGGTTCGGAATAACTCCCCGAAAGGGGTGCTAATACTGGATGATG
TCGAAAGACCAAAGATTTATCGCCCTGAGATGAGCCCGCGTAGGATTAGCTAGTTGGTGTGGTAAAGG
CGCACCAAGGCGACGATCCTTAGCTGGTCTGAGAGGATGATCAGCCACACTGGGACTGAGACACGGCT
>Zotu6404
GAACGCTGGCGGCGTGCCTAACACATGCAAGTCGTACGCTCGCAAGAGCGTGGCGAACGGGTGAGTAA
TAGCAAGGAACGTGCCTTATCCCGGGAAATACCCCACCGAAAGGTGGGCTAATGTCCCATAGTCTCGC
AAGAGTAAAGCTCCGGCGGGATAAGATCGGCCTTGTTCCTATCAGCTTGTTGGTGGGGTAATGGCCTA
CCAAGGCGACGACGGGTAGCTGGTCTGAGAGGATGATCAGCCACAATGGGACTGAGACACGGCC
>Zotu6406
GAACGTTAGCGGCGCGCCTAACACATGCAAGTCGCACGAGAAAGGGCTTCGGCCCCGGTACAGTGGCG
CACGGGTGAGTAACACGTAGGTAATCTGCCCTTGAGTGGTGGATAACCCTCCGAAAGGAGGGCTAATA
CAGCATGAGACCACGGCATCTCCGGATGCAGGGGTCAAAGTGGGCCTCTTTATAAAAGCTCACGCTTG
AGGATGAACCTGCGGCCCATCAGCTAGTTGGTAAGGTAACGGCTTACCAAGGCAAAGACGGGTAGCTG
GTCTGAGAGGATGAACAGCCACACTGGAACTGAGACACGGTC
>Zotu6407
GAACGCTGGCGGCGTGCCTAATACATGCAAGTCGAACGGTCTTTTCTATGGAAGCTTGCTTCCACTGA
GAAGATAGTGGCGGACGGGTGAGTAACACGTGGGTAACCTGCCCATAAGAGGGGGATAACATCCGGAA
ACGGGTGCTAATACCGCATAGTTTTCTTGATCGCATGATCGGGAAAGGAAAGACGGCCTTTGTGCTGT
CGCTTATGGATGGACCCGCGGCGTATTAGTTAGTTGGTGAGGTAATGGCTCACCAAGACGATGATACG
TAGCCGACCTGAGAGGGTGATCGGCCACATTGGGACTGAGACACGGCC
>Zotu6408
GAACGCTGGCGGCGTGCTTAACACATGCAAGTCGAGCGGAAAGGCCCTTCGGGGTACTCGAGCGGCGA
ACGGGTGAGTAACACGTGAGTAATCTGCCCTTGGCTTTGGGATAACTACCGGAAACGGTGGCTAATAC
CGGATACGACCGATCCCTGCATGGGGTGTTGGTGGAAAGTTTTTCGGCCAGGGATGTGCTCGCGGCC
TATCAGCTTGTTGGTGGGGTAATGGCCTACCAAGGCTTCGACGGGTAGCCGGCCTGAGAGGGTGACCG
GCCACACTGGGACTGAGACACGGCC
>Zotu6410
GAACGCTGGCGGCGTGCTTAACACATGCAAGTCGAACGGAAAGGCCCAGCTTGCTGGGTACTCGAGTG
GCGAACGGGTGAGTAACACGTGGGTGATCTGCCTTGCACTCTGGGATAAGCCTGGGAAACTGGGTCTA
ATACCGGATAGGACCTCTGGCTGCATGGTTGGGGGTGGAAAGCTTTTGCGGTGTGAGATGGGCCCGCG
GCCTATCAGCTTGTTGGTGGGGTAATGGCCTACCAAGGCGACGACGGGTAGCCGACCTGAGAGGGTGA
TCGGCCACACTGGGACTGAGACACGGCC
>Zotu6411
GAACGCTGGCGGCGTGCTTAACACATGCAAGTCGAACGATGACCCCGTGCTTGCACGGGTGATGAGTG
GCGAACGGGTGAGTAACACGTGAGTAACCTGCCCCTGACTCTGGGATAAGCCCGGGAAACTGGGTCTA
ATACCGGATATGACTACTGGCCGCATGGCCTGGTGGTGGAAAGAATTTCGGTTGGGGATGGACTCGCG
GCCTATCAGCTTGTTGGTGAGGTAGTGGCTCACCAAGGCGACGACGGGTAGCCGGCCTGAGAGGGTGA
CCGGCCACACTGGGACTGAGACACGGCC
>Zotu6412
GAACGTTAGCGGCGCGCCTAACACATGCAAGTCGCACGAGAAAGGGCTTCGGCCCCGGTACAGTGGCG
CACGGGTGAGTAACACGTAGATCATCTCCCCTCGAGTGGTGGATAACTCTCCGAAAGGAGAGCTAATA

CAGCATGAGACCACGGCTTCGTTGAAGCTGCGGCCAAAGCAGCCCTCTACACGTAAGGTTGCGCTTGA
GGATGAGTCTGCGGCCCATCAGCTAGTTGGTAGGGTAATGGCCTACCAAGGCTAAGACGGGTAGCTGG
TCTGAGAGGATGAACAGCCACACTGGAACTGAGACACGGTC
>Zotu6413
GAACGCTGGCGGCGTGCCTAATACATGCAAGTCGAACGCTTCACTTCGGTGAAGAGTGGCGAACGGGT
GAGTAATACATAAGTAACCTGGCATCTACAGGGGGATAACTGATGGAAACGTCAGCTAAGACCGCATA
GGTGTAGAGATCGCATGAACTCTATATGAAAAGTGCTACGGGACTGGTAGATGATGGACTTATGGCGC
ATTAGCTTGTTGGTAGGGTAACGGCCTACCAAGGCGACGATGCGTAGCCGACCTGAGAGGGTGACCGG
CCACACTGGGACTGAGACACGGCC
>Zotu6414
GAACGCTGGCGGCAGGCTTAACACATGCAAGTCGAACGCCCCGCAAGGGGAGTGGCAGACGGGTGAGT
AACGCGTGGGAACCTACCTAGAACTTCGGAATAACACATGGAAACGTGTGCTAATACCGGATACGCCC
TACGGGGGAAAGATTTATCGGTTTTAGATGGGCCCGCGTAAGATTAGCTAGTTGGTGAGGTAATGGCT
CACCAAGGCGACGATCTTTAGCTGGTCTGAGAGGATGATCAGCCACACTGGGACTGAGACACGGCC
>Zotu6415
GAACGCTGGCGGCAGGCTTAACACATGCAAGTCGAACGAAGGCTTCGGCCTTAGTGGCGCACGGGTGA
GTAACACGTGGGAATATACCTCTCAGTGGGGGATAACGTCGGGAAACTGACGCTAATACCGCATACGC
CCTTCGGGGGAAAGATTTATCGCTGAGAGATTAGCCCGCGTCCGATTAGCTAGTTGGTGAGGTAATGG
CTCACCAAGGCGACGATCGGTAGCTGGTCTGAGAGGATGATCAGCCACACTGGGACTGAGACACGGCC
>Zotu6419
GAACGCTGGCGGCATGCTTAACACATGCAAGTCGCACGGGCAGCAATGTCAGTGGCGGACGGGTGAGT
AACGCGTAGGAACATGTCCAGGGGTGGGGGATAACGCTGGGAAACTGGCGCTAATACCGCATGTGAGC
TGAGGCTCAAAGCCGTGAGGCGCCTTTGGAGTGGCCTGCGTCCGATTAGCTTGTTGGTGGGGTAATGG
CTTACCAAGGCGACGATCGGTAGCTGGTCTGAGAGGACGACCAGCCACACTGGGACTGAGACACGGCC
>Zotu6420
GAACGCTGGCGGTATGCCTTACACATGCAAGTCGTACGAGAGTTTTTAACTCAAGTGGCGGACGGGTG
AGTAACACGTAAGAATTTGACTTTAGGAGGGGGACAACAACTGGAAACGGTTGCTAATACCCCATATG
CTTTCGAGTGAAACGGATTTTTCCGCCTAAAGAGAAGCTTGCGGCTGATTAGCTTGTTGGTGAGGTAA
GGGCTCACCAAGGCGACGATCAGTATCTGGTTTGAGAGGACGATCAGACACACTGGAACTGAGACACG
GTC
>Zotu6422
CAACGCTGGCGGCGTGCCTAACACATGCAAGTCGCACGAGAAAGGGACTTCGGTCCTGAGTAAAGTGG
CGCACGGGTGAGTAACACGTGACTAATCTACCCTCGAGTGGGGAATAACCTAGGGAAACCTGGGCTAA
TACCGCATAACACTTACGAGTCAAAGCAGCAATGCGCTTGGGGAGGAGGTCGCAGCAGATTAGCTAGT
TGGCGGGGTAATGGCCCACCAAGGCGATGATCTGTATCCGGCCTGAGAGGGCGCACGGACACACTGGA
ACTGAAACACGGTC
>Zotu6423
GAACGCTGGCGGTATGCTTAACACATGCAAGTCGAACGAATGAGGGTCCTAGCTTGCTAGGGCCTAAG
TTAGTGGCAAACGGGTGAGTAACACGTGGGAATCTACCCAGTAGTACAGAATAACAGTTAGAAATGAC
TGCTAATACTGTATAGTATCGAGAGATTAAAGATTTATCGCTACTGGATGAGCCCGCGTCAGATTAGG
TAGTTGGTGGGGTAATGGCCTACCAAGCCAAAGATCTGTAGCTGGTCTGAGAGGATGATCAGCCACAC
TGGAACTGAGACACGGTC
>Zotu6424
GAACGCTAGCGGCAGGCTTAACACATGCAAGTCGAGGGGTATAGTAGCAATACTAGAGACCGGCGCAC
GGGTGCGTAACGCGTATGCAATCTACCTTTTACAGAGGGATAGCCCAGAGAAATTTGGATTAATACCT
CATAGTATATAGACTTCGCATGGAGATTATATTAAAGTCACAACGGTAAAAGATGAGCATGCGTCCCA
TTAGCTAGTTGGTAAGGTAACGGCTTACCAAGGCGACGATGGGTAGGGGTCCTGAGAGGGAGATCCCC
CACACTGGTACTGAGACACGGAC
>Zotu6425
GAACGCTGGCGGCGTGCTTAACACATGCAAGTCGAACGGATCCCAAGTGTCAGCAATGATGTGGAAGG
GTTAGTGGCGAACGGGTGAGTAACACGTGAGGAACCTGCCCCGGACTCAGGGATAACGGCTGGAAACG
GCTGCTAATACCTGATGCCGTCGTGTCATCGCATGGTGATGTGACGAAAGAATCTCGGTTCGGGAGGG
TCTCGCGGCCTATCAGCTTGTTGGTGGGGTAATGGCCTACCAAGGCGTCGACGGGTAGCTGGTCTGAG
AGGATGATCAGCCACACTGGGACTGAGACACGGCC

>Zotu6426
GAACGCTGGCGGCGTGCTTAACACATGCAAGTCGAGCGGAAAGGCCACTTCGGTGGTACTCGAGCGGC
GAACGGGTGAGTAACACGTGAGTAATCTGCCCTTGGCTTTGGGATAGCCACCGGAAACGGTGATTAAT
ACCGGATACGACAACTTCTCGCATGGGATGGTTGTGGAAAGTTTCTCGGCCAGGGATGTGCTCGCGGC
CTATCAGCTTGATGGTGAGGTAATGGCTCACCATGGCTTCGACGGGTAGCCGGCCTGAGAGGGTGACC
GGCCACACTGGGACTGAGACACGGCC
>Zotu6427
GAACGCTGGCGGCGTGGATAAGGCATGCAAGTCAAGGGGGCCCGCAAGGGCAACCGGCGAACGAGGTA
GTAACACGTAGGTACACACCCTAAAGTCAGGGATAACTAGTCGAAAGATTAGATAATACTTGATAGTC
TCTTTGGAGTAAAGATTTATCGCTTTAGGAGTGGCCTGCGGGATATCAGCTAGTTGGTAGTGTAATGG
ACTACCAAGGCTATGACGTCTAGGGGAGCTGAGAGGCTGACCCCCACCGATGGTACTGAGACACGGAC
>Zotu6428
GAACGCTGGCGGCAGGCTTAACACATGCAAGTCGAGCGGATGAAAGTAGCTTGCTACTGGATTCAGCG
GCGGACGGGTGAGTAATGCTTAGGAATCTGCCTATTAGTGGGGGACAACATCTCGAAAGGGATGCTAA
TACCGCATACGTCCTACGGGAGAAAGCAGGGGATCTTCGGACCTTGCGCTAATAGATGAGCCTAAGTC
GGATTAGCTAGTTGGTGGGGTAAAGGCCTACCAAGGCGACGATCTGTAGCGGGTCTGAGAGGATGATC
CGCCACACTGGGACTGAGACACGGCC
>Zotu6429
GAACGCTGGCGGCGTGGATAAGTCATGCAAGTAGAACGCCCCGCAAGGGGAGTTGCGAACGAGGTAGT
AACACGTAGGTACGTCCCCCGAAGTCGGGCATAGCCAGTCGAAAGACTGGGTAATTCCCGATAGTCCC
GAAAGGGTAAAGGTTTTTCGCTTCGGGAGCGGCCTGCGTAGTATCAGCTAGTTGGTAGGGTAATGGCC
TACCAAGGCTACGACGCTTAGGGGGGCTGAGAGGCCGATCCCCACCGATGGAACTGCGACACGGTC
>Zotu6431
GAACGCTGGCGGCGTGCCTAATACATGCAAGTCGAGCGGAACTGGGGGAGCTTGCTCCTTCCAGTTTA
GCGGCGGACGGGTGAGTAACACGTGGGCAACCTGCCCTGCAGATCGGGATAACTCCGGGAAACCGGTG
CTAATACCGAATAGTTTGAGGCCTCTCCTGAGGCTTCACGGAAAGACGGTTTCGGCTGTCACTGCAGG
ATGGGCCCGCGGCGCATTAGCTAGTTGGTGGGGTAACGGCCCACCAAGGCCACGATGCGTAGCCGACC
TGAGAGGGTGACCGGCCACACTGGGACTGAGACACGGCC
>Zotu6432
GAACGCTGGCGGCGTGCCTAAGACATGCAAGTCGAGTGGGTTTAGACCCACGGCGAACGGGTGAGTAA
TACCTAGGAACTTTCTTTAAGTGGAGCATAACCCATAGAAATGTGGGATAATACTCCATGTGACCGCA
AGGTCAAAGTCGCAAGACGCTTAAAGTGAGGCCTGGGCACCATCAGCTAGTTGGTAAGGTAATGGCTT
ACCAAGGCTATGACGGTTAGGGGTCCCGAGAGGGATAACCCCCACACTGGAACTGCGACACGGTC
>Zotu6437
GAACGCTGGCGGCGTGCCTAATACATGCAAGTCGAGCGAATCTGAGGGAGCTTGCTCCCAAAGATTAG
CGGCGGACGGGTGAGTAACACGTGGGCAACCTGCCTGTAAGACTGGGATAACTTCGGGAAACCGGAGC
TAATACCGGATAATTCTTATTAACTCATGTTAATAAGCTAAAAGACGGCGTAAGCTGTCACTTACAGA
TGGGCCCGCGGCGCATTAGCTAGTTGGTGAGGTAACGGCTCACCAAGGCAACGATGCGTAGCCGACCT
GAGAGGGTGATCGGCCACACTGGGACTGAGACACGGCC
>Zotu6438
GAACGCTAGCGGCAGGCCTAACACATGCAAGTCGAACGGTAACAGGTGTAGCAATACATGCTGACGAG
TGGCGCACGGGTGCGTAACACGTATGTAACTTGCCTCTAATTGGGAAATAGCTTCGAGAAATCGAAAT
TAATGTCCCATAATAATTAGACTAGGCATCTAGTTTAATTTAAAGCTCAGGCGGTTAGAGATAGACAT
GCGGCCTATTAGTTTGTTGGTGAGGTAACGGCTCACCAAGGCTACGATAGGTAGGGGGCCTGAGAGGG
TTATCCCCCACACTGGAACTGAGACACGGTC
>Zotu6439
GAACGCTGGCGGCGCGCTTAACACATGCAAGTCGAGCGAGAACCAAGCCTTCGGGCTTGGGGACAGCG
GCGAACGGGTGAGTAACACGTGGGTAATCAACCCTCGACACTGGGATAGCCCGGGGAAACCCGGATTA
ATACCGGATACCCCATCTGGCCTTCGGGCCGGCTGGAAAAGGTAGCTTTGGCCTCCGGTCGAGGACGA
GCCCGCGGCGGATTAGCTTGTTGGCGGGGTAAAAGCCCACCAAGGCGACGATCCGTAGCTGGTCTGAG
AGGACGATCAGCCACACTGGGACTGAGACACGGCC
>Zotu6440
GAACGCTGGCGGCAGGCTTAACACATGCAAGTCGAACGATGACTCTCTAGCTTGCTAGAGATGATTAG
TGGCGGACGGGTGAGTAACATTTAGGAATCTGCCTAGTAGTGGGGGATAGCTCGGGGAAACTCGAATT

AATACCGCATACGACCTACGGGTGAAAGGGGGCGCAAGCTCTTGCTATTAGATGAGCCTAAATCAGAT
TAGCTAGTTGGTGGGGTAAAGGCCCACCAAGGCGACGATCTGTAACTGGTCTGAGAGGATGATCAGTC
ACACCGGAACTGAGACACGGTC
>Zotu6442
GAACGCTGGCGGCAGGCTTAACACATGCAAGTCGAACGCCCCGCAAGGGGAGTGGCAGACGGGTGAGT
AACGCGTGGGAATCTACCTAGAACTACGGAACAACAGTTGGAAACGGCTGCTAATACCGTATACGCCC
TACGGGGGAAAGATTTATCGGTTTTAGATGAGCCCGCGTAAGATTAGCTAGTTGGTGGGGTAATGGCC
TACCAAGGCGACGATCTTTAGCTGGTCTGAGAGGATGATCAGCCACACTGGGACTGAGACACGGCC
>Zotu6443
GAACGCTGGCGGCGTGCTTAACACATGCAAGTCGAACGATGAAGCGGTGCTTGCGCCGTGGATTAGTG
GCGAACGGGTGAGTAACACGTGGGTGATCTGCCCCGTACTCTGGGATAAGCCTGGGAAACTGGGTCTA
ATACTGGATAGGACCATGCTCTAGTGTGTCATGGTGGAAAGTTTTTTCGGTACGGGATGAGCTCGCGG
CCTATCAGCTTGTTGGTGGGGTAATGGCCTACCAAGGCGTCGACGGGTAGCCGGCCTGAGAGGGTGAT
CGGCCACATTGGGACTGAGATACGGCC
>Zotu6444
GAACGCTGGCGGCGTGCTTAACACATGCAAGTCGAACGGGCCCGCCCTCTTTTTTGGGGGTGGGTTGA
GTGGCGAACGGGTGAGTATCACGTGAGTAACCTGCCCCCTGCTCCTGGATAACTGCTTGAAAGGGCGG
CTAATACGGGGTGTGCCGGCTGGTCCGCATGGGCCTGGTCGGGATAGTTTCATCTTCGGGTGTTTCGG
TGGGGGATGGGCTCGCGGCCTATCAGCTTGTTGGTGGGGTGATGGCCCACCAAGGCGGTGACGGGTAG
CCGGCCTGAGAGGGTGGGCGGCCACACTGGGACTGAGACACGGCC
>Zotu6445
GAACGCTGGCGGCAGGCTTAACACATGCAAGTCGAGCGCGTAGCAATACGAGCGGCAGACGGGTGAGT
AACGCGTGGGAACCTACCCTTTTCTACGGAATAACGCATGGAAACGTGTGCTAATACCGTATACGCCC
TTTGGGGGAAAGATTTATCGGAGAAGGATGGGCCCGCGTTGGATTAGCTAGTTGGTGGGGTAAAGGCC
TACCAAGGCGACGATCCATAGCTAGTCTGAGAGGATGATCAGCCACATTGGGACTGAGACACGGCC
>Zotu6448
GAACGCTAGCGGCAGGCTTAATACATGCAAGTCGAGGGGCAGCAGGGATAGCAATATTCGCTGGCGAC
CGGCGCACGGGTGCGTAACACGTATACAATCTGCCTTTAACAGGGGGATAGCCCGGAGAAATCCGGAT
TAATACCCCATAACAGTGATTCTGGCATCAGAGACACTTAAAAGCTCCGGCGGTTAAAGATGAGTATG
CGTCTGATTAGCTAGTTGGTGAGGTAACGGCTCACCAAGGCGACGATCAGTAGGGGATCTGAGAGGAT
TAACCCCCACACGGATACTGAGACACGGAT
>Zotu6450
GAACGCTGGCGGCGTGCCTAATACATGCAAGTCGAACGAACGAATAAGAGCTTGCTCTTATAAGTTAG
TGGCGCACGGGTGAGTAATATATAGCTAATCTGCCTCTTAGTAGAGGATAACAGTCGGAAACGATTGC
TAATACTCTATACTTCTGCTTTACATAAGTAAAGTAGAGAAAGTTTTTCGCTAAGAGATGAGGCTATA
CTGTATCAGCTAGTTGGTAAGGTAACGGCTTACCAAGGCTATGACGCATAACTGGTCTGAGAGGATGA
TCAGTCACATTGGAACTGAGACACGGTC
>Zotu6452
GAACGCTGGCGGCGTGCTTAACACATGCAAGTCGAACGGTGACCTGAGGTGCTTGCACCTTGGTGATC
AGTGGCGAACGGGTGAGTAACACGTGAGTAACCTGCCCCAGACTCTGGAATAACCCCGGGAAACCGGA
GCTAATACCGGATACGACATCAGGCCGCATGGTCATGGTGTGGAAAGTTTTTCGGTCTGGGATGGGCT
CGCGGCCTATCAGCTTGTTGGTGAGGTAGTGGCTCACCAAGGCGACGACGGGTAGCCGGCCTGAGAGG
GCGACCGGCCACACTGGGACTGAGACACGGCC
>Zotu6453
GAACGCTAGCGGTATGCTTAACACATGCAAGTCGTACGGAACAGCAATGTTTAGTGGCGAACGGGTGA
GTAACGCGTGAGAATTTGCCTTTAGGAAGAGGACAACAATTAGAAATAATTGCTAACACTCTATATGC
CGTTTGGTGAAAAAAGCAATTTGCCTAAAGAGAAGCTCGCGTCTGATTAGCTAGTTGGTGGGGTAAAG
GCCTACCAAGGCGACGATCAGTAGCTGGTCTGAGAGGACGATCAGCCACACTGGAACTGAGACACGGT
C
>Zotu6454
GAACGCTGGCGGCGTGCTTAACACATGCAAGTCGAGCGGAAAGGCCACTTCGGTGGTACTCGAGCGGC
GAACGGGTGAGTAACACGTGAGTAATCTGCCCTTGGCTTTGGGATAGCCACCGGAAACGGTGATTAAT
ACCGGATACGACAACTTCTCGCATGGGATGGTTGTGGAAAGTTTTTCGGCCAGGGATGTGCTCGCGGC
CTATCAGCTTGATGGTGAGGTAATGGCTCACCATGGCTTCGACGGGTAGCCGGCCTGAGAGGGTGACC

GGCCACACTGGGACTGAGACACGGCC
>Zotu6456
GAACGCTGGCGGCATGCCTAACACATGCAAGTCGAACGAAGGCTTCGGCCTTAGTGGCGCACGGGTGC
GTAACGCGTGGGAATCTGCCCCTTGGTTCGGAATAACAGTTAGAAATGACTGCTAATACCGGATGATG
ACGAAAGTCCAAAGATTTATCGCCAGGGGATGAGCCCGCGTAGGATTAGGTAGTTGGTGGGGTAATGG
CCTACCAAGCCGACGATCCTTAGCTGGTCTGAGAGGATGATCAGCCACACTGGGACTGAGGCACGGCC
>Zotu6457
GAACGCTGGCGGCGTGCCTAATACATGCAAGTCGAGCGAACTTGCGGGAGCTTGCTCCCAAAAGTTAG
CGGCGGACGGGTGAGTAACACGTGGGCAACCTACCTATAAGACTGGGATAACTTCGGGAAACCGGAGC
TAATACCGGATAATTCTTTTCTGCACATGTAGAAAAGCTAAAAGACGGTTTACGCTGTCACTTATAGA
TGGGCCCGCGGCGCATTAGCTAGTTGGTGAGGTAACGGCTCACCAAGGCCACGATGCGTAGCCGACCT
GAGAGGGTGATCGGCCACACTGGGACTGAGACACGGCC
>Zotu6458
GAACGCTAGCGGCAGGCTTAACACATGCAAGTCGAGGGGTATAGTAGCAATACTAGAGACCGGCGCAC
GGGTGCGTAACGCGTATGCAATCTACCTTTTGCAGAGGGATAGCCCAGAGAAATTTGGATTAATACCT
CATAGTATAGTGGTACCGCATGGTACTATTATTAAAGTCACAACGGCAAAAGATGAGCATGCGTCCCA
TTAGCTAGATGGTAAGGTAACGGCTTACCATGGCTACGATGGGTAGGGGTCCTGAGAGGGAGATCCCC
CACACTGGTACTGAGACACGGAC
>Zotu6459
GAACGCTGGCGGCGTGCCTAACACATGCAAGTCGAACGGGGTGTCGAGAGCTTGCTCTTGACACTTAG
TGGCGAACGGGTGAGTAACGCGTGAGAAACCTGCCTCTCAGTGGGGGACAACAGTTGGAAACGACTGC
TAATACCGCATAATGTATCCGGAAGGCATCTTCTGGATACCAAAGCTTTATGTGCTGAGAGATGGTCT
CGCGTCTGATTAGCTAGATGGCGGGGTAACGGCCCACCATGGCGACGATCAGTAGCCGGTCTGAGAGG
ATGAACGGCCACATTGGGACTGAGATACGGCC
>Zotu6460
AAACGCTGGCGGCGTGCCTAATACATGCAAGTCGAACGAGGTAGCAATACCAAGTGGCGCACGGGTGA
GTAACACGTAGATAATCTGCCTCGAAGTGGGGAACAACTCAGCGAAAGTTGAGCTAACACCGCATGAG
ACCACAATAGCTGCGGCTAAAGGGGTTAAAAATTTATTGCTTCGAGATGAGTCTGCGTCAGATTAGCT
AGTTGGTGAGGTAATGGCTCACCAAGGCTACGATCTGTAGCTGGTCTGAGAGGATGATCAGCCACACT
GGAACTGAGACACGGTC
>Zotu6461
CAACGCTGGCGGCGTGCCTAACACATGCAAGTCGAACGCGAAAAGGACTTCGGTCCTGAGTAGAGTGG
CGTACGGGTGAGTAACACGTGGGTAATCTACCTTTGGGTGGGGAATAACTCAGGGAAACTTGAGCTAA
TACCGCATAATGCTACGGCTCGACATCGAGTCGTTGTTAAAGTAGCAATACGCCTGAAGAGGAGCCTG
CGCCTGATTAGCTAGTTGGTAGGGTAACGGCCTACCAAGGCTTCGATCAGTAGCTGGTCTGAGAGGAC
GGCCAGCCACAATGGCACTGAAACACGGGC
>Zotu6462
GAACGCTGGCGGCGTGCCTAATACATGCAAGTCGAGCGGATCTTTAGAAGCTTGCTTCTAAAGATTAG
CGGCGGACGGGTGAGTAACACGTGGGCAACCTGCCTGTAAGACTGGGATAACTTCGGGAAACCGAGGC
TAATACCGGATAATCCTTTTCCTCACATGAGGGAAAGTTGAAAGACGGCGCAAGCTGTCACTTACAGA
TGGGCCCGCGGCGCATTAGCTAGTTGGTGAGGTAATGGCTCACCAAGGCAACGATGCGTAGCCGACCT
GAGAGGGTGATCGGCCACACTGGGACTGAGACACGGCC
>Zotu6463
GAACGCTGGTAACGTGGATACCGGCATGCAAGTCACGGGGGCCGTAAGGCAACCGGCGAACGGCTTAG
TAACACGTAGATACATACCCAAAAGTGGGGCATAGCGTCGAAAGACGAGTCAATTCCCCATAGTCTCG
CAAGAGTAAAGCAGCAATGCGCTTTTGGAATGGTCTGCGGCCTATCAGCTTGTTGGTGAGGTAACGGC
TCACCAAGGCTATGACGGGTAGGGGGCGTGAGAGCGCGACCCCCAAGAATGGCACTGCGACACGGGC
>Zotu6465
GAACGCTGGCGGCGTGCTTAACACATGCAAGTCGAACGGTGAAGCGCTTGCCTTTTTGGTGGGTGTGG
ATGAGTGGCGAACGGGTGAGTAACACGTGAGTAACCTGCCCTCTTCTTCTGGATAACCTCATGAAAGT
GGGGCTAATACGGGGTATTCCGGGTCTGTCGCATGGCGGGTTTGGGAAAGATTGCGCCTTTGGGTGTT
TTCGGTGGGGGATGGGCTCGCGGCCTATCAGCTTGTTGGTGGGGTGATGGCCTACCAAGGCGGTGACG
GGTAGCCGGCCTGAGAGGGTGGACGGCCACACTGGGACTGAGACACGGCC
>Zotu6466

GAACGCTGGCGGCGTGCTTAACACATGCAAGTCGAACGGTAAGGCCCCTTCGGGGGTACACGAGTGGC
GAACGGGTGAGTAACACGTGAGCAACCTGCCCTTGACTCTGGAATAACAGTTGGAAACAGCTGCTAAT
ACCGGATATGCACCGGACCTCTCCTGGGGTGTGGTGGAAAGCTTTGGCGGTCAGGGATGGGCTCGCGG
CCTATCAGCTTGTTGGTGAGGTGATGGCTCACCAAGGCTTCGACGGGTAGCCGGCCTGAGAGGGCGAC
CGGCCACACTGGGACTGAGATACGGCC
>Zotu6467
GAACGCTGGCGGCGTGCTTAACACGTGCAAGTGGAGCGACGAACCAGGGCTTGCCCTGGGGCAGAGCC
GCGAACGGGTGAGTAACACGTGGGTAACCTACCCCGATGATTGGGACAACCCGAGGAAACTCGGGCTA
ATACCAAATGTGCTCGAAGTCCATGAGGACTCCGAGTAAAGATAGCTTCGGCCTTCGCATCGGGACGG
GCCCGCGGCCCATTAGCTTGTTGGTGGGGTAACGGCCTACCAAGGCGACGATGGGTAGCTGGTCTGAG
AGGACGATCAGCCACACTGGGACTGAGACACGGCC
>Zotu6468
GAACGCTGGCGGCAGGCTTAACACATGCAAGTCGAACGGTTGTAGCAATACAGCAGTGGCGCACGGGT
GAGTAACGCGTGGGAACATACCTTAGAGTTCGGAATAACAGCGGGAAACTGCTGCTAATACCGGATAT
TCTCTCCGGAGGAAAGATTTATCGCTCTAAGATTGGCCCGCGTCAGATTAGCTAGTTGGTGGGGTAAT
GGCCTACCAAGGCTCCGATCTGTAGCTGGTCTGAGAGGACGACCAGCTACACTGGAACTGAGACACGG
TC
>Zotu6469
GAACGCTAGCGGCAGGCCTAACACATGCAAGTCGAGGGGCAGCACAAGTAAAGCTTGCTTTATTGGTG
GCGACCGGCGCACGGGTGAGTAACGCGTATGCAACCTACCTTATACTGAGGGATAGCCCGAAGAAATT
CGGATTAATACCTCATAGTATTATCGAGAGGCATTTTTTGATAATTAAAGCTCCGGCGGTATAAGATG
GGCATGCGTCCCATTAGCTAGATGGTGAGGTAACGGCTCACCATGGCAACGATGGGTAGGGGGCCTGA
GAGGGTGGTCCCCCACACTGGTACTGAGACACGGAC
>Zotu6470
GAACGCTAGCGGCAGGCCTAATACATGCAAGACGAACGGTAATCGGTGTAGCAATACATCGGCAAGAG
TGTCGTAAGGGTGCGTAACACGTATGCAATCTGCCCTCAACAGGAGGATAGCTCCCCGAAAGGGGAAT
TAATACTCCATAAGATCACGCGAAGGCATCTGAGAGTGATTAAATCTACGGAGGTTGAGGATGAGCAT
GCGGATGATTAGCTAGTTGGCGGTGTAACGGACCACCAAGGCAATGATCAATAGGGGAACTGAGAGGT
TTATCCCCCACACTGGCACTGAGATACGGGC
>Zotu6471
GAACGCTGGCGGCGTGCTTAACACATGCAAGTCGAACGGTGACCCGGAGCTTGCTCCGGCGATCAGTG
GCGAACGGGTGAGTAACACGTGAGCAACCTGCCCTTCACTCTGGGATAACTCCGGGAAACCGGGGCTA
ATACCGGATACGACCCGAGTGGGCATCCACATCGGGTGGAAAGTTTTTCGGTGAAGGATGGGCTCGCG
GCCTATCAGCTTGTTGGTGAGGTAATGGCTCACCAAGGCGACGACGGGTAGCCGGCCTGAGAGGGCGA
CCGGCCACACTGGGACTGAGACACGGCC
>Zotu6473
GAATGCTGGCGGCGTGCCTAACACATGCAAGTCGAGCGGTAACAGGAGAATTTATTCTTGCTGACGAG
CGGCGGACGGCTGAGTAACGCGTGGGAACATGCCCCAAAGTGAGGGATAAGCTCCAGAAATGGAGTCT
AATACCGCATATGATCTTCGGATTAAAGGATTTATCCGCTTTGGGGGTGGCCTGCGTAAGATTAGATA
GTTGGTGAGGTAATGGCTCACCAAGTCTACGATCTTTAGCTGGTCTGAGAGGATGATCAGCCAGACTG
GAACTGAGACACGGTC
>Zotu6474
GAACGCTAGCTACAGGCTTAACACATGCAAGTCGAGGGGCAGCATGACGATAGCTTGCTATTGTTGAT
GGCGACCGGCGCACGGGTGAGTAACGCGTATCCAACCTTCCCATTACTCAGGTATAGCCCAGTGAAAA
CTGGATTAACCCCTGATGGTCTTATCCTTCCGCATGGTTGGGTGAGTAAAGTTCACGGTAATGGATGG
GGATGCGTCTGATTAGGTAGTAGGCGGGGTAACGGCCCACCTAGCCGACGATCAGTAGGGGTTCTGAG
AGGAAGGTCCCCCACATTGGAACTGAGACACGGTC
>Zotu6475
GAACGCTAGCGGCAGGCCTAATACATGCAAGTCGAACGGGATCCGTCGGAGAGCTTGCTCGAAGACGG
TGAGAGTGGCGCACGGGTGCGTAACGCGTGAGCAACCTACCTCTATCAGGGGGATAGCCTCTCGAAAG
AGAGATTAACACCGCATAACATATCTGACCGGCATCGGTTAGCTATTAAATATTTATAGGATAGAGAT
GGGCTCGCGTGACATTAGCTAGTTGGTAGGGTAACGGCTTACCAAGGCGACGATGTCTAGGGGCTCTG
AGAGGAGAATCCCCCACACCGGTACTGAGACACGGAC
>Zotu6476

GAACGCTGGCGGCAGGCTTAACACATGCAAGTCGAGCGCCCACTTCGGTGGGAGCGGCAGACGGGTGA
GTAACACGTGGGAATCTACCCATCTCTACGGAACAACTCCGGGAAACTGGAGCTAATACCGTATACGC
CCTTTGGGGGAAAGATTTATCGGAGATGGATGAGCCCGCGTTGGATTAGCTAGTTGGTGGGGTAAAGG
CCTACCAAGGCGACGATCCATAGCTGGTCTGAGAGGATGATCAGCCACACTGGGACTGAGACACGGCC
>Zotu6477
GAACGCTGGCGGCGTGCTTAACACATGCAAGTGGAGCGACGAACCGGGGCTTGCCCCGGGGCAGAGCC
GCGAACGGGTGAGTAACACGTGGGTTACCTACCCCGATGATTGGGACAACCCGAGGAAACTCGGGCTA
ATACCAAATGTGCTCCTGGCCGTGATGGCCTTGAGGAAAGGAAGCTTCGGCCTCCGCATCGGGATGGG
CCCGCGGCCCATTAGCTAGTTGGTGGGGTAACGGCCTACCAAGGCGACGATGGGTAGCTGGTCTGAGA
GGACGATCAGCCACACTGGGACTGAGACACGGCC
>Zotu6478
GAACGCTGGCGGCATGCCTTACACATGCAAGTCGAACGGCAGCACGGGAGCAATCCTGGTGGCGAGTG
GCGAACGGGTGAGTAATACATCGGAACGTGCCCAGTAGTGGGGGATAGCCCGGCGAAAGCCGGATTAA
TACCGCATACGACCTACGGGTGAAAGCGGGGGATCGCAAGACCTCGCGCTATTGGAGCGGCCGATGGC
AGATTAGGTTGTTGGTGAGGTAAAAGCTCACCAAGCCGACGATCTGTAGCTGGTTTGAGAGGACGACC
AGCCACACTGGGACTGAGACACGGCC
>Zotu6479
GAACGCTGGCGGCGTGCCTAATACATGCAAGTCGAGCGGTAACACTTCGGGTGACGAGCGGCGGACGG
CTGAGTAACGCGTAGGAACGTACCCCAAACTGAGGGATAAGCACCGGAAACGGTGTCTAATACCGCAT
GTGATCTTCGGATTAAAGCTTCGGCGGTTTGGGAACGGCCTGCGTATGATTAGGTTGTTGGTGAGGTA
ATGGCTCACCAAGCCTACGATCATTAGCTGGTCTGAGAGGATGATCAGCCAGACTGGGACTGAGACAC
GGCC
>Zotu6480
GAACGCTGGCGGCGTGCCTAATACATGCAAGTCGAGCGGTAAGGTTCTCACTCTGTATTCAACTAGTT
TGAATGTGGTGTGAGAATACACGAGCGGCGGACGGCAGAGTAACGCGTAGGAACGTACCCCATAGTGA
GGGATAAGCACCGGAAACGGTGTCTAATACCGCATAATCTCTTCGGAGCAAAGCCTTGTGCGCTTTGG
GAGCGGCCTGCGTATGATTAGTTTGTTGGTGAGGTAACGGCTCACCAAGACTACGATCATTAGCTGGT
CTGAGAGGATGATCAGCCTGACTGGAACTGAGAACGGTC
>Zotu6482
GAACGCTGGCGGCGTGCTTAACACATGCAAGTCGAACGGTAAGGCCCTTCGGGGTACACGAGTGGCGA
ACGGGTGAGTAACACGTGGGTAATCTGCCCTGCACTTCGGGATAAGCCTGGGAAACTGGGTCTAATAC
CGGATATGAGCTCCTGCCGCATGGTGGGGGTTGGAAAGTTTTTCGGTGCAGGATGTGCCCGCGGCCTA
TCAGCTTGTTGGTGGGGTAATGGCCTACCAAGGCGACGACGGGTAGCCGGCCTGAGAGGGTGATCGGC
CACATTGGGACTGAGACACGGCC
>Zotu6483
GAACGCTGGCGGCATGCCTTACACATGCAAGTCGAACGGTAACAGGGAGCTTGCTCCGCTGACGAGTG
GCGAACGGGTGAGTAATATATCGGAACGTACCTTTGAGTGGGGGATAACTAGTCGAAAGATTAGCTAA
TACCGCATACGATCTACGGATGAAAGCGGGGGACCCGTAAGGGCCTCGTGCTCATAGAGCGGCCGATA
TCTGATTAGCTAGTTGGTGAGGTAAAGGCTCACCAAGGCATCGATCAGTAGCTGGTCTGAGAGGACGA
CCAGCCACACTGGGACTGAGACACGGCC
>Zotu6484
GAACGCTGGAAATGTGGATACCGGCATGCAAGTCAAGGGGCCGCAAGGCACCGGCGAACGGCTTAGTA
ACACGTAGATACATACCCAAAAGTGGGGCACAGCTCGTCGAAAGACGGGGTAATTTCCCATAGCACCG
CAAGGTTAAACACGCAAGTGGCTTTTGGAATGGTCTGCGGTCTATCAGCTAGTTGGCGAGGTAAAAGC
TCACCAAGGCTATGACGGATAGGGGGCGTGAGAGCGCGACCCCCAAGAATGGCACTGCGACACGGGC
>Zotu6485
GAACGCTGGCGGCATGCTTTACACATGCAAGTCGAACGGCAGCACGGACTTCGGTCTGGTGGCGAGTG
GCGAACGGGTGAGTAATACATCGGAACGTGCCCAGTTGTGGGGGATAACTACTCGAAAGAGTAGCTAA
TACCGCATGAGAACTGAGGTTGAAAGCAGGGGATCGTAAGACCTTGCGCAACTGGAGCGGCCGATGGC
AGATTAGGTAGTTGGTGGGATAAAAGCTTACCAAGCCGACGATCTGTAGCTGGTCTGAGAGGACGACC
AGCCACACTGGGACTGAGACACGGCC
>Zotu6487
GAACGCTGGCGGCGTGCTTAACACATGCAAGTCGAACGCTGAAGCTTGGGTGCTTGCATCTGGGTGGA
TGAGTGGCGAACGGGTGAGTAATACGTGAGTAACCTGCCCTTGACTCTGGGATAAGCCTGGGAAACTG

GGTCTAATACTGGATACTACATTCTCTCGCATGGGGGTGTGTGGAAAGGGTTTTACTGGTTTTGGATG
GGCTCACGGCCTATCAGCTTGTTGGTGGGGTAATGGCCTACCAAGGCGACGACGGGTAGCCGGCCTGA
GAGGGTGACCGGCCACACTGGGACTGAGACACGGCC
>Zotu6488
GAACGCTGGCGGCGTGCTTAACACATGCAAGTCGAACGGAGTACCCGAGATAGAAGCTTCGGCAGAAA
TTTTGGAATACTTAGTGGCGGACGGGTGAGTAACACGTGAGTAACCTGCCTTCGAGTGGGGAATAACA
CACCGAAAGGTGTGCTAATACCGCATAACATATGCGTACCGCATGATATGCATATCAAAGATTTATCG
CTCGAAGATGGACTCGCGTCCAATTAGGTAGTTGGTGAGGTAACGGCCCACCAAGCCGACGATTGGTA
GCCGGACTGAGAGGTTGAACGGCCACATTGGGACTGAGACACGGCC
>Zotu6489
GAACGCTGGCGGCGTGCCTAACACATGCAAGTCGAGCGGAGGTACCCAAAGAGCAATCTGAGGGTACT
TTAGCGGCGGACGGGTGAGTAACGCGTGAGCAACCTGCCTTTCACAGGGGGATAACGCACCGAAAGGT
GCACTAATACCGCATGAGACCACAGAGCCGAGTGGCAAAGGGGTCAAAGGAGTAATCCGGTGAGAGAT
GGGCTCGCGTCCGATTAGATAGTTGGTGGGGTAACGGCCTACCAAGTCAACGATCGGTAGCCGACCTG
AGAGGGTGATCGGCCACACTGGAACTGAGAAACGGTC
>Zotu6490
GAACGCTGGCGGCGCGCCTAACACATGCAAGTCGAACGAGCGAGAGAGAGCTTGCTTTCTCGAGCGAG
TGGCGAACGGGTGAGTAACGCGTGAGGAACCTGCCTCAAAGAGGGGGACAACAGTTGGAAACGACTGC
TAATACCGCATAAGCCCACGGTGCCGCATGGCACAGAGGGAAAAGGAGCAATCCGCTTTGAGATGGCC
TCGCGTCCGATTAGCTGGTTGGTGAGGTAATGGCCCACCAAGGCGACGATCGGTAGCCGGACTGAGAG
GTTGAACGGCCACATTGGGACTGAGACACGGCC
>Zotu6495
GAACGCTGGCGGCGTGCTTTACACATGCAAGTCGAGCGGCAGCATGACCTTCGGGTCGATGGCGAGCG
GCGAACGGGTGAGTAATACATCGGAACGTACCTTTTAGTGGTGGATAACGCATCGAAAGATGTGCTAA
TACAGCATAATCTCTGAGGAGGAAAGGCGGGGATCACGCAAGTGACCTGTCGCTGAAAGAGCGGCCGA
TGGTCGATTAGCTAGTTGGCGGGGTAAAAGCCCACCAAGGCGATGATCGATAGCGGGTCTGAGAGGAC
GATCCGTCACACTGGGACTGAGACACGGCC
>Zotu6496
GAACGCTGGCGGCATGCCTAACACATGCAAGTCGAACGAGACCTTCGGGTCTAGTGGCGCACGGGTGC
GTAACGCGTGGGAACCTGCCTTGAGGTTCGGAATAACTCAGAGAAATTTGAGCTAATACCGGATAATG
TCTTCGGACCAAAGATTTATCGCCTTTAGATGGGCCCGCGTTGGATTAGCTAGTTGGTGGGGTAAAGG
CCTACCAAGGCGACGATCCATAGCTGGTCTTAGAGGATGATCAGCCACACTGGGACTGAGACACGGCC
>Zotu6497
GAACGCTGGCGGCGTGCTTAACACATGCAAGTCGAACGAGATCCAACGAGTGGCAACACTCGTGAAGA
TCTAGTGGCGAACGGGTGCGTAACACGTGAGAAACCAACCCCGAACTTCGGGATAACAGGTGGAAACG
CCTGCTAATACCGGATACCTCCGCCGAGTCGCATGGCTTGGCGAAGAAAGGTCCGCCGGTTCGGGACG
GTCTCGCGGCCTATCAGCTTGTTGGTGGGGTAATGGCCTACCAAGGCATCGACGGGTAGCTGGTCTGA
GAGGATGATCAGCCACACTGGGACTGAGACACGGCC
>Zotu6498
GAACGCTGGCGGCGTGCTTAACACATGCAAGTCGAACGGTGAAGCAGAGCTTGCTCTGTGGATCAGTG
GCGAACGGGTGAGTAACACGTGAGCAACCTGCCCTGGACTCTGGGATAAGCGCTGGAAACGGCGTCTA
ATACTGGATATGAGCCTCTTCCGCATGGTGGGGGTTGGAAAGATTTTTCGGTCTGGGATGGGCTCGCG
GCCTATCAGCTTGTTGGTGAGGTAATGGCTCACCAAGGCGTCGACGGGTAGCCGGCCTGAGAGGGTGA
CCGGCCACACTGGGACTGAGACACGGCC
>Zotu6500
CAACGCTGGCGGCGCGCCTAACACATGCAAGTCGAACGAGAAAGTGGAGCAATCCATGAGTAAAGTGG
CGTACGGGTGAGTAACACGTGGATAACCTGCCCTTTAGTGGGGAATACCGCTGGGAAACCGGCGACAA
TACCGCATAAGCTCGAGAGAGGAAAGCAGCAATGCGCTGGAGGAGGGGTCCGCGGTCGATTAGCTAGT
TGGTAGGGTAATGGCCTACCAAGGCTGTGATCGATAGCCGGCCTGAGAGGGCACACGGCCACACTGGC
ACTGAAACACGGGC
>Zotu6501
GAACGCTGGCGGCGTGCTTAACACATGCAAGTCGAACGGGGTCCATCCTTCGGGAGAAGACCTAGTGG
CGAACGGGTGAGTAACACGTGAGCAACCTGTCCCAAAGACTGGGATAAGCCAGGGAAACTTGGTCTAA
TACCGGATGCCCTCTTCGAGTCATAAGGCTTGATGAGGAAATGATTTATTGCTTTGGGAGGGGCTCGC

GGCCTATCAGCTTGTTGGTGGGGTAATGGCCTACCAAGGCTTCGACGGGTAGCTGGTCTGAGAGGATG
ATCAGCCACACTGGGACTGAGACACGGCC
>Zotu6504
GAACGCTGGCGGCGTGCCTAACACATGCAAGTCGAGCGATGAAGCTCCTTCGGGAGTGGATTAGCGGC
GGACGGGTGAGTAACACGTGGGTAACCTGCCTCATAGAGGGGAATAGCCTCCCGAAAGGGAGATTAAT
ACCGCATAAAGTATGAAAGTCGCATGACTTTTATACCAAAGGAGTAATCCGCTATGAGATGGACCCGC
GGCGCATTAGCTAGTTGGTGAGGTAACGGCTCACCAAGGCGACGATGCGTAGCCGACCTGAGAGGGTG
ATCGGCCACATTGGAACTGAGACACGGTC
>Zotu6505
GAACGCTGGCGGCACGCCTAACACATGCAAGTCGAGCGGTAGCAGGGTTAGTAGCAATACTAACTGCT
GACGAGCGGCGGACGGGTGAGTAATATGTGGGAACTTACCCTTCAGAGGGGGATAACCAAGGGAAACT
TTGGCTAATACCGCGTAATCTCGAGAGAGGAAAGCTGCCGCAAGGTGGCGCTGAAGGAGAGGCCTGCA
TCCGATTAGCTAGTTGGTAAGGTAAAAGCTTACCAAGGCGATGATCGGTAACTGGTCTGAGAGGACGA
CCAGTCACACTGGGACTGAGACACGGCC
>Zotu6506
GAACGCTGATGGCGTGCCTCATACATGCAAGTCGAGCGCGTAGCAATACGAGCGGCGGACGGGTGAGT
AACACGTAAACAATCTGCCCTAAAATGGGGAATAACTATCCCAACGGATAGCTAATACCGCATAATGA
TGGAGACGGCATCGTCTCTTCTCAAAAGGCTTCGGCCGTTTTAGGATGAGTTTGCGGCCCATTAGCTT
GTTGGCGGGGTAACGGCCCACCAAGGCTACGATGGGTATCCGGCCTGAGAGGGTGTACGGACACATTG
GGACTGAGATACGGCC
>Zotu6507
GAACGCTGGCGGCGTGCTTAACACATGCAAGTCGAACGAGCCTTGCAACCACTGAGGTGCTTGCACCG
AAGTGATTGCGCTGGCGAGTGGCGGACGGGTGAGTAACGCGTGGGTAACCTGCCTCGTACAGGGGGAT
ACCAGTTGGAAACGACTGTTAATACCGCATAAGCGCACAGGATCGCATGATCTAGTGTGAAAAACTCC
GGTGGTACGAGATGGACCCGCGTCTGATTAGCTAGTTGGTGAGGTAACGGCCCACCAAGGCGACGATC
AGTAGCCGGCCTGAGAGGGTGAACGGCCACATTGGGACTGAGACACGGCC
>Zotu6508
GAACGCTGGCGGCGTGCCTAATACATGCAAGTCGAGCGGTAAGGCCCATCCAGATTATTCGATCACGA
CAACTTCGGTTGTCATAGCAAACCAGGAAAGTCCCCGGCTAGGGAAACAGTGGTGCGGGAAGATCGAG
TTATGTGGTTGGGTACACGAGCGGCGGACGGCTGAGTAACACGTAGGAACGTACCCCAAAGTGAGGGA
TAAGCACCAGAAATGGTGTCTAATACCGCATGTGATCTTCGGATTAAAGCTTCGGCGCTTTGGGAACG
GCCTGCGCATGATTAGTTTGTTGGTGAGGTAATGGCTCACCAAGACTACGATCATTAGCTGGTCTGAG
AGGATGATCAGCCAGACTGGGACTGAGACACGGCC
>Zotu6509
GAACGCTGGCGGCGTGCTTAACACATGCAAGTCGAACGATGAAGGGAGCTTGCTCCTGGATTAGTGGC
GAACGGGTGAGTAACACGTGAGTAACCTGCCCTTGACTCTGGGATAAGCCTGGGAAACTGGGTCTAAT
ACCGGATATGACCTTCCATCGCATGGTGGTTGGTGGAAAGCTTTTGTGGTTTTGGATGGACTCGCGGC
CTATCAGCTTGTTGGTGGGGTAATGGCCTACCAAGGCGACGACGGGTAGCCGGCCTGAGAGGGTGACC
GGCCACACTGGGACTGAGACACGGCC
>Zotu6510
GAACGCTGGCGGCGTGCCTAATACATGCAAGTCGAACGGTCTTTTCTATGGAAGCTTGCTTCCGCTGA
GAAGATAGTGGCGAACGGGTGAGTAACACGTGGGTAACCTGCCCATAAGAGGGGGATAACATCCGGAA
ACGGGTGCTAATACCGCATAGTTTTCTTGATCGCATGATTGAGAAAGGAAAGACGGCCTTTGTGCTGT
CGCTTATGGATGGACCCGCGGCGTATTAGTTAGTTGGTGAGGTAACGGCTCACCAAGACGATGATACG
TAGCCGACCTGAGAGGGTGATCGGCCACATTGGGACTGAGACACGGCC
>Zotu6514
GAACGCTAGCGGCAGGCTTAATACATGCAAGTCGTGGGGCATCACAGTGTAGCAATACATGGGTGGCG
ACCGGCAAACGGGTGCGTAACACGTACGCAACTTTCCTTCGAGCGGGGAATAGCCCAGAGAAATTTGG
ATTAATACCCCATAAGATAAAGATGTGGCATCACATTTTTATTAAAGCTCCGGCACTTGAAGATGGGC
GTGCGGCTGATTAGGTAGTTGGTGGGGTAACGGCCCACCAAGCCAACGATCAGTAGCTGATGTGAGAG
CATGATCAGCCACACGGGCACTGAGACACGGGC
>Zotu6515
GAACGCTGGCGGCGTGCTTAACACATGCAAGTCGAACGATGAAGCCCAGCTTGCTGGGCGGATTAGTG
GCGAACGGGTGAGTAACACGTGAGTAACCTACCCTTGACTCTGGGATAAGCCCGGGAAACTGGGTCTA

ATACCGGATATGAGCGTCCACCGCATGGTGGGTGTTGGAAAGAATTTCGGTCATGGATGGACTCGCGG
CCTATCAGCTTGTTGGTGAGGTAATGGCTCACCAAGGCGACGACGGGTAGCCGGCCTGAGAGGGTGAC
CGGCCACACTGGGACTGAGACACGGCC
>Zotu6517
GAACGCTGGCGGCAGGCCTAACACATGCAAGTCGAACGCCCCGCAAGGGGAGTGGCAGACGGGTGAGT
AACACGTGGGAACATACCCTTTGGTTCGGAACAACTCAGGGAAACTTGAGCTAATACCGGATAAGTGC
GAGAGCAGAAAGATTTATCGCCGAAGGATTGGCCCGCGTCTGATTAGCTTGTTGGTGAGGTAACGGCT
CACCAAGGCGACGATCAGTAGCTGGTCTGAGAGGATGATCAGCCACATTGGGACTGAGACACGGCC
>Zotu6522
GAACGCTGGCGGCGTGCTTAACACATGCAAGTCGAACGGTGACCTCGGAGCTTGCTCCGGGCGATCAG
TGGCGAACGGGTGAGTAACACGTGAGTAACCTGCCCCAGACTCTGGGATAACCCCGGGAAACCGGAGC
TAATACCGGATACGACACGAAGAGGCATCTCTATCGTGTGGAAAGTTCTTCGGTCTGGGATGGACTCG
CGGCCTATCAGCTAGTTGGTGAGGTAACGGCTCACCAAGGCGACGACGGGTAGCCGGCCTGAGAGGGC
GACCGGCCACACTGGGACTGAGACACGGCC
>Zotu6524
GAACGCTGGCGGCAGGCTTAACACATGCAAGTCGAACGCCCCGCAAGGGGAGTGGCAGACGGGTGAGT
AACGCGTGGGAATCTACCTTTTGCTACGGGATAGCTCCGGGAAACTGGAATTAATACCGTATGTGCCC
TTCGGGGGAAAGATTTATCGGCAAAGGATGAGCCCGCGTTGGATTAGCTAGTTGGTGGGGTAAAGGCC
TACCAAGGCGACGATCCATAGCTGGTCTAAGAGGATGATCAGCCACATTGGGACTGAGACACGGCC
>Zotu6525
GAACGCTGGCGGCGTGCTTAACACATGCAAGTCGAACGGAAAGGCCCTTCGGGGTGCTCGAGTGGCGA
ACGGGTGAGTAACACGTGGGTGATCTGCCCTGCACTTTGGGATAAGCCTGGGAAACTGGGTCTAATAC
CGAATATGATCATGCCTCTCCTGGGGTGTGGTGGAAAGCTTTTGCGGTGTGGGATGGGCCCGCGGCCT
ATCAGCTTGTTGGTGGGGTGATGGCCTACCAAGGCGACGACGGGTAGCCGGCCTGAGAGGGTGTCCGG
CCACACTGGGACTGAGATACGGCC
>Zotu6526
GAACGCTGGCGGCGCGCTTAATACATGCAAGTCGAGCGAGAACCATGGCTTCGGCCATGGGGACAGCG
GCGAACGGGTGAGTAACACGTGGGTAATCAACCCCCGACACCGGGATAGCCCAGGGAAACTTGGATTA
ATACCGGATACCCCATCCGGCTCTCGAGCCGGCTGGAAAAGGGTAGCTTCGGCCTCCGGTCGGGGACG
AGCCCGCGGCGGATTAGCTTGTTGGTGAGGTAAAAGCTCACCAAGGCGACGATCCGTAGCTGGTCTGA
GAGGACGATCAGCCACACTGGGACTGAGACACGGCC
>Zotu6528
GAACGCTGGCGGCATGCCTTACACATGCAAGTCGAACGGCAGCGCGGGAGCAATCCTGGCGGCGAGTG
GCGAACGGGTGAGTAATATATCGGAACGTGCCCAGTAGTGGGGGATAGCCCGGCGAAAGCCGGATTAA
TACCGCATGAGACCTGAGGGTGAAAGCGGGGGATCGCAAGACCTCGCGCTACTGGAGCGGCCGATATC
AGATTAGCTAGTTGGTGGGGTAAAGGCCTACCAAGGCAACGATCTGTAGTTGGTCTGAGAGGACGACC
AGCCACACTGGGACTGAGACACGGCC
>Zotu6530
CGAACGGGTGAGTAACACGTGAGCAACCTGCCCCTCACTCTGGGATAAGCGCTGGAAACGGCGTCTAA
TACTGGATACGAGGCACAACCGCATGGTTAGTGTCTGGAAAGATTTTTTGGTGGGGGATGGGCTCGCG
GCCTATCAGCTTGTTGGTGAGGTAATGGCTCACCAAGGCGTCGACGGGTAGCCGGCCTGAGAGGGTGA
CCGGCCACACTGGGACTGAGACACGGCC
>Zotu6532
GAACGCTGGCGGCAGGCCTAATACATGCAAGTCGAACGGATCCTTCGGGATCAGTGGCGGACGGGTGA
GTAACACGTGGGAACGTGCCTTTTGGTTCGGAACAAGCCGGGGAAACCTGGTCTAATGCCGAATGTGC
CCTTCGGGGGAAAGATTTATCGCCATTAGAGCGGCCCGCGTCTGATTAGCTAGTTGGTGGGGTAAAGG
CCTACCAAGGCTACGATCAGTAGCTGGTCTGAGAGGATGATCAGCCACACTGGGACTGAGACACGGCC
>Zotu6533
GAACGCTGGCGGCGTGCCTAATACATGCAAGTCGAGCGGACTGATTAAGAGCTTGCTCTTATGACGTT
AGCGGCGGACGGGTGAGTAACACGTGGGCAACCTGCCCTGTAGATCGGGATAACATCGAGAAATCGGT
GCTAATACCGGGTAATATCTGAGATCGCATGATCTTCGGTTAAAAGATGGCTCCGGCTATCACTACAG
GATGGGCCCGCGGCGCATTAGCTAGTTGGTAAGGTAATGGCTTACCAAGGCGACGATGCGTAGCCGAC
CTGAGAGGGTGATCGGCCACACTGGGACTGAGACACGGCC
>Zotu6534

GAACGCTGGCGGAATGCTTTACACATGCAAGTCGAACGATGAACCTTAGCTTGCTAGGGGGATTAGTG
GCGAACGGGTGAGTAATATATCGGAACGTGCCTTGTAATGGGGATAACTAGTCGAAAGATTAGCTAA
TACCGCATACGCCCTGAGGGGGAAAGTAGGGGATCTTCGGACCTTACGTTATAAGAGCGGCCGATATC
TGATTAGCTAGTTGGTGGGGTAATGGCCTACCAAGGCTTCGATCAGTAGCTGGTCTGAGAGGACGACC
AGCCACACTGGAACTGAGACACGGTC
>Zotu6535
GAACGCTGGCGGCGTGCCTAATACATGCAAGTCGAGCGGACAGATGGGAGCTTGCTCCCTGATGTCAG
CGGCGGACGGGTGAGTAACACGTGGGTAACCTGCTTGTAAGACTGGGATAACTCCGGGAAACCGGGGC
TAATACCGGATGCTTGTTTGAACCGCATGGTTCAAACATAAAAGGTGGCTTCGGCTACCACTTACAGA
TGGACCCGCGGCGCATTAGCTAGTTGGTGAGGTAACGGCTCACCAAGGCAACGATGCGTAGCCGACCT
GAGAGGGTGATCGGCCACACTGGGACTGAGACACGGCC
>Zotu6536
GAACGCTGGCGGCGTGCTTAACACATGCAAGTCGAACGGAAAGGCCAGTGCTTGCACTGGTACTCGAG
TGGCGAACGGGTGAGTAACACGTGGGTGATCTGCCCTGCACTTCGGGATAAGCCTGGGAAACTGGGTC
TAATACCGGATAGGACGATGGTTTGGATGCCGTTGTGGAAAGTTTTTTCGGTGTGGGATGAGCTCGCG
GCCTATCAGCTTGTTGGTGGGGTAATGGCCTACCAAGGCGTCGACGGGTAGCCGGCCTGAGAGGGTGT
ACGGCCACATTGGGACTGAGATACGGCC
>Zotu6537
GAACGCTGGCGGCGTGCCTAAGACATGCAAGTCGAGTGGGTTTAGACCCACGGCGAACGGGTGAGTAA
TACCTAGGAACTTTTCTTTAGTTGAGGATATCCCACCGAAAGGTGGAGCAATACTCAATGTGGCCGCA
AGGCTAAAGTCGCAAGACGCTAAAGGTGAGGCCTGGGCACCATCAGCTAGTTGGTAAGGTAAAAGCTT
ACCAAGGCTATGACGGTTAGGGGCCCCGAGAGGGGAAACCCCCACACTGGGACTGCGACACGGCC
>Zotu6538
GAACGCTGGCGGCGTGCCTAATACATGCAAGTCGAGCGGTAAGGCCCTTCGGGGTACACGAGCGGCGG
ACGGCTGAGTAACGCGTAGGAACGTACCCCAAACTGAGGGATAAGCACCAGAAATGGTGTCTAATACC
GCATGTGCTCCACGGAGTAAAGCTTCGGCGGTTTGGGAACGGCCTGCGTACGATTAGATTGTTGGTGA
GGTAATGGCTCACCAAGTCGACGATCGTTAGCTGGTCTGAGAGGATGATCAGCCAGACTGGGACTGAG
AACGGCC
>Zotu6539
GAACGCTAGCGGCAGGCTTAATACATGCAAGTCGTGGGGCAGCGCAGGTAGCAATACTGGGCGGCGAC
CGGCAAACGGGTGCGGAACACGTACACAACCTTCCTACAAGTGGGGAATAGCCCAGAGAAATTTGGAT
TAATACCCCGTAACATATAGATGTGGCATCATATTTATATTATAGCTTCGGCGCTTGTTGATGGGTGT
GCGGCTGATTAGATAGTTGGCGGGGTAACGGCCCACCAAGTCTACGATCAGTAGCTGATGTGAGAGCA
TGATCAGCCACACGGGCACTGAGACACGGGC
>Zotu6540
GAACGCTGGCGGCGTGCCTAATACATGCAAGTCGAGCGCGTGAAATTCGCGGATCTCTTCGGAGTGAT
GCGGATGGATCGAGCGGCGGACGGGTGAGTAACACGTGGGCAACCTGCCCGTAAGACTGGGATAACTT
CGGGAAACCGAAGCTAATACCGGATAGGCTTTCGCACCGCATGGTGCGAAAGGGAAAGATGCTTCTGG
CATCACTTACGGATGGGCCCGCGGCGCATTAGCTTGTTGGTGAGGTAACGGCTCACCAAGGCGACGAT
GCGTAGCCGACCTGAGAGGGTGATCGGCCACACTGGGACTGAGACACGGCC
>Zotu6541
GAACGCTAGCGGCAGGCCTAATACATGCAAGGCGAGGGGGCAGCAATGTCACCGTCGTACGGGTGCGT
AACGCGTATGCAACCTACCTATCACTGGGGGATAGCCCGGGGAAACCCGGATTAATACCGCATGACAC
ATTAGGTCCTCATGGGCTATATGTTAAAGATTTATTGGTGATAGATGGGCATGCGTTCGATTAGCTAG
TTGGTATGGGTAACGGCCTACCAAGGCGACGATCGATAGGGGAGCTGAGAGGTTGATCCCCCACACGG
GCACTGAGATACGGGC
>Zotu6542
GAACGCTGGCGGCAGGCCTAACACATGCAAGTCGAACGCTCCGCAAGGAGAGTGGCAGACGGGTGAGT
AACACGTGGGAATCTTCCTATCGGTACGGAATAGCTCAGGGAAACTTGAGGTAATACCGCATACGCCC
TTCGGGGGAAAGATTTATCGCCGATAGATGAGCCCGCGTCTGATTAGCTAGTTGGTGGGGTAATGGCC
TACCAAGGCGACGATCAGTAGCTGGTCTGAGAGGATGACCAGCCACACTGGGACTGAGACACGGCC
>Zotu6543
GAACGCTGGCGGCGTGCCTAAGACATGCAAGTCGAGTGGGTTTAGACCCACGGCGAACGGGTGAGTAA
TACCTAGGAACTTTCTTTAAGTGGAGCATATCCTACCGAAAGGTAGAGCAATTCTCCATGTGATCGCA

AGATTAAAGTCGCAAGACGCTTAAAGTGAGGCCTGGGCACCATCAGCTAGTTGGTAAGGTAATGGCTT
ACCAAGGCTATGACGGTTAGGGGTCCCGAGAGGGAGAACCCCCACACTGGGACTGCGACACGGCC
>Zotu6546
GAACGCTGGCGGCATGCTTTACACATGCAAGTCGGACGGCAGCACAGAGAAGCTTGCTTCTTGGGTGG
CGAGTGGCGAACGGGTGAGTAATATATCGGAACGTACCGAGTAATGGGGGATAACTAATCGAAAGATT
AGCTAATACCGCATATTCTCTGAGGAGGAAAGCAGGGGACCTTCGGGCCTTGCGTTATTCGAGCGGCC
GATATCTGATTAGCTAGTTGGTGGGGTAAAGGCCTACCAAGGCGACGATCAGTAGCGGGTCTGAGAGG
ATGATCCGCCACACTGGGACTGAGACACGGCC
>Zotu6550
GAACGCTGGCGGCGTGCCTAATACATGCAAGTCGAGCGGACAGAAGGGAGCTTGCTCCCGGAAGTCAG
CGGCGGACGGGTGAGTAACACGTGGGTAACCTGCCTGTAAGACTGGGATAACTCCGGGAAACCGGGGC
TAATACCGGATAATATCAAGAGCTGCATGGCTCTTGATTGAAAGGCGGCTTCGGCTGTCACTTACAGA
TGGACCCGCGGCGCATTAGCTAGTTGGTGAGGTAACGGCTCACCAAGGCGACGATGCGTAGCCGACCT
GAGAGGGTGATCGGCCACACTGGGACTGAGACACGGCC
>Zotu6551
GAACGCTGGCGGCGTGCTTAACACATGCAAGTCGAACGATGAACAGGAGCTTGCTCCTGGGATTAGTG
GCGAACGGGTGAGTAACACGTGAGTAACCTGCCCTTGACTCTGGGATAAGCGTTGGAAACGACGTCTA
ATACCGGATATGACCCTCGCTGGCATCGGCTGGGGGTGGAAAGATTTTTTGGTCAAGGATGGACTCGC
GGCCTATCAGGTAGTTGGTGAGGTAATGGCTCACCAAGCCGACGACGGGTAGCCGGCCTGAGAGGGTG
ACCGGCCACACTGGGACTGAGACACGGTC
>Zotu6552
GAACGCTAGCGGCAGGCTTAATACATGCAAGTCGAGGGGCAGCGGGGGTAGCAATACCTGCCGGCGAC
CGGCAAACGGGTGCGGAACACGTACGCAACCTTCCCAAAACAGGTGAATAGCCCTCCGAAAGGAGGAT
TAATACACCGTACCATAATGAGGTGGCATCACTTTATTATTATAGATAGCAATATCGGTTTTGGATGG
GCGTGCGGCTGATTAGGTAGTTGGCGGGGTAACGGCCCACCAAGCCTCCGATCAGTAACTGGTGTGAG
AGCACGACCAGTCACACGGGCACTGAGACACGGGC
>Zotu6555
GAACGCTGGCGGTAGGCCTAACACATGCAAGTCGAACGGCAGCACAGGAGAGCTTGCTCTCTGGGTGG
CGAGTGGCGGACGGGTGAGGAATACATCGGAATCTACCTTTTCGTGGGGGATAACGTAGGGAAACTTA
CGCTAATACCGCATACGACCTACGGGTGAAAGTGGGGGACCGCAAGGCCTCACGCGATTAGATGAGCC
GATGTCCGATTAGCTAGTTGGCGGGGTAATGGCCCACCAAGGCGACGATCGGTAGCTGGTCTGAGAGG
ATGATCAGCCACACTGGAACTGAGACACGGTC
>Zotu6557
GAACGCTGGCGGCGCGCTTAACACATGCAAGTCGAGCGAGAACCAGGGCTTCGGCCCTGGGGACAGCG
GCGAACGGGTGAGTAACACGTGGGTAATCTGCCCTCGGCACTGGGATAACCCGGGGAAACCCGGACTA
ATACCGGATGGCCTCTCGAGCTTTCGGGCTTGGGAGAAAAGGTAGCTTTGGCCTCCGGCCGAGGATGA
GCCCGCGGCCGATTAGCTTGTTGGCGGGGTAACGGCCCACCAAGGCGACGATCGGTAGCTGGTCTGAG
AGGACGATCAGCCACACTGGGACTGAGACACGGCC
>Zotu6558
GAACGCTGGCGGCATGCCTTACACATGCAAGTCGAACGCGAAAGCGGGGCAACCCGCAAGTAGAGTGG
CGAACGGGTGAGTAATACATCGGAACATTTCCAGTAGCGGGGGATAACCTGTCGAAAGATAGGCTAAT
ACCGCATACGACCTACGGGTGAAAGCGGAGGACCGCAAGGCTTCGCACTATTGGAGTGGCCGATGTCG
GATTAGCTAGTTGGCGGGGTAAAAGCCCACCAAGGCTACGATCCGTAGCTGGTCTGAGAGGACGATCA
GCCACACTGGAACTGAGACACGGTC
>Zotu6559
GAACGCTGGCGGCATGCCTAACACATGCAAGTCGAACGAGACCTTCGGGTCTAGTGGCGCACGGGTGC
GTAACGCGTGGGAATCTGCCCTTGGGTTCGGAATAACTCGCCGAAAGGCGTGCTAATACCGGATGATG
TCGCAAGACCAAAGATTTATCGCCCAAGGATGAGCCCGCGTAGGATTAGCTAGTTGGTGAGGTAAAGG
CTCACCAAGGCGACGATCCTTAGCTGGTCTGAGAGGATGATCAGCCACACTGGGACTGAGACACGGCC
>Zotu6560
GAACGCTGGCGGCAGGCCTAACACATGCAAGTCGAGCGGCAGCACGGGAGCTTGCTCCTGGTGGCGAG
CGGCGGACGGGTGAGTAATGCCTAGGAATCTGCCTGGTAGTGGGGGATAACGTTCGGAAACGGACGCT
AATACCGCATACGTCCTACGGGAGAAAGTGGGGGACCTTCGGGCCTCACGCTATCAGATGAGCCTAGG
TCGGATTAGCTAGTTGGTAGGGTAAAGGCCTACCAAGGCGACGATCCGTAACTGGTCTGAGAGGATGA

TCAGTCACACTGGAACTGAGACACGGTC
>Zotu6562
GAACGCTGGAAATGTGGATACCGGCATGCAAGTCAAGGGGCCGCAAGGCACCGGCGAACGGCTTAGTA
ACACGTGGATACATACCGCAAAGTGGGGCATAGCTCGTTGAAAAACGGGGTAATTTCCCATAGTCCCG
CAAGGGTAAAGGCGCAAGCCGCTTTGTGAATGGTCCGCGGTCTATCAGCTAGTTGGTGAGGTAATGGC
TCACCAAGGCTATGACGGATAGGGGGCGTGAGAGCGCGACCCCCAAGAATGGCACTGCGACACGGGC
>Zotu6563
GAACGCTGGCGGCGTGCCTAATACATGCAAGTCGAGCGGACTTGACAGAGCTTGCTCTGTTCAAGTTA
GCGGCGGACGGGTGAGTAACACGTGGGTAACCTGCCTGTAAGACTGGGATAACTCCGGGAAACCGGGG
CTAATACCGGATAATATCAAGAACCGCATGGTTCTTGATTGAAAGGCGGCTTCGGCTGTCACTTACAG
ATGGACCCGCGGCGCATTAGCTAGTTGGTGAGGTAACGGCTCACCAAGGCGACGATGCGTAGCCGACC
TGAGAGGGTGATCGGCCACACTGGGACTGAGACACGGCC
>Zotu6564
GAACGCTGGCGGCGTGCTTAACACATGCAAGTCGAACGGACTAAGGGGCTTGCTCCTTAGTTAGTGGC
GCACGGGTGAGTAACGCGTGGATAATCTACCCGATACTCTGGGATAACATCTCGAAAGGGGTGCTAAT
ACCGGATAAGCCTACGAGAGCTGCGGCTCATGTAGGAAAAGGTGGGGACCTTCGGGCCTGCCGATATC
GGATGAGTCCGCGTCCCATTAGCTAGTTGGCGGGGTAAAGGCCCACCAAGGCTACGATGGGTAGCTGG
TCTGAGAGGATGATCAGCCACACTGGAACTGAGACACGGTC
>Zotu6565
GAACGCTGGCAGTGTGCCTAAGACATGCAAGTCGAACGGAAGTCTTCGGACTTTAGTGGCGAACGGGT
GAGTAACACGTGGGCGACCTACCTCCAAGTACGGCATAACCCCGGGAAACCGGAGCTAATTCCGTATG
TGCTCGTATCTTGTTTGATACGCGTAAAGATTTATCGCTTGGAGATGGGCCCGCGGCGCATCAGCTAG
TTGGTGGGGTAACGGCCTACCAAGGCGACGACGCGTAGCCGACCTGAGAGGGTGACCGGCCACAGGGG
CACTGAGACACGGGC
>Zotu6566
GAATGCTGGCGGCGTGGATAAGTCATGCAAGTCAAGGGGGCCCGCAAGGGCAACCGGCAAACGAGGTA
GTAACACGTAGGTACACCCCCCGAAGTCGAGCATAGCTAGCCGAAAGGTTAGGTAATTCTCGATAGTC
CCGCAAGGGTAAAGTTTTTCGCTTCGGGAGTGGCCTGCGTAGTATCAGCTAGTTGGTAGGGTAATGGC
CTACCAAGGCTACGACGCTTAGGGGAGCTGAGAGGCTGACCCCCACCGATGGAACTGCGACACGGTC
>Zotu6567
GAACGCTGGCGGCGTGCTTAACACATGCAAGTCGAACGCTGAAGCTTGGGTGCTTGCATCTGGGTGGA
TGAGTGGCGAACGGGTGAGTAATACGTGAGTAACCTGCCCTTGACTCTGGGATAAGCCTGGGAAACTG
GGTCTAATACTGGATACTACACTCTCTCGCATGGGGGTGTGTGGAAAGGGTTTTACTGGTTTTGGATG
GGCTCACGGCCTATCAGCTTGTTGGTGGGGTAATGGCCTACCAAGGCGACGACGGGTAGCCGGCCTGA
GAGGGTGACCGGCCACACTGGGACTGAGACACGGCC
>Zotu6568
GAACGCTGGCGGCAGGCCTAACACATGCAAGTCGAACGAACTCTTCGGAGTTAGTGGCGGACGGGTGA
GTAACACGTGGGAACGTGCCTTTAGGTTCGGAATAACTCAGGGAAACTTGTGCTAATACCGAATGTGC
CCTTCGGGGGAAAGATTTATCGCCTTTAGAGCGGCCCGCGTCTGATTAGCTAGTTGGTAGTGTAATGG
ACTACCAAGGCGACGATCAGTAGCTGGTCTGAGAGGATGATCAGCCACATTGGGACTGAGACACGGCC
>Zotu6569
GAACGCTGGCGGCGTGCCTAATACATGCAAGTCGAGCGGTAAGGCCTCTTCGGAGGTACACGAGCGGC
GGACGGCTGAGTAACACGTAGGAACGTACCCCAAACTGAGGGATAAGCACCAGAAATGGTGTCTAATA
CCGCATGTGATCTTCGGATTAAAGCTTCGGCGGTTTGGGAACGGCCTGCGCATGATTAGCTTGTTGGT
GAGGTAAAAGCTCACCAAGGCGACGATCATTAGCTGGTCTGAGAGGACGATCAGCCACACTGGGACTG
AGACACGGCC
>Zotu6570
GAACGCTGGCGGCGTGCCTAAGACATGCAAGTCGAGTGGGTTTAGGCCCACGGCGAACGGGTGAGTAA
TACCTAGGAACTTTCTTTAAGTGGAGCATAACCCATAGAAATGTGGGATAATACTCCATGTGATAGCA
ATATCAAAGTCGCAAGACGCTTAAAGTGAGGCCTGGGCACCATCAGCTAGTTGGTAAGGTAATGGCTT
ACCAAGGCTATGACGGTTAGGGGCCCCGAGAGGGGAGACCCCCACACTGGAACTGCGACACGGTC
>Zotu6573
GAACGCTGGCGGCGTGCCTAAGACATGCAAGTCGAGTGGGTTTAGACCCACGGCGAACGGGTGAGTAA
TACCTAGGTACTTTCTTTAAGTGGAGCATAACCAGTCGAAAGATTGGATAATACTTCATGTGCCCCGG

AGTTAAATCCGGGGTAAAGTCGCAAGACGCTTAAAGTGAGGCCTGGGCACCATCAGCTAGTTGGTAAG
GTAATGGCTTACCAAGGCTATGACGGTTAGGGGTCCCGAGAGGGATAACCCCCACACTGGAACTGCGA
CACGGTC
>Zotu6574
GAACGCTAGCGGCAGGCCTAATACATGCAAGTCGAACGGTCGTAGTAATACGATAGTGGCGCACGGGT
GCGTAACACGTATGCAACCTACCTTATAGGGGGGGATAGCCTTTGGAAACGGAGATTAATACCCCATA
ATATATTGACTCGGCATCGAGTTATTATTAAAGATTTATCGCTATAAGCTGGGCATGCGTCCAATTAG
TTAGTTGGCGGGGTAACGGCTCACCAAGACGATGATTGGTAGGGGAACTGAGAGGTTAATCCCCCACA
CTGGCACTGAGATACGGGC
>Zotu6575
GAATGCTGGCGGCGTGCCTAACACATGCAAGTCGAGCGGCAGCGCGAGTAGTTTACTACTTGGCGGCG
AGCGGCGGACGGCTGAGTAACGCGTGGGAACGTGCCCCAAAGTGAGGAATAACTGCCCGAAAGGGTAG
CTAATGCCGCATATGCTCTTAGGAGTAAAGTCTTCGGACGCTTTGGGAGCGGCCCGCGTCGGATTAGG
TTGTTGGTGAGGTAATGGCTCACCAAGCCGACGATCCGTAGCTGGTCTGAGAGGATGATCAGCCAGAC
TGGAACTGAGACACGGTC
>Zotu6576
GAACGCTGGCGGCGTGCTTAACACATGCAAGTCGAACGATGAAAGAGGAGCTTGCTCCTCTGGATTAG
TGGCGAACGGGTGAGTAACACGTGAGCAATCTGCCCCTGACTCCGGAATAACTACGGGAAACTGTAGC
TAATACCGGATACGCGCCGAACAGGCATCTGTATAGGTGGGAAAGAATTTCGGTCAGGGATGAGCTCG
CGGCCTATCAGCTTGTTGGTGAGGTAACGGCTCACCAAGGCGACGACGGGTAGCCGGCCTGAGAGGGT
GACCGGCCACATTGGGACTGAGGCACGGCC
>Zotu6577
GAACGCTGGCGGCGTGCCTAACACATGCAAGTCGAACGGAAGTTAGAGCTTCGGTTTTAACTTTAGTG
GCGAACGGGTGAGTAACGCGTGAGGAACCTGCCTTTCAGTGGGGGACAACAGTTGGAAACGACTGCTA
ATACCGCATGATGCTTTTGGGAGACATCTCCTGGAAGCCAAAGCTTTATGTGCTGAAAGATGGCCTCG
CGTCTGATTAGCTAGTTGGTGAGGTAACGGCTCACCAAGGCGACGATCAGTAGCCGGTCTGAGAGGAT
GAACGGCCACATTGGGACTGAGATACGGCC
>Zotu6578
GAACGCTGGCGGCGTGCTTAACACATGCAAGTCGAACGGTGAACATGGAGCTTGCTCTGTGGGATCAG
TGGCGAACGGGTGAGTAACACGTGAGCAACCTGCCCCTGACTCTGGGATAAGCGCTGGAAACAGCGTC
TAATACTGGATACGAACAACGATCGCATGGTCAGTTGTTGGAAAGATTTTTTGGTTGGGGATGGGCTC
GCGGCCTATCAGCTTGTTGGTGAGGTAATGGCTCACCAAGGCGTCGACGGGTAGCCGGCCTGAGAGGG
TGACCGGCCACACTGGGACTGAGACACGGCC
>Zotu6579
GAACGCTGGCGGCATGCTTAACACATGCAAGTCGAACGGATTAATATGGATTGCCATATTAGTTAGTG
GCAAACGGGTGAGTAACGCGTGGGAATCTACCTAGAAGTATGGAATAACAATTAGAAATGATTGCTAA
TACCGTATGTGCTTTGAGAAGTAAAGATTTATCGCTTCTAGATGAGCCCGCGTTAGATTAGCTAGTTG
GTGGGGTAATGGCCTACCAAGGCAACGATCTATAGCTGGTCTGAGAGGATGATCAGCCACACTGGGAC
TGAGACACGGCC
>Zotu6581
GAACGCTGGCGGCGTACTTAACACATGCAAGTCGTACGAGAAAGGGGACTTCGGTCCCTGAGTACAGT
GGCGAACGGGTGCGTAACACGTAAGTAATCTACCCCAAACACTGGGATAACCCTCCGAAAGGAGGGCT
AAAACTGGATAGGGTCCCCGAGGTATAAGTCGCGGGGGGAAAACGGGCCTCTGTTTCATGCTCGTGGT
TTGGGATGAGCTTGCGGCCCATTAGCTAGTTGGTAGGGTAATGGCCTACCAAGGCAACGATGGGTAGC
TGGTCTGAGAGGATGATCAGCCACACTGGCACTGAGACACGGGC
>Zotu6582
GAACGCTAGCGGCAGGCCTAATACATGCAAGTCGGACGGGATTCAGGGAGCAGCTTGCTACGAACTGA
TGAGAGTGGCGCACGGGTGCGTAACGCGTGAGCAACCTACCCATATCAGGGGGATAGCCTCTCGAAAG
AGAGATTAACACCGCATAACATCATTAACCGGCATCGGATTATGATCAAATATTTATAGGATATGGAT
GGGCTCGCGTGACATTAGCTAGTTGGAGAGGTAACGGCTCACCAAGGCTACGATGTCTAGGGGCTCTG
AGAGGAGAATCCCCCACACTGGTACTGAGACACGGAC
>Zotu6583
GAACGCTGGCGGCGTGCCTAACACATGCAAGTCGAACGCGAAAGTCCTTCGGGATGAGTAAAGTGGCG
CACGGGTGAGTAACACGTAGGTGACCTGCCTTCTAGAGGGGAATAACCAGATGAAAATTTGGCTAATG

CCGCATACGATCCACAAATCTTAGAGTAGTGGATGAAAGAGTGCCTCTGTATATGCACTTACTAGTAG
ATGGGCCTGCGGTACATTAGCTTGTTGGTGGGGTAATGGCCTACCAAGGCGACGATGTATATCCGGTC
TGAGAGGATGATCGGACACACTGGAACTGAGACACGGTC
>Zotu6584
GAACGCTGGCGGCAGGCTTAACACATGCAAGTCGAGCGGGCCCTTCGGGGTCAGCGGCAGACGGGTGA
GTAACACGTGGGAACGTGCCCTTCGGTTCGGAATAACGCAGGGAAACTTGCGCTAATACCGGATACGC
CCTTTTGGGGAAAGGTTTACTGCCGAAGGATCGGCCCGCGTCTGATTAGCTAGTTGGTGGGGTAATGG
CCCACCAAGGCGACGATCAGTAGCTGGTCTGAGAGGATGATCAGCCACACTGGGACTGAGACACGGCC
>Zotu6586
GAACGCTGGCGGCGTGCCTAATACATGCAAGTCGAGCGGACTTTTAAAAGCTTGCTTTTAAAAGTTAG
CGGCGGACGGGTGAGTAACACGTGGGTAACCTGCCTGTAAGACTGGGATAACTCCGGGAAACCGGGGC
TAATACCGGATAACTTCTTTCCTCGCATGAGGAAAGATTGAAAGATGGCTTCGGCTATCACTTACAGA
TGGACCCGCGGCGCATTAGCTAGTTGGTGAGGTAACGGCTCACCAAGGCAACGATGCGTAGCCGACCT
GAGAGGGTGATCGGCCACACTGGGACTGAGACACGGCC
>Zotu6587
GAACGCTGGCGGCGTGCTTAACACATGCAAGTCGAACGGTAAGGCCCTTTCGGGGGTACACGAGTGGC
GAACGGGTGAGTAACACGTGGGTGACCTGCCCCTTACTCTGGGATAAGCCTGGGAAACTGGGTCTAAT
ACCGGATAGGACTTCATGCTGCATGGTGTGGGGTTGAAAGTTTTTCGGTGGGGGATGGGCCCGCGGCC
TATCAGCTTGTTGGTGGGGTGATGGCCTACCAAGGCTTCGACGGGTAGCCGGCCTGAGAGGGCGTCCG
GCCACACTGGGACTGAGACACGGCC
>Zotu6588
GAACGCTTGCGGCAGGCTTAACACATGCAAGTCGAGGGGTATAGTTCTTCGGAACTAGAGACCGGCGC
ACGGGTGCGTAACGCGTATGCAATCTACCTTTCACAGAGGGATAGCCCAGAGAAATTTGGATTAATAC
CTCATAGTATAATGAGTTGGCATCAACACATTATTAAAGTCACAACGGTGAAAGATGAGCATGCGTCC
CATTAGCTAGATGGTAAGGTAACGGCTTACCATGGCTACGATGGGTAGGGGTCCTGAGAGGGAGATCC
CCCACACTGGTACTGAGACACGGAC
>Zotu6589
GAACGCTGGCGGCGTGCCTAACACATGCAAGTCGAACGTGAAAGGGAGCAATCCTGAGTAAAGTGGCG
TACGGGTGAGTAACGCGTAGGTAATCTACCCCAGAGTCTGGAATAACCTGCCGAAAGGCGGGCTAATA
CTAGGTAGAGCGATGGAATCCGTAATTGGGTTTTTCGGGAAAGGGAGCTCGTAAGAGCCCTCGCTTTG
GGATGAGCCTGCGTCTCATTAGCTAGTTGGTAGGGTAATGGCCTACCAAGGCTTTGATGGGTAGCTGG
TCTGAGAGGATGATCAGCCACACTGGAACTGAAACACGGTC
>Zotu6590
GAACGCTGGCGGCATGCCTAACACATGCAAGTCGAACGAAGGCTTCGGCCTTAGTGGCGCACGGGTGC
GTAACGCGTGGGAACCTGCCCTTTGGTTCGGAATAACCAGGAGAAATTCTGGCTAATACCGGATGATG
TCGAAAGACCAAAGATTTATCGCCATAGGATGGGCCCGCGTAGGATTAGCTAGTTGGTGAGGTAAAGG
CTCACCAAGGCGACGATCCTTAGCTGGTCTGAGAGGATGATCAGCCACACTGGGACTGAGACACGGCC
>Zotu6591
GAACGCTGGCGGCGTGCTTAACACATGCAAGTCGAACGGTGAAGCCAAGCTTGCTTGGTGGATCAGTG
GCGAACGGGTGAGTAACACGTGAGCAATCTGCCCTTGACTCTGGGATAAGCGCTGGAAACGGCGTCTA
ATACTGGATATGAGCTGTGGCCGCATGGTCGGCAGTTGGAAAGAATTTCGGTCAAGGATGAGCTCGCG
GCCTATCAGCTTGTTGGTGAGGTAATGGCTCACCAAGGCGTCGACGGGTAGCCGGCCTGAGAGGGTGA
CCGGCCACACTGGGACTGAGACACGGCC
>Zotu6592
GAACGCTGGCGGCATGCCTAACACATGCAAGTCGAACGATGTCTTCGGACATAGTGGCGCACGGGTGC
GTAACGCGTGGGAATCTGCCCTTAGGTTCGGAATAACCATTGGAAACGATGGCTAATACCGGATGATG
ACGTAAGTCCAAAGATTTATCGCCTGAGGATGAGCCCGCGTAGGATTAGCTAGTTGGTGTGGTAAAGG
CGCACCAAGGCGACGATCCTTAGCTGGTCTGAGAGGATGATCAGCCACACTGGGACTGAGACACGGCC
>Zotu6593
GAACGCTGGCGGCATGCCTTACACATGCAAGTCGAACGGTAACAGGTTAAGCTGACGAGTGGCGAACG
GGTGAGTAATATATCGGAACGTGCCCAATCGTGGGGGATAACGCAGCGAAAGTTGTGCTAATACCGCA
TAAGATCTACGGATGAAAGCGGGGGATCGCAAGACCTCGCGCGAGTGGAGCGGCCGATATCAGATTAG
CTAGTTGGTGGGGTAAAGGCCCACCAAGGCGACGATCTGTAGCTGGTCTGAGAGGACGACCAGCCACA
CTGGGACTGAGACACGGCC

>Zotu6595
GAACGCTGGCGGTGCGCCTAACACATGCAAGTCGCGCGAGGAGCAATCCTAGCGGCGAACGGCTGCGT
AACACGTTGGTACATGCCCCGAACTCAGGGATAACCCTCCGAAAGGAGGGCTAATACCGGATGGTCCC
GTGAGGGTAAAGCTTCGGCGGTTCGGGAGTGGCCTGCGGCCTATCAGATAGTTGGTAAGGTAACGGCT
TACCAAGTCGATGACGGGTAGCTGATTTGAGAGAATGATCAGCCAGAATGGAACTGAGACACGGTC
>Zotu6596
GAACGCTGGCGGCGTGCTTAACACATGCAAGTCGAACGATGAAGCCAGCTTGCTGGTGGATTAGTGGC
GAACGGGTGAGTAACACGTGGGCAACCTGCCCTCAGCTCTGGGATAACTCCAAGAAATTGGTGCTAAT
ACCGGATATTCATGTGACCTCGCATGGGGTTGTGTGGAAAGTTTTTCGGCTGGGGATGGGCCCGCGGC
CTATCAGCTTGTTGGTGGGGTGATGGCCTACCAAGGCGACGACGGGTAGCCGGCCTGAGAGGGTGACC
GGCCACACTGGGACTGAGACACGGCC
>Zotu6597
GAACGCTGGCGGCGTGCTTAACACATGCAAGTCGAACGGTGAACACGGAGCTTGCTCTATGGGATCAG
TGGAGAACGGGTGAGTAACACGTGAGCAACCTGCCCCTGACTCTGGAATAAGCGCTGGAAACGGCGTC
TAATACTGGATATGTGACGTGACCGCATGGTCTGCGTTTGGAAAGATTTTTCGGTTGGGGATGGGCTC
GCGGCCTATCAGCTTGTTGGTGAGGTAATGGCTCACCAAGGCGTCGACGGGTAGCCGGCCTGAGAGGG
TGACCGGCCACACTGGGACTGAGACACGGCC
>Zotu6599
GAACGCTGGCGGCGTGCTTAACACATGCAAGTCGAGCGGTAAGGCCCTTCGGGGTACACGAGCGGCGA
ACGGGTGAGTAACACGTGGGTGACCTGCCTCTAGCTCTGGGATAAGCCTGGGAAACTGGGTCTAATAC
CGGATATGACTTCACGGCGCATGTCGTGGGGTGGAAAGCCTTGAGTGGCTAGAGATGGGCCCGCGGCC
TATCAGCTTGTTGGTGGGGTGATGGCCTACCAAGGCGACGACGGGTAGCCGGCCTGAGAGGGCGACCG
GCCACACTGGGACTGAGACACGGCC
>Zotu6600
GAACGCTAGCGGCAGGCCTAATACATGCAAGTCGAACGATAGTCGGGGCTTGCTCCGACGAAAAGTGG
CGCACGGGTGCGTAACGCGTATGCAACCTACCTTAATCAGGGGGATAGCCCGAAGAAATTCGGATTAA
CACCGCATAAAAACACAGGATAGCATTATCCAATGTTCAAATATTTATAGGATTAAGATGGGCATGCG
TGTCATTAGCTAGTTGGCGGGGTAACGGCCCACCAAGGCGACGATGACTAGGGGATCTGAGAGGATGA
CCCCCCACACTGGTACTGAGACACGGAC
>Zotu6601
GAACGCTGGCGGCGTGCCTAAGACATGCAAGTCGAGTGGGTTTAGACCCACGGCGAACGGGTGAGTAA
TACCTAGGAACTTTCTTTAAGTGGAGCATAACTAGTCGAAAGATTAGATAATTCTCCATGTGGCAGTA
ATGCTAAAGTCGCAAGACGCTTAAAGTGAGGCCTGGGCACCATCAGCTAGTTGGTAAGGTAATGGCTT
ACCAAGGCTATGACGGTTAGGGGTCCCGAGAGGGATAACCCCCACACTGGAACTGCGACACGGTC
>Zotu6603
GAACGCTGGCGGCGTGCTTAACACATGCAAGTCGAACGATGAACTCCAGCTTGCTGGGGGGATTAGTG
GCGAACGGGTGAGTAACACGTGAGTAACCTGCCCTTGACTCTGGGATAAGCGTTGGAAACGACGTCTA
ATACCGGATACGACCAGCCTCGGCATCGAGTGTTGGTGGAAAGATTTTTTGGTCAAGGATGGACTCGC
GGCCTATCAGGTAGTTGGTGAGGTAATGGCTCACCAAGCCTACGACGGGTAGCCGGCCTGAGAGGGTG
ACCGGCCACACTGGGACTGAGACACGGCC
>Zotu6604
CAACGCTGGCGGCGTGCCTAACACATGCAAGTCGTACGAGAAAGTGGAGCAATCCATGAGTAAAGTGG
CGACCGGGTGAGTAACACGTGACTACCTACCTTCTAGTGGGGGATAACTCCGGGAAACCGGAGCTAAT
ACCGCATAACATCGCAAGATCAAAGCAGCAATGCGCTGGAAGAGGGGGTCGCGGCTGATTAGCTAGTT
GGTAGGGTAATGGCCTACCAAGGCAAAGATCGGTATCCGGCCTGAGAGGGCGCACGGACACACTGGAA
CTGAAACACGGTC
>Zotu6606
GAACGCTGGCGGCGTGCCTAAAACATGCAAGTCGAGCGGTAAGGCCCTTCGGGGTACACGAGCGGCGG
ACGGGTGAGTAACACGTGGGAATCTACCCCAAACTGAGGGATAAGCACCGGAAACGGTGTCTAATACC
GCATATGGTCTTCGGATTAAAGCTTCGGCGGTTTGGGAAGAGCCCGCGTAAGATTAGCTAGTTGGTGA
GGTAAAGGCTCACCAAGGCGACGATCTTTAGCTGGTTTGAGAGGATGATCAGCCACACTGGAACTGAG
ACACGGTC
>Zotu6607
GAACGCTAGCGGCGTGCCTAAGGTATGCAAGTCGAGCGAGAGCCTTCGGGCTCTAGCGGCAAACGGTT

GAGTAACACGTAGACAACCTGCCCCAAAGACGGGGATAACTCGGGGAAACTCGGGCTAATACCGGATG
TGGAGGGAAGCGGCATCGCTTCTTATCTAAACGAGCAATCGGCTTTGGGATGGGTCTGCGGCCTATCA
GCTAGCTGGTGGGGTAACGGCCTACCAGGGCGACGACGGGTAGCTGGTCTGAGAGGATGACCAGCCAG
ACGGGGACTGAGACACGGCC
>Zotu6608
GAACGCTGGCGGCGTGCTTAACACATGCAAGTCGAGCGATGAAACACCTTCGGGTGTGAATTAGCGGC
GAACGGGTGAGGAACACGTGAGAAATCTGCCTTCAACTCTGGGATAACTCCGGGAAACCGGGGCTAAT
ACCGGATATGAGCCTCGATCGCATGATCAGGGCTGGAAAGTTTTTCGGTTGAAGATGATCTCGCGGCC
TATCAGCTTGTTGGTGAGGTAATGGCTCACCAAGGCGACGACGGGTAGCCGGCCTGAGAGGGCGACCG
GCCACACTGGGACTGAGACACGGCC
>Zotu6609
GAACGCTGGCGGCGTGCCTTAAGCATGCAAGTCAAGGGGGGCTCAGCAATGAGTCAACCGGCGGACGG
GTGAGTAATGCGTAGGAATCTTCCTCGAAGTTGGACATAGCTAGCCGAAAGGTTGGGTAATTTCCAAT
GTGACCGTAAGGTCAAAGCAGCAATGCGCTTTGAGATGAGCCTGCGTCCTATCAGCTAGTTGGTGGGG
TAAAAGCCTACCAAGGCGACGACGGGTAGCTGGTCTGAGAGGATGGTCAGCCACAATGGGACTGCGAC
ACGGCC
>Zotu6610
GAACGCTGGCGGCATGCCTAAGACATGCAAGTCGAACGAAGTGGCCCAATGAAGATGAAGTGCTTGCA
CTGATTTGGAAATGGATTCCCACTTAGTGGCAGACGGGTGAGTAACACGTGGGTAACCTACCCCTTAG
TCTGGGACAACTATTGGAAACGATAGCTAATACCGGATGATTCACAATTAGATAACTTTTTGTGCTAA
AAGGAGCTTCGGCTTCGCTTTGGGATGGGCTTGCGGTGTATTAGCTAGTTGGTAGGGTAATGGCCTAC
CAAGGCTACGATGCATATCCGAGCTGAGAGGCTGATCGGGCACATTGGAACTGAGATACGGTC
>Zotu6611
GAACGCTGGCGGCGTGCCTAACACATGCAAGTCGAACGTGAAATGGAGCAATCCAAAGTAAAGTGGCG
TACGGGTGAGTAACGCGTAGGTAATCTACCCCAAAGTTTGGAATAACCTGCCGAAAGGCGGGCTAATA
CTGGATAGAGCGACGGAACTCGTAACGAGTTTTTCGGGAAAGGGAGCCCGCAAGGGCCTTCGCTTTGG
GATGAGCCTGCGTCTCATTAGCTAGTTGGTAGGGTAATGGCCTACCAAGGCATTGATGGGTAGCTGGT
CTGAGAGGATGATCAGCCACACTGGAACTGAAACACGGTC
>Zotu6615
GAACGCTGGCGGCATGCCTTACACATGCAAGTCGAACGGCAGCACGGGAGCAATCCTGGTGGCGAGTG
GCGAACGGGTGAGTAATATATCGGAACGTGCCCAGTCGTGGGGGATAACGTAGCGAAAGCTACGCTAA
TACCGCATACGATCTACGGATGAAAGCGGGGGACTCGCAAGAGCCTCGCGCGATTGGAGCGGCCGATA
TCAGATTAGGTTGTTGGTGAGGTAAAAGCTCACCAAGCCTGCGATCTGTAGCTGGTCTGAGAGGACGA
CCAGCCACACTGGAACTGAGACACGGTC
>Zotu6616
CAACGCTGGCGGCGCGCTTAACACATGCAAGTCGCACGAGAAAGCCTGGCAACGGGTGAGTAAAGTGG
CGTACGGGTGAGTAACACGTGGGTATCCACCTCTTAGTGGGGAATAACCCTGGGAAACCGGGGCTAAT
ACCGCATAAGTCCGTGAGGAGAAAGCAGCAATGCGCTGAGAGAGGAGCCCGCGGCCGATTAGCTAGTT
GGTAGGGTAATGGCCTACCAAGGCAACGATCGGTAGCCGGCCTGAGAGGGCACACGGCCACACTGGCA
CTGAAACACGGGC
>Zotu6619
GAACGCTAGCGACAGGCTTAACACATGCAAGTCGAGGGGCAGCGGGGTAGAGAGCTTGCTCTCTACTG
CCGGCGACCGGCGCACGGGTGAGTAACACGTATGCAACCTGCCTCAAACCGGGGGATAAGCGGCGGAA
ACGCCGTCTAATACCGCGTATGACTTCGGGGGGCATCCCCCGGGGTTGAAAGATTAGATTCGGTTTGA
GATGGGCATGCGGCGCATTAGTTAGTTGGCGGGGTAACGGCCCACCAAGGCGACGATGCGTAGGGGTT
CTGAGAGGAAGGTCCCCCACACTGGTACTGAGACACGGAC
>Zotu6620
GAACGCTGGCGGCGTGCTTAACACATGCAAGTGGAGCGACGAACCGGGCTTCGGCCCGGGGCAGAGCC
GCGAACGGGTGAGTAACACGTGGGTTACCTGCCCCATTGACAGGGACAACCCGGGGAAACCCGGGCTA
ATACCTGATGTGCCCCTCGGGGGAAAGGAAGCTTCGGCCTCCGCAATGGGATGGGCCCGCGGCCCATT
AGCTTGTTGGTGGGGTAACGGCCTACCAAGGCGACGATGGGTAGCTGGTCTGAGAGGACGATCAGCCA
CACTGGGACTGAGACACGGCC
>Zotu6621
GAACGCTGGCGGTATGCCTAATACATGCAAGTCGTGCGGAAAGGCCCTTCGGGGTACTCGAGCGGCGG

ACGGCAGAGTAACGCGTAGGAACATACCCCAAACTGAGGGATAACTGCTCGAAAGAGTAGCTAATACC
GCATGTGGTCTTCGGACTAAAGCTTCGGCGGTTTGGGAATGGCCTGCGTTCGATTAGCTAGTTGGTGA
GGTAAATGCTCACCAAGGCGACGATCGATAGCTGGTCTGAGAGGATGATCAGCCTGACTGGAACTGAG
ACACGGTC
>Zotu6622
GAACGCTGGCGGCATGCCTAACACATGCAAGTCGAACGAAGGCTTCGGCCTTAGTGGCGCACGGGTGC
GTAACGCGTGGGAATCTGCCCCTTGGTTCGGAATAACAGTTGGAAACGACTGCTAATACCGGATGACG
ACGAAAGTCCAAAGATTTATCGCCGAGGGATGAGCCCGCGTAGGATTAGCTAGTTGGTGTGGTAAAGG
CGCACCAAGGCGACGATCCTTAGCTGGTCTGAGAGGATGATCAGCCACACTGGGACTGAGACACGGCC
>Zotu6623
GAATGCTGGCGGCGTGGATAAGTCATGCAAGTCAAGGGGGCCCGTAAGGGCAACCGGCAGACGAGGTA
GTAACACGTAGGTACGTCCCCCGAAGTCGGGCATAGCCAGCCGAAAGGTTGGGTAATTCCCGATAGTC
CCGCAAGGGTAAAGGTTTTTCGCTTCGGGAGCGGCCTGCGTAGTATCAGCTAGTTGGTAGGGTAACGG
CCTACCAAGGCTACGACGCTTAGGGGAGCTGAGAGGCTGACCCCCACCGATGGAACTGCGACACGGTC
>Zotu6624
GAACGCTGGCGGCGTGCTTAACACATGCAAGTCGAACGGTGAAGGGACCGGCTTTTGCTGGTTCTGGA
TGAGTGGCGAACGGGTGAGTAACACGTGAGTAACCTGCCCCCTTCTTCTGGATAACCGCATGAAAGTG
TGGCTAATACGGGATATTCTGGGTCTGTCGCATGGCGGGCCTGGGAAAGATGCGCCTTTGGGTGTTTT
TGGTGGGGGATGGGCTCGCGGCCTATCAGCTTGTTGGTGGGGTGATGGCCTACCAAGGCGGTGACGGG
TAGCCGGCCTGAGAGGGTGGACGGTCACACTGGGACTGAGACACGGCC
>Zotu6625
GAACGCTGGCGGCGTGCCTAATACATGCAAGTCGAACGAAACTTTCTTACACCGAATGCTTGCATTCA
CCGTAAGAAGTTGAGTGGCGGACGGGTGAGTAACACGTGGGTAACCTGCCTAAAAGAAGGGGATAACA
CTTGGAAACAGGTGCTAATACCGTATATCTTTAAGGATCGCATGATCCTTAGATGAAAGATGGTTCTG
CTATCGCTTTTAGATGGACCCGCGGCGTATTAACTAGTTGGTGGGGTAACGGCCTACCAAGGTGATGA
TACGTAGCCGAACTGAGAGGTTGATCGGCCACATTGGGACTGAGACACGGCC
>Zotu6627
GAACGCTGGCGGCATGCCTTACACATGCAAGTCGAACGGTAACAGGTCTTCGGATGCTGACGAGTGGC
GAACGGGTGAGTAATACATCGGAACGTGCCCGATCGTGGGGGATAACGAGGCGAAAGCTTTGCTAATA
CCGCATACGATCTACGGATGAAAGCAGGGGATCAGTAATGACCTTGCGCGGACGGAGCGGCCGATGGC
AGATTAGGTAGTTGGTGGGATAAAAGCTTACCAAGCCGACGATCTGTAGCTGGTCTGAGAGGATGATC
AGCCACACTGGGACTGAGACACGGCC
>Zotu6628
GAACGCTGGCGGCATGCCTAACACATGCAAGTCGAACGAGATTGTTACTTCGGTTTCAATCTAGTGGC
GCACGGGTGCGTAACGCGTGGGAATCTACCCTTGGGTTCGGAATAACAGTTAGAAATGACTGCTAATA
CCGGATGATGACGTAAGTCCAAAGATTTATCGCCCAGGGATGAGCCCGCGTAGGATTAGCTAGTTGGT
GAGGTAAAGGCTCACCAAGGCTACGATCCTTAGCTGGTCTGAGAGGATGATCAGCCACACTGGGACTG
AGACACGGCC
>Zotu6630
GAACGCTGGCGGCAGGCTTAACACATGCAAGTCGAACGCCCCGCAAGGGGAGTGGCAGACGGGTGAGT
AACGCGTGGGAATCTACCTAGTTCTGCGGAACAACAGTTGGAAACGACTGCTAATACCGCATACGCCC
TACGGGGGAAAGATTTATCGGAATTAGATGAGCCCGCGTAAGATTAGCTAGTTGGTGGGGTAATGGCC
TACCAAGGCGACGATCTTTAGCTGGTCTGAGAGGATGATCAGCCACACTGGGACTGAGACACGGCC
>Zotu6631
GAACGCTGGCGGCGTGCCTAAGACATGCAAGTCGAGTGGTCACTTTACTGTGACCACGGCGAACGGGT
GAGTAATACCTAGGAACTTTCTTTAAGTGGAGGATAACCAGTCGAAAGATTGGATAATACTTCATGTG
GCAGTAATGCTAAAGTCGCAAGACGCTTAAAGTGAGGCCTGGGCACCATCAGCTAGTTGGTAAGGTAA
TGGCTTACCAAGGCTATGACGGTTAGGGGTCCCGAGAGGGAGAACCCCCACACTGGAACTGCGACACG
GTC
>Zotu6632
GAACGCTAGCTGCAGGCCTAATACATGCAAGTCGAACGGTGTCTTCGGACATAGTGGCGCACGGGTGC
GTAACACGTATGCAACCTACCTTACATTGGGGGATAGCCTTTCGAAAGGGAGATTAATACCGCATAAG
ACAGTAACTAGGCATCTAGTAGCTGTTAAAGATTTATCGATGTAAGCTGGGCATGCGTCTAATTAGCT
AGTTGGTGAGGTAATGGCTCACCAAGGCAATGATTGGTAGGGGAACTGAGAGGTCAATCCCCCACACT

GGCACTGAGATACGGGC
>Zotu6634
GAACGCTGGCGGCGTGCCTAATACATGCAAGTCGAGCGAACTTGAGGGAGCTTGCTCCCAAGAGTTAG
CGGCGGACGGGTGAGTAACACGTGGGCAACCTACCTGTAAGACTGGGATAACTTCGGGAAACCGGAGC
TAATACCGGATAACTTCTTTCCTCGCATGGGGAGAGGTTGAAAGACGGCTTCGGCTGTCACTTACAGA
TGGGCCCGCGGCGCATTAGCTAGTTGGTGAGGTAACGGCTCACCAAGGCGACGATGCGTAGCCGACCT
GAGAGGGTGATCGGCCACACTGGGACTGAGACACGGCC
>Zotu6635
GAACGCTGGCGGCAGGCTTAACACATGCAAGTCGAACGCATCCTTCGGGGTGAGTGGCGGACGGGTGA
GTAACACGTGGGAACGTACCCTTTGGTTCGGGACAACTCTGGGAAACTGGAGCTAATACCGGATACGG
CCGAGAGGCGAAAGGTTTACTGCCGAAGGATCGGCCCGCGTCTGATTAGCTAGTTGGTGGGGTAACGG
CCTACCAAGGCGACGATCAGTAGCTGGTCTGAGAGGATGATCAGCCACACTGGGACTGAGACACGGCC
>Zotu6636
GAACGCTGGCGGCGTGCCTAAGACATGCAAGTCGAGTGGGTTTAGACCCACGGCAAACGGGTGAGTAA
TACCTAGGAACTTTCTTTAAGTGGAGCATAATCAGCCGAAAGGTTGGATAATTCTTCATGTGGCAGCA
ATGCTAAAGTCGCAAGACGCTTAAAGTGAGGCCTGGGCACCATCAGCTAGTTGGTAAGGTAATGGCTT
ACCAAGGCTATGACGGTTAGGGGCCCTCAGAGGGGAGTCCCCCACACTGGAACTGCGACACGGTC
>Zotu6637
GAACGCTGGCGGCGTGCTTAACACATGCAAGTCGAACGGAAAGGCCCCTTCGGGGGTACTCGAGTGGC
GAACGGGTGAGTAACACGTGGGCAACCTGCCCCTAGCTCTGGGATAACTCCGGGAAACCGGTGCTAAT
ACCGGATATCGACCTTCGCGGGCATCCGCGACTGTCAAAAGTTTTTCGGCTAGGGATGGGCCCGCGGC
CTATCAGCTTGTTGGTGGGGTAATGGCCTACCAAGGCGACGACGGGTAGCCGGCCTGAGAGGGCGACC
GGCCACACTGGGACTGAGACACGGCC
>Zotu6638
CAACGCTGGCGGCGTGCCTAACACATGCAAGTCGAACGCGAAAGCCCTTTCGGGGGTAAGTAGAGTGG
CGTCCGGGTGAGTAACACGTGGGTAATCTGCCTTCGGGTGGGGAATAACTCGCCGAAAGGTGAGCTAA
TACCGCATAATGCAGCGGCACCAAATGGTGATTAGTTGTTAAAGCGGGGGACCCTTCGGGGCCTCGCG
CCTGAAGAGGAGCCTGCGGCAGATTAGCTAGTTGGTGGGGTAATGGCTCACCAAGGCTTCGATCTGTA
GCTGGTCTGAGAGGACGGCCAGCCACACTGGCACTGACACACGGGC
>Zotu6639
GAACGCTGGCGGCAGGCTTAACACATGCAAGTCGAGCGGGGGTAGCAATACCCTAGCGGCGAACGGGT
GAGGAATGCTTGGGAATCTGCCTTGTAGTGGGGGATAACGTCTCGAAAGGGACGCTAATACCGCATAC
GTCCTACGGGAGAAAGGGGGGGATCTTCGGACCTCTCGCTATAAGATGAGCCCAAGTCGGATTAGCTA
GTTGGTGGGGTAAAGGCCTACCAAGGCGACGATCCGTAGCTGGTCTGAGAGGATGATCAGCCACACTG
GAACTGAGACACGGTC
>Zotu6640
GAACGCTGGCGGCAGGCTTAACACATGCAAGTCGAGCGGAGCGAGGGTGCTTGCACCTTAGCTTAGCG
GCGGACGGGTGAGTAATGCTTAGGAATCTGCCTATTAGTGGGGGACAACATTCCGAAAGGAATGCTAA
TACCGCATACGCCCTACGGGGGAAAGCAGGGGATCTTCGGACCTTGCGCTAATAGATGAGCCTAAGTC
AGATTAGCTAGTTGGTGGGGTAAAGGCCTACCAAGGCGACGATCTGTAGCGGGTCTGAGAGGATGATC
CGCCACACTGGGACTGAGACACGGCC
>Zotu6641
GAACGCTAGCGGCAGGCCTAATACATGCAAGTCGAGGGGCAGCAGGTGTAGCAATACATGCTGGCGAC
CGGCGCACGGGTGCGTAACACGTATGCAACTTACCTTTAACTGGAGGATAGCCGCCCGAAAGGGTGAT
TAATACTCCATAATATTATGAAATAGCATTATTTTATAATTAAAGCTCCGGCGGTTAAAGATGGGCAT
GCGTCCGATTAGCTAGTTGGCGAGGTAAAAGCTCACCAAGGCTACGATCGGTAGGGGTTCTGAGAGGA
TATACCCCCACACTGGTACTGAGACACGGAC
>Zotu6642
GAACGCTGGCTGCGTGCTTAACACATGCAAGTCGAACGCTGAAGCCCCAGCTTGCTGGGGTGGATGAG
TGGCGAACGGGTGAGTAACACGTGAGTAACCTGCCCCTGACTTCGGGATAAGCCCGGGAAACTGGGTC
TAATACCGGATACGACCGTCGGACGCATGTCTGGTGGTGGAAAGTTTTTTCGGTTGGGGATGGGCTCG
CGGCCTATCAGTTTGTTGGTGAGGTAATGGCTCACCAAGACGACGACGGGTAGCCGGCCTGAGAGGGC
GACCGGCCACACTGGGACTGAGACACGGCC
>Zotu6643

GAACGCTGGCGGCACGTTTTAAGCATGCAAGTCGAACGGCAGTGGGGGCTTGCTCCCATGAGAGTGGC
GAACGGGTGAGTAACACGTAGGTGATCTGCCTTGAGGATGGGGATAGCTTGTGGAAACACAGGGTAAT
ACCGAATGAGCTGACAGGGTTTTGGCCCAGTTAGTAAAGGTGCTACGGCACCGCCTTAAGAGGAGCCT
GCGGCTGATTAGCTAGTTGGTGGGGTAAAGGCCTACCAAGGCGACGATCAGTAGCCGGCCTGAGAGGG
TGTACGGCCACACTGGAACTGAGACACGGTC
>Zotu6644
GAACGCTGGCGGTGCGCCTAAAACATGCAAGTCGAGTGGGTTTAGACCCACGGCAGACGGCTGCGTAA
CACATGGGTACCTACCCCGAACTCAGGGATATCCCGCCGAAAGGCGGAGTAATACCGGATAGTCCCGC
AAGGGTAAAGCTTTCGCGGTTCGGGAGGGGCCTGTGCCCTATCAGTTAGTTGGTGAGGTAACGGCTCA
CCAAGACAATGACGGGTAGCTGGTTTGAGAGAACGACCAGCCAGAATGGAACTGAGACACGGTC
>Zotu6645
GAACGCTGGCGGCGTGCCTAAGACATGCAAGTCGAGTGGGTTTAGGCCCACGGCGAACGGGTGAGTAA
TACCTAGGAACGTATCTTGAGTTGAGCATAACCCATCGAAAGGTGGGATAATTCTCAATGTGGCAGTA
ATGCTAAAGTCGTAAGACGCTCAAGAAGCGGCCTGGGCACCATCAGCTAGTTGGTAAGGTAATGGCTT
ACCAAGGCTATGACGGTTAGGGGCCCCGAGAGGGGAGACCCCCACACTGGAACTGCGACACGGTC
>Zotu6646
GAACGCTGGCGGCGTGCTTAACACATGCAAGTCGAACGGGGATTACCGAACGGAAGGGCTTCGGTCCG
GAAGGTTGGTAATTCTAGTGGCGGACGGGTGAGTAACGCGTGAGCAACCTGTCCCAGACAGGGGGATA
ACACTTGGAAACAGGTGCTAATACCGCATAAGACCACGGGGAGACATCTACCTGGGGTAAAAGGTGCA
AACCGGTTTGGGGTGGGCTCGCGTCCGATTAGATAGTTGGTGAGGTAACGGCCCACCAAGTCGACGAT
CGGTAGCCGATCTGAGAGGATGACCGGCCACATTGGAACTGAGAGACGGTC
>Zotu6648
GAACGCTGGCGGCGTGCCTAACACATGCAAGTCGAACGAGAGCCCGTGGAAGGAGACTTCGGTCAACG
GAAGCGGGGGACAGTGGCGAACGGGTGAGTAACGCGTGAGGAACCTGCCTTTCAGAGGGGGACAACAG
TTGGAAACGACTGCTAATACCGCATAACACAGCGAGATCGCATGATTTTGCTGTCAAAGATTTATCGC
TGAAAGATGGCCTCGCGTCTGATTAGATAGTTGGTGGGGTAACGGCCTACCAAGTCTGCGATCAGTAG
CCGGACTGAGAGGTTGAACGGCCACATTGGGACTGAGATACGGCC
>Zotu6650
GAACGCTGGCGGCGTGCTTAACACATGCAAGTCGAGCGGAAAGGCCCTTCGGGGTACTCGAGCGGCGA
ACGGGTGAGTAACACGTGAGCAACCTGCCCTAGGCTTTGGGATAACCCTCGGAAACGGGGGCTAATAC
CGAATATGATGCTTCACCGCATGGTGGGGTGTGGAAAGTTTTTCGGCCTGGGATGGGCTCGCGGCCTA
TCAGCTTGTTGGTGGGGTGATGGCCTACCAAGGCGACGACGGGTAGCCGGCCTGAGAGGGCGACCGGC
CACACTGGGACTGAGACACGGCC
>Zotu6651
GAACGCTGGCGGCGTGCCTAACACATGCAAGTCGAGCGGCAGCGCGGGAGCAATCCTGGCGGCGAGCG
GCGGACGGGTGAGCAATGCTTGGGAATCTGCCCATCAGTGGGGGACAACCCGGGGAAACTCGGGCTAA
TACCGCATACGTTCTTTGGAAGAAAGCAGGGGACCGCAAGGCCTTGCGCTGATGGATGAGCCCAAGTC
GGATTAGCTAGTTGGTGAGGTAACGGCTCACCAAGGCGACGATCCGTAGCTGGTCTGAGAGGACGACC
AGCCACACCGGAACTGAGACACGGTC
>Zotu6652
GAACGCTGGCGGCGTGCCTAACACATGCAAGTCGAACGAGGTCCATCCGGTAGCAATACCGGTGAAGA
TCGAGTGGCGAACGGGTGAGTAGCACGTGAGCAACCTGCCCCGAAGACCGGGACAACACCGGGAAACC
GGTGCTAATACCGGATACCCCCACCGAGTCGCATGGCTCGGTGAGGAAATGGATTCCGCTTCGGGAGG
GGCTCGCGGCCTATCAGCTTGTTGGTGAGGTAACGGCTCACCAAGGCGTCGACGGGTAGCTGGTCTGA
GAGGACGATCAGCCACACTGGGACTGAGACACGGCC
>Zotu6655
GAACGCTGGCGGCGTGCCTAAGACATGCAAGTCGAGTGGGTTTAGACCCACGGCGAACGGGTGAGTAA
TACCTAGGAACTTTCTTTAAGTGGAGCATAACCAGTCGAAAGATTGGACAATTCTTCATGTGGCAGTA
ATGCTAAAGTCGCAAGACGCTTAAAGTGAGGCCTGGGCACCATCAGCTAGTTGGTAAGGTAATGGCTT
ACCAAGGCTATGACGGTTAGGGGTCCCGAGAGGGATAACCCCCACACTGGAACTGCGACACGGTC
>Zotu6656
GAACGCTAGCGATAGGCCTAACACATGCAAGTCGAGGGGCAGCACATGACGTAGCAATACAGATGGTG
GCGACCGGCGCACGGGTGAGTAACACGTATGCAACCTACCTTCAACAGGAGAATAACCCGTCGAAAGA
CGGACTAATACTCCATAACACAGGGATCCCGCATGGGAATATTTGTTAAAGATTTATCTGTTGAAGAT

GGGCATGCGCTCCATTAGCTAGTTGGTGAGGTAACGGCTCACCAAGTCAGCGATGGATAGGGGAACTG
AGAGGTTTATCCCCCACACTGGTACTGAGACACGGAC
>Zotu6657
GAACGCTGGCGGCGTGCTTAACACATGCAAGTCGAGCGGTAAGGCCCTTCGGGGTACACGAGCGGCGA
ACGGGTGAGTAACACGTGAGTAATCTGCCCCTCACTTCGGGATAAGCCTCGGAAACGGGGTCTAATAC
CGGATACGACACAGACCTGCATGGGTTCTGTGTGGAAAGTTTTTCGGTGGGGGATGTGCTCGCGGCCT
ATCAGCTTGTTGGTGGGGTAATGGCCTACCAAGGCTTCGACGGGTAGCCGGCCTGAGAGGGTGACCGG
CCACACTGGGACTGAGACACGGCC
>Zotu6658
GAACGCTGGCGGCGTGCCTAATACATGCAAGTAGAACGCTGAAGAGAGGAGCTTGCTCTTCTTGGATG
AGTTGCGAACGGGTGAGTAACGCGTAGGTAACATGCCTGGTAGCGGGGGATAACTATTGGAAACGATA
GCTAATACCGCATGATATTAATTGTCGCATGATAATTAATTGAAAGATGCAATTGCATCACTACCAGA
TGGACCTGCGTTGTATTAGCTAGTTGGTGAGGTAACGGCTCACCAAGGCGACGATACATAGCCGACCT
GAGAGGATGATCGGCCACACTGGGACTGAGACACGGCC
>Zotu6662
GAACGCTGGCGGCGTGCTTAACACATGCAAGTCGAGCGATGAAGCTCCTTCGGGAGTGGATTAGCGGC
GGACGGGTGAGTAACACGTGGGTAACCTGCCTCATAGAGGGGAATAGCCTTCCGAAAGGAAGATTAAT
ACCGCATAAGATTGTAGTTCCGCATGGAATAGCAATTAAAGGAGTAATCCGCTATGAGATGGACCCGC
GTCGCATTAGCTAGTTGGTGAGGTAACGGCTCACCAAGGCGACGATGCGTAGCCGACCTGAGAGGGTG
ATCGGCCACATTGGGACTGAGACACGGCC
>Zotu6663
GAACGCTGGCGGCGTGCTTAACACATGCAAGTCGAACGATGATCCCCAGCTTGCTGGGGGGATTAGTG
GCGAACGGGTGAGTAACACGTGAGTAACCTGCCCTTGACTCTGGGATAAGCCTGGGAAACTGGGTCTA
ATACCGGATATGACCTTCCATCGCATGGTGGTTGGTGGAAAGCTTTTGTGGTTTTGGATGGACTCGCG
GCCTATCAGCTTGTTGGTGGGGTAATGGCCTACCAAGGCGACGACGGGTAGCCGGCCTGAGAGGGTGA
CCGGCCACACTGGGACTGAGACACGGCC
>Zotu6664
GAACGCTGGCGGCATGCTTAACACATGCAAGTCGAACGGTAGCAGGCCCTTCGGGGTGCTGACGAGTG
GCGGACGGGTGAGTAATGCGTAGGAATCTACCTAATTGTGTGGGATAGCCTAGGGAAACTTAGGGTAA
TACCGCATAAGCCCTACGGGGGAAAGCAGGGGACTGTTAAAGGCCTTGCGCGATTAGATGAGCTTACG
TCGGATTAGCTAGTTGGTGGGGTAAAGGCCTACCAAGGCGATGATCCGTAGCTGGTCTGAGAGGACGA
CCAGCCACACTGGGACTGAGACACGGCC
>Zotu6667
GAACGCTGGCGGCAGGCCTAACACATGCAAGTCGAACGCTGCAGCAATGCAGAGTGGCAGACGGGTGA
GTAACGCGTGGGAACCTTCCCAGTGGTACGGAATAGCTCAGGGAAACTTGAGGTAATACCGTATACGC
CCCTAGGGGGAAAGATTTATCGCCATTGGATGGGCCCGCGTCGGATTAGCTAGTTGGTGAGGTAACGG
CTCACCAAGGCGACGATCCGTAGCTGGTTTGAGAGAATGACCAGCCACACTGGGACTGAGACACGGCC
>Zotu6669
CGGCGCGCTCTAGGAGAGTACCCTGACCCTTTAAGAAGAGTGGAAGGATCCGAGGGATCACATCACCA
CTTGAGAGGGAGTCAGAGGGCTCTCGCCGGT
>Zotu6670
GAACGCTGGCGGCAGGCCTAACACATGCAAGTCGAACGCACCTTTCGGGGTGAGTGGCAGACGGGTGA
GTAACACGTGGGAACGTGCCCTTCAGTTCGGAATAACCCAGGGAAACTTGGGCTAAGACCGGATACGC
CCTTTTTGGGGAAAGATTGATCGCTGAAGGATCGGCCCGCGTCTGATTAGCTAGTTGGTGAGGTAATG
GCTCACCAAGGCGACGATCAGTAGCTGGTCTGAGAGGATGATCAGCCACACTGGGACTGAGACACGGC
C
>Zotu6672
GAACGCTAGCGGGAGGCCTAATACATGCAAGTCGAACGGTAACAGGATAGCAATATCGCTGACGAGTG
GCGTACGGGTGCGTAACGCGTACACAATCTGCCTTATACTGGAGCATACCCCCAAGAAATTGGGACTA
ATTCTCCATAATATATTTGGATGGCATCATTTGAATATTAAAACTCCGGTGGTATAAGATGAGTGTGC
GTCCGATTAGGTAGTTGGTGAGGTAACGGCTCACCAAGCCGACGATCGGTAGCTGGTCTGAGAGGACG
ATCAGCCACACGGGCACTGAGACACGGGC
>Zotu6673
GAACGCTGGCGGCGTGCTTAACACATGCAAGTCGAACGATGAAGCTCCAGCTTGCTGGGGTGGATTAG

TGGCGAACGGGTGAGTAACACGTGAGTAACCTGCCCCTGACTTCGGGATAACCACGAGAAATCGTGGC
TAATACTGGATGTCCACATGTGCCTGCATGGGTGTGTGGAAAGATTTATCGGTTGGGGATGGGCTC
GCGGCCTATCAGCTTGTTGGTGGGGTGATGGCCTACCAAGGCGACGACGGGTAGCCGGCCTGAGAGGG
TGACCGGCCACACTGGGACTGAGACACAGCC
>Zotu6674
GAACGCTGGCGGCGTGCCTAACACATGCAAGTCGAGCGAGAAAGTTCCTTCGGGAGCGAGTAAAGCGG
CGCACGGGTGAGTAACACGTAGGTAACCTGCCCCTTAGTCTGGAACAACTAGAGGAAACTCTAGCTAA
TGCCGGATGAGACCACGGTCCCCTCGGGGACTGCGACCAAAGATGGCCTCTACTTGTAAGCTATCGCT
GAGGGATGGGCCTGCGGGCCATTAGCTTGTTGGTGAGGTAACGGCTCACCAAGGCTATGATGGCTAGC
TGGTCTGAGAGGATGACCAGCCACACTGGGACTGAGACACGGCC
>Zotu6675
GAACGCTGGCGGCGTGCCTAACACATGCAAGTCTAACGGGGCAGCAATGCCCAGTGGCGAACGGGTGC
GTAACACGTGAGGAACTTGTCCATCGGCGGGGGATAGCCGGCCCAACGGCCGGGTAATACCGCGTACG
TCGGCGAGACCGCATGGTCTTGCCGGGAAAGCAGCAATGCACCGATGGAGAGCCTCGCGGCCTATCAG
CTAGTTGGTGAGGTAACGGCTCACCAAGGCGACGACGGGTAGCTGGTCTGAGAGGATGATCAGCCACA
TTGGGACTGAGACACGGCC
>Zotu6676
GAACGCTGGCGGCATGCCTAACACATGCAAGTCGAACGAGACCTTCGGGTCTAGTGGCGCACGGGTGC
GTAACGCGTGGGAATCTGCCCTTGGGTTCGGAATAACAGTTGGAAACGACTGCTAATACCGGATGATG
ACTTCGGTCCAAAGATTTATCGCCCAGGGATGAGCCCGCGTCGGATTAGCTAGTTGGTGAGGTAAAGG
CTCACCAAGGCAACGATCCGTAGCTGGTCTGAGAGGATGATCAGCCACACTGGGACTGAGACACGGCC
>Zotu6678
GAACGCTGGCGGCAGGCTTAACACATGCAAGTCGAGCGATGACGACCTAGCTTGCTAGGTTTGATTAG
CGGCGGACGGGTGAGTAAAGTTTAGGAATCTGCCTTATAGTGGGGGATAGCTTGGGGAAACCCAAATT
AATACCGCATACGTCCTACGGGAGAAAGGTGCCTTAGGGTATCGCTATGAGATGAGCCTAAACCAGAT
TAGCTAGTTGGTGGGGTAAAGGCCTACCAAGGCGACGATCTGTAGCTGGTCTGAGAGGATGATCAGCC
ACACCGGAACTGAGACACGGTC
>Zotu6679
GAACGCTGGCGGCGTGCCTAATACATGCAAGTCGAACGAACCGCGACTGAGTGCTTGCACTTGGTCAA
GGTGAGTGGCGAACGGGTGAGTAACACGTGGGTAACCTACCTCATAGTGGGGGATAACAGTCGGAAAC
GACTGCTAATACCGCATAGGACATAGGATCACATGATCCAGTGAGGAAAGGTGGCGCAAGCTATCGCT
AAGAGATGGACCCGCGGTGCATTAGCTAGTTGGTAGGGTAACGGCCTACCAAGGCGATGATGCATAGC
CGACCTGAGAGGGTGATCGGCCACATTGGGACTGAGACACGGCC
>Zotu6681
GAACGCTAGCTATATGCTTAACACATGCAAGTCGAACGTCGTTTTCGGGAAGCTGGGCAGAAGGAAAA
TAGGCTCCTAGCTAAAGGTAGCTTGTCTCGCCCAGGAGGTGAGAACAGTTGAGAACAAAGTGGCGAAC
GGGTGCGTAACGCGTGGGAATCTGCCGAACAGTTCGGGCCAAATCCTGAAGAAAGCTAAAAAGCGCTG
TTTGATGAGCCCGCGTAGTATTAGGTAGTTGGTCAGGTAAAGGCTGACCAAGCCAATGATGCTTAGCT
GGTCTTTTCGGATGATCAGCCACACTGGGACTGAGACACGGCC
>Zotu6682
GAACGCTGGCGGCGTGCCTAACACATGCAAGTCGAGCGATCTCTTCGGAGAGAGCGGCGGACGGGTGA
GTAACGCGTGGGTAACCTGCCCTGTACACACGGATAACATACCGAAAGGTATGCTAATACGGGATAAT
ATATAAGAGTCGCATGACTTTTATATCAAAGATTTTTCGGTACAGGATGGACCCGCGTCTGATTAGCT
TGTTGGCGGGGTAACGGCCCACCAAGGCGACGATCAGTAGCCGACCTGAGAGGGTGATCGGCCACATT
GGAACTGAGACACGGTC
>Zotu6684
GAACGCTGGCGGCAGGCCTAACACATGCAAGTCGAGCGGGCACCTTCGGGTGTCAGCGGCAGACGGGT
GAGTAACACGTGGGAACGTGCCCTTTGGTTCGGAACAACTCCGGGAAACTGGAGCTAATACCGGATAA
GCCCTTCGGGGGAAAGATTTATCGCCAAAGGATCGGCCCGCGTCTGATTAGCTAGTTGGTAGGGTAAT
GGCCTACCAAGGCGACGATCAGTAGCTGGTCTGAGAGGATGATCAGCTACACTGGGACTGAGACACGG
CC
>Zotu6685
GAACGCTGGCGGCGTGCCTAATACATGCAAGTCGAGCGAACAGAATTGGAGCTTGCTCCAATGACGTT
AGCGGCGGACGGGTGAGTAACACGTGGGTAACCTGCCCTACAGACTGGGATAACTTCGGGAAACCGGA

GCTAATACCAGATAATACTTTTTAACACATGTTAGAAAGTTGAAAGATGGTTCTGCTATCACTGTAGG
ATGGACCCGCGGCGCATTAGCTAGTTGGTGAGGTAACGGCTCACCAAGGCAACGATGCGTAGCCGACC
TGAGAGGGTGATCGGCCACACTGGGACTGAGACACGGCC
>Zotu6686
GAACGCTGGCGGTATGCTTAACACATGCAAGTCGAGCGCGAAAGTGGCTTCGGCCACGAGTAGAGCGG
CGGACGGGTGAGTAACACGTGGATAATCTGCCTTCAGGACTGGGATAACTCTGGGAAACTAGGGCTAA
TACCGGATAAGACCACAGGTTTTATGGAACCAGGGGTAAAAGGCGGCCTTCGGGCTGTCACCTAAAGA
TGAGTCCGCGTCCCATCAGGTAGTTGGTGAGGTAACGGCTCACCAAGCCGAAGACGGGTAGCCGACCT
TAGCGGGTGATCGGCCACACGGGGACTGAGACACGGCC
>Zotu6687
GAATGCTGGCGGCATGCTTTACACATGCAAGTCGAACGGCAGCGCGGACTTCGGTCTGGCGGCGAGTG
GCGAACGGGTGAGTAATACATCGGAACGTACCCAGAAGTGGGGGATAACGCAGCGAAAGTTGCGCTAA
TACCGCATACGTTCTACGGAAGAAAGTGGGGGATCGCAAGACCTCATGCTTTTGGAGCGGCCGATGTC
TGATTAGCTAGTTGGTAGGGTAAAGGCCTACCAAGGCGACGATCAGTAGCTGGTCTGAGAGGACGACC
AGCCACACTGGAATTGAGACACGGTC
>Zotu6688
GAACGTTAGCGGCACGCCTAACACATGCAAGTCGCACGAGAAAGGGAGCAATCCTGAGTAGAGTGGCG
CACGGGTGCGTAACACGTAGATAACCTGCCCTTTGGAGGGGGATAACGTTCCGAAAGGAGCGCTAATA
CCGCATAACGCTTTTTCCGGTTTGCCGGAGATTGACAAAGATAGGGACCCGCAAGGGCCTATCGCCAG
AGGAGGGGTCTGCGGCCCATCAGCTAGTTGGGGAGGTAATGGCTCACCAAGGCAGAGACGGGTAGCTG
GTCTGAGAGGATGATCAGCCACACTGGTACTGGAACACGGAC
>Zotu6689
GAACGCTGGCGGCATGCCTTACACATGCAAGTCGAACGGCAGCACGGGGGGTAACCCCTGGTGGCGAG
TGGCGAACGGGTGAGTAATATATCGGAACGTGCCCTGTAGCGGGGGATAACCTATCGAAAGATGGACT
AATACCGCATACGCTCTACGGAGGAAAGCGGGGGTCGCAAGACCTCGCACTATAGGAGCGGCCGATAT
CGGATTAGCTAGTTGGCGGGGTAAAAGCCCACCAAGGCGACGATCCGTAGCTGGTCTGAGAGGATGAT
CAGCCACACTGGAACTGAGACACGGTC
>Zotu6691
GAATGCTGGCGGCGTGGATAAGTCATGCAAGTCAAGGGGGCCCGCAAGGGCAACCGGCAGACGAGGTA
GTAACACGTAGGTACGTCCCCCGAAGTCGGGCATAGCCATCCGAAAGGATGGGTAATTCCCGATAGTC
CCGCAAGGGTAAAGGTTTTTCGCTTCGGGAGCGGCCTGCGTAATATCAGCTAGTTGGTGAGGTAATGG
CTCACCAAGGCTACGACGTTTAGGGGAGCTGAGAGGCTGACCCCCACCGATGGAACTGCGACACGGTC
>Zotu6692
GAACGCTAGCGGCAGGCCTAACACATGCAAGTCGAGGGGTATAGTAGCAATACTAGAGACCGGCGCAC
GGGTGCGTAACGCGTATGCAATCTACCTTTTACAGGGGAATAGCCCAGAGAAATTTGGATTAATGCCC
CATGGTATTATCGGATGGCATCATTTGATAATTAAAGTTCCAACGGTAAAAGATGAGCATGCGTCCTA
TTAGCCAGTTGGTAAGGTAACGGCTTACCAAGGCTACGATAGGTAGGGGTCCTGAGAGGGAGATCCCC
CACACTGGTACTGAGACACGGAC
>Zotu6693
TAACGCTGGCGGCGTGCATGAGACATGCAAGTCATACGGGCGACCAGGGCTTGCTCTGGTTGCTAGTG
GCGGACGGCTGAGTAACGCGTAGGTGACCTGCCCCGAAGTGGGGAATAACTGCCCGAAAGGGTGGCTA
ATACCGCATGTGGTGATGCGTTCATTCGCATCAGTAAAGCCGCAAGGCGCTTCGGGAGGGGCCTGCGT
CCGATTAGCTCGTTGGTGGGGTAACGGCTCACCAAGGCGACGATCGGTAGGTGGTCTGAGAGGACGAT
CACCCAGACTGGGACTGAGACACGGCC
>Zotu6694
GAACGCTGGCGGCAGGCTTAACACATGCAAGTCGAACGCTCCGCAAGGAGAGTGGCAGACGGGTGAGT
AACGCGTGGGAACCTTCCTATTGGTTCGGAATAGCTCAGGGAAACTTGGGGTAATACCGGATACGCCC
TATGGGGGAAAGATTTATCGCCAATAGATGGGCCCGCGTCTGATTAGCTAGTTGGTGAGGTAATGGCT
CACCAAGGCGACGATCAGTAGCTGGTTTGAGAGAATGACCAGCCACACTGGGACTGAGACACGGCC
>Zotu6696
GAACGCTGGCGGCGTGCCTAATACATGCAAGTAGAACGCTGAAGGAATAGCTTGCTATTCTGGAAGAG
TTGCGAACGGGTGAGTAACGCGTAGGTAACCTACCTCATAGCGGGGGATAACTATTGGAAACGATAGC
TAATACCGCATAAGAATACTTATTGCATGATAAGTATTTAAAAGGAACAACTGTTTCGCTGTGAGATG
GACCTGCGTTGTATTAGCTAGTTGGTAAGGTAACGGCTTACCAAGGCGACGATACATAGCCGACCTGA

GAGGGTGATCGGCCACACTGGGACTGAGACACGGCC
>Zotu6697
GAACGCTGGCGGCGTGCCTAACACATGCAAGTCGAGCGAGAGACTTCGGTCTCTAGCGGCGGACGGGT
GAGTAACACGTGGGTAACCTGCCTCGTAGAGGGGGATAGCCTTCCGAAAGGAAGATTAATACCGCATA
ACGTAGTTTAGTCGCATGACTAGATTATCAAAGGAGTAATCCGGTTTGAGATGGACCCGCGGCGCATT
AGCTAGTTGGTGAGGTAACGGCTCACCAAGGCGACGATGCGTAGCCGACCTGAGAGGGTGATCGGCCA
CATTGGAACTGAGATACGGTC
>Zotu6698
GAACGCTAGCGGCAGGCCTAACACATGCAAGTCGAGGGGTAGCAGGTTCAAGCTTGCTTGAATGCTGA
CGACCGGCGCACGGGTGCGTAACGCGTATGCAACCTACCTTGTACTGGGGTATAGCCCGGAGAAATTC
GGATTAATCCCCCATAGTATTATTGGATGGCATCATTTGATAATTAAAGGTTACGGTACAGGATGGGC
ATGCGTCCCATTAGCTAGATGGTGAGGTAACGGCTCACCATGGCGACGATGGGTAGGGGGCCTGAGAG
GGTGGTCCCCCACACTGGTACTGAGACACGGAC
>Zotu6699
GAACGCTGGCGGCGTGCTTAACACATGCAAGTCGAACGGTGAAGCAGGAGCTTGCTCTTGTGGATCAG
TGGCGAACGGGTGAGTAACACGTGAGTAACCTGCCCTTGACTCTGGGATAAGCGTTGGAAACGACGTC
TAATACCGGATATTTCAACCGACGGCATCGTCTGGTTGTGGAAAGATTTTTTGGTCAAGGATGGACTC
GCGGCCTATCAGCTTGTTGGTGAGGTAATGGCTCACCAAGGCGACGACGGGTAGCCGGCCTGAGAGGG
TGACCGGCCACACTGGGACTGAGACACGGCC
>Zotu6700
GAACGCTGGCGGCAGGCTTAACACATGCAAGTCGAACGCATCCTTCGGGATGAGTGGCAGACGGGTGA
GTAACACGTGGGAACGTGCCCTTCGGTTCGGAATAACTCTGGGAAACTAGAGCTAATACCGGATACGC
CCTTTTGGGGAAAGGTTTACTGCCGAAGGATCGGCCCGCGTCTGATTAGCTAGTTGGTGGGGTAATGG
CCTACCAAGGCGACGATCAGTAGCTGGTCTGAGAGGATGATCAGCCACACTGGGACTGAGACACGGCC
>Zotu6704
GAACGCTGGCGGCATGCCTTACACATGCAAGTCGAACGGTAGAGTAGCAATACTCGAGAGTGGCGAAC
GGGTGAGTAATATATCGGAACGTGCCCAGTCGTGGGGGATAACGTAGAGAAATTTACGCTAATACCGC
ATACGATCTAAGGATGAAAGCGGGGGATCGCAAGACCTCGCGCGATTGGAGCGGCTGATATCAGATTA
GGTTGTTGGTGAGGTAAAAGCTCACCAAGCCGACGATCTGTAGCTGGTTTGAGAGAACGACCAGCCAC
ACTGGGACTGAGACACGGCC
>Zotu6705
GAACGCTGGCGGCATGCTTAACACATGCAAGTCGAACGAGACCTTCGGGTCTAGTGGCGCACGGGTGC
GTAACGCGTGGGAATCTGCCCTTAGGTACGGAATAACTCAGAGAAATTTGTGCTAATACCGTATGATG
ACGTAAGTCCAAAGATTTATCGCCTAAGGATGAGCCCGCGTAGGATTAGCTTGTTGGTGAGGTAAAAG
CTCACCAAGGCGACGATCCTTAGCTGGTCTGAGAGGATGATCAGCCACACTGGGACTGAGACACGGCC
>Zotu6706
GAACGCTGGCGGCAGGCCTAACACATGCAAGTCGAACGGATCCTTTTAGGATTAGTGGCGGACGGGTG
AGTAACACGTGGGAACGTGCCTTTTGGTTCGGAATAACTCAGGGAAACCTGTGCTAATACCGGATGTG
CCCTTCGGGGGAAAGATTTATCGCCATTAGAGCGGCCCGCGTCTGATTAGCTTGTTGGTGAGGTAAAG
GCTCACCAAGGCGACGATCAGTAGCTGGTCTGAGAGGATGATCAGCCACACTGGGACTGAGACACGGC
C
>Zotu6709
GAACGCTGGCGGCAGGCTTAACACATGCAAGTCGAGCGCATCCTTCGGGGTGAGCGGCAGACGGGTGA
GTAACGCGTGGGAATCTACCTTTTGCTACGGAATAGCTCCGGGAAACTGGAATTAATACCGTATGTGC
CCTTAGGGGGAAAGATTTATCGGCAAGAGATGAGCCCGCGTTGGATTAGCTAGTTGGTGGGGTAAAGG
CCTACCAAGGCGACGATCCATAGCTGGTCTGAGAGGATGATCAGCCACATTGGGACTGAGACACGGCC
>Zotu6710
AAACGCTGGCGGTGTGCTTAACACATGCAAGTCGAACGAGAAAATTCCTTCGGGGATGAGTACAGTGG
CGAACGGGTGAGTAACACGTGAGAATCTGCCCATTAGTGGAGAATATCTTCGAGAAATCGAGGTTAAT
ACTGCATAAGTCCAGCTTGCTGGAGAAAGACGGCCTCTTTGCTGTCGCTGATGGATGAGCTCGCGTCC
TATTAGTTAGTTGGTGAGGTAAAGGCTCACCAAGACGAAGATGGGTAGCCGGCCTGAGAGGGTGTACG
GCCACATTGGGACTGAGATACGGCC
>Zotu6711
GAACGCTGGCGGCGTGCTTAACACATGCAAGTCGAACGATGAAGCAGGAGCTTGCTCCTGTGGATTAG

TGGCGAACGGGTGAGTAACACGTGAGTAATCTGCCCCTGACTCCGGGATAAGCACTGGAAACGGTGTC
TAATACCGGATACGAGCTGCGAAGGCATCTTCAGCAGCTGGAAAGAACTTCGGTCAGGGATGAACTCG
CGTCCTATCAGCTAGTTGGTGAGGTAAAGGCTCACCAAGGCGACGACGGGTAGCCGGCCTGAGAGGGT
GACCGGCCACACTGGGACTGAGACACGGCC
>Zotu6714
GAACGTTAGCGGCGCGCTTAACACATGCAAGTCGAGCGAGAAAGGGGCAACCCCGGTAAAGCGGCGCA
CGGGTGAGTAACACGTAGGTAATCTACCCCCAGGTGGTGGATAACCTTCCGAAAGGAAGGCTAATACA
GCATGGGACCACGGCTCCGCAAGGAGTTGAGGTGAAAGTCGGCCTCTTCACGAAAGCCGACGCCAGGG
GATGAGCCTGCGGCCCATCAGCTAGTTGGTAGGGTAATGGCCTACCAAGGCAAAGACGGGTAGCTGGT
CTGAGAGGATGATCAGCCACACTGGAACTGAGACACGGTC
>Zotu6715
GAACGCTGGCGGCATGCCTTACACATGCAAGTCGAACGGCAGCGCGGGGCAACCTGGCGGCGAGTGGC
GAACGGGTGAGTAATATATCGGAACGTACCCTGGAGTGGGGGATAACGTAGCGAAAGTTACGCTAATA
CCGCATACGATCTAAGGATGAAAGTGGGGGATCGCAAGACCTCATGCTCCTGGAGCGGCCGATATCTG
ATTAGCTAGTTGGTGGGGTAAAGGCCTACCAAGGCATCGATCAGTAGCTGGTCTGAGAGGACGACCAG
CCACACTGGAACTGAGACACGGTC
>Zotu6716
GAACGCTGGCGGCGTGCCTAATACATGCAAGTCGAGCGGACAGATGGGAGCTTGCTCCCTGAAGTTAG
CGGCGGACGGGTGAGTAACACGTGGGCAACCTGCCCTGCAGATGGGGATAACTCCGGGAAACCGGGGC
TAATACCGAATAATCCATTCTCCCACATGGGAGAATGCTGAAAGACGGTTTCGGCTGTCACTGCAGGA
TGGGCCCGTGGCGCATTAGCTAGTTGGTGGGATAACGGCCTACCAAGGCAACGATGCGTAGCCGACCT
GAGAGGGTGATCGGCCACACTGGGACTGAGACACGGCC
>Zotu6718
GAACGCTAGCGGGAGGCCTAACACATGCAAGCCGAGCGGTATTTGTCTTTCGGGACGGAGAGAGCGGC
GTACGGGTGCGGAACACGTGTGCAACCTGCCTTTATCAGGGGGATAGCCTTTCGAAAGGAAGATTAAT
ACCCCATAATATATTAATTGGCATCAATTGATATTGAAAACTACGGTGGATAGAGATGGGCACGCGCA
AGATTAGATAGTTGGTAGGGTAACGGCCTACCAAGTCAGTGATCTTTAGGGGGCCTGAGAGGGTGATC
CCCCACACTGGTACTGAGACACGGAC
>Zotu6719
GAACGCTGGCGGCGTGCCTAACACATGCAAGTCGAACGGGACTGTTTAGTGACGATTTCGGTCTGAAC
TTTACAGTTTAGTGGCGGACGGGTGAGTAACGCGTGGATAACCTGCCTTTCACAGGGGGATAGCAGCT
GGAAACGGCTGGTAATACCGCATACGCGAGAGATGCCGCATGACATCACTCGGAAAGATTTATCGGTG
AAAGATGGATCCGCGTCTGATTAGCCAGTTGGCGGGGTAACGGCCCACCAAAGCGACGATCAGTAGCC
GGCCTGAGAGGGCGGACGGCCACATTGGGACTGAGACACGGCC
>Zotu6721
GAACGCTGGCGGCGTGCTTAACACATGCAAGTCGAACGGTGAACTTGGGAGCTTGCTTCCGGGGGATC
AGTGGCGAACGGGTGAGTAACACGTGAGTAACCTGCCCCAGACTCTGGGATAAGCGTTGGAAACGACG
TCTAATACCGGATACGAGCTTCAGCCGCATGGCTAGGAGTTGGAAAGAATTTCGGTTTGGGATGGACT
CGCGGCCTATCAGCTAGTTGGTGAGGTAATGGCTCACCAAGGCGACGACGGGTAGCCGGCCTGAGAGG
GTGACCGGCCACACTGGGACTGAGACACGGCC
>Zotu6722
GAACGTTGGCGGCGCGCTTAACACATGCAAGTCGCACGAGAAAGGGGGCAACCCTGAGTAAAGTGGCG
CACGGGTGCGTAACACGTAGGCAATCTACTCTCAGGAGGGGGATAACTACCCGAAAGGGTAGCTAATA
CCGCGTAAGACCACGGCTTCTCTGGAAGCTGCGGTTAAAGGTGGCCTCTACTTGTAAGCTATCACCTG
AGAATGAGCCTGCGGCCCATCAGCTAGTTGGTAAGGTAACGGCTTACCAAGGCTACGACGGGTAGCTG
GTCTGAGAGGATGATCAGCCACACTGGCACTGAAACACGGGC
>Zotu6723
GAACGCTGGCGGCGTGCTTAACACATGCAAGTCGAGCGGAAAGGCCCTTCGGGGTACTCGAGCGGCGA
ACGGGTGAGTAACACGTGGGCAACCTGCCCTCAGCTCTGGGATAACTCCGGGAAACCGGGGCTAATAC
CGGATACGACTTCCCTGGGCATCCTGGGGGGTGGAAAGTTCTTCGGCTGAGGATGGGCCCGCGGCCTA
TCAGCTTGTTGGTGGGGTGATGGCCTACCAAGGCGACGACGGGTAGCCGGCCTGAGAGGGCGACCGGC
CACACTGGGACTGAGACACGGCC
>Zotu6724
GAACGCTAGCGGCATGCCTAACACATGCAAGTCGAACGCGGACTTCGGTCCGAGTGGCGAACGGGTGC

GTAACACGTAGGTGACCTGCCCACCGGTGGGGGATAACCGCGAGAAATCGTGGCTAATACCGCATGCG
ATCCTGGTCTTTCGAAAGCCAGGAGGAAAGCTTCATGTGCCGGTGGAGGGGCCTGCGGCCTATCAGCT
AGTTGGTGAGGTAACGGCTCACCAAGGCGATGACGGGTAGCTGACCTGAGAGGGTGGTCAGCCACAGG
GGAACTGAGAGACGGTC
>Zotu6725
GAACGCTGGCGGCGTGCCTAATACATGCAAGTCGAGCGAATGATGAAGAAGCTTGCTTCTTCTGATTT
AGCGGCGGACGGGTGAGTAACACGTGGGCAACCTGCCCTGTAGATTGGGATAACTCCGGGAAACCGGG
GCTAATACCGAATAATCCATTACCTCACATGAGGAAATGTTAAAAGACGGTTTCGGCTGTCACTACAG
GATGGGCCCGCGGCGCATTAGCTAGTTGGTGAGGTAACGGCTCACCAAGGCGACGATGCGTAGCCGAC
CTGAGAGGGTGATCGGCCACACTGGGACTGAGACACGGCC
>Zotu6729
GAACGTTGGCGGCGTGCCTTAAGCATGCAAATCAAGGGGGTCCCGCAAGGGACTACCGGTGGACGGGT
GAGTAATACGTGGGAATCTACCTCGAAGTTGGACATAGCTAGCCGAAAGGCTGGGTAATTTCCAATGT
GAACGTAAGTTCAAAGCCGCAAGGCGCTTTGAGATGAGCCTGCGTCCTATCAGCTAGTTGGTGGGGTA
ATGGCCTACCAAGGCATCGACGGGTAGCTGGACTGAGAGGTCGGCCAGCCACAATGGGACTGCGACAC
GGCC
>Zotu6730
AAACGCTGGCGGCGTGCCTAACACATGCAAGTCGAACGTCCTTGCTGTTTTCGCAAGAGGACAGCAAG
GGAGTGGCGGACGGGTGAGTAACACGTGGGTACCTACCCTGGGGTGAGGAATACCGGCGAGAAATTGC
CGACAAGACCGCATAGGCTCTCCTACGAGAGCAAAGCCGAAAGGCGCCCTGGGATGGGCCTGCGGCCG
ATTAGCTGGTTGGTGAGGTAAGAGCTCACCAAGGCGACGATCGGTAGCTGGTCTGAGAGGACGAACAG
CCACACTGGGATTGAGAACGGCC
>Zotu6735
GAACGCTGGCGGCATGCCTAACACATGCAAGTCGAACGAACCCTTCGGGGTTAGTGGCGCACGGGTGC
GTAACGCGTGGGAATCTGCCCTTTGCTTCGGAATAACAGTTAGAAATGACTGCTAATACCGGATGATG
TCGCAAGACCAAAGATTTATCGGCAAAGGATGAGCCCGCGTAGGATTAGGTAGTTGGTGGGGTAATGG
CCTACCAAGCCGACGATCCTTAGCTGGTCTGAGAGGATGATCAGCCACACTGGGACTGAGACACGGCC
>Zotu6736
GAACGCTGGCGGCGTGCTTAACACATGCAAGTCGAGCGGTAAGGCCCCTTCGGGGGTACACGAGCGGC
GAACGGGTGAGTAACACGTGAGCAATCTGCCCTCCACATCGGGATAACCCCCGGAAACGGGAGCTAAT
ACCGGATACGACCACTTCAGGCATCTGATGGTGGTGGAAAGTTCCGGCGGTGGAGGATGAGCTCGCGG
CCTATCAGCTTGTTGGTGGGGTAACGGCCCACCAAGGCTTCGACGGGTAGCCGGCCTGAGAGGGTGAC
CGGCCACACTGGGACTGAGACACGGCC
>Zotu6737
GAACGCTGGCGGCGTGCCTAATACATGCAAGTCGAGCGAACCTTTGGGAGCTTGCTCCCAAAGGTTAG
CGGCGGACGGGTGAGTAACACGTGGGCAACCTGCCTATAAGGCTGGGATAACTTCGGGAAACCGGAGC
TAATACCGGATAATTCTTTTCTACTCATGTAGAAAAGCTAAAAGACGGCTTACGCTGTCACTTATAGA
TGGGCCCGCGGCGCATTAGCTAGTTGGTGAGGTAACGGCTCACCAAGGCCACGATGCGTAGCCGACCT
GAGAGGGTGATCGGCCACACTGGGACTGAGACACGGCC
>Zotu6740
GAACGCTGGCGGCGTGCCTAATACATGCAAGTCGAGCGAATCTTTGGGAGCTTGCTCCTAAAGGTTAG
CGGCGGACGGGTGAGTAACACGTGGGCAACCTGCCTATAAGACTGGGATAACTTCGGGAAACCGGAGC
TAATACCGGATCATTCTTTTCTACACATGTAGAAAAGCTAAAAGACGGTTTACGCTGTCACTTATAGA
TGGGCCCGCGGCGCATTAGCTAGTTGGTGAGGTAACGGCTCACCAAGGCCACGATGCGTAGCCGACCT
GAGAGGGTGATCGGCCACACTGGGACTGAGACACGGCC
>Zotu6742
GAACGCTGGCGGCGTGCCTAATACATGCAAGTCGAGCGGAACATTTATTGGTGCTTGCACCGATGAAT
GTTTAGCGGCGGACGGGTGAGTAACACGTGGGCAACCTGCCCTGTAGATTGGGATAACTCCGGGAAAC
CGGGGCTAATACCGAATAATACTTTTTAACTCATGTTAGAAAGTTGAAAGATGGTTCTGCTATCACTA
CAGGATGGGCCCGCGGCGCATTAGCTAGTTGGTGAGGTAACGGCTCACCAAGGCGACGATGCGTAGCC
GACCTGAGAGGGTGATCGGCCACACTGGGACTGAGACACGGCC
>Zotu6744
GAACGCTGGCGGCAGGCTTAACACATGCAAGTCGAGCGCCCCGCAAGGGGAGCGGCAGACGGGTGAGT
AACACGTGGGAATCTACCCATCCCTACGGAACAACTCCGGGAAACTGGAGCTAATACCGTATACGCCC

TTCGGGGGAAAGATTTATCGGGGATGGATGAGCCCGCGTTGGATTAGCTAGTTGGTGGGGTAAAGGCC
TACCAAGGCGACGATCCATAGCTGGTCTGAGAGGATGATCAGCCACATTGGGACTGAGACACGGCC
>Zotu6745
GCCGTGTCTCAGTCCCAGTGTGGCTGATCATCCTCTCAGACCAGCTACAGATCGTCGGCTTGGTGAGC
CATTACCTCACCAACTACCTAATCTGACGCGGGCTCATCCATCAGCGATAAATCTTTCCTCCGTAGAG
AATATACGGTATTAGCTTTTATTTCTAAAAGTTATTCCGTACTGATGGGTAGATTCCCACATGTTACT
CACCCGTCTGCCACTAACTAATTGGAGCAAGCCCCAATTAGTCCGTTCGACTTGCATGTGTTAAGCAT
ACCGATAGCGTTCG
>Zotu6747
CAACGCTGGCGGCGTGCCTAACACATGCAAGTCGAACGCGAAAGGGACTTCGGTTCTTAGTAGAGTGG
CGTACGGGTGAGTAACGCGTGGGTCATCTGCCCTCGGGTGGGGAATAACTAACCGAAAGGTTAGCTAA
TACCGCATAACACAGTGATGTCAAAGCAGCAATGTGCCTGAGGAGGAGCCTGCGTCTGATTAGGTAGT
TGGTGGGGTAACGGCCTACCAAGCCTGCGATCAGTAGCTGGTCTGAGAGGACGGCCAGCCACAATGGC
ACTGAAACACGGGC
>Zotu6749
GAACGCTAGCGGCAGGCCTAATACATGCAAGTCGAGGGGCAGCACGGTAGCAATACTGGTGGCGACCG
GCGCACGGGTGCGTAACACGTATACAATCTGCCTTTAACAGGGGGATAGCCCGGAGAAATCCGGATTA
ATACCCCATAACAATGGGATCAGCATTGATCTTATTTAAAAGCTCCGGCGGTTAGAGATGAGTATGCG
TCTGATTAGCTAGATGGTGAGGTAACGGCTCACCATGGCGACGATCAGTAGGGGATCTGAGAGGATTA
ACCCCCACACGGATACTGAAACACGGAT
>Zotu6750
CAACGTTGGCGGCGCGCCTAACACATGCAAGTCGAACGAGAAAGTGGAGCAATCCATGAGTAAAGTGG
CGTACGGGTGAGTAACACGTGGGTAATCCACCTCTTGATGGGGAATAACGCTGGGAAACTGGCGCTAA
TACCGCATAAGTTCGTAAGAAGAAAGTCGCAAGACGCCGAGAGAGGAGCCCGCGGCCGATTAGCTAGT
TGGTAGGGTAAAAGCCTACCAAGGCTATGATCGGTAGCCGGCCTGAGAGGGCACACGGCCACACTGGC
ACTGAAACACGGGC
>Zotu6751
GAATGCTAGCGGTGTGCCTGAGATATGCAAGTCGAGCGATTGTTTGACCGCAAGGTTGAATAGTAGCG
GCGGACGGCTGAGTAATGTACAGGAATCTGCCCCTTAGTGGAACATAGCTCCGGGAAACCGGGGGTAA
TTTTCCATGGTTTCACCTTTTTGGTGAATAAAAGCCTTTACCGGTTGCTAAGGGATGAGCCTGTATCC
TATCAGGTAGTTGGTGGGGTAAAAGCCTACCAAGCCTATGACGGGTAGCTGATCTGGGAGGATGATCA
GCCAGAGGGGGACTGAGACACGGCC
>Zotu6752
GAACGCTGGCGGCGTGCCTAATACATGCAAGTCGAGCGGAACTTTCATTGGAGCTTGCTCCTTTGACA
GTTTAGCGGCGGACGGGTGAGTAACACGTGGGTAACCTACCCTGTAGATTGGGATAACTTCGGGAAAC
CGAGGCTAATACCGAATAATACTTTTAATCTCATGGTTGAAAGTTGAAAGATGGTTCTGCTATCACTA
CAGGATGGGCCCGCGGTGCATTAGCTAGTTGGTGAGGTAACGGCTCACCAAGGCCACGATGCATAGCC
GACCTGAGAGGGTGATCGGCCACACTGGGACTGAGACACGGCC
>Zotu6753
GAACGCTGGCGGTGTGCTTAACACATGCAAGTGGAGCGACGAACCAGAGCTTGCTCTGGGGCAGAGCC
GCGAACGGGTGAGTAACACGTGGGTAACGTACCCCAATAACCGGGACAACCCGAGGAAACTCGGGCTA
ATACCGGATGTGCTCCTCCCTCATCAGAGGGTTGAGTAAAGGAAGCTTCGGCCTCCGTATTGGGAGCG
GCCCGCGGGCTATTAGTTTGTTGGTGGGGTAACGGCCTACCAAGACTGCGATAGCTAGCTGGTCTGAG
AGGACGATCAGCCACACTGGGACTGAGACACGGCC
>Zotu6755
GAACGCTGGCGGCAGGCTTAACACATGCAAGTCGAACGATGACTAACTAGCTTGCTAGTTATGATTAG
TGGCGGACGGGTGAGTAACATTTAGGAATCTGCCTAGTAGTGGGGGATAGCTCGGGGAAACTCGAATT
AATACCGCATACGACCTACGGGTGAAAGGGGGCGCAAGCTCTTGCTATTAGATGAGCCTAAATCAGAT
TAGCTAGTTGGTGGGGTAAAGGCCTACCAAGGCGACGATCTGTAACTGGTCTGAGAGGATGATCAGTC
ACACCGGAACTGAGACACGGTC
>Zotu6756
GAACGCTGGCGGCGTGCTTAACACATGCAAGTCGAACGGGAAATACTTTATTGAAACTTCGGTCGATT
TAATCTATTTCTAGTGGCGGACGGGTGAGTAACGCGTGGGTAACCTGCCTTATACTGGGGGATAACAG
CCAGAAATGGCTGCTAATACCGCATAAGCGCACGGGGCCGCATGGTCCTGTGTGAAAAACTCCGGTGG

TATAAGATGGACCCGCGTTGGATTAGCTAGTTGGCAGGGCAGCGGCCTACCAAGGCGACGATCCATAG
CCGGCCTGAGAGGGTGAACGGCCACATTGGGACTGAGACACGGCC
>Zotu6757
GAACGCTGGCGGCGTGCCTAATACATGCAAGTCGAGCGAATCAAAGGGAGCTTGCTCCCTTTTGGTTA
GCGGCGGACGGGTGAGTAACACGTGGGCAACCTGCCTATAAGACTGGGATAACTTCGGGAAACCGGAG
CTAATACCGGATAACTCATATCTACTCATGTAGATATGCTAAAAGACGGTTTACGCTGTCACTTATAG
ATGGGCCCGCGGCGCATTAGCTAGTTGGTGAGGTAACGGCTCACCAAGGCGACGATGCGTAGCCGACC
TGAGAGGGTGATCGGCCACACTGGGACTGAGACACGGCC
>Zotu6758
GAACGCTAGCTACAGGCTTAACACATGCAAGTCGAGGGGCAGCATGGTCTTAGCTTTCTAAGGCTGAT
GGCGACCGGCGCACGGGTGAGTAACATGTATCCAACCTGCCGTCTACTCTTGGCCAGCCTTCTGAAAG
GAAGATTAATCCAGGATGGGATCATGAGTTCACATGTCCGCATGATTAAAGGTATTTTCCGGTAGACG
ATGGGGATGCGTTCCATTAGATAGTAGGCGGGGTAACGGCCCACCTAGTCAACGATGGATAGGGGTTC
TGAGAGGAAGGTCCCCCACATTGGAACTGAGACACGGTC
>Zotu6759
GAACGCTGGCGGCAGGCTTAACACATGCAAGTCGAGCGGGGGAAAGTAGCTTGCTACTGGACCTAGCG
GCGGACGGGTGAGTAATGCTTAGGAATCTGCCTATTAGTGGGGGACAACATTCCGAAAGGAATGCTAA
TACCGCATACGTCCTACGGGAGAAAGCAGGGGACCTTCGGGCCTTGCGCTAATAGATGAGCCTAAGTC
GGATTAGCTAGTTGGTGGGGTAAAGGCCTACCAAGGCGACGATCTGTAGCGGGTCTGAGAGGATGATC
CGCCACACTGGGACTGAGACACGGCC
>Zotu6760
GAACGCTGGTGGCGTGCTTAACACATGCAAGTCGAGCGAGGCGTCCGCTTTCGGGTGGATAGTCGAGC
GGCGAACGGGTGAGTAACACGTGAGTAATCTTCCCTGGGCTCGGGGATAACCTTGGGAAACTGAGGCT
AATACCGGATGCGCGTTGTTTGGGGCTTCCTGGATGACGGAAAGGTTCCATCTCGTGAGGGGTGGTTC
CGGCCTTGGATGAGCTCGCGGCCTATCAGCTTGTTGGTGCGGTGAGGGCGTACCAAGGCGACGACGGG
TAGCCGGCCTGAGAGGGTGACCGGCCACACTGGGACTGAGATACGGCC
>Zotu6761
GAACGCTGGCGGCGTGCTTAACACATGCAAGTCGAGCGGTAAGGCCTTTCGGGGTACACGAGCGGCGA
ACGGGTGAGTAACACGTGGGTGATCTGCCCTGCACTCTGGGATAAGCCTGGGAAACTGGGTCTAATAC
TGGATATGACCTCAGCATGCATGTGCTGGGGTGGAAAGCTTTTGTGGTGCAGGATGGGCCCGCGGCCT
ATCAGCTTGTTGGTGGGGTAATGGCCTACCAAGGCGACGACGGGTAGCCGACCTGAGAGGGTGACCGG
CCACACTGGGACTGAGACACGGCC
>Zotu6763
GAACGCTGGCGGCGTGCTTAACACATGCAAGTCGAACGGAAAGGCCCTTCGGGGTACTCGAGTGGCGA
ACGGGTGAGTAACACGTGGGTGATCTGCCCTGCACTTCGGGATAAGCCTGGGAAACTGGGTCTAATAC
CGGATATGACCACATGATGCATGTCGTGTGGTGGAAAGCTTTTGCGGTGTGGGATGGGCCCGCGGCCT
ATCAGCTTGTTGGTGGGGTCACGGCCTACCAAGGCGACGACGGGTAGCCGGCCTGAGAGGGTGACCGG
CCACACTGGGACTGAGATACGGCC
>Zotu6766
GAACGCTAGCGGCAGGCCTAATACATGCAAGTCAAGGGGCCCAGCAATGGGCACCGGCGGACGGGTGC
GTAACGCGTACACAATCTACCCTGGAGCGGGGGATAGCATCGGGAAACTGGTGGTAATACCCCATAGT
TTTGTGAGTTGGCATCAGCTTACAAATAAAGTTCCGGCGCTCTGGGATGAGTGTGCGTCCGATTAGCT
AGTTGGTAGGGTAACGGCCTACCAAGGCGACGATCGGTAGGGGGTGTGAGAGCATGACCCCCCACACG
GGTACTGAGACACGGAC
>Zotu6767
GAACGCTAGCGGCAGGCCTAATACATGCAAGTCGAGGGGTAGTGGTTGCTTGCAACCATGAGACCGGC
GCACGGGTGCGTAACGCGTATGCAACCTACCTCTATCTGGGGGATAGCCCGAAGAAATTCGGATTAAC
ACCGCATAAAATCACAGTACGGCATCGTACAATGATCAAATATTTATAGGATGGAGATGGGCATGCGT
GTCATTAGTTAGTTGGCGGGGTAACGGCCCACCAAGACCACGATGACTAGGGGATCTGAGAGGATGAC
CCCCCACACTGGTACTGAGACACGGAC
>Zotu6768
GAACGCTGGCGGCGTGCTTAACACATGCAAGTCGAACGGTAAGGCCTTTCGGGGTACACGAGTGGCGA
ACGGGTGAGTAACACGTGGGTGATCTGCCCTGCACTTCGGGATAAGCTTGGGAAACTGGGTCTAATAC
CGGATATGACTACGAATCGCATGGTTTGTGGTGGAAAGCTTTTGCGGTGTGGGATGGGCCCGCGGCCT

ATCAGCTTGTTGGTGGGGTGATGGCCTACCAAGGCGACGACGGGTAGCCGGCCTGAGAGGGTGTCCGG
CCACACTGGGACTGAGATACGGCC
>Zotu6769
GAACGCTGGCGGCGTGCTTAACACATGCAAGTCGAACGGTAAGGCCCTTTCGGGGGTACACGAGTGGC
GAACGGGTGAGTAACACGTGGGTAATCTGCCCTGCACTTCGGGATAAGCCTGGGAAACTGGGTCTAAT
ACCGGATATTCAGCTCCTGCCGCATGGTGGGGGTTGGAAAGTTTTTCGGTGCAGGATGAGCCCGCGGC
CTATCAGCTTGTTGGTGGGGTAATGGCCTACCAAGGCGACGACGGGTAGCCGGCCTGAGAGTGTGATC
GGCCACACTGGGACTGAGACACGGCC
>Zotu6770
GAACGCTAGCGACAGGCCTAACACATGCAAGTCGAGGGGCAGCATAAAGGTAGCAATACTTTGGTGGC
GACCGGCGCACGGGTGAGTAACACGTATGCAACCTACCTGTAAGAGGGGAATAACCCGGAGAAATTCG
GACTAATACCGCATAATACCACGAAGCTGCATGGCTTTGTGTTTAAACATTTATGGCTTACAGATGGG
CATGCGTATGATTAGATAGTTGGTAGGGTAACGGCCTACCAAGTCAACGATCATTAGGGGTTCTGAGA
GGAAGGTCCCCCACACTGGTACTGAGACACGGAC
>Zotu6771
GAACGCTGGCGGCGTGCCTAATACATGCAAGTCGAGCGCGTTTCGAAGGTGCTTGCACCTTCAATGAA
ACGAGCGGCGGACGGGTGAGTAACACGTAGGTAACCTACCTTTATGACTGGGATAACCACGGGAAACC
GGGGCTAATACCGGATAAGATTTAGCTTCGCATGAAGCAGAAGTAAAAGGCAGTTTCGGCTGTCACAT
AAAGATGGACCTGCGGCGCATTAGCTAGTTGGAGAGGTAAGAGCTCCCCAAGGCGACGATGCGTAGCC
GACCTGAGAGGGTGATCGGCCACACTGGGACTGAGACACGGCC
>Zotu6772
GAACGCTGGCGGCGTGCTTAACACATGCAAGTCGAGCGGTAAGGCACTTCGGTGTACACGAGCGGCGA
ACGGGTGAGTAACACGTGAGTAATCTGCCCTTCACTCTGGGATAAGCACTCGAAAGGGTGTCTAATAC
CAGATACGACACAGACCTGCATGGGTTCTGTGTGGAAACTTTTGTGGGTGGGGGATGTGCTCGCGGCC
TATCAGCTTGTTGGTGGGGTAATGGCCTACCAAGGCTTTGACGGGTAGCCGGCCTGAGAGGGTGACCG
GCCACACTGGGACTGAGACACGGCC
>Zotu6773
GAACGCTGGCGGCGTGCTTAACACATGCAAGTCGAGCGAGGTCCAACTGGTGGCAACACCAGGGAAGG
CCTAGCGGCGAACGGGTGAGTAACACGTGAGCAACCTGCCCCGAAGACTGGGATAACACCGGGAAACC
GGTGCTAATACCGGATATCCCCATCTGGCCGCATGACCGGATGAGGAAATGAATTTCGCTTCGGGAGG
GGCTCGCGGGCTATCAGCTTGTTGGTGGGGTAACGGCTCACCAAGGCGACGACGGCTAGCTGGTCTGA
GAGGACGACCAGCCACACTGGGACTGAGACACGGCC
>Zotu6775
GAACGCTGGCGGCGTGCTTAACACATGCAAGTCGAACGGTGAACACGGAGCTTGCTCTGTGGGATCAG
TGGCGAACGGGTGAGTAACACGTGAGCAACCTGCCCCTGACTCTGGGATAAGCGCTGGAAACGGCGTC
TAATACTGGATACGAGACGTGATCGCATGGTCAACGTCTGGAAAGAATTTCGGTTGGGGATGGGCTCG
CGGCCTATCAGCTTGTTGGTGAGGTAATGGCTCACCAAGGCGTCGACGGGTAGCCGGCCTGAGAGGGT
GACCGGCCACACTGGGACTGAGACACGGCC
>Zotu6776
GAACGTTGGCGGCGTGCCTTAAGCATGCAAATCAAGGGGGTCCCGCAAGGGACTACCGGTGGACGGGT
GAGTAATGCGTGGGAATGTACCTCGAAGTTGGATATAGCTAGCCGAAAGGTTGGGTAATCTCCAATGT
GAACGAAAGTTTAGAGCCGCAAGGCGCTTTGAGATCAGCCCGCGTCCTATCAGCTTGTTGGTGGGGTA
ATGGCCTACCAAGGCGACGACGGGTAGCTGGACTGAGAGGTCGGTCAGCCACAATGGGACTGCGACAC
GGCC
>Zotu6777
GAACGCTGGCGGCGTGGATTAGGCATGCAAGTCGCGCGACAGTACGGGTTCGCCCGTATATGGAGCGG
CGAACGGGTGAGTAACACGTGAACAATCTGTCCCTACATTCCGGATAATTGGCCGAAAGGCCTTGTAA
TACGGGGGAGGACGGTGATGAGGCATCTCATTATCGGGGAAGGCGCAAGCCGTGCAGGGAGGAGTTCG
CGGATTATCAGCTTGTTGGTGGGGTAATGGCCTACCAAGGCGACGACGATTAGCTGGTCTGAGAGGAT
GGTCAGCCACATTGGAACTGAGACACTGTC
>Zotu6778
GAACGCTGGCGGCAGGCTTAACACATGCAAGTCGAACGCCCCGCAAGGGGAGTGGCAGACGGGTGAGT
AACACGTGGGAACATACCCTTCGGTTCGGAACAACGCCGGGAAACTGGCGCTAATACCGGATACGGCT
TACGGGCGAAAGATTGATCGCCGAAGGATTGGCCCGCGTTCGATTAGCTTGTTGGTGAGGTAACGGCT

CACCAAGGCGACGATCGATAGCTGGTCTGAGAGGATGGCCAGCCACATTGGGACTGAGACACGGCC
>Zotu6779
GAACGCTGGCGGCGTGCTTAACACATGCAAGTCGAACGAGAATCTCTGGATAGAGGCTTCGGCCAATT
GAAAGAGAGGACAGTGGCGGACTGGTGAGTAACGCGTGAGGAACCTGCCTTTCAGAGGGGGACAACAG
TTGGAAACGACTGCTAATACCGCATGACACATTCGAGCGGCATCGCTTGAATGTCAAAGATTTATCGC
TGAAAGATGGCCTCGCGTCTGATTAGCTAGTTGGTGAGGTAACGGCCCACCAAGGCGACGATCAGTAG
CCGGACTGAGAGGTTGACCGGCCACATTGGGACTGAGATACGGCC
>Zotu6781
GAACGCTGGCGGCAGGCCTAACACATGCAAGTCGAACGGGTCCTTCGGGACTAGTGGCGGACGGGTGA
GTAACGCGTGGGAACATACCCTTTGGTACGGAATAGCCTCGGGAAACTGAGAGTAATACCGTATGTGC
CCTTCGGGGGAAAGATTTATCGCCAAAGGATTGGCCCGCGTTGGATTAGGTAGTTGGTGGGGTAATGG
CCTACCAAGCCGACGATCCATAGCTGGTTTGAGAGGATGATCAGCCACACTGGGACTGAGACACGGCC
>Zotu6783
GAACGCTGGCGGCGTGCCTAATACATGCAAGTCGAGCGAGCAGAACCAGCAGATTTACTTCGGTAATG
ACGCTGGGGACGCGAGCGGCGGATGGGTGAGTAACACGTGGGGAACCTGCCCCATAGTCTGGGATACC
ACTTGGAAACAGGTGCTAATACCGGATAAGAAAGCAGATCGCATGATCAGCTTATAAAAGGCGGCGTA
AGCTGTCGCTATGGGATGGCCCCGCGGTGCATTAGCTAGTTGGTAAGGTAACGGCCTACCAAGGCGAT
GATGCATAGCCGAGTTGAGAGACTGATCGGCCACATTGGGACTGAGACACGGCC
>Zotu6785
GAACGCTGGCGGCATGCCTAACACATGCAAGTTGAACGAGAAGTTCTGGAACAAGTCTTCGGACAAGG
GAAGGGACGGGAAGTAGCGAACGGGTGAGTAACGCGTGGGCAACCAACCTTACACAGGGGGATAACAC
AGAGAAATTTGTGCTAATACCGCATAAGCGCACAGTACCGCATGGTACGGTGTGAAAAACTCCGGTGG
TGTAAGACGGGCCCGCGTCTGATTAGCTAGTTGGTGAGGTAAGAGCTCACCAAGGCGACGATCAGTAG
CCGACCTGAGAGGGTGATCGGCCACATTGGGACTGAGACACGGCC
>Zotu6787
GAACGCTGGCGGCAGGCTTAACACATGCAAGTCGAGCGAGATCTTCGGGTCTAGCGGCGGACGGGTGA
GTAACGCGTGGGAACGTGCCCTTTGCTACGGAATAGTCCTGGGAAACTGGGGGTAATACCGTATACGC
CCTTTTGGGGAAAGATTTATCGGCGAAGGATCGGCCCGCGTTGGATTAGGTAGTTGGTGGGGTAATGG
CCTACCAAGCCGACGATCCATAGCTGGTTTGAGATGATGATCAGCCACACTGGGACTGAGACACGGCC
>Zotu6789
AAACGCTGGCGGCGTGCCTAATACATGCAAGTCGAACGAATGTAGCAATACATGAGTGGCGTACGGGT
GAGTAACACGTGGATAATCTTCCTTTAAGTGGGGAATAACCAATCGAAAGATTGGCTAATACCGCATA
AGACCACAGTTTCTTCGGAGACAGAGGTTAAAGGCTTCGGCCGCTTTTAGATGAGTCTGCGTCCGATT
AGCTAGTTGGTGAGGTAATGGCTCACCAAGGCGACGATCGGTAACTGGTCTGAGAGGATGACCAGTCA
CACTGGGACTGAGACACGGCC
>Zotu6793
GAACGCTGGCGGCGTGCTTAACACATGCAAGTCGAACGGTAAGGCCCTTCGGGGTACACGAGTGGCGA
ACGGGTGAGTAACACGTGGGTGATCTGCCCTGCACTTTGGGATAAGCCTGGGAAACTGGGTCTAATAC
CGAATAGGATCATGGCCTTCATGGGTTGTGGTGGAAAGCTTTTGCGGTGTGGGATGGGCCCGCGGCCT
ATCAGCTTGTTGGTGGGGTAATGGCCTACCAAGGCGACGGCGGGTAGCCGGCCTGAGAGGGTGTCCGG
CCACACTGGGACTGAGATACGGCC
>Zotu6795
GAACGCTGGCGGTGTGCTTCACACATGCAAGTCGAACGGTAGCAATACAGTGGCGGACGGGTGAGTAA
CGCGTGGGAATCTGCCCTGGGGTGGGGGATAGCAGACCGAAAGGTCTATTAATACCGCATATGCCGAA
AGGTAAAAGGAGTAATCCGCCCGAGGATGAGCCTGCGTCCGATTAGCTAGTTGGTGGGGTAAAAGCCT
ACCAAGGCTACGATCGGTAGCTGGTCTGAGAGGATGATCAGCCACAATGGGACTGAGACACGGCC
>Zotu6798
GAACGCTAGCGGCATGCCTAACACATGCAAGTCGAGGGGTAGAAGTCTTCGGACTTTGAGACCGGCGC
ACGGGTGCGTAACGCGTATGCAATCTACCTTGTACTGAGGGATAGCCCAGAGAAATTTGGATTAATAC
CTCATAGTATTTTCGGGTGACATCACCTGATTATTAAAGTTCCAACGGTACAAGATGAGCATGCGTCC
CATTAGCTAGTTGGTATGGTAACGGCATACCAAGGCTATGATGGGTAGGGGTCCTGAGAGGGAGATCC
CCCACACTGGTACTGAGACACGGAC
>Zotu6799
GAACGCTAGCGGCAGGCCTAATACATGCAAGTCGAACGGGTCGCAAGGTCAGTGGCGAACGGGTGCGT

AACGCGTAAGCAACCTGCCCACAACTGGGGGATAGCCTTGCGAAAGCGAGGGTAAACCCGCATGGTCC
TTTGGAGTCACCTGGCTTTTAAGGTAAACATTTATGGGTTGTGGAGGGGCTTGCGTCTGATTAGCTAG
TTGGTGGGGTAACGGCCCACCAAGGCGATGATCAGTAGGGGTTCTGAGAGGATTGGCCCCCACATGGG
TACTGAGACACGGAC
>Zotu6800
GAACGCTAGCGGCAGGCTTAATACATGCAAGTCGAGGGGCAGCACAGATAGCAATATTGGGTGGCGAC
CGGCAAACGGGTGCGGAACACGTACACAACTTACCTTTTGGTGGGGGATAGCCTTTGGAAACGGAGAT
TAATACCCCATAAGATATTATGGTCGCATGATTGTGATATTAAAGATTTATTGCCAGAAGATGGGTGT
GCGTATGATTAGGTAGTTGGTGAGGTAACGGCTCACCAAGCCCACGATCATTAACTGGTGTGAGAGCA
CGACCAGTCACACGGGCACTGAGACACGGGC
>Zotu6801
GAATGCTGGCGGCGTGGATAAGTCATGCAAGTCAAGGGGGCCCGCAAGGGCAACCGGCAGACGAGGTA
GTAACACGTAGGTAGATCCCCCGAAGTCGGGCATAGCCAGCCGAAAGGCTGGGTAATTCCCGATAGTC
CCGCAAGGGTAAAGGTTTTTCGCTTCGGGAGTCGCCTGCGTAGTATCAGCTAGTTGGTAGGGTAACGG
CCTACCAAGGCTACGACGCTTAGGGGAGCTGAGAGGCTGACCCCCACCGATGGAACTGCGACACGGTC
>Zotu6803
GAACGCTGGCGGCAGGCTTAACACATGCAAGTCGAACGCCCCGCAAGGGGAGTGGCAGACGGGTGAGT
AACGCGTGGGAATCTACCTAGTTCTACGGAACAACTGATGGAAACGTCAGCTAATACCGTATACGCCC
TACGGGGGAAAGATTTATCGGAATTAGATGAGCCCGCGTAAGATTAGCTAGTTGGTGGGGTAATGGCC
TACCAAGGCGACGATCTTTAGCTGGTCTGAGAGGATGATCAGCCACACTGGGACTGAGACACGGCC
>Zotu6804
GAACGCTGGCGGCGTGCCTAATACATGCAAGTCGAGCGGAGCTTATGAGGTGCTTGCACCGAGTAAGC
TTAGCGGCGAACGGGTGAGTAACACGTGGGCAACCTGCCTGTAAGACCGGGATAACTCACGGAAACGT
GGGCTAATACCGGATAGTTGAGCACCCCGCCTGGGGAGCTTGGGAAAGGCGGAGCAATCTGCCGCTTA
CAGATGGGCCCGCGGCGCATTAGCTAGTTGGTGGGGTAAAGGCCTACCAAGGCGACGATGCGTAGCCG
ACCTGAGAGGGTGATCGGCCACACTGGGACTGAGACACGGCC
>Zotu6805
GAACGCTGGCGGCGTGCCTAACACATGCAAGTCGAACGTGAACGGAGCAATCCTAGTAAAGTGGCGTA
CGGGTGAGTAACACGTAGGTAATCTACCTTACAGTTTGGAATAACCTGACGAAAGTCGGGCTAATACT
AGATAGAGCGACGGAAGCCGTAACGGTTTTTTCGGGAAAGGGAGCTCGATAAGAGCCTTCGCTGTAAG
ATGAGCCTGCGTCTGATTAGTTAGTTGGTGGGGTAATGGCCTACCAAGACGATGATCAGTAGCTGGTC
TGAGAGGATGATCAGCCACACTGGAACTGAAACACGGTC
>Zotu6806
GAACGCTGGCGGCGTGCTTAACACATGCAAGTCGAACGGTGACGGAGAGCTTGCTCTCCTGATCAGTG
GCGAACGGGTGAGTAACACGTGGGCAACCTGCCCCAGACTTCGGGATAACGCTTCGAAAGAGGTGCTA
ATACCGGATACGAACCTCCCTGGCATCTGGGAGGTTGGAAAGTTTTTCGGTCTGGGATGGGCCCGCGG
CCTATCAGCTTGTTGGTGAGGTAACGGCTCACCAAGGCGACGACGGGTAGCCGGCCTGAGAGGGCGAC
CGGCCACACTGGGACTGAGACACGGCC
>Zotu6807
GAATGCTGGCGGCGTGGATAAGGCATGCAAGTCGAACGGTCCGCAAGGACAGTGGCAGACGAGGTAGT
AACACGTACGAACGCACCCCATAGTCTGGCATAGCTTACCGAAAGGTGAGGTAATTCCGGATGGTCCC
TAACAAGGGTAAAGTTTTTTCGCTATGGGAGCGGCGTGCGTAATATCAGCTAGTTGGTAGGGTAATGG
CCTACCAAGGCTACGACGTTTAGGGGAGCTGAGAGGCTGACCCCCACCGATGGGACTGAGACACGGCC
>Zotu6808
GAACGCTGGCGGCATGCCTAACACATGCAAGTCGAACGAGATCTTTCGGGATCTAGTGGCGCACGGGT
GCGTAACGCGTGGGAATCTGCCCTGGGGTTTGGGATAACAGTTGGAAACGACTGCTAATACCAAATGA
CGTCGATAAGACCAAAGATTTATCGCCCTAGGATGAGCCCGCGTCGGATTAGCTAGTTGGTGGGGTAA
AGGCCTACCAAGGCGACGATCCGTAGCTGGTCTGAGAGGATGATCAGCCACACTGGGACTGAGACACG
GCC
>Zotu6809
GAACGCTGGCGGCAGGCTTAACACATGCAAGTCGAACGGGCACTTCGGTGCTAGTGGCAGACGGGTGA
GTAACACGTGGGAACGTACCTTTCGGTTCGGAATAATACAGGGAAACTTGTACTAATACCGGATACGC
CCTTCGGGGGAAAGATTTATCGCCGATTGATCGGCCCGCGTCTGATTAGCTAGTTGGTGAGGTAATGG
CTCACCAAGGCGACGATCAGTAGCTGGTCTGAGAGGATGATCAGCCACACTGGGACTGAGACACGGCC

>Zotu6810
GAACGCTGGCGGCGTGCTTAAGACATGCAAGTCGCACGAGTCATTTCGGTGGCTAGTGGCGCACGGGT
GCGTAACGCGTGACTGACCTACCTCCAAGTTCTGAATAACCTTCCGAAAGGAGGGCTAATACAGGATG
TGAAGCCCCATTCTGATGGGTCTTTAAAGGTTTACCGCTTGGAGATGGGGTCGCGTCCCATCAGCTTG
TTGGTGGGGTAATGGCTTACCAAGGCTACGACGGGTAGCCGGCCTGAGAGGGTGGCCGGCCACAAGGG
CACTGAGACACGGGC
>Zotu6811
GAACGCTGGCGGCATGCCTTACACATGCAAGTCGAACGGCAGCACGGGAGCAATCCTGGTGGCGAGTG
GCGAACGGGTGAGTAATATATCGGAACGTGCCCAGTAGTGGGGGATAGCCCGGCGAAAGCCGGATTAA
TACCGCATACGACCTGTGGGTGAAAGCGGGGGATCGCAAGACCTCGCGCTATTGGAGCGGCCGATATC
AGATTAGCTAGTTGGTGGGGTAAAAGCCCACCAAGGCGACGATCTGTAGCTGGTCTGAGAGGACGACC
AGCCACACTGGGACTGAGACACGGCC
>Zotu6812
CAACGCTGGCGGCGCGCTTAACACATGCAAGTCGCACGAGAAAGTGGAGCAATCCATGAGTAAAGTGG
CGTACGGGTGAGTAACACGTGGATGATCTACCTCTTGGTGGGGAATAACCCTGGGAAACCGGGGCTAA
TACCGCATAAGCCTGCAAAGGGAAAGCAGCAATGCGCCGAGAGAGGAGTCCGCGGCCGATTAGCTAGT
TGGTAGGGTAATGGCCTACCAAGGCAATGATCGGTAGCCGGCCTGAGAGGGCACACGGCCACACTGGC
ACTGAAACACGGGC
>Zotu6814
GAACGCTGGCGGCGTGCTTAACACATGCAAGTCGAACGGTGACCTCGAGAGCTTGCTCTTGGGTGATC
AGTGGCGAACGGGTGAGTAACACGTGAGTAACCTGCCCCAGACTCTGGAATAACCCCGGGAAACCGGA
GCTAATACCGGATACGAAGCGGAGCCGCATGGCTACCACTTGGAAAGTTTTTCGGTCTGGGATGGACT
CGCGGCCTATCAGCTAGTTGGTGAGGTAACGGCTCACCAAGGCGACGACGGGTAGCCGGCCTGAGAGG
GCGACCGGCCACACTGGGACTGAGACACGGCC
>Zotu6816
GAACGCTGGCGGCAGGCTTAACACATGCAAGTCGAGCGGGCCCTTCGGGGTCAGCGGCAGACGGGTGA
GTAACGCGTGGGAACGTACCATTTGCTACGGAATAACTCAGGGAAACTTGTGCTAATACCGTATGAGC
CCCCCTTTAAAATTTCAGGAATCATAAAATGCCCTGGCATTTTATGGGGGGGAAAGATTTATCGGCAA
ATGATCGGCCCGCGTTGGATTAGCTAGTTGGTGGGGTAAAGGCCTACCAAGGCGACGATCCATAGCTG
GTCTGAGAGGATGATCAGCCACACTGGGACTGAGACACGGCC
>Zotu6817
GAACGCTGGCGGCGCGCTTAACACATGCAAGTCGAGCGAGAACCAGGGCTTCGGCCCTGGGGACAGCG
GCGAACGGGTGAGTAACACGTGGATAACCTGCCCTCGGCACCGGGATAGCCCGGGGAAACCCGGATTA
ATACCGGATAGCCCTTTCGGCTTTCGAGCCGGTCGGAAAAGGTAGCTTCGGCCTCTGGCCGAGGATGG
GTCCGCGGCGGATTAGCTTGTTGGTGGGGTAATGGCCTACCAAGGCACCGATCCGTAGCTGGTCTGAG
AGGACGATCAGCCACACTGGGACTGAGACACGGCC
>Zotu6818
GAACGCTTGCGGCGTGCCTAAGAAATGCAAGTCGAGCGGGGGAGCAATCCCTAGCGGCGAACGGTCGC
GTAACACGTAGGCAACCTGCCCGAAAGACCGGGACAACACTTCGAAAGGGGTGCTAATACCGGATGTG
CCCGCTTCTTCACCTGGGGAGGCGAGTAAATCCTCGCAAGGGGGCTTTTGGAGGGTCCTGCGGCCTAT
CAGCTAGATGGTGAGGTAACGGCCCACCATGGCGACGACGGGTAGCTGGTCTGAGAGGATGGTCAGCT
GGACTGGGACTGAGATACGGCC
>Zotu6819
GAACGCTAGCGATGTGCCTAAGGTATGCAAGTCGAACGATGAATTTCTTGTAGCAATACTTGAAATTC
GTAGTGGCAGACGGCTGAGTAATGCGTAGGAATCTACCTTGAAGACGGGTATAATATTGCGAAAGCGG
TACTAATCCCCGATAGTCTCGAAAGAGTAAAGCTTTTGCGCTTCAAGATGAGCCTGCGTCCTATCAGG
TAGTTGGTGGGGTAATTGCCTACCAAGCCTATGACGGGTAGCTGGTCTGAGAGGATGGACAGCCAGAC
TGGGACTGAGAAACGGCC
>Zotu6820
GAACGCTGGCGGCGTGGCTAATACATGCAAGTCGAACGCATTTTCTTTCACCGTAGCTTGCTACACCG
AAAGAAAATGAGTGGCGAACGGGTGAGTAACACGTGGGTAACCTGCCCATCAGCGGGGGATAACACTT
GGAAACAGGTGCTAATACCGCATAATTCCATTTACCGCATGGTAGATGGGTGAAAGGCGCTTTTGCGT
CACTGATGGATGGACCCGCGGTGCATTAGCTAGTTGGTGGGGTAACGGCCTACCAAGGCTGCGATGCA
TAGCCGACCTGAGAGGGTGATCGGCCACACTGGGACTGAGACACGGCC

>Zotu6822
GAACGCTGGCGGCGTGCTTAACACATGCAAGTCGAACGGAAAGGTCTCTTCGGAGACACTCGAGTGGC
GAACGGGTGAGTAACACGTGGGCAATCTGCCCTGCACTTCGGGATAAGCCTGGGAAACTGGGTCTAAT
ACCGGATAGGACCTTTAGACGCATGTCTTTTGGTGGAAAGCTTTTGCGGTGTGGGATGGGCCCGCGGC
CTATCAGCTTGTTGGTGGGGTGATGGCCTACCAAGGCGACGACGGGTAGCCGGCCTGAGAGGGTGTCC
GGCCACACTGGGACTGAGATACGGCC
>Zotu6823
GAACGCTGGCGGCGTGCTTAACACATGCAAGTCGAACGATGAAGCACCAGCTTGCTGGTGTGGATTAG
TGGCGAACGGGTGAGTAACACGTGAGTAACCTGCCCCCTACTCTGGGATAAGCCTGGGAAACTGGGTC
TAATACTGGATATGACCAATCACTGCATGGTGTGTTGGTGGAAAGATTTTTTGGTGGGGGATGGACTC
GCGGCCTATCAGCTTGTTGGTGGGGTAATGGCCTACCAAGGCGACGACGGGTAGCCGGCCTGAGAGGG
CGACCGGCCACACTGGGACTGAGACACGGCC
>Zotu6824
GAACGCTGGCGGCAGGCTTAACACATGCAAGTCGAACGGGCGTAGCAATACGTCAGTGGCAGACGGGT
GAGTAACACGTGGGAACGTACCTTTTGGTTCGGAACAACACAGGGAAACTTGTGCTAATACCGGATAA
GCCCTTACGGGGAAAGATTTATCGCCAAAAGATCGGCCCGCGTCTGATTAGCTAGTTGGTGAGGTAAT
GGCTCACCAAGGCGACGATCAGTAGCTGGTCTGAGAGGATGATCAGCCACATTGGGACTGAGACACGG
CC
>Zotu6825
GAACGCTGGCGGCGTGCTTAACACATGCAAGTCGAACGATGAACTAGGAGCTTGCTCTTGGGGATTAG
TGGCGAACGGGTGAGTAACACGTGAGTAACCTGCCCTTGACTCTGGGATAAGCGTTGGAAACGACGTC
TAATACCGGATACGAGCTTCCACCGCATGGTGAGTTGCTGGAAAGAATTTTGGTCAAGGATGGACTCG
CGGCCTATCAGCTTGTTGGTGAGGTAATGGCTCACCAAGGCGACGACGGGTAGCCGGCCTGAGAGGGT
GACCGGCCACACTGGGACTGAGACACGGCC
>Zotu6826
GAACGCTGGCGGCATGCCTTACACATGCAAGTCGAACGGTAACGGGTTAAGCCGACGAGTGGCGAACG
GGTGAGTAATATATCGGAACGTGCCCAGTCGTGGGGGATAACGTAGAGAAATTTACGCTAATACCGCA
TACGATCTAAGGATGAAAGCGGGGGATCGCAAGACCTCGCGCGATTGGAGCGGCCGATATCAGATTAG
GTTGTTGGTGAGGTAAAAGCTCACCAAGCCGACGATCTGTAGCTGGTTTGAGAGAACGACCAGCCACA
CTGGGACTGAGACACGGCC
>Zotu6827
GAACGCTGGCGGCAGGCTTAACACATGCAAGTCGAACGGAGAGAGGTAGCTTGCTACTGATCTTAGCG
GCGGACGGGTGAGTAATGCTTAGGAATCTGCCTATTAGTGGGGGACAACATTTCGAAAGGAATGCTAA
TACCGCATACGTCCTACGGGAGAAAGCAGGGAATCTTCGGACCTTGCGCTAATAGATGAGCCTAAGTC
GGATTAGCTAGTTGGTGGGGTAAAGGCCTACCAAGGCGACGATCTGTAGCGGGTCTGAGAGGATGATC
CGCCACACTGGGACTGAGACACGGCC
>Zotu6828
GAACGCTGGCGGCGTGCTTAACACATGCAAGTGGAGCGACGAACCAGGGCTTGCCCTGGGGCAGAGCC
GCGAACGGGTGAGTAACACGTGGGTCACCTGCCTCGATGACCGGGACAACCCGAGGAAACTCGGGCTA
ATACCGGATGTGCTCTTCGGAGGAAAGGTAGCTTCGGCTTCCGCATCGAGATGGGCCCGCGGCGCATT
AGCTTGTTGGTGAGGTAACGGCTCACCAAGGCGACGATGCGTAGCTGGTCTGAGAGGACGATCAGCCA
CACTGGGACTGAGACACGGCC
>Zotu6829
GAACGCTGGCGGCGTGCCTAACACATGCAAGTCTACGAGAAAGGGGTAGCAATATCCCGAGTAAAGTG
GCGCACGGGTGAGTAACACGTAGGCAATCTGCCTTTAGGACGGGGATAATCACGCGAAAGCGTGACTA
ATACCCGATGATGCAGCGGCACCGCATGGTGACAGTTGTTAAAAGCCCGCAAGGGCGCCTAGAGATGA
GCCTGCGTCCCATTAGGTAGTTGGCGGGGTAACGGCCCACCAAGCCTTCGATGGGTAGCTGGTCTGAG
AGGATGATCAGCCACACTGGAACTGAGACACGGTC
>Zotu6830
GAACGCTGGCGGCGCGCTTAACACATGCAAGTCGAGCGAGAACCAGTCCTTCGGGGCTGGGGACAGCG
GCGAACGGGTGAGTAACACGTGGGTAATCTGCCCTCGGCACCGGAATAGCCCGGGGAAACCCGGATTA
ATGCCGGATGGCCTCGCCGGCCTTCGGGCTGGCGAGAAAAGGTAGCTTCGGCCTCCGGCCGAGGATGA
GCCCGCGGCCGATTAGCTTGTTAGTGGGGTAACGGCTCACCAAGGCGACGATCGGTAGCTGGTCTGAG
AGGACGATCAGCCACACTGGGACTGAGACACGGCC

>Zotu6831
GAACGCTGGCGGCGTGCCTAATACATGCAAGTTGAGCGGATCTTGTCCTTCGGGATAAGGTTAGCGGC
GGACGGGTGAGTAACACGTAGGCAACCTGCCCTCGAGACTGGGATAACCTCCGGAAACGGATGCTAAT
ACCAGATACGCGATCTTCCCTCCTGGGGAGATCGGGAAAGACGGAGCAATCTGTTACTCGGGGATGGG
CCTGCGGCGCATTAGCTAGTTGGTGAGGTAACGGCTCACCAAGGCGACGATGCGTAGCCGACCTGAGA
GGGTGATCGGCCACACTGGGACTGAGACACGGCC
>Zotu6834
GAACGCTGGCGGCAGGCTTAACACATGCAAGTCGAACGCTCGTAGCAATACGGGAGTGGCAGACGGGT
GAGTAACGCGTGGGAACATACCCTCCGGTTCGGAATAGCTCAGGGAAACTTGGATTAATACCGGATAC
GGCCGAGAGGCGAAAGATTTATCGCCGGAGGATTGGCCCGCGTTCGATTAGCTAGTTGGTGAGGTAAC
GGCTCACCAAGGCGATGATCGATAGCTGGTCTGAGAGGATGATCAGCCACATTGGGACTGAGACACGG
CC
>Zotu6835
GAACGCTGGCGGCATGCCTAACACATGCAAGTCGAACGAAGCCTTCGGGCTTAGTGGCGCACGGGTGC
GTAACGCGTGGGAATCTGCCCTTAGGTACGGAATAACTCATGGAAACGTGTGCTAATACCGTATGATG
TCTTCGGACCAAAGATTTATCGCCTAGGGATGAGCCCGCGTTGGATTAGCTAGTTGGTGAGGTAAAGG
CTCACCAAGGCGACGATCCATAGCTGGTCTGAGAGGATGATCAGCCACACTGGGACTGAGACACGGCC
>Zotu6836
GAACGCTGGCGGTATGCTTAACACATGCAAGTCGAACGGAATGCTTCGGCATTTAGTGGCGGACGGGT
GAGTAACGCGTGAGAATCTGGCTTTAGGTTCGGGACAACAGTGGGAAACTGCTGCTAATACCGAATGT
GCCTTAATCGGTGAAAGATTAATTGCCTGAAGATGAGCTCGCGTCTGATTAGCTAGTTGGTGAGGTAA
AGGCTCACCAAGGCGACGATCAGTAACTGGTCTGAGAGGATGACCAGTCACACTGGGACTGAGACACG
GCC
>Zotu6837
GAACGCTGGCGGCGTGCCTAAGACATGCAAGTCGAATGTGCTCGCAAGAGTAACATGGCGAACGGGTG
AGTAACACGTGGGAACTTCCATTAAGTTTAGAATAACTTTCCGAAAGGAAAGATAATACTAAATGTTA
TCGCAAGATAAAAGGAGCAATCCGCTTAATGCAAGGCCCGCGGTCCATCAGCTAGTTGGTAGGGTAAA
AGCCTACCAAGGCTATGACGGATAGGGGAGCCGAGAGGCCTAACCCCCACACTGGGACTGCGACACGG
CC
>Zotu6839
GAACGCTGGCGGCGTGCTTAACACATGCAAGTCGAACGGAAAGGCCCTTCGGGGTACTCGAGTGGCGA
ACGGGTGAGTAACACGTGGGTGATCTGCCCTGCACTTCGGGATAAGCCTGGGAAACTGGGTCTAATAC
CGGATATACCTCATGGATTGCATGGTTTGTGGGGGAAAGCTTTTGCGGTGTGGGATGGGCCCGCGGCC
TATCAGCTTGTTGGTGGGGTGATGGCCTACCAAGGCGACGACGGGTAGCCGGCCTGAGAGGGTGTCCG
GCCACACTGGGACTGAGATACGGCC
>Zotu6840
GAATGCTGGCGGTATGGATAAGGCATGCAAGTCGAACGGCCCGCAAGGGCAGTGGCAGACGAGGTAGT
AACACGTACGAACGCACCCCAAAGTCAGGGATAGCCAGCCGAAAGGTTGGGTAATACTTGATGGTCCC
GAAAGGGTAAAGATTTATCGCTTTGGGAGCGGCGTGCGCAATATCAGCTAGTTGGTAAGGTAACGGCT
TACCAAGGCTACGACGTTTAGGGGAGCTGAGAGGCTGACCCCCACCGATGGGACTGAGACACGGCC
>Zotu6841
GAACGCTGGCGGTGTGCCTAAAACATGCAAGTCGAACGGGGTCCTTCGGGACTCAGTGGCGAACGGCG
GAGTAATACATGAACAACGTGCCCTTAAGCCTGGGATATACAGAGGAAACTCTGGGCAATACCAGATG
TGGTGAAGTCTTGGCATCAAGACATCATTAAACGGTTTTTCGCTTTTGGAGCGGTTCATGGCCTATCA
GGTAGTTGGTGAGGTAACGGCTCACCAAGCCGACGACGGGTAGCGGGTCTGAGAGGATGGTCCGCTCG
AGTGGGACTGAGACACGGCC
>Zotu6846
GAACGCTGGCGGCGTGCCTAATACATGCAAGTCGAGCGGATCTTTAGAAGCTTGCTTCTAAAGATTAG
CGGCGGACGGGTGAGTAACACGTGGGCAACCTGCCTGTAAGACTGGGATAACTGCGGGAAACCGAGGC
TAATACCGGATAATCCTTTTCCTCACATGAGGAAAAGCTGAAAGACGGCGCAAGCTGTCACTTACAGA
TGGGCCCGCGGCGCATTAGCTAGTTGGTGAGGTAATGGCTCACCAAGGCAACGATGCGTAGCCGACCT
GAGAGGGTGATCGGCCACACTGGGACTGAGACACGGCC
>Zotu6847
GAACGCTAGCTACAGGCTTAACACATGCAAGTCGAGGGGCATCAGTTTGGTTTGCTTGCAAACCAAAG

CTGGCGACCGGCGCACGGGTGAGTAACACGTATCCAACCTGCCTCATACTCGGGGATAGCCTTTCGAA
AGAAAGATTAATATCCGATAGCATATATTTCCCGCATGGGTTTTATATTAAAGAAATTCGGTATGAGA
TGGGGATGCGTTCCATTAGTTTGTTGGCGGGGTAACGGCCCACCAAGACTACGATGGATAGGGGTTCT
GAGAGGAAGGTCCCCCACATTGGAACTGAGACACGGTC
>Zotu6848
GAACGTTAGCGGCGCGCCTAAGACATGCAAGTCGAGCGAGAAAGGGCTTCGGCCCCGGTACAGCGGCG
CACGGGTGAGTAACACGTAGGTAATCAACCCTCGAGTGGTGGACAACTCTCCGAAAGGAGAGCTAATA
CAGCATGACGTGGCGGTCTTCCGAGATCGTTTCCAAAGCGGGCCTCTTCATGAAAGCTCGCGCTCGAG
GATGAGCCTGCGGCCCATCAGCTAGTTGGCGGGGTAACAGCCCACCAAGGCGTAGACGGGTAGCTGGT
CTGAGAGGATGAACAGCCACACTGGAACTGAGACACGGTC
>Zotu6849
GAACGCTGGCGGCGTGCTTAACACATGCAAGTCGAACGGTGACGAGCAGCTTGCTGCTCTGATCAGTG
GCGAACGGGTGAGTAACACGTGAGCAACCTACCCTTGACTCCGGGATAACAGCTGGAAACAGCTGCTA
ATACCGGATATGACCTCGACCGACATCAGTTGAGGTGGAAAGTTCTGACGGTCAAGGATGGGCTCGCG
GCCTATCAGCTAGTTGGTGAGGTAACGGCTCACCAAGGCTTCGACGGGTAGCCGGCCTGAGAGGGCGA
CCGGCCACACTGGGACTGAGATACGGCC
>Zotu6851
GAACGCTGGCGGCGTGCTTAACACATGCAAGTCGAACGATGATGGCCCGGGCTTGCCCGGGTCTGATT
AGTGGCGAACGGGTGAGTAACACGTGAGTAACCTGCCCCTGACTTCGGGATAACCGCGAGAAATCGTG
GCTAATACCGGATATGACACCCCGGCGCATGGTGGGGTGTGGAAAGACTGTGTCGGTTGGGGATGGGC
TCGCGGCCTATCAGCTTGTTGGTGGGGTGATGGCCTACCAAGGCGACGACGGGTAGCCGGCCTGAGAG
GGTGACCGGCCACACTGGGACTGAGACACGGCC
>Zotu6852
GAACGCTGGCGGCATGCCTAACACATGCAAGTCGAACGGGGTTTCTTCGGAAACCTAGTGGCGAACGG
GTGAGTAACGCGTAGGCAACCTGCCCTCCGGATGGGGACAACATTCCGAAAGGAGTGCTAATACCGAA
TGTGAAGCGGTTATCGCATGATGACCGTTTGAAAGATGGCCTCTATTTATAAGCTATCGCCGGGGGAT
GGGCCTGCGTCTGATTAGCCAGTTGGCGGGGTAACGGCCCACCAAAGCGACGATCAGTAGCCGGTCTG
AGAGGATGAACGGCCACATTGGGACTGAGACACGGCC
>Zotu6853
GAACGCTGGCGGCATGCCTAACACATGCAAGTCGAACGAGACCTTCGGGTCTAGTGGCGCACGGGTGC
GTAACGCGTGGGAATCTGCCCTTGGGTACGGAATAACAGTTAGAAATGACTGCTAATACCGTATAATG
ACTTCGGTCCAAAGATTTATCGCCCAGGGATGAGCCCGCGTAGGATTAGCTTGTTGGTGAGGTAAAAG
CTCACCAAGGCGACGATCCTTAGCTGGTCTGAGAGGATGATCAGCCACACTGGGACTGAGACACGGCC
>Zotu6854
GAACGCTGGCGGCATGCCTAACACATGCAAGTCGAACGAGACCTTCGGGTCTAGTGGCGCACGGGTGC
GTAACGCGTGGGAACCTGCCTTTAGGTTCGGAATAACAGTGAGAAATTACTGCTAATACCGGATAATG
TCTTCGGACCAAAGATTTATCGCCTTTAGATGGGCCCGCGTTGGATTAGCTAGTTGGTGGGGTAAAGG
CCTACCAAGGCGACGATCCATAGCTGGTCTGAGAGGATGATCAGCCACACTGGGACTGAGACACGGCC
>Zotu6855
GAACGCTGGCGGCGTGCCTAATACATGCAAGTCGAACGCTGGAGATCTAGCTTGCTAGATCGAAGGAG
TGGCGAACGGGTGAGTAATACATAAGCAACCTACCCACGAAGACTGGGATAATCTCTGGAAACGGGGA
CTAATACCGGATAGGTAATCGGAAGGCATCTTCTGGTTATTAAAGGTTAAAAACACTGGTGGATGGGC
TTATGGCGCATTAGTTAGTTGGTGAGGTAACGGCCCACCAAGACGATGATGCGTAGCCGGCCTGAGAG
GGTGAACGGCCACATTGGGACTGAGACACGGCC
>Zotu6857
GAACGCTAGCGGGAGGCTTAACACATGCAAGCCGAGGGGTAGAATTAGCTTGCTAATTTGAGACCGGC
GCACGGGTGCGTAACGCGTATGCAACTTGCCCTACTGAAAAGGATAGCCCAGAGAAATTTGGATTAAT
ACTTTATAATAGAAGACTTGGCATCAAGTTTTTTTGAAAGATTTATCGCAGTAGGATAGGCATGCGTA
AGATTAGATAGTTGGTGAGGTAACGGCTCACCAAGTCGACGATCTTTAGGGGGCCTGAGAGGGTGAAC
CCCCACACTGGTACTGAGACACGGAC
>Zotu6859
GAACGCTGGCGGCGTGCCTAATACATGCAAGTCGAACGGACATAGCAATATGTTAGTGGCGACCGGGT
GCGTAACACGTAGCTGACCTACCCTGGCGCGGGGGACAACAGTTCGAAAGGACTGCTAATACCGCATA
ATCCGGCAGGAACTAGACCCTTGTCGGGAAACTTCCGGGGCGCTGGGAGGGGGCTGCAGCCCATCAGG

TAGTTGGTGAGGTAATGGCTCACCAAGCCGACGACGGGTAGGGGGCCTGAGAGGGTGGTCCCCCACAC
TGGGACTGAAAGACGGCC
>Zotu6861
GAACGCTGGCGGCGTGCTTAACACATGCAAGTCGAACGGAAAGGCCCCCACCTTGGTGGGGGTGCTCG
AGTGGCGAACGGGTGAGTAACACGTGGGTGATCTGCCCTGTACTTCGGGATAAGCTTGGGAAACTGGG
TCTAATACCGGATAGGACCATGGTTTAGTGTCTCATGGTGGAAAAATTATTGTTTTGGTGCAGGATGA
GCTCGCGGCCTATCAGCTTGTTGGTGGGGTAATGGCCTACCAAGGCGTCGACGGGTAGCCGGCCTGAG
AGGGTGGACGGCCACATTGGGACTGAGATACGGCC
>Zotu6862
GAACGCTGGCGGCGTGCTTAACACATGCAAGTGGAGCGACGAACCAGGGCTTGCCCTGGGGCAGAGCC
GCGAACGGGTGAGTAACACGTGGGTAACCTACCCCGATGACCGGGACAACCCGAGGAAACTCGGGCTA
ATACCGGATGTGCTCTTGGTTCATGAGGACCTTGAGTAAAGATAGCTTCGGCCTTCGCATCGGGACGG
GCCCGCGGCCCATTAGCTTGTTGGTGGGGTAACGGCCTACCAAGGCGACGATGGGTAGCTGGTCTGAG
AGGACGATCAGCCACACTGGGACTGAGACACGGCC
>Zotu6863
GAACGCTGGCGGCATGCCTTACACATGCAAGTCGAACGGTAACAGCTCTTCGGAGGCTGACGAGTGGC
GAACGGGTGAGTAATACATCGGAACGTGCCCGATCGTGGGGGATAACGGAGCGAAAGCTTTGCTAATA
CCGCATACGATCTACGGATGAAAGCAGGGGACCCTCGGGCCTTGCGCGAACGGAGCGGCCGATGGCAG
ATTAGGTAGTTGGTGGGATAAAAGCTTACCAAGCCGACGATCTGTAGCTGGTCTGAGAGGACGACCAG
CCACACTGGGACTGAGACACGGCC
>Zotu6865
GAACGCTAGCGGCAGGCTTAACACATGCAAGTCGAGGGGTATGGTTCTTCGGAACCAGAGACCGGCGC
ACGGGTGCGTAACGCGTATGCAATCTGCCTTTCACAAAGGAATAGCCCAGAGAAATTTGGATTAATGC
CTTATAGCATTACGACTTGGCATCAAGATGTAATTAAAGATTTATCGGTGAAAGATGAGCATGCGTCC
CATTAGCTAGTTGGTAAGGTAACGGCTTACCAAGGCGATGATGGGTAGGGGTCCTGAGAGGGAGATCC
CCCACACTGGTACTGAGACACGGAC
>Zotu6868
GAACGCTGGCGGTGTGCTTCACACATGCAAGTCGAACGGGGTAGCAATACCCAGTGGCGGACGGGTGA
GTAACGCGTAGAAATCTGCCTTGAGATGGGGGATAACAGTCCGAAAGGATTGCTAATACCGCATATGT
ATGTGCCCGGACACGGGCGTATATGAAAGGAGTAATCCGTCTTAAGATGAGTCTGCGTCCGATTAGCT
AGTTGGTAGGGTAAAGGCCTACCAAGGCGACGATCGGTAGCTGGTCTGAGAGGATGATCAGCCACAAT
GGGACTGAGACACGGCC
>Zotu6869
GAACGCTGGCGGCGTGCCTAATACATGCAAGTCGAGCGAGCTTGCCTAGATGATTTTAGTGCTTGCAC
TAAATGAAACTAGATACAAGCGAGCGGCGGACGGGTGAGTAACACGTGGGTAACCTGCCCAAGAGACT
GGGATAACACCTGGAAACAGATGCTAATACCGGATAACAACACTAGACGCATGTCTAGAGTTTGAAAG
ATGGTTCTGCTATCACTCTTGGATGGACCTGCGGTGCATTAGCTAGTTGGTAAGGTAACGGCTTACCA
AGGCAATGATGCATAGCCGAGTTGAGAGACTGATCGGCCACATTGGGACTGAGACACGGCC
>Zotu6872
GAACGCTGGCGGCGTGCTTAACACATGCAAGTCGAACGGAAAGGCCGAAGCTTGCTTCGGTACTCGAG
TGGCGAACGGGTGAGTAACACGTGGGTGATCTGCCCTGTACTTCGGGATAAGCTTGGGAAACTGGGTC
TAATACCGGATATGACGATCTCTTGGATGGGGTTGTGGAAAGCTTTTGCGGTATGGGATGAGCTCGCG
GCCTATCAGCTTGTTGGTGGGGTAATGGCCTACCAAGGCGTCGACGGGTAGCCGGCCTGAGAGGGTGT
ACGGCCACATTGGGACTGAGATACGGCC
>Zotu6874
GAACGCTGGCGGCGTGCATAACACATGCAAGTCGAACGATGAAACTCTAAAGATTTCTTCGGAATGAA
TTAGAATGGATTAGTGGCGAACGGGTGAGTAACACGTGAGTAACCTGCCTTAGAGAAGGGGATAGCCT
TTGGAAACGAAGAATAATACCCTATGAAATGATAATATCGCATGATAGTATCATCAAAGATTTATCGC
TCTAAGATGGACTTGCGCCTGATTAGCTAGTTGGTGGAGTAACAGCCCACCAAGGCAACGATCAGTAG
CCGGCTTGAGAGAGTGTACGGCCACATTGGGACTGAGACACGGCC
>Zotu6876
GAACGCTAGCGGCATGCCTAAGGTATGCAAGTCGTGCGCAAAACCACCTTCGGGTGGTGGAAAGCGGC
GGACGGGTGAGTAACGCGTAGAAACATACCCCAGAGTGAGGGATAATTCCGGGAAACCGGGACTAATA
CCGCATATTGTCTAAGGACAAAAGATTTATCGCTTTGGGAATGGTCTGCGTCCGATTAGCTTGTTGGT

GAGGTAAAGGCTCACCAAGGCTATGATCGGTAGCTGGTTTGAGAGGATGATCAGCCACACTGGAACTG
AGACACGGTC
>Zotu6877
GAACGCTGGCGGCGTGCTTAACACATGCAAGTCGAGCGGTAAGGCTCCTTCGGGAGTACACGAGCGGC
GAACGGGTGAGTAACACGTGAGTAATCTGCCCCTCACTTCGGGATAAGCCTCGGAAACGAGGTCTAAT
ACCGGATACGACCAACTCTTGCATGAGATGTTGGTGGAAAGTTTTTTTCGGTGGGGGATGTGCTCGCG
GCCTATCAGCTTGTTGGTGGGGTAATGGCCTACCAAGGCTTCGACGGGTAGCCGGCCTGAGAGGGTGA
CCGGCCACACTGGGACTGAGACACGGCC
>Zotu6878
GAACGCTAGCGGCAGGCCTAATACATGCAAGTCGAGCGGATTGTAGCAATACAGTTAGCGGCGCACGG
GTGAGTAACGCGTACACAATCTACCTTCAACAGGGGAATAGCCTCGGGAAACTGGGAGTAATACCCCA
TAGCATCTTTGGATCGCATGATCTGATTATTAAAGTTACGGCGGTTGAAGATGAGTGTGCGTCTGATT
AGATTGTTGGTGAGGTAACGGCTCACCAAGTCGATGATCAGTAAGGGGCGTGAGAGCGTGGCCCCTCA
CATGGACACTGAGACACGGGT
>Zotu6879
GAACGCTGGCAGTGTGCCTAAGACATGCAAGTCGAACGGAGGTCTTCGGACCTTAGTGGCGAACGGGT
GAGTAACACGTGGGCGACCTACCTCCAAGTACGGCATAACCCCGGGAAACCGGAGCTAATTCCGTATG
TGCTCGTATCTCGTTTGATACGCGTAAAGATTTATCGCTTGGAGATGGGCCCGCGGCGCATCAGCTTG
TTGGTGGGGTAACGGCCTACCAAGGCGACGACGCGTAGCCGACCTGAGAGGGTGACCGGCCACAGGGG
CACTGAGACACGGGC
>Zotu6880
GAACGCTGGCGGCGTGCTTAACACATGCAAGTCGAACGGTGACAGGGGAGCTTGCTCCCCTCGATCAG
TGGCGAACGGGTGAGTAACACGTGAGTAACCTGCCCCAGACTCTGGGATAACATCGGGAAACCGGTGC
TAATACCGGATATGAGACGAGACTGCATGGTCATCGTCTGGAAAGATTTATCGGTCTGGGATGGACTC
GCGGCCTATCAGCTAGTTGGTGAGGTAACGGCTCACCAAGGCGACGACGGGTAGCCGGCCTGAGAGGG
CGAACGGCCACACTGGAACTGAGACACGGTC
>Zotu6882
CAACGCTGGCGGCGCGCTTAACACATGCAAGTCGAACGAGAAAGTGGAGCAATCCATGAGTAAAGTGG
CGCACGGGTGAGTAACACGTGGGTAATCTGCCTCTGGGTGGGGAATAACTCTGGGAAACCGGAGCTAA
TACCGCATAAGCCCCCATTGAATAAATGGACGGGAAAGGTGAAAATCGCCGAGAGAGGAGCCCGCGGT
CGATTAGCTAGTTGGTGAGGTAAAGGCTCACCAAGGCTGTGATCGATAGCCGGCCTGAGAGGGCACAC
GGCCACACTGGCACTGAAACACGGGC
>Zotu6883
GAACGCTGGTGGAGTGTTTTATACATGCAAGTCGAGCGAGGACAGTAGCAATACTGAGCCGAGCGGCG
AACGGGTGAGTAACACGTAAGCAATCTGCCCTGTAATCGGGAACAACCGGGCCAACGCCCGGCTAATG
CCGGATATCGTGGCGAGTCCCCTGACTCGTTGACGAAAGATTTATCGTTACAGGATGAGCTTGCGTCT
GATTAGCTAGTTGGCGGGGTAACGGCCCACCAAGGCGACGATCAGTAGCCGGCCTGAGAGGGTGATCG
GCCACATTGGGACTGAGATACGGCC
>Zotu6886
GAACGCTGGCGGTGTGCCTAATACATGCAAGTCGAACGAGACCTTCGGGTCTAGTGGCGGACGGGTGA
GTAACACGTTGGTACCTACCCCTAAGTGGGGAATAACTGGCCGAAAGGTCGGCTAATACCGCATAATC
CCGCAAGGGTAAAGCAGTAATGCGCTTAGGGAGGGGCCTGCGTCTGATTAGCTAGTTGGTAGGGTAAA
AGCCTACCAAGGCGACGATCAGTAGCTGGATCGAGAGGTTGACCAGCCACACTGGGACTGAGATACGG
CC
>Zotu6887
GAACGCTGGCGGCGTGCTTAACACATGCAAGTGGAGCGACGAACCAGGGCTTGCCCTGGGGCAAAGCC
GCGAACGGGTGAGTAACACGTGGGTAACCCACCCCGATGATCGGGACAACCCGAGGAAACTCGGGCTA
ATACCGAATGTGCCTGAAGTCCATGAGGACTCCAAGTAAAGATAGCTTCGGCCTTCGCATCGGGACGG
GCCCGCGGCCCATTAGCTTGTTGGTGAGGTAACGGCTCACCAAGGCGACGATGGGTAGCTGGTCTGAG
AGGACGATCAGCCACACTGGGACTGAGACACGGCC
>Zotu6888
GAACGCTAGCGGCAGGCTTAACACATGCAAGTCGAGGGGTAGTATAGCAATATATGAGACCGGCGCAC
GGGTGCGTAACGCGTATGCAATCTACCTTTTACAGGGGAATAGCCCAGAGAAATTTGGATTAATGCCC
CATGGTATTATAGAGTGGCATCACTTTATAATTAAAGTTCCAACGGTAAAAGATGAGCATGCGTCCCA

TTAGTTAGTTGGTAAGGTAACGGCTTACCAAGACGATGATGGGTAGGGGTCCTGAGAGGGAGATCCCC
CACACTGGTACTGAGACACGGAC
>Zotu6889
GAACGCTGGCGGCGTGGATAAGGCATGCAAGTCGAACGGTGCAAGGTAGCAATACTTTGCATAGTGGC
GAACGAGTTAGTAACACCTTGGTACATACCTCGAAGTCGGGCACAACTAGTCGAAAGATTAGCTAATT
CCCGATAGTCTCGCAAGAGTAAAGTCGCAAGACGCTTTGAGAGTGGCCTAGGTCCTATCAGCTTGTTG
GCGGGGTAAAAGCCCACCAAGGCTATGACGGGTAGGGGGCGTGAGAGCGCGACCCTCAACGACGAAAC
TGAGATACGGTT
>Zotu6890
TAACGCTGGCGGTATGCCTAACACATGCAAGTCGAACGGACCTTTATTTTTGAGAATCTCTCTTTATT
GGGGCGTCTCATAAATAATGGTTAGTGGCGGACGGGTGAGTAACACGTTGGTACCTGCCCTCTAGTGG
GGAATAATTCGCCGAAAGGCGGACTAATACCGCATGTGATCTACGGATCAAAGCCTTCGGGCGCTAGA
GGAGGGGCCTGCGGTTGATTAGCTAGTTGGTGAGGTAAAAGCTCACCAAGGCGACGATCAATAGCTGG
ATCGAGAGGTTGACCAGCCACACTGGGACTGAGATACGGCC
>Zotu6891
GAACGCTGGCGGCGTGCTTAACACATGCAAGTCGAACGATGATGCTCAAGCTTGCTTGGGTTGATTAG
TGGCGAACGGGTGAGTAACACGTGGGTGATCTGCCCTGCACTTCGGGATAAGCCTGGGAAACTGGGTC
TAATACCGGATATGACCCTCCTTTAGTGTGGGGGGTGTAAAGCTTTTGCGGTGTGGGATGAGCCTGCG
GCCTATCAGCTTGTTGGTGGGGTAATGGCCTACCAAGGCGTCGACGGGTAGCCGGCCTGAGAGGGTGA
TCGGCCACATTGGGACTGAGACACGGCC
>Zotu6892
GAACGCTGGCGGAATGCTTTACACATGCAAGTCGAACGGCAGCACGAGGGCAACCTTGGTGGCGAGTG
GCGAACGGGTGAGTAATGTATCGGAACGTGCCCAGTCGTGGGGGATAACGTAGCGAAAGCTACGCTAA
TACCGCATACGATCTATGGATGAAAGCGGGGGATCGCAAGACCTCGCGCGATTGGAGCGGCCGATATC
AGATTAGGTAGTTGGTGGGGTAAAGGCCTACCAAGCCGACGATCTGTAGCTGGTCTGAGAGGACGACC
AGCCACACTGGGACTGAGACACGGCC
>Zotu6893
GAACGCTGGCGGCGTGCTTAACACATGCAAGTCGAGCGGTAAGGCCCTTCGGGGTACACGAGCGGCGA
ACGGGTGAGTAACACGTGGGTGACCTGCCCTCAGCTCTGGGATAAGCCTGGGAAACCGGGTCTAATAC
CGGATAGGACTACATGCTTCATGGTGTGTGGTGGAAAGTTTTTTCGGCTGGGGATGGGCCCGCGGCCT
ATCAGCTTGTTGGTGGGGTGATGGCCTACCAAGGCGACGACGGGTAGCCGGCCTGAGAGGGCGACCGG
CCACACTGGGACTGAGACACGGCC
>Zotu6894
GAACGCTGGCGGCATGCCTAACACATGCAAGTCGAACGAGACCTTCGGGTCTAGTGGCGCACGGGTGC
GTAACGCGTGGGAATCTGCCCTTGGGTTCGGAATAACTCAGAGAAATTTGAGCTAATACCGGATGATG
ACGTAAGTCCAAAGATTTATCGCCCAGGGATGAGCCCGCGTAGGATTAGCTAGTTGGTGGGGTAAAGG
CCTACCAAGGCGACGATCCTTAGCTGGTCTGAGAGGATGATCAGCCACACTGGGACTGAGACACGGCC
>Zotu6896
GAACGCTATCGGTATGCTTAACACATGCAAGTTGAACGGATTATTTAGGGCTTGCTCTAAATAATTAG
TAGCAAACGGGTGAGTAACACGTGGGAATCTGCCCATCAGTACGGAATAACACTTAGAAATAAATGCT
AATACCGTATATTCTCTTAGGAGGAAAGATTTATCGCTGATGGATGAGCCCGCGTCAGATTAGGTAGT
TGGTAGGGTAATGGCCTACCAAGCCAACGATCTGTAGCTGGTCTGAGAGGATGATCAGCCACACTGGG
ACTGAGACACGGCC
>Zotu6897
GAACGCTGGCGGCGTGCTTAACACATGCAAGTCGAACGGTGAAGCCCAGCTTGCTGGGTGGATCAGTG
GCGAACGGGTGAGTAACACGTGAGCAATCTGCCCCTGACTCTGGGATAAGCGCTGGAAACGGCGTCTA
ATACTGGATACGAGCCGTGAAGGCATCTTCAACGGTTGGAAAGATTTTTCGGTCAGGGATGAGCTCGC
GGCCTATCAGCTTGTTGGTGAGGTAATGGCTCACCAAGGCGTCGACGGGTAGCCGGCCTGAGAGGGTG
ACCGGCCACACTGGGACTGAGACACGGCC
>Zotu6900
CAACGCTGGCGGCGTGCCTAACACATGCAAGTCGAACGCGAAAGGGACTTCGGTTCTTAGTAGAGTGG
CGTACGGGTGAGTAACGCGTGGGTAATCTACCCTTGGGTGGGGAATAACTAACCGAAAGGTTAGCTAA
TACCGCATAATGCAGCAATGTTAAAGCAGCAATGCGCCTAAGGAGGAGCCTGCGTCTGATTAGCTAGT
TGGTGAGGTAATGGCTCACCAAGGCTTCGATCAGTAGCTGGTCTGAGAGGACGGCCAGCCACAATGGC

ACTGAAACACGGGC
>Zotu6901
GAACGCTGGCAGCATGCTTTACACATGCAAGTCGAACGGTAACAGGTCTTCGGATGCTGACGAGTGAC
GAACGGGTGAGTAATACATCGGAACGTGCCTAGTAGTGGGGGATAACTACTCGAAAGAGTAGCTAATA
CCGCATGAGATCTACGGATGAAAGCAGGGGACCTTCGGGCCTTGTGCTACTAGAGCGGCTGATGGCAG
ATTAGGTAGTTGGTGGGGTAAAGGCTTACCAAGCCTGCGATCTGTAGCTGGTCTGAGAGGACGACCAG
CCACACTGAGACTGAGACACGGCC
>Zotu6902
GAACGCTGGCGGCGTGCCTAATACATGCAAGTCGAGCGGAGTTATTCCTTCGGGGATAGCTTAGCGGC
GGACGGGTGAGTAACACGTAGGCAACCTGCCTGCAAGACCGGGATAACATTCGGAAACGGATGCTAAT
ACCGGATACGCGATTTCCTCGCATGGGGGAATCGGGAAAGGCGGCGCAAGCTGTCACTTGCAGATGGG
CCTGCGGCGCATTAGCTAGTTGGTGGGGTAACGGCTCACCAAGGCGACGATGCGTAGCCGACCTGAGA
GGGTGATCGGCCACACTGGGACTGAGACACGGCC
>Zotu6903
GAACGCTAGCGATAGGCTTAACACATGCAAGTCGAGGGGCAGCAGGGACTTCGGTCTGCTGGCGACCG
GCGCACGGGTGCGTAACGCGTATGCAACTTGCCCTGTAGAGGAGGATAACCCGCTGAAAGGCGGACTA
ATACTGCGTACTCTCATTGAACGGCATCGTTTGATGAGGAAAGAAATTCGCTACAGGATAGGCATGCG
TCCCATTAGCTAGTTGGTGAGGTAACGGCTCACCAAGGCGACGATGGGTAGGGGAACTGAGAGGTTTA
TCCCCCACACTGGTACTGAGACACGGAC
>Zotu6904
GAACGCTGGCGGCGCGCCTAACACATGCAAGTCGAACGGAGCTTAGAGAGCTTGCTTTTTAAGCTTAG
TGGCGAACGGGTGAGTAACGCGTGAGTAGCCTGCCCTAGAGTGGGGGACAACAGTTGGAAACGACTGC
TAATACCGCATAAGCCCACGGTACCGCATGATACTGAGGGAAAAGGATTTATTCGCTTTAGGATGGAC
TCGCGTCCAATTAGCTAGTTGGTGAGGTAACGGCTCACCAAGGCGACGATTGGTAGCCGGACTGAGAG
GTTGAACGGCCACATTGGGACTGAGACACGGCC
>Zotu6905
GAACGCTGGCGGCGTGCTTAACACATGCAAGTCGAGCGACGCGCCTTCGGGTGACGGAGCGGCGAACG
GGTGAGTAACACGTGGGCAACCTGCCCTCAGCTCTGGGATAACTCCGGGAAACCGGGGCTAATACCGG
ATACGACCTCGGAGGGCATCTTCTGGGGTGGAAAGTTCTTCGGCTGGGGATGGGCCCGCGGCCTATCA
GCTTGTTGGTGGGGTGATGGCTCACCAAGGCGACGACGGGTAGCCGGCCTGAGAGGGCGACCGGCCAC
ACTGGGACTGAGACACGGCC
>Zotu6906
GAACGCTGGCGGCGTGCCTAATACATGTAAGTCGAGCGAACGGACAGGAGTGCTTGCACTCCTTGAAG
TTAGCGGCGGACGGGTGAGTAACACGTGGGTAACCTACCTGTAAGACTGGGATAACTTCGGGAAACCG
GAGCTAATACCGGATAATATTTTCCACCTCATGGTGGAATAGTGAAAGACGGTTTCGGCTGTCACTTA
CAGATGGACCCGCGGCGCATTAGCTAGTTGGTGAGGTAACGGCTCACCAAGGCGACGATGCGTAGCCG
ACCTGAGAGGGTGATCGGCCACACTGGGACTGAGACACGGCC
>Zotu6907
GAACGCTGGCGGCGTGCCTAATACATGCAAGTCGAACGAGAAAGTGTAGCAATACATTAGTAAAGTGG
CAGACGGGTGAGTAATACATAGGTAACCTACCTCCGAGATGGGGATAACCTTCCGAAAGGAGGGCTAA
TACCGAATGAGACCACGAGTACTCTGTACTTGAGGCCAAAGGCGACCTCTACTTGTAAGTTGTCGCTT
GGAGATGGGCTTATGCACCATTAGCTTGTTGGTGAGGTAACGGCTCACCAAGGCTGTGATGGTTAGCT
GATCTGAGAGGTTGATCAGCCACACTGGAACTGAGACACGGTC
>Zotu6908
GAACGCTGGCGGCGTGCCTAATACATGCAAGTCGAGCGAATTGATGAGAAGCTTGCTTCTCTGATGTT
AGCGGCGGACGGGTGAGTAACACGTGGGCAACCTGCCCTATAGTTGGGGATAACTCCGGGAAACCGGG
GCTAATACCGAATAATACATTTCATCTCCTGGTGAAATGTTGAAAGATGGCTTCTTGCTATCACTATA
GGATGGGCCCGCGGCGCATTAGCTAGTTGGTGAGGTAATGGCTCACCAAGGCGACGATGCGTAGCCGA
CCTGAGAGGGTGATCGGCCACACTGGGACTGAGACACGGCC
>Zotu6911
GAACGCTGGCGGCGCGTTTTAAGCATGCAAGTCGGACGGCAAGGGCTGTAGCAATATGGCCCCTAGAG
TGGCGAACGGGTGAGTAACACGTAGGTGATCTACCTCTGGATGGGGGATAGCCCGTGGAAACACGGGG
TAATACCGCATGAGGCCTTGTGGGTAAAGCCATGAGGTGAAAGGTGCAATTGCACCGTCTGGGGATGA
GCCTGCGGTCCATTAGTTAGTTGGCGGGGTAAAGCCCACCAAGACTCCGATGGATAGCCGGCCTGAG

AGGGTGTACGGCCACACTGGAACTGAGACACGGTC
>Zotu6912
GAACGCTGGCGGCATGCCTAACACATGCAAGTCGAACGGCAGCACAAAAGGGGTAACCCTTTGGGTGG
CGAGTGGCGAACGGGTGCGTAACGCGTAGGAATCTACCTTTTAGTGGGGGATAACCCGAGGAAACTCG
GGCTAATACCGCATATTCTCTAAGGAGGAAAGCAGGGGACCTTTCGGGGCCTTGCGCTATTAGATGAG
CCTGCGTCGGATTAGCTAGTTGGTGAGGTAATGGCTCACCAAGGCGATGATCCGTAGCTGGTTCGAGA
GGACGACCAGCCACACTGGGACTGAGACACGGCC
>Zotu6913
GAACGCTGGCGGCATGCCTAACACATGCAAGTCGAACGAAGGCTTCGGCCTTAGTGGCGCACGGGTGC
ATAACGCGTGGGAATCTGCCCCTTGGTTCGGAATAACAGTTAGAAATGACTGCTAATACCGGATGATG
ACGTAAGTCCAAAGATTTATCGCCGAGGGATGAGCCCGCGTAGGATTAGCTAGTTGGTGTGGTAAGAG
CGCACCAAGGCGACGACCCTTAGCTGGTCTGAGAGGATGATCAGCCACACTGAGACTGAGACACGGCC
>Zotu6915
GAACGCTAGCGGCAGGCATAATACATGCAAGGCGAGGGGGTAGCAATACCACCGTCGTACGGGTGCGT
AACACGTATGCAACCTGCCCTTCACTGGGGGATAGCCTGGGGAAACTCGGATTAATACCGCATAAGAC
AGTAGACTGGCATCAGTTAGCTGTTAAAGATTTATTGGTGAAGGATGGGCATGCGTCTGATTAGCTAG
TTGGCGGGGTAAAGGCCCACCAAGGCTACGATCAGTAGGGGTTCTGAGAGGATTGGCCCCCACACTGG
CACTGAGATACGGGC
>Zotu6917
GAACGCTGGCGGCGTGCCTAACACATGCAAGTCGAGCGAGAAAAGGGGGGCAACTCCCTGAGTAAAGC
GGCGAACGGGTGAGTAACACGTAGGTGATCTACCCTCGAGTCCAGGATAACCTACCGAAAGGTGGGCT
AATACCGGATATGACCACGGGAGCGACGGCTCCTGCGGTGAAAGGTGGCCTCTGCTTGCAAGCTACCG
CTCAAGGATGAGCCTGCGCGCCATTAGCTAGTTGGCGGGGTAACGGCCCACCAAGGCGATGATGGCTA
GCTGGTCTGAGAGGATGGCCAGCCACACTGGGACTGGAACACGGCC
>Zotu6918
GAACGCTGGCGGCAGGCTTAACACATGCAAGTCGAACGCTCATCTTCGGATGGGAGTGGCAGACGGGT
GAGTAACACGTGGGAACGTACCCTTCGATCTGGGATAACTTCGGGAAACTGGAGCTAATACCGGATAC
GCCCTTTTGGGGAAAGGTTTACTGTCGAAGGATCGGCCCGCGTCTGATTAGCTAGTTGGTGGGGTAAC
GGCCTACCAAGGCGACGATCAGTAGCTGGTCTGAGAGGATGATCAGCCACACTGGGACTGAGACACGG
CC
>Zotu6919
GAACGCTGGCGGCGTGCCTAATACATGCAAGTCGAGCGGATCTTACCTTCGGGTAAGGTTAGCGGCGG
ACGGGTGAGTAACACGTGGGTAACCTGCCCATAAGACTGGGATAACATTCGGAAACGAATGCTAATAC
CGGATACGCGAAACGGTCGCATGATCGGATCGGGAAAGGCGGAGCAATCTGCCACTTATGGATGGACC
CGCGGCGCATTAGCTAGTTGGTGGGGTAACGGCTCACCAAGGCGACGATGCGTAGCCGACCTGAGAGG
GTGATCGGCCACACTGGGACTGAGACACGGCC
>Zotu6920
GAACGCTGGCGGCAGGCCTAACACATGCAAGTCGAGCGGATGAGTGGAGCTTGCTCCATGATTCAGCG
GCGGACGGGTGAGTAATGCCTAGGAATCTGCCTGGTAGTGGGGGACAACGTTTCGAAAGGAACGCTAA
TACCGCATACGTCCTACGGGAGAAAGCAGGGGACCTTCGGGCCTTGCGCTATCAGATGAGCCTAGGTC
GGATTAGCTAGTAGGTGAGGTAATGGCTCACCTAGGCGACGATCCGTAACTGGTCTGAGAGGATGATC
AGTCACACTGGAACTGAGACACGGTC
>Zotu6921
GAACGCTGGCGGCGTGCTTAACACATGCAAGTCGAACGCTGAAGCTCCAGCTTGCTGGGGTGGATGAG
TGGCGAACGGGTGAGTAATACGTGAGTAACCTGCCCTTGACTCTGGGATAAGCCTGGGAAACCGGGTC
TAATACTGGATACGACTCCTCATCGCATGGTGGGGTGTGGAAAGGGTTTTACTGGTTTTGGATGGGCT
CACGGCCTATCAGCTTGTTGGTGGGGTAATGGCCTACCAAGGCGACGACGGGTAGCCGGCCTGAGAGG
GTGACCGGCCACACTGGGACTGAGACACGGCC
>Zotu6922
GAACGCTGGCGGCGTGCTTAACACATGCAAGTCGAACGGTGACCTTGGAGCTTGCTCCGAGCGATCAG
TGGCGAACGGGTGAGTAACACGTGAGTAACCTGCCCCAGACTCTGGGATAACCTCGGGAAACCGGGGC
TAATACCGGATATGAGATGCTTCTGCATGGAATGCGTCTGGAAAGATTTATCGGTCTGGGATGGACTC
GCGGCCTATCAGCTTGTTGGTGAGGTAATGGCTCACCAAGGCGACGACGGGTAGCCGGCCTGAGAGGG
CGACCGGCCACACTGGAACTGAGACACGGTC

>Zotu6923
GAACGCTGGCGGCATGCCTAACACATGCAAGTCGAACGCTCCGCAAGGGGAGTGGCGAACGGGTGAGT
AACGCGTGGGAACCTGCCCAGTAGTTGGGGATAACCGCGGGAAACTGCGGCTAATACCGAATACGCCC
TTCGGGGGAAAGATTTATCGCTATTGGAGGGGCCCGCGTCGGATTAGCTAGTTGGTGAGGTAACGGCT
CACCAAGGCGACGATCCGTAGCTGGTCTGAGAGGATGATCAGCCACACTGGGACTTAGACACGGCC
>Zotu6930
GAACGCTAGCGGCAGGCCTAATACATGCAAGTCGAACGGTTGTAGCAATACAATAGTGGCGCACGGGT
GCGTAACACGTATGCAACCTACCTTATAGATGGGGATAGCCTTTGGAAACGGAGATTAATACCCGATA
GTATATATTAGTGGCATCATTGATATATTAAAGATTTATCGCTATAAGCTGGGCATGCGTCCAATTAG
TTAGTTGGCGGGGTAACGGCTCACCAAGACTTTGATTGGTAGGGGAACTGAGAGGTTAATCCCCCACA
CTGGCACTGAGATACGGGC
>Zotu6931
GAACGCTGGCGGCACGCCTAACACATGCAAGTCGAACGGAGAAAATGATTTCGGTTATTTTCTTAGTG
GCGGACGGGTGAGTAACACGTGAGGAACCTGCCTCTGAGAGTGGGATAGCTTCTGGAAACGGATGGTA
ATACCGCATAACATAGCGAAACCGCATGATTTTGCTATCAAAGTATTTCGCTCAGAGATGGCCTCGCG
TCTGATTAGATAGTTGGTGAGGTAACGGCTCACCAAGTCGACGATCAGTAGCCGGACTGAGAGGTTGA
ACGGCCACATTGGGACTGAGACACGGCC
>Zotu6932
GAACGCTGGCGGCGTGCTTAACACATGCAAGTCGAGCGGTAAGGCACTTCGGTGTACACGAGCGGCGA
ACGGGTGAGTAACACGTGAGTAATCTGCCCTCCACTCTGGGATAAGCACTCGAAAGGGTGTCTAATAC
CGGATACGACCGATCACTGCATGGTGTGTTGGTGGAAACTTTTATGGGTGGGGGATGTGCTCGCGGCC
TATCAGCTTGTTGGTGGGGTAATGGCCTACCAAGGCTTCGACGGGTAGCCGGCCTGAGAGGGTGACCG
GCCACACTGGGACTGAGACACGGCC
>Zotu6935
GAACGCTGGCGGCATGCCTTACACATGCAAGTCGAACGGTAACAGGTCTTCGGATGCTGACGAGTGGC
GAACGGGTGAGTAATGCATCGGAACGTGCCCAGTCGTGGGGGATAACTGCTCGAAAGAGCAGCTAATA
CCGCATACGACCTGAGGGTGAAAGCGGGGGACCGTAAGGCCTCGCGCGATTGGAGCGGCCGATGTCAG
ATTAGGTAGTTGGTGGGGTAAAAGCTCACCAAGCCAACGATCTGTAGCTGGTCTGAGAGGACGACCAG
CCACACTGGGACTGAGACACGGCC
>Zotu6936
GAACGCTGGCGGCATGCTTTACACATGCAAGTCGAACGGCAGCGCGGGGCAACCTGGCGGCGAGTGGC
GAACGGGTGAGTAATATATCGGAACGTACCCAAGAGTGGGGGATAACGTAGCGAAAGTTACGCTAATA
CCGCATACGATCTAAGGATGAAAGCAGGGGACCGCAAGGCCTTGTGCTCCTGGAGCGGCCGATATCTG
ATTAGCTAGTTGGTAGGGTAAAGGCCTACCAAGGCATCGATCAGTAGCTGGTCTGAGAGGACGACCAG
CCACACTGGAACTGAGACACGGTC
>Zotu6937
GAACGCTAGCGGCAGGCTTAATACATGCAAGTCAAGGGGGTAGCAATACAACCGGCGCACGGGTGAGT
AACGCGTACACAACATACCATATACTGGGGTATAGCTTTGGGAAACTGAAGGTAATCCCCCATAACAT
TGCGAGAGGGCATCTGATTGCAATTAAAGTTTCGACGGTATATGATTGGTGTGCGTTTGATTAGCTAG
TTTGTAGGGTAACGGCCTACCAAGGCGACGATCAATAGCTGGCGTGAGAGCGCGACCAGCCACACTGG
AACTGAGACACGGTC
>Zotu6938
GAACGCTGGCGGCGTGCTTAACACATGCAAGTCGAACGAGGAATTTTAAATGAGACTTCGGTGGATTT
TGAAATTCTTAGTGGCGGACGGGTGAGTAACGCGTGGGCAACCTGCCCTGTACAGGGGGACAACAGCT
GGAAACGGCTGCTAATACCGCATAAGCGCACAGCATCGCATGATGCCGTGTGAAAAACTCCGGTGGTA
CAGGATGGGCCCGCGTTGGATTAGGTAGTTGGTGGGGTAACGGCCCACCAAGCCGACGATCCATAGCC
GACCTGAGAGGGTGACCGGCCACATTGGGACTGAGACACGGCC
>Zotu6939
GAACGCTGGCGGCGTGCCTAATACATGCAAGTAGAACGCTGAAGGAGGAGCTTGCTCTTTCCGGATGA
GTTGCGAACGGGTGAGTAACGCGTAGGTAACCTGCCTGGTAGCGGGGGATAACTATTGGAAACGATAG
CTAATACCGCATAACAGTAGATATCGCATGATAGCTTCTTGAAAGGTGCAACTGCACCACTACCAGAT
GGACCTGCGTTGTATTAGCTAGTTGGTGAGGTAACGGCTCACCAAGGCAACGATACATAGCCGACCTG
AGAGGGTGATCGGCCACACTGGGACTGAGACACGGCC
>Zotu6941

GAACGCTGGCGGCGTGCTTAACACATGCAAGTCGAGCGGATTGAAGACTTCGGTCTGATATTAGCGGC
GGACGGGTGAGTAACGCGTGGGTGACCTGCCTTATACAGGGGGATAACAGTGAGAAATCACTGCTAAT
ACCGCATAAGACCACACTACCACATGGTAGAGAGGTAAAAGGAGCAATCCGGTATGAGATGGGCCCGC
GTCTGATTAGCTAGTTGGAGAGGTAATGGCTCATCAAGGCGACGATCAGTAGCCGACCTGAGAGGGTG
ATCGGCCACATTGGGACTGAGACACGGCC
>Zotu6942
GAACGCTGGCGGCATGCCTAATACATGCAAGTCGAACGCTGAAGCTCTCTGCTTGCAGAGAGTGGATG
AGTGGCGAACGGGTGAGTAACACGTGGGGAACCTGCCCATGAGAGGGGGATAACATTCGGAAACGGAT
GCTAATACCGCATAGGTACAAGACACGCATGTGTCCTGTATGAAAGACGGCTTCGGCTGTCGCTCATG
GATGGCCCCGCGGCGTATTAGCTAGTTGGTAAGGTAATGGCTTACCAAGGCATTGATACGTAGCCGAC
CTGAGAGGGTGATCGGCCACACTGGGACTGAGACACGGCC
>Zotu6943
GAACGCTGGCGGCATGCTTTACACATGCAAGTCGAACGGCAGCGCGGGCTTCGGCCTGGCGGCGAGTG
GCGAACGGGTGAGTAATATATCGGAACGTGCCCAAGAGTGGGGGATAACGTAGCGAAAGTTACGCTAA
TACCGCATACGATCTAAGGATGAAAGCAGGGGACCTTCGGGCCTTGTGCTCCTGGAGCGGCCGATATC
TGATTAGCTAGTTGGTGAGGTAAAGGCTCACCAAGGCGACGATCAGTAGCTGGTCTGAGAGGACGACC
AGCCACACTGGAACTGAGACACGGTC
>Zotu6944
GAACGCTGGCGGCGTGCTTAACACATGCAAGTCGAACGAGAATCTACTGAAAGAGTTTTCGGACAATG
GAAGTAGAGGAAAGTGGCGGACGGGTGAGTAACGCGTGAGGAACCTGCCTTGAAGAGGGGGACAACAG
TTGGAAACGACTGCTAATACCGCATGACGCATAGAGGTCGCATGATCTTTATGCCAAAGATTTATCGC
TTCAAGATGGCCTCGCGTCTGATTAGCTAGTTGGCGGGGTAACGGCCCACCAAGGCGACGATCAGTAG
CCGGACTGAGAGGTTGAACGGCCACATTGGGACTGAGATACGGCC
>Zotu6945
GAACGCTGGCGGCGTGCCTAATACATGCAAGTCGAGCGGACGGATGGGAGCTTGCTCCCTGAAGTTAG
CGGCGGACGGGTGAGTAACACGTGGGCAACCTGCCTGTAAGACTGGGATAACTCCGGGAAACCGGGGC
TAATACCGGATAATTCTTATCCTCTCATGAGGATAAGCCGAAAGACGGTTTCGGCTGTCACTTACAGA
TGGGCCCGCGGCGCATTAGCTAGTTGGTGAGGTAACGGCTCACCAAGGCAACGATGCGTAGCCGACCT
GAGAGGGTGATCGGCCACACTGGGACTGAGACACGGCC
>Zotu6946
GAACGCTGGCGGCGTGCCTAACACATGCAAGTCGAGCGGGGATGTTGGAGAGCTTGCTCAAAAACATT
CTAGCGGCGGACGGGTGAGTAACGCGTGAGCAACCTGCCTATCACAGGGGAATAACGCACCGAAAGGT
GTGCTAATACCGCATGAGACCACAGGACCGCATGGTCAAGGGGTCAAAGATTTATCGGTGATAGATGG
GCTCGCGTCTGATTAGCTGGTTGGTGAGGTAACGGCCCACCAAGGCGACGATCAGTAGCCGACCTGAG
AGGGTGATCGGCCACATTGGAACTGAGAAACGGTC
>Zotu6948
GAACGCTGGCGGCGTGCCTAATACATGCAAGTCGAGCGCAGGAAGCAGACTGATCTCCTTCGGGAGTG
ATGTTTGTGGAATGAGCGGCGGACGGGTGAGTAACACGTGGGCAACCTGCCTGTAAGACCGGGATAAC
TTGCGGAAACGTGAGCTAATACCGGATAAAGCTCCATGCCGCATGGCATGGGGATGAAAGACGGCTTA
GCTGTCGCTTGCAGATGGGCCCGCGGCGCATTAGCTAGTTGGTGGGGTGATGGCCTACCAAGGCGACG
ATGCGTAGCCGACCTGAGAGGGTGACCGGCCACACTGGGACTGAGACACGGCC
>Zotu6949
GAACGCTGGCGGCGTGCCTAACACATGCAAGTCGAGCGATAGATATGTAAGTGAGTTTTCGGATGAGC
GAGCATATTGAAAGCGGCGGACGGGTGAGTAACGCGTAGGCAACCTGGCTCATACTAAGGGATAGCTC
CGGGAAACCGGGATTAATACCTCATAACACCGAAGTGTCACATGGCAATTTGGTCAAAGATTTATCGG
TATGAGATGGGCCTGCGTCTGATTAGCTAGTTGGTAGGGTAACGGCCTACCAAGGCAACGATCAGTAG
CCGACCTGAGAGGGTGATCGGCCACACTGGAACTGAGACACGGTC
>Zotu6950
GAACGCTGGCGGCGTGCTTAACACATGCAAGTCGAGCGGAAAGGCCCTTTCGGGGGTACTCGAGCGGC
GAACGGGTGAGTAACACGTGAGTAATCTGCCCCTGGCTTTGGGATAGCCACCGGAAACGGTGATTAAT
ACCGAATATGCACTACTTCTCGCATGGGTTGTGGTGGAAAGTTTTTCGGCCAGGGATGTGCTCGCGGC
CTATCAGCTTGATGGTGAGGTAATGGCTCACCATGGCTTCGACGGGTAGCCGGCCTGAGAGGGTGACC
GGTCACACTGGGACTGAGACACGGCC
>Zotu6951

CAACGCTGGCGGCGTGCCTAATGCATGCAAGTCGAACGGGGTTCACTTCGGTGAACTTACGTGGCGGA
CGGGTGAGGAGCACGTGGGCAACCTGCCCTTTGGTGGAGGATAACCGCTGGAAACAGTGGGTAATACT
GCATGAGACATTCTGACGAGTGGGTTGGAATGGGAAAGCCGAGAGGCGCCGAAGGAGGGGCCTGCGTC
CGATTAGCTAGACGGTGGGGTAACGGCCTACCGTGGCAGTGATCGGTAGCTGGTTTGAGAGGATGATC
AGCCACACGGGGACTGAGACACGGCC
>Zotu6952
GAACGCTGGCGGCATGCCTAACACATGCAAGTCGAACGAAGCCTTCGGGTTTAGTGGCGCACGGGTGC
GTAACGCGTGGGAATCTGCCCTTGGGTTCGGAATAACAGCGAGAAATTGCTGCTAATACCGGATGATG
ACGAAAGTCCAAAGATTTATCGCCCAGGGATGAGCCCGCGTAGGATTAGCTAGTTGGTGAGGTAAAGG
CTCACCAAGGCGACGATCCTTAGCTGGTCTGAGAGGATGATCAGCCACACTGGGACTGAGACACGGCC
>Zotu6953
AAATGCTGGCGGCGTGCCTAATACATGCAAGTCGAGCGGTAAGGCTTTCACAGATTATTTAAACGGAA
AAGTCCAAGTAATTGGATACTATCCTTCAACGTTTTACTCCTGGCTAGGAGAGAAATGGTTGAATAAT
GTGTGAAAGTACACGAGCGGCGAACGGCTGAGTAACGCGTAGGAATGTACCCCAAAGTGAGGAATAAG
CACCGGAAACGGTGTCTAATGCCGCATGTGGTCTAAGGACTAAAGCCTTCGGGCGCTTTGGGAGCAGC
CTGCGTAAGATTAGTTTGTTGGTGAGGTAATGGCTCACCAAGACTACGATCTTTAGCTGGTCTGAGAG
GATGATCAGCCAGACTGGAACTGAGACACGGTC
>Zotu6955
GAACGCTGGCGGCGTGCCTAATACATGCAAGTCGAGCGAACTTTTGGGAGCTTGCTCCCAAAAGTTAG
CGGCGGACGGGTGAGTAACACGTGGGCAACCTGCCTATAAGACTGGGATAACTTCGGGAAACCGGAGC
TAATACCGGATAATTCTTTTCTACTCATGTAGAAAAGCTAAAAGACGGTTTACACTGTCACTTATAGA
TGGGCCCGCGGCGCATTAGCTAGTTGGTGAGGTAACGGCTCACCAAGGCCACGATGCGTAGCCGACCT
GAGAGGGTGATCGGCCACACTGGGACTGAGACACGGCC
>Zotu6957
GAACGCTGGCGGCATGCCTAACACATGCAAGTCGAACGATCACTTCGGTGATAGTGGCGCACGGGTGC
GTAACGCGTGGGAATCTACCCTTGGGTTCGGAATAACTCAGAGAAATTTGAGCTAATACCGGATGATG
ACGTAAGTCCAAAGATTTATCGCCCAAGGATGAGCCCGCGTAGGATTAGCTAGTTGGTGAGGTAAAGG
CTCACCAAGGCGACGATCCTTAGCTGGTCTGAGAGGATGATCAGCCACACTGGGACTGAGACACGGCC
>Zotu6958
GAACGCTGGAAATGTGGATACCGGCATGCAAGTCAAGGGGCCGCAAGGCACCGGCGAACGGCTTAGTA
ACACGTGGATACATACCTTAAAGTGGGGCATAGCTCGTCGAAAGACGGGGTAATTTCCCATAGTCTCG
CAAGAGTAAAGGCGCAAGCCGCTTTAAGAATGGTCCGCGGTCTATCAGCTAGTTGGTGAGGTAATGGC
TCACCAAGGCTATGACGGATAGGGGGCGTGAGAGCGCGACCCCCAAGAATGGCACTGCGACACGGGC
>Zotu6959
GAACGCTAGCGGCGGGCTTAATACATGCAAGTCAAGGGGTAGCAATACACCGGCGGACGGGTGCGTAA
CGCGTATGCAACCTACCTTTATCTGGGGGATAACTCGGGGAAACTCGAGCTAATACCGCATAAGAAAG
TGATCTGGCATCAGATAGCTTTTAAAGATTTATTGGATAAAGATGGGCATGCGTAAGATTAGATAGTT
GGTGAGGTAACGGCTCACCAAGTCTACGATCTTTAGGGGCTCTGAGAGGAGAATCCCCCACACTGGCA
CTGAGATACGGGC
>Zotu6960
GAACGCTGGCGGCACGCCTAACACATGCAAGTCGAACGGACTTAAAAGAGCTTGCTTTTTTAAGTTAG
TGGCGGACGGGTGAGTAACACGTGAGCAACCTGCCTCTGAGAGGGGGATAGCTTCTGGAAACGGATGG
TAATACCCCATAAAATATAAATATCGCATGATAGATATATCAAAGAAATTCGCTCAGAGATGGGCTCG
CGTCTGATTAGATAGTTGGTGAGGTAACGGCTCACCAAGTCGACGATCAGTAGCCGGACTGAGAGGTT
GAACGGCCACATTGGGACTGAGACACGGCC
>Zotu6962
GAACGCTGGCTGCGTGCTTAACACATGCAAGTCGAACGCTGAAGCCCTGGTGCTTGCACCGGGGTGGA
TGAGTGGCGAACGGGTGAGTAACACGTGAGTAACCTGCCCCCGACTTCGGGATAAGCCCGGGAAACTG
GGTCTAATACCGGATATGACGACCGAAGGCATCTTTGGTTGTGGAAAGTTTTTTCGGTTGGGGATGGG
CTCGCGGCCTATCAGTTTGTTGGTGAGGTAATGGCTCACCAAGACGACGACGGGTAGCCGGCCTGAGA
GGGCGACCGGCCACACTGGGACTGAGACACGGCC
>Zotu6963
GAACGCTGGCGGCGTGCCTAATACATGCAAGTCGAACGCATTTTCTTTCACCGTAGCTTGCTACACCG
AAAGAAAATGAGTGGCGAACGGGTGAGTAACACGTGGGTAACCTGCCCATCAGCGAGGGATAACACTT

GGAAACAGGTGCTAATACCGCATAATTCCATTTACCGCATGGTAGATGGATGAAAGGCGCTTTTGCGT
CACTGATGGATGGACCCGCGGTGCATTAGCTAGTTGGTGGGGTAACGGCCTACCAAGGCTGCGATGCA
TAGCCGACCTGAGAGGGTGATCGGCCACACTAGGACTGAGACACGGCC
>Zotu6964
GAACGCTGGCGGCATGCCTAACACATGCAAGTCGAACGAGACCTTCGGGTCTAGTGGCGCACGGGTGC
GTAACGCGTGGGAACCTGCCTTGAGGTTCGGAATAACTCCCCGAAAGGGGTGCTAATACCGGATGATG
TCTTCGGACCAAAGATTTATCGCCTTTAGATGGGCCCGCGTTGGATTAGCTAGTTGGTGGGGTAAAGG
CCTACCAAGGCGACGATCCATAGCTGGTCTGAGAGGATGATCAGCCACACTGGGACTGAGACACGGCC
>Zotu6966
GAACGCTAGCGGCATGCCTAACACATGCAAGTCGAACGCCCCTTTCGGGGGGAGTGGCAGACGGGTGA
GTAACACGTGGGAACATACCCTTTGGTTCGGAACAACGTTTGGAAACGGACGCTAATACCGGATACGC
CCTTTGGGGGAAAGGCTTTGCTGCCGAAGGATTGGCCCGCGTTCGATTAGCTTGATGGCGGGGTAACG
GCCCACCATGGCGACGATCGATAGCTGGTCTGAGAGGATGGCCAGCCACATTGGGACTGAGACACGGC
C
>Zotu6968
GAACGCTGGCGGAATGCTTTACACATGCAAGTCGAACGGCAGCACGGGGGCAACCCTGGTGGCGAGTG
GCGAACGGGTGAGTAATACATCGGAACGTGCCCAGTCGTGGGGGATAACGCAGCGAAAGCTGTGCTAA
TACCGCATACGATCTACGGATGAAAGCGGGGGATCGCAAGACCTCGCGCGATTGGAGCGGCCGATGGC
AGATTAGGTAGTTGGTGGGGTAAAGGCTCACCAAGCCGACGATCTGTAGCTGGTCTGAGAGGACGACC
AGCCACACTGGGACTGAGACACGGCC
>Zotu6971
GAACGCTGGCGGCATGCTTTACACATGCAAGTCGAACGGCAGCGGGGGCTTCGGCCTGCCGGCGAGTG
GCGAACGGGTGAGTAATGCATCGGAACGTGCCCATGTCGTGGGGGATAACGTATCGAAAGGTACGCTA
ATACCGCATACGCCCTGAGGGGGAAAGCGGGGGATTCTTCGGAACCTCGCGCGATTGGAGCGGCCGAT
GTCGGATTAGCTAGTAGGTGAGGTAAAGGCTCACCTAGGCGACGATCCGTAGCGGGTCTGAGAGGATG
ATCCGCCACACTGGGACTGAGACACGGCC
>Zotu6972
GAACGCTGGCGGCGTGCTTAACACATGCAAGTCGAACGGAAAGGTCTCTTCGGAGATACTCGAGTGGC
GAACGGGTGAGTAACACGTGGGTGATCTGCCCTGCACTTCGGGATAAGCTTGGGAAACTGGGTCTAAT
ACCGGATATGATCATGCGCCTCATGGTGTGTGGTGGAAAGCTTTTGCGGTGTGGGATGGGCCCGCGGC
CTATCAGCTTGTTGGTGGGGTGATGGCCTACCAAGGCGACGACGGGTAGCCGGCCTGAGAGGGTGTCC
GGCCACACTGGGACTGAGATACGGCC
>Zotu6975
GAACGCTGGAAATGTGGATACCGGCATGCAAGTCAAGGGGCCGCAAGGCACCGGCGAACGGCTTAGTA
ACACGTAGATACATACCTTCAATTGGAGCATAGCTCGTCGAAAGACGGGGTAATTCTCCATAGTCTCG
CAAGAGTAAAGGCGCAAGCCGATTGAAGAATGGTCTGCGGTCTATCAGCTAGTTGGTGAGGTAATGGC
TCACCAAGGCTATGACGGATAGGGGGCGTGAGAGCGCGACCCCCAAGAATGGCACTGCGACACGGGC
>Zotu6977
GAACGCTAGCGGCAGGCCTAACACATGCAAGTCGAGGGAAAGTAGTAGCAATACTATGAAACCGGCGC
ACGGGTGCGCAACGCGTATGCAACCTTTCTATAACTGGGGCATAGCTCGTCGAAAGGCGAATTAATTC
CCCATAAGATCATGGTCTGGCATCAGATAGTGATTAAAGCTCCGGCGGTTATAGGTGGGCATGCGTGT
CATTAGCTAGTTGGTAAGGTAACGGCTTACCAAGGCTACGATGACTAGGGGGTCTGAGAGGATGATCC
CCCACACTGGAACTGAGACACGGTC
>Zotu6979
GAACGCTGGCGGCGTGCTTAACACATGCAAGTCGAACGAGAACGGATTATAGCTTGCTATAATTGTCA
GCTAAGTGGCGCACGGGTGAGTAATATATAGGTAATGTGCCCTAGAGAAGAGGATAACAGTTGGAAAC
GACTGCTAAGACTCTATATGCCTTTAAGACAGAAGTCTGCAAGGGAAATATTTATAGCTCTAGGATCA
GCCTGTACAGTATCAGCTAGTTGGTGAGGTAATGGCTCACCAAGGCAATGACACTTAACTGGTTTGAG
AGGATGATCAGTCACACTGGAACTGAGACACGGTC
>Zotu6981
GAACGCTGGCGGCGTGCTTAACACATGCAAGTGGAGCGACGAACCAGGGCTTGCCCTGGGGCAGAGCC
GCGAACGGGTGAGTAACACGTGGGTAACGTGCCCCGATGACTGGGACAACCCGGGGAAACCCGGGCTA
ATACCGGATGTGCTCCCTCACCTCGGTGAGACGAGTAAAGGAAGCTTCGGCCTCCGCATCGGGATCGG
CCCGCGGCCCATTAGCTTGTTGGTGAGGTAACGGCTCACCAAGGCCGCGATGGGTAGCTGGTCTGAGA

GGACGATCAGCCACACTGGGACTGAGACACGGCC
>Zotu6983
GAACGCTGGCGGCGTGCTTAACACATGCAAGTCGAGCGGAAAGGCCACTTCGGTGGTACTCGAGCGGC
GAACGGGTGAGTAACACGTGAGTAATCTGCCCCATGCTCCGGAATAGCCAGCCGAAAGGCTGATTAAT
GCCGGATATGACATCGTCAGGCATCTGGTGGTGTGGAAAGTTTTTTCGGCGTGGGATGTGCTCGCGGC
CTATCAGCTTGTTGGTGGGGTAATGGCCTACCAAGGCTTCGACGGGTAGCCGGCCTGAGAGGGTGACC
GGCCACACTGGGACTGAGACACGGCC
>Zotu6984
CAACGCTGGCGGCGCGCCTAACACATGCAAGTCGAACGAGAAAGTGGAGCAATCCATGAGTACAGTGG
CGTACGGGTGAGTAACACGTGGGTAATCTACCTTTTAGTGAGGAATAACCAAGGGAAACTTTGGCTAA
TACCGCATAAGCCTGAGAAGGGAAAGCAGAAATGCGCTGAAAGAGGAGCCCGCGGCCGATTAGCTAGT
TGGTGGGGTAAGAGCCCACCAAGGCAGAGATCGGTAGCCGGCCTGAGAGGGCACACGGCCACACTGGC
ACTGAAACACGGGC
>Zotu6985
GAACGCTAGCGGCAGGCCTAACACATGCAAGTCGAGGGGTAGAGGCTTTCGGGCCTTGAGACCGGCGC
ACGGGTGCGTAACGCGTATGCAATCTGCCTTGTACGGGGGGATAGCCCAGAGAAATTTGGATTAATAC
CCCATGGTATTTTAGGGTGGCATCACCTTATTATTAAAGTTCCAACGGTACAAGATGAGCATGCGTCC
CATTAGCTAGTTGGTATGGTAACGGCATACCAAGGCAATGATGGGTAGGGGTCCTGAGAGGGAGATCC
CCCACACTGGTACTGAGACACGGAC
>Zotu6986
GAACGCTGGCGGCAGGCTTAACACATGCAAGTTGAACGTGATTTAATTTGTGCTTGCACAGATTAATG
AAAGTGGCGCACGGGTGAGTAACACGTGGGAACATACCGATTAGTGGGGGATAACTATTGGAAACGAT
AGCTAATACCGCATGAGCCCTGAGGGGGAAAGATTTATCGCTAATTGAATGGCCCGCGGAAGATTAGA
TAGTTGGTAGGGTAATGGCCTACCAAGTCTACGATCTATAGCTGGTCTGAGAGGACGATCAGCCACAC
TGGAACTGAGACACGGTC
>Zotu6988
GAACGCTGGCGGCGTGCCTAACGCATGCAAGTCGAACGAGGCCTTCGGGCCTAGTGGCGGACGGGTGA
GTAACACGTAGGAACCTGCCCGGAAGTGGGGGACAACTCCACGAAAGCGGGGCTAATACCGCATACGG
TCTACGGACCAAAGCCTTCGGGCGCTTTCGGAGGGGCCTGCGGCGGATTAGCTAGTTGGTGGGGTAAC
GGCCTACCAAGGCGACGATCCGTAGCTGGTCTGAGAGGATGATCAGCCACACTGGGACTGAGACACGG
CC
>Zotu6990
GAACGCTAGCGGCAGGCTTAATACATGCAAGGCGAGGGGGCAGCAATGTCACCGTCGTACGGGTGCGT
AACGCGTATGCAACCTACCTTTTACTGGGGGATAGCCCGGGGAAACCCGGATTAATACCGCATAACAC
ATTTATATCACCTGATATTTATTGTTAAAGATTTATTGGTAAAAGATGGGCATGCGTTCGATTAGCTA
GTTGGCAGGGTAACGGCCTACCAAGGCGACGATCGATAGGGGAGCTGAGAGGTTGATCCCCCACACGG
GCACTGAGATACGGGC
>Zotu6992
GAACGCTGGCGGCGTGCTTAACACATGCAAGTCGAACGGATCATCTTACACTTGTGTTTGGTGGTTAG
TGGCGAACGGGTGAGTAACACGTGAGTAACCTGCCCTTCACTGTGGGATAACGGAGGGAAATTTCTGC
TAATACCGCATATGACATTGCCTCGCATGGGGTGGTGTGGAAAGTTTTTTCGGTGGGGGATGGACTCG
CGGCCTATCAGCTTGTTGGTGGGGTAGTGGCTCACCAAGGCTTTGACGGGTAGCCGGCCTGAGAGGGC
GACCGGCCACACTGGGACTGAGATACGGCC
>Zotu6993
AAGCAGAGCCCAGATCCCCGGAGGCCAGAGCTGCCACACGTGCTGCTGGGCCATTTAGGACGTGCGAG
AAGCAACAGCATGCACGCAACACTGAGATCTCTGACCTGAGCAACTGAAAACAAGGGCAGTCG
>Zotu6997
GAACGCTAGCGGGATGCTTTACACATGCAAGTCGAACGGCAGCACGAGAGAGCTTGCTCTCTTGGTGG
CGAGTGGCGGACGGGTGAGTAATATATCGGAACGTGCCCAGTAGCGGGGGATAACTACTCGAAAGAGT
GGCTAATACCGCATACGCCCTACGGGGGAAAGGGGGGGATCGCAAGACCTCTCACTATTGGAGCGGCC
GATATCGGATTAGCTAGTTGGTGGGGTAAAGGCTCACCAAGGCAACGATCCGTAGCTGGTTTGAGAGG
ACGACCAGCCACACTGGGACTGAAACACGACC
>Zotu6999
GAACGCTGGCGGCATGCCTTACACATGCAAGTCGAACGGCAGCACGGGAGCAATCCTGGTGGCGAGTG

GCGAACGGGTGAGTAATATATCGGAACGTGCCCAGTCGTGGGGGATAACGTAGCGAAAGCTACGCTAA
TACCGCATACGATCTATGGATGAAAGCGGGGGATCGCAAGACCTCGCGCGATTGGAGCGGCCGATATC
AGATTAGCTAGTTGGTGGGGTAAAAGCTCACCAAGGCGACGATCTGTAGCTGGTCTGAGAGGACGACC
AGCCACACTGGAACTGAGACACGGTC
>Zotu7000
GAACGCTGGCGGCATGCCTAATACATGCAAGTCGAACGGGAAGCACGAAAGTGCGGAGAGTGGCGAAC
GGGTGAGTAACACGTAAGTGACCTGCCTTCAAGACGGGGATAACTGTTGGAAACGGCAGCTAATACCG
GATAGGATACCGTGCGTAGGGCAAGGTATTGAAAGGAAGCAAATGCTTCGCTTGAAGATGGGCTTGCG
GCGCATTAGCTAGTTGGAGTGATAAGAGCACACCAAGGCGATGATGCGTAGCCGACCTGAGAGGGTGT
ACGGCCACACTGGGACTGAGACACGGCC
>Zotu7001
GAACGCTGGCGGCATGCCTAACACATGCAAGTCGAACGAGACCTTCGGGTCTAGTGGCGCACGGGTGC
GTAACGCGTGGGAATCTACCCCTTGGTTCGGAATAACTCAGAGAAATTTGAGCTAATACCGGATGATG
ACGTAAGTCCAAAGATTTATCGCCGAGGGATGAGCCCGCGTAGGATTAGCTAGTTGGTGAGGTAAAGG
CTCACCAAGGCGACGATCCTTAGCTGGTCTGAGAGGATGATCAGCCACACTGGGACTGAGACACGGCC
>Zotu7002
GAACGCTGGCGGCGTGCCTAATACATGCAAGTCGAGCGAACTTTTGGGGGCTTGCTCCCAAAAGTTAG
CGGCGGACGGGTGAGTAACACGTGGGCAACCTGCCTATAAGACTGGGATAACTTCGGGAAACCGGAGC
TAATACCGGATAATTCTTTTCTACACATGTAGAAAAGCTAAAAGACGGTTTACGCTGTCACTTATAGA
TGGGCCCGCGGCGCATTAGCTAGTTGGTGAGGTAACGGCTCACCAAGGCCACGATGCGTAGCCGACCT
GAGAGGGTGATCGGCCACACTGGGACTGAGACACGGCC
>Zotu7003
GAACGCTGGCGGCATGCTTTACACATGCAAGTCGAACGGTAACAGGGAGCTTGCTCCGCTGACGAGTG
GCGAACGGGTGAGTAATGCATCGGAACGTACCCAGTCGTGGGGGATAACGTAGCGAAAGTTACGCTAA
TACCGCATACGTCCTGAGGGAGAAAGCGGGGGACCGTAAGGCCTCGCGCGATTGGAGTGGCCGATGTC
GGATTAGCTAGTTGGTGGGGTAAAGGCCTACCAAGGCGACGATCCGTAGCGGGTCTGAGAGGATGATC
CGCCACACTGGGACTGAGACACGGCC
>Zotu7004
GAACGTTGGCGGCGTGCCTTAAGCATGCAAATCAAGTGGGTCCCGCAAGGGACTACCGGTGGACGGGT
GAGTAATGCGTGGGAATCTACCTCGAAGTGGGATATAGCCAGCCGAAAGGTTGGGTAATCTCCCATGT
GATCGTAAGATCAAAGTCGCAAGACGCTTTTAGATGAGCCCACGTCCTATCAGCTAGTTGGTGGGGTA
ATGGCCTACCAAGGCAACGACGGGTAGCTGGACTGAGAGGTCGGTCAGCCACAATGGGACTGCGACAC
GGCC
>Zotu7007
GAACGCTGGCGGCGTGCCTAATACATGCAAGTCGAGCGTCCTTGACAGCGCATTTCTTTCCTTCGTTT
TAGACGGGGGGAATCAAGGAATGTAGTGTCAAGGGAGCGGCGGACGGCTGAGTAACGCGTAGGAACGT
ACCCCAAAGTGAGGGATAAGCACCGGAAACGGTGTCTAATACCGCATAATATCTCCGGATTAAAGCTT
CGGCGCTTTGGGAGCGGCCTGCGTATGATTAGCTTGTTGGTGAGGTAATGGCTCACCAAGGCGACGAT
CATTAGCTGGTCTGAGAGGATGACCAGCCAGACTGGAACTGAGACACGGTC
>Zotu7009
GAACGCTGGCGGCGTGCTTAACACATGCAAGTCGAACGGTGAACCAGGGCTTGCCCTGGGAATCAGTG
GCGAACGGGTGAGTAACACGTGGGCAACCTGCCCCTGACTCTGGGATAACTCCGGGAAACCGGGGCTA
ATACCGGATACGACCCTGGAAGGCATCTTCCGGGGTGGAAAGTTTTTCGGTCAGGGATGGGCCCGCGG
CCTATCAGCTTGTTGGTGGGGTAATGGCCTACCAAGGCGACGACGGGTAGCCGGCCTGAGAGGGCGAC
CGGCCACACTGGGACTGAGACACGGCC
>Zotu7011
GAACGCTGGCGGCGTGCCTAATACATGCAAGTCGAGCGAACTTGCGGGAGCTTGCTCCCAAAAGTTAG
CGGCGGACGGGTGAGTAACACGTGGGCAACCTGCCTATAAGACTGGGATAACTTCGGGAAACCGGAGC
TAATACCGGATAATCCTTTTCTACACATGTAGGAAAGCTGAAAGACGGTTTACGCTGTCACTTATAGA
TGGGCCCGCGGCGCATTAGCTAGTTGGTGAGGTAACGGCTCACCAAGGCCACGATGCGTAGCCGACCT
GAGAGGGTGATCGGCCACACTGGGACTGAGACACGGCC
>Zotu7014
GAACGCTGGCGGCAGGCCTAACACATGCAAGTCGAACGCCCCGCAAGGGGAGTGGCAGACGGGTGAGT
AACACGTGGGAACCTTCCCTATAGTACGGAATAGCCCAGGGAAACTTGGAGTAATACCGTATACGCCC

GAAAGGGGAAAGAATTTCGCTATAGGATGGGCCCGCGTAGGATTAGCTAGTTGGTGAGGTAATGGCTC
ACCAAGGCGACGATCCTTAGCTGGTTTGAGAGAACGACCAGCCACACTGGGACTGAGACACGGCC
>Zotu7015
GAACGCTGGCGGCAGGCTTAACACATGCAAGTCGAGCGGGCCCTTCGGGGTCAGCGGCAGACGGGTGA
GTAACACGTGGGAACGTGCCCTTCGGTTCGGAATAACTCAGGGAAACTTGAGCTAATACCGGATACGC
CCTTTTGGGGAAAGGTTTACTGCCGAAGGATCGGCCCGCGTCTGATTAGCTTGTTGGTGGGGTAACGG
CCTACCAAGGCGACGATCAGTAGCTGGTCTGAGAGGATGATCAGCCACACTGGGACTGAGACACGGCC
>Zotu7016
CAACGCTGGCGGCGCGCTTAACACATGCAAGTCGAACGAGAAAGGGAGCAATCCTGAGTAAAGTGGCG
CACGGGTGAGTAACACGTGGGTAATCTACCCTCTGGTGGGGAATAACTCTGGGAAACCGGAGCTAATA
CCGCATAAGACCGGGAGGTGAAAGGGCGAAAGTTCGCCGGAGGAGGAGCCCGCGGCTGATTAGCTAGT
TGGTAGGGTAAGAGCCTACCAAGGCTGAGATCGGTAGCCGGCCTGAGAGGGCACACGGCCACACTGGC
ACTGAAACACGGGC
>Zotu7018
GAACGCTGGCGGCGTGCTTAACACATGCAAGTCGAACGGTGATCTCGCAGCTTGCTGCGGGTGATCAG
TGGCGAACGGGTGAGTAACACGTGAGTAACCTGCCCTTGACTCTGGGATAACTCCGGGAAACCGGTGC
TAATACTGGATATGACTCTGGCCCGCATGGGTTGGGGTGGAAAGTTTTTCGGTCAAGGATGGACTCGC
GGCCTATCAGCTTGTTGGTGGGGTAATGGCCTACCAAGGCGACGACGGGTAGCCGGCCTGAGAGGGCG
ACCGGCCACACTGGGACTGAGACACGGCC
>Zotu7019
GAACGCTGGCGGCGTGCCTAATACATGCAAGTCGAGCGGACTTGATTGGAGCTTGCTCTGATCAAGTT
AGCGGCGGACGGGTGAGTAACACGTGGGTAACCTGCCTGTAAGACTGGGATAACTTCGGGAAACCGGA
GCTAATACCGGATAATCCGTTTCACCGCATGGTGAAACGTTGAAAGATGGCTTCGGCTATCACTTACA
GATGGACCCGCGGCGCATTAGCTAGTTGGTGGGGTAATGGCCCACCAAGGCGACGATGCGTAGCCGAC
CTGAGAGGGTGATCGGCCACACTGGGACTGAGACACGGCC
>Zotu7020
GAACGCTGGCGGCAGGCCTAACACATGCAAGTCGAGCGCATCCTTCGGGGTGAGCGGCGGACGGGTTA
GTAACGCGTGGGAACGTACCTTTTGCTACGGAATAGCCCTGGGAAACCGGGGTAATGCCGTATGTGC
CCTTTGGGGGAAAGATTTATCGGCAAGAGATCGGCCCGCGTTGGATTAGGTAGTTGGTGGGGTAATGG
CTCACCAAGCCTACGATCCATAGCTGGTTTGAGAGGATGATCAGCAACACTGGGACTGAGACACGGCC
>Zotu7021
GAACGCTGGCGGCGTGCTTAACACATGCAAGTCGAACGGTGAACCTCGCTTGCGGGGGGATCAGTGGC
GAACGGGTGAGTAACACGTGGGCAACCTGCCCTCAGCTCTGGGATAACTCCAAGAAATTGGGGCTAAT
ACCGGATATGACCGCTGACCGCATGGTCTGGTGGTGGAAAGATTTATCGGCTGGGGATGGGCCCGCGG
CCTATCAGCTTGTTGGTGGGGTAGTGGCCTACCAAGGCGACGACGGGTAGCCGGCCTGAGAGGGTGAC
CGGCCACACTGGGACTGAGACACGGCC
>Zotu7022
GAACGCTGGCGGCAGGCCTAACACATGCAAGTCGAGCGGTAGCACAAGGGAGCTTGCTCCCTGGGTGA
CGAGCGGCGGACGGGTGAGTAATGTCTGGGAAACTGCCTGATGGAGGGGGATAACTACTGGAAACGGT
AGCTAATACCGCATAACGTCGCAAGACCAAAGAGGGGGACCTTCGGGCCTCTTGCCATCAGATGTGCC
CAGATGGGATTAGCTAGTAGGTGGGGTAATGGCTCACCTAGGCGACGATCCCTAGCTGGTCTGAGAGG
ATGACCAGCCACACTGGAACTGAGACACGGTC
>Zotu7026
GAACGCTGGCGGCATGCCTTACACATGCAAGTCGAACGGCAGCGCGGACTTCGGTCTGGCGGCGAGTG
GCGAACGGGTGAGTAATACATCAGAACGTACCCTGTTGTGGGGGATAACTAGTCGAAAGATTAGCTAA
TACCGCATACAACCTGAGGGTGAAAGCGGGGAACCGTAAGACCTCGCGCAGCAGGAGCGGCCGATGTC
TGATTAGCTAGTTGGTGGGGTAAAGGCCCACCAAGGCGACGATCAGTAGCTAGTCTGAGAGGACGATC
AGCCACACTGGGACTGAGACACGGCC
>Zotu7027
GAACGCTGGCGGCAGGCTTAACACATGCAAGTCGAACGCCCTGGCAACGGGGAGTGGCAGACGGGTGA
GTAACACGTGGGAACATACCCTTCGGTTCGGAACAACGCCGGGAAACTGGCGCTAATACCGGATACGG
CTTACGGGCGAAAGATTGATCGCCGAAGGATTGGCCCGCGTTCGATTAGCTTGTTGGTGAGGTAACGG
CTCACCAAGGCGACGATCGATAGCTGGTCTGAGAGGATGGCCAGCCACATTGGGACTGAGACACGGCC
>Zotu7028

GAACGCTGGCGGCGTGCTTAACACATGCAAGTCGAACGGTGACGAGGGAGCTTGCTCCCTCTGATCAG
TGGCGAACGGGTGAGTAACACGTGAGTAACCTGCCCCAGACTCTGGGATAACATCGGGAAACCGGTGC
TAATACCGGATATGAGACGAGACCGCATGGTCATCGTCTGGAAAGATTTATCGGTCTGGGATGGACTC
GCGGCCTATCAGCTAGTTGGTGAGGTAATGGCTCACCAAGGCGACGACGGGTAGCCGGCCTGAGAGGG
CGAACGGCCACACTGGAACTGAGACACGGTC
>Zotu7030
GAACGCTGGCGGCGTGCTTAACACATGCAAGTCGAGCGGAAAGGCCACTTCGGTGGTACTCGAGCGGC
GAACGGGTGAGTAACACGTGAGTAATCTGCCCTTCACTTCGGGATAGCTCCGGGAAACCGGGATTAAT
ACCGGATATGACTACCTTCGGCATCGGGGGTGGTGGAAAGTTTTTCGGTGGGGGATGTGCTCGCGGC
CTATCAGCTTGATGGTGGGGTAATGGCCTACCATGGCTTCGACGGGTAGCCGGCCTGAGAGGGTGACC
GGCCACACTGGGACTGAGACACGGCC
>Zotu7031
GAACGCTGGCGGCAGGCCTAACACATGCAAGTCGAGCGGATGACGGGAGCTTGCTCCTTGATTCAGCG
GCGGACGGGTGAGTAATACCTAGGAATCTGCCTGGTAGTGGGGGACAACGTTTCGAAAGGAACGCTAA
TACCGCATACGTCCTACGGGAGAAAGCAGGGGACCTTCGGGCCTTGCGCTATCAGATGAGCCTAGGTC
GGATTAGCTGGTTGGTGAGGTAATGGCTCACCAAGGCGACGATCCGTAACTGGTCTGAGAGGATGATC
AGTCACACTGGAACTGAGACACGGTC
>Zotu7032
GAACGCTGGCGGCGTGCCTAATACATGCAAGTCGAGCGGAGCTGATGAGGTGCTTGCACCTCTGATGC
TTAGCGGCGGACGGGTGAGTAACACGTAGGTAACCTGCCTGTAAGACCGGGATAACATTCGGAAACGA
ATGCTAATACCGGATACGCGATCCTCTCGCATGAGAGAATCGGGAAAGACGGAGCAATCTGTAACTTA
CGGATGGACCTGCGGCGCATTAGCTAGTTGGTGAGGTAACGGCTCACCAAGGCGACGATGCGTAGCCG
ACCTGAGAGGGTGATCGGCCACACTGGGACTGAGACACGGCC
>Zotu7033
GAACGCTAGCGGCAGGCCTAACACATGCAAGCCGAGGGGCATCGCGGGGTGGCAACACTCTGGCGGCG
ACCGGCGGAAGGGTGCGTAACACGTGAGCAACATTCCCGGATCAGGGACATAACCCGGAGAAATCCGG
ACTAATTTCCCATGGTTCCTTCAGGGGGCATCCCCTGGGGGGTAAAGATACGTCGGATCCGGATTGGC
TCGCGGGACATTAGCTAGACGGCGGGGTAACGGCCCACCGTGGCGACGATGTCTAGGGGCTCTGAGAG
GAGTGACCCCCACATTGGAACTGAGACACGGTC
>Zotu7034
GAACGCTGGCGGCGTGCTTAAGACATGCAAGTCGAACGCAGTCCCTTCGGGGGCTGAGTGGCGCACGG
GTGAGTAACACGTAACTGACCTGCCCAGAAGTTCGGAATAACTGGTCGAAAGATCAGCTAATACCGAA
TGTGCAGTGCCGCTGTGGCGGCACTGTAAAGGTCCACGCTTCTGGATGGGGTTGCGTTCCATCAGCTA
GATGGTGGGGTAAAGGCCTACCATGGCGACGACGGATAACCGGCCTGAGAGGGTGGCCGGTCACAGGG
GCACTGAGACACGGGC
>Zotu7036
GAATGCTGGCGGCGTGGATAAGGCATGCAAGTCAAGGGGGCCCGCAAGGGCAACCGGCAGACGAGGTA
GTAACACGTAGGTACATCCCCCGAAGTCGGGCATAGCTAGCCGAAAGGCTAGGTAATTCCCGATAGTC
CCGCAAGGGTAAAGGTTTTTCGCTTCGGGAGTGGCCTGCGCAGTATCAGCTAGTTGGTAGGGTAACGG
CCTACCAAGGCTACGACGCTTAGGGGAGCTGAGAGGCTGACCCCCACCGATGGGACTGAGAGACGGCC
>Zotu7037
GAACGCTGGCAGCATGCCTTACACATGCAAGTCGAACGGCAGCGCGGAAGCAATCCTGGCGGCGAGTG
GCGAACGGGTGAGTAATACATCGGAACGTGCCCAATCGTGGGGGATAACGCAGCGAAAGCTGTGCTAA
TACCGCATACGATCTACAGATGAAAGCAGGGGATCGCAAGACCTTGCGCGAATGGAGCGGCCGATGGC
AGATTAGGTAGTTGGTGAGGTAAAGGCTCACCAAGCCTTCGATCTGTAGCTGGTCTGAGAGGACGACC
AGCCACACTGGGACTGAAACACGGCC
>Zotu7038
GAACGCTGGCGGCAGGCTTAACACATGCAAGTCGAGCGCGTAGCAATACGAGCGGCAGACGGGTGAGT
AACGCGTGGGAATCTACCTTTTGCTACGGAACAACTCTGGGAAACTGGAGCTGATACCGTATGTGTCC
GCAAGGAGAAAGATTTATCGGCAAAAGATGAGCCCGCGTCCGATTAGCTAGTTGGTGGGGTAACGGCC
TACCAAGGCGACGATCGGTAGCTGGTCTGAGAGGATGATCAGCCACACTGGGACTGAGACACGGCC
>Zotu7039
GAACGCTAGCGGCAGGCTTAATACATGCAAGTCGAGGGGCAACAGGGGTAGCAATACCTGCTGGCGAC
CGGCAAACGGGTGCGGAACACGTACAGAACCTTCCTTTAAGCGGGGGATAGCCCATAGAAATGTGGAT

TAATACCCCATAGTATTATTGATTGGCATCAGTTAATAATTAAAGATTTATCACTTAAAGATGGCTGT
GCGTCTGATTAGGTAGTTGGTCGGGGTAACGGCCCACCAAGCCAACGATCAGTAACTGGTGTGAGAGC
ACGACCAGTCACACGGGCACTGAGACACGGGC
>Zotu7040
GAACGCTGGTAACGTGGATACCGGCATGCAAGTCAAGGGGGCCCGCAAGGGCAACCGGCGAAAGGCAT
AGTAACACGTAGGTACATACCCCAAAATGGACGACAGCTCGTCGAAAGACGGGGTAATACTCCATAAT
CTCGCAAGAGCAAAGTCGTAAGACGTTTTGGGAATGGCCTGCGGCCTATCAGATAGTTGGCGGTGTAA
TAGACCACCAAGTCGATGACGGGTAGGGGGTGTGAGAGCACGACCCCCATGAATGGCACTGCGACACG
GGC
>Zotu7041
GAACGCTGGCGGCGTGCCTAATACATGCAAGTAGAACGCTGAAGCTTGGTGCTTGCACCGAGTGGATG
AGTTGCGAACGGGTGAGTAACGCGTAGGTAACCTGCCTCTTAGCGGGGGATAACTATTGGAAACGATA
GCTAATACCGCATAACAATGAATGACACATGTCATTTATTTGAAAGGGGCAATTGCTCCACTACAAGA
TGGACCTGCGTTGTATTAGCTAGTAGGTGAGGTAACGGCTCACCTAGGCGACGATACATAGCCGACCT
GAGAGGGTGATCGGCCACACTGGGACTGAGACACGGCC
>Zotu7043
GAACGCTAGCGGCGGGCCTAACACATGCAAGTCGAGCGGGGAGCTTCGGCTCTTTAGCGGCGAACGGG
TGAGTAACACGTAGGTAATGTGTCCTCTGGTGGGGAATAACCGTGGGAAACCATGGCTAATACCGCAT
ATCGCGATTCGTCGTGAAAGCCTTCGGGCGCCAGTTGAGCAGCCTGCGTCCCATTATTTAGTCCTAGT
TGGTAGGGTAATGGCCTACCAAGGGTACGATGGGTAGCTGGTCTGAGAGGATGATCAGCCACACTGGA
ACTGAGACACGGTC
>Zotu7044
GAACGCTGGCGGCGCGCCTAAAACATGCAAGTCGAGCGAGAACCAGGACTTCGGTCCTGGGGACAGCG
GCGAACGGGTGCGTAACACGTGAGCAACCTGCCCCGAAGACCGGGACAAGCCGGGGAAACCTGGTCTA
ATACCGGATGCCCTCACCAGATCGCATGGTCAAGTGAGGAAATGGATTCCGCTTCGGGATGGGCTCGC
GGTCTATCAGCTTGTTGGTGGGGTAACGGCCCACCAAGGCGACGACGGATAGCTGGTCTGAGAGGACG
ATCAGCCACACTGGGACTGAGACACGGCC
>Zotu7047
GAACGCTGGCGGCGTGCCTAACACATGCAAGTCGAGCGAGATCCATCCAGTGGCAACACTGGTGAAGA
TCTAGCGGCGAACGGGTGAGTAACACGTGAGAAACCTGCCCCGGAGACCGGGATAACCCGAGGAAACT
CGGGCTAATACCGGATACCCCCACCGAACCGCATGATTTGGTGAGGAAATGCTCCGACGCTCCGGGAG
GGTCTCGCGGCCTATCAGCTAGTTGGTGAGGTAATGGCTCACCAAGGCTAAGACGGGTAGCTGGTCTG
AGAGGACGATCAGCCACACTGGGACTGAGACACGGCC
>Zotu7048
GAACGCTGGCGGCATGCCTTACACATGCAAGTCGAACGGTAACGGGCCTTCGGGCGCCGACGAGTGGC
GAACGGGTGAGTAATGCATCGGAACGTGCCCAGTAGTGGGGGATAGCCCGGCGAAAGCCGGATTAATA
CCGCATGAGACCTGAGGGTGAAAGCGGGGGATCGCAAGACCTCGCGCTACTGGAGCGGCCGATGTCAG
ATTAGCTAGTTGGTGGGGTAAAGGCCTACCAAGGCAACGATCTGTAGTTGGTCTGAGAGGACGACCAG
CCACACTGGGACTGAGACACGGCC
>Zotu7050
GAACGCTAGCGGCAGGCCTAATACATGCAAGTCGAACGGTGGTAGCAATACCATAGTGGCGCACGGGT
GCGTAACACGTAACTAACCTACCCACAACTGGGGGATAGCCCGCCGAAAGGCGGATTAATACCGCATA
AGCTCTTTGGCTGGCATCAGCTGATTAGGAAAGATTTATTGGTTGTGGATGGGGTTGCGGGTCATTAG
CTAGTTGGGTGGGTAACGGCTGCCCAAGGCGACGATGACTAGGGGAGCTGAGAGGCTGGTCCCCCACA
CGGGCACTGAGATACGGGC
>Zotu7051
GAACGCTAGCGGCAGGCTTAACACATGCAAGTCGAGGGGTAAGGCAGCAATGTCTGAGACCGGCGCAC
GGGTGCGTAACGCGTATGCAATCTACCTTTTACAGGGGAATAGCCCAGAGAAATTTGGATTAATGCCC
CATGGTATAATGAGTTGGCATCAACACATTATTAAAGTTCCAACGGTAAAAGATGAGCATGCGTCCCA
TTAGTTAGTTGGTAAGGTAACGGCTTACCAAGACGATGATGGGTAGGGGTCCTGAGAGGGAGATCCCC
CACACTGGTACTGAGACACGGAC
>Zotu7052
GAACGCTAGCGGCAGGCTTAATACATGCAAGTCGAGGGGGCAGCAATGTCACCGGCGAACGGGTGCGT
AACACGTATGCAACCTACCTATATCAGGGGGATAGCCCGAGGAAACTCGGATTAATACCGCATAACAT

TAGGATCGGCATCGATTTTAATTGAAAGATTTATTGGATATAGATGGGCATGCGTAGGATTAGCTAGT
TGGTAAGGTAACGGCTTACCAAGGCTACGATCCTTAGGGGCTCTGAGAGGAGTATCCCCCACACTGGC
ACTGAGATACGGGC
>Zotu7053
AGCCGTTGGAAATCGCTGTATTCAACTCAGATACACGGGAGTTCAGGCCCAGTGCTCACACATGCTTT
TCCCGCAACCTCTCCCAGCCACTAACATAAGGATGTTAGCA
>Zotu7055
GAACGCTGGCGGCGTGCTTAACACATGCAAGTCGAGCGGAAAGGCCACTTCGGTGGTACTCGAGCGGC
GAACGGGTGAGTAACACGTGAGTAATCTGCCCCTGGCTTCGGGATAGCCACCGGAAACGGTGATTAAT
ACCGGATATGACAACTTGTTGCATGATGGGTTGTGGAAAGTTTTTCGGCCAGGGATGTGCTCGCGGCC
TATCAGCTTGATGGTGAGGTAATGGCTCACCATGGCTTCGACGGGTAGCCGGCCTGAGAGGGTGACCG
GCCACACTGGGACTGAGACACGGCC
>Zotu7056
GAACGCTGGCGGCAGGCCTAATACATGCAAGTCGAACGGATGTAGCAATACATTAGTGGCGCACGGGT
GCCTAACGCGTGGGAACATACCCCTTGGTAAGGAATAATGTCTGGAAACGGACACTAATACATTATAA
TGACTTCGGTCCAAAGATTTATCGCCAGGGGATTGGCCCGCGTCTGATTAGCTAGTTGGTGAGGTAAC
GGCTCACCAAGGCGACGATCAGTAGCTGGTCTGAGAGGATGATCAGCCACACTGGGACTGAGACACGG
CC
>Zotu7058
GAACGCTGGCGGCATGCCTTACACATGCAAGTCGAACGGTAACGGGTCCTTCGGGATGCCGACGAGTG
GCGAACGGGTGAGTAATATATCGGAACGTGCCCAGTAGTGGGGGATAACTGCTCGAAAGAGCAGCTAA
TACCGCATACGACCTGAGGGTGAAAGGGGGGGATCGCAAGACCTCTCGCTATTGGAGCGGCCGATATC
AGATTAGCTAGTTGGTGGGGTAAAGGCCTACCAAGGCAACGATCTGTAGTTGGTCTGAGAGGACGACC
AGCCACACTGGGACTGAGACACGGCC
>Zotu7060
GAACGCTGGCGGCGTGCTTAACACATGCAAGTTGAGCGGTAAGGCCCCTTCGGGGGTACACGAGCGGC
GAACGGGTGAGTAACACGTGAGCAATCTGCCCTTCACTTTGGGATAACTCTGGGAAACGAGAGCTAAT
ACCGAATACGACCACTTCAGGCATCTGATGGTGGTGGAAAGTTCCGGCGGTGAAGGATGAGCTCGCGG
CCTATCAGCTAGTTGGTGGAGTAACGGCCCACCAAGGCTTCGACGGGTAGCCGGCCTGAGAGGGTGAC
CGGCCACACTGGGACTGAGACACGGCC
>Zotu7061
GAACGCTGGCGGCATGCCTAACACATGCAAGTCGAACGCTCGTCTTCGGACGGGAGTGGCGCACGGGT
GAGTAACGCGTGGGAACCTGCCCCGGGGTTCGGGATAACCGATGGAAACGTCGGCTAATACCGGATGC
GTCCTAAGGGAGAAAGATTTATCGCCCTGGGAGGGGCCCGCGTCGGATTAGCTAGTTGGTGGGGTAAA
GGCCTACCAAGGCGATGATCCGTAGCTGGTCTGAGAGGATGATCAGCCACACTGGGACTGAGACACGG
CC
>Zotu7062
GAACGCTGGCGGCAGGCTTAACACATGCAAGTCGAGCGGGCACTTCGGTGTCAGCGGCAGACGGGTGA
GTAACACGTGGGAACGTACCCTTCGGTTCGGAATAACGCTGGGAAACTAGCGCTAATACCGGATACGC
CCCTATGGGGAAAGGTTTACTGCCGAAGGATCGGCCCGCGTCTGATTAGCTAGTTGGTGGGGTAATGG
CCTACCAAGGCGACGATCAGTAGCTGGTCTGAGAGGATGATCAGCCACACTGGGACTGAGACACGGCC
>Zotu7064
GAACGCTGGCGGCGTGCCTAATACATGCAAGTCGAGCGATCCTTTTATGATTCACTTTGGTTTTTTCA
ATGTCTCTCGGTTTTTCTTTAAGAAAATCAGCTTTGCTGAACCGAGCGATGAGAAAAAACCTTAAGAT
TTGTTTCGTAATTGGATAGCGGCGGACGGGTGAGTAACACGTGGGTACTTGCCTCGAAGTGGAGGATA
GCTCCGGGAAACCGGAATTAATACTCCATGGTCTCTTTGCGCAAGCATTGAGTAAAGATTTATCGCTT
CGAGATGGTCCCGCGGCCGATTAGCTAGTTGGTGAGGTAATGGCTTACCAAGGCAATGATCGGTAGCT
GGTCTGAGAGGACGATCAGCCACACTGGGACTGAGATACGGCC
>Zotu7065
CAACGCTGGCGGCGTGCCTCAGACATGCAAGTCGAACGGTTAAACTTTCCTTCGGGAAAGATATAAAG
TGGCGCACGGGTGAGTAACACGTAGGTAATGTACCTTTGGGTGGGGAATAACTTAGGGAAACTTAAGC
TAATACCGCATAATGCAGCGGCTCCTTCGGGAGACAGTTGTTAAAGATTTATCGCCTAAAGAGCAGCC
TGCGGCAGATTAGCTAGTTGGTAAGGTAACGGCTTACCAAGGCTACGATCTGTATCCGACCTGAGAGG
GTGGTCGGACACACTGACACTGAATAACGGGT

>Zotu7066
GAACGCTGGCGGCATGCTTTACACATGCAAGTCGAACGGCAGCACGGGCTTCGGCCTGGTGGCGAGTG
GCGAACGGGTGAGTAATACATCGGAACGTACCCAGAAGTGGGGGATAACGCAGCGAAAGTTGCGCTAA
TACCGCATACGTTCTACGGAAGAAAGTGGGGGATCTTCGGACCTCATGCTTTTGGAGCGGCCGATGTC
TGATTAGCTAGTTGGTGGGGTAAAGGCTCACCAAGGCGACGATCAGTAGCTGGTCTGAGAGGACGACC
AGCCACACTGGAACTGAGACACGGTC
>Zotu7067
GAACGCTGGCGGCAGGCTTAACACATGCAAGTCGAACGGGCACCTTCGGGTGTCAGTGGCAGACGGGT
GAGTAACACGTGGGAACGTACCCTTCGGTTCGGAATAACACAGGGAAACTTGTGCTAATACCGGATAC
GCCCTTGGCCTCTGGCCGTGGGGAAAGGTTTACTGCCGGAGGATCGGCCCGCGTCTGATTAGCTAGTT
GGTGAGGTAACGGCTCACCAAGGCGACGATCAGTAGCTGGTCTGAGAGGATGATCAGCCACACTGGGA
CTGAGACACGGCC
>Zotu7068
GAACGCTGGCGGCGTGCCTAACACATGCAAGTCGAACGTGAACGGGAGCAATCCTAAGTAAAGTGGCG
TACGGGTGAGTAACACGTAGGTAATCTACCTTACAGTTCGGAATAACCGGCCGAAAGGCTGGCTAATA
CTGGATAGAGCGACGGAAATCTTAATGATTTTTTCGGGAAAGGGAGCCTGATAAGGGCCCTCGCTGTA
AGATGAGCCTGCGTCCCATTAGCTAGTTGGTAGGGTAACGGCCTACCAAGGCAGTGATGGGTAGCTGG
TCTGAGAGGATGATCAGCCACACTGGAACTGAAACACGGTC
>Zotu7071
GAACGCTGGCGGTGTGCTTAACACATGCAAGTGGAGCGACGAACCAGAGCTTGCTCTGGGGCAGAGCC
GCGAACGGGTGAGTAACACGTGGGTAACGTACCCCGATCACCGGGACAACCCGAGGAAACTCGGGCTA
ATACCGGATGTGTCCGTGAATCATGAGGTTCACGTGCAAAGGAAGCTTCGGCCTCCGGATCGGGAGCG
GCCCGCGGCCCATTAGTTTGTTGGTGGGGTAATGGCCTACCAAGACCGCGATGGGTAGCTGGTCTGAG
AGGACGATCAGCCACACTGGGACTGAGACACGGCC
>Zotu7075
GAACGCTGGCGGCGTGCCTAATACATGCAAGTCGAGCGGAGTTGAATAAGTGCTTGCACTAATTCAAC
TTAGCGGCGAACGGGTGAGTAACACGTAAAGAACCTGCCTCATAGACTGGGACAACTACTGGAAACGG
TAGCTAATACCGGATAACATATTTTTCTGCATGGAAGAATATTAAAAGTCGGTCATGCTGACACTATG
AGATGGCTTTGCGGTGCATTAGCTAGTTGGTAGGGTAAAGGCCTACCAAGGCGACGATGCATAGCCGA
CCTGAGAGGGTGATCGGCCACACTGGGACTGAGACACGGCC
>Zotu7078
GAACGCTGGCGGCGTGCTTAACACATGCAAGTCGAGCGGAAAGGCCCCTTCGGGGGTACTCGAGCGGC
GAACGGGTGAGTAACACGTGGGTAATCTGCCCGGGACACAGGAATAACCAGTGGAAACGCTGGCTAAT
GCCTGATGACGTGCCGGCCCCTCGGGGCCGTCACCAAAGGTAGCTTCGGCCTCCGGTCCCGGATGAGC
CCGCGGCCCATTAGCTTGTTGGCGGGGTAACGGCCCACCAAGGCGTCGATGGGTAGCTGGTCTGAGAG
GACGATCAGCCACACTGGGACTGAGACACGGCC
>Zotu7079
GAACGCTGGCGGCGTGCTTAACACATGCAAGTCGAGCGGTAAGGCCCTTCGGGGTACACGAGCGGCGA
ACGGGTGAGTAACACGTGAGCAATCTGCCCTTCACTTTGGGATAACTCTCGGAAACGGGAGCTAATAC
CGGATATGACCACTTCGCGCATGTGATGGTGGTGGAAAGTTCTGGCGGTGGAGGATGAGCTCGCGGCC
TATCAGCTTGTTGGTGGGGTAATGGCCTACCAAGGCTTCGACGGGTAGCCGGCCTGAGAGGGTGACCG
GCCACACTGGGACTGAGACACGGCC
>Zotu7080
GAACGCTGGCGGCGTGCCTAATACATGCAAGTCGAGCGGACTTTCATCGGTGCTTGCACCTTTGAAAG
TTAGCGGCGGACGGGTGAGTAACACGTGGGTAACCTACCCTATAGCTTGGGATAACTCCGGGAAACCG
GGGCTAATACCGAATAATACGTTTTCCTTCATGGGAAGACGTTGAAAGATGGTTTTGCTATCACTATA
GGATGGGCCCGCGGCGTATTAGCTAGTTGGTGAGGTAATGGCTCACCAAGGCAACGATACGTAGCCGA
CCTGAGAGGGTGATCGGCCACACTGGGACTGAGACACGGCC
>Zotu7081
GAACGCTGGCGGCAGGCTTAACACATGCAAGTCGAACGCATCCTTCGGGGTGAGTGGCAGACGGGTGA
GTAACACGTGGGAACGTACCCTTCGATCTGGGATAACTTCGGGAAACTGGAGCTAATACCGGATACGC
CCTTTTGGGGAAAGGTTTACTGTCGAAGGATCGGCCCGCGTCTGATTAGCTAGTTGGTGGGGTAACGG
CCTACCAAGGCGACGATCAGTAGCTGGTCTGAGAGGATGATCAGCCACACTGGGACTGAGACACGGCC
>Zotu7082

GAACGCTGGCGGCGTGCCTAATACATGCAAGTCGAACGGGCTGCCATAAGGCAGCCAGTGGCGAACGG
GTGAGGAGCACATGGGGAACCTGCCCGTCAGAGGGGGACAACAGCTGGAAACGGCTGCTAAGACCGCA
TAGGCTCAAGGAGGGCATCCTCGTTGAGTTAAAGTGTCCTTTCGGGGACAGCTGACGGATGGACCTGT
GGCGCATTAGCTAGTTGGCGGGGTAAAGGCCCACCAAGGCGACGATGCGTAGCCGGCCTGAGAGGGCG
GACGGCCACACTGGGACTGAGACACGGCC
>Zotu7083
GAACGCTGGCGGCAGGCTTAACACATGCAAGTCGAACGCACCTTTCGGGGTGAGTGGCAGACGGGTGA
GTAACGCGTGGGAACGTGCCCTTCAGTTCGGGATAACCCAGGGAAACTTGGGCTAATACCGGATACGT
GCGAGAGCAGAAAGATTGATCGCTGAAGGATCGGCCCGCGTCTGATTAGCTGGTTGGTGGGGTAATGG
CCCACCAAGGCGACGATCAGTAGCTGGTCTGAGAGGATGATCAGCCACATTGGGACTGAGACACGGCC
>Zotu7084
GAACGCTAGCTACAGGCTTAACACATGCAAGTCGAGGGGTAACATGACGGTGGCTTGCCATCGTTGAT
GACGACCGGCGCACGGGTGAGTAACGCGTATCCAACCTGGCCCACACTGGGGAATAGTCCCTCGAAAG
AGAGCTTAATGCCCCATGTTGCAGCATTGTCACATGTTGATGCTGCCAAAGGCTTGCCGGTATGGGAT
GGGGATGCGTCCGATCAGCTAGTTGGCGGGGTAACGGCCCACCAAGGCGACGATCGGTAGGGGTTCTG
AGAGGAAGGTCCCCCACATTGGTACTGAGACACGGAC
>Zotu7085
GAACGCTGGCGGCGTGCCTAATACATGCAAGTCGAACGGTGGCAGCAATGTCATAGTGGCGACCGGGT
GCGTAACACGTAGCTGACCTGCCCTTCAGCGGGGGACAACAGTTCGAAAGGACTGCTAATACCGCATG
AACTTGGGATGACTAGAATGTTCCAAGGAAACTTCTGGGGCTGAAGGAGGGGGCTGCGGCCCATCAGG
TAGTTGGTGAGGTAACGGCTCACCAAGCCAACGACGGGTAGGGGGCCTGAGAGGGTGGTCCCCCACAC
TGGGACTGACAAACGGCC
>Zotu7086
GAACGCTGGCGGCGTGCCTAAGACATGCAAGTCGAGCGGGTTTAGACCCGCGGCAAACGGGTGAGTAA
TATCTAGGAACATACCTCGAGTTGAGCATAACCTACCGAAAGGTAGAGCAATTCTCAATGTGGCAGCA
ATGCTAAAGTCGCAAGACGCTCGAGAAGTGGCCTGGACACCATCAGCTAGTTGGTAAGGTAATGGCTT
ACCAAGGCTTTGACGGTTAGGGGTCCCGAGAGGGAAAACCCCCACACTGGAACTGAGACACGGTC
>Zotu7087
GAACGCTAGCGGCAGGCCTAATACATGCAAGTCGAACGGGCTGGAGTAGCAATACTCCAGTTAGTGGC
GCACGGGTGCGTAACGCGTATGCAACCTACCTTTTATTGGGGGATAGCCCGAAGAAATTCGGATTAAT
ACCGCATAATATCATTTGAGGGCATCTGAGGATGATTAAAGCTACGGCGATAAAAGATGGGCATGCGT
GTCATTAGCTAGTTGGTGGTGGTAACGGCACACCAAGGCGACGATGACTAGGGGTTCTGAGAGGATGA
TCCCCCACACTGGTACTGAGATACGGAC
>Zotu7088
CAACGCTGGCGGCGCGCCTAACACATGCAAGTCGAACGAGAAAGTGGAGCAATCCATGAGTAAAGTGG
CGTACGGGTGAGTAACACGTGGGTGATCTACCTCTTAGTGAGGAATAACCCTGGGAAACCGGGGCTAA
TACCGCATAAGCCTTAGCAGGGAAAGTCGAAAGACGCTGAGAGAGGAGCCCGCGGCCGATTAGCTAGT
TGGTGGGGTAAAAGCCCACCAAGGCAGAGATCGGTAGCCGGCCTGAGAGGGCACACGGCCACACTGGC
ACTGAAACACGGGC
>Zotu7089
GAACGCTGGCGGCGTGCCTAATACATGCAAGTCGAGCGGTAAGGCTCCTCCAGGTTATTTGATTGTAT
AAGCAGCATCTAGGCTAGATGTTGTTGATCGAATAATGTGGTGGAGTACACGAGCGGCGGACGGCTGA
GTAACGCGTAGGAACATACCCCAAACTGAGGGATAACTGCTCGAAAGAGTAGCTAATACCGCATGTGG
TCTTCGGATTAAAGCTTCGGCGGTTTGGGAGTGGCCTGCGTATGATTAGATTGTTGGTGAGGTAACGG
CTCACCAAGTCGACGATCATTAGCTGGTCTGAGAGGATGATCAGCCAGACTGGGACTGAGAACGGCC
>Zotu7092
GAACGCTGGCGGAATGCTTTACACATGCAAGTCGAGCGGCAGCGCGGGGCAACCTGGCGGCGAGCGGC
GAACGGGTGAGTAACATATCGGAACGTACCCAGTCGTGGGGGATAACTATCCGAAAGGATAGCTAATA
CCGCATACGACCTGAGGGTGAAAGGGGGGGATCGCAAGACCTCTCGCGATTGGAGCGGCCGATATCGG
ATTAGCTAGTTGGTGAGGTAAAGGCTCACCAAGGCGACGATCCGTAGCTGGTCTGAGAGGACGACCAG
CCACACTGGAACTGAGACACGGTC
>Zotu7094
GAACGCTGGCGGCGTGCCTAACACATGCAAGTCGAACGAAGCTTGAATTATGATTTCTTCGGAATGAA
TAATGATATGACTGAGTGGCGGACGGGTGAGTAACGCGTGAGCAACCTGCCCTTCGGAGGGGGATAGT

GTCTGGAAACGGACAGTAATACCGCATAATGTATTTTTACCGCATGATAGGAATACCAAAACTGAGGT
GCCGAAGGATGGGCTCGCGTTGGATTAGATAGTTGGTGGGGTAACGGCCTACCAAGTCGACGATCCAT
AGCCGGACTGAGAGGTTGAACGGCCACATTGGGACTGAGACACGGCC
>Zotu7095
GAACGCTGGCGGCGCGCTTAACACATGCAAGTCGAGCGAGAACCAGTCCTTCGGGACTGGGGACAGCG
GCGAACGGGTGAGTAACACGTGGGTAATCTGCCCTCGATTCTGGGATAGCCCGGGGAAACCCGGATTA
ATACCGGATAGCCTCTCGAGCTTACGAGCTCGTGAGAAAAGGTAGCTTCGGCCTCCGATCGAGGATGA
GCCCGCGGCGGATTAGCTTGTTGGTGGGGTAATGGCCTACCAAGGCGACGATCCGTAGCTGGTCTGAG
AGGACGATCAGCCACACTGGGACTGAGACACGGCC
>Zotu7097
GAACGCTGGCGGCGTGCTTAACACATGCAAGTCGAACGGTGAAAGAGAGCTTGCTCTCTGGATCAGTG
GCGAACGGGTGAGTAACACGTGAGCAATCTGCCCTTGACTCTGGGATAAGCGTTGGAAACGACGTCTA
ATACCGGATAAGAGCTGTGAAGGCATCTTCAACAGCTGGAAAGAACTTCGGTCAAGGATGAGCTCGCG
GCCTATCAGCTAGTTGGTGAGGTAATGGCTCACCAAGGCGTCGACGGGTAGCCGGCCTGAGAGGGTGA
CCGGCCACACTGGGACTGAGACACGGCC
>Zotu7098
GAACGTTGATGGCGTGCCTCATACATGCAAGTCGAGCGCCCCGCAAGGGGAGCGGCGGACGGGTGAGT
AACACGTAAAGAATCTGCCCTGAAGCGGGGAATAACTATCCCAACGGATAGCTAATACCGCATAATGA
TGGAGGAGGCATCTCCTCTTCTCAAAAGGCTTCGGCCACTTCAGGATGACTTTGCGTCCCATTAGCTT
GTTGGCGGGGTAACGGCCCACCAAGGCTACGATGGGTATCCGGCCTGAGAGGGTGTACGGACACATTG
GGACTGAGATACGGCC
>Zotu7099
GAACGCTGGCGGCAGGCTTAACACATGCAAGTCGAACGCCCCGCAAGGGGAGTGGCAGACGGGTGAGT
AACGCGTGGGAATCTACCTTGAACTACGGGATAGCTCCGGGAAACTGGAATTAATACCGTATGTGCCC
TTAGGGGGAAAGATTTATCGGTTCAAGATGAGCCCGCGTTGGATTAGCTAGTTGGTGGGGTAAAGGCC
TACCAAGGCGACGATCCATAGCTGGTCTGAGAGGATGACCAGCCACATTGGGACTGAGACACGGCC
>Zotu7100
CAACGCTGGCGGCGTGCCTAACACATGCAAGTCGCACGAGAAAGGGGCTTCGGTCCTGAGTAAAGTGG
CGCACGGGTGAGTAACACGTGACTAACCTACCTCCGAGTGGGGAATAACCTAGAGAAATCTGGGCTAA
TACCGCATAACACTTACGAGTCAAAGCAGCAATGCGCTTGGAGAGGGGGTCGCGGTCGATTAGCTAGT
TGGCGGGGTAATGGCCCACCAAGGCGATGATCGATATCCGGCCTGAGAGGGCGCACGGACACACTGGA
ACTGAAACACGGTC
>Zotu7101
GAACGCTGGCGGCATGCCTTACACATGCAAGTCGAACGGCAGCATGGGTGCTTGCACCTGATGGCGAG
TGGCGAACGGGTGAGTAATGCATCGGAACGTACCCGGAAGTGGGGGATAACGTAGCGAAAGTTACGCT
AATACCGCATATTCTGTGAGCAGGAAAGAGGGGGATCGCAAGACCTCTCGCTTTCGGAGCGGCCGATG
TCGGATTAGCTAGTTGGCGGGGTAAAGGCCCACCAAGGCGACGATCCGTAGCGGGTCTGAGAGGATGA
TCCGCCACACTGGGACTGAGACACGGCC
>Zotu7104
GAACGCTGGCGGCATGCTTTACACATGCAAGTCGAACGGCAGCGCGGGGCAACCTGGCGGCGAGTGGC
GAACGGGTGAGTAATATATCGGAACGTACCCAAGAGTGGGGGATAACGTAGCGAAAGTTACGCTAATA
CCGCATACGATCTAAGGATGAAAGCAGGGGATCTTCGGACCTTGTGCTCCTGGAGCGGCCGATATCTG
ATTAGCTAGTTGGTGAGGTAAAGGCTCACCAAGGCCACGATCAGTAGCTGGTCTGAGAGGACGACCAG
CCACACTGGAACTGAGACACGGTC
>Zotu7106
GAACGCTGGCGGCGTGCTTAACACATGCAAGTCGAACGGTAAGGCCCTTCGGGGTACACGAGTGGCGA
ACGGGTGAGTAACACGTGAGTGACCTGCCCTCTACTTCGGGATAACTTCCAGAAATGGTGGCTAATAC
CGGATATTCACATGTTCCTGCATGGGGGTGTGTGGAAAGCCTTGTGCGGTAGGGGATGGACTCGCGGC
CTATCAGCTTGTTGGTGAGGTAGTGGCTCACCAAGGCGTCGACGGGTAGCCGGCCTGAGAGGGTGACC
GGCCACATTGGGACTGAGATACGGCC
>Zotu7107
CAACGCTGGCGGCGTGCCTAATGCATGCAAGTCGAACGGGGTCCCCTTCGGGGGGCTTGCGTGGCGGA
CGGGTGAGGAACACGTGGCCAACCTGCCCACTGGCGGGGGGTAGCCCGCGGAAACGCGGGGTAATTCC
GCATGAACGGCGCGACCGGGTAGGGTCGTGGCGCAAAGGTCCTTCGGGGCCGCCGGTGGAGGGGGCTG

CGGCCGATTAGCTAGTTGGGGGGGTAACGGCCTCCCAAGGCGATGATCGGTAGCTGGTCTGAGAGGAT
GATCAGCCACACGGGGACTGAGACACGGCC
>Zotu7108
GAACGCTGGCGGCGTGCCTAATACATGCAAGTCGAGCGGTAAGGCCCCTTCGGGGGTACACGAGCGGC
GGACGGCTGAGTAACACGTAGGAACGTACCCCAAAGTGAGGGATAAGCACCAGAAATGGTGTCTAATA
CCGCATGTGCTCTAAGGAGTAAAGCTTCGGCGCTTTGGGAACGGCCTGCGTCCGATTAGCTTGTTGGT
GAGGTAATAGCTCACCAAGGCGACGATCGGTAGCTGGTCTGAGAGGATGATCAGCCAGACTGGGACTG
AGACACGGCC
>Zotu7110
GAACGCTGGCGGCGTGCCTTAAGCATGCAAGTCGAATGGTCGCAAGACAATGGCGGACGGGTGAGTAA
TACATGGGAATCTACCTTGAAATGGGGCATAGCCCTCCGAAAGGAGGGGTAATACCGCATGTGATCGC
AAGATTAAAGTCGCAAGACGTTTCAAGATGAGCCTGTGTCCTATCAGCTTGTTGGTGGGGTAATGGCC
TACCAAGGCAACGACGGGTAGCTGGTCTGAGAGGATGATCAGCCACAATGGCACTGAGACACGGGC
>Zotu7111
GAACGCTGGCGGCAGGCCTAACACATGCAAGTCGAACGCTCGTAGCAATACGGGAGTGGCAGACGGTT
GAGTAACGCGTGGGAACCTTCCCAGTGGTACGGAATAGCTCAGGGAAACTTGAGGTAATACCGTATAC
GCCCCTAGGGGGAAAGATTTATCGCCATTGGATGGGCCCGCGTCGGATTAGCTAGTTGGTGAGGTAAC
GGCTCACCAAGGCGACGATCCGTAGCTGGTTTGAGAGAATGACCAGCCACACTGGGACTGAGACACGG
CC
>Zotu7114
GAACGCTGGCGGCAGGCTTAACACATGCAAGTCGAACGCCCCGCAAGGGGAGTGGCAGACGGGTGAGT
AACACGTGGGAACATACCCTTCGGTTCGGAACAACGCCGGGAAACTGGCGCTAATACCGGATACGGCC
TTTGGGCGAAAGATTGATCGCCGAAGGATTGGCCCGCGTTCGATTAGCTTGTTGGTGAGGTAACGGCT
CACCAAGGCGACGATCGATAGCTGGTCTGAGAGGATGGCCAGCCACATTGGGACTGAGACACGGCC
>Zotu7116
GAACGCTGGCGGCGTGCTTAACACATGCAAGTCGAACGGTGAAGCAGGAGCTTGCTCCTGTGGATCAG
TGGCGAACGGGTGAGTAACACGTGAGTAACCTGCCCCAGACTCTGGGATAAGCGTTGGAAACGACGTC
TAATACCGGATACGAGACGAGAAGGCATCTTCATCGTCTGGAAAGAACTTCGGTCTGGGATGGACTCG
CGGCCTATCAGGTAGTTGGTGAGGTAACGGCCCACCAAGCCTACGACGGGTAGCCGGCCTGAGAGGGT
GACCGGCCACACTGGGACTGAGACACGGCC
>Zotu7117
TCCGTATCTCAGTACCCATGTGGGGGCCAATCCTCTCAGAACCCCTACTGATCATCGCCTTGGTGGGC
CGTTACCCCCCCAACTAGCTAATCAGACGCAAGCCCCTCTCATACCCATAAATGTTTACCAATATCAT
CAGGTGATGACACTGGACCGTGCGGGTTTACCCCAGCTTTCGCCGGGCTATCCCCCAGTATGAGGCAG
GTTGCTTACGCGTTACGCACCCGTTTGCCACTGTCCTTGCGAACCGTTCGACTTGCATGTATTAGGCC
TGCCGCTAGCGTTCA
>Zotu7119
GAACGCTGGCGGCATGCCTAACACATGCAAGTCGAACGAAGGCTTCGGCCTTAGTGGCGCACGGGTGC
GTAACGCGTGGGAATCTGCCCTTGGGTTCGGAATAACAGTTGGAAACGACTGCTAATACCGGATGATG
ACGTAAGTCCAAAGATTTATCGCCCAAGGATGAGCCCGCGTAGGATTAGCTAGTTGGTGGGGTAAAGG
CCCACCAAGGCGACGATCCTTAGCTGGTCTGAGAGGATGATCAGCCACACTGGGACTGAGACACGGCC
>Zotu7120
GAACGCTGGCGGCGTGCCTAATACATGCAAGTCGAGCGGTAAGGCTTCCACTCTGTACTTTAGTTTCT
AGAGTATGGTGTGGAAGTACACGAGCGGCGGACGGCTGAGTAACGCGTAGGAACGTACCCCAAAGTGA
GGGATAGCCCACCGAAAGGTGGATTAATACCGCATGTGATCTTCGGATTAAAGCTTTACGCGCTTTGG
GAACGGCCTGCGTATGATTAGCTTGTTGGTGAGGTAATGGCTCACCAAGGCGACGATCATTAGCTGGT
CTGAGAGGATGATCAGCCAGACTGGGACTGAGACACGGCC
>Zotu7122
GAACGCTGGCGGCGTGCTTAACACATGCAAGTCGAACGGAAAGGTCTCTTCGGAGATACTCGAGTGGC
GAACGGGTGAGTAACACGTGGGTGATCTGCCCTGCACTTCGGGATAAGCTTGGGAAACTGGGTCTAAT
ACCGGATAGGACTACGGACTGCATGGTCTGTGGTGGAAAGCTTTTGCGGTGTGGGATGGGCCCGCGGC
CTATCAGCTTGTTGGTGGGGTGATGGCCTACCAAGGCGACGACGGGTAGCCGGCCTGAGAGGGTGTCC
GGCCACACTGGGACTGAGATACGGCC
>Zotu7123

GAACGCTGGCGGCGTGCTTAAGACATGCAAGTCGAACGGCCTGAAGCTTGCTTCAGGCAGTGGCGCAC
GGGTGAGTAGCGCGTGACTGACCTGCCCCAAAGTCCTCGAATAACTGGCTGAAAGGTCAGCTAATACG
GGATGTGCAGCACCCTCGTGTGGGTGTTGTAAAGGCTATGACCGCTTTGGGATGGGGTTGCGTTCCAT
CAGCTAGTTGGTAGGGTAAAGGCCTACCAAGGCGACGACGGATCACCGGCCTGAGAGGGTGGCCGGTC
ACAGGGGCACTGAGACACGGGT
>Zotu7125
CAACGCTGGCGGCGTGCCTAACACATGCAAGTCGAACGAGAAAGTGGAGCAATCCATGAGTAAAGTGG
CGACCGGGTGAGTAACACGTGACTAACCTACCCTACAGTGGGGGATAACCTCGGGAAACCGGGGCTAA
TACCGCATAACATCGCAAGATCAAAGAAGCAATTCGCTAAAGGAGGGGGTCGCGGCTGATTAGTTAGT
TGGTGAGGTAACGGCCCACCAAGACGAAGATCAGTAGCCGGCCTGAGAGGGCGCACGGCCACACTGGA
ACTGAAACACGGTC
>Zotu7126
GAACGCTGGCGGCAGGCTTAACACATGCAAGTCGAACGCCCCGCAAGGGGAGTGGCAGACGGGTGAGT
AACACGTGGGAACATACCCTTTGGTTCGGAATAACTCAGGGAAACTTGAGCTAATACCGGATACGTCC
GCGAGGAGAAAGATTCATCGCCGAAGGATTGGCCCGCGTCCGATTAGCTAGTTGGTGTGGTAATGGCG
CACCAAGGCGACGATCGGTAGCTGGTCTGAGAGGATGATCAGCCACATTGGGACTGAGACACGGCC
>Zotu7128
GAACGCTGGCGGCATGCCTAACACATGCAAGTCGAACGATCCCTTCGGGGATAGTGGCGCACGGGTGC
GTAACGCGTGGGAATCTGCCCTTGGGTTCGGAATAACAGTGAGAAATTACTGCTAATACCGGATGATG
TCTTCGGACCAAAGATTTATCGCCCAGGGATGAGCCCGCGTAGGATTAGCTAGTTGGTGGGGTAATGG
CCTACCAAGGCGACGATCCTTAGCTGGTCTGAGAGGATGATCAGCCACACTGGGACTGAGACACGGCC
>Zotu7129
GAACGCTGGCGGCGTGCTTAACACATGCAAGTCGAACGGTGAACCTTGGAGCTTGCTCTAAGGGGATC
AGTGGCGAACGGGTGAGTAACACGTGAGTAACCTGCCCCGGACTCTGGAATAAGCGCTGGAAACGGCG
TCTAATACCGGATACGAGACGCAACCGCATGGTTAGTGTCTGGAAAGTTTTTCGGTCTGGGATGGACT
CGCGGCCTATCAGCTTGTTGGTGAGGTAATGGCTCACCAAGGCGACGACGGGTAGCCGGCCTGAGAGG
GCGACCGGCCACACTGGGACTGAGACACGGCC
>Zotu7131
GAACGCTGGCGGCGTGCTTAAGACATGCAAGTCGGACGCATCCCCCTCGGGGGATGAGTGGCGCACGG
GTGAGTAACGCGTAACTGACCTACCCTCAAGTCCTGACCAACTCCTCGAAAGAGGAGCTAATGTGGGA
TGTGCTGCCTCACCGTGGTGGGGCAGTAAAGGCTATGACCGCTTGAGGATGGGGTTGCGTTTCATCAG
CTAGATGGTGGGGTAAAGGCCTACCATGGCGACGACGAATAGCCGGCCTGAGAGGGTGGCCGGCCACA
GGGGCACTGAGACACGGGC
>Zotu7132
GAACGCTGGCGGCAGGCTTAACACATGCAAGTCGAGCGCCCCGCAAGGGGAGCGGCAGACGGGTGAGT
AACGCGTGGGAATCTACCTTTTGCTACGGAATAACGCATGGAAACGTGTGCTAATACCGTATGTGCCC
TTCGGGGGAAAGATTTATCGGCAAAGGATGAGCCCGCGTTGGATTAGCTAGTTGGTGGGGTAAAGGCC
TACCAAGGCGACGATCCATAGCTGGTCTGAGAGGATGATCAGCCACATTGGGACTGAGACACGGCC
>Zotu7133
GAACGCTGGCGGCGTGCTTAACACATGCAAGTCGAACGGAAAGGCCCTTCGGGGTACTCGAGTGGCGA
ACGGGTGAGTAACACGTGGGTGATCTGCCCTGCACTTTGGGATAAGCCTGGGAAACTGGGTCTAATAC
CGAATACACCTCTCCGGCTGCATGGTTGGTGGGGGAAAGCTTTTGCGGTGTGGGATGGGCCCGCGGCC
TATCAGCTTGTTGGTGAGGTTACGGCTCACCAAGGCGACGACGGGTAGCCGGCCTGAGAGGGTGACCG
GCCACACTGGGACTGAGATACGGCC
>Zotu7134
GAACGCTGGCGGTGTGCCTAATACATGCAAGTCGAGCGGTAAGGCCCCTTCGGGGGTACACGAGCGGC
GGACGGCTGAGTAACACGTAGGAACGTACCCCAAAGTGAGGGATAAGCACCAGAAATGGTGTCTAATA
CCGCATGTGCTCTAAGGAGTAAAGCTTTATGCGCTTTGGGAACGGCCTGCGCATGATTAGCTTGTTGG
TGAGGTAAAAGCTCACCAAGGCGACGATCATTAGCTGGTCTGAGAGGATGATCAGCCAGACTGGGACT
GAGACACGGCC
>Zotu7135
GAATGCTGGCGGCGCGCCTAACACATGCAAGTCGAGCGGTAACAGGGGTAATTTATTACCTGCTGACG
AGCGGCGGACGGCTGAGTAACGCGTGGGAACGTACCCCAAAGTGAGGAATAACCGCCCGAAAGGGTGG
CTAATGCCACATGTGATCTTCGGATTAAAGGATTTATCCGCTTTGGGAACGGCCTGCGTATGATTAGA

TAGTTGGTGAGGTAAAGGCTCACCAAGTCTACGATCATTAACTGGTCTGAGAGGATGATCAGTCAGAC
TGGAACTGAGACACGGTC
>Zotu7137
GAACGCTAGCGGGAGGCCTAATACATGCAAGTCGAACGGTAACAGGGTAGCAATACCGCTGACGAGTG
GCGCACGGGTGAGTAACACGTACACAATCTGCCTTATACTGGAGCATACCCCCAAGAAATTGGACTA
ATTCTCCATAACATATTGAAAAGGCATCTTTTTAATATTAAAACTCAGGTGGTATAAGATGAGTGTGC
GTCTGATTAGCTAGTTGGTAAGGTAACGGCTTACCAAGGCAACGATCAGTAACTGGTCTGAGAGGATG
ATCAGTCACACGGATACTGAGACACGGGT
>Zotu7138
GAATGCTGGCGGCGTGCCTAATACATGCAAGTCGAGCGGACCTGAATGGAGCTTGCTCCAGACAGGTT
AGCGGCGGACGGGTGAGTAACACGTAGGCAACCTGCCCGCAAGACCGGGATAACGTAGGGAAACTTAC
GCTAATACCGGATAGGGCACTCTCTCGCATGAGAGGGTGCGGAAAGGTGGCGCAAGCTACCACTTGCG
GATGGGCCTGCGGCGCATTAGCTAGTTGGTGGGGTAACGGCCTACCAAGGCGACGATGCGTAGCCGAC
CTGAGAGGGTGACCGGCCACACTGGGACTGAGACACGGCC
>Zotu7139
TAACGCTGGCGGCGTGCATAACACATGCAAGTCGAACGATGAAACTTAATTGATCTCTTCGGAGTGAT
AATAAGCGGATTAGTGGCGAACGGGTGAGTAACACGTGAGTAACCTGCCTTAGAGAAGGGGATAGCCT
TTGGAAACGAAGAATAATACCCTATGAAATGATAATATCGCATGATAGTATCATCAAAGATTTATCGC
TCTAAGATGGACTTGCGCCTGATTAGCTAGTTGGTGGAGTAACAGCCCACCAAGGCAACGATCAGTAG
CCGGCTTGAGAGAGTGTACGGCCACATTGGGACTGAGACACGGCC
>Zotu7141
GAACGCTGGCGGCGTGCTTAACACATGCAAGTCGAACGAACGGAGGGAGCTTGCTCCCCGAGTTAGTG
GCGGACGGGTGAGTAACACGTGAGCAACCTGCCCTATACACCGGGATAACATAGAGAAATTTGTGCTA
ATACCGGATGAGACCACACCGAGGCATCTCGGAGGGGTTAAAGCTCAGGCGGTATAGGAGGGGCTCGC
GGCCCATTAGATAGTTGGTGAGGTAACGGCCCACCAAGTCAGCGATGGGTAGCCGACCTGAGAGGGTG
ATCGGCCACATTGGAACTGAGATACGGTC
>Zotu7142
GAACGCTGGCGGCATGCCTAACACATGCAAGTCGAACGAGGGTAGCAATACCTTAGTGGCGGACGGGT
GAGGAATACATAGGAATTTGCCCTTAAGTGGGGGATAGCCCGAGGAAACCCGGATTAATACCGCATGA
ACTCGTGAGAGCAAAGTCGGGGACCTTCGGGCCTGGCGCTTTTGGAGAAGCCTATGTCGGATTAGCTA
GTTGGTGAGGTAATGGCTCACCAAGGCTGCGATCCGTAACTGGTCTGAGAGGATGATCAGTCACACCG
GAACTGAGACACGGTC
>Zotu7143
GAACGCTGGCGGCAGGCCTAACACATGCAAGTCGAGCGATCGCAGCAATGCGGGAGCGGCAGACGGGT
GAGTAACACGTGGGAACGTACCCTTCGGTTCGGAATAACTCAGGGAAACTTGAGCTAATACCGGATAC
GTCCGAGAGGAGAAAGATTTATCGCCGAAGGATCGGCCCGCGTCTGATTAGCTAGTTGGTGAGGTAAC
GGCTCACCAAGGCGACGATCAGTAGCTGGTCTGAGAGGATGATCAGCCACATTGGGACTGAGACACGG
CC
>Zotu7144
GAACGCTGGCGGCGTGCTTAACACATGCAAGTCGAACGGGCGTAGCAATACGTCAGTGGCGGACGGGT
GAGTAACGCGTGAGCAATCTGCCTCTGTCAGGGGGATAACACTTAGAAATAGGTGCTAATACCGCATA
AGACCACGTTTCGGCATCGGAGAGTGGTAAAAGATTTATCGGACAGAGATGAGCTCGCGTCCCATTAG
CTAGTTGGTAAGGTAACGGCCTACCAAGGCGACGATGGGTAGCCGACCTGAGAGGGTGATCGGCCACA
CTGGAACTGAGAAACGGTC
>Zotu7145
GAACGCTGGCGGCGTGCTTAACACATGCAAGTCGAGCGGAAAGGCCACTTCGGTGGTACTCGAGCGGC
GAACGGGTGAGTAACACGTGAGTAATCTGCCCTTGGCTTCGGGATAGCCACCGGAAACGGTGATTAAT
ACCGGATATGACCACTTCGTGCATGCGATGGTGGTGGAAAGTTTTTCGGCCAGGGATGTGCTCGCGGC
CTATCAGCTTGATGGTGAGGTAATGGCTCACCATGGCTTCGACGGGTAGCCGGCCTGAGAGGGTGACC
GGTCACACTGGGACTGAGACACGGCC
>Zotu7147
GAACGCTGGCGGCGTGCTTAACACATGCAAGTCGAACGGAAAGGCCCTTCGGGGTACTCGAGTGGCGA
ACGGGTGAGTAACACGTGGGTGATCTGCCCTGCACTTTGGGATAAGCCTGGGAAACTGGGTCTAATAC
CGGATATGATCACGGGCTTCATGGTCTGTGGTGGAAAGCTTTTGCGGTGTGGGATGGGCCCGCGGCCT

ATCAGCTTGTTGGTGGGGTAATGGCCTACCAAGGCGTCGACGGGTAGCCGGCCTGAGAGGGTGTCCGG
CCACACTGGGACTGAGATACGGCC
>Zotu7148
GAACGCTGGCGGTATGCTTTACACATGCAAGTCGTACGGGAGTTTTTAACTCAAGTGGCGGACGGGTG
AGTAACACGTAAGAATTTGCCTTTAGGAGGGGGATAACAACTGGAAACGGTTGCTAATACCCCATATG
CTTTTGAGTGAAATGGAATTTCCGCCTAAAGAGAAGCTTGCGGCTGATTAGCTTGTTGGTGAGGTAAT
GGCTTACCAAGGCGACGATCAGTATCTGGTTTGAGAGGACGATCAGACACACTGGAACTGAGACACGG
TC
>Zotu7149
GAACGCTAGCGGCAGGCTTAACACATGCAAGTCGAGGGGTAGAAGGAGCTTGCTCCTTTGAGACCGGC
GCACGGGTGCGTAACGCGTATGCAATTTACCTTTTACAGAGGGATAGCCCAGAGAAATTTGGATTAAT
ACCTCATAGTATTATGAAATGGCATCATTTTATAATTAAAGTTCCAACGGTAAAAGATGAGCATGCGT
CCCATTAGCTAGTTGGTGTGGTAACGGCACACCAAGGCAACGATGGGTAGGGGTCCTGAGAGGGAGAT
CCCCCACACTGGTACTGAGACACGGAC
>Zotu7151
GAACGCTGGCGGCGTGCTTAACACATGCAAGTCGAACGGAAAGGCCCTGCTTGCAGGGTGCTCGAGTG
GCGAACGGGTGAGTAACACGTGGGTGATCTGCCCCTGACTTTGGGATAAGCCTGGGAAACTGGGTCTA
ATACCGGATATGACCAAGGATTGCATGGTTCTTGGTGGAAAGCTTTTGCGGTTGGGGATGGCCCGCG
GCCTATCAGCTTGTTGGTGGGGTAATGGCCTACCAAGGCGACGACGGGTAGCCGACCTGAGAGGGTGA
TCGGCCACACTGGGACTGAGACACGGCC
>Zotu7152
GAACGCTGGCGGCGTGCCTAATACATGCAAGTCGAGCGAATCTTTGGGAGCTTGCTCCTAAAGGTTAG
CGGCGGACGGGTGAGTAACACGTGGGCAACCTGCCTATAAGACTGGGATAACTTCGGGAAACCGGAGC
TAATACCGGATAATTCTTTTCTACACATGTAGGGGAGCTAAAAGACGGTTTACGCTGTCACTTATAGA
TGGGCCCGCGGCGCATTAGCTAGTTGGTGAGGTAACGGCTCACCAAGGCCACGATGCGTAGCCGACCT
GAGAGGGTGATCGGCCACACTGGGACTGAGACACGGCC
>Zotu7153
GAACGCTGGCGGCAGGCTTAACACATGCAAGTCGAGCGCCCCGCAAGGGGAGCGGCAGACGGGTGAGT
AACGCGTGGGAACGTACCCTTTACTACGGAATAACTCAGGGAAACTTGTGCTAATACCGTATGTGCCC
TTCGGGGGAAAGATTTATCGGTAAAGGATCGGCCCGCGTTGGATTAGCTAGTTGGTGGGGTAAAGGCC
TACCAAGGCGACGATCCATAGCTGGTCTGAGAGGATGATCAGCCACACTGGGACTGAGACACGGCC
>Zotu7154
CAACGCTGGCGGCGTGCCTCAGACATGCAAGTCGAACGATTAAACTTTCCTTCGGGGAAGATATAAAG
TGGCGCACGGGTGAGTAACACGTAGGTAATGTACCTTTGGGTGGGGAATAACTTAGGGAAACTTAAGC
TAATACCGCATAATGCAGCGGCTTCTTCGGAAGACAGTTGTTAAAGATTTATCGCCTAAAGAGCAGCC
TGCGGCAGATTAGCTAGTTGGTAAGGTAACGGCTTACCAAGGCTACGATCTGTATCCGACCTGAGAGG
GTGGTCGGACACACTGACACTGAATAACGGGT
>Zotu7155
GAACGCTGGCGGCGTGCTTAACACATGCAAGTCGAACGAAGTCCATGGAGCTTGCTCCGGAAGACTGA
GTGGCGAACGGGTGCGTAACACGTGAGAAACCTGCCCCGGACTTTGGGATAACAGTTGGAAACGACTG
CTAATACCGAATACCACCATCTCCTCGCATGGGGAGTTGATGAAAGGTCCGCCGGTCCGGGAGGGTCT
CACGGCCTATCAGCTTGTTGGAGAGGTAACGGCTCACCAAGGCATCGACGGGTAGCTGGTCTGAGAGG
ATGATCAGCCACACTGGGACTGAGACACGGCC
>Zotu7156
GAACGCTGGCGGCGTGCTTAACACATGCAAGTCGAACGGTGAAGGGAAAGCTTGCTTTTCTGGATCAG
TGGCGAACGGGTGAGTAACACGTGAGTAACCTGCCCTTGACTCTGGGATAAGCGTTGGAAACGACGTC
TAATACCGGATATGAGCCTCCTCCGCATGGTGAGGGGTTGGAAAGATTTTTTGGTCAAGGATGGACTC
GCGGCCTATCAGCTTGTTGGTGAGGTAATGGCTCACCAAGGCGACGACGGGTAGCCGGCCTGAGAGGG
TGACCGGCCACACTGGGACTGAGACACGGCC
>Zotu7157
GAACGCTGGCGGCATGCCTAACACATGCAAGTCGAACGAGACCTTCGGGTCTAGTGGCGCACGGGTGC
GTAACGCGTGGGAATCTACCCTTGGGTTCGGAATAACTCAGAGAAATTTGAGCTAATACCGGATGATG
ACGTAAGTCCAAAGATTTATCGCCCAAGGATGAGCCCGCGTAGGATTAGCTAGTTGGTGAGGTAAAGG
CTCACCAAGGCGACGATCCTTAGCTGGTCTGAGAGGATGATCAGCCACACTGGGACTGAGACACGGCC

>Zotu7158
GAACGCTGGCGGCGTGCTTAACACATGCAAGTCGAACGGTGATCTTGGGAGCTTGCTCCTTAAGTGAT
CAGTGGCGAACGGGTGAGTAACACGTGAGTAACCTGCCCCAGACTCTGGAATAACCCCGGGAAACCGG
AGCTAATACCGGATATGACACCAATCCGCATGGGTATGGTGTGGAAAGTTTTTCGGTCTGGGATGGAC
TTGCGGCCTATCAGCTTGTTGGTGAGGTAATGGCTCACCAAGGCGACGACGGGTAGCCGGCCTGAGAG
GGCGACCGGCCACACTGGGACTGAGACACGGCC
>Zotu7159
TAACGCTGGCGGTATGCCTAACACATGCAAGTCGAACGAGATCTTCGGATCTAGTGGCGGACGGGTTA
GTAACACGTTGGAACCTACCCCGAAGTGGGGAATAACTGGCCGAAAGGTCGGCTAATACCCCATAGTC
TTATACTCGTTATAAGTAAAGAATTTCGCTTCGGGAGGGGCCTGCGTAGCATTAGCTTGTTGGTGAGG
TAAGAGCTCACCAAGGCTACGATGCTTAGCTGGATCGAGAGGTTGACCAGCCACACTGGGACTGAGAT
ACGGCC
>Zotu7160
GAACGCTGGCGGCGTGCCTAATACATGCAAGTCGAGCGGTAAGGCCTCTTCGGAGGTACACGAGCGGC
GGACGGCTGAGTAACACGTAGGAACGTACCCCAAACTGAGGGATAAGCACCAGAAATGGTGTCTAATA
CCGCATGTGATCTTCGGATTAAAGCTTCGGCGGTTTGGGAACGGCCTGCGCATGATTAGCTTGTTGGT
GAGGTAAAAGCTCACCAAGGCGACGATCCGTAGCTGGTCTGAGAGGACGATCAGCCACACTGGGACTG
AGACACGGCC
>Zotu7162
GAACGCTGGCGGCAGGCTTAACACATGCAAGTTGAACGGGATTTGTGTGGTGCTTGCACCATATAATG
AGAGTAGCGCACTGGTGAGTAACACGTGGGAACGTACCTTTTGGTGGGGGACAACATCTGGAAACGGA
TGCTAATACCGCATAAGACCTGAGGGTGAAAGATTTATCGCCGAAAGAACGGCCCGCGGAAGATTAGG
CAGTTGGTGGGGTAAAGGCCTACCAAACCTACGATCTATAGCTGGTCTGAGAGGACGATCAGCCACAC
TGGAACTGAGACACGGTC
>Zotu7163
GAACGCTGGCGGCATGCCTTACACATGCAAGTCGAACGGCAGCGCGGGGCAACCTGGCGGCGAGTGGC
GAACGGGTGAGTAATATATCGGAACGTACCCTGGAGTGGGGGATAACGTAGCGAAAGTTACGCTAATA
CCGCATACGATCCATGGATGAAAGCAGGGGACCGCAAGGCCTTGTGCTCCAGGAGCGGCCGATATCTG
ATTAGCTAGTTGGCGGGGTAAAAGCCCACCAAGGCATCGATCAGTAGCTGGTCTGAGAGGACGACCAG
CCACACTGGAACTGAGACACGGTC
>Zotu7164
GAACGCTAGCGGCAGGCCTAACACATGCAAGTCGAGGGGTAGAAGGCTTCGGCCTTTGAGACCGGCGC
ACGGGTGCGTAACGCGTATGCAATCTGCCTTACACTAAGGGATAGCCCAGAGAAATTTGGATTAATAC
CTTATAGTATATTTTTGTGGCATCACAAGATTATTAAAGATTTATTGGTGTAAGATGAGCATGCGTCC
CATTAGCTAGTTGGTATGGTAACGGCATACCAAGGCTATGATGGGTAGGGGTCCTGAGAGGGAGATCC
CCCACACTGGTACTGAGACACGGAC
>Zotu7165
GAACGCTAGCGGCAGGCTTAATACATGCAAGTCGAGGGGCAGCACGGGGGCAACCCTGGTGGCGACCG
GCAAACGGGTGCGGAACACGTACGCAACTTTCCCAAAACTGGTGAATAGCCTTCCGAAAGGGAGATTA
ATACACCGTAAGATATAGGAGTGGCATCACTTTTATATTATAGCTCCGGCGGTTTTGGATGGGCGTGC
GCCTGATTAGGTAGTTGGCGGGGTAACGGCCCACCAAGCCTGCGATCAGTAACTGGTGTGAGAGCACG
ACCAGTCACACGGGCACTGAGACGCGGGC
>Zotu7167
GAACGCTGGCGGCAGGCTTAACACATGCAAGTCGAACGCTCCGCAAGGAGAGTGGCAGACGGGTGAGT
AACACGTGGGAACGTACCCTTTACTACGGAATAACGCATGGAAACGTGTGCTAATACCGTATGTGCCC
TTTGGGGGAAAGATTTATCGGTAAAGGATCGGCCCGCGTTGGATTAGCTAGTTGGTGGGGTAAAGGCC
TACCAAGGCGACGATCCATAGCTGGTCTGAGAGGATGATCAGCCACATTGGGACTGAGACACGGCC
>Zotu7168
GAACGCTAGCGACAGGCTTAACACATGCAAGTCGAGGGGCAGCATTTTATTAGCTTGCTAATAAAGAT
GGCGACCGGCGCACGGGTGAGTAACGCGTATGCAACCTACCTATCAGAGGGGGATAGCCCATTGAAAG
ATGGATTAATACCGCATAAAACAGGGATCCCGCATGGGAATATTTGTTAAAGATTTATTGCTGATAGA
TGGGCATGCGTTCCATTAGCTTGTTGGTGAGGTAACGGCTCACCAAGGCATCGATGGATAGGGGTTCT
GAGAGGAAGGTCCCCCACACTGGTACTGAGACACGGAC
>Zotu7170

GAACGCTGGCGGCATGCCTTACACATGCAAGTCGAGCGGCAGCGCGGGGGCAACCCTGGCGGCGAGCG
GCGAACGGGTGAGTAATACATCGGAACGTGTCCTGTCGTGGGGGATAACCAGTCGAAAGACTGGCTAA
TACCGCATGAGATCGAAAGATGAAAGCGGGGGACCGCAAGGCCTCGCGCGATTGGAGCGGCCGATGTC
GGATTAGCTAGTTGGCGGGGTAAAAGCCCACCAAGGCGACGATCCGTAGCTGGTTTGAGAGAACGACC
AGCCACACTGGGACTGAGACACGGCC
>Zotu7171
GAACGCTGGCGGCATGCCTAACACATGCAAGTCGAACGAGACCTTCGGGTCTAGTGGCGCACGGGTGC
GTAACGCGTGGGAATCTGCCCAAAGCTACGGAATAACTCCTCGAAAGGGGTGCTAATACCGTATGATG
TCGCAAGACCAAAGATTTATCGGCTTTGGATGAGCCCGCGTAGGATTAGCTTGTTGGTGAGGTAAAGG
CTCACCAAGGCGACGATCCTTAGCTGGTCTGAGAGGATGATCAGCCACACTGGGACTGAGACACGGCC
>Zotu7172
GAACGCTGGCGGCGTGCTTAACACATGCAAGTCGAACGATGAAGCCCTTCGGGGTGGATTAGTGGCGA
ACGGGTGAGTAACACGTGGGCAATCTGCCCTTCACTCTGGGACAAGCCCTGGAAACGGGGTCTAATAC
CGGATAATACTTTCTCTCTCATGGGGGAAGGTTAAAAGCTCCGGCGGTGAAGGATGAGCCCGCGGCCT
ATCAGCTAGTTGGTGGGGTAATGGCCTACCAAGGCGACGACGGGTAGCCGGCCTGAGAGGGCGACCGG
CCACACTGGGACTGAGACACGGCC
>Zotu7173
GAACGCTGGCGGCGTGCCTAATACATGCAAGTCGAACGCTTCCTATCGGTGCTTGCACCGAGAAAGAA
GAGTGGCGGACGGGTGAGTAACACGTGGGTAACCTACCTATAAGCGGGGGATAACATTCGGAAACGGA
TGCTAATACCGCATAGATCTTGATGCCTCCTGGCATCGGGAAGAAAGACGGCTTCGGCTGTCACTTGT
AGATGGACCCGCGGCGCATTAGTTAGTTGGTGAGGTAATGGCTCACCAAGACGATGATGCGTAGCCGA
CCTGAGAGGGTGATCGGCCACACTGGGACTGAGACACGGCC
>Zotu7174
GAACGCTAGCGGCGGGCTTAATACATGCAAGTTAGGGGGGCAGCAATGTTCACCGGCGTACGGGTGCG
TAACACGTATATAACATACCCTTTAGGGGAGGATACATCGAAGAAATTCGGGATAAAACTCCGTAAGC
TAATGTCAGGGCATCCTGGTATTAGGAAAATTTCTAAAGGATTGGTATGCGTCTGATTAGCTAGTTGG
CGAGGTAACGGCTCACCAAGGCGACGATCAGTAGCTGGTCTTAGAGGATGATCAGCCACACTGGCACT
GAGACACGGGC
>Zotu7175
GAACGCTGGCGGCGTGCCTAACACATGCAAGTCGAACGTGAAAGGGAGCAATCCTGAGTAAAGTGGCG
TACGGGTGAGTAACGCGTAGGTAATCTACCCCAAAGTTTGGAATAACCTGCCGAAAGGCGGGCTAATA
CTGGATAGAGCGACGGAATCCGTAATTGGGTTTTTCGGGAAAGGGAGGAAGCAATTCCCTTCGCTTTG
GGATGAGCCTGCGTCTCATTAGCTAGTTGGCGGGGTAAAGGCCCACCAAGGCTATGATGGGTAGCTGG
TCTGAGAGGATGATCAGCCACACTGGAACTGAAACACGGTC
>Zotu7176
GAACGCTAGCGGCAGGCCTAACACATGCAAGTCGAGGGGTATTGTAGCAATACAAGAGACCGGCGCAC
GGGTGCGTAACGCGTATGCAATCTACCTTTTACAGAGGGATAGCCCAGAGAAATTTGGATTAATACCT
CATAGTATATTTTAGTGGCATCACTAGATTATTAAAGTCACAACGGTAAAAGATGAGCATGCGTCCCA
TTAGCTAGTTGGTAAGGTAACGGCTTACCAAGGCTACGATGGGTAGGGGTCCTGAGAGGGAGATCCCC
CACACTGGTACTGAGACACGGAC
>Zotu7178
GAACGCTGGCGGAATGCTTTACACATGCAAGTCGAACGGCAGCACGGGAGCTTGCTCCTGGTGGCGAG
TGGCGAACGGGTGAGTAATATATCGGAACGTATCCAATAATGGGGGATAACTAACCGAAAGGTTGGCT
AATACCGCATACGCCCTACGGGGGAAAGCAGGGGATCGTAAGACCTTGCGTTAATGGAGCGGCCGATA
TCTGATTAGCTAGTTGGTGGGGTAAAGGCTCACCAAGGCTTTGATCAGTAGCTGGTCTGAGAGGACGA
CCAGCCACACTGGAACTGAGACACGGTC
>Zotu7181
GAACGCTGGCGGCATGCCTTACACATGCAAGTCGAACGGCAGCACGGGAGCAATCCTGGTGGCGAGTG
GCGAACGGGTGAGTAAAGTATCGGAACGTGCCCAGTCGTGGGGGATAACGTAGTGAAAATTACGCTAA
TACCGCATACGATCTATGGATGAAAGCGGGGGATCGCAAGACCTCGCGCGATTGGAGCGGCCGATATC
AGATTAGGTAGTTGGTGGGGTAAAGGCTCACCAAGCCAACGATCTGTAGCTGGTCTGAGAGGACGACC
AGCCACACTGGAACTGAGACACGGTC
>Zotu7182
GAACGCTGGCGGCGTGCTTAACACATGCAAGTCGAACGATGACGGTGGTGCTTGCACTGCCTGATTAG

TGGCGAACGGGTGAGTAACACGTGAGTAACCTGCCCTTCACTTCGGGATAACCTCGGGAAATCGTGGC
TAATACCGGATATGAACTCCTGCCGCATGGTGGGGGTTGGAAAGATTTATCGGTGAAGGATGGACTCG
CGGCCTATCAGTTTGTTGGTGAGGTAATGGCTCACCAAGACGATGACGGGTAGCCGGCCTGAGAGGGC
GACCGGCCACACTGGGACTGAGACACGGCC
>Zotu7183
GAACGCTGGCGGCAGGCCTAACACATGCAAGTCGAGCGGATGAAGTAAGCTTGCTTACGGATTCAGCG
GCGGACGGGTGAGTAATGCCTAGGAATCTGCCTGGTAGTGGGGGATAACGTTTCGAAAGGAGCGCTAA
TACCGCATACGTCCTACGGGAGAAAGCAGGGGACCTTCGGGCCTTGCGCTATCAGATGAGCCTAGGTC
GGATTAGCTAGTTGGTGAGGTAATGGCTCACCAAGGCTACGATCCGTAACTGGTCTGAGAGGATGATC
AGTCACACTGGAACTGAGACACGGTC
>Zotu7184
GAACGCTGGCGGCAGGCCTAACACATGCAAGTCGAACGGGTGTAGCAATACATCAGTGGCGCACGGGT
GAGTAACGCGTGGGAACGTGCCCTTCACTACGGAACAACGTCGGGAAACTGACGCTAATACCGTATAA
TGTCTCCGGACCAAAGATTTATCGGTGAAGGATCGGCCCGCGTCTGATTAGCTAGTTGGTGAGGTAAA
AGCTCACCAAGGCGACGATCAGTAGCTGGTTTGAGAGAATGATCAGCCACACTGGGACTGAGACACGG
CC
>Zotu7185
GAACGCTGGCGGCGTGCTTAACACATGCAAGTCGAACGGGTTTTGACAGAGTATCGTCAAAATTAGTG
GCAGACGGGTGAGTATCACATGGGTCATTTACCCTCGAGTGGAAAATAACTCCGAGAAATCGGAGCTA
ATGTTCCATAAGACCACGGCCTTTCCTACGAAAGGCTGGGGTCAAAGCTCCGCAAGGGGCGCTTGAGG
ATGAGCCTGTGTCCTATCAGCTAGTTGGTGAGGTAACGGCCCACCAAGGCTAAGACGGGTAGCCGGCC
TGAAAGGGCGATCGGCCACACTGGAACTGAGACACGGTC
>Zotu7187
GAACGCTGGCGGCAGGCCTAACACATGCAAGTCGAGCGGCAGCACGGCAGTAGCAATACTGTGGGTGG
CGAGCGGCGGACGGGTGAGGAACACATCGGAATCTACCCTGTCGTGGGGGACAACGTAGGGAAACTTA
CGCTAATACCGCATACGACCTTCGGGTGAAAGTGGGGGACCGCAAGGCCTCACGCGATTGGATGAGCC
GATGTCCGATTAGCTAGTTGGCGGGGTAAAGGCCCACCAAGGCGACGATCGGTAGCTGGTCTGAGAGG
ACGACCAGCCACACTGGAACTGAGACACGGTC
>Zotu7188
GAACGCTGGCTGCGTGCTTAACACATGCAAGTCGAACGCTGAAGCCTGGTGCTTGCACCGGGTGGATG
AGTGGCGAACGGGTGAGTAACACGTGAGTAACCTGCCCCCGATTTCGGGATAAGCCCGGGAAACTGGG
TCTAATACCGGATATGACCAGAGGACGCATGTTCTGTGGTGGAAAGTTTTTCGATCGGGGATGGGCTC
GCGGCCTATCAGCTTGTTGGTGGGGTAATGGCCTACCAAGGCGACGACGGGTAGCCGGCCTGAGAGGG
CGACCGGCCACACTGGGACTGAGACACGGCC
>Zotu7190
GAACGCTAGCGGTAGGCTTAATACATGCAAGTCGTACGGGATTATTCTTCGGAATATGAGAGTGGCGC
ACGGGTGCGTAACACGTATGTAACCTACCTTGTAGAGAGGGATAGCCCGAAGAAATTTGGATTAATAC
CTCGTGGTCTTATGAGAAGGCATCTTTTTATAAGTAAAGGAGCAATTTGCTACAAGATGGACATGCGC
AAGATTAGATTGTTGGTGAGGTAACGGCTCACCAAGTCAATGATCTTTAGGGGTTCTGAGAGGAAGGT
CCCCCACACTGGCACTGAGATACGGGC
>Zotu7191
GAACGCTGGCGGCAGGCTTAACACATGCAAGTCGAACGCATCCTTCGGGGTGAGTGGCAGACGGGTGA
GTAACACGTGGGAACGTACCCTTTGGTTCGGAATAACCCTGGGAAACTAGGGCTAATACCGGATACGG
CCGTGAGGCGAAAGGTTTACTGCCGAAGGAACGGCCCGCGTCCGATTAGCTAGTTGGTGTGGTAATGG
CGCACCAAGGCGACGATCGGTAGCTGGTCTGAGAGGATGACCAGCCACATTGGGACTGAGACACGGCC
>Zotu7193
GAACGCTAGCTACAGGCTTAACACATGCAAGTCGAGGGGCAGCATGGTCTTAGCTTGCTAAGGCTGAT
GGCGACCGGCGCACGGGTGAGTAACATGTATCCAACCTGCCGTCTACTCTTGGCCAGCCTTCTGAAAG
GAAGATTAATCCAGGATGGGATCATGAGTTCACATGTCCGCATGATTAAAGGTATTTTCCGGTAGACG
ATGGGGATGCGTTCCATTAGATAGTAGGCGGGGTAACGGCCCACCTAGTCAACGATGGATAGGGGTTC
TGAGAGGAAGGTCCCCCACATTGGAACTGAGACACGGTC
>Zotu7194
GAACGCTAGCGGCAGGCTTAATACATGCAAGTCGAGGGGTAGCATGGGTAGCAATACCTGATGACGAC
CGGCAAACGGGTGCGGAACACGTACGCAACCTTCCCATAACTGGTGAATAGCCTTCCGAAAGGGAGAT

TAATACACCGTAACATAACGAAGCGGCATCGCTTTGTTATTATAGGCTTCGGCCGGTTTTGGATGGGC
GTGCGCCTGATTAGGTAGTTGGCGGGGTAACGGCCCACCAAGCCTGCGATCAGTAACTGGTGTGAGAG
CACGACCAGTCACACGGGCACTGAGACACGGGC
>Zotu7195
GAACGCTAGCTACAGGCTTAACACATGCAAGTCGAGGGGAAACGACATCGAAAGCTTGCTTTTGATGG
GCGTCGACCGGCGCACGGGTGAGTAACGCGTATCCAACCTGCCCACCACTTGGGAATAACCTTGCGAA
AGTAAGACTAATACCCAATGATATCTCTAGAAGACATCTGAAGGAGATTAAAGATTTATCGGTGATGG
ATGGGGATGCGTCTGATTAGCTTGTTGGCGGGGTAACGGCCCACCAAGGCAACGATCAGTAGGGGTTC
TGAGAGGAAGGTCCCCCACATTGGAACTGAGACACGGTC
>Zotu7196
GAACGCTGGCGGCGTGCCTAATACATGCAAGTCGAGCGGATCTTCAGGAGCTTGCTCCTGAAGATTAG
CGGCGGACGGGTGAGTAACACGTGGGCAACCTGCCTGTAAGACTGGGATAACTTCGGGAAACCGAGGC
TAATACCGGATAATCCTTTTCCTCACATGAGGGAAAGCTGAAAGACGGCGCAAGCTGTCACTTACAGA
TGGGCCCGCGGCGCATTAGCTAGTTGGTGAGGTAACGGCTCACCAAGGCGACGATGCGTAGCCGACCT
GAGAGGGTGATCGGCCACACTGGGACTGAGACACGGCC
>Zotu7199
GAACGCTGGCGGCGTGCCTAACACATGCAAGTCGGATGTGCCGCAAGGTGCATGGCAGACGGGTGAGT
AACGCGTAGGGAATCTACCTTTTAGTGGGGAATAACGCTCCGAAAGGAGTGGTAATACCGCATGAGAC
CGCTAGCTGGGACGCTTGTGGTGAAACCTGGGGACCGCAAGGCCTGGGGCTAGAAGAGGATCCTGCGT
CTGATTAGCTAGTTGGCGGGGTAATGGCCCACCAAGGCGACGATCAGTAGCCGGCCTGAGAGGGTGAT
CGGCCACACTGGAACTGAGACACGGTC
>Zotu7200
AAACGTTGGCGGCGTGCTTAACACATGCAAGTCGCGCGAGAAAGCTTCTTCGGAAGCGTGTAAAGCGG
CGGACGGGTGCGTAACACGTGAGAATTTCCCCTTCAGTGAGGAATACCCACGAGAAATTGTGGTTAAT
ACTGCATACGTTCCTACGGGAAGAAAGGTGGCTTTATGCTGCCGCTGAAGGATAAGCTTGCGGGCTAT
TAGCTAGTTGGTAGGGTAAAGGCCTACCAAGGCGATGATGGCTAGCCGGCCTGAGAGGGTGTACGGCC
ACATTGGGACTGAGATACGGCC
>Zotu7205
GAACGCTGGCGGCGTGCTTAACACATGCAAGTCGAACGATGAAGCCAGCTTGCTGGTGGATTAGTGGC
GAACGGGTGAGTAACACGTGGGTGATCTGCCCCAAACTTTGGGATAAGCCTGGGAAACTGGGTCTAAT
ACCGAATATGACCGATACTCGCATGGGTGTTGGTGGAAAGCTCCGGCGGTTTGGGATGGGCCCGCGGC
CTATCAGCTTGTTGGTGGGGTAATGGCCTACCAAGGCGACGACGGGTAGCCGGCCTGAGAGGGCGACC
GGCCACACTGGGACTGAGACACGGCC
>Zotu7207
GAACGCTGGCGGCGTGCTTAACACATGCAAGTCGAGCGGTAAGGCCTCTTCGGGGGTACACGAGCGGC
GAACGGGTGAGTAACACGTGGGTGACCTGCCCTCAGCTCTGGGATAAGCCTGGGAAACTGGGTCTAAT
ACCGGATATGACCTGGCCTCGCATGGGGTTGGGTGGAAAGTTTTTCGGCTGGGGATGGGCCCGCGGCC
TATCAGCTTGTTGGTGGGGTGATGGCCTACCAAGGCGACGACGGGTAGCCGGCCTGAGAGGGCGACCG
GCCACACTGGGACTGAGATACGGCC
>Zotu7209
GAACGCTGGCGGCGTGCTTAACACATGCAAGTCGAACGGTGAAGGTGGGAGCTTGCTCCCGCCGGATC
AGTGGCGAACGGGTGAGTAACACGTGAGCAATCTGCCCCGGACTCTGGGATAAGCGTTGGAAACGACG
TCTAATACCGGATACGACGCGCGAAGGCATCTTCTGCGCGTGGAAAGAACTTCGGTTCGGGATGAGCT
CGCGGCCTATCAGCTAGTTGGTGAGGTAATGGCCCACCAAGGCGACGACGGGTAGCCGGCCTGAGAGG
GTGACCGGCCACACTGGGACTGAGACACGGCC
>Zotu7210
GAACGCTGGCGGCAGGCCTAACACATGCAAGTCGAACGCCCTAGCAATAGGGAGTGGCAGACGGGTGA
GTAACGCGTGGGAACCTCTCCATTGGTACGGAATAGCCCAGGGAAACTTGGAGTAATACCGTATAAGC
CCTTCGGGGGAAAGATTTATCGCCATTGGGTGGGCCCGCGTCGGATTAGCTAGTTGGTGAGGTAACGG
CTCACCAAGGCTACGATCCGTAGCTGGTTTGAGAGAATGACCAGCCACACTGGGACTGAGACACGGCC
>Zotu7211
GAACGCTGGCGGCGTGCTTAACACATGCAAGTCGAACGGTGAAGCAGGAGCTTGCTCCTGTGGATCAG
TGGCGAACGGGTGAGTAACACGTGAGCAATCTGCCCTGGACTCTGGGATAAGCGTTGGAAACGACGTC
TAATACCGGATACGAGACGCGAAGGCATCTTCAGCGTCTGGAAAGAACTTCGGTCCAGGATGAGCTCG

CGGCCTATCAGGTAGTTGGTGAGGTAATGGCTCACCAAGCCTACGACGGGTAGCCGGCCTGAGAGGGT
GACCGGCCACACTGGGACTGAGACACGGCC
>Zotu7214
GAACGCTAGCGGCAGGCCTAATACATGCAAGTCGGACGGGATTGGAGAGCTTGCTTTCCATGAGAGTG
GCGCACGGGTGCGTAACACGTATGTAACCTACCTTCGTCAGGGGGATAGCCTCTCGAAAGAGAGATTA
AGACCGCATAACATTATGGAATAGCATTATTTTATAATCAAATATTTATAGGACGAAGATGGGCATGC
GGAACATTAGCTAGTTGGTAAGGTAACGGCTTACCAAGGCTACGATGTTTAGGGGATCTGAGAGGATG
ACCCCCCACACTGGTACTGAGACACGGAC
>Zotu7215
GAACGCTGGCGGCGTGCCTAACACATGCAAGTCGTACGGGATTTGGGGGCAACTCCAAGTTCAGTGGC
AGACGGGTGCGTAACACGTGAGCAACTTGTCCGACGGCGGGGGATAGCCGGTCTAACGACCGGGTAAT
ACCGCGTACGCTCATTTGGGGACATCCCTGGGTGAGGAAAGCCGCAAGGCACCGACGGAGAGGCTCGC
GACCTATCAGCTAGTTGGCGGGGTAACGGCCCACCAAGGCTACGACGGGTAGCTGGTCTGAGAGGACG
ATCAGCCACATTGGGACTGAGACACGGCC
>Zotu7216
AAACGCTGGCGGCGTGCATGAGACATGCAAGTCGAACGGACGATCAGGGCTTGCTCTGATCGTTAGTG
GCGGACGGCTGAGTAACGCGTGGGTGACCTGCCCCGAAGTGGGGAATAACTGCCCGAAAGGGTGGCTA
ATACCGCATGTGGTGGTGCGTTCGATCGCACCAGTAAAGCCGCAAGGCGCTTCGGGAGGGGCCTGCGT
CCGATTAGCTCGTTGGTGGGGTAACGGCTCACCAAGGCGACGATCGGTAGGTGGTCTGAGAGGACGAT
CACCCAGACTGGGACTGAGACACGGCC
>Zotu7217
GAACGCTGGCGGCATGCTTTACACATGCAAGTCGAACGGCAGCACGGGCTTCGGTCTGGTGGCGAGTG
GCGAACGGGTGAGTAATATATCGGAACGTACCCAGAAGTGGGGGATAACGTAGCGAAAGTTACGCTAA
TACCGCATACGATCTACGGATGAAAGTGGGGGACCTTCGGGCCTCATGCTTTTGGAGCGGCCGATATC
TGATTAGCTAGTTGGTAGGGTAAAGGCCTACCAAGGCGACGATCAGTAGCTGGTCTGAGAGGACGACC
AGCCACACTGGAACTGAGACACGGTC
>Zotu7219
GAACGCTGGCGGCGTGCTTAACACATGCAAGTCGAACGGAAAGGCCCGCTTGCGGGTACTCGAGTGGC
GAACGGGTGAGTAACACGTGGGCAACCTGCCCTCAGCTCTGGGATAACACCGGGAAACCGGTGCTAAT
ACTGGATATCAACTTCTGTCCGCATGGGTGGTTGTTGAAAGTTTTTCGGCTGGGGATGGGCCCGCGGC
CTATCAGCTTGTTGGTGGGGTAATGGCCTACCAAGGCGACGACGGGTAGCCGGCCTGAGAGGGCGACC
GGCCACACTGGGACTGAGACACGGCC
>Zotu7221
GTTGCAACTTCACGCGTGGAATTTGTGATGAGGATGCCGTGGAACCAGGTTTCGGAGCATCTTGAAGA
GCCCCCTATGGTGGGAATCAATTGAAGCTGTACGCTTCGCAAAGTTTAGTTGAACTATGATAATTTAG
TTGGGGTGAAAAAAAGAACATTTGATGGCGTGTATGACATTTTTGCATTGAATGTGCACGGTGGAGAG
GTTCGTCATCCACCGGAATATAAAAATATCGAGGCCTTAATTAAAAGCTAGGTAGGGTAAAGGTAACA
AAACGTAAATGACAACTTAAGCAGCTTGACTGATTCGCTTTATTCTAGCAGCGTTAAAACGTCGCTAC
TATAGACGTTATAGATGATATACGATCAGTGTTTACCTATGCTTCCTTGAATGGCACCGAACGGCACC
GGGCAAATGTAGCGCAGAAAGG
>Zotu7222
GAACGCTGGCGGCGTGCTTAAGACATGCAAGTCGAACGGACAGAGCTTGCTCTGTCAGTGGCGCACGG
GTGAGTAACGCGTAACTGACCTACCCCCAAGTCGCGGATAACGCGCCGAAAGGCACGCTAATACGTGA
TGTGCACGCAGGTTTCTGGCCTGCTGTGTAAAGACTGGATCGCTTGAGGATGGGGTTGCGTTCCATCA
GCTAGATGGTGGGGTAACGGCCCACCATGGCGACGACGGATCGCCGGCCTGAGAGGGTGGCCGGCCAC
AGGGGCACTGAGACACGGGT
>Zotu7223
GAACGCTGGCGGCGTGCTTAAGACATGCAAGTCGTACGGATACCTTCGGGTATTAGTGGCGCACGGGT
GAGTAACGCGTAACTGACCTGCCCCCAAGTCGCGGATAACGTCTCGAAAGAGACGCTAATACGTGATG
TGCAGTACCGCTGTGGCGGTACTGTAAAGACTGGATCGCTTGGGGATGGGGTTGCGTTTCATCAGCTA
GTTGGTGGGGTAAAGGCCTACCAAGGCGACGACGAATAGCCGGCCTGAGAGGGTGGCCGGCCACAGGG
GCACTGAGACACGGGC
>Zotu7224
GAACGCTGGCGGCGTGCCTAATACATGCAAGTCGAGCGCCAGGGGTGCTCCCTTACGGGAGAGCCCCT

GGAGCGGCGGACGGGTGAGTAACACGTAGGCAACCTGCCTGTAAGATCGGGATAACTACCGGAAACGG
TAGCTAAGACCGGATAGCTGGTTTCTCCGCATGGAGGGATCATGAAACACGGGGCAACCTGTGGCTTA
CAGATGGGCCTGCGGCGCATTAGCTAGTTGGTGGGGTAACGGCTCACCAAGGCGACGATGCGTAGCCG
ACCTGAGAGGGTGATCGGCCACACTGGGACTGAGACACGGCC
>Zotu7225
GAACGCTGGCGGCATGCCTAACACATGCAAGTCGAACGAAGGCTTCGGCCTTAGTGGCGCACGGGTGC
GTAACGCGTGGGAATCTGCCCTTGGGTTCGGAATAACAGTGAGAAATTACTGCTAATACCGGATGATG
ACTTCGGTCCAAAGATTTATCGCCCAAGGATGAGCCCGCGTAAGATTAGCTAGTTGGTGAGGTAAAGG
CTCACCAAGGCGACGATCTTTAGCTGGTCTGAGAGGATGATCAGCCACACTGGGACTGAGACACGGCC
>Zotu7226
GAACGCTGGCGGCGTGCCTAACACATGCAAGTCGAACGTGAAAGGGGGCAACCCTGAGTAAAGTGGCG
CACGGGTGAGTAACGCGTAGGTAATCTACCCCAGAGTCTGGAATAACCTGCCGAAAGGCGGGCTAATA
CTGGATAGAGCGACGGAACCCGTAATCGGGTTTTTCGGGAAAGGGAGGAAGCAATTCCCCTTGCTCTG
GGATGAGCCTGCGTCTCATTAGCTAGTTGGTGAGGTAACGGCTCACCAAGGCATTGATGGGTAGCTGG
TCTGAGAGGATGATCAGCCACACTGGAACTGAAACACGGTC
>Zotu7227
GAACGCTGGCGGCGCGCCTAATACATGCAAGTCGAGCGAGAAGGTGTAGCAATACACTTGTAAAGCGG
CGAACGGGTGAGGAACACATGGGTAACCTACCATCGAGTGGGGAATAACCAGCCGAAAGGTTGGCTAA
TACCGCGTACGCCTGCGGGTCTCCGGATTCGCAGGGAAAGCCACACTGAGAGTGTGGCGCTCATTGAT
GGGCTCATGTCCTATCAGCTTGTTGGTAGGGTAACGGCCTACCAAGGCTTTGACGGGTAGCTGGTCTG
AGAGGACGATCAGCCACACTGGCACTGCGACACGGGC
>Zotu7228
GAACGCTGGCGGCGTGCTTAACACATGCAAGTCGAACGGTGATGCAGAGCTTGCTCTGCGGATCAGTG
GCGAACGGGTGAGTAACACGTGAGTAACCTGCCCTTGACTCTGGGATAAGCGTTGGAAACGACGTCTA
ATACCGGATACGACCCGTGGAGGCATCTCCTACGGGTGGAAAGAACTTCGGTTGAGGATGGACTCGCG
GCCTATCAGCTTGTTGGTGAGGTAATGGCTCACCAAGGCGACGACGGGTAGCCGGCCTGAGAGGGTGA
CCGGCCACACTGGGACTGAGACACGGCC
>Zotu7229
CAACGCTGGCGGCGTGCCTAACACATGCAAGTCGAACGCGAAAGTCCCGCAAGGGATTAGTAGAGTGG
CAAACGGGTGAGTAACACGTGGGTGACCTGCCTTCGAGCGGGGGATAACGTCCAGAAAGGGACGCTAA
TACCGCATAACATCCTGCCTTTTAAGAGGTGGAGATCAAAGCCGGGGATCGCAAGACCTGGCACTTGA
AGAGGGGCCCGCGCCTGATTAGCTAGTTGGTGAGGTAATGGCTCACCAAGGCAACGATCAGTATCCGG
CCTGAGAGGGCGGACGGACACACTGGGACTGAGACACGGCC
>Zotu7230
GAACGCTGGCGGCGTGCTTAACACATGCAAGTGGAGCGACGAACCGGGGCTTGCCCCGGGGCAGAGCC
GCGAACGGGTGAGTAACACGTGGGTAACCCACCTTGATGACTGGGACAACCCGAGGGAACTCGGGCTA
ATACCGGATGTGATCCTCTTTCGTAAGGAAGATGATGAAAGATAGCTTCGGCTTTCGCATCAAGACGG
GCCCGCGGCCCATTAGCTTGTTGGTGGGGTAATGGCCTACCAAGGCTTCGATGGGTAGCTGGTCTGAG
AGGACGATCAGCCACACTGGGACTGAGACACGGCC
>Zotu7234
GAACGCTAGCGGCAGGCCTAACACATGCAAGTCGAGGGAGGAGCTTTCGGGCTTCGACCGGCGTACGG
GTGCGTAACGCGTATACAATCTGCCTTTCACATAGGGATAGCCCGAAGAAATTTGGATTAATACCTAA
TGGTATATAGGAGTGGCATCACATTTATATTAAAGCTGAGGCGGTGAAAGATGAGTATGCGTTCTATT
AGCTAGTTGGAGAGGTAACGGCTCCCCAAGGCTACGATAGATAGGGGTTCTGAGAGGGATATCCCCCA
CACTGGTACTGAGATACGGAC
>Zotu7236
GAACGCTGGCGGCAGGCCTAACACATGCAAGTCGAACGAAGCCTTCGGGCTTAGTGGCGGACGGGTGA
GTAACGCGTGGGAACATACCCTTTGGTACGGAATAGCCTTGGGAAACTGGGAGTAATACCGTATGTGC
CCTTCGGGGGAAAGATTTATCGCCAAAGGATTGGCCCGCGTTGGATTAGGTAGTTGGTGGGGTAATGG
CCTACCAAGCCGACGATCCATAGCTGGTTTGAGAGGATGATCAGCCACACTGGGACTGAGACACGGCC
>Zotu7238
GAACGCTAGCGATAGGCTTAACACATGCAAGTCGAGGGGCAGCAGGGACTTCGGTCCGCTGGCGACCG
GCGCACGGGTGCGTAACGCGTATGCAACTTGCCCTGTAGAGGAGGATAACCCGCTGAAAGGCGGACTA
ATACTGCGTACTCTCATCGGACGGCATCGTTTGATGAGGAAAGAAATTCGCTACAGGATAGGCATGCG

TCCCATTAGTTAGTTGGTGAGGTAACGGCTCACCAAGGCGACGATGGGTAGGGGAACTGAGAGGTTTA
TCCCCCACACTGGTACTGAGACACGGAC
>Zotu7240
GAACGCTGGCGAGATGCTTTACACATGCAAGTCGTACGAGAGCTTCAAACTCAAGTGGCGGACGGGTG
AGTAACACGTAAGAATTTGCCTTTAGGAGGGGGACAACAACTGGAAACGGTTGCTAATACCCCATATG
CTTTCGAGTGAAACGGATTTTTCCGCCTAAAGAGAAGCTTGCGGCTGATTAGCTTGTTGGTGAGGTAA
TGGCTCACCAAGGCGACGATCAGTATCTGGTTTGAGAGGACGATCAGACACACTGGAACTGAGACACG
GTC
>Zotu7241
GAACGCTGGCGGCATGCCTAAGACATGCAAGTCGAACGAGAAGGCCCAAAGAAGATGGAGTGCTTGCA
CAAAGTTGGAAATGGATCACCTTCTAGTGGCAAACGGGTGAGTAACACGTGGGTTACCTACCTCTAAG
ACGGGGATAACAGTTAGAAATGATTGCTAATACCGGATGTGCTCTACGGAGTAAAGGAGCCTTTAAAG
CTTCGCTTAGAGATGGGCCTGCGGTGCATTAGCTAGTTGGTGGGGTAACGGCCTACCAAGGCGACGAT
GCATAGCCGAACTGAGAGGTTAATCGGCCACACTGGGACTGAGACACGGCC
>Zotu7243
GAACGCTGGCGGCATGCCTAACACATGCAAGTCGAACGAGATCTTCGGATCTAGTGGCGCACGGGTGC
GTAACGCGTGGGAATCTGCCCTTGGGTTCGGAATAACAGTTAGAAATGACTGCTAATACCGGATGATG
TCTTCGGACCAAAGATTTATCGCCCAAGGATGAGCCCGCGTAGGATTAGCTAGTTGGTGAGGTAAAGG
CTCACCAAGGCGACGATCCTTAGCTGGTCTGAGAGGATGATCAGCCACACTGGGACTGAGACACGGCC
>Zotu7245
GAACGCTGATGGCGTGCCTCATACATGCAAGTCGAGCGGAATAGCAATATTCAGCGGCGGACGGGTGA
GTAACGCGTAAACAATCTGCCCTAAAGTGGGGAATAACTATCCCAACGGATAGCTAATACCGCATAAT
GATGAAGAGGACATCCTCTTTTCTCAAAAACCTTCGGGTGCTTTAGGATGAGTTTGCGTCCCATTAGC
TTGTTGGCGGTGTAACGGACCACCAAGGCGACGATGGGTAGCCGGCCTGAGAGGGTGTACGGCCACAT
TGGGACTGAGATACGGCC
>Zotu7247
GAACGCTGGCGGCGTGCTTAACACATGCAAGTCGAACGGAAAGGCCTCAGCTTGCTGGGGTACTCGAG
TGGCGAACGGGTGAGTAACACGTGGGTGATCTGCCCTGCACTTTGGGATAAGCTTGGGAAACTGGGTC
TAATACCGAATATGACCACTTCTTGGATGTTGTGGTGGAAAGCTTTTGCGGTGTGGGATGAGCCTGCG
GCCTATCAGCTTGTTGGTGGGGTAATGGCCTACCAAGGCGGCGACGGGTATCCGGCCTGAGAGGGTGT
ACGGACACATTGGGACTGAGATACGGCC
>Zotu7248
GAACGCTGGCGGCGTGCCTTAGGCATGCAAGTCGAATGGTTCCGCAGCAATGCGGAGTAATGGCAGAC
GGGTGAGGAACGCGTAGGAATCTACCCCTGGGTGGACCATAGCTCATCGAAAGATGGGGTAATAGTCC
ATGGTCTCGAAAGAGTAAAGCACATAACTGTGCGCCGAGGGAGGAGCCTGCGTCCTATCAGCTTGTTG
GTGGGGTAATGGCCTACCAAGGCAATGACGGGTAGCTGGACTGAGAGGTCGATCAGCCACAATAGCAC
TGAGACACGGGC
>Zotu7249
GAACGCTAGCGGCAGGCCTAACACATGCAAGTCGAGGGGTAGAAGTCTTCGGACTTTGAGACCGGCGC
ACGGGTGCGTAACGCGTATGCAATCTGCCTTACACTAAGGGATAGCCCAGAGAAATTTGGATTAATAC
CTTATAGTATATGATGGGGACATCCCCGACATATTAAAGTTTCAACGGTGTAAGATGAGCATGCGTCC
CATTAGTTAGTTGGTGTGGTAACGGCACACCAAGACAATGATGGGTAGGGGTCCTGAGAGGGAGATCC
CCCACACTGGTACTGAGACACGGAC
>Zotu7250
GAACGCTGGCGGCGTGCCTAACACATGCAAGTGGAGCGACGAACCGGGGCTTGCCCCGGGGCAGAGCC
GCGAACGGGTGAGTAACACGTGGGTTACCTGCCCCGATGACTGGGACAACCCGTCGAAAGACGGGCTA
ATACCGGATGTGCCCTTTGGGGGAAAGGAAGCTTCGGCCTCCGCATCGGGATGGGCCCGCGGCGCATT
AGCTTGTTGGTGGGGTAATGGCTTACCAAGGCGACGATGCGTAGCTGGTCTGAGAGGACGATCAGCCA
CACTGGGACTGAGACACGGCC
>Zotu7251
GAACGCTGGCGGCGTGCCTAATACATGCAAGTAGAACGCTGAAGCTTGGTGCTTGCACCGAGCGGATG
AGTTGCGAACGGGTGAGTAACGCGTAGGTAACCTGCCTGGTAGCGGGGGATAACTATTGGAAACGATA
GCTAATACCGCATAACAGTCGACATTGCATGATGTTGATTTGAAAGGGGCAAATGCTCCACTACCAGA
TGGACCTGCGTTGTATTAGCTAGTTGGTGAGGTAACGGCTCACCAAGGCGACGATACATAGCCGACCT

GAGAGGGTGATCGGCCACACTGGGACTGAGACACGGCC
>Zotu7252
GAACGTTGGCGGCATGTCTTACACATGCAAGTCGAACGGCAGCACGGGTGCTTGCATCTGGTGGCGAG
TGGCGAACGGGTGAGTAAAGTATCGGAACGTGCCTGGTAGTGGGGGATAACTAGTCGAAAGACTAGCT
AATACCGCATACGATTTAAGGATGAAAGCAGGGGACCTTCGGGCCTTGTGCTATCAGAGCGGCCGATA
TCTGATTAGCTAGTTGGTGGGGTAAGAGCCCACCAAGGCGACGATCAGTAGCTGGTCTGAGAGGACGA
CCAGCCACACTGGAACTGAGACACGGTC
>Zotu7253
GAACGCTGGCGGCGTGCTTAACACATGCAAGTCGAGCGGTAAGGCCCCTTCGGGGGTACACGAGCGGC
GAACGGGTGAGTAACACGTGAGCAATCTGCCCTCCACATCGGGATAACCCCCGGAAACGGGAGCTAAT
ACCGGATACGACCACCTCAGGCATCTGATGGTGGTGGAAAGTTCCGGCGGTGGAGGATGAGCTCGCGG
CCTATCAGCTTGTTGGTGGGGTAACGGCCCACCAAGGCTTCGACGGGTAGCCGGCCTGAGAGGGTGAC
CGGCCACACTGGGACTGAGACACGGCC
>Zotu7255
GAACGCTGGCGGCGTGCTTAACACATGCAAGTCGAACGCTGAAGCCCAGCTTGCTGGGTGGATGAGTG
GCGAACGGGTGAGTAACACGTGAGTAACCTGCCCCCTTCTTTGGGATAACGCCCGGAAACGGGTGCTA
ATACTGGATATTCACTGATCTTCGCATGGGGGTTGGTGGAAAGGTTTGTTCTGGTGGGGGATGGGCTC
GCGGCCTATCAGCTTGTTGGTTGGGTGATGGCCTACCAAGGCTTTGACGGGTAGCCGGCCTGAGAGGG
TGACCGGTCACATTGGGACTGAGATACGGCC
>Zotu7256
GAACGCTGGCGGCGTGCCTAATACATGCAAGTCGAGCGAAGTGGAGAGAGCTTGCTCTCAAAGCTTAG
CGGCGGACGGGTGAGTAACACGTAGGCAACCTGCCTGTAAGTCTGGGATAACTCCGGGAAACCGGAGC
TAATACCGGATAACTTGTAAGACCGCATGGTCTTACACTAAAAGGACATGATCCTCTTACAGATGGGC
CTGCGGCGCATTAGCTAGTTGGTGAGGTAACGGCTCACCAAGGCAACGATGCGTAGCCGACCTGAGAG
GGTGACCGGCCACACTGGGACTGAGACACGGCC
>Zotu7257
GAACGTTAGCGGCGCGCTTAACACATGCAAGTCGAGCGAGAAAGGGCGCAAGCCCCGGTAAAGCGGCG
CACGGGTGAGTAACACGTAGGTAATCTACCCCCAGGCGGTGGATAACCTTCCGAAAGGAGGGCTAATA
CAGCATGGGACCACGGTGCCGAAAGGCATTGAGGTGAAAGACGGCCTCTATTCATAAGCTGTCACCAG
GGGATGAGCCTGCGGCCCATCAGCTAGTTGGTAGGGTAACGGCCTACCAAGGCGAAGACGGGTAGCTG
GTCTGAGAGGATGATCAGCCACACTGGAACTGAGACACGGTC
>Zotu7258
GAACGCTGGCGGCATGCCTAACACATGCAAGTCGAACGAGACCTTCGGGTCTAGTGGCGCACGGGTGC
GTAACGCGTGGGAATCTGCCCTTAGGTTCGGAATAACTCAGGGAAACTTGAGCTAATACCGGATAATG
ACTTCGGTCCAAAGATTTATCGCCTAGGGATGAGCCCGCGTCGGATTAGCTAGTTGGTGAGGTAAAAG
CTCACCAAGGCGACGATCCGTAGCTGGTCTGAGAGGATGATCAGCCACACTGGGACTGAGACACGGCC
>Zotu7260
GAACGCTGGCGGCGTACCTGATGTATGCAAGTCGAGCGGTAAGGCTCCTACAGGCTATTTGATTGTTA
AGGCAACGATGGCTATCGTTTCTAGATCAAATTGCGTGTTGGAGTACACGAGCGGCGGACGGGTGAGT
AACGCGTAGGAACGTACCCCAAAGTGAGGGATAAGCTCCGGAAACGGAGTCTAATACCGCATGTGATC
TTCGGATTAAAGTATTTATACGCTTTAGGAGCGGCCTGCGCCCGATTAGCTTGTTGGCGGAGTAAAAG
CCCACCAAGGCGATGATCGGTAGCTGGTCTGAGAGGACGATCAGCCACACTGGGACTGAGACACGGCC
>Zotu7261
GAACGCTGGCGGCATGCTTAACACATGCAAGTCGAACGGATTTATGGAGCTTGCTTCATAAGTTAGTG
GCAGACGGGTGAGTAACGCGTGGGAATGTACCTTTTGGTTCGGAATAACTGCGGGAAACTGTGGCTAA
TACCGGATAAGCCCGTGAGGGGAAAGATTTATCGCCAAAAGAGCAGCCCGCGTTAGATTAGGTAGTTG
GTGGGGTAATGGCCTACCAAGCCTGCGATCTATAGCTGGTCTGAGAGGATGATCAGCCACACTGGGAC
TGAGACACGGCC
>Zotu7262
GAACGCTGGCGGCGTGCTTAACACATGCAAGTCGAACGGTGAAAGAGGGAGCTTGCTTCCTCTGGATC
AGTGGCGAACGGGTGAGTAACACGTGAGTAACCTGCCCTTGACTCTGGGATAAGCGTTGGAAACGACG
TCTAATACCGGATACGAGCTTCAGCCGCATGGCTAGGAGCTGGAAAGAACTTCGGTCAAGGATGGACT
CGCGGCCTATCAGCTAGTTGGTGAGGTAACGGCCCACCAAGGCGACGACGGGTAGCCGGCCTGAGAGG
GTGACCGGCCACACTGGGACTGAGACACGGCC

>Zotu7264
GAATGCTGGCGGCGTGGATAAGTCATGCAAGTCAAGGGGGCCCGCAAGGGCAACCGGCAAACGAGGTA
GTAACACGTAGGTAGACCCCCCGAAGTCGAGCATAGCCAGCCGAAAGGTTGGGTAATTCTCGATAGTC
CCGCAAGGGTAAAGCCGTTGTATTTATATAACTGGCGCTTCGGGAGTTGCCTGCGTAGTATCAGCTAG
TTGGTAGGGTAATGGCCTACCAAGGCTACGACGCTTAGGGGAGCTGAGAGGCTGACCCCCACCGATGG
AACTGCGACACGGTC
>Zotu7265
GAACGCTGGCGGCGTGCCTTAAGCATGCAAGTAGAACGAGTTCTATCTTGCCGCAAGGCAAGCTTGGA
ACTAGTTGCGAACGGCTGAGTAATCAATAGGAATCTTTCCTTTGGTGGGGAATACCTCGTCGAAAGAC
GAGCTAATACCGCATAATATCTACGGATGAAAGCGCATAACTGCGCGCCGATTGAGGAGCCTATTGCC
CATCAGGCAGTTGGTAGGGTAATGGCCTACCAAACCTACGACGGGTACCCGATCTGAGAGGATGACCG
GGCAGAATGGGACTGAGACACGGCC
>Zotu7266
GAACGCTGGCGGCAGGCCTAACACATGCAAGTCGAGCGGATGATGGGAGCTTGCTCCCGGATTCAGCG
GCGGACGGGTGAGTAATGCCTAGGAATCTGCCTGGTAGTGGGGGACAACGTTTCGAAAGGAACACTAA
TACCGCATACGTCCTACGGGAGAAAGCAGGGGACCTTCGGGCCTTGCGCTATCAGATGAGCCTAGGTC
AGATTAGCTAGTTGGTGGGGTAAAGGCCTACCAAGGCGACGATCCGTAACTGGTCTGAGAGGATGATC
AGTCACACTGGAACTGAGACACAGTC
>Zotu7267
GAACGCTGGCTGTATGGATAAGGCATGCAAGTCGAACGGCCCGCAAGGGCAGTGGCAGACGAGGTAGT
AACACGTACGAACGTACTCCCAAGTCAGGAATAGCTCACCGAAAGGTGGGGTAATACCTGATGGTCCC
TACGGGGTAAAGATTTATCGCTTGGGAAGCGGCGTGCGTAGTATCAGCTTGTTGGTAAGGTAATGGCT
TACCAAGGCTACGACGCTTAGGGGAGCTGAGAGGCTGACCCCCACCGATGGGACTGAGACACGACC
>Zotu7269
GAACGCTGGCGGCAGGCTTAACACATGCAAGTCGAGCGGGCATCTTCGGGTGTCAGCGGCGGACGGGT
GAGTAACGCGTGGGAACGTGCCCTCTGGTTCGGAATAACTCAGGGAAACTTGAGCTAATACCGGATAC
GCCCTTTTGGGGAAAGGCTTGCTGCCGGAGGATCGGCCCGCGTCTGATTAGCTAGTTGGTGAGGTAAC
GGCTCACCAAGGCGACGATCAGTAGCTGGTCTGAGAGGATGATCAGCCACACTGGGACTGAGACACGG
CC
>Zotu7270
GAACGCTAGCGGTATGCTTAACACATGCAAGTCGTACGGAGAAGCAATTCTTAGTGGCGAACGGGTGA
GTAACGCGTAAGTATTTGCCTTTAGGAAGAGGATAACATTTAGAAATAACTGCTAAAACTCTATATGC
CGTTTGGTGAAAAAAGCAATTTGCCTAAAGAGAAGCTTGCGTCTGATTAGCTAGTTGGTGGGGTAATG
GCCTACCAAGGCGACGATCAGTAGCTGGTCTGAGAGGACGATCAGCCACACTGGAACTGAGACACGGT
C
>Zotu7271
GAACGCTGGCGGCGTGCTTAACACATGCAAGTCGAACGGGGCGAATGAAAGCTTGCTTTTATGAGCTT
AGTGGCAAACGGGTGAGTAACACGTAGGCAACCTGCCGACAGGATGGGGACAACATTCCGAAAGGAAT
GCTAATACCGAATGAAGTCGAGGGAAGGCATCTTCCTTCGATGAAAGATGGCCTCTATTTATAAGCTA
TCACCTGTCGATGGGCCTGCGTCTGATTAGCTCGTTGGTGAGGTAACGGCTCACCAAGGCGACGATCA
GTAGCCGGTCTGAGAGGATGAACGGCCACATTGGGACTGAGACACGGCC
>Zotu7273
GAACGCTGGCGGCAGGCTTAACACATGCAAGTCGAGCGGGCATCCTTGTGGTGTCAGCGGCAGACGGG
TGAGTAACGCGTGGGAACGTGCCCTTCGGTTCGGAATAACTCAGGGAAACTTGAGCTAATACCGGATA
CGTGCGAGAGCAGAAAGGTTTATCTGCCGAAGGATCGGCCCGCGTCTGATTAGCTAGTTGGTGAGGTT
ATGGCTCACCAAGGCGACGATCAGTAGCTGGTCTGAGAGGATGATCAGCCACACTGGGACTGAGACAC
GGCC
>Zotu7274
GAACGCTGGCGGCGTGCTTAACACATGCAAGTCGAACGAGAATCTACTGAAGGAGGATTCGTCCAACG
GAAGTAGAGGAAAGTGGCGGACGGGTGAGTAACGCGTGAGGAACCTGCCTTGAAGAGGGGGACAACAG
TTGGAAACGACTGCTAATACCGCATGACGCATAGGGGTCGCATGATTCTTATGCCAAAGATTTATCGC
TTCAAGATGGCCTCGCGTCTGATTAGCTAGTTGGCGGGGTAACGGCCCACCAAGGCGACGATCAGTAG
CCGGACTGAGAGGTTGAACGGCCACATTGGGACTGAGATACGGCC
>Zotu7278

GAACGCTGGCGGCGTGCCTAACACATGCAAGTCGAACGGAGATCTTATGGAATGCGGCTTCGGCCAAA
TGAAGTTTGATCTTAGTGGCGAACGGGTGAGTAACGCGTGAGTAACCTGCCCTTCAGAGGGGGACAAC
AGTTGGAAACGACTGCTAATACCGCATAATGTATTTTTAAGGCATCTTAGAAATACCAAAGGAGCAAT
CCGCTGAAGGATGGACTCGCGTCCGATTAGATAGTTGGTGAGGTAACGGCCCACCAAGTCGACGATCG
GTAGCCGGACTGAGAGGTTGATCGGCCACATTGGGACTGAGACACGGCC
>Zotu7279
GAACGCTGGCGGCATGCCTAATACATGCAAGTCGAACGATGATATCACTGCTTGCAGTGATTGATTAG
TGGCGAACGGGTGAGTAACACGTGAGGAACTTGCCCATGAGCGGGGGACAACATTCGGAAACGGATGC
TAATACCCCATAGGTGGATTGGTCGCATGACGAATTCATTAAAGGTGGCTTTGCTACCACTCATGGAT
AGCCTCGCGGCGTATTAGCTAGTTGGTAAGGTAATGGCTTACCAAGGCAGTGATACGTAGCCGACTTG
AGAGGGTGATCGGCCACACTGGGACTGAGACACGGCC
>Zotu7280
GAACGCTGGCGGCATGCCTAACACATGCAAGTCGAACGCTCTTGGGGCTTCGGTTTCAAGGGAGTGGC
GCACGGGTGAGTAGCGCGTGGGAACCTGCCCTGTTGTGGGGGATAACCGAGGGAAACTTCGGCTAATA
CCGCATACGCCGGGAAACCGGGAAAGATTTATCGCAGCAGGAGGGGCCCGCGTCGGATTAGGTAGTTG
GTGGGGTAACGGCCCACCAAGCCTTTGATCCGTAGCTGGTCTGAGAGGATGATCAGCCACACTGGGAC
TGAGACACGGCC
>Zotu7281
GAACGCTGGCGGCAGGCCTAACACATGCAAGTCGAACGGCAGCACAGGAGAGCTTGCTCTCTGGGTGG
CGAGTGGCGGACGGGTGAGGATAGCGTCGGAATCTGCCTATTTGTGGGGGATAACCTCGGGAAACCGG
GACTAATACCGCATACGACCTACGGGTGAAAGCGGAGGACCTTCGGGCTTCGCGCAGATAGATGAGCC
GACGTCGGATTAGCTAGTTGGCGGGGTAATGGCCCACCAAGGCGACGATCCGTAGCTGGTCTGAGAGG
ATGATCAGCCACACTGGAACTGAGACACGGTC
>Zotu7282
GAACGCTGGCGGCAGGCCTCAAACATGCAAGTCTAGGGGGTGTAGCAATACATCACCGGCGGACGGGT
GCGTAACGCGTGGGAATATACCTATTGGTTCGGAACAACTGAGGGAAACTTCAGCTAATACCGAATGT
GCCCGTAAGGGGAAAGATTTATCGCCAATGGATTAGCCCGCGTAGGATTAGCTAGTTGGTGGGGTAAT
GGCCCACCAAGGCTCCGATCCTTAGCTGTTCTGAGAGGAAGATCAGCCACACTGGGACTGAGACACGG
CC
>Zotu7283
GAACGCTGGCGGCAGGCCTAACACATGCAAGTCGAGCGGACATCTTCGGATGTTAGCGGCGGACGGGT
GAGTAACGCGTGGGAACGTACCTTTTGCTTCGGAATAGCCTCGGGAAACTGGGAGTAATACCGAATGT
GCTCTTCGGAGGAAAGATTTATCGGCAAAAGATCGGCCCGCGTTGGATTAGGTAGTTGGTGGGGTAAT
GGCCTACCAAGCCGACGATCCATAGCTGGTTTGAGAGGATGATCAGCCACACTGGGACTGAGACACGG
CC
>Zotu7285
GAACGCTGGCGGCAGGCCTAACACATGCAAGTCGAACGCCGTAGCAATACGGAGTGGCAGACGGGTGA
GTAACACGTGGGAACGTGCCCTTTGGTTCGGAACAACTCCGGGAAACTGGAGCTAATACCGGATAAGC
CCTAACGGGGAAAGATTTATCGCCGAAGGATCGGCCCGCGTCTGATTAGCTAGTTGGCGGGGTAATGG
CCCACCAAGGCGACGATCAGTAGCTGGTCTGAGAGGATGATCAGCCACATTGGGACTGAGACACGGCC
>Zotu7286
GAACGCTGGCGGCGTGCTTAACACATGCAAGTCGAACGGAAAGGCCCTTTCGGGGGTACTCGAGTGGC
GAACGGGTGAGTAACACGTGGGTGATCTGCCCTGCACTTCGGGATAAGCCTGGGAAACTGGGTCTAAT
ACCGGATATACCTCATGGATTGCATGGTTTGTGGGGGAAAGCTTTTGCGGTGTGGGATGGGCCCGCGG
CCTATCAGCTTGTTGGTGGGGTGATGGCCTACCAAGGCGTCGACGGGTAGCCGGCCTGAGAGGGTGTC
CGGCCACACTGGGACTGAGATACGGCC
>Zotu7288
GAACGCTGGCGGCATGCTTAACACATGCAAGTCGAACGGCAGCACGAACTAGTTTACTAGTTGGGTGG
CGAGTGGCGGACGGGTGAGTAACGCGTAGGAATATGCCTTGAAGAGGGGGATAACTTGGGGAAACTCA
AGCTAATACCGCATAAACTCTAAGGAGCAAAGCTGGGGACCTTCGGGCCTGGCGCTTCAAGAATAGCC
TGCGTCTGATTAGCTAGTTGGCGGGGTAAGGGCCCACCAAGGCGACGATCAGTAGCTGGTCTGAGAGG
ATGATCAGCCACACTGGGACTGAGACACGGCC
>Zotu7289
GAACGCTGGCGGCAGGCTTAACACATGCAAGTCGAACGCATCGCAAGATGAGTGGCAGACGGGTGAGT

AACGCGTGGGAATCTACCCAACCCTACGGAACAACTCCGGGAAACTGGAGCTAATACCGTATACGCCC
TTTGGGGGAAAGATTTATCGGGGATGGATGAGCCCGCGTTGGATTAGCTAGTTGGTGGGGTAAAGGCC
TACCAAGGCGACGATCCATAGCTGGTCTGAGAGGATGATCAGCCACATTGGGACTGAGACACGGCC
>Zotu7290
TGTCCGAGAACTGCTCGGGAGGAGGCGAGCCGTCAGGGATGCGGCAAGAAACATGGCACGACGTAAGG
AAAATGTGCCGAAGGCGTGGTGTATGAAATTCCCCTTTCTTGTC
>Zotu7291
AATTTTATCGCTAAAAAATAATAAGAATAAACGCAACACGTTCTCACGTGATGTGCGTGTTGTCGCAT
TTGGCGTATCTCTTTGTTCCACTTTTTACACCGTAATTGCATTATTATTACGACGGGGCAGAGCGCGG
AAAAAGAGGCAACACTCACCCCCTTCCATGGACTGAAACACGTGTATTTTTTATATTAAGAGCGT
>Zotu7292
AAACGCTGGCGGCGCACATAAGACATGCAAGTCGAACGAACTTAATTTTAATATTTATATTAAAAAGC
GGTTAGTGGCGGACTGGTGAGTAACGCGTAAGCAATCTGCCTATCAGAGGGGAATAACAGTGAGAAAT
CATTGCTAATACCGCATATGCCATAAGAATCACATGATTCTAGTGGGAAAGGAGCAATCCACTGATAG
ATGAGCTTGCGTCTGATTAGCTAGTTGGTGGGGTAACGGCCTACCAAGGCGACGATCAGTAGCCGGAC
TGAGAGGTTGAACGGCCACATTGGGACTGAGATACGGCC
>Zotu7293
GAACGCTGGCGGCGTGCTTAACACATGCAAGTCGAACGAGATCCAATGTGTAGCAATACACAGGAAGA
TCTAGTGGCGAACGGGTGAGGAACACGTGAGAAACGTACCCCAGACTCTGGGATAACAATCCGAAAGG
ATTGCTAATACCGGATGACGTCGGAAGTCCGCATGGACATCTGACGAAAGGGATTCCGGTTTGGGAGC
GTCTCGCGGACCTATCAGCTAGTTGGAGGGGTAACGGCCCACCAAGGCAACGACGGGTAGCTGGTCTG
AGAGGATGAACAGCCACACTGGGACTGAGACACGGCC
>Zotu7295
GAACGCTGGCGGCGTGCTTAACACATGCAAGTCGAACGGTAAGGCCCTTCGGGGTACACGAGTGGCGA
ACGGGTGAGTAACACGTGGGTGATCTGCCCTGCACTTCGGGATAAGCCCGGGAAACTGGGTCTAATAC
CGGATAGGACCACAGCATGCATGTGTTGTGGTGGAAAGATTTATCGGTGCAGGATGGGCCCGCGGCCT
ATCAGCTTGTTGGTGGGGTAATGGCCTACCAAGGCGACGACGGGTAGCCGGCCTGAGAGGGCGACCGG
CCACACTGGGACTGAGACACGGCC
>Zotu7296
GAACGCTGGCGGCGTGCCTAATACATGCAAGTCGAACGCACTGACAGAGAACTTGTTCTCTTGACGTG
AGTGGCGAACGGGTGAGTAACACGTGGGAAACCTACCTTCTAGCGGGGGATAACAGTCGGAAACGACT
GCTAATACCGCATAGTGCTTTTCTTGGCATCAAGGAAAGAGGAAAGATGGTTCTGCTATCACTAGAAG
ATGGTCCTGCGGTGCATTAGCTAGTTGGTAAGGTAACGGCTTACCAAGGCGATGATGCATAGCCGACC
TGAGAGGGTAATCGGCCACATTGGGACTGAGACACGGCC
>Zotu7297
GAACGCTGGTAACGTGGATACCGGCATGCAAGTCACGGGGGCCGCAAGGCAACCGGCGAACGGCTTAG
TAACACGTGGATACATACCCCAAAGTGGAGTACAGCTCGTCGAAAGACGGGGTAATCCTCCATAGTCT
CCTCTTTCGCTTTTTGTTTCACAAAAGCTTCAGAAGGACAAAGTCGCAAGGCAAATACGCTTTGCAGA
GCAAGACTTGTCCATCCGAAGCTCTGGTGGAACGCAGAGCGAAGGAGGAGTAAAGGCGCAAGCCGCTT
TGGGAATGGTCTGCGGTCTATCAGCTAGTTGGTGAGGTAATGGCTCACCAAGGCTATGACGGATAGGG
GGCGTGAGAGCGCGACCCCCAAGAATGGCACTGCGACACGGGC
>Zotu7298
GAACGCTGGCGGCATGCCTTACACATGCAAGTCGAACGGTAACGCGGGGGCAACCCTGGCGACGAGTG
GCGAACGGGTGAGTAATACATCGGAACGTACCCAGTAGTGGGGGATAGCTCGGCGAAAGCCGGATTAA
TACCGCATACGACCTATGGGTGAAAGCGGGGGATCGCAAGACCTCGCGCTATTGGAGCGGCTGATGTC
GGATTAGCTAGTTGGTGAGGTAAAGGCTCACCAAGGCGACGATCCGTAGCTGGTCTGAGAGGACGACC
AGCCACACTGGGACTGAGACACGGCC
>Zotu7299
GAACGCTAGCGGCGTGCTTAACACATGCAAGTCGAACGCTGAAGCCCGCTTCGGTGGGTGGATGAGTG
GCGAACGGGTGAGTAACACGTGGGCAATCTACCCCGCACTCGGGGATAAGCCTTGGAAACGAGGTCTA
ATACCCGATAGGACATGCCATCGCATGGTGGTGTGTGGAAAGTGTGTAGCGGTGTGGGATGAGCCCGC
GGCCTATCAGCTTGTTGGTGGCGGTGATGGCGCATCAAGGCGACGACGGGTAGCCGGCCTGAGAGGGT
GACCGGCCACACTGGGACTGAGACACGGCC
>Zotu7301

GAACGCTGGCGGCATGCCTAATACATGCAAGTCGAACGAGCTGACAGAGTGCTTGCACTCTCGACGCG
AGTGGCGGACGGGTGAGTAACACGTGGGAAACCTACCTGTAAGTATGGGATAACATTCGGAAACGGAT
GCTAATACCATATAGATTTTAGTTCCGCATGGAACTAAAAGAAAAGACGGCTTTGCTGTCGCTTACAG
ATGGTCCCGCGGCGCATTAGCTAGTTGGTGGGATAAAAGCTCACCAAGGCCATGATGCGTAGCCGACC
TGAGAGGGTAACCGGCCACACTGGGACTGAGACACGGCC
>Zotu7302
GAACGTTAGCGGCGCGCCTAACACATGCAAGTCGCACGCGAAAGGGGCAACCCAAGTAAAGTGGCGCA
CGGGTGCGTAACACGTAGATAACCTGCCCTTTGGAGGGGGATAACGTTCCGAAAGGAGCGCTAATACC
GCATACAGCATTCTGGGGTTTGCTCCGGGATGGCAAAGGTAGGGACCCGCAAGGGCCTATCGCCGAAG
GAGGGGTCTGCGGCCCATCAGCTAGTTGGCGGGGTAATGGCCCACCAAGGCAAAGACGGGTAGCTGGT
CTGAGAGGATGATCAGCCACACTGGTACTGGAACACGGAC
>Zotu7303
GAACGCTAGCGGCAGGCCTAACACATGCAAGTCGAGGGGTAGAAGTCTTCGGACTTTGAGACCGGCGC
ACGGGTGCGTAACGCGTATGCAATCTACCTTGTACTGAGGGATAGCCCAGAGAAATTTGGATTAATAC
CTCATAGTATTTTCGGGTGACATCACCTGATTATTAAAGTTCCAACGGTACAAGATGAGCATGCGTCC
CATTAGCTAGTTGGTATGGTAACGGCATACCAAGGCTATGATGGGTAGGGGTCCTGAGAGGGAGATCC
CCCACACTGGTACTGAGACACGGCC
>Zotu7304
GAACGCTGGCGGCATGCCTAACACATGCAAGTCGAACGAGACCTTCGGGTCTAGTGGCGCACGGGTGC
GTAACACGTGGGAACCTGCCCTTAGGTTCGGAATAACCAGGAGAAATCCTGGCTAATACCGGATAATG
TCTTCGGACCAAAGATTTATCGCCTTTGGATGGGCCCGCGTAGGATTAGCTAGTTGGTGGGGTAATGG
CCTACCAAGGCGACGATCCTTAGCTGGTCTGAGAGGATGATCAGCCACACTGGGACTGAGACACGGCC
>Zotu7307
GAACGCTGGCGGCAGGCCTAACACATGCAAGTCGAACGGCAGCGGGTAGGAAGCTTGCTTCCTATGCC
GGCGAGTGGCGGACGGGTGAGGAACACATCGGAATCTACTCCGTCGTGGGGGATAACGTAGGGAAACT
TACGCTAATACCGCATACGACCTAAGGGTGAAAGTGGGGGACCGCAAGGCCTCACGCGATGGAATGAG
CCGATGTCCGATTAGCTAGTTGGCGGGGTAAAGGCCCACCAAGGCGACGATCGGTAGCTGGTCTGAGA
GGATGATCAGCCACACTGGGACTGAGACACGGCC
>Zotu7308
GAACGCTGGCGGCGTGCCTAACACATGCAAGTCGAACGGGGTTGAGGTCTTCGGACCTTAACTTAGTG
GCGAACGGGTGAGTAACGCGTGAGAAACCTGCCTTTCAGTGGGGGACAACAGTTGGAAACGACTGCTA
ATACCGCATAATGTTATTTGGGGGCATCCCCGAATAACCAAAGCTTTATGTGCTGAAAGATGGTCTCG
CGTCTGATTAGCTAGATGGCGGGGTAACGGCCCACCATGGCGACGATCAGTAGCCGGTCTGAGAGGAT
GAACGGCCACATTGGGACTGAGATACGGCC
>Zotu7310
GAACGCTGGCGGCATGCTTAACACATGCAAGTCGAACGAGACCTTCGGGTCTAGTGGCGCACGGGTGC
GTAACGCGTGGGAATCTGCCCTTAGGTACGGAATAACTCAGAGAAATTTGTGCTAATACCGTATGATG
TCGAAAGACCAAAGATTTATCGCCTAAGGATGAGCCCGCGTAAGATTAGCTAGTTGGTAGGGTAAAGG
CCTACCAAGGCTACGATCTTTAGCTGGTCTGAGAGGATGATCAGCCACACTGGGACTGAGACACGGCC
>Zotu7311
TAACGCTGGCGGCGTGCATGAGACATGCAAGTCGAACGGGTGACTCGGGCTTGCTCGAGTCACTAGTG
GCGAACGGCTGAGTAACACGTAGGTGACCTGCCCCGAAGTGGGGAATAACTGCCCGAAAGGGTGGCTA
ATACCGCATGTGGTCGCACGTTCATTCGTGCGAGTAAAGCCGCAAGGCGCTTCGGGAGGGGCCTGCGT
CCGATTAGCTCGTTGGTGGGGTAATGGCTCACCAAGACGACGATCGGTAGGTGGTCTGAGAGGACGAT
CACCCAGACTGGGACTGAGACACGGCC
>Zotu7312
GAACGCTGGCGGCAGGCTTAACACATGCAAGTCGAGCGGGGAGATGTAGCTTGCTACATTTCCTAGCG
GCGGACGGGTGAGTAATGCTTAGGAATCTGCCTATTAGTGGGGGACAACATTCCGAAAGGAATGCTAA
TACCGCATATGCCCTACGGGGGAAAGCAGGGGATCTTCGGACCTTGCGCTAATAGATGAGCCTAAGTT
AGATTAGCTAGTTGGTGGGGTAAAGGCCTACCAAGGCGACGATCTGTAGCGGGTCTGAGAGGATGATC
CGCCACACTGGGACTGAGACACGGCC
>Zotu7313
GAACGCTAGCGGCAGGCCTAATACATGCAAGTCGAACGGTAAGTTGAATAGCAATATTTGACCTAGAG
TGACGTACGGGTGCGTAACACGTATGCAACCTACCCTTTACAGGAGGATAGCTCGGGGAAACTCGAAT

TAACCCTCCATGAGATCATAGAGTAGCATTACTCAGTGATTAAAACTCAGGTGGTAAAGGATGGGCAT
GCGTCTGATTAGTTAGTTGGCGGGGTAACGGCCCACCAAGACGATGATCAGTAGGGGAACTGAGAGGT
TGATCCCCCACACTGGCACTGAGATACGGGC
>Zotu7314
GAACGCTGGCGGCATGCTTAACACATGCAAGTCGCACGGGTAGTTTTCGGACTGTCAGTGGCGGACGG
GTGAGTAACGCGTAGGGATTTATCCATGGGTGGGGGATAACCGCGGGAAACTGTGGCTAATACCGCAT
GAAGCCTGAGGGCCAAAGGCAGCGATGTCGCCTGTGGAGGAGCCTGCGTTCGATTAGCTTGTTGGTGG
GGTAAAGGCCTACCAAGGCGATGATCGATAGCTGGTCTGAGAGGATGATCAGCCACACTGGGACTGAG
ACACGGCC
>Zotu7315
GAACGCTGGCGGCGTGCTTAACACATGCAAGTCGAACGATGAAGCCAGAGCTTGCTCTGGTGGATTAG
TGGCGAACGGGTGAGTAACACGTGAGTAACCTACCCTTGACTCTGGGATAAGCCCGGGAAACTGGGTC
TAATACCGGATATGAGTCATCACCGCATGGTGGTGGTTTGAAAGATTTATCGGTCTTGGATGGACTCG
CGGCCTATCAGCTTGTTGGTGAGGTAATGGCTCACCAAGGCGACGACGGGTAGCCGACCTGAGAGGGT
GACCGGCCACACTGGGACTGAGACACGGCC
>Zotu7317
GAACGCTGGCGGCAGGCCTAACACATGCAAGTCGAGCGGTAACAGGGGAAAGCTTGCTTTCCCGCTGA
CGAGCGGCGGACGGGTGAGTAATGTATGGGGATCTGCCCGATAGAGGGGGATAACTACTGGAAACGGT
GGCTAATACCGCATAATCTCTAAGGAGCAAAGCAGGGGAACTTCGGTCCTTGCGCTATCGGATGAACC
CATATGGGATTAGCTAGTAGGTGAGGTAATGGCTCACCTAGGCGACGATCCCTAGCTGGTCTGAGAGG
ATGATCAGCCACACTGGGACTGAGACACGGCC
>Zotu7319
GAACGCTGGCGGCATGCTTTACACATGCAAGTCGAACGGCAGCACGGACTTCGGTCTGGTGGCGAGTG
GCGAACGGGTGAGTAATACATCGGAACGTGCCCAGTTGTGGGGGATAACTACTCGAAAGAGTAACTAA
TACCGCATGAAAACTGAGGTTGAAAGCAGGGGATCGCAAGACCTTGCGCAACTGGAGCGGCCGATGGC
AGATTAGGTAGTTGGTGGGATAAAAGCTTACCAAGCCGACGATCTGTAGCTGGTCTGAGAGGACGACC
AGCCACACTGGGACTGAGACACGGCC
>Zotu7322
CAACGCTGGCGGCGCGCCTAACACATGCAAGTCGAACGAGAAAGTGGAGCAATCCATGAGTACAGTGG
CGTACGGGTGAGTAACACGTGGGTAATCTACCTTTTAGTGAGGAATAACCAAGGGAAACCATGGCTAA
TACCGCATAAGCCTGAGAAGGGAAAGCAGCAATGCGCTGAAAGAGGAGCCCGCGGCCGATTAGCTAGT
TGGTGGGGTAAGAGCCCACCAAGGCAGAGATCGGTAGCCGGCCTGAGAGGGCACACGGCCACACTGGC
ACTGAAACACGGGC
>Zotu7323
GAACGCTAGCGGCAGGCCTAATACATGCAAGTCGAGGGGCAGCAGGATAGCAATATCGCTGGCGACCG
GCGCACGGGTGCGTAACACGTATATAATCTGCCTTTAACAGGGGGATAGCCCGGAGAAATCCGGATTA
ATACCCCATAATAGATCGAATGGCATCATTTGGTTTTGAAAGCTCCGGCGGTTAAAGATGAGTATGCG
TCTGATTAGCTAGTTGGTGAGGTAACGGCTCACCAAGGCGACGATCAGTAGGGGATCTGAGAGGATTA
ACCCCCACACGGATACTGAGACACGGAT
>Zotu7324
GAACGCTGGCGGCGTGCTTAACACATGCAAGTCGAGCGAGATTCAACCGGTGGCAACACCGGGGAAGA
TCGAGCGGCGAACGGGTGAGTAACACGTGAGCAACCTGCCCCGAAGACTGGGATAACACCGGGAAACC
GGTGCTAATACCGGATACCCCCACCAGGCCGCATGACCAGGTGAGGAAATGGCTGGGTCTAATAAACT
CAGTCCGCTTCGGGAGGGGCTCGCGGGCTATCAGCTTGTTGGTGGGGTAACGGCCTACCAAGGCGACG
ACGGCTAGCTGGTCTGAGAGGACGACCAGCCACACTGGGACTGAGACACGGCC
>Zotu7325
CAACGCTGGCGGCGTGCCTAACACATGCAAGTCGAACGCGAAAGCCCCGCAAGGGGTAAGTAGAGTGG
CAAACGGGTGAGTAACACGTGGGTGACCTGCCTTCGAGTGGGGGATAACGTCCCGAAAGGGACGCTAA
TACCGCATGACATCCTGCTCTTGGACGAGCGGAGATCAAAGCCGGGGATCGCAAGACCTGGCGCTTGA
AGAGGGGCCCGCGCCTGATTAGCTAGTTGGTGAGGTAATGGCTCACCAAGGCGACGATCAGTATCCGG
CCTGAGAGGGCGGACGGACACACTGGGACTGAGACACGGCC
>Zotu7330
GAACGCTGGCGGCATGCCTTACACATGCAAGTCGAACGGATTCAAGGAGCTTGCTCCTTGAGTTAGTG
GCGAACGGGTGAGTAAAGCATCGGAACGTACCCAGTAGTGGGGGATAACGTAGCGAAAGTTACGCTAA

TACCGCATACGACCTGAGGGTGAAAGCAGGGGATCGCAAGACCTTGCGCTATTGGAGCGGCCGATGTC
GGATTAGCTAGTTGGTAGGGTAAAAGCCTACCAAGGCGACGATCCGTAGCTGGTCTGAGAGGACGACC
AGCCACACTGGGACTGAGACACGGCC
>Zotu7332
GAACGCTGGCGGCAGGCTTAACACATGCAAGTCGAGCGGGCGAGGTTGCTTCGGTAACTTAGCTAGCG
GCGGACGGGTGAGTAATGCTTAGGAATCTGCCTATTAGTGGGGGACAACATTCCGAAAGGGATGCTAA
TACCGCATACGTCCTACGGGAGAAAGCAGGGGCTCTTTATGACCTTGCGCTAATAGATGAGCCTAAGT
CGGATTAGCTAGTTGGTGGGGTAAAGGCCTACCAAGGCGACGATCTGTAGCGGGTCTGAGAGGATGAT
CCGCCACACTGGGACTGAGACACGGCC
>Zotu7334
GAACGCTGGCGGCGTGCTTAACACATGCAAGTCGAACGAAGAGCGACGGAAGCTTGCTTCTATCAATC
TTAGTGGCGAACGGGTGAGTAACGCGTAATCAACCTGCCCTTCAGAGGGGGACAACAGTTGGAAACGA
CTGCTAATACCGCATACGATCTAATCTCGGCATCGAGGATAGATGAAAGGTGGCCTCTATGTATAAGC
TATCACTGAAGGAGGGGATTGCGTCTGATTAGCTAGTTGGAGGGGTAACGGCCCACCAAGGCGATGAT
CAGTAGCCGGTCTGAGAGGATGAACGGCCACATTGGGACTGAGACACGGCC
>Zotu7335
GAACGCTGGCGGCGTGCCTAATGCATGCAAGTCGCACGCAGGGTGCTTGCACTCTGAGTGGCGCACGG
CTGAGGATCACGTGGGAACATGCCTCTTGGTGGGGGACAACAGCGCGAAAGCGATGCTAATCCCGCAT
ACGCTCGGGAACGAGGAAAGCGGCAACGCGCCGGGAGAGTGGCCCGCGGCCCATCAGGTCGTAGGTGG
GGTAATGGCCTACCTAGCCGATGACGGGTAGCTGGTCTGTGAGGACAGACAGCCAGACTGGGACTGAG
ACACGGCC
>Zotu7337
GAACGCTGGCGGCAGGCTTAACACATGCAAGTCGAGCGGGCCCTTCGGGGTCAGCGGCAGACGGGTGA
GTAACACGTGGGAACGTACCCTTCGGTTCGGAATAACCCTGGGAAACTAGGGCTAATACCGGATACGT
CCGAGAGGAGAAAGGTTTACTGCCGAAGGATCGGCCCGCGTCTGATTAGCTAGTTGGTGGGGTAATGG
CCTACCAAGGCGACGATCAGTAGCTGGTCTGAGAGGATGATCAGCCACATTGGGACTGAGACACGGCC
>Zotu7338
GAACGTTAGCGGCGCGCCTAACACATGCAAGTCGCGCGAGAAAGGGCTTCGGCCCCAGTAAAGCGGCG
CACGGGTGAGTAACACGTAGGTCATCTTCCCTTGAGTGGTGGATAACTCTCCGAAAGGAGAGCTAATA
CAGCATAAGACCACGACCTCGCTGAGGTTGCGGCCAAAGCAGACCTCTTCACGAAAGTTTGCGCTTAA
GGATGAGCCTGCGGCCCATCAGGTAGTTGGTAGGGTAATGGCCTACCAAGCCTAAGACGGGTAGCTGG
TCTGAGAGGATGAACAGCCACACTGGAACTGAGACACGGTC
>Zotu7339
GAACGCTGGCGGCATGCCTAACACATGCAAGTCGAACGAAGGCTTCGGCCTTAGTGGCGCACGGGTGC
GTAACGCGTGGGAATCTACCCTTAGGTTCGGAATAACAGTTGGAAACGACTGCTAATACCGGATGATG
ACGTAAGTCCAAAGATTTATCGCCTAGGGATGAGCCCGCGTAGGATTAGCTAGTTGGTGTGGTAAAGG
CGCACCAAGGCGACGATCCTTAGCTGGTCTGAGAGGATGATCAGCCACACTGGGACTGAGACACGGCC
>Zotu7342
GAACGCTGGCGGCAGGCTTAACACATGCAAGTCGAGCGGGCACTTCGGTGTCAGCGGCAGACGGGTGA
GTAACACGTGGGAACGTGCCCTTCGGTTCGGAATAACTCAGGGAAACTTGAGCTAATACCGGATACGC
CCTTATGGGGAAAGGTTTACTGCCGAAGGATCGGCCCGCGTCTGATTAGCTTGTTGGTGGGGTAACGG
CCTACCAAGGCGACGATCAGTAGCTGGTCTGAGAGGATGATCAGCCACACTGGGACTGAGACACGGCC
>Zotu7344
GAACGCTGGCGGCATGCCTTACACATGCAAGTCGAACGGTAGAGGGGCAACCCTTGAGAGTGGCGAAC
GGGTGAGTAATATATCGGAACGTGCCCAGTCGTGGGGGATAACGTAGAGAAATTTACGCTAATACCGC
ATACGATCTAAGGATGAAAGCGGGGGACTCGCAAGAGCCTCGCGCGACTGGAGCGGCTGATATCAGAT
TAGCTTGTTGGTGAGGTAAAAGCTCACCAAGGCGACGATCTGTAGCTGGTTTGAGAGAACGACCAGCC
ACACTGGGACTGAGACACGGCC
>Zotu7345
GAACGCTGGCGGCATGCTTTACACATGCAAGTCGAACGGCAGCGCGGGGCAACCTGGCGGCGAGTGGC
GAACGGGTGAGTAATATATCGGAACGTACCCAGAAGTGGGGGATAACGTAGCGAAAGTTACGCTAATA
CCGCATACGATCTAAGGATGAAAGTGGGGGATCGCAAGACCTCATGCTTTTGGAGCGGCCGATATCTG
ATTAGCTAGTTGGTGAGGTAAAGGCTCACCAAGGCGACGATCAGTAGCTGGTCTGAGAGGACGACCAG
CCACACTGGGACTGAGACACGGCC

>Zotu7347
GAACGCTGGCGGCGTGCCTAATACATGCAAGTCGAGCGGTAACACTTCGGGTGACGAGCGGCGGACGG
GTGAGTAATGCATGGGAACGTGCCCCAAAGTGAGGAATAAGCACCAGAAATGGTGTCTAATACCGCAT
ATTATCTTAGGATCAAAGCTTCGGCGCTTTGGGAGCGGCCCATGTCTGATTAGCTAGTTGGTAGGGTA
AAAGCCTACCAAGGCGACGATCAGTAGCTGGTTTGAGAGGATGATCAGCCACACTGGAACTGAGACAC
GGTC
>Zotu7348
GAACGCTGGCGGCGTGCCTAATACATGCAAGTCGAGCGGAGTTGATGAGGTGCTTGCACCTCTGATAC
TTAGCGGCGGACGGGTGAGTAACACGTAGGTAACCTGCCTGTAAGACTGGGATAACATTCGGAAACGG
ATGCTAATACCGGATACGCGGATTGATCGCATGATCAAACTGGGAAAGATGGAGCGATCTATCACTTA
CAGATGGACCTGCGGCGCATTAGCTAGTTGGTGAGGTAACGGCTCACCAAGGCGACGATGCGTAGCCG
ACCTGAGAGGGTGATCGGCCACACTGGGACTGAGACACGGCC
>Zotu7350
GAACGCTAGCGGCAGGCTTAACACATGCAAGTCGAGGGGTATAAGGGAGCTTGCTCCTTTAGAGACCG
GCAAACGGGTGCGGAACACGTACACAACCTACCCTTATCTTGGGAATAGCCCATAGAAATGTGGATTA
ATACCCAATGGTATATTAAAGTGGCATCACTTAAATATTAAAGAACTTCGGATAAGGATGGGTGTGCG
TAAGATTAGGTAGTTGGTGAGGTAACGGCTCACCAAGCCAGCGATCTTTAGCTGATGTGAGAGCATGA
TCAGCCACACGGGCACTGAGACACGGGC
>Zotu7351
GAACGCTGGCGGTATGCTTTACACATGCAAGTCGAACGCAAGTTTTTAACTTGAGTGGCGGACGGGTG
AGTAACACGTGAGAATTCGCCTTTAGGCGGGGAATAACTATGGGAAACCATAGCTAATGCCCCATATG
CTCGCAAGAGTGAAAGAAGCAATTCACCCAAAGAGAAGCTCGCGGCTGATTAGCTAGTTGGTAAGGTA
ATGGCTTACCAAGGCGACGATCAGTAGCTGGTTTGAGAGAACGATCAGCCACACTGGAACTGAGACAC
GGTC
>Zotu7352
GAACGCTAGCGATAGGCTTAACACATGCAAGTCGAGGGGCAGCATGGTCTTAGCTTGCTAAGACTGAT
GGCGACCGGCGCACGGGTGCGTAACGTGTATGCAACTTGCCTCACAGAGGGGGATAACCCGTCGAAAG
ACGGACTAATACCGCGTACACTCCAGTCTGGGCATCCAGGCTAGAGGAAATGAATTTCGCTGTGAGAT
AGGCATGCGTCCCATTAGCTAGTTGGTGAGGTAACGGCTCACCAAGGCAACGATGGGTAGGGGAACTG
AGAGGTTGAACCCCCACACTGGTACTGAGACACGGAC
>Zotu7353
GAATGCTGGCGGTATGGATAAGGCATGCAAGTCGAACGGCCCGCAAGGGCAGTGGCAGACGAGGTAGT
AACACGTAGGAACGTACACCGAAGTCAGGTATAGCTCGTCGAAAGGCGGGGTAATCCCTGATGGTCCC
GCGAGGGTAAAGATTTATCGCTTCGGTAGCGGCCTGCGCAATATCAGCTTGTTGGTGAGGTAACGGCT
CACCAAGGCTACGACGTTTAGGGGCGCTGAGAGGCGGACCCCCACCGATGGGACTGAGATACGGCC
>Zotu7354
AAACGCTGGCGGCGTGCCTAATACATGTAAGTCGAACGAAGGGTTTGTAGCAATACTTACCCTTAGTG
GCGAATGGGTGAGTAACGCGTTGGTGACCTACCCCGAAGAGGGGGATAACAGTTCGAAAGGACTGCTA
ATACCCCATGTGGTCAATGGATTTAGAAACTGTTGTCTAAAGGAGAAATCCGCTTCGGGAGGGGCCTG
CGTCCCATCAGCTAGTTGGTAGGGTAATGGCCTACCAAGGCTACGACGGGTAGGGGGCCTGAGAGGGT
GGCCCCCCACATTGGCACTGAAACACGGGC
>Zotu7355
GAACGCTGGCGGCAGGCCTAACACATGCAAGTCGAGCGGTAACAGGGGGTGCTTGCACCCCGCTGACG
AGCGGCGGACGGGTGAGTAATGCATAGGAATCTGCCCGATAGTGGGGGATAACCTGGGGAAACCCAGG
CTAATACCGCATACGTCCTACGGGAGAAAGGGGGCTCCGGCTCCCGCTATCGGATGAGCCTATGTCGG
ATTAGCTGGTTGGTGAGGTAAAGGCTCACCAAGGCGACGATCCGTAGCTGGTCTGAGAGGATGATCAG
CCACATCGGGACTGAGACACGGCC
>Zotu7357
GAACGCTGGCGGCGTGCTTAACACATGCAAGTCGAACGAGACCCTGCCTTCGGGTGGGTGTTCTAGTG
GCGAACGGGTGAGTAACACGTGGGCAACCTGCCCCCGGCTCTGGGATAACTCCAAGAAATTGGGGCTA
ATACCGGATATGACCGCTGACCGCATGGTCTGGTGGTGGAAAGTTTTTCGGCTGGGGATGGGCCCGCG
GCCTATCAGCTTGTTGGTGGGGTAGTGGCCTACCAAGGCGACGACGGGTAGCCGGCCTGAGAGGGTGA
CCGGCCACACTGGGACTGAGACACGGCC
>Zotu7359

GAACGCTGGCGGCAGGCCTAACACATGCAAGTCGAGCGGACCCTTCGGGGTTAGCGGCGGACGGGTGA
GTAACGCGTGGGAATATGCCCTTCTCTACGGAATAGTCCTGGGAAACTGGGGGTAATACCGTATACGC
CTTTTGGGGGAAAGATTTATCGGGGAAGGATTAGCCCGCGTTGGATTAGGTAGTTGGTGGGGTAATGG
CTCACCAAGGCTACGATCCGGAGCTGGTCTGAGAGGACGACCAGCCACACCGGAACTGAGACACGGTC
>Zotu7360
GAACGCTGGCGGCGTGCTTAACACATGCAAGTCGAACGATGAAGTTCCTTCGGGGACGGAATAGTGGC
GAACGGGTGAGGAACACGTGAAGAATCTGCCCTCCACTTTGGGATAACTCCGGGAAACCGGGGCTAAT
ACCGAATATTGAAACATAAGTGGCATCACTGAGTTTTGAAAGAATTTCGGTGGAGGATGACTTCGCGG
CCTATCAGCTTGTTGGTGAGGTAATGGCTCACCAAGGCGACGACGGGTAGCCGGCCTGAGAGGGCGAC
CGGCCACACTGGGACTGAGACACGGCC
>Zotu7362
GAACGCTGGCGGCAGGCTTAACACATGCAAGTCGAGCGGGGAAATGTAGCTTGCTACATTACCTAGCG
GCGGACGGGTGAGTAATGCTTAGGAATCTGCCTATTAGTGGGGGACAACATTTCGAAAGGAATGCTAA
TACCGCATACGCCCTACGGGGGAAAGCAGGGGATCTTCGGACCTTGCGCTAATAGATGAGCCTAAGTC
GGATTAGCTAGTTGGTGGGGTAAAGGCCTACCAAGGCGACGATCTGTAGCGGGTCTGAGAGGATGATC
CGCCACACTGGGACTGAGACACGGCC
>Zotu7363
GAACGCTGGCGGCGTGCTTAACACATGCAAGTCGAACGGTGATGATGGAGCTTGCTCTGTCGGATCAG
TGGCGAACGGGTGAGTAACACGTGAGTAACCTGCCCTCAACTCTGGGATAACTTCCCGAAAGGGAAGC
TAATACTGGATATGAACTGCACGGGCATCTGTGTGGTTGTAAAGATTTTTTGGTTGAGGATGGACTCG
CGGCCTATCAGCTTGTTGGTGGGGTAATGGTCTACCAAGGCGACGACGGGTAGCCGGCCTGAGAGGGC
GACCGGCCACACTGGGACTGAGACACGGCC
>Zotu7364
GAACGCTGGCGGCAGGCCTAACACATGCAAGTCGAGCGGTAGCACAGAGAGCTTGCTCTCGGGTGACG
AGCGGCGGACGGGTGAGTAATGTCTGGGAAACTGCCTGATGGAGGGGGATAACTACTGGAAACGGTAG
CTAATACCGCATAACGTCGCAAGACCAAAGAGGGGGACCTTCGGGCCTCTTGCCATCAGATGTGCCCA
GATGGGATTAGCTAGTAGGTGGGGTAATGGCTCACCTAGGCGACGATCCCTAGCTGGTCTGAGAGGAT
GACCAGCCACACTGGAACTGAGACACGGTC
>Zotu7366
GAACGCTGGCGGCATGCCTTACACATGCAAGTCGAACGGTAACGGGCCTTCGGGTGCCGACGAGTGGC
GAACGGGTGAGTAATATATCGGAACGTGCCCAGTAGTGGGGGATAGCCCGGCGAAAGCCGGATTAATA
CCGCATACGACCTGAGGGTGAAAGCGGGGGATCGCAAGACCTCGCGCTATTGGAGCGGCCGATATCAG
ATTAGCTAGTTGGTGGGGTAAAGGCCTACCAAGGCGACGATCTGTAGCTGGTCTGAGAGGACGACCAG
CCACACTGGGACTGAGACACGGCC
>Zotu7367
GAACGCTGGCGGCGTGCCTAATACATGCAAGTCGAGCGAACCAAAAAGGAGCTTGCTCCTTTACGGTT
AGCGGCGGACGGGTGAGTAACACGTGGGTAACCTGCCCTCAAAACTGGGATAACATCGAGAAATCGGT
GCTAATACCGGATAATTAATAAGACCGCATGGTCTTATTATAAAAGGTGGCTTCGGCTACTATTTGAG
GATGGGCCCGCGGCGCATTAGCTAGTTGGTAAGGTAACAGCTTACCAAGGCGACGATGCGTAGCCGAC
CTGAGAGGGTGATCGGCCACATTGGGACTGAGACACGGCC
>Zotu7368
GAACGCTGGCGGCGTGCCTAATACATGCAAGTCGAGCGAGCAGAACCAGCAGATTTACTTCGGTAATG
ACGCTGGGGACGCGAGCGGCGGATGGGTGAGTAACACGTGGGGAACCTGCCCCATAGTCTGGGATACC
ACTTGGAAACAGGTGCTAATACCGGATAAGAAAGCAGATCGTATGATCAGCTTATAAAAGGCGGCGTA
AGCTGTCGCTATGGGATGGCCCCGCGGTGCATTAGCTAGTTGGTAAGGTAACGGCTTACCAAGGCGAT
GATGCATAGCCGAGTTGAGAGACTGATCGGCCACATTGGGACTGAGACACGGCC
>Zotu7370
GAACGCTGGCGGCGTGCTTAACACATGCAAGTCGAGCGGAAAGGCCCACTTGTGGGTACTCGAGCGGC
GAACGGGTGAGTATCACGTGAGTAATCTGCCCTTCACTTTTGGATAACCCTCGGAAACGGGGGCTAAT
ACGGAATATTCAGTGCTGCCTGCATGGGTGGTGTTGGAAAGATTTTTTGGTGGGGGATGAGCTCGCGG
CCTATCAGCTTGATGGTGGGGTAATGGCCTACCATGGCTTTGACGGGTAGCCGGCCTGAGAGGGTGTC
CGGCCACACTGGGACTGAGACACGGCC
>Zotu7371
GAACGCTAGCGAAATGCCTAACACATGCAAGTCGAACGGATCTTGCCTCGTAGTAATACGGAGTAAGA

TTAGTGGCAAACGAGCGAGTAACGCGTGCTTAACCTGCCCCGAAGATGGGTATAACTGCAGAGATGTA
GCTAATTCCCAATATGCTCACGATGGTGAAATTCCAATGTGAGGAAAGATTTATCGCTTCGGGAGGGG
GGTGCGTCCTATCAGGTAGTTGGTGGGGTAATGGCCTACCAAGCCTATGACGGGTAACCGGTCTGAGA
TGATGTCCGGTCGCGATGGGACTGAGATACGGCC
>Zotu7372
GAACGCTAGCGACAGGCTTAACACATGCAAGTCGAGGGGCAGCATGATTTGTAGCAATACAGATTGAT
GGCGACCGGCGCACGGGTGAGTAACGCGTATGCAACTTACCTATCAGAGGGGGATAGCCCGGCGAAAG
TCGGATTAATACCCCATAAAACAGGGGTCCCGCATGGGAATATTTGTTAAAGATTCATCGCTGATAGA
TAGGCATGCGTTCCATTAGGCAGTTGGCGGGGTAACGGCCCACCAAACCGACGATGGATAGGGGTTCT
GAGAGGAAGGTCCCCCACATTGGTACTGAGACACGGAC
>Zotu7373
GAACGCTGACAGAATGCTTAACACATGCAAGTCTACTTGAACTTCGGTTTGGGTGGCGGACGGGTGAG
TAACGCGTAAAGAACTTGCCTCACAGACTGGGACAACATTTGGAAACGAATGCTAATACTAGATATTA
TGAGATATGCGCATGCATAACTTATGAAAGCTATATGCACTGTGAGAGAGCTTTGCGTCCCATTAGCT
AGTTGGAGAGGTAACGGCTCACCAAGGCGATGATGGGTAGCCGGCCTGAGAGGGTGAACGGCCACAAG
GGGACTGAGACACGGCC
>Zotu7374
GAACGCCGGCGGTGTGCCTAATACATGCAAGTCGTACGCACTGGCCCAACTGATTGATGGTGCTTGCA
CCTGATTGACGATGGATCACCAGTGAGTGGCGGACGGGTGAGTAACACGTAGGTAACCTGCCCCGGAG
CGGGGGATAACATTTGGAAACAGATGCTAATACCGCATAACAATAAAAGCCGCATGGCTTTTATTTGA
AAGATGGCTTTGGCTATCACTCTGGGATGGACCTGCGGTGCATTAGCTAGTTGGTAAGGTAACGGCTT
ACCAAGGCGATGATGCATAGCCGAGTTGAGAGACTGATCGGCCACAATGGAACTGAGACACGGTC
>Zotu7376
GAACGCTGGCGGCGTGCTTAACACATGCAAGTCGAACGGAAAGGCCCAAGCTTGCTTGGGTACTCGAG
TGGCGAACGGGTGAGTAACACGTGGGTGATCTGCCCTGCACTTCGGGATAAGCCTGGGAAACTGGGTC
TAATACTGGATAGGACCACTTCTTGGATGTTGTGGTGGAAAGTTTTTTCGGTGTGGGATGAGCTCGCG
GCCTATCAGCTTGTTGGTGGGGTAATGGCCTACCAAGGCGTCGACGGGTAGCCGGCCTGAGAGGGTGT
ACGGCCACATTGGGACTGAGATACGGCC
>Zotu7377
GAACGCTGGCGGCGTGCTTAATACATGCAAGTCGAACGGGTATAGCAATATACTAGTGGCGACCGGGT
GCGTAACACGTAGTTGACCTGCCCCTCAGCGGGGGATAACGTTTCGAAAGAGACGCTAATACCGCATG
ATCTCGATGGGATTAGAAGCCATTGAGGAAACTTCAGGGGTTGAGGGAGGGGACTGCGGCCCATCAGG
TAGTTGGTGAGGTAACGGCTCACCAAGCCAATGACGGGTAGGGGGCCTGAGAGGGTGGTCCCCCACAC
TGGAACTGACACACGGTC
>Zotu7378
GAATGCTGGCGGCGTGGATAAGGCATGCAAGTCGAACGGTCCGCAAGGACAGTGGCAGACGAGGTAGT
AACACGTACGAACGCACCCCAGAGTCTGGCATAGCTCGTCGAAAGACGGGGTAATTCCGGATGGTCCC
GAAAGGGTAAAGTTTTTCGCTCTGGGAGCGGCGTGCGTAATATCAGCTAGTTGGTAAGGTAATGGCT
TACCAAGGCTACGACGTTTAGGGGAGCTGAGAGGCTGACCCCCACCGATGGGACTGAGACACGGCC
>Zotu7380
GAACGCTGGCGGCGTGCTTAACACATGCAAGTCGAGCGAAGCGCAGAAACGGATTTCTTCGGATTGAA
GGTTCTGTGACTGAGCGGCGGACGGGTGAGTAACGCGTGGGGAACCTGCCCTGTACCGGGGGATAACA
GACAGAAATGTCTGCTAATACCGCATAAGCGCACGAGAAGGCATCTTCTTGTGTGAAAAACTCCGGTG
GTACAGGATGGACCCGCGTTGGATTAGCCAGTTGGCGGGGTAACGGCCCACCAAAGCGACGATCCATA
GCCGGCCTGAAAGGGCGACCGGCCACATTGGGACTGAGACACGGCC
>Zotu7383
GAACGCTGGCGGTGTGCCTTAAGCATGCAAGTCGAGCGGGTCTAGCAATAGACCAGCGGCAGACGGGT
GAGTAACGCGTAGGAATCTACCTCAAAGTGGGGAATAGCCTTCCGAAAGGGAGGGTAATACCGCATGT
GATCGCAAGATCAAAGCCGTAAGGCGCTTTGAGAGGAGCCTGCGTCCTATCAGTTAGTTGGTGGGGTA
ATGGCCTACCAAGACTATGACGGGTAGCCGATGTGAGAGCATGATCGGCCACAAGGTTACTGAGACAC
GGAA
>Zotu7385
GAACGCTGGCGGCGTGCCTAACACATGCAAGTCGAGCGAGGCCCCTTCCTCCGGGGAGGGAGCCGAGC
GGCGAACGGGTGAGTAACACGTGAGCAACCTGCCCCTGGCACTGGGATAACTCCGGGAAACCGGGGCT

AATACCGGATATCCATCCGGGTGGGCATCCGCCTGGATGAAAAGGGTTCCGTCGCGCAAGCGGCTGTT
CCGGCCAGGGAGGGGCTCGCGGCCTATCAGCTTGTTGGTGAGGTAACGGCTCACCAAGGCGATGACGG
GTAGCCGGCCTGAGAGGGTGACCGGCCACGCTGGGACTGAGACACGGCC
>Zotu7386
GAACGCTGGCGGCGTGCTTAACACATGCAAGTCGAACGATGAAGCTGGTGCTTGCACTGGTGGATTAG
TGGCGAACGGGCGAGTAACACGTGAGTAACCTGTCCTCTTCTTTGGGATAACGGCTGGAAACGGCTGC
TAATACTGGATATTCTGACCTTGTCGCATGGTGGGGTTGGGAAAGCTTTTGCGGTAGAGGGTGGGCTC
GCGGCCTATCAGCTTGTTGGTGGGGTGATGGCTTACCAAGGCTTTGACGGGTAGCCGGCCTGAGAGGG
TGGTCGGTCGCACTGGGACTGAGATACGGCC
>Zotu7387
GAACGCTGGCGGCATGCCTTACACATGCAAGTCGAACGGCAGCACGGGAGCAATCCTGGTGGCGAGTG
GCGAACGGGTGAGTAATATATCGGAACGTGCCCAGTAGTGGGGGATAGCCCGGCGAAAGCCGGATTAA
TACCGCATACGACCTGAGGGTGAAAGCGGGGGATCGCAAGACCTCGCGCTATTGGAGCGGCCGATATC
AGATTAGCTAGTTGGTGGGGTAAAGGCCTACCAAGGCAACGATCTGTAGTTGGTCTGAGAGGACGACC
AGCCACACTGGGACTGAGACACGGCC
>Zotu7388
GAACGCTGGCGGCATGCCTAACACATGCAAGTCGAACGAGGGCTTCGGCCCTAGTGGCGCACGGGTGA
GTAACGCGTGGGAACCTGCCCTGTGGTACGGAATAACTCCGGGAAACTGGAGCTAATACCGTATGCGT
CCTGCGGGACAAAGATTTATCGCCATGGGATGGGCCCGCGTAGGACTAGCTTGTTGGTGGGGTAACGG
CCTACCAAGGCTTCGATCCTTAGCTGGTCTGAGAGGATGATCAGCCACACTGGGACTGAGACACGGCC
>Zotu7389
GAACGCTGGCGGCGTGCCTAACACATGCAAGTCGAACGGAACTTCTTTAAAGGATTTCTTCGGAATGA
ATTTGATTAAGTTTAGTGGCGGACGGGTGAGTAACGCGTGAGTAGCCTGCCTCTAAGAGGGGAATAAC
ATTCTGAAAAGAATGCTAATACCGCATAACATATATTTATCGCATGGTAGATATATCAAAGATTTATC
GCTTAGAGATGGACTCGCGTCCGATTAGTTAGTTGGTGAGGTAACGGCTCACCAAGACCGCGATCGGT
AGCCGGACTGAGAGGTTGAACGGCCACATTGGGACTGAGACACGGCC
>Zotu7390
GAACGCTAGCGATGTGCTTTAAGCATGCAAGTAGGGCGGGTTGTAGCAATACAACCAGCTGCGAACGG
CTGAGTATAAAATAGGTATCTACCTCATGGTGGTGAATACCCCGTCGAAAGACGGGCTAATACAGCAT
AAGATTCTAAATGAAAGCGTGTAACTACGCGCCGAGAGAGGAGCCTATTTCCCATCAGCTTGTTGGTA
GGGTAATGGCCTACCAAGGCTACGACGGGTAGGGGGTGTGAGAGCATGACCCCCCAGAATGGTACTGA
GACACGGAC
>Zotu7391
GAACGCTGGCGGCGTGCTTAACACATGCAAGTCGAACGGAAAGGCCCTTCGGGGTACTCGAGTGGCGA
ACGGGTGAGTAACACGTGGGTGATCTGCCCTGCACTTTGGGATAAGCCTGGGAAACTGGGTCTAATAC
CGGATAGGACCACGGCATTCATGTGCTGTGGTGGAAAGCTTTTGCGGTGTGGGATGGGCCCGCGGCCT
ATCAGCTTGTTGGTGGGGTAATGGCCTACCAAGGCGACGACGGGTAGCCGGCCTGAGAGGGTGACCGG
CCACACTGGGACTGAGATACGGCC
>Zotu7395
GAACGCTAGCGGCAGGCCTAACACATGCAAGTCGAGGGGTAAGGAGAGCTTGCTCTCCCTAGACCGGC
GCACGGGTGCGTAACACGTGTACAATCTACCTTTTGCTAAGGGATAGCCCGAAGAAATTTGGATTAAT
ACCTTATAGTATTGTTTGGTGGCATCACTGAATAATTAAAGCTCTGGTGGCAAAAGATGAGTACGCGT
CCCATTAGCTAGTTGGTGTGGTAACGGCATACCAAGGCTACGATGGGTAGGGGTCCTGAGAGGGAGGT
CCCCCACACTGGTACTGAGACACGGAC
>Zotu7396
GAACGCTGGCGGCGTGCTTAACACATGCAAGTCGAACGGTGATCTTGGGAGCTTGCTCTTGGGTGATC
AGTGGCGAACGGGTGAGTAACACGTGAGTAACCTGCCCCTGACTCTGGAATAACTTCGGGAAACCGAT
GCTAATACCGGATATGACACAGAATCGCATGGTTTCTGTGTGGAAAGTTTTTCGGTTGGGGATGGACT
CGCGGCCTATCAGCTTGTTGGTGAGGTAATGGCTCACCAAGGCGACGACGGGTAGCCGGCCTGAGAGG
GCGATCGGCCACACTGGGACTGAGACACGGCC
>Zotu7398
GAACGCTGGCGGCGTGCTTAAGACATGCAAGTCGAACGAGGGCTTCTTGTAGCAATACGGGGAGTTCC
TAGTGGCGAATGGGTGCGTAACACGTAGGTGACCTGCCCTGAAGAGGAGGATAACAGTCCGAAAGGAC
TGCTAATACTCCATGTGGTCCTGGAGGTTAGAGGCTCCAGGTCTAAAGTTGCAAAACGCTTCAGGAGG

GGCCTGCGGCCCATCAGCTTGTTGGTAGGGTAATGGCCTACCAAGGCGACGACGGGTAGGGGACCTGA
GAGGGTGGCCCCCCACATTGGCACTGACACACGGGC
>Zotu7399
GAACGCTGGCGGCGTGCTTAACACATGCAAGTCGAACGATTAAAGCCCCTTCGGGGGTGTATAGAGTG
GCGAACGGGTGAGTAACACGTGGGCAACCTGCCCTGTGCTTCGGGATAACCTAGGGAAACCTGGGCTA
ATACCGGATACTCCGTAAGGATCGCATGGTCCACGCGGGAAAGTTCCGGCGGCACAGGATGGGCCCGC
GGCCCATTAGCTTGTTGGTGGGGTAACGGCCCACCAAGGCAACGATGGGTAGCCGAGCTGAGAGGCTG
ATCGGCCACACTGGGACTGAGACACGGCC
>Zotu7400
GAACGCTAGCTACAGGCTTAACACATGCAAGTCGAGGGGAAACGACATCGAAAGCTTGCTTTTGATGG
GCGTCGACCGGCGCACGGGTGAGTAACGCGTATCCAACCTGCCCACCACTTGGGGATAACCTTGCGAA
AGTAAGACTAATACCCAATGACGTCTCTAGAAGACATCTGAAAGAGATTAAAGATTTATCGGTGATGG
ATGGGGATGCGTCTGATTAGCTTGTTGGCGGGGTAACGGCCCACCAAGGCGACGATCAGTAGGGGTTC
TGAGAGGAAGGTCCCCCACATTGGAACTGAGACACGGTC
>Zotu7401
GAACGCTAGCTACAGGCTTAACACATGCAAGTCGAGGGGAAACGACATCGAAAGCTTGCTTTTGATGG
GCGTCGACCGGCGCACGGGTGAGTAACGCGTATCCAACCTGCCCACCACTTGGGAATAACCTTGCGAA
AGTAAGACTAATACCCAATGATATCTCTAGAAGACATCTGAAAGAGATTAAAGGTTTATCGGTGATGG
ATGGGGATGCGTCTGATTAGCTTGTTGGCGGGGTAACGGCCCACCAAGTCAACGATCAGTAGGGGTTC
TGAGAGGAAGGTCCCCCACATTGGAACTGAGACACGGTC
>Zotu7403
GAACGCTGGCGGCGTGCTTAACACATGCAAGTCGAGCGGAAAGGCCCTTCGGGGTACTCGAGCGGCGA
ACGGGTGAGTAACACGTGGGCAACCTGCCCTCAGCTCTGGGATAACTCCGGGAAACCGGGGCTAATAC
CGGATATGACTGTCCTGGGCATCTGGGGTGGTGGAAAGTTTCTCGGCTGAGGATGGGCCCGCGGCCTA
TCAGCTTGTTGGTGGGGTGATGGCCTACCAAGGCGACGACGGGTAGCCGGCCTGAGAGGGCGACCGGC
CACACTGGGACTGAGACACGGCC
>Zotu7406
GAACGCTGGCGGCAGGCTTAACACATGCAAGTCGAGCGCCCACTTCGGTGGGAGCGGCAGACGGGTGA
GTAACACGTGGGAATCTACCCATCTCTACGGAACAACTCCGGGAAACTGGAGCTAATACCGTATACGC
CCTTTGGGGGAAAGATTTATCGGAGATGGATGAGCCCGCGTTGGATTAGCTAGTTGGTGGGGTAAGGG
CCTACCAAGGCGACGATCCATAGCTGGTCTGAGAGGATGATCAGCCACACTGGGACTGAGACACGGCC
>Zotu7410
GAACGCTAGCGGCAGGCTTAACACATGCAAGTCGAGGGGTATGGTAGCAATACCAGAGACCGGCGCAC
GGGTGCGTAACGCGTATGCAATCTACCTTTTACAGAGGGATAGCCCAGAGAAATTTGGATTAATACCT
CATAGTATAGCTGTACCGCATGGTACTACTATTAAAGTTACAACGGTAAAAGATGAGCATGCGTCCCA
TTAGCTAGTTGGTAAGGTAACGGCTTACCAAGGCTACGATGGGTAGGGGTCCTGAGAGGGAGATCCCC
CACACTGGTACTGAGACACGGAC
>Zotu7411
GAACGCTGGCGGCGTGCTTAATACATGCAAGTCGAACGCTCTCGACCTTCGGGTCACATGAGGGAGTG
GCGAACGGGTGCGTAACACGTGAGAAACCTACCCCGGAGTTGGGAATAACTCGAGGAAACTCGAGCTA
ATACCCGATGCCCTCACCGGATCGCATGATCTGGTGAGGAAAGTTCCGGCGCTCCGGGACGGTCTCGC
GGCCTATCAGCTTGTTGGTGAGGTAACGGCTCACCAAGGCTTCGACGGGTAGCTGGTCTGAGAGGACG
ATCAGCCACACTGGGACTGAGACACGGCC
>Zotu7414
GAACGCTGGCGGCATGCCTAACACATGCAAGTCGAACGAGACCTTCGGGTCTAGTGGCGCACGGGTGC
GTAACGCGTGGGAATCTGCCCTTGGGTTCGGGATAACAGTTGGAAACGACTGCTAATACCGGATGATG
ACTTCGGTCCAAAGATTTATCGCCCAAGGATGAGCCCGCGTAGGATTAGCTTGTTGGTGAGGTAAAAG
CTCACCAAGGCGACGATCCTTAGCTGGTCTGAGAGGATGATCAGCCACACTGGGACTGAGACACGGCC
>Zotu7418
GAACGCTGGCGGCATGCCTAACACATGCAAGTCGAACGAGACCTTTGGGTCTAGTGGCGCACGGGTGC
GTAACGCGTGGGAATCTGCCCTTGGGTTCGGAATAACTGGGAGAAATTCCAGCTAATACCGGATGATG
TCTTCGGACCAAAGATTTATCGCCCAAGGATGAGCCCGCGTAGGATTAGCTAGTTGGTGAGGTAAAGG
CTCACCAAGGCGACGATCCTTAGCTGGTCTGAGAGGATGATCAGCCACACTGGGACTGAGACACGGCC
>Zotu7421

GAACGCTGGCGGCAGGCTTAACACATGCAAGTCGAGCGGAGAGAGGTAGCTTGCTACTGATCTTAGCG
GCGGACGGGTGAGTAATGCTTAGGAATCTGCCTATTAGTGGGGGACAACATTTCAAAGGAATGCTAA
TACCGCATACGTCCTACGGGAGAAAGCAGGGGATCTTCGGACCTTGCGCTAATAAATGAGCCTAAGTC
GGATTAGCTAGTTGGTGGGGTAAAGGCCTACCAAGGCGACGATCTGTAGCGGGTCTGAGAGGATGATC
CGCCACACTGGGACTGAGACACGGCC
>Zotu7424
GAACGCTGGCGGCAGGCTTAACACATGCAAGTCGAACGCCCCGCAAGGGGAGTGGCAGACGGGTGAGT
AACGCGTGGGAATCTACCCAGATCTACGGAACAACAGTTGGAAACGACTGCTAATACCGTATACGCCC
TACGGGGGAAAGATTTATCGGATTTGGATGAGCCCGCGTAAGATTAGCTAGTTGGTGAGGTAATGGCT
CACCAAGGCGACGATCTTTAGCTGGTCTGAGAGGATGATCAGCCACACTGGGACTGAGACACGGCC
>Zotu7425
GAACGCTGGCGGCAGGCTTAACACATGCAAGTCGAGCGCCCCGCAAGGGGAGCGGCAGACGGGTGAGT
AACACGTGGGAACGTACCTTTTGCTACGGAATAGCTCCGGGAAACTGGAATTAATACCGTATGTGCCC
TTCGGGGGAAAGATTTATCGGCAAAAGATCGGCCCGCGTCTGATTAGCTAGTTGGTGGGGTAATGGCC
TACCAAGGCGACGATCAGTAGCTGGTCTGAGAGGATGATCAGCCACACTGGGACTGAGACACGGCC
>Zotu7427
GAACGCTAGCGGTATGCCTAATACATGCAAGTCGTACGCGATCACTTTTTTGCTCGCAGCAGTGGTTA
TTTATAACCACCTTTGCGAGCAGAATTATGGTGATTGAGTGGCGGACGGGTGAGTAACACGTTGGTAC
CTGCCCCAAAGTGGGGAATAATTCACCGAAAGGTGGACTAATACCCCATAGTCTCCGGTTTATCCGGA
GTAAAGCTTTAGCGCTTTGGGAGGGGCCTGCGTCCGATTAGCTAGTTGGTAGAGTAACGGTTTACCAA
GGCTACGATCGGTAGCTGGATCGAGAGGTTGACCAGCCACACTGGGACTGAGATACGGCC
>Zotu7429
GAACGCTAGCGGCAGGCTTAACACATGCAAGTCGAGGGGTATATGTCTTCGGATATAGAGACCGGCGC
ACGGGTGCGTAACGCGTATGCAATCTACCTTTTACAGGGGAATAGCCCAGAGAAATTTGGATTAATGC
CCCATAGTATTATAGAATGGCATCATTTTATAATTAAAGTCACAACGGTAAAAGATGAGCATGCGTCC
CATTAGTTAGTTGGTAAGGTAACGGCTTACCAAGACAATGATGGGTAGGGGTCCTGAGAGGGAGATCC
CCCACACTGGTACTGAGACACGGAC
>Zotu7430
GAACGCTGGCGGCGTGCTTAACACATGCAAGTCGAACGATGATGCCCAGCTTGCTGGGCGGATTAGTG
GCGAACGGGTGAGTAACACGTGAGTAACCTGCCCCTGACTCTGGGATAAGCGTTGGAAACGACGTCTA
ATACTGGATATGATCACTGGTCGCATGGCCTGGTGGTGGAAAGATTTTTTGGTTGGGGATGGACTCGC
GGCCTATCAGCTTGTTGGTGAGGTAATGGCTCACCAAGGCGACGACGGGTAGCCGGCCTGAGAGGGTG
ACCGGCCACACTGGGACTGAGACACGGCC
>Zotu7432
GAACGCTGGCGGCGTGCCTAATACATGCAAGTCGAACGGGGTGCAGCAATGTACCAAGTGGCGATCGG
GTGCGTAACACGTAGCTGACCTACCTCTTGGCGGGGGACAACAGGCCGAAAGGCTTGCTAATACCGCA
TAATTTCGGGGAAACCAGAATACCCTGAAGAAACGCGCAAGCGGCCGAGAGAGGGGGCTGCGGCCCAT
CAGGTAGTTGGTGAGGTAACGGCTCACCAAGCCGACGACGGGTAGGGGGCCTGAGAGGGTGACCCCCC
ACACTGGGACTGACAGACGGCC
>Zotu7433
CAACGCTGGCGGCGTGCCTAACACATGCAAGTCGAACGCGAAAGGGGCTTCGGTCCTGAGTAGAGTGG
CGAACGGGTGAGTAACGCGTGGGTGACCTACCCTCGAGTGGGGGATAACGTTCCGAAAGGGACGCTAA
TACCGCATGACATTCTGGGTTTGAAGACCTGGAAATCAAAGCCGGGGACCGCAAGGCCTGGCGCTTGA
GGAGGGGCCCGCGTCTGATTAGCTAGTTGGCGGGGTAACGGCCCACCAAGGCGACGATCAGTAGCCGG
CCTGAGAGGGCGGACGGCCACACTGGGATTGAGACACGGCC
>Zotu7434
GAACGCTGGCGGCGTGCCTAACACATGCAAGTCGAGCGCGGTTGTGCTTAGTATTGAGTGTTTTATTG
ATATAAAACATTGAATTCTATGCACAACTGAGCGGCGGACGGGTGAGTAACGCGTGGGTAACCTGCCC
TATACACATGGATAACATACTGAAAAGTTTACTAATACATGATAAAATAGTTTTTCGGCATCGAAGAA
TTATCAAAGTGTTAGCGGTATAGGATGGACCCGCGTCTGATTAGCTAGTTGGTGAGATAACTGCCCAC
CAAGGCGACGATCAGTAGCCGACCTGAGAGGGTGATCGGCCACATTGGAACTGAGACACGGTC
>Zotu7435
CAACGCTGGCGGCGTGCCTAACACATGCAAGTCGAACGAGAAAGGGCTTTCGGGCCTGAGTAAAGTGG
CGCACGGGTGAGTAACACGTGACTAACCTACCTTTGAGCGGGGGATAACCTAGGGAAACCTGGGCTAA

TACCGCATAATACCTACGGGTCAAAGCGAGCAATCGCACTTAAAGAGGGGGTCGCGGCAGATTAGCTA
GTTGGTAAGGTAATGGCTTACCAAGGCGATGATCTGTATCCGGCCTGAGAGGGCGCACGGACACACTG
GAACTGAAACACGGTC
>Zotu7437
GAACGCTGGCGGCGTGCTTAACACATGCAAGTCGAGCGACGCTCTTCCTTCGGGAAGATGACGGAGCG
GCGAACGGGTGAGTAACACGTGGGCAACCTGCCCCTAGCTCTGGGACAACACCGGGAAACCGGTGCTA
ATACCGGATACGACATCGAGCGGCATCTCTCGATGTGGAAAGTTTTTCGGCTAGGGATGGGCCCGCGG
CCTATCAGCTTGTTGGTGGGGTAATGGCCCACCAAGGCGACGACGGGTAGCCGGCCTGAGAGGGCGAC
CGGCCACACTGGGACTGAGACACGGCC
>Zotu7438
GAACGCTGGCGGCGTGCTTAACACATGCAAGTCGAACGGAGCGAATGAAAGCTTGCTTTTATAAGCTT
AGTGGCAAACGGGTGAGTAACACGTAGACAACCTGCCGACAGGATGGGGACAACATTCCGAAAGGAAT
GCTAATACCGAATGAAGCGCAGGAGAGGCATCTCTCCTGCGTGAAAGATGGCCTCTATATATAAGCTA
TCACCTGTTGATGGGTCTGCGTCTGATTAGCTGGTAGGTGAGGTAACGGCTCACCTAGGCGACGATCA
GTAGCCGGTCTGAGAGGATGAACGGCCACATTGGGACTGAGACACGGCC
>Zotu7441
GAACGCTGGCGGCGTGCTTAACACATGCAAGTCGAGCGGAAAGGCCACTTCGGTGGTACTCGAGCGGC
GAACGGGTGAGTAACACGTGAGTAATCTGCCCCTGGCTTTGGGATAGCCACCGGAAACGGTGATTAAT
ACCGGATATGACCGACTTCCGCATGGTTGTTGGTGGAAAGTTTTTCGGCCAGGGATGTGCTCGCGGCC
TATCAGCTTGATGGTGAGGTAATGGCTCACCATGGCTTCGACGGGTAGCCGGCCTGAGAGGGTGACCG
GTCACACTGGGACTGAGACACGGCC
>Zotu7442
GAACGTTGATGGCGTGCCTCATACATGCAAGTCGAGCGCCCCGCAAGGGGAGCGGCGGACGGGTGAGT
AACACGTAAAGAATCTGCCCTGAAGCGGGGAATAACTATCCCAACGGATAGCTAATACCGCATAATGA
TGGAGGAGGCATCTCCTTTTCTCAAAAGGCTTCGGCCACTTCAGGATGACTTTGCGTCCCATTAGCTT
GTTGGCGGGGTAACGGCCCACCAAGGCTACGATGGGTATCCGGCCTGAGAGGGTGTACGGACACATTG
GGACTGAGATACGGCC
>Zotu7444
GAACGCTGGCGGCGTGCCTAATACATGCAAGTCGAGCGAACCTTTTAAAAGCTTGCTTTTGAAAGGTT
AGCGGCGGACGGGTGAGTAACACGTGGGCAACCTGCCCTATAGTTTGGGATAACTCCGGGAAACCGGG
GCTAATACCGGATAATACAATTTAACTCCTGTTAGATTGTTGAAAGATGGTTTCGGCTATCTCTATAG
GATGGGCCCGCGGCGCATTAGCTAGTTGGTAGGGTAACGGCCTACCAAGGCGACGATGCGTAGCCGAC
CTGAGAGGGTGATCGGCCACACTGGGACTGAGACACGGCC
>Zotu7445
GAACGCTAGCGGCAGGCTTAATACATGCAAGTCGTGGGGCAGCGCAGTGTAGCAATACATGGGCGGCG
ACCGGCAAACGGGTGCGGAACACGTACGCAACCTTCCTTTAAGCGGGGGATAGCCCAGGGAAACTTGG
ATTAATACCCCATAAGATTATGGTGTGGCATCACACAGTAATTAAAGCTCCGGCACTTAAAGATGGGC
GTGCGGCTGATTAGGTAGTTGGCGGAGTAACAGCCCACCAAGCCAACGATCAGTAACTGGTGTGAGAG
CACGACCAGTCACACGGGCACTGAGACACGGGC
>Zotu7446
GAACGCTAGCGGCAGGCTTAATACATGCAAGTCGTGGGGCAGCATGGTTGTAGCAATACAACTGATGG
CGACCGGCAAACGGGTGCGGAACACGTACGCAATCTACCCAGAACTGGCGAATAGCCTTCCGAAAGGA
AGATTAATACGCCGTAACATTATAAAGTGGCATCACTTAATAATTAAAGCTCCGGCGGTTTTGGATGA
GCGTGCGCCCCATTAGGTAGTTGGTTGAGGTAATGGCTCACCAAGCCTGCGATGGGTAACTGGTGTGA
GAGCACGACCAGTCACACGGGCACTGAGACACGGGC
>Zotu7447
TAGCGCTGGCGGCGTGCCTAATACATGCAAGTCGAACGGACCTTTATTTTTGAGAGTTCCTTTATAGG
ATTTCTCATAAATACTGGTTAGTGGCGGACGGGTGAGTAACACGTTGGTACATGCCCTCTAGTGGGGA
ATAACTGGCCGAAAGGTCGGCTAATACCGCATGTGGTCTACGGATCAAAGCAGTAATGCGCTAGAGGA
GTGGCCTGCGTCTGATTAGCTAGTTGGTAGTGTAATGGACTACCAAGGCAACGATCAGTAGCTGGTTC
GAGAGGATGACCAGCCACACTGGGACTGAGATACGGCC
>Zotu7448
GAACGCTGGCGGCAGGCCTAACACATGCAAGTCGAGCGGATGACGGGAGCTTGCTCCTTGATTCAGCG
GCGGACGGGTGAGTAATGCCTAGGAATCTGCCTGGTAGTGGGGGACAACGTTCCGAAAGGGGCGCTAA

TACCGCATACGTCCTACGGGAGAAAGTGGGGGATCTTCGGACCTCACGCTATCAGATGAGCCTAGGTC
GGATTAACTAGTTGGTGAGGTAATGGCTCACCAAGGCGACGATCCGTAACTGGTCTGAGAGGATGATC
AGTCACACTGGAACTGAGACACGGTC
>Zotu7449
GAACGCTGGCGGCATGCTTTACACATGCAAGTCGAACGGCAGCGCGGGGCAACCTGGCGGCGAGTGGC
GAACGGGTGAGTAATACATCGGAACGTACCCAGAAGTGGGGGATAACGTAGCGAAAGTTACGCTAATA
CCGCATACGTTCTACGGAAGAAAGTGGGGGATCTTCGGACCTCATGCTTTTGGAGCGGCCGATGTCTG
ATTAGCTAGTTGGTAGGGTAAAGGCCTACCAAGGCGACGATCAGTAGCTGGTCTGAGAGGACGACCAG
CCACACTGGGACTGAGACACGGCC
>Zotu7452
GAACGCTGGCGGCGTGCATAACACATTCAAGTCGAACGGAGCTTTGGGGATTAATCCTTCGGGAAGCG
TTTTCAAAGCTTAGTGGCGGACGGGTGAGTAACGCGTGAGCAACCTGCCTTTCAAAGCGGGATAACGT
CTGGAAACGGACGCTAATACCGCATAACGCATCGACATCACATGGTGATGATGCCAAAGGGGCAACCC
GATGAAAGATGGGCTCGCGTCTGATTAGATAGTTGGTGAGGTAACGGCTCACCAAGTCGACGATCAGT
AGCCGGACTGAGAGGTTGAACGGCCACATTGGAACTGAGATACGGTC
>Zotu7453
GCCGTGTCTCAGTCCCAGTGTGGCTGATCATCCTCTCAGACCAGCTACAGATCGTCGGCTTGGTGAGC
CATTACCTCACCAACTACCTAATCTGACGCGGGCTCACCCATCAGCGATAAATCTTTCCTCCGTAGAG
AATATACGGTATTAGCTTTTATTTCTAAAAGTTATTCCGTACTGATGGACAGATTCCCACGTGTTACT
CACCCGTCTGCCACTAACTAATTGGAGCAAGCCCCAATTAGTCCGTTCGACTTGCATGTGTTAAGCAT
ACCGATAGCGTTCG
>Zotu7455
GAACGCTGGCGGAATGCTTAACACATGCAAGTCGCACGGTCAGCAATGGCAGTGGCGGACGGGTGAGT
AACGCGTAGGAATATGTCCAGAGGTGGGGGACAACCCCGGGAAACTGGGGCTAATACCGCATATGAGC
TGAGGCTCAAAGCAGTGATGCGCCTTTGGAGTAGCCTGCGTCCGATTAGCTTGTTGGTGGGGTAATGG
CCTACCAAGGCGACGATCGGTAGCTGGTCTGAGAGGACGACCAGCCACACTGGGACTGAGACACGGCC
>Zotu7456
GAATGCTGGCGGCGTGCCTAACACATGCAAGTCGAACGGTAACAGAGATAATTTATTATTTGCTGACG
AGTGGCGGACGGCTGAGTAACGCGTGGGAACATGCCCCAAAGTGAGGGATAAGCTCCAGAAATGGAGT
CTAATACCGCATATGATCTTCGGATTAAAGGATTTATCCGCTTTGGGAGTGGCCTGCGTACGATTAGA
TAGTTGGTGAGGTAATGGCTTACCAAGTCTACGATCGTTAGCTGGTCTGAGAGGATGACCAGCCAGAC
TGGAACTGAGACACGGTC
>Zotu7458
GAACGCTGGCGGCGTGCTTAACACATGCAAGTCGAACGAGGTCCATCCACCCGCAAGGGTGGTGAAGA
CCTAGTGGCGAACGGGTGAGTAACACGTGAGAAACCTGCCCCGAAGACCGGGATAACCGGCCGAAAGG
TCGGCTAATACCGGATACCTTCACGACATCTCATGGTGATGTGAAGAAATGGATTCCGCTTCGGGAGG
GTCTCGCGGCCTATCAGCTTGTTGGTGAGGTAACGGCTCACCAAGGCGACGACGGGTAGCTGTTCTGA
GAGGAAGATCAGCCACACTGGGACTGAGACACGGCC
>Zotu7459
GAACGCTGGCGGTCTGCTTAACACATGCAAGTCGAACGGAGTAGAGATACTTAGTGGCGGACGGGTGA
GTAACGCGTGAGAATCTGCCTTCAGGACGGAGACAACAGTTGGAAACGACTGCTAAGCCCCGATGTAC
CGAAAGGGAAAATATTTATAGCCTGAAGATGAGCTCGCGTCCGATTAGCTAGTTGGAGAGGTAAAAGC
TCACCAAGGCGACGATCGGTAGCTGGTCTGAGAGGACGATCAGCCACACTGGGACTGAGACACGGCC
>Zotu7460
GAACGCTAGCGGGAGGCCTAATACATGCAAGTCGAGCGGTTCAGCAATGGATAGCGGCAGACGGGTGC
GTAACGCGTATGAAACTTACCCTACACAGGAGGATAGCCTTGGGAAACTGAGAGTAACCCTCCATAGT
CTTGAGATACCGCATGGTATTTTAAGTAAAACTTAGGTGGTGTAGGATAGTCGTGCGTCCGATTAGCT
AGTTGGTGAGGTAATGGCTCACCAAGGCGACGATCGGTAGCTGGTCTGAGAGGATGATCAGCCACACG
GGCACTGAGACACGGGC
>Zotu7461
GAACGCTGACAGAATGCTTAACACATGCAAGTCTTTGGCAAATCTGTGCTTGCACAGCCTAGCCAAGG
CGGACGGGTGAGTAACGCGTAAAGAACTTGCCCTGCAGACAGGGATAACAGACGGAAACGACTGATAA
TACCTGATACAATTGCCAGCACGCATGTGCCCGGCAATGAAAAGTGATGCTGCAGGAGAGCTTTGCGT
CCTATTAGCTTGTTGGTGAGGTAACGGCTCACCAAGGCGATGATAGGTAGCCGGCCTGAGAGGGTGAA

CGGCCACAAGGGGACTGAGATACGGCC
>Zotu7463
GAACGCTGGCGGCGTGCCTAATACATGCAAGTCGAGCGAACTTTTGGGAGCTTGCTCCCAAAAGTTAG
CGGCGGACGGGTGAGTAACACGTGGGCAACCTGCCTATAAGACTGGGATAACTTCGGGAAACCGGAGC
TAATACCGGATAATTCTTTTCTACTCATGTAGAAAAGCTAAAAGACGGTTTATGCTGTCACTTATAGA
TGGGCCCGCGGCGCATTAGCTAGTTGGTGAGGTAACGGCTCACCAAGGCCACGATGCGTAGCCGACCT
GAGAGGGTGATCGGCCACACTGGGACTGAGACACGGCC
>Zotu7465
GAACGCTGGCGGCGTGCTTAACACATGCAAGTCGAACGAGGTCCATCCACCCGCAAGGGTGGTGAAGA
CCTAGTGGCGAACGGGTGAGTAACACGTGAGGAACCTGCCCCGGAGACCGGGATAACTCACCGAAAGG
TGAGCTAATACCGGATACCTTCACGATGTCGCATGGCGACGTGAAGAAATGGATTCCGCTTCGGGAGG
GTCTCGCGGCCTATCAGCTTGTTGGTGAGGTAACGGCTCACCAAGGCGACGACGGGTAGCTGTTCTGA
GAGGAAGATCAGCCACACTGGGACTGAGACACGGCC
>Zotu7468
GAACGCTGGCGGCGTGCTTAACACATGCAAGTCGAACGCTGAAGCCCAGCTTGCTGGGTGGATGAGTG
GCGAACGGGTGAGTAACACGTGAGTAACCTGCCCCCTTCTTTGGGATAACGCCCGGAAACGGGTGCTA
ATACTGGATATTCACTGATCTTCGCATGGGGGTTGGTGGAAAGGTTTGTTCTGGTGAGGGATGGGCTC
GCGGCCTATCAGCTTGTTGGTGGGGTGATGGCCTACCAAGGCTTTGACGGGTAGCCGGCCTGAGAGGG
TGACCGGTCACATTGGGACTGAGATACGGCC
>Zotu7469
GAACGCTGGCGGTATGCTTAACACATGCAAGTCGAACGGTGTTTTAGGACACAGTGGCGGACGGGTGA
GTAACGCGTGAGAATCTGGCTTCAGGTCTGGGACAACCATTGGAAACGGTGGCTAATACCGGATGTGC
CGAGAGGTAAAAGGCTTGCTGCCTGAAGATGAGCTCGCGTCTGATTAGCTAGTAGGTGGTGTAAGGGA
CTACCTAGGCGACGATCAGTAGCTGGTCTGAGAGGATGATCAGCCACACTGGGACTGAGACACGGCC
>Zotu7470
GAACGCTGGCGGCGCGTTTTAAGCATGCAAGTCGAGCGGCAAGGGTACTTCGGTACCCCTAGAGCGGC
GGACGGGTGAGTAACACGTGGACAACCTGCCCCCGGGAGAGGGATAGCCCAGGGAAACCTGGATCAAT
ACCTCGTAAGACCACACACTGTAGGGTGAGTGGGAAAAGGTGCTTCGGCACCGCCTGGGGATGGATCC
GCGTCCCATTAGCTAGATGGTAGGGTAAGAGCCTACCATGGCGACGATGGGTAGCCGGCCTGAGAGGG
TGAACGGCCACACTGGAACTGAGACACGGTC
>Zotu7472
GAACGCTGGCGGCAGGCCTAACACATGCAAGTCGAACGATCCTTCGGGATAGTGGCGGACGGGTGAGT
AACACGTGGGAACGTACCTTTAGGTTCGGAACAACACAGGGAAACTTGTGCTAATACCGAATGTGCCC
TTCGGGGGAAAGATTTATCGCCTTTAGAGCGGCCCGCGTTAGATTAGCTAGTTGGTGGGGTAATGGCC
CACCAAGGCTCCGATCTATAGCTGGTCTGAGAGGATGATCAGCCACATTGGGACTGAGACACGGCC
>Zotu7473
GAACGCTAGCGGGAGGCCTAATACATGCAAGTCGAACGGTAACAGGTAGCAATACGCTAACGAGTGGC
GCACGGGTGAGTAACACGTACACAATCTGCCTATAACAGGGGCATAACTTCGGGAAACTGAAGCTAAT
TCCCCATAAGACTGTAGAAAGGCATCTTTCAGCAGTTAAAGCTCCGGCGGTTATAGATGAGTGTGCGT
TTGATTAGCTAGTTGGTAAGGTAACGGCTTACCAAGGCTACGATCAATAACTGGTCTGAGAGGATGAT
CAGTCACACGGGCACTGAGACACGGGC
>Zotu7474
GAACGCTGGTGGTATGCTTAACACATGCAAGTCGAACGGACTTAGAGAAGCTTGCTTCTCGAAAGTTA
GTGGCAAACGGGTGAGTAATACATAGGAATCTACCTAGCAGTATGGAATAACCCTGGGAAACCAGAGC
TAATACCATATATTACCGATTTATCGGGAAAGATTTATCGCTGTTAGATGAGCCTGTGGCGGATTAGC
TTGTTGGTAGGGTAATGGCCTACCAAGGCGATGATCTGTAGCTGGTCTGAGAGGATGATCAGCCACAC
TGGGACTGCGACACGGCC
>Zotu7475
GAACGCTGGCGGCGTGCCTAACACATGCAAGTCGAGCGGTAACAGGCGTAGCAATACGTGCTGACGAG
CGGCGAACGGGTGAGTAATGCTTCGGAATCTACCCAGTAGTGGGGAATAACCAACCGAAAGGTTGGCT
AATACCGCATACGTCCTACGGGAGAAAGCGGGGGATCGTAAGACCTCGCGCTATCGGATGAGCCGAAG
CCGGATTAGCTAGTTGGTAGGGTAACGGCCTACCAAGGCGACGATCCGTAGCTGGTCTGAGAGGACGA
CCAGCCACACTGGGACTGAGACACGGCC
>Zotu7476

GAACGCTAGCGGCAGGCTTAATACATGCAAGTCGAACGGATGTAGCAATACATTAGTGGCGCACGGGT
GAGTAACGCGTACATAACATGCCTTTAGCTTAGGGATAGCCTTGGGAAACTGGGAGTAATACCTAATA
GTATTTTTGGATGGCATCATCTGGAAATTAAAGTTACGGCGGCTAAAGATTGATGTGCGTAAGATTAG
GTAGTTGGTAAGGTAACGGCTTACCAAGCCTACGATCTTTAGGGGGCGTGAGAGCGTGACCCCCCACA
CGGGTACTGAGACACGGAC
>Zotu7481
GAACGCTGGCGGCGTGCTTAACACATGCAAGTCACGGGGGCCCGCAAGGGCAACCGGCGAACGGGTGC
GTAACACGTGAGCAACCTGCCGATCTGAGGGGGATAGCCGGCCCAACGGCCGGGTAATACCGCATACG
TTCATGGGGAGGAGTCTCCTTATGAAGAAACCTCCGGGGCAGATCGAGGGGCTCGCGGCCTATCAGCT
AGTTGGCGGTGTAATGGACCACCAAGGCGACGACGGGTAGCTGGTCTGAGAGGATGGCCAGCCACATT
GGGACTGAGAGACGGCC
>Zotu7482
GAACGCTGGCGGCGTGCCTAACACATGCAAGTCGAGCGGAGATTGCAGCAATGTAATTTTAGCGGCGG
ACTGGTGAGTAACGCGTGGGCAACCTGCCTCACACAGGGGGATAACACAGGGAAACTTGTGCTAATAC
CGCATAACATCATGGAGGGACATCCCTTTATGATCAAAGGAGCAATCCGGTGTGAGATGGGCCTGCGT
CCGATTAGTTAGTTGGCGGTGTAACGGACCACCAAGACGACGATCGGTAGCCGAACTGAGAGGTTGAT
CGGCTACATTGGAACTGAGATACGGTC
>Zotu7483
GAACATTGGCGGTAGGCTTAACACATGCAAGTCGAGCGAGGCGTAATAGTCGAGCGGCGAACGGGTGA
GTAATACATGGAAATCTACCCTGAAGGGGGGGATAACGTTCAGAAATGAACGCTAATACCGCATAATC
CATATTGGGAAAGCGTTGCAGCGCTTTAGGATGAGTCCATGGCTGATTAGCTTGTTGGTGAGGTAATG
GCTCACCAAGGCGACGATCAGTAGCTGGTCTGAGAGGATGACCAGCCACACTGGAACTGAGACACGGT
C
>Zotu7484
GAACGCTGGCGGCGTGCTTAACACATGCAAGTCGAACGGTGAAAGAGGAGCTTGCTCCTCTGGATCAG
TGGCGAACGGGTGAGTAACACGTGAGTAACCTGCCCCAGACTCTGGGATAAGCACTGGAAACGGTGTC
TAATACCGGATACGAGCTTCAACCGCATGGTTAGGAGCTGGAAAGAATTTCGGTCTGGGATGGACTCG
CGGCCTATCAGCTAGTTGGTGAGGTAACGGCCCACCAAGGCGACGACGGGTAGCCGGCCTGAGAGGGT
GACCGGCCACACTGGGACTGAGACACGGCC
>Zotu7485
GAACGCTGGTGGTATGCTTAACACATGCAAGTCGAACGTGCTTAAAGGGGCAACTCTTTAAGTCAGTG
GCAAACGGGTGAGGAATATATAGGAATCTTCCCAGCAGTAAGGAATAACATTGGGAAACCAATGCTAA
TGCCATATATTACCGATTTATCGGGAAAGATTTATCGCTGTTGGATGAGCCTGTATCGGATTAGCTAG
TTGGTAGGGTAATGGCCTACCAAGGCAATGATCCGTAGCTGGTCTGAGAGGATGATCAGCCACATTGG
GACTGCGACACGGCC
>Zotu7489
GAACGCTAGCGGCAGGCTTAATACATGCAAGTCGAGGGGCAGCATGAGTGTAGCAATACATTTGATGG
CGACCGGCAAACGGGTGCGGAACACGTACAGAACCTTCCTTCGAGCGGGGAATAGCCCAGAGAAATTT
GGATTAATACCCCATAGTATTATGAAGTGGCATCATTTTATAATTAAAGATTTATCACTTGAAGATGG
CTGTGCGGCTGATTAGATAGTTGGTGAGGTAACGGCTCACCAAGTCTACGATCAGTAACTGGTGTGAG
AGCACGACCAGTCACACGGGCACTGAGACACGGGC
>Zotu7491
GAACGCTGGCGGCGTGCCTAATACATGCAAGTCGAGCGGATCAACAGGAGCTTGCTCCTGTTGATTAG
CGGCGGACGGGTGAGTAACACGTGGGCAACCTGCCTGTAAGACTGGGATAACACCGGGAAACCGGTGC
TAATACCGGATAATCCTTTTCCTCTCATGAGGAAAAGCTGAAAGTCGGTTTCGGCTGACACTTACAGA
TGGGCCCGCGGCGCATTAGCTAGTTGGTGAGGTAACGGCTCACCAAGGCGACGATGCGTAGCCGACCT
GAGAGGGTGATCGGCCACACTGGGACTGAGACACGGCC
>Zotu7496
GAACGCTGGCGGCGTGCCTAATACATGCAAGTCGGGCGGACCGAACGAGGGCTTGCTCTTGTTTGGTC
AGCGGCGGACGGGTGAGTAACACGTGGGCAACCTGCCCGCAAGACCGGGATAACTCCGGGAAACCGGA
GTTAATACCGGATAACACCGAAGACCGCATGGTCTTCGGTTGAAAGGCGGCCTTTGGGCTGTCACTTG
CGGATGGGCCCGCGGCGCATTAGCTAGTTGGTGAGGTAACGGCTCACCAAGGCGACGATGCGTAGCCG
GCCTGAGAGGGTGACCGGCCACACTGGGACTGAGACACGGCC
>Zotu7498

GAACGCTGGTAACGTGGATACCGGCATGCAAGTCACGGGGGCAGCAATGCAACCGGCGAACGGCTTAG
TAACACGTGGGTACATACCTTAAACTGGAGCACAGCTCGTCGAAAGACGGGGTAATTCTCCATAGTCC
CGCAAGGGTAAAGCAGCAATGCGGTTTAAGAATGGCCTGCGGCCTATCAGGTAGTTGGCGGTGTAATA
GACCACCAAGCCTATGACGGGTAGGGGGCGTGAGAGCGCGACCCCCAAGAATGGCACTGCGACACGGG
C
>Zotu7500
GAACGCTGGCGGCAGGCCTAACACATGCAAGTCGAGCGGGGGAGCGATCCCTAGCGGCGGACGGGTGA
GTAACGCGTGGGAACATACCCTTTGGTACGGAATAGCCCCGGGAAACTGGGAGTAATACCGTATGTGC
TCTTCGGAGGAAAGATTTATCGCCAAAGGATTGGCCCGCGTTGGATTAGGTAGTTGGTGGGGTAATGG
CCTACCAAGCCGACGATCCATAGCTGGTTTGAGAGGATGATCAGCCACACTGGGACTGAGACACGGCC
>Zotu7501
GAACGCTGGCGGCATGCTTTACACATGCAAGTCGGACGGCAGCGGGGAAGTGCTTGCACTTCTGCCGG
CGAGTGGCGAACGGGTGAGTAATGCATTGGAACGTACCGGGTAATGGGGGATAACTTGCCGAAAGGTA
AGCTAATACCGCATACGCTTTAAGAAGGAAAGCGGGGGATCATCTGACCTCGCGTTATCCGAGCGGCC
AATGTCTGATTAGCTAGTTGGTGGGGTAAAAGCCTACCAAGGCAACGATCAGTAGCGGGTCTGAGAGG
ATGATCCGCCACACTGGGACTGAGACACGGCC
>Zotu7502
GAACGCTGGCGGCGTGCTTAACACATGCAAGTCGAACGGTGAAAGAGGAGCTTGCTCCTCTGGATCAG
TGGCGAACGGGTGAGTAACACGTGAGTAACCTGCCCCAGACTCTGGGATAAGCGTTGGAAACGACGTC
TAATACCGGATACGAGCTTCAGTCGCATGACTAGGAGCTGGAAAGAATTTCGGTCTGGGATGGACTCG
CGGCCTATCAGGTAGTTGGTGAGGTAACGGCCCACCAAGCCTACGACGGGTAGCCGGCCTGAGAGGGT
GACCGGCCACACTGGGACTGAGACACGGCC
>Zotu7503
GAACGCTGGCGGCGTGCTTAATACATGCAAGTCGAGCGGTAACACTTCGGGTGACGAGCGGCGGACGG
CTGAGTAACACGTAGGAACGTACCCCAAACTGAGGGATAAGCACCAGAAATGGTGTCTAATACCACAT
GTGATCTAAGGATTAAAGCTCCGGCGGTTTGGGAACGGCCTGCGCATGATTAGCTTGTTGGTGAGGTA
AAAGCTCACCAAGGCTACGATCATTAGCTGGTCTGAGAGGATGACCAGCCAGACTGGGACTGAGACAC
GGCC
>Zotu7505
GAACGCTGGCGGCGTGCTTAACACATGCAAGTCGAACGATGAAAGTCGTTTTGATATCTTCGGATTGA
TAAACTTCTGGATTAGTGGCGGACGGGTGAGTAACGCGTGAGCAATCTGCCTTACACAAAGGGATAGC
CTCGGGAAACCGGGATTAATACCATATGACATCTATTAACCGCATGGTTTATAGATTAAAGAATTTCG
GTGTAAGATGAGCTCGCGTCCCATTAATTAGTTGGTGAGGGTAACGGCCCACCAAGATAGCGATGGGT
AGCCGATCTAAGAGGATGACCGGCCACATTGGAACTGAGAAACGGTC
>Zotu7508
GAACGCTGGCGGCAGGCTTAACACATGCAAGTCGAGCGGATGAAAGTAGCTTGCTACTGGATTCAGCG
GCGGACGGGTGAGTAATGCTTAGGAATCTGCCTATTAGTGGGGGACAACGTTCCGAAAGGAGCGCTAA
TACCGCATACGTCCTACGGGAGAAAGCAGGGGACCTTTGGGCCTTGCGCTAATAGATGAGCCTAAGTC
GGATTAGCTAGTTGGTAGGGTAAAGGCCTACCAAGGCGACGATCTGTAGCGGGTCTGAGAGGATGATC
CGCCACACTGGGACTGAGACACGGCC
>Zotu7510
AAACGCTGGCGGCGTGCATGAGACATGCAAGTCGAACGAATTTCTCGGGCTTGCTCGAGAAGTTAGTG
GCGGACGGCTGAGTAACGCGTAGGTGACCTGCCCCGAAGTGGGGAATAACTGCCCGAAAGGGTGGCTA
ATACCGCATGTGATCGTTCATTCATTTGTTCGATCAAAGCAGTAATGCGCTTTGGGAGGGGCCTGCGT
CCGATTAGCTCGTTGGTGGGGTAATGGCTCACCAAGGCGACGATCGGTAGGTGGTCTGAGAGGACGAT
CACCCAGACTGGGACTGAGACACGGCC
>Zotu7511
GGTTCAGTCTTGGTTTCGCTTGGTTAACCTTTGATTTAGCTGTATTGCCAACCACGACTCAAAGCCAA
TCGGTACCGTGCAACGCTCACGGGAAACGGAGCGTCACAGAAATGCATCGTGCAAAGTGTACCTCGAA
AGTTGTATC
>Zotu7512
GAACGCTAGCGGCAGGCCTAATACATGCAAGTCGAGGGCATCAGCAATGGTGACCGGCGCACGGGTGC
GTAACGCGTATGCAACCTGCCCCGTACTTGGGGATAGCCTTTCGAAAGAGAGATTAACACCCAATAGA
CTAGCAGGGAGACATCTTCCAACTAGTAAAGGTTACGGTACGGGATGGGCATGCGTCCGATTAGCTAG

ATGGCGGGGTAACGGCCCACCATGGCTACGATCGGTAGGGGTTCTGAGAGGATGGTCCCCCACACTGG
CACTGAGATACGGGC
>Zotu7513
GAACGCTGGCGGCAGGCCTAACACATGCAAGTCGAACGCCCTGGCAACAGGGAGTGGCAGACGGGTGA
GTAACGCGTGGGAATCTACCTTGTGGTACGGAACAACCAAGGGAAACTTTGGCTAATACCGTATGAGC
CCTTCGGGGGAAAGATTTATCGCCATAAGATGAGCCCGCGTAGGATTAGCTAGTTGGTGAGGTAATGG
CTCACCAAGGCGACGATCCTTAGCTGGTCTGAGAGGATGACCAGCCACACTGGGACTGAGACACGGCC
>Zotu7516
GAACGCTAGCGGCAGGCCTAACACATGCAAGTCGAGGGGTATTGTAGCAATACAAGAGACCGGCGCAC
GGGTGCGTAACGCGTATGCAATCTACCTTTTACAGAGGGATAGCCCAGAGAAATTTGGATTAATACCT
CATAGTATATTTTAGTGGCATCACTAGATTATTAAAGTCACAACGGTAAAAGATGAGCATGCGTCCCA
TTAGCTAGTTGGTAAGGTAACGGCTTACCAAGGCTACGATGGGTAGGGGTCCTGAGAGGGAGATCCCC
CACACTGGTACTGAAACACGGAC
>Zotu7517
GAACGCTAGCGGCAGGCTTAACACATGCAAGTCGAGGGGTATATTTCTTCGGAGATAGAGACCGGCGC
ACGGGTGCGTAACGCGTATGCAATCTACCTTTCACAGAGGGATAGCCCAGAGAAATTTGGATTAATAC
CTCATAGTATAATTGAATGGCATCATTTAATTATTAAAGTCACAACGGTGAAAGATGAGCATGCGTCC
CATTAGCTTGTTGGTAAGGTAACGGCTTACCAAGGCTACGATGGGTAGGGGTCCTGAGAGGGAGATCC
CCCACACTGGTACTGAGACACGGAC
>Zotu7518
GAACGCTGGCGGCGTGCCTAACACATGCAAGTCGAACGGGGTGATTTCCTTCGGGAGATCACTTAGTG
GCGAACGGGTGAGTAACGCGTGAGAAACCTGCCTTTCAGTGGGGGACAACAGTTGGAAACGACTGCTA
ATACCGCATAATGTATTTTGGGGGCATCCCTGAAATACCAAAGCTTTATGTGCTGAAAGATGGTCTCG
CGTCTGATTAGCTAGATGGCGGGGTAACGGCCCACCATGGCGACGATCAGTAGCCGGTCTGAGAGGAT
GAACGGCCACATTGGGACTGAGATACGGCC
>Zotu7519
GAACGCTAGCGGCAGGCTTAACACATGCAAGTCGAGGGGTATAGTTTTTCGGAACTAGAGACCGGCGC
ACGGGTGCGTAACGCGTATGCAATCTACCTTTTGCAGAGGGATAGCCCAGAGAAATTTGGATTAATAC
CTCATAGTATGTGAGAATGGCATCATTTTCACATTAAAGTCACAACGGCAAAAGATGAGCATGCGTAC
CATTAGCTAGTTGGTAAGGTAACGGCTTACCAAGGCAACGATGGGTAGGGGTCCTGAGAGGGAGATCC
CCCACACTGGTACTGAGACACGGAC
>Zotu7520
GAACGCTGGCGGCAGGCCTAACACATGCAAGTCGAACGGCAGCATGAGAGAGCTTGCTCTCTTGATGG
CGAGTGGCGGACGGGTGAGGAACACATCGGAATCTGCCCAGTCGTGGGGGATAACGTAGGGAAACTTA
CGCTAATACCGCATACGACCTACGGGTGAAAGCAGGGGATCTTCGGACCTTGCGCGATTGGATGAGCC
GATGTCGGATTAGCTAGTTGGCGGGGTAAAGGCCCACCAAGGCGACGATCCGTAGCTGGTCTGAGAGG
ATGATCAGCCACACTGGAACTGAGACACGGTC
>Zotu7521
GAACGCTAGCGGCAGGCTTAATACATGCAAGGCGAGGGGGCAGCAATGTCACCGTCGTACGGGTGCGT
AACGCGTATGCAACCTACCTTCAACTGGGGGATAGCCCGGGGAAACCCGGATTAATACCGCATAAGAC
ATTTGGGCCACCTGGTTTGATTTGTTAAAGATTTATTGGTTGAAGATGGGCATGCGTTCGATTAGCTA
GTTGGCAGGGTAACGGCCTACCAAGGCGACGATCGATAGGGGAGCTGAGAGGTTGATCCCCCACACGG
GCACTGAGATACGGGC
>Zotu7523
GAACGCTGGCGGCGTGCTTAACACATGCAAGTCGAGCGACAGCGCTCTTCGGGGTGCATGGAGCGGCG
AACGGGTGAGTAACACGTGGGCAACCTGCCCTCAGCTCTGGGACAACTCCGGGAAACCGGGGCTAATA
CCGGATACGACCTTCCCTCGCATGGGGGTCGGTGGAAAGTTTTTCGGCTGAGGATGGGCCCGCGGCCT
ATCAGCTTGTTGGTGGGGTGATGGCCTACCAAGGCGACGACGGGTAGCCGGCCTGAGAGGGCGACCGG
CCACACTGGGACTGAGACACGGCC
>Zotu7526
GAACGCTGGCGGCGTGCTTAACACATGCAAGTCGAACGGTGAAAGAGGAGCTTGCTCCTCTGGATCAG
TGGCGAACGGGTGAGTAACACGTGAGCAATCTGCCCCGAACTCTGGGATAAGCACTGGAAACGGTGTC
TAATACCGGATACGACGCGGGAGGGCATCCTCTCCGCGTGGAAAGAATTTCGGTTCGGGATGAGCTCG
CGGCCTATCAGCTTGTTGGTGAGGTAATGGCTCACCAAGGCGACGACGGGTAGCCGGCCTGAGAGGGT

GACCGGCCACACTGGGACTGAGACACGGCC
>Zotu7527
GAACGCTAGCGGCAGGCTTAATACATGCAAGGCGAAGGGGCATTTATGTCACTGTCGTACGGGTGCGT
AACGCGTATGCAACCTACCTATCACTGGGGGATAGCCCGGGGAAACCCGGATTAATACCGCATAAAAC
AGGGGCACCGCATGGTGATATTTGTTAAAGATTTATTGGTGATAGATGGGCATGCGTTCGATTAGCTA
GTTGGTATGGGTAACGGCCTACCAAGGCGACGATCGATAGGGGAGCTGAGAGGTTGATCCCCCACACG
GGCACTGAGATACGGGC
>Zotu7528
GAACGCTAGCGGCAGGCTTAACACATGCAAGTCGAGGGGTATAGTAGCAATACTAGAGACCGGCGCAC
GGGTGCGTAACGCGTATGCAATCTACCTTTTGCAGAGGGATAGCCCAGAGAAATTTGGATTAATACCT
CATAGTATATAGAGTTGGCATCAACACTATATTAAAGTCACAACGGCAAAAGATGAGCATGCGTCCCA
TTAGCTTGTTGGTAAGGTAACGGCTTACCAAGGCAACGATGGGTAGGGGTCCTGAGAGGGAGATCCCC
CACACTGGTACTGAGACACGGAC
>Zotu7530
GAACGCTGGCGGCGTGCCTAACACATGCAAGTCGAACGGAGCCTTCGCAAGAGGGCTGAGTGGCGCAC
GGGTGAGGAACACGTAGCTGACCTGCCTTCCAGCGGGGGATAACGTCCCGAAAGGGATGCTAAGACCG
CATAAGCTCACAGGGCTTAGAAACCTGCGAGAAAAAGCTTCGGCTACTGGAAGAGGGGGCTGCGGCCC
ATCAGGTAGTTGGCGGGGTAACGGCCCACCAAGCCGACGACGGGTCGGGGATCTGAGAGGATGATCCC
CCACACTGGGACTGAGACACGGCC
>Zotu7531
GAACGCTGGCGGCGCGCTTAACACATGCAAGTCGAGCGGGAACCGAGCCTTCGGGCTCGGGGAAAGCG
GCGAACGGGTGAGTAACACGTGGGTAATCTGCCCTCGGCACCGGGATAGCCCGGGGAAACTCGGATTA
ATACCGGATAGGCTCCGGAGCCTTCGGGCACTGGAGAAAAGGTGGCTTCGGCTTCCGGCTGAGGATGA
GCCCGCGGCGGATTAGCTTGTTGGCGGGGTAACGGCCCACCAAGGCGACGATCCGTAGCTGGTCTGAG
AGGACGATCAGCCACACTGGGACTGAGACACGGCC
>Zotu7532
GAACGCTGGCGGCGTGCTTAACACATGCAAGTCGAACGGAAAGGCCCTTCGGGGTACTCGAGTGGCGA
ACGGGTGAGTAACACGTGGGTGATCTGCCCTGCACTTCGGGATAAGCCTGGGAAACTGGGTCTAATAC
CGGATATACCTCATGGATTGCATGATTTGTGGGGGAAAGCTTTTGCGGTGTGGGATGGGCCCGCGGCC
TATCAGCTTGTTGGTGGGGTGATGGCCTACCAAGGCGTCGACGGGTAGCCGGCCTGAGAGGGTGTCCG
GCCACACTGGGACTGAGATACGGCC
>Zotu7534
GAACGCTGGCGGCGTGCTTAACACATGCAAGTGGAGCGACGAACCGGGGCTTGCCCCGGGGCAGAGCC
GCGAACGGGTGAGTAACACGTGGGTTACCTGCCCCGATGATTGGGACAACCCGAGGAAACTCGGGCTA
ATACCAAATGTGCTCCTGGCCATAATGGCCTTGAGGAAAGGAAGCTTCGGCCTCCGCATCGGGATGGG
CCCGCGGCCCATTAGCTAGTTGGTGGGGTAACGGCCTACCAAGGCTACGATGGGTAGCTGGTCTGAGA
GGACGATCAGCCACACTGGGACTGAGACACGGCC
>Zotu7535
GAACGCTGGCGGCGTGCCTAACACATGCAAGTCGAACGCGAAAGGGAGCAATCCCCGGTAGCGTGGCA
GACGGGTGAGTAACACGTGGGTAACCTACCCGGAGGTGGGGGATAACCCTTCGAAAGGAGGGCTAATA
CCGCATGACACCGTGGGGACCTCGGTCCTTGCGATCAAAGCTGGCCTCTGTTTACAAGCTAGCGCCAC
CGGATGGGCCCGCGCCCCATTAGCTAGTTGGTGAGGTAACGGCTCACCAAGGCGACGATGGGTAGCCG
GCCTGAGAGGGCGATCGGCCACACTGGAACTGCGACACGGTC
>Zotu7536
GAACGCTAGCGGCAGGCTTAATACATGCAAGGCGAGGGGGCAGCAATGTCACCGTCGTACGGGTGCGT
AACGCGTATGCAACCTACCTATCACTGGGGGATAGCCCGGGGAAACCCGGATTAATACCGCATGACAC
ATTTGTATAGCATTATACGATTTGTTAAAGATTTATTGGTGATAGATGGGCATGCGTTCGATTAGCTA
GTTGGCAGGGTAACGGCCTACCAAGGCAACGATCGATAGGGGAGCTGAGAGGTTGATCCCCCACACGG
GCACTGAGATACGGGC
>Zotu7538
GAACGCTAGCGGCAGGCTTAATACATGCAAGTCGAGGGGCAGCACAGATAGCAATATTGGGTGGCGAC
CGGCAAACGGGTGCGGAACACGTACGCAACCTTCCCGAAACAGGTGAATAGCCCTCCGAAAGGAGGAT
TAATACACCGTGAGATAATGAGATGGCATCATTTTATTATTATAGATAGCAATATCGGTTTTGGATGG
GCGTGCGCCTGATTAGGTAGTTGGGGGGGTAACGGCCCACCAAGCCAACGATCAGTAACTGGTGTGAG

AGCACGACCAGTCACACGGGCACTGAGACACGGGC
>Zotu7539
GAACGCTGGCGGCATGCTTAACACATGCAAGTCGAACGCACCGCAAGGTGAGTGGCGCACGGGTGAGT
AACGCGTGGGAATATGCCTAGCAGTGGGGAATAACCAAGGGAAACTTTGGCTAATACCGCATACGCCC
TTCGGGGGAAAGATTTATCGCTGTTAGATTAGCCCGCGTCCGATTAGCTAGTTGGTGAGGTAATGGCT
CACCAAGGCTCCGATCGGTAGCTGGTCTGAGAGGATGATCAGCCACACTGGGACTGAGACACGGCC
>Zotu7540
GAACGCTGGCGGCGCGCTTAACACATGCAAGTCGAGCGAGAACCAGCCCTTCGGGGCTGGGGACAGCG
GCGAACGGGTGAGTAACACGTGGGTAATCTGCCCTCGGTTCTGGGATAGCCCGGGGAAACCCGGATTA
ATACCGGATGGCCTCTCGAGCTTTCGGGCTCGCGAGAAAAGGTAGCTTCGGCCTCCGACCGAGGATGA
GCCCGCGGCGGATTAGCTTGTTGGTGGGGTAACGGCCCACCAAGGCGACGATCCGTAGCTGGTCTGAG
AGGACGATCAGCCACACTGGGACTGAGACACGGCC
>Zotu7542
GAACGCTGGAAATGTGGATACTGGTATGCAAGTCAAGGGGCCGCAAGGCACCGGCGAACTGCTTAGTA
ACACGTAGATACATACCGTAAAGTGGAACATAGCTCGTCGAAAGACGGGGTAATTTTCCATGTGAACG
CAAGTTTAAAGGCGCAAGCCGCTTTACGAATGGTCTGCGGTCTATCAGCTAGTTGGTGAGGTAAAGGC
TCACCAAGGCTATGACGGATAGGGGGCGTGAGAGCGCGACCCCCAAGAATGGCACTGCGACACGGGC
>Zotu7543
GAACGCTGGCGGCGTGCTTAACACATGCAAGTCGAACGATGATGCCGTGCTTGCACGGTTGATTAGTG
GCGAACGGGTGAGTAACACGTGAGTAACCTGCCCCTCACTTTGGGATAACTCCGGGAAACCGGGGCTA
ATACTGGATATGAGCCTTCCTCGCATGGGGTTGGTTGGAAAGGGTTTCTGGTGGGGGATGGGCTCGCG
GCCTATCAGCTTGTTGGTGGGGTGATGGCCTACCAAGGCTTCGACGGGTAGCCGGCCTGAGAGGGCGA
CCGGCCACATTGGGACTGAGATACGGCC
>Zotu7544
GAACGCTAGCGGCAGGCTTAATACATGCAAGTCGAACGGCAGCACGTTAAGGTGGCGAGTGGCAGACG
GGTGAGTAACGCGTACATAACCTACCTTTGGCAGGGGGAAAGTCTAGGGAAACTTGGAATAATCCCGC
ATGAGATTATCGGGTGGCATCACCTGGTAATGAAAGGAGTAATCCGGCTGAAGATGGATGTGCGTCTC
ATTAGGTAGTTGGTAGGGTAACGGCCTACCAAGCCAGCGATGAGTAGGGGGCGTGAGAGCGCGAACCC
CCACACGGGTACTGAGAACGGAC
>Zotu7545
GAATGCTGGCGGCGTGCCTAATACATGCAAGTCGAGCGGTAACAGGCTTTCACTGTTTACTTCTCTCG
ATTCGGAAACGAAACGAGAATGAGGAGTAAAGAGTGAAAGTGCTGACGAGCGGCGGACGGCTGAGTAA
CGCGTGGGAACATGCCCCAAAGTGAGGGATAAGCATCGGAAACGGTGTCTAATACCGCATATGGTCTT
CAGATTAAAGGATTTATCCGCTTTGGGAGTGGCCCGCGTACGATTAGGTTGTTGGTGAGGTAATGGCT
CACCAAGCCTACGATCGTTAACTGGTCTGAGAGGATGATCAGTCAGACTGGAACTGAGACACGGTC
>Zotu7546
GAACGCTGGCGGCGTGCCTAACACATGCAAGTCGGATGTGCCCGCAAGGGTGCATGGCAGACGGGTGA
GTAACGCGTGGGGAACCTACCTTTTTGTGGGGGATAACGCTCCGAAAGGAGCGGTAATACCGCATAAG
ACCATGAGCTGGAATGCTGATGGTGAAAGCCGGGGATCGAAAGACCTGGCGCAAGGAGAGGGTCCTGC
GTCTGATTAGCTAGTTGGCGGGGTAACGGCCCACCAAGGCGACGATCAGTAGCCGGCCTGAGAGGGTG
ATCGGCCACACTGGAACTGAGACACGGTC
>Zotu7547
GAACGCTTGCGGCGTGCCTAAGAAATGCAAGTCGAGCGGAGTTAGAGACTTCGGTTTCTAACTTAGCG
GCGAACGGTCGCGTAACACGTAGGCAACCTGCCCCAAAGACCGGGACAACAGGGGGAAACTCTTGCTA
ATACCGGATGTGGCCACCCGACTGCCTGGTTGGGTGATTAAATCCTCGCAAGGGGGCTTTGGGAGGGG
CCTGCGGCCTATCAGCTAGTAGGTGAGGTAACGGCTCACCTAGGCGACGACGGGTAGCTGGTCTGAGA
GGATGGTCAGCCGGACTGGGACTGAGACACGGCC
>Zotu7548
CAACGCTGGCGGCGCGCTTAACACATGCAAGTCGAGCGAGAAAGGGGAGCAATCCCTGAGTAAAGCGG
CGTACGGGTGAGTAACACGTGGATGATCTACCTTTTAGTGGGGAATAACCCTGGGAAACCGGGGCTAA
TACCGCATAAGCCTGAGAAGGGAAAGCAGCAATGCGCTGAAAGAGGAGTCCGCGGCCGATTAGCTAGT
TGGTGAGGTAATGGCTCACCAAGGCGGTGATCGGTAGCCGGCCTGAGAGGGCACACGGCCACACTGGC
ACTGAAACACGGGC
>Zotu7549

GAACGCTGGCGGCATGCCTAACACATGCAAGTCGAACGAAGCCTTCGGGCTTAGTGGCGCACGGGTGC
GTAACGCGTGGGAATCTGCCCTTGGGTTCGGAATAACAGTTAGAAATGACTGCTAATACCGGATGATG
TCTTCGGACCAAAGATTTATTGCCCAAGGATGAGCCCGCGTCGGATTAGCTAGTTGGTGGGGTAAAGG
CCCACCAAGGCGACGATCCGTAGCTGGTCTGAGAGGATGATCAGCCACACTGGGACTGAGACACGGCC
>Zotu7550
GAACGCTGGCGGCAGGCTTAACACATGCAAGTCGAGCGCGTAGCAATACGAGCGGCAGACGGGTGAGT
AACGCGTGGGAACCTACCCTTTTCTACGGAATAACGCATGGAAACGTGTGCTAATACCGTATACGCCC
TTTGGGGGAAAGATTTATCGGAGAAGGATGGGCCCGCGTTGGATTAGCTAGTTGGTGGGGTAAAGGCC
TACCAAGGCGACGATCCATAGCTGGTCTGAGAGGATGATCAGCCACATTGGGACTGAGACACGGCC
>Zotu7551
GAACGCTAGCGGCAGGCTTAATACATGCAAGTCGAGGGGCAGCATGGTTGTAGCAATACAACTGATGG
CGACCGGCAAACGGGTGCGGAACACGTACGCAACCTACCCATAACTGGGGAATAGCCCGGGGAAACCC
GGATTAATACCTCGTAACCTTATGAAGTGGCATCGCTTTATAAGTATAGCTCCGGCGGTTATGGATGG
GCGTGCGCCTGATTAGGTAGTTGGCGGGGTAACGGCCCACCAAGCCTGCGATCAGTAACTGGTGTGAG
AGCACGACCAGTCACACGGGCACTGAGACACGGGC
>Zotu7552
GAACGCTGGCGGCGTGCCTAATACATGCAAGTCGTGCGGACTTATCTTCGGATAAGTTAGCGGCGGAC
GGGTGAGTAACACGTGGGCAACCTGCCTGTAAGACTGGGATAACTCCGGGAAACCGGTGCTAATACCA
GATACAATCAAAGACTGCCTGGTTTTTGATGGAAAGGTCTTGTACTGCTTACAGATGGGCCCGCGGCG
CATTAGCTAGTTGGTGGGGTAATGGCTCACCAAGGCGACGATGCGTAGCCGACCTGAGAGGGTGATCG
GCCACACTGGGACTGAGACACGGCC
>Zotu7553
GAACGCTGGCGGCGTACCTGATGTATGCAAGTCGAGCGGTAAGGTCCCTCTCATTTTCTAGACTAACC
ACAGTTTTAAAAGATTGTGACAGCTTAGAGAAAATGAGTGGGATACACGAGCGGCGGACGGGTGAGTA
ACGCGTAGGAACGTACCCTAAAGTGAGGCATAAGCTCCGGAAACGGAGTCTAATACCGCATGTGATCT
TCGGATTAAAGTATTTATACGCTTTAGGAGCGGCCTGCGCCCGATTAGCTTGTTGGCGGAGTAAAAGC
CCACCAAGGCGACGATCGGTAGCTGGTCTGAGAGGACGATCAGCCACACTGGGACTGAGACACGGCC
>Zotu7556
GAACGCTAGCGGCAGGCCTAATACATGCAAGTCGAACGGTAAATGTCCCTTTGGGGATATGAGAGTGG
CGCACGGGTGCGTAACGCGTATGCAACCCACCCGCTGTCGGGGGATAGCCCGTAGAAATGCGAATTAA
TACCCCATGAGGAGATAGGGCGGCATCGTCCTTTTTTCAAAACTACGGTGACGGCGGACGGGCATGCG
TGCCATTAGATAGTTGGCGGGGTAACGGCCCACCAAGTCGACGATGGCTAGGGGATCTGAGAGGACGG
TCCCCCACACTGGTACTGAGACACGGAC
>Zotu7557
GAACGCTGGCGGCATGCCTTACACATGCAAGTCGAACGGTAACAGGTCTTCGGATGCTGACGAGTGGC
GAACGGGTGAGTAATACATCGGAACGTGCCCGATCGTGGGGGATAACGCAGCGAAAGCTGTGCTAATA
CCGCATACGATCTACGGATGAAAGCAGGGGACCGCAAGGCCTTGCGCGGACGGAGCGGCCGATGGCAG
ATTAGGTAGTTGGTGGGATAAAAGCTTACCAAGCCGACGATCTGTAGCTGGTCTGAGAGGACGACCAG
CCACACTGGGACTGAGACACGGCC
>Zotu7558
GAACGCTGGCGGCATGCCTAACACATGCAAGTCGAACGATCCCTTCGGGGATAGTGGCGCACGGGTGC
GTAACGCGTGGGAATCTGCCCTTGGGTTCGGAATAACAGTGAGAAATTACTGCTAATACCGGATGATG
TCTTCGGACCAAAGATTTATCGCCCAAGGATGAGCCCGCGTAGGATTAGCTAGTTGGTGGGGTAATGG
CCTACCAAGGCGACGATCCTTAGCTGGTCTGAGAGGATGATCAGCCACACTGGGACTGAGACACGGCC
>Zotu7561
GAACGCTGGCGGCGTGCTTAACACATGCAAGTCGAGCGGTAAGGCTCCTTCGGGAGTACACGAGCGGC
GAACGGGTGAGTAACACGTGAGTAATCTGCCCTTCACTTCGGGATAACCGCCGGAAACGGTGGCTAAT
ACCGGATACGACGCACTCTTGCATGAGATGTGCGTGGAAAGTTTTCTCGGTGAAGGATGTGCTCGCGG
CCTATCAGCTTGTTGGTGAGGTAATGGCTCACCAAGGCTTCGACGGGTAGCCGGCCTGAGAGGGTGAC
CGGCCACACTGGGACTGAGACACGGCC
>Zotu7563
GAACGCTAGCGGGAGGCCTAACACATGCAAGCCGAGCGGGATTTGTTGGTTAGCTTGCTAACTAACAA
TGAGAGCGGCGTACGGGTGCGTAACACGTGTGCAACCTGCCTTTATCTGGGGGATAGCCTTTCGAAAG
GAAGATTAATACCCCATAATATATTAATTGGCATCAATTAATATTGAAAGCTCCGGCGGATAGAGATG

GGCACGCGTAAGATTAGCTAGTTGGTGAGGTAACGGCTCACCAAGGCTGCGATCTTTAGGGGGCCTGA
GAGGGTGATCCCCCACACTGGTACTGAGACACGGAC
>Zotu7564
GAACGCTGGCGGCGTGCTTAACACATGCAAGTCGAGCGGAAAGGCTTCCTTCGGGAAGTACTCGAGCG
GCGAACGGGTGAGTAACACGTGGATAATCAACCTCTTGCTCTGGAATACCCCGGGAAACTCGGATCA
ATGCCAGATAAAGTGCGGTGCTGCAGGGTGCCGTACCAAAGATAGCTTCGGCCTTCGGCGAGAGACGA
GTCCGCGGCCCATTAGCTTGTTGGCGGGGTAACGGCCCACCAAGGCTACGATGGGTAGCTGGTCTGAG
AGGATGATCAGCCACACTGGGACTGAGACACGGCC
>Zotu7565
GAACGCTGGCGGCGTGCCTAACACATGCAAGTCGAGCGAGAAAGTCTTCGGATGAGTAAAGCGGCGGA
CGGGTGAGTAACGCGTAGGTAATCTACCTTCAAGTGGGGGATAACGTTCCGAAAGGAACGCTAATACC
GCATGTGAATCCGGACTGATGAGTCTGTATTCAAAGGGGGCTCGTAAGGGCTTTCGCTAGAAGATGAG
CCTGCGTCCCATTAGCTAGTTGGTGAGGTAACGGCTCACCAAGGCGACGATGGGTAGCTGGTCTGAGA
GGACGACCAGCCACACTGGAACTGACACACGGTC
>Zotu7568
GAACGCTGGCGGCGTGCTTAACACATGCAAGTGGAGCGACGAACCAGGGCTTGCCCTGGGGCAGAGCC
GCGAACGGGTGAGTAACACGTGGGTTACCTGCCCCGATGATTGGGACAACCCGAGGAAACTCGGGCTA
ATACCAAATGTGCTCGTGTCCGTGATGGACTCGAGGAAAGGAAGCTTCGGCCTCCGCATCGGGATGGG
CCCGCGGCCCATTAGCTAGTTGGTGGGGTAATGGCCTACCAAGGCGACGATGGGTAGCTGGTCTGAGA
GGACGATCAGCCACACTGGGACTGAGACACGGCC
>Zotu7569
ACGGTCACTGGATAGGGACTTAATGTGCACGGCGGCCACCTCTTTGTAGAGTTGACATGTTGCTGTAC
GTCTCGGAACTCCAAGTGCTGTCTTCAAGCCTGCACGGTGAA
>Zotu7570
GAACGCTGGCGGAATGCTTTACACATGCAAGTCGAACGGCAGCACGGACTTCGGTCTGGTGGCGAGTG
GCGAACGGGTGAGTAATATATCGGAACGTGCCCAGTCGTGGGGGATAACGTAGTGAAAATTACGCTAA
TACCGCATACGATCTAAGGATGAAAGCGGGGGATCGCAAGACCTCGCGCGATTGGAGCGGCCGATATC
AGATTAGGTAGTTGGTGAGGTAAAGGCTCACCAAGCCAACGATCTGTAGCTGGTCTGAGAGGACGACC
AGCCACACTGGAACTGAGACACGGTC
>Zotu7571
GAACGCTAGCGGTATGCCTAACACATGCAAGTCGTACGGAAAAGAAATTTTTAGTGGCGCACGGGTGA
GTAATACATGAGAATTCACCTTTAAGGAAGAGGATAACAATTAGAAATAGTTGCTAAAACTCTATATA
CCATTCTATGGTAAAAAAAGTAATTTGCCTAAAGAGAAGCTCATGCCTGATTAGCTAGTTGGTAGGGT
AAAGGCCTACCAAGGCGATGATCAGTAGTTGATCTGAGAGGATGATCAGCCACACTGGAACTGAGACA
CGGTC
>Zotu7572
CCCGTGTCTCAGTGCCCGTGTGACTGATCATCCTCTCAGACCAGTTACTGATCGTTGCCTTGGTGGGC
CGTTACCCCGCCAACTAGCTAATCAGACGCACACCCATCTTATACCATCGGAATTTTAATTGCAAAAT
GATGCCATTTCGCAATGTTATGGGGAATTAATCTTAGTTTCCCAAGGCTATGCCCCAGTATAAGGCAG
GTTGTGTACGTGTTACTCACCCGTGCGCCACTCGTCAGCAGTATTGCTACCCTGTTACCGTTCGACTT
GCATGTATTAGGCCTCCCGCTAGCGTTCA
>Zotu7575
GAACGCTGGCGGCAGGCTTAACACATGCAAGTCGAGCGGGCGTAGCAATACGTCAGCGGCAGACGGGT
GAGTAACGCGTGGGAACGTACCTTTTGGTTCGGAACAACTGAGGGAAACTTCAGCTAATACCGGATAA
GCCCTTACGGGGAAAGATTTATCGCCGAAAGATCGGCCCGCGTCTGATTAGCTAGTTGGTGAGGTAAC
GGCTCACCAAGGCGACGATCAGTAGCTGGTCTGAGAGGATGATCAGCCACATTGGGACTGAGACACGG
CC
>Zotu7576
GAACGCTAGCGGGAGGCCTAACACATGCAAGCCGAGCGGGATTTGTTGGTTAGCTTGCTAACTAATAA
TGAGAGCGGCGTACGGGTGCGTAACACGTGTGCAACCTGCCTTTATCTGGGGGATAGCCTTTCGAAAG
GAAGATTAATACCCCATAATATATTGATTGGCATCAGTTAATATTGAAAGCTCCGGCGGATAGAGATG
GGCACGCGTAAGATTAGCTAGTTGGTGAGGTAACGGCTCACCAAGGCTGCGATCTTTAGGGGGCCTGA
GAGGGTGATCCCCCACACTGGTACTGAGACACGGAC
>Zotu7577

GAACGCTGGCGGCAGGCCTAATACATGCAAGTCGAGCGCCCCTTCGGGGGAGCGGCGGACGGGTGAGT
AACGCGTGGGAACGTGCCCTTTGGTTCGGAACAACACAGGGAAACTTGTGCTAATACCGAATGTGCCC
TTCGGGGGAAAGATTTATCGCCATTGGAGCGGCCCGCGTTGGATTAGGTAGTTGGTGGGGTAAAGGCC
CACCAAGCCTACGATCCATAGCTGGTCTGAGAGGATGATCAGCCACACTGGGACTGAGACACGGCC
>Zotu7578
GAACGCTGGCGGCAGGCCTAACACATGCAAGTCGAACGAGAAGCCACCTTCGGGTGGTGGACAGTGGC
AGACGGGTGAGTAACGCGTGGGAACGTGCCTTTCGGTTCGGAATAACCCAGGGAAACTTGGGCTAATA
CCGGATACGCCCTTCGGGGGAAAGATTCATTGCCGAAAGATCGGCCCGCGTCGGATTAGCTAGTTGGT
GGGGTAATGGCTCACCAAGGCGACGATCCGTAGCTGGTCTGAGAGGATGACCAGCCACACTGGGACTG
AGACACGGCC
>Zotu7579
GAACGCTGGCGGCGTGCTTAACACATGCAAGTCGAGCGGTAAGGCTCCTTCGGGGGTACACGAGCGGC
GAACGGGTGAGTAACACGTGAGTAATCTGCCCTTCACTTCGGGATAAGCTTCGGAAACGGGGTCTAAT
ACCGGATATGACACCTTGATGCATGTTGGGGTGTGGAAAGTTTTTCGGTGGGGGATGTGCTCGCGGC
CTATCAGCTTGTTGGTGGGGTAATGGCCTACCAAGGCTTTGACGGGTAGCCGGCCTGAGAGGGTGACC
GGCCACACTGGGACTGAGACACGGCC
>Zotu7580
GAACGCTGGCGGCATGCTTAACACATGCAAGTCGAACGGCAGCACAGCAGAGCTTGCTCTGTGGGTGG
CGAGTGGCGGACGGGTGAGTAATGCATCGGGACCTACCCAGACGTGGGGGATAACGTAGGGAAACTTA
CGCTAATACCGCATACGTCCTACGGGAGAAAGCGGGGGATCGCAAGACCTCGCGCGGTTGGATGGACC
GATGTGCGATTAGCTAGTTGGTAAGGTAACGGCTTACCAAGGCGACGATCGCTAGCTGGTCTGAGAGG
ATGATCAGCCACACTGGGACTGAGACACGGCC
>Zotu7581
GAACGCTGGTAACGTGGATACCGGCATGCAAGTCACGGGGGCCGCAAGGCAACCGGCGAACGGCTTAG
TAACACGTGGGTACATACCTTAAATTGGAGCACAGCTCGTCGAAAGACGGGGTAATTCTCCATAGTCC
CGCAAGGGTAAAGCAGCAATGCGATTTAAGAATGGCCTGCGGCCTATCAGATAGTTGGCGGTGTAATA
GACCACCAAGTCTATGACGGGTAGGGGGCGTGAGAGCGCGACCCCCAAGAATGGCACTGCGACACGGG
C
>Zotu7582
GAACGCTGGCGGCATGCCTAATACATGCAAGTCGTACGGACATTTCTAATGTCAGTGGCGAACGGGTG
AGTAACACGTGGACAACCTACCTGTGCTTGGGGGATACGCTTTGGAAACGAAGTTTAATACCCCATGT
ATTTGGAAGAGTCATCTCTTCCTCTTTAAAGAGGCCTTCAAGCCTCGAGCATAGATGGGTCCGCGGTG
CATTAGTTTGTTGGCGAGGTAACGGCTCACCAAGACCATGATGCATAGCCGGCCTGAGAGGGCGATCG
GCCACACTGGGACTGAGACACGGCC
>Zotu7583
GAACGCTAGCTACAGGCTTAACACATGCAAGTCGAGGGGTTTTATTTGAGAAGCTTGCTTTTCGGATG
AAACGACCGGCGCACGGGTGAGTAACGCGTATCCAACCTGCCTTTCATATTGGGATAGCCTTTCGAAA
GAGAGATTAATACCGAATAACATATTGTGATTGCATGATAACAATATGAAAGCTCCGGCGATGAAAGA
TGGGGATGCGTTTCATTAGGTAGTAGGCGGGGTAACGGCCCACCTAGCCAACGATGAATAGGGGTTCT
GAGAGGAAGGTCCCCCACACTGGAACTGAGACACGGTC
>Zotu7585
GAACGCTGGCGGCGTGCCTAAGACATGCAAGTCGAATGTCCGCAAGGACATGGCGAACGGGTGAGTAA
TACCTAGGAACTTTCTTATAGCCGGGCATAACTCCGCGAAAGCGGAGATAATTCCCGATATTATCGCA
AGATCAAAGTAGCAATACACTATAAGTGAGGCCTGGGCACCATCAGCTAGTTGGTAAGGTAACGGCTT
ACCAAGGCTATGACGGTTAGGGGCCCCGAGAGGGGAAACCCCCACACTGGAACTGCGACACGGTC
>Zotu7586
GCCGTGTCTCAGTCCCAGTGTGGCTGATCATCCTCTCAGACCAGCTACAGATCGTCGGCTTGGTGAGC
CATTACCTCACCAACTACCTAATCTGACGCGGGCTCATCCATCAGCGATAAATCTTTCCTCCGCAGAG
AATATACGGTATTAGCTTTTATTTCTAAAAGTTATTCCGTACTGATGGGTAGATTCCCACGTGTTACT
CACCCGTCTGCCACTAACTAATTGGAGCAAGCCCCAATTAGTCCGTTCGACTTGCATGTGTTAAGCAT
ACCGATAGCGTTCG
>Zotu7587
GAACGCTGGCGGCGTGCTTTACACATGCAAGTCGAGCGGCAGCATGGTCTTCGGACTGATGGCGAGCG
GCGAACGGGTGAGTAATACATCGAAACGTGCCTTTTAGTGGGGGATAACCCATCGAAAGATGGGCTAA

TACCGCATAATCTCTGAGGAGGAAAGCTGGGGACTCGCAAGGGCCTGGCGCTGAAAGAGCGGTCGATG
GTCGATTAGTTAGTTGGTGAGGTAAAAGCTCACCAAGGCAATGATCGATAGCTGTTCTGAGAGGAAGA
TCAGCCACACTGGAACTGAGACACGGTC
>Zotu7590
GAACGCTGGCGGCGTGCTTAACACATGCAAGTCGAACGAGTAAGTGCTGTAGCAATACAGATTACTGC
TAGTGGCAGACGGGTGAGTAATACATGGGAATCTGCCTCGGAGTGTGGGATAACAGTTGGAAACGACT
GCTAATACCGCATATTGCCGAAAGGTGAAAGATTTTTCGCTCCGAGATGAGCCCATGCAAGATTAGCT
TGTTGGTGGGGTAAAAGCCTACCAAGGCGACGATCTTTAGCTGGTCTGAGAGGATGACCAGCCACACT
GGAACTGAGACACGGTC
>Zotu7592
GAACGCTGGCGGCGTGCCTTAAGCATGCAAGTAGAACGAGTTCTATCTTGCCGCAAGGCAAGCTTAGA
ACTAGTTGCGAACGGCTGAGTAATGAGTAAGAATCTTTCCTTTAGTGGGGAATACCTCATCGAAAGAT
GAGCTAATACCGCATAATACCTCCGGGTGAAAGCGATTAACTTCGCGCTGATTGATGAGCTTACTCCC
TATCAGGTAGTTGGTGGGGTAATAGCCTACCAAGCCTACGACGGGTACCCGATCTGAGAGGATGGACG
GGCAGAATGGGACTGAGACACGGCC
>Zotu7594
GAACGCTAGCGGCATGCCTAACACATGCAAGTCGAACGAAGGCTTCGGCCTTAGTGGCGCACGGGTGC
GTAACGCGTGGGAATCTGCCCCTTGGTTCGGAATAACAGTTGGAAACGACTGCTAATACCGGATGACG
ACGTAAGTCCAAAGATTTATCGCCGAGGGATGAGCCCGCGTAGGATTAGCTAGTTGGTGTGGTAAGAG
CGCACCAAGGCGACGATCCTTAGCTGGTCTGAGAGGATGATCAGCCACACTGGGACTGAGACACGGCC
>Zotu7595
GAACGCTGGCGGCGTGGATGAGGCATGCAAGTCGAATGGCCTAGTAGCAATACTAGAAACCATGGCAG
ACGAGGTAGGAACACGTAGGTAACGTACCCCAAAGTCGGGCATAACCGGTCGAAAGATTGGATAATTC
TCGATGGTCTCGGAGATTTATTTCTCCGAGTAAAGCGAATTATTTATTTAGTTTACGCTTTGGGAACG
GCCTGCGGGCTATCAGCTTGTTGGTAAGGTAATGGCTTACCAAGGCTACGACGGCTAGGGGAGGTGAG
AGCCTGACCCCCACCGGTGGAACTGCGACACGGTC
>Zotu7596
GAACGCTGGCGGCGTGCCTAATACATGCAAGTCGAGCGAATCTGAGGGAGCTTGCTCCCAAAGATTAG
CGGCGGACGGGTGAGTAACACGTGGGCAACCTGCCTGTAAGACTGGGATAACTTCGGGAAACCGGAGC
TAATACCGGATAATCCCTTTCCTCACATGAGGAAAGGCTGAAAGACGGTTTCGGCTGTCACTTACAGA
TGGGCCCGCGGCGCATTAGCTAGTTGGTGAGGTAACGGCTCACCAAGGCCACGATGCGTAGCCGACCT
GAGAGGGTGATCGGCCACACTGGGACTGAGACACGGCC
>Zotu7598
GAACGCTGGCGGCAGGCCTAACACATGCAAGTCGAACGGGATCTTCGGATCTAGTGGCGGACGGGTGA
GTAACGCGTGGGAACGTGCCCTTTGGTTCGGAATAGCCCTGGGAAACTGGGAGTAATACCGGATACGG
TCGAGAGACGAAAGATTTATCGCCGAAGGATCGGCCCGCGTTGGATTAGGTAGTTGGTGGGGTAATGG
CCTACCAAGCCGACGATCCATAGCTGGTTTGAGAGGATGATCAGCCACACTGGGACTGAGACACGGCC
>Zotu7599
GAACGCTGGCGGCATGCCTAACACATGCAAGTCGAACGAAGCCTTCGGGCTTAGTGGCGCACGGGTGC
GTAACGCGTGGGAATCTGCCCTTGGGTTCGGAATAACTCAGAGAAATTTGAGCTAATACCGGATGATG
ACGTAAGTCCAAAGATTTATCGCCCAGGGATGAGCCCGCGTAGGATTAGCTAGTTGGTGAGGTAAAGG
CTCACCAAGGCGACGATCCTTAGCTGGTCTGAGAGGATGATCAGCCACACTGGGACTGAGACACGGCC
>Zotu7600
GAACGCTGGCGGCATGCCTAACACATGCAAGTCGAACGAGATCTTCGGATCTAGTGGCGCACGGGTGC
GTAACGCGTGGGAATCTGCCCCTTGCTTCGGAATAACAGTGAGAAATTACTGCTAATACCGGATGATG
TCTTCGGACCAAAGATTTATCGGCAAGGGATGAGCCCGCGTAGGATTAGGTAGTTGGTGGGGTAATGG
CCTACCAAGCCGACGATCCTTAGCTGGTCTGAGAGGATGATCAGCCACACTGGGACTGAGACACGGCC
>Zotu7601
CAACGCTGGCGGCGTGCCTAACACATGCAAGTCGAACGCGAAAGGGGGCAACCCTGAGTAGAGTGGCG
TCCGGGTGAGTAACACGTGAATAATCTACCTATGGGTGGGGAATAACTTCTCGAAAGGGAAGCTAATA
CCGCATAAAATCTAGTGGGGGTGGCCCTGTTAGATCAAAGAGGGGGATCGCAAGACCTCTCGCCAATA
GAGGAGTTCGCGGCTGATTAGCTAGTTGGCGGGGTAACGGCCCACCAAGGCAACGATCAGTAGCCGGC
CTGAGAGGGCGGTCGGCCACACTGGCACTGAAACACGGGC
>Zotu7602

GAACGCTGGCGGCGTGCTTAACACATGCAAGTCGAACGGTGAAAGCGGAGCTTGCTCTGCTGGATCAG
TGGCGAACGGGTGAGTAACACGTGAGCAATCTGCCCCTGACTCTGGGATAAGCGCTGGAAACGGCGTC
TAATACTGGATACGAGCTGCGAAGGCATCTTCAGCAGCTGGAAAGAACTTCGGTCAGGGATGAGCTCG
CGGCCTATCAGCTTGTTGGTGAGGTAACGGCTCACCAAGGCGTCGACGGGTAGCCGGCCTGAGAGGGT
GACCGGCCACACTGGGACTGAGACACGGCC
>Zotu7603
GAACGCTGGCGGCATGCTTTACACATGCAAGTCGAACGGCAGCGCGGGGCAACCTGGCGGCGAGTGGC
GAACGGGTGAGTAATATATCGGAACGTACCCTGGAGTGGGGGATAACGTAGCGAAAGTTACGCTAATA
CCGCATACGATCTAAGGATGAAAGTGGGGGATTCGCAAGAACCTCATGCTCCTGGAGCGGCCGATATC
TGATTAGCTAGTTGGTAGGGTAAAGGCCTACCAAGGCAACGATCAGTAGCTGGTCTGAGAGGACGACC
AGCCACACTGGAACTGAGACACGGTC
>Zotu7604
GAACGCTGGCGGCGTGGATTAGGCATGCAAGTCGAACGAGAAAGGGTTTTCGGACCTGAGTAAAGTGG
CGAACGGGTGAGTAACGCGTAGATAACCTACCTTTAAGCGGGGGATAATCCAGGGAAACTTGGCTTAA
TACCGCATGTGGTCGAGAGACTAAAGATGGCCTCTGTTTACAAGCTGTCGTTTAGAGATGGGTCTGCG
TCTTATTAGTTAGTTGGTGGGGTAATGGCCTACCAAGGCAATGATAAGTAGGCGGCCTTAACGGGTGG
TCGCCCACACTGGGATTGAGATACGGCC
>Zotu7605
GAACGCTGGCGGCGTGCCTAATACATGCAAGTCGAGCGGACAGATGGGAGCTTGCTCCCTGATGTTAG
CGGCGGACGGGTGAGTAACACGTGGGTAACCTGCCTGTAAGACTGGGATAACTCCGGGAAACCGGGGC
TAATACCGGATGGTTGTTTGAACCGCATGGTTCAAACATAAAAGGTGGCTTCGGCTACCACTTACAGA
TGGACCCGCGGCGCATTAGCTAGTTGGTGAGGTAACGGCTCACCAAGGCGACGATGCGTAGCCGACCT
GAGAGGGTGATCGGCCACACTGGGACTGAGACACGGCC
>Zotu7607
GAACGCTGGCAGCAGGCCTAACACATGCAAGTCGAGCGGTAACGCGGGGCAACCTGGCGACGAGCGGC
GGACGGGTGAGTAACGCGTAGGAATCAACCCAGTAGCGGGGGATAGCCCGGGGAAACTCGGATTAATA
CCGCATACGCCCTAAGGGGGAAAGCAGGGGATCGTAAGACCTTGCACTATTGGATGAGCCTGCGTTGG
ATTAGCTAGTTGGTAGGGTAAAGGCCTACCAAGGCGACGATCCATAACTGGTCTGAGAGGATGATCAG
TCACACCGGAACTGAGACACGGTC
>Zotu7610
GAACGCTGGCGGCATGCCTTACACATGCAAGTCGAGGGGTATGGTTCTTCGGAACCAGAGACCGGCGC
ACGGGTGCGTAACGCGTATGCAATCTGCCTTTCACAAAGGAATAGCCCAGAGAAATTTGGATTAATGC
CTTATAGCATTACGACTTGGCATCAAGATGTAATTAAAGATTTATCGGTGAAAGATGAGCATGCGTCC
CATTAGCTAGTTGGTAAGGTAACGGCTTACCAAGGCGATGATGGGTAGGGGTCCTGAGAGGGAGATCC
CCCACACTGGTACTGAGACACGGAC
>Zotu7611
GAACGCTGGCGGCATGCCTAACACATGCAAGTCGAACGAGACCTTCGGGTCTAGTGGCGCACGGGTGC
GTAACGCGTGGGAATCTGCCCTTGGGTTCGGAATAACAGTTAGAAATGACTGCTAATACCGGATGATG
TCTTCGGACCAAAGATTTATCGCCCAAGGATGAGCCCGCGTAAGATTAGCTAGTTGGTGGGGTAATGG
CCTACCAAGGCGACGATCTTTAGCTGGTCTGAGAGGATGATCAGCCACACTGGGACTGAGACACGGCC
>Zotu7612
GAACGCTGGCGGCAGGCCTAACACATGCAAGTCGAACGGATCCTTCGGGATTAGTGGCGGACGGGTGA
GTAACGCGTGGGAACGTGCCCTTTGGTTCGGAATAGCCTCGGGAAACTGGGAGTAATACCGGATAAGC
CCTTCGGGGGAAAGATTTATCGCCAAAGGATCGGCCCGCGTTGGATTAGGTAGTTGGTGGGGTAATGG
CCTACCAAGCCGACGATCCATAGCTGGTTTGAGAGGATGATCAGCCACACTGGGACTGAGACACGGCC
>Zotu7613
GAACGCTGGTAACGTGGATACCGGCATGCAAGTCGAGCGGCCCGCAAGGGCAGCGGCGAACGGCTTAG
TAACACGTGGATACATACCCCAAAGTGGAGAACAGCTCGTCGAAAGACGGGGTAATGCTCCATAATAC
CGCAAGGTTAAAGCAGCAATGCGCTTTGGGAATGGTCCGCGGCCTATCAGCTTGTTGGCGGGGTAAAA
GCCCACCAAGGCTATGACGGGTAGGGGGTGTGAGAGCACGGCCCCCAAGAATTGGACTGCGACACGGC
C
>Zotu7614
GAACGCTGGCGGCGTGCCTAAGACATGCAAGTCGAGGGGGTTTAGACCCCCGGCGAACGGGTGAGTAA
CACGTGGGAACGATCAATAAGTTCGGGATAACTCTCCGAAAGGAGAGACAATACCGGATGTGGAAGCA

ATTCTAAAGATTTATCGCTTATTGTTCGGCCCGCGTCCCATCAGCTAGTTGGTAAGGTAATGGCTTAC
CAAGGCTATGACGGGTAGCGGAACCGAGAGGTTGATCCGCCACACTGGGACTGCGACACGGCC
>Zotu7615
GAACGCTGGCGGCAGGCTTAACACATGCAAGTCGAACGCCCCGCAAGGGGAGTGGCAGACGGGTGAGT
AACACGTGGGAACATACCCTTCGGTTCGGAACAACGCCGGGAAACTGGCGCTAATACCGGATACGGCC
TGTGGGCGAAAGATTGATCGCCGAAGGATTGGCCCGCGTTCGATTAGCTTGTTGGTGAGGTAACGGCT
CACCAAGGCGACGATCGATAGCTGGTCTGAGAGGATGGCCAGCCACATTGGGACTGAGACACGGCC
>Zotu7616
GAACGCTGGCGGCGTGCCTAACACATGCAAGTCGAACGGACGAGGCTTCTTCGGAGGCCAAGTTAGTG
GCGGACGGGTGAGTAACACGTGAGCAACCTGCCCTTCAGAGGGGGACAACAGTTGGAAACGACTGCTA
ATACCGCATGATACATTTGAGAGGCATCTCTTGAATGTCAAAGATTTTATCGCTGAAGGATGGGCTCG
CGTCTGATTAGATAGTTGGTGGGGTAACGGCTCACCAAGTCGACGATCAGTAGCCGGACTGAGAGGTT
GAACGGCCACATTGGGACTGAGATACGGCC
>Zotu7618
GAACGCTGGCGGCAGGCTTAACACATGCAAGTCGAACGCCCCGCAAGGGGAGTGGCAGACGGGTGAGT
AACGCGTGGGAATCTACCTTGAACTACGGGATAGCTCCGGGAAACTGGAATTAATACCGTATGTGCCC
TTAGGGGGAAAGATTTATCGGTTCAGGATGAGCCCGCGTTGGATTAGCTAGTTGGTGGGGTAACGGCC
CACCAAGGCGACGATCCATAGCTGGTCTGAGAGGATGACCAGCCACATTGGGACTGAGACACGGCC
>Zotu7619
GAACGCTGGCGGCGTGCCTAACACATGCAAGTCGAACGAGATCCATCCAGTGGCAACACTGGTGAAGA
TCTAGTGGCGAACGGGTGAGTAACACGTGAGGAACCTGCCCCGAAGACCGGGATAACCCTCCGAAAGG
AGAGCTAATACCGGATACCCCCAACTGATCGCATGGTCGGTTGAGGAAATGGATTCCGCTTCGGGAGG
GCCTCGCGGCCTATCAGCTAGTTGGTGAGGTAATGGCTCACCAAGGCATCGACGGGTAGCTGGTCTGA
GAGGACGATCAGCCACACTGGGACTGAGACACGGCC
>Zotu7620
GAACGCTAGCGGCAGGCCTAATACATGCAAGTCGAACGGTTCGCAAGGACAGTGGCAAACGGGTGCGT
AACGCGTAAGCAACCTGCCTCATACTGGGGGATAGCCCGGCGAAAGCTGGGGTAAACCCGCACGGTCC
AGTTTCATCACCTGGTGAGATTGGTAAACATTTATGGGTATGAGAGGGGCTTGCGTCTGATTAGTTAG
TTGGCAGGGTAACGGCCTACCAAGACGATGATCAGTAGGGGTTCTGAGAGGATTGGCCCCCACATGGG
TACTGAGATACGGAC
>Zotu7621
GAACGCTGGCGACGTGCTTAACACATGCAAGTCGAACGGTGAACCAGGGCTTGCCCTGGGAATCAGTG
GCGAACGGGTGAGTAACACGTGGGCAACCTGCCCCTGACTCTGGGATAACTCCGGGAAACCGGAGCTA
ATACCGGATATGACCTCTGACGGCATCGTCGGAGGTGGAAAGTTTTTCGGTCAGGGATGGGCCCGCGG
CCTATCAGCTTGTTGGTGGGGTAATGGCCCACCAAGGCGACGACGGGTAGCCGGCCTGAGAGGGCGAC
CGGCCACACTGGGACTGAGACACGGCC
>Zotu7622
GAACGCTGGCGGCGTGCCTAATACATGCAAGTCGAGCGAACCCTTTGGGGTTAGCGGCGGACGGGTGA
GTAACACGTGGGCAACCTACCTTGCAGATGGGGATAACACCGAGAAATCGGTGCTAATACCGAATAAC
ACTTCACTTCACATGAAGAGAAGTTAAAAGTTGGCTTCGGCTAACACTGTGAGATGGGCCCGCGGCGC
ATTAGCTAGTTGGTGAGGTAACGGCTCACCAAGGCAACGATGCGTAGCCGACCTGAGAGGGTGAACGG
CCACACTGGGACTGAGACACGGCC
>Zotu7623
GAACGCTGGCGGCGTGCTTAACACATGCAAGTCGAACGATGAAAGAGGAGCTTGCTCCTCTGGATTAG
TGGCGAACGGGTGAGTAACACGTGAGCAATCTGCCCCTGACTCCGGAATAACTACGGGAAACTGTAGC
TAATACCGGATACGCGCCGAACAGGCATCTGTATAGGTGGGAAAGAATTTCGGTCAGGGATGAGCTCG
CGGCCTATCAGCTTGTTGGTGAGGTAACGGCTCACCAAGGCGACGACGGGTAGCCGGCCTGAGAGGGT
GACCGGCCACATTGGGACTGAGGCACGACC
>Zotu7624
CAACGCTGGCGGCGCGCTTAACACATGCAAGTCGCACGAGAAAGGGAGCAATCCTGAGTAAAGTGGCG
TACGGGTGAGTAACACGTGGATCATCTACCTTTTAGTGGGGAATACCTCCGGGAAACTGGAGACAATA
CCGCATAAGCTCGTAAGAGGAAAGCAGCAATGCGCTGAAAGAGGAGTCCGCGGCTGATTATGCTAGTT
GGCGGGGTAACGGCCCACCAAGGCGAAGATCGGTAGCCGGCCTGAGAGGGCTCACGGCCACACTGGCA
CTGAAACACGGGC

>Zotu7625
GAACGCTGGCGGCGTGCTTAACACATGCAAGTCGAACGAAGCAGCAATGCTTAGTGGCGGACGGGTGA
GTAACGCGTAGGTAACCTGCCTCATAGTGGGGGATACCCATCCGAAAGGATGGCTAATACCGCATAAG
CTGGTTCTTTGGAATAGGGATCAGGAAAGATTTATTGCTATGAGAGGGGCCTGCGTTCGATTAGCTAG
TTGGTGAGGTAATGGCTCACCAAGGCGACGATCGATAGCTGGTCTGAGAGGACGATCAGCCACACTGG
AACTGAGACACGGTC
>Zotu7629
CAACGCTGGCGGCGTGCCTAACACATGCAAGTCGAACGCAGTTACTCGCAAGGGTAACTGAGTGGCGA
ACGGGTGAGTAACGCGTGGGTGACCTACCTTCGAGTGGGGGATAACGTCCCGAAAGGGACGCTAATAC
CGCATGACATCCTGCCTTTGAACAGGCGGAGATCAAAGCCGGGGATCGCAAGACCTGGCGCTTGATGA
GGGGCCCGCGTCTGATTAGCTAGTTGGTGAGGTAACGGCTCACCAAGGCGACGATCAGTATCCGGCCT
GAGAGGGCGGACGGACACACTGGGACTGAGACACGGCC
>Zotu7633
GAACGCTGGCGGCATGCCTAATACATGCAAGTCGAGCGAATGACGAAGAAGCTTGCTTCTTCTGATTT
AGCGGCGGACGGGTGAGTAACACGTGGGCAACCTGCCCTGTAGATTGGGATAACTCCGGGAAACCGGG
GCTAATACCGAATAATCCATTTCCTCACATGGGGAGATGTTAAAAGACGGCATCTCGCTGTCACTACA
GGATGGGCCCGCGGCGCATTAGCTAGTTGGTGAGGTAACGGCTCACCAAGGCGACGATGCGTAGCCGA
CCTGAGAGGGTGATCGGCCACACTGGGACTGAGACACGGCC
>Zotu7636
GAATGCTGGCGGCGTGGATAAGTCATGCAAGTCAAGGGGGCCCGCAAGGGCAACCGGCAGACGAGGTA
GTAACACGTAGGTAGATCCCCCAGAGTCGGGCATAGCCAGTCGAAAGACTGGGTAATTCCCGATGGTC
CCGCAAGGGTAAAGGTTTTTCGCTCTGGGAGTTGCCTGCGTAGTATCAGCTAGTTGGTAAGGTAACGG
CTTACCAAGACTACGACGCTTAGGGGAGCTGAGAGGCTGACCCCCACCGATGGAACTGCGACACGGTC
>Zotu7637
GAACGCTGGCGGCGTGCTTTACACATGCAAGTCGAGCGGCAGCGCGACCTTCGGGTTGGCGGCGAGCG
GCGAACGGGTGAGTAATACATCGGAACGTACCTTTTAGTGGGGGATAACGCATCGAAAGATGTGCTAA
TACCGCATAATCTCTGAGGAGGAAAGGCGGGGATCACGCAAGTGACCTGTCGCTGAAAGAGCGGCCGA
TGGTCGATTAGCTAGTTGGCGGGGTAAAAGCCCACCAAGGCGATGATCGATAGCGGGTCTGAGAGGAC
GATCCGTCACACTGGGACTGAGACACGGCC
>Zotu7638
GAACGCTGGCGGCGTGCTTAACACATGCAAGTCGAACGATGAAGCCCAGCTTGCTGGGTGGATTAGTG
GCGAACGGGTGAGTAACACGTGAGTAACCTGCCCCCGACTCTGGGATAAGCCCGGGAAACTGGGTCTA
ATACCGGATATTACCTTTGACCGCATGGTTGTTGGTGGAAAGATTTATCGGTGGGGGATGGACTCGCG
GCCTATCAGCTTGTTGGTGAGGTAATGGCTCACCAAGGCGACGACGGGTAGCCGGCCTGAGAGGGTGA
CCGGCCACACTGGGACTGAGACACGGCC
>Zotu7639
GAACGCTGGCGGCGTGCTTAACACATGCAAGTCGAACGGGAAATACTTTATTGAAACTTCGGTGGATT
TAATTTATTTCTAGTGGCGGACGGGTGAGTAACGCGTGGGTAACCTGCCTTATACTGGGGGATAACAG
CCAGAAATGGCTGCTAATACCGCATAAGCGCACAGAACCGCATGGTTCGGTGTGAAAAACTCCGGTGG
TATAAGATGGACCCGCGTTGGATTAGCTAGTTGGCAGGGCAGCGGCCTACCAAGGCGACGATCCATAG
CCGGCCTGAGAGGGTGAACGGCCACATTGGGACTGAGACACGGCC
>Zotu7640
GAACGCTGGCGGCAGGCTTAACACATGCAAGTCGAGCGGGCATTTCGGTGTCAGCGGCAGACGGGTGA
GTAACACGTGGGAACGTGCCCTTCGGTTCGGAATAACTCAGGGAAACTTGAGCTAATACCGGATACGC
CCTTATGGGGAAAGGTTTACTGCCGAAGGATCGGCCCGCGTCTGATTAGCTTGTTGGTGGGGTAACGG
CCTACCAAGGCGACGATCAGTAGCTGGTCTGAGAGGATGATCAGCCACACTGGGACTGAGACACGGCC
>Zotu7641
GAACGCTGGCGGCAGGCTTAACACATGCAAGTCGAGCGGGCATAGCAATATGTCAGCGGCAGACGGGT
GAGTAACGCGTGGGAACGTACCTTTTGGTTCGGAACAACTGAGGGAAACTTCAGCTAATACCGGATAA
GCCCTTACGGGGAAAGATTTATCGCCGAAAGATCGGCCCGCGTCTGATTAGCTAGTTGGTGAGGTAAT
GGCTCACCAAGGCGACGATCAGTAGCTGGTCTGAGAGGATGATCAGCCACATTGGGACTGAGACACGG
CC
>Zotu7643
GAACGCTGGCGGCATGCCTAACACATGCAAGTCGAACGGGATCCTGGCTTCGGCCAGGTGAGAGTGGC

GGACGGGTGAGTAACGCGTGGGAACCTGCCCAGTGGTGGGGGATAACTGCGGGAAACTGCAGCTAATA
CCGCATGAGCCCGAGAGGGGAAAGATTTATCGCCATTGGAGGGGCCCGCGTTCGATTAGGTAGTTGGT
GAGGTAACGGCTCACCAAGCCGACGATCGATAGCTGGTCTGAGAGGATGATCAGCCACACTGGGACTG
AGATACGGCC
>Zotu7644
GAACGCTGGCGGCATGCTTTACACATGCAAGTCGAACGGTAACAGGTCTTCGGATGCTGACGAATGGC
GAACGGGTGAGTAATACATCGGAACGTGCCTAATAGTAGGGGATAACTACTCGAAAGAGTAGCTAATA
CCGCATGAGATCTACGGATGAAAGCAGGGGACCTTCGGGCCTTGTGCTACTAGAGCGGCTGATGGCAG
ATTAGGTAGTTGGTGGGGTAAAAGCTTACCAAGCCTGCGATCTGTAGCTGGTCTGAGAGGACGACCAG
CCACACTGGGACTGAGACACGGCC
>Zotu7645
GAACGCTGGCGGCGTGGATAAGGCATGCAAGTCAAGGGGGCCCGCAAGGGCAACCGGCAGACGAGGTA
GTAACGCGCAGGGAGATCCCCCAAAGTCAGGCATAGCCAGCCGAAAGGTTGGGTAATTCCTGATAGTC
CCGCAAGGGTAAAGATTTATCGCTTTGGGATTCACCTACGTAATATCAGCTAGTTGGTGAGGTAATGG
CTCACCAAGGCTACGACGTTTAGGGGAGCTGAGAGGCTGACCCCCACCGATGGGACTGCGACACGGCC
>Zotu7646
GAACGCTGGCGGCGTGCCTAATACATGCAAGTCGAGCGGAGTTGATGAGGTGCTTGCACCTCTGATAC
TTAGCGGCGGACGGGTGAGTAACACGTAGGTAACCTGCCTGTAAGACCGGGATAACATTCGGAAACGA
ATGCTAATACCGGATACGCGATTTTCTCGCATGAGAGAATCGGGAAAGACGGAGCAATCTGTCGCTTA
CGGATGGACCTGCGGCGCATTAGCTAGTTGGTGAGGTAACGGCTCACCAAGGCGACGATGCGTAGCCG
ACCTGAGAGGGTGATCGGCCACACTGGGACTGAGACACGGCC
>Zotu7648
GAACGCTGGCGGCAGGCCTAACACATGCAAGTCGAACGTGAAGCCACCTTCGGGTGGTGGAAAGTGGC
AGACGGGTGAGTAACGCGTGGGAATCTACCCATTGGTACGGAATAACTCAGGGAAACTTGAGCTAATA
CCGTATGTGCCCGAAAGGGGAAAGATTTATCGCCATTGGATGAGCCCGCGTCTGATTAGCTAGTTGGT
GGGGTAACGGCCCACCAAGGCGACGATCAGTAGCTGGTCTGAGAGGATGATCAGCCACACTGGGACTG
AGACACGGCC
>Zotu7651
GAACGCTGGCGGCAGGCCTAATACATGCAAGTCGGACGGGATTGGAGAGCTTGCTTTCCATGAGAGTG
GCGCACGGGTGCGTAACACGTATGTAACCTACCTTCGTCAGGGGGATAGCCTCTCGAAAGAGAGATTA
AGACCGCATAACATTATTGGATAGCATTATTTAATAATCAAATATTTATAGGACGAAGATGGGCATGC
GGAACATTAGCTAGTTGGTAAGGTAACGGCTTACCAAGGCTACGATGTTTAGGGGATCTGAGAGGATG
ACCCCCCACACTGGTACTGAGACACGGAC
>Zotu7653
GAACGCTAGCGATAGGCCTAACACATGCAAGTCGAGGGGCAGCGATGGTAGCAATACCAGTCGGCGAC
CGGCGGATGGGTGAGTAACACGTATGCAACCTGCCTTCAACATTGGAATAACCCGTTGAAAGACGGAC
TAATGCCTTATAACACAGGGGCGCCGCATGGCGATATTTGTTAAAGATTTTATCGGTTGAAGATGGGC
ATGCGCATGATTAGTTAGTTGGTAAGGTAACGGCTTACCAAGACAACGATCATTAGGGGTTCTGAGAG
GAAGGTCCCCCACACTGGTACTGAGACACGGAC
>Zotu7654
GAACGCTGGCGGCGTGCCTAATACATGCAAGTCGAACGCACTCTCGTTTAGATTGAAGGAGCTTGCTC
CTGATTGATAAACATTTGAGTGAGTGGCGGACGGGTGAGTAACACGTGGGTAACCTGCCCTAAAGTGG
GGGATAACATTTGGAAACAGATGCTAATACCGCATAAAACCTAACACCGCATGGTGTAGGGTTGAAAG
ATGGTTTCGGCTATCACTTTAGGATGGACCCGCGGTGCATTAGTTAGTTGGTGAGGTAAAGGCTCACC
AAGACCGTGATGCATAGCCGACCTGAGAGGGTAATCGGCCACACTGGGACTGAGACACGGCC
>Zotu7655
GAACGCTGGCGGCAGGCCTAACACATGCAAGTCGAGCGGATGACGGGAGCTTGCTCCTTGATTCAGCG
GCGGACGGGTGAGTAATACCTAGGAATCTGCCTGGTAGTGGGGGACAACGTTTCGAAAGGAACGCTAA
TACCGCATACGTCCTACGGGAGAAAGCAGGGGACCTTCGGGCCTTGCGCTATCAGATGAGCCTAGGTC
GGATTAACTAGTTGGTGAGGTAATGGCTCACCAAGGCGACGATCCGTAACTGGTCTGAGAGGATGATC
AGTCACACTGGAACTGAGACACGGTC
>Zotu7656
GAACGCTGGCGGCGTGCTTAACACATGCAAGTCGAACGTGAGAATAGCAATATTTGAAAGTGGCAAAC
GGGTGAGTAACACGTGGGTATCTGCCTTTGAGTGGGGGATAACGACTGGAAACGGTCGCTAATACCGC

ATACGTTCACATTTTGTGGAGAGATGTGAAGAAAGATATATTGTTCAAAGAGGAGCCTGCGTCCGATT
AGCTAGTTGGTGGGGTAAAGGCCTACCAAGGCGATGATCGGTAGCTGGTCTGAGAGGATGGTCAGCCA
CACGGGAACTGAGAAACGGTC
>Zotu7659
GAACGCTAGCGGCAGGCCTAACACATGCAAGTCGAGGGGTATAGTAGCAATACTAGAGACCGGCGCAC
GGGTGCGTAACGCGTATGCAATCTACCTTTTACAGGGGAATAGCCCAGAGAAATTTGGATTAATGCCC
CATGGTATTATCGGATGGCATCATTTGATAATTAAAGTTCTAACGGTAAAAGATGAGCATGCGTCCTA
TTAGCTAGTTGGTAAGGTAACGGCTTACCAAGGCTACGATAGGTAGGGGTCCTGAGAGGGAGATCCCC
CACACTGGTACTGAGACACGGAC
>Zotu7660
GAACGCTGGCGGCGTGCCTAACACATGCAAGTCGAGCGATGAACCCTTCGGGGGGATTAGCGGCGGAC
GGGTGAGTAACACGTGGGTAACCTGCCTCAAAGAGGGGGATAGCCTCCCGAAAGGGAGATTAATACCG
CATAACATCGAGCTATCGCATGGTAGTTTAATCAAAGGAGTAATCCGCTTTGAGATGGACCCGCGGCG
CATTAGCTAGTTGGTGAGGTAACGGCTCACCAAGGCGACGATGCGTAGCCGACCTGAGAGGGTGATCG
GCCACATTGGAACTGAGACACGGTC
>Zotu7664
GAACGCTGGCGGCGTGCCTAATACATGCAAGTCGAGCGGTAAGGCTCCTACAGGTTATTTGATCGGGT
TTATCCTAAGTTTTCTCTGGCTAGAGAAGGCAAAAGATCGAATAATGTGTTGGAGTACACGAGCGGCG
GACGGCTGAGTAACGCGTAGGAACGTACCCCAAAGTGAGGGATAAGCACCGGAAACGGTGTCTAATAC
CGCATGTGGTCTTCGGATTAAAGCTTCGGCGCTTTGGGAACGGCCTGCGTATGATTAGCTTGTTGGTG
AGGTAAAAGCTCACCAAGGCGACGATCATTAGCTGGTCTGAGAGGATGATCAGCCAGACTGGGACTGA
GACACGGCC
>Zotu7666
GAACGCTGGCGGCGTGCTTAACACATGCAAGTCGAACGGTAAGGCCCTTCGGGGTACACGAGTGGCGA
ACGGGTGAGTAACACGTGGGTGATCTGCCCTGTACTTTGGGATAAGCCTGGGAAACTGGGTCTAATAC
CGAATATGACCTTCTCCTGCATGGGGGTTGGTGGAAAGCTTTTGCGGTACAGGATGGGCCCGCGGCCT
ATCAGCTTGTTGGTGGGGTAATGGCCTACCAAGGCGACGACGGGTAGCCGGCCTGAGAGGGCGACCGG
CCACACTGGGACTGAGACACGGCC
>Zotu7667
CAACGCTGGCGGCGCGCTTAACACATGCAAGTCGAACGAGAAAGTGGAGCAATCCATGAGTAAAGTGG
CGTACGGGTGAGTAACACGTGGGTAATCTGACTCTTTGTGAGGAATAACCCTGGGAAACCGGGGCTAA
TACCGCATAAGCCCGAGAGGGGAAAGTCGCAAGACGCTGAGAGAGGAGCCCGCGGCCGATTAGCTAGT
TGGCAGGGTAAAAGCCTACCAAGGCATAGATCGGTAGCCGGCCTGAGAGGGCACACGGCCACACTGGC
ACTGAAACACGGGC
>Zotu7669
GAACGCTGGCGGCGTGCTTAACACATGCAAGTCGAACGAGGTATAAGGACGGAAGTTTTCGGACGGAA
GATCTTGTACTGAGTGGCGGACGGGTGAGTAACGCGTGGGAAACCTACCCTGTTCCGGGGGATACCAG
TTGGAAACGACTGTTAATACCGCATAAGCGCACAGCACCGCATGGTGCAGTGTGAAAAACTCCGGTGG
AACAGGATGGTCCCGCGTCTGATTAGGCAGTTGGCAGGGTAACGGCCTACCAAACCGACGATCAGTAG
CCGGCCTGAGAGGGTGAACGGCCACATTGGGACTGAGACACGGCC
>Zotu7670
GAACGCTGGCGGCGTGCCTAATACATGCAAGTCGAGCGAATCTTTTAGGAGCTTGCTCCTAAAAGGTT
AGCGGCGGACGGGTGAGTAACACGTGGGCAACCTGCCTGTAAGACTGGGATAACTTCGGGAAACCGGA
GCTAATACCGGATAATTTCTTCCCTCTCCTGAGGGAAGGTTGAAAGACGGCTTCGGCTGTCACTTACA
GATGGGCCCGCGGCGCATTAGCTAGTTGGTGAGGTAACGGCTCACCAAGGCAACGATGCGTAGCCGAC
CTGAGAGGGTGATCGGCCACACTGGGACTGAGACACGGCC
>Zotu7671
GAACGTTGGCGGTGCGCCTAATACATGCAAGTCGCGGGGTCTGGAGCAATCCAGACACCGGCAAACGG
GTCCGTAACACGTTGGAACCTGCCCTGAAGTGAGGGATAACCCCGAGAAATCGGGGCTAATACCGCAT
ATGGCCGCAAGGTTAAAGCAGCAATGCGCTTCAGGAGGGGCCTGCGTCCTATCAGCTTGTTGGCGAGG
TAATGGCTCACCAAGGCTTTGACGGGTAGCTGGTCTGAGAGGATGATCAGCGACAGGGAGACTGAGAC
ACGGCT
>Zotu7672
GAACGCTGGCGGCGTGCTTAACACATGCAAGTCGAACGATGAAACAATTCTTGATGTCTTCGGACTGA

TTGAGCTGTGGATTAGTGGCGGACGGGTGAGTAACGCGTGAGCAATCTGCCTTACACAAAGGGATAGC
CTCGGGAAACCGGGATTAATACCATATGATATCTATTAACCGCATGGCCTATAGATTAAAGAATTTCG
GTGTAAGATGAGCTCGCGTCCCATTAATTAGTTGGTGAGGGTAACGGCCCACCAAGATAGCGATGGGT
AGCCGATCTAAGAGGATGACCGGCCACATTGGAACTGAGAAACGGTC
>Zotu7673
CAACGCTGGCGGCGCGCCTAATGCATGCAAGTCGGACGGGGTCGCCTTCGGGCGGCTCACGTGGCGGA
CGGGCGAGGAACACGTGGGCAACCTGCCCCGGGGCGGGGGGTAGCCCCCGGAAACGGGGGGTAATTCC
GCGTACGCTCACCCTGCTGTGGGCGGGGTGAGGAAAGGCGCGAGCCACCCCGGGAGGGGCCTGCGGCG
GATTAGCTTGTTGGCGGGGTAACGGCCCACCAAGGCGGTGATCCGTAGCTGGTCTGAGAGGACGGCCA
GCCGCAGGGGGACTGAGACACGGCC
>Zotu7674
GAACGCTGGCGGCGTGCTTAACACATGCAAGTCGAACGATGAAAGAGAATCCGATCTCTTCGGAGTGA
AGAATCTCTGGATTAGTGGCGGACGGGTGAGTAACGCGTGAGCAATCTGCCTTACACAAAGGGATAGC
CTCGGGAAACCGGGATTAATACCATATGATATCTACAAGCCGCATGGCCTATAGATTAAAGAATTTCG
GTGTAAGATGAGCTCGCGTCCCATTAATTAGTTGGTGAGGGTAACGGCCCACCAAGATAGCGATGGGT
AGCCGATCTAAGAGGATGACCGGCCACATTGGAACTGAGAAACGGTC
>Zotu7675
ACCGTGTCTCAGTTCCAGTGTGACTGATCATCCTCTCAGACCAGTTACGGATCGTCGCCTTGGTGAGC
CATTACCTCACCAACTAGCTAATCCGACCTAGGCTCATCTGATAGCGCAAGGCCCGAAGGTCCCCTGC
TTTCTCCCGTAGGACGTATGCGGTATTAGCGTTCCTTTCGAAACGTTGTCCCCCACTACCAGGCAGAT
TCCTAGGCATTACTCACCCGTCCGCCGCTGAATCCAGGAGCAAGCTCCTCTCATCCGCTCGACTTGCA
TGTGTTAGGCCTGCCGCCAGCGTTCA
>Zotu7678
GAACGCTAGCTACAGGCTTAACACATGCAAGTCGAGGGGAAACGACATCGAAAGCTTGCTTTTGATGG
GCGTCGACCGGCGCACGGGTGAGTAACGCGTATCCAACCTGCCCACCACTTGGGGATAACCTTGCGAA
AGTAAGACTAATACCCAATGATATCTCTAGAAGGCATCTGAAAGAGATTAAAGATTTACCGGTGATGG
ATGGGGATGCGTCTGATTAGCTTGTTGGCGGGGTAACGGCCCACCAAGGCAACGATCAGTAGGGGTTC
TGAGAGGAAGGTCCCCCACATTGGAACTGAGACACGGTC
>Zotu7680
GAACGCTGGCGGCAGGCTTAACACATGCAAGTCGAACGCCCCGCAAGGGGAGTGGCAGACGGGTGAGT
AACGCGTGGGAACGTGCCCTTCGCTTCGGAATAGCCCCGGGAAACTGGGAGTAATACCGGATACGCCG
TATTGGGAAAGATTTATCGGCGAAGGATCGGCCCGCGTTAGATTAGCTAGTTGGCGGTGTAATGGACC
ACCAAGGCTACGATCTATAGCTGGTCTGAGAGGATGATCAGCCACACTGGGACTGAGACACGGCC
>Zotu7682
GAACGCTGGCGGCATGCCTAACACATGCAAGTCGAACGAGACCTTCGGGTCTAGTGGCGCACGGGTGC
GTAACGCGTGGGAATCTGCCCAAAGGTTCGGAATAACTCGGGGAAACCCGTGCTAATACCGGATGATA
TCGCGAGATCAAAGATTTATCGCCTTTGGATGAGCCCGCGTAGGATTAGCTTGTTGGTGGGGTAAAGG
CCTACCAAGGCGACGATCCTTAGCTGGTCTGAGAGGATGATCAGCCACACTGGGACTGAGACACGGCC
>Zotu7683
GAACGCTGGCGGCGTGCCTAACACATGCAAGTCGAACGCGAACGGTGCTTTCGGGTGCCTAGTAGAGT
GGCGCACGGGTGAGTAACACGTAGACAACCTGCCTTCGAGCCCGGAATAACCTTTCGAAAGAAGGGCT
AATACCGGATAACATCCCGGGTTGCATGACTTGGGATTGAAAGGCTTGCTACTCGAAGAGGGGTCTGC
GGGACATTAGCTAGTTGGTGGGGTAACGGCCTACCAAGGCGACGATGTCTAGCGGGTCTGAGAGGATG
ATCCGCCACACTGGAACTGAGACACGGTC
>Zotu7684
GAACGCTAGCGGCATGCTTAACACATGCAAGTCGAACGGCAGCATAGGAGCTTGCTCCTGATGGCGAG
TGGCGAACGGGTGAGTAATACGTAGGAATCTACCTTATAGAGGGGGACAACCCGGGGAAACTCGGGCT
AATACCGCATGATCTCTATGGAGTAAAGCGGGGGATCTTCTGACCTCGCGCTATAAGATGAGCCTATG
TCGGATTAGCTTGTTGGTGGGGTAATTGCCTACCAAGGCGACGATCCGTAGCTGGTCTGAGAGGATGA
TCAGCCACACTGGGACTGAGACACGGCC
>Zotu7685
GAACGCTGGCGGCGTGCTTAACACATGCAAGTCGAGCGGTAAGGCCCTTCGGGGTACACGAGCGGCGA
ACGGGTGAGTAACACGTGAGCAATCTGCCCATCACATCGGGATAACTCCGGGAAACCGGTGCTAATAC
CGAATACGACCACTTCAGGCATCTGACGGTGGTGGAAAGTTCCGGCGGTGATGGATGAGCTCGCGGCC

TATCAGCTTGTTGGTGGGGTAACGGCCCACCAAGGCTTCGACGGGTAGCCGGCCTGAGAGGGTGACCG
GCCACACTGGGACTGAGACACGGCC
>Zotu7686
GAACGCTGGCGGCGTGCTTAACACATGCAAGTCGAGCGAAGCACTTGTTTGGAACCCTCCGGGAGGAA
GAACAATTGACTGAGCGGCGGACGGGTGAGTAACGCGTGGGGAACCTGCCTTATACCGGGGGATAACA
GCTGGAAACGGCTGCTAATACCGCATAAGCGCACGGCACCGCATGGTGCAGTGTGAAAAACTCCGGTG
GTATAAGATGGACCCGCGTTAGATTAGCTAGTCGGCGGGGTAACGGCCCACCAAGGCGACGATCTATA
GCCGGCCTGAGAGGGCGACCGGCCACATCGGGACTGAGACACGGCC
>Zotu7689
GAACGCTGGCGGCGTGCCTAATACATGCAAGTCGAGCGAATCTCTTCGGAGATTAGCGGCGGACGGGT
GAGTAACACGTGGGCAACCTGCCTATAAGACTGGGATAACTTCGGGAAACCGGAGCTAATACCGGATA
ACATCAAGGACCACATGGTCTTCGATTAAAAGATGGCGTCAAGCTATCACTTATAGATGGGCCCGCGT
CGCATTAGCTAGTTGGTGAGGTAACGGCTCACCAAGGCGACGATGCGTAGCCGACCTGAGAGGGTGAT
CGGCCACACTGGGACTGAGACACGGCC
>Zotu7691
GAACGCTGGCGGCATGCCTTACACATGCAAGTCGAACGGCAGCACGGGGGCAACCCTGGTGGCGAGTG
GCGAACGGGTGAGTAATACATCGGAACGTGTCCTGTAGTGGGGGATAGCCCGGCGAAAGCCGGATTAA
TACCGCATACGATCTACGGAAGAAAGTGGGGGATCTTCGGACCTCGCGCTGTAGGGGCGGCCGATGGC
AGATTAGCTAGTTGGTGGGGTAAAGGCCTACCAAGGCGACGATCTGTAGCTGGTCTGAGAGGACGACC
AGCCACACTGGGACTGAGACACGGCC
>Zotu7693
GAACGCTGGCGGCATGCTTTACACATGCAAGTCGAACGGTAACAGGGAGCTTGCTCCGCTGACGAGTG
GCGAACGGGTGAGTAATATATCGGAACGTACCCAGAAGTGGGGGATAACGTAGCGAAAGTTACGCTAA
TACCGCATACGATCCACGGATGAAAGTAGGGGACCTTCGGGCCTTATGCTTTTGGAGCGGCCGATATC
TGATTAGCTAGTTGGTGAGGTAAAGGCTCACCAAGGCGACGATCAGTAGCTGGTCTGAGAGGACGACC
AGCCACACTGGAACTGAGACACGGTC
>Zotu7695
GAACGCTGGTAACGTGGATACCGGCATGCAAGTCAAGGGGACCCGCAAGGGTAACCGGCGAACGGCTT
AGTAATACGTAGATACATACCGTAAAGTGGGGCATAGCTCGTTGAAAAACGGGGTAATTTCCCATAGT
CTCGCAAGAGTAAAGGCGCAAGCCGCTTTATGAATGGTCTGCGGTCTATCAGCTAGTTGGTGGGGTAA
AAGCCTACCAAGGCTATGACGGATAGGGGGCGTGAGAGCGCGACCCCCAAGAATGGCACTGCGACACG
GGC
>Zotu7697
GAACGCTGGCGGCGCGTCTTAAGCATGCAAGTCGAACGGCAAGAGAGGAGCTTGCTTCTCTCCTAGAG
TGGCGGACTGGTGAGGAACACGTGGGTAATCTACCCTTAAGATGGGGATAGCTGCTAGAAATAGCAGG
TAATACCGAATACACTCAGTGCTTCACAAGAGGTAGTGAGGAAAGGAAGCTACGGCTTCGCTTGAGGA
TGAGCTTGCGTCCCATTAGCTTGTTGGTGAGGTAAAGGCCCACCAAGGCGACGATGGGTATCCGGCCT
GAGAGGGTGAACGGACACATTGGGACTGAGATACGGCC
>Zotu7699
GAACGCTAGCGGCAGGCCTAATACATGCAAGTCGAACGGAGTAGTAATACTTAGTGGCAAACGGGTGC
GTAACGCGTAAGCAACCTGCCTTCTACTGGGGGATAGCCCGGCGAAAGCTGGGGTAAACCCGCACGGT
CCAGAAGAATCACCTGTTTTTTCTGGTAAACATTTATGGGTAGAAGAGGGGCTTGCGTCTGATTAGCT
AGTTGGCGGGGTAACGGCCCACCAAGGCGATGATCAGTAGGGGTTCTGAGAGGATTGGCCCCCACATG
GGTACTGAGATACGGAC
>Zotu7700
GAACGCTGGCGGTGTGGATAAGACATGCAAGTCGTGCGGTTTTCCGACGAGTCGCAAGACCTGTTGGA
AGATAGCGGCGAACGGGTTAGTAACACGTAGGTATTTGCCCCAAAGACGGGAATAACCAGCCGAAAGG
TTGGCTAATACCCGATGGTCTCGCAAGAGTAAAGGCGAAAGCCGCTTTGGGAGAAGCCTGCGGGCTAT
CAGCTAGTTGGTGGGGTAACGGCCTACCAAGGCGATGACGGCTAGGGGGTGTGAGAGCGCGACCCCCA
ACATTGGGACTGAGACACGGCC
>Zotu7701
CAACGCTGGCGGCGTGCCTCAGACATGCAAGTCGAACGATTAAGACTCCCTTCGGGGAGTGTATAAAG
TGGCGCACGGGTGAGTAACACGTAAGTAATCTACCTTCGAGTGGGGAATAACGTTCGGAAACGGACGC
TAATACCGCATAACGCAGCGGCATCGCAAGATGACAGTTGTTAAAGGAGCAATCCGCTTGAAGAGGAG

CTTGCGGCAGATTAGCTTGTTGGTAAGGTAACGGCTTACCAAGGCTACGATCTGTATCCGGCCTGAGA
GGGCGGTCGGACACACTGACACTGAATAACGGGT
>Zotu7702
GAACGCTAGCGGCAGGCCTAATACATGCAAGTCGGACGGGATAGAAGAGCTTGCTTTTCTTGAGAGTG
GCGCACGGGTGCGTAACACGTATGTAACCTACCTTGTTCAGGGGGATAGCCTCTCGAAAGAGAGATTA
AGACCGCATAACGTTATTGAATGGCATCATTTAATAACCAAATATTTATAGGAACAAGATGGGCATGC
GGAACATTAGCTAGTTGGTAAGGTAATGGCTTACCAAGGCTACGATGTTTAGGGGATCTGAGAGGATG
GCCCCCCACACTGGTACTGAGACACGGAC
>Zotu7703
GAACGCTGGTGGCGTGCTTAACACATGCAAGTCGAGCGAGGCGTCCGCCTCCGGGCGGTCAGCCGAGC
GGCGAACGGGTGAGTAACACGTGAGCAATCTTCCCAAGGCTCGGGGATAACCTCGGGAAACTGAGGCT
AATACCGGATGAGCGTTTTTCGGGACATCCCGGAAAACGAAAAGGTTTCGCACTGCGAGGTGCGTTTC
CGGCCTTGGATGAGCTCGCGGCCTATCAGCTTGTTGGTGCGGTAAATGCGCACCAAGGCGACGACGGG
TAGCCGGCCTGAGAGGGTGATCGGCCACACTGGGACTGAGACACGGCC
>Zotu7704
GAACGCTGGCGGCATGCCTAACACATGCAAGTCGAACGAGACCTTCGGGTCTAGTGGCGCACGGGTGC
GTAACGCGTGGGAATCTGCCCTTGGGTACGGAATAACAGCGAGAAATTGCTGCTAATACCGTATAATG
ACGTAAGTCCAAAGATTTATCGCCCAGGGATGAGCCCGCGTAGGATTAGCTTGTTGGTGAGGTAAAAG
CTCACCAAGGCGACGATCCTTAGCTGGTCTGAGAGGATGATCAGCCACACTGGGACTGAGACACGGCC
>Zotu7706
GAACGCTAGCGGCAGGCTTAACACATGCAAGTCGAGGGGTATATTTCTTCGGAGATAGAGACCGGCGC
ACGGGTGCGTAACGCGTATGCAATCTACCTTTTACAGGGGAATAGCCCAGAGAAATTTGGATTAATGC
CCCATAGTATATATGAATCGCATGGTTTATATATTAAAGTCACAACGGTAAAAGATGAGCATGCGTCC
CATTAGTTAGTTGGTAAGGTAACGGCTTACCAAGACAATGATGGGTAGGGGTCCTGAGAGGGAGATCC
CCCACACTGGTACTGAGACACGGAC
>Zotu7707
GAACGCTGGCGGCGTGCTTAACACATGCAAGTCGAACGGAAAGGCCCTTCGGGGTACTCGAGTGGCGA
ACGGGTGAGTAACACGTGGGTGATCTGCCCTGCACTTCGGGATAAGCTTGGGAAACTGGGTCTAATAC
CGGATAGGATCATGGCTCTCATGGGTTGTGGTGGAAAGCTTTTGCGGTGTGGGATGGGCCCGCGGCCT
ATCAGCTTGTTGGTGGGGTGATGGCCTACCAAGGCGACGACGGGTAGCCGGCCTGAGAGGGTGTCCGG
CCACACTGGGACTGAGATACGGCC
>Zotu7708
GAATGCTGGCGGCGTGGATAAGGCATGCAAGTCGAGTGGGTTTAGACCCACGGCAGACGAGGTAGTAA
CACGTAGGAACGTACCCCAAAGTCAGGGATATTCTGTCGAAAGACAGCGCAATACCTGATAGTCTCTA
AGGAGTAAAGATTTATCGCTTTGGGAGCGGCCTGCGTAATATCAGCTAGTTGGTGAGGTAATGGCTCA
CCAAGGCTATGACGTTTAGGGGAGCTGAGAGGCTGACCCCCACCGATGGAACTGAGATACGGTC
>Zotu7710
GAACGCTGGCGGCAGGCCTAACACATGCAAGTCGAGCGGTAGCACAAGGGAGCTTGCTCCCTGGGTGA
CGAGCGGCGGACGGGTGAGTAATGTCTGGGAAACTGCCTGATGGAGGGGGATAACTACTGGAAACGGT
AGCTAATACCGCATAACGTCGCAAGACCAAAGAGGGGGACCTTCGGGCCTCTTGCCATCGGATGTGCC
CAGATGGGATTAGCTAGTAGGTGGGGTAACGGCTCACCTAGGCGACGATCCCTAGCTGGTCTGAGAGG
ATGACCAGCCACACTGGAACTGAGACACGGTC
>Zotu7711
GAACGCTGGCGGCAGGCTTAACACATGCAAGTCGAACGATCCTTCGGGATAGTGGCGCACGGGTGAGT
AACGCGTGGGAATGTACCATATAGTGGGGAATAACGTTGGGAAACTAACGCTAATACCGCATACGCCC
TTCGGGGGAAAGATTTATCGCTATATGATCAGCCCGCGTGAGATTAGGTAGTTGGTGGGGTAATGGCC
TACCAAGCCTACGATCTCTAGCTGGTCTGAGAGGATGATCAGCCACACTGGAACTGAGACACGGTC
>Zotu7714
GAACGCTAGCGGCAGGCTTAACACATGCAAGTCGAGGGGTATAGTAGCAATACTAGAGACCGGCGCAC
GGGTGCGTAACGCGTATGCAATCTACCTTTAGCAGGGGCATAGCCCAGAGAAATTTGGATTAATTCCC
CATAGTATTATAGTATGGCATCATATTATAATTAAAGTAACAACGGCTAAAGATGAGCATGCGTCCCA
TTAGCTAGTTGGTATGGTAACGGCATACCAAGGCAATGATGGGTAGGGGTCCTGAGAGGGAAATCCCC
CACACTGGTACTGAGACACGGAC
>Zotu7717

GAACGCTAGCGGCAGGCCTAACACATGCAAGTCGAGGGGTAATATAGCAATATATGAGACCGGCGCAC
GGGTGCGTAACGCGTATGCAACCTACCTTTTACAGGGGAATAGCCCAGAGAAATTTGGATTAATGCCC
CATGGTGTATTTGAGTGGCATCACTTGATTACTAAAGTTCCAACGGTAAAAGATGGGCATGCGTCCTA
TTAGCTAGATGGTAAGGTAACGGCTTACCATGGCTACGATAGGTAGGGGTCCTGAGAGGGAGATCCCC
CACACTGGTACTGAGACACGGAC
>Zotu7720
GAACATTGGCGGAAAGCTTAACACATGCAAGTCGAGCGAGGCTTAGAGCCTAGCGGCGAACGGGTGCG
TAATACATAGGAATCTGCCCCGAAGTGGGGGACAACAACCAGAAATGGTTGCTAATACCCCATAATCC
ATATTGGGAAAGCGCGCAGCGCTTTGGGATGAGCCTATGGTCGATTAGCTAGTTGGTGGGGTAAAAGC
CTACCAAGGCGACGATCGATAGCTGTTCTGAGAGGATGATCAGCCACACTGGAACTGAGACACGGTC
>Zotu7721
GAACGCTGGCGGCGTGGATAAGACATGCAAGTCGAACGGGATCATTGGAGTAGCAATATTTTGATGGT
CTAGTGGCGAACGGGTGCGTAACACGTGGATACATTCCGGAAAGCGGGGGATAGCCCAGGGAAACTTG
GATTAATACCGCATGTGGTCGAGAGACTAAAGGCAGGGACCGCAAGGCCTGCCACTTTCCGATTGGTC
CGCGGCCTATCAGCTAGTTGGCGGGGTAAAAGCCCACCAAGGCTACGACGGGTAGCTGGTCTTAGAGG
ACGACCAGCCACACTGGAACTGAGACACGGTC
>Zotu7722
GAACGTTAGCGGCGCGCCTAACACATGCAAGTCGCACGAGAGGGGGCAACCCTGAAAGTGGCGCACGG
GTGCGTAACACGTAGATAACCTGCCCTTTGGAGGGGGATAACGTTCCGAAAGGAGCGCTAATACCGCA
TACAGCATTCTGGGGTTTGCTCTGGGATGGCAAAGGTAGGGACCCGCAAGGGCCTATCGCCGAAGGAG
GGGTCTGCGGCCCATCAGCTAGTTGGGGAGGTAATGGCTCACCAAGGCGAAGACGGGTAGCTGGTCTG
AGAGGATGATCAGCCACACTGGTACTGGAACACGGAC
>Zotu7723
GAACGTTGGCGGCGTGCCTAACACATGCAAGTTGAGCGTGGCCTTAGTTTACTAAGGTCTAGCAGCGG
ACGGCAGAGTAATGCACAGGAACGTACCTCGAGGTGGGGGATAGCCCGTCGAAAGACGGGGTAATACC
GCATATGTTCGCAAGAACAAAGCCGCAAGGCGCCATGAGAACGGCCTGTGTCCTATCAGGTAGTTGGT
GGGGTAAACGCCTACCAAGCCTATGACGGGTAGCTGGTCTGAGAGGATGATCAGCCTGATCGGGACTG
AGACACGGCC
>Zotu7724
GAACGCTGGCGGCGTGCTTAACACATGCAAGTCGAACGGTGAAAGAGAGCTTGCTCTCTGGATCAGTG
GCGAACGGGTGAGTAACACGTGAGCAATCTGCCCCTGACTCTGGGATAACAGCTGGAAACAGCTGCTA
ATACCGGATACGAGCTGCGAAGGCATCTTCAGCAGCTGGAAAGAATTTCGGTCAGGGATGAGCTCGCG
GCCTATCAGCTAGTTGGTGAGGTAATGGCTCACCAAGGCGTCGACGGGTAGCCGGCCTGAGAGGGTGA
CCGGCCACACTGGGACTGAGACACGGCC
>Zotu7726
GAACGCTGGCGGCGTGCCTAACACATGCAAGTCAACTTGAAGGAAAGAGCAACCTGACCTGATGGTGG
CGCAAGGGTGCGTAACGCGTAAGTAACTTGCCCCAAGGTAAGGAATAACGTCGAGAAATCGGCGCTAA
TACCTCATAATGCAGCGGATCCGCATGGATATGTTGTTAAAGGTTTACCGCCTTGGGATAGGCTTGCG
TCTGATTAGCTAGTTGGTGTGGTAACGGCGCACCAAGGCTACGATCAGTAACTAGTCTGAGAGGATGA
TCAGTCACACTGGAACTGAGACACGGTC
>Zotu7728
GAATGCTGGCGGCGTGGATAAGTCATGCAAGTCAAGGGGACCGCAAGGTAACCGGCAAACGAGGTAGT
AACACGTAGGTAGACCCCCTCTAGTCGAGCATAGCCAGCCGAAAGGTTGGGTAATTCTCGATAGTCCC
GCAAGGGTAAAGATTTATCGCTAGAGGAGTTGCCTGCGTAGTATCAGCTAGTTGGTAGGGTAATGGCC
TACCAAGGCTACGACGCTTAGGGGAGCTGAGAGGCTGACCCCCACCGATGGAACTGCGACACGGTC
>Zotu7729
GAACGCTAGCGGCAGGCTTAATACATGCAAGTCGAGGGGCAGCATAGGTAGCAATACTTGATGGCGAC
CGGCAAACGGGTGCGGAACACGTACGCAACTTTCCTTCAAATGGTGAATAGCCTCTCGAAAGAGAGAT
TAATACATCGTAACATATAGACAAGGCATCTTGTTTATATTATAGCTCCGGCGTTTGAAGATGGGCGT
GCGGCTGATTAGGTAGTTGGTAGGGTAACGGCCTACCAAGCCTGCGATCAGTAACTGGCGTGAGAGCG
CGACCAGTCACACGGGCACTGAGACACGGGC
>Zotu7730
GAACGCTAGCGGCAGGCTTAACACATGCAAGTCGAGGGGTATAGTTCTTCGGAATTAGAGACCGGCGC
ACGGGTGCGTAACGCGTATTCAATCTACCTTTCACAGAGGGATAGCCCAGAGAAATTTGGATTAATAC

CTCATAGTATTACGAGATGGCATCATTTTGTAATTAAAGTCACAACGGTGAAAGATGAGCATGCGTCC
CATTAGCTTGTTGGTAAGGTAACGGCTTACCAAGGCTACGATGGGTAGGGGTCCTGAGAGGGAGATCC
CCCACACTGGTACTGAGACACGGAC
>Zotu7731
TAACGCTAGCGGCAGGCATAATACATGCAAGTTGAACGGGATTAGTTGTAGTAATACGACTATGAGAG
TAGCGCACGGGTGCGTAACACGTATGCAATCTGCCCCTATCTGTGGAATAGCCCCTCGAAAGAGGGAT
TAATACCACATAATATAGTGAGTAGGCATCTACTTACTATCAAAGCTCCGGCGGATAGGGATGAGCAT
GCGGCTGATTAGGTAGTTGGTGAGGTAATGGCTCACCAAGCCGACGATCAGTAGGGGGTCTGAGAGGA
TGTCCCCCCACACTGGTACTGAGATACGGAC
>Zotu7732
GAACGCTAGCGGCATGCTTAACACATGCAAGTCGAACGGTAACAGGGAGCTTGCTCCGCTGACGAGTG
GCGAACGGGTGAGTAATATATCGGAACGTGCCCAAGAGTGGGGGATAACGTAGCGAAAGTTACGCTAA
TACCGCATACGATCCAAGGATGAAAGCAGGGGACCTTCGGGCCTTGTGCTCCTGGAGCGGCCGATATC
TGATTAGCTAGTTGGTGAGGTAAAGGCTCACCAAGGCGACGATCAGTAGCTGGTCTGAGAGGACGACC
AGCCACACTGGAACTGAGACACGGTC
>Zotu7733
GAACGCTGGCGGTGTGCCTAATACATGCAAGTCGAACGAGACCTTCGGGTCTAGTGGCGGACGGGTGA
GTAACACGTTGGTACCTACCCCGAAGTGGGGAATAACTGCCCGAAAGGGTGGCTAATACCGCATAATA
CCGCAAGGTTAAAGCAGTAATGCGCTTCGGGAGGGGCCTGCGTCCCATTAGCTTGTTGGTAGTGTAAT
GGACTACCAAGGCGACGATGGGTAGCTGGTTCGAGAGGATGACCAGCCACACTGGGACTGAGATACGG
CC
>Zotu7736
GAACGCTAGCGGCAGGCTTAATACATGCAAGTCGAGGGGCAGCACGGGAGCAATCCTGGTGGCGACCG
GCAAACGGGTGCGGAACACGTACGCAACCTTCCCATAACTGGTGAATAGCCTTTCGAAAGAGAGATTA
ATACACCGTAACATAACGAAGAGGCATCTCTTTGTTATTATAGCCGCAAGGCGGTTTTGGATGGGCGT
GCGCCTGATTAGGTATTTGGCGGGGTAACGGCCCACCAAGCCTGCGATCAGTAACTGGTGTGAGAGCA
CGACCAGTCACACGGGCACTGAGACACGGGC
>Zotu7737
GAACGCTGGCGGCGTGCCTAAGACATGCAAGTCGAGTGGGTTTAGACCCACGGCGAACGGGTGAGTAA
TACCTAGGAACTTTCTTTAAGTGGAGCATAACTAGTCGAAAGATTAGATAATTCTCCATGTGACCGCA
AGGTTAAAGTCGCAAGACGCTTAAAGTGAGGCCTGGGCACCATCAGCTAGTTGGTAAGGTAATGGCTT
ACCAAGGCTATGACGGTTAGGGGTCCCGAGAGGGATAACCCCCACACTGGAACTGCGACACGGTC
>Zotu7739
GAACGCTGGCGGCGCGCCTAACACATGCAAGTCGAACGGCACCTATCTTCGGATAGAAGCGAGTTGCG
AACGGCTGAGTAACACGTGGAGAACCTGCCCCCTCCCCCGGGATAGCCGCCCGAAAGGACGGGTAATA
CCGGATACCCCGGGGTGCCGCATGGCACCCCGGCTAAAGCCCCGACGGGAGGGGATGGCTCCGCGGCC
CATCAGGTAGACGGCGGGGTGACGGCCCACCGTGCCGACAACGGGTAGCCGGGTTGAGAGACCGACCG
GCCAGATTGGGACTGAGACACGGCC
>Zotu7741
CAACGCTGGCGGCGTGCCTCAGACATGCAAGTCGAACGATTAAAGCTCTCTTCGGAGAGTGCATAAAG
TGGCGCACGGGTGAGTAACACGTAAGTAATCTACCTTCGAGTGGGGAATAACAGTCGGAAACGACTGC
TAATACCGCATAATGCAGCGGCATCTATGATGACAGTTGTTAAAGATTTATCGCTTGAAGAGGAGCTT
GCGGCAGATTAGCTAGTTGGTAAGGTAACGGCTTACCAAGGCGACGATCTGTATCCGACCTGAGAGGG
TGGTCGGACACACTGACACTGAATAACGGGT
>Zotu7742
TAACGTTGGCGGCGTGCATAACACATGCAAGTTGAGCGTGGCTTTAGTTTACTAAAGTCTAGCAGCGG
ACGGCTGAGTAATGCACAGGAATGTACCTCGAAGTGGGGGATAGCCCATCGAAAGATGGGGTAATACC
GCATGTGATCGCAAGATCAAAGCCGCAAGGCGCTTTGAGAGCAGCCTGTGTCCTATCAGATTGTTGGT
GGGGTAATGGCCTACCAAGTCTATGACGGGTAGCTGGTCTGAGAGGATGACCAGCCAGATCGGGACTG
AGACACGGCC
>Zotu7748
GAACGCTGGCGGCAGGCCTAACACATGCAAGTCGAGCGGTAGCACAAGGGAGCTTGCTCCCTGGGTGA
CGAGCGGCGGACGGGTGAGTAATGTCTGGGAAACTGCCTGATGGAGGGGGATAACTACTGGAAACGGT
AGCTAATACCGCATAACGTCGCAAGACCAAAGAGGGGGACCTTCGGGCCTCTTGCCATCAGATGTGCC

CAGATGGGATTAGCTAGTAGGTGGGGTAACGGCTCACCTAGGCGACGATCCCTAGCTGGTCTGAGAGG
ATGACCAGCCACACTGGAACTGAGACACGGTC
>Zotu7749
GAACGCTGGCGGCGTGCCTAACACATGCAAGTCGAGCGGCAGCGCACCCTTCGGGGTGGCGGCGAGCG
GCGGACGGCTGAGTAACACGTGGGAATGTACCCCAAAGTGAGGAATAACTGCCCGAAAGGGTAGCTAA
TGCCGCATAAGCTCTTCGGAGTAAAGGCTTCGGCCGCTTTGGGAACAGCCCGCGTACGATTAGATAGT
TGGTGAGGTAATGGCTCACCAAGTCGACGATCGTTAGCTGGTCTGAGAGGATGACCAGCCAGACTGGA
ACTGAGACACGGTC
>Zotu7750
GAACGCTGGCGGCGTGCTTAACACATGCAAGTCTACGAGAATAAGGTAGCAATATCTTTAGTAAAGTG
GCGCACGGGTGCGTAACACGTAAGCAATCTACCTTTGGGTCTGGAATAACTTCGAGAAATCGAGGCTA
ATACCGGATAATGCAGCGGCACCGCATGGTGATGTTGTTAAAGTTTAAGTAATGCCTATAGATGAGCT
TGCGCCTGATTAGCTAGTTGGTAGGGTAAAGGCCTACCAAGGCTACGATCAGTAGCTGGTCTGAGGGG
ATGATCAGCCACACTGGAACTGAGACACGGTC
>Zotu7751
GAACGCTGGCGGCATGCTTTACACATGCAAGTCGAACGGTAACAGGGAGCTTGCTCCGCTGACGAGTG
GCGAACGGGTGAGTAATATATCGGAACGTGCCCAAGAGTGGGGGATAACGTAGCGAAAGTTACGCTAA
TACCGCATACGATCCAAGGATGAAAGCAGGGGACCTTCGGGCCTTGTGCTCCTGGAGCGGCCGATATC
TGATTAGCTAGTTGGTGAGGTAAAGGCTCACCAAGGCGACGATCAGTAGCTGGTCTGAGAGGATGATC
AGCCACACTGGGACTGAGACACGGCC
>Zotu7752
TAACGCTAGCGGCAGGCCTAATACATGCAAGTCGAACGGTAACAGGTCCAGCAATGGATGCTGACGAG
TGGCGCACGGGTGCGTAACACGTATGCAATCTACCCTTAACTGGGGGATAGCCCCGAGAAATCGGGAT
TAATACCCCATAACATTATGAAGTGGCATCATTTTATAATTAAAGCTCCGGCGGTTTTGGATGAGCAT
GCGCGACATTAGCTAGTTGGTGAGGTAACGGCTCACCAAGGCTGCGATGTCTAGGGGTTCTGAGAGGA
TTCACCCCCACACTGGTACTGAGACACGGAC
>Zotu7753
GAACGCTGGCGGCGTGCTTAACACATGCAAGTCGAACGGTGATCTTGAGAGCTTGCTCTTGGGTGATC
AGTGGCGAACGGGTGAGTAACACGTGAGGAACCTGCCCCAGACTCTGGAATAACCCCGGGAAACCGGA
GCTAATACCGGATATGACACCAGATCGCATGGTCATGGTGTGGAAAGTTTTTCGGTCTGGGATGGCCT
CGCGGCCTATCAGCTTGTTGGTGAGGTAGTGGCTCACCAAGGCGACGACGGGTAGCCGGCCTGAGAGG
GCGACCGGCCACACTGGGACTGAGACACGGCC
>Zotu7754
GAACGCTGGCGGCAGGCTTAACACATGCAAGTCGAACGCCCCGCAAGGGGAGTGGCAGACGGGTGAGT
AACACGTGGGAACGTGCCCAAAGGTTCGGAACAACTCCGGGAAACTGGAGCTAATACCGAATGTGTCC
GTAAGGAGAAAGATTTATCGCCTTTGGATCGGCCCGCGTCAGATTAGCTAGTTGGTGGGGTAATGGCC
TACCAAGGCGACGATCTGTAGCTGTTCTGAGAGGAAGATCAGCCACACTGGGACTGAGACACGGCC
>Zotu7755
GAACGCTGGCGGCGTGCTTAACACATGCAAGTCGAACGGTAAGGCCCTTTCGGGGGTACACGAGTGGC
GAACGGGTGAGTAACACGTGAGTAACCTGCCCCTAACTCTGGGATAACTCCTGGAAACAGGTGCTAAT
ACCGGATATTTAGCTGGGACTGCATGGTCCTGGTTGGAAAGCTCCGGCGGTTAGGGATGGACTCGCGG
CCTATCAGCTTGTTGGTGAGGTAATGGCTCACCAAGGCTTCGACGGGTAGCCGGCCTGAGAGGGTGAC
CGGCCACATTGGGACTGAGATACGGCC
>Zotu7756
GAACGCTGGCGGCGTGCTTAACACATGCAAGTCGAACGGGGTCCAAGCGTTAGCAATAACGTGGAAGA
CCTAGTGGCGAACGGGTGAGTAACACGTGAGGAACCTACCCCGGACTCAGGGATAACGGCTGGAAACG
GCTGCTAATACCTGATGCCGTCGGAGCATCACATGGTGCACTGACGAAAGAATCTCGGTTCGGGAGGG
TCTCGCGGCCTATCAGCTAGTTGGTGGGGTAACGGCCTACCAAGGCATCGACGGGTAGCTGGTCTGAG
AGGATGATCAGCCACACTGGGACTGAGACACGGCC
>Zotu7757
GAACGCTGGCGGCGTGCCTAATACATGCAAGTCGAGCGAATCTTTGGGAGCTTGCTCCCATTGGTTAG
CGGCGGACGGGTGAGTAACACGTGGGCAACCTGCCTGTAAGACTGGGATAACTTCGGGAAACCGGAGC
TAATACCGGATAATCCATTTCCTCTCATGGGGAAATGCTGAAAGACGGTTTCGGCTGTCACTTACAGA
TGGGCCCGCGGCGCATTAGCTAGTTGGTGAGGTAACGGCTCACCAAGGCGACGATGCGTAGCCGACCT

GAGAGGGTGATCGGCCACACTGGGACTGAGACACGGCC
>Zotu7758
GAACGCTGGCGGCGTGCTTAATACATGCAAGTCGAGCGAAGCACTTTAACGGAATTCTTCGGAAGGAA
GATAAAGTGACTGAGCGGCGGACGGGTGAGTAACGCGTGGGTAACCTGCCTTATACAGGGGGATAACA
GTTAGAAATGGCTGCTAATACCGCATAAGCGCACAGGACCGCATGGTCTGGTGTGAAAAACTCCGGTG
GTATAAGATGGACCCGCGTCTGATTAGCTAGTTGGTGGGGTAACGGCTCACCAAGGCGACGATCAGTA
GCCGGCCTGAGAGGGTGAACGGCCACATTGGGACTGAGACACGGCC
>Zotu7759
GAACGCTGGCGGCGCGCCTAACACATGCAAGTCGAACGCGAAAGGGAGCAATCCTGAGTAAAGTGGCG
CACGGGTGAGTAACGCGTAGGTAATCTACCTTTGCGACCGGGATAACCAGCCGAAAGGTTGGCTAATA
CTGGATAGAGCGCACGATCCATAAAGGATTGTGCGGGAAAGGGTGGCGTAGCAATACGCTACTGCGCT
GAGATGAGCCTGCGTCTCATTAGCTAGTTGGTAGGGTAAAGGCCTACCAAGGCGATGATGGGTAGCTG
GTCTGAGAGGACGATCAGCCACACTGGAACTGAAACACGGTC
>Zotu7760
GAACGCTGGCGGCAGGCTTAACACATGCAAGTCGAGCGGAGAGAGGTAGCTTGCTACTGATCTTAGCG
GCGGACGGGTGAGTAATGCTTAGGAATCTGCCTATTAGTGGGGGACAACATTTCGAAAGGAATGCTAA
TACCGCATACGTCCTACGGGAGAAAGCAGGGAATCTTCGGACCTTGCGCTAATAGATGAGCCTAAGTC
GGATTAGCTAGTTGGTAGGGTAAAGGCCTACCAAGGCGACGATCTGTAGCGGGTCTGAGAGGATGATC
CGCCACACTGGGACTGAGACACGGCC
>Zotu7762
GAACGCTGGCGGCATGCCTAACACATGCAAGTCGAACGCCATGGTTTCGGCCATGGAGTGGCGCACGG
GTGAGTAACGCGTGGGAACCTGCCTAGCAGTATGGGACAACCGCGGGAAACTGCGGCTAATACCGTAT
ACGCCCGTCTTTGGGAGACCAGGGGCGGGGAAAGATTGATCGCTGTTAGAGGGGCCCGCGTCGGATTA
GGTAGTTGGTGGGGTAAAGGCCTACCAAGCCTGCGATCCGTAGCTGGTCTGAGAGGATGATCAGCCAC
ACTGGGACTGAGACACGGCC
>Zotu7763
GAACGCTGGCGGCAGGCTTAACACATGCAAGTCGAGCGCATCCTTCGGGGTGAGCGGCAGACGGGTGA
GTAACGCGTGGGAATCTACCTTTTGCTACGGAATAGCTCCGGGAAACTGGAATTAATACCGTATGTGC
CCTTTGACGGTGGACGTTGGAGGGGAAAGATTTATCGGCAAAGGATGAGCCCGCGTTGGATTAGCTAG
TTGGTGGGGTAAAGGCCTACCAAGGCGACGATCCATAGCTGGTCTGAGAGGATGATCAGCCACATTGG
GACTGAGACACGGCC
>Zotu7764
GAACGCTGGCGGCATGCTTAACACATGCAAGTCGAACGGTAACAGGCCCTTCGGGGTGCTGACGAGTG
GCGGACGGGTGAGTAAAGCGTAGGAATCTATCTAGTGGAGTGGGATAACCTAGGGAAACTTAGGGTAA
TACCGCATAAGTTCTAAGGAATAAAGCAGGGGACCTTCGGGCCTTGCGCTATTAGCTGAGCCTACGTC
GGATTAGCTAGTTGGTGGGGTAAAGGCCTACCAAGGCGATGATCCGTAGCTGGTTTGAGAGGACGACC
AGCCACACTGGGACTGAGACACGGCC
>Zotu7769
GAACGCTAGCTACAGGCTTAACACATGCAAGTCGAGGGGAAACGATATTGGAAGCTTGCTTCCGATAG
GCGTCGACCGGCGCACGGGTGAGTAACGCGTATCCAACCTGCCCACCACTTGGGGATAACCTTGCGAA
AGTAAGACTAATACCCAATGACGTCTCTAGAAGACATCTGAAAGAGATTAAAGATTCATCGGTGATGG
ATGGGGATGCGTCTGATTAGCTTGTTGGCGGGGTAACGGCCCACCAAGGCGACGATCAGTAGGGGTTC
TGAGAGGAAGGTCCCCCACATTGGAACTGAGACACGGTC
>Zotu7770
GAACGCTGGCGGAATGCTTTACACATGCAAGTCGAACGGCAGCACGGGGGCAACCCTGGTGGCGAGTG
GCGAACGGGTGAGTAATACATCGGAACGTGCCCAGTCGTGGGGGATAACGTAGCGAAAGCTACGCTAA
TACCGCATACGAACTCTGGTTGAAAGCGGGGGATCGCAAGACCTCGCGCGATTGGAGCGGCCGATGGC
AGATTAGGTAGTTGGTGGGGTAAAGGCTCACCAAGCCGACGATCTGTAGCTGGTCTGAGAGGACGACC
AGCCACACTGGGACTGAGACACGGCC
>Zotu7772
GAACGCTAGCGGCAGGCTTAATACATGCAAGTCGAACGGTAACAGGTGTAGCAATACATGCTGACGAG
TGGCAGACGGGTGAGGAACACGTACACAACCTCCCTCTTACTGGGGCACAACTCCGAGAAATCGGGGC
TAATTCCCCATAACATTTCGAAAGGGCATCCAATTGAAATTAAAACTCCGGTGGTAAGAGATGGGTGT
GCGTCTGATTAGCTAGTTGGTAGGGTAACGGCCTACCAAGGCAACGATCAGTAACTGGTGTGAGAGCA

CGACCAGTCACACGGGCACTGAGATACGGGC
>Zotu7774
CAACGCTGGCGGCGCGCTTAACACATGCAAGTCGCACGAGAAAGGGGGCAACCCTGAGTACAGTGGCG
TACGGGTGAGTAACACGTGGATTATCTACCTTTTAGTGGGGAATACCCCTGGGAAACCGGGGACAATA
CCGCATAAGCCCGCAAGGGGAAAGCAGCAATGCGCTGAAAGAGGAGTCCGCGGCTGATTAGCTAGTTG
GCGGGGTAACGGCCCACCAAGGCGAAGATCGGTAGCCGGCCTGAGAGGGCACACGGCCACACTGGCAC
TGAAATACGGGC
>Zotu7775
GAACGCTGGCGGCATGCCTTACACATGCAAGTCGAACGGCAGCACGGGAGCAATCCTGGTGGCGAGTG
GCGAACGGGTGAGTAATATATCGGAACGTGCCCAGTCGTGGGGGATAACGTAGCGAAAGCTACGCTAA
TACCGCATACGATCTACGGATGAAAGCGGGGGACTCGTAAGGGCCTCGCGCGATTGGAGCGGCCGATA
TCAGATTAGGTTGTTGGTGAGGTAAAAGCTCACCAAGCCTGCGATCTGTAGCTGGTCTGAGAGGACGA
CCAGCCACACTGGAACTGAGACACGGTC
>Zotu7776
GAACGCTGGCGGCGTGCTTAACACATTCAAGTCGAACGGTGACGAGAGACTTCGGTCTCTCCGATCAG
TGGCGGACGGGTGAGTAATGCATGAGCAACCTGCCTCGAAGTGGGGAATAACACAGAGAAATTTGTGC
TAATACCGCATAATGTGTTCGGGAGGCATCTCCTGAACAAGAAAGGATTTATTCGCTTTGAGATGGGC
TCATGTCTGATTAGGTAGTTGGTGGGGTAACGGCCTACCAAGCCTGCGATCAGTAGCCGGACTGAGAG
GTTGAACGGCCACATTGGGACTGAGATACGGCC
>Zotu7780
GAACGCTGGCGGTATGCTTTACACATGCAAGTCGTACGAGAGTTTTTAACTCAAGTGGCGGACGGGTG
AGTAACACGTAAGAATTTGCCTTTAGGAGGGGGATAACAACTGGAAACGGTTGCTAATACCCCATATG
CTTTCGAGTGAAATGGATTTTTCCGCCTAAAGAGAAGCTTGCGGCTGATTAGCTTGTTGGTAAGGTAA
TGGCTTACCAAGGCGACGATCAGTATCTGGTTTGAGAGGACGATCAGACACACTGGAACTGAGACACG
GTC
>Zotu7781
GAACGCTGGCGGCAGGCTTAACACATGCAAGTCGAGCGCCCCGCAAGGGGAGCGGCAGACGGGTGAGT
AACGCGTGGGAATCTACCCATCTCTACGGAATAACTCAGGGAAACTTGTGCTAATACCGTATACGCCC
TTTGGGGGAAAGATTTATCGGAGATGGATGAGCCCGCGTTGGATTAGCTAGTTGGTGGGGTAAAGGCC
TACCAAGGCGACGATCCATAGCTGGTCTGAGAGGATGATCAGCCACACTGGGACTGAGACACGGCC
>Zotu7782
GAACGCTGGCGGCGTGCTTAACACATGCAAGTCGAACGGTGAAGCAGAGCTTGCTCTGTGGATCAGTG
GCGAACGGGTGAGTAACACGTGAGTAACCTGCCCTTGACTCTGGGATAAGCGTTGGAAACGACGTCTA
ATACCGGATACGAGCTGAGACCGCATGGTCATCAGTTGGAAAGATTTTTTGGTCAAGGATGGACTCGC
GGCCTATCAGCTTGTTGGTGAGGTAATGGCTCACCAAGGCGACGACGGGTAGCCGGCCTGAGAGGGTG
ACCGGCCACACTGGGACTGAGACACGGCC
>Zotu7783
GAACGTTAGCGGCGCGCCTAAGACATGCAAGTCGAGCGAGAAAGGGCTTCGGCCCCGGTAAAGCGGCG
CACGGGTGAGTAACACGTAGGTAATCAACCCTCGAGTGGTGGACAACTCTCCGAAAGGAGAGCTAATA
CAGCATGATGGCGCGACTTTACGAAGTTGTGCCTAAAGCGGGCCTCTCCACGGAAGCTCGCGCTTGAG
GATGAGCCTGCGGCCCATCAGCTAGTTGGTTGGGGTAATGGCCCACCAAGGCTACGACGGGTAGCTGG
TCTGAGAGGATGAACAGCCACACTGGAACTGAGACACGGTC
>Zotu7785
GAACGCTAGCGGCAGGCTTAACACATGCAAGTCGAGGGGTAGTATAGCAATATATGAGACCGGCGCAC
GGGTGCGTAACGCGTATGCAATCTACCTTTTACAGAGGGATAGCCCAGAGAAATTTGGATTAATACCT
CATAGTATATTTGAGTGGCATCACTTGATTATTAAAGTTCCAACGGTAAAAGATGAGCATGCGTCCTA
TTAGCTAGATGGTAAGGTAACGGCTTACCATGGCTACGATAGGTAGGGGTCCTGAGAGGGAGATCCCC
CACACTGGTACTGAGACACGGAC
>Zotu7789
GAACGCTGGCGGCGTGCTTAACACATGCAAGTCGAACGGGCTTTTCGGAGCTAGTGGCGGACGGGTGA
GTAATACATAGGTAATCTGCCTTTAAGTGGGGGACAACCAGGCGAAAGTTTGGCTAATACCGCGTAAT
GTCGTTGGTTGAGATACGAACGAAGAAAGATTTATTGCTTTTAGAGGAGCCTATGTTTGATTAGCTAG
TTGGTGTGGTAAAGGCTCACCAAGGCGACGATCAATAGCTGGTCTGAGAGGACGATCAGCCACACTGG
GACTGAGACACGGCC

>Zotu7791
GAACGCTGGCGGCGCGTCTTAAGCATGCAAGTCGGACGGTAGAGTAGCAATACTTTAGAGTGGCGAAC
GGGTGAGTAACACGTAAATGATCTGCCCCCTGCACTGGGATAGCCTATGGAAACATAGGGTAAAACCG
GATGAGCTGGTATATTTGGAAATTTATCAGGAAAATGGCTACGGCCATGACAGGGGATGAGTTTGCGG
CTGATTAGCTAGTTGGTGAGGTAAAGGCCCACCAAGGCGACGATCAGTAGCCGGCCTGAGAGGGTGAT
CGGCCACATTGGGACTGAGACACGGCC
>Zotu7792
GAACGCTGGCGGCATGCCTAACACATGCAAGTCGAACGATGCCTTCGGGCATAGTGGCGCACGGGTGC
GTAACGCGTGGGAATCTGCCCTTGGGTTCGGAATAACTCGCCGAAAGGCGTGCTAATACCGGATAATG
ACGTAAGTCCAAAGATTTATCGCCCAAGGATGAGCCCGCGTAGGATTAGCTAGTTGGTGGGGTAAAGG
CCCACCAAGGCGACGATCCTTAGCTGGTCTGAGAGGATGATCAGCCACACTGGGACTGAGACACGGCC
>Zotu7793
GAACGCTGGCGGCATGCCTAATACATGCAAGTCGAGCGAATGATGAAGAAGCTTGCTTCTTCTGATTT
AGCGGCGGACGGGTGAGTAACACGTGGGCAACCTACCCTATAGATTGGGATAACACAGGGAAACCTGT
GCTAATACCGAATAATACATTTTCTTTCATGAGAAGATGTTGAAAGATGGTCTCGGCTATCACTATAG
GATGGGCCCGCGGCGCATTAGCTAGTTGGTGAGGTAATGGCTCACCAAGGCAACGATGCGTAGCCGAC
CTGAGAGGGTGATCGGCCACACTGGGACTGAGACACGGCC
>Zotu7795
GAACGCTGGCGGCGTGCTTAAGACATGCAAGTCGAACGCACCTCTTCGGAGGTGAGTGGCGCACGGGT
GAGTAACGCGTAACTGACGTACCTCGAAGTCTGGGATAACGTCCCGAAAGGGACGCTAATACCGGATG
TGCAGTCATCTTTTGGGATGCCTGTAAAGCCTTGAGTGCTTCGAGCTCGGGTTGCGTTCCATCAGCTA
GTAGGTAGGGTAAAGGCCTACCTAGGCAACGACGGATAGCCGGCCTGAGAGGGTGGCCGGCCACAGGG
GCACTGAGACACGGGC
>Zotu7796
GAACGCTAGCGGCAGGCTTAATACATGCAAGTCGAGGGGCAGCACAGGTAGCAATACTGGGTGGCGAC
CGGCAAACGGGTGCGGAACACGTACACAACCTTCCTTTAAGTGGGGGATAGCCCAGAGAAATTTGGAT
TAATACCCCGTAATATTGTTGAGTGGCATCACTTAATAATTAAAGCTCCGGCGCTTAGAGATGGGTGT
GCGTCTGATTAGGTAGTTGGTAGGGGTAACGGCCCACCAAGCCAGCGATCAGTAACTGGTGTGAGAGC
ACGACCAGTCACACGGGCACTGAGACACGGGC
>Zotu7798
GAACATTGGCGGAAAGCTTAACACATGCAAGTCGAGCGAGGCTTAGAGCCTAGCGGCGAACGGGTGCG
TAATACATAGGAATCTGCCCCGAAGTGGGGGACAACAACCAGAAATGGTTGCTAATACCCCATAATCC
ATATTGGGAAAGCGCGCAGCGCTTTGGGATGAGCCTATGGTCGATTAGCTAGTTGGTGGGGTAAAAGC
CTACCAAGGCGACGATCGATAGCTGGTCTGAGAGGATGATCAGCCACACTGGAACTGAGACACGGTC
>Zotu7799
GAACGCTAGCGGCATGCCTAACACATGCAAGTCGAGCGGATGAGAGGAGCTTGCTCCTGGATTCAGCG
GCGGACGGGTGAGTAATGCCTAGGAATCTGCCTGGTAGTGGGGGACAACGTTTCGAAAGGAACGCTAA
TACCGCATACGTCCTACGGGAGAAAGCAGGGGACCTTCGGGCCTTGCGCTATCAGATGAGCCTAGGTC
GGATTAGCTAGTTGGTAGGGTAATGGCCTACCAAGGCGACGATCCGTAGCTGGTCTGAGAGGACGATC
AGCCACACTGGGACTGAGACACGGCC
>Zotu7803
GAACGCTGGCGGCAGGCCTAACACATGCAAGTCGAGCGCGCCCTTTCGGGGGTGAGCGGCGGACGGGT
GAGTAACGCGTGGGAATATGCCCTTCTCTACGGAATAGTCCTGGGAAACTGGGGGTAATACCGTATAC
GCCCTTTGGGGGAAAGATTTATCGGGGAAGGATTAGCCCGCGTTGGATTAGGTAGTTGGTGGGGTAAT
GGCCCACCAAGCCGACGATCCATAGCTGGTTTGAGAGGATGATCAGCCACACTGGGACTGAGACACGG
CC
>Zotu7804
GAACGCTGGCGGCGTGCTTAACACATGCAAGTCGAACGGTGATGCAGAGCTTGCTCTGCGGATCAGTG
GCGAACGGGTGAGTAACACGTGAGTAACCTGCCCTTGACTCTGGGATAAGCGTTGGAAACGACGTCTA
ATACCGGATACGACCCGTGGAGGCATCTCCAACGGGTGGAAAGAATTTTGGTTGAGGATGGACTCGCG
GCCTATCAGCTTGTTGGTGAGGTAATGGCTCACCAAGGCGACGACGGGTAGCCGGCCTGAGAGGGTGA
CCGGCCACACTGGGACTGAGACACGGCC
>Zotu7805
GAACGCTGGCGGCGTGCTTAATACATGCAAGTAGAACGCTGAAGGAATAGCTTGCTATTCTGGAAGAG

TTGCGAACGGGTGAGTAACGCGTAGGTAACCTACCTCATAGCGGGGGATAACTATTGGAAACGATAGC
TAATACCGCATAAGAATACTTATTGCATGATAAGTGTTTAAAAGGAACAACTGTTTCACTGTGAGATG
GACCTGCGTTGTATTAGCTAGTTGGTAAGGTAACGGCTTACCAAGGCGACGATACATAGCCGACCTGA
GAGGGTGATCGGCCACACTGGGACTGAGACACGGCC
>Zotu7807
GAACGCTGGCGGTATGCCTTATACATGCAAGTCGTACGGGAGTTTTTAACTCAAGTGGCGGACGGGTG
AGTAACACGTAAGAATTTGCCTTCAGGAGGGGGATAACAACTGGAAACGGTTGCTAATACCCCATATG
CTTTAGAGTGAAACGGTTTAAACTCCGCCTGAAGAGAAGCTTGCGGCTGATTAGCTTGTTGGTGAGGT
AATGGCTCACCAAGGCGACGATCAGTATCTGGTTTGAGAGGACGATCAGACACACTGGAACTGAGACA
CGGTC
>Zotu7808
GAACGCTGGCGGCGTGCCTAATACATGCAAGTCGAGCGAATGGATGGGAGCTTGCTCCCAGAAGTTAG
CGGCGGACGGGTGAGTAACACGTGGGCAACCTACCTACAAGATTGGGATAACTTCGGGAAACCGGAGC
TAATACCGGGTAATACATTGGATCGCATGGTCCGATGTTGAAAGATGGCTTCGGCTATCACTTGTAGA
TGGGCCCGCGACGCATTAGCTAGTTGGTGAGGTAACGGCTCACCAAGGCCACGATGCGTAGCCGACCT
GAGAGGGTGATCGGCCACACTGGGACTGAGACACGGCC
>Zotu7810
TAAAACATCATCAACATCTGCGGAAGTCGCAGCTACTAAACAAGATATACAACACGTTTCGAAAGGGC
GGCCACAACATTGGACTATTTTTA
>Zotu7811
GAACGCTGGCGGCGTGCCTAATACATGCAAGTCGATCGGAATGTAGTAATACATTAAGAGGCGAACGG
GTGAGTAACACGTAGGTAATCTGCCCCAAGGACTGGGATACCCAAGCGAAAGTTTGGCTAATACCGGA
TATGAGGAAACTTGAAAGCAGCGTTAGCTGCACCATGGGATGAGCCTGCGGCGCATTAGCTAGTTGGT
GAGATAAGAGCCCACCAAGGCAATGATGCGTAGCCGACCTGAGAGGGTGAACGGCCACATTGGAACTG
AGACACGGTC
>Zotu7814
GAACGCTGGCGGCGTGCCTAACACATGCAAGTCGAACGGGACCAGGGGCAACTCTGGTTCAGTGGCGG
ACGGGTGCGTAACACGTGAGGAACATGACTTTCGGTGGGGGATAGCCGGCCCAACGGCCGGGTAATAC
CGCGTACGACCTTTTGGGGACATCCCTGATTGGTGAAAGCAGCAATGCGCCGAAAGAGTGCCTCGCGG
CCTATCAGCTAGTTGGTGGGGTAACGGCTCACCAAGGCGACGACGGGTAGCTGGTCTGAGAGGATGGC
CAGCCACATTGGGACTGAGACACGGCC
>Zotu7816
TAACGCTGGCGGCGTGCCTAACACATGCAAGTCGAACGAGGTAGCAATACCTAGTGGCAGACGGGTGA
GTAACGCGTAGATAATGTGCCCTCTTGCGGGGGATACCGTTTCGAAAGGAACGTTAATACCGCATAAC
ACGGCTTAGGGGCCTCCCTAGGCCGTCAAACTTCCGGGGCGGGAGGAGCAGTCTGCGTTCTATCAGCT
AGTTGGTAAGGTAATGGCTTACCAAGGCTACGACGGATAACTGGTCTGAGAGGATGATCAGTCACACT
GGAACTGAGACACGGTC
>Zotu7817
GAACGCTGGCGGCAGGCCTAATACATGCAAGTCGGACGAGTAAGAGCCCGCAAGGGAGTTCTGCTAGT
GGCAAACGGGTGCGGAACACGTGGGAATCTACCCAAAAGTATGGAACAACAGTTGGAAACGACTGCTA
ATACCATATACGCTCGAAAGAGGAAAGATTTATCGCTTTTGGATGAGCCCGCGTAAGATTAGCTAGTT
GGTGAGGTAATGGCTCACCAAGGCAACGATCTTTAGCTGGTCTGAGAGGACGATCAGCCACACTGGGA
CTGAGATACGGCC
>Zotu7818
GAACGCTGGCGGCATGCCTTACACATGCAAGTCGAACGGTAACAGGTTAAGCTGACGAGTGGCGAACG
GGTGAGTAATGTATCGGAACGTGCCCAGTCGTGGGGGATAACTGCTCGAAAGAGCAGCTAATACCGCA
TACGACCTGAGGGTGAAAGCGGGGGATCGCAAGACCTCGCGCGATTGGAGCGGCCGATATCAGATTAG
CTAGTTGGTGGGGTAAGAGCCCACCAAGGCGACGATCTGTAGCTGGTCTGAGAGGACGACCAGCCACA
CTGGGACTGAGACACGGCC
>Zotu7819
GAACGCTGGCGGCGTGCTTAACACATGCAAGTCGAACGGTGACCTCGAGAGCTTGCTCTTGGGTGAAC
AGTGGCGAACGGGTGAGTAACACGTGAGTAACCTGCCCCTGACTCTGGAATAAGCGCTGGAAACGGCG
TCTAATACCGGATATGACACACCCTCGCATGGGGAGTGTGTGGAAAGTTTTTCGGTCTGGGATGGACT
CGCGGCCTATCAGCTTGTTGGTGAGGTAATGGCTCACCAAGGCGATGACGGGTAGCCGGCCTGAGAGG

GCGACCGGCCACACTGGGACTGAGACACGGCC
>Zotu7821
GAACGCTGGCGGCGTGGATAAGACATGCAAGTCGAACGGGATATGTTTTGTAGCAATACAAAATATGT
CTAGTGGCGCAAGGGTGCTTAACACGTGGGCAATCTGCCGAGATGTGGGGGATAGCTCGCCGAAAGGC
GAATTAATACCGCATGTGGCCTGTGAAGACATCTTCACTCCGCCAAAGCCGGGGCAACCTGGCGCATC
TTGATGAGCCCGCGGCCTATCAGCTAGTTGGTGAGGTAATGGCTCACCAAGGCTATGACGGGTAGCTG
GTCTGAGAGGACGACCAGCCACACTGGAACTGAGACACGGTC
>Zotu7822
GAACGCTGGCGGCGTGCTTAACACATGCAAGTCGAGCGGTAAGGCCCTTCGGGGTACACGAGCGGCGA
ACGGGTGAGTAACACGTGAGCAATCTGCCCTTCACTTTGGGATAACCCCCGGAAACGGGAGCTAATAC
CGAATATGACCCTGCCATGCATGTGGTGGGGTGGAAAGTTCTGGCGGTGGAGGATGAGCTCGCGGCCT
ATCAGCTTGTTGGTGGGGTAATGGCCTACCAAGGCTTCGACGGGTAGCCGGCCTGAGAGGGTGACCGG
CCACACTGGGACTGAGACACGACC
>Zotu7826
GAACGCTAGCGGCAGGCCTAATACATGCAAGTCGAACGGTCCTAGCAATAGGATAGTGGCGCACGGGT
GCGTAACGCGTAACCAACCTACCTGGATCTGGGGGATAGCCCGCCGAAAGGCGGATTAATACCGCATA
AGCTAAATGACCGGCATCGGTTGTTTCGTAAAGATTTATTGGATTCAGATGGGGTTGCGTAGCATTAG
CTAGATGGCGGGGTAACGGCCCACCATGGCGACGATGCTTAGGGGACCTGAGAGGGTGATCCCCCACA
CTGGCACTGAGATACGGGC
>Zotu7827
GAACGCTGGCGGCAGGCTTAACACATGCAAGTCGAGCGCCCCGCAAGGGGAGCGGCAGACGGGTGAGT
AACGCGTGGGAATCTACCCATCTCTACGGAACAACTCCGGGAAACTGGAGCTAATACCGTATACGTCC
TGAGGGAGAAAGATTTATCGGAGATGGATGAGCCCGCGTCGGATTAGCTAGTTGGTGGGGTAATGGCC
TACCAAGGCGACGATCCGTAGCTGGTCTGAGAGGATGATCAGCCACACTGGGACTGAGACACGGCC
>Zotu7828
GAATGCTGGCGGCGTGGATAAGGCATGCAAGTCAAGGGGACCGCAAGGTAACCGGCAGACGAGGTAGT
AACACGTACGAACGCACCCCAGAGTCTGGCATAGCTCGTCGAAAGACGGGGTAATTCCGGATGGTCCC
GTAAGGGTAAAGTTTTTTCGCTCTGGGAGCGGCGTGCGTAATATCAGCTAGTTGGTAGGGTAATGGCC
TACCAAGGCTACGACGTTTAGGGGAGCTGAGAGGCTGACCCCCACCGATGGGACTGAGACACGGCC
>Zotu7830
GAACGCTGGCGGCATGCTTTACACATGTAAGTCGAACGGCAGCACGGACTTCGGTCTGGTGGCGAGTG
GCGAACGGGTGAGTAATACATCGGAACGTGCCCAGTTGTGGGGGATAACTACTCGAAAGAGTAGCTAA
TACCGCATGAGAACTGAGGTTGAAAGCAGGGGATCGCAAGACCTTGCGCAACTGGAGCGGCCGATGGC
AGATTAGGTAGTTGGTGGGATAAAAGCTTACCAAGCCGACGATCTGTAGCTGGTCTGAGAGGACGACC
AGCCACACTGGGACTGAGACACGGCC
>Zotu7831
GAACGCTAGCGGCAGGCTTAATACATGCAAGTCGAGGGGCAGCACGAATAGCAATATTTGGTGGCGAC
CGGCAAACGGGTGCGGAACACGTACAGAACCTTCCTTCGAGCGGGGAATAGCCCGGAGAAATCCGGAT
TAATACCCCATAGTAAATTAGAGAGGCATCTCTTTGATTTTAAAGATTTATCACTTGAAGATGGCTGT
GCGCCTGATTAGGTAGTTGGCGGGGTAATGGCCCACCAAGCCTTCGATCAGTAACTGGCGTGAGAGCG
CGACCAGTCACACGGGCACTGAGACACGGGC
>Zotu7832
GAACGCTTGCGGCGTGCCTAAGAAATGCAAGTCGAACGGATCACCTTCGGGTGGTTAGTGGCGAACGG
TCGCGTAACACGTAGGCAACCTGCCTCAAAGTGGGGGACAACAGCCCGAAAGGGCTGCTAATACCGCA
TGTGATTCCTTGGAGGCATCTCTCTGGAATCAAAGCCTGAGTTTACTTAGGCGCTTTGAGATGGGCTT
GCGGCCTATCAGCTTGTTGGTGAGGTAATGGCTTACCAAGGCGATGACGGGTAGCTGGTCTGAGAGGA
CGATCAGCCGGATTGGGACTGAGATACGGCC
>Zotu7833
GAACGCTGGCGGCGTGCCTAACACATGCAAGTCGAACGCGAAAGTCCTTCGGGATGAGTAAAGTGGCG
CACGGGTGAGTAACACGTAGGTGACCTGCCTTTTAGAGGGGAATAACCAGAAGAAATTTTGGCTAATG
CCGCATACGAAATACAAAAGTAAAAATAGTATTTGAAAGGATGCCTCTGTATATGCATCCACTAAAAG
ATGGGCCTGCGGAACATTAGCTAGTTGGTGGGGTAAAGGCCTACCAAGGCAACGATGTTTATCCGGTC
TGAGAGGATGATCGGACACACTGGAACTGAGACACGGTC
>Zotu7835

GAACGCTGGCGGCAGGCTTAACACATGCAAGTCGAGCGGAGATGAGGTGCTTGCACCTTATCTTAGCG
GCGGACGGGTGAGTAATGCTTAGGAATCTGCCATTTAGTGGGGGACAACATCTCGAAAGGGATGCTAA
TACCGCATACGCCCTACGGGGGAAAGCAGGGGATCTTCGGACCTTGCGCTAAATGATGAGCCTAAGTC
GGATTAGCTAGTTGGTGGGGTAAAGGCCTACCAAGGCGACGATCTGTAGCGGGTCTGAGAGGATGATC
CGCCACACTGGGACTGAGACACGGCC
>Zotu7836
GAACGCTGGCGGCGTGCTTAACACATGCAAGTCGAACGGTGAAACAGGAGCTTGCTCCTGCGGATCAG
TGGCGAACGGGTGAGTAACACGTGAGCAATCTGCCCTGGACTCTGGGATAAGCGTTGGAAACGACGTC
TAATACCGGATACGAGCTGCGAAGGCATCTTCAGCAGCTGGAAAGAACTTCGGTCCAGGATGAGCTCG
CGGCCTATCAGCTAGTTGGTGAGGTAACGGCCCACCAAGGCGACGACGGGTAGCCGGCCTGAGAGGGT
GACCGGCCACACTGGGACTGAGACACGGCC
>Zotu7837
GAACGCTGGCGGCGTGCTTAACACATGCAAGTCGAACGAGGTCCATGGAGCTTGCTCCGGAAGATCTA
GTGGCGAACGGGTGCATAACACGTGAGAAACCTGCCTCAGTCTCTGGGATAACAGTTGGAAACGACTG
CTAATACCGGATGCCGTCACACCATCACATGGTGGAGTGACGAAAGATTTATCGGATTGAGAGGGTCT
CGCGGCCTATCAGCTAGTTGGAGAGGTAACGGCTCACCAAGGCATCGACGGGTAGCTGGTCTGAGAGG
ATGATCAGCCACACTGGGACTGAGACACGGCC
>Zotu7839
GAACGCTGGCGGCAGGCTTAACACATGCAAGTCGAACGCCCCGCAAGGGGAGTGGCAGACGGGTGAGT
AACGCGTGGGAACATGCCTTTCGGTTCGGAATAGTCCCGGGAAACTGGGGTTAATACCGGATGCGCCC
TTCGGGGGAAAGATTTATCGCCGAAAGATTGGCCCGCGTCTGATTAGCTAGTTGGCGGTGTAATGGAC
CACCAAGGCGACGATCAGTAGCTGGTCTGAGAGGATGATCAGCCACACTGGGACTGAGACACGGCC
>Zotu7840
GAACGCTGGCGGCATGCCTAATACATGCAAGTCGAACGAACCATCCTGATGATTGAAGCTTGCTTCAT
GATTCAGATTTTGGTGAGTGGCGGACGGGTGAGTAACACGTGGGTAACCTGCCCAGAAGTGGGGGATA
ACATTTGGAAACAAGTGCTAATACCGCATAACAACTTAGATCACATGATCTTTGTTTAAAAGATGGTT
TTGCTATCTCTTCTGGATGGACCCGCGGCGTATTAGCTAGTTGGTGAGGTAATAGCTCACCAAGGCGA
TGATACGTAGCCGACCTGAGAGGGTAATCGGCCACATTGGGACTGAGACACGGCC
>Zotu7841
[truncated: 462,440 more chars]
