# Supplementary material for: Illuminating the bacterial microbiome of Australian ticks with 16S and Rickettsia-specific next-generation sequencing
Source: Curr Res Parasitol Vector Borne Dis. 2021 Jun 11;1:100037. doi: 10.1016/j.crpvbd.2021.100037 (PMC8906098; doi:10.1016/j.crpvbd.2021.100037)
Supplement: Multimedia component 11 — Additional file 11.List of 16S NGS ZOTUs denoised with UNOISE3 in USEARCH v10.0 with chimeras removed using UCHIME2. [file mmc11.pdf]

Additional file 11. List of *16S* NGS ZOTUs denoised with UNOISE3 in USEARCH v10.0 with chimeras removed using UCHIME2.

>Zotu1
GAACGCTGGCGGCGTGCTTAACACATGCAAGTCGAACGAGCAAGAGCTGTAGCAATACAGGGTTCTGCTA
GTGGCAGACGGGTGAGTAATACATGGGAATCTACCTTGCAGTGCGAAATAACAGTTGGAAGCAATTGCTA
ATGTCGCATATAGCCGAGAGGTGAAAGGTTTACTGCTGCAAGATGGGCCCATGCAGGATTAGCTAGTTGG
TGAGGTAATGGCTCACCAAGGCAACGATCTTTAGTTGGTCTGAGAGGATGATCAGCCACACTGGAACTGA
GACACGGTC
>Zotu3
GAACGCTAGCGGCATGCCTAACACATGCAAGTCGAACGGCAGCGGGGGGAGCTTGCTCCCTGACGGCGA
GTGGCGGACGGGTGAGTAACACGTAGGAATCTACCTTAATTATAATAGTTAGTGGGGGATAACCCGGGGA
AACTCGGGCTAATACCGCATAATCTCTTCAAAGCAAAGCGGGGGATCTTCGGACCTCGTGCTATGAGATGA
GCCTGCGTCGGATTAGCTAGTTGGTAGGGTAATGGCCTACCAAGGCGACGATCCGTAGCTGGTCTGAGAG
GACGATCAGCCACACTGGGACTGAGACACGGCC
>Zotu5
GAACGCTAGCGGCAGGCTTAATACATGCAAGTCGTGGGGCAGCATGAGTAGCAATACTTGATGGCGACCG
GCAAACGGGTGCGGAACACGTACACAACCTTCCGTCAAGCGGGGAATAGCCCAGAGAAATTTGGATTAAT
ACCCCATAATATATTTGATAAGGCATCTTATTAATATTAAAGCTTCGGCACTTGATGATGGGTGTGCGGCTG
ATTAGATAGTTGGTGAGGTAACGGCTCACCAAGTCTACGATCAGTAGCTGATGTGAGAGCATGATCAGCCA
CACGGGCACTGAGACACGGGC
>Zotu7
GAACGCTGGCGGTATGCTTAACACATGCAAGTCGAACGGCAGCATGTTCCTCACTCAAATCACTTTAATTTC
TTTTTGGAAAAGAAATACGGAAAGTGATTTGAGTGAGGAATGATGGCAAGTGGCGGACGGGTGAGTAAT
AGGTAGGAATCTGTCCTAGAGAGGGGGATAACTTTCGGAAACGAGAGCTAATACCACATGATATTGAAAA
ATCAAAGGCAGGGGACCTTTCGAGGCCTTGCGCTCTAGGGTGAGCCTGCATCGGATTAGCTAGTTGGTAG
GGTAAAAGCCTACCAAGGCGACGATCCGTAGCTGGTCTGAGAGGATGACCAGCCACACTGGGACTGAGAC
ACGGCC
>Zotu9
GAACGCTATCGGTATGCTTAACACATGCAAGTCGAACGGACTAATTGGGGCTTGCTCCAATTAGTTAGTGG
CAGACGGGTGAGTAACACGTGGGAATCTACCCATCAGTACGGAATAACTTTTAGAAATAAAAGCTAATACC
GTATATTCTCTGCGGAGGAAAGATTTATCGCTGATGGATGAGCCCGCGTCAGATTAGGTAGTTGGTGAGGT
AATGGCTCACCAAGCCGACGATCTGTAGCTGGTCTGAGAGGATGATCAGCCACACTGGGACTGAGACACG
GCC
>Zotu11
GAACGCTATCGGTATGCTTAACACATGCAAGTCGAACGGACTAATTGGGGCTTGCTCCAATTAGTTAGTGG
CAGACGGGTGAGTAACATGTGGGAATCTACCCATCAGTACGGAATAACTTTTAGAAATAAAAGCTAATACC
GTATATTCTCTACGGAGGAAAGATTTATCGCTGATGGATGAGCCCGCGTCAGATTAGGTAGTTGGTGAGGT
AATGGCTCACCAAGCCGACGATCTGTAGCTGGTCTGAGAGGATGATCAGCCACACTGGGACTGAGACACG
GCC
>Zotu13
GAACGCTGGTGGCATGCTTAACACATGCAAGTCGAACGGTACAGGACTAGCTTGCTAGTTGCTGACGAGT
GGCGGACGGGTGAGTAACGCGTAGGAATCTGCCCATCTGAGGGGGATACCAGTTGGAAACGACTGTTAAT
ACCGCATAGTATCTGTGGATTAAAGGTGGCTTTTAGGCTGTCGCAGATGGATGAGCCTGCGTTAGATTAGC
TAGTTGGTGGGGTAAGGGCCTACCAAGGCTACGATCCATAGCTGATTTGAGAGGATGATCAGCCACATTG
GGACTGAGACACGGCC
>Zotu15
GAACGCTAGCGGCAGGCTTAATACATGCAAGTCGTGGGGCAGCACAGGGTAGCAATACTTGGGTGGCGA
CCGGCAAACGGGTGCGGAACACGTACACAACCTTCCTACAAGTGGGGAATAGCCCAGAGAAATTTGGATT
AATACCCCGTAACATATAGAGATGGCATCATTTTTATATTATAGCTACGGCGCTTGTTGATGGGTGTGCGGC
TGATTAGGTAGTTGGCGGGGTAACGGCCCACCAAGCCTTCGATCAGTAGCTGATGTGAGAGCATGATCAG
CCACACGGGCACTGAGACACGGGC
>Zotu17

GAACGCTGGCGGCATGCCTAACACATGCAAGTCGAACGGCAGCACAGTAAAGATTTCGGTCTTTACGGGT
GGCGAGTGGCGGACGGGTGAGTAAAGCGTAGGAATCTGTCCTGGAGAGTGGGATAACCCGAGGAAACTC
GGGCTAATACCGCATGATATCGAGAGATCAAAGCAGGGGACCCTTCGGGGCCTTGCGCTCTAGGATGAGC
CTACGTCGGATTAGCTAGTTGGTGGGGTAAAGGCCTACCAAGGCGACGATCCGTAGCTGGTCTGAGAGGA
TGACCAGCCACACTGGGACTGAGACACGGCC
>Zotu19
GAACGCTGGCGGCAAGCTTAACACATGCAAGTCGAACGGATTTTTGTCGTAGCTTGCTATGATAAAAATTA
GTGGCAGACGGGTGAGTAATGCATAGGAATCTACCTAGTAGTATGGGATAGCCACTAGAAATGGTGGGTA
ATACTGTATAATCCCTGCGGGGGAAAGATTTATCGCTATTAGATGAGCCTATGTTAGATTAGCTAGTTGGTA
GGGTAAAGGCCTACCAAGGCAGTGATCTATAGCTGGTCTGAGAGGATGATCAGCCACACTGGAACTGAGA
TACGGTC
>Zotu21
GAACGCTAGCGGGATGCCTTACACATGCAAGTCGAACGGCAGCACGGACTTCGGTCTGGTGGCGAGTGGC
GAACGGGTGAGTAATGTATCGGAACGTGCCCAGTAGCGGGGGATAACTACGCGAAAGCGTAGCTAATACC
GCATACGCCCTACGGGGGAAAGCAGGGGATCGCAAGACCTTGCACTATTGGAGCGGCCGATATCGGATTA
GCTAGTTGGTGGGGTAACGGCTCACCAAGGCGACGATCCGTAGCTGGTTTGAGAGGACGACCAGCCACAC
TGGGACTGAGACACGGCC
>Zotu24
GAACGCTGGCGGCAGGCCTAACACATGCAAGTCGAGCGGATGAGAGGAGCTTGCTCCTGGATTCAGCGGC
GGACGGGTGAGTAATGCCTAGGAATCTGCCTGGTAGTGGGGGACAACGTTTCGAAAGGAACGCTAATACC
GCATACGTCCTACGGGAGAAAGCAGGGGACCTTCGGGCCTTGCGCTATCAGATGAGCCTAGGTCGGATTA
GCTAGTTGGTGAGGTAATGGCTCACCAAGGCGACGATCCGTAACTGGTCTGAGAGGATGATCAGTCACAC
TGGAACTGAGACACGGTC
>Zotu26
GAACGCTGGCGGCATGCCTAACACATGCAAGTCGAACGAGCCCTTCGGGGTTAGTGGCGCACGGGTGCGT
AACGCGTGGGAATCTGCCCTTGGGTTCGGAATAACTCGCCGAAAGGCGTGCTAATACCGGATGATGTCGA
AAGACCAAAGATTTATCGCCCAAGGATGAGCCCGCGTAAGATTAGGTAGTTGGTGAGGTAAAGGCTCACC
AAGCCGACGATCTTTAGCTGGTCTGAGAGGATGATCAGCCACACTGGGACTGAGACACGGCC
>Zotu28
AAACGCTGGCGGCATGCCTAACACATGCAAGTCGAACGGCAGCACAGTAAAGATTTCGGTCTTTACGGGT
GGCGAGTGGCGGATGGGTGAGTAAAGCGTAGGAATCTGTCCTGGAGAGTGGGATAACCCGAGGAAACTC
GGGCTAATACCGCATGATATCGAGAGATCAAAGCAGGGGACCCTTCGGGGCCTTGCGCTCTAGGATGAGC
CTACGTCGGATTAGCTAGTTGGTGGGGTAAAGGCCTACCAAGGCGACGATCCGTAGCTGGTCTGAGAGGA
TGACCAGCCACACTGGGACTGAGACACGTCC
>Zotu30
GAACGCTGGCGGCATGCCTAACACATGCAAGTCGAACGGCAGCACAGTAAAGATTTCGGTCTTTACGGGT
GGCGAGTGGCGGACGGGTGAGTAAAGCGTAGGAATCTGTCCTGGAGAGTGGAATAACCCGAGGAAACTC
GGGCTAATACCGCATGATATCGAGAGATCAAAGCAGGGGACCCTTCGGGGCCTTGCGCTCTAGGATGAGC
CTACGTCGGATTAGCTAGTTGGTGGGGTAAAGGCCTACCAAGGCGACGATCCGTAGCTGGTCTGAGAGGA
TGACCAGCCACACTGGGACTGAGACACGGCC
>Zotu32
GAACGCTGGCGGCGTGCTTAACACATGCAAGTCGAACGGAAAGGCCCTGCTTGCAGGGTACTCGAGTGGC
GAACGGGTGAGTAACACGTGGGTGATCTGCCCTGCACTTCGGGATAAGCCTGGGAAACTGGGTCTAATAC
CGGATAGGAGCCATTTTTAGTGTGATGGTTGGAAAGTTTTTTCGGTGTAGGATGAGCTCGCGGCCTATCAG
CTTGTTGGTGGGGTAATGGCCTACCAAGGCGGCGACGGGTAGCCGGCCTGAGAGGGTGGACGGCCACAT
TGGGACTGAGATACGGCC
>Zotu34
GAACGCTGGCGGCATGCCTTACACATGCAAGTCGAACGGCAGCATGATCTAGCTTGCTAGATTGATGGCG
AGTGGCGAACGGGTGAGTAATACATCGGAACGTGCCCTGTAGTGGGGGATAACTAGTCGAAAGATTAGCT
AATACCGCATACGACCTGAGGGTGAAAGTGGGGGACCGCAAGGCCTCATGCTATAGGAGCGGCCGATGTC

TGATTAGCTAGTTGGTGAGGTAAAGGCTCACCAAGGCGACGATCAGTAGCTGGTCTGAGAGGACGATCAG
CCACACTGGGACTGAGACACGGCC
>Zotu36
GAACGCTGGTGGCATGCTTAACACATGCAAGTCGAACGGTAACAGGACTAGCTTGCTAGTTGCTGACGAG
TGGCGGACGGGTGAGTAACACGTAGGAATCTGCCCATCTGAGGGGGATACCAGTTGGAAACGACTATTAA
TACCGCATAGTATCTGTGGATTAAAGGTGGCTTTTGAGCTGTCGCATATGGATGAGCCTGCGTTGGATTAG
CTAGTTGGTGAGGTAAGGGCCTACCAAGGCTACGATCCATAGTTGATTTGAGAGGATGATCAGCCACATTG
GGACTGAGACACGGCC
>Zotu38
GAACGCTGGCGGCATGCCTAACACATGCAAGTCGAACGAGACCTTCGGGTCTAGTGGCGCACGGGTGCGT
AACGCGTGGGAACCTGCCCTTAGGTTCGGAATAACTCCCCGAAAGGGGTGCTAATACCGGATAATGTCTTC
GGACCAAAGATTTATCGCCTTTGGATGGGCCCGCGTTGGATTAGCTAGTTGGTAGGGTAAAAGCCTACCAA
GGCGACGATCCATAGCTGGTCTGAGAGGATGATCAGCCACACTGGGACTGAGACACGGCC
>Zotu40
GAACGCTGGCGGCAAGCCTAACACATGCAAGTCGAACGAATTATCTTTATAGTTTACTATAAGGATAATTA
GTGGCAGACGGGTGAGTAATGCATAGGAATCTACCTAGTAGTATGGAATAGCTGTTAGAAATGACAGGTA
ATACCGTATAATCCCTGAGGGGGAAAGATTTATCGCTATTAGATGAGCCTATGTTAGATTAGCTAGTTGGT
GAGGTAATGGCTTACCAAGGCTGTGATCTATAGCTGGTCTGAGAGGACGATCAGCCACACTGGAACTGAG
ATACGGTC
>Zotu42
GAACGCTGGTGGCATGCTTAACACATGCAAGTCGAACGGTAACAGGACTAGCTTGCTAGTTGCTGACGAG
TGGCGGACGGGTGAGTAACACGTAGGAATCTGCCCATCTGAGGGGGATACCAGTTGGAAACGACTATTAA
TACCGCATAGTATCTGTGGATTAAAGATGGCTTTTAGGCTGTCGCGGATGGATGAGCCTGCGTTGGATTAG
CTAGTTGGTGAGGTAAGGGCCTACCAAGGCTACGATCCATAGTTGATTTGAGAGGATGATCAGCCACATTG
GGACTGAGACACGGCC
>Zotu44
GAACGCTAGCGGGAGGCCTAACACATGCAAGCCGAGCGGTATTGTTTCTTCGGAAATGAGAGAGCGGCGT
ACGGGTGCGTAACACGTGTGCAACCTGCCTTTATCTGGGGGATAGCCTTTCGAAAGGAAGATTAATACTCC
ATAATATATTGAACGGCATCGTTTAATATTGAAAGCTCCGGCGGATAGAGATGGGCACGCGCAAGATTAGC
TAGTTGGTGAGGTAACGGCTCACCAAGGCGATGATCTTTAGGGGGCCTGAGAGGGTGATCCCCCACACTG
GTACTGAGACACGGAC
>Zotu46
GAACGCTGGCGGCATGCCTAACACATGCAAGTCGAACGGCAGCACAGTAAAGATTTCGGTCTTTATGGGT
GGCGAGTGGCGGACGGGTGAGTAATGCGTAGGGATCTGTCCTGGAGAGAGGGATAACCCGAGGAAACTC
GGGCTAATACCTCATGATATTGAGAGATCAAAGGCAGGGGACCGAAAGGCCTTGCGCTCTGGGGTGAGCC
TACGTCGGATTAGCTAGTTGGTGGGGTAAGAGCCTACCAAGGCGACGATCCGTAGCTGGTCTGAGAGGAT
GACCAGCCACACTGGGACTGAGACACGGCC
>Zotu48
GAACGCTGGCGGCGTGCTTAACACATGCAAGTCGAACGGAAAGGCCCTTTCGGGGGTACTCGAGTGGCGA
ACGGGTGAGTAACACGTGGGTGATCTGCCCTGCACTTCGGGATAAGCTTGGGAAACTGGGTCTAATACCG
GATAGGACTACGGACTGCATGGTCTGTGGTGGAAAGCTTTTGCGGTGTGGGATGGGCCCGCGGCCTATCA
GCTTGTTGGTGGGGTGATGGCCTACCAAGGCGACGACGGGTAGCCGGCCTGAGAGGGTGTCCGGCCACA
CTGGGACTGAGATACGGCC
>Zotu50
GAACGCTGGCGGCATGCCTTACACATGCAAGTCGAACGGCAGCATGATCTAGCTTGCTAGATTGATGGCG
AGTGGCGAACGGGTGAGTAATACATCGGAACGTGCCCTGTAGTGGGGGATAACTAGTCGAAAGATTAGCT
AATACCGCATACGACCTGAGGGTGAAAGTGGGGGACCGCAAGGCCTCATGCTATAGGAGCGGCCGATGTC
TGATTAGCTAGTTGGTGGGGTAAAGGCCCACCAAGGCGACGATCAGTAGCTGGTCTGAGAGGACGATCAG
CCACACTGGGACTGAGACACGGCC
>Zotu52

GAACGCTGGCGGCGTGCCTAATACATGCAAGTAGAACGCTGAAGGAGGAGCTTGCTCTTCTGGATGAGTT
GCGAACGGGTGAGTAACGCGTAGGTAACCTGCCTGGTAGCGGGGGATAACTATTGGAAACGATAGCTAAT
ACCGCATAATAGTAGATGTTGCATGACATTTGCTTAAAAGGTGCAATTGCATCACTACCAGATGGACCTGC
GTTGTATTAGCTAGTTGGTGAGGTAACGGCTCACCAAGGCAACGATACATAGCCGACCTGAGAGGGTGAT
CGGCCACACTGGGACTGAGACACGGCC

>Zotu54

GAACGCTGGCGGCATGCTTAACACATGCAAGTCGGACGGGAAGTGGTGTTTCCAGTGGCGGACGGGTGA
GTAACGCGTAAGAACCTGCCCTTGGGAGGGGAACAACAGCTGGAAACGGCTGCTAATACCCCGTAGGCTG
AGGAGCAAAAGGAGGAATCCGCCCGAGGAGGGGCTCGCGTCTGATTAGCTAGTTGGTGAGGCAATAGCT
TACCAAGGCGATGATCAGTAGCTGGTCCGAGAGGATGATCAGCCACACTGGGACTGAGACACGGCC

>Zotu56

GAACGCTGGCGGCATGCCTAACACATGCAAGTCGAACGAAGGCTTCGGCCTTAGTGGCGCACGGGTGCGT
AACGCGTGGGAATCTGCCCCTTGGTTCGGAATAACAGTTAGAAATGACTGCTAATACCGGATGATGACTTC
GGTCCAAAGATTTATCGCCGAGGGATGAGCCCGCGTAGGATTAGGTAGTTGGTGGGGTAAAGGCCTACCA
AGCCGACGATCCTTAGCTGGTCTGAGAGGATGATCAGCCACACTGGGACTGAGACACGGCC

>Zotu58

GAACGCTGGCGGCAAGCCTAACACATGCAAGTCGAACGGGGTTATATATAGCTTGCTATAGTGTAGCCTAG
TGGCAGACGGGTGAGTAATGTATAGGAATCTACCTAGTAGTACGGAATAATTACTGGAAACGGCAACTAA
TACCGTATACGCCCTATGGGGGAAAGATTTATTGCTATTAGATGAGCCTATATTAGATTAGCTAGTTGGTAG
GGTAATGGCCTACCAAGGCTATGATCTATAGCTGATCTGAGAGGATGATCAGCCACACTGGAACTGAGATA
CGGTC

>Zotu61

GAACGCTGGCGGCGTGCCTAATACATGCAAGTAGAACGCTGAAGAGAGGAGCTTGCTCTTCTTGGATGAG
TTGCGAACGGGTGAGTAACGCGTAGGTAACCTGCCTTGTAGCGGGGGATAACTATTGGAAACGATAGCTA
ATACCGCATAACAATGGATGACACATGTCATTTATTTGAAAGGGGCAATTGCTCCACTACAAGATGGACCT
GCGTTGTATTAGCTAGTAGGTGAGGTAATGGCTCACCTAGGCGACGATACATAGCCGACCTGAGAGGGTG
ATCGGCCACACTGGGACTGAGACACGGCC

>Zotu63

GAACGCTGGCGGCGTGCCTAATACATGCAAGTAGAACGCTGAGGACTGGTGCTTGCACCGGTCCAAGGAG
TTGCGAACGGGTGAGTAACGCGTAGGTAACCTACCTCATAGCGGGGGATAACTATTGGAAACGATAGCTA
ATACCGCATAAAAGTGTTTAACACATGTTAAACATTTAAAAGATGCAACTGCATCACTATGAGATGGACCTG
CGTTGTATTAGCTAGTTGGTGAGGTAACGGCTCACCAAGGCGACGATACATAGCCGACCTGAGAGGGTGA
TCGGCCACACTGGGACTGAGACACGGCC

>Zotu65

GAACGCTGGCGGCGTGCCTAATACATGCAAGTCGAGCGAACAGATAAGGAGCTTGCTCCTTTGACGTTAG
CGGCGGACGGGTGAGTAACACGTGGATAACCTACCTATAAGACTGGGATAACTTCGGGAAACCGGAGCTA
ATACCGGATAACATATTGAACCGCATGGTTCAATAGTGAAAGGCGGCTTTGCTGTCACTTATAGATGGATC
CGCGCCGTATTAGCTAGTTGGTAAGGTAACGGCTTACCAAGGCAACGATACGTAGCCGACCTGAGAGGGT
GATCGGCCACACTGGAACTGAGACACGGTC

>Zotu67

AAACGCTGGCGGCATGCCTAACACATGCAAGTCGAACGGCAGCACAGTAAAGATTTCGGTCTTTACGGGT
GGCGAGTGGCGGATGGGTGAGTAAAGCGTAGGAATCTGTCCTGGAGAGTGGGATAACCCGAGGAAACTC
GGGCTAATACCGCATGATATCGAGAGATCAAAGCAGGGGACCCTTCGGGGCCTTGCGCTCTAGGATGAGC
CTACGTCGGATTAGCTAGTTGGTGGGGTAAAGGCCTACCAAGGCGACGATCCGTAGCTGGTCTGAGAGGA
TGACCAGCCACACTGGGACTGAGACACGGCC

>Zotu69

GAACGCTGGCGGCGTGCCTAATACATGCAAGTCGAGCGAACAGACGAGGAGCTTGCTCCTCTGACGTTAG
CGGCGGACGGGTGAGTAACACGTGGATAACCTACCTATAAGACTGGGATAACTTCGGGAAACCGGAGCTA
ATACCGGATAACATGTTGAACCGCATGGTTCAACAGTGAAAGACGGTCTTGCTGTCACTTATAGATGGATC
CGCGCCGCATTAGCTAGTTGGTAAGGTAACGGCTTACCAAGGCAACGATGCGTAGCCGACCTGAGAGGGT
GATCGGCCACACTGGAACTGAGACACGGTC

>Zotu71
GAACGCTGGCGGCGTGCCTAATACATGCAAGTCGAGCGAACAGATAAGGAGCTTGCTCCTTTGAAGTTAG
CGGCGGACGGGTGAGTAACACGTGGGTAACCTACCTATAAGACTGGGATAACTTCGGGAAACCGGAGCTA
ATACCGGATAACATTTAGAACCGCATGGTTCTAAAGTGAAAGATGGTTTTGCTATCACTTATAGATGGACCC
GCGCCGTATTAGCTAGTTGGTAAGGTAACGGCTTACCAAGGCGACGATACGTAGCCGACCTGAGAGGGTG
ATCGGCCACACTGGAACTGAGACACGGTC
>Zotu73
GAACGCTGGCGGCGTGCCTAATACATGCAAGTAGAACGCTGAAGGAAGGAGCTTGCTCTTTCCGGATGAG
TTGCGAACGGGTGAGTAACGCGTAGGTAACCTGCCTGGTAGCGGGGGATAACTATTGGAAACGATAGCTA
ATACCGCATAACAGTAGATATTGCATGATATCTGCTTGAAAGGTGCAATTGCACCACTACCAGATGGACCT
GCGTTGTATTAGCTAGTTGGTGAGGTAACGGCTCACCAAGGCGACGATACATAGCCGACCTGAGAGGGTG
ATCGGCCACACTGGGACTGAGACACGGCC
>Zotu75
GAACGCTGGCGGCATGCCTTACACATGCAAGTCGAACGGCATCGCGGGAAAGCTTGCTTTCCTGGCGGCG
AGTGGCGAACGGGTGAGTAACACATCGGAACGTACCGGCTAGTGGGGGATAACCCGTCGAAAGACGGGC
TAATACCGCATACGCTCTTCGGAGGAAAGCAGGGGACCTTTGGGCCTTGCGCTAGCCGAGCGGCCGATGT
CCGATTAGCTAGTTGGTGGGGTAAAGGCCTACCAAGGCGACGATCGGTAGCGGGTCTGAGAGGACGATCC
GCCACACTGGGACTGAGACACGGCC
>Zotu77
AAACGCTGGCGGCATGCCTAACACATGCAAGTCGAACGGCAGCACAGTAAAGATTTCGGTCTTTACGGGT
GGCGAGTGGCGGACGGGTGAGTAAAGCGTAGGAATCTGTCCTGGAGAGTGGGATAACCCGAGGAAACTC
GGGCTAATACCGCATGATATCGAGAGATCAAAGCAGGGGACCCTTCGGGGCCTTGCGCTCTAGGATGAGC
CTACGTCGGATTAGCTAGTTGGTGGGTGGGGTAAAGGCCTACCAAGGCGACGATCCGTAGCTGGTCTGAG
AGGATGACCAGCCACACTGGGACTGAGACACGTCC
>Zotu79
GAACGCTGGCGGCATGCCTAACACATGCAAGTCGAACGGCAGCACAGTAAAGATTTCGGTCTTTACGGGT
GGCGAGTGGCGGACGGGTGAGTAAAGCGTAGGAATCTGTCCTGGAGAGTGGGATAACCCGAGGAAACTC
GGGCTAATACCGCATGATATCGAGAGATCAAAGCAGGGGACCCTTCGGGGCCTTGCGCTCTAGGATGAGC
CTACGTCGGATTAGCTAGTTGGTGGGTGGGGTAAAGGCCTACCAAGGCGACGATCCGTAGCTGGTCTGAG
AGGATGACCAGCCACACTGGGACTGAGACACGTCC
>Zotu83
GAACGCTGGCGGCATGCCTAACACATGCAAGTCGAACGGCAGCACAGTAAAGATTTCGGTCTTTACGGGT
GGCGAGTGGCGGATGGGTGAGTAAAGCGTAGGAATCTGTCCTGGAGAGTGGGATAACCCGAGGAAACTC
GGGCTAATACCGCATGATATCGAGAGATCAAAGCAGGGGACCCTTCGGGGCCTTGCGCTCTAGGATGAGC
CTACGTCGGATTAGCTAGTTGGTGGGGTAAAGGCCTACCAAGGCGACGATCCGTAGCTGGTCTGAGAGGA
TGACCAGCCACACTGGGACTGAGACACGTCC
>Zotu85
GAACGCTGGCGGCGTGCTTAACACATGCAAGTCGAACGGAAAGGCCCTTTCGGGGGTACTCGAGTGGCGA
ACGGGTGAGTAACACGTGGGTGATCTGCCCTGCACTTCGGGATAAGCTTGGGAAACTGGGTCTAATACCG
GATAGGACTACGAATCGCATGGTTTGTGGTGGAAAGCTTTTGCGGTGTGGGATGGGCCCGCGGCCTATCA
GCTTGTTGGTGGGGTGATGGCCTACCAAGGCGACGACGGGTAGCCGGCCTGAGAGGGTGTCCGGCCACA
CTGGGACTGAGATACGGCC
>Zotu87
GAACGCTGGCGGCAGGCCTAACACATGCAAGTCGAACGGTAACAGGAAACAGCTTGCTGTTTCGCTGACG
AGTGGCGGACGGGTGAGTAATGTCTGGGAAACTGCCTGATGGAGGGGGATAACTACTGGAAACGGTAGC
TAATACCGCATAACGTCGCAAGACCAAAGAGGGGGACCTTCGGGCCTCTTGCCATCGGATGTGCCCAGAT
GGGATTAGCTAGTAGGTGGGGTAACGGCTCACCTAGGCGACGATCCCTAGCTGGTCTGAGAGGATGACCA
GCCACACTGGAACTGAGACACGGTC
>Zotu89

GAACGCTGGCGGCAGGCTTAACACATGCAAGTCGAACGGGCACTTCGGTGCTAGTGGCAGACGGGTGAG
TAACACGTGGGAACGTACCTTTCGGTTCGGAATAATTCAGGGAAACTTGGACTAATACCGGATACGCCCTT
CGGGGGAAAGATTTATCGCCGATAGATCGGCCCGCGTCTGATTAGCTAGTTGGTGAGGTAATGGCTCACC
AAGGCGACGATCAGTAGCTGGTCTGAGAGGATGATCAGCCACATTGGGACTGAGACACGGCC
>Zotu91
GAACGCTGGCGGCAGGCTTAACACATGCAAGTCGAACGCGTGTAGCAATACACGAGTGGCGCACGGGTG
AGTAACGCGTGGATATCTGCCTTTTGGTTCGGAATAACCCCGGGAAACTGGGGCTAATACCGGATGGTTCC
TTCGGGATAAAGATTTATCGCCAAAAGATGAGTCCGCGTACGATTAGCTAGTTGGTGAGGTAATGGCTCAC
CAAGGCGACGATCGTTAGCTGGTCTGAGAGGACGATCAGCCACACTGGGACTGAGACACGGCC
>Zotu93
GAACGCTGGCGGCGTGCTTAACACATGCAAGTCGAACGGAAAGGCCCTTCGGGGTACTCGAGTGGCGAAC
GGGTGAGTAACACGTGGGTGATCTGCCCTGCACTCTGGGATAAGCCTGGGAAACTGGGTCTAATACCGGA
TAGGACCACACACTTCATGGTGAGTGGTGCAAAGCTTTTGCGGTGTGGGATGAGCCCGCGGCCTATCAGCT
TGTTGGTGGGGTAATGGCCCACCAAGGCGACGACGGGTAGCCGGCCTGAGAGGGTGACCGGCCACACTG
GGACTGAGATACGGCC
>Zotu95
GAACGCTGGCGGCGTGCCTAATACATGCAAGTAGAACGCAGAGGTCAGGTGCTTGCACTGGACTGATGAG
TTGCGAACGGGTGAGTAACGCGTAGGTAACCTACCTGATAGCGGGGGATAACTATTGGAAACGATAGCTA
ATACCGCATAAAAGCCAATGACCCATGTCATTGGCTTGAAAGAAGCAAACGCTTCACTATGAGATGGACCT
GCGTTGTATTAGCTAGTTGGTAGGGTAAAGGCCTACCAAGGCGACGATACATAGCCGACCTGAGAGGGTG
AACGGCCACACTGGGACTGAGACACGGCC
>Zotu97
GAACGCTGGTGGCATGCTTAACACATGCAAGTCGAACGGTAACAGGACTAGCTTGCTAGTTGCTGACGAG
TGGCGGACGGGTGAGTAACACGTAGGAATCTGCCCATCTGAGGGGGATACCAGTTGGAAACGACTATTAA
TACCGCATAGTATCTGTGGATTAAAAATGGCTTTTAGGCTGTCGCGGATGGATGAGCCTGCGTTGGATTAG
CTAGTTGGTGAGGTAAGGGCCTACCAAGGCTACGATCCATAGTTGATTTGAGAGGATGATCAGCCACATTG
GGACTGAGACACGGCC
>Zotu99
GAACGCTGGCGGCATGCCTAACACATGCAAGTCGAACGCATCGCAAGATGAGTGGCGCACGGGTGAGTA
ACGCGTGGGAACCTGCCCTATGGTGCGGAATAACACCGGGAAACTGGTGCTAATACCGCATGTGTCCTTCG
GTCGTGGAGATCGGAGGAGAAAGATTTATCGCCATAGGAGGGGCCCGCGTCCGATTAGGTAGTTGGTGG
GGTAATGGCCTACCAAGCCTTCGATCGGTAGCTGGTCTGAGAGGATGATCAGCCACACTGGGACTGAGAC
ACGGCC
>Zotu101
GAACGCTGGCGGCGTGCCTAATACATGCAAGTAGAACGCTGAAGGAGGAGCTTGCTCTTCTGGATGAGTT
GCGAACGGGTGAGTAACGCGTAGGTAACCTGCCTGGTAGCGGGGGATAACTATTGGAAACGATAGCTAAT
ACCGCATAAGAGTAGATGTTGCATGACATTTGCTTAAAAGGTGCAATTGCATCACTACCAGATGGACCTGC
GTTGTATTAGCTAGTTGGTGAGGTAACGGCTCACCAAGGCAACGATACATAGCCGACCTGAGAGGGTGAT
CGGCCACACTGGGACTGAGACACGGCC
>Zotu103
GAACGCTGGCGGCATGCTTTACACATGCAAGTCGAACGGCAGCGCGGGGCAACCTGGCGGCGAGTGGCG
AACGGGTGAGTAATATATCGGAACGTACCCAAGAGTGGGGGATAACGTAGCGAAAGTTACGCTAATACCG
CATACGATCTAAGGATGAAAGTGGGGGATCGCAAGACCTCATGCTCCTGGAGCGGCCGATATCTGATTAG
CTAGTTGGTGAGGTAAAGGCTCACCAAGGCGACGATCAGTAGCTGGTCTGAGAGGACGACCAGCCACACT
GGGACTGAGACACGGCC
>Zotu106
GAACGCTGGCGGCATGCTTAACACATGCAAGTCGAACGGGAAGTGGTGTTTCCAGTGGCGAACGGGTGA
GTAACGCGTAAGAACCTGCCCTTGGGAGGGGAACAACAACTGGAAACGGTTGCTAATACCCCGTAGGCTG
AGGAGCAAAAGGAGAAATCCGCCCAAGGAGGGGCTCGCGTCTGATTAGCTAGTTGGTGAGGTAATAGCTT
ACCAAGGCGATGATCAGTAGCTGGTCCGAGAGGATGATCAGCCACACTGGGACTGAGACACGGCC

>Zotu108
GAACGCTGGCGGCAGGCTTAACACATGCAAGTCGAACGCCCCGCAAGGGGAGTGGCAGACGGGTGAGTA
ACGCGTGGGAACATACCCTTTCCTGCGGAATAGCTCCGGGAAACTGGAATTAATACCGCATACGCCCTACG
GGGGAAAGATTTATCGGGGAAGGATTGGCCCGCGTTGGATTAGCTAGTTGGTGGGGTAAAGGCCTACCAA
GGCGACGATCCATAGCTGGTCTGAGAGGATGATCAGCCACATTGGGACTGAGACACGGCC
>Zotu110
GAACGCTGGCGGCGTGCTTAACACATGCAAGTCGAACGGAAAGGCCTCTTTCGGGGGGTACTCGAGTGGC
GAACGGGTGAGTAACACGTGAGTAACCTGCCCTTGACTTTGGGATAACTTCAGGAAACTGGGGCTAATACC
GGATAGGAGCTCCTGCTGCATGGTGGGGGTTGGAAAGTTTCGGCGGTTGGGGATGGACTCGCGGCTTATC
AGCTTGTTGGTGGGGTAGTGGCTTACCAAGGCTTTGACGGGTAGCCGGCCTGAGAGGGTGACCGGCCACA
TTGGGACTGAGATACGGCC
>Zotu112
GAACGCTGGCGGCGTGCCTAATACATGCAAGTAGAACGCTGAAGGAGGAGCTTGCTTCTCCGGATGAGTT
GCGAACGGGTGAGTAACGCGTAGGTAACCTGCCTGGTAGCGGGGGATAACTATTGGAAACGATAGCTAAT
ACCGCATAACAGTAGATGTTGCATGACATTTGCTTAAAAGGTGCAATTGCATCACTACCAGATGGACCTGC
GTTGTATTAGCTAGTTGGTGGGGTAACGGCTCACCAAGGCGACGATACATAGCCGACCTGAGAGGGTGAT
CGGCCACACTGGGACTGAGACACGGCC
>Zotu114
GAACGCTGGCGGCGTGCTTAACACATGCAAGTCGAACGGTGAAGTCCCTTCGGGGGCGGATCAGTGGCGA
ACGGGTGAGTAACACGTGGGCAATCTGCCCCTCACTGTGGGATAACTCCGGGAAACCGGGGCTAATACCG
CATACGAAGCCTGGCGGCATCGCCGGGTTTGGAAAGTTTTTCGGTGAGGGATGAGCCCGCGGCCTATCAG
CTTGTTGGTGAGGTAATGGCTCACCAAGGCGACGACGGGTAGCCGGCCTGAGAGGGCGACCGGCCACACT
GGGACTGAGACACGGCC
>Zotu116
GAACGCTGGCGGCGTGCCTAATACATGCAAGTAGAACGCTGAAGAGAGGAGCTTGCTCTTCTTGGATGAG
TTGCGAACGGGTGAGTAACGCGTAGGTAACCTGCCTTGTAGCGGGGGATAACTATTGGAAACGATAGCTA
ATACCGCATAACAATGAATGACACATGTCATTTATTTGAAAGGGGCAATTGCTCCACTACAAGATGGACCT
GCGTTGTATTAGCTAGTAGGTGAGGTAACGGCTCACCTAGGCGACGATACATAGCCGACCTGAGAGGGTG
ATCGGCCACACTGGGACTGAGACACGGCC
>Zotu118
GAACGCTGGCGGCGTGCCTAATACATGCAAGTCGAGCGAACTGAAGAGGAGCTTGCTCCTTTGACGTTAG
CGGCGGACGGGTGAGTAACACGTGGGTAACCTACCTATAAGACTGGAATAACTCCGGGAAACCGGGGCTA
ATGCCGGATAATATGTTGAACCGCATGGTTCAACAGTGAAAGACGGTTTTGCTGTCACTTATAGATGGACC
CGCGCCGCATTAGCTAGTTGGTGAGGTAACGGCTCACCAAGGCGACGATGCGTAGCCGACCTGAGAGGGT
GATCGGCCACACTGGAACTGAGACACGGTC
>Zotu120
GAACGCTGGCGGCAGGCCTAACACATGCAAGTCGAACGGTAACAGGAAGCAGCTTGCTGCTTCGCTGACG
AGTGGCGGACGGGTGAGTAATGTCTGGGAAACTGCCTGATGGAGGGGGATAACTACTGGAAACGGTAGC
TAATACCGCATAACGTCGCAAGACCAAAGAGGGGGACCTTCGGGCCTCTTGCCATCGGATGTGCCCAGAT
GGGATTAGCTAGTAGGTGGGGTAACGGCTCACCTAGGCGACGATCCCTAGCTGGTCTGAGAGGATGACCA
GCCACACTGGAACTGAGACACGGTC
>Zotu122
GAACGCTGGCGGTGTGCTTAATACATGCAAGTCGAACGAAGAGGCCCTTTCGGGGGTACTTCAGTGGCGG
ACGGCTGAGTAATGCATAGGTAACGTGCCCTTAACTGGGGGATAACACCGGGAAACTGGTGCTAATACCG
CATAAGTGCCAGGTTGAAATACCTGTCATTAAAGAATTTCGGTTAAGGATCGGCCTGTGTCGGATTAGCTT
GTTGGTAGGGTAATGGCCTACCAAGGCGACGATCCGTAGTTGGTCTGAGAGGATGACCAGCCAGAGTGG
GACTGAGAACGGCC
>Zotu124
GAACGCTGGCGGCGTGCCTAATACATGCAAGTAGAACGCTGAAGGAGGAGCTTGCTTCTCTGGATGAGTT
GCGAACGGGTGAGTAACGCGTAGGTAACCTGCCTGGTAGCGGGGGATAACTATTGGAAACGATAGCTAAT

ACCGCATAAGAGTAGATGTTGCATGACATTTGCTTAAAAGGTGCAATTGCATCACTACCAGATGGACCTGC
GTTGTATTAGCTAGTTGGTGGGGTAACGGCTCACCAAGGCGACGATACATAGCCGACCTGAGAGGGTGAT
CGGCCACACTGGGACTGAGACACGGCC
>Zotu126
GAACGCTGGCGGCAGGCTTAACACATGCAAGTCGAGCGGGCGTAGCAATACGTCAGCGGCAGACGGGTG
AGTAACGCGTGGGAACGTACCTTTTGGTTCGGAACAACACAGGGAAACTTGTGCTAATACCGGATAAGCCC
TTACGGGGAAAGATTTATCGCCGAAAGATCGGCCCGCGTCTGATTAGCTAGTTGGTGAGGTAACGGCTCAC
CAAGGCGACGATCAGTAGCTGGTCTGAGAGGATGATCAGCCACATTGGGACTGAGACACGGCC
>Zotu128
GAACGCTAGCGGCAGGCTTAATACATGCAAGTCGTGGGGCAGCATGTCCCGACGCAAGTCGGGATGATGG
CGACCGGCAAACGGGTGCGGAACACGTACACAACCTTCCTTTAAGTGGGGAATAGCCCAGGGAAACTTGG
ATTAATACCCCGTAATATTATGAAGTGGCATCATTTTATAATTATAGCTCCGGCGCTTGAAGATGGGTGTGC
GGCTGATTAGGTAGTTGGCGGGGTAACGGCCCACCAAGCCTACGATCAGTAACTGGTGTGAGAGCACGAC
CAGTCACACGGGCACTGAGACACGGGC
>Zotu130
GAACGCTGGCGGCGTGCCTAATACATGCAAGTCGTACGCAATTGATCCACCGAGTGCTTGCACTCACCGGA
TCGTTGAGTGGCGCACGGGTGAGTAACACGTGGGTAACCTGCCCACCAGAGGGGGATAACAGTCGGAAA
CGATTGCTAATACCGCATAGGTTGAACGACCGCATGGTCGCTCAAGGAAAGACGGCTTTGGCTGTCACTGG
TGGATGGACCCGCGGCGTATTAGTTAGTTGGTGAGGTAACGGCTCACCAAGACAATGATACGTAGCCGAC
CTGAGAGGGTGATCGGCCACATTGGGACTGAGACACGGCC
>Zotu132
GAACGCTGGCGGCATGCTTTACACATGCAAGTCGAGCGGCAGCGCGGGGGCAACCCTGGCGGCGAGCGG
CGAACGGGTGAGTAATGCATCGGAACGTATCCTGTAGTGGGGGATAACCCAGCGAAAGTTGGGCTAATAC
CGCATGAGCTCTGAGGAGGAAAGCGGGGGACCTGAAAGGGCCTCGCGCTGCCGGAGCGGCCGATGTCCG
ATTAGCTAGTTGGTGGGGTAAAGGCTTACCAAGGCGACGATCGGTAGCTGGTCTGAGAGGATGATCAGCC
ACACTGGGACTGAGACACGGCC
>Zotu134
GAACGCTAGCGGCAGGCTTAATACATGCAAGTCGTGGGGCAGCGCAGGTAGCAATACTGGGCGGCGACC
GGCAAACGGGTGCGGAACACGTACACAACCTTCCTATAAGTGGGGAATAGCCCAGAGAAATTTGGATTAA
TACCCCGTAACATAATGATGTGGCATCACATTGTTATTATAGCTTCGGCGCTTATTGATGGGTGTGCGGCTG
ATTAGATAGTTGGCGGGGTAACGGCCCACCAAGTCTACGATCAGTAGCTGATGTGAGAGCATGATCAGCC
ACACGGGCACTGAGACACGGGC
>Zotu136
GAACGCTAGCGGGAGGCCTAACACATGCAAGCCGAGCGGTATTTGTTCTTCGGAACAGAGAGAGCGGCGC
ACGGGTGCGGAACACGTGTGCAACCTGCCTTTATCTGGGGGATAGCCTTTCGAAAGGAAGATTAATACCCC
ATAATATATTGAGTGGCATCATTTGATATTGAAAACTCCGGTGGATAGAGATGGGCACGCGCAAGATTAGA
TAGTTGGTGAGGTAACGGCTCACCAAGTCAATGATCTTTAGGGGGCCTGAGAGGGTGATCCCCCACACTG
GTACTGAGACACGGAC
>Zotu138
GAACGCTGGCGGCGTGCCTAATACATGCAAGTCGAGCGAACAGACGAGGAGCTTGCTCCTTTGACGTTAG
CGGCGGACGGGTGAGTAACACGTGGGTAACCTACCTATAAGACTGGGATAACTTCGGGAAACCGGAGCTA
ATACCGGATAATATTTCGAACCGCATGGTTCGATAGTGAAAGATGGCTCTGCTATCACTTATAGATGGACCT
GCGCCGTATTAGCTAGTTGGTAAGGTAACGGCTTACCAAGGCAACGATACGTAGCCGACCTGAGAGGGTG
ATCGGCCACACTGGAACTGAGACACGGTC
>Zotu140
GAACGCTAGCGGGAGGCCTAACACATGCAAGCCGAGCGGTAGAGTTCCTTCGGGAACTTGAGAGCGGCG
TACGGGTGCGTAACACGTGTGCAACCTACCTTTATCTGGGGGATAGCCTTTCGAAAGGAAGATTAATACCC
CATAATATAATGATTGGCATCAATTATTATTGAAAACTCCGGTGGATAGAGATGGGCACGCGCAAGATTAG
ATAGTTGGTGAGGTAACGGCTCACCAAGTCAATGATCTTTAGGGGTCCTGAGAGGGAGATCCCCCACACTG
GTACTGAGACACGGAC

>Zotu142
GAACGCTGGCGGCGTGCCTAATACATGCAAGTCGAGCGAACAGACGAGGAGCTTGCTCCTTTGACGTTAG
CGGCGGACGGGTGAGTAACACGTGGGTAACCTACCTATAAGACTGGGATAACTTCGGGAAACCGGAGCTA
ATACCGGATAATATTTCGAACCGCATGGTTCGATAGTGAAAGATGGCTTTGCTATCACTTATAGATGGACCT
GCGCCGTATTAGCTAGTTGGTAAGGTAACGGCTTACCAAGGCAACGATACGTAGCCGACCTGAGAGGGTG
ATCGGCCACACTGGAACTGAGACACGGTC
>Zotu144
GAACGCTGGCGGCGTGCCTAATACATGCAAGTAGAACGCTGAAGGAGGAGCTTGCTCTTTCCGGATGAGT
TGCGAACGGGTGAGTAACGCGTAGGTAACCTGCCTGGTAGCGGGGGATAACTATTGGAAACGATAGCTAA
TACCGCATAACAGTAGATATTGCATGATATCTACTTGAAAGGTGCAATTGCATCACTACCAGATGGACCTGC
GTTGTATTAGCTAGTTGGTGAGGTAACGGCTCACCAAGGCGACGATACATAGCCGACCTGAGAGGGTGAT
CGGCCACACTGGGACTGAGACACGGCC
>Zotu146
GAACGCTGGCGGTCTGCTTAACACATGCAAGTCGAACGGTGTCTTCGGACACAGTGGCGGACGGGTGAGT
AACGCGTGAGAATCTACCCTCAGGTCGGGGATAACTCCTGGAAACGGGAGCTAATACCGGATGTGCCGAA
AGGTGAAAGGTTAACTGCCTGGGGAGGAGCTCGCGTCTGATTAGCTAGTTGGTGGGTTAAAAGCTTACCA
AGGCGACGATCAGTAGCTGGTCTGAGAGGATGAGCAGCCACACTGGGACTGAGACACGGCC
>Zotu148
GAACGCTGGCGGCATGCTTAACACATGCAAGTCGGACGGGAAGTGGTGTTTCCAGTGGCGGACGGGTGA
GTAACGCGTAAGAACCTGCCCTTGGGAGGGGAACAACAACTGGAAACGGTTGCTAATACCCCGTAGGCTG
AGGAGCAAAAGGAGGAATCCGCCCGAGGAGGGGCTTGCGTCTGATTAGCTAGTTGGTGAGGTAATAGCTT
ACCAAGGCGATGATCAGTAGCTGGTCCGAGAGGATGATCAGCCACACTGGGACTGAGACACGGCC
>Zotu150
GAACGCTGGCGGCAGGCCTAACACATGCAAGTCGAACGGTAGCACAGAGGAGCTTGCTCCTTGGGTGACG
AGTGGCGGACGGGTGAGTAATGTCTGGGAAACTGCCCGATGGAGGGGGATAACTACTGGAAACGGTAGC
TAATACCGCATAACGTCGCAAGACCAAAGAGGGGGACCTTCGGGCCTCTTGCCATCGGATGTGCCCAGAT
GGGATTAGCTAGTAGGTGGGGTAACGGCTCACCTAGGCGACGATCCCTAGCTGGTCTGAGAGGATGACCA
GCCACACTGGAACTGAGACACGGTC
>Zotu152
GAACGCTGGCGGCGTGCTTAACACATGCAAGTCGAACGGTGACGAAGAGCTTGCTCTTCTGATCAGTGGC
GAACGGGTGAGTAACACGTGAGCAACCTACCCTTGACACTGGGATAACAGTTGGAAACAGCTGCTAATAC
CGGATATGAACTCAGCAGGCATCTGCCGAGTTGGAAAGCTCCGACGGTCAAGGATGGGCTCGCGGCCTAT
CAGCTCGTTGGTGAGGTAACGGCTCACCAAGGCGACGACGGGTAGCCGGCCTGAGAGGGCGACCGGCCA
CACTGGGACTGAGATACGGCC
>Zotu154
GAACGCTGGCGGCGTGCTTAACACATGCAAGTCGAACGAAGAGCGATGGAAGCTTGCTTCTATCAATCTTA
GTGGCGAACGGGTGAGTAACGCGTAATCAACCTGCCCTTCAGAGGGGGACAACAGTTGGAAACGACTGCT
AATACCGCATACGATCCAATCTCGGCATCGAGGATGGATGAAAGGTGGCCTCTATTTATAAGCTATCACTG
AAGGAGGGGATTGCGTCTGATTAGCTAGTTGGAGGGGTAACGGCCCACCAAGGCAATGATCAGTAGCCG
GTCTGAGAGGATGAACGGCCACATTGGGACTGAGACACGGCC
>Zotu156
GAACGCTGGCGGCATGCTTAACACATGCAAGTCGAACGGCAGCACAGTAGAGCTTGCTCTATGGGTGGCG
AGTGGCGGACGGGTGAGTAATGCATCGGGACCTACCCAGACGTGGGGGATAACGTAGGGAAACTTACGC
TAATACCGCATACGTCCTACGGGAGAAAGCGGGGGATCGAAAGACCTCGCGCGGTTGGATGGACCGATGT
GCGATTAGCTTGTTGGTGAGGTAACGGCTCACCAAGGCGACGATCGCTAGCTGGTCTGAGAGGATGATCA
GCCACACTGGGACTGAGACACGGCC
>Zotu159
GAACGCTGGCGGCATGCCTAACACATGCAAGTCGAACGAGCCCTTCGGGGTTAGTGGCGCACGGGTGCGT
AACGCGTGGGAATCTGCCCTTTGCTTCGGAATAACTTCGGGAAACTGAAGCTAATACCGGATGATGTCCTC
ATTAGAGATATTGGGGACCAAAGATTTATCGGCAAAGGATGAGCCCGCGTAGGATTAGGTAGTTGGTGGG

GTAAAGGCCTACCAAGCCGACGATCCTTAGCTGGTCTGAGAGGATGATCAGCCACACTGGGACTGAGACA
CGGCC
>Zotu161
GAACGCTGGCGGCATGCCTAACACATGCAAGTCGAACGAAGGCTTCGGCCTTAGTGGCGCACGGGTGCGT
AACGCGTGGGAATCTGCCCTTGGGTTCGGGATAACAGTGAGAAATTACTGCTAATACCGGATGATGACTTC
GGTCCAAAGATTTATCGCCCAGGGATGAGCCCGCGTTGGATTAGCTAGTTGGTGGGGTAAAGGCCTACCA
AGGCGACGATCCATAGCTGGTCTGAGAGGATGATCAGCCACACTGGGACTGAGACACGGCC
>Zotu163
GAACGCTGGCGGCGTGCCTAATACATGCAAGTCGAGCGAATGGATTGAGAGCTTGCTCTCAAGAAGTTAG
CGGCGGACGGGTGAGTAACACGTGGGTAACCTGCCCATAAGACTGGGATAACTCCGGGAAACCGGGGCT
AATACCGGATAACATTTTGAACTGCATGGTTCGAAATTGAAAGGCGGCTTCGGCTGTCACTTATGGATGGA
CCCGCGTCGCATTAGCTAGTTGGTGAGGTAACGGCTCACCAAGGCAACGATGCGTAGCCGACCTGAGAGG
GTGATCGGCCACACTGGGACTGAGACACGGCC
>Zotu165
GAACGCTGGCGGTGTGCCTAATACATGCAAGTCGAGCGGCAGCACGAATAGTTTACTATTTGGTGGCGAG
CGGCGGACGGCTGAGTAACACGTAGGAACATACCCCAAAGTGAGGGATAACTGCTCGAAAGAGTAGCTAA
TACCGCATGTGATCTAAGGATTAAAGCTTCGGCGCTTTGGGAATGGCCTGCGTTCGATTAGCTAGTTGGTA
AGGTAAAAGCTTACCAAGGCGACGATCGATAGCTGGTCTGAGAGGATGATCAGCCAGACTGGGACTGAG
ACACGGCC
>Zotu168
GAACGCTGGCGGCGTGCTTAACACATGCAAGTCGAACGGAAAGGCCCAGCTTGCTGGGTGCTCGAGTGGC
GAACGGGTGAGTAACACGTGGGTGATCTGCCTCTAACTTCGGGATAAGCTTGGGAAACTGGGTCTAATAC
CGGATAGGACAATCGTTTAGTGTCGGTTGTGGAAAGTTTTTTCGGTTAGGGATGAGCCCGCGGCCTATCAG
CTTGTTGGTGGGGTAATGGCCTACCAAGGCGTCGACGGGTAGCCGGCCTGAGAGGGTGGACGGCCACATT
GGGACTGAGATACGGCC
>Zotu170
GAACGCTTGCGGCGTGCCTAAGAAATGCAAGTCGAGCGGGGGAGCAATCCCTAGCGGCGAACGGTCGCG
TAACACGTAGGCAACCTGCCCCAGAGACCGGGACAACACTCCGAAAGGGGTGCTAATACCGGATGCGGCC
GGCGGAAGACCTCTTCTGCCGATTAAAGCCCTTCGGGGGCTCTGGGAGGGGCCTGCGGCCTATCAGCTAG
ATGGTGGGGTGATAGCCTACCATGGCGACGACGGGTAGCTGGTCTGAGAGGATGGTCAGCCGGACTGGG
ACTGAGATACGGCC
>Zotu172
GAACGCTGGCGGCGTGCCTAATACATGCAAGTCGAGCGAACAGATAAGGAGCTTGCTCCTTTGACGTTAG
CGGCGGACGGGTGAGTAACACGTGGGTAACCTACCTATAAGACTGGAATAACTCCGGGAAACCGGGGCTA
ATGCCGGATAACATTTAGAACCGCATGGTTCTAAAGTGAAAGATGGTTTTGCTATCACTTATAGATGGACCC
GCGCCGTATTAGCTAGTTGGTAAGGTAACGGCTTACCAAGGCAACGATACGTAGCCGACCTGAGAGGGTG
ATCGGCCACACTGGAACTGAGACACGGTC
>Zotu174
GAACGCTGGCGGCGTGCCTAATACATGCAAGTAGAACGCTGAAGAGAGGAGCTTGCTCTTCTTGGATGAG
TTGCGAACGGGTGAGTAACGCGTAGGTAACCTGCCTGGTAGCGGGGGATAACTATTGGAAACGATAGCTA
ATACCGCATAAAATTGATTATTGCATGATAATTAATTGAAAGATGCAATTGCATCACTACCAGATGGACCTG
CGTTGTATTAGCTAGTTGGTGAGGTAACGGCTCACCAAGGCGACGATACATAGCCGACCTGAGAGGGTGA
TCGGCCACACTGGGACTGAGACACGGCC
>Zotu176
GAACGCTGGCGGCGTGCTTAACACATGCAAGTCGAACGATGATCCGGTGCTTGCACCGGGGATTAGTGGC
GAACGGGTGAGTAACACGTGAGTAACCTGCCCTTAACTCTGGGATAAGCCTGGGAAACTGGGTCTAATAC
CGGATATGACTCCTCATCGCATGGTGGGGGGTGGAAAGCTTTTGTGGTTTTGGATGGACTCGCGGCCTATC
AGCTTGTTGGTGAGGTAATGGCTCACCAAGGCGACGACGGGTAGCCGGCCTGAGAGGGTGACCGGCCAC
ACTGGGACTGAGACACGGCC
>Zotu178

GAACGCTGGCGGCGTGCCTAATACATGCAAGTAGAACGCTGAAGGAAGGAGCTTGCTCTTTCCGGATGAG
TTGCGAACGGGTGAGTAACGCGTAGGTAACCTGCCTGGTAGCGGGGGATAACTATTGGAAACGATAGCTA
ATACCGCATAACAGTAGATATTGCATGATATCTGCTTGAAAGGTGCAAATGCATCACTACCAGATGGACCT
GCGTTGTATTAGCTAGTTGGTGAGGTAACGGCTCACCAAGGCGACGATACATAGCCGACCTGAGAGGGTG
ATCGGCCACACTGGGACTGAGACACGGCC
>Zotu180
GAACGCTGGCGGCGTGCCTAATACATGCAAGTCGAGCGAACAGATAAGGAGCTTGCTCCTTTGACGTTAG
CGGCGGACGGGTGAGTAACACGTGGGTAACCTACCTATAAGACTGGAATAACTCCGGGAAACCGGGGCTA
ATGCCGGATAATACACAGAACCGCATGGTTCTGTGATGAAAGACGGTTTTGCTGTCACTTATAGATGGACC
CGCGGCGTATTAGCTAGTTGGTAAGGTAACGGCTTACCAAGGCAACGATACGTAGCCGACCTGAGAGGGT
GATCGGCCACACTGGAACTGAGACACGGTC
>Zotu182
GAACGCTATCGGTATGCTTAACACATGCAAGTCGGACGAATTAATTAGGGCTTGCCCTAATTAATTAGTGG
CAGACGGGTGAGTAACACGTGGGAATCTACCCATCAGTACGGAATAACTTTTAGAAATAAAAGCTAATACC
GTATATTCTCTAAGGAGGAAAGATTTATCGCTGATGGATGAGCCCGCGCCAGATTAGGTAGTTGGTGAAGT
AATGGCTCACCAAGCCAACGATCTGTAGCTGGTCTGAGAGGATGATCAGCCACACTGGGACTGAGACACG
GCC
>Zotu184
GAACGCTGGCGGCGTGCCTAATACATGCAAGTAGAACGCTGAAGGAGGAGCTTGCTTCTCTGGATGAGTT
GCGAACGGGTGAGTAACGCGTAGGTAACCTGCCTGGTAGCGGGGGATAACTATTGGAAACGATAGCTAAT
ACCGCATAAGAGTAGATGTTGCATGACATTTGCTTAAAAGGTGCAATTGCATCACTACCAGATGGACCTGC
GTTGTATTAGCTAGTTGGTGAGGTAACGGCTCACCAAGGCAACGATACATAGCCGACCTGAGAGGGTGAT
CGGCCACACTGGGACTGAGACACGGCC
>Zotu188
GATAATAAGCAAACAAAGAGTAAAAGAATCCCTGAATCAGCCGACGGTAATTCCATGACGTGGGTCAGCG
TTAAATTATTGACCAAAGCGATTGATCGCTGTATCTGGCGTTATATATGAAATGAACATAACAAACATCAAG
TCGCTGTTTGGCAAGATGAGAAGCAGAACTTGAAGTCACATCCTGATTCGTTCTTTAAGGTGGGGGT
>Zotu190
GAACGCTGGCGGCGTGCCTAATACATGCAAGTCGAACGAACAGACTTGGAAGCTTGCTTCCAATGACGTTA
GTGGCGCACGGGTGAGTAACACGTGGAGAACCTACCCTTTAGCGGGGGATAACAGTCGGAAACGACTGCT
AAGACCGCATAGAACGTTAAGTCGCATGACAAAACGAGGAAAAATGGCTTCGGCTATTACTAAAGGATGG
CTCCGCGGTGCATTAGTTAGTTGGTAAGGTAACGGCTTACCAAGACAGTGATGCATAGCCGACCTGAGAG
GGTGATCGGCCACATTGGGACTGAGACACGGCC
>Zotu193
GAACGCTGGCGGCGTGCCTAATACATGCAAGTCGAGCGGATTGAACGGGAGCTTGCTTCCGTTCGGTTAG
CGGCGGACGGGTGAGTAACACGTGGGCAACCTGCCCGTAAGACCGGGATAACTTCGGGAAACCGGAGCT
AATACCGGATAACACCGAAGACCGCATGGTCTTCGGTTGAAAGGCGGCTTTGGCTGTCACTTACGGATGG
GCCCGCGGCGCATTAGCTAGTTGGTGAGGTAACGGCTCACCAAGGCGACGATGCGTAGCCGACCTGAGAG
GGTGATCGGCCACACTGGGACTGAGACACGGCC
>Zotu195
GAACGCTGGCGGCGTGCCTAATACATGCAAGTCGAGCGAATGGATTAAGAGCTTGCTCTTATGAAGTTAGC
GGCGGACGGGTGAGTAACACGTGGGTAACCTACCCATAAGACTGGGATAACTCCGGGAAACCGGGGCTA
ATACCGGATAATATTTTGAACTGCATAGTTCGAAATTGAAAGGCGGCTTCGGCTGTCACTTATGGATGGAC
CCGCGTCGCATTAGCTAGTTGGTGAGGTAACGGCTCACCAAGGCGACGATGCGTAGCCGACCTGAGAGGG
TGATCGGCCACACTGGGACTGAGACACGGCC
>Zotu197
GAACGCTGGCGGCGTGCCTAATACATGCAAGTAGAACGCTGAAGGAGGAGCTTGCTTCTCTGGATGAGTT
GCGAACGGGTGAGTAACGCGTAGGTAACCTGCCTGGTAGCGGGGGATAACTATTGGAAACGATAGCTAAT
ACCGCATAATAGCAGTTATTGCATGATAACTGTTTGAAAGGTGCAATTGCACCACTACCAGATGGACCTGC
GTTGTATTAGCTAGTTGGTGGGGTAACGGCTCACCAAGGCGACGATACATAGCCGACCTGAGAGGGTGAT
CGGCCACACTGGGACTGAGACACGGCC

>Zotu199
GAACGCTGGCGGCGTGCTTAACACATGCAAGTCGAACGGAAAGGCCAGTGCTTGCACTGGTGCTCGAGTG
GCGAACGGGTGAGTAACACGTGGGTGATCTGCCCTGCACTTTGGGATAAGCCTGGGAAACTGGGTCTAAT
ACCAGATAGGACCATGCTTTGGATGGTGTGGTGGAAAGTTTTTTCGGTGTGGGATGAGCTTGCGGCCTATC
AGCTTGTTGGTGGGGTAATGGCCTACCAAGGCGTCGACGGGTAGCCGGCCTGAGAGGGTGTACGGCCAC
ATTGGGACTGAGATACGGCC
>Zotu201
GAACGCTGGCGGCATGCCTAACACATGCAAGTCGAACGAGATCTTCGGATCTAGTGGCGCACGGGTGCGT
AACGCGTGGGAATCTGCCTTGGGGTTCGGAATAACTCCCCGAAAGGGGTGCTAATACCGGATGATGTCGA
AAGACCAAAGATTTATCGCCCTGAGATGAGCCCGCGTAGGATTAGCTAGTTGGTGTGGTAAAGGCGCACC
AAGGCGACGATCCTTAGCTGGTCTGAGAGGATGATCAGCCACACTGGGACTGAGACACGGCC
>Zotu203
GAACGCTGGCGGCGTGCTTAACACATGCAAGTCGAGCGGTAAGGCCCTTCGGGGTACACGAGCGGCGAA
CGGGTGAGTAACACGTGGGTGATCTGCCTCGCACTCTGGGATAAGCCTGGGAAACTGGGTCTAATACCGG
ATATGAACATGGTCTGCATGGACTGTGTTGGAAAGGTTTACTGGTGCGAGATGGGCCCGCGGCCTATCAG
CTTGTTGGTGGGGTAATGGCCTACCAAGGCGACGACGGGTAGCCGACCTGAGAGGGTGACCGGCCACACT
GGGACTGAGACACGGCC
>Zotu205
GAACGCTGGCGGCGTGCTTAACACATGCAAGTCGAACGCTGAAGCTCAGCTTGCTGGGTGGATGAGTGGC
GAACGGGTGAGTAACACGTGAGTAACCTGCCCCCTTCTTTGGGATAACGCCCGGAAACGGGTGCTAATACT
GGATATTCACTGATCTTCGCATGGGGGTTGGTGGAAAGGTTTTTCTGGTGGGGGATGGGCTCGCGGCCT
ATCAGCTTGTTGGTGGGGTGATGGCCTACCAAGGCTTTGACGGGTAGCCGGCCTGAGAGGGTGACCGGTC
ACATTGGGACTGAGATACGGCC
>Zotu207
GAACGCTGGCGGCGTGCTTAACACATGCAAGTCGAACGGAAAGGCCCAAGCTTGCTTGGGTACTCGAGTG
GCGAACGGGTGAGTAACACGTGGGTGATCTGCCCTGCACTTCGGGATAAGCCTGGGAAACTGGGTCTAAT
ACCGGATAGGACCACATTTTGGATGGTGTGGTGGAAAGTTTTTCGGTGTGGGATGAGCTCGCGGCCTATCA
GCTTGTTGGTGGGGTAATGGCCTACCAAGGCGTCGACGGGTAGCCGGCCTGAGAGGGTGTACGGCCACAT
TGGGACTGAGATACGGCC
>Zotu209
GAATGCTGGCGGCGTGCCTAACACATGCAAGTCGAGCGGTAACAGGAGAATTTATTCTTGCTGACGAGCG
GCGGACGGCTGAGTAACGCGTGGGAACATGCCCCAAAGTGAGGGATAAGCTCCAGAAATGGAGTCTAAT
ACCGCATATGATCTTCGGATTAAAGGATTTATCCGCTTTGGGAGTGGCCTGCGTACGATTAGATAGTTGGT
GAGGTAATGGCTTACCAAGTCTACGATCGTTAGCTGGTCTGAGAGGATGACCAGCCAGACTGGAACTGAG
ACACGGTC
>Zotu211
GAACGCTAGCGGGAGGCCTAACACATGCAAGCCGAGCGGTAGGGTTTCTTCGGAAACCTGAGAGCGGCG
CACGGGTGCGGAACACGTGTGCAACCTACCTTTATCAGGGGGATAGCCTTTCGAAAGGAAGATTAATACCC
CATAATATATTAGATGGCATCATTTGATATTGAAAACTCCGGTGCATAGAGATGGGCACGCGCAAGATTAG
ATAGTTGGTGAGGTAACGGCTCACCAAGTCAGTGATCTTTAGGGGGCCTGAGAGGGTGATCCCCCACACT
GGTACTGAGACACGGAC
>Zotu213
GAACGCTGGCGGCGTGCTTAACACATGCAAGTCGAACGGTGAAGGGATCAGTTTTCTGGTTCTGGATGAG
TGGCGAACGGGTGAGTAACACGTGAGTAACCTGCCCCCTTCTTCTGGATAACCGCATGAAAGTGTGGCTAA
TACGGGATATTCTGCGCCTGCTGCATGGTGGGTGTGGGAAAGATATTGTTTCGGTGGGGGATGGGCTCGC
GGCCTATCAGCTTGTTGGTGGGGTGATGGCCTACCAAGGCGGTGACGGGTAGCCGGCCTGAGAGGGTGG
ACGGTCACACTGGGACTGAGACACGGCC
>Zotu215
GAACGCTAGCGGCAGGCCTAACACATGCAAGTCGAGGGAGAAGCCCTTCGGGGCGGAAACCGGCGCACG
GGTGCGTAACGCGTATGCAACCTACCTTTCACAGGGGGATAGCCCGGAGAAATTTGGATTAATATCCCATA

ATATTGTTGGATGGCATCATTTGGCAATTAAAGTTACGATGGTGGAAGATGGGCATGCGTCCTATTAGCTA
GTTGGCGAGGTAACGGCTCGCCAAGGCGACGATAGGTAGGGGTCCTGAGAGGGAGATCCCCCACACTGG
TACTGAGACAGGGAC
>Zotu217
GAACGCTGGCGGCATGCCTAATACATGCAAGTTGAACGATGATATTCTCTGCTTGCAGAGAATTGAAGAGT
AGCGAACGGGTGAGCAACACGTGGGGAACCTGCCCATGAGAGGGGGATAACATTCGGAAACGGATGCTA
ATACCGCATAATGCTTTGGACCGCCTGGTCCAGAGACGAAAGACGGCTTTTGCTGTCACTCATGGATGGCC
CCGCGCCGTATTAGCTAGTTGGTGAGGTAATGGCTCACCAAAGCTGTGATACGTAGCCGACCTGAGAGGG
TGATCGGCCACACTGGGACTGAGACACGGCC
>Zotu219
GAACGCTGGCGGCGTGCTTAACACATGCAAGTCGAACGGAAAGGCTCCTGCTTGCAGGGGTACTCGAGTG
GCGAACGGGTGAGTAACACGTGGGTGATCTGCCCTGCACTTCGGGATAAGCCTGGGAAACTGGGTCTAAT
ACCGGATAGGACCACATTTTGGATGGTGTGGTGGAAAGTTTTTCGGTGTGGGATGAGCTCGCGGCCTATCA
GCTTGTTGGTGGGGTAATGGCCTACCAAGGCGTCGACGGGTAGCCGGCCTGAGAGGGTGTACGGCCACAT
TGGGACTGAGATACGGCC
>Zotu222
GAACGCTGGCGGCATGCCTAACACATGCAAGTCGAACGAACCCTTCGGGGTTAGTGGCGCACGGGTGCGT
AACGCGTGGGAATCTGCCCTTTGCTTCGGAATAACTCAGGGAAACTTGTGCTAATACCGGATGATGTCTTC
GGACCAAAGATTTATCGGCAAGGGATGAGCCCGCGTAGGATTAGGTAGTTGGTGGGGTAAAGGCCTACCA
AGCCGACGATCCTTAGCTGGTCTGAGAGGATGATCAGCCACACTGGGACTGAGACACGGCC
>Zotu224
GAACGCTATCGGTATGCTTAACACATGCAAGTCGGACGAATTAATTAGGGCTTGCCCTAATTAATTAGTGG
CAGACGGGTGAGTAACACGTGGGAATCTACCCATCAGTACGGAATAACTTTTAGAAATAAAAGCTAATACC
GTATATTCTCTAAGGAGGAAAGATTTATCGCTGATGGATGAGCCCGCGCCAGATTAGGTAGTTGGTGAAGT
AATGGCTCACCAAGCCAACAATCTGTAGCTGGTCTGAGAGGATGATCAGCCACACTGGGACTGAGACACG
GCC
>Zotu226
GAACGCTGGCGGCGTGCTTAACACATGCAAGTCGAACGGGATTTAGTAAACAGAAGCCTCGGTGGAAGAT
TACTAATGAGAGTGGCGAACGGGTGAGTAACGCGTGAGCAACCTGCCTATGACAGTGGGATAGCCTCGGG
AAACCGGGATTAATACCGCATAAAATCGTAGAAACACATGTTTTAACGGTCAAAGATTTATCGGTCATAGA
TGGGCTCGCGTCTGATTAGCTAGTTGGTGAGATAACAGCCCACCAAGGCGACGATCAGTAGCCGGTCTGA
GAGGATGAACGGCCACATTGGAACTGAGACACGGTC
>Zotu228
GAACGCTGGCGGCGTGCCTAATACATGCAAGTAGAACGCTGAAGGAGGAGCTTGCTTCTCTGGATGAGTT
GCGAACGGGTGAGTAACGCGTAGGTAACCTGCCTGGTAGCGGGGGATAACTATTGGAAACGATAGCTAAT
ACCGCATAACAGTAGATGTTGCATGACATTTGCTTAAAAGGTGCAATTGCATCACTACCAGATGGACCTGC
GTTGTATTAGCTAGTTGGTGGGGTAACGGCTCACCAAGGCGACGATACATAGCCGACCTGAGAGGGTGAT
CGGCCACACTGGGACTGAGACACGGCC
>Zotu230
GAACGCTGGTGGCATGCTTAACACATGCAAGTCGAACGGTACAGGACTAGCTTGCTAGTTGCTGACGAGT
GGCGGACGGGTGAGTAACGCGTAGGAATCTGCCCATCTGAGGGGGATACCAGTTGGAAACGACTGTTAAT
ACCGCATAGTATCTGTGGATTAAAGGTGGCTTTTGGGCTGTCGCAGATGGATGAGCCTGCGTTGGATTAGC
TAGTTGGTGGGGTAAGGGCCTACCAAGGCTACGATCCATAGCTGATTTGAGAGGATGATCAGCCACATTG
GAACTGAGACACGGCC
>Zotu232
GAACGCTGGCGGCGTGCTTAACACATGCAAGTCGAACGGAAAGGCCCTGCTTGCAGGGTGCTCGAGTGGC
GAACGGGTGAGTAACACGTGGGTGATCTGCCCCTTATTTTGGGATAAGCCTGGGAAACTGGGTCTAATACT
GGATAGGACCATGCTGTAGGTGGTGTGGTGGAAAGATTAGTTTCGGTAAGGGATGAGCTCGCGGCCTATC
AGCTTGTTGGTGGGGTAATGGCCTACCAAGGCGTCGACGGGTAGCCGGCCTGAGAGGGTGGACGGCCAC
ATTGGGACTGAGATACGGCC

>Zotu234
GAACGCTGGCGGCAGGCCTAACACATGCAAGTCGAGCGAGCATTTCGGTGCTAGCGGCGGACGGGTGAG
TAACGCGTGGGAACGTGCCCTTTTCTGCGGAATAGTCCTGGGAAACTGGGGGTAATACCGCATACGCCCTT
TTGGGGAAAGATTTATCGGAGAAGGATCGGCCCGCGTTGGATTAGGTAGTTGGTGGGGTAATGGCCCACC
AAGCCGACGATCCATAGCTGGTTTGAGAGGATGATCAGCCACACTGGGACTGAGACACGGCC
>Zotu236
AAACGCAAGAGACGTGCCTAACACATGCAAATTAAATAGTTAATAGTAACTATAGTGAATTGGTGAGTAAT
ATATGAAAATTTAAATTTAGATTAGAAGTAATCCTAATAAATTAGGAGTAATCTCTTATAAGTGCATAAGTG
CTTTTAAAAGTTAATACTGTCTAAAGAAAAGTTCATATCTGATTAGCTAGTTAGTAGGGTAAAGGCCTACTA
AGGCGAAGATCAGTAGCTACCCTGAGAGGGGGAATAGCCACATTGGGATTGAAATACAGCC
>Zotu238
GAACGCTGGCGGCGTGCCTAATACATGCAAGTAGAACGCTGAAGGAGGAGCTTGCTTCTCTGGATGAGTT
GCGAACGGGTGAGTAACGCGTAGGTAACCTGCCTGGTAGCGGGGGATAACTATTGGAAACGATAGCTAAT
ACCGCATAAGAGTAGATGTTGCATGACATTTGCTTAAAAGGTGCAATTGCATCACTACCAGATGGACCTGC
GTTGTATTAGCTAGTTGGTGAGGTAACGGCTCACCAAGGCGACGATACATAGCCGACCTGAGAGGGTGAT
CGGCCACACTGGGACTGAGACACGGCC
>Zotu241
GAACGCTGGCGGCATGCCTTACACATGCAAGTCGAACGGTAACAGGTCTTCGGATGCTGACGAGTGGCGA
ACGGGTGAGTAATACATCGGAACGTGCCCGAGAGTGGGGGATAACGAAGCGAAAGCTTTGCTAATACCGC
ATACGATCTCAGGATGAAAGCAGGGGACCGCAAGGCCTTGCGCTCACGGAGCGGCCGATGGCAGATTAG
GTAGTTGGTGGGATAAAAGCTTACCAAGCCGACGATCTGTAGCTGGTCTGAGAGGACGACCAGCCACACT
GGGACTGAGACACGGCC
>Zotu243
GAACGCTGGCGGCATGCCTAACACATGCAAGTCGAACGAGACCTTCGGGTCTAGTGGCGCACGGGTGCGT
AACGCGTGGGAATCTGCCTTTCGGTTCGGAATAACTCGGGGAAACCCGTGCTAATACCGGATGATGTCGCA
AGACCAAAGATTTATCGCCGAAAGATGAGCCCGCGTCGGATTAGCTAGTTGGTGGGGTAAAGGCCTACCA
AGGCGACGATCCGTAGCTGGTCTGAGAGGATGATCAGCCACACTGGGACTGAGACACGGCC
>Zotu245
GAACGCTGGCGGCAGGCTTAACACATGCAAGTCGAGCGCCCCGCAAGGGGAGCGGCAGACGGGTGAGTA
ACGCGTGGGAACATGCCCTTCGGTTCGGAATAACTCAGGGAAACTTGAGCTAATACCGGATACGTCCGTAA
GGAGAAAGATTTATTGCCGAAGGATTGGCCCGCGTCCGATTAGCTAGTTGGTGAGGTAACGGCTCACCAA
GGCGACGATCGGTAGCTGGTCTGAGAGGATGATCAGCCACACTGGGACTGAGACACGGCC
>Zotu247
GAACGCTGGCGGCGTGCTTAACACATGCAAGTCGAACGGTGAACACGGAGCTTGCTCTGTGGGATCAGTG
GCGAACGGGTGAGTAACACGTGAGCAACCTGCCCCTGACTCTGGGATAAGCGCTGGAAACGGCGTCTAAT
ACTGGATATGTGACGTGACCGCATGGTCTGCGTCTGGAAAGAATTTCGGTTGGGGATGGGCTCGCGGCCT
ATCAGCTTGTTGGTGAGGTAATGGCTCACCAAGGCGTCGACGGGTAGCCGGCCTGAGAGGGTGACCGGCC
ACACTGGGACTGAGACACGGCC
>Zotu249
CGTCAGGTCCCAGTGCGCTTGGTTAATTACACCTTAGAAGACCTCTCCCTGCCGCGAAGAAGGGCAGTAGC
GCAATTCATCCCGGCGAGCATTACAGAAATCGGACAAACGGAGAGTATCGGACTAACAGGA
>Zotu251
GAACGCTGGCGGCATGCCTAATACATGCAAGTCGAGCGAACAGATGAAGTGCTTGCACTTCTGACGTTAGC
GGCGAACGGGTGAGTAACACGTAAGGAATCTACCTATAAGCGGGGGATAACATTCGGAAACGGGTGCTA
ATACCGCATAATATCTTCTTCCGCATGGAAGAAGATTGAAAGACGGCTCTGCTGTCACTTATAGATGACCTT
GCGGTGCATTAGTTAGTTGGTGGGGTAATGGCCTACCAAGACGATGATGCATAGCCGACCTGAGAGGGTG
ATCGGCCACATTGGGACTGAGACACGGCC
>Zotu253
GAACGCTGGCGGCATGCTTAACACATGCAAGTCGGACGGGAAGTGGTGTTTCCAGTGGCGGACGGGTGA
GTAACGCGTAAGAACCTGCCCTTGGGAGGGGAACAACAGCTGGAAACGGCTGCTAATACCCCATAGGCTG

AGGAGCAAAAGGAGGAATCCGCCCAAGGAGGGGCTCGCGTCTGATTAGTTAGTTGGTGAGGCAATGGCT
TACCAAGGCGACGATCAGTAGCTGGTCCGAGAGGATGATCAGCCACACTGGGACTGAGACACGGCC
>Zotu256
GAACGCTGGCGGCGTGCTTAACACATGCAAGTCGAACGATGAACCTCCTTCGGGAGGGGATTAGTGGCGA
ACGGGTGAGTAACACGTGGGCAATCTGCCCTTCACTCTGGGACAAGCCCTGGAAACGGGGTCTAATACCG
GATACGACTACCGACCGCATGGTCTGGTGGTGGAAAGCTCCGGCGGTGAAGGATGAGCCCGCGGCCTATC
AGCTTGTTGGTGGGGTGATGGCCTACCAAGGCGACGACGGGTAGCCGGCCTGAGAGGGCGACCGGCCAC
ACTGGGACTGAGACACGGCC
>Zotu258
GAACGCTGGCGGTATGCTTAACACATGCAAGTCGAACGGTGCTTTAGGGCACAGTGGCGGACGGGTGAGT
AACGCGTGAGAATCTGGCTTCAGGTCTGGGACAACCACTGGAAACGGTGGCTAATACCGGATGTGCCGAG
AGGTGAAAGGTTAACTGCCTGAAGATGAGCTCGCGTCTGATTAGCTAGTTGGAAGTGTAATGGACTCCCAA
GGCGACGATCAGTAGCTGGTCTGAGAGGACGATCAGCCACACTGGGACTGAGACACGGCC
>Zotu260
GAACGCTAGCGGCAGGCTTAACACATGCAAGTCGAGGGGTATAGTTCTTCGGAGCTAGAGACCGGCGCAC
GGGTGCGTAACGCGTATGCAATCTACCTTTTACAGAGGGATAGCCCAGAGAAATTTGGATTAATACCTCAT
AGTATGTGAGAATCGCATGGTTTTCACATTAAAGTCACAACGGTAAAAGATGAGCATGCGTCCCATTAGCT
TGTTGGTAAGGTAACGGCTTACCAAGGCAACGATGGGTAGGGGTCCTGAGAGGGAGATCCCCCACACTGG
TACTGAGACACGGAC
>Zotu262
GAACGCTGGCGGCGTGCCTAATACATGCAAGTCGAGCGGATGAATCGAAAGCTTGCTTTTGATTCGTTAGC
GGCGGACGGGTGAGTAACACGTGGGCAACCTGCCCTGTAGACGGGGATAACACCGAGAAATCGGTGCTA
ATACCGGATAACACGAAATGTCGCATGACGTTTCGTTGAAAGACGGCGCAAGCTGTCGCTACAGGATGGG
CCCGCGGCGCATTAGCTAGTTGGTGAGGTAACGGCTCACCAAGGCGACGATGCGTAGCCGACCTGAGAGG
GTGATCGGCCACACTGGGACTGAGACACGGCC
>Zotu264
GAACGCTGGCGGCGTGCTTAACACATGCAAGTCGAACGGAAAGGCCCTTCGGGGTGCTCGAGTGGCGAAC
GGGTGAGTAACACGTGGGTGATCTGCCCTGCACTTTGGGATAAGCCTGGGAAACTGGGTCTAATACCGAA
TATGATCATCGGCTTCATGGTCGGTGGTGGAAAGCTTTTGCGGTGTGGGATGGGCCCGCGGCCTATCAGCT
TGTTGGTGGGGTAATGGCCTACCAAGGCGTCGACGGGTAGCCGGCCTGAGAGGGTGTCCGGCCACACTG
GGACTGAGATACGGCC
>Zotu267
GAACGCTGGCGGCGTGCCTAATACATGCAAGTCGAGCGGACCGAACGAGAGCTTGCTCTTGTTTGGTCAG
CGGCGGACGGGTGAGTAACACGTGGGCAACCTGCCCGCAAGACCGGGATAACTCCGGGAAACCGGAGCT
AATACCGGATAACACCAAAGACCGCATGGTCTTTGGTTGAAAGGCGGCTTCGGCTGTCACTTGCGGATGG
GCCCGCGGCGCATTAGCTAGTTGGTGAGGTAACGGCTCACCAAGGCGACGATGCGTAGCCGGCCTGAGAG
GGTGACCGGCCACACTGGGACTGAGACACGGCC
>Zotu269
GAACGCTGGCGGCATGCTTAACACATGCAAGTCGAACGCAGTACTCAAGCTTGCTTGAGTAGCTGAGTGG
CAGACGGGTGAGTAACACGTGGGAACTTACCTTTTAGACTGGGACAACCACTGGAAACGGTGGCTAATAC
CGGATACGCCCTGAGGGGGAAAGCAGAAATGCGCTAAAGGAGAGGCCCGCGGCGGATTAGGTAGTTGGT
GGGGTAAAGGCCTACCAAGCCGATGATCTGTAGCTGGTTTGAGAGGATGATCAGCCACACTGGGACTGAG
ACACGGCC
>Zotu271
GAACGCTAGCTACAGGCTTAACACATGCAAGTCGAGGGGAAACGGCATTGAGTGCTTGCACTCTTTGGAC
GTCGACCGGCGCACGGGTGAGTAACGCGTATCCAACCTTCCCATTACTGTGGGATAACCTGCCGAAAGGCA
GACTAATACCGCATAGTCTTCGATGACGGCATCAGATTTGAAGTAAAGATTTATCGGTAATGGATGGGGAT
GCGTCTGATTAGCTTGTTGGCGGGGTAACGGCCCACCAAGGCAACGATCAGTAGGGGTTCTGAGAGGAAG
GTCCCCCACATTGGAACTGAGACACGGTC
>Zotu273

GAACGCTGGCGGTATGCTTTACACATGCAAGTCGTACGAGAATTTTTAATTCAAGTGGCGGACGGGTGAGT
AACACGTAAGAATTTGCCTTTAGGAGGGGGATAACAACTGGAAACGGTTGCTAATACCCCATATGCTTTCG
AGTGAAATGGAGTTTTCCGCCTAAAGAGAAGCTTGCGGCTGATTAGCTTGTTGGTAAGGTAATGGCTTACC
AAGGCGACGATCAGTATCTGGTTTGAGAGGACGATCAGACACACTGGAACTGAGACACGGTC
>Zotu275
GAACGCTGGCGGCGTGCCTAATACATGCAAGTCGAGCGGACCGAACGAGAGCTTGCTCTTGTTTGGTCAG
CGGCGGACGGGTGAGTAACACGTGGGCAACCTGCCCGCAAGACCGGGATAACTCCGGGAAACCGGAGCT
AATACCGGATAACACCAAAGACCGCATGGTCTTTGGTTGAAAGGCGGCTTCGGCTGCCACTTGCGGATGG
GCCCGCGGCGCATTAGCTAGTTGGTGAGGTAACGGCTCACCAAGGCGACGATGCGTAGCCGGCCTGAGAG
GGTGACCGGCCACACTGGGACTGAGACACGGCC
>Zotu277
GAACGCTGGCGGTGTGCTTAACACATGCAAGTCGAACGGTAAGGCCCTTTCGGGGGTACACGAGTGGCGA
ACGGGTGAGTAACACGTGAGTAACCTGCCCTCATCTCTGGGATAACAGTCGGAAACGGTTGCTAATACTGG
ATATGAGCTTGCAAGGCATCTTGTGGGTTGGAAAGATTTTTTTGGTTGGGGATGGACTCGCGGCCTATCAG
CTTGTTGGTGAGGTAGTGGCTCACCAAGGCTTCGACGGGTAGCCGGCCTGAGAGGGCGACCGGCCACATT
GGGACTGAGATACGGCC
>Zotu279
GAACGCTAGCGGCAGGCCTTACACATGCAAGTCGAGGGGTAACAGGGAAAAGCTTGCTTTTCCGCTGACG
ACCGGCGCACGGGTGCGTAACACGTATGCAACCTACCTCTTACTGAGGAATAGCCCAGAGAAATTTGGATT
AATACCTCATAGTATCACCAGTTCGCATGTTCAGGTGATTAAAGGTTACGGTAAGAGATGGGCATGCGCCC
CATTAGCTAGTTGGTGAGGTAATGGCTCACCAAGGCAACGATGGGTAGGGGGTCTGAGAGGACTATCCCC
CACACTGGTACTGAGACACGGAC
>Zotu281
GAACGCTGGCGGCGTGCCTAATACATGCAAGTCGAGCGGACCGAATAGAAGCTTGCTTCTGTTCGGTTAGC
GGCGGACGGGTGAGTAACACGTGGGCAACCTGCCCGTAAGACCGGGATAACTTCGGGAAACCGGAGCTA
ATACCGGATAACACCGAAGACCGCATGGTCTTCGGTTGAAAGGCGGCTTTGGCTGTCACTTACGGATGGGC
CCGCGGCGCATTAGCTAGTTGGTGAGGTAACGGCTCACCAAAGCGACGATGCGTAGCCGACCTGAGAGGG
TGATCGGCCACACTGGGACTGAGACACGGCC
>Zotu283
GAACGCTGGCGGCGTGCCTAATACATGCAAGTCGAGCGAACAGATAAGGAGCTTGCTCCTTTGACGTTAG
CGGCGGACGGGTGAGTAACACGTGGGTAACCTACCTATAAGACTGGAATAACTCCGGGAAACCGGGGCTA
ATGCCGGATAATATTTAGAACCGCATGGTTCTAAAGTGAAAGATGGTTTTGCTATCACTTATAGATGGACCC
GCGCCGTATTAGCTAGTTGGTGGGGTAATGGCTTACCAAGGCAACGATACGTAGCCGACCTGAGAGGGTG
ATCGGCCACACTGGAACTGAGACACGGTC
>Zotu285
GAACGCTGGCGGCGTGCTTAACACATGCAAGTCGAACGATGAAAGAGGAGCTTGCTCCTCTGGATTAGTG
GCGAACGGGTGAGTAACACGTGAGCAATCTGCCCCTGACTCCGGAATAACTACGGGAAACTGTAGCTAAT
ACCGGATATGCGCCGAACAGGCATCTGTATCGGTGGGAAAGAATTTCGGTCAGGGATGAGCTCGCGGCCT
ATCAGCTTGTTGGTGAGGTAACGGCTCACCAAGGCGACGACGGGTAGCCGGCCTGAGAGGGTGACCGGC
CACATTGGGACTGAGACACGGCC
>Zotu287
GAACGCTGGCGGCGTGCTTAACACATGCAAGTCGAACGGAAAGGCCCAAGCTTGCTTGGGTACTCGAGTG
GCGAACGGGTGAGTAACACGTGGGTGATCTGCCCTGCACTTCGGGATAAGCCTGGGAAACTGGGTCTAAT
ACCGGATAGGACCGCATTTTGGATGGTGTGGTGGAAAGTTTTTCGGTGTGGGATGAGCTCGCGGCCTATC
AGCTTGTTGGTGGGGTAATGGCCTACCAAGGCGTCGACGGGTAGCCGGCCTGAGAGGGTGTACGGCCAC
ATTGGGACTGAGATACGGCC
>Zotu289
GAACGCTGGCGGCATGCTTTACACATGCAAGTCGAACGGCAGCGCGGGGCAACCTGGCGGCGAGTGGCG
AACGGGTGAGTAATATATCGGAACGTACCCAAGAGTGGGGGATAACGTAGCGAAAGTTACGCTAATACCG
CATACGATCCAAGGATGAAAGCAGGGGACCGCAAGGCCTTGTGCTCCTGGAGCGGCCGATATCTGATTAG

CTAGTTGGTGAGGTAAAGGCTCACCAAGGCGACGATCAGTAGCTGGTCTGAGAGGACGACCAGCCACACT
GGAACTGAGACACGGTC
>Zotu291
GAACGCTGGCGGCAGGCCTAACACATGCAAGTCGAGCGGATGAGAAGAGCTTGCTCTTCGATTCAGCGGC
GGACGGGTGAGTAATACCTAGGAATCTGCCTGGTAGTGGGGGACAACGTTTCGAAAGGAACGCTAATACC
GCATACGTCCTACGGGAGAAAGCAGGGGACCTTCGGGCCTTGCGCTATCAGATGAGCCTAGGTCGGATTA
GCTAGTTGGTGAGGTAATGGCTCACCAAGGCTACGATCCGTAACTGGTCTGAGAGGATGATCAGTCACACT
GGAACTGAGACACGGTC
>Zotu293
GAACGCTAGCGGCAGGCATAATACATGCAAGGCGAAGGGGCAGCAATGTCACTGTCGTACGGGTGCGTA
ACGCGTATGCAACCTACCTATCACTGGGGGATAGCCCGGGGAAACCCGGATTAATACCGCATAAAACAGG
GGCACCGCATGGTGATATTTGTTAAAGATTTATTGGTGATAGATGGGCATGCGTTCGATTAGCTAGTTGGC
GGGGTAACGGCCCACCAAGGCGACGATCGATAGGGGAGCTGAGAGGTTGGTCCCCCACACGGGCACTGA
GATACGGGC
>Zotu295
GAACGCTGGCGGTATGCTTAACACATGCAAGTCGAACGGTAGCAGGCCTTCGGGCGCTGACGAGTGGCGG
ACGGGTGAGTAACGCGTAGGAATCTGCCTCATAGTGGGGGACAACGTGGGGAAACTCACGCTAATACCGC
ATACGCCCTACGGGGGAAAGCGGGGGACCTTCGGGCCTCGCGCTATGAGATGAGCCTGCGTTAGATTAGC
TAGTTGGTGGGGTAAAGGCCTACCAAGGCGACGATCTATAGCTGGTCTGAGAGGACGATCAGCCACACTG
GGACTGAGACACGGCC
>Zotu297
GAACGCTGGCGGCGTGCCTAATACATGCAAGTCGAACGCTTTTTCTTTCACCGGAGCTTGCTCCACCGGAA
GAAAAGGAGTGGCGAACGGGTGAGTAACACGTGGGTAACCTGCCCATCAGAAGGGGATAACACTTGGAA
ACAGGTGCTAATACCGTATAACAATAGAAACCGCATGGTTTCTATTTGAAAGGCGCTTTTGCGTCACTGATG
GATGGACCCGCGGTGCATTAGCTAGTTGGTGAGGTAACGGCTCACCAAGGCAACGATGCATAGCCGACCT
GAGAGGGTGATCGGCCACATTGGGACTGAGACACGGCC
>Zotu299
GAACGCTGGCGGCGTGCCTAATACATGCAAGTCGAACGAACAGCGACTTGAACTTGTTCAAGTCAATGTTA
GTGGCGCACGGGTGAGTAACACGTGGAGAACCTACCCTTAAGCGGGGGATAACCATTGGAAACGATGACT
AATACCGCATAGAATCACAGATCGCATGATCAATGAAGGAAAGACGGCTTCGGCTGTCACTTAAGGATGG
CTCCGCGGTGCATTAGCTAGATGGTGGGGTAAAGGCCTACCATGGCAATGATGCATAGCCGACCTGAGAG
GGTGATCGGCCACATTGGGACTGAGACACGGCC
>Zotu301
GAACGCTGGCGGCGTGCCTAATACATGCAAGTCGAGCGAATGGATTGAGAGCTTGCTCTCAAGAAGTTAG
CGGCGGACGGGTGAGTAACACGTGGGTAACCTGCCCATAAGACTGGGATAACTCCGGGAAACCGGGGCT
AATACCGGATAACATTTTGAACCGCATGGTTCGAAATTGAAAGGCGGCTTCGGCTGTCACTTATGGATGGA
CCCGCGTCGCATTAGCTAGTTGGTGAGGTAACGGCTCACCAAGGCAACGATGCGTAGCCGACCTGAGAGG
GTGATCGGCCACACTGGGACTGAGACACGGCC
>Zotu303
GAACGCTGGCGGCGTGCCTAATACATGCAAGTCGAGCGAACAGATAAGGAGCTTGCTCCTTTGACGTTAG
CGGCGGACGGGTGAGTAACACGTGGGTAACTTACCTATAAGACTGGAATAACTCCGGGAAACCGGGGCTA
ATGCCGGATAACATGTTGAACCGCATGGTTCTACAGTGAAAGGCGGCTTTGCTGTCACTTATAGATGGACC
CGCGCCGTATTAGCTAGTTGGTGAGGTAACAGCTCACCAAGGCAACGATACGTAGCCGACCTGAGAGGGT
GATCGGCCACACTGGAACTGAGACACGGTC
>Zotu305
CAACGCTGGCGGCGCGCTTAACACATGCAAGTCGAACGAGAAAGTGGAGCAATCCATGAGTAAAGTGGCG
CACGGGTGAGTAACACGTGGATCATCTGCCCTCTTGTGGGGAATACCGCTGGGAAACCGGCGACAATACC
GCATAAGCTCGGAAGAGGAAAGCCGAAAGGCGCAGGAGGAGGAGTCCGCGGTCGATTAGTTAGTTGGCA
GGGTAACGGCCTACCAAGACTGTGATCGATAGCCGGCCTGAGAGGGCACACGGCCACACTGGCACTGAAA
CACGGGC

>Zotu307
GAACGCTGGCGGCAGGCTTAACACATGCAAGTCGAACGGGCACCTTCGGGTGTCAGTGGCAGACGGGTG
AGTAACACGTGGGAACGTACCCTTCGGTTCGGAATAACTCAGGGAAACTTGAGCTAATACCGGATACGCCC
TTTTGGGGAAAGGTTTACTGCCGAAGGATCGGCCCGCGTCTGATTAGCTTGTTGGTGGGGTAACGGCCTAC
CAAGGCGACGATCAGTAGCTGGTCTGAGAGGATGATCAGCCACACTGGGACTGAGACACGGCC
>Zotu309
CAACGCTGGCGGCGCGCTTAACACATGCAAGTCGCACGAGAAAGGGGAGCAATCCCTGAGTAAAGTGGC
GTACGGGTGAGTAACACGTGGATGATCTACCTTCTAGTGGGGAATAACCCTGGGAAACTGGGGCTAATAC
CGCATAAGCTCGAGAGAGGAAAGCAGCAATGCGCTGGAAGAGGAGTCCGCGGCCGATTAGCTAGTTGGC
AGGGTAAAAGCCTACCAAGGCAGAGATCGGTAGCCGGCCTGAGAGGGCACACGGCCACACTGGCACTGA
AACACGGGC
>Zotu311
GAACGCTGGCGGCGTGCCTAATACATGCAAGTAGAACGCTGAAGGAGGAGCTTGCTTCTCCGGATGAGTT
GCGAACGGGTGAGTAACGCGTAGGTAACCTGCCTGGTAGCGGGGGATAACTATTGGAAACGATAGCTAAT
ACCGCATAATAGTAGATGTTGCATGACATTTACTTAAAAGGTGCAATTGCATCACTACCAGATGGACCTGC
GTTGTATTAGCTAGTTGGTGGGGTAACGGCTCACCAAGGCGACGATACATAGCCGACCTGAGAGGGTGAT
CGGCCACACTGGGACTGAGACACGGCC
>Zotu313
GAACGTTAGCGGCACGCCTAACACATGCAAGTCGCACGAGAAAGGGGCAACCTGAGTAAAGTGGCGCAC
GGGTGCGTAACACGTAGATAACCTGCCCTTCGAAGGGGGATAACGTTCCGAAAGGAGCGCTAATACCGCA
TACAGCATCATCCGGTTCGCCGGGAGATGGCAAAGGTAGGGACCGGCAACGGCCTATCGTCGAAGGAGG
GGTCTGCGGCCCATCAGCTAGTTGGGGAGGTAATGGCTCACCAAGGCAGAGACGGGTAGCTGGTCTGAG
AGGATGATCAGCCACACTGGTACTGGAACACGGAC
>Zotu315
GAACGCTGGCGGCGTGCTTAACACATGCAAGTCGAACGGAAAGGCCCAGCTTGCTGGGTACTCGAGTGGC
GAACGGGTGAGTAACACGTGGGTGATCTGCCCTGCACTCTGGGATAAGCCTGGGAAACTGGGTCTAATAC
CGGATATGACCATGCCATGCATGTGGTGTGGTGGAAAGCTTTTGCGGTGTGGGATGGGCCCGCGGCCTAT
CAGCTTGTTGGTGGGGTAATGGCCTACCAAGGCGACGACGGGTAGCCGACCTGAGAGGGTGATCGGCCA
CACTGGGACTGAGACACGGCC
>Zotu317
GAACGCTAGCGGCAGGCCTAATACATGCAAGACGAACGGCAAGAATGATAGCAATATTGTTCCTAGAGTG
TCGTAAGGGTGCGTAACACGTATGCAATCTACCCGAAACTGGAGCATAGCTCCCCGAAAGGGGAATTAAC
ACTCCATAAGATCATAGGATGGCATCATCTAGTGATTAAAACTGAGGTGGTTTCGGATGAGCATGCGTTTG
ATTAGTTAGTTGGTGAGGTAACGGCTCACCAAGACGATGATCAATAGGGGAACTGAGAGGTTGATCCCCC
ACACTGGCACTGAGATACGGGC
>Zotu319
GAACGCTGGCGGCATGCTTAACACATGCAAGTCGAACGGGAAGTGGTGTTTCCAGTGGCGAACGGGTGA
GTAATGCGTAAGAACCTGCCCTTGGGAGGGGAACAACAGCTGGAAACGGTTGCTAATACCCCATAGAATT
TAGTACCTCGTAGAGGTAGGCTGAGGAGCAAAAGGGAGGAATCCGCCCAAGGAGGGGCTCACGTCTGAT
TAGCTAGTTGGTGAGGCAATGGCTTACCAAGGCTCCGATCAGTAGTTGGTCTGAGAGGATGATCAGCCAC
ACTGGGACTGAGACACGGCC
>Zotu321
GAACGCTGGCGGCAGGCTTAACACATGCAAGTCGAACGCATCCTTCGGGGTGAGTGGCAGACGGGTGAG
TAACGCGTGGGAACGTGCCCTTCAGTTCGGAATAACCCAGGGAAACTTGGGCTAATACCGGATACGTGCG
AGAGCAGAAAGATTTATCGCTGAAGGATCGGCCCGCGTCTGATTAGCTAGTTGGTGGGGTAATGGCCCAC
CAAGGCGACGATCAGTAGCTGGTCTGAGAGGATGATCAGCCACATTGGGACTGAGACACGGCC
>Zotu324
GAACGCTGGCGGCGTGCCTAATACATGCAAGTCGAACGCTTCCTACCGGTGCTTGCACCGGGAAAGAAGA
GTGGCGGACGGGTGAGTAACACGTGGGTAACCTACCTACAAGCGGGGGATAACATTCGGAAACGGATGC
TAATACCGCATAGATCTTTTTGTCTCCTGGCAGAAAGAAGAAAGACGGTTTCGGCTGTCACTTGTAGATGG

ACCCGCGGCGCATTAGTTAGTTGGTGAGGTAATGGCTCACCAAGACGATGATGCGTAGCCGACCTGAGAG
GGTGATCGGCCACACTGGGACTGAGACACGGCC
>Zotu326
GAACGCTAGCTACAGGCTTAACACATGCAAGTCGAGGGGAAACGATGAGAGAGCTTGCTCTCTTAGGCGT
CGACCGGCGCACGGGTGAGTAACGCGTATCCAACCTGCCCATTACAGGGGAATAACCTTGCGAAAGTAAG
ACTAATGCCCCATGGTATTCTCAGCAGACATCTAATGAGGATTAAAGATTTATCGGTAATGGATGGGGATG
CGTCTGATTAGCTTGTTGGCGGGGTAACGGCCCACCAAGGCGACGATCAGTAGGGGTTCTGAGAGGAAGG
TCCCCCACATTGGAACTGAGACACGGTC
>Zotu328
GAACGCTGGCGGCGTGCTTAACACATGCAAGTCGAACGATGAAGCCCAGCTTGCTGGGCGGATTAGTGGC
GAACGGGTGAGTAACACGTGAGTAACCTGCCCTTGACTCTGGGATAAGCCCGGGAAACTGGGTCTAATAC
CGGATAGGAGCGCCTGTCGCATGACGGGTGCTGGAAAGATCTTTCGGTCATGGATGGACTCGCGGCCTAT
CAGCTTGTTGGTGAGGTAACGGCTCACCAAGGCGACGACGGGTAGCCGGCCTGAGAGGGTGACCGGCCA
CACTGGGACTGAGACACGGCC
>Zotu330
GAACGCTAGCGGCAGGCTTAATACATGCAAGTCGAGGGGCAGCAGGATAGCAATATTGCTGGCGACCGG
CGCACGGGTGCGTAACACGTATACAATCTACCTTTAACAGGGGGATAGCCCGAAGAAATTCGGATTAATAC
CCCATAATAGTTTTCTTGGCATCAAGAGGATTTGAAAGCTCCGGCGGTTAGAGATGAGTATGCGGTCTATT
AGCTAGTAGGTGAGGTAACGGCTCACCTAGGCTACGATAGATAGGGGATCTGAGAGGACTACCCCCCACA
CTGGTACTGAGACACGGAC
>Zotu332
GAACGCTGGCGGCGTGCTTAACACATGCAAGTCGAACGGAAAGGCCCTTTCGGGGGTACTCGAGTGGCGA
ACGGGTGAGTAACACGTGGGTGATCTGCCCTGCACTTCGGGATAAGCTTGGGAAACTGGGTCTAATACCG
GATATGATCACGGGCTTCATGGTCTGTGGTGGAAAGCTTTTGCGGTGTGGGATGGGCCCGCGGCCTATCA
GCTTGTTGGTGGGGTAATGGCCTACCAAGGCGACGACGGGTAGCCGGCCTGAGAGGGTGTCCGGCCACA
CTGGGACTGAGATACGGCC
>Zotu334
GAACGCTTGCGGCGTGCCTAAGAAATGCAAGTCGAACGGATAGTCCAGCAATGGTCTATTAGTGGCGAAC
GGTCGCGTAACACGTAGGCAACCTGCCCGGAAGTGGGGGACAACAGCCTGAAAGGGCTGCTAATACCGC
ATGTGGCCGCCTGGTGGCATCACCGGGTGTCTAAAGCTTTATGCGCTTCTGGATGGGCTTGCGGCCTATCA
GGTAGTTGGTGGGGTAATGGCCTACCAAGCCGACGACGGGTAGCTGGTCTGAGAGGACGATCAGCCGGA
TTGGGACTGAGATACGGCC
>Zotu336
GAACGCTAGCGGCAGGCTTAACACATGCAAGTCGAGGGGTAGAGTTAGCTTGCTAACTTGAGACCGGCGC
ACGGGTGCGTAACGCGTATGCAATCTACCTTTTACAGAGGGATAGCCCAGAGAAATTTGGATTAATACCTC
ATAGTATAATGACTCGGCATCGAGATATTATTAAAGTCACAACGGTAAAAGATGAGCATGCGTCCCATTAG
CTAGTTGGTGTGGTAACGGCACACCAAGGCAACGATGGGTAGGGGTCCTGAGAGGGAGATCCCCCACACT
GGTACTGAGACACGGAC
>Zotu338
GAACGCTGGAAATGTGGATACCGGCATGCAAGTCAAGGGGCCGCAAGGCACCGGCGAACGGCTTAGTAA
CACGTAGATACATACCCAAAAGTGGGGCATAGCTCGTCGAAAGACGGGGTAATTTCCCATGTTCCCGAAAG
GGCAAAGCAGCAATGCGCTTTTGGAATGGTCTGCGGTCTATCAGCTAGTTGGTGAGGTAATGGCTCACCAA
GGCTATGACGGATAGGGGGCGTGAGAGCGCGACCCCCAAGAATGGCACTGCGATACGGGC
>Zotu340
GAACGCTGGCGGCGTGCTTAACACATGCAAGTCGAACGGAAAGGCCCGCTTGCGGGTACTCGAGTGGCGA
ACGGGTGAGTAACACGTGGGCAACCTGCCCTCAGCTCTGGGATAACTCCGGGAAACCGGTGCTAATACTG
GATATCGACTTCTGCCTCGCATGGGGTGTTGTTGAAAGTTTTTCGGCTGGGGATGGGCCCGCGGCCTATCA
GCTTGTTGGTGGGGTGATGGCCTACCAAGGCGACGACGGGTAGCCGGCCTGAGAGGGCGACCGGCCACA
CTGGGACTGAGACACGGCC
>Zotu343

GAACGCTGGCGGCGTGCCTAATACATGCAAGTAGAACGCTGAAGACTTTAGCTTGCTAGAGTTGGAAGAG
TTGCGAACGGGTGAGTAACGCGTAGGTAACCTGCCTACTAGCGGGGGATAACTATTGGAAACGATAGCTA
ATACCGCATAATAGTATTTAACACATGTTAGAGACTTGAAAGATGCAATTGCATCACTAGTAGATGGACCT
GCGTTGTATTAGCTAGTTGGTGGGGTAACGGCCTACCAAGGCGACGATACATAGCCGACCTGAGAGGGTG
ATCGGCCACACTGGGACTGAGACACGGCC
>Zotu346
GAACGCTGGCGGCAGGCTTAACACATGCAAGTCGAGCGGGGAAGGGTAGCTTGCTACCTGACCTAGCGGC
GGACGGGTGAGTAATGCTTAGGAATCTGCCTATTAGTGGGGGACAACATTCCGAAAGGAATGCTAATACC
GCATACGCCCTACGGGGGAAAGCAGGGGATCTTCGGACCTTGCGCTAATAGATGAGCCTAAGTCAGATTA
GCTAGTTGGTGGGGTAAAGGCCTACCAAGGCGACGATCTGTAGCGGGTCTGAGAGGATGATCCGCCACAC
TGGGACTGAGACACGGCC
>Zotu349
GAACGCTAGCGGCAGGCCTAATACATGCAAGTCAAGGGGTAGCAATACACCGGCGGACGGGTGCGTAAC
GCGTACACAACATACCTTTAAGCGGGGGATAGCATTGGGAAACTGGTGGTAATACCCCATAACATTGCGG
GTTGGCATTAACTTGCAATTAAAGTTTCGGCGCTTAAAGATTGGTGTGCGTCTGATTAGCTAGTTGGCGGG
GTAACGGCCCACCAAGGCGACGATCAGTAGGGGGTGTGAGAGCATGACCCCCCACATGGGTACTGAGAC
ACGGAC
>Zotu351
GAACGCTGGCGGCGTGCTTAACACATGCAAGTCGAACGGTGAACTCCGCTTGCGGGGGGATCAGTGGCGA
ACGGGTGAGTAACACGTGGGCAACCTGCCCCTGGCTCTGGGATAACTCCAAGAAATTGGGGCTAATACCG
GATATGACCGCTGACCGCATGGTCTGGTGGTGGAAAGATTTATCGGCTGGGGATGGGCCCGCGGCCTATC
AGCTTGTTGGTGGGGTAGTGGCCTACCAAGGCGACGACGGGTAGCCGGCCTGAGAGGGTGACCGGCCAC
ACTGGGACTGAGACACGGCC
>Zotu353
GAACGCTGGCGGCAGGCTTAACACATGCAAGTCGAACGAGTGTAGCAATACACTAGTGGCGCACGGGTGA
GTAACACGTGGATATCTGCCTTTTGGTTCGGAATAACCCTGGGAAACTAGGGCTAATACCGAATGGTTCCT
ACGGGATAAAGATTTATCGCCAAAAGATGAGTCCGCGTCCGATTAGCTAGTTGGTGAGGTAATGGCTCACC
AAGGCGACGATCGGTAGCTGGTCTGAGAGGACGATCAGCCACACTGGGACTGAGACACGGCC
>Zotu355
GAACGCTGGCGGCGTGCTTAACACATGCAAGTCGAGCGATGAAGCACCTTCGGGTGTGAATTAGCGGCGA
ACGGGTGAGGAACACGTGAAGAATCTGCCTTCAACTCTGGGATAACTCCGGGAAACCGGGGCTAATACCG
GATATGAGCCTTGATCGCATGATCGGGGCTGGAAAGTTTTTCGGTTGAAGATGACTTCGCGGCCTATCAGC
TTGTTGGTGAGGTAATGGCTCACCAAGGCGACGACGGGTAGCCGGCCTGAGAGGGCGACCGGCCACACT
GGGACTGAGACACGGCC
>Zotu357
GAACGCTGGCGGCGTGCCTAATACATGCAAGTCGAACGAGAGCGACCGGTGCTTGCACTGGTCAATCTAG
TGGCGAACGGGTGAGTAACACGTGGGTAACCTGCCCATCAGAGGGGGATAACATCCGGAAACGGATGCT
AAAACCGCATAGGTCTTCGAGCCGCATGGCTTGAAGAGGAAAAGAGGCGCAAGCTTCTGCTGATGGATGG
ACCCGCGGTGCATTAGCTAGTTGGTGAGGTAACGGCTCACCAAGGCCGTGATGCATAGCCGACCTGAGAG
GGTGATCGGCCACATTGGGACTGAGACACGGCC
>Zotu359
AAACGCTGGCGGCATGCCTAACACATGCAAGTCGAACGGCAGCACAGTAAAGATTTCGGTCTTTACGGGT
GGCGAGTGGCGGACGGGTGAGTAAAGCGTAGGAATCTGTCCTGGAGAGTGGGATAACCCGAGGAAACTC
GGGCTAATACCGCATGATATCGAGAGATCAAAGCAGGGGACCCTTCGGGGCCTTGCGCTCTAGGATGAGC
CTACGTTGGATTAGCTAGTTGGTGGGTGGGGTAAAGGCCTACCAAGGCGACGATCCGTAGCTGGTCTGAG
AGGATGACCAGCCACACTGGGACTGAGACACGTCC
>Zotu361
GAACGCTGGCGGCATGCCTTACACATGCAAGTCGAACGGTAACAGGTTAAGCTGACGAGTGGCGAACGG
GTGAGTAATATATCGGAACGTGCCCAGTCGTGGGGGATAACTACTCGAAAGAGTAGCTAATACCGCATAC
GACCTGAGGGTGAAAGCGGGGGATCGCAAGACCTCGCGCGATTGGAGCGGCCGATATCAGATTAGGTAG

TTGGTGGGGTAAAAGCCCACCAAGCCAACGATCTGTAGCTGGTCTGAGAGGACGACCAGCCACACTGGGA
CTGAGACACGGCC
>Zotu363
GAACGCTGGCGGCGTGCCTAATACATGCAAGTCGAGCGAACAGACGAGGAGCTTGCTCCTTTGACGTTAG
CGGCGGACGGGTGAGTAACACGTGGGTAACCTACCTATAAGACTGGGATAACTTCGGGAAACCGGAGCTA
ATACCGGATAATATTTCGAACCGCATGGTTCGATAGTGAAAGATGGCTCTGCTATCACTTATAGATGGACCT
GCGCCGTATTAGCTAGTTGGTAAGGTAACAGCTTACCAAGGCAACGATACGTAGCCGACCTGAGAGGGTG
ATCGGCCACACTGGAACTGAGACACGGTC
>Zotu365
GAACGCTGGCGGCGTGCTTAACACATGCAAGTCGAACGATGATGCCCAGCTTGCTGGGTGGATTAGTGGC
GAACGGGTGAGTAACACGTGAGTAACCTGCCCCTGACTCTGGGATAAGCGTTGGAAACGACGTCTAATAC
TGGATATGATCACTGGCCGCATGGTCTGGTGGTGGAAAGATTTTTTGGTTGGGGATGGACTCGCGGCCTAT
CAGCTTGTTGGTGAGGTAATGGCTCACCAAGGCGACGACGGGTAGCCGGCCTGAGAGGGTGACCGGCCA
CACTGGGACTGAGACACGGCC
>Zotu367
GAACGCTGGCGGCGTGCCTAATACATGCAAGTCGAGCGCCCTGCACTTTGCCCATCAGCCCAAACAACGGA
GCTGGTGAGCAGCGTGTAGGGAGCGGCGGACGGCTGAGTAACACGTAGGAACGTACCCCAAAGTGAGGC
ATAAGCACCAGAAATGGTGTCTAATACCGCATGTGCTCTTAGGAGTAAAGCTTCGGCGCTTTGGGAACGGC
CTGCGCATGATTAGCTTGTTGGTGAGGTAAAAGCTCACCAAGGCGACGATCATTAGCTGGTCTGAGAGGAT
GACCAGCCAGACTGGGACTGAGAACGGCC
>Zotu369
GAACGCTAGCGGGAGGCCTAACACATGCAAGCCGAGCGGTATTTGTTCTTCGGAACAGAGAGAGCGGCGC
ACGGGTGCGGAACACGTGTGCAACCTGCCTTTATCTGGGGGATAGCCTTTCGAAAGGAAGATTAATACCCC
ATAATATATTGAGTGGCATCACTTAATATTGAAAACTCCGGTGGATAGAGATGGGCACGCGCAAGATTAGA
TAGTTGGTGAGGTAACGGCTCACCAAGTCAATGATCTTTAGGGGGCCTGAGAGGGTGATCCCCCACACTG
GTACTGAGACACGGAC
>Zotu371
GAACGCTGGCGGCAGGCTTAACACATGCAAGTCGAGCGGGCCCTTCGGGGTCAGCGGCAGACGGGTGAG
TAACGCGTGGGAACGTGCCCTTCGGTTCGGAATAACTCAGGGAAACTTGAGCTAATACCGGATACGCCCTT
TTGGGGAAAGGCTTGACTGCCGAAGGATCGGCCCGCGTCTGATTAGCTAGTTGGTGAGGTTATGGCTCAC
CAAGGCGACGATCAGTAGCTGGTCTGAGAGGATGATCAGCCACACTGGGACTGAGACACGGCC
>Zotu374
GAACGCTGGCGGCGTGCTTAACACATGCAAGTCGAGCGAGAAGTTTTGAAAAGATTCTTCGGATGAAATTT
AAAATGGAAAGCGGCGGACGGGTGAGTAACGCGTAGGCAACCTGCCCCATACAAAGGGATAGCATTTGG
AAACGAATATTAATACCTTATGAAACTTAACTAGTGCATGCTAGGTAGGTCAAAGATTTATCGGTATGGGA
TGGGCCTGCGTCTGATTAGCTAGTTGGTGAGGTAACGGCTCACCAAGGCGACGATCAGTAGCCGACCTGA
GAGGGTGAACGGCCACATTGGAACTGAGACACGGTC
>Zotu376
GAACGCTAGCGACAGGCTTAACACATGCAAGTCGAGGGGCATCATGATTTGTAGCAATACAAATTGATGG
CGACCGGCGCACGGGTGAGTAACGCGTATGCAACCTACCTATCAGAGGGGGATAGCCCATTGAAAGATGG
ATTAATACCGCATAAAACAGGGGTCCCGCATGGGAATATTTGTTAAAGATTTATTGCTGATAGATGGGCAT
GCGTTCCATTAGCTGGTTGGTAAGGTAACGGCTTACCAAGGCATCGATGGATAGGGGTTCTGAGAGGAAG
GTCCCCCACACTGGTACTGAGACACGGAC
>Zotu379
GAACGCTGGCGGCAGGCCTAACACATGCAAGTCGAACGGTAACAGGAAGAAGCTTGCTTCTTTGCTGACG
AGTGGCGGACGGGTGAGTAATGTCTGGGAAACTGCCTGATGGAGGGGGATAACTACTGGAAACGGTAGC
TAATACCGCATAACGTCGCAAGACCAAAGAGGGGGACCTTCGGGCCTCTTGCCATCGGATGTGCCCAGAT
GGGATTAGCTTGTTGGTGGGGTAACGGCTCACCAAGGCGACGATCCCTAGCTGGTCTGAGAGGATGACCA
GCCACACTGGAACTGAGACACGGTC
>Zotu381

GAACGCTAGCGGCAGGCTTAATACATGCAAGTCGAACGGTTACAGCAATGTGACGAGTGGCGCACGGGTG
AGTAACGCGTACATAACATACCTTTAACTGGCGAATAGCCTTGGGAAACTGAGAGTAATACGCCATAACAT
TGTGATTCGGCATCGGATTACAATTAAAGTTCCGGCGGTTAGAGATTGATGTGCGTTGGATTAGGTAGTTG
GTGAGGTAACGGCTCACCAAGCCTTCGATCCATAGGGGGCGTGAGAGCGTGGTCCCCCACACGGGTACTG
AGACACGGAC
>Zotu383
TAATGTTGGCAGTGTGCCTAATACATGCAAGTCGAGCGATATAGCGGCGGACGAGAGAGTAATAGGAAG
GAACATGCCCCGAACTCAAGGATAGCCACCCGAAAGGGTGGGTAATCCTGGATAGTCCCGCAAGGGTAAA
GCTTTATGCGGTTCGGGAGTGGCCTTCCTCCTATCAGCTTGTTGGTGAGGTAATAGCTCACCAAGGCGATG
ACGGGTAGCTGGTCTGAGAGGATGACCAGCCTCGATGGGACTGAGACACGGCC
>Zotu385
GAACGCTGGCGGCAGGCTTAACACATGCAAGTCGAACGGGTGTAGCAATACATCAGTGGCAGACGGGTG
AGTAACACGTGGGAACCTTCCTCGTTGTACGGAACAACTCAGGGAAACTTGAGCTAATACCGTATACGTCC
GAGAGGAGAAAGATTTATCGCAATGAGACGGGCCCGCGTCGGATTAGCTAGTTGGTGGGGTAATGGCCTA
CCAAGGCGACGATCCGTAGCTGATCTGAGAGGATGATCAGCCACACTGGGACTGAGACACGGCC
>Zotu387
GAACGCTGGCGGCGTGCCTAATACATGCAAGTCGAGCGAATCAGATGGGAGCTTGCTCCCGGATGATTAG
CGGCGGACGGGTGAGTAACACGTGGGCAACCTGCCCGTAAGATTGGGATAACTCCGGGAAACCGGTGCT
AATACCGAATAGTTTCTGACACCTCATGGTGTCAGAGGGAAAGTTGGCTTCGGCTAACACTTACGGATGGG
CCCGCGGCGCATTAGCTAGTTGGTGAGGTAACGGCTCACCAAGGCGACGATGCGTAGCCGACCTGAGAGG
GTGATCGGCCACACTGGGACTGAGACACGGCC
>Zotu389
GAACGCTGGCGGCGTGCCTAATACATGCAAGTCGAGCGAACAGATAAGGAGCTTGCTCCTTTGACGTTAG
CGGCGGACGGGTGAGTAACACGTGGGTAACTTACCTATAAGACTGGAATAACTCCGGGAAACCGGGGCTA
ATGCCGGATAACATGTTGAACCGCATGGTTCTACAGTGAAAGGCGGCTTTGCTGTCACTTATAGATGGACC
CGCGCCGTATTAGCTAGTTGGTGAGGTAACGGCTCACCAAGGCAACGATACGTAGCCGACCTGAGAGGGT
GATCGGCCACACTGGAACTGAGACACGGTC
>Zotu391
GAACGCTGGCGGCGCGCCTAAAACATGCAAGTCGAACGAGAAGCCCGGGCTTGCCCGGGTGGACAGTGG
CGAACGGGTGAGTAACACGTGAGGAACCTGCCCCGAAGACCGGGACAACACCGGGAAACCGGTGCTAAT
ACCGGATACCTTCACCGGATCGCATGGTCTGGTGAAGAAATGGATTCCGCTTCGGGATGGCCTCGCGGCCT
ATCAGCTAGTTGGTGAGGTAACGGCTCACCAAGGCGACGACGGGTAGCTGGTCTGAGAGGATGTCCAGCC
ACACTGGGACTGAGACACGGCC
>Zotu393
GAACGCTGGCGGCGTGCCTAATACATGCAAGTAGAACGCTGAAGCTTGGTGCTTGCGCCGAGCGGATGAG
TTGCGAACGGGTGAGTAACGCGTAGGTAACCTGCCTGGTAGCGGGGGATAACTATTGGAAACGATAGCTA
ATACCGCATAATAGTAGATGTTGCATGACATTTGCTTAAAAGGTGCAATTGCATCACTACCAGATGGACCTG
CGTTGTATTAGCTAGTTGGTGAGGTAACGGCTCACCAAGGCAACGATACATAGCCGACCTGAGAGGGTGA
TCGGCCACACTGGGACTGAGACACGGCC
>Zotu395
GAACGCTGGCGGCGTGCTTAACACATGCAAGTCGAGCGGTAAGGCCTTTCGGGGTACACGAGCGGCGAAC
GGGTGAGTAACACGTGGGTGATCTGCCCTGCACTCTGGGATAAGCTTGGGAAACTGGGTCTAATACCGGA
TATGACCACAGCATGCATGTGTTGTGGTGGAAAGATTTATCGGTGCAGGATGGGCCCGCGGCCTATCAGCT
TGTTGGTGGGGTAATGGCCTACCAAGGCGACGACGGGTAGCCGACCTGAGAGGGTGACCGGCCACACTG
GGACTGAGACACGGCC
>Zotu397
GAACGCTGGCGGCGTGCTTAACACATGCAAGTCGAGCGAAGCGGCTGAATTGATTCTTCGGATGATTTTCA
GTTGACTGAGCGGCGGACGGGTGAGTAACGCGTGGGTGACCTGCCCCATACCGGGGGATAACAGCTGGA
AACGGCTGCTAATACCGCATAAGCGCACAGAGCTGCATGGCTCCGTGTGAAAAACTCTGGTGGTATGGGA
TGGGCCCGCGTCTGATTAGGCAGTTGGCGGGGTAACGGCCCACCAAACCGACGATCAGTAGCCGGCCTGA
GAGGGCGACCGGCCACATTGGGACTGAGACACGGCC

>Zotu399
GAACGCTGGCGGCGTGCTTAACACATGCAAGTCGAACGGAAAGGCTCCTGCTTGCAGGGGTACTCGAGTG
GCGAACGGGTGAGTAACACGTGGGTGATCTGCCCTGCACTTCGGGATAAGCCTGGGAAACTGGGTCTAAT
ACCGGATAGGACGATGGTTTGGATGCCATTGTGGAAAGTTTTTTCGGTGTGGGATGAGCTCGCGGCCTATC
AGCTTGTTGGTGGGGTAATGGCCTACCAAGGCGTCGACGAGTAGCCGGCCTGAGAGGGTGTACGGCCACA
TTGGGACTGAGATACGGCC
>Zotu401
GAACGCTGGCGGCGTGCTTAACACATGCAAGTCGAACGGAAAGGCCAGTGCTTGCACTGGTACTCGAGTG
GCGAACGGGTGAGTAACACGTGGGTGATCTGCCCTGCACTTCGGGATAAGCCTGGGAAACTGGGTCTAAT
ACTGGATAGGACCACTTCTTGGATGGTGTGGTGGAAAGTTTTTTCGGTGTGGGATGAGCTCGCGGCCTATC
AGCTTGTTGGTGGGGTAATGGCCTACCAAGGCGTCGACGGGTAGCCGGCCTGAGAGGGTGTACGGCCAC
ATTGGGACTGAGATACGGCC
>Zotu403
GAACGCTGGCGGCAGGCCTAACACATGCAAGTCGAGCGGATGAGGGGAGCTTGCTCCCTGATTTAGCGGC
GGACGGGTGAGTAATGTATAGGGAGCTGCCCGATAGAGGGGGATACCAGTTGGAAACGACTGTTAATAC
CGCATAATGTCTACGGACCAAAGTGTGGGACCTTCGGCCACATGCTATCGGATGCACCTATATGGGATTA
GCTAGTTGGTGGGGTAACGGCTCACCAAGGCGACGATCCCTAGCTGGTTTGAGAGGATGATCAGCCACAC
TGGAACTGAGACACGGTC
>Zotu405
GAACGCTGGCGGCGTGCTTAACACATGCAAGTCGAACGGGAAACCTTTTATTGAAGCTTCGGCAGATTTAG
CTGGTTTCTAGTGGCGGACGGGTGAGTAACGCGTGGGTAACCTGCCTTATACAGGGGGATAACAGTCAGA
AATGACTGCTAATACCGCATAAGCGCACAGAGCTGCATGGCTCGGTGTGAAAAACTCCGGTGGTATAAGA
TGGACCCGCGTTGGATTAGCTAGTTGGTGAGGTAACGGCCCACCAAGGCGACGATCCATAGCCGGCCTGA
GAGGGTGAACGGCCACATTGGGACTGAGACACGGCC
>Zotu407
GAACGCTGGCGGCGTGCCTAACACATGCAAGTCGAACGGAGATTTCAGCAATGGAATCTTAGTGGCGAAC
GGGTGAGTAACGCGTAGACAACCTACCTTTTAGATGGGGACAACACTGCGAAAGTGGTGCTAATACCGAA
TGTTGCATTTTGGTTGCATAACCGAAATACTAAAGATGGCCTCTATTTATAAGCTATCGCTAAAAGATGGGT
CTGCGTCTGATTAGCTAGTTGGTGAGGTAATGGCTCACCAAGGCGACGATCAGTAGCCGGTCTGAGAGGA
TGAACGGCCACACTGGGACTGAGACACGGCC
>Zotu409
GAACGCTGGCGGCAGGCTTAACACATGCAAGTCGAGCGAAGGCTTCGGCCTTAGCGGCGGACGGGTGAG
TAACGCGTGGGAACGTGCCCTTTTCTTCGGAATAGCCCAGGGAAACTTGGATTAATACCGGATACGCCCTT
CGGGGGAAAGATTTATCGGAGAAGGATCGGCCCGCGTTGGATTAGGTAGTTGGTGGGGTAATGGCCTACC
AAGCCGACGATCCATAGCTGGTTTGAGAGGATGATCAGCCACACTGGGACTGAGACACGGCC
>Zotu411
GAACGCTGGCGGCATGCCTAATACATGCAAGTTGAACGATGATATTCTCTGCTTGCAGAGAATTGAAGAGT
AGCGAACGGGTGAGTAACACGTGGGGAACCTGCCCATGAGAGGGGGATAACATTCGGAAACGGATGCTA
ATACCGCATAATGCTTTGGACCGCCTGGTCCAGAGACGAAAGACGGCTTTTGCTGTCACTCATGGATGGCC
CCGCGCCGTATTAGCTAGTTGGTGAGGTAATGGCTCACCAAAGCTGTGATACGTAGCCGACCTGAGAGGG
TGATCGGCCACACTGGGACTGAGACACGGCC
>Zotu413
GAACGCTGACAGAATGCTTAACACATGCAAGTCAACTTGAACTTCGGTTTGGGTGGCGGACGGGTGAGTA
ACGCGTAAAGAACTTGCCTCACAGCTAGGGACAACATTTGGAAACGAATGCTAATACCTGATATTATGATT
ATATGGCATCGTATAATTATGAAAGCTATATGCGCTGTGAGAGAGCTTTGCGTCCCATTAGCTAGTTGGAG
AGGTAACGGCTCACCAAGGCGATGATGGGTAGCCGGCCTGAGAGGGTGATCGGCCACAAGGGGACTGAG
ACACGGCC
>Zotu415
GAACGCTAGCGGCAGGCCTAATACATGCAAGTCGAACGGGCGGTAGCAATACCGTTAGTGGCGCACGGGT
GCGTAACGCGTAACCAACCTACCTGGATCTGGGGGATAGCCCGCCGAAAGGCGGATTAATACCGCATAAG

CTACGTGACCGGCATCGGTTGGGTAGTAAAGATTTATTGGATTCAGATGGGGTTGCGTAGCATTAGCTAGA
TGGCGGGGTAACGGCCCACCATGGCGACGATGCTTAGGGGACCTGAGAGGGTGATCCCCCACACTGGCAC
TGAGATACGGGC
>Zotu417
GAACGCTGGCGGCGTGCCTAATACATGCAAGTTGAGCGCTGAAGGTTGGTACTTGTACCGACTGGATGAG
CAGCGAACGGGTGAGTAACGCGTGGGGAATCTGCCTTTGAGCGGGGGACAACATTTGGAAACGAATGCT
AATACCGCATAAAAACTTTAAACACAAGTTTTAAGTTTGAAAGATGCAATTGCATCACTCAAAGATGATCCC
GCGTTGTATTAGCTAGTTGGTGAGGTAAAGGCTCACCAAGGCGATGATACATAGCCGACCTGAGAGGGTG
ATCGGCCACATTGGGACTGAGACACGGCC
>Zotu419
GAACGCTGGCGGCGTGCCTAATACATGCAAGTAGAACGCTGAAGAGAGGAGCTTGCTCTTCTTGGATGAG
TTGCGAACGGGTGAGTAACGCGTAGGTAACCTGCCTGGTAGCGGGGGATAACTATTGGAAACGATAGCTA
ATACCGCATAAAATTGATTATTGCATGATAATTAATTGAAAGATGCAACTGCATCACTACCAGATGGACCTG
CGTTGTATTAGCTAGTTGGTGAGGTAACGGCTCACCAAGGCGACGATACATAGCCGACCTGAGAGGGTGA
TCGGCCACACTGGGACTGAGACACGGCC
>Zotu421
GAACGCTGGCGGCGTGCCTAATACATGCAAGTCGAGCGAGCTGAATTCAAAGATTCCTTCGGGATGATTTG
TTGGACGCTAGCGGCGGATGGGTGAGTAACACGTGGGCAATCTGCCCTAAAGACTGGGATACCACTTGGA
AACAGGTGCTAATACCGGATAACAACATGAATCGCATGATTCAAGTTTGAAAGGCGGCGTAAGCTGTCACT
TTAGGATGAGCCCGCGGCGCATTAGCTAGTTGGTGGGGTAAAGGCCTACCAAGGCAATGATGCGTAGCCG
AGTTGAGAGACTGATCGGCCACATTGGGACTGAGACACGGCC
>Zotu423
GAACGCTGGCGGCATGCTTAACACATGCAAGTTGAACGCAGGATAAGCCTTCGGGTTTATTGCTGAGTAGC
AAACGGGTGAGTAACGCGTGGGAATCTGCCTTTATGTGGGGGATAACTAAGGGAAACTTTAGCTAATACC
GCATACGCTCTACGGAGGAAAGACTTCGGTCGCATAAAGAGGAGCCCGCGTCAGATTAGGTAGTTGGTAG
GGTAAAGGCCTACCAAGCCAATGATCTGTAGCTGGTCTGAGAGGATGATCAGCCACACTGGGACTGAGAC
ACGGCC
>Zotu425
GAACGCTAGCGGGAGGCCTAACACATGCAAGCCGAGCGGTAGAAGATCTTCGGATCTTTGAGAGCGGCGT
ACGGGTGCGGAACACGTGTGCAACCTGCCTTTATCAGGGGGATAGCCTTTCGAAAGGAAGATTAATACCCC
ATAATATATTGATTGGCATCAGTTAATATTGAAAACTCCGGTGGATAGAGATGGGCACGCGCAAGATTAGA
TAGTTGGTAGGGTAACGGCCTACCAAGTCAGTGATCTTTAGGGGGCCTGAGAGGGTGATCCCCCACACTG
GTACTGAGACACGGAC
>Zotu427
GAACGCTAGCGGCAGGCTTAATACATGCAAGGCGAGGGGGCAGCAATGTCACCGTCGTACGGGTGCGTA
ACGCGTATGCAACCTACCTATCACTGGGGGATAGCCCGGGGAAACCCGGATTAATACCGCATAATACATTC
GGGCCTCATGGCCTGATTAGTTAAAGATTTATTGGTGACAGATGGGCATGCGTTCGATTAGCTAGTTGGTG
CAGGTAACGGCTCACCAAGGCGACGATCGATAGGGGAGCTGAGAGGTTGATCCCCCACACGGGCACTGA
GATACGGGC
>Zotu429
GAACGCTGGCGGCGTGCTTAACACATGCAAGTCGAACGGAAAGGCCCTTTCGGGGGTACTCGAGTGGCGA
ACGGGTGAGTAACACGTGGGTGATCTGCCCTGCACTTCGGGATAAGCTTGGGAAACTGGGTCTAATACCG
GATAGGACCATGCCATGCATGTGGTGTGGTGGAAAGCTTTTGCGGTGTGGGATGGGCCCGCGGCCTATCA
GCTTGTTGGTGGGGTGATGGCCTACCAAGGCGACGACGGGTAGCCGGCCTGAGAGGGTGTCCGGCCACA
CTGGGACTGAGATACGGCC
>Zotu431
GAACGCTGGCGGCGTGCTTAACACATGCAAGTCGAACGATGAAGCCCTTTCGGGGGTGGATTAGTGGCGA
ACGGGTGAGTAACACGTGGGCAATCTGCCCTGCACTTCGGGACAAGCCCTGGAAACGGGGTCTAATACCG
GATATGACCATCGGGTGCATGCTCTGATGGTGGAAAGCTCCGGCGGTGCAGGATGAGCCCGCGGCCTATC
AGCTTGTTGGTGGGGTGATGGCCTACCAAGGCGACGACGGGTAGCCGGCCTGAGAGGGCGACCGGCCAC
ACTGGGACTGAGACACGGCC

>Zotu433
GAACGCTGGCGGCATGCCTAACACATGCAAGTCGAACGAAGGCTTCGGCCTTAGTGGCGCACGGGTGCGT
AACGCGTGGGAATCTGCCCCTTGGTTCGGAATAACAGTTAGAAATGACTGCTAATACCGGATGATGACGTA
AGTCCAAAGATTTATCGCCGAGGGATGAGCCCGCGTAGGATTAGCTAGTTGGTGTGGTAAGAGCGCACCA
AGGCGACGATCCTTAGCTGGTCTGAGAGGATGATCAGCCACACTGGGACTGAGACACGGCC
>Zotu436
GAACGCTAGCGGGAGGCCTAACACATGCAAGTCGAACGGGATTGGGAGCTTGCTCCCATGAGAGTGGCG
CACGGGTGAGTAACGCGTATGCAACCTACCTTTAACAGAGGGATAGCCTATAGAAATGTGGATTAATACCT
CATAGTTCTGAGAAGCCGCATGGTTTTTCAGATAAAGCTCCGGCGGTTAAAGATGGGCATGCGTGACATTA
GCTAGTTGGTAAGGTAACGGCTTACCAAGGCTACGATGTCTAGGGGTTCTGAGAGGATGATCCCCCACACT
GGTACTGAGACACGGAC
>Zotu438
GAACGCTGGCGGCGTGCCTAATACATGCAAGTCGAGCGAACGGATAAGGAGCTTGCTCCTTTGAAGTTAG
CGGCGGACGGGTGAGTAACACGTGGGTAACCTACCTATAAGACTGGGATAACTTCGGGAAACCGGAGCTA
ATACCGGATAACATTTAGAACCGCATGGTTCTAAAGTGAAAGATGGTTTTGCTATCACTTATAGATGGACCC
GCGCCGTATTAGCTAGTTGGTAAGGTAATGGCTTACCAAGGCGACGATACGTAGCCGACCTGAGAGGGTG
ATCGGCCACACTGGAACTGAGACACGGTC
>Zotu440
GAACGCTAGCGGGAGGCTTAACACATGCAAGTCGAGGGGTACAGTAGCAATACTGGAGACCGGCGCACG
GGTGAGTAACGCGTATGCAACCTGCCCTTTACAGGAGGATAGCCCGCTGAAAGGCGGATTAATACTGCAT
AAGATTGTACTGCTGCATGGCAGAGCAATAAAAGCTGAGGCGGTAAAGGATGGGCATGCGTGACATTAGC
TAGTTGGTGGGGTAACGGCCCACCAAGGCAACGATGTCTAGGGGTTCTGAGAGGAAGGTCCCCCACACTG
GTACTGAGACACGGAC
>Zotu442
GAACGCTGGCGGCGTGCCTAATACATGCAAGTCGAGCGAATGGATTAAGAGCTTGCTCTTATGAAGTTAGC
GGCGGACGGGTGAGTAACACGTGGGTAACCTGCCCATAAGACTGGGATAACTCCGGGAAACCGGGGCTA
ATACCGGATAATATTTTGAACTGCATGGTTCGAAATTGAAAGGCGGCTTCGGCTGTCACTTATGGATGGAC
CCGCGTCGCATTAGCTAGTTGGTGAGGTAACGGCTCACCAAGGCAACGATGCGTAGCCGACCTGAGAGGG
TGATCGGCCACACTGGGACTGAGACACGGCC
>Zotu444
GAACGTTAGCGGCGCGCTTAACACATGCAAGTCGCACGAGAAAGGGCTTCGGCCCCGGTACAGTGGCGCA
CGGGTGAGTAACACGTGGGTAATCTTCCCTCTGGCGGGGGATAACCTTCCGAAAGGAGGGCTAATACCGC
ATAAGACCACGACTCCGAAAGGAGACGAGGTTAAAGCTGGCCTCTATACATAAGCTAGCACCAGAGGATG
AGCCCGCGGCCCATCAGGTAGTTGGTAGGGTAACGGCCTACCAAGCCTAAGACGGGTAGCTGGTCTGAGA
GGATGATCAGCCACACTGGAACTGAGACACGGTC
>Zotu446
GAACGCTGGCGGCATGCCTAATACATGCAAGTCGAACGCTGAAGCACTCTGCTTGCAGAGTGCGGATGAG
TGGCGAACGGGTGAGTAACACGTGGGAAATCTGCCTATAAGAAGGGGATAACATTCGGAAACGGATGCTA
ATACCGCATATATTCTTGGACCGCCTGGCCCTTGAAAGAAAGACGGCTTTGCTGTCGCTTATAGATGATCCC
GCGGCGTATTAGTTAGTTGGTGGGGTAAAGGCCTACCAAGACAATGATACGTAGCCGACCTGAGAGGGTG
ATCGGCCACACTGGGACTGAGACACGGCC
>Zotu448
TAACGCTAGCGGCAGGCATAATACATGCAAGTTGAACGAGATAAGTTGTAGCAATATGACTTTGAAAGTG
GCGCACGGGTGCGTAACACGTATGCAACCTGCCCATATCTGGGGAATAGCCTCTCGAAAGAGAGATTAAT
ACCCCATACCACAATGTTAAGGCATCTTAATATTGTGAAAGCTCAGGCGGATATGGATGGGCATGCGGCTG
ATTAGCTAGTTGGTAGGGTAACGGCCTACCAAGGCTACGATCAGTAGGGGGTCTGAGAGGATGTCCCCCC
ACACTGGTACTGAGATACGGAC
>Zotu450
GAACGCTGGCGGCAGGCTTAACACATGCAAGTCGAGCGCCCCGCAAGGGGAGCGGCAGACGGGTGAGTA
ACGCGTGGGAATCTACCCTGATCTGCGGGATAGCTCCGGGAAACTGGAATTAATACCGCATGTGCCCTGAG

GGGCAAAGATTTATCGGATTGGGATGAGCCCGCGTCGGATTAGCTAGTTGGTGGGGTAAAGGCCTACCAA
GGCGACGATCCGTAGCTGGTCTAAGAGGATGATCAGCCACATTGGGACTGAGACACGGCC
>Zotu452
TAACGCTAGCGGCAGGCCTAATACATGCAAGTCGAACGGCAGCACGAATAGCAATATTTGGTGGCGAGTG
GCGCACGGGTGCGTAACGCGTATGCAATCTACCTATAACTGGGGAATAACCCCGAGAAATCGGGGCTAAT
ACCGCATAATACTACTGAACGGCATCGCTTGGTAGTCAAATCTCCGGAGGTTATAGATGAGCATGCGTGAC
ATTAGCTTGTTGGTGAGGTAACGGCTCACCAAGGCTACGATGTCTAGGGGGTCTGAGAGGATGATCCCCC
ACACTGGTACTGAGACACGGAC
>Zotu454
GAACGCTGGCGGTATGCCTAAGACATGCAAGTCGCACGATCTGTTTCGGACGGGTAGTGGCGGACGGGT
GAGTAACACGTGGGTGATCTGCCCAGAAGTCCGGGACAACTAGGCGAAAGCTTAGCTAATCCGGGATGTG
GACCGAGATTTTGATCTTTGTCTAAAGCTTCGGCGCTTCTGGATGAGCCTGCGGCGCATCAGCTGGTTGGC
GGGGTAACGGCCCACCAAGGCGACGACGCGTAGCTGGTCTGAGAGGACGACCAGCCACAGGGGTACTGA
GACACGGAC
>Zotu456
GAACGCTAGCGGCAGGCTTAACACATGCAAGTCGAGGGGTAGAAGCAGCTTGCTGCTTTGAGACCGGCGC
ACGGGTGCGTAACGCGTATACAATCTACCTTTTACAGAGGGATAGCCCAGAGAAATTTGGATTAATACCTC
ATAGTATTTTCGAATGGCATCATTTGATTATTAAAGTTCCAACGGTAAAAGATGAGTATGCGTCCCATTAGC
TAGATGGTAAGGTAACGGCTTACCATGGCAACGATGGGTAGGGGTCCTGAGAGGGAGATCCCCCACACTG
GTACTGAGACACGGAC
>Zotu458
GAACGCTGGCGGCGTGCCTAATACATGCAAGTCGAGCGGACGAATCGAAAGCTTGCTTTTGATTCGTTAGC
GGCGGACGGGTGAGTAACACGTGGGCAACCTGCCCTGTAGACGGGGATAACACCGAGAAATCGGTGCTA
ATACCGGATAACACGAAATGTCGCATGACGTTTCGTTGAAAGACGGCGTAAGCTGTCGCTACAGGATGGG
CCCGCGGCGCATTAGCTAGTTGGTGAGGTAACGGCTCACCAAGGCGACGATGCGTAGCCGACCTGAGAGG
GTGATCGGCCACACTGGGACTGAGACACGGCC
>Zotu460
TAACGTTGGCGGCGTGCATAACACATGCAAGTTGAGCGTGGCTTTAGTTTACTAAAGTCTAGCAGCGGACG
GCTGAGTAATGCACAGGAACGTACCCGGAAGTGGGGAATAGCCCATCGAAAGATGGGGTAATACCGCAT
GTGCTCGCAAGAGTAAAGCCGCAAGGCGCTACCGGAACGGCCTGTGTCCTATCAGGTTGTTGGTGGGGTA
AAAGCCTACCAAGCCTATGACGGGTAACTGGTCTGAGAGGATGACCAGTCAGATCGGGACTGAGACACGG
CC
>Zotu462
GAACGCTGGCGGCATGCTTTACACATGCAAGTCGAACGGCAGCACGGGTGCTTGCACCTGGTGGCGAGTG
GCGAACGGGTGAGTAATGCACCGGAACGTATCCAATAATGGGGGATAACGCACCGAAAGGTGTGCTAATA
CCGCATACGCCCTGAGGGGGAAAGCGGGGGATCGAAAGACCTCGCGTTAATGGAGCGGCCGATGTCGGA
TTAGCTAGTTGGTGAGGTAAAGGCTCACCAAGGCGACGATCCGTAGCTGGTCTGAGAGGACGACCAGCCA
CACTGGAACTGAGACACGGTC
>Zotu464
GAACGTTGGCGGCGTGCCTAATACATGCAAGTCGAACGCGGTCTCCTAATTGAAAAACCGTGCTTGCACGG
GTTGGATTTTAGATCGGACCGAGTGGCGAACTGGTGAGTAACACGTGGGTAACCTGCCCAGAAGTAGGGG
ATAACACCTGGAAACAGATGCTAATACCGTATAACAACTAAAACTACATGGTTTTAGTTTGAAAGCTGGCCT
TGGCGCTAGTGCTTTTGGATGGACCCGCGGCGTATTAGCTAGTTGGTGAGATAATAGCTCACCAAGGCGAT
GATACGTAGCAGACCTGAGAGGGTAATCTGCCACAATGGGACTGAGACACGGCC
>Zotu466
CGTCAGGTCCCAGTGCGTTTGGTTAATTACACCTTAGAAGACCTTTCACTGCCGCGAAAAAGGGTAGTAGC
GCAATTCATCCCGGCGAACATTACAGAAAGCGGACAAACAGAAAGTATCGGACTAACAGGA
>Zotu468
GAACGCTGGCGGCGTGCCTAATACATGCAAGTAGAACGCTGAAGGAGGAGCTTGCTCTTCTGGATGAGTT
GCGAACGGGTGAGTAACGCGTAGGTAACCTGCCTGGTAGTGGGGGATAACTATTGGAAACGATAGCTAAT

ACCGCATAATAACAGTTGTTGCATGACAACTGTTTGAAAGGTGCAATTGCATCACTACCAGATGGACCTGC
GTTGTATTAGCTAGTTGGTGGGGTAACGGCTCACCAAGGCGACGATACATAGCCGACCTGAGAGGGTGAT
CGGCCACACTGGGACTGAGACACGGCC
>Zotu470
GAACGCTAGCGGCAGGCTTAACACATGCAAGTCGAGGGGCAGCGCAGGTAGCAATACTGGGCGGCGACC
GGCAAACGGGTGCGGAACACGTACAGAATCTACCTTGATCTGGGGCATAGCCCGGAGAAATCCGGATTAA
CTCCCCATAACATATATGAGTGGCATCACTTGAATATTAAAAATTTATTGGATCGAGATGACTGTGCGTCCC
ATTAGCTAGTTGGTAGGGTAACGGCCTACCAAGGCGACGATGGGTAGCTGGTGTGAGAGCACGACCAGCC
ACACGGGCACTGAGACACGGGC
>Zotu472
GAACGCTGGCGGCATGCCTAACACATGCAAGTCGAACGAGACCTTCGGGTCTAGTGGCGCACGGGTGCGT
AACGCGTGGGAATCTGCCCTTGGGTGCGGAATAACTTCTCGAAAGGGATGCTAATACCGCATAATGTCGTA
AGACCAAAGATTTATCGCCCAAGGATGAGCCCGCGTAAGATTAGCTAGTTGGTGGGGTAAAAGCCTACCA
AGGCGACGATCTTTAGCTGGTCTGAGAGGATGATCAGCCACACTGGGACTGAGACACGGCC
>Zotu474
GAACGCTGGCGGCAGGCCTAACACATGCAAGTCGAACGGATCCTTCGGGATTAGTGGCGGACGGGTGAG
TAACACGTGGGAACGTGCCCTTTGGTTCGGAACAACTCAGGGAAACTTGAGCTAATACCGGATGTGCCCTT
CGGGGGAAAGATTTATCGCCATTGGAGCGGCCCGCGTCTGATTAGCTAGTTGGTGGGGTAAAGGCCCACC
AAGGCGACGATCAGTAGCTGGTCTGAGAGGATGATCAGCCACATTGGGACTGAGACACGGCC
>Zotu476
GAACGCTGGCGGCGTGCTTAACACATGCAAGTCGAACGGAAAGGCCCTTCGGGGTACTCGAGTGGCGAAC
GGGTGAGTAACACGTGGGTGATCTGCCCTGCACTTTGGGATAAGCCTGGGAAACTGGGTCTAATACCGAA
TATGATCACGCTTCTCATGGGGTGTGGTGGAAAGCTTTTGCGGTGTGGGATGGGCCCGCGGCCTATCAGCT
TGTTGGTGGGGTAATGGCCTACCAAGGCGACGACGGGTAGCCGGCCTGAGAGGGTGTCCGGCCACACTG
GGACTGAGATACGGCC
>Zotu478
GAACGCTGGCGGCGTGCCTAATACATGCAAGTCGAACGCGAGCGATTGGAGCTTGCTCTAATCAATCGAG
TGGCGAACGGGTGAGTAACACGTGGGCAACCTGCCCATCAGCCTGGGATAACCACGGGAAACCGTGGCTA
ATACCGGATAGACCGAGTGACAGAAGTCACAATGGAGAAAGTCGGTTTTACTGACACTGATGGATGGGCC
TGCGGCGCATTAGCTAGTTGGAGAGGTAGTGGCTCCCCAAGGCAACGATGCGTAGCCGACCTGAGAGGGT
GAACGGCCACACTGGAACTGAGACACGGTC
>Zotu480
GAACGCTAGCGGCAGGCCTAATACATGCAAGTCGAACGCCCAGCAATGGGAGTGGCGCACGGGTGCGTA
ACGCGTATGCAACCTACCTTAAACTGGGGGATAGCCTTCCGAAAGGAAGATTAATACCCCATAGTATCACG
AGAGGGCATCTGATTGTGATTAAAGATTTATCGGTTTAAGATGGGCATGCGTGACATTAGCTAGTTGGTGA
GGTAACGGCTCACCAAGGCTACGATGTCTAGGGGTTCTGAGAGGATGATCCCCCACACTGGTACTGAGAT
ACGGAC
>Zotu482
GAACGCTAGCGGCAGGCCTAATACATGCAAGTCGAACGGTAAATATTCAGCAATGGATATGAGAGTGACG
TACGGGTGCGTAACACGTATGCAACCTACCCTTTACAGGCGGATAGCTCGGGGAAACTCGGATTAACCCGT
CATAATATAATTGGATGGCATCATTTGATTATTAAATCTACGGAGGTAAAGGATGGGCATGCGTCTGATTA
GTTAGTTGGCGGTGTAACGGACCACCAAGACGATGATCAGTAGGGGAACTGAGAGGTTGATCCCCCACAC
TGGCACTGAGATACGGGC
>Zotu484
GAACGCTGGCGGCGTGCTTAACACATGCAAGTCGAACGGGGTGTTTATTTCGGTAAACACCAAGTGGCGA
ACGGGTGAGTAACGCGTAAGCAATCTACCTTCAAGATGGGGACAACACTTCGAAAGGGGTGCTAATACCG
AATGAATGTAAGAGTATCGCATGAGACACTTACTAAAGGAGGCCTCTGACATAAGCTTCCGCTTGAAGATG
AGCTTGCGTCTGATTAGCTAGTTGGTGAGGGTAAAGGCCCACCAAGGCGACGATCAGTAGCCGGTCTGAG
AGGATGAACGGCCACATTGGGACTGAGACACGGCC
>Zotu486

GAACGCTGGCGGCAGGCTTAACACATGCAAGTCGAACGCCCGAGCAATCGGGAGTGGCAGACGGGTGAG
TAACGCGTGGGAACATACCCTTTGGACTGGAATAACTGGGGGAAACTTCAGCTAAGACCGGATACGTGCG
AGAGCAGAATGATTTATCGCCGAAGGATTGGCCCGCGTCCGATTAGCTAGTTGGTGTTGGTAATGGCTCAC
CAAGGCGACGATCGGTAGCTGGTCTGAGAGGATGACCAGCCACATTGGGACTGAGACACGGCC

>Zotu488
CAACGCTGGCGGCGCGCTTAACACATGCAAGTCGAACGAGAAAGGGAGCAATCCTGAGTAAAGTGGCGTA
CTGGTGAGTAACACGTGGATATCTACCTCTTGGTGGGGAATAACCTGGGGAAACCTGGGCTAATACCGCAT
AAGTTCGAGAGAAGAAAGGCGAAAGTCGCCGAGAGAGGAGTCCGCGGTCGATTAGGTAGTTGGCAGGGT
AAAGGCCTACCAAGCCGAAGATCGATAGCCGGCCTGAGAGGGCACACGGCCACACTGGCACTGAAACACG
GGC

>Zotu490
GAACGCTGGCGGCGTGCCTTATACATGCAAGTCAAACGGGTGTAGCAATACACCAGTGGCGAACGGGTGA
GTAATACGTAGGTACTTTGCTTAAGTTGGGCATAACTAGCCGAAAGGTTAGCTAATTCCCGATGTGATCGT
AAGATCAAAGTCGCAAGACGCTTAAGAATAGGCCTGCGTCCTATCAGCTTGTTGGCGGGGTAAAAGCCCA
CCAAGGCTATGACGGGTAGCTGGTCTGAGAGGATGATCAGCCACAATGGGACTGCGACACGGCC

>Zotu492
GAACGCTGGCGGCGTGCCTAATACATGCAAGTCGAGCGAACAGATGAGAAGCTTGCTTCTCTGATGTTAGC
GGCGGACGGGTGAGTAACACGTGGGTAACCTACCTATAAGACTGGGATAACTCCGGGAAACCGGGGCTA
ATACCGGATAATATTTTGAACCGCATGGTTCAATAGTGAAAGACGGTTTCGGCTGTCACTTATAGATGGAC
CCGCGCCGTATTAGCTAGTTGGTAAGGTAACGGCTTACCAAGGCGACGATACGTAGCCGACCTGAGAGGG
TGATCGGCCACACTGGAACTGAGACACGGTC

>Zotu494
GAACGCTGGCGGCATGCCTAACACATGCAAGTCGAACGAGACCTTCGGGTCTAGTGGCGCACGGGTGCGT
AACGCGTGGGAACCTGCCCTTAGGTTCGGAATAACTCCTCGAAAGGGGTGCTAATACCGGATAATGTCTTC
GGACCAAAGATTTATCGCCTTTGGATGGGCCCGCGTTGGATTAGCTAGTTGGTGAGGTAAAGGCTCACCAA
GGCGACGATCCATAGCTGGTCTTAGAGGATGATCAGCCACACTGGGACTGAGACACGGCC

>Zotu496
GAACGCTGACAGAATGCTTAACACATGCAAGTCAACTTGAATTTGGGTTTTTAACTTAGGTTTGGGTGGCG
GACGGGTGAGTAACGCGTAAAGAACTTGCCTCACAGGTAGGGACAACATTTGGAAACGAATGCTAATACC
TAATATTATGATAATAGGGCATCCTATAATTATGAAAGCTAAAAGCACTGTGAGAGAGCTTTGCGTCCCATT
AGCTAGTTGGAGAGGTAACGGCTCACCAAGGCGATGATGGGTAGCCGGCCTGAGAGGGTGATCGGCCAC
AAGGGGACTGAGACACGGCC

>Zotu498
GAACGCTGGCGGCAGGCCTAACACATGCAAGTCGAGCGGATGATGGGAGCTTGCTCCCGGATTCAGCGGC
GGACGGGTGAGTAATGCCTAGGAATCTGCCTGGTAGTGGGGGACAACGTTTCGAAAGGAACGCTAATACC
GCATACGTCCTACGGGAGAAAGCAGGGGACCTTCGGGCCTTGCGCTATCAGATGAGCCTAGGTCGGATTA
GCTAGTTGGTGGGGTAAAGGCCTACCAAGGCGACGATCCGTAACTGGTCTGAGAGGATGATCAGTCACAC
TGGAACTGAGACACGGTC

>Zotu500
GAACGCTGGCGGCGTGCTTAACACATGCAAGTCGAGCGGAAAGGCCCTTTCGGGGGTACTCGAGCGGCG
AACGGGTGAGTAACACGTGAGTAATCTGCCCTTCACTTTGGGATAGCTCCCGGAAACGGGGATTAATACCG
GATATGACCATGGGTCGCATGATCTTGTGGTGGAAAGTTTTTTCGGTGGGGGATGTGCTCGCGGCCTATCA
GCTTGTTGGTGGGGTAATGGCCTACCAAGGCTTCGACGGGTAGCCGGCCTGAGAGGGTGACCGGCCACAC
TGGGACTGAGACACGGCC

>Zotu502
GAACGCTGGCGGCATGCTTAACACATGCAAGTCGAACGGCAGCACAGCAGAGCTTGCTCTGTGGGTGGCG
AGTGGCGGACGGGTGAGTAATGCATCGGGACCTACCCAGACGTGGGGGATAACGTAGGGAAACTTACGC
TAATACCGCATACGTCCTACGGGAGAAAGCGGGGGATCTTCGGACCTCGCGCGGTTGGATGGACCGATGT
GCGATTAGCTAGTTGGTGAGGTAACGGCTCACCAAGGCGACGATCGCTAGCTGGTCTGAGAGGATGATCA
GCCACACTGGGACTGAGACACGGCC

>Zotu504
GAATGCTGGCGGCGCGCCTAACACATGCAAGTCGAGCGGTAACATGGACTTCGGTCTGATGACGAGCGGC
GGACGGCTGAGTAACGCGTGGGAACATGCCCCAAAGTGAGGAATAAGCTCCAGAAATGGAGTCTAATGCC
GCATATGGTCTTCGGATTAAAGGATTTATCCGCTTTGGGAGTGGCCTGCGTATGATTAGATAGTTGGTGAG
GTAATGGCTCACCAAGTCTACGATCATTAACTGGTCTGAGAGGATGATCAGTCAGACTGGAACTGAGACAC
GGTC
>Zotu506
GAACGCTGGAAACGTGGATACCGGCATGCAAGTCGAGCGGACCCGCAAGGGTCAGCGGCGAACGGCTTA
GTAACACGTGGGTACATACCCTAAACTGGAGGACAGCTCGTCGAAAGACGGGGTAATACTCCATAGTCTC
GCAAGAGTAAAGTCGTAAGACGGTTTAGGAATGGCCTGCGGCCTATCAGATAGTTGGCGAGGTAATGGCT
CACCAAGTCTATGACGGGTAGGGGGCGTGAGAGCGCGACCCCCAAGAATGGCACTGCGACACGGGC
>Zotu508
GAACGCTGGAAATGTGGATACCGGCATGCAAGTCAAGGGGCCGCAAGGCACCGGCGAACGGCTTAGTAA
CACGTAGATACATACCGTAAAGTGGGGCACAGCTCGTCGAAAGACGGGGTAATTTCCCATGTGACCGCAA
GGTTAAAGGCGCAAGCCGCTTTATGAATGGTCTGCGGTCTATCAGCTAGTTGGTGAGGTAATGGCTCACCA
AGGCTATGACGGATAGGGGGCGTGAGAGCGCGACCCCCAAGAATGGCACTGCGACACGGGC
>Zotu510
GAACGCTGGCGGCGTGCTTAACACATGCAAGTCGAACGGAAAGGCCCTTCGGGGTACTCGAGTGGCGAAC
GGGTGAGTAACACGTGGGTGATCTGCCTCGTACTCTGGGATAAGCCTGGGAAACTGGGTCTAATACCGGA
TAGGACCTCTGGTCGCATGGCTGGTGGTGGAAAGTTTTTCGGTACGAGATGGGCCCGCGGCCTATCAGCTT
GTTGGTGGGGTAATGGCCTACCAAGGCGACGACGGGTAGCCGGCCTGAGAGGGCGACCGGCCACACTGG
GACTGAGACACGGCC
>Zotu512
GAACGCTGGCGGCGTGCTTAACACATGCAAGTCGAACGGTGAAGCTCCAGCTTGCTGGAGTGGATCAGTG
GCGAACGGGTGAGTAACACGTGAGCAACCTGCCCCTGACTCTGGGATAACAGCCGGAAACGGCTGCTAAT
ACCGGATATGCACCATGAACGCATGTTCTGTGGTGGGAAAGATTTTTCGGTTGGGGATGGGCTCGCGGCCT
ATCAGCTTGTTGGTGAGGTAATGGCTCACCAAGGCGTCGACGGGTAGCCGGCCTGAGAGGGTGACCGGCC
ACACTGGGACTGAGACACGGCC
>Zotu514
GAATGCTGGCGGCGTGGATAAGTCATGCAAGTCAAGGGGGCCCGCAAGGGCAACCGGCAGACGAGGTAG
TAACACGTAGGTAGATCCCCAGAAGTCGGGCATAGCCAGTCGAAAGACTGGGTAATTCCCGATAGTCCCG
CAAGGGTAAAGGTTTTTCGCTTCTGGAGTTGCCTGCGTAGTATCAGCTAGTTGGTAGGGTAATGGCCTACC
AAGGCTACGACGCTTAGGGGGGCTGAGAGGCCGATCCCCACCGATGGAACTGCGACACGGTC
>Zotu517
GAACGCTGGCGGCGTGCCTAACACATGCAAGTCGAACGGAGCACCTTCGGGTGCTTAGTGGCGAACGGGT
GAGTAACGCGTGAGAAACCTGCCTTTCAGTGGGGGACAACAGTTGGAAACGACTGCTAATACCGCATAAT
GTCATTTGGGGGCATCCCTGAATGACCAAAGATTTATTGCTGAAAGATGGTCTCGCGTCTGATTAGCTAGTT
GGTGAGGTAACGGCTCACCAAGGCGACGATCAGTAGCCGGTCTGAGAGGATGAACGGCCACATTGGGAC
TGAGATACGGCC
>Zotu519
GAACGCTGGCGGCAGGCTTAACACATGCAAGTCGAACGCTCCGCAAGGAGAGTGGCAGACGGGTGAGTA
ACACGTGGGAACGTACCCTTTACTACGGAACAACTCCGGGAAACTGGAGCTAATACCGTATGTGCCCTTCG
GGGGAAAGATTTATCGGTAAAGGATCGGCCCGCGTTGGATTAGCTAGTTGGTGGGGTAAAGGCCTACCAA
GGCGACGATCCATAGCTGGTCTGAGAGGATGATCAGCCACATTGGGACTGAGACACGGCC
>Zotu521
GAACGCTAGCGGGAGGCCTAACACATGCAAGCCGAGCGGTAGAGATCTTTCGGGATCTTGAGAGCGGCGT
ACGGGTGCGGAACACGTGTGCAACCTGCCTTTATCTGGGGGATAGCCTTTCGAAAGGAAGATTAATACCCC
ATAATATGTTGGATGGCATCATTCGACATTGAAAACTCCGGTGGATAGAGATGGGCACGCGCAAGATTAG
ATAGTTGGTGAGGTAACGGCTCACCAAGTCTGCGATCTTTAGGGGGCCTGAGAGGGTGATCCCCCACACT
GGTACTGAGACACGGAC

>Zotu524
GAACGCTAGCTACAGGCTTAACACATGCAAGTCGAGGGGCAGCATGGAAGAAGCTTGCTTCTTCTGATGG
CGACCGGCGCACGGGTGCGTAACGCGTATCAAACCTGCCTCATACTCGGGGATAGCCTTGCGAAAGTAAG
ATTAATACCCGATGTTGTTATGTTACCGCATGGTGATATAACCAAAGATTTATCGGTATGAGATGGTGATGC
GTCCGATTAGGTAGTAGGCGGGGTAACGGCCCACCTAGCCTTCGATCGGTAGGGGTTCTGAGAGGAAGGT
CCCCCACACTGGAACTGAGACACGGTC
>Zotu526
GAACGCTGGCAGCGTGCTTAACACATGCAAGTCGAACGGTGAAGCCAAGCTTGCTTGGTGGATCAGTGGC
GAACGGGTGAGTAACACGTGAGCAACCTGCCCCGGACTCTGGGATAAGCGCTGGAAACGGCGTCTAATAC
CGGATATGAACCACAGCCGCATGGTTGGTGGTTGGAAAGATTTTTTGGTTCGGGATGGGCTCGCGGCCTAT
CAGCTTGTTGGTGAGGTAATGGCTCACCAAGGCGTCGACGGGTAGCCGGCCTGAGAGGGTGACCGGCCAC
ACTGGGACTGAGACACGGCC
>Zotu528
TAACGTTGGCAGCGTGCCTAATACATGCAAGTCGCGCGATATAGCGGCGGACGAGAGAGTAATAGTAAGG
AACTTACCCCTGAGTGAAGGATAGCCATCCGAAAGGATGGGTAATCCTGCATAATCCCGCAAGGGCAAAG
CCGCAAGGCGCTTAGGGAGAGGCCTTACTCCTATCAGCTTGTTGGTGAGGTAAAGGCTCACCAAGGCGAT
GACGGGTAGCTGGTCTGAGAGGATGACCAGCCTCGATGGGACTGAGACACGGCC
>Zotu530
GAACGCTGGCGGCATGCCTAACACATGCAAGTCGAACGAAGGCTTCGGCCTTAGTGGCGCACGGGTGCGT
AACGCGTGGGAATCTGCCCTTAGGTTCGGAATAACAGCTGGAAACGGCTGCTAATACCGGATGATATCGC
GAGATCAAAGATTTATCGCCTGAGGATGAGCCCGCGTTGGATTAGGTAGTTGGTGGGGTAAAGGCCTACC
AAGCCGACGATCCATAGCTGGTCTGAGAGGATGATCAGCCACACTGGGACTGAGACACGGCC
>Zotu533
GAACGCTAGCGGGAGGCCTAACACATGCAAGCCGAGCGGTAGAAGATCTTCGGATCTTTGAGAGCGGCGC
ACGGGTGCGGAACACGTGTGCAACCTGCCTTTATCTGGGGGATAGCCTTTCGAAAGGAAGATTAATACCCC
ATAATATATTTTACGGCATCGTAAGATATTGAAAACTCCGGTGGATAGAGATGGGCACGCGCAAGATTAGT
TAGTTGGTGAGGTAACGGCTCACCAAGACGATGATCTTTAGGGGGCCTGAGAGGGTGATCCCCCACACTG
GTACTGAGACACGGAC
>Zotu535
GAACGCTGGCGGCGTGCTTAACACATGCAAGTCGAGCGGTAAGGCTCCTTCGGGAGTACACGAGCGGCGA
ACGGGTGAGTAACACGTGAGCAATCTGCCCTTCACTTCGGGATAACTCCGGGAAACCGGTGCTAATACCGA
ATACGACCACTTCACGCATGTGATGGTGGTGGAAAGTTCCGGCGGTGGAGGATGAGCTCGCGGCCTATCA
GCTTGTTGGTGGGGTAATGGCCCACCAAGGCAACGACGGGTAGCCGGCCTGAGAGGGTGACCGGCCACA
CTGGGACTGAGACACGGCC
>Zotu537
GAACGCTGGCGGCGTGCTTAACACATGCAAGTCGAACGATGAAAGAGAATCCGATCTCTTCGGAGTGAAG
AATCTCTGGATTAGTGGCGGACGGGTGAGTAACGCGTGAGCAATCTGCCTTACACAAAGGGATAGCCTCG
GGAAACCGGGATTAATACCATATGATATCTATTAACCGCATGGCCTATAGATTAAAGAATTTCGGTGTAAG
ATGAGCTCGCGTCCCATTAATTAGTTGGTGAGGGTAACGGCCCACCAAGATAGCGATGGGTAGCCGATCTA
AGAGGATGACCGGCCACATTGGAACTGAGAAACGGTC
>Zotu539
GAACGCTAGCGGGAGGCCTAACACATGCAAGCCGAGCGGTATTGTTTCTTCGGAAATGAGAGAGCGGCGT
ACGGGTGCGGAACACGTGTGCAACCTGCCTTTATCTGGGGGATAGCCTTTCGAAAGGAAGATTAATACCCC
ATAATATACTGAATGGCATCATTCGGTATTGAAAACTCCGGTGGATAGAGATGGGCACGCGCAAGATTAG
ATAGTTGGTGAGGTAACGGCTCACCAAGTCTGCGATCTTTAGGGGGCCTGAGAGGGTGATCCCCCACACT
GGTACTGAGACACGGAC
>Zotu541
GAACGCTGGCGGCGTGCTTAACACATGCAAGTCGAGCGGTAAGGCCCCTTCGGGGGTACACGAGCGGCG
AACGGGTGAGTAACACGTGAGTAATCTGCCCTTCAGTTCGGGATAACCACCGGAAACGGTGGCTAATACC
GGATACGACCAGCCTCGGCATCGGGTGCTGGTGGAAAGTTTTTTCGCTGGAGGATGTGCTCGCGGCCTATC

AGCTTGTTGGTGAGGTAACGGCTCACCAAGGCTTCGACGGGTAGCCGGCCTGAGAGGGTGACCGGCCACA
CTGGGACTGAGACACGGCC
>Zotu543
GAACGCTGGCGGCGTGCTTAACACATGCAAGTCGAGAGGTAAGGCCTTTCGGGGTACACGAGCGGCGAA
CGGGTGAGTAACACGTGGGTGATCTGCCCTGCACTTCGGGATAAGCCTGGGAAACTGGGTCTAATACCGG
ATATGACTTCAGGTTGCATGACTTGGGGTGGAAAGATTTATCGGTGCAGGATGGGCCCGCGGCCTATCAG
CTTGTTGGTGGGGTAATGGCCTACCAAGGCGACGACGGGTAGCCGACCTGAGAGGGTGACCGGCCACACT
GGGACTGAGACACGGCC
>Zotu545
GAACGCTAGCGACAGGCCTAACACATGCAAGTCGAGGGGCAGCACGGTGTAGCAATACACGGGTGGCGA
CCGGCGCACGGGTGAGTAACGCGTATGCAACCTGCCTACTACAAGGGGATAACCCGTTGAAAGACGGACT
AATACCCTATAACACAGGGGATCCGCATGGATATATTTGTTAAAGATTTATCGGTAGTAGATGGGCATGCG
TTCCATTAGCTTGTTGGTGAGGTAACGGCTCACCAAGGCAACGATGGATAGGGGAACTGAGAGGTTGATC
CCCCACACTGGAACTGAGACACGGTC
>Zotu547
GAACGCTGGCGGCGTGCTTAACACATGCAAGTCGAACGGTGAAACAGGAGCTTGCTCCTGCGGATCAGTG
GCGAACGGGTGAGTAACACGTGAGAAATCTGCCCTGGACTCTGGGATAAGCGTTGGAAACGACGTCTAAT
ACCGGATACGAGCTGCGAAGGCATCTTCAGCAGCTGGAAAGAACTTCGGTCCAGGATGATCTCGCGGCCT
ATCAGCTAGTTGGTGAGGTAACGGCTCACCAAGGCGACGACGGGTAGCCGGCCTGAGAGGGTGACCGGC
CACACTGGGACTGAGACACGGCC
>Zotu549
GAACGCTGGCGGCAGGCCTAACACATGCAAGTCGAGCGCGCCCTTCGGGGTGAGCGGCGGACGGGTGAG
TAACGCGTGGGAACGTGCCCTTTTCTACGGAATAGCCTCGGGAAACTGAGAGTAATACCGTATACGCCCTT
TGGGGGAAAGATTTATCGGAAAAGGATCGGCCCGCGTTGGATTAGGTAGTTGGTGGGGTAATGGCCTACC
AAGCCGACGATCCATAGCTGGTTTGAGAGGATGATCAGCCACACTGGGACTGAGACACGGCC
>Zotu551
GAACGCTGGCGGCGTGCTTAACACATGCAAGTCGAGCGGGGCACTTCTGGTGTCCAGCGGCGAACGGGTG
AGTAACACGTGGGCAACCTGCCCTCAGCTCTGGGATAAGCCGGGGAAACCCGGTCTAATACCGGATATGA
CATGCTCTCGCATGGGGGTGTGTGGAAAGATTTATCGGCTGGGGATGGGCCCGCGGCCTATCAGCTTGTT
GGTGGGGTGATGGCCTACCAAGGCGACGACGGGTAGCCGGCCTGAGAGGGCGATCGGTCACACTGGGAC
TGAGACACGGCC
>Zotu553
GAACGCTAGCGGCAGGCCTAATACATGCAAGTCGAGGGGTAGAGTAGGCTTGCTTACTTGAGACCGGCGC
ACGGGTGCGTAACGCGTATGCAACCTACCTTTATCTGGGGGATAGCCCGGAGAAATCCGGATTAATACCGC
ATAAAATCACAGAATGGCATTATTCAATGATCAAACATTAATGGGATAGAGATGGGCATGCGTGTCATTAG
CTAGTTGGCGGGGTAACGGCCCACCAAGGCGACGATGACTAGGGGATCTGAGAGGATGACCCCCCACACT
GGTACTGAGACACGGAC
>Zotu555
GAACGCTAGCGGCAGGCTTAATACATGCAAGTCGTGGGGCAGCAGGAGTGGTAGCAATACCACTTGCTGG
CGACCGGCAAACGGGTGCGGAACACGTACGCAACTTACCCTTAAGTGGGGGATAGCCCGGGGAAACCCG
GATTAATACCTCGTAATACTTAGGTGTGGCATCACACTTTAGTTATAGCCTTCGGGCGCTTAAGGATGGGCG
TGCGTCCCATTAGGTAGTTGGTTGAGGTAACGGCTCACCAAGCCTGCGATGGGTAACTGGTGTGAGAGCA
CGACCAGTCACACGGGCACTGAGACACGGGC
>Zotu557
GAACGCTAGCGGCAGGCTTAATACATGCAAGTCGTGGGGCAGCATGATGTAGCAATACATTGATGGCGAC
CGGCAAACGGGTGCGGAACACGTACACAACCTTCCTACAAGTGGGGAATAGCCCAGAGAAATTTGGATTA
ATACCCCGTAACATATTTGAGATGGCATCATCTTAATATTATAGCTTCGGCGCTTGTTGATGGGTGTGCGGC
TGATTAGATAGTTGGCGGGGTAACGGCCCACCAAGTCTACGATCAGTAGCTGATGTGAGAGCATGATCAG
CCACACGGGCACTGAGACACGGGC
>Zotu559

GAACGCTGGCGGCGTGCTTAACACATGCAAGTCGAACGGTAAGGCCCTTTTGGGGGTACACGAGTGGCGA
ACGGGTGAGTAACACGTGAGTAACCTGCCCTTGACTCTGGGATAATTCTTCGAAAGAGGTTGTAATACTGG
ATATGAGCTTCTGCCGCATGGTGGGGGTTGGAAAGTTCTGGCGGTCAGGGATGGGCTCGCGGCCTATCAG
CTTGTTGGTGAGGTAGTGGCTTACCAAGGCGACGACGGGTAGCCGGCCTGAGAGGGCGACCGGCCACACT
GGGACTGAGATACGGCC
>Zotu561
GAACGCTGGCGGCGTGCTTAACACATGCAAGTCGAACGATGAACCTCACTTGTGGGGGGATTAGTGGCGA
ACGGGTGAGTAACACGTGAGTAACCTGCCCTTGACTCTGGGATAAGCCTGGGAAACCGGGTCTAATACTG
GATACGACCTTCTGGCGCATGCCATGTTGGTGGAAAGCTTTTGTGGTTTTGGATGGACTCGCGGCCTATCA
GCTTGTTGGTGGGGTAATGGCCTACCAAGGCGACGACGGGTAGCCGGCCTGAGAGGGTGACCGGCCACA
CTGGGACTGAGACACGGCC
>Zotu563
GAACGCTAGCGGCAGGCCTAATACATGCAAGTCGAGGGGTAGAGGTTGCTTGCAATCTTGAGACCGGCGC
ACGGGTGCGTAACGCGTATGTAACCTACCTTTATCTGGGGGATAGCCCGGAGAAATCCGGATTAATACCGC
ATAAAATCACAGTATTGCATAATACAATGATCAAACATTTATGGGATGAAGATGGGCATGCGTGTCATTAG
CTAGTTGGCGGGGTAACGGCCCACCAAGGCGACGATGACTAGGGGATCTGAGAGGATGACCCCCCACACT
GGTACTGAGACACGGAC
>Zotu566
GAACGCTGGCGGGATGCTTTACACATGCAAGTCGAACGGCAACGCGGACTTCGGTCTGGCGGCGAGTGGC
GAACGGGTGAGTAATGTATCGGAACGTACCCAGTAGAGGGGGATAACTACGCGAAAGCGTAGCTAATACC
GCATAAGCCCTAAGGGGGAAAGCGGGGGATCTTCGGACCTCGCGCTATTGGAGCGGCTGATATCAGATTA
GCTAGTTGGTGGGGTAAAGGCCTACCAAGGCGACGATCTGTAGCTGGTTTGAGAGGACGACCAGCCACAC
TGGGACTGAGACACGGCC
>Zotu568
AAACGCTGGCGGCGTGCATGAGACATGCAAGTCGAACGAACGAGCTTGCTCGTTAGTGGCGGACGGCTGA
GTAACGCGTAGGTGACCTGCCCCGAAGTGGGGGATAACTGCCCGAAAGGGTGGCTAATACCGCATGTGGC
GACACTTTTATTGGTGTCATCAAAGCCGCGAGGCGCTTCGGGAGGGGCCTGCGTCCGATTAGCTCGTTGGT
GGGGTAATGGCTCACCAAGGCGACGATCGGTAGGTGGTCTGAGAGGACGATCACCCAGACTGGGACTGA
GACACGGCC
>Zotu570
GAACGCTGGCGGCGTGCCTAATACATGCAAGTCGAGCGAACTGAAAAGGAGCTTGCTCCTTTGACGTTAG
CGGCGGACGGGTGAGTAACACGTGGGTAACCTACCTATAAGACTGGAATAACTCCGGGAAACCGGGGCTA
ATGCCGGATAATATGTTGAACCGCATGGTTCAACAGTGAAAGACGGTTTTGCTGTCACTTATAGATGGACC
CGCGCCGCATTAGCTAGTTGGTGAGGTAACGGCTCACCAAGGCGACGATGCGTAGCCGACCTGAGAGGGT
GATCGGCCACACTGGAACTGAGACACGGTC
>Zotu572
GAACGCTAGCGGCAGGCCTAATACATGCAAGTCGAGGGGTAGAGTTAGCTTGCTAACTTGAGACCGGCGC
ACGGGTGCGTAACGCGTATGCAACCTACCTTTATCTGGGGGATAGCCCGAAGAAATTCGGATTAACACCGC
ATAAAATCACAGTATCGCATGATACAATGATCAAATATTTATAGGATAAAGATGGGCATGCGTGTCATTAG
TTAGTTGGCGGGGTAACGGCCCACCAAGACCACGATGACTAGGGGATCTGAGAGGATGACCCCCCACACT
GGTACTGAGACACGGAC
>Zotu574
GAACGCTGGCGGCGTGCCTAATACATGCAAGTCGAGCGAACGGACAAGGAGCTTGCTCCTTTGAAGTTAG
CGGCGGACGGGTGAGTAACACGTGGGTAACCTACCTATAAGACTGGAATAACTCCGGGAAACCGGGGCTA
ATGCCGGATAACATGTTGAACCGCATGGTTCAACAGTGAAAGACGGTCTTGCTGTCACTTATAGATGGACC
CGCGCCGTATTAGCTAGTTGGTGGGGTAACGGCCTACCAAGGCGACGATACGTAGCCGACCTGAGAGGGT
GATCGGCCACACTGGAACTGAGACACGGTC
>Zotu577
GAACGCTGGCGGCGTGCCTAATACATGCAAGTCGAGCGGACCGGATCGGAGCTTGCTCTGGTTTGGTCAG
CGGCGGACGGGTGAGTAACACGTGGGCAACCTGCCCGCAAGACCGGGATAACTCCGGGAAACCGGAGCT
AATACCGGATAACACCGAAGACCGCATGGTCTTTGGTTGAAAGGCGGCCTTTGGCTGTCACTTGCGGATGG

GCCCGCGGCGCATTAGCTAGTTGGTGAGGTAACGGCTCACCAAGGCGACGATGCGTAGCCGGCCTGAGAG
GGTGACCGGCCACACTGGGACTGAGACACGGCC
>Zotu579
GAACGCTGGCGGCGTGGATAAGGCATGCAAGTCAAGCGGGTCCCTCACCTTTCGCAAGAAAGGTGAGGG
ATACCGCGGCGAACGAGTTAGTAACACCTTGGTATCTGCCTCAAAGACGGGCATAACCAGTCGAAAGATTG
GCTAATTCCCGATGGTCCCTTACGGGTAAAGGAGCAATCCGCTTTGAGAGGAGCCTAGGTCCTATCAGCTT
GTTGGCGGGGTAACGGCCCACCAAGGCTATGACGGGTAGGAGGTGTGAGAGCACGACCTTCAACGACGA
AACTGAGATACGGTT
>Zotu581
GAACGCTGGCGGCATGCCTTACACATGCAAGTCGAACGGCAGCACGGACTTCGGTCTGGTGGCGAGTGGC
GAACGGGTGAGTAATACATCGGAACGTGTCCTGTAGTGGGGGATAGCCCGGCGAAAGCCGGATTAATACC
GCATACGCTCTACGGAGGAAAGGGGGGGATCGCAAGACCTCCCGCTACAGGGGCGGCCGATGGCAGATT
AGCTAGTTGGTGGGGTAAAGGCCTACCAAGGCGACGATCTGTAGCTGGTCTGAGAGGACGACCAGCCACA
CTGGGACTGAGACACGGCC
>Zotu583
GAACGCTGGCGGCAGGCTTAACACATGCAAGTCGAACGAGTGTAGCAATACACTAGTGGCGCACGGGTGA
GTAACACGTGGATATCTGCCTTTTGGTTCGGGATAACCCTGGGAAACTAGGGCTAATACCGAATGGTTCCT
ACGGGATAAAGATTTATCGCCAAAAGATGAGTCCGCGTCCGATTAGCTAGTTGGTGAGGTAATGGCTCACC
AAGGCGACGATCGGTAGCTGGTCTGAGAGGACGATCAGCCACACTGGGACTGAGACACGGCC
>Zotu585
GAACGCTGGCGGCGTGCTTAACACATGCAAGTCGAACGGTAAGGCCCCTTCGGGGGTACACGAGTGGCGA
ACGGGTGAGTAACACGTGAGTAACCTGCCCTTGACTCTGGGATAACAGCTGGAAACAGCTGCTAATACCG
GATATCAGCCTCGATGGCATCTTCGAGGCTTGAAAGCTCCGGCGGTCATGGATGGACTCGCGGCCTATCAG
CTTGTTGGTGAGGTAACGGCTCACCAAGGCGACGACGGGTAGCCGGCCTGAGAGGGCGACCGGCCACATT
GGGACTGAGATACGGCC
>Zotu587
GAACGCTAGCGGCAGGCCTAATACATGCAAGTCGAGCGGCAAGTCGAGGAGTAATCTAAGACCTAGAGCG
GCGTACGGGTGCGTAACGCGTATGCAACCTACCTCATACAGGGGGATAGCCCGGAGAAATCTGGATTAAT
ACCCCATGGCACAGCACAAGGGCATCTGCGAGCTGTTAAAGATTCATCGGTATGAGATGGGCATGCGTCT
GATTAGCTAGTTGGCGGTGTAACGGACCACCAAGGCGACGATCAGTAGGGGTTCTGAGAGGAAGGTCCCC
CACACTGGCACTGAGATACGGGC
>Zotu589
GAACGCTAGCGGCAGGCTTAATACATGCAAGTCGAGGGGCAGCAGGGCTGTAGCAATACAGTTGCTGGC
GACCGGCAAACGGGTGCGGAACACGTACGCAACTTACCCAGAACTGGGGCATAGCCCTCCGAAAGGAGG
ATTAACACCCCATAAGATAGTAGAGTGGCATCACTCAGCTATTAAAACTCCGGTGGTTTTGGATGGGCGTG
CGGCCCATTAGATAGTTGGTGAGGTAACGGCTCACCAAGTCTGCGATGGGTAACTGGTGTGAGAGCACGA
CCAGTCACACGGGCACTGAGACACGGGC
>Zotu591
GAACGCTGGCGGCATGCTTTACACATGCAAGTCGAACGGCAGCGCGGGGCAACCTGGCGGCGAGTGGCG
AACGGGTGAGTAATATATCGGAACGTACCCAAGAGTGGGGGATAACGTAGCGAAAGTTACGCTAATACCG
CATACGATCTAAGGATGAAAGTGGGGGATCTTCGGACCTCATGCTCCTGGAGCGGCCGATATCTGATTAGC
TAGTTGGTGAGGTAAAGGCTCACCAAGGCGACGATCAGTAGCTGGTCTGAGAGGACGACCAGCCACACTG
GAACTGAGACACGGTC
>Zotu593
GAACGCTGGCGGCGTGCTTAACACATGCAAGTCGAGCGGTAAGGCCTTTCGGGGTACACGAGCGGCGAAC
GGGTGAGTAACACGTGGGTGATCTGCCCTGCACTCTGGGATAAGCTTGGGAAACTGGGTCTAATACCGGA
TATGACCGCAGCATGCATGTGTTGTGGTGGAAAGATTTATCGGTGCAGGATGGGCCCGCGGCCTATCAGCT
TGTTGGTGGGGTAATGGCCTACCAAGGCGACGACGGGTAGCCGACCTGAGAGGGTGACCGGCCACACTG
GGACTGAGACACGGCC
>Zotu595

GAACGCTGGTAACGTGGATACCGGCATGCAAGTCACGGGGGCCGCAAGGCAACCGGCGAACGGCTTAGT
AACACGTGGGTACATACCTTAGAGTGGAGCATAGCTCGTCGAAAGACGGGGTAATTCTCCATAGCATCGCA
AGATTAAACCCGTAAGGGGCTCTAAGAATGGCCTGCGGCCTATCAGTTAGTTGGCGAGGTAATAGCTCACC
AAGACGATGACGGGTAGGGGGCGTGAGAGCGCGACCCCCAAGAATGGCACTGCGACACGGGC

>Zotu598
GAACGCTGGCGGCGTGCCTAATACATGCAAGTCGAGCGAGCTGAATTCAAAGATCCCTTCGGGGTGATTT
GTTGGACGCTAGCGGCGGATGGGTGAGTAACACGTGGGCAATCTGCCCTAAAGACTGGGATACCACTTGG
AAACAGGTGCTAATACCGGATAACAACATGAATCGCATGATTCAAGTTTGAAAGGCGGCGCAAGCTGTCAC
TTTAGGATGAGCCCGCGGCGCATTAGCTAGTTGGTGGGGTAAAGGCCTACCAAGGCAATGATGCGTAGCC
GAGTTGAGAGACTGATCGGCCACATTGGGACTGAGACACGGCC

>Zotu600
GAACGCTGGCGGCGTGCTTAACACATGCAAGTCGAACGGTGAAGCCCTTCGGGGTGGATCAGTGGCGAAC
GGGTGAGTAACACGTGAGCAACCTGCCCCTGGCTTCGGGATAACTCCGGGAAACCGGGGCTAATACCGAA
TATGACACGCTCTCGCATGGGATGTGTGTGGAAAGTTTTTCGGCTGGGGATGGGCTCGCGGCCTATCAGCT
TGTTGGTGGGGTGATGGCCTACCAAGGCGACGACGGGTAGCCGGCCTGAGAGGGCGACCGGCCACACTG
GGACTGAGACACGGCC

>Zotu602
GAACGCTAGCGGCAGGCCTAATACATGCAAGTCGGACGGGATTGGAGAGCTTGCTTTCCATGAGAGTGGC
GCACGGGTGCGTAACACGTATGTAACCTACCTTATCCAGGGGGATAGCCTCTCGAAAGAGAGATTAACACC
GCATAACATTGAGACACAGCATTGTGATTCAATCAAATATTTATAGGGATAAGATGGGCATGCGGGACATT
AGCTAGTTGGCGGGGTAACGGCCCACCAAGGCGACGATGTCTAGGGGATCTGAGAGGATGACCCCCCATA
CTGGTACTGAGACACGGAC

>Zotu604
GAACGCTGGCGGCGTGCCTAATACATGCAAGTAGAACGCTGAAGGAGGAGCTTGCTTCTCCGGATGAGTT
GCGAACGGGTGAGTAACGCGTAGGTAACCTGCCTGGTAGCGGGGGATAACTATTGGAAACGATAGCTAAT
ACCGCATAAGAGTAGATGTTGCATGACATTTGCTTAAAAGGTGCAATTGCATCACTACCAGATGGACCTGC
GTTGTATTAGCTAGTTGGTGGGGTAACGGCTCACCAAGGCGACGATACATAGCCGACCTGAGAGGGTGAT
CGGCCACACTGGGACTGAGACACGGCC

>Zotu606
CAACGCTGGCGGCGCGCTTAACACATGCAAGTCGAACGAGAAAGGGGAGCAATCCCTGAGTACAGTGGC
GTACGGGTGAGTAACACGTGGATGATCTACCTTTTGGTGGGGAATACCTCTGGGAAACCGGAGACAATAC
CGCATAAGCTCGGAAGAGGAAACCCGCAAGGGGCCGAAAGAGGAGTCCGCGGCCGATTAGCTAGTTGGT
GGGGTAAAAGCTCACCAAGGCAAAGATCGGTAGCCGGCCTGAGAGGGCACACGGCCACACTGGCACTGA
AACACGGGC

>Zotu608
GAATGCTGGCGGCGTGGATAAGTCATGCAAGTCAAGGGGGCCCGCAAGGGCAACCGGCAGACGAGGTAG
TAACACGTAGGTACGTCCCCCGAAGTCGGGCATAGCCATCCGAAAGGATGGGTAATTCCCGATAGTCCCGC
AAGGGTAAAGGTTTTTCGCTTCGGGAGCGGCCTGCGTAGTATCAGCTAGTTGGTAGGGTAATGGCCTACCA
AGGCTACGACGCTTAGGGGAGCTGAGAGGCTGACCCCCACCGATGGAACTGCGACACGGTC

>Zotu610
GAACGCTGGCGGCAGGCCTAACACATGCAAGTCGAACGCCTTAGCAATAAGGAGTGGCAGACGGGTGAG
TAACACGTGGGAACATACCCTTCGGTTCGGAATAACTCAGGGAAACTTGTGCTAATACCGGATACGTCAGT
GATGAGAAAGATTTATCGCCGAAGGATTGGCCCGCGTCTGATTAGCTAGTTGGTGAGGTAATGGCTCACCA
AGGCGACGATCAGTAGCTGGTCTGAGAGGATGACCAGCCACATTGGGACTGAGACACGGCC

>Zotu612
TAACGCTGGCGGCGTGCATAACACATGCAAGTCGAACGATGAAACTTAAAAGATTTCTTCGGAATGACCTT
AAGTGGATTAGTGGCGAACGGGTGAGTAACGCGTGAGTAACCTGCCTTACACAAGGGGATAGCCTCTGGA
AACGGAGAATAATACCCTATGAAACTATCTTGTCGCATGAAAAGATAGTCAAAGTGTATTAGCGGTGTAAG
ATGGACTTGCGTCTGATTAGCTAGTTGGTGAGATAACAGCCCACCAAGGCAACGATCAGTAGCCGGCTTGA
GAGAGTGTACGGCCACATTGGGACTGAGACACGGCC

>Zotu614
GAACGCTGACAGAATGCTTAACACATGCAAGTCAACTTGAACTTCGGTTTGGGTGGCGGACGGGTGAGTA
ACGCGTAAAGAACTTGCCTCACAGATAGGGACAACATTTGGAAACGAATGCTAATACCTGATATTATGATT
TTAGGGCATCCTAGAATTATGAAAGCTATATGCGCTGTGAGAGAGCTTTGCGTCCCATTAGCTAGTTGGAG
AGGTAACGGCTCACCAAGGCGATGATGGGTAGCCGGCCTGAGAGGGTGATCGGCCACAAGGGGACTGAG
ACACGGCC
>Zotu616
GAACGCTGGCGGCGTGCCTAATACATGCAAGTCGAACGCACAGCGAAAGGTGCTTGCACCTTTCAAGTGA
GTGGCGAACGGGTGAGTAACACGTGGACAACCTGCCTCAAGGCTGGGGATAACATTTGGAAACAGATGCT
AATACCGAATGAAACTTAGTGTCGCATGATACGAAGTTAAAAGGCGCTTTGGCGTCACCTAGAGATGGATC
CGCGGTGCATTAGTTAGTTGGTGGGGTAAAGGCCTACCAAGACAATGATGCATAGCCGAGTTGAGAGACT
GATCGGCCACATTGGGACTGAGACACGGCC
>Zotu618
GAACGCTAGCGGCAGGCTTAACACATGCAAGTCGAGCGGTAGATGGACTTCGGTTCATTGAGAGCGGCAA
ACGGGTGCGGAACACGTACGCAACATACCCTTCTCTGGGGGATAGCCTTCTGAAAAGGAGATTAATACCCC
ATAAGATAATAGAGTGGCATCACTTAGTTATTAAAGATTTATTGGAGAGGGATTGGCGTGCGTCCTATTAG
CTAGTTGGTAAGGTAATGGCTTACCAAGGCGACGATAGGTAGCTGGTGTGAGAGCACGACCAGCCACACG
GGCACTGAGACACGGGC
>Zotu620
GAACGCTGGCGGCGTGCCTAAAACATGCAAGTCGAACGGGGGGTAACCCCAGTGGCGAACGGCGGAGTA
ATACATAAGCAACGTGCCCTAAAGATCGGGATAGTCAGTGGAAACGCTGGGTAATACCGGATGTGACGGA
AAGAAGGCATCTTCATTCCATTAAATGGTTTTTCGCTTTAGGAGCGGCTTATGGCCTATCAGGTAGTTGGTG
AGGTAACGGCTCACCAAGCCGACGACGGGTAGCGGGTCTGAGAGGACGACCCGCTCGAGTGGGACTGAG
ACACGGCC
>Zotu623
GAACGCTGGCGGCAGGCTTAACACATGCAAGTCGAGCGGGCGCTTTCGGGCGTCAGCGGCGGACGGGTT
AGTAACGCGTGGGAACGTGCCTTCTGCTCTGGAATAGCCCTGGGAAACTGGGGGTAATACCGGATGTGCC
CTGAGGGGGAAAGATTTATCGGCAGGAGATCGGCCCGCGTCTGATTAGGTAGTTGGTGGGGTAATGGCTC
ACCAAGCCGACGATCGGTAGCTGGTTTGAGAGGATGATCAGCAACACTGGGACTGAGACACGGCC
>Zotu625
GAACGCTAGCGGCAGGCCTAATACATGCAAGTCGAACGAGATTAACCAGCTTGCTGGTTATGAAAGTGGC
GCACGGGTGCGTAACGCGTATGCAACCTACCTTATTCAGGGGGATAGCCCGAAGAAATTCGGATTAACACC
GCATAAAATCACAGTATAGCATTATACAATGATCAAATATTTATAGGAATAAGATGGGCATGCGTGTCATTA
GCTAGTTGGCGGGGTAATGGCCCACCAAGGCGACGATGACTAGGGGATCTGAGAGGATGACCCCCCACAC
TGGTACTGAGACACGGAC
>Zotu627
GAACGCTGGCGGCGTGCTTAACACATGCAAGTCGAACGATGATGCCCTGCTTGCAGGGTGGATTAGTGGC
GAACGGGTGAGTAACACGTGGGTGATCTGCCCCGCACTTCGGGATAAGCCTGGGAAACTGGGTCTAATAC
CGGATACGACCCCCTGGTAGGTCAGGGGGTGGAAAGATTTATCGGTGTGGGATGAGCCTGCGGCCTATCA
GCTTGTTGGTGGGGTAATGGCCTACCAAGGCGTCGACGGGTAGCCGGCCTGAGAGGGTGATCGGCCACAT
TGGGACTGAGACACGGCC
>Zotu629
GAACGCTGGCGGCAGGCTTAACACATGCAAGTCGAACGGGCGTAGCAATACGTCAGTGGCAGACGGGTG
AGTAACACGTGGGAACGTACCTCTTGGTTCGGAACAACACAGGGAAACTTGTGCTAATACCGGATAAGCCC
TTACGGGGAAAGATTTATCGCCGAGAGATCGGCCCGCGTCTGATTAGCTAGTTGGTAGGGTAATGGCCTAC
CAAGGCGACGATCAGTAGCTGGTCTGAGAGGATGATCAGCCACATTGGGACTGAGACACGGCC
>Zotu631
GAACGCTGGCGGCAGGCCTAACACATGCAAGTCGAGCGAGACCTTCGGGTCTAGCGGCGGACGGGTGAG
TAACGCGTGGGAATATGCCCTTTTCTACGGAATAGTCCTGGGAAACTGGGGGTAATACCGTATACGCCCTT

TGGGGGAAAGATTTATCGGAGAAGGATTAGCCCGCGTTGGATTAGGTAGTTGGTGGGGTAATGGCCTACC
AAGCCGACGATCCATAGCTGGTTTGAGAGGATGATCAGCCACACTGGGACTGAGACACGGCC
>Zotu633
GAACGCTAGCGGGAGGCCTAACACATGCAAGCCGAGCGGTAGAAAGTAGCTTGCTACTTTTGAGAGCGGC
GTACGGGTGCGTAACACGTGTGCAACCTGCCTTTATCTGGAGAATAGCCTTTCGAAAGGAAGATTAATGCT
CCATAACATATTGAATGGCATCATTTAATATTGAAAGCTCCGGCGGATAGAGATGGGCACGCGCAAGATTA
GTTAGTTGGTGAGGTAACGGCTCACCAAGGCGATGATCTTTAGGGGGCCTGAGAGGGTGATCCCCCACAC
TGGTACTGAGACACGGAC
>Zotu635
GAACGCTGGCGGCGTGCTTAACACATGCAAGTCGAGCGGAAAGGCCCTTCGGGGTACTCGAGCGGCGAA
CGGGTGAGTAACACGTGAGTAATCTGCCCTGTGCTTTGGGATAGCCACCGGAAACGGTGATTAATACCGG
ATATGACCAGGTACCGCATGGTGTTTGGTGGAAAGTTTTTCGGCGCAGGATGTGCTCGCGGCCTATCAGCT
TGTTGGTGGGGTAATGGCCTACCAAGGCTTCGACGGGTAGCCGGCCTGAGAGGGTGACCGGCCACACTGG
GACTGAGACACGGCC
>Zotu637
GAACGCTGGCGGCACGCCTAACACATGCAAGTCGAACGGCAGCACGGGGTAGATTTATCTATTTGGGTGG
CGAGTGGCGGACGGGTGAGTAACGCGTAGGAATATGCCTTTAAGACTGGGATAGCCTTGGGAAACTGAG
GGTAATACCGGATGAGCTCTGCGGAGGAAAGCTGGGGACCAGAGATGGCCTGGCGCTTAAAGAATAGCC
TGCGTCTGATTAGCTAGTTGGTGGGGTAAAGGCCTACCAAGGCGACGATCGGTAGCTGGACTGAGAGGTT
GGACAGCCACACTGGGACTGAGACACGGCC
>Zotu640
GAACGCTGGCGGCATGCCTAACACATGCAAGTCGAACGAAGGCTTCGGCCTTAGTGGCGCACGGGTGCGT
AACGCGTGGGAATCTGCCCTTGGGTTCGGAATAACAGTTAGAAATGACTGCTAATACCGGATGATGACGA
AAGTCCAAAGATTTATCGCCCAGGGATGAGCCCGCGTAGGATTAGGTAGTTGGTGGGGTAAAGGCCTACC
AAGCCGACGATCCTTAGCTGGTCTGAGAGGATGATCAGCCACACTGGGACTGAGACACGGCC
>Zotu642
GAATGCTGGCGGCGTGGATAAGTCATGCAAGTCAAGGGGGCCGCAAGGCAACCGGCAGACGAGGTAGTA
ACACGTAGGTAGATCCCCCGAAGTCGGGCATAGCCGGTCGAAAGACCGGGTAATTCCCGATAGTCCCGCA
AGGGTAAAGTTTTTTCGCTTCGGGAGTTGCCTGCGTAGTATCAGCTAGTTGGTAGGGTAATGGCCTACCAA
GGCAACGACGCTTAGGGGGGCTGAGAGGCCGATCCCCACCGATGGAACTGCGACACGGTC
>Zotu644
GAACGCTGGCGGCAGGCTTAACACATGCAAGTCGAGCGCCCCGCAAGGGGAGCGGCAGACGGGTGAGTA
ACACGTGGGAACGTACCCTTTGGTTCGGAACAACCCAGGGAAACTTGGGCTAATACCGGATAAGTCCGTA
AGGAGAAAGATTTATCGCCAAAGGATCGGCCCGCGTCTGATTAGCTAGTTGGTAGGGTAATGGCCTACCA
AGGCTACGATCAGTAGCTGGTCTGAGAGGATGATCAGCCACATTGGGACTGAGACACGGCC
>Zotu646
GAACGCTGGCGGCATGCTTAACACATGCAAGTCGCACGGGCAGCAATGTCAGTGGCGGACGGGTGAGTA
ACGCGTAGGAACATGTCCATGAGGTGGGGGACAACCCTGGGAAACTGGGGCTAATACCGCATATCGGCTG
AGGCCGAAAGCCTTCGGGCGCCTTTGGAGTGGCCTGCGTTCGATTAGCTTGTTGGTGGGGTAATGGCCTAC
CAAGGCTACGATCGATAGCTGGTCTGAGAGGACGACCAGCCACACTGGGACTGAGACACGGCC
>Zotu648
GAACGCTGGCGGCAGGCCTAACACATGCAAGTCGAACGGCAGCACAGAAGAGCTTGCTCTTTGGGTGGCG
AGTGGCGGACGGGTGAGTAATGTCTGGGAAACTGCCCGATGGAGGGGGATAACTACTGGAAACGGTAGC
TAATACCGCATAACGTCGCGAGACCAAAGTGGGGGACCTTCGGGCCTCACACCATCGGATGTGCCCAGAT
GGGATTAGCTAGTAGGTGGGGTAATGGCTCACCTAGGCGACGATCCCTAGCTGGTCTGAGAGGATGACCA
GCCACACTGGAACTGAGACACGGTC
>Zotu650
GAACGCTGGCGGCGCGCCTAACACATGCAAGTCGAACGATTAAAGCACCTTCGGGTGTGTATAAAGTGGC
GAACGGCTGAGTAACACGTGGGCAACCTGCCCCTCTCATTGGGATAGCCACGGGAAACCGTGGATAATAC
CGAATACTTCGAGTCTCCCGCATGGAAGACTCGAGAAAGCTCCGGCGGAGAGGGATGGGCCCGCGGCCTG

TTAGCTTGTTGGTGGGGTAACGGCCTACCAAGGCAATGATGGGTAGCTGGGTTGAGAGACCGACCAGCCA
GATTGGGACTGAGACACGGCC
>Zotu652
GAACGCTGGCGGCGTGCCTAAGACATGCAAGTCGAGTGGGTTTAGACCCACGGCGAACGGGTGAGTAAT
ACCTAGGAACTTTCTTTAAGTGGAGGATAACCAGTCGAAAGATTGGATAATACTTCATGTGGCAGTAATGC
TAAAGCAGCAATGCGCTTAAAGTGAGGCCTGGGCACCATCAGCTAGTTGGTAAGGTAATGGCTTACCAAG
GCTATGACGGTTAGGGGTCCCGAGAGGGATAACCCCCACACTGGAACTGCGACACGGTC
>Zotu654
GAACGCTGGTGGCGTGCTTAACACATGCAAGTCGAGCGAGGCGTCCGCTTTCGGGTGGATAGTCGAGCGG
CGAACGGGTGAGTAACACGTGAGTAATCTTCCCTGGGCTCGGGGATAACCTCGGGAAACTGAGGGTAATA
CCGGATGTGCGTTTTCTGGCGCCTGCTGGGAGACGGAAAGGTTCCATCTCGTGAGGGGTGGTTCCGGCCTT
GGATGAGCTCGCGGCCTATCAGCTTGTTGGTGCGGTGAGGGCGTACCAAGGCGACGACGGGTAGCCGGC
CTGAGAGGGTGACCGGCCACACTGGGACTGAGATACGGCC
>Zotu656
GAACGCTGGCGGCGTGCCTAATACATGCAAGTCGAGCGGACCGAATAGAAGCTTGCTTCTGTTCGGTTAGC
GGCGGACGGGTGAGTAACACGTGGGCAACCTGCCCGTAAGACCGGGATAACTTCGGGAAACCGGAGCTA
ATACCGGATAACACCGAAGACCGCATGGTCTTTGGTTGAAAGACGGCTTTGGCTGTCACTTACGGATGGGC
CCGCGGCGCATTAGCTAGTTGGTGAGGTAACGGCTCACCAAGGCGACGATGCGTAGCCGACCTGAGAGG
GTGATCGGCCACACTGGGACTGAGACACGGCC
>Zotu658
GAACGCTGGCGGCGTGCTTAACACATGCAAGTCGAACGAAGAGCGATGGAAGCTTGCTTCTATCAATCTTA
GTGGCGAACGGGTGAGTAACGCGTAATCAACCTGCCCTTCAGAGGGGGACAACAGTTGGAAACGACTGCT
AATACCGCATACGATTTAATCTCGGCATCGAGGATAGATGAAAGGTGGCCTCTACATGTAAGCTATCACTG
AAGGAGGGGATTGCGTCTGATTAGCTAGTTGGAGGGGTAACGGCCCACCAAGGCGATGATCAGTAGCCG
GTCTGAGAGGATGAACGGCCACATTGGGACTGAGACACGGCC
>Zotu660
GAACGCTGGCGGCATGCCTAATACATGCAAGTCGAACGAGATCTTCGGATCTAGTGGCGCACGGGTGCGT
AACGCGTGGGAATCTGCCCTTGGGTTCGGAATAACTTCTGGAAACGGAAGCTAATACCGGATGATGACGT
AAGTCCAAAGATTTATCGCCCAAGGATGAGCCCGCGTAGGATTAGCTAGTTGGTGAGGTAAAGGCTCACC
AAGGCGACGATCCTTAGCTGGTCTGAGAGGATGATCAGCCACACTGGGACTGAGACACGGCC
>Zotu662
AAACGCTGGCGGCGTGCGTAACACATGCAAGTCGAACGAGAATGTGCTGACAGATCCTTAGGGTGAAGAT
AGCACAGGACAGTGGCGGATGGGTGAGTAACGCGTAAGAAACCTGCCTTTCACACCGGGATAGCAGCTG
GAAACGGCTATTAATACCGGATGACACTTTTTCCCCGCATGAGGAAGAGGTTAAAGAATTTCGGTGAAAGA
TGGTCTTGCGTCTGATTAGCTAGTTGGTGGGGTAACGGCCTACCAAGGCGACGATCAGTAGCCGGCCTGA
GAGGGTGTACGGCCACATTGGGACTGAGACACGGCC
>Zotu664
GAACGCTGGCGGCGTGCCTAATACATGCAAGTCGAGCGAACAGATAAGGAGCTTGCTCCTTTGACGTTAG
CGGCGGACGGGTGAGTAACACGTGGGTAACCTACCTATAAGACTGGAATAACTCCGGGAAACCGGGGCTA
ATGCCGGATAATATTTAGAACCGCATGGTTCTAAAGTGAAAGATGGTTTTACTATCACTTATAGATGGACCC
GCGCCGTATTAGCTAGTTGGTGGGGTAATGGCTTACCAAGGCAACGATACGTAGCCGACCTGAGAGGGTG
ATCGGCCACACTGGAACTGAGACACGGTC
>Zotu666
GAACGCTGGAAACGTGGATACCGGCATGCAAGTCGAGCGGCCAGCAATGGCAGCGGCGAACGGCTTAGT
AACACGTAGGTACATACCCCAAAGTGGAGGACAGCTCGTCGAAAGACGGGGTAATACTCCATAGCATCGC
AAGATTAAAGTAGCAATACGCTTTGGGAATGGCCTGCGACCTATCAGATAGTTGGCGAGGTAATAGCTCAC
CAAGTCTATGACGGGTAGGGGGCGTGAGAGCGCGACCCCCAAGAATGGCACTGCGACACGGGC
>Zotu668
GAACGCTAGCTACAGGCTTAACACATGCAAGTCGAGGGGCAGCATGGTGGATGCTTGCATTCACCGATGG
CGACCGGCGAATGGGTGAGTAACGCGTATCCAACCTGGCGTGCACTCCGGCACAGCCCCTGGAAACGGGG

ATTAATTCCGGATGGTGTGTTATCAGCGCATGCTGTTATCACTAAAGGTCACGGTGCACGATGGGGATGCG
TTCCATTAGATTGTTGGCGGGGTAACGGCCCACCAAGTCATCGATGGATAGGGGTTCTGAGAGGAAGGTC
CCCCACACTGGAACTGAGACACGGTC

>Zotu670

GAACGCTGGCGGCGTGCTTAACACATGCAAGTCGAACGGAAAGGCCCAAGCTTGCTTGGGTACTCGAGTG
GCGAACGGGTGAGTAACACGTGGGTGATCTGCCCTGCACTTCGGGATAAGCCTAGGAAACTGGGTCTAAT
ACCGGATAGGACCACTTCTTGGATGTTGTGGTGGAAAGTTTTTTCGGTGTGGGATGAGCTCGCGGCCTATC
AGCTTGTTGGTGGGGTAATGGCCTACCAAGGCGTCGACGGGTAACCGGCCTGAGAGGGTGTACGGCCACA
TTGGGACTGAGATACGGCC

>Zotu673

GAACGCTGGCGGCGTGCCTAATACATGCAAGTCGAACGAATCTTCTTCGGAAGATGAGTGGCGAACGGGT
GAGTAATACATAGGTAACCTGCCCCTGTGCGGGGGATAACAGGAGGAAACTCCTGCTAATACCGCATAGC
CATGAGCACCGCATGGAGCTCATGCCAAATATCCTTTCGGGGATAGCGCAGGGATGGACCTATGGCGCATT
AGCTTGTTGGCGGGGTAACGGCCCACCAAGGCAACGATGCGTAGCCGGCCTGAGAGGGCGGACGGCCAC
ATTGGGACTGAGACACGGCC

>Zotu675

GAACGCTAGCGATAGGCTTAACACATGCAAGTCGAGGGGCAGCAGGGACTTCGGTCTGCTGGCGACCGGC
GCACGGGTGCGTAACGCGTATGCAACTTGCCTTATAGAGGGGGATAACCCGTTGAAAGACGGACTAATAC
CGCGTATTCTTATTTAGTGGCATCATTAGATAAGGAAAGATTAATTGCTATAAGATAGGCATGCGTCCCATT
AGCTAGTTGGTGAGGTAACGGCTCACCAAGGCGACGATGGGTAGGGGAACTGAGAGGTTGAACCCCCAC
ACTGGTACTGAGACACGGAC

>Zotu677

GAACGCTAGCTACAGGCTTAACACATGCAAGTCGAGGGGAAACGACATTGAAGCTTGCTTCGATGGTCGT
CGACCGGCGCACGGGTGAGTAACGCGTATCCAACCTGCCTCTGACCAAGGGATAACCCGTCGAAAGTCGG
ACTAATACCTTATGCAGTCGTCGGAAGTCATCTGATGACGACGAAAGATTTCATCGGTGAGAGATGGGGAT
GCGTCTGATTAGCTTGTTGGTGGGGTAACGGCCCACCAAGGCGACGATCAGTAGGGGTTCTGAGAGGAAG
GTCCCCCACATTGGAACTGAGACACGGTC

>Zotu679

GAACGCTGGCGGCGTGCCTAATACATGCAAGTCGAGCGAACAGATAAGGAGCTTGCTCCTTTGACGTTAG
CGGCGGACGGGTGAGTAACACGTGGGTAACCTACCTATAAGACTGGAATAACTCCGGGAAACCGGGGCTA
ATGCCGGATAACATGTTGAACCGCATGGTTCTACAGTGAAAGGCGGCTTTGCTGTCACTTATAGATGGACC
CGCGCCGTATTAGCTAGTTGGTGAGGTAACGGCTCACCAAGGCAACGATACGTAGCCGACCTGAGAGGGT
GATCGGCCACACTGGAACTGAGACACGGTC

>Zotu682

GAACGCTGGCGGCGTGCTTAACACATGCAAGTCGAACGGTGAAAGGGAGCTTGCTCTCTGGATCAGTGGC
GAACGGGTGAGTAACACGTGAGCAATCTGCCCTTGACTCTGGGATAAGCGTTGGAAACGACGTCTAATAC
CGGATACGAGCTGTGAAGGCATCTTCAACAGCTGGAAAGAACTTCGGTCAAGGATGAGCTCGCGGCCTAT
CAGCTAGTTGGTGAGGTAATGGCTCACCAAGGCGTCGACGGGTAGCCGGCCTGAGAGGGTGACCGGCCA
CACTGGGACTGAGACACGGCC

>Zotu684

GAACGCTGGCGGCGTGCCTAATACATGCAAGTCGAACGGTCTTTTCTATGGAAGCTTGCTTCCACTGAGAA
GATAGTGGCGAACGGGTGAGTAACACGTGGGTAACCTGCCCATAAGAGGGGGATAACATCCGGAAACGG
GTGCTAATACCGCATAGTTTTCTTGATCGCATGATCGGGAAAGGAAAGACGGCCTTTGTGCTGTCGCTTAT
GGATGGACCCGCGGCGTATTAGTTAGTTGGTGAGGTAACGGCTCACCAAGACGATGATACGTAGCCGACC
TGAGAGGGTGATCGGCCACATTGGGACTGAGACACGGCC

>Zotu686

GAACGCTGGCGGCGTGCCTAATACATGCAAGTCGAGCGCCTGCACAGTGCTCATTAACTAAACTTAGTGAG
TAATGTGCAGGAGCGGCGGACGGCTGAGTAACGCGTAGGAACGTACCCCAGAGTGAGGGATAAGCCCGA
GAAATCGGGTCTAATACCGCATAATCTCTTCGGAGCAAAGCTTTATGCGCTTTGGGAACGGCCTGCGTATG
ATTAGCTTGTTGGTGAGGTAATGGCTCACCAAGGCAATGATCATTAGCTGGTCTGAGAGGATGATCAGCCA
GACTGGGACTGAGACACGGCC

>Zotu688
GAACGCTAGCGGCAGGCCTAATACATGCAAGTCGAACGGGTGTAGCAATACACTAGTGGCGCACGGGTGC
GTAACGCGTAACCAACCTACCGACAACTGGGGGATAGCCCGCCGAAAGGCGGATTAATACCGCATATACC
GGCAGCCTGGCATCAGGTAACTGGGAAAGATTTATTGGTTGTCGATGGGGTTGCGTAGCATTAGCTAGAT
GGCGGGGTAACGGCCCACCATGGCGACGATGCTTAGGGGACCTGAGAGGGTGATCCCCCACACTGGCACT
GAGATACGGGC
>Zotu690
GAACGCTGGCGGCGTGCTTAACACATGCAAGTCGAACGGTGATGCTCCAGCTTGCTGGAGTGGATCAGTG
GCGAACGGGTGAGTAACACGTGAGCAACCTGCCCCAGACTCTGGAATAAGCGCTGGAAACGGCGTCTAAT
ACTGGATACGAAACTGGCCCGCATGGGCATGGTTTGGAAAGTTTTTCGGTTTGGGATGGGCTCGCGGCCTA
TCAGCTTGTTGGTGAGGTAATGGCTCACCAAGGCGACGACGGGTAGCCGGCCTGAGAGGGCGACCGGCC
ACACTGGGACTGAGACACGGCC
>Zotu692
GAACGCTGGCGGCGTGCTTAACACATGCAAGTCGAACGGACCCTTTGGGGTTAGTGGCGAACGGGTGAGT
AACACGTGGGTGATCTGCCCTGCACTTTGGGATAAGCCTGGGAAACTGGGTCTAATACCGGATAGGACTAC
GCACTGCATGGTGTGTGGTGGAAAGCTTTTGCGGTGTGGGATGGGCCCGCGGCCTATCAGCTTGTTGGTG
GGGTGATGGCCTACCAAGGCGACGACGGGTAGCCGGCCTGAGAGGGTGACCGGCCACACTGGGACTGAG
ATACGGCC
>Zotu694
GAACGCTGGCGGCGTGCTTAACACATGCAAGTCGAACGATGAAGCCCTTCGGGGTGGATTAGTGGCGAAC
GGGTGAGTAACACGTGGGCAATCTGCCCTTCACTCTGGGACAAGCCCTGGAAACGGGGTCTAATACCGGA
TAACACTCTGTCCCGCATGGGACGGGGTTGAAAGCTCCGGCGGTGAAGGATGAGCCCGCGGCCTATCAGC
TTGTTGGTGGGGTAATGGCCTACCAAGGCGACGACGGGTAGCCGGCCTGAGAGGGCGACCGGCCACACT
GGGACTGAGACACGGCC
>Zotu697
GAACGCTGGCGGCATGCTTTACACATGCAAGTCGAACGGCAGCGCGGGGCAACCTGGCGGCGAGTGGCG
AACGGGTGAGTAACATATCGGAACGTACCCAAGAGTGGGGGATAACGTAGCGAAAGTTACGCTAATACCG
CATACGATCCAAGGATGAAAGTGGGGGATCGCAAGACCTCATGCTCCTGGAGCGGCCGATATCTGATTAG
CTAGTTGGTGAGGTAAAGGCTCACCAAGGCGACGATCAGTAGCTGGTCTGAGAGGACGACCAGCCACACT
GGAACTGAGACACGGTC
>Zotu699
GAACGCTAGCGGCAGGCTTAACACATGCAAGTCGAGGGGTAGAAGTTTTCGGAATTTGAGACCGGCGCAC
GGGTGCGTAACGCGTATGCAATCTACCTTTTACAGAGGGATAGCCCAGAGAAATTTGGATTAATACCTCAT
AGTGTTATAGAGTGGCATCACTTTATAACTAAAGTCACAACGGTAAAAGATGAGCATGCGTCCCATTAGCT
AGATGGTAAGGTAACGGCTTACCATGGCTACGATGGGTAGGGGTCCTGAGAGGGAGATCCCCCACACTGG
TACTGAGACACGGAC
>Zotu701
GAACGCTGGCGGCATGCCTAACACATGCAAGTCGAACGAGATCTTCGGATCTAGTGGCGCACGGGTGCGT
AACGCGTGGGAATCTGCCTTGGGGTTCGGAATAACTCCCCGAAAGGGGTGCTAATACCGGATGATGTCGA
GAGACCAAAGATTTATCGCCCTGAGATGAGCCCGCGTAGGATTAGCTAGTTGGTGTGGTAAAGGCGCACC
AAGGCGACGATCCTTAGCTGGTCTGAGAGGATGATCAGCCACACTGGGACTGAGACACGGCC
>Zotu703
GAACGCTGGCGGCATGCCTAACACATGCAAGTCGAACGAGATCTTCGGGTCTAGTGGCGCACGGGTGCGT
AACGCGTGGGAATCTGCCCTTTGGTTCGGAATAACAGTTGGAAACGACTGCTAATACCGGATGATGACGTA
AGTCCAAAGATTTATCGCCAGAGGATGAGCCCGCGTAGGATTAGCTAGTTGGTGTGGTAAGAGCGCACCA
AGGCGACGATCCTTAGCTGGTCTGAGAGGATGATCAGCCACACTGGGACTGAGACACGGCC
>Zotu705
GAACGCTGGCGGTGTGCTTCACACATGCAAGTCGAACGAGGTAGCAATACCTAGTGGCGGACGGGTGAGT
AACGCGTGGGAATCTGCCTTTAGATGGGGGATAACGGGCCGAAAGGTCCGCTAATACCGCATATGCCGCA

AGGTGAAAGGAGTAATCCGTCTAAAGATGAGCCCGCGTCCGATTAGCTAGTTGGTAGAGTAAAAGCCTAC
CAAGGCGACGATCGGTAGCTGGTCTGAGAGGATGATCAGCCACAATGGGACTGAGACACGGCC
>Zotu707
GAACGCTGGCGGCGTGCTTAACACATGCAAGTCGAGCGGTAAGGCCCTTCGGGGTACACGAGCGGCGAA
CGGGTGAGTAACACGTGAGCAATCTGCCCTTCACTCTGGGATAGCCTCGGGAAACTGAGATTAATACCGGA
TACGACCACTTCGGGCATCCGATGGTGGTGGAAAGTTCCGGCGGTGAAGGATGAGCTCGCGGCCTATCAG
CTTGTTGGCGGGGTAATGGCCCACCAAGGCTTCGACGGGTAGCCGGCCTGAGAGGGTGACCGGCCACACT
GGGACTGAGACACGGCC
>Zotu709
GAACGCTAGCTACAGGCTTAACACATGCAAGTCGAGGGGCAGCGGGAAGCAAGCTTGCTTGCTTTGCCGG
CGACCGGCGTACGGGTGCGTAACGCGTACCGAACCTACCTCACACTCCGGGATAGCCCTGCGAAAGCAGG
ATTAATACCGGGTAGCCTCACTATATCGCATGATATTATGAGTAAAGATTCATTGGTGTGAGATGGCGGTG
CGTCCCATTAGTTAGTTGGCGGGGTAACGGCCCACCAAGACGACGATGGGTAGGGGTTCTGAGAGGAAG
GTCCCCCACATTGGAACTGAGACACGGTC
>Zotu711
GAACGCTGGCGGCATGCTTAACACATGCAAGTCGGACGGGAAGTGGTGTTTCCAGTGGCGGACGGGTGA
GTAACGCGTAAGAACCTGCCCTTGGGAGGGGAACAACAGCTGGAAACGGCTACTAATACCCCATAGGCTG
AGGAGCAAAAGGAGGAATCCGCCCAAGGAGGGGCTCGCGTCTGATTAGTTAGTTGGTGAGGCAATGGCT
TACCAAGGCGACGATCAGTAGCTGGTCCGAGAGGATGATCAGCCACACTGGGACTGAGACACGGCC
>Zotu714
AAACGCTGGCGGGGTGCATGAGACATGCAAGTCGAACGAGGTGCCATCGGGCTTGCTCGATGGCTACCTA
GTGGCGGACGGCTGAGTAACGCGTAGGTGACCTACCCGGAAGTGGGGAATAACTGCCCGAAAGGGTGGC
TAATACCGCATGTGGTCGACGATTCATTTCGTCGTCTAAAGGAGCAATTCGCTTCCGGAGGGGCCTGCGTC
CGATTAGCTAGTTGGTGGGGTAACGGCTCACCAAGGCGACGATCGGTAGCTGGTCTGAGAGGATGATCAG
CCAGACTGGGACTGAGAACGGCC
>Zotu716
CAACGCTGGCGGCGTGCCTAACACATGCAAGTCGAACGAGAAAGTGGAGCAATCCATGAGTAAAGTGGCG
CACGGGTGAGTAACACGTGACTAACCTACCTTTGAGCGGGGGATAACCTAGGGAAACCTGGGCTAATACC
GCATAATACCTACGGGTCAAAGCGAGCAATCGCACTTAAAGAGGGGGTCGCGGCAGATTAGCTAGTTGGT
GAGGTAATGGCTCACCAAGGCGATGATCTGTATCCGGCCTGAGAGGGCGCACGGACACACTGGAACTGAA
ACACGGTC
>Zotu718
GAACGCTAGCGGCAGGCTTAATACATGCAAGTCGAGGGGCAGCGGGAGTAGCAATACTTGCCGGCGACC
GGCGCACGGGTGCGTAACACGTATACAATCTACCCTTTACAGGGGGATAGCCCTCCGAAAGGAGGATTAA
TACCCCATAATAAATTGAATGGCATCATTTGATTTTGAAAGATTTATCGGTAAAGGATGAGTATGCGTCTGA
TTAGCTAGTTGGAGAGGTAACGGCTCCCCAAGGCGACGATCAGTAGGGGATCTGAGAGGACTACCCCCCA
CACTGGTACTGAGACACGGAC
>Zotu720
GAACGCTAGCGGCAGGCCTAATACATGCAAGTCGAACGGTGAGTAGCAATACTCATAGTGGCGCACGGGT
GCGTAACACGTAGGTAACCTACCTTTTACCGGGGGATAACGATGGGAAACCATCGCTAATACCGCATAAGA
TAGCAGAGGGGCATCTCTTAGTTATTAAAGATTTATTGGTAGAAGATGGGCCTGCGTCCGATTAGCTAGTT
GGTGGTGTAACAGACCACCAAGGCGATGATCGGTAGGGGTTCTGAGAGGAAGATCCCCCACACTGGCACT
GAGATACGGGC
>Zotu722
GAACGCTGGCGGCATGCTTTACACATGCAAGTCGAACGGTAACAGGGAGCTTGCTCCGCTGACGAGTGGC
GAACGGGTGAGTAATATATCGGAACGTGCCCAAGAGTGGGGGATAACGTAGCGAAAGTTACGCTAATACC
GCATACGATCTATGGATGAAAGCAGGGGACCTTCGGGCCTTGTGCTCCTGGAGCGGCCGATATCTGATTAG
CTAGTTGGTGAGGTAAAGGCTCACCAAGGCGACGATCAGTAGCTGGTCTGAGAGGACGACCAGCCACACT
GGAACTGAGACACGGTC
>Zotu724

GAACGCTGGCGGCGTGCCTAATACATGCAAGTAGAACGCTGAAGGAGGAGCTTGCTTCTCTGGATGAGTT
GCGAACGGGTGAGTAACGCGTAGGTAACCTGCCTGGTAGCGGGGGATAACTATTGGAAACGATAGCTAAT
ACCGCATAAGAGTAGATGTTGCATGACATTTGCTTAAAAGGTGCAATTGCATCACTACCAGATGGACCTGC
GTTGTATTAGCTAGTTGGTGAGGTAACGGCTCACCAAGGCGACGATACATAGCCGACCTGAGAGGGTGAT
CGGCCACACTGAGACTGAGACACGGCC

>Zotu726

CAACGCTGGCGGCGCGCTTAACACATGCAAGTCGAACGAGAAAGGGGAGCAATCCCTGAGTAAAGTGGC
GCACGGGTGAGTAACACGTGGATCATCTACCCTCTTGTGGGGAATACCGATGGGAAACCGTCGACAATAC
CGCATAAGCTCGAGAGAGGAAAGCCGCAAGGCGCAGGAGGAGGAGTCCGCGGTCGATTAGTTAGTTGGC
AGGGTAAAAGCCTACCAAGACTGTGATCGATAGCCGGCCTGAGAGGGCACACGGCCACACTGGCACTGAA
ACACGGGC

>Zotu728

GAATGCTGGCGGCGTGCCTAACACATGCAAGTCGAGCGGCAGCGAGGGGTGCTTGCACCCTGTCGGCGA
GCGGCGAACGGCTGAGTAACGCGTAGGAATTTGCCCCAAAGTGAGGAATAACTGCCCGAAAGGGTAGCT
AATGCCGCATATGGTCTTCGGATTAAAGGATTTATCCGCTTTGGGAGAAGCCTGCGTTGGATTAGGTAGTT
GGTAGGGTAATGGCCTACCAAGCCGACGATCCATAGCTGGTCTGAGAGGATGATCAGCCAGACTGGAACT
GAGACACGGTC

>Zotu731

GAATGCTGGCGGCGTGCCTAACACATGCAAGTCGAGCGGCATCGCGGTCTTCGGATTGGCGGCGAGCGGC
GGACGGCTGAGTAACGCGTGGGAACATACCCCAAAGTGAGGGATAACCGCCCGAAAGGGTGGCTAATAC
CGCATATGGTCTTCGGATTAAAGGATTTATCCGCTTTGGGAATGGCCTGCGTACGATTAGATAGTTGGTAG
GGTAAAGGCCTACCAAGTCGACGATCGTTAACTGGTCTGAGAGGATGATCAGTCAGACTGGAACTGAGAC
ACGGTC

>Zotu733

TAACGCTAGCGGCAGGCCTAATACATGCAAGTCGAACGGCAGCACGGGTAGCAATACCTGGTGGCGAGTG
GCGCACGGGTGCGTAACGCGTATGCAATCTACCTGTAACTGGGGAATAGCCTTCCGAAAGGAAGATTAAC
ACCGCATAATATCACGAAGCGGCATCGCTTTGTGATCAAATCTCCGGAGGTTACAGATGAGCATGCGTGAC
ATTAGCTAGTTGGTGAGGTAACGGCTCACCAAGGCGACGATGTCTAGGGGGTCTGAGAGGATGATCCCCC
ACACTGGTACTGAGACACGGAC

>Zotu735

GAACGCCGGCGGTGTGCCTAATACATGCAAGTCGAACGCGTTGGCCCAATTGATTGACGGTGCTTGCACCT
GATTGATTTTGGTCGCCAACGAGTGGCGGACGGGTGAGTAACACGTAGGTAACCTGCCCAGAAGCGGGG
GACAACATTTGGAAACAGATGCTAATACCGCATAACAACGTTGTTCGCATGAACAACGCTTAAAAGATGGC
TTCTCGCTATCACTTCTGGATGGACCTGCGGTGCATTAGCTTGTTGGTGGGGTAATGGCCTACCAAGGCGA
TGATGCATAGCCGAGTTGAGAGACTGATCGGCCACAATGGGACTGAGACACGGCC

>Zotu737

GAACGCTAGCTATAGGCTTAACACATGCAAGTCGAGGGGAAACGGCAAAGAGTGCTTGCACACTTTGGAC
GTCGACCGGCGCACGGGTGAGTAACGCGTATCCAACCTGCCCTTTACTTGGGGATAACCCGTTGAAAGACG
GACTAATACCCAATGATATTCATTGATGACATCTGATTTGAATTAAAGATTTATCGGTATAGGATGGGGATG
CGTCTGATTAGCTTGTTGGTGAGGTAACGGCTCACCAAGGCAACGATCAGTAGGGGTTCTGAGAGGAAGG
TCCCCCACATTGGAACTGAGACACGGTC

>Zotu739

GAACGCTGGCGGCGTGCTTAACACATGCAAGTCGAGCGGAAAGGCCCTTCGGGGTACTCGAGCGGCGAA
CGGGTGAGTAACACGTGAGTAACCTGCCCTGGACTTTGGGATAACCCTCGGAAACGGGGGCTAATACCGG
ATATGACTTTTCCTCGCATGGGGATTGGTGGAAAGTTTTTCGGTCTGGGATGGGCTCGCGGCCTATCAGCT
TGTTGGTGGGGTAATGGCCTACCAAGGCGACGACGGGTAGCCGGCCTGAGAGGGCGACCGGCCACACTG
GGACTGAGACACGGCC

>Zotu741

GAACGCTAGCGGCAGGCTTAACACATGCAAGTCGAGGGGTATAAGGAGCTTGCTCCTTAGAGACCGGCGC
ACGGGTGCGTAACGCGTATGCAACCTACCTTGAACAGGGGGATAGCCCAGAGAAATTTGGATTAATACCC
CATGGTATATTTGAGTGGCATCACTTGATTATTAAAGTTCCAACGGTTCAAGATGGGCATGCGTCCCATTAG

CTAGATGGTAAGGTAACGGCTTACCATGGCGACGATGGGTAGGGGTCCTGAGAGGGAGATCCCCCACACT
GGTACTGAGACACGGAC
>Zotu743
GAACGCTGGCGGCGTGCCTAACACATGCAAGTCGAGCGAGAGACTTCGGTCTCTAGCGGCGGACGGGTG
AGTAACACGTGGGTAACCTGCCTCGTAGAGGGGGATAGCCTTCCGAAAGGAAGATTAATACCGCATAACG
TATATCTGGCGCATGCCAGATATATCAAAGGAGTAATCCGCTATGAGATGGACCCGCGGCGCATTAGCTAG
TTGGTGAGGTAACGGCTCACCAAGGCGACGATGCGTAGCCGACCTGAGAGGGTGATCGGCCACATTGGAA
CTGAGATACGGTC
>Zotu745
GAACGCTGGCGGCGTGCTTAACACATGCAAGTCGAGCGGAAAGGCTCCTTCGGGGGTACTCGAGCGGCG
AACGGGTGAGTAACACGTGAGTAATCTGCCCCTGGCTTTGGGATAGCCACCGGAAACGGTGATTAATACC
GGATACGACCACTTCTCGCATGGGATGGTGGTGGAAAGTTTTTCGGCCAGGGATGTGCTCGCGGCCTATCA
GCTTGATGGTGAGGTAATGGCTCACCATGGCTTCGACGGGTAGCCGGCCTGAGAGGGTGACCGGCCACAC
TGGGACTGAGACACGGCC
>Zotu747
GAACGCTGGCGGCGTGCTTAACACATGCAAGTCGAACGGTAAGGCCCTTCGGGGTACACGAGTGGCGAAC
GGGTGAGTAACACGTGGGTAATCTGCCCTGCACTTCGGGATAAGCCTGGGAAACTGGGTCTAATACCGGA
TATTCAGCTTCTGCCGCATGGTGGTGGTTGGAAAGTTTTTCGGTGCAGGATGAGCCCGCGGCCTATCAGCT
TGTTGGTGGGGTAATGGCCTACCAAGGCGACGACGGGTAGCCGGCCTGAGAGGGTGATCGGCCACACTG
GGACTGAGACACGGCC
>Zotu749
GAACGCTGGCGGCGTGCTTAACACATGCAAGTCGAACGGTGAAGGGACCAGCTTGCTGGTTCTGGATGAG
TGGCGAACGGGTGAGTAACACGTGAGTAACCTGCCCCCTTCTTCTGGATAACCGCATGAAAGTGTGGCTAA
TACGGGATATTCTGGGTCTGTCGCATGACGGGCCCGGGAAAGATATTGTTTCGGTGGGGGATGGGCTCGC
GGCCTATCAGCTTGTTGGTGGGGTGATGGCCTACCAAGGCGGTGACGGGTAGCCGGCCTGAGAGGGTGG
ACGGTCACACTGGGACTGAGACACGGCC
>Zotu751
GAACGCTGGCGGCGTGCCTAACACATGCAAGTCGAACGAGGTCCATCCGGTGGCAACACCGGTGAAGACC
TAGTGGCGAACGGGTGAGTAACACGTGAGGAACCTGCCCCGAAGACCGGGATAACCCTCCGAAAGGAGA
GCTAATACCGGATACCCCCACCGGATCGCATGATCTGGTGAGGAAATGGATTCCGCTTCGGGAGGGCCTC
GCGGCCTATCAGCTAGTTGGTGGGGTAACGGCCCACCAAGGCTTCGACGGGTAGCTGGTCTGAGAGGACG
ATCAGCCACACTGGGACTGAGACACGGCC
>Zotu753
GAACGCTGGCGGCGTGCTTAACACATGCAAGTCGAACGCTGAAGCCCGAGCTTGCTCGGGTGGATGAGTG
GCGAACGGGTGAGTAACACGTGAGTAACCTGCCCCGAACTCTGGGATAAGCGCTGGAAACGGCGTCTAAT
ACTGGATATGTCCTGTCACCGCATGGTGTGCAGGTGGAAAGATTTATCGGTTCGGGATGGACTCGCGGCCT
ATCAGCTTGATGGTGAGGTAATGGCTCACCATGGCGACGACGGGTAGCCGGCCTGAGAGGGTGACCGGC
CACACTGGGACTGAGACACGGCC
>Zotu755
GAACGCTAGCGGCAGGCTTAACACATGCAAGTCGAGGGGTAGAAGCAGCTTGCTGCTTTGAGACCGGCGC
ACGGGTGCGTAACGCGTATGCAATCTGCCTTTCACAAAGGGATAGCCCAGAGAAATTTGGATTAATACCTT
ATAGTATATTGATGTGGCATCACATTATTATTAAAGATTTATCGGTGAAAGATGAGCATGCGTCCCATTAGC
TAGTTGGTAAGGTAACGGCTTACCAAGGCAACGATGGGTAGGGGTCCTGAGAGGGAGATCCCCCACACTG
GTACTGAGACACGGAC
>Zotu757
GAACGCTGGCGGCGTGCCTAATACATGCAAGTAGAACGCTGAAGGAGGAGCTTGCTTCTCTGGATGAGTT
GCGAACGGGTGAGTAACGCGTAGGTAACCTGCCTGGTAGCGGGGGATAACTATTGGAAACGATAGCTAAT
ACCGCATAATAGTAGATGTTGCATGACATTTGCTTAAAAGGTGCAATTGCATCACTACCAGATGGACCTGC
GTTGTATTAGCTAGTTGGTGGGGTAACGGCTCACCAAGGCGACGATACATAGCCGACCTGAGAGGGTGAT
CGGCCACACTGGGACTGAGACACGGCC

>Zotu759
GAACGCTGGCGGCGTGCTTAACACATGCAAGTCGAACGGTGAACCTGCTTCGGTGGGGGATCAGTGGCGA
ACGGGTGAGTAACACGTGGGCAACCTGCCCCTGGCTCTGGGATAACTCCAAGAAATTGGGGCTAATACCG
GATATGACCGCTGACCGCATGGTCTGGTGGTGGAAAGATTTATCGGCTGGGGATGGGCCCGCGGCCTATC
AGCTTGTTGGTGGGGTAGTGGCCTACCAAGGCGACGACGGGTAGCCGGCCTGAGAGGGTGACCGGCCAC
ACTGGGACTGAGACACGGCC
>Zotu761
GAACGCTGGTGGCGTGCTTAACACATGCAAGTCGAGCGAGGCGTCCGCTTTCGGGTGGATAGTCGAGCGG
CGAACGGGTGAGTAACACGTGAGTAATCTTCCCTGGGCTCGGGGATAACCTCGGGAAACTGAGGCTAATA
CCGGATGTGCGTTGTTTGGGGCTTCCTGGATGACGGAAAGGTTCCATCTCGTGAGGGGTGGTTCCGGCCTT
GGATGAGCTCGCGGCCTATCAGCTTGTTGGTGCGGTGATGGCGTACCAAGGCGACGACGGGTAGCCGGCC
TGAGAGGGTGACCGGCCACACTGGGACTGAGATACGGCC
>Zotu763
GAACGCTGGCGGTATGCTTAACACATGCAAGTCGAACGGGGTACTTTCGGGTACCTAGTGGCGGACGGGT
GAGTAACGCGTGAGAACATGCCTTTAGATTGGGGACAACAGTTGGAAACGACTGCTAATACCGAATGTGC
CGAGAGGTGAAAGATTAATTGTCTAAAGATTGGCTCGCGTCAGATTAGGTAGTTGGAGTGGTAACGGCAC
ACCAAGCCTACGATCTGTACTTGGTCTGAGAGGATGACCAGGCACACTGGAACTGAGACACGGTC
>Zotu765
GAACGCTGGCGGCGTGCCTAACACATGCAAGTCGAGCGAGGTCCATTCGGTAGCAATACCGATGAAGACC
TAGCGGCGAACGGGTGAGTAACACGTGAGAAACCTGCCCCAAAGTCTGGGATAACCCGGGGAAACCCGA
GCTAATACCGGATACCTTCTTTTGATCGCATGGTCAGCTGAAGAAAGTTTTTTCGCTTTGGGAGGGTCTCGC
GGCCTATCAGTTTGTTGGTGGGGTAACGGCCTACCAAGACATCGACGGGTAGCTGGTCTGAGAGGACGAT
CAGCCACACTGGGACTGAGACACGGCC
>Zotu770
GAACGCTGGCGGTGTGCTTCACACATGCAAGTCGAACGAGGTAGCAATACCTAGTGGCGGACGGGTGAGT
AACGCGTGGGAATCTGCCTTTAGATGGGGGATAACGGGCCGAAAGGTCCGCTAATACCGCATATGCCGAG
AGGTGAAAGGAGTAATCCGTCTAAAGATGAGCCCGCGTCCGATTAGCTAGTTGGTAGAGTAAGAGCCTAC
CAAGGCGACGATCGGTAGCTGGTCTGAGAGGATGATCAGCCACAATGGGACTGAGACACGGCC
>Zotu772
GAACGCTGGCGGCGTGCCTAATACATGCAAGTCGAGCGAACAGATAAGGAGCTTGCTCCTTTGACGTTAG
CGGCGGACGGGTGAGTAACACGTGGATAACCTACCTATAAGACTGGGATAACTTCGGGAAACCGGAGCTA
ATACCGGATAACATATTGAACCGCATGGTTCAATAGTGAAAGACGGCCTTGCTGTCACTTATAGATGGATC
CGCGCCGTATTAGCTAGTTGGTAAGGTAACGGCTTACCAAGGCAACGATACGTAGCCGACCTGAGAGGGT
GATCGGCCACACTGGAACTGAGACACGGTC
>Zotu774
GAACGCTAGCTATAGGCTTAACACATGCAAGTCGAGGGGCAGCGAATAGATAGCTTGCTATTTATGTCGGC
GACCGGCGCACGGGTGAGTAACGCGTATCCAACCTGCCCATAACTAAGGGATAACCCAGCGAAAGTTGGA
CTAATACCTTATGTATTCGTTTGATCTCATGAGATTACGAATAAAGATTTATCGGTTATGGATGGGGATGCG
TCTGATTAGCTTGTTGGCGGGGTAACGGCCCACCAAGGCAACGATCAGTAGGGGTTCTGAGAGGAAGGTC
CCCCACATTGGAACTGAGACACGGTC
>Zotu777
GAACGCTGGCGGCGTGCTTAACACATGCAAGTCGAACGATGAACCTCCCTCGGGAGGGGATTAGTGGCGA
ACGGGTGAGTAACACGTGGGCAATCTGCCCTGCACTCTGGGATAACTCCGGGAAACCGGAGCTAATACCG
GATACGATCACCGCGGGCATCCGTGGTGGTGGAAAGCTCCGGCGGTGCAGGATGAGCCCGCGGCCTATCA
GCTTGTTGGTGGGGTGACGGCCCACCAAGGCGACGACGGGTAGCCGGCCTGAGAGGGCGACCGGCCACA
CTGGGACTGAGACACGGCC
>Zotu779
GAACGCTGGCGGCATGCTTAACACATGCAAGTCGAACGGCAGCATGACCTAGCTTGCTAGGTTGATGGCG
AGTGGCGGACGGGTGAGTAACGCGTAGGAATATGCCTTAGAGAGGGGGATAACTTGGGGAAACTCAAGC
TAATACCGCGTGATGTCTTAGGACTAAAGCTGGGGACCGCAAGGCCTGGCGCTTTAAGATTAGCCTGCGTC

CGATTAGCTAGTTGGTGGGGTAAGGGCTTACCAAGGCGACGATCGGTAGCTGGTCTGAGAGGATGATCAG
CCACACTGGGACTGAGACACGGCC
>Zotu781
GAACGCTGGCGGCGTGCCTAATACATGCAAGTAGAACGCTGAAGCTTGGTGCTTGCACCGAGCGGATGAG
TTGCGAACGGGTGAGTAACGCGTAGGTAACCTGCCTGGTAGCGGGGGATAACTATTGGAAACGATAGCTA
ATACCGCATAAGAGTAGATGTTGCATGACATTTGCTTAAAAGGTGCAATTGCATCACTACCAGATGGACCT
GCGTTGTATTAGCTAGTTGGTGAGGTAACGGCTCACCAAGGCAACGATACATAGCCGACCTGAGAGGGTG
ATCGGCCACACTGGGACTGAGACACGGCC
>Zotu783
GAACGCTGGCGGCGTGCTTAACACATGCAAGTCGAGCGAGGCCCTCCTTCGGGGGGTGCCCTAGCGGCGA
ACGGGTGAGTAACACGTGGGCAACCTGCCCTCAGCTCTGGGATAACTCCAAGAAATTGGTGCTAATACCG
GATGTGACCGCTGACCGCATGGTCTGGTGGTGGAAAGATTCATCGGCTGAGGATGGGCCCGCGGCCTATC
AGCTTGTTGGTGGGGTCACGGCCCACCAAGGCGACGACGGGTAGCCGGCCTGAGAGGGTGACCGGCCAC
ACTGGGACTGAGACACGGCC
>Zotu786
GAACGCTGACAGAATGCTTAACACATGCAAGTCAACTTGAACTTCGGTTTGGGTGGCGGACGGGTGAGTA
ACGCGTAAAGAACTTGCCTCACAGCTAGGGACAACATTTGGAAACGAATGCTAATACCTGATATTATGATA
ATAGGGCATCCTATAATTATGAAAGCTATATGCGCTGTGAGAGAGCTTTGCGTCCCATTAGCTAGTTGGAG
AGGTAACGGCTCACCAAGGCGATGATGGGTAGCCGGCCTGAGAGGGTGATCGGCCACAAGGGGACTGAG
ACACGGCC
>Zotu788
GAACGCTTGCGGCGTGCCTAAGAAATGCAAGTCGAACGGAAAGGTCCTTCGGGACACTCGAGTGGCGAAC
GGTCGCGTAACACGTAAGCAACCTGCCTCAAAGTGGGGGACAACAGTCTGAAAAGACTGCTAATACCGCA
TGTGGTCAACTAAGGGCATCCTTGGTTGTCTAAAGGATTTATTCGCTTTGAGATGGGCTTGCGGCCTATCAG
GTAGTTGGTGGGGTAACGGCCTACCAAGCCTTCGACGGGTAGCTGGTCTGAGAGGACGATCAGCCGGATT
GGGACTGAGATACGGCC
>Zotu790
GAACGCTGGCGGCGTGCTTAACACATGCAAGTCGAACGGTGAAGCCAAGCTTGCTTGGTGGATCAGTGGC
GAACGGGTGAGTAACACGTGAGCAACCTGCCCTGGACTCTGGGATAAGCGCTGGAAACGGCGTCTAATAC
TGGATATGAGCTCCTATCGCATGGTGGGGGTTGGAAAGATTTTTCGGTCTGGGATGGGCTCGCGGCCTATC
AGCTTGTTGGTGAGGTAATGGCTCACCAAGGCGTCGACGGGTAGCCGGCCTGAGAGGGTGACCGGCCAC
ACTGGGACTGAGACACGGCC
>Zotu792
GAACGCTGGCGGCGTGCTTAACACATGCAAGTCGAACGGAAAGGCCCTGCTTGCAGGGTGCTCGAGTGGC
GAACGGGTGAGTAACACGTGGGTGATCTGCCCCTTACTTTGGGATAAGCCTGGGAAACTGGGTCTAATACT
GGATAGGACCATGCTGTAGGTGGTGTGGTGGAAAGATTTTTTCGGTAAGGGATGAGCTCGCGGCCTATCA
GCTTGTTGGTGGGGTAATGGCCTACCAAGGCGTCGACGGGTAGCCGGCCTGAGAGGGTGGACGGCCACA
TTGGGACTGAGATACGGCC
>Zotu794
GAACGCTGGCGGCGTGCTTAACACATGCAAGTCGAACGATGAACCGCTTTCGGGCGGGGATTAGTGGCGA
ACGGGTGAGTAACACGTGGGCAATCTGCCCTGCACTCTGGGACAAGCCCTGGAAACGGGGTCTAATACCG
GATATGATTGTCCGCCGCATGGTGGATGGTGTAAAGCTCCGGCGGTGCAGGATGAGCCCGCGGCCTATCA
GCTTGTTGGTGAGGTAGTGGCTCACCAAGGCGACGACGGGTAGCCGGCCTGAGAGGGCGACCGGCCACA
CTGGGACTGAGACACGGCC
>Zotu796
GAACGCTGGCGGCGTGCTTAACACATGCAAGTCGAACGGGAAGAGATGAAGAGCTTGCTCTTTATCAAAT
CCAGTGGCAAACGGGTGAGTAACACGTAAACAACCTGCCTTCAGGATGGGGACAACAGACGGAAACGACT
GCTAATACCGAATACGTTCCACGGGCCGCATGACCTGTGGAAGAAAGGGTAGCCTCTACCTGTAAGCTATC
GCCTGAAGAGGGGTTTGCGTCTGATTAGGCAGTTGGTGGGGTAACGGCTCACCAAACCAACGATCAGTAG
CCGGTCTGAGAGGATGAACGGCCACACTGGAACTGAGACACGGTC

>Zotu798
GAACGCTGGCGGCGTGCCTAACACATGCAAGTCGAGCGGAGTTGTTATGAGATTCATTAGATGATTATTTC
AACTTAGCGGCGGATGGGTGAGTAACACGTGGGTAACCTGCCCTGCACTGGGGGATAACAGCTGGAAAC
GGCTGTTAATACCGCATATGCACACACCGGCGCATGCCGGAGTGTGGAAAGATTCATCGGTGCAGGATGG
GCCCGCGCCGGATTAACTTGTTGGCAGGGTAATGGCCTACCAAGGTGATGATCCGTAGCCGGCCTGAGAG
GGCGACCGGCCACATTGGGACTGAGACACGGCC
>Zotu800
GAACGCTGGCGGCATGCTTAACACATGCAAGTCGCACGGTCGGCGCAAGTCGGCAGTGGCGGACGGGTG
AGGAACGCGTAGGAACATGTCCTGAGGTGGGGGATAACGCTGGGAAACTGGCGCTAATACCGCATATGA
GCTGAGGCTCAAAGCCGCGAGGCGCCTTGGGAGTGGCCTGCGTCCGATTAGGTAGTTGGTGGGGTAATG
GCTTACCAAGCCTGCGATCGGTAGCTGGTCTGAGAGGACGACCAGCCACACTGGGACTGAGACACGGCC
>Zotu802
GAACGCTGGCGGCGTGCTTAACACATGCAAGTCGAACGCTGAAGCACCAGCTTGCTGGTGTGGATGAGTG
GCGAACGGGTGAGTAATACGTGAGTAACCTGCCCTTGACTCTGGGATAAGCCCGGGAAACTGGGTCTAAT
ACTGGATGCTACATGTCACCGCATGGTGGTGTGTGGAAAGGGTTTACTGGTCTTGGATGGGCTCACGGCCT
ATCAGCTTGTTGGTGAGGTAATGGCTCACCAAGGCGACGACGGGTAGCCGGCCTGAGAGGGTGACCGGC
AACACTGGGACTGAGACACGGCC
>Zotu805
GAACGCTGGCGGCATGCTTTACACATGCAAGTCGAACGGTAACAGGTCTTCGGATGCTGACGAGTGGCGA
ACGGGTGAGTAACACATCGGAACGTGCCTAGTAGTGGGGGATAACTACTCGAAAGAGTAGCTAATACCGC
ATGAGATCTAAGGATGAAAGCAGGGGACCTTCGGGCCTTGCGCTACTAGAGCGGCTGATGGCAGATTAGG
TAGTTGGTGGGATAAAAGCTTACCAAGCCGACGATCTGTAGCTGGTCTGAGAGGACGACCAGCCACACTG
GGACTGAGACACGGCC
>Zotu807
GAATGCTGGCGGTGTGGATAGGGCATGCAAGTCAAGGGGACCGCAAGGTAACCGGCAGACGAGATAGTA
ACGCGTAGGAACGTACCCAGAAGTCGGGCACAGCTCGCCGAAAGGCGGGGTAATTCCCGATGGTGTCGCA
AGACTAAAGATTTATCGCTTCTGGAGCGGCCTGCGTAATATCAGCTTGTTGGTGAGGTAATGGCTCACCAA
GGCTATGACGTTTAGGGGAGCTGAGAGGCTGACCCCCATCGATGGAATTGAGATACGGTC
>Zotu809
GAACGCTGGCGGCGTGCTTAACACATGCAAGTCGAACGAGATCCATTCAGTAGCAATACTGATGAAGATCT
AGTGGCGAACGGGTGCGTAACACGTGAGGAACTTGTCCCGGTCTCTGGGATAACAGTTGGAAACGACTGC
TAATACCGGATGACGTCGGAACTTCGCATGGAGTACTGACGAAAGGGTTACTGGATCGGGAGAGCCTCGC
GGCCTATCAGCTTGTTGGTGGGGTAACGGCCTACCAAGGCATCGACGGGTAGCTGGTCTGAGAGGATGAT
CAGCCACACTGGGACTGAGACACGGCC
>Zotu811
GAACGCTAGCGGCAGGCCTAACACATGCAAGTCGAGGGAGGAGCTTTCGGGCTTCGACCGGCGTACGGG
TGCGTAACGCGTATACAATCTGCCTTTCACATAGGGATAGCCCGAAGAAATTTGGATTAATACCTAATGGTA
TATAGGGATGGCATCATTTTTATATTAAAGCTGAGGCGGTGAAAGATGAGTATGCGTTCTATTAGCTAGTT
GGAGAGGTAACGGCTCCCCAAGGCGACGATAGATAGGGGTTCTGAGAGGGATATCCCCCACACTGGTACT
GAGATACGGAC
>Zotu813
GAACGCTGGCGGCATGCCTTACACATGCAAGTCGAACGGCAGCACGGAGCTTGCTCTGGTGGCGAGTGGC
GAACGGGTGAGTAATATATCGGAACGTACCCTGGAGTGGGGGATAACGTAGCGAAAGTTACGCTAATACC
GCATACGATCTAAGGATGAAAGTGGGGGATCGCAAGACCTCATGCTCGTGGAGCGGCCGATATCTGATTA
GCTAGTTGGTAGGGTAAAAGCCTACCAAGGCATCGATCAGTAGCTGGTCTGAGAGGACGACCAGCCACAC
TGGAACTGAGACACGGTC
>Zotu815
AAACGCTGGCGGCGTGCCTAATGCATGCAAGTCGAACGGGGTCGCTTCGGCGGCTTACGTGGCGGACGG
GTGAGGAACACGTGGGCAATCTGCCGTCGGGTGGGGGGTAGCCGCTGGAAACAGCGGGTAATTCCGCAT
GTGCTTACCGATCTGTGGGGTTGGTAAGGAAAGCCTTCGGGCGCCCGACGAGGAGCCTGCGGCCGATTAG

CTAGCTGGTGGGGTAACGGCCTACCAGGGCGATGATCGGTAGCTGGTCTGAGAGGACGGCCAGCCACAG
GGGAACTGAGACACGGTC

>Zotu817

GAACGCTGGCGGCGTGCCTAATACATGCAAGTCGAGCGAAGTTTTTCTGGTGCTTGCACTAGAAAAACTTA
GCGGCGAACGGGTGAGTAACACGTAAAGAACCTGCCTCATAGACTGGGACAACTATTGGAAACGATAGCT
AATACCGGATAACAGCATTAACTGCATGGTTGATGTTTGAAAGTTGGTTTTGCTAACACTATGAGATGGCTT
TGCGGTGCATTAGCTAGTTGGTGGGGTAAAGGCCTACCAAGGCGACGATGCATAGCCGACCTGAGAGGGT
GATCGGCCACACTGGGACTGAGACACGGCC

>Zotu819

GAACGCTGGCGGCGTGCCTAATACATGCAAGTCGAGCGAATGGATTAAGAGCTTGCTCTTATGAAGTTAGC
GGCGGACGGGTGAGTAACACGTGGGTAACCTGCCCATAAGACTGGGATAACTCCGGGAAACCGGGGCTA
ATACCGGATAATATTTTGAACCGCATGGTTCGAAATTGAAAGGCGGCTTCGGCTGTCACTTATGGATGGAC
CCGCGTCGCATTAGCTAGTTGGTGAGGTAACGGCTCACCAAGGCAACGATGCGTAGCCGACCTGAGAGGG
TGATCGGCCACACTGGGACTGAGACACGGCC

>Zotu821

GAACGCTGGCGGCAGGCCTAACACATGCAAGTCGAACGATGTAGCAATACATAGTGGCGCACGGGTGAG
GAACACGTGGGAATCTCCCTTCTAGTACGGGATAACACCGGGAAACTGGTGCTAATACCGTATGACCCCTC
CGGGGGAAAGATTTATCGCTAGAAGATGAGCCCGCGTAAGATTAGCTAGTTGGTGAGGTAATGGCTCACC
AAGGCTACGATCTTTAGCTGGTCTTAGAGGACGATCAGCCACACTGGAACTGAGACACGGTC

>Zotu823

GAACGCTGGCGGCGTGCTTAACACATGCAAGTCGAACGAACTCGACCTTCGGGTCAATGAGTTAGTGGCG
AACGGGTGAGTAACACGTGAGAAACCTACCCTGGAGATGGGAATAACTCGGGGAAACCCGGGCTAATACT
CGATACCTTCTCCCGGTCGCATGACCGGGAGAAGAAAGCTCAGGCGCTTTAGGATGGTCTCGCGGCCTATC
AGCTAGTTGGTGAGGTAACGGCTCACCAAGGCGACGACGGGTAGCTGGTCTGAGAGGACGATCAGCCAC
ACTGGGACTGAGACACGGCC

>Zotu825

GAACGCTGGCGGCGTGCCTAATACATGCAAGTCGAGCGGACAGATGGGAGCTTGCTCCCTGATGTTAGCG
GCGGACGGGTGAGTAACACGTGGGTAACCTGCCTGTAAGACTGGGATAACTCCGGGAAACCGGGGCTAA
TACCGGATGGTTGTTTGAACCGCATGGTTCAGACATAAAAGGTGGCTTCGGCTACCACTTACAGATGGACC
CGCGGCGCATTAGCTAGTTGGTGAGGTAACGGCTCACCAAGGCAACGATGCGTAGCCGACCTGAGAGGGT
GATCGGCCACACTGGGACTGAGACACGGCC

>Zotu827

GAACGCTGACAGAATGCTTAACACATGCAAGTCTTTGGCAAATCTGTGCTTGCACAGCCTAGCCAAGGCGG
ACGGGTGAGTAACGCGTAAAGAACTTACCCTGCAGACAGGGATAACAGACGGAAACGACTGATAATACCT
GATATAATTGCCAGCACGCATGTGCCCGGCAATGAAAAGTAATGCTGCAGGAGAGCTTTGCGTCCTATTAG
CTTGTTGGTGAGGTAACGGCTCACCAAGGCGATGATAGGTAGCCGGCCTGAGAGGGTGAACGGCCACAA
GGGGACTGAGATACGGCC

>Zotu829

GAACGCTGGCGGCAGGCCTAACACATGCAAGTCGAGCGAGATCTTCGGGTCTAGCGGCGGACGGGTGAG
TAACGCGTGGGAATATGCCTCTTGGTACGGAATAGCCTCGGGAAACTGGGAGTAATACCGTATGTGCCCTT
TGGGGGAAAGATTTATCGCCAAGAGATTAGCCCGCGTTGGATTAGGTAGTTGGTGGGGTAATGGCCTACC
AAGCCGACGATCCATAGCTGGTTTGAGAGGATGATCAGCCACACTGGGACTGAGACACGGCC

>Zotu831

GAACGCTGGCGGCAGGCCTAACACATGCAAGTCGAACGAGCTGGGACCTTCGGGTCCCGATGCTAGTGGC
GCACGGGTGAGTAACGCGTGGGAACGTACCCTTTAGTACGGAATAACCCAGGGAAACTTGGGCTAATACC
GTATGACTCCTTCGGGAGAAAGATTTATCGCTAAAGGATCGGCCCGCGTCTGATTAGGTAGTTGGTAGGGT
AATGGCCTACCAAGCCTACGATCAGTAGCTGGTCTGAGAGGATGATCAGCCACACTGGGACTGAGACACG
GCC

>Zotu833

GAACGCTGGCGGCGTGCTTAACACATGCAAGTCGAACGATGAAGCCCAGCTTGCTGGGTGGATTAGTGGC
GAACGGGTGAGTAACACGTGAGCAACCTGCCCCTGACTCTGGGATAAGCGCTGGAAACGGCGTCTAATAC
TGGATATGTCCCGTCACCGCATGGTGTGCGGGTGGAAAGATTTTTCGGTTGGGGATGGGCTCGCGGCCTA
TCAGCTTGTTGGTGAGGTAATGGCTCACCAAGGCGTCGACGGGTAGCCGGCCTGAGAGGGTGACCGGCCA
CACTGGGACTGAGACACGGCC

>Zotu835

GAACGCTAGCGGCAGGCTTAACACATGCAAGTCGAGGGGCAGCGCGGTAGCAATACTGGCGGCGACCGG
CAAACGGGTGCGGAACACGTACAGAACCTACCTTGATCTGGGGCATAGCCCGGAGAAATCCGGATTAACT
CCCCATAATATACATGAGTGGCATCACTTGAGTATTAAAAATTTATTGGATCGAGATGGCTGTGCGTCCCAT
TAGCTAGTTGGTAGGGTAACGGCCTACCAAGGCAACGATGGGTAGCTGGTGTGAGAGCACGACCAGCCAC
ACGGGCACTGAGACACGGGC

>Zotu837

GAACGCTGGCGGCGTGCTTAACACATGCAAGTCGAACGGTGAACTCCGCTTGCGGGGGGATCAGTGGCGA
ACGGGTGAGTAACACGTGGGCAACCTGCCCTCAGCTCTGGGATAACTCCAAGAAATTGGGGCTAATACCG
GATATGACTGCTGACCGCATGGTCTGGTGGTGGAAAGATTTATCGGCTGAGGATGGGCCCGCGGCCTATC
AGCTTGTTGGTGGGGTAGTGGCCTACCAAGGCGACGACGGGTAGCCGGCCTGAGAGGGTGACCGGCCAC
ACTGGGACTGAGACACGGCC

>Zotu839

TAACGCTGGCGGCATGCCTAACACATGCAAGTCGAATGTTGCCGCAAGGTAAACATGGCAGACGGCTGAG
TAACGCGTGGGGACTTACCCCGAAGTCGAGCATAACTCGCCGAAAGGCGAGATAATTCTCGATGTTGTCGA
AAGACTAAAGATTTATCGCTTCGGGAGAGACCTGCGTCCGATTAGCTTGTTGGTGAGGTAACGGCTCACCA
AGGCTTTGATCGGTAGGGGGCCTGAGAGGGTGACCCCCCAGACTGGGACTGAGACACGGCC

>Zotu842

GAACGCTAGCGGCAGGCTTAATACATGCAAGTCGAGGGACGGTAGCAATACTGGACCGGCAAACGGGTG
CGGAACACGTACGCAACCTACCCCAAACAGGTGAATAGCCTTCTGAAAAGGAGATTAATACACCGTAAAAT
ATATGGATGGCATCATTTGTATATAATAGCTCAGGCGGTTTGGGATGGGCGTGCGCCTGATTAGGTAGTTG
GTAGGGTAACGGCCTACCAAGCCAGCGATCAGTAACTGGTGTGAGAGCACGACCAGTCACACGGGCACTG
AGACACGGGC

>Zotu844

GAACGCTGGCGGCGTGCTTAACACATGCAAGTCGAACGATGACGACCGGGCTTGCCTGGTCTGATTAGTG
GCGAACGGGTGAGTAACACGTGAGTAACCTGCCCTTCACTCTGGGATAACCTCGGGAAATCGGGGCTAAT
ACCGGATATGAGTTTCCATCGCATGGTGGTTTCTGGAAAGTTTTTCGGTGAAGGATGGACTCGCGGCCTAT
CAGTTTGTTGGTGAGGTAATGGCTCACCAAGGCGATGACGGGTAGCCGGCCTGAGAGGGCGACCGGCCA
CACTGGGACTGAGACACGGCC

>Zotu848

GAACGCTGGCGGCGTGCCTAACACATGCAAGTCGAGCGGTAACAGGCGTAGCAATACGTGCTGACGAGC
GGCGAACGGGTGAGCAATGCTTCGGAATCTGCCCAATGGTGGGGAATAACCAGTCGAAAGATTGGCTAAT
ACCGCATACGCCCTTCGGGGGAAAGCGGGGGTCGCAAGACCTCGCGCCGATGGATGAGCCGAAGTCGGA
TTAGCTAGTTGGTAGGGTAACGGCCTACCAAGGCGACGATCCGTAGCTGGTCTGAGAGGACGACCAGCCA
CACTGGGACTGAGACACGGCC

>Zotu850

GGCAAGAATGTTGAAGAGCTAAGTACTGCTCACATGCACTGTCCTCCACAAAGGACGCTAACCGTGTGTGT
TGAGATTTCAGCACACATTAAAGAACCCCCAGGCGGGCAAAATTAAC

>Zotu852

AAACGCTGGCGGCGTGCCTAACACATGCAAGTCGAGCGAGAAAGGTCTTCGGACTGAGTACAGCGGCGG
ACGGGTGAGTAACGCGTAGGAATCTGCCCTTTAGTGAGGAATACCATCGAGAAATCGGTGTTAATACTGCA
TACGTTCCGAAAGGAAGAAAGGTGGCCTCTTTGCTACCGCTAAAGGATGAGCCTGCGTACTATTAGCTAGT
TGGTAGGGTAAAGGCCTACCAAGGCGATGATGGTTAGCCGGCCTGAGAGGGTGTACGGCCACATTGGAA
CTGAGACACGGTC

>Zotu854

GAACGCTGGCGGCAGGCTTAACACATGCAAGTCGAGCGCCCCGCAAGGGGAGCGGCAGACGGGTGAGTA
ACGCGTGGGAATCTACCTTTTGCTACGGAATAACGCAGGGAAACTTGTGCTAATACCGTATGTGCCCTTTG
GGGGAAAGATTTATCGGCAAAGGATGAGCCCGCGTTGGATTAGCTAGTTGGTGGGGTAAAGGCCTACCAA
GGCGACGATCCATAGCTGGTCTGAGAGGATGATCAGCCACATTGGGACTGAGACACGGCC

>Zotu856

GAACGCTGGCGGCATGCTTTACACATGCAAGTCGAACGGTAACAGGCCTTCGGGTGCTGACGAGTGGCGA
ACGGGTGAGTAATGCATCGGAACGTACCCAGAGGTGGGGGATAACGCAGCGAAAGCTGTGCTAATACCG
CATGTGATCTGAGGATGAAAGCGGGGGACCGAGCAGCAATGTTTGGCCTCGCGCCTTTGGAGCGGCCGAT
GTCGGATTAGCTAGTTGGTGGGGTAAAGGCCTACCAAGGCGACGATCCGTAGCTGGTCTGAGAGGACGAC
CAGCCACACTGGGACTGAGACACGGCC

>Zotu859

GAACACTGGCGGCATGCCTAACACATGCAAGTCGAACGAGATCTTCGGGTCTAGTGGCGCACGGGTGCGT
AACGCGTGGGAATCTGCCCTTTGGTTCGGAATAACAGTTGGAAACGACTGCTAATACCGGATGATGACGA
AAGTCCAAAGATTTATCGCCAGAAGATGAGCCCGCGTAGGATTAGCTAGTTGGTGTGGTAAAGGCGCACC
AAGGCGACGATCCTTAGCTGGTCTGAGAGGATGATCAGCCACACTGGGACTGAGACACGGCC

>Zotu861

GAACGCTGGCGGCGTGCCTAACACATGCAAGTCGAGCGAACCCTTCGGGGTGAGCGGCGGACGGGTGAG
TAACGCGTGGGTAACCTGCCCTGTACACACGGATAACATACCGAAAGGTATGCTAATACGAGATAAAATGT
AATGATCGCATGGTCGATACATCAAAGCTCCGGCGGTACAGGATGGACCCGCGTCTGATTAGCTAGTTGGT
GAGGTAATGGCTCACCAAGGCGACGATCAGTAGCCGACCTGAGAGGGTGATCGGCCACATTGGAACTGA
GACACGGTC

>Zotu863

GAATGCTGGCGGCGTGCCTAACACATGCAAGTCGAGCGGTAAGGCCCCTTCGGGGGTACACGAGCGGCG
GACGGCTGAGTAACGCGTGGGAACATGCCCCAAAGTGAGGGATAAGCTCCAGAAATGGAGTCTAATACCG
CATATGGTCTTCGGATTAAAGGATTTATCCGCTTTGGGAGTGGCCTGCGTACGATTAGATAGTTGGTGAGG
TAATGGCTTACCAAGTCTACGATCGTTAACTGGTCTGAGAGGATGATCAGTCAGACTGGAACTGAGACACG
GTC

>Zotu865

GAACGCTGGCGGCGTGCCTAATACATGCAAGTCGAGCGAATCGGAGGGAGCTTGCTCCCAAAGATTAGCG
GCGGACGGGTGAGTAACACGTGGGCAACCTGCCTGTAAGACTGGGATAACTTCGGGAAACCGGAGCTAAT
ACCGGATAATCCTTTTCCTCTCATGAGGAAAAGCTGAAAGACGGTTTCGGCTGTCACTTACAGATGGGCCC
GCGGCGCATTAGCTAGTTGGTGAGGTAACGGCTCACCAAGGCAACGATGCGTAGCCGACCTGAGAGGGT
GATCGGCCACACTGGGACTGAGACACGGCC

>Zotu867

GAACGCTGGCGGCGTGCCTAATACATGCAAGTCGAGCGAACCAATAAGAAGCTTGCTTTTTGTTGGTTAGC
GGCGGACGGGTGAGTAACACGTGGGTAACCTGCCTGTAAGACCGGGATAACTCCGGGAAACCGGTGCTA
ATACCGGATAGATTATCTTTCCGCCTGGAGAGATAAGGAAAGATGGCTTTTGCCATCACTTACAGATGGGC
CCGCGGCGCATTAGCTAGTTGGTGAGGTAACGGCTCACCAAGGCGACGATGCGTAGCCGACCTGAGAGG
GTGATCGGCCACACTGGGACTGAGACACGGCC

>Zotu869

GAACGCTAGCGGCAGGCCTAATACATGCAAGTCGAACGGTGTCTTCGGACATAGTGGCGCACGGGTGCGT
AACACGTATGCAACCTACCTTACATTGGGGGATAGCCTTTCGAAAGGGAGATTAATACCGCATAAGACAGT
AACTAGGCATCTAGTAGCTGTTAAAGATTTATCGATGTAAGCTGGGCATGCGTCCAATTAGCTAGTTGGTG
AGGTAATGGCTCACCAAGGCAATGATTGGTAGGGGAACTGAGAGGTCAATCCCCCACACTGGCACTGAGA
TACGGGC

>Zotu871

GAACGCTGGCGGCGTGCCTAACACATGCAAGTGGAGCGACGAACCGGGCTTCGGCCCGGGGCAAAGCCG
CGAACGGGTGAGTAACACGTGGGTAACCTGCCCTGATGATTGGGACAACCCGAGGAAACTCGGGCTAATA
CCAAATGTGCCCCAAGACCTTAAGGTCTACGGGGAAAGGAAGCTTCGGCCTCCGCATCGGGATGGGCCCG
CGGCCCATTAGCTTGTTGGTGAGGTAATGGCTCACCAAGGCAACGATGGGTAGCTGGTCTGAGAGGACGA
TCAGCCACACTGGGACTGAGACACGGCC

>Zotu873
GAACGCTGGCGGCGCGCCTAACACATGCAAGTCGAGCGATAAGCCACCTTCGGGTGGTGGAAAGCGGCG
AACGGGTGAGTAACACGTGAGGAACCTGCCCCGGACTCTGGGATAACAGTGGGAAACCGCTGCTAATACC
GGATACACCATCTCTACGGCATCGCAGAAATGGGAAAGATTTATCGGTCTGGGATGGCCTCGCGGCCTATC
AGCTAGTTGGTGGGGTAACGGCCTACCAAGGCAACGACGGGTAGCTGGCGTGAGAGCGTGGCCAGCCAC
ACTGGGACTGAGATACGGCC
>Zotu875
TAACGCTGGCGGCGTGCCTAACACATGCAAGTCGAACGGACGTAGCAATACGTTAGTGGCAGACGGGTGA
GTAACACGTAGATAACGTACCCCTCCGCTGGGGATAGCCACTGGAAACGGTGGGTAAAACCGAATATTCTT
CAATATGGGCATCTATGTTGAAGGAAAACTTTGGTGTGGAGGGATCGGTTTGCGACCTATCAGCTAGTTGG
TGAGGTAATGGCTCACCAAGGCGACGACGGGTAACCGGTCTGAGAGGACGGTCGGTCACACTGGCACTG
AGATACGGGC
>Zotu878
GAACACTAGCGGCGTGCCTGAGATATGCAAGTCGAATGTCTTTTTCTCCGCAAGGAGAGAAGGTAATGGC
GGACGGCTGAGTAACACGTGGGGACCTGCCCTAAAGTAGGGAATAGCTCACCGAAAGGTGGGGTAATAC
TCTATATCCAGTTTACTGGAAAGAGCTTAACTGCTCGCTTAAGGATGGACCTGCGTCCTATCAGATAGTTGG
TGAGGTAAAGGCTTACCAAGTCTATGACGGGTAGCTGGCCTGAGAAGGTGGTCAGCCAGAGGAGCACTG
AGATACGGGC
>Zotu882
GAACGCTAGCGGTATGCTTAACACATGCAAGTCGTACGGAAGAGCAATCTTTAGTGGCGAACGGGTGAGT
AACGCGTGAGAATTTGCCTTTAGGAAGAGGATAACAATTGGAAACAGTTGCTAAAACTCTATATGCCGTTT
ACGGTGAAAAAAGTAATTTGCCTAAAGAGAAGCTCGCGTCTGATTAGCTAGTTGGTGGGGTAAAGGCCTA
CCAAGGCGACGATCAGTAGCTGGTCTGAGAGGACGATCAGCCACACTGGAACTGAGACACGGTC
>Zotu885
GAACGCTGGCGGCGTGCTTAACACATGCAAGTCGAACGATGATGCCCAGCTTGCTGGGTGGATTAGTGGC
GAACGGGTGAGTAACACGTGAGTAACCTGCCCTTTCCTCTGGGATAACTCCGGGAAACCGGTGCTAATACT
GGATATGACTCCAACCTGCATGGGTATGGGGTGGAAAGATTTATCGGGATGGGATGGACTCGCGGCCTAT
CAGCTTGTTGGTGGGGTAATGGCCTACCAAGGCGACGACGGGTAGCCGGCCTGAGAGGGTGACCGGCCA
CACTGGGACTGAGACACGGCC
>Zotu888
GAACGCTGGCGGCGTGCCTAATACATGCAAGTCGAGCGAAGTTTTTCTGGTGCTTGCACTAGAAAAACTTA
GCGGCGAACGGGTGAGTAACACGTAAAGAACCTGCCTCATAGACGGGGACAACTATTGGAAACGATAGCT
AATACCGGATAACAGCATAAATCGCATGATATATGTTTAAAAGTTGGTTTCGGCTAACACTATGAGATGGC
TTTGCGGTGCATTAGCTAGTTGGTGGGGTAAAGGCCTACCAAGGCGACGATGCATAGCCGACCTGAGAGG
GTGATCGGCCACACTGGGACTGAGACACGGCC
>Zotu890
GAACGCTGGCGGCGTGCCTAAGACATGCAAGTCGAGTGGGTTTAGACCCACGGCGAACGGGTGAGTAAT
ACCTAGGAACTTTCTTTAAGTGGAGCATAACCAGTCGAAAGATTGGATAATACTTCATGTGGCAGTAATGC
TAAAGTCGCAAGACGCTTAAAGTGAGGCCTGGGCACCATCAGCTAGTTGGTAAGGTAATGGCTTACCAAG
GCTATGACGGTTAGGGGTCCCGAGAGGGATAACCCCCACACTGGAACTGCGACACGGTC
>Zotu892
GAACGCTGGCGGCGTGCCTTAAGCATGCAAGTCGAACGGTCCTCGCAAGAGGGCAGTGGCGGACGGGTG
AGTAACGCGTGAGAATCTACCTTAAAGTGGGACATAGCTAGCCGAAAGGTTGGGTAATTTCCCATAGTCCC
CGATTTAATCGGGGTAAAGCCGCAAGGCGCTTTAAGATGAGCTCGCGTCCTATCAGCTTGTTGGTGGGGTA
AAAGCCTACCAAGGCGATGACGGGTAGCTGGTCTGAGAGGATGGTCAGCCACAATGGAACTGCGACACG
GTC
>Zotu894
GAACGCTAGCGGGAGGCCTAACACATGCAAGCTGAGCGGTATTTGTCTTTCGGGACAGAGAGAGCGGCGT
ACGGGTGCGGAACACGTGTGCAACCTGCCTTTATCTGGGGGATAGCCTTTCGAAAGGGAGATTAATACCCC
ATAATATAATGAGTGGCATCACTGATTATTGAAAACTCCGGTGGATAGAGATGGGCACGCGCAAGATTAG

ATAGTTGGTGAGGTAACGGCTCACCAAGTCGATGATCTTTAGGGGGCCTGAGAGGGTGATCCCCCACACT
GGTACTGAGACACGGAC
>Zotu896
GAACGCTGGCGGCGTGCCTAACACATGCAAGTCGAGCGAGAGACTTCGGTCTCTAGCGGCGGACGGGTG
AGTAACACGTGGGTAACCTGCCTCGTAGAGGGGGATAGCCTTCCGAAAGGAAGATTAATACCGCATAACG
TAGTTTAGTCGCATGACTAGATTATCAAAGGAGTAATCCGCTATGAGATGGACCCGCGGCGCATTAGCTAG
TTGGTGAGGTAACGGCTCACCAAGGCGACGATGCGTAGCCGACCTGAGAGGGTGATCGGCCACATTGGAA
CTGAGATACGGTC
>Zotu898
GAACGCTGGCGGCGTGCTTAACACATGCAAGTCGAACGCTGAAGCACCAGCTTGCTGGTGTGGATGAGTG
GCGAACGGGTGAGTAATACGTGAGTAACCTGCCCTTGACTCTGGGATAAGCCCGGGAAACTGGGTCTAAT
ACTGGATGCTACATGTCACCGCATGGTGGTGTGTGGAAAGGGTTTACTGGTCTTGGATGGGCTCACGGCCT
ATCAGCTTGTTGGTGAGGTAATGGCTCACCAAGGCGACGACGGGTAGCCGGCCTGAGAGGGTGACCGGC
CACACTGGGACTGAGACACGGCC
>Zotu900
GAACGCTGGCGGCGTGCCTAATACATGCAAGTCGAGCGAAGTTTGATTGGTGCTTGCACCGATTAAACTTA
GCGGCGAACGGGTGAGTAACACGTAAAGAACCTGCCTCATAGACTGGGACAACTACTGGAAACGGTAGCT
AATACCGGATAATATATTTTACTGCATGGTAAAATATTGAAAGTCGGTCATGCTGACACTATGAGATGGCTT
TGCGGTGCATTAGCTAGTTGGTAGGGTAAAGGCCTACCAAGGCGACGATGCATAGCCGACCTGAGAGGGT
GATCGGCCACACTGGGACTGAGACACGGCC
>Zotu902
GAACGCTGGCGGCGTGCCTAATACATGCAAGTGGAACGCATTGATATCACCGGAGCTTGCTCCACCGATAT
AAATGAGTCGCGAACGGGTGAGTAACGCGTAGGTAACCTGCCTGATAGCGGGGGATAACTATTGGAAAC
GATAGCTAATACCGCATGAGAGTGTTTAACACATGTTAGAGACTTAAAAGATACAATTGTATCACTATCAGA
TGGACCTGCGTTGTATTAGCTAGTTGGTAGGGTAGCGGCCTACCAAGGCAACGATACATAGCCGACCTGA
GAGGGTGATCGGCCACACTGGGACTGAGACACGGCC
>Zotu904
GAACGCTGGCGGCGTGCTTAACACATGCAAGTCACGGGGGCCCGCAAGGGTAACCGGCGAACGGGTGCG
TAACACGTGAGCAATCTGCCGTCCACTGGGGGATAGCCGGCCCAACGGCCGGGTAATACCGCATACGCTT
GCCGGGTGGCATCACCTGGCAAGGAAACCTCCGGGGGTGGACGAGGAGCTCGCGGCCTATCAGCTAGTT
GGTGGGGTCACGGCCCACCAAGGCGATGACGGGTAGCTGGTCTGAGAGGATGGCCAGCCACATTGGGAC
TGAGATACGGCC
>Zotu907
GAACGCTAGCTACAGGCTTAACACATGCAAGTCGAGGGGAAACGGCATTTGGTGCTTGCACCATTTGGAC
GTCGACCGGCGCACGGGTGAGTAACGCGTATCCAACCTGCCCATAAGTAGGGTATAACCCGCAGAAATGC
GGACTAATCCCCTATGTTGTTCAAAGACGGCATCAGATTTGAACCAAAGGCTTGCCGCTTATGGATGGGGA
TGCGTCCGATTAGCTTGTTGGTGAGGTAACGGCTCACCAAGGCTTCGATCGGTAGGGGTTCTGAGAGGAA
GGCCCCCCACATAGGAACTGAGACACGGTC
>Zotu909
GAACGCTGGCGGCGTGCTTAACACATGCAAGTCGAACGCTGAAGCTCCAGCTTGCTGGGGTGGATGAGTG
GCGAACGGGTGAGTAATACGTGAGTAACCTGCCCTTGACTCTGGGATAAGCCTGGGAAACCGGGTCTAAT
ACTGGATACGACGTCCTACCGCATGGTGGGGCGTGGAAAGGGTTTTACTGGTTTTGGATGGGCTCACGGC
CTATCAGCTTGTTGGTGGGGTAATGGCCTACCAAGGCGACGACGGGTAGCCGGCCTGAGAGGGTGACCG
GCCACACTGGGACTGAGACACGGCC
>Zotu911
GAACGCTGGCGGCATGCCTAATACATGCAAGTCGAACGCTGAAGCACTCTGCTTGCAGAGTGCGGATGAG
TGGCGAACGGGTGAGTAACACGTGGGAAATCTGCCTATAAGAGGGGGATAACATTCGGAAACGGATGCT
AATACCGCATATATTCTTGGACCGCCTGGTCCTTGAAAGAAAGACGGCTTTGCTGTCGCTTATAGATGATCC
CGCGGCGTATTAGTTAGTTGGTGGGGTAAAGGCCTACCAAGACAATGATACGTAGCCGACCTGAGAGGGT
GATCGGCCACACTGGGACTGAGACACGGCC

>Zotu913
GAACGCTAGCGGCGAGCCTAACACATGCAAGTCGAGCGGGGAGCAATCCCAGCGGCGAACGGGTGAGTA
ACACGTAGGTGACGTGTCCTTTGGTGGGGAACAACCGTGGGAAACCATGGCTAATTCCGCATATGTCTTTC
GGGATGAAAGCCTTTGGGCGCCAGAGGAGCGGCCTGCGGCCGATTATTTAGTCCTAGTTGGTAGGGTAAT
GGCCTACCAAGGGTACGATCGGTAGCTGGTCTGAGAGGATGATCAGCCACACTGGAACTGAGACACGGTC
>Zotu915
GAACGCTGGCGGCGTGCTTAACACATGCAAGTCGAACGGTGAACCCGCTTCGGTGGGGATCAGTGGCGA
ACGGGTGAGTAACACGTGGGCAACCTGCCCCCGGCTCTGGGATAACTCCAAGAAATTGGGGCTAATACCG
GATATGACCGCTGACCGCATGGTCTGGTGGTGGAAAGATTTATCGGCTGGGGATGGGCCCGCGGCCTATC
AGCTTGTTGGTGGGGTAGTGGCCTACCAAGGCGACGACGGGTAGCCGGCCTGAGAGGGTGACCGGCCAC
ACTGGGACTGAGACACGGCC
>Zotu917
GAACGCTGGCGGCAGGCTTAACACATGCAAGTCGAGCGGGCCCTTCGGGGTCAGCGGCGGACGGGTTAG
TAACGCGTGGGAACGTGCCTCTTGCTCTGGAATAGCCCCGGGAAACTGGGGGTAATACCGGATGTGCCCT
GAGGGGGAAAGGTTTACCGGCAAGAGATCGGCCCGCGTCTGATTAGGTAGTTGGTGGGGTAACGGCCCA
CCAAGCCGACGATCGGTAGCTGGTTTGAGAGGATGATCAGCAACACTGGGACTGAGACACGGCC
>Zotu919
GAATGTTGGCGGCGTGCTTGAGATATGCAAGTCGAACGATGTCCCTGCCGCAAGGTAGGGATAAAGTGGC
GGACGGCTGAGTAACACGTAGATATCTACCTCAAAGCGAATGATAGCTCATCGAAAGATGGGGTAACCATT
CATGTGTCACATACCGCAAGGTATTTGATAAAGGGCATAACTGCTCACTTTAAGATGAGTCTGCGGCCTATC
AGGTAGTTGGTAAGGTAATGGCTTACCAAGCCTATGACGGGTAGCTGGCCTGAGAGGGTGGTCAGCCAGA
GGGGCACTGAGATACGGGC
>Zotu921
GAACGCTGGCGGCGTGCTTAACACATGCAAGTCGAACGGAAAGGCCCCTGCTTGCAGGGGTACTCGAGTG
GCGAACGGGTGAGTAACACGTGGGTGATCTGCCTTGCACTCTGGGATAAGCTTGGGAAACTGGGTCTAAT
ACCGGATAGGAACCGTCTTTAGTGTGGTGGTTGGAAAGTTTTTTTTCGGTGCAAGATGAGCTCGCGGCCTA
TCAGCTTGTTGGTGGGGTAATGGCCTACCAAGGCGTCGACGGGTAGCCGGCCTGAGAGGGTGTACGGCCA
CATTGGGACTGAGATACGGCC
>Zotu923
GAACGCTGGCGGCGCGCCTAAAACATGCAAGTCGAACGAGAAGCCCGGGCTTGCCCGGGTGGACAGTGG
CGAACGGGTGAGTAACACGTGAGGAACCTGCCCCGAAGACCGGGACAACACCGGGAAACCGGTGCTAAT
ACCGGATACCTTCGCCGGATCGCATGGTCTGGTGAAGAAATGGATTCCGCTTCGGGATGGCCTCGCGGCCT
ATCAGCTTGTTGGTGAGGTAACGGCTCACCAAGGCAACGACGGGTAGCTGGTCTGAGAGGATGTCCAGCC
ACACTGGGACTGAGACACGGCC
>Zotu925
GAACGCTGGCGGCACGCCTAATACATGCAAGTCGAGCGCGTGAAGCTTTCCGAAGCCTTCGGGCGGACGA
AAGTGGATCGAGCGGCGGACGGGTGAGTAACACGTAGGCAACCTGCCTGTAAGACCGGGATAACTCCGG
GAAACCGGGGCTAATACCGGATAGGATCTCTTTTCGCATGAGAAGGGATTGAAAGGATGGCCGCAAGGCT
ATCGCTTACAGATGGGCCTGCGGCGCATTAGCTGGTTGGTGAGGTAACGGCTCACCAAGGCGACGATGCG
TAGCCGGCCTGAGAGGGTGACCGGCCACACTGGGACTGAGACACGGCC
>Zotu927
GAACGCTGGTAACGTGGATACCGGCATGCAAGTCAAGGGGCCGCAAGGCACCGGCGAACGGCTTAGTAA
CACGTAGATACATACCGTAAAGTGGGGCACAGCTCGTCGAAAGACGGGGTAATTCTCCATAGTTCTCGCAA
GAGTAAAGGCGCAAGCCGCTTTATGAATGGTCTGCGGTCTATCAGCTAGTTGGTGAGGTAATGGCTCACCA
AGGCTATGACGGATAGGGGGCGTGAGAGCGCGACCCCCAAGAATGGCACTGCGATACGGGC
>Zotu929
GAACGCTGGCGGCGTGCTTAACACATGCAAGTCGAACGATGAAGCCCTTCGGGGTGGATTAGTGGCGAAC
GGGTGAGTAACACGTGGGCAATCTGCCCTGCACTCTGGGACAAGCCCTGGAAACGGGGTCTAATACCGGA
TGATATCCCCTCTCGCATGGGAGGGGGTTGAAAGCTCCGGCGGTGCAGGATGAGCCCGCGGCCTATCAGC

TAGTTGGTGAGGTAGAAGCTCACCAAGGCGACGACGGGTAGCCGGCCTGAGAGGGCGACCGGCCACACT
GGGACTGAGACACGGCC
>Zotu932
GAACGCTGGCGGCGTGCCTAATACATGCAAGTCGAGCGAATCTTTGGGAGCTTGCTCCTAAAGGTTAGCG
GCGGACGGGTGAGTAACACGTGGGCAACCTGCCTGTAAGACTGGGATAACTTCGGGAAACCGGAGCTAAT
ACCGGATAATCCTTTTCTTCTCATGAAGAAAAGCTGAAAGATGGCGCAAGCTATCACTTACAGATGGGCCC
GCGGCGCATTAGCTAGTTGGTGAGGTAATGGCTCACCAAGGCGACGATGCGTAGCCGACCTGAGAGGGT
GATCGGCCACACTGGGACTGAGACACGGCC
>Zotu934
GAACGCTGGCGGCATGCCTAACACATGCAAGTCGAACGAGACCTTCGGGTCTAGTGGCGCACGGGTGCGT
AACGCGTGGGAACCTGCCTTTAGGTTCGGGATAACAGTTAGAAATGACTGCTAATACCGGATGATGTCTTC
GGACCAAAGATTTATCGCCTTTAGATGGGCCCGCGTTGGATTAGCTAGTTGGTGGGGTAAAGGCCTACCAA
GGCGACGATCCATAGCTGGTCTGAGAGGATGATCAGCCACACTGGGACTGAGACACGGCC
>Zotu937
GAACGCTGGCGGCATGCTTAACACATGCAAGTCGCACGGGAACCGAGGGCTTGCTCTCGGGGAGAGTGG
CGCACGGGTGAGTAACACGTAGATAACGTTCCATTTGGTCTGGGATAACCTGCCGAAAGGCGGGCTAATA
CCGGATACGTTTCTTTGAGATTCGTCTTGGAGAAAGAAAGCAGGGCAACTTGCGCCAGTTGATCGGTCTGC
GTCCCATCAGCTAGTTGGTGAGGTAACGGCTCACCAAGGCGACGACGGGTAGCTGGTCTGAGAGGATGAT
CAGCCACACTGGGACTGAGACACGGCC
>Zotu939
TAACGCTAGCGGCAGGCCTAATACATGCAAGTCGAACGGTAACACGGGTAGCAATACCTGGTGACGAGTG
GCGCACGGGTGCGTAACGCGTATGTAATCTACCTCTAACTGGGGAATAGCCTTCCGAAAGGAAGATTAATA
CCGCATAATATAAAGGAGAGACATCTCCCCTTTATCAAAACTCAGGTGGTTAGAGATGAACATGCGTGACA
TTAGCTAGTTGGTAAGGTAACGGCTTACCAAGGCTACGATGTCTAGGGGGTCTGAGAGGATGATCCCCCAC
ACTGGTACTGAGACACGGAC
>Zotu941
GAACGCTGGCGGCGTGCCTAACACATGCAAGTCGAGCGGTAACAGGTGTAGCAATACATGCTGACGAGCG
GCGAACGGGTGAGTAATGCTTCGGAATCTACCTTATGGTGGGGAATAACTAGCCGAAAGGTTAGCTAATA
CCGCATACGTCCTACGGGAGAAAGCAGGGGATCGCAAGACCTTGCGCCAAAAGATGAGCCGAAGTCGGAT
TAGCTAGTTGGTAGGGTAACGGCCTACCAAGGCGACGATCCGTAGCTGGTCTGAGAGGACGACCAGCCAC
ACTGGGACTGAGACACGGCC
>Zotu943
GAACGCTGGCGGCGTGCCTAATACATGCAAGTAGAACGCTGAAGAGAGGAGCTTGCTCTTCTTGGATGAG
TTGCGAACGGGTGAGTAACGCGTAGGTAACCTGCCTGGTAGCGGGGGATAACTATTGGAAACGATAGCTA
ATACCGCATAATAGTAGATGTTGCATGACATTTGCTTAAAAGGTGCAAATGCATCACTACCAGATGGACCT
GCGTTGTATTAGCTAGTTGGTGGGGTAACGGCTCACCAAGGCGACGATACATAGCCGACCTGAGAGGGTG
ATCGGCCACACTGGGACTGAGACACGGCC
>Zotu945
GAACGCTGGCGGAATGCTTTACACATGCAAGTCGAACGGCAGCACGGGTGCTTGCACCTGGTGGCGAGTG
GCGAACGGGTGAGTAACGCATCGGAACGTGCCTTTTAGTGGGGGATAACTATCCGAAAGGATAGCTAATA
CCGCATACGCCCTGAGGGGGAAAGGGGGGGATCGCAAGACCTCTCGCTAAAAGAGCGGCCGATGTCCGA
TTAGCTAGTTGGTGAGGTAAAGGCTCACCAAGGCGACGATCGGTAGCTGGTCTGAGAGGATGATCAGCCA
CACTGGAACTGAGACACGGTC
>Zotu948
GAACGCTAGCGGCAGGCCTAATACATGCAAGTCGAACGGTGTCTTCGGACATAGTGGCGCACGGGTGCGT
AACACGTATGCAACCTACCTTACATTGGGGGATAGCCTTTCGAAAGGGAGATTAATACCGCATAAGACAGT
AGCTGGGCATCCAGCAGCTGTTAAAGATTTATCGATGTAAGCTGGGCATGCGTCCAATTAGCTAGTTGGTG
AGGTAATGGCTCACCAAGGCAATGATTGGTAGGGGAACTGAGAGGTCAATCCCCCACACTGGCACTGAGA
TACGGGC
>Zotu950

GAACGCTAGCGGCAGGCTTAATACATGCAAGTCGAGGGGCAACAGGGGTAGCAATACCTGCTGGCGACC
GGCAAACGGGTGCGGAACACGTACAGAACCTTCCTTTAAGCGGGGGATAGCCCATAGAAATGTGGATTAA
TACCCCATAGTAATGTTGACTGGCATCAGTTGATATTTAAAGATTTATCACTTAAAGATGGCTGTGCGGCTG
ATTAGGTAGTTGGTCGGGGTAACGGCCCACCAAGCCAACGATCAGTAACTGGTGTGAGAGCACGACCAGT
CACACGGGCACTGAGACACGGGC
>Zotu952
GAACGCTGGCGGCAGGCCTAACACATGCAAGTCGAACGGAGTTATATTGTAGCTTGCTATAGTATAACTTA
GTGGCAGACGGGTGAGTAATGTATAGGAATCTACCTAGTAGTACGGAATAATTGTTGGAAACGGCAACTA
ATACCGTATACGCCCTACGGGGGAAAAATTTATTGCTATTAGATGAGCCTTATTAGATTAGCTAGTTGGTGG
AGTAATAGCCTACCAAGGCAATGATCTATAGCTGATCTGAGAGGATGATCAGCCACACTGGAACTGAGATA
CGGTC
>Zotu955
GAACGCTGGCGGCATGCCTAACACATGCAAGTCGAACGGCAGCACAGTAAAGATTTCGGTCTTTATGGGT
GGCGAGTGGCGGACGGGTGAGTAATGCGTAGGAATCTGTCTTGGAGCGTGGGATAACCCGAGGAAACTC
GGGCTAATACCACATGATATCGAGAGATCAAAAGCAGGGGACCGAAAGGCCTTGCGCTCTGGGGTGAGCC
TACGTCGGATTAGCTAGTTGGTGGGGTAAAAGCCTACCAAGGCGACGATCCGTAGCTGGTCTGAGAGGAT
GACCAGCCACACTGGGACTGAGACACGGCC
>Zotu957
GAACGCTGGCGGCGTGCCTAACACATGCAAGTCGAACGAGGCCCAAGCAGTGGCAACACTGCGGAAGGC
CTAGTGGCGAACGGGTGAGTAACACGTGAGGAACCTGCCCCGAAGACCGGGATAACCCCCTGAAAAGGG
AGCTAATACCGGATGCCCCCACCGGACCGCATGGTCTGGTGAGGAAATGGATTCCGCTTCGAGAGGGCCT
CGCGGCCTATCAGCTTGTTGGTGAGGTAACGGCTCACCAAGGCATCGACGGGTAGCTGGTCTGAGAGGAC
GATCAGCCACACTGGGACTGAGACACGGCC
>Zotu959
GAACGCTAGCGGCAGGCTTAACACATGCAAGTCGAGGGGTATAGTTCTTCGGAACTAGAGACCGGCGCAC
GGGTGCGTAACGCGTATGCAATCTACCTTTTACAGAGGGATAGCCCAGAGAAATTTGGATTAATACCTCAT
AGTATCTTCGATCCGCATGGATTGATTATTAAAGTCACAACGGTAAAAGATGAGCATGCGTCCCATTAGCTT
GTTGGTAAGGTAACGGCTTACCAAGGCAACGATGGGTAGGGGTCCTGAGAGGGAGATCCCCCACACTGGT
ACTGAGACACGGAC
>Zotu961
CAACGCTGGCGGCGCGCTTAACACATGCAAGTCGAACGAGAAAGGGTAGCAATACCTGAGTAAAGTGGCG
TACGGGTGAGTAACACGTGGATAATCTACCTCTTGGCGGGGAATACCTCTGGGAAACCGGAGACAATACC
GCATAAGCTCGAGAGAGGAAACCCGCAAGGGGCCGAGAGAGGAGTCCGCGGCCGATTAGCTAGTTGGTG
AGGTAAAGGCTCACCAAGGCGAAGATCGGTAGCCGGCCTGAGAGGGCACACGGCCACACTGGCACTGAG
ACACGGGC
>Zotu963
GAACGCTGGCGGCAGGCCTAACACATGCAAGTCGAACGCTGTAGCAATACAGAGTGGCAGACGGGTGAG
TAACACGTGGGAATCTTCCTATCGGTACGGAATAGCTCAGGGAAACTTGGGGTAATACCGCATACGCCCTT
CGGGGGAAAGATTTATCGCCGATAGATGAGCCCGCGTCTGATTAGCTAGTTGGTGAGGTAATGGCTCACC
AAGGCGACGATCAGTAGCTGGTCTGAGAGGATGACCAGCCACACTGGGACTGAGACACGGCC
>Zotu965
GAACGCTGGCGGCGTGCCTAATACATGCAAGTCGAGCGGACTTAAAAAGCTTGCTTTTTAAGTTAGCGGCG
GACGGGTGAGTAACACGTGGGCAACCTGCCTGTAAGACTGGGATAACTTCGGGAAACCGGAGCTAATACC
GGATAATCCTTTCCTACACATGTAGGGAAGTCTGAAAGACGGTTTACGCTGTCGCTTACAGATGGGCCCGC
GGCGCATTAGCTGGTTGGTGAGGTAACGGCTCACCAAGGCGACGATGCGTAGCCGACCTGAGAGGGTGA
TCGGCCACACTGGGACTGAGACACGGCC
>Zotu968
GAACGCTAGCTACAGGCTTAACACATGCAAGTCGAGGGGAAACGGCATTAAGTGCTTGCACTTTTTGGACG
TCGACCGGCGCACGGGTGAGTAACGCGTATCCAACCTTCCCATGACTAAGGGATAACCCGCCGAAAGGCA
GACTAATACCTTATGGTCTTCACTGACGGCATCAGATGTGAAGTAAAGATTTATCGGTTATGGATGGGGAT

GCGTCTGATTAGCTTGTTGGCGGGGTAACGGCCCACCAAGGCAACGATCAGTAGGGGTTCTGAGAGGAAG
GTCCCCCACATTGGAACTGAGACACGGTC
>Zotu971
GAACGCTGGCGGCATGCCTTACACATGCAAGTCGAACGGCAGCGCGGACTTCGGTCTGGCGGCGAGTGGC
GAACGGGTGAGTAATACATCGGAACGTACCCTGTTGTGGGGGATAACTAGTCGAAAGATTAGCTAATACC
GCATACGACCTGAGGGTGAAAGTGGGGGACCGCAAGGCCTCACGCAGCAGGAGCGGCCGATGTCTGATT
AGCTAGTTGGTGGGGTAAAGGCCCACCAAGGCGACGATCAGTAGCTGGTCTGAGAGGACGATCAGCCAC
ACTGGGACTGAGACACGGCC
>Zotu973
GAACGCTGGCGGCGTGCCTAATACATGCAAGTCGAACGAGAGCGACCGGTGCTTGCACTGGTCAATCTAG
TGGCGAACGGGTGAGTAACACGTGGGTAACCTGCCCATCAGAGGGGGATAACATTCGGAAACGGATGCT
AAAACCGCATAGGTCCTTTAACCGCATGGTTGAAGGAGGAAAAGAGGCGCAAGCTTCTGCTGATGGATGG
ACCCGCGGTGCATTAGCTAGTTGGTGAGGTAACGGCTCACCAAGGCCGTGATGCATAGCCGACCTGAGAG
GGTGATCGGCCACATTGGGACTGAGACACGGCC
>Zotu975
CAACGCTGGCGGCGTGCCTAATGCATGCAAGTCGAACGGGGTCATCTTCGGATGGCTTACGTGGCGGACG
GGTGAGGAACACGTGGGTAACCTGCCCACTGGTGGGGGCTAGCCTTCGGAAACGGAGGGTAATTCCGCAT
GAACTCGGATCCCGCGTGGGGGATGTGAGGAAAGCCGAGAGGCGCCAGTGGAGGGGCCCGCGTCCGATT
AGTTAGTTGGTGGGGTAACGGCTCACCAAGGCGATGATCGGTAGCTGGTTTGAGAGGATGATCAGCCACA
CGGGGACTGAGACACGGCC
>Zotu977
GAACGCTGGCGGCGTGCCTAATACATGCAAGTCGAGCGGACCGAACGAGAGCTTGCTCTTGTTCGGTCAG
CGGCGGACGGGTGAGTAACACGTGGGCAACCTGCCCGCAAGACCGGGATAACTCCGGGAAACCGGAGCT
AATACCGGATAACACCAAAGACCGCATGGTCTTTGGTTGAAAGGCGGCTTCGGCTGCCACTTGCGGATGG
GCCCGCGGCGCATTAGCTAGTTGGTGAGGTAACGGCTCACCAAGGCGACGATGCGTAGCCGGCCTGAGAG
GGTGACCGGCCACACTGGGACTGAGACACGGCC
>Zotu979
GAACGCTAGCGATAGGCTTAACACATGCAAGTCGAGGGGCAGCATTATTTTAGCTTGCTAAGATAGATGGC
GACCGGCGCACGGGTGCGTAACGCGTATGCAACCTGCCTGTAATTAGGGAATAACCCGGTGAAAGTCGGA
CTAATACCCTATATTCTTCTTTCTCCGCATGGAGGAGGACGGAAAGATTTATTGATTACAGATGGGCATGCG
TCCCATTAGCTAGTTGGTAAGGTAACGGCTTACCAAGGCAACGATGGGTAGGGGGACTGAGAGGTTGACC
CCCCACACTGGTACTGAGACACGGAC
>Zotu981
GAACGCTGGCGGCGTGCTTAACACATGCAAGTCGAACGGTGACCCAGGGCTTGCCCTGGTGATCAGTGGC
GAACGGGTGAGTAACACGTGGGCAACCTGCCCCTGACTTCGGGATAACTCCTCGAAAGAGGAGCTAATAC
CGGATACGACCACTTCGGACATCCGATGGTGGTGGAAAGTTTTTCGGTCAGGGATGGGCCCGCGGCCTAT
CAGCTAGTTGGTGGGGTAATGGCCTACCAAGGCGACGACGGGTAGCCGGCCTGAGAGGGCGACCGGCCA
CACTGGGACTGAGACACGGCC
>Zotu983
GAACGCTGGCGGCGTGCCTAATACATGCAAGTCGAACGCTTCCTACCGGTGCTTGCACCGGGAAAGAAGA
GTGGCGGACGGGTGAGTAACACGTGGGTAACCTACCTACAAGCGGGGGATAACATTCGGAAACGGATGC
TAATACCGCATAGATCTTTTTGTCTCCTGACAGAAAGAAGAAAGACGGTTTCGGCTGTCACTTGTAGATGG
ACCCGCGGCGCATTAGTTAGTTGGTGAGGTAATGGCTCACCAAGACGATGATGCGTAGCCGACCTGAGAG
GGTGATCGGCCACACTGGGACTGAGACACGGCC
>Zotu985
GAACGCTGGCGGCGTGCTTAACACATGCAAGTCGAGCGGTAAGGCCCTTCGGGGTACACGAGCGGCGAA
CGGGTGAGTAACACGTGGGTGACCTGCCCTCCACTCTGGGATAAGCCTGGGAAACCGGGTCTAATACCGG
ATAGGACTCCTGGATGCATGTCCGGGGGTGGAAAGTTTTTTCGGTGGGGGATGGGCCCGCGGTCTATCAG
CTTGTTGGTGGGGTGATGGCCTACCAAGGCGACGACGGATAGCCGGCCTGAGAGGGTGATCGGCCACACT
GGGACTGAGACACGGCC

>Zotu987
GAACGCTGGCGGCGTGCTTAACACATGCAAGTCGAACGGTAAGGCTCCTTCGGGAGTACACGAGTGGCGA
ACGGGTGAGTAACACGTGAGCAACCTGCCCTTGACTTTGGGATACCAGCTGGAAACAGCTGCTAATACCG
GATATGACCCCTTTACTCATGTTTTGGGGTGGAAAGCTCCGGCGGTCAGGGATGGGCTCGCGGCCTATCAG
CTTGTTGGTGAGGTAACGGCTCACCAAGGCTTCGACGGGTAGCCGGCCTGAGAGGGCGACCGGCCACACT
GGGACTGAGATACGGCC
>Zotu989
GAACGCTAGCGGCAGGCTTAATACATGCAAGTCGAGGGATGCCAGCAATGGTAGACCGGCAAACGGGTG
CGTAACACGTACACAACTTTCCGTCAAGTGGGGCATAGCCCGGGGAAACCCGGATTAATACCCCATAAAAT
ATAAGCTGCGGCATCGCAGTAATATTAAAGCTCCGGCGCTTGATGATGGGTGTGCGGCTGATTAGGTAGTT
GGCGGGGTAACGGCCCACCAAGCCTTCGATCAGTAGCTGATGTGAGAGCATGATCAGCCACACGGGCACT
GAGACACGGGC
>Zotu991
GAACGCTGGCGGCGTGCCTAATACATGCAAGTCGAGCGAATGGATTAAGAGCTTGCTCTTATGAAGTTAGC
GGCGGACGGGTGAGTAACACGTGGGTAACCTGCCCATAAGACTGGGATAACTCCGGGAAACCGGGGCTA
ATACCGGATAACATTTTGAACTGCATGGTTCGAAATTGAAAGGCGGCTTCGGCTGTCACTTATGGATGGAC
CCGCGTCGCATTAGCTAGTTGGTGAGGTAACGGCTCACCAAGGCAACGATGCGTAGCCGACCTGAGAGGG
TGATCGGCCACACTGGGACTGAGACACGGCC
>Zotu993
GAACGCTAGCGGCAGGCTTAACACATGCAAGTCGAGGGGTATATTTCTTCGGAGATAGAGACCGGCGCAC
GGGTGCGTAACGCGTATGCAATCTACCTTTTACAGAGGGATAGCCCAGAGAAATTTGGATTAATACCTCAT
AGTATAGCAGCTTCGCATGAAGCCACTATTAAAGTCACAACGGTAAAAGATGAGCATGCGTCCCATTAGCT
AGTTGGTAAGGTAACGGCTTACCAAGGCTACGATGGGTAGGGGTCCTGAGAGGGAGATCCCCCACACTGG
TACTGAGACACGGAC
>Zotu995
GAACGCTGGCGGCAGGCCTAACACATGCAAGTCGAGCGGATGAGAGGAGCTTGCTCTTCGATTCAGCGGC
GGACGGGTGAGTAATGCCTAGGAATCTGCCTAGTAGTGGGGGACAACGTTTCGAAAGGAACGCTAATACC
GCATACGTCCTACGGGAGAAAGTGGGGGATCTTCGGACCTCACGCTATTAGATGAGCCTAGGTCGGATTA
GCTAGTTGGTAGGGTAAAGGCCTACCAAGGCGACGATCCGTAACTGGTCTGAGAGGATGATCAGTCACAC
TGGAACTGAGACACGGTC
>Zotu997
GAACGCTAGCGGGAGGCCTAACACATGCAAGCCGAGCGGTATTTGTTCTTCGGAACAGAGAGAGCGGCGT
ACGGGTGCGGAACACGTGTGCAACCTGCCTTTATCAGGGAGATAGCCTTTCGAAAGGAAGATTAATATCCC
ATAATATTTAGACTGGCATCAGTATAAATTGAAAGCTCCGGCGGATAGAGATGGGCACGCGCAAGATTAG
ATAGTTGGCGGGGTAACGGCCCACCAAGTCAATGATCTTTAGGGGGCCTGAGAGGGTGATCCCCCACACT
GGTACTGAGACACGGAC
>Zotu999
TAACGCTGGCGGTATGCCTAACACATGCAAGTCGAACGGAATCTTATTTGTTCTACGCCTCAAACTAGCAAT
AGTATGCGTCGTGGAACTGTAAGATTTAGTGGCGAACGGGTGAGTAACACGTTGGTACCTGCCCTCTAGTG
GGGAATAACTAGCCGAAAGGTTGGCTAATACCGCATGTGATCTTCGGATCAAAGCCTTCGGGCGCTAGAG
GAGGGGCCTGCGGATGATTAGCTAGTTGGTGAGGTAAAAGCTCACCAAGGCGACGATCATTAGCTGGATC
GAGAGGTTGACCAGCCACACTGGGACTGAGATACGGCC
>Zotu1001
GAACGCTGGCGGCGTGCTTAACACATGCAAGTCGAACGGAAAGGCCCTTTCGGGGGTACTCGAGTGGCGA
ACGGGTGAGTAACACGTGGGTGATCTGCCCTGCACTTCGGGATAAGCCTGGGAAACTGGGTCTAATACCG
GATATGATCATGCTCTTCATGGGGTGTGGTGGAAAGCTTTTGCGGTGTGGGATGGGCCCGCGGCCTATCA
GCTTGTTGGTGGGGTAATGGCCTACCAAGGCGACGACGGGTAGCCGGCCTGAGAGGGTGTCCGGCCACA
CTGGGACTGAGATACGGCC
>Zotu1003

GAACGCTGGCGGCAGGCCTAACACATGCAAGTCGAGCGGATGAAGGGAGCTTGCTCCTGGATTCAGCGGC
GGACGGGTGAGTAATGCCTAGGAATCTGCCTGGTAGTGGGGGATAACGTCCGGAAACGGGCGCTAATAC
CGCATACGTCCTGAGGGAGAAAGTGGGGGATCTTCGGACCTCACGCTATCAGATGAGCCTAGGTCGGATT
AGCTAGTTGGTGGGGTAAAGGCCTACCAAGGCGACGATCCGTAACTGGTCTGAGAGGATGATCAGTCACA
CTGGAACTGAGACACGGTC
>Zotu1005
GAACGCTGGCGGCGTGCTTAACACATGCAAGTCGAACGGAAAGGCTCCTGCTTGCAGGGGTACTCGAGTG
GCGAACGGGTGAGTAACACGTGGGTGATCTGCCTTGTACTCTGGGATAAGCTTGGGAAACTGGGTCTAAT
ACCGGATAGGAACTGCCTTTAGTGTGGTGGTTGGAAAGTTTTTTCGGTGCAAGATGAGCTCGCGGCCTATC
AGCTTGTTGGTGGGGTAATGGCCTACCAAGGCGTCGACGGGTAGCCGGCCTGAGAGGGTGTACGGCCAC
ATTGGGACTGAGATACGGCC
>Zotu1007
GAACGCTAGCGGTGTGCCTGAGATATGCAAGTCGAGCGACGTGTCTTTGTAGCTTGCTACAAAGATAACGA
GCGGCGTCCGGCTGAGTAACGCGTAGGAATCTGCCCCCGACTGGGGAATAGCCAACCGAAAGGTTGGGTA
ATACCCCATAGTCTCTTTCTTTATTGAGAGAGTAAAGGCCTTTACCGGCCGGTCAGGGATGAGCCTGCGTCC
TATCAGGTAGTTGGTGAGGTAACGGCTCACCAAGCCAATGACGGGTAGCTGGTCTCATGAGGACGACCAG
CCAGAGGGGGACTGAGACACGGCC
>Zotu1009
GAACGCTGGCGGCGTGCTTAACACATGCAAGTCGAACGAAGAGCGATGGAAGCTTGCTTCTATCAATCTTA
GTGGCGAACGGGTGAGTAACGCGTAATCAACCTGCCCTTCAGAGGGGGACAACAGTTGGAAACGACTGCT
AATACCGCATACGATCTAACCTCGGCATCGAGGATAGATGAAAGGTGGCCTCTATTTATAAGCTATCACTG
AAGGAGGGGATTGCGTCTGATTAGCTAGTTGGAGGGGTAATGGCCCACCAAGGCGATGATCAGTAGCCG
GTCTGAGAGGATGAACGGCCACATTGGGACTGAGACACGGCC
>Zotu1011
GAACGTTGGCGGCGTGCCTAACACATGCAAGTCGAGCGAGAAAGGTCGAAAGGCTGAGTACAGCGGCGA
ACGGGTGAGTAACGCGTGAGAATCTACCCTTTAGTGGAGAATACCCTGGAGAAATTCAGGTTAATACTGCA
TATTCTCGGAAACGAGTAAAGATGGCCTAATTAGCTGTCGCTAGAGGATGAGCTTGCGTACTATTATGCTA
GTTGGTGGGGTAATGGCCTACCAAGGCGTTGATGGTTAGCCGGCCTGAGAGGGTGTACGGCCACACTGGA
ACTTAGACACGGTC
>Zotu1014
CTGACACTTATCAGTAGATTACATTTTAAGCCTACCAAATCCAAAATGCTATGTAAATGATATTCTTCAGTTC
CCAAGTTAACCTTGCACCTGGCAACCGTTGTCCCAAACAGCTTCCTCCTTCAGAAGGAGCTATTGATTAAAA
GTCAGGATTGACAATATTTCTTCA
>Zotu1016
GAACGCTGGCGGCGTGCCTAATACATGCAAGTCGAGCGAATAGATTAAGAGCTTGCTCTTATGAAGTTAGC
GGCGGACGGGTGAGTAACACGTGGGTAACCTACCCATAAGACTGGGATAACTCCGGGAAACCGGGGCTA
ATACCGGATAATATTTTGAACTGCATAGTTCGAAATTGAAAGGCGGCTTCGGCTGTCACTTATGGATGGAC
CCGCGTCGCATTAGCTAGTTGGTGAGGTAACGGCTCACCAAGGCGACGATGCGTAGCCGACCTGAGAGGG
TGATCGGCCACACTGGGACTGAGACACGGCC
>Zotu1018
GAACGCTGGCGGCGTGCTTAACACATGCAAGTCGAACGCTGAAGCCCAGCTTGCTGGGTGGATGAGTGGC
GAACGGGTGAGTAACACGTGAGTAACCTGCCCCCTTCTTTGGGATAACGCCCGGAAACGGGTGCTAATACT
GGATATTCACTGATCTTCGCATGGGGGTTGGTGGAAAGGTTTTTTCTGGTGGGGGATGGGCTCGCGGCCT
ATCAGCTTGTTGGTGGGGTGATGGCCTACCAAGGCTTTGACGGGTAGCCGGCCTGAGAGGGTGACCGGTC
ACATTGGGACTGAGATACGGCC
>Zotu1020
GAACGCTGGCGGCGTGCCTAATACATGCAAGTCGAGCGAATCGGAGGGAGCTTGCTCCCGAAGATTAGCG
GCGGACGGGTGAGTAACACGTGGGCAACCTGCCTGTAAGACTGGGATAACTTCGGGAAACCGGAGCTAAT
ACCGGATAATCCTTTTCCTCTCATGAGGAAAAGCTGAAAGACGGTTTCGGCTGTCACTTACAGATGGGCCC
GCGGCGCATTAGCTAGTTGGTGAGGTAACGGCTCACCAAGGCAACGATGCGTAGCCGACCTGAGAGGGT
GATCGGCCACACTGGGACTGAGACACGGCC

>Zotu1023
GAACGCTGGCGGCGTGCTTAACACATGCAAGTCGAACGGTGAAGGTGGGAGCTTGCTCCCGCCGGATCAG
TGGCGAACGGGTGAGTAACACGTGAGCAATCTGCCCCGAACTCTGGGATAAGCACTGGAAACGGTGTCTA
ATACTGGATACGACGCGAGAGGGCATCCTCATCGCGTGGAAAGAATTTCGGTTCGGGATGAGCTCACGGC
CTATCAGCTAGTTGGTGAGGTAATGGCTCACCAAGGCGACGACGGGTAGCCGGCCTGAGAGGGTGACCG
GCCACACTGGGACTGAGACACGGCC
>Zotu1025
GAACGCTGGCGGCGTGCCTAATACATGCAAGTCGAGCGCGGGAAGCAGGCAGAACTCCTTCGGGAGGGA
CGCTTGTGGAACGAGCGGCGGACGGGTGAGTAACACGTGGGCAACCTACCTGTAAGACTGGGATAACTCC
GGGAAACCGGGGCTAATACCGGATAGGCACCATAGGCGCATGCATGTGGTGTGAAAGGCGGGGCGACCT
GCCGCTTACAGATGGGCCTGCGGCGCATTAGCTAGTTGGTGAGGTAACGGCTCACCAAGGCGACGATGCG
TAGCCGACCTGAGAGGGTGACCGGCCACACTGGGACTGAGACACGGCC
>Zotu1027
GAACGCTGGCGGCATGCCTAATACATGCAAGTCGAACGGTCTTGATGGGCTTGCTCATCAAGGTAGTGGC
GGACGGGTGAGTAACGCGTGGGAACCTGCCTGGCAGTGGGGAATAACCGCGGGAAACTGCGGCTAATAC
CGCATACGCCCCCTGGACTTCGGTTTAGGGGGGAAAGATTTATCGCTGTCGGAGGGGCCCGCGTCGGATT
AGCTAGTTGGCGGGGTAACGGCCCACCAAGGCGACGATCCGTAGCTGGTCTGAGAGGATGATCAGCCACA
CTGGGACTTAGACACGGCC
>Zotu1029
GAACGCTGGCGGCAGGCCTAACACATGCAAGTCGAACGGTAACAGGAAGCAGCTTGCTGCTTCGCTGACG
AGTGGCGGACGGGTGAGTAATGTCTGGGAAACTGCCTGATGGAGGGGGATAACTACTGGAAACGGTAGC
TAATACCGCATAACGTCGCAAGACCAAAGAGGGGGACCTTCGGGCCTCTTGCCATCGGATGTGCCCAGAT
GGGATTAGCTTGTTGGTGGGGTAACGGCTCACCAAGGCGACGATCCCTAGCTGGTCTGAGAGGATGACCA
GCCACACTGGAACTGAGACACGGTC
>Zotu1031
GAATGCTGGCGGCGTGCCTAACACATGCAAGTCGAGCGGCAGCGCGAGGAGTTTACTCCTTGGCGGCGAG
CGGCGGACGGCTGAGTAACGCGTGGGAACGTGCCCCAAAGTGAGGAATAACTGCCCGAAAGGGTGGCTA
ATGCCGCATATGATCTTCGGATTAAAGGATTTATCCGCTTTGGGAGCGGCCCGCGTCGGATTAGGTAGTTG
GTGAGGTAATGGCTCACCAAGCCGACGATCCGTAGCTGGTCTGAGAGGATGATCAGCCAGACTGGAACTG
AGACACGGTC
>Zotu1033
GAACGCTGGCGGTATGCTTAACACATGCAAGTCGAACGAATGAGATTTTAAGCTTGCTTAAGATCAAGTTA
GTGGCAAACGGGTGAGTAACACGTGGGAATCTACCCAGTAGTACGGAATAACGGTTAGAAATGACCGCTA
ATACCGTATAGTATCGAGAGATTAAAGATTTATCGCTACTGGATGAGCCCGCGTCAGATTAGGTAGTTGGT
GGGGTAAAAGCCTACCAAGCCCAAGATCTGTAGCTGGTCTGAGAGGATGACCAGCCACACTGGAACTGAG
ACACGGTC
>Zotu1035
GAACGCTGGCGGCAGGCCTAACACATGCAAGTCGGACGGTAGCACAGAGAGCTTGCTCTTGGGTGACGA
GTGGCGGACGGGTGAGTAATGTCTGGGGATCTGCCCGATAGAGGGGGATAACCACTGGAAACGGTGGCT
AATACCGCATAACGTCGCAAGACCAAAGAGGGGGACCTTCGGGCCTCTCACTATCGGATGAACCCAGATG
GGATTAGCTAGTAGGCGGGGTAATGGCCCACCTAGGCGACGATCCCTAGCTGGTCTGAGAGGATGACCAG
CCACACTGGAACTGAGACACGGTC
>Zotu1037
GAACGCTAGCGGCAGGCCTAATACATGCAAGCCGGACGGCAAGTTGAATAGCAATATTTGACCTAGAGTG
GCGCAAGGGTGCGTAACACGTATGCAACCTACCCAGAACCGGGGCATAGCCCTCCGAAAGGAGGATTAAC
TTCCCATAGGAAGACGAATTGGCATCAATTTGTTTTTAAACTACGGGGTTCTGGATGGGCATGCGTTTGATT
AGCTAGTTGGCGGGGTAATGGCCCACCAAGGCGATGATCAATAGGGGACCTGAGAGGGTGATCCCCCACA
CTGGCACTGAGATACGGGC
>Zotu1039

GAACGCTGGCGGCGTGCTTAACACATGCAAGTCGAACGATGAAGCCAGCTTGCTGGTGGATTAGTGGCGA
ACGGGTGAGTAACACGTGGGCAACCTGCCCTCAGCTCTGGGATAACTCCAAGAAATTGGTGCTAATACCG
GATATTCTTCTTTCACCGCATGGTGGTTGGAGGAAAGTTTTTCGGCTGGGGATGGGCCCGCGGCCTATCAG
CTTGTTGGTGGGGTGATGGCCTACCAAGGCGACGACGGGTAGCCGGCCTGAGAGGGTGACCGGTCACACT
GGGACTGAGACACGGCC
>Zotu1041
GAACGCTGGCGGCGTGCCTAATACATGCAAGTCGAGCGAACAGATAAGGAGCTTGCTCCTTTGACGTTAG
CGGCGGACGGGTGAGTAACACGTGGGTAACCTACCTATAAGACTGGGATAACTTCGGGAAACCGGAGCTA
ATACCGGATAACATTTGGAACCGCATGGTTCTAAAGTGAAAGATGGTTTTGCTATCACTTATAGATGGACCC
GCGCCGTATTAGCTAGTTGGTAAGGTAACGGCTTACCAAGGCAACGATACGTAGCCGACCTGAGAGGGTG
ATCGGCCACACTGGAACTGAGACACGGTC
>Zotu1043
GAACGCTGGCGGCGTGCTTAACACATGCAAGTCGAACGATGAACCACTTCGGTGGGGATTAGTGGCGAAC
GGGTGAGTAACACGTGGGCAATCTGCCCTTCACTCTGGGACAAGCCCTGGAAACGGGGTCTAATACCGGA
TACCACTACCGCAGGCATCTGTGGTGGTTGAAAGCTCCGGCGGTGAAGGATGAGCCCGCGGCCTATCAGC
TTGTTGGTGAGGTAATGGCTCACCAAGGCGACGACGGGTAGCCGGCCTGAGAGGGCGACCGGCCACACT
GGGACTGAGACACGGCC
>Zotu1045
GAACGTTAGCGGCGCGCCTAAGACATGCAAGTCGAGCGAGAAAGGGCTTCGGCCCCAGTAAAGCGGCGC
ACGGGTGAGTAACACGTAGGTTATCAACCCCTGAGTGGTGGACAACCCTCCGAAAGGAGGGCTAATACAG
CATAATGCCAGCAATGGCCAAAGCGGGCCTCTTCATGAAAGCTCGCGCTCGGGGATGAGCCTGCGGCCCA
TCAGGTAGTTGGTTGGGGTAACGGCCCACCAAGCCTACGACGGGTAGCTGGTCTGAGAGGATGAACAGCC
ACACTGGAACTGAGACACGGTC
>Zotu1047
GAACGCTGGCGGCATGCCTTACACATGCAAGTCGAACGGCAGCGCGGGAGCAATCCTGGCGGCGAGTGG
CGAACGGGTGAGTAATATATCGGAACGTGCCCATTAGTGGGGGATAGCCCGGCGAAAGCCGGATTAATAC
CGCATACGACCTGAGGGTGAAAGCGGGGGATCGCAAGACCTCGCGCTATTGGAGCGGCCGATATCAGATT
AGCTAGTTGGTGGGGTAAAGGCCTACCAAGGCAACGATCTGTAGTTGGTCTGAGAGGACGACCAGCCACA
CTGGGACTGAGACACGGCC
>Zotu1049
GAACGCTGGCGGCGTGCTTAACACATGCAAGTGGAGCGACGAAGCGGACTTCGGTCCGTGGCAGAGCCG
CGAACGGGTGAGTAACACGTGGGTAACCCACCCCGATGACCGGGACAACCCGAGGAAACTCGGGCTAATA
CCGGATGTTCTCCTTTCTCATAAGGGAATCGAGCAAAGATAGCTTTTAGCCTTCGCATCGGGACGGGCCCG
CGGCCCATTAGCTTGTTGGTGAGGTAACGGCTCACCAAGGCGACGATGGGTAGCTGGTCTGAGAGGACGA
TCAGCCACACTGGGACTGAGACACGGCC
>Zotu1051
GAACGCTAGCGGGAGGCCTAACACATGCAAGCCGAGCGGTATTTGTCTTTCGGGACAGAGAGAGCGGCGT
ACGGGTGCGTAACACGTGTGCAACCTACCTTTATCAGGGGGATAGCCTTTCGAAAGGAAGATTAATACCCC
ATAATATATTGAATGGCATCATTAAATATTGAAAACTCCGGTGGATAAAGATGGGCACGCGCAAGATTAGA
TAGTTGGTGAGGTAACGGCTCACCAAGTCGATGATCTTTAGGGGTCCTGAGAGGGAGATCCCCCACACTG
GTACTGAGACACGGAC
>Zotu1053
GAACGCTGGCGGCGTGCTTAACACATGCAAGTCGAACGATGACCCAGAGCTTGCTCTGGTGATTAGTGGA
GAACGGGTGAGTAACACGTGAGTAACCTGCCCTTGACTCTGGGATAAGCACTGGAAACGGTGTCTAATAC
CGGATACGACAACCGACGGCATCGTCTGGTTGTGGAAAGAATTTTGGTCAAGGATGGACTCGCGGCCTAT
CAGCTAGTTGGTGAGGTAATGGCCCACCAAGGCGACGACGGGTAGCCGGCCTGAGAGGGTGACCGGCCA
CACTGGGACTGAGACACGGCC
>Zotu1056
GAACGCTGGCGGCAGGCTTAATACATGCAAGTCGAGCGGGCACTTCGGTGTCAGCGGCGCACGGGTGCGT
AACGCGTGGGAACATGCCTTTAGGTACGGAACAACCAAGGGAAACTTTGGCTAATACCGTATAATATCTCT

GGATCAAAGATTTATCGCCTAAAGATTGGCCCGCGTTAGATTAGGTAGTTGGTGGGGTAATGGCCTACCAA
GCCGACGATCTATAGGGGGTCTGAGAGGATGATCCCCCACACTGGAACTGAGACACGGTC
>Zotu1058
GAACGCTGGCGGCGTGCCTAATACATGCAAGTCGAGCGGATCTCTTCGGAGATCAGCGGCGGACGGGTGA
GTAACACGTGGGCAACCTGCCTATAAGACTGGGATAACTTCGGGAAACCGGAGCTAATACCGGATAACAT
CAAGGACCACATGGTCTTCGATTAAAAGATGGCGTCAAGCTATCACTTATAGATGGGCCCGCGTCGCATTA
GCTAGTTGGTGAGGTAACGGCTCACCAAGGCGACGATGCGTAGCCGACCTGAGAGGGTGATCGGCCACAC
TGGGACTGAGACACGGCC
>Zotu1060
GAACGCTGGCGGCGTGCCTAATACATGCAAGTCGAGCGAGCTTGCCTAGATGAATTTGGTGCTTGCACCAA
ATGAAACTAGATACAAGCGAGCGGCGGACGGGTGAGTAACACGTGGGTAACCTGCCCAAGAGACTGGGA
TAACACCTGGAAACAGATGCTAATACCGGATAACAACACTAGACGCATGTCTAGAGTTTAAAAGATGGTTC
TGCTATCACTCTTGGATGGACCTGCGGTGCATTAGCTAGTTGGTAAGGTAACGGCTTACCAAGGCAATGAT
GCATAGCCGAGTTGAGAGACTGATCGGCCACATTGGGACTGAGACACGGCC
>Zotu1062
GAACGCTGGCGGCATGCCTAATACATGCAAGTCGAACGCTGAAGCACTCTGCTTGCAGAGTGCGGATGAG
TGGCGAACGGGTGAGTAACACGTGGGAAATCTGCCTAAAAGTGGGGGATAACATTCGGAAACGGATGCT
AATACCGCATAGATTCTTGAACCGCATGGTTCTTGAAAGAAAGACGGCTTTGCTGTCGCTTTTAGATGATCC
CGCGGCGTATTAGTTAGTTGGTGGGGTAAAGGCCTACCAAGACCATGATACGTAGCCGACCTGAGAGGGT
GATCGGCCACACTGGGACTGAGACACGGCC
>Zotu1066
GAACGCTGGCGGCAGGCTTAACACATGCAAGTCGAGCGGGGTGATGTAGCTTGCTACCTGACCTAGCGGC
GGACGGGTGAGTAATGCTTAGGAATCTGCCTATTAGTGGGGGACAACATTCCGAAAGGAATGCTAATACC
GCATACGCCCTACGGGGGAAAGCAGGGGATCTTCGGACCTTGCGCTAATAGATGAGCCTAAGTCAGATTA
GCTAGTTGGTGGGGTAAAGGCCTACCAAGGCGACGATCTGTAGCGGGTCTGAGAGGATGATCCGCCACAC
TGGGACTGAGACACGGCC
>Zotu1068
GAACGCTGGCGGCAGGCCTAACACATGCAAGTCGAACGGTAGCACAGAGGAGCTTGCTCCTTGGGTGACG
AGTGGCGGACGGGTGAGTAATGTCTGGGAAACTGCCCGATGGAGGGGGATAACTACTGGAAACGGTAGC
TAATACCGCATAACGTCGCAAGACCAAAGAGGGGGACCTTCGGGCCTCTTGCCATCGGATGTGCCCAGAT
GGGATTAGCTAGTAGGCGGGGTAACGGCTCACCTAGGCGACGATCCCTAGCTGGTCTGAGAGGATGACCA
GCCACACTGGAACTGAGACACGGTC
>Zotu1070
GAACGCTGGCGGCGCGCCTAACACATGCAAGTCGAGCGATGAAAGTTGTTCAGATCCTTCGGGTGACGAA
CTTCTGGATTAGCGGCGAACGGGTGAGTAACGCGTGAGGAACCTGCCTCTCACAACGGGATAGCCTCGGG
AAACCGGGATTAATACCGTATGACACTATCAGATGGCATCATCAGATAGTTAAAGTGCTAGCGGTGAGAGA
TGGCCTCGCGTCTGATTAGTTAGTTGGTGGGGTAACGGCCTACCAAGACGACGATCAGTAACCGGCCTGA
GAGGGTGAACGGTCACATTGGAACTGAGACACGGTC
>Zotu1072
GAACGCTAGCGGGATGCTTTACACATGCAAGTCGAACGGCAGCGCGAGAGAGCTTGCTCTCTTGGCGGCG
AGTGGCGGACGGGTGAGTAATATATCGGAACGTGCCCAGTAGCGGGGGATAACTACTCGAAAGAGTGGC
TAATACCGCATACGCCCTACGGGGGAAAGGGGGGGATCGCAAGACCTCTCACTATTGGAGCGGCCGATAT
CGGATTAGCTAGTTGGTGGGGTAAAGGCTCACCAAGGCAACGATCCGTAGCTGGTTTGAGAGGACGACCA
GCCACACTGGGACTGAGACACGGCC
>Zotu1075
GAACGCTGGCGGCATGCCTTACACATGCAAGTCGAACGGCAGCACGGGAGCAATCCTGGTGGCGAGTGG
CGAACGGGTGAGTAATGTATCGGAACGTGCCCAGTCGTGGGGGATAACGTAGCGAAAGCTACGCTAATAC
CGCATACGATCTATGGATGAAAGCGGGGGACCGTAAGGCCTCGCGCGATTGGAGCGGCCGATATCAGATT
AGGTAGTTGGTGGGGTAAAGGCCCACCAAGCCGACGATCTGTAGCTGGTCTGAGAGGACGACCAGCCACA
CTGGGACTGAGACACGGCC

>Zotu1077
GAACGCTGGCGGTATGGATGAGGCATGCAAGTCGAATGCATTTAGTTGCATGGCAGACGAGGTAGTAACA
CGTAGGAACGTACCCAGAAGTCGGGCATAACCTGTCGAAAGGCAGAATAATTCTCGATAGTCTCGCAAGA
GTAAAGTTTTTCGCTTCTGGAGCGGCCTGCGCAATATCAGCTAGTTGGTGAGGTAATGGCTCACCAAGGCT
ATGACGTTTAGGGGAGCTGAGAGGCTGACCCCCACCGATGGGACTGAGACACGGCC
>Zotu1080
GAACGCTGGCGGCGCGCTTAACACATGCAAGTCGAGCGAGAACCCTCCTTCGGGAGGGGGAAAGCGGCG
AACGGGTGAGTAACACGTGAGTAATCCACCCTCGGTACCGGGATAGCCCGGGGAAACCCGGATTAATACC
GGATGGCCCAGCAGCCCTGCGGGGCGGCTGGAAAAGGTAGCTTCGGCCTCCGACCGAGGACGAGCTCGC
GGCGGATTAGCTTGTTGGTGGGGTAACGGCCTACCAAGGCGACGATCCGTAGCTGGTCTGAGAGGACGAT
CAGCCACACTGGGACTGAGACACGGCC
>Zotu1082
GAACGCTGGCGGCATGCCTAATACATGCAAGTCGAGCGAACAGACGAGGTGCTTGCACCTCTGACGTTAG
CGGCGGATGGGTGAGTAACACGTGGGCAACCTGCCCTGCAGATGGGGATAACTCCGGGAAACCGGGGCT
AATACCGAATAAACCATTGAACCTCCTGGTTCGATGTTGAAAGACGGTTTCGGCTGTCACTGCAGGATGGG
CCCGCGGCGCATTAGCTAGTTGGTGGGATAACGGCCTACCAAGGCAACGATGCGTAGCCGACCTGAGAGG
GTGATCGGCCACACTGGGACTGAGACACGGCC
>Zotu1084
GAACGCTGGCGGCGTGCTTAACACATGCAAGTCGAACGGTGAAGGGACCAGCTTGCTGGTTCTGGATGAG
TGGCGAACGGGTGAGTAACACGTGAGTAACCTGCCCCCTTCTTCTGGATAACCGCATGAAAGTGTGGCTAA
TACGGGATATTCTGCGCCTGCTGCATGGTGGGTGTGGGAAAGATATTGTTTCGGTGGGGGATGGGCTCGC
GGCCTATCAGCTTGTTGGTGGGGTGATGGCCTACCAAGGCGGTGACGGGTAGCCGGCCTGAGAGGGTGG
ACGGTCACACTGGGACTGAGACACGGCC
>Zotu1086
CAACGCTGGCGGCGTGCCTAATGCATGCAAGTTGAGCGGGGTCGTTCTTCGGAGGGGCTTACGCGGCGGA
CGGGTGCGTAACACGTGGGGAACCTGCCCTGGGGTGGGGGATAGCCGAGGGAAACCTTGGGTAATTCCG
CATACGCTTGCGTACCGGAAGGGTACGTGAGGAAAGGGTCTTTCGGGACTCGCCTTGGGAGGGGCCTGCG
GCCGATTAGCTAGACGGTGGGGTAACGGCTCACCGTGGCGATGATCGGTAGCTGGTCTGAGAGGACGGC
CAGCCACACGGGGACTGAGAGACGGCC
>Zotu1089
GAACGCTGGAAATGTGGATACCGGCATGCAAGTCAAGGGGCCGCAAGGCACCGGCGAACGGCTTAGTAA
CACGTAGATACATACCGCAAAGTGGAGCATAGCTCGTCGAAAGACGGGGTAATTTTCCATAGTCCCGCAAG
GGTAAAGGCGCAAGCCGCTTTGTGAATGGTCTGCGGTCTATCAGCTAGTTGGTGAGGTAATGGCTCACCAA
GGCTATGACGGATAGGGGGCGTGAGAGCGCGACCCCCAAGAATGGCACTGCGACACGGGC
>Zotu1091
GAACGCTGGCGGCATGCCTTACACATGCAAGTCGAACGGCAGCACGGACTTCGGTCTGGTGGCGAGTGGC
GAACGGGTGAGTAATACATCGGAACGTGCCCTGTTGTGGGGGATAACTAGTCGAAAGATTAGCTAATACC
GCATACGACCTGAGGGTGAAAGCGGGGGACCGCAAGGCCTCGCGCAATAGGAGCGGCCGATGTCTGATT
AGCTAGTTGGTGGGGTAAAGGCCTACCAAGGCGACGATCAGTAGCTGGTCTGAGAGGACGATCAGCCACA
CTGGGACTGAGACACGGCC
>Zotu1093
GAACGCTGGCGGCGTGCTTAACACATGCAAGTCGAACGAAGAGCGATGGAAGCTTGCTTCTATCAATCTTA
GTGGCGAACGGGTGAGTAACGCGTAATCAACCTGCCCTTCAGAGGGGGACAACAGTTGGAAACGACTGCT
AATACCGCATACGATCTAACCTCGGCATCGAGGATAGATGAAAGGTGGCCTCTATTTATAAGCTATCACTG
AAGGAGGGGATTGCGTCTGATTAGCTAGTTGGAGGGGTAACGGCCCACCAAGGCGATGATCAGTAGCCG
GTCTGAGAGGATGAACGGCCACATTGGGACTGAGACACGGCC
>Zotu1095
GAACGCTGGCGGCATGCCTAATACATGCAAGTCGAGCGAACAGACGAAGTGCTTGCACTTCTGACGTTAG
CGGCGAACGGGTGAGTAACACGTAAGGAATCTACCTATAAGCGGGGGATAACATTCGGAAACGGGTGCT
AATACCGCATAATATCTTCTTCCGCATGGAAGAAGATTGAAAGACGGCTCTGCTGTCACTTATAGATGACCT

TGCGGTGCATTAGTTAGTTGGTGGGGTAATGGCCTACCAAGACGATGATGCATAGCCGACCTGAGAGGGT
GATCGGCCACATTGGGACTGAGACACGGCC
>Zotu1097
GAACGCTAGCGGCAGGCTTAACACATGCAAGTCGAGGGGCAGCACGATGTAGCAATACATGGGTGGCGA
CCGGCGCACGGGTGCGTAACACGTATGCAATCTTCCCTTCACTGGGGCATAGCCCGGAGAAATCCGGATTA
ATACCCCTTAATAGCTCGAGTGGCATCACTTGGGTTTGAAAGTTCCGACGGTGAAGGATGAGCATGCGCCC
GATTAGCTAGTTGGTGAGGTAATGGCTCACCAAGGCTACGATCGGTAGGGGGCCTGAGAGGGTTATCCCC
CACACTGGTACTGAGACACGGAC
>Zotu1099
GAACGCTGGCGGCGTGCCTAAGACATGCAAGTCGAGTGGGTTTAGACCCACGGCGAACGGGTGAGTAAT
ACCTAGGAACTTTCTTTAAGTAGAGGATAACTCCGCGAAAGCGGAGATAATACTCTATGTGATAGTAATAT
CAAAGTAGCAATACGCTTAAAGTGAGGCCTGGGCACCATCAGCTAGTTGGTAAGGTAACGGCTTACCAAG
GCTATGACGGTTAGGGGTCCCGAGAGGGATAACCCCCACACTGGAACTGCGACACGGTC
>Zotu1101
GAACGCTGGCGGCGTGCTTAACACATGCAAGTCGAACGGTGAAGGGACTGGTTTTTGCTGGTTCTGGATG
AGTGGCGAACGGGTGAGTAACACGTGAGTAACCTGCCCCCTTCTTCTGGATAACCGCATGAAAGTGTGGCT
AATACGGGATATTCTGCGCCTGCTGCATGGTGGGTGTGGGAAAGATATTGTTTCGGTGGGGGATGGGCTC
GCGGCCTATCAGCTTGTTGGTGGGGTGATGGCCTACCAAGGCGGTGACGGGTAGCCGGCCTGAGAGGGT
GGACGGTCACACTGGGACTGAGACACGGCC
>Zotu1103
GAACGCTAGCGGCAGGCTTAATACATGCAAGTCGAACGGTAACAGGTGTAGCAATACGCGCTGACGAGTG
GCAGACGGGTGAGGAACACGTACGCAATCTCCCTCTTTCTGGTGAATAGCCCTGAGAAATTAGGATTAATA
CACCATAGTATTACGAAACGGCATCGTTTTGTAATTAAAACTCCGGTGGAAAGAGATGAGCGTGCGACTGA
TTAGCTAGTTGGTAAGGTAACGGCTTACCAAGGCAACGATCAGTAACTGGTGTGAGAGCACGACCAGTCA
CACGGGCACTGAGATACGGGC
>Zotu1105
GAACGCTGGCGGCATGCCTTACACATGCAAGTCGAACGGCAGCACGGGTGCTTGCACCTGGTGGCGAGTG
GCGAACGGGTGAGTAATACATCGGAACATGTCCTGTAGTGGGGGATAGCCCGGCGAAAGCCGGATTAATA
CCGCATACGATCTACGGATGAAAGCGGGGGACCTTCGGGCCTCGCGCTATAGGGTTGGCCGATGGCTGAT
TAGCTAGTTGGTGGGGTAAAGGCCTACCAAGGCGACGATCAGTAGCTGGTCTGAGAGGACGACCAGCCAC
ACTGGGACTGAGACACGGCC
>Zotu1107
AAACGCTGGCGGCGTGCCTAATGCATGCAAGTCGAACGGGAGCCCCTTTGGGGGTCGACCGTGGCGGAC
GGGTGAGGAACACGTAGCTAACCTGCCCAGGTATGGGGGATATGCGCTGGAAACGGCGTGCAATACCGC
ATACGTTCATCGGTCGGGAGACGGATGAGGAAAGCGCTTCGGCGTGTACCTGGAGGGGGCTGCGGCCGA
TTAGCTAGTTGGAGTGGTAAGAGCACCCCAAGGCGATGATCGGTAGCTGGTCTGAGAGGACGATCAGCCA
CACGGGGACTGAGACACGGCC
>Zotu1109
GAACGCTGGCGGCGTGCCTAATACATGCAAGTAGAACGCACAGTTTATACCGTAGCTTGCTACACCATAGA
CTGTGAGTTGCGAACGGGTGAGTAACGCGTAGGTAACCTGCCTGGTAGCGGGGGATAACTATTGGAAACG
ATAGCTAATACCGCATAATATTAATTATTGCATGATAATTAATTGAAAGGTGCAATTGCACCACTACCAGAT
GGACCTGCGTTGTATTAGCTAGTAGGTGAGGTAACGGCTCACCTAGGCGACGATACATAGCCGACCTGAG
AGGGTGATCGGCCACACTGGGACTGAGACACGGCC
>Zotu1112
GAACGCTAGCGGCAGGCTTAACACATGCAAGTCGAGGGGCAGCAGGGACTTCGGTCCGCTGGCGACCGG
CAAACGGGTGCGGAACACGTACGCAACATACCTCTATCTGGGGCATAGCCCGGTGAAAACCGGATTAACTC
CCCATAGTATTTTTTGGTGGCATCACCAGTTAATTAAAAATTCATTGGATAGAGATTGGCGTGCGTCCTATT
AGCTAGTTGGCGGGGTAAAGGCCCACCAAGGCGACGATAGGTAGCTGATGTGAGAGCATGATCAGCCAC
ACGGGCACTGAGACACGGGC
>Zotu1114

GAACGCTGGCGGCAGGCTTAACACATGCAAGTCGAGCGCCCCGCAAGGGGAGCGGCAGACGGGTGAGTA
ACGCGTGGGAATCTACCCTCTACTACGGAATAACGCAGGGAAACTTGTGCTAATACCGTATGTGTCCTTTTT
GGAGAAAGATTTATCGGTAGGGGATGAGCCCGCGTTGGATTAGCTAGTTGGTGGGGTAAAGGCCTACCAA
GGCGACGATCCATAGCTGGTCTGAGAGGATGATCAGCCACATTGGGACTGAGACACGGCC
>Zotu1118
GAACGCTAGCGGCAGGCCTAACACATGCAAGTCGAGCGGGATTCGGTGGCTTGCCACCGATGAGAGCGG
CGCACGGGTGCGTAACGCGTATACAACCTACCTTTTGCTACGGGATAGCCCAGAGAAATTTGGATTAACAC
CGTATGGTATAAGGACCCGGCATCGGGATCTTATTAAAGGCTACGGCAAAAGATGGGTATGCGTCCTATTA
GCTAGATGGTGCGGTAACGGCGCACCATGGCAACGATAGGTAGGGGCCCTGAGAGGGGGATCCCCCACA
CTGGTACTGAGACACGGAC
>Zotu1120
GAACGTTGGCGGCGTGGATTAGGCATGCAAGTCGTACGCACCGCAAGGTGAGTGGCGCAAGGGTGAGTA
AGGCGAAGGTAACCAACCCCGAGATTGGGTATAGCCGTGGGAAACCGCGGGTAATCCCCAGCGATGTTGC
TGAGTGGCATCACTCGACAACCAAAGCTCCGGCGTCTTGGGACGGGCCTTCGTGGTATTAGGTTGTTGGCG
GGGTAATGGCCCACCAAGCCTCTGATGCCTACCGGGCGTGCGAGCGTGGCCCGGCACACTGGGACTGAGA
CACTGCC
>Zotu1122
GAACGCTGGCGGCGTGCTTAACACATGCAAGTCGAACGATGAAGCTCCAGCTTGCTGGGGTGGATTAGTG
GCGAACGGGTGAGTAACACGTGAGTAACCTGCCCTTCACTCTGGGATAAGCCTTGGAAACGAGGTCTAAT
ACTGGATATTCATACATGGTCGCATGGTTGTGTGTGGAAAGATTTTTTGGTGGGGGATGGACTCGCGGCCT
ATCAGCTTGTTGGTGAGGTAGTGGCTCACCAAGGCTTTGACGGGTAGCCGGCCTGAGAGGGTGACCGGCC
ACACTGGGACTGAGACACGGCC
>Zotu1124
TAACGTTGGCAGTGTGCCTAACACATGCAAGTCGAGCGGCTCGCAAGAGCAGCGGCAGACGAGAGAGTA
ATAACAAGGAACTTACCCCCGAGTGAAGGATAGCCATCCGAAAGGATGGGTAATCCTGCATAATATCGAA
AGATCAAAGCCTTCGGGCGCTTGGGGAGAGGCCTTGTCCCTATCAGCTAGTTGGTGAGGTAATGGCTCACC
AAGGCGATGACGGGTAGCTGGACTGAGAGGTTGACCAGCCTCGATGGGACTGAGACACGGCC
>Zotu1126
GAACGCTGGCGGCATGCCTTACACATGCAAGTCGAACGGTAACAGGTCTTCGGACGCTGACGAGTGGCGA
ACGGGTGAGTAATACATCGGAACGTGCCCGATCGTGGGGGATAACGAGGCGAAAGCTTTGCTAATACCGC
ATACGATCTACGGATGAAAGCGGGGGATCTTCGGACCTCGCGCGGACGGAGCGGCCGATGGCAGATTAG
GTAGTTGGTGGGATAAAAGCTTACCAAGCCGACGATCTGTAGCTGGTCTGAGAGGATGATCAGCCACACT
GGGACTGAGACACGGCC
>Zotu1128
GAACGCTGGCGGCGTGCTTAACACATGCAAGTCGAACGGTGAAGCGGGTGCTTGCACTCGTGGATCAGTG
GCGAACGGGTGAGTAACACGTGAGTAACCTGCCCATCACTCTGGGATAAGCGCTGGAAACGGCGTCTAAT
ACTGGATACGACCAACCCTTGCATGGGGTGTTGGTGGAAAGATTTATCGGTGGTGGATGGACTCGCGGCC
TATCAGCTTGTTGGTGGGGTAATGGCCTACCAAGGCGACGACGGGTAGCCGGCCTGAGAGGGCGACCGG
CCACACTGGGACTGAGACACGGCC
>Zotu1130
GAACGCTAGCGGCAGGCTTAACACATGCAAGTCGAGGGGTAAGGTAGCAATATCTGAGACCGGCGCACG
GGTGCGTAACGCGTATGCAATCTACCTTTTGCAGAGGGATAGCCCAGAGAAATTTGGATTAATACCTCATA
GTATATAGAGTTGGCATCAACACTATATTAAAGTCACAACGGCAAAAGATGAGCATGCGTCCCATTAGTTA
GTTGGTAAGGTAACGGCTTACCAAGACTACGATGGGTAGGGGTCCTGAGAGGGAGATCCCCCACACTGGT
ACTGAGACACGGAC
>Zotu1132
GAACGCTGGCGGCATGCCTAATACATGCAAGTCGAACGCACGAAGTTGAAAAGCTTGCTTTTCGACCAAGT
GAGTGGCGGACGGGTGAGTAACACGTGGGTAACCTGCCCATAAGAGGGGGATAACATTCGGAAACGGAT
GCTAATACCGCATATTTCAAGTGACCGCATGGTCGCTTGATGAAAGGTGGCTTCGGCTACCACTTATGGAT
GGACCCGCGGTGCATTAGTTAGTTGGTGAGGTAATGGCTCACCAAGACCATGATGCATAGCCGACCTGAG
AGGGTGATCGGCCACACTGGGACTGAGACACGGCC

>Zotu1135
GAACGCTGGCGGCGTGCCTAATACATGCAAGTCGAGCGAACCACTTCGGTGGGAAGCGGCGAACGGGTG
AGTAACACGTAGGTAATCTGCCCATCAGACGGGGACAACGATTGGAAACGATCGCTAATACCGGATAGGA
CGAAAGTTTAAAGATGCTCCTGGCATCACTGATGGATGAGCCTGCGGCGCATTAGCTAGTTGGTGGGGTA
AAGGCCTACCAAGGCGACGATGCGTAGCCGACCTGAGAGGGTGAACGGCCACACTGGGACTGAGACACG
GCC
>Zotu1137
GAACGCTGGCGGCGTGCTTAACACATGCAAGTCGAGCGGAAAGGCCTCTTCGGGGGTACTCGAGCGGCG
AACGGGTGAGTAACACGTGAGTAACCTGCCCCAGTCTTTGGGATAACCCTCGGAAACGGGGGCTAATACC
GGATATTACTGCCGGCCGCATGGTTGGTGGTGGAAAGTTTTTTCGGTCTGGGATGGGCTCGCGGCCTATCA
GCTTGTTGGTGGGGTGATGGCCTACCAAGGCGACGACGGGTAGCCGGCCTGAGAGGGCGACCGGCCACA
CTGGGACTGAGACACGGCC
>Zotu1139
GAACGCTGGCGGCGTGCCTAACACATTCAAGTCGAACGGAGTTAAGTTGAAAGCTTGCTTTTGACTTAGCT
TAGTGGCGGACGGGTGAGTAATGCATGAGCAACCTGCCTTTAAGAGGGGAATAACACAGAGAAATTTGTG
CTAATACCGCATAGAACACTGAGAGGGCATCCTCATAGTGGGAAAGAAGCAATTCGCTTAAAGATGGGCT
CATGTCTGATTAGATAGTTGGTGAGGTAACGGCTCACCAAGTCTGCGATCAGTAGCCGGACTGAGAGGTT
GAACGGCCACATTGGAACTGAGATACGGTC
>Zotu1141
GAACGCTGGCGGCATGCCTAACACATGCAAGTCGAACGAGACCTTCGGGTCTAGTGGCGCACGGGTGCGT
AACGCGTGGGAATCTACCCTTGGGTTCGGAATAACAGTTAGAAATGACTGCTAATACCGGATGATGACGTA
AGTCCAAAGATTTATCGCCCAGGGATGAGCCCGCGTAGGATTAGGTAGTTGGTGGGGTAAAGGCTCACCA
AGCCGACGATCCTTAGCTGGTCTGAGAGGATGATCAGCCACACTGGGACTGAGACACGGCC
>Zotu1143
GAACGCTGGCGGCATGCTTTACACATGCAAGTCGAACGGCAGCGAAGAGAAGCTTGCTTCTCTGTCGGCG
AGTGGCGAACGGGTGAGTATAGCATCGGAACGTGCCAAGTAGTGGGGGATAACCAAGCGAAAGTTTGGC
TAATACCGCGTAAGCTCTAAGGAGGAAAGTAGGGGACCGCAAGGCCTTACGCTATTTGATCGGCCGATGT
CGGATTAGCTAGTTGGTGAGGTAATGGCTCACCAAGGCGATGATCCGTAGCGGGTCTGAGAGGACGATCC
GCCACACTGGGACTGAGACACGGCC
>Zotu1145
GAACGCTGGCGGCGTGCCTAATACATGCAAGTCGAACGCGCGGATCAGGAGCTTGCTCCTGTGACGCGAG
TGGCGGACGGGTGAGTAACACGTAGGCAACCTGCCCATCAGACCGGGATAACCACGGGAAACCGTGGCT
AATACCGGATAATCCTTTTCCACACAGGTGGAAAAGTTGAAAGGCGGTCTTTTGGCTGTCACTGATGGATG
GGCCTGCGGCGCATTAGCTGGTTGGTGGGGTAACGGCCCACCAAGGCGACGATGCGTAGCCGACCTGAG
AGGGTGATCGGCCACACTGGGACTGAGACACGGCC
>Zotu1147
GAACGCTGGCGGCGCGCTTAATACATGCAAGTCGAGCGAGAACCATGGCTTCGGCCATGGGGACAGCGG
CGAACGGGTGAGTAACACGTGGGTAATCAACCCTCGACACTGGGATAGCCCGGGGAAACCCGGATTAATA
CCGGATGTCCCATCTGGCCTTCGGGCCGGCTGGAAAAGGTAGCTTCGGCCTCCGGTCGGGGACGAGCCCG
CGGCGGATTAGCTTGTTGGTGGGGTAACGGCCCACCAAGGCGACGATCCGTAGCTGGTCTGAGAGGACGA
TCAGCCACACTGGGACTGAGACACGGCC
>Zotu1150
GAACGCTGGCGGCGTGCCTAATACATGCAAGTCGAACGAACCGCGACTAGGTGCTTGCACTTGGTCAAGG
TGAGTGGCGAACGGGTGAGTAACACGTGGGTAACCTACCTCATAGTGGGGGATAACAGTCGGAAACGACT
GCTAATACCGCATAGGACATGGAATCACATGATTCAGTGAGGAAAGGTGGCGCAAGCTATCGCTAAGAGA
TGGACCCGCGGTGCATTAGCTAGTTGGTAGGGTAACGGCCTACCAAGGCGATGATGCATAGCCGACCTGA
GAGGGTGATCGGCCACATTGGGACTGAGACACGGCC
>Zotu1153
GAACGCTGGCGACGCGTCTTAAGCATGCAAGTCGAACGGCAACACGGGGCAACCTGGTGGCGAGTGGCG
AACGGGTGAGTAACACGTAGGTGATCTGCCTTTGGGATGGGGATAGCCGTTAGAAATAGCGGGTAATACC

GAATGAAGTCATTCGACTATGGTTGGATGATGAAAGGGGCTAAGGCCTCGCCTGGAGATGAGCCTGCGGC
TGATTAGCTAGTTGGTGGGGTAAGAGCCTACCAAGGCGACGATCAGTAGCCGGCCTGAGAGGGTGGCCG
GCCACACTGGGACTGAGATACGGCC
>Zotu1155
GAACGCTGGCGGCGTGCTTAACACATGCAAGTCGAACGATGAAGACCGGTGCTTGCACTGGTTGGATTAG
TGGCGAACGGGTGAGTAATACGTGAGTAACCTGCCTTTGACTCTGGGATAAGCCTGGGAAACTGGGTCTA
ATACCGGATACGACACGAAACCGCATGGTTTGGTGTGGAAAGGGTTTGTACTGGTTTTAGATGGGCTCACG
GCCTATCAGCTTGTTGGTGAGGTAACGGCTCACCAAGGCGACGACGGGTAGCCGGCCTGAGAGGGTGACC
GGCCACACTGGGACTGAGACACGGCC
>Zotu1157
GAACGCTGGCGGCGTGCTTAACACATGCAAGTCGAACGATGAAGCCTAGCTTGCTAGGTGGATTAGTGGC
GAACGGGTGAGTAATACGTGAGTAACCTACCTTTAACTCTGGGATAAGCCCGGGAAACTGGGTCTAATACC
GGATACGACCAATCTCCGCATGGGGTGTTGGTGGAAAGCGTTATGTAGTGGTTATAGATGGGCTCACGGC
CTATCAGCTTGTTGGTGAGGTAACGGCTCACCAAGGCGACGACGGGTAGCCGGCCTGAGAGGGTGACCG
GCCACACTGGGACTGAGACACGGCC
>Zotu1159
GAACGCTAGCGGCAGGCCTAATACATGCAAGTCGAGGGGTATAGGGAGCTTGCTCCCTTGAGACCGGCGC
ACGGGTGCGTAACGCGTATGCAACCTACCCAAACCTGGGGGATAGCCCGGAGAAATCCGGATTAATACCG
CATAAAATCACAGCAGGGCATCCTGCAATGATCAAACATTCATGGGGTTTGGATGGGCATGCGTGCCATTA
GTTAGTTGGCGGGGTAACGGCCCACCAAGACTTCGATGGCTAGGGGATCTGAGAGGATGGCCCCCCACAC
TGGTACTGAGACACGGAC
>Zotu1161
GAACGCTGGCGGCGCGCCTAACACATGCAAGTCGAACGGAGCTTAGAGAGCTTGCTTTTTAAGCTTAGTG
GCGAACGGGTGAGTAACGCGTGAGTAACCTGCCCTAGAGTGGGGGACAACAGTTGGAAACGACTGCTAA
TACCGCATAAGCCCACGGTACCGCATGGTACTGAGGGAAAAGGATTTATTCGCTTTAGGATGGACTCGCGT
CCAATTAGCTAGTTGGTGAGGTAACGGCCCACCAAGGCGACGATTGGTAGCCGGACTGAGAGGTTGAACG
GCCACATTGGGACTGAGACACGGCC
>Zotu1163
GAACGCTGGCGGCGTGCTTAACACATGCAAGTGGTGCGACGAACCAGGGCTTGCCCTGGGGCCAAGCCGC
GAACGGGTGAGTAACACGTGAGTAACCTGCCCCGATGACCGGGACAACCCGAGGAAACTCGGGCTAATAC
CGGATGTGGTGCACGGATTTAAGTCTGTGTACTAAAGGAAGCTTCGGCCTCCGCATTGGGAGGGGCTCGC
GGCCCATTAGCTTGTTGGTGGGGTAACGGCCTACCAAGGCAACGATGGGTAGCTGGTCTGAGAGGACGAT
CAGCCACACTGGGACTGAGACACGGCC
>Zotu1165
GAACGCTGGCGGCGTGCTTAACACATGCAAGTCGAGCGGGGTCCATCCAGTGGCAACACTGGGGAAGACC
TAGCGGCGAACGGGTGAGTAACACGTGAGAAACCTGCCCCGAAGTCTGGGATAACCCGAGGAAACTCGA
GCTAATACCGGATACCTTCCACTGATCGCATGGTCGGTGGAAGAAAGAATTTCGCTTCGGGAGGGTCTCGC
GGCCTATCAGCTTGTTGGCGGGGTAACGGCCCACCAAGGCGTCGACGGGTAGCTGGTCTGAGAGGACGAT
CAGCCACACTGGGACTGAGACACGGCC
>Zotu1168
GAATGCTGGCGGCGCGCCTAACACATGCAAGTCGAGCGGTAACAGGAGTAATTTATTACTTGCTGACGAG
CGGCGGACGGCTGAGTAACGCGTGGGAACATGCCCCAAAGTGAGGAATAACTACTCGAAAGAGTAGCTA
ATGCCGCATATGATCTTCGGATTAAAGGATTTATCCGCTTTGGGAGTGGCCCGCGTACGATTAGGTAGTTG
GTGAGGTAATGGCTCACCAAGCCTACGATCGTTAACTGGTCTGAGAGGATGATCAGTCAGACTGGAACTG
AGACACGGTC
>Zotu1170
GAACGCTGGCGGCAGGCTTAACACATGCAAGTCGAGCGGAGAGAGGTAGCTTGCTACTGATCTTAGCGGC
GGACGGGTGAGTAATGCTTAGGAATCTGCCTATTAGTGGGGGACAACATTTCGAAAGGAATGCTAATACC
GCATACGTCCTACGGGAGAAAGCAGGGGATCTTCGGACCTTGCGCTAATAGATGAGCCTAAGTCGGATTA
GCTAGTTGGTGGGGTAAAGGCCTACCAAGGCGACGATCTGTAGCGGGTCTGAGAGGATGATCCGCCACAC
TGGGACTGAGACACGGCC

>Zotu1172
GAACGCTGGCGGCGTGCCTAATACATGCAAGTCGAGCGAACAGATAAGGAGCTTGCTCCTTTGACGTTAG
CGGCGGACGGGTGAGTAACACGTGGGTAACTTACCTATAAGACTGGAATAACTCCGGGAAACCGGGGCTA
ATGCCGGATAACATTTAGAACCGCATGGTTCTAAAGTGAAAGATGGTTTTGCTATCACTTATAGATGGACCC
GCGCCGTATTAGCTAGTTGGTAAGGTAACGGCTTACCAAGGCAACGATACGTAGCCGACCTGAGAGGGTG
ATCGGCCACACTGGAACTGAGACACGGTC
>Zotu1174
GAACGCTGGCGGCGTGCCTAATACATGCAAGTCGAACGAACGGATAAAGAGCTTGCTCTTTTGAAGTTAGT
GGCGGACGGGTGAGTAACACGTGGGTAACCTACCTCACAGCTGGGGATAACATCGAGAAATCGATGCTAA
TACCGAATGTGCTGAACATCATAAGATGTTCAAGTGAAAGACGGTTTCGGCTGTCACTGTGAGATGGACCC
GCGCTGGATTAGCTAGTTGGTAAGGTAATGGCTTACCAAGGCGACGATCCATAGCCGACCTGAGAGGGTG
ATCGGCCACATTGGGACTGAGACACGGCC
>Zotu1176
GAACGCTGGCGGCGTGCTTAACACATGCAAGTCGAACGGAAAGGCCCTTCGGGGTACTCGAGTGGCGAGC
GGGTGAGTAACACGTGGGTGATCTGCCCTGCACTTTGGGATAAGCCTGGGAAACTGGGTCTAATACCGAA
TATGATCACGCTTCTCATGGGGTGTGGTGGAAAGCTTTTGCGGTGTGGGATGGGCCCGCGGCCTATCAGCT
TGTTGGTGGGGTAATGGCCTACCAAGGCGACGACGGGTAGCCGGCCTGAGAGGGTGTCCGGCCACACTG
GGACTGAGATACGGCC
>Zotu1178
GAACGCTGGCGGCGTGCCTAATACATGCAAGTCGAACGGAGCAGCAATGCTTAGTGGCGACCGGGTGAGT
AACACGTAGTTGACCTGCCCCTCAGCGGGGGACAACAGTTCGAAAGGACTGCTAATACCGCATACATTGGT
CGAGATTAGAAGTCGATCAAGAAACTTACGGGGTTGAGGGAGGGGACTGCGGCCCATCAGGTAGTTGGT
GAGGTAGAAGCTCACCAAGCCGACGACGGGTAGGGGGCCTGAGAGGGTGACCCCCCACACTGGAACTGA
CATACGGTC
>Zotu1180
GAACGTTGGCGGCATGCCTAACACATGCAAGTCGAACGCGGACTTCGGTCCGAGTGGCGAACGGGTGCGT
AACACGTAGGTGACCTGCCCACCGGTGGGGGATAACCGCGAGAAATCGTGGCTAATACCGCATGCGATCC
TGGTCTTTCGAAAGCCAGGAGGAAAGCTTCATGTGCCGGTGGAGGGGCCTGCGGCCTATCAGCTAGTTGG
TGAGGTAACGGCTCACCAAGGCGATGACGGGTAGCTGACCTGAGAGGGTGGTCAGCCACAGGGGAACTG
AGAGACGGTC
>Zotu1182
GAACGCTGGCGGCGTGCTTAACACATGCAAGTGGAGCGACGAACCAGGGCTTGCCCTGGGGCAAAGCCG
CGAACGGGTGAGTAACACGTGGGTAACCTGCCCCGACGACTGGGACAACCCGAGGAAACTCGGGCTAATA
CCGGATAATTTCACCACTCATAAGTGTGGTGATTAAAGGAGGCTTCGGCTTCCGCATCGGGAGGGGCCCGC
GGCCCATTAGCTTGTTGGTGAGGTAACGGCTCACCAAGGCGACGATGGGTAGCTGGTCTGAGAGGACGAT
CAGCCACACTGGGACTGAGACACGGCC
>Zotu1184
GAACGCTGGCGGCGTGCCTAATACATGCAAGTAGAACGCTGAAGAGAGGAGCTTGCTCTTCTTGGATGAG
TTGCGAACGGGTGAGTAACGCGTAGGTAACCTGCCTGGTAGCGGGGGATAACTATTGGAAACGATAGCTA
ATACCGCATGATATTAATTGTCGCATGATAATTAATTGAAAGATGCAATTGCATCACTACCAGATGGACCTG
CGTTGTATTAGCTAGTTGGTGAGGTAACGGCTCACCAAGGCGACGATACATAGCCGACCTGAGAGGGTGA
TCGGCCACACTGGGACTGAGACACGGCC
>Zotu1186
GAACGCTGGCGGCGTGCTTAACACATGCAAGTCGAACGGGCCCGCTCCTTTTTTTGGGGGCGGGTTGAGT
GGCGAACGGGTGAGTATCACGTGAGCAACCTGCCCTCCTCTCCTGGATAACCGCTTGAAAGGGCGGCTAAT
ACGGGGTGCTCCGGTTGGTCCGCATGGGCTGGTCGGGAAAGATTCATCGTTGTGGTGTTTCGGTGGGGGA
TGGGCTCGCGGCCTATCAGCTTGTTGGTGGGGTGATGGCCCACCAAGGCGGTGACGGGTAGCCGGCCTGA
GAGGGTGGGCGGCCACACTGGGACTGAGACACGGCC
>Zotu1188

GAATGTTGGCGGCGTGCCTGAGATATGCAAGTCGAGCGAACTTGTTTCCGCAAGGGAACGAGTCAGCGGC
GGACGGCTGAGTAACACGTAGGTATCTCCCCCTATCTGGGGAATAGCTCCGCGAAAGCGGAGATAATACC
CCATGATTCCGTCCTTTGGACGGACAAAGAGAGTAACTTTTCGGAAAGGGATGAACCTGCGGCTTATCAGA
TAGTTGGTGGGGTAACGGCCTACCAAGTCTACGACGAGTAGCTGGCTTGAGAGAGTGGTCAGCCAGAGG
GGCACTGAGACACGGGC
>Zotu1190
GAACGCTGGCGGCGTGCTTAACACATGCAAGTCGAGCGGAAAGGCCACTTCGGTGGTACTCGAGCGGCGA
ACGGGTGAGTAACACGTGAGTAATCTGCCCTTGGCTTTGGGATAGCCACCGGAAACGGTGATTAATACCG
GATACGACCACTCCTGGCATCAGGTGGTGGTGGAAAGTTTTTCGGCCAGGGATGTGCTCGCGGCCTATCAG
CTTGATGGTGAGGTAATGGCTCACCATGGCTTCGACGGGTAGCCGGCCTGAGAGGGTGACCGGTCACACT
GGGACTGAGACACGGCC
>Zotu1192
GAACGCTAGCGGCAGGCTTAACACATGCAAGTCGAGGGGTAGAAGTTTTCGGACTTTGAGACCGGCGCAC
GGGTGCGTAACGCGTATGCAATCTACCTTTTACAGAGGGATAGCCCAGAGAAATTTGGATTAATACCTCAT
AGTGTTATAGAGTGGCATCACTTTATAACTAAAGTCACAACGGTAAAAGATGAGCATGCGTCCCATTAGCT
AGATGGTAAGGTAACGGCTTACCATGGCTACGATGGGTAGGGGTCCTGAGAGGGAGATCCCCCACACTGG
TACTGAGACACGGAC
>Zotu1194
GAACGCTAGCGGCAGGCTTAATACATGCAAGTCAAGGGGGTAGCAATACAACCGGCGCACGGGTGAGTA
ACGCGTACACAACATACCATATACTGGAGTATAGCTTTGGGAAACTGAAGGTAATCCTCCATAACATTGCG
AGAAGGCATCTAATTGCAATTAAAGTTTCGACGGTATATGATTGGTGTGCGTTTGATTAGCTAGTTGGTAG
GGTAACGGCCTACCAAGGCGACGATCAATAGCTGGCGTGAGAGCGCGACCAGCCACACTGGAACTGAGAC
ACGGTC
>Zotu1196
GAACGCTTGCGGCGCGCCTAATACATGCAAGTCGAACGAGGTAGCAATACCTAGTGGCGCACGGGTGAGT
AACGCGTGAATAATCTGCCTACCAGTGGGGGATAACTAACCGAAAGGTTAGCTAATACCGCATGAGGCCA
CAATGTCTGCGGACAAAGGGGTTAAAGATTTATCGCTGGTAGATGAGTTTGCGTGGGATTAGCTAGTTGGT
GGGGTAATGGCCTACCAAGGCAACGATCTCTAACTGGTCTGAGAGGATGGCCAGTCACACTGGAACTGAG
ACACGGTC
>Zotu1198
GAACGCTGGCGGCGTGCTTAACACATGCAAGTGGAGCGACGAACCGGGGCTTGCCCCGGGGCAGAGCCG
CGAACGGGTGAGTAACACGTGGGTTACGTGCCCCGATGATTGGGACAACCCGAGGAAACTCGGGCTAATA
CCAAATGTGCTCGTGTCCGTGATGGACTCGAGGAAAGGAAGCTTCGGCCTCCGCATCGGGATCGGCCCGC
GGCCCATTAGCTAGTTGGTGGGGTAATGGCCTACCAAGGCGACGATGGGTAGCTGGTCTGAGAGGACGAT
CAGCCACACTGGGACTGAGACACGGCC
>Zotu1200
GAACGCTGGCGGCGTGCCTAATACATGCAAGTCGAGCGAATCGATGGGAGCTTGCTCCCTGAGATTAGCG
GCGGACGGGTGAGTAACACGTGGGTAACCTGCCTGTAAGATTGGGATAACTCCGGGAAACCGGAGCTAAT
ACCGGATAACATTTTGAACCGCATGGTTCAAAATTGAAAGATGGCTTCTAGCTATCACTTACAGATGGACCC
GCGGCGCATTAGCTAGTTGGTGAGGTAATGGCTCACCAAGGCAACGATGCGTAGCCGACCTGAGAGGGTG
ATCGGCCACACTGGGACTGAGACACGGCC
>Zotu1202
GAACGCTAGCTACAGGCTTAACACATGCAAGTCGTGGGGCAGCGGATGCTTAGCTTGCTAAGTATGCCGG
CGACCGGCGCACGGGTGAGTAACGCGTACCGAACCTGCCCATCACACAGGGATAGGCTTGCGAAAGCAAG
ATTAATACCTGATGGTCTCAATTTTATGCATGTATGATTGAGTAAAGCCTTCGGGCGGTGATGGATGGCGG
TGCGTCCCATTAGGAAGTTGGCGGGGTAACGGCCCACCAATCCTTCGATGGGTAGGGGTTCTGAGAGGAA
GGTCCCCCACATTGGAACTGAGATACGGTC
>Zotu1204
GAACGCTGGCGGCGTGCTTAACACATGCAAGTCGAACGATGATGCCCAGCTTGCTGGGTGGATTAGTGGC
GAACGGGTGAGTAACACGTGAGTAACCTGCCCCTGACTCTGGGATAAGCGTTGGAAACGACGTCTAATAC
TGGATATGACTGCCGGCCGCATGGTCTGGTGGTGGAAAGATTTTTTGGTTGGGGATGGACTCGCGGCCTA

TCAGCTTGTTGGTGAGGTAATGGCTCACCAAGGCGACGACGGGTAGCCGGCCTGAGAGGGTGACCGGCC
ACACTGGGACTGAGACACGGCC
>Zotu1207
GAACGCTGGCGGCAGGCCTAACACATGCAAGTCGAACGGACCCTTCGGGGTTAGTGGCGGACGGGTGAG
TAACACGTGGGAACGTGCCTTTAGGTTCGGAATAGCTCCTGGAAACGGGTGGTAATGCCGAATGTGCCCTT
CGGGGGAAAGATTTATCGCCTTTAGAGCGGCCCGCGTCTGATTAGCTAGTTGGTGAGGTAACGGCTCACCA
AGGCGACGATCAGTAGCTGGTCTGAGAGGATGACCAGCCACACTGGGACTGAGACACGGCC
>Zotu1209
GAACGCTGGCGGCAGGCTTAACACATGCAAGTCGAACGGTCTCTTCGGAGGCAGTGGCAGACGGGTGAG
TAACGCGTGGGAATCTACCTAGTTCTACGGAACAACAGAGGGAAACTTCTGCTAATACCGTATACGCCCTA
CGGGGGAAAGATTTATCGGAATTAGATGAGCCCGCGTAAGATTAGCTAGTTGGTGGGGTAATGGCCTACC
AAGGCGACGATCTTTAGCTGGTCTGAGAGGATGATCAGCCACACTGGGACTGAGACACGGCC
>Zotu1212
GAACGCTGGCGGCAGGCCTAACACATGCAAGTCGAACGCTGTAGCAATACAGAGTGGCAGACGGGTGAG
TAACGCGTGGGAACATACCCTTTGGTTCGGAATAACTCAGGGAAACTTGAGCTAATACCGGATACGTCCGC
GAGGAGAAAGATTCATCGCCGAAGGATTGGCCCGCGTTCGATTAGCTAGTTGGTGAGGTAACGGCTCACC
AAGGCGACGATCGATAGCTGGTCTGAGAGGATGATCAGCCACATTGGGACTGAGACACGGCC
>Zotu1214
GAACGCTGGCGGCGTGCCTAACACATGCAAGTCGAACGGGACTCGGGACTTCGGTTCTGAGTTTAGTGGC
GAACGGGTGAGTAACGCGTGAGGAACCTGCCTTTCAGTGGGGGACAACAGTTGGAAACGACTGCTAATAC
CGCATAATGTGTTTTGGGGGCATCCCCGAGACACCAAAGCTTTATGTGCTGAAAGATGGCCTCGCGTCTGA
TTAGATAGTTGGTGAGGTAACGGCTCACCAAGTCTGCGATCAGTAGCCGGTCTGAGAGGATGAACGGCCA
CATTGGGACTGAGATACGGCC
>Zotu1216
GAACGCTAGCGGCAGGCTTAATACATGCAAGTCGAGGGGCAGCACGAATAGCAATATTTGGTGGCGACCG
GCAAACGGGTGCGGAACACGTACGCAACCTTCCCATAACTGGTGAATAGCCTTCCGAAAGGGAGATTAAT
ACACCGTAACATAAAGAGTAGGCATCTATTTTTTATTATAGCTTCGGCGGTTTTGGATGGGCGTGCGCCTGA
TTAGGTAGTTGGCGGGGTAACGGCCCACCAAGCCTTCGATCAGTAACTGGTGTGAGAGCACGACCAGTCA
CACGGGCACTGAGACACGGGC
>Zotu1218
GAACGCTGGCGGCGTGCTTAACACATGCAAGTCGAGCGGAAAGGCCCTTCGGGGTACTCGAGCGGCGAA
CGGGTGAGTAACACGTGAGTAACCTGCCCTAGGCTCTGGGATAACTGTCGGAAACGATGGCTAATACCGG
ATATTCATCTCCTGCCGCATGGTGGGGGTTGGAAAGTTTTTCGGCTTGGGATGGGCTCGCGGCCTATCAGC
TTGTTGGTGGGGTGATGGCCTACCAAGGCTTCGACGGGTAGCCGGCCTGAGAGGGCGACCGGCCACACTG
GGACTGAGACACGGCC
>Zotu1220
GAACGCTGGCGGCGTGCTTAACACATGCAAGTCGAACGGTGACGCTAGAGCTTGCTCTGGTTGATCAGTG
GCGAACGGGTGAGTAACACGTGAGTAACCTGCCCTTGACTCTGGGATAACTCCGGGAAACCGGGGCTAAT
ACCGGATACGAGACGCGATCGCATGGTCGGCGTCTGGAAAGTTTTTCGGTCAAGGATGGACTCGCGGCCT
ATCAGCTTGTTGGTGAGGTAACGGCTCACCAAGGCGTCGACGGGTAGCCGGCCTGAGAGGGCGACCGGC
CACACTGGGACTGAGACACGGCC
>Zotu1222
TAACGCTAGCGGCAGGCATAATACATGCAAGTTGAACGGGATTAATTGTAGCAATACGATTATGAGAGTA
GCGCACGGGTGCGTAACGCGTATGCAACCTGCCTTCATCTGGGGAATAGCCCCTCGAAAGAGGGATTAAT
ACCGCATAAAATAGTGTATAGGCATCTATTTACTATTAAAACTATGGTGGATGAAGATGGGCATGCGTGAC
ATTAGCTAGTTGGTAGGGTAATGGCCTACCAAGGCTACGATGTCTAGGGGGTCTGAGAGGATGTCCCCCC
ACACTGGTACTGAGATACGGAC
>Zotu1225
GAACGCTAGCGGCAGGCTTAATACATGCAAGTCGAGGGGCAGCGGAGTAGCAATACTGCCGGCGACCGG
CGCACGGGTGCGTAACACGTATACAATCTACCTTAAACAGGGGGATAGCCTTTCGAAAGAGAGATTAATAC

CCCGTAATAAATTGAATGGCATCATTTGATTTTGAAAGCTCCGGCGGTTTGAGATGAGTATGCGTCTGATTA
GCTAGTTGGTGAGGTAACGGCTCACCAAGGCGACGATCAGTAGGGGATCTGAGAGGACTAACCCCCACAC
TGGTACTGAGACACGGAC
>Zotu1227
GAACGCTAGCGGCAGGCTTAACACATGCAAGTCGAGGGGTATAGTAGCAATACTAGAGACCGGCGCACG
GGTGCGTAACGCGTATGCAATCTACCTTTTACAGAGGGACAGCCCAGAGAAATTTGGATTAATACCTCATA
GTATTATACAATGGCATCATTATATAATTAAAGTCACAACGGTAAAAGATGAGCATGCGTCCCATTAGCTAG
TTGGTAAGGTAACGGCTTACCAAGGCAACGATGGGTAGGGGTCCTGAGAGGGAGATCCCCCACACTGGTA
CTGAGACACGGAC
>Zotu1229
GAACGCTAGCGGCAGGCCTAATACATGCAAGTCGAACGGATACCTTCGGGTATTAGTGGCGTACGGGTGC
GTAACGCGTAAACAATCTACCTCATACTGGGGGATAGCCCTGGGAAACCGGGAGTAAGCCCGCATGGCAT
CGTTGGTCTTCATGGACTGATGATTAAAGATTTATTGGTATGAGATGAGTTTGCGTCTGATTAGTTAGTTGG
CGGGGTAACGGCCCACCAAGACGATGATCAGTAGGGGCTCTGAGAGGAGCGGCCCCCACACTGGCACTG
AGACACGGGC
>Zotu1231
GAACGCTGGCGGCGTGCTTTAGGGATGCGAGTCGAGCGGTTTTTTAGGTGAAATGATGTTTCGCAAGAAA
TTGAAGCGATCTTAAAAAATAGCGGCAGACGACTGAGTAACACGTAGACAACTTACCCATAACACAGAGAT
AGCCACGCGAAAGCGTGGGTAATATCTGATAGCCCATATAGGTGAAATTCCGGTATGGTAAAGGCGCAAG
CCGGTTATGGAGGGGTCTGCGGCCCATCAGCTAGTTGGCAGGATAACAGCCTACCAAGGCGAGGACGGGT
AGGGGGCGTGAGAGCGCGACCCCCCAGGATGGCACTGAGATACGGGC
>Zotu1233
GAACGCTGGCGGCGTGCCTAATACATGCAAGTCGAGCGGACCGAACGAGAGCTTGCTCTTGTTCGGTCAG
CGGCGGACGGGTGAGTAACACGTGGGCAACCTGCCCGCAAGACCGGGATAACTCCGGGAAACCGGAGCT
AATACCGGATAACACCAAAGACCGCATGGTCTTTGGTTGAAAGGCGGCTTCGGCTGTCACTTGCGGATGG
GCCCGCGGCGCATTAGCTAGTTGGTGAGGTAACGGCTCACCAAGGCGACGATGCGTAGCCGGCCTGAGAG
GGTGACCGGCCACACTGGGACTGAGACACGGCC
>Zotu1235
GAACGCTAGCGGCAGGCTTAACACATGCAAGTCGAGGGGCAGCATGAGGAGTAGCAATACACCTTGATG
GCGACCGGCGCAAGGGTGCGTAACGCGTGAGCAACATGCCCGTATCAGGGACATAACCGGTGGAAACGC
CGCCTAATTTCCCATAACATCAGAGGTGACATCGTCTTTGGTTGAAAGATTCGTCGGATACGGATTGGCTCG
CGTGATATTAGCTGGTTGGTGAGGTAACGGCTCACCAAGGCTACGATATCTAGGGGTTCTGAGAGGAAGG
TCCCCCACATTGGAACTGAGACACGGTC
>Zotu1237
GAACGCTGGCGGCGTGCCTAATACATGCAAGTCGAGCGGATCTTGTCCTTCGGGATAAGGTTAGCGGCGG
ACGGGTGAGTAACACGTAGGCAACCTGCCCTCGAGACTGGGATAACCTCCGGAAACGGATGCTAATACCA
GATACGCGATCTTCCCACCTGGGGAGATCGGGAAAGACGGAGCAATCTGTTACTCGGGGATGGGCCTGCG
GCGCATTAGCTAGTTGGTGAGGTAACGGCTCGCCAAGGCGACGATGCGTAGCCGACCTGAGAGGGTGATC
GGCCACACTGGGACTGAGACACGGCC
>Zotu1241
GAACGCTGGCGGCGTGCTTAACACATGCAAGTCGAACGGTGACCTCGGAGCTTGCTCCGGGCGATCAGTG
GCGAACGGGTGAGTAACACGTGAGTAACCTGCCCCTGGCTCTGGGATAACTGTGGGAAACCGCAGCTAAT
ACCGGATACGACACAGACAGGCATCTGTACTGTGTGGAAAGTTTTTCGGCCAGGGATGGACTCGCGGCCT
ATCAGCTTGTTGGTGGGGTAACGGCCCACCAAGGCGACGACGGGTAGCCGGCCTGAGAGGGCGACCGGC
CACACTGGGACTGAGACACGGCC
>Zotu1243
GAACGCTGGCAGCGTGCTTAACACATGCAAGTCGAACGATGATGCCCAGCTTGCTGGGTTGATTAGTGGC
GAACGGGTGAGTAACACGTGAGTAACCTGCCCTTGACTTCGGGATAACTCCGGGAAACCGGGGCTAATAC
CGGATATGAGCCGTCCTCGCATGGGGGTGGTTGGAAAGTTTTTCGGTCAGGGATGGGCTCGCGGCCTATC
AGCTTGTTGGTGGGGTGATGGCCTACCAAGGCGACGACGGGTAGCCGGCCTGAGAGGGCGACCGGCCAC
ACTGGGACTGAGACACGGCC

>Zotu1246
GAACGCTAGCGGCAGGCCTAATACATGCAAGTCGAACGGACAGTAGCAATACTGTTAGTGGCGCACGGGT
GCGTAACACGTAACTAACCTACCCACAACTGGGGGATAGCCCGCCGAAAGGCGGATTAATACCGCATAAG
ACTATAGAGTGGCATCACTTAGTAGTTAAAGATTTATTGGTTGTGGATGGGGTTGCGGGTCATTAGCTAGT
TGGTTGGGTAACGGCTGACCAAGGCGACGATGACTAGGGGAGCTGAGAGGCTGGTCCCCCACACGGGCA
CTGAGATACGGGC
>Zotu1249
GAACGCTGGCGGCATGCCTTACACATGCAAGTCGAACGGTAACGCGGGGGCAACCCTGGCGACGAGTGG
CGAACGGGTGAGTAATACATCGGAACATATCCAGTAGCGGGGGATAACCTATCGAAAGATGGACTAATAC
CGCATACGATCTACGGATGAAAGCAGGGGACCGCAAGGCCTTGCACTATTGGAGTGGCCGATGTCGGATT
AGCTAGTTGGTGGGGTAAAAGCTTACCAAGGCGACGATCCGTAGCTGGTCTGAGAGGACGATCAGCCACA
CTGGAACTGAGACACGGTC
>Zotu1252
GAACGCTGGCGGCGTGCCTAATACATGCAAGTCGAGCGGACCGGATCGGAGCTTGCTCTGATTTGGTCAG
CGGCGGACGGGTGAGTAACACGTGGGCAACCTGCCCGCAAGACCGGGATAACTCCGGGAAACCGGAGCT
AATACCGGATAACACCGAAGACCGCATGGTCTTTGGTTGAAAGGCGGCCTTTGGCTGTCACTTGCGGATGG
GCCCGCGGCGCATTAGCTAGTTGGTGAGGTAACGGCTCACCAAGGCGACGATGCGTAGCCGGCCTGAGAG
GGTGACCGGCCACACTGGGACTGAGACACGGCC
>Zotu1254
GAACGCTAGCGGGAGGCTTAATACATGCAAGTCGAGCGGTAATTTGTTTATAGCAATATAGATGAGCTAG
AGCGGCGCACGGGTGAGTAACGCGTACATGACCTACCCTATACTGGGGGATAGTCTTGGGAAACTGAGAA
TAATCCCGCATGTGATTATTGGACTGCATGGTTTGATAATTAAAGCTACGGCGGTAAAGGATGGATGTGCG
TCTGATTAGCTAGTTGGTAGGGTAACGGCCTACCAAGGCGATGATCAGTAGGGGGCGTGAGAGCGTGAAC
CCCCACACGGGTACTGAGACACGGAC
>Zotu1256
GAACGCTAGCGGCAGGCCTAACACATGCAAGTCGAGGGGTAGAGGGTGCTTGCACCCTTGAGACCGGCG
CACGGGTGCGTAACGCGTATGCAACCTGCCTTCCACAGGGGAATAGCCCAGAGAAATTTGGATTAATGCCC
CATGGCACAGCGAGACGGCATCGTCTTGCTGTTAAAGTAACAACGGTGGAAGATGGGCATGCGTCCCATT
AGCTAGTTGGTAGGGTAACGGCCTACCAAGGCTACGATGGGTAGGGGTCCTGAGAGGGAGATCCCCCACA
CTGGTACTGAGACACGGAC
>Zotu1258
GAACGCTGGCGGCGTGCCTAATACATGCAAGTCGTGCGGGTGAAGCCGACCACCCCTTCGGGGGCGGATG
GCGGAACCAGCGGCGGACGGGTGAGTAACACGTGGGCAACCTGCCTGCAAGACCGGGATAACTGCGGGA
AACCGGAGCTAATACCGGATAAGAGGCTTTTCCGCATGGGGGAGCCTTAAAAGGCGGCGGAAGTCGTCAC
TTGCAGATGGGCCCGCGGCGCATTAGCTAGTTGGTGAGGTAAGGGCTCACCAAGGCGACGATGCGTAGCC
GACCTGAGAGGGTGACCGGCCACACTGGGACTGAGACACGGCC
>Zotu1260
GAACGCTGGCGGCGTGCTTAACACATGCAAGTCGAACGGAGGATTTATTTCGGTAGATCCTTAGTGGCGA
ACGGGTGAGTAACGCGTGGGCAATCTGCCCTTCAGATGGGGACAACATCCCGAAAGGGGTGCTAATACCG
AATACGATCCTTCCACCGCATGGAGGGAGGATGAAAGATGGCCTCTATACATAAGCTATCGCTGAAGGAT
GAGCCTGCGTCTGATTAGCTTGTTGGTGGGGTAACGGCCTACCAAGGCGATGATCAGTAGCCGGTCTGAG
AGGATGAACGGCCACATTGGGACTGAGACACGGCC
>Zotu1262
GAACGCTGGCGGCGCGCTTAACACATGCAAGTCGAGCGATGAAATCTTGACAGATCCCTTCGGGGTGAAG
ATAAGATGGATTAGCGGCGGACGGGTGAGTAACACGTGAGTAACCTGCCTTAGACATTGGGATAGCCTCG
GGAAACCGGGATTAATACCAAATGAAATCATAGGTGCCCCTGCACTAATGATCAAAACTCCGGTGGTCTAA
GATGGACTCGCGTCCCATTAGCTAGTTGGTGAGGTAACGGCCCACCAAGGCGACGATGGGTAGCCGGCCT
GAGAGGGTGAACGGCCACATTGGAACTGAGAAACGGTC
>Zotu1265

GAACGCTAGCGGGAGGCCTAATACATGCAAGTCGAACGGTAACAGGTAGCAATACGCTGACGAGTGGCG
CACGGGTGAGTAACACGTACACAATCTGCCTTATACTGGGGCATATCCTCGCGAAAGTGAGAGCAATTCCC
CATAATATATTTGAATGGCATCATTTGAATATTAAAGCTCAGGCGGTATAAGATGAGTGTGCGTCTGATTAG
GTAGTTGGTGAGGTAATGGCTCACCAAGCCTGCGATCAGTAGCTGGTCTGAGAGGATGATCAGTCACACG
GACACTGAGACACGGGT
>Zotu1267
GAACGCTGGCGGCGTGCCTAATACATGCAAGTCGAGCGAATCAGATGGGAGCTTGCTCCCGTATGATTAG
CGGCGGACGGGTGAGTAACACGTGGGCAACCTGCCCGTAAGATTGGGATAACTCCGGGAAACCGGTGCT
AATACCGAATAGTTTCTGACACCTCATGGTATCAGAGGGAAAGTTGGCTTTGGCTAACACTTACGGATGGG
CCCGCGGCGCATTAGCTAGTTGGTGAGGTAACGGCTCACCAAGGCAACGATGCGTAGCCGACCTGAGAGG
GTGATCGGCCACACTGGGACTGAGACACGGCC
>Zotu1270
TAACGCTAGCGGCAGGCCTAATACATGCAAGTTGAACGGGATTTTGGGTAGCAATATCTGAATGAGAGTA
GCGCACGGGTGCGTAACGCGTATGCAACCTACCTTTGTCTGGGGGATAGCCTTCCGAAAGGAAGATTAATA
CCGCATAACGTATTGATGTAGCATTACATTAATACCAAACCTCCGGGGGACAAAGATGGGCATGCGTTCGA
TTAGCTAGTTGGTGAGGTAACGGCTCACCAAGGCTACGATCGATAGGGGGTCTGAGAGGATGATCCCCCA
CACTGGTACTGAGACACGGAC
>Zotu1272
GAACGCTAGCGGCAGGCCTAATACATGCAAGACGAACGGTAAGTTTGATAGCAATATTGAACCTAGAGTG
TCGTAAGGGTGCGTAACACGTATGCAATCTACCCGAAACTGGAGCATAGCTCCCCGAAAGGGGAATTAAC
ACTCCATAAGATCATGGTGCGGCATCGCAGAGTGATTAAAACTCCGGTGGTTTCGGATGAGCATGCGTTTG
ATTAGTTAGTTGGTGAGGTAACGGCTCACCAAGACGATGATCAATAGGGGAACTGAGAGGTTGATCCCCC
ACACTGGCACTGAGATACGGGC
>Zotu1275
GAACGCTAGCGGCAGGCCTAACACATGCAAGTCGAGGGGTAGAGGGGAGCTTGCTCTCCTTGAGACCGGC
GGACGGGTGAGTAGCGCGTATGGAATCTGCCTTGTACAGGGGAATAGCCCAGGGAAACTTGGATTAATGC
CCCATAGTGTTGAGGGGCGGCATTGTTCTTCAACTAAAGATTTATCGGTACAAGATGACCATGCGTCCCATT
AGCTTGTTGGTGTGGTAACGGCACACCAAGGCTACGATGGGTAGGGGCCCTGAGAGGGGGATCCCCCACA
CTGGTACTGAGACACGGAC
>Zotu1277
GAACGCTGGCGGCGCGCTTAACACATGCAAGTCGAACGGTGAACGGGGCTTCGGCCCCCGGATCAGTGGC
GAACGGGTGCGTAACACGTGGGCAACCTGCCCCAGACACTGGGATAACTCCGGGAAACCGGGGCTAATAC
CGGATACTCCGATCGGATCGCATGGTCTGATCGGGAAAGCTTTTGCGGTCTGGGATGGGCCCGCGGCCTAT
CAGCTTGTTGGTGAGGTAACGGCTCACCAAGGCGACGACGGGTAGCTGGTCTGAGAGGACGACCAGCCAC
ACTGGGACTGAGATACGGCC
>Zotu1279
GAACGCTGGCGGCGTGCTTAACACATGCAAGTCGAACGGAAAGGCCCTGCTTGCAGGGTACTCGAGTGGC
GAACGGGTGAGTAACACGTGGGTGATCTGCCCTGTACTTTGGGATAAGCCTGGGAAACTGGGTCTAATAC
CGAATATGACCACTTCTTGGATGGGGTGGTGGAAAGCTTTTGCGGTATGGGATGAGCTCGCGGCCTATCA
GCTTGTTGGTGGGGTAATGGCCTACCAAGGCGTCGACGGGTAGCCGGCCTGAGAGGGTGTACGGCCACAT
TGGGACTGAGATACGGCC
>Zotu1281
GAACGCTAGCGGCAGGCCTAATACATGCAAGTCGAACGGGATTTGGGAGCTTGCTCCTGATGAGAGTGGC
GCACGGGTGCGTAACACGTATGTAACCTACCTTAATCTGGGGGATAGCCTCTCGAAAGAGAGATTAACACC
GCATAACATTATCGGACAGCATTTTCTGATAATCAAATATTTATAGGATTAAGATGGGCATGCGGAACATTA
GCTAGTTGGTAAGGTAATGGCTTACCAAGGCTACGATGTTTAGGGGATCTGAGAGGATGACCCCCCACACT
GGTACTGAGACACGGAC
>Zotu1283
GAACGCTAGCGGCAGGCTTAATACATGCAAGTCAAGGGGGTAGCAATACAACCGGCGCACGGGTGAGTA
ACGCGTACACAACATGCCATATACTGGAGTATAGCTTTGGGAAACTGAAGGTAATCCTCCATAACATTTCG
GATAGGCATCTATTTGAAATTAAAGTTTCGACGGTATATGATTGGTGTGCGTTTGATTAGCTAGTTGGTAG

GGTAACGGCCTACCAAGGCTACGATCAATAGCTGGCGTGAGAGCGCGACCAGCCACACTGGAACTGAGAC
ACGGTC
>Zotu1285
GAACGCTAGCGGCAGGCCTAATACATGCAAGTCGAACGATAGTCCCAGCTTGCTGGGGCAAAAGTGGCGC
ACGGGTGCGTAACGCGTATGCAACCTACCTTAATCAGGGGGATAGCCCGAAGAAATTCGGATTAACACCG
CATAAAATCACAGCACAGCATTGTGCAATGATCAAATATTTATAGGATTAAGATGGGCATGCGTGACATTA
GTTAGTTGGCGGGGTAACGGCCCACCAAGACCACGATGTCTAGGGGATCTGAGAGGATGACCCCCCACAC
TGGTACTGAGACACGGAC
>Zotu1288
GAACGCTGGCGGCGTGCTTAACACATGCAAGTCGAACGGTGAAACAGGAGCTTGCTCCTGTGGATCAGTG
GCGAACGGGTGAGTAACACGTGAGTAACCTGCCCCAGACTCTGGGATAAGCACTGGAAACGGTGTCTAAT
ACCGGATACGAGCTTCAGCCGCATGGCTAGGAGCTGGAAAGAATTTCGGTCTGGGATGGACTCGCGGCCT
ATCAGCTAGTTGGTGAGGTAACGGCCCACCAAGGCGACGACGGGTAGCCGGCCTGAGAGGGTGACCGGC
CACACTGGGACTGAGACACGGCC
>Zotu1290
GAACGCTGGCGGCGTGCCTAATACATGCAAGTCGAGCGAACTTGCGGGAGCTTGCTCCCAAAAGTTAGCG
GCGGACGGGTGAGTAACACGTGGGCAACCTGCCTGTAAGACTGGGATAACTTCGGGAAACCGGAGCTAAT
ACCGGATAACTCTTATCAACTCATGTTGGTAAGCTAAAAGACGGTTTCGGCTGTCACTTACAGATGGGCCC
GCGGCGCATTAGCTAGTTGGTGAGGTAACGGCTCACCAAGGCAACGATGCGTAGCCGACCTGAGAGGGT
GATCGGCCACACTGGGACTGAGACACGGCC
>Zotu1292
GAACGCTGGCGGCGTGCCTAATACATGCAAGTCGAACGAATCTTCTTCGGAAGATGAGTGGCGAACGGGT
GAGTAATACATAGGTAACCTGCCCCTGTGCGGGGGATAACAGGAGGAAACTCCTGCTAATACCGCATAGC
CATGAGCACCGCATGGAGCTCATGCCAAATATCCTTTCGGGGATAGCGCAGGGATGGACCTATGGCGCATT
AGCTAGTTGGCGGGGCAACGGCCCACCAAGGCAACGATGCGTAGCCGGCCTGAGAGGGCGGACGGCCAC
ATTGGGACTGAGACACGGCC
>Zotu1294
GAACGCTGGCGGCGTGCTTAACACATGCAAGTCACGGGGGTCCCGCAAGGGATAACCGGCGAACGGGTG
CGTAACACGTGACCAACCTGCCCTCGTGTGGGGGATAGCCGGGCTAACGCCCGGGTAATACCGCATACGTT
CCTCGAGCGGAGTCGCTTGGGGAGGAAAGCTCCGGCGCACGGGGAGGGGGTCGCGGCCTATCAGCTCGT
TGGCGGGGTAACGGCCCACCAAGGCGACGACGGGTAGCTGGTCTGAGAGGATGGCCAGCCACATTGGGA
CTGAGAGACGGCC
>Zotu1296
GAACGCTGGCGGCGTGCCTAACACATGCAAGTCGAGCGAGAGACTTCGGTCTCTAGCGGCGGACGGGTG
AGTAACACGTGGGTAACCTGCCTCAAACAGGGGGATAGCCTTCCGAAAGGAAGATTAATACCGCATAACG
TAGTTTAGTCGCATGACTAGATTATCAAAGGAGTAATCCGGTTTGAGATGGACCCGCGGCGCATTAGCTAG
TTGGTGAGGTAACGGCTCACCAAGGCGACGATGCGTAGCCGACCTGAGAGGGTGATCGGCCACATTGGAA
CTGAGATACGGTC
>Zotu1298
GAACGCTGGCGGCGTGTTTAACACATGCAAGTCGAGCGAGAAACTTCTCAGTTGATTCTTCGGATGAAACA
GAGTTGTGGAAAGCGGCGGACGGGTGAGTAACGCGTAGGCAACCTGCCCCTTGCAGAGGGATAGCCTCG
GGAAACCGGGATTAAAACCTCATAATGCGGCACTGAGACATCTCGGAGCCGCCAAAGATTTATCGGCAAG
GGATGGGCCTGCGTCTGATTAGTTAGTTGGTGGGGTAACGGCCTACCAAGGCGACGATCAGTAGCCGACC
TGAGAGGGTGATCGGCCACATTGGAACTGAGACACGGTC
>Zotu1300
GAACGCTGGTGGCGTGCTTAACACATGCAAGTCGAGCGAGGCGTCCGCTTTCGGGTGGATAGTCGAGCGG
CGAACGGGTGAGTAACACGTGAGTAATCTTCCCTGGGCTCGGGGATAACCTCGGGAAACTGAGGCTAATA
CCGGATGTGCGTTGTTTGGGGCTTCCTGGATGACGGAAAGGTTGCGTGCCGTGAGGTGCGTTTCCGGCCTT
GGATGAGCTCGCGGCCTATCAGCTTGTTGGTGCGGTGATGGCGTACCAAGGCGACGACGGGTAGCCGGCC
TGAGAGGGTGACCGGCCACACTGGGACTGAGATACGGCC

>Zotu1303
GAACGCTGGCGGCGTGCCTAACACATGCAAGTCGAGCGATTCTCTTCGGAGAATAGCGGCGGACGGGTGA
GTAACGCGTGGGTAACCTGCCCTGTACACACGGATAACATACCGAAAGGTATGCTAATACGGGATAATATA
TTTTGATGGCATCATCGAAATATCAAAGCTCCGGCGGTACAGGATGGACCCGCGTCTGATTAGCTAGTTGG
TGAGGTAATGGCTCACCAAGGCGACGATCAGTAGCCGACCTGAGAGGGTGATCGGCCACATTGGAACTGA
GACACGGTC
>Zotu1305
GAACGCTGGCGGCGTGCTTAACACATGCAAGTCGAACGATGAAGCCTAGCTTGCTGGGTGGATTAGTGGC
GAACGGGTGAGTAACACGTGAGTAACCTGCCCTTGACTCTGGGATAAGCACTGGAAACGGTGTCTAATAC
CGGATATTTCAGCTGGCCGCATGGTCTGGTTGTGGAAAGAATTTTGGTCAAGGATGGACTCGCGGCCTATC
AGGTTGTTGGTGAGGTAATGGCTCACCAAGCCTACGACGGGTAGCCGGCCTGAGAGGGTGACCGGCCACA
CTGGGACTGAGACACGGCC
>Zotu1307
GAACGCTGGCGGCGTGCCTAATACATGCAAGTCGAACGAGAGCGACCGGTGCTTGCACTGGTTAATCTAG
TGGCGAACGGGTGAGTAACACGTGGGTAACCTGCCCATCAGAGGGGGATAACATCCGGAAACGGATGCT
AAAACCGCATAGGTCTTCGAGCCGCATGGCTTGAAGAGGAAAGAGGCGCAAGCTTCTGCTGATGGATGG
ACCCGCGGTGCATTAGCTAGTTGGTGAGGTAACGGCTCACCAAGGCCGTGATGCATAGCCGACCTGAGAG
GGTGATCGGCCACATTGGGACTGAGACACGGCC
>Zotu1309
GAACGCTAGCGGCAGGCTTAACACATGCAAGTCGAGGGGTAACAGGGTCTTCGGACCGCTGACGACCGGC
AAACGGGTGCGGAACACGTACGCAACCTACCCTTTACTGGAGCATAGCCCGTAGAAATGCGGATTAACACT
CCATAGTATTCAAGAGTGGCATCACTCAGGAATTAAAAATTTATTGGTAAAGGATGGGCGTGCGTCCCATT
AGCTAGTTGGTATGGGTAATGGCCTACCAAGGCGACGATGGGTAGCTGATGTGAGAGCATGATCAGCCAC
ACGGGCACTGAGACACGGGC
>Zotu1312
GAACGCTGGCGGCATGCCTAATACATGCAAGTCGAACGCTGAAGCACTCTGCTTGCAGAGTGCGGATGAG
TGGCGAACGGGTGAGTAACACGTGGGAAATCTGCCTAAGAGTGGGGGATAACATTCGGAAACGGATGC
TAATACCGCATAGATTCTTGGACCGCATGGTTCTGGAAAGAAAGACGGCTTTGCTGTCGCTTTTAGATGATC
CCGCGGCGTATTAGTTAGTTGGTGGGGTAACGGCCTACCAAGACCATGATACGTAGCCGACCTGAGAGGG
TGATCGGCCACACTGGGACTGAGACACGGCC
>Zotu1314
AAACGCTGGCGGCGCACATAAGACATGCAAGTCGAACGGAAGTCGTTATAATGATTTAGATTTTGATTTGA
GGATTTATCCAATTTGATGATGACGAAGATTTATAACAATGGCTTTAGTGGCGGACTGGTGAGTAACGCGT
AAGCAATCTGCCTATTAGAGGGGAATAACAGTGAGAAATCATTGCTAATACCGCATATGCCATAATTATCG
CATGATAGTAGTGGGAAAGGAGTAATCCGCTAATAGATGAGCTTGCGTCTGATTAGCTAGTTGGTGGGGT
AACGGCCTACCAAGGCGACGATCAGTAGCCGGACTGAGAGGTTGAACGGCCACATTGGGACTGAGATAC
GGCC
>Zotu1317
GAACGCTGGCGGCGCGCTTAACACATGCAAGTCGAGCGATGAAATTTTGACAGATCCCTTCGGGGTGAAG
ACAAAATGGATTAGCGGCGGACGGGTGAGTAACACGTGAGTAACCTGCCTTAGACATTGGGATAGCCTCG
GGAAACCGGGATTAATACCGAATAAAATCATAGGTGCACATGCACAAATGATCAAAACTCCGGTGGTCTAA
GATGGACTCGCGTCCCATTAGCTAGTTGGTAGGGTAACGGCCTACCAAGGCGACGATGGGTAGCCGGCCT
GAGAGGGTGAACGGCCACATTGGAACTGAGAAACGGTC
>Zotu1319
GAACGCTGGCGGCGTGCCTAATACATGCAAGTCGAGCGAATCAGATGGGAGCTTGCTCCCGGATGATTAG
CGGCGGACGGGTGAGTAACACGTGGGCAACCTGCCCGTAAGATTGGGATAACTCCGGGAAACCGGTGCT
AATACCGAATAGTTTCTGACACCTCATGGTGTCAGAGGGAAAGTTGGCTTCGGCTAACACTTACGGATGGG
CCCGCGGCGCATTAGCTAGTTGGTGAGGTAACGGCTCACCAAGGCAACGATGCGTAGCCGACCTGAGAGG
GTGATCGGCCACACTGGGACTGAGACACGGCC
>Zotu1322

GCCGGCAATGTTGAAGAGGTAAGTACTGCTCACATGCACTGTCCTCCACGGAGGACGCTAACCATGTGTGT
CAAGATCTCAGCACACATTAAAGAACCCTCAGGCGGGCCAAAATTGAC
>Zotu1324
GAACGCTGGCGGCGTGCCTAATACATGCAAGTCGAGCGCGACTGATTACACCGTTCTTCGGAACACCGTAA
TCATCGAGCGGCGGACGGGTGAGTAACACGTGGGCAACCTACCCTTAAGACTGGGATAACCGCGGGAAAC
CGTGGCTAATACCGGATAATACTTTTATACAACGGTATAGAAGTTGAAAGGTGGATTTCTATCACTTAAGG
ATGGGCCTGCGGCGCATTAGCTGGTTGGTAAGGTAACGGCTTACCAAGGCGACGATGCGTAGCCGACCTG
AGAGGGTGATCGGCCACACTGGAACTGAGACACGGTC
>Zotu1326
GAACGCTAGCTACAGGCTTAACACATGCAAGTCGAGGGGAAACGGCATTAAGTGCTTGCACTTTTTGGACG
TCGACCGGCGCACGGGTGAGTAACGCGTATCCAACCTGCCCCTTACCAGGGAATAACCCGTTGAAAGACG
GACTAATGCCCTATGCAGTCCTTTGACGGCATCAGATTAGGACTAAAGATTCATCGGTATGGGATGGGGAT
GCGTCTGATTAGCTTGTTGGCGGGGTAACGGCCCACCAAGGCATCGATCAGTAGGGGTTCTGAGAGGAAG
GTCCCCCACATAGGAACTGAGACACGGTC
>Zotu1328
GAACGCTGGCGGAATGCTTTACACATGCAAGTCGAACGGCAGCACGGGGGCAACCCTGGTGGCGAGTGG
CGAACGGGTGAGTAATACATCGGAACGTGCCCAGTCGTGGGGGATAACTACGCGAAAGCGTAGCTAATAC
CGCATACGATCTACGGATGAAAGCGGGGGACCGTAAGGCCTCGCGCGATTGGAGCGGCCGATGGCAGAT
TAGGTAGTTGGTGGGGTAAAGGCCTACCAAGCCGACGATCTGTAGCTGGTCTGAGAGGACGACCAGCCAC
ACTGGGACTGAGACACGGCC
>Zotu1332
GAACGCTGGCGGCGTGCTTAACACATGCAAGTGGAGCGACGAAGCGGGCTTCGGCCCGTGGCAGAGCCG
CGAACGGGTGAGTAACACGTGGGTAACGTGCCCCAATGACTGGGACAACCCGGGGAAACCCGGGCTAAT
ACCGGATGTGCTCCTTCCACATCAGTGGATTGAGTAAAGGTAGCTTCGGCTTCCGCATTGGGATCGGCCCG
CGGCGCATTAGCTTGTTGGTGAGGTAACGGCTCACCAAGGCTTCGATGCGTAGCTGGTCTGAGAGGACGA
TCAGCCACACTGGGACTGAGACACGGCC
>Zotu1334
GAACGCTAGCGGCAGGCTTAATACATGCAAGTCGAGGGGTAACATGGGTAGCAATACCTGATGACGACCG
GCAAACGGGTGCGGAACACGTACGCAACCTTCCCATAACTGGTGAATAGCCCTCCGAAAGGAGGATTAAT
ACATCGTAATATAATGGAGTGGCATCACTCTATTATCATAGGCTTCGGCCGGTTTTGGATGGGCGTGCGCCT
GATTAGGTAGTTGGCGGGGTAACGGCCCACCAAGCCTGCGATCAGTAACTGGTGTGAGAGCACGACCAGT
CACACGGGCACTGAGACACGGGC
>Zotu1337
GAACGCTTGCGACGTGCCTAAGAAATGCAAGTCGAACGGGGGAGCAATCCCTAGTGGCGAACGGTCGCGT
AACACGTGGGCAACCTGCCCTGAAGCGGGGGACAACAGTCCGAAAGGGCTGCTAATACCGCATGTGAAG
GGAGATGGCATCATCTCTTGTTCAAAGCTTTATGCACTTCAGGATGGGCCCGCGGCCTATCAGCTAGTTGGT
GGGGTAACGGCCTACCAAGGCTTCGACGGGTAGCTGGTCTGAGAGGACGGCCAGCCGGATTGGGACTGA
GATACGGCC
>Zotu1339
GAACGCTGGCGGCATGCCTAATACATGCAAGTTGAACGATGGTATTCTCTGCTTGCAGAGAATTGAAGAGT
AGCGAACGGGTGAGCAACACGTGGGGAACCTGCCCATGAGAGGGGGATAACATTCGGAAACGGATGCTA
ATACCGCATAATGCTTTGGACCGCCTGGTCCAGAGACGAAAGACGGCTTTTGCTGTCACTCATGGATGGCC
CCGCGCCGTATTAGCTAGTTGGTGAGGTAATGGCTCACCAAAGCTGTGATACGTAGCCGACCTGAGAGGG
TGATCGGCCACACTGGGACTGAGACACGGCC
>Zotu1343
GAACGCTAGCTACAGGCTTAACACATGCAAGTCGAGGGGCAGCATGGTCTTAGCTTGCTAAGACTGATGG
CGACCGGCGCACGGGTGAGTAACACGTATCCAACCTGCCGTCTACTCTTGGACAGCCTTCTGAAAGGAAGA
TTAATACAAGATGGCATCATGAGTCCGCATGTTCACATGATTAAAGGTATTCCGGTAGACGATGGGGATGC
GTTCCATTAGATAGTAGGCGGGGTAACGGCCCACCTAGTCTTCGATGGATAGGGGTTCTGAGAGGAAGGT
CCCCCACATTGGAACTGAGACACGGTC

>Zotu1345
GAACGCTGGCGGCGTGCTTAACACATGCAAGTCGAACGGTGAACGAGGGAGCTTGCTCCCTCCGGATCAG
TGGCGAACGGGTGAGTAACACGTGAGTAACCTGCCCTTGACTCTGGGATAAGCGTTGGAAACGACGTCTA
ATACCGGATACGACCTTCGGAGGCATCTCCTGGGGGTGGAAAGAATTTCGGTCAAGGATGGACTCGCGGC
CTATCAGCTAGTTGGTGAGGTAATGGCTCACCAAGGCGACGACGGGTAGCCGGCCTGAGAGGGTGACCG
GCCACACTGGGACTGAGACACGGCC
>Zotu1347
GAACGCTGGCGGCAGGCTTAACACATGCAAGTCGAACGAGCTGGGTCCTTCGGGGCCCGATGCTAGTGGC
GCACGGGTGAGTAACACGTGGGAATATGCCCTTTAGTACGGAATAACCAAGGGAAACTTTGGCTAATACC
GTATACGCCCTTCGGGGGAAAGATTTATCGCTAAAGGATTAGCCCGCGTCTGATTAGGTAGTTGGTGAGGT
AATGGCTCACCAAGCCGACGATCAGTAGCTGGTCTGAGAGGATGATCAGCCACACTGGGACTGAGACACG
GCC
>Zotu1349
GAACGCTGGCGGCGTGCTTAACACATGCAAGTCGAACGGTGAAGCACCTTCCTTTTGGTGGGTGTGGATG
AGTGGCGAACGGGTGAGTAACACGTGAGTAACCTGCCCCCTTCTTCTGGATAACCGCATGAAAGTGTGGCT
AATACGGGATATTCTGCGCCTGCTGCATGGTGGGTGTGGGAAAGATGCGCCTTTGGGTGTTTTTGGTGGG
GGATGGGCTCGCGGCCTATCAGCTTGTTGGTGGGGTGATGGCCTACCAAGGCGGTGACGGGTAGCCGGC
CTGAGAGGGTGGACGGTCACACTGGGACTGAGACACGGCC
>Zotu1353
GAACGCTGGCGGCGTGCTTAACACATGCAAGTCGAGCGGAAAGGCCACTTCGGTGGTACTCGAGCGGCGA
ACGGGTGAGTAACACGTGAGTAATCTGCCCCTGGCTTTGGGATAGCCACCGGAAACGGTGATTAATACCG
GATATGACGACTTCTCGCATGAGTTGTTGTGGAAAGTTTTTCGGCCAGGGATGTGCTCGCGGCCTATCAGC
TTGATGGTGAGGTAATGGCTCACCATGGCTTCGACGGGTAGCCGGCCTGAGAGGGTGACCGGCCACACTG
GGACTGAGACACGGCC
>Zotu1355
GAACGCTGGCGGCGTGCCTAAGACATGCAAGTCGAGTGGGTTTAGACCCACGGCGAACGGGTGAGTAAT
ACCTAGGAACTTTACTTTAGTCGGGCATAACCAGTCGAAAGATTGGACAATTCCCGATATTATCGCAAGAT
GAAAGCAGCAATGCGCTAAAGTTGAGGCCTGGGCACCATCAGCTAGTTGGTAAGGTAATGGCTTACCAAG
GCTATGACGGTTAGGGGTCCCGAGAGGGATAACCCCCACACTGGAACTGCGACACGGTC
>Zotu1357
GAACGCTGGCGGCAGGCTTAACACATGCAAGTCGAGCGGGGAGATGTAGCTTGCTACATTTCCTAGCGGC
GGACGGGTGAGTAATGCTTAGGAATCTGCCTATTAGTGGGGGACAACATTCCGAAAGGAATGCTAATACC
GCATACGCCCTACGGGGGAAAGCAGGGGATCTTCGGACCTTGCGCTAATAGATGAGCCTAAGTCAGATTA
GCTAGTTGGTGGGGTAAAGGCCTACCAAGGCGACGATCTGTAGCGGGTCTGAGAGGATGATCCGCCACAC
TGGGACTGAGACACGGCC
>Zotu1360
GAACGCTGGCGGCGTGCCTAATACATGCAAGTAGAACGCTGAAGGAGGAGCTTGCTTCTCTGGATGAGTT
GCGAACGGGTGAGTAACGCGTAGGTAACCTGCCTGGTAGCGGGGGATAACTATTGGAAACGATAGCTAAT
ACCGCATAATAGTAGTTGTTGCATGACAACTGTTTGAAAGGTGCAATTGCATCACTACCAGATGGACCTGC
GTTGTATTAGCTAGTTGGTGGGGTAACGGCTCACCAAGGCGACGATACATAGCCGACCTGAGAGGGTGAT
CGGCCACACTGGGACTGAGACACGGCC
>Zotu1362
GAACGCTAGCGGCAGGCTTAACACATGCAAGTCGAGGGGTATAGTAGCAATACTAGAGACCGGCGCACG
GGTGCGTAACGCGTATGCAATCTACCTTTTGCAGGGGAATAGCCCAGAGAAATTTGGATTAATGCCCCATA
GTATAGTACAATCGCATGATTGAACTATTAAAGTTACAACGGCAAAAGATGAGCATGCGTCCCATTAGCTA
GTTGGTAAGGTAACGGCTTACCAAGGCGACGATGGGTAGGGGTCCTGAGAGGGAGATCCCCCACACTGGT
ACTGAGACACGGAC
>Zotu1364
GAACGCTGGCGGCGTGCTTAACACATGCAAGTCGAACGATGATGCCCAGCTTGCTGGGTGGATTAGTGGC
GAACGGGTGAGTAACACGTGAGTAACCTGCCCCTGACTCTGGGATAAGCGTTGGAAACGACGTCTAATAC

TGGATACGACTGCCGGCCGCATGGTCTGGTGGTGGAAAGATTTTTTGGTTGGGGATGGACTCGCGGCCTA
TCAGCTTGTTGGTGAGGTAATGGCTCACCAAGGCGACGACGGGTAGCCGGCCTGAGAGGGTGACCGGCC
ACACTGGGACTGAGACACGGCC

>Zotu1366

GAACGCTGGCGGCGTGCTTAACACATGCAAGTCGAACGATGATGCCCAGCTTGCTGGGTTGATTAGTGGC
GAACGGGTGAGTAACACGTGAGTAACCTGCCCTTGACTTCGGGATAACTCCGGGAAACCGGGGCTAATAC
CGGATATGAGCCGTCCTCGCATGGGGGTGGTTGGAAAGTTTTTCGGTCAGGGATGGGCTCGCGGCCTATC
AGCTTGTTGGTGGGGTGATGGCCTACCAAGGCGACGACGGGTAGCCGGCCTGAGAGGGCGACCGGCCAC
ACTGGGACTGAGACACGGCC

>Zotu1368

CAACGCTGGCGGCGCGCTTAACACATGCAAGTCGAGCGAGAAAGTGGAGCAATCCATGAGTAAAGCGGC
GTACGGGTGAGTAACACGTGGGTAATCTACCTCTTAGTGGGGAATAACCCTGGGAAACCGGGGCTAATAC
CGCATAATCTCGAGAGAGGAAAGCAGCAATGCGCTGAGAGAGGAGCCCGCGGCCGATTAGCTAGTTGGC
AGGGTAAAAGCCTACCAAGGCATAGATCGGTAGCCGGCCTGAGAGGGCACACGGCCACACTGGCACTGA
AACACGGGC

>Zotu1371

GAACGCTAGCTACAGGCTTAACACATGCAAGTCGAGGGGCAGCATGAACTTAGCTTGCTAAGTTTGATGGC
GACCGGCGCACGGGTGAGTAACACGTATCCAACCTGCCGATGACTCGGGGATAGCCTTTCGAAAGAAAGA
TTAATACCCGATGGCATAGTTCTTCCGCATGGTAGAACTATTAAAGGATTTCGGTCATCGATGGGGATGCG
TTCCATTAGGTTGTTGGCGGGGTAACGGCCCACCAAGCCTTCGATGGATAGGGGTTCTGAGAGGAAGGTC
CCCCACATTGGAACTGAGACACGGTC

>Zotu1373

GAACGCTAGCGGCAGGCCTAATACATGCAAGTCGAACGCGATTGGGGAGCTTGCTCCTCATGAAAGTGGC
GCACGGGTGCGTAACGCGTATGCAACCTACCTTAATCAGGGGGATAGCCCGGAGAAATCCGGATTAACAC
CGCATAAAATCACAGTCTGGCATCAGATAATGATCAAATATTTATAGGATTAAGATGGGCATGCGTGACAT
TAGTTAGTTGGTAAGGTAACGGCTTACCAAGACGACGATGTCTAGGGGATCTGAGAGGATGACCCCCCAC
ACTGGTACTGAGACACGGAC

>Zotu1376

GAACGCTGGCGGCGTGCCTAACACATGCAAGTCGAGCGATTCTCTTCGGAGAAGAGCGGCGGACGGGTG
AGTAACGCGTGGGTAACCTGCCCTGTACACACGGATAACATACCGAAAGGTATGCTAATACGAGATAATAT
GCTTTTATCGCATGGTAGAAGTATCAAAGCTTTTGCGGTACAGGATGGACCCGCGTCTGATTAGCTAGTTG
GTAAGGTAACGGCTTACCAAGGCGACGATCAGTAGCCGACCTGAGAGGGTGATCGGCCACATTGGAACTG
AGACACGGTC

>Zotu1379

AAACGCTGGCGGCGTGCATGAGACATGCAAGTCGAACGAACGAGCTTGCTCGTTAGTGGCGGACGGCTGA
GTAACGCGTAGGTGACCTGCCCTGAAGTGGGGGATAACTGCCCGAAAGGGTGGCTAATACCGCATGTGGC
GACACGTTCATTTGTGTCATCAAAGCCGCGAGGCGCTTCGGGAGGGGCCTGCGTCCGATTAGCTCGTTGGT
GGGGTAATGGCTCACCAAGGCGACGATCGGTAGGTGGTCTGAGAGGACGATCACCCAGACTGGGACTGA
GACACGGCC

>Zotu1382

GAACGCTAGCGGCAGGCTTAATACATGCAAGGCGAAGGGGCAGCAATGTCACTGTCGTACGGGTGCGTAA
CGCGTATGCAACCTACCTATCACTGGGGGATAGCCCGGGGAAACCCGGATTAATACCGCATAACACAGGG
GTCCCGCATGGGTACTATTTGTTAAAGATTTATTGGTGGTAGATGGGCATGCGTTCGATTAGCTAGTTGGT
ATGGGTAACGGCCTACCAAGGCGACGATCGATAGGGGAGCTGAGAGGTTGATCCCCCACACGGGAACTG
AGATACGGGC

>Zotu1384

GAACGCTAGCGGCAGGCTTAACACATGCAAGTCGAGGGGCAGCACGATGTAGCAATACATTGGTGGCGAC
CGGCGCACGGGTGCGTAACACGTATGGAATCTGCCCTTTACTGGAGGATAGCCCGGAGAAATCCGGATTA
ATACTCCATAACAGTTCGAGTGGCATCACTTGGATTTGAAAGCTCCGGCGGTAAAGGATGACCATGCGCAC
GATTAGCTAGTAGGTGAGGTAACGGCTCACCTAGGCTACGATCGTTAGGGGGCCTGAGAGGGTTATCCCC
CACACTGGTACTGAGACACGGAC

>Zotu1387
GAACGCTGGCGGCAGGCCTAACACATGCAAGTCGAGCGAGGTTTTCGGACCTAGCGGCGGACGGGTGAG
TAATGCGTAGGAAGCTGCCCGATAGAGGGGGATACCAGTTGGAAACGACTGTTAATACCGCATAATGTCT
ACGGACCAAAGTGTGGGACCTTCGGGCCACATGCTATCGGATGCGCCTACGTGGGATTAGCTAGTTGGTG
GGGTAATGGCTCACCAAGGCGACGATCCCTAGCTGGTTTGAGAGGATGATCAGCCACACTGGGACTGAGA
CACGGCC
>Zotu1389
GAACGCTGGCGGCGTGCTTAACACATGCAAGTCGAACGATGAAGCCCAGCTTGCTGGGTGGATTAGTGGC
GAACGGGTGAGTAACACGTGAGTAACCTGCCCTTGACTCTGGGATAAGCCTGGGAAACTGGGTCTAATAC
TGGATATGACTTCTCACCGCATGGTGGGGGGTGGAAAGATTTATTGGTCTTGGATGGACTCGCGGCCTATC
AGCTTGTTGGTGAGGTAATGGCTCACCAAGGCGACGACGGGTAGCCGGCCTGAGAGGGTGACCGGCCAC
ACTGGGACTGAGACACGGCC
>Zotu1391
GAATGCTGGCGGCGTGCCTAATACATGCAAGTCGAGCGGTAAGGCCCTTCGGGGTACACGAGCGGCGGA
CGGCTGAGTAACGCGTGGGAACATGCCCCAAAGTGAGGGATAAGCACCGGAAACGGTGTCTAATACCGCA
TATGGTCTTCGGATTAAAGGATTTATCCGCTTTGGGAGTGGCCCGCGTACGATTAGTTTGTTGGTGAGGTA
ATGGCTTACCAAGACTACGATCGTTAACTGGTCTGAGAGGATGATCAGTCAGACTGGAACTGAGACACGG
TC
>Zotu1393
GAACGCTGGCGGCATGCCTTACACATGCAAGTCGAACGGCAGCACGGGGGCAACCCTGGTGGCGAGTGG
CGAACGGGTGAGTAATATATCGGAACGTACCCAGTAGTGGGGGATAACTATCCGAAAGGATAGCTAATAC
CGCATACGATCTGTGGATGAAAGCAGGGGATCTTCGGACCTTGTGCTATTGGAATGGCCGATATCTGATTA
GCTAGTTGGTAGGGTAAAAGCCTACCAAGGCGACGATCAGTAGCTGGTCTGAGAGGACGACCAGCCACAC
TGGAACTGAGACACGGTC
>Zotu1396
GAACGCTGGCGGCAGGCTTAACACATGCAAGTCGAGCGCGCCCTTCGGGGTGAGCGGCGGACGGGTGAG
TAACGCGTGGGAACGTGCCCTTTGGTTCGGAATAGCCCCGGGAAACTGGGAGTAATGCCGAATAAGCCCT
TGGCAGCGATGTCTTGGGGAAAGATTTATCGCCAAAGGATCGGCCCGCGTCGGATTAGGTAGTTGGTGGG
GTAACGGCCTACCAAGCCGACGATCCGTAGCTGGTTTGAGAGGATGACCAGCCACACTGGGACTGAGACA
CGGCC
>Zotu1398
GAACGCTAGCGGCAGGCCTAATACATGCAAGTCGAACGGGTCGCAAGGCCAGTGGCAAACGGGTGCGTA
ACGCGTAAGCAACCTGCCTCATACTGGGGGATAGCCCGGCGAAAGCTGGGGTAAACCCGCACGGTCCAGT
TTTATCACCTGGTAAGATTGGTAAACATTTATGGGTATGAGAGGGGCTTGCGTCTGATTAGTTAGTTGGCA
GGGTAACGGCCTACCAAGACGATGATCAGTAGGGGTTCTGAGAGGATTGGCCCCCACATGGGTACTGAGA
TACGGAC
>Zotu1400
GAACGCTGGCGGCGCGCTTAACACATGCAAGTCGAGCGAGAACCAGGGCTTCGGCCCTGGGGACAGCGG
CGAACGGGTGAGTAACACGTGGATAACCTGCCCTCGGCACCGGGATAGCCCGGGGAAACCCGGATTAATA
CCGGATAGCCACTCCGACTCTCGGGTCGGTCTGAAAAGGTAGCTTCGGCCTCTGGCCGAGGATGGGTCCG
CGGCGGATTAGCTTGTTGGTGGGGTAACGGCCTACCAAGGCTCCGATCCGTAGCTGGTCTGAGAGGACGA
TCAGCCACACTGGGACTGAGACACGGCC
>Zotu1402
GAACGCTGGCGGCGTGCCTAATACATGCAAGTCGAGCGGACAGATGGGAGCTTGCTCCCTGATGTTAGCG
GCGGACGGGTGAGTAACACGTGGGTAACCTGCCTGTAAGACTGGGATAACTCCGGGAAACCGGGGCTAA
TACCGGATGCTTGTTTGAACCGCATGGTTCAGACATAAAAGGTGGCTTCGGCTACCACTTACAGATGGACC
CGCGGCGCATTAGCTAGTTGGTGAGGTAACGGCTCACCAAGGCAACGATGCGTAGCCGACCTGAGAGGGT
GATCGGCCACACTGGGACTGAGACACGGCC
>Zotu1404

GAACGCTGGCGGTATGCTTTACACATGCAAGTCGTACGAGAGTTTTTAACTCAAGTGGCGGACGGGTGAG
TAACACGTAAGAATTTGCCTTTAGGAGGGGGATAACAACTGGAAACGGTTGCTAATACCCCATATGCTTTC
GAGTAAAATGGATTTTCCGCCTAAAGAGAAGCTTGCGGCTGATTAGCTTGTTGGTGAGGTAATGGCTCACC
AAGGCGACGATCAGTATCTGGTTTGAGAGGATGATCAGACACACTGGAACTGAGACACGGTC
>Zotu1406
GAACGCTGGCGGCAGGCTTAACACATGCAAGTCGAACGATGAAACTCTAGCTTGCTAGAGATGATTAGTG
GCGGACGGGTGAGTAACATTTAGGAATCTGCCTAGTAGTGGGGGATAGCTCGGGGAAACTCGAATTAATA
CCGCATACGACCTACGGGTGAAAGGGGGCGCAAGCTCTTGCTATTAGATGAGCCTAAATCAGATTAGCTA
GTTGGTGGGGTAAAGGCCTACCAAGGCGACGATCTGTAACTGGTCTGAGAGGATGATCAGTCACACCGGA
ACTGAGACACGGTC
>Zotu1408
GAACGCTGGCGGCGCGCTTAACACATGCAAGTCGAGCGAGAACCAGGGCTTCGGCCCTGGGGACAGCGG
CGAACGGGTGAGTAACACGTGGATAACCTGCCCTCGGCACCGGGATAGCCCGGGGAAACCCGGATTAATA
CCGGATAGCCTTTCCGACTCTCGAGTCGGTCCGAAAAGGTAGCTTCGGCCTCTGGCCGAGGATGGGTCCGC
GGCGGATTAGCTTGTTGGTGGGGTAACGGCCTACCAAGGCTCCGATCCGTAGCTGGTCTGAGAGGACGAT
CAGCCACACTGGGACTGAGACACGGCC
>Zotu1410
GAACGCTGGCGGCGTGCTTAACACATGCAAGTCGAACGGTAAGGCCCTTCGGGGTACACGAGTGGCGAAC
GGGTGAGTAACACGTGGGTAATCTGCCCTGCACTTCGGGATAAGCCTGGGAAACCGGGTCTAATACCGGA
TATTCAGTTCCTGCCGCATGGTGGGGGTTGGAAAGTTTTTCGGTGCAGGATGAGCCCGCGGCCTATCAGCT
TGTTGGTGGGGTAATGGCCTACCAAGGCGACGACGGGTAGCCGGCCTGAGAGGGTGATCGGCCACACTG
GGACTGAGACACGGCC
>Zotu1412
GAACGCTAGCGGCAGGCTTAACACATGCAAGTCGAGGGGCAGCGCGGTAGCAATACTGGCGGCGACCGG
CAAACGGGTGCGGAACACGTACAGAACCTACCTTTACCTGGGGCATAGCCCGGAGAAATCCGGATTAACTC
CCCATAATATAATTGGATGGCATCATTTGGTTATTAAAAATTTATTGGGTAAAGATGGCTGTGCGTCCCATT
AGCTAGTTGGTAGGGTAACGGCCTACCAAGGCGACGATGGGTAGCTGGTGTGAGAGCACGACCAGCCAC
ACGGGCACTGAGACACGGGC
>Zotu1414
GAACGCTGGCGGCGTGCTTAACACATGCAAGTCGAGCGGTAAGGCCCTTTCGGGGGTACACGAGCGGCG
AACGGGTGAGTAACACGTGAGTAATCTGCCCTTCACTTCGGGATAAGCCTCGGAAACGGGGTCTAATACCG
GATATGACCACTTCGGGCATCCGATGGTGGTGGAAAGTTTTTTCGGTGGGGGATGTGCTCGCGGCCTATCA
GCTTGTTGGTGGGGTAATGGCCTACCAAGGCTTCGACGGGTAGCCGGCCTGAGAGGGTGACCGGCCACAC
TGGGACTGAGACACGGCC
>Zotu1418
GAACGCTGGCGGCAGGCCTAACACATGCAAGTCGAGCGGATGAGTGGAGCTTGCTCCATGATTCAGCGGC
GGACGGGTGAGTAATGCCTAGGAATCTGCCTGGTAGTGGGGGACAACGTTTCGAAAGGAACGCTAATACC
GCATACGTCCTACGGGAGAAAGCAGGGGACCTTCGGGCCTTGCGCTATCAGATGAGCCTAGGTCGGATTA
GCTAGTTGGTGGGGTAAAGGCCTACCAAGGCGACGATCCGTAACTGGTCTGAGAGGATGATCAGTCACAC
TGGAACTGAGACACGGTC
>Zotu1421
GAACGCTAGCGGCAGGCTTAATACATGCAAGTCGAGGGGCAGCGCGGGTAGCAATACCTGGCGGCGACC
GGCAAACGGGTGCGGAACACGTACGCAACCTTCCTTCAAGTGGGGAATAGCCCAGGGAAACTTGGATTAA
TACCCCTTAATATTATTGAGTGGCATCACTTGATAATTAAAGCTCCGGCGCTTGAAGATGGGCGTGCGCCTG
ATTAGGTAGTTGGCGGGGTAACAGCCCACCAAGCCTACGATCAGTAACTGGTGTGAGAGCACGACCAGTC
ACACGGGCACTGAGACACGGGC
>Zotu1424
GAACGCTGGCGGCGTGCTTAACACATGCAAGTCGAACGGTAAGGCCCTTCGGGGTACACGAGTGGCGAAC
GGGTGAGTAACACGTGAGTGACCTGCCCTTTACTTTGGGATAACCACCAGAAATGGTGGCTAATACCGAAT
ATGACTCCTGCCTGCATGGGTGGGGGTGGAAAGCTCCGGCGGTAAGGGATGGACTCGCGGCCTATCAGCT

TGTTGGTGAGGTAGTGGCTCACCAAGGCTTCGACGGGTAGCCGGCCTGAGAGGGTGACCGGCCACATTGG
GACTGAGATACGGCC
>Zotu1426
GAACGCTAGCGGCAGGCCTAATACATGCAAGTCGAACGGGCTTTGGTTTTCGGATCAGGGCTAGTGGCGC
ACGGGTGCGTAACGCGTACACAACATACCCTTTACTGGGGGATAGCGGTGGGAAACTGCCAGTAATACCC
CATGCCCTTACGAGGTGGCATTACCTTGTGAGGAAAGTTACGGCGGTAAAGGATTGGTGTGCGTCCGATTA
GCTAGTTGGCGGGGTAACGGCCCACCAAGGCGACGATCGGTAGGGGGTGTGAGAGCATGGCCCCCCACA
CGGGTACTGAGACACGGAC
>Zotu1428
GAACGCTAGCGGCAGGCCTAATACATGCAAGTCGAACGGTGTCTTCGGACATAGTGGCGCACGGGTGCGT
AACACGTATGCAACCTACCTTACATTGGGGGATAGCCTTTCGAAAGGGAGATTAATACCGCATAAGACAGT
AGCTGGGCATCCAGCAGCTGTTAAAGATTTATCGATGTAAGCTGGGCATGCGTCCAATTAGCTAGTTGGTG
AGGTAACGGCTCACCAAGGCTATGATTGGTAGGGGAACTGAGAGGTCAATCCCCCACACTGGCACTGAGA
TACGGGC
>Zotu1430
TAACGCTGGCGGTATGCCTAATACATGCAAGTCGAACGCGCACTTCGGTGCGAGTGGCGGACGGGTGAGT
AACACGTTGGAACCTGCCCCGAAGTGGGGAATAACCCATCGAAAGATGGGCTAATACCCCATAGTCTTATG
CTTGTCATAAGTAAAGTTTTTCGCTTCGGGAGGGGCCTGCGCAACATTAGCTAGTTGGTGAGATAAAAGCC
CACCAAGGCGACGATGTTTAGCTGGATCGAGAGGTTGACCAGCCACACTGGGACTGAGATACGGCC
>Zotu1433
GAACGCTGGCGGCGCGCCTAAAACATGCAAGTCGAACGAGAAACCCGGGCTTGCCCGGGTGGACAGTGG
CGAACGGGTGAGTAACACGTGAGGAACCTGCCCCGAAGACCGGGACAACACCGGGAAACCGGTGCTAAT
ACCGGATGTCCTCACCGGATCGCATGATCTGGTGAGGAAATGGATTCCGCTTCGGGATGGCCTCGCGGCCT
ATCAGCTAGTTGGTGGGGTAACGGCCCACCAAGGCGACGACGGGTAGCTGGTCTGAGAGGATGTCCAGCC
ACACTGGGACTGAGACACGGCC
>Zotu1436
GAACGCTGGAAATGTGGATACCGGCATGCAAGTCAAGGGGCCGCAAGGCACCGGCGAACGGCTTAGTAA
CACGTGGATACATACCGCAAAGTGGGGCATAGCTCGTCGAAAGACGGGGTAATTTCCCATAGTCCCGCAA
GGGTAAAGGCGCAAGCCGCTTTGTGAATGGTCCGCGGCCTATCAGATAGTTGGCGAGGTAAAAGCTCACC
AAGTCTATGACGGGTAGGGGGCGTGAGAGCGCGACCCCCAAGAATGGCACTGCGACACGGGC
>Zotu1439
GAATGCTGGCGGCGTGCCTAATACATGCAAGTCGAGCGGTAAGGCCCTTCGGGGTACACGAGCGGCGGA
CGGCTGAGTAACGCGTGGGAACATGCCCCAAAGTGAGGGATAACCTCGAGAAATCGAGGCTAATACCGCA
TATGGTCTTCGGATTAAAGGATTTATCCGCTTTGGGAGTGGCCCGCGTACGATTAGTTTGTTGGTGAGGTA
ATGGCTCACCAAGACTACGATCGTTAACTGGTCTGAGAGGATGATCAGTCAGACTGGAACTGAGACACGG
TC
>Zotu1441
GAACGCTGGCGGCGCGCCTAACACATGCAAGTCGAACGGAACTTGAGAGAGCTTGCTTTTTCAAGTTTAGT
GGCGAACGGGTGAGTAACGCGTGAGTAACCTGCCCTGGAGTGGGGGACAACAGTTGGAAACGACTGCTA
ATACCGCATAAGCCCACGGTGCCGCATGGCACAGCGGGAAAAGGATTTATTCGCTTCAGGATGGACTCGC
GTCCAATTAGCTAGTTGGTGAGGTAACGGCCCACCAAGGCGACGATTGGTAGCCGGACTGAGAGGTTGAA
CGGCCACATTGGGACTGAGACACGGCC
>Zotu1443
GAACGCTGGCGGCGTGCCTAACACATGCAAGTCGAGCGATTTACTTCGGTAAAGAGCGGCGGACGGGTGA
GTAACGCGTGGGTAACCTGCCCTGTACACACGGATAACATACCGAAAGGTATGCTAATACGAGATAATATA
CTTTTATCGCATGGTAGAAGTATCAAAGCTCCGGCGGTACAGGATGGACCCGCGTCTGATTAGCTAGTTGG
TAAGGTAACGGCTTACCAAGGCGACGATCAGTAGCCGACCTGAGAGGGTGATCGGCCACATTGGAACTGA
GACACGGTC
>Zotu1445

TAACGCTGGCGGCATGCCTAATACATGCAAGTCGAACGAGAGTACTTGTACTCTAGTGGCGAACGGGTGA
GTAACACGTGGGCAACCTGTCCTAAAGACGAAGATAACAGTTGGAAACGACTGATAATACTGGATAGTTTA
ACGAAACGCATGTTTTGTTAATTAAAGGTGGTAACACCGCTTTAGGAGGGGCCCGCGGTGCATTAGCTAGT
TGGTGAGGTAACGGCCCACCAAGGCTACGATGCATAGCTGTGCTGAGAGGCAAAACAGCCACACTGGGAC
TGAGACACGGCC
>Zotu1447
GAACGCTGGCGGCGTGCCTAATACATGCAAGTAGAACGCTGAAGGAGGAGCTTGCTTCTCCGGATGAGTT
GCGAACGGGTGAGTAACGCGTAGGTAACCTGCCTGGTAGCGGGGGATAACTATTGGAAACGATAGCTAAT
ACCGCATAAGAGTAGATGTTGCATGACATTTGCTTAAAAGGTGCAATTGCATCACTACCAGATGGACCTGC
GTTGTATTAGCTAGTTGGTGGGGTAACGGCTTACCAAGGCGACGATACATAGCCGACCTGAGAGGGTGAT
CGGCCACACTGGGACTGAGACACGGCC
>Zotu1449
GAACGCTGGCGGCGTGCTTAACACATGCAAGTCGAACGGTGATGGCCAGCTTGCTGGTCTGATCAGTGGC
GAACGGGTGAGTAACACGTGAGCAACCTGCCCTTCACTCTGGGATAAGCCCTGGAAACGGGGTCTAATAC
CGGATATGAGACGCACACGCATGTGTAGTGTCTGGAAAGATTTATCGGTGGGGGATGGGCTCGCGGCCTA
TCAGCTTGTTGGTGGGGTAGTGGCCTACCAAGGCGACGACGGGTAGCCGGCCTGAGAGGGCGACCGGCC
ACACTGGGACTGAGACACGGCC
>Zotu1451
GAACGCTGGCGGCAGGCTTAACACATGCAAGTCGAGCGCCCCGCAAGGGGAGCGGCAGACGGGAGAGTA
ACACGTGGGAACGTACCCTTCAGTACGGAACAACCAAGGGAAACTTTGGCTAATACCGTATACGTCCGTAA
GGAGAAAGATTTATCGCTGAAGGATCGGCCCGCGTCTGATTAGCTAGTTGGTGGGGTAATGGCCCACCAA
GGCGACGATCAGTAGCTGGTCTGAGAGGATGATCAGCCTCACTGGGACTGAGACACGGCC
>Zotu1454
GAATGCTGGCGGCGTGGATAAGTCATGCAAGTCAAGGGGGCCCGCAAGGGCAACCGGCAGACGAGGTAG
TAACACGTAGGTAGATCCCCCGAAGTCGGGCATAGCCAGTCGAAAGACTGGGTAATTCCCGATAGTCCCGC
AAGGGTAAAGGTTTTTCGCTTCGGGAGTTGCCTGCGTAGTATCAGCTAGTTGGTAGGGTAATGGCCTACCA
AGGCTACGACGCTTAGGGGAGCTGAGAGGCTGACCCCCACCGATGGAACTGCGACACGGTC
>Zotu1456
GAACGCTGGCGGCGTGCTTAACACATGCAAGTCGAACGGTGAAGGTGGAGCTTGCTCTGCTGGATCAGTG
GCGAACGGGTGAGTAACACGTGAGCAATCTGCCCCTGACTCTGGGATAACAGTTGGAAACAGCTGCTAAT
ACTGGATATGAACTGTGAAGGCATCTTCAGCAGTTGGAAAGAATTTCGGTCAGGGATGAGCTCGCGGCCT
ATCAGCTTGTTGGTGAGGTAATGGCTCACCAAGGCGTCGACGGGTAGCCGGCCTGAGAGGGTGACCGGCC
ACACTGGGACTGAGACACGGCC
>Zotu1458
GAACGCTGGCGGCAGGCCTAACACATGCAAGTCGAGCGGATGAAGGGAGCTTGCTCCCTGATTTAGCGGC
GGACGGGTGAGTAATGCCTAGGAATCTGCCTGGTAGTGGGGGATAACGTTCCGAAAGGAACGCTAATACC
GCATACGTCCTACGGGAGAAAGCAGGGGACCTTCGGGCCTTGCGCTATCAGATGAGCCTAGGTCGGATTA
GCTAGTTGGTGAGGTAATGGCTCACCAAGGCGACGATCCGTAACTGGTCTGAGAGGATGATCAGTCACAC
TGGAACTGAGACACGGTC
>Zotu1461
GAACGCTGGCGGCAGGCCTAACACATGCAAGTCGAACGCGACCTTCGGGTCGAGTGGCGCACGGGTGAG
TAACGCGTGGGAACCTGTCCTGTGGTACGGAATAACATCGGGAAACTGGTGCTAATACCGTATATCCCCTG
AGGGGGAAAGATTTATCGCCATAGGAGGGGCCCGCGTAGGATTAGCTAGTTGGTGAGGTAACGGCTCACC
AAGGCGACGATCCTTAGCTGGTCTGAGAGGATGATCAGCCACACTGGGACTGAGACACGGCC
>Zotu1464
GAACGCTGGCGGCAGGCTTAACACATGCAAGTCGAACGGTCCTTCGGGATAGTGGCGGACGGGTGAGTA
ACACGTGGGAACGTACCCTTAGGTTCGGAACAACACAGGGAAACTTGTGCTAATACCGGATAAGCCCTTCG
GGGGAAAGATTTATCGCCTTTGGAGCGGCCCGCGTCTGATTAGCTTGTTGGTGAGGTAATGGCTCACCAAG
GCTACGATCAGTAGCTGGTCTGAGAGGATGGCCAGCCACACTGGGACTGAGACACGGCC
>Zotu1466

GAACGCTGGCGGCGTGCCTAATACATGCAAGTCGAGCGAACAGACGAGGAGCTTGCTCCTTTGATGTTAG
CGGCGGACGGGTGAGTAACACGTAGGTAACCTACCTATAAGACTGGGATAACTTCGGGAAACCGGAGCTA
ATACCGGATAATATTTCGAACCGCATGGTTCGATAGTGAAAGATGGCTTTGCTATCACTTATAGATGGACCT
GCGCCGTATTAGCTAGTTGGTAAGGTAACGGCTTACCAAGGCAACGATACGTAGCCGACCTGAGAGGGTG
ATCGGCCACACTGGAACTGAGACACGGTC
>Zotu1468
GAACGCTGGCTGCGTGCTTAACACATGCAAGTCGAACGCTGAAGCCGACAGCTTGCTGTTGGTGGATGAG
TGGCGAACGGGTGAGTAACACGTGAGTAACCTGCCCCTGATTTCGGGATAAGCCTGGGAAACTGGGTCTA
ATACCGGATACGACCTCCTGACGCATGTTGGGTGGTGGAAAGTTTTTCGATCGGGGATGGGCTCGCGGCCT
ATCAGCTTGTTGGTGGGGTAATGGCCTACCAAGGCGACGACGGGTAGCCGGCCTGAGAGGGCGACCGGC
CACACTGGGACTGAGACACGGCC
>Zotu1470
GAACGCTGGCGGCGTGCTTAACACATGCAAGTCGAACGAGATCCATTCAGTAGCAATACTGATGAAGATCT
AGTGGCGAACGGGTGCGTAACACGTGAGGAACTTGTCCCGGTCTCTGGGATAACAGTTGGAAACGACTGC
TAATACCGGATGACGTCGGAACTTCGCATGGAGTACTGACGAAAGGGTTACTGGATCGGGAGAGCCTCGC
GGCCTATCAGCTTGTTGGTGGGGTAATGGCCTACCAAGGCATCGACGGGTAGCTGGTCTGAGAGGATGAT
CAGCCACACTGGGACTGAGACACGGCC
>Zotu1473
GAACGCTGGCGGCGTGGATAAGGCATGCAAGTCGAAAGGGTTTAGGCCCTTGGCAAACGGGTTAGTAAT
GCATAGGAACTTTCCTTGAAGTCGTGAATAATCTGGAGAAATCCAGGATAATACACGATGTGCTCTACGGA
GTAAAGATTTATCGCTTCAAGAGAGGCCTATGTCCTATCAGCTAGTTGGTAGTGTAATGGACTACCAAGGC
TATGACGGGTAGGGGGTGTGAGAGCACGGCCCCCAACATTGGGACTGAGAACTGCC
>Zotu1475
GAACGCTGGCGGCGTGCCTAACACATGCAAGTCGAACGGGGTCCATCTGGTGGCAACACCGGGGAAGAC
CTAGTGGCGAACGGGTGAGTAACACGTGAGAAACCCACCCCGATCTCTGGGATAACAGGTGGAAACACCT
GCTAATACCGGATGCCTTCACCTCGTCGCATGGCGGGGTGAAGAAAGGGCTCTACCGGATCGGGACGGTC
TCGCGGCCTATCAGCTTGTTGGTGGGGTAATGGCCCACCAAGGCTTCGACGGGTAGCTGGTCTGAGAGGA
CGATCAGCCACACTGGGACTGAGACACGGCC
>Zotu1479
GAACGCTGGCGGCGTGCCTAATACATGCAAGTCGAGCGGACTGAATGAAAGCTTGCTTTCATTCGGTTAGC
GGCGGACGGGTGAGTAACACGTGGGCAACCTGCCCGTAAGACCGGGATAACTTCGGGAAACCGGAGCTA
ATACCGGATAACACCGAAGATCGCATGATCTTTGGTTGAAAGGCGGTTTTGGCTGTCACTTACGGATGGGC
CCGCGGCGCATTAGTTAGTTGGTGAGGTAACGGCTCACCAAGGCGACGATGCGTAGCCGACCTGAGAGGG
TGATCGGCCACACTGGGACTGAGACACGGCC
>Zotu1481
GAACGCTAGCAATATGCTTTACACATGCAAGTCAAATGTGTCGTTTATATTAAACGCTACATGGCAAACGG
GTGAGTAACGCGTAGGAATCTACCTAACAGTACGGAATAAGTTGCACAGGTATCCTGTGAAATCAATACCG
TATATAAAAGATATATATCGCTGTTAGATGAGCCTGCGTAAGATTAGGTAGTTGGTAAGGTAATGGCTTAC
CAAGCCTTGGATCTTTAGCTGGTCTGAGAGGATGATCAGCCACACTAGGACTGAGACACGGCC
>Zotu1483
GAACGCTGGCGGCAGGCCTAACACATGCAAGTCGAGCGGTAGCACAGAGAGCTTGCTCTCGGGTGACGA
GCGGCGGACGGGTGAGTAATGTCTGGGAAACTGCCTGATGGAGGGGGATAACTACTGGAAACGGTAGCT
AATACCGCATAATGTCGCAAGACCAAAGTGGGGGACCTTCGGGCCTCATGCCATCAGATGTGCCCAGATG
GGATTAGCTAGTAGGTGGGGTAACGGCTCACCTAGGCGACGATCCCTAGCTGGTCTGAGAGGATGACCAG
CCACACTGGAACTGAGACACGGTC
>Zotu1485
GAACGCTAGCGGCAGGCCTAACACATGCAAGTCGAGGGAGAGGTTACCTTCGGGTAACCAAAACCGGCGC
ACGGGTGCGTAACGCGTATACAATCTGCCTTTCACATAGGGATAGCCCGAAGAAATTTGGATTAATACCTA
ATGGTATATAAGAGCGGCATCGTTTTTATATTAAAGCTACGGTGGTGAAAGATGAGTATGCGTTCTATTAG
CTAGTTGGAGAGGTAACGGCTCCCCAAGGCGACGATAGATAGGGGTTCTGAGAGGGATGTCCCCCACACT
GGTACTGAGATACGGAC

>Zotu1487
GAACGCTAGCGGCAGGCCTAATACATGCAAGTCGAACGGTTGTATTTATACAATAGTGGCGCACGGGTGC
GTAACACGTATGAAACCTACCTTATAGACGGGGATAGCCTTTGGAAACGGAGATTAATACCCGATGGTATA
TATTGATGGCATCATTAATATATTAAAGATTTATCGCTATAAGCTGGTCATGCGTCCAATTAGTTAGTTGGC
GGGGTAATGGCTCACCAAGACGATGATTGGTAGGGGAACTGAGAGGTTAATCCCCCACACTGGCACTGAG
ATACGGGC
>Zotu1489
GAACGCTAGCGGCAGGCCTAATACATGCAAGTCGGACGGGATTGGAGAGCTTGCTTTCCATGAGAGTGGC
GCACGGGTGCGTAACACGTATGTAACCTACCCTTATCAGGGGGATAGCCTCTCGAAAGAGAGATTAAGCCC
GCATAATATGAAGACACAGCATTGTGATTTCATCAAATATTTATAGGATAAGGATGGGCATGCGGAACATT
AGCTAGTTGGTAAGGTAATGGCTTACCAAGGCTACGATGTTTAGGGGATCTGAGAGGATGACCCCCCACA
CTGGTACTGAGACACGGAC
>Zotu1491
GAACGCTGGCGGCGTGCCTAATACATGCAAGTCGAGCGAACCTTTGGGAGCTTGCTCCCAAAGGTTAGCG
GCGGACGGGTGAGTAACACGTGGGCAACCTGCCTATAAGACTGGGATAACTTCGGGAAACCGGAGCTAAT
ACCGGATAATTCTTTTCTACTCATGTAGAAAAGCTAAAAGACGGTTTACGCTGTCACTTATAGATGGGCCCG
CGGCGCATTAGCTAGTTGGTGAGGTAACGGCTCACCAAGGCCACGATGCGTAGCCGACCTGAGAGGGTGA
TCGGCCACACTGGGACTGAGACACGGCC
>Zotu1493
GAACGCTGGCGGCGTGCCTAATACATGAAAGTAGAACGCTGAAGACTTTAGCTTGCTAGAGTTGGAAGAG
TTGCGAACGGGTGAGTAACGCGTAGGTAACCTGCCTACTAGCGGGGGATAACTATTGGAAACGATAGCTA
ATACCGCATAATAGTATTTAACACATGTTAGAGACTTGAAAGATGCAATTGCATCACTAGTAGATGGACCT
GCGTTGTATTAGCTAGTTGGTGGGGTAACGGCCTACCAAGGCGACGATACATAGCCGACCTGAGAGGGTG
ATCGGCCACACTGGGACTGAGACACGGCC
>Zotu1496
GAACGCTGGCGGCGTGCTTAACACATGCAAGTCGAACGATGAAGCCCAGCTTGCTGGGTGGATTAGTGGC
GAACGGGTGAGTAACACGTGAGTAACCTGCCCTTGACTCTGGGATAAGCCTGGGAAACTGGGTCTAATAC
CGGATAGGAACGTCCACCGCATGGTGGGTGTTGGAAAGATTTATCGGTCATGGATGGACTCGCGGCCTAT
CAGCTTGTTGGTGAGGTAATGGCTCACCAAGGCGACGACGGGTAGCCGGCCTGAGAGGGTGACCGGCCA
CACTGGGACTGAGACACGGCC
>Zotu1498
CAACGCTGGCGGCGCGCTTAACACATGCAAGTCGCACGAGAAAGTGGAGCAATCCATGAGTACAGTGGCG
TACGGGTGAGTAACACGTGGATCATCTACCTCTTGGTGGGGAATAACCCTGGGAAACCGGGGCTAATACC
GCATAAGCCTGCAAAGGGAAAGCAGCAATGCGCCGAGAGAGGAGTCCGCGGCCGATTAGCTGGTTGGTA
GGGTAACGGCCTACCAAGGCAATGATCGGTAGCCGGCCTGAGAGGGCACACGGCCACACTGGCACTGAA
ACACGGGC
>Zotu1501
GAACGCTGGCGGCGTGCCTAAGACATGCAAGTCGAGTGGGTTTAGACCCACGGCGAACGGGTGAGTAAT
ACCTAGGAACGTCCCTTAAGTTGAGGATATCCCATCGAAAGATGGTGCAATACTCAATGTGATCGCAAGAT
CAAAGTCGCAAGACGCTTAAGGTGCGGCCTGGGCACCATCAGCTAGTTGGTAAGGTAATGGCTTACCAAG
GCTATGACGGTTAGGGGCCCCGAGAGGGGAGACCCCCACACTGGAACTGCGACACGGTC
>Zotu1503
GAACGCTAGCGGCAGGCCTAATACATGCAAGTCGGACGGGATTCCGTTGTAGCTTGCTATGACGGATGAG
AGTGGCGCACGGGTGCGTAACGCGTGAGCAACCTGCCCATGTCAGGGGGATGGCCCGTTGAAAGACGGA
TTAACACCGCATAATACATTATGCCCGCCTGGGTATTCTGTTAAATATTTATAGGACATGGATGGGCTCGCG
TGGCATTAGCTTGTTGGTAAGGTAACGGCTTACCAAGGCTATGATGCCTAGGGGCTCTGAGAGGAGAATC
CCCCACACTGGTACTGAGACACGGAC
>Zotu1505
GAACGCTGGCGGCAGGCTTAACACATGCAAGTCGAGCGGGCGTAGCAATACGTCAGCGGCAGACGGGTG
AGTAACGCGTGGGAACGTACCTTTTGGTTCGGAACAACACAGGGAAACTTGTGCTAATACCGGATAAGCCC

TTACGGGGAAAGATTTATCGCCGAAAGATCGGCCCGCGTCTGATTAGCTAGTTGGTGGGGTAATGGCCCA
CCAAGGCGACGATCAGTAGCTGGTCTGAGAGGATGATCAGCCACATTGGGACTGAGACACGGCC
>Zotu1507
GAACGCTGGCGGCGTGCTTAACACATGCAAGTCGAGCGGAGTTAATTGAAAGCTTGCTTTTGATTAACTTA
GCGGCGGACGGGTGAGTAACGTGTGGGCAACCTGCCCTGTACTGTGGAATAATCACTTGAAAAGGTGACT
AATACCGCATGTGGTCCCTGGAAGGCATCTTCTGGGGAAGAAAGGATTTATTCGGTACAGGATGGGCCCG
CATCTGATTAGCTAGTTGGTGAGATAACAGCCCACCAAGGCGACGATCAGTAGCCGACCTGAGAGGGTGA
TCGGCCACATTGGGACTGAGACACGGCC
>Zotu1509
GAACGCTGGCGGCAGGCCTAACACATGCAAGTCGAGCGCGCCCTTCGGGGTGAGCGGCGGACGGGTGAG
TAACGCGTGGGAACGTGCCCTTCTCTGCGGAATAGCCTCGGGAAACTGAGAGTAATACCGCATACGCCCTT
TGGGGGAAAGATTTATCGGAGAAGGATCGGCCCGCGTTGGATTAGGTAGTTGGTGGGGTAATGGCCCACC
AAGCCGACGATCCATAGCTGGTTTGAGAGGATGATCAGCCACACTGGGACTGAGACACGGCC
>Zotu1512
GAACGCTGGCGGCATGCCTAACACATGCAAGTCGGACGGGAAGTGGTGTTTCCAGTGGCGGACGGGTGA
GTAACGCGTAAGAACCTGCCCTTGGGAGGGGAACAACAACTGGAAACGGCTGCTAATACCCCGTAGGCTG
AGGAGCAAAAGGAGGAATCTGCCCGAGGAGGGGCTCGCGTCTGATTAGCTAGTTGGTGAGGCAATAGCT
TACCAAGGCGATGATCAGTAGCTGGTCCGAGAGGATGATCAGCCACACTGGGACTGAGACACGGCC
>Zotu1514
GAACGCTGGCGGCGTGCCTAACACATGCAAGTCGAACGGAGATTTCATTTATGGAATCTTAGTGGCGAAC
GGGTGAGTAACGCGTAGACAACCTACCTTTTAGATGGGGACAACACTGCGAAAGTGGTGCTAATACCGAA
TGTTGCATTTTGGTTGCATAACCGAAATACTAAAGATGGCCTCTATTTATAAGCTATCGCTAAAAGATGGGT
CTGCGTCTGATTAGCTAGTTGGTGAGGTAATGGCTCACCAAGGCGACGATCAGTAGCCGGTCTGAGAGGA
TGAACGGCCACACTGGGACTGAGACACGGCC
>Zotu1517
CAACGCTGGCGGCGTGCCTAATGCATGCAAGTCGAACGGGGTCATCTTCGGATGGCTTACGTGGCGGACG
GGTGAGGAGCACGTGGGCAACCTGCCCTTTGGTGGAGGATAACCGCTGGAAACAGTGGGTAATACTGCAT
AACGCATTTTGACGAGTGGGTTGGAATGTAAAAGTCGCAAGACGCCGAAGGAGGGGCCTGCGTCCGATTA
GCTAGACGGTGGGGTAACGGCCTACCGTGGCGACGATCGGTAGCTGGTTTGAGAGGATGATCAGCCACAC
GGGGACTGAGACACGGCC
>Zotu1519
GAACGCTGGCGGCGTGCCTAATACATGCAAGTCGAGCGGAAATTTTATTGGTGCTTGCACCTTTAAAATTTT
AGCGGCGGACGGGTGAGTAACACGTGGGTAACCTACCTTATAGATTGGAATAACTCCGGGAAACCGGGGC
TAATACCGAATAATACTTTTTAACACATGTTTGAAAGTTGAAAGACGGTTTCGGCTGTCACTATAAGATGGA
CCCGCGGCGCATTAGCTAGTTGGTGAGGTAACGGCTCACCAAGGCAACGATGCGTAGCCGACCTGAGAGG
GTGATCGGCCACACTGGGACTGAGACACGGCC
>Zotu1521
TAACGCTGGCGGCGTGCATAACACATGCAAGTCGAACGATGAAACTTAAAAGATTTCTTCGGAATGAACTT
AAGTGGATTAGTGGCGAACGGGTGAGTAACGCGTGAGTAACCTGCCTTACACAAGGGGATAGCCTCTGGA
AACGGAGAATAATACCCTATGAAACTATCTTATCGCATGAAAAGATAGTCAAAGTGTTTTAGCGGTGTAAG
ATGGACTTGCGTCTGATTAGCTAGTTGGTGAGATAACAGCCCACCAAGGCAACGATCAGTAGCCGGCTTGA
GAGAGTGTACGGCCACATTGGGACTGAGACACGGCC
>Zotu1523
GAACGCTGGCGGAATGCTTTACACATGCAAGTCGAACGGCAGCACGAGGGCAACCTTGGTGGCGAGTGG
CGAACGGGTGAGTAATATATCGGAACGTGCCCAGTCGTGGGGGATAACGTAGTGAAAATTACGCTAATAC
CGCATACGATCTATGGATGAAAGCGGGGGATCGCAAGACCTCGCGCGATTGGAGCGGCCGATATCAGATT
AGGTAGTTGGTGGGGTAAAGGCTCACCAAGCCAACGATCTGTAGCTGGTCTGAGAGGACGACCAGCCACA
CTGGAACTGAGACACGGTC
>Zotu1525

GAACGCTAGCTACAGGCTTAACACATGCAAGTCGAGGGGAAACGATGAGAGAGCTTGCTCTCTTAGGCGT
CGACCGGCGCACGGGTGAGTAACGCGTATCCAACCTGCCCATTACAGGGGAATAACCTTGCGAAAGTAAG
ACTAATGCCCCATGGTATTCTCAGCAGACATCTAATGAGGATTAAAGATTTATCGGTAATGGATGGGGATG
CGTCTGATTAGCTTGTTGGCGGGGTAACGGCCCACCAAGGCAACGATCAGTAGGGGTTCTGAGAGGAAGG
TCCCCCACATTGGAACTGAGACACGGTC

>Zotu1527

GAACGCTGGCGGCATGCCTAACACATGCAAGTCGAACGAAGGCTTCGGCCTTAGTGGCGCACGGGTGCGT
AACGCGTGGGAATCTGCCCTTGGGTCTGGGATAACTCAGAGAAATTTGAGCTAATACCGGATGATGACTTC
GGTCCAAAGATTTATCGCCCGAGGATGAGCCCGCGTTGGATTAGCTAGTTGGTGGGGTAAAGGCCTACCA
AGGCGACGATCCATAGCTGGTCTGAGAGGATGATCAGCCACACTGGGACTGAGACACGGCC

>Zotu1529

GAACGCTGGCGGCATGCTTAACACATGCAAGTCGCACGGTCAGGTTTCGGCCTGGCAGTGGCGGACGGGT
GAGTAACGCGTAGGTATCTATCCATGGGTGGGGGATAACGGTTGGAAACGACTGCTAATACCGCATGATA
CCCGAGGGTCAAAGGCGCAAGTCGCCTGTGGAGGAGCCTGCGTACGATTAGCTAGTTGGTGGGGTAAAG
GCCTACCAAGGCGACGATCGTTAGCTGGTCTGAGAGGATGATCAGCCACACTGGGACTGAGACACGGCC

>Zotu1531

GAACGCTGGCGGCAGGCCTAACACATGCAAGTCGAGCGCCCCGCAAGGGGAGCGGCAGACGGGAGAGTA
ACACGTGGGAACGTACCCTTCAGTTCGGAACAACCCAGGGAAACTTGGGCTAATACCGGATACGTCCGTAA
GGAGAAAGATTTATCGCTGAAGGATCGGCCCGCGTCTGATTAGCTAGTTGGTGAGGTAACGGCTCACCAA
GGCGACGATCAGTAGCTGGTCTGAGAGGATGATCAGCCTCACTGGGACTGAGACACGGCC

>Zotu1533

GAACGCTGGCGGCGTGCTTAACACATGCAAGTCGAGCGGTAAGGCCCTTCGGGGTACACGAGCGGCGAA
CGGGTGAGTAACACGTGAGCAATCTGCCCTCCACATCGGGATAACCCCCGGAAACGGGAGCTAATACCGG
ATACGACCACTTCAGGCATCTGATGGTGGTGGAAAGTTCCGGCGGTGGAGGATGAGCTCGCGGCCTATCA
GCTTGTTGGTGAGGTAATGGCCCACCAAGGCTTCGACGGGTAGCCGGCCTGAGAGGGTGACCGGCCACAC
TGGGACTGAGACACGGCC

>Zotu1536

GAACGCTGGCGGCGTGCCTAATACATGCAAGTCGAACGGTAACACCTCGGGTGACGAGTGGCGGACGGCT
GAGTAACGCGTAGGAATGTACCCCAAAGTGAGGGATAATCCACCGAAAGGTGGACTAATACCGCATATTA
TCTTCGGATCAAAGCAGCAATGCGCTTTGGGAACAGCCTGCGTCTGATTAGCTAGTTGGTGAGGTAATGGC
TCACCAAGGCGACGATCGGTAGCTGGTCTGAGAGGATGATCAGCCAGACTGGGACTGAGACACGGCC

>Zotu1538

GAACGCTAGCGGGAGGCCTAACACATGCAAGCCGAGCGGTATTGCTTCTTCGGAGGTGAGAGAGCGGCG
TACGGGTGCGGAACACGTGTGCAACCTGCCCTTATCAAGGGGATAGCCCAGAGAAATTTGGATTAATACCC
TATAACATATAGAGTGGCATCACTTTATATTAAAAACTACGGTGGATAAGGATGGGCACGCGCAAGATTAG
CTAGTTGGTGAGGTAACGGCTCACCAAGGCGACGATCTTTAGGGGGCCTGAGAGGGTGATCCCCCACACT
GGGACTGAGACACGGCC

>Zotu1540

GAACGCTAGCGGGAGGCCTAACACATGCAAGCTGAGCGGTAGAGTTCCTTCGGGGACTTGAGAGCGGCG
TACGGGTGCGGAACACGTGTGCAACCTGCCTTTATCAGGGGGATAGCCTTTCGAAAGGAAGATTAATACCC
CATAATATAATTGATGGCATCATTGATTATTGAAAACTCCGGTGGATAAAGATGGGCACGCGCAAGATTAG
ATAGTTGGTGAGGTAACGGCTCACCAAGTCGATGATCTTTAGGGGGCCTGAGAGGGTGATCCCCCACACT
GGTACTGAGACACGGAC

>Zotu1543

GAACGCTAGCTACAGGCTTAACACATGCAAGTCGAGGGGCAGCATTGGAGTAGCAATACTTCAGATGGCG
ACCGGCGCACGGGTGAGTAACACGTATCCAACCTGTCGCTTACTCGGGGATAGCCTTTCGAAAGAAAGATT
AATACCCGATGGCATAAAGAGTTCGCATGTTCTCTTTATTAAAGAATTTCGGTAAGCGATGGGGATGCGTT
CCATTAGATAGTTGGCGGGGTAACGGCCCACCAAGTCTACGATGGATAGGGGTTCTGAGAGGAAGGTCCC
CCACATTGGAACTGAGACACGGTC

>Zotu1545

GAACGCTAGCGGGAGGCCTAACACATGCAAGCCGAGCGGTATTGTTTCTTCGGAAATGAGAGAACGGCGT
ACGGGTGCGGAACACGTGTGCAACCTGCCTTTATCTGGGGGATAGCCTTTCGAAAGGAAGATTAATACTCC
ATAACATATTGATTGGCATCAATTAATATTGAAAGCTCCGGCGGATAGAGATGGGCACGCGCAAGATTAGC
TAGTTGGTGAGGTAACGGCTCACCAAGGCGATGATCTTTAGGGGGCCTGAGAGGGTGATCCCCCACACTG
GTACTGAGACACGGAC
>Zotu1547
GAACGCTGGCGGCGTGCCTAACACATGCAAGTCGAGCGAACCCTTCGGGGTGAGCGGCGGACGGGTGAG
TAACGCGTGGGTAACCTGCCCTGTACACACGGATAACATACCGAAAGGTATGCTAATACGAGATAACATGT
AATGATCGCATGGTCGATACATCAAAGCTCCGGCGGTACAGGATGGACCCGCGTCTGATTAGCTAGTTGGT
GAGGTAATGGCTCACCAAGGCGACGATCAGTAGCCGACCTGAGAGGGTGATCGGCCACATTGGAACTGA
GACACGGTC
>Zotu1549
GAACGCTGGCGGCGTGCTTAACACATGCAAGTCGAACGATGAACCTGGAGCTTGCTCTAGGGAATTAGTG
GCGAACGGGTGAGTAACACGTGAGTAACCTGCCCTTGACTCTGGGATAACCTCCGGAAACGGAAGCTAAT
ACCGGATATGACGCACGGAGGCATCTCCTGTGCGTGGAAAGAACTTCGGTCAAGGATGGACTCGCGGCCT
ATCAGGTAGTTGGTGAGGTAACGGCTCACCAAGCCTACGACGGGTAGCCGGCCTGAGAGGGTGACCGGC
CACACTGGGACTGAGACACGGCC
>Zotu1551
GAACGCTGGCGGCATGCCTAACACATGCAAGTCGAACGAAGGCTTCGGCCTTAGTGGCGCACGGGTGCGT
AACGCGTGGGAATCTGCCCTCAGGTTCGGAATAACAACGAGAAATTGCTGCTAATACCGGATGATATCGCG
AGATCAAAGATTTATCGCCTGAGGATGAGCCCGCGTAGGATTAGCTAGTTGGTGTGGTAAAGGCGCACCA
AGGCGACGATCCTTAACTGGTCTGAGAGGATGATCAGCCACACTGGGACTGAGACACGGCC
>Zotu1553
CAACGCTGGCGGCGTGCATGAGACATGCAAGTCGAACGGACGACAAAGCTTGCTTTGTCGTTAGTGGCGG
ACGGCTGAGTAACGCGTAGGTGACCTGCCTCGAAGTGGGGAATAACTGCCCGAAAGGGTGGCTAATACCG
CATGTGGTGGTGTATTCGATTACACCAGTAAAGCCGCAAGGCGCTTCGAGAGGGGCCTGCGTCCGATTAG
CTCGTTGGTGGGGTAACGGCTCACCAAGGCGACGATCGGTAGGTGGTCTGAGAGGACGATCACCCAGACT
GGGACTGAGACACGGCC
>Zotu1555
GAACGCTGGCGGCAGGCCTAACACATGCAAGTCGAACGCACCGCAAGGTGAGTGGCAGACGGGTGAGTA
ACGCGTGGGAACCTTCCCTTTGGTTCAGGATAGCCCCGGGAAACTGGGATTAATACTGGATAAGATCTACG
GATGAAAGATTTATCGCCGAAGGATGGGCCCGCGTAAGATTAGCTAGTTGGTGAGGTAATGGCTCACCAA
GGCTTTGATCTTTAGCTGGTCTGAGAGGATGATCAGCCACACTGGAACTGAGACACGGTC
>Zotu1557
GAACGCTGGCGGCGTGCCTAAGACATGCAAGTCGAGTGGGTTTAGACCCACGGCGAACGGGTGAGTAAT
ACCTAGGAACTTTCTTTAAGTGGAGGATAACCAGTCGAAAGATTGGATAATACTTCATGTGGCAGTAATGC
TAAAGTCGCAAGACGCTTAAAGTGAGGCCTGGGCACCATCAGCTAGTTGGTAAGGTAATGGCTTACCAAG
GCTATGACGGTTAGGGGTCCCGAGAGGGATAACCCCCACACTGGAACTGCGACACGGTC
>Zotu1559
GAACGCTGGCGGCGTGCTTAACACATGCAAGTCGAACGTGAACGGAGCAATCCTAGTAAAGTGGCGTACG
GGTGAGTAACACGTAGGTAATCTACCTTAGAGTTCGGAATAACCTGCCGAAAGGCGGGCTAATACTGGAT
AGAGCGACGGAATTCGTAAAGAATTTTTCGGGAAAGGGAGCTTGATAAGAGCCCTCGCTTTAAGATGAGC
CTGCGTCTGATTAGTTAGTTGGTAAGGTAACGGCTTACCAAGACGATGATCAGTAGCTGGTCTGAGAGGAT
GATCAGCCACACTGGAACTGAAACACGGTC
>Zotu1561
GAACGCTGACGGTGTGCTTTAGACATGCAAGTCGAACGAGAAAGCCCTTCGGGGTTAGTAAAGTGGCGAA
CGGGTGAGTAACACGTGGGCAATCTGCCCCTCGCAGGGGGACAACCGGAGGAAACTCCGGCTAATACCCC
GTACGCTTGTCGGACCGCATGGTACTGACAAGGAAAGGTAGCTTCGGCCATCCGGCGAGGGATGAGCCCG
CGGTACATTAGCTAGTTGGTGGGGTAACGGCCCACCAAGGCGACGATGTATAGCTGGTCTGAGAGGATGA
TCAGCCACACTGGGACTGAGACACGGCC

>Zotu1563
GAACGCTGGCGGCGTGCTTAACACATGCAAGTCGAACGGAAAGGCCCTTCGGGGTGCTCGAGTGGCGAAC
GGGTGAGTAACACGTGGGTGATCTGCCCTGCACTTTGGGATAAGCCTGGGAAACTGGGTCTAATACCGAA
TATGATCATGCCTCTCCTGGGGTGTGGTGGAAAGCTTTTGCGGTGTGGGATGGGCCCGCGGCCTATCAGCT
TGTTGGTGGGGTAATGGCCTACCAAGGCGTCGACGGGTAGCCGGCCTGAGAGGGTGTCCGGCCACACTG
GGACTGAGATACGGCC
>Zotu1566
GAACGCTGGCGGCGTGCTTAACACATGCAAGTCGAACGGTGACGCTGGAGCTTGCTCCAGCTAATCAGTG
GCGAACGGGTGAGTAACACGTGAGCAACCTGCCCTTGACTCTGGGATAACCCCGGGAAACCGGAGCTAAT
ACCGGATACGAACCGCACAGGCATCTGCGTGGTTGGAAAGTTTTTCGGTCAAGGATGGGCTCGCGGCCTA
TCAGCTTGTTGGTGGGGTAACGGCCCACCAAGGCGACGACGGGTAGCCGGCCTGAGAGGGCGACCGGCC
ACACTGGGACTGAGACACGGCC
>Zotu1568
GAACGCTAGCGGCAGGCTTAATACATGCAAGTCGTGGGGCAGCAGCTTTGTAGCAATACAGAGGCTGGCG
ACCGGCAAACGGGTGCGGAACACGTACACAACCTTCCTTTAAGTGGGGGATAGCCCAGGGAAACTTGGAT
TAATACCCCGTAATACTATGAAGTGGCATCACTTTATATTTATAGCTCCGGCGCTTAATGATGGGTGTGCGG
CTGATTAGGTAGTTGGCGGGGTAACGGCCCACCAAGCCTGCGATCAGTAACTGGTGTGAGAGCACGACCA
GTCACACGGGCACTGAGACACGGGC
>Zotu1570
GAATGCTGGCGGCGCGCCTAACACATGCAAGTCGAGCGGAAAGGCCCTTCGGGGTACTCGAGCGGCGGA
CGGCTGAGTAACGCGTGGGAACATGCCCCAAAGTGAGGAATAACTGCCCGAAAGGGTAGCTAATGCCGCA
TATGATCTTCGGATTAAAGGATTTATCCGCTTTGGGAGTGGCCCGCGTACGATTAGGTAGTTGGTGAGGTA
AAGGCTCACCAAGCCCACGATCGTTAACTGGTCTGAGAGGATGACCAGTCAGACTGGAACTGAGACACGG
TC
>Zotu1573
GAACGCTAGCTACAGGCTTAACACATGCAAGTCGAGGGGAAACGACATCGAAAGCTTGCTTTTGATGGGC
GTCGACCGGCGCACGGGTGAGTAACGCGTATCCAACCTGCCCACCACTTGGGGATAACCTTGCGAAAGTA
AGACTAATACCCAATGATATCTCTAGAAGACATCTGAAAGAGATTAAAGATTTATCGGTGATGGATGGGGA
TGCGTCTGATTAGCTTGTTGGCGGGGTAACGGCCCACCAAGGCAACGATCAGTAGGGGTTCTGAGAGGAA
GGTCCCCCACATTGGAACTGAGACACGGTC
>Zotu1575
GAACGCTAGCGGCAGGCCTAATACATGCAAGTCGAACGATATTATCCAGCTTGCTGGATATGAAAGTGGC
GCACGGGTGCGTAACGCGTATGCAACCTACCTTAATCAGGGGGATAGCCCGAAGAAATTCGGATTAAGAC
CGCATAAAAACACAGGATAGCATTATCCAATGTTCAAATATTTATAGGATTAAGATGGGCATGCGTGACAT
TAGTTAGTTGGCGGGGTAACGGCCCACCAAGACCACGATGTCTAGGGGATCTGAGAGGATGACCCCCCAC
ACTGGTACTGAGACACGGAC
>Zotu1577
GAACGCTGGCGGCACGTTTTAAGCATGCAAGTCGAACGGCAGTGGGGGTAACCCCATGAGAGTGGCGAA
CGGGTGAGTAACACGTAGGTGATCTGCCTTGAGGATGGGGATAGCTTGTGGAAACACAGGGTAATACCGA
ATAAGCTGGCGGGATTTTGGTCCTGTTAGTAAAGGTGCTACGGCACCGCCTTGAGAGGAGCCTGCGGCTG
ATTAGCTAGTTGGTGGGGTAAAGGCCTACCAAGGCGACGATCAGTAGCCGGCCTGAGAGGGTGTACGGCC
ACACTGGAACTGAGACACGGTC
>Zotu1580
GAACGCTGGCGGCGTGCTTAACACATGCAAGTCGAACGGTAAGGCCCTTTCGGGGGTACACGAGTGGCGA
ACGGGTGAGTAACACGTGGGTGATCTGCCCTGCACTTTGGGATAAGCCTGGGAAACTGGGTCTAATACCG
AATAGGACCATGCCATGCATGTGGTGTGGTGGAAAGCTTTTGCGGTGTGGGATGGGCCCGCGGCCTATCA
GCTTGTTGGTGAGGTTACGGCTCACCAAGGCGACGACGGGTAGCCGGCCTGAGAGGGTGACCGGCCACA
CTGGGACTGAGATACGGCC
>Zotu1582

GAACGCTGGCGGCGCGCTTAACACATGCAAGTCGTACGGAGTTGTTCCTTCGGGAGCAGCTCAGTGGCGA
ACGGGTGCGTAACACGTGAGCAACCTGCCTGCCGGTGGGGGATAACCCGGGGAAACCCGGGCTAATACC
GCATTCGCTCTTTCTCTCGATTGGGGGATTGAGGAAAGGTGCTGCAAGGCACTGCCGGCAGAGGGGCTCG
CGGCGTATCAGCTAGCTGGTGAGGTAACGGCTCACCAGGGCAACGACGCGTAGCCGATCTGAGAGGATG
ATCGGCCACATTGGGACTGAGATACGGCC
>Zotu1585
GAACGCTGGCGGCATGCCTAACACATGCAAGTCGAACGAGGTTTCCGCTTCGGCGGTTGCCTAGTGGCGC
ACGGGTGAGTAACGCGTGGGAACCTGCCCCGTGGTACGGAATAACTCCGGGAAACTGGAGCTAAGACCGT
ATGTGTCCCGAGGGACAAAGATTCATCGCCATGGGATGGGCCCGCGTAGGATTAGCTTGTTGGCGGGGTA
ACGGCCCACCAAGGCGACGATCCTTAGCTGGTCTGAGAGGATGATCAGCCACACTGGGACTGAGACACGG
CC
>Zotu1587
GAACGCTAGCGGCAGGCATAATACATGCAAGGCGAAGGGGCAGCAATGTCACTGTCGTACGGGTGCGTA
ACGCGTATGCAACTTATCTATCACTGGGGGATAGCCCGGGGAAACCCGGATTAATACCGCATAAAACAGG
GGCACCGCATGGTGATATTTGTTAAAGATTTATTGGTGATAGATGGGCATGCGTTCGATTAGCTAGTTGGC
GGGGTAACGGCCCACCAAGGCGACGATCGATAGGGGAGCTGAGAGGTTGGTCTCCCACACGGGCACTGA
GATACGGGC
>Zotu1590
GAATGCTGGCGACGTGGATAAGGCATGCAAGTCAAGGGGGCCGCAAGGCAACCGGCAGACGAGGTAGTA
ACACGTACGAACGCACCCCGAAGTCTGGTATAGCCGGCCGAAAGGCCGGGTAATCCCGGATGGTACCGCA
AGGTTAAAGATTTATCGCTTCGGGAGCGGCGTGCGCAATATCAGCTAGTTGGTAGGGTAATGGCCTACCAA
GGCTACGACGTTTAGGGGAGCTGAGAGGCTGACCCCCACCGATGGGACTGAGACACGGCC
>Zotu1592
GAACGCTGGCGGCGTGCTTCACACATGCAAGTCGAACGAGGTCCAATCAGTCGCAAGACTGGTGAAGACC
GAGTGGCGAACGGGTGCGTAACACGTGAGGAACCTGTCCCGGTCTCCGGAATAACAAATGGAAACACTTG
CTAATACGGGATGACGTCGGAACGTCGCATGACGAACTGACGAAAGGGTTACCGGACCGGGAGGGCCTC
GCGGCCTATCAGCTCGTTGGTGGGGTAACGGCCCACCAAGGCTTCGACGGGTAGCTGGTCTGAGAGGATG
ATCAGCCACACTGGGACTGAGACACGGCC
>Zotu1595
GAACGCTGGCGGTATGCTTTACACATGCAAGTCGTACGAGAGTTTTTAACTCAAGTGGCGGACGGGTGAG
TAACACGTAAGAATTTGCCTTTAGGAGGGGGATAACAACTGGAAACGGTTGCTAATACCCCATATGCTTTC
GAGTGAAATGGATTTTTCCGCCTAAAGAGAAGCTTGCGGCTGATTAGCTTGTTGGTGAGGTAATGGCTTAC
CAAGGCGACGATCAGTATCTGGTTTGAGAGGACGATCAGACACACTGGAACTGAGAGACGGTC
>Zotu1597
GAACGCTGGCGGCATGCTTAACACATGCAAGTCGAACGGTAACGCGGGGGTAACCCTGGCGACGAGTGG
CGGACGGGTGAGTAATACTTAGGAATCTGCCCAGTAGTGGGGGACAACCCGGGGAAACCCGGGCTAATA
CCGCATACGCCCCATGGGGGAAAGCGGGGGACCGCAAGGCCTCGCGCTATTGGATGAGCCTAAGTCCGAT
TAGCTAGTTGGTAGGGTAACGGCCTACCAAGGCGACGATCGGTAGCTGGTCTGAGAGGATGATCAGCCAC
ACTGGGACTGAGACACGGCC
>Zotu1599
GAACGCTAGCGGCAGGCCTAATACATGCAAGTCGAGGGGTAGCAATACCACTGGCGCACGGGTGCGTAAC
ACGTTTGAAATCTGCCCTTTATTGGGGGATAGCCCGCCGAAAGGCGGATTAATACCGCATATGCCGGTGGA
GTGGCATCACTCAGCCGGGAAAGGCTTCGGCCGATAGAGGATGATCATGCGTCTGATTAGCTAGTTGGCG
GGGTAGAGGCCCACCAAGGCGACGATCAGTAGGGGACCTGAGAGGGCGATCCCCCACACGGACACTGAG
ATACGGGT
>Zotu1601
GAACGCTGGCGGCGTGCTTAACACATGCAAGTCGAACGGAAAGGCCCTGCTTGCAGGGTACTCGAGTGGC
GAACGGGTGAGTAACACGTGGGTGATCTGCCTCGTACTTCGGGATAAGCCTGGGAAACTGGGTCTAATAC
CGGATAGGACCTTCTTTTAGTGTGGAAGGTGGAAAGTTTTTCGGTACGAGATGAGCCCGCGGCCTATCAGC
TTGTTGGTGGGGTAATGGCCTACCAAGGCGACGACGGGTAGCCGGCCTGAGAGGGTGTACGGCCACATTG
GGACTGAGACACGGCC

>Zotu1604
GAACGCTGGCGGCAGGCTTAACACATGCAAGTCGAGCGCCCCGCAAGGGGAGCGGCAGACGGGTGAGTA
ACGCGTGGGAATCTACCGTGCCCTACGGAATAGCTCCGGGAAACTGGAATTAATACCGTATACGCCCTACG
GGGGAAAGATTTATCGGGGTATGATGAGCCCGCGTTGGATTAGCTAGTTGGTGGGGTAAAGGCCTACCAA
GGCGACGATCCATAGCTGGTCTGAGAGGATGATCAGCCACATTGGGACTGAGACACGGCC
>Zotu1606
GCCGGCAATGTTGAAGAGGTAAGAACTGCTCACATGCACTGTCCTCCCCGGAGGACGCTAACCATGTGTGT
CGAGATTTCGGCACACGTTAAAGAACCCTCGGGTAGGCAAAATTAAC
>Zotu1608
GAACGCTGGCGGCGTGCTTAACACATGCAAGTCAAAGGGTAGCAATATCCTTGGCGCACGGGTGAGTAAC
GCGTAGATTATCTGCCCCATACACAGACATAACTTTTCGAAAGAGACGATAATATCTGATGAGGTGCTTCG
GCATCAAAGTAGCAATACGGTATGGGATGAGTCTGCGTCTGATTAGCTAGTTGGCGAGGTAATGGCTCACC
AAGGCGACGATCAGTAGCTGGTCTGAGAGGACGATCAGCCACACTGGAACTGAGACACGGTC
>Zotu1610
GAACGCTGGCGGCGTGCCTAACACATGCAAGTCGAGCGATTCTCTTCGGAGAAGAGCGGCGGACGGGTG
AGTAACGCGTGGGTAACCTGCCCTGTACACACGGATAACATACCGAAAGGTATGCTAATACGAGATAACAT
ACTTTTATCGCATGGTAGAAGTATCAAAGCTCCGGCGGTACAGGATGGACCCGCGTCTGATTAGCTAGTTG
GTAAGGTAACGGCTTACCAAGGCGACGATCAGTAGCCGACCTGAGAGGGTGATCGGCCACATTGGAACTG
AGACACGGTC
>Zotu1612
GAACGCTGGCGGCGTGCTTAACACATGCAAGTCGAACGATGAAGCTGGAGCTTGCTCTGGTGGATTAGTG
GCGAACGGGTGAGTAACACGTGAGCAACCTGCCCCTGACTCTGGGATAACAGCCGGAAACGGCTGCTAAT
ACCGGATATGCACCATGAACGCATGTTCTGTGGTGGGAAAGATTTTTCGGTTGGGGATGGGCTCGCGGCCT
ATCAGCTTGTTGGTGAGGTAATGGCTCACCAAGGCGTCGACGGGTAGCCGGCCTGAGAGGGTGACCGGCC
ACACTGGGACTGAGACACGGCC
>Zotu1614
GAACGCTAGCGGCAGGCTTAATACATGCAAGTCGTGGGGCAGCAGGGGTAGCAATACTCGCTGGCGACC
GGCAAACGGGTGCGGAACACGTACACAACCTTCCTTTAAGCGGGGAATAGCCCAGAGAAATTTGGATTAA
TACCCCATAAGATATAATATAGGCATCTATAATATATTAAAGCTTCGGCACTTGATGATGGGTGTGCGTATG
ATTAGGTAGTTGGTAAGGTAACGGCTTACCAAGCCTACGATCATTAGCTGATGTGAGAGCATGATCAGCCA
CACGGGCACTGAGACACGGGC
>Zotu1616
GAACGCTGGCGGCGTGCCTAATACATGCAAGTCGAGCGGACTGAATGAAAGCTTGCTTTCATTCGGTTAGC
GGCGGACGGGTGAGTAACACGTGGGCAACCTGCCCGTAAGACCGGGATAACTTCGGGAAACCGGAGCTA
ATACCGGATAACACCGAAGATCGCATGATCTTTGGTTGAAAGGCGGCTTTGGCTGTCACTTACGGATGGGC
CCGCGGCGCATTAGCTAGTTGGTGAGGTAACGGCTCACCAAGGCGACGATGCGTAGCCGACCTGAGAGG
GTGATCGGCCACACTGGGACTGAGACACGGCC
>Zotu1619
CAACGCTGGCGGCGCGCTTAACACATGCAAGTCGAACGAGAAAGGGAGCAATCCTGAGTAAAGTGGCGTA
CGGGTGAGTAACACGTGGATCATCTGCCCTTTGGTGGGGAATAACTCCTGGAAACGGGAGATAATACCGC
ATAAGTCCGAGAGGAGAACGCCGAAAGGCGCCGAAGGAGGAGTCCGCGGCTGATTAGCTAGTTGGTAGG
GTAATGGCCTACCAAGGCGACGATCGGTAGCCGGCCTGAGAGGGCACACGGCCACACTGGCACTGAGAC
ACGGGC
>Zotu1622
GAACGCTGGCGACAGGCTTAACACATGCAAGTCGAACGCCCCACAAGGGAAGTGGCAGACGGGTGAGTA
ACGCGTGGGAACATACCCTTTCCTGCGGAATAGCTCTGGGAAACTGGAATTAATACCGCATACGCCCTACG
GGGGAAAGATTTATCGGGGAAGGATTGGCCCGCGTTGGATTAGCTAGTTGGTGGGGTAAAGGCCTACCAA
GGCGACAATCCATAGCTGGTCTGAGAGGATGATCAGCCACATTGGGACTGAGACACGGCC
>Zotu1624

GAACGCTGGCGGCAGGCTTAACACATGCAAGTCGAACGCCCCGCAAGGGGAGTGGCAGACGGGTGAGTA
ACGCGTGGGAATCTACCTAGTTCTACGGAACAACACAGGGAAACTTGTGCTAATACCGTATACGCCCTACG
GGGGAAAGATTTATCGGAATTAGATGAGCCCGCGTAAGATTAGCTAGTTGGTGGGGTAATGGCCTACCAA
GGCGACGATCTTTAGCTGGTCTGAGAGGATGATCAGCCACACTGGGACTGAGACACGGCC
>Zotu1626
GAACGCTAGCGGGAGGCCTAACACATGCAAGCCGAGCGGTAGAGTTCCTTCGGGAACTTGAGAGCGGCG
TACGGGTGCGTAACACGTGTGCAACCTGCCTTTATCTGGGAGATAGCCCTCCGAAAGGGGGATTAATATTC
CATAATATATCAGTTGGCATCAATTGATATTGAAAGCTCCGGCGGATAGAGATGGGCACGCGCAAGATTAG
ATAGTTGGTGAGGTAACGGCTCACCAAGTCTGCGATCTTTAGGGGGCCTGAGAGGGTGATCCCCCACACT
GGTACTGAGACACGGAC
>Zotu1628
GAACGCTGGCGGCGTGCCTAATACATGCAAGTCGAGCGAATCAATGGGAGCTTGCTCCCTGAGATTAGCG
GCGGACGGGTGAGTAACACGTGGGTAACCTGCCTGTAAGACTGGGATAACTTCGGGAAACCGGAGCTAAT
ACCGGATAATTTCTTTTCTCGCATGAGAAGAGATGGAAAGATGGTTTTGCTATCACTTACAGATGGACCCGC
GGCGCATTAGCTAGTTGGTGAGGTAACGGCTCACCAAGGCCACGATGCGTAGCCGACCTGAGAGGGTGAT
CGGCCACACTGGGACTGAGACACGGCC
>Zotu1630
CAACGCTGGCGGCGCGCCTAACACATGCAAGTCGAACGAGAAAGTGGAGCAATCCATGAGTACAGTGGCG
TACGGGTGAGTAACACGTGGGTAATCTACCTCTTAGTGGGGAATAACCAGGGGAAACTCTGGCTAATACC
GCATAAGCCTGAGAAGGGAAAGCAGCAATGCGCTGAGAGAGGAGCCCGCGGCCGATTAGCTAGTTGGTG
GGGTAAGAGCCCACCAAGGCAGAGATCGGTAGCCGGCCTGAGAGGGCACACGGCCACACTGGCACTGAA
ACACGGGC
>Zotu1632
GAATGCTGGCGGCGTGGATGAGGCATGCAAGTCGAATGAGTTTATACTCATGGCAGACGAGGTAGGAAC
ACGTGGGTACGTACCCAAGAGTCAGGGATAATCCGTCGAAAGGCGGGATAATACTTGATAGTCCCTTCGG
GGTAAAGATTTATCGCTCTTGGAACGGCCCGCGGGCTATCAGCTTGTTGGTAAGGTAACGGCTTACCAAGG
CTACGACGGCTAGGGGAGGTGAGAGCCTGACCCCCACCGATGGAACTGCGACACGGTC
>Zotu1634
GAACGCTAGCGGCAGGCCTAATACATGCAAGTCGAACGGACCTTGTTCCTTCGGGGACTTGGTTAGTGGC
GCACGGGTGCGTAACGCGTATGCAACCTACCTTCTATTGGGGGATAGCCCCGGGAAACCGGGATTAATAC
CGCATGAGATCACTTGAGGGCATCCGAAGGTGATTAAAGCCGAGGCGATAGAAGATGGGCATGCGTGCC
ATTAGCTAGTTGGCGGGGTAAGGGCCCACCAAGGCGACGATGGCTAGGGGTTCTGAGAGGATGGTCCCCC
ACACTGGTACTGAGACACGGAC
>Zotu1636
TAACGTTGGCAGTGTGCCTAACACATGCAAGTCGCGCGATATAGCGGCAGACGAGAGAGTAATAACAAGG
AACTTACCCCCGAGTGAAGGATAGCCCACCGAAAGGTGGGGTAACCCTGCATAACATCGAAAGATCAAAG
CCGTAAGGCGCTTGGGGAGAGGCCTTGTTCCTATCAGCTAGTTGGTGAGGTAATGGCTCACCAAGGCGAT
GACGGGTAGCTGGACTGAGAGGTTGACCAGCCTCGATGGGACTGAGACACGGCC
>Zotu1638
GAACGCTAGCGGCAGGCCTAATACATGCAAGTCGAACGGTTCGCAAGAATAGTGGCAAACGGGTGCGTAA
CGCGTAAGCAACCTGCCTCAAACTGGGGGATAGCCCGGCGAAAGCTGGGGTAAACCCGCACGGTTCCCAT
TTATCACCTGGTAGTTGGGGTAAACATTTATGGGTTTGAGAGGGGCTTGCGTCTGATTAGTTAGTTGGCAG
GGTAACGGCCTACCAAGACGATGATCAGTAGGGGTTCTGAGAGGATTGGCCCCCACATGGGTACTGAGAT
ACGGAC
>Zotu1641
GAACGCTGGCGGCATGCCTAACACATGCAAGTCGAACGAAGTGCCTTCGGGTGCTTAGTGGCGCACGGGT
GCGTAACGCGTGGGAATCTACCCCTTGGTTCGGAATAACAGTTGGAAACGACTGCTAATACCGGATGATGA
CGTAAGTCCAAAGATTTATCGCCGAGGGATGAGCCCGCGTAGGATTAGGTAGTTGGTGTGGTAAAGGCGC
ACCAAGCCGACGATCCTTAGCTGGTCTGAGAGGATGATCAGCCACACTGGGACTGAGACACGGCC
>Zotu1643

GAACGCTGGCGGCATGCCTAACACATGCAAGTCGAACGAAGCCTTCGGGCTTAGTGGCGCACGGGTGCGT
AACGCGTGGGAATCTGCCCTTGGGTTCGGAATAACAGTTAGAAATGACTGCTAATACCGGATGATGTCTTC
GGACCAAAGATTTATCGCCCAGGGATGAGCCCGCGTAGGATTAGGTAGTTGGTGGGGTAAAGGCCTACCA
AGCCGACGATCCTTAGCTGGTCTGAGAGGATGATCAGCCACACTGGGACTGAGACACGGCC
>Zotu1645
GAACGCTAGCGGCAGGCTTAATACATGCAAGTCGAACGGGATTTAGGAGCTTGCTTCTAATGAGAGTGGC
GCACGGGTGAGTAACACGTACACAATCTACCTTGTACAGGGGGATAGCCTTGGGAAACTGAGAATAATAC
CCCATAGTTTTAATTTATCGCATGATAGATTAAATAAAGCTGAGGCGGTACAAGATGAGTGTGCGGATGAT
TAGGTAGTTGGTAGGGTAACGGCCTACCAAGCCGACGATCAATAGCTGGCGTGAGAGCGCGACCAGCCAC
ACTGGAACTGAGACACGGTC
>Zotu1647
GAACGCTAGCGGCAGGCTTAACACATGCAAGTCGAGGGGCAGCATGAGTGTTAGCAATAACACTTGATGG
CGACCGGCGCAAGGGTGCGTAACGCGTGAGCAACTTGCCCGTTTCAGGGGGATAACCGGTGGAAACGCC
GACTAATACCCCATGGCATTGGTTTGAGGCATCTCTTACCAATTAAAGAACTTCGGAAACGGATAGGCTCG
CGTGACATTAGCTAGATGGCGGGGTAACGGCCCACCATGGCGACGATGTCTAGGGGTTCTGAGAGGAAG
GTCCCCCACACTGGAACTGAGACACGGTC
>Zotu1649
GAACGCTGGCGGCGCGCTTAACACATGCAAGTCGAGCGATGAAACTTTAACAGAACCCTTCGGGGCGAAG
ATAAAGTGGATTAGCGGCGGACGGGTGAGTAACACGTGAGTAACCTGCCTTAGACATTGGGATAGCCTCG
GGAAACCGGGATTAATACCAAATAAAATCATAATGACACATGTCATAACGATCAAAACTCCGGTGGTCTAA
GATGGACTCGCGTCCCATTAGCTAGTTGGTAGAGTAAAAGCCTACCAAGGCGACGATGGGTAGCCGGCCT
GAGAGGGTGAACGGCCACATTGGAACTGAGAAACGGTC
>Zotu1651
GAACGCTAGCGGCAGGCTTAATACATGCAAGTCGAACGGTAACAGGTGTAGCAATACACGCTGACGAGTG
GCGCACGGGTGCGTAACGCGTATGCAACTTACCCTTAACTGGAGGATAGCTCGAAGAAATTCGAATTAATA
CTCCATAGTATTGTGATCCCGCATGGGATTGCTCTTAAAGCTCCGGCGGTTAAGGATGGGCATGCGTGACA
TTAGCTAGTTGGTGAGGTAAAAGCTCACCAAGGCAACGATGTCTAGGGGATCTGAGAGGATTATCCCCCAC
ACTGGTACTGAGACACGGAC
>Zotu1653
GAACGCTGGCGGCGTGCTTAACACATGCAAGTCGAACGGTGAACCCGCTTCGGTGGGGGATCAGTGGCGA
ACGGGTGAGTAACACGTGGGCAACCTGCCCCCGGCTCTGGGATAACTCCAAGAAATTGGGGCTAATACCG
GATATGACCGCTGGCCGCATGGTCTGGTGGTGGAAAGATTTATCGGCTGGGGATGGGCCCGCGGCCTATC
AGCTTGTTGGTGGGGTAGTGGCCTACCAAGGCGACGACGGGTAGCCGGCCTGAGAGGGTGACCGGCCAC
ACTGGGACTGAGACACGGCC
>Zotu1655
GAACGCTGACAGAATGCTTAACACATGCAAGTCTTTGGCGAATCTGTGCTTGCACAGGCTAGCCAAGGCGG
ACGGGTGAGTAACGCGTAAAGAACTTGCCCTGCAGACAGGGATAACAGACGGAAACGACTGATAACACCT
GATACAATTGCCGGCACGCATGTGCCTGGCAATGAAAAGAGATGCTGCGGGAGAGCTTTGCGTCCTATTA
GCTTGTTGGTGAGGTAACGGCTCACCAAGGCGATGATAGGTAGCCGGCCTGAGAGGGTGAACGGCCACA
AGGGGACTGAGATACGGCC
>Zotu1657
GAACGCTGGCGGCGTGCTTAACACATGCAAGTCGAACGGTGACGGTGGAGCTTGCTCCACCTGATCAGTG
GCGAACGGGTGAGTAACACGTGAGTAACCTGCCCCTTACTTCGGGATAACCTCGGGAAATCGTGGCTAATA
CCGGATATGAGACCTTGCTGCATGGCTGGGTCTGGAAAGATTTATCGGTGAGGGATGGACTCGCGGCCTA
TCAGCTTGTTGGTGAGGTAATGGCTCACCAAGGCGACGACGGGTAGCCGGCCTGAGAGGGCGACCGGCC
ACACTGGGACTGAGATACGGCC
>Zotu1659
GAACGCTGGCGGCGTGCCTAATACATGCAAGTCGAGCGGATCTCGTCAAGAGATCAGCGGCGGACGGGT
GAGTAACACGTGGGCAACCTGCCTATAAGACTGGGATAACTTCGGGAAACCGGAGCTAATACCGGATAAC
ATCAAGGACCACATGGTCTTCGATTAAAAGATGGCGTCAAGCTATCACTTATAGATGGGCCCGCGTCGCAT

TAGCTAGTTGGTGAGGTAACGGCTCACCAAGGCGACGATGCGTAGCCGACCTGAGAGGGTGATCGGCCAC
ACTGGGACTGAGACACGGCC
>Zotu1661
GAACGCTGGCGGCGTGCCTAATACATGCAAGTCGAGCGCAGGAAATCGACGGAACCCTTCGGGGGGAAG
TCGACGGAATGAGCGGCGGACGGGTGAGTAACACGTAAAGAACCTGCCCTCAGGTCTGGGATAACCACG
AGAAATCGGGGCTAATACCGGATGGGTCATCGGACCGCATGGTCCGAGGATGAAAGGCGCTTCGGCGTC
GCCTGGGGATGGCTTTGCGGTGCATTAGCTAGTTGGTGGGGTAATGGCCCACCAAGGCGACGATGCATAG
CCGACCTGAGAGGGTGATCGGCCACACTGGGACTGAGACACGGCC
>Zotu1663
GAACGCTAGCGGCAGGCCTAACACATGCAAGTCGAGGGGCAGCACAGTGTAGCAATACATGGGTGGCGA
CCGGCGCACGGGTGAGTAACGCGTATGCAACCTACCCTATACTGGGGGATAGCTCAGAGAAATCTGAATT
AAACCCGCATAAAGCAGGGGTACCGCATGGTACTATTTGCTAAAACTCCGGTGGTATAGGATGGGCATGC
GTGTCATTAGCTAGTTGGTGAGGTAACGGCTCACCAAGGCGACGATGACTAGGGGGTCTGAGAGGATGAT
CCCCCACACTGGTACTGAGACACGGAC
>Zotu1665
GAACGCTAGCGGCAGGCCTAATACATGCAAGTCGAACGCGATTATCCAGCTTGCTGGATATGAAAGTGGC
GCACGGGTGCGTAACGCGTGAGCAACCTACCTCTGTCAGGGGGATAGCCCGGCGAAAGTCGGATTAACAC
CGCATGACATTATTGATGTGGCATCACATTATAATCAAATATTTATAGGACAGAGATGGGCTCGCGTGACA
TTAGCTAGTTGGAGAGGTAACGGCTCACCAAGGCAACGATGTCTAGGGGCTCTGAGAGGAGAATCCCCCA
CACTGGTACTGAGACACGGAC
>Zotu1668
GAACGCTGGCGGTATGCCTAATACATGCAAGTCGAACGAGATCTTCGGATCTAGTGGCGCACGGGTGCGT
AACGCGTGGGAATCTGCCCTTGGGTTCGGAATAACGTCGGGAAACTGACGCTAATACCGGATGATGACGA
AAGTCCAAAGATTTATCGCCCAGGGATGAGCCCGCGTAGGATTAGCTAGTTGGTGAGGTAAAGGCTCACC
AAGGCTACGATCCTTAGCTGGTCTGAGAGGATGATCAGCCACACTGGGACTGAGACACGGCC
>Zotu1670
GAACGCTGGCGGCGTGCTTAACACATGCAAGTCGAACGGTGAACACGGAGCTTGCTCTGTGGGATCAGTG
GCGAACGGGTGAGTAACACGTGAGCAACCTGCCCCTGACTCTGGGATAAGCGCTGGAAACGGCGTCTAAT
ACTGGATACGAGTAGCGGCCGCATGGTCAGTTACTGGAAAGATTTATTGGTTGGGGATGGGCTCGCGGCC
TATCAGCTTGTTGGTGAGGTAATGGCTCACCAAGGCGTCGACGGGTAGCCGGCCTGAGAGGGTGACCGGC
CACACTGGGACTGAGACACGGCC
>Zotu1672
TAACGCTGGCGGCATGCCTAATACATGCAAGTCGAACGAAAGTAGCAATCTTTAGTGGCGAACGGGTGAG
TAACACGTATCTAACCTACCATTTAGTGGGGAATAACTGTTGGAAACGGCAGCTAATACCGCATATGTTATT
TATTTGCAATGATAAATAATGAAAGGAGCATTTGCTTCGCTAATTGATGGGGATGCGGCGCATTAACTAGT
TGTGGTAAGGTAATGGCTTACCAAGGTAACGACGCGTAGCCGATCTGAGAGGATGAACGGCCACATTGGG
ACTGAGACACGGCC
>Zotu1674
GAACGCTGGCGGCGCGCCTAATACATGCAAGTCGAGCGAGAAGACGTAGCAATACGTTTGTAAAGCGGCG
AACGGGTGAGGAATACATGGGTAACCTACCCTCGAGTGGGGAATAACTAGCCGAAAGGTTAGCTAATACC
GCATACGACTCTTGGTCTGCGGATCGAGAGGGAAAGCGATACCGTGGGTATCGCGCTCATGGATGGGCTC
ATGTCCTATCAGCTTGTTGGTGAGGTAACGGCTCACCAAGGCTTCGACGGGTAGCTGGTCTGAGAGGACG
ATCAGCCACACTGGCACTGCGACACGGGC
>Zotu1677
GAACGCTGGCGGCGTGCTTAACACATGCAAGTCGAACGGTGAACCCCTTCGGGGGGGATCAGTGGCGAAC
GGGTGAGTAACACGTGAGCAACCTGCCCCTGGCTCTGGGACAACCACTGGAAACGGTGGCTAATACCGGA
TACGACTCATCACCGCATGGTGTGTGGGTGGAAAGTTTTTCGGCTGGGGATGGGCTCGCGGCCTATCAGCT
TGTTGGTGGGGTAATGGCCTACCAAGGCGACGACGGGTAGCCGGCCTGAGAGGGCGACCGGCCACACTG
GGACTGAGACACGGCC
>Zotu1680

GAACGCTGGCGGCATGCCTTACACATGCAAGTCGAACGGCAGCACGGGAGCAATCCTGGTGGCGAGTGG
CGAACGGGTGAGTAATACATCGGAACGTGCCCAATCGTGGGGGATAACGCAGCGAAAGCTGTGCTAATAC
CGCATACGATCTACGGATGAAAGCAGGGGACCGCAAGGCCTTGCGCGAATGGAGCGGCCGATGGCAGAT
TAGGTAGTTGGTGGGGTAAAGGCTCACCAAGCCTGCGATCTGTAGCTGGTCTGAGAGGACGACCAGCCAC
ACTGGGACTGAGACACGGCC
>Zotu1682
GAACGCTGGCGGCGTGCTTAACACATGCAAGTCGAACGGTGAAGGAGAGCTTGCTCTCTGGATCAGTGGC
GAACGGGTGAGTAACACGTGAGCAATCTGCCCCTGACTCTGGGATAAGCGTTGGAAACGACGTCTAATAC
CGGATACGAGCTGCGAAGGCATCTTCAGCAGCTGGAAAGAACTTCGGTCAGGGATGAGCTCGCGGCCTAT
CAGCTAGTTGGTGAGGTAATGGCTCACCAAGGCGTCGACGGGTAGCCGGCCTGAGAGGGTGACCGGCCA
CACTGGGACTGAGACACGGCC
>Zotu1684
GAACGCTGGCGGCGTGCTTAACACATGCAAGTCGAGCGGAAAGGCCCACTTGTGGGTACTCGAGCGGCGA
ACGGGTGAGTAACACGTGAGTAATCTGCCCTCCACTCTGGGATAACGTTCAGAAATGGGTGCTAATACCGG
ATGTACACAGACTGGCGCATGTTGGTGTGTGGAAAGGTTTTTCTGGTGGGGGATGTGCTCGCGGCCTATCA
GCTTGTTGGTGGGGTAATGGCCTACCAAGGCTTTGACGGGTAGCCGGCCTGAGAGGGTGACCGGCCACAC
TGGGACTGAGACACGGCC
>Zotu1686
GAACGCTGGCGGCGTGCTTAACACATGCAAGTCGAACGATGATGCCCAGCTTGCTGGGTGGATTAGTGGC
GAACGGGTGAGTAACACGTGAGTAATCTGCCCTTCACTTCGGGATAAGCCTCGGAAACGGGGTCTAATACT
GGATATGACCTTCTCTCGCATGGGGGTTGGTGGAAAGTTTTTCGGTGGGGGATGTGCTCGCGGCCTATCAG
CTTGTTGGTGAGGTAATGGCTCACCAAGGCGACGACGGGTAGCCGGCCTGAGAGGGTGATCGGCCACACT
GGGACTGAGACACGGCC
>Zotu1688
GAACGCTGGCGGCGCGCTTAACACATGCAAGTCGAGCGAGAACCTTGCCTTCGGGCTTGGGGACAGCGGC
GAACGGGTGAGTAACACGTGGGTAATCAACCCTCGACACTGGGATAGCCCGGGGAAACCCGGATTAATAC
CGGATACCCCATCTGGCCTTCGGGCCGGCTGGAAAAGGTAGCTTTGGCCTCCGGTCGAGGACGAGCCCGC
GGCGGATTAGCTTGTTGGTGGGGTAAAGCCCACCAAGGCGACGATCCGTAGCTGGTCTGAGAGGACGAT
CAGCCACACTGGGACTGAGACACGGCC
>Zotu1690
GAACGCTGGCGGCGTGCTTAACACATGCAAGTCGAACGGTGAACCTGCTTCGGTGGGGGATCAGTGGCGA
ACGGGTGAGTAACACGTGGGCAACCTGCCCTCAGCTCTGGGATAACTCCAAGAAATTGGGGCTAATACCG
GATATGACCGCTGACCGCATGGTCTGGTGGTGGAAAGATTTATCGGCTGGGGATGGGCCCGCGGCCTATC
AGCTTGTTGGTGGGGTAGTGGCCTACCAAGGCGACGACGGGTAGCCGGCCTGAGAGGGTGACCGGCCAC
ACTGGGACTGAGACACGGCC
>Zotu1692
GAATGCTGGCGGCGTGGATGAGGCATGCAAGTCGAATGGGTTTAGGCCCATGGCAGACGAGGTAGGAAC
ACGTAGGTACGTACCCCAAAGTCAGGGATAACCCGGAGAAATCCGGGATAATACTTGATAGTCTCTTCGGA
GTAAAGATTTATCGCTTTGGGAACGGCCTGCGGGCTATCAGCTTGTTGGTAAGGTAACGGCTTACCAAGGC
TACGACGGCTAGGGGAGGTGAGAGCCTGACCCCCACCGATGGAACTGCGACACGGTC
>Zotu1694
GAACGCTGGCGGCGTGCTTAACACATGCAAGTCGAACGGAAAGGCCCTTCGGGGTACTCGAGTGGCGAAC
GGGTGAGTAACACGTGGGTGATCTGCCTCGTACTTTGGGATAAGCCTGGGAAACTGGGTCTAATACCGAA
TATGACCTTGGGATGCATGTCTCTTGGTGGAAACTTTTTGGGTACGAGATGGGCCCGCGGCCTATCAGCTT
GTTGGTGGGGTAATGGCCTACCAAGGCGACGACGGGTAGCCGGCCTGAGAGGGCGACCGGCCACACTGG
GACTGAGACACGGCC
>Zotu1696
GAACGCTAGCGGCAGGCCTAATACATGCAAGTCGAACGGTAAGTGGAATAGCAATATTCTACCTAGAGTG
ACGTACGGGTGCGTAACACGTATGCAACCTACCCGCAACTGGGGTATAGCTCGGTGAAAACCGGATTAAC
CCCCCATAAGACCACGCGAGGGCATCTGAGCGTGGTAAAAACTGAGGTGGTTGCGGATGGGCATGCGGAT

GATTAGCTAGTTGGCGGTGTAACGGACCACCAAGGCGATGATCATTAGGGGAACTGAGAGGTTGATCCCC
CACACTGGCACTGAGATACGGGC
>Zotu1698
GAACGCTGGCGGCGTGCCTAATACATGCAAGTCGAGCGAATTTTTGGGAGCTTGCTCCCAAAAATTAGCGG
CGGACGGGTGAGTAACACGTGGGCAACCTGCCTATAAGACTGGGATAACTTCGGGAAACCGGAGCTAATA
CCGGATAATTCTTTTCCTCTCATGAGGAAAAGCTGAAAGACGGTTTACGCTGTCACTTATAGATGGGCCCGC
GGCGCATTAGCTAGTTGGTGAGGTAACGGCTCACCAAGGCAACGATGCGTAGCCGACCTGAGAGGGTGAT
CGGCCACACTGGGACTGAGACACGGCC
>Zotu1700
GAACGCTGGCGGCGTGCCTAATACATGCAAGTCGAGCGAACAGATAAGGAGCTTGCTCCTTTGACGTTAG
CGGCGGACGGGTGAGTAACACGTGGGTAACCTACCTATAAGACTGGAATAACTCCGGGAAACCAGGGCTA
ATGCCGGATAACATTTAGAACCGCATGGTTCTAAAGTGAAAGATGGTTTTGCTATCACTTATAGATGGACCC
GCGCCGTATTAGCTAGTTGGTAAGGTAATGGCTTACCAAGGCAACGATACGTAGCCGACCTGAGAGGGTG
ATCGGCCACACTGGAACTGAGACACGGTC
>Zotu1703
GAACGCTGGCGGCGTGCTTAACACATGCAAGTCGAACGGTGAAGGAGAGCTTGCTCTCTGGATCAGTGGC
GAACGGGTGAGTAACACGTGAGCAATCTGCCCTTGACTCTGGGATAAGCGCTGGAAACGGCGTCTAATAC
TGGATACGAGCTGCGAAGGCATCTTCAGCAGCTGGAAAGAACTTCGGTCAAGGATGAGCTCGCGGCCTAT
CAGCTTGTTGGTGAGGTAATGGCTCACCAAGGCGTCGACGGGTAGCCGGCCTGAGAGGGTGACCGGCCAC
ACTGGGACTGAGACACGGCC
>Zotu1705
GAACGCTGGCGGCGTGCCTAATACATGCAAGTCGAGCGGTAAGGTTCTCACTCTGTATTTAACTAGTTTGA
ATATAGTGTGAGAATACACGAGCGGCGGACGGCTGAGTAACACGTAGGAACGTACCCCAAAGTGAGGGA
TAAGCATCGGAAACGATGTCTAATACCGCATGTGATCTTCGGATTAAAGCTTCGGCGCTTTGGGAACGGCC
TGCGCATGATTAGCTTGTTGGTGAGGTAATAGCTCACCAAGGCGATGATCATTAGCTGGTCTGAGAGGATG
ACCAGCCAGACTGGGACTGAGAACGGCC
>Zotu1708
GAACGCTAGCGGCAGGCTTAACACATGCAAGTCGAGGGGCAGCACAGGTAGCAATACCGGGTGGCGACC
GGCAAACGGGTGCGGAACACGTACTGAATCTACCTTTGTCTGGGGCATAGCCCGAAGAAATTCGGATTAA
CTCCCCATAATATAGATGAGTGGCATCACTCGACTATTAAAAATTTATTGGACAGAGATGACAGTGCGTCCC
ATTAGCTAGTTGGTAGGGTAACGGCCTACCAAGGCGACGATGGGTAGCTGGTGTGAGAGCACGACCAGCC
ACACGGGCACTGAGACACGGGC
>Zotu1710
GAACGCTAGCGACAGGCTTAACACATGCAAGTCGAGGGGCAGCGGAGGTAAAGGCTTGCCTTTACCTGCC
GGCGACCGGCGCACGGGTGAGTAACACGTATGCAACCTGGCCGTCACAGGAGGATAACCGGGAGAAATT
CCGCCTAATACTCCATAACACCGGAGGGAGACATCTCCCACCGGTCAAAGGAATTTATTCCGGTGACGGAT
GGGCATGCGGCGCATTAGCTAGTCGGCGGGGTAACGGCCCACCGAGGCGACGATGCGTAGGGGTTCTGA
GAGGAAGGTCCCCCACATTGGAACTGAGACACGGTC
>Zotu1712
GAACGCTGGCGGCGTGCTTAACACATGCAAGTCGAACGATGAAGCCCACTTGTGGGTGGATTAGTGGCGA
ACGGGTGAGTAACACGTGAGTAACCTGCCCTTGACTCTGGGATAAGCCTGGGAAACTGGGTCTAATACCG
GATATGACTCCTCATCGCATGGTGGGGGGTGGAAAGCTTTTTGTGGTTTTGGATGGACTCGCGGCCTATCA
GCTTGTTGGTGAGGTAATGGCTTACCAAGGCGACGACGGGTAGCCGGCCTGAGAGGGTGACCGGCCACA
CTGGGACTGAGACACGGCC
>Zotu1714
GAACGCTGGCGGCAGGCCTAACACATGCAAGTCGAGCGCGTGTAGCAATACACGAGCGGCGCACGGGTG
AGTAACGCGTGGGAATCTGCCCTACAGTGGGGAACAACCACGGGAAACTGTGGCTAATACCGCATACGTC
CTTCGGGAGAAAGGCTTAGGCCGCTGTAGGAGGAGCCCGCGTCCGATTAGCTAGTTGGTGAGGTAATGGC
TCACCAAGGCGACGATCGGTAACTGGTCTGAGAGGACGACCAGTCACACTGGGACTGAGACACGGCC
>Zotu1716

GAACGCTGGCGGCGTGCTTAACACATGCAAGTCGAACGGGATTTAGTAGACAGAAACCTCGGTGGAAGAT
TACTAATGAGAGTGGCGAACGGGTGAGTAACGCGTGAGCAACCTGCCTATGACAGTGGGATAGCCTCGGG
AAACCGGGATTAATACCGCATAAAATCGTAGAAACACATGTTTCAACGGTCAAAGATTTATCGGTCATAGA
TGGGCTCGCGTCTGATTAGCTAGTTGGTGAGATAACAGCCCACCAAGGCGACGATCAGTAGCCGGTCTGA
GAGGATGAACGGCCACATTGGAACTGAGACACGGTC

>Zotu1718
GAACGCTGGCGGCGCGCTTAACACATGCAAGTCGAGCGAGAACCAGGACTTCGGTCCTGGGGACAGCGG
CGAACGGGTGAGTAACACGTGGGTAATCTGCCCTCGATTCTGGGATAGCCCGGGGAAACTCGGATTAATA
CCGGATAGCCTTTCAAGCTTTCGGGCGTGAAAGAAAAGGTAGCTTCGGCCTCCGATCGAGGATGAGCCCG
CGGTGGATTAGCTTGTTGGCGGGGTAATGGCCCACCAAGGCGACGATCCATAGCTGGTCTGAGAGGACGA
TCAGCCACACTGGGACTGAGACACGGCC

>Zotu1720
GAACGCTGGCGGCATGCCTAACACATGCAAGTCGAACGATGCTTTCGGGCATAGTGGCGCACGGGTGCGT
AACGCGTGGGAATCTGCCCTTGGGTCTGGGATAACAGTTGGAAACGACTGCTAATACCGGATGATATCGC
GAGATCAAAGATTTATCGCCCGAGGATGAGCCCGCGTCAGATTAGCTAGTTGGTGGGGTAAAGGCCTACC
AAGGCGACGATCTGTAGCTGGTCTGAGAGGATGATCAGCCACACTGGGACTGAGACACGGCC

>Zotu1724
GAACGCTGGCGGCGTGCCTAATACATGCAAGTAGAACGCTGAAGGAGGAGCTTGCTTCTCCGGATGAGTT
GCGAACGGGTGAGTAACGCGTAGGTAACCTGCCTGGTAGTGGGGGATAACTATTGGAAACGATAGCTAAT
ACCGCATAACAGCAGTTGTTGCATGACAACTGTTTGAAAGGTGCAATTGCACCACTACCAGATGGACCTGC
GTTGTATTAGCTAGTTGGTGGGGTAACGGCTCACCAAGGCGACGATACATAGCCGACCTGAGAGGGTGAT
CGGCCACACTGGGACTGAGACACGGCC

>Zotu1726
GAACGCTGGCGGCAGGCCTAACACATGCAAGTCGAGCGGTAGAGAGAAGCTTGCTTCTCTTGAGAGCGGC
GGACGGGTGAGTAATGCCTAGGAATCTGCCTGGTAGTGGGGGATAACGTTCGGAAACGGACGCTAATACC
GCATACGTCCTACGGGAGAAAGCAGGGGACCTTCGGGCCTTGCGCTATCAGATGAGCCTAGGTCGGATTA
GCTAGTTGGTGAGGTAATGGCTCACCAAGGCTACGATCCGTAACTGGTCTGAGAGGATGATCAGTCACACT
GGAACTGAGACACGGTC

>Zotu1728
AAACGCTGGCGGCGTGCATGAGACATGCAAGTCGAACGAGTTCCTGGAGCTTGCTCCAGGCGCTAGTGGC
GGACGGCTGAGTAACGCGTGGGTGACCTACCCCGAAGTGGGGAATAACTGCCCGAAAGGGTGGCTAATA
CCGCATGTGATCGTTCTTTCAGTTGATCGATCAAAGCAGCAATGCGCTTCGGGAGGGGCCTGCGTCCGATT
AGCTCGTTGGTGGGGTAATGGCTCACCAAGGCGACGATCGGTAGGTGGTCTGAGAGGACGATCACCCAGA
CTGGGACTGAGACACGGCC

>Zotu1730
GAACGCTGGCGGCGTGCTTAACACATGCAAGTCGAACGAAGAGCGATGGAAGCTTGCTTCTATCAATCTTA
GTGGCGAACGGGTGAGTAACGCGTAATCAACCTGCCCTTCAGAGGGGGACAACAGTTGGAAACGACTGCT
AATACCGCATACGATCCAATCTCGGCATCGAGACTGGATGAAAGGTGGCCTCTATTTATAAGCTATCACTG
AAGGAGGGGATTGCGTCTGATTAGCTAGTTGGAGGGGTAACGGCCCACCAAGGCGATGATCAGTAGCCG
GTCTGAGAGGATGAACGGCCACATTGGGACTGAGACACGGCC

>Zotu1732
GAACGCTGGCGGCACGCTTAACACATGCAAGTCGAACGAGAACTTCGGAGCTTGCTCCGAGGGGACAGTG
GCGAACGGGTGAGTAACACGTGAGGAACCTGCCCCGAAGACCGGGATAACACCGGGAAACCGGTGCTAA
TACCGGATGCCCTCTCCGGATCGCATGATCTGGCGAGGAAATGGATTCCGCTTCGGGATGGCCTCGCGGCC
TATCAGCTTGTTGGTGGGGTAACGGCCTACCAAGGCTTCGACGGGTAGCTGGTCTGAGAGGATGTCCAGC
CACACTGGGACTGAGACACGGCC

>Zotu1734
GAACGCTGGCGGCGTGCTTAACACATGCAAGTCGAACGATGAAGCCTAGCTTGCTGGGTGGATTAGTGGC
GAACGGGTGAGTAACACGTGAGCAACCTGCCCCTCACTCTGGGATAAGCGCTGGAAACGGCGTCTAATAC
TGGATACGAAACGCAACCGCATGGTTAGTGTTTGGAAAGATTTTTGGTGGGGGATGGGCTCGCGGCCTA

TCAGCTTGTTGGTGAGGTAATGGCTCACCAAGGCGTCGACGGGTAGCCGGCCTGAGAGGGTGACCGGCCA
CACTGGGACTGAGACACGGCC
>Zotu1736
GAACGCTGGCGGCAGGCTTAACACATGCAAGTCGAGCGGGGAAGAGTAGCTTGCTACTTTACCTAGCGGC
GGACGGGTGAGTAATGCTTAGGAATCTGCCTATTAGTGGGGGACAACATCTCGAAAGGGATGCTAATACC
GCATACGTCCTACGGGAGAAAGCAGGGGACCTTCGGGCCTTGCGCTAATAGATGAGCCTAAGTCGGATTA
GCTAGTTGGTGGGGTAAAGGCCTACCAAGGCGACGATCTGTAGCGGGTCTGAGAGGATGATCCGCCACAC
TGGGACTGAGACACGGCC
>Zotu1738
GAACGCTGGCGGCGTGCTTAACACATGCAAGTCGAACGGAAAGGCCTTGTGCTTGCACAAGGTACTCGAG
TGGCGAACGGGTGAGTAACACGTGGGTGATCTGCCCTGCACTGTGGGATAAGCCTGGGAAACTGGGTCTA
ATACCATATAGGACCGCATCTTGGATGGTGTGGTGGAAAGCTTTTGCGGTGTGGGATGAGCCTGCGGCCT
ATCAGCTTGTTGGTGGGGTAATGGCCTACCAAGGCGGCGACGGGTATCCGGCCTGAGAGGGTGTACGGAC
ACATTGGGACTGAGATACGGCC
>Zotu1741
GAACGCTGGCGGCGTGCTTAACACATGCAAGTCGAACGGAAAGGCCCTTTCGGGGGTACTCGAGTGGCGA
ACGGGTGAGTAACACGTGGGTGATCTGCCCTGCACTTCGGGATAAGCTTGGGAAACTGGGTCTAATACCG
GATAGGACCATGGCATGCATGTGCTGTGGTGGAAAGCTTTTGCGGTGTGGGATGGGCCCGCGGCCTATCA
GCTTGTTGGTGGGGTGATGGCCTACCAAGGCGACGACGGGTAGCCGGCCTGAGAGGGTGTCCGGCCACA
CTGGGACTGAGATACGGCC
>Zotu1743
GAACGCTGGCGGCGCGCCTAAAACATGCAAGTCGAACGAGAAACCCGGGCTTGCCCGGGTGGACAGTGG
CGAACGGGTGAGTAACACGTGAGGAACCTGCCCCGAAGACCGGGACAACACCGGGAAACCGGTGCTAAT
ACCGGATGTCCTCATCGGACCGCATGATCTGATGAGGAAATGGATTCCGCTTCGGGATGGCCTCGCGGCCT
ATCAGCTAGTTGGTGGGGTAACGGCCCACCAAGGCGACGACGGGTAGCTGGTCTGAGAGGATGTCCAGCC
ACACTGGGACTGAGACACGGCC
>Zotu1746
CAACGCTGGCGGCGTGCATGAGACATGCAAGTCGAACGGACGACTCGGGCTTGCCTGAGTCGTTAGTGGC
GGACGGCTGAGTAACGCGTGGGTGACCTGCCCCGAAGTGGGGAATAACTGCCCGAAAGGGTGGCTAATA
CCGCATGTGACGGTGCGTTCGATCGCATCATCAAAGCAGCAATGCGCTTCGGGAGGGGCCTGCGTTCGATT
AGCTCGTTGGTGGGGTAACGGCTCACCAAGGCGACGATCGATAGGTGGTCTGAGAGGACGATCACCCAGA
CTGGGACTGAGACACGGCC
>Zotu1749
GAACGCTGGCGGCGTGCCTAACACATGCAAGTCGAACGGGGGTAGCAATACCCCAGTGGCGAACGGGTG
CGTAACACGTGAGGAACATTCCTTCCGGTGGGGGATAGCCGGCCTAACGGCCGGGTAATACCGCATACGC
TGTTCGGGGTGCATACCCTGGATCGGAAAGCAGCAATGCGCCGGAAAGGTGCCTCGCGGCCTATCAGCTA
GTTGGTGAGGTAATGGCTCACCAAGGCGACGACGGGTAGCTGGTCTGAGAGGATGGCCAGCCACATTGG
GACTGAGACACGGCC
>Zotu1753
GAACGCTGGCGGCAGGCCTAACACATGCAAGTCGAGCGGATGAGAGGAGCTTGCTCTTCGATTCAGCGGC
GGACGGGTGAGTAATGCCTAGGAATCTGCCTAGTAGTGGGGGACAACGTTTCGAAAGGAACGCTAATACC
GCATACGTCCTACGGGAGAAAGTGGGGGATCTTCGGACTTTACGCTATTAGATGAGCCTAGGTCGGATTA
GCTAGTTGGTAGGGTAAAGGCCTACCAAGGCGACGATCCGTAACTGGTCTGAGAGGATGATCAGTCACAC
TGGAACTGAGACACGGTC
>Zotu1756
CAACGCTGGCGGCGTGCCTAATGCATGCAAGTCGGACGTGGCCCTTTCGGGGGTGCGCGTGGCGGACGG
GTGAGGAACGCGTGGGGAACCTGCCTGTTAGTGGGGGGTAGCCATCGGAAACGGTGGGTAATTCCGCAT
GAGCTCAGTTGCCGCGTGGGGTGATTGAGGAAAGCCTTCGGGCGCTGACAGATGGTCCTGCGGCCGATTA
GCTCGTTGGTGGGGTAACGGCCTACCAAGGCGATGATCGGTAGCTGGTCTGAGAGGACGGCCAGCCACAT
GGGGACTGAGACACGGCC

>Zotu1758
GAACGCTGGCGGCGTGCCTAATACATGCAAGTCGAACGCATTTTCTTTCACCGTAGCTTGCTACACCGAAA
GAAAATGAGTGGCGAACGGGTGAGTAACACGTGGGTAACCTGCCCATCAGCGGGGGATAACACTTGGAA
ACAGGTGCTAATACCGCATAATTCCATTTACCGCATGGTAGATAGATGAAAGGCGCTTTTGCGTCACTGAT
GGATGGACCCGCGGTGCATTAGCTAGTTGGTGGGGTAACGGCCTACCAAGGCTGCGATGCATAGCCGACC
TGAGAGGGTGATCGGCCACACTGGGACTGAGACACGGCC
>Zotu1760
GAACGCTAGCGGCAGGCTTAATACATGCAAGTCGTGGGGCAGCGCAGTGTAGCAATACATGGGCGGCGA
CCGGCAAACGGGTGCGGAACACGTACGCAACCTTCCTTTAAGTGGAGAATAGCCCAGGGAAACTTGGATT
AATACTCCGTAACATTACGAAGTGGCATCACTTTGTAATTATAGCAGCAATGCGCTTGAAGATGGGCGTGC
GCCTGATTAGGTAGTTGGCGGGGTAACGGCCCACCAAGCCTACGATCAGTAACTGGTGTGAGAGCACGAC
CAGTCACACGGGCACTGAGACACGGGC
>Zotu1762
GAACGCTGGCGGCGTGCTTAACACATGCAAGTCGAACGGAAAGGCCCTTTCGGGGGTACTCGAGTGGCGA
ACGGGTGAGTAACACGTGGGTGATCTGCCCTGCACTTCGGGATAAGCTTGGGAAACTGGGTCTAATACCG
GATAGGATCACGCTCTTCATGGGGTGTGGTGGAAAGCTTTTGCGGTGTGGGATGGGCCCGCGGCCTATCA
GCTTGTTGGTGGGGTAATGGCCTACCAAGGCGACGACGGGTAGCCGGCCTGAGAGGGTGTCCGGCCACA
CTGGGACTGAGATACGGCC
>Zotu1764
CAACGCTGGCGGCGTGCCTCAGACATGCAAGTCGTACGCGAAAGGCCCCTTCGGGGGCTGAGTAGAGTGG
CGTCCGGGTGAGTAACACGTGGGCAATCTGCCTCTGGGTGGGGAATAACCCGGGGAAACCCGGGCTAATA
CCGCATACGGCGGCGCAGCCTGCGGGCCGCGTCGTGAAAGCGGGGGACCTCGGCAACGAGGCCTCGCGC
CCGGAGAGGAGCCTGCGGCGGATTAGCTAGTTGGTGAGGTAACGGCTCACCAAGGCGACGATCCGTAGC
CGGCCTGAGAGGGCGGTCGGCCACACTGGCACTGACACACGGGC
>Zotu1766
GAACGCTGGCGGCGTGCCTAATACATGCAAGTCGAGCGAACCAATAGGAGCTTGCTCCTGTTGGTTAGCG
GCGGACGGGTGAGTAACACGTGGGCAACCTGCCTGTAAGACTGGGATAACACCGGGAAACCGGTGCTAA
TACCGGATAATCCTTTTCCTCTCATGAGGAAAAGCTGAAAGTCGGTGCAAGCTGACACTTACAGATGGGCC
CGCGGCGCATTAGCTAGTTGGTGAGGTAACGGCTCACCAAGGCGACGATGCGTAGCCGACCTGAGAGGGT
GATCGGCCACACTGGGACTGAGACACGGCC
>Zotu1768
GAACGCTGGCGGCATGCCTAATACATGCAAGTCGAACGCTGAAGCACTCTGCTTGCAGAGTGCGGATGAG
TGGCGAACGGGTGAGCAACACGTGGGAAATCTGCCTATAAGTGGGGGATAACATTCGGAAACGGATGCT
AATACCGCATATATTCTTGGACCGCCTGGTCCTTGAAAGAAAGACGGCTTTGCTGTCGCTTATAGATGATCC
CGCGGCGTATTAGTTAGTTGGTGGGGTAAAGGCCTACCAAGACAATGATACGTAGCCGACCTGAGAGGGT
GATCGGCCACACTGGGACTGAGACACGGCC
>Zotu1770
GAACGCTAGCGACAGGCTTAACACATGCAAGTCGAGGGGCAGCAATTTGTAGCAATACAAAGTTGGCGAC
CGGCGCACGGGTGAGTAACGCGTATGCAACCTACCTACAACAGAAGAATAATCCGGCGAAAGTCGGTCTA
ATACTTCATAAAACAGGGACATCGCATGATGTTATTTGTTAAAGATTTATCGGTTGTAGATGGGCATGCGTA
CCATTAGGTAGTTGGTGAGGTAACGGCCCACCAAGCCTACGATGGTTAGGGGTTCTGAGAGGAAAGTCCC
CCACACTGGTACTGAGACACGGAC
>Zotu1772
CAACGCTGGCGGCGCGCCTAACACATGCAAGTCGAACGAGAAAGTGGAGCAATCCATGAGTAAAGTGGC
GTACGGGTGAGTAACACGTGGATCATCTACCCTCTAGTGGGGAATACCGCTGGGAAACCGGCGACAATAC
CGCATAAGCTCGAGAGAGGAAAGCAGCAATGCGCTGGAGGAGGAGTCCGCGGTCGATTAGTTAGTTGGC
GGGGTAAAGGCCCACCAAGACTGTGATCGATAGCCGGCCTGAGAGGGCACACGGCCACACTGGCACTGA
AACACGGGC
>Zotu1774

GAACGCTGGCGGCGTGCCTTAGGCATGCAAATCAAGGGGGGCTCGTAAGAGTCAACCGGTGGACGGGTG
AGTAACGCGTGAGAATCTACCTTAAAGTGGGGCATAGCTTCTCGAAAGAGAAGGTAATTTCCCATGTGATC
GCAAGATCAAAGCCGCAAGGCGCTTTAAGATGAGCTCGCGTCCTATCAGCTTGTTGGTGGGGTAATGGCCT
ACCAAGGCGACGACGGGTAGCTGGTCTGAGAGGATGGTCAGCCACAATGGAACTGCGACACGGTC
>Zotu1776
GAACGCTGGCGGCGTGCTTAACACATGCAAGTCGAGCGATGAAGCACCTTCGGGTGTGAATTAGCGGCGA
ACGGGTGAGGAACACGTGAGAAATCTGCCTTCAACTCTGGGATAACTCCGTGAAAACGGGGCTAATACCG
GATATGAAACCTTCAGGCATCTGAGGGTTTGGAAAGTTTTTCGGTTGAAGATGATCTCGCGGCCTATCAGC
TTGTTGGTGAGGTAATGGCTCACCAAGGCGACGACGGGTAGCCGGCCTGAGAGGGCGACCGGCCACACT
GGGACTGAGACACGGCC
>Zotu1778
GAACGCTGGCGGCGTGCCTAATACATGCAAGTCGTACGCATTGATAATGGAACTTGTTCCATTTGACGTGA
GTGGCGCACGGGTGAGTAACACGTGGGAAACCTACCCTTCAGCGGGGGATAACCGTCGGAAACGATGGC
TAATACCGCATAGGTCTTTGAGTCACCTGACTTAAAGAGGAAAGACGGCTTCGGCTGTCACTGAAGGATGG
TCCCGCGGTGCATTAGCTAGTTGGTAAGGTAATGGCTTACCAAGGCGATGATGCATAGCCGACCTGAGAG
GGTGATCGGCCACATTGGGACTGAGACACGGCC
>Zotu1780
GAACGCTGGCGGCACGCCTAATGCATGCAAGTCGAACGACGCACCTTGGAGGAGTAATCCTCTGGGGTGA
CGGAGTGGCGAACGGGTGCGTAACACGTGAGCAACCTGCCCCGAAGATCGGAATAGCCCAAGGAAACTT
GGATTAATACCGAATACCCTCATCCTTCCGCATGGTCGGATGAGAAAAGGCCTTTGCGCTTCGGGATGGGC
TCGCGGCCTATCAGCTAGTTGGTGAGGTAACGGCTCACCAAGGCAACGACGGGTAGCTGGTCTGAGAGGA
CGATCAGCCACACTGGGACTGAGACACGGCC
>Zotu1782
GAACGCTGGCGGCGTGCTTAACACATGCAAGTCGAACGATGACAGTGGTGCTTGCACCGCCTGATTAGTG
GCGAACGGGTGAGTAACACGTGAGTAACCTGCCCTCCACTTCGGGATAACCTCGGGAAATCGTGGCTAAT
ACCGGATACGAGCACTCATCGCATGGTGGGTGTTGGAAAGATTTATCGGTGGGGGATGGACTCGCGGCCT
ATCAGTTTGTTGGTGAGGTGATGGCTCACCAAGACGATGACGGGTAGCCGGCCTGAGAGGGCGACCGGCC
ACACTGGGACTGAGACACGGCC
>Zotu1784
GAACGCTAGCGGCATGCTTTACACATGCAAGTCGAACGGTAACAGGTCTTCGGATGCTGACGAGTGGCGA
ACGGGTGAGTAATACATCGGAACGTGCCTAGTAGTGGGGGATAACTACTCGAAAGAGTAGCTAATACCGC
ATGAGATCTACGGATGAAAGCAGGGGACCTTCGGGCCTTGTGCTACTAGAGCGGCTGATGGCAGATTAGG
TAGTTGGTGGGGTAAAGGCTTACCAAGCCTGCGATCTGTAGCTGGTCTGAGAGGACGACCAGCCACACTG
GGACTGAGACACGGCC
>Zotu1786
GAACGCTGGCGGCAGGCTTAACACATGCAAGTCGAACGGGCACCTTCGGGTGTCAGTGGCAGACGGGTG
AGTAACACGTGGGAACGTACCCTTCGGTTCGGAATAACTCAGGGAAACTTGAGCTAATACCGGATACGCCC
TTTTGAGGAAAGGTTTACTGCCGAAGGATCGGCCCGCGTCTGATTAGCTTGTTGGTGGGGTAACGGCCTAC
CAAGGCGACGATCAGTAGCTGGTCTGAGAGAATGATCAGCCACACTGGGACTGAGACACGGCC
>Zotu1788
GAACGCTGGCGGCAGGCCTAACACATGCAAGTCGAGCGGCAGCGGGTCCTTCGGGATGCCGGCGAGCGG
CGGACGGGTGAGGAATGCGTCGGAATCTGCCTTTTTGTGGGGGATAACGTAGGGAAACTTACGCTAATAC
CGCATACGACCTACGGGTGAAAGTGGGGGACCTTCGGGCCTCACGCAGAAAGATGAGCCGACGCCGGATT
AGCTAGTTGGCGGGGTAAAGGCCCACCAAGGCGACGATCCGTAGCTGGTCTGAGAGGATGATCAGCCACA
CTGGAACTGAGACACGGTC
>Zotu1790
GAACGCTGGCGGCGTGCTTAACACATGCAAGTCGAACGGAAAGGCCCTTTCGGGGGTACTCGAGTGGCGA
ACGGGTGAGTAACACGTGGGTGATCTGCCCTGCACTTTGGGATAAGCCTGGGAAACTGGGTCTAATACCG
GATAGGACTACGCGATGCATGTCGTGTGGTGGAAAGCTTTTGCGGTGTGGGATGGGCCCGCGGCCTATCA
GCTTGTTGGTGGGGTGATGGCCTACCAAGGCGACGACGGGTAGCCGGCCTGAGAGGGTGACCGGCCACA
CTGGGACTGAGATACGGCC

>Zotu1792
GAACGCTGGCGGCGTGCTTAACACATGCAAGTGGAGCGACGAACCCGAGCTTGCTCGGGGGCAAAGCCG
CGAACGGGTGAGTAACACGTGGGTAATCTGCCCTGATGATTGGGACAACCCCGGGAAACCGGGGCTAATA
CCAAATGTGCTCTTTCCACATCAGTGGATCGAGGAAAGGAGGCTTCGGCTTCCGCATCGGGATGAGCCCGC
GGCCCATTAGCTTGTTGGTGGGGTAATGGCCTACCAAGGCTCCGATGGGTAGCTGGTCTGAGAGGACGAT
CAGCCACACTGGGACTGAGACACGGCC
>Zotu1794
GAACGCTGGCGGCGTGCTTAACACATGCAAGTCGAACGATGATGCTGGTGCTTGCACTGGTGGATTAGTG
GCGAACGGGTGAGTAACACGTGAGTAACCTGCCCCTGACTTCGGGATAAGCCCGGGAAACTGGGTCTAAT
ACCGGATATGACTTCCTGCTGCATGGCAGGGGGTGGAAAGATTTATCGGTTGGGGATGGACTCGCGGCCT
ATCAGCTTGTTGGTGAGGTAATGGCTCACCAAGGCGACGACGGGTAGCCGGCCTGAGAGGGTGACCGGC
CACACTGGGACTGAGACACGGCC
>Zotu1797
GAACGCTGGCGGCGTGCTTAACACATGCAAGTCGAACGATGAAGCCAGCTTGCTGGTGGATTAGTGGCGA
ACGGGTGAGTAACACGTGGGTGATCTGCCCCAAACTTTGGGATAAGCCTGGGAAACTGGGTCTAATACCG
AATATGACCACTGGATGCATGTCTGGTGGTGGAAAGCTCCGGCGGTTTGGGATGGGCCCGCGGCCTATCA
GCTTGTTGGTGGGGTAATGGCCTACCAAGGCGACGACGGGTAGCCGGCCTGAGAGGGCGACCGGCCACA
CTGGGACTGAGACACGGCC
>Zotu1800
GAACGCTGGCGGCGTGCCTAATACATGCAAGTCGAGCGAATCAATGGGAGCTTGCTCCCTGAGATTAGCG
GCGGACGGGTGAGTAACACGTGGGTAACCTGCCTGTAAGACTGGGATAACTTCGGGAAACCGGAGCTAAT
ACCGGATAATTTCTTTTCTCGCATGAGGAAAGATGGAAAGATGGTTCTGCTATCACTTACAGATGGACCCG
CGGCGCATTAGCTAGTTGGTGAGGTAATGGCTCACCAAGGCGACGATGCGTAGCCGACCTGAGAGGGTGA
TCGGCCACACTGGGACTGAGACACGGCC
>Zotu1802
GAACGCTGGCGGCGTGCCTAATACATGCAAGTCGAACGGTTTAGTTCCAAGAGGCTTGCCTTTTGGGACTA
GACAGTGGCGGACGGGTGAGTAACACGTAGGCAACCTACCTGTCAAACCGGGATAACCTCGGGAAACCGA
AGCTAAACCCGGATAAGCTCCTTTTTCGCATGAAGGGGGAGTGAAAAGGTGCGACCTGATGACAGATGGG
CCTGCGGCGCATTAGCTAGTAGGTAGGGTAACGGCCTACCTAGGCGATGATGCGTAGCCGACCTGAGAGG
GCGTACGGCCACACTGGGACTGAGATACGGCC
>Zotu1804
GAACGCTGGCGGCGTGCCTAATACATGCAAGTCGAACGCTTCACTTCGGTGAAGAGTGGCGAACGGGTGA
GTAATACATAAGTAACCTGACATCTACAGGGGGATAACTGATGGAAACGTCAGCTAAGACCGCATAGGTG
TAGAGATCGCATGAACTCTATATGAAAAGTGCTACGGGACTGGTAGATGATGGACTTATGGCGCATTAGCT
TGTTGGTAGGGTAACGGCCTACCAAGGCGACGATGCGTAGCCGACCTGAGAGGGTGACCGGCCACACTG
GGACTGAGACACGGCC
>Zotu1806
GAACGCTAGCTACAGGCTTAACACATGCAAGTCGAGGGGAAACGACATTGAAGCTTGCTTCGATGGTCGT
CGACCGGCGCACGGGTGAGTAACGCGTATCCAACCTGCCTCTGACTAAGGGATAACCCGGCGAAAGTCGG
ACTAATACCTTATGAGGTTTTCAGCAGACATCTAATGAAAACGAAAGATTTATCGGTCAGTGATGGGGATG
CGTCTGATTAGCTTGTTGGCGGGGTAACGGCCCACCAAGGCAACGATCAGTAGGGGTTCTGAGAGGAAGG
TCCCCCACATTGGAACTGAGACACGGTC
>Zotu1808
GAACGCTAGCGGCAGGCTTAACACATGCAAGTCGAGGGGCAGCGAGATGTAGCAATACATTGTCGGCGAC
CGGCGCACGGGTGCGTAACGCGTATGCAACCTACCATATACAGGAGGATAACCATTCGAAAGAATGACTA
ATACTCCATAGTATTATTTTGAGGCATCTTAGAATAATTAAAGAATTTCGGTATATGCTGGGCATGCGTGAC
ATTAGCTAGTTGGAGAGGTAACGGCTCACCAAGGCTACGATGTCTAGGGGTTTTGAGAGGAAGATCCCCC
ACACTGGTACTGAGACACGGAC
>Zotu1810

GAACGCTGGCGGCAGGCTTAACACATGCAAGTCGAACGGATCCTTCGGGATTAGTGGCAGACGGGTGAGT
AACGCGTGGGAACGTACCCTTTGCTACGGAATAACTCAGGGAAACTTGAGCTAATACCGTATGTGCCCTAC
GGGGGAAAGATTTATCGGCAAAGGATCGGCCCGCGTTGGATTAGCTAGTTGGTGGGGTAATGGCCTACCA
AGGCGACGATCCATAGCTGGTCTGAGAGGATGATCAGCCACACTGGGACTGAGACACGGCC
>Zotu1813
GAACGCTGGCGGCGTGCTTAACACATGCAAGTCGAGCGGAAAGGCCACTTCGGTGGTACTCGAGCGGCGA
ACGGGTGAGTAACACGTGAGTAATCTGCCCCTGGCTTTGGGATAGCCACCGGAAACGGTGATTAATACCG
GATATGACATGGTCTCGCATGGGATTGTGTGGAAAGTTTTTCGGCCAGGGATGTGCTCGCGGCCTATCAGC
TTGATGGTGAGGTAATGGCTCACCATGGCTTCGACGGGTAGCCGGCCTGAGAGGGTGACCGGTCACACTG
GGACTGAGACACGGCC
>Zotu1816
GAACGCTAGCTGCAGGCTTAACACATGCAAGTCGAGGGGCAGCGGGAGTAGCAATACTTGCCGGCGACC
GGCGGAAGGGTGCGTAACGCGTGAGCAACATTCCCGTATCAGGGGGATAACCGGTGGAAACGCCGACTA
ATACCCCATAAGAACATCTGCGGCATCAAAGGTGTTTGAAAGATTCGTCGGATGCGGATTGGCTCGCGTGA
CATTAGCTAGTTGGTGAGGTAACGGCCCACCAAGGCGACGATGTCTAGGAGTTCTGAGAGGAAGATCCCC
CACATTGGAACTGAGACACGGTC
>Zotu1818
AAACGCTGGCGGCGTGCATGAGACATGCAAGTCGAACGGACGATTCGGGCTTGCTCGAATCGTTAGTGGC
GGACGGCTGAGTAACGCGTGGGTGACCTGCCCCGAAGTGGGGAATAACTGCCCGAAAGGGTGGCTAATA
CCGCATGTGGTCGCACGTTCAATCGTGCGAGTAAAGCCGCAAGGCGCTTCGGGAGGGGCCTGCGTCCGAT
TAGCTCGTTGGTGGGGTAATGGCTCACCAAGGCGACGATCGGTAGGTGGTCTGAGAGGACGATCACCCAG
ACTGGGACTGAGACACGGCC
>Zotu1820
GAACGCTGGCGGCAGGCCTAACACATGCAAGTCGAGCGCACCCTTCGGGGTGAGCGGCGGACGGGTGAG
TAACGCGTGGGAACGTGCCCTTCTCTGCGGAATAGCCTCGGGAAACTGAGAGTAATACCGCATACGCCCTT
TGGGGGAAAGATTTATCGGAGAAGGATCGGCCCGCGTTGGATTAGGTAGTTGGTGGGGTAATGGCCCACC
AAGCCGACGATCCATAGCTGGTTTGAGAGGATGATCAGCCACACTGGGACTGAGACACGGCC
>Zotu1822
GAACGCTGGCGGAATGCTTTACACATGCAAGTCGAACGGCAGCACGGGGCAACCTGGTGGCGAGTGGCG
AACGGGTGAGTAATATATCGGAACGTGCCCAATCGTGGGGGATAACGTAGCGAAAGTTACGCTAATACCG
CATACGATCTACGGATGAAAGCGGGGGATCGCAAGACCTCGCGCGAATGGAGCGGCCGATATCAGATTAG
GTAGTTGGTGGGGTAAAGGCTCACCAAGCCAACGATCTGTAGCTGGTCTGAGAGGACGACCAGCCACACT
GGAACTGAGACACGGTC
>Zotu1824
GAACGCTGACAGAATGCTTAACACATGCAAGTCGATGGGGAAGTGGTGCTTGCACCATGGTAACCATGGC
GGACGGGTGAGTAACGCGTAAGGAACTTGCCTGCGGGACTGGGATAACAGCAGGAAACTGCTGATAATA
CCGGATAAGGTCATTAAGCTGCATAGCTTAGTGATGAAAGGAGACGCCTGCGGAGAGCCTTGCGTCCTATT
AGCTAGTTGGAGGGGTAACGGCCCACCAAGGCGAAGATAGGTAGCCGGCCTGAGAGGGTGGACGGCCAC
AAGGGGACTGAGATACGGCC
>Zotu1826
CAACGCTGGCGGCGCGCTTAACACATGCAAGTCGAGCGAGAAAGGGGGCAACCCTGAGTAAAGCGGCGT
ACGGGTGAGTAACACGTGGATTATCTACCTTTTAGTGGGGAATAACTTTGGGAAACCGAAGCTAATACCGC
ATAAGCCTGTAAAGGGAAAGCAGCAATGCGCTGGAAGAAGAGTCCGCGGCCGATTAGCTAGTTGGTGAG
GTAACGGCCCACCAAGGCGACGATCGGTAGCCGGCCTGAGAGGGCACACGGCCACACTGGCACTGAAAC
ACGGGC
>Zotu1829
GAACGCTGGCGGCGTGCCTAATACATGCAAGTCGAGCGAACTGATTAAGAAGCTTGCTTCTTATGACGTTA
GCGGCGGACGGGTGAGTAACACGTGGGCAACCTGCCCTATAGTTGGGGATAACTCCGGGAAACCGGGGC
TAATACCGAATGATACATTTTAACTCCTGTTAGAATGTTGAAAGATGGTTTACGCTATCGCTATAGGATGGG
CCCGCGGCGCATTAGCTAGTTGGTGAGGTAACGGCTCACCAAGGCGACGATGCGTAGCCGACCTGAGAGG
GTGATCGGCCACACTGGGACTGAGACACGGCC

>Zotu1831
GAACGCTGGCGGCGTGCTTAACACATGCAAGTCGAGCGGTAAGGCCCTTCGGGGTACACGAGCGGCGAA
CGGGTGAGTAACACGTGAGTAATCTGCCCTTCACTTCGGGATAACCACCGGAAACGGTGGCTAATACCGG
ATATGACTTCTCTTCGCATGTTGAGAGGTGGAAAGTTCTGGCGGTGGAGGATGTGCTCGCGGCCTATCAGC
TTGTTGGTGAGGTAATGGCTCACCAAGGCTTCGACGGGTAGCCGGCCTGAGAGGGTGACCGGCCACACTG
GGACTGAGACACGGCC
>Zotu1833
GAACGCTGGCGGCATGCTTAACACATGCAAGTCGCACGAGGGGGCAACCCCTAGTGGCGGACGGGTGAG
TAACGCGTAGGTATCTATCCTGAGGTGGGGGATAACAGCGGGAAACTGCTGCTAATACCGCATGACACCT
GTGGGTCAAAGGCGCAAGTCGCCTTGGGAGGAGCCTGCGTCCGATTAGCTAGTTGGTGGGGTAAAGGCCT
ACCAAGGCTTCGATCGGTAGCTGGTCTGAGAGGATGATCAGCCACACTGGGACTGAGACACGGCC
>Zotu1835
GAACGCTGGCGGCAGGCTTAACACATGCAAGTCGAACGCCCCGCAAGGGGAGTGGCAGACGGGTGAGTA
ACGCGTGGGAACGTACCTATCGGTTCGGAATAACCCAGGGAAACTTGGACTAATACCGGATACGCCCTTTT
GGGGGAAAGATTTATCGCCGATAGATCGGCCCGCGTCTGATTAGCTAGTTGGTGGGGTAATGGCCTACCA
AGGCGACGATCAGTAGCTGGTCTGAGAGGATGATCAGCCACACTGGGACTGAGACACGGCC
>Zotu1837
GAACGCTAGCGGCAGGCCTAATACATGCAAGTCGAACGAGATTATCCAGCTTGCTGGATATGAAAGTGGC
GCACGGGTGCGTAACGCGTATGCAACCTACCTTTATCTGGGGGATAGCCCGGAGAAATCCGGATTAATACC
GCATAAAATCACAGGATGGCATTATCCAATGATCAAACATTTATGGGATGAAGATGGGCATGCGTGTCATT
AGCTAGTTGGCGGGGTAACGGCCCACCAAGGCGACGATGACTAGGGGATCTGAGAGGATGGCCCCCCAC
ACTGGTACTGAGACACGGAC
>Zotu1840
GAACGCTGGCGGCGTGCCTAATACATGCAAGTCGAGCGAACAGATGAGGAGCTTGCTCCTCTGATGTTAG
CGGCGGACGGGTGAGTAACACGTGGGCAACCTGCCCTATAGTTTGGGATAACTTGCGGAAACGTGAGCTA
ATACCGAATAATACAACGAAGCTCCTGCTTCGATGTTGAAAGATGGCTTTGCTATCGCTATAGGATGGGCC
CGCGGCGCATTAGCTAGTAGGTGAGGTAACGGCTCACCTAGGCAACGATGCGTAGCCGACCTGAGAGGGT
GATCGGCCACACTGGGACTGAGACACGGCC
>Zotu1842
GAACGCTGGCGGCATGCCTAATACATGCAAGTCGAACGCTGAAGTACTCTGCTTGCAGAGTGCGGATGAG
TGGCGAACGGGTGAGTAACACGTGGGGAATCTGCCTATAAGTGGGGGATAACATTCGGAAACGGATGCT
AATACCGCATATATTCTTGGACCGCCTGATCCTTGAAAGAAAGACGGCTTTGCTGTCGCTTATAGATGATCC
CGCGGCGTATTAGTTAGTTGGTGGGGTAAAGGCCTACCAAGACAATGATACGTAGCCGACCTGAGAGGGT
GATCGGCCACACTGGGACTGAGACACGGCC
>Zotu1844
GAACGCTGGCGGCGTGCTTAACACATGCAAGTGGAGCGACGAAGCGGACTTCGGTCCGTGGCAGAGCCG
CGAACGGGTGAGTAACACGTGGATAACCTACCCCAATGACCGGGACAACCCGAGGAAACTCGAGCTAATA
CCGGATGTTCTCTCTTCCTCTAAGGGAATTGAGCAAAGATAGCTTCGGCCTTCGCATTGGGACGGGTCCGC
GGCCCATTAGCTTGTTGGTGAGGTAACGGCTCACCAAGGCGACGATGGGTAGCTGGTCTGAGAGGACGAT
CAGCCACACTGGGACTGAGACACGGCC
>Zotu1846
GAACGCTGGCGGCGTGCCTAACACATGCAAGTCGAGCGAGAGACTTCGGTCTCTAGCGGCGGACGGGTG
AGTAACACGTGGGTAACCTGCCTCGTAGAGGGGGATAGCCTTCCGAAAGGAAGATTAATACCGCATAACA
TATATCTGGCGCATGCCAGATATATCAAAGGAGTAATCCGCTATGAGATGGACCCGCGGCGCATTAGCTAG
TTGGTGAGGTAACGGCTCACCAAGGCGACGATGCGTAGCCGACCTGAGAGGGTGATCGGCCACATTGGAA
CTGAGATACGGTC
>Zotu1848
GAACGCTGGCGGCATGCCTAACACATGCAAGTCGAACGGAATCTTATATGTTCCTCGTTTCGGATTTTTAAT
CCGCGTTGAGGAACAGTAAGATTTAGTGGCGGACGGGTGAGTAACACGTTGGTACCTACCCTCTAGTGGG
GAATAACTTCGAGAAATCGAGGCTAATACCGCATAATATCTACGGATTAAAGCATTTATGCGCTAGAGGAG

GGGCCTGCGTCTGATTAGCTAGTTGGTGAGGTAAAAGCTCACCAAGGCGACGATCAGTAGCTGGATCGAG
AGGTTGACCAGCCACACTGGGACTGAGATACGGCC
>Zotu1850
GAACGCTAGCGGCAGGCCTAATACATGCAAGTCGAACGGGTTGAGGTAGCAATACTTCAATTAGTGGCGC
ACGGGTGCGTAACGCGTATGCAACCTACCTTTTATTGGGGGATAGCCCGAGGAAACTCGGATTAATACCGC
ATAACATCATTTGAGGGCATCTGAAGATGATTAAAGCTAAGGCGATAAAAGATGGGCATGCGTGTCATTA
GCTAGTTGGTTGAGGTAAAGGCTCACCAAGGCGACGATGACTAGGGGTTCTGAGAGGATGATCCCCCACA
CTGGTACTGAGATACGGAC
>Zotu1852
GAACGCTGGCGGCGTGCTTAACACATGCAAGTCGAGCGGTAAGGCTCCTTCGGGGGTACACGAGCGGCG
AACGGGTGAGTAACACGTGAGTAATCTGCCCTTGGCTTTGGGATAAGCACCGGAAACGGTGTCTAATACC
GGATATACACGCATTGTCGCATGGTGGTGTGTGGAAAGTTTTTCGGCCTTGGATGTGCTCGCGGCCTATCA
GCTTGATGGTGGGGTAATGGCCTACCATGGCTTCGACGGGTAGCCGGCCTGAGAGGGTGACCGGTCACAC
TGGGACTGAGACACGGCC
>Zotu1854
GAACGCTGGCGGCGTGCTTAACACATGCAAGTCGAACGGAAAGGCCTCTGCTTGCAGAGGTACTCGAGTG
GCGAACGGGTGAGTAACACGTGGGTGATCTGCCTTGCACTTCGGGATAAGCCTGGGAAACTGGGTCTAAT
ACCGGATAGGACCATGCTTTAGTGTGTGTGGTGGAAAGTTTTTCGGTGCGAGATGAGCCCGCGGCCTATCA
GCTTGTTGGTGGGGTAATGGCCTACCAAGGCGACGACGGGTAGCCGGCCTGAGAGGGTGTACGGCCACA
TTGGGACTGAGACACGGCC
>Zotu1857
GAACGCTAGCGGCAGGCCTAATACATGCAAGTTGAACGGTTAGTAGCAATACTGATAGTGGCGCACGGGT
GCGTAACACGTAGCTAACCTGCCCAGAACTGGGGGATAGCCCGCCGAAAGGCGGATTAACACCGCATAAT
ATCAGTACCTGGCATCAGGTGGTGATTAAATCTACGGAGGTTCTGGATGGGGCTGCGGGTCATTAGCTAGT
TGGTCAGGTAACGGCTTACCAAGGCGACGATGACTAGGGGAGCTGAGAGGCTGGTCCCCCACACGGGCA
CTGAGATACGGGC
>Zotu1859
GAACGCTGGCGGCGTGCTTAACACATGCAAGTCGAACGGTGAAAGAGGAGCTTGCTCCTCTGGATCAGTG
GCGAACGGGTGAGTAACACGTGAGTAACCTGCCCCAGACTCTGGGATAAGCGTTGGAAACGACGTCTAAT
ACCGGATACGAGCTTCAGCCGCATGGCTAGGAGCTGGAAAGAATTTCGGTCTGGGATGGACTCGCGGCCT
ATCAGGTAGTTGGTGAGGTAACGGCCCACCAAGCCTACGACGGGTAGCCGGCCTGAGAGGGTGACCGGC
CACACTGGGACTGAGACACGGCC
>Zotu1861
GAACGCTAGCGGGAGGCCTAACACATGCAAGCCGAGCGGTAGAAGATCTTCGGATCTTTGAGAGCGGCGT
ACGGGTGCGGAACACGTGTGCAACCTGCCTTTATCAGGGGGATAGCCTTTCGAAAGGAAGATTAATACCCC
ATAATATAATTGATGGCATCATTAATTATTGAAAACTCCGGTGGATAGAGATGGGCACGCGCAAGATTAGA
TAGTTGGTAGGGTAACGGCCTACCAAGTCAGTGATCTTTAGGGGGCCTGAGAGGGTGATCCCCCACACTG
GTACTGAGACACGGAC
>Zotu1863
GAACGCTGGCGGCATGCTTTACACATGCAAGTCGAACGGCAACGAGGAGAAGCTTGCTTCTCTGTCGGCG
AGTGGCGAACGGGTGAGTATAGCATCGGAACGTGCCAAGTAGTGGGGGATAACCAAACGAAAGTTTGGC
TAATACCGCGTAAGCTCCAAGGAGGAAAGTAGGGGACCTAAATAAGGCCTTACGCTATTTGATCGGCCGA
TGTCGGATTAGCTAGTTGGTGGGGTAATGGCTCACCAAGGCAATGATCCGTAGCGGGTCTGAGAGGACGA
TCCGCCACACTGGGACTGAGACACGGCC
>Zotu1865
GAACGCTGGCGGCGTGCTTTAGGCATGCAAGTCGAACGGTTTCCGCAAGGAAGCAGTGGCAGACGGGTG
AGTAATACGTAGGAATCTACCCCTCGGTGGACCATAGCTCATCGAAAGATGGGGTAATAGTTCATGGTCTC
GTAAGAGTAAAGCGATTAACTTCGCGCCAAGGGAGGAGCCTGCGTCCTATCAGCTAGTTGGTGGGGTAAA
AGCCTACCAAGGCAATGACGGGTAGCTGGTCTGAGAGGATGGTCAGCCACAACTGCACTGAGACACGGGC
>Zotu1868

GAACGCTGGCGGCGTGCTTAACACATGCAAGTCGAGCGGAAAGGCCACTTCGGTGGTACTCGAGCGGCGA
ACGGGTGAGTAACACGTGAGTAATCTGCCCCTGGCTTCGGGATAGCCACCGGAAACGGTGATTAATACCG
GATATGACATGGACTCGCATGGGTTTGTGTGGAAAGTTTTTCGGCCAGGGATGTGCTCGCGGCCTATCAGC
TTGATGGTGAGGTAATGGCTCACCATGGCTTCGACGGGTAGCCGGCCTGAGAGGGTGACCGGCCACACTG
GGACTGAGACACGGCC
>Zotu1870
GAACGCTGGCGGCGCGCCTAATACATGCAAGTCGAGCGAGAACCGGGACTTCGGTCCCGGGGAGAGCGG
CGAACGGGTGCGTAACACGTGAGCAACCTGCCCCAAAGACCGGGACAACCCGGGGAAACCCGAGCTAATA
CCGGATGTCCTCATCGGATCGCATGGTCTGGTGAGGAAATGGATTCCGCTTTGGGATGGGCTCGCGGTCTA
TCAGCTTGTTGGTGGGGTAACGGCTCACCAAGGCGACGACGGATAGCTGGTCTGAGAGGACGATCAGCCA
CACTGGGACTGAGACACGGCC
>Zotu1872
GAACGCTGGCGGCGTGCCTAATACATGCAAGTCGAGCGGATCGAACGGGAGCTTGCTTCCGTTCGGTTAG
CGGCGGACGGGTGAGTAACACGTGGGCAACCTGCCCGTAAGGCCGGGATAACTTCGGGAAACCGGAGCT
AATACCGGATAACACCGAAGACCGCATGGTCTTCGGTTGAAAGGCGGCTTTGGCTGTCACTTACGGATGG
GCCCGCGGCGCATTAGCTAGTTGGTGAGGTAACGGCTCACCAAGGCGACGATGCGTAGCCGACCTGAGAG
GGTGATCGGCCACACTGGGACTGAGACACGGCC
>Zotu1874
GAACGCTGGCGGCGTGCTTAACACATGCAAGTCGAACGGAAAGGCCCAGCTTGCTGGGTACTCGAGTGGC
GAACGGGTGAGTAACACGTGGGTGATCTGCCCCAAACTTCGGGATAAGCCTGGGAAACTGGGTCTAATAC
CGGATATGACCTGCTCCTGCATGGGGGTTGGTGGAAAGCTTTTGCGGTTTGGGATGGGCCCGCGGCCTAT
CAGCTTGTTGGTGGGGTAATGGCCTACCAAGGCGACGACGGGTAGCCGACCTGAGAGGGTGATCGGCCA
CACTGGGACTGAGACACGGCC
>Zotu1876
GAACGCTGGCGGCGTGCGTAACACATGCAAGTCGGACGGACGGAGTAATCCGTCAGTGGCGGACGGGTG
CGTAACACGTGGCTACCAACCCATCGCTGGGGGATACCCGGGGGAAACTCTGGTTAAGACCGCATACGCT
CGAAAAGAGGAAAGCCCGCAAGGGCGGCGATGGCTGGGGCTGCGGCCGATTAGGTAGATGGTGGGGTA
AGGGCCTACCATGCCGACGATCGGTAGCTGGTCTGAGAGGATGGCCAGCCACACTGGGATTGAGAACGGC
C
>Zotu1878
GAACGCTAGCGGCAGGCTTAATACATGCAAGTCGAGGGGCAGCAGGGGTAGCAATACTCGCTGGCGACC
GGCAAACGGGTGCGGAACACGTACAGAACCTTCCTTTAAGCGGGGGATAGCCCAGGGAAACCTGGATTAA
TACCCCATAGTATTGTTGGGTGGCATCACTTAATAATTAAAGATTTATCACTTAAAGATGGCTGTGCGTATG
ATTAGGTAGTTGGCGGGGTAACGGCCCACCAAGCCTTCGATCATTAACTGGTGTGAGAGCACGACCAGTC
ACACGGGCACTGAGACACGGGC
>Zotu1880
GAATGATGGCGGCGTGGATGAGGCATGCAAGTCGAATGAGTTTATACTCATGGCAGACGAGGTAGGAAC
ACGTGGGTACGTACCCAAGAGTCAGGGATAATCCGTCGAAAGGCGGGATAATACTTGATAGTCCCTTCGG
GGTAAAGATTTATCGCTCTTGGAACGGCCCGCGGGCTATCAGCTTGTTGGTAAGGTAACGGCTTACCAAGG
CTACGACGGCTAGGGGAGGTGAGAGCCTGACCCCCACCGATGGAACTGCGACACGGTC
>Zotu1883
GAACGCTGGCGGCAGGCTTAACACATGCAAGTCGAGCGGGGGAAGGTAGCTTGCTACTGGACCTAGCGG
CGGACGGGTGAGTAATGCTTAGGAATCTGCCTATTAGTGGGGGACAACATCTCGAAAGGGATGCTAATAC
CGCATACGTCCTACGGGAGAAAGCAGGGGATCTTCGGACCTTGCGCTAATAGATGAGCCTAAGTCGGATT
AGCTAGTTGGTGGGGTAAAGGCCTACCAAGGCGACGATCTGTAGCGGGTCTGAGAGGATGATCCGCCACA
CTGGGACTGAGACACGGCC
>Zotu1885
GAACGCTGGCGGTAGGCCTAACACATGCAAGTCGAACGGCAGCACAGTAAGAGCTTGCTCTTACGGGTGG
CGAGTGGCGGACGGGTGAGGAATACATCGGAATCTACTTTTTCGTGGGGGATAACGTAGGGAAACTTACG
CTAATACCGCATACGACCTACGGGTGAAAGCAGGGGATCTTCGGACCTTGCGCGATTGAATGAGCCGATG

TCGGATTAGCTAGTTGGCGGGGTAAAGGCCCACCAAGGCGACGATCCGTAGCTGGTCTGAGAGGATGATC
AGCCACACTGGAACTGAGACACGGTC
>Zotu1888
GAACGCTGGCGGCGTGCCTAATACATGCAAGTAGAACGCTGAAGCTTGGTGCTTGCACCGAGCGGATGAG
TTGCGAACGGGTGAGTAACGCGTAGGTAACCTGCCTCTTAGCGGGGGATAACTATTGGAAACGATAGCTA
ATACCGCATAAAAGTCGACATTGCATGATGTTGACTTGAAAGGTGCAATTGCATCACTAAGAGATGGACCT
GCGTTGTATTAGCTAGTTGGTGAGGTAACGGCTCACCAAGGCGACGATACATAGCCGACCTGAGAGGGTG
ATCGGCCACACTGGGACTGAGACACGGCC
>Zotu1890
GAACGCTGGCGGCGTGCTTAACACATGCAAGTCGAACGATGAAGCCCAGCTTGCTGGGTGGATTAGTGGC
GAACGGGTGAGTAACACGTGAGTAACCTGCCCTTGACTCTGGGATAAGCACTGGAAACGGTGTCTAATAC
CGGATATTTCAGCTGGCCGCATGGTCTGGTTGTGGAAAGAATTTTGGTCAAGGATGGACTCGCGGCCTATC
AGGTTGTTGGTGAGGTAATGGCTCACCAAGCCTACGACGGGTAGCCGGCCTGAGAGGGTGGCCGGCCAC
ACTGGGACTGAGACACGGCC
>Zotu1892
GAACGCTGGCGGCATGCCTAACACATGCAAGTCGAACGAGACCTTCGGGTCTAGTGGCGCACGGGTGCGT
AACGCGTGGGAATCTGCCCTTCGGTTGGGAATAACACAGAGAAATTTGTGCTAATACCTTATAATGTCTTCG
GACCAAAGATTTATCGCCGAGGGATGAGCCCGCGTAGGATTAGCTAGTTGGTGGGGTAATGGCCTACCAA
GGCGACGATCCTTAGCTGGTCTGAGAGGATGATCAGCCACACTGGGACTGAGACACGGCC
>Zotu1894
GAACGCTAGCGGCAGGCTTAATACATGCAAGTCGAACGGTAAGTATGGTAGCAATATTATACGAGAGTGA
CGTACGGGTGCGTAACACGTATGCAACCTACCTTGTACAGGAGGATAGCTCGGGGAAACTCGAATTAACCC
TCCATAAGATAGTGGTGTGGCATCACACAGCTATAAAAACTTTATTGTGGTACAAGATGGGCATGCGTCTG
ATTAGCTAGTTGGCGGGGTAATGGCCCACCAAGGCGATGATCAGTAGGGGACCTGAGAGGGTGATCCCCC
ACACTGGCACTGAGATACGGGC
>Zotu1897
GAACGCTGGCGGCGTGCTTAACACATGCAAGTCGAACGGTGACGGAGAGCTTGCTCTCCTGATCAGTGGC
GAACGGGTGAGTAACACGTGAGCAACCTACCCTTGACTCTGGGATAACAGTTGGAAACAGCTGCTAATACC
GGATACGACCCTGGCCGGCATCGGCCGGGGTGGAAAGCTCCGACGGTCAAGGATGGGCTCGCGGCCTAT
CAGCTTGTTGGTGAGGTAACGGCTCACCAAGGCGACGACGGGTAGCCGGCCTGAGAGGGCGACCGGCCA
CACTGGGACTGAGATACGGCC
>Zotu1899
GAACGCTGGCGGCGTGCTTAACACATGCAAGTCGAGCGGTAAGGCACTTCGGTGTACACGAGCGGCGAAC
GGGTGAGTAACACGTGAGTAATCTGCCCTTCACTTTGGGATAAGCCTCGGAAACGGGGTCTAATACCGGAT
ACGACCACTTCGGGCATCTGATGGTGGTGGAAAGTTTTTTCGGTGGGGGATGTGCTCGCGGCCTATCAGCT
TGTTGGTGGGGTAATGGCCTACCAAGGCTTCGACGGGTAGCCGGCCTGAGAGGGTGACCGGCCACACTGG
GACTGAGACACGGCC
>Zotu1901
GAATGCTGGCGGCGTGGATAAGGCATGCAAGTCGAACGGTCCGCAAGGACAGTGGCAGACGAGGTAGTA
ACACGTAGGTACTCCCCCCAAAGTCTGGTATAGCTCGTCGAAAGACGGGGTAATCCCGGATGGTCCCGAAA
GGGTAAAGATTTATCGCTTTGGGAGAGGCCTGCGTAATATCAGCTTGTTGGTGAGGTAATGGCTCACCAAG
GCTACGACGTTTAGGGGCGCTGAGAGGCGGACCCCCACCGATGGGACTGAGACACGGCC
>Zotu1903
GAACGCTGGCGGCGTGCTTAACACATGCAAGTCGAACGGTGAAGCGCCTTCCTTTGTGGTGGGTGTGGAT
GAGTGGCGAACGGGTGAGTAACACGTGAGTAACCTGCCCCCTTCTTCTGGATAACCGCATGAAAGTGTGG
CTAATACGGATATTCTGGGTCTGTCGCATGGTGGGCCTGGGAAAGATTGCGCCTTTTGGGTGTTTTTGGT
GGGGGATGGGCTCGCGGCCTATCAGCTTGTTGGTGGGGTGATGGCCTACCAAGGCGGTGACGGGTAGCC
GGCCTGAGAGGGTGGACGGTCACACTGGGACTGAGACACGGCC
>Zotu1906

GAACGCTGGCGGCGTGCCTAATACATGCAAGTGGAACGCATGATTGATACCGGAGCTTGCTCCACCATTAA
TCATGAGTCGCGAACGGGTGAGTAACGCGTAGGTAACCTACCTCATAGCGGGGGATAACTATTGGAAACG
ATAGCTAATACCGCATAACAGTATTTATCGCATGGTAAATGCTTGAAAGGAGCAACTGCTTCACTATGAGAT
GGACCTGCGTTGTATTAGCTAGTTGGTGGGGTAACGGCTCACCAAGGCCTCGATACATAGCCGACCTGAGA
GGGTGATCGGCCACACTGGGACTGAGACACGGCC

>Zotu1908
GAACGCTTGCTGCGTGCCTGAGAAATGCAAGTCGAACGAGATTGAGCTTTCGGGTTCAGTCTAGTGGCGA
ACGGTCGAGTAACACGTAAGCAACCTGCCCATCCGCGGGGGACAAGCCGTCGAAAGACGGGGTAATACCG
CATACGATCTACGGAGACATCTCCGGAGATGAAAGATTCATCACGGATGGAGGGGCTTGCGGCCTATCAG
CTAGTTGGTTGGGGTAACGGCTCACCAAGGCGACGACGGGTAGCGGGTCTGAGAGGACGACCCGCCGGA
CTGGGACTGAGACACGGCC

>Zotu1910
GAACGCTGGCGGCGTGCTTAACACATGCAAGTCGAACGATGACGGTGGTGCTTGCATCACCTGATTAGTG
GCGAACGGGTGAGTAACACGTGAGTAACCTGCCCTTCACTTCGGGATAACCTCGGGAAATCGTGGCTAATA
CCGGATATGAGCTCCTTCCGCATGGCGGGGGTTGGAAAGATTTATCGGTGAAGGATGGACTCGCGGCCTA
TCAGTTTGTTGGTGAGGTAATGGCTCACCAAGGCGATGACGGGTAGCCGGCCTGAGAGGGCGACCGGCCA
CACTGGGACTGAGACACGGCC

>Zotu1912
GAACGCTGGCGGCGTGCCTAATACATGCAAGTCGAGCGGATTGATGGGAGCTTGCTCCCTGATATCAGCG
GCGGACGGGTGAGTAACACGTGGGCAACCTGCCTATAAGACTGGGATAACTTCGGGAAACCGGAGCTAAT
ACCGGATAATCCCTTTCCTCTCATGAGGAAAGGCTGAAAGATGGTTTACGCTATCACTTATAGATGGGCCC
GCGGCGCATTAGCTAGTTGGTGAGGTAACGGCTCACCAAGGCAACGATGCGTAGCCGACCTGAGAGGGT
GATCGGCCACACTGGGACTGAGACACGGCC

>Zotu1914
GAACGTTAGCGGCAGGCTTAACACATGCAAGTCGAGCGAGAACGGGACTTCGGTCCTTAGTAAAGCGGCG
GACGGGTGAGTAACACGTGGGTAATGTGCCCTTCAGCGGGGGATAACAGTCCGAAAGGATTGCTAATACC
GCATACGTCAGCCGGGTCTTCGGATCTGGTTGGGAAAGGTGGCCTCTCCATGGAAGCTACCACTGAAGGA
GCAGCCCGCGGCCCATCAGCTAGTTGGCGGGGTAACGGCCCACCAAGGCAAAGACGGGTAGCTGGTCTG
AGAGGATGATCAGCCACACTGGAACTGAGACACGGTC

>Zotu1916
GAACGCTGGCGGCGTGCCTAATACATGCAAGTCGAGCGGATCTTTAGAAGCTTGCTTCTAAAGATTAGCGG
CGGACGGGTGAGTAACACGTGGGCAACCTGCCTGTAAGACTGGGATAACTTCGGGAAACCGAGGCTAATA
CCGGATAATCCTTTTCCTCACATGAGGGGAAGCTGAAAGACGGCGTAAGCTGTCACTTACAGATGGGCCCG
CGGCGCATTAGCTAGTTGGTGAGGTAACGGCTCACCAAGGCAACGATGCGTAGCCGACCTGAGAGGGTGA
TCGGCCACACTGGGACTGAGACACGGCC

>Zotu1918
GAACGCTAGCAGAACGCTTAACACATGCAAGTCGAACGAATGTATTGTCTTCGGATAATATGTTAGTGGCA
AACGGGCGAGTAACGCGTATCTAACCTGCCCTTGTGTGAGGCAAAACTATGGAGACATAGCTAATTCCTCA
TACGCTCTAAGAAATGAAAATACTTAGAGGAAAGATTTATCGCACAAGGAGGGGGATGCGTCCTATCAGG
TAGTTGGTGAGGTAACTGCTCACCAAGCCTATGACGGGTAGCCGGTCTGAGAGGATGTCCGGCCGCAATG
GAACTGAGACACGGTC

>Zotu1920
GAACGCTAGCGGCAGGCCTAATACATGCAAGTCGGACGGGATTTAGGAGCTTGCTTCTAATGAGAGTGGC
GCACGGGTGCGTAACACGTATGTAACCTACCTTCTTCAGGGGGATAGCCTCTCGAAAGAGAGATTAAGACC
GCATAAAATCACAATGTGGCATCACAAAATGATCAAATATTTATAGGAAGAAGATGGGCATGCGGAACATT
AGCTAGTTGGTAAGGTAACGGCTTACCAAGGCTACGATGTTTAGGGGATCTGAGAGGATGACCCCCCACA
CTGGTACTGAGACACGGAC

>Zotu1922
GAATGCTGGCTGCGTGCTTAACACATGCAAGTCGAACGGTGAAGGAGGGCTTGCCCTCCGGATCAGTGGC
GAACGGGTGAGTAACACGTGGGCAACCTGCCCCTCACTCTGGGATAACACCGGGAAACCGGTGCTAATAC
CGGATACGACCAGGCGCGACATCTCGCCTGGTGGAAAGAACTTCGGTGAGGGATGGGCCCGCGGCCTATC

AGCTTGTTGGTGAGGTAACGGCTCACCAAGGCGACGACGGGTAGCCGGCCTGAGAGGGCGACCGGCCAC
ACTGGGACTGAGACACGGCC

>Zotu1924

TAACGCTGACGGTGTGCCTAATACATGCAAGTCGAACGAGCAGTATGTTGGTGGCAACACCTTTATACTGC
TAGTGGCGAACGGGTGAGTAACGCGTGGGTAATCTGCCTTAAAGACTGGGATAACTCCTCGAAAGAGGTG
CTAATACCGGATGGTCTATATTGATCTCATGATTTATATAGTAAAGGTAGCCGCAAGGCCTCTGCTTTAAGA
TGAGCTCGCGTCCTATTAGCTAGTTGGTGGGGTAATGGCCTACCAAGGCAATGATGGGTAGCCGGTCTGA
GAGGATGGTCGGCCACACTGGGACTGAGATACGGCC

>Zotu1926

GAACGCTGGCGGCGTGCTTAACACATGCAAGTCGAACGAGGTCCACCGCGTAGCAATACAAGGGAAGACC
TAGTGGCGAACGGGTGCGTAACACGTGAGGAACTTGTCCCGGTCTCTGGGATAACAGTTGGAAACGACTG
CTAATACCGGATGACGTCGGAATCTCGCATGGGATACTGACGAAAGGGTTACTGGATCGGGAGAGCCTCG
CGGCCTATCAGCTTGTTGGTGAGGTAACGGCTCACCAAGGCATCGACGGGTAGCTGGTCTGAGAGGATGA
TCAGCCACACTGGGACTGAGACACGGCC

>Zotu1928

GAACGCTGGTGGCGTGCCTAATACATGCAAGTCGAGCGAACGGACGAGAAGCTTGCTTCTCTGATGTTAG
CGGCGGACGGGTGAGTAACACGTGGATAACCTACCTATAAGACTGGGATAACTTCGGGAAACCGGAGCTA
ATACCGGATAATATTTTGAACCGCATGGTTCAAAAGTGAAAGACGGTCTTGCTGTCACTTATAGATGGATCC
GCGCTGCATTAGCTAGTTGGTAAGGTAACGGCTTACCAAGGCAACGATGCATAGCCGACCTGAGAGGGTG
ATCGGCCACACTGGAACTGAGACACTGTC

>Zotu1930

GAACGCTGGCGGCGTGCCTAACACATGCAAGTCGAGCGCGAATAGGGGCAACCCTTAGTAGAGCGGCGC
ACGGGTGCGTAACACGTGGATAATCTGCCTGGATGCTCGGGATAACCAGTCGAAAGATTGGCTAATACCG
GATAAGCCCACGGTTTCTTCGGAGACTGAGGGAAAAGGTGGCCTCTGTATACAAGCTATCACAACCAGATG
AGTCCGCGGCCCATCAGCTAGTTGGCGGGGTAATGGCCCACCAAGGCAACGACGGGTAGCTGGTCTGAGA
GGACGATCAGCCACACTGGAACTGAGACACGGTC

>Zotu1932

GAACGCTGGCGGCGTGCTTAACACATGCAAGTCGAACGATGATCTCCCGCTTGCGGGGGTGATTAGTGGC
GAACGGGTGAGTAATACGTGAGTAACCTGCCCTTGACTCTGGGATAAGCCTGGGAAACCGGGTCTAATAC
TGGATACTACTTCCTGCCGCATGGTGGGGGGTGGAAAGGGTTTTACTGGTTTTGGATGGGCCCACGGCCTA
TCAGCTTGTTGGTGGGGTAATGGCTCACCAAGGCGACGACGGGTAGCCGGCCTGAGAGGGTGACCGGCC
ACACTGGGACTGAGACACGGCC

>Zotu1934

GAACGCTGGCGGCGTGCTTAACACATGCAAGTCGAACGATGAAGCCCAGCTTGCTGGGTGGATTAGTGGC
GAACGGGTGAGTAACACGTGAGTAACCTGCCCTTGACTCTGGGATAAGCGCTGGAAACGGCGTCTAATAC
CGGATACGACCTGCCCCGGCATCGGGTGCGGGTGGAAAGTTTTTCGGTCAAGGATGGACTCGCGGCCTAT
CAGGTAGTTGGTGAGGTAATGGCTCACCAAGCCGACGACGGGTAGCCGGCCTGAGAGGGTGACCGGCCA
CACTGGGACTGAGACACGGCC

>Zotu1936

GAACGCTGGCGGCGTGCTTAACACATGCAAGTCGAACGGAAAGGCCAGTGCTTGCACTGGTGCTCGAGTG
GCGAACGGGTGAGTAACACGTGGGTGATCTGCCTTGCACTCTGGGATAAGCTTGGGAAACTGGGTCTAAT
ACCGGATAGGAACCGTCTTTAGTGTGGTGGTTGGAAAGTTTTTTCGGTGCAAGATGAGCTCGCGGCCTATC
AGCTTGTTGGTGGGGTAATGGCCTACCAAGGCGTCGACGGGTAGCCGGCCTGAGAGGGTGTACGGCCAC
ATTGGGACTGAGATACGGCC

>Zotu1939

GGTGGTGTAGCTCCTGGAGGTGTCAATGAAGCCGTCGAAAGGCTCGGTGCTTGACCGAGCAGAGATGGG
CGAAGAGGTGGACAATCATCCGACATGGGGCTGTTACGCCTATCGCTGGAGAACGTCAGTCGTGGCCAAA
AATATTAGGAATATTTGATCATACT

>Zotu1942

GAACGCTGGCGGCGTGCCTAATACATGCAAGTCGAGCGGGCCGAACGAGAGCTTGCTCTTGTTCGGTCAG
CGGCGGACGGGTGAGTAACACGTGGGCAACCTGCCCGCAAGACCGGGATAACTCCGGGAAACCGGAGCT
AATACCGGATAACACCAAAGACCGCATGGTCTTTGGTTGAAAGGCGGCTTCGGCTGCCACTTGCGGATGG
GCCCGCGGCGCATTAGCTAGTTGGTGAGGTAACGGCTCACCAAGGCGACGATGCGTAGCCGGCCTGAGAG
GGTGACCGGCCACACTGGGACTGAGACACGGCC
>Zotu1944
GAACGCTGGCGGCGTGCCTAACACATGCAAGTTGAGCGACGAACCAGGGCTTGCCCTGGGGCAAAGCAG
CGAACGGGTGAGTAACACGTGGGTAACCTGCCCCGATGACCGGGACAACCCGAGGAAACTCGGGCTAATA
CCGGATGTGCCCTCCGGACACAAGTCCGGCGGGTAAAGGAAGCTTCGGCCTCCGTATCGGGAGGGGCCCG
CGGCCCATTAGCTAGTTGGTGAGGTAACGGCTCACCAAGGCCGCGATGGGTAGCTGGTCTGAGAGGACGA
TCAGCCACACTGGGACTGAGACACGGCC
>Zotu1947
GAACGCTGGCGGCATGCCTAACACATGCAAGTCGAACGAAGTCCTTCGGGGCTTAGTGGCGCACGGGTGC
GTAACGCGTGGGAATCTGCCCTTGGGTTCGGAATAACAGTTAGAAATGACTGCTAATACCGGATGATGTCG
CAAGACCAAAGATTTATCGCCCAGGGATGAGCCCGCGTAGGATTAGGTAGTTGGTGGGGTAAAGGCTCAC
CAAGCCGACGATCCTTAGCTGGTCTGAGAGGATGATCAGCCACACTGGGACTGAGACACGGCC
>Zotu1949
GAACGCTGGCGGCGTGCCTAATACATGCAAGTCGAGCGAGGGTCTTCGGACCCTAGCGGCGGACGGGTG
AGTAACACGTAGGCAACCTGCCTGTAAGACTGGGATAACATAGGGAAACTTATGCTAATACCAGATAGCGT
CTTCTCTTGCATGAGAGGAGACGGAAAGATGGCGCAAGCTATCACTTACAGATGGGCCTGCGGCGCATTA
GCTAGTTGGTGGGGTAATGGCCTACCAAGGCGACGATGCGTAGCCGACCTGAGAGGGTGACCGGCCACA
CTGGGACTGAGACACGGCC
>Zotu1951
GAACGCTGGCGGCAGGCTTAACACATGCAAGTCGAACGCATCCTTCGGGGTGAGTGGCAGACGGGTGAG
TAACGCGTGGGAACGTGCCCTTCAGTTCGGAATAACCCAGGGAAACTTGGGCTAATACCGGATACGTGCG
AGAGCAGAAAGATTTATCGCTGAAGGATCGGCCCGCGTCTGATTAGCTAGTTGGTGAGGTAATGGCTCAC
CAAGGCGACGATCAGTAGCTGGTCTGAGAGGATGATCAGCCACATTGGGACTGAGACACGGCC
>Zotu1954
GAACGCTGGCGGCGTGCTTAACACATGCAAGTCGAACGGTAAGGCTCCTTCGGGAGTACACGAGTGGCGA
ACGGGTGAGTAACACGTGAGCAACCTGCCCTTGACTTTGGGATACCAGCTGGAAACAGCTGCTAATACCG
GATACGACCCCTTGACGCATGTCTTGGGGTGGAAAGCTCCGGCGGTCAGGGATGGGCTCGCGGCCTATCA
GCTTGTTGGTGAGGTAATGGCTCACCAAGGCTTCGACGGGTAGCCGGCCTGAGAGGGCGACCGGCCACAC
TGGGACTGAGATACGGCC
>Zotu1956
GAACGCTGGCGGCGTGCTTAACACATGCAAGTCGAGCGGAAAGGCCACTTCGGTGGTACTCGAGCGGCGA
ACGGGTGAGTAACACGTGAGTAATCTGCCCTGGACTTTGGGATAGCCACCGGAAACGGTGATTAATACCG
GATATGACCGCGCATCGCATGGTGCGTGGTGGAAAGTTTTTCGGTTCAGGATGTGCTCGCGGCCTATCAGC
TTGATGGTGGGGTAATGGCCTACCATGGCTTCGACGGGTAGCCGGCCTGAGAGGGTGACCGGCCACACTG
GGACTGAGACACGGCC
>Zotu1958
GAACGCTGGCGGCAGGCTTAACACATGCAAGTCGAACGCATCCTTCGGGGTGAGTGGCAGACGGGTGAG
TAACGCGTGGGAACGTGCCCTTCAGTTCGGGATAACCCAGGGAAACTTGGGCTAATACCGGATACGTGCG
AGAGCAGAAAGATTTATCGCTGAAGGATCGGCCCGCGTCTGATTAGCTAGTTGGTGAGGTAATGGCTCAC
CAAGGCGACGATCAGTAGCTGGTCTGAGAGGATGATCAGCCACATTGGGACTGAGACACGGCC
>Zotu1961
GAACGCTGGCGGCGTGCTTAACACATGCAAGTCGAACGGAAAGGCCAGTGCTTGCACTGGTACTCGAGTG
GCGAACGGGTGAGTAACACGTGGGTGATCTGCCCCGCACTTCGGGATAAGCCTGGGAAACTGGGTCTAAT
ACCGGATAGGACCACTTCTTAGAGGTTGTGGTGGAAAGTTTTTTCGGTGTGGGATGAGCTCGCGGCCTATC
AGCTTGTTGGTGGGGTAATGGCCTACCAAGGCGTCGACGGGTAGCCGGCCTGAGAGGGTGTACGGCCAC
ATTGGGACTGAGATACGGCC

>Zotu1964
GAACGCTGGCGGCGTGCCTAACACATGCAAGTCGAGCGATTCTCTTCGGAGAAGAGCGGCGGACGGGTG
AGTAACGCGTGGGTAACCTGCCCTGTACACACGGATAACATATCGAAAGGTATGCTAATACGGGATAACAT
ATTTTGATGGCATCATCGAAATATCAAAGCTCCGGCGGTACAGGATGGACCCGCGTCTGATTAGCTAGTTG
GTGAGGTAATGGCTCACCAAGGCGACGATCAGTAGCCGACCTGAGAGGGTGATCGGCCACATTGGAACTG
AGACACGGTC
>Zotu1966
GAACGCTGGCGGCGTGCTTAACACATGCAAGTCGAACGATGAAGCCCAGCTTGCTGGGTGGATTAGTGGC
GAACGGGTGAGTAACACGTGAGCAACGTGCCCATAACTCTGGGATAACCTCCGGAAACGGTGGCTAATAC
TGGATATCTAACACGATCGCATGGTCTGTGTTTGGAAAGATTTTTTGGTTATGGATCGGCTCACGGCCTATC
AGCTTGTTGGTGAGGTAATGGCTCACCAAGGCGACGACGGGTAGCCGGCCTGAGAGGGTGACCGGCCAC
ACTGGGACTGAGACACGGCC
>Zotu1968
GAACGCTGGCGGCAGGCTTAACACATGCAAGTCGAGCGGAAACGATAGTAGCTTGCTACTAGGCGTCGAG
CGGCGGACGGGTGAGTAATACTTAGGAATCTGCCCAGTAGTGGGGGATAGCTCGGGGAAACTCGAATTAA
TACCGCATACACCCTACGGGGAAAAGGGGGCGCTTGCGCTCTCGCTATTGGATGAGCCTAAGTCGGATTA
GCTAGTTGGTGGGGTAAAGGCCTACCAAGGCGACGATCTGTAGCTGGTCTGAGAGGATGATCAGCCACAC
CGGAACTGAGACACGGTC
>Zotu1970
CAACGCTGGCGGCGTGCCTAATGCATGCAAGTCGCACGGGGTGGCCTTCGGGTCGCTTACGTGGCGGACG
GGTGAGGAACACGTGGGCAACCTGCCCGGTGGTGGGGGCTAGCCCTCGGAAACGAGGGGTAATTCCGCA
TGAGCTCATTTTCCGAGTGGGGAGGATGAGGAAAGCTCTTCGGAGCGCCATCGGAGGGGTCTGCGTCCGA
TTAGTTAGCTGGTGGGGTAACGGCCTACCAGGGCGATGATCGGTAGCTGGTTTGAGAGGATGATCAGCCA
CACGGGGACTGAGACACGGCC
>Zotu1972
GAACGCTGGCGGCGTGCTTAACACATGCAAGTCGAACGATGAAGCCTGGTGCTTGCACCGGGTGGATTAG
TGGCGAACGGGTGAGTATCACGTGAGTAACCTGCCCTTGACTCTGGGATAAGCCCGGGAAACTGGGTCTA
ATACCGGATATGACCAGTCACCGCATGGTGTGCTGGTGGAAAGCTTTAGCGGTTTTGGATGGACTCGCGGC
CTATCAGCTAGACGGTGAGGTAATGGCTCACCGTGGCGATGACGGGTAGCCGGCCTGAGAGGGTGACCG
GCCACACTGGGACTGAGACACGGCC
>Zotu1975
GAATGCTGGCGGCGTGGATAAGTCATGCAAGTCAAGGGGGCCCGCAAGGGCAACCGGCAGACGAGGTAG
TAACACGTAGGTACGTCCCCCGAAGTCGGGCATAGCCATCCGAAAGGATGGGTAATTCCCGATAGTCCCGC
AAGGGTAAAGGTTTTTCGCTTCGGGAGCGGCCTGCGTAGTATCAGCTAGTTGGTAGGGTAACGGCCTACC
AAGGCTACGACGCTTAGGGGAGCTGAGAGGCTGACCCCCACCGATGGAACTGCGACACGGTC
>Zotu1978
GAACGCTGGCGGCATGCCTAACACATGCAAGTCGAACGAAGGCTTCGGCCTTAGTGGCGCACGGGTGCGT
AACGCGTGGGAATCTGCCCTTGGGTTCGGAATAACAGCGAGAAATTGCTGCTAATACCGGATGAAGACGA
AAGTCCAAAGATTTATCGCCTGAGGATGAGCCCGCGTTGGATTAGGTAGTTGGTGGGGTAAAGGCCTACC
AAGCCGACGATCCATAGCTGGTCTGAGAGGATGATCAGCCACACTGGGACTGAGACACGGCC
>Zotu1980
GAACGCTGGCGGCAGGCCTAACACATGCAAGTCGAGCGGCAGCACGGGAGCTTGCTCCTGGTGGCGAGC
GGCGGACGGGTGAGTAATGCCTAGGAATCTGCCTGTTAGTGGGGGATAACTCGGGGAAACTCGAGCTAAT
ACCGCATACGTCCTACGGGAGAAAGTGGGGGACCTTCGGGCCTCACGCTAACAGATGAGCCTAGGTCGGA
TTAGCTAGTTGGTGGGGTAAAGGCCTACCAAGGCGACGATCCGTAGCTGGTCTGAGAGGATGATCAGCCA
CACTGGAACTGAGACACGGTC
>Zotu1982
GAACGCTGGCGGCAGGCTTAACACATGCAAGTCGAGCGGGCCCTTCGGAGTCAGCGGCGGACGGGTGAG
TAACGCGTGGGAACGTGCCTTCTGGTCTGGAATAACCCTGGGAAACTAGGGCTAATACCGGATACGCCCTT

TTGGAGAAAGGTTTACTGCCGGAAGATCGGCCCGCGTCTGATTAGCTAGTTGGTGGGGTAACGGCCTACC
AAGGCGACGATCAGTAGCTGGTCTGAGAGGATGATCAGCCACACTGGGACTGAGACACGGCC
>Zotu1984
GAACGCTGGTGGCGTGCCTAATACATGCAAGTCGAGCGAATCTGAGGGAGCTTGCTCCCAAAGATTAGCG
GCGGACGGGTGAGTAACACGTGGGCAACCTGCCTGTAAGACTGGGATAACTTCGGGAAACCGGAGCTAAT
ACCGGATAACATATGAGACCACATGGTCTTATATTAAAAGATGGCTTTTAGCTATCACTTACAGATGGGCCC
GCGGCGCATTAGCTAGTTGGTGAGGTAACGGCTCACCAAGGCAACGATGCGTAGCCGACCTGAGAGGGT
GATCGGCCACACTGGGACTGAGACACGGCC
>Zotu1986
GAACGCTGGCGGCGTGCTTAACACATGCAAGTCGAGCGGAAAGGCTCCCTTCGGGGGGTACTCGAGCGGC
GAACGGGTGAGTAACACGTGAGTGATCTGCCCCTCACTTCGGGATAACTGTCTGAAAGGGTGGCTAATACC
GGATATTCTTTCATTGCTGCATGGTGGTGGAGGGAAAGTTTTTGCGGTGGGGGATGTGCTCGCGGCCTATC
AGCTTGTTGGTGGGGTAATGGCCTACCAAGGCTTTGACGGGTAGCCGGCCTGAGAGGGTGACCGGCCACA
CTGGGACTGAGACACGGCC
>Zotu1988
GAACGCTGGCGGCGTGCTTAACACATGCAAGTCGAACGATGATCAGGAGCTTGCTCCTGTGATTAGTGGC
GAACGGGTGAGTAACACGTGAGTAACCTGCCCCTGACTCTGGGATAAGCGTTGGAAACGACGTCTAATAC
TGGATATGATCACTGGCCGCATGGTCTGGTGGTGGAAAGATTTTTTGGTTGGGGATGGACTCGCGGCCTAT
CAGCTTGTTGGTGAGGTAATGGCTCACCAAGGCGACGACGGGTAGCCGGCCTGAGAGGGTGACCGGCCA
CACTGGGACTGAGACACGGCC
>Zotu1991
GAACGCTGGCGGTAGGCCTAACACATGCAAGTCGAACGGCAGCACAGGAGAGCTTGCTCTCTGGGTGGCG
AGTGGCGGACGGGTGAGGAATACATCGGAATCTACTCTGTCGTGGGGGATAACGTAGGGAAACTTACGCT
AATACCGCATACGACCTACGGGTGAAAGCAGGGGATCTTCGGACCTTGCGCGATTGAATGAGCCGATGTC
GGATTAGCTAGTTGGCGGGGTAAAGGCCCACCAAGGCGACGATCCGTAGCTGGTCTGAGAGGATGATCA
GCCACACTGGAACTGAGACACGGTC
>Zotu1993
GAACGCTGGCGGCGTGCCTAACACATGCAAGTCGAGCGGTAACGCGGGAGCAATCCTGGCGACGAGCGG
CGAACGGGTGAGTAACGCTTCGGAATCTACCCAGTAGTGGGGAATAACCAACCGAAAGGTTGGCTAATGC
CGCATACGACCTGAGGGTGAAAGGGGGGGATCGCAAGACCTCTCGCTATTGGATGAGCCGAAGTCGGATT
AGCTAGTTGGTAGGGTAACGGCCTACCAAGGCGACAATCCGTAGCTGGTCTGAGAGGACGACCAGCCACA
CTGGGACTGAGACACGGCC
>Zotu1995
GAACGCTAGCGGGAGGCCTAACACATGCAAGCCGAGCGGTAGAAAGTAGCTTGCTACTTTTGAGAGCGGC
GTACGGGTGCGGAACACGTGTGCAACCTGCCTTTATCTGGGGAATAGCCTTTCGAAAGGAAGATTAATGCC
CCATAACATATTGATTGGCATCAATTAATATTGAAAGCTCCGGCGGATAGAGATGGGCACGCGCAAGATTA
GCTAGTTGGTGAGGTAACGGCTCACCAAGGCGATGATCTTTAGGGGGCCTGAGAGGGTGATCCCCCACAC
TGGTACTGAGACACGGAC
>Zotu1997
GAACGCTGGCGGCATGCCTAACACATGCAAGTCGAACGAAGCCTTCGGGCTTAGTGGCGCACGGGTGCGT
AACGCGTGGGAATCTGCCCCTTGCTTCGGAATAACAGTTAGAAATGACTGCTAATACCGGATGATGTCTTC
GGACCAAAGATTTATCGGCAAGGGATGAGCCCGCGTAGGATTAGGTAGTTGGTGGGGTAAAGGCCTACCA
AGCCGACGATCCTTAGCTGGTCTGAGAGGATGATCAGCCACACTGGGACTGAGACACGGCC
>Zotu1999
GAACGCTGGCGGCGTGCTTAACACATGCAAGTCGAACGGAAAGGCTCCAGCTTGCTGGGGTGCTCGAGTG
GCGAACGGGTGAGTAACACGTAAGTGATCTGCCCTGCACTGGGGGATAAGCTTGGGAAACTGGGTCTAAT
ACCCTATAGGACCGCATCGTGGTTGGTGTGGTGGAAAGGTTTTTCTGGTGTGGGATGAGCTTGCGGCCTAT
CAGCTTGTTGGTGGGGTAATGGCCTACCAAGGCGGCGACGGGTAGCCGGCCTGAGAGGGTGTGCGGCCA
CATTGGGACTGAGATACGGCC
>Zotu2001

GAACGCTGGCGGCGTGCTTAACACATGCAAGTCGAACGGAGAAGGAGGAGCTTGCTCCTCTGGATCAGTG
GCGAACGGGTGAGTAACACGTGAGTAACCTGCCCCAGACTCTGGGATAAGCGTTGGAAACGACGTCTAAT
ACCGGATACGAGCTTCAGCCGCATGGCTAGGAGCTGGAAAGAACTTCGGTCTGGGATGGACTCGCGGCCT
ATCAGGTAGTTGGTGAGGTAACGGCCCACCAAGCCTACGACGGGTAGCCGGCCTGAGAGGGTGACCGGC
CACACTGGGACTGAGACACGGCC
>Zotu2003
GAACGCTGGCGGCATGCCTTACACATGCAAGTCGAACGGTAACAGGTCTTCGGATGCTGACGAGTGGCGA
ACGGGTGAGTAATACATCGGAACGTGCCCGATCGTGGGGGATAACGAAGCGAAAGCTTTGCTAATACCGC
ATAAGATCTACGGATGAAAGCAGGGGACCGCAAGGCCTTGCGCGAACGGAGCGGCCGATGGCAGATTAG
GTAGTTGGTGGGATAAAAGCTTACCAAGCCGACGATCTGTAGCTGGTCTGAGAGGACGACCAGCCACACT
GGGACTGAGACACGGCC
>Zotu2005
GAACGCTGGCGGCGTGCCTAACACATGCAAGTCGAGCGGCAGCGCGGGAGCAATCCTGGCGGCGAGCGG
CGGACGGGTGAGCAATGCTTGGGAATCTGCCCATCAGTGGGGGACAACCCGGGGAAACTCGGGCTAATA
CCGCATACGTTCTGAGGAAGAAAGCAGGGGATCGCAAGACCTTGCGCTGATGGATGAGCCCAAGTCGGAT
TAGCTAGTTGGTGAGGTAACGGCTCACCAAGGCGACGATCCGTAGCTGGTCTGAGAGGACGACCAGCCAC
ACCGGAACTGAGACACGGTC
>Zotu2007
GAACGCTGGCGGCGTGCCTAATACATGCAAGTAGAACGCTGAAGGAGGAGCTTGCTCTTCTGGATGAGTT
GCGAACGGGTGAGTAACGCGTAGGTAACCTGCCTGGTAGCGGGGGATAACTATTGGAAACGATAGCTAAT
ACCGCATAATAGTAGATGTTGCATGATATTTACTTAAAAGGTGCAATTGCATCACTACCAGATGGACCTGCG
TTGTATTAGCTAGTTGGTGGGGTAACGGCTCACCAAGGCGACGATACATAGCCGACCTGAGAGGGTGATC
GGCCACACTGGGACTGAGACACGGCC
>Zotu2009
TAACGCTGGCAGAGTGCATAACACATGCAAGTCGAGCGGAACTGTTTGAGATTACATCAAGGGCAGTTTA
GCGGCAGACGAGTGAGTAACACGTAAGCAATCTACCCTCGAATGGCGAATAACCAGTCGAAAGGCTGGCT
AATACGCCGTATTCTCCCCAGAAGGCATCTTAAGGGGAGGAGAGACCCGCAAGGGTCGTTCAAGGAGGAG
CTTGCGGCCCATCAGCTAGTTGGCGGTGTAACGGACCACCAAGGCTACGACGGGTACCCGGCCTGAAAGG
GCGGACGGGCACGATGGCACTGAGACACGGGC
>Zotu2012
GAACGCTGGCGGCAGGCTTAACACATGCAAGTCGAACGCGTGTAGCAATACACGAGTGGCGCACGGGTG
AGTAACGCGTGGATATCTGCCTTTTGGTTCGGAATAACCCCGGGAAACTGGGGCTAATACCGGATGGTTCC
TACGGGATAAAGATTTATCGCCAAAAGATGAGTCCGCGTCCGATTAGCTAGTTGGTGGGGTAATGGCCCAC
CAAGGCGACGATCGGTAGCTGGTCTGAGAGGACGATCAGCCACACTGGGACTGAGACACGGCC
>Zotu2015
GAACGCTGGCGGCGTGCCTAATACATGCAAGTCGAGCGGAAGTTGGAGAGCTTGCTCTTCAACTTTAGCG
GCGGACGGGTGAGTAACACGTGGGCAACCTGCCCTGCAGATCGGGATAACTCCGGGAAACCGGTGCTAAT
ACCGAATAGTTTTCGCCACCTCCTGGTGGCGTACGGAAAGACGGTTTCGGCTGTCACTGCAGGATGGGCCC
GCGGCGCATTAGCTAGTTGGTGGGGTAACGGCCCACCAAGGCAACGATGCGTAGCCGACCTGAGAGGGT
GATCGGCCACACTGGGACTGAGACACGGCC
>Zotu2017
GAACGCTAGCGGCAGGCCTAATACATGCAAGTCGAACGGAGCAGCAATGCTTAGTGGCGCACGGGTGCGT
AACGCGTATGAAACCTGCCCTTCACTGGGGGATAACCTCTCGAAAGAGAGGCTAATACCGCATAAGATAGT
AGAACGGCATCGTTCAGCTCTTAAAACTCCGGTGGTGAAGGATGGTCATGCGTCCAATTAGTTAGTTGGTA
GGGTAACGGCCTACCAAGACGATGATTGGTAGGGGGTCTGAGAGGATGATCCCCCACACTGGTACTGAGA
TACGGAC
>Zotu2020
GAACGCTGGCGGCAGGCCTAACACATGCAAGTCGAGCGCCCCGCAAGGGGAGCGGCGGACGGGTGAGTA
ACGCGTGGGAACGTGCCCTTTGCTTCGGAATAGCCCCGGGAAACTGGGAGTAATACCGAATGTGCCCCCC
ATTTTGGAGATGGCGGGGGAAAGATTTATCGGCAAAGGATCGGCCCGCGTTGGATTAGGTAGTTGGTGGG

GTAACGGCCTACCAAGCCGACGATCCATAGCTGGTTTGAGAGGATGATCAGCCACACTGGGACTGAGACA
CGGCC
>Zotu2023
AAACGCTGGCGGCGTGCTTAACACATGCAAGTCGAACGAAGTTTTTCATTCGTGGAAAACTTAGTGGCGGA
CGGGTGAGTAACGCGTGGGTAACCTGCCCTGTACAGGGGGACAACAGCTGGAAACGGCTGCTAATACCGC
ATAAGCCCTTAGCACTGCATGGTGCATAGGGAAAAGGAGTAATCCGGTACAGGATGGACCCGCGTCTGAT
TAGCCAGTTGGCAGGGTAACGGCCTACCAAAGCAACGATCAGTAGCCGATCTGAGAGGATGTACGGCCAC
ATTGGGACTGAGACACGGCC
>Zotu2025
GAACGCTGGCGGCGTGCCTAATACATGCAAGTAGAACGCACAGGATGCACCGTAGTTTACTACACCGTATT
CTGTGAGTTGCGAACGGGTGAGTAACGCGTAGGTAACCTGCCTGGTAGCGGGGGATAACTATTGGAAACG
ATAGCTAATACCGCATAAGAACATTTACTGCATGGTAGATGTTTAAAAGGTGCAAAAGCATCACTACCAGA
TGGACCTGCGTTGTATTAGCTAGTTGGTGAGGTAACGGCTCACCAAGGCAACGATACATAGCCGACCTGAG
AGGGTGATCGGCCACACTGGGACTGAGACACGGCC
>Zotu2028
GAACGCTGGCGGCGTGCTTAACACATGCAAGTCGAACGGAAAGGCCCTTCGGGGTACTCGAGTGGCGAAC
GGGTGAGTAACACGTGGGTGATCTGCCCTGCACTTTGGGATAAGCCTGGGAAACTGGGTCTAATACCGAA
TATGATCACTGGCTTCATGGTTGGTGGTGGAAAGCTTTTGCGGTGTGGGATGGGCCCGCGGCCTATCAGCT
TGTTGGTGGGGTAATGGCCTACCAAGGCGTCGACGGGTAGCCGGCCTGAGAGGGTGTCCGGCCACACTG
GGACTGAGATACGGCC
>Zotu2030
GAACGCTGGCGGTATGCTTAACACATGCAAGTCGTACGAAAATCTTTGATTTTAGTGGCGGACGGGTGAGT
AACACGTGAGAATCTGCCCTTAGGAAGAGGATAACAGCTGGAAACGGTTGCTAATACTCTATACGCCGAG
AGGTAAAAGGATCTATCCACCTTAGGAAGAGCTCGCGTCTGATTAGCTTGTTGGTAGGGTAATGGCCTACC
AAGGCGACGATCAGTAGCTGGTTTGAGAGGACGACCAGCCACACTGGGACTGAGACACGGCC
>Zotu2032
GAACGCTGGCGGCATGCTTAACACATGCAAGTCGAACGAGATAAAACTCATGTTAAAGTGGGTTTTATCTA
GTGGCGGACGGGTGAGTAACGCGTAAGAACCTACCCTTAGGAGGGGGACAACAATTGGAAACGGTTGCT
AATACCCCATATGCTGAGGAGTAAAACGGTAACAACCGCCTAGGGATGGGCTTGCGTCTGATTAGCTTGTT
GGTGGGGTAAATGCTTACCAAGGCAACGATCAGTAGTTGGTCTGAGAGGATGACCAGCCACACTGGGACT
GAGACACGGCC
>Zotu2035
GAACGCTGGTAACGTGGATACCGGCATGCAAGTCACGGGGGCCGCAAGGCAACCGGCGAACGGCTTAGT
AACACGTAGATACATACCGTAAAGAGGGGCATAGCTCGTTGAAAAACGGGGTAATTTCCCATAGTCCCGCA
AGGGTAAAGCAGCAATGCGCTTTATGAATGGTCTGCGGTCTATCAGCTTGTTGGTGAGGTAAAGGCTTACC
AAGGCTATGACGGATAGGGGGCGTGAGAGCGCGACCCCCAAGAATGGCACTGCGACACGGGC
>Zotu2037
GAACGCTGGCGGCATGCTTTACACATGCAAGTCGAACGGTAACAGGGAGCTTGCTCCGCTGACGAGTGGC
GAACGGGTGAGTAATATATCGGAACGTGCCCAAGAGTGGGGGATAACGTAGCGAAAGTTACGCTAATACC
GCATACGATCTAAGGATGAAAGCAGGGGACCTTCGGGCCTTGTGCTCCTGGAGCGGCCGATATCTGATTA
GCTAGTTGGTGAGGTAAAGGCTCACCAAGGCGACGATCAGTAGCTGGTCTGAGAGGACGACCAGCCACAC
TGGAACTGAGACACGGTC
>Zotu2040
GAACGCTGGCGGTATGCCTAACACATGCAAGTCGAACGGAACCTTCGGGTTTAGTGGCGGACGGGTGAGT
AACGCGTGAGAATCTGCCTCTAAGATGGGGACAACAGTCGGAAACGACTGCTAATACCCGATATGGCGAA
AGCTGAAAGATTTATCGCTTAGAGATGAGCTCGCGTTGGATTAGCTAGTTGGTAGTGTAATGGACTACCAA
GGCGACGATCCATAGCTGGTCTGAGAGGATGAGCAGCCACACTGGGACTGAGACACGGCC
>Zotu2042
AAACGTTGGCGGCGTGCTTAACACATGCAAGTCGAGCGAGAAAAGTTTTCGGACTTAGTACAGCGGCGAA
CGGGTGAGTAACGCGTGAGAATCTGCCTTTTAGTGGAGAATACACTCGAGAAATCGAGATCAATACTGCAT

AAGTCCGGTAGCGGAGAAAGATGGCTTTAATGCTGTCGCTAGAAGATGAGCTTGCGTACTATTAGCTAGTT
GGTGGGGTAAAAGCCTACCAAGGCGATGATGGTTAGCCGGCCTGAGAGGGTGTGCGGCCACACTGGAAC
TTAGACACGGTC
>Zotu2044
GAACGCTAGCGGCAGGCTTAACACATGCAAGTCGAGGGGCAGCGGGGGTAGCAATACCTGCCGGCGACC
GGCAAACGGGTGCGGAACACGTACGCAACATACCTCTTACTGGGGCATAGCCTTCTGAAAAGGAGATTAA
CACCCCATAGTATTATTAGCTGGCATCAGTTTGATAATTAAAAATTTATTGGTAAGAGATTGGCGTGCGTCC
CATTAGCTAGTTGGTAGGGTAACGGCCTACCAAGGCGACGATGGGTAGCTGGTGTGAGAGCACGACCAGC
CACACGGGCACTGAGACACGGGC
>Zotu2046
GAACGCTGGCGGCGTGCCTAATACATGCAAGTCGAGCGAATCTTTAAAAGCTTGCTTTTGAAGATTAGCGG
CGGACGGGTGAGTAACACGTGGGCAACCTGCCTGTAAGACTGGGATAACTTCGGGAAACCGGAGCTAATA
CCGGATGATCTTTTCTCTCGCATGAGGGAATTCTGAAAGACGGCGCAAGCTGTCACTTACAGATGGGCCCG
CGGCGCATTAGCTAGTTGGTGAGGTAACGGCTCACCAAGGCGACGATGCGTAGCCGACCTGAGAGGGTG
ATCGGCCACACTGGGACTGAGACACGGCC
>Zotu2048
GAACGCTGGCGGCGTGCCTAATACATGCAAGTCGAGCGGACCACAAAGAAGCTTGCTTCTGAGTGGTTAG
CGGCGGACGGGTGAGTAACACGTGGGCAACCTGCCCGTAAGACCGGGATAACTTCGGGAAACCGGAGCT
AATACCGGATAACACCGAAGATCGCATGATCTTTGGTTGAAAGGCGGCTTTGGCTGTCACTTACGGATGGG
CCCGCGGCGCATTAGCTAGTTGGTGAGGTAACGGCTCACCAAGGCGACGATGCGTAGCCGACCTGAGAGG
GTGATCGGCCACACTGGGACTGAGACACGGCC
>Zotu2050
GAACGCTGGCGGCGTGCCTAACACATGCAAGTCGAGCGATTCTCTTCGGAGAAGAGCGGCGGACGGGTG
AGTAACGCGTGGGTAACCTGCCCTGTACACACGGATAACATACCGAAAGGTATGCTAATACGGGATAACAT
ATTTTGATGGCATCATCGAAATATCAAAGCTCCGGCGGTACAGGATGGACCCGCGTCTGATTAGCTAGTTG
GTGAGGTAATGGCTCACCAAGGCAACGATCAGTAGCCGACCTGAGAGGGTGATCGGCCACATTGGAACTG
AGACACGGTC
>Zotu2052
GAACGCTGGCGGCGTGCTTAACACATGCAAGTCGAACGCTGAAACCAGATGCTTGCATTTGGTGGATGAG
TGGCGAACGGGTGAGTAACACGTGGGTGATCTGCCCTACACTTTGGGATAAGCCTGGGAAACTGGGTCTA
ATACCGAATATTCACACCACCGTAGGGGTGGTGTGGAAAGCTTTATGCGGTGTGGGATGAGCCTGCGGCC
TATCAGCTTGTTGGTGGGGTAATGGCCTACCAAGGCGTCGACGGGTAGCCGGCCTGAGAGGGTGTACGGC
CACATTGGGACTGAGACACGGCC
>Zotu2054
GAACGCTGGCGGCGTGCCTAATACATGCAAGTCGAGCGGGCCGAATGGAAGCTTGCTTTCATTCGGTTAG
CGGCGGACGGGTGAGTAACACGTGGGTAACCTGCCCGTAAGACCGGGATAACTCCGGGAAACCGGGGCT
AATACCGGATAACACCGAAGACCGCATGGTCTTCGGTTGAAAGGTGGCTTCGGCTACCACTTACGGATGG
GCCCGCGGCGCATTAGCTAGTTGGTGAGGTAACGGCTCACCAAGGCGACGATGCGTAGCCGGCCTGAGAG
GGTGACCGGCCACACTGGGACTGAGACACGGCC
>Zotu2057
TCTTTTGGTGGGGTCTCGTCTGTGTTTGAGTCACCTCTGGGGTGTGTGGAGTTTGGGGTTGGGAAAGGCCA
GTTCTCCCTCTTCCTCTGTCTACTCCC
>Zotu2061
GAACGCTGGCGGCATGCCTTACACATGCAAGTCGAACGGCAGCGCGGACTTCGGTCTGGCGGCGAGTGGC
GAACGGGTGAGTAATACATCGGAACGTACCCTGTTGTGGGGGATAACTAGTCGAAAGATTAGCTAATACC
GCATACGACCTGAGGGTGAAAGTGGGGGACCGCAAGGCCTCACGCAGCAGGAGCGGCCGATGTCTGATT
AGCTAGTTGGTGGGGTAAGAGCCTACCAAGGCGACGATCAGTAGCTGGTCTGAGAGGACGATCAGCCACA
CTGGGACTGAGACACGGCC
>Zotu2063

GAACGCTGGCGGCAGGCCTAATACATGCAAGTCGAGCGGATGTAGCAATACATCAGCGGCGCACGGGAG
AATAACGCGTGGGAACCTTCCTTTTGGTTCGGAATAACACCGGGAAACTGGTGCTAATACCGAATAAGCCC
TTCGGGGGAAAGATTTATTGCCGAAAGATGGGCCCGCGTCTGATTAGCTAGTTGGTGAGGTAACGGCTCA
CCAAGGCTACGATCAGTAGCTGATCTGAGAGGATGATCAGCCTCACTGGGACTGAGACACGGCC
>Zotu2066
GCGATCCTGGAGACCCGGGATCGAATCCCACGTCGGGCTCCCGGTGCATGGAGCCTGCTGCTCCCTCTGCC
TGTGTCTCTGCCTCTCTCTGTGTGTCTTCCATGAATAAATGAAATATTTTTTTTAAATAAATAAAGTTCCCAG
GCCTTCACA
>Zotu2069
GAACGCTGGCGGCGTGCTTAACACATGCAAGTCGAGCGGTAAGGCCCTTCGGGGTACACGAGCGGCGAA
CGGGTGAGTAACACGTGGGTGATCTGCCCTGTACTTCGGGATAAGCCTGGGAAACTGGGTCTAATACCGG
ATATGACCTTACATCGCATGGTGTTTGGTGGAAAGATTTATCGGTACAGGATGGGCCCGCGGCCTATCAGC
TTGTTGGTGGGGTAATGGCCTACCAAGGCGACGACGGGTAGCCGGCCTGAGAGGGCGACCGGCCACACT
GGGACTGAGACACGGCC
>Zotu2071
GAACGCTGGCGGCAGGCTTAACACATGCAAGTCGAGCGGGGAAAGGTAGCTTGCTACCTGACCTAGCGGC
GGACGGGTGAGTAATGCTTAGGAATCTGCCTATTAGTGGGGGACAACATTCCGAAAGGAATGCTAATACC
GCATACGCCCTACGGGGGAAAGCAGGGGATCTTCGGACCTTGCGCTAATAGATGAGCCTAAGTCAGATTA
GCTAGTTGGTGGGGTAAAGGCCTACCAAGGCGACGATCTGTAGCGGGTCTGAGAGGATGATCCGCCACAC
TGGGACTGAGACACGGCC
>Zotu2073
GAACGCTGGCGGCAGGCTTAACACATGCAAGTCGAGCGGAGTGATGGTGCTTGCACTATCACTTAGCGGC
GGACGGGTGAGTAATGCTTAGGAATCTGCCATTTAGTGGGGGACAACATTCCGAAAGGAATGCTAATACC
GCATACGTCCTACGGGAGAAAGCAGGGGATCTTCGGACCTTGCGCTAAATGATGAGCCTAAGTCGGATTA
GCTAGTTGGTGGGGTAAAGGCCTACCAAGGCGACGATCTGTAGCGGGTCTGAGAGGATGATCCGCCACAC
TGGGACTGAGACACGGCC
>Zotu2076
GAACGCTAGCGGCAGGCCTAATACATGCAAGTCGGACGGGATCCATCGGAGAGCTTGCTCGAAGATGGTG
AGAGTGGCGCACGGGTGCGTAACGCGTGAGCAACCTACCTCTATCAGGGGGATAGCCTCTCGAAAGAGAG
ATTAACACCGCATAACATCAACAGTTCGCATGTTCGGTTGATTAAATATTTATAGGATAGAGATGGGCTCGC
GTGACATTAGCTAGTTGGTAGGGTAACGGCCTACCAAGGCGACGATGTCTAGGGGCTCTGAGAGGAGAAT
CCCCCACACTGGTACTGAGACACGGAC
>Zotu2078
GAACGCTAGCGGCAGGCTTAACACATGCAAGTCGAGGGGTATAATTCTTCGGAATTAGAGACCGGCGCAC
GGGTGCGTAACGCGTATGCAATCTACCTTTTGCAGAGGGATAGCCCAGAGAAATTTGGATTAATACCTCAT
AGTATAGTACCTTGGCATCAAGGAACTATTAAAGTCACAACGGCAAAAGATGAGCATGCGTCCCATTAGCT
TGTTGGTAAGGTAACGGCTTACCAAGGCAACGATGGGTAGGGGTCCTGAGAGGGAGATCCCCCACACTGG
TACTGAGACACGGAC
>Zotu2081
GAACGCTAGCGATAGGCTTAACACATGCAAGTCGAGGGGCAGCATGGTCTTAGCTTGCTAAGACTGATGG
CGACCGGCGCACGGGTGCGTAACGCGTATGCAACTTGCCTCACAGAGGGGGATAACCCGTCGAAAGACG
GACTAATACCGCGTACACTCTATGTTGGGCATCCAATGTAGAGGAAATGAAATTCGCTGTGAGATAGGCAT
GCGTCCCATTAGCTAGTTGGTGAGGTAACGGCTCACCAAGGCGACGATGGGTAGGGGAACTGAGAGGTT
GAACCCCCACACTGGTACTGAGACACGGAC
>Zotu2083
GAACGCTGGCGGCATGCCTAACACATGCAAGTCGAACGAGGTTTCCGCTTCGGTGGGACCCTAGTGGCGC
ACGGGTGAGTAACGCGTGGGAACCTGCCCTGTGGTACGGAATAACTCCGGGAAACTGGAGCTAATACCGT
ATGCGTCCTTTGGGACAAAGATTTATCGCCATGGGATGGGCCCGCGTAGGATTAGCTTGTTGGTGGGGTAA
CGGCCTACCAAGGCAACGATCCTTAGCTGGTCTGAGAGGATGATCAGCCACACTGGGACTGAGACACGGC
C

>Zotu2087
GAACGCCGGCGGTGTGCCTAATACATGCAAGTCGTACGCACTGGCCCAACTGATTGATGGTGCTTGCACCT
GATTGACGATGGATCACCAGTGAGTGGCGGACGGGTGAGTAACACGTAGGTAACCTGCCCCGGAGCGGG
GGATAACATTTGGAAACAGATGCTAATACCGCATAACAACAAAAGCCGCATGGTTTTTGTTTGAAAGATGG
CTTTGGCTATCACTCTGGGATGGACCTGCGGTGCATTAGCTAGTTGGTAAGGTAACGGCTTACCAAGGCGA
TGATGCATAGCCGAGTTGAGAGACTGATCGGCCACAATGGAACTGAGACACGGTC
>Zotu2090
GAACGCTAGCGGCAGGCTTAATACATGCAAGTCGAACGGTCTTAGCAATAAGACGAGTGGCGCACGGGTG
AGTAACGCGTACATAACATACCTTTAACTGGCGAATAGCCTTGGGAAACTGAGAGTAATACGCCATAACAT
TGTGAAACGGCATCGTTTTACAATTAAAGTTCCGGCGGTTAGAGATTGATGTGCGTTGGATTAGGTAGTTG
GCGAGGTAACGGCTCACCAAGCCGACGATCCATAGGGGGCGTGAGAGCGTGGTCCCCCACACGGGTACT
GAGACACGGAC
>Zotu2092
GAACGCTGGCGGCGTGCCTAAGACATGCAAGTCGAGTGGGTTTAGACCCACGGCAAACGGGTGAGTAATA
TCTAGGAACGTACCTCTAGTTGAGCATAACCTACCGAAAGGTAGAGCAATTCTCAATGTGACCGCAAGGTT
AAAGTCGCAAGACGCTAGAGAAGCGGCCTGGACACCATCAGCTTGTTGGTAAGGTAAAAGCTTACCAAGG
CTATGACGGTTAGGGGCCCCGAGAGGGGAAACCCCCACACTGGAACTGAGACACGGTC
>Zotu2094
GAACGCTAGCGACAGGCCTAACACATGCAAGTCGAGGGGCAGCACATGAGTAGCAATACGATGGTGGCG
ACCGGCGCACGGGTGAGTAACACGTATGCAACCTACCTGTAAATGGGGAATAACCCGGAGAAATTCGGAC
TAACACCGCATAACACTGTTTTTTCGCATGAAAGGACAGTTAAATATTTATAGTTTACAGATGGGCATGCGT
ATGATTAGATAGTTGGTAGGGTAACGGCCTACCAAGTCTACGATCATTAGGGGTTCTGAGAGGAAGGTCC
CCCACACTGGTACTGAGACACGGAC
>Zotu2097
GAACGCTGGCGGCGTGCTTAACACATGCAAGTCGAACGGTGAAGGGGAGCTTGCTTCCTGGATCAGTGGC
GAACGGGTGAGTAACACGTGAGCAATCTGCCCCTGACTCTGGGATAAGCGCTGGAAACGGCGTCTAATAC
TGGATACGAGCTGCGATCGCATGGTCAGCAGTTGGAAAGAATTTCGGTCAGGGATGAGCTCGCGGCCTAT
CAGCTTGTTGGTGAGGTAATGGCTCACCAAGGCGTCGACGGGTAGCCGGCCTGAGAGGGTGACCGGCCAC
ACTGGGACTGAGACACGGCC
>Zotu2099
GAACGCTGGCGGCGTGCTTAACACATGCAAGTCGAACGGTGAAGCTCCAGCTTGCTGGAGTGGATCAGTG
GCGAACGGGTGAGTAACACGTGAGCAACCTGCCCCTGACTCTGGGATAACAGCCGGAAACGGCTGCTAAT
ACCGGATATGCACCATGAACGCATGTTCTGTGGTGGGAAAGATTTTTCGGTTGGGGATGGGCTCGCGGCCT
ATCAGCTTGTTGGTGAGGTAATGGCTCACCAAGGCGTCGACGGGTAGCCGGCCTGAGGGGGTGACCGGCC
ACACTGGGACTGAGACACGGCC
>Zotu2101
GAACGCTGGCGGTATGCTTAACACATGCAAGTCGAACGGCAGCACGTTGCTTCTTTTCGGAGAAGTAATGG
TGGCGAGTGGCGGACGGGTGAGTAACGCGTAGGAATCTACCCTAAAGTGGGGGATAACCCGGGGAAACT
CGGGCTAATACCGCATAATCTCTACGGAGCAAAGCTGGGGACCAGAGTTTACTTTGGCCTGGCGCTATAGG
ATGAGCCTGCGTCGGATTAGCTAGTTGGTGGGGTAATGGCCTACCAAGGTAACGATCCGTAGCTGGTCTG
AGAGGATGATCAGCCACACTGGGACTGAGACACGGCC
>Zotu2103
GAACGCTAGCGGCAGGCCTAATACATGCAAGTCGAACGGAATATAGTTTTCGGATTATATTTAGTGGCGCA
CGGGTGAGTAACACGTACATAACTTACCCTCAAGAGGGGGATAGCCTTGGGAAACTGAGCGTAATACCCC
ATAGTATTATTGAACGGCATCGTTTGATAATTAAAGCTACGGCGCTTGAGGATAGATGTGCGTCTGATTAG
CTAGTTGGTAAGGTAACGGCTTACCAAGGCGACGATCAGTAGGGGGCGTGAGAGCGTGGCCCCCCACACG
GGTACTGAGACACGGAC
>Zotu2105
GAACGCTAGCGGCATGCTTAACACATGCAAGTCGAACGGACTAATTGGGGCTTGCTCCAATTAGTTAGTGG
CAGACGGGTGAGTAACACGTGGGAATCTACCCATCAGTACGGAATAACTTTTAGAAATAAAAGCTAATACC

GTATATTCTCTACGGAGGAAAGATTTATCGCTAATGGATGAGCCCGCGTCAGATTAGGTAGTTGGTGAGGT
AACGGCTCACCAAGCCGACGATCTGTAGCTGGTCTGAGAGGATGATCAGCCACACTGGGACTGAGACACG
GCC
>Zotu2108
GAACGCTGGCGGCGTGCTTAACACATGCAAGTCGAACGGTGAACCTGCTTCGGTGGGGATCAGTGGCGA
ACGGGTGAGTAACACGTGGGCAACCTGCCCCCGGCTCTGGGATAACTCCAAGAAATTGGGGCTAATACCG
GATGTTCACGGCTGGCCGCATGGTCGGTGGTGGAAAGATTTATCGGCTGGGGATGGCCCGCGGCCTATC
AGCTTGTTGGTGGGGTAGTGGCCTACCAAGGCGACGACGGGTAGCCGGCCTGAGAGGGTGACCGGCCAC
ACTGGGACTGAGACACGGCC
>Zotu2111
GAACGCTGGCGGAGTGCTTTAAGCATGCAAGTAGAACGGGTTTTGATAGCAATATTAAGATCAGTTGCGA
ACGGCTGAGTAATAAGTAAGAATCTTTCCTTTGGTGGAGAATACCTCGTCGAAAGACGAGCTAATACTGCA
TAAGACCTACGGGTGAAAGCGCATAACTGCGCGCCGACTGAGGAGCTTACTTCCCATCAGGCAGTTGGTA
GGGTAATGGCCTACCAAACCTACGACGGGTACCCGATCTGAGAGGATGGACGGGCAGAATGGGACTGAG
ACACGGCC
>Zotu2113
GAACGCTGGCGGCAGGCTTAACACATGCAAGTCGAACGCACCTTCGGGTGAGTGGCAGACGGGTGAGTA
ACACGTGGGAACATACCTCTTTGTTCGGAATAACGCATGGAAACGTGCGCTAATACCGGATACGCCCTTAT
GGGGAAAGATTTATCGCAGAGAGATTGGCCCGCGTCTGATTAGCTAGTTGGTGAGGTAATGGCTCACCAA
GGCGACGATCAGTAGCTGGTCTGAGAGGATGATCAGCCACACTGGGACTGAGACACGGCC
>Zotu2115
GAACGCTGGCGGCGTGCTTAACACATGCAAGTCGAACGGAAAGGCCCTGCTTGCAGGGTACTCGAGTGGC
GAACGGGTGAGTAACACGTGGGTGATCTGCCCTGTACTTTGGGATAAGCCTGGGAAACTGGGTCTAATAC
CGAATATGACAATTGTTTGGATGCGATTGTGGAAAGCTTTTGCGGTATGGGATGAGCTCGCGGCCTATCAG
CTTGTTGGTGGGGTAATGGCCTACCAAGGCGTCGACGGGTAGCCGGCCTGAGAGGGTGTACGGCCACATT
GGGACTGAGATACGGCC
>Zotu2118
GAACGCTGGCGGCGTGCTTAACACATGCAAGTCGAACGGAAAGGCCCCGCTCGCGGGGTACTCGAGTGGC
GAACGGGTGAGTAACACGTGGGTGATCTGCCCTGCACTTCGGGATAAGCCTGGGAAACTGGGTCTAATAC
CGGATAGGACCCTCCTTTAGTGTGGGGGGTGGAAAGTTTTTCGGTGCAGGATGAGCCCGCGGCCTATCAG
CTTGTTGGTGGGGTAATGGCCTACCAAGGCGTCGACGGGTAGCCGGCCTGAGAGGGTGGACGGCCACATT
GGGACTGAGATACGGCC
>Zotu2120
AAACGCTGGCGGCGTGCTTTAAGCATGCAAGTCGCACGGATGCTCGCAAGAGTGTGAGTGGCAGACGGCT
GAGGAAGACGGAAGGAACCTGCCCTTCAGAGGGGGATAACTCCGCGAAAGCGGGGCTAAGACCGCGTAC
GCTCGCCCGGGGGAGAGCCGGGTGAGGAAAACCGCGAGGTGCTGAAGGAGGGGCTTCCGGCCCATCAG
GTAGTTGGGGGGGTAACGGCCCACCAAGCCGATGACGGGTCGCTGGTCTAAGAGGATGATCAGCCAGAC
TGGGACTGAGACACGGCC
>Zotu2122
GAACGCTGGCGGCGTGCTTAACACATGCAAGTCGAACGGAAAGGTCTCTTCGGAGATACTCGAGTGGCGA
ACGGGTGAGTAACACGTGGGTGATCTGCCCTGCACTTCGGGATAAGCTTGGGAAACTGGGTCTAATACCG
GATATGATCATGCGCCTCATGGTGTGTGGTGGAAAGCTTTTGCGGTGTGGGATGGGCCCGCGGCCTATCA
GCTTGTTGGTGGGGTAATGGCCTACCAAGGCGACGACGGGTAGCCGGCCTGAGAGGGTGTCCGGCCACA
CTGGGACTGAGATACGGCC
>Zotu2124
GAACGCTGGCGGCGTGCTTAACACATGCAAGTCGAACGGTAAGGCTCCAGCTTGCTGGGGTACACGAGTG
GCGAACGGGTGAGTAACACGTGGGTGACCTGCCCTGCACTTCGGGATAAGCCTGGGAAACTGGGTCTAAT
ACCGGATAGGACCGCATCGTAGGTGGTGTGGTGGAAAGTTTTTTCGGTGTGGGATGGGCCCGCGGCCTAT
CAGCTTGTTGGTGGGGTAATGGCCTACCAAGGCGGCGACGGGTAGCCGGCCTGAGAGGGTGGACGGCCA
CATTGGGACTGAGACACGGCC

>Zotu2126
GAACGCTGGCGGCATGCCTAATACATGCAAGTCGAACGCCAAATCTTCCTGCTTGCAGGAAGAGGCGGAG
TGGCGAACGGGTGAGTAACACGTGAGGAACTTGCCCATAAGAGGGGGATAACACCCGGAAACGGGTGCT
AATACCGCATAGGTTTTCCCAGTGCATGCTGGGAAAATAAAAGGTGGCTCTGCTACCGCTTATGGATAGCC
TCGCGGCGCATTAGCTAGTTGGTAAGGTAAAGGCCTACCAAGGCCATGATGCGTAGCCGACTTGAGAGGG
TGATCGGCCACACTGGAACTGAGACACGGTC
>Zotu2130
GAACGCTGGCGGCATGCCTAATACATGCAAGTCGAGCGAATGAAGAAGAAGCTTGCTTCTTCTGATTTAGC
GGCGGACGGGTGAGTAACACGTGGGTAACCTACCCTATAGATTGGGATAACTCCGGGAAACCGGGGCTAA
TACCGAATAATACCTTTCCTCTCATGAGGAGATGTTGAAAGATGGCTTCTCGCTATCACTATAGGATGGGCC
CGCGGCGCATTAGCTAGTTGGTGAGGTAACGGCTCACCAAGGCAACGATGCGTAGCCGACCTGAGAGGGT
GATCGGCCACACTGGGACTGAGACACGGCC
>Zotu2132
GAATGCTGGCGACATGGATAAGGCATGCAAGTCAAGGGGGGCCGCAAGGTCAACCGGCAGACGAGGTAG
TAACACGTAAGTACGTACCCCATAGTCTGGTATAGCTCGTCGAAAGACGGGGTAATCCCGGATGGTCCCGC
AAGGGTAAAGATTTATCGCTATGGGAGCGGCTTGCGTAATATCAGCTTGTTGGTGAGGTAATGGCTCACCA
AGGCTACGACGTTTAGGGGAGCTGAGAGGCTGACCCCCACCGATGGGACTGAGACACGGCC
>Zotu2134
GAACGCTGGCGGCGTGCCTAATACATGCAAGTCGAGCGAACAGAGAAGGAGCTTGCTCCTTCGACGTTAG
CGGCGGACGGGTGAGTAACACGTGGGCAACCTACCCTATAGTTTGGGATAACTCCGGGAAACCGGGGCTA
ATACCGAATCATCTCTTTTACTTCATGGTAAAAGACTAAAAGACGGTTTCGGCTGTCGCTATAGGATGGGCC
CGCGGCGCATTAGCTAGTTGGTGAGGTAATGGCTCACCAAGGCGACGATGCGTAGCCGACCTGAGAGGGT
GATCGGCCACACTGGGACTGAGACACGGCC
>Zotu2136
GAACGCTGGCGGCGTGCTTAACACATGCAAGTCGAGCGGAAAGGCCCTTCGGGGTACTCGAGCGGCGAA
CGGGTGAGTAACACGTGAGGAACCTGCCCTGGACTTTGGGATAACCCTCGGAAACGGGGGCTAATACCGG
ATACGACCTTCAACCGCATGGTTTGTTGGTGGAAAGTTTTTCGGTCTGGGATGGTCTCGCGGCCTATCAGCT
TGTTGGTGGGGTAATGGCCTACCAAGGCGACGACGGGTAGCCGGCCTGAGAGGGCGACCGGCCACACTG
GGACTGAGACACGGCC
>Zotu2138
GAACGCTGGCGGTATGCCTAAGACATGCAAGTCGCACGGTTTAGCAATAAACAGTGGCGGACGGGTGAGT
AACACGTGGGAGATCTGCCCCGTAGTAGAGGAAAACTGAGGGAAACTTCAGCTAATCCCCTATGTGGATG
CTGGTTCTGACCGGTATCTAAAGCTTTGGCGCTATGGGATGAGCCTGCGGCCCATCAGGTAGTTGGCGGG
GTAAAAGCCCACCAAGCCGACGACGGGTAGCTGGTCTGAGAGGACGACCAGCCACAGGGGTACTGAGAC
ACGGAC
>Zotu2140
GAACGCTGGCGGCAGGCCTAACACATGCAAGTCGAGCGGTAGCACGGGAGAGCTTGCTCTCTGGGTGAC
GAGCGGCGGACGGGTGAGTAATGTCTGGGAAACTGCCTGATGGAGGGGGATAACTACTGGAAACGGTAG
CTAATACCGCATGACCTCGCAAGAGCAAAGTGGGGGACCTTCGGGCCTCACGCCATCGGATGTGCCCAGA
TGGGATTAGCTAGTAGGTGGGGTAATGGCTCACCTAGGCGACGATCCCTAGCTGGTCTGAGAGGATGACC
AGCCACACTGGAACTGAGACACGGTC
>Zotu2142
GAACGCTGGCGGCATGCTTTACACATGCAAGTCGAACGGCAGCGCGGACTTCGGTCTGGCGGCGAGTGGC
GAACGGGTGAGTAATACATCGGAACGTACCCAGAAGTGGGGGATAACGCAGCGAAAGTTGCGCTAATACC
GCATACGTTCTACGGAAGAAAGTGGGGGATCGCAAGACCTCATGCTTTTGGAGCGGCCGATGTCTGATTA
GCTAGTTGGTAGGGTAAAGGCCTACCAAGGCGACGATCAGTAGCTGGTCTGAGAGGACGACCAGCCACAC
TGGAACTGAGACACGGTC
>Zotu2144
GAACGCTGGCGGCGTGCTTAACACATGCAAGTCGAGCGGAAAGGCCCTTCGGGGTACTCGAGCGGCGAA
CGGGTGAGTAACACGTGAGCAACCTGCCCTTAGCTTTGGGATAACCCTCGGAAACGGGGGCTAATACCGA

ATGATCACTGCCGGGCGCATGTTTGGTGGTGGAAAGTTTTTCGGCTGGGGATGGGCTCGCGGCCTATCAG
CTTGTTGGTGGGGTGATGGCCTACCAAGGCGATGACGGGTAGCCGGCCTGAGAGGGCGACCGGCCACAC
TGGGACTGAGACACGGCC

>Zotu2147

GAACGCTGGCGGCGTGCCTAATACATGCAAGTCGAGCGGACTGAATGGAAGCTTGCTTCCGTTCGGTTAG
CGGCGGACGGGTGAGTAACACGTGGGCAACCTGCCCGTAAGACCGGGATAACTTCGGGAAACCGGAGCT
AATACCGGATAACACCGAAGATCGCATGATCTTTGGTTGAAAGGCGGCTTTGGCTGTCACTTACGGATGGG
CCTGCGGCGCATTAGCTAGTTGGTGAGGTAACGGCTCACCAAGGCGACGATGCGTAGCCGACCTGAGAGG
GTGATCGGCCACACTGGGACTGAGACACGGCC

>Zotu2150

TAACGCTGGCGGCGTGCATAACACATGCAAGTCGAACGATGAAACTTAAATGATTTCTTCGGAATGAACTT
AAGTGGATTAGTGGCGGACGGGTGAGTAACGCGTGAGTAACCTGCCTTACACAAGGGGATAGCCTCTGGA
AACGGAGAATAATACCCTATAAAACCATCATATCGCATGAAGTGATGGTCAAAGTGTTAGCGGTGTAAGAT
GGACTTGCGTCTGATTAGCTAGTTGGTGAGAAAAAAGCCCACCAAGGCAACGATCAGTAGCCGGCTTGAG
AGAGTGTACGGCCACATTGGGACTGAGACACGGCC

>Zotu2154

GAACGCTAGCTACAGGCTTAACACATGCAAGTCGAGGGGCAGCATTGGAGTAGCAATACTTCAGATGGCG
ACCGGCGCACGGGTGAGTAACACGTATCCAACCTGTCGCTTACTCGGGGATAGCCTTTCGAAAGAAAGATT
AATACCCGATGGCATAAGGAGTTCGCATGTTCTCTTTATTAAAGAATTTCGGTAAGCGATGGGGATGCGTT
CCATTAGATAGTTGGCGGGGTAACGGCCCACCAAGTCTACGATGGATAGGGGTTCTGAGAGGAAGGTCCC
CCACATTGGAACTGAGACACGGTC

>Zotu2156

CAACGTTGGCGGCGCGCCTAACACATGCAAGTCGAACGAGAAAGTGGAGCAATCCATGAGTAAAGTGGCG
TACGGGTGAGTAACACGTGGGTAATCTACCTCTTGATGGGGAATAACGCTGGGAAACTGGCGCTAATACC
GCATAAGTTCGTAAGAAGAAAGTCGCAAGACGCCGAGAGAGGAGCCCGCGGCCGATTAGCTAGTTGGTA
GGGTAAAAGCCTACCAAGGCTATGATCGGTAGCCGGCCTGAGAGGGCACACGGCCACACTGGCACTGAAA
CACGGGC

>Zotu2158

GAACGCTAGCGGCAGGCCTAATACATGCAAGTCGAACGGGCCGCAAGGTCAGTGGCGAACGGGTGCGTA
ACGCGTAAGCAACCTGCCCACAACTGGGGGATAGCCTTGCGAAAGCGAGGGTAAACCCGCATGGTCCTTT
GGAGTCACCTGGCTTTTAAGGTAAACATTTATGGGTTGTGGAGGGGCTTGCGTCTGATTAGCTAGTTGGTG
GGGTAACGGCCCACCAAGGCGATGATCAGTAGGGGTTCTGAGAGGATTGGCCCCCACATGGGTACTGAGA
CACGGAC

>Zotu2160

GAACGCTGGCGGCGTGCCTAATACATGCAAGTCGAGCGGACTTGACAGAGCTTGCTCTGTTCAAGTTAGCG
GCGGACGGGTGAGTAACACGTGGGTAACCTGCCTGTAAGACTGGGATAACTCCGGGAAACCGGGGCTAA
TACCGGATAATATCAAGAGCTGCATGGCTCTTGATTGAAAGGCGGCTTCGGCTGTCACTTACAGATGGACC
CGCGGCGCATTAGCTAGTTGGTGAGGTAACGGCTCACCAAGGCGACGATGCGTAGCCGACCTGAGAGGGT
GATCGGCCACACTGGGACTGAGACACGGCC

>Zotu2162

GAACGCTGGCGGCGTGCCTAATACATGCAAGTCGAGCGAATGACGAGGAGCTTGCTCCTCTGATTTAGCG
GCGGACGGGTGAGTAACACGTGGGTAACCTGCCCTGTAGACTGGGATAACTTCGGGAAACCGGAGCTAAT
ACCGGATAATTCTTTTAACCTCATGGTTTTAAGCTGAAAGGCGCTTCGGCGTCACTACAGGATGGACCCGC
GGTGCATTAGCTAGTTGGTGAGGTAACGGCTCACCAAGGCAACGATGCATAGCCGACCTGAGAGGGTGAT
CGGCCACATTGGGACTGAGACACGGCC

>Zotu2164

GAACGCTAGCGGCAGGCTTAATACATGCAAGTCGAGGGGCAGCAGGGTGTAGCAATACATCGCTGGCGA
CCGGCAAACGGGTGCGGAACACGTACGCAACTTACCCATAACTGGTGAATAGCCCTCCGAAAGGAGGATT
AATACACCGTAACCTTATGAAGTGGCATCACTTTATAAGTATAGCTCCGGCGGTTATGGATGGGCGTGCGC
CTGATTAGGTAGTTGGTGAGGGTAACGGCCCACCAAGCCTTCGATCAGTAACTGGTGTGAGAGCACGACC
AGTCACACGGGCACTGAGACACGGGC

>Zotu2166
GAACGCTAGCGGGAGGCCTAACACATGCAAGCCGAGCGGTAGGGTTTCTTCGGAAACCTGAGAGCGGCG
CACGGGTGCGGAACACGTGTGCAACCTACCTTTATCAGGGGGATAGCCTTTCGAAAGGAAGATTAATACCC
CATAATATATTGAATGGCATCATTTGATATTGAAAACTCCGGTGGATAGAGATGGGCACGCGCAAGATTAG
ATAGTTGGTGAGGTAACGGCTCACCAAGTCAATGATCTTTAGGGGGCCTGAGAGGGTGATCCCCCACACT
GGTACTGAGACACGGAC
>Zotu2168
GAACGCTAGCGGCAGGCCTAATACATGCAAGCCGAACGGCAAGCTTGGTAGCAATATCAAGCCTAGAGTG
GCGTAAGGGTGCGTAACACGTATGCAATCTGCCCGCAACAGGAGGATAGCTCCCCGAAAGGGGAATTAAT
ACTCCATAAGATCACGAGAGGGCATCTGATTGTGATTAAACTACAAGGGGTTGCGGATGAGCATGCGGTT
GATTAGCTAGTTGGTAAGGTAACGGCTTACCAAGGCGATGATCAATAGGGGAACTGAGAGGTTTATCCCC
CACACTGGCACTGAGATACGGGC
>Zotu2170
GAACGCTGGCGGCATGCCTAACACATGCAAGTCGAACGTGAAAGTACTTCGGTATGAGTAAAGTGGCGCA
CGGGTGAGTAACACGTAGGTGACCTGCCTATTAATGGGGAATAACCAGAAGAAATTTTGGCTAATACCGC
ATACGAAGCACAAATCTTAGAGTAGTGCTTGAAAGGATGCCTCTGTATATGCATTCGTTGATAGATGGGCC
TGCGGTACATTAGCTAGTTGGTGGGGTAAAGGCCTACCAAGGCAACGATGTATATCCGGTCTGAGAGGAT
GATCGGACACACTGGAACTGAGACACGGTC
>Zotu2172
GAACGCTAGCGGCAGGCCTAATACATGCAAGTCGAGGGGTAGTGGAAGCTTGCTTCCATGAGACCGGCGC
ACGGGTGCGTAACGCGTATGCAACCTACCTTCATCTGGGGGATAGCCCGGAGAAATCCGGATTAACACCG
CATAAAATCACAGTACGGCATCGTACAATGATCAAATATTTATAGGATGGAGATGGGCATGCGTGTCATTA
GTTAGTTGGCGGGGTAACGGCCCACCAAGACCGCGATGACTAGGGGATCTGAGAGGATGGCCCCCCACAC
TGGTACTGAGACACGGAC
>Zotu2174
GAACGCTGGCGGCGTGCCTAATACATGCAAGTCGAGCGGTAAGGCCCCTTCGGGGGTACACGAGCGGCG
GACGGCTGAGTAACACGTAGGAACGTACCCCAAACTGAGGGATAAGCACCAGAAATGGTGTCTAATACCG
CATGTGGTCTTCGGATTAAAGCTTCGGCGGTTTGGGAACGGCCTGCGCATGATTAGCTTGTTGGTGAGGTA
ATAGCTCACCAAGGCGACGATCATTAGCTGGTCTGAGAGGATGATCAGCCAGACTGGGACTGAGAACGGC
C
>Zotu2176
GAACGCTGGCGGCAGGCTTAACACATGCAAGTCGAGCGCGCCCTTCGGGGTGAGCGGCGAACGGGTGAG
TAACGCGTGGGAACGTGCCCCTTTCTTCGGAATAGCCTCGGGAAACTGGGAGTAATGCCGAATACGCCCCT
TACGGGGGAAAGATTTATCGGAGAGGGATCGGCCCGCGTTGGATTAGGTAGTTGGTGGGGTAATGGCCT
ACCAAGCCGACGATCCATAGCTGGTTTGAGAGGATGATCAGCCACACTGGGACTGAGACACGGCC
>Zotu2178
GAACGCTAGCGGCAGGCCTAATACATGCAAGTCGAACGGTTGTAGTAATACAATAGTGGCGCACGGGTGC
GTAACACGTATGCAACCTACCTTATAGCGGGGGATAGCCTTTGGAAACGGAGATTAATACCCCATAATATA
TTGACTCGGCATCGGGTTATTATTAAAGATTTATCGCTATAAGCTGGGCATGCGTCCAATTAGTTAGTTGGC
GGGGTAATGGCTCACCAAGACGATGATTGGTAGGGGAACTGAGAGGTTAATCCCCCACACTGGCACTGAG
ATACGGGC
>Zotu2180
GAACGCTGGCGGCATGCCTAACACATGCAAGTCGAACGAAGCCTTCGGGCTTAGTGGCGCACGGGTGCGT
AACGCGTGGGAATCTGCCCTTGGGTTCGGAATAACAGTTAGAAATGACTGCTAATACCGGATGATGACGTA
AGTCCAAAGATTTATCGCCCAAGGATGAGCCCGCGTAGGATTAGCTTGTTGGTGAGGTAAAAGCTCACCAA
GGCGACGATCCTTAGCTGGTCTGAGAGGATGATCAGCCACACTGGGACTGAGACACGGCC
>Zotu2182
GAACGCTGGCGGCAGGCTTAACACATGCAAGTCGAGCGGGCGAGGTTGCTTCGGTAACTGAGCTAGCGGC
GGACGGGTGAGTAATGCTTAGGAATCTGCCTATTAGTGGGGGACAACATTCCGAAAGGAATGCTAATACC
GCATACGCCCTACGGGGGAAAGCAGGGGATCTTCGGACCTTGCGCTAATAGATGAGCCTAAGTCAGATTA

GCTAGTTGGTGGGGTAAAGGCCTACCAAGGCGACGATCTGTAGCGGGTCTGAGAGGATGATCCGCCACAC
TGGGACTGAGACACGGCC
>Zotu2184
GAACGCTGGCGGCGTGCCTAAGACATGCAAGTCGAGTGGGTTTAGACCCACGGCGAACGGGTGAGTAAT
ACCTAGGAACTTTCTTTAAGTAGAGGATAACCAGCCGAAAGGTTGGATAATACTCTATGTGATAGTAATAT
CAAAGTCGCAAGACGCTTAAAGTGAGGCCTGGGCACCATCAGCTAGTTGGTAAGGTAATGGCTTACCAAG
GCTATGACGGTTAGGGGTCCCGAGAGGGATAACCCCCACACTGGAACTGCGACACGGTC
>Zotu2186
GAACGCTGGCGGCGTGCTTAACACATGCAAGTCGAGCGGTAAGGCTCCTTCGGGAGTACACGAGCGGCGA
ACGGGTGAGTAACACGTGAGTAACCTGCCCCATTCTTCGGAATAACCAGTGGAAACGCTGGCTAATGCCGA
ATACGACCATCGAAGGCATCTTCTGGTGGTGGAAAGCTCCGGCGGAATGGGATGGACTCGCGGCCTATCA
GCTTGTTGGTGAGGTAATGGCTCACCAAGGCGACGACGGGTAGCCGGCCTGAGAGGGTGACCGGCCACA
CTGGGACTGAGACACGGCC
>Zotu2188
GAACGCTGGCGGCGTGCCTAATACATGCAAGTCGAGCGAAGCAAACGCTTTTCTTCGGAAAAGCGTAAGC
TTAGCGGCGGACGGGTGAGTAACACGTGGGTAACCTGCCCATAAGCCTGGGATAACATTCGGAAACGAAT
GCTAATACCGGATATGCAACATCATCGCCTGGTGGTGTTGGGAAAGGCGGAGCAATCTGCCACTTATGGAT
GGACCCGCGGTGCATTAGCTAGTTGGTGAGGTAAAGGCTCACCAAGGCGACGATGCATAGCCGACCTGAG
AGGGTGATCGGCCACACTGGGACTGAGACACGGCC
>Zotu2191
GAACGCTGGTGGCGTGCTTAACACATGCAAGTCGAGCGAGGCGTCCGCTTTCGGGTGGATAGTCGAGCGG
CGAACGGGTGAGTAACACGTGAGTAATCTTCCCTGGGCTCGGGGATAACCTTGGGAAACTGAGGCTAATA
CCGGATGTGCGTTGTTTGGGGCTTCCTGGATGACGGAAAGGTTGCGTGCCGTGAGGTGCGTTTCCGGCCTT
GGATGAGCTCGCGGCCTATCAGCTTGTTGGTGCGGTGATGGCGTACCAAGGCGACGACGGGTAGCCGGCC
TGAGAGGGTGACCGGCCACACTGGGACTGAGATACGGCC
>Zotu2193
GAACGCTAGCGGCAGGCCTAATACATGCAAGCCGAACGGCAAGTGTTTTAGCAATAGAACACCTAGAGTG
GCGTAAGGGTGCGTAACACGTATGCAATCTGCCCTCAACAGGAGGATAGCTCCCCGAAAGGGGAATTAAG
ACTTCATAGGATCATAGAGTGGCATCACTTAGTGATTAAATTACAAGAGGTTGAGGATGAGCATGCGCATG
ATTAGTTAGTTGGCGGGGTAATGGCCCACCAAGACGATGATCATTAGGGGAACTGAGAGGTTGATCCCCC
ACACTGGCACTGAGATACGGGC
>Zotu2195
TAACGCTAGCGGCAGGCCTAATACATGCAAGTCGAACGGCAGCACGGGTAGCAATATCTGGTGGCGAGTG
GCGCACGGGTGCGTAACGCGTATGCAATCTACCTGTAACTGGGGAATAGCCTTCCGAAAGGAAGATTAAT
ACCGCATAATATCACGAAACGGCATCGTTTTGTGATCAAATCTCCGGAGGTTACAGATGAGCATGCGTGAC
ATTAGCTAGTTGGTGAGGTAACGGCTCACCAAGGCTACGATGTCTAGGGGGTCTGAGAGGATGATCCCCC
ACACTGGTACTGAGACACGGAC
>Zotu2198
GAACGCTAGCGGCAGGCTTAACACATGCAAGTCGAGGGGTAGTCTAGCAATAGATGAGACCGGCGCACG
GGTGCGTAACGCGTATGCAATCTACCTTTTACAGAGGGATAGCCCAGAGAAATTTGGATTAATACCTCATA
GCATAGCAGCTTCGCATGAAGTCACTATTAAAGTTCCAACGGTAAAAGATGAGCATGCGTCCTATTAGCTA
GATGGTAAGGTAACGGCTTACCATGGCTACGATAGGTAGGGGTCCTGAGAGGGAGATCCCCCACACTGGT
ACTGAGACACGGAC
>Zotu2200
GAACGCTGGCGGCAGGCTTAACACATGCAAGTCGAACGCCCCTTTCGGGGGGAGTGGCAGACGGGTGAG
TAACACGTGGGAACATACCCTTTGGTTCGGAACAACGTTTGGAAACGGACGCTAATACCGGATACGCCCTT
TGGGGGAAAGGCTTTGCTGCCGAAGGATTGGCCCGCGTTCGATTAGCTTGATGGCGGGGTAACGGCCCAC
CATGGCGACGATCGATAGCTGGTCTGAGAGGATGGCCAGCCACATTGGGACTGAGACACGGCC
>Zotu2203

GAACGCTGGCGGCGTGCTTAACACATGCAAGTCGAGCGGAAAGGCCACTTCGGTGGTACTCGAGCGGCGA
ACGGGTGAGTAACACGTGAGTAATCTGCCCCTGGCTTTGGGATAGCCACCGGAAACGGTGATTAATACCG
GATACGACCGCTCCTCGCATGAGGTGGTGGTGGAAAGTTTTTCGGCCAGGGATGTGCTCGCGGCCTATCA
GCTTGATGGTGAGGTAATGGCTCACCATGGCTTCGACGGGTAGCCGGCCTGAGAGGGTGACCGGTCACAC
TGGGACTGAGACACGGCC
>Zotu2206
AAACGCTGGCGGCGTGCCTAATGCATGCAAGTCGAACGGGATCACCCCGTTGCCAGTCCTTCGGGGCTGG
TGGGGTGAGGACCGTGGCGGACGGGTGAGGAACGCGTGGCCGACCTGCCCTGATCACGGGGATAACCCG
GGGAAACCCGGGCGAATACCCGATGCCCTGGGGGATGGGGACATCCTTCAGGAAAGCGCTATGGAGCGG
ATCAGGAGGGGGCTGCGCCCGATTAGCTAGCTGGTGGGGTAACGGCCTACCAGGGCGACGATCGGTCGC
TGGTCTGAGAGGATGATCAGCCACACGGGGACTGAGACACGGCC
>Zotu2208
GAACGCTGGCGGCGTGCTTAACACATGCAAGTCGAACGATGAAGCTGCCTGCTTGCAGGTGGTGGATTAG
TGGCGAACGGGTGAGTATCACGTGAGTAACCTGCCCTTGACTCTGGGATAAGCCCGGGAAACTGGGTCTA
ATACCGGATATGACCTCTCATCGCATGGTGGGGGGTGGAAAGTTTTTAACGGTCTTGGATGGGCTCGCGGC
CTATCAGCTTGACGGTGGGGTAGTGGCCTACCGTGGCTTTGACGGGTAGCCGGCCTGAGAGGGTGACCGG
CCACACTGGGACTGAGACACGGCC
>Zotu2210
GAACGCTGGCGGCGTGCTTAATACATGCAAGTCGAACGAGGTTGATCCAGTGGAAACACTGGTGACTGCC
TAGTGGCGAACGGGTGAGTAACACGTGAGCAACCTGCCCCGAAGACCGGGATAACAGTGGGAAACCGCT
GCTAATACCGGATACCCCCACAGGGTCACATGGCCTGGTGAGGAAATGCCTGATTCTTTGGAGTCATACGC
TTCGGGAGGGGCTCGCGGCCTATCAGCTTGTTGGTGGGGTAACGGCCTACCAAGGCAACGACGGGTAGCT
GGTCTGAGAGGACGATCAGCCACACTGGGACTGAGACACGGCC
>Zotu2212
GAACGCTAGCGGCAGGCCTAATACATGCAAGTCGAACGGTAACATGAGTAGCAATACTTGATGACGAGTG
GCGCACGGGTGCGTAACGCGTATGCAATCTACCTTTAACTGGTGAATAGCCCGAAGAAATTCGGATTAATA
CACCATAGTATATTTGAATGGCATCATTTAAATATTAAAGTAACAACGGTTAGAGATGAGCATGCGTGACA
TTAGCTAGTTGGTGAGGTAATGGCTCACCAAGGCTACGATGTCTAGGGGATCTGAGAGGATTAACCCCCAC
ACTGGTACTGAGACACGGAC
>Zotu2214
GAACGCTGGCGGCGTGCTTAACACATGCAAGTCGAACGAGGCGCTTGCGCCTAGTGGCGAACGGGTGAGT
AACACGTGAGCAACCTGCCCCTGGCTCTGGGATAGCCTCCGGAAACGGGGATTAATACCGGACATGACTG
CTGCCGGCATCGGCGGCGGTGGAAAGTTTTTCGGCCAGGGAGGGGCTCGCGGCCTATCAGCTTGTTGGGG
GGGTAACGGCCCACCAAGGCGACGACGGGTAGCCGGCCTGAGAGGGCGACCGGCCACACTGGGACTGAG
ACATGGCC
>Zotu2216
GAACGCTAGCGGAAGGCCTAATACATGCAAGTCGAACGGAGATATTATTTCGGTAATATTTTAGTGGCGCA
CGGGTGAGTAACGCGTACACAACCTACCTTATATTGGGGGATAGCCCTGGGAAACTGGGAGTAATACCGC
ATAATATTATAAAGCGGCATCGTTTAATAATTAAAGTTACGGCGATATAAGATGGGTGTGCGTCTGATTAG
GTAGTTGGTAGGGTAATGGCCTACCAAGCCGACGATCAGTAGGGGGTGTGAGAGCATGGCCCCCCACACG
GGTACTGAGACACGGAC
>Zotu2219
GAATGCTGGCGGCGTGGATAAGTCATGCAAGTCAAGGGGGCCCGCAAGGGCAACCGGCAGACGAGGTAG
TAACACGTAGGTACGTCCCCCGAAGTCGGGCATAGCCAGTCGAAAGATTGGGTAATTCCCGATAGTCCTGT
AAAGGTAAAGTTTTTCGCTTCGGGAGCGGCCTGCGTAGTATCAGCTAGTTGGTAGGGTAACGGCCTACCAA
GGCTACGACGCTTAGGGGAGCTGAGAGGCTGACCCCCACCGATGGAACTGCGACACGGTC
>Zotu2221
GAACGCTGGCGGCATGCCTAACACATGCAAGTCGAACGAGAGCTTTCGGGCTCTAGTGGCGCACGGGTGC
GTAACGCGTGGGAATCTGCCCTTTGGTTCGGGATAACTCAGGGAAACTTGAGCTAATACCGGATGATGTCG
CGAGACCAAAGATTTATCGCCGGAGGATGAGCCCGCGTCGGATTAGCTAGTTGGTGGGGTAAAGGCCTAC
CAAGGCGACGATCCGTAGCTGGTCTGAGAGGATGATCAGCCACACTGGGACTGAGACACGGCC

>Zotu2223
GAACGCTGGCGACATGGATAAGGCATGCAAGTCAAGGGGGCCCGCAAGGGCAACCGGCAGACGAGGTA
GTAACACGTAGGTACTCCCCCCGAAGTCCGGCATAGCTCGTCGAAAGACGGGGTAATTCCGGATAGTCCCG
CAAGGGTAAAGTTTTTCGCTTCGGGAGAGGCCTGCGTAGTATCAGCTTGTTGGTAAGGTAACGGCTTACCA
AGGCTACGACGCTTAGGGGAGCTGAGAGGCTGACCCCCACCGATGGGACTGAGACACGGCC
>Zotu2225
AAACGCTGGCGGCGTGCTTAACACATGCAAGTCGAGCGAGAAAGCTCTTTCGGGAGTGAGTAAAGCGGCG
GACGAGTTAGTAACACGTGAGAATCTACCCCTCAGTGAAGAATACCCTCGAGAAATCGAGGCTAATGCTGC
ATACGTCTCAACAGAGAGATCTGTTTAGAGAAAGGTGGCCTCTTTGCTGCCGCTGGGGGATGAGCTTGCGC
ACTATTAGCTAGTTGGTGGGGTAAAGGCCTACCAAGGCTATGATGGTTAGCCGGCCTGAGAGGGTGTACG
GCAACGCTGGAACTTAGACACGGTC
>Zotu2228
GAACGCTAGCGGCAGGCCTAATACATGCAAGTCGAGGGGCAGCGGGAGTAGCAATACTTGCCGGCGACC
GGCGCACGGGTGCGTAACACGTATACAATCTGCCCATGTCTGGGGGATAGCCCGGAGAAATCCGGATTAA
TACCCCATAATAAATGAAAGGGCATCCTTTTATTTTGAAAGCTCCGGCGGACATGGATGAGTATGCGTCTG
ATTAGTTAGTTGGCGAGGTAACGGCTCACCAAGACCACGATCAGTAGGGGATCTGAGAGGATTAACCCCC
ACACGGATACTGAGACACGGAT
>Zotu2231
GAACGCTGGCGGCAGGCTTAACACATGCAAGTCGAGCGGGGAGATGTAGCTTGCTACATTTCCTAGCGGC
GGACGGGTGAGTAATGCTTAGGAATCTGCCTATTAGTGGGGGACAACATTCCGAAAGGAATGCTAATACC
GCATACGCCCTACGGGGGAAAGCAGGGGATCTTCGGACCTTGCGCTAATAGATGAGCCTAAGTCAGATTA
GCTAGTTGGTGGGGTAAAGGCCTACCAAGGCGACGATCTGTAACGGGTCTGAGAGGATGATCCGCCACAC
TGGGACTGAGACACGGCC
>Zotu2233
GAACGCTGGCGGCGTGCTTAACACATGCAAGTCGAACGGTAAGGCCCTTCGGGGTACACGAGTGGCGAAC
GGGTGAGTAACACGTGAGTAACCTGCCCTTGACTCTGGGATAACAGTTGGAAACAGCTGCTAATACCGGAT
ACGAACCCTTGTCGCATGATGTGGGTTGGAAAGCTCCGGCGGTCAGGGATGGACTCGCGGCCTATCAGCT
TGTTGGTGAGGTAGTGGCTCACCAAGGCTTCGACGGGTAGCCGGCCTGAGAGGGCGACCGGCCACATTGG
GACTGAGATACGGCC
>Zotu2235
GAACGCTGGCGGCGTGCCTAATACATGCAAGTCGAACGAGAGCGACCGGTGCTTGCACTGGTCAATCTAG
TGGCGAACGGGTGAGTAACACGTGGGTAACCTGCCCATCAGAGGGGGATAACATTCGGAAACGGATGCT
AAAACCGCATAGGTCTTCGAACCGCATGGTTTGAAGAGGAAAAGAGGCGCAAGCTTCTGCTGGTGGATGG
ACCCGCGGTGCATTAGCTAGTTGGTGAGGTAACGGCTCACCAAGGCCGTGATGCATAGCCGACCTGAGAG
GGTGATCGGCCACATTGGGACTGAGACACGGCC
>Zotu2239
GAACGCTAGCTACAGGCTTAACACATGCAAGTCGAGGGGCAGCATGAATATTGGCTTGCCAATATTTGATG
GCGACCGGCGCACGGGTGAGTAACACGTATCCAACCTTCCGGTTACTCGGGGATAGGCTTTCGAAAGAAA
GATTAATACCCGATGTTGTGTATCTTTCTCCTGATTGATACGCCAAAGGATTCCGGTAACCGATGGGGATGC
GTTCCATTAGGCAGTTGGCGGGGTAACGGCCCACCAAACCTTCGATGGATAGGGGTTCTGAGAGGAAGGT
CCCCCACATTGGAACTGAGACACGGTC
>Zotu2241
GAACGCTGGCGGCGTGCCTAATACATGCAAGTAGAACGCTGAAGCTTGGTGCTTGCACCGAGTGGATGAG
TTGCGAACGGGTGAGTAACGCGTAGGTAACCTGCCTCTTAGCGGGGGATAACTATTGGAAACGATAGCTA
ATACCGCATAAAAGTCGATATCGCATGATGTTGACTTGAAAGGTGCAATTGCATCACTAAGAGATGGACCT
GCGTTGTATTAGCTAGTTGGTGAGGTAACGGCTCACCAAGGCGACGATACATAGCCGACCTGAGAGGGTG
ATCGGCCACACTGGGACTGAGACACGGCC
>Zotu2243
GAACGCTGGCGGCGTGCCTAATACATGCAAGTAGAACGCACAGGATACACCGTAGTTTACTACACCGTATT
CTGTGAGTTGCGAACGGGTGAGTAACGCGTAGGTAACCTGCCTGGTAGCGGGGGATAACTATTGGAAACG

ATAGCTAATACCGCATAAGAACATTTACTGCATGGTAGATGTTTAAAAGGTGCAAATGCATCACTACCAGAT
GGACCTGCGTTGTATTAGCTAGTAGGTGAGGTAACGGCTCACCTAGGCGACGATACATAGCCGACCTGAG
AGGGTGATCGGCCACACTGGGACTGAGACACGGCC

>Zotu2245

TAACGTTGGCGGCGTGCATAACACATGCAAGTTGAGCGTGGCTCTAGGTTTACTTAGAGTCTAGCAGCGGA
CGGCTGAGTAATGCACAGGAACATACCTCGCAGTGGGGAATAGCCCTTCGAAAGAAGGGGTAATACCGCA
TAGTCTCGCAAGAGTAAAGCCGCAAGGCGCTGTGAGAATGGCCTGTGTCCTATCAGGTAGTTGGTGTGGG
TAATGGCCTACCAAGCCTATGACGGGTAGCTGGTCTGAGAGGATGACCAGCCAGATCGGGACTGAGACAC
GGCC

>Zotu2247

GAACGCTGGCGGCGTGCCTAATACATGCAAGTCGAGCGGAAGTTGGAGAGCTTGCTCTTCAACTTTAGCG
GCGGACGGGTGAGTAACACGTGGGCAACCTGCCCTGCAGATCGGGATAACTCCGGGAAACCGGTGCTAAT
ACCGAATAGTTTTCGCCACCTCCTGGTGGCGTACGGAAAGACGGTTTCGGCTGTCACTGCAGGATGGGCCC
GCGGCGCATTAGCTAGTTGGTGGGGTAACGGCCCACCAAGGCGACGATGCGTAGCCGACCTGAGAGGGT
GATCGGCCACACTGGGACTGAGACACGGCC

>Zotu2249

GAACGCTGGCGGCATGCTTAACACATGCAAGTCGCACGGGCAGTGGTAACATTGTCAGTGGCGGACGGGT
GAGTAACGCGTAGGAATCTGTCCTTGGGTGGGGGATAACAGTGGGAAACTACTGCTAATACCGCATGACA
ACTGAGGTTTAAAGGCGTGAGTCGCCTGAGGAGGAGCCTGCGTTCGATTAGCTAGTTGGTGGGGTAAAGG
CCTACCAAGGCTACGATCGATAGCTGGTCTGAGAGGATGATCAGCCACACTGGGACTGAGACACGGCC

>Zotu2251

AAATGCTGGCGGCGTGCCTAATACATGCAAGTCGAGCGGTAAGGCTTTCACAGATTATTTAAACGGAAAA
GTCCATTTATGGATACTATCCTTCAACGTTTCTTCACCCGGCTAGGTGATATATAAATGGTTGAATAATGTGT
GAAAGTACACGAGCGGCGGACGGCTGAGTAACGCGTAGGAATGTACCCCAAAGTGAGGGATAAGCTCTG
GAAACAGAGTCTAATACCGCATGTGATCTAAGGATTAAAGCCTTCGGGCGCTTTGGGAGCAGCCTGCGTAA
GATTAGTTTGTTGGTGAGGTAATGGCTCACCAAGACTACGATCTTTAGCTGGTCTGAGAGGATGATCAGCC
AGACTGGGACTGAGACACGGCC

>Zotu2253

GAACGCTGGCGGCGCGCTTAACACATGCAAGTCGAGCGAGAACCAGGACTTCGGTCCTGGGGACAGCGG
CGAACGGGTGAGTAACACGTGGGTAATCTGCCCTCGATTCTGGGATAGCCCGGGGAAACCCGGATTAATA
CCGGATAGCCTTTCGAGCTTTCGGGCTTGGAAGAAAAGGTAGCTTCGGCCTCCGATCGAGGATGAGCCCG
CGGTGGATTAGCTTGTTGGCGGGGTAATGGCCCACCAAGGCGACGATCCATAGCTGGTCTGAGAGGACGA
TCAGCCACACTGGGACTGAGACACGGCC

>Zotu2257

GAACGCTAGCGGCAGGCTTAACACATGCAAGTCGAGGGGTATAGTAGCAATACTAGAGACCGGCGCACG
GGTGCGTAACGCGTATGCAATCTACCTTTTGCAGGGGAATAGCCCAGAGAAATTTGGATTAATGCCCCATA
GTATAGTGACTCGGCATCGAGATACTATTAAAGTTACAACGGCAAAAGATGAGCATGCGTCCCATTAGCTA
GTTGGTAAGGTAACGGCTTACCAAGGCGACGATGGGTAGGGGTCCTGAGAGGGAGATCCCCCACACTGGT
ACTGAGACACGGAC

>Zotu2260

GAACGCTGGCGGCGCGCTTAACACATGCAAGTCGAGGGAGAACCGGCTTTCGGGTCGGGGAGACCGGCG
AACGGGTGAGTAACACGTGAGGAACCTGCCCTGTAGATCGGAATAACCATGGGAAACTGTGGCTAATGCC
GAATATGCTCTTCGCATCGCATGGTGTGGGGAGGAAATGGGTTTCTGCTACGGGATGGCCTCGCGGCCTAT
CAGCTTGTTGGTGGGGTGATGGCCTACCAAGGCGACGACGGGTAGCTGGCGTGAGAGCGCGACCAGCCA
CACTGGGACTGAGACACGGCC

>Zotu2263

GAACGCTGGCGGCGTGCTTAACACATGCAAGTCGAACGATGAAGAAAAGAATTGAAGCTTCGGCAGAAAT
TCATTTCGGATTAGTGGCAGACGGGTGAGTAACGCGTGAGCAACCTACCTCTTACAAAGGGATAGCCTCGG
GAAACCGGGATTAATACCATATAACATTGCGACGAAGCATTTCGATGCAATCAAAGCTACGGCGGTAAGA
GATGGGCTCGCGTCCCATTAACCAGATGGTGAGGTAACGGCTCACCATAGTAACGATGGGTAACCGGCCT
GAGAGGGTGAACGGTCACACTGGAACTGAGACACGGTC

>Zotu2265
GAACGCTGGCGGCGCGCTTAACACATGCAAGTCGAGCGAGAACCAGGACTTCGGTCCTGGGGACAGCGG
CGAACGGGTGAGTAACACGTGGGTAATCTGCCCTCGGTTCCGGGATAGCCCGGGGAAACTCGGATTAATA
CCGGATAGCCTCACGAACCTTCGGGCGAGTGAGAAAAGGTAGCTTTGGCCTCTGACCGAGGATGAGCCCG
CGGTGGATTAGCTTGTTGGCGGGGTAACGGCCCACCAAGGCGACGATCCATAGCTGGTCTGAGAGGACGA
TCAGCCACACTGGGACTGAGACACGGCC
>Zotu2267
GAACGCTGGCAGCGTGCTTAACACATGCAAGTCGAACGGTGAAGCCCAGCTTGCTGGGTGGATCAGTGGC
GAACGGGTGAGTAACACGTGAGCAATCTGCCCCTGACTCTGGGATAAGCGCTGGAAACGGCGTCTAATAC
TGGATATGAACCGTGGAGGCATCTTCAACGGTTGGAAAGATTTTTGGTCAGGGATGAGCTCGCGGCCTAT
CAGCTTGTTGGTGAGGTAATGGCTCACCAAGGCGTCGACGGGTAGCCGGCCTGAGAGGGTGACCGGCCAC
ACTGGGACTGAGACACGGCC
>Zotu2270
GAACGCTGGCGGCGTGCTTAACACATGCAAGTCGAACGCTGAAGCCTAGCTTTTGTTGGGTGGATGAGTG
GCGAACGGGTGAGTAACACGTGAGTAACCTGCCCCCTTCTTTGGGATAACGCCCGGAAACGGGTGCTAAT
ACTGGATATTCACTGATCTTCGCATGGGGGTTGGTGGAAAGGTTTTTTCTGGTGGGGGATGGGCTCGCGG
CCTATCAGCTTGTTGGTGGGGTGATGGCCTACCAAGGCTTTGACGGGTAGCCGGCCTGAGAGGGTGACCG
GTCACATTGGGACTGAGATACGGCC
>Zotu2272
GAACGCTGGCGGCGTGCTTAACACATGCAAGTCGAACGATGAAGCCCAGCTTGCTGGGTGGAAGAGTGGC
GAACGGGTGAGTAACACGTGAGTAACCTGCCCTGAACTCTGGGATAAGCACTGGAAACGGTGTCTAATAC
TGGATACGACCTATCACCGCATGGTGTGTGGGTGGAAAGATTTATCGGTTTTGGATGGACTCGCGGCCTAT
CAGCTAGATGGTGAGGTAATGGCTCACCATGGCGACGACGGGTAGCCGGCCTAAGAGGGTGACCGGCCA
CACTGGGACTGAGACACGGCC
>Zotu2274
GAACGCTGGCGGCGTGCTTAATACATGCAAGTCGAACGAGGTTGATCCAGTGGTAACACTGGTGACTACCT
AGTGGCGAACGGGTGAGTAACACGTGAGCAACCTGCCCCGAAGACCGGGATAACAGTGGGAAACCGCTG
CTAATACCGGATACCCCCACCGGATCACATGGTCTGGTGAGGAAATGGATTCCGCTTCGGGAGGGGCTCG
CGGCCTATCAGCTTGTTGGTGGGGTAACGGCCTACCAAGGCTTCGACGGGTAGCTGGTCTGAGAGGACGA
TCAGCCACACTGGGACTGAGACACGGCC
>Zotu2276
GAACGCTGGCGGCGTGGATAAGGCATGCAAGTCGAACGGAACTTATTTAGCTCGAGATGCGATTACATCA
AGTCAAGAATTTTGTAAGTTTAGTGGCAGACGAGGTAGGAATACATATGTACATACCCCCGAGACGGGCA
CAACACCGCGAAAGCGGTGCTAATTCCCGATATTGTGGTAACACGAAAGTCGCAAGACGCTCAGGGATTG
GCGTATGGGCCATCAGCTTGTTGGTAGGGTAACGGCCTACCAAGGCTATGACGGCTAGGAGGCGTGAGAG
CGTGACCTCCACCGACGGAACTGAGACACGGTC
>Zotu2278
TAACGCTGGCGGCGTGCATAACACATGCAAGTCGAACGATGAAACTTTTTTGATTTCTTCGGAATGAAAAA
AAGTGGATTAGTGGCGGACGGGTGAGTAACGCGTGAGTAACCTGCCTTAGAGAAGGGGATAGCCTTTGG
AAACGAAGAATAATACCCTATGAAACCATAACCTCGCATGAGGCCTTGGTCAAAGATTTATCGCTCTAAGAT
GGACTTGCGTCTGATTAGTTAGTTGGTGGGGTAACGGCCTACCAAGACAGCGATCAGTAGCCGGCTTGAG
AGAGTGTACGGCCACATTGGGACTGAGACACGGCC
>Zotu2280
GAACGCTGGCGGCGTGGATAAGGCATGCAAGTCGAGTGGGTTTAGACCCACGGCAGACGAGGTAGGAAC
ACGTAGGTATGTACCCTCAAGTCGGAGATAACCCATCGAAAGGTGGAGTAATATTCGATAGTCCCTCCGGG
GTAAAGGCGCAAGCCGCTTGAGGAACAGCCTGCGGGCTATCAGCTAGTTGGTAGGGTAATGGCCTACCAA
GGCGATGACGGCTAGGGGAGCTGAGAGGCTGACCCCCACCGATGGAACTGAGACACGGTC
>Zotu2283
GAACGCTGGCGGCGTGCTTAACACATGCAAGTCGAACGGATCGCATGTTTTCGGATGTGTGGTGAGTGGC
GAACGGGTGAGTAACACGTGAGTAACCTGCCCCGAACTCTGGGATAACGACTCGAAAGGGTTGCTAATAC

CGGATATTGAACTGTTGCCGTGTGGTGGTGGTTTGAAAGTTTTTCGGTTCGGGATGGGCTCGCGGCCTATC
AGCTTGTTGGTGGGGTGATGGCCTACCAAGGCGTCGACGGGTAGCCGGCCTGAGAGGGCGACCGGCCAC
ACTGGGACTGAGATACGGCC
>Zotu2288
GAACGCTAGCGGGAGGCTTAATACATGCAAGTCGAGCGGTAAGTTATTTATAGCAATATAGATGACCTAG
AGCGGCGCACGGGTGAGTAACGCGTACATAACCTACCCTTTACTGGGGGAAAGTCACGGGAAACTGTGAA
TAATACCGCATAGTATTAATTAACTGCATGGTTGATTAATTAAAGATTTATTGGTAAGGGATGGGTGTGCGT
CTGATTAGCTAGTTGGTAGGGTAACGGCCTACCAAGGCGATGATCAGTAGGGGGCGTGAGAGCGTGATCC
CCCACACGGGTACTGAGACACGGAC
>Zotu2290
GAACGCTGGCGGCAGGCTTAACACATGCAAGTCGAACGCTCGCAGCAATGCGGGAGTGGCAGACGGGTG
AGTAACGCGTGGGAACATACCCTTTGGTTCGGAATAACTGAGAGAAATTTCAGCTAATACCGGATGTGCCC
TTCGGGGGAAAGATTTATCGCCAAAGGATTGGCCCGCGTCTGATTAGCTAGTTGGTGAGGTAACGGCTCAC
CAAGGCGACGATCAGTAGCTGGTCTGAGAGGATGATCAGCCACACTGGGACTGAGACACGGCC
>Zotu2292
GAACGCTGGCGGCGTGCTTAACACATGCAAGTCGAACGGAAAGGCCGAAGCTTGCTTTGGTACTCGAGTG
GCGAACGGGTGAGTAACACGTGGGTGATCTGCCCTGCACTTTGGGATAAGCCTGGGAAACTGGGTCTAAT
ACCGGATAGGACCACGGTGTGGTGATCGTGGTGGAAAGTTTTTCGGTGTGGGATGAGCTCGCGGCCTAT
CAGCTTGTTGGTGGGGTAATGGCCTACCAAGGCGTCGACGGGTAGCCGGCCTGAGAGGGTGTACGGCCAC
ATTGGGACTGAGATACGGCC
>Zotu2295
GAACGCTAGCGGCAGGCTTAATACATGCAAGTCGTGGGACGATAGCAATATTGGACCGGCAAACGGGTGC
GTAACACGTACACAACTTTCCGTCAAGTGGGGGATAGCCTAAAGAAATTTGGATTAATACCCCATAGAATA
ATGATGTGGCATCATATTATTATTAAAGCTCCGGCGCTTGATGATGGGTGTGCGGCTGATTAGGTAGTTGG
CGGGGTAACGGCCCACCAAGCCTTCGATCAGTAGCTGATGTGAGAGCATGATCAGCCACACGGGCACTGA
GACACGGGC
>Zotu2297
GAACGCTGGCGGTATGCCTAACACATGCAAGTCGAACGGAATCTTCGGATTTAGTGGCGGACGGGTGAGT
AACGCGTGAGAATCTGCCTTCAGGATGGGGACAACAGTTGGAAACGACTGCTAATACCCGATATGCAGCG
ATGTGAAAGATTTATCGCCTGGAGATGAGCTCGCGTTGGATTAGCTAGATGGTGTGGTAATGGCGCACCAT
GGCGACGATCCATAGCTGGTCTGAGAGGATGAGCAGCCACACTGGGACTGAGACACGGCC
>Zotu2299
GAACGCTGGCGGCGTGCTTAACACATGCAAGTCGAGCGAACCTTCGGGTTAGCGGCGAACGGGTGAGTAA
CACGTGGGCAACCTGCCCCGAGCTCTGGGATAACCCCGGGAAACCGGGGCTAATACCGGATACGACACCG
ACAGACATCTGTCGGTGTGGAAAGTTTTTCGGCTCGGGATGGGCCCGCGGCCTATCAGCTTGTTGGTGGG
GTAACGGCCTACCAAGGCGACGACGGGTAGCCGGCCTGAGAGGGCGACCGGCCACACTGGGACTGAGAC
ACGGCC
>Zotu2301
GAACGCTGGCGGCGTGCTTAAGACATGCAAGTCGAACGGGCTCTTCGGAGCCAGTGGCGCACGGGTGAG
TAACGCGTAACTGACCTACCCAGAAGTCCGGAATAACGGGTCGAAAGATCCGCTAATACTCGATGTGCAGT
GTCGCTGTGGCGACACTGTAAAGATTGATCGCTTGTGGATGGGGTTGCGTTTCATCAGCTAGTTGGTGGGG
TAAAGGCCTACCAAGGCGACGACGAATAGCCGGCCTGAGAGGGTGGCCGGCCACAGGGGCACTGAGACA
CGGGT
>Zotu2303
GAACGCTGGCGGTATGCTTTACACATGCAAGTCATACGAGGATTTTTAATTCAAGTGGCGGACGGGTGAGT
AACACGTAAGAATTTACCTTTAGGAGGGGGATAACAACTGGAAACGGTTGCTAATACCCCATATGCTTTCG
AGTGAAATGGAGTTTTCCGCCTAGAGAGAAGCTTGCGGCTGATTAGCTTGTTGGTGAGGTAATGGCTTACC
AAGGCGACGATCAGTATCTGGTTTGAGAGGACGATCAGACACACTGGAACTGATACACGGTC
>Zotu2305

GAACGCTGGCGGCGTGCTTAACACATGCAAGTCGAACGATGAAGCCCAGCTTGCTGGGTGGAAGAGTGGC
GAACGGGTGAGTAACACGTGAGTAACCTGCCCTGAACTCTGGGATAAGCACTGGAAACGGTGTCTAATAC
TGGATACGACCTATCACCGCATGGTGTGTGGGTGGAAAGATTTATCGGTTTTGGATGGACTCGCGGCCTAT
CAGCTAGATGGTGAGGTAATGGCTCACCATGGCGACGACGGGTAGCCGGCCTGAGAGGGTGACCGGCCA
CACTGGGATTGAGACACGGCC

>Zotu2308

GAACGCTGGCGGCGTGCCTAATACATGCAAGTCGAACGAACCGACGGAGAACTTGTTCTCTTGACGTGAG
TGGCGGACGGGTGAGTAACACGTGGGAAACCTACCCTTCAGCGGGGGATAACGGTTCGAAAGAATCGCTA
ATACCGCATAAAAGGATCAGTCGCCTGGCTGATTCGTAAAAGATGGCTCTGCTATCACTGAAGGATGGGCC
CGCGGCGCATTAGCTAGTTGGTAAGGTAACGGCTTACCAAGGCGATGATGCGTAGCCGACCTGAGAGGGT
AATCGGCCACATTGGGACTGAGACACGGCC

>Zotu2310

GAACGCTGGCGGTGTGCCTAATACATGCAAGTCGAGCGGTAAGGCCCCTTCGGGGGTACACGAGCGGCG
GACGGCTGAGTAACACGTAGGAACGTACCCCAAAGTGAGGGATAAGCACCAGAAATGGTGTCTAATACCG
CATGTGCTCTAAGGAGTAAAGCTTTATGCGCTTTGGGAACGGCCTGCGCATGATTAGCTTGTTGGTAAGGT
AAAAGCTTACCAAGGCTACGATCATTAGCTGGTCTGAGAGGATGATCAGCCAGACTGGGACTGAGACACG
GCC

>Zotu2312

TAACGCTGGCAGCGTGCATAACACATGCAAGTCGAGCGGAGTTGTTTGAGATTATATCAAAGACAATTTAG
CGGCAGACGAGTGAATAACACGTAAGGAATCTACCCCGGAGCGAGGAACAACCAGCCGAAAGGTTGGCT
AATACCTCGTACGACCCGAGAAAGGCATCTTTTTCGGTTGAAAGGCGTAAGCCGCTCTGGGAGGATCTTGC
GGCCTATCAGCTAGTTGGCGGTGTAACGGACCACCAAGGCGACGACGGGTAGCCGGCCTGAAAGGGCGA
TCGGCCACGGAGGCACTGAGACACGGGC

>Zotu2314

GAACGCTGGCGGCGTGCCTAACACATGCAAGTCGAACGAGGCCCAAGCAGTGGCAACACTGCGGAAGGC
CTAGTGGCGAACGGGTGAGTAACACGTGAGGAACCTGCCCCGAAGACCGGGATAACCCCCTGAAAAGGG
AGCTAATACCGGATGCCCCCACCGGACCGCATGGTCTGGTGAGGAAATGGATTCCGCTTCGGGAGGGCCT
CGCGGCCTATCAGCTTGTTGGTGAGGTAACGGCTCACCAAGGCGTCGACGGGTAGCTGGTCTGAGAGGAC
GATCAGCCACACTGGGACTGAGACACGGCC

>Zotu2316

GAACGCTAGCGGCAGGCCTAATACATGCAAGTCGGACGGGATTTAGGAGCTTGCTTCTAATGAGAGTGGC
GCACGGGTGCGTAACACGTATGTAACCTACCTTGTTCAGGGGGATAGCCTCTCGAAAGAGAGATTAAGAC
CGCATAAAATCACAATGTGGCATCACAAAATGATCAAATATTTATAGGAACAAGATGGGCATGCGGAACAT
TAGCTAGTTGGTAAGGTAACGGCTTACCAAGGCTACGATGTTTAGGGGATCTGAGAGGATGACCCCCCAC
ACTGGTACTGAGACACGGAC

>Zotu2318

GAACGCTGGCGGCGTGCTTAACACATGCAAGTCGAACGGAAAGGCCTGCTTTCCCTTGTGGATTGCGGGT
GCTCGAGTGGCGAACGGGTGAGTAACACGTGAGTGATCTGCCCCCAACTTGGGTATAAGCCTGGGAAACT
GGGTCTAATTCCCGATAGGACTGCAGGGTGGTGCCTGTGGTGGAAAACGATTTTCTAGTGGTTGGGGATG
AGCTCGCGGCCTATCAGCTTGTTGGTGGGGTAATGGCCTACCAAGGCGGCGACGGGTAGCCGGCCTGAGA
GGGTGGACGGCCACATTGGGACTGAGATACGGCC

>Zotu2320

GAACGCTAGCTACAGGCTTAACACATGCAAGTCGAGGGGAAACGGCATTGAGTGCTTGCACTCTTTGGAC
GTCGACCGGCGCACGGGTGAGTAACGCGTATCCAACCTTCCCATTACTGTGGGATAACCTGCCGAAAGGCA
GACTAATATCGCATAGTCTTCGATGACGGCATCAGATTTGAAGTAAAGATTTATCGGTAATGGATGGGGAT
GCGTCTGATTAGCTTGTTGGCGGGGTAACGGCCCACCAAGGCGACGATCAGTAGGGGTTCTGAGAGGAAG
GTCCCCCACATTGGAACTGAGACACGGTC

>Zotu2322

GAACGCTGGCGGCGTGCTTAACACATGCAAGTCGAACGGTGAAGCCAAGCTTGCTTGGTGGATCAGTGGC
GAACGGGTGAGTAACACGTGAGCAACCTGCCCTGGACTCTGGGATAAGCGCTGGAAACGGCGTCTAATAC
TGGATATGAGCCCCTATCGCATGGTGGGGGTTGGAAAGATTTTTTGGTCTGGGATGGGCTCGCGGCCTATC

AGCTTGTTGGTGAGGTAATGGCTCACCAAGGCGTCGACGGGTAGCCGGCCTGAGAGGGTGACCGGCCAC
ACTGGGACTGAGACACGGCC
>Zotu2324
GAACGCTGGCGGCGTGCCTAATACATGCAAGTCGAGCGAATCAAAGGGAGCTTGCTCCCTGAGGTTAGCG
GCGGACGGGTGAGTAACACGTGGGCAACCTGCCTATAAGACTGGGATAACTTCGGGAAACCGGAGCTAAT
ACCGGATAATTCATATCTACTCATGTAGGTATGCTGAAAGACGGTTTACGCTGTCACTTATAGATGGGCCCG
CGGCGCATTAGCTAGTTGGTGAGGTAACGGCTCACCAAGGCAACGATGCGTAGCCGACCTGAGAGGGTGA
TCGGCCACACTGGGACTGAGACACGGCC
>Zotu2326
GAACGCTGGCGGCGTGCTTAACACATGCAAGTCGAGCGATGAAGTTTCCTTCGGGAAACGGATTAGCGGC
GGACGGGTGAGTAACACGTGGGTAACCTGCCTCATAGAGTGGAATAGCCTTCCGAAAGGAAGATTAATAC
CGCATAACGTTGAAAGATGGCATCATCATTTAACCAAAGGAGTAATCCGCTATGAGATGGACCCGCGGCGC
ATTAGCTAGTTGGTGGGGTAACGGCCTACCAAGGCGACGATGCGTAGCCGACCTGAGAGGGTGATCGGCC
ACATTGGGACTGAGACACGGCC
>Zotu2329
GAACGCTAGCGGGAGGCCTAACACATGCAAGCCGAGCGGTAGGGTTTCTTCGGAAACCTGAGAGCGGCG
CACGGGTGCGGAACACGTGTGCAACCTACCTTTATCAGGGGGATAGCCTTTCGAAAGGAAGATTAATACCC
CATAATATATTAGATGGCATCATTTGATATTGAAAACTCCGGTGGATAGAGATGGGCACGCGCAAGATTAG
ATAGTTGGTGAGGTAACGGCTCACAAAGTCAGTGATCTTTAGGGGGCCTGAGAGGGTGATCCCCCACACT
GGTACTGAGACACGGAC
>Zotu2331
CGCAAGCCTTGGGGAACTGTGCAGCTGGTTTGATCGGGAACGCGAGTACCGCCCAGGTAGCTCAGGCTAG
TGTCATGACCGACTGTCCAGCACGAGAACAGCTCAACGGCATCTTCTTCCTTCGCCGCCCCACAATATACGC
GCGTAATCGGCCGCGCACTTCAAATAGCATAAATTCCTATCACAATA
>Zotu2334
GAACGCTGGCGGCGTGCCTAATACATGCAAGTCGAACGCATTTTCTTTCACCGTAGCTTGCTACACCGAAA
GAAAATGAGTGGCGAACGGGTGAGTAACACGTGGGTAACCTGCCCATCAGCGGGGGATAACACTTGGAA
ACAGGTGCTAATACCGCATAATTCCATTTACCGCATGGTAGATGGATGAAAGGCGCTTTTGCGTCACTGAT
GGATGGACCCGCGGTGCATTAGCTAGTTGGTGGGGTAACGGCCTACCAAGGCTGCGATGCATAGCCGACC
TGAGAGGGTGATCGGCCACACTGGGACTGAGACACGGCC
>Zotu2336
GAACGCTGGCGGCATGCCTAATACATGCAAGTCGAACGAGACCTTCGGGTCTAGTGGCGCACGGGTGCGT
AACGCGTGGGAATCTGCCCTTGGGTTCGGAATAACAGTTAGAAATGACTGCTAATACCGGATGATGACGTA
AGTCCAAAGATTTATCGCCCAAGGATGAGCCCGCGTAGGATTAGCTAGTTGGTGAGGTAAAGGCTCACCA
AGGCTACGATCCTTAGCTGGTCTGAGAGGATGATCAGCCACACTGGGACTGAGACACGGCC
>Zotu2339
GAACGCTGGCGGCGTGCCTAACACATGCAAGTCGAGCGAGAGACTTCGGTCTCTAGCGGCGGACGGGTG
AGTAACACGTGGGTAACCTGCCTCAAACAGGGGGATAGCCTTCCGAAAGGAAGATTAATACCGCATAACG
TATATCTGGCGCATGCCAGATATATCAAAGGAGTAATCCGGTTTGAGATGGACCCGCGGCGCATTAGCTAG
TTGGTGAGGTAACGGCTCACCAAGGCGACGATGCGTAGCCGACCTGAGAGGGTGATCGGCCACATTGGAA
CTGAGATACGGTC
>Zotu2341
GAACGCTGGCGGCGTGCTTAACACATGCAAGTCGAGCGGTAAGGCCCTTCGGGGTACACGAGCGGCGAA
CGGGTGAGTAACACGTGGGTGACCTGCCCTCCACTTCGGGATAAGCCTGGGAAACTGGGTCTAATACCGG
ATAGGACCTCCTGGTGCATGCCGGGTGGTGGAAAGTTTTTCGGTGGGGGATGGGCCCGCGGTCTATCAGC
TTGTTGGTGGGGTGATGGCCTACCAAGGCGACGACGGATAGCCGGCCTGAGAGGGTGATCGGCCACACT
GGGACTGAGACACGGCC
>Zotu2343
GAACGCTGGCGGCAGGCTTAACACATGCAAGTCGAACGATGACTAACTAGCTTGCTAGTTATGATTAGTGG
CGGACGGGTGAGTAACATTTAGGAATCTGCCTAGTAGTGGGGGATAGCTCGGGGAAACTCGAATTAATAC

CGCATACGACCTACGGGTGAAAGGGGGCGCAAGCTCTTGCTATTAGATGAGCCTAAATCAGATTAGCTAGT
TGGTGGGGTAAAGGCCCACCAAGGCGACGATCTGTAACTGGTCTGAGAGGATGATCAGTCACACCGGAAC
TGAGACACGGTC
>Zotu2345
GAACGCTAGCGGCAGGCCTAACACATGCAAGTCGAGGGGCAGCACGATTGAGCTTGCTTAATTGGTGGCG
ACCGGCGCACGGGTGCGTAACGCGTATGCAACCTACCTCATACTGGGGTATAGCCCGAAGAAATTCGGATT
AACCCCCCATAGTATTATAGAATGGCATCATTTTATAATTAAAGGTTACGGTATGAGATGGGCATGCGTCCC
ATTAGCTAGATGGTGAGGTAACGGCTCACCATGGCAACGATGGGTAGGGGGCCTGAGAGGGTGGTCCCC
CACACTGGTACTGAGACACGGAC
>Zotu2348
GAACGCTGGCGGCGTGCCTAACACATGTAAGTCGAACGGGGCTGGGGGGGCAACTCTCCAGTTCAGTGGC
AGACGGGTGCGTAACACGTGAGAAACTTGTCCATCGGTGGGGGATAGCCGGCCCAACGGCCGGTTAATAC
CGCGTACGCTCGTTTGGGGGCATCCCTGAACGATGAAAGCATCGCAAGATGCGCCGGCGGAGAGTCTCGC
GGCCTATCAGCTAGTTGGCGGGGTAACGGCCCACTAAGGCGACGACGGGTAGCTGGTCTGAGAGGATGG
CCAGCCACATTGGGACTGAGACACGGCC
>Zotu2350
GAACGCTGGCGGCGTGCCTAAGACATGCAAGTCGAATGCTCCGCAAGGGGCATGGCGAACGGGTGAGTA
ATACCTAGGAACTTTCTTTAAGTGGAGCATAACCAGTCGAAAGATTGGATAATACTCCATGTGGCAGTAAT
GCTAAAGTCGCAAGACGCTTAAAGTGAGGCCTGGGCACCATCAGCTAGTTGGTAAGGTAATGGCTTACCA
AGGCTATGACGGTTAGGGGCCCCGAGAGGGGAAACCCCCACACTGGAACTGCGACACGGTC
>Zotu2352
GAACGCTAGCGGGAGGCCTAACACATGCAAGCCGAGCGGTATTACTTCTTCGGAGGTGAGAGAGCGGCGT
ACGGGTGCGGAACACGTGTGCAACCTGCCCTTATCAAGGGGATAGCCCAGAGAAATTTGGATTAATACCCT
ATAACATATAGAGTGGCATCACTTTATATTAAAAACTACGGTGGATAAGGATGGGCACGCGCAAGATTAGC
TAGTTGGTGAGGTAACGGCTCACCAAGGCGACGATCTTTAGGGGGCCTGAGAGGGTGATCCCCCACACTG
GGACTGAGACACGGCC
>Zotu2355
GAACGCTGGCGGCGTGCCTAATACATGCAAGTCGAGCGAACTCTTTAAAAGCTTGCTTTTGAAGAGTTAGC
GGCGGACGGGTGAGTAACACGTGGGCAACCTGCCTATAAGACTGGGATAACTTCGGGAAACCGGAGCTA
ATACCAGATAATTCTTTTCTACACATGTAGGAAAGCTGAAAGACGGTTTACGCTGTCACTTATAGATGGGCC
CGCGGCGCATTAGCTAGTTGGTGAGGTAACGGCTCACCAAGGCCACGATGCGTAGCCGACCTGAGAGGGT
GATCGGCCACACTGGGACTGAGACACGGCC
>Zotu2358
GAACGCTGGCGGCGTGCTTAACACATGCAAGTCGAACGATGAAGCCCAGCTTGCTGGGTGGATTAGTGGC
GAACGGGTGAGTAACACGTGAGCAACCTGCCCCTGACTCTGGGATAAGCGCTGGAAACGGCGTCTAATAC
TGGATACGAACAAGAATCGCATGGTTACTTGTTGGAAAGATTTTTTGGTTGGGGATGGGCTCGCGGCCTAT
CAGCTTGTTGGTGAGGTAATGGCTCACCAAGGCGTCGACGGGTAGCCGGCCTGAGAGGGTGACCGGCCAC
ACTGGGACTGAGACACGGCC
>Zotu2360
GAACGCTGGCGGCAGGCTTAACACATGCAAGTCGAGCGCCCCGCAAGGGGAGCGGCAGACGGGTGAGTA
ACGCGTGGGAATCTACCTTTTGCTACGGAATAACTCAGGGAAACTTGTGCTAATACCGTATGTGCCCTTCGG
GGGAAAGATTTATCGGCAAAGGATGAGCCCGCGTTGGATTAGCTAGTTGGTGAGGTAAAGGCTCACCAAG
GCGACGATCCATAGCTGGTCTGAGAGGATGATCAGCCACACTGGGACTGAGACACGGCC
>Zotu2364
GAACGCTGGCGGCAGGCTTAACACATGCAAGTCGAACGCATCCTTCGGGGTGAGTGGCAGACGGGTGAG
TAACGCGTGGGAACGTGCCCTTCAGTTCGGAATAACCCAGGGAAACTTGGGCTAATACCGGATACGTGCG
AGAGCAGAAAGATTTATCGCTGAAGGATCGGCCCGCGTCTGATTAGCTAGTTGGTAGGGTAATGGCCTAC
CAAGGCGACGATCAGTAGCTGGTCTGAGAGGATGATCAGCCACATTGGGACTGAGACACGGCC
>Zotu2366

GAACGCTGGCGGCGTGCCTAATACATGCAAGTCGAGCGGAGTTGATGAGGTGCTTGCACCTCTGATGCTTA
GCGGCGGACGGGTGAGTAACACGTAGGTAACCTGCCTGTAAGACTGGGATAACATTCGGAAACGAATGCT
AATACCGGATACGCGATTTTCTCGCATGAGAGAACCGGGAAAGACGGAGCAATCTGTCACTTACGGATGG
ACCTGCGGCGCATTAGCTAGTTGGTGAGGTAACGGCTCACCAAGGCGACGATGCGTAGCCGACCTGAGAG
GGTGATCGGCCACACTGGGACTGAGACACGGCC

>Zotu2368

GAACGCTGGCGGCAGGCCTCAAACATGCAAGTCTAGGGGGTGTAGCAATACATCACCGGCGGACGGGTG
CGTAACACGTGGGAATATACCTTTCGGTACGGAACAACTGCGGGAAACTGCAGCTAATACCGTATACGTCC
TTGCGGAGAAAGATTTATCGCCGAAGGATTAGCCCGCGTTCGATTAGCTAGTTGGTGAGGTAATGGCTCAC
CAAGGCGACGATCGATAGCTGTTCTGAGAGGAAGATCAGCCACACTGGGACTGAGACACGGCC

>Zotu2370

GAACGCTGGCGACATGCCTAATACATGCAAGTCGAACGCTGAAGCACTCTGCTTGCAGAGTGCGGATGAG
TGGCGAACGGGTGAGTAACACGTGGGGAATCTGCCTATAAGTGGGGGATAACATTCGGAAACGGATGCT
AATACCGCATATATTCTTGGACCGCCTGGTCCTTGAAAGAAAGACGGCTTTGCTGTCGCTTATAGATGATCC
CGCGGCGTATTAGTTAGTTGGTGGGGTAAAGGCCTACCAAGACAATGATACGTAGCCGACCTGAGAGGGC
GATCGGCCACACTGGGACTGAGACACGGCC

>Zotu2372

CAACGCTGGCGGCGTGCCTAATGCATGCAAGTCGGACGGGCTTGATCTTTGGATCGAGTCACGTGGCGGA
CGGGTGCGGAGCACGTGGGCAAGCTGCCCCGGGGCGGGGGATAGCCGGCGGAAACGCCGGGCAATTCC
GCGTACGTTGGCCGGTCGGTGGGACTGGTCAGGAAAGGGTCTTGGGACTCACCCTGGGAGGCGCCTGCG
GCCGATTAGCTAGTTGGGGGGGTAACGGCTTCCCAAGGCGATGATCGGTAGCTGGTCTGAGAGGACGGCC
AGCCACACGGGGACTGAGACACGGCC

>Zotu2374

GAATGCTGGCTGTATGGATAAGGCATGCAAGTCGAACGGTCCGCAAGGACAGTGGCAGACGAGGTAGTA
ACACGTAAGTACGTACCCTGTAGTCTGGCATAGCTCGTCGAAAGACGGGGTAATACCGGATGGTCCCGTAA
GGGTAAAGTTTTTCGCTACAGGAGCGGCTTGCGTAATATCAGCTTGTTGGTGAGGTAATGGCTCACCAAGG
CTACGACGTTTAGGGGAGCTGAGAGGCTGACCCCCACCGATGGGACTGAGACACGGCC

>Zotu2376

GAACGCTGGCGGCAGGCTTAACACATGCAAGTCGAACGGTTGTAGCAATACAGCAGTGGCGCACGGGTG
AGTAACGCGTGGGAACGTACCTCTTAGTTCGGAATAACTATTGGAAACGATAGCTAATACCGGATACTCCC
TTCGGGGGAAAGATTTATCGCTGAGAGATCGGCCCGCGTAAGATTAGGTAGTTGGTAGGGTAATGGCCTA
CCAAGCCTACGATCTTTAGCTGGTCTGAGAGGATGATCAGCCACACTGGAACTGAGACACGGTC

>Zotu2378

GAACGCTGGCGGCGTGCTTAACACATGCAAGTCGAACGGTGAAGCCAAGCTTGCTTGGTGGATCAGTGGC
GAACGGGTGAGTAACACGTGAGCAACCTGCCCTGGACTCTGGGATAAGCGCTGGAAACGGCGTCTAATAC
TGGATATGAGACGTGATCGCATGGTCGTGTTTGGAAAGATTTTTCGGTCTGGGATGGGCTCGCGGCCTATC
AGCTTGTTGGTGAGGTAATGGCTCACCAAGGCGTCGACGGGTAGCCGGCCTGAGAGGGTGACCGGCCAC
ACTGGGACTGAGACACGGCC

>Zotu2380

GAACGCTGGCGGCGTGCTTAACACATGCAAGTCGAACGGGAAGCTTTGACAAGTTTTACTTTGATGAGTGG
ACAGTGGCGAACGGGTGAGTAACGCGTAGGTAACCAACCTTATGCTGGGGGATAGCCTTTGGAAACGAAG
AGTAATACCGCATAAGACCACACTGCCGCATGGCAGAGGGGTAAAAGATTTATTGGCATGAGACGGACCT
GCGTACCATTAGATAGTTGGTGGGGTAACGGCCTACCAAGTCGACGATGGTTAGCCGACCTGAGAGGGTG
ATCGGCCACATTGGGACTGAGACACGGCC

>Zotu2384

GAACGCTGGCGGCGTGCTTAACACATGCAAGTCGAACGGTGAAGCAGGAGCTTGCTCCTGTGGATCAGTG
GCGAACGGGTGAGTAACACGTGAGCAATCTGCCCTGGACTCTGGGATAAGCGTTGGAAACGACGTCTAAT
ACCGGATACGAGACGCGAAGGCATCTTCAGCGTCTGGAAAGAACTTCGGTCCAGGATGAGCTCGCGGCCT
ATCAGCTAGTTGGTGAGGTAATGGCTCACCAAGGCGACGACGGGTAGCCGGCCTGAGAGGGTGACCGGC
CACACTGGGACTGAGACACGGCC

>Zotu2387
GAACGCTGGCGGTGTGCTTCACACATGCAAGTCGAACGAGGTAGCAATACCTAGTGGCGGACGGGTGAGT
AACGCGTGGGAATCTGCCTTTAGATGGGGGATAACGGGTCGAAAGACTCGCTAATACCGCATATGACGAA
AGTTGAAAGGAGTAATCCGTCTAAAGATGAGCCCGCGTCCGATTAGCTAGTTGGTAGAGTAAAAGCCTACC
AAGGCGACGATCGGTAGCTGGTCTGAGAGGATGATCAGCCACAATGGGACTGAGACACGGCC
>Zotu2392
TAACGCTAGCGGCAGGCCTAATACATGCAAGTCGAGGGGTAACAGGGTAGCAATACCGCTGACGACCGGC
GCACGGGTGAGTAACACGTATGTAACCTACCTATAACTGGGGAATAGCCTTCCGAAAGGAAGATTAACACC
GCATAATATCACGAGACGGCATCGTTTTGTGATCAAACCTCCGGGGGTTATAGATGGACATGCGTAACATT
AGCTAGTTGGTGAGGTAACGGCTCACCAAGGCTACGATGTTTAGGGGGTCTGAGAGGATGATCCCCCACA
CTGGTACTGAGACACGGAC
>Zotu2395
GAACGCTAGCGGCAGGCCTAACACATGCAAGTCGAGGGGCATCAGAGGTAGCAATACTTGCTGGCGACCG
GCAAACGGGTGCGTAACACTTACGCAATCTTCCCCTAACTGGAGGATAGCCTCTCGAAAGAGAGATTAATA
CTCCGTAACATTAAGATGTGGCATCACATTTTAATTATAGCTCCGGCGGTTAGGGATGAGCGTGAGGCTGA
TTAGATAGTTGGTGAGGTAACGGCTCACCAAGTCAACGATCAGTAGCTGATGTGAGAGCATGATCAGCCA
CACGGGCACTGAGACACGGGC
>Zotu2397
GAACGCTGGCGGCATGCCTAACACATGCAAGTCGAACGAAGCCTTCGGGCTTAGTGGCGCACGGGTGCGT
AACGCGTGGGAATCTGCCCCTCGGTTCGGAATAACAGTTAGAAATGACTGCTAATACCGGATGATGACGA
AAGTCCAAAGATTTATCGCCGAGGGATGAGCCCGCGTAGGATTAGCTAGTTGGTGAGGTAATGGCTCACC
AAGGCGACGATCCTTAGCTGGTCTGAGAGGATGATCAGCCACACTGGGACTGAGACACGGCC
>Zotu2399
GAACGCTGGCGGCGTGCTTAACACATGCAAGTCGAACGAAGCGCTTTGTTTTGATTCCTTCGGGATGAAGA
ACTTAGTGACTGAGTGGCGGACGGGTGAGTAACGCGTGGGTAACCTGCCTCATACAGGGGGATAACAGCT
GGAAACGGTTGCTAATACCGCATAACCCGCTAGGGCCACATGGCCCGGACGGAAAAGATTTTATCGGTAT
GAGATGGACCCGCGTCTGATTAGCTAGTTGGAGGGGTAACGGCCCACCAAGGCGACGATCAGTAGCCGAC
TTGAGAGAGTGATCGGCCACATTGGGACTGAGACACGGCC
>Zotu2402
TAACGCTGGCGGCGTGCCTAATACATGCAAGTCGAACGCGAAAGTCCTTCGGGATGAGTAGAGTGGCGCA
CGGGTGAGTAACACGTGGGTAATCTGCCTTAAGATGGGGAATAACCCCTCGAAAGAGGGGCTAATACCGC
ATAACATCGCAGCTACTGTGGTAGCAGTGATTAAACGTGACCTCTATTTATAAGTTACGGTCTTAAGATGAG
CCTGCGCACTATTAGCTAGTTGGTGGGGTAATGGCCTACCAAGGCTACGATAGTTAGCTGGTCTGAGAGGA
TGATCAGCCACACTGGAACTGAGACACGGTC
>Zotu2405
GAACGCTGGCGGCGTGCCTAACACATGCAAGTCGAGCGATCTTCTTCGGAAGAGAGCGGCGGACGGGTG
AGTAACGCGTGGGTAACCTGCCCTGTACACACGGATAACATACCGAAAGGTATGCTAATACGAGATAATAT
GCTTTTATCGCATGGTAGAAGTATCAAAGCTCTGGCGGTACAGGATGGACCCGCGTCTGATTAGCTAGTTG
GTAAGGTAACGGCTTACCAAGGCGACGATCAGTAGCCGACCTGAGAGGGTGATCGGCCACATTGGAACTG
AGACACGGTC
>Zotu2407
GAATGTTGGCGGTGCGTCTAATACATGCAAGTCGAGCGAGGCTTCGAGCCGAGCGGCGAACGGCTGAGTA
ACACGTTGGTAACTGCCCCATACTCAGGGATAACCATGAGAAATCATGGCTAATACCGGATGGTCCTGCAA
AGGTAAAGCTTTTGCGGTATGGGAGGTGCCTGCGGCCTATCAGTTTGTTGGTGAGGTAATGGCTCACCAAG
ACTATGACGGGTAACTGGTCTGAGAGGATGATCAGTCAGAGGGAAACTGAGACACGGTT
>Zotu2409
GAACGCTAGCTACAGGCTTAACACATGCAAGTCGAGGGGCAGCATGGTCTTAGCTTGCTAAGGCTGATGG
CGACCGGCGCACGGGTGAGTAACACGTATCCAACCTGCCGTCTACTCTTGGCCAGCCTTCTGAAAGGAAGA
TTAATCCAGGATGGGATCATGAGTTCACATGTCCGCATGATTAAAGGTATTTTCCGGTAGACGATGGGGAT

GCGTTCCATTAGATAGTAGGCGGGGTAACGGCCCACCTAGTCAACGATGGATAGGGGTTCTGAGAGGAAG
GTCCCCCACATTGGAACTGAGACACGGTC

>Zotu2411

GAACGCTGGCGGCGCGCTTAACACATGCAAGTCGAGCGAGAACCAGGGCTTCGGCCCTGGGGACAGCGG
CGAACGGGTGAGTAACACGTGGGTAATCAACCCTCGACATCGGGATAGCCCGGGGAAACCCGGATTAATA
CCGAATGCCCCGGCCGGCTCTCGGGCTGGACGGAAAAGGTAGCTTCGGCCTCCGGTCGGGGACGAGCCCG
CGGCGGATTAGCTTGTTGGCGGGGTAACGGCCCACCAAGGCGACGATCCGTAGCTGGTCTGAGAGGACG
ATCAGCCACACTGGGACTGAGACACGGCC

>Zotu2413

GAACGCTGGCGGCGTGCTTAACACATGCAAGTCGAACGGTGATGTCAGAGCTTGCTCTGGCGGATCAGTG
GCGAACGGGTGAGTAACACGTGAGTAACCTGCCCCCGACTCTGGGATAACTGCTAGAAATGGTAGCTAAT
ACCGGATATGACGATTGGCCGCATGGTCTGGTCGTGGAAAGAATTTCGGTTGGGGATGGACTCGCGGCCT
ATCAGGTTGTTGGTGAGGTAATGGCTCACCAAGCCTACGACGGGTAGCCGGCCTGAGAGGGTGACCGGCC
ACACTGGGACTGAGACACGGCC

>Zotu2417

GAACGCTGGCGGCGTGCCTAATACATGCAAGTGGGACGCAAGGGAACACACTGTGCTTGCACACCGTGTT
TTCTTGAGTCGCGAACGGGTGAGTAACGCGTAGGTAACCTGCCTATTAGCGGGGGATAACTATTGGAAAC
GATAGCTAATACCGCATAATATTAATTATTGCATGATAATTGATTGAAAGATGCAAGCGCATCACTAGTAGA
TGGACCTGCGTTGTATTAGCTAGTTGGTAAGGTAAGAGCTTACCAAGGCGACGATACATAGCCGACCTGAG
AGGGTGATCGGCCACACTGGGACTGAGACACGGCC

>Zotu2419

GAACGCTGGCGGCGTGCTTAACACATGCAAGTCGAACGATGAAGGGGGAGCTTGCTCCTCCGGATTAGTG
GCGAACGGGTGAGTAATACGTGAGCAATGTGCCCTTGACTCTGGGATAAGCGCTGGAAACGGCGTCTAAT
ACCGGATACGACCATCGGAGGCATCTCCTGGTGGTGGAAAGAACTTCGGTCAAGGATCAGCTCACGGCCT
ATCAGCTTGTTGGTGAGGTAATGGCTCACCAAGGCGACGACGGGTAGCCGGCCTGAGAGGGTGACCGGC
CACACTGGGACTGAGACACGGCC

>Zotu2422

GAACGCTGGCGGCGTGCTTAACACATGCAAGTCGAACGGTGAAGGGACCGGTTTTTGCTGGTTCTGGATG
AGTGGCGAACGGGTGAGTAACACGTGAGTAACCTGCCCCCTTCTTCTGGATAACCGCATGAAAGTGTGGCT
AATACGGGATATTCTGCGCCTGCTGCATGGTGGGTGTGGGAAAGATATTGTTTCGGTGGGGGATGGGCTC
GCGGCCTATCAGCTTGTTGGTGGGGTGATGGCCTACCAAGGCGGTGACGGGTAGCCGGCCTGAGAGGGT
GGACGGTCACACTGGGACTGAGACACGGCC

>Zotu2424

GAACGCTGGCGGCGTGCTTAACACATGCAAGTCGAACGATGAAGCCCAGCTTGCTGGGTGGATTAGTGGC
GAACGGGTGAGTAACACGTGAGTAACCTGCCCCTGACTCTGGGATAAGCACTGGAAACGGTGTCTAATAC
CGGATACGAACTTTGACCGCATGGTCAGGAGTTGGAAAGAATTTTGGTCTGGGATGGACTCGCGGCCTATC
AGCTTGTTGGTGAGGTAATGGCTCACCAAGGCGACGACGGGTAGCCGGCCTGAGAGGGTGACCGGCCAC
ACTGGGACTGAGACACGGCC

>Zotu2426

GAACGCTAGCGGCAGGCTTAACACATGCAAGTCGAGGGGCAGCAGGGATTTCGGTCCGCTGGCGACCGG
CAAACGGGTGCGGAACACGTACGCAACATACCTCTATCTGGGGCATAGCCTTCTGAAAAGAAGATTAACAC
CCCATAAGATCATAGAATGGCATCATTTAGTGATTAAAAATTTATTGGATAGAGATTGGCGTGCGTCCTATT
AGCTAGTTGGTGCGGGTAAAGGCCCACCAAGGCGACGATAGGTAGCTGGTGTGAGAGCACGACCAGCCA
CACGGGCACTGAGACACGGGC

>Zotu2429

GAACGCTGGCGGCGTGCCTAACACATGCAAGTCGAACGAAGAGGCCCCTTCGGGGGTACTTCAGTGGCGG
ACGGGTGAGTACAACATAGGTGACCTGCCCCAAAGCGGGGGATAACAGTCCGAAAGGATTGCTAATACCC
CATGTGGTCCTTGAGATTTGAGGCTCAATGACTAAAGGAGTAATCCGCTTTGGGAGGGGCCTGTGGCCCAT
CAGCTTGTTGGTAGGGTAATGGCCTACCAAGGCTACGACGGGTAGGGGGCCTGAGAGGGTGACCCCCCAC
ATTGGCACTGACACACGGGC

>Zotu2431
GAACGCTAGCGGCAGGCCTAACACATGCAAGTCGAGGGAGGAGCTTTCGGGCTTCGACCGGCGTACGGG
TGCGTAACGCGTATACAATCTGCCTTTCACATAGGGATAGCCCGAAGAAATTTGGATTAATACCTAATGGTA
TATAGATGTGGCATCACATTTATATTAAAGCTGAGGCGGTGAAAGATGAGTATGCGTTCTATTAGCTAGTT
GGAGAGGTAACGGCTCCCCAAGGCTACGATAGATAGGGGTTCTGAGAGGGATATCCCCCACACTGGTACT
GAGATACGGAC
>Zotu2433
GAACGCTGGCGGCGTGCTTAACACATGCAAGTCGAGCGGTAAGGCCTTTCGGGGTACACGAGCGGCGAAC
GGGTGAGTAACACGTGGGTGATCTGCCCTGCACTTCGGGATAAGCCTGGGAAACTGGGTCTAATACCGGA
TATGACCTCAGGTTGCATGACTTGGGGTGGAAAGATTTATCGGTGCAGGATGGGCCCGCGGCCTATCAGCT
TGTTGGTGGGGTAATGGCCTACCAAGGCGACGACGGGTAGCCGACCTGAGAGGGTGACCGGCCACACTG
GGACTGAGACACGGCC
>Zotu2435
GAACGCTGGCGGCGTGCCTAATACATGCAAGTCGAGCGAATCTTTGGGAGCTTGCTCCTAAAGATTAGCG
GCGGACGGGTGAGTAACACGTGGGCAACCTGCCTATAAGACTGGGATAACTTCGGGAAACCGGAGCTAAT
ACCGGATAATTCTTTTCTACTCATGTAGGAAAGCTAAAAGACGGTTTACGCTGTCACTTATAGATGGGCCCG
CGGCGCATTAGCTAGTTGGTGAGGTAACGGCTCACCAAGGCCACGATGCGTAGCCGACCTGAGAGGGTGA
TCGGCCACACTGGGACTGAGACACGGCC
>Zotu2437
GAACGCTGGCGGCGTGCTTAACACATGCAAGTGGAGCGACGAAGCGGGCTTCGGCCCGTGGCAGAGCCG
CGAACGGGTGAGTAACACGTGGGTAACGTGCCCCAATGACTGGGACAACCCGGGGAAACCCGGGCTAAT
ACCAGATGTGGTCTCTTCACATCAGTGAGGTGACTAAAGGTAGCTTCGGCTTCCGCATTGGGATCGGCCCG
CGGCGCATTAGCTAGTTGGTGAGGTAAAGGCTTACCAAGGCAACGATGCGTAGCTGGTCTGAGAGGACGA
TCAGCCACACTGGGACTGAGACACGGCC
>Zotu2440
GAACGCTTGCGATGTGTCTAAGGCATGCAAGTCGAATGCTAGTAGCAATACTGGCATGGCAAACGGGAGA
GTAACACATTGGTAACTTACCTCGAAGACGGGCATAACCCGGAGAAATCCGGAGTAATTCCCGATGGTCGC
GGGGGGACACAAGTCATTCCCACGTAAAGTTTTTCGCTTCGAGAGAGACCTATGTTCTATCAGCTAGTTG
GTGAGGTAAAGGCTCACCAAGGCGACGACGGATAGCGGGTGTGAGAGCATGGCCCGCCTAACTGGGACT
GAGAAACTGCC
>Zotu2442
GAATGCTGGCGGCGTGGATAAGGCATGCAAGTCGAACGGTCCGCAAGGACAGTGGCAGACGAGGTAGTA
ACACGTAGGTACTCCCCCCGAAGTCTGGTATAGCTCGTCGAAAGACGGGGTAATCCCGGATGGTCCCGAA
AGGGTAAAGATTTATCGCTTCGGGAGAGGCCTGCGTAATATCAGCTTGTTGGTAAGGTAATGGCTTACCAA
GGCTACGACGTTTAGGGGAGCTGAGAGGCTGACCCCCACCGATGGGACTGAGACACGGCC
>Zotu2445
GAACGCTGGCGGAGTGCCTTAAGCATGCAAGTAGAACGAGTTCTATTGTGCCGCAAGGCACGATTGAACT
AGTTGCGAACGGCTGAGTAATCAATAGGAATCTTTCCTTTGGTGGGGAATACCTCGTCGAAAGACGAGCTA
ATACCGCATAAAATCTACGGATAAAAGCGCATAACTGCGCGCCGATTGAGGAGCCTATTGCCCATCAGGTA
GTTGGTAGGGTAATGGCCTACCAAGCCTACGACGGGTACCCGATCTGAGAAGATGACCGGGCAGAATGG
GACTGAGACACGGCC
>Zotu2447
GAACGCTGGCGGCGTGCCTAATACATGCAAGTCGAGCGGACCGAACGAGGGCTTGCTCTTGTTTGGTCAG
CGGCGGACGGGTGAGTAACACGTGGGCAACCTGCCCGCAAGACCGGGATAACTCCGGGAAACCGGAGCT
AATACCGGATAACACCGAAGACCGCATGGTCTTCGGTTGAAAGGCGGCCTTTGGGCTGTCACTTGCGGATG
GGCCCGCGGCGCATTAGCTAGTTGGTGAGGTAACGGCTCACCAAGGCGACGATGCGTAGCCGGCCTGAGA
GGGTGACCGGCCACACTGGGACTGAGACACGGCC
>Zotu2449
GAACGCTGACGGCGTGCTTAACACATGCAAGTCGAACGATGAAGCCCAGCTTGCTGGGTGGAAGAGTGGC
GAACGGGTGAGTAACACGTGAGTAACCTGCCCCGGACTCTGGGATAAGCGCTGGAAACGGCGTCTAATAC

TGGATATGACCCATACCGGCATCGGTTGTGGGTGGAAAGATTTATCGGTTCGGGATGGGCTCGCGGCCTAT
CAGCTGGATGGTGAGGTAACGGCTCACCATGGCGACGACGGGTAGCCGGCCTGAGAGGGTGACCGGCCA
CACTGGGACTGAGACACGGCC
>Zotu2451
GAACGCTGGCGGCAGGCCTAACACATGCAAGTCGAACGCCGTAGCAATACGGAGTGGCAGACGGGTGAG
TAACGCGTGGGAACCTACCTTACGGTCGGGAATAGCTCAGGGAAACTTGGGGTAATACCGGATACGCCCT
TACGGGGAAAGATTTATCGCCGTTAGATGGGCCCGCGTAGGATTAGCTAGTTGGTGAGGTAATGGCTCAC
CAAGGCGACGATCCTTAGCTGGTCTGAGAGGATGATCAGCCACACTGGGACTGAGACACGGCC
>Zotu2453
GAACGCTGGCGGCATGCCTAACACATGCAAGTCGAACGAGGGTAGCAATACTCTAGTGGCGGACGGGTG
AGGAATACGTAGGAATCTGCCCTTAAGTGGGGGATAACCCGGGGAAACCCGGATTAATACCGCATGATCT
CGAAAGAGCAAAGCAGGGGATCGCAAGACCTTGTGCTTTTGGAGGAGCCTACGTCGGATTAGCTAGTTGG
TGAGGTAAAGGCTCACCAAGGCAACGATCCGTAACTGGTCTGAGAGGATGATCAGTCACACCGGAACTGA
GACACGGTC
>Zotu2455
GAACGCTGGCGGCGTGCCTAACACATGCAAGTCGAACGAGGTCCAACCAATGGCAACATTGGGGAATACC
TAGTGGCGAACGGGTGAGTAACACGTGAGGAACCTGCCCCGAAGACCGGGATAACGCTCCGAAAGGAAC
GCTAATACCGGATACCTTCACCGGACCGCATGATCTGGTGAAGAAATGGATTCCGCTTCGGGAGGGTCTCG
CGGCCTATCAGCTTGTTGGTGGGGTAATGGCCCACCAAGGCTTCGACGGGTAGCTGGTCTGAGAGGACGA
TCAGCCACACTGGGACTGAGACACGGCC
>Zotu2458
GAACGCTAGCGGCAGGCTTAACACATGCAAGTCGAGGGGTAGAAGGAGCTTGCTCCTTTGAGACCGGCGC
ACGGGTGCGTAACGCGTATGCAATCTACCTTATACAGGGGAATAGCCCAGAGAAATTTGGATTAATGCCCC
ATGGTATTATAGAATGGCATCATTTTATAATTAAAGTTCCAACGGTATAAGATGAGCATGCGTCCCATTAGT
TAGTTGGTAAGGTAACGGCTTACCAAGACGATGATGGGTAGGGGTCCTGAGAGGGAGATCCTCCACACTG
GTACTGAGACACGGAC
>Zotu2460
GAACGCTGGCGGCGTGCTTAACACATGCAAGTCGAACGATGATGCCCAGCTTGCTGGGTTGATTAGTGGC
GAACGGGTGAGTAATACGTGAGTAACCTGCCCTTGACTCTGGGATAAGCCTGGGAAACCGGGTCTAATAC
TGGATGCGACGGGCCATCGCATGGTGGTTCGTGGAAAGGGTTTCTGGTCTTGGATGGGCTCACGGCCTAT
CAGCTGGTTGGTGGGGTAATGGCTCACCAAGGCGACGACGGGTAGCCGGCCTGAGAGGGTGACCGGCCA
CACTGGGACTGAGACACGGCC
>Zotu2462
GAACGCTGGCGGCGTGCTTAACACATGCAAGTCGCACGGGACTGGGGGCAACTCCAGTTCAGTGGCGGAC
GGGTGCGTAACACGTAAGCAACATGTCCAAGGGTGGGGGATAGCCGGCCCAACGGCCGGGTAATACCGC
ATGCAGTCACCGCTGGCATCTACGGTGACGAAAGCCGGGGCAACCTGGCGCCCATGGAGTGGCTTGCGGC
CTATCAGCTAGTTGGTGGGGTAACGGCTCACCAAGGCGACGACGGGTAGCTGGTCTGAGAGGATGACCAG
CCACATTGGGACTGAGACACGGCC
>Zotu2464
GAACGCTGGCGGCATGCCTAACACATGCAAGTCGAACGAAGCCTTCGGGCTTAGTGGCGCACGGGTGCGT
AACGCGTGGGAATCTGCCCTTGGGTTCGGAATAACAGTTAGAAATGACTGCTAATACCGGATGATGTCTTC
GGACCAAAGATTTATCGCCCAAGGATGAGCCCGCGTCGGATTAGCTAGTTGGTGAGGTAAAGGCTCACCA
AGGCGACGATCCGTAGCTGGTCTGAGAGGATGATCAGCCACACTGGGACTGAGACACGGCC
>Zotu2467
GAACGCTGGCGGCATGCCTAACACATGCAAGTCGAACGAGACCTTCGGGTCTAGTGGCGCACGGGTGCGT
AACGCGTGGGAACCTGCCCTTAGGTTCGGAATAACTCAGAGAAATTTGAGCTAATACCGGATAATGTCTTC
GGACCAAAGATTTATCGCCTTTGGATGGGCCCGCGTAGGATTAGCTTGTTGGTGGGGTAATGGCCTACCAA
GGCGACGATCCTTAGCTGGTCTGAGAGGATGATCAGCCACACTGGGACTGAGACACGGCC
>Zotu2469

GAACGCTGGCGGCGTGCCTAATACATGCAAGTCGAACGCACTGACAGAGAACTTGTTCTCTTGACGTGAGT
GGCGAACGGGTGAGTAACACGTGGGAAACCTACCTTCTAGCGGGGGATAACAGTCGGAAACGACTGCTA
ATACCGCATAGTGCTTTTCTTGGCATCAAGGAAAGAGGAAAGATGGTTCTGCTATCACTAGAAGATGGTCC
CGCGGTGCATTAGCTAGTTGGTAAGGTAACGGCTTACCAAGGCGATGATGCATAGCCGACCTGAGAGGGT
AATCGGCCACATTGGGACTGAGACACGGCC

>Zotu2471

GAACGCTGGCGGCGTGCCTAATACATGCAAGTCGAGCGGAGTTGATGAGGTGCTTGCACCTCTGATGCTTA
GCGGCGGACGGGTGAGTAACACGTAGGTAACCTGCCTGTAAGACTGGGATAACATTCGGAAACGAATGCT
AATACCGGATACGCGATCCTCTCGCATGAGGGGATCGGGAAAGACGGAGCAATCTGTCGCTTACGGATGG
ACCTGCGGCGCATTAGCTAGTTGGTGAGGTAACGGCTCACCAAGGCGACGATGCGTAGCCGACCTGAGAG
GGTGATCGGCCACACTGGGACTGAGACACGGCC

>Zotu2474

GAACGCTGGCGGCGTGCTTAACACATGCAAGTCGAACGGTGAAGCCCCTTCGGGGGTGGATCAGTGGCGA
ACGGGTGAGTAACACGTGGGCAACCTACCCTTGACTCTGGGATAACCCCGGGAAACCGGAGCTAATACCG
GATACGACGCGGATCGACATCCTTCCGCGTGGAAAGTTTTTCGGTCAGGGATGGGCCCGCGGCCTATCAGC
TTGTTGGTGGGGTAATGGCCTACCAAGGCGACGACGGGTAGCCGGCCTGAGAGGGCGACCGGCCACACT
GGGACTGAGACACGGCC

>Zotu2476

GAACGCTGGCGGCGTGCCTAATACATGCAAGTCGAGCGGCAGCGCGAATAGTTTACTATTTGGCGGCGAG
CGGCGGACGGCTGAGTAACACGTAGGAACGTACCCCAAAGTGAGGAATAACTGCCCGAAAGGGTGGCTA
ATGCCGCATGTGCTCTAAGGAGTAAAGCTTCGGCGCTTTGGGAGCGGCCTGCGTCTGATTAGTTTGTTGGT
GAGGTAATGGCTCACCAAGACTACGATCGGTAGCTGGTCTGAGAGGATGATCAGCCAGACTGGGACTGAG
ACACGGCC

>Zotu2478

AAACGCTGGCGGCGTACCTAAGACATGCAAGTCGAACGGGACTTTCCGCTTCGGCGGGGAGTCGAGTGGC
GGACGGGTGAGTAACGCGTAGGAACCTGTCGCACAGTGGGGGATAACACGGGGAAACCGGTGCTAATAC
CGCATGTGGTCAGAGATGACGAAAGCAGCAATGCGCTGTGTGAGGGGCCTGCGTCCGATTAGCTGGTTGG
TGGGGTAAAGGCCTACCAAGGCGACGATCGGTAGCTGGTCTGAGAGGATGGTCAGCCACACTGGGACTG
AGATACGGCC

>Zotu2480

GAACGCTGGCGGCGTGCTTAACACATGCAAGTCGAACGATGAAGCCGGAGCTTGCTCCGGCGGATTAGTG
GCGAACGGGTGAGTAACACGTGAGTAACCTGCCCCAGACTCTGGGATAAGCACTGGAAACGGTGTCTAAT
ACCGGATACGAGCTTCAGCCGCATGGCTAGGAGCTGGAAAGAATTTCGGTCTGGGATGGACTCGCGGCCT
ATCAGGTAGTTGGTGAGGTAAAGGCTCACCAAGCCGACGACGGGTAGCCGGCCTGAGAGGGTGACCGGC
CACACTGGGACTGAGACACGGCC

>Zotu2482

GAACGCTAGCGGCAGGCTTAACACATGCAAGTCGAGGGGTATAGTAGCAATACTAGAGACCGGCGCACG
GGTGCGTAACGCGTATGCAATCTACCTTTCACAGAGGGATAGCCCAGAGAAATTTGGATTAATACCTCATA
GTATTATGATGTGGCATCACATTATAATTAAAGTCACAACGGTGAAAGATGAGCATGCGTCCCATTAGCTT
GTTGGTAAGGTAACGGCTTACCAAGGCAACGATGGGTAGGGGTCCTGAGAGGGAGATCCCCCACACTGGT
ACTGAGACACGGAC

>Zotu2484

GAACGCTGGCGGCATGCCTAACACATGCAAGTCGAACGAGACCTTCGGGTCTAGTGGCGCACGGGTGCGT
AACGCGTGGGAATCTGCCCTTGGGTCCGGAATAACAGTTAGAAATGACTGCTAATACCGGATGATGTCTTC
GGACCAAAGATTTATCGCCCAGGGATGAGCCCGCGTAGGATTAGCTTGTTGGTGAGGTAAAGGCTCACCA
AGGCGACGATCCTTAGCTGGTCTGAGAGGATGATCAGCCACACTGGGACTGAGACACGGCC

>Zotu2486

GAACGCTGGCGGCGTGCTTAACACATGCAAGTCGAACGAGAAGCGGGAGCTTGCTCCCGCAGACAGTGGC
GAACGGGTGAGTAACACGTGAGTAACCTGCCCTTGACTCTGGGACAACTCTGGGAAACTGGAGCTAATAC
CGGATACGAGACGCTCAGGCATCTGAAGCGTCTGGAAAGAATTTCGGTCAAGGATGGACTCGCGGCCTAT

CAGCTTGTTGGCGGGGTAATGGCCCACCAAGGCGACGACGGGTAGCCGGCCTGAGAGGGCGACCGGCCA
CACTGGAACTGAGACACGGTC
>Zotu2488
GAACGCTGGCGGCGCGCCTAACACATGCAAGTCGAACGGAGCTTAGAGAGCTTGCTTTTTAAGCTTAGTG
GCGAACGGGTGAGTAACGCGTGAGTAACCTGCCCTAGAGTGGGGGACAACAGTTGGAAACGACTGCTAA
TACCGCATAAGCCCACGGTACCGCATGATACTGAGGGAAAAGGATTTATTCGCTTTAGGATGGACTCGCGT
CCAATTAGCTAGTTGGTGAGGTAACGGCTCACCAAGGCGACGATTGGTAGCCGGACTGAGAGGTTGAACG
GCCACATTGGGACTGAGACACGGCC
>Zotu2490
GAACGCTGGCGGCGTGCCTAATACATGCAAGTCGAGCGAACTTGCGGGAGCTTGCTCCCAAAAGTTAGCG
GCGGACGGGTGAGTAACACGTGGGCAACCTGCCTATAAGACTGGGATAACTTCGGGAAACCGGAGCTAAT
ACCAGATAATTCTTTTCTACACATGTAGAAAAGCTAAAAGACGGTTTACGCTGTCACTTATAGATGGCCCG
CGGCGCATTAGCTAGTTGGTGAGGTAACGGCTCACCAAGGCCACGATGCGTAGCCGACCTGAGAGGGTGA
TCGGCCACACTGGGACTGAGACACGGCC
>Zotu2492
GAACGCTGGCGGCGTGCCTAACACATGCAAGTCGAACGGAACTGTTTTGAAAGATTTCTTCGGAATGAATT
TGATTTAGTTTAGTGGCGGACGGGTGAGTAACGCGTGAGTAACCTGCCTTCAAGAGGGGGATAACATTCT
GAAAAGAATGCTAATACCGCATGACATATCGGAACCACATGGTTCTGGTATCAAAGATTTTATCGCTTGAA
GATGGACTCGCGTCCGATTAGTTAGTTGGTGAGGTAACGGCTCACCAAGACCGCGATCGGTAGCCGGACT
GAGAGGTTGAACGGCCACATTGGGACTGAGACACGGCC
>Zotu2494
GAACGCTAGCGGCAGGCCTAATACATGCAAGTCGAGGGGCAGCGGGGGTAGCAATACCTGCCGGCGACC
GGCAAACGGGTGCGGAACACGTACGCAACCTTCCTTTAAGCGGGGAATAGCCCAGAGAAATTTGGATTAA
TACCCCATAAGAATGTGGATTGGCATCAGTCAGCATTTAAAGATTTATCACTTAGAGATGGGCGTGCGTCT
GATTAGGTAGTTGGTGAGGTAACGGCTCACCAAGCCGACGATCAGTAACTGGCGTGAGAGCGCGACCAGT
CACACGGGCACTGAGACACGGGC
>Zotu2496
GAACGCTGGCGGCATGCCTAACACATGCAAGTCGAACGAAGGCTTCGGCCTTAGTGGCGCACGGGTGCGT
AACGCGTGGGAATCTGCCCCTCGGTTCGGAATAACAGTTAGAAATGACTGCTAATACCGGATAATGACGAA
AGTCCAAAGATTTATCGCCGAGGGATGAGCCCGCGTAGGATTAGGTAGTTGGTAGGGTAAAGGCCTACCA
AGCCGACGATCCTTAGCTGGTCTGAGAGGATGATCAGCCACACTGGGACTGAGACACGGCC
>Zotu2498
CAACGCTGGCGGCGTGCCTAACACATGCAAGTCGAACGCGGTCACCCCGCAAGGGGTGACTGAGTGGCAA
ACGGGTGAGTAACACGTGGGTGACCTGCCTCTGAGTGGGGGATAACGTTTCGAAAGGAACGCTAATACCG
CATGACATCCTGCCTTTGGACAGGCGGAGATCAAAGCCGGGGATCGCAAGACCTGGCGCTTGGAGAGGG
GCCCGCGTCCGATTAGCTAGTTGGTGAGGTAACGGCTCACCAAGGCGACGATCGGTATCCGGCCTGAGAG
GGCGGACGGACACACTGGGACTGAGACACGGCC
>Zotu2500
GAACGCTGGCGGCGTGCCTAAGACATGCAAGTCGAGTGGGTTTAGACCCACGGCAAACGGGTGAGTAATA
CCTAGGAACTTTCTTTAAGTGGAGCATAACCAGCCGAAAGGTTGGATAATACTCCATACCATCGCAAGATG
AAAGTCGCAAGACGCTTAAAGTGAGGCCTGGGCACCATCAGCTAGTTGGTAAGGTAATGGCTTACCAAGG
CTATGACGGTTAGGGGCCCCGAGAGGGGAGTCCCCCACACTGGAACTGCGACACGGTC
>Zotu2502
GAACGCTGGCGGCATGCCTTACACATGCAAGTCGAACGGCAGCACGGGGGCAACCCTGGTGGCGAGTGG
CGAACGGGTGAGTAACATATCGGAACGTGCCCTGGAGTGGGGGATAACTAGTCGAAAGACTAGCTAATAC
CGCATACGATCTACGGATGAAAGTGGGGGATCGCAAGACCTCATGCTCGTGGAGCGGCCGATATCTGATT
AGCTAGTTGGTGGGGTAAAGGCCTACCAAGGCATCGATCAGTAGCTGGTCTGAGAGGACGACCAGCCACA
CTGGGACTGAGACACGGCC
>Zotu2505

GAACGCTGGCGGTATGTCTAACACATGCAAGTCGAACGAGATCTTTTTTTGTTTACAAGAAAAAGATTGC
AGTGGCGGACGGGTGAGGAACACGTAAGAACTTACCTTTTGGCGGGGGATAACATCGGGAAACTGTTGCT
AATACCCCATAATGCTGAGGAGCGAAAGGGAAACTGCCAAAAGAGAGGCTTGCGTCTGATTAGCTAGTTG
GTGGAGGTAAATGCTCCCCAAGGCGACGATCAGTAGCTGGTCTGAGAGGATGATCAGCCACACTGGGACT
GAGACACGGCC
>Zotu2507
GAACGCTAGCGGGAGGCCTAACACATGCAAGCCGAGCGGTAGAAGTCCTTCGGGACTTTGAGAGCGGCG
TACGGGTGCGGAACACGTGTGCAACCTGCCTTTATCAGGGGGATAGCCTTTCGAAAGGAAGATTAATACCC
CATAATATAATGAACGGCATCGTTTATTATTGAAAACTCCGGTGGATAGAGATGGGCACGCGCAAGATTAG
ATAGTTGGTAGGGTAACGGCCTACCAAGTCAGTGATCTTTAGGGGGCCTGAGAGGGTGATCCCCCACACT
GGTACTGAGACACGGAC
>Zotu2509
GAACGCTAGCGGCATGCTTAACACATGCAAGTCGGACGGGAAGTGGTGTTTCCAGTGGCGGACGGGTGA
GTAACGCGTAAGAACCTGCCCTTGGGAGGGGAACAACAACTGGAAACGGTTGCTAATACCCCGTAGGCTG
AGGAGCAAAAGGAGGAATCCGCCCGAGGAGGGGCTCGCGTCTGATTAGCTAGTTGGTGAGGCAATAGCT
TACCAAGGCGATGATCAGTAGCTGGTCCGAGAGGATGATCAGCCACACTGGGACTGAGACACGGCC
>Zotu2511
GAACGCTGGCGGCGTGCCTAACACATGCAAGTCGAACGAGGTCCATGCAGTGGCAACACTGCGGAAGACC
TAGTGGCGAACGGGTGAGTAACACGTGAGGAACTTGCCCCGGAGACCGAGATAACCTTCCGAAAGGATG
GCTAATACCGGATATCGTCACCGAGCCGCATGGCTTGGTGACGAAATGGATTCCGCTCCGGGAGAGCCTC
GCGGCCTATCAGCTAGTTGGTGGGGTAACGGCCCACCAAGGCATCGACGGGTAGCTGGTCTGAGAGGAC
GATCAGCCACACTGGGACTGAGACACGGCC
>Zotu2513
GAACGCTGGCGGCGTGCCTAAGACATGCAAGTCGAGTGGGTTTAGACCCACGGCGAACGGGTGAGTAAT
ACCTAGGAACTTTCTTTAAGTGGAGCATAACCCATAGAAATGTGGGATAATTCTCCATGTGCCCCGGAGTTA
CATCCGGGGCAAAGTCGCAAGACGCTTAAAGTGAGGCCTGGGCACCATCAGCTAGTTGGTAAGGTAATGG
CTTACCAAGGCTATGACGGTTAGCGGTCCCGAGAGGGAGAACCGCCACACTGGAACTGCGACACGGTC
>Zotu2515
GAACGCTGGCGGCGTGCTTAACACATGCAAGTCGAGCGACAGCACTCTTCGGGGTGCATGGAGCGGCGAA
CGGGTGAGTAACACGTGGGCAACCTGCCCTCAGCTCTGGGACAACTCCGGGAAACCGGGGCTAATACCGG
ATACGACCTTCCCTCGCATGGGGGTCGGTGGAAAGTTTTTCGGCTGAGGATGGGCCCGCGGCCTATCAGCT
TGTTGGTGGGGTGATGGCCTACCAAGGCGACGACGGGTAGCCGGCCTGAGAGGGCGACCGGCCACACTG
GGACTGAGACACGGCC
>Zotu2517
GAACGCTGGCGGCGTGCTTAACACATGCAAGTCGAACGGAAAGGCCCTTCGGGGTACTCGAGTGGCGAAC
GGGTGAGTAACACGTGGGTGATCTGCCCTGCACTTCGGGATAAGCCTGGGAAACTGGGTCTAATACCGGA
TATACCTCATGGATTGCATGGTTTGTGGGGGAAAGCTTTTGCGGTGTGGGATGGGCCCGCGGCCTATCAGC
TTGTTGGTGGGGTGATGGCCTACCAAGGCGTCGACGGGTAGCCGGCCTGAGAGGGTGTCCGGCCACACTG
GGACTGAGATACGGCC
>Zotu2519
GAACGCTGGCGGCATGCCTAACACATGCAAGTCGAACGAGACCTTCGGGTCTAGTGGCGCACGGGTGCGT
AACGCGTGGGAATCTGCCCTTGGGTTCGGAATAACAGTGAGAAATTACTGCTAATACCGGATGATGTCTTC
GGACCAAAGATTTATCGCCCAGGGATGAGCCCGCGTAGGATTAGCTAGTTGGTGGGGTAATGGCCTACCA
AGGCGACGATCCTTAGCTGGTCTGAGAGGATGATCAGCCACACTGGGACTGAGACACGGCC
>Zotu2521
GAACGCTGGCGGCGTGCTTAACACATGCAAGTCGAACGGAAAGGCCCTTCGGGGTACTCGAGTGGCGAAC
GGGTGAGTAACACGTGGGTGATCTGCCCTGCACTTTGGGATAAGCCTGGGAAACTGGGTCTAATACCGAA
TAGGACTACGCTCTTCATGGGGTGTGGTGGAAAGCTTTTGCGGTGTGGGATGGGCCCGCGGCCTATCAGC
TTGTTGGTGGGGTAATGGCCTACCAAGGCGACGACGGGTAGCCGGCCTGAGAGGGTGACCGGCCACACT
GGGACTGAGATACGGCC

>Zotu2523
GAACGCTGGCGGCATGCCTAACACATGCAAGTCGAACGGCAGCACAGCAGTAGCAATACTGTGGGTGGCG
AGTGGCGGACGGGTGAGTAATGCATCGGGACCTACCTGAACGTGGGGGATAACGTAGGGAAACTTACGC
TAATACCGCATACGTCCTACGGGAGAAAGCGGGGGACCTTCGGGCCTCGCGCGGTCAGACGGACCGATGT
TCGATTAGCTAGTTGGTAAGGTAACGGCTTACCAAGGCGACGATCGATAGCTGGTCTGAGAGGATGATCA
GCCACACTGGAACTGAGACACGGTC
>Zotu2525
GAACGCTGGCGGCAGGCCTAAAACATGCAAGTCGAACGAACCTTCGGGTTAGTGGCGCACGGGTGAGTA
ACGCGTGGGAATGTACCTCTGGGTGCGGAACAACGTCGGGAAACTGACGCTAATACCGCATAATGTCTTC
GGACCAAAGATTTATCGCCTAGAGATCAGCCCGCGTTAGATTAGCTAGTTGGTGAGGTAAGAGCTCACCAA
GGCTACGATCTATAGCTGGTCTGAGAGGATGATCAGCCACACTGGAACTGAGACACGGTC
>Zotu2528
GAACGCTAGCGGCAGGCCTAATACATGCAAGTCGAACGGTGGTAGCAATATCATAGTGGCAAACGGGTGC
GTAACGCGTAAGGAACCTGCCCCTGATTGGGGGATAGCCCGGCGAAAGCTGGGGTAAACCCGCATGGTTC
AGTTGGATGGCATCATTTGACTGATAAATATTTATAGATCAGGGAGGGTCTTGCGTCTGATTAGTTAGTTG
GTGGGGTAACGGCTCACCAAGACGATGATCAGTAGGGGCTCTGAGAGGAGCGGCCCCCACATGGACACT
GAGACACGGGT
>Zotu2530
GAACGCTGGCGGCGTGCTTAACACATGCAAGTCGAACGAGGTCCAGCCGAGCTTTCGGGCAGAGCGAAG
ACCTAGTGGCGAACGGGTGCGTAACACGTGAGGAACCTGCCCCGGACTTGGGAATAACAGTCGGAAACG
GCTGCTAATACCGAATATCTCCGTAGCGTCGCATGGCGCATCGAAGAAAGCTTTTGCGGTCTGGGAGGGCC
TCGCGGCCTATCAGCTAGTTGGCGAGGTAACGGCTCACCAAGGCATCGACGGGTAGCTGGTCTGAGAGGA
TGATCAGCCACACTGGGACTGAGACACGGCC
>Zotu2532
GAACGCTGGCGGCGTGCTTAACACATGCAAGTCGAACGGTAAGGCCCCTTCGGGGGTACACGAGTGGCGA
ACGGGTGAGTAACACGTGGGTAATCTGCCCTGCACTTCGGGATAAGCTTGGGAAACCGGGTCTAATACCG
GATATGAACTCCTGCCGCATGGTGGGGGTTGGAAAGTTTTTGGTGCAGGATGAGCCCGCGGCCTATCAG
CTTGTTGGTGGGGTAATGGCTTACCAAGGCGACGACGGGTAGCCGGCCTGAGAGGGTGATCGGCCACACT
GGGACTGAGACACGGCC
>Zotu2534
GAACGCTAGCTACAGGCTTAACACATGCAAGTCGCAGGGTAACGTGAGGGAAGCTTGCTTCCCTTGACGA
CGACTGGCGCACGGGTGAGTAACGCGTATCCAACCTTCCCATGACCACGGGATAACCCGTTGAAAGACGG
ACTAATACCGTATGACGTCGTTTGCTGACATCAAATAACGATTAAAGGTTTGGCGGTGATGGATGGGGATG
CGTCTGATTAGCTTGTTGGCGGGGTAACGGCCCACCAAGGCGACGATCAGTAGGGGTTCTGAGAGGAAGG
TCCCCCACATTGGAACTGAGACACGGTC
>Zotu2536
GAACGCTGACAGAATGCTTAACACATGCAAGTCGATGGGGAAGCGGCGCTTGCGCCGTGGCAACCATGGC
GGACGGGTGAGTAACGCGTAAAGAACTTGCCGGATGGACCGGGATAACAGCTGGAAACGGCTGGCAAGA
CCGGATACTGTGGGCTGGCCGCATGGCTGGCCCATGAAAAGGGATGCCGTCCGAGAGCTTTGCGTCCTATT
AGCTGGTTGGTAAGGTGACGGCTTACCAAGGCGATGATAGGTAGCCGGCCTGAGAGGGTGGACGGCCAC
AAGGGGACTGAGATACGGCC
>Zotu2538
GAACGCTAGCGGCAGGCTTAATACATGCAAGTCGAGGGAGATTTTAGCTTCGGCTAAATGGAACCGGCGC
ACGGGTGCGTAACGCGTACACAATCTGCCTAATACTGGAGTATAGCCTCGGGAAACTGGGAGTAATCCTCC
ATAACATTATCGGGTGGCATCACTTGATAATTAAAGCTCAGGCGGTATTAGATGAGTGTGCGTATGATTAG
CTAGTTGGTAGGGTAACGGCCTACCAAGGCGACGATCATTAGCTGGCGTGAGAGCGCGACCAGCCACACT
GGAACTGAGACACGGTC
>Zotu2540
GAACGCTGGCGGCGTGCTTAACACATGCAAGTCGAACGATGATGCCCAGCTTGCTGGGCGGATTAGTGGC
GAACGGGTGAGTAACACGTGAGTAACCTGCCCCTGACTCTGGGATAAGCGTTGGAAACGACGTCTAATAC

TGGATATGATCGCCGACCGCATGGTCTGGTGGTGGAAAGATTTTTTGGTTGGGGATGGACTCGCGGCCTAT
CAGCTTGTTGGTGAGGTAATGGCTCACCAAGGCGACGACGGGTAGCCGGCCTGAGAGGGTGACCGGCCA
CACTGGGACTGAGACACGGCC

>Zotu2542

GAACGCTGGCGGCAGGCTTAACACATGCAAGTCGAGCGCCCCGCAAGGGGAGCGGCAGACGGGTGAGTA
ACGCGTGGGAATCTACCCATCTCTACGGAATAACGCATGGAAACGTGTGCTAATACCGTATACGTCCTTTTG
GAGAAAGATTTATCGGAGATGGATGAGCCCGCGTTGGATTAGCTAGTTGGTGGGGTAAAGGCCTACCAAG
GCGACGATCCATAGCTGGTCTGAGAGGATGATCAGCCACACTGGGACTGAGACACGGCC

>Zotu2545

GAACGCTGGCGGCGTGCCTAACACATGCAAGTCGAGCGAACCCTTCGGGGTGAGCGGCGGACGGGTGAG
TAACGCGTGGGTAACCTGCCCTGTACACACGGATAACATACCGAAAGGTATGCTAATACGAGATAATATGT
AATGATCGCATGATCGATACATCAAAGCTCCGGCGGTACAGGATGGACCCGCGTCTGATTAGCTAGTTGGT
GAGGTAATGGCTCACCAAGGCGACGATCAGTAGCCGACCTGAGAGGGTGATCGGCCACATTGGAACTGA
GACACGGTC

>Zotu2547

GAACGCTAGCGGCGTGCTTAACACATGCAAGTCGAACGCTGAAGCCCGCTTCGGTGGGTGGATGAGTGGC
GAACGGGTGAGTAACACGTGGGCAATCTACCCCGCACTCGGGGATAAGCCTTGGAAACGAGGTCTAATAC
CCGATAGGACATGCCATCGCATGGTGGTGTGTGGAAAGTGTGTAGCGGTGTGGGATGAGCCCGCGGCCTA
TCAGCTTGTTGGTGGCGGTGATGGCGCACCAAGGCGACGACGGGTAGCCGGCCTGAGAGGGTGACCGGC
CACACTGGGACTGAGACACGGCC

>Zotu2550

GAACGCTGGCGGCGTGCCTAATACATGCAAGTCGAGCGCCTTCACTGTGTGCTTCTCGGTAACGAGGAACA
TATTGTGAAGGAGCGGCGGACGGCTGAGTAACGCGTGGGAACATACCCCAAAGTGAGGGATAAGCACCG
GAAACGGTGTCTAATACCGCATATGATCTTCGGATTAAAGCCTTCGGGCGCTTTGGGAGTGGCCTGCGTAA
GATTAGCTAGTTGGTGAGGTAAAGGCTCACCAAGGCGACGATCTTTAGTTGGTCTGAGAGGATGACCAGC
CAGACTGGAACTGAGACACGGTC

>Zotu2552

GAACGCTGGCGGCGTGCTTAACACATGCAAGTCGAGCGGAAAGGCCCCTTTGGGGGTACTCGAGCGGCG
AACGGGTGAGTATCACGTGAGTAATCTGCCCTTCACTTTTGGATAACCCTCGGAAACGGGGGCTAATACGG
AATATTCAGTGCTGCCTGCATGGGTGGTGTTGGAAAGATTTTTTGGTGGGGGATGAGCTCGCGGCCTATCA
GCTTGATGGTGGGGTAATGGCCTACCATGGCTTTGACGGGTAGCCGGCCTGAGAGGGTGTCCGGCCACAC
TGGGACTGAGACACGGCC

>Zotu2554

GAACGCTGGCGGCGTGCTTAACACATGCAAGTCGAACGGAAAGGCCCTTCGGGGTACTCGAGTGGCGAAC
GGGTGAGTAACACGTGGGTGATCTGCCTCGTACTTTGGGATAAGCCTGGGAAACTGGGTCTAATACCGAA
TATGACCTTGAGATGCATGTCTCTTGGTGGAAACTTTTTGGGTACGAGATGGGCCCGCGGCCTATCAGCTT
GTTGGTGGGGTAATGGCCTACCAAGGCGACGACGGGTAGCCGGCCTGAGAGGGCGACCGGCCACACTGG
GACTGAGACACGGCC

>Zotu2557

GAACGCTGGCGGCGTGCCTAATACATGCAAGTCGAGCGAACGGATAGGAGCTTGCTCCTTGAAGTTAGCG
GCGGATGGGTGAGTAACACGTGGGCAACCTGCCTGTAAGACTGGGATAACTTCGGGAAACCGGAGCTAAT
ACCGGATAATTTCTTTTTCCTCCTGGGGGAAGGTTGAAAGACGGCTTCGGCTGTCACTTACAGATGGGCCC
GCGGCGCATTAGCTAGTTGGTGGGATAACGGCCTACCAAGGCGACGATGCGTAGCCGACCTGAGAGGGT
GATCGGCCACACTGGGACTGAGACACGGCC

>Zotu2559

GAACGCTAGCGGCAGGCCTAATACATGCAAGTCGAGGGGCAGCACGGGTAGCAATATCTGGTGGCGACC
GGCGCACGGGTGCGTAACGCGTATGCAATCTACCTATTACTGGAGAATAGCCTTCCGAAAGGGAGATTAAT
GCTCCATAACATTATGATGTGGCATCACATTATAATTAAAGTTCCGGCGGTAATAGATGAGCATGCGTGAC
ATTAGCTAGTTGGTGAGGTAATGGCTCACCAAGGCTACGATGTCTAGGGGCTCTGAGAGGATTGTCCCCCA
CACTGGTACTGAGACACGGAC

>Zotu2561
GAACGCTGGCGGCGTGCCTAATACATGCAAGTCGAGCGAACAGATAAGGAGCTTGCTCCTTTGACGTTAG
CGGCGGACGGGTGAGTAACACGTGGGTAACCTACCTATAAGACTGGGATAACTTCGGGAAACCGGAGCTA
ATACCGGATAATATTTCGAACCGCATGGTTCGATAGTGAAAGATGGCCCTGCTATCACTTATAGATGGACCT
GCGCCGTATTAGCTAGTTGGTAAGGTAACGGCTTACCAAGGCAACGATACGTAGCCGACCTGAGAGGGTG
ATCGGCCACACTGGAACTGAGACACGGTC
>Zotu2563
GAACGCTAGCGGGAGGCTTAACACATGCAAGTCGAGGGGCAGCACGAGGTAGCAATACTTTGGTGGCGA
CCGGCGCACGGGTGAGTAACGCGTATGCAACCTACCCTGTACAGGGGGATAGCCCGGAGAAATTCGGATT
AATACCCCATAAAGATATCTGGAGGCATCTTTGGATATTTAAAGATTTATCGGTACGGGATGGGCATGCGT
AACATTAGCTAGTTGGTAGGGTAACGGCCTACCAAGGCAACGATGTTTAGGGGTCCTGAGAGGGTGATCC
CCCACACTGGTACTGAGACACGGAC
>Zotu2566
GAACGCTGGCGGCATGCCTAACACATGCAAGTCGAACGATGCCTTCGGGCATAGTGGCGCACGGGTGCGT
AACGCGTGGGAATCTGCCCTTGGCTTCGGAATAACAGTTAGAAATGACTGCTAATACCGGATGATGTCCCC
CTGAGAGATCTTGGGGACCAAAGATTTATCGGCCAGGGATGAGCCCGCGTAGGATTAGGTAGTTGGTGGG
GTAAAGGCCTACCAAGCCGACGATCCTTAGCTGGTCTGAGAGGATGATCAGCCACACTGGGACTGAGACA
CGGCC
>Zotu2569
GAACGCTGGCGGCGTGCCTAATACATGCAAGTCGAACGCACAGCGAAAGGTGCTTGCACCTTTCAAGTGA
GTGGCGAACGGGTGAGTAACACGTGGACAACCTGCCTCAAGGCTGGGGATAACATTTGGAAACAGATGCT
AATACCGAATAAAACTTAGTATCGCATGATACCAGGTTAAAAGGCGCTACGGCGTCACCTAGAGATGGATC
CGCGGTGCATTAGTTAGTTGGTGGGGTAAAGGCCTACCAAGACAATGATGCATAGCCGAGTTGAGAGACT
GATCGGCCACATTGGGACTGAGACACGGCC
>Zotu2571
GAACGCTGGCGGCGTGCCTAATACATGCAAGTAGAACGCTGAAGGGGGAGCTTGCTTCTCCGGATGAGTT
GCGAACGGGTGAGTAACGCGTAGGTAACCTGCCTGGTAGCGGGGGATAACTATTGGAAACGGTAGCTAAT
ACCGCATAAGAGTAGATGTTGCATGACATTTACTTAAAAGGTGCAATTGCATCACTACCAGATGGACCTGC
GTTGTATTAGCTAGTTGGTGGGGTAACGGCTCACCAAGGCGACGATACATAGCCGACCTGAGAGGGTGAT
CGGCCACACTGGGACTGAGACACGGCC
>Zotu2573
GAACGCTGGCGGCGTGCTTAACACATGCAAGTCGAGCGGTAAGGCCCTTTCGGGGGTACACGAGCGGCG
AACGGGCGAGTAACACGTGAGTAATCTGCCCTTCACTTGGGGATAAGCCTCGGAAACGGGGTCTAATACCT
GATATGACGGCCGTCTGCATGGGCTGGTCGTGGAAAGTTTTTTCGGTGGGGGATGTGCTCGCGGCCTATC
AGCTTGTTGGTGGGGTAATGGCCTACCAAGGCTTCGGCGGGTAGCCGGCCTGAGAGGGTGACCGGCCACA
CTGGGACTGAGACACGGCC
>Zotu2577
GAACGCTAGCGGCAGGCCTAATACATGCAAGTCGAGGGGTAGAGAGAGCTTGCTTTCTTGAGACCGGCGC
ACGGGTGCGTAACGCGTATGCAACCTACCTTTATCTGGGGGATAGCCCGAAGAAATTCGGATTAACACCGC
ATAAAATCACAGTATCGCATGGTACAATGATCAAATATTTATAGGATAAAGATGGGCATGCGTGTCATTAG
TTAGTTGGCGGGGTAACGGCCCACCAAGACCACGATGACTAGGGGATCTGAGAGGATGACCCCCCACACT
GGTACTGAGACACGGAC
>Zotu2579
GAACGCTAGCTACAGGCTTAACACATGCAAGTCGAGGGGCATCAGTTTGGTTTGCTTGCAAACCAAAGCTG
GCGACCGGCGCACGGGTGAGTAACACGTATCCAACCTGCCTCATACTCGGGGATAGCCTTTCGAAAGAAA
GATTAATATCCGATAGCATATATTTCCCGCATGGGTTTTATATTAAAGAAATTCGGTATGAGATGGGGATGC
GTTCCATTAGTTTGTTGGGGGGGTAACGGCCCACCAAGACTACGATGGATAGGGGTTCTGAGAGGAAGGT
CCCCCACATTGGAACTGAGACACGGTC
>Zotu2581

GAACGCTAGCGGCAGGCCTAACACATGCAAGTCGAGGGGTAGATATCTTCGGGTATTGAGACCGGCGCAC
GGGTGCGTAACGCGTATGCAATCTACCTTTTACTAAGGGATAGCCCAGAGAAATTTGGATTAATACCTTATA
GTATGTTTGAATGGCATCGTTTGAACATTAAAGATTTATTGGTAAAAGATGAGCATGCGTCCCATTAGTTAG
TTGGTAAGGTAACGGCTTACCAAGACGATGATGGGTAGGGGTCCTGAGAGGGAGATCCCCCACACTGGTA
CTGAGACACGGAC
>Zotu2585
GAACGCTGGCGGCAGGCTTAACACATGCAAGTCGAGCGCCCCGCAAGGGGAGCGGCAGACGGGTGAGTA
ACACGTGGGAATCTACCCATCCCTACGGAACAACTCCGGGAAACTGGAGCTAATACCGTATACGCCCTTCG
GGGGAAAGATTTATCGGGGATGGATGAGCCCGCGTTGGATTAGCTAGTTGATGGGGTAAAGGCCTACCAA
GGCGACGATCCATAGCTGGTCTGAGAGGATGATCAGCCACATTGGGACTGAGACACGGCC
>Zotu2587
AAACGCTGGCGGCGTGCTTAACACATGCAAGTCGAACGGACTGATTTTGGTGCTTGCACCGAAAGAAAGTT
AGTGGCGGACGGGTGAGTAACACGTGAGTAACCTGCCTTTCAGAGGGGAACAACAGTTGGAAACGACTG
CTAATACCGCATAACGTACAGGTGTCGCATGGCACATGTACCAAAGATTTATCGCTGAGGGATGGGCTCGC
GTCCGATTAGGCAGTTGGCGGGGTAAAGGCCCACCAAACCGACGATCGGTAGCCGGACTGAGAGGTTGA
ACGGCCACATTGGGACTGAGACACGGCC
>Zotu2590
GAACGCTGGCGGCGTGCTTAACACATGCAAGTCGAACGGAAAGGCCCTGCTTGCAGGGTACTCGAGTGGC
GAACGGGTGAGTAACACGTGGGTGATCTGCCCTGTACTTTGGGATAAGCCTGGGAAACTGGGTCTAATAC
CGGATATGACAATTGTTTGGATGCGATTGTGGAAAGCTTTTGCGGTATGGGATGAGCTCGCGGCCTATCAG
CTTGTTGGTGGGGTAATGGCCTACCAAGGCGTCGACGGGTAGCCGGCCTGAGAGGGTGTACGGCCACATT
GGGACTGAGATACGGCC
>Zotu2594
GAACGCTGGCGGCAGGCTTAACACATGCAAGTCGAACGCATCCTTCGGGGTGAGTGGCAGACGGGTGAG
TAACGCGTGGGAATCTACCTCTTGCTACGGGATAGCTCCGGGAAACTGGAATTAATACCGTATGTGCCCCTT
CGGGGGAAAGATTTATCGGCAAGGGATGAGCCCGCGTTGGATTAGCTAGTTGGTGGGGTAAAGGCCTACC
AAGGCGACGATCCATAGCTGGTCTGAGAGGATGATCAGCCACATTGGGACTGAGACACGGCC
>Zotu2596
GAACGCTAGCGGCAGGCTTAATACATGCAAGTCGAGGGGGCAGCAATGTCACCGGCGAACGGGTGCGTA
ACGCGTATGCAACCTACCTACATCAGGGGGATAGCCCTAGGAAACTGGGATTAACACCGCATAACAATAGA
GTTGGCATCAACTTTATTTGAAATATTTATAGGATGTAGATGGGCATGCGTAGGATTAGCTAGTTGGTAAG
GTAACGGCTTACCAAGGCTACGATCCTTAGGGGCTCTGAGAGGAGTATCCCCCACACTGGCACTGAGATAC
GGGC
>Zotu2598
GAACGCTAGCGGCAGGCTTAACACATGCAAGTCGAGGGGTATAAGGAGCTTGCTCCTTAGAGACCGGCGC
ACGGGTGCGTAACGCGTATGCAACCTACCTTGAACAGGGGGATAGCCCAGAGAAATTTGGATTAATACCC
CATGGTATATTTGAGTGGCATCACTTGATTATTAAAGTTCCAACGGTTCAAGATGGGCATGCGTCCCATTAG
CTAGATGGTAAGGTAACGGCTTACCATGGCGACGATGGGTAAGGGTCCTGAGAGGGAGATCCCCCACACT
GGTACTGAGACACGGAC
>Zotu2601
GAACGCTGGCGGCATGCCTTACACATGCAAGTCGAACGGCAGCGCGGGCTTCGGCCTGGCGGCGAGTGG
CGAACGGGTGAGTAATACATCGGAACGTGCCCTGTTGTGGGGGATAACTAGTCGAAAGATTAGCTAATAC
CGCATACGACCTGAGGGTGAAAGCGGGGGACCGTAAGGCCTCGCGCAATAGGAGCGGCCGATGTCTGAT
TAGCTAGTTGGTGGGGTAAAGGCCTACCAAGGCGACGATCAGTAGCTGGTCTGAGAGGACGATCAGCCAC
ACTGGGACTGAGACACGGCC
>Zotu2604
GAACGCTAGCGGCAGGCCTAATACATGCAAGTCGAGGGGTAACAGGGTAGCAATACCGCTGACGACCGG
CGCACGGGTGCGTAACACGTATGCAATCTACCTCTAACAGAGGGATAGCCCGAAGAAATTCGGATTAATAC
CTCATAACATCACGGGAAGGCATCTTCCTGTGCTTAAAGATTTATCGGTTAGAGATGAGCATGCGTCCGATT
AGCTAGTTGGTGAGGTAATGGCTCACCAAGGCTACGATCGGTAGGGGGTCTGAGAGGATGATCCCCCACA
CTGGTACTGAGATACGGAC

>Zotu2606
GAACGCTGGCGGCGTGCTTAACACATGCAAGTCGAACGGAAAGGCCCTGCTTGCAGGGTACTCGAGTGGC
GAACGGGTGAGTAACACGTGGGTGATCTGCCCTGCACTTCGGGATAAGCTTGGGAAACTGGGTCTAATAC
CGGATAGGACCGCACTTTAGTGTGTGTGGTGGAAAGTTTTTCGGTGTAGGATGAGCTCGCGGCCTATCAG
CTTGTTGGTGGGGTAATGGCCTACCAAGGCGGCGACGGGTAGCCGGCCTGAGAGGGTGTACGGCCACATT
GGGACTGAGATACGGCC
>Zotu2609
GAACGCTGGCTGCGTGCTTAACACATGCAAGTCGAACGCTGAAGCCTGGTGCTTGCACCGGGTGGATGAG
TGGCGAACGGGTGAGTAACACGTGAGTAACCTGCCCCTGACTTCGGGATAAGCCCGGGAAACTGGGTCTA
ATACCGGATACAACTGCCGGACGCATGTCTGGTGGTGGAAAGTTTTTCGGTTGGGGATGGGCTCGCGGC
CTATCAGTTTGTTGGTGAGGTAATGGCTCACCAAGACGACGACGGGTAGCCGGCCTGAGAGGGCGACCGG
CCACACTGGGACTGAGACACGGCC
>Zotu2611
GAACGCTTGCGGCGTACCTAAGAAATGCAAGTCGAGCGGGGGAGCAATCCCTAGCGGCGAACGGTCGCG
TAACACGTAGGCAACCTGCCCGAGAGACCGGGACAACACTTCGAAAGAGGTGCTAATACCGGATGTGGCC
GGCGGGAGACATCTCCTGCCGATTAAACCCCTTCGGGGGCTCTTGGAGGGGCCTGCGGCCTATCAGCTAG
ATGGTGGGGTAACGGCCTACCATGGCGACGACGGGTAGCTGGTCTGAGAGGATGGTCAGCCGGATTGGG
ACTGAGATACGGCC
>Zotu2613
GAACGCTAGCGGCAGGCCTAACACATGCAAGTCGAGGGGTATAGTAGCAATACTAGAGACCGGCGCACG
GGTGCGTAACGCGTATGCAATCTACCTTTTACAGGGGAATAGCCCAGAGAAATTTGGATTAATGCCCCATG
GTATTATCGGATGGCATCATTTGATAATTAAAGTTCCAACGGTAAAAGATGAGCATGCGTCCTATTAGCTAG
TTGGTAAGGTAACGGCTTACCAAGGCTACGATAGGTAGGGGTCCTGAGAGGGAGATCCCCCACACTGGTA
CTGAGACACGGAC
>Zotu2617
ATGATCCCAGAGTCCTGGGATTGAGTGCCACATTGGGATCCCTGCTCAGCAGGGAGTCTGCTTCTCCCTCTG
CTTCTCACCCCACTCGTGCTTTCTCTCTCACTCTCAAATAAATAAATAAGTAAAATCTTAAA
>Zotu2619
GAACGCTGGCGGCGTGCCTAACACATGCAAGTCGAACGGGGTAGCAATACCCAGTGGCGAACGGGTGCG
TAACACGTGAGGAACATACCCTTCGGCGGGGGATAGCCGGCCCAACGGCCGGGTAATACCGCGTACGATC
TTCGGGTGGCATCACCTGGGGATGAAAGCAGCAATGCGCCGATGGAGTGCCTCGCGGCCTATCAGCTAGT
TGGTGAGGTAATGGCTCACCAAGGCGACGACGGGTAGCTGGTCTGAGAGGATGATCAGCTACATTGGGAC
TGAGACACGGCC
>Zotu2622
GAACGCTGGCGGCGTGCCTAATACATGCAAGTCGAGCGGACTTGATGAGGAGCTTGCTCCTCTGAGAGTT
AGCGGCGGACGGGTGAGTAACACGTAGGCAACCTGCCCTCAAGACTGGGATAACTACCGGAAACGGTAG
CTAATACCGGATAATTCGTGCTGCTGCATGGCAGCGTGATGAAAGACGGAGCAATCTGTCACTTGAGGATG
GGCCTGCGGCGCATTAGCTAGTTGGTGGGGTAATGGCCTACCAAGGCGACGATGCGTAGCCGACCTGAGA
GGGTGATCGGCCACACTGGGACTGAGACACGGCC
>Zotu2624
GAACGCTGGCGGCGTGCTTAACACATGCAAGTCGAACGGAAAGGCCACAGCTTGCTGTGGTACTCGAGTG
GCGAACGGGTGAGTAACACGTGGGTGATCTGCCCTGTACTTCGGGATAAGCTTGGGAAACTGGGTCTAAT
ACCGGATAGGAATCACATGTGGGTGTGTGGTTGGAAAGCTTTTGCGGTATGGGATGAGCTCGCGGCCTAT
CAGCTTGTTGGTGGGGTAATAGCCTACCAAGGCGTCGACGGGTAGCCGGCCTGAGAGGGTGTACGGCTAC
ATTGGGACTGAGATACGGCC
>Zotu2626
GAACGCTGGCGGCGTGCTTAACACATGCAAGTCGAGCGGTAAGGCCCTTCGGGGTACACGAGCGGCGAA
CGGGTGAGTAACACGTGGGTGACCTGCCCCCTACTCTGGGATAAGCCTGGGAAACTGGGTCTAATACCGG
ATATGACTACATGTCGCATGGTGTGTGGTGGAAAGTTTTTCGGTGGGGGATGGGCCCGCGGCCTATCAGC

TTGTTGGTGGGGTGATGGCCTACCAAGGCGACGACGGGTAGCCGGCCTGAGAGGGCGACCGGCCACACT
GGGACTGAGACACGGCC
>Zotu2628
GAACGCTGGCGGCGTGCCTAATACATGCAAGTCGAGCGGGGATGCTTCCGGAAGCCCTTCGGGGCGGAA
GGCTGCATCCTAGCGGCGGACGGGTGAGTAACGCGTAGGGAACCTGCCCCCAACACCGGGATAACCCGG
GGAAACTCGGGCTAATACCGGATGCGGCCGACGAATCGCATGGTTTGTCGGTCAAAGGGCTTACCGGTTG
GGGATGGCCCTGCGTCCCATTAGCTAGTTGGTGAGGTAACGGCTCACCAAGGCGACGATGGGTAGCCGGC
CTGAGAGGGTGACCGGCCACACTGGCACTGAGACACGGGC
>Zotu2630
GAACGCTGGCGGCGTGCCTAATACATGCAAGTCGAGCGCAGGAAGCTGACGGAACTCTTCGGAGGGAAG
GCAGTGGAATGAGCGGCGGACGGGTGAGTAACACGTAAGGAACCTGCCTCAAGGATTGGGATAACTCCG
AGAAATCGGAGCTAATACCGGATAGTTCAACGGACCGCATGGTCCGCTGATGAAAGGCGCTTCGGCGTCA
CCTTGAGATGGCCTTGCGGTGCATTAGCTAGTTGGTGGGGTAACGGCCCACCAAGGCGACGATGCATAGC
CGACCTGAGAGGGTGATCGGCCACACTGGGACTGAGACACGGCC
>Zotu2632
GAACGCTGGCGGCGTGCCTAAGACATGCAAGTCGAGTGGGTTTAGGCCCACGGCGAACGGGTGAGTAAT
ACCTAGGAACGTATCTTTAGTTGAGGATATCCCGCCGAAAGGCGGAGCAATACTCAATGTCATCGTAAGAT
GAAAGTCGCAAGACGCTAAAGAAGCGGCCTGGGCACCATCAGCTAGTTGGTAAGGTAAAGGCTTACCAAG
GCTATGACGGTTAGGGGCCCCGAGAGGGGAGACCCCCACACTGGAACTGCGACACGGTC
>Zotu2634
AAACGCTGGCGGCGTGCCTTATGCATGCAAGTCGAACGAGGCTAGGACTTCGGTCCGAAACCTAGTGGCG
GACGGGTGAGTAACACGTACGTGACCTGTCCGGAAGTGGGGAATAACCAGCCGAAAGGTTGGCTAATACC
GCATACGGTCACCGCTTGGAAGCGGATGACCAAAGCCGAAAGGCGCTTCCGGAGGGGCGTGCGGCGGAT
TAGCTAGTTGGTGTGGTAACGGCGCACCAAGGCGATGATCCGTAGCTGGTCTGAGAGGATGGTCAGCCAC
ACTGGGACTGAGACACGGCC
>Zotu2636
GAACGCTGGCGGCGTGCCTAATACATGCAAGTCGAGCGAACTGACGAGGAGCTTGCTCCTTTGACGTTAG
CGGCGGACGGGTGAGTAACACGTGGGTAACCTACCTATAAGACTGGAATAACTCCGGGAAACCGGGGCTA
ATGCCGGATAACATATCGAACCGCATGGTTCGATAGTGAAAGACGGTCTTGCTGTCACTTATAGATGGACC
CGCGCCGTATTAGCTAGTTGGTGAGGTAACGGCTCATCAAGGCGACGATACGTAGCCGACCTGAGAGGGT
GATCGGCCACACTGGAACTGAGACACGTTC
>Zotu2638
GAACGCTGGCGGCGTGCTTAACACATGCAAGTCGAGCGGAAAGGCCCCTTCGGGGGTACTCGAGCGGCG
AACGGGTGAGTAACACGTGGGTAATCTGCCCTAGACACAGGAATAACCAGTGGAAACGCTGGCTAATGCC
TGATGATGTGCCGGCCTCACGGGGCTGGTACCAAAGGTAGCTTCGGCTTCCGGTCTTGGATGAGCCCGCG
GCCCATTAGCTAGTTGGCGGGGTAATGGCCCACCAAGGCTTCGATGGGTAGCTGGTCTGAGAGGACGATC
AGCCACACTGGGACTGAGACACGGCC
>Zotu2640
GAACGCTGGCGGCGTGCTTAACACATGCAAGTCGAGCGAAGCACTCTGACTTCTTCGGAATGATGAGTGA
CTGAGCGGCGGACGGGTGAGTAACGCGTGGGTAACCTGCCTCACACAGGGGGATAACAGTTAGAAATGA
CTGCTAATACCGCATAAGACCACAGCACCGCATGGTGCAGGGGTAAAAACTCCGGTGGTGTGAGATGGAC
CCGCGTCTGATTAGCTAGTTGGTGAGGTAACGGCTCACCAAGGCGACGATCAGTAGCCGACCTGAGAGGG
TGACCGGTCACATTGGGACTGAGACACGGCC
>Zotu2642
GAACGCTGGCGGCATGCCTAACACATGCAAGTCGAACGAAGCCTTCGGGCTTAGTGGCGCACGGGTGCGT
AACGCGTGGGAATCTGCCCCTCGGTTCGGAATAACAGTTAGAAATGACTGCTAATACCGGATAATGACGTA
AGTCCAAAGATTTATCGCCGAGGGATGAGCCCGCGTAGGATTAGCTTGTTGGTGGGGTAATGGCCCACCA
AGGCGACGATCCTTAGCTGGTCTGAGAGGATGATCAGCCACACTGGGACTGAGACACGGCC
>Zotu2646

GAACGCTAGCGGCAGGCCTAATACATGCAAGTCGAGCGGAGAGTAGCAATACTTTTAGCGGCGGACGGGT
GAGTAACGCGTACACAACATGCCTTTGGTTGGGGGATAGCCAGTGGAAACGCTGAGTAATACCGCATAAG
ACTACGTTTCGGCATCGGAGAGTAGTTAAAGTTACGGCGACCAGAGATTGGTGTGCGTTCGATTAGCTAGT
TGGCGGGGTAACGGCCCACCAAGGCGACGATCGATAGGGGGTGTGAGAGCATGGCCCCCCACACGGGTA
CTGAGACACGGAC

>Zotu2648

GAACGCTGGCGGCGTGCCTAATACATGCAAGTCGAGCGAAAGTTTCAAAAGCTTGCTTTTGGAACTTTAGC
GGCGGACGGGTGAGTAACACGTGGGCAACCTGCCTGTAAGACTGGGATAACTCCGGGAAACCGGGGCTA
ATACCGGATAATCCTTTTCCTTGCATAGGGAAAAGCTGAAAGACGGTTTCGGCTGTCACTTACAGATGGGC
CCGCGGCGCATTAGCTAGTTGGTGGGGTAACGGCCCACCAAGGCGACGATGCGTAGCCGACCTGAGAGG
GTGATCGGCCACACTGGGACTGAGACACGGCC

>Zotu2650

TAACGCTGGCGGCGTGCATAACACATGCAAGTCGAACGATGAAACTTAAATGATCTCTTCGGAGTGATTCT
AAGTGGATTAGTGGCGGACGGGTGAGTAACGCGTGAGTAACCTGCCTTAGAGAAGGGGATAGCCTTTGG
AAACGAAGAATAATACCCTATGATACCAACCCTACGCATGTAGAGTTGGTCAAAGATTTATCGCTCTAAGAT
GGACTTGCGTCTGATTAGTTAGTTGGTGAGGTAACGGCCCACCAAGACAGCGATCAGTAGCCGGCTTGAG
AGAGTGTACGGCCACATTGGGACTGAGACACGGCC

>Zotu2652

GAACGCTGGCGGCGTGCTTAACACATGCAAGTCGAACGATGATCAGGAGCTTGCTCCTGTGATTAGTGGC
GAACGGGTGAGTAACACGTGAGTAACCTGCCCCTGACTCTGGGATAAGCGTTGGAAACGACGTCTAATAC
TGGATATGATCACTGGTCGCATGGCCTGGTGGTGGAAAGATTTTTTGGTTGGGGATGGACTCGCGGCCTAT
CAGCTTGTTGGTGAGGTAATGGCTCACCAAGGCGACGACGGGTAGCCGGCCTGAGAGGGTGACCGGCCA
CACTGGGACTGAGACACGGCC

>Zotu2654

GAACGCTGGCGGCGTGCTTAACACATGCAAGTCGAGCGGTAAGGCCTTTCGGGGTACACGAGCGGCGAAC
GGGTGAGTAACACGTGGGTGATCTGCCCTGCACTTCGGGATAAGCCTGGGAAACTGGGTCTAATACCGGA
TATGACTTCAGGTTGCATGACTTGGGGTGGAAAGATTTATCGGTGCAGGATGGGCCCGCGGCCTATCAGCT
TGTTGGTGGGGTAATGGCCTACCAAGGCGACGACGGGTAGCCGACCTGAGAGGGTGACCGGCCACACTG
GGACTGAGACACGGCC

>Zotu2656

GAACGCTGGCGGCAGGCTTAACACATGCAAGTCGAACGATGACTCTCTAGCTTGCTAGAGATGATTAGTG
GCGGACGGGTGAGTAACATTTAGGAATCTACCTAGTAGTGGGGGATAGCTCGGGGAAACTCGAATTAATA
CCGCATACGACCTACGGGTGAAAGGGGGCGCAAGCTCTTGCTATTAGATGAGCCTAAATCAGATTAGCTA
GTTGGTGGGGTAAAGGCCCACCAAGGCGACGATCTGTAACTGGTCTGAGAGGATGATCAGTCACACCGGA
ACTGAGACACGGTC

>Zotu2660

GAATGCTGGCGGCGTGGATAAGTCATGCAAGTCAAGGGGGCCGCAAGGCAACCGGCAGACGAGGTAGTA
ACACGTAGGTAGATCCCCCGAAGTCGGGCATAGCCATCCGAAAGGATGGGTAATTCCCGATAGTCCCGCA
AGGGTAAAGGTTTTTCGCTTCGGGAGTTGCCTGCGTAGTATCAGCTAGTTGGTAGGGTAACGGCCTACCAA
GGCTACGACGCTTAGGGGAGCTGAGAGGCTGACCCCCACCGATGGAACTGCGACACGGTC

>Zotu2662

GAACGCTAGCGGCAGGCCTAATACATGCAAGTCGAACGGGATGGAGTAGCAATACTCTATCTAGTGGCGC
ACGGGTGCGTAACGCGTATGCAACCTACCTTTTATTGGGGGATAGCCCGAGGAAACTCGGATTAATACCGC
ATGATACTATTTGAGGGCATCTTCATTATAGTTAAAGTTAAGGCGATAAAAGATGGGCATGCGTGACATTA
GCTAGTTGGTAGGGGTAACGGCCCACCAAGGCGACGATGTCTAGGGGTTCTGAGAGGATGGTCCCCCACA
CTGGTACTGAGATACGGAC

>Zotu2664

GAACGCTGGTAACGTGGATACCGGCATGCAAGTCAAGGGGCCGCAAGGCACCGGCAAACGGCTTAGTAA
CACGTGGATACATACCCAAAAGTGGGGCACAGCTCGTCGAAAGACGGGGTAATTTCCCATAGTCTCGCAA
GAGTAAAGCAGCAATGCGCTTTTGGAATGGTCTGCGGTCTATCAGCTAGTTGGTGAGGTAATGGCTCACCA
AGGCTATGACGGATAGGGGGCGTGAGAGCGCGACCCCCAAGAATGGCACTGCGACACGGGC

>Zotu2666
GAACGCTGGCGGCGTGCTTAACACATGCAAGTCGAGCGGAAAGGCTCCTTTCGGGGGTACTCGAGCGGC
GAACGGGTGAGTAACACGTGAGTAATCTGCCCCTCACTCTGGGATAACTGCCTGAAAGGGTGGCTAATACC
GGATGCGACCGTCCCCTGCATGGGGTGGTGGTGGAAAGGGTTTCTGGTGGGGGATGTGCTCGCGGCCTAT
CAGCTTGTTGGTGGGGTAATGGCCTACCAAGGCTTTGACGGGTAGCCGGCCTGAGAGGGTGACCGGCCAC
ACTGGGACTGAGACACGGCC
>Zotu2669
GAACGCTGGCGGCGTGCCTAATACATGCAAGTAGAACGCTGAAGAGAGGAGCTTGCTCTTCTTGGATGAG
TTGCGAACGGGTGAGTAACGCGTAGGTAACCTGCCTTGTAGCGGGGGATAACTATTGGAAACGATAGCTA
ATACCGCATAACAATGGATGACCCATGTCATTTATTTGAAAGGGGCAAATGCTCCACTACAAGATGGACCT
GCGTTGTATTAGCTAGTAGGTGAGGTAACGGCTCACCTAGGCGACGATACATAGCCGACCTGAGAGGGTG
ATCGGCCACACTGGGACTGAGACACGGCC
>Zotu2671
GAACGCTGGCGGCGTGCCTAATACATGCAAGTCGAGCGGACTTGATGGAGTGCTTGCACTCCTGAGAGTT
AGCGGCGGACGGGTGAGTAACACGTAGGTAACCTGCCCATAAGACCGGGATAACATTCGGAAACGAATGC
TAATACCGGATATGCAATTCTTTCGCATGAGAGAGTTGGGAAAGACGGAGCAATCTGTCACTTATGGATGG
ACCTGCGGTGCATTAGCTAGTTGGTGAGGTAATGGCTCACCAAGGCGACGATGCGTAGCCGACCTGAGAG
GGTGATCGGCCACACTGGGACTGAGACACGGCC
>Zotu2673
GAACGCTGGCGGCGTGCTTAACACATGCAAGTCGAACGGAAAGGCCCTTCGGGGTACTCGAGTGGCGAAC
GGGTGAGTAACACGTGGGTGATCTGCCCTGCACTTCGGGATAAGCCTGGGAAACTGGGTCTAATACCGGA
TAGGACCACGGTCTGCATGGACTGTGGTGGAAAGCTTTTGCGGTGTGGGATGGGCCCGCGGCCTATCAGC
TTGTTGGTGGGGTGATGGCCTACCAAGGCGACGACGGGTAGCCGGCCTGAGAGGGTGACCGGCCACACT
GGGACTGAGATACGGCC
>Zotu2675
GAACGCTGGCGGCAGGCTTAACACATGCAAGTCGAACGCTTCATCTTCGGGTGGGGAGTGGCAGACGGGT
GAGTAACGCGTGGGAACGTACCCTTCGGTTCGGAATAACTCAGGGAAACTTGAGCTAATACCGGATAAGC
CCTTTTTGGGGAAAGGTTGATCTGCCGAAGGATCGGCCCGCGTCCGATTAGCTAGTTGGTGTTGGTAATGG
CTCACCAAGGCGACGATCGGTAGCTGGTCTGAGAGGATGGCCAGCCACATTGGGACTGAGACACGGCC
>Zotu2679
TAAAGTTGGCGGCGTGCCTAAGGTATGCAAGTCGCGCGGTTTTTATTGATTCGCAAGATGATTTAAGAATA
GCGGCGAACGGCTGAGTAACGCGTGGGAATCTCCCTCGAAGTCAAGGATAGCCCACCGAAAGGTGGAGT
AATCCTGGATGTGAACTGTGGTTTAAAGCCGCAAGGCGCTTCGAGATGAGCCTGCGTCCTATCAGGTTGTT
GGCGGGGTAAATGCCCACCAAGCCTATGACGGGTAGCTGGTCTGAGAGGATGACCAGCCAGAATGGGAC
TGAGACACGGCC
>Zotu2681
GAACGCTGGCGGCGTGCTTAACACATGCAAGTCGAGCGGAAAGGCTCCTTCGGGGGTACTCGAGCGGCG
AACGGGTGAGTAACACGTGAGTAATCTGCCCTGGACTTCGGGATAGCCCACCGAAAGGTGGATTAATACC
GGATATGACGCGTCCTCGCATGGGGGTGTGTGGAAAGTTTTTCGGTTCAGGATGTGCTCGCGGCCTATCA
GCTTGATGGTGAGGTAATGGCTCACCATGGCTTCGACGGGTAGCCGGCCTGAGAGGGTGACCGGCCACAC
TGGGACTGAGACACGGCC
>Zotu2683
TGTCCGAGAACTGCTCGGGAGGAGGCGAGCCGACAGGGATGCGGCAAGAAACATGGCACGACGTACGG
GAAATGTGTCAAAGGCGTGGTGTATGAAATTACCCTTTCTTGTG
>Zotu2687
GAACGCTAGCGGCAGGCCTAATACATGCAAGTCGAACGGGATCCACTGGAGAGCTTGCTCGAAGGTGGTG
AGAGTGGCGAATGGGTGCGTAACGCGTGAGCAACTTACCCTTGTCAGGGGGATAGCCCGGCGAAAGTCG
GATTAACACCGCATAACATCATAGGATGGCATCATTTTATGATCAAATATTTATAGGACAAGGATGGGCTC
GCGTGACATTAGCTAGTTGGTAAGGTAACGGCTTACCAAGGCTTCGATGTCTAGGGGCTCTGAGAGGAGA
ATCCCCCACACTGGTACTGAGACACGGAC

>Zotu2689
GAACGCTGGCGGCGTGCTTAACACATGCAAGTCGAACGGTGAACCAGAGCTTGCTCTGGGGATCAGTGGC
GAACGGGTGAGTAACACGTGGGCAACCTGCCCCAGACTCTGGGATAACCTCGGGAAACCGATGCTAATAC
CGGATACGACCACTTCGGACATCCGACGGTGGTGGAAAGTTTTTCGGTCTGGGATGGGCCCGCGGCCTATC
AGCTAGTTGGTGAGGTAATGGCCTACCAAGGCGACGACGGGTAGCCGGCCTGAGAGGGCGACCGGCCAC
ACTGGAACTGAGACACGGTC
>Zotu2691
GAACGCTGGCGGCGTGCTTAACACATGCAAGTCGAGCGGAAAGGCTCCTTCGGGGGTACTCGAGCGGCG
AACGGGTGAGTAACACGTGAGTAATCTGCCCCTGGCTTTGGGATAGCCACCGGAAACGGTGATTAATACC
GGATATGACCACTTCACGCATGTGATGGTGGTGGAAAGTTTTTCGGCCAGGGATGTGCTCGCGGCCTATCA
GCTTGATGGTGAGGTAATGGCTCACCATGGCTTCGACGGGTAGCCGGCCTGAGAGGGTGACCGGTCACAC
TGGGACTGAGACACGGCC
>Zotu2693
GAACGCTAGCGGCAGGCTTAATACATGCAAGTCGAGGGGCAGCACGGGTAGTAATACCTGGTGACGACC
GGCAAACGGGTGCGGAACACGTACGCAACCTTCCCATAACTGGTGAATAGCCCTCCGAAAGGAGGATTAA
TACACCGTAACATAATGAAGTGGCATCACTTTATTATTATAGGCAGCAATGTCGGTTTTGGATGGGCGTGC
GCCTGATTAGGTAGTTGGCGGGGTAACGGCCCACCAAGCCTGCGATCAGTAACTGGTGTGAGAGCACGAC
CAGTCACACGGGCACTGAGACACGGGC
>Zotu2695
GAACGCTGGCGGCGTGCTTAACACATGCAAGTCGAACGCACCTTCGGGTGAGTGGCGGACGGGTGAGTA
ACGCGTGGGAATCTGCCCTCAGGAGGGGGATAACGACTGGAAACGGTCGCTAATACCCCATATGCCGAGA
GGTGAAATGAATTTCGCCTGAGGATGAGCCCGCGTCTGATTAGCTAGTTGGTGGGGTAAGAGCTCACCAA
GGCGACGATCAGTAGCTGGTCTGAGAGGATGATCAGCCACACTGGGACTGAGACACGGCC
>Zotu2697
GAACGCTGGCGGCGTGCTTAACACATGCAAGTCGAACGAAAAGACGGAAAGAGCTTGCTCTTTTCAGAAT
TGAGTGGCAAACGGGTGAGTAACACGTAAACAACCTGCCTTCAGGATGGGGACAACAGACGGAAACGACT
GCTAATACCGAATAAGTTCCAAGGGCCGCATGGCCCATGGAAGAAAAGGTGGCCTCTACCTGTAAGCTATC
GCCTGAAGAGGGGTTTGCGTCTGATTAGCTGGTTGGAGGGGTAACGGCCCACCAAGGCGACGATCAGTAG
CCGGTCTGAGAGGATGAACGGCCACACTGGAACTGAGACACGGTC
>Zotu2699
GAACGCTGGCGGCATGCCTAACACATGCAAGTCGAACGAAGCCTTCGGGCTTAGTGGCGCACGGGTGCGT
AACGCGTGGGAATCTGCCCCTTGCTTCGGAATAACAGTTAGAAATGACTGCTAATACCGGATGAAGTCTTC
GGACCAAAGATTTATCGGCAAGGGATGAGCCCGCGTAGGATTAGGTAGTTGGTGGGGTAAAGGCCTACCA
AGCCGACGATCCTTAGCTGGTCTGAGAGGATGATCAGCCACACTGGGACTGAGACACGGCC
>Zotu2701
GAACGCTGGCGGCATGCCTAACACATGCAAGTCGGACGGGAAGCATCCTCGTGGTGTTTCCAGTGGCGGA
CGGGTGAGTAACGCGTAAGAACTTGCCCTTGGGAGGGGGACAACAATTGGAAACGGTTGCTAATACCCCG
TAAGCTGAGGAGCAAAAGGGAGGGATCCGCCCAAGGATGGGCTTGCGTCTGATTAGCTGGTTGGTGAGG
CAATGGCTTACCAAGGCAACGATCAGTAGCTGGTCTGAGAGGATGATCAGCCACACTGGGACTGAGACAC
GGCC
>Zotu2705
GAACGCTGGCGGCGTGCCTAATACATGCAAGTCGAACGGTTTAGTTCCAAGAGGCTTGCCTGTTGGAACTA
GACAGTGGCGGACGGGTGAGTAACACGTAGGCAACCTACCTGTCAAACCGGGATAACCTCGGGAAACCGA
AGCTAAACCCGGATAAGCTCCTTTTTCGCATGAAGGGGGAGTGAAAAGGTGCGACCTGATGACAGATGGG
CCTGCGGCGCATTAGCTAGTAGGTAGGGTAACGGCCTACCTAGGCGATGATGCGTAGCCGACCTGAGAGG
GCGTACGGCCACACTGGGACTGAGATACGGCC
>Zotu2708
GAACGCTGGCGGCGTGCCTAATACATGCAAGTCGAGCGGATCTTTAGAAGCTTGCTTCTAAAGATTAGCGG
CGGACGGGTGAGTAACACGTGGGCAACCTGCCTGTAAGACTGGGATAACTTCGGGAAACCGAGGCTAATA
CCGGATAATCCTTTTTTTCACATGAGGGAAAGCTGAAAGACGGCGCAAGCTGTCACTTACAGATGGGCCCG

CGGCGCATTAGCTAGTTGGTGAGGTAACGGCTCACCAAGGCAACGATGCGTAGCCGACCTGAGAGGGTGA
TCGGCCACACTGGGACTGAGACACGGCC
>Zotu2712
GAACGCTGGAAATGTGGATACCGGCATGCAAGTCAAGGGGCCGCAAGGCACCGGCGAACGGCTTAGTAA
CACGTAGATACATACCGCAAAGTGGGGCATAGCTCGTTGAAAAACGGGGTAATTTCCCATAGTTCCCGCAA
GGGTAAAGACGCAAGTCGCTTTGTGAATGGTCTGCGGTCTATCAGCTAGTTGGTGGGGTAAAAGCCTACC
AAGGCTATGACGGATAGGGGGCGTGAGAGCGCGACCCCCAAGAATGGCACTGCGACACGGGC
>Zotu2714
CAACGCTGGCGGCGCGCCTAACACATGCAAGTCGAACGAGAAAGCCGCAAGGCAAGTAAAGTGGCGTAC
GGGTGAGTAACACGTGGATAATCTGCCTTTTAGTGGGGAATACCGTTGGGAAACCGACGACAATACCGCA
TAATCCCGAGAGGGGAAAGCCGTAAGGCGCTGAAAGAGGAGTCCGCGGTCGATTAGGTAGTTGGCGGGG
TAAAAGCCCACCAAGCCTGTGATCGATAGCCGGCCTGAGAGGGCACACGGCCACACTGGCACTGAAACAC
GGGC
>Zotu2717
GAACGCTGGCGGCGTGCTTAACACATGCAAGTCGAACGGTGACGAGCAGCTTGCTGCTCTGATCAGTGGC
GAACGGGTGAGTAACACGTGAGCAACCTACCCTTGACTCCGGGATAACAGCTGGAAACAGCTGCTAATAC
CGGATACGACCTCGACCGACATCAGTTGAGGTGGAAAGTTCTGGCGGTCAAGGATGGGCTCGCGGCCTAT
CAGCTAGTTGGTGAGGTAACGGCTCACCAAGGCTTCGACGGGTAGCCGGCCTGAGAGGGCGACCGGCCA
CACTGGGACTGAGATACGGCC
>Zotu2719
GAACGCTGGCGGCGTGCCTAATACATGCAAGTCGTGCGGAAAGGCCTCTTCGGAGGTACTCGAGCGGCGG
ACGGCTGAGTAACGCGTAGGAACGTACCCCAAACTGAGGGATAACTGCTCGAAAGAGTAGCTAATACCGC
ATGTGCTCTAAGGAGTAAAGCTTCGGCGGTTTGGGAACGGCCTGCGTAAGATTAGCTTGTTGGTGAGGTA
ATGGCTCACCAAGGCGACGATCTTTAGCTGGTCTGAGAGGATGATCAGCCAGACTGGGACTGAGACACGG
CC
>Zotu2721
GAACGCTGGCGGCGCGCCTAATACATGCAAGTCGAGCGAGAAGACGTAGCAATACGTTTGTAAAGCGGCG
AACGGGTGAGGAATACATGGGTAACCTACCCTCGAGTGGGGAATAACTAGCCGAAAGGTTAGCTAATACC
GCGTACGCTCCTTGGACTTCGGTTCAGGGAGGAAAGCGATACCGTGGGTATCGCGCTCTTGGATGGGCTC
ATGTCCTATCAGCTTGTTGGTGAGGTAACGGCTCACCAAGGCTTCGACGGGTAGCTGGTCTGAGAGGACG
ATCAGCCACACTGGCACTGCGACACGGGC
>Zotu2723
GAACGCTAGCGGCAGGCTTAATACATGCAAGTCGAGGGGCAGCACGGATAGCAATATTTGGTGGCGACCG
GCGCACGGGTGCGTAACACGTATACAACCTACCTATAACAGGGGGATAGCCTTTCGAAAGAGAGATTAAT
ACCCCGTAATACAAGACTCGGCATCGAGCTTTGTTGAAAGCTCCGGCGGTTATAGATGGGTATGCGTTTGA
TTAGCTAGTTGGTGAGGTAACGGCTCACCAAGGCTACGATCAATAGGGGATCTGAGAGGACTAACCCCCA
CACTGGTACTGAGACACGGAC
>Zotu2726
GAACGCTGGCGGTATGCTTAACACATGCAAGTCGAACGGAACCTTTCGGGGTTTAGTGGCGGACGGGTGA
GTAACGCGTGAGAATCGGCCCTCAGGTTGGGGACAACCATTGGAAACGGTGGCTAATACCGAATAAGCCG
AGAGGTGAAAGATTAATTGCCTGGGGAAGAGCTCGCGTCTGATTAGCTAGTTGGTGGGGTAAAGGCCTAC
CAAGGCACCGATCAGTAACTGGTCTGAGAGGATGGCCAGTCACACTGGGACTGAGACACGGCC
>Zotu2729
GAACGCTGGCGGCGTGCCTAATACATGCAAGTCGAGCGGAGTTTCAAGAAGCTTGCTTTTTGAAACTTAGC
GGCGGACGGGTGAGTAACACGTGGGCAACCTGCCCCTTAGACTGGGATAACTCCGGGAAACCGGAGCTA
ATACCGGATAATCCCTTTCTCCACCTGGAGAGAGGGTGAAAGATGGCTTCGGCTATCACTAGGGGATGGG
CCCGCGGCGCATTAGCTAGTTGGTAAGGTAACGGCTTACCAAGGCGACGATGCGTAGCCGACCTGAGAGG
GTGATCGGCCACACTGGGACTGAGACACGGCC
>Zotu2732

GAACGCTGGCGGCGTGCTTAACACATGCAAGTCGAGCGAAGCAGTTAAGTTGATTTCTTCGGATTGATTCT
TGACTGACTGAGCGGCGGACGGGTGAGTAACGCGTGGGTGACCTGCCCCATACCGGGGGATAACAGCTG
GAAACGGCTGCTAATACCGCATAAGCGCACAGAGCTGCATGGCTCCGTGTGAAAAACTCCGGTGGTATGG
GATGGGCCCGCGTCTGATTAGGCAGTTGGCGGGGTAACGGCCCACCAAACCGACGATCAGTAGCCGGCCT
GAGAGGGCGACCGGCCACATTGGGACTGAGACACGGCC

>Zotu2734
GAACGCTGGCGGCGTGCCTAATACATGCAAGTCGAGCGGACTTGACAGAGCTTGCTCTGTTCAAGTCAGC
GGCGGACGGGTGAGTAACACGTGGGTAACCTGCCTGTAAGACTGGGATAACTCCGGGAAACCGGGGCTA
ATACCGGATAACATCAAGAGCTGCATGGCTCTTGATTGAAAGGCGGCTTCGGCTGTCACTTACAGATGGAC
CCGCGGCGCATTAGCTAGTTGGTGAGGTAACGGCTCACCAAGGCGACGATGCGTAGCCGACCTGAGAGG
GTGATCGGCCACACTGGGACTGAGACACGGCC

>Zotu2736
GAATGCTGGCGGCGTGCCTAACACATGCAAGTCGAGCGGTAACAGGTCTAGTTTACTAGATGCTGACGAG
CGGCGGACGGCTGAGTAACGCGTGGGAACGTGCCCCAAAGTGAGGAATAACTGCCCGAAAGGGTAGCTA
ATGCCGCATATGATCTTCGGATTAAAGTCTTCGGACGCTTTGGGAGCGGCCCGCGTTAGATTAGGTAGTTG
GTAGGGTAATGGCCTACCAAGCCGACGATCTATAGCTGGTCTGAGAGGATGATCAGCCAGACTGGGACTG
AGACACGGCC

>Zotu2738
GAACGCTGGCGGCGTGCCTAACACATGCAAGTCGAGCGATTCTCTTTTGAGAAGAGCGGCGGACGGGTGA
GTAACGCGTGGGTAACCTGCCCTGTACACACGGATAACATACCGAAAGGTATGCTAATACGGGATAATATA
TTTTGATGGCATCATCGAAATATCAAAGCTCCGGCGGTACAGGATGGACCCGCGTCTGATTAGCTAGTTGG
TGAGGTAATGGCTCACCAAGGCAACGATCAGTAGCCGACCTGAGAGGGTGATCGGCCACATTGGAACTGA
GACACGGTC

>Zotu2740
GAACGCTGGCGGCGTGCTTAACACATGCAAGTCGAACGAACCTTCGGGTTAGTGGCGAACGGGTGAGTAA
CACGTGGGCAACCTGCCTCGAGCTCTGGGATAACTCCGGGAAACCGGGGCTAATACCGGATATGACCTGC
TGAGACATCTTGGTGGGTGGAAAGTTTTTCGGCTCGAGATGGGCCCGCGGCCTATCAGCTTGTTGGTGGG
GTAATGGCCTACCAAGGCGACGACGGGTAGCCGGCCTGAGAGGGCGACCGGCCACACTGGGACTGAGAC
ACGGCC

>Zotu2742
GAACGCTGGCGGCGTGCCTAACACATGCAAGTCGAGCGGCAGCGAGTTTTACACTAAGTTCTGGAAGATA
TTCGAGAATTTTGTGTAAAATGTCGGCGAGCGGCGGACGGCTGAGTAACGCGTGGGAACATACCCCAAAG
TGAGGGATAACTGCCCGAAAGGGTGGCTAATACCGCATATGATCTTCGGATTAAAGCATTTATGCGCTTTG
GGAATGGCCTGCGTCGGATTAGGTAGTTGGTGAGGTAAAGGCTCACCAAGCCGACGATCCGTAACTGGTT
TGAGAGGATGACCAGTCAGATTGGAACTGAGACACGGTC

>Zotu2745
GAACGCTAGCAGAACGCCTAACACATGCAAGTCGAACGAACCAGCAATGGTTAGTGGCAAACGGGCGAGT
AACGCGTATCTAACCTACCCTTGAGTGAGGCACAACTACAGAGATGTAGCTAATTCCTCATACGTTCTAGAG
AATGAGAATATCTAGAAGAAAGATTTATCGCTCAGGGAGGGGGGTGCGTCCTATCAGGTAGTTGGTGAGG
TAATAGCTCACCAAGCCTATGACGGGTAGCCGGTCTGAGAGGATGTCCGGCCGCAATGGAACTGAGAACG
GTC

>Zotu2747
GAACGCTGGCGGCATGCTTAACACATGCAAGTCGAACGGTAGCAGGCCCTTCGGGGTGCTGACGAGTGGC
GGACGGGTGAGTAATGCGTAGGAATCTACCTAGTTGAGTGGGATAGCCTAGGGAAACTTAGGGTAATACC
GCGTAAGCCCGAGAGGGGAAAGCGGGGGACCGAAAGGCCTTGCGCGATTAGATGAGCCTACGTCGGATT
AGCTTGTTGGTGGGGTAAAGGCCTACCAAGGCGATGATCCGTAGCTGGTCTGAGAGGACGACCAGCCACA
CTGGGACTGAGACACGGCC

>Zotu2749
TCGAAATCGCGTAGACCTGGAGTCCACCATTGCTTGGTCGCGGAAAATTTTGTCCCAAAAATCCAACTTTTG
GCTTTATTTTGTCTTCAATATCTTTTGTTCATATAG

>Zotu2751
GAACGCTGGCGGCAGGCCTAACACATGCAAGTCGAGCGCGAAAGTCTTCGGATGAGTAGAGCGGCGAAC
GGGTGAGTAACGCGTGGGAATCTGCCTAGTAGTGGGGGACAACGTTCCGAAAGGAACGCTAATACCGCAT
ACGCCCTACGGGGGAAAGGGGGGGATCTTCGGACCTCTCGCTATTAGATGAGCCCGCGTCGGATTAGCTA
GTTGGTAGGGTAAAGGCCTACCAAGGCTACGATCCGTAACTGGTCTGAGAGGATGATCAGTCACACTGGA
ACTGAGACACGGTC
>Zotu2753
GAACGCTGGCGGCGTGCTTAACACATGCAAGTCGAACGATGAAAGCGGGAGCTTGCTCCTGCTGGATTAG
TGGCGAACGGGTGAGTAACACGTGAGTAACCTGCCCTTGACTCTGGGATAAGCGTTGGAAACGACGTCTA
ATACCGGATACGAGCTTCGGTCGCATGACCAGGAGCTGGAAAGAACTTCGGTCAAGGATGGACTCGCGGC
CTATCAGCTAGTTGGTGAGGTAACGGCTCACCAAGGCGACGACGGGTAGCCGGCCTGAGAGGGTGACCG
GCCACACTGGGACTGAGACACGGCC
>Zotu2756
GAACGCTAGCGGGAGGCTTAACACATGCAAGCCGAGGGGTATAGTTCTTTCGGGAACTAGAGACCGGCGC
ACGGGTGCGTAACGCGTATGCAACTTACCCTACTGAAAAGGATAGCCCTTCGAAAGGAGGATTAATACTTT
ATAACAGATTTAATGGCATCATTAGATTTTGAAAGATTTATCGCAGTAGGATAGGCATGCGTAAGATTAGTT
AGTTGGTGAGGTAACGGCTCACCAAGACGATGATCTTTAGGGGGCCTGAGAGGGTGAACCCCCACACTGG
TACTGAGACACGGAC
>Zotu2758
GAACGCTGGCGGTGTGCTTCACACATGCAAGTCGAACGAGGTAGCAATACCTAGTGGCGGACGGGTGAGT
AACGCGTGGGAATCTGCCTTTAGATGGGGGATAACGAGCCGAAAGGTTCACTAATACCGCATATGCCGCA
AGGTGAAAGGAGTAATCCGTCTAAAGATGAGCCCGCGTCCAATTAGCTAGTTGGTGGGGTAAAGGCCTAC
CAAGGCGACGATCGGTAGCTGGTCTGAGAAGATGATCAGCCACAATGGGACTGAGACACGGCC
>Zotu2760
GAACGTTGGCGGCAGGCTTAACACATGCAAGTCGAGCGGAGAGAGGTAGCTTGCTACTGATCTTAGCGGC
GGACGGGTGAGTAATGCTTAGGAATCTGCCTATTAGTGGGGGACAACATTTCGAAAGGAATGCTAATACC
GCATACGTCCTACGGGAGAAAGCAGGGGATCTTCGGACCTTGCGCTAATAGATGAGCCTAAGTCGGATTA
GCTAGTTGGTGGGGTAAAGGCCTACCAAGGCGACGATCTGTAGCGGGTCTGAGAGGATGATCCGCCACAC
TGGGACTGAGACACGGCC
>Zotu2762
GAACGCTAGCGGCAGGCCTAACACATGCAAGTCGAGGGGTAGAAGTCTTCGGACTTTGAGACCGGCGCAC
GGGTGCGTAACGCGTATGCAATCTACCTTGTACTGAGGGATAGCCCAGAGAAATTTGGATTAATACCTCAT
AGTATTTTCGGGTGACATCACCTGATTATTAAAGTTCCAACGGTACAAGATGAGCATGCGTCCCATTAGCTA
GTTGGTATGGTAACGGCATACCAAGGCTATGATGGGTAGGGGTCCTGAGAGGGAGATCCCCCACACTGGT
ACTGAGACACGGAC
>Zotu2764
GAACGCTGGCGGCGTGCCTAATACATGCAAGTCGAGCGCCCTTGACAGCGCATTTCTTTCCTTCGTTTTAGA
CGGGGGGAATCAAGGAATGTAGTGTCAAGGGAGCGGCGGACGGCTGAGTAACGCGTAGGAACGTACCCC
AAAGTGAGGGATAAGCACCGGAAACGGTGTCTAATACCGCATAATATCTCCGGATTAAAGCTTCGGCGCTT
TGGGAGCGGCCTGCGTATGATTAGCTTGTTGGTGAGGTAATGGCTCACCAAGGCGACGATCATTAGCTGG
TCTGAGAGGATGACCAGCCAGACTGGAACTGAGACACGGTC
>Zotu2766
GAACGCTGGCGGCGTGCCTAATACATGCAAGTCGAGCGAACGGACAGGAGTGCTTGCACTCCTTGAAGTT
AGCGGCGGACGGGTGAGTAACACGTGGGTAACCTACCTGTAAGACTGGGATAACTTCGGGAAACCGGAG
CTAATACCGGATAATATTTTCCACCTCATGGCGGAATAGTGAAAGACGGTTTCGGCTGTCACTTACAGATG
GACCCGCGGCGCATTAGCTAGTTGGTGAGGTAACGGCTCACCAAGGCGACGATGCGTAGCCGACCTGAGA
GGGTGATCGGCCACACTGGGACTGAGACACGGCC
>Zotu2768
GAACGCTGGCGGCGTGCTTAACACATGCAAGTCGAACGCTGAAGCCCAGCTTGCTGGGTGGATGAGTGGC
GAACGGGTGAGTAACACGTGAGTAACCTGCCCCCTTCTTTGGGATAACGCCCGGAAACGGGTGCTAATACT

GGATATTCACTGATCTTCGCATGGGGGTTGGTGGAAAGGTTTGTTCTGGTGGGGGATGGGCTCGCGGCCT
ATCAGCTTGTTGGTGGGGTGATGGCCTACCAAGGCTTTGACGGGTAGCCGGCCTGAGAGGGTGACCGGTC
ACATTGGGACTGAGATACGGCC
>Zotu2770
GAACGCTAGCGGCAGGCCTAATACATGCAAGTCGGACGGGATTGAGGAGCTTGCTCTTCATGAGAGTGGC
GCACGGGTGCGTAACACGTATGTAACCTACCTTGTTCAGGGGGATAGCCTCTCGAAAGAGAGATTAACACC
GCATAAAATCAATTTATGGCATCATAGAATGATCAAATATTTATAGGAACAAGATGGGCATGCGGAACATT
AGCTAGTTGGTAAGGTAACGGCTTACCAAGGCTACGATGTTTAGGGGATCTGAGAGGATGACCCCCCACA
CTGGTACTGAGACACGGAC
>Zotu2772
GAACGCTGGCGGCGTGCCTAATACATGCAAGTCGAGCGGACGTTTTATTGGTGCTTGCACCGATTTAACGT
TAGCGGCGGACGGGTGAGTAACACGTGGGCAACCTACCCTGTAGATTGGGATAACTCCGGGAAACCGGG
GCTAATACCGAATAATACTTTTTAACTCATGTTAGAAAGTTGAAAGATGGTTTACGCTATCACTACAGGATG
GGCCCGCGGCGCATTAGCTAGTTGGTGAGGTAATGGCTCACCAAGGCCACGATGCGTAGCCGACCTGAGA
GGGTGATCGGCCACACTGGGACTGAGACACGGCC
>Zotu2774
TAACGCTAGCGGCAGGCCTAATACATGCAAGTCGAACGGTAACATGGGTAGCAATATCTGATGACGAGTG
GCGCACGGGTGCGTAACACGTATGCAATCTACCTCTAACTGGGGAATAGCCTTCCGAAAGGGAGATTAAA
ACCGCATAATATCACGAGACGGCATCGCCTTGTGATTAAATCTACGGAGGTTAGAGATGAGCATGCGTCCG
ATTAGCTAGTTGGTAGAGTAACAGCCTACCAAGGCTACGATCGGTAGGGGGTCTGAGAGGATGATCCCCC
ACACTGGTACTGAGACACGGAC
>Zotu2776
GAACGCTGGCGGCGTGCTTAACACATGCAAGTCGAACGGGAAATACTTTATTGAAACTTCGGTGGATTTAA
TTTATTTCTAGTGGCGGACGGGTGAGTAACGCGTGGGTAACCTGCCTTATACTGGGGGATAACAGCCAGA
AATGACTGCTAATACCGCATAAGCGCACAGAACCGCATGGTTCGGTGTGAAAAACTCCGGTGGTATAAGAT
GGACCCGCGTTGGATTAGCTAGTTGGCAGGGCAGTGGCCTACCAAGGCGACGATCCATAGCCGGCCTGAG
AGGGTGAACGGCCACATTGGGACTGAGACACGGCC
>Zotu2781
GAACGCTGGCGGCGTGCTTAACACATGCAAGTCGAACGGAAAGGCCCTGCTTGCAGGGTACTCGAGTGGC
GAACGGGTGAGTAACACGTGGGTGATCTGCCCTGCACTTCGGGATAAGCCTGGGAAACTGGGTCTAATAC
CGGATAGGACCATCGTTTAGTGTCGGTGGTGGAAAGTTTTTCGGTGCAGGATGAGCCCGCGGCCTATCAG
CTTGTTGGTGGGGTAATGGCCTACCAAGGCGTCGACGGGTAGCCGGCCTGAGAGGGTGGACGGCCACATT
GGGACTGAGACACGGCC
>Zotu2783
GAACGCTGGCGGCGTGCCTAATACATGCAAGTCGAGCGAATCGATGGGAGCTTGCTCCCTGAGATTAGCG
GCGGACGGGTGAGTAACACGTGGGTAACCTGCCTGTAAGACTGGGATAACTTCGGGAAACCGGAGCTAAT
ACCGGATAGTTTCTTTTCTCGCATGAGAAGAGATGGAAAGATGGTTCTGCTATCACTTACAGATGGACCCG
CGGCGCATTAGCTAGTTGGTGAGGTAACGGCTCACCAAGGCGACGATGCGTAGCCGACCTGAGAGGGTG
ATCGGCCACACTGGGACTGAGACACGGCC
>Zotu2785
GAACGCTGGCGGCAGGCCTAACACATGCAAGTCGAACGGACTCTTCGGAGTTAGTGGCGGACGGGTGAG
TAACACGTGGGAACGTGCCTTTAGGTTCGGAATAAGCCCGGGAAACTGGGTCTAATACCGAATGTGCCCTT
CGGGGGAAAGATTTATCGCCTTTAGAGCGGCCCGCGTCTGATTAGCTAGTTGGTGGTGTAATGGACCACCA
AGGCGACGATCAGTAGCTGGTCTGAGAGGATGACCAGCCACATTGGGACTGAGACACGGCC
>Zotu2788
GAACGCTAGCGGCAGGCCTAATACATGCAAGTCGAACGGTGATAGCAATATCATAGTGGCGCACGGGTGC
GTAACACGTATGCAACCTACCTTATACTGAGGGATAGCCTTTGGAAACGGAGATTAATACCTCATAGGACA
GTTGACTTGGCATCAAGTTACTGTTAAAGATTTATTGGTATAAGATGGGCATGCGTCCAATTAGTTAGTTGG
CGAGGTAACGGCTCACCAAGACGATGATTGGTAGGGGAACTGAGAGGTTAATCCCCCACACTGGCACTGA
GATACGGGC

>Zotu2791
GAACGCTGGCGGCGCGCTTAATACATGCAAGTCGAGCGAGAACCGGCCCTTCGGGGCCGGGGAAAGCGG
CGAACGGGTGAGTAACACGTGGGTAATCTGCCCTCGGCACCGGAATAGCCCGGGGAAACCCGGATTAATG
CCGGATGGCCTCTCGAGCTCTTGGGCTCGTGAGAAAAGGTAGCTTCGGCCTCCGGCCGAGGAGGAGCCCG
CGGCGGATTAGCTTATTGGTGGGGTAACGGCCCACCAAGGCGACGATCCGTAGCTGGTCTGAGAGGACGA
TCAGCCACACTGGGACTGAGACACGGCC
>Zotu2794
GAACGCTGGCGGCGTGCCTAACACATGCAAGTCGAACGAGGTCCATCCTTCGGGAGAAGACCTAGTGGCG
AACGGGTGAGTAACACGTGAGGAACCTGCCCCGAAGACCGGGATAACCCTCCGAAAGGAGAGCTAATACC
GGATACCCCCACCGAACCGCATGGTTTGGTGAGGAAATGGATTCCGCTTCGGGAGGGCCTCGCGGCCTAT
CAGCTAGTTGGTGGGGTAACGGCCCACCAAGGCATCGACGGGTAGCTGGTCTGAGAGGACGATCAGCCAC
ACTGGGACTGAGACACGGCC
>Zotu2799
GAACGCTGGCGGCGTGGATAAGGCATGCAAGTCAAGGGGGTTTCGCAGCAATGCGAAGCAACCGGCGAA
CGAGTTAGTAACACGTAGGTATCTACCCCAAAGTGGGACATAGCCCCGCGAAAGCGGGGGTAATTTCCCAT
GGTCTCGCAAGAGTAAAGTCGCAAGACGCTTTGGGATGAGCCTGCGTCCTATCAGCTAGTTGGTGAGGTA
ATGGCTCACCAAGGCAATGACGGGTAGGGGGTGTGAGAGCACGACCCCCAACGATGGGACTGAGATACG
GCC
>Zotu2801
GAACGCTGGCGGCGTGCCTAATACATGCAAGTCGAACGGTTTAGTTCCAAGAGGCGTGCCTGTTGGGACT
AGGCAGTGGCGGACGGGTGAGTAACACGTAGGCAACCTACCTGTCAAACCGGGATAACCTCGGGAAACC
GAAGCTAAACCCGGATAAGCTCCTTTTTCGCATGAAGGGGGAGTGAAAAGGTGCGACCTGATGACAGATG
GGCCTGCGGCGCATTAGCTAGTAGGTAGGGTAACGGCCTACCTAGGCGATGATGCGTAGCCGACCTGAGA
GGGCGTACGGCCACACTGGGACTGAGATACGGCC
>Zotu2804
GAACGCTGGCGGCATGCCTTACACATGCAAGTCGAACGGTAGAGGAGCAATCCTCGAGAGTGGCGAACG
GGTGAGTAATATATCGGAACGTGCCCAGTAGTGGGGGATAGCCCGGCGAAAGCCGGATTAATACCGCATG
AGACCTGAGGGTGAAAGCGGGGGACTCGCAAGAGCCTCGCGCTACTGGAGCGGCCGATATCAGATTAGG
TAGTTGGTGGGGTAAAAGCCTACCAAGCCTGCGATCTGTAGCTGGTCTGAGAGGACGACCAGCCACACTG
GGACTGAGACACGGCC
>Zotu2806
GAACGCTGGCGGCGTGCCTAATACATGCAAGTCGAGCGGACAGACAGGAGTGCTTGCACTCCTTGAAGTC
AGCGGCGGACGGGTGAGTAACACGTGGGTAACCTACCTATAAGACTGGGATAACTTCGGGAAACCGGAG
CTAATACCGGATAATATGTTTCACCTCATGGTGAAACAGTGAAAGACGGTTCTGCTGTCACTTATAGATGGA
CCCGCGGCGCATTAGCTAGTTGGTGAGGTAACGGCTCACCAAGGCGACGATGCGTAGCCGACCTGAGAGG
GTGATCGGCCACACTGGGACTGAGACACGGCC
>Zotu2808
GAACGCTGGCGGCGTGCCTAATACATGCAAGTGGAACGCATTGATTTCACCGGAGCTTGCTCCACCGAAAT
TAGTGAGTCGCGAACGGGTGAGTAACGCGTAGGTAACCTACCTCTTAGCGGGGGATAACTATTGGAAACG
ATAGCTAATACCGCATAACAATTGGATACACATGTACCCTATTTAAAAGGAGCAACTGCTTCACTAAGAGAT
GGACCTGCGTTGTATTAGCTAGTTGGTAGGGTAACGGCCTACCAAGGCATCGATACATAGCCGACCTGAGA
GGGTGATCGGCCACACTGGGACTGAGACACGGCC
>Zotu2810
GAACGCTGGCGGCGTGCCTAATACATGCAAGTCGAGCGGTAAGGCCTCTTCGGAGGTACACGAGCGGCG
GACGGCTGAGTAACACGTAGGAACGTACCCCAAAGTGAGGGATAAGCACCAGAAATGGTGTCTAATACCG
CATGTGATCTTCGGATTAAAGCTCCGGCGCTTTGGGAACGGCCTGCGCATGATTAGCTTGTTGGTGAGGTA
ATAGCTCACCAAGGCGACGATCATTAGCTGGTCTGAGAGGATGACCAGCCAGACTGGGACTGAGAACGGC
C
>Zotu2812

GAACGCTAGCGGCAGGCTTAACACATGCAAGTCGAGGGGTAGGGTAGCAATACCTGAGACCGGCGCACG
GGTGCGTAACGCGTATGCAATCTACCTTATACAGAGGGATAGCCCAGAGAAATTTGGATTAATACCTCATG
GTATTTTCGAATGGCATCATTTGATTATTAAAGTTCCAACGGTATAAGATGAGCATGCGTCCCATTAGCTAG
ATGGTAAGGTAACGGCTTACCATGGCTACGATGGGTAGGGGTCCTGAGAGGGAGATCCCCCACACTGGTA
CTGAGACACGGAC
>Zotu2814
GAACGCTGGCGGCGTGCCTAACACATGCAAGTCGAACGTGAAAATCTTCGGATGAGTAAAGTGGCGTACG
GGTGAGTAACACGTGGGTAATCTACCTTAAAGTTTGGAATAACCTGCCGAAAGGCGGGCTAATACCAGAT
AGAGCGAGAAAGACGTGAAGTCTTTTTCGGGAAAGGGAGCTCGCAAGAGCCCTTGCTTTAAGATGAGCCT
GCGCTCCATTAGCTAGTTGGTAGGGTAAAAGCCTACCAAGGCGATGATGGATAGCTGATTTAAGAGGATG
ATCAGCCACACTGGAACTGAAACACGGTC
>Zotu2817
GAACGCTGGCGGCGTGCCTAATACATGCAAGTCGAGCGAATCAATAGGTGCTTGCACCTATTGATTAGCGG
CGGACGGGTGAGTAACACGTGGGCAACCTGCCTGTAAGACTGGGATAACACCGGGAAACCGGTGCTAATA
CCGGATAATCCTTTTCCTCTCATGAGGAAAAGCTGAAAGTCGGTTTCGGCTGACACTTACAGATGGGCCCG
CGGCGCATTAGCTAGTTGGTGAGGTAACGGCTCACCAAGGCGACGATGCGTAGCCGACCTGAGAGGGTG
ATCGGCCACACTGGGACTGAGACACGGCC
>Zotu2820
GAACGCTGGCGGCGTGCCTAATACATGCAAGTCGAGCGGAGTCATTCCTTCGGGGATGGCTTAGCGGCGG
ACGGGTGAGTAACACGTAGGCAACCTGCCTGCAAGATCGGGATAACATTCGGAAACGAATGCTAATACCG
GATACACAATTTCCTCGCATGGGGAGATTGGGAAAGACGGCGCAAGCTGTCACTTGCAGATGGGCCTGCG
GTGCATTAGCTAGTTGGTGGGGTAACGGCTCACCAAGGCGACGATGCATAGCCGACCTGAGAGGGTGATC
GGCCACACTGGGACTGAGACACGGCC
>Zotu2822
GAACGCTGGCGGCGTGCTTAACACATGCAAGTGGAGCGACGAACCAGGGCTTGCCCTGGGGCAGAGCCG
CGAACGGGTGAGTAACACGTGGGTAACCTACCCCGAAGACTGGGACAACCCGAGGAAACTCGGGCTAATA
CCAGATGTGGTCTCTCTTCATAAGTAGAGCGACTAAAGGGAGCTTCGGCCCCCGCTTTGGGAGGGGCCCGC
GGCCCATTAGCTTGTTGGTGAGGTAACGGCTCACCAAGGCTTCGATGGGTAGCTGGTCTGAGAGGACGAT
CAGCCACACTGGGACTGAGACACGGCC
>Zotu2824
GAACGCTGGCGGCGTGCCTAATACATGCAAGTCGAGCGAACAGATAAGGAGCTTGCTCCTTTGACGTTAG
CGGCGGACGGGTGAGTAACATGTGGGTAACCTACCTATAAGACTGGAATAACTCCGGGAAACCGGGGCTA
ATGCCGGATAACATATAGAACCGCATGGTTCTATAGTGAAAGATGGTTTTGCTATCACTTATAGATGGACCC
GCGCCGTATTAGCTAGTTGGTAAGGTAATGGCTTACCAAGGCGACGATACGTAGCCGACCTGAGAGGGTG
ATCGGCCACACTGGAACTGAGACACGGTC
>Zotu2826
GAACGCTGGCGGCGTGCTTAACACATGCAAGTCGAACGGAAAGGCCCTTCGGGGTACTCGAGTGGCGAAC
GGGTGAGTAACACGTGGGTGATCTGCCCTGCACTTTGGGATAAGCCTGGGAAACTGGGTCTAATACCGAA
TAGGACCACGCGCTTCATGGTGTGTGGTGGAAAGCTTTTGCGGTGTGGGATGGGCCCGCGGCCTATCAGC
TTGTTGGTGGGGTAATGGCCTACCAAGGCGACGACGGGTAGCCGGCCTGAGAGGGTGACCGGCCACACT
GGGACTGAGATACGGCC
>Zotu2829
GAACGCTGGCGGCGTGCCTAATACATGCAAGTAGAACGCTGAAGCTTGGTGCTTGCACCGAGCGGATGAG
TTGCGAACGGGTGAGTAACGCGTAGGTAACCTGCCTCTTAGCGGGGGATAACTATTGGAAACGATAGCTA
ATACCGCATAAAAGACGATATCGCATGATATTGATTTGAAAGGTGCAATTGCATCACTAAGAGATGGACCT
GCGTTGTATTAGCTAGTTGGTGAGGTAACGGCTCACCAAGGCGACGATACATAGCCGACCTGAGAGGGTG
ATCGGCCACACTGGGACTGAGACACGGCC
>Zotu2831
GAACGCTGGCGGCATGCCTTACACATGCAAGTCGAACGGTAACAGGTCTTCGGACGCTGACGAGTGGCGA
ACGGGTGAGTAACACATCGGAACGTGCCCGATCGTGGGGGATAACGGAGCGAAAGCTTTGCTAATACCGC
ATACGATCTACGGATGAAAGCAGGGGACCGCAAGGCCTTGCGCGGACGGAGCGGCCGATGGCAGATTAG

GTAGTTGGTGGGATAAAAGCTTACCAAGCCGACGATCTGTAGCTGGTCTGAGAGGATGATCAGCCACACT
GGGACTGAGACACGGCC
>Zotu2834
GAACGCTGGCGGCAGGCTTAACACATGCAAGTCGAGCGGGGGAGATTGCTTCGGTGATTGACCTAGCGGC
GGACGGGTGAGTAATACTTAGGAATCTGCCTATTAATGGGGGACAACATCTCGAAAGGGATGCTAATACC
GCATACGCCCTACGGGGGAAAGCAGGGGATCACTTGTGACCTTGCGTTAATAGATGAGCCTAAGTCGGAT
TAGCTAGTTGGTGGGGTAAAGGCCTACCAAGGCGACGATCTGTAGCGGGTCTGAGAGGATGATCCGCCAC
ACTGGGACTGAGACACGGCC
>Zotu2836
GAATGCTGGCGGTGTGGATAGGGCATGCAAGTCAAGGGGACTCAGTTAGTAATAATTGGGTAACCGGCA
GACGAGATAGTAACGCGTAGGAACGTACCCAGAAGTCGGGCACAGCTCGCCGAAAGGCGGGGTAATTCC
CGATGGTGACGAAAGTCTAAAGATTTATCGCTTCTGGAGCGGCCTGCGTAATATCAGCTTGTTGGTGAGGT
AATGGCTCACCAAGGCTATGACGTTTAGGGGAGCTGAGAGGCTGACCCCCATCGATGGAATTGAGATACG
GTC
>Zotu2838
AAACGCTGGCGGCGTGCCTAATACATGCAAGTCGATCGCTGGAATTCCAGCTTGCTGGAATGAAGGAGAG
GCGAACGGGTGAGTAATACATAAGCAACCTGCCCACGAAGACTGGGATAATTCCTGGAAACGGGGACTAA
TACCGGATAGGTAGTGTGATCGCATGATCGCACTATTAAAGGTTCAAGACACTGGTGGATGGGCTTATGGC
GCATTAGCTGGTTGGTGAGGTAACGGCCCACCAAGGCGATGATGCGTAGCCGGCCTGAGAGGGTGAACG
GCCACATTGGGACTGAGACACGGCC
>Zotu2840
GAACGCTGGCGGCATGCCTTACACATGCAAGTCGAACGCGAAAGGGGGGGCAACTCCCCAAGTAGAGTG
GCGAACGGGTGAGTAATACATCGGAACGTGTCCAGTAGCGGGGGATAACCTCTCGAAAGAGAGGCTAAT
ACCGCATACGTCCTACGGGAGAAAGAGGGGGATCGCAAGACCTCTCACTATTGGAGCGGCCGATGTCGGA
TTAGCTAGTTGGTGGGGTAAAAGCCCACCAAGGCGACGATCCGTAGCTGGTCTGAGAGGACGACCAGCCA
CACTGGAACTGAGACACGGTC
>Zotu2842
GAACGTTAGCGGCAGGCCTAACACATGCAAGTCGAGCGAGAATAGGGCTTCGGCTCTTAGTAAAGCGGCG
GACGGGTGCGTAACACGTAGATAATCTGCCCTCTAGCGGGGAACAACAGTCCGAAAGGATTGCTAATGCC
GCATGTCACTGCGGGGACTCCGGTCCCTGTTGGAAAAGGGGCCCGCAAGGGTCTCACTAGAGGATGAGTC
TGCGGCCCATCAGCTTGTTGGCGGGGTAATGGCCCACCAAGGCTAAGACGGGTAGCTGGTCTGAGAGGAT
AACCAGCCACACTGGGACTGAGACACGGCC
>Zotu2845
GAACGCTGGCGGCAGGCCTAACACATGCAAGTCGAGCGGTAGAGAGGAGCTTGCTCCTCTTGAGAGCGGC
GGACGGGTGAGTAATGCCTAGGAATCTGCCTGGTAGTGGGGGATAACGTTCGGAAACGGACGCTAATACC
GCATACGTCCTACGGGAGAAAGCAGGGGACCTTCGGGCCTTGCGCTATCAGATGAGCCTAGGTCGGATTA
GCTAGTTGGTGAGGTAATGGCTCACCAAGGCGACGATCCGTAACTGGTCTGAGAGGATGATCAGTCACAC
TGGAACTGAGACACGGTC
>Zotu2848
GAATGCTGGCGGCGTGGATGAGGCATGCAAGTCGAATGGGTTTAGACCCATGGCAGACGAGGTAGGAAC
ACGTAGGTACGTACCGGAGAGTCAGGGATAACCTGGAGAAATCCAGAATAATACTTGATGGTCCCGAGAG
GGTAAAGATTTATCGCTCTCTGAACGGCCTGCGTTCTATCAGCTTGTTGGTAAGGTAACGGCTTACCAAGGC
TACGACGGATAGGGGAGGTGAGAGCCTGACCCCCACCGATGGAACTGCGACACGGTC
>Zotu2850
GAACGCTGGCGGCGTGCTTAACACATGCAAGTCGAACGATGAAGCCCAGCTTGCTGGGTGGAAGAGTGGC
GAACGGGTGAGTAACACGTGAGTAACCTGCCCCGGACTCTGGGATAAGCGCTGGAAACGGCGTCTAATAC
TGGATATGACCCATACCGGCATCGGTTGTGGGTGGAAAGATTTATCGGTTCGGGATGGGCTCGCGGCCTAT
CAGCTGGATGGTGAGGTAACGGCTCACCATGGCGACGACGGGTAGCCGGCCTGAGAGGGTGACCGGCCA
CACTGGGACTGAGACACGGCC
>Zotu2852

GAACGCTGGCGGTATGCTTAACACATGCAAGTCGAACGGAGCTTTTCGGAGCTTAGTGGCGGACGGGTGA
GTAACGCGTGAGAATCTGCCTTTTGGACCGGGACAACTGCGGGAAACGGCAGCTAAGACCGGATGTGCCT
TCGGGTGAAATATTCATAGCCAAAAGAGGAGCTCGCGACCGATTAGCTAGTTGGTGGGGTAAGAGCCTAC
CAAGGCTGCGATCGGTAGCCGGTTTGAGAGGACGACCGGCCACACTGGGACTGAGACACGGCC
>Zotu2854
GAACGCTAGCGGCAGGCCTAATACATGCAAGTCGAACGGTCAGTAGCAATACTGATAGTGGCGCACGGGT
GCGTAACACGTAACTAACCTACCTATAACTGGGGGATAGCCCGCCGAAAGGCGGATTAATACCGCATAAGC
TAGTTAAACGGCATCGTTTGATTAGTAAAGATTTATTGGTTATAGATGGGGTTGCGGGTCATTAGCTAGTT
GGTAAGGTAACGGCTTACCAAGGCGACGATGACTAGGGGAGCTGAGAGGCTGGTCCCCCACACGGGCAC
TGAGATACGGGC
>Zotu2857
GAACGCTGGCGGTATGCCTTAAGCATGCAAGTCGAGCGCATTGGAAGTCGCAAGATTTTCAGTGAGCGGC
GGACGGGTGAGTAATAGTAAGGAACGTACCCCGAAGTGGAGGATAGCTCGCCGAAAGGCGGGGTAAGCC
TGCATAGTATCGTAAGATTAAAGAGCGTAACTGCTCGCTTTGGGAGCGGCCTTATTCCTATCAGCTTGTTGG
TGAGGTAATAGCTCACCAAGGCTATGACGGGTAGGGGGCGTGAGAGCGTGACCCCCCACAATGGCACTGA
GACACGGGC
>Zotu2859
GAACGCTGGCGGCGTGCTTAACACATGCAAGTCGAACGGAAAGGCCCTTCGGGGTACTCGAGTGGCGAAC
GGGTGAGTAACACGTGGGTGATCTGCCCTGCACTTCGGGATAAGCTTGGGAAACTGGGTCTAATACCGGA
TAGGATCATGGCTCTCATGGGTTGTGGTGGAAAGCTTTTGCGGTGTGGGATGGGCCCGCGGCCTATCAGC
TTGTTGGTGGGGTAATGGCCTACCAAGGCGTCGACGGGTAGCCGGCCTGAGAGGGTGTCCGGCCACACTG
GGACTGAGATACGGCC
>Zotu2861
GAACGCTGGCGGCATGCCTTACACATGCAAGTCGAACGGTAACGCGGGGCAACCTGGCGACGAGTGGCG
AACGGGTGAGTAATACATCGGAACGTGCCCAGTAGTGGGGGATAGCCCGGCGAAAGCCGGATTAATACC
GCATACGACCTACGGGTGAAAGCGGGGGATCGCAAGACCTCGCGCTACTGGAGCGGCCGATGTCAGATTA
GGTAGTTGGTGGGGTAAAGGCCTACCAAGCCGACGATCTGTAGCTGGTCTGAGAGGACGACCAGCCACAC
TGGGACTGAGACACGGCC
>Zotu2863
GAACGCTGGCGGCGTGCTTAACACATGCAAGTCGAGCGGAAAGGCCCTTTGGGGTACTCGAGCGGCGAAC
GGGTGAGTAACACGTGGGTAACCTGCCCCTTGCTCTGGGATAACTCCGGGAAACCGGTGCTAATACTGGAT
ATTCATGCTTGCAGGCATCTGTGGGTGTGGAAAGTTTTTTCGGCGAGGGATGGACCCGCGGCCTATCAGCT
TGTTGGTGGGGTGATGGCCTACCAAGGCTTTGACGGGTAGCCGGCCTGAGAGGGCGACCGGCCACACTG
GGACTGAGACACGGCC
>Zotu2866
GAACGCTAGCGGCAGGCCTAATACATGCAAGTCGAGGGGCAGCACGATCTTCGGATTGGTGGCGACCGGC
GCACGGGTGCGTAACGCGTATGCAACCTACCTATTACTAAGGGATAGCCCGGGGAAACTCGGATTAATACC
TTATAGCATTACAAGATGGCATCTGAAAGTAATTAAAGATTTATCGGTAATAGATGGGCATGCGTATCATTA
GTTAGTTGGTGAGGTAATGGCTCACCAAGACCGCGATGATTAGGGGGCCTGAGAGGGTGATCCCCCACAC
TGGTACTGAGATACGGAC
>Zotu2868
GAACGCTGGCGGCGTGCCTAACACATGCAAGTCGAACGAGAAAGGGGCTTCGGCTCCGAGTAAAGTGGC
GCACGGGTGAGTAACACGTAGGTAACCTGCCTCTAAGATTGGGATAACCTGTCGAAAGACGAGCTAATAC
CAAATAAGACCACGGTCTCCTCGGGGACAGGGGTCAAAGGTAGCCTCTATTTATAAGCTATCGCTGAGAGA
TGGGCCTGCGTACCATTAGCTAGTTGGTGAGGTAACGGCTCACCAAGGCAAAGATGGTTAGCTGGTCTGA
GAGGATGATCAGCCACACTGGAACTGAGACACGGTC
>Zotu2871
GAACGCTGGCGGCGTGCCTAACACATGCAAGTCGAGCGATTCTCTTCGGAGAAGAGCGGCGGACGGGTG
AGTAACGCGTGGGTAACTTGCCCTGTACACACGGATAACATACCGAAAGGTATGCTAATACGAGATAATAT
ACTTTTATCGCATGGTAGAAGTATCAAAGCTCCGGCGGTACAGGATGGACCCGCGTCTGATTAGCTAGTTG

GTAAGGTAACGGCTTACCAAGGCGACGATCAGTAGCCGACCTGAGAGGGTGATCGGCCACATTGGAACTG
AGACACGGTC
>Zotu2873
GAACGCTAGCGGCAGGCTTAACACATGCAAGTCGAGGGGTAGAAGCAGCTTGCTGCTTTGAGACCGGCGC
ACGGGTGCGTAACGCGTATACAATCTACCTTTTACAGAGGGATAGCCCAGAGAAATTTGGATTAATACCTC
ATAGTATTTTCGAATCGCATGATTTGATTATTAAAGTTCCAACGGTAAAAGATGAGTATGCGTCCCATTAGC
TAGATGGTAAGGTAACGGCTTACCATGGCAACGATGGGTAGGGGTTCTGAGAGGGAGATCCCCCACACTG
GTACTGAGACACGGAC
>Zotu2878
GAACGCTGGCGGCGTGCCTAACACATGCAAGTCGAGCGGGGTGAGTTCTTCGGAACGATCCTAGCGGCGG
ACGGGTGAGTAACACGTGGGTAACCTGCCTCAAAGTGGGGGATAGCCTTTCGAAAGGAAGATTAATACCG
CATAACCCATAGCTTCCGCATGGAAGCAATGGTAAAGATTTATCGCTTTGAGATGGACCCGCGGCGCATTA
GCTAGTTGGTGAGGTAACGGCTCACCAAGGCCACGATGCGTAGCCGACCTGAGAGGGTGATCGGCCACAT
TGGAACTGAGACACGGTC
>Zotu2880
TAACGCTGGCGGTATGCCTAACACATGCAAGTCGAACGGAATGCTATTTAAGAAAATCTCACACTAGTGAG
GGATTTTCACGAGTAGTATTTAGTGGCGGACGGGTGAGTAACACGTTGGAACCTGCCCCAAAGTGGGGAA
TAACTGCCCGAAAGGGTAGCTAATACCCCATAGTATCCAGTTTATCTGGATTAAAGATTTATCGCTTTGGGA
GGGGCCTGCGTCTGATTAGCTAGTTGGTGAGGTAAAAGCTCACCAAGGCGACGATCAGTAGCTGGTTCGA
GAGGATGACCAGCCACACTGGGACTGAGATACGGCC
>Zotu2883
GAACGCTGGCGGCGTGCCTAATACATGCAAGTCGAGCGAACAGATAAGGAGCTTGCTCCTTTGACGTTAG
CGGCGGACGGGTGAGTAACACGTGGGTAACCTACCTATAAGACTGGAATAACTCCGGGAAACCGGGGCTA
ATGCCGGATAACATATAGAACCGCATGGTTCTATAGTGAAAGATGGTTTTGCTATCACTTATAGATGGACCC
GCGCCGTATTAGCTAGTTGGTAAGGTAACGGCTTACCAAGGCGACGATACGTAGCCGACCTGAGAGGGTG
ATCGGCCACACTGGAACTGAGACACGGTC
>Zotu2885
GAACGCTGGCGGCGTGCTTAACACATGCAAGTGGAGCGACGAACCGGGGCTTGCCCCGGGGCAGAGCCG
CGAACGGGTGAGTAACACGTGGGTAACCCACCTCGATGACCGGGACAACCCGAGGAAACTCGGGCTAATA
CCGGATGTGATCCTCTTCCGCAAGGAAGTTGATGAAAGATAGCTTCGGCCTTCGCATCGAGACGGGCCCGC
GGCCCATTAGCTTGTTGGTGGGGTAACGGCCTACCAAGGCTTCGATGGGTAGCTGGTCTGAGAGGACGAT
CAGCCACACTGGGACTGAGACACGGCC
>Zotu2888
GAACGCTGGCGGCAGGCTTAACACATGCAAGTCGAACGGGCACTTCGGTGCTAGTGGCAGACGGGTGAG
TAACACGTGGGAACGTACCTTTCGGTTCGGAATAATTCAGGGAAACTTGGACTAATACCGGATACGCCCTT
CGGGGGAAAGATTTATCGCCGATAGATCGGCCCGCGTCTGATTAGCTAGTTGGTGAGGTAATGGCTCACC
AAGGCGACGATCAGTAGCTGGTCTGAGAGGATGGCCAGCCACACTGGGACTGAGACACGGCC
>Zotu2890
GAACGCTGGCGGCATGCTTAACACATGCAAGTTGAACGCAGAATAAACCTTCGGGTTTATTGCTGAGTAGC
AAACGGGTGAGTAACGCGTGGGAATCTGCCTTTTTGTGGGGGATAACTGAGGGAAACTTCAGCTAATACC
GCATACGCTCTACGGAGGAAAGACTTCGGTCGCAAAAAGAGGAGCCCGCGTCAGATTAGGTAGTTGGTAG
GGTAAAGGCCTACCAAGCCAATGATCTGTAGCTGGTCTGAGAGGATGATCAGCCACACTGGGACTGAGAC
ACGGCC
>Zotu2892
GAACGCTGGCGGCGTGCCTAATACATGCAAGTCGAGCGAACTGATGAGAAGCTTGCTTCTCTGACGTTAGC
GGCGGACGGGTGAGTAACACGTGGGCAACCTGCCCTATAGTTGGGGATAACTCCGGGAAACCGGGGCTA
ATACCGAATGATACATTTTAACTCCTGTTAGAATGTTGAAAGATGGTTTACGCTATCGCTATAGGATGGGCC
CGCGGCGCATTAGCTAGTTGGTGAGGTAACGGCTCACCAAGGCGACGATGCGTAGCCGACCTGAGAGGGT
GATCGGCCACACTGGGACTGAGACACGGCC
>Zotu2894

GAACGCTAGCGGCAGGCCTAATACATGCAAGTCGAACGGTCAGCAATGACAGTGGCGCATGGGTGCGTAA
CGCGTATGCAACCTACCCATCAAAGGGGGATAGCCCGGGGAAACCCGGATTAATACCGCATAAGATAGTA
TTCTGGCATCAGAGAGCTATTAAACATTTATGGTTGATGGATGGGCATGCGTTAGATTAGCTAGTTGGTGT
AGTAACGGTGCACCAAGGCGATGATCTATAGGGGTTCTGAGAGGAAGATCCCCCACACTGGTACTGAGAT
ACGGAC
>Zotu2896
GAACGCTAGCGGCAGGCCTAATACATGCAAGTCGAACGGTTGTAGCAATACAATAGTGGCGCACGGGTGC
GTAACACGTATGCAACCTACCTTATAGAGGGGGATAGCCTTTGGAAACGGAGATTAATACCCCATAACACA
TTGATACGGCATCGTATTATTGTTAAAGATTTATCGCTATAAGCTGGGCATGCGTCCAATTAGTTAGTTGGT
GAGGTAATGGCTCACCAAGACTTTGATTGGTAGGGGAACTGAGAGGTTAATCCCCCACACTGGCACTGAG
ATACGGGC
>Zotu2898
CAACGCTGGCGGCGTGCCTAACACATGCAAGTCGAACGAGAAAGGGCTTTCGGGCCTGAGTAAAGTGGCG
CACGGGTGAGTAACACGTGACTAACCTACCCTTGAGCGGGGGATAACCTAGGGAAACCTGGGCTAATACC
GCATAACACCTACGGGTCAAAGCGAGCAATCGCACTTAAGGAGGGGGTCGCGGCAGATTAGCTAGTTGGT
GAGGTAATGGCTCACCAAGGCGATGATCTGTATCCGGCCTGAGAGGGCGCACGGACACACTGGAACTGAA
ACACGGTC
>Zotu2900
GAACGCTGGCGGCGTGCATAACACATTCAAGTCGAACGGTGATGAGGACTTCGGTCCTCTGATCAGTGGC
GGACGGGTGAGTAACGCGTGAGCAACCTGCCCTTGTGTGGGGGATAACGTCCGGAAACAGACGCTAATAC
CGCATAACGTATCGAGACCGCATGGTCATGATACCAAAGATTTATTGCGCAAGGATGGGCTCGCGTTGGAT
TAGATAGTTGGTGGGGTAACGGCCTACCAAGTCAACGATCCATAGCCGGACTGAGAGGTTGAACGGCCAC
ATTGGAACTGAGAAACGGTC
>Zotu2902
GAACGCTAGCGGCAGGCCTAATACATGCAAGTCGGACGGGATCCATCGGAGAGCTTGCTCGAGGATGGTG
AGAGTGGCGCACGGGTGCGTAACGCGTGAGCAACCTACCTCTATCAGGGGGATAGCCTCTCGAAAGAGAG
ATTAACACCGCATAACATCAACAGTTCGCATGTTCGGTTGATTAAATATTTATAGGATAGAGATGGGCTCGC
GTGACATTAGCTAGTTGGTAGGGTAACGGCTTACCAAGGCGACGATGTCTAGGGGCTCTGAGAGGAGAAT
CCCCCACACTGGTACTGAGACACGGAC
>Zotu2904
GAACGCTGGCGGCATGCCTAACACATGCAAGTCGAACGAGATCTTCGGATCTAGTGGCGCACGGGTGCGT
AACGCGTGGGAATCTGCCCTTGGGTTCGGAATAACAGTGAGAAATTACTGCTAATACCGGATGATGTCTTC
GGACCAAAGATTTATCGCCCAAGGATGAGCCCGCGTAGGATTAGGTAGTTGGTGGGGTAATGGCCTACCA
AGCCGACGATCCTTAGCTGGTCTGAGAGGATGATCAGCCACACTGGGACTGAGACACGGCC
>Zotu2906
GAACGCTGGCGGCAGGCCTCAAACATGCAAGTCGAGCGCCCAGCAATGGGAGCGGCGGACGGGTGCGTA
ACACGTGGGAATGTACCTATTGGTTCGGAACAACGCAGGGAAACTTGCGCTAATACCGAATGTGCCCTTAC
GGGGAAAGATTCATCGCCAATAGATCAGCCCGCGTCCGATTAGCTAGTTGGTGAGGTAAAGGCTCACCAA
GGCGACGATCGGTAGCTGTTCTGAGAGGAAGATCAGCCACACTGGGACTGAGACACGGCC
>Zotu2908
GAACGCTGGCGGCGTGCCTAACACATGCAAGTCGAGCGAACCCTTCGGGGTGAGCGGCGGACGGGTGAG
TAACGCGTGGGTAACCTGCCCTGTACACACGGATAACATACCGAAAGGTATGCTAATACGAGATAATATAT
TTTAATCGCATGGTTAAGATATCAAAGCTCCGGCGGTACAGGATGGACCCGCGTCTGATTAGCTAGTTGGT
GAGGTAATGGCTCACCAAGGCGACGATCAGTAGCCGACCTGAGAGGGTGATCGGCCACACTGGAACTGA
GACACGGTC
>Zotu2913
GAACGCTGGTGGTATGCTTAACACATGCAAGTCGAACGGGCTTGGAGGGGCAACTCTTTAAGTCAGTGGC
AAACGGGTGAGGAATATATAGGAATCTCCCCAACAGTAAGGAATAACATTGGGAAACCAATGCTAATGCC
ATATATTACCGATTTATCGGGAAAGATTTATCGCTGTTGGATGAGCCTTTATCGGATTAGCTAGTTGGTAGG
GTAATGGCCTACCAAGGCAATGATCCGTAGCTGGTCTGAGAGGATGATCAGCCACATTGGGACTGCGACA
CGGCC

>Zotu2915
GAACGCTGGCGGCAGGCTTAACACATGCAAGTCGAACGGGCTTCTTCGGAAGTCAGTGGCAGACGGGTGA
GTAACACGTGGGAACGTGCCCTTCGGTTCGGAATAACTCAGGGAAACTTGAGCTAATACCGGATACGCCCT
TTTGGGGAAAGGTTTACTGCCGAAGGATCGGCCCGCGTCTGATTAGCTTGTTGGTGGGGTAACGGCCTACC
AAGGCGACGATCAGTAGCTGGTCTGAGAGGATGATCAGCCACACTGGGACTGAGACACGGCC
>Zotu2917
GAACGCTGGCGGCAGGCTTAACACATGCAAGTCGAGCGGGCACTTCGGTGTCAGCGGCAGACGGGTGAG
TAACACGTGGGAACGTACCCTTCGGTTCGGAATAACGCTGGGAAACTAGCGCTAATACCGGATACGCCCTT
ATGGGGAAAGGTTTACTGCCGAAGGATCGGCCCGCGTCTGATTAGCTAGTTGGTGGGGTAATGGCCTACC
AAGGCGACGATCAGTAGCTGGTCTGAGAGGATGATCAGCCACACTGGGACTGAGACACGGCC
>Zotu2920
GAACGCTGGCGGTATGCTTAACACATGCAAGTCGAACGGGGTGCTTCGGCACCTAGTGGCGGACGGGTGA
GTAACGCGTGAGAATCTGACTCGAGGACGGGGACAACCACTGGAAACGGTGGCTAAGACCCGATATGCC
GAGAGGTGAAAATAATTTAGCCTTGAGAGGAGCTCGCGTCTGATTAGCTAGTTGGTGGGGTAAGAGCCTA
CCAAGGCGACGATCAGTAGCTGGTCTGAGAGGATGATCAGCCACACTGGGACTGAGACACGGCC
>Zotu2922
GAACGCTGGCGGCACGCTTAAAACATGCAAGTCGAACGTGAAACCCGGGCTTGCCTGGGTGGAAAGTGGC
GAACGGGTGCGTAACACGTGAGCAACCTGCCCCGAAGACTGGGATAACACCGGGAAACCGGTGCTAATAC
CGGATGCCTTCACCACGTCGCATGGCGAGGTGAAGAAATGGATTCCGCTTCGGGATGGGCTCGCGGCCTA
TCAGCTTGTTGGTGGGGTAACGGCCTACCAAGGCAACGACGGGTAGCTGGTCTGAGAGGATGTCCAGCCA
CACTGGGACTGAGACACGGCC
>Zotu2926
GAACGCTGGCGGCGCGCTTAACACATGCAAGTCGAGCGATGAAATCTTGACAGATCCCTTCGGGGTGAAG
ATAAGATGGATTAGCGGCGGACGGGTGAGTAACACGTGAGTAACCTGCCTTAGACATTGGGATAGCCTCG
GGAAACCGGGATTAATACCAAATGAAATCATAGGTGCCCATGCACTAATGATCAAAACTCCGGTGGTCTAA
GATGGACTCGCGTCCCATTAGCTAGTTGGTGAGGTAACGGCCCACCAAGGCGACGATGGGTAGCCGGCCT
GAGAGGGTGAACGGCCACATTGGAACTGAGAAACGGTC
>Zotu2928
GAACGCTGGCGGCGTGCTTAACACATGCAAGTCGAGGGGGCGGGAGGGGCAACCCTGTCGTCACCGGCG
AACGGGTGCGTAACACGTGAGCGACCTACCTTTCTGCGGGGGATAGCCGGCCCAACGGCCGGGTAATACC
GCATACGCTCTTCTTCGGGCATCCGGGGGGGAGGAAACTTTCGAGGCAGAGAGAGGGGCTCGCGGCCTAT
CAGCTAGTTGGCGGGGTAACGGCCCACCAAGGCAGTGACGGGTAGCTGGTCTGAGAGGATGGCCAGCCA
CATTGGGACTGAGAGACGGCC
>Zotu2930
GAACGCTAGCGGTATGCTTAACACATGCAAGTCGTACGGAAAAGCAATTTTTAGTGGCGAACGGGTGAGT
AACGCGTGAGAATTTGCCTTTAGGAAGAGGATAACAATTGGAAACAGTTGCTAAAACTCTATATGCCGTTT
ACGGTGAAAAAAGTAATTTGCCTAAAGAGAAGCTCGCGTCTGATTAGCTAATTGGTGGGATAAAGGCCTA
CCAAGGCGACGATCAGTAGCTGGTCTGAGAGGACGATCAGCCACACTGGAACTGAGACACGGTC
>Zotu2932
GAACGCTGGCGGCGTGCCTAACACATGCAAGTCGAACGGAGTCAATTGAGGAAGTTTTTGGATGGAATCA
GCTGACTTAGTGGCGGACGGGTGAGTAACGCGTGGGAAACCTGCCCTGTACTGGGGGATAACACTTAGAA
ATAGGTGCTAAAACCGCATAAGCGCACGGGATCGCATGGTCTTGTGTGAAAAACTCCGGTGGTACAGGAT
GGTCCCGCGTCTGATTAGCCAGTTGGCAGGGTAACGGCCTACCAAAGCGACGATCAGTAGCCGGCCTGAG
AGGGTGGACGGCCACATTGGGACTGAGACACGGCC
>Zotu2935
GAACGCTGGCGGCGTGCCTAATACATGCAAGTCGAGCGAACCAATGGGAGCTTGCTCCCTGAGGTTAGCG
GCGGACGGGTGAGTAACACGTGGGTAACCTGCCTGTAAGATTGGGATAACTCCGGGAAACCGGAGCTAAT
ACCGGATAACATTTCGAACCGCATGGTTCGAAATTGAAAGATGGCTTCGGCTATCACTTACAGATGGACCC
GCGGCGCATTAGCTAGTTGGTGAGGTAACGGCTCACCAAGGCAACGATGCGTAGCCGACCTGAGAGGGT
GATCGGCCACACTGGGACTGAGACACGGCC

>Zotu2937
GAACGCTAGCGGCAGGCTTAACACATGCAAGTCGAGGGGTAGAAGGAGCTTGCTCCTTTGAGACCGGCGC
ACGGGTGCGTAACGCGTATGCAATCTACCTTTTACAGAGGGATAGCCCAGAGAAATTTGGATTAATACCTC
ATAGTATTATGAAATGGCATCATTTTATAATTAAAGTTCCAACGGTAAAAGATGAGCATGCGTCCCATTAGC
TAGTTGGTGTGGTAACGGCACACCAAGGCAACGATGGGTAGGGGTCCTGAGAGGGAGATCCCCCACACTG
GTACTGAGACACGGAC
>Zotu2940
GAACGCTGGCGGCGTGCCTAATACATGCAAGTAGAACGCTGAAGCTTGGTGCTTGCACCGAGCGGATGAG
TTGCGAACGGGTGAGTAACGCGTAGGTAACCTGCCTGGTAGCGGGGGATAACTATTGGAAACGATAGCTA
ATACCGCATGACAACAGTTATTGCATGATAACTGTTTAAAAGGGGCAACTGCTCCACTACCAGATGGACCT
GCGTTGTATTAGCTAGTTGGTGAGGTAACGGCTCACCAAGGCGACGATACATAGCCGACCTGAGAGGGTG
ATCGGCCACACTGGGACTGAGACACGGCC
>Zotu2942
GAACGCTGGCGGCGTGCCTAATACATGCAAGTCGAGCGGACAGACGAGGTGCTTGCACCTCTGAAGTCAG
CGGCGGACGGGTGAGTAACACGTGGGTAACCTACCTGTAAGACTGGGATAACTTCGGGAAACCGGAGCTA
ATACCGGATAATATTTTCCACCTCATGGTGGAATAGTGAAAGACGGTTTTGCTGTCACTTACAGATGGACCC
GCGGCGCATTAGCTAGTTGGTGAGGTAACGGCTCACCAAGGCGACGATGCGTAGCCGACCTGAGAGGGT
GATCGGCCACACTGGGACTGAGACACGGCC
>Zotu2945
GAACGCTGGCGGCGTGCCTAACACATGCAAGTCGAGCGGTAACAGGCGTAGCAATACGTGCTGACGAGC
GGCGAACGGGTGAGCAATGCTTCGGAATCTGCCCAATGATGGGGAATAACCAGTCGAAAGATTGGCTAAT
ACCGCATACGCCCTTCGGGGGAAAGCAGGGGATCGCAAGACCTTGCGTCGATGGATGAGCCGAAGTCGG
ATTAGCTAGTTGGTAGGGTAACGGCCTACCAAGGCGACGATCCGTAGCTGGTCTGAGAGGACGACCAGCC
ACACTGGGACTGAGACACGGCC
>Zotu2947
GAACGCTGGCGGCATGCCTAACACATGCAAGTCGAACGGGCACCTTCGGGTGCTAGTGGCGCACGGGTGC
GTAACGCGTGGGAATCTGCCCTTGGGTTCGGAATAACTCAGCGAAAGCTGTGCTAATACCGGATGATGAC
GTAAGTCCAAAGATTTATCGCCCGAGGATGAGCCCGCGTTGGATTAGCTAGTTGGTGGGGTAAAGGCCTA
CCAAGGCGACGATCCATAGCTGGTCTGAGAGGATGATCAGCCACACTGGGACTGAGACACGGCC
>Zotu2950
GAACGCTGGCGGCGTGCCTAATACATGCAAGTCGAGCGAATTTTTGGGAGCTTGCTCCCGAAAATTAGCG
GCGGACGGGTGAGTAACACGTGGGCAACCTGCCTATAAGACTGGGATAACTTCGGGAAACCGGAGCTAAT
ACCGGATAATTCTTTTCCTCTCATGAGGAAAAGCTGAAAGACGGTTTCGGCTGTCACTTATAGATGGGCCC
GCGGCGCATTAGCTAGTTGGTGAGGTAACGGCTCACCAAGGCAACGATGCGTAGCCGACCTGAGAGGGT
GATCGGCCACACTGGGACTGAGACACGGCC
>Zotu2953
CAACGCTGGCGGCGTGCCTAACACATGCAAGTCGAACGAGAAAGTGGGGGCAACCCTGCGAGTACAGTG
GCAGACGGGTGAGTAACACGTGAACAACCTACCCTTGAGTGGGGAATAACACCGCGAAAGCGGTGCTAAT
ACCGCATAACATCTTTGGGTCGCATGACCTGGGGATCAAAAGCGCAAGTTGCTTGAGGAGGGGTTCGCGG
CTGATTAGCTAGTTGGTGAGGTAACGGCCCACCAAGGCAACGATCAGTAGCCGGCCTGAGAGGGCGAACG
GCCACACTGGAACTGAGACACGGTC
>Zotu2956
GAACGCTGGCGGCGTGCCTAACACATGCAAGTCGCGCGAGAAAGTCCTTCGGGATGAGTAGAGCGGCGC
ACGGGTGAGTAATACATAGGAACGTACCTTTCGGTGGGGGATAACCACGGGAAACTGTGGCTAATACCGC
ATAAAGACGTGAGTCTAAAGCAGGCCTCTATTTATAAGCTTGTGCCGGTAGAGCGGCCTATGTCCCATTAG
CTAGTTGGTAGGGTAATGGCCTACCAAGGCGACGATGGGTAGCTGGTCTGAGAGGATGATCAGCCACACT
GGGACTGAGACACGGCC
>Zotu2959
GAACGCTGGCGGCGGGCCTCATACATGCAAGTCGAGCGCGAAAGTCCGCAAGGATGAGTAAAGCGGCGC
ACGGGTGAGTAACACGTGGGTAATCTACCTTTTGGATTGGAATAACCTGTCGAAAGACGGGCTAATGCCG

AGTAAGCTTTCTTGAACGTGAAGTTCGAGCAAGGAAAGATGGCGCAAGCTATCGCCGAAAGATGAGCCTG
CGTCCCATTAGTTAGTTGGTGGGGTAATGGCTTACCAAGACAATGATGGGTACCTGGTTTGAGAGAATGAT
CAGGCACACTGGAACTGAAACACGGTC

>Zotu2963
GAACGCTTGCGGCAGGCTTAATACATGCAAGTCGGACGGTAACAGGGTCTTCGGACTGCTGACGAGTGGC
GGATGGGTGAGTAACGCGTATGCAACCTTCCTCACACCGAGAAAAAGTCGACTGAAAAGTTGAATAAAGT
CTCGTGGTGTGCATTTCTCGCATGAGATGTGTATGATAGAAGCAATTTGGTGTGAGATGGGCATGCGTATC
ATTAGCTAGTTGGTAGGGTAACGGCTTACCAAGGCGACGATGATTAGGGGGCCTGAGAGGGTGGTCCCCC
ACACTGGGACTGAGACACGGCC

>Zotu2965
GAACGCTGGCGGCGCGCCTAACACATGCAAGTCGAGCGCTCACCTTCGGGTGGGAGCGGCGGACGGGTG
AGTAACGCGTGGGAACCTGCCCTGGAGTTCGGGACAACCGCGGGAAACTGCGGCTGACACCGGATACGCC
CCTTTGGGGGGAAAGTTTACGCTCTGGGATGGGCCCGCGTCCGATTAGGTAGTTGGTGGGGTAATGGCCT
ACCAAGCCAATGATCGGTAGCCGGTCTGAGAGGACGATCGGCCACACTGGGACTGAGACACGGCC

>Zotu2967
GAACGCTGGCGGCAGGCCTAACACATGCAAGTCGAACGGATCCTTCGGGATTAGTGGCGGACGGGTGAG
TAACACGTGGGAACGTGCCCTTTGGTTCGGAACAACTCAGGGAAACTTGAGCTAATACCGGATGTGCCCTT
CGGGGGAAAGATTTATCGCCATTGGAGCGGCCCGCGTCTGATTAGCTAGTTGGTGAGGTAAAGGCTCACC
AAGGCGACGATCAGTAGCTGGTCTGAGAGGATGATCAGCCACATTGGGACTGAGACACGGCC

>Zotu2969
GAACGCTGGCGGCGTGCTTAACACATGCAAGTCGAACGGTGAAGGAGAGCTTGCTCTTTGGATCAGTGGC
GAACGGGTGAGTAACACGTGAGCAATCTGCCCTTGACTCTGGGATAAGCGTTGGAAACGACGTCTAATAC
CGGATACGAGCTGTGACCGCATGGTCAACAGTTGGAAAGAACTTCGGTCAAGGATGAGCTCGCGGCCTAT
CAGCTTGTTGGTGAGGTAATGGCTCACCAAGGCGTCGACGGGTAGCCGGCCTGAGAGGGTGACCGGCCAC
ACTGGGACTGAGACACGGCC

>Zotu2971
GAACGTTAGCGGCGCGCTTAACACATGCAAGTCGAACGAGAAAGGGCTTCGGCCCCGGTAAAGTGGCGCA
CGGGTGAGTAACACGTGGGTAAACTTCCCTTGAGCGGGGGATAACATTCCGAAAGGAGTGCTAATACCGC
ATAAGACCACGGAGTCGAAAGATTCTGAGGTAAAAGCAGGCCTCTATACATAAGCTTGCACTCGAGGATGT
GCCCGCGGCCCATCAGGTAGTTGGTAGGGTAATGGCCTACCAAGCCAAAGACGGGTAGCTGGTCTGAGAG
GATGATCAGCCACACTGGAACTGAGACACGGTC

>Zotu2973
GAACGTTAGCGGCGCGCCTAATACATGCAAGTCGTGCGAGAAAGGGCTTCGGCCCCGGTACAGCGGCGCA
CGGGTGAGTAACACGTAGGTAATCTTCCCTCGAATGGTGGATAACCCTCCGAAAGGAGAGCTAATACAGC
ATAAGACCACGACCTCGTTGAGGTTGCGGCCAAAGCAGGCCTCTACTTGTAAGCTTGCGTTTGAGGATGAG
CCTGCGGCCCATCAGCTAGTTGGTAGGGTAATGGCCTACCAAGGCTAAGACGGGTAGCTGGTCTGAGAGG
ATGAACAGCCACACTGGAACTGAGACACGGTC

>Zotu2975
GAACGCTGGCGGCGTGCCTAATACATGCAAGTCGAGCGAACCAATAAGAAGCTTGCTTTTTGTTGGTTAGC
GGCGGACGGGTGAGTAACACGTGGGTAACCTGCCTGTAAGACCGGGATAACTCCGGGAAACCGGTGCTA
ATACCGGATAGATTATCTTTCCGCCTGGAGAGATAAGGAAAGATGGCTTTCGCCATCACTTACAGATGGGC
CCGCGGCGCATTAGCTAGTTGGTGAGGTAACGGCTCACCAAGGCGACGATGCGTAGCCGACCTGAGAGG
GTGATCGGCCACACTGGGACTGAGACACGGCC

>Zotu2978
GAACGTTGGCGGCGTGTCTAAGGCATGCAAGTCGAGGGACAGATCTAAGGGTAGCAATATTTTTAGGTAC
TGACCGGCAGACGGGTGCGTAACACATTGGTAACCTGCCTTCGAGACGGGGATAGCTTTCCGAAAGGAAA
ATTAATACCCGATGGTCATGAGGGGTCGTATGACATTCTCATGTAAATGATTTATCGCTCGAAGAGGGGCC
TATGGCCTATCAGCTAGTTGGTGAGGTAATGGCTCACCAAGGCTATGACGGGTAGGGGATGTGAGAGCAT
GATCCCCCTCACTGGGACTGAGACACTGCC

>Zotu2980

GAACGCTGGCGGCGTGCTTAACACATGCAAGTCGAACGGAAAGGCCTCTTGCTTGCAGGAGGTACTCGAG
TGGCGAACGGGTGAGTAACACGTGGGTGATCTGCCTTGCACTCTGGGATAAGCTTGGGAAACTGGGTCTA
ATACCGGATATGAACTGCCTTTAGTGTGGTGGTTGGAAAGTTTTTCGGTGCAAGATGAGCTCGCGGCCTA
TCAGCTTGTTGGTGGGGTAATGGCCTACCAAGGCGTCGACGGGTAGCCGGCCTGAGAGGGTGTACGGCCA
CATTGGGACTGAGATACGGCC
>Zotu2982
GAACGCTGGCGGCGTGCTTAACACATGCAAGTCGAACGAGAACGGATTATAGCTTGCTATAATTGTCAGCT
AAGTGGCGCACGGGTGAGTAATGTATAGGTAATATGCCTCTTACTAAGGGATAACAATTGGAAACGATTG
CTAATACCTTATACTCCTTGTTAACTTAAGTTAATAAGGGAAAGATTTATTGGTAAGAGATTAGCCTGTATT
GTATCAGTTAGTTGGTGGGGTAATGGCCTACCAAGACTATGACACATAACTGGTTTGAGAGGATGATCAGT
CACACTGGAACTGAGACACGGTC
>Zotu2984
GAACGCTGGCGGCATGCCTAACACATGCAAGTCGAACGAGACCTTCGGGTCTAGTGGCGCACGGGTGCGT
AACGCGTGGGAATCTGCCCTTGGGTGCGGAATAACTTTCCGAAAGGAATGCTAATACCGCATAATGTCGTA
AGACCAAAGATTTATCGCCCAAGGATGAGCCCGCGTAAGATTAGCTAGTTGGTGGGGTAAAAGCCTACCA
AGGCGACGATCTTTAGCTGGTCTGAGAGGATGATCAGCCACACTGGGACTGAGACACGGCC
>Zotu2986
GAACGCTGGCGGCGTGCTTAACACATGCAAGTCGAACGGTGAAAGAGAGCTTGCTCTCTGGATCAGTGGC
GAACGGGTGAGTAACACGTGAGCAATCTGCCCTTGACTCTGGGATAAGCGTTGGAAACGACGTCTAATAC
CGGATACGAGCTGTGAAGGCATCTTCAACAGCTGGAAAGAACTTCGGTCAAGGATGAGCTCGCGGCCTAT
CAGCTAGTTGGTGAGGTAATGGCTCACCAAGGCGTCGACGGGTAGCCGGCCTGAGAGGGTGACCGGCCA
CACTGGGACTGAGACACGGCC
>Zotu2988
GAACGCTAGCGGCAGGCCTAATACATGCAAGTCGAGGGGTTAGCAATAACACCGGCGCACGGGTGCGTA
ACACGTATGCAACCTACCTTATACAGGGGGATAGCCTTTGGAAACGGAGATTAATACCCCATAGTATTAAA
TGATGGCATCATTATTTAATTAAAGATTTATTGGTATAAGATGGGCATGCGTCCAATTAGTTAGTTGGTTAG
GTAACGGCTGACCAAGACGATGATTGGTAGGGGAACTGAGAGGTTAATCCCCCACACTGGCACTGAGATA
CGGGC
>Zotu2990
GAACGCTAGCGGCAGGCTTAATACATGCAAGTCGAGGGGCAGCACAGGTAGCAATACTGGGTGGCGACC
GGCAAACGGGTGCGGAACACGTACGCAACCTTCCTTTAAGTGGGGGATAGCCCAGGGAAACTTGGATTAA
TACCCCTTAACATTATGAGATGGCATCATTTTATAATTAAAGCTCCGGCGCTTAAAGATGGGCGTGCGCCTG
ATTAGGTAGTTGGCGGGGTAACAGCCCACCAAGCCTACGATCAGTAACTGGTGTGAGAGCACGACCAGTC
ACACGGGCACTGAGACACGGGC
>Zotu2992
GAACGCTGGCGGCGTGCCTAACACATGCAAGTCGAACGGAGAGATACGAGAGCTTGCTCTTGTAAATCTT
AGTGGCAAACGGGTGAGTAACGCGTAAACAACCTGCCCAACGGATGGGGACAACAGACGGAAACGACTG
CTAATACCCAATAGACTCCGGTTTCGCATGAAACCGACAGGAAAGGTGGCCTCTCGAAGAAGCTACCGCCG
CAGGAGGGGTTTGCGTTTGATTAGTTAGTTGGTGGGGTAACGGCCTACCAAGACGATGATCAATAGCCGG
TCTGAGAGGATGAACGGCCACACTGGAACTGAGACACGGTC
>Zotu2995
GAACGCTGGCGGCGTGCCTAATACATGCAAGTCGAGCGAACGGAGGAGAGCTTGCTCTCCGAAGTTAGCG
GCGGACGGGTGAGTAACACGTGGGCAACCTGCCTATAAGACTGGGATAACTTCGGGAAACCGGAGCTAAT
ACCGGATAGATTCTTTTCTCTCATGAGGAAAGAAGGAAAGATGGTTTCGGCTATCACTTATAGATGGGCCC
GCGGCGCATTAGCTAGTTGGTAAGGTAACGGCTTACCAAGGCGACGATGCGTAGCCGACCTGAGAGGGTG
ATCGGCCACACTGGGACTGAGACACGGCC
>Zotu2997
GAACGCTGGCGGCAGGCTTAACACATGCAAGTCGAGCGCCCTCTTCGGAGGGAGCGGCGGACGGGTGAG
TAACACGTGGGAACGTGCCCTTCGGTTCGGAATAACTCAGGGAAACTTGAGCTAACACCGGATACGCCCTT
CGGGGGAAAGGTTACGCCGATGGAGCGGCCCGCGTTGGATTAGCTAGTTGGTGAGGTAAAGGCTCACCA
AGGCTACGATCCATAGCTGGTCTGAGAGGATGATCAGCCACACTGGGACTGAGACACGGCC

>Zotu2999
GAACGCTAGCGGCAGGCTTAACACATGCAAGTCGAGGGGCAGCGCGGGTAGCAATACCTGGCGGCGACC
GGCAAACGGGTGCGGAACACGTACAGAACCTACCTTTACCTGGGGCATAGCCCGGAGAAATCCGGATTAA
CTCCCCATAACATAAGAGAGTGGCATCACTCATTTATTAAAAATTTATTGGGTAAAGATGGCTGTGCGTCCC
ATTAGCTAGTTGGTAGGGTAACGGCCTACCAAGGCGACGATGGGTAGCTGGTGTGAGAGCACGACCAGCC
ACACGGGCACTGAGACACGGGC
>Zotu3002
GAACGCTGGCGGCGTGCTTAACACATGCAAGTCGAGCGGTAAGGCCCTTCGGGGTACACGAGCGGCGAA
CGGGTGAGTAACACGTGGGTGACCTGCCCTCCACTCTGGGATAAGCCTGGGAAACTGGGTCTAATACCGG
ATATGACCACATGTCGCATGGTGTGTGGTGGAAAGCTTTACGTGGTGGGGGATGGACCCGCGGCCTATCA
GCTTGTTGGTGGGGTAATGGCCTACCAAGGCGACGACGGGTAGCCGGCCTGAGAGGGCGACCGGCCACA
CTGGGACTGAGACACGGCC
>Zotu3004
GAACGCTGGCGGCGTGCCTAACACATGCAAGTGGAGCGACGGACCAGGGCTTGCCCTGGGGCCAAGCCG
CGAACGGGTGAGTAACACGTGGGTAACCTGCCCCGATGACCGGGACAACCCGAGGAAACTCGGGCTAATA
CCGGATGTGCTCGCCGCACTCAAGTGTGGTGAGTAAAGGAAGCTTCGGCCTCCGCTTCGGGAGGGGCCCG
TGGCCCATTAGCTTGTTGGTGAGGTAACGGCTCACCAAGGCAACGATGGGTAGCTGGTCTGAGAGGACGA
TCAGCCACACTGGGACTGAGACACGGCC
>Zotu3006
GAACGCTGGCGGCGTGCTTAACACATGCAAGTCGAGCGGAAAGGCCACTTCGGTGGTACTCGAGCGGCGA
ACGGGTGAGTAACACGTGAGTAATCTGCCCTTGGCTTTGGGATAGCCACCGGAAACGGTGATTAATACCG
AATATGCACTACTTCTCGCATGGGTTGTGGTGGAAAGTTTTTCGGCCAGGGATGTGCTCGCGGCCTATCAG
CTTGATGGTGAGGTAATGGCTCACCATGGCTTCGACGGGTAACCGGCCTGAGAGGGTGACCGGTCACACT
GGGACTGAGACACGGCC
>Zotu3008
GAACGCTGGCGGCGTGCTTAACACATGCAAGTCGAACGATGATCAGGAGCTTGCTTCTGTGATTAGTGGC
GAACGGGTGAGTAACACGTGAGTAACCTGCCCCTGACTCTGGGATAAGCGTTGGAAACGACGTCTAATAC
TGGATAGGACTACTGGCCGCATGGTCTGGTGGTGGAAAGATTTTTTGGTTGGGGATGGACTCGCGGCCTA
TCAGCTTGTTGGTGAGGTAATGGCTCACCAAGGCGACGACGGGTAGCCGGCCTGAGAGGGTGACCGGCC
ACACTGGGACTGAGACACGGCC
>Zotu3010
GAACGCTGGCGGCGTGCCTAACACATGCAAGTCGAACGGAATGAGGGGGCTTGCTCCTTCATTTAGTGGC
GCACGGGTGAGTAACGCGTAGATAATCTGCCTGATGATCTGGGATAACACTTCGAAAGAGGTGCTAATAC
CGGATAAGCCCACGGAGTCTTTGGATTCTGCGGGAAAAGGGGGGGACCTTCGGGCCTTCTGTCATCAGAT
GAGTCTGCGTACCATTAGCTAGTTGGTAGGGTAATGGCCTACCAAGGCTACGATGGTTAGCTGGTCTGAGA
GGATGATCAGCCACACTGGAACTGAGACACGGTC
>Zotu3012
GAACGCTGGCGGCGTGCCTAACACATGCAAGTTGAGCGACGAACCGGGGCTTGCCCCGGGGCAAAGCAG
CGAACGGGTGAGTAACACGTGGGGAACCTGCCCCGAAGACCGGGACAACCCGAGGAAACTCGGGCTAAT
ACCGGATATGTCCAACGAGCATGAGCTCATTGGGCAAAGGAAGCTTCGGCCTCCGCTTCGGGAGGGTCCC
GCGGCCCATTAGCTAGTCGGTGAGGTAACGGCTCACCGAGGCAACGATGGGTAGCTGGTCTGAGAGGAC
GATCAGCCACACTGGGACTGAGACACGGCC
>Zotu3015
GAACGCTGGCGGCGTGCTTAATACATGCAAGTCGAACGAGGTCCTTCGGGACCTAGTGGCGAACGGGTGA
GTAACACGTGAGCAACCTGCCCCGAAGACCGGGATAACAGTGGGAAACCGCTGCTAATACCGGATACCCC
CACCGGATCACATGGTCTGGTGAGGAAATGCCTGATTCTTCTAGATTCAGACGCTTCGGGAGGGGCTCGCG
GCCTATCAGCTTGTTGGTGGGGTAACGGCCTACCAAGGCTTCGACGGGTAGCTGGTCTGAGAGGACGATC
AGCCACACTGGGACTGAGACACGGCC
>Zotu3017

GAACGCTGGCGGCGTGCTTAACACATGCAAGTCGAACGGATCCCATGGCTTAGCAATAAGTCGGAAGGGT
TAGTGGCGAACGGGTGAGTAACACGTGAGGAACCTGCCCCGGACTCAGGGATAACGGCTGGAAACGGCT
GCTAATACCTGATGCCGTCGTATCATCGCATGGTGGTGTGACGAAAGAATCTCGGTTCGGGAGGGTCTCGC
GGCCTATCAGCTTGTTGGTGGGGTAATGGCCTACCAAGGCGTCGACGGGTAGCTGGTCTGAGAGGATGAT
CAGCCACACTGGGACTGAGACACGGCC
>Zotu3019
GAACGCTGGCGGTGTGCTTAATACATGCAAGTCGAGCGGTAAGGCCCCTTCGGGGGTACACGAGCGGCG
GACGGCTGAGTAACGCGTGGGAACGTACCCCAAAGTGAGGGATAAGCACCAGAAATGGTGTCTAATACCG
CATGTGCTCTACGGAGTAAAGCTTCGGCGCTTTGGGAACGGCCCGCGTCCGATTAGCTAGTTGGTGAGGTA
ATAGCTTACCAAGGCTACGATCGGTAGCTGGTCTGAGAGGATGACCAGCCAGACTGGGACTGAGAACGGC
C
>Zotu3023
CCCACACGCTATTGGATGACTTTTCTGACGACCTTTACACCACATTTCAGCATAATCAGTTCGCGTGTGCGCT
CTTCTTGAATGTGGCGAAAGGTTTCGATTCTCTTAGTCATAAAGTTCTATTAAGTGAGCTTTATTATTACGGT
TTCCGCGGCCCTTTTTTCTCATTGCTCAAGAGCTTCTTAACTGATAGGTTCCAGCAGGTGTCCATAGGCGGT
GCAGTAAGTTCCAAAATACATTTGAAGGCTGGGGTGCCTCTGGGTTCAGTGCTATCTACACTTTTGTTTAAT
CTATATGTCAATGACCTCTCCCTTTCGACATCTACTTGTATTGTAAATCAATATGCGGATGATACTCTACTGTT
ATCGCGGCATGTGAATAACGGTGACGCGTT
>Zotu3025
GAACGCTGGCGGCGTGCCTAATACATGCAAGTCGAGCGGAGCACTTCGGTGCTTAGCGGCGGACGGGTG
AGTAACACGTAGGCAACCTGCCTGTAAGACCGGGATAACTACCGGAAACGGTAGCTAAGACCGGATAACT
GGTTTCTCCGCATGGAGGGATCATGAAACACGGTGCAAGCTGTGGCTTACGGATGGGCCTGCGGCGCATT
AGCTAGTTGGTGGGGTAACGGCCTACCAAGGCGACGATGCGTAGCCGACCTGAGAGGGTGAACGGCCAC
ACTGGGACTGAGACACGGCC
>Zotu3027
GAACGCTGGCGGCGTGCTTAACACATGCAAGTCGAACGAGATTCAGTCAGTAGCAATACTGACGAAAATCT
AGTGGCGAACGGGTGCGTAACACGTGAGGAACTTGTCCCGGTCTCTGGGATAACAGTTGGAAACGACTGC
TAATACCGGATGACGTCGGAACTTCGCATGGAGTACTGACGAAAGGGTTACTGGATCGGGAGAGCCTCGC
GGCCTATCAGCTTGTTGGTGGGGTAACGGCCTACCAAGGCATCGACGGGTAGCTGGTCTGAGAGGATGAT
CAGCCACACTGGGACTGAGACACGGCC
>Zotu3029
GAACGCTGGCGGCACGTTTTAAGCATGCAAGTCGAACGGCAGTGGGGGCTTGCTCCCATGAAAGTGGCGA
ACGGGTGAGTAACACGTAGGTGATCTGCCTTGAGGATGGGGATAGCCTGTGGAAACACAGGGTAATACCG
AATGAGCTGACGACACTTTGGTGATGTTAGTAAAGGTGCTACGGCACCGCCTTAAGAGGAGCCTGCGGCT
GATTAGCTAGTTGGTGGGGTAAAGGCCTACCAAGGCGACGATCAGTAGCCGGCCTGAGAGGGTGTACGG
CCACACTGGAACTGAGACACGGTC
>Zotu3032
TAATGCTGGTGGTATGCATAACACATGCAAGTTGAACGAAGAGGGCTTGCCCTCTTAGTGGCGAACGGGC
GAGTAACGCATATTTAACTTACTTACGCGAGGAGAATAGCAATCCGAAAGGGTTATTAATATTTCATAGGTT
TAAGACTTAAATTAAAGGGGGCGTTCCTTCGGGAGCCCCGCGCGTAAATAGGAATATGTCCTATTAGGTTG
TTGGTAGGGTAAATGCCTACCAAGCCGTTGATGGGTAGCTGGGCTGAGAGGCCGACCAGCCGCAATGGGA
TTGAAATATGGCC
>Zotu3034
GAACGCTGGCGGCAGGCCTAACACATGCAAGTCGAGCGCCCCGCAAGGGGAGCGGCAGACGGGAGAGTA
ACACGTGGGAACATGCCCTTCAGTACGGAACAACCAAGGGAAACTTTGGCTAATACCGTATACGTCCGTAA
GGAGAAAGATTTATCGCTGAAGGATTGGCCCGCGTCTGATTAGCTAGTTGGTGAGGTAATGGCTCACCAA
GGCGACGATCAGTAGCTGGTCTGAGAGGATGATCAGCCTCACTGGGACTGAGACACGGCC
>Zotu3037
GAACGCTGGCGGCGCGCCTAACACATGCAAGTCGAACGAGCGAGAGAGAGCTTGCTTTCTCGAGCGAGTG
GCGAACGGGTGAGTAACGCGTGAGGAACCTGCCTCAAAGAGGGGGACAACAGTTGGAAACGACTGCTAA
TACCGCATAAGCCCACGACCCGGCATCGGGAAGAGGGAAAAGGAGCAATCCGCTTTGAGATGGCCTCGCG

TCCGATTAGCTAGTTGGTGAGGTAACGGCCCACCAAGGCGACGATCGGTAGCCGGACTGAGAGGTTGAAC
GGCCACATTGGGACTGAGACACGGCC
>Zotu3039
GAACGCTGGCGGCGCGCCTAACACATGCAAGTCGAACGAGAGATGAGGAGCTTGCTCTTCAAATCGAGTG
GTGAACGGGTGAGTAACGCGTGAGGAACCTGCCTCAAAGAGGGGGACAACAGTTGGAAACGACTGCTAA
TACCGCATAAGCCCACGGCTCGGCATCGAGCAGAGGGAAAAGGAGCAATCCGCTTTGAGATGGCCTCGCG
TCCGATTAGCTAGTTGGTGAGGTAACGGCCCACCAAGGCGACGATCGGTAGCCGGACTGAGAGGTTGAAC
GGCCACATTGGGACTGAGACACGGCC
>Zotu3041
GAACGCTGGCGGCGTGCTTAACACATGCAAGTCGAACGATGAACCTGTGCTTGCACGGGGGATTAGTGGC
GAACGGGTGAGTAACACGTGAGTAACCTGCCCTTGACTTCGGGATAAGCCTGGGAAACTGGGTCTAATAC
TGGATACGACCTCTCATCGCATGGTGTGGGGGTGGAAAGTTTTTGCGGTTTTGGATGGACTCGCGGCCTAT
CAGCTTGTTGGTGAGGTAATGGCTCACCAAGGCGACGACGGGTAGCCGGCCTGAGAGGGTGACCGGCCA
CACTGGGACTGAGACACGGCC
>Zotu3043
GAACGCTGGCGGCGTGCTTAACACATGCAAGTCGAGCGGTAAGGCTCCTTCGGGAGTACACGAGCGGCGA
ACGGGTGAGTAACACGTGAGTAATCTGCCCCTCACTTCGGGATAAGCCTCGGAAACGAGGTCTAATACCG
GATACGACCACTTCTTGCATGAGATGGTGGTGGAAAGTTTTTCGGTGGGGGATGTGCTCGCGGCCTATCAG
CTTGTTGGTGGGGTAATGGCCTACCAAGGCTTCGACGGGTAGCCGGCCTGAGAGGGTGACCGGCCACACT
GGGACTGAGACACGGCC
>Zotu3045
GAACGCTGGCGGCATGCCTTACACATGCAAGTCGAACGCGAAAGCGGAGCAATCCGCAAGTAGAGTGGC
GAACGGGTGAGTAATACATCGGAACATTTCCAGTAGCGGGGGATAACCTGTCGAAAGATAGGCTAATACC
GCATACGCCCCACGGGGGAAAGCGGAGGACCGCAAGGCTTCGCACTATTGGAGTGGCCGATGTCGGATTA
GCTAGTTGGCGGGGTAAAAGCCCACCAAGGCTACGATCCGTAGCTGGTCTGAGAGGACGATCAGCCACAC
TGGAACTGAGACACGGTC
>Zotu3047
GAACGCTGGCGGCGTGCTTAACACATGCAAGTCGAGCGGTAAGGCCCTTCGGGGTACACGAGCGGCGAA
CGGGTGAGTAACACGTGGGTGACCTGCCCTCAGCTCTGGGATAAGCCTGGGAAACCGGGTCTAATACCGG
ATACGACCATTGGACGCATGTCTGGTGGTGGAAAGTTTTTCGGCTGGGGATGGGCCCGCGGCCTATCAGCT
TGTTGGTGGGGTGATGGCCTACCAAGGCGGTGACGGGTAGCCGGCCTGAGAGGGCGACCGGCCACACTG
GGACTGAGACACGGCC
>Zotu3049
GAACGCTGGCGGCAGGCCTAACACATGCAAGTTGAACGATGTAGCAATACAGAGTAGCGCACGGGTGCGT
AACGCGTGGGAATATGCCCTTCACTACGGAACAACGTTTGGAAACGAACGCTAATACCGTATAATGACTTC
GGTCCAAAGATTTATCGGTGAAGGATTAGCCCGCGTCTGATTAGCTAGTTGGTGAGGTAATGGCTCACCAA
GGCGACGATCAGTAGCTGGTCTGAGAGGATGATCAGCCACACTGGGACTGAGACACGGCC
>Zotu3051
GAACGCTGGCGGCAGGCCTAACACATGCAAGTCGAACGGCAGCACAGCAGTAGCAATACTGTGGGTGGC
GAGTGGCGGACGGGTGAGGAATACATCGGAATCTGCCTAGTCGTGGGGGATAACGTAGGGAAACTTACG
CTAATACCGCATACGACCTACGGGTGAAAGCGGGGGATCGCAAGACCTCGCGCGATTAGATGAGCCGATG
TCCGATTAGCTAGTTGGCGGGGTAATGGCCCACCAAGGCGACGATCGGTAGCTGGTCTGAGAGGATGATC
AGCCACACTGGAACTGAGACACGGTC
>Zotu3053
GAACGCTGGCGGCGTGCCTAATACATGCAAGTAGAACGCTGAAGGAATAGCTTGCTATTCTGGAAGAGTT
GCGAACGGGTGAGTAACGCGTAGGTAACCTACCTCATAGCGGGGGATAACTATTGGAAACGATAGCTAAT
ACCGCATAAGAATACTTATTGCATGATAAGTATTTAAAAGGAACAACAGTTTCACTGTGAGATGGACCTGC
GTTGTATTAGCTAGTTGGTAAGGTAACGGCTTACCAAGGCGACGATACATAGCCGACCTGAGAGGGTGAT
CGGCCACACTGGGACTGAGACACGGCC
>Zotu3055

GAACGCTGGCGGCGTGCCTAATACATGCAAGTCGAGCGAACAGATGAGGAGCTTGCTCCTCTGATGTTAG
CGGCGGACGGGTGAGTAACACGTGGGCAACCTGCCCTATAGTTTGGGATAACTCGCGGAAACGCGAGCTA
ATACCGAATAATACATTGAAGCTCCTGCTTTAATGTTGAAAGATGGTTTCGGCTATCGCTATAGGATGGGCC
CGCGGCGCATTAGCTAGTAGGTGAGGTAACGGCTCACCTAGGCAACGATGCGTAGCCGACCTGAGAGGGT
GATCGGCCACACTGGGACTGAGACACGGCC
>Zotu3058
GAACGCTGGCGGCGTGCTTAACACATGCAAGTCGAGCGGAAAGGCCCTTTCGGGGGTACTCGAGCGGCG
AACGGGTGAGTAACACGTGAGCAACCTGCCCCAGGCTTTGGGATAACCCTCGGAAACGGGGGCTAATACC
GGATAGGACCTGTCATCGCATGGTGATGGGTGGAAAGTTTTTCGGCTTGGGATGGGCTCGCGGCCTATCA
GCTTGTTGGTGGGGTGATGGCCTACCAAGGCTTCGACGGGTAGCCGGCCTGAGAGGGCGACCGGCCACAC
TGGGACTGAGACACGGCC
>Zotu3061
GAACGCTGGCGGCATGCCTAACACATGCAAGTCGAACGAAGCCTTCGGGCTTAGTGGCGCACGGGTGCGT
AACGCGTGGGAATCTGCCCTTCGGTTCGGAACAACAGTTAGAAATGACTGCTAATACCGGATAATGACGTA
AGTCCAAAGATTTATCGCCGAAGGATGAGCCCGCGTAGGATTAGCTAGTTGGTAAGGTAAAAGCTTACCA
AGGCGACGATCCTTAGCTGGTCTGAGAGGATGATCAGCCACACTGGGACTGAGACACGGCC
>Zotu3064
GAACGCTGGCGGCGTGCTTAACACATGCAAGTCGAACGGTGATGATCAAGCTTGCTTGGTCGGATCAGTG
GCGAACGGGTGAGTAACACGTGAGCAACCTGCCCCAGACTCTGGGATAACTTCGGGAAACCGGAGCTAAT
ACCGGATATGACACTCGCACGCATGTGCTGGGTGTGGAAAGTTTTTCGGTCTGGGATGGGCTCGCGGCCTA
TCAGCTTGTTGGTGAGGTAGTGGCTCACCAAGGCGACGACGGGTAGCCGGCCTGAGAGGGCGACCGGCC
ACACTGGGACTGAGACACGGCC
>Zotu3066
GAACGCTGGCGGCAGGCTTAACACATGCAAGTCGAACGCCCCGCAAGGGGAGTGGCAGACGGGTGAGTA
ACGCGTGGGAATCTACCTAGTTCTACGGAACAACAGTTGGAAACGACTGCTAATACCGTATACGCCCTACG
GGGGAAAGATTTATCGGAATTAGATGAGCCCGCGTAAGATTAGCTAGTTGGTGGGGTAATGGCCTACCAA
GGCGACGATCTTTAGCTGGTCTGAGAGGATGATCAGCCACACTGGGACTGAGACACGGCC
>Zotu3068
GAACGCTGGCGGCAGGCTTAACACATGCAAGTCGAGCGGGGAAAGGTAGCTTGCTACCGGACCTAGCGG
CGGACGGGTGAGTAATGCTTAGGAATCTGCCTATTAGTGGGGGACAACATCTCGAAAGGGATGCTAATAC
CGCATACGTCCTACGGGAGAAAGCAGGGGATCTTCGGACCTTGCGCTAATAGATGAGCCTAAGTCGGATT
AGCTAGTTGGTGGGGTAAAGGCCTACCAAGGCGACGATCTGTAGCGGGTCTGAGAGGATGATCCGCCACA
CTGGGACTGAGACACGGCC
>Zotu3070
GAACGCTGGCGGCGTGCTTTACACATGCAAGTCGAACGGCAGCATAGATAGTAGCAATACTATTGGGTGG
CGAGTGGCGGACGGGTGAGTAATACATCGAGACGTGCCTAATAGATTGGGATAACCTGTCGAAAGATAGG
CTAATACCGGATAGTCTCTGAGGAGGAAAGCTGGGGACTTAGGAATAAGCCTGGCGCTAATAGAGCGATC
GATGGCCGATTAGTTAGTTGGTGGGGTAAAGGCTTACCAAGGCGATGATCGGTAGCTGTTCTGAGAGGAA
GATCAGCCACACTGGAACTGAGAGACGGTC
>Zotu3073
GAACGCTGGCGGTATGCCTAACACATGCAAGTCGAACGGTCTCTTCGGAGATAGTGGCGGACGGGTGAGT
AACGCGTGAGAATCTGCCTTCAGGATGGGGACAACAGAGGGAAACTTCTGCTAAAACCCAATGTGCCGAA
AGGTGAAATATTTATAGCCTGAAGAGGAGCTCGCGTCCGATTAGTTAGTTGGTGGGGTAAAGGCCTACCA
AGGCAGCGATCGGTAGCTGGTCTGAGAGGATGAGCAGCCACACTGGGACTGAGACACGGCC
>Zotu3075
GAACGCTGGCGGCATGCCTAACACATGCAAGTCGAACGAGACCTTCGGGTCTAGTGGCGCACGGGTGCGT
AACGCGTGGGAACCTGCCTTTAGGTTCGGAATAACTCCTCGAAAGGGGTGCTAATACCGAATAATGTCTTC
GGACCAAAGATTTATCGCCTTTAGATGGGCCCGCGTTGGATTAGCTAGTTGGTGGGGTAAAGGCTCACCAA
GGCGACGATCCATAGCTGGTCTTAGAGGATGATCAGCCACACTGGGACTGAGACACGGCC
>Zotu3077

GAACGCTGGCGGCGTGCCTAATACATGCAAGTCGAGCGAGAAAGTCTTCGGATGAGTAAAGCGGCGGAC
GGGTGAGTAACACGTGGGTAATCAACCTTCTAGTGGGGAATAACGTTTCGAAAGGAGCGCTAATACCGCA
TGTGAATACGAGTCGTTGGATTTGTATTCAAAGGGGGGTCGTAAGATCTCTCGCTAGAAGACGAGCCTGCG
TCCCATTAGCTAGTTGGTGAGGTAAAGGCTCACCAAGGCATCGATGGGTAGCTGGTCTGAGAGGACGACC
AGCCACACTGGAACTGACACACGGTC
>Zotu3079
GAACGCTGGCGGCGTGCTTAACACATGCAAGTCGAACGGTGAAGCAAGAGCTTGCTCTTGTGGATCAGTG
GCGAACGGGTGAGTAACACGTGAGTAACCTGCCCCAGACTCTGGGATAACCTCGGGAAACCGTGGCTAAT
ACCGGATACGAAACGAGATCGCATGATCATCGTTTGGAAAGAATTTCGGTCTGGGATGGACTCGCGGCCT
ATCAGCTTGTTGGTGAGGTAATGGCTCACCAAGGCGACGACGGGTAGCCGGCCTGAGAGGGCGACCGGC
CACACTGGAACTGAGACACGGTC
>Zotu3081
GAACGCTGGCGGCGTGCCTAACACATGCAAGTCGAGCGGGATCCAACCAGTGGCAACACTGGGGAAGAT
CTAGCGGCGAACGGGTGAGTAACACGTGAGAAACTTGCCCCGGAGTTCGGGATAACCCGAGGAAACTCG
AGCTAATACCGAATGCCCTCTTCTGATCGCATGGTCGGATGAGGAAAGATTTATCGCTCCGGGAGAGTCTC
GCGGCCTATCAGCTAGTTGGCGGGGTAACGGCCCACCAAGGCATCGACGGGTAGCTGGTCTGAGAGGAC
GATCAGCCACACTGGGACTGAGACACGGCC
>Zotu3083
GAACGCTGGCGGCGTGCTTAACACATGCAAGTCGAACGGTGATCCTGGTGCTTGCACCGGGTGATCAGTG
GCGAACGGGTGAGTAACACGTGAGCAACCTGCCCCTGACTCTGGGATAACCCCGAGAAATCGGAGCTAAT
ACCGGATACGAGACGCACAGGCATCTGTAGCGTCTGGAAAGAATTTTGGTCAGGGATGGGCTCGCGGCCT
ATCAGCTTGTTGGTGGGGTAACGGCCCACCAAGGCGACGACGGGTAGCCGGCCTGAGAGGGCGACCGGC
CACACTGGGACTGAGACACGGCC
>Zotu3086
GAACGCTGGCGGCATGCCTAACACATGCAAGTCGAACGAAGGCTTCGGCCTTAGTGGCGCACGGGTGCGT
AACGCGTGGGAATCTGCCCTTGGGTTCGGAATAACAGTTAGAAATGACTGCTAATACCGGATGATGACGA
AAGTCCAAAGATTTATCGCCCAGGGATGAGCCCGCGTAGGATTAGGTAGTTGGTGGGGTAAAGGCTCACC
AAGCCGACGATCCTTAGCTGGTCTGAGAGGATGATCAGCCACACTGGGACTGAGACACGGCC
>Zotu3088
GAACGCTGGCGGCAGGCTTAACACATGCAAGTCGAACGATGTAGCAATACATAGTGGCGCACGGGTGAGT
AACGCGTGGGAACATGCCCTTCAGTTCAGAATAACAGCGGGAAACTGCTGCTAATACTGGATATTCCCTCC
GGGGGAAAGATTTATCGCTGAAGGATTGGCCCGCGTAAGATTAGGTAGTTGGTGGGGTAATGGCCTACCA
AGCCTACGATCTTTAGCTGGTCTGAGAGGATGATCAGCCACACTGGAACTGAGACACGGTC
>Zotu3090
GAACGCTGGCGGCGTGCCTAACACATGCAAGTGGAGCGACGAACCCGGACTTGTCCGGGGGCAAAGCCG
CGAACGGGTGAGTAACACGTGGATTATCTGCCCCAATGACCGGGACAACTCGAGGAAACTCGAGCTAATA
CCGGATGTGCTCTTCGGAGGAAAGGAAGCTTCGGCCTCCGCATTGGGATGAGTCCGCGGCGCATTAGCTA
GTTGGTGAGGTAAAGGCTCACCAAGGCGACGATGCGTAGCTGGTCTGAGAGGACGATCAGCCACACTGG
AACTGAGACACGGTC
>Zotu3092
GAACGCTGGCGGCGTGCTTAACACATGCAAGTCGAACGGTGAAGCTCCAGCTTGCTGGAGTGGATCAGTG
GCGAACGGGTGAGTAACACGTGAGCAACCTGCCCCTGACTCTGGAATAAGCGCTGGAAACGGCGTCTAAT
ACTGGATACGAGAAGGGTCCGCATGGGCACTTTCTGGAAAGTTTTTCGGTTGGGGATGGGCTCGCGGCCT
ATCAGCTTGTTGGTGAGGTAATGGCTCACCAAGGCGACGACGGGTAGCCGGCCTGAGAGGGCGACCGGC
CACACTGGGACTGAGACACGGCC
>Zotu3095
GAACGCTGGCGGCGTGCCTAATACATGCAAGTCGAGCGAATCTTGAGGTGCTTGCACCTCTTGGTTAGCGG
CGGACGGGTGAGTAACACGTGGGCAACCTGCCTGTAAGACTGGGATAACACCGGGAAACCGGTGCTAATA
CCGGATAATCCTTTTCCTCTCATGAGGAAAAGCTGAAAGTCGGTTTACGCTGACACTTACAGATGGGCCCG
CGGCGCATTAGCTAGTTGGTGAGGTAACGGCTCACCAAGGCGACGATGCGTAGCCGACCTGAGAGGGTG
ATCGGCCACACTGGGACTGAGACACGGCC

>Zotu3097
GAACGCTGGCGGCAGGCCTAACACATGCAAGTCGAGCGGTCGTAGCAATACGGCAGCGGCAGACGGGAG
AGTAACACGTGGGAACGCGCCCTTCGGTTCGGAATAACTCAGGGAAACTTGGGCTAATACCGGATACGCC
CTTACGGGGAAAGATTTATTGCCGAAGGAACGGCCCGCGTCCGATTAGCTTGTTGGTGAGGTAATGGCTCA
CCAAGGCGACGATCGGTAGCTGGTCTGAGAGGATGACCAGCCTCACTGGGACTGAGACACGGCC
>Zotu3099
GAACGCTGGCGGCATGCCTAACACATGCAAGTCGAACGAAACCTTCGGGTTTAGTGGCGCACGGGTGCGT
AACGCGTGGGAATCTGCCCCTTGGTTCGGAATAACAGTTGGAAACGACTGCTAATACCGGATGACGACGT
AAGTCCAAAGATTTATCGCCGAGGGATGAGCCCGCGTAGGATTAGGTAGTTGGTGTGGTAAAGGCGCACC
AAGCCGACGATCCTTAGCTGGTCTGAGAGGATGATCAGCCACACTGGGACTGAGACACGGCC
>Zotu3102
GAACGCTAGCTACAGGCTTAACACATGCAAGTCGAGGGGCAGCATGGTCTTAGCTTGCTAAGGCTGATGG
CGACCGGCGCACGGGTGAGTAACACGTATCCAACCTGCCGTCTACTCTTGGCCAGCCTTCTGAAAGGAAGA
TTAATCCAGGATGGCATCATGAGTTCACATGTCCGCATGATTAAAGGTATTTTCCGGTAGACGATGGGGAT
GCGTTCCATTAGATAGTAGGCGGGGTAACGGCCCACCTAGTCAACGATGGATAGGGGTTCTGAGAGGAAG
GTCCCCCACATTGGAACTGAGACACGGTC
>Zotu3105
GAACGCTGGCGGCGTGCTTAACACATGCAAGTCGAACGGGATCCCAGGAGCTTGCTCCTGGGTGAGAGTG
GCGAACGGGTGAGTAATGCGTGACCGACCTGCCCCATGCACCGGAATAGCTCCTGGAAACGGGTGGTAAT
GCCGGATGCTCCATCACACTGCATGGTGTGTTGGGAAAGCCTTTGCGGCATGGGATGGGGTCGCGTCCTAT
CAGCTTGATGGCGGGGTAACGGCCCACCATGGCTTCGACGGGTAGCCGGCCTGAGAGGGCGACCGGCCA
CATTGGGACTGAGATACGGCC
>Zotu3107
GAACGCTGGCGGCGTGCCTAACACATGCAAGTCGAGCGATTTACTTCGGTAAAGAGCGGCGGACGGGTGA
GTAACGCGTGGGTAACCTGCCCTGTACACACGGATAACATACCGAAAGGTATGCTAATACGAGATAATATG
TTTTTATCGCATGGTAGAAGTATCAAAGCTTTTGCGGTACAGGATGGACCCGCGTCTGATTAGCTAGTTGGT
AAGGTAACGGCTTACCAAGGCGACGATCAGTAGCCGACCTGAGAGGGTGATCGGCCACATTGGAACTGAG
ACACGGTC
>Zotu3110
GAACGCTAGCGGCAGGCCTAACACATGCAAGTCGAGGGGTATAGTAGCAATACTAGAGACCGGCGCACG
GGTGCGTAACGCGTATGCAATCTACCTTTTACAGGGGAATAGCCCAGAGAAATTTGGATTAATGCCCCATG
GTATATATGAATGGCATCATTTATATATTAAAGTTCCAACGGTAAAAGATGAGCATGCGTCCCATTAGTTAG
TTGGTAAGGTAACGGCTTACCAAGACGATGATGGGTAGGGGTCCTGAGAGGGAGATCCCCCACACTGGTA
CTGAGACACGGAC
>Zotu3113
GAACGCTGGCGGCGTGCTTAACACATGCAAGTGGAGCGACGAACCAGGGCTTGCCCTGGGGCAGAGCCG
CGAACGGGTGAGTAACACGTGGGTAACCTACCTTGATGACCGGGACAACCCGAGGAAACTCGGGCTAATA
CCGGATGTGCTCCTTCCACATCAGTGGATTGAGGAAAGATAGCTTCGGCCTTCGCATCAAGATGGGCCCGC
GGCGCATTAGCTTGTTGGTGGGGTAACGGCCTACCAAGGCTTCGATGCGTAGCTGGTCTGAGAGGACGAT
CAGCCACACTGGGACTGAGACACGGCC
>Zotu3115
GAACGCTGGCGGCGTGCTTAACACATGCAAGTCGAGCGGTAAGGCCCTTCGGGGTACACGAGCGGCGAA
CGGGTGAGTAACACGTGGGTGACCTGCCCTCCACTTCGGGATAAGCCCGGGAAACTGGGTCTAATACCGG
ATATGACCTTGAAGCGCATGTTTCTTGGTGGAAAGTTTTTTCGGTGGGGGATGGGCCCGCGGTCTATCAGC
TTGTTGGTGGGGTGATGGCCTACCAAGGCGACGACGGATAGCCGGCCTGAGAGGGTGATCGGCCACACT
GGGACTGAGACACGGCC
>Zotu3117
GAACGCTGGCGGCGTGCTTAACACATGCAAGTCGAACGGAAAGGCCAGTGCTTGCACTGGTACTCGAGTG
GCGAACGGGTGAGTAACACGTGGGTGATCTGCCTTGTACTCTGGGATAAGCTTGGGAAACTGGGTCTAAT
ACCGGATAGGAACTGCCTTTAGTGTGGTGGTTGGAAAGTTTTTTCGGTGCAAGATGAGCTCGCGGCCTATC

AGCTTGTTGGTGGGGTAATGGCCTACCAAGGCGTCGACGGGTAGCCGGCCTGAGAGGGTGTACGGCCAC
ATTGGGACTGAGATACGGCC
>Zotu3119
GAACGCTGGCGGCGTGCTTAACACATGCAAGTCGAACGGTGAAGGTCAGCTTGCTGACCGGATCAGTGGC
GAACGGGTGAGTAACACGTGAGCAACCTGCCCTCCACTCTGGGATAAGCCTTGGAAACGAGGTCTAATACT
GGATACGAGACGCCCGGGCATCTGGAGCGTCTGGAAAGATTTATCGGTGGGGGATGGGCTCGCGGCCTAT
CAGCTTGTTGGTGGGGTGATGGCCTACCAAGGCGACGACGGGTAGCCGGCCTGAGAGGGCGACCGGCCA
CACTGGGACTGAGACACGGCC
>Zotu3122
GAACGCTGGCGGCGTGCTTAACACATGCAAGTCGAGGGGGCGGGAGGGGCAACCCTGTCGTCACCGGCG
AACGGGTGCGTAACACGTGAGCGACCTACCTTTTTGCGGGGGATAGCCGGCCCAACGGCCGGGTAATACC
GCATACGCTCTTCTTCGGGCATCCGGGGGGGAGGAAACTTTCGAGGCAGAGAGAGGGGCTCGCGGCCTAT
CAGCTAGTTGGCGGGGTAACGGCCCACCAAGGCAGTGACGGGTAGCTGGTCTGAGAGGATGGCCAGCCA
CATTGGGACTGAGAGACGGCC
>Zotu3124
GAACGCTGGCGGCGTGCTTAACACATGCAAGTCGAACGATGAAGCTGGAGCTTGCTCCGGTGGATTAGTG
GCGAACGGGTGAGTAACACGTGAGCAATCTGCCCTTGACTCTGGGATAACTGCGGGAAACTGTAGCTAAT
ACCGGATACGAGGCTTGGAGGCATCTCCAGGCCTGGAAAGAATTTCGGTCAAGGATGAGCTCGCGGCCTA
TCAGCTAGTTGGTGAGGTAATGGCTCACCAAGGCGACGACGGGTAGCCGGCCTGAGAGGGTGACCGGCC
ACACTGGGACTGAGACACGGCC
>Zotu3126
GAACGCTGGCGGCGTGCCTAATACATGCAAGTCGAGCGGTAAGGCCACTTCGGTGGTACACGAGCGGCG
GACGGCTGAGTAACGCGTAGGAACGTACCCCAAACTGAGGGATAACTGCTCGAAAGAGTAGCTAATACCG
CATGTGCTCTACGGAGTAAAGCTTCGGCGGTTTGGGAACGGCCTGCGTATGATTAGCTTGTTGGTGAGGTA
ATGGCTCACCAAGGCGACGATCATTAGCTGGTCTGAGAGGATGATCAGCCAGACTGGGACTGAGACACGG
CC
>Zotu3129
GAACGCTGGCGGCGTGCATAACACATTCAAGTCGAACGGTGAAGCACCTTCGGGTGTGGATCAGTGGCGG
ACGGGTGAGTAACGCGTGAGCAATCTGCCCTGCACTGAGGGATAACGACCGGAAACAGTCGCTAATACCT
CATGATGTTTTCTTACCGCATGGTAGGACTACCAAAGATTTATTGGTGCAGGATGAGCTCGCGTCCGATTAG
ATAGTTGGCGGGGCAACGGCCCACCAAGTCTGCGATCGGTAGCCGGACTGAGAGGTTGAACGGCCACATT
GGAACTGAGAAACGGTC
>Zotu3131
GAACGCTGGCGGCGTGCTTAACACATGCAAGTCGAACGGTGACCTCGAGAGCTTGCTCTTGGGTGATCAG
TGGCGAACGGGTGAGTAACACGTGAGTAACCTGCCCCAGACTCTGGAATAACCCCGGGAAACCGGAGCTA
ATACCGGATACGAGACGGAGCCGCATGGCTACCGTTTGGAAAGTTCTTCGGTCTGGGATGGACTCGCGGC
CTATCAGCTAGTTGGTGAGGTAACGGCTCACCAAGGCGACGACGGGTAGCCGGCCTGAGAGGGCGACCG
GCCACACTGGGACTGAGACACGGCC
>Zotu3133
GAACGCTGGCGGCAGGCTTAACACATGCAAGTCGAGCGCCCCGCAAGGGGAGCGGCAGACGGGTGAGTA
ACGCGTGGGAATCTACCCTTTACTACGGAATAACGCATGGAAACGTGTGCTAATACCGTATGTGTCCTTCG
GGAGAAAGATTTATCGGTAAAGGATGAGCCCGCGTTGGATTAGCTAGTTGGTGGGGTAAAGGCCTACCAA
GGCGACGATACATAGCTGGTCTGAGAGGATGATCAGCCACATTGGGACTGAGACACGGCC
>Zotu3135
GAACGCTGGCGGCAGGCTTAACACATGCAAGTCGAGCGCCCCGCAAGGGGAGCGGCAGACGGGTGAGTA
ACGCGTGGGAATCTACCTAACTCTACGGAATAACCCAGGGAAACTTGGACTAATACCGTATACGCCCCTCG
GGGGAAAGATTTATCGGAGTTGGATGAGCCCGCGTTGGATTAGCTAGTTGGTGGGGTAATGGCCTACCAA
GGCGACGATCCATAGCTGGTCTGAGAGGATGATCAGCCACACTGGGACTGAGACACGGCC
>Zotu3138

GAACGCTGGCGGCGGGTTTAACACATGCAAGTCGAGGGACGAAGCCTTTCTTCGGAGAGGTGGAGACCG
GCGAACGGGTGAGTAACACGTGAGGAACCTACCCGGTACTTCGGGATAACCGCGGGAAACCGTGGCTAAT
ACCGGATATTCCATGTCTGGCGCATGTCAGGTTTGGGAAAGTTCAGGCGGTATCGGACGGCCTCGCGGCG
TATCAGCTTGTTGGTGGGGTAATGGCTTACCAAGGCGTCGACGCGTAGCTGGAGTGAGAGCTCGATCAGC
CACACTGGGACTGAGACACGGCC
>Zotu3140
GAACGCTAGCGGCAGGCCTAATACATGCAAGTCGAACGGTCGTAGTGATACGATAGTGGCGCACGGGTGC
GTAACACGTATGCAACCTACCTTATAGAGGGGGATAGCCTTTGGAAACGGAGATTAATACCCCATAATACA
TTTGATAGGCATCTATTTAATGTTAAAGATTTATCGCTATAAGCTGGGCATGCGTCCAATTAGTTAGTTGGC
GGGGTAATGGCTCACCAAGACTTTGATTGGTAGGGGAACTGAGAGGTTAATCCCCCACACTGGCACTGAG
ATACGGGC
>Zotu3142
GAACGCTGGCGGCGTGCTTAACACATGCAAGTCGAACGAAGCATTCGCAGCTGAGGCCCTTCGGGGTTGA
CGCAGCGAATGACTGAGTGGCGGACGGGTGAGTAACACGTGAGTAACCTGCCTTTCACCGTGGAATAACC
ATTGGAAACGATGGCTAATACCGCATAATGCTACATGACGGCATCGTCGTGTATCCAAAGATTTTATCAGT
GAAAGATGGACTCGCGCCCGATTAGATAGTTGGTGAGGTAACGGCTCACCAAGTCTGCGATCGGTAGCCG
AACTGAGAGGTTGATCGGCCACATTGGGACTGAGACACGGCC
>Zotu3144
GAACGCTGGCGGCATGCCTAATACATGCAAGTCGAACGAAGTTATGATGGAGCTTGCTCCTGATTAGCTTA
GTGGCGGACGGGTGAGTAACACGTGGGCAACCTGCCTACAAGTTGGGGATAACTAGCGGAAACGTTAGCT
AATACCGAATGGTCAAGGATGAGGCATCTTATCCTTGGGAAAGACGGAGCAATCTGTCACTTGTAGATGG
GCCCGCGGTGCATTAGCTAGTTGGTGAGGTAACGGCTCACCAAGGCGACGATGCATAGCCGACCTGAGAG
GGTGAACGGCCACACTGGGACTGAGACACGGCC
>Zotu3146
GAACGCTGGCGGCAGGCTTAACACATGCAAGTCGAACGCCCCGCAAGGGGAGTGGCAGACGGGTGAGTA
ACGCGTGGGAATCTACCGTGCCCTGCGGAATAGCTCCGGGAAACTGGAATTAATACCGCATACGCCCTACG
GGGGAAAGATTTATCGGGGTATGATGAGCCCGCGTTGGATTAGCTAGTTGGTGGGGTAAAGGCCTACCAA
GGCGACGATCCATAGCTGGTCTGAGAGGATGATCAGCCACATTGGGACTGAGACACGGCC
>Zotu3148
GAACGCTGGCGGCGTGCCTAACACATGCAAGTCGAACGGATGCTTTCGGGCATTAGTGGCGCACGGGTGA
GTAACACGTGGTTGACCTACCTCAAAGTAAGGGATAACGTCTCGAAAGGGATGCTAATACCTTATAAGCTC
CCGGCATTTAGAAAGTTGGGAGAAAAGTTCCGGCGCTTTGAGAGGGGACTGCGAGCCATCAGCTAGTTGG
TGAGGTAATAGCTCACCAAGGCGACGACGGCTAGGAGGCCTGAGAGGGTGACCTCCCACACTGGAACTGA
GACACGGTC
>Zotu3151
GAACGCTGGCGGCGTGCTTAACACATGCAAGTCGAACGATGATCCCCAGCTTGCTGGGGGGATTAGTGGC
GAACGGGTGAGTAACACGTGAGTAACCTGCCCTTAACTCTGGGATAAGCCTGGGAAACTGGGTCTAATAC
CGGATATGACTGATCATCGCATGGTGGTTGGTGGAAAGCTTTTTGTGGTTTTGGATGGACTCGCGGCCTAT
CAGCTTGTTGGTGAGGTAATGGCTTACCAAGGCGACGACGGGTAGCCGGCCTGAGAGGGTGACCGGCCA
CACTGGGACTGAGACATGGCC
>Zotu3153
GAACGCTAGCGGCAGGCTTAACACATGCAAGTCGAGGGGTAGAGTTCTTCGGAACTTGAGACCGGCGCAC
GGGTGCGTAACGCGTATGCAATCTACCTTTCACAGAGGGATAGCCCAGAGAAATTTGGATTAATACCTCAT
AGTATAACGACCTGGCATCAGGATGTTATTAAAGTCACAACGGTGAAAGATGAGCATGCGTCCCATTAGCT
AGTTGGTAAGGTAACGGCTTACCAAGGCTACGATGGGTAGGGGTCCTGAGAGGGAGATCCCCCACACTGG
TACTGAGACACGGAC
>Zotu3155
CAACGCTGGCGGCGCGCTTAACACATGCAAGTCGCACGAGAAAGCCCGCAAGGGTGAGTAAAGTGGCGC
ACGGGTGAGTAACACGTGGATAATCTACCTCTTAGTGGGGAATAACTTTGGGAAACCGAAGCTAATACCGC
ATAAGCTCGGAAGAGGAAAGCAGCAATGCGCTGAGAGAGGAGTCCGCGGCCGATTAGCTAGTTGGTAGG

GTAAAGGCCTACCAAGGCAATGATCGGTAGCCGGCCTGAGAGGGCACACGGCCACACTGGCACTGAAACA
CGGGC
>Zotu3157
GAACGCTGGCGGCGTGCCTAATACATGCAAGTCGAACGGTCTTTTCTATGGAAGCTTGCTTCCACTGAGAA
GATAGTGGCGGACGGGTGAGTAACACGTGGGTAACCTGCCCATAAGAGGGGGATAACATCCGGAAACGG
GTGCTAATACCGCATAGTTTTCTTGATCGCATGATCGAGAAAGGAAAGACGGCCTTTGTGCTGTCGCTTATG
GATGGACCCGCGGCGTATTAGTTAGTTGGTGAGGTAATGGCTCACCAAGACGATGATACGTAGCCGACCT
GAGAGGGTGATCGGCCACATTGGGACTGAGACACGGCC
>Zotu3159
GAACGCTGGCGGCGTGCCTAATACATGCAAGTCGAGCGGACTTGATGGAGAGCTTGCTCTCCTGATGGTTA
GCGGCGGACGGGTGAGTAACACGTAGGCAACCTGCCTGCAAGACCGGGATAACCCACGGAAACGTGAGC
TAATACCGGATATCTCATTTCCTCTCCTGAGGGGATGATGAAAGACGGAGCAATCTGTCACTTGCGGATGG
GCCTGCGGCGCATTAGCTAGTTGGTGAGGTAACGGCTCACCAAGGCGACGATGCGTAGCCGACCTGAGAG
GGTGAACGGCCACACTGGGACTGAGACACGGCC
>Zotu3161
GAACGCTGGCGGCGTGCCTAATACATGCAAGTCGAGCGGACCGACGGGAGCTTGCTCCCTTAGGTCAGCG
GCGGACGGGTGAGTAACACGTGGGTAACCTGCCTGTAAGACTGGGATAACTCCGGGAAACCGGGGCTAA
TACCGGATGCTTGATTGAACCGCATGGTTCCAATCATAAAAGGTGGCTTTTAGCTACCACTTACAGATGGAC
CCGCGGCGCATTAGCTAGTTGGTGAGGTAACGGCTCACCAAGGCGACGATGCGTAGCCGACCTGAGAGG
GTGATCGGCCACACTGGGACTGAGACACGGCC
>Zotu3163
GAACGCTGGCGGCATGCCTAATACATGCAAGTCGAACGCTGAAGCACTCTGCTTGCAGAGTGCGGATGAG
TGGCGAACGGGTGAGTAACACGTGGGAAATCTGCCTAAGAGTGGGGGATAACATTCGGAAACGGATGCT
AATACCGCATATATTCTTGAACCGCATGGTTCTGGAAAGAAAGACGGCTTTGCTGTCGCTTTTAGATGATCC
CGCGGCGTATTAGTTAGTTGGTGGGGTAAAGGCCTACCAAGACCATGATACGTAGCCGACCTGAGAGGGT
GATCGGCCACACTGGGACTGAGACACGGCC
>Zotu3165
GAACGCTGGCGGCAGGCTTAACACATGCAAGTCGAACGCCCGAGCAATCGGGAGTGGCAGACGGGTGAG
TAACACGTGGGAACATACCCTTCGGTTCGGAACAACGTTTGGAAACGAACGCTAATACCGGATACGGCTCG
AGGGCGAAAGATTCATCGCCGAAGGATTGGCCCGCGTTCGATTAGCTAGATGGCGGGGTAACGGCCCACC
ATGGCGACGATCGATAGCTGGTCTGAGAGGATGGCCAGCCACATTGGGACTGAGACACGGCC
>Zotu3167
GAACGCTGGCGGCAGGCCTAACACATGCAAGTCGAGCGGCAGCGGGTCCTTCGGGATGCCGGCGAGCGG
CGGACGGGTGAGGAATGCGTCGGAATCTGCCTTTTTGTGGGGGATAACGTAGGGAAACTTACGCTAATAC
CGCATACGACCTACGGGTGAAAGTGGGGGACCGCAAGGCCTCACGCAGAAAGATGAGCCGACGCCGGAT
TAGCTAGTTGGCGGGGTAAAGGCCCACCAAGGCGACGATCCGTAGCTGGTCTGAGAGGATGATCAGCCAC
ACTGGAACTGAGACACGGTC
>Zotu3169
GAATGCTGGCGGCGTGGATAAGTCATGCAAGTCAAGGGGGCCCGCAAGGGCAACCGGCAAACGAGGTAG
TAACACGTAGGTAGACCCCCCGAAGTCGGGCATAGCCCTCCGAAAGGAGGGGTAATTCCCGATAGTCCCG
CAAGGGTAAAGTTTTTCGCTTCGGGAGTCGCCTGCGTAGTATCAGCTAGTTGGTAGGGTAACGGCCTACCA
AGGCTACGACGCTTAGGGGAGCTGAGAGGCTGACCCCCACCGATGGGACTGCGACACGGCC
>Zotu3171
GAACGCTGGCGGCGTGCTTAACACATGCAAGTCGAACGATGAACCCCAGCTTGCTGGGGGGATTAGTGGC
GAACGGGTGAGTAACACGTGAGTAACCTGCCCCTGACTCTGGGATAAGCGCTGGAAACGGCGTCTAATAC
CGGATATGACAACCGATGGCATCGTCTGGTTGTGGAAAGAATTTCGGTTGGGGATGGACTCGCGGCCTAT
CAGGTTGTTGGTGAGGTAATGGCTCACCAAGCCTACGACGGGTAGCCGGCCTGAGAGGGTGACCGGCCAC
ACTGGGACTGAGACACGGCC
>Zotu3174

GAACGCTGGCGGCATGCCTAACACATGCAAGTCGAACGAAGCCTTCGGGCTTAGTGGCGCACGGGTGCGT
AACGCGTGGGAACCTGCCTTTAGGTTCGGAATAACAGTTGGAAACGACTGCTAATACCGGATGATGTCTTC
GGACCAAAGATTTATCGCCTTTAGATGGGCCCGCGTTGGATTAGCTAGTTGGTGGGGTAAAGGCCTACCAA
GGCGACGATCCATAGCTGGTCTGAGAGGATGATCAGCCACACTGGGACTGAGACACGGCC
>Zotu3176
GAACGCTGGCGGCATGCCTAACACATGCAAGTCGAACGAGACCTTCGGGTCTAGTGGCGCACGGGTGCGT
AACGCGTGGGAATCTGCCCCTTGGTTCGGGATAACAGTTGGAAACGACTGCTAATACCGGATGATGACGA
AAGTCCAAAGATTTATCGCCGAGGGATGAGCCCGCGTAGGATTAGGTAGTTGGTGGGGTAAAGGCCTACC
AAGCCGACGATCCTTAGCTGGTCTGAGAGGATGATCAGCCACACTGGGACTGAGACACGGCC
>Zotu3180
GAACGCTGGCGGAATGCTTTACACATGCAAGTCGAGCGGCAGCGCGGGGGCAACCCTGGCGGCGAGCGG
CGAACGGGTGAGTAATGTATCGGAACGTACCCAGTCGTGGGGGATAACTATCCGAAAGGATAGCTAATAC
CGCATACGACCTGAGGGTGAAAGCGGGGGACCGAAAGGCCTCGCGCGATTGGAGCGGCCGATATCGGAT
TAGCTAGTTGGTGAGGTAAAGGCTCACCAAGGCGACGATCCGTAGCTGGTCTGAGAGGACGACCAGCCAC
ACTGGAACTGAGACACGGTC
>Zotu3182
GAACGCTGGCGGCGTGCCTAATACATGCAAGTCGAACGAGGTGCTTTTGTAGCAATACGAGAGTATCCTA
GTGGCGAACGGGTGAGTAACGCGTGGGTGACCTGCCCCAAAGTGTGGGATAACAGTCCGAAAGGATTGC
TAATACCTCATGTGGTTGTTGGGTTTAGAAACTGACAACTAAAGGAGTAATCCGCTTTGGGAGGGACCTGC
GTCCCATCAGCTAGTTGGTGAGGTAATGGCTCACCAAGGCTACGACGGGTAGGGGACCTGAGAGGGTGG
CCCCCCACAATGGAACTGAAACACGGTC
>Zotu3184
GAACGCTGGCGGCGTGCTTAACACATGCAAGTCGAGCGGTAAGGCCCTTCGGGGTACACGAGCAGCGAAC
GGGTGAGTAACACGTGGGTGATCTGCCCTGTACTTCGGGATAAGCCTGGGAAACTGGGTCTAATACCGGA
TATGACCTTACATCGCATGGTGTTTGGTGGAAAGATTTATCGGTACAGGATGGGCCCGCGGCCTATCAGCT
TGTTGGTGGGGTAATGGCCTACCAAGGCGACGACGGGTAGCCGGCCTGAGAGGGCGACCGGCCACACTG
GGACTGAGACACGGCC
>Zotu3188
GAACGCTGGCGGTCTGCTTAACACATGCAAGTCGAACGGAGTAGAAATACTTAGTGGCGGACGGGTGAGT
AACGCGTGAGAATCTGCCTTCAGGACGGAGACAACAGTTGGAAACGACTGCTAAGCCCCGATGAACCGAA
AGGGAAAATATTTATAGCCTGAAGATGAGCTCGCGTCCGATTAGCTAGTTGGCGGAGTAAAAGCCCACCA
AGGCGACGATCGGTAGCTGGTCTGAGAGGACGATCAGCCACACTGGGACTGAGACACGGCC
>Zotu3190
GAACGCTGGCGGCGTGCCTAACACATGCAAGTCGAACGATCAGATGTCTTCGGACATTTGGGAGTGGCGC
ACGGGTGAGTAATGCATAGGAACATACCTAAAGGCGGGGAATAACTGCGGGAAACTTCAGCTAATACCCC
ATGAAGTCGCAAGACCAAAGGAGGCCTCTACTTGTAAGCTTTCACCTTTAGAATGGCCTATGTCCCATTAGC
TAGTTGGTAGGGTAAAGGCCTACCAAGGCGACGATGGGTAGCTGGTCTGAGAGGACGATCAGCCACACTG
GAACTGAGACACGGTC
>Zotu3192
GAACGCTGGCGGCGCGCTTAACACATGCAAGTCGAGCGAGAACCGGGGCTTCGGCTCCGGGGAAAGCGG
CGAACGGGTGAGTAACACGTGGGTAACCTGCCCTCGGCACCGGGATAGCCCGGGGAAACCCGGATTAATA
CCGGACGGCCTCCGGAGCCTTCGGGCGCCGGAGAGAAGGTGGCTTCGGCTTCCGGCCGAGGAGGGGCCT
GCGGCGGATTAGCTTGTTGGCGGGGTAACGGCCCACCAAGGCGACGATCCGTAGCTGGTCTGAGAGGAC
GATCAGCCACACTGGGACTGAGACACGGCC
>Zotu3194
GAACGCTAGCGGCAGGCCTAATACATGCAAGTCGAACGCGATTAGGGAGCTTGCTCCCTATGAAAGTGGC
GCACGGGTGCGTAACGCGTATGCAACCTACCTTAATCAGGGGGATAGCCCGGAGAAATCCGGATTAACAC
CGCATAAAATCACACTTCGGCATCGAGGAATGATCAAATATTTATAGGATTAAGATGGGCATGCGTGACAT
TAGTTAGTTGGTGAGGTAACGGCTCACCAAGGCGACGATGTCTAGGGGATCTGAGAGGATGACCCCCCAC
ACTGGTACTGAGACACGGAC

>Zotu3196
GAACGCTGGCGGCGTGCTTAACACATGCAAGTCGAGCGGAAAGGCCCTTCGGGGTACTCGAGCGGCGAA
CGGGTGAGTAACACGTGAGTAATCTGCCCTTGGCTTCGGGATAGCCACCGGAAACGGTGATTAATACCGG
ATATGACTACTTCCTGCATGGGGTGGTAGTGGAAAGTTTTTCGGCCAGGGATGTGCTCGCGGCCTATCAGC
TTGATGGTGAGGTAATGGCTCACCATGGCTTCGACGGGTAGCCGGCCTGAGAGGGTGACCGGTCACACTG
GGACTGAGACACGGCC
>Zotu3198
GAACGCTAGCGGCAGGCCTAATACATGCAAGTCGAACGAGATTAGTAGCAATACTATGAAAGTGGCGCAC
GGGTGCGTAACACGTATGCAATCTACCTTTAATTGGAGGATAGCCTCGAGAAATCGGGATTAATACTCCAT
AACATTATGAAATGGCATCATTTTATAATTAAACCTCCGGGGATTAAAGATGAGCATGCGCAACATTAGCTA
GTTGGTAAGGTAATGGCTTACCAAGGCTACGATGTTTAGGGGGTCTGAGAGGATGATCCCCCACACTGGT
ACTGAGACACGGAC
>Zotu3202
GAACGCTGGCGGCGTGCTTAACACATGCAAGTCGAACGGAAAGGCCCTTCGGGGTACTCGAGTGGCGAAC
GGGTGAGTAACACGTGGGTGATCTGCCCTGCACTTTGGGATAAGCCTGGGAAACTGGGTCTAATACCGAA
TACACCTCATTGACTGCATGGTTGGTGGGGGAAAGCTTTTGCGGTGTGGGATGGGCCCGCGGCCTATCAG
CTTGTTGGTGAGGTTACGGCTCACCAAGGCGACGACGGGTAGCCGGCCTGAGAGGGTGACCGGCCACACT
GGGACTGAGATACGGCC
>Zotu3204
GAACGCTAGCGGCAGGCCTAATACATGCAAGTCGGACGGGATAGAAGAGCTTGCTTTTCTTGAGAGTGGC
GCACGGGTGCGTAACACGTATGTAACCTACCTTATCCAGGGGGATAGCCTCTCGAAAGAGAGATTAAGAC
CGCATAATATTATTGAATAGCATTATTTAATAATCAAATATTTATAGGGATAAGATGGGCATGCGGGACATT
AGCTAGTTGGCGGGGTAACGGCCCACCAAGGCGACGATGTCTAGGGGATCTGAGAGGATGACCCCCCACA
CTGGTACTGAGACACGGAC
>Zotu3206
CAACGCTGGCGGCGCGCTTAACACATGCAAGTCGCACGAGAAAGGGAGCAATCCTGAGTAAAGTGGCGTA
CGGGTGAGTAACACGTGGATCATCTACCTTTTAGTGGGGAATACCCCCGGGAAACTGGGGACAATACCGC
ATAAGCTCGTGAGAGGAAAGCCGAAAGGCGCTGAAAGAGGAGTCCGCGGCTGATTAGCTAGTTGGTGAG
GTAACGGCCCACCAAGGCGAAGATCGGTAGCCGGCCTGAGAGGGCACACGGCCACACTGGCACTGAAAT
ACGGGC
>Zotu3209
CAACGCTAGCGGCGTGCCTAACACATGCAAGTCGAACGCGAAAGGGACTTCGGTTCTTAGTAGAGTGGCG
TACGGGTGAGTAACGCGTGGGTCATCTGCCCTCGGGTGGGGAATAACTAACCGAAAGGTTAGCTAATACC
GCATAACACAGTGATGTCAAAGCAGCAATGTGCCTGAGGAGGAGCCTGCGTCTGATTAGGTAGTTGGTGG
GGTAACGGCCTACCAAGCCTGCGATCAGTAGCTGGTCTGAGAGGACGGCCAGCCACAATGGCACTGAAAC
ACGGGC
>Zotu3211
GAACGCTGGCGGCGTGCTTAACACATGCAAGTCGAACGCTGAAGCCCAGCTTGCTGGGTGGATGAGTGGC
GAACGGGTGAGTAACACGTGAGCAACCTGCCCTTCACTCTGGGATAACTCCGGGAAACCGGGGCTAATAC
CGGATGCGAGCACTGCACGCATGTGCGGGTGCTGGAAAGTTTTTCGGTGAGGGATGGGCTCGCGGCCTAT
CAGCTTGTTGGTGAGGTAGTGGCTCACCAAGGCGACGACGGGTAGCCGGCCTGAGAGGGCGACCGGCCA
CACTGGGACTGAGACACGGCC
>Zotu3213
GAACGCTGGCGGCGTGCCTAATACATGCAAGTCGAGCGAACAGATAAGGAGCTTGCTCCTTTGACGTTAG
CGGCGGACGGGTGAGTAACACGTGGGTAACCTACCTATAAGACTGGAATAACTCCGGGAAACCGGGGCTA
ATGCCGGATAATATTTAGAACCGTATGGTTCTAAAGTGAAAGATGGTTTTACTATCACTTATAGATGGACCC
GCGCCGTATTAGCTAGTTGGTGGGGTAATGGCTTACCAAGGCAACGATACGTAGCCGACCTGAGAGGGTG
ATCGGCCACACTGGAACTGAGACACGGTC
>Zotu3216

GAACGCTGGCGGCGTGCCTAATACATGCAAGTCGAGCGGACTTTTAAAAGCTTGCTTTTAAAAGTTAGCGG
CGGACGGGTGAGTAACACGTGGGTAACCTGCCTGTAAGACTGGGATAACTCCGGGAAACCGGGGCTAATA
CCGGATAACTTTTTTCCTCGCATGAGGAAAAATTGAAAGATGGCTTCGGCTATCACTTACAGATGGACCCGC
GGCGCATTAGCTAGTTGGTGAGGTAACGGCTCACCAAGGCAACGATGCGTAGCCGACCTGAGAGGGTGAT
CGGCCACACTGGGACTGAGACACGGCC
>Zotu3220
GAACGCTGGCGGCGTGCCTAATACATGCAAGTCGTGCGAACTGATTAGAAGCTTGCTTCTATGACGTTAGC
GGCGGACGGGTGAGTAACACGTGGGCAACCTGCCTGTAAGACTGGGATAACTTCGGGAAACCGAAGCTA
ATACCGGATAGGATCTTCTCCTTCATGGGAGATGATTGAAAGATGGTTTCGGCTATCACTTACAGATGGGC
CCGCGGTGCATTAGCTAGTTGGTGAGGTAACGGCTCACCAAGGCAACGATGCATAGCCGACCTGAGAGGG
TGATCGGCCACACTGGGACTGAGACACGGCC
>Zotu3222
GAACGCTGGCGGCGTGCTTAACACATGCAAGTCGAACGCTGAAGACCTGGCTTTTGTTGGGTTGGATGAG
TGGCGAACGGGTGAGTAACACGTGAGTAACCTGCCCTCTTCTTTGGGATAACGCCCGGAAACGGGTGCTA
ATACTGGATATTCACTGATCTTCGCATGGGGGTTGGTGGAAAGGTTTGTTCTGGTGGGGGATGGGCTCGC
GGCCTATCAGCTTGTTGGTGGGGTGATGGCCTACCAAGGCTTTGACGGGTAGCCGGCCTGAGAGGGTGAC
CGGTCACATTGGGACTGAGATACGGCC
>Zotu3225
GAACGCTAGCGGCAGGCCTAATACATGCAAGTCGGACGGGATCCGTCGGAGAGCTTGCTCGAAGACGGT
GAGAGTGGCGCACGGGTGCGTAACGCGTGAGCAACCTACCTCTATCAGGGGGATAGCCTCTCGAAAGAGA
GATTAACACCGCATAATATCAACAGTTCGCATGTTCGGTTGATTAAATATTTATAGGATAGAGATGGGCTCG
CGTGACATTAGCTAGTTGGTAGGGTAACGGCTTACCAAGGCGACGATGTCTAGGGGCTCTGAGAGGAGAA
TCCCCCACACTGGTACTGAGACACGGAC
>Zotu3227
GAACGCTGGCGGTATGCTTAACACATGCAAGTCGAACGGAGTACTTCGGTACTTAGTGGCGGACGGGTGA
GTAACTCGTGAGAAACTGCCCCTTGGACTGGGACAACAGCTGGAAACGGCTGCTAACACCGGATGTGCCG
AAAGGTGAAATATTTATAGCCAGGGGATGTGCTCGCGTCTGATTAGCTAGTAGGTGAGGTAAAGGCTTAC
CTAGGCGACGATCAGTAGCTGGTCTGAGAGGATGATCAGCCACACTGGGACTGAGACACGGCC
>Zotu3230
GAACGCTGGCGGCGTGCCTAATACATGCAAGTCGAGCGGAAGTTGGAGAGCTTGCTCTTCAACTTTAGCG
GCGGACGGGTGAGTAACACGTGGGCAACCTGCCCTGCAGATCGGGATAACTCCGGGAAACCGGTGCTAAT
ACCGAATAGTTTTCGCCACCTCCTGGTGGCGTACGGAAAGACGGTTTCGGCTGTCACTGCAGGATGGGCCC
ACGGCGCATTAGCTAGTTGGTGGGGTAACGGCCCACCAAGGCAACGATGCGTAGCCGACCTGAGAGGGT
GATCGGCCACACTGGGACTGAGACACGGCC
>Zotu3232
GAACGCTAGCGGCAGGCCTAATACATGCAAGTCTAGGGGGCAGCAATGTCACCGGCGCACGGGTGCGTA
ACACGTATGCAACCTACCTTCTACAGGGGTACAGCCTTCCGAAAGGGAGATTAATACCGCATGAGACCACA
CCTATTCATATAGGAGGGGTTAAAGATTTATCGGTAGAAGATGGGCATGCGTCTGATTAGTTAGTTGGCGG
GGTAACGGCTCACCAAGACGATGATCAGTAGGGGATCTGAGAGGACTATCCCCCACACTGGCACTGAGAT
ACGGGC
>Zotu3234
GAACGCTAGCGGCAGGCTTAACACATGCAAGTCGAGGGGTAGGGTAGCAATACCTGAGACCGGCGCACG
GGTGCGTAACGCGTATGCAATCTACCTTTTACAGAGGGATAGCCCAGAGAAATTTGGATTAATACCTCATA
GCATAGCAGCTTCGCATGAAGTCACTATTAAAGTTCCAACGGTAAAAGATGAGCATGCGTCCTATTAGCTA
GATGGTAAGGTAACGGCTTACCATGGCTACGATAGGTAGGGGTCCTGAGAGGGAGATCCCCCACACTGGT
ACTGAGACACGGAC
>Zotu3236
GAACGCTAGCGACAGGCTTAACACATGCAAGTCGAGGGGCAGCATAAAAGTAGCAATACTTTGGTGGCGA
CCGGCGCACGGGTGAGTAACGCGTATGCAACCTACCTATCAGAGGGGAATAACCCGGCGAAAGTCGGACT
AATACCGCATAAAACAGGGGCACCGCATGGTGCTATTTGTTAAAAATTTATTGCTGATAGATGGGCATGCG

TTCCATTAGGTAGTTGGAGAGGTAACGGCTCACCAAGCCTACGATGGATAGGGGAACTGAGAGGTTGGTC
CCCCACACTGGTACTGAGACACGGAC

>Zotu3238

GAACGCTGGCGGCAGGCTTAACACATGCAAGTCGAACGATGACTCTCTAGCTTGCTAGAGAAGATTAGTG
GCGGACGGGTGAGTAACATTTAGGAATCTGCCTAGTAGTGGGGGATAGCTCGGGGAAACTCGAATTAATA
CCGCATACGACCTACGGGTGAAAGGGGGCGCAAGCTCTTGCTATTAGATGAGCCTAAATCAGATTAGCTA
GTTGGTGGGGTAAAGGCCCACCAAGGCGACGATCTGTAACTGGTCTGAGAGGATGATCAGTCACACCGGA
ACTGAGACACGGTC

>Zotu3241

GAACGCTGGCGGCGTGCCTAATACATGCAAGTCGAGCGGTAAGGTTCTGCACTTAGTATTTGACCCAGTAA
GTCCCTCAGAGGATACTGCTTGGAAAATCGAGCTCTAGGCTAGAGTAAGATGTCGAATGTTGAGTGTAGA
ATACACGAGCGGCGGACGGCTGAGTAACGCGTAGGAATGTACCCCAAAGTGAGGGATAAGCACCGGAAA
CGGTGTCTAATACCGCATGTGATCTTCGGATTAAAGCTTTATGCGCTTTGGGAGCGGCCTGCGTAAGATTA
GCTTGTTGGTGAGGTAAAGGCTCACCAAGGCGACGATCTTTAGCTGGTCTGAGAGGATGACCAGCCAGAC
TGGGACTGAGACACGGCC

>Zotu3244

GAACGCTGGCGGCGCGCTTAACACATGCAAGTCGAGCGAGAACCATTCCTTCGGGAATGGGGACAGCGGC
GAACGGGTGAGTAACACGTGGGTAATCTGCCCTCGATTCTGGGATAGCCCGGGGAAACCCGGATTAATAC
CGGATAGCCTCTCGAGCTTTCGAGCTCGTGAGAAAAGGTAGCTTCGGCCTCCGATCGAGGATGAGCCCGC
GGCGGATTAGCTTGTTGGTGGGGTAATGGCCCACCAAGGCGACGATCCGTAGCTGGTCTGAGAGGACGAT
CAGCCACACTGGGACTGAGACACGGCC

>Zotu3246

GAACGCTAGCGGGAGGCTTAACACATGCAAGCCGAGGGGTATTCAGTTGCTTGCAACTGGAGAGACCGGC
GTACGGGTGAGTAACGCGTATGTAACTTGCCTCATTCAACGGGATAGCCCGGGGAAACCCGGATTAATATC
GTATAACAGCAATTCGGGCATCCGGAATGTTTGAAAGATTTATCGGAATGAGATAGACATGCGTATGATTA
GTTAGTTGGCGGGGTAACGGCCCACCAAGACGATGATCATTAGGGGTCCTGAGAGGGTGATCCCCCACAC
TGGTACTGAGACACGGAC

>Zotu3248

GAACGCTGGCGGCGTGCCTAATACATGCAAGTCGAGCGCGAAGATCAGGAGCTTGCTCCTGAGATTCGAG
CGGCGGACGGGTGAGTAACACGTAGGCAACCTACCCTTGAGATTGGGATAACTACCGGAAACGGTAGCTA
ATACCGGATACGACATTCCTGCATAAGTAAGAATGTTAAAAGGCGGATTTATCTGCCGCTCATGGATGGGC
CTGCGGTGCATTAGCTAGTTGGTGAGATAGTAGCTCACCAAGGCGACGATGCATAGCCGACCTGAGAGGG
TGATCGGCCACACTGGGACTGAGACACGGCC

>Zotu3250

GAACGCTGGCGGCGTGCTTAACACATGCAAGTCGAGCGGTAAGGCCTTTCGGGGTACACGAGCGGCGAAC
GGGTGAGTAACACGTGGGTGACCTGCCCTCCACTCTGGGATAAGCCTGGGAAACTGGGTCTAATACTGGA
TATGACTTCTGGGTGCATGCCTGGTTGTGGAAAGATTTATCGGTGGGGGATGGGCCCGCGGTCTATCAGCT
TGTTGGTGGGGTGATGGCCTACCAAGGCGACGACGGATAGCCGGCCTGAGAGGGTGATCGGCCACACTG
GGACTGAGACACGGCC

>Zotu3253

GAACGCTGGCGGTATGCTTAACACATGCAAGTCGTACGAGAGTTTTTAACTCTAGTGGCGGACGGGTGAG
TAACACGTGAGAATCTACCCCAAGGAGGGGAATAACAGCTGGAAACGGTTGCTAATACCCCATATGCCGA
AAGGTAAAAGGATGTATCCACCTTAGGAAGAGCTCGCGTCTGATTAGCTAGTTGGTGAGGTAATAGCTCAC
CAAGGCCATGATCAGTAGCTGGTTTGAGAGGACGATCAGCCACACTGGGACTGAGACACGGCC

>Zotu3255

GAATGCTGGCGGCGTGGATAAGGCATGCAAGTCAAGGGGGCCCGCAAGGGCAACCGGCAAACGAGGTAA
TAACACGTGGGTACGTACCTGGAAGTCAGGCATAGCTCATCGAAAGGTGAGGTAATTCCTGATAGTCTCTT
CGGAGTAAAGTTTTTTCGCTTCCAGAGCGGCCTGCGGGATATCAGCTAGTTGGTAGGGTAATGGCCTACCA
AGGCGATGACGTCTAGGGGAGCTGAGAGGCTGACCCCCACCGATGGAACTGAGACACGGTC

>Zotu3257

GAACGCTAGCGATAGGCCTAACACATGCAAGTCGAGGGGCAGCATAGAGGTAGTAATACTTTGGTGGCGA
CCGGCGCACGGGTGAGTAACACGTATGCAACCTGCCTTCAACACGGGGATAACCCGATGAAAGTCGGACT
AATATTCGATAACACAGGGGCACCGCATGGTGATATTTGTTAAAGATTAATTGGTTGAAGATGGGCATGCG
CATGATTAGTTAGTTGGTAGGGTAACGGCCTACCAAGGCAACGATCATTAGGGGTTCTGAGAGGAAGATC
CCCCACACTGGTACTGAGACACGGAC
>Zotu3259
GAACGCTAGCGGCAGGCCTAATACATGCAAGTCGAACGGTTGTATTTATACAATAGTGGCGCACGGGTGC
GTAACACGTATGCAACCTACCTTATAGACGGGGATAGCCTTTGGAAACGGAGATTAATACCCGATAGTACT
GTTGACTGGCATCAGTTAATATTTAAAGATTTATCGCTATAAGCTGGGCATGCGTCCAATTAGTTAGTTGGT
GAGGTAACGGCTCACCAAGACGATGATTGGTAGGGGAACTGAGAGGTTAATCCCCCACACTGGCACTAAG
ATACGGGC
>Zotu3261
GAATGCTGGCGATGTGGATAAGTCATGCAAGTCAAGGGGGCCCGCAAGGGCAACCGGCAGACGAGGTAG
TAACACGTAGGTACGTCCCCTTTAGTCGGGCATAGCCAGTCGAAAGATTGGGTAATTCCCGATGGTCCCGC
AAGGGTAAAGGTTTTCCGCTAGAGGAGCGGCCTGCGTAGTATCAGCTAGTTGGTAGGGTAATGGCCTACC
AAGGCTACGACGCTTAGGGGAGCTGAGAGGCTGACCCCCACCGATGGGACTGCGACACGGCC
>Zotu3263
GAACGCTGGCGGCGTGCCTAACACATGCAAGTCGAGCGAGAAAGTAGCAATACGAGTAAAGCGGCGAAC
GGGTGAGTAACACGTAGGTAATCTACCTTTTAGTGGGGAATACCGTTTCGAAAGGAACGTTAATACCGCAT
GTGGATCCTTTCCGATGGGAGAGTATCTAAAGGGGGGTGTAGCAATACATCTCTCGCTAATCGATGAGCCT
GCGTCCCATTAGTTAGTTGGTAAGGTAATGGCTTACCAAGACTTCGATGGGTAGCTGGTCTGAGAGGACGA
CCAGCCACACTGGAACTGACACACGGTC
>Zotu3265
GAACGCTGGAAATGTGGATACCGGCATGCAAGTCAAGGGGCCGCAAGGCACCGGCGAACGGCTTAGTAA
CACGTGGATACATACCGCAAAGCGGAGCACAGCTCGTCGAAAGACGGGGTAATTCTCCATGATAACGCAA
GTTTAAAGGCGCAAGCCACTTTGTGAATGGTCCGCGGTCTATCAGCTAGTTGGTGAGGTAATGGCTCACCA
AGGCTATGACGGATAGGGGGCGTGAGAGCGCGACCCCCAAGAATGGCACTGCGACACGGGC
>Zotu3267
GAACGCTGGCGGCGTGCCTAACACATGCAAGTCGAGCGAAGCACTCTGCTTCAATTTCTTCGGATTGAGAA
GTAGTTTGACTGAGCGGCGGACGGGTGAGTAACGCGTGGGTAATCTGCCTCACACAGGGGGATAACAGTT
AGAAATGACTGCTAATACCGCATAAGGCCACAGCACCGCATGGTGCAGGGGTAAAAACTCCGGTGGTGTG
AGATGAGCCCGCGTCTGATTAGCCAGTTGGCGGGGTAACGGCCCACCAAAGCGACGATCAGTAGCCGGCC
TGAGAGGGTGATCGGCCACATTGGGACTGAGACACGGCC
>Zotu3270
GAACGCTGGCGGTATGCTTTACACATGCAAGTCGTACGAGAGTTTTGAACTCAAGTGGCGGACGGGTGAG
TAACACGTAAGAATTTGCCTTTAGGAGGGGGACAACAACTGGAAACGGTTGCTAATACCCCATATGCTTTC
GAGTGAAACGGTTTTTCCGCCTAAAGAGAAGCTTGCGGCTGATTAGCTTGTTGGTAAGGTAATGGCTTACC
AAGGCGACGATCAGTATCTGGTTTGAGAGGACGATCAGACACACTGGAACTGAGACACGGTC
>Zotu3273
GAACGCTGGCGGCGTGCTTAACACATGCAAGTCGAGCGGTAAGGCCCTTCGGGGTACACGAGCGGCGAA
CGGGTGAGTAACACGTGGGTGATCTGCCCTGCACTTCGGGATAAGCCTGGGAAACTGGGTCTAATACCGG
ATATGACCTCCGATCGCATGGTTGGTGGTGGAAAGATTTATCGGTGCAGGATGGGCCCGCGGCCTATCAG
CTTGTTGGTGGGGTAATGGCCTACCAAGGCGACGACGGGTAGCCGACCTGAGAGGGTGACCGGCCACACT
GGGACTGAGACACGGCC
>Zotu3275
GAACGCTGGCGGCGTGCTTAACACATGCAAGTCGAGCGGTAAGGCACCTTCGGGTGTACACGAGCGGCGA
ACGGGTGAGTAACACGTGAGCAATCTGCCCTTCTCATCGGAATAAGCGCGGGAAACTGCGTCTAATGCCG
AATATGACCTCCTTTCGCATGATCGGAGGTGGAAAGCTCCGGCGGAGAAGGATGAGCTCGCGGCCTATCA
GCTAGTTGGTGAGGTAACGGCCCACCAAGGCGACGACGGGTAGCCGGCCTGAGAGGGTGACCGGCCACA
CTGGGACTGAGACACGGCC

>Zotu3278
GAACGCTGGCGGCATGCCTAACACATGCAAGTCGAACGAAGGCTTCGGTCTTAGTGGCGCACGGGTGCGT
AACGCGTGGGAATCTGTCCCTCGGTTCGGAATAACAGTTAGAAATGACTGCTAATACCGGATGATGACGTA
AGTCCAAAGATTTATCGCCGAGGGATGAGCCCGCGTAGGATTAGCTAGTTGGTGTGGTAAGAGCGCACCA
AGGCGACGATCCTTAGCTGGTCTGAGAGGATGATCAGCCACACTGGGACTGAGACACGGCC
>Zotu3281
GAACGCTAGCGGCAGGCCTAATACATGCAAGTCGAACGGTAACATGAGTAGCAATACTTGATGACGAGTG
GCGCACGGGTGCGTAACGCGTATGCAATCTACCTTCAACTGGTGAATAGCCCGAAGAAATTCGGATTAATA
CACCATAGTATTGTTTGTTGGCATCAACAGACAATTAAAACTCCGGTGGTTGAAGATGAGCATGCGTGACA
TTAGCTAGTTGGTGAGGTAATGGCTCACCAAGGCTACGATGTCTAGGGGATCTGAGAGGATTAACCCCCAC
ACTGGTACTGAGACACGGAC
>Zotu3283
GAACGCTGGCGGCGTGCTTAACACATGCAAGTCGAACGATGATGCTCAAGCTTGCTTGGGTTGATTAGTGG
CGAACGGGTGAGTAACACGTGGGTGATCTGCCCTGCACTTCGGGATAAGCCTGGGAAACTGGGTCTAATA
CCGGATATGACCCTCCTTTAGTGTGGGGGGTGGAAAGCTTTTGCGGTGTGGGATGAGCCTGCGGCCTATC
AGCTTGTTGGTGGGGTAATGGCCTACCAAGGCGTCGACGGGTAGCCGGCCTGAGAGGGTGATCGGCCAC
ATTGGGACTGAGACACGGCC
>Zotu3285
GAACGCTGGCGGCGTGCTTAACACATGCAAGTCGAGCGAGGCCCTCCTTCGGGGGGTGCCCTAGCGGCGA
ACGGGTGAGTAACACGTGGGCAACCTGCCCTCAGCTCTGGGATAACTCCAAGAAATTGGTGCTAATACCG
GATGTGACCGCTGACCGCATGGTCTGGTGGTGGAAAGATTCATCGGCTGAGGATGGGCCCGCGGCCTATC
AGCTTGTTGGTGGGGTGATGGCCTACCAAGGCGACGACGGGTAGCCGGCCTGAGAGGGTGACCGGCCAC
ACTGGGACTGAGACACGGCC
>Zotu3287
GAATGCTGGCGGCGTGCTTAACACATGCAAGTCGAGCGGTAAGGCCCTTCGGGGTACACGAGCGGCGGA
CGGCTGAGTAACGCGTGGGAACATGCCCCAAAGTGAGGAATAACTGCCCGAAAGGGTGGCTAATGCCGC
ATATGATCTTCGGATTAAAGGATTTATCCGCTTTGGGAGTGGCCTGCGTACGATTAGGTAGTTGGTGAGGT
AATGGCTCACCAAGCCGACGATCGTTAGCTGGTCTGAGAGGATGACCAGCCAGACTGGAACTGAGACACG
GTC
>Zotu3289
GAACGCTGGCGGCGTGCTTAACACATGCAAGTCGAACGCTGAAGCTCCCAGCTTGCTGGGGGTGGATGAG
TGGCGAACGGGTGAGTATCACGTGAGGAACCTGCCCTGAACTTCGGGATAAGCCTGGGAAACTGGGTCTA
ATACCGGATAGGACATTCTACCGCATGGTGGGGTGTGGAAAGTTTTAACGGTTTGGGATGGTCTCGCGGC
CTATCAGCTTGTTGGTGGGGTAATGGCCTACCAAGGCGATGACGGGTAGCCGGCCTGAGAGGGTGACCGG
CCACACTGGGACTGAGACACGGCC
>Zotu3291
TAACGCTGGCGGCGTGCATAACACATGCAAGTCGAACGATGAAACTTAAATGATTTCTTCGGAATGAACTT
AAGTGAATTAGTGGCGAACGGGTGAGTAACGCGTGAGTAACCTGCCTTACACAAGGGGATAGCCTCTGGA
AACGGAGAATAATACCCTATGACATTAATACTTCGCATGAAGCATTAATCAAAGTGTTAGCGGTGTAAGAT
GGACTTGCGTCTGATTAGCTAGTTGGTGAGATAACAGCCCACCAAGGCAACGATCAGTAGCCGGCTTGAG
AGAGTGTACGGCCACATTGGGACTGAGACACGGCC
>Zotu3293
GAACGCTGGCGGCAGGCTTAACACATGCAAGTCGAGCGGGCGTAGCAATACGTCAGCGGCAGACGGGTG
AGTAACGCGTGGGAACGTACCCTTTGGTTCGGAACAACACAGGGAAACTTGTGCTAATACCGGATAAGCC
CTTACGGGGAAAGATTTATCGCCGAAGGATCGGCCCGCGTCTGATTAGCTAGTTGGTGAGGTAATGGCTCA
CCAAGGCGACGATCAGTAGCTGGTCTGAGAGGATGATCAGCCACACTGGGACTGAGACACGGCC
>Zotu3295
GAACGCTGGCGGCGTGCTTAACACATGCAAGTCGAACGGTGAAGCGCCTTCCTTTGTGGTGGGTGTGGAT
GAGTGGCGAACGGGTGAGTAACACGTGAGTAACCTGCCCCCTTCTTCTGGATAACCGCATGAAAGTGTGG
CTAATACGGGATATTCTGTGCCTGCTGCATGGTGGGTGTGGGAAAGATTGCGCCTTTGGGTGTTTTTGGTG

GGGGATGGGCTCGCGGCCTATCAGCTTGTTGGTGGGGTGATGGCCTACCAAGGCGGTGACGGGTAGCCG
GCCTGAGAGGGTGGACGGTCACACTGGGACTGAGACACGGCC
>Zotu3298
GAACGCTAGCGGCAGGCCTAATACATGCAAGTCGAGGGGTATAGGAGGCTTGCTTCCTAGAGACCGGCGC
ACGGGTGCGTAACGCGTATGCAACCTACCCTTTTCTGGGGGATAGCCCGAAGAAATTCGGATTAATACCGC
ATAAGACCACACGATGGCATCATCGAGAGGTCAAACATTTATGGGAGAAGGATGGGCATGCGTGCCATTA
GCTAGTTGGCGGGGTAACGGCCCACCAAGGCGACGATGGCTAGGGGATCTGAGAGGATGACCCCCCACA
CTGGTACTGAGACACGGAC
>Zotu3300
GAACGCTGGCGGCGCGCCTAATACATGCAAGTCGAACGGGCACACTTCGGTGTGTCAGTGGCGGACGGGT
GAGTAACACGTGAGTGACCTGCCTCCTAGTACGGAATAACTCTGGGAAACCAGAGCTAATACCGCATGTGC
TTAGTGTTTCATGATGCTAAGTAAAGATTTATTCGCTAGGAGAGGGGCTTGCGGCGGATTAGCTAGTCGGT
GGGGTAACGGCCTACCGAGGCGACGATCCGTAGCTGGTCTGAGAGGACGACCAGCCACACTGGGACTGA
GACACGGCC
>Zotu3302
GAACGCTAGCGGCAGGCCTAATACATGCAAGCCGAACGGCAAGTCTGATAGCAATATTGGACCTAGAGTG
GCGCAAGGGTGCGTAACACGTATGCAATCTGCCCACGACAGGAGTATAGCTCCCCGAAAGGGGAATTAAC
CCTCCATAACACCGCGCGAGGGCATCCGAGAGCGGTTAAACCAAAAGGGGTCGTGGATGAGCATGCGGTT
GATTAGCTAGTTGGTAGGGTAACGGCCTACCAAGGCGATGATCAATAGGGGTTCTGAGAGGAAGGTCCCC
CACACTGGCACTGAGATACGGGC
>Zotu3304
GAACGCTGGTGGCGTGCTTAACACATGCAAGTCGAGCGAGGCGTCCGCCTCCGGGCGGTCAGCCGAGCG
GCGAACGGGTGAGTAACACGTGAGCAATCTTCCCTCGGCTCGGGGATAACCTCGGGAAACTGAGGCTAAT
ACCGGATGAACGTTTTTCGGGGCATCCCGGAAAACGAAAAGGTTTCGCACCGCGAGGTGCGTTTCCGGCC
GGGGATGAGCTCGCGGCCTATCAGTTTGTTGGTGTGGTAAATGCGCACCAAGACGACGACGGGTAGCCGG
CCTGAGAGGGTGATCGGCCACACTGGGACTGAGACACGGCC
>Zotu3307
GAACGCTGGCGGCGTGCTTAACACATGCAAGTCGAACGATGAACCTCCTTCGGGAGGGGATTAGTGGCGA
ACGGGTGAGTAACACGTGGGCAATCTGCCCTGCACTCTGGGACAAGCCCTGGAAACGGGGTCTAATACCG
GATACTGATCATCTTGGGCATCCTTGGTGATCGAAAGCTCCGGCGGTGCAGGATGAGCCCGCGGCCTATCA
GCTTGTTGGTGAGGTAATGGCTCACCAAGGCGACGACGGGTAGCCGGCCTGAGAGGGCGACCGGCCACA
CTGGGACTGAGACACGGCC
>Zotu3309
TAACGCTGGCGGCGTGCATAACACATGCAAGTCGAACGATGAAACTTAAATGATTTCTTCGGAATGAACTT
AAGTGGATTAGTGGCGGACGGGTGAGTAACGCGTGAGTAACCTGCCTTACACAAGGGGATAGCCTCTGGA
AACGGAGAATAATACCCTATGATGTTTCTATCTCGCATGAGATATTAACCAAAGTGTTAGCGGTGTAAGAT
GGACTTGCGTCTGATTAGCTAGTTGGTGAGAAAAAAGCCCACCAAGGCAACGATCAGTAGCCGGCTTGAG
AGAGTGTACGGCCACATTGGGACTGAGACACGGCC
>Zotu3314
GAACGCTGGCGGCATGCCTTACACATGCAAGTCGAACGGCAGCATGACGTGTAGCAATACACGTTGATGG
CGAGTGGCGAACGGGTGAGTAATATATCGGAACATGTCCTGTAGCGGGGGATAACCTATCGAAAGATGGA
CTAATACCGCATACGCTCTACGGAGGAAAGCGGGGGTCGCAAGACCTCGCACTATAGGAGTGGCCGATAT
CGGATTAGCTAGTTGGCGGGGTAAAAGCCCACCAAGGCGACGATCCGTAGCTGGTCTGAGAGGATGATCA
GCCACACTGGAACTGAGACACGGTC
>Zotu3318
GAACGCTGGCGGTGTGCTTAACACATGCAAGTGGAGCGACGAACCAGGGCTTGCCCTGGGGCAGAGCCG
CGAACGGGTGAGTAACACGTGGGTAACCTACCCCGATGACTGGGACAACCCGAGGAAACTCGGGCTAATA
CCGGATGTGCTCGGAGATCACGAGATTCTCGAGTAAAGGAAGCTTCGGCCTCCGCATTGGGAGGGGCCCG
CGGCCCATTAGCTTGTTGGTGGGGTAACGGCCTACCAAGGCTGCGATGGGTAGCTGGTCTGAGAGGACGA
TCAGCCACACTGGGACTGAGACACGGCC

>Zotu3321
GAACGCTGGCGGCGTGCTTAACACATGCAAGTCGAGCGAGGCCCTCTCTTCGGGGGGGTGCCCTAGCGGC
GAACGGGTGAGTAACACGTGGGCAACCTGCCCTCAGCTCTGGGATAACTCCAAGAAATTGGTGCTAATACC
GGATGTGACCGCTGACCGCATGGTCTGGTGGTGGAAAGATTCATCGGCTGGGGATGGGCCCGCGGCCTAT
CAGCTTGTTGGTGGGGTCACGGCCCACCAAGGCGACGACGGGTAGCCGGCCTGAGAGGGTGACCGGCCA
CACTGGGACTGAGACACGGCC
>Zotu3324
GAACGCTGGCGGCGCGCCTAAAACATGCAAGTCGAACGAGAAACCCGGGCTTGCCCGGGTGGACAGTGG
CGAACGGGTGAGTAACACGTGAGGAACCTGCCCCGAAGACCGGGACAACACCGGGAAACCGGTGATAAT
ACCGGATGTCCTCACCGGATCGCATGATCTGGTGAGGAAATGGATTCCGCTTCGGGATGGCCTCGCGGCCT
ATCAGCTAGTTGGTGGGGTAACGGCCCACCAAGGCGACGACGGGTAGCTGGTCTGAGAGGATGTCCAGCC
ACACTGGGACTGAGACACGGCC
>Zotu3327
GAACGCTGGCGGCGTGCCTAATACATGCAAGTCGAACGCTTTTTCCAATCATCGTAGCTTGCTACACCGATT
GGAAATTGAGTGGCGAACGGGTGAGTAACACGTGGGTAACCTGCCCAAAAGAGGGGGATAACACTTGGA
AACAGGTGCTAATACCGCATAACCATGATGACCGCATGGTCATTATGTAAAAGATGGTTTCGGCTATCACTT
TTGGATGGACCCGCGGCGTATTAACTTGTTGGTGGGGTAACGGCCTACCAAGGTAATGATACGTAGCCGA
ACTGAGAGGTTGATCGGCCACATTGGGACTGAGACACGGCC
>Zotu3330
GAACGCTGGCGGCGTGCCTAATACATGCAAGTCGAACGAAGTTATGAGAGAGCTTGCTCTCGATTAGCTTA
GTGGCGGACGGGTGAGTAACACGTAGGCAACCTGCCCCTAAGATCGGGATAACATTCGGAAACGAATGCT
AAGACCGGATAAACAATCTACCCGCATGGGTGGATTGGGAAACACGGAGCAATCTGTGGCTTAGGGATGG
GCCTGCGGCGCATTAGCTAGTTGGTGGGGTAACGGCTCACCAAGGCGACGATGCGTAGCCGACCTGAGAG
GGTGAACGGCCACACTGGGACTGAGACACGGCC
>Zotu3335
GAACGCTAGCGGCAGGCCTAATACATGCAAGTCGAACGGGCTTGGGTTCTTCGGGACTCGAGTCAGTGGC
GCACGGGTGCGTAACGCGTATGCAACCTACCTTCCACAGGGGGATAGCCCCGGGAAACCGGGATTAATAC
CGCATGACACCATTTTGTGGCATCACAGAATGGTCAAAGTTTCGGCGGTGGAAGATGGGCATGCGTGCCAT
TAGCTAGTTGGCGGGGTAGAGGCCCACCAAGGCTACGATGGCTAGGGGTTCTGAGAGGATGGTCCCCCAC
ACTGGTACTGAGACACGGAC
>Zotu3337
GAACGCTGGCGGCGTGCTTAACACATGCAAGTCGAACGAGGCCCTCTCTTCGGAGGGGTGTTCTAGTGGC
GAACGGGTGAGTAACACGTGGGCAACCTGCCCCCGGCTCTGGGATAACTCCAAGAAATTGGGGCTAATAC
CGGATATGACCGCTGACCGCATGGTCTGGTGGTGGAAAGTTTTTCGGCTGGGGATGGGCCCGCGGCCTAT
CAGCTTGTTGGTGGGGTAGTGGCCTACCAAGGCGACGACGGGTAGCCGGCCTGAGAGGGTGACCGGCCA
CACTGGGACTGAGACACGGCC
>Zotu3339
GAACGCTGGCGGCGTGCTTAACACATGCAAGTGGTGCGACGAACCAGGGCTTGCCCTGGGGCAAAGCCGC
GAACGGGTGAGTAACACGTGAGTAACCTGCCCCGATGACCGGGACAACCCGAGGAAACTCGGGCTAATAC
CGGATTTGGCGCACAGATTTAAGTTTGTGCGCTAAAGGAAGCTTCGGCCTCCGCATTGGGAGGGGCTCGC
GGCCCATTAGCTTGTTGGTGGGGTAACGGCCTACCAAGGCAACGATGGGTAGCTGGTCTGAGAGGACGAT
CAGCCACACTGGGACTGAGACACGGCC
>Zotu3341
GGACGCTGGCGGCGTGCTTAACACATGCAAGTCGAACGATGATCCCCAGCTTGCTGGGGGGATTAGTGGC
GAACGGGTGAGTAACACGTGAGTAACCTGCCCCTGACTCTGGGATAAGCGCTGGAAACGGCGTCTAATAC
CGGATATGACAGCCGACGGCATCGTCTGGTTGTGGAAAGATTTTTCGGTTGGGGATGGACTCGCGGCCTAT
CAGGTTGTTGGTGAGGTAATGGCTCACCAAGCCTACGACGGGTAGCCGGCCTGAGAGGGTGACCGGCCAC
ACTGGGACTGAGACACGGCC
>Zotu3345

GAACGCTGGCGGCGTGCTTAACACATGCAAGTCGAGCGGAAAGGCCCTTCGGGGTACTCGAGCGGCGAA
CGGGTGAGTAACACGTGAGTAATCTGCCCTTCACTCTGGGATAGCCACCGGAAACGGTGATTAATACCGGA
TATGACACGTCCCTGCATGGGGTGCGTGTGGAAACTTTTTTGGGTGGGGGATGTGCTCGCGGCCTATCAGC
TTGTTGGTGGGGTAATGGCCTACCAAGGCTTCGACGGGTAGCCGGCCTGAGAGGGTGACCGGCCACACTG
GGACTGAGACACGGCC
>Zotu3347
GAACGCTGGCGGCGTGCTTAACACATGCAAGTCGAACGGAAAGGCCTGCTTTCCCTTGTGGATTGCGGGT
ACTCGAGTGGCGAACGGGTGAGTAACACGTGAGTGATCTGCCCCCAACTTGGGTATAAGCCTGGGAAACT
GGGTCTAATTCCCGATAGGACTGCAGGGTGGTGCCTGTGGTGGAAAACGATTTTTTAGTGGTTGGGGATG
AGCTCGCGGCCTATCAGCTTGTTGGTGGGGTAATGGCCTACCAAGGCGGCGACGGGTAGCCGGCCTGAGA
GGGTGGACGGCCACATTGGGACTGAGATACGGCC
>Zotu3349
GAACGCTGGCGGCGTGCCTAAGACATGCAAGTCGAACGGGTTTAGACCCGTGGCGAACGGGTGAGTAAC
GCGTGGGTACTTCCATTAAGTTTGGAATAACCTTTCGAAAGAGAGGACAATACCAAATGTGGTCGCAAGAC
TAAAGCCGCAAGGCGCTTAATGCGAGGCCCGCGTCCCATCAGCTAGTTGGTAGGGTAAAGGCCTACCAAG
GCTATGACGGGTAGCGGAACCGAGAGGTTGATCCGCCACACTGGGACTGCGACACGGCC
>Zotu3354
GAACGCTGGCGGCGTGCCTAATACATGCAAGTAGAACGCTGAAGGAATAGCTTGCTATTCTGGAAGAGTT
GCGAACGGGTGAGTAACGCGTAGGTAACCTACCTCATAGCGGGGGATAACTATTGGAAACGATAGCTAAT
ACCGCATAAGAATACTTATTGCATGATAAGTGTTTAAAAGGAACAACTGTTTCACTGTGAGATGGACCTGC
GTTGTATTAGCTAGTTGGTAAGGTAACGGCTTACCAAGGCGACGATACATAGCCGACCTGAGAGGGTGAT
CGGCCACACTGGGACTGAGACACGGCC
>Zotu3357
GAACGCTAGCGGCAGGCCTAATACATGCAAGACGAACGGCAAGAATTGTAGCAATACGATTCCTAGAGTG
TCGCACGGGTGCGTAACACGTATGCAACCTACCTTGTACAGGAGTATAGCTCGGGGAAACTCGAATTAACC
CTCCATAATATGATTGAATGGCATCATTTAATTATTAAAACTTCGGTGGTACAAGATGGGCATGCGTTTGAT
TAGCTAGTTGGCGGGGTAATGGCCCACCAAGGCGATGATCAATAGGGGAACTGAGAGGTTGATCCCCCAC
ACTGGCACTGAGATACGGGC
>Zotu3359
GAACGCTGGCGGCGTGCTTAACACATGCAAGTCGAGCGGTAAGGCCCTTTCGGGGGTACACGAGCGGCG
AACGGGTGAGTAACACGTGAGTAATCTGCCCTTCACTTCGGGATAACCACCGGAAACGGTGGCTAATACCG
GATATGACCATTCCCGGCATCGGGTGGTGGTGGAAAGTTCTGGCGGTGGAGGATGAGCTCGCGGCCTATC
AGCTTGTTGGTGGGGTAGTGGCCTACCAAGGCGACGACGGGTAGCCGGCCTGAGAGGGTGACCGGCCAC
ACTGGGACTGAGACACGGCC
>Zotu3361
GAACGCTGGCGGCAGGCCTAACACATGCAAGTCGAACGGATTCTTCGGGATTAGTGGCGGACGGGTGAGT
AACACGTGGGAACGTGCCTTTTGGTTCGGAACAACTCAGGGAAACTTGAGCTAATACCGGATAAGCCTTTC
GAGGGAAAGATTTATCGCCATTAGAGCGGCCCGCGTAGGATTAGCTAGTTGGTGGGGTAAAGGCCCACCA
AGGCTACGATCCTTAGCTGGTCTGAGAGGATGATCAGCCACATTGGGACTGAGACACGGCC
>Zotu3364
GAACGCTGGCGGCGTGCTTCACACATGCAAGTCGAGCGAGGCCCTTCCTTCGGGAAGGAGCCCTAGCGGC
GAACGGGTGAGTAACACGTGGGCAACCTGCCCTCAGCTCTGGGATAAGCCCGGGAAACCGGGTCTAATAC
CGGATATGACTCCGACAGGCATCTTGCGGAGTGGAAAGATTCATCGGCTGAGGATGGGCCCGCGGCCTAT
CAGCTTGTTGGTGGGGTAACGGCCTACCAAGGCGACGACGGGTAGCCGGCCTGAGAGGGCGACCGGCCA
CACTGGGACTGAGACACGGCC
>Zotu3367
GAACGCTGGCGGCAGGCTTAACACATGCAAGTCGAACGATGAAACTCTAGCTTGCTAGAGATGATTAGTG
GCGGACGGGTGAGTAACATTTAGGAATCTGCCTAGTAGTGGGGGATAGCTCGGGGAAACTCGAATTAATA
CCGCATACGACCTACGGGTGAAAGGGGGCGCAAGCTCTTGCTATTAGATGAGCCTAAATCAGATTAGCTA
GTTGGTGGGGTAAAGGCCCACCAAGGCGACGATCTGTAACTGGTCTGAGAGGATGATCAGTCACACCGGA
ACTGAGACACGGTC

>Zotu3369
CAACGCTGGCGGCGTGCCTAATGCATGCAAGTCGAACGGGGTGTCCTTCGGGGCACTTACGTGGCGGACG
GGTGAGGAACACGTGGGTAACCTGCCCACTGGTGGGGGCTAGCCTTCGGAAACGGAGGGTAATTCCGCAT
GATCTCGCAGCCCGCGTGGGGGTTGTGAGGAAAGCCTTCGGGCGCCAGTGGAGGGGCCCGCGCCCGATT
AGCTTGTTGGTGGGGTAACGGCTTACCAAGGCGATGATCGGTAGCTGGTTTGAGAGGATGATCAGCCACA
CGGGGACTGAGACACGGCC
>Zotu3371
GAACGCTGGCGGCGTGCTTAACACATGCAAGTCGAACGGAAAGGCCCTGCTTGCAGGGTACTCGAGTGGC
GAACGGGTGAGTAACACGTGGGTGATCTGCCCTGCACTTCGGGATAAGCCTGGGAAACTGGGTCTAATAC
CGGATAGGAACCATCTTTAGTGTGGTGGTTGGAAAGTTTTTCGGTGTAGGATGAGCTCGCGGCCTATCAG
CTTGTTGGTGGGGTAATGGCCTACCAAGGCGGCGACGGGTAGCCGGCCTGAGAGGGTGTACGGCCACATT
GGGACTGAGATACGGCC
>Zotu3373
GAACGCTGGCGGCGTGCTTAACACATGCAAGTCGAACGGTGAAGATCCGGCTTTGGTCGGGTTGGATCAG
TGGCGAACGGGTGAGTAACACGTGAGTAACCTGCCCTTGACTCTGGGATAACTCCGGGAAACCGGTGCTA
ATACTGGATATGACGCTGCCCTGCCTGGGGTGGTGTGGAAAGATTTATCGGTTGGGGATGGACTCGCGGC
CTATCAGCTTGTTGGTGAGGTAGTGGCTTACCAAGGCGACGACGGGTAGCCGGCCTGAGAGGGTGATCGG
CCACACTGGGACTGAGACACGGCC
>Zotu3375
GAACGCTGGCGGCGTGCCTAATACATGCAAGTAGAACGCTGAAGCTTGGTGCTTGCACCGAGCGGATGAG
TTGCGAACGGGTGAGTAACGCGTAGGTAACCTGCCTCTTAGCGGGGGATAACTATTGGAAACGATAGCTA
ATACCGCATAAAAGTCGATATCGCATGATATTGATTTGAAAGGTGCAAATGCATCACTAAGAGATGGACCT
GCGTTGTATTAGCTAGTTGGTGAGGTAACGGCTCACCAAGGCGACGATACATAGCCGACCTGAGAGGGTG
ATCGGCCACACTGGGACTGAGACACGGCC
>Zotu3377
GAACGCTGGAAATGTGGATACCGGCATGCAAGTCAAGGGGCCGCAAGGCACCGGCGAACGGCTTAGTAA
CACGTAGATACATACCCAAAAGTGGGGCATAGCTCGTCGAAAGACGGGGTAATTTCCCATGTGCTCGCAA
GAGTAAAGACGCAAGTCGCTTTTGGAATGGTCTGCGGTCTATCAGCTAGTTGGCGGGGTAAAAGCCCACC
AAGGCTATGACGGATAGGGGGCGTGAGAGCGCGACCCCCAAGAATGGCACTGCGACACGGGC
>Zotu3379
GAACGCTGGCGGCATGCCTAACACATGCAAGTCGAACGAAGGCTTCGGCCTTAGTGGCGCACGGGTGCGT
AACGCGTGGGAATCTGCCCCTTGGTTCGGAATAACAGTTGGAAACGACTGCTAATACCGGATGATGACGTT
AAGTCCAAAGATTTATCGCCGAGGGATGAGCCCGCGTAGGATTAGCTAGTTGGTGTGGTAAGAGCGCACC
AAGGCGACGATCCTTAGCTGGTCTGAGAGGATGATCAGCCACACTGGGACTGAGACACGGCC
>Zotu3381
GAACGCTGGCGGCGTGCTTAACACATGCAAGTCGAGCGAGGCCCTCCTTCGGGGGGTGCCCTAGCGGCGA
ACGGGTGAGTAACACGTGGGCAACCTGCCCTCAGCTCTGGGATAACTCCAAGAAATTGGTGCTAATACCG
GATGTGACCGCTGACCGCATGGTCTGGTGGTGGAAAGATTCATCGGCTGAGGATGGGCCCGCGGCCTATC
AGCTTGTTGGTGGGGTAACAGCCTACCAAGGCGACGACGGGTAGCCGGCCTGAGAGGGTGACCGGCCAC
ACTGGGACTGAGACACGGCC
>Zotu3383
GAACACTGGCGGCAGGCCTAACACATGCAAGTCGAGCGGATGACGGGAGCTTGCTCCTTGATTCAGCGGC
GGACGGGTGAGTAATGCCTAGGAATCTGCCTGGTAGTGGGGGACAACGTTTCGAAAGGAACGCTAATACC
GCATACGTCCTACGGGAGAAAGCAGGGGACCTTCGGGCCTTGCGCTATCAGATGAGCCTAGGTCGGATTA
GCTAGTTGGTGAGGTAATGGCTCACCAAGGCGACGATCCGTAACTGGTCTGAGAGGATGATCAGTCACAC
TGGAACTGAGACACGGTC
>Zotu3385
GAACGCTAGCGGCAGGCTTAACACATGCAAGTCGAGGGGTATATTTCTTCGGAGATAGAGACCGGCGCAC
GGGTGCGTAACGCGTATGCAATCTACCTTTTACAGAGGGATAGCCCAGAGAAATTTGGATTAATACCTCAT
AGTATTATGAAATGGCATCATTTTATAATTAAAGTCACAACGGTAAAAGATGAGCATGCGTCCCATTAGCTA

GTTGGTAAGGTAACGGCTTACCAAGGCTACGATGGGTAGGGGTCCTGAGAGGGAGATCCCCCACACTGGT
ACTGAGACACGGAC
>Zotu3387
GAACGCTAGCGGCAGGCCTAATACATGCAAGTCGAACGGGGTAGCAATACCCAGTGGCGCACGGGTGCG
TAACGCGTATGCAACCTACCTTTTGCTGGGGGATAGCCCGGAGAAATCCGGATTAATACCGCATAACACCT
TTTGATGGCATCTGAGGAAGGTTAAAGATTTATCGGCAAAAGATGGGCATGCGTGCCATTAGCTAGTTGGT
GGGGTAACAGCCCACCAAGGCGACGATGGCTAGGGGGTCTGAGAGGATGGTCCCCCACACTGGCACTGA
GATACGGGC
>Zotu3390
GAACGCTGGCGGCGCGCTTAACACATGCAAGTCGAACGGAGGGCTTCGGCCCTCAGTGGCGAACGGGTG
CGTAACACGTGAGCAACCTGCCCCTGTGCGGGGGATAACCGGGGGAAACCCCGGCTAATACCGCATGAGA
CCACGACCTCCGTGGAGGTCGTGCTTAAAGGTCCTTTCGGGGGCTGCGCAGGGAGGGGCTCGCGGCGTAT
CAGCTAGCTGGTGAGGTAACGGCTCACCAGGGCAACGACGCGTAGGGGGTCTGAGAGGATGGCCCCCCA
CATTGGGACTGAGATACGGCC
>Zotu3392
GAACGCTGACAGAATGCTTAACACATGCAAGTCTACTTGAATTTGGGTTTTTAACTTAGATTTGGGTGGCG
GACGGGTGAGTAACGCGTAAAGAACTTGCCTCACAGCTAGGGACAACATTTAGAAATGAATGCTAATACCT
AATATTATGATAATAGGGCATCCTATAATTATGAAAGCTATATGCGCTGTGAGAGAGCTTTGCGTCCCATTA
GCTAGTTGGAGAGGTAACGGCTCACCAAGGCGATGATGGGTAGCCGGCCTGAGAGGGTGATCGGCCACA
AGGGGACTGAGACACGGCC
>Zotu3394
GAACGCTGGCGGCATGCCTAACACATGCAAGTCGAACGAGATCTTCGGGTCTAGTGGCGCACGGGTGCGT
AACGCGTGGGAATCTGCCCTTTGGTTCGAAATAACAGTTGGAAACGACTGCTAATACCGGATGATGACGAA
AGTCCAAAGATTTATCGCCAGAAGATGAGCCCGCGTAGGATTAGCTAGTTGGTGTGGTAAAGGCGCACCA
AGGCGACGATCCTTAGCTGGTCTGAGAGGATGATCAGCCACACTGGGACTGAGACACGGCC
>Zotu3396
GAACGCTGGCGGCATGCCTTACACATGCAAGTCGAACGGTAGAGGGGGCAACCCCTTGAGAGTGGCGAA
CGGGTGAGTAATGCATCGGAACGTGCCCAGTCGTGGGGGATAACTATCCGAAAGGATGGCTAATACCGCA
TGAGATCTGTGGATGAAAGCGGGGGACCTGAAAGGGCCTCGCGCGATTGGAGCGGCCGATGTCAGATTA
GCTAGTTGGTGGGGTAAAGGCCCACCAAGGCGACGATCTGTAACTGGTTTGAGAGAACGACCAGTCACAC
TGGGACTGAGACACGGCC
>Zotu3398
GAACGCTGGCGGCGCACCTAAGACATGCAAGTCGAACGGGGGCTTCGGCCCCAGTGGCGGACGGGTGAG
TAACACGTAGGGGACCTGCCCCGTGGCGGGGGATAACACCGGGAAACCGGTGCTAAGACCGCATACGTCC
GATGGCATAGCGGCCATCGGGGAAATGCGCGAGCAGCCACGGGAGGGCCTTGCGGCGGATTAGGTAGTT
GGTGGGGTAGAAGCCTACCAAGCCGACGATCCGTAGCTGGTCTGAGAGGACGACCAGCCACACCGGGAC
TGAGACACGGCC
>Zotu3401
GAACGCTGGCGGCGTGCCTAACACATGCAAGTGGAGCGACGAACCGGGGCTTGCCCCGGGGCAGAGCCG
CGAACGGGTGAGTAACACGTGGGTTACCTGCCTCGATGACTGGGACAACCCGTCGAAAGACGGGCTAATA
CCGGATGTGCCCCTTGGGGGAAAGGAAGCTTTGGCCTCCGCATCGGGATGGGCCCGCGGCGCATTAGCTT
GTTGGTGCGGTAATGGCGTACCAAGGCGACGATGCGTAGCTGGTCTGAGAGGACGATCAGCCACACTGG
GACTGAGACACGGCC
>Zotu3404
GAACGCTGGCGGCGTGCCTAACACATGCAAGTGGAGCGACGACCCGGACTTCGGTCCGGTGCAAAGCCGC
GAACGGGTGAGTAACACGTGGGTTACCTGCCCCGATGACAGGGACAACCTCGGGAAACCGAGGCTAATAC
CTGATGAGCCCGCAAGGGGAAAGGAAGCTTCGGCCTCCGCATCGGGATGGGCCCGCGGCGCATTAGCTTG
TTGGTGGGGTAAAGGCCTACCAAGGCGACGATGCGTAGCTGGTCTGAGAGGACGATCAGCCACACTGGG
ACTGAGACACGGCC
>Zotu3406

GAACGCTAGCTACAGGCTTAACACATGCAAGTCGAGGGGCAGCATGAACTTAGCTTGCTAAGTTTGATGGC
GACCGGCGCACGGGTGAGTAACACGTATCCAACCTGCCGATGACTCGGGGATAGCCTTTCGAAAGAAAGA
TTAATACCCGATGGCATAGTTCTTCCGCATGGTAGAACTATTAAAGAATTTCGGTCATCGATGGGGATGCGT
TCCATTAGGTTGTTGGCGGGGTAACGGCCCACCAAGCCTTCGATGGATAGGGGTTCTGAGAGGAAGGTCC
CCCACATTGGAACTGAGACACGGTC
>Zotu3408
GAACGCTGGCGGCGTGCTTAACACATGCAAGTCGAGCGGTAAGGCCCTTTCGGGGGTACACGAGCGGCG
AACGGGTGAGTAACACGTGAGTAATCTGCCCTTCACTCTGGGATAAGCACTCGAAAGGGTGTCTAATACCG
GATACGACACAGACTGGCATCGGTTCTGTGTGGAAACTTTTGTGGGTGGGGGATGTGCTCGCGGCCTATCA
GCTTGTTGGTGGGGTAATGGCCTACCAAGGCTTTGACGGGTAGCCGGCCTGAGAGGGTGACCGGCCACAC
TGGGACTGAGACACGGCC
>Zotu3410
GAACGCTGGCGGCGTGCTTAACACATGCAAGTCGAGCGGTAAGGCTCCTTCGGGGGTACACGAGCGGCG
AACGGGTGAGTAACACGTGAGTAATCTGCCCTTCACTTCGGGATAACCATCGGAAACGGTGGCTAATACCG
GATACGACGTACTCCTGCATGGGATGTGCGTGGAAAGTTTTTTCGGTGGGGGATGTGCTCGCGGCCTATCA
GCTTGTTGGTGAGGTAATGGCTCACCAAGGCTTCGACGGGTAGCCGGCCTGAGAGGGTGACCGGCCACAC
TGGGACTGAGACACGGCC
>Zotu3412
GAACGCTGGCGGCGTGCCTTATACATGCAAGTCACACGGGTGTAGCAATACACCAGTGGCGAACGGCTGA
GTAACACGTGGGAACTTCCTTTAAGTTCGGAATAACCCGCCGAAAGGCGGACTAATACCGGATGTGCCCGA
AAGGGTAAAGCCGCAAGGCGCTTAAAGCGAGGCCCGCGCACCATCAGGTAGTTGGTAGGGTAATGGCCT
ACCAAGCCTATGACGGTTAGAGGGCCCGAGAGGGTGTACCTCCAGACGGGAACTGCGACACGGTC
>Zotu3414
GAACGCTAGCGATAGGCTTAACACATGCAAGTCGAGGGGCAGCATTATTTTAGCTTGCTAAGATAGATGGC
GACCGGCGCACGGGTGCGTAACGCGTATGCAACCTGCCTGTAATTAGGGAATAACCCGGTGAAAGTCGGA
CTAATACCCTATACTCTTCTTTCTCCGCATGGAGGAGGACGGAAAGATTTATTGATTACAGATGGGCATGCG
TCCCATTAGCTAGTTGGTAAGGTAACGGCTTACCAAGGCAACGATGGGTAGGGGGACTGAGAGGTTGACC
CCCCACACTGGTACTGAGACACGGAC
>Zotu3416
GAACGCTGGCGGCGTGCTTAAGACATGCAAGTCGAACGATCGTCTTCGGACGGTAGTGGCGCACGGGTGA
GTAACACGTAACTGACCTGCCCCCAAGTTCCGAATAACCCTCCGAAAGGAGGGCTAATACGGAATGTGCTG
TCACCTCCTGTGGTGCCAGTAAAGATTGATCGCTTGGGGATGGGGTTGCGTTCCATCAGCTTGTTGGTGGG
GTAAAGGCCCACCAAGGCAACGACGGATAACCGGCCTGAGAGGGTGGCCGGTCACAGGGGCACTGAGAC
ACGGGT
>Zotu3418
GAACGCTGGCGGCGTGCTTAACACATGCAAGTCGAGCGATTTACTTCGGTAAAGAGCGGCGGACGGGTGA
GTAACGCGTGGGTAACCTGCCCTGTACACACGGATAACATACCGAAAGGTATGCTAATACGAGATAATATG
CTTTTATCGCATGGTAGAAGTATCAAAGCTTTTGCGGTACAGGATGGACCCGCGTCTGATTAGCTAGTTGGT
AAGGTAACGGCTTACCAAGGCGACGATCAGTAGCCGACCTGAGAGGGTGATCGGCCACATTGGAACTGAG
ACACGGTC
>Zotu3420
GAACGCTGGCGGTGTGCTTAACACATGCAAGTCGAACGGGCAAGAGCTGTAGCAATACAGAATTCTGCTA
GTGGCAAACGGGTGAGTAATACATGGGAATCTACCTTGCAGTGCGAAATAACAGTTGGAAGCAATTGCTA
ATGTCGCATATAGCCGAGAGGTGAAAGATTTATCGCTGCAAGACGAGCCCATGCAGGATTAGCTAGTTGG
TGGGGTAACGGCCTACCAAGGCGACGATACGTAGCCGACCTGAGAGGGTGATCGGCCACACTGGAACTG
AGACACGGTC
>Zotu3422
TAACGCTGGCGGCGTGCCTAATACATGCAAGTCGAACGGATAGTAATATCAGTGGCGAACGGGTGAGTAA
TACGTGGGTAATCTACCTTCTCATGGGGAATAACTCTCCGAAAGGAGAGCTAATACCGCATAAGACCACGG
GTGCTGCGGCACCTGAGGTTAAAGGCGACCTCTACTTGTAAGTTGTCGTGAGAAGATGAGCTTGCGTCTGA

TTAGCTAGTTGGTGAGGTAAAAGCTTACCAAGGCTCTGATCAGTAGCTGGTCTGAGAGGATGGTCAGCCAC
ACTGGAACTGAGACACGGTC
>Zotu3424
GAACGCTGGCGGCGTGCTTAACACATGCAAGTCGAACGGGATTTAGTAGACAGAAGCTTCGGTGGAAGAT
TACTAATGAGAGTGGCGAACGGGTGAGTAACGCGTGAGCAACCTGCCTATGACAGTGGGATAGCCTCGGG
AAACCGGGATTAATACCGCATAAAATCGTAGAAACACATGTTTTAACGGTCAAAGATTTATCGGTCATAGA
TGGGCTCGCGTCTGATTAGCTAGTTGGTGAGATAACAGCCCACCAAGGCGACGATCAGTAGCCGGTCTGA
GAGGATGAACGGCCACATTGGAACTGAGACACGGTC
>Zotu3427
GAACGCTGGCGGCATGCCTAACACATGCAAGTCGAACGAAGCCTTCGGGCTTAGTGGCGCACGGGTGCGT
AACACGTGGGAATCTGCCCTTCGGTTCGGAATAACAGTGGGAAACTACTGCTAATACCGGATAATGACGTA
AGTCCAAAGATTTATCGCCGAAGGATGAGCCCGCGTAGGATTAGGTAGTTGGTGGGGTAATGGCCTACCA
AGCCGACGATCCTTAGCTGGTCTGAGAGGATGATCAGCCACACTGGGACTGAGACACGGCC
>Zotu3429
GAACGCTGGCGGCGTGCCTAATACATGCAAGTCGAGCGAGGTTCTTCGGAACCTAGCGGCGGACGGGTGA
GTAACACGTGGGTAACCTGCCTGTAAGACCGGGATAACTTCGGGAAACCGAAGCTAATACCGGATACTTTC
GAGCATCGCATGATGCTTGATGGAAAGACGGCTTCGGCTGTCACTTACAGATGGACCCGCGTCGCATTAGC
TAGTTGGTGAGGTAACGGCTCACCAAGGCGACGATGCGTAGCCGACCTGAGAGGGTGATCGGCCACACTG
GGACTGAGACACGGCC
>Zotu3431
GAACGCTGGCGGCATGCTTTACACATGCAAGTCGAACGGCAGCGAAGAGAAGCTTGCTTCTCTGTCGGCG
AGTGGCGAACGGGTGAGTATAGCATCGGAACGTGCCAAGTAGTGGGGGATAACCAAACGAAAGTTTGGC
TAATACCGCATAAGCTCTAAGGAGGAAAGTAGGGGACCGTAAGGCCTTACGCTATTTGATCGGCCGATGTC
GGATTAGCTAGTTGGTGAGGTAATGGCTCACCAAGGCGATGATCCGTAGCGGGTCTGAGAGGACGATCCG
CCACACTGGGACTGAGACACGGCC
>Zotu3433
GAACGCTGGCGGCGTGCTTAACACATGCAAGTCGAACGATGACGGAGGAGCTTGCTCCTCCTGATTAGTG
GCGAACGGGTGAGTAACACGTGAGCAATGTGCCCTTGACTCTGGGATAAGCGCTGGAAACGGCGTCTAAT
ACCGGATACGACCACCGGAGGCATCTCCTGGTGGTGGAAAGAACTTCGGTCAAGGATCAGCTCACGGCCT
ATCAGCTAGTTGGTGAGGTAATGGCTCACCAAGGCGACGACGGGTAGCCGGCCTGAGAGGGTGACCGGC
CACACTGGGACTGAGACACGGCC
>Zotu3437
GAACGCTGGCGGCGTGCCTAATACATGCAAGTCGAGCGGTAAGGCTCTGCACTTAGTATTTGATCGTAAAG
TCCTCGGACTTCTAAGCAGATTGATTCGACCCTAGGCTAGGGAACATGATACGAGTAGAGATCGAATAATG
AGTGTAGAGTACACGAGCGGCGGACGGCTGAGTAACACGTAGGAACATACCCCAAAGTGAGGGATAACT
GCTCGAAAGAGTAGCTAATACCGCATGTGCTCTAAGGAGTAAAGCTTCGGCGCTTTGGGAATGGCCTGCG
CATGATTAGCTTGTTGGTGAGGTAAAAGCTCACCAAGGCGACGATCATTAGCTGGTCTGAGAGGATGATCA
GCCAGACTGGGACTGAGACACGGCC
>Zotu3440
AAACGCTGGCGGCGTGCTTAACACATGCAAGTCGAACGGTAAAGCCCTTCGGGGTACACGAGTGGCGAAC
GGGTGAGTAACGCGTGAGTAACCTGCCCTTCACTCTGGGATAACAGTTGGAAACGGCTGCTAATACCGGAT
ATTCAGCTCCTGTCGCATGGTGGGGGTTGGAAAGTTTTGGTGGTGGAGGATGGGCTCGCGTCCTATCAGCT
TGTTGGTGGGGTGATGGCCTACCAAGGCGTCGACGGGTAGCCGGCCTGAGAGGGCGACCGGCCACATTG
GGACTGAGATACGGCC
>Zotu3442
GAACGCTGGCGGCGTGCTTAACACATGCAAGTCGAGCGGAAAGGCCCACTTGTGGGTACTCGAGCGGCGA
ACGGGTGAGTAACACGTGAGTAATCTGCCCTCCACTCTGGGATAACGTTCAGAAATGGGTGCTAATACCGG
ATGTACACGTGCTGACGCATGTTGGTGTGTGGAAAGGTTTTCTGGTGGGGGATGTGCTCGCGGCCTATCA
GCTTGTTGGTGGGGTAATGGCCTACCAAGGCTTTGACGGGTAGCCGGCCTGAGAGGGTGACCGGCCACAC
TGGGACTGAGACACGGCC

>Zotu3446
GAACGCTGGCGGCGTGCCTAACACATGCAAGTCGAACGGGGTAGAGAGCTTCGGTTTTCTACTTAGTGGC
GAACGGGTGAGTAACGCGTGAGAAACCTGCCTTTCAGTGGGGGACAACAGTTGGAAACGACTGCTAATAC
CGCATAATGTTATTTGGGGGCATCCCCGAATAACCAAAGCTTTATGTGCTGAAAGATGGTCTCGCGTCTGAT
TAGCTAGATGGCGGGGTAACGGCCCACCATGGCGACGATCAGTAGCCGGTCTGAGAGGATGAACGGCCA
CATTGGGACTGAGATACGGCC
>Zotu3448
GAACGCTGGCGGCGTGCTTAACACATGCAAGTCGAGCGGAAAGGCCCTTCGGGGTACTCGAGCGGCGAA
CGGGTGAGTAACACGTGAGTAATCTGCCCTTGGCTTCGGGATAGCCACCGGAAACGGTGATTAATACCGG
ATACGACAACTTCTCGCATGGGATGGTTGTGGAAAGTTTTTCGGCCAGGGATGTGCTCGCGGCCTATCAGC
TTGATGGTGAGGTAATGGCTCACCATGGCTTCGACGGGTAGCCGGCCTGAGAGGGTGACCGGTCACACTG
GGACTGAGACACGGCC
>Zotu3450
GAACGCTGGCGGCAGGCTTAACACATGCAAGTCGAACGCCCCGCAAGGGGAGTGGCAGACGGGTGAGTA
ACACGTGGGAACATACCCTTCGGTTCGGAACAACGTTTGGAAACGAACGCTAATACCGGATACGGCTTACG
AGCGAAAGATTGATCGCCGAAGGATTGGCCCGCGTTCGATTAGCTTGATGGTGAGGTAACGGCTCACCAT
GGCGACGATCGATAGCTGGTCTGAGAGGATGGCCAGCCACATTGGGACTGAGACACGGCC
>Zotu3453
GAACGCTAGCGGGAGGCTTAACACATGCAAGTCGAGCGGGATTCCCTTCGGGGATGAGAGCGGCGCACG
GGTGCGTAACGCGTATGCAACCTGCCTTATACAGGGGGATAGCCCATGGAAACGTGGATTAATATCCCATA
GGATTACAAATTCGCATGGAAAAGTAATTAAAACTACGGTGGTATAAGATGGGCATGCGTCTGATTAGGTA
GTTGGTAGGGTAACGGCCTACCAAGCCGACGATCAGTAGGGGTTCTGAGAGGATTACCCCCCACACTGGT
ACTGAGACACGGAC
>Zotu3459
GAACGCTGGCGGCGTGCCTAATACATGCAAGTCGAGCGAATCAATAGGAGCTTGCTCCTGTTGATTAGCG
GCGGACGGGTGAGTAACACGTGGGCAACCTGCCTGTAAGACTGGGATAACTTCGGGAAACCGGAGCTAAT
ACCGGATAATCCTTTTCTACTCATGTAGAAAAGCTGAAAGACGGTTTCGGCTGTCACTTACAGATGGGCCC
GCGGCGCATTAGCTAGTTGGTGAGGTAACGGCTCACCAAGGCGACGATGCGTAGCCGACCTGAGAGGGT
GATCGGCCACACTGGGACTGAGACACGGCC
>Zotu3461
GAACGCTGGCGGCAGGCCTAACACATGCAAGTCGAGCGGATGAAAGGAGCTTGCTCCTGGATTCAGCGGC
GGACGGGTGAGTAATGCCTAGGAATCTGCCTGGTAGTGGGGGACAACGTTTCGAAAGGAACGCTAATACC
GCATACGTCCTACGGGAGAAAGCAGGGGACCTTCGGGCCTTGCGCTATCAGATGAGCCTAGGTCGGATTA
GCTAGTTGGTGGGGTAAAGGCCTACCAAGGCGACGATCCGTAACTGGTCTGAGAGGATGATCAGTCACAC
TGGAACTGAGACACGGTC
>Zotu3466
GAACACTGGCGACGTGCTTAACACATGCAAGTCGAACGGAAAGGCCCTGCTTGCAGGGTACTCGAGTGGC
GAACGGGTGAGTAACACGTGGGTGATCTGCCCTGCACTTCGGGATAAGCCTGGGAAACTGGGTCTAATAC
CGGATAGTACCATCGTTTAGTGTCGGTGGTGGAAAGTTTTTCGGTGCAGGATGAGCCCGCGGCCTATCAGC
TTGTTGGTGGGGTAATGGCCTACCAAGGCGTCGACGGGTAGCCGGCCTGAGAGGGTGGACGGCCACATT
GGGACTGAGACACGGCC
>Zotu3468
GAACGCTGGCGGCGCGCTTAACACATGCAAGTCGTACGGGGGTCTTCGGACCTCAGTGGCGAACGGGTGC
GTAACACGTGAGCAACCTGCCTGGTCGTGGGGGATAACCAGTGGAAACGCTGGCTAATACCGCATGAGCC
CTTTCTGCCGATTGGTGGGATGAGGAAAGCTCCTGCAAGGGAGTGCGACCAGAGGGGCTCGCGGCGTATC
AGCTAGTTGGAGGGGTAACGGCCCACCAAGGCGATGACGCGTAGCCGATCTGAGAGGATGATCGGCCAC
ATTGGGACTGAGATACGGCC
>Zotu3470
GAACGTTGGTAACGTGGATACCGGCATGCAAGTCAAGGGGCCCGCAAGGGCACCGGCGAACGGCTTAGT
AACACGTGGATACATACCGTAAAGTGGGGCATAGCTCGTCGAAAGACGGGGTAATTTCCCATGTGAACGC

AAGTTTAAACACGCAAGTGGCTTTACGAATGGTCTGCGGTCTATCAGCTAGTTGGTGGGGTAATGGCCTAC
CAAGGCTATGACGGATAGGGGGCGTGAGAGCGCGACCCCCAAGAATGGAACTGCGACACGGTC
>Zotu3473
AAACGCTGGCGGCGTGCATGAGACATGCAAGTCGAACGAACGAGCTTGCTCGTTAGTGGCGGACGGCTGA
GTAACGCGTAGGTGACCTGCCCTGAAGTGGGGGATAACTGCCCGAAAGGGTGGCTAATACCGCATGTGGC
GACACGTTCATTCGTGTCATCAAAGCCGCGAGGCGCTTCGGGAGGGGCCTGCGTCCGATTAGCTCGTTGGT
GGGGTAATGGCTCACCAAGGCGACGATCGGTAGGTGGTCTGAGAGGACGATCACCCAGACTGGGACTGA
GACACGGCC
>Zotu3476
GAACGCTGGCGGCGTGCCTAATACATGCAAGTCGAGCGGACCGGATGGAAGCTTGCTTTCGTTCGGTTAG
CGGCGGACGGGTGAGTAACACGTGGGCAACCTGCCCGTAAGACCGGGATAACTTCGGGAAACCGGAGCT
AATACCGGATAACACTGAAGACCGCATGGTCTTTGGTTGAAAGGCGGCTTTAGCTGTCACTTACGGATGGG
CCCGCGGCGCATTAGCTAGTTGGTGAGGTAACGGCTCACCAAGGCGACGATGCGTAGCCGACCTGAGAGG
GTGATCGGCCACACTGGGACTGAGACACGGCC
>Zotu3480
GAACGCTGGCGGCGTGCTTAACACATGCAAGTCGAACGATGAAGCCCTGCTTGCAGGGTGGATTAGTGGC
GAACGGGTGAGTAACACGTGGGTGATCTGCCCTGCACTTCGGGATAAGCCTGGGAAACTGGGTCTAATAC
CGGATATGACCCTCCTTTAGTGTGGGGGGTGGAAAGCTTTTGCGGTGTGGGATGAGCCTGCGGCCTATCA
GCTTGTTGGTGGGGTAATGGCCTACCAAGGCGTCGACGGGTAGCCGGCCTGAGAGGGTGATCGGCCACAT
TGGGACTGAGACACGGCC
>Zotu3484
GAACGCTAGCGGGATGCCTTACACATGCAAGTCGAACGGCAGCACGGACTTCGGTCTGGTGGCGAGTGGC
GAACGGGTGAGTAATGTATCGGAACGTGCCCAGTAGCGGGGGATAACTACGCGAAAGCGTAGCTAATACC
GCATACGCCCTTAGGGGGAAAGCAGGGGATCGCAAGACCTTGCACTATTGGAGCGGCCGATATCGGATTA
GCTAGTTGGTGAGGTAATGGCTCACCAAGGCCGCGATCCGTAGCTGGTTTGAGAGGACGACCAGCCACAC
TGGGACTGAGACACGGCC
>Zotu3486
GAACGCTGGCGGCGTGCTTAACACATGCAAGTCGAACGATGAACCTCCTTCGGGAGGGGATTAGTGGCGA
ACGGGTGAGTAACACGTGGGCAATCTGCCCTGCACTCTGGGATAACTCCGGGAAACCGGAGCTAATACCG
GATACGACCACTGCGGGCATCCGTGGTGGTGGAAAGCTCCGGCGGTGCAGGATGAGCCCGCGGCCTATCA
GCTTGTTGGTGGGGTGATGGCCTACCAAGGCGACGACGGGTAGCCGGCCTGAGAGGGCGACCGGCCACA
CTGGGACTGAGGCACGGCC
>Zotu3491
TCGCATTTCGATGGAGGCGAAATGGTAGAGGCCCGTGCACTGTGCGATGTCAGTGCATATTAAAGAACAC
CAGATGGTCGAAACTTCCGGAGCCTCCCACTACGGCGTGCTCCATAACCATATCCTGATATTATTAGTATAT
TCTCAAACGTACAGTCAGTGTCAAAGCTTCAAAGACCTCCGGACACAGGAAAAAAACTGAAAGTT
>Zotu3494
GAACGCTGGCGGCGTGCCTAATACATGCAAGTCGAGCGAGAAAGTCCTTCGGGATGAGTACAGCGGCGCA
CGGGTGAGTAACACGTAGGTACCTGCCCTTTAGTGGGGAATAACACCGGGAAACCGGTGCTAATACCGCA
TATGTAGGAGACTACAAAGGGGGCGTAGCAATACGCTCTCGCTAGAGGAGGGACCTGCGCGACATTAGGT
AGTTGGTGGGGTAATGGCCCACCAAGCCGACGATGTCTATCCGGTCTGAGAGGATGATCGGACACACTGG
AACTGAGACACGGTC
>Zotu3496
GAACGCTGGCGGCATGCCTAACACATGCAAGTCGAACGGTAGCATGAACTAGCTTGCTAGTTTGATGACG
AGTGGCGAACGGGTGAGTAACGCGTAGGAATACATCCTAGAGAGGGGGACAACCTGGGGAAACTCAGGC
TAATACCGCATGACATCTGAGGATTAAAGGCTGGGGACCTTCGGGCCTGGCGCTTTAGGGGTAGCCTGCG
TACGATTAGCTAGTTGGTGGGGTAAGGGCCTACCAAGGCGACGATCGTTAGCTGGTCTGAGAGGACGAAC
AGCCACACTGGGACTGAGACACGGCC
>Zotu3499

GAACGCTGGCGGCGTGCCTAACACATGCAAGTCGAACGTGAAATCGAAAGAAAGTAAAGTGGCGTACGG
GTGAGTAACACGTAGGTAATCTACCTAATAGTTTGGAATAACCTGCCGAAAGGCGGGCTAATACTGGATAG
AGCGAAGGACTCGTGAGGAGTTTTTCGGGAAAGGGAGGTCGTAAAGATCCCTCGCTATTGGATGAGCCTG
CGTCTCATTAGCTAGTTGGTAGGGTAAAGGCCTACCAAGGCTTTGATGGGTAGCTGGTCTGAGAGGATGAT
CAGCCACACTGGAACTGAAACACGGTC
>Zotu3502
GAACGCTGGCGGTATGCCTTATACATGCAAGTCGTACGAGAGTTTTTAACTCTAGTGGCGGACGGGTGAGT
AACACGTAAGAATTTGCCTTTAGGAGGGGGACAACAACTGGAAACGGTTGCTAATACCCCATATGCTTTCG
AGTGAAATGGATTTCCGCCTAAAGAGAAGCTTGCGGCTGATTAGCTTGTTGGTAGGGTAATGGCCTACCAA
GGCGACGATCAGTATCTGGTTTGAGAGGACGATCAGACACACTGGAACTGAGACACGGTC
>Zotu3504
GAATGCTGGCGATGTGGATAAGTCATGCAAGTCGAACGGTCCGCAAGGACAGTGGCAGACGAGGTAGTA
ACACGTAGGTACACCCCCCGAAGTCGGCATAGCCAGCCGAAAGGTTGGGTAATTCCCGATAGTCGCGCA
AGCGTAAAGATTTATCGCTTCGGGAGTGGCCTGCGTAATATCAGCTAGTTGGTAAGGTAACGGCTTACCAA
GGCTACGACGTTTAGGGGAGCTGAGAGGCTGACCCCCACCGATGGGACTGCGATACGGCC
>Zotu3506
GAACGCTGGCGGCGTGCCTAATACATGCAAGTCGAACGCATTTTCTTTCACCGTAGCTTGCTACACCGAAA
GAAAATGAGTGGCGAACGGGTGAGTAACACGTGGGTAACCTGCCCATCAGCGGGGGATAACACTTGGAA
ACAGGTGCTAATACCGCATAATTCCATTTACCGCATGGTAGATGGGTGAAAGGCGCTTTTGCGTCACTGAT
GGATGGACCCGCGGTGCATTAGCTAGTTGGTGGGGTAACGGCCTACCAAGGCTGCGATGCATAGCCGACC
TGAGAGGGTGATCGGCCACACTGGGACTGAGACACGGCC
>Zotu3509
GAACGCTGGCGGCGTGCTTAACACATGCAAGTCGAACGGTGAAGCCAAGCTTGCTTGGTGGATCAGTGGC
GAACGGGTGAGTAACACGTGAGCAACCTGCCCTGGACTCTGGGATAAGCGCTGGAAACGGCGTCTAATAC
TGGATATGAGCTCTCATCGCATGGTGGGGGTTGGAAAGATTTTTCGGTCTGGGATGGGCTCGCGGCCTATC
AGCTTGTTGGTGAGGTAATGGCTCACCAAGGCGTCGACGGGTAGCCGGCCTGAGAGGGTGACCGGCCAC
ACTGGGACTGAGACACGGCC
>Zotu3513
GAACGCTGGCGGCGTGCTTAACACATGCAAGTCGAACGGTGAAGGGGCCTGCTTTTGTGGGTCCTGGATG
AGTGGCGAACGGGTGAGTAACACGTGAGTAACCTGCCCCCTTCTTCTGGATAACCGCATGAAAGTGTGGCT
AATACGGGATATTCTGTGCCTGCTGCATGGTGGGTGTGGGAAAGATTGCGCTTTTGGGTGTTTTTGGTGGG
GGATGGGCTCGCGGCCTATCAGCTTGTTGGTGGGGTGATGGCCTACCAAGGCGGTGACGGGTAGCCGGC
CTGAGAGGGTGGACGGTCACACTGGGACTGAGACACGGCC
>Zotu3516
GAACGCTGGCGGCGTGCTTAACACATGCAAGTCGAACGGAAAGGCTCCTGCTTTTGTGGGGGTGCTCGAG
TGGCGAACGGGTGAGTAACACGTGGGTGATCTGCCCTCAACTTCGGGATAAGCTTGGGAAACTGGGTCTA
ATACCGGATAGGACCATGCAGTGGTGTGTGTGGTGGAAAGATGTTTTTTGGTTGGGGATGAGCTCGCGGC
CTATCAGCTTGTTGGTGGGGTAATGGCCTACCAAGGCGTCGACGGGTAGCCGGCCTGAGAGGGTGGACG
GCCACATTGGGACTGAGATACGGCC
>Zotu3518
GAACGCTGGCGGCATGCCTTACACATGCAAGTCGAACGGCAGCACGGGAGCAATCCTGGTGGCGAGTGG
CGAACGGGTGAGTAAAGCATCGGAACGTGCCCAGTCGTGGGGGATAACGTAGCGAAAGCTACGCTAATA
CCGCATACGATCTGTGGATGAAAGCGGGGGACCGTAAGGCCTCGCGCGATTGGAGCGGCCGATGTCAGAT
TAGGTAGTTGGTGGGGTAAAGGCTCACCAAGCCGACGATCTGTAGCTGGTCTGAGAGGACGACCAGCCAC
ACTGGGACTGAGACACGGCC
>Zotu3520
GAACGCTGGCGGCGTGCTTAACACATGCAAGTCGAGCGAACCCTTCGGGGTGAGCGGCGGACGGGTGAG
TAACGCGTGGGTAACCTGCCCTGTACACACGGATAACATACCGAAAGGTATGCTAATACGAGATAATATGT
AATGATCGCATGATCGATACATCAAAGCTCCGGCGGTACAGGATGGACCCGCGTCTGATTAGCTAGTTGGT
GAGGTAATGGCTCACCAAGGCGACGATCAGTAGCCGACCTGAGAGGGTGATCGGCCACATTGGAACTGA
GACACGGTC

>Zotu3522
GAACGCTGGCGGCATGCCTAACACATGCAAGTCGAACGAGACCTTCGGGTCTAGTGGCGCACGGGTGCGT
AACGCGTGGGAACCTGCCCTTAGGTTCGGAATAACTTCCCGAAAGGGATGCTAATACCGGATAATGTCTTC
GGACCAAAGATTTATCGCCTTTGGATGGGCCCGCGTAGGATTAGCTTGTTGGTGGGGTAATGGCCTACCAA
GGCGACGATCCTTAGCTGGTCTGAGAGGATGATCAGCCACACTGGGACTGAGACACGGCC
>Zotu3525
GAACGCTGGCGGCGTGCTTAACACATGCAAGTCGAGCGAGAAAGCCCCTTCGGGGGTGAGTACAGCGGC
GAACGGGTGAGTAACACGTGGGTAACCTGCCTTGAGGAGCGGGACAACCCGAGGAAACTCGGGCTAATA
CCGCATGACTCGTCGCGTCCCAAGGCGCGACGGCAAATGGTAGCTTCGGCCTCCGCCTCAAGATGGGCCC
GCGCCGCATTAGCTTGTTGGTGGGGTAATGGCTCACCAAGGCGACGATGCGTAGCTGGTCTGAGAGGACG
ATCAGCCACACTGGGACTGAGACACGGCC
>Zotu3528
GAACGCTGGCGGCGTGCTTAACACATGCAAGTCGAACGGTGAAGCAGAGCTTGCTCTGTGGATCAGTGGC
GAACGGGTGAGTAACACGTGAGCAATCTGCCCCTGACTCTGGGATAAGCGCTGGAAACGGCGTCTAATAC
CGGATACGAGACGTGAAGGCATCTTCAACGTCTGGAAAGAACTTCGGTCAGGGATGAGCTCGCGGCCTAT
CAGCTAGTTGGTGAGGTAATGGCTCACCAAGGCGTCGACGGGTAGCCGGCCTGAGAGGGTGACCGGCCA
CACTGGGACTGAGACACGGCC
>Zotu3530
GAACGCTAGCGGCAGGCTTAATACATGCAAGTCGAGGGGCAGCACGAATAGCAATATTTGGTGGCGACCG
GCAAACGGGTGCGGAACACGTACGCAACCTTCCCATAACTGGTGAATAGCCTTCCGAAAGGGAGATTAAT
ACACCGTAACATAGTTGAGTGGCATCACTTGACTATTATAGCTTCGGCGGTTTTGGATGGGCGTGCGCCTG
ATTAGGTAGTTGGCGGGGTAACGGCCCACCAAGCCTTCGATCAGTAACTGGTGTGAGAGCACGACCAGTC
ACACGGGCACTGAGACACGGGC
>Zotu3533
GAACGCTGGCGGCGTGCTTAACACATGCAAGTCGAACGATGAAGCCCTTCGGGGTGGATTAGTGGCGAAC
GGGTGAGTAACACGTGGGCAATCTGCCCTTCACTCTGGGACAAGCCCTGGAAACGGGGTCTAATACCGGA
TAACACTCTGTCCCGCATGGGGCGGGGTTGAAAGCTCCGGCGGTGAAGGATGAGCCCGCGGCCTATCAGC
TTGTTGGTGGGGTAATGGCCTACCAAGGCGACGACGGGTAGCCGGCCTGAGAGGGCGACCGGCCACACT
GGGACTGAGACACGGCC
>Zotu3535
GAACGCTGGCGGCATGCCTTACACATGCAAGTCGAACGGCAGCATAGGAGCTTGCTCCTGATGGCGAGTG
GCGAACGGGTGAGTAATATATCGGAACGTACCCAAGAGTGGGGGATAACGTAGCGAAAGTTACGCTAATA
CCGCATACGATCTAAGGATGAAAGTGGGGGATCGCAAGACCTCATGCTCCTGGAGCGGCCGATATCTGAT
TAGCTAGTTGGTGGGGTAAAAGCTCACCAAGGCGACGATCAGTAGCTGGTCTGAGAGGACGACCAGCCAC
ACTGGGACTGAGACACGGCC
>Zotu3539
GAACGCTGGCGGCGTGCCTAATACATGCAAGTCGAGCGGACTGATGGGAGCTTGCTCCCTGATGTTAGCG
GCGGACGGGTGAGTAACACGTGGGCAACCTGCCCTATAGCTTGGGATAACTCCGGGAAACCGGGGCTAAT
ACCGGATAATACAATCTAGCTCCTGCTAGATTGTTGAAAGATGGTTTCGGCTATCGCTATAGGATGGGCCC
GCGGCGCATTAGCTAGTTGGTAGGGTAATGGCCTACCAAGGCGACGATGCGTAGCCGACCTGAGAGGGT
GATCGGCCACACTGGGACTGAGACACGGCC
>Zotu3541
GAACGCTAGCGGCAGGCCTAATACATGCAAGTCGAACGGTAACAGGGTAGCAATACCGCTGACGAGTGGC
GCACGGGTGCGTAACGCGTATGCAATCTACCTTCAACTAGTGAATAGCTCGAAGAAATTCGGATTAATACA
CCATAGAATTATTGATTGGCATCAATTAATAATTAAAGCTCAGGCGGTTGAAGATGAGCATGCGTGACATT
AGCTAGTTGGTGAGGTAACGGCTCACCAAGGCTACGATGTCTAGGGGATCTGAGAGGATTAACCCCCACA
CTGGTACTGAGACACGGAC
>Zotu3543
GAACGCTGGCGGCGTGCTTAACACATGCAAGTCGAACGTGAGGGTGTAGCAATATGCCTGAAAGTGGCGA
ACGGGTGAGTAACACGTAGGTAACCTGCCTTGAAGCGGGGGATAACGGCTGGAAACGGCTGCTAATACCG

CATATTGTGAGTGGAGTGGGAACTTCACTTGTGAAAATTTTGGTGCTTCAAGAGGGGCCTGCGTCCGATTA
GCTAGTTGGTGGGGTAAGAGCCTACCAAGGCGATGATCGGTAGCTGGTCTGAGAGGACGATCAGCCACAC
GGGAACTGAGAAACGGTC

>Zotu3545

GAACGCTGGCGGCATGCCTTACACATGCAAGTCGAACGGCAGCGCGGGAGCAATCCTGGCGGCGAGTGG
CGAACGGGTGAGTAATATATCGGAACGTGCCCATTAGTGGGGGATAGCCCGGCGAAAGCCGGATTAATAC
CGCATACGACCTGAGGGTGAAAGCGGGGGATCGCAAGACCTCGCGCTATTGGAGCGGCCGATATCAGATT
AGCTAGTTGGTGGGGTAAAAGCCTACCAAGGCAACGATCTGTAGTTGGTCTGAGAGGACGACCAGCCACA
CTGGGACTGAGACACGGCC

>Zotu3547

AAACGCTAGCGGCAGGCCTAATACATGCAAGTCGAACGGGGTCCTTCGGGGCCCAGTGGCGCACGGGTGC
GTAACGCGTATGCAACCTACCTTGAACTGGGGGATAGCCCGGAGAAATCCGGATTAATACCGCATAAGACC
ACGTGAGAACATCCGAGTGTGGTTAAAGATTCATCGGTTCAAGATGGGCATGCGTGCCATTAGCTAGTTAG
TGGTGTAACGGACCACCAAGGCGACGATGGCTAGGGGTTCTGAGAGGACGGTCCCCCACACTGGCACTGA
GATACGGCC

>Zotu3550

GAACGCTAGCTACAGGCTTAACACATGCAAGTCGAGGGGTAACATGACGGTGGCTTGCCATCGTTGATGA
CGACCGGCGCACGGGTGAGTAACGCGTATCCAACCTGGCCCACACTGGGGAATAGTCCCTCGAAAGAGAG
CTTAATGCCCCATGTTGCAGCATTGTCACATGTTGATGCTGCCAAAGGCTTGCCGGTATGGGATGGGGATG
CGTCCGATTAGCTAGTTGGCGGGGTAACGGCCCACCAAGGCGACGATCGGTAGGGGTTCTGAGAGGAAG
GTCCCCCACATTGGTACTGAGACACGGAC

>Zotu3552

GAACGCTGGCGGCATGCCTAACACATGCAAGTCGAACGAAGGCTTCGGCCTTAGTGGCGCACGGGTGCGT
AACGCGTGGGAATCTGCCCTTGGGTTCGGAATAACTCCCCGAAAGGGGTGCTAATACCGGATGATGTCGC
AAGACCAAAGATTTATCGCCCAAGGATGAGCCCGCGTTGGATTAGCTAGTTGGTGAGGTAAAAGCTCACC
AAGGCGACGATCCATAGCTGGTCTGAGAGGATGATCAGCCACACTGGGACTGAGACACGGCC

>Zotu3556

GAACGCTGGCGGCATGCTTAACACATGCAAGTCGCACGCCCAGCAATGGGAGTGGCGGACGGGTGAGTA
ACGCGTAGGAACGTATCCAGAGGTGGGGGACAACCCCGGGAAACTGGGGCTAATACCGCATATGGGCTG
AGGCCCAAAGCAGTGATGCGCCTTTGGAGCGGCCTGCGTCCGATTAGGTTGTTGGTGGGGTAATGGCCTA
CCAAGCCTGCGATCGGTAGCTGGTCTGAGAGGATGATCAGCCACACTGGGACTGAGACACGGCC

>Zotu3558

GAACGCTGGCGGCGTGCTTAACACATGCAAGTCGAGCGGTAAGGCTCCTTCGGGAGTACACGAGCGGCGA
ACGGGTGAGTAACACGTGAGCAATCTGCCCTTCACACTGGGATAACCTTCGGAAACGAGGACTAATACCG
GATACGACCACTTCAGACATCTGATGGTGGTGGAAAGCTCCGGCGGTGGAGGATGAGCTCGCGGCCTATC
AGCTTGTTGGTGAGGTAACGGCTCACCAAGGCTTCGACGGGTAGCCGGCCTGAGAGGGTGACCGGCCACA
CTGGGACTGAGACACGGCC

>Zotu3560

GAACGCTGGCGGCAGGCTTAACACATGCAAGTCGAACGCCCCGCAAGGGGAGTGGCAGACGGGTGAGTA
ACGCGTGGGAATCTACCCAGTTCTACGGAACAACTGAGGGAAACTTCAGCTAATACCGTATACGCCCTACG
GGGGAAAGATTTATCGGAATTGGATGAGCTCGCGTTGGATTAGCTAGTTGGTGGGGTAATGGCCTACCAA
GGCGACGATCCATAGCTGGTCTGAGAGGATGATCAGCCACACTGGGACTGAGACACGGCC

>Zotu3562

GAACGCTAGCTACAGGCTTAACACATGCAAGTCGAGGGGAAACGGCATTTGGTGCTTGCACCATTTGGAT
GTCGACCGGCGCACGGGTGAGTAACGCGTATCCAACCTGCCCATAAGTAGGGTATAACCCGCAGAAATGC
GGACTAATCCCCTATGTTGTTCAAAGACGGCATCAGATTTGAACCAAAGGCTTGCCGCTTATGGATGGGGA
TGCGTCCGATTAGCTTGTTGGTGAGGTAACGGCTCACCAAGGCTTCGATCGGTAGGGGTTCTGAGAGGAA
GGCCCCCCACATAGGAACTGAGACACGGTC

>Zotu3565

GAACGCTGGCGGCATGCCTAACACATGCAAGTCGAACGAGAGGACCCCGCAAGGGGTTAGTAAAGTGGC
GCACGGGTGAGTAACACGTGGGTAATCTGCCCTTTAGTTTGGGATAACCCTTCGAAAGAAGGGCTAATACC
GGAGAGACCACAGGGACATCAGTCCTAGGGGTTGAAGATGGGGACCGCAAGGCCTATCGCTAAAGGATG
AGCCCGCATACTATCAGCTAGTTGGTGAGGTAATGGCTCACCAAGGCTATGACGGTTAGCTGGTCTGAGAG
GATGATCAGCCACACTGGAACTGAGACACGGTC

>Zotu3568

GAACGCTGGCGGCGTGCTTAACACATGCAAGTCGAACGGAAAGGCCCTTCGGGGTACTCGAGTGGCGAAC
GGGTGAGTAACACGTGGGTGATCTGCCCTGCACTTCGGGATAAGCCTGGGAAACTGGGTCTAATACCGGA
TAGGACTACGGCCTGCATGGGTTGTGGTGGAAAGCTTTTGCGGTGTGGGATGGGCCCGCGGCCTATCAGC
TTGTTGGTGGGGTGATGGCCTACCAAGGCGACGACGGGTAGCCGGCCTGAGAGGGTGACCGGCCACACT
GGGACTGAGATACGGCC

>Zotu3570

GAACGCTGGCGGCATGCCTAATACATGCAAGTCGAGCGAATGATGAAGAAGCTTGCTTCTTCTGATTTAGC
GGCGGACGGGTGAGTAACACGTGGGTAACCTACCTTACAGATTGGGATAACTTCGGGAAACCGGAGCTAA
TACCGAATAACACATTTCTTCTCAGGAAGAGATGTTGAAAGACGGTTTCGGCTGTCACTGTAAGATGGGCC
CGCGGCGCATTAGCTAGTTGGTGAGGTAACGGCTCACCAAGGCAACGATGCGTAGCCGACCTGAGAGGGT
GATCGGCCACACTGGGACTGAGACACGGCC

>Zotu3572

GAACGCTGACAGAATGCTTAACACATGCAAGTCAACTTGAATTTGGGTTTTTAACTTAGATTTGGGTGGCG
GACGGGTGAGTAACGCGTAAAGAACTTGCCTCACAGCTAGGGACAACATTTAGAAATGAATGCTAATACCT
GATATTATGATTTTAAGGCATCTTAGAATTATGAAAGCTATAAGCACTGTGAGAGAGCTTTGCGTCCCATTA
GCTAGTTGGAGAGGTAACAGCTCACCAAGGCGATGATGGGTAGCCGGCCTGAGAGGGTGAACGGCCACA
AGGGGACTGAGACACGGCC

>Zotu3576

GAACGCTGGCGGCGTGCTTAACACATGCAAGTCGAGCGGAAAGGCCCTTCGGGGTACTCGAGCGGCGAA
CGGGTGAGTAACACGTGAGTAACCTGCCCTGGACTTTGGGGTAACCCTCGGAAACGGGGGCTAATACCGG
ATATGACTTTTCCTCGCATGGGGATTGGTGGAAAGTTTTTCGGTCTGGGATGGGCTCGCGGCCTATCAGCT
TGTTGGTGGGGTAATGGCCTACCAAGGCGACGACGGGTAGCCGGCCTGAGAGGGCGACCGGCCACACTG
GGACTGAGACACGGCC

>Zotu3578

GAATGCTGGCGGTGTGGATAGGGCATGCAAGTCAAGGGGACCGCAAGGTAACCGGCAGACGAGATAGTA
ACGCGTAGGAACGTACCCAGAAGTCGGGCACAGCTCGCCGAAAGGCGGGGTAATTCCCGATGGTGACGA
AAGTCTAAAGATTTATCGCTTCTGGAGCGGCCTGCGTAATATCAGCTAGTTGGTGAGGTAATGGCTCACCA
AGGCTATGACGTTTAGGGGAGCTGAGAGGCTGACCCCCATCGATGGAATTGAGATACGGTC

>Zotu3580

GAACGCTGGCGGCAGGCTTAACACATGCAAGTCGAGCGCACTCATTTAGAGTGAGCGGCAGACGGGTGA
GTAACGCGTGGGAATCTACCCTTTTCTACGGAATAACACAGAGAAATTTGTGCTAATACCGTATACGTCCTA
CTGGAGAAAGATTTATCGGAGAAGGATGAGCCCGCGTTGGATTAGCTAGTTGGTGAGGTAAAGGCTCACC
AAGGCGACGATCCATAGCTGGTCTGAGAGGATGATCAGCCACACTGGGACTGAGACACGGCC

>Zotu3582

GAACGCTAGCGGCAGGCTTAACACATGCAAGTCGAGGGGTATATCATTTCGGTGATAGAGACCGGCGCAC
GGGTGCGTAACGCGTATGCAATCTACCTTTTACAGGGGAATAGCCCAGAGAAATTTGGATTAATGCCCCAT
AGTATTATGAAATGGCATCATTTTATAATTAAAGTTCCAACGGTAAAAGATGAGCATGCGTCCCATTAGTTA
GTTGGTAAGGTAACGGCTTACCAAGACGATGATGGGTAGGGGTCCTGAGAGGGAGATCCCCCACACTGGT
ACTGAGACACGGAC

>Zotu3585

GAATGCTGGCGGTGTGGATAGGGCATGCAAGTCAAGGGGACTCAGTTAGTAATAATTGGGTAACCGGCA
GACGAGATAGTAACGCGTAGGAACGTACCCAGAAGTCGGGCACAGCTCGCCGAAAGGCGGGGTAATTCC
CGATGGTGTCGCAAGACTAAAGATTTATCGCTTCTGGAGCGGCCTGCGTAATATCAGCTTGTTGGTGAGGT
AATGGCTCACCAAGGCTATGACGTTTAGGGGAGCTGAGAGGCTGACCCCCATCGATGGAATTGAGATACG
GTC

>Zotu3587
GAACGCTGGCGGCGTGCCTAATACATGCAAGTCGAGCGAATCTTTGGGAGCTTGCTCCCGAAGGTTAGCG
GCGGACGGGTGAGTAACACGTGGGCAACCTGCCTGTAAGACTGGGATAACACCGGGAAACCGGTGCTAA
TACCGGATAATTCTTTTCCTCTCATGAGGAAAAGCTGAAAGTCGGTTTCGGCTGACACTTACAGATGGGCCC
GCGGCGCATTAGCTAGTTGGTGAGGTAACGGCTCACCAAGGCGACGATGCGTAGCCGACCTGAGAGGGT
GATCGGCCACACTGGGACTGAGACACGGCC
>Zotu3590
GAACGCTGGCGGCATGCTTAACACATGCAAGTCGAACGGGAAGTGGTGTTTCCAGTGGTGAACGGGTGAG
TAACGCGTAAGAACCTGCCCTTGGGAGGGGAACAACAACTGGAAATGGTTGCTAATACCCTATAGGCTGA
GGAGCAAAAGGAGAAATCCGCCCAAGGAGGGGCTCGCGTCTGATTAGCTAGTTGGTGAGGTAATAGCTTA
CCAAGGCGATGATCAGTAGCTGGTCCGAGAGGATGATCAGCCACAATGGGACTGAGACACGGCC
>Zotu3592
TAACGCTAGCAGGATGCCTAACACATGCAAGTCGAACGAGGTAGCAATACCTAGTGGCAAACGGGTGAGT
AATACATGCTCAATCTACCCATAAGAGGGAGATAACAGCTCGAAAGAGTTGCTAATATCCCATAACATGTA
GAAACATAAGTTTTTACATCTAAAGTTGCGTATGCAACACTTTTGGATGAGGGTGTGTCTTATCAGATAGTT
GGTGAGGTAACGGCCCACCAAGTCGATGACGAGTAGCTGTGCTGAGAGGCAGAACAGCCACAATGGGAC
TGAGACACGGTC
>Zotu3594
GAACGCTAGCGGCAGGCTTAATACATGCAAGTCGAGGGGCAGCATGAGTGTAGCAATACATTTGATGGCG
ACCGGCAAACGGGTGCGGAACACGTACAGAACCTTCCTTCGAGCGGGGAATAGCCCAGAGAAATTTGGAT
TAATACCCCATAGTATATTGAAGAGGCATCTCTTTAATATTAAAGATTTATCACTTGAAGATGGCTGTGCGG
CTGATTAGATAGTTGGCGGGGTAATGGCCCACCAAGTCTACGATCAGTAACTGGTGTGAGAGCACGACCA
GTCACACGGGCACTGAGACACGGGC
>Zotu3598
GAACGCTGGCGGCGTGCCTAATACATGCAAGTCGAGCGGTAAGGCTCTCACTCTGTACTTTAGTTTCTAGA
GTATGGTGTGAGAGTACACGAGCGGCGGACGGCTGAGTAACGCGTAGGAACGTACCCCAAAGTGAGGGA
TAGCCCACCGAAAGGTGGATTAATACCGCATGTGCTCTTCGGAGTAAAGCTTTATGCGCTTTGGGAACGGC
CTGCGTATGATTAGTTTGTTGGTGAGGTAATGGCTCACCAAGACTACGATCATTAGCTGGTCTGAGAGGAT
GATCAGCCAGACTGGGACTGAGACACGGCC
>Zotu3600
GAACGCTGGCGGCGTGCTTAACACATGCAAGTCGAACGGAAAGGCCTGTGCTTGCACGGGTACTCGAGTG
GCGAACGGGTGAGTAACACGTGGGTGATCTGCCCTTCACTTCGGGATAAGCCTGGGAAACTGGGTCTAAT
ACTGGATAGGACCATCGTTTAGTGTCGGTGGTGGAAAGTTTTTTCGGTGGAGGATGAGCTCGCGGCCTATC
AGCTTGTTGGTGGGGTAATGGCCTACCAAGGCGACGACGGGTAGCCGGCCTGAGAGGGTGGACGGCCAC
ATTGGGACTGAGACACGGCC
>Zotu3602
GGTGGTGCTCCCCGCAGCTCACCTGGCAGCCCCAGGGCACCTGCTGTGCCTCCCTGGTCTCTTCGTCCCACC
GCCCTTGCCAGCTCCGCATTGGGTGGCACAT
>Zotu3604
GAACGCTGGCGGCGTGCCTAATACATGCAAGTCGAGCGAATCTGAGGGAGCTTGCTCCCAAAGATTAGCG
GCGGACGGGTGAGTAACACGTGGGCAACCTGCCTGTAAGACTGGGATAACTTCGGGAAACCGGAGCTAAT
ACCGGATAACATATGAGACCACATGGTCTTATATTAAAAGATGGCTTTTAGCTATCACTTACAGATGGGCCC
GCGGCGCATTAGCTAGTTGGTGAGGTAACGGCTCACCAAGGCAACGATGCGTAGCCGACCTGAGAGGGT
GATCGGCCACACTGGGACTGAGACACGGCC
>Zotu3607
GAACGCTGGCGGCGTGCCTAATACATGCAAGTCGAGCGGATCTTGTCCTTCGGGATAAGGTTAGCGGCGG
ACGGGTGAGTAACACGTGGGTAACCTGCCCATAAGACTGGGATAACATTCGGAAACGAATGCTAATACCG
GATACGCGAAGCGGTCGCATGATCGAATCGGGAAAGGCGGAGCAATCTGCCACTTATGGATGGACCCGCG
GCGCATTAGCTAGTTGGTGGGGTAACGGCTCACCAAGGCGACGATGCGTAGCCGACCTGAGAGGGTGATC
GGCCACACTGGGACTGAGACACGGCC

>Zotu3609
GAACGCTGGCGGCAGGCTTAACACATGCAAGTCGAACGGTAACATAAAGAAGCTTGCTTCTTTGATGACG
AGTGGCGGACGGGTGAGTAATGCTTGGGAATCTAGCTTATGGAGGGGGATAACTACGGAAACTGTAGC
TAATACCGCGTAATATCGGAAGATTAAAGTGTGGGACCTTCGGGCCACATGCCATAGGATGAGCCCAAGT
GGGATTAGGTAGTTGGTGAGGTAAAGGCTCACCAAGCCGACGATCTCTAGCTGGTCTGAGAGGATGACCA
GCCACACTGGGACTGAGACACGGCC
>Zotu3611
GAACGCTGGCGGCGTGCCTAATACATGCAAGTAGAACGCTGAAGAGAGGGGCTTGCTCTTCTTGGATGAG
TTGCGAACGGGTGAGTAACGCGTAGGTAACCTGCCTGGTAGCGGGGGATAACTATTGGAAACGATAGCTA
ATACCGCATAAAATGGATTATCGCATGATAATCCATTGAAAGGTGCAAATGCATCACTACCAGATGGACCT
GCGTTGTATTAGCTAGTTGGTGGGGTAACGGCTCACCAAGGCGACGATACATAGCCGACCTGAGAGGGTG
ATCGGCCACACTGGGACTGAGACACGGCC
>Zotu3613
GAACGCTGGCGGCGTGCTTAACACATGCAAGTCGAACGGGCCGTTTTCGGACGGTCAGTGGCAGACGGGT
GCGTAACACGTGGGTGACCTGCCCTCAAGTGGGGAACAACGCGACTAACGTCGCGCTAATACCGCATAAG
TTCGCTTCTTCGGTGGGGCGAGTAAAGCAGCAATGCGCTTGGGGATGGGCCTGCGGCGGATTAGGTAGTT
GGTGGGGTAACGGCCTACCAAGCCGATGATCCGTAGGGGGCGTGAGAGCGTGACCCCCCACATTGGCACT
GAGACACGGGC
>Zotu3617
GAACGCTGGCGGCGTGCTTAACACATGCAAGTCGAGCGGTAAGGCCCTTCGGGGTACACGAGCGGCGAA
CGGGTGAGTAACACGTGAGCAATCTGCCCTTCACATCGGGATAACCCCCGGAAACGGGAGCTAATACCGG
ATACGACCACTTCAGGCATCTGATGGTGGTGGAAAGTTCCGGCGGTGGAGGATGAGCTCGCGGCCTATCA
GCTTGTTGGTGAGGTAATGGCTCACCAAGGCTTCGACGGGTAGCCGGCCTGAGAGGGTGACCGGCCACAC
TGGGACTGAGACACGGCC
>Zotu3619
GAACGCTAGCGGCAGGCCTAACACATGCAAGTCGAGGGAGGAGCCTTCGGGCTTCGACCGGCGTACGGG
TGCGTAACGCGTATACAATCTGCCTTTCACATAGGGATAGCCCGAAGAAATTTGGATTAATACCTAATGGTA
TATAGGAGTGGCATCACATTTATATTAAAGCTGAGGCGGTGAAAGATGAGTATGCGTTCTATTAGCTAGTT
GGAGAGGTAACGGCTCCCCAAGGCTACGATAGATAGGGGTTCTGAGAGGGATATCCCCCACACTGGTACT
GAGATACGGAC
>Zotu3623
GAACGCTGGCGGCGTGCTTAACACATGCAAGTCGAACGATGATGCCCAGCTTGCTGGGTGGATTAGTGGC
GAACGGGTGAGTAACACGTGAGTAACCTGCCCCTGACTCTGGGATAAGCGTTGGAAACGACGTCTAATAC
TGGATACGACTACCGGCCGCATGGTCTGGTGGTGGAAAGATTTTTTGGTTGGGGATGGACTCGCGGCCTA
TCAGCTTGTTGGTGAGGTAATGGCTCACCAAGGCGACGACGGGTAGCCGGCCTGAGAGGGTGACCGGCC
ACACTGGGACTGAGACACGGCC
>Zotu3625
GAACGCTAGCTACAGGCTTAACACATGCAAGTCGAGGGGAAACGACATCGAAAGCTTGCTTTTGATGGGC
GTCGACCGGCGCACGGGTGAGTAACGCGTATCCAACCTGCCCACCACTTGGGGATAACCTTGCGAAAGTA
AGACTAATACCCAATGACGTCTCTAGAAGACATCTGAAAGAGATTAAAGATTTATCGGTGATGGATGGGGA
TGCGTCTGATTAGCTTGTTGGCGGGGTAACGGCCCACCAAGGCAACGATCAGTAGGGGTTCTGAGAGGAA
GGTCCCCCACATTGGAACTGAGACACGGTC
>Zotu3627
GAACGCTGGCGGCAGGCTTAACACATGCAAGTCGAGCGCCTCGCAAGAGGAGCGGCAGACGGGTGAGTA
ACGCGTGGGAATCTACCCATCTCTACGGAACAACTCCGGGAAACTGGAGCTAATACCGTATACGTCCTTCG
GGAGAAAGATTTATCGGAGATGGATGAGCCCGCGTTGGATTAGCTAGTTGGTGGGGTAATGGCCTACCAA
GGCGACGATCCATAGCTGGTCTGAGAGGATGATCAGCCACATTGGGACTGAGACACGGCC
>Zotu3629
GAACGCTGGCGGCATGCCTAACACATGCAAGTCGAACGAGACCTTCGGGTCTAGTGGCGCACGGGTGCGT
AACGCGTGGGAATCTGCCCTTAGGTTCGGAATAACAGTTAGAAATGACTGCTAATACCGGATAATGACGTA

AGTCCAAAGATTTATCGCCTAGGGATGAGCCCGCGTAAGATTAGCTTGTTGGTGAGGTAAAGGCTCACCAA
GGCGACGATCTTTAGCTGGTCTGAGAGGATGATCAGCCACACTGGGACTGAGACACGGCC
>Zotu3631
GAACGCTGGCGGCATGCCTAACACATGCAAGTCGAACGAGACCTTCGGGTCTAGTGGCGCACGGGTGCGT
AACGCGTGGGAATCTACCCTTGGGTTCGGAATAACAGCTAGAAATGGCTGCTAATACCGGATGATGTCTTC
GGACCAAAGATTTATCGCCCAGGGATGAGCCCGCGTAGGATTAGCTTGTTGGTGAGGTAAAAGCTCACCA
AGGCGACGATCCTTAGCTGGTCTGAGAGGATGATCAGCCACACTGAGACTGAGACACGGCC
>Zotu3633
GAACGCTGGCGGCGTGCCTAATACATGCAAGTCGAACGCTTCACTTCGGTGAAGAGTGGCGAACGGGTGA
GTAATACATAAGTAACCTGGCATCTACAGGGGGATAACTGATGGAAACGTCAGCTAAGACCGCATAGGTG
TAGAGATCGCATGAACTCTATATGAAAAGTGCTACGGGACTGGTAGATGATGGACTTATGGCGCATTAGCT
AGTTGGTAGGGTAACGGCCTACCAAGGCGACGATGCGTAGCCGACCTGAGAGGGTGACCGGCCACACTG
GGACTGAGACACGGCC
>Zotu3635
GAACGCTGGCGGAATGCTTTACACATGCAAGTCGAGCGGCAGCACGGGGGCAACCCTGGTGGCGAGCGG
CGAACGGGTGAGTAATACATCGGAACATGCCCAGTCGTGGGGGATAACACTTCGAAAGAAGTGCTAATAC
CGCATACGTCCTGAGGGAGAAAGCGGGGGATCGCAAGACCTCGCGCGATTGGAGTGGCCGATGTCAGAT
TAGCTAGTTGGTGGGGTAAAGGCCTACCAAGGCAACGATCTGTAGCTGGTCTGAGAGGATGATCAGCCAC
ACTGGGACTGAGACACGGCC
>Zotu3637
GAACGCTTGCGACATGCCTAAGAAATGCAAGTCGAGCGGGGGTACGGCTTCGGTTGTACCTTTAGCGGCG
AACGGTCGCGTAACACGTAGGCAACCTGCCTGCAAGACCGGGACAACAGTTCGAAAGGACTGCTAATACC
GGATGTGTTCACGGGGAGGCATCTTCCTGTGAATAAATCCCTTACGGGGCTTGTGGAGGGGCCTGCGGCC
TATCAGCTAGATGGTGGGGTAACAGCCTACCATGGCGACGACGGGTAGCTGGTCTGAGAGGATGGTCAGC
CGGATTGGGACTGAGACACGGCC
>Zotu3640
GAACGCTAGCGAGAGGCCTAACACATGCAAGTCGAGGGGTAACAGGGGAGCTTGCTCCCGCTGACGACT
GGCGGATGGGTGAGTAACGCGTATGCAACCTGCCTTTAACTGTGGGATAGCTCCCTGAAAGGGGAATTAA
TACCGCATAAGACCACGTTGTTTCATGACAGAGTGGTAAAATATTTATAGGTTAAAGATGGGCATGCGTGA
CATTAGCTAGTTGGTGAGGTAACGGCTCACCAAAGCTACGATGTCTAGGGGTTCTGAGAGGAAGGTCCCC
CACACTGGTACTGAGACACGGAC
>Zotu3642
GAACGCTGGCGGCGTGGATTAGGCATGCAAGTCGAACGAGAAAGGGTTCGCAAGAACCCGAGTAGAGTG
GCGAAAGGGTGAGTAGTACCTAGGTAACCTGCCCGGAAGACGGGGACAACGTCGTGAAAACGACGCTAA
TACCCGATATGTCGGCGAGATCGCATGGTATTGCCGGCAAACGGTAGCCTCTATTTATAAGCTTCCGCTTTC
GGAGGGGCCTCGGGCCTATCAGCTAGTTGGCGGGGTAACGGCCCACCAAGGCAGGGACGGGTAGCCGGT
CTGAGAGGATGGTCGGCCACACTGGGACTGAGACACTGCC
>Zotu3645
GAACGCTGGCGGCGTGCCTTAAGCATGCAAGTCGTACGGCTCCGCAAGGAGCAGTGGCGGACGGTCGAG
TAACGCGTGGGGATCTACCTTGAAGTTGGACATAGCTCGTCGAAAGGCGGGGTAATTTCCAATGTGAACG
CAAGTTCAAAGCCGCAAGGCGCTTTAAGATGAACCCGCGTCCTATCAGCTAGTTGGTGAGGTAAAGGCTCA
CCAAGGCATCGACGGGTAGCTGGTCTGAGAGGATGATCAGCCGGAATAGCACTGAGACACGGGC
>Zotu3647
GAACGCTAGCGGCAGGCCTAATACATGCAAGTCGGACGGGATCCAGGAGCTTGCTTCTGGTGAGAGTGGC
GCACGGGTGCGTAACACGTATGTAACCTACCCTTGTCAGGGGGATAGCCTCTCGAAAGAGAGATTAAGAC
CGCATAACATTATGATGTGGCATCACAAAATAATCAAATATTTATAGGACAAGGATGGGCATGCGGAACAT
TAGCTAGTTGGTAAGGTAACGGCTTACCAAGGCTACGATGTTTAGGGGATCTGAGAGGATGACCCCCCAC
ACTGGTACTGAGACACGGAC
>Zotu3651

GAACGCTGGTAACGTGGATACCGGCATGCAAGTCACGGGGGCCGCAAGGCAACCGGCGAACGGCTTAGT
AACACGTAGATACATACCGCAAAGTGGAGCATAGCTCGTCGAAAGACGGGGTAATTCTCCATAGTCCCGCA
AGGGTAAAGCAGCAATGCGCTTTGTGAATGGTCTGCGGCCTATCAGATAGTTGGCGAGGTAAAAGCTCAC
CAAGTCTATGACGGGTAGGGGGCGTGAGAGCGCGACCCCCAAGAATGGCACTGCGACACGGGC
>Zotu3653
GAACGCTGGCGGCATGCCTAATACATGCAAGTCGAGCGAATGATGAAGAAGCTTGCTTCTTCTGATTTAGC
GGCGGACGGGTGAGTAACACGTGGGTAACCTACCTTACAGATTGGGATAACTTCGGGAAACCGGAGCTAA
TACCGAATAATACATTTCTTTTCATGAAGAGATATTGAAAGACGGTTTCGGCTGTCACTGTAAGATGGGCCC
GCGGCGCATTAGCTAGTTGGTGAGGTAACGGCTCACCAAGGCAACGATGCGTAGCCGACCTGAGAGGGT
GATCGGCCACACTGGGACTGAGACACGGCC
>Zotu3656
GAACGCTGGCGGCGTGCTTAACACATGCAAGTCGAACGGGGACCATGAAGTGGCAACACTTCGGAAGTCC
GAGTGGCGAACGGGTGAGTAACACGTGAGGAACTTGTCCCGGTCTCTGGGATAACAGTTGGAAACGACTG
CTAATACCGGATGACGTCGGAGCACCGCATGGTGCACTGACGAAAGGGTTACTGGATCGGGAGAGCCTCG
CGACCTATCAGCTAGTTGGTGGGGTAACGGCCTACCAAGGCATCGACGGGTAGCTGGTCTGAGAGGATGA
TCAGCCACACTGGGACTGAGACACGGCC
>Zotu3663
GAACGCTAGCGGCAGGCTTAACACATGCAAGTCGAGGGGCAGCGCGGAGTAGCAATACTTTGGCGGCGA
CCGGCGGAAGGGTGCGTAACGCGTGAGCAACATGCCCGTCTTTGGGAGATAACCGATGGAAACGTCGCCT
AATATCCCATAACAACTGAGGAGGCATCTTTTCAGTTTGAAAGTTTCGATGAAGACGGATTGGTTCGCGTG
ACATTAGCTAGTTGGAGTGGTAACGGCACACCAAGGCGACGATGTCTAGGGGTTCTGAGAGGAAGGTCCC
CCACACTGGAACTGAGACACGGTC
>Zotu3666
GAACGCTAGCGGCAGGCTTAACACATGCAAGTCGAGGGGTATGTTTCTTCGGAAGCAGAGACCGGCGCAC
GGGTGCGTAACGCGTATGCAATCTACCTTTTACAGAGGGATAGCCCAGAGAAATTTGGATTAATACCTCAT
AGTATTATGAAATGGCATCATTTTATAATTAAAGTCACAACGGTAAAAGATGAGCATGCGTCCCATTAGCTA
GTTGGTAAGGTAACGGCTTACCAAGGCTACGATGGGTAGGGGTCCTGAGAGGGAGATCCCCCACACTGGT
ACTGAGACACGGAC
>Zotu3668
GAACGCTGGCGGCATGCCTAATACATGCAAGTCGAGCGGAACACTTGGAGCTTGCTCCAAGCGTTTAGCG
GCGGACGGGTGAGTAACACGTGGGCAACCTGCCCTGCAGATCGGGATAACTCCGGGAAACCGGTGCTAAT
ACCGAATAGTTTTCGGCCCCTCCTGGGGCCGTACGGAAAGACGGCATCTCGCTGTCACTGCAGGATGGGCC
CGCGGCGCATTAGCTAGTTGGTGGGGTAACGGCCCACCAAGGCGACGATGCGTAGCCGACCTGAGAGGG
TGATCGGCCACACTGGGACTGAGACACGGCC
>Zotu3671
GAACGCTGGCGGCGTGCCTAATACATGCAAGTCGAGCGGACAGATGGGAGCTTGCTCCCTGATGTTAGCG
GCGGACGGGTGAGTAACACGTGGGTAACCTGCCTGTAAGACTGGGATAACTCCGGGAAACCGGGGCTAA
TACCGGATGGTTGTTTGAACCGCATGGTTCAAACATAAAAGGTGGCTTCGGCTACCACTTACAGATGGACC
CGCGGCGCATTAGCTAGTTGGTGAGGTAACGGCTCACCAAGGCAACGATGCGTAGCCGACCTGAGAGGGT
GATCGGCCACACTGGGACTGAGACACGGCC
>Zotu3674
GAACGCTGGCGGCGTGCTTAACACATGCAAGTCGAGCGGTAAGGCCCTTCGGGGTACACGAGCGGCGAA
CGGGTGAGTAACACGTGAGCAATCTGCCCTTCACTCTGGGATAACCCCCGGAAACGGGAGCTAATACCGG
ATATGACCACTACACGCATGTGTTGGTGGTGGAAAGTTCTGGCGGTGGAGGATGAGCTCGCGGCCTATCA
GCTTGTTGGTGGGGTAATGGCCCACCAAGGCTTCGACGGGTAGCCGGCCTGAGAGGGTGACCGGCCACAC
TGGGACTGAGACACGGCC
>Zotu3676
GAACGCTGGCGGCAGGCCTAACACATGCAAGTCGAACGCCCTAGCAATAGGGAGTGGCAGACGGGTGAG
TAACGCGTGGGAACGTACCCTTCGGTTCGGAATAAGCCGGGGAAACCTGGTCTAATACCGGATACGTGCG
AGAGCAGAAAGATTTATCGCCGAAGGATCGGCCCGCGTCCGATTAGCTAGTTGGTGAGGTAATGGCTCAC
CAAGGCGACGATCGGTAGCTGGTCTGAGAGGATGATCAGCCACACTGGGACTGAGACACGGCC

>Zotu3679
GCCGTGTCTCAGTCCCAGTGTGGCTGATCGTCCTCTCAGACCAGCTACGGATCGTCGCCTTGGTAGGCCATT
ACCCTACCAACTAGCTAATCCGACGCAGGCTCATCTCATAGCACGAGGTCCGAAGATCCCCCGCTTTGCTTT
GAAGAGATTATGCGGTATTAGCCCGAGTTTCCCCGGGTTATCCCCCACTAACTATTATAATTAAGGTAGATT
CCTACGTGTTACTCACCCGTCCGCCACTCGCCGTCAGGGAGCAAGCTCCCCCCGCTGCCGTTCGACTTGCAT
GTGTTAGGCATGCCGCTAGCGTTCA
>Zotu3681
GAACGCTGGCGGCAGGCCTAACACATGCAAGTCGAGCGGATGACGGGAGCTTGCTCCTTGATTCAGCGGC
GGACGGGTGAGTAATGCCTAGGAATCTGCCTGGTAGTGGGGGACAACGTTCCGAAAGGAGCGCTAATACC
GCATACGTCCTACGGGAGAAAGTGGGGGATCTTCGGACCTCACGCTATCAGATGAGCCTAGGTCGGATTA
GCTAGTTGGTGAGGTAAAGGCTCACCAAGGCGACGATCCGTAACTGGTCTGAGAGGATGATCAGTCACAC
TGGAACTGAGACACGGTC
>Zotu3683
GAACGCTAGCTACAGGCCTAACACATGCAAGTCGTGGGGCAGCGGGTATGCTTGTTTTCAAGTATATGCCG
GCGACCGGCGCACGGGTGAGTAACGCGTATCCAACCTGCCCATCACAAGGGAATAACCCGTCGAAAGTCG
GCCTAATGCCCTATGTATTCCGATGAAGTCCTCTGAATCGGAATAAAGCATTTGCGGTGATGGATGGGGAT
GCGTCTGATTAGCTTGTCGGCGGGGTAACGGCCCACCGAGGCGACGATCAGTAGGGGTTCTGAGAGGAA
GGTCCCCCACATTGGAACTGAGACACGGTC
>Zotu3685
GAACGCTGGCGGCGTGCTTAACACATGCAAGTCGAGCGGAAAGGCCTCCTTCGGGGGGTACTCGAGCGGC
GAACGGGTGAGTAACACGTGAGTAATCTGCCCTTCACTTCGGGATAGCTCTCGGAAACGGGGATTAATACC
GGATATGACTGCGCTACGCATGTGGTGTGGTGGAAAGTTTTTTTCGGTGGGGGATGTGCTCGCGGCCTATC
AGCTTGTTGGTGGGGTAATGGCCTACCAAGGCTTCGACGGGTAGCCGGCCTGAGAGGGTGACCGGCCACA
CTGGGACTGAGACACGGCC
>Zotu3687
GAACGCTGGCGGCGTGCTTAACACATGCAAGTCGAACGGTGAAAGAGAGCTTGCTCTCTGGATCAGTGGC
GAACGGGTGAGTAACACGTGAGCAATCTGCCCCTGACTCTGGGATAACAGTTGGAAACAGCTGCTAATAC
CGGATACGAGACGTGAAGGCATCTTCAACGTCTGGAAAGAACTTCGGTCAGGGATGAGCTCGCGGCCTAT
CAGCTAGTTGGTGAGGTAATGGCTCACCAAGGCGTCGACGGGTAGCCGGCCTGAGAGGGTGACCGGCCA
CACTGGGACTGAGACACGGCC
>Zotu3689
GAACGCTGGCGGCGTGCCTAATACATGCAAGTCGAGCGAATTGATGGGAGCTTGCTCCCTGATATTAGCG
GCGGACGGGTGAGTAACACGTGGGCAACCTGCCCTGCAGATGGGGATAACTCCGGGAAACCGGGGCTAA
TACCGAATAATCGGTTCTTCCGCATGGAAGAACTCTGAAAGACGGTTTCGGCTGTCACTGCAGGATGGGCC
CGCGGCGCATTAGCTAGTTGGTGGGGTAATGGCCTACCAAGGCGACGATGCGTAGCCGACCTGAGAGGGT
GATCGGCCACACTGGGACTGAGACACGGCC
>Zotu3691
GAACGCTGGCGGCAGGCTTAACACATGCAAGTCGAACGAACCCTTCGGGGTTAGTGGCGGACGGGTGAG
TAACACGTGGGAACGTGCCCTTAGGTTCGGAACAACACAGGGAAACTTGTGCTAATACCGGATGTGCCCCT
CACGGGGGAAAGATTTATCGCCTTTGGAGCGGCCCGCGTCTGATTAGCTTGTTGGTGAGGTAAAGGCTCAC
CAAGGCTACGATCAGTAGCTGGTCTGAGAGGATGGCCAGCCACATTGGGACTGAGACACGGCC
>Zotu3695
GAACGCTAGCGGCAGGCCTAATACATGCAAGTCGAACGGACCTTGTTCCTTCGGGGACTTGGTTAGTGGC
GCACGGGTGCGTAACGCGTATGCAACCTACCTTCTATTGGGGGATAGCCCCGGGAAACCGGGATTAATAC
CGCATGAGATCACTTGAGGGCATCCGAAGGTGATTAAAGCTGAGGCGATAGAAGATGGGCATGCGTGCCA
TTAGCTAGTTGGCGGGGTAAGGGCCCACCAAGGCGACGATGGCTAGGGGTTCTGAGAGGATGGTCCCCCA
CACTGGTACTGAGACACGGAC
>Zotu3699
GAATGCTGGCGGTGTGGATAGGGCATGCAAGTCAAGGGGACCCGCAAGGGTAACCGGCAGACGAGATAG
TAACGCGTAGGAACGTACCCAGAAGTCGGGCACAGCTCGCCGAAAGGCGGGGTAATTCCCGATGGTGAC

GAAAGTCTAAAGATTTATCGCTTCTGGAGCGGCCTGCGTAATATCAGCTTGTTGGTGAGGTAATGGCTCAC
CAAGGCTATGACGTTTAGGGGAGCTGAGAGGCTGACCCCCATCGATGGAATTGAGATACGGTC
>Zotu3702
GAACGTTGGCGGCGTGCCTTAAGCATGCAAATCAAGGGGGTCCCGCAAGGGACTACCGGTGGACGGGTG
AGTAATGCGTGGGAATGTACCTCGAAGTTGGATATAGCTAGCCGAAAGGTTGGGTAATCTCCAATGTGAA
CGAAAGTTTAAAGCCGCAAGGCGCTTTGAGATCAGCCCGCGTCCTATCAGCTTGTTGGTGGGGTAATGGCC
TACCAAGGCGACGACGGGTAGCTGGACTGAGAGGTCGGTCAGCCACAATGGGACTGCGACACGGCC
>Zotu3704
GAACGCTGGCGGCAGGCTTAACACATGCAAGTCGAGCGGATGAAAGTAGCTTGCTACTGAATTCAGCGGC
GGACGGGTGAGTAATGCTTAGGAATCTGCCCATTAATGGGGGACAACAGTTGGAAACGACTGCTAATACC
GCATACGCCCTACGGGGGAAAGCAGGGGATCTTCGGACCTTGCGTTAATGGATGAGCCTAAGTCGGATTA
GCTAGTTGGTGGGGTAAAGGCCTACCAAGGCGACGATCTGTAGCGGGTCTGAGAGGATGATCCGCCACAC
TGGGACTGAGACACGGCC
>Zotu3706
GAACGCTAGCGGCAGGCTTAATACATGCAAGTCGAGGGGCAGCAAGGGTAGCAATACCTGTTGGCGACC
GGCAAACGGGTGCGGAACACGTACAGAACCTTCCTTTAAGCGGGGGATAGCCCATAGAAATGTGGATTAA
TACCCCATAGCAATATTTGATGGCATCATTAGATATTTAAAGATTTATCACTTAAAGATGGCTGTGCGGCTG
ATTAGGTAGTTGGTGTGGGTAACGGCCCACCAAGCCAGCGATCAGTAACTGGTGTGAGAGCACGACCAGT
CACACGGGCACTGAGACACGGGC
>Zotu3709
GAACGCTGGCGGCGCGCTTAACACATGCAAGTCGTACGGAGTTGTCCCTTCGGGGGCGGCTCAGTGGCGA
ACGGGTGCGTAACACGTGAGCAACCTGCCTGCCGGTGGGGGATAACCCGGGGAAACCCGGGCTAATACC
GCATTCGCTCTTTCTCTCGATTGGGAGATTGAGGAAAGGTGCTGCAAGGCACTGCCGGCAGAGGGGCTCG
CGGCGTATCAGCTAGCTGGTGGGGTAACGGCCTACCAGGGCAACGACGCGTAGCCGATCTGAGAGGATG
ATCGGCCACATTGGGACTGAGATACGGCC
>Zotu3711
GAACGCTGGCGGCATGCTTAACACATGCAAGTCGAACGGAATTAAGTTTTACATTTTGAATCCTCAAAATG
CGAGCTTAATTTAGTGGCGGACGGGTGAGTAACGCGTAAGAACCTACCTTTAGGAGGGGGACAACAGCTG
GAAACGGCTGCTAATACCCCATATGCTGAGGAGTAAAATGGTGTAACAACCGCCTAAAGATGGGCTTGCG
TCTGATTAGCTTGTTGGTAAGGTAAAGGCTTACCAAGGCAACGATCAGTAGCTGGTCTGAGAGGATGATCA
GCCACACTGGGACTGAGACACGGCC
>Zotu3715
GAACGCTGGCGGCATGCTTTACACATGCAAGTCGAACGGCAGCGCGGGGCAACCTGGCGGCGAGTGGCG
AACGGGTGAGTAATACATCGGAACGTACCCAGAAGTGGGGGATAACGTAGCGAAAGTTACGCTAATACCG
CATACGTTCTACGGAAGAAAGCGGGGGATCGCAAGACCTCGTGCTTTTGGAGCGGCCGATGTCTGATTAG
CTAGTTGGTGAGGTAAAGGCTCACCAAGGCGACGATCAGTAGCTGGTCTGAGAGGACGACCAGCCACACT
GGGACTGAGACACGGCC
>Zotu3717
GAACGCTGGCGGCAGGCTTAACACATGCAAGTCGAGCGGGGCCTTTCGGGGCTCAGCGGCGGACGGGTT
AGTAACGCGTGGGAACGTGCCTTTTGCTTCGGAATAGCCCTGGGAAACTGGGGGTAATACCGGATGTGCC
CTGAGGGGGAAAGATTTATCGGCAAGAGATCGGCCCGCGTCTGATTAGGTAGTTGGTGGGGTAATGGCCC
ACCAAGCCGACGATCGGTAGCTGGTTTGAGAGGATGATCAGCAACACTGGGACTGAGACACGGCC
>Zotu3719
GAATGCTGGCGGCGTGGATAAGCCATGCAAGTCGAACGGTCCGCAAGGACAGTGGCAGACGAGGTAGTA
AGGCATAGGGAGATCCCCCAGAGTCAGGGATAGCCAGTCGAAAGATTGGGTAATACTTGATGGTCCGGTG
ACGGTAAAGATTTATCGCTCTGGGAGTCACCTATGTAATATCAGCTAGTTGGTGAGGTAATGGCTCACCAA
GGCTACGACGTTTAGGGGAGCTGAGAGGCTGACCCCCACCGATGGGACTGCGATACGGCC
>Zotu3721
GAACGCTAGCGGGAGGCTTAACACATGCAAGTCGAGGGGCATCACGAGGTAACAATACTTTGGTGGCAAC
CGGCGCACGGGTGAGTAACGCGTATGCAACCTACCCTGTACAGGGGGATAGCCCGGAGAAATTCGGATTA

ATACCCCATAAAGATATTTGAAGGCATCTTTAGATATTTAAAGTTTCGACGGTACGGGATGGGCATGCGTA
ACATTAGCTAGTTGGTAGGGTAACGGCCTACCAAGGCAACGATGTTTAGGGGTCCTGAGAGGGTGATCCC
CCACACTGGTACTGAGACACGGAC

>Zotu3723
GAACGCTGGCGGCGTGCTTAACACATGCAAGTCGAACGGTGAAGCCCAGCTTGCTGGGTGGATCAGTGGC
GAACGGGTGAGTAACACGTGAGCAACCTGCCCCTGACTCTGGGATAAGCGCTGGAAACGGCGTCTAATAC
TGGATACGAGTAGCGACCGCATGGTCAGTTACTGGAAAGATTTATTGGTTGGGGATGGGCTCGCGGCCTA
TCAGCTTGTTGGTGAGGTAATGGCTCACCAAGGCGTCGACGGGTAGCCGGCCTGAGAGGGTGACCGGCCA
CACTGGGACTGAGACACGGCC

>Zotu3725
GAACGCTGGCGGCATGCTTAACACATGCAAGTCGGACAGGAAGTGGTGTTTCCAATGGCGGACGGGTGA
GTAACGCGTAAGAACCTGCCCTTGGGAGGGGAACAACAGCTAGAAACGGCTACTAATACCCCGTAGGCTG
AGGAGCAAAAGGAGATATCCGCCTGAGGAGGGGCTCGCGTCTGATTAGCTAGTTGGTGAGGCAATAGCTT
ACCAAGGCGATGATCAGTAGCTGGTTCGAGAGGATGATCAGCCACACTGGGACTGAGACACGGCC

>Zotu3727
GAACGCTGGCGGCGTGCCTAATACATGCAAGTCGAGCGAATCAATGGGAGCTTGCTCCCTGAGATTAGCG
GCGGACGGGTGAGTAACACGTGGGTAACCTGCCTGTAAGACTGGGATAACTTCGGGAAACCGGAGCTAAT
ACCGGATAATTTCTTTTCTCGCATGAGGGAAGATGGAAAGATGGTTCTGCTATCACTTACAGATGGACCCG
CGGCGCATTAGCTAGTTGGTGAGGTAATGGCTCACCAAGGCAACGATGCGTAGCCGACCTGAGAGGGTGA
TCGGCCACACTGGGACTGAGACACGGCC

>Zotu3729
GAACGCTGGCGGCGTGCCTAATACATGCAAGTCGTGCGAGGCTCTTCGGAGTCGAGCGGCGGACGGGTG
AGTAACACGTGGGCAACCTGCCCGACAGATCGGGATAACGCTTGGAAACGAGTGCTAATACCGGATACGC
TTTTGAGGCGCATGCCTCGAAAGGGAAAGTTGCTTTTGCAGCACTGTCGGATGGGCCCGCGGCGCATTAGC
TAGTTGGTGAGGTAACGGCTCACCAAGGCGACGATGCGTAGCCGACCTGAGAGGGTGACCGGCCACACTG
GGACTGAGACACGGCC

>Zotu3731
GAACGCTGGCGGCGTGCCTAATACATGCAAGTCGAGCGGTAAGGCTTCCACAGTTTGTATCTCCCTTATAA
CAAAGTTATGAGATACGGAGGTGCAAAGCGTGGAAGTACACGAGCGGCGGACGGCTGAGTAACGCGTAG
GAACGTACCCCAGAGTGAGGGATAAGCACCGGAAACGGTGTCTAATACCGCATACTACCTTCGGGTCAAA
GCTTTAGCGCTTTGGGAACGGCCTGCGTATGATTAGCTTGTTGGTGAGGTAATGGCTTACCAAGGCGACGA
TCATTAGCTGGTCTGAGAGGATGATCAGCCAGACTGGGACTGAGACACGGCC

>Zotu3733
GAACGCTGGCGGCAGGCTTAACACATGCAAGTCGAGCGGAAACGATGGGAGCTTGCTCCCAGGCGTCGA
GCGGCGGACGGGTGAGTAATACTTAGGAATCTGCCCAGTAGTGGGGGATAGCTCGGGGAAACTCGAATT
AATACCGCATACACCCTACGGGGAAAAGGGGGCGCTTGCGCTCTCGCTATTGGATGAGCCTAAGTCGGATT
AGCTAGTTGGTGGGGTAAAGGCCTACCAAGGCGACGATCTGTAGCTGGTCTGAGAGGATGATCAGCCACA
CCGGGACTGAGACACGGCC

>Zotu3736
GAACGCTGGCGGCAGGCCTAACACATGCAAGTCGAACGGATCCTTCGGGATTAGTGGCGGACGGGTGAG
GAACACGTGGGAACGTGCCTTTTGGTTCGGAACAACTCAGGGAAACTTGAGCTAATACCGGATAAGCCCTT
CGGGGGAAAGATTTATCGCCATTAGAGCGGCCCGCGTCTGATTAGCTAGTTGGTGAGGTAAAGGCTCACC
AAGGCGACGATCAGTAGCTGGTCTGAGAGGATGATCAGCCACATTGGGACTGAGACACGGCC

>Zotu3738
GAACGCTGGCGGCGTGCCTAATACATGCAAGTCGAGCGAATCTTCAGGAGCTTGCTCCTGAAGATTAGCG
GCGGACGGGTGAGTAACACGTGGGCAACCTGCCTGTAAGACTGGGATAACTTCGGGAAACCGAGGCTAAT
ACCGGATAATCCTTTTCCTCACATGAGGGGAAGCTGAAAGACGGCGCAAGCTGTCACTTACAGATGGGCCC
GCGGCGCATTAGCTAGTTGGTGAGGTAACGGCTCACCAAGGCGACGATGCGTAGCCGACCTGAGAGGGT
GATCGGCCACACTGGGACTGAGACACGGCC

>Zotu3741

GAATGCTGGCGGCGTGGATAAGTCATGCAAGTAGAACGCCCCGCAAGGGGAGTTGCGAACGAGGTAGTA
ACACGTAGGTACGTCCCCCGAAGTCGGGCATAGCCAGTCGAAAGACTGGGTAATTCCCGATAGTCCCGAA
AGGGTAAAGGTTTTTCGCTTCGGGAGCGGCCTGCGTAGTATCAGCTAGTTGGTAGGGTAATGGCCTACCAA
GGCTACGACGCTTAGGGGGGCTGAGAGGCCGATCCCCACCGATGGAACTGCGACACGGTC

>Zotu3743

GAACGCTGGCGGCATGCCTAACACATGCAAGTCGAACGAGACCTTCGGGTCTAGTGGCGCACGGGTGCGT
AACGCGTGGGAATCTGCCCTTGGGTTCGGAATAACTCGCCGAAAGGCGTGCTAATACCGGATGATGTCGT
AAGACCAAAGATTTATCGCCCAGGGATGAGCCCGCGTAAGATTAGCTAGTTGGTGAGGTAAAGGCTCACC
AAGGCGACGATCTTTAGCTGGTCTGAGAGGATGATCAGCCACACTGGGACTGAGACACGGCC

>Zotu3746

AAACGCTGGCGGCGTGCCTAATACATGCAAGTCAAACGGGGAAAGGCTTCGGCCGAGTACTAGTGGCGCA
CGGGTGAGTAACACGTGGGTAATCTGCCTGGAAGTGGGGGATAACCAGTCGAAAGATTGGTTAATACCGC
ATAAGACCACAGAGTCTAAGGACTCAGGGGTCAAAGGTTTTTCGCTTCCAGATGAGCCTGCGCGGGATTA
GCTAGTTGGTGAGGTAACGGCTCACCAAGGCTATGATCTCTAGCTGGTCTGAGAGGATGATCAGCCACACT
GGAACTGAGACACGGTC

>Zotu3748

GAACGCTAGCGACAGGCTTAACACATGCAAGTCGAGGGGCAGCGGGGGAGTGGCTTGCCACTCCCGCCG
GCGACCGGCGCACGGGTGAGTAACACGTATGCAACCTGCCCTTGACAGGGGAATAAGCGGCGGAAACGC
CGTCTAATTCCGCGTACACCGCGGGAGGGGCATCCCTCCGGCGGAAAAGGAGGCGACTCCGGTCAAGGAT
GGGCATGCGGCGCATTAGCTAGACGGCGGGGTAACGGCCCACCGTGGCGACGATGCGTAGGGGTTCTGA
GAGGAAGGTCCCCCACATTGGAACTGAGACACGGTC

>Zotu3750

GAACGCTAGCGGCAGGCCTAACACATGCAAGTCGAGGGGCAGCGCGAGGTAGCAATACCTTGGCGGCGA
CCGGCGGACGGGTGCGTAACGCGTATGGAACCAACCCGTCACAGGGGGACAAGCCAGGGAAACTTGGTC
TAATACCCCATATTACTGTTTGACCGCATGATTAGACAGTGAAAGCCTACGGGCGGTGACGGACGGGCATG
CGTGACATTAGCTAGTTGGTAAGGCAACGGCTTACCAAGGCGACGATGTCTAGGGGAGCTGAGAGGCTAA
TCCCCCACACTGGTACTGAGACACGGAC

>Zotu3752

AAACGCTGACAGAATGCTTAACACATGCAAGTCTATGAGGAAGTTTAGCTTGCTAAATGGACTCATGGCGG
ACGGGTGAGTAACGCGTAAAGAACTTGCCCTTTAGACTGGGATAACAGAGGGAAACTTCTGATAATACTG
GATAAGTTAGTATATCGCATGATATGCAAATGAAAGCTACGGCACTAAAGGAGAGCTTTGCGTCCTATTAG
CTAGTTGGTAAGGTAAGAGCTTACCAAGGCGATGATAGGTAGCCGGCCTGAGAGGGTGGACGGCCACAA
GGGGACTGAGATACGGCC

>Zotu3754

GAACGCTGGCGGCAGGCTTAACACATGCAAGTCGAACGCCCCGCAAGGGGAGTGGCAGACGGGTGAGTA
ACGCGTGGGAATCTACCCTGATCTGCGGGATAGCTCCGGGAAACTGGAATTAATACCGCATGTGCCCTGAG
GGGCAAAGGTTTACCGGATTGGGATGAGCCCGCGTCCGATTAGCTAGTTGGTGGGGTAAAGGCCTACCAA
GGCGACGATCGGTAGCTGGTCTAAGAGGATGATCAGCCACATTGGGACTGAGACACGGCC

>Zotu3756

GAACGCTAGCGGCAGGCCTAATACATGCAAGTCGAACGGTAACATGAATAGCAATATTTGATGACGAGTG
GCGCACGGGTGCGTAACGCGTATGCAATCTACCTTCAACTGGTGAATAGCCCGAAGAAATTCGGATTAACA
CACCATAGTATAATAGAATGGCATCATTTTATTATTAAAACTCCGGTGGTTGAAGATGAGCATGCGTGACAT
TAGCTAGTTGGTGAGGTAATGGCTCACCAAGGCTACGATGTCTAGGGGATCTGAGAGGATTAACCCCCAC
ACTGGTACTGAGACACGGAC

>Zotu3758

GAACGCTGGCGGCGTGCCTAATACATGCAAGTCGAGCGGACAGATGGGAGCTTGCTCCCTGATGTTAGCG
GCGGACGGGTGAGTAACACGTGGGTAACCTGCCTGTAAGACTGGGATAACTCCGGGAAACCGGGGCTAA
TACCGGATGGTTGTTTGAACCGCATGGTTCAGACATAAAAGGTGGCTTCGGCTATCACTTACAGATGGACC
CGCGGCGCATTAGCTAGTTGGTGAGGTAACGGCTCACCAAGGCGACGATGCGTAGCCGACCTGAGAGGGT
GATCGGCCACACTGGGACTGAGACACGGCC

>Zotu3760
GAACGCTAGCGGCAGGCTTAATACATGCAAGTCGAACGGATGTAGCAATACATTAGTGGCGCACGGGTGA
GTAACGCGTACATAACATGCCTTTAGCTTAGGAATAGCCTTGGGAAACTGGGAGTAATACCTAATAGTATT
TTTGGATGGCATCATCTGGAAATTAAAGTTACGGCGGCTAAAGATTGATGTGCGTAAGATTAGCTAGTTGG
CGAGGTAACGGCTCACCAAGGCGACGATCTTTAGGGGGCGTGAGAGCGTGACCCCCCACACGGGTACTGA
GACACGGAC
>Zotu3763
GAACGCTGGCGGCGTGCTTAACACATGCAAGTCGAGCGAGGTTCAACTGGTGGCAACACCAGGGAAGACC
GAGCGGCGAACGGGTGAGTAACACGTGAGCAACCTGTCCCGAAGACCGGGATAACACCGGGAAACCGGT
GCTAATACCGGATGTCCCCACCGGACCACATGGTTCGGTGAGGAAATGGATTCCGCTTCGGGAGGGGCTC
GCGGGCTATCAGCTTGTTGGTGGGGTAACGGCCTACCAAGGCAACGACGGCTAGCTGGTCTGAGAGGACG
ACCAGCCACACTGGGACTGAGACACGGCC
>Zotu3765
GAACGCTAGCTACAGGCTTAACACATGCAAGTCGAGGGGCAGCATGGTCTTAGCTTGCTAAGACTGATGG
CGACCGGCGCACGGGTGAGTAACACGTATCCAACCTGCCGTCTACTCTTGGACAGCCTTCTGAAAGGAAGA
TTAATACAAGATGGCATCATGAGTCCACATGTTCACATGATTAAAGGTATTCCGGTAGACGATGGGGATGC
GTTCCATTAGATAGTAGGCGGGGTAACGGCCCACCTAGTCTTCGATGGATAGGGGTTCTGAGAGGAAGGT
CCCCCACATTGGAACTGAGACACGGTC
>Zotu3767
GAACGCTGGCGGCGTGCTTAACACATGCAAGTCGAACGGAAAGGCCCTTCGGGGTACTCGAGTGGCGAAC
GGGTGAGTAACACGTGGGTGATCTGCCCTGCACTCTGGGATAAGCCTGGGAAACTGGGTCTAATACCGGA
TAGGACCGCATGCTTCATGGTGTGTGGTGCAAAGCTTTTGCGGTGTGGGATGAGCCCGCGGCCTATCAGCT
TGTTGGTGGGGTAATGGCCTACCAAGGCGACGACGGGTAGCCGGCCTGAGAGGGTGACCGGCCACACTG
GGACTGAGATACGGCC
>Zotu3769
GAACGCTGGCGGCAGGCTTAACACATGCAAGTCGAACGGGCACCTTCGGGTGTCAGTGGCAGACGGGTG
AGTAACACGTGGGAACGTGCCCTTCGGTTCGGAATAACTCAGGGAAACTTGAGCTAATACCGGATACGCCC
TTATGGGGAAAGGTTTACTGCCGAAGGATCGGCCCGCGTCTGATTAGCTTGTTGGTGGGGTAACGGCCTAC
CAAGGCGACGATCAGTAGCTGGTCTGAGAGGATGATCAGCCACACTGGGACTGAGACACGGCC
>Zotu3772
AAACGCTGGCGGTGTGCCTGAGATATGCAAGTCGAGCGGGAATCATTGAAGTCGCAAGATGGATATGATT
TCAGCGGCGGACGGCTGAGTAACACGTAAGAATCTCCCCCAAAGTCTCACATAGCTCACCGAAAGGTGGG
GTAATTTGAGATAGTCTCCCCTCGCAAGAGGGGTGTAAAGCCGCAAGGCGCTTCGGGATGAGCTTGCGTC
CTATCAGGTAGTTGGTGGGGTAATGGCCCACCAAGCCTACGACGGGTAGCTGGCCTCATGAGGGTGGTCA
GCCAGAAGGGGACTGAGATACGGCC
>Zotu3774
GAACGCTGGCGGCGGGCTTAACACATGCAAGTCGAACGCGAAAGCCCCTTCGGGGGTGAGTAGAGTGGC
GCACGGGTGAGTATCGCGTAGACAACCTGCCCTTTGCTGGGGAATAACCACGGGAAACCGTGGCTAATAC
CGCATATTGTCTTAACCAGGCATCTGGATTAGATGAAAGCCTTCGGGCGGCAAAGGATGGGTCTGCGTCG
GATTAGCTAGTAGGTAGGGTAAAAGCCTACCTAGGCGACGATCCGTAGCTGGTCTGAGAGGATGATCAGC
CACACTGGCACTGAGACACGGGC
>Zotu3777
GAACGCTGGCGGCGTGCTTAACACATGCAAGTCGAACGATGAAGCCAGCTTGCTGGTGGATTAGTGGCGA
ACGGGTGAGTAACACGTGAGTAACCTGCCCTTGACTCTGGGATAAGCCTGGGAAACCGGGTCTAATACTG
GATACGACCTTCCCACGCATGTGGTGTTGGTGGAAAGCTTTTGTGGTTTTGGATGGACTCGCGGCCTATCA
GCTTGTTGGTGGGGTAATGGCCTACCAAGGCGACGACGGGTAGCCGGCCTGAGAGGGTGACCGGCCACA
CTGGGACTGAGACACGGCC
>Zotu3779
GAACGCTGGCGGCATGCTTAACACATGCAAGTCGAACGGTAACAGGTCTAGCTTGCTAGATGCTGACGAG
TGGCGGACGGGTGAGTAACGCGTAGGAATCTACCCCGCGGTGGGGGATAACACGAGGAAACTCGTGCTA

ATACCGCATACGCTCTACGGAGGAAAATTTTTTTGCCGTGGGATGAGCCTGCGTTAGATTAGCTTGTTGGTA
GGGTAATGGCCTACCAAGGCGATGATCTATAGCTGTTCTGAGAGGAAGATCAGCCACACTGGGACTGAGA
CACGGCC

>Zotu3781

GAACGCTGGCGGCGTGCCTAATACATGCAAGTCGAGCGGAAGTTGAAGAGCTTGCTCTTCAACTTTAGCG
GCGGACGGGTGAGTAACACGTGGGCAACCTGCCCTGCAGATCGGGATAACTCCGGGAAACCGGTGCTAAT
ACCGAATAGTTTTGGGTCCCTCCTGGGACCCAACGGAAAGACGGTTTCGGCTGTCACTGCAGGATGGGCCC
GCGGCGCATTAGCTAGTTGGTGGGGTAACGGCCCACCAAGGCCACGATGCGTAGCCGACCTGAGAGGGT
GATCGGCCACACTGGGACTGAGACACGGCC

>Zotu3783

GAACGCTAGCGGCAGGCTTAATACATGCAAGTCGAGGGGTAACAGGGGTAGCAATACCTGCTGACGACCG
GCAAACGGGTGCGGAACACGTACGCAACCTTCCCATAACTGGTGAATAGCCCTCCGAAAGGAGGATTAAT
ACACCGTAACATAATGAAGTGGCATCACTTTATTATTATAGGCTTCGGCCGGTTTTGGATGGGCGTGCGCCT
GATTAGGTAGTTGGCGGGGTAACGGCCCACCAAGCCTGCGATCAGTAACTGGTGTGAGAGCACGACCAGT
CACACGGGCACTGAGACACGGGC

>Zotu3785

GAACGCTGGCGGCGTGGATAAGTCATGCAAGTCAAGGGGACCGCAAGGTAACCGGCAAACGAGGTAGTA
ACACGTAGGTACTCCCCCCGAAGTTTGGCATAGCTCGTCGAAAGACGGGGTAATTCCAAATAGTCCCGCAA
GGGTAAAGATTTATCGCTTCGGGAGAGGCCTGCGTAGTATCAGTTAGTTGGTAGGGTAACGGCCTACCAA
GGCTACGACGCTTAGGGGAGCTGAGAGGCTGACCCCCACCGATGGGACTGCGACACGGCC

>Zotu3787

GAATGCTGGCGGCGTGCCTAATACATGCAAGTCGAGCGGTAACAGGGCTTCACCGTTTCCTTGACACACCT
AGCGCTTTATCGTAGGTTGGTTAGATTCGAGGGAACAGTGAAGCTGCTGACGAGCGGCGGACGGCTGAGT
AACGCGTGGGAACGGACCCCAAACTGAGGGATAACGCACCGAAAGGTGTGCTAATACCGCATGTGATCTT
CGGATTAAAGTCTTCGGACGGTTTGGGACTGGCCTGCGTACGATTAGATAGTTGGTAGGGTAATGGCCTAC
CAAGTCGACGATCGTTAGATGGTTTGAGAAGATGATCATCCAGACTGGGACTGAGACACGGCC

>Zotu3789

GAACGCTGGCGGCGTGCCTAACACATGCAAGTCGAGCGATCTTCTTCGGAAGAGAGCGGCGGACGGGTG
AGTAACGCGTGGGTAACCTGCCCTGTACACACGGATAACATACCGAAAGGTATGCTAATACGAGATAACAT
ACTTTTATCGCATGGTAGAAGTATCAAAGCTCCGGCGGTACAGGATGGACCCGCGTCTGATTAGCTAGTTG
GTAAGGTAACGGCTTACCAAGGCGACGATCAGTAGCCGACCTGAGAGGGTGATCGGCCACATTGGAACTG
AGACACGGTC

>Zotu3792

GAATGCTGGCGGCGTGCCTAATACATGCAAGTCGAGCGGTAACAGGGGGTCTCACCGTTTCCTTGACACAC
CTAGCGCTATGATCGTAGGTTGTTGACTTCGAGGGAACAGTGAGATCCTGCTGACGAGCGGCGGACGGCT
GAGTAACGCGTGGGAACGGACCCCAAACTGAGGGATAACGCACCGAAAGGTGTGCTAATACCGCATGTGA
TCTTCGGATTAAAGGATTTATCCGGTTTGGGACTGGCCTGCGTACGATTAGATAGTTGGTAGGGTAACGGC
CTACCAAGTCGACGATCGTTAGATGGTTTGAGAAGATGATCATCCAGACTGGGACTGAGACACGGCC

>Zotu3794

GAACGCTAGCGGGAGGCCTAACACATGCAAGCCGGGCGGGATCCCTTCGGGGTGAGAGCGGCGTACGGG
TGAGGAGCACGTGTGCAACCTGCCCCTGTCTGGGGGATAGCCCTCCGAAAGGAGGATTAACACCCCATAG
GATGTTCGCCGGCATCGGCGGTCATTGAAAGGTTACGGACGGGGATGGGCACGCGCAGGATTAGGTAGTT
GGCAGGGTAACGGCCTACCAAGCCTTTGATCCTTAGGGGGCCTGAGAGGGTGATCCCCCACACTGGTACT
GAGACACGGAC

>Zotu3796

GAACGCTGGCGGCATGCTTTACACATGCAAGTCGAACGGCAGCGCGGGGCAACCTGGCGGCGAGTGGCG
AACGGGTGAGTAATATATCGGAACGTACCCAAGAGTGGGGGATAACGTAGCGAAAGTTACGCTAATACCG
CATACGATCCAAGGATGAAAGTGGGGGATCTTCGGACCTCATGCTCCTGGAGCGGCCGATATCTGATTAGC
TAGTTGGTGAGGTAAAGGCTCACCAAGGCGACGATCAGTAGCTGGTCTGAGAGGACGACCAGCCACACTG
GGACTGAGACACGGCC

>Zotu3798
GAATGCTGGCGACGTGGATAAGGCATGCAAGTCAAGGGGGCCGCAAGGCAACCGGCAGACGAGGTAGTA
ACACGTACGAACGCACCCCGAAGTCTGGAATAGCTCGTCGAAAGACGGGGTAATACCGGATGGTACCGCA
AGGTTAAAGATTTATCGCTTCGGGAGCGGCGTGCGCAATATCAGCTAGTTGGTGAGGTAATGGCTCACCAA
GGCTACGACGTTTAGGGGAACTGAGAGGTTGACCCCCACCGATGGGACTGAGACACGGCC
>Zotu3800
GAACGCTAGCGGGAGGCCTAACACATGCAAGCCGAGCGGTATTTGTCTTTCGGGACGAAGAGAGCGGCGT
ACGGGTGCGGAACACGTGTGCAACCTGCCTTTATCAGGGGGATAGCCTTTCGAAAGGAAGATTAATACCCC
ATAATATATTAATTGGCATCAATTGATATTGAAAACTACGGTGGATAGAGATGGGCACGCGCAAGATTAGA
TAGTTGGTAGGGTAACGGCCTACCAAGTCAGTGATCTTTAGGGGGCCTGAGAGGGTGATCCCCCACACTG
GTACTGAGACACGGAC
>Zotu3804
GAACGCTGGCGGCAGGCCTAACACATGCAAGTCGAACGATAAGCCACCTTCGGGTGGTGGACAGTGGCA
GACGGGTGAGTAACGCGTGGGAACGTGCCTTTCGGTTCGGGATAACCCAGGGAAACTTGGGCTAATACCG
GATACGCCCTTCGGGGGAAAGATTCATTGCCGAAAGATCGGCCCGCGTCGGATTAGCTTGTTGGTGGGGT
AATGGCCTACCAAGGCGACGATCCGTAGCTGGTCTGAGAGGATGACCAGCCACACTGGGACTGAGACACG
GCC
>Zotu3806
GAACGCTGGCGGCGTGCTTAACACATGCAAGTCGAGCGGTAAGGCCCTTCGGGGTACACGAGCGGCGAA
CGGGTGAGTAACACGTGGGTGACCTGCCCTCCACTTCGGGATAAGCCTGGGAAACCGGGTCTAATACCGG
ATATGACTTCTGGGTGCATGCCTGGTTGTGGAAAGTTTTTCGGTGGGGGATGGGCCCGCGGTCTATCAGC
TTGTTGGTGGGGTGATGGCCTACCAAGGCGACGACGGATAGCCGGCCTGAGAGGGTGATCGGCCACACT
GGGACTGAGACACGGCC
>Zotu3809
GAACGCTGGCGGAATGCTTAACACATGCAAGTCGCACGGGGGCTTTCGGGCTCTAGTGGCGGACGGGTGA
GTAACGCGTAGGAACGTGTCTTGGGGTGGGGGATAACGCTGGGAAACTGGCGCTAATACCGCATATCTGC
TGTGGCAGAAAGCCTTCGGGTGCCTTGAGAGCGGCCTGCGTCCGATTAGCTTGTTGGTGGGGTAATGGCT
TACCAAGGCGACGATCGGTAGCTGGTCTGAGAGGACGATCAGCCACACTGGGACTGAGACACGGCC
>Zotu3815
TAACGCTGGCGGCGTGCTTAACACATGCAAGTCGAACGAGGGCCTTTCTCCCGCAAGGGAGGGGGGTTCC
TAGTGGCGAACGGGTGAGTAACACGTGGGCAACCTGTCCTTTCGTGGGGGATAACACCGGGAAACCGGTG
CTAATACCGCGTACGGTCGCCGAAGGCATCTTCGGTGAAGAAAGCAGCAATGCGCGAAAGGGTGGGCCC
GCGGCCTATCAGCTAGTCGGTGGGGTAACGGCCTACCGAGGCTTTGACGGGTAGCTGGTCTGAGAGGACG
ACCAGCCACACGGGGACTGAGACACGGCC
>Zotu3818
GAACGCTGGCGGCGTGCCTAATACATGCAAGTCGAGCGGAGCACTTCGGTGCTTAGCGGCGGACGGGTG
AGTAACACGTAGGCAACCTGCCTATAAGACTGGGATAACTATCGGAAACGATAGCTAAGACCGGATAACT
GGTTTTCTCGCATGAGAGAATCATGAAACACGGAGCAATCTGTGGCTTATAGATGGGCCTGCGGCGCATTA
GCTAGTTGGTGAGGTAACGGCTCACCAAGGCGACGATGCGTAGCCGACCTGAGAGGGTGAACGGCCACA
CTGGGACTGAGACACGGCC
>Zotu3820
GAATGCTGGCGGCGCGCCTAACACATGCAAGTCGAGCGGTAACAGGTCTAGTTTACTAGATGCTGACGAG
CGGCGGACGGCTGAGTAACGCGTGGGAACATGCCCCAAAGTGAGGAATAAGCTCCAGAAATGGAGTCTA
ATGCCGCATATGGTCTTCGGATTAAAGGATTTATCCGCTTTGGGAGTGGCCTGCGTATGATTAGATAGTTG
GTGAGGTAATGGCTCACCAAGTCTACGATCATTAACTGGTCTGAGAGGATGATCAGTCAGACTGGAACTGA
GACACGGTC
>Zotu3822
GAACGCTGGCGGCGTGCCTAACACATGCAAGTCGCGGGAGAAAGTCTTCGGATGAGTAAACCGGCGCAC
GGGTGAGTAACACGTGGGTAATCTTCCTTCAGGCCTGGAATAACCCGGGGAAACTCGGGCTAATACCGGA
TAAGACCACGCAACGAGAGTTGTGTGAGAAAAGATGAGGACCTCTTCGGAGGCTTTTCACCAGAAGATGA

GCCCGCGGCCCATCAGCTAGTTGGCGGGGTAACGGCCCACCAAGGCTAAGACGGGTAGCTGGTCTGAGA
GGACGACCAGCCACACTGGAACTGAGACACGGTC
>Zotu3824
GAACGCTGGCGGCGTGCTTAACACATGCAAGTCGAGCGAAGCAGTTAAGTTGATTTCTTCGGATTGATTCT
TGACTGACTGAGCGGCGGACGGGTGAGTAACGCGTGGGTGACCTGCCCCATACCGGGGGATAACAGCTG
GAAACGGCTGCTAATACCGCATAAGCGCACAGAGCTGCATGGCTCGGTGTGAAAAACTCCGGTGGTATGG
GATGGGCCCGCGTCTGATTAGGCAGTTGGCGGGGTAACGGCCCACCAAACCGACGATCAGTAGCCGGCCT
GAGAGGGCGACCGGCCACATTGGGACTGAGACACGGCC
>Zotu3827
GAACGCTGGCGGCGTGCTTAACACATGCAAGTCGAACGATGAAGCCAGCTTGCTGGTGGATTAGTGGCGA
ACGGGTGAGTAACACGTGGGTGATCTGCCCCAAACTTTGGGATAAGCCTGGGAAACTGGGTCTAATACCG
AATATTACCTTCTCCTGCATGGGGGTTGGTGGAAAGCTCCGGCGGTTTGGGATGGGCCCGCGGCCTATCAG
CTTGTTGGTGGGGTAATGGCCTACCAAGGCGACGACGGGTAGCCGGCCTGAGAGGGCGACCGGCCACAC
TGGGACTGAGACACGGCC
>Zotu3829
GAACGCTGGTGGCGTGCTTAACACATGCAAGTCGAACGGTGAAGCCCAGCTTGCTGGGTGGATCAGTGGC
GAACGGGTGAGTAACACGTGAGCAACCTGCCCTCCACTTCGGGATAAGCCTTGGAAACGAGGTCTAATAC
CGGATACGAGCACTTACCGCATGGTGGGTGTTGGAAAGTTTTTCGGTGGGGGATGGGCTCGCGGCCTATC
AGCTTGTTGGTGGGGTAATGGCCTACCAAGGCGTCGACGGGTAGCCGGCCTGAGAGGGCGACCGGCCAC
ACTGGGACTGAGACACGGCC
>Zotu3831
AAACGCTGGCGGCGTACCTAAAACATGCAAGTCGAACGGCTCACTTCGGTGGGTAGTGGCGAACGGCTGA
GTAACACGTGGGTGACCTGCCTTCGAACGGGGGATAACTGCCCGAAAGGGTGGCTAATACCGCAGATGTC
GGTTCTTCGTTGAGCTGACTAAACCGTTTAGGGTTCGAAGAGGGGCCTGCGTCCGATTAGCTAGCTGGTGG
GGTAAAGGCCTACCAGGGCGACGATCGGTAGCTGGTCTGAGAGGATGATCAGCCAGACTGGGACTGCGA
GACGGCC
>Zotu3833
GAACGCTGGCGGCATGCCTAACACATGCAAGTCGAACGAGACCTTCGGGTCTAGTGGCGCACGGGTGCGT
AACGCGTGGGAATCTGCCCTTGGGTTCGGGATAACAGTTGGAAACGACTGCTAATACCGGATGATGACTTC
GGTCCAAAGATTTATCGCCCAGGGATGAGCCCGCGTAGGATTAGCTAGTTGGTGGGGTAAAAGCTCACCA
AGGCGACGATCCTTAGCTGGTCTGAGAGGATGATCAGCCACACTGGGACTGAGACACGGCC
>Zotu3835
GAACGCTAGCTATATGCTTAACACATGCAAGTCGAACGTTGTTTTCGGGGAGCTGGGCAGAAGGAAAAGA
GGCTCCTAGCTAAAGGTAGCTTGTCTCGCCCAGGAGGTGGGAACAGTTGAGAACAAAGTGGCGAACGGGT
GCGTAACGCGTGGGAATCTGCCGAACAGTTCGGGCCAAATCCTGAAGAAAGCTAAAAAGCGCTGTTTGAT
GAGCCTGCGTAGTATTAGGTAGTTGGTCAGGTAAAGGCTGACCAAGCCAATGATGCTTAGCTGGTCTTTTC
GGATGATCAGCCACACTGGGACTGAGACACGGCC
>Zotu3837
GAACGTTGATGGCGTGCCTCATACATGCAAGTCGAGCGCCCCGCAAGGGGAGCGGCGGACGGGTGAGTA
ACACGTAAAGAATCTGCCCTGAAGCGGGGAATAACTATCCCAACGGATAGCTAATACCGCATAATGATGGA
GGAGGCATCTCCTTTTCTCAAAAGGCTTCGGCCACTTCAGGATGACTTTGCGTCCCATTAGCTTGTTGGCGG
TGTAACGGACCACCAAGGCTACGATGGGTATCCGGCCTGAGAGGGTGTACGGACACATTGGGACTGAGAT
ACGGCC
>Zotu3839
GAACGCTGGCGGCGTGCTTAACACATGCAAGTCGAGCGGAAAGGCCACTTCGGTGGTACTCGAGCGGCGA
ACGGGTGAGTAACACGTGAGTAATCTGCCCTTGGCTTTGGGATAGCCACCGGAAACGGTGATTAATACCG
GATACGACAACTACTCGCATGGGTTGGTTGTGGAAAGTTTTTCGGCCAGGGATGTGCTCGCGGCCTATCAG
CTTGATGGTGAGGTAATGGCTCACCATGGCTTCGACGGGTAGCCGGCCTGAGAGGGTGACCGGTCACACT
GGGACTGAGACACGGCC
>Zotu3841

GAACGCTGGCGGCGTGCCTAACACATGCAAGTCGAGCGAGTGGAGTTCTTCGGAACAAAGCTAGCGGCG
GACGGGTGAGTAACACGTGGGCAACCTGCCTCATAGAGGGGAATAGCCTCCCGAAAGGGAGATTAATACC
GCATAAGATTGTAGCTTCGCATGAAGTAGCAATTAAAGGAGCAATCCGCTATGAGATGGGCCCGCGGCGC
ATTAGCTAGTTGGTGAGGTAGCGGCTCACCAAGGCGACGATGCGTAGCCGACCTGAGAGGGTGATCGGCC
ACATTGGGACTGAGACACGGCC
>Zotu3844
GAACGCTAGCGGCAGGCCTAATACATGCAAGCTGAACGGCAAGAGTTGTAGCAATACGATTCCTAGAGTA
GCGTAAGGGTGTGTAACACGTATGCAATCTACCCACAACAGGAGGATAGCTCCCCGAAAGGGGAATTAAG
ACTCCATAATATCATATGAGGGCATCTGAGTATGATTAAAGTACAAGCGGTTGTGGATGAGCATGCGTCTG
ATTAGTTAGTTGGCGGGGTAACGGCCCACCAAGACGATGATCAGTAGGGGAACTGAGAGGTTGGTCCCCC
ACACTGGCACTGAGATACGGGC
>Zotu3846
AAACGCTGGCGGCGTGCCTAACACATGCAAGTCGAACGCCCTATGCTTCGGTGTAGGGAGTGGCGGACGG
GTGAGTAACACGTGGGATATCTACCCAGAGGTGAGGAATACCGGCGAGAAATCGCCGACAATACCGCATG
CGCTTCGCAAGAAGCAAAGCCTGGCAACAGGCGCCACTGGACGATCCTGCGGTCGATTAGCTAGTTGGCG
GGGTCAAAGCCCACCAAGGCGACGATCGATAGCTGGTCTGAGAGGACGATCAGCCACACTGGGATTGAG
AACGGCC
>Zotu3848
GAACGCTGGCGGCGTGCTTAACACATGCAAGTCGCGTGCGCAGTTCGCGTAGCAATACGTGGATGGGCGC
ACGGCGCACGGGTGCGTAACACGTGGGCAATCTGCCCTCCGATGGGGAATAACCCCGCGAAAGCGGGGC
TAATACCGCATAATATTCCGAGGACTTCGGTTCTTGGATTCAAAGCAGTGATGCGTCGGAGGAGGAGCCCG
CGCCCGATTAGCTAGTTGGTAGGGTAACGGCCTACCAAGGCGATGATCGGTAGCTGGTCTGAGAGGATGA
TCAGCCACACTGGAACTGAGACACGGTC
>Zotu3851
GAACGCTAGCGGCAGGCCTAATACATGCAAGTCGGACGGGATTGCAGTATAGCTTGCTATACTGCATGAG
AGTGGCGCACGGGTGCGTAACGCGTGAGCAACCTGCCCATGTCAGGGGGATAGCCCGTTGAAAGACGGA
TTAATACCGCATAACACATAGAGACCACCTGGTTTCTATGTCAAATATTTATAGGACATGGATGGGCTCGCG
TGGCATTAGTTAGTTGGTAAGGTAACGGCTTACCAAGACGATGATGCCTAGGGGCTCTGAGAGGAGTGAC
CCCCACACTGGTACTGAGACACGGAC
>Zotu3853
GAACGCTGGCGGCAGGCCTAACACATGCAAGTCGAGCGCCCCGCAAGGGGAGCGGCGGACGGGTGAGTA
ACACGTGGGAATTTTCCTCAAGGTACGGAACAACTCAGGGAAACTTGGGCTAATACCGTATGATGCAGAG
ATGCCAAAGATTTATCGCCTTGGGATAAGCCCGCGTCAGATTAGGTAGTTGGTGGGGTAATGGCCTACCAA
GCCTGTGATCTGTAGCTGGTCTGAGAGGACGATCAGCCACATTGGGACTGAGACACGGCC
>Zotu3856
GAACGCTAGCGGTGCGCCTAACACATGCAAGTCGAGTGCCCCGCCTCTGGTGGGGCACGGCAAACGGGTG
AGTAACACGTTGGTAACTTACCCCGGAGTGAGGAATAACCCGCCGAAAGGCGGGCTAATGCCGCATAAGA
AAGGGGGGACGAAAGTCATTCCCTTTTAAAGTCGTAAGACGCTTCGGGAGAGGCCTGCGTAGTATCAGCT
AGTTGGTAGGGTAACGGCCTACCAAGGCTATGACGCTTAGTTGGTCTGAGAGGACGACCAGCCACAATGG
GACTGAGACACGGCC
>Zotu3858
GAACGCTGGCGGCGTGCTTAACACATGCAAGTCGAGCGGTAAGGCTCCTTCGGGAGTACACGAGCGGCGA
ACGGGTGAGTAACACGTGAGTAATCTGCCCTCCACTTCGGGATAAGCGCCGGAAACGGCGTCTAATACCG
AATACGACGCACTCTCGCATGAGATGTGCGTGGAAAGTTCTATCGGTGGAGGATGTGCTCGCGGCCTATCA
GCTTGTTGGTGAGGTAACGGCTCACCAAGGCTTCGACGGGTAGCCGGCCTGAGAGGGTGACCGGCCACAC
TGGGACTGAGACACGGCC
>Zotu3860
GAACGCTGGCGGCATGCCTAACACATGCAAGTCGAACGAAGCCTTCGGGCTTAGTGGCGCACGGGTGCGT
AACGCGTGGGAATCTGCCCTTGGGTTCGGAATAACGTCGGGAAACTGACGCTAATACCGGATAATATCGC
GAGATCAAAGATTTATCGCCTGAGGATGAGCCCGCGTAGGATTAGCTAGTTGGTGGGGTAAAGGCCTACC
AAGGCGACGATCCTTAGCTGGTCTGAGAGGATGATCAGCCACACTGGGACTGAGACACGGCC

>Zotu3862
GAACGCTGGCGGCGTGCCTAACACATGCAAGTCGAGCGATCTCTTCGGAGAGAGCGGCGGACGGGTGAG
TAACGCGTGGGTAACCTGCCCTGTACACACGGATAACATACCGAAAGGTATACTAATACGGGATAACATAC
GAAAGTCGCATGGCTTTTGTATCAAAGCTCCGGCGGTACAGGATGGACCCGCGTCTGATTAGCTAGTTGGT
AAGGTAATGGCTTACCAAGGCAACGATCAGTAGCCGACCTGAGAGGGTGATCGGCCACACTGGAACTGAG
ACACGGTC
>Zotu3865
TAACGTTGGCAGCGTGCCTAATACATGCAAGTCGAGCGGCCCGCAAGGGCAGCGGCAGACGAGAGAGTA
ATAGTAAGGAACTTACCCCCGAGTGAAGGATAGCCATCCGAAAGGATGGGTAATCCTGCATAATATCGAA
AGATCAAAGCCGCAAGGCGCTTGGGGAGAGGCCTTACACCTATCAGCTTGTTGGCGAGGTAAATGCTCAC
CAAGGCGATGACGGGTAGCTGGTCTGAGAGGATGACCAGCCTCGATGGGACTGAGACACGGCC
>Zotu3869
GAACGCTGGCGGCGTGCCTAATACATGCAAGTCGAGCGAAGTTTTTCTGGTGCTTGCACTAGAAAAACTTA
GCGGCAAACGGGTGAGTAACACGTAAAGAACCTGCCTCATAGACTGGGACAACTATTGGAAACGATAGCT
AATACCGGATAACAGCATTAACTGCATGGTTGATGTTTAAAAGTTGGTTTTGCTAACACTATGAGATGGCTT
TGCGGTGCATTAGCTAGTTGGTGGGGTAAAGGCCTACCAAGGCGACGATGCATAGCCGACCTGAGAGGGT
GATCGGCCACACTGGGACTGAGACACGGCC
>Zotu3872
GAACGCTAGCGATAGGCTTAACACATGCAAGTCGAGGGGTAACGTGTTGGAAGCTTGCTTCCGATGACGA
CGACCGGCGGATGGGTGCGTAACGCGTATGCAACTTGCCTCACAGCGGAGAATAACCCGGAGAAATCCGG
ACTAATGCTCCATACACTCTTAGGGACCCATGTCCCTGAGAGGAAAGATTTATCGCTGTGAGATAGGCATG
CGTCCTATTAGGTAGTTGGTGAGGTAACGGCTCACCAAGCCGACGATAGGTAGGGGTGCTGAGAGGCAGA
TCCCCCACATTGGGACTGAGACACGGCC
>Zotu3878
GAACGCTGGCGGCGTGCCTAACACATGCAAGTCGAACGGAACTGTTTTGAAAGATTTCTTCGGAATGAATT
TGATTTAGTTTAGTGGCGGACGGGTGAGTAACGCGTGAGTAACCTGCCTTCAAGAGGGGGATAACATTCT
GAAAAGAATGCTAATACCGCATGACATATCGGAACCACATGGTTTTGATATCAAAGATTTTATCGCTTGAA
GATGGACTCGCGTCCGATTAGTTAGTTGGTGAGGTAACGGCTCACCAAGACCGCGATCGGTAGCCGGACT
GAGAGGTTGAACGGCCACATTGGGACTGAGACACGGCC
>Zotu3880
GAACGCTAGCGGCAGGCCTAATACATGCAAGTCGAGGGGTAGTGTAGCAATACATGAGACCGGCGCACG
GGTGCGTAACGCGTATGAAAGGTGTCCAAATCTCTGGGATAGCCCTCGGAAACGGGGATTAATACCGGAT
GGTCCGTAGTAGTGGCATCACTTATACGGTAAAGCTCAGGCGGATTTGGGTCCTCATGCGTACGATTAGCT
AGTTGGCGAGGTAACGGCTCACCAAGGCGACGATCGTTAGGGGTCCTGAGAGGGAGATCCCCCACACTGG
TACTGAGACACGGGC
>Zotu3882
GAACGCTAGCGGCAGGCCTAATACATGCAAGTCGGACGGGATTGGAGAGCTTGCTTTCCATGAGAGTGGC
GCACGGGTGCGTAACACGTATGTAACCTACCTTCGTCAGGGGGATAGCCTCTCGAAAGAGAGATTAAGAC
CGCATAACATTATTGGATAGCATTATTTAATAATCAAATATTTATAGGACGAAGATGGGCATGCGGAACATT
AGCTAGTTGGTAAGGTAACGGCTTACCAAGGCTACGATGTTTAGGGGATCTGAGAGGATGACCCCCCACA
CTGGTACTGAGACACGGAC
>Zotu3884
GAACGCTGGCGGCGTGCCTAATACATGCAAGTCGAACGAAGGTACTTGTACCTTAGTGGCGAACGGGTGA
GTAACACGTAGGTAACCTGCCCTCAAGACGAGGATAACAGTTGGAAACGACTGCTAATACTCGATAGGTAA
TCACTAGGCATCTAGAGATTATTAAAGATCCGTTTGGATTACTTGAGGAGGGACCTGCGCCGCATTAGTTA
GTTGGTAAGGTAATGGCTTACCAAGACCATGATGCGTAGCCGGACTGAGAGGTTGAACGGCCACACTGGG
ACTGAGACACGGCC
>Zotu3886
GAACGCTAGCGGCAGGCCTGATACATGCAAGTCGGACGGGATTGGAGGGCTTGCTTTCCATGAGAGTGGC
GCACGGGTGCGTAACACGTATGTAACCTACCCATATCAGGGGGATAGCCTCTCGAAAGAGAGATTAAGCC

CGCATAACATCACGGGGCAGCATTGTACTGTGATCAAATATTTATAGGATATGGATGGGCATGCGGAACAT
TAGCTAGTTGGCGGGGTAACGGCCCACCAAGGCGACGATGTTTAGGGGATCTGAGAGGATGGCCCCCCAC
ACTGGTACTGAGACACGGAC
>Zotu3888
TAACGCTGGCGGCGTGCCTAATACATGCAAGTCGAACGGAACACTATTTTTGAGAACCTCGCTCCTTTGCG
GGATTGTCATCAGTAGTGTTTAGTGGCGGACGGGTGAGTAACACGTTGGTACCTGCCCTTTAGTGGGGAAT
AACTCGGCGAAAGTCGGGCTAATACCGCATGTGATCCTTCGGGATTAAAGCCTTCGGGCGCTAAAGGAGG
GGCCTGCGTCTGATTAGCTTGTTGGTAAGGTAATGGCTTACCAAGGCGATGATCAGTAGCTGGTTCGAGAG
GATGATCAGCCACACTGGAACTGAGATACGGTC
>Zotu3890
GAACGCTGGCGGCGTGCTTAACACATGCAAGTCGAACGATGAACCACCTTCGGGTGGGGATTAGTGGCGA
ACGGGTGAGTAACACGTGGGCAATCTGCCCTGCACTCTGGGACAAGCCCTGGAAACGGGGTCTAATACCG
GATACTGACCTGCCAAGGCATCTTGGCGGGTCGAAAGCTCCGGCGGTGCAGGATGAGCCCGCGGCCTATC
AGCTTGTTGGTGAGGTAATGGCTCACCAAGGCGACGACGGGTAGCCGGCCTGAGAGGGCGACCGGCCAC
ACTGGGACTGAGACACGGCC
>Zotu3893
GAACGCTGGCGGCGTGCTTAACACATGCAAGTCGAGCGATGAAGTTTCCTTCGGGAAACGGATTAGCGGC
GGACGGGTGAGTAACACGTGGGTAACCTGCCTCATAGAGTGGGATAGCCTTCCGAAAGGAAGATTAATAC
CGCATAATGTTGAAAGATGGCATCATCATTTAACCAAAGGAGCAATCCGCTATGAGATGGACCCGCGGCGC
ATTAGCTAGTTGGTGGGGTAACGGCCTACCAAGGCGACGATGCGTAGCCGACCTGAGAGGGTGATCGGCC
ACATTGGGACTGAGACACGGCC
>Zotu3897
GAACGCTGGCGGCAGGCCTAACACATGCAAGTCGAGCGGCAGCGGACCTTCGGGTGCCGGCGAGCGGCG
GACGGGTGAGTAAGGCATCGGAATCTGCCCTGTTGTGGGGGATAACGTAGGGAAACTTACGCTAATACCG
CATACGACCTACGGGTGAAAGCGGGGGACCTTCGGGCCTCGCGCAGCAGGATGAGCCGATGTCCGATTAG
CTAGTTGGTGGGGTAAAGGCCCACCAAGGCGACGATCGGTAGCCGGTCTGAGAGGATGATCGGCCACACT
GGGACTGAGACACGGCC
>Zotu3900
GAACGCTAGCGGCAGGCTTAATACATGCAAGTCGAACGGCAAGGATCTTCGGATCCCTAGAGTGGCGCAC
GGGTGAGTAACACGTACACAACATACCCTATATTGGAGGATAGCCTTGGGAAACTGGGGGTAATATTCCAT
AGTATTTATTGGAGGCATCTCCTGATAAATTAAAGAGCAATCGATATAGGATTGGTGTGCGTCCCATTAGCT
AGTTGGTAGGGTAACGGCCTACCAAGGCTACGATGGGTAGCTGGCGTGAGAGCGCGACCAGCCACACTG
GAACTGAGACACGGTC
>Zotu3902
CAACGCTGGCGGCGTGCCTCAGACATGCAAGTCGAACGATTAAGACTTCCTTCGGGAAGTGTATAAAGTG
GCGCACGGGTGAGTAACACGTAAGTAATCTACCTTCGAGTGGGGAATAACCCGGGGAAACCCGAGCTAAT
ACCGCATAACGCAGCGGCATCGCAAGATGACAGTTGTTAAAGGAATTTATTTCGCTTGAAGAGGAGCTTGC
GGCAGATTAGCTAGTTGGTAAGGTAACGGCTTACCAAGGCTACGATCTGTATCCGGTCTAAGAGGACGGT
CGGACACACTGACACTGAATAACGGGT
>Zotu3904
GAACGCTGGCGGCAGGCCTAATACATGCAAGTCGAACGCTGTAGCAATACAGAGTGGCGCACGGGTGAG
TAATGCATGGGAACATACCCCTTAGTACGGAACAACACCGGGAAACTGGTGCTAATACCGTATAATGTCTA
CGGACCAAAGATTTATCGCTGAGGGATTGGCCCATGTTGGATTAGCTTGTTGGCGAGGTAATGGCTCACCA
AGGCGACGATCCATAGCTGGTCTGAGAGGATGATCAGCCACACTGGGACTGAGACACGGCC
>Zotu3909
AAACGCTGGCGGCGTGCCTAACACATGCAAGTCGAACGCACTCTCGCGAGAGAGTGAGTGGCGGACGGG
TGAGTAACACGTGGGTACCTACCGTGAGGTGAGGAATACCACCGAGAAATCGGTGACAAGACCGCATATG
CTCGCAAGAGCAAAGTTTCTTTTCGGAGAGACGCCACACGATGGGCCTGCGCCTGATTAGCTGGTTGGTGA
GGTAATGGCTCACCAAGGCGACGATCAGTAGCTGGTCTGAGAGGACGATCAGCCACACTGGGATTGAGAA
CGGCC

>Zotu3911
GAACGCTGGCGGCGTGCTTAACACATGCAAGTCGAACGGTGAAAGAGAGCTTGCTCTCTGGATCAGTGGC
GAACGGGTGAGTAACACGTGAGCAATCTGCCCCTGACTCTGGGATAAGCGTTGGAAACGACGTCTAATAC
CGGATACGAGCTGCGAAGGCATCTTCAGCAGCTGGAAAGAACTTCGGTCAGGGATGAGCTCGCGGCCTAT
CAGCTAGTTGGTGAGGTAATGGCTCACCAAGGCGTCGACGGGTAGCCGGCCTGAGAGGGTGACCGGCCA
CACTGGGACTGAGACACGGCC
>Zotu3913
GAACGCTGGCGGCAGGCTTAACACATGCAAGTCGAACGGGCGTAGCAATACGTCAGTGGCAGACGGGTG
AGTAACACGTGGGAACGTACCTTTTGGTTCGGAACAACACAGGGAAACTTGTGCTAATACCGGATAAGCCC
TTACGGGGAAAGATTTATCGCCGAAAGATCGGCCCGCGTCTGATTAGCTAGTTGGTAGGGTAACGGCCTAC
CAAGGCGACGATCAGTAGCTGGTCTGAGAGGATGATCAGCCACATTGGGACTGAGACACGGCC
>Zotu3915
GAACGCTGGCGGCGTGCCTAATACATGCAAGTCGAGCGGACCGGATTGGGGCTTGCCTTGATTCGGTCAG
CGGCGGACGGGTGAGTAACACGTGGGCAACCTGCCCGCAAGACCGGGATAACTCCGGGAAACCGGAGCT
AATACCGGATAACACCGAAGACCGCATGGTCTTCGGTTGAAAGGCGGCCTTTGGGCTGTCACTTGCGGATG
GGCCCGCGGCGCATTAGCTAGTTGGTGAGGTAACGGCTCACCAAGGCGACGATGCGTAGCCGGCCTGAGA
GGGTGACCGGCCACACTGGGACTGAGACACGGCC
>Zotu3917
GAACGCTGGCGGCATGCCTAACACATGCAAGTCGAACGAGACCTTCGGGTCTAGTGGCGCACGGGTGCGT
AACGCGTGGGAATCTGCCCTTGGGTTCGGAATAACTCGCCGAAAGGCGTGCTAATACCGGATGATGTCGT
AAGACCAAAGATTTATCGCCCAGGGATGAGCCCGCGTAAGATTAGGTAGTTGGTGAGGTAAAGGCTCACC
AAGCCGACGATCTTTAGCTGGTCTGAGAGGATGATCAGCCACACTGGGACTGAGACACGGCC
>Zotu3919
GAACGCTGGCGGCATGCTTTACACATGCAAGTCGAACGGCAGCGCGGGGCAACCTGGCGGCGAGTGGCG
AACGGGTGAGTAATATATCGGAACGTACCCAAGAGTGGGGGATAACGTAGCGAAAGTTACGCTAATACCG
CATACGATCTAAGGATGAAAGCAGGGGACCGCAAGGCCTTGTGCTCCTGGAGCGGCCGATATCTGATTAG
CTAGTTGGTGAGGTAAAGGCTCACCAAGGCGACGATCAGTAGCTGGTCTGAGAGGACGACCAGCCACACT
GGGACTGAGACACGGCC
>Zotu3923
GAACGCTGGCGGCGTGCCTAATACATGCAAGTCGAACGAAACTTCTTTATCACCGAGTGCTTGCACTCACC
GATAAAGAGTTGAGTGGCGAACGGGTGAGTAACACGTGGGCAACCTGCCCAAAAGAGGGGGATAACACT
TGGAAACAGGTGCTAATACCGCATAACCATAGTTACCGCATGGTAACTATGTAAAAGGTGGCTATGCTACC
GCTTTTGGATGGGCCCGCGGCGCATTAGCTAGTTGGTGGGGTAAAGGCTTACCAAGGCAATGATGCGTAG
CCGAACTGAGAGGTTGATCGGCCACATTGGGACTGAGACACGGCC
>Zotu3926
GAACGCTAGCGGCAGGCTTAACACATGCAAGTCGAGGGGCAGCGCGGTAGCAATACTGGCGGCGACCGG
CAAACGGGTGCGGAACACGTACAGAACCTACCTTGATCTGGGGCATAGCCCGGAGAAATCCGGATTAACT
CCCCATAATATACACGAGTGGCATCACTTGAGTATTAAAAATTTATTGGATCGAGATGGCTGTGCGTCCCAT
TAGCTAGTTGGTAGGGTAACGGCCTACCAAGGCAACGATGGGTAGCTGGTGTGAGAGCACGACCAGCCAC
ACGGGCACTGAGACACGGGC
>Zotu3930
GAACGCTGGCGGCGTGCTTAACACATGCAAGTCGAACGATGAAGCCGGAGCTTGCTCTGGTGGATTAGTG
GCGAACGGGTGAGTAACACGTGAGTAACCTACCCTTGACTCTGGGATAAGCCCGGGAAACTGGGTCTAAT
ACCGGATATGAGTCATCACCGCATGGTGGTGGTTTGAAAGATTTATCGGTCTTGGATGGACTCGCGGCCTA
TCAGCTTGTTGGTGAGGTAATGGCTCACCAAGGCGACGACGGGTAGCCGGCCTGAGAGGGTGACCGGCC
ACACTGGGACTGAGACACGGCC
>Zotu3932
GAACGCTGGCGGCAGGCCTAACACATGCAAGTCGAACGCTGTAGCAATACAGAGTGGCAGACGGGTGAG
TAACACGTGGGAACATGCCCTTCGGTTCGGAATAACTCAGGGAAACTTGAGCTAATACCGGATACGTCCGC

AAGGAGAAAGATTTATCGCCGAAGGATTGGCCCGCGTCTGATTAGCTTGTTGGTGAGGTAATGGCTCACCA
AGGCGACGATCAGTAGCTGGTCTGAGAGGATGATCAGCCACATTGGGACTGAGACACGGCC
>Zotu3935
GAACGCTGGCGGCATGCCTTACACATGCAAGTCGAACGGTAACGCGGGGCAACCTGGCGACGAGTGGCG
AACGGGTGAGTAATATATCGGAACGTGCCCAGTAGTGGGGGATAGCCCGGCGAAAGCCGGATTAATACC
GCATGAGACCTGAGGGTGAAAGCGGGGGATCGCAAGACCTCGCGCTATTGGAGCGGCCGATATCAGATT
AGCTAGTTGGTGGGGTAAAGGCCTACCAAGGCAACGATCTGTAGTTGGTCTGAGAGGACGACCAGCCACA
CTGGGACTGAGACACGGCC
>Zotu3937
GAACGCTGGCGGCGTGCTTAACACATGCAAGTCGAGCGGTAAGGCCCTTCGGGGTACACGAGCGGCGAA
CGGGTGAGTAACACGTGGGTGACCTGCCCTCCACTTCGGGATAAGCCTGGGAAACTGGGTCTAATACCGG
ATAGGACCATTCCATGCATGTGGTTTGGTGGAAAGTTTTTTCGGTGGGGGATGGGCCCGCGGTCTATCAGC
TTGTTGGTGGGGTGATGGCCTACCAAGGCGACGACGGATAGCCGGCCTGAGAGGGTGATCGGCCACACT
GGGACTGAGACACGGCC
>Zotu3939
GAACGCTGGCGGCAGGCTTAACACATGCAAGTCGAACGCCCGAGCAATCGGGAGTGGCAGACGGGTGAG
TAACACGTGGGAACATACCCTTCGGTTCGGAACAACGTTTGGAAACGAACGCTAATACCGGATACGGCCTC
TGGGCGAAAGATTCATTGCCGAAGGATTGGCCCGCGTTCGATTAGCTTGTTGGTGAGGTAACGGCTCACCA
AGGCGACGATCGATAGCTGGTCTGAGAGGATGGCCAGCCACATTGGGACTGAGACACGGCC
>Zotu3942
GAACGCTGGCAGTGTGCCTAAGACATGCAAGTCGAACGGGAGTCTTCGGACTCTAGTGGCGAACGGGTGA
GTAACACGTGGGCGACCTACCCCCAAGTACGGCATAACCCCGGGAAACCGGAGCTAATTCCGTATGTGCTT
ACGTCTCATTTGACGTACGTAAAGATTTATCGCTTGGGGATGGGCCCGCGGCGCATCAGCTAGTTGGTGGG
GTAACGGCCTACCAAGGCGACGACGCGTAGCCGACCTGAGAGGGTGACCGGCCACAGGGGCACTGAGAC
ACGGGC
>Zotu3944
GAACGCTGGCGGTATGCTTAACACATGCAAGTCGAACGGATATAGCAATATATTAGTGGCGAACGGGTGA
GTAATGCAAAGGAATCTACCTAGCAGTAAGGGATAACTTTGGGAAACCAAAGCTAATACCATATATTACCG
CCTGATTAACTTCAGGGGGGAAAGATTTATCGCTGTTAGATGAGCCTTTGTCGGATTAGCTAGTTGGTAGG
GTAATGGCCTACCAAGGCTATGATCCGTAGCTGGTCTGAGAGGATGATCAGCCACACTGGAACTGAGACA
CGGTC
>Zotu3946
GAACGCTGGCGGCGTGCCTAATACATGCAAGTCGAGCGGACGAATTTTTGGTGCTTGCACCAGACTTTCGT
TAGCGGCGGACGGGTGAGTAACACGTGGGTAACCTACCCTATAGACTGGGATAACTTCGGGAAACCGAGG
CTAATACCGGATAATACTTGAAACCACATGGTTTCTTGTTAAAAGATGGTTCTGCTATCACTATAGGATGGA
CCCGCGGCGCATTAGCTAGTTGGTGAGGTAACGGCTCACCAAGGCGACGATGCGTAGCCGACCTGAGAGG
GTGATCGGCCACACTGGGACTGAGACACGGCC
>Zotu3948
GAACGCTGGCGGCATGCCTTACACATGCAAGTCGAACGGTAACAGGCCGCAAGGTGCTGACGAGTGGCG
AACGGGTGAGTAATGCATCGGAACGTGCCCAGTAGTGGGGGATAGCCCGGCGAAAGCCGGATTAATACC
GCATACGACCTGAGGGTGAAAGGGGGGGATCGCAAGACCTCTCGCTATTGGAGCGGCCGATGTCAGATTA
GCTAGTTGGTGGGGTAAAGGCCTACCAAGGCAACGATCTGTAGTTGGTCTGAGAGGACGACCAGCCACAC
TGGGACTGAGACACGGCC
>Zotu3950
GAACGCTGGCGGCGTGCCTAATACATGCAAGTCGAGCGAGCAGAACCAGCAGATTTACTTCGGTAATGAC
GCTGGGGACGCGAGCGGCGGATGGGTGAGTAACACGTGGGGAACCTGCCCCATAGTCTGGGATACCACT
TGGAAACAGGTGCTAATACCGGATAAGAAAGCAGATCGCATGATCAGCTTATAAAAGGCGGCGTAAGCTG
TCGCTATGGGATGGCCCCGCGGTGCATTAGCTAGTTGGTAAGGTAACGGCTTACCAAGGCAATGATGCATA
GCCGAGTTGAGAGACTGATCGGCCACATTGGGACTGAGACACGGCC
>Zotu3952

GAACGCTGGCGGCGTGCCTAAGACATGCAAGTCGAGTGGGTTTAGGCCCACGGCAAACGGGTGAGTAAT
ATCTAGGAACGTACCACTAGTTGAGCATAACCTACCGAAAGGTAGAGCAATTCTCAATGTGACCGCAAGGT
TAAAGTCGCAAGACGCTAGTGAAGCGGCCTGGACACCATCAGCTTGTTGGTAAGGTAAAAGCTTACCAAG
GCTATGACGGTTAGGGGCCCCGAGAGGGGAAACCCCCACACTGGAACTGAGACACGGTC
>Zotu3954
GAACGCTGGCGGCATGCCTAATACATGCAAGTCGAACGATGATATCACTGCTTGCAGTGATTGATTAGTGG
CGAACGGGTGAGTAACACGTGAGGAACTTGCCCATGAGCGGGGGACAACATTCGGAAACGGATGCTAAT
ACCCCATAGGTGGATTGGTCGCATGACTAATTCATTAAAGGTGGCTCTGCTACCACTCATGGATAGCCTCGC
GGCGTATTAGCTAGTTGGTAAGGTAATGGCTTACCAAGGCAGTGATACGTAGCCGACTTGAGAGGGTGAT
CGGCCACACTGGGACTGAGACACGGCC
>Zotu3956
GAACGCTAGCTACAGGCTTAACACATGCAAGTCGAGGGGCAGCATGACGATAGCTTGCTATTGTTGATGG
CGACCGGCGCACGGGTGAGTAACGCGTATCCAACCTGCCCTTGTCCATCGGATAACCCGTCGAAAGGCGG
CCTAACACGATATGCAGTTCTCAGCAGGCATCTAACGAGAACGAAATGTGAAGGAGAAGGATGGGGATGC
GTCTGATTAGCTTGTTGGCGGGGTAACGGCCCACCAAGGCGACGATCAGTAGGGGTTCTGAGAGGAAGGT
CCCCCACATTGGAACTGAGACACGGTC
>Zotu3959
GAACGCTGGCGGCATGCCTAACACATGCAAGTCGAACGAAGGCTTCGGCCTTAGTGGCGCACGGGTGCGT
AACGCGTGGGAATCTGCCCCTCGGTTCGGAATAACAGCGAGAAATTGCTGCTAATACCGGATAATGACGTA
AGTCCAAAGATTTATCGCCGAGGGATGAGCCCGCGTAGGATTAGCTAGTTGGTGTGGTAAAGGCGCACCA
AGGCGACGATCCTTAGCTGGTCTGAGAGGATGATCAGCCACACTGGGACTGAGACACGGCC
>Zotu3961
GAACGCTAGCTACAGGCTTAACACATGCAAGTCGAGGGGCAGCATGTCGGTTGCTTGCAACCGACGATGG
CGACCGGCGCACGGGTGAGTAACGCGTATCCAACCTACCCTTGTCCATCGGATAACCCGTCGAAAGGCGG
CCTAACACGATATGCGGTTCTCAGCAGGCATCTAACGAGAACGAAATGTGAAGGAGAAGGATGGGGATGC
GTCTGATTAGCTTGTTGGCGGGGTAACGGCCCACCAAGGCGACGATCAGTAGGGGTTCTGAGAGGAAGGT
CCCCCACATTGGAACTGAGACACGGTC
>Zotu3963
GAACGCTGGCGGCGTGCTTCATACATGCAAGTCGAACGAGAAGTTACCTTCGGGTGATGGAAAGTGGCGG
ACGGGTGAGTAATATGTAGAGAATCTGCCCTTGAGAGGGGGACAACAGAGGGAAACTTCTGCTAATACCC
CATATGAGCGTAGCTGAAATGCTATTCTTGAAAACTCCGGTGCTCAAGGATGAGTCTGCATCTGATTAGCTA
GTTGGTGGTGTAATGGACTACCAAGGCGACGATCAGTAGCTGGTTTGAGAGGATGATCAGCCACAATGGG
ACTGAGACACGGCC
>Zotu3967
GAACGCTAGCGGCAGGCTTAACACATGCAAGTCGAGGGGTATAGTTCTTCGGAATTAGAGACCGGCGCAC
GGGTGCGTAACGCGTATGCAATCTACCTTTCACAGAGGGATAGCCCAGAGAAATTTGGATTAATACCTCAT
AGTATATAGAGTTGGCATCAACATTATATTAAAGTCACAACGGTGAAAGATGAGCATGCGTCCCATTAGCT
AGTTGGTAAGGTAACGGCTTACCAAGGCTACGATGGGTAGGGGTCCTGAGAGGGAGATCCCCCACACTGG
TACTGAGACACGGAC
>Zotu3969
GAACGCTGGCGGCAGGCTTAACACATGCAAGTCGAACGCCCCGCAAGGGGAGTGGCAGACGGGTGAGTA
ACGCGTGGGAATCTACCCAGTTCTACGGAACAACTGAGGGAAACTTCAGCTAATACCGTATACGCCCTACG
GGGGAAAGATTTATCGGAATTGGATGAGCCCGCGTTGGATTAGCTAGTTGGTGGGGTAATGGCCTACCAA
GGCGACGATCCATAGCTGGTCTGAGAGGATGATCAGCCACACTGGGACTGAGACACGGCC
>Zotu3973
GAACGCTAGCGGCAGGCCTAATACATGCAAGTCGAACGAGCTACAGGACTTCGGTTCTGTGGTTAGTGGC
GCACGGGTGCGTAACGCGTATGCAACTTACCTTCCACAGGGGGATAGCCCACCGAAAGGTGGATTAATAC
CGCATAACATCATTTGAGGGCATCCGAAGATGATCAAAGCTGCGGCGGTGGAAGATGGGCATGCGTGCCA
TTAGCTAGTTGGTAGGGTAACGGCCTACCAAGGCGACGATGGCTAGGGGTTCTGAGAGGATGGTCCCCCA
CACTGGTACTGAGACACGGAC

>Zotu3976
GAACGCTGGCGGCGTGCTTAACACATGCAAGTCGAACGGAAAGGCTCTCTTCGGAGAGTACTCGAGTGGC
GAACGGGTGAGTAACACGTGGGTAACCTACCTTTGACTGGGGGACAACCGGGGGAAACTCCGGCTAATAC
CGCATAACGTGCGACGTCATAAGGCGACGTACCAAAGATAGCTTCGGCTTTCGGTTAAAGATGGGCCCGC
GGCGGATTAGCTTGTTGGTGGGGTAACGGCCTACCAAGGCTACGATCCGTAGCTGGTCTGAGAGGACGAT
CAGCCACACTGGAACTGAGACACGGTC
>Zotu3979
GAACGCTGGCGGCGTGCCTAACACATGCAAGTCGTGCGGGAATACGGGAGCAATCCTGTTAGTACAGCGG
CGCACGGGTGAGTAACACGTAGGTAACCTGCCTTGGAGCCTGGGATAACCTGTCGAAAGATGGGCTAATA
CCAGATAAGACCACGAGAACCTCGGTTCTTGGGGTAAAAGATGGCCTCTGTTTACAAGCTATCACTCGGAG
ATGGGCCTGCGTGCCATTAGCTAGTTGGTGAGGTAACGGCTCACCAAGGCAGAGATGGCTAGCTGGTCTG
AGAGGATGGCCAGCCACACTGCGACTGAGACACGGCC
>Zotu3981
GAACGCTGGCGGCGTGCTTAACACATGCAAGTCGAGCGGAAAGGCCTCTTCGGGGGTACTCGAGCGGCG
AACGGGTGAGTAACACGTGAGTAATCTGCCCTTCACACATGGATAACCACCGGAAACGGTGGCTAATACAT
GATACGACACGTCTTCGCATGAGGTGCGTGTGGAAAGTTTTTTCGGTGGAGGATGTGCTCGCGGCCTATCA
GCTTGTTGGTGGGGTAATGGCCTACCAAGGCTTCGACGGGTAGCCGGCCTGAGAGGGTGACCGGCCACAC
TGGGACTGAGACACGGCC
>Zotu3983
GAACGCTGGCGGCATGCTTTACACATGCAAGTCGAACGGCAGCGCGGGGCAACCTGGCGGCGAGTGGCG
AACGGGTGAGTAATATATCGGAACGTACCCAAGAGTGGGGGATAACGTAGCGAAAATTACGCTAATACCG
CATAAGATCTAAGGATGAAAGCGGGGGATCGCAAGACCTCGTGCTCCTGGAGCGGCCGATATCTGATTAG
CTAGTTGGTGAGGTAAAGGCTCACCAAGGCGACGATCAGTAGCTGGTCTGAGAGGACGACCAGCCACACT
GGAACTGAGACACGGTC
>Zotu3985
GAACGCTGGCGGCGTGCTTAACACATGCAAGTCGAGCGGAAAGGCCCTTTCGGGGGTACTCGAGCGGCG
AACGGGTGAGTAACACGTGAGTAATCTGCCCTTCACTTGGGGATAACCTCCGGAAACGGTGGCTAATACCC
GATACGACCATGGATCGCATGATCTTGTGGTGGAAAGTTTTTTCGGTGGGGGATGTGCTCGCGGCCTATCA
GCTTGTTGGTGGGGTAATGGCCTACCAAGGCTTCGACGGGTAGCCGGCCTGAGAGGGTGACCGGCCACAC
TGGGACTGAAACACGGCC
>Zotu3989
GAACGCTGGCGGCGTGCCTAATACATGCAAGTCGAGCGGAACTTTGATTGGTGCTTGCACCGATTAAAGTT
TAGCGGCGGACGGGTGAGTAACACGTGGGTAACCTGCCCTACAGACTGGGATAACTTCGGGAAACCGAG
GCTAATACCGGATAATACTTTTTAACACATGTTAGAAAGTTGAAAGATGGTTTCGGCTATCACTGTAGGATG
GACCCGCGGCGCATTAGCTAGTTGGTGAGGTAACGGCTCACCAAGGCAACGATGCGTAGCCGACCTGAGA
GGGTGATCGGCCACACTGGGACTGAGACACGGCC
>Zotu3992
GAACGCTGGCGGCGTGCTTAACACATGCAAGTCGAGCGGTAAGGCCCTTCGGGGTACACGAGCGGCGAA
CGGGTGAGTAACACGTGGGTGACCTGCCCTCAGCTCCGGGATAAGCCTGGGAAACCGGGTCTAATACCGG
ATACGACCATTGGATGCATGTCCGGTGGTGGAAAGTTTTTCGGCTGGGGATGGGCCCGCGGCCTATCAGCT
TGTTGGTGGGGTGATGGCCTACCAAGGCGGTGACGGGTAGCCGGCCTGAGAGGGCGACCGGCCACACTG
GGACTGAGACACGGCC
>Zotu3996
GAACGCTGGCGGCGCGCTTAACACATGCAAGTCGAGCGATCGGCCGACCCTTCGGGGACGGTACGGAGC
GGCGAACGGGTGAGTAACACGTGGGTAATCTGCCCTCGGCACTGGGATAGCCCGGGGAAACCCGGATTA
ATACCGGATGGTCTCCGGAGCCTGCGGGCGCCGGAGAAAAGGAAGCTTCGGCCTCCGACCGAGGATGAG
CCCGCGGCGGATTAGCTTGTTGGTGGGGTAAAGGCCCACCAAGGCGACGATCCGTAGCTGGTGTGAGAGC
ATGACCAGCCACACTGGGACTGAGACACGGCC
>Zotu3998

GAACGCTGGCGGCGTGCCTAATACATGCAAGTCGAGCGGACCTGAATGGAGCTTGCTCCAGACAGGTTAG
CGGCGGACGGGTGAGTAACACGTAGGCAACCTGCCCGCAAGACCGGGATAACGTAGGGAAACTTACGCT
AATACCGGATAGGGCACTCTATCGCATGAGAGAGTGCGGAAAGGTGGCGCAAGCTACCACTTGCGGATGG
GCCTGCGGCGCATTAGCTAGTTGGTGGGGTAACGGCCTACCAAGGCGACGATGCGTAGCCGACCTGAGAG
GGTGACCGGCCACACTGGGACTGAGACACGGCC

>Zotu4000
GAACGCTAGCGGCAGGCCTAATACATGCAAGTCGAACGATAGTGAAGGGCTTGCTCTTCACGAAAAGTGG
CGCACGGGTGCGTAACGCGTATGCAACCTACCTTAATCAGGGGGATAGCCCGAAGAAATTCGGATTAACA
CCGCATAAAATCACAGTATAGCATTATACAATGATCAAATATTTATAGGATTAAGATGGGCATGCGTGACAT
TAGTTAGTTGGCGAGGTAACGGCTCACCAAGACCACGATGTCTAGGGGATCTGAGAGGATGACCCCCCAC
ACTGGTACTGAGACACGGAC

>Zotu4003
GAACGCTGGCGGCAGGCTTAACACATGCAAGTCGAGCGAGCCCTTCGGGGTCAGCGGCGGACGGGTGAG
TAACGCGTGGGAACGTGCCTTCTGGTCTGGAATAACCCTGGGAAACTAGGACTAATACCGGATACGCCCTT
TTGGGGAAAGGTTTACTGCCGGAAGATCGGCCCGCGTCTGATTAGCTAGTTGGTGGGGTAACGGCCTACC
AAGGCGACGATCAGTAGCTGGTCTGAGAGGATGATCAGCCACACTGGGACTGAGACACGGCC

>Zotu4005
GAACGCTGGCGGCGTGGATTAGGCATGCAAGTCGAGCGATCTCGCAAGAGATAGCGGCAAACGGGTGAG
GAACACGTAAATAATCTTCCCCCTATCTGGACATAGCCCTATGAAAATAGGAGTAATATCCAATGACAACAT
TTAGCAGCATTGCTTTATGTTTAAAGACAGGGACCGCAAGGCCTGTCGGTAAGGGATGAGTTTGCGTAGCA
TCAGCTAGTTGGTAGGGTAATGGCCTACCAAGGCTATGACGCTTAGCTGGTCTGAGAGGATGGTCAGCCA
CACTGGGACTGAGACACTGCC

>Zotu4008
GAACGCTGGCGGCGTGCCTAACACATGCAAGTCGAGCGATTCTCTTCGGAGAAGAGCGGCGGACGGGTG
AGTAACGCGTGGGTAACCTGCCCTGTACACACGGATAACATACCGAAAGGTATGCTAATACGAGATAATAT
ACTTTTATCGCATGATAGAAGTATCAAAGCTCCGGCGGTACAGGATGGACCCGCGTCTGATTAGCTAGTTG
GTAAGGTAACGGCTTACCAAGGCGACGATCAGTAGCCGACCTGAGAGGGTGATCGGCCACATTGGAACTG
AGACACGGTC

>Zotu4010
AAACGCTGGCGGCGTGCATAACACATGCAAGTCGAACGATGAAACTTAAATGATTTCTTCGGAATGAAATT
AAGTGGATTAGTGGCGAACGGGTGAGTAACACGTGAGTAACCTGCCTTACACAAGGGGATAGCCTTTGGA
AACGAAGAATAATACCCTATAAAGTCAAAATATCGCATGATAAAGTGACCAAAGATTTATCGGTGTAAGAT
GGACTTGCGTCTGATTAGCTAGTTGGTGAGATAAAAGCCCACCAAGGCGACGATCAGTAGCCGGCTTGAG
AGAGTGTACGGCCACATTGGGACTGAGACACGGCC

>Zotu4012
GAACGCTGGCGGCGTGCCTAATACATGCAAGTCGAGCGAATTTTTGGGAGCTTGCTCCCGAAGGTTAGCG
GCGGACGGGTGAGTAACACGTGGGCAACCTGCCTATAAGACTGGGATAACTTCGGGAAACCGGAGCTAAT
ACCGGATAATTCTTTTCTACACATGTAGAAAAGCTAAAAGACGGTTTACGCTGTCACTTATAGATGGGCCCG
CGGCGCATTAGCTAGTTGGTGAGGTAACGGCTCACCAAGGCCACGATGCGTAGCCGACCTGAGAGGGTGA
TCGGCCACACTGGGACTGAGACACGGCC

>Zotu4015
GAACGCTGGCGGCATGCCTAACACATGCAAGTCGAACGAGACCTTCGGGTCTAGTGGCGCACGGGTGCGT
AACGCGTGGGAATCTGCCCTTGGGTACGGAATAACTCTCCGAAAGGAGTGCTAATACCGTATGATGTCGCA
AGACCAAAGATTTATCGCCCAGGGATGAGCCCGCGTAGGATTAGCTAGTTGGTGAGGTAAAGGCTCACCA
AGGCGACGATCCTTAGCTGGTCTGAGAGGATGATCAGCCACACTGGGACTGAGACACGGCC

>Zotu4020
GAACGCTAGCTACAGGCTTAACACATGCAAGTCGAGGGGAAACGACATCGAAAGCTTGCTTTTGATGGGC
GTCGACCGGCGCACGGGTGAGTAACGCGTATCCAACCTGCCCATCACTTGGGGATAACCTTGCGAAAGTA
AGACTAATACCCAATGATATCTCTAGAAGACATCTGAAAGGGATTAAAGATTTATCGGTGATGGATGGGGA
TGCGTCTGATTAGCTTGTTGGCGGGGTAACGGCCCACCAAGGCGACGATCAGTAGGGGTTCTGAGAGGAA
GGTCCCCCACATTGGAACTGAGACACGGTC

>Zotu4022
AAACGCTGGCGGCGTGCTTAACACATGCAAGTCGAACGAAGTTTTTCATTCGTGGAAAACTTAGTGGCGGA
CGGGTGAGTAACGCGTGGGTAACCTGCCCTGTACAGGGGGACAACAGCTGGAAACGGCTGCTAATACCGC
ATAAGCCCTTAGCACTGCATGGTGCATAGGGAAAAGGAGCAATCCGGTACAGGATGGACCCGCGTCTGAT
TAGCCAGTTGGCAGGGTAACGGCCTACCAAAGCGACGATCAGTAGCCGATCTGAGAGGATGTACGGCCAC
ATTGGGACTGAGACACGGCC
>Zotu4024
GAACGCTGGCGGCGTGCTTAACACATGCAAGTCGAGCGGTAAGGCACTTCGGTGTACACGAGCGGCGAAC
GGGTGAGTAACACGTGAGTAATCTGCCCTTCACTCTGGGATAAGCACTAGAAATGGTGTCTAATACCGGAT
ACGACCAATCCCTGCATGGGGTGTTGGTGGAAACTTTTGTGGTGGGGGATGTGCTCGCGGCCTATCAGC
TTGTTGGTGGGGTAATGGCCTACCAAGGCTTCGACGGGTAGCCGGCCTGAGAGGGTGACCGGCCACACTG
GGACTGAGACACGGCC
>Zotu4026
GAATGCTGGCGGCATGCCTTACACATGCAAGTCGAACGGCAGCGCGGACTTCGGTCTGGCGGCCAGTGGC
GAACGGGTGAGTAATACATCGGAACGTACCCTGTTGTGGGGGATAACTAGTCGAAAGATTAGCTAATACT
GCATATGACCTGAGGGTGAAAGCGGGGGACCGTAAGGCCTCGCGCAGCAGGAGCGGCCGATGTCTGATT
AGCTAGTTGGTGGGGTAAAGGCCCACCAAGGCGACGATCAGTAGCTGGTCTGAGAGGACGATCAGCCAC
ACTGGGACTGAGACACGGCC
>Zotu4028
GAACGCTGGCGGCGTGCCTAATACATGCAAGTCGAACGGTGCAGCAATGTACAGTGGCGACCGGGTGAGT
AACACGTAGTTGACCTACCCTTCCGCGGGGGACAACAGTTCGAAAGGACTGCTAATACCGCATACATTGGC
AGAGTTTAGAAGTCTGTCAAGAAACTTCAAGGGCGGAAGGAGGGGGCTGCGGCCCATCAGGTAGTTGGT
GAGGTAGTGGCTCACCAAGCCGACGACGGGTAGGGGGCCTGAGAGGGTGACCCCCCACACTGGAACAGA
CATACGGTC
>Zotu4030
GAACGCTGGCGGCGTGCTTAACACATGCAAGTCGAGCGAGGCCCGTTCCTTCGGGAACGGTGCCCTAGCG
GCGAACGGGTGAGTAACACGTGGGCAACCTGCCCCTAGCACTGGGATAACTCCGGGAAACCGGGGCTAAT
ACCGGATACGACCTCTGAGGGCATCCTCGGAGGTGGAAAGGATTCTGGCTAGGGATGGGCCCGCGGCCTA
TCAGCTTGTTGGTGGGGTAATGGCCTACCAAGGCGACGACGGGTAGCCGGCCTGAGAGGGCGACCGGCC
ACACTGGGACTGAGACACGGCC
>Zotu4032
GAATGCTGGCGGTGTGCCTTAAGCATGCAAGTCGAGCGGCTCGCAAGAGCAGCGGCAGACGGGTGAGTA
ACACGTAGGAATCTACCTCAAAGTGGGGAATAGCCAGCCGAAAGGTTGGGTAATACCGCATGTGATCGAA
AGATTAAAGCAGCAATGCGCTTTGAGAGGAGCCTGCGTCCTATCAGCTAGTTGGTGAGGTAATAGCTTACC
AAGGCAATGACGGGTAGCCGATGTGAGAGCATGATCGGCCACAAGGTCACTGAGACACGGGA
>Zotu4037
GAACGCTGGCGGCATGCCTAACACATGCAAGTCGAACGAGACCTTCGGGTCTAGTGGCGCACGGGTGCGT
AACGCGTGGGAATCTGCCCCTTGGTTCGGAATAACAGTTAGAAATGACTGCTAATACCGGATGATGACTTC
GGTCCAAAGATTTATCGCCAAGGGATGAGCCCGCGTAGGATTAGCTAGTTGGTGGGGTAAAGGCCTACCA
AGGCGACGATCCTTAGCTGGTCTGAGAGGATGATCAGCCACACTGGGACTGAGACACGGCC
>Zotu4039
GAACGCTGGCGGCGTGCCTAATACATGCAAGTCGAGCGAACCTTTGGGAGCTTGCTCCCAAAGGTTAGCG
GCGGACGGGTGAGTAACACGTGGGCAACCTGCCTATAAGACTGGGATAACTTCGGGAAACCGGAGCTAAT
ACCGGATAACTCATATCTACTCATGTAGATATGCTAAAAGACGGTTTCGGCTGTCACTTATAGATGGGCCCG
CGGCGCATTAGCTAGTTGGTGAGGTAACGGCTCACCAAGGCGACGATGCGTAGCCGACCTGAGAGGGTG
ATCGGCCACACTGGGACTGAGACACGGCC
>Zotu4043
GAACGCTAGCGGCAGGCTTAATACATGCAAGTCGAGGGGCAGCATGGTGTAGCAATACACTGATGGCGAC
CGGCAAACGGGTGCGGAACACGTACGCAACCTTCCTTCAACTGAGGAATAGCCCAGGGAAACTTGGATTA
ATACCTCGTAACATTATGAAGTGGCATCACTTTATAATTATAGCTCAGGCGGTTGAAGATGGGCGTGCGAC

TGATTAGGTAGTTGGCGGAGTAACAGCCCACCAAGCCTGCGATCAGTAACTGGTGTGAGAGCACGACCAG
TCACACGGGCACTGAGACACGGGC
>Zotu4046
GAACGCTGGCGGCGTGCCTAATACATGCAAGTCGAGCGAATCCGAGGGAGCTTGCTCCCAAAGATTAGCG
GCGGACGGGTGAGTAACACGTGGGCAACCTGCCTGTAAGACTGGGATAACTTCGGGAAACCGGAGCTAAT
ACCGGACATTTCTTTTCTTCACATGAAGGAAAGTTGAAAGACGGCGCAAGCTGTCACTTACAGATGGGCCC
GCGGCGCATTAGCTAGTTGGTGAGGTAACGGCTCACCAAGGCGACGATGCGTAGCCGACCTGAGAGGGT
GATCGGCCACATTGGGACTGAGACACGGCC
>Zotu4049
GAACGCTGGCGGCATGCCTAACACATGCAAGTCGAACGGCAGCACAGCAGTAGCAATACTGTGGGTGGCG
AGTGGCGAACGGGTGAGGAATGCGTCGGAATCTGCCCAGTTGTGGGGGATAACTAGCCGAAAGGTTAGC
TAATACCGCATACGACCGAGAGGTGAAAGCGGGGGACCGTAAGGCCTCGCGCGATTGGATGAGCCGACG
CCGGATTAGCTTGTTGGTGGGGTAAAGGCCTACCAAGGCGACGATCCGTAGCTGGTCTGAGAGGATGATC
AGCCACACTGGAACTGAGACACGGTC
>Zotu4051
TAACGCTAGCGGCAGGCCTAACACATGCAAGTCGAGGGGCAACAGTTGTAGCAATACAAGCTGGCGACCG
GCGCACGGGTGCGTAACGCGTATGCAACCTACCTTTTACAGGGGGATAGTCTGAAGAAATTTAGAATAATA
CCCCATAGTATTATGTATTGGCATCAATATATAATTAAAGATTTATCGGTAAAAGATGGGCATGCGTATCAT
TAGCTAGTTGGAGAGGTAACGGCTCACCAAGGCTATGATGATTAGGGGTTCTGAGAGGATTTACCCCCACA
CTGGTACTGAGACAGGGAC
>Zotu4054
GAACGCTGGCGGTGTGCCTAATACATGCAAGTCGAGCGGTAACACGTCATGGTGACGAGCGGCGGACGG
CTGAGTAACGCGTAGGAACATACCCCAAACTGAGGGATAACTGCCCGAAAGGGTAGCTAATACCGCATGT
GATCTTCGGATTAAAGCTTCGGCGGTTTGGGAATGGCCTGCGTTCGATTAGGTAGTTGGTGAGGTAAAAGC
TCACCAAGCCGACGATCGATAGCTGGTCTGAGAGGATGATCAGCCAGACTGGGACTGAGACACGGCC
>Zotu4058
GAACGCTGGCGGCAGGCTTAACACATGCAAGTCGAGCGGGCGATGTTGCTTCGGTAACTGAGCTAGCGGC
GGACGGGTGAGTAAAGCTTAGGAATCTGCCTATTAGTGGGGGACAACATTCCGAAAGGGATGCTAATACC
GCATACGTCCTACGGGAGAAAGCAGGGGATCACTTGTGACCTTGCGCTAATAGATGAGCCTAAGTCGGAT
TAGCTAGTTGGTGGGGTAAAGGCCTACCAAGGCGACGATCTGTAGCGGGTCTGAGAGGATGATCCGCCAC
ACTGGGACTGAGACACGGCC
>Zotu4060
GAACGCTGGCGGCGTGCTTAACACATGCAAGTCGAACGATGACCTCCAGCTTGCTGGGGTGATTAGTGGC
GAACGGGTGAGTAACACGTGAGTAACCTGCCCTTGACTCTGGGATAAGCACTGGAAACGGTGTCTAATAC
CGGATACGACAACCGACGGCATCGTCTGGTTGTGGAAAGAATTTTGGTCAAGGATGGACTCGCGGCCTAT
CAGCTAGTTGGTGAGGTAATGGCCCACCAAGGCGACGACGGGTAGCCGGCCTGAGAGGGTGACCGGCCA
CACTGGGACTGAGACACGGCC
>Zotu4062
GAACGCTGGCGGCGTGCTTAACACATGCAAGTCGAGCGGAAAGGCCCTTTCGGGGGTACTCGAGCGGCG
AACGGGTGAGTAACACGTGAGTAATCTGCCTCTGGCTTCGGGATAGCCACCGGAAACGGTGATTAATACC
GGATATGACACATGGTCGCATGGTTGTGTGTGGAAAGTTTTTTCGGCCAGGGATGTGCTCGCGGCCTATCA
GCTTGATGGTGAGGTAATGGCTCACCATGGCTTCGACGGGTAGCCGGCCTGAGAGGGTGACCGGCCACAC
TGGGACTGAGACACGGCC
>Zotu4065
GAACGCTGGCGGCGTGCCTAATACATGCAAGTCGAGCGGATCTTTGGGAGCTTGCTCCCAAAGGTTAGCG
GCGGACGGGTGAGTAACACGTGGGCAACCTGCCTGTAAGACTGGGATAACTTCGGGAAACCGGAGCTAAT
ACCGGATAACTTCTTTTCTCGCATGAGGAGAGGTTGAAAGATGGCTTCGGCTATCACTTACAGATGGGCCC
GCGGCGCATTAGCTAGTTGGTGAGGTAACGGCTCACCAAGGCAACGATGCGTAGCCGACCTGAGAGGGT
GATCGGCCACACTGGGACTGAGACACGGCC
>Zotu4067

GAACGCTGGCGGCGTGCTTAACACATGCAAGTCGAACGGTGACGACGGGGCTTGCCCTGTCTGATCAGTG
GCGAACGGGTGAGTAACACGTGAGTAACCTGCCCTTGACTCTGGGATAACCGCGGGAAACGGCGGCTAAT
ACCGGATATGAGACGTACAGGCATCTGTTGCGTCTGGAAAGATTTATCGGTCAAGGATGGGCTCGCGGCC
TATCAGCTTGTTGGTGGGGTGATGGCCTACCAAGGCGACGACGGGTAGCCGGCCTGAGAGGGCGACCGG
CCACACTGGGACTGAGACACGGCC
>Zotu4069
GAATGCTGGCGGTATGCTTAACACATGCAAGTCGAACGGAACCTTTCGGGGTTTAGTGGCGGACGGGTGA
GTAACGCGTGAGAATCGGCCCTCAGGTTGGGGACAACCATTGGAAACGGTGGCTAATACCGAATAAGCCG
AAAGGTGAAAGATTAATCGCCTGGGGAAGAGCTCGCGTCTGATTAGCTAGTTGGTGGGGTAAAGGCCTAC
CAAGGCACCGATCAGTAACTGGTCTGAGAGGATGGCCAGTCACACTGGGACTGAGACACGGCC
>Zotu4071
CAACGCTGGCGGCGTGCCTAACACATGCAAGTCGAACGAGAAAGGGACTTCGGTCCTGAGTAAAGTGGCG
CACGGGTGAGTAACACGTGACTAACCTACCCTTGAGTGGGGAATAACTTCGAGAAATCGAGGCTAATACC
GCATAACACCTACGGGTCAAAGGAGCAATTCGCTTAAGGAGGGGGTCGCGGCAGATTAGCTAGTTGGCGG
GGTAATGGCCCACCAAGGCAGTGATCTGTATCCGGCCTGAGAGGGCGCACGGACACACTGGAACTGAAAC
ACGGTC
>Zotu4073
GAACGCTGGCGGCGTGCTTAACACATGCAAGTCGAACGGTGACCTCGAGAGCTTGCTCTTGGGTGATCAG
TGGCGAACGGGTGAGTAACACGTGAGTAACCTGCCCCAGACTCTGGAATAACCCCGGGAAACCGGAGCTA
ATACCGGATACGAGACGGAGCCGCATGGCTACCGTCTGGAAAGTTTTTCGGTCTGGGATGGACTCGCGGC
CTATCAGTTAGTTGGTGAGGTAACGGCTCACCAAGACGACGACGGGTAGCCGGCCTGAGAGGGCGACCG
GCCACACTGGGACTGAGACACGGCC
>Zotu4075
GAACGCTGGCGGCAGGCTTAACACATGCAAGTCGAGCGGGCACTTTCGGGTGTCAGCGGCGGACGGGTT
AGTAACGCGTGGGAACGTGCCCTTTGCTCTGGAATAGCCCTGGGAAACTGGGGGTAATACCGGATGTGCC
CTGAGGGGGAAAGATTTATCGGCAAGGGATCGGCCCGCGTCTGATTAGGTAGTTGGTGAGGTAACGGCTC
ACCAAGCCGACGATCGGTAGCTGGTTTGAGAGGATGATCAGCAACACTGGGACTGAGACACGGCC
>Zotu4077
GAACGCTGGCGGCGTGCTTAACACATGCAAGTCGAGCGGTAAGGCCCTTTCGGGGGTACACGAGCGGCG
AACGGGTGAGTAACACGTGAGTAATCTGCCCTTCACTTCGGGATAACCGCCGGAAACGGTGGCTAATACC
GGATACGACGCACTCCTGCATGGGATGTGTGTGGAAAGTTTTTTCGGTGGGGGATGTGCTCGCGGCCTATC
AGCTTGTTGGTGAGGTAACGGCTCACCAAGGCTTCGACGGGTAGCCGGCCTGAGAGGGTGACCGGCCACA
CTGGGACTGAGACACGGCC
>Zotu4080
GAACGCTGGCGGCATGCCTTACACATGCAAGTCGAACGGCAGCACGGGAGCAATCCTGGTGGCGAGTGG
CGAACGGGTGAGTAAAGTATCGGAACGTGCCCAGTCGTGGGGGATAACGTAGCGAAAGCTACGCTAATAC
CGCATACGATCTATGGATGAAAGCGGGGGACCGTAAGGCCTCGCGCGATTGGAGCGGCCGATATCAGATT
AGGTAGTTGGTGGGGTAAAGGCCCACCAAGCCGACGATCTGTAGCTGGTCTGAGAGGACGACCAGCCACA
CTGGGACTGAGACACGGCC
>Zotu4082
GAACGCTGGCGGCAGGCCTAACACATGCAAGTCGAGCGAACCCTTCGGGGTTAGCGGCGGACGGGTGAG
TAACGCGTGGGAACGTGCCCTTTGCTACGGAATAGCCCCGGGAAACTGGGAGTAATACCGTATACGCCCTT
AGGGGGAAAGATTTATCGGCAAGGGATCGGCCCGCGTTGGATTAGGTAGTTGGTGGGGTAATGGCCTACC
AAGCCGACGATCCATAGCTGGTTTGAGAGGATGATCAGCCACACTGGGACTGAGACACGGCC
>Zotu4084
AAACGCTGGCGGCGTGCTTAACACATGCAAGTCGAACGTGAAAGTTTCTTCGGAAATGAGTAAAGTGGCG
AACGGGTGAGTAACGCGTGAGAATCTGCCCTTTAGTGAAGGATACCATCGAGAAATCGATGTTAATACTGC
ATATCTTTGCAAAAAGAAAGGTGGCCTATAAGCTGCCGCTAAAGGATGAGCTTGCGTGCTATTAGCTAGTT
GGTGGGGTAATGGCCTACCAAGGCGATGATGGCTAGCCGGCCTGAGAGGGTGTACGGCCACATTGGAAC
TGAGAAACGGTC

>Zotu4086
GAACGCTGACGGCGTGCTTAACACATGCAAGTCGAACGGGATCCATTAGCGCTTTTGTGTTTTTGGTGAGA
GTGGCGAACGGGTGAGTAACACGTGAGTAACCTGCCCTTTTCTTTGGGATAAGCCTGGGAAACTGGGTCTA
ATACTGGATGTTCCGGCTTCCTCGCATGGGGTTGTTGGGAAAGGTTTTTTCTGGATTGGGATGGGCTCGCG
GCTTATCAGCTTGTTGGTGGGGTGATGGCTTACCAAGGCTTTGACGGGTAGCCGGCCTGAGAGGGTGGTC
GGTCACACTGGGACTGAGATACGGCC
>Zotu4089
GAACGCTGGCGGCACGCCTAACACATGCAAGTCGAGCGGTAGCAGGATTAGTAGCAATACTAATCGCTGA
CGAGCGGCGGACGGGTGAGTAATACGTAGGAACCTGCCCTTAAGCGGGGGATAACTAAGGGAAACTTTA
GCTAATACCGCATACACTCGAGAGAGGAAAGCTGCCGTAAGGTGGCACTTGAGGAGGGGCCTGCGTCCGA
TTAGCTAGTTGGTAGGGTAAGAGCCTACCAAGGCAATGATCGGTAACTGGTCTGAGAGGACGACCAGTCA
CACTGGGACTGAGACACGGCC
>Zotu4091
GAACGCTGGCGGCGTGCTTAACACATGCAAGTCGAACGGTGACAGGGAGCTTGCTCCCTCGATCAGTGGC
GAACGGGTGAGTAACACGTGAGTAACCTGCCCCAGACTCTGGGATAACGTCGAGAAATTGGCGCTAATAC
CGGATACGAGACGAGACCGCATGGTCATTGTCTGGAAAGAATTTCGGTCTGGGATGGACTCGCGGCCTAT
CAGCTTGTTGGTGAGGTAATGGCTCACCAAGGCGACGACGGGTAGCCGGCCTGAGAGGGCGACCGGCCA
CACTGGAACTGAGACACGGTC
>Zotu4093
GAACGCTGGCGGCGTGCTTAACACATGCAAGTCGAACGATTAAACCGGCTTCGGTCGGATATAGAGTGGC
GAACGGGTGAGTAACACGTGACCAACCTACCCCTTACACTGGGACAACCACTGGAAACGGTGGCTAATACC
GGATACTCCGAGTATCTCGCATGAGGTATTCGGGAAAGCCTTCACGGTAAGGGATGGGGTCGCGGCCCAT
TAGGTAGACGGCGGGGTAACGGCCCACCGTGCCTGCGATGGGTAGCCGGGCTGAGAGGCTGATCGGCCA
CATTGGGACTGAGATACGGCC
>Zotu4095
GAACGTTAGCGGCGCGCCTAACACATGCAAGTCGCACGAGAAAGGGCTTCGGCCCCGGTAAAGTGGCGCA
CGGGTGAGTAACACGTGGGTAATCTTCCTCCGAGCGGGGGATAACATTCCGAAAGGAGTGCTAATACCGC
ATAAGACCACGGTGTCGAAAGACGCTGAGGTAAAAGCTGGCCAATTCATGAAAGCTAGCACTTGGAGATG
AGCCCGCGGCCCATCAGGCAGTTGGCAGGGTAATGGCCTACCAAACCGAAGACGGGTAGCTGGTCTGAGA
GGATGATCAGCCACACTGGAACTGAGACACGGTC
>Zotu4097
GAACGCTGGCGGCGTGCTTAACACATGCAAGTCGAACGGTGAACACGGAGCTTGCTCTGTGGGATCAGTG
GCGAACGGGTGAGTAACACGTGAGCAACCTGCCCCTGACTCTGGGATAAGCGCTGGAAACGGCGTCTAAT
ACTGGATACGAGTAGCGACCGCATGGTCAGTTACTGGAAAGATTTATTGGTTGGGGATGGGCTCGCGGCC
TATCAGCTTGTTGGTGAGGTAATGGCTCACCAAGGCGTCGACGGGTAGCCGGCCTGAGAGGGTGACCGGC
CACACTGGGACTGAGACACGGCC
>Zotu4099
GAACGCTGGCGGCATGCCTTACACATGCAAGTCGAACGGCAGCACGGGGGCAACCCTGGTGGCGAGTGG
CGAACGGGTGAGTAATACATCGGAACGTGTCCTGTAGTGGGGGATAGCCCGGCGAAAGCCGGATTAATAC
CGCATACGATCTACGGAAGAAAGCGGGGGATCCTTCGGGACCTCGCGCTATAGGGGCGGCCGATGGCAG
ATTAGCTAGTTGGTGGGGTAAAGGCCTACCAAGGCGACGATCTGTAGCTGGTCTGAGAGGACGACCAGCC
ACACTGGGACTGAGACACGGCC
>Zotu4102
GAACGCTGGCGGCGTGCTTAACACATGCAAGTCGAACGGTAAGGCCCCTTCGGGGGTACACGAGTGGCGA
ACGGGTGAGTAACACGTGAGCAACCTACCCTTGACTCTGGAATAACGGTTGGAAACAGCCGCTAATACCG
GATACGACCTTGAGCGTCATCGCTCGAGGTGGAAAGCTCCGGCGGTCAGGGATGGGCTCGCGGCCTATCA
GCTAGTTGGTGAGGTAACGGCTCACCAAGGCTTCGACGGGTAGCCGGCCTGAGAGGGCGACCGGCCACA
CTGGGACTGAGATACGGCC
>Zotu4104

GAACGCTGGCGGCGTGCCTTAAGCATGCAAGTCAAGGGGGGCTCGCAAGAGTCAACCGGCGGACGGGTG
AGTAACGCGTGAGAATCTACCTTAAAGTGGGACATAGCCCTCCGAAAGGAGGGGTAATTTCCCATGTGATC
CGGCTTTGCCGGATTAAAGCCGCAAGGCGCTTTAAGATGAGCTCGCGTCCTATCAGCTAGTTGGTGGGGTA
AAAGCCTACCAAGGCGACGACGGGTAGCTGGACTGAGAGGTTGGTCAGCCACAATGGCACTGCGACACG
GGC
>Zotu4107
GAACGCTGGCGGCATGCCTAACACATGCAAGTCGAACGATGCTTTCGGGCATAGTGGCGCACGGGTGCGT
AACGCGTGGGAATCTGCCCTTGGGTCTGGGATAACAGCGGGAAACTGCTGCTAATACCGGATGATGACGT
TAAGTCCAAAGATTTATCGCCCAGGGATGAGCCCGCGTAGGATTAGCTAGTTGGTGGGGTAAAGGCCTAC
CAAGGCGACGATCCTTAGCTGGTCTGAGAGGATGATCAGCCACACTGGGACTGAGACACGGCC
>Zotu4109
GAACGCTAGCGGCAGGCCTAATACATGCAAGTCGGACGGGATTTGGGAGCTTGCTCCTGATGAGAGTGGC
GCACGGGTGCGTAACACGTATGTAACCTACCTTAATCTGGGGGATAGCCTCTCGAAAGAGAGATTAACACC
GCATAACATTATCGGATAGCATTTTTTGATAATCAAATATTTATAGGATTAAGATGGGCATGCGGAACATTA
GCTAGTTGGTAAGGTAATGGCTTACCAAGGCTACGATGTTTAGGGGATCTGAGAGGATGACCCCCCACACT
GGTACTGAGACACGGAC
>Zotu4111
GAACGCTGGCGGCGTGCTTAACACATGCAAGTCGAACGAAGAGCGACGGAAGCTTGCTTCTATCAATCTTA
GTGGCGAACGGGTGAGTAACGCGTAATCAACCTGCCCTTCAGAGGGGGACAACAGTTGGAAACGACTGCT
AATACCGCATACGATCTAATCTCGGCATCGAGGATGGATGAAAGATGGCCTCTACATGTAAGCTATCACTG
AAGGAGGGGATTGCGTCTGATTAGCTAGTTGGAGGGGTAACGGCCCACCAAGGCGATGATCAGTAGCCG
GTCTGAGAGGATGAACGGCCACATTGGGACTGAGACACGGCC
>Zotu4113
GAACGCTGGCGGCGTGCCTAATACATGCAAGTCGAGCGGACTTTGCCTTCGGGACAAGATTAGCGGCGGA
CGGGTGAGTAACACGTGGGTAACCTGCCCATAAGACTGGGATAACATTCGGAAACGAATGCTAATACCGG
ATACGCGAAACGGTCGCATGATCGAATCGGGAAAGGCGGAGCAATCTGCCACTTATGGATGGACCCGCGG
CGCATTAGCTAGTTGGTGGGGTAACGGCTCACCAAGGCGACGATGCGTAGCCGACCTGAGAGGGTGATCG
GCCACACTGGGACTGAGACACGGCC
>Zotu4115
GAACGCTGGCGGCGTGCCTAACACATGCAAGTCGAACGGAACTTCTTTAAAGGATTTCTTCGGAATGAATT
TGATTAAGTTTAGTGGCGGACGGGTGAGTAACGCGTGAGTAACCTGCCTCTAAGAGGGGAATAACATTCT
GAAAAGAATGCTAATACCGCATAATATATATTTATCGCATGGTAGATATATCAAAGATTTATCGCTTAGAGA
TGGACTCGCGTCCGATTAGTTAGTTGGTGAGGTAACGGCTCACCAAGACCGCGATCGGTAGCCGGACTGA
GAGGTTGAACGGCCACATTGGGACTGAGACACGGCC
>Zotu4118
GAACGCTGGCGGCATGCCTAACACATGCAAGTCGAACGAGATCTTCGGATCTAGTGGCGCACGGGTGCGT
AACGCGTGGGAATCTGCCCTTGGGTTCGGAATAACAGTGGGAAACTACTGCTAATACCGGATAATGACGT
AAGTCCAAAGATTTATCGCCCTGGGATGAGCCCGCGTAGGATTAGCTAGTTGGTGGGGTAAAGGCCTACC
AAGGCGACGATCCTTAGCTGGTCTGAGAGGATGATCAGCCACACTGGGACTGAGACACGGCC
>Zotu4120
GAACGCTGGCGGCGTGCCTAAGACATGCAAGTCGAGTGGGTTTAGACCCACGGCGAACGGGTGAGTAAT
ACCTAGGAACTTTCTTTAAGTGGAGCATAACCAGTCGAAAGATTGGGCAATTCTTCATGTGGCAGTAATGC
TAAAGTCGCAAGACGCTTAAAGTGAGGCCTGGGCACCATCAGCTAGTTGGTAAGGTAATGGCTTACCAAG
GCTATGACGGTTAGGGGTCCCGAGAGGGATAACCCCCACACTGGAACTGCGACACGGTC
>Zotu4124
GAATGCTGGCGGTGTGGATAAGTCATGCAAGTCGAACGGTCCGCAAGGACAGTGGCAGACGAGGTAGTA
ACACGTAGGTACACCCCCCGAAGTCGGGCATAGCCAGCCGAAAGGTTGGGTAATTCCCGATAGTCGCGCA
AGCGTAAAGATTTATCGCTTCGGGAGTGGCCTGCGTAATATCAGCTAGTTGGTAAGGTAACGGCTTACCAA
GGCTACGACGTTTAGGGGAGCTGAGAGGCTGACCCCCACCGATGGGACTGCGATACGGCC
>Zotu4132

GAATGCTGGCGGCGTGCCTAACACATGCAAGTCGAGCGGCAGCGAGTTTTACACTGAATTCTGGAAGCTTC
TGGGTAATATAGAAGATCTATTCAAGAATTTTGTGTAAAATGTCGGCGAGCGGCGGACGGCTGAGTAACG
CGTAGGAATTTGCCCCAAAGTGAGGAATAACTGCCCGAAAGGGTAGCTAATGCCGCATATGGTCTTCGGAT
TAAAGCCTTCGGGCGCTTTGGGAGAAGCCTGCGTTGGATTAGGTAGTTGGTAGGGTAATGGCCTACCAAG
CCGACGATCCATAGCTGGTCTGAGAGGATGATCAGCCAGACTGGAACTGAGACACGGTC
>Zotu4134
GAATGCTGGCGATGTGGATAAGTCATGCAAGTCAAGGGGGCCGCAAGGCAACCGGCAGACGAGGTAGTA
ACACGTACGAACGCACCCCGAAGTCTGGTATAGCTCACCGAAAGGTGAGGTAATCCCGGATGGTACCGCA
AGGTTAAAGATTTATCGCTTCGGGAGCGGCGTGCGCAATATCAGCTAGTTGGTAGGGTAATGGCCTACCAA
GGCTACGACGTTTAGGGGAGCTGAGAGGCTGACCCCCACCGATGGGACTGAGACACGGCC
>Zotu4136
GAACACTGGCGGCAGGCTTAACACATGCAAGTCGAGCGGGGTGATGTAGCTTGCTACCTGACCTAGCGGC
GGACGGGTGAGTAATGCTTAGGAATCTGCCTATTAGTGGGGGACAACATTCCGAAAGGAATGCTAATACC
GCATACGCCCTACGGGGGAAAGCAGGGGATCTTCGGACCTTGCGCTAATAGATGAGCCTAAGTCAGATTA
GCTAGTTGGTGGGGTAAAGGCCTACCAAGGCGACGATCTGTAGCGGGTCTGAGAGGATGATCCGCCACAC
TGGGACTGAGACACGGCC
>Zotu4139
GAACGCTGGCGGCATGCTTTACACATGCAAGTCGAACGGTAACAGGGAGCTTGCTCCGCTGACGAGTGGC
GAACGGGTGAGTAATATATCGGAACGTACCCAAGAGTGGGGGATAACGTAGCGAAAGTTACGCTAATACC
GCATACGATCTAAGGATGAAAGCAGGGGATCTTCGGACCTTGTGCTCCTGGAGCGGCCGATATCTGATTAG
CTAGTTGGTGAGGTAAAGGCTCACCAAGGCGACGATCAGTAGCTGGTCTGAGAGGACGACCAGCCACACT
GGAACTGAGACACGGTC
>Zotu4143
GAACGCTGGCGGCGCGCCTAATACATGCAAGTCGAACGAAAACCTCGGACTTCGGTCTGGGGGGACAGTG
GCGAACGGGTGCGTAACACGTGAGCAACCTGCCTCGGAGACCGGGACAACGCGGGGAAACCCGTGCTAA
TACCGGATACCCTCCTCGGATCGCATGGTCTGATGAGGAAATGGATTCCGCTCCGAGATGGGCTCGCGGCC
TATCAGCTTGTTGGTGAGGTAACGGCTCACCAAGGCTTCGACGGGTAGCTGGTCTGAGAGGACGATCAGC
CACACTGGGACTGAGACACGGCC
>Zotu4146
GAACGCTGGCGGCGTGCCTAATACATGCAAGTCGAGCGGAGTTACAGGAGCTTGCTCCTGTAACTTAGCG
GCGGACGGGTGAGTAACACGTGGGCAACCTGCCTATAAGACTGGGATAACTTCGGGAAACCGGAGCTAAT
ACCGGATAATTCTTTTCCTCTCATGAGGAAAAGCTGAAAGACGGTTTCGGCTGTCACTTATAGATGGGCCC
GCGGCGCATTAGCTAGTTGGTGAGGTAACGGCTCATCAAGGCAACGATGCGTAGCCGACCTGAGAGGGTG
AACGGCCACACTGGGACTGAGACACGGCC
>Zotu4149
GAACGCTGGCGGCGTGCCTAACACATGCAAGTCGGATGTGCCCGCAAGGGTGCATGGCAGACGGGTGAG
TAACGCGTGGGAAACCTACCTCTTTGTGGGGAATAACGCTCCGAAAGGAGTGGTAATACCGCATGAGACC
ATGAGCTGGGACGCTCATGGTGAAACCTGGGGACCGCAAGGCCTGGGGCAAGGAGAGGGTCCTGCGTCT
GATTAGCTAGTTGGTGGGGTAACGGCCTACCAAGGCAACGATCAGTAGCCGGCCTGAGAGGGTGATCGGC
CACACTGGAACTGAGACACGGTC
>Zotu4151
GAACGCTGGCGGCGTGCCTAATACATGCAAGTCGAGCGGACTGAATGGAAGTTTGCTTTCGTTCGGTTAGC
GGCGGACGGGTGAGTAACACGTGGGCAACCTGCCCGTAAGACCGGGATAACTTCGGGAAACCGGAGCTA
ATACCGGATAACACCGAAGATCGCATGGTCTTTGGTTGAAAGGCGGCTTTGGCTGTCACTTACGGATGGGC
CCGCGGCGCATTAGCTAGTTGGTGAGGTAACGGCTCACCAAGGCGACGATGCGTAGCCGACCTGAGAGG
GTGATCGGCCACACTGGGACTGAGACACGGCC
>Zotu4155
GAACGCTGGCGGCGTGCTTAACACATGCAAGTGGAGCGACGAACCAGGGCTTGCCCTGGGGCAGAGCCG
CGAACGGGTGAGTAACACGTGGGTAACCTGCCCCGATCACTGGGACAACCCGAGGAAACTCGGGCTAATA
CCGGATGTGATCCCGTCACACCAGTGACGTGATGAAAGGTAGCTTCGGCTTCCGGATTGGGATGGGCCCG

CGGCCCATTAGCTCGTTGGTGGGGTAACGGCCTACCAAGGCGACGATGGGTAGCTGGTCTGAGAGGACGA
TCAGCCACACTGGGACTGAGACACGGCC
>Zotu4157
GATCGCTGGCGGCGTGCTTAACACATGCAAGTCGAGCGAATTCTAGCAGCGTATCGCTAGAGTTAGCGGC
AGACGGGTGAGTAATACTTGGGCAATCTGCCCTCGAGAGGGAAACAACCGGCCGAAAGGCTGGCTAATGT
CCCATAAGACCACAGGGATGCATATTCCAGGGGTCAAAGCTCCGCAAGGGGCGCTTGAGGAGGAGCCCG
AGTCCTATCATTGGTAGTTGGTGAGGTAACGGCTCACCAAGCCTCTGACGGGTAGCCGGCCTGAAAGGGT
GACCGGCCACACTGGCACTGAGACACGGGC
>Zotu4162
GAATGCTGGCGGCGCGCCTAACACATGCAAGTCGAGCGGTAACATGTTTCTCACTGTCTTCTTCTGTTTTTA
CAGGAGGGAATTGTGAGAAATGATGACGAGCGGCGGACGGCTGAGTAACGCGTGGGAACATGCCCCAAA
GTGAGGAATAACTGCCCGAAAGGGTGGCTAATGCCGCATATGGTCTTCGGATTAAAGGATTTATCCGCTTT
GGGAGTGGCCTGCGTATGATTAGATAGTTGGTGAGGTAATGGCTCACCAAGTCTACGATCATTAACTGGTC
TGAGAGGATGATCAGTCAGACTGGAACTGAGACACGGTC
>Zotu4164
GAACGCTGGCGGCGTGCCTTAAGCATGCAAGTCGTATGATCGCAAGATAATGGCGGACGGGTGAGTAACG
CGTGAGAATCTGCCTTGAAGTGGGGCATAGCCCTCCGAAAGGAGGGGTAATACCGCATGTGATAGCAATA
TCAAAGCAGCAATGCGCTTTAAGATGAGCTCGCGTAAGATTAGCTAGTTGGTGGGGTAAAGGCCTACCAA
GGCGACGATCTTTAGCTGGTCTGAGAGGATGGTCAGCCACAATGGAACTGAGAACGGTC
>Zotu4167
GAACGCTAGCGGCAGGCCTAACACATGCAAGTCGAGGGGTAGTATAGCAATATATGAGACCGGCGCACG
GGTGCGTAACGCGTATGCAATCTACCTTTTACAGAGGGATAGCCCAGAGAAATTTGGATTAATACCTCATA
GCATTGCAGAATGGCATCATTCAGCAATTAAAGTCACAACGGTAAAAGATGAGCATGCGTCCTATTAGCTA
GTTGGTAAGGTAACGGCTTACCAAGGCTACGATAGGTAGGGGTCCTGAGAGGGAGATCCCCCACACTGGT
ACTGAGACACGGAC
>Zotu4169
TAACGCTGGCGGCGTGCATAACACATGCAAGTCGAACGATGAAACTCTAAAGATTTCTTCGGAATGAATTA
GAATGGATTAGTGGCGAACGGGTGAGTAACACGTGAGTAACCTGCCTTAGAGAAGGGGATAGCCTTTGGA
AACGAAGAATAATACCCTATGAAATGATAATATCGCATGATAGTATCATCAAAGATTTATCGCTCTAAGATG
GACTTGCGCCTGATTAGCTAGTTGGTGGAGTAACAGCCCACCAAGGCAACGATCAGTAGCCGGCTTGAGA
GAGTGTACGGCCACATTGGGACTGAGACACGGCC
>Zotu4172
GAACGCTGGCGGCATGCCTAACACATGCAAGTCGAACGAGACCTTCGGGTCTAGTGGCGCACGGGTGCGT
AACGCGTGGGAATCTGCCCTTGGGTTCGGAATAACTCCTCGAAAGGGGTGCTAATACCGGATGATGTCGC
AAGACCAAAGATTTATCGCCCAAGGATGAGCCCGCGTAGGATTAGCTTGTTGGTGAGGTAAAAGCTCACC
AAGGCGACGATCCTTAGCTGGTCTGAGAGGATGATCAGCCACACTGGGACTGAGACACGGCC
>Zotu4178
AAACGCTGGCGGCGTGCCTAACACATGCAAGTCGAACGCCCTTAGCAATAAGGGAGTGGCGGACGGGTG
AGGAACACGTGGGTGACCTACCTCGAAGTGAGGAATACCGGCGAGAAATCGCCGACAATACCGCATACGC
TTCGCAAGAAGCAAAGTCGCAAGACGCTTTGAGATGGGCCCGCGGCCGATTAGCTAGTTGGTGGGGTAAT
GGCCTACCAAGGCGACGATCGGTAGCTGGTCTGAGAGGACGATCAGCCACACTGGGATTGAGAACGGCC
>Zotu4182
GAATGCTGGCGGCGTGGATAAGGCATGCAAGTCGAACGGTCCGCAAGGACAGTGGCAGACGAGGTAGTA
ACACGTACGAACGCACCCCATAGTCTGGCATAGCTCGTCGAAAGACGGGGTAATTCCGGATGGTCCCTACC
AAGGGTAAAGTTTTTTCGCTATGGGAGCGGCGTGCGTAATATCAGCTTGTTGGTAAGGTAATGGCTTACCA
AGGCTACGACGTTTAGGGGAGCTGAGAGGCTGACCCCCACCGATGGGACTGAGACACGGCC
>Zotu4185
GAACGCTAGCGGCAGGCCTAATACATGCAAGTCGAACGGGATCCGTCGGAGAGCTTGCTCGAAGACGGTG
AGAGTGGCGCACGGGTGCGTAACGCGTGAGCAACCTACCTCTATCAGGGGGATAGCCTCTCGAAAGAGAG
ATTAACACCGCATAACATATCTGACCGGCATCGGTTAGCTATTAAATATTTATAGGATAGAGATGGGCTCGC

GTGACATTAGCTAGTTGGTAGGGTAACGGCTTACCAAGGCGACGATGTCTAGGGGCTCTGAGAGGAGAAT
CCCCCACACTGGTACTGAGACACGGAC

>Zotu4187

GAACGCTGGCGGCGTGCCTAATACATGCAAGTCGAGCGAATCAATAGGAGCTTGCTCCTGTTGGTTAGCG
GCGGACGGGTGAGTAACACGTGGGCAACCTGCCTGTAAGACTGGGATAACACCGGGAAACCGGTGCTAA
TACCGGATAATCCTTTTCCTCTCATGAGGAAAAGCTGAAAGTCGGTGCAAGCTGACACTTACAGATGGGCC
CGCGGCGCATTAGCTAGTTGGTGAGGTAACGGCTCACCAAGGCGACGATGCGTAGCCGACCTGAGAGGGT
GATCGGCCACACTGGGACTGAGACACGGCC

>Zotu4190

GAACGCTAGCGGCAGGCCTAATACATGCAAGTCGGACGGGATTGGAGAGCTTGCTTTCCATGAGAGTGGC
GCACGGGTGCGTAACACGTATGTAACCTACCTTCACCAGGGGGATAGCCTCTCGAAAGAGAGATTAAGAC
CGCATAAAATTGTTTAATGGCATCGTTGAACAATCAAATATTTATAGGGTGAAGATGGGCATGCGGAACAT
TAGCTAGTTGGTAAGGTAACGGCTTACCAAGGCTACGATGTTTAGGGGATCTGAGAGGATGACCCCCCAC
ACTGGTACTGAGACACGGAC

>Zotu4193

GAATGCTGGCGGCGTGCCTAACACATGCAAGTCGAGCGGTAACAGGAGTAATTTATTACTTGCTGACGAG
CGGCGGACGGCTGAGTAACGCGTGGGAACGTGCCCCAAAGTGAGGAATAAGCTCCAGAAATGGAGTCTA
ATGCCGCATATGATCTTCGGATTAAAGGATTTATCCGCTTTGGGAGCGGCCCGCGTTAGATTAGGTAGTTG
GTAGGGTAATGGCCTACCAAGCCGACGATCTATAGCTGGTCTGAGAGGATGATCAGCCAGACTGGGACTG
AGACACGGCC

>Zotu4195

GAACGCTGGCGGCGTGCCTAATACATGCAAGTCGAGCGGACCGAATAGAAGCTTGCTCCTGTTCGGTTAG
CGGCGGACGGGTGAGTAACACGTGGGCAACCTGCCCGTAAGACCGGGATAACTTCGGGAAACCGGAGCT
AATACCGGATAACACCGAAGACCGCATGGTCTTCGGTTGAAAGGCGGCTTTGGCTGTCACTTACGGATGG
GCCCGCGGCGCATTAGCTAGTTGGTGAGGTAACGGCTCACCAAGGCGACGATGCGTAGCCGACCTGAGAG
GGTGATCGGCCACACTGGGACTGAGACACGGCC

>Zotu4197

GAACGCTGGCGGCGTGCTTAACACATGCAAGTCGAACGATGACGGTGGTGCTTGCACCACCTGATTAGTG
GCGAACGGGTGAGTAACACGTGAGTAACCTGCCCCCACTTCGGGATAACCTCGGGAAATCGTGGCTAAT
ACCGGATACGAGCACTCATCGCATGGTGGGTGCTGGAAAGATTTATCGGTGGGGGATGGACTCGCGGCCT
ATCAGTTTGTTGGTGAGGTGATGGCTCACCAAGACGATGACGGGTAGCCGGCCTGAGAGGGCGACCGGCC
ACACTGGGACTGAGACACGGCC

>Zotu4199

GAACGCTAGCGATAGGCCTAACACATGCAAGTCGAGGGGCAGCACATGACGTAGCAATACAGATGGTGGC
GACCGGCGCACGGGTGAGTAACACGTATGCAACCTACCTTCAACAGGAGAATAACCCGTCGAAAGACGGA
CTAATACTCCATAACACAGGGATCCCGCATGGGAATATTTGTTAAAGATTTATCGGTTGAAGATGGGCATG
CGCTCCATTAGCTAGTTGGTGAGGTAACGGCTCACCAAGGCAACGATGGATAGGGGAACTGAGAGGTTTA
TCCCCCACACTGGTACTGAGACACGGAC

>Zotu4201

GAACGCTGGCGGCGTGCTTAACACATGCAAGTCGAACGGTGAGGGAGAGCTTGCTCTCTGGATCAGTGGC
GAACGGGTGAGTAACACGTGGGCAATCTGCCCCTGACTCTGGGATAAGCGCTGGAAACGGCGTCTAATAC
CGGATACGAGCTGCGAAGGCATCTTCAGCAGCTGGAAAGAACTTCGGTCAGGGATGAGCTCGCGGCCTAT
CAGCTAGTTGGTGAGGTAACGGCTCACCAAGGCGTCGACGGGTAGCCGGCCTGAGAGGGTGACCGGCCA
CACTGGGACTGAGACACGGCC

>Zotu4203

GAACGCTGGCGGCGTGCTTAACACATGCAAGTCGAACGGTGAAGGACGGAGCTTGCTCCGACCGGATCAG
TGGCGAACGGGTGAGTAACACGTGAGTAACCTGCCCCAGACTCTGGAATAACCCCGGGAAACCGGAGCTA
ATACCGGATACGAAGTAGAGCTGCATGGCTACTACTTGGAAAGTTTTTCGGTCTGGGATGGACTCGCGGCC
TATCAGCTAGTTGGTGAGGTAAAGGCTCACCAAGGCGACGACGGGTAGCCGGCCTGAGAGGGCGACCGG
CCACACTGGGACTGAGACACGGCC

>Zotu4205
GAACGCTGGCGGCAGGCTTAACACATGCAAGTCGAGCGCCCCGCAAGGGGAGCGGCAGACGGGTGAGTA
ACGCGTGGGAATCTACCCAATACTACGGAATAGCTCCGGGAAACTGGAATTAATACCGTATGTGCCCGACA
GGGGAAAGATTTATCGGTATTGGATGAGCCCGCGTTGGATTAGCTAGTTGGTGGGGTAATGGCCTACCAA
GGCGACGATCCATAGCTGGTCTGAGAGGATGATCAGCCACACTGGGACTGAGACACGGCC
>Zotu4207
GAACGCTGGCGGCAGGCTTAACACATGCAAGTCGAGCGGACCTTTCGGGGTCAGCGGCGGACGGGTGAG
TAACGCGTGGGAACGTGCCTTCTGGTTCGGAATAACCCTGGGAAACTAGGGCTAATACCGGATACGCCCTT
ATGGGGAAAGGTTTACTGCCGGAAGATCGGCCCGCGTCTGATTAGCTAGTTGGTGGGGTAACGGCCTACC
AAGGCGACGATCAGTAGCTGGTCTGAGAGGATGATCAGCCACACTGGGACTGAGACACGGCC
>Zotu4209
GAACGCTAGCGGCAGGCTTAATACATGCAAGTCGAGGGGGCAGCAATGTCACCGGCGAACGGGTGCGTA
ACGCGTATGCAACCTACCTACATCAGGGGGATAGCCCTAGGAAACTGGGATTAACACCGCATAATATTATT
AATGGCATCATTGGTAATTGAAATATTTATAGGATGTAGATGGGCATGCGTAGGATTAGCTAGTTGGTAAG
GTAACGGCTTACCAAGGCTACGATCCTTAGGGGCTCTGAGAGGAGTATCCCCCACACTGGCACTGAGATAC
GGGC
>Zotu4212
GAACGCTGGCGGCGTGCCTAATACATGCAAGTCGAGCGAATTTTTTGGAGCTTGCTCCTAAAAATGAGCGG
CGGACGGGTGAGTAACACGTGGGCAACCTGCCTGTAAGACTGGGATAACTTCGGGAAACCGGAGCTAATA
CCGGATAATTCTTTCTCTCACATGAGGGAAAGCTGAAAGACGGTTTCGGCTGTCACTTACAGATGGACCCG
CGGCGCATTAGCTAGTTGGTGAGGTAACGGCTCACCAAGGCGACGATGCGTAGCCGACCTGAGAGGGTG
ATCGGCCACATTGGGACTGAGACACGGCC
>Zotu4216
GAACGCTGGCGGCGTGCTTAACACATGCAAGTCGAACGGAAAGGCCCTTCGGGGTACTCGAGTGGCGAAC
GGGTGAGTAACACGTGGGTGATCTGCCCTGCACTTTGGGATAAGCCTGGGAAACTGGGTCTAATACCGAA
TACACCCTTCTGGCTGCATGGTCTGGGAGGGGAAAGCTTTTGCGGTGTGGGATGGGCCCGCGGCCTATCA
GCTTGTTGGTGAGGTTATGGCTTACCAAGGCGACGACGGGTAGCCGGCCTGAGAGGGTGACCGGCCACAC
TGGGACTGAGATACGGCC
>Zotu4218
GAACGCTGGCGGCGTGTCTAAGGCATGCAAGTCGAACGGGCCCTCGGAGTTAGCAATAATTTTGAGGGCT
AGTGGCAAACGGGTGAGTAACACATTGGTAACCTGCCCCGAAGTTCGGAATAACGGTCCGAAAGGACCGC
TAATACCGGATGTGATCCCCTGTCATATGACGGGTGAGGAAAGCCCAAAAGGCGCTTCGGGAGGGACCTA
TGGTCTATCAGCTAGTTGGTGCGGGTAATGGCCCACCAAGGCTACGACGGATAGCAGGCGTGAGAGCGCG
ACCTGCCTCAGTGGAACTGAGACACTGTC
>Zotu4220
GAACGCTAGCGGCAGGCCTAATACATGCAAGCCGAACGGCAAGATTTTCAGCAATGAGAATCCTAGAGTG
GCGTAAGGGTGCGTAACACGTATGCAATCTACCCTCAACAGGAGGATAGCTCCCCGAAATGGGAATTAAG
ACTCCATAAGATCACACGAAGGCATCTGAGAGTGATTAAAGTACAAGCGGTTGAGGATGAGCATGCGTCT
GATTAGTTAGTTGGCGGGGTAACGGCCCACCAAGACGATGATCAGTAGGGGAACTGAGAGGTTGATCCCC
CACACTGGCACTGAGATACGGGC
>Zotu4222
GAACGCTGGCGGTGCGCCTAAAACATGCAAGTCGAGTGGGTTTAGACCCACGGCGAACGGCTGCGTAACA
CGTGGATACTTGCCTCGTACTCAGGGATACCTCTCCGAAAGGAGAGCTAATACCGGATGGTCCCGAAAGG
GTAAAGATTTATCGGTACGAGAGAGGTCTGCGGCCTATCAGATAGTTGGCAGGGTAATGGCCTACCAAGT
CGATGACGGGTAGCTGGATTGAGAGATCGACCAGCCAGAATGGAACTGAGACACGGTC
>Zotu4224
AAACGCTGGCGGCGTGCATGAGACATGCAAGTCGAACGAACGAGCTTGCTCGTTAGTGGCGAACGGCTGA
GTAACGCGTAGGTGACCTGCCCCGAAGTGGGGGATAACTGCCCGAAAGGGTGGCTAATACCGCATGTGGC
GACGCTTTCATTGGCGTCATCAAAGCCGCGAGGCGCTTCGGGAGGGGCCTGCGTCCGATTAGCTCGTTGGT

GGGGTAATGGCTCACCAAGGCGACGATCGGTAGGTGGTCTGAGAGGACGATCACCCAGACTGGGACTGA
GACACGGCC
>Zotu4228
GAACGCTGGCGGCGTGCTTAACACATGCAAGTCGAGCGGTAAGGCTCCTTCGGGAGTACACGAGCGGCGA
ACGGGTGAGTAACACGTGAGTAATCTGCCCTCCACTTTGGGATAAGCCTCGGAAACGAGGTCTAATACCGA
ATACGACCACTTCCTGCATGGGATGGTGGTGGAAAGTTTTTCGGTGGGGGATGTGCTCGCGGCCTATCAG
CTTGTTGGTGGGGTAATGGCCTACCAAGGCTTCGACGGGTAGCCGGCCTGAGAGGGTGACCGGCCACACT
GGGACTGAGACACGGCC
>Zotu4231
GAACGCTGGCGGCATGCCTAACACATGCAAGTCGAACGAGACCTTCGGGTCTAGTGGCGCACGGGTGCGT
AACGCGTGGGAATCTGCCCTTGGGTACGGAATAACTCAGAGAAATTTGAGCTAATACCGTATAATGACTTC
GGTCCAAAGATTTATCGCCCAAGGATGAGCCCGCGTAAGATTAGCTAGTTGGTGGGGTAAAGGCCTACCA
AGGCGACGATCTTTAGCTGGTCTGAGAGGATGATCAGCCACACTGGGACTGAGACACGGCC
>Zotu4233
GAACGCTGGCGGCGTGCTTAACACATGCAAGTCGAACGGAAAGGCCCTTCGGGGTACTCGAGTGGCGAAC
GGGTGAGTAACACGTGGGTGATCTGCCCTGCACTTCGGGATAAGCCTGGGAAACTGGGTCTAATACCGGA
TATACCTCATGGACTGCATGGTTCGTGGGGGAAAGCTTTTGCGGTGTGGGATGGGCCCGCGGCCTATCAG
CTTGTTGGTGGGGTGATGGCCTACCAAGGCGTCGACGGGTAGCCGGCCTGAGAGGGTGTCCGGCCACACT
GGGACTGAGATACGGCC
>Zotu4236
GAACGCTGGCGGCGTGCTTAACACATGCAAGTGGAGCGACGAACCAGGGCTTGCCCTGGGGCAGAGCCG
CGAACGGGTGAGTAACACGTGGGTAACCTGCCCAGATGACCGGGACAACCCGAGGAAACTCGGGCTAATA
CCGGATGTGCTCCTTCCACATCAGTGGATTGAGGAAAGGTAGCTTCGGCTTCCGCATCTGGATGGGCCCGC
GGCGCATTAGCTTGTTGGTGAGGTAACGGCTCACCAAGGCGACGATGCGTAGCTGGTCTGAGAGGACGAT
CAGCCACACTGGGACTGAGACACGGCC
>Zotu4238
GAACGCTGGCGGCGTGCCTAATACATGCAAGTCGAGCGAACTTGAGGGAGCTTGCTCCCAAAAGTTAGCG
GCGGACGGGTGAGTAACACGTGGGCAACCTGCCTATAAGACTGGGATAACTTCGGGAAACCGGAGCTAAT
ACCGGATAATTCATATCTACTCATGTAGATATGCTGAAAGACGGTTTACGCTGTCACTTATAGATGGGCCCG
CGGCGCATTAGCTAGTTGGTGAGGTAACGGCTCACCAAGGCAACGATGCGTAGCCGACCTGAGAGGGTGA
TCGGCCACACTGGGACTGAGACACGGCC
>Zotu4240
GAACGCTGGCGGCATGCTTTACACATGCAAGTCGAACGGTAACAGGTCTTCGGATGCTGACGAGTGGCGA
ACGGGTGAGTAATGCATCGGAACGTGCCCAGTCGTGGGGGATAACGCAGCGAAAGCTGTGCTAATAACGC
ATACGATCTATGGATGAAAGCGGGGGATCGCAAGACCTCGCGCGATTGGAGCGGCCGATGTCAGATTAGC
TAGTTGGTGGGGTAAAGGCCCACCAAGGCGACGATCTGTAGCTGGTCTGAGAGGACGACCAGCCACACTG
GGACTGAGACACGGCC
>Zotu4243
GAACGCTGGCGGCGTGCTTAACACATGCAAGTCGAACGGTGAAGCCCAGCTTGCTGGGCGGATCAGTGGC
GAACGGGTGAGTAACACGTGAGCAACCTGCCCTCCACTTCGGGATAAGCCTTGGAAACGGGGTCTAATAC
CGGATATGAGCGCTCACCGCATGGTGGGTGTTGGAAAGTTTTTCGGTGGGGGATGGGCTCGCGGCCTATC
AGCTTGTTGGTGGGGTGATGGCCTACCAAGGCGTCGACGGGTAGCCGGCCTGAGAGGGCGACCGGCCAC
ACTGGGACTGAGACACGGCC
>Zotu4247
GAACGCTGGCGGCGTGCCTAATACATGCAAGTCGTGCGGAACCTTCGGGTTCAGCGGCGGACGGGTGAGT
AACACGTGGGCAACCTGCCCGACAGATCGGGATAACGCTTGGAAACGAGTGCTAATACCGGATACGCTTTT
GAGGCGCATGCCTCGAAAGGGAAAGTTGCTTTTGCAGCACTGTCGGATGGGCCCGCGGCGCATTAGCTAG
TTGGTGAGGTAACGGCTCACCAAGGCGACGATGCGTAGCCGACCTGAGAGGGTGACCGGCCACACTGGG
ACTGAGACACGGCC
>Zotu4250

GAACGCTGGCGGCAGGCTTAACACATGCAAGTCGAACGCCCCGCAAGGGGAGTGGCAGACGGGTGAGTA
ACGCGTGGGAATCTACCCTTTACTACGGAATAACGCATGGAAACGTGTGCTAATACCGTATGTGTCCTTCG
GGAGAAAGATTTATCGGTAAAGGATGAGCCCGCGTTGGATTAGCTAGTTGGTGGGGTAAAGGCCTACCAA
GGCGACGATCCATAGCTGGTCTGAGAGGATGATCAGCCACATTGGGACTGAGACACGGCC
>Zotu4252
AAATGCTGGCGGCGTGCCTAATACATGCAAGTCGTGCGGAAACACTTCGGGTGTCGAGCGGCGGACGGCT
GAGTAACGCATAGGAATGTACCCCAAAGTGAGGGATAACTCCGAGAAATCGGGCTAATACCGCATATGC
TCTAAGGAGTAAAGCTTCGGCGCTTTGGGAACAGCCTATGTCCGATTAGCTAGTTGGTGAGGTAAAGGCTT
ACCAAGGCGACGATCGGTAGCTGGTCTGAGAGGATGATCAGCCAGACTGGGACTGAGACACGGCC
>Zotu4254
GAACGCTGGCGGCATGCCTAACACATGCAAGTCGAACGATGCTTTCGGGCATAGTGGCGCACGGGTGCGT
AACGCGTGGGAATCTGCCCTTGGGTCTGGGATAACAGTTGGAAACGACTGCTAATACCGGATGATATCGC
GAGATCAAAGATTTATCGCCCGAGGATGAGCCCGCGTAGGATTAGCTAGTTGGTGGGGTAAAGGCCTACC
AAGGCGACGATCCTTAGCTGGTCTGAGAGGATGATCAGCCACACTGGGACTGAGACACGGCC
>Zotu4257
GAACGCTGGCGGCGTGCTTAACACATGCAAGTCGAACGGAAAGGCCCCCACCATTTTTGGTGGGGGTGCT
CGAGTGGCGAACGGGTGAGTAACACGTGGGTGATCTGCCCTGTACTTCGGGATAAGCTTGGGAAACTGGG
TCTAATACCGGATAGGACCATGGTTTAGTGTCTCATGGTGGAAAGATTATTGTTTTGGTGCAGGATGAGCT
CGCGGCCTATCAGCTTGTTGGTGGGGTAATGGCCTACCAAGGCGTCGACGGGTAGCCGGCCTGAGAGGGT
GGACGGCCACATTGGGACTGAGATACGGCC
>Zotu4260
GAACGCTGGTAACGTGGATACCGGCATGCAAGTCAAGGGGGCCCGCAAGGGCAACCGGCGAACGGCATA
GTAACACGTGGGTACATACCCTAAAGTGGAGGACAGCTCGTCGAAAGACGGGGTAATACTCCATAGTCTC
GCAAGAGTAAAGTCGCAAGACGCTTTAGGAATGGCCTGCGGCCTATCAGGTAGTTGGCGGTGTAATAGAC
CACCAAGCCTATGACGGGTAGGGGGCGTGAGAGCGCGACCCCCATGAATGGCACTGCGACACGGGC
>Zotu4262
GAACGCTGGCGGCGTGCCTAATACATGCAAGTCGAGCGAACTCTTTAAAAGCTTGCTTTTGAAGAGTTAGC
GGCGGACGGGTGAGTAACACGTGGGCAACCTGCCTATAAGACTGGGATAACTTCGGGAAACCGGAGCTA
ATACCGGATAATTCTTTTCTACACATGTAGGAAAGCTGAAAGACGGTTTACGCTGTCACTTATAGATGGGCC
CGCGGCGCATTAGCTAGTTGGTGAGGTAACGGCTCACCAAGGCTACGATGCGTAGCCGACCTGAGAGGGT
GATCGGCCACACTGGGACTGAGACACGGCC
>Zotu4265
GAACGCTGGCGGCGTGCATAACACATTCAAGTCGAACGGAGATACATTGATTAATCCTTCGGGAAGCGTTG
ATGTATTTTAGTGGCGGACGGGTGAGTAACGCGTGAGCAACCTGCCCTTCATTGAGGGATAACGTCTGGA
AACGGACGCTAATACCTCATGACACATAGTTACCGCATGGTAGGTATGTTAAAGGAATCTGATGAAGGATG
GGCTCGCGTATGATTAGACAGTTGGCGGGGTAACGGCCCACCAAATCAACGATCATTAGCCGAACTGAGA
GGTTGAACGGCCACATTGGAACTGAGAAACGGTC
>Zotu4267
GAACGCTGGCGGCATGCCTAACACATGCAAGTCGAACGAGGCCTTCGGGTCTAGTGGCGCACGGGTGCGT
AACGCGTGGGAATCTGCCCCTTGGTTCGGAATAACAGCGAGAAATTGCTGCTAATACCGGATAATGACGTA
AGTCCAAAGATTTATCGCCAAGGGATGAGCCCGCGTAGGATTAGCTAGTTGGTGTGGTAAAGGCGCACCA
AGGCGACGATCCTTAGCTGGTCTGAGAGGATGATCAGCCACACTGGGACTGAGACACGGCC
>Zotu4269
GAACGCTGGCGGCAGGCTTAACACATGCAAGTCGAACGCCCCGCAAGGGGAGTGGCAGACGGGTGAGTA
ACGCGTGGGAATCTACCCAGATCTACGGAACAACAGAGGGAAACTTCTGCTAATACCGTATACGCCCTACG
GGGGAAAGATTTATCGGATTTGGATGAGCCCGCGTAAGATTAGCTAGTTGGTGAGGTAATGGCTCACCAA
GGCGACGATCTTTAGCTGGTCTGAGAGGATGATCAGCCACACTGGGACTGAGACACGGCC
>Zotu4273
GAACGCTGGCGGCATGCCTAACACATGCAAGTCGAACGAGACCTTCGGGTCTAGTGGCGCACGGGTGCGT
AACGCGTGGGAATCTGCCCTTCGGTTGGGAATAACTCAGGGAAACTTGAGCTAATACCTTATAATGTCTTC

GGACCAAAGATTTATCGCCGAGGGATGAGCCCGCGTAGGATTAGCTAGTTGGTGGGGTAATGGCCTACCA
AGGCGACGATCCTTAGCTGGTCTGAGAGGATGATCAGCCACACTGGGACTGAGACACGGCC
>Zotu4275
GAACGCTGGCGGCAGGCTTAACACATGCAAGTCGAGCGGGCCCTTCGGGGTCAGCGGCGGACGGGTTAG
TAACGCGTGGGAACGTGCCCTTTGCTTCGGAATAGCCCTGGGAAACTGGGGGTAATACCGGATGTGCCCT
GAGGGGGAAAGATTTATCGGCAAGGGATCGGCCCGCGTCTGATTAGGTAGTTGGTGCGGTAACGGCGCA
CCAAGCCGACGATCGGTAGCTGGTTTGAGAGGATGATCAGCAACACTGGGACTGAGACACGGCC
>Zotu4277
GAACGCTGGCGGCGTGCTTAACACATGCAAGTCGAACGGAAAGGCCTGTTTTCCCTTGTGGATTGCGGGTA
CTCGAGTGGCGAACGGGTGAGTAACACGTGAGTGATCTGCCCCCAACTTGGGTATAAGCCTGGGAAACTG
GGTCTAATTCCCGATAGGACTGCAGGGTGGTGCCTGTGGTGGAAAACGATTTTTTAGTGGTTGGGGATGA
GCTCGCGGCCTATCAGCTTGTTGGTGGGGTAATGGCCTACCAAGGCGGCGACGGGTAGCCGGCCTGAGAG
GGTGGACGGCCACATTGGGACTGAGATACGGCC
>Zotu4279
GAACGCTGGCGGCGTGCTTAACACATGCAAGTCACGGGGGCCCGCAAGGGCAACCGGCGAACGGGTGCG
TAACACGTGAGCGACCTGCCGAGGTGTGGGGGATAGCCGGGCCAACGCCCGGGTAATACCGCATACGATC
ACCGGGGGGCATCCCCTGGTGATGAAACCTCCGGGGCACTTCGAGGGGCTCGCGGCTTATCAGCTAGTTG
GCGGGGTAACGGCCCACCAAGGCGACGACGAGTAGCTGGTCTGAGAGGATGGCCAGCCACATTGGGACT
GAGAGACGGCC
>Zotu4281
GAACGCTGGCGGCAGGCCTAACACATGCAAGTCGAGCGGCAGCGGGAAAGTAGCTTGCTACTTTTGCCGG
CGAGCGGCGGACGGGTGAGTAATGCCTGGGAAATTGCCCAGTCGAGGGGGATAACAGTTGGAAACGACT
GCTAATACCGCATACGCCCTACGGGGGAAAGCAGGGGACCTTCGGGCCTTGCGCGATTGGATATGCCCAG
GTGGGATTAGCTAGTTGGTGAGGTAATGGCTCACCAAGGCGACGATCCCTAGCTGGTCTGAGAGGATGAT
CAGCCACACTGGAACTGAGACACGGTC
>Zotu4283
GAACGCTGGCGGCGTGCCTAAGACATGCAAGTCGAGTGGGTTTAGACCCACGGTAAACGGGTGAGTAATA
CCTAGGAACTTTCTTTAAGTGGAGCATAACCAGCCGAAAGGTTGGATAATACTCCATACCATCGCAAGATG
AAAGTCGCAAGACGCTTAAAGTGAGGCCTGGGCACCATCAGCTAGTTGGTAAGGTAATGGCTTACCAAGG
CTATGACGGTTAGGGGCCCCGAGAGGGGAGTCCCCCACACTGGAACTGCGACACGGTC
>Zotu4286
GAACGCTGGCGGCAGGCTTAACACATGCAAGTCGAACGGTTGTAGCAATACAGCAGTGGCGCACGGGTG
AGTAACGCGTGGGAACATACCTTAGAGTTCGGAATAACAGCGGGAAACTGCTGCTAATACCGGATATTCTC
TCCGGAGGAAAGATTTATCGCTCTAAGATTGGCCCGCGTCAGATTAGCTAGTTGGTGGGGTAATGGCCTAC
CAAGGCTCCGATCTGTAGCTGGTCTGAGAGGACGACCAGCCACACTGGAACTGAGACACGGTC
>Zotu4288
GAACGCTGGCGGCGTGCCTAATACATGCAAGTCGAACGCATTTTCTTTCACCGTAGCTTGCTACACCGAAA
GAAAATGAGTGGCGAACGGGTGAGTAACACGTGGGCAACCTGCCCATCAGCGGGGGATAACACTTGGAA
ACAGGTGCTAATACCGCATAATTCCATTTACCGCATGGTAGAGGGATGAAAGGCGCTTTTGCGTCACTGAT
GGATGGACCCGCGGTGCATTAGCTAGTTGGTGGGGTAACGGCCTACCAAGGCTGCGATGCATAGCCGACC
TGAGAGGGTGATCGGCCACACTGGGACTGAGACACGGCC
>Zotu4291
GAACGCTGGCGGCGTGCTTAACACATGCAAGTCGAACGATTGAAGCTGGAGCTTGCTCCGGCCGATTTAGT
GGCGGACGGGTGAGTAACGCGTGGGTAACCTGCCTCATACAGGGGGATAGCAGTTGGAAACGACTGGTA
AAACCGCATAAGCGCACGGTGTCGCATGACACAGTGTGAAAACACTCCGGTGGTATGAGATGGACCCGCG
TCTGATTAGCTAGTTGGTGAGGTAACGGCCCACCAAGGCGACGATCAGTAGCCGGCCTGAGAGGGTGAAC
GGCCACATTGGGACTGAGACACGGCC
>Zotu4293
GAACGCTGGCGGCGTGGATAAGACATGCAAGTCGAACGGGATCATTGAGGTAGCAATATTTCGATGGTCT
AGTGGCGAACGGGTGCGTAACACGTGGATACATTCCGGGAAGCGGGGGATAGCCCAGGGAAACTTGGAT

TAATACCGCATGTGGTCGAGAGACTAAAGGCAGGGACCGCAAGGCCTGCCACTTTCCGATTGGTCCGCGG
CCTATCAGCTAGTTGGCGGGGTAAAAGCCCACCAAGGCTACGACGGGTAGCTGGTCTTAGAGGACGACCA
GCCACACTGGAACTGAGACACGGTC
>Zotu4295
GAACGCTGGCGGCAGGCTTAACACATGCAAGTCGAGCGGACCTTTCGGGGTCAGCGGCGGACGGGTGAG
TAACGCGTGGGAACGTGCCTTCTGGTTCGGAATAACCCTGGGAAACTAGGGCTAATACTGGATACGCCCTT
ATGGGGAAAGGTTTACTGCCGGAAGATCGGCCCGCGTCTGATTAGCTAGTTGGTGGGGTAACGGCCTACC
AAGGCGACGATCAGTAGCTGGTCTGAGAGGATGATCAGCCACACTGGGACTGAGACACGGCC
>Zotu4298
GAACGCTGGCGGAATGCTTTACACATGCAAGTCGAACGGCAGCACGGGGGCAACCCTGGTGGCGAGTGG
CGAACGGGTGAGTAATACATCGGAACGTGCCCAGTCGTGGGGGATAACGTAGCGAAAGCTACGCTAATAC
CGCATACGAACTGAGGTTGAAAGCGGGGGATCGCAAGACCTCGCGCGATTGGAGCGGCCGATGGCAGAT
TAGGTAGTTGGTGGGGTAAAGGCCTACCAAGCCGACGATCTGTAGCTGGTCTGAGAGGACGACCAGCCAC
ACTGGGACTGAGACACGGCC
>Zotu4300
GAACGCTGGCGGCATGCCTAACACATGCAAGTCGAACGAAGCCTTCGGGCTTAGTGGCGCACGGGTGCGT
AACGCGTGGGAATCTGCCCTTGGGTTCGGAATAACAGTTAGAAATGACTGCTAATACCGGATGATGTCTTC
GGACCAAAGATTTATCGCCCAGGGATGAGCCCGCGTAGGATTAGGTAGTTGGTGGGGTAATGGCCTACCA
AGCCGACGATCCTTAGCTGGTCTGAGAGGATGATCAGCCACACTGGGACTGAGACACGGCC
>Zotu4302
GAACGCTGGCGGCGTGCCTAATACATGCAAGTCGAGCGGAACATTTATTGGTGCTTGCACCGATAAATGTT
TAGCGGCGGACGGGTGAGTAACACGTGGGCAACCTGCCCTGTAGATTGGGATAACTCCGGGAAACCGGG
GCTAATACCGAATAATACTTTTTAACTCATGTTAGAAAGTTGAAAGATGGTTCTGCTATCACTACAGGATGG
GCCCGCGGCGCATTAGCTAGTTGGTGAGGTAACGGCTCACCAAGGCGACGATGCGTAGCCGACCTGAGAG
GGTGATCGGCCACACTGGGACTGAGACACGGCC
>Zotu4304
GAACGCTGGCGGCAGGCTTAACACATGCAAGTCGAGCGGAGATGAGGTGCTTGCACCTTATCTTAGCGGC
GGACGGGTGAGTAATGCTTAGGAATCTGCCTATTAGTGGGGGACAACATTCCGAAAGGAATGCTAATACC
GCATACGTCCTACGGGAGAAAGCAGGGAATCTTCAGACCTTGCGCTAATAGATGAGCCTAAGTCGGATTA
GCTAGTTGGTGGGGTAAAGGCCTACCAAGGCGACGATCTGTAGCGGGTCTGAGAGGATGATTCGCCACAC
TGGGACTGAGACACGGCC
>Zotu4306
GAACGCTGGCGGCGTGCCTAATACATGCAAGTCGAGCGGACAGATGGGAGCTTGCTCCCTGATGTTAGCG
GCGGACGGGTGAGTAACACGTGGGTAACCTGCCTGTAAGACTGGGATAACTCCGGGAAACCGGGGCTAA
TACCGGATGCTTGATTGAACCGCATGGTTCAATTATAAAAGGTGGCTTTTAGCTACCACTTACAGATGGACC
CGCGGCGCATTAGCTAGTTGGTGAGGTAACGGCTCACCAAGGCGACGATGCGTAGCCGACCTGAGAGGGT
GATCGGCCACACTGGGACTGAGACACGGCC
>Zotu4308
GAACGCTGGCGGCAGGCTTAACACATGCAAGTCGAGCGCCGTAGCAATACGGAGCGGCAGACGGGTGAG
TAACACGTGGGAACGTACCTTTTGGTTCGGAACAACTGAGGGAAACTTCAGCTAATACCGGATAAGCCCTT
ACGGGGAAAGATTTATCGCCGAAAGATCGGCCCGCGTCTGATTAGCTAGTTGGTGAGGTAACGGCTCACC
AAGGCGACGATCAGTAGCTGGTCTGAGAGGATGATCAGCCACACTGGGACTGAGACACGGCC
>Zotu4311
GAACGCTGGCGGCATGCCTAACACATGCAAGTCGAACGAGACCTTCGGGTCTAGTGGCGCACGGGTGCGT
AACGCGTGGGAATCTGCCCTTAGGTTCGGGATAACTCAGGGAAACTTGAGCTAATACCGGATGACGACTTC
GGTCCAAAGATTTATCGCCTAAGGATGAGCCCGCGTAGGATTAGCTTGTTGGTGAGGTAAAGGCTCACCAA
GGCGACGATCCTTAGCTGGTCTGAGAGGATGATCAGCCACACTGGGACTGAGACACGGCC
>Zotu4313
GAACGCTGGCGGCGTGCTTAACACATGCAAGTCGAACGGAAAGGCCCTGCTTGCAGGGTACTCGAGTGGC
GAACGGGTGAGTAACACGTGGGTGATCTGCCCTGTACTTTGGGATAAGCCTGGGAAACTGGGTCTAATAC

CGGATAGGACCACATCGTAGGGGTGTGGTGGAAAGATTTTTCGGTGCAGGATGAGCCCGCGGCCTATCAG
CTTGTTGGTGGGGTAATGGCCTACCAAGGCGTCGACGGGTAGCCGGCCTGAGAGGGTGTACGGCCACATT
GGGACTGAGACACGGCC
>Zotu4315
GAACGCTGGCGGCGTGCATAACACATGCAAGTCGAACGATGAAACTTAGTAGACTTCTTCGGAAAGACACT
AAGCGGATTAGTGGCGAACGGGTGAGTAACGCGTGAGTAACCTGCCTTACACAAGGGGATAGCCTTTGGA
AACGAAGAATAATACCCTATAAAACCATAAAAGCACATGCTAGAATGGTCAAAGTGATAGCGGTGTAAGA
TGGACTTGCGTCTGATTAGCTAGTTGGTGAGATAAAAGCTCACCAAGGCAACGATCAGTAGCCGGCTTGAG
AGAGTGTACGGCCACATTGGGACTGAGACACGGCC
>Zotu4318
GAACGCTGGCGGCGTGCCTAATACATGCAAGTCGAACGCATTTTCTTTCACCGTAGCTTGCTACACCGAAA
GAAAATGAGTGGCGAACGGGTGAGTAACACGTGGGTAACCTGCCCATCAGCGGGGGATAACACTTGGAA
ACAGGTGCTAATACCGCATAATTCCATTTACTGCATGGTAGATGGATGAAAGGCGCTTTTGCGTCACTGAT
GGATGGACCCGCGGTGCATTAGCTAGTTGGTGGGGTAACGGCCTACCAAGGCTGCGATGCATAGCCGACC
TGAGAGGGTGATCGGCCACACTGGGACTGAGACACGACC
>Zotu4320
GAACGCTGGCGGCGTGCCTAACACATGCAAGTCGAACGAGGTCCATCCTTCGGGAGAAGACCTAGTGGCG
AACGGGTGAGTAACACGTGAGGAACCTGCCCCGAAGACCGGGATAACTCTCCGAAAGGATAGCTAATACC
GGATACCCCCACCGAGCCGCATGGTTTGGTGAGGAAATGGATTCCGCTTCGGGAGGGCCTCGCGGCCTAT
CAGCTAGTTGGTGGGGTAACGGCCCACCAAGGCATCGACGGGTAGCTGGTCTGAGAGGACGATCAGCCAC
ACTGGGACTGAGACACGGCC
>Zotu4325
GAACGCTAGCGGCAGGCCTAATACATGCAAGTCGAGCGGGTGTTAGCAATAACATTAGCGGCGGACGGGT
GAGTAACGCGTACACAACATGCCTTTGGTTGGGGGATAGCCAGTGGAAACGCTGAGTAATACCGCATAAG
ACTCCGGCACGGCATCGTGCAGAAGTTAAAGTTACGGCGACCAGAGATTGGTGTGCGTTCGATTAGCTAGT
TGGCGGGGTAACGGCCCACCAAGGCGACGATCGATAGGGGGTGTGAGAGCATGGCCCCCACACGGGTA
CTGAGACACGGAC
>Zotu4327
GAACGCTGGCGGCGTGCTTAACACATGCAAGTCGAACGGGATCCAAAGGCTTTTTGTTTTTGGTGAGAGTG
GCGAACGGGTGAGTAACACGTGAGTAACCTGCCCCCTTCTTTTGGATAACCGCATGAAAGTGTGGCTAATA
CAGGATATTCCAGGCTGGTCGCATGATTGGTTTGGGAAATGGTTTGGTCTGGTGGGGGATGGGCTCGCGG
CCTATCAGCTTGTTGGTGGGGTGATGGCCTACCAAGGCGGTGACGGGTAGCCGGCCTGAGAGGGTGGGC
GGTCACACTGGGACTGAGATACGGCC
>Zotu4329
GAACGCTGGCGGCAGGCCTAACACATGCAAGTCGAACGGATCCTTCGGGATTAGTGGCAGACGGGTGAGT
AACACGTGGGAACGTGCCCTTTGGTTCGGAACAACTCAGGGAAACTTGAGCTAATACCGGATGTGCCCTTC
GGGGGAAAGATTTATCGCCATTGGAGCGGCCCGCGTCTGATTAGGTAGTTGGTGGGGTAAAGGCCCACCA
AGCCTACGATCAGTAGCTGGTCTGAGAGGATGATCAGCCACACTGGGACTGAGACACGGCC
>Zotu4331
GAACGCTGGCGGCGTGCCTAACACATGCAAGTCGAACGGGTGTAGCAATACATCAGTGGCGGACGGGTG
AGTATAGCATGGGTATCTGCCTTGTGGAGGGGGAATAGCTGCGGGAAGCTGCAGGTAAGTCCGCGTAAGCA
AAGTGTACGAGAGTGCATTTTGGAAAGTATCGGGAACAGTAATGTCCGGTAGCCATGAGATGAGCCCATG
TCCCATTAGCTAGTTGGTAGGGTAAAGGCCTACCAAGGTAAAGATGGTTAGCTGGTCTGAGAGGACGACC
AGCCACACTGGAACTGAGATACGGTC
>Zotu4335
GAACGCTGGCGGCATGCCTAACACATGCAAGTCGAACGAAGGCTTCGGCCTTAGTGGCGCACGGGTGCGT
AACGCGTGGGAATCTGCCTTGGGGTTCGGAATAACTCCCCGAAAGGGGTGCTAATACCGGATGATGTCGA
AAGACCAAAGATTTATCGCCCTGAGATGAGCCCGCGTAAGATTAGCTAGTTGGTGGGGTAAAGGCCTACC
AAGGCGACGATCTTTAGCTGGTCTGAGAGGATGATCAGCCACACTGGGACTGAGACACGGCC
>Zotu4337

GAACGCTGGCGGCGTGCTTAACACATGCAAGTCGAGCGGTAAGGCCCTTCGGGGTACACGAGCGGCGAA
CGGGTGAGTAACACGTGAGCAATCTGCCCTTCACTCTGGGATAGCCTCGGGAAACTGAGATTAATACCGGA
TACGACCACTTCAGGCATCTGATGGTGGTGGAAAGTTCCGGCGGTGAAGGATGAGCTCGCGGCCTATCAG
CTTGTTGGCGGGGTAATGGCCCACCAAGGCTTCGACGGGTAGCCGGCCTGAGAGGGTGACCGGCCACACT
GGGACTGAGACACGGCC
>Zotu4339
GAACGCTGGCGGCGTGCCTAATACATGCAAGTCGAGCGAACCTCAAGAAGCTTGCTTCTTGAGGTTAGCG
GCGGACGGGTGAGTAACACGTGGGCAACCTGCCTATAAGACTGGGATAACTTCGGGAAACCGGAGCTAAT
ACCGGATAATTCTTCTTTACACATGTAGAGAAGCTGAAAGGCGGCTTCGGCTGTCACTTATAGATGGGCCC
GCGGCGCATTAGCTAGTTGGTGAGGTAACGGCTCACCAAGGCAACGATGCGTAGCCGACCTGAGAGGGT
GATCGGCCACACTGGGACTGAGACACGGCC
>Zotu4341
CAACGCTGGCGGCGTGCCTAATGCATGCAAGTCGAGCGGGGTGCCTTTCGGGGCACTTACGCGGCGGACG
GGTGCGGAACACGTGGGCAACCTGCCCCGAGGTGGGGGATAGCCGGTGGAAACACCGGGTAATTCCGCA
TGAGGTCACTGGCTGGAGGGCTGGTGAGGAAAGGGTCTTTGACCCGCCTTGGGAGGGGCCTGCGGCCGA
TTAGCTAGACGGTGGGGTAACGGCCTACCGTGGCGATGATCGGTAGCTGGTCTGAGAGGACGGCCAGCCA
CACGGGGACTGAGACACGGCC
>Zotu4343
GAACGCTAGCGGCAGGCTTAACACATGCAAGTCGAGGGGTATAAGGAGCTTGCTCCTTAGAGACCGGCGC
ACGGGTGCGTAACGCGTATGCAACCTACCTTGAACAGGGGAATAGCCCAGAGAAATTTGGATTAATGCCC
CATGGTATTCTGAGATGGCATCATCTTATTATTAAAGATTCATCGGTTCAAGATGGGCATGCGTCCCATTAG
CTTGTTGGTGAGGTAACGGCTCACCAAGGCGATGATGGGTAGGGGTCCTGAGAGGGAGATCCCCCACACT
GGTACTGAGACACGGAC
>Zotu4346
GAACGCTGGCGGCATGCTTAACACATGCAAGTCGCACGAACCTTTCGGGGTTAGTGGCGGACGGGTGAGT
AACGCGTAGGGATCTGTCCATGGGTGGGGGATAACACTGGGAAACTGGTGCTAATACCGCATGACACCTG
AGGGTCAAAGGCGAGAGTCGCCTGTGGAGGAACCTGCGTTCGATTAGCTAGTTGGTGGGGTAACTGCCTA
CCAAGGCGATGATCGATAGCTGGTCTGAGAGGATGATCAGCCACACTGGGACTGAGACACGGCC
>Zotu4350
GAACGCTGGCGGCAGGCTTAACACATGCAAGTCGAGCGGGGAGATGTAGCTTGCTACATTTCCTAGCGGC
GGACGGGTGAGTAATGCTTAGGAATCTGCCTATTAGTGGGGGACAACATTCCGAAAGGAATGCTAATACC
GCATACGCCCTACGAGGGAAAGCAGGGGATCTTCGGACCTTGCGCTAATAGATGAGCCTAAGTCAGATTA
GCTAGTTGGTGGGGTAAAGGCCTACCAAGGCGACGATCTGTAGCGGGTCTGAGAGGATGATCCGCCACAC
TGGGACTGAGACACGGCC
>Zotu4352
GAACGCTAGCGGCAGGCTTAACACATGCAAGTCGAGGGGCATCGGGGATAGCAATATCCGCCGGCGACC
GGCGAAAGGGTGCGTAACACGTGAGCAACATTCCCGTATCTGGGAGATAACCGATGGAAACGTCGACTAA
CATCCCATAACAACCATGAAGGCATCTTCGAGGTTTGAAAACTCCGGTGGATACGGATTGGCTCGCGTCCC
ATTAGCTAGTTGGCGTGGTAACGGCACACCAAGGCGACGATGGGTAGGGGTTCTGAGAGGAAGGTCCCCC
ACATTGGAACTGAGACACGGTC
>Zotu4354
GAACGCTAGCGGCAGGCCTAATACATGCAAGTCGAACGGTGGCAGCAATGCCATAGTGGCGCACGGGTG
CGTAACGCGTAACCAACCTACCCAGAACTGGGGGATAGCCCGCCGAAAGGCGGATTAATACCGCATAAGC
TCTGCTTCCGGCATCGGAAGCTTAGTAAAGATTTATTGGTTTTGGATGGGGTTGCGTGCCATTAGCTAGTTG
GTGGGGTAACGGCCCACCAAGGCGACGATGGCTAGGGGAGCTGAGAGGCTGGTCCCCCACACGGGCACT
GAGATACGGGC
>Zotu4357
GAACGCTAGCGGCAGGCTTAATACATGCAAGTCGAACGGTTACAGCAATGTGACGAGTGGCGCACGGGTG
AGTAACGCGTACATAACATACCTTTAACTGGCGAATAGCCTTGGGAAACTGAGAGTAATACGCCATAACAT
TGTGATTCGGCATCGGATTACAATTAAAGTTCCGGCGGTTAGAGATTGATGTGCGTTGGATTAGGTAGTTG

GTGAGGTAACGGCTCACCAAGCCAGCGATCCATAGGGGGCGTGAGAGCGTGGTCCCCCACACGGGTACTG
AGACACGGAC
>Zotu4360
GAACGCTAGCGGCAGGCTTAACACATGCAAGTCGAGGGGTATAGTTCTTCGGAGCTAGAGACCGGCGCAC
GGGTGCGTAACGCGTATGCAATCTACCTTTTACAGAGGGATAGCCCAGAGAAATTTGGATTAATACCTCAT
AGTATAGTACAATCGCATGATTGAACTATTAAAGTCACAACGGTAAAAGATGAGCATGCGTCCCATTAGCT
TGTTGGTAAGGTAACGGCTTACCAAGGCTACGATGGGTAGGGGTCCTGAGAGGGAGATCCCCCACACTGG
TACTGAGACACGGAC
>Zotu4366
GAACGCTGGCGGCGTGCCTAATACATGCAAGTCGAGCGGATCTCTTCGGAGATCAGCGGCGGACGGGTGA
GTAACACGTGGGCAACCTGCCTATAAGACTGGGATAACTTCGGGAAACCGGAGCTAATACCGGATAACAT
CAAGGGCCACATGGTCTTCGATTAAAAGATGGCGTCAAGCTATCACTTATAGATGGGCCCGCGTCGCATTA
GCTAGTTGGTGAGGTAACGGCTCACCAAGGCGACGATGCGTAGCCGACCTGAGAGGGTGATCGGCCACAC
TGGGACTGAGACACGGCC
>Zotu4370
GAACGCTGGCGGCGTGCCTAATACATGCAAGTCGAGCGAATCAAAGGGAGCTTGCTCCCTTTTGGTTAGCG
GCGGACGGGTGAGTAACACGTGGGCAACCTGCCTATAAGACTGGGATAACTTCGGGAAACCGGAGCTAAT
ACCGGATAATTCATATCTACTCATGTAGGTATGCTGAAAGACGGTTTCGGCTGTCACTTATAGATGGGCCCG
CGGCGCATTAGCTAGTTGGTGAGGTAACGGCTCACCAAGGCAACGATGCGTAGCCGACCTGAGAGGGTGA
TCGGCCACACTGGGACTGAGACACGGCC
>Zotu4374
GAACGCTAGCGGCAGGCCTAATACATGCAAGTCGAACGGGATTGGGGAGCTTGCTCCCCATGAGAGTGGC
GCACGGGTGCGTAACACGTATGCAACCTACCTTTACCAGGGGGATAGCCTCTCGAAAGAGAGATTAACACC
GCATAACATTGAGCTATGGCATCATAGTTTAATCAAATATTTATAGGGTAAAGATGGGCATGCGGAACATT
AGCTAGTTGGTGAGGTAACGGCTCACCAAGGCGACGATGTTTAGGGGATCTGAGAGGATGACCCCCCACA
CTGGTACTGAGACACGGAC
>Zotu4377
GAACGCTGGCGGCGTGCCTAATACATGCAAGTCGAGCGAATGAAGAGAGAAGCTTGCTTCTCTCGGATTT
AGCGGCGGACGGGTGAGTAACACGTGGGTAACCTACCTTATAGTTTGGGATAACTCCGGGAAACCGGGGC
TAATACCGAATAATACTTTTAAACGCATGTTTGAAAGTTGAAAGACGGTCTCGGCTGTCACTATAAGATGG
ACCCGCGGCGCATTAGCTAGTTGGTGAGGTAATGGCTCACCAAGGCCACGATGCGTAGCCGACCTGAGAG
GGTGATCGGCCACACTGGGACTGAGACACGGCC
>Zotu4379
GAACGCTGGCGGCATGCCTTACACATGCAAGTCGAACGGCAGCGCAGGAGCAATCCTGGCGGCGAGTGG
CGAACGGGTAAGTAATACATCGGAACGTGCCCAATCGTGGGGGATAACGCAGCGAAAGCTGTGCTAATAC
CGCATACGATCTACAGATGAAAGCAGGGGATCGCAAGACCTTGCGCGAATGGAGCGGCCGATGGCAGATT
AGGTAGTTGGTGAGGTAAAGGCTCACCAAGCCTTCGATCTGTAGCTGGTCTGAGAGGACGACCAGCCACA
CTGGGACTGAGACACGGCC
>Zotu4384
GAACGCTATCGGTATGCTTAACACATGCAAGTCGAACGGCAGCACAGTAAAGATTTCGGTCTTTACGGGTG
GCGAGTGGCGGACGGGTGAGTAAAGCGTAGGAATCTGTCCTGGAGAGTGGGATAACCCGAGGAAACTCG
GGCTAATACCGCATGATATCGAGAGATCAAAGCAGGGGACCCTTCGGGGCCTTGCGCTCTAGGATGAGCC
TACGTCGGATTAGCTAGTTGGTGGGGTAAAGGCCTACCAAGGCGACGATCCGTAGCTGGTCTGAGAGGAT
GACCAGCCACACTGGGACTGAGACACGTCC
>Zotu4386
GAACGCTAGCTGCAGGCTTAACACATGCAAGTCGAGGGGCAGCGGGAGTAGCAATACTTGCCGGCGACC
GGCGGAAGGGTGCGTAACGCGTGAGCAACATTCCCGTATCAGGGGGATAACCGGTGGAAACGCCGACTA
ATACCCCATAAGAACATCTGCGGCATCAAAGGTGTTTGAAAGATTCGTCGGATGCGGATTGGCTCGCGTGA
CATTAGCTAGTTGGTGAGGTAACGGCCCACCAAGGCGACGATGTCTAGGGGTTCTGAGAGGAAGATCCCC
CACATTGGAACTGAGACACGGTC

>Zotu4388
GAACGCTGGCGGCGTGGATGAGGCATGCAAGTCGAATGGGCTTAGACCCATGGCAGACGAGGTAGGAAC
ACGTAGGTACATACCCTAAAGTCACGGATAACCCGGCGAAAGTCGGAATAATACGTGATAGTCCCTTCGGG
GTAAAGATTTATCGCTTTAGGAATGGCCTGCGTTCTATCAGCTTGTTGGTAAGGTAACGGCTTACCAAGGCT
ACGACGGATAGGGGAGGTGAGAGCCTGACCCCCACCGATGGAACTGCGACACGGTC
>Zotu4390
GAACGCTGGCGGCGTGCCTAATACATGCAAGTCGAGCGGACTTTAAAAGCTTGCTTTTAAAGTTAGCGGCG
GACGGGTGAGTAACACGTGGGCAACCTGCCTGTAAGACTGGGATAACTTCGGGAAACCGGAGCTAATACC
GGATAATCCTTTTCCTCTCATGAGGAAAAGCTGAAAGACGGTTTACGCTGTCACTTACAGATGGGCCCGCG
GCGCATTAGCTAGTTGGTGAGGTAACGGCTCACCAAGGCGACGATGCGTAGCCGACCTGAGAGGGTGATC
GGCCACACTGGGACTGAGACACGGCC
>Zotu4392
GAACGCTGGCGGCGTGCTTAACACATGCAAGTCGAGCGAAGCAGTTAAGAAGATTCTTCGGATGATTCTTA
ACTGACTGAGCGGCGGACGGGTGAGTAACGCGTGGGTGACCTGCCCCATACCGGGGGATAACAGCTGGA
AACGGCTGCTAATACCGCATAAGCGCACAGAGCTGCATGGCTCGGTGTGAAAAACTCCGGTGGTATGGGA
TGGGCCCGCGTCTGATTAGGCAGTTGGCGGGGTAACGGCCCACCAAACCGACGATCAGTAGCCGGCCTGA
GAGGGCGACCGGCCACATTGGGACTGAGACACGGCC
>Zotu4394
GAACGCTGGCGGCGTGCTTAACACATGCAAGTCGAACGGAAAGGCCTCTGCTTGCAGAGGTACTCGAGTG
GCGAACGGGTGAGTAACACGTGGGTGATCTGCCCTGCACTTCGGGATAAGCCTGGGAAACTGGGTCTAAT
ACCGGATAGGACGATGGTTTGGATGCCATTGTGGAAAGTTTTTCGGTGTGGGATGAGCTCGCGGCCTATCA
GCTTGTTGGTGGGGTAATGGCCTACCAAGGCGTCGACGGGTAGCCGGCCTGAGAGGGTGTACGGCCACAT
TGGGACTGAGATACGGCC
>Zotu4396
GAACGCTGGCGGCGTGCTTAACACATGCAAGTCGAACGGTGAACCCTCTTCGGAGGGGGATCAGTGGCGA
ACGGGTGAGTAACACGTGGGCAACCTGCCCTCAGCTCTGGGATAACTCCGGGAAACCGGGGCTAATACCG
GATACGACTTCCTGTGGCATCTCGGGAAGTGGAAAGTTTTTCGGCTGAGGATGGGCCCGCGGCCTATCAGC
TTGTTGGTGGGGTAATGGCCCACCAAGGCGACGACGGGTAGCCGGCCTGAGAGGGCGATCGGCCACACT
GGGACTGAGACACGGCC
>Zotu4398
GAACGCTGGCGGCGTGCTTAACACATGCAAGTCGAACGGGAAACCTTTTATTGAAGCTTCGGCAGATTTAG
ATTGTTTCTAGTGGCGGACGGGTGAGTAACGCGTGGGTAACCTGCCTTGTACAGGGGGATAACAGTCAGA
AATGACTGCTAATACCGCATAAGCGCACAGGACCGCATGGTCCGGTGTGAAAAACTCCGGTGGTATAAGA
TGGACCCGCGTTGGATTAGCTAGTTGGTGAGGTAACGGCCCACCAAGGCGACGATCCATAGCCGGCCTGA
GAGGGTGAACGGCCACATTGGGACTGAGACACGGCC
>Zotu4400
GAACGCTGGAAATGTGGATACCGGCATGCAAGTCAAGGGGCCGCAAGGCACCGGCAAACGGCTTAGTAA
CACGTAGATACATACCGCAAAGTGGGGCATAGCTCGTCGAAAGACGGGGTAATTTCCCATAGTCCCGCAA
GGGTAAAGGCGCAAGCCGCTTTGTGAATGGTCTGCGGTCTATCAGCTAGTTGGTGAGGTAAAGGCTTACC
AAGGCTATGACGGATAGGGGGCGTGAGAGCGCGACCCCCAAGAATGGCACTGCGACACGGGC
>Zotu4402
GAGCGCTGGCGGCGTGCTTAACACATGCAAGTCGAGCGGAAAGGCCCTTCGGGGTACTCGAGCGGCGAA
CGGGTGAGTAACACGTGAGTAATCTGCCCTTGGCTTCGGGATAGCCACCGGAAACGGTGATTAATACCGG
ATACGACAACTTCTCGCATGGGATGGTTGTGGAAAGTTTTTCGGCCAGGGATGTGCTCGCGGCCTATCAGC
TTGATGGTGAGGTAATGGCTCACCATGGCTTCGACGGGTAGCCGGCCTGAGAGGGTGACCGGTCACACTG
GGACTGAGACACGGCC
>Zotu4404
GAACGCTGGCGGCAGGCTTAACACATGCAAGTCGAACGATGATTTCCCAGCTTGCTGGGAATGATTAGTG
GCGGACGGGTGAGTAACATTTAGGAATCTGCCTAGTAGTGGGGGATAGCTCGGGGAAACTCGAATTAATA
CCGCATACGACCTACGGGTGAAAGGGGGCGCAAGCTCTTGCTATTAGATGAGCCTAAATCAGATTAGCTA

GTTGGTGGGGTAAAGGCCCACCAAGGCGACGATCTGTAACTGGTCTGAGAGGATGATCAGTCACACCGGA
ACTGAGACACGGTC
>Zotu4406
GAACGCTGGCGGTATGCCTAACACATGCAAGTCGAACGAAGCCTTCGGGCTTAGTGGCGCACGGGTGCGT
AACGCGTGGGAATCTGCCCTTGGGTTCGGAATAACAGTTAGAAATGACTGCTAATACCGGATGATGACGTA
AGTCCAAAGATTTATCGCCCAAGGATGAGCCCGCGTAGGATTAGCTTGTTGGTGAGGTAAAAGCTCACCAA
GGCGACGATCCTTAGCTGGTCTGAGAGGATGATCAGCCACACTGGGACTGAGACACGGCC
>Zotu4408
GAACGCTGGCGGCATGCCTAACACATGCAAGTCGAACGAGATCTTCGGATCTAGTGGCGCACGGGTGCGT
AACGCGTGGGAATCTGCCCTTGGGTTCGGAATAACTCAGAGAAATTTGAGCTAATACCGGATGATGACGTA
AGTCCAAAGATTTATCGCCCAAGGATGAGCCCGCGTAGGATTAGCTAGTTGGTGAGGTAAAGGCTCACCA
AGGCGACGATCCTTAGCTGGTCTGAGAGGATGATCAGCCACACTGGGACTGAGACACGGCC
>Zotu4412
AAACGCTGGCGGCGTGCTTAACACATGCAAGTCGAACGGAAAGGCCCTTTCGGGGGTACTCGAGTGGCGA
ACGGGTGAGTAACACGTGGGTGATCTGCCCTGCACTTCGGGATAAGCTTGGGAAACTGGGTCTAATACCG
GATAGGACTACGCACTGCATGGTGTGTGGTGGAAAGCTTTTGCGGTGTGGGATGGGCCCGCGGCCTATCA
GCTTGTTGGTGGGATGATGGCCTACCAAGGCGACGACGGGTAGCCGGCCTGAGAGGGTGTCCGGCCACA
CTGGGACTGAGATACGGCC
>Zotu4414
GAACGCTAGCGGCAGGCCTAATACATGCAAGTCGAACGGTCAGTAGCAATACTGATAGTGGCGCACGGGT
GCGTAACACGTAACTAACCTACCCACAACTGGGGGATAGCCCGCCGAAAGGCGGATTAATACCGCATAAG
CTCTTGACCCGGCATCGGGTTATTAGTAAAGATTTATTGGTTGTGGATGGGGTTGCGGGTCATTAGCTAGT
TGGTTGGGTAACGGCTGACCAAGGCGACGATGACTAGGGGAGCTGAGAGGCTGGTCCCCCACACGGGCA
CTGAGATACGGGC
>Zotu4417
GAACGCTGGCGGCATGCCTAACACATGCAAGTCGAACGAGATCCTTCGGGGTCTAGTGGCGCACGGGTGC
GTAACGCGTGGGAATCTGCCCTTGGGTTCGGAATAACACAGAGAAATTTGTGCTAATACCGGATGATGTCT
TCGGACCAAAGATTTATCGCCCAAGGATGAGCCCGCGTAGGATTAGGTAGTTGGTGGGGTAATGGCCTAC
CAAGCCGACGATCCTTAGCTGGTCTGAGAGGATGATCAGCCACACTGGGACTGAGACACGGCC
>Zotu4419
GAACGCTAGCGGCAGGCCTAATACATGCAAGTCGAACGGCTCGCAAGAGCAGTGGCAAACGGGTGCGTA
ACGCGTAAGCAACCTGCCTCAAACTGGGGGATAGCCCGGCGAAAGCTGGGGTAAACCCGCACGGTCCCCT
TGAGTCACCTGGCTTATTGGGTAAACATTTATGGGTTTGAGAGGGGCTTGCGTCTGATTAGTTAGTTGGCA
GGGTAACGGCCTACCAAGACGATGATCAGTAGGGGTTCTGAGAGGATTGGCCCCCACATGGGTACTGAGA
TACGGAC
>Zotu4421
GAACGCTGGCGGCGTGCTTAACACATGCAAGTCGAACGATGACGGTGGTGCTTGCACCACCTGATTAGTG
GCGAACGGGTGAGTAACACGTGAGTAACCTGCCCCTCACTTCGGGATAACCTCGGGAAATCGTGGCTAAT
ACCGGATATGAACTCCTGCCGCATGGTGGGTGTTGGAAAGATTTATCGGTGAGGGATGGACTCGCGGCCT
ATCAGTTTGTTGGTGAGGTAATGGCTCACCAAGACGATGACGGGTAGCCGGCCTGAGAGGGCGACCGGCC
ACACTGGGACTGAGACACGGCC
>Zotu4424
GAACGCTGGCGGCAGGCCTAACACATGCAAGTCGAACGCCCCGCAAGGGGAGTGGCGGACGGGTGAGTA
ACACGTGGGAATTTTCCTCAAGGTACGGAACAACTCAGGGAAACTTGGGCTAATACCGTATGATGCAGAG
ATGCCAAAGATTTATCGCCTTGGGATAAGCCCGCGTCAGATTAGGTAGTTGGTGGGGTAATGGCCTACCAA
GCCTGTGATCTGTAGCTGGTCTGAGAGGACGATCAGCCACATTGGGACTGAGACACGGCC
>Zotu4426
ACCGTGTCTCAGTTCCAGTGTGGCTGATCATCCTCTCAGACCAACTAAAGATCGTCGCCTTGGTGAGCCATT
ACCCCACCAACTAGCTAATCCTGCATGGGCTCGTCTTGCAGCGATAAATCTTTCACCTCTCGGCTATATGCG

ACATTAGCAATTGCTTCCAACTGTTATTTCGCACTGCAAGGTAGATTCCCATGTATTACTCACCCGTCTGCCA
CTAGCAGAATTCTGTATTGCTACAGCTCTTGCCCGTTCGACTTGCATGTGTTAAGCACACCGCCAGCGTTCA
>Zotu4428
GAACGCTGGCGGCAGGCTTAACACATGCAAGTCGAACGCTCCGCAAGGAGAGTGGCAGACGGGTGAGTA
ACGCGTGGGAACGTACCTTTCAGTTCGGAATAACTCAGGGAAACTTGTGCTAATACCGGATACGCCCGCAA
GGGGAAAGATTTATCGCTGAAAGATCGGCCCGCGTTGGATTAGCTAGTTGGTGGGGTAAAGGCCTACCAA
GGCGACGATCCATAGCTGGTCTGAGAGGATGATCAGCCACACTGGGACTGAGACACGGCC
>Zotu4430
CAACGCTGGCGGCGTGCCTAACACATGCAAGTCGAACGAGAAAGGGTGAGTGTAGCAATACATTTGCCTG
AGTAAAGTGGCGACCGGGTGAGTAACACGTGGCCAACCTACCCTCGAACGGGGGATAACCTGGGGAAAC
TCGGGCTAATACCGCATAACGTCCCGGCGACATAAGCCGCTGGGACCAAAGGCCCGCAAGGGTCACTTGA
GGAGGGGGCTGCGGCCGATTAGCTAGTTGGTGAGGTAACGGCCCACCAAGGCAATGATCGGTAGCCGGC
CTGAGAGGGCGCACGGTCACACTGGCACTGAAACACGGGC
>Zotu4432
GAACGCTGGCGGCGTGCCTAATACATGCAAGTCGAACGAGAAAGTGGCTTCGGCCATGAGTACAGTGGCA
GACGGGTGAGTAATACATAGGTAACCTTCCCTCAAGATGGGGACAACCTTCCGAAAGGAGGGCTAATACC
GAATGAGACCACAGTGACATATGTCACAGGGGTTAAAGACGGCCTCTATTTATAAGCTTTCGCTTGAGGAT
GGGCTTATGCACCATTAGCTAGTTGGTAGGGTAACGGCCTACCAAGGCTGCGATGGTTAGCTGATCTGAGA
GGTTGATCAGCCACACTGGAACTGAGACACGGTC
>Zotu4434
GAACGCTGGCGGCGTGCCTAACACATGCAAGTCGAACGGGTGTAGCAATACATCAGTGGCGGACGGGTG
AGTATAGCATGGGTATCTGCCTTGTGGAGGGGAATAGCTGCGGGAAGCTGCAGGTAAGTCCGCGTAAGCA
AAGTGTACGAGAGTGCATTTTGGAAAGTATCGGGAACAGTAATGTCCGGTAGCCATGAGATGAGCCCATG
TCCCATTAGCTAGTTGGTAGGGTAAAGGCCTACCAAGGTAAAGATGGGTAGCTGGTCTGAGAGGACGACC
AGCCACACTGGAACTGAGATACGGTC
>Zotu4436
GAACGCTAGCGGCAGGCCTAATACATGCAAGTCGAGGGGCAGCACGGGTAGTAATACTTGGTGGCGACC
GGCGCACGGGTGCGTAACGCGTATGCAACCTACCTTATACAGGGGGATAGCCCAGAGAAATTTGGATTAA
TACCCCATAGTATCATTGAGAGGCATCTTGATATGATTAAAGATTTATTGGTATAAGATGGGCATGCGTCTG
ATTAGTTAGTTGGTGAGGTAACGGCTCACCAAGACATCGATCAGTAGGGGTTCTGAGAGGATGATCCCCCA
CACTGGCACTGAGATACGGGC
>Zotu4438
GAACGCTAGCGGCAGGCCTAATACATGCAAGTCGAGCGGATTGTAGCAATACAGTTAGCGGCGCACGGGT
GAGTAACGCGTACACAATCTACCTTCAACTGGGGAATAGCCTCGGGAAACTGGGAGTAATACCCCATAGTA
TAACAGAGCGGCATCGCTTTGTTATTAAAGTTCCGGCGGTTGAAGATGGGTGTGCGTCTGATTAGATTGTT
GGTGAGGTAACGGCTCACCAAGTCGATGATCAGTAAGGGGCGTGAGAGCGTGGCCCCTCACATGGACACT
GAGACACGGGT
>Zotu4440
GAACGCTGGCGGCGCGCCTAACACATGCAAGTCGAACGAGCGAGAGAGAGCTTGCTTTCTCGAGCGAGTG
GCGAACGGGTGAGTAACGCGTGAGGAACCTGCCTCAAAGAGGGGGACAACAGTTGGAAACGACTGCTAA
TACCGCATAAGCCCACGGCTCGGCATCGAGCAGAGGGAAAAGGAGCAATCCGCTTTGAGATGGCCTCGCG
TCCGATTAGCTAGTTGGTGAGGTAACGGCCCACCAAGGCGACGATCGGTAGTCGGACTGAGAGGTTGAAC
GGCCACATTGGGACTGAGACACGGCC
>Zotu4443
GAACGCTGGAAATGTGGATACTGGCATGCAAGTCAAGGGGACCGCAAGGTCACCGGCGAACGGCTTAGT
AACACGTAGATACATACCGCAAAGTGGGGCATAGCTCGTTGAAAAACGGGGTAATTTCCCATAGTCTCGCA
AGAGTAAACACGCAAGTGGCTTTGTGAATGGTCTGCGGTCTATCAGCTAGTTGGTGAGGTAAAGGCTCACC
AAGGCTATGACGGATAGGGGGCGTGAGAGCGCGACCCCCAAGAATGGCACTGCGACACGGGC
>Zotu4446

GAACGCTGGCGGCGTGCCTAATACATGCAAGTCGAGCGGACGGATGGGAGCTTGCTCCCTGAAGTTAGCG
GCGGACGGGTGAGTAACACGTGGGCAACCTGCCCTGCAGATGGGGATAACTCCGGGAAACCGGGGCTAA
TACCGAATAATCAGTTTCTCCGCATGGAGAAACTCTGAAAGACGGTTTCGGCTGTCACTGCAGGATGGGCC
CGCGGCGCATTAGCTAGTTGGTGGGATAACGGCCTACCAAGGCAACGATGCGTAGCCGACCTGAGAGGGT
GATCGGCCACACTGGGACTGAGACACGGCC
>Zotu4448
GAACGCTGGCGGCGTGCTTAACACATGCAAGTCGAACGGAAAGGCCCACCACCTTGGTGGTGGGTACTCG
AGTGGCGAACGGGTGAGTAACACGTGGGTGATCTGCCCCGCACTGTGGGATAAGCCTGGGAAACTGGGT
CTAATACCACATACGACCACACTGTAGACAGTGTGGTGGAAAGCCTTGTGCGGTGTGGGATGAGCCCGCG
GCCTATCAGCTTGTTGGTGGGGTAATGGCCTACCAAGGCGGCGACGGGTAGCCGGCCTGAGAGGGTGGA
CGGCCACATTGGGACTGAGACACGGCC
>Zotu4452
GAACGCTGGCGGCGTGCCTAATACATGCAAGTCGAGCGGACCTGAATGGAGCTTGCTCCAGACAGGTTAG
CGGCGGACGGGTGAGTAACACGTAGGCAACCTGCCCGCAAGACCGGGATAACGTAGGGAAACTTACGCT
AATACCGGATAGGGCACTCTCTCGCATGAGAGAGTGCGGAAAGGTGGCGCAAGCTACCACTTGCGGATGG
GCCTGCGGCGCATTAGCTAGTTGGTGGGGTAACGGCCTACCAAGGCGACGATGCGTAGCCGACCTGAGAG
GGTGACCGGCCACACTGGGACTGAGACACGGCC
>Zotu4454
GAACGCTGGCGGCGTGCCTAATACATGCAAGTCGAGCGGACCTCTTCGGAGGTCAGCGGCGGACGGGTG
AGGAACACGTGGGTAATCTGCCTTTCAGACCGGAATAACGCCCGGAAACGGGCGCTAATGCCGGATACGC
CCGCGAGGAGGCATCTTCTTGCGGGGAAAGGCCCAACTGGGCCGCTGAGAGAGGAGCCCGCGGCGCATT
AGCTGGTTGGCGGGGTAACGGCCCACCAAGGCGACGATGCGTAGCCGACCTGAGAGGGTGACCGGCCAC
ACTGGGACTGAGACACGGCC
>Zotu4458
GAACGCTGGCGGCGTGCTTAACACATGCAAGTCGAGCGGTAAGGCCCTTCGGGGTACACGAGCGGCGAA
CGGGTGAGTAACACGTGGGTGATCTGCCTCGTACTTCGGGATAAGCCTGGGAAACTGGGTCTAATACCGG
ATATGACCACGGACTGCATGGTTTGTGGTGGAAAGATTTATCGGTGCGAGATGGGCCCGCGGCCTATCAG
CTTGTTGGTGGGGTAATGGCCTACCAAGGCGACGACGGGTAGCCGACCTGAGAGGGTGACCGGCCACACT
GGGACTGAGACACGGCC
>Zotu4461
CAACGCTGGCGGCGTGCCTAACACATGCAAGTCGAACGAGAAAGTGGAGCAATCCATGAGTAAAGTGGCG
ACCGGGTGAGTAACACGTGACTAACCTACCCTAGAGTGGGGGATAACCTCGGGAAACCGGGGCTAATACC
GCATAACATCACGAGGGGAAGATCCTTGTGATCAAAGGAGCAATTCGCTCAAGGAGGGGGTCGCGGCTG
ATTAGCTAGTTGGTGGGGTAACGGCCCACCAAGGCAAAGATCAGTAGCCGGCCTGAGAGGGCGCACGGC
CACACTGGAACTGAAACACGGTC
>Zotu4464
GAACGCTGGCGGCATGCCTTACACATGCAAGTCGAACGGCAGCACGGGAGCTTGCTCCTGGTGGCGAGTG
GCGAACGGGTGAGTAATGCATCGGAACGTGCCCTGAAGTGGGGGATAACGTAGCGAAAGTTACGCTAAT
ACCGCATATTCTGTGAGCAGGAAAGCAGGGGATCTTCGGACCTTGCGCTTTTGGAGCGGCCGATGTCGGA
TTAGCTAGTTGGTGGGGTAAAGGCTCACCAAGGCAACGATCCGTAGCGGGTCTGAGAGGATGATCCGCCA
CACTGGAACTGAGACACGGTC
>Zotu4466
GAACGCTGGCGGCATGCCTAACACATGCAAGTCGAACGAAGTCCTTCGGGGCTTAGTGGCGCACGGGTGC
GTAACGCGTGGGAATCTGCCCTTAGGTTCGGAATAACAGTGGGAAACTACTGCTAATACCGGATGATGAC
GTAAGTCCAAAGATTTATCGCCTAGGGATGAGCCCGCGTAAGATTAGCTAGTTGGTGGGGTAAAGGCCTA
CCAAGGCTACGATCTTTAGCTGGTCTGAGAGGATGATCAGCCACACTGGGACTGAGACACGGCC
>Zotu4471
GAACGCTAGCGACAGGCTTAACACATGCAAGTCGAGGGGCAGCGGGGGATAAGCTTGCTTTTCCTGCCGG
CGACCGGCGCACGGGTGAGTAACACGTATGCAACCTGCCCCTGTCAGAGGGATAATCCGGAGAAATCCGG
TCTAATATCGCATAAAGCGTTTGCGGGGCATCCTGCGAGCGCCAAAGGAGTAATCCGGACAGGGATGGGC

ATGCGGCGCATTAGCTAGTTGGGGGGGTAACGGCCCACCAAGGCGACGATGCGTAGGGGTTCTGAGAGG
AAGGTCCCCCACATTGGTACTGAGACACGGAC
>Zotu4473
GAACGCTGGCGGCGTGCCTAATACATGCAAGTCGAGCGCTTGCACAAGAAAGCTTGCTTTCTTGTTGCTTG
AGCGGCGGACGGGTGAGTAACACGTGGGCAACCTGCCTATAAGACTGGGATAACTTCGGAAAACCGGAG
CTAATACCGGATAATGCTTTTGAACACATGTTTGAAAGCTGAAAGATGGTTTCGGCTATCACTTATAGATGG
GCCCGCGGCGCATTAGCTAGTTGGTGAGGTAACGGCTCACCAAGGCGACGATGCGTAGCCGACCTGAGAG
GGTGATCGGCCACACTGGGACTGAGACACGGCC
>Zotu4476
GAACGCTGGCGGCGTGCCTAACACATGCAAGTCGAACGAGGCCCAAGCAGTGGCAACACTGCGGAAGGC
CTAGTGACGAACGGGTGAGTAACACGTGAGGAACCTGCCCCGAAGACCGGGATAACCCCCTGAAAAGGG
AGCTAATACCGGATGCCCCCACCGGACCGCATGGTCTGGTGAGGAAATGGATTCCGCTTCGGGAGGGCCT
CGCGGCCTATCAGCTTGTTGGTGAGGTAACGGCTCACCAAGGCGTCGACGGGTAGCTGGTCTGAGAGGAC
GATCAGCCACACTGGGACTGAGACACGGCC
>Zotu4479
GAACGCTGGCGGCGTGCCTAATACATGCAAGTGGAACGCATGATTGATACCGGAGCTTGCTCCACCATTAA
TCATGAGTCGCGAACGGGTGAGTAACGCGTAGGTAACCTGCCTGATAGTGGGGGATAACTATTGGAAACG
ATAGCTAATACCGCATAAGAGTCGTTAACACATGTTAATGATTTAAAAGGAGCAATTGCTTCACTATCAGAT
GGACCTGCGTTGTATTAGCTAGTTGGTGAGGTAACGGCTCACCAAGGCCTCGATACATAGCCGACCTGAGA
GGGTGATCGGCCACACTGGGACTGAGACACGGCC
>Zotu4481
GAACGCTGGCGGCGTGCTTAACACATGCAAGTCGAGCGGAAAGGCCCTTCGGGGTACTCGAGCGGCGAA
CGGGTGAGTAACACGTGAGTAACCTGCCCCTGACTCTGGGATAAGCCTGGGAAACTGGGTCTAATACCGG
ATACGACCATTTCTCGCATGGGATGGTGGTGGAAAGTCTTTTCGGTTGGGGATGGGCTCGCGGCCTATCAG
CTTGTTGGTGGGGTGATGGCCTACCAAGGCGACGACGGGTAGCCGGCCTGAGAGGGCGACCGGCCACAC
TGGGACTGAGACACGGCC
>Zotu4484
GAACGCTGGCGGCGTGCCTAATACATGCAAGTCGAGCGAACCAACAGGAGCTTGCTCCTGTTGGTTAGCG
GCGGACGGGTGAGTAACACGTGGGCAACCTGCCTGTAAGACTGGGATAACACCGGGAAACCGGTGCTAA
TACCGGATAATCCTTTTCCTCTCATGAGGAAAAGCTGAAAGTCGGTTTCGGCTGACACTTACAGATGGGCCC
GCGGCGCATTAGCTAGTTGGTGAGGTAACGGCTCACCAAGGCGACGATGCGTAGCCGACCTGAGAGGGT
GATCGGCCACACTGGGACTGAGACACGGCC
>Zotu4486
GAACGCTGGCGGTATGCTTAACACATGCAAGTCGAACGAGTAGCTACTTCGGTAGCGAGAGTGGCGGACG
GGTGAGTAACGCGTAGGAATCTGCCTGTTAGTGGGGGACAACGTGGGGAAACGCACGCTAATACCGCATA
CGCCCTACGGGGGAAAGGAGGGGACCTTCGGGCCTTTCGCTAACAGATGAGCCTGCGTGGGATTAGCTAG
TTGGTAGGGTAAAGGCCTACCAAGGCGACGATCCCTAGCTGGTCTGAGAGGACGATCAGCCACACTGGGA
CTGAGACACGGCC
>Zotu4488
GAACGCTGGCGGCGTGCTTAACACATGCAAGTCGAACGGTGAAACAGGAGCTTGCTCCTGCGGATCAGTG
GCGAACGGGTGAGTAACACGTGAGTAACCTGCCCTTGACTCTGGGATAAGCGTTGGAAACGACGTCTAAT
ACCGGATACGAGCTTCAGTCGCATGGCTAGGAGCTGGAAAGAACTTCGGTCAAGGATGGACTCGCGGCCT
ATCAGCTAGTTGGTGAGGTAACGGCCCACCAAGGCGACGACGGGTAGCCGGCCTGAGAGGGTGACCGGC
CACACTGGGACTGAGACACGGCC
>Zotu4490
GAACGCTGGCGGCGTGCTTAACACATGCAAGTCACGGGGGCCCGCAAGGGCAACCGGCGAACGGGTGCG
TAACACGTGAGCAATTTGCCGATTTCTGGGGGATAGCCGGCCCAACGGCCGGGTAATACCGCATACGCTCA
GTGGGGGGAGTCCCCTGCTGAGGAAACCTCCGGGGGAAATCGAGGAGCTCGCGGCCTATCAGCTAGTTG
GCGGGGTAACGGCCCACCAAGGCGACGACGGGTAGCTGGTCTGAGAGGATGGCCAGCCACATTGGGACT
GAGAGACGGCC

>Zotu4492
GAACGCTGGCGGCGTGCTTAACACATGCAAGTCGAACGGAAAGGCCCTTCGGGGTACTCGAGTGGCGAAC
GGGTGAGTAACACGTGGGTGATCTGCCCTGCACTTCGGGATAAGCCTGGGAAACTGGGTCTAATACCGGA
TAGGACTACGCGATGCATGTCGTGTGGTGGAAAGCTTTTGCGGTGTGGGATGGGCCCGCGGCCTATCAGC
TTGTTGGTGGGGTGATGGCCTACCAAGGCGACGACGGGTAGCCGGCCTGAGAGGGTGTCCGGCCACACT
GGGACTGAGATACGGCC
>Zotu4496
GAACGCTGGCGGCGTGCTTAACACATGCAAGTCGAGTGCGCGTCTGTAGCAATACAGATGGCGCACGGCG
CACGGGTGCGTAACACGTGGGTAATCTGCCCTTCGAAGGGGAATAACTCGCCGAAAGGCGAGCTAATTCC
GCATAACATTCCGAGAACCTTGGTTTTTGGATTCAAAGGCGTAAGTCGTCGGAGGAGGAGCCCGCGCACG
ATTAGCTAGTTGGTAGGGTAACGGCCTACCAAGGCTATGATCGTTAGCTGGTCTGAGAGGATGGCCAGCC
ACACTGGAACTGAGACACGGTC
>Zotu4499
GAACGCTGGCGGCGTGCCTAATACATGCAAGTCGAGCGAATCTTTGGGAGCTTGCTCCCAAAGATTAGCG
GCGGACGGGTGAGTAACACGTGGGCAACCTGCCTATAAGACTGGGATAACTTCGGGAAACCGGAGCTAAT
ACCGGATAATTCATATCTACTCATGTAGATATGCTGAAAGACGGTTTACGCTGTCACTTATAGATGGGCCCG
CGGCGCATTAGCTAGTTGGTGAGGTAACGGCTCACCAAGGCAACGATGCGTAGCCGACCTGAGAGGGTGA
TCGGCCACACTGGGACTGAGACACGGCC
>Zotu4503
GAACGCTAGCGGCAAGCTTAATACATGCAAGTCGAGGGGCAGCGCAGTGTAGCAATACATGGGCGGCGA
CCGGCAAACGGGTGCGGAACACGTACAGAACCTTCCTTCGAGCGGGGAATAGCCCAGAGAAATTTGGATT
AATACCCCATAGCATATTGAAGTGGCATCACTTTAATATTAAAGATTTATCACTTGAAGATGGCTGTGCGGC
TGATTAGATAGTTGGCGGGGTAACGGCCCACCAAGTCTGCGATCAGTAACTGGTGTGAGAGCACGACCAG
TCACACGGGCACTGAGACACGGGC
>Zotu4505
GAACGCTGGCGGCGTGCCTAATACATGCAAGTTGAACGATGATTTCTGGTACTTGTATCAGAATGAAGAGT
AGCGAACGGGTGAGTAACGCGTGGGTAACCTACCTCTTAGCGGGGGACAACTATTGGAAACGATAGCTAA
TACCGCATAACAATGCTTAACACATGTTAAGGATTTGAAAGTACCAATTGGTACACTAGGAGATGGACCCG
CGTTGTATTAGCTAGTTGGTAGTGTAATGGACTACCAAGGCGATGATACATAGCCGACCTGAGAGGGTGAT
CGGCCACATTGGGACTGAGACACGGCC
>Zotu4507
GAACGCTGGCGGCGTGCCTAACACATGCAAGTCGAGCGGAGTTTAGCGGGCGGCGACTTCGGTCAAAGCC
TGTTAAACTTAGCGGCGGACGGGTGAGTAACACGTAGGCAACCTGCCCATGACACGGGGATAGCTTCGGG
AAGCCGGAGGTAATACCCGATACCTTTACTTATCGCATGGTAAGTAAAGAAAGCGACTTAGCGGTCATGGA
TGGGCCTGCGTCCCATTAGCTAGTTGGTGAGATAACAGCCCACCAAGGCGACGATGGGTAGCCGACCTGA
GAGGGTGACCGGCCACACTGGAACTTAGACACGGTC
>Zotu4509
GAACGCTGGCGGCGTGCTTAACACATGCAAGTCGAACGGAAAGGCCCTTCGGGGTACTCGAGTGGCGAAC
GGGTGAGTAACACGTGGGTGATCTGCCCTGCACTTCGGGATAAGCTTGGGAAACTGGGTCTAATACCGGA
TATGATCATGCTCTTCATGGGGTGTGGTGGAAAGCTTTTGCGGTGTGGGATGGGCCCGCGGCCTATCAGCT
TGTTGGTGGGGTAATGGCCTACCAAGGCGACGACGGGTAGCCGGCCTGAGAGGGTGTCCGGCCACACTG
GGACTGAGATACGGCC
>Zotu4511
GAACGCTGGCGGCATGCCTTACACATGCAAGTCGAACGCGAAAGGGGGCAACCCTGAGTAGAGTGGCGA
ACGGGTGAGTAACACATCGGAACATACCCAGTAGTGGGGGATAACCAGTCGAAAGACTGGCTAATACCGC
ATACGACCTACGGGTGAAAGCGGGGGACCGCAAGGCCTCGCGCCATTGGATTGGCCGATGTCCGATTAGC
TAGTTGGCGGGGTAAAAGCCCACCAAGGCGACGATCGGTAGCTGGTCTGAGAGGATGATCAGCCACACTG
GGACTGAGACACGGCC
>Zotu4514

GAACGCTGGCGGCGTGCCTAATACATGCAAGTCGAGCGAATTGATGGGAGCTTGCTCCCTGATGTTAGCG
GCGGACGGGTGAGTAACACGTGGGCAACCTGCCCTGCAGATGGGGATAACTCCGGGAAACCGGGGCTAA
TACCGAATAATCGGTTCTTCCGCATGGAAGAACTCTGAAAGACGGTTTCGGCTGTCACTGCAGGATGGGCC
CGCGGCGCATTAGCTAGTTGGTGGGGTAATGGCCTACCAAGGCGACGATGCGTAGCCGACCTGAGAGGGT
GATCGGCCACACTGGGACTGAGACACGGCC
>Zotu4516
GAATGCTGGCGGTATGGATAAGGCATGCAAGTCAAGGGGGGCCGCAAGGTCAACCGGCAGACGAGGTAG
TAACACGTACGAACGCGCCCTGTAGTCTGGCATAGCTCGTCGAAAGACGGGGTAATTCCGGATGGTCCCG
AAAGGGTAAAGATTTATCGCTACAGGAGCGGCGTGCGTAGTATCAGCTAGTTGGTAAGGTAATGGCTTAC
CAAGGCTACGACGCTTAGGGGGGGTGAGAGCCCGATCCCCACCGATGGGACTGAGACACGGCC
>Zotu4519
GAACGCTGGCGGCGTGCCTAACACATGCAAGTCGAACGGTCCTTCGGGACAGTGGCGCACGGGGGAGGA
ACACGTGGCTGACCTGCCCTCAAGTGCGGGATAACTTCGGGAAACCGAAGCTAATACCGCGTAAGCTCAC
GGTCGTTAGGGGGCTGTGAGAAAAGGCTTCGGTCGCTTGAGGAGGGGGCTGCGGCGCATCAGGTAGATG
GTGGGGTAACGGCCTACCATGCCGACGACGCGTCGGGGGTCTGAGAGGATGATCCCCCACACTGGGACTG
AGACACGGCC
>Zotu4521
GAACGCTGGCGGCGCGCTTAACACATGCAAGTCGAACGGTTAAGGCCCCCTCGGGGGCGAATAGAGTGG
CGAACGGCTGAGTAACACGTGGGCAACCTGCCCCCCGCACCGGGATAGCCTCGGGAAACCGTGGGTAATA
CCGGATACTCCCCCACCGGCGCATGCCGGAGGGGGGAAAGCGTACACGGCGGGGGATGGGCCCGCGGCC
TGTTAGCTCGTTGGCGGGGTAACGGCCCACCAAGGCGATTATGGGTAGCCGGGTTGAGAGACCGACCGGC
CAGATTGGGACTGAGACACGGCC
>Zotu4524
GAACGCTGGCGGCATGCTTAACACATGCAAGTCGAACGGTTATATGGGGCTTGCTCCATATGATAGTGGCA
GACGGGTGAGGAACGCGTGGGAACGTACCCCAAGGTTCGGAATAACAGTTGGAAACGACTGCTAATACC
GGATAAGTCCGAGAGGAGAAAGGTTTACCGCCTTGGGAGCGGCCCGCGTTAGATTAGGTAGTTGGTAGG
GTAAAGGCCTACCAAGCCGATGATCTATAGCTGGTTTGAGAGGATGATCAGCCACACTGGGACTGAGACA
CGGCC
>Zotu4526
GAACGCTGGCGGCGTGCCTAACACATGCAAGTCGAACGAGATCCATGGAGTGCTTGCACTCCGGAAGATC
TAGTGGCGAACGGGTGAGTAACACGTGAGAAACCTGCCCCAGACTCTGGGATAACCTCGGGAAACCGAAG
CTAATACCGGATATTCTCCTTTGGTCGCATGGCCAGTTGAGGAAAGTTTTTCGGTCTGGGAGGGTCTCGCG
GCCTATCAGCTAGTTGGTGGGGTAACGGCCCACCAAGGCGACGACGGGTAGCTGGTTTGAGAGAACGATC
AGCCACACTGGAACTGAGACACGGTC
>Zotu4528
GAACGCTGGCGGCGTGCTTAACACATGCAAGTCGAACGGGATCCATCAGGCTTTGCTTGGTGGTGAGAGT
GGCGAACGGGTGAGTAATGCGTGACCGACCTGCCCCATACACCGGAATAGCTCCTGGAAACGGGTGGTAA
TGCCGGATGCTCCAGTTGATCGCATGGTCTTCTGGGAAAGCTTTCGCGGTATGGGATGGGGTCGCGTCCTA
TCAGCTTGACGGCGGGGTAACGGCCCACCGTGGCTTCGACGGGTAGCCGGCCTGAGAGGGCGACCGGCC
ACATTGGGACTGAGATACGGCC
>Zotu4532
GAACGCTGGCGGCGTGGATAAGGCATGCAAGTCGAGAGGGTTAGACCCTCGGCAAACGGGTTAGTAATG
CATAGGAATTTACCCTGAAGTCGTGGATAATCCGGAGAAATCCGGAATAATACACGATATGTTCTACGGAA
TAAAGATTTATCGCTTCAGGAGAAGCCTATGTCCTATCAGCTTGTTGGTGGTGTAAAGGACCACCAAGGCT
ATGACGGGTAGGGGGTGTGAGAGCACGGCCCCCAACATTGGAACTGCGACACTGTC
>Zotu4537
GAACGCTAGCGGCAGGCCTAATACATGCAAGTCGAGGGGCAGCGGGGTAGCAATACTGCCGGCGACCGG
CGCACGGGTGCGTAACACGTATGCAACCTACCTTAAACAGGGGGATAGCCCGGGGAAACTCGGATTAATA
CCCCATAACACGGTGATACCACATGGTAATGTCGTTAAAGATTTATCGGTTTAAGCTGGGCATGCGTTCGAT
TAGCTAGTTGGTAAGGTAATGGCTTACCAAGGCTACGATCGATAGGGGGTCTGAGAGGATGATCCCCCAC
ACTGGTACTGAGATACGGAC

>Zotu4539
GAACGCTGGCGGCAGGCTTAACACATGCAAGTCGAGCGGGGAAAGGTAGCTTGCTACCTGACCTAGCGGC
GGACGGGTGAGTAATGCTTAGGAATCTGCCTATTAGTGGGGGACAACATTCCGAAAGGAATGCTAATACC
GCATACGCCCTACGGGGGAAAGCAGGGGATCTTCGGACCTTGCGCTAATAGATGAGCCTAAGTCAGATTA
GCTAGTTGGTGGGGTAAAGGCCTACCAAGGCGACGATCTGTAGCGGGTCTGAGAGGATGATCCGCCACAC
TGGGACTGAGACACAGCC
>Zotu4543
GAACGCTAGCGGCGTGCTTAACACATGCAAGTGGAGCGACGAACCAGGGCTTGCCCTGGGGCAGAGCCG
CGAACGGGTGAGTAACACGTGGGTAACCTACCCCGATGATTGGGACAACCCGGGGAAACCCGGGCTAATA
CCAAATGTGGTCTCTCTTCATAAGTAGAGCGACTAAAGGAAGCTTCGGCCTCCGCATTGGGAGGGGCCCGC
GGCCCATTAGCTTGTTGGTGAGGTAACGGCTCACCAAGGCTTCGATGGGTAGCTGGTCTGAGAGGACGAT
CAGCCACACTGGGACTGAGACACGGCC
>Zotu4545
GAACGCTAGCGGCAGGCTTAATACATGCAAGTCGAGGGGCAGCGCGGGTAGCAATACCTGGCGGCGACC
GGCAAACGGGTGCGGAACACGTACGCAACCTTCCCAAAACTGGTGAATAGCCCTCCGAAAGGAGGATTAA
TACACCGTACCATAACGAAGAGGCATCTCTTTGTTATTATAGTAGCAATACGGTTTTGGATGGCGTGCGCC
TGATTAGGTAGTTGGCGGGGTAACGGCCCACCAAGCCTGCGATCAGTAACTGGTGTGAGAGCACGACCAG
TCACACGGGCACTGAGACACGGGC
>Zotu4547
GAACGCTGGCGGCGTGCCTTAGGCATGCAAGTCGAACGGTTTCCGCAAGGAAGCAGTGGCAGACGGGTG
AGTAACGCGTAGAAATCTACCCCTAGGAGGACCATAGCTCATCGAAAGATGGGGTAATAGTCCATGGTCTC
GCAAGAGTAAAGCAATTAACTTTGCGCCAAGGGAGGAGTCTGCGTCCTATCAGCTTGTTGGTGGGGTAAT
GGCCTACCAAGGCAATGACGGGTAGCTGGACTGAGAGGTCGATCAGCCACAACTGCACTTAGACACGGGC
>Zotu4549
GAATGCTGGCGGCGTGGATAAGTCATGCAAGTCAAGGGGGCCTGTAAGGGCAACCGGCAGACGAGGTAG
TAACACGTAGGTACGTCCCCCGAAGTCGGGCATAGCCATCCGAAAGGATGGGTAATTCCCGATAGTCCCGC
AAGGGTAAAGGTTTTTCGCTTCGGGAGCGGCCTGCGTAGTATCAGCTAGTTGGTAGGGTAATGGCCTACCA
AGGCTACGACGCTTAGGGGAGCTGAGAGGCTGACCCCCACCGATGGAACTGCGACACGGTC
>Zotu4551
GAACGCTGGCGGCAGGCTTAACACATGCAAGTCGAGCGGGCTCTTTCGGGGGTCAGCGGCGGACGGGTT
AGTAACGCGTGGGAACGTGCCTTTTGCTTCGGAATAGCCCTGGGAAACTGGGGGTAATACCGGATGTGCC
CTGAGGGGGAAAGATTTATCGGCAAGAGATCGGCCCGCGTCTGATTAGGTAGTTGGTGTGGTAACGGCGC
ACCAAGCCGACGATCGGTAGCTGGTTTGAGAGGATGATCAGCAACACTGGGACTGAGACACGGCC
>Zotu4554
GAACGCTGGCGGCGTGCCTAATACATGCAAGTCGAGCGGACTTTTAAAAGCTTGCTTTTAAAAGTTAGCGG
CGGACGGGTGAGTAACACGTGGGCAACCTGCCTGTAAGACTGGGATAACTTCGGGAAACCGGAGCTAATA
CCGGATAATGTTTTTTCTCTCATGAGGAGAAACTGAAAGATGGTTTACGCTATCACTTACAGATGGGCCCGC
GGCGCATTAGCTAGTTGGTGAGGTAACGGCTCACCAAGGCGACGATGCGTAGCCGACCTGAGAGGGTGAT
CGGCCACACTGGGACTGAGACACGGCC
>Zotu4556
CAACGCTGGCGGCGTGCCTAACACATGCAAGTCGAACGCGGTTGCCCCGCAAGGGGCAACTGAGTGGCGA
ACGGGTGAGTAACGCGTGGGTGATCTACCTTCGAGTGGGGGATAACGTCCCGAAAGGGACGCTAATACCG
CATGACATCCTGCCTTTGAACAGGCGGAGATCAAAGCCGGGGATCGCAAGACCTGGCGCTTGTTGAGGAG
CCCGCGTCCGATTAGCTAGTTGGTGGGGTAATGGCCCACCAAGGCGACGATCGGTAGCCGGCCTGAGAGG
GCGGACGGCCACACTGGGACTGAGACACGGCC
>Zotu4558
GAACGCTGGCGGCATGCCTAACACATGCAAGTCGAACGAGACCTTCGGGTCTAGTGGCGCACGGGTGCGT
AACGCGTGGGAATCTGCCCTTTGCTTCGGAATAACTCAGGGAAACTTGAGCTAATACCGGATAATGTCGCA
AGACCAAAGATTTATCGGCAAAGGATGAGCCCGCGTAGGATTAGCTTGTTGGTGGGGTAATGGCCCACCA
AGGCGACGATCCTTAGCTGGTCTGAGAGGATGATCAGCCACACTGGGACTGAGACACGGCC

>Zotu4560
GAACGCTAGCGGCAGGCTTAACACATGCAAGTCGAGGGGTATAGTAGCAATACTAGAGACCGGCGCACG
GGTGCGTAACGCGTATGCAATCTACCTTTTGCAGGGGAATAGCCCAGAGAAATTTGGATTAATGCCCCATA
GTATAGTACAATCGCATGATTGAACTATTAAAGTCACAACGGCAAAAGATGAGCATGCGTCCCATTAGCTA
GTTGGTAAGGTAACGGCTTACCAAGGCGACGATGGGTAGGGGTCCTGAGAGGGAGATCCCCCACACTGGT
ACTGAGACACGGAC
>Zotu4564
GAACGCTGGCGGCATGCCTAACACATGCAAGTCGAACGAACTCTTCGGAGTTAGTGGCGCACGGGTGCGT
AACGCGTGGGAATCTGCCCTTGGGTTCGGAATAACAGTTAGAAATGACTGCTAATACCGGATGATGTCTTC
GGACCAAAGATTTATCGCCCAGGGATGAGCCCGCGTAGGATTAGGTAGTTGGTGGGGTAAAGGCCTACCA
AGCCGACGATCCTTAGCTGGTCTGAGAGGATGATCAGCCACACTGGGACTGAGACACGGCC
>Zotu4566
GAACGCTGGCGGCATGCCTAACACATGCAAGTCGAACGAGATCTTCGAGTCTAGTGGCGCACGGGTGCGT
AACGCGTGGGAATCTGCCCTTTGGTTCGGAATAACAGTTGGAAACGACTGCTAATACCGGATGATGACGA
AAGTCCAAAGATTTATCGCCAGAGGATGAGCCCGCGTAGGATTAGCTAGTTGGTGTGGTAAAGGCGCACC
AAGGCGACGATCCTTAGCTGGTCTGAGAGGATGATCAGCCACACTGGGACTGAGACACGGCC
>Zotu4568
GAACGCTGGCGGCGTGCCTTAAGCATGCAAGTCGAACGGACCGCAAGGTCAGTGGCGGACGGGTGAGTA
ACGCGTGGGAATCTGCCTTAAAGTTGGACATAGCTCATCGAAAGATGGGGTAATTTCCAATGTGGATGTAA
ATCTAAAGTCGCAAGACGCTTTAAGATGAGCCCGCGTCCTATCAGCTAGTTGGTGGGGTAATGGCCTACCA
AGGCGACGACGGGTAGCTGGTCTGAGAGGATGGTCAGCCACAATAGCATTGAGATACGGGC
>Zotu4571
GAACGCTGGCGGCGGGCCTCATACATGCAAGTCGAGCGCGAAAGTCCGCAAGGATGAGTAAAGCGGCGC
ACGGGTGAGTAACACGTGGATAATCTACCCTTTGGATTGGAATAACCTGTCGAAAGACGGGCTAATGCCG
AGTAAGCCCTCGAGAGCGTGAAGCTCTTGCGGGGAAAGATGGCGCAAGCTATCGCCGATGGATGAGTCTG
CGTCCCATTAGTTAGTTGGTGAGGTAACGGCTTACCAAGACAATGATGGGTACCTGGTCTGAGAGGATGAC
CAGGCACACTGGAACTGAAACACGGTC
>Zotu4573
GAACGCTGGAAATGTGGATACTGGCATGCAAGTCAAGGGGCCGCAAGGCACCGGCGAACGGCTTAGTAA
CACGTAGATACATACCGTAAAGTGGGGCATAGCTCGTCGAAAGACGGGGTAATTTCCCATGTGAACGCAA
GTTTAAAGGCGCAAGCCGCTTTATGAATGGTCTGCGGTCTATCAGCTAGTTGGTGAGGTAAAGGCTTACCA
AGGCTATGACGGATAGGGGGCGTGAGAGCGCGACCCCCAAGAATGGCACTGCGACACGGGC
>Zotu4577
GAATGCTGGCGGCGTGCCTAACACATGCAAGTCGAGCGGCAGCGCGAGTAGTTTACTACTTGGCGGCGAG
CGGCGGACGGCTGAGTAACGCGTGGGAACGTGCCCCAAAGTGAGGAATAACTGCCCGAAAGGGTAGCTA
ATGCCGCATATGGTCTTCGGATTAAAGTATTTATACGCTTTGGGAGCGGCCCGCGTCGGATTAGGTTGTTG
GTGAGGTAATGGCTCACCAAGCCGACGATCCGTAGCTGGTCTGAGAGGATGATCAGCCAGACTGGAACTG
AGACACGGTC
>Zotu4580
GAACGCTGGCGGCATGCCTTACACATGCAAGTCGAACGGCAGCGCGGGGCAACCTGGCGGCGAGTGGCG
AACGGGTGAGTAATATATCGGAACGTACCCTGGAGTGGGGGATAACGTAGCGAAAGTTACGCTAATACCG
CATACGATCTAAGGATGAAAGTGGGGGATGCGTAAGCACCTCATGCTCCTGGAGCGGCCGATATCTGATT
AGCTAGTTGGTGGGGTAAAGGCCCACCAAGGCATCGATCAGTAGCTGGTCTGAGAGGACGACCAGCCACA
CTGGAACTGAGACACGGTC
>Zotu4582
GAACGCTGGCGGCGTGCCTAACACATGCAAGTCGAACGGTCCATTTCGGTGGATAGTGGCGCACGGGTGA
GTAACACGTGGTTGACCTACCTCAGAGTAAGGGATAACGTCTCGAAAGGGATGCTAATACCTTATAAGCTT
CCGGTATTTAGAAAACTGGGAGAAAAGTTCCGGCGCTTTGAGAGGGGACTGCGAGCCATCAGCTAGTTGG
TGTGGTAATGGCACACCAAGGCGACGACGGCTAGGGGGCCTGAGAGGGTGACCCCCCACACTGGAACTG
AGACACGGTC

>Zotu4585
GAACGCTGGCGGCGTGCTTAACACATGCAAGTCGAACGGTGACGGAGAGCTTGCTCTCCTGATCAGTGGC
GAACGGGTGAGTAACACGTGGGCAACCTGCCCCTGACTTCGGGATAACGCTTCGAAAGAGGTGCTAATAC
CGAATACGAACTCCCTTGGCATCTTGGGAGTTGGAAAGTTTTTCGGTCAGGGATGGGCCCGCGGCCTATCA
GCTTGTTGGTGAGGTAATGGCTCACCAAGGCGACGACGGGTAGCCGGCCTGAGAGGGCGACCGGCCACA
CTGGGACTGAGACACGGCC
>Zotu4587
GAACGCTGGCGGCATGCCTAACACATGCAAGTCGAACGAGACCTTCGGGTCTAGTGGCGCACGGGTGCGT
AACGCGTGGGAATCTGCCCCTTGATTCGGAATAACTCAGAGAAATTTGAGCTAATACCGGATGATGACGTA
AGTCCAAAGATTTATCGCCAAGGGATGAGCCCGCGTAGGATTAGCTAGTTGGTGAGGTAAAGGCTCACCA
AGGCGACGATCCTTAGCTGGTCTGAGAGGATGATCAGCCACACTGGGACTGAGACACGGCC
>Zotu4589
GAACGCTGGCGGCGTGCTTAACACATGCAAGTCGAGCGGAAAGGCCCTTTCGGGGGTACTCGAGCGGCG
AACGGGTGAGTAACACGTGAGTAATCTGCCCCTGGCTTTGGGATAGCCACCGGAAACGGTGATTAATACC
GGATATGACCACTTTTCGCATGGATGGTGGTGGAAAGTTTTTCGGCCAGGGATGTGCTCGCGGCCTATCAG
CTTGATGGTGAGGTAATGGCTCACCATGGCTTCGACGGGTAGCCGGCCTGAGAGGGTGACCGGCCACACT
GGGACTGAGACACGGCC
>Zotu4592
GAACGCTGGCGGCGTGCTTAACACATGCAAGTCGAACGAGGTCCATCCGGTAGCAATACCGGAGAAGACC
TAGTGGCGAACGGGTGAGTAACACGTGAGGAACCTGCCCCGAAGACCGGGATAACAGCGGGAAACCGCT
GCTAATACCGGATACCCCCATCGGACCACATGGCCTGATGAGGAAATGGATTCCGCTTCGGGAGGGCCTC
GCGGCCTATCAGCTTGTTGGTGAGGTGACGGCTCACCAAGGCGACGACGGGTAGCTGGTCTGAGAGGAC
GATCAGCCACACTGGGACTGAGACACGGCC
>Zotu4596
GAACGCTGGCGGCAGGCTTAACACATGCAAGTCGAACGCCCCGCAAGGGGAGTGGCAGACGGGTGAGTA
ACACGTGGGAACATACCCTTCGGTTCGGAACAACGCCGGGAAACTGGCGCTAATACCGGATACGGCTTGA
GGGCGAAAGATTCATCGCCGAAGGATTGGCCCGCGTTCGATTAGCTTGTTGGTGAGGTAACGGCTCACCA
AGGCGACGATCGATAGCTGGTCTGAGAGGATGGCCAGCCACATTGGGACTGAGACACGGCC
>Zotu4598
GAACGCTGGCGGCAGGCTTAACACATGCAAGTCGAGCGGGGAAAGGTAGCTTGCTACCTAACCTAGCGGC
GGACGGGTGAGTAATGCTTAGGAATCTGCCTATTAGTGGGGGACAACATTCCGAAAGGAATGCTAATACC
GCATACGCCCTACGGGGAAAAGCAGGGGATCTTCGGACCTTGCGCTAATAGATGAGCCTAAGTCAGATTA
GCTAGTTGGTGGGGTAAAGGCCTACCAAGGCGACGATCTGTAGCGGGTCTGAGAGGATGATCCGCCACAC
TGGGACTGAGACACGGCC
>Zotu4600
GAACGCTGGCGGCGTGCCTAATACATGCAAGTCGAACGGTAAGGCTTTCACAGTTTGCTGGTAGTACGTAA
GTACTCGAGCAAAGCGTGAAAGTACACGAGTGGCGGACGGCAGAGTAACGCGTAGGAATGTACCCCAAA
GTGAGGGATAACTGCTCGAAAGAGTAGCTAATACCGCATGTGGTCTTAGGATTAAAGCTTCGGCGCTTTGG
GAACAGCCTGCGTAAGATTAGCTAGTTGGTAGTGTAATGGACTACCAAGGCGACGATCTTTAGCTGGTCTG
AGAGGATGATCAGCCTGACTGGAACTGAGACACGGTC
>Zotu4603
GAACGCTGGTGGTATGCTTAACACATGCAAGTCGAACGGGCATAGCAATATGTCAGTGGCAAACGGGTGA
GGAATATATAGGAATCTACCTAGCAGTAAGGAATAACCTTGGGAAATCAAGGCTAATGCCATATATTACCG
ATTTATCGGGAAAGATTTATCGCTGTTAGATGAGCCTGTATCAGATTAGCTAGTTGGTGGGGTAATGGCCT
ACCAAGGCAATGATCTGTAGCTGGTCTGAGAGGATGATCAGCCACACTGGAACTGCGACACGGTC
>Zotu4605
GAACGCTGGCGGCGTGCTTAACACATGCAAGTCGAACGGTGAAAGAGGAGCTTGCTCCTCTGGATCAGTG
GCGAACGGGTGAGTAACACGTGAGTAACCTGCCCCAGACTCTGGGATAAGCGTTGGAAACGACGTCTAAT
ACCGGATACGAGCTTCAGCCGCATGGCTAGGAGCTGGAAAGAATTTCGGTCTGGGATGGACTCGCGGCCT

ATCAGGTAGTTGGTGAGGTAAAGGCCCACCAAGCCTACGACGGGTAGCCGGCCTGAGAGGGTGACCGGC
CACACTGGGACTGAGACACGGCC

>Zotu4608

GAACGCTGGCGGCAGGCTTAACACATGCAAGTCGAGCGCCCCGCAAGGGGAGCGGCAGACGGGTGAGTA
ACGCGTGGGAATCTACCCATCTCTACGGAACAACTCCGGGAAACTGGAGCTAATACCGTATACGCCCTTCG
GGGGAAAGATTTATCGGAGATGGATGAGCCCGCGTTGGATTAGGTAGTTGGTGGGGTAATGGCCTACCAA
GCCGACGATCCATAGCTGGTCTGAGAGGATGATCAGCCACATTGGGACTGAGACACGGCC

>Zotu4610

GAACGCTAGCGGGAGGCCTAATACATGCAAGTCGAACGGTAACAGGGGTAGCAATACCTGCTGACGAGT
GGCGTACGGGTGCGTAACACGTACACAACCTGCCTTATACTGGAGCATACCCCCAAGAAATTGGGACTAAT
TCTCCATAATATATTTGAAAGGCATCTTTTGAATATTAAAACTCCGGTGGTATAAGATGGGTGTGCGTCCGA
TTAGATAGTTGGTGAGGTAACGGCTCACCAAGTCAACGATCGGTAGCTGGTCTGAGAGGACGATCAGCCA
CACGGGCACTGAGACACGGGC

>Zotu4612

GAACGCTGGCGGCAGGCCTAACACATGCAAGTCGAACGAGCTGGGACCTTCGGGTCCCGATGCTAGTGGC
GCACGGGTGAGTAACACGTGGGAATCTACCCAATAGTACGGAATAACTGAGGGAAACTTTGGCTAATACC
GTATACGCCCTTCGGGGGAAAGATTTATCGCTATTGGATGAGCCCGCGTCTGATTAGGTAGTTGGTGAGGT
AACGGCTCACCAAGCCGACGATCAGTAGCTGGTCTGAGAGGATGATCAGCCACACTGGGACTGAGACACG
GCC

>Zotu4614

GCCGTGTCTCAGTCCCAGTGTGGCTGGTCATCCTCTCAGACCAGCTACGGATCGTCGCCTTGGTAGGCTTTT
ACCCTACCAACTAGCTAATCCGATGCAGGCTCACCCTAGAGCGCAAGGCCTCGAAAGGTCCCCTGCCTTTG
ATTTTTCAATATCATGTGGTATTAGCTCTCGTTTCCGAAAGTTATCCCCCTCTCTAGGACAGATTCCTACCTAT
TACTCACCCGTCCGCCACTTGCCATCATTCCTCACTCAAATCACTTTCCGTATTTCTTTTCCAAAAAGAAATTA
AAGTGATTTGAGTGAGGAACATGCTGCCGTTCGACTTGCATGTGTTAAGCATACCGCCAGCGTTCA

>Zotu4617

GAACGCTAGCGGCAGGCCTAATACATGCAAGTCGAACGGTAAATATTCAGCAATGGATATGAGAGTGACG
TACGGGTGCGTAACACGTATGCAACCTACCCTTTACAGGCGGATAGCTCGGGGAAACTCGAATTAACCCGT
CATAAGATCATAGAGTGGCATCACTTAGTGATTAAAATTACGGTGGTAAAGGATGGGCATGCGTCTGATTA
GTTAGTTGGCGGGGTAACGGCCCACCAAGACGATGATCAGTAGGGGAACTGAGAGGTTGATCCCCCACAC
TGGCACTGAGATACGGGC

>Zotu4619

GAACGCTGGCGGCGTGCCTAATACATGCAAGTCGAGCGGAGTTGATGAGGTGCTTGCACCTCTGATGCTTA
GCGGCGGACGGGTGAGTAACACGTAGGTAACCTGCCTGTAAGACTGGGATAACATTCGGAAACGAATGCT
AATACCGGATACGCGATTCTCTCGCATGGGAGAATCGGGAAAGACGGAGCAATCTGTCACTTACGGATGG
ACCTGCGGCGCATTAGCTAGTTGGTGAGGTAACGGCTCACCAAGGCGACGATGCGTAGCCGACCTGAGAG
GGTGATCGGCCACACTGGGACTGAGACACGGCC

>Zotu4621

GAACGCTGGCTGCGTGCTTAACACATGCAAGTCGAACGATGACGGTGGTGCTTGCATCACCTGATTAGTGG
CGAACGGGTGAGTAACACGTGAGTAACCTGCCCTTCACTTCGGGATAACCTCGGGAAATCGTGGCTAATAC
CGGATATGAGCTCCTTCCGCATGGCGGGGGTTGGAAAGATTTATCGGTGAAGGATGGACTCGCGGCCTAT
CAGTTTGTTGGTGAGGTAATGGCTCACCAAGGCGATGACGGGTAGCCGGCCTGAGAGGGCGACCGGCCA
CACTGGGACTGAGACACGGCC

>Zotu4624

GAACGCTGGCGGCGTGCCTAATACATGCAAGTCGAGCGAGTCCCTTCGGGGGCTAGCGGCAGACGGGTG
AGTAACACGTAGGCAACCTGCCCGTAAGATCGGGATAACATAGGGAAACTTATGCTAATACCGAATAGAG
TCTTTTCTCGCATGAGGAGAGACGGAAAGATGGCGCAAGCTATCACTTACGGATGGGCCTGCGGCGCATT
AGCTAGTTGGTGGGGTAACGGCCTACCAAGGCGACGATGCGTAGCCGACCTGAGAGGGTGACCGGCCAC
ACTGGGACTGAGACACGGCC

>Zotu4626

GAACGCTGGCGGCAGGCCTAACACATGCAAGTCGAGCGCCGTAGCAATACGGAGCGGCAGACGGGTGAG
TAACGCGTGGGAACGTGCCCTTCAGTTCGGAATAACCCAGGGAAACTTGGGCTAATACCGGATACGTCCGT
AAGGAGAAAGATTTATCGCTGAAGGATCGGCCCGCGTAAGATTAGCTAGTTGGTGAGGTAACGGCTCACC
AAGGCTATGATCTTTAGCTGTTCTGAGAGGAAGACCAGCCACACTGGGACTGAGACACGGCC
>Zotu4628
GAACGCTGGCGGCGTGCCTAATACATGCAAGTCGAGCGAATCGATGGGAGCTTGCTCCCTGAGATTAGCG
GCGGACGGGTGAGTAACACGTGGGCAACCTGCCTATAAGACTGGGATAACTTCGGGAAACCGGAGCTAAT
ACCGGATACGTTCTTTTCTCGCATGAGAGAAGATGGAAAGACGGTTTACGCTGTCACTTATAGATGGGCCC
GCGGCGCATTAGCTAGTTGGTGAGGTAATGGCTCACCAAGGCGACGATGCGTAGCCGACCTGAGAGGGT
GATCGGCCACACTGGGACTGAGACACGGCC
>Zotu4630
GAACGCTGGCGGCGTGCTTAACACATGCAAGTCGAACGGAAAGGCCCTTCGGGGTACTCGAGTGGCGAAC
GGGTGAGTAACACGTGGGTGATCTGCCCTGCACTTCGGGATAAGCTTGGGAAACTGGGTCTAATACCGGA
TATGACTACTGGCTTCATGGTTGGTGGTGGAAAGCTTTTGCGGTGTGGGATGGGCCCGCGGCCTATCAGCT
TGTTGGTGGGGTAATGGCCTACCAAGGCGACGACGGGTAGCCGGCCTGAGAGGGTGTCCGGCCACACTG
GGACTGAGATACGGCC
>Zotu4632
AAACGCTGGCGGCGTGCATAAAACATGCAAGTCGAACGGACTTCAGTCCGCAAGGACTGAAGTGAGTGGC
GAACGGCTGAGTAACACGTAGGTGACCTGCCCCGAAGTTTGGGATAACAACTCGAAAGAGTTGCTAATAC
CGGATGTGACCACCGATTCATTTCGGTGCTTAAAGTCGTAAGACGCTTTGGGAGGGGCCTGCGGCGGATTA
GCTAGTTGGTGTGGTAACGGCATACCAAGGCTTCGATCCGTAGCTGGTCTGAGAGGATGGCCAGCCAGAC
TGGGACTGAGACACGGCC
>Zotu4635
GAACGCTGGCGACAGGCTTAACACATGCAAGTCGAACGATGACTAACTAGCTTGCTAGATATGATTAGTGG
CGGACGGGTGAGTAACATTTAGGAATCTGCCTAGTAGTGGGGGATAGCTCGGGGAAACTCGAATTAATAC
CGCATACGACCTACGGGTGAAAGGGGGCGCAAGCTCTTGCTATTAGATAAGCCTAAATCAGATTAGCTAGT
TGGTGGGGTAAAGGCCCACCAAGGCGACGATCTGTAACTGGTCTGAGAGGATGATCAGTCACACCGGAAC
TGAGACACGGTC
>Zotu4637
GAACGCTAGCGGCAGGCCTAATACATGCAAGTCGAACGGTAACATGAGTAGCAATACTTGATGACGAGTG
GCGCACGGGTGCGTAACGCGTATGCAATCTACCTTTAACCGGTGAATAGCCCGAAGAAATTCGGATTAATA
CACCATAGTATGTTTGAGTGGCATCACTTAAACATTAAAACTCCGGTGGTTAAAGATGAGCATGCGTGACA
TTAGCTAGTTGGTGAGGTAATGGCTCACCAAGGCTACGATGTCTAGGGGATCTGAGAGGATTAACCCCCAC
ACTGGTACTGAGACACGGAC
>Zotu4639
GAACGCTGGCGGTATGCTTTACACATGCAAGTCGAACGGGGGTTTTTAACTCTAGTGGCGGACGGGTGAG
TAACACGTAAGAATTTGCCTTTAGGAGGGGGACAACAGCTGGAAACGGCTGCTAATACCCCATATGCTTTC
GAGTGAAATGGATTTCCGCCTGAAGAGAAGCTTGCGGCTGATTAGCTTGTTGGTGAGGTAATGGCTCACCA
AGGCGACGATCAGTATCTGGTTTGAGAGGACGATCAGACACACTGGAACTGAGACACGGTC
>Zotu4642
GAACGCTAGCGGCAGGCCTAATACATGCAAGTCGAGGGGTAACAGGAATAGCAATATTTGCTGACGACCG
GCGCACGGGTGCGTAACACGTATGTAATCTGCCCATCACTGGAGGATAGCCCGGAGAAATCCGGATTAAT
ACTCCATAATAAAAGAGAAAGCATTTTCTTTTTTTGAAAGCTCCGGCGGTGATGGATGAACATGCGACTGA
TTAGTTTGTTGGTGAGGTAACGGCTCACCAAGACTTCGATCAGTAGGGGTACTGAGAGGTTAAACCCCCAC
ACTGGTACTGAGACACGGAC
>Zotu4645
GAACGCTGGCGGCGTGCCTAATACATGCAAGTCGAGCGAATTTTTGGGAGCTTGCTCCTAAAAGTTAGCGG
CGGACGGGTGAGTAACACGTGGGCAACCTGCCTATAAGACTGGGATAACTTCGGGAAACCGGAGCTAATA
CCGGATAATTCTTTTCTACACATGTAGAAAAGCTGAAAGACGGTTTACGCTGTCACTTATAGATGGGCCCGC
GGCGCATTAGCTAGTTGGTGAGGTAACGGCTCACCAAGGCGACGATGCGTAGCCGACCTGAGAGGGTGAT
CGGCCACACTGGGACTGAGACACGGCC

>Zotu4648
GAACGCTGGCGGCGTGCTTAACACATGCAAGTCGAGCGGTAAGGCCTTTCGGGGTACACGAGCGGCGAAC
GGGTGAGTAACACGTGGGTGATCTGCCCTGCACTCTGGGATAAGCCTGGGAAACTGGGTCTAATACTGGA
TATGACCGTGGGATGCATGTTTCATGGTGGAAAGCTTTTGTGGTGCAGGATGGGCCCGCGGCCTATCAGCT
TGTTGGTGGGGTAATGGCCTACCAAGGCGACGACGGGTAGCCGACCTGAGAGGGTGACCGGCCACACTG
GGACTGAGACACGGCC
>Zotu4651
GAACGCTGGCGGCGTGCCTAACACATGCAAGTCGAACGAGGTAGCAATACCGAGTGGCGGACGGGTGAG
TAATACATAGGCAACCTGCCCAACAGTGGGGGATACCCAGTCGAAAGATTGGCTAATACCGCATACGATCC
CTGGTTGGAATACCTGGGATGAAAGATTTATCGCTGTTGGATGGGCCTGTGGTGGATTAGCTAGTTGGTG
GGGTAACGGCCTACCAAGGCGACGATCCATAGCTGGTCTGAGAGGATGATCAGCCACACTGGGACTGAGA
CACGGCC
>Zotu4653
GAACGCTGGCGGCGTGCTTAACACATGCAAGTCGAACGATGAAGCCCAGCTTGCTGGGTGGAAGAGTGGC
GAACGGGTGAGTAACACGTGAGTAACCTGCCCCGGACTCTGGGATAAGCGCTGGAAACGGCGTCTAATAC
TGGATATGACCCATACCGGCATCGGTTGTGGGTGGAAAGATTTATCGGTTCGGGATGGGCTCGCGGCCTAT
CAGCTGGATGATGAGGTAACGGCTCACCATGGCGACGACGGGTAGCCGGCCTGAGAGAGTGACCGGCCA
CACTGGGACTGAGACACGGCC
>Zotu4655
GAACGCTGGCGGCGTGCTTAACACATGCAAGTCAAGGGGGCCCGCAAGGGCAACCGGCGAACGGGTGCG
TAACACGTGAGCGACCTGCCCGCTTGTGGGGGATAGCCGGCCCAACGGCCGGGTAATACCGCATACGCTT
TCCCTGGGGCATCTTGGGGATCGGAAAGATTTATCGCAGGCGGAGGGGCTCGCGGCCTATCAGCTAGTTG
GCGAGGTAACGGCTCACCAAGGCGACGACGGGTAGCTGGTCTGAGAGGATGGCCAGCCACATTGGGACT
GAGAGACGGCC
>Zotu4658
GAACATTGGCGGTAGGCTTAACACATGCAAGTCGAGCGAGGCGTAATAGTCGAGCGGCGAACGGGTGAG
TAATACATGGGAATCTACCTCGAAGTGGGGGATAACGTTCAGAAATGGACGCTAATACCGCATAATCCGGA
AACGGGAAAGCGCGCTGCGCTTTGAGATGAGCCCATGGCTGATTAGCTAGTTGGTAGGGTAAAGGCTTAC
CAAGGCGACGATCAGTAGCTGGTCTGAGAGGATGATCAGCCACACTGGAACTGAGACACGGTC
>Zotu4660
GAACGCTGGCGGCGTGCCTAATACATGCAAGTCGTGCGGGGTCTTGAAGGGCTTGCCCTTTAAGACCCAG
CGGCGAACGGGTGAGTAACACGTGGGTAACCTGCCCGCAAGACCGGGATAACTCCGGGAAACCGGGGCT
AATACCGGATAAGACTCCGGATCGCATGATCCGAAGTGGAAAGGCGGCGTCAAGCTGCCACTTGCGGATG
GGCCCGCGGCGCATTAGCTGGTTGGTGAGGTAACGGCTCACCAAGGCGACGATGCGTAGCCGACCTGAGA
GGGTGATCGGCCACACTGGGACTGAGACACGGCC
>Zotu4662
GAACGCTGGCGGCGTGCCTAACACATGCAAGTCGAGCGAAGTTTTTAAAGTGAATCCCTTCGGGGTGAAA
CTTTAATTATCTTAGCGGCGGACGGGTGAGTAACGCGTGAGAAACCTGCCTTTCACAAAGGGATAGCCTCG
GGAAACTGGGATTAATACCTTATGATACTTTTACTTCACATGAAGAAAAAGTTAAAGCGTAAAGCGGTGAA
AGATGGTCTCGCGTCCCATTAGCTAGTTGGTAAGGTAATAGCTTACCAAGGCGACGATGGGTAGCCGGCCT
GAGAGGGTGAACGGCCACACTGGAACTGAGACACGGTC
>Zotu4667
GAACGCTGGCGGCATGCCTAACACATGCAAGTCGAACGAGACCTTCGGGTCTAGTGGCGCACGGGTGCGT
AACGCGTGGGAATCTACCCTTGGGTTCGGAATAACAGTTGGAAACGACTGCTAATACCGGATGATGACGT
AAGTCCAAAGATTTATCGCCCAAGGATGAGCCCGCGTAGGATTAGCTTGTTGGTGAGGTAAAAGCTCACCA
AGGCGACGATCCTTAGCTGGTCTGAGAGGATGATCAGCCACACTGGGACTGAGACACGGCC
>Zotu4669
GAACGCTGGCGGCGTGCCTAACACATGCAAGTCGAGCGATGAAGTTTCCTTCGGGAAACGGATTAGCGGC
GGACGGGTGAGTAACACGTGGGTAACCTGCCTCATAGAGGGGAATAGCCTCCCGAAAGGGAGATTAATAC
CGCATAACATTGCAGTTTCGCATGAAACAGCAATTAAAGGAGCAATCCGCTATGAGATGGACCCGCGGCGC

ATTAGCTAGTTGGTAAGGTAATGGCTTACCAAGGCGACGATGCGTAGCCGACCTGAGAGGGTGATCGGCC
ACATTGGGACTGAGACACGGCC
>Zotu4671
GAACGCTGGCGGCATGCTTTACACATGCAAGTCGAACGGCAGCGCGGGGCAACCTGGCGGCGAGTGGCG
AACGGGTGAGTAATATATCGGAACGTACCCAGAAGTGGGGGATAACGTAGCGAAAGTTACGCTAATACCG
CATACGATCTACGGATGAAAGTGGGGGACCGCAAGGCCTCATGCTTTTGGAGCGGCCGATATCTGATTAG
CTAGTTGGTGAGGTAAAGGCTCACCAAGGCGACGATCAGTAGCTGGTCTGAGAGGACGACCAGCCACACT
GGGACTGAGACACGGCC
>Zotu4674
GAACGCTAGCGGCAGGCCTAATACATGCAAGTCGAGGGGTAGTGTAGCAATACATGAGACCGGCGCACG
GGTGCGTAACGCGTATGAAAGGTGTCCAAATCTCTGGGATAGCCCTCGGAAACGGGGATTAATACTGGAT
GGTCTATATGTGTGGCATCACACTTATAGTAAAGCTCAGGCGGATTTGGGTCCTCATGCGTACGATTAGCTA
GTTGGCGGGGTAATGGCCCACCAAGGCGACGATCGTTAGGGGTCCTGAGAGGGAGATCCCCCACACTGGT
ACTGAGACACGGGC
>Zotu4678
GAACGCTGGCGGCAGGCCTAACACATGCAAGTCGAACGGATGTAGCAATACATTAGTGGCGCACGGGAG
AGTAACGCGTGGGAATATACCTTTTGGTTTGGGATAACACCGGGAAACTGGTGCTAATACCGGATAAGCCC
TTCGGGGGAAAGATTTATCGCCGAAAGATTAGCCCGCGTAGGATTAGCTAGTTGGTGAGGTAAAGGCTCA
CCAAGGCTACGATCCTTAGCTGGTCTAAGAGGATGATCAGCCTCACTGGGACTGAGACACGGCC
>Zotu4680
GAACGCTGGCGGCGCGCTTAACACATGCAAGTCGAGCGAGAACCAGTCCTTCGGGGCTGGGGACAGCGG
CGAACGGGTGAGTAACACGTGGGTAATCTGCCCTCGGCACCGGAATAGCCCGGGGAAACCCGGATTAATG
CCGGATGGCCTCGCCGGCCTTCGGGCTGGCGAGAAAAGGTAGCTTCGGCCTCCGGCCGAGGATGAGCCCG
CGGCCGATTAGCTTGTTGGTGGGGTAACGGCTCACCAAGGCGACGATCGGTAGCTGGTCTGAGAGGACGA
TCAGCCACACTGGGACTGAGACACGGCC
>Zotu4683
GAACGCTAGCGGCAGGCCTAATACATGCAAGTCGAACGAGATTGAAGAGCTTGCTTTTCATGAAAGTGGC
GCACGGGTGCGTAACGCGTATGCAACCTACCTTCTTCAGGGGGATAGCCTCTCGAAAGAGAGATTAACACC
GCATAATATTATTGGATGGCATCATTTAATAATCAAATATTTATAGGAAGAAGATGGGCATGCGTGACATT
AGTTAGTTGGCGGGGTAATGGCCCACCAAGACCACGATGTCTAGGGGATCTGAGAGGATGACCCCCCACA
CTGGTACTGAGACACGGAC
>Zotu4687
GAACGCTGGCGGCGTGCTTAACACATGCAAGTCGAACGATGAAGCCCAGCTTGCTGGGTGGATTAGTGGC
GAACGGGTGAGTAACACGTGAGTAACCTGCCCTTCACTCTGGGATAAGCCTTGGAAACGAGGTCTAATACT
GGATATGACCTGTCACCGCATGGTGGTGGGTGGAAAGTTTTTCGGTGGGGGATGGACTCGCGGCCTATCA
GCTTGTTGGTGAGGTAGTGGCTCACCAAGGCGACGACGGGTAGCCGGCCTGAGAGGGTGATCGGCCACA
CTGGGACTGAGACACGGCC
>Zotu4691
GAACGCTGGCGGTATGGATAAGGCATGCAAGTCGAGCGCCCCGCAAGGGGAGCGGCAGACGATGTAGTA
ACACGTAGGTACATCCCCCAAAGTCAGGAATAGCTAGTCGAAAGATTAGGTAATACTTGATATGCTGCAAA
GTGAAAGATTTATCGCTTTGGGAGTGGCCTGCGTCGTATCAGCTAGTTGGTGAGGTAATGGCTCACCAAGG
CTATGACGCGTAGGGGAGCTGAGAGGCTGACCCCCACAGATGAGACTGAGACACGGCT
>Zotu4694
GAACGCTGGCGGCGTGCCTAATACATGCAAGTCGAACGCAACACCTTCGGGTGTTGAGTGGCGGACGGGT
GAGTAACACGTGGGTAACCTGCCCATCAGCCTGGGATAACCACCGGAAACGGTGGCTAATACCGGATATG
TCTACGGATGAAAGTCGGATTATCTGACGCTGATGGATGGACCTGCGGCGCATTAGCTAGTTGGTGAGGT
AGAGGCTCACCAAGGCGACGATGCGTAGCCGACCTGAGAGGGTGATCGGCCACACTGGGACTGAGACAC
GGCC
>Zotu4697

GAACGCTGGCGGCGTGCTTAACACATGCAAGTCGAGGGAACCTGGGGGGCAACCTTCAGGTGACCGGCG
AACGGGTGCGTAACACGTGAGCGACCTACCGTTTTGCGGGGGATAGCCGGCCCAACGGCCGGGTAATACC
GCATACGCTCATCGGGCGGCATCGTCTGGTGAGGAAACCTCAGGGGCGGAACGAGGGGCTCGCGGCCTA
TCAGCTAGTTGGTGAGGTAACGGCTCACCAAGGCAACGACGGGTAGCTGGTCTGAGAGGATGGCCAGCCA
CATTGGGACTGCGACACGGCC
>Zotu4700
GAACGCTAGCGGCAGGCCTAATACATGCAAGTCGAGGGGCAGCGCAGTGTAGCAATACATGGGCGGCGA
CCGGCAAACGGGTGCGGAACACGTACGCAACCTTCCTTCAAGCGGGGAATAGCCCAGAGAAATTTGGATT
AATACCCCATAGCACTGTCCGGTGGCATCATTGGACAGTTAAAGATTTATCACTTGAAGATAGGCGTGCGT
CTGATTAGGTAGTTGGTGAGGTAACGGCTCACCAAGCCGACGATCAGTAACTGGCGTGAGAGCGCGACCA
GTCACACGGGCACTGAGACACGGGC
>Zotu4703
GAACGCTAGCGGCAGGCTTAACACATGCAAGTCGAGGGGTATAGTAGCAATACTAGAGACCGGCGCACG
GGTGCGTAACGCGTATGCAATCTACCTTTAGCAGGGGCATAGCCCAGAGAAATTTGGATTAATTCCCCATA
GTATTATAGTATGGCATCATATTATAATTAAAGTAACAACGGCTAAAGATGAGCATGCGTCCCATTAGCTAG
TTGGTATGGTAACGGCATACCAAGGCAATGATGGGTAGGGGTCCTGAGAGGGAGATCCCCCACACTGGTA
CTGAGACACGGAC
>Zotu4705
GAACGCTGGCGGCGTGCTTAACACATGCAAGTCGAACGGTGACCTCGGAGCTTGCTCCGGGTGATCAGTG
GCGAACGGGTGAGTAACACGTGAGTAACCTGCCCCAGACTCTGGGATAACCCCGGGAAACCGGAGCTAAT
ACCGGATACGACGCGAACAGGCATCTGTATCGCGTGGAAAGTTCTTCGGTCTGGGATGGACTCGCGGCCT
ATCAGCTTGTTGGTGAGGTAACGGCTCACCAAGGCGACGACGGGTAGCCGGCCTGAGAGGGCGACCGGC
CACACTGGGACTGAGACACGGCC
>Zotu4707
GAACGCTGGCGGCGTGCTTCATACATGCAAGTCGAACGAGAAGCTGACTTCGGTCAGTGGAAAGTGGCGG
ACGGGTGAGTAATATGTAGGAAATCTGCCTTAGAGAGGGGGACAACAGAGGGAAACTTCTGCTAATACCC
CATATGAGCGTACCTGAAATGGTATTCTTGAAAACTCCGGTGCTCTAAGATGAGCCTGCATCTGATTAGCTT
GTTGGTGGGGTAATGGCCTACCAAGGCGACGATCAGTAGCTGGTTTGAGAGGATGATCAGCCACAATGGG
ACTGAGACACGGCC
>Zotu4710
GAACGCTAGCGGGAGGCCTAACACATGCAAGCCGAGCGGTAGGGTTTCTTCGGAAACCTGAGAGCGGCG
CACGGGTGCGGAACACGTGTGCAACCTACCTTTATCAGGGGGATAGCCTTTCGAAAGGAAGATTAATACCC
CATAATATATTAGATGGCATCATTTGATATTGAAAACTCCGGTGGATAGAGATGGGCACGCGCAAGATTAG
ATAGTTGGTGAGGTAACGGCTCACCAAGTCCATGATCTTTAGGGGGCCTGAGAGGGTGATCCCCCACACTG
GTACTGAGACACGGAC
>Zotu4713
GAACGCTGGCGGCATGCTTTACACATGCAAGTCGGACGGCAGCACAGAGAAGCTTGCTTCTTGGGTGGCG
AGTGGCGAACGGGTGAGTAACATATCGGAACGTACCGGGCAGTGGGGGATAACTAATCGAAAGATTAGC
TAATACCGCATATATTCTGAGGAAGAAAGCAGGGGACCATTTGGCCTTGCGCTGTTTGAGCGGCCGATATC
TGATTAGCTGGTTGGTGGGGTAAAGGCCTACCAAGGCGACGATCAGTAGCGGGTCTGAGAGGATGATCCG
CCACACTGGGACTGAGACACGGCC
>Zotu4720
GAACGCTGGCGGCGTGCTTAACACATGCAAGTCGAGCGGTAAGGCACCTTCGGGTGTACACGAGCGGCGA
ACGGGTGAGTAACACGTGAGTAATCTGCCCTTCACTTCGGGATAACCGCCGGAAACGGTGGCTAATACCG
GATACGACGCACTCTCGCATGGGATGTGCGTGGAAAGTCTTATCGGTGGAGGATGTGCTCGCGGCCTATC
AGCTTGTTGGTGAGGTAACGGCTCACCAAGGCTTCGACGGGTAGCCGGCCTGAGAGGGTGACCGGCCACA
CTGGGACTGAGACACGGCC
>Zotu4722
GAACGCTGGCGGCGTGCTTAACACATGCAAGTCGAACGGTGAAGCCCAGCTTGCTGGGTGGATCAGTGGC
GAACGGGTGAGTAACACGTGAGCAATCTGCCCCTGACTCTGGGATAAGCGCTGGAAACGGCGTCTAATAC
TGGATACGAACAGCGACCGCATGGTCAGTTGTTGGAAAGATTTTTCGGTCAGGGATGAGCTCGCGGCCTAT

CAGCTTGTTGGTGAGGTAATGGCTCACCAAGGCGTCGACGGGTAGCCGGCCTGAGAGGGTGACCGGCCAC
ACTGGGACTGAGACACGGCC

>Zotu4725

GAACGCTGGCGGCATGCCTTACACATGCAAGTCGAACGGCAGCGCGGGCTTCGGCCTGGCGGCGAGTGG
CGAACGGGTGAGTAACATATCGGAACGTGCCCTGGAGTGGGGGATAACTAGTCGAAAGATTAGCTAATAC
CGCATACGATCTACGGAGGAAAGTGGGGGACCGCAAGGCCTCACGCTCGTGGAGCGGCCGATATCTGATT
AGCTAGTTGGTGGGGTAAAGGCCTACCAAGGCATCGATCAGTAGCTGGTCTGAGAGGACGACCAGCCACA
CTGGGACTGAGACACGGCC

>Zotu4730

CAACGCTGACGGCGTGCCTAACACATGCAAGTCGAACGTGAAAGGGGCTTCGGCCCTGAGTAAAGTGGCA
AACGGGTGAGTAACGCGTGGGTGATCTGCCTCTGAGTGGGGGATAACCTTCCGAAAGGAGGGCTAATACC
GCATGACATCCTGTTCTTGGATGAACGGTGATCGAAGCTGGGGACCGCAAGGCCTGGCGCTTGGAGAGGA
GCCCGCGTCCGATTAGCTAGTTGGTGGGGTAATGGCCTACCAAGGCGACGATCGGTAGCCGGCCTGAGAG
GGCGGACGGCCACACTGGGACTGAGACACGGCC

>Zotu4732

GAACGCTGGCGGCGTGCCTAACACATGCAAGTCGAACGAGATGCGTATCGGAAAGCTTCGGCCGGAAGAT
ACGTTATCTAGTGGCGGACGGGTGAGTAACACGTGGGTAACCTGCCTTATGGAGGGGGATAACAGAGAG
AAATCACTGCTAATACCGCATAAGCACACAGTACCGCATGGTACAGGGTGAAAAGATTTATCGCCATAAGA
TGGACCCGCGTCTGATTAGCCAGTTGGCAGGGTAAAAGCCTACCAAAGCAACGATCAGTAGCCGATCTGA
GAGGATGACCGGCCACATTGGGACTGAGACACGGCC

>Zotu4734

GAACGCTGGCGGCGTGCCTAATACATGCAAGTCGAGCGAACAGATGAGGAGCTTGCTCCTCTGATGTTAG
CGGCGGACGGGTGAGTAACACGTGGGCAACCTGCCCTATAGTTTGGGATAACTTGCGGAAACGTGAGCTA
ATACCGGATAATACAACGAAGCTCCTGCTTCGTTGTTGAAAGATGGCTTTGCTATCGCTATAGGATGGGCC
CGCGGCGCATTAGCTAGTAGGTGAGGTAACGGCTCACCTAGGCAACGATGCGTAGCCGACCTGAGAGGGT
GATCGGCCACACTGGGACTGAGACACGGCC

>Zotu4737

GAACGCTGGCGGCGTGCCTAACACATGCAAGTCAAGGGTTTGATTTATCAGACACTGGCATCCGGGTGAG
TAACGCGTAGGTCATCTGCCCCTATCACAGACATAACAGTTCTAACGGACTGCTAATATCTGATGAGGCGA
GCAATCGTCAAAGCTTAGGCGGATAGGGATGAGCCTGCGTCTGATTAGGTAGTTGGTGAGGTAATGGCTC
ACCAAGCCTTCGATCAGTAGCTGGTCTGAGAGGACGAACAGCCACACTGGAACTGAGACACGGTC

>Zotu4740

GAACGCTAGCGGCAGGCTTAACACATGCAAGTCGAGGGGTATAGTTAGCTTGCTAATTAGAGACCGGCGC
ACGGGTGCGTAACGCGTATGCAATCTACCTTTCACAGAGGGATAGCCCAGAGAAATTTGGATTAATACCTC
ATAGTATTATAGAATGGCATCATTTTATAATTAAAGTCACAACGGTGAAAGATGAGCATGCGTCCCATTAGC
TATTTGGTATGGTAACGGCATACCAAGGCTACGATGGGTAGGGGTCCTGAGAGGGAGATCCCCCACACTG
GTACTGAGACACGGAC

>Zotu4743

GAACGCTGGCGGCGTGCTTAACACATGCAAGTCGAACGATGAAGCCCAGCTTGCTGGGTGAAAGAGTGGC
GAACGGGTGAGTAACACGTGAGTAACCTGCCCTGAACTCTGGGATAAGCACTGGAAACGGTGTCTAATAC
TGGATACGACCTATCACCGCATGGTGTGTGGGTGGAAAGATTTATCGGTTTTGGATGGACTCGCGGCCTAT
CAGCTAGATGGTGAGGTAATGGCTCACCATGGCGACGACGGGTAGCCGGCCTGAGAGGGTAACCGGCCA
CACTGGGACTGAGACACAGCC

>Zotu4745

GAACGCTGGCGGCAGGCCTAACACATGCAAGTCGAACGGGTGTCTTCGGGCACTAGTGGCGGACGGGTG
AGTAACGCGTGGGAACATACCCCTTTCTTCGGAATAGCCTCGGGAAACTGGGAGTAATACCGGATACGCCC
TTCGGGGGAAAGATTTATCGGTAAGGGATTGGCCCGCGTTGGATTAGGTAGTTGGTGGGGTAATGGCCTA
CCAAGCCGACGATCCATAGCTGGTTTGAGAGGATGATCAGCCACACTGGGACTGAGACACGGCC

>Zotu4748

GAACGCTGGCGGCGTGCCTAATACATGCAAGTCGAGCGAATCACTGGGAGCTTGCTCCCGGTGGTTAGCG
GCGGACGGGTGAGTAACACGTGGGCAACCTGCCTGTAAGACTGGGATAACTTCGGGAAACCGGAGCTAAT
ACCGGATAATTTCTTCCCTCGCATGAGGGAAGGTTGAAAGTCGGTTTCGGCTGACACTTACAGATGGGCCC
GCGGCGCATTAGCTAGTTGGTGAGGTAACGGCTCACCAAGGCGACGATGCGTAGCCGACCTGAGAGGGT
GATCGGCCACACTGGGACTGAGACACGGCC
>Zotu4750
GAACGCTGGCGGCATGCCTAACACATGCAAGTCGAACGAGACCTTCGGGTCTAGTGGCGCACGGGTGCGT
AACGCGTGGGAACCTGCCCTTAGGTTCGGAATAACTCAGAGAAATTTGAGCTAATACCGGATAATGTCTTC
GGACCAAAGATTTATCGCCTTTGGATGGGCCCGCGTTGGATTAGCTAGTTGGTGGGGTAATGGCCTACCAA
GGCGACGATCCATAGCTGGTCTGAGAGGATGATCAGCCACACTGGGACTGAGACACGGCC
>Zotu4754
GAACGCTAGCGGCAGGCCTAATACATGCAAGTCGAGCGGATTGTAGCAATACAGTTAGCGGCGCACGGGT
GAGTAACGCGTACACAATCTACCTTCAACAGGGGAATAGCCTCGGGAAACTGGGAGTAATACCCCATAGC
ATCTTTGGGTCGCATGACCTGATTATTAAAGTTACGGCGGTTGAAGATGAGTGTGCGTCTGATTAGATTGTT
GGTGAGGTAACGGCTCACCAAGTCGATGATCAGTAAGGGGCGTGAGAGCGTGGCCCCTCACATGGACACT
GAGACACGGGT
>Zotu4756
GAACGCTGGCGGCATGCCTAACACATGCAAGTCGAACGAGACCTTCGGGTCTAGTGGCGCACGGGTGCGT
AACGCGTGGGAATCTGCCCTTGGGTTCGGAACAACAGTTGGAAACGACTGCTAATACCGGATGATGACTTC
GGTCCAAAGATTTATCGCCCAAGGATGAGCCCGCGTAGGATTAGCTTGTTGGTGAGGTAAAAGCTCACCAA
GGCGACGATCCTTAGCTGGTCTGAGAGGATGATCAGCCACACTGGGACTGAGACACGGCC
>Zotu4759
GAACGCTGGCGGCAGGCTTAACACATGCAAGTCGAACGCCCCGCAAGGGGAGTGGCAGACGGGTGAGTA
ACGCGTGGGAATCTACCCAGATCTTCGGAACAACTGAGGGAAACTTCAGCTAATACCGGATACGCCCTACG
GGGGAAAGATTTATCGGTTCTGGATGAGCCCGCGTTGGATTAGCTAGTTGGTGGGGTAATGGCCTACCAA
GGCGACGATCCATAGCTGGTCTGAGAGGATGATCAGCCACACTGGGACTGAGACACGGCC
>Zotu4761
GAACGCTGGCGGCATGCCTAACACATGCAAGTCGAACGAAGGCCTTCGGGCTTTAGTGGCGCACGGGTGC
GTAACGCGTGGGAATCTGCCCTTAGGTTCGGAATAACAGCTGGAAACGGCTGCTAATACCGGATGAAGAC
GAAAGTCCAAAGATTTATCGCCTGAGGATGAGCCCGCGTTGGATTAGGTAGTTGGTGGGGTAAAGGCCTA
CCAAGCCGACGATCCATAGCTGGTCTGAGAGGATGATCAGCCACACTGGGACTGAGACACGGCC
>Zotu4763
GAACGCTGGCGGCGTGCTTAACACATGCAAGTCGAACGAACCTTCGGGTTAGTGGCGGACGGGTGAGTAA
CGCGTGGGAATCTGCCCTCAGGAGGGGGATAACGGCTGGAAACGGCCGCTAATACCCCATATGCCGAGAG
GTGAAACGAATTTCGCCTGAGGATGAGCCCGCGTCTGATTAGCTAGTTGGTGGGGTAAGAGCCTACCAAG
GCGACGATCAGTAGCTGGTCTGAGAGGATGATCAGCCACACTGGGACTGAGACACGGCC
>Zotu4765
GAACGCTGGCGGCGTGCCTAATACATGCAAGTCGAGCGAACCACTTCGGTGGTGAGCGGCGAACGGGTG
AGTAACACGTAGGTGATCTGCCCATCAGACGGGGACAACGATTGGAAACGATCGCTAATACCGGATAGGA
CGAAAGTTTAAAGGTGCTTCTGGCACCGCTGATGGATGAGCCTGCGGCGCATTAGCTAGTTGGTAGGGTA
AAGGCCTACCAAGGCGACGATGCGTAGCCGACCTGAGAGGGTGAACGGCCACACTGGGACTGAGACACG
GCC
>Zotu4767
GAACGCTGGCGGCGTGCCTAATACATGCAAGTTGAGCGGCAGCGAGTTTTGCACTTATCCTTCCGACCCAT
ACGTGGGTGGAAGGATGTGTGCAGAATGTCGGCGAGCGGCGGACGGCAGAGTAACGCGTAGGAATGTAC
CCCAAAGTGAGGGATAACTGCTCGAAAGAGTAGCTAATACCGCATGTGATCTTCGGATTAAAGCTTTATGC
GCTTTGGGAACAGCCTGCGTAAGATTAGCTAGTTGGTGAGGTAATGGCTCACCAAGGCGACGATCTTTAGC
TGGTCTGAGAGGATGATCAGCCTGACTGGAACTGAGACACGGTC
>Zotu4769

GAACGCTGGCGGCAGGCTTAACACATGCAAGTCGAACGCCCCGCAAGGGGAGTGGCAGACGGGTGAGTA
ACGCGTGGGAATCTACCTAGAACTACGGAACAACAGTTGGAAACGACTGCTAATACCGTATACGCCCTACG
GGGGAAAGATTTATCGGTTTTAGATGAGCCCGCGTAAGATTAGCTAGTTGGTGGGGTAATGGCCTACCAA
GGCGACGATCTTTAGCTGGTCTGAGAGGATGATCAGCCACACTGGGACTGAGACACGGCC

>Zotu4771

GAACGCTGGCGGCGTGCTTAACACATGCAAGTCGAGCGACGCTCCTCCTTCGGGAGGATGACGGAGCGGC
GAACGGGTGAGTAACACGTGGGCAACCTGCCCCTAGCTCTGGGATAAGCCCGGGAAACCGGGTCTAATAC
CGGATATGAGTTCGGAAGGCATCTTCTGACCTGGAAAGTTTTTCGGCTAGGGATGGGCCCGCGGCCTATCA
GCTTGTTGGTGAGGTAACGGCCCACCAAGGCGACGACGGGTAGCCGGCCTGAGAGGGCGACCGGCCACA
CTGGGACTGAGACACGGCC

>Zotu4773

GAACGCTGGCGGCGTGCTTAACACATGCAAGTGGAGCGACGAACCGGGGCTTGCCCCGGGGCAGAGCCG
CGAACGGGTGAGTAACACGTGGGTAACCCACCTCGATGACCGGGACAACCCGAGGAAACTCGGGCTAATA
CCGGATGTGATCCTCTTTCGTAAGGAAGATGATGAAAGATAGCTTCGGCCTTCGCATCGAGACGGGCCCGC
GGCCCATTAGCTTGTTGGTGGGGTAATGGCCTACCAAGGCTTCGATGGGTAGCTGGTCTGAGAGGACGAT
CAGCCACACTGGGACTGAGACACGGCC

>Zotu4775

GAACGCTGGCGGCGTGCCTAATACATGCAAGTCGAGCGGACAGATGGGAGCTTGCTCCCTGATGTTAGCG
GCGGACGGGTGAGTAACACGTGGGTAACCTGCCTGTAAGACTGGGATAACTCCGGGAAACCGGGGCTAA
TACCGGATGCTTGTTTGAACCGCATGGTTCAAACATAAAAGGTGGCTTCGGCTACCACTTACAGATGGACC
CGCGGCGCATTAGCTAGTTGGTGAGGTAATGGCTCACCAAGGCAACGATGCGTAGCCGACCTGAGAGGGT
GATCGGCCACACTGGGACTGAGACACGGCC

>Zotu4778

GAACGCTGGCGGCGTGCCTAATACATGCAAGTCGAGCGGACTTGTGGGAGCTTGCTCCCGCAAGTTAGCG
GCGGACGGGTGAGTAACACGTGGGCAACCTGCCTGTAAGACTGGGATAACTTCGGGAAACCGGAGCTAAT
ACCGGATAACTCCTTTCCTCACCTGGGGAAAGGTTGAAAGATGGCTTCGGCTATCACTTACAGATGGGCCC
GCGGCGCATTAGCTAGTTGGTGAGGTAACGGCTCACCAAGGCGACGATGCGTAGCCGACCTGAGAGGGT
GATCGGCCACACTGGGACTGAGACACGGCC

>Zotu4781

GAACGCTAGCGGGAGGCTCAACACATGCAAGCCGAGGGGTATATAATTGCTTGCAATTATAGAGACCGGC
GGATGGGTGAGTAACGCGTATGTAACTTACCCTATTCATTGGGATAGCCCGAGGAAACTCGGATTAATACC
AGATAACACTAAGGTCGGCATTGACTTTAGTTGAAAGATTTATCGGAATAGGATAGACATGCGTCAGATTA
GCTAGTTGGTGAGATAGAGGCTCACCAAGGCAATGATCTGTAGGGGGCCTGAGAGGGTGATCCCCCACAC
TGGTACTGAGACACGGAC

>Zotu4788

GAACGCTAGCGGCAGGCCTAACACATGCAAGTCGAGCGGGATTCGGTGGCTTGCCACCGATGAGAGCGG
CGCACGGGTGCGTAACGCGTATACAGCCTACCTTTTGCTACGGGATAGCCCAGAGAAATTTGGATTAACAC
CGTATGGTATAAGGACCCGGCATCGGGATCTTATTAAAGGCTACGGCAAAAGATGGGTATGCGTCCTATTA
GCTAGATGGTGCGGTAACGGCGCACCATGGCAACGATAGGTAGGGGCCCTGAGAGGGGGATCCCCCACA
CTGGTACTGAGACACGGAC

>Zotu4790

CAACGCTGGCGGCGCGCTTAACACATGCAAGTCGAGCGAGAAAGGGGGCAACCCTGAGTAAAGCGGCGT
ACGGGTGAGTAACACGTGGATTATCTACCTTTTAGTGGGGAATAACTTTGGGAAACCGAAGCTAATACCGC
ATAAGCCTGTAAAGGGAAAGCAGCAATGCGCTGGAAGAAGAGTCCGCGGCCGATTAGCTAGTTGGTGAG
GTAACGGCCCACCAAGGCGATGATCGGTAGCCGGCCTGAGAGGGCACACGGCCACACTGGCACTGAAACA
CGGGC

>Zotu4792

GAACGCTGACAGAATGCTTAACACATGCAAGTCTACTTGAAGTAACTTCGGTTATGGATGGTGGCGGACG
GGTGAGTAACGCGTAAATAACTTGCCCCCTAGTCTGGGACAACTGTTGGAAACGACAGCTAATACTAGATA
TTATGAGACCTTGGCATCAAGGATTTATGAAAGCTATATGCGCTAGGGGAGAGATTTGCGTCCTATTAGCT

AGTTGGTGAGGTAATGGCTCACCAAGGCGACGATAGGTAGCCGGCCTGAGAGGGTGAACGGCCACAAGG
GGACTGAGACACGGCC
>Zotu4796
GAACGCTGGCGGCGTGCCTAATACATGCAAGTCGAGCGCCCCTTTAAGAGGTGACAAAGAGATTTGTCGG
TTCCTATGGGGAGCGGCGGACGGCTGAGTAACGCGTGGGAACGTGCCCCAAAGTGAGGGATAAGCACCG
GAAACGGTGTCTAATACCGCATATGATCTTCGGATTAAAGCCTTGTGCGCTTTGGGAGCGGCCTGCGTCCT
ATTAGATAGTTGGTGAGGTAAAGGCTCACCAAGTCAACGATGGGTAGCTGGTCTGAGAGGATGACCAGCC
AGACTGGAACTGAGACACGGTC
>Zotu4800
GAACGCTGGCGGCATGCTTAACACATGCAAGTCGAACGGTAACAGGCCCGCAAGGGTGCTGACGAGTGG
CGGACGGGTGAGTAAAGCGTAGGAATCTATCTAGTGGAGTGGGATAACCTAGGGAAACTTAGGGTAATAC
CGCATAAGTTCTAAGGAATAAAGCAGGGGACCTTCGGGCCTTGCGCTATTAGCTGAGCCTACGTCGGATTA
GCTAGTTGGTGGGGTAAAGGCCTACCAAGGCGATGATCCGTAGCTGGTTTGAGAGGACGACCAGCCACAC
TGGGACTGAGACACGGCC
>Zotu4802
CAACGCTGGCGGCGTGCCTCAGACATGCAAGTCGAACGATTAAACTTTCCTTCGGGGAAGATATAGAGTG
GCGCACGGGTGAGTAACACGTAGGTAATCTACCTCTGGGTGGGGAATAACTTGTGGAAACGCAAGCTAAT
ACCGCATAATGCAGCGGCACCGAATGGTGACAGTTGTTAAAGCTTTATGTGCCTGGAGAGGAGCCTGCGG
CGGATTAGGTAGTTGGTAAGGTAACGGCTTACCAAGCCTGCGATCCGTATCCGGTCTAAGAGGACGGTCG
GACACACTGACACTGAATAACGGGT
>Zotu4805
GAACGCTGGCGGCATGCCTTACACATGCAAGTCGAACGGTAACAGGTCTTCGGATGCTGACGAGTGGCGA
ACGGGTGAGTAATACATCGGAACGTGCCCAATCGTGGGGGATAACACATCGAAAGATGTGCTAATACCGC
ATAAGATCTACGGATGAAAGCGGGGGACTCGCAAGAGCCTCGCGCGAATGGAGCGGCCGATGGCAGATT
AGGTAGTTGGTGTGGTAAAAGCGCACCAAGCCGACGATCTGTAGCTGGTCTGAGAGGACGACCAGCCACA
CTGGGACTGAGACACGGCC
>Zotu4808
CAACGCTGGCGGCGTGCCTCAGACATGCAAGTCGAACGCGAAAGTTTCCTTCGGGAGACGAGTAGAGTGG
CGCACGGGTGAGTAACACGTAGGTAATTTGCCTTCGGGTGGGGAACAACTCAGGGAAACTTGAGCTAATA
CCGCATAATGCAGCGGCATTTAATTATGACAGTTGTTAAAGCGGAGGATCGAAAGACTTCGCGCCTGAAGA
GAAGCCTGCGGCAGATTAGCTAGTTGGTAAGGTAATGGCTTACCAAGGCGACGATCTGTAACCGGTCTAA
GAGGACGGTCGGTCACACTGACACTGAATAACGGGT
>Zotu4811
GAACGCTGGCGGCGTGCCTAATACATGCAAGTCGAGCGAATCTTCAGGAGCTTGCTCCTGAAGATTAGCG
GCGGACGGGTGAGTAACACGTGGGCAACCTGCCTGTAAGACTGGGATAACTTCGGGAAACCGAGGCTAAT
ACCGGATAATCCTTTTTCTCACATGAGGGAAAGCTGAAAGACGGCGCAAGCTGTCACTTACAGATGGGCCC
GCGGCGCATTAGCTAGTTGGTGAGGTAACGGCTCACCAAGGCAACGATGCGTAGCCGACCTGAGAGGGT
GATCGGCCACACTGGGACTGAGACACGGCC
>Zotu4813
GAACGCTAGCGGCAGGCTTAACACATGCAAGTCGAGGGGTAGTAGGAGCTTGCTTCTATGAGACCGGCGC
ACGGGTGCGTAACGCGTATGCAATCTGCCTCTTACTGGGGGATAGCCCGGAGAAATCCGGATTAATACCCC
ATGGTATTTTGGAATGGCATCATTCTATTATTAAAGATTTATCGGTAAGAGATGAGCATGCGTCCCATTAGT
TAGTTGGTGTGGTAACGGCACACCAAGACGATGATGGGTAGGGGTCCTGAGAGGGAGATCCCCCACACTG
GTACTGAGACACGGAC
>Zotu4815
GAACGCTAGCGGCAGGCTTAACACATGCAAGTCGAGGGGTATAGTTCTTCGGAACTAGAGACCGGCGCAC
GGGTGCGTAACGCGTATGCAATCTACCTTTCACAAAGGGATAGCCCAGAGAAATTTGGATTAATACCTTAT
AGTATAGCGACTTCGCATGGAGCTGCTATTAAAGATTTATCGGTGAAAGATGAGCATGCGTCCCATTAGCT
AGTTGGTAAGGTAACGGCTTACCAAGGCTACGATGGGTAGGGGTCCTGAGAGGGAGATCCCCCACACTGG
TACTGAGACACGGAC

>Zotu4818
AAACGCTGGCGGCGTGCCTAATACATGCAAGTCGAACGGGGAAAGTCTTCGGATGAGTACTAGTGGCGAA
CGGGAGAGGAACACGTGGGTAATCTACCTTTAGTTGAAGAATAACTAGTCGAAAGATTAGCTAATACTTCG
TAAGACCACAGTATCTAATGATACAGGGGTTATAGGAGCAATCCGACTTTAGATGAGCCCGCGTCCGATTA
GCTAGTTGGTGAGGTAATGGCTCACCAAGGCTGTGATTGGTAGCTGGTCTGAGAGGATGACCAGCCTCAC
TGGAACTGACACACGGTC
>Zotu4820
GAACGCTGGCGGCGTGCCTAAAACATGCAAGTCGAACGAGAAAGTGGGCAACCATGAGTATAGTGGCGC
ACGGGTGAGTAACACGTGGATAACCTACCTTAAGAACTGGGATAACAAGCCGAAAGGTTTGCTAATACCG
GATGAAGTGTCGGAGGCATTGGGGCCTCTGCCATGAAAACGGGCGCAAGCTACGGTCTTGAGAGGGGTCT
GCGTCCCATTAGCTAGTTGGTGAGGTAAAAGCTTATCCAAGGCGAAGATGGGTAGCTGGTCTGAGAGGAC
GACCAGCCACACTGGAACTGAAACACGGTC
>Zotu4823
GAACGCTGGCGGCGTGCCTAATACATGCAAGTCGAGCGGACGTTTTATTGGTGCTTGCACCGATTTAACAT
TAGCGGCGGACGGGTGAGTAACACGTGGGCAACCTACCCTGTAGATTGGGATAACTCCGGGAAACCGGG
GCTAATACCGAATAATACTTTTTAACTCATGTTAGAAAGTTGAAAGATGGTTTACGCTATCACTACAGGATG
GGCCCGCGGCGCATTAGCTAGTTGGTGAGGTAATGGCTCACCAAGGCCACGATGCGTAGCCGACCTGAGA
GGGTGATCGGCCACACTGGGACTGAGACACGGCC
>Zotu4825
GAACGCTGGCGGCGTGCCTAACACATGCAAGTCGAACGGGGTTAAGTGAAATTTTCGGATGGAGCTTAAC
TTAGTGGCGGACGGGTGAGTAACGCGTGGATAACCTGCCTCACACTGGGGGATAGCAGCTGGAAACGGCT
GGTAATACCGCATAAGACCACAGCACCGCATGGTGCAGGGGTAAAAGATTTATCGGTGTGAGATGGATCC
GCGTCTGATTAGCCAGTTGGCGGGGTAACGGCCCACCAAAGCGACGATCAGTAGCCGGCCTGAGAGGGT
GAACGGCCACATTGGGACTGAGACACGGCC
>Zotu4829
GAACGCTGGCGGCGTGCCTAATACATGCAAGTCGAGCGAATCTCGAGGTGCTTGCACCTCTTGGTTAGCGG
CGGACGGGTGAGTAACACGTGGGCAACCTGCCTGTAAGACTGGGATAACACCGGGAAACCGGTGCTAATA
CCGGATAATTCTTTTCCTCTCATGAGGAAAAACTGAAAGTCGGCTTACGCTGACACTTACAGATGGGCCCGC
GGCGCATTAGCTAGTTGGTGAGGTAACGGCTCACCAAGGCGACGATGCGTAGCCGACCTGAGAGGGTGAT
CGGCCACACTGGGACTGAGACACGGCC
>Zotu4831
GAACGCTGGCGGCGTGCTTAACACATGCAAGTCGAGCGGAAAGGCCACTTCGGTGGTACTCGAGCGGCGA
ACGGGTGAGTAACACGTGAGTAATCTGCCCCCCACTTTGGGATAGCCACCGGAAACGGTGATTAATACCG
GATATGACCCTTGGCCGCATGGTTGTGGGTGGAAAGTTTTTCGGTGGGGGATGTGCTCGCGGCCTATCAG
CTTGATGGTGGGGTAATGGCCTACCATGGCTTCGACGGGTAGCCGGCCTGAGAGGGTGACCGGCCACACT
GGGACTGAGACACGGCC
>Zotu4834
GAACGCTGGCGGCGTGCTTAACACATGCAAGTCGAGCGGAAAGGCCACTTCGGTGGTACTCGAGCGGCGA
ACGGGTGAGTAACACGTGAGTAATCTGCCCTTCACTTGGGGATAACTACCGGAAACGGTGGCTAATACCCG
ATACGACCATGGATCGCATGATCTTGTGGTGGAAAGTTTTTTCGGTGGAGGATGTGCTCGCGGCCTATCAG
CTTGTTGGTGGGGTAATGGCCTACCAAGGCTTCGACGGGTAGCCGGCCTGAGAGGGTGACCGGCCACACT
GGGACTGAGACACGGCC
>Zotu4836
GAACGCTGGCGGCGTGCTTAACACATGCAAGTCGAACGGTGACGGAGAGCTTGCTCTCCTGATCAGTGGC
GAACGGGTGAGTAACACGTGGGCAACCTGCCCCTGACTTCGGGATAACGCTTCGAAAGAGGTGCTAATAC
CGAATACGAACTTCCTTGGCATCTGGGAAGTTGGAAAGTTTTTCGGTCAGGGATGGGCCCGCGGCCTATCA
GCTTGTTGGTGAGGTAATGGCTCACCAAGGCGACGACGGGTAGCCGGCCTGAGAGGGCGACCGGCCACA
CTGGGACTGAGACACGGCC
>Zotu4838

GAACGCTGGCGGCAGGCTTAACACATGCAAGTCGAGCGGGGATAGGGTGCTTGCACCTGATTCCTAGCGG
CGGACGGGTGAGTAATGCTTAGGAATCTGCCTATTAGTGGGGGACAACGTTCCGAAAGGGACGCTAATAC
CGCATACGTCCTACGGGAGAAAGCAGGGGATCTTCGGACCTTGCGCTAATAGATGAGCCTAAGTCGGATT
AGCTAGTTGGTGGGGTAAAGGCCTACCAAGGCGACGATCTGTAGCGGGTCTGAGAGGATGATCCGCCACA
CTGGGACTGAGACACGGCC

>Zotu4840

GAACGCTGGCGGCGTGCTTAACACATGCAAGTCGAACGGTAAGGCCCTTCGGGGTACACGAGTGGCGAAC
GGGTGAGTAACACGTGGGTAATCTGCCCTGCACTTCGGGATAAGCCTGGGAAACTGGGTCTAATACCGGA
TATTCAGCTCCTGCCGCATGGTGGGGGTTGGAAAGTTTTTCGGTGCAGGATGAGCCCGCGGCCTATCAGCT
TGTTGGTGGGGTAATGGCCCACCAAGGCGACGACGGGTAGCCGGCCTGAGAGGGTGATCGGCCACACTG
GGACTGAGACACGGCC

>Zotu4843

GAACGCTGGCTGCGTGCTTAACACATGCAAGTCGAACGCTGAAGCCCTGGTGCTTGCACCGGGGTGGATG
AGTGGCGAACGGGTGAGTAACACGTGAGTAACCTGCCCCCGACTTCGGGATAAGCCCGGGAAACTGGGTC
TAATACCGGATATGACCATGACTCGCATGAGTGATGGTGGAAAGTTTTTTCGGTTGGGGATGGGCTCGCG
GCCTATCAGTTTGTTGGTGAGGTAATGGCTCACCAAGACGACGACGGGTAGCCGGCCTGAGAGGGCGACC
GGCCACACTGGGACTGAGACACGGCC

>Zotu4845

GAACGCTGGCGGCGTGCTTAACACATGCAAGTCGAGCGGTAAGGCCCTAGCTTGCTGGGGTACACGAGCG
GCGAACGGGTGAGTAACACGTGAGTAATCTGCCCTTCACTCTGGGATAAGCACTCGAAAGGGTGTCTAATA
CCGGATATGACTGCTTCCTGCATGGGATGGTGGTGGAAACTTTTGTGGGTGGGGGATGTGCTCGCGGCCT
ATCAGCTTGTTGGTGGGGTAATGGCCTACCAAGGCTTCGACGGGTAGCCGGCCTGAGAGGGTGACCGGTC
ACACTGGGACTGAGACACGGCC

>Zotu4847

GAACGCTGGCGGCGTGCTTAACACATGCAAGTCGAACGTGAAATGGGGCAACCCAAAGTAAAGTGGCGTA
CGGGTGAATAACGCGTAGGTAATCTACCCCAAAATCTGGAATAACCGGCCGAAAGGCTGGCTAATACTAG
ATAGAGCGATGGAATCCGTAATTGGGTTTTTCGGGAAAGGGAGCTCGCAAGAGCCTTCGTTTTGGGATGA
GCCTGCGTCTCATTAGCTAGTTGGCGAGGTAACGGCTCACCAAGGCATTGATGAGTAGCTGGTCTGAGAG
GATGATCAGCCACACTGGAACTGAAACACGGTC

>Zotu4855

GAACACTGGCGGCAGGCCTAACACATGCAAGTCGAGCGGATGAGTGGAGCTTGCTCCATGATTCAGCGGC
GGACGGGTGAGTAATGCCTAGGAATCTGCCTGGTAGTGGGGGACAACGTTTCGAAAGGAACGCTAATACC
GCATACGTCCTACGGGAGAAAGCAGGGGACCTTCGGGCCTTGCGCTATCAGATGAGCCTAGGTCGGATTA
GCTAGTTGGTGGGGTAAAGGCCTACCAAGGCGACGATCCGTAACTGGTCTGAGAGGATGATCAGTCACAC
TGGAACTGAGACACGGTC

>Zotu4857

GAACGCTAGCGGCAGGCCTAATACATGCAAGTCGAACGGTGGTAGCGATACCATAGTGGCGCACGGGTGC
GTAACGCGTAACCAACCTGCCCTTGACTGGGGGATAGCCCGCCGAAAGGCGGATTAATACCGCATAAGAC
TCGTTCCTGGCATCAGGGGTTAGTTAAAGATTTATTGGTCAAGGATGGGGTTGCGTGACATTAGCTGGTTG
GCGGGGTAACGGCCCACCAAGGCGACGATGTCTAGGGGAGCTGAGAGGCTGGTCCCCCACACGGGCACT
GAGATACGGGC

>Zotu4859

GAACGCTGGCGGCGTGCTTAACACATGCAAGTCGAGCGAAGCAGTTAAGTTGATTTCTTCGGATTGATTCT
TAATTGACTGAGCGGCGGACGGGTGAGTAACGCGTGGGTGACCTGCCCCATACCGGGGGATAACAGCTG
GAAACGGCTGCTAATACCGCATAAGCGCACAGAGCTGCATGGCTCAGTGTGAAAAACTCCGGTGGTATGG
GATGGGCCCGCGTCTGATTAGGCAGTTGGCGGGGTAACGGCCCACCAAACCGACGATCAGTAGCCGGCCT
GAGAGGGCGACCGGCCACATTGGGACTGAGACACGGCC

>Zotu4861

GAACGCTGGCGGCGTGCTTAACACATGCAAGTCGAACGAGAACGGATTATAGCTTGCTATAATTGTCAGCT
AAGTGGCGCACGGGTGAGTAATATATAGGTAATGTGCCCTAGAGAAGAGGATAACAGTTGGAAACGACTG
CTAAGACTCTATATGCCTTTAAGGCAGAAGTCTGCAAGGGAAATATTTATAGCTCTAGGATCAGCCTGTACA

GTATCAGTTAGTTGGTGAGGTAATGGCTCACCAAGACAATGACGCTTAACTGGTTTGAGAGGATGATCAGT
CACACTGGAACTGAGACACGGTC
>Zotu4863
TAACGCTGGCGGCGTGCCTAACACATGCAAGTCGAACGGTTCTAAGCGCGAGCTTAGGATAGTGGCGAAC
GGGTGAGTAAAAGGTAGGAATCTACCCCTTGGACGGAGATAGCTACTGGAAACGGTAGGTAAACTTCGAT
AAGCCCGAGAGGGGAAAGTGGTGAGACAGCCGAGGGATGAGCCTACTCTCCATCAGGTAGTTGGTGAGA
TAAAAGCTTACCAAGCCGATGACGGATAACCGGTGTGAGAGCATGAACGGTCACAAGGGCACTGAGAGAC
GGGC
>Zotu4865
GAACGCTAGCGGCAGGCCTAACACATGCAAGTCGAGGGGTAGTATAGCAATATATGAGACCGGCGCACG
GGTGCGTAACGCGTATGCAATCTACCTTTTACAGAGGGATAGCCCAGAGAAATTTGGATTAATACCTCATA
GTGTATTTGAGTGGCATCACTTGATTACTAAAGTCACAACGGTAAAAGATGAGCATGCGTCCCATTAGCTT
GTTGGTAAGGTAACGGCTTACCAAGGCTACGATGGGTAGGGGTCCTGAGAGGGAGATCCCCCACACTGAT
ACTGAGACACGGAC
>Zotu4867
GAACGCTGGCGGCGTGCTTAACACATGCAAGTCGAACGGTAAGGCCCCTTCGGGGGTACACGAGTGGCGA
ACGGGTGAGTAACACGTGAGCAACCTGCCCTTGACACTGGAATAACAGTTGGAAACAGCTGCTAATGCCG
GATATGACCTTCGGTGACATCACCTGGAGGTGGAAAGTGCTTAGCGGTCAAGGATGGGCTCGCGGCCTAT
CAGCTAGTTGGTGAGGTAACGGCTCACCAAGGCTTCGACGGGTAGCCGGCCTGAGAGGGCGACCGGCCA
CACTGGGACTGAGATACGGCC
>Zotu4870
GAACGCTAGCGGCAGGCCTAATACATGCAAGTCGAACGAGATTAGTAGCAATACTATGAAAGTGGCGCAC
GGGTGCGTAACACGTATGCAATCTACCTTTAATTGGAGCATAGCCTCGAGAAATCGGGATTAATACTCCAT
AACATTATGAAATGGCATCATTTTATAATTAAACCTCCGGGGATTAAAGATGAGCATGCGCAACATTAGCTA
GTTGGTAAGGTAATGGCTTACCAAGGCTACGATGTTTAGGGGGTCTGAGAGGATGATCCCCCACACTGGT
ACTGAGACACGGAC
>Zotu4872
CAACGCTGGCGGCGTGCCTCAGACATGCAAGTCGAACGATTAAGACTCCCTTCGGGGAGTGTATAAAGTG
GCGTACGGGTGAGTAACACGTAAGTAATCTACCTTCGAGTGGGGAATAACAGTCGGAAACGGCTGCTAAT
ACCGCATAATGCAGCGGCATCGAAAGATGACAGTTGTTAAAGGAGCAATTCGCTTGAAGAGGAGCTTGCG
GCAGATTAGCTAGTTGGTAAGGTAATGGCTTACCAAGGCTACGATCTGTATCCGGTCTAAGAGGACGGTCG
GACACACTGACACTGAATAACGGGT
>Zotu4875
GAATGCTGGCGGCGTGGATAAGGCATGCAAGTCGAGTGGGTTTAGACCCACGGCAGACGAGGTAGTAAC
ACGTAGGAACGTACCCCAAAGTCCGGGATATTCCATCGAAAGATGGCGCAATACCGGATGGTCTCTAAGG
AGTAAAGATTTATCGCTTTGGGAGCGGCCTGCGCAGTATCAGCTAGTTGGTAGGGTAATGGCCTACCAAG
GCTATGACGCTTAGGGGAGCTGAGAGGCTGACCCCCACCGATGGAACTGAGATACGGTC
>Zotu4877
GAATGCTGTCGGCGTGGATAAGGCATGCAAGTCAAGGGGGCCGCAAGGCAACCGGCAGACGAGGTAGTA
ACACGTAGGTAGATACCCTGGAGTCGGGCATAGCCAGTCGAAAGACTGGGTAATTCCCGATGGTCTCGCA
AGAGTAAAGGTTTTTCGCTCCGGGAGTCGCCTGCGCAATATCAGCTAGTTGGTGAGGTAATGGCTCACCAA
GGCTACGACGTTTAGGGGAGCTGAGAGGCTGACCCCCACCGATGGGACTGAGATACGGCC
>Zotu4879
GAACGCTAGCGGGAGGCCTAACACATGCAAGCCGAGCGGTATTTCTTCTTCGGAAGAGAGAGAGCGGCGT
ACGGGTGCGTAACACGTGTGCAACCTACCTTTATCTGGGGGATAGCCTTTCGAAAGGAAGATTAATACCCC
ATAATATATTGAATGGCATCATTTAATATTGAAAACTCCGGTGGATAGAGATGGGCACGCGCAAGACTAGA
TAGTTGGTGAGGTAACGGCTCACCAAGTCTGTGATCTTTAGGGGTCCTGAGAGGGAGATCCCCCACACTGG
TACTGAGACACGGAC
>Zotu4881

GAACGCTGGCGGCAGGCTTAACACATGCAAGTCGAGCGGGGAGATGTAGCTTGCTACATTTCCTAGCGGC
GGACGGGTGAGTAATGCTTAGGAATCTGCCTATTAGTGGGGGACAACATTCCGAAAGGAATGCTAATACC
GCATACGCCCTACGGGAGAAAGCAGGGGATCTTCGGACCTTGCGCTAATAGATGAGCCTAAGTCAGATTA
GCTAGTTGGTGGGGTAAAGGCCTACCAAGGCGACGATCTGTAGCGGGTCTGAGAGGATGATCCGCCACAC
TGGGACTGAGACACGGCC

>Zotu4883
GAACGCTAGCGGCAGGCCTAACACATGCAAGTCGAGGGGTATCGGGGGCTTGCCCCCGAGAGACCGGCG
CACGGGTGAGCAACGCGTATAGAACCTACCCGTTACAGGGGGATAGCCCAGAGAAATTTGGATCAATACC
CCGTGGGGCCCTTGACCGGCATCGGTCTTGGGCTAAAGAACCTCGGTAACGGATGGCTATGCGTCCCATTA
GCTAGTTGGTAGGGTAACGGCCTACCAAGGCCGCGATGGGTAGGGGCCCTGAGAGGGGGATCCCCCACA
CTGGTACTGAGACACGGAC

>Zotu4885
GAACGCTGGCGGCGTGCCTAATACATGCAAGTCGAACGGTCTTTTCTATGGAAGCTTGCTTCCATAGAAAA
GATAGTGGCGAACGGGTGAGTAACACGTGGGTAACCTGCCCATAAGAGGGGGATAACATCCGGAAACGG
GTGCTAATACCGCATAGTTTTCTTGATCGCATGATCGGGAAAGGAAAGACGGCCTTTGTGCTGTCGCTTAT
GGATGGACCCGCGGCGTATTAGTTAGTTGGTGAGGTAACGGCTCACCAAGACGATGATACGTAGCCGACC
TGAGAGGGTGATCGGCCACATTGGGACTGAGACACGGCC

>Zotu4887
GAACGCTGGCGGCGTGCCTAATACATGCAAGTCGAGCGAATCAAAGGGAGCTTGCTCCCTTTGATTAGCG
GCGGACGGGTGAGTAACACGTGGGCAACCTGCCTATAAGACTGGGATAACTTCGGGAAACCGGAGCTAAT
ACCGGATAATTCATATCTACTCATGTAGATATGCTGAAAGACGGTTCACGCTGTCACTTATAGATGGGCCCG
CGGCGCATTAGCTAGTTGGTGAGGTAACGGCTCACCAAGGCGACGATGCGTAGCCGACCTGAGAGGGTG
ATCGGCCACACTGGGACTGAGACACGGCC

>Zotu4891
GAACGCTGGCGGCGTGCCTAATACATGCAAGTCGAGCGAACCGTAAAGGAGCTTGCTCCTTTACGGTTAGC
GGCGGACGGGTGAGTAACACGTGGGTAACCTGCCCTCAAAACTGGGATAACATCGAGAAATCGGTGCTAA
TACCGGATAATCAATAAGACCGCATGGTCTTATTATAAAAGGTGGCTTCGGCTACTATTTGAGGATGGGCC
CGCGGCGCATTAGCTAGTTGGTAAGGTAACAGCTTACCAAGGCGACGATGCGTAGCCGACCTGAGAGGGT
GATCGGCCACATTGGGACTGAGACACGGCC

>Zotu4894
GAACGCTGGCGGCGTGCCTAATACATGCAAGTCGAGCGAACGGACAAGGAGCTTGCTCCTTTGAAGTTAG
CGGCGGACGGGTGAGTAACACGTGGGTAACCTACCTATAAGACTGGAATAACTCCGGGAAACCGGGGCTA
ATGCCGGATAATATGTTGAACCGCATGGTTCAACAGTGAAAGACGGTCTTGCTGTCACTTATAGATGGACC
CGCGCCGTATTAGCTAGTTGGTGGGGTAACGGCCTACCAAGGCAACGATACGTAGCCGACCTGAGAGGGT
GATCGGCCACACTGGAACTGAGACACGGTC

>Zotu4896
GAACGCTGGCGGCGTGCTTAACACATGCAAGTCGAACGGAAAGGCCCTTCGGGGTACTCGAGTGGCGAAC
GGGTGAGTAACACGTGGGTGATCTGCCCTGCACTTCGGGATAAGCCTGGGAAACTGGGTCTAATACCGGA
TATGACCTTGGGATGCATGTCCTTTGGTGGAAAGCTTTTGCGGTGTGGGATGGGCCCGCGGCCTATCAGCT
TGTTGGTGGGGTTAAGGCCTACCAAGGCGACGACGGGTAGCCGGCCTGAGAGGGTGACCGGCCACACTG
GGACTGAGATACGGCC

>Zotu4898
TAACGCTGGCGGCGTGCCTAACACATGCAAGTCGAACGGTGCAGCAATGCACAGTGGCAGACGGGTGAGT
AACGCGTAGATAACGTGCCCTCTTGCGGGGGATACCAGTTCGAAAGGACTGTTAATACCGCATAACATCTC
CGAGGGGCATCCTTTGGAGAGCAAACTGATTCAGGGCGAGAGGAGCGGTCTGCGTACTATCAGGTAGTTG
GTGAGGTAATGGCTCACCAAGCCTACGACGGTTAACTGGTCTGAGAGGATGATCAGTCACACTGGAACTG
AGACACGGTC

>Zotu4900
GAACGCTGGCGGCATGCTTAACACATGCAAGTCGAACGGCAGCATGGTTTAGTTTACTAGACTGATGGCG
AGTGGCGGACGGGTGAGTAACGCGTAGGAATATGCCTTGAAGAGGGGGATAACTTGGGGAAACTCAAGC
TAATACCGCATGAGATCTTAGGATGAAAGCTGGGGACCGTAAGGCCTGGCGCTTTAAGAATAGCCTGCGT

CCGATTAGCTAGTTGGTGGGGTAATGGCCTACCAAGGCGACGATCGGTAGCTGGTCTGAGAGGATGGCCA
GCCACACTGGGACTGAGACACGGCC
>Zotu4903
CAACGCTGGCGGCGTGCCTAACACATGCAAGTCGAACGAGAAAGTCCCGCAAGGGATGAGTAAAGTGGC
GACCGGGTGAGTAACACGTGACTAACCTACCCTAGAGTGGGGAATAACCTCGGGAAACCGGGGCTAATAC
CGCATAACATCGCAGAGTGAAAGTCTTTGCGATCAAAGGAGTAATTCGCTCAAGGAGGGGGTCGCGGCTG
ATTAGTTAGTTGGCGGGGTAACGGCCCACCAAGACTTAGATCAGTAGCCGGCCTGAGAGGGCGCACGGCC
ACACTGGAACTGAAACACGGTC
>Zotu4907
GAACGCTGGCGGTATGCTTAACACATGCAAGTCGTACAAAAATCTTTGATTTTAGTGGCGGACGGGTGAGT
AACACGTGAGAATCTGCCCTTAGGAAGAGGATAACAGCTGGAAACGGTTGCTAATACTCTATACGCCGAG
AGGTAAAAGGATCTATCCACCTTAGGAAGAGCTCGCGTCTGATTAGCTTGTTGGTAGGGTAATGGCCTACC
AAGGCGACGATCAGTAGCTGGTTTGAGAGGACGACCAGCCACACTGGGACTGAGACACGGCC
>Zotu4911
GAACGCTGGCGGCGTGCTTAACACATGCAAGTCGAACGAGGTCCATGCAGTCGCAAGACTGCGGAAGACC
GAGTGGCGAACGGGTGCGTAACACGTGAGGAACTTGTCCCGGTCTCTGGAATAACAAGTGGAAACACTTG
CTAATACCGGATGCCGTCGGGCCATCGCATGGTGGTCTGACGAAAGGGTTACCGGATCGGGAGAGCCTCG
CGGCCTATCAGCTTGTTGGTGAGGTAACGGCTCACCAAGGCATCGACGGGTAGCTGGTCTGAGAGGATGA
TCAGCCACACTGGGACTGAGACACGGCC
>Zotu4913
GAACGCTGGCGGTATGCCTTACACATGCAAGTCGAACGGTAGAGGAGCAATCCTCGAGAGTGGCGAACG
GGTGAGTAATACATCGGAACGTGCCCAGTCGTGGGGGATAACGTAGCGAAAGCTACGCTAATACCGCATA
CGACCCGAGGGTGAAAGCGGGGGACTCGCAAGAGCCTCGCGCGATTGGAGCGGCCGATGGCAGATTAGG
TAGTTGGTGGGGTAAAGGCCCACCAAGCCTGCGATCTGTAGCTGGTCTGAGAGGACGACCAGCCACACTG
GGACTGAGACACGGCC
>Zotu4916
GAACGCTGGCGGCAGGCTTAACACATGCAAGTCGAACGGATCCTTCGGGATTAGTGGCAGACGGGTGAGT
AACGCGTGGGAACGTACCCTTTGCTACGGAATAACTCAGGGAAACTTGAGCTAATACCGTATGTGCCCTAC
GGGGGAAAGATTTATCGGCAAAGGATCGGCCCGCGTTGGATTAGCTAGTTGGTGGGGTAATGGCCTACCA
AGGCGACGATCCATAGCTGGTCTGAGAGGATGATCAGTCACACTGGGACTGAGACACGGCC
>Zotu4919
GAACGCTGGCGGCATGCCTAACACATGCAAGTCGAACGAGATCTTCGGATCTAGTGGCGCACGGGTGCGT
AACGCGTGGGAATCTGCCCTTGGGTTCGGAATAACAGTGAGAAATTACTGCTAATACCGGATGAAGTCTTC
GGACCAAAGATTTATCGCCCAGGGATGAGCCCGCGTAGGATTAGGTAGTTGGTGGGGTAATGGCCTACCA
AGCCGACGATCCTTAGCTGGTCTGAGAGGATGATCAGCCACACTGGGACTGAGACACGGCC
>Zotu4922
GAACGCTGGCGGCATGCCTTACACATGCAAGTCGAACGGCAGCACGGGAGCAATCCTGGTGGCGAGTGG
CGAACGGGTGAGTAATATATCGGAACGTGCCCAGTCGTGGGGGATAACGCAGCGAAAGCTGTGCTAATAC
CGCATACGATCTCTGGATGAAAGCGGGGGACCTGCAAGGGCCTCGCGCGATTGGAGCGGCCGATATCAGA
TTAGCTAGTTGGTGGGGTAAAAGCCCACCAAGGCGACGATCTGTAGCTGGTCTGAGAGGACGACCAGCCA
CACTGGAACTGAGACACGGTC
>Zotu4924
GAACGCTGGCGGCAGGCTTAACACATGCAAGTCGAACGGGCCCTTCGGGGTCAGTGGCAGACGGGTGAG
TAACACGTGGGAACGTGCCCTTCAGTTCGGAATAACCCAGGGAAACTTGGGCTAATACCGGATACGCCCGC
AAGGGGAAAGATTTTATCGCTGAAGGATCGGCCCGCGTCTGATTAGCTAGTTGGTGAGGTAAAGGCTCAC
CAAGGCGACGATCAGTAGCTGGTCTGAGAGGATGATCAGCCACACTGGGACTGAGACACGGCC
>Zotu4926
GAACGCTGGCGGCATGCTTTACACATGCAAGTCGAACGGCAGCGAGGAGGAGCTTGCTTCTCTGTCGGCG
AGTGGCGAACGGGTGAGTATAGCATCGGAACGTGCCAAGTAGTGGGGGATAACCAAGCGAAAGTTTGGC
TAATACCGCATAAGCTCTAAGGAGGAAAGTAGGGGACCGTAAGGCCTTACGCTATTTGATCGGCCGATGTC

GGATTAGCTAGTTGGTGAGGTAATGGCTCACCAAGGCGATGATCCGTAGCGGGTCTGAGAGGACGATCCG
CCACACTGGGACTGAGACACGGCC
>Zotu4928
GAACGCTGGCGGCGTGCTTAACACATGCAAGTCGAGCGAATTTTGACAGCGTATCGTCAGAGTTAGCGGC
AGACGGGTGAGTAATACTTGGGCAATCTGCCCTTGAGAGGGAAACAACCTGCCGAAAGGTGGGCTAATGT
CCCGTAAGACCACAAGATGGCATCATCTAGGGGTCAAAGCTCCGCAAGGGGCGCTCGAGGAGGAGCCTAA
GTTCTATCATGGTAGTTGGCGGGGTAACGGCCCACCAAGCCTTTGACGGATAGCCGGCCTGAAAGGGTGA
TCGGCCACACTGGCACTGAGACACGGGC
>Zotu4934
GAACGCTGGCGGCGTGCTTAACACATGCAAGTCGAACGGTGAACTCCGCTTGCGGGGGGATCAGTGGCGA
ACGGGTGAGTACACGTGGGCAACCTGCCCTCAGCTCTGGGATAACTCCAAGAAATTGGGGCTAATACCGG
ATATGACCGCTGACCGCATGGTCTGGTGGTGGAAAGATTTATCGGCTGGGGATGGGCCCGCGGCCTATCA
GCTTGTTGGTGGGGTAGTGGCCTACCAAGGCGACGACGGGTAGCCGGCCTGAGATGGTGACCGGCCACA
CTGGGACTGAGACACGGCC
>Zotu4937
GAACGCTGGCGGTATGCTTTACACATGCAAGTCGTACGAGAGTTTTTAACTCAAGTGGCGGACGGGTGAG
TAACACGTAAGAATTTGCCTTTAGGAGGGGGATAACAACTGGAAACGGTTGCTAATACCCCATATGCTTTC
GAGTGAAACGGATTTTCCGCCTAAAGAGAAGCTTGCGGCTGATTAGCTTGTTGGTGAGGTAATGGCTCACC
AAGGCGACGATCAGTATCTGGTTTGAGAGGATGATCAGACACACTGGAACTGAGACACGGTC
>Zotu4939
GAACGCTGGCGGCGTGCCTAATACATGCAAGTCGAACGAAGAGTATTTGTAGTAATACAGATGCTTCTTAG
TGGCGAACGGGTGAGTAACGCGTTGGTGACCTGCCCCAAAGTGTGGGATAACAGTCCGAAAGGATTGCTA
ATACCTCATGTGGTCTTCGGAATTAGAAACCGATTTCTAAAGGAGTAATCCGCTTTGGGAGGGGCCTGCGT
CCCATCAGCTAGTTGGTGAGGTAACGGCTCACCAAGGCTTCGACGGGTAAGGGGCCTGAGAGGGTGGCCC
CTCACAATGGAACTGAAACACGGTC
>Zotu4941
GAACGCTGGCGGCGTGCTTAACAAATGCAAGTCGAACGAGGTAAGAAGCTTCGGTTTTTACCGAGTGGC
GGACTGGTGAGTAACACGTGAGTAACCAACCTTATACAGGGGGATACCACAAAGAAATTTGTGCTAATACC
GCATAATACAAGAAAGCCACATGGCAGAGAAGTTAAAGGAGAAATCCGGTATAAGACGGACTCGCGCATC
ATTAGCTAGTTGGTGAGGTAACGGCCCACCAAGGCAACGATGATTAGCCGGCCTGAGAGGGTGTACGGCC
ACATTGGGACTGAGATACGGCC
>Zotu4943
GAACACTGGCGGCATGCCTAACACATGCAAGTCGAACGATTGCCTTCGGGTGATAGTGGCGCACGGGTGC
GTAACACGTGGGAATCTGCCCTTAGGTTCGGAATAACAGCTGGAAACGGCTGCTAATACCGGATGATATCG
CGAGATCAAAGATTTATCGCCTGAGGATGAGCCCGCGTTGGATTAGGTAGTTGGTAGGGTAAAGGCCTAC
CAAGCCGACGATCCATAGCTGGTCTGAAAGGATGATCAGCCACACTGGGACTGAGACACGGCC
>Zotu4945
GAACGCTGGCGGCGTGCTTAACACATGCAAGTCGAACGGTGAAAGAGGAGCTTGCTCCTCTGGATCAGTG
GCGAACGGGTGAGTAACACGTGAGAAATGTGCCCTGGACTCTGGGATAAGCGTTGGAAACGACGTCTAAT
ACCGGATACGCGACGCGAAGGCATCTTCAGCGTCGGGAAAGAACTTCGGTCCAGGATCATCTCGCGGCCT
ATCAGCTAGTTGGTGAGGTAACGGCCCACCAAGGCGACGACGGGTAGCCGGCCTGAGAGGGTGACCGGC
CACACTGGGACTGAGACACGGCC
>Zotu4947
CAACGCTGGCGGCGCGCCTAACACATGCAAGTCGCACGAGAAAGGGAGCAATCCTGAGTAAAGTGGCGTA
CGGGTGAGTAACACGTGGGAATCTACCTCTTGGCGGGGAATAACCCTGGGAAACCGGGGCTAATACCGCA
TAAGCCCGTGAGGGGAAAGCAGCAATGCACCGAGAGAGGAGCCCGCGGCCGATTAGCTAGTTGGTGAGG
TAATGGCTCACCAAGGCGAAGATCGGTAGCCGGCCTGAGAGGGCACACGGCCACACTGGCACTGAAACAC
GGGC
>Zotu4951

GAACGCTGGCGGCAGGCCTAACACATGCAAGTCGAGCGGTAGAGAGTGCTTGCACTCTTGAGAGCGGCG
GACGGGTGAGTAATGCCTAGGAATCTGCCTGGTGGTGGGGGATAACGTTCGGAAACGGACGCTAATACCG
CATACGTCCTACGGGAGAAAGCGGGGGATCTTCGGACCTCGCGCCATCAGATGAGCCTAGGTCGGATTAG
CTAGTTGGTGGGGTAATGGCTCACCAAGGCGACGATCCGTAACTGGTCTGAGAGGATGATCAGTCACACT
GGAACTGAGACACGGTC

>Zotu4953
GAACGCTAGCGGCAGGCTTAACACATGCAAGTCGAGGGGTAGGGCAGCAATGCTTGAGACCGGCGCACG
GGTGCGTAACGCGTATGCAATCTACCTTTTGCAGAGGGATAGCCCAGAGAAATTTGGATTAATACCTCATA
GTAATATGACTTCGCATGGAGATATATTTAAAGTAACAACGGCAAAAGATGAGCATGCGTATCATTAGCTA
GATGGTAAGGTAACGGCTTACCATGGCTACGATGATTAGGGGTCCTGAGAGGGAGATCCCCCACACTGGT
ACTGAGACACGGAC

>Zotu4956
GAACGCTGGCGGCGTGCTTAACACATGCAAGTCGAGCGGTAAGGCCCTTCGGGGTACACGAGCGGCGAA
CGGGTGAGTAACACGTGAGCAACCTGCCCCTGACTCCGGGATAAGCGGTGGAAACGCCGTCTAATACCGG
ATATGACACGCCACCTCATGGTGGTGTGTGGAAAGTTTTTCGGTCAGGGATGGGCTCGCGGCCTATCAGCT
TGTTGGTGGGGTAACGGCCTACCAAGGCGATTACGGGTAGCCGGCCTGAGAGGGCGACCGGCCACACTG
GGACTGAGACACGGCC

>Zotu4958
GAACGCTGGCGGCGTGCTTAACACATGCAAGTCGAACGGTGACCTCGAGAGCTTGCTCTTGGGTGATCAG
TGGCGAACGGGTGAGTAACACGTGAGTAACCTGCCCCAGACTCTGGAATAACCCCGGGAAACCGGAGCTA
ATACCGGATACGAGACGGAGCCGCATGGCTACCGTTTGGAAAGTTTTTCGGTCTGGGATGGACTCGCGGC
CTATCAGCTAGTTGGTGAGGTAACGGCTCACCAAGGCGACGACGGGTAGCCGGCCTGAGAGGGCGACCG
GCCACACTGGGACTGAGACACGGCC

>Zotu4960
GAACGCTGGCGGCGTGCCTAACACATGCAAGTCGAACGGAAGTATACGAAGTCTTCGGACGGAATATACT
TTAGTGGCGGACGGGTGAGTAACGCGTGGGTAACCTGCCTCATACAGGGGGATAACAGCTGGAAACGGC
TGCTAAAACCGCATAAGCATACACTGCCGCATGGCAGAGTGTGAAAATACTTGTAGGTATGAGATGGGCC
CGCGTCTGATTAGCTAGTTGGTAGGGTAACGGCCTACCAAGGCGACGATCAGTAGCCGGCTTGAGAGAGT
GACCGGCCACATTGGGACTGAGACACGGCC

>Zotu4962
GAACGCTGGCGGCATGCCTAACACATGCAAGTCGAACGAGACCTTCGGGTCTAGTGGCGCACGGGTGCGT
AACGCGTGGGAATCTGCCCTTGGGTTCGGGATAACAGTTGGAAACGACTGCTAATACCGGATGATGACTTC
GGTCCAAAGATTTATCGCCCAGGGATGAGCCCGCGTAGGATTAGCTAGTTGGTGAGGTAAAGGCTCACCA
AGGCGACGATCCTTAGCTGGTCTGAGAGGATGATCAGCCACACTGGGACTGAGACACGGCC

>Zotu4964
GAACGCTGGCGGCGTGCTTAACACATGCAAGTCGAACGATGATGCCCAGCTTGCTGGGTGGATTAGTGGC
GAACGGGTGAGTAACACGTGAGTAACCTGCCCCTGACTCTGGGATAAGCGTTGGAAACGACGTCTAATAC
TGGATATGACTACCGGCCGCATGGTCTGGTGGTGGAAAGATTTCTTGGTTGGGGATGGACTCGCGGCCTA
TCAGCTTGTTGGTGAGGTAATGGCTCACCAAGGCGACGACGGGTAGCCGGCCTGAGAGGGTGACCGGCC
ACACTGGGACTGAGACACGGCC

>Zotu4966
GAACGCTGGCGGCGTGCCTAACACATGCAAGTCGAACGGAATATTTCGGATGAGGCCTTCGGGCGGATGA
AGAGATATTTAGTGGCGGACGGGTGAGTAACGCGTGGGTAACCTGCCCTGTACCGGGGGATAACAGTTGG
AAACGACTGCTAATACCGCATAAGCACACGGTGCCGCATGGCACAGGGTGAAAAACTCCGGTGGTACAGG
ATGGACCCGCGTCTGATTAGCTTGTTGGTGAGGTAACGGCCCACCAAGGCGACGATCAGTAGCCGACTTG
AGAGAGTGATCGGCCACATTGGGACTGAGACACGGCC

>Zotu4969
GAACGCTGGCGGCGTGCCTAATACATGCAAGTCGAACGATGGCGATTTTCTCCTTACGGAGAAAACGCCGT
AGTGGCGGACGGGTGAGTAACACGTGGGCAACCTGCCTGTAAGCTGGGGATAACTAGCGGAAACGTTAG
CTAATACCGAATGGTCAAGAGGAAGGCATCTTCCACTTGGGAAAGATGGAGCAATCTATCGCTTACAGATG

GGCCCGCGTCGCATTAGCTAGTAGGTGAGGTAACGGCTCACCTAGGCGACGATGCGTAGCCGACCTGAGA
GGGTGAACGGCCACACTGGGACTGAGACACGGCC

>Zotu4971

GAACGCTGGCGGCGTGCTTAACACATGCAAGTCGAGCGAGGCTCCCTCTTCGGAGGGATGTCCTAGCGGC
GAACGGGTGAGTAACACGTGGGCAACCTGCCCCTAGCACTGGGATAACCCCGGGAAACCGGGGCTAATAC
CGGATATGACCTCGATGGGCATCCGTCGAGGTGGAAAAGGTTACTGGCTAGGGATGGGCCCGCGGCCTAT
CAGCTTGTTGGTGGGGTAACGGCCTACCAAGGCGACGACGGGTAGCCGGCCTGAGAGGGCGACCGGCCA
CACTGGGACTGAGACACGGCC

>Zotu4973

GAACGCTGGCGGCGTGCCTAAGACATGCAAGTCGAGTGGGTTTAGACCCACGGCGAACGGGTGAGTAAT
ACCTAGGAACGTACCTTTAGTCGAGCATACGCAGCCGAAAGGTTGTTTAATTCTCGATGTGACCGCAAGGT
TAAAGTCGTAAGACGCTAAAGAATCGGCCTGGACACCATCAGCTAGTTGGTAGGGTAAAAGCCTACCAAG
GCGACGACGGTTAGGGGTCCCGAGAGGGATAACCCCCACACTGGGACTGCGACACGGCC

>Zotu4975

GAACGCTGGCGGCATGCCTTACACATGCAAGTCGAACGGCAGCATAGGAGCTTGCTCCTGATGGCGAGTG
GCGAACGGGTGAGTAATACATCGGAACGTACCCTGTTGTGGGGGATAACTAGTCGAAAGACTAGCTAATA
CCGCATACGATCTACGGATGAAAGTGGGGGATCGCAAGACCTCATGCTCCTGGAGCGGCCGATATCTGATT
AGCTAGTTGGTGGGGTAAAAGCTCACCAAGGCGACGATCAGTAGCTGGTCTGAGAGGACGACCAGCCACA
CTGGGACTGAGACACGGCC

>Zotu4980

GAACGCTAGCGGCAGGCTTAACACATGCAAGTCGAGGGGTATAGTTCTTCGGAACTAGAGACCGGCGCAC
GGGTGCGTAACGCGTATGCAATCTACCTTTCACAGAGGGATAGCCCAGAGAAATTTGGATTAATACCTCAT
AGTATGCAGAGTTGGCATCAACGCTGCATTAAAGTCACAACGGTGAAAGATGAGCATGCGTCCCATTAGCT
AGTTGGTAAGGTAACGGCTTACCAAGGCTACGATGGGTAGGGGTCCTGAGAGGGAGATCCCCCACACTGG
TACTGAGACACGGAC

>Zotu4984

GAACGCTAGCGGCAGGCTTAACACATGCAAGTCGAGGGGTAGTAGGAGCTTGCTTCTATGAGACCGGCGC
ACGGGTGCGTAACGCGTATGCAATCTGCCTCTCACTGGGGGATAGCCCGGAGAAATCCGGATTAATACCCC
ATGGTATTTTGGAATGGCATCATTCTATTATTAAAGATTTATCGGTGAGAGATGAGCATGCGTCCCATTAGT
TAGTTGGTGTGGTAACGGCACACCAAGACGATGATGGGTAGGGGTCCTGAGAGGGAGATCCCCCACACTG
GTACTGAGACACGGAC

>Zotu4986

GAACGCTGGCGGCGTGCCTAATACATGCAAGTCGAGCGAATCTTTGGGAGCTTGCTCCTAAAGGTTAGCG
GCGGACGGGTGAGTAACACGTGGGCAACCTGCCTATAAGACTGGGATAACTTCGGGAAACCGGAACTAAT
ACCGGATAATTCTTTTCTACACATGTAGAAAAGCTAAAAGACGGTTTACGCTGTCACTTATAGATGGGCCCG
CGGCGCATTAGCTAGTTGGTGAGGTAACGGCTCACCAAGGCCACGATGCGTAGCCGACCTGAGAGGGTGA
TCGGCCACACTGGGACTGAGACACGGCC

>Zotu4988

GAACGCTGGCGGCAGGCTTAACACATGCAAGTCGAGCGGGCATCCTTGTGGTGTCAGCGGCAGACGGGT
GAGTAACGCGTGGGAACGTGCCCTTCGGTTCGGAATAACTCAGGGAAACTTGAGCTAATACCGGATACGT
GCGAGAGCAGAAAGGTTTATCTGCCGAAGGATCGGCCCGCGTCTGATTAGCTAGTTGGTGAGGTTACGGC
TCACCAAGGCGACGATCAGTAGCTGGTCTGAGAGGATGATCAGCCACACTGGGACTGAGACACGGCC

>Zotu4991

CAACGCTGGCGGCGTGCCTAACACATGCAAGTCGCACGAGAAAGGGGCTTCGGCCCTGAGTAAAGTGGCG
CACGGGTGAGTAACACGTGAATCATCTACCTTCGAGTGGGGAATAACCTAGAGAAATCTGGGCTAATACC
GCATAACACTTACGAGTCAAAGCAGCAATGCGCTTGAAGAGGAGTTCGCGGCAGATTAGTTAGTTGGTGG
GGTAATGGCCTACCAAGACGATGATCTGTATCCGGCCTGAGAGGGCGCACGGACACACTGGAACTGAAAC
ACGGTC

>Zotu4993

GAACGCTGGCGGCGTGCCTAATACATGCAAGTCGAGCGAATCTTTGGGAGCTTGCTCCCAAAGATTAGCG
GCGGACGGGTGAGTAACACGTGGGCAACCTGCCTATAAGACTGGGATAACTTCGGGAAACCGGAGCTAAT
ACCGGATAATTCTTTTCTACTCATGTAGGAAAGCTAAAAGACGGTTTACGCTGTCACTTATAGATGGGCCCG
CGGCGCATTAGCTAGTTGGTGAGGTAACGGCTCACCAAGGCCACGATGCGTAGCCGACCTGAGAGGGTGA
TCGGCCACACTGGGACTGAGACACGGCC
>Zotu4995
GAACGCTGGCGGCAGGCCTAACACATGCAAGTCGAACGGACCCTTCGGGGTTAGTGGCGGACGGGTGAG
TAACACGTGGGAACGTGCCTTTAGGTTCGGAATAGCTCCTGGAAACGGGTGGTAATGCCGAATGTGCCCTT
CGGGGGAAAGATTTATCGCCTTTAGAGCGGCCCGCGTCTGATTAGCTAGTTGGTGAGGTAAAGGCTCACC
AAGGCTACGATCAGTAGCTGGTCTGAGAGGATGACCAGCCACATTGGGACTGAGACACGGCC
>Zotu4998
GAACGCTAGCGGCAGGCTTAATACATGCAAGTCGTACGGGATCCGAGGTAGCAATACTTTGGTGAGAGTG
GCGCACGGGTGCGTAACACGTATGCAACCTACCTTTAACCGGGACACAACCTCGAGAAATTGTGGCTAATA
TCCCATAACACTGCGAAGTGGCATCACTTTGCAGTTAAAACTCCGGTGGTTATTGATGGGCATGCGCGACA
TTAGCTAGTTGGTGAGGTAACGGCCCACCAAGGCTACGATGTCTAGGGGTTCTGAGAGGATTATCCCCCAC
ACTGGTACTGAGACACGGAC
>Zotu5002
GAACGCTGGCGGCGTGCTTAACACATGCAAGTCGAACGGTGACGAGCAGCTTGCTGCTCTGATCAGTGGC
GAACGGGTGAGTAACACGTGAGCAACCTACCCTTGACTCTGGGATAACAGCTGGAAACAGCTGCTAATACC
GGATACGACCTCGACCGACATCTGTTGAGGTGGAAAGTTCTGGCGGTCAAGGATGGGCTCGCGGCCTATC
AGCTAGTTGGTGAGGTAACGGCTCACCAAGGCTTCGACGGGTAGCCGGCCTGAGAGGGCGACCGGCCAC
ACTGGGACTGAGATACGGCC
>Zotu5004
GAACGCTAGCGGCAAGCTTAATACATGCAAGTCGAGGGGCAGCGCAGTGTAGCAATACATGGGCGGCGA
CCGGCAAACGGGTGCGGAACACGTACAGAACCTTCCTTCGAGCGGGGAATAGCCCAGAGAAATTTGGATT
AATACCCCATAGTATATTGGAGTGGCATCACTTTAATATTAAAGATTTATCACTTGAAGATGGCTGTGCGGC
TGATTAGATAGTTGGCGGGGTAACGGCCCACCAAGTCTGCGATCAGTAACTGGTGTGAGAGCACGACCAG
TCACACGGGCACTGAGACACGGGC
>Zotu5006
GAACGCTGGCGGCAGGCTTAACACATGCAAGTCGAACGGGCACTTCGGTGCTAGTGGCAGACGGGTGAG
TAACGCGTGGGAACGTGCCTTTCGGTTCGGAATAATCCAGGGAAACTTGGACTAATACCGGATACGCCCTT
CGGGGGAAAGATTTATCGCCGAAAGATCGGCCCGCGTCTGATTAGCTAGTTGGTGAGGTAAAGGCTCACC
AAGGCGACGATCAGTAGCTGGTCTGAGAGGATGATCAGCCACATTGGGACTGAGACACGGCC
>Zotu5008
GAACGCTGGCGGCATGCCTAAAACATGCAAGTCGAGCGGTAAGGCCCCTTCGGGGGTACACGAGCGGCG
GACGGGTGAGTAACGCGTGGGAATCTACCCCAAACTGAGGGATAAGCATTGGAAACGATGTCTAATACCG
CATATGGTCTTCGGATTAAAGCTTCGGCGGTTTGGGAAGAGCCCGCGTAAGATTAGCTAGTTGGTGAGGTA
AAGGCTCACCAAGGCGACGATCTTTAACTGGTTTGAGAGAATGATCAGTCACACTGGAACTGAGACACGG
TC
>Zotu5011
GAACGCTGGCGGCGTGCTTAACACATGCAAGTCGAACGGTGATGATGGAGCTTGCTCTGTCGGATCAGTG
GCGAACGGGTGAGTAACACGTGAGTAACCTGCCCTCAACTCTGGGATAACTTCCCGAAAGGGAAGCTAAT
ACTGGATATGAACTGCACGGGCATCTGTGTGGTTGTAAAGATTTTTTGGTTGAGGATGGACTCGCGGCCTA
TCAGCTTGTTGGTGGGGTAATGGCCTACCAAGGCGACGACGGGTAGCCGGCCTGAGAGGGCGACCGGCC
ACACTGGGACTGAGACACGGCC
>Zotu5013
GAACGCTAGCGGGAGGCCTAACACATGCAAGCCGAGCGGTAGAAGTCCTTCGGGACTTTGAGAGCGGCG
TACGGGTGCGGAACACGTGTGCAACCTGCCTTTATCAGGGGGATAGCCTTTCGAAAGGAAGATTAATACCC
CATAATATAATGAACGGCATCGCTTATTATTGAAAACTCCGGTGGATAGAGATGGGCACGCGCAAGATTAG
ATAGTTGGTAGGGTAACGGCCTACCAAGTCAGTGATCTTTAGGGGGCCTGAGAGGGTGATCCCCCACACT
GGTACTGAGACACGGAC

>Zotu5015
GAACGCTGGCGGCGTGCTTAACACATGCAAGTCGAACGATGAAGCTCCAGCTTGCTGGGGTGGAAGAGTG
GCGAACGGGTGAGTAACACGTGAGTAACCTGCCCTGAACTCTGGGATAAGCGCTGGAAACGGTGTCTAAT
ACTGGATACGACCCATGGGGGCATCTTCTGTGGGTGGAAAGATTTATCGGTTTGGGATGGGCTCGCGGCC
TATCAGCTGGATGGTGGGGTAACGGCTCACCATGGCGACGACGGGTAGCCGGCCTGAGAGGGTGACCGG
CCACACTGGGACTGAGACACGGCC
>Zotu5019
GAACGCTGGCGGCGTGCCTAACACATGCAAGTCGAACGTGAACGGGGCAACCCTAGTAAAGTGGCGTACG
GGTGAGTAACGCGTAGGTAATCTACCCCAGAGTTTGGAATAACCGGCCGAAAGGCTGGCTAATACTGGAT
AGAGCGACGGAATCCGTAATTGGGTTTTTCGGGAAAGGGAGCTTGATAAGAGCCCTCGCTTTGGGATGAG
CCTGCGTCTCATTAGCTAGTTGGTGGGGTAATGGCCCACCAAGGCTTTGATGGGTAGCTGGTCTGAGAGGA
TGATCAGCCACACTGGAACTGAAACACGGTC
>Zotu5023
CAACGCTGGCGGCGTGCCTAACACATGCAAGTCGAACGCGAAAGTCCCGCAAGGGATCAGTAGAGTGGCA
AACGGGTGAGTAACACGTGGGCGACCTACCTTCGAGTGGGGGATAACCTTCCGAAAGGAGGGCTAATACC
GCATGACATCCCATGTTTGGATACATGGACATCAAAGCCGGGGATCGCAAGACCTGGCGCTTGAAGAGGG
GCCTGCGCCCGATTAGCTAGTTGGTGGGGTCATGGCTCACCAAGGCTTCGATCGGTATCCGGCCTGAGAG
GGCGGACGGACACACTGGAACTGAGACACGGTC
>Zotu5026
GAACGCCGGCGGTGTGCCTAATACATGCAAGTCGAACGCGTTGGCCCAACTGATTGATTGTGCTTGCACAG
GATTGACGTTGGTTTCCCAACGAGTGGCGGACGGGTGAGTAACACGTAGGCAACCTGCCCCGAAGCGGGG
GACAACATTTGGAAACAAGTGCTAATACCGCATAACAGCTTTGACCGCCTGGTCAAAGCTTAAAAGATGGT
TTCGGCTATCACTTCGGGATGGGCCTGCGGCGCATTAGCTTGTTGGTGGGGTAACGGCCTACCAAGGCGAT
GATGCGTAGCCGAGTTGAGAGACTGATCGGCCACAATGGGACTGAGACACGGCC
>Zotu5028
GAACGCTAGCGGCAGGCCTAATACATGCAAGTCGAACGCGATTGCGGTGCTTGCACCGCATGAGAGTGGC
GTACGGGTGCGTAACGCGTGAGCAACCTACCGTTGTCTGGGGGATAGCCCGGAGAAATCCGGATTAATAC
CGCATAATATTAGAGAGCAGCATTGCTTTCTGATCAAACATTTATGGGACAGCGATGGGCTCGCGTGACAT
TAGCTAGTTGGAGTGGTAACGGCACACCAAGGCGACGATGTCTAGGGGATCTGAGAGGATGACCCCCCAC
ACTGGGACTGAGACACGGCC
>Zotu5030
GAACGCTGGCGGCGCGCCTAATACATGCAAGTCGAGCGAGAAGACGTAGCAATACGTTTGTAAAGCGGCG
AACGGGTGAGGAATACATGGGTAACCTACCCTCGAGTGGGGAATAACTAGCCGAAAGGTTAGCTAATACC
GCATACGATTCCCGGACTGCGGTTCGGGAAGGAAAGCGATACCGTGGGTATCGCGCTCTTGGATGGGCTC
ATGTCCTATCAGCTTGTTGGTGAGGTAATGGCTCACCAAGGCTTCGACGGGTAGCTGGTCTGAGAGGACG
ATCAGCCACACTGGCACTGCGACACGGGC
>Zotu5033
GAACGCTAGCGGCAGGCTTAACACATGCAAGTCGAGGGGTATAGTTCTTCGGAATTAGAGACCGGCGCAC
GGGTGCGTAACGCGTATGCAATCTACCTTTCACAGAGGGATAGCCCAGAGAAATTTGGATTAATACCTCAT
AGTATAACGACTCGGCATCGAGATGTTATTAAAGTCACAACGGTGAAAGATGAGCATGCGTCCCATTAGCT
AGTTGGTAAGGTAACGGCTTACCAAGGCTATGATGGGTAGGGGTCCTGAGAGGGAGATCCCCCACACTGG
TACTGAGACACGGAC
>Zotu5040
GAACGCTGGCGGCATGCTTAACACATGCAAGTCGAACGAGACCTTCGGGTCTAGTGGCGCACGGGTGCGT
AACGCGTGGGAATCTACCCTTTGCTACGGAATAACTCAGAGAAATTTGTGCTAATACCGTATAATGTCGCA
AGACCAAAGATTTATCGGCAGAGGACGAGCCCGCGTAAGATTAGCTTGTTGGTGAGGTAAAGGCTCACCA
AGGCGACGATCTTTAGCTGGTCTGAGAGGATGATCAGCCACACTGGGACTGAGACACGGCC
>Zotu5043
GAACGCTGGCGGCGTGCTTAACACATGCAAGTCGAGCGGTAAGGCTCTTCGGAGTACACGAGCGGCGAAC
GGGTGAGTAACACGTGAGCAACCTGCCCATGACACTGGGATAACGATTGGAAACAGTCGCTAATACCGGA

TACGACCTTGGTGGACATCTACACGAGGTGGAAAGCTCCGGCGGTCATGGATGGGCTCGCGGCCTATCAG
CTTGTTGGTGAGGTAACGGCTCACCAAGGCGTCGACGGGTAGCCGGCCTGAGAGGGCGACCGGCCACACT
GGGACTGAGATACGGCC
>Zotu5046
GAACGCTGGCGGCATGCCTAACACATGCAAGTCGAACGAGACCTTCGGGTCTAGTGGCGCACGGGTGCGT
AACGCGTGGGAACCTGCCTTTAGGTTCGGAATAACTCCCCGAAAGGGGTGCTAATACCGGATAATGTCTTC
GGACCAAAGATTTATCGCCTTTAGATGGGCCCGCGTTGGATTAGCTAGTTGGTGGGGTAAAGGCCTACCAA
GGCGACGATCCATAGCTGGTCTGAGAGGATGATCAGCCACACTGGGACTGAGACACGGCC
>Zotu5048
GAACGCTGGCGGCGTGCTTAACACATGCAAGTCGAACGGTGATGGAGGAGCTTGCTCTTCTTGATCAGTG
GCGAACGGGTGAGTAACACGTGAGTAACCTGCCCTTGACTCTGGGATAACTCCGGGAAACCGGGGCTAAT
ACTGGATACGAAGCGCCTCGGCATCGGGTGCGCTTGGAAAGTTTTTCGGTCAAGGATGGACTCGCGGCCT
ATCAGCTTGTTGGCGGGGTAACGGCCCACCAAGGCGTCGACGGGTAGCCGGCCTGAGAGGGCGACCGGC
CACACTGGGACTGAGACACGGCC
>Zotu5050
GAACGCTGGCGGCATGCCTAACACATGCAAGTCGAACGAGACCTTCGGGTCTAGTGGCGCACGGGTGCGT
AACGCGTGGGAATCTGCCCTTGGGTTCGGAATAACAGTTAGAAATGACTGCTAATACCGGATGATGTCTTC
GGACCAAAGATTTATCGCCCAAGGATGAGCCCGCGTAGGATTAGGTAGTTGGTGAGGTAAAGGCTCACCA
AGCCGACGATCCTTAGCTGGTCTGAGAGGATGATCAGCCACACTGGGACTGAGACACGGCC
>Zotu5052
CCCGTGTCTCAGTGCCCGTGTGGCTGATCATGCTCTCACATCAGCTACTGATCGTAGACTTGGTGAGCCGTT
ACCTCACCAACTATCTAATCAGCCGCACACCCATCATCAAGTGCCGAAGCTTTAATATTAATAAGATGCCTTA
TCAAATATATTATGGGGTATTAATCCAAATTTCTCTGGGCTATTCCCCGCTTGACGGAAGGTTGTGTACGTG
TTCCGCACCCGTTTGCCGGTCGCCATCAAGTATTGCTACTCATGCTGCCCCACGACTTGCATGTATTAAGCCT
GCCGCTAGCGTTCA
>Zotu5055
GAATGCTGGCGGCGTGGATAAGTCATGCAAGTCAAGGGGGCCCGCAAGGGCAACCGGCAGACGAGGTAG
TAACACGTAGGTACGTCCCCCGAAGTCGGGCATAGCCAGCCTAAAGGTTGGGTAATTCCCGATAGTCCCGC
AAGGGTAAAGGTTTTTCGCTTCGGGAGCGGCCTGCGTAGTATCAGCTAGTTGGTAGGGTAACGGCCTACC
AAGGCTACGACGCTTAGGGGAGCTGAGAGGCTGACCCCCACCGATGGAACTGCGACACGGTC
>Zotu5057
GAACGCTGGCGGCAGGCCTAACACATGCAAGTCGAGCGGATGACAGGAGCTTGCTCCTGAATTCAGCGGC
GGACGGGTGAGTAATGCCTAGGAATCTGCCTGGTAGTGGGGGACAACGTCTCGAAAGGGACGCTAATACC
GCATACGTCCTACGGGAGAAAGTGGGGGATCTTCGGACCTCACGCTATCAGATGAGCCTAGGTCGGATTA
GCTAGTTGGTGAGGTAATGGCTCACCAAGGCGACGATCCGTAACTGGTCTGAGAGGATGATCAGTCACAC
TGGAACTGAGACACGGTC
>Zotu5061
GAACGCTAGCGGCAGGCTTAACACATGCAAGTCGAGGGGTATAGTAGCAATACTAGAGACCGGCGCACG
GGTGCGTAACGCGTATGCAATCTACCTTTTACAGAGGGATAGCCCAGAGAAATTTGGATTAATACCTCATA
GTATAATGACTCGGCATCGAGATGTTATTAAAGTTACAACGGTAAAAGATGAGCATGCGTCCCATTAGCTA
GTTGGTGTGGTAACGGCACACCAAGGCAACGATGGGTAGGGGTCCTGAGAGGGAGATCCCCCACACTGG
TACTGAGACACGGAC
>Zotu5064
GAACGCTGGCGGCGTGCCTAATACATGCAAGTCGAGCGAACTTTTGAGAGCTTGCTCTCAAAAGTTAGCGG
CGGACGGGTGAGTAACACGTGGGCAACCTACCTATAAGACTGGGATAACTTACGGAAACGTGAGCTAATA
CCGGATAATTCTTTCTAACACATGTTAGGAAGTTGAAAGATGGCTTCTCGCTATCACTTATAGATGGGCCCG
CGGCGCATTAGCTAGTTGGTGGGGTAATGGCCTACCAAGGCAACGATGCGTAGCCGACCTGAGAGGGTGA
TCGGCCACACTGGGACTGAGACACGGCC
>Zotu5068

GAACGCTAGCGGCAGGCCTAACACATGCAAGTCGAGGGGTAGAGCTACTTTAGTAGTTTGAGACCGGCGC
ACGGGTGCGTAACGCGTATGCAATCTACCTTATACAGAGGGATAGCCCAGAGAAATTTGGATTAATACCTC
ATAGTATATTTGAGTGGCATCATTTAGATATTAAAGATTTATCGGTATAAGATGAGCATGCGTCCCATTAGT
TAGTTGGTGTGGTAACGGCATACCAAGACGATGATGGGTAGGGGTCCTGAGAGGGAGATCCCCCACACTG
GTACTGAGACACGGAC
>Zotu5070
GAACGCTAGCGGCAGGCCTAATACATGCAAGTCGAGGGGCATCAGGCTGTAGCAATACAGTGCTGGCGAC
CGGCAAACGGGTGCGAAACACGTACATAACTTTCCGATAACTGGGGCATAACATTAAGAAATTGGTGCTAA
TTCCCCATAGCATTGTTGAGAGGCATCTCTTGGCAATTAAAGCTCAGGCGGTTATTGATAGATGTGCGGCT
GATTATGGTAGTTGGTGAGGTAACGGCTCACCAAGCCTACGATCAGTAACTGGTGTGAGAGCACGACCAG
TCACACGGGCACTGAGACACGGGC
>Zotu5072
GAACGCTGGCGGCGTGCCTAACACATGCAAGTCGAGCGATTTACTTCGGTAAAGAGCGGCGGACGGGTGA
GTAACGCGTGGGTAACCTGCCTCATACACATGGATAACATACCGAAAGGTATGCTAATACAAGATAATATA
AGAGATTCGCATGGATTTCTTATCAAAGCTCCGGCGGTATGAGATGGACCCGCGTCTGATTAGCTAGTTGG
TAAGGTAACGGCTTACCAAGGCGACGATCAGTAGCCGACCTGAGAGGGTGATCGGCCACATTGGAACTGA
GACACGGTC
>Zotu5074
GAACGCTGGCGGCATGCCTAACACATGCAAGTCGAACGGCAGCATGAATGGGGGTAACCCTATTTGATGG
CGAGTGGCGGACGGGTGAGTAATATGTAGGAATCTACCTAGAGGAGGGGGATAACTTGGGGAAACCCAA
GCTAATACCACATAAGCCCTACGGGGGAAAGCAGGGGACCGCAAGGCCTTGCGCCTTATGATGAGCCTAC
ATCGGATTAGCTAGTTGGTGGGGTAAAGGCCTACCAAGGCGACGATCCGTAGCTGGTCTGAGAGGATGAT
CAGCCACACTGGGACTGAGACACGGCC
>Zotu5077
GAACGCTGGCGGCAGGCTTAACACATGCAAGTCGAGCGGGCACTTCGGTGTCAGCGGCAGACGGGTGAG
TAACGCGTGGGAACGTGCCCTTCGGTTCGGAATAACTCAGGGAAACTTGAGCTAATACCGGATACGCCCTT
TTGGGGAAAGGTTTACTGCCGAAGGATCGGCCCGCGTCTGATTAGCTAGTTGGTGAGGTTACGGCTCACCA
AGGCGACGATCAGTAGCTGGTCTGAGAGGATGATCAGCCACACTGGGACTGAGACACGGCC
>Zotu5081
CAACGCTGGCGGCGCGCCTAACACATGCAAGTCGAACGAGAAAGCAGGAGCAATCTTGTGAGTAAAGTGG
CGTACGGGTGAGTAACACGTGGATTATCTACCTCTTTGTGGGGGATAACCCTGGGAAACCGGGGCTAATAC
CGCATAAGTCCGAGAGGAGAAAGCAGCAATGCGCAGAGAGAGGAGTCCGCGGCCGATTAGCTAGTTGGT
AGGGTAAAAGCCTACCAAGGCGACGATCGGTAGCCGGCCTGAGAGGGCACACGGCCACACTGGCACTGA
AACACGGGC
>Zotu5085
GAACGCTAGCGGCAGGCCTAACACATGCAAGTCGAGGGGTAACAGGGTAGCAATATCGCTGACGACCGG
CGCACGGGTGAGTAACGCGTATGCAACCTTCCTTTAACTGGGGGATAGCCCGGAGAAATTCGGATTAATAC
CCCATAAGATCATAGAAAGACATCTTTTAGTGATTAAAGCTCCGGCGGTTAAAGATGGGCATGCGTGCCAT
TAGCTAGTTGGTGAGGTAACGGCTCACCAAGGCTACGATGGTTAGGGGGTCTGAGAGGATGGTCCCCCAC
ACTGGTACTGAGACACGGAC
>Zotu5087
GAACGCTGGCGGCGTGCTTAATACATGCAAGTCGAACGGACAATATTAGATAGCTTGCTATTTGATATTGT
TAGTGGCGAACGGGTGAGTAATACATAGGTAATCTGCCCTGAAGAGCGGGATAGCTGTTGGAAACGACAA
TTAATACCGCATAGGTAATCGGGAGGCATCTTCTGATTATTAAAGATCCGTTTGGATCACTACAGGATGAG
CTTATGGTGTATTAGCTAGTTGGCAGGGTAAAGGCCTACCAAGGCGATGATACATAGCCGGCTTGAGAGG
GTGAACGGCCACATTGGGACTGAGACACGGCC
>Zotu5089
GAACGCTAGCGACAGGCCTAACACATGCAAGTCGAGGGGCAGCACATGAGTAGCAATACGATGGTGGCG
ACCGGCGCACGGGTGAGTAACACGTATGCAACCTACCTGTAAGAGGGGAATAACCCGGAGAAATTCGGAC
TAATACCGCATAATACCTTGAAGCTGCATGGCTTTGAGGTTAAACATTTATGGCTTACAGATGGGCATGCGT

ATGATTAGCTAGTTGGTGAGGTAACGGCTCACCAAGGCAACGATCATTAGGGGTTCTGAGAGGAAGGTCC
CCCACACTGGTACTGAGACACGGAC
>Zotu5091
GAACGCTGGCGGCGCGCCTAACACATGCAAGTCGAACGGAGTCAAGAGGAGCTTACTTTTCTTGACTTAGT
GGCGAACGGGTGAGTAACGCGTGAGTAACCTGCCCTGGAGTGGGGGACAACAGTTGGAAACGACTGCTA
ATACCGCATAAGCCCACGGATTCGCATGGATCTGCGGGAAAAGGATTTATTTGCTTCAGGATGGACTCGCG
TCCAATTAGCTAGTTGGTGAGGTAACGGCCCACCAAGGCGACGATTGGTAGCCGGACTGAGAGGTTGAAC
GGCCACATTGGGACTGAGACACGGCC
>Zotu5093
GAACGCTGGCGGCGTGCCTAAGACATGCAAGTCGTACGTGAAAGTAGCAATATGAGTAAAGTGGCGCACG
GGTGAGTAATAACTAGGTAATGCGCCCATTCGCGGGGAATAACCAGGCGAAAGCTTGGCTAATGCCGCAT
GAGTCGGGAGGTCTCCGGATCTTTTTGACAAAGCCGTAAGGCGCGAATGGAGTGGCCTAGTCCCTATCAG
CTAGTTGGTGAGGTAACGGCTCACCAAGGCTTCGACGGGTAGCTGGTTTGAGAGGACGATCAGCCACACT
GGTACTGGAACACGGAC
>Zotu5098
GAACGCTGGCGGCGTGCTTAACACATGCAAGTCGAACGGAAAGGCCCTTCGGGGTACTCGAGTGGCGAAC
GGGTGAGTAACACGTGGGTGATCTGCCCTGCACTTTGGGATAAGCCTGGGAAACTGGGTCTAATACCGGA
TAGGACCACGCGCTTCATGGTGTGTGGTGGAAAGCTTTTGCGGTGTGGGATGGGCCCGCGGCCTATCAGC
TTGTTGGTGGGGTAATGGCCTACCAAGGCGACGACGGGTAGCCGGCCTGAGAGGGTGACCGGCCACACT
GGGACTGAGATACGGCC
>Zotu5101
GAACGCTGGCGGCGTGCTTAACACATGCAAGTCGAGCGGAAAGGCTCCTTCGGGGGTACTCGAGCGGCG
AACGGGTGAGTAACACGTGAGTAATCTGCCCTCCACTTCGGGATAGCCACCGGAAACGGTGATTAATACCG
GATATGACTACTGCTCGCATGAGTTGGTGGTGGAAAGTTTTTCGGTGGGGGATGTGCTCGCGGCCTATCAG
CTTGTTGGTGAGGTAATGGCTCACCAAGGCTTCGACGGGTAGCCGGCCTGAGAGGGTGACCGGTCACACT
GGGACTGAGACACGGCC
>Zotu5104
GAACGATGGCGAGATGCCTTACACATGCAAGTCGTACGGGAATTTTTAATTCAAGTGGCGGACGGGTGAG
TAACACGTAAGAATTTGCCTTTAGGAGGGGGACAACAACTGGAAACGGTTGCTAATACCCCATATGCTTTC
GAGTGAAATGGATTTTTCCGCCTAAAGAGAAGCTTGCGGCTGATTAGCTTGTTGGTGAGGTAATGGCTCAC
CAAGGCGACGATCAGTATCTGGTTTGAGAGGATGATCAGACACACTGGAACTGAGACACGGTC
>Zotu5106
GAACGCTGGCGGCATGCCTTACACATGCAAGTCGAACGGCAGCATGACGTGTAGCAATACACGTTGATGG
CGAGTGGCGAACGGGTGAGTAATATATCGGAACGTGCCCTGTAGCGGGGGATAACCTATCGAAAGATGG
ACTAATACCGCATACGCTCTACGGAGGAAAGCGGGGGTCGCAAGACCTCGCACTATAGGAGCGGCCGATA
TCGGATTAGCTAGTTGGCGGGGTAAAAGCCCACCAAGGCGACGATCCGTAGCTGGTCTGAGAGGATGATC
AGCCACACTGGAACTGAGACACGGTC
>Zotu5108
TAACGCTAGCGGCAGGCCTAATACATGCAAGTCGAACGGTAACATGGGTAGCAATATCTGATGACGAGTG
GCGCACGGGTGCGTAACGCGTATGTAATCTACCTCTAACTGGGGGATAGCCTTCCGAAAGGAAGATTAATA
CCGCATAATATATTAAAGAGGCATCTCTTTGTTATCAAAACTCTGGTGGTTAGAGATGAACATGCGTGACAT
TAGCTAGTTGGTGAAGTAATAGCTCACCAAGGCTACGATGTCTAGGGGGTCTGAGAGGATGATCCCCCAC
ACTGGTACTGAGACACGGAC
>Zotu5111
GAACGCTGGCGGCATGCCTAACACATGCAAGTCGAACGGAGATTATTTCGGTAATCTTAGTGGCGAACGG
GTGAGTAACGCGTAGGCAACCTGCCCTTTAGTTGGGGACAACATCCCGAAAGGGGTGCTAATACCGAATG
TGATCGCTTTCTCGCATGAGAGAGCGATGAAAGATGGCCTCTATTTATAAGCTATCGCTAAAGGATGGGCC
TGCGTCTGATTAGCTAGTTGGTGGGGTAACGGCCTACCAAGGCGATGATCAGTAGCCGGTCTGAGAGGAT
GAACGGCCACATTGGGACTGAGACACGGCC
>Zotu5113

GAACGCTGGCGGCGTGCATAACACATGCAAGTCGAACGATGAAACTTTTTTGATTTCTTCGGAATGAAAAA
AAGTGGATTAGTGGCGGACGGGTGAGTAACGCGTGAGTAACCTGCCTTAGAGAAGGGGATAGCCTTTGG
AAACGAAGAATAATACCCTATGAAACCATAACCTCGCATGAGGCCATGGTCAAAGATTTATCGCTCTAAGA
TGGACTTGCGTCTGATTAGTTAGTTGGTGGGGTAACGGCCCACCAAGACAGCGATCAGTAGCCGGCTTGA
GAGAGTGTACGGCCACATTGGGACTGAGACACGGCC

>Zotu5115
GAACGCTGGCGGCGTGCCTAATACATGCAAGTCGAGCGAACTTGCGGGAGCTTGCTCCCAAAAGTTAGCG
GCGGACGGGTGAGTAACACGTGGGCAACCTGCCTATAAGACTGGGATAACTTCGGGAAACCGGAGCTAAT
ACCGGATAATTCTTTTCTACACATGTAGGAAAATTAAAAGACGGTTTACGCTGTCACTTATAGATGGGCCCG
CGGCGCATTAGCTAGTTGGTGAGGTAACGGCTCACCAAGGCCACGATGCGTAGCCGACCTGAGAGGGTGA
TCGGCCACACTGGGACTGAGACACGGCC

>Zotu5118
GAACGCTGGCGGCATGCCTTACACATGCAAGTCGAGCGGCAGCGCGGGGGCAACCCTGGCGGCGAGCGG
CGAACGGGTGAGTAATACATCGGAACGTGTCCAGTTGTGGGGGATAACCAGTCGAAAGACTGGCTAATAC
CGCATACGATCTACGGATGAAAGCGGGGGATCGCAAGACCTCGCGCTATTGGAGCGGCCGATGTCCGATT
AGCTAGTTGGCGGGGTAACGGCCCACCAAGGCGACGATCGGTAGCTGGTCTGAGAGGATGATCAGCCAC
ACTGGAACTGAGACACGGTC

>Zotu5122
GAACGCTGGCGGCGTGCCTAATACATGCAAGTCGAGCGAATCTTTGGGAGCTTGCTCCCGAAGATTAGCG
GCGGACGGGTGAGTAACACGTGGGCAACCTGCCTATAAGACTGGGATAACTTCGGGAAACCGGAGCTAAT
ACCGGATAATTCTTTTTCTCTCATGAGGAAAAGCTGAAAGACGGTTTACGCTGTCACTTATAGATGGGCCCG
CGGCGCATTAGCTAGTTGGTGAGGTAACGGCTCACCAAGGCAACGATGCGTAGCCGACCTGAGAGGGTGA
TCGGCCACACTGGGACTGAGACACGGCC

>Zotu5124
GAACGCTGGCGGCAGGCCTAACACATGCAAGTCGAGCGGATGATGGGAGCTTGCTCCCGGATTCAACGGC
GGACGGGTGAGTAATGCCTAGGAATCTGCCTAGTAGTGGGGGACAACGTTTCGAAAGGAACGCTAATACC
GCATACGTCCTACGAGAGAAAGCAGGGGACCTTCGGGCCTTGCACTATCAGATGAGCCTAGGTCGGATTA
GCTAGTTGGTGGGGTAAAGGCCTACCAAGGCGACGATCCGTAACTGGTCTGAGAGGATGATCAGTCACAC
TGGAACTGAGACACGGTC

>Zotu5126
GAACGCTGGCGGCGTGCTTAACACATGCAAGTCGAACGATGAAGACCGGTGCTTGCACTGGTTGGATTAG
TGGCGAACGGGTGAGTAATACGTGAGTAACCTGCCTTTGACTCTGGGATAAGCCTTGGAAACGAGGTCTA
ATACCGGATATGACACAGTTTCGCATGAAATCTGTGTGGAAAGGGTTTGTACTGGTTTTAGATGGGCTCAC
GGCCTATCAGCTTGTTGGTGAGGTAATGGCTCACCAAGGCGACGACGGGTAGCCGGCCTGAGAGGGTGAC
CGGCCACACTGGGACTGAGACACGGCC

>Zotu5128
GAACGCTAGCGGCAGGCTTAATACATGCAAGTCGTGGGGCAGCACAGAGTAGCAATACTTGGGTGGCGAC
CGGCAAACGGGTGCGGAACACGTACGCAACCTTCCTTCAGGCGGGGAATAGCCCAGAGAAATTTGGATTA
ATACCCCATAAGATTTTGAGATGGCATCATCTTATTATTAAAGATTTATCACCTGAAGATGGGCGTGCGGCT
GATTAGGTAGTTGGCGGGGTAACGGCCCACCAAGCCTACGATCAGTAGCTGATGTGAGAGCATGATCAGC
CACACGGGCACTGAGACACGGGC

>Zotu5130
CAACGCTGGCGGCGTGCCTAACACATGCAAGTCGAGCGAGAGGAGGTAGCAATACCTTAGTAAAGCGGC
GCACGGGTGAGTAACACGTGGATAACCTGCCTCGAAGTGGGGAATAACCCCGGGAAACCGGAGCTAATAC
CGCATATTCTGACGAGATCTGCGGATTTTGTCAGGAAAGGAGCAATTCGCTTTGAGAGGGGTCCGCGGCT
GATTAGCTAGTTGGTGAGGTAATGGCTCACCAAGGCCACGATCAGTAGCCGGTCTGAGAGGACGGTCGGC
CACACTGGAACTGAGACACGGTC

>Zotu5133
GAACGCTGGCGGCATGCCTAACACATGCAAGTCGAACGAGATCTTCGGATCTAGTGGCGCACGGGTGCGT
AACGCGTGGGAATCTGCCCTGGGGTACGGAATAACTCAGAGAAATTTGAGCTAATACCGTATGATGACGT

AAGTCCAAAGATTTATCGCCCTGGGATGAGCCCGCGTAGGATTAGCTAGTTGGTGGGGTAAAGGCCTACC
AAGGCGACGATCCTTAGCTGGTCTGAGAGGATGATCAGCCACACTGGGACTGAGACACGGCC
>Zotu5135
GAACGCTGGCGGCAGGCTTAACACATGCAAGTCGAGCGGACCTTTCGGGGTCAGCGGCGGACGGGTGAG
TAACGCGTGGGAACGTGCCTTCCGGTTCGGAATAACCCTGGGAAACTAGGGCTAATACCGGATACGCCCTT
ATGGGGAAAGGTTTACTGCCGGAAGATCGGCCCGCGTCTGATTAGCTAGTTGGTGGGGTAACGGCCTACC
AAGGCGACGATCAGTAGCTGGTCTGAGAGGATGATCAGCCACACTGGGACTGAGACACGGCC
>Zotu5137
GAACGCTGGCGGCGTGCCTAACACATGCAAGTCGAGCGATCTTCTTCGGAAGAGAGCGGCGGACGGGTG
AGTAACGCGTGGGTAACCTGCCCTGTACACACGGATAACATACCGAAAGGTATGCTAATACGAGATAATAT
GCTTTTATCGCATGGTAGAAGTATCAAAGCTTTTGCGGTACAGGATGGACCCGCGTCTGATTAGCTAGTTG
GTGAGGTAACGGCTCACCAAGGCGACGATCAGTAGCCGACCTGAGAGGGTGATCGGCCACATTGGAACTG
AGACACGGTC
>Zotu5139
GAACGCTGGCGGCAGGCCTAACACATGCAAGTCGAGCGCCCCGCAAGGGGAGCGGCAGACGGGAGAGTA
ACACGTGGGAACGTACCCTTCAGTTCGGAACAACCCAGGGAAACTTGGGCTAATACCGAATACGTCCGTAA
GGAGAAAGATTTATCGCTGAAGGATCGGCCCGCGTCTGATTAGCTAGTTGGTGAGGTAACGGCTCACCAA
GGCGACGATCAGTAGCTGGTCTGAGAGGATGATCAGCCTCACTGGGACTGAGACACGGCC
>Zotu5141
GAATGCTGGCGGCGTGGATGAGTCATGCAAGCCGAACGGCCCGCAAGGGCAGTGGCAGACGAGGTAGTA
ACACGTAGGTAGATACCCCAGAGTCGGGCATAGCCAGCCGAAAGGTTGGGTAATTCCCGATAGTCTTGTG
AAAGTAAAGGAGCAATCCGCTCTGGGAGTCGCCTGCGTAATATCAGCTAGTTGGTGAGGTAATGGCTCAC
CAAGGCTACGACGTTTAGGGGAGCTGAGAGGCTGACCCCCACCGATGGGACTGAGATACGGCC
>Zotu5143
GAACGCTAGCGGCAGGCCTAATACATGCAAGTCGAGGGGCAGCACGGGTAGCAATACCTGGTGACGACC
GGCAAACGGGTGCGGAACACGTACGCAACCTTCCCATAACTGGTGAATAGCCCTCCGAAAGGAGGATTAA
TACACCGTAACATAATGAAGTGGCATCACTTTATTATTATAGGCAGCAATGTCGGTTTTGGATGGGCGTGC
GCCTGATTAGGTAGTTGGCGGGGTAACGGCCCACCAAGCCTGCGATCAGTAACTGGTGTGAGAGCACGAC
CAGTCACACGGGCACTGAGACACGGGC
>Zotu5145
GAACGCTGGCGGCGTGCCTAATACATGCAAGTCGAACGAGAAAGTGTAGCAATACATTAGTAAAGTGGCA
GACGGGTGAGTAATACGTAGATAATCTTCCTCCAAGATGGGGATAACCTTCCGAAAGGAGGGCTAATACC
GAATGAGACCACACTGACTATGGTCAGAGCGGTCAAAGGTGACCTCTATTTATAAGTTATCGCTTGGAGAT
GAGTTTATGCACCATCAGGTAGTTGGTAAGGTAATGGCTTACCAAGCCTTTGACGGTTAGCTGATCTGAGA
GGTTGATCAGCCACACTGGAACTGAGACACGGTC
>Zotu5147
GAACGCTGGCGGCAGGCTTAACACATGCAAGTCGAGCGGGCCCTTTCGGGGGTCAGCGGCAGACGGGTG
AGTAACACGTGGGAACGTACCCTTTGGTTCGGAATAACGCTGGGAAACTAGCGCTAATACCGGATACGCCC
TTGGCCTTTGGCCCTGGGGAAAGGCTTGTCTGCCGAAGGATCGGCCCGCGTCTGATTAGCTAGTTGGTGG
GGTAATGGCCTACCAAGGCGACGATCAGTAGCTGGTCTGAGAGGATGATCAGCCACACTGGGACTGAGAC
ACGGCC
>Zotu5149
GAACGCTGGCGGCATGCTTAACACATGCAAGTTGAACGCAGAATAGGCCTTCGGGTCTATTGCTGAGTAGC
AAACGGGTGAGTAACGCGTGGGAATCTACCTTTATGTGGGGGATAACTGAGGGAAACTTCAGCTAATACC
GCATACGCTCTACGGAGGAAAAGCTTCACTTTTCAGTAATGAGTAATTGTATTGCTCATTACTGAGTAGTGA
AGTTGCATAAAGAGGAGCCCGCGTCAGATTAGGTAGTTGGTGGGGTAAAGGCCTACCAAGCCAATGATCT
GTAGCTGGTCTGAGAGGATGATCAGCCACACTGGGACTGAGACACGGCC
>Zotu5152
GAACGCTGGCGGCATGCTTAACACATGCAAGTCGCACGGGCACTTTCGGGTGTTAGTGGCGGACGGGTGA
GTATCGCGTAGGGATTTATCGTTGGGTGGGGGATAACCTTGGGAAACTGGGGCTAATACCGCATGACGCC

TGGGGGCCAAAGCTACGGCGCCTGACGAGGAGCCTGCGTCCGATTAGGTAGTTGGTTGGGTAATGGCTGA
CCAAGCCTGCGATCGGTAGCTGGTCTGAGAGGATGATCAGCCACACTGGGACTGAGACACGGCC
>Zotu5154
GAACGCTGGCGGCGTGCTTAACACATGCAAGTCGAACGATGAAGCTTCCTGCTTGCAGGAAGTGGATTAG
TGGCGAACGGGTGAGTATCACGTGAGCAACCTGCCCTTGACTCTGGGATAAGCCTGGGAAACTGGGTCTA
ATACCGGATATAACCTCTTACCGCATGGTGAGTGGTGGAAAGCTTTTGCGGTCTTGGATGGGCTCGCGGCC
TATCAGCTTGACGGTGAGGTAGTGGCTCACCGTGGCGATGACGGGTAGCCGGCCTGAGAGGGTGACCGG
CCACACTGGGACTGAGACACGGCC
>Zotu5156
GAACGCTGGCGGCGTGCTTAACACATGCAAGTCGAGCGGTAAGGCTCCTTCGGGGGTACACGAGCGGCG
AACGGGTGAGTAACACGTGAGTAATCTGCCCATGGCTCTGGGATAAGCACTCGAAAGGGTGTCTAATACC
GGATATGACAATCCTCCGCATGGGGTGGTTGTGGAAAGTTTTTCGGCTATGGATGTGCTCGCGGCCTATC
AGCTTGTTGGTGGGGTAATGGCCTACCAAGGCTTTGACGGGTAGCCGGCCTGAGAGGGTGACCGGTCACA
CTGGGACTGAGACACGGCC
>Zotu5158
GAACGCTGGCGGCGTGCCTAACACATGCAAGTCGAACGAAGCGATTTGGAGGAAGTTTTCGGATGGAATC
TGAATTGACTGAGTGGCGGACGGGTGAGTAACGCGTGGGTAACCTGCCTCACACTGGGGGACAACAGCTG
GAAACGGCTGCTAATACCGCATAAGCGCACAGCTTCGCATGAAGCAGTGTGAAAAACTCCGGTGGTGTGA
GATGGACCCGCGTCTGATTAGCTAGTTGGTAAGGTAACGGCTTACCAAGGCGACGATCAGTAGCCGACCT
GAGAGGGTGACCGGCCACATTGGGACTGAGACACGGCC
>Zotu5162
GAACGCTGGCGGCGTGCCTAATACATGCAAGTCGAGCGAAGTGGAGGAAGCTTGCTTCCAAAGCTTAGCG
GCGAACGGGTGAGTAACACGTGGGCAACCTGCCTGACAGACTGGGATAACTTGGGGAAACTCAAGCTAAT
ACCGGATAGTATCGCAAACTGCATGGTTTGCGATGGAAAGTCTGAACGACACTGTCAGATGGGCCCGCGG
CGCATTAGCTAGTTGGTGGGGTAATGGCCTACCAAGGCGACGATGCGTAGCCGACCTGAGAGGGTGACCG
GCCACACTGGGACTGAGACACGGCC
>Zotu5165
GAACGCTGGAAATGTGGATACCGGCATGCAAGTCAAGGGGCCGCAAGGCACCGGCGAACGGCTTAGTAA
CACGTAGATACATACCGTGAAGTGGGGCACAGCTCGTCGAAAGACGGGGTAATTTCCCATAGTCTCGCAA
GAGTAAACTCGCAAGAGGCTTCATGAATGGTCTGCGGTCTATCAGCTAGTTGGTGAGGTAATGGCTCACCA
AGGCTATGACGGATAGGGGGCGTGAGAGCGCGACCCCCAAGAATGGCACTGCGACACGGGC
>Zotu5169
GAACGCTGGCGGCGTGCTTAACACATGCAAGTCGAACGGAAAGGCCTCTTCGGAGGTACTCGAGTGGCGA
ACGGGTGAGTAACACGTGGGTGATCTGCCCTGCACTCTGGGATAAGCCTGGGAAACTGGGTCTAATACCG
GATAGGACCATGCTTTAGTGTGTGTGGTGGAAAGTTTTTTCGGTGTAGGATGAGCCCGCGGCCTATCAGCT
TGTTGGTGGGGTAATGGCCTACCAAGGCGGCGACGGGTAGCCGGACTGAGAGGTTGGTCGGCCACATTG
GGACTGAGATACGGCC
>Zotu5171
GAACGCTGGCGGCGTGCCTAATACATGCAAGTCGAGCGAGTCCCTTCGGGGGCTAGCGGCGGACGGGTG
AGTAACACGTAGGCAACCTGCCCGTAAGATCGGGATAACATAGGGAAACTTATGCTAATACCGAATAGAG
TCTTTTCTCGCATGAGGAGAGACGGAAAGATGGCGCAAGCTATCACTTACGGATGGGCCTGCGGCGCATT
AGCTAGTTGGTGGGGTAACGGCCTACCAAGGCGACGATGCGTAGCCGACCTGAGAGGGTGACCGGCCAC
ACTGGGACTGAGACACGGCC
>Zotu5173
GAACGCTAGCGGGAGGCCTAATACATGCAAGTCGAGCGTCCAGCAATGGAAGCGGCAGACGGGTGCGTA
ACGCGTATGAAACTTGCCCTACACAGGAGAATAGCCTTGGGAAACTGAGAGTAATACTCCATAGCATTATA
GAGTCGCATGATTTTATAATTAAAGCTCCGGCGGTGTAGGATAGTCGTGCGTCCGATTAGCTAGTTGGCGG
GGTAACGGCCCACCAAGGCGACGATCGGTAGCTGGTCTGAGAGGATGATCAGCCACACGGGCACTGAGA
CACGGGC
>Zotu5176

GAACGCTGGCGGCGCGCCTAACACATGCAAGTCGAACGAGAGATGAGGAGCTTGCTCTTCAGATCGAGTG
GCGAACGGGTGAGTAACGCGTGAGGAACCTGCCTCAAAGAGGGGGACAACAGTTGGAAACGACTGCTAA
TACCGCATAAGCCCACAGCTCGGCATCGAGCAGAGGGAAAAGGAGTGATCCGCTTTGAGATGGCCTCGCG
TCCGATTAGCTGGTTGGTGAGGTAACGGCCCACCAAGGCGACGATCGGTAGCCGGACTGAGAGGTTGAAC
GGCCACATTGGGACTGAGACACGGCC

>Zotu5178
GAACGCTGGCGGCATGCCTTACACATGCAAGTCGAACGGCAGCACGGGGGCAACCCTGGTGGCGAGTGG
CGAACGGGTGAGTAATACATCGGAACGTGCCCTGAAGTGGGGGATAACGTAGCGAAAGTTACGCTAATAC
CGCATATTCTGTGAGCAGGAAAGCAGGGGATCGCAAGACCTTGCGCTTTAGGAGCGGCCGATGTCGGATT
AGCTAGTTGGTGAGGTAAAGGCTCACCAAGGCGACGATCCGTAGCGGGTCTGAGAGGATGATCCGCCACA
CTGGGACTGAGACACGGCC

>Zotu5180
GAACGCTGGCGGCAGGTCTTAGGCATGCAAGTCGAACGGGCGTAGCAATACGTCAGTGGCGCACGGGTG
AGTAACGCGTGGGAATATGCCCATTACTGGGGGATAACGTTTGGAAACGAACGCTAATACCGCATAAGCC
GGAAACGGGAAAGATTTATCGGTAATGGATTAGCCCGCGTTGGATTAGCTTGTTGGTGGGGTAACGGCCT
ACCAAGGCGATGATCCATAGCTGGTCTGAGAGGACGATCAGCCACACTGGAACTGAGACACGGTC

>Zotu5183
AGACATTTGTTCTGCGCGCAGACGCATCGTCTACCAGTGTGGGAGCCGTGCTCATGCGGAATTATGACGGT
GTTCTCCATCCAGTCGCCTACGCCAGCAGGAA

>Zotu5185
GAACGCTAGCGGCAGGCTTAACACATGCAAGTCGAGGGGTATAGTAGCAATACTAGAGACCGGCGCACG
GGTGCGTAACGCGTATGCAATCTACCTTTTACAGAGGGATAGCCCAGAGAAATTTGGATTAATACCTTATA
GTATATATGAATGGCATCATTTATATATTAAAGTTCCAACGGTAAAAGATGAGCATGCGTCCCATTAGCTAG
ATGGTAAGGTAACGGCTTACCATGGCTACGATGGGTAGGGGTCCTGAGAGGGAGATCCCCCACACTGGTA
CTGAGACACGGAC

>Zotu5187
GAACGCTGGCGGCATGCCTAACACATGCAAGTCGAACGAAGGCTTCGGCCTTAGTGGCGCACGGGTGCGT
AACGCGTGGGAATCTGCCCCTTGGTTCGGAATAACAGTTGGAAACGACTGCTAATACCGGATGACGACGT
AAGTCCAAAGATTTATCGCCGAGGGATGAGCCCGCGTAGGATTAGCTAGTTGGTGTGGTAAAGGCGCACC
AAGGCGACGATCCTTAGCTGGTCTGAGAGGATGATCAGCCACACTGGGACTGAGACACGGCC

>Zotu5189
GAACGCTGGCGGCAGGCCTAACACATGCAAGTCGAGCGCGCCTTCGGGTGAGCGGCGGACGGGTGAGTA
ACACGTGGGAATATGCCTTTCGGTGCGGAATAGTCTTGGGAAACTGAGGGTAATACCGCATACGCCCCCTG
GGGGAAAGATTTATCGCCGAAAGATTAGCCCGCGTTGGATTAGGTAGTTGGTGGGGTAATGGCCTACCAA
GCCGACGATCCATAGCTGGTTTGAGAGGATGATCAGCCACACTGGGACTGAGACACGGCC

>Zotu5192
GAACGCTGGAAATGTGGATACCGGCATGCAAGTCAAGGGGCCGCAAGGCACCGGCGAACGGCTTAGTAA
CACGTAGATACATACCGTAAAGTGGGGCATAGCTCGTCGAAAGACGGGGTAATTTCCCATGGTCCCGCAA
GGGTAAAGGCGCAAGCCGCTTTATGAATGGTCTGCGGTCTATCAGCTAGTTGGTGAGGTAATGGCTCACCA
AGGCTATGACGGATAGGGGGCGTGAGAGCGCGACCCCCAAGAATGGCACTGCGACACGGGC

>Zotu5196
GAACGCTGGCGGCGTGCTTAACACATGCAAGTCGAACGGAAAGGCCTTGTGCTTGCACAAGGTACTCGAG
TGGCGAACGGGTGAGTAACACGTGGGTGATCTGCCCTGCACTGTGGGATAAGCCCGGGAAACTGGGTCTA
ATACCATATAGGACCACTTCTTGGATGTTGTGGTGGAAAGCTTTTGCGGTGTGGGATGAGCCTGCGGCCTA
TCAGCTTGTTGGTGGGGTAATGGCCTACCAAGGCGGCGACGGGTATCCGGCCTGAGAGGGTGTACGGACA
CATTGGGACTGAGATACGGCC

>Zotu5198
GAACGCCGGCGGTGTGCCTAATACATGCAAGTCGAACGCGTTGGCCCAATTGATTGATGGTGCTTGCACCT
GATTGATTTTGGTCGCCAACGAGTGGCGGACGGGTGAGTAACACGTAGGTAACCTGCCCAGAAGCGGGG
GACAACATTTGGAAACAGATGCTAATACCGCATAACAGCGTTGTTCGCATGAACAACGCTTAAAAGATGGC

TTCTCGCTATCACTTCTGGATGGACCTGCGGTGCATTAGCTTGTTGGTGGGGTAACGGCCTACCAAGGCGA
TGATGCATAGCCGAGTTGAGAGACTGATCGGCCACAATGGGACTGAGACACGGCC
>Zotu5200
GAACGCTGGCGGCGCGCCTAACGCATGCAAGTCGAACGAGGCTGACAAGGTGCTTGCACCTTCGATGCCT
AGTGGCGAACGGGTGAGGAACACGTGAGAAACCTGCCCCAAACACGGGAATAACCCGAGGAAACTCTGG
CTAATGCCCGATACCCTCCGCAGTTCGCATGAACAGCGAAGGAAAGATCAGTCGGTTTGGGAGGGTCTCG
CGTCCTATCAGCTAGTTGGTGAGGTAACGGCTCACCAAGGCGACGACGGGTAGCTGGTTTGAGAGAACGA
TCAGCCACACTGGAACTGAGACACGGTC
>Zotu5202
GAACGCTAGCGGCAGGCCTAACACATGCAAGTCGAGGGGCAGCGGGTTTTAGCAATAAGATGCCGGCGA
CCGGCGCACGGGTGCGTAACGCGTATACAATTTGCCTTATACTGGGAGATAGCCCGGAGAAATTCGGATTA
ATATCCTATGATAATGTTGAATCGCATGGTTTGACATTTAAAGCTACGGCGGTATAAGATGAGTATGCGTCT
GATTAGCTAGTTGGTGAGGTAATGGCTCACCAAGGCGACGATCAGTAGGGGGCCTGAGAGGGTTATCCCC
CACACTGGTACTGAGACACGGAC
>Zotu5204
GAACGCTGGCGGCGTGCCTAATACATGCAAGTCGAGCGAACAGATAAAGAGCTTGCTCCTTTGACGTTAGC
GGCGGACGGGTGAGTAACACGTGGGTAACCTACCTATAAGACTGGAATAACTCCGGGAAACCGGGGCTA
ATGCCGGATAATACACAGAACCGCATGGTTCTGTGATGAAAGACGGTTTTGCTGTCACTTATAGATGGACC
CGCGGCGTATTAGCTAGTTGGTAAGGTAACGGCTTACCAAGGCAACGATACGTAGCCGACCTGAGAGGGT
GATCGGCCACATTGGGACTGAGACACGGCC
>Zotu5206
GAACGCTGGCGGCGTGCATAACACATTCAAGTCGAACGGTGCGAGGGAGCAATCCCTCAATCAGTGGCGG
ACGGGTGAGTAACGCGTGAGCAACCTGCCCTTATGTGTGGGATAACGTTTGGAAACGGACGCTAATACCG
CATAATGCATCTGAATCGCATGTTTCGGATGCCAAAGATTTATTGCATAAGGATGGGCTCGCGTCTGATTAG
ATAGTTGGTGGGGTAACGGCCTACCAAGTCTGCGATCAGTAGCCGGACTGAGAGGTTGAACGGCCACATT
GGAACTGAGAAACGGTC
>Zotu5209
GAACGCTGGCGGCGTGCTTCACACATGCAAGTCGAACGAGGTCCAATCAGTCGCAAGACTGGTGAAGACC
GAGTGGCGAACGGGTGCGTAACACGTGAGGAACCTGTCCCGGTCTCCGGAATAACAAGTGGAAACACTTG
CTAATACGGGATGACGTCGGAACGTCGCATGACGAACTGACGAAAGGGTTACCGGACCGGGAGGGCCTC
GCGGCCTATCAGCTCGTTGGTGGGGTAACGGCCCACCAAGGCTTCGACGGGTAGCTGGTCTGAGAGGATG
ATCAGCCACACTGGGACTGAGACACGGCC
>Zotu5211
GAACGCTGGCGGCAGGCTTAACACATGCAAGTCGAGCGGATACTTCGGTATCAGCGGCAGACGGGTGAGT
AACGCGTGGGGATCTGCCTAGTGGTACGGAACAACACAGGGAAACTTGTGCTAATACCGTATAAGTCCTTC
GGGAGAAAGATTTATCGCCATTAGATGAACCCGCGTCTGATTAGCTAGTTGGTGAGGTAATGGCTCACCAA
GGCGACGATCAGTAGCTGGTCTGAGAGGATGATCAGCCACACTGGGACTGAGACACGGCC
>Zotu5213
GAACGCTGGCGGCGTGGCTAAGACATGCAAGTCGCACGGTGTAGCAATACACAGTGGCGAACGGGAGAG
TAATGAATCGCTAATGTACCCCGGACTCTGGGATAGCTATCCGAAAGGGTAGGTAATACCAGATGAGACCA
CGGCAGGGCATCCTGCTGTGCTCAAAGGTCCGCCGGTCTGGGAGCAGGCGATTTCGTATCAGCTAGTTGGT
GAGGTAACGGCTCACCAAGGCGACGACGCGTAGCCGGACTGAGAGGTTGGCCGGCCTCATTGGGACTGA
GACACTGCC
>Zotu5215
GAACGCTGGCGGCGTGCTTAACACATGCAAGTCGAACGGAAAGGCCCGCTTGCGGGTACTCGAGTGGCGA
ACGGGTGAGTAACACGTGGGCAACCTGCCCCCGGCTTTGGGATAACACAAAGAAATTTGTGCTAATACCG
AATATTGACAACTTGAGGCATCTTTTGTTGTTGAAAGTTTTTTCGGCTGGGGATGGGCCCGCGGCCTATCAG
CTTGTTGGTGGGGTAATGGCCTACCAAGGCGACGACGGGTAGCCGGCCTGAGAGGGCGACCGGCCACAC
TGGAACTGAGACACGGTC
>Zotu5218

GAACGCTGGCGGCGTGCTTAACACATGCAAGTCGAACGATGAAGCTCCAGCTTGCTGGAGTGGATTAGTG
GCGAACGGGTGAGTAACACGTGAGTAACCTGCCCTTGACTCTGGGATAAGCCTTGGAAACGAGGTCTAAT
ACCGGATAGGAGCACTCATCGCATGGTGGGTGTTGGAAAGAATTTCGGTCATGGATGGACTCGCGGCCTA
TCAGCTTGTTGGTGAGGTAATGGCTCACCAAGGCGACGACGGGTAGCCGGCCTGAGAGGGTGACCGGCC
ACACTGGGACTGAGACACGGCC
>Zotu5221
CAACGCTGGCGGCGTGCCTAATGCATGCAAGTCGTGCGGGCTGCCTTCGGGCAGTCACGCGGCGGACGGG
TGCGGAACACGTGGGCAACCTGCCCTGGGGCGGGGGATATTCCCCGGAAACGGGGAGTAATACCGCATA
CGCTCGATCCTCCGTGGGAGGGTTGAGGAAAGGCGAAAGCCACCCCTGGAGGGGCCTGCGGCCGATTAG
CTAGACGGCGGGGTAACGGCCCACCGTGGCTGTGATCGGTAGCTGGTCTGAGAGGACGGCCAGCCACAC
GGGGACTGAGACACGGCC
>Zotu5223
GAACGCTGGCGGCAGGCTTAACACATGCAAGTCGAACGGGCACCTTCGGGTGTCAGTGGCAGACGGGTG
AGTAACACGTGGGAACGTACCCTTCGGTTCGGAATAACTCAGGGAAACTTGAGCTAATACCGGATACGCCC
TTACGGGGAAAGGTTTACTGCCGAAGGATCGGCCCGCGTCTGATTAGCTAGTTGGTGGGGTAATGGCCTA
CCAAGGCGACGATCAGTAGCTGGTCTGAGAGGATGATCAGCCACACTGGGACTGAGACACGGCC
>Zotu5225
GAACGCTGGCGGCGTGCCTAATACATGCAAGTCGAGCGAACGGACAGGAGTGCTTGCACTCCTTGAAGTT
AGCGGCGGACGGGTGAGTAACACGTGGGTAACCTACCTTTAAGACTGGGATAACTTCGGGAAACCGGAGC
TAATACCGGATAATATTTTCCACCTCATGGTGGAATAGTGAAAGACGGTTTCGGCTGTCACTTACAGATGG
ACCCGCGGCGCATTAGCTAGTTGGTGAGGTAACGGCTCACCAAGGCGACGATGCGTAGCCGACCTGAGAG
GGTGATCGGCCACACTGGGACTGAGACACGGCC
>Zotu5229
TAACGCTAGCGGCAGGCCTAATACATGCAAGTCGAGGGGTAACAGGGTAGCAATACCGCTGACGACCGGC
GCACGGGTGAGTAACGCGTATGTAACCTACCTATAACTGGGGAATAGCCTTCCGAAAGGAAGATTAACACC
GCATAATATCACGAGACGGCATCGTTTTGTGATCAAACCTCCGGGGGTTATAGATGGACATGCGTAACATT
AGCTAGTTGGTGAGGTAACGGCTCACCAAGGCGACGATGTTTAGGGGGTCTGAGAGGATGATCCCCCACA
CTGGTACTGAGACACGGAC
>Zotu5231
GAACGCTGGCGGCGTGCTTAACACATGCAAGTCGAACGAGGATGTTGGCTTCGGCTGACATTCGAGTGGC
GGACGGGTGAGTAACGCGTGAGCAATCTGTCTCAGAGAGGGGGATAACAACTGGAAACAGTTGCTAATAC
CGCATAAGACCACGAAGGGGCATCCCTTTGAGGTAAAAGCAGCAATGCGCTTTGAGGTGAGCTCGCGTCC
CATTAGATAGTTGGTGAGGTAACGGCCCACCAAGTCAGCGATGGGTAGCCGACCTGAGAGGGTGACCGGC
CACATTGGGACTGAGACACGGCC
>Zotu5233
GAACGCTAGCGGCAGGCTTAATACATGCAAGTCAAGGGGGTAGCAATACAACCGGCGCACGGGTGAGTA
ACGCGTACACAACATACCATATACTGGGGTATAGCTTTGGGAAACTGAAGGTAATCCCCCATAACATTGCG
AGAGGGCATCTGATTGCAATTAAAGTTTCGACGGTATATGATTGGTGTGCGTTTGATTAGCTAGTTGGTAG
GGTAACGGCCTACCAAGGCGACGATCAATAGCTGGCGTGAGAGCGCGACCAGCCACACTGGAACTGAGAC
ACGGTC
>Zotu5236
GAACGCTAGCGGCAGGCTTAATACATGCAAGTCGAACGGCAAGGATTCTTCGGAATCCCTAGAGTGGCGC
ACGGGTGAGTAACACGTACACAACATGCCCTATATTGGGAGATAGCATCGGGAAACTGGTGGTAATACCC
CATAGTGTTTATGATAGGCATCTATTTTAAACTAAAGATTTATTGATATAGGATTGGTGTGCGTCCCATTAG
CTAGTTGGTAGGGTAACGGCCTACCAAGGCGACGATGGGTAGCTGGCGTGAGAGCGCGACCAGCCACACT
GGAACTGAGACACGGTC
>Zotu5240
GAACGCTGGCGGCATGCTTAACACATGCAAGTCGAACGGCAGCACAAACTAGTTTACTAGTTGGGTGGCG
AGTGGCGAACGGGTGAGTAACGCGTAGGAACATGCCTTAAAGAGTGGGACAACCTGGGGAAACTCAGGC
TAATACCGCATAACCTCCGAGGAGAGAAGCTGGGGACCTTAGGGCCTGGCGCTTTAAGATTGGCCTGCGT

CTGATTAGCTAGTTGGTGGGGTAAGGGCCTACCAAGGCGACGATCAGTAGCTGGTCTGAGAGGATGACCA
GCCACACTGGAACTGAGACACGGTC

>Zotu5242

GAACGCTGGCGGCGTGCCTAATACATGCAAGTCGAGCGGATCTTTTCCTTCGGGGAAAGGTTAGCGGCGG
ACGGGTGAGTAACACGTGGGTAACCTGCCCATAAGACTGGGATAACATTCGGAAACGAATGCTAATACCG
GATACGCGAATTGGTCGCATGGCCGAATCGGGAAAGGCGGAGTAATCTGCCACTTATGGATGGACCCGCG
GCGCATTAGCTAGTTGGTGAGGTAACGGCTCACCAAGGCGACGATGCGTAGCCGACCTGAGAGGGTGATC
GGCCACACTGGGACTGAGACACGGCC

>Zotu5246

GAACGCTAGCGGCAGGCCTAATACATGCAAGTCGAGGGGTATTGTAGCAATACGAGAGACCGGCGCACG
GGTGCGTAACGCGTATGAAAGGTGTCCAAATCACTAGGATAGCCCTCGGAAACGGGGATTAATACTGGAT
GGTCTGTAGGGGTGGCATCATCTTTACGGTAAAGCTCAGGCGGATTTGGGTCCTCATGCGTACGATTAGCT
AGTTGGCGAGGTAACGGCTCCCCAAGGCGACGATCGTTAGGGGTCCTGAGAGGGAGATCCCCCACACTGG
TACTGAGACACGGGC

>Zotu5248

GAACGCTGGCGGCGTGCTTAACACATGCAAGTCGAGTGCGCGCCCGTAGCAATACGGGTGGCGCACGGC
GCACGGGTGCGTAACACGTGGGCAATCTGCCCTCTGATGGGGAATAACCCGCCGAAAGGCGGGCTAATAC
CGCATAACATTCCGAGAACTCTGGTTTTTGGATTCAAAGGCGTAAGTCGTCAGAGGAGGAGCCCGCGCAC
GATTAGCTAGTTGGCAGGGTAACGGCCTACCAAGGCTTTGATCGTTAGCTGGTCTGAGAGGATGGCCAGC
CACACTGGAACTGAGACACGGTC

>Zotu5251

GAACGCTGGCGGCGTGCCTAATACATGCAAGTCGAACGAGAAAGTGTAGCAATACATTAGTAAAGTGGCA
GACGGGTGAGTAATACATAGGTAACCTCCCCTCGAGATGGGAATAACCTTCCGAAAGGAGGGCTAATACC
AAATGAGACCACGGGAACTAAGGTTCCTGAGGTTAAAGATGACCTCTATTTATAAGTTATCGCTTGAGGAT
GGGCTTATGCACCATCAGCTAGTTGGTAAGGTAACGGCTTACCAAGGCTTTGACGGTTAGCTGATCTGAGA
TGTTGATCAGCCACACTGGAACTGAGACACGGTC

>Zotu5253

GAACGCTGGCGGCGTGCCTAATACATGCAAGTCGAGCGAATTTTTGGGAGCTTGCTCCTAAAAGTTAGCGG
CGGACGGGTGAGTAACACGTGGGCAACCTACCTATAAGACTGGGATAACTTCGGGAAACCGGAGCTAATA
CCGGATAATTCATATTTACTCATGTAGATATGCTGAAAGACGGTTTACGCTGTCACTTATAGATGGGCCCGC
GGCGCATTAGCTAGTTGGTGAGGTAACGGCTCACCAAGGCGACGATGCGTAGCCGACCTGAGAGGGTGAT
CGGCCACACTGGGACTGAGACACGGCC

>Zotu5259

GAACGCTGGCGGCATGCCTTACACATGCAAGTCGAACGGCAGCATAGGAGCTTGCTCCTGATGGCGAGTG
GCGAACGGGTGAGTAATATATCGGAACGTACCCAAGAGTGGGGGATAACGTAGCGAAAGTTACGCTAATA
CCGCATACGATCTACGGATGAAAGTGGGGGATCGCAAGACCTCATGCTCCTGGAGCGGCCGATATCTGATT
AGCTAGTTGGTGGGGTAAAAGCTCACCAAGGCGACGATCAGTAGCTGGTCTGAGAGGACGACCAGCCACA
CTGGGACTGAGACACGGCC

>Zotu5263

GAACGCTGGCGGCGTGGATAAGGCATGCAAGTCGAGTGGGTTTAGGCCCACGGCAAACGGGTTAGTAAT
GCATAGGAATTTTCCCTGAAGTCGTGAATAATCTGGAGAAATCCAGGATAATACACGATGTGCTCTACGGA
GTAAAGATTTATCGCTTCAGGAGAAGCCTATGTCCTATCAGCTAGTTGGTAGTGTAATGGACTACCAAGGC
TATGACGGGTAGGGGGTGTGAGAGCACGGCCCCCAACATTGGGACTGAGAACTGCC

>Zotu5265

GAACGCTGGCGGCGTGCTTAACACATGCAAGTCGAACGAAGCACTTTTTATAAAGTTTTCGGATGGAGTAA
AAAGTGACTGAGTGGCGGACGGGTGAGTAACGCGTGGGTAACCTGCCTTATACAGGGGGATAACAGTTG
GAAACGACTGCTAATACCGCATAAGCGCACAATGCTGCATGGCATCGTGTGAAAAGATTTATCGGTATAAG
ATGGACCCGCGTCTGATTAGCTGGTTGGTGAGGTAACGGCCCACCAAGGCGACGATCAGTAGCCGGCTTG
AGAGAGTGAACGGCCACATTGGGACTGAGACACGGCC

>Zotu5268

GAACGCTGGCGGCGTGCTTAACACATGCAAGTCGAGCGGTAAGGCCCCTTCGGGGGTACACGAGCGGCG
AACGGGTGAGTAACACGTGAGTAATCTGCCCTTCACTTCGGGATAACCGCCGGAAACGGTGGCTAATACC
GGATACGACGCACTCTCGCATGAGATGTGCGTGGAAAGTCTTCTCGGTGAAGGATGTGCTCGCGGCCTATC
AGCTTGTTGGTGGGGTAACGGCCTACCAAGGCTTCGACGGGTAGCCGGCCTGAGAGGGTGACCGGCCACA
CTGGGACTGAGACACGGCC
>Zotu5270
GAACGCTGGCGGCGTGCCTAACACATGCAAGTCGAACGAGGTCCATGAGAGCTTGCTCTCGGACGACCTA
GTGGCGAACGGGTGAGTAACACGTGAGAAACCTGCCTCGGAGTGGGGAATAACTCGGGGAAACTCGAGC
TAATACCGCATACCTTCAGATGATCGCATGGTCGACTGAAGAAAGAATTTCGCTCTGAGAGGGTCTCGCGG
CCTATCAGCTAGTTGGTGGGGTAACGGCCTACCAAGGCAACGACGGGTAGCTGGATTGAGAGATCGATCA
GCCACACTGGAACTGAGACACGGTC
>Zotu5272
GAACGCTGGCGGCGTGCCTAATACATGCAAGTCGAGCGAACTTGCGGGAGCTTGCTCCCAAAAGTTAGCG
GCGGACGGGTGAGTAACACGTGGGCAACCTACCTATAAGACTGGGATAACTTCGGGAAACCGGAGCTAAT
ACCGGATAATTCTTTTCTACACATGTGGAAAAGCTAAAAGACGGTTTACGCTGTCACTTATAGATGGGCCCG
CGGCGCATTAGCTAGTTGGTGAGGTAACGGCTCACCAAGGCCACGATGCGTAGCCGACCTGAGAGGGTGA
TCGGCCACACTGGGACTGAGACACGGCC
>Zotu5274
GAACGCTGGCGGCGTGCCTAACACATGCAAGTCGAACGAGAAAGCCGCAAGGTGAGTAAAGTGGCGGAC
GGGTGAGTAACGCGTAGGTAATCTACCTTTTAGTGGGGAATAGCCATTGGAAACGATGGATAATACCGCA
TAAGATTTATGGTTGCAAAACTGTAAAAGAAAGGGGGGTCGCAAGATCTTTCGCTAGAAGATGAGCCTGC
GTCCCATTAGCTAGTTGGTGGGGTAAGAGCCTACCAAGGCAACGATGGGTAGCTGGTCTGAGAGGATGAT
CAGCCACACTGGAACTGACACACGGTC
>Zotu5277
GAACGCTGGCGGCGTGCTTAACACATGCAAGTCGAACGCTGAACTCCGCTTGCGGAGGGATGAGTGGCGA
ACGGGTGAGTAACACGTGGGCAACCTGCCCCCGGCTCTGGGATAACTCCAAGAAATTGGGGCTAATACCG
GATGGTCACCGGCTACCGCATGGTGGTGGGTGGAAAGGATTTCCGGCTGGGGATGGGCCCGCGGCCTATC
AGCTTGTTGGTGGGGTAGTGGCCTACCAAGGCGACGACGGGTAGCCGGCCTGAGAGGGTGACCGGCCAC
ACTGGGACTGAGACACGGCC
>Zotu5279
GAACGCTGGCGGCGTGCTTAACACATGCAAGTCGAACGGTGAAAGAGGGAGCTTGCTCCTTCTGGATCAG
TGGCGAACGGGTGAGTAACACGTGAGTAACCTGCCCTTGACTCTGGGATAAGCGTTGGAAACGACGTCTA
ATACCGGATACGACCTTCGGAGGCATCTCCTGAAGGTGGAAAGAATTTCGGTCAAGGATGGACTCGCGGC
CTATCAGCTAGTTGGTGAGGTAATGGCCCACCAAGGCGACGACGGGTAGCCGGCCTGAGAGGGTGACCG
GCCACACTGGGACTGAGACACGGCC
>Zotu5281
CAACGCTGGCGGCGCGCTTAACACATGCAAGTCGAACGAGAAAGGGGAGCAATCCCTGAGTAAAGTGGC
GTACGGGTGAGTAACACGTGGGTAATCTACCTTTTGGTGGGGAATAACGCTGGGAAACCGGCGCTAACAC
CGCATAAGCTCGAAAGAGGAAAAGAGAAATCTGCCGAAAGAGGAGCCCGCGGCTGATTAGCTAGTTGGT
GGGGTAAAGGCTCACCAAGGCTTTGATCGGTAGCCGGCCTGAGAGGGCACACGGCCACACTGGCACTGAA
ACACGGGC
>Zotu5285
GAACGCTAGCGGCAGGCCTAATACATGCAAGTCGAGGGGCAGCGGGAATAGCAATATTTGCCGGCGACC
GGCGCACGGGTGCGTAACACGTATACAATCTGCCTATAACAGGGGGATAGCCCGGAGAAATCCGGATTAA
CCCCCCATAACAATAGAAGAAGCATTTCTTTTATTTGAAAACTACGGTGGTTATAGATGAGTATGCGTATGA
TTAGCTAGTTGGTAGTGTAATGGACTACCAAGGCGACGATCATTAGGGGTACTGAGAGGTTAAACCCCCAC
ACGGATACTGAGACACGGAT
>Zotu5289
CAACGCTGGCGGCGTGCCTCAGACATGCAAGTCGAACGATTAAAGCTCTCTTCGGAGAGTGCATAGAGTG
GCGCACGGGTGAGTAACACGTAAGTAATCTACCCTCGAGTGGGGAATAACGTCCGGAAACGGACGCTAAT
ACCGCATAATGCAGCGGCACCGCAAGGTGACAGTTGTTAAAGATTTATCGCTTGAGGAGGAGCTTGCGGC

AGATTAGCTAGTTGGTAAGGTAATGGCTTACCAAGGCTACGATCTGTAACCGGTCTAAGAGGACGGTCGG
TCACACTGACACTGAATAACGGGT

>Zotu5291

GAACGCTGGCGGCATGCCTTACACATGCAAGTCGAACGGTAACAGGTTAAGCTGACGAGTGGCGAACGG
GTGAGTAATATATCGGAACGTGCCCAGTTGTGGGGGATAACTACTCGAAAGAGTGGCTAATACCGCATAC
GACCTGAGGGTGAAAGCGGGGGATCGCAAGACCTCGCGCAATTGGAGCGGCCGATATCAGATTAGGTAG
TTGGTGGGGTAAAGGCTCACCAAGCCAACGATCTGTAGCTGGTCTGAGAGGACGACCAGCCACACTGGGA
CTGAGACACGGCC

>Zotu5294

GAACGCTGGCGGCGTGCTTAACACATGCAAGTCGAACGGTGAACCCTTCGGGGGGATCAGTGGCGAACG
GGTGAGTAACACGTGAGCAACCTGCCCTTGGCTCTGGGACAACCACTGGAAACGGTGGCTAATACCGGAT
ACGACCCATGCAGGCATCTGTTGTGGGTGGAAAGATTTTTCGGCTGGGGATGGGCTCGCGGCCTATCAGCT
TGTTGGTGGGGTAATGGCCTACCAAGGCGACGACAGGTAGCCGGCCTGAGAGGGCGACCGGCCACACTG
GGACTGAGACACGGCC

>Zotu5296

CAACGCTGGCGGCGTGCCTAACACATGCAAGTCGAACGAGAAAGTGGAGCAATCCATGAGTAAAGTGGCG
ACCGGGTGAGTAACACGTGACTAACCTACCCTAGAGTGGGGAATAACCTCGGGAAACCGGGGCTAATACC
GCATAACATCGCGAGGTGTAAATTCCTTGTGATCAAAGGAGCAATTCGCTCAAGGAGGGGGTCGCGGCTG
ATTAGCTAGTTGGTGAGGTAACGGCCCACCAAGGCTAAGATCAGTATCCGGCCTGAGAGGGCGCACGGAC
ACACTGGAACTGAAACACGGTC

>Zotu5300

GAACGCTGGCGGCATGCCTAACACATGCAAGTCGAACGAGATTATTGACTTCGGTTGATAGTCTAGTGGCG
CACGGGTGCGTAACGCGTGGGAATCTGCCCTTAGGTTCGGAATAACTCAGAGAAATTTGAGCTAATACCG
GATAATGACGTAAGTCCAAAGATTTATCGCCTAGGGATGAGCCCGCGTAAGATTAGCTAGTTGGTGGGGT
AAAGGCCTACCAAGGCGACGATCTTTAGCTGGTCTGAGAGGATGATCAGCCACACTGGGACTGAGACACG
GCC

>Zotu5302

GAACGCTGGCGGCGTGCTTAACACATGCAAGTCGAACGAGACAATTAAAAGCTTGCTTTTAAACGTCGAGT
GGCAAACGGGTGAGTAACGCGTAGACAACCTGCCGCAAAGATGGGGACAACAGTTCGAAAGGACTGCTA
ATACCGAATGTTCTGGAAGTTCCGCATGGAAACTCCAGCAAAGATGGCCTCTACTTGTAAGCGATCGCTTT
GCGATGGGTCTGCGTCTGATTAGCTAGTTGGTGGGGCAAGGGCCTACCAAGGCGACGATCAGTAGCCGGT
CTGAGAGGATGAACGGCCACATTGGGACTGAGACACGGCC

>Zotu5304

GAACGCTGGCGGCATGCCTAACACATGCAAGTCGAACGAGACCTTCGGGTCTAGTGGCGCACGGGTGCGT
AACGCGTGGGAATCTGCCCTTGGGTTCGGAATAACAGTTAGAAATGACTGCTAATACCGGATGATGTCTTC
GGACCAAAGATTTATCGCCCAAGGATGAGCCCGCGTAGGATTAGCTAGTTGGTGAGGTAAAGGCTCACCA
AGGCGACGATCCTTAGCTGGTCTGAGAGGATGATCAGCCACACTGGGACTGAGACACGGCC

>Zotu5306

AAACGCTGGCGGCGTGCTTAACACATGCAAGTCGAACGAAGTTTTTCTTTCGGGAGAAACTTAGTGGCGGA
CGGGTGAGTAACGCGTGGGTAACCTGCCCTGTACAGGGGGACAACAGCTGGAAACGGCTGCTAATACCGC
ATAAGCCCTTAGCACTGCATGGTGCATAGGGAAAAGGAGTAATCCGGTACAGGATGGACCCGCGTCTGAT
TAGCCAGTTGGCAGGGTAACGGCCTACCAAAGCGACGATCAGTAGCCGATCTGAGAGGATGTACGGCCAC
ATTGGGACTGAGACACGGCC

>Zotu5308

GAACGCTGGCGGCATGCCTTACACATGCAAGTCGAACGGCAGCACGGAGCTTGCTCTGGTGGCGAGTGGC
GAACGGGTGAGTAATATATCGGAACGTACCCAAGAGTGGGGGATAACGTAGCGAAAGTTACGCTAATACC
GCATACGATCTAAGGATGAAAGTGGGGGATCGCAAGACCTCATGCTCCTGGAGCGGCCGATATCTGATTA
GCTAGTTGGTGAGGTAAAGGCTCACCAAGGCATCGATCAGTAGCTGGTCTGAGAGGACGACCAGCCACAC
TGGAACTGAGACACGGTC

>Zotu5312

GAACGCTGGCGGCATGCCTAACACATGCAAGTCGAACGAAGCCTTCGGGTTTAGTGGCGCACGGGTGCGT
AACGCGTGGGAATCTGCCCTTTGGTTCGGAATAACAGTTGGAAACGACTGCTAATACCGGATGATGACGA
AAGTCCAAAGATTTATCGCCAGAGGATGAGCCCGCGTAGGATTAGCTAGTTGGTGTGGTAAAGGCGCACC
AAGGCGACGATCCTTAGCTGGTCTGAGAGGATGATCAGCCACACTGGGACTGAGACACGGCC
>Zotu5315
GAACGCTAGCGGCAGGCCTAATACATGCAAGTCGGACGGGATTTTGGAGCTTGCTTCAAATGAGAGTGGC
GCACGGGTGCGTAACACGTATGTAACCTACCTTGTTCAGGGGGATAGCCTCTCGAAAGAGAGATTAAGAC
CGCATAAAATCACAATGTGGCATCACAAAATGATCAAATATTTATAGGAACAAGATGGGCATGCGGAACAT
TAGCTAGTTGGTAAGGTAACGGCTTACCAAGGCTACGATGTTTAGGGGATCTGAGAGGATGACCCCCCAC
ACTGGTACTGAGACACGGAC
>Zotu5317
GAACGCTGGCGGCGTGCTTAACACATGCAAGTCGAACGGTGAAGGACGGAGCTTGCTCCGACCGGATCAG
TGGCGAACGGGTGAGTAACACGTGAGTAACCTGCCCCAGACTCTGGAATAACCCCGGGAAACCGGAGCTA
ATACCGGATACGAAGTGGAGCCGCATGGCTACCACTTGGAAAGTTTTTCGGTCTGGGATGGACTCGCGGC
CTATCAGCTAGTTGGTGAGGTAACGGCTCACCAAGGCGACGACGGGTAGCCGGCCTGAGAGGGCGACCG
GCCACACTGGGACTGAGACACGGCC
>Zotu5321
GAACGCTAGCGGCAGGCTTAATACATGCAAGTCGAGGGGCAGCACAGATAGCAATATTGGGTGGCGACC
GGCAAACGGGTGCGGAACACGTACACAACCTTCCTTTCGGTGGGGGATAGCCTTTGGAAACGAAGATTAA
TACCCCATAAAACATATTTACCGCATGGTATGATATGTTAAAGATTTATTGCCGGAAGATGGGTGTGCGTAT
GATTAGGTAGTTGGTAGGGTAATGGCCTACCAAGCCGACGATCATTAACTGGTGTGAGAGCACGACCAGT
CACACGGGCACTGAGACACGGGC
>Zotu5323
GAACGCTGGCGGCGTGCCTAATACATGCAAGTCGAACGAAGTTATGAGAGAGCTTGCTCTCGATTAGCTTA
GTGGCGGACGGGTGAGTAACACGTAGGCAACCTGCCCCTAAGATCGGGATAACATTCGGAAACGAATGCT
AAGACCGGATAAACAATCTGCCCGCATGGGCGGATTGGGAAACACGGAGCAATCTGTGGCTTAGGGATG
GGCCTGCGGCGCATTAGCTAGTTGGTGGGGTAACGGCTCACCAAGGCGACGATGCGTAGCCGACCTGAGA
GGGTGAACGGCCACACTGGGACTGAGACACGGCC
>Zotu5325
CAACGCTGGCGGCGTGCCTAACACATGCAAGTCGCACGAGAAAGGGGCTTCGGCCCTGAGTACAGTGGCG
CACGGGTGAGTAACACGTGAATTATCTACCTCCGAGTGGGGAATAACTGAGAGAAATCTTAGCTAATACCG
CATAACACTTACGAGTCAAAGCAGCAATGCGCTTGGAGAGGAGTTCGCGGCAGATTAGTTAGTTGGCGGG
GTAATGGCCCACCAAGACGATGATCTGTATCCGGCCTGAGAGGGCGCACGGACACACTGGAACTGAAACA
CGGTC
>Zotu5329
AAACGCTGGCGGCGCACATAAGACATGCAAGTCGAACGGAAGTCGTTGTAATGAAATAGAAACGGACAG
AGAACTTGTTCGAAGAAAGTGGAAGTAGACTTACAACAATGGCTTTAGTGGCGGACTGGTGAGTAACGCG
TAAGGAACCTGCCTTCTAGAGGGGAATAACAATGAGAAATCATTGCTAATACCGCATATGCCGAGAGAATC
ACATGGTACAATCGGGAAAAGAGTAATCTGCTAGAAGATGGCCTTGCGTCTGATTAGATAGTTGGTGGGG
TAACGGCCTACCAAGTCGACGATCAGTAGCCGGACTGAGAGGTTGAACGGCCACATTGGGACTGAGATAC
GGCC
>Zotu5331
GAACGCTGGCGGCAGGCCTAACACATGCAAGTCGAACGGATGTAGCAATACATTAGTGGCGCACGGGAG
AGTAACGCGTGGGAACCTACCTTTTGGTTTGGGATAACATCGGGAAACTGGTGCTAATACCGGATAAGCCC
TTCGGGGGAAAGATTTATCGCCGAAAGATGGGCCCTCGTTGGATTAGCTAGTTGGTGAGGTAACGGCTCA
CCAAGGCTACGATCCATAGCTGGTCTGAGAGGATGATCAGCCTCACTGGGACTGAGACACGGCC
>Zotu5333
GAACGCTGGCGGCGTGCCTAAGACATGCAAGTCGAGTGGGTTTAGACCCACGGCGAACGGGTGAGTAAT
ACCTAGGAACTTTCTTTAAGTGGAGCATAACCAGTCGAAAGATTGGATAATACTCCATGCCATCGCAAGAT
GAAAGTCGCAAGACGCTTAAAGTGAGGCCTGGGCACCATCAGCTAGTTGGTAAGGTAATGGCTTACCAAG
GCTATGACGGTTAGGGGTCCCGAGAGGGAGAACCCCCACACTGGAACTGCGACACGGTC

>Zotu5335
GAACGCTGGCGGCAGGCTTAACACATGCAAGTCGAACGCATCCTTCGGGGTGAGTGGCAGACGGGTGAG
TAACGCGTGGGAACGTGCCCTTCAGTTCGGGATAACCCAGGGAAACTTGGGCTAATACCGGATACGTGCG
AGAGCAGAAAGATTTATCGCTGAAGGATCGGCCCGCGTCTGATTAGCTAGTTGGTGAGGTAATGGCTCAC
CAAGGCGACGATCAGTAGCTGGTCTGAGAGGATGATCAGCCACACTGGGACTGAGACACGGCC
>Zotu5338
GAACGCTGGCGGTATGCTTAACACATGCAAGTCGAACGGTGTCTTCGGACACAGTGGCGGACGGGTGAGT
AACGCGTGAGAATCTGGCTTTAGGTCTGGGACAACCACTGGAAACGGTGGCTAATACCGGATGTGCCGAG
AGGTGAAAGGTTAACTGCCTGAAGATGAGCTCGCGTCTGATTAGCTAGTTGGTGGTGTAATGGACTACCAA
GGCGACGATCAGTAGCTGGTCTGAGAGGACGATCAGCCACACTGGGACTGAGACACGGCC
>Zotu5341
GCCGTGTCTCAGTCCCAGTGTGGCTGATCGTCCTCTCAGACCAGCTACGGATCGTCGCCTTGGTAGGCCATT
ACCCTACCAACTAGCTAATCCGACGCAGGCTCATCTCATAGCACGAGGTCCGAAGATCCCCCGCTTTGCTTT
GAAGAGATTATGCGGTATTAGCCCGAGTTTCCCCGGGTTATCCCCCACTAACTATTATAATTAAGGTAGATT
CCTACGTGTTACTCCCCCGTCCGCCACTCGCCGTCAGGGAGCAAGCTCCCCCCGCTGCCGTTCGACTTGCAT
GTGTTAGGCATGCCGCTAGCGTTCA
>Zotu5343
GAACGTTGGCGGTGCGCTTAACACATGCAAGTCGAGCGAGAAAGATCTTCGGATTGAGTACAGCGGCAGA
CGGGTGAGTAACGCGTGGGAATCTACCTTCTAGTGAGGAATACCTCTGAGAAATCGGAGTTAATACTGCAT
AAGTCCCTTCGGGGAGAAAGATGGCCTCTTTGCTGTCGCTAGAAGACGAGCCTGCGTACTATTAGCTAGTT
GGTGGGGTAAAGGCCTACCAAGGCAATGATGGTTAACCGGCCTGAGAGGGTGATCGGTCACATTGGAACT
GAGATACGGTC
>Zotu5345
GAACGCTGGCGGCGTGCTTAAGACATGCAAGTCGAACGGCCTTCTTCGGAAGGCAGTGGCGCACGGGTGA
GTAACGCGTAACTGACCTACCCTCAAGTCTGGAATAACGGGTCGAAAGATCCGCTAATACTGGATGTGCAG
TGTCGCTGTGGCGACACTGTAAAGATTGATCGCTTGAGGATGGGGTTGCGTTTCATCAGCTGGTTGGTGGG
GTAAAGGCCTACCAAGGCGACGACGAATAGCCGGCCTGAGAGGGTGGCCGGCCACAGGGGCACTGAGAC
ACGGGT
>Zotu5347
GAACGCTGATGGCGTGCCTCATACATGCAAGTCGAGCGCTGTAGCAATACGGAGCGGCGGACGGGTGAG
TAACACGTAAACAATCTGCCCTCAAGTGGGGAATAACTATCCCAACGGATAGCTAATACCGCATAAGGATG
GAGGTGGCATCACCTCTTCTCGAAAGGCTTCGGCCGCTTAAGGATGAGTTTGCGTCCCATTAGCTTGTTGG
CGGGGTAACGGCCCACCAAGGCTACGATGGGTATCCGGCCTGAGAGGGTGTACGGACACATTGGGACTG
AGATACGGCC
>Zotu5351
GAACGCTAGCGGCAGGCCTAATACATGCAAGTCGGACGGGATTGGAGAGCTTGCTTTCCATGAGAGTGGC
GCACGGGTGCGTAACACGTATGTAACCTACCTTAATCTGGGGGATAGCCTCTCGAAAGAGGGATTAACACC
GCATAACATGTTTGAATAGCATTATTTAAACATCAAATATTTATAGGATTAAGATGGGCATGCGGAACATTA
GCTAGTTGGTAAGGTAATGGCTTACCAAGGCTACGATGTTTAGGGGATCTGAGAGGATGACCCCCCACACT
GGTACTGAGACACGGAC
>Zotu5354
CGTCAGGTCCCAGTGCGCTTGGTTAATTACACCTTAGAAGACCTTTCCCTGCCGCGAAAAAGGGTAGTAGC
GCAATTCATCCCGGCGAACATTACAGAAAGCGGACAAACGGAGAGTATCGGACTAACAGGA
>Zotu5356
GAACGCTGGCGGCGTGCCTAATACATGCAAGTCGAGCGGATCTTTAGGAGCTTGCTCCTAAAGATTAGCG
GCGGACGGGTGAGTAACACGTGGGCAACCTACCTGTAAGACTGGGATAACTTCGGGAAACCGGAGCTAAT
ACCGGATAATTTCTTTCTCCACATGGAGAAAGGTTGAAAGACGGCTCACGCTGTCACTTACAGATGGGCCC
GCGGCGCATTAGCTAGTTGGTGAGGTAACGGCTCACCAAGGCAACGATGCGTAGCCGACCTGAGAGGGT
GATCGGCCACACTGGGACTGAGACACGGCC
>Zotu5358

GAACGCTGGCGGCGTGCTTAACACATGCAAGTCGAACGCTGAAGCCTGGTGCTTGCACTGGGTGGATGAG
TGGCGAACGGGTGAGTAACACGTGAGTAACCTGCCCTTGACTTCGGGATAAGCCCGGGAAACTGGGTCTA
ATACTGGATATGACTCCTTACCGCATGGTGGGGGGTGGAAAGATTTATCGGTTGGGGATGGACTCGCGGC
CTATCAGTTTGTTGGTGGGGTAATGGCCTACCAAGACGACGACGGGTAGCCGGCCTGAGAGGGTGACCGG
CCACACTGGGACTGAGACACGGCC
>Zotu5360
GAACGCTGGCGGCACGCTTAACACATGCAAGTCGAACGGAGAATTTTGATTTATTGAAATTCTTAGTGGCG
GACGGGTGAGTAACACGTGAGCAACCTGCCTCTGAGAGAGGAATAATTTCTGGAAACGGATTGTAATACC
TCATAACATACAGTTATCGCATGGTGATTGTATCAAAGATTTATCACTCAGAGATGGGCTCGCGTCTGATTA
GATAGTTGGTGAGGTAACGGCTCACCAAGTCGACGATCAGTAGCCGGACTGAGAGGTTGAACGGCCACAT
TGGGACTGAGACACGGCC
>Zotu5362
GAATGCTGGCGGCGTGCCTAACACATGCAAGTCGAGCGGCAGCGCGAATAGTTTACTATTTGGCGGCGAG
CGGCGGACGGCTGAGTAACGCGTGGGAATTTGCCCCAAAGTGAGGAATAACTGCCCGAAAGGGTAGCTA
ATACCGCATATGGTCTTCGGATTAAAGCTTTCGAGCGCTTTGGGAGAAGCCCGCGTCGGATTAGATAGTTG
GTGAGGTAATGGCTCACCAAGTCGACGATCCGTAGCTGGTCTGAGAGGATGATCAGCCAGACTGGAACTG
AGACACGGTC
>Zotu5364
GAATGCTGGCGGCGCGCCTAACACATGCAAGTCGAGCGGTAACAGGGGTAATTTATTACCTGCTGACGAG
CGGCGGACGGCTGAGTAACGCGTGGGAACGTACCCCAAAGTGAGGAATAACTGCCCGAAAGGGTGGCTA
ATGCCGCATATGATCTTCGGATTAAAGGATTTATCCGCTTTGGGAACGGCCTGCGTAAGATTAGATAGTTG
GTGAGGTAAAGGCTCACCAAGTCAACGATCTTTAACTGGTCTGAGAGGATGACCAGTCAGACTGGAACTG
AGACACGGTC
>Zotu5367
GAACGCTGGCGGCAGGCTTAACACATGCAAGTCGAACGCCCCGCAAGGGGAGTGGCAGACGGGTGAGTA
ACGCGTGGGAATCTACCTTTTGCTACGGAATAACTCAGGGAAACTTGTGCTAATACCGTATGTGCCCTTCGG
GGGAAAGATTTATCGGCAAAGGATGAGCCCGCGTTGGATTAGCTAGTTGGTGGGGTAAAGGCCTACCAAG
GCGACGATCCATAGCTGGTCTGAGAGGATGATCAGCCACATTGGGACTGAGACACGGCC
>Zotu5369
GAACGCTGGCGGCAGGCCTAACACATGCAAGTCGAGCGAGGACTTCGGTCCTAGCGGCGGACGGGTGAG
TAACGCGTGGGAACGTGCCCTTTGCTTCGGAATAGCCCTGGGAAACTGGGAGTAATACCGAATGTGCCCTT
AGGGGGAAAGATTTATCGGCAAAGGATCGGCCCGCGTTGGATTAGGTAGTTGGTGGGGTAACGGCCTACC
AAGCCGACGATCCATAGCTGGTTTGAGAGGATGATCAGCCACACTGGGACTGAGACACGGCC
>Zotu5371
GAACGTTAGCGGCGCGCTTAATACATGCAAGTCGTGCGAGAAAGGGCTTCGGCCCCGGTACAGCGGCGCA
CGGGTGAGTAACACGTAGATCATCTCCCCTCGAGTGGTGGATAACCCTCCGAAAGGAGGGCTAATACAGC
ATGAGACCACGACCTCGCTGAGGTTGCGGCCAAAGTAGGCCTCTACTTGTAAGCTTACGCTTGAGGATGAG
TCTGCGGCCCATCAGCTAGTTGGTAAGGTAACGGCTTACCAAGGCGAAGACGGGTAGCTGGTCTGAGAGG
ATGAACAGCCACACTGGAACTGAGACACGGTC
>Zotu5373
AGTAGATTAAGTGTGATTACTACCGTCAAATATAGCGAGCGTCTCAGGGTCTGGCAACGTTCGTTTTTCTCC
GTCATGGCGGAACCCATGCGGTTAGAGGTTTCGATGTATGGGCCCTATGGCGAGCTTTTCCGCTAGAGTCA
GTATATTAACCCTATGGACACGACGATGCACGCGGCGACAGCAGACGACGATACACCACCGACAAGCCGA
GACACTAAGGTGCTTCGCCCAAAATAAACAGGTGGTGTACACACTTGTGTGCATGACGCATCAAGAGG
>Zotu5376
GAACGCTGGCGGCAGGCCTAACACATGCAAGTCGAGCGGGGGTAGCAATACCCTAGCGGCGAACGGGTG
AGGAATGCTTGGGAATCTGCCTGGTAGTGGGGGATAACGTTCCGAAAGGAACGCTAATACCGCATACGTC
CTACGGGAGAAAGGGGGGGATCTTCGGACCTCTCGCTATCAGATGAGCCCAAGCGGGATTAGCTAGTTGG
TGAGGTAAAGGCTCACCAAGGCGACGATCCCTAGCTGGTCTGAGAGGATGATCAGCCACACTGGAACTGA
GACACGGTC

>Zotu5379
GAACGCTAGCGGCAGGCTTAACACATGCAAGTCGAGGGGCAGCGGGAGTAGCAATACTTGCCGGCGACC
GGCGGAAGGGTGCGTAACGCGTGAGCAACATTCCCGTATCAGGGGGATAACCGGTGGAAACGCCGACTA
ATACCCCATAAGAACATCTGCGGCATCAAAGGTGTTTGAAAGATTCGTCGGATGCGGATTGGCTCGCGTGA
CATTAGCTAGTTGGTGAGGTAACGGCCCACCAAGGCGACGATGTCTAGGGGTTCTGAGAGGAAGATCCCC
CACATTGGAACTGAGACACGGTC
>Zotu5381
GAACGCTAGCGGGAGGCTTAACACATGCAAGCCGAGGGGTATATAATTGCTTGCAATTATAGAGACCGGC
GGATGGGTGAGTAACGCGTATGTAACTTACCCTATTCATTGGGATAGCCCGAGGAAACTCGGATTAATACC
AGATAACACTAAGGTCGGCATTGACTTTAGTTGAAAGATTTATCGGAATAGGATAGACATGCGTCAGATTA
GCTAGTTGGTGAGATAGAGGCTCACCAAGGCAATGATCTGTAGGGGGCCTGAGAGGGTGATCCCCCACAC
TGGTACTGAGACACGGAC
>Zotu5383
GAACGCTGGCGGCGTGCTTAACACATGCAAGTCGAACGATGAAGGGGGAGCTTGCTTCCCTGGATTAGTG
GCGAACGGGTGAGTAACACGTGAGCAATCTGCCCTTGACTCTGGGATAAGCGTTGGAAACGACGTCTAAT
ACCGGATATGAGCTGTGAAGGCATCTTCAACAGCTGGAAAGAACTTCGGTCAAGGATGAGCTCGCGGCCT
ATCAGGTAGTTGGTGAGGTAATGGCTCACCAAGCCTACGACGGGTAGCCGGCCTGAGAGGGTGACCGGCC
ACACTGGGACTGAGACACGGCC
>Zotu5385
GAACGCTGGCGGCGTGCTTAACACATGCAAGTCGAACGGGATCCATCCAGTGGCAACACTGGTGAAGATC
TAGTGGCGAACGGGTGAGTAACACGTGAGAAACCTGTCCCGGTCTCTGGAATAACAGTGGGAAACCACTG
CTAATACCGGATACCTTCACTGCGTCGCATGGCGCGGTGAAGAAAGGCTTGCCGGATTGGGAGGGTCTCG
CGGCCTATCAGCTAGTTGGTGGGGTAACGGCCTACCAAGGCGTCGACGGGTAGCTGGTCTGAGAGGACGA
TCAGCCACACTGGGACTGAGACACGGCC
>Zotu5387
GAACGCTGGCGGCGTGCTTAACGCATGCAAGTCGAACGGTGAACCTGCTTCGGTGGGGGATCAGTGGCGA
ACGGGTGAGTAACACGTGGGCAACCTGCCCTCAGCTCTGGGATAACTCCAAGAAATTGGGGCTAATACCG
GATATGACCGCTGACCGCATGGTCTGGTGGTGGAAAGATTTATCGGCTGGGGATGGGCCCGCGGCCTATC
AGCTTGTTGGTGGGGTAGTGGCCTACCAAGGCGACGACGGGTAGCCGGCCTGAGAGGGTGACCGGCCAC
ACTGGGACTGAGACACGGCC
>Zotu5390
GAACGCTAGCGGGAGGCTTAATACATGCAAGTCGTACGGTAACAGGTGTAGCAATACATGCTGACGAGTG
GCGCACGGGTGAGGAACACGTACACAATCTGCCTTTGACTGGGGGATAGCCTTGGGAAACTAAGATTAAT
ACCCCATAATATTACGATGTGGCATCACATTGTAATTAAACCTCCGGGGGTCAAAGATGAGTGTGCGTCTG
ATTAGGTAGTTGGTAGGGTAACGGCCTACCAAGCCAACGATCAGTAGCTGGCGTGAGAGCGCGACCAGCC
ACACGGGAACTGAGACACGGTC
>Zotu5393
GAACGCTGGCGGCATGCTTTACACATGCAAGTCGAACGGCAGCGCGGGGCAACCTGGCGGCGAGTGGCG
AACGGGTGAGTAATATATCGGAACGTACCCAAGAGTGGGGGATAACGTAGCGAAAGTTACGCTAATACCG
CATACGATCTACGGATGAAAGTGGGGGATCGCAAGACCTCATGCTCCTGGAGCGGCCGATATCTGATTAG
CTAGTTGGTAGGGTAATGGCCTACCAAGGCGACGATCAGTAGCTGGTCTGAGAGGACGACCAGCCACACT
GGAACTGAGACACGGTC
>Zotu5395
GAACGCTAGCGACAGGCCTAACACATGCAAGTCGAGGGGCAACAGGAAGTAGCAATACTTTGCTGGCGAC
CGGCGCACGGGTGAGTAACGCGTATGCAACCTGCCTACTACAGGGGGATAACCCGTCGAAAGACGGCCTA
ATACCGCATAATACAGGTTTACTGCATGGTAAATTTGTTAAAGAACTTCGGTAGTAGATGGGCATGCGTTCC
ATTAGCTAGTTGGTGAGGTAACGGCTCACCAAGGCAACGATGGATAGGGGAACTGAGAGGTCGATCCCCC
ACACTGGAACTGAGACACGGAC
>Zotu5397

GAACGCTAGCGATAGGCCTAACACATGCAAGTCGAGGGGCAGCACATGAAGTAGCAATACGGATGGTGG
CGACCGGCGCACGGGTGAGTAACGCGTATGCAATCTACCTACAACAAGGGAATAACTCGTTGAAAGACGG
ACTAATACCCTATAAAACAGGGGTCCCGCATGGGAATATTTGTTAAAGATTTATCGGTTGTAGATGAGCAT
GCGTTCCATTAGCTTGTTGGTGAGGTAACGGCCCACCAAGGCAACGATGGATAGGGGAACTGAGAGGTTT
ATCCCCCACACTGGTACTGAGACACGGAC
>Zotu5399
CAACGCTGGCGGCGTGCCTAACACATGCAAGTCGAACGCGAAAGTGGAGCAATCCATGAGTAGAGTGGCA
AACGGGTGAGTAACGCGTGGGTGACCTACCTCCGAGTGGGGGATAACGTCTCGAAAGGGACGCTAATACC
GCATAACATCCCGTCTTTTAAGAGATGGCGATCAAAGCCTGGGACCGCAAGGCCAGGCGCTTGAAGAGGG
GCCCGCGTCTGATTAGCTAGTTGGTGGGGTAATGGCTCACCAAGGCAACGATCAGTATCCGGCCTGAGAG
GGCGGACGGACACACTGGGACTGAGACACGGCC
>Zotu5401
GAACGCTAGCGGCAGGCTTAATACATGCAAGTCGTGGGGCAGCGCAGTGTAGCAATACATGGGCGGCGA
CCGGCAAACGGGTGCGGAACACGTACACAACCTTCCTTCAAATGGGGAATAGCCCAGGGAAACTTGGATT
AATACCCCGTAATATTATGGAGTGGCATCACTTTATAATTATAGCTCCGGCGTTTGAAGATGGGTGTGCGG
CTGATTAGGTAGTTGGCGGGGTAACGGCCCACCAAGCCTTCGATCAGTAACTGGTGTGAGAGCACGACCA
GTCACACGGGCACTGAGACACGGGC
>Zotu5403
GAACGCTGGCGGCGCGCTTAACACATGCAAGTCGAGCGAGAACCATGGCTTCGGCTATGGGGACAGCGG
CGAACGGGTGAGTAACACGTGGGTAATCAACCCTCGACACTGGGATAGCCCGGGGAAACTCGGATTAATA
CCGGATACCCCATCTGGCCTTCGGGCCGGCTGGAAAAGGTAGCTTTGGCCTCCGGTCGAGGACGAGCCCG
CGGCGGATTAGCTTGTTGGTGGGGTAAAAGCCCACCAAGGCGACGATCCGTAGCTGGTCTGAGAGGACGA
TCAGCCACACTGGGACTGAGACACGGCC
>Zotu5406
GAACGCTGGCGGCGTGCTTAACACATGCAAGTCGAGCGGTAAGGCCCTTCGGGGTACACGAGCGGCGAA
CGGGTGAGTAACACGTGGGTGATCTGCCCTGTACTTCGGGATAAGCCTGGGAAACTGGGTCTAATACCGG
ATATGACTCCAGGATGCATGTCTTGGGGTGGAAAGATTTATCGGTACAGGATGGGCCCGCGGCCTATCAG
CTTGTTGGTGGGGTAATGGCCTACCAAGGCGACGACGGGTAGCCGACCTGAGAGGGTGACCGGCCACACT
GGGACTGAGACACGGCC
>Zotu5409
GAACGCTGGCGGCGTGCCTAATACATGCAAGTCGAGCGAATTTTTGGGAGCTTGCTCCCAAAAATTAGCGG
CGGACGGGTGAGTAACACGTGGGCAACCTGCCTGTAAGACTGGGATAACTTCGGGAAACCGGAGCTAATA
CCAGATAATTCTTATCAACTCATGTTGATAAGCTGAAAGACGGTTTCGGCTGTCACTTACAGATGGGCCCGC
GGCGCATTAGCTAGTTGGTGAGGTAACGGCTCACCAAGGCAACGATGCGTAGCCGACCTGAGAGGGTGAT
CGGCCACACTGGGACTGAGACACGGCC
>Zotu5411
GAACGCTGGCGGCAGGCCTAACACATGCAAGTCGAACGGCAGCACAGGAGAGCTTGCTCTCTGGGTGGC
GAGTGGCGGACGGGTGAGGAATACATCGGAATCTACCTTGTCGTGGGGGATAACGTAGGGAAACTTACG
CTAATACCGCATACGACCTACGGGTGAAAGCAGGGGACCTTCGGGCCTTGCGCGATTGGATGAGCCGATG
TCGGATTAGCTAGTTGGCGGGGTAAAGGCCCACCAAGGCGACGATCCGTAGCTGGTCTGAGAGGATGATC
AGCCACACTGGAACTGAGACACGGTC
>Zotu5413
GAACGCTGGCGGCATGCTTTACACATGCAAGTCGAACGGTAACAGGTCTTCGGATGCTGACGAGTGGCGA
ACGGGTGAGTAATACATCGGAACGTGCCTAGTAGTGGGGGATAACTACTCGAAAGAGTAGCTAATACTGC
ATGAGATCTACGGATGAAAGCAGGGGACCTTCGGGCCTTGTGCTACTAGAGCGGCTGATGGCAGATTAGG
TAGTTGGTGGGGTAAAAGCTTACCAAGCCTGCGATCTGTAGCTGGTCTGAGAGGACGACCAGCCACACTG
GGACTGAGACACGGCC
>Zotu5416
GAACGCTAGCGGCAGGCTTAATACATGCAAGTCGAGGGGCAGCACGGGTAGCAATACCTGGTGACGACC
GGCAAACGGGTGCGGAACACGTACGCAACCTTCCCATAACTGGTGAATAGCCCTCCGAAAGGAGGATTAA
TACACCGTAACATAATGAAGTGGCATCACTTTATTATTATAGGCAGCAATGTCGGTTTTGGATGGGCGTGC

GCCTGATTAGGTAGTTGGCGGGGTAACGGCCCACCAAGCCTGCGATCAGTAACTGGTGTGAGAGCACGAC
CAGTCACACGGGCACTGAGACACGGGC
>Zotu5418
GAACGCTGGCGGCATGCTTTACACATGCAAGTCGAACGGCAGCGGGGAGGAGCTTGCTTCTCTGCCGGCG
AGTGGCGAACGGGTGAGTAATACATCGGAACGTGTCGAGAAGAGGGGGATAACCACCCGAAAGGGTGGC
TAATACCGCATAAATTCTGAGGAAGAAAGCTGGGGACCTAGGAATAGGCCTAGCGCTTTTTGAGCGGCCG
ATGTCGGATTAGCTAGTTGGTGAGGTAAAGGCTCACCAAGGCGAAGATCTGTAGCGGGTCTGAGAGGATG
ATCCGCCACACTGGGACTGAGACACGGCC
>Zotu5420
GAACGCTAGCGGCAGGCTTAACACATGCAAGTCGAGGGGTATAGTAGCAATACTAGAGACCGGCGCACG
GGTGCGTAACGCGTATGCAATCTACCTTTTGCAGGGGAATAGCCCAGAGAAATTTGGATTAATGCCCCATA
GTATATAGAGTTGGCATCAACATTATATTAAAGTCACAACGGCAAAAGATGAGCATGCGTCCCATTAGCTA
GTTGGTAAGGTAACGGCTTACCAAGGCGACGATGGGTAGGGGTCCTGAGAGGGAGATCCCCCACACTGGT
ACTGAGACACGGAC
>Zotu5423
GAACGCTGGCGGCATGCCTAACACATGCAAGTCGAACGGACATCTTCGGATGTTAGTGGCGCACGGGTGC
GTAACGCGTGGGAATCTACCCCTGGGTTCGGAATAACGGTTGGAAACGACTGCTAATACCGGATGATGAC
GTAAGTCCAAAGATTTATCGCCCAGGGATGAGCCCGCGTAGGATTAGGTAGTTGGTGTGGTAAAGGCGCA
CCAAGCCGACGATCCTTAGCTGGTCTGAGAGGATGATCAGCCACACTGGGACTGAGACACGGCC
>Zotu5426
GAACGCTGGCGGCATGGATAAGGCATGCAAGTCGAGTGGGTTTAGACCCACGGCAAACGGGTTAGTAAT
GCGTAGGAATGTCCCTCAAAGTCGTGAATAATCCGTCGAAAGGCGGAAGAATACACGATGTGCTCCACGG
AGTAAAGATTTATCGCTTTGAGAGCAGCCTGCGTCCTATCAGCTAGTTGGTGGGGTAAAAGCCTACCAAGG
CTTTGACGGGTAGGGGGTGTGAGAGCATGGCCCCCAACATTGGGACTGAGACACTGCC
>Zotu5429
GAACGCTGGCGGCGTGCCTAAGACATGCAAGTCGAGTGGGTTTAGACCCACGGCGAACGGGTGAGTAAT
ACCTAGGAACTTTCTCTAAGTGGAGCATAACCAGTCGAAAGATTGGATAATACTTCATGTGACCGCAAGGT
TAAAGTCGCAAGACGCTTAAAGTGAGGCCTGGGCACCATCAGCTAGTTGGTAAGGTAATGGCTTACCAAG
GCTATGACAGTTAGGGGTCCCGAGAGGGATAACCCCCACACTGGAACTGCGACACGGTC
>Zotu5431
GAACGCTGGCGGCAGGCCTAACACATGCAAGTCGAGCGCCCCGCAAGGGGAGCGGCAGACGGGAGAGTA
ACACGTGGGAACGTACCCTTCAGTACGGAACAACCAAGGGAAACTTTGGCTAATACCGTATACGTCCGTAA
GGAGAAAGATTTATCGCTGAAGGATCGGCCCGCGTCTGATTAGCTAGTTGGTGAGGTAACGGCTCACCAA
GGCGACGATCAGTAGCTGGTCTGAGAGGATGATCAGCCTCATTGGGACTGAGACACGGCC
>Zotu5433
GAACGCTAGCGGCAGGCTTAACACATGCAAGTCGAGGGGTATGGTTCTTCGGAACCAGAGACCGGCGCAC
GGGTGCGTAACGCGTATGCAATCTACCTTTTACAGAGGGATAGCCCAGAGAAATTTGGATTAATACCTCAT
AGTATATAGAGTTGGCATCAACACTATATTAAAGTTCCAACGGTAAAAGATGAGCATGCGTCCCATTAGCT
AGATGGTAAGGTAACGGCTTACCATGGCAACGATGGGTAGGGGTCCTGAGAGGGAGATCCCCCACACTG
GTACTGAGACACGGAC
>Zotu5435
GAACGCTGGCGGCATGCCTAACACATGCAAGTCGAACGAGACCTTCGGGTCTAGTGGCGCACGGGTGCGT
AACGCGTGGGAACCTGCCCTTAGGTTCGGAATAACTCAGAGAAATTTGAGCTAATACCGGATAATGTCTTC
GGACCAAAGATTTATCGCCTTTGGATGGGCCCGCGTAGGATTAGCTAGTTGGTGGGGTAATGGCCTACCAA
GGCGACGATCCTTAGCTGGTCTGAGAGGATGATCAGCCACACTGGGACTGAGACACGGCC
>Zotu5437
GAACGCTGGCGGCGTGCCTAACACATGCAAGTCGAACGGGATCCATGAGAGCTTGCTCTCGGAAGATCTA
GTGGCGAACGGGTGAGTAACACGTGAGAAACCTGCCTCGGAGTGGGGAATAACTCGGGGAAACTCGAGC
TAATACCGCATACCTTCAGATGATCGCATGGTCGACTGAAGAAAGAATTTCGCTCTGAGAGGGTCTCGCGG

CCTATCAGCTAGTTGGTGGGGTAACGGCCTACCAAGGCAACGACGGGTAGCTGGATTGAGAGATCGATCA
GCCACACTGGAACTGAGACACGGTC
>Zotu5440
GAACGCTGGCGGCAGGCCTAATACATGCAAGTCGAACGGCAGCACAGCAGAGCTTGCTCTGTGGGTGGCG
AGTGGCGGACGGGTGAGGAATACATGGGAATCTGCCCAGTCGTGGGGGATAACGTAGGGAAACTTACGC
TAATACCGCATGCGCCCTACGGGGGAAAGCAGGGGATCTTCGGACCTTGTGCGATTGGATGAGCCCATGT
TCGATTAGCTAGTTGGCGGGGTAACGGCCCACCAAGGCGACGATCGATAGCTGGTCTGAGAGGATGATCA
GCCACACTGGAACTGAGACACGGTC
>Zotu5443
GAACGCTAGCGGCGGGCTTAATACATGCAAGTCGAGCGGTAACAGGTGTAGCAATACATGCTGACGAGCG
GCAGACGGGTGAGGAACACGTACGCAACCTCCCTCCAACTGGGGCACAACTCCGTGAAAACGGGGCTAAT
TCCCCATAACATTGCGAAAGGGCATCTGATTGCAATTAAAGATTCGTCGGTTGGAGATGGGCGTGCGACTG
ATTAGCCAGTTGGTAGGGTAACGGCCTACCAAAGCAACGATCAGTAGCTGGTGTGAGAGCACGACCAGCC
ACACGGGCACTGAGATACGGGC
>Zotu5445
GAACGCTGGCGGCGTGCTTAACACATGCAAGTCGAACGAAGCGCTTAGATCTCTTCGGAGTGATGAGTGA
CTGAGTGGCGGACGGGTGAGTAACGCGTGGGTAACCTGCCTCGTACAGGGGGACAACAGTTGGAAACGA
CTGCTAATACCGCATAAGCGCACGGTATCGCATGATACAGTGTGAAAAGCTGAGGCGGTACGAGATGGAC
CCGCGTTGGATTAGCTAGTTGGTGGGGTAACGGCCTACCAAGGCGACGATCCATAGCCGACCTGAGAGGG
TGACCGGCCACATTGGGACTGAGACACGGCC
>Zotu5448
GAACGCTGGCGGCATGCTTTACACATGCAAGTCGAACGGCAGCGCGGGGCAACCTGGCGGCGAGTGGCG
AACGGGTGAGTAATATATCGGAACGTACCCAGAAGTGGGGGATAACGTAGCGAAAGTTACGCTAATACCG
CATACGATCTACGGATGAAAGTGGGGGACCTTCGGGCCTCATGCTTTTGGAGCGGCCGATATCTGATTAGC
TAGTTGGTGAGGTAAAGGCTCACCAAGGCGACGATCAGTAGCTGGTCTGAGAGGACGACCAGCCACACTG
GGACTGAGACACGGCC
>Zotu5450
GCCGTGTCTCAGTCCCAGTGTGGCTGGTCGTCCTCTCAGACCAGCTACTGATCGTCGCCTTGGTGAGCTTTT
ACCCCACCAACTAGCTAATCAGATATCGGCCGCTCCAGGAGCATGAGGTCTTGCGATCCCCCACTTTCATCC
GTAGATCGTATGCGGTATTAGCTAGTCTTTCGACTAGTTATCCCCCACTCTAGGGCACGTTCCGATATATTAC
TCACCCGTTCGCCACTCGCCATCAGGAGCAAGCTCCTATGCTGCCGTTCGACTTGCATGTGTAAGGCATGCC
GCCAGCGTTCA
>Zotu5452
GAACGCTGGCGGCGTGCCTAATACATGCAAGTCGAGCGGTAAGGCTACTTCGGTAGTACACGAGCGGCGG
ACGGCTGAGTAACACGTAGGAACATACCCCAAACTGAGGGATAACTGCTCGAAAGAGTAGCTAATACCGC
ATGTGCTCTTCGGAGTAAAGCTACGGCGGTTTGGGAATGGCCTGCGTAAGATTAGCTAGTTGGTGAGGTA
ATAGCTCACCAAGGCGACGATCTTTAGCTGGTCTGAGAGGATGATCAGCCAGACTGGGACTGAGAACGGC
C
>Zotu5454
GAACGCTGGCGGCGTGCCTAACACATGCAAGTCGAGCGGCAGCGCGGGAGCAATCCTGGCGGCGAGCGG
CGGACGGGTGAGCAATGCTTGGGAATCTGCCCATCAGTGGGGGACAACCCGGGGAAACTCGAGCTAATAC
CGCATACGTTCTTTGGAAGAAAGCAGGGGACCGCAAGGCCTTGCGCTGATGGATGAGCCCAAGTCGGATT
AGCTAGTTGGTGAGGTAACGGCTCACCAAGGCGACGATCCGTAGCTGGTCTGAGAGGACGACCAGCCACA
CCGGAACTGAGACACGGTC
>Zotu5458
GAACGCTGGCGGCGTGCCTAATACATGCAAGTCGAGCGAATCTTTGGGAGCTTGCTCCCAAAGATTAGCG
GCGGACGGGTGAGTAACACGTGGGCAACCTGCCTATAAGACTGGGATAACTTCGGGAAACCGGAGCTAAT
ACCGGATAATTCATATCTACTCATGTAGGTATGCTGAAAGACGGTTTACGCTGTCACTTATAGATGGGCCCG
CGGCGCATTAGCTAGTTGGTGAGGTAACGGCTCACCAAGGCAACGATGCGTAGCCGACCTGAGAGGGTGA
TCGGCCACACTGGGACTGAGACACGGCC

>Zotu5460
GAACGCTGGCGGCATGCTTAACACATGCAAGTCTCACGGGCAGCAATGTCAGTGGCGGACGGGTGAGTAA
CGCGTAGGTATCTATCCTGAGGTGGGGGACAACCGTGGGAAACTACGGCTAATACCGCATAATGCCTGAG
GGCCAAAGGCGCGAGTCGCCACAGGAGGAGCCTGCGTACGATTAGCTAGTTGGTGGGGTAAAGGCCTAC
CAAGGCGACGATCGTTAGCTGGTCTGAGAGGATGATCAGCCACACTGGGACTGAGACACGGCC
>Zotu5463
GAACGCTAGCGGCAGGCCTAATACATGCAAGTCGGACGGGATTGAGGAGCTTGCTTCTCATGAGAGTGGC
GCACGGGTGCGTAACACGTATGTAACCTACCTTCGTCAGGGGGATAGCCTCTCGAAAGAGAGATTAAGAC
CGCATAACATTGAGACACAGCATTGTGATTCAATCAAATATTTATAGGACGAAGATGGGCATGCGGAACAT
TAGCTAGTTGGTAAGGTAACGGCTTACCAAGGCTACGATGTTTAGGGGATCTGAGAGGATGACCCCCCAC
ACTGGTACTGAGACACGGAC
>Zotu5465
GAACGCTGGCGGCATGCCTAACACATGCAAGTCGAACGAGACCTTCGGGTCTAGTGGAGCACGGGTGCGT
AACGCGTGGGAATCTGCCCTTCGGTTGGGAATAACTCAGGGAAACTTGAGCTAATACCTTATAATGTCTTC
GGACCAAAGATTTATCGCCGAGGGATGAGCCCGCGTAGGATTAGCTAGTTGGTGGGGTAATGGCCTACCA
AGGCAACGATCCTTAGCTGGTCTGAGAGGATGATCAGCCACACTGGGACTGAGACACGGCC
>Zotu5468
GAACGCTGGCGGCGTGCTTAACACATGCAAGTCGAGCGGTAAGGCTCCTTCGGGAGTACACGAGCGGCGA
ACGGGTGAGTAACACGTGAGCAATCTGCCCTTCTCATCGGAATAACCATTGGAAACGATGGCTAATGCCGA
ATATGACCTCGGATCACATGTTCTGAGGTGGAAAGCTCCGGCGGAGAAGGATGAGCTCGCGGCCTATCAG
CTTGTTGGTGAGGTAATGGCTCACCAAGGCAATGACGGGTAGCCGGCCTGAGAGGGTGACCGGCCACACT
GGGACTGAGACACGGCC
>Zotu5470
GAACGCTGGCGGCGTGCCTAATACATGCAAGTCGAGCGGTGAAGCACCTTCGGGTGTGGATCAGCGGCGA
ACGGGTGAGTAACACGTGGGCAACCTGCCCTCAAGCCTGGGATAACTCCGGGAAACCGGGGCTAACACCA
GATAATCTTCTTGGACGCATGTGTGAGAAGTAAAAGTTTACACTTGAGGATGGGCCCGCGGTGCATTAGCT
AGTTGGTGGGGTAACGGCCTACCAAGGCGACGATGCATAGCCGACCTGAGAGGGTGACCGGCCACACTG
GGACTGAGACACGGCC
>Zotu5473
GAACGCTGGCGGCGTGCCTAACACATGCAAGTCGAGCGGGGATGAGGGAGCTTGCTTCCGAATCATAGCG
GCGGACGGGTGAGTAACGCGTGAGCAACCTGCCTCTTACAGGGGGATAACGCATCGAAAGATGTGCTAAT
ACCGCGTAAGACCACAGTACCGCATGGTACAGGGGTCAAAGGAGCAATCCGGTAAGAGATGGGCTCGCG
TCCGATTAGCTAGTTGGTGAGATAAAAGCCCACCAAGGCGACGATCGGTAGCCGACCTGAGAGGGTGATC
GGCCACATTGGAACTGAGAAACGGTC
>Zotu5475
AAACGCTGGCGGCGTGCATAACACATGCAAGTCGAACGATGAAGATTAAATGATTTCTTCGGAATGAACTT
AATTGGATTAGTGGCGGACGGGTGAGTAACGCGTGAGTAACCTGCCTTGTACAAGGGGATAGCCGTTGGA
AACGACGAATAATACCCTATGACATCAAATAATCGCATGATAATTTGATCAAAGATTTATCGGTATAAGATG
GACTCGCGTCTGATTAGCTAGTTGGTAGGGTAAAAGCCTACCAAGGCGACGATCAGTAGCCGGCTTGAGA
GAGTGTACGGCCACATTGGGACTGAGACACGGCC
>Zotu5478
GAACGCTAGCGACAGGCCTAACACATGCAAGTCGAGGGGTAACAGGGAGCTTGCTCCGCTGACGACCGGC
GCACGGGTGAGTAACGCGTATGCAACCTGCCTATTACAGGGGGATAACCCGGAGAAATTCGGATTAATAC
CGCATAACACTATTGAGTGGCATCATTTAATAGTTAAAGATTTATTGGTAATAGATGGGCATGCGTTCCATT
AGTTAGTTGGTGAGGTAACGGCTCACCAAGACGTCGATGGATAGGGGTTCTGAGAGGAAGGTCCCCCACA
CTGGTACTGAGACACGGAC
>Zotu5482
GAACGCTGGCGGCGTGCCTAATACATGCAAGTCGAGCGGTAAGGCCACTTCGGTGGTACACGAGCGGCG
GACGGCTGAGTAACGCGTAGGAACATACCCCAAAGTGAGGGATAACTGCTCGAAAGAGTAGCTAATACCG
CATATGGTCTAAGGATTAAAGCTTCGGCGCTTTGGGAATGGCCTGCGTATGATTAGTTTGTTGGTGAGGTA

ATGGCTCACCAAGACTACGATCATTAGCTGGTCTGAGAGGATGATCAGCCAGACTGGGACTGAGACACGG
CC
>Zotu5485
GAACGCTGGCGGCGTGCCTAACACATGCAAGTCGAGCGCGATTTATGAAGACGAAACTTCGGTCTAGGAA
TCATAATGAAAGCGGCGGACGGGTGAGTAACGCGTAGGCAACCTGCCCCAAACACAGGGATAGCCTCGG
GAAACCGGGATTAAAACCTGATAATGCCAAAGAGTCACATGGCTCGTTGGCCAAAGATTTATCGGTTTGGG
ATGGGCCTGCGTCTGATTAGTTTGTTGGTGGGGTAACGGCCTACCAAGACTACGATCAGTAGCCGACCTGA
GAGGGTGATCGGCCACATTGGAACTGAGACACGGTC
>Zotu5487
GAACGCTGGCGGCGTGCCTAAGACATGCAAGTCGAGCGGGGCAGGTTTATACCTGTCCAGCGGCGGACG
GGTGAGTAACGCGTGGGTGACCTACCCGGAAGAGGCGGACAACCTGGGGAAACCCAGGCTAATCCGCCA
TGTGGTCCTGTCCTGTGGGGCAGGACTAAAGGGTGAATAGCCCGCTTCCGGATGGGCCCGCGTCCCATCA
GCTAGTTGGTGGGGTAAAGGCCCACCAAGGCGACGACGGGTAGCCGGTCTGAGAGGATGGCCGGCCACA
GGGGCACTGAGACACGGGC
>Zotu5489
GAACGCTGGCGGCATGCTTAACACATGCAAGTCGAACGAGCTAATGGGTAACTATTAGTATAGTGGCGGA
CGGGTGCGTAACGCGTAAGAACCTACCTTTTGGAGGGGGATAACATCGGGAAACCTTTGGTAATACCCCAT
AATGCTGAGGAGTTAAAGCAGCGAGAGCTGGCCAAAAGAGGGGCTTGCGTCTGATTAGCTAGTTGGTGG
GGGTAAAAGCCTCCCAAGGCCACGATCAGTAGCTGGTCTGAGAGGATGATCAGCCACACTGGGACTGAGA
CACGGCC
>Zotu5495
GAACGCTGGCGGCAGGCTTAACACATGCAAGTCGAGCGGGCGAGGTTGCTTCGGTAACTGAGCTAGCGGC
GGACGGGTGAGTAAAGCTTAGGAATCTGCCTATTAGTGGGGGACAACATTTCGAAAGGGATGCTAATACC
GCATACGTCCTACGGGAGAAAGCAGGGGACCTTCGGGCCTTGCGCTAATAGATGAGCCTAAGTCGGATTA
GCTAGTTGGTGGGGTAAAGGCCTACCAAGGCGACGATCTGTAGCGGGTCTGAGAGGATGATCCGCCACAC
TGGGACTGAGACACGGCC
>Zotu5498
GAACGCTGGCGGCGTGCTTAACACATGCAAGTCGAGCGGAAAGGCCACTTCGGTGGTACTCGAGCGGCGA
ACGGGTGAGTAACACGTGAGTAATCTGCCCCTGGCTTTGGGATAGCCACCGGAAACGGTGATTAATACCG
GATACGACAACTTCTCGCATGGGATGGTTGTGGAAAGTTTTTCGGCCAGGGATGTGCTCGCGGCCTATCAG
CTTGATGGTGAGGTAATGGCTCACCATGGCTTCGACGGGTAGCCGGCCTGAGAGGGTGACCGGCCACACT
GGGACTGAGACACGGCC
>Zotu5502
GAACGCTGGCGGCGTGCCTAATACATGCAAGTCGAGCGAATCAAAGGGAGCTTGCTCCCTTTTGATTAGCG
GCGGACGGGTGAGTAACACGTGGGCAACCTGCCTATAAGACTGGGATAACTTCGGGAAACCGGAGCTAAT
ACCGGATAATTCTTTTCTACACATGTAGGAAAGCTGAAAGACGGTTTACGCTGTCACTTATAGATGGGCCC
GCGGCGCATTAGCTAGTTGGTGAGGTAACGGCTCACCAAGGCGACGATGCGTAGCCGACCTGAGAGGGT
GATCGGCCACACTGGGACTGAGACACGGCC
>Zotu5504
GAACGCTGGCGGCGTGCTTAACACATGCAAGTCGAACGATGATCAGGAGCTTGCTCCTGTGATTAGTGGC
GAACGGGTGAGTAACACGTGAGTAACCTGCCCCTGACTCTGGGATAAGCGTTGGAAACGACGTCTAATAC
TGGATACGACTACTGGCCGCATGGTCTGGTGGTGGAAAGATTTTTTGGTTGGGGATGGACTCGCGGCCTAT
CAGCTTGTTGGTGAGGTAATGGCTCACCAAGGCGACGACGGGTAGCCGGCCTGAGAGGGTGACCGGCCA
CACTGGGACTGAGACACGGCC
>Zotu5507
GAACATTGGCGGTAGGCTTAACACATGCAAGTCGAGCGAGGCGTAATAGTCGAGCGGCGAACGGGTGAG
TAATACATGGAAATCTACCCTGAAGTGGGGGATAACGTTCAGAAATGAACGCTAATACCGCATAATCCATA
TTGGGAAAGCGTTGCAGCGCTTTAGGATGAGTCCATGGCTGATTAGCTTGTTGGTGAGGTAATGGCTCACC
AAGGCGACGATCAGTAGCTGGTCTGAGAGGATGACCAGCCACACTGGAACTGAGACACGGTC
>Zotu5510

GAACGCTGGCGGCGTGCTTAACACATGCAAGTCGAACGGTGAACTCAGCTTGCGGGGGGATCAGTGGCG
AACGGGTGAGTAACACGTGGGCAACCTGCCCTCAGCTCTGGGATAACTCCAAGAAATTGGGGCTAATACT
GGATATGACCGCTGACCGCATGGTCTGGTGGTGGAAAGATTTATCGGCTGGGGATGGGCCCGCGGCCTAT
CAGCTTGTTGGTGGGGTAGTGGCCTACCAAGGCGACGACGGGTAGCCGGCCTGAGAGGGTGACCGGCCA
CACTGGGACTGAGACACGGCC
>Zotu5514
GAACGCTGGCGGCGCGCCTAAAACATGCAAGTCGAACGAGAAGCCTGGGCTTGCCCGGGTGGACAGTGG
CGAACGGGTGAGTAACACGTGAGGAACCTGCCCCGAAGACCGGGACAACACCGGGAAACCGGTGCTAAT
ACCGGATACCTTCACCGAATCGCATGGTCTGGTGAAGAAATGGATTCCGCTTCGGGATGGCCTCGCGGCCT
ATCAGCTTGTTGGTGAGGTAATGGCTCACCAAGGCGACGACGGGTAGCTGGTCTGAGAGGATGTCCAGCC
ACACTGGGACTGAGACACGGCC
>Zotu5517
GAACGCTGGCGGCATGCCTTACACATGCAAGTCGAACGGCAGCACGGGTGCTTGCACCTGGTGGCGAGTG
GCGAACGGGTGAGTAACGCATCGGAACGTGCCTTTTAGTGGGGGATAACTATCCGAAAGGATAGCTAATA
CCGCATACGCCCTGAGGGGGAAAGGGGGGGATCGCAAGACCTCTCGCTAAAAGAGCGGCCGATGTCCGA
TTAGCTAGTTGGTGAGGTAAAGGCTCACCAAGGCGACGATCGGTAGCTGGTCTGAGAGGATGATCAGCCA
CACTGGAACTGAGACACGGTC
>Zotu5519
GAACGCTGGCGGCGTGCCTAATACATGCAAGTCGAGCGAATCTTTGGGAGCTTGCTCCCTATAGATTAGCG
GCGGACGGGTGAGTAACACGTGGGTAACCTGCCTGTAAGACTGGGATAACTTCGGGAAACCGGAGCTAAT
ACCGGATAGTTTCTTTTCTCGCATGAGAAGAGATGGAAAGATGGTTCTGCTATCACTTACAGATGGACCCG
CGGCGCATTAGCTAGTTGGTGAGGTAACGGCTCACCAAGGCGACGATGCGTAGCCGACCTGAGAGGGTG
ATCGGCCACACTGGGACTGAGACACGGCC
>Zotu5521
GAACGCTGGCGGCATGCCTAACACATGCAAGTCGAACGAAGGCTTCGGCCTTAGTGGCGCACGGGTGCGT
AACGCGTGGGAATCTGCCCTTAGGTTCGGAATAACAGCTGGAAACGGCTGCTAATACCGGATGAAGACGA
AAGTCCAAAGATTTATCGCCTGAGGATGAGCCCGCGTTGGATTAGGTAGTTGGTGGGGTAAAGGCCTACC
AAGCCGACGATCCATAGCTGGTCTGAGAGGATGATCAGCCACACTGGGACTGAGACACGGCC
>Zotu5523
GATCATTGGCGGCAGGCTTAACACATGCAAGTCGAGCGAGGCTAAGAGCCGAGCGGCGAACGGGTGAGT
AATACTTGGGAATCTACCCTGGAGTGGGGGATAACAACCAGAAATGGTTGCTAATACCGCATAATCCATAT
TGGGAAAGCGCTTAGCGCTCTAGGATGAGCCCAAGTCTGATTAGCTAGTTGGGGGGGTAATGGCCCACCA
AGGCGACGATCAGTAGCTGGTTTGAGAGGATGACCAGCCACACTGGAACTGAGACACGGTC
>Zotu5525
GAACGCTGGCGGCATGCCTAACACATGCAAGTCGAACGATGCCTTCGGGCATAGTGGCGCACGGGTGCGT
AACGCGTGGGAATCTGCCCTTTGGTTCGGGATAACTGTTGGAAACGACAGCTAATACCGGATGATGACGTA
AGTCCAAAGATTTATCGCCAGAGGATGAGCCCGCGTCGGATTAGCTAGTTGGTGGGGTAAAGGCCTACCA
AGGCGACGATCCGTAGCTGGTCTGAGAGGATGATCAGCCACACTGGGACTGAGACACGGCC
>Zotu5531
GAACGCTGGCGGCGTGCTTAACACATGCAAGTCGAACGGTAAGGCCCTTCGGGGTACACGAGTGGCGAAC
GGGTGAGTAACACGTGGGTAATCTGCCCTGCACTTCGGGATAAGCCTGGGAAACTGGGTCTAATACCGGA
TATTCAGTTCCTGCCGCATGGTGGGGGTTGGAAAGTTTTTCGGTGCAGGATGAGCCCGCGGCCTATCAGCT
TGTTGGTGGGGTAATGGCCTACCAAGGCGACGACGGGTAGCCGGCCTGAGAGGGTGATCGGCCACACTG
GGACTGAGACACGGCC
>Zotu5534
GAACGCTGGCGGCAGGCTTAACACATGCAAGTCGAGCGGGCCCTTCGGGGTCAGCGGCAGACGGGTGAG
TAACACGTGGGAACGTGCCCTTCGGTTCGGAATAACTCAGGGAAACTTGAGCTAATACCGGATACGCCCTT
TTGGGGAAAGGTTTACTGCCGAAGGATCGGCCCGCGTCTGATTAGCTAGTTGGTGGGGTAATGGCCCACC
AAGGCGACGATCAGTAGCTGGTCTGAGAGGATGATCAGCCACACTGGGACTGAGACACGGCC
>Zotu5536

GAACGCTGGCGGCAGGCTTAACACATGCAAGTCGAGCGGGGAAGGTTGCTTCGGTAACTGACCTAGCGGC
GGACGGGTGAGTAATGCTTAGGAATCTGCCTATTAGTGGGGGACAACATTCCGAAAGGAATGCTAATACC
GCATACGTCCTACGGGAGAAAGCAGGGGATCATTTGACCTTGCGCTAATAGATGAGCCTAAGTCGGATTA
GCTAGTTGGTGGGGTAAAGGCCTACCAAGGCGACGATCTGTAGCGGGTCTGAGAGGATGATCCGCCACAC
TGGGACTGAGACACGGCC

>Zotu5539

TAACGCTGGCGGCGTGCCTAACACATGCAAGTCGAACGGACCTTTATTTTTGAGACGCCCCATTTCCTTGGA
GCGCCCCATAAATAATGGTTAGTGGCGAACGGGTGAGTAACACGTTGGTACCTGCCCTCTAGTGGGGGAT
AACTGCCCGAAAGGGTAGCTAATACCGCATGTGATCTACGGATCAAAGCAGCAATGCGCTAGAGGAGGGG
CCTGCGGCTGATTAGCTTGTTGGTAGGGTAATGGCCTACCAAGGCGACGATCAGTAGCTGGATCGAGAGG
TTGACCAGCCACACTGGGACTGAGATACGGCC

>Zotu5541

GAACGCTGGCGGCGTGCTTAACACATGCAAGTCGAACGATGACGGTGGTGCTTGCACTGCCTGATTAGTG
GCGAACGGGTGAGTAACACGTGAGTAACCTGCCCTTCACTTCGGGATAACCTCGGGAAATCGTGGCTAATA
CCGTATATGAACTCCTGCCGCATGGTGGGGGTTGGAAAGATTTATCGGTGAAGGATGGACTCGCGGCCTA
TCAGTTTGTTGGTGAGGTAATGGCTCACCAAGACGATGACGGGTAGCCGGCCTGAGAGGGCGACCGGCCA
CACTGGGACTGAGACACGGCC

>Zotu5543

GAACGCTAGCGGGATGCCTTACACATGCAAGTCGAACGGCAGCACGGACTTCGGTCTGGTGGCGAGTGGC
GAACGGGTGAGTAATGTATCGGAACGTGCCTAGTAGCGGGGGATAACTACGCGAAAGCGTAGCTAATACC
GCATACGCCCTACGGGGGAAAGCGGGGGATCGTAAGACCTCGCACTATTAGAGCGGCCGATATCGGATTA
GCTAGTTGGTGAGGTAAAGGCTCACCAAGGCGACGATCCGTAGCTGGTTTGAGAGGACGACCAGCCACAC
TGGGACTGAGACACGGCC

>Zotu5545

GAACGCTGGCGGCGTGCTTAATACATGCAAGTCGAACGAGGTCCTTCGGGACCTAGTGGCGAACGGGTGA
GTAACACGTGAGCAACCTGCCCCGAAGACCGGGATAACAGTGGGAAACCGCTGCTAATACCGGATACCCC
CACCGGATCACATGGTCTGGTGAGGAAATGCTGACTTTCGAGTCATACGCTTCGGGAGGGGCTCGCGGCC
TATCAGCTTGTTGGTGGGGTAACGGCCTACCAAGGCAACGACGGGTAGCTGGTCTGAGAGGACGATCAGC
CACACTGGGACTGAGACACGGCC

>Zotu5548

GAATGCTGGCGGCGTGCTTAACACATGCAAGTCGAGCGGTAAGGCCCCTTCGGGGGTACACGAGCGGCG
GACGGCTGAGTAACGCGTAGGAACATGCCCCAAAGTGAGGAATAAGCTCCAGAAATGGAGTCTAATGCCG
CATATGATCTTCGGATTAAAGGATTTATCCGCTATGGGAGTGGCCTGCGTATGATTAGGTAGTTGGTGAGG
TAAAGGCTCACCAAGCCGACGATCATTAGCTGGTCTGAGAGGATGACCAGCCAGACTGGAACTGAGACAC
GGTC

>Zotu5550

GAACGTTGGCGGCGTGCCTAACACATGCAAGTCGAACGGAAATAGCAATATTTGAGTGGCGCACGGGTGA
GTAACACGTAGCTAATCTACCTCAAAGCGCGGGATAACGTGTCGAAAGACAGGCTAATACCGCATAAGCTC
GCGACCTGTAGAGGGTTGCGAGAAAAGGCGAAAGTCACTTTGAGATGAGGCTGCGGCCCATCAGCTAGTT
GGGGTGGTAAAAGCACACCAAGGCAACGACGGGTAGGGGACCTGAGAGGGTGACCCCCCACACTGGGAC
TGAGACACGGCC

>Zotu5552

GAACGCTAGCGGCAGGCCTAATACATGCAAGCTGAACGGCAAGTTGAATAGCAATATTTGACCTAGAGTA
GCGTAAGGGTGCGTAACACGTATGCAATCTGCCCGCAACAGGAGGATAGCTTGATGAAAGTTGAGATAAG
ACTCCATAAGATCACACGAGGGCATCTGAGGGTGATTAAACTACAAGGGGTTGCGGATGAGCATGCGTCT
GATTAGTTAGTTGGCGGGGTAACGGCCCACCAAGACGATGATCAGTAGGGGAACTGAGAGGTTGGTCCCC
CACACTGGCACTGAGATACGGGC

>Zotu5554

GAACGCTGGCGGCGCGCCTAAAACATGCAAGTCGAACGAGAAACTCGGGCTTGCTCGGGTGGACAGTGG
CGAACGGGTGAGTAACACGTGAGGAACCTGCCCCGAAGACCGGGACAACACCGGGAAACCGGTGCTAAT
ACCGGATGTCCTCATCGGACCGCATGATCTGATGAGGAAATGGATTCCGCTTCGGGATGGCCTCGCGGCCT

ATCAGCTAGTTGGTGGGGTAACGGCCCACCAAGGCGACGACGGGTAGCTGGTCTGAGAGGATGTCCAGCC
ACACTGGGACTGAGACACGGCC
>Zotu5558
GATCGCTGGCGGCGTGCTTAACACATGCAAGTCGAGCGGATTTCTGCAGTGTATCGCAGGAATTAGCGGC
AGACGGGTGAGTAATACTTGGGCAATCTGCCCTTGAGTGGGAAACAACCGGCCGAAAGGCTGGCTAATGT
CCCATAAGACCACAGGATGGCATCATCCAGGGGTCAAAGCTCCGTAAGGGGCGCTTGAGGAGGAGCCTGA
GTCCTATCATGGTAGTTGGTGAGGTAACGGCTCACCAAGCCTTTGACGGGTAGCCGGCCTGAAAGGGTGA
TCGGCCACACTGGCACTGAGACACGGGC
>Zotu5561
CAACGCTGGCGGCGTGCCTAACACATGCAAGTCGAACGCGAAAGTGGAGCAATCCATGAGTAGAGTGGCA
AACGGGTGAGTAACACGTGGGTGACCTGCCTTCGAGTGGGGGATAACGTCCCGAAAGGGACGCTAATACC
GCATGACATCCTGCCTTTGAACAGGCGGAGATCAAAGCCGGGGATCGCAAGACCTGGCGCTTGAAGAGGG
GCCCGCGTCCGATTAGCTAGTTGGTGAGGTAATGGCTCACCAAGGCAACGATCGGTAGCCGGCCTGAGAG
GGCGGACGGCCACACTGGGACTGAGACACGGCC
>Zotu5564
GAACGCTAGCGGCAGGCTTAATACATGCAAGGCGAGGGGGCAGCAATGTCACCGTCGTACGGGTGCGTA
ACGCGTATGCAACCTACCTTCAACTGGGGGATAGCCCGGGGAAACCCGGATTAATACCGCATAATACATTG
GTGTAGCATTACATTATTTGTTAAAGATTTATTGGTTGAAGATGGGCATGCGTTCGATTAGCTAGTTGGCAG
GGTAACGGCCTACCAAGGCGACGATCGATAGGGGAGCTGAGAGGTTGATCCCCCACACGGGCACTGAGA
TACGGGC
>Zotu5566
GAACGCTGGCGGCGTGCTTAACACATGCAAGTCGAACGAATAACCCTTCTTCGGAAGGTTATAGAGTGGC
GAACGGGTGAGTAACACGTGACCAACCTGCCTCTTAGATTGGGATAACTCAGAGAAATTTGAGCTAATACC
GAATACTCCGATATCTTCGCATGGAGATGTCGGGAAAGCTTTTGCGCTAAGAGATGGGGTCGCGGCCTATT
AGATAGTTGGTGGGGTAACGGCCTACCAAGTCAACGATAGGTAGCCGGGTTGAGAGACTGATCGGCCACA
TTGGGACTGAGATACGGCC
>Zotu5569
GAACGCTGGCGGCATGCTTTACACATGCAAGTCGAACGGCAGCGCGGGGCAACCTGGCGGCGAGTGGCG
AACGGGTGAGTAATATATCGGAACGTACCCAGAAGTGGGGGATAACGTAGCGAAAGTTACGCTAATACCG
CATACGATCTACGGATGAAAGTGGGGGACCTTCGGGCCTCATGCTTGTGGAGCGGCCGATATCTGATTAGC
TAGTTGGTGGGGTAAAGGCTCACCAAGGCAACGATCAGTAGCTGGTCTGAGAGGACGACCAGCCACACTG
GGACTGAGACACGGCC
>Zotu5575
GAACGCTGGCGGCGTGCTTAACACATGCAAGTCGAACGGTGATCTCGGTGCTTGCACCGGGTGATCAGTG
GCGAACGGGTGAGTAACACGTGAGCAACCTGCCCCTGACTCTGGGATAACCCCGAGAAATCGGAGCTAAT
ACCGGATACGAGACGCACGGGCATCCGTAGCGTCTGGAAAGAATTTTGGTCAGGGATGGGCTCGCGGCCT
ATCAGCTTGTTGGTGGGGTAACGGCCCACCAAGGCGACGACGGGTAGCCGGCCTGAGAGGGCGACCGGC
CACACTGGGACTGAGACACGGCC
>Zotu5584
GAACGCTAGCGGCAGGCCTAATACATGCAAGTCGAACGGTAACATGAGTAGCAATACTTGATGACGAGTG
GCGCACGGGTGCGTAACGCGTATGCAATCTACCTTTAACAGGTGAATAGCCCGAAGAAATTCGGATTAAAA
CACCATAGTATTGTTTGTTGGCATCAACAGACAATTAAAACTCCGGTGGTTAAAGATGAGCATGCGTGACA
TTAGCTAGTTGGTGAGGTAATGGCTCACCAAGGCAACGATGTCTAGGGGATCTGAGAGGATTAACCCCCA
CACTGGTACTGAGACACGGAC
>Zotu5588
GAACGCTAGCGGCAGGCCTAATACATGCAAGGCGAACGGCAAGTTTGATAGCAATATTGAACCTAGAGTG
TCGTAAGGGTGCGTAACACGTATGCAATCTGCCCTGTACAGGAGTATAGCTCCCCGAAAGGGGAATTAACA
CTGCATAAGATTAAGGAGTGGCATCACTTTTTAATTAAAACTGAGGTGGTACAGGATGAGCATGCGCATGA
TTAGTTAGTTGGCGGGGTAACGGCCCACCAAGACGATGATCATTAGGGGAACTGAGAGGTTGATCCCCCA
CACTGGCACTGAGATACGGGC

>Zotu5592
CGTCAGGTCCCAGTGCGCTTGGTTAATTACACCTTAGAAGACCTTTCCCTGCCGCAAAAAAGGGTAGTAGC
GCAATTCATCCCGGCGAACATTACAGAAAGCGGGCAAAGGGAGAGTATCGGACTAACAGGA
>Zotu5594
GAACGCTGGCGGCAGGCCTAACACATGCAAGTCGAACGAACTCTTCGGAGTTAGTGGCGGACGGGTGAGT
AACACGTGGGAACGTGCCTTTTGGTTCGGAATAACTCAGGGAAACTTGTGCTAATACCGAATGTGCCCTTC
GGGGGAAAGATTTATCGCCATTAGAGCGGCCCGCGTCTGATTAGCTAGTTGGTGAGGTAAAGGCTCACCA
AGGCTACGATCAGTAGCTGGTCTGAGAGGATGACCAGCCACATTGGGACTGAGACACGGCC
>Zotu5596
GAACGCTGGCGGCGTGCCTAATACATGCAAGTCGAGCGAATCAAAGGGAGCTTGCTCCCAATGATTAGCG
GCGGACGGGTGAGTAACACGTGGGCAACCTGCCCGTAAGATTGGGATAACTCCGGGAAACCGGAGCTAA
TACCGAATAGGTTCTTTCTTTGCATAAAGGAAGAAGGAAAGTTGGCCTCGGCTAACACTTACGGATGGGCC
CGCGGCGCATTAGCTAGTTGGTGAGGTAACGGCTCACCAAGGCAACGATGCGTAGCCGACCTGAGAGGGT
GATCGGCCACACTGGGACTGAGACACGGCC
>Zotu5599
GAACGCTGGCGGCGTGCCTAATACATGCAAGTCGAACGGGCTGCCATAAGGCAGCCAGTGGCGAACGGG
TGAGGAGCACATGGGGAACCTGCCCGTCAGAGGGGGACAACAGCTGGAAACGGCTGCTAAGACCGCATA
GGCTCAAGGAGGGCATCCTCGTTGAGTTAAAGTGTCCTTTCGGGGACAGCTGACGGATGGACCTGTGGCG
CATTAGCCAGTTGGCGGGGTAAAGGCCCACCAAGGCGACGATGCGTAGCCGGCCTGAGAGGGCGGACGG
CCACACTGGGACTGAGACACGGCC
>Zotu5601
GAACGCTGGCGGCGTGCTTAACACATGCAAGTCGAACGCTGAAGCCCAGCTTGCTGGGTGGATGAGTGGC
GAACGGGTGAGTAACACGTGAGTAACCTGCCCTTCACTCTGGGATAACTCCGGGAAACCGGTGCTAATACT
GGATAGGACTCCTGCCTGCATGGGTGGGGGTGGAAAGATTTATCGGTGATGGATGGACTCGCGGCCTATC
AGCTTGTTGGTGGGGTAATGGCCTACCAAGGCGACGACGGGTAGCCGGCCTGAGAGGGTGACCGGCCAC
ACTGGGACTGAGACACGGCC
>Zotu5605
GAACGCTTGCGGCGTGCCTAAGAAATGCAAGTCGAACGGACAGTGGCTTCGGTTACTGTTAGTGGCGAAC
GGTCGCGTAACACGTAAGAAACCTGCCTTGAAGCGGGGGACAACAGTCCGAAAGGGCTGCTAATACCGCA
TGTGGCCAGTGAGGGGCATCCCTTACTGTCTAAAGATTTATCACTTCAAGATGGTCTTGCGGCCTATCAGCT
AGTTGGTGAGGTAACGGCTTACCAAGGCGACGACGGGTAGCTGGTCTGAGAGGACGATCAGCCGGATTG
GGACTGAGATACGGCC
>Zotu5608
GAACGCTGGCGGCAGGCTTAACACATGCAAGTCGAGCGGGCGATGTTGCTTCGGTAACTGAGCTAGCGGC
GGACGGGTGAGTAAAGCTTAGGAATCTGCCTATTAGTGGGGGACAACATTTCGAAAGGGATACTAATACC
GCATACGTCCTACGGGAAAAAGCAGGGGACCTTCGGGCCTTGTGCTAATAGATGAGCCTAAGTCGGATTA
GCTAGTTGGTGGGGTAAAGGCCTACCAAGGCGACGATCTGTAGCGGGTCTGAGAGGATGATCCGCCACAC
TGGGACTGAGACACGGCC
>Zotu5611
GAACGTTGGCGGCGTGCCTAATACATGCAAGTCGAACGCGGTCTCCTAATTGAAAAACCGTGCTTGCACGG
GTTGGATTTTAGATCGGACCGAGTGGCGAACTGGTGAGTAACACGTGGGTAACCTGCCCAGAAGTAGGGG
ATAACACCTGGAAACAGATGCTAATACCGTATAACAACTAAAACCACATGGTTTTAGTTTGAAAGCTGGCCT
TTGCGCTAGTGCTTTTGGATGGACCCGCGGCGTATTAGCTAGTTGGTGAGATAATAGCTCACCAAGGCGAT
GATACGTAGCAGACCTGAGAGGGTAATCTGCCACAATGGGACTGAGACACGGCC
>Zotu5613
GAATGCTGGCGGCGTGGATAAGTCATGCAAGTCAAGGGGGCCCGCAAGGGCAACCGGCAGACGAGGTAG
TAACACGTAGGTACGTCCCCCGAAGTCGGGCATAGCCAATCGAAAGGTTGGGTAATTCCCGATAGTCCCGC
AAGGGTAAAGGTTTTTCGCTTCGGGAGCGGCCTGCGTAGTATCAGCTAGTTGGTAGGGTAATGGCCTACCA
AGGCGACGACGCTTAGGGGAGCTGAGAGGCTGACCCCCACCGATGGAACTGCGACACGGTC
>Zotu5617

GAACGCTGGCGGCGTGCTTAACACATGCAAGTCGAACGATGAAGCCCAGCTTGCTGGGTGGAAGAGTGGC
GAACGGGTGAGTAACACGTGAGTAACCTGCCCCGGACTCTGGGATAAGCGCTGGAAACGGCGTCTAATAC
TGGATATGACCCATACCGGCATCGGTTGTGGGTGGAAAGATTTATCGGTTCGGGATGGGCTCGCGGCCTAT
CAGCTGGATGGTGAGGTAACGGCTCACCATGGCAACAACGGGTAGCCGGCCTGAGAGGGTGACCGGCCA
CACTGGGACTGAGACACGGCC
>Zotu5620
GAACGCTGGCGGCGTGCTTAACACATGCAAGTCGAACGGTGAAGGGACCGGCTTTTGCTGGTTCTGGATG
AGTGGCGAACGGGTGAGTAACACGTGAGTAACCTGCCCCCTTCTTCTGGATAACCGCATGAAAGTGTGGCT
AATACGGGATATTCTGGGTCTGTCGCATGGCGGGCCTGGGAAAGATGCGCCTTTGGGTGTTTTTGGTGGG
GGATGGGCTCGCGGCCTATCAGCTTGTTGGTGGGGTGATGGCCTACCAAGGCGGTGACGGGTAGCCGGC
CTGAGAGGGTGGACGGTCACACTGGGACTGAGACACGGCA
>Zotu5622
GAACGCTGGCGGCGTGCTTAACACATGCAAGTCGAACGGATCCTCGGCTTCGGTTGGGGGTTAGTGGCGA
ACGGGTGAGTAACACGTGAGCAACCTGCCCTTCACTCTGGGATAACTCCGGGAAACCGGGGCTAATACCG
GATAGGACTCCTCGTCGCATGGTGGGGGGTGGAAAGGTGCCCTGTTGGGGGTTTCTGGTGGGGGATGGG
CTCGCGGCCTATCAGCTTGTTGGTGGGGTGATGGCCTACCAAGGCGACGACGGGTAGCCGGCCTGAGAGG
GTGACCGGCCACACTGGGACTGAGACACGGCC
>Zotu5626
GAACGCTGGCGGTATGCCTAATACATGCAAGTCGTACGCGATCACTTTTTTGCTCGCAGCAGTGGTTATTTA
TAACCACCTTTGCGAGCAGAATTATGGTGATTGAGTGGCGGACGGGTGAGTAACACGTTGGTACCTGCCCC
AAAGTGGGGAATAATTCACCGAAAGGTGGACTAATACCCCATAGTCTCCGGTTTATCCGGAGTAAAGCTTT
AGCGCTTTGGGAGGGGCCTGCGTCCGATTAGCTAGTTGGTAGAGTAACGGTTTACCAAGGCTACGATCGG
TAGCTGGATCGAGAGGTTGACCAGCCACACTGGGACTGAGATACGGCC
>Zotu5629
GAACGCTGGCGGCGTGCTTAAGGTATGCAAGTCGAACGAGAATTTTGCAGTAGCAATATTGTAAAATTCTA
GTGGCAAACGGGTGAGTAATGCGTAGATAATCTACCCTTAACATTGAAATAACTTTGCGAAAGTAAGGCTA
ATGTCATGTGTGGTGTATAAGTGAAATTCTTATATATTAAAGGAGCAATCCGGTTAAGGATGAGTCTGCGT
CCTATCAGCTAGTTGGTGAGGTAAATGCTCACCAAGGCGACGACGGGTAGCTGGTCTGAGAGGATGATCA
GCCACATTGGAACTGCGACACGGTC
>Zotu5631
GAACGCTGGCGGCATGCCTTACACATGCAAGTCGAACGGCAGCACGGGAGCAATCCTGGTGGCGAGTGG
CGAACGGGTGAGTAATATATCGGAACGTGCCCAGTCGTGGGGGATAACGTAGCGAAAGCTACGCTAATAC
CGCATACGATCTATGGATGAAAGCGGGGGATCGCAAGACCTCGCGCGATTGGAGCGGCCGATATCAGATT
AGCTAGTTGGTGGGGTAAAGGCCCACCAAGGCGACGATCTGTAGCTGGTCTGAGAGGACGACCAGCCACA
CTGGGACTGAGACACGGCC
>Zotu5635
GAACGCTGGCGGCGTGCCTAATACATGCAAGTCGAGCGGACTTGATGGAGAGCTTGCTCTCCTGATAGTTA
GCGGCGGACGGGTGAGTAACACGTAGGCAACCTGCCTGCAAGACCGGGATAACCCACGGAAACGTGAGC
TAATACCGGATATCTCATTTCCTCTCCTGAGGAGATGATGAAAGACGGAGCAATCTGTCACTTGCGGATGG
GCCTGCGGCGCATTAGCTAGTTGGTGAGGTAACGGCTCACCAAGGCGACGATGCTTAGCCGACCTGAGAG
GGTGAACGGCCACACTGGGACTGAGACACGGCC
>Zotu5638
GAACGCTAGCTACAGGCTTAACACATGCAAGTCGTGGGGCAGCGGATACTTAGCTTGCTAAGTATGCCGG
CGACCGGCGCACGGGTGAGTAACGCGTACCGAACCTGCCCATCACACAGGGATAGGCTTGCGAAAGCAAG
ATTAATACCTGATGGTCTCAGTTGTATGCATGTATAATTGAGTAAAGCCTTCGGGCGGTGATGGATGGCGG
TGCGTCCCATTAGGAAGTTGGCGGGGTAACGGCCCACCAATCCTTCGATGGGTAGGGGTTCTGAGAGGAA
GGTCCCCCACATTGGAACTGAGATACGGTC
>Zotu5640
GAACGCTGGAAATGTGGATACCGGCATGCAAGTCAAGGGGCCGCAAGGCACCGGCGAACGGCTTAGTAA
CACGTAGATACATACCTTAAAGTGGGGCATAGCTCGTCGAAAGACGGGGTAATTTCCCATAGTCTCGCAAG

AGTAAAGGCGCAAGCCGCTTTAAGAATGGTCTGCGGTCTATCAGCTAGTTGGTGAGGTAATGGCTCACCAA
GGCTATGACGGATAGGGGGCGTGAGAGCGCGACCCCCAAGAATGGCACTGCGACACGGGC
>Zotu5642
GAACGCTGGCGGCGTGCTTAACACATGCAAGTCGAGCGGAAAGGCCCTTTCGGGGGTACTCGAGCGGCG
AACGGGTGAGTAACACGTGAGTAATCTGCCCTTGGCTTTGGGATAACCACCGGAAACGGTGGCTAATACC
GGATACGACCAATCTCTGCATGGAGTGTTGGTGGAAAGTTTTTTCGGCCAGGGATGTGCTCGCGGCCTATC
AGCTTGTTGGTGGGGTAATGGCCTACCAAGGCTTCGACGGGTAGCCGGCCTGAGAGGGTGACCGGCCACA
CTGGGACTGAGACACGGCC
>Zotu5645
GAACGCTGGCGGCGCGCTTAACACATGCAAGTCGAGCGAGAACCAGGCCTTCGGGCCTGGGGACAGCGG
CGAACGGGTGAGTAACACGTGGGTAATCAACCCTCGGTACTGGGATAGCCCGGGGAAACCCGGATTAATA
CCGGATGGCCCTTCGGATCTTCGGATCTGACGGAAAAGGTAGCTTCGGCCTCCGACCGAGGACGAGCCCG
CGGCGGATTAGCTTGTTGGTGGGGTAACGGCTCACCAAGGCGACGATCCGTAGCTGGTCTGAGAGGACGA
TCAGCCACACTGGGACTGAGACACGGCC
>Zotu5647
GAACGCTGGCGGCATGCTTAACACATGCAAGTCGAACGAGCTAATGGGTAACTATTAGTATAGTGGCGGA
CGGGTGAGTAACGCGTAAGAACCTACCTTTTGGAGGGGGATAACAGCGGGAAACTGTTGCTAATACCCCA
TAATGCTGAGGAGTTAAAGCAGTGAAAACTGGCCAAAAGAGGGGCTTGCGTCTGATTAGCTAGTTGGTGG
GGTAAAAGCCTACCAAGGCCACGATCAGTAGTCGGTCTGAGAGGACTAACGGCCACACTGGGACTGAGAC
ACGGCC
>Zotu5649
GAACGCTAGCGGCAGGCCTAATACATGCAAGTCGAACGGGATTCCGGTTTAGCTTGCTAAGCTGGATGAG
AGTGGCGTACGGGTGCGTAACGCGTGAGCAACCTGCCCATGTCAGGGGTATAGCCCGTTGAAAGACGGAT
TAACACCGCATAATACATTTGAATCGCATGGTTTTGATGTTAAATATTTATAGGACATGGATGGGCTCGCGT
GGCATTAGCTAGTTGGTATGGTAACGGCATACCAAGGCTATGATGCCTAGGGGCTCTGAGAGGAGAATCC
CCCACACTGGTACTGAGACACGGAC
>Zotu5653
GAACGCTGGCGGCATGCTTAACACATGCAAGTCGAACGAATGATTTCTTCTACTTGTAGAAGAAATTAATG
AGTGGCGGACGGGTGAGTAACACGTAAGAACCTACCTTTTGGAGAGGGACAACCACTGGAAACGGTGGC
TAAAACCTCATAAAAAGCTGAGAAGTGAAAAGATGAAAAATCGCCAAAAGATGGGCTTGCGGCTGATTAG
CTTGTTGGTGAGGTAATGGCTTACCAAGGCAATGATCAGTAGCTGGTCTGAGAGGATGATCAGCCACACTG
GGACTGAGACACGGCC
>Zotu5655
CAACGCTGGCGGCGTGCCTAACACATGCAAGTCGAACGAGAAAGTGGAGCAATCCATGAGTAAAGTGGCG
ACCGGGTGAGTAACACGTGACTAACCTACCTCCGAGTGGGGAATAACTCCGGGAAACCGGGGCTAATACC
GCATAACATCGCAAGATCAAAGCAGCAATGCGCTTGGAGAGGGGGTCGCGGCTGATTAGCTAGTTGGCGG
GGTAACGGCCCACCAAGGCGAAGATCGGTATCCGGCCTGAGAGGGCGCACGGACACACTGGAACTGAAA
CACGGTC
>Zotu5658
AAACGCTGGCGGCGTGCCTAACACATGCAAGTCGAACGCCCTTAGCAATAAGGGAGTGGCGGACGGGTG
AGGAACACGTGGGTGACCTACCTCGAAGTGAGGAATACCGGCGAGAAATCGCCGACAATACCGCATACGC
TTCGCAAGAAGCAAAGTCGCAAGACGCTTTGAGATGGGCCCGCGGCCGATTAGCTGGTTGGTGGGGTAAT
GGCCTACCAAGGCGACGATCGGTAGCTGGTCTGAGAGGACGATCAGCCACACTGGGATTGAGAACGGCC
>Zotu5661
GAACGCTAGCGACAGGCTTAACACATGCAAGTCGAGGGGTAACAGGGGAGCTTGCTTCCGCTGACGACCG
GCGCACGGGTGAGTAACGCGTATGCAACCTACCTATAAGAGGGGAATAACCCGGAGAAATTCGGACTAAT
ACCGCATAATAACAAATTAAGGCATCTTGATTTGTTTAAATATTTATAGCTTATAGATGGGCATGCGTCTGA
TTAGTTTGTTGGTGAGGTAACGGCTCACCAAGACTACGATCAGTAGGGGTTCTGAGAGGAAGGTCCCCCAC
ACTGGTACTGAGACACGGAC
>Zotu5664

GAACGCTGGTAACGTGGATACCGGCATGCAAGTCACGGGGGCCGCAAGGCAACCGGCGAACGGCTTAGT
AACACGTAGATATATACCTTAAAGTGGAGCATAGCTCGTTGAAAAGCGGGGTAATTCTCCATAGTCCCGCA
AGGGTAAAGGCGCAAGCCGCTTTAAGAATAGTCTGCGGTCTATCAGCTAGTTGGTGAGGTAATGGCTCAC
CAAGGCTATGACGGATAGGGGGCGTGAGAGCGCGACCCCCAAGAATGGCACTGCGACACGGGC
>Zotu5667
GAACGCTGGCGGCGTGCTTAACACATGCAAGTCGAACGCTGAAGCCCAGCTTGCTGGGTGGATGAGTGGC
GAACGGGTGAGTAACACGTGGGTAATCTGCCCCGTACTCCGGGATAAGCCCGGGAAACTGGGTCTAATAC
CGGATAGGACACGCTATCGCATGGTGGTGTGTGGAAAGCTCCGGCGGTACGGGATGAGCCCGCGGCCTAT
CAGCTTGTTGGTGGGGTGATGGCCTACCAAGGCGACGACGGGTAGCCGGCCTGAGAGGGTGACCGGCCA
CACTGGGACTGAGACACGGCC
>Zotu5669
GAATGCTGGCGGCGTGCCTAATACATGCAAGTCGAGCGGTAACAGGGCTTCACCGTTTCCTTGACACACCT
GACCATTGTGTAGGTTGTTTGATTTCGAGGGAACAGTGAAGCTGCTGACGAGCGGCGGACGGCTGAGTAA
CGCGTGGGAACATACCCCAAACTGAGGGATAACTAGTCGAAAGATTAGCTAATACCGCATGTGATCTTCGG
ATTAAAGCTTTCGGGCGGTTTGGGAATGGCCTGCGTACGATTAGATAGTTGGTGAGGTAATGGCTCACCAA
GTCGACGATCGTTAGATGGTTTGAGAGGATGATCATCCAGACTGGGACTGAGACACGGCC
>Zotu5672
GAACGCTGGCGGTATGCCTAACACATGCAAGTCGTACGGAAAAGAAATTTTTAGTGGCGAACGGGTGAGT
AACGCGTGAGAATTTGCTTCTAGGTAGAGGATAACCAATAGAAATATTGGCTAAAACTCTATAATTCTATTA
AAAAAATAGCTAAAAAAAGAAATTTGCCTAGAAAAAAGCTCGCGTCTGATTAGCTAGTTGGTAAGGGAAA
GGCTTACCAAGGCGACGATCAGTAGCTGATTTGAGAGGATGATCAGCCACACTGGAACTGAGACACGGTC
>Zotu5675
GAACGCTAGCGATAGGCTTAACACATGCAAGTCGAGGGGCAGCATAGAGTCAGCTTGCTGATTTGGATGG
CGACCGGCGGATGGGTGCGTAACGCGTATGCAACTTACCTGTCAGAGGGGGATAACCCGGAGAAATTCGG
ACTAATACCGCATATACTTGAGATGCTGCATGGTATTTCAAGGAAATATTTATAGCTGACAGATAGGCATGT
GTTCCATTAGCTAGTAGGTGAGGTAACGGCTCACCTAGGCGACGATGGATAGGGGAACTGAGAGGTTAAA
CCCCCACACTGGTACTGAGACACGGAC
>Zotu5677
GAACGCTGGCGGCGTGCCTAATACATGCAAGTCGAGCGGATTCATTCCTTCGGGGATGGGTTAGCGGCGG
ACGGGTGAGTAACACGTAGGCAACCTGCCTATAAGCCTGGGATAACTTCCGGAAACGGATGCTAATACCG
GATACGTTGAATTCTCGCATGAGGATTCATGGAAAGACGGCGCAAGCTGTCACTTATGGATGGGCCTGCG
GCGCATTAGCTAGTTGGTGGGGTAACGGCTCACCAAGGCGACGATGCGTAGCCGACCTGAGAGGGTGATC
GGCCACACTGGGACTGAGACACGGCC
>Zotu5680
GAACGCTGGCGGAATGCTTTACACATGCAAGTCGAACGGCAGCACGGGAGCTTGCTCCTGGTGGCGAGTG
GCGAACGGGTGAGTAACATATCGGAACGTATCCAATAATGGGGGATAACTAATCGAAAGGTTGGCTAATA
CCGCATACGCCCTGAGGGGGAAAGCTGGGGATCTTCGGACCTAGCGTTAATGGAGCGGCCGATATCTGAT
TAGCTAGTTGGTGGGGTAAAGGCCCACCAAGGCGACGATCAGTAGCTGGTCTGAGAGGACGACCAGCCAC
ACTGGAACTGAGACACGGTC
>Zotu5683
GAACGCTGGCGGCGTGCCTTAAGCATGCAAGTCGAACGAATCGTAAGATTAGTGGCGGACGGTCGAGTAA
CGCGTAGGGATCTGCCTTAAAGTTGGACATAGCTCATCGAAAGGTGGGGTAATTTCCAATGTGAATGCAAA
TTTAAAGCCGCAAGGCGCTTTAAGATGAACCTGCGTCCTATCAGCTAGTTGGTGAGGTAATGGCTCACCAA
GGCATCGACGGGTAGCTGGTCTGAGAGGACGACCAGCCGGAATAGCACTGAGACACGGGC
>Zotu5685
GAACGCTGGCGGCAGGCCTAACACATGCAAGTCGAACGCCCCGCAAGGGGAGTGGCAGACGGGTGAGTA
ACACGTGGGAACCTTCCCAACGGTACGGAATAGCCCAGGGAAACTTGGAGTAATACCGTATACGCCCCTAG
GGGGAAAGATTTATCGCCGTTGGATGGGCCCGCGTCTGATTAGCTAGTTGGTGAGGTAACGGCTCACCAA
GGCGACGATCAGTAGCTGGTCTGAGAGGATGATCAGCCACACTGGGACTGAGACACGGCC
>Zotu5687

GAACGCTGGCGGCAGGCTTAACACATGCAAGTCGAACGCCCCGCAAGGGGAGTGGCAGACGGGTGAGTA
ACGCGTGGGAATCTACCCAGTTCTACGGAACAACTGAGGGAAACTTCAGCTAATACCGTATACGCCCTACG
GGGGAAAGATTTATCGGAATTGGATGAGCCCGCGTTGGATTAGCTAGTTGGTGAGGTAACGGCTCACCAA
GGCGACGATCCATAGCTGGTCTGAGAGGATGATCAGCCACACTGGGACTGAGACACGGCC

>Zotu5690

GAACGTTGGCGGCGTGGATTAGGCATGCAAGTCGTACGGGCCGCAAGGCCAGTGGCGCAAGGGTGAGTT
AGGCGATGGCAACCAACCCCGCGAATGGGCATAGCCACGGGAAACTGTGGGTAATTCCCAGCGATGTTCG
AGATGGGCATCCGTCTTGAACCAAAGGTGCGATTCCGTCGCGGGACGGCCATCGTGATATTAGATTGTTG
GCGGGGTAACGGCCCACCAAGTCTATGATGTCTACCGGGCGTGTGAGCGTGGCCCGGCACACTGGGACTG
AGACACTGCC

>Zotu5694

GAACGCTAGCGGCAGGCCTAATACATGCAAGTCGAGGGGTAGGGTCGGAGTAATCTGACCTGAGACCGG
CGCACGGGTGCGTAACACGTATGCAACCTACCTTTTACTGGGGTATAGCTCAGAGAAATCTGAATTAATCC
CCCATAATGTTATTGAATGGCATCATTTAATAACTAAAGCTGAGGCGGTAAAAGATGGGCATGCGTTGTAT
TAGCTAGTTGGTGAGGTAATGGCTCACCAAGGCTACGATACATAGGGGTTCTGAGAGGATGATCCCCCAC
ACTGGTACTGAGACACGGAC

>Zotu5698

GAACGCTAGCGGCAGGCTTAACACATGCAAGTCGAGGGGTATAGTTCTTCGGAGCTAGAGACCGGCGCAC
GGGTGCGTAACGCGTATGCAATCTACCTTTTACAGAGGGATAGCCCAGAGAAATTTGGATTAATACCTCAT
AGCATTATGATATGGCATCGTATTATAATTAAAGTTCCAACGGTAAAAGATGAGCATGCGTCCCATTAGCTA
GTTGGTGTGGTAACGGCACACCAAGGCTACGATGGGTAGGGGTCCTGAGAGGGAGATCCCCCACACTGGT
ACTGAGACACGGAC

>Zotu5700

GAACGCTGGCGGCAGGCTTAACACATGCAAGTCGAACGCTCGCAGCAATGCGGGAGTGGCAGACGGGTG
AGTAACGCGTGGGAACATACCCTTTGGTTCGGAATAACTGAGAGAAATTTCAGCTAATACCGGATGTGCCC
TTCGGGGGAAAGATTTATCGCCAAAGGATTGGCCCGCGTCTGATTAGCTAGTTGGTGAGGTAACGGCTCAC
CAAGGCGACGATCAGTAGCTGGTCTGAGAGGATGATCAGCTACACTGGGACTGAGACACGGCC

>Zotu5702

CAACGCTGGCGGCGCGCTTAACACATGCAAGTCGCACGAGAAAGGGAGCAATCCTGAGTATAGTGGCGCA
CGGGTGAGTAACACGTGGATGATCTACCTCCTTGTGGGGAATAACCCTGGGAAACCGGGGCTAATACCGC
ATACGTCCGAGAGGAGAAAGCCGAAAGGCGCAGGGAGAGGAGTCCGCGGTCGATTAGCTAGTTGGCAGG
GTAATGGCCTACCAAGGCGACGATCGATAGCCGGCCTGAGAGGGCACACGGCCACACTGGCACTGAAACA
CGGGC

>Zotu5704

GAACGCTGGCGGCGCGCCTAACACATGCAAGTCGAGCGGGGACATCTGGAGAGTAATCAAGAGATGTTCT
AGCGGCGGACGGGTGAGTAACGCGTGAGTGACCTGCCTTACACAGGGGGATACCGTACCGAAAGGTACA
CTAATACCGCATGAGACCACAGCAGCGCATGCTGCAGAGGTCAAAGGAGCAATCCGGTGTAAGATGGACT
CGCGTCCGATTAGATAGTTGGCGGGGTAACGGCCCACCAAGTCAGTGATCGGTAGCCGACCTGAGAGGGT
GATCGGCCACATTGGAACTGAGAGACGGTC

>Zotu5706

GAACGCTGGCGGCAGGCCTAACACATGCAAGTCGAGCGAGATCTTCGGATCTAGCGGCGGACGGGTGAG
TAACGCGTGGGAACGTACCTCTTGCTACGGAATAGCCTCGGGAAACTGGGAGTAATACCGTATGTGCCCTA
CGGGGGAAAGATTTATCGGCAAGAGATCGGCCCGCGTTGGATTAGGTAGTTGGTGAGGTAACGGCTCACC
AAGCCGACGATCCATAGCTGGTTTGAGAGGATGATCAGCCACACTGGGACTGAGACACGGCC

>Zotu5709

AAACGCTGGCGGCGCACATAAGACATGCAAGTCGAACGGAAGTCGTTATGAAATGAATTGAAGATTTATCT
AATTGAAGTTTCATAACAATGGCTTTAGTGGCGGACTGGTGAGTAACACGTAAGCAATCTACCTATTAGAG
GGGAATAACAATGAGAAATCATTGCTAATACCGCATAAGCTTGTAGCATCGCATGATGCAGCAAGAAAAG
GAGTAATCCGCTAATAGATGAGCTTGCGGCTGATTAGCTAGTTGGTAAGGTAACGGCTTACCAAGGCAAC
GATCAGTAGCCGGGCTGAGAGGCTCTGCGGCCACATTGGGACTGAGATACGGCC

>Zotu5716
GAACGCTGGCGGCAGGCTTAACACATGCAAGTCGAACGCATCCTTCGGGGTGAGTGGCAGACGGGTGAG
TAACGCGTGGGAACATGCCCTTCAGTTTGGAATAACCCAGGGAAACTTGGGCTAATACCGGATACGCCCTT
AGGGGGAAAGATTTATCGCTGAAGGAGTGGCCCGCGTCTGATTAGCTAGTTGGTGAGGTAATGGCTCACC
AAGGCGACGATCAGTAGCTGGTCTGAGAGGATGATCAGCCACACTGGGACTGAGACACGGCC
>Zotu5718
GAACGCTGGCGGCGTGCCTAATACATGCAAGTCGAGCGCGAAGATCAGGAGCTTGCTCCTGAGATTTGAG
CGGCGGACGGGTGAGTAACACGTAGGCAACCTACCCTTGAGATTGGGATAACTACCGGAAACGGTAGCTA
ATACCGGATACGACTTTTTCACATAAGTGGAACTGTTAAAAGGCGGATTTATCTGCCGCTCATGGATGGGC
CTGCGGTGCATTAGCTAGTTGGTGAGATAGTAGCTCACCAAGGCGACGATGCATAGCCGACCTGAGAGGG
TGATCGGCCACACTGGGACTGAGACACGGCC
>Zotu5720
GAACGCTAGCTACAGGCTTAACACATGCAAGTCGCAGGGTAACGTGAGGGAAGCTTGCTTCCCTTGACGA
CGACTGGCGCACGGGTGAGTAACGCGTATCCAACCTTCCCATGACCACGGGATAACCCGTTGAAAGACGG
ACTAATACCGTATGACGTCGTTTGCTGACATCAAATAACGATTAAAGGTTTAGCGGTGATGGATGGGGATG
CGTCTGATTAGCTTGTTGGCGGGGTAACGGCCCACCAAGGCGACGATCAGTAGGGGTTCTGAGAGGAAGG
TCCCCCACATTGGAACTGAGACACGGTC
>Zotu5726
GAACGCTGGCGGCGTGCCTAATACATGCAAGTCGAGCGAATCAAAGGGAGCTTGCTCCCTGAGATTAGCG
GCGGACGGGTGAGTAACACGTGGGCAACCTGCCTATAAGACTGGGATAACTTCGGGAAACCGGAGCTAAT
ACCGGATAATTCTTATCAACTCATGTTGGTAAGCTGAAAGACGGTTTACGCTGTCACTTATAGATGGGCCCG
CGGCGCATTAGCTAGTTGGTGAGGTAACGGCTCACCAAGGCGACGATGCGTAGCCGACCTGAGAGGGTG
ATCGGCCACACTGGGACTGAGACACGGCC
>Zotu5729
GAACGCTGGCGGCGTGCTTAACACATGCAAGTCGAACGATGAAGCCCTTCGGGGTGGATTAGTGGCGAAC
GGGTGAGTAACACGTGGGCAATCTGCCCTGCACTCTGGGACAAGCCCTGGAAACGGGGTCTAATACCGGA
TATGACCTTTCCCCTCCTGGGGTTTGGTGGAAAGCTCCGGCGGTGCAGGATGAGCCCGCGGCCTATCAGCT
TGTTGGTGGGGTAATGGCCTACCAAGGCGACGACGGGTAGCCGGCCTGAGAGGGCGACCGGCCACACTG
GGACTGAGACACGGCC
>Zotu5732
GAACGCTGGCGGCGTGCTTAACACATGCAAGTCAAGGGGGCCCGCAAGGGTAACCGGCGAACGGGTGCG
TAACACGTGAGCAATCTGCCTAGATGTGGGGGATAGCCGGCCCAACGGCCGGGTAATACCGCATACGCTC
TCTGGTCGGCATCGACTGGAGAGGAAACATTCGTGGCATCTAGAGGAGCTCGCGGCCTATCAGCTAGTTG
GTGGGGTAACGGCCTACCAAGGCTTTGACGGGTAGCTGGTCTGAGAGGATGGCCAGCCACATTGGGACTG
AGATACGGCC
>Zotu5734
GAACGCTAGCTGCAGGCTTAACACATGCAAGTCGAGGGGCAGCGGGAGTAGCAATACTTGCCGGCGACC
GGCGGAAGGGTGCGTAACGCGTGAGCAACATTCCCGTATCAGGGGGATAACCGGTGGAAACGCCGACTA
ATACCCCATAAGAACATCTGCGGCACCAAAGGTGTTTGAAAGATTCGTCGGATGCGGATTGGCTCGCGTGA
CATTAGCTAGTTGGTGAGGTAACGGCCCACCAAGGCGACGATGTCTAGGGGTTCTGAGAGGAAGATCCCC
CACATTGGAACTGAGACACGGTC
>Zotu5736
GAACGCTGGCGGTGTGCCTAATACATGCAAGTCGAACGGACCTTCGGGTTAGTGGCGAACGGGTGAGTAA
CACGTTGGTACCTGCCCTCTAGTGGGGAATAATTCGCCGAAAGGCGGACTAATACCGCATATTGCCGCAAG
GTGAAAGCAGTAATGCGCTAGAGGAGGGGCCTGCGGCCCATTAGCTTGTTGGTAGTGTAAAAGACTACCA
AGGCTACGATGGGTAGCTGGATCGAGAGGTTGACCAGCCACACTGGGACTGAGATACGGCC
>Zotu5738
GAACGCTGGCGGCGTGCTTAACACATGCAAGTCGAACGGTGAACCAGGAGCTTGCTCTTGGGGATCAGTG
GCGAACGGGTGAGTAACACGTGAGCAATCTGCCCCTGACTCTGGGATAAGCGCTGGAAACGGCGTCTAAT
ACTGGATACGAGCCGTGAAGGCATCTTCAACGGTTGGAAAGATTTTTCGGTCAGGGATGAGCTCGCGGCC

TATCAGCTTGTTGGTGAGGTAATGGCTCACCAAGGCGTCGACGGGTAGCCGGCCTGAGAGGGTGACCGGC
CACACTGGGACTGAGACACGGCC
>Zotu5740
GAACGCTAGCGGCAGGCCTAACACATGCAAGTCGAGGGGTAGAGGAAGCTTGCTTCCTTGAGACCGGCGC
ACGGGTGCGTAACGCGTATGCAATCTACCTTGTACAGAGGGATAGCCCAGAGAAATTTGGATTAATACCTC
ATGGTATTATTGAATGGCATCATTTGATAATTAAAGTTCCAACGGTACAAGATGAGCATGCGTCCTATTAGC
TAGATGGTAAGGTAACGGCTTACCATGGCTACGATAGGTAGGGGTCCTGAGAGGGAGATCCCCCACACTG
GTACTGAGACACGGAC
>Zotu5742
GAACGCTGGCGGCGTGCCTAATACATGCAAGTCGAGCGGTAACAGGTCTAGTTTACTAGATGCTGACGAG
CGGCGGACGGCTGAGTAACACGTAGGAACGTACCCCAAAGTGAGGGATAACTGCTCGAAAGAGTAGCTA
ATACCGCATGTGATCCACGGATTAAAGCTTTATGCGCTTTGGGAACGGCCTGCGTTCGATTAGCTAGATGG
TAGTGTAATGGACTACCATGGCGACGATCGATAGCTGGTCTGAGAGGATGATCAGCCAGACTGGGACTGA
GACACGGCC
>Zotu5744
GAACGCTGGCGGCATGCCTAACACATGCAAGTCGAACGAGATCTTTCGGGGTCTAGTGGCGCACGGGTGC
GTAACGCGTGGGAATCTGCCTTGGGGTTCGGAATAACGTCGGGAAACTGACGCTAATACCGGATGATGAC
GTAAGTCCAAAGATTTATCGCCCTGAGATGAGCCCGCGTCCGATTAGCTAGTTGGTGGGGTAAAGGCCTAC
CAAGGCGACGATCGGTAGCTGGTCTGAGAGGATGATCAGCCACACTGGGACTGAGACACGGCC
>Zotu5746
GAACGCTGGCGGCGTGCCTAAAACATGCAAGTCGTGCGGTAACAGGCCCTTCGGGGTGCTGACGAGCGGC
GGACGGCTGAGTAACGCGTGGGAATCTGCCCCAAAGTGAGGGATAACTGCCCGAAAGGGTGGCTAATAC
CGCATATGGTCTTCGGATTAAAGGAGTAATCCGCTTTGGGAAGAGCCCGCGTAAGATTAGATAGTTGGTGA
GGTAATGGCTCACCAAGTCGACGATCTTTAACTGGTTTGAGAGGATGATCAGTCAGACTGGAACTGAGACA
CGGTC
>Zotu5748
GAACGCTGGCGGCGTGCCTAATACATGCAAGTCGAACGGTCTTTTCTATGGAAGCTTGCTTCCGCTGAGAA
GATAGTGGCGGACGGGTGAGTAACACGTGGGTAACCTGCCCATAAGAGGGGGATAACATCCGGAAACGG
GTGCTAATACCGCATAGTTTTTCTGATCGCATGATCGGGAAAGGAAAGACGGCCTTTGTGCTGTCGCTTAT
GGATGGACCCGCGGCGTATTAGTTAGTTGGTGAGGTAATGGCTCACCAAGACGATGATACGTAGCCGACC
TGAGAGGGTGATCGGCCACATTGGGACTGAGACACGGCC
>Zotu5751
GAACGCTGGCGGCGTGCTTAACACATGCAAGTCGAACGGTGAAGGGACTGGTTTTGCTGGTTCTGGATGA
GTGGCGAACGGGTGAGTAACACGTGAGTAACCTGCCCCCTTCTTCTGGATAACCGCATGAAAGTGTGGCTA
ATACGGGATATTCTGGGTCTGTCGCATGGTGGGCCTGGGAAAGATTGCGCTTTTTTGGTGTTTTTGGTGGG
GGATGGGCTCGCGGCCTATCAGCTTGTTGGTGGGGTGATGGCCTACCAAGGCGGTGACGGGTAGCCGGC
CTGAGAGGGTGGACGGTCACACTGGGACTGAGACACGGCC
>Zotu5753
GAACGCTGGCGGCGTGCTTAACACATGCAAGTCGAACGGATCACTTTTACTTCGGTTTTGGTGGTTAGTGG
CGAACGGGTGAGTAACACGTGAGTAACCTGCCCTTCACTGTGGGATAACGAGAAGAAATTCTTGCTAATAC
CGCATATGACGCGTCTCCGCATGGGGGTGTGTGGAAAGTTTTTTCGGTGGGGGATGGACTCGCGGCCTAT
CAGCTTGTTGGTGGGGTAATGGCTCACCAAGGCTTTGACGGGTAGCCGGCCTGAGAGGGCGACCGGCCAC
ACTGGGACTGAGATACGGCC
>Zotu5755
GAACGCTGGCGGCAGGCTTAACACATGCAAGTCGAACGATGATTACCTAGCTTGCTAGGTATGATTAGTGG
CGAACGGGTGAGTAATGCTTAGGAATCTGCCTGATAGTGGGGGACAACTTGGGGAAACCCAAGCTAATAC
CGCATACGTCCTACGGGAGAAAGGGGGCTTTTAGCTCTCGCTATCAGATGAGCCTAAGTCGGATTAGCTAG
TTGGTGGGGTAAAGGCCTACCAAGGCGACGATCTGTAGCTGGTCTTAGAGGATGATCAGCCACACTGGGA
CTGAGACACGGCC
>Zotu5760

GAACGCTAGCGGCAGGCTTAACACATGCAAGTCGAGGGGCAGCGCGGGTAGCAATACCTGGCGGCGACC
GGCAAACGGGTGCGGAACACGTACAGAACCTACCTTTGTCTGGGGCATAGCCCGGAGAAATCCGGATTAA
CTCCCCATAATATAATTGAGTGGCATCACTTGACTATTAAAAATTTATTGGACAGAGATGGCTGTGCGTCCC
ATTAGCTAGTTGGTAGGGTAACGGCCTACCAAGGCAACGATGGGTAGCTGGTGTGAGAGCACGACCAGCC
ACACGGGCACTGAGACACGGGC
>Zotu5764
GAACGCTGGCGGCATGCCTAACACATGCAAGTCGAACGAAACCTTCGGGTTTAGTGGCGCACGGGTGCGT
AACGCGTGGGAATCTGCCTCTCGGTTCGGAATAACAGTTAGAAATGACTGCTAATACCGGATGATGACGAA
AGTCCAAAGATTTATCGCCGAGAGATGAGCCCGCGTAGGATTAGCTAGTTGGTGGGGTAAAGGCCTACCA
AGGCGACGATCCTTAGCTGGTCTGAGAGGATGATCAGCCACACTGGGACTGAGACACGGCC
>Zotu5766
GAACGCTGGCGGCAGGCCTAACACATGCAAGTCGAACGCCCCGCAAGGGGAGTGGCAGACGGGTGAGTA
ACACGTGGGAACCTTCCCTTTGGTACGGAATAACCCACCGAAAGGTGGATTAATGCCGCATAAGCCCGTGA
GGGGAAAGATTTATCGCCAAAGGATGGGCCCGCGTAGGATTAGCTAGTTGGTGAGGTAATGGCTCACCAA
GGCGACGATCCTTAGCTGGTCTGAGAGGACGACCAGCCACACTGGGACTGAGACACGGCC
>Zotu5771
GAACGCTGGCGGCAGGCTTAACACATGCAAGTCGAGCGGGGAAAGGTAGCTTGCTACCTAACCTAGCGGC
GGACGGGTGAGTAATGCTTAGGAATCTGCCTATTAGTGGGGGACAACATCTCGAAAGGGATGCTAATACC
GCATACGTCCTACGGGAGAAAGCAGGGGACCTTCGGGCCTTGCGCTAATAGATGAGCCTAAGTCGGATTA
GCTAGTTGGTGGGGTAAAGGCCTACCAAGGCGACGATCTGTAGCGGGTCTGAGAGGATGATCCGCCACAC
TGGGACTGAGACACGGCC
>Zotu5773
GAACGCTGGCGGCGTGCTTAACACATGCAAGTCGAACGGGAATTACTTTATTGAAACTTCGGTCGATTTAA
TTTAATTCTAGTGGCGGACGGGTGAGTAACGCGTGGGTAACCTGCCTTATACAGGGGGATAACAGTCAGA
AATGGCTGCTAATACCGCATAAGCGCACAGAGCTGCATGGCTCAGTGTGAAAAACTCCGGTGGTATAAGA
TGGACCCGCGTTGGATTAGCTAGTTGGTGGGGTAACGGCCCACCAAGGCGACGATCCATAGCCGGCCTGA
GAGGGTGAACGGCCACATTGGGACTGAGACACGGCC
>Zotu5777
GAACGCTGGCGGCAGGCCTAACACATGCAAGTCGAACGGTAACACAGAGAGCTTGCTCTCGGGTGACGAG
TGGCGGACGGGTGAGTAATGTCTGGGAAACTGCCTGATGGAGGGGGATAACTACTGGAAACGGTAGCTA
ATACCGCATAACGTCGCAAGACCAAAGAGGGGGACCTTCGGGCCTCTTGCCATCGGATGTGCCCAGATGG
GATTAGCTAGTAGGTGGGGTAACGGCTCACCTAGGCGACGATCCCTAGCTGGTCTGAGAGGATGACCAGC
CACACTGGAACTGAGACACGGTC
>Zotu5781
GAATGGTGGCAGCGTGGATGAGTCATGCAAGTCAAGGGGGCCGCAAGGCAACCGGCAAACGAGGTAGTA
ACACGTAGGTAGATCCCCCCGAGTCGGGCATAGCTTGCCGAAAGGCAAGGTAATTCCCGATGGTCCCGCA
AGGGTAAAGTTTTTCGCTCGGGGAGTCGCCTGCGTAGTATCAGCTAGTTGGTAGGGTAACGGCCTACCAA
GGCTACGACGCTTAGGGGGGGTGAGAGCCCGATCCCCACCGATGGGACTGCGACACGGCC
>Zotu5785
GAACGCTGGCGGCGTGCCTAACACATGCAAGTCGAGCGATTTACTTCGGTAAAGAGCGGCGGACGGGTGA
GTAACGCGTGGGTAACCTGCCTCATACACATGGATAACATACCGAAAGGTATGCTAATACAAGATGATATA
AGAGATTCGCATGGATTTCTTATCAAAGCTTTTGCGGTATGAGATGGACCCGCGTCTGATTAGCTAGTTGGT
AAGGTAACGGCTTACCAAGGCGACGATCAGTAGCCGACCTGAGAGGGTGATCGGCCACATTGGAACTGAG
ACACGGTC
>Zotu5787
GAACGCTGGCGGCATGCCTAACACATGCAAGTCGAACGAGACCTTCGGGTCTAGTGGCGCACGGGCGCGT
AACGCGTGGGAACCTGCCTTTAGGTTCGGAATAACTCCTCGAAAGGGGTGCTAATACCGAATAATGTCTTC
GGACCAAAGATTTATCGCCTTTAGATGGGCCCGCGTTGGATTAGCTAGTTGGTGGGGTAAAGGCTCACCAA
GGCGACGATCCATAGCTGGTCTTAGAGGATGATCAGCCACACTGGGACTGAGACACGGCC
>Zotu5790

GAACGCTGGAAATGTGGATACTGGCATGCAAGTCAAGGGGCCGCAAGGCACCGGCGAACTGCTTAGTAAC
ACGTAGATACATACCGTAAAGTGGAACATAGCTCGTCGAAAGACGGGGTAATTTTCCATGTGAACGCAAGT
TTAAAGGCGCAAGCCGCTTTACGAATGGTCTGCGGTCTATCAGCTAGTTGGTGAGGTAAAGGCTCACCAAG
GCTATGACGGATAGGGGGCGTGAGAGCGCGACCCCCAAGAATGGCACTGCGACACGGGC
>Zotu5793
GAACGCTGGCGGCGTGCCTAATACATGCAAGTCGAGCGGAGATTTGAAAAGCTTGCTTTTCAAATCTTAGC
GGCGGACGGGTGAGTAACACGTGGGCAACCTGCCCTTGAGACGGGGATAACTCCGGGAAACCGGAGCTA
ATACCGGATAACACATATCTTCGCATGAGGATATGTTAGAAGGTGGCTTTTAGCTACCACTCAAGGATGGG
CCCGCGGCGCATTAGCTAGTTGGTGAGGTAACGGCTCACCAAGGCGACGATGCGTAGCCGACCTGAGAGG
GTGATCGGCCACACTGGGACTGAGACACGGCC
>Zotu5796
GAACGCTGGCGGCGTGCCTAACACATGCAAGTCGAGCGAGATCCATCCAGTGGCAACACTGGTGAAGATC
TAGCGGCGAACGGGTGAGTAACACGTGAGAAACCTGCCCCGGAGACCGGGATAACCCGAGGAAACTCGG
GCTAATACCGGATACCCCCACCGAACCGCATGATTTGGTGAGGAAATGCTCCGACGCTCCGGGAGGGTCTC
GCGGCCTATCAGCTAGTTGGTGAGGTAACGGCTCACCAAGGCTAAGACGGGTAGCTGGTCTGAGAGGACG
ATCAGCCACACTGGGACTGAGACACGGCC
>Zotu5798
GAACGCTGGCGGCGCGTCTTAAGCATGCAAGTCGAACGGCAACACGGGGCAACCTGGTGGCGAGTGGCG
AACGGGTGAGTAACACGTAGGTGATCTGCCTTTGGGCTGGGGATAGCCATTAGAAATAGTGGGTAATACC
GAATGAAGTCACGGAGCTTTGGCACTGTGATGAAAGGGGCTACGGCCTCGCCTGGAGATGAGCCTGCGTC
TGATTAGCTAGTTGGTGGGGTAAGAGCCTACCAAGGCGACGATCAGTAGCCGGCCTGAGAGGGTGGTCG
GCCACACTGGGACTGAGATACGGCC
>Zotu5800
GAACGCTAGCGGCAGGCTTAACACATGCAAGTCGAGGGGTAGTCTAGCAATAGATGAGACCGGCGCACG
GGTGCGTAACGCGTATGCAATCTACCTTTTACAGAGGGATAGCCCAGAGAAATTTGGATTAATACCTCATA
GTATAGTACAATCGCATGATTGAACTATTAAAGTTACAACGGTAAAAGATGAGCATGCGTCCCATTAGCTA
GTTGGTAAGGTAACGGCTTACCAAGGCGACGATGGGTAGGGGTCCTGAGAGGGAGATCCCCCACACTGGT
ACTGAGACACGGAC
>Zotu5802
GAACGCTGGCGGCAGGCTTAACACATGCAAGTCGAGCGGGGAAAGGTAGCTTGCTACCTGACCTAGCGGC
GGACGGGTGAGTAATGCTTAGGAATCTGCCTATTAGTGGGGGACAACATTCCGAAAGGAATGCTAATACC
GCATACGTCCTACGGGAGAAAGCAGGGGATCTTCGGACCTTGCGCTAATAGATGAGCCTAAGTCAGATTA
GCTAGTTGGTGGGGTAAAGGCCTACCAAGGCGACGATCTGTAGCGGGTCTGAGAGGATGATCCGCCACAC
TGGGACTGAGACACGGCC
>Zotu5805
GAACGCTGGCGGCATGCCTTACACATGCAAGTCGAACGGTAACAGGGAGCTTGCTCCGCTGACGAGTGGC
GAACGGGTGAGTAATATATCGGAACGTACCTTTGAGTGGGGGATAACTAGTCGAAAGATTAGCTAATACC
GCATACGATCTACGGATGAAAGCGGGGGATCGCAAGACCTCGTGCTCATAGAGCGGCCGATATCTGATTA
GCTAGTTGGTGAGGTAAAGGCTCACCAAGGCATCGATCAGTAGCTGGTCTGAGAGGACGACCAGCCACAC
TGGGACTGAGACACGGCC
>Zotu5807
GAACGCTGGCGGCGTGCCTAATACATGCAAGTCGAGCGGATCTTTGGGAGCTTGCTCCCAAAGATTAGCG
GCGGACGGGTGAGTAACACGTGGGCAACCTGCATATGAGACTGGGATAACTCCGGGAAACCGGGGCTAA
TACCGGATAATTCTTTTCTACACATGTAGAAAAGCTGAAAGATGGTTTCGGCTATCACTCATAGATGGGCCC
GCGGCGCATTAGCTAGTTGGTGAGGTAACGGCTCACCAAGGCGACGATGCGTAGCCGACCTGAGAGGGT
GATCGGCCACACTGGGACTGAGACACGGCC
>Zotu5809
GAATGGTGGCGATGTGGATGAGTCATGCAAGTCAAGGGGGCCTTCGGGCAACCGGCAGACGAGGTAGTA
ACACGTAGGTAGATCCCCCGGAGTCGGGCATAGCCATTCGAAAGGATGGGTAATTCCCGATGGTCTCGTG
AGAGTAAAGCCGTTGTATTTATATAACTGGTCGCTCCGGGAGTCGCCTGCGTAGTATCAGCTAGTTGGTAG

GGTAATGGCCTACCAAGGCTACGACGCTTAGGGGAGCTGAGAGGCTGACCCCCACCGATGGGACTGAGAC
ACGGCC
>Zotu5811
GAACGCTGGCGGCGTGCTTAACACATGCAAGTCGAACGGTGATCCTGGTGCTTGCACCGGGTGATCAGTG
GCGAACGGGTGAGTAACACGTGAGCAACCTGCCCCCGACTCTGGGATAACTTCGGGAAATCGGAGCTAAT
ACCGGATACGAGACGCACGGGCATCCGTAGCGTCTGGAAAGAATTTCGGTCGGGGATGGGCTCGCGGCCT
ATCAGCTTGTTGGTGGGGTAACGGCCTACCAAGGCGACGACGGGTAGCCGGCCTGAGAGGGCGACCGGC
CACACTGGGACTGAGACACGGCC
>Zotu5815
GAACGCTGGCGGCGTGCCTAACACATGCAAGTCGAACGGAGTCAAGAAGCTTGCTTTTTGACTTAGTGGC
GGACGGGTGAGTAACGCGTGAGTAACCTGCCTCTGAGAGGGGAATAACGTTCTGAAAAGAACGCTAATAC
CGCATGACGTATCGAAGCCGCATGACTTTGATACCAAAGATTTTATCGCTCGGAGATGGACTCGCGTCCGA
TTAGTTAGTTGGTGAGGTAACGGCTCACCAAGACCGCGATCGGTAGCCGGACTGAGAGGTTGATCGGCCA
CATTGGGACTGAGACACGGCC
>Zotu5818
GAACGCTGGCGGCGTGCTTAACACATGCAAGTCGAACGGTAAGGCCCTAGCTTGCTGGGGTACACGAGTG
GCGAACGGGTGAGTAACACGTGGGTGACCTGCCCTGCACTTCGGGATAAGCCTGGGAAACTGGGTCTAAT
ACCGGATAGGACCGCATCGTAGGTGGTGTGGTGGAAAGTTTTTTCGGTGTGGGATGGGCCCGCGGCCTAT
CAGCTTGTTAGTGGGGTAATGGCCTACCAAGGCGGCGACGGGTAGCCGGCCTGAGAGGGTGGACGGCCA
CATTGGGACTGAGACACGGCC
>Zotu5822
GAACGCTGGCGGTATGCTTTACACATGCAAGTCGAACGGAGTTGGAGCTTGCTCCAATTTAGTGGCGAACG
GGTGAGTAATATATCGGAACATATCCGGAAGTGGGGGATAACGTAGCGAAAGTTACGCTAATACCGCATA
TGCCCTGAGGGGGAAAGGGGGGGATCGCAAGACCTCTCGCTTTCGGAGTGGCCGATACCGGATTAGCTAG
ATGGTGAGGTAAAGGCTCACCATGGCGACGATCCGTAGCTGGTCTGAGAGGACGACCAGCCACACTGGAA
CTGAGACACGGTC
>Zotu5825
GAACGCTGGCGGCGCGCCTAACACATGCAAGTCGAACGGCACCCCTCTCCGGAGGGAAGCGAGTGGCGA
ACGGCTGAGTAACACGTGGAGAACCTGCCCCCTCCCCCGGGATAGCCGCCCGAAAGGACGGGTAATACCG
GATACCCCCGGGCGCCGCATGGCGCCCGGGCTAAAGCCCCGACGGGAGGGGATGGCTCCGCGGCCCATC
AGGTAGACGGCGGGGTGACGGCCCACCGTGCCGACAACGGGTAGCCGGGTTGAGAGACCGACCGGCCAG
ATTGGGACTGAGACACGGCC
>Zotu5827
GAACGCTGGCGGCGCGCCTAATACATGCAAGTCGAGCGAGAAGGTGTAGCAATACACTTGTAAAGCGGCG
AACGGGTGAGGAACACATGGGTAACCTACCATCGAGTGGGGAATAACCAGCCGAAAGGTTGGCTAATACC
GCGTACGCCTGCGGGTCTTCGGATTCGCAGGGAAAGCCACACTGAGAGTGTGGCGCTCATTGATGGGCTC
ATGTCCTATCAGCTTGTTGGTAGGGTAACGGCCTACCAAGGCTTTGACGGGTAGCTGGTCTGAGAGGACG
ATCAGCCACACTGGCACTGCGACACGGGC
>Zotu5829
GAACGCTGGCGGCAGGCTTAACACATGCAAGTCGAGCGGGGAAAAGTAGCTTGCTACTTGACCTAGCGGC
GGACGGGTGAGTAATGCTTAGGAATCTGCCTATTAGTGGGGGACAACATCTCGAAAGGGATGCTAATACC
GCATACGTCCTACGGGAGAAAGCAGGGGACCTTCGGGCCTTGCGCTAATAGATGAGCCTAAGTCGGATTA
GCTAGTTGGTGGGGTAAAGGCCTACCAAGGCGACGATCTGTAGCGGGTCTGAGAGGATGATCCGCCACAC
TGGGACTGAGACACGGCC
>Zotu5831
GAACGCTGGCGGCGTGCCTAACACATGCAAGTCGAGCGGGAAGTCCTTAAACGAAACTTCGGTCGAGTGA
GAGGATGGATAGCGGCGGACGGGTGAGTAACGCGTAGGTAAGCTGCCCCACGCACAGGGATAGCCTCGG
GAAACTGGGATTAATACCTGATAATGCGGAGAGATCACATGGTCACTCCGCCAAAGATTTATCGGCATGGG
ATGCACCTGCGTCTGATTAGTTAGTTGGTGAGGTAACGGCCCACCAAGACAGCGATCAGTAGCCGACCTGA
GAGGGTAATCGGCCACATTGGAACTGAGACACGGTC

>Zotu5833
GAACGCTAGCTACAGGCTTAACACATGCAAGTCGAGGGGCATCGCGGCGTAGCAATACGCTGGCGGCGAC
CGGCGCACGGGTGAGTAACGCGTATCCGACCTGGCCTTCACTCTGGGATAGCCCCTCGAAAGAGGGATTA
ATACCGGATATGTGGCAGAGTTACATGGCACTGCCACGAAAGATTTATCGGTGAAGGATGGGGATGCGTC
CCATTAGCTTGTTGGCGGGGTAACGGCCCACCAAGGCGACGATGGGTAGGGGTACTGAGAGGTAGGTCCC
CCACATTGGAACTGAGACACGGTC
>Zotu5835
GAACGCTGGCGGCGTGCTTAACACATGCAAGTCGAACGGGAAACATTTTATTGAAGCTTCGGCAGATTTAG
CTTGTTTCTAGTGGCGGACGGGTGAGTAACGCGTGGGTAACCTGCCTTATACTGGGGGATAACAGCCAGA
AATGACTGCTAATACCGCATAAGCGCACAGGACCGCATGGTTCGGTGTGAAAAACTCCGGTGGTATAAGA
TGGACCCGCGTTGGATTAGCTAGTTGGCAGGGCAGCGGCCTACCAAGGCGACGATCCATAGCCGGCCTGA
GAGGGTGAACGGCCACATTGGGACTGAGACACGGCC
>Zotu5837
GAACGCTGGCGGCGTGCTTAACACATGCAAGTCGAACGGAAAGGCCCCTTTTTGGGGTACTCGAGTGGCG
AACGGGTGAGTAACACGTGGGTGATCTGCCCTCAACTTCGGGATAAGCTTGGGAAACTGGGTCTAATACC
GGATAGGACTCCTCTTTAGTGTGAGGGGTGGAAAGTTTTTTCGGTTGGGGATGAGCTCGCGGCCTATCAGC
TTGTTGGTGGGGTAATGGCCTACCAAGGCGTCGACGGGTAGCCGGCCTGAGAGGGTGTACGGCCACATTG
GGACTGAGATACGGCC
>Zotu5839
GAACGCTGGCGGCATGCCTTACACATGCAAGTCGAACGGCAGCACGGGAGCAATCCTGGTGGCGAGTGG
CGAACGGGTGAGTAATACATCGGAACGTGTCCTGTCGTGGGGGATAGCCCGGCGAAAGCCGGATTAATAC
CGCATACGACCTACGGGTGAAAGCGGGGGATCGCAAGACCTCGCGCGATTGGAGCGGCCGATGTCAGATT
AGCTTGTTGGTGGGGTAAAAGCCTACCAAGGCAACGATCTGTAGCTGGTCTGAGAGGACGACCAGCCACA
CTGGGACTGAGACACGGCC
>Zotu5841
GAACGCTGGCGGTATGCTTAACACATGCAAGTCGAACGGACTTAGGGAGCTTGCTCCCGAAAGTTAGTGG
CAAACGGGTGAGTAACGCGTGGGAATCTCCCCATCAGTACGGAACAACATTTGGAAACAAATGCTAATACC
GTATATTCTCGAGAGAGGAAAGATTTATCGCTGATGGATGAGCCCGCGTCGGATTAGGTAGTTGGTGAGG
TAATGGCTCACCAAGCCGACGATCCGTAGCTGGTCTGAGAGGATGATCAGCCACACTGGGACTGAGACAC
GGCC
>Zotu5843
GAACGCTGACAGAATGCTTAACACATGCAAGTCTACTTGAATTTGGGTTTTTTAACTTAGATTTGGGTGGCG
GACGGGTGAGTAACGCGTAAAGAACTTGCCTCACAGCTAGGGACAACATTTGGAAACGAATGCTAATACC
TGATATTATGATTATAAGGCATCTTAGAATTATGAAAGCTATATGCGCTGTGAGAGAGCTTTGCGTCCCATT
AGCTAGTTGGAGAGGTAACGGCTCACCAAGGCGATGATGGGTAGCCGGCCTGAGAGGGTGAACGGCCAC
AAGGGGACTGAGACACGGCC
>Zotu5845
GAACGCTGGCGGCGTGCCTAATACATGCAAGTCGAGCGAATCTTTGGGAGTTTGCTCCTAAAGGTTAGCG
GCGGACGGGTGAGTAACACGTGGGCAACCTGCCTATAAGACTGGGATAACTTCGGGAAACCGGAGCTAAT
ACCGGATAATTCTTTTCTACACATGTAGAAAAGCTAAAAGACGGTTTACGCTGTCACTTATAGATGGGCCCG
CGGCGCATTAGCTAGTTGGTGAGGTAACGGCTCACCAAGGCCACGATGCGTAGCCGACCTGAGAGGGTGA
TCGGCCACACTGGGACTGAGACACGGCC
>Zotu5849
GAACGCTGGCGGCGTGCCTAAAACATGCAAGTCGAACGAGGGAGCAATCCCTAGTGGCGAACGGCGGAG
TAAGACATGAACAACGTGCCCTAAGGTCCGGGACAAACGCAGAAATGCCATAAATACCGGATGTGGCCCC
GATTCGGCATCGAGTTGGGATTAAACGGATTTTCGCCTTAGGAGCGGTTCATGGCCTATCAGGTAGTTGGT
GGGGTAACGGCCCACCAAGCCGACGACAGGTAGCGGGTCTGAGAGGATGATCCGCCCGAGTGGGACTGA
GACACGGCC
>Zotu5855

GAGCGCTGGCGGCGTGCTTAACACATGCAAGTCGAGCGGAAAGGCCCTTCGGGGTACTCGAGCGGCGAA
CGGGTGAGTAACACGTGAGTAATCTGCCCTTGGCTTCGGGATAGCCACCGGAAACGGTGATTAATACCGG
ATACGACAACTTCTCGCATGGGATGGTTGTGGAAAGTTTTTCGGCCAGGGATGTGCTCGCGGCCTATCAGC
TTGATGGTGAGGTAATGGCTCACCATGGCTTCGACGGGTAGCCGGCCTGAGAGGGTGACCGGTCACACTG
GGACTGGGACACGGCC

>Zotu5857
GAACGCTGGCGGCATGCCTAATACATGCAAGTCGAACGATCACTTCGGTGGTAGTGGCGCACGGGTGCGT
AACGCGTGGGAATCTGCCCTTGGGTTCGGAATAACTTCTGGAAACGGAAGCTAATACCGGATGATGACGT
AAGTCCAAAGATTTATCGCCCAGGGATGAGCCCGCGTAGGATTAGCTAGTTGGTGGGGTAAAGGCTTACC
AAGGCGACGATCCTTAGCTGGTCTGAGAGGATGATCAGCCACACTGGGACTGAGACACGGCC

>Zotu5859
GAACGCTGGCGGCATGCTTTACACATACAAGTCGAACGGTAACAGGTCTTCGGATGCTGACGAGTGGCGA
ACGGGTGAGTAATACATCGGAACGTGCCTAGTAGTGGGGGATAACTACTCGAAAGAGTAGCTAATACCGC
ATGAGATCTACGGATGAAAGCAGGGGACCTTCGGGCCTTGTACTACTAGAGCGGCTGATGGCAGATTAGG
TAGTTGGTGGGGTAAAGGCTTACCAAGCCTGCGATCTGTAGCTGGTCTGAGAGGACAACCAGCCACACTG
GGACTGAGACACGGCC

>Zotu5861
GAACGCTGGCGGCAGGCCTAACACATGCAAGTCGAACGGTAACACAGAGAGCTTGCTCTCGGGTGACGAG
TGGCGGACGGGTGAGTAATGTCTGGGAAACTGCCTGATGGAGGGGGATAACTACTGGAAACGGTAGCTA
ATACCGCATAACGTCGCAAGACCAAAGAGGGGGACCTTCGGGCCTCTTGCCATCAGATGTGCCCAGATGG
GATTAGCTAGTAGGTGGGGTAACGGCTCACCTAGGTGACGATCCCTAGCTGGTCTGAGAGGATGACCAGC
CACACTGGAACTGAGACACGGTC

>Zotu5865
GAACGCTGGCGGCAGGCTTAACACATGCAAGTCGAGCGGGTGTAGCAATACATCAGCGGCAGACGGGTG
AGTAACGCGTGGGAACGTACCTTTTGGTTCGGAACAACACAGGGAAACTTGTGCTAATACCGGATAAGCCC
TTACGGGGAAAGATTTATCGCCGAAAGATCGGCCCGCGTCTGATTAGCTAGTTGGTGAGGTAATGGCTCAC
CAAGGCGACGATCAGTAGCTGGTCTGAGAGGATGATCAGCCACATTGGGACTGAGACACGGCC

>Zotu5867
GAACGCTGGCGGCATGCTTAACACATGCAAGTCGAACGCAGCATCAGGGCTTGCTCTGATGGCTGAGTGG
CAAACGGGTGAGTAACACGTGGGAACTTACCCTTTAGACTGGGACAACCACTGGAAACGGTGGCTAATAC
CGGATACGCCCTGAGGGGGAAAGCAGCAATGCGCTAAGGGAGAGGCCCGCGGCAGATTAGGTAGTTGGT
GGGGTAAAGGCCTACCAAGCCTGTGATCTGTAGCTGGTTTGAGAGGATGATCAGCCACACTGGGACTGAG
ACACGGCC

>Zotu5869
GAACGCTGGCGGTGTGCTTAATACATGCAAGTCGAGCGGTAAGGCCTCTTCGGAGGTACACGAGCGGCGG
ACGGCTGAGTAACGCGTAGGAATCTACCCCGAACTGAGGAATAAGCACCAGAAATGGTGTCTAATGCCGC
ATATGCTCTTAGGAGTAAAGCTTCGGCGGTTTGGGAAGAGCCTGCGTTCGATTAGCTTGTTGGTAAGGTAA
CGGCTTACCAAGGCGACGATCGATAGCTGGTCTGAGAGGATGACCAGCCAGACTGGGACTGAGAACGGC
C

>Zotu5872
GAACGCTGGCGGCGTGCTTAACACATGCAAGTCGAACGGTGAAGCAGAGCTTGCTCTGTGGATCAGTGGC
GAACGGGTGAGTAACACGTGAGCAACCTGCCCCTGACTCTGGGATAAGCGCTGGAAACGGCGTCTAATAC
TGGATATGAGCTGCGACCGCATGGTCAGCAGTTGGAAAGATTTTTCGGTCTGGGATGGGCTCACGGCCTAT
CAGCTTGTTGGTGAGGTAATGGCTCACCAAGGCGTCGACGGGTAGCCGGCCTGAGAGGGTGACCGGCCAC
ACTGGGACTGAGACACGGCC

>Zotu5874
GAATGCTGGCGGCGCGCCTAACACATGCAAGTCGAGCGGTAACATGTTTCTCACTGTCTTCTTCTTTTTATA
AAAGGAGGGAATTGTGAGAAATGATGACGAGCGGCGGACGGCTGAGTAACGCGTGGGAACATGCCCCAA
AGTGAGGGATAAGCTCCAGAAATGGAGTCTAATACCGCATATGATCTTAGGATTAAAGGATTTATCCGCTT
TGGGAGTGGCCTGCGTACGATTAGATAGTTGGTGAGGTAAAGGCTCACCAAGTCTACGATCGTTAACTGTT
CTGAGAGGATGATCAGTCAGACTGGAACTGAGACACGGTC

>Zotu5879
GAACGCTGGCGGTGTGCCTAATACATGCAAGTCGAGCGGTAAGGCCCCTTCGGGGGTACACGAGCGGCG
GACGGCTGAGTAACACGTAGGAACGTACCCCAAACTGAGGGATAAGCACCAGAAATGGTGTCTAATACCG
CATGTGCTCTAAGGAGTAAAGCTTCGGCGGTTTGGGAGCGGCCTGCGTCCGATTAGCTAGTTGGTGAGGT
AATAGCTTACCAAGGCGACGATCGGTAGCTGGTCTGAGAGGATGATCAGCCAGACTGGGACTGAGAACG
GCC
>Zotu5882
GAACGCTGGCGGCGTGCCTAATACATGCAAGTCGAGCGAAGTTGAGGGAGCTTGCTCCTGAGACTTAGCG
GCGAACGGGTGAGTAACACGTGGGCAACCTGCCCGATAGACCGGGATAACTTGGGGAAACCCAAGCTAAT
ACCGGATAGGATCATTGACCGCCTGGTTGATGATGGAAAGTCTGAACGACACTATCGGATGGGCCCGCGG
CGCATTAGCTAGTTGGTGGGGTAACGGCCTACCAAGGCGACGATGCGTAGCCGACCTGAGAGGGTGACCG
GCCACACTGGGACTGAGACACGGCC
>Zotu5885
GAACGCTGGCGGCATGCCTTACACATGCAAGTCGAACGGCAGCGCGGGGCAACCTGGCGGCGAGTGGCG
AACGGGTGAGTAATATATCGGAACGTACCCTGGAGTGGGGGATAACGTAGCGAAAGTTACGCTAATACCG
CATACGATCTAAGGATGAAAGCAGGGGACCGCAAGGCCTTGTGCTCCTGGAGCGGCCGATATCTGATTAG
CTAGTTGGTGGGGTAAAGGCCCACCAAGGCATCGATCAGTAGCTGGTCTGAGAGGACGACCAGCCACACT
GGAACTGAGACACGGTC
>Zotu5889
GAACGCTGGCGGCAGGCTTAACACATGCAAGTCGAACGCCCCGCAAGGGGAGTGGCAGACGGGTGAGTA
ACGCGTGGGAATCTACCTAGTTCTGCGGAACAACAGAGGGAAACTTCTGCTAATACCGCATACGCCCTACG
GGGGAAAGATTTATCGGAATTAGATGAGCCCGCGTAAGATTAGCTAGTTGGTGGGGTAATGGCCTACCAA
GGCGACGATCTTTAGCTGGTCTGAGAGGATGATCAGCCACACTGGGACTGAGACACGGCC
>Zotu5891
GAACGCTGGCGGCATGCCTAACACATGCAAGTCGAACGAGACCTTCGGGTCTAGTGGCGCACGGGTGCGT
AACGCGTGGGAATCTGCCCTTGGGTTCGGAATAACAGTTAGAAATGACTGCTAATACCGGATAATGACGTA
AGTCCAAAGATTTATCGCCCAAGGATGAGCCCGCGTAGGATTAGCTAGTTGGTGGGGTAAAGGCCCACCA
AGGCGACGATCCTTAGCTGGTCTGAGAGGATGATCAGCCACACTGGGACTGAGACACGGCC
>Zotu5894
GAACGCTAGCGGCAGGCCTAATACATGCAAGTCGAGGGGCAGCAGGGTAGCAATATCGCTGGCGACCGG
CGCACGGGTGCGTAACACGTATACAATCTGCCTTTAACAGGGGGATAGCCTTTCGAAAGAGAGATTAATAC
CCCATAACACATGAAATAGCATTATTTTATGTTAAAAGCTCCGGCGGTTAGAGATGAGTATGCGTCTGATTA
GCTAGTAGGTGAGGTAACGGCTCACCTAGGCGACGATCAGTAGGGGATCTGAGAGGATTAACCCCCACAC
GGATACTGAGACACGGAT
>Zotu5897
TAACGCTAGCGGCAGGCCTAATACATGCAAGTCGAACGGGATTGATTGTAGCAATATGATCATGAGAGTG
GCGCACGGGTGCGTAACACGTATGCAACCTACCTATAACTGGGGGATAGCCTCCCGAAAGGGAGATTAAC
ACCGCATAACATTATGAAGTGGCATCACTTTATAATTAAATCTACGGAGGTTATAGATGGGCATGCGTCCG
ATTAGCTAGTTGGTGAGGTAACGGCTCACCAAGGCTCCGATCGGTAGGGGGTCTGAGAGGATGATCCCCC
ACACTGGTACTGAGACACGGAC
>Zotu5899
GAACGCTGGCGGCGCGCTTAACACATGCAAGTCGAACGGGTGTCCTTCGGGGCATCAGTGGCGGACGGGT
GCGTAACACGTAAGCGACCTGCCTTCAGGTGGGGGATAACCCCGGGAAACCGGGGCTAATACCGCATGAG
CTCTTCGGGCTGCATGGTCTGAAGAGGAAAGCTTCTGTAAGGGAGCGCTTGAAGAGGGGCTTGCGGCGTA
TCAGCTAGTTGGCGAGGTAACGGCTCACCAAGGCGACGACGCGTAGGGGGTCTGAGAGGACGGCCCCCC
ACATTGGGACTGAGATACGGCC
>Zotu5901
GAACGCTGGCGGCAGGCTTAACACATGCAAGTCGAGCGGGCATCCTTGTGGTGTCAGCGGCAGACGGGT
GAGTAACGCGTGGGAACGTGCCCTTCGGTTCGGAATAACCCAGGGAAACTTGAGCTAATACCGGATACGT

GCGAGAGCAGAAAGGTTTATCTGCCGAAGGATCGGCCCGCGTCTGATTAGCTAGTTGGTGAGGTTACGGC
TCACCAAGGCGACGATCAGTAGCTGGTCTGAGAGGATGATCAGCCACACTGGGACTGAGACACGGCC
>Zotu5903
GAACGCTGGCGGCGTGCCTAATACATGCAAGTCGAGCGGACAGATGGGAGCTTGCTCCCTGATGTCAGCG
GCGGACGGGTGAGTAACACGTGGGCAACCTGCCTGTAAGACTGGGATAACTCCGGGAAACCGGGGCTAA
TACCGGATAATTCTTTTCTCTTCATGGAGAAAAGCTGAAAGACGGTTTCGGCTGTCACTTACAGATGGGCCC
GCGGCGCATTAGCTAGTTGGTGAGGTAACGGCTCACCAAGGCGACGATGCGTAGCCGACCTGAGAGGGT
GATCGGCCACACTGGGACTGAGACACGGCC
>Zotu5905
GAACGCTAGCGGCAGGCTTAACACATGCAAGTCGAGGGGTAGAAGGAGCTTGCTTCTTTGAGACCGGCGC
ACGGGTGCGTAACGCGTATGCAATCTACCTTATACAGGGGAATAGCCCAGAGAAATTTGGATTAATGCCCC
ATGGTATTATAGAATGGCATCATTTTATAATTAAAGTTCCAACGGTATAAGATGAGCATGCGTCCCATTAGT
TAGTTGGTAAGGTAACGGCTTACCAAGACGATGATGGGTAGGGGTCCTGAGAGGGAGATCCCCCACACTG
GTACTGAGACACGGAC
>Zotu5908
GAACGCTGGCGGCGTGCTTAACACATGCAAGTCGAACGGTGATGGGGAGCTTGCTCCTCTGATCAGTGGC
GAACGGGTGAGTAACACGTGAGTAACCTGCCCTCGACTCTGGGATAAGCGTTGGAAACGACGTCTAATAC
CGGATACGACCTCGGATGGCATCTTCCGGGGTGGAAAGAGTTTCGGTCGAGGATGGACTCGCGGCCTATC
AGCTTGTTGGTGAGGTAGTGGCTCACCAAGGCGACGACGGGTAGCCGGCCTGAGAGGGTGACCGGCCAC
ACTGGGACTGAGACACGGCC
>Zotu5911
GAACGCTGGCGGCGTGCCTAATACATGCAAGTCGAGCGAATCTTTGGGAGCTTGCTCCCATTGGTTAGCGG
CGGACGGGTGAGTAACACGTGGGCAACCTGCCTGTAAGACTGGGATAACTTCGGGAAACCGGAGCTAATA
CCGGATAATCCATTTCCTCTCATGGGGAGATGCTGAAAGACGGTTTCGGCTGTCACTTACAGATGGGCCCG
CGGCGCATTAGCTAGTTGGTGAGGTAACGGCTCACCAAGGCGACGATGCGTAGCCGACCTGAGAGGGTG
ATCGGCCACACTGGGACTGAGACACGGCC
>Zotu5914
GAACGCTAGCGGGAGGCCTAACACATGCAAGCTGAGCGGTATAGTTTCTTCGGAAATGAGAGAGCGGCGT
ACGGGTGCGGAACACGTGTGCAACCTGCCTTTATCTAGGGGATAGCCTTTCGAAAGGAAGATTAATACTCC
ATAATATATTGAACGGCATCGTTTAATATTGAAAGCTCCGGCGGATAGAGATGGGCACGCGCAAGATTAGC
TAGTTGGTGAGGTAACGGCTCACCAAGGCGATGATCTTTAGGGGGCCTGAGAGGGCGATCCCCCACACTG
GTACTAAGACACGGAC
>Zotu5916
GAACGCTGGCGGCGTGCCTAACACATGCAAGTCGAACGGGGGTAGCAATACCCCAGTGGCGAACGGGTG
CGTAACACGTGAGGAACATTCCTTCCGGTGGGGGATAGCCGGCCTAACGGCCGGGTAATACCGCATACGC
TGTTCGGGGTGCATACCCCGGATCGGAAAGCAGCAATGCGCCGGAAAGGTGCCTCGCGGCCTATCAGCTA
GTTGGTGAGGTAATGGCTCACCAAGGCGACGACGGGTAGCTGGTCTGAGAGGATGGCCAGCCACATTGG
GACTGAGACACGGCC
>Zotu5921
GAACGCTGGCGGCAGGCTTAACACATGCAAGTCGAGCGCCCCGCAAGGGGAGCGGCAGACGGGTGAGTA
ACGCGTGGGAATCTACCTTTTGCTACGGAATAACTCAGGGAAACTTGTGCTAATACCGTATGTGCCCTTTGG
GGGAAAGATTTATCGGCAAAGGATGAGCCCGCGTTGGATTAGGTAGTTGGTGGGGTAATGGCCTACCAAG
CCGACGATCCATAGCTGGTCTGAGAGGATGATCAGCCACATTGGGACTGAGACACGGCC
>Zotu5923
GAACGCTGGCGGCAGGCCTAATACATGCAAGTCGAGCGGTCCCTTCGGGGGCAGCGGCGGACGGGTGAG
TAACGCGTGGGAACGTGCCCTTTGGTACGGAACAACTGAGGGAAACTTCAGCTAATACCGTATGTGCCCTT
TGGGGGAAAGATTTATCGCCATTGGAGCGGCCCGCGTTAGATTAGCTAGTTGGTAGGGTAATGGCCTACC
AAGGCGACGATCTATAGCTGGTCTGAGAGGATGATCAGCCACACTGGGACTGAGACACGGCC
>Zotu5926

GAACGCTGGCGGCATGCCTAACACATGCAAGTCGAACGAGATCCTTCGGGGTCTAGTGGCGCACGGGTGC
GTAACGCGTGGGAATCTGCCCTTAGGTTCGGAATAACAGTTAGAAATGACTGCTAATACCGGATGATGACG
TTAAGTCCAAAGATTTATCGCCTGAGGATGAGCCCGCGTAGGATTAGCTAGTTGGTGTGGTAAAGGCGCAC
CAAGGCGACGATCCTTAGCTGGTCTGAGAGGATGATCAGCCACACTGGGACTGAGACACGGCC

>Zotu5929

GAACGCTGGCGGCAGGCTTAACACATGCAAGTCGAGCGAAGGCTTCGGCCTTAGCGGCGGACGGGTGAG
TAACGCGTGGGAACGTGCCCTTCACTACGGAATAGGCCCGGGAAACTGGGTTTAATACCGTATACGCCCTT
TGGGGGAAAGATTTATCGGTGAAGGATCGGCCCGCGTTGGATTAGGTAGTTGGTGGGGTAATGGCCTACC
AAGCCGACGATCCATAGCTGGTTTGAGAGGATGATCAGCCACACTGGGACTGAGACACGGCC

>Zotu5931

GAACGCTGGCGGAATGCTTTACACATGCAAGTCGAACGGCAGCACGGACTTCGGTCTGGTGGCGAGTGGC
GAACGGGTGAGTAATATATCGGAACGTGCCCAGTCGTGGGGGATAACGTAGCGAAAGCTACGCTAATACC
GCATACGATCTATGGATGAAAGCGGGGGATCGCAAGACCTCGCGCGATTGGAGCGGCCGATATCAGATTA
GGTAGTTGGTGGGGTAAAGGCCTACCAAGCCGACGATCTGTAGCTGGTCTGAGAGGACGACCAGCCACAC
TGGGACTGAGACACGGCC

>Zotu5933

GAACGCTGGCGGCATGCCTAACACATGCAAGTCGAACGAAGCCTTCGGGCTTAGTGGCGCACGGGTGCGT
AACGCGTGGGAATCTGCCCTTGGGTTCGGAATAACAGTTAGAAATGACTGCTAATACCGGATGATGTCTTC
GGACCAAAGATTTATCGCCCAAGGATGAGCCCGCGTAGGATTAGCTTGTTGGTGAGGTAAAGGCTCACCA
AGGCGACGATCCTTAGCTGGTCTGAGAGGATGATCAGCCACACTGGGACTGAGACACGGCC

>Zotu5935

GAACGCTGGCGGCGTGCCTAATACATGCAAGTCGAACGGTTTAGTTCCAAGAGGCTTGCCTTTTGGAACTA
AGCAGTGGCGGACGGGTGAGTAACACGTAGGCAACCTACCTGTCAAACCGGGATAACCTCGGGAAACCGA
AGCTAAACCCGGATAAGCTCCTTTTTCGCATGAAGGGGGAGTGAAAAGGTGCGACCTGATGACAGATGGG
CCTGCGGCGCATTAGCTAGTAGGTAGGGTAACGGCCTACCTAGGCGATGATGCGTAGCCGACCTGAGAGG
GCGTACGGCCACACTGGGACTGAGATACGGCC

>Zotu5937

GAACGCTGGCGGCGTGCTTAACACATGCAAGTCGAACGGTGAAGGAGGAGCTTGCTCTTCTGGATCAGTG
GCGAACGGGTGAGTAACACGTGAGCAATCTGCCCTTGACTCTGGGATAACAGTTGGAAACAGCTGCTAAT
ACCGGATACGAGACGTGACCGCATGGTCAACGTCTGGAAAGAACTTCGGTCAAGGATGAGCTCACGGCCT
ATCAGCTAGTTGGTGAGGTAATGGCTCACCAAGGCGACGACGGGTAGCCGGCCTGAGAGGGTGACCGGC
CACACTGGGACTGAGACACGGCC

>Zotu5940

GAACGCTGGCGGCAGGCTTAACACATGCAAGTCGAGCGGGCACTTTCGGGTGTCAGCGGCGGACGGGTG
AGTAACGCGTGGGAACGTGCCCTCTGGTTCGGAATAACTCAGGGAAACTTGAGCTAATACCGGATACGCC
CTTTTGGGGAAAGGCTTGCTGCCGGAGGATCGGCCCGCGTCTGATTAGCTAGTTGGTGAGGTAACGGCTC
ACCAAGGCGACGATCAGTAGCTGGTCTGAGAGGATGATCAGCCACACTGGGACTGAGACACGGCC

>Zotu5945

GAACGCTGGCGGCGTGCTTAACACATGCAAGTCGAGCGGTAAGGCTCCTTCGGGAGTACACGAGCGGCGA
ACGGGTGAGTAACACGTGAGCAATCTGCCCTTCACTTTGGGATAACCTCCGGAAACGGTGGCTAATACCGA
ATATGACCACTTCACGCATGTGATGGTGGTGGAAAGTTCTGGCGGTGGAGGATGAGCTCGCGGCCTATCA
GCTTGTTGGTGAGGTAATGGCTCACCAAGGCTTCGACGGGTAGCCGGCCTGAGAGGGTGACCGGCCACAC
TGGGACTGAGACACGGCC

>Zotu5947

GAACGCTAGCTACAGGCTTAACACATGCAAGTCGAGGGGAAACGACATCGAAAGCTTGCTTTTGATGGGC
GTCGACCGGCGCACGGGTGAGTAACGCGTATCCAACCTGCCCATCACTTGGGGATAACCTTGCGAAAGTA
AGACTAATACCCAATGATATCTCTAGAAGACATCTGAAAGGGATTAAAGATTTATCGGTGATGGATGGGGA
TGCGTCTGATTAGCTTGTTGGCGTGGTAACGGCCCACCAAGGCGACGATCAGTAGGGGTTCTGAGAGGAA
GGTCCCCCACATTGGAACTGAGACACGGTC

>Zotu5950

GAACGCTGGCGGCAGGCCTAACACATGCAAGTCGAACGCTCGTAGCAATACGGGAGTGGCAGACGGGTG
AGTAACGCGTGGGAACCTTCCCAGTGGTACGGAATAGCTCAGGGAAACTTGAGGTAATACCGTATACGCC
CCTAGGGGGAAAGATTTATCGCCATTGGATGGGCCCGCGTCGGATTAGCTAGTTGGTGAGGTAACGGCTC
ACCAAGGCGACGATCCGTAGCTGGTTTGAGAGAATGACCAGCCACACTGGGACTGAGACACGGCC
>Zotu5952
GAACGCTGGCGGCGTGCCTAATACATGCAAGTCGAGCGGTAAGGCTCCTACAGTTTATTTGATCATAATGA
CTTCGGTCATTGCAGCAAACCACCTGCGACCCCGGCTAGGGAAGTAGTGGCGAGGGAAGATCGAATGAAG
TGTTGGAGTACACGAGCGGCGGACGGCTGAGTAACACGTAGGAACGTACCCCAAAGTGAGGCATAAGCA
CCAGAAATGGTGTCTAATACCGCATGTGCTCTTCGGAGTAAAGCTACGGCGCTTTGGGAACGGCCTACGCA
TGATTAGCTTGTTGGTGAGGTAAAAGCTCACCAAGGCGACGATCATTAGCTGGTCTGAGAGGATGATCAG
CCAGACTGGGACTGAGAACGGCC
>Zotu5954
GAACGCTGGCGGCATGCCTAACACATGCAAGTCGAACGAGACCTTCGGGTCTAGTGGCGCACGGGTGCGT
AACGCGTGGGCATCTACCCTTGGGTTCGGAATAACTCAGAGAAATTTGAGCTAATACCGGATGATGTCGTA
AGACCAAAGATTTATCGCCCAGGGATGAGCCCGCGTAAGATTAGCTAGTTGGTGGGGTAAAGGCCTACCA
AGGCGACGATCTTTAGCTGGTCTGAGAGGATGATCAGCCACACTGGGACTGAGACACGGCC
>Zotu5956
GAATGCTGGCGATGTAGATGAGTCATGCAAGTCAAGGGGGCCCGCAAGGGCAACCGGCAGACGAGGTAG
TAACGCGTAGGTAGACCCCCCGAAGTCGGGCATAGCCAGCCGAAAGGCTGGGTAATTCCCGATAGTCCCG
CAAGGGTAAAGGTTTTTCGCTTCGGGAGTCGCCTGCGCAGTATCAGCTAGTTGGTAAGGTAACGGCTTACC
AAGGCTACGACGCTTAGGGGAGCTGAGAGGCTGACCCCCACCGATGGGACTGCGACACGGCC
>Zotu5959
GAACGCTGGCGGCATGCTTAATACATGCAAGTCGAACGAGACCTTCGGGTCTAGTGGCGCACGGGTGCGT
AACGCGTGGGAATCTACCCTTTGCTACGGAATAACTCAGAGAAATTTGTGCTAATACCGTATAATGTCGCA
AGACCAAAGATTTATCGGCAGAGGACGAGCCCGCGTAAGATTAGCTTGTTGGTGAGGTAAAGGCTCACCA
AGGCGACGATCTTTAGCTGGTCTGAGAGGATGATCAGCCACACTGGGACTGAGACACGGCC
>Zotu5965
GCCGTATCTCAGTCCCAATGTGGCCGGTCACCCTCTCAGGCCGGCTACCCGTCAAAGCCTTGGTAAGCCACT
ACCCCACCAACAAGCTGATAAGCCGCGAGTCCATCCCCAACCGCCGAAACTTTCCAACCCCCACCATGCAGC
AGGAGCTCCTATCCGGTATTAGCCCCAGTTTCCTGAAGTTATCCCAAAGTCAAGGGCAGGTTACTCACGTGT
TACTCACCCGTTCGCCACTCGAGCACCCCACAAAAGCAGGGCCTTTCCGTTCGACTTGCATGTGTTAAGCAC
GCCGCCAGCGTTCG
>Zotu5967
GAACGCTGGCGGCGTGCCTAATACATGCAAGTCGTGCGGACTTATCTTCGGATAAGTTAGCGGCGGACGG
GTGAGTAACACGTGGGCAACCTGCCTGTAAGACTGGGATAACTCCGGGAAACCGGTGCTAATACCAGATA
CAATCAAAGACTGCCTGGTTTTTGATGGAAAGGTCTTGTACCGCTTACAGATGGGCCCGCGGCGCATTAGC
TAGTTGGTGGGGTAATGGCTCACCAAGGCGACGATGCGTAGCCGACCTGAGAGGGTGATCGGCCACACTG
GGACTGAGACACGGCC
>Zotu5970
GAACGCTGGCGGCATGCCTAACACATGCAAGTCGAACGAGACCTTCGGGTCTAGTGGCGCACGGGTGCGT
AACGCGTGGGAATCTGCCCTTAGGTTCGGAATAACAGTTAGAAATGACTGCTAATACCGGATAATGACGTA
AGTCCAAAGATTTATCGCCTAGGGATGAGCCCGCGTAGGATTAGCTAGTTGGTGGGGTAAAAGCCTACCA
AGGCGACGATCCTTAGCTGGTCTGAGAGGATGATCAGCCACACTGGGACTGAGACACGGCC
>Zotu5972
GAACGCTGGCGGCGTGCCTAACACATGCAAGTCGAGCGATTTACTTCGGTAAAGAGCGGCGGACGGGTGA
GTAACGCGTGGGTAACCTGCCCTGTACACACGGATAACATACCGAAAGGTATGCTAATACGAGATAATATG
CTTTTATCGCATGGTAGAAGTATCAAAGCTTTTGCGGTACAGGATGGACCCGCGTCTGATTAGCTAGTTGGT
GAGGTAACGGCTCACCAAGGCGACGATCAGTAGCCGACCTGAGAGGGTGATCGGCCACATTGGAACTGA
GACACGGTC
>Zotu5974

GAACGCTGGCGGCGTGCCTAATACATGCAAGTCGAGCGAATCAAAGGGAGCTTGCTCCCTTTTGGTTAGCG
GCGGACGGGTGAGTAACACGTGGGCAACCTGCCTATAAGACTGGGATAACTTCGGGAAACCGGAGCTAAT
ACCGGATCATTCATATCTACTCATGTGGATATGCTGAAAGACGGTTTACGCTGTCACTTATAGATGGGCCCG
CGGCGCATTAGCTAGTTGGTGAGGTAACGGCTCACCAAGGCGACGATGCGTAGCCGACCTGAGAGGGTG
ATCGGCCACACTGGGACTGAGACACGGCC
>Zotu5977
GAACGCTAGCGGCAGGCCTAATACATGCAAGTCGAGGGGTATTGTAGCAATACGAGAGACCGGCGCACG
GGTGCGTAACGCGTATGAAAGGTGTCCAAATCACTGGGATAGCCCTCGGAAACGGGGATTAATACCGGAT
GGTCTCTTTATGTGGCATCACATTAAGAGTAAAGCTCAGGCGGATTTGGGTCCTCATGCGTACGATTAGCTA
GTTGGCGGGGTAATGGCCCACCAAGGCGACGATCGTTAGGGGTCCTGAGAGGGAGATCCCCCACACTGGT
ACTGAGACACGGGC
>Zotu5979
GAACGCTAGCGGGAGGCCTAATACATGCAAGTCGAGCGGTAACTTTCTTCGGGAAGCTAGAGCGGCGCAC
GGGTGAGTAACGCGTACATGACCTACCTTTAAGACTGTGATAGCCCTGGGAAACTGGGATTAATAACGGAT
GTGATAATAGGTATAAGGATTTATTATTAAAGATGAGTCGCTTAGAGATGGGTGTGCGTCTGATTAGCTAG
TTGGTAGGGTAACGGCCTACCAAGGCGACGATCAGTAGGGGGCGTGAGAGCGTGACCCCCCACACGGGT
ACTGAGACACGGAC
>Zotu5982
GAACGCTGGCGGCAGGCTTAACACATGCAAGTCGAGCGGGCATCCTTGTGGTGTCAGCGGCAGACGGGT
GAGTAACGCGTGGGAACGTGCCCTTCGGTTCGGAATAACTCAGGGAAACTTGAGCTAATACCGGATACGC
CCTTATGGGGAAAGGTTTACTGCCGAAGGATCGGCCCGCGTCTGATTAGCTGGTTGGTGAGGTTACGGCTC
ACCAAGGCGACGATCAGTAGTTGGTCTGAGAGGATGATCAGCCACACTGGGACTGAGACACGGCC
>Zotu5987
GAACGCTGGCGGCATGCCTAACACATGCAAGTCGAACGAAGGCTTCGGCCTTAGTGGCGCACGGGTGCGT
AACGCGTGGGAATCTGCCCTTGGGTTCGGAATAACAGTTAGAAATGACTGCTAATACCGGATGATGACGTA
AGTCCAAAGATTTATCGCCCAGGGATGAGCCCGCGTAGGATTAGCTAGTTGGTGAGGTAAAGGCTCACCA
AGGCGACGATCCTTAGCTGGTCTGAGAGGATGATCAGCCACACTGGGACTGAGACACGGCC
>Zotu5989
GAACGCTAGCGGCAGGCCTAACACATGCAAGTCGAGGGGCAGCATAAAACAAGCTTGCTTGTTTGGTGGC
GACCGGCGCACGGGTGCGTAACGCGTATGCAACCTACCCTGTACTGAGGGATAGCCCGGAGAAATTCGGA
TTAACACCTCATAGGATCTTAGATCGGCATCGATTAGAGATTAAAGGTTACGGTACAGGATGGGCATGCGT
CCCATTAGCTAGATGGTAAGGTAACGGCTTACCATGGCAACGATGGGTAGGGGGCCTGAGAGGGTGGTCC
CCCACACTGGTACTGAGACACGGAC
>Zotu5991
GAACGCTGGCGGCATGCCTTACACATGCAAGTCGAACGGCAGCGCGGGAACAATCCTGGCGGCGAGTGG
CGAACGGGTGAGTAATACATCAGAACGTGCCCAATCGTGGGGGATAACGCAGCGAAAGCTGTGCTAATAC
CGCATACGATCTACGGATGAAAGCAGGGGATCACAAGACCTTGCGCGAATGGAGCGGCCGATGGCAGATT
AGGTAGTTGGTGAGGTAAAGGCTCACCAAGCCTTCGACCTGTAGCTGGTCTGAGAGGACGACCAGCCACA
CTGGGACTGAGACACGGCC
>Zotu5993
GAACGCTGGCGGCAGGCTTAACACATGCAAGTCGAGCGCCCCGCAAGGGGAGCGGCAGACGGGTGAGTA
ACGCGTGGGAATCTACCGTACCCTACGGAATAACGCATGGAAACGTGTGCTAATACCGTATACGCCCTACG
GGGGAAAGATTTATCGGGGTATGATGAGCCCGCGTTGGATTAGCTAGTTGGTGGGGTAAAGGCCTACCAA
GGCGACGATCCATAGCTGGTCTGAGAGGATGATCAGCCACATTGGGACTGAGACACGGCC
>Zotu5996
GAACGCTGGCGGCGTGCCTAATACATGCAAGTCGAGCGGACCTGAATGGAGCTTGCTCCAGACAGGTTAG
CGGCGGACGGGTGAGTAACACGTAGGCAACCTGCCCGCAAGACCGGGATAACGTAGGGAAACTTACGCT
AATACCGGATAGGGCACTCTCTCGCATGAGAGGGTGCGGAAAGGTGGCGCAAGCTACCACTTGCGGATGG
GCCTGCGGCGCATTAGCTAGTTGGTGGGGTAACGGCCTACCAAGGCGACGATGCGTAGCCGACCTGAGAG
GGTGACCGGCCACACTGGGACTGAGACACGGCC

>Zotu5998
GAACGCTGGCGGCATGCCTAACACATGCAAGTCGAACGAAGGCTTCGGCCTTAGTGGCGCACGGGTGAGT
AACACGTGGGAACCTGCCTTTCGGTTCGGAATAACGTTTGGAAACGAACGCTAACACCGGATACGCCCTTC
GGGGGAAAGTTTACGCCGAGAGAGGGGCCCGCGTCCGATTAGGTAGTTGGTGAGGTAACGGCTCACCAA
GCCGACGATCGGTAGCTGGTCTGAGAGGATGATCAGCCACACTGGGACTGAGACACGGCC
>Zotu6000
GAACGCTGGCGGCGTGCTTAACACATGCAAGTCGAGCGGAAAGGCCACTTCGGTGGTACTCGAGCGGCGA
ACGGGTGAGTAACACGTGAGTAATCTGCCCTCCACTTTGGGATAGCCACCGGAAACGGTGATTAATACCGG
ATATGACCTTTGCCTGCATGGGTTTTGGTGGAAAGTTTTTCGGTGGGGGATGTGCTCGCGGCCTATCAGCT
TGATGGTGGGGTAATGGCCTACCATGGCTTCGACGGGTAGCCGGCCTGAGAGGGTGACCGGCCACACTGG
GACTGAGACACGGCC
>Zotu6002
GAACGCTGGCGGCGTGCTTAACACATGCAAGTCAAGCGGTAAGGCCCTTTCGGGGGTACACGAGCGGCGA
ACGGGTGAGTAACACGTGAGTAATCTGCCCTTCACTTGGAAATAAGCCTCGGAAACGGGGTCTAATACCTG
ATATGACGGCCGTCTGCATGGGCTGGTCGTGGAAAGTTTTTCGGTGGGGGATGTGCTCGCGGCCTATCA
GCTTGTTGGTGGGGTAATGGCCTACCAAGGCTTCGACGGGTAGCCGGCCTGAGAGGGTGACCGGCCACAC
TGGGACTGAGACACGGCC
>Zotu6004
GAACGCTGGCGGCGTGCCTAATACATGCAAGTCGAGCGGTAAGGTTCTGCACTCAGTATTTGACCAGATAA
GTTTCGAAAGAAATACTGTCTGACGCAAGGTGACTCCCCGGCGGGAGGATCCGAGTCGAATCATGAGTGT
AGAATACACGAGCGGCGGACGGCTGAGTAACACGTAGGAACGTACCCCAAACTGAGGGATAAGCACCAG
AAATGGTGTCTAATACCGCATGTGATCTTCGGATTAAAGCTTCGGCGGTTTGGGAACGGCCTGCGCATGAT
TAGCTTGTTGGTGAGGTAATGGCTCACCAAGGCGACGATCATTAGCTGGTCTGAGAGGATGATCAGCCAG
ACTGGAACTGAGAACGGTC
>Zotu6007
GAACGCTGGCGGCAGGCCTAACACATGCAAGTCGAACGGTAGCGGGAAGAAGCTTGCTTCTTTGCCGACG
AGTGGCGGACGGGTGAGTAATGTCTGGGGATCTGCCCGATGGAGGGGGATAACCACTGGAAACGGTGGC
TAATACCGCATAACGTCGCAAGACCAAAGTGGGGGACCTTCGGGCCTCACACCATCGGATGAACCCAGAT
GGGATTAGCTAGTAGGTGGGGTAACGGCTCACCTAGGCGACGATCCCTAGCTGGTCTGAGAGGATGACCA
GCCACACTGGAACTGAGACACGGTC
>Zotu6009
GAACGCTGGCGGCGTGCCTAATACATGCAAGTCGAGCGAATGGATGAAGAAAGCTTGCTTTCTTCTGATGT
TAGCGGCGGACGGGTGAGTAACACGTGGGCAACCTGCCCTTTAGATTGGGATAACTTCGGGAAACCGGAG
CTAATACCAAATAATACTTTCGAATTCATGTTTGGAAGTTGAAAGACGGTTTCGGCTGTCACTAAAGGATGG
GCCCGCGGTGCATTAGCTAGTTGGTGAGGTAACGGCTCACCAAGGCAACGATGCATAGCCGACCTGAGAG
GGTGATCGGCCACACTGGGACTGAGACACGGCC
>Zotu6011
GAACGCTGGCGGCGTGCCTAATACATGCAAGTAGAACGCTGAAGGGAGAGCTTGCTCTTCTGGATGAGTT
GCGAACGGGTGAGTAACGCGTAGGTAACCTGCCTGGTAGCGGGGGATAACTATTGGAAACGATAGCTAAT
ACCGCATAATAGTAGATGTTGCATGACATTTGCTTGAAAGGTGCAATTGCATCACTACCAGATGGACCTGC
GTTGTATTAGCTAGTTGGTGGGGTAACGGCTCACCAAGGCGACGATACATAGCCGACCTGAGAGGGTGAT
CGGCCACACTGGGACTGAGACACGGTC
>Zotu6018
GAACGCTGGCGGCAGGCCTAACACATGCAAGTCGAGCGAAGTCTTCGGACTTAGCGGCGGACGGGTGAG
TAACGCGTGGGAACATACCCAAAGGTACGGAATAGCCTCGGGAAACTGGGAGTAATACCGTATGTGCCCT
TCGGGGGAAAGATTTATCGCCTTTGGATTGGCCCGCGTTGGATTAGGTAGTTGGTGGGGTAATGGCCTACC
AAGCCGACGATCCATAGCTGGTTTGAGAGGATGATCAGCCACACTGGGACTGAGACACGGCC
>Zotu6020
GAACGCTGGCGGCATGCCTAATACATGCAAGTCGTGCGGACAGATGGAAGGCTTGCCTTCCTGAAGTCAG
CGGCGGACGGGTGAGTAACACGTGGGCAACCTGCCCGCAAGACCGGGATAACTGCGGGAAACCACAGCT

AATACCGGATACGACCTCTTCTCGCATGAGCGGAGGTGGAAAGGCGGCGTCAAGCTGTCACTTGCGGATG
GGCCCGCGGCGCATTAGCTAGTTGGTAAGGTAGCGGCTTACCAAGGCGACGATGCGTAGCCGACCTGAGA
GGGTGACCGGCCACACTGGGACTGAGACACGGCC
>Zotu6025
GAACGCTAGCGGCAGGCCTAATACATGCAAGTCGGACGGGATTGAGAAGCTTGCTTTTCATGAGAGTGGC
GCACGGGTGCGTAACACGTATGTAACCTACCTTTATCAGGGGGATAGCCTCTCGAAAGAGAGATTAAGCCC
GCATAACATGGAGATGCAGCATTGCATTTCCATCAAATATTTATAGGATAAAGATGGGCATGCGGAACATT
AGCTAGTTGGTAAGGTAATGGCTTACCAAGGCTACGATGTTTAGGGGATCTGAGAGGATGACCCCCCACA
CTGGTACTGAGACACGGAC
>Zotu6029
GAACGCTGGCGTCGTGCCTAATACATGCAAGTAGAACGCTGAAGGAGGAGCTTGCTTCTCCGGATGAGTT
GCGAACGGGTGAGTAACGCGTAGGTAACCTGCCTGGTAGCGGGGGATAACTATTGGAAACGATAGCTAAT
ACCGCATAAGAGTAGATATTGCATGATATCTGCTTGAAAGGTGCAATTGCATCACTACCAGATGGACCTGC
GTTGTATTAGCTAGTTGGTGAGGTAACGGCTCACCAAGGCGACGATACATAGCCGACCTGAGAGGGTGAT
CGGCCACACTGGGACTGAGACACGGCC
>Zotu6031
GAACGTTGGCGGCGTGGATTAGGCATGCAAGTCGTACGGGCCGCAAGGCCAGTGGCGCAAGGGTGAGTA
AGGCGACGGTAACCAACCCCAGGACTGGGTATAGCCGCGGGAAACTGCGGGTAATCCCCAGCGACGTCGC
GGGGGGGCATCCCCCCGCGACCAAAGCTCCGGCGTCGTGGGACGGGCCGTCGTGGTATTAGGTTGTTGGC
GGGGTAACGGCCCACCAAGCCGGCGATGCCTACCGGGCGTGCGAGCGTGGCCCGGCACACTGGGACTGA
GACACGGCC
>Zotu6035
GAACGCTGGCGGCATGCCTAAGACATGCAAGTCGAACGAGACGACCTTTGAAAGACTGAGTGCTTGCACA
AAGTTGGAAGAAGATCATCGTCTAGTGGCAGACGGGTGAGTAACACGTGGGTAACCTACCTTTAAGCTGG
GGATAACAGTTGGAAACGACTGATAATACCGAATAAGATATAGTAATATATTGAAAGAAGCCTTTAAAGCT
TCACTTAAAAATGGGCCTGCGGCGTATTAACTAGTTGGTGGGGTAATGGCTCACCAAGGTAACGATGCGTA
GCCGAACTGAGAGGTTGAACGGCCACACTGGGACTGAGACACGGCC
>Zotu6038
GAACGCTGGCGGCGTGCTTAAGACATGCAAGTCGGACGCATCCCCCTCGGGGGATGAGTGGCGCACGGG
TGAGTAACGCGTAACTGACCTACCCTCAAGTCCTGACCAACTCCTCGAAAGAGGAGCTAATGTGGGATGTG
CTGCCTCACCGTGGTGAGGCAGTAAAGGCTATGACCGCTTGAGGATGGGGTTGCGTTTCATCAGCTAGATG
GTGGGGTAAAGGCCTACCATGGCGACGACGAATAGCCGGCCTGAGAGGGTGGCCGGCCACAGGGGCACT
GAGACACGGGC
>Zotu6040
GAACGCTGGCGGCGTGCCTAATACATGCAAGTCGAGCGGATCTTTAGAAGCTTGCTTCTAAAGATTAGCGG
CGGACGGGTGAGTAACACGTGGGCAACCTGCCTGTAAGACTGGGATAACTTCGGGAAACCGAGGCTAATA
CCGGATAATCCTTTTCCTCACATGAGGGAAAGCTGAAAGACGGCCTTAAAAGCTGTCACTTACAGATGGGC
CCGCGGCGCATTAGCTAGTTGGTGAGGTAACGGCTCACCAAGGCAACGATGCGTAGCCGACCTGAGAGGG
TGATCGGCCACACTGGGACTGAGACACGGCC
>Zotu6043
GAACGCTGGCGGCAGGCCTAACACATGCAAGTCGAGCGGATGAGAGGAGCTTGCTCCTGGATTCAGCGGC
GGACGGGTGAGTAATGCCTAGGAATCTGCCTGGTAGTGGGGGATAACGTCCGGAAACGGGCGCTAATAC
CGCATACGTCCTGAGGGAGAAAGTGGGGGATCTTCGGACCTCACGCTATCAGATGAGCCTAGGTCGGATT
AGCTAGTTGGTGGGGTAAAGGCCTACCAAGGCGACGATCCGTAACTGGTCTGAGAGGATGATCAGTCACA
CTGGAACTGAGACACGGTC
>Zotu6045
GAACGCTGGCGGCGTGCTTAACACATGCAAGTCGAACGATGAAGCCCAGCTTGCTGGGCGGATTAGTGGC
GAACGGGTGAGTAACACGTGAGTAACCTGCCCTTGACTCTGGGATAAGCCCGGGAAACTGGGTCTAATAC
CGGATAGGAGCGCCTGTCGCATGACGGGTGTTGGAAAGATTTTTCGGTCATGGATGGACTCGCGGCCTAT
CAGCTTGTTGGTGAGGTAATGGCTCACCAAGGCGACGACGGGTAGCCGGCCTGAGAGGGTGACCGGCCA
CACTGGGACTGAGACACGGCC

>Zotu6048
GAACGCTGGCGGCGTGCTTAACACATGCAAGTCGAGCGGAAAGGCCACTTCGGTGGTACTCGAGCGGCGA
ACGGGTGAGTAACACGTGAGTAATCTGCCCCTGGCTTTGGGATAGCCACCGGAAACTGTGATTAATACCGG
ATACGACAACTTCTCGCATGGGATGGTTGTGGAAAGTTTTTCGGCCAGGGATGTGCTCGCGGCCTATCAGC
TTGATGGTGAGGTAACGGCTCACCATGGCTTCGACGGGTAGCCGGCCTGAGAGGGTGACCGGCCACACTG
GGACTGAGACACGGCC
>Zotu6050
GAACGCTGGCGGCGTGCTTAACACATGCAAGTCGAGCGGAAAGGCCCTTCGGGGTACTCGAGCGGCGAA
CGGGTGAGTAACACGTGAGCAACCTGCCCTTGGCTCTGGGATAGCCTCCGGAAACGGGGATTAATACCGG
ACATGCACTTCCCGCGGCATCGCGGGGGGTGGAAAGTTTTTCGGCCGGGGAGGGGCTCGCGGCCTATCA
GCTTGTTGGGGGGGTAACGGCCCACCAAGGCGACGACGGGTAGCCGGCCTGAGAGGGCGACCGGCCACA
CTGGGACTGAGACACGGCC
>Zotu6053
GAACGCTGGCGGCGTGCCTAACACATGCAAGTCGAACGGAGTAATTACTTCGGTGATTACTTAGTGGCGA
ACGGGTGAGTAACGCGTGAGAAACCTGCCTTTCAGTGGGGGACAACAGTTGGAAACGACTGCTAATACCG
CATAATGTTATTTGGGGGCATCCCTGAATAACCAAAGCTTTATGTGCTGAAAGATGGTCTCGCGTCTGATTA
GCTAGTTGGTGAGGTAACGGCTCACCAAGGCGACGATCAGTAGCCGGTCTGAGAGGATGAACGGCCACAT
TGGGACTGAGATACGGCC
>Zotu6055
GAACGCTGGCGGCGTGCTTAACACATGCAAGTCGAACGGAAAGGCCCTTCGGGGTACTCGAGTGGCGAAC
GGGTGAGTAACACGTGGGTGATCTGCCCTGCACTTCGGGATAAGCCTGGGAAACTGGGTCTAATACCGGA
TAGGACCACGCGCTTCATGGTGTGTGGTGGAAAGCTTTTGCGGTGTGGGATGGGCCCGCGGCCTATCAGC
TTGTTGGTGGGGTAATGGCCTACCAAGGCGTCGACGGGTAGCCGGCCTGAGAGGGTGTCCGGCCACACTG
GGACTGAGATACGGCC
>Zotu6058
GAACGCTGGCGGCGTGCTTAACACATGCAAGTCGAACGGATCACTCTTTGGGGTGGTTAGTGGCGAACGG
GTGAGTAACACGTGAGCAACCTGCCCTTCACTCTGGGATAACTGTGGGAAACTTCAGCTAATACCGGATAT
GACCTGTCACTGCATGGTGGTGGGTGGAAAGATTTTTTGGTGGGGGATGGGCTCGCGGCCTATCAGCTTG
TTGGTGGGGTGATGGCCTACCAAGGCTTTGACGGGTAGCCGGCCTGAGAGGGCGACCGGCCACACTGGG
ACTGAGATACGGCC
>Zotu6060
GAACGCTGGCGGCGTGCCTAATACATGCAAGTCGAGCGAATCTTTGGGAGCTTGCTCCCAAAGATTAGCG
GCGGACGGGTGAGTAACACGTGGGCAACCTGCCTGTAAGACTGGGATAACTTCGGGAAACCGGAGCTAAT
ACCGGATAATTCTTTTCTACTCATGTAGGAAAGCTGAAAGACGGTTTCGGCTGTCACTTACAGATGGGCCC
GCGGCGCATTAGCTAGTTGGTGAGGTAACGGCTCACCAAGGCGACGATGCGTAGCCGACCTGAGAGGGT
GATCGGCCACACTGGGACTGAGACACGGCC
>Zotu6062
GAACGTTAGCGGCGCGCCTAACACATGCAAGTCGCACGAGAAAGGGCTTCGGCCCCGGTACAGTGGCGCA
CGGGTGAGTAACACGTAGATCATCTCCCCTCGAGTGGTGGATAACTCTCCGAAAGGAGAGCTAATACAGC
ATGAGACCACGGCTTCGTTGAAGCTGCGGCCAAAGCAGGCCTCTACACGTAAGCTTGCGCTTGAGGATGA
GTCTGCGGCCCATCAGCTAGTTGGTAGGGTAATGGCCTACCAAGGCTAAGACGGGTAGCTGGTCTGAGAG
GATGAACAGCCACACTGGAACTGAGACACGGTC
>Zotu6064
GAACGCTGGCGGCGTGCCTAATACATGCAAGTCGAGCGGACTTAAAAGGAGCTTGCTCCTATTAAGTTAGC
GGCGGACGGGTGAGTAACACGTGGGCAACCTGCCCTATAGTTTGGGATAACTCCGGGAAACCGGGGCTAA
TACCGAATGATACATTTTAACTCCTGTTAGAATGTTGAAAGATGGTTCTGCTATCGCTATAGGATGGGCCCG
CGGCGCATTAGCTAGTAGGTGAGGTAACGGCTCACCTAGGCGACGATGCGTAGCCGACCTGAGAGGGTG
ATCGGCCACACTGGGACTGAGACACGGCC
>Zotu6069

GAACGCTAGCTACAGGCTTAACACATGCAAGTCGAGGGGCAGCATTTTAGTTTGCTTGCAAACTAAAGATG
GCGACCGGCGCACGGGTGAGTAACACGTATCCAACCTGCCGATAACTCGGGGATAGCCTTTCGAAAGAAA
GATTAATATCCGATGGTATATTTTCTCCGCATGGTGAGAATATTAAAGAATTTCGGTTATCGATGGGGATGC
GTTCCATTAGTTTGTTGGCGGGGTAACGGCCCACCAAGACCACGATGGATAGGGGTTCTGAGAGGAAGGT
CCCCCACATTGGAACTGAGACACGGTC
>Zotu6071
GAACGCTGGCGGCGTGCTTAACACATGCAAGTCGAACGGAAAGGCCCTTCGGGGTACTCGAGTGGCGAAC
GGGTGAGTAACACGTGGGTGATCTGCCCTGCACTTTGGGATAAGCCTGGGAAACTGGGTCTAATACCGAA
TAGGACCACGGCATTCATGTGCTGTGGTGGAAAGCTTTTGCGGTGTGGGATGGGCCCGCGGCCTATCAGC
TTGTTGGTGGGGTAATGGCCTACCAAGGCGACGACGGGTAGCCGGCCTGAGAGGGTGACCGGCCACACT
GGGACTGAGATACGGCC
>Zotu6073
GAACGCTAGCGGCAGGCCTAATACATGCAAGTCGAGGGGCAGCGATGTAGCAATACAGTCGGCGACCGG
CGCACGGGTGCGTAACACGTATGCAACCTGCCTTCAACTGGGGGATAGCCCGGGGAAACTCGGATTAATA
CCCCATAACATGGTGAAACAGCATTGTTTTATCATTAAAGCTCCGGCGGTTGAAGATGGGCATGCGTTCGA
TTAGCTAGTTGGTGTAGGTAACGGCTCACCAAGGCTACGATCGATAGGGGGTCTGAGAGGATGATCCCCC
ACACTGGTACTGAGATACGGAC
>Zotu6075
GAACGCTGGCGGCGTGCCTAATACATGCAAGTCGAGCGAACTTTTGAGAGCTTGCTCTCAAAAGTTAGCGG
CGGACGGGTGAGTAACACGTGGGCAACCTACCTATAAGACTGGGATAACTTACGGAAACGTGAGCTAATA
CCGGATAATTCTTTCTAACACATGTTAGGAAGTTGAAAGATGGCTTCTCGCTATCACTTATAGATGGGCCCG
CGGCGCATTAGCTAGTTGGTGGGGTAATGGCCTACTAAGGCAACGATGCGTAGCCGACCTGAGAGGGTGA
TCGGCCACACTGGGACTGAGACACGGCC
>Zotu6080
GAATGCTGGCGGCGTGGATGAGGCATGCAAGTCGAATGGGTTTAGACCCATGGCAGACGAGGTAGTAAC
ACGTAGGTACGTACCCCAAAGTCGGGCATAATCCGCCGAAAGGCGGGATAATTCCCGATGGTCCCGCAAG
GGTAAAGATTTATCGCTTTGGGAACGGCCTGCGACGTATCAGCTAGTTGGTGAGGTAATGGCTCACCAAG
GCGACGACGCGTAGGGGAGCTGAGAGGCTGACCCCCACCGATGGCACTGCGACACGGGC
>Zotu6084
GAACGCTGGCGGCGTGCTTAACACATGCAAGTCGAGTGCGCAGTCGTTTGTAGCAATACAAATGATGGGC
GCACGGCGCACGGGTGCGTAACACGTGGGTAATTTGCCCTCGAGTGGGGAATAACCCGCCGAAAGGCGG
GCTAATACCGCATAACACTCCCCGGACTACGGTCTAGGGATTCAAAGCCCGCAAGGGCGCTTGAGGAGAA
ACCCGCGGCCGATTAGCTAGTTGGCGAGGTAACGGCTCACCAAGGCAATGATCGGTAGCTGGTCTGAGAG
GATGGCCGGCCACACTGGAACTGAGATACGGTC
>Zotu6087
GAACGCTGGCGGCGTGCCTAATACATGCAAGTCGAGCGAATTGATGAGAAGCTTGCTTCTCTGAAGTTAGC
GGCGGACGGGTGAGTAACACGTGGGCAACCTGCCCTATAGTTTGGGATAACTCCGGGAAACCGGGGCTAA
TACCGAATAATACATTTTAACTCCTGTTAGAATGTTGAAAGATGGTTTACGCTATCGCTATAGGATGGGCCC
GCGGCGCATTAGCTAGTAGGTGAGGTAACGGCTCACCTAGGCGACGATGCGTAGCCGACCTGAGAGGGT
GATCGGCCACACTGGGACTGAGACACGGCC
>Zotu6089
GAACGCTAGCGGCAGGCCTAACACATGCAAGTCGAGGGGTAGAAGGAGCTTGCTCCTTTGAGACCGGCGC
ACGGGTGCGTAACGCGTATGCAACCTACCTTTCACAGGGGAATAGCCCAGAGAAATTTGGATTAATGCCCC
ATGGCACAGCGAGACGGCATCGTCTTGCTGTTAAAGTAACAACGGTGAAAGATGGGCATGCGTCCCATTA
GCTAGTTGGTAAGGTAACGGCTTACCAAGGCTATGATGGGTAGGGGTCCTGAGAGGGAGATCCCCCACAC
TGGTACTGAGACACGGAC
>Zotu6091
GAACGCTAGCGGCAGGCTTAATACATGCAAGGCGAGGGGGCAGCAATGTCACCGTCGTACGGGTGCGTA
ACGCGTATGCAACCTACCTATCACTGGGGGATAGCCCGGGGAAACCCGGATTAATACCGCATAATACATAT
GGTCCACCTGGATTGTTTTGTTAAAGATTTATTGGTGATAGATGGGCATGCGTTCGATTAGCTAGTTGGTAT

GGGTAACGGCCTACCAAGGCAACGATCGATAGGGGAGCTGAGAGGTTGATCCCCCACACGGGCACTGAG
ATACGGGC
>Zotu6093
GAACGCTGGCGGCGTGCCTAACACATGCAAGTCGAGCGGGAAAGTGGGGGCAACTCCACGAGTACAGCG
GCGCACGGGTGAGTAACACGTAGGTAACCTACCTACGAGACCGGGATAACCTGCCGAAAGGCGGGCTAAT
ACCGGATAAGACCACGGGAATCGCGGTTCCTGGGGTAAAAGGTGGCCTCTCCTTGGAAGCTACTACTCGA
AGATGGGCCTGCGCGCCATTAGCTAGTTGGCGGGGTAATGGCCCACCAAGGCAAAGATGGCTAGCTGGTC
TGAGAGGATGGCCAGCCACACTGGGACTGAGACACGGCC
>Zotu6095
CAACGCTGGCGGCGTGCCTCAGACATGCAAGTCGAACGATTAAACTTTCCTTCGGGGAAGATATAAAGTG
GCGCACGGGTGAGTAACACGTAAGTAATCTACCTTCGAGTGGGGAATAACGTTCGGAAACGAACGCTAAT
ACCGCATAATGCAGCGGCATCGCGAGATGACAGTTGTTAAAGATTTATCGCTTGAAGAGGAGCTTGCGGC
AGATTAGCTAGTTGGTAAGGTAACGGCTTACCAAGGCGACGATCTGTATCCGGTCTAAGAGGACGGTCGG
ACACACTGACACTGAATAACGGGT
>Zotu6097
GAACGCTGGCGGCGTGCCTAATACATGCAAGTCGAGCGGAGTTACAGGAGCTTGCTCCTGTAACTTAGCG
GCGGACGGGTGAGTAACACGTGGGCAACCTGCCTATAAGACTGGGATAACTTCGGGAAACCGGAGCTAAT
ACCGGATAATTCTTTTCCTCTCATGGGGAAAAGCTGAAAGACGGTTTACGCTGTCACTTATAGATGGGCCC
GCGGCGCATTAGCTAGTTGGTGAGGTAACGGCTCACCAAGGCAACGATGCGTAGCCGACCTGAGAGGGT
GATCGGCCACACTGGGACTGAGACACGGCC
>Zotu6099
GAACGCTAGCGGCAGGCCTAATACATGCAAGCCGAACGGCAAGATTTTCAGCAATGAGAATCCTAGAGTG
GCGTAAGGGTGCGTAACACGTATGCAATCTACCCTCAACAGGAGGATAGCTCCCCGAAAGGGGAATTAAG
ACTCCATAAGATCACACGAAGGCATCTGAGAGTGATTAAAGTACAAGCGGTTGAGGATGAGCATGCGTCT
GATTAGTTAGTTGGCGGGGTAACGGCCCACCAAGACGATGATCAGTAGGGGAACTGAGAGGTTGATCCCC
CACACTGGCACTGAGATACGGGC
>Zotu6101
GAACGCTGGCGGCGTGCCTAATACATGCAAGTCGTGCGGTAACAGGTTCTCACTATTTGCTTCTCTCTCAAT
CAGTAAAACGATCGGGACTCAGAGGAGTAAGTTGTGAGAATGCTGACGAGCGGCGGACGGCTGAGTAAC
GCGTAGGAACGTGCCCCAAAGTGAGGGATAAGCTCCAGAAATGGAGTCTAATACCGCATATGGTCTTCGG
ATTAAAGCTTCGGCGCTTTGGGAGCGGCCTGCGTTTGATTAGATAGTTGGTGAGGTAAAGGCTCACCAAGT
CGACGATCAATAGCTGGTCTGAGAGGATGATCAGCCAGACTGGAACTGAGACACGGTC
>Zotu6103
GAACGCTGGCGGCATGCTTAACACATGCAAGTCGAACGCGGTGTCCTGGTTCGCCGGGGCACTGAGTGGC
GCACGGGTGAGTAGCGCGTGGGAACCTGCCCAGCAGTGGGGGATAACCGAGGGAAACCTCGGCTAATAC
CGCATGCGCCGGAGGTAACTCCGGGAAAGATTTATCGCTGTTGGAGGGGCCCGCGTCGGATTAGCTAGTT
GGTGGGGTAACGGCCCACCAAGGCGATGATCCGTAGCTGGTCTGAGAGGATGATCAGCCACACTGGGACT
GAGACACGGCC
>Zotu6105
GAACGCTGGCGGCGTGCCTAATACATGCAAGTCGAGCGAACAAGGAAGAAACTTGTTTCTTCCTTTGTTAG
CGGCGGATGGGTGAGTAACACGTGGGCAACCTGCCCTGCAGTTGGGGATAACTCCGGGAAACCGGGGCT
AATACCGAATAATCAGTTCCTTCGCATGAAGGAACTCTGAAAGACGGCTATGCTGTCACTGCAGGATGGGC
CCGCGGCGCATTAGCTAGTTGGTGGGGTAACGGCTTACCAAGGCGACGATGCGTAGCCGACCTGAGAGG
GTGATCGGCCACACTGGGACTGAGACACGGCC
>Zotu6107
GAACGCTGGCGGCATGCCTAACACATGCAAGTCGAACGGCATCACGTACTAGTTTACTAGTATGGTGGCGA
GTGGCGCACGGGTGAGTAACGCGTAGGAATATGCCTTAAAGAGGGGGACAACCTGGGGAAACTCAGGCT
AATACCGCATACGCTCTAAGGAGGAAAGCTGGGGACCTTCGGGCCTGGCGCTTTAAGAATAGCCTGCGTA
CGATTAGCTAGTTGGTGGGGTAAAGGCCTACCAAGGCGACGATCGTTAGCTGGTCTGAGAGGATGGCCAG
CCACACTGGGACTGAGACACGGCC

>Zotu6110
GAACGCTAGCGGCAGGCTTAACACATGCAAGTCGAGGGGTAGAGTAGCAATACTTGAGACCGGCGCACG
GGTGCGTAACGCGTATGCAACCTACCTTTTACAGGGGAATAGCCCAGAGAAATTTGGATTAATGCCCCATG
GTATATTTTAGTGGCATCACTGGATTATTAAAGTTCCAACGGTAAAAGATGGGCATGCGTCCTATTAGCTAG
ATGGTAAGGTAACGGCTTACCATGGCTACGATAGGTAGGGGTCCTGAGAGGGAGATCCCCCACACTGGTA
CTGAGACACGGAC
>Zotu6113
GAACGCTGGCGGCAGGCTTAACACATGCAAGTCGAACGGTAACATAAAGAAGCTTGCTTCTTTGATGACG
AGTGGCGGACGGGTGAGTAATGCTTGGGAATCTGGCTTATGGAGGGGGATAACTACGGAAACTGTAGC
TAATACCGCGTAGAATCGGGAGATGAAAGTGTGGGACCTTCGGGCCACATGCCATAGGATGAGCCCAAGT
GGGATTAGGTAGTTGGTGAGGTAAAGGCTCACCAAGCCGACGATCTCTAGCTGGTCTGAGAGGATGACCA
GCCACACTGGGACTGAGACACGGCC
>Zotu6115
GAACGCTAGCGGCAGGCTTAATACATGCAAGTCGTGGGGCAGCATGGTGTAGCAATACACTGATGGCGAC
CGGCAAACGGGTGCGGAACACGTACGCAACCTTCCTTTAACTGGAGAATAGCCCGGGGAAACCCGGATTA
ATACTCCGTAACATTATGAAGTGGCATCACTTTATAATTATAGCTCCGGCGGTTGAAGATGGGCGTGCGTCT
GATTAGGTAGTTGGCGGGGTAACGGCCCACCAAGCCTACGATCAGTAACTGGTGTGAGAGCACGACCAGT
CACACGGGCACTGAGACACGGGC
>Zotu6117
GAACGCTGGCGGCAGGCTTAACACATGCAAGTCGAGCGGGCCTCTTCGGGGGTCAGCGGCGGACGGGTT
AGTAACGCGTGGGAACGTGCCTTCTGCTTCGGAATAGCCCTGGGAAACCGGGGTAATACCGGATGTGCC
CTGAGGGGGAAAGATTTATCGGCAGGAGATCGGCCCGCGTTGGATTAGGTAGTTGGTGGGGTAACGGCC
CACCAAGCCGACGATCCATAGCTGGTTTGAGAGGATGATCAGCAACACTGGGACTGAGACACGGCC
>Zotu6119
CAACGCTGGCGGCGTGCCTAACACATGCAAGTCGCACGAGAAAGTGGGGGCAACTCCGTGAGTAAAGTG
GCGCACGGGTGAGTAACACGTGACTAACCTACCTTCGAATGGGGAATAACCTCGGGAAACCGGGACTAAT
ACCGCATAATATCCGCAGTTGAGATGCTACGGATCAAACGCGCAAGCGGTTTGAAGAGGGGGTCGCGGCT
GATTAGCTAGTTGGTGAGGTAATGGCCCACCAAGGCAATGATCAGTATCCGGCCTGAGAGGGCGCACGGA
CACACTGGAACTGAAACACGGTC
>Zotu6121
GAACGCTGGCGGCAGGCTTAACACATGCAAGTCGAACGGTAACATAAAGAAGCTTGCTTCTTTGATGACG
AGTGGCGGACGGGTGAGTAATGCTTGGGAATCTAGCTTATGGAGGGGGATAACTACGGAAACTGTAGC
TAATACCGCGTAGAATCGGGAGATGAAAGTGTGGGACCTTCGGGCCACATGCCATAGGATGAGCCCAAGT
GGGATTAGGTAGTTGGTGAGGTAAAGGCTCACCAAGCCGACGATCTCTAGCTGGTCTGAGAGGATGACCA
GCCACACTGGGACTGAGACACGGCC
>Zotu6125
AAACGTTGGCGGTGCGTCTTAGACATGCAAGTCGAGCGTGAAAGCCCGCAAGGGTGAGTAAAGCGGCGA
ACGGGTGAGTAATACTTGGGTAACGTACCTCCTAGATCGGGATAACGCGCCGAAAGGCGTGCTAATACCG
GATGTGACCACGGTTCCTACGGGAGCAGGGGTAAAAGATGGCGCAAGCTATCACTGGGAGATCGGCTCAA
GCACCATTAGCTAGTTGGCGGGGTAACGGCCCACCAAGGCTACGATGGTTAGCCGGCCTGAGAGGGTGAT
CGGCCACACTGGGACTGAGATACGGCC
>Zotu6127
TAACGCTGGCGGCGTGCCTAACACATGCAAGTCGAACGGCCCAGCACCTTTTGCAGCATCCAGTGTCGCAG
CGATATTTCGTTGCGCTTTGGTTTATGATGGTACAAAAGGTGCTGGGTAGTGGCGAACGGGTGAGTAACAC
GTTGGTACCTGCCCTCTAGTGGGGAATAATTCGCCGAAAGGCGGACTAATACCGCATGTGATCTACGGATC
AAAGCAGCAATGCGCTAGAGGAGGGGCCTGCGGCTGATTAGCTTGTTGGTAGGGTAATGGCCTACCAAGG
CGACGATCAGTAGCTGGATCGAGAGGTTGACCAGCCACACTGGGACTGAGATACGGCC
>Zotu6129
GAACGCTAGCGGCAGGCCTAATACATGCAAGTCGAACGGTAACAGGGTAGCAATACCGCTGACGAGTGGC
GCACGGGTGCGTAACGCGTATGCAATCTACCTTTAACTAGTGAATAGCTCGAAGAAATTCGAATTAATACA

CCATAGTATTATTAATTGGCATCAATTGATAATTAAAGCTCCGGCGGTTAAAGATGAGCATGCGTGACATTA
GCTAGTTGGTGAGGTAACGGCTCACCAAGGCTACGATGTCTAGGGGATCTGAGAGGATTAACCCCCACAC
TGGTACTGAGACACGGAC
>Zotu6131
GAACGCTGGCGGCAGGCTTAACACATGCAAGTCGAGCGCCCCGCAAGGGGAGCGGCAGACGGGTGAGTA
ACGCGTGGGAACGTACCTTTTGGTTCGGAACAACTCCGGGAAACTGGAGCTAATACCGGATAAGCCCTAAC
GGGGAAAGATTTATCGCCGAAAGATCGGCCCGCGTCTGATTAGCTAGTTGGTGAGGTAACGGCTCACCAA
GGCGACGATCAGTAGCTGGTCTGAGAGGATGATCAGCCACACTGGGACTGAGACACGGCC
>Zotu6133
GAACGCTGGCGGCGTGCTTAACACATGCAAGTCGAACGAAGCACTTTTACAGATTTCTTCGGAATGAAGTT
TTAGTGACTGAGTGGCGGACGGGTGAGTAACGCGTGGGTAACCTGCCTCACACAGGGGGATAACAGTTG
GAAACGGCTGCTAATACCGCATAAGCGCACAGTACCGCATGGTACAGTGTGAAAAACTCTGGTGGTGTGA
GATGGACCCGCGTCTGATTAGCTAGTTGGCAGGGTAACGGCCTACCAAGGCGACGATCAGTAGCCGACCT
GAGAGGGTGACCGGCCACATTGGGACTGAGACACGGCC
>Zotu6135
GAACGCTGGCGGCGCGCCTAACACATGCAAGTCGAGCGAGAACCAGGGCTTGCCCTGGGGACAGCGGCG
AACGGGTGCGTAACACGTGAGCAACCTGCCCCAAAGACCGGGACAACCCGAGGAAACTCGGACTAATACC
GGATGCCCTCACCGGGTCGCATGGTCTGGTGAGGAAATGGATTCCGCTTTGGGATGGGCTCGCGGTCTAT
CAGCTTGTTGGTGGGGTAACGGCCTACCAAGGCGACGACGGATAGCTGGTCTGAGAGGACGATCAGCCAC
ACTGGGACTGAGACACGGCC
>Zotu6137
GAACGCTGGCGGCGTGCTTAACACATGCAAGTCGAACGATGACGGTGGTGCTTGCATCACCTGATTAGTG
GCGAACGGGTGAGTAACACGTGAGTAACCTGCCCTTCACTTCGGGATAACCTCGGGAAATCGTGGCTAATA
CCGGATATGAGCTCCTTCCGCATGGCGGGGGTTGGAAAGATTTATCGGCGAAGGATGGACTCGCGGCCTA
TCAGTTTGTTGGTGAGGTAATGGCTCACCAAGGCGATGACGGGTAGCCGGCCTGAGAGGGCGACCGGCCA
CACTGGGACTGAGACACGGCC
>Zotu6139
GAACACTGGCGGCATGCCTAACACATGCAAGTCGAACGAAATCTTCGGATCTAGTGGCGCACGGGTGCGT
AACGCGTGGGAATCTGCCTTGGGGTTCGGAATAACTCCCCGAAAGGGGTGCTAATACCGGATGATGTCGA
AAGACCAAAGATTTATCGCCCTGAGATAAGCCCGCGTAGGATTAGCTAGTTGGTGTGGTAAAGGCGCACC
AAGGCGACGATCCTTAGCTGGTCTGAGAGGATGATCAGCCACACTGGGACTGAGACACGGCC
>Zotu6142
GAACGCTGGCGGCGTGCTTAATACATGCAAGTCGAACGTGATTGTAGCGATACATGAAAGTGGCAAACGG
GTGAGTAACACGTAGGAACCTTCCTTGAAGTGGGGGATAACCATTGGAAACGGTGGCTAATACCGCATAG
TTTCACTCAAATGGGAATAGAGTGAATAAAGATTTATTGCTTTAAGAGGGGCCTGCGGTCGATTAGCTAGT
TGGTGAGGTAAAGGCTCACCAAGGCAGTGATCGATAGCTGGTCTGAGAGGATGATCAGCCACACGGGAA
CTGAGAAACGGTC
>Zotu6145
GAACGCTGGCGGCAGGCCTAAAACATGCAAGTCGAACGAACCTTCGGGTTAGTGGCGCACGGGTGCGTAA
CGCGTGGGAATATGCCTCTTGGTGCGGAACAACGTCTGGAAACGGACGCTAATACCGCATAATATCTACGG
ATCAAAGATTTATCGCCAAGAGATTAGCCCGCGTCTGATTAGCTAGTTGGTGAGGTAATGGCTCACCAAGG
TGACGATCAGTAGCTGGTCTGAGAGGATGATCAGCCACACTGGAACTGAGACACGGTC
>Zotu6148
GAACGCTGGCGGCAGGCTTAACACATGCAAGTCGAGCGGGCATCCTTGTGGTGTCAGCGGCAGACGGGT
GAGTAACGCGTGGGAACGTGCCCTTCGGTTCGGAATAACTCAGGGAAACTTGAGCTAATACCGGATACGC
CCTTTTGGGGAAAGGCTTGACTGCCGAAGGATCGGCCCGCGTCTGATTAGCTAGTTGGTGAGGTTACGGCT
CACCAAGGCGACGATCAGTAGCTAGTCTGAGAGGATGATCAGCCACACTGGGACTGAGACACGGCC
>Zotu6153
GAACGCTGGCGGCGTGCTTCATACATGCAAGTCGAACGATAAGGTCTCTTCGGAGATACATAAGTGGCGG
ACGGGTGAGTAATATGTAGGAAATCTGCCCTAGAGAAGGGGACAACAGAGGGAAACTTCTGCTAATACCC

TATATGAGCGTAGTTGAAATACTATTCTTGAAATGAATTTCGCTCTAGGATGAGCCTGCATCTGATTAGTTT
GTTGGCGGTGTAATGGACCACCAAGGCGACGATCAGTAGCTGGTTTGAGAGGATGATCAGCCACAATGGG
ACTGAGACACGGCC

>Zotu6155

GAACGCTGGCGGCGTGCCTAATACATGCAAGTCGAGCGAATCATTAGGAGCTTGCTCCTAATGATTAGCGG
CGGACGGGTGAGTAACACGTGGGCAACCTGCCTATAAGACTGGGATAACTTCGGGAAACCGGAGCTAATA
CCGGATAATCCTTTTCCTCTCATGAGGAAAAGCTGAAAGACGGTTTTTAGCTGTCACTTATAGATGGGCCCG
CGGCGCATTAGCTAGTTGGTGAGGTAATGGCTCACCAAGGCAACGATGCGTAGCCGACCTGAGAGGGTGA
TCGGCCACACTGGGACTGAGACACGGCC

>Zotu6160

GAACGCTGGCGGCAGGCCTAATACATGCAAGTCGAACGCCCCGCAAGGGGAGTGGCGCACGGGTGAGTA
ACGCGTGGGAATGTGCCCTTAGGTGCGGAACAACGTTTGGAAACGAACGCTAATACCGCATAATATCTCCG
GATCAAAGATTTATCGCCTAGGGATCAGCCCGCGTTGGATTAGCTAGTTGGTGGGGTAACGGCCTACCAAG
GCGACGATCCATAGCTGGTCTGAGAGGATGATCAGCCACACTGGGACTGAGACACGGCC

>Zotu6163

GAACGCTGGCGGCATGCCTAACACATGCAAGTCGAACGAGACCTTCGGGTCTAGTGGCGCACGGGTGCGT
AACGCGTGGGAATCTGCCCTTGGGTTCGGAATAACTCAGAGAAATTTGAGCTAATACCGGATGATGTCGAA
AGACCAAAGATTTATCGCCTGAGGATGAGCCCGCGTTGGATTAGCTAGTTGGTGGGGTAAAGGCCTACCA
AGGCGACGATCCATAGCTGGTCTGAGAGGATGATCAGCCACACTGGGACTGAGACACGGCC

>Zotu6167

GAACGCTGGCGGCGTGCTTAACACATGCAAGTCGAACGATGAAGCCGGAGCTTGCTCCGGTGGATTAGTG
GCGAACGGGTGAGTAACACGTGAGCAATCTGCCCTTGACTCTGGGATAACTGCGGGAAACTGTAGCTAAT
ACCGGATACGACACCTTGGGGCATCCCAGGGTGTGGAAAGAATTTCGGTCAAGGATGAGCTCGCGGCCTA
TCAGCTAGTTGGTGAGGTAATGGCTCACCAAGGCGACGACGGGTAGCCGGCCTGAGAGGGTGACCGGCC
ACACTGGAACTGAGACACGGTC

>Zotu6170

GAACGCTGGCGGCGTGCCTAATACATGCAAGTCGTGCGGACTTATCTTCGGATAAGTTAGCGGCGGACGG
GTGAGTAACACGTGGGCAACCTGCCTGTAAGACTGGGATAACTCCGGGAAACCGGTGCTAATACCAGATA
CAATCAAAGACTGCCTGGTTTTTGATAGAAAGGTCTTGTACCGCTTACAGATGGGCCCGCGGCGCATTAGC
TAGTTGGTGGGGTAACGGCTCACCAAGGCGACGATGCGTAGCCGACCTGAGAGGGTGATCGGCCACACTG
GGACTGAGACACGGCC

>Zotu6172

GAACGCTGGCGGCAGGCCTAACACATGCAAGTCGAGCGGCCGTAGCAATACGGCAGCGGCAGACGGGAG
AGTAACACGTGGGAACGCGCCCTTTGGTTCGGAATAACTCAGGGAAACTTGAGCTAATACCGGATAAGCC
CTTACGGGGAAAGATTTATCGCCAAAGGAACGGCCCGCGTCCGATTAGCTAGTTGGTGAGGTAACGGCTC
ACCAAGGCGACGATCGGTAGCTGGTCTGAGAGGATGACCAGCCTCACTGGGACTGAGACACGGCC

>Zotu6175

GAACGCTAGCGGCGGGCCTAATACATGCAAGTCGAGGGGCATCGGGAGTAGCAATACTTGCCGGCGACC
GGCGCACGGGTGCGTAACGCGTATGCAATCTACCTCTGACATGGGGATAGCCCGAAGAAATTCGGATTAA
TACCCAATGGTATCTTAATATGACCTCATATAAAGATTAAAGATTTATCGGTCAGAGATGAGCATGCGTCTG
ATTAGCTAGTTGGTGATGTAATTGCTCACCAAGGCAATGATCAGTAGGGGTTCTGAGAGGATTATCCCCCA
CACCGGTACTGAGACAGGGAC

>Zotu6179

GAACGCTGGCGGCGTGCTTAACACATGCAAGTCGAACGGAAAGGCCTCTGCTTGCAGGGGTGCTCGAGTG
GCGAACGGGTGAGTAACACGTGGGTGATCTGCCCTGCACTTTGGGATAAGCCTGGGAAACTGGGTCTAAT
ACCAGATAGGACCACGTCTTGGATGGTGTGGTGGAAAGTTTTTTCGGTGTGGGATGAGCTCGCGGCCTATC
AGCTTGTTGGTGGGGTAATGGCCTACCAAGGCGTCGACGGGTAGCCGGCCTGAGAGGGTGTACGGCCAC
ATTGGGACTGAGATACGGCC

>Zotu6182

GAACGCTGGCGGCGTGCTTAACACATGCAAGTCGAACGGTGAACCAGGGCTTGCCTTGGGGATCAGTGGC
GAACGGGTGAGTAACACGTGGGCAACCTGCCCCAGACTCTGGGATAACTCCGGGAAACCGGGGCTAATAC
CGGATACGAATCGAGTTGGCATCTTCTCGATTGGAAAGTTTTTCGGTCTGGGATGGGCCCGCGGCCTATCA
GCTTGTTGGTGGGGTAATGGCCTACCAAGGCGACGACGGGTAGCCGGCCTGAGAGGGCGAACGGCCACA
CTGGGACTGAGACACGGCC
>Zotu6184
GAACGCTGGCGGCGTGCCTAATACATGCAAGTCGAACGAAGGACGCTGACGGAATTCTTCGGAAGGAAGT
CGGCATTCCTGAGTGGCGGACGGGTGAGTAACACGTAGGCAACCTGCCTGTAAGTCGGGGATAACTTCGG
GAAACCGGAGCTAATACCGGATAAGGCAAACCGATCGCATGGTTGGTTTGGTAAAGGAGGTACGCTTCCG
CTTACAGATGGGCCTGCGGCGCATTAGCTTGTTGGTGAGGTAACGGCTCACCAAGGCGACGATGCGTAGC
CGACCTGAGAGGGTGACCGGCCACACTGGGACTGAGACACGGCC
>Zotu6188
GAACGCTGGCGGCGTGCCTAATACATGCAAGTCGAGCGAATCAATAGGAGCTTGCTCCTGTTGGTTAGCG
GCGGACGGGTGAGTAACACGTGGGCAACCTGCCTGTAAGACTGGGATAACACCGGGAAACCGGTGCTAA
TACCGGATAATCCTTTTTCTCTCATGAGGAAGAGCTGAAAGTCGGTTTCTGCTGACACTTACAGATGGGCCC
GCGGCGCATTAGCTAGTTGGTGAGGTAACGGCTCACCAAGGCGACGATGCGTAGCCGACCTGAGAGGGT
GATCGGCCACACTGGGACTGAGACACGGCC
>Zotu6192
GAACGCTGGCGGCGTGCTTAACACATGCAAGTCGAACGGAAAGGCCCTTTCGGGGGTACTCGAGTGGCGA
ACGGGTGAGTAACACGTGGGTGATCTGCCCTGCACTTCGGGATAAGCTTGGGAAACTGGGTCTAATACCG
GATAGGATCATGGCTCTCATGGGTTGTGGTGGAAAGCTTTTGCGGTGTGGGATGGGCCCGCGGCCTATCA
GCTTGTTGGTGGGGTAATGGCCTACCAAGGCGTCGACGGGTAGCCGGCCTGAGAGGGTGTCCGGCCACAC
TGGGACTGAGATACGGCC
>Zotu6194
GAACGCTGGCGGTATGCCTAATACATGCAAGTCGTGCGGAAAGGCCCTTCGGGGTACTCGAGCGGCGGAC
GGCAGAGTAACGCGTAGGAACGTACCCCAAAGTGAGGAATAACTGCTCGAAAGAGTAGCTAATGCCGCAT
GTGGTCTTCGGACTAAAGCCTTCGGGCGCTTTGGGAACGGCCTGCGTTCGATTAGCTAGTTGGTGAGGTAA
ATGCTCACCAAGGCGATGATCGATAGCTGGTCTGAGAGGATGATCAGCCTGACTGGAACTGAGACACGGT
C
>Zotu6198
GAACGCTGGCGGCGTGCTTAACACATGCAAGTCGAACGGAAAGGCCTCTAGCTTGCTAGAGGTACTCGAG
TGGCGAACGGGTGAGTAACACGTGGGTGATCTGCCCTGCACTGTGGGATAAGCCTGGGAAACTGGGTCTA
ATACCATATAGGACCGCACTTTGGATGGTGTGGTGGAAAGCTTTTGCGGTGTGGGATGAGCCTGCGGCCT
ATCAGCTTGTTGGTGGGGTAATGGCCTACCAAGGCGGCGACGGGTATCCGGCCTGAGAGGGTGTACGGAC
ACATTGGGACTGAGATACGGCC
>Zotu6202
GAACGCTGGCGGCGTGCTTAACACATGCAAGTCGAGCGGTAAGGCTCCTTCGGGAGTACACGAGCGGCGA
ACGGGTGAGTAACACGTGAGCAATCTGCCCTTCACTTTGGGATAACCCCGGGAAACCGGGGCTAATACCG
AATACGACCACCGATGGCATCGTCTGGTGGTGGAAAGTTCCGGCGGTGAAGGATGAGCTCGCGGCCTATC
AGCTTGTTGGTGGGGTAACGGCCCACCAAGGCATTGACGGGTAGCCGGCCTGAGAGGGTGACCGGCCAC
ACTGGGACTGAGACACGGCC
>Zotu6204
GAACGCTGACAGAATGCTTAACACATGCAAGTCGACTTGAATTTGGGTTTTTAACTTAGGTTTGGGTGGCG
GACGGGTGAGTAATGCGTAAAGAACTTGCCTCACAGCTAGGGACAACATTTGGAAACGAATGCTAATACCT
AATATTATGATAATAGGGCATCTTATAATTATGAAAGCTATAAGCGCTGTGAGAGAGCTTTGCGTCCCATTA
GCTAGTTGGAGAGGTAACAGCTCACCAAGGCGATGATGGGTAGCCGGCCTGAGAGGGTGATCGGCCACA
AGGGGACTGAGACACGGCC
>Zotu6206
GAACGCTGGCGGCAGGCTTAACACATGCAAGTCGAACGCTCCGCAAGGAGAGTGGCAGACGGGTGAGTA
ACGCGTGGGAACGTACCTTTCAGTTCGGAATAACTCAGGGAAACTTGTGCTAATACCGGATACGCCCGTAA

GGGGAAAGATTTATCGCTGAAAGATCGGCCCGCGTTGGATTAGCTAGTTGGTGGGGTAAAGGCCTACCAA
GGCGACGATCCATAGCTGGTCTGAGAGGATGATCAGCCACACTGGGACTGAGACACGGCC
>Zotu6209
GAACGCTGGCGGCGTGCCTAAGACATGCAAGTCGAGTGGGTTTAGACCCACGGCAAACGGGTGAGTAATA
CCTAGGAACATACCTTTAGTTGAGCATAACCTACCGAAAGGTAGGACAATTCTCAATGTGGCAGCAATGCT
AAAGTCGCAAGACGCTAAAGAAGTGGCCTGGGCACCATCAGCTAGTTGGTAAGGTAAAGGCTTACCAAGG
CTATGACGGTTAGGGGCCCCGAGAGGGGAAACCCCCACACTGGAACTGAGACACGGTC
>Zotu6213
GAACGCTGGCGGCGTGCCTAAGACATGCGAGTCGAGTGGGTTTAGACCCACGGCAAACGGGTGAGTAAT
ACCTAGGAACTTTCTTTAAGTGGAGCATAACCAGCCGAAAGGTTGGATAATACTCCATACCATCGCAAGAT
GAAAGTCGCAAGACGCTTAAAGTGAGGCCTGGGCACCATCAGCTAGTTGGTAAGGTAATGGCTTACCAAG
GCTATGACGGTTAGGGGCCCCGAGAGGGGAGTCCCCCACACTGGAACTGCGACACGGTC
>Zotu6215
GAACGCTGGCGGCGTGCTTAACACATGCAAGTCGAACGATGAAGCGCCCAGCTTGCTGGGTGTGGATTAG
TGGCGAACGGGTGAGTATCACGTGAGTAACCTGCCCTTGACTCTGGGATAAGCCTGGGAAACTGGGTCTA
ATACCGGATACAACTGCCTACCGCATGGTGGGTGGTGGAAAGCTTTTGCGGTTTTGGATGGACTCGCGGCC
TATCAGCTTGACGGTGAGGTAATGGCTCACGGTGGCTTTGACGGGTAGCCGGCCTGAGAGGGTGACCGGC
CACACTGGGACTGAGACACGGCC
>Zotu6217
GAACGCTGGCGGCGTGCTTAACACATGCAAGTCGAGCGGAAAGGCCCCTTTGGGGGTACTCGAGCGGCG
AACGGGTGAGTAACACGTGAGTAATCTGCCCTTCACTTTGGGATAACCCCCGGAAACGGGAGCTAATACCG
GATACGACCACTCCGGGCATCTGGTGGTGGTGGAAACTTTTTGGGTGGGGGATGTGCTCGCGGCCTATCA
GCTGGTTGGTGGGGTAATGGCCTACCAAGGCTTTGACGGGTAGCCGGCCTGAGAGGGTGACCGGCCACAC
TGGGACTGAGACACGGCC
>Zotu6220
GAACGCTGGCGGCGTGCTTAACACATGCAAGTCACGGGGGCCCGCAAGGGCAACCGGCGAACGGGTGCG
TAACACGTGAGCAATCTGCCGATCTCTGGGGGATAGCCGGCCCAACGGCCGGGTAATACCGCATACGTTCT
CGAGGGGGAGTCCCCTTGAGAAGAAACCTCCGGGGGAGATCGAGGAGCTCGCGACCTATCAGCTAGTTG
GCGGGGTAACGGCCCACCAAGGCATCGACGGGTAGCTGGTCTGAGAGGATGGCCAGCCACATTGGGACT
GCGACACGGCC
>Zotu6222
GAACGCTGGCGGCGTGCCTAATACATGCAAGTCGAGCGGACAGATAAGGAGCTTGCTCCTTTGACGTTAG
CGGCGGACGGGTGAGTAACACGTGGGCAACCTGCCCCTTAGACTGGGATAACTCCGGGAAACCGGAGCTA
ATACCGGATAATCCCTTTCTCCACCTGGAGAGAGGGTGAAAGATGGCTTCGGCTATCACTAAGGGATGGGC
CCGCGGCGCATTAGCTAGTTGGTAAGGTAACGGCTTACCAAGGCAACGATGCGTAGCCGACCTGAGAGGG
TGATCGGCCACACTGGGACTGAGACACGGCC
>Zotu6224
GAACGCTGGCGGCATGCTTAACACATGCAAGTCGAACGGCAGCATAGCTTGGTTTACCAAGTGGATGGCG
AGTGGCGAACGGGTGAGTAACGCGTAGGAATATACCTTAAAGAGGGGGATAACTTGGGGAAACTCAAGC
TAATACCGCGTAATATCTGAGGATAAAAGCTGGGGACCTTCGGGCCTGGCGCTTTAAGAATAGCCTGCGTC
CGATTAGCTAGTTGGTGGGGTAAAGGCCTACCAAGGCGACGATCGGTAGCTGGTCTGAGAGGATGATCAG
CCACACTGGGACTGAGACACGGCC
>Zotu6226
GAACGCTGGCGGAATGCTTTACACATGCAAGTCGAACGGCAGCACGGACTTCGGTCTGGTGGCGAGTGGC
GAACGGGTGAGTAATATATCGGAACGTGTCCAATAATGGGGGATAACTAATCGAAAGGTTGGCTAATACC
GCATACGCCCTACGGGGGAAAGCAGGGGATCTTCGGACCTTGCGTTAATGGAGCGGCCGATATCTGATTA
GCTAGTTGGTGGGGTAAAGGCCCACCAAGGCGACGATCAGTAGCTGGTCTGAGAGGACGACCAGCCACA
CTGGAACTGAGACACGGTC
>Zotu6228

GAACGCTGGCGGCATGCTTAACACATGCAAGTCGAACGAGACCTTCGGGTCTAGTGGCGCACAGGTGCGT
AACGCGTGGGAATCTGCCCTTAGGTACGGAATAACTCAGAGAAATTTGTGCTAATACCGTATGATGTCGAA
AGACCAAAGATTTATCGCCTAAGGATGAGCCCGCGTAAGATTAGCTTGTTGGTGAGGTAAAAGCTCACCAA
GGCGACGATCTTTAGCTGGTCTGAGAGGATGATCAGCCACACTGGGACTGAGACACGGCC

>Zotu6230

TAACGCTGGCGGTGTGCCTAATACATGCAAGTCGAACGGACCTTTATTTTTGAGAACTCCCTTATAGGATGT
CTCACAAATAATGGTTAGTGGCGGACGGGTGAGTAACACGTTGGTACCTGCCCTCTAGTGGGGAATAACT
GGCCGAAAGGTCGGCTAATACCGCATGTGATCGCAAGATCAAAGCAGCAATGCGCTAGAGGAGGGGCCT
GCGTCTGATTAGCTAGTTGGTAGTGTAATGGACTACCAAGGCGACGATCAGTAGCTGGTTCGAGAGGATG
ACCAGCCACACTGGGACTGAGATACGGCC

>Zotu6234

GAACGCTGGCGGTGTGCTTAACACATGCAAGTCGAACGGGCAAGAGCTGTAGCAATACAGAATTCTGCTA
GTGGCAAACGGGTGAGTAATACATGGGAATCTACCTTGCAGTGCGAAATAACAGTTGGAAGCAATTGCTA
ATGTCGCATATAGCCGAGAGGTGAAAGATTTATCGCTGCAAGACGAGCCCATGCAGGATTAGCTAGTTGG
TAAGGTAACGGCTTACCAAGGCAACGATACGTAGCCGACCTGAGAGGGTGATCGGCCACACTGGAACTGA
GACACGGTC

>Zotu6236

GAACGCTAGCGGCAGGCTTAATACATGCAAGTCGAACGGTAAGTGGTGTAGCAATACATCACCAAGAGTG
ACGTACGGGTGCGTAACACGTATGCAACCTACCTATAACAGGAGTATAGCTCGGGGAAACTCGAATTAACC
CTCCATAATATGATTGAATGGCATCATTTGATTATTAAAACTGAGGTGGTTATAGATGGGCATGCGTCTGAT
TAGCTAGTTGGCGGGGTAATGGCCCACCAAGGCGATGATCAGTAGGGGAACTGAGAGGTTGATCCCCCAC
ACTGGCACTGAGATACGGGC

>Zotu6239

GAACGCTGGCGGCGTGCCTAATACATGCAAGTCGAGCGGATCTTTTAAAAGCTTGCTTTTAAAAGATTAGC
GGCGGACGGGTGAGTAACACGTGGGCAACCTGCCTGTAAGACTGGGATAACTCCGGGAAACCGGGGCTA
ATACCGGATAATTCTTGTCCCCGCGTGGGGGCAAGCTGAAAGACGGCTTATGCTGTCACTTACAGATGGGC
CCGCGGCGCATTAGCTAGTTGGTGAGGTAATGGCTCACCAAGGCAACGATGCGTAGCCGACCTGAGAGGG
TGATCGGCCACACTGGGACTGAGACACGGCC

>Zotu6241

GAACGCTAGCGACAGGCCTAACACATGCAAGTCGAGGGGCAGCGAGTTTGTAGCAATACGAATGTCGGCG
ACCGGCGCACGGGTGCGTAACAGGTGTGCAATCTGTCCATAGTCGGGGAATAGCCCAGCGAAAGTTGGAT
TAATGCTCCATGTGTCATGATAGGGCATCCTATTATGATGAAACGAGAGGACTATGGGTGAGCACGCTTCT
GATTAGATAGTTGGTGAGGTAACGGCTCACCAAGTCGACGATCAGTAGGGGTTCTGAGAGGAAGGTCCCC
CACATTGGAACTGAGACACGGTC

>Zotu6244

GAACGCTGGCGGCATGCCTAACACATGCAAGTCGAACGAAGGCTTCGGCCTTAGTGGCGCACGGGTGCGT
AACGCGTGGGAATCTGCCCCTCGGTTCGGAATAACAGAGAGAAATTTCTGCTAATACCGGATAATGACGTA
AGTCCAAAGATTTATCGCCGAGGGATGAGCCCGCGTAGGATTAGCTAGTTGGTGTGGTAAAGGCGCACCA
AGGCGACGATCCTTAGCTGGTCTGAGAGGATGATCAGCCACACTGGGACTGAGACACGGCC

>Zotu6246

GAACGCTGGCGGCGTGCTTAACACATGCAAGTCGAGGGTAGCAATACACTGGCGCAAGGGTGCGTAACGC
GTAGATTATCTGCCCCATACACTAACATAACGCTTGGAAACGGGTGCTAATATTAGATGAGGTGCTTCGGC
ATCAAAGTTTCGACGGTATGGGATGAGTCTGCGTCTGATTAGCTAGTTGGTAGTGTAATGGACTACCAAGG
CGACGATCAGTAGCTGGTCTGAGAGGACGATCAGCCACACTGGAACTGAGACACGGTC

>Zotu6248

GAACGCTGGCGGCATGCCTAACACATGCAAGTCGAACGAGGGCTTCGGCCCTAGTGGCGCACGGGTGAGT
AACGCGTGGGAACCTGCCCTGTGGTACGGAATAACTCCGGGAAACTGGAGCTAATACCGTATGTGTCCTGT
GGGACAAAGATTTATCGCCATGGGATGGGCCCGCGTAGGATTAGCTTGTTGGTGGGGTAACGGCCTACCA
AGGCAACGATCCTTAGCTGGTCTGAGAGGATGATCAGCCACACTGGGACTGAGACACGGCC

>Zotu6251

GAACGCTAGCGGCAGGCCTAACACATGCAAGTCGAGGGGTAACAGGGTAGCAATATCGCTGACGACCGG
CGCACGGGTGAGTAACGCGTATGCAACCTTCCTTTAACTGGGGGATAGCCCGGAGAAATTCGGATTAATAC
CCCATAACATCATAGAAAGGCATCTTTCAGTGATTAAAGCTCCGGCGGTTAAAGATGGGCATGCGTGCCAT
TAGCTAGTTGGTGAGGTAACGGCTCACCAAGGCTACGATGGTTAGGGGGTCTGAGAGGATGATCCCCCAC
ACTGGTACTGAGACACGGAC
>Zotu6254
GAACGCTAGCGGCAGGCCTAATACATGCAAGTCGAGGGGCAGCAGGGATAGCAATATTCGCTGGCGACC
GGCGCACGGGTGCGTAACACGTATACAATCTGCCTTTAACAGGGGGATAGCCCGGAGAAATCCGGATTAA
TACCCCATAACACAAGAAACGGCATCGTTTTTGTTAAAAGCTCCGGCGGTTAGAGATGAGTATGCGTCTG
ATTAGCTAGTTGGTGAGGTAACGGCTCACCAAGGCAATGATCAGTAGGGGATCTGAGAGGATTAACCCCC
ACACGGATACTGAGACACGGAT
>Zotu6256
GAACGCTAGCGGGAGGCCTAACACATGCAAGCCGAGCGGTAGAGATTCTTCGGAATCTTGAGAGCGGCGC
ACGGGTGCGGAACACGTGTGCAACCTGCCTTTATCTGGGGGATAGCCTTTCGAAAGGAAGATTAATACCCC
ATAATATATTGAATGGCATCATTTGATATTGAAAACTCCGGTGGATAGAGATGGGCACGCGCAGGATTAGA
TAGTTGGTGAGGTAACGGCTCACCAAGTCAACGATCCTTAGGGGGCCTGAGAGGGTGATCCCCCACACTG
GTACTGAGACACGGAC
>Zotu6258
GAACGCTAGCGGCAGGCCTAATACATGCAAGTCGAACGGGCGATAGCAATATCGCTAGTGGCGCACGGGT
GCGTAACGCGTAACCAACCTGCCCTGAACTGGGGGATAGCCCGCCGAAAGGCGGATTAATACCGCATAAA
CCAACAGTGTGGCATCACATAATTGGTAAAGATTTATTGGTTCAGGATGGGGTTGCGTAGCATTAGCTAGT
TGGCGGGGTAATAGCCCACCAAGGCGACGATGCTTAGGGGACCTGAGAGGGTGATCCCCCACACTGGCAC
TGAGATACGGGC
>Zotu6262
CAACGCTGGCGGCGTGCCTAACACATGCAAGTCGAACGCGAAAGTGGAGCAATCCATGAGTAGAGTGGC
GAACGGGTGAGTAACACGTGGGTGACCTACCTTCGAATGGGGGATAACGTCCCGAAAGGGACGCTAATAC
CGCATAACATCCTGTCCTTGAACGGATGGAGATCAAAGCCGGGGATCGCAAGACCTGGCGTTTGAAGAGG
GGCCCGCGTCCGATTAGCTAGTTGGTGAGGTAATGGCTCACCAAGGCGACGATCGGTAACCGGCCTGAGA
GGGCGGACGGTCACACTGGGACTGAGACACGGCC
>Zotu6264
GAACGCTGGCGGCGTGCCTAACACATGCAAGTCGGATGTGCCGCAAGGTGCATGGCAGACGGGTGAGTA
ACGCGTGGGCAATCTACCTATCAGTGGGGAATAACGATCCGAAAGGATTGGTAATACCGCATGTGACCATC
GGCTGGGATGCTGGTGGTGAAACCCGGGGACCGCAAGGCCTGGGGCTGATAGAGGAGCCTGCGTCTGAT
TAGCTAGTTGGCGGGGTAACGGCCCACCAAGGCGACGATCAGTAGCCGGCCTGAGAGGGTGATCGGCCA
CACTGGAACTGAGACACGGTC
>Zotu6269
GAACGCTGGCGGCAGGCTTAACACATGCAAGTCGAACGCCCCGCAAGGGGAGTGGCAGACGGGTGAGTA
ACACGTGGGAACGTACCCTTTACTACGGAATAACGCATGGAAACGTGTGCTAATACCGTATGTGCCCTTCG
GGGGAAAGATTTATCGGTAAAGGATCGGCCCGCGTTGGATTAGCTAGTTGGTGGGGTAAAGGCCTACCAA
GGCGACGATCCATAGCTGGTCTGAGAGGATGATCAGCCACATTGGGACTGAGACACGGCC
>Zotu6271
GAACGCTGGCGGCGTGCCTAACACATGCAAGTCGAGCGATCTTCTTCGGAAGAGAGCGGCGGACGGGTG
AGTAACGCGTGGGTAACCTGCCCTGTACACACGGATAACATACCGAAAGGTATGCTAATACGAGATAATAT
GCTTTTATCGCATGGTAGAAGTATCAAAGCTCCGGCGGTACAGGATGGACCCGCGTCTGATTAGCTAGTTG
GTGAGGTAACGGCTCACCAAGGCGACGATCAGTAGCCGACCTGAGAGGGTGATCGGCCACATTGGAACTG
AGACACGGTC
>Zotu6275
TAACGCTGGCGGCGCGCCTAATACATGCAAGTCGAACGATGTAGCAATACATAGTGGCGCACGGGAGAGT
AACACGTGGATAATCTCCCCTCGAGAGAAGAATAACTAGTCGAAAGATTGGCTAATACTTCATAAGACCAC
AGAGACTAATGTCTCAGAGGTAAAAGTCGTAAGACGCTTGAGGATGAGTCTGCGTCCCATTAGCTAGTTGG

TGAGGTAATGGCTCACCAAGGCGACGATGGGTAGCTGGTCTGAGAGGATGATCAGCCTCACTGGAACTGA
CACACGGTC
>Zotu6277
GAACGCTAGCGGCAGGCTTAACACATGCAAGTCGAGGGGTATAGTAGCAATACTAGAGACCGGCGCACG
GGTGCGTAACGCGTATGCAATCTACCTTTTGCAGGGGAATAGCCCAGAGAAATTTGGATTAATGCCCCATA
GTATAATGCCTCGGCATCGAGATATTATTAAAGTCACAACGGCAAAAGATGAGCATGCGTCCCATTAGCTA
GTTGGTAAGGTAACGGCTTACCAAGGCGACGATGGGTAGGGGTCCTGAGAGGGAGATCCCCCACACTGGT
ACTGAGACACGGAC
>Zotu6281
GAACGCTGGCGGCGTGCTTAACACATGCAAGTCGAGCGAAGCGCAGAAACGGATTTCTTCGGATTGAAGG
TTCTGTGACTGAGCGGCGGACGGGTGAGTAACGCGTGGGGAACCTGCCCTGTACCGGGGGATAACAGAC
AGAAATGTCTGCTAATACCGCATAAGCGCACGAGAAGGCATCTTCTTGTGTGAAAAACTCCGGTGGTACAG
GATGGACCCGCGTTGGATTAGCCAGTTGGCGGGGTAACGGCCCACCAAAGCGACGATCCATAGCCGGCCT
GAGAGGGCGACCGGCCACATTGGGACTGAGACACGGCC
>Zotu6285
GAACGCTGGCGGCGTGCTTAACACATGCAAGTCGAACGGAAAGGCCCCCAGCTTGCTGGGGGTACTCGAG
TGGCGAACGGGTGAGTAACACGTGGGTGATCTGCCCTGCACTTCGGGATAAGCCTGGGAAACTGGGTCTA
ATACCGGATAGGAGCCATCTTTGGTGTGATGGTTGGAAAGTTTTTCGGTGTAGGATGAGCCTGCGGCCTA
TCAGCTTGTTGGTGGGGTAATGGCCTACCAAGGCGTCGACGGGTAGCCGGCCTGAGAGGGTGTACGGCCA
CATTGGGACTGAGATACGGCC
>Zotu6287
CAACGCTGGCGGCGCGCTTAACACATGCAAGTCGCACGAGAAAGGGGAGCAATCCCTGAGTACAGTGGC
GTACGGGTGAGTAACACGTGGATCATCTACCTTCTAGTGGGGAATAACCCTGGGAAACCGGGGCTAATAC
CGCATAAGCTCGAGAGAGGAAAGCAGCAATGCGCTGGAAGAGGAGTCCGCGGCCGATTAGCTAGTTGGC
AGGGTAAAAGCCTACCAAGGCATCGATCGGTAGCCGGCCTGAGAGGGCACACGGCCACACTGGCACTGAA
ACACGGGC
>Zotu6289
GAACGCTGGCGGCGTGCCTAACACATGCAGGTCGAGCGAGTGAATCTCCTTCGGGAGTGAAGCTAGCGGC
GGACGGGTGAGTAACACGTGGGCAACCTGCCTCATAGAGGGGAATAGCCTTTCGAAAGGAAGATTAATAC
CGCATAAGATTGTAGCTTCGCATGAAGTAGCAATTAAAGGAGCAATCCGCTATGAGATGGGCCCGCGGCG
CATTAGCTAGTTGGTGAGGTAACGGCTCACCAAGGCGACGATGCGTAGCCGACCTGAGAGGGTGATCGGC
CACATTGGGACTGAGACACGGCC
>Zotu6293
CAACGCTGGCGGCGCGCTTAACACATGCAAGTCGCACGAGAAAGGGTAGCAATATCTGAGTAAAGTGGCG
TACGGGTGAGTAACACGTGGATCATCTACCTTTTGGCGGGGAATACCTCCTGGAAACGGGAGACAATACC
GCATAAGCTCGAAAGAGGAAAGCAGCAATGCACCGAAAGAGGAGTCCGCGGTCGATTAGCTAGATGGTG
AGGTAATGGCTCACCATGGCGAAGATCGATAGCCGGCCTGAGAGGGCTCACGGCCACACTGGCACTGAAA
CACGGGC
>Zotu6296
GAACGCTGGCGGCGCGCTTAACACATGCAAGTCGAGCGAGAACCAGGCCTTCGGGCCTGGGGACAGCGG
CGAACGGGTGAGTAACACGTGGGTAATCAACCCTCGATACTGGGATAGCCCGGGGAAACCCGGATTAATA
CCGGATGGCCCTTTGGATCCTCGGATCTGAAGGAAAAGGTAGCTTCGGCCTCCGATCGAGGACGAGCCCG
CGGCGGATTAGCTTGTTGGTGAGGTAACGGCCCACCAAGGCGACGATCCGTAGCTGGTCTGAGAGGACGA
TCAGCCACACTGGGACTGAGACACGGCC
>Zotu6298
CAACGCTGGCGGCGTGCCTAACACATGCAAGTCGAACGCGAAAGTGGAGCAATCCATGAGTAGAGTGGCA
AACGGGTGAGTAACACGTGGGTGACCTGCCTTCGAGCGGGGGATAACGTCCCGAAAGGGACGCTAATACC
GCATAACATCCTGCCTTTTAAGAGGTGGAGATCAAAGCCGGGGATCGCAAGACCTGGCACTTGAAGAGGG
GCCCGCGCCTGATTAGCTAGTTGGTGGGGTAATGGCCTACCAAGGCAACGATCAGTATCCGGCCTGAGAG
GGCGGACGGACACACTGGGACTGAGACACGGCC

>Zotu6300
GAACGCTGGCGGCGTGCTTAACACATGCAAGTCGAGCGAAGCAGTTAAGAAGATTCTTCGGATGAAACTT
GATTGACTGAGCGGCGGACGGGTGAGTAACGCGTGGGTGACCTGCCCCATACCGGGGGATAACAGCTGG
AAACGGCTGCTAATACCGCATAAGCGCACAGAGCTGCATGGCTCAGTGTGAAAAACTCCGGTGGTATGGG
ATGGGCCCGCGTCTGATTAGGCAGTTGGCGGGGTAACGGCCCACCAAACCGACGATCAGTAGCCGGCCTG
AGAGGGCGACCGGCCACATTGGGACTGAGACACGGCC
>Zotu6302
GAACGCTGGCGGCATGCCTAACACATGCAAGTCGAACGAGACCTTCGGGTCTAGTGGCGCACGGGTGCGT
AACGCGTGGGAATCTGCCCTTGGGTTCGGAATAACTCAGAGAAATTTGAGCTAATACCGGATGATGACGTA
AGTCCAAAGATTTATCGCCCAGGGATGAGCCCGCGTAAGATTAGCTAGTTGGTGGGGTAAAGGCCTACCA
AGGCGACGATCTTTAGCTGGTCTGAGAGGATGATCAGCCACACTGGGACTGAGACACGGCC
>Zotu6307
GAACGCTGGCGGCGTGCCTAACACATGCAAGTCGAACGGGATCCATGGAGTGCTTGCACTCCGGAAGATC
TAGTGGCGAACGGGTGAGTAACACGTGAGAAACCTGCCCTAGACTCTGGGATAACCTCGGGAAACCGAAG
CTAATACCGGATATTCTCCTTTGGTCGCATGGCCAGTTGAGGAAAGTTTTTCGGTCTGGGAGGGTCTCGCG
GCCTATCAGCTAGTTGGTGGGGTAACGGCCCACCAAGGCTACGACGGGTAGCTGGTTTGAGAGAACGATC
AGCCACACTGGAACTGAGACACGGTC
>Zotu6310
GAACGCTGGCGGCGTGCTTAACACATGCAAGTCGAACGGAAAGGCCTCTTGCTTGCAGGAGGTACTCGAG
TGGTGAACGGGTGAGTAACACGTGGGTGATCTGCCTTGCACTCTGGGATAAGCTTGGGAAACTGGGTCTA
ATACCGGATATGAACTGCCTTTAGTGTGGTGGTTGGAAAGTTTTTCGGTGCAAGATGAGCTCGCGGCCTA
TCAGCTTGTTGGTGGGGTAATGGCCTACCAAGGCGTCGACGGGTAGCCGGCCTGAGAGGGTGTACGGCCA
CATTGGGACTGAGATACGGCC
>Zotu6312
GAACGCTGGCGGCGTGCCTAAGACATGCAAGTCGAGTGGGTTTAGACCCACGGCGAACGGGTGAGTAAT
ACCTAGGAACTTTCTTTAAGTGGAGCATAACTAGTCGAAAGATTAGATAATTCTCCATGTGGCAGCAATGCT
AAAGTCGCAAGACGCTTAAAGTGAGGCCTGGGCACCATCAGCTAGTTGGTAAGGTAATGGCTTACCAAGG
CTATGACGGTTAGGGGTCCCGAGAGGGATAACCCCCACACTGGAACTGCGACACGGTC
>Zotu6316
GAACGCTAGCGGCGTGCCTAACACATGCAAGTCGAGCGATCTTCTTCGGAAGAGAGCGGCGGACGGGTG
AGTAACGCGTGGGTAACCTGCCCTGTACACACGGATAACATACCGAAAGGTATGCTAATACGAGATAATAT
GCTTTTATCGCATGGTAGAAGTATCAAAGCTCTGGCGGTACAGGATGGACCCGCGTCTGATTAGCTAGTTG
GTAAGGTAACGGCTTACCAAGGCGACGATCAGTAGCCAACCTGAGAGGGTGATCGGCCACATTGGAACTG
AGACACGGTC
>Zotu6319
GAACGCTGGCGGCGTGCTTAACACATGCAAGTGGAGCGACGAACCCGGACTTGTCCGGGGGCAGAGCCG
CGAACGGGTGAGTAACACGTGGGTAACCCACCTCGATGACCGGGACAACCCGAGGAAACTCGGGCTAATA
CCGGATGTGATGGTTCTCTATAAGGAGAACTAGGAAAGATAGCTTCGGCCTTCGCATCGAGACGGGCCCG
CGGCCCATTAGCTTGTTGGTGGGGTAATGGCCTACCAAGGCTTCGATGGGTAGCTGGTCTGAGAGGACGA
TCAGCCACACTGGGACTGAGACACGGCC
>Zotu6322
GAACGCTGGCGGCGTGCCTAATACATGCAAGTCGAGCGGACCTCTTTTGAGGTCAGCGGCGGACGGGTGA
GGAACACGTGGGTAATCTGCCTTTCAGACCGGAATAACGCCCGGAAACGGGCGCTAATGCCGGATACGCC
CGCGAGGAGGCATCTTCTTGCGGGGAAAGGCCCGATTGGGCCGCTGAGAGAGGAGCCCGCGGCGCATTA
GCTAGTTGGCGGGGTAACGGCCCACCAAGGCGACGATGCGTAGCCGACCTGAGAGGGTGACCGGCCACA
CTGGGACTGAGACACGGCC
>Zotu6324
GAACGCTGGCGGCGTGCTTAACACATGCAAGTCGAACGGTAAGGCCCTTCGGGGTACACGAGTGGCGAAC
GGGTGAGTAACACGTGGGTAATCTGCCCTGCACATCGGGATAAGCCTGGGAAACTGGGTCTAATACCGAA
TAGGACCACAGGACACATGTCCTGTGGTGGAAAGCTTTTGCGGTGTGGGATGGGCCCGCGGCCTATCAGC

TTGTTGGTGGGGTGATGGCCTACCAAGGCGACGACGGGTAGCCGGCCTGAGAGGGTGTCCGGCCACACT
GGGACTGAGATACGGCC
>Zotu6328
GAACGCTGGCGGCATGCCTAACACATGCAAGTCGAACGAGACCTTCGGGTCTAGTGGCGCACGGGTGCGT
AACGCGTGGGGATCTGCCCTTTGCTTCGGAATAACAGTTAGAAATGACTGCTAATACCGGATGATGTCGTA
AGACCAAAGATTTATCGGCAAAGGATGAACCCGCGTAGGATTAGCTAGTTGGTAGGGTAAAGGCCTACCA
AGGCGACGATCCTTAGCTGGTCTGAGAGGATGATCAGCCACACTGGGACTGAGACACGGCC
>Zotu6332
GAACGCTGGCGGCGTGTTTAACACATGCAAGTCGAGCGAGAAGCTTTCCAGTCGAATCTTCGGATGAAAC
AGGATTGCGGAAAGCGGCGGACGGGTGAGTAACGCGTAGGCAACCTGCCCCCTGCAGAGGGATAGCCTC
GGGAAACCGGGATTAAAACCTCATAATGCGCGAGTGAGACATCTCACATGCGCCAAAGATTTATCGGCAG
GGGATGGGCCTGCGTCTGATTAGTTAGTTGGTGGGGTAACGGCCTACCAAGGCGACGATCAGTAGCCGAC
CTGAGAGGGTGATCGGCCACATTGGAACTGAGACACGGTC
>Zotu6336
GAACGCTGGCGGCGTGCTTAACACATGCAAGTCGAGCGGTAAGGCCCTTTCGGGGGTACACGAGCGGCG
AACGGGTGAGTAACACGTGAGTAATCTGCCCTTCACTTCGGGATAAGCTTCGGAAACGGGGTCTAATACCG
GATATGACACAGTTTCGCATGAGATCTGTGTGGAAAGTTTTTTCGGTGGGGGATGTGCTCGCGGCCTATCA
GCTTGTTGGTGGGGTAATGGCCTACCAAGGCTTCGACGGGTAACCGGCCTGAGAGGGTGACCGGTCACAC
TGGGACTGAGACACGGCC
>Zotu6339
GAACGCTGGCGGCATGCTTAACACATGCAAGTCGAACGAGCTAATGGGTAACTATTAGTGTAGTGGCGGA
CGGGTGCGTAACGCGTAAGAACCTACCTTTTGGAGGGGGATAACATCGGGAAACTGTTGGTAATACCCCA
TAATGCTGAGGAGTTAAAGCAGCGAGAGCTGGCCAAAAGAGGGGCTTGCGTCTGATTAGCTAGTTGGTGG
GGGTAAAAGCCTCCCAAGGCCACGATCAGTAGCTGGTTTGAGAGGATGATCAGCCACACTGGGACTGAGA
CACGGCC
>Zotu6341
GAACGCTGGCGGCGTGCTTAACACATGCAAGTCGAACGCTGAGGCTCCCAGCTTGCTGGGGGTGGATGAG
TGGCGAACGGGTGAGTATCACGTGAGGAACCTGCCCTGAACTTCGGGATAAGCCTGGGAAACTGGGTCTA
ATACCGGATAGGACATTCTACCGCATGGTGGGGTGTGGAAAGTTTTAACGGTTTGGGATGGTCTCGCGGC
CTATCAGCTTGTTGGTGGGGTAATGGCCTACCAAGGCGATGACGGGTAGCCGGCCTGAGAGGGTGACCGG
CCACACTGGGACTGAGACACGGCC
>Zotu6347
TAACGCTAGCGGCAGGCCTAATACATGCAAGTCGAACGGCAGCACGGGTAGCAATACCTGGTGGCGAGTG
GCGCACGGGTGCGTAACGCGTATGCAATCTACCTGTAACTGGGGAATAGCCTTCCGAAAGGAAGATTAAT
ACCGCATAATATCACGAGACGGCATCGCCTTGTGATCAAATCTCCGGAGGTTACAGATGAGCATGCGTGAC
ATTAGCTAGTTGGTGAGGTAACGGCTCACCAAGGCTACGATGTCTAGGGGGTCTGAGAGGATGATCCCCC
ACACTGGTACTGAGACACGGAC
>Zotu6350
GAACGCTGGCGGCGTGCCTAATACATGCAAGTCGAGCGCCCCTTTAAGAGGTGATAATCGAAAGATTATC
GGTTCCTATGGGGAGCGGCGGACGGCTGAGTAACGCGTGGGAACGTGCCCCAAAGTGAGGGATAAGCAC
CGGAAACGGTGTCTAATACCGCATATGATCTTCGGATTAAAGCTTTATGCGCTTTGGGAGCGGCCTGCGTC
CTATTAGATAGTTGGTGAGGTAAAGGCTCACCAAGTCAACGATGGGTAGCTGGTCTGAGAGGATGACCAG
CCAGACTGGAACTGAGACACGGTC
>Zotu6353
GAACGCTGGCGGCAGGCTTAACACATGCAAGTCGAGCGGTAGCACAGGGGAGCTTGCTCCCTGGGTGAC
GAGCGGCGGACGGGTGAGTAATGTCTGGGAAACTGCCTGATGGAGGGGGATAACTACTGGAAACGGTAG
CTAATACCGCATAACGTCGCAAGACCAAAGAGGGGGACCTTCGGGCCTCTTGCCATCAGATGTGCCCAGAT
GGGATTAGCTAGTAGGTGGGGTAACGGCTCACCTAGGCGACGATCCCTAGCTGGTCTGAGAGGATGACCA
GCCACACTGGAACTGAGACACGGTC
>Zotu6355

GAACGCTGGCGGCATGCCTAACACATGCAAGTCGAACGGGATGGAGCCTTCGGGCTCCGGCGAGTGGCG
AACGGGTGAGTAACACGTGGGTAACCTGCCCTTCACTGGGGGACAACCGAGGGAAACTTCGGCTAATACC
GCATGATGTGCGTTGTCTCAAGGCGACGTACCAAAACTCCGGTGGTGAAGGATGGGCCCGCGGCGGATTA
GCTAGTTGGTGGGGTAATGGCCTACCAAGGCGACGATCCGTAGCTGGTCTGAGAGGACGATCAGCCACAC
TGGGACTGAGACACGGCC

>Zotu6357
GAACGCTGGCGGCGCACTTAACACATGCAAGTCGAGCGAGAACCAGGGCTTCGGCCCTGGGGACAGCGG
CGAACGGGTGAGTAACACGTGGATAACCTGCCCTCGGCACCGGGATAGCCCGGGGAAACCCGGATTAATA
CCGGATAGCCATTCCGACTCTCGGGTCGGTCTGAAAAGGTAGCTTCGGCCTCTGGCCGAGGATGGGTCCGC
GGCGGATTAGCTTGTTGGTGGGGTAACGGCCTACCAAGGCACCGATCCGTAGCTGGTCTGAGAGGACGAT
CAGCCACACTGGGACTGAGACACGGCC

>Zotu6361
GAACGCTGGCGGCATGCCTAGCACATGCAAGTTGAACGAGATCTTCGGGTCTAGTGGCGCACGGGTGCGT
AACGCGTGGGAATCTGCCCTTTGGTTCGGAATAACAGTTGGAAACGACTGCTAATACCGGATGATGACGA
AAGTCCAAAGATTTATCGCCAGAGGATGAGCCCGCGTAGGATTAGCTAGTTGGTGTGGTAAAGGCGCACC
AAGGCGACGATCCTTAGCTGGTCTGAGAGGATGATCAGCCACACTGGGACTGAGACACGGCC

>Zotu6363
GAACGCTATCGGTATGCTTAACACATGCAAGTTGAACGGATTATTTAGGGCTTGCTCTAAATAATTAGTAGC
AAACGGGTGAGTAACACGTGGGAATCTACCCATCAGTACGGAATAACATTTAGAAATAAATGCTAATACCG
TATATTCTCTTAGGAGGAAAGATTTATCGCTGATGGATGAGCCCGCGTCAGATTAGGTAGTTGGTAGGGTA
ATGGCCTACCAAGCCAACGATCTGTAGCTGGTCTGAGAGGATGATCAGCCACACTGGGACTGAGACACGG
CC

>Zotu6365
GAACGCTAGCGGCAGGCCTAATACATGCAAGTCAAGGGGTAGCAATACACCGGCGGACGGGTGCGTAAC
GCGTACATAACATGCCCTTAGGTGGGGGATAGTATTGGGAAACTGATAGTAATACCCCATAGTATTGATTT
ATGGCATCATGAGTCAATTAAAGGTTACGCCTAAGGATTGATGTGCGTATGATTAGCTAGTTGGTGGGGTA
ACGGCTCACCAAGGCGACGATCATTAGGGGGTGTGAGAGCATGACCCCCCACACGGGTACTGAGACACGG
AC

>Zotu6369
GAACGTTAGCGGCGCGCCTAACACATGCAAGTCGAGCGAGAAAGGGCTTCGGCCCCGGTAAAGCGGCGC
ACGGGTGAGTAACACGTAGGTAATCTGCCCCTGAGCGGGGGATAACCTTCCGAAAGGAGGGCTAATACCG
CATAAGACCACGCCTGCGAGAGCAGGAGGGGTCAAAGCCGGCCTCTATACATAAGCTGGCACTTGGGGAT
GAGCCTGCGGCCCATCAGCTAGTTGGTGGTGTAACGGACCACCAAGGCGAAGACGGGTAGCTGGTCTGAG
AGGATGATCAGCCACACTGGAACTGAGACACGGTC

>Zotu6372
CAACGCTGGCGGCGCGCTTAACACATGCAAGTCGCACGAGAAAGGGAGCAATCCTAAGTAAAGTGGCGTA
CGGGTGAGTAACACGTGGATCATCTACCTTTTGGCGGGGAATACCTCCTGGAAACGGGAGACAATACCGC
ATAAGCTCGAAAGAGGAAAGCAGCAATGCACCGAAAGAGGAGTCCGCGGTCGATTAGCTAGATGGTGAG
GTAATGGCTCACCATGGCGAAGATCGATAGCCGGCCTGAGAGGGCTCACGGCCACACTGGCACTGAAACA
CGGGC

>Zotu6376
GAACGCTGGTGGCGTGTTTTATACATGCAAGTCGAGCGAGGACGGTAGCAATACCGAGCCGAGCGGCGA
ACGGGTGAGTAACGCGTAAGCAATCTGCCCCACAATCGGGAACAACCGTGCCAACGCGCGGCTAATGCCG
GATATTGTAGTCTACTGCATAGTAGGCCAACGAAAGATTTATTGTTGTGGGATGAGCTTGCGTCCGATTAG
CTAGTTGGCGGGGTAATAGCCCACCAAGGCGACGATCGGTAGCCGGCCTGAGAGGGTGATCGGCCACATT
GGGACTGAGATACGGCC

>Zotu6378
GAACGCTGGCGGCGTGGTTAAGGAATGCAAGTCGAACGGTAAGCAGGTAATTTATTATTTGCTTATAGTG
GCAAACGGGTTAGTAACGCGTAGGTAACTTACCTTGAAGCCCCGAATAATTCGCCGAAAGGCGAACTAATA
CGGGATGGTAATGAGGGGGCGAAAGCCATTCTCATTTAAAGATTCATCACTTCAAGAGAGGCCTGCGTCCT

ATCAGCTAGTTGGCGAGGTAATTGCTCACCAAGGCTATGACGGGTAGGGGGACTGAGAGGTTGGCCCCCA
ACATCGGAACTGAGACACTGTC
>Zotu6380
GAACGCTGGCGGCATGCCTAACACATGCAAGTCGAACGAAGGCCCGATTTCGGTTGGGTCCTTAGTGGCG
CACGGGTGCGTAACGCGTGGGAATCTGCCCCTCGGTTCGGAATAACAGTTAGAAATGACTGCTAATACCG
GATGATGACGTAAGTCCAAAGATTTATCGCCGAGGGATGAGCCCGCGTAGGATTAGCTAGTTGGTGTGGT
AAGAGCGCACCAAGGCGACGATCCTTAGCTGGTCTGAGAGGATGATCAGCCACACTGGGACTGAGACACG
GCC
>Zotu6384
GAACGCTGGCGGCGTGCCTAATACATGCAAGTCGAGCGGACCTCTTCGGAGGTCAGCGGCGGACGGGTG
AGTAACACGTGGGCAACCTGCCTATAAGACTGGGATAACTTCGGGAAACCGGAGCTAATACCGGATAACA
TCAAGGACCACATGGTCTTCGATTAAAAGATGGCGTCAAGCTATCACTTATAGATGGGCCCGCGTCGCATT
AGCTAGTTGGTGAGGTAACGGCTCACCAAGGCGACGATGCGTAGCCGACCTGAGAGGGTGATCGGCCAC
ACTGGGACTGAGACACGGCC
>Zotu6386
GAACGCTGGCGGCGTGCCTAATACATGCAAGTCGAGCGAATCAATGGGAGCTTGCTCCCATTGATTAGCG
GCGGACGGGTGAGTAACACGTGGGTAACCTGCCTGTAAGACTGGGATAACTTCGGGAAACCGGAGCTAAT
ACCGGATAGTTTCTTTTCTCGCATGAGAGAAGATGGAAAGATGGTTCTGCTATCACTTACAGATGGACCCG
CGGCGCATTAGCTAGTTGGTGAGGTAACGGCTCACCAAGGCCACGATGCGTAGCCGACCTGAGAGGGTGA
TCGGCCACACTGGGACTGAGACACGGCC
>Zotu6389
GAACGTTGGCGGCGTGGATTAGGCATGCAAGTCGAACGCCCCGCAAGGGGAGTGGCGCAAGGGTGAGTA
AGGCGATGGTAACCAACCCCGAGGTCGGGCATAGCCGAGGGAAACCTCGGGTAATTCCCGGCGATGCAG
GAGACTGGCATCAGTTTCCTGTCAAAGGCTTCGGCCGCCGCGGGACGGGCCGTCGTGGTATTAGGTAGTT
GGTGGGGTGACGGCTCACCAAGCCTGCGATGCCTACCGGGTGTGAGAGCATGGCCCGGCACACTGGGACT
GAGACACTGCC
>Zotu6391
GAACGCTAGCGGGAGGCCTAATACATGCAAGTCGAGCGGTAACTTTCTTCGGGAAGCTAGAGCGGCGCAC
GGGTGAGTAACGCGTACATGACCTACCTTTAAGACCGGGATAGCCCTGGGAAACTGGGATTAATACCGGA
TGTGATAATAGATATAAAGATTTATTATTAAAGTTCCGGCGCTTAGAGATGGGTGTGCGTCTGATTAGCTA
GTTGGTAGGGTAACGGCCTACCAAGGCGACGATCAGTAGGGGGCGTGAGAGCGTGGCCCCCACACGGG
TACTGAGACACGGAC
>Zotu6393
GAACGCTGGCGGCATGCCTAACACATGCAAGTCGAACGGCAGCACAGTAAAGATTTCGGTCTTTATGGGT
GGCGAGTGGCGGACGGGTGAGTAATACGTAGGAATCTGTCCTGGAGAGGGGGATAACCCGAGGAAACTC
GGGCTAATACCGCATGATATTGAGAAATCAAAAGCAGGGGACCTGAAAGGGCCTTGCGCTCTGGGTTGAG
CCTACGTCGGATTAGCTAGTTGGTGGGGTAAAGGCCTACCAAGGCAACGATTCGTAGTTGGTCTGAGAGG
ATGACCAGCCACACTGGGACTGAGACACGGCC
>Zotu6395
GAACGCTGGCGGCATGCCTAATACATGCAAGTCGAACGCTGAAGCACTCTGCTTGCAGAGTGCGGATGAG
TGGCGAACGGGTGAGTAACACGTGGGAAATCTGCCTAAGAGTGGGGGATAACATTCGGAAACGGATGC
TAATACCGCATAGATTCTTGGACCGCATGGTTCTGGAAAGAAAGACGGCTTTGCTGTCGCTTTTAGATGATC
CCGCGGCGTATTAGTTAGTTGGTGGGGTAATGGCTTACCAAGACCATGATACGTAGCCGACCTGAGAGGG
TGATCGGCCACACTGGGACTGAGACACGGCC
>Zotu6398
GAACGCTGGCAGCGTGCCTAACACATGCAAGTCGAACGGAACTTCTTTAAAGGATTTCTTCGGAATGAATT
TGATTAAGTTTAGTGGCGGACGGGTGAGTAACGCGTGAGTAACCTGCCTCTAAGAGGGGAATAACATTCT
GAAAAGAATGCTAATACCGCATAATATATATTTATCGCATGGTAGATATATCAAAGATTTATCGCTTAGAGA
TGGACTCGCGTCCGATTAGTTAGTTGGTGAGGTAACGGCTCACCAAGACCGCGATCGGTAGCCGGACTGA
GAGGTTGAACGGCCACATTGGGACTGAGACACGGCC

>Zotu6400
GAACGCTGGCGGCATGCTTAACACATGCAAGTCGAACGGCAGCACGGTGTGTAGCGATACACATGGGTGG
CGAGTGGCGGACGGGTGAGTAATGCGTAGGAATCTGCCTTAAAGAGTGGGACAACCCGAGGAAACTCGG
GCTAATACCGCATGAAGTCGGAAGACTAAAGGCTGGGGTAACCTGGCGCTTTGAGAGGAGCCTGCGTCTG
ATTAGCTAGTTGGTGGGGTAAGAGCTTACCAAGGCGATGATCGGTAGCTGGTCTGAGAGGATGATCAGCC
ACACTGGGACTGAGACACGGCC
>Zotu6403
GAACGCTGGCGGTATGCCTAACACATGCAAGTCGAACGAGATCTTCGGATCTAGTGGCGCACGGGTGCGT
AACGCGTGGGAATCTGCCTTGGGGTTCGGAATAACTCCCCGAAAGGGGTGCTAATACTGGATGATGTCGA
AAGACCAAAGATTTATCGCCCTGAGATGAGCCCGCGTAGGATTAGCTAGTTGGTGTGGTAAAGGCGCACC
AAGGCGACGATCCTTAGCTGGTCTGAGAGGATGATCAGCCACACTGGGACTGAGACACGGCT
>Zotu6407
GAACGCTGGCGGCGTGCCTAATACATGCAAGTCGAACGGTCTTTTCTATGGAAGCTTGCTTCCACTGAGAA
GATAGTGGCGGACGGGTGAGTAACACGTGGGTAACCTGCCCATAAGAGGGGGATAACATCCGGAAACGG
GTGCTAATACCGCATAGTTTTCTTGATCGCATGATCGGGAAAGGAAAGACGGCCTTTGTGCTGTCGCTTAT
GGATGGACCCGCGGCGTATTAGTTAGTTGGTGAGGTAATGGCTCACCAAGACGATGATACGTAGCCGACC
TGAGAGGGTGATCGGCCACATTGGGACTGAGACACGGCC
>Zotu6410
GAACGCTGGCGGCGTGCTTAACACATGCAAGTCGAACGGAAAGGCCCAGCTTGCTGGGTACTCGAGTGGC
GAACGGGTGAGTAACACGTGGGTGATCTGCCTTGCACTCTGGGATAAGCCTGGGAAACTGGGTCTAATAC
CGGATAGGACCTCTGGCTGCATGGTTGGGGGTGGAAAGCTTTTGCGGTGTGAGATGGGCCCGCGGCCTAT
CAGCTTGTTGGTGGGGTAATGGCCTACCAAGGCGACGACGGGTAGCCGACCTGAGAGGGTGATCGGCCA
CACTGGGACTGAGACACGGCC
>Zotu6412
GAACGTTAGCGGCGCGCCTAACACATGCAAGTCGCACGAGAAAGGGCTTCGGCCCCGGTACAGTGGCGCA
CGGGTGAGTAACACGTAGATCATCTCCCCTCGAGTGGTGGATAACTCTCCGAAAGGAGAGCTAATACAGC
ATGAGACCACGGCTTCGTTGAAGCTGCGGCCAAAGCAGCCCTCTACACGTAAGGTTGCGCTTGAGGATGA
GTCTGCGGCCCATCAGCTAGTTGGTAGGGTAATGGCCTACCAAGGCTAAGACGGGTAGCTGGTCTGAGAG
GATGAACAGCCACACTGGAACTGAGACACGGTC
>Zotu6414
GAACGCTGGCGGCAGGCTTAACACATGCAAGTCGAACGCCCCGCAAGGGGAGTGGCAGACGGGTGAGTA
ACGCGTGGGAACCTACCTAGAACTTCGGAATAACACATGGAAACGTGTGCTAATACCGGATACGCCCTACG
GGGGAAAGATTTATCGGTTTTAGATGGGCCCGCGTAAGATTAGCTAGTTGGTGAGGTAATGGCTCACCAA
GGCGACGATCTTTAGCTGGTCTGAGAGGATGATCAGCCACACTGGGACTGAGACACGGCC
>Zotu6420
GAACGCTGGCGGTATGCCTTACACATGCAAGTCGTACGAGAGTTTTTAACTCAAGTGGCGGACGGGTGAG
TAACACGTAAGAATTTGACTTTAGGAGGGGGACAACAACTGGAAACGGTTGCTAATACCCCATATGCTTTC
GAGTGAAACGGATTTTTCCGCCTAAAGAGAAGCTTGCGGCTGATTAGCTTGTTGGTGAGGTAAGGGCTCAC
CAAGGCGACGATCAGTATCTGGTTTGAGAGGACGATCAGACACACTGGAACTGAGACACGGTC
>Zotu6423
GAACGCTGGCGGTATGCTTAACACATGCAAGTCGAACGAATGAGGGTCCTAGCTTGCTAGGGCCTAAGTT
AGTGGCAAACGGGTGAGTAACACGTGGGAATCTACCCAGTAGTACAGAATAACAGTTAGAAATGACTGCT
AATACTGTATAGTATCGAGAGATTAAAGATTTATCGCTACTGGATGAGCCCGCGTCAGATTAGGTAGTTGG
TGGGGTAATGGCCTACCAAGCCAAAGATCTGTAGCTGGTCTGAGAGGATGATCAGCCACACTGGAACTGA
GACACGGTC
>Zotu6425
GAACGCTGGCGGCGTGCTTAACACATGCAAGTCGAACGGATCCCAAGTGTCAGCAATGATGTGGAAGGGT
TAGTGGCGAACGGGTGAGTAACACGTGAGGAACCTGCCCCGGACTCAGGGATAACGGCTGGAAACGGCT
GCTAATACCTGATGCCGTCGTGTCATCGCATGGTGATGTGACGAAAGAATCTCGGTTCGGGAGGGTCTCGC

GGCCTATCAGCTTGTTGGTGGGGTAATGGCCTACCAAGGCGTCGACGGGTAGCTGGTCTGAGAGGATGAT
CAGCCACACTGGGACTGAGACACGGCC
>Zotu6428
GAACGCTGGCGGCAGGCTTAACACATGCAAGTCGAGCGGATGAAAGTAGCTTGCTACTGGATTCAGCGGC
GGACGGGTGAGTAATGCTTAGGAATCTGCCTATTAGTGGGGGACAACATCTCGAAAGGGATGCTAATACC
GCATACGTCCTACGGGAGAAAGCAGGGGATCTTCGGACCTTGCGCTAATAGATGAGCCTAAGTCGGATTA
GCTAGTTGGTGGGGTAAAGGCCTACCAAGGCGACGATCTGTAGCGGGTCTGAGAGGATGATCCGCCACAC
TGGGACTGAGACACGGCC
>Zotu6432
GAACGCTGGCGGCGTGCCTAAGACATGCAAGTCGAGTGGGTTTAGACCCACGGCGAACGGGTGAGTAAT
ACCTAGGAACTTTCTTTAAGTGGAGCATAACCCATAGAAATGTGGGATAATACTCCATGTGACCGCAAGGT
CAAAGTCGCAAGACGCTTAAAGTGAGGCCTGGGCACCATCAGCTAGTTGGTAAGGTAATGGCTTACCAAG
GCTATGACGGTTAGGGGTCCCGAGAGGGATAACCCCCACACTGGAACTGCGACACGGTC
>Zotu6438
GAACGCTAGCGGCAGGCCTAACACATGCAAGTCGAACGGTAACAGGTGTAGCAATACATGCTGACGAGTG
GCGCACGGGTGCGTAACACGTATGTAACTTGCCTCTAATTGGGAAATAGCTTCGAGAAATCGAAATTAATG
TCCCATAATAATTAGACTAGGCATCTAGTTTAATTTAAAGCTCAGGCGGTTAGAGATAGACATGCGGCCTAT
TAGTTTGTTGGTGAGGTAACGGCTCACCAAGGCTACGATAGGTAGGGGGCCTGAGAGGGTTATCCCCCAC
ACTGGAACTGAGACACGGTC
>Zotu6440
GAACGCTGGCGGCAGGCTTAACACATGCAAGTCGAACGATGACTCTCTAGCTTGCTAGAGATGATTAGTG
GCGGACGGGTGAGTAACATTTAGGAATCTGCCTAGTAGTGGGGGATAGCTCGGGGAAACTCGAATTAATA
CCGCATACGACCTACGGGTGAAAGGGGGCGCAAGCTCTTGCTATTAGATGAGCCTAAATCAGATTAGCTA
GTTGGTGGGGTAAAGGCCCACCAAGGCGACGATCTGTAACTGGTCTGAGAGGATGATCAGTCACACCGGA
ACTGAGACACGGTC
>Zotu6444
GAACGCTGGCGGCGTGCTTAACACATGCAAGTCGAACGGGCCCGCCCTCTTTTTTGGGGGTGGGTTGAGT
GGCGAACGGGTGAGTATCACGTGAGTAACCTGCCCCCTGCTCCTGGATAACTGCTTGAAAGGGCGGCTAA
TACGGGGTGTGCCGGCTGGTCCGCATGGGCCTGGTCGGGATAGTTTCATCTTCGGGTGTTTCGGTGGGGG
ATGGGCTCGCGGCCTATCAGCTTGTTGGTGGGGTGATGGCCCACCAAGGCGGTGACGGGTAGCCGGCCTG
AGAGGGTGGGCGGCCACACTGGGACTGAGACACGGCC
>Zotu6448
GAACGCTAGCGGCAGGCTTAATACATGCAAGTCGAGGGGCAGCAGGGATAGCAATATTCGCTGGCGACC
GGCGCACGGGTGCGTAACACGTATACAATCTGCCTTTAACAGGGGGATAGCCCGGAGAAATCCGGATTAA
TACCCCATAACAGTGATTCTGGCATCAGAGACACTTAAAAGCTCCGGCGGTTAAAGATGAGTATGCGTCTG
ATTAGCTAGTTGGTGAGGTAACGGCTCACCAAGGCGACGATCAGTAGGGGATCTGAGAGGATTAACCCCC
ACACGGATACTGAGACACGGAT
>Zotu6452
GAACGCTGGCGGCGTGCTTAACACATGCAAGTCGAACGGTGACCTGAGGTGCTTGCACCTTGGTGATCAG
TGGCGAACGGGTGAGTAACACGTGAGTAACCTGCCCCAGACTCTGGAATAACCCCGGGAAACCGGAGCTA
ATACCGGATACGACATCAGGCCGCATGGTCATGGTGTGGAAAGTTTTTCGGTCTGGGATGGGCTCGCGGC
CTATCAGCTTGTTGGTGAGGTAGTGGCTCACCAAGGCGACGACGGGTAGCCGGCCTGAGAGGGCGACCG
GCCACACTGGGACTGAGACACGGCC
>Zotu6454
GAACGCTGGCGGCGTGCTTAACACATGCAAGTCGAGCGGAAAGGCCACTTCGGTGGTACTCGAGCGGCGA
ACGGGTGAGTAACACGTGAGTAATCTGCCCTTGGCTTTGGGATAGCCACCGGAAACGGTGATTAATACCG
GATACGACAACTTCTCGCATGGGATGGTTGTGGAAAGTTTTTCGGCCAGGGATGTGCTCGCGGCCTATCAG
CTTGATGGTGAGGTAATGGCTCACCATGGCTTCGACGGGTAGCCGGCCTGAGAGGGTGACCGGCCACACT
GGGACTGAGACACGGCC
>Zotu6457

GAACGCTGGCGGCGTGCCTAATACATGCAAGTCGAGCGAACTTGCGGGAGCTTGCTCCCAAAAGTTAGCG
GCGGACGGGTGAGTAACACGTGGGCAACCTACCTATAAGACTGGGATAACTTCGGGAAACCGGAGCTAAT
ACCGGATAATTCTTTTCTGCACATGTAGAAAAGCTAAAAGACGGTTTACGCTGTCACTTATAGATGGGCCCG
CGGCGCATTAGCTAGTTGGTGAGGTAACGGCTCACCAAGGCCACGATGCGTAGCCGACCTGAGAGGGTGA
TCGGCCACACTGGGACTGAGACACGGCC

>Zotu6459

GAACGCTGGCGGCGTGCCTAACACATGCAAGTCGAACGGGGTGTCGAGAGCTTGCTCTTGACACTTAGTG
GCGAACGGGTGAGTAACGCGTGAGAAACCTGCCTCTCAGTGGGGGACAACAGTTGGAAACGACTGCTAAT
ACCGCATAATGTATCCGGAAGGCATCTTCTGGATACCAAAGCTTTATGTGCTGAGAGATGGTCTCGCGTCT
GATTAGCTAGATGGCGGGGTAACGGCCCACCATGGCGACGATCAGTAGCCGGTCTGAGAGGATGAACGG
CCACATTGGGACTGAGATACGGCC

>Zotu6461

CAACGCTGGCGGCGTGCCTAACACATGCAAGTCGAACGCGAAAAGGACTTCGGTCCTGAGTAGAGTGGCG
TACGGGTGAGTAACACGTGGGTAATCTACCTTTGGGTGGGGAATAACTCAGGGAAACTTGAGCTAATACC
GCATAATGCTACGGCTCGACATCGAGTCGTTGTTAAAGTAGCAATACGCCTGAAGAGGAGCCTGCGCCTGA
TTAGCTAGTTGGTAGGGTAACGGCCTACCAAGGCTTCGATCAGTAGCTGGTCTGAGAGGACGGCCAGCCA
CAATGGCACTGAAACACGGGC

>Zotu6463

GAACGCTGGTAACGTGGATACCGGCATGCAAGTCACGGGGGCCGTAAGGCAACCGGCGAACGGCTTAGT
AACACGTAGATACATACCCAAAAGTGGGGCATAGCGTCGAAAGACGAGTCAATTCCCCATAGTCTCGCAAG
AGTAAAGCAGCAATGCGCTTTTGGAATGGTCTGCGGCCTATCAGCTTGTTGGTGAGGTAACGGCTCACCAA
GGCTATGACGGGTAGGGGGCGTGAGAGCGCGACCCCCAAGAATGGCACTGCGACACGGGC

>Zotu6466

GAACGCTGGCGGCGTGCTTAACACATGCAAGTCGAACGGTAAGGCCCCTTCGGGGGTACACGAGTGGCGA
ACGGGTGAGTAACACGTGAGCAACCTGCCCTTGACTCTGGAATAACAGTTGGAAACAGCTGCTAATACCG
GATATGCACCGGACCTCTCCTGGGGTGTGGTGGAAAGCTTTGGCGGTCAGGGATGGGCTCGCGGCCTATC
AGCTTGTTGGTGAGGTGATGGCTCACCAAGGCTTCGACGGGTAGCCGGCCTGAGAGGGCGACCGGCCACA
CTGGGACTGAGATACGGCC

>Zotu6468

GAACGCTGGCGGCAGGCTTAACACATGCAAGTCGAACGGTTGTAGCAATACAGCAGTGGCGCACGGGTG
AGTAACGCGTGGGAACATACCTTAGAGTTCGGAATAACAGCGGGAAACTGCTGCTAATACCGGATATTCTC
TCCGGAGGAAAGATTTATCGCTCTAAGATTGGCCCGCGTCAGATTAGCTAGTTGGTGGGGTAATGGCCTAC
CAAGGCTCCGATCTGTAGCTGGTCTGAGAGGACGACCAGCTACACTGGAACTGAGACACGGTC

>Zotu6470

GAACGCTAGCGGCAGGCCTAATACATGCAAGACGAACGGTAATCGGTGTAGCAATACATCGGCAAGAGTG
TCGTAAGGGTGCGTAACACGTATGCAATCTGCCCTCAACAGGAGGATAGCTCCCCGAAAGGGGAATTAAT
ACTCCATAAGATCACGCGAAGGCATCTGAGAGTGATTAAATCTACGGAGGTTGAGGATGAGCATGCGGAT
GATTAGCTAGTTGGCGGTGTAACGGACCACCAAGGCAATGATCAATAGGGGAACTGAGAGGTTTATCCCC
CACACTGGCACTGAGATACGGGC

>Zotu6473

GAATGCTGGCGGCGTGCCTAACACATGCAAGTCGAGCGGTAACAGGAGAATTTATTCTTGCTGACGAGCG
GCGGACGGCTGAGTAACGCGTGGGAACATGCCCCAAAGTGAGGGATAAGCTCCAGAAATGGAGTCTAAT
ACCGCATATGATCTTCGGATTAAAGGATTTATCCGCTTTGGGGGTGGCCTGCGTAAGATTAGATAGTTGGT
GAGGTAATGGCTCACCAAGTCTACGATCTTTAGCTGGTCTGAGAGGATGATCAGCCAGACTGGAACTGAG
ACACGGTC

>Zotu6475

GAACGCTAGCGGCAGGCCTAATACATGCAAGTCGAACGGGATCCGTCGGAGAGCTTGCTCGAAGACGGTG
AGAGTGGCGCACGGGTGCGTAACGCGTGAGCAACCTACCTCTATCAGGGGGATAGCCTCTCGAAAGAGAG
ATTAACACCGCATAACATATCTGACCGGCATCGGTTAGCTATTAAATATTTATAGGATAGAGATGGGCTCGC
GTGACATTAGCTAGTTGGTAGGGTAACGGCTTACCAAGGCGACGATGTCTAGGGGCTCTGAGAGGAGAAT
CCCCCACACCGGTACTGAGACACGGAC

>Zotu6478
GAACGCTGGCGGCATGCCTTACACATGCAAGTCGAACGGCAGCACGGGAGCAATCCTGGTGGCGAGTGG
CGAACGGGTGAGTAATACATCGGAACGTGCCCAGTAGTGGGGGATAGCCCGGCGAAAGCCGGATTAATA
CCGCATACGACCTACGGGTGAAAGCGGGGGATCGCAAGACCTCGCGCTATTGGAGCGGCCGATGGCAGA
TTAGGTTGTTGGTGAGGTAAAAGCTCACCAAGCCGACGATCTGTAGCTGGTTTGAGAGGACGACCAGCCA
CACTGGGACTGAGACACGGCC
>Zotu6480
GAACGCTGGCGGCGTGCCTAATACATGCAAGTCGAGCGGTAAGGTTCTCACTCTGTATTCAACTAGTTTGA
ATGTGGTGTGAGAATACACGAGCGGCGGACGGCAGAGTAACGCGTAGGAACGTACCCCATAGTGAGGGA
TAAGCACCGGAAACGGTGTCTAATACCGCATAATCTCTTCGGAGCAAAGCCTTGTGCGCTTTGGGAGCGGC
CTGCGTATGATTAGTTTGTTGGTGAGGTAACGGCTCACCAAGACTACGATCATTAGCTGGTCTGAGAGGAT
GATCAGCCTGACTGGAACTGAGAACGGTC
>Zotu6483
GAACGCTGGCGGCATGCCTTACACATGCAAGTCGAACGGTAACAGGGAGCTTGCTCCGCTGACGAGTGGC
GAACGGGTGAGTAATATATCGGAACGTACCTTTGAGTGGGGGATAACTAGTCGAAAGATTAGCTAATACC
GCATACGATCTACGGATGAAAGCGGGGGACCCGTAAGGGCCTCGTGCTCATAGAGCGGCCGATATCTGAT
TAGCTAGTTGGTGAGGTAAAGGCTCACCAAGGCATCGATCAGTAGCTGGTCTGAGAGGACGACCAGCCAC
ACTGGGACTGAGACACGGCC
>Zotu6485
GAACGCTGGCGGCATGCTTTACACATGCAAGTCGAACGGCAGCACGGACTTCGGTCTGGTGGCGAGTGGC
GAACGGGTGAGTAATACATCGGAACGTGCCCAGTTGTGGGGGATAACTACTCGAAAGAGTAGCTAATACC
GCATGAGAACTGAGGTTGAAAGCAGGGGATCGTAAGACCTTGCGCAACTGGAGCGGCCGATGGCAGATT
AGGTAGTTGGTGGGATAAAAGCTTACCAAGCCGACGATCTGTAGCTGGTCTGAGAGGACGACCAGCCACA
CTGGGACTGAGACACGGCC
>Zotu6488
GAACGCTGGCGGCGTGCTTAACACATGCAAGTCGAACGGAGTACCCGAGATAGAAGCTTCGGCAGAAATT
TTGGAATACTTAGTGGCGGACGGGTGAGTAACACGTGAGTAACCTGCCTTCGAGTGGGGAATAACACACC
GAAAGGTGTGCTAATACCGCATAACATATGCGTACCGCATGATATGCATATCAAAGATTTATCGCTCGAAG
ATGGACTCGCGTCCAATTAGGTAGTTGGTGAGGTAACGGCCCACCAAGCCGACGATTGGTAGCCGGACTG
AGAGGTTGAACGGCCACATTGGGACTGAGACACGGCC
>Zotu6490
GAACGCTGGCGGCGCGCCTAACACATGCAAGTCGAACGAGCGAGAGAGAGCTTGCTTTCTCGAGCGAGTG
GCGAACGGGTGAGTAACGCGTGAGGAACCTGCCTCAAAGAGGGGGACAACAGTTGGAAACGACTGCTAA
TACCGCATAAGCCCACGGTGCCGCATGGCACAGAGGGAAAAGGAGCAATCCGCTTTGAGATGGCCTCGCG
TCCGATTAGCTGGTTGGTGAGGTAATGGCCCACCAAGGCGACGATCGGTAGCCGGACTGAGAGGTTGAAC
GGCCACATTGGGACTGAGACACGGCC
>Zotu6496
GAACGCTGGCGGCATGCCTAACACATGCAAGTCGAACGAGACCTTCGGGTCTAGTGGCGCACGGGTGCGT
AACGCGTGGGAACCTGCCTTGAGGTTCGGAATAACTCAGAGAAATTTGAGCTAATACCGGATAATGTCTTC
GGACCAAAGATTTATCGCCTTTAGATGGGCCCGCGTTGGATTAGCTAGTTGGTGGGGTAAAGGCCTACCAA
GGCGACGATCCATAGCTGGTCTTAGAGGATGATCAGCCACACTGGGACTGAGACACGGCC
>Zotu6498
GAACGCTGGCGGCGTGCTTAACACATGCAAGTCGAACGGTGAAGCAGAGCTTGCTCTGTGGATCAGTGGC
GAACGGGTGAGTAACACGTGAGCAACCTGCCCTGGACTCTGGGATAAGCGCTGGAAACGGCGTCTAATAC
TGGATATGAGCCTCTTCCGCATGGTGGGGGTTGGAAAGATTTTTCGGTCTGGGATGGGCTCGCGGCCTATC
AGCTTGTTGGTGAGGTAATGGCTCACCAAGGCGTCGACGGGTAGCCGGCCTGAGAGGGTGACCGGCCAC
ACTGGGACTGAGACACGGCC
>Zotu6501
GAACGCTGGCGGCGTGCTTAACACATGCAAGTCGAACGGGGTCCATCCTTCGGGAGAAGACCTAGTGGCG
AACGGGTGAGTAACACGTGAGCAACCTGTCCCAAAGACTGGGATAAGCCAGGGAAACTTGGTCTAATACC

GGATGCCCTCTTCGAGTCATAAGGCTTGATGAGGAAATGATTTATTGCTTTGGGAGGGGCTCGCGGCCTAT
CAGCTTGTTGGTGGGGTAATGGCCTACCAAGGCTTCGACGGGTAGCTGGTCTGAGAGGATGATCAGCCAC
ACTGGGACTGAGACACGGCC

>Zotu6505

GAACGCTGGCGGCACGCCTAACACATGCAAGTCGAGCGGTAGCAGGGTTAGTAGCAATACTAACTGCTGA
CGAGCGGCGGACGGGTGAGTAATATGTGGGAACTTACCCTTCAGAGGGGGATAACCAAGGGAAACTTTG
GCTAATACCGCGTAATCTCGAGAGAGGAAAGCTGCCGCAAGGTGGCGCTGAAGGAGAGGCCTGCATCCG
ATTAGCTAGTTGGTAAGGTAAAAGCTTACCAAGGCGATGATCGGTAACTGGTCTGAGAGGACGACCAGTC
ACACTGGGACTGAGACACGGCC

>Zotu6508

GAACGCTGGCGGCGTGCCTAATACATGCAAGTCGAGCGGTAAGGCCCATCCAGATTATTCGATCACGACA
ACTTCGGTTGTCATAGCAAACCAGGAAAGTCCCCGGCTAGGGAAACAGTGGTGCGGGAAGATCGAGTTAT
GTGGTTGGGTACACGAGCGGCGGACGGCTGAGTAACACGTAGGAACGTACCCCAAAGTGAGGGATAAGC
ACCAGAAATGGTGTCTAATACCGCATGTGATCTTCGGATTAAAGCTTCGGCGCTTTGGGAACGGCCTGCGC
ATGATTAGTTTGTTGGTGAGGTAATGGCTCACCAAGACTACGATCATTAGCTGGTCTGAGAGGATGATCAG
CCAGACTGGGACTGAGACACGGCC

>Zotu6510

GAACGCTGGCGGCGTGCCTAATACATGCAAGTCGAACGGTCTTTTCTATGGAAGCTTGCTTCCGCTGAGAA
GATAGTGGCGAACGGGTGAGTAACACGTGGGTAACCTGCCCATAAGAGGGGGATAACATCCGGAAACGG
GTGCTAATACCGCATAGTTTTCTTGATCGCATGATTGAGAAAGGAAAGACGGCCTTTGTGCTGTCGCTTATG
GATGGACCCGCGGCGTATTAGTTAGTTGGTGAGGTAACGGCTCACCAAGACGATGATACGTAGCCGACCT
GAGAGGGTGATCGGCCACATTGGGACTGAGACACGGCC

>Zotu6515

GAACGCTGGCGGCGTGCTTAACACATGCAAGTCGAACGATGAAGCCCAGCTTGCTGGGCGGATTAGTGGC
GAACGGGTGAGTAACACGTGAGTAACCTACCCTTGACTCTGGGATAAGCCCGGGAAACTGGGTCTAATAC
CGGATATGAGCGTCCACCGCATGGTGGGTGTTGGAAAGAATTTCGGTCATGGATGGACTCGCGGCCTATC
AGCTTGTTGGTGAGGTAATGGCTCACCAAGGCGACGACGGGTAGCCGGCCTGAGAGGGTGACCGGCCAC
ACTGGGACTGAGACACGGCC

>Zotu6522

GAACGCTGGCGGCGTGCTTAACACATGCAAGTCGAACGGTGACCTCGGAGCTTGCTCCGGGCGATCAGTG
GCGAACGGGTGAGTAACACGTGAGTAACCTGCCCCAGACTCTGGGATAACCCCGGGAAACCGGAGCTAAT
ACCGGATACGACACGAAGAGGCATCTCTATCGTGTGGAAAGTTCTTCGGTCTGGGATGGACTCGCGGCCTA
TCAGCTAGTTGGTGAGGTAACGGCTCACCAAGGCGACGACGGGTAGCCGGCCTGAGAGGGCGACCGGCC
ACACTGGGACTGAGACACGGCC

>Zotu6525

GAACGCTGGCGGCGTGCTTAACACATGCAAGTCGAACGGAAAGGCCCTTCGGGGTGCTCGAGTGGCGAAC
GGGTGAGTAACACGTGGGTGATCTGCCCTGCACTTTGGGATAAGCCTGGGAAACTGGGTCTAATACCGAA
TATGATCATGCCTCTCCTGGGGTGTGGTGGAAAGCTTTTGCGGTGTGGGATGGGCCCGCGGCCTATCAGCT
TGTTGGTGGGGTGATGGCCTACCAAGGCGACGACGGGTAGCCGGCCTGAGAGGGTGTCCGGCCACACTG
GGACTGAGATACGGCC

>Zotu6528

GAACGCTGGCGGCATGCCTTACACATGCAAGTCGAACGGCAGCGCGGGAGCAATCCTGGCGGCGAGTGG
CGAACGGGTGAGTAATATATCGGAACGTGCCCAGTAGTGGGGGATAGCCCGGCGAAAGCCGGATTAATA
CCGCATGAGACCTGAGGGTGAAAGCGGGGGATCGCAAGACCTCGCGCTACTGGAGCGGCCGATATCAGA
TTAGCTAGTTGGTGGGGTAAAGGCCTACCAAGGCAACGATCTGTAGTTGGTCTGAGAGGACGACCAGCCA
CACTGGGACTGAGACACGGCC

>Zotu6532

GAACGCTGGCGGCAGGCCTAATACATGCAAGTCGAACGGATCCTTCGGGATCAGTGGCGGACGGGTGAG
TAACACGTGGGAACGTGCCTTTTGGTTCGGAACAAGCCGGGGAAACCTGGTCTAATGCCGAATGTGCCCTT
CGGGGGAAAGATTTATCGCCATTAGAGCGGCCCGCGTCTGATTAGCTAGTTGGTGGGGTAAAGGCCTACC
AAGGCTACGATCAGTAGCTGGTCTGAGAGGATGATCAGCCACACTGGGACTGAGACACGGCC

>Zotu6534
GAACGCTGGCGGAATGCTTTACACATGCAAGTCGAACGATGAACCTTAGCTTGCTAGGGGGATTAGTGGC
GAACGGGTGAGTAATATATCGGAACGTGCCTTGTAATGGGGGATAACTAGTCGAAAGATTAGCTAATACC
GCATACGCCCTGAGGGGGAAAGTAGGGGATCTTCGGACCTTACGTTATAAGAGCGGCCGATATCTGATTA
GCTAGTTGGTGGGGTAATGGCCTACCAAGGCTTCGATCAGTAGCTGGTCTGAGAGGACGACCAGCCACAC
TGGAACTGAGACACGGTC
>Zotu6536
GAACGCTGGCGGCGTGCTTAACACATGCAAGTCGAACGGAAAGGCCAGTGCTTGCACTGGTACTCGAGTG
GCGAACGGGTGAGTAACACGTGGGTGATCTGCCCTGCACTTCGGGATAAGCCTGGGAAACTGGGTCTAAT
ACCGGATAGGACGATGGTTTGGATGCCGTTGTGGAAAGTTTTTTCGGTGTGGGATGAGCTCGCGGCCTATC
AGCTTGTTGGTGGGGTAATGGCCTACCAAGGCGTCGACGGGTAGCCGGCCTGAGAGGGTGTACGGCCAC
ATTGGGACTGAGATACGGCC
>Zotu6539
GAACGCTAGCGGCAGGCTTAATACATGCAAGTCGTGGGGCAGCGCAGGTAGCAATACTGGGCGGCGACC
GGCAAACGGGTGCGGAACACGTACACAACCTTCCTACAAGTGGGGAATAGCCCAGAGAAATTTGGATTAA
TACCCCGTAACATATAGATGTGGCATCATATTTATATTATAGCTTCGGCGCTTGTTGATGGGTGTGCGGCTG
ATTAGATAGTTGGCGGGGTAACGGCCCACCAAGTCTACGATCAGTAGCTGATGTGAGAGCATGATCAGCC
ACACGGGCACTGAGACACGGGC
>Zotu6541
GAACGCTAGCGGCAGGCCTAATACATGCAAGGCGAGGGGGCAGCAATGTCACCGTCGTACGGGTGCGTA
ACGCGTATGCAACCTACCTATCACTGGGGGATAGCCCGGGGAAACCCGGATTAATACCGCATGACACATTA
GGTCCTCATGGGCTATATGTTAAAGATTTATTGGTGATAGATGGGCATGCGTTCGATTAGCTAGTTGGTAT
GGGTAACGGCCTACCAAGGCGACGATCGATAGGGGAGCTGAGAGGTTGATCCCCCACACGGGCACTGAG
ATACGGGC
>Zotu6543
GAACGCTGGCGGCGTGCCTAAGACATGCAAGTCGAGTGGGTTTAGACCCACGGCGAACGGGTGAGTAAT
ACCTAGGAACTTTCTTTAAGTGGAGCATATCCTACCGAAAGGTAGAGCAATTCTCCATGTGATCGCAAGATT
AAAGTCGCAAGACGCTTAAAGTGAGGCCTGGGCACCATCAGCTAGTTGGTAAGGTAATGGCTTACCAAGG
CTATGACGGTTAGGGGTCCCGAGAGGGAGAACCCCCACACTGGGACTGCGACACGGCC
>Zotu6550
GAACGCTGGCGGCGTGCCTAATACATGCAAGTCGAGCGGACAGAAGGGAGCTTGCTCCCGGAAGTCAGC
GGCGGACGGGTGAGTAACACGTGGGTAACCTGCCTGTAAGACTGGGATAACTCCGGGAAACCGGGGCTA
ATACCGGATAATATCAAGAGCTGCATGGCTCTTGATTGAAAGGCGGCTTCGGCTGTCACTTACAGATGGAC
CCGCGGCGCATTAGCTAGTTGGTGAGGTAACGGCTCACCAAGGCGACGATGCGTAGCCGACCTGAGAGG
GTGATCGGCCACACTGGGACTGAGACACGGCC
>Zotu6555
GAACGCTGGCGGTAGGCCTAACACATGCAAGTCGAACGGCAGCACAGGAGAGCTTGCTCTCTGGGTGGCG
AGTGGCGGACGGGTGAGGAATACATCGGAATCTACCTTTTCGTGGGGGATAACGTAGGGAAACTTACGCT
AATACCGCATACGACCTACGGGTGAAAGTGGGGGACCGCAAGGCCTCACGCGATTAGATGAGCCGATGTC
CGATTAGCTAGTTGGCGGGGTAATGGCCCACCAAGGCGACGATCGGTAGCTGGTCTGAGAGGATGATCAG
CCACACTGGAACTGAGACACGGTC
>Zotu6559
GAACGCTGGCGGCATGCCTAACACATGCAAGTCGAACGAGACCTTCGGGTCTAGTGGCGCACGGGTGCGT
AACGCGTGGGAATCTGCCCTTGGGTTCGGAATAACTCGCCGAAAGGCGTGCTAATACCGGATGATGTCGC
AAGACCAAAGATTTATCGCCCAAGGATGAGCCCGCGTAGGATTAGCTAGTTGGTGAGGTAAAGGCTCACC
AAGGCGACGATCCTTAGCTGGTCTGAGAGGATGATCAGCCACACTGGGACTGAGACACGGCC
>Zotu6562
GAACGCTGGAAATGTGGATACCGGCATGCAAGTCAAGGGGCCGCAAGGCACCGGCGAACGGCTTAGTAA
CACGTGGATACATACCGCAAAGTGGGGCATAGCTCGTTGAAAAACGGGGTAATTTCCCATAGTCCCGCAA

GGGTAAAGGCGCAAGCCGCTTTGTGAATGGTCCGCGGTCTATCAGCTAGTTGGTGAGGTAATGGCTCACC
AAGGCTATGACGGATAGGGGGCGTGAGAGCGCGACCCCCAAGAATGGCACTGCGACACGGGC
>Zotu6564
GAACGCTGGCGGCGTGCTTAACACATGCAAGTCGAACGGACTAAGGGGCTTGCTCCTTAGTTAGTGGCGC
ACGGGTGAGTAACGCGTGGATAATCTACCCGATACTCTGGGATAACATCTCGAAAGGGGTGCTAATACCG
GATAAGCCTACGAGAGCTGCGGCTCATGTAGGAAAAGGTGGGGACCTTCGGGCCTGCCGATATCGGATGA
GTCCGCGTCCCATTAGCTAGTTGGCGGGGTAAAGGCCCACCAAGGCTACGATGGGTAGCTGGTCTGAGAG
GATGATCAGCCACACTGGAACTGAGACACGGTC
>Zotu6566
GAATGCTGGCGGCGTGGATAAGTCATGCAAGTCAAGGGGGCCCGCAAGGGCAACCGGCAAACGAGGTAG
TAACACGTAGGTACACCCCCCGAAGTCGAGCATAGCTAGCCGAAAGGTTAGGTAATTCTCGATAGTCCCGC
AAGGGTAAAGTTTTTCGCTTCGGGAGTGGCCTGCGTAGTATCAGCTAGTTGGTAGGGTAATGGCCTACCAA
GGCTACGACGCTTAGGGGAGCTGAGAGGCTGACCCCCACCGATGGAACTGCGACACGGTC
>Zotu6568
GAACGCTGGCGGCAGGCCTAACACATGCAAGTCGAACGAACTCTTCGGAGTTAGTGGCGGACGGGTGAGT
AACACGTGGGAACGTGCCTTTAGGTTCGGAATAACTCAGGGAAACTTGTGCTAATACCGAATGTGCCCTTC
GGGGGAAAGATTTATCGCCTTTAGAGCGGCCCGCGTCTGATTAGCTAGTTGGTAGTGTAATGGACTACCAA
GGCGACGATCAGTAGCTGGTCTGAGAGGATGATCAGCCACATTGGGACTGAGACACGGCC
>Zotu6573
GAACGCTGGCGGCGTGCCTAAGACATGCAAGTCGAGTGGGTTTAGACCCACGGCGAACGGGTGAGTAAT
ACCTAGGTACTTTCTTTAAGTGGAGCATAACCAGTCGAAAGATTGGATAATACTTCATGTGCCCCGGAGTTA
AATCCGGGGTAAAGTCGCAAGACGCTTAAAGTGAGGCCTGGGCACCATCAGCTAGTTGGTAAGGTAATGG
CTTACCAAGGCTATGACGGTTAGGGGTCCCGAGAGGGATAACCCCCACACTGGAACTGCGACACGGTC
>Zotu6575
GAATGCTGGCGGCGTGCCTAACACATGCAAGTCGAGCGGCAGCGCGAGTAGTTTACTACTTGGCGGCGAG
CGGCGGACGGCTGAGTAACGCGTGGGAACGTGCCCCAAAGTGAGGAATAACTGCCCGAAAGGGTAGCTA
ATGCCGCATATGCTCTTAGGAGTAAAGTCTTCGGACGCTTTGGGAGCGGCCCGCGTCGGATTAGGTTGTTG
GTGAGGTAATGGCTCACCAAGCCGACGATCCGTAGCTGGTCTGAGAGGATGATCAGCCAGACTGGAACTG
AGACACGGTC
>Zotu6577
GAACGCTGGCGGCGTGCCTAACACATGCAAGTCGAACGGAAGTTAGAGCTTCGGTTTTAACTTTAGTGGCG
AACGGGTGAGTAACGCGTGAGGAACCTGCCTTTCAGTGGGGGACAACAGTTGGAAACGACTGCTAATACC
GCATGATGCTTTTGGGAGACATCTCCTGGAAGCCAAAGCTTTATGTGCTGAAAGATGGCCTCGCGTCTGAT
TAGCTAGTTGGTGAGGTAACGGCTCACCAAGGCGACGATCAGTAGCCGGTCTGAGAGGATGAACGGCCAC
ATTGGGACTGAGATACGGCC
>Zotu6579
GAACGCTGGCGGCATGCTTAACACATGCAAGTCGAACGGATTAATATGGATTGCCATATTAGTTAGTGGCA
AACGGGTGAGTAACGCGTGGGAATCTACCTAGAAGTATGGAATAACAATTAGAAATGATTGCTAATACCGT
ATGTGCTTTGAGAAGTAAAGATTTATCGCTTCTAGATGAGCCCGCGTTAGATTAGCTAGTTGGTGGGGTAA
TGGCCTACCAAGGCAACGATCTATAGCTGGTCTGAGAGGATGATCAGCCACACTGGGACTGAGACACGGC
C
>Zotu6582
GAACGCTAGCGGCAGGCCTAATACATGCAAGTCGGACGGGATTCAGGGAGCAGCTTGCTACGAACTGATG
AGAGTGGCGCACGGGTGCGTAACGCGTGAGCAACCTACCCATATCAGGGGGATAGCCTCTCGAAAGAGA
GATTAACACCGCATAACATCATTAACCGGCATCGGATTATGATCAAATATTTATAGGATATGGATGGGCTC
GCGTGACATTAGCTAGTTGGAGAGGTAACGGCTCACCAAGGCTACGATGTCTAGGGGCTCTGAGAGGAGA
ATCCCCCACACTGGTACTGAGACACGGAC
>Zotu6584
GAACGCTGGCGGCAGGCTTAACACATGCAAGTCGAGCGGGCCCTTCGGGGTCAGCGGCAGACGGGTGAG
TAACACGTGGGAACGTGCCCTTCGGTTCGGAATAACGCAGGGAAACTTGCGCTAATACCGGATACGCCCTT

TTGGGGAAAGGTTTACTGCCGAAGGATCGGCCCGCGTCTGATTAGCTAGTTGGTGGGGTAATGGCCCACC
AAGGCGACGATCAGTAGCTGGTCTGAGAGGATGATCAGCCACACTGGGACTGAGACACGGCC
>Zotu6587
GAACGCTGGCGGCGTGCTTAACACATGCAAGTCGAACGGTAAGGCCCTTTCGGGGGTACACGAGTGGCGA
ACGGGTGAGTAACACGTGGGTGACCTGCCCCTTACTCTGGGATAAGCCTGGGAAACTGGGTCTAATACCG
GATAGGACTTCATGCTGCATGGTGTGGGGTTGAAAGTTTTTCGGTGGGGGATGGGCCCGCGGCCTATCAG
CTTGTTGGTGGGGTGATGGCCTACCAAGGCTTCGACGGGTAGCCGGCCTGAGAGGGCGTCCGGCCACACT
GGGACTGAGACACGGCC
>Zotu6589
GAACGCTGGCGGCGTGCCTAACACATGCAAGTCGAACGTGAAAGGGAGCAATCCTGAGTAAAGTGGCGT
ACGGGTGAGTAACGCGTAGGTAATCTACCCCAGAGTCTGGAATAACCTGCCGAAAGGCGGGCTAATACTA
GGTAGAGCGATGGAATCCGTAATTGGGTTTTTCGGGAAAGGGAGCTCGTAAGAGCCCTCGCTTTGGGATG
AGCCTGCGTCTCATTAGCTAGTTGGTAGGGTAATGGCCTACCAAGGCTTTGATGGGTAGCTGGTCTGAGAG
GATGATCAGCCACACTGGAACTGAAACACGGTC
>Zotu6591
GAACGCTGGCGGCGTGCTTAACACATGCAAGTCGAACGGTGAAGCCAAGCTTGCTTGGTGGATCAGTGGC
GAACGGGTGAGTAACACGTGAGCAATCTGCCCTTGACTCTGGGATAAGCGCTGGAAACGGCGTCTAATAC
TGGATATGAGCTGTGGCCGCATGGTCGGCAGTTGGAAAGAATTTCGGTCAAGGATGAGCTCGCGGCCTAT
CAGCTTGTTGGTGAGGTAATGGCTCACCAAGGCGTCGACGGGTAGCCGGCCTGAGAGGGTGACCGGCCAC
ACTGGGACTGAGACACGGCC
>Zotu6595
GAACGCTGGCGGTGCGCCTAACACATGCAAGTCGCGCGAGGAGCAATCCTAGCGGCGAACGGCTGCGTA
ACACGTTGGTACATGCCCCGAACTCAGGGATAACCCTCCGAAAGGAGGGCTAATACCGGATGGTCCCGTG
AGGGTAAAGCTTCGGCGGTTCGGGAGTGGCCTGCGGCCTATCAGATAGTTGGTAAGGTAACGGCTTACCA
AGTCGATGACGGGTAGCTGATTTGAGAGAATGATCAGCCAGAATGGAACTGAGACACGGTC
>Zotu6597
GAACGCTGGCGGCGTGCTTAACACATGCAAGTCGAACGGTGAACACGGAGCTTGCTCTATGGGATCAGTG
GAGAACGGGTGAGTAACACGTGAGCAACCTGCCCCTGACTCTGGAATAAGCGCTGGAAACGGCGTCTAAT
ACTGGATATGTGACGTGACCGCATGGTCTGCGTTTGGAAAGATTTTTCGGTTGGGGATGGGCTCGCGGCCT
ATCAGCTTGTTGGTGAGGTAATGGCTCACCAAGGCGTCGACGGGTAGCCGGCCTGAGAGGGTGACCGGCC
ACACTGGGACTGAGACACGGCC
>Zotu6600
GAACGCTAGCGGCAGGCCTAATACATGCAAGTCGAACGATAGTCGGGGCTTGCTCCGACGAAAAGTGGCG
CACGGGTGCGTAACGCGTATGCAACCTACCTTAATCAGGGGGATAGCCCGAAGAAATTCGGATTAACACC
GCATAAAAACACAGGATAGCATTATCCAATGTTCAAATATTTATAGGATTAAGATGGGCATGCGTGTCATT
AGCTAGTTGGCGGGGTAACGGCCCACCAAGGCGACGATGACTAGGGGATCTGAGAGGATGACCCCCCAC
ACTGGTACTGAGACACGGAC
>Zotu6604
CAACGCTGGCGGCGTGCCTAACACATGCAAGTCGTACGAGAAAGTGGAGCAATCCATGAGTAAAGTGGCG
ACCGGGTGAGTAACACGTGACTACCTACCTTCTAGTGGGGGATAACTCCGGGAAACCGGAGCTAATACCG
CATAACATCGCAAGATCAAAGCAGCAATGCGCTGGAAGAGGGGGTCGCGGCTGATTAGCTAGTTGGTAGG
GTAATGGCCTACCAAGGCAAAGATCGGTATCCGGCCTGAGAGGGCGCACGGACACACTGGAACTGAAACA
CGGTC
>Zotu6607
GAACGCTAGCGGCGTGCCTAAGGTATGCAAGTCGAGCGAGAGCCTTCGGGCTCTAGCGGCAAACGGTTGA
GTAACACGTAGACAACCTGCCCCAAAGACGGGGATAACTCGGGGAAACTCGGGCTAATACCGGATGTGGA
GGGAAGCGGCATCGCTTCTTATCTAAACGAGCAATCGGCTTTGGGATGGGTCTGCGGCCTATCAGCTAGCT
GGTGGGGTAACGGCCTACCAGGGCGACGACGGGTAGCTGGTCTGAGAGGATGACCAGCCAGACGGGGA
CTGAGACACGGCC
>Zotu6609

GAACGCTGGCGGCGTGCCTTAAGCATGCAAGTCAAGGGGGGCTCAGCAATGAGTCAACCGGCGGACGGG
TGAGTAATGCGTAGGAATCTTCCTCGAAGTTGGACATAGCTAGCCGAAAGGTTGGGTAATTTCCAATGTGA
CCGTAAGGTCAAAGCAGCAATGCGCTTTGAGATGAGCCTGCGTCCTATCAGCTAGTTGGTGGGGTAAAAG
CCTACCAAGGCGACGACGGGTAGCTGGTCTGAGAGGATGGTCAGCCACAATGGGACTGCGACACGGCC
>Zotu6611
GAACGCTGGCGGCGTGCCTAACACATGCAAGTCGAACGTGAAATGGAGCAATCCAAAGTAAAGTGGCGTA
CGGGTGAGTAACGCGTAGGTAATCTACCCCAAAGTTTGGAATAACCTGCCGAAAGGCGGGCTAATACTGG
ATAGAGCGACGGAACTCGTAACGAGTTTTTCGGGAAAGGGAGCCCGCAAGGGCCTTCGCTTTGGGATGAG
CCTGCGTCTCATTAGCTAGTTGGTAGGGTAATGGCCTACCAAGGCATTGATGGGTAGCTGGTCTGAGAGGA
TGATCAGCCACACTGGAACTGAAACACGGTC
>Zotu6616
CAACGCTGGCGGCGCGCTTAACACATGCAAGTCGCACGAGAAAGCCTGGCAACGGGTGAGTAAAGTGGC
GTACGGGTGAGTAACACGTGGGTATCCACCTCTTAGTGGGGAATAACCCTGGGAAACCGGGGCTAATACC
GCATAAGTCCGTGAGGAGAAAGCAGCAATGCGCTGAGAGAGGAGCCCGCGGCCGATTAGCTAGTTGGTA
GGGTAATGGCCTACCAAGGCAACGATCGGTAGCCGGCCTGAGAGGGCACACGGCCACACTGGCACTGAA
ACACGGGC
>Zotu6620
GAACGCTGGCGGCGTGCTTAACACATGCAAGTGGAGCGACGAACCGGGCTTCGGCCCGGGGCAGAGCCG
CGAACGGGTGAGTAACACGTGGGTTACCTGCCCCATTGACAGGGACAACCCGGGGAAACCCGGGCTAATA
CCTGATGTGCCCCTCGGGGGAAAGGAAGCTTCGGCCTCCGCAATGGGATGGGCCCGCGGCCCATTAGCTT
GTTGGTGGGGTAACGGCCTACCAAGGCGACGATGGGTAGCTGGTCTGAGAGGACGATCAGCCACACTGG
GACTGAGACACGGCC
>Zotu6622
GAACGCTGGCGGCATGCCTAACACATGCAAGTCGAACGAAGGCTTCGGCCTTAGTGGCGCACGGGTGCGT
AACGCGTGGGAATCTGCCCCTTGGTTCGGAATAACAGTTGGAAACGACTGCTAATACCGGATGACGACGA
AAGTCCAAAGATTTATCGCCGAGGGATGAGCCCGCGTAGGATTAGCTAGTTGGTGTGGTAAAGGCGCACC
AAGGCGACGATCCTTAGCTGGTCTGAGAGGATGATCAGCCACACTGGGACTGAGACACGGCC
>Zotu6624
GAACGCTGGCGGCGTGCTTAACACATGCAAGTCGAACGGTGAAGGGACCGGCTTTTGCTGGTTCTGGATG
AGTGGCGAACGGGTGAGTAACACGTGAGTAACCTGCCCCCTTCTTCTGGATAACCGCATGAAAGTGTGGCT
AATACGGGATATTCTGGGTCTGTCGCATGGCGGGCCTGGGAAAGATGCGCCTTTGGGTGTTTTTGGTGGG
GGATGGGCTCGCGGCCTATCAGCTTGTTGGTGGGGTGATGGCCTACCAAGGCGGTGACGGGTAGCCGGC
CTGAGAGGGTGGACGGTCACACTGGGACTGAGACACGGCC
>Zotu6627
GAACGCTGGCGGCATGCCTTACACATGCAAGTCGAACGGTAACAGGTCTTCGGATGCTGACGAGTGGCGA
ACGGGTGAGTAATACATCGGAACGTGCCCGATCGTGGGGGATAACGAGGCGAAAGCTTTGCTAATACCGC
ATACGATCTACGGATGAAAGCAGGGGATCAGTAATGACCTTGCGCGGACGGAGCGGCCGATGGCAGATT
AGGTAGTTGGTGGGATAAAAGCTTACCAAGCCGACGATCTGTAGCTGGTCTGAGAGGATGATCAGCCACA
CTGGGACTGAGACACGGCC
>Zotu6630
GAACGCTGGCGGCAGGCTTAACACATGCAAGTCGAACGCCCCGCAAGGGGAGTGGCAGACGGGTGAGTA
ACGCGTGGGAATCTACCTAGTTCTGCGGAACAACAGTTGGAAACGACTGCTAATACCGCATACGCCCTACG
GGGGAAAGATTTATCGGAATTAGATGAGCCCGCGTAAGATTAGCTAGTTGGTGGGGTAATGGCCTACCAA
GGCGACGATCTTTAGCTGGTCTGAGAGGATGATCAGCCACACTGGGACTGAGACACGGCC
>Zotu6632
GAACGCTAGCTGCAGGCCTAATACATGCAAGTCGAACGGTGTCTTCGGACATAGTGGCGCACGGGTGCGT
AACACGTATGCAACCTACCTTACATTGGGGGATAGCCTTTCGAAAGGGAGATTAATACCGCATAAGACAGT
AACTAGGCATCTAGTAGCTGTTAAAGATTTATCGATGTAAGCTGGGCATGCGTCTAATTAGCTAGTTGGTG
AGGTAATGGCTCACCAAGGCAATGATTGGTAGGGGAACTGAGAGGTCAATCCCCCACACTGGCACTGAGA
TACGGGC

>Zotu6637
GAACGCTGGCGGCGTGCTTAACACATGCAAGTCGAACGGAAAGGCCCCTTCGGGGGTACTCGAGTGGCGA
ACGGGTGAGTAACACGTGGGCAACCTGCCCCTAGCTCTGGGATAACTCCGGGAAACCGGTGCTAATACCG
GATATCGACCTTCGCGGGCATCCGCGACTGTCAAAAGTTTTTCGGCTAGGGATGGGCCCGCGGCCTATCAG
CTTGTTGGTGGGGTAATGGCCTACCAAGGCGACGACGGGTAGCCGGCCTGAGAGGGCGACCGGCCACAC
TGGGACTGAGACACGGCC
>Zotu6639
GAACGCTGGCGGCAGGCTTAACACATGCAAGTCGAGCGGGGGTAGCAATACCCTAGCGGCGAACGGGTG
AGGAATGCTTGGGAATCTGCCTTGTAGTGGGGGATAACGTCTCGAAAGGGACGCTAATACCGCATACGTC
CTACGGGAGAAAGGGGGGGATCTTCGGACCTCTCGCTATAAGATGAGCCCAAGTCGGATTAGCTAGTTGG
TGGGGTAAAGGCCTACCAAGGCGACGATCCGTAGCTGGTCTGAGAGGATGATCAGCCACACTGGAACTGA
GACACGGTC
>Zotu6641
GAACGCTAGCGGCAGGCCTAATACATGCAAGTCGAGGGGCAGCAGGTGTAGCAATACATGCTGGCGACC
GGCGCACGGGTGCGTAACACGTATGCAACTTACCTTTAACTGGAGGATAGCCGCCCGAAAGGGTGATTAA
TACTCCATAATATTATGAAATAGCATTATTTTATAATTAAAGCTCCGGCGGTTAAAGATGGGCATGCGTCCG
ATTAGCTAGTTGGCGAGGTAAAAGCTCACCAAGGCTACGATCGGTAGGGGTTCTGAGAGGATATACCCCC
ACACTGGTACTGAGACACGGAC
>Zotu6643
GAACGCTGGCGGCACGTTTTAAGCATGCAAGTCGAACGGCAGTGGGGGCTTGCTCCCATGAGAGTGGCGA
ACGGGTGAGTAACACGTAGGTGATCTGCCTTGAGGATGGGGATAGCTTGTGGAAACACAGGGTAATACCG
AATGAGCTGACAGGGTTTTGGCCCAGTTAGTAAAGGTGCTACGGCACCGCCTTAAGAGGAGCCTGCGGCT
GATTAGCTAGTTGGTGGGGTAAAGGCCTACCAAGGCGACGATCAGTAGCCGGCCTGAGAGGGTGTACGG
CCACACTGGAACTGAGACACGGTC
>Zotu6646
GAACGCTGGCGGCGTGCTTAACACATGCAAGTCGAACGGGGATTACCGAACGGAAGGGCTTCGGTCCGG
AAGGTTGGTAATTCTAGTGGCGGACGGGTGAGTAACGCGTGAGCAACCTGTCCCAGACAGGGGGATAAC
ACTTGGAAACAGGTGCTAATACCGCATAAGACCACGGGGAGACATCTACCTGGGGTAAAAGGTGCAAACC
GGTTTGGGGTGGGCTCGCGTCCGATTAGATAGTTGGTGAGGTAACGGCCCACCAAGTCGACGATCGGTAG
CCGATCTGAGAGGATGACCGGCCACATTGGAACTGAGAGACGGTC
>Zotu6652
GAACGCTGGCGGCGTGCCTAACACATGCAAGTCGAACGAGGTCCATCCGGTAGCAATACCGGTGAAGATC
GAGTGGCGAACGGGTGAGTAGCACGTGAGCAACCTGCCCCGAAGACCGGGACAACACCGGGAAACCGGT
GCTAATACCGGATACCCCCACCGAGTCGCATGGCTCGGTGAGGAAATGGATTCCGCTTCGGGAGGGGCTC
GCGGCCTATCAGCTTGTTGGTGAGGTAACGGCTCACCAAGGCGTCGACGGGTAGCTGGTCTGAGAGGACG
ATCAGCCACACTGGGACTGAGACACGGCC
>Zotu6656
GAACGCTAGCGATAGGCCTAACACATGCAAGTCGAGGGGCAGCACATGACGTAGCAATACAGATGGTGGC
GACCGGCGCACGGGTGAGTAACACGTATGCAACCTACCTTCAACAGGAGAATAACCCGTCGAAAGACGGA
CTAATACTCCATAACACAGGGATCCCGCATGGGAATATTTGTTAAAGATTTATCTGTTGAAGATGGGCATGC
GCTCCATTAGCTAGTTGGTGAGGTAACGGCTCACCAAGTCAGCGATGGATAGGGGAACTGAGAGGTTTAT
CCCCCACACTGGTACTGAGACACGGAC
>Zotu6658
GAACGCTGGCGGCGTGCCTAATACATGCAAGTAGAACGCTGAAGAGAGGAGCTTGCTCTTCTTGGATGAG
TTGCGAACGGGTGAGTAACGCGTAGGTAACATGCCTGGTAGCGGGGGATAACTATTGGAAACGATAGCTA
ATACCGCATGATATTAATTGTCGCATGATAATTAATTGAAAGATGCAATTGCATCACTACCAGATGGACCTG
CGTTGTATTAGCTAGTTGGTGAGGTAACGGCTCACCAAGGCGACGATACATAGCCGACCTGAGAGGATGA
TCGGCCACACTGGGACTGAGACACGGCC
>Zotu6663

GAACGCTGGCGGCGTGCTTAACACATGCAAGTCGAACGATGATCCCCAGCTTGCTGGGGGGATTAGTGGC
GAACGGGTGAGTAACACGTGAGTAACCTGCCCTTGACTCTGGGATAAGCCTGGGAAACTGGGTCTAATAC
CGGATATGACCTTCCATCGCATGGTGGTTGGTGGAAAGCTTTTGTGGTTTTGGATGGACTCGCGGCCTATC
AGCTTGTTGGTGGGGTAATGGCCTACCAAGGCGACGACGGGTAGCCGGCCTGAGAGGGTGACCGGCCAC
ACTGGGACTGAGACACGGCC
>Zotu6667
GAACGCTGGCGGCAGGCCTAACACATGCAAGTCGAACGCTGCAGCAATGCAGAGTGGCAGACGGGTGAG
TAACGCGTGGGAACCTTCCCAGTGGTACGGAATAGCTCAGGGAAACTTGAGGTAATACCGTATACGCCCCT
AGGGGGAAAGATTTATCGCCATTGGATGGGCCCGCGTCGGATTAGCTAGTTGGTGAGGTAACGGCTCACC
AAGGCGACGATCCGTAGCTGGTTTGAGAGAATGACCAGCCACACTGGGACTGAGACACGGCC
>Zotu6670
GAACGCTGGCGGCAGGCCTAACACATGCAAGTCGAACGCACCTTTCGGGGTGAGTGGCAGACGGGTGAG
TAACACGTGGGAACGTGCCCTTCAGTTCGGAATAACCCAGGGAAACTTGGGCTAAGACCGGATACGCCCTT
TTTGGGGAAAGATTGATCGCTGAAGGATCGGCCCGCGTCTGATTAGCTAGTTGGTGAGGTAATGGCTCACC
AAGGCGACGATCAGTAGCTGGTCTGAGAGGATGATCAGCCACACTGGGACTGAGACACGGCC
>Zotu6673
GAACGCTGGCGGCGTGCTTAACACATGCAAGTCGAACGATGAAGCTCCAGCTTGCTGGGGTGGATTAGTG
GCGAACGGGTGAGTAACACGTGAGTAACCTGCCCCTGACTTCGGGATAACCACGAGAAATCGTGGCTAAT
ACTGGATGTCCACATGTGCCTGCATGGGTGTGTGTGGAAAGATTTATCGGTTGGGGATGGGCTCGCGGCC
TATCAGCTTGTTGGTGGGGTGATGGCCTACCAAGGCGACGACGGGTAGCCGGCCTGAGAGGGTGACCGG
CCACACTGGGACTGAGACACAGCC
>Zotu6675
GAACGCTGGCGGCGTGCCTAACACATGCAAGTCTAACGGGGCAGCAATGCCCAGTGGCGAACGGGTGCG
TAACACGTGAGGAACTTGTCCATCGGCGGGGGATAGCCGGCCCAACGGCCGGGTAATACCGCGTACGTCG
GCGAGACCGCATGGTCTTGCCGGGAAAGCAGCAATGCACCGATGGAGAGCCTCGCGGCCTATCAGCTAGT
TGGTGAGGTAACGGCTCACCAAGGCGACGACGGGTAGCTGGTCTGAGAGGATGATCAGCCACATTGGGA
CTGAGACACGGCC
>Zotu6678
GAACGCTGGCGGCAGGCTTAACACATGCAAGTCGAGCGATGACGACCTAGCTTGCTAGGTTTGATTAGCG
GCGGACGGGTGAGTAAAGTTTAGGAATCTGCCTTATAGTGGGGGATAGCTTGGGGAAACCCAAATTAATA
CCGCATACGTCCTACGGGAGAAAGGTGCCTTAGGGTATCGCTATGAGATGAGCCTAAACCAGATTAGCTA
GTTGGTGGGGTAAAGGCCTACCAAGGCGACGATCTGTAGCTGGTCTGAGAGGATGATCAGCCACACCGGA
ACTGAGACACGGTC
>Zotu6681
GAACGCTAGCTATATGCTTAACACATGCAAGTCGAACGTCGTTTTCGGGAAGCTGGGCAGAAGGAAAATA
GGCTCCTAGCTAAAGGTAGCTTGTCTCGCCCAGGAGGTGAGAACAGTTGAGAACAAAGTGGCGAACGGGT
GCGTAACGCGTGGGAATCTGCCGAACAGTTCGGGCCAAATCCTGAAGAAAGCTAAAAAGCGCTGTTTGAT
GAGCCCGCGTAGTATTAGGTAGTTGGTCAGGTAAAGGCTGACCAAGCCAATGATGCTTAGCTGGTCTTTTC
GGATGATCAGCCACACTGGGACTGAGACACGGCC
>Zotu6684
GAACGCTGGCGGCAGGCCTAACACATGCAAGTCGAGCGGGCACCTTCGGGTGTCAGCGGCAGACGGGTG
AGTAACACGTGGGAACGTGCCCTTTGGTTCGGAACAACTCCGGGAAACTGGAGCTAATACCGGATAAGCC
CTTCGGGGGAAAGATTTATCGCCAAAGGATCGGCCCGCGTCTGATTAGCTAGTTGGTAGGGTAATGGCCTA
CCAAGGCGACGATCAGTAGCTGGTCTGAGAGGATGATCAGCTACACTGGGACTGAGACACGGCC
>Zotu6686
GAACGCTGGCGGTATGCTTAACACATGCAAGTCGAGCGCGAAAGTGGCTTCGGCCACGAGTAGAGCGGC
GGACGGGTGAGTAACACGTGGATAATCTGCCTTCAGGACTGGGATAACTCTGGGAAACTAGGGCTAATAC
CGGATAAGACCACAGGTTTTATGGAACCAGGGGTAAAAGGCGGCCTTCGGGCTGTCACCTAAAGATGAGT
CCGCGTCCCATCAGGTAGTTGGTGAGGTAACGGCTCACCAAGCCGAAGACGGGTAGCCGACCTTAGCGGG
TGATCGGCCACACGGGGACTGAGACACGGCC

>Zotu6688
GAACGTTAGCGGCACGCCTAACACATGCAAGTCGCACGAGAAAGGGAGCAATCCTGAGTAGAGTGGCGC
ACGGGTGCGTAACACGTAGATAACCTGCCCTTTGGAGGGGGATAACGTTCCGAAAGGAGCGCTAATACCG
CATAACGCTTTTTCCGGTTTGCCGGAGATTGACAAAGATAGGGACCCGCAAGGGCCTATCGCCAGAGGAG
GGGTCTGCGGCCCATCAGCTAGTTGGGGAGGTAATGGCTCACCAAGGCAGAGACGGGTAGCTGGTCTGA
GAGGATGATCAGCCACACTGGTACTGGAACACGGAC
>Zotu6691
GAATGCTGGCGGCGTGGATAAGTCATGCAAGTCAAGGGGGCCCGCAAGGGCAACCGGCAGACGAGGTAG
TAACACGTAGGTACGTCCCCCGAAGTCGGGCATAGCCATCCGAAAGGATGGGTAATTCCCGATAGTCCCGC
AAGGGTAAAGGTTTTTCGCTTCGGGAGCGGCCTGCGTAATATCAGCTAGTTGGTGAGGTAATGGCTCACCA
AGGCTACGACGTTTAGGGGAGCTGAGAGGCTGACCCCCACCGATGGAACTGCGACACGGTC
>Zotu6693
TAACGCTGGCGGCGTGCATGAGACATGCAAGTCATACGGGCGACCAGGGCTTGCTCTGGTTGCTAGTGGC
GGACGGCTGAGTAACGCGTAGGTGACCTGCCCCGAAGTGGGGAATAACTGCCCGAAAGGGTGGCTAATA
CCGCATGTGGTGATGCGTTCATTCGCATCAGTAAAGCCGCAAGGCGCTTCGGGAGGGGCCTGCGTCCGATT
AGCTCGTTGGTGGGGTAACGGCTCACCAAGGCGACGATCGGTAGGTGGTCTGAGAGGACGATCACCCAG
ACTGGGACTGAGACACGGCC
>Zotu6696
GAACGCTGGCGGCGTGCCTAATACATGCAAGTAGAACGCTGAAGGAATAGCTTGCTATTCTGGAAGAGTT
GCGAACGGGTGAGTAACGCGTAGGTAACCTACCTCATAGCGGGGGATAACTATTGGAAACGATAGCTAAT
ACCGCATAAGAATACTTATTGCATGATAAGTATTTAAAAGGAACAACTGTTTCGCTGTGAGATGGACCTGC
GTTGTATTAGCTAGTTGGTAAGGTAACGGCTTACCAAGGCGACGATACATAGCCGACCTGAGAGGGTGAT
CGGCCACACTGGGACTGAGACACGGCC
>Zotu6698
GAACGCTAGCGGCAGGCCTAACACATGCAAGTCGAGGGGTAGCAGGTTCAAGCTTGCTTGAATGCTGACG
ACCGGCGCACGGGTGCGTAACGCGTATGCAACCTACCTTGTACTGGGGTATAGCCCGGAGAAATTCGGAT
TAATCCCCCATAGTATTATTGGATGGCATCATTTGATAATTAAAGGTTACGGTACAGGATGGGCATGCGTCC
CATTAGCTAGATGGTGAGGTAACGGCTCACCATGGCGACGATGGGTAGGGGGCCTGAGAGGGTGGTCCC
CCACACTGGTACTGAGACACGGAC
>Zotu6700
GAACGCTGGCGGCAGGCTTAACACATGCAAGTCGAACGCATCCTTCGGGATGAGTGGCAGACGGGTGAGT
AACACGTGGGAACGTGCCCTTCGGTTCGGAATAACTCTGGGAAACTAGAGCTAATACCGGATACGCCCTTT
TGGGGAAAGGTTTACTGCCGAAGGATCGGCCCGCGTCTGATTAGCTAGTTGGTGGGGTAATGGCCTACCA
AGGCGACGATCAGTAGCTGGTCTGAGAGGATGATCAGCCACACTGGGACTGAGACACGGCC
>Zotu6705
GAACGCTGGCGGCATGCTTAACACATGCAAGTCGAACGAGACCTTCGGGTCTAGTGGCGCACGGGTGCGT
AACGCGTGGGAATCTGCCCTTAGGTACGGAATAACTCAGAGAAATTTGTGCTAATACCGTATGATGACGTA
AGTCCAAAGATTTATCGCCTAAGGATGAGCCCGCGTAGGATTAGCTTGTTGGTGAGGTAAAAGCTCACCAA
GGCGACGATCCTTAGCTGGTCTGAGAGGATGATCAGCCACACTGGGACTGAGACACGGCC
>Zotu6709
GAACGCTGGCGGCAGGCTTAACACATGCAAGTCGAGCGCATCCTTCGGGGTGAGCGGCAGACGGGTGAG
TAACGCGTGGGAATCTACCTTTTGCTACGGAATAGCTCCGGGAAACTGGAATTAATACCGTATGTGCCCTTA
GGGGGAAAGATTTATCGGCAAGAGATGAGCCCGCGTTGGATTAGCTAGTTGGTGGGGTAAAGGCCTACCA
AGGCGACGATCCATAGCTGGTCTGAGAGGATGATCAGCCACATTGGGACTGAGACACGGCC
>Zotu6711
GAACGCTGGCGGCGTGCTTAACACATGCAAGTCGAACGATGAAGCAGGAGCTTGCTCCTGTGGATTAGTG
GCGAACGGGTGAGTAACACGTGAGTAATCTGCCCCTGACTCCGGGATAAGCACTGGAAACGGTGTCTAAT
ACCGGATACGAGCTGCGAAGGCATCTTCAGCAGCTGGAAAGAACTTCGGTCAGGGATGAACTCGCGTCCT
ATCAGCTAGTTGGTGAGGTAAAGGCTCACCAAGGCGACGACGGGTAGCCGGCCTGAGAGGGTGACCGGC
CACACTGGGACTGAGACACGGCC

>Zotu6715
GAACGCTGGCGGCATGCCTTACACATGCAAGTCGAACGGCAGCGCGGGGCAACCTGGCGGCGAGTGGCG
AACGGGTGAGTAATATATCGGAACGTACCCTGGAGTGGGGGATAACGTAGCGAAAGTTACGCTAATACCG
CATACGATCTAAGGATGAAAGTGGGGGATCGCAAGACCTCATGCTCCTGGAGCGGCCGATATCTGATTAG
CTAGTTGGTGGGGTAAAGGCCTACCAAGGCATCGATCAGTAGCTGGTCTGAGAGGACGACCAGCCACACT
GGAACTGAGACACGGTC
>Zotu6718
GAACGCTAGCGGGAGGCCTAACACATGCAAGCCGAGCGGTATTTGTCTTTCGGGACGGAGAGAGCGGCG
TACGGGTGCGGAACACGTGTGCAACCTGCCTTTATCAGGGGGATAGCCTTTCGAAAGGAAGATTAATACCC
CATAATATATTAATTGGCATCAATTGATATTGAAAACTACGGTGGATAGAGATGGGCACGCGCAAGATTAG
ATAGTTGGTAGGGTAACGGCCTACCAAGTCAGTGATCTTTAGGGGGCCTGAGAGGGTGATCCCCCACACT
GGTACTGAGACACGGAC
>Zotu6721
GAACGCTGGCGGCGTGCTTAACACATGCAAGTCGAACGGTGAACTTGGGAGCTTGCTTCCGGGGGATCAG
TGGCGAACGGGTGAGTAACACGTGAGTAACCTGCCCCAGACTCTGGGATAAGCGTTGGAAACGACGTCTA
ATACCGGATACGAGCTTCAGCCGCATGGCTAGGAGTTGGAAAGAATTTCGGTTTGGGATGGACTCGCGGC
CTATCAGCTAGTTGGTGAGGTAATGGCTCACCAAGGCGACGACGGGTAGCCGGCCTGAGAGGGTGACCG
GCCACACTGGGACTGAGACACGGCC
>Zotu6723
GAACGCTGGCGGCGTGCTTAACACATGCAAGTCGAGCGGAAAGGCCCTTCGGGGTACTCGAGCGGCGAA
CGGGTGAGTAACACGTGGGCAACCTGCCCTCAGCTCTGGGATAACTCCGGGAAACCGGGGCTAATACCGG
ATACGACTTCCCTGGGCATCCTGGGGGGTGGAAAGTTCTTCGGCTGAGGATGGGCCCGCGGCCTATCAGC
TTGTTGGTGGGGTGATGGCCTACCAAGGCGACGACGGGTAGCCGGCCTGAGAGGGCGACCGGCCACACT
GGGACTGAGACACGGCC
>Zotu6725
GAACGCTGGCGGCGTGCCTAATACATGCAAGTCGAGCGAATGATGAAGAAGCTTGCTTCTTCTGATTTAGC
GGCGGACGGGTGAGTAACACGTGGGCAACCTGCCCTGTAGATTGGGATAACTCCGGGAAACCGGGGCTA
ATACCGAATAATCCATTACCTCACATGAGGAAATGTTAAAAGACGGTTTCGGCTGTCACTACAGGATGGGC
CCGCGGCGCATTAGCTAGTTGGTGAGGTAACGGCTCACCAAGGCGACGATGCGTAGCCGACCTGAGAGG
GTGATCGGCCACACTGGGACTGAGACACGGCC
>Zotu6730
AAACGCTGGCGGCGTGCCTAACACATGCAAGTCGAACGTCCTTGCTGTTTTCGCAAGAGGACAGCAAGGG
AGTGGCGGACGGGTGAGTAACACGTGGGTACCTACCCTGGGGTGAGGAATACCGGCGAGAAATTGCCGA
CAAGACCGCATAGGCTCTCCTACGAGAGCAAAGCCGAAAGGCGCCCTGGGATGGGCCTGCGGCCGATTAG
CTGGTTGGTGAGGTAAGAGCTCACCAAGGCGACGATCGGTAGCTGGTCTGAGAGGACGAACAGCCACACT
GGGATTGAGAACGGCC
>Zotu6736
GAACGCTGGCGGCGTGCTTAACACATGCAAGTCGAGCGGTAAGGCCCCTTCGGGGGTACACGAGCGGCG
AACGGGTGAGTAACACGTGAGCAATCTGCCCTCCACATCGGGATAACCCCCGGAAACGGGAGCTAATACC
GGATACGACCACTTCAGGCATCTGATGGTGGTGGAAAGTTCCGGCGGTGGAGGATGAGCTCGCGGCCTAT
CAGCTTGTTGGTGGGGTAACGGCCCACCAAGGCTTCGACGGGTAGCCGGCCTGAGAGGGTGACCGGCCAC
ACTGGGACTGAGACACGGCC
>Zotu6740
GAACGCTGGCGGCGTGCCTAATACATGCAAGTCGAGCGAATCTTTGGGAGCTTGCTCCTAAAGGTTAGCG
GCGGACGGGTGAGTAACACGTGGGCAACCTGCCTATAAGACTGGGATAACTTCGGGAAACCGGAGCTAAT
ACCGGATCATTCTTTTCTACACATGTAGAAAAGCTAAAAGACGGTTTACGCTGTCACTTATAGATGGGCCCG
CGGCGCATTAGCTAGTTGGTGAGGTAACGGCTCACCAAGGCCACGATGCGTAGCCGACCTGAGAGGGTGA
TCGGCCACACTGGGACTGAGACACGGCC
>Zotu6744

GAACGCTGGCGGCAGGCTTAACACATGCAAGTCGAGCGCCCCGCAAGGGGAGCGGCAGACGGGTGAGTA
ACACGTGGGAATCTACCCATCCCTACGGAACAACTCCGGGAAACTGGAGCTAATACCGTATACGCCCTTCG
GGGGAAAGATTTATCGGGGATGGATGAGCCCGCGTTGGATTAGCTAGTTGGTGGGGTAAAGGCCTACCAA
GGCGACGATCCATAGCTGGTCTGAGAGGATGATCAGCCACATTGGGACTGAGACACGGCC
>Zotu6747
CAACGCTGGCGGCGTGCCTAACACATGCAAGTCGAACGCGAAAGGGACTTCGGTTCTTAGTAGAGTGGCG
TACGGGTGAGTAACGCGTGGGTCATCTGCCCTCGGGTGGGGAATAACTAACCGAAAGGTTAGCTAATACC
GCATAACACAGTGATGTCAAAGCAGCAATGTGCCTGAGGAGGAGCCTGCGTCTGATTAGGTAGTTGGTGG
GGTAACGGCCTACCAAGCCTGCGATCAGTAGCTGGTCTGAGAGGACGGCCAGCCACAATGGCACTGAAAC
ACGGGC
>Zotu6750
CAACGTTGGCGGCGCGCCTAACACATGCAAGTCGAACGAGAAAGTGGAGCAATCCATGAGTAAAGTGGCG
TACGGGTGAGTAACACGTGGGTAATCCACCTCTTGATGGGGAATAACGCTGGGAAACTGGCGCTAATACC
GCATAAGTTCGTAAGAAGAAAGTCGCAAGACGCCGAGAGAGGAGCCCGCGGCCGATTAGCTAGTTGGTA
GGGTAAAAGCCTACCAAGGCTATGATCGGTAGCCGGCCTGAGAGGGCACACGGCCACACTGGCACTGAAA
CACGGGC
>Zotu6752
GAACGCTGGCGGCGTGCCTAATACATGCAAGTCGAGCGGAACTTTCATTGGAGCTTGCTCCTTTGACAGTT
TAGCGGCGGACGGGTGAGTAACACGTGGGTAACCTACCCTGTAGATTGGGATAACTTCGGGAAACCGAGG
CTAATACCGAATAATACTTTTAATCTCATGGTTGAAAGTTGAAAGATGGTTCTGCTATCACTACAGGATGGG
CCCGCGGTGCATTAGCTAGTTGGTGAGGTAACGGCTCACCAAGGCCACGATGCATAGCCGACCTGAGAGG
GTGATCGGCCACACTGGGACTGAGACACGGCC
>Zotu6755
GAACGCTGGCGGCAGGCTTAACACATGCAAGTCGAACGATGACTAACTAGCTTGCTAGTTATGATTAGTGG
CGGACGGGTGAGTAACATTTAGGAATCTGCCTAGTAGTGGGGGATAGCTCGGGGAAACTCGAATTAATAC
CGCATACGACCTACGGGTGAAAGGGGGCGCAAGCTCTTGCTATTAGATGAGCCTAAATCAGATTAGCTAGT
TGGTGGGGTAAAGGCCTACCAAGGCGACGATCTGTAACTGGTCTGAGAGGATGATCAGTCACACCGGAAC
TGAGACACGGTC
>Zotu6757
GAACGCTGGCGGCGTGCCTAATACATGCAAGTCGAGCGAATCAAAGGGAGCTTGCTCCCTTTTGGTTAGCG
GCGGACGGGTGAGTAACACGTGGGCAACCTGCCTATAAGACTGGGATAACTTCGGGAAACCGGAGCTAAT
ACCGGATAACTCATATCTACTCATGTAGATATGCTAAAAGACGGTTTACGCTGTCACTTATAGATGGGCCCG
CGGCGCATTAGCTAGTTGGTGAGGTAACGGCTCACCAAGGCGACGATGCGTAGCCGACCTGAGAGGGTG
ATCGGCCACACTGGGACTGAGACACGGCC
>Zotu6759
GAACGCTGGCGGCAGGCTTAACACATGCAAGTCGAGCGGGGGAAAGTAGCTTGCTACTGGACCTAGCGG
CGGACGGGTGAGTAATGCTTAGGAATCTGCCTATTAGTGGGGGACAACATTCCGAAAGGAATGCTAATAC
CGCATACGTCCTACGGGAGAAAGCAGGGGACCTTCGGGCCTTGCGCTAATAGATGAGCCTAAGTCGGATT
AGCTAGTTGGTGGGGTAAAGGCCTACCAAGGCGACGATCTGTAGCGGGTCTGAGAGGATGATCCGCCACA
CTGGGACTGAGACACGGCC
>Zotu6761
GAACGCTGGCGGCGTGCTTAACACATGCAAGTCGAGCGGTAAGGCCTTTCGGGGTACACGAGCGGCGAAC
GGGTGAGTAACACGTGGGTGATCTGCCCTGCACTCTGGGATAAGCCTGGGAAACTGGGTCTAATACTGGA
TATGACCTCAGCATGCATGTGCTGGGGTGGAAAGCTTTTGTGGTGCAGGATGGGCCCGCGGCCTATCAGCT
TGTTGGTGGGGTAATGGCCTACCAAGGCGACGACGGGTAGCCGACCTGAGAGGGTGACCGGCCACACTG
GGACTGAGACACGGCC
>Zotu6766
GAACGCTAGCGGCAGGCCTAATACATGCAAGTCAAGGGGCCCAGCAATGGGCACCGGCGGACGGGTGCG
TAACGCGTACACAATCTACCCTGGAGCGGGGGATAGCATCGGGAAACTGGTGGTAATACCCCATAGTTTTG
TGAGTTGGCATCAGCTTACAAATAAAGTTCCGGCGCTCTGGGATGAGTGTGCGTCCGATTAGCTAGTTGGT

AGGGTAACGGCCTACCAAGGCGACGATCGGTAGGGGGTGTGAGAGCATGACCCCCCACACGGGTACTGA
GACACGGAC
>Zotu6768
GAACGCTGGCGGCGTGCTTAACACATGCAAGTCGAACGGTAAGGCCTTTCGGGGTACACGAGTGGCGAAC
GGGTGAGTAACACGTGGGTGATCTGCCCTGCACTTCGGGATAAGCTTGGGAAACTGGGTCTAATACCGGA
TATGACTACGAATCGCATGGTTTGTGGTGGAAAGCTTTTGCGGTGTGGGATGGGCCCGCGGCCTATCAGCT
TGTTGGTGGGGTGATGGCCTACCAAGGCGACGACGGGTAGCCGGCCTGAGAGGGTGTCCGGCCACACTG
GGACTGAGATACGGCC
>Zotu6770
GAACGCTAGCGACAGGCCTAACACATGCAAGTCGAGGGGCAGCATAAAGGTAGCAATACTTTGGTGGCGA
CCGGCGCACGGGTGAGTAACACGTATGCAACCTACCTGTAAGAGGGGAATAACCCGGAGAAATTCGGACT
AATACCGCATAATACCACGAAGCTGCATGGCTTTGTGTTTAAACATTTATGGCTTACAGATGGGCATGCGTA
TGATTAGATAGTTGGTAGGGTAACGGCCTACCAAGTCAACGATCATTAGGGGTTCTGAGAGGAAGGTCCC
CCACACTGGTACTGAGACACGGAC
>Zotu6772
GAACGCTGGCGGCGTGCTTAACACATGCAAGTCGAGCGGTAAGGCACTTCGGTGTACACGAGCGGCGAAC
GGGTGAGTAACACGTGAGTAATCTGCCCTTCACTCTGGGATAAGCACTCGAAAGGGTGTCTAATACCAGAT
ACGACACAGACCTGCATGGGTTCTGTGTGGAAACTTTTGTGGGTGGGGGATGTGCTCGCGGCCTATCAGCT
TGTTGGTGGGGTAATGGCCTACCAAGGCTTTGACGGGTAGCCGGCCTGAGAGGGTGACCGGCCACACTGG
GACTGAGACACGGCC
>Zotu6776
GAACGTTGGCGGCGTGCCTTAAGCATGCAAATCAAGGGGGTCCCGCAAGGGACTACCGGTGGACGGGTG
AGTAATGCGTGGGAATGTACCTCGAAGTTGGATATAGCTAGCCGAAAGGTTGGGTAATCTCCAATGTGAA
CGAAAGTTTAGAGCCGCAAGGCGCTTTGAGATCAGCCCGCGTCCTATCAGCTTGTTGGTGGGGTAATGGCC
TACCAAGGCGACGACGGGTAGCTGGACTGAGAGGTCGGTCAGCCACAATGGGACTGCGACACGGCC
>Zotu6778
GAACGCTGGCGGCAGGCTTAACACATGCAAGTCGAACGCCCCGCAAGGGGAGTGGCAGACGGGTGAGTA
ACACGTGGGAACATACCCTTCGGTTCGGAACAACGCCGGGAAACTGGCGCTAATACCGGATACGGCTTAC
GGGCGAAAGATTGATCGCCGAAGGATTGGCCCGCGTTCGATTAGCTTGTTGGTGAGGTAACGGCTCACCA
AGGCGACGATCGATAGCTGGTCTGAGAGGATGGCCAGCCACATTGGGACTGAGACACGGCC
>Zotu6781
GAACGCTGGCGGCAGGCCTAACACATGCAAGTCGAACGGGTCCTTCGGGACTAGTGGCGGACGGGTGAG
TAACGCGTGGGAACATACCCTTTGGTACGGAATAGCCTCGGGAAACTGAGAGTAATACCGTATGTGCCCTT
CGGGGGAAAGATTTATCGCCAAAGGATTGGCCCGCGTTGGATTAGGTAGTTGGTGGGGTAATGGCCTACC
AAGCCGACGATCCATAGCTGGTTTGAGAGGATGATCAGCCACACTGGGACTGAGACACGGCC
>Zotu6785
GAACGCTGGCGGCATGCCTAACACATGCAAGTTGAACGAGAAGTTCTGGAACAAGTCTTCGGACAAGGGA
AGGGACGGGAAGTAGCGAACGGGTGAGTAACGCGTGGGCAACCAACCTTACACAGGGGGATAACACAGA
GAAATTTGTGCTAATACCGCATAAGCGCACAGTACCGCATGGTACGGTGTGAAAAACTCCGGTGGTGTAA
GACGGGCCCGCGTCTGATTAGCTAGTTGGTGAGGTAAGAGCTCACCAAGGCGACGATCAGTAGCCGACCT
GAGAGGGTGATCGGCCACATTGGGACTGAGACACGGCC
>Zotu6789
AAACGCTGGCGGCGTGCCTAATACATGCAAGTCGAACGAATGTAGCAATACATGAGTGGCGTACGGGTGA
GTAACACGTGGATAATCTTCCTTTAAGTGGGGAATAACCAATCGAAAGATTGGCTAATACCGCATAAGACC
ACAGTTTCTTCGGAGACAGAGGTTAAAGGCTTCGGCCGCTTTTAGATGAGTCTGCGTCCGATTAGCTAGTT
GGTGAGGTAATGGCTCACCAAGGCGACGATCGGTAACTGGTCTGAGAGGATGACCAGTCACACTGGGACT
GAGACACGGCC
>Zotu6795
GAACGCTGGCGGTGTGCTTCACACATGCAAGTCGAACGGTAGCAATACAGTGGCGGACGGGTGAGTAAC
GCGTGGGAATCTGCCCTGGGGTGGGGGATAGCAGACCGAAAGGTCTATTAATACCGCATATGCCGAAAGG

TAAAAGGAGTAATCCGCCCGAGGATGAGCCTGCGTCCGATTAGCTAGTTGGTGGGGTAAAAGCCTACCAA
GGCTACGATCGGTAGCTGGTCTGAGAGGATGATCAGCCACAATGGGACTGAGACACGGCC
>Zotu6799
GAACGCTAGCGGCAGGCCTAATACATGCAAGTCGAACGGGTCGCAAGGTCAGTGGCGAACGGGTGCGTA
ACGCGTAAGCAACCTGCCCACAACTGGGGGATAGCCTTGCGAAAGCGAGGGTAAACCCGCATGGTCCTTT
GGAGTCACCTGGCTTTTAAGGTAAACATTTATGGGTTGTGGAGGGGCTTGCGTCTGATTAGCTAGTTGGTG
GGGTAACGGCCCACCAAGGCGATGATCAGTAGGGGTTCTGAGAGGATTGGCCCCCACATGGGTACTGAGA
CACGGAC
>Zotu6801
GAATGCTGGCGGCGTGGATAAGTCATGCAAGTCAAGGGGGCCCGCAAGGGCAACCGGCAGACGAGGTAG
TAACACGTAGGTAGATCCCCCGAAGTCGGGCATAGCCAGCCGAAAGGCTGGGTAATTCCCGATAGTCCCG
CAAGGGTAAAGGTTTTTCGCTTCGGGAGTCGCCTGCGTAGTATCAGCTAGTTGGTAGGGTAACGGCCTACC
AAGGCTACGACGCTTAGGGGAGCTGAGAGGCTGACCCCCACCGATGGAACTGCGACACGGTC
>Zotu6804
GAACGCTGGCGGCGTGCCTAATACATGCAAGTCGAGCGGAGCTTATGAGGTGCTTGCACCGAGTAAGCTT
AGCGGCGAACGGGTGAGTAACACGTGGGCAACCTGCCTGTAAGACCGGGATAACTCACGGAAACGTGGG
CTAATACCGGATAGTTGAGCACCCCGCCTGGGGAGCTTGGGAAAGGCGGAGCAATCTGCCGCTTACAGAT
GGGCCCGCGGCGCATTAGCTAGTTGGTGGGGTAAAGGCCTACCAAGGCGACGATGCGTAGCCGACCTGA
GAGGGTGATCGGCCACACTGGGACTGAGACACGGCC
>Zotu6806
GAACGCTGGCGGCGTGCTTAACACATGCAAGTCGAACGGTGACGGAGAGCTTGCTCTCCTGATCAGTGGC
GAACGGGTGAGTAACACGTGGGCAACCTGCCCCAGACTTCGGGATAACGCTTCGAAAGAGGTGCTAATAC
CGGATACGAACCTCCCTGGCATCTGGGAGGTTGGAAAGTTTTTCGGTCTGGGATGGGCCCGCGGCCTATCA
GCTTGTTGGTGAGGTAACGGCTCACCAAGGCGACGACGGGTAGCCGGCCTGAGAGGGCGACCGGCCACA
CTGGGACTGAGACACGGCC
>Zotu6808
GAACGCTGGCGGCATGCCTAACACATGCAAGTCGAACGAGATCTTTCGGGATCTAGTGGCGCACGGGTGC
GTAACGCGTGGGAATCTGCCCTGGGGTTTGGGATAACAGTTGGAAACGACTGCTAATACCAAATGACGTC
GATAAGACCAAAGATTTATCGCCCTAGGATGAGCCCGCGTCGGATTAGCTAGTTGGTGGGGTAAAGGCCT
ACCAAGGCGACGATCCGTAGCTGGTCTGAGAGGATGATCAGCCACACTGGGACTGAGACACGGCC
>Zotu6810
GAACGCTGGCGGCGTGCTTAAGACATGCAAGTCGCACGAGTCATTTCGGTGGCTAGTGGCGCACGGGTGC
GTAACGCGTGACTGACCTACCTCCAAGTTCTGAATAACCTTCCGAAAGGAGGGCTAATACAGGATGTGAAG
CCCCATTCTGATGGGTCTTTAAAGGTTTACCGCTTGGAGATGGGGTCGCGTCCCATCAGCTTGTTGGTGGG
GTAATGGCTTACCAAGGCTACGACGGGTAGCCGGCCTGAGAGGGTGGCCGGCCACAAGGGCACTGAGAC
ACGGGC
>Zotu6812
CAACGCTGGCGGCGCGCTTAACACATGCAAGTCGCACGAGAAAGTGGAGCAATCCATGAGTAAAGTGGCG
TACGGGTGAGTAACACGTGGATGATCTACCTCTTGGTGGGGAATAACCCTGGGAAACCGGGGCTAATACC
GCATAAGCCTGCAAAGGGAAAGCAGCAATGCGCCGAGAGAGGAGTCCGCGGCCGATTAGCTAGTTGGTA
GGGTAATGGCCTACCAAGGCAATGATCGGTAGCCGGCCTGAGAGGGCACACGGCCACACTGGCACTGAA
ACACGGGC
>Zotu6816
GAACGCTGGCGGCAGGCTTAACACATGCAAGTCGAGCGGGCCCTTCGGGGTCAGCGGCAGACGGGTGAG
TAACGCGTGGGAACGTACCATTTGCTACGGAATAACTCAGGGAAACTTGTGCTAATACCGTATGAGCCCCC
CTTTAAAATTTCAGGAATCATAAAATGCCCTGGCATTTTATGGGGGGGAAAGATTTATCGGCAAATGATCG
GCCCGCGTTGGATTAGCTAGTTGGTGGGGTAAAGGCCTACCAAGGCGACGATCCATAGCTGGTCTGAGAG
GATGATCAGCCACACTGGGACTGAGACACGGCC
>Zotu6818

GAACGCTTGCGGCGTGCCTAAGAAATGCAAGTCGAGCGGGGGAGCAATCCCTAGCGGCGAACGGTCGCG
TAACACGTAGGCAACCTGCCCGAAAGACCGGGACAACACTTCGAAAGGGGTGCTAATACCGGATGTGCCC
GCTTCTTCACCTGGGGAGGCGAGTAAATCCTCGCAAGGGGGCTTTTGGAGGGTCCTGCGGCCTATCAGCTA
GATGGTGAGGTAACGGCCCACCATGGCGACGACGGGTAGCTGGTCTGAGAGGATGGTCAGCTGGACTGG
GACTGAGATACGGCC
>Zotu6820
GAACGCTGGCGGCGTGGCTAATACATGCAAGTCGAACGCATTTTCTTTCACCGTAGCTTGCTACACCGAAA
GAAAATGAGTGGCGAACGGGTGAGTAACACGTGGGTAACCTGCCCATCAGCGGGGGATAACACTTGGAA
ACAGGTGCTAATACCGCATAATTCCATTTACCGCATGGTAGATGGGTGAAAGGCGCTTTTGCGTCACTGAT
GGATGGACCCGCGGTGCATTAGCTAGTTGGTGGGGTAACGGCCTACCAAGGCTGCGATGCATAGCCGACC
TGAGAGGGTGATCGGCCACACTGGGACTGAGACACGGCC
>Zotu6823
GAACGCTGGCGGCGTGCTTAACACATGCAAGTCGAACGATGAAGCACCAGCTTGCTGGTGTGGATTAGTG
GCGAACGGGTGAGTAACACGTGAGTAACCTGCCCCCTACTCTGGGATAAGCCTGGGAAACTGGGTCTAAT
ACTGGATATGACCAATCACTGCATGGTGTGTTGGTGGAAAGATTTTTTGGTGGGGGATGGACTCGCGGCCT
ATCAGCTTGTTGGTGGGGTAATGGCCTACCAAGGCGACGACGGGTAGCCGGCCTGAGAGGGCGACCGGC
CACACTGGGACTGAGACACGGCC
>Zotu6825
GAACGCTGGCGGCGTGCTTAACACATGCAAGTCGAACGATGAACTAGGAGCTTGCTCTTGGGGATTAGTG
GCGAACGGGTGAGTAACACGTGAGTAACCTGCCCTTGACTCTGGGATAAGCGTTGGAAACGACGTCTAAT
ACCGGATACGAGCTTCCACCGCATGGTGAGTTGCTGGAAAGAATTTTGGTCAAGGATGGACTCGCGGCCT
ATCAGCTTGTTGGTGAGGTAATGGCTCACCAAGGCGACGACGGGTAGCCGGCCTGAGAGGGTGACCGGC
CACACTGGGACTGAGACACGGCC
>Zotu6827
GAACGCTGGCGGCAGGCTTAACACATGCAAGTCGAACGGAGAGAGGTAGCTTGCTACTGATCTTAGCGGC
GGACGGGTGAGTAATGCTTAGGAATCTGCCTATTAGTGGGGGACAACATTTCGAAAGGAATGCTAATACC
GCATACGTCCTACGGGAGAAAGCAGGGAATCTTCGGACCTTGCGCTAATAGATGAGCCTAAGTCGGATTA
GCTAGTTGGTGGGGTAAAGGCCTACCAAGGCGACGATCTGTAGCGGGTCTGAGAGGATGATCCGCCACAC
TGGGACTGAGACACGGCC
>Zotu6829
GAACGCTGGCGGCGTGCCTAACACATGCAAGTCTACGAGAAAGGGGTAGCAATATCCCGAGTAAAGTGGC
GCACGGGTGAGTAACACGTAGGCAATCTGCCTTTAGGACGGGGATAATCACGCGAAAGCGTGACTAATAC
CCGATGATGCAGCGGCACCGCATGGTGACAGTTGTTAAAAGCCCGCAAGGGCGCCTAGAGATGAGCCTGC
GTCCCATTAGGTAGTTGGCGGGGTAACGGCCCACCAAGCCTTCGATGGGTAGCTGGTCTGAGAGGATGAT
CAGCCACACTGGAACTGAGACACGGTC
>Zotu6831
GAACGCTGGCGGCGTGCCTAATACATGCAAGTTGAGCGGATCTTGTCCTTCGGGATAAGGTTAGCGGCGG
ACGGGTGAGTAACACGTAGGCAACCTGCCCTCGAGACTGGGATAACCTCCGGAAACGGATGCTAATACCA
GATACGCGATCTTCCCTCCTGGGGAGATCGGGAAAGACGGAGCAATCTGTTACTCGGGGATGGGCCTGCG
GCGCATTAGCTAGTTGGTGAGGTAACGGCTCACCAAGGCGACGATGCGTAGCCGACCTGAGAGGGTGATC
GGCCACACTGGGACTGAGACACGGCC
>Zotu6835
GAACGCTGGCGGCATGCCTAACACATGCAAGTCGAACGAAGCCTTCGGGCTTAGTGGCGCACGGGTGCGT
AACGCGTGGGAATCTGCCCTTAGGTACGGAATAACTCATGGAAACGTGTGCTAATACCGTATGATGTCTTC
GGACCAAAGATTTATCGCCTAGGGATGAGCCCGCGTTGGATTAGCTAGTTGGTGAGGTAAAGGCTCACCA
AGGCGACGATCCATAGCTGGTCTGAGAGGATGATCAGCCACACTGGGACTGAGACACGGCC
>Zotu6837
GAACGCTGGCGGCGTGCCTAAGACATGCAAGTCGAATGTGCTCGCAAGAGTAACATGGCGAACGGGTGA
GTAACACGTGGGAACTTCCATTAAGTTTAGAATAACTTTCCGAAAGGAAAGATAATACTAAATGTTATCGCA
AGATAAAAGGAGCAATCCGCTTAATGCAAGGCCCGCGGTCCATCAGCTAGTTGGTAGGGTAAAAGCCTAC
CAAGGCTATGACGGATAGGGGAGCCGAGAGGCCTAACCCCCACACTGGGACTGCGACACGGCC

>Zotu6840
GAATGCTGGCGGTATGGATAAGGCATGCAAGTCGAACGGCCCGCAAGGGCAGTGGCAGACGAGGTAGTA
ACACGTACGAACGCACCCCAAAGTCAGGGATAGCCAGCCGAAAGGTTGGGTAATACTTGATGGTCCCGAA
AGGGTAAAGATTTATCGCTTTGGGAGCGGCGTGCGCAATATCAGCTAGTTGGTAAGGTAACGGCTTACCA
AGGCTACGACGTTTAGGGGAGCTGAGAGGCTGACCCCCACCGATGGGACTGAGACACGGCC
>Zotu6846
GAACGCTGGCGGCGTGCCTAATACATGCAAGTCGAGCGGATCTTTAGAAGCTTGCTTCTAAAGATTAGCGG
CGGACGGGTGAGTAACACGTGGGCAACCTGCCTGTAAGACTGGGATAACTGCGGGAAACCGAGGCTAAT
ACCGGATAATCCTTTTCCTCACATGAGGAAAAGCTGAAAGACGGCGCAAGCTGTCACTTACAGATGGGCCC
GCGGCGCATTAGCTAGTTGGTGAGGTAATGGCTCACCAAGGCAACGATGCGTAGCCGACCTGAGAGGGTG
ATCGGCCACACTGGGACTGAGACACGGCC
>Zotu6848
GAACGTTAGCGGCGCGCCTAAGACATGCAAGTCGAGCGAGAAAGGGCTTCGGCCCCGGTACAGCGGCGC
ACGGGTGAGTAACACGTAGGTAATCAACCCTCGAGTGGTGGACAACTCTCCGAAAGGAGAGCTAATACAG
CATGACGTGGCGGTCTTCCGAGATCGTTTCCAAAGCGGGCCTCTTCATGAAAGCTCGCGCTCGAGGATGAG
CCTGCGGCCCATCAGCTAGTTGGCGGGGTAACAGCCCACCAAGGCGTAGACGGGTAGCTGGTCTGAGAGG
ATGAACAGCCACACTGGAACTGAGACACGGTC
>Zotu6851
GAACGCTGGCGGCGTGCTTAACACATGCAAGTCGAACGATGATGGCCCGGGCTTGCCCGGGTCTGATTAG
TGGCGAACGGGTGAGTAACACGTGAGTAACCTGCCCCTGACTTCGGGATAACCGCGAGAAATCGTGGCTA
ATACCGGATATGACACCCCGGCGCATGGTGGGGTGTGGAAAGACTGTGTCGGTTGGGGATGGGCTCGCG
GCCTATCAGCTTGTTGGTGGGGTGATGGCCTACCAAGGCGACGACGGGTAGCCGGCCTGAGAGGGTGACC
GGCCACACTGGGACTGAGACACGGCC
>Zotu6853
GAACGCTGGCGGCATGCCTAACACATGCAAGTCGAACGAGACCTTCGGGTCTAGTGGCGCACGGGTGCGT
AACGCGTGGGAATCTGCCCTTGGGTACGGAATAACAGTTAGAAATGACTGCTAATACCGTATAATGACTTC
GGTCCAAAGATTTATCGCCCAGGGATGAGCCCGCGTAGGATTAGCTTGTTGGTGAGGTAAAAGCTCACCA
AGGCGACGATCCTTAGCTGGTCTGAGAGGATGATCAGCCACACTGGGACTGAGACACGGCC
>Zotu6855
GAACGCTGGCGGCGTGCCTAATACATGCAAGTCGAACGCTGGAGATCTAGCTTGCTAGATCGAAGGAGTG
GCGAACGGGTGAGTAATACATAAGCAACCTACCCACGAAGACTGGGATAATCTCTGGAAACGGGGACTAA
TACCGGATAGGTAATCGGAAGGCATCTTCTGGTTATTAAAGGTTAAAAACACTGGTGGATGGGCTTATGGC
GCATTAGTTAGTTGGTGAGGTAACGGCCCACCAAGACGATGATGCGTAGCCGGCCTGAGAGGGTGAACG
GCCACATTGGGACTGAGACACGGCC
>Zotu6861
GAACGCTGGCGGCGTGCTTAACACATGCAAGTCGAACGGAAAGGCCCCCACCTTGGTGGGGGTGCTCGAG
TGGCGAACGGGTGAGTAACACGTGGGTGATCTGCCCTGTACTTCGGGATAAGCTTGGGAAACTGGGTCTA
ATACCGGATAGGACCATGGTTTAGTGTCTCATGGTGGAAAAATTATTGTTTTGGTGCAGGATGAGCTCGCG
GCCTATCAGCTTGTTGGTGGGGTAATGGCCTACCAAGGCGTCGACGGGTAGCCGGCCTGAGAGGGTGGAC
GGCCACATTGGGACTGAGATACGGCC
>Zotu6863
GAACGCTGGCGGCATGCCTTACACATGCAAGTCGAACGGTAACAGCTCTTCGGAGGCTGACGAGTGGCGA
ACGGGTGAGTAATACATCGGAACGTGCCCGATCGTGGGGGATAACGGAGCGAAAGCTTTGCTAATACCGC
ATACGATCTACGGATGAAAGCAGGGGACCCTCGGGCCTTGCGCGAACGGAGCGGCCGATGGCAGATTAG
GTAGTTGGTGGGATAAAAGCTTACCAAGCCGACGATCTGTAGCTGGTCTGAGAGGACGACCAGCCACACT
GGGACTGAGACACGGCC
>Zotu6868
GAACGCTGGCGGTGTGCTTCACACATGCAAGTCGAACGGGGTAGCAATACCCAGTGGCGGACGGGTGAG
TAACGCGTAGAAATCTGCCTTGAGATGGGGGATAACAGTCCGAAAGGATTGCTAATACCGCATATGTATGT
GCCCGGACACGGGCGTATATGAAAGGAGTAATCCGTCTTAAGATGAGTCTGCGTCCGATTAGCTAGTTGGT

AGGGTAAAGGCCTACCAAGGCGACGATCGGTAGCTGGTCTGAGAGGATGATCAGCCACAATGGGACTGA
GACACGGCC
>Zotu6872
GAACGCTGGCGGCGTGCTTAACACATGCAAGTCGAACGGAAAGGCCGAAGCTTGCTTCGGTACTCGAGTG
GCGAACGGGTGAGTAACACGTGGGTGATCTGCCCTGTACTTCGGGATAAGCTTGGGAAACTGGGTCTAAT
ACCGGATATGACGATCTCTTGGATGGGGTTGTGGAAAGCTTTTGCGGTATGGGATGAGCTCGCGGCCTATC
AGCTTGTTGGTGGGGTAATGGCCTACCAAGGCGTCGACGGGTAGCCGGCCTGAGAGGGTGTACGGCCAC
ATTGGGACTGAGATACGGCC
>Zotu6876
GAACGCTAGCGGCATGCCTAAGGTATGCAAGTCGTGCGCAAAACCACCTTCGGGTGGTGGAAAGCGGCG
GACGGGTGAGTAACGCGTAGAAACATACCCCAGAGTGAGGGATAATTCCGGGAAACCGGACTAATACC
GCATATTGTCTAAGGACAAAAGATTTATCGCTTTGGGAATGGTCTGCGTCCGATTAGCTTGTTGGTGAGGT
AAAGGCTCACCAAGGCTATGATCGGTAGCTGGTTTGAGAGGATGATCAGCCACACTGGAACTGAGACACG
GTC
>Zotu6878
GAACGCTAGCGGCAGGCCTAATACATGCAAGTCGAGCGGATTGTAGCAATACAGTTAGCGGCGCACGGGT
GAGTAACGCGTACACAATCTACCTTCAACAGGGGAATAGCCTCGGGAAACTGGGAGTAATACCCCATAGC
ATCTTTGGATCGCATGATCTGATTATTAAAGTTACGGCGGTTGAAGATGAGTGTGCGTCTGATTAGATTGTT
GGTGAGGTAACGGCTCACCAAGTCGATGATCAGTAAGGGGCGTGAGAGCGTGGCCCCTCACATGGACACT
GAGACACGGGT
>Zotu6880
GAACGCTGGCGGCGTGCTTAACACATGCAAGTCGAACGGTGACAGGGGAGCTTGCTCCCCTCGATCAGTG
GCGAACGGGTGAGTAACACGTGAGTAACCTGCCCCAGACTCTGGGATAACATCGGGAAACCGGTGCTAAT
ACCGGATATGAGACGAGACTGCATGGTCATCGTCTGGAAAGATTTATCGGTCTGGGATGGACTCGCGGCC
TATCAGCTAGTTGGTGAGGTAACGGCTCACCAAGGCGACGACGGGTAGCCGGCCTGAGAGGGCGAACGG
CCACACTGGAACTGAGACACGGTC
>Zotu6883
GAACGCTGGTGGAGTGTTTTATACATGCAAGTCGAGCGAGGACAGTAGCAATACTGAGCCGAGCGGCGAA
CGGGTGAGTAACACGTAAGCAATCTGCCCTGTAATCGGGAACAACCGGGCCAACGCCCGGCTAATGCCGG
ATATCGTGGCGAGTCCCCTGACTCGTTGACGAAAGATTTATCGTTACAGGATGAGCTTGCGTCTGATTAGCT
AGTTGGCGGGGTAACGGCCCACCAAGGCGACGATCAGTAGCCGGCCTGAGAGGGTGATCGGCCACATTG
GGACTGAGATACGGCC
>Zotu6887
GAACGCTGGCGGCGTGCTTAACACATGCAAGTGGAGCGACGAACCAGGGCTTGCCCTGGGGCAAAGCCG
CGAACGGGTGAGTAACACGTGGGTAACCCACCCCGATGATCGGGACAACCCGAGGAAACTCGGGCTAATA
CCGAATGTGCCTGAAGTCCATGAGGACTCCAAGTAAAGATAGCTTCGGCCTTCGCATCGGGACGGGCCCG
CGGCCCATTAGCTTGTTGGTGAGGTAACGGCTCACCAAGGCGACGATGGGTAGCTGGTCTGAGAGGACGA
TCAGCCACACTGGGACTGAGACACGGCC
>Zotu6890
TAACGCTGGCGGTATGCCTAACACATGCAAGTCGAACGGACCTTTATTTTTGAGAATCTCTCTTTATTGGGG
CGTCTCATAAATAATGGTTAGTGGCGGACGGGTGAGTAACACGTTGGTACCTGCCCTCTAGTGGGGAATAA
TTCGCCGAAAGGCGGACTAATACCGCATGTGATCTACGGATCAAAGCCTTCGGGCGCTAGAGGAGGGGCC
TGCGGTTGATTAGCTAGTTGGTGAGGTAAAAGCTCACCAAGGCGACGATCAATAGCTGGATCGAGAGGTT
GACCAGCCACACTGGGACTGAGATACGGCC
>Zotu6892
GAACGCTGGCGGAATGCTTTACACATGCAAGTCGAACGGCAGCACGAGGGCAACCTTGGTGGCGAGTGG
CGAACGGGTGAGTAATGTATCGGAACGTGCCCAGTCGTGGGGGATAACGTAGCGAAAGCTACGCTAATAC
CGCATACGATCTATGGATGAAAGCGGGGGATCGCAAGACCTCGCGCGATTGGAGCGGCCGATATCAGATT
AGGTAGTTGGTGGGGTAAAGGCCTACCAAGCCGACGATCTGTAGCTGGTCTGAGAGGACGACCAGCCACA
CTGGGACTGAGACACGGCC

>Zotu6894
GAACGCTGGCGGCATGCCTAACACATGCAAGTCGAACGAGACCTTCGGGTCTAGTGGCGCACGGGTGCGT
AACGCGTGGGAATCTGCCCTTGGGTTCGGAATAACTCAGAGAAATTTGAGCTAATACCGGATGATGACGTA
AGTCCAAAGATTTATCGCCCAGGGATGAGCCCGCGTAGGATTAGCTAGTTGGTGGGGTAAAGGCCTACCA
AGGCGACGATCCTTAGCTGGTCTGAGAGGATGATCAGCCACACTGGGACTGAGACACGGCC
>Zotu6897
GAACGCTGGCGGCGTGCTTAACACATGCAAGTCGAACGGTGAAGCCCAGCTTGCTGGGTGGATCAGTGGC
GAACGGGTGAGTAACACGTGAGCAATCTGCCCCTGACTCTGGGATAAGCGCTGGAAACGGCGTCTAATAC
TGGATACGAGCCGTGAAGGCATCTTCAACGGTTGGAAAGATTTTTCGGTCAGGGATGAGCTCGCGGCCTAT
CAGCTTGTTGGTGAGGTAATGGCTCACCAAGGCGTCGACGGGTAGCCGGCCTGAGAGGGTGACCGGCCAC
ACTGGGACTGAGACACGGCC
>Zotu6901
GAACGCTGGCAGCATGCTTTACACATGCAAGTCGAACGGTAACAGGTCTTCGGATGCTGACGAGTGACGA
ACGGGTGAGTAATACATCGGAACGTGCCTAGTAGTGGGGGATAACTACTCGAAAGAGTAGCTAATACCGC
ATGAGATCTACGGATGAAAGCAGGGGACCTTCGGGCCTTGTGCTACTAGAGCGGCTGATGGCAGATTAGG
TAGTTGGTGGGGTAAAGGCTTACCAAGCCTGCGATCTGTAGCTGGTCTGAGAGGACGACCAGCCACACTG
AGACTGAGACACGGCC
>Zotu6903
GAACGCTAGCGATAGGCTTAACACATGCAAGTCGAGGGGCAGCAGGGACTTCGGTCTGCTGGCGACCGGC
GCACGGGTGCGTAACGCGTATGCAACTTGCCCTGTAGAGGAGGATAACCCGCTGAAAGGCGGACTAATAC
TGCGTACTCTCATTGAACGGCATCGTTTGATGAGGAAAGAAATTCGCTACAGGATAGGCATGCGTCCCATT
AGCTAGTTGGTGAGGTAACGGCTCACCAAGGCGACGATGGGTAGGGGAACTGAGAGGTTTATCCCCCACA
CTGGTACTGAGACACGGAC
>Zotu6905
GAACGCTGGCGGCGTGCTTAACACATGCAAGTCGAGCGACGCGCCTTCGGGTGACGGAGCGGCGAACGG
GTGAGTAACACGTGGGCAACCTGCCCTCAGCTCTGGGATAACTCCGGGAAACCGGGGCTAATACCGGATA
CGACCTCGGAGGGCATCTTCTGGGGTGGAAAGTTCTTCGGCTGGGGATGGGCCCGCGGCCTATCAGCTTG
TTGGTGGGGTGATGGCTCACCAAGGCGACGACGGGTAGCCGGCCTGAGAGGGCGACCGGCCACACTGGG
ACTGAGACACGGCC
>Zotu6907
GAACGCTGGCGGCGTGCCTAATACATGCAAGTCGAACGAGAAAGTGTAGCAATACATTAGTAAAGTGGCA
GACGGGTGAGTAATACATAGGTAACCTACCTCCGAGATGGGGATAACCTTCCGAAAGGAGGGCTAATACC
GAATGAGACCACGAGTACTCTGTACTTGAGGCCAAAGGCGACCTCTACTTGTAAGTTGTCGCTTGGAGATG
GGCTTATGCACCATTAGCTTGTTGGTGAGGTAACGGCTCACCAAGGCTGTGATGGTTAGCTGATCTGAGAG
GTTGATCAGCCACACTGGAACTGAGACACGGTC
>Zotu6912
GAACGCTGGCGGCATGCCTAACACATGCAAGTCGAACGGCAGCACAAAAGGGGTAACCCTTTGGGTGGCG
AGTGGCGAACGGGTGCGTAACGCGTAGGAATCTACCTTTTAGTGGGGGATAACCCGAGGAAACTCGGGCT
AATACCGCATATTCTCTAAGGAGGAAAGCAGGGGACCTTTCGGGGCCTTGCGCTATTAGATGAGCCTGCGT
CGGATTAGCTAGTTGGTGAGGTAATGGCTCACCAAGGCGATGATCCGTAGCTGGTTCGAGAGGACGACCA
GCCACACTGGGACTGAGACACGGCC
>Zotu6915
GAACGCTAGCGGCAGGCATAATACATGCAAGGCGAGGGGGTAGCAATACCACCGTCGTACGGGTGCGTA
ACACGTATGCAACCTGCCCTTCACTGGGGGATAGCCTGGGGAAACTCGGATTAATACCGCATAAGACAGTA
GACTGGCATCAGTTAGCTGTTAAAGATTTATTGGTGAAGGATGGGCATGCGTCTGATTAGCTAGTTGGCGG
GGTAAAGGCCCACCAAGGCTACGATCAGTAGGGGTTCTGAGAGGATTGGCCCCCACACTGGCACTGAGAT
ACGGGC
>Zotu6918
GAACGCTGGCGGCAGGCTTAACACATGCAAGTCGAACGCTCATCTTCGGATGGGAGTGGCAGACGGGTGA
GTAACACGTGGGAACGTACCCTTCGATCTGGGATAACTTCGGGAAACTGGAGCTAATACCGGATACGCCCT

TTTGGGGAAAGGTTTACTGTCGAAGGATCGGCCCGCGTCTGATTAGCTAGTTGGTGGGGTAACGGCCTACC
AAGGCGACGATCAGTAGCTGGTCTGAGAGGATGATCAGCCACACTGGGACTGAGACACGGCC
>Zotu6920
GAACGCTGGCGGCAGGCCTAACACATGCAAGTCGAGCGGATGAGTGGAGCTTGCTCCATGATTCAGCGGC
GGACGGGTGAGTAATGCCTAGGAATCTGCCTGGTAGTGGGGGACAACGTTTCGAAAGGAACGCTAATACC
GCATACGTCCTACGGGAGAAAGCAGGGGACCTTCGGGCCTTGCGCTATCAGATGAGCCTAGGTCGGATTA
GCTAGTAGGTGAGGTAATGGCTCACCTAGGCGACGATCCGTAACTGGTCTGAGAGGATGATCAGTCACAC
TGGAACTGAGACACGGTC
>Zotu6922
GAACGCTGGCGGCGTGCTTAACACATGCAAGTCGAACGGTGACCTTGGAGCTTGCTCCGAGCGATCAGTG
GCGAACGGGTGAGTAACACGTGAGTAACCTGCCCCAGACTCTGGGATAACCTCGGGAAACCGGGGCTAAT
ACCGGATATGAGATGCTTCTGCATGGAATGCGTCTGGAAAGATTTATCGGTCTGGGATGGACTCGCGGCCT
ATCAGCTTGTTGGTGAGGTAATGGCTCACCAAGGCGACGACGGGTAGCCGGCCTGAGAGGGCGACCGGC
CACACTGGAACTGAGACACGGTC
>Zotu6930
GAACGCTAGCGGCAGGCCTAATACATGCAAGTCGAACGGTTGTAGCAATACAATAGTGGCGCACGGGTGC
GTAACACGTATGCAACCTACCTTATAGATGGGGATAGCCTTTGGAAACGGAGATTAATACCCGATAGTATA
TATTAGTGGCATCATTGATATATTAAAGATTTATCGCTATAAGCTGGGCATGCGTCCAATTAGTTAGTTGGC
GGGGTAACGGCTCACCAAGACTTTGATTGGTAGGGGAACTGAGAGGTTAATCCCCCACACTGGCACTGAG
ATACGGGC
>Zotu6932
GAACGCTGGCGGCGTGCTTAACACATGCAAGTCGAGCGGTAAGGCACTTCGGTGTACACGAGCGGCGAAC
GGGTGAGTAACACGTGAGTAATCTGCCCTCCACTCTGGGATAAGCACTCGAAAGGGTGTCTAATACCGGAT
ACGACCGATCACTGCATGGTGTGTTGGTGGAAACTTTTATGGGTGGGGGATGTGCTCGCGGCCTATCAGCT
TGTTGGTGGGGTAATGGCCTACCAAGGCTTCGACGGGTAGCCGGCCTGAGAGGGTGACCGGCCACACTGG
GACTGAGACACGGCC
>Zotu6936
GAACGCTGGCGGCATGCTTTACACATGCAAGTCGAACGGCAGCGCGGGGCAACCTGGCGGCGAGTGGCG
AACGGGTGAGTAATATATCGGAACGTACCCAAGAGTGGGGGATAACGTAGCGAAAGTTACGCTAATACCG
CATACGATCTAAGGATGAAAGCAGGGGACCGCAAGGCCTTGTGCTCCTGGAGCGGCCGATATCTGATTAG
CTAGTTGGTAGGGTAAAGGCCTACCAAGGCATCGATCAGTAGCTGGTCTGAGAGGACGACCAGCCACACT
GGAACTGAGACACGGTC
>Zotu6938
GAACGCTGGCGGCGTGCTTAACACATGCAAGTCGAACGAGGAATTTTAAATGAGACTTCGGTGGATTTTGA
AATTCTTAGTGGCGGACGGGTGAGTAACGCGTGGGCAACCTGCCCTGTACAGGGGGACAACAGCTGGAA
ACGGCTGCTAATACCGCATAAGCGCACAGCATCGCATGATGCCGTGTGAAAAACTCCGGTGGTACAGGAT
GGGCCCGCGTTGGATTAGGTAGTTGGTGGGGTAACGGCCCACCAAGCCGACGATCCATAGCCGACCTGAG
AGGGTGACCGGCCACATTGGGACTGAGACACGGCC
>Zotu6941
GAACGCTGGCGGCGTGCTTAACACATGCAAGTCGAGCGGATTGAAGACTTCGGTCTGATATTAGCGGCGG
ACGGGTGAGTAACGCGTGGGTGACCTGCCTTATACAGGGGGATAACAGTGAGAAATCACTGCTAATACCG
CATAAGACCACACTACCACATGGTAGAGAGGTAAAAGGAGCAATCCGGTATGAGATGGGCCCGCGTCTGA
TTAGCTAGTTGGAGAGGTAATGGCTCATCAAGGCGACGATCAGTAGCCGACCTGAGAGGGTGATCGGCCA
CATTGGGACTGAGACACGGCC
>Zotu6943
GAACGCTGGCGGCATGCTTTACACATGCAAGTCGAACGGCAGCGCGGGCTTCGGCCTGGCGGCGAGTGGC
GAACGGGTGAGTAATATATCGGAACGTGCCCAAGAGTGGGGGATAACGTAGCGAAAGTTACGCTAATACC
GCATACGATCTAAGGATGAAAGCAGGGGACCTTCGGGCCTTGTGCTCCTGGAGCGGCCGATATCTGATTA
GCTAGTTGGTGAGGTAAAGGCTCACCAAGGCGACGATCAGTAGCTGGTCTGAGAGGACGACCAGCCACAC
TGGAACTGAGACACGGTC

>Zotu6945
GAACGCTGGCGGCGTGCCTAATACATGCAAGTCGAGCGGACGGATGGGAGCTTGCTCCCTGAAGTTAGCG
GCGGACGGGTGAGTAACACGTGGGCAACCTGCCTGTAAGACTGGGATAACTCCGGGAAACCGGGGCTAA
TACCGGATAATTCTTATCCTCTCATGAGGATAAGCCGAAAGACGGTTTCGGCTGTCACTTACAGATGGCCC
GCGGCGCATTAGCTAGTTGGTGAGGTAACGGCTCACCAAGGCAACGATGCGTAGCCGACCTGAGAGGGT
GATCGGCCACACTGGGACTGAGACACGGCC
>Zotu6948
GAACGCTGGCGGCGTGCCTAATACATGCAAGTCGAGCGCAGGAAGCAGACTGATCTCCTTCGGGAGTGAT
GTTTGTGGAATGAGCGGCGGACGGGTGAGTAACACGTGGGCAACCTGCCTGTAAGACCGGGATAACTTGC
GGAAACGTGAGCTAATACCGGATAAAGCTCCATGCCGCATGGCATGGGGATGAAAGACGGCTTAGCTGTC
GCTTGCAGATGGGCCCGCGGCGCATTAGCTAGTTGGTGGGGTGATGGCCTACCAAGGCGACGATGCGTAG
CCGACCTGAGAGGGTGACCGGCCACACTGGGACTGAGACACGGCC
>Zotu6950
GAACGCTGGCGGCGTGCTTAACACATGCAAGTCGAGCGGAAAGGCCCTTTCGGGGGTACTCGAGCGGCG
AACGGGTGAGTAACACGTGAGTAATCTGCCCCTGGCTTTGGGATAGCCACCGGAAACGGTGATTAATACC
GAATATGCACTACTTCTCGCATGGGTTGTGGTGGAAAGTTTTTCGGCCAGGGATGTGCTCGCGGCCTATCA
GCTTGATGGTGAGGTAATGGCTCACCATGGCTTCGACGGGTAGCCGGCCTGAGAGGGTGACCGGTCACAC
TGGGACTGAGACACGGCC
>Zotu6952
GAACGCTGGCGGCATGCCTAACACATGCAAGTCGAACGAAGCCTTCGGGTTTAGTGGCGCACGGGTGCGT
AACGCGTGGGAATCTGCCCTTGGGTTCGGAATAACAGCGAGAAATTGCTGCTAATACCGGATGATGACGA
AAGTCCAAAGATTTATCGCCCAGGGATGAGCCCGCGTAGGATTAGCTAGTTGGTGAGGTAAAGGCTCACC
AAGGCGACGATCCTTAGCTGGTCTGAGAGGATGATCAGCCACACTGGGACTGAGACACGGCC
>Zotu6955
GAACGCTGGCGGCGTGCCTAATACATGCAAGTCGAGCGAACTTTTGGGAGCTTGCTCCCAAAAGTTAGCG
GCGGACGGGTGAGTAACACGTGGGCAACCTGCCTATAAGACTGGGATAACTTCGGGAAACCGGAGCTAAT
ACCGGATAATTCTTTTCTACTCATGTAGAAAAGCTAAAAGACGGTTTACACTGTCACTTATAGATGGGCCCG
CGGCGCATTAGCTAGTTGGTGAGGTAACGGCTCACCAAGGCCACGATGCGTAGCCGACCTGAGAGGGTGA
TCGGCCACACTGGGACTGAGACACGGCC
>Zotu6958
GAACGCTGGAAATGTGGATACCGGCATGCAAGTCAAGGGGCCGCAAGGCACCGGCGAACGGCTTAGTAA
CACGTGGATACATACCTTAAAGTGGGGCATAGCTCGTCGAAAGACGGGGTAATTTCCCATAGTCTCGCAAG
AGTAAAGGCGCAAGCCGCTTTAAGAATGGTCCGCGGTCTATCAGCTAGTTGGTGAGGTAATGGCTCACCA
AGGCTATGACGGATAGGGGGCGTGAGAGCGCGACCCCCAAGAATGGCACTGCGACACGGGC
>Zotu6960
GAACGCTGGCGGCACGCCTAACACATGCAAGTCGAACGGACTTAAAAGAGCTTGCTTTTTTAAGTTAGTGG
CGGACGGGTGAGTAACACGTGAGCAACCTGCCTCTGAGAGGGGGATAGCTTCTGGAAACGGATGGTAAT
ACCCCATAAAATATAAATATCGCATGATAGATATATCAAAGAAATTCGCTCAGAGATGGGCTCGCGTCTGA
TTAGATAGTTGGTGAGGTAACGGCTCACCAAGTCGACGATCAGTAGCCGGACTGAGAGGTTGAACGGCCA
CATTGGGACTGAGACACGGCC
>Zotu6963
GAACGCTGGCGGCGTGCCTAATACATGCAAGTCGAACGCATTTTCTTTCACCGTAGCTTGCTACACCGAAA
GAAAATGAGTGGCGAACGGGTGAGTAACACGTGGGTAACCTGCCCATCAGCGAGGGATAACACTTGGAA
ACAGGTGCTAATACCGCATAATTCCATTTACCGCATGGTAGATGGATGAAAGGCGCTTTTGCGTCACTGAT
GGATGGACCCGCGGTGCATTAGCTAGTTGGTGGGGTAACGGCCTACCAAGGCTGCGATGCATAGCCGACC
TGAGAGGGTGATCGGCCACACTAGGACTGAGACACGGCC
>Zotu6966
GAACGCTAGCGGCATGCCTAACACATGCAAGTCGAACGCCCCTTTCGGGGGGAGTGGCAGACGGGTGAGT
AACACGTGGGAACATACCCTTTGGTTCGGAACAACGTTTGGAAACGGACGCTAATACCGGATACGCCCTTT

GGGGGAAAGGCTTTGCTGCCGAAGGATTGGCCCGCGTTCGATTAGCTTGATGGCGGGGTAACGGCCCACC
ATGGCGACGATCGATAGCTGGTCTGAGAGGATGGCCAGCCACATTGGGACTGAGACACGGCC
>Zotu6971
GAACGCTGGCGGCATGCTTTACACATGCAAGTCGAACGGCAGCGGGGGCTTCGGCCTGCCGGCGAGTGGC
GAACGGGTGAGTAATGCATCGGAACGTGCCCATGTCGTGGGGGATAACGTATCGAAAGGTACGCTAATAC
CGCATACGCCCTGAGGGGGAAAGCGGGGGATTCTTCGGAACCTCGCGCGATTGGAGCGGCCGATGTCGG
ATTAGCTAGTAGGTGAGGTAAAGGCTCACCTAGGCGACGATCCGTAGCGGGTCTGAGAGGATGATCCGCC
ACACTGGGACTGAGACACGGCC
>Zotu6975
GAACGCTGGAAATGTGGATACCGGCATGCAAGTCAAGGGGCCGCAAGGCACCGGCGAACGGCTTAGTAA
CACGTAGATACATACCTTCAATTGGAGCATAGCTCGTCGAAAGACGGGGTAATTCTCCATAGTCTCGCAAG
AGTAAAGGCGCAAGCCGATTGAAGAATGGTCTGCGGTCTATCAGCTAGTTGGTGAGGTAATGGCTCACCA
AGGCTATGACGGATAGGGGGCGTGAGAGCGCGACCCCCAAGAATGGCACTGCGACACGGGC
>Zotu6979
GAACGCTGGCGGCGTGCTTAACACATGCAAGTCGAACGAGAACGGATTATAGCTTGCTATAATTGTCAGCT
AAGTGGCGCACGGGTGAGTAATATATAGGTAATGTGCCCTAGAGAAGAGGATAACAGTTGGAAACGACTG
CTAAGACTCTATATGCCTTTAAGACAGAAGTCTGCAAGGGAAATATTTATAGCTCTAGGATCAGCCTGTACA
GTATCAGCTAGTTGGTGAGGTAATGGCTCACCAAGGCAATGACACTTAACTGGTTTGAGAGGATGATCAGT
CACACTGGAACTGAGACACGGTC
>Zotu6983
GAACGCTGGCGGCGTGCTTAACACATGCAAGTCGAGCGGAAAGGCCACTTCGGTGGTACTCGAGCGGCGA
ACGGGTGAGTAACACGTGAGTAATCTGCCCCATGCTCCGGAATAGCCAGCCGAAAGGCTGATTAATGCCG
GATATGACATCGTCAGGCATCTGGTGGTGTGGAAAGTTTTTTCGGCGTGGGATGTGCTCGCGGCCTATCAG
CTTGTTGGTGGGGTAATGGCCTACCAAGGCTTCGACGGGTAGCCGGCCTGAGAGGGTGACCGGCCACACT
GGGACTGAGACACGGCC
>Zotu6985
GAACGCTAGCGGCAGGCCTAACACATGCAAGTCGAGGGGTAGAGGCTTTCGGGCCTTGAGACCGGCGCA
CGGGTGCGTAACGCGTATGCAATCTGCCTTGTACGGGGGGATAGCCCAGAGAAATTTGGATTAATACCCCA
TGGTATTTTAGGGTGGCATCACCTTATTATTAAAGTTCCAACGGTACAAGATGAGCATGCGTCCCATTAGCT
AGTTGGTATGGTAACGGCATACCAAGGCAATGATGGGTAGGGGTCCTGAGAGGGAGATCCCCCACACTGG
TACTGAGACACGGAC
>Zotu6988
GAACGCTGGCGGCGTGCCTAACGCATGCAAGTCGAACGAGGCCTTCGGGCCTAGTGGCGGACGGGTGAG
TAACACGTAGGAACCTGCCCGGAAGTGGGGGACAACTCCACGAAAGCGGGGCTAATACCGCATACGGTCT
ACGGACCAAAGCCTTCGGGCGCTTTCGGAGGGGCCTGCGGCGGATTAGCTAGTTGGTGGGGTAACGGCCT
ACCAAGGCGACGATCCGTAGCTGGTCTGAGAGGATGATCAGCCACACTGGGACTGAGACACGGCC
>Zotu6992
GAACGCTGGCGGCGTGCTTAACACATGCAAGTCGAACGGATCATCTTACACTTGTGTTTGGTGGTTAGTGG
CGAACGGGTGAGTAACACGTGAGTAACCTGCCCTTCACTGTGGGATAACGGAGGGAAATTTCTGCTAATAC
CGCATATGACATTGCCTCGCATGGGGTGGTGTGGAAAGTTTTTTCGGTGGGGGATGGACTCGCGGCCTATC
AGCTTGTTGGTGGGGTAGTGGCTCACCAAGGCTTTGACGGGTAGCCGGCCTGAGAGGGCGACCGGCCACA
CTGGGACTGAGATACGGCC
>Zotu6997
GAACGCTAGCGGGATGCTTTACACATGCAAGTCGAACGGCAGCACGAGAGAGCTTGCTCTCTTGGTGGCG
AGTGGCGGACGGGTGAGTAATATATCGGAACGTGCCCAGTAGCGGGGGATAACTACTCGAAAGAGTGGC
TAATACCGCATACGCCCTACGGGGGAAAGGGGGGGATCGCAAGACCTCTCACTATTGGAGCGGCCGATAT
CGGATTAGCTAGTTGGTGGGGTAAAGGCTCACCAAGGCAACGATCCGTAGCTGGTTTGAGAGGACGACCA
GCCACACTGGGACTGAAACACGACC
>Zotu7000

GAACGCTGGCGGCATGCCTAATACATGCAAGTCGAACGGGAAGCACGAAAGTGCGGAGAGTGGCGAACG
GGTGAGTAACACGTAAGTGACCTGCCTTCAAGACGGGGATAACTGTTGGAAACGGCAGCTAATACCGGAT
AGGATACCGTGCGTAGGGCAAGGTATTGAAAGGAAGCAAATGCTTCGCTTGAAGATGGGCTTGCGGCGCA
TTAGCTAGTTGGAGTGATAAGAGCACACCAAGGCGATGATGCGTAGCCGACCTGAGAGGGTGTACGGCCA
CACTGGGACTGAGACACGGCC

>Zotu7002

GAACGCTGGCGGCGTGCCTAATACATGCAAGTCGAGCGAACTTTTGGGGGCTTGCTCCCAAAAGTTAGCG
GCGGACGGGTGAGTAACACGTGGGCAACCTGCCTATAAGACTGGGATAACTTCGGGAAACCGGAGCTAAT
ACCGGATAATTCTTTTCTACACATGTAGAAAAGCTAAAAGACGGTTTACGCTGTCACTTATAGATGGGCCCG
CGGCGCATTAGCTAGTTGGTGAGGTAACGGCTCACCAAGGCCACGATGCGTAGCCGACCTGAGAGGGTGA
TCGGCCACACTGGGACTGAGACACGGCC

>Zotu7004

GAACGTTGGCGGCGTGCCTTAAGCATGCAAATCAAGTGGGTCCCGCAAGGGACTACCGGTGGACGGGTG
AGTAATGCGTGGGAATCTACCTCGAAGTGGGATATAGCCAGCCGAAAGGTTGGGTAATCTCCCATGTGATC
GTAAGATCAAAGTCGCAAGACGCTTTTAGATGAGCCCACGTCCTATCAGCTAGTTGGTGGGGTAATGGCCT
ACCAAGGCAACGACGGGTAGCTGGACTGAGAGGTCGGTCAGCCACAATGGGACTGCGACACGGCC

>Zotu7009

GAACGCTGGCGGCGTGCTTAACACATGCAAGTCGAACGGTGAACCAGGGCTTGCCCTGGGAATCAGTGGC
GAACGGGTGAGTAACACGTGGGCAACCTGCCCCTGACTCTGGGATAACTCCGGGAAACCGGGGCTAATAC
CGGATACGACCCTGGAAGGCATCTTCCGGGGTGGAAAGTTTTTCGGTCAGGGATGGGCCCGCGGCCTATC
AGCTTGTTGGTGGGGTAATGGCCTACCAAGGCGACGACGGGTAGCCGGCCTGAGAGGGCGACCGGCCAC
ACTGGGACTGAGACACGGCC

>Zotu7014

GAACGCTGGCGGCAGGCCTAACACATGCAAGTCGAACGCCCCGCAAGGGGAGTGGCAGACGGGTGAGTA
ACACGTGGGAACCTTCCCTATAGTACGGAATAGCCCAGGGAAACTTGGAGTAATACCGTATACGCCCGAAA
GGGGAAAGAATTTCGCTATAGGATGGGCCCGCGTAGGATTAGCTAGTTGGTGAGGTAATGGCTCACCAAG
GCGACGATCCTTAGCTGGTTTGAGAGAACGACCAGCCACACTGGGACTGAGACACGGCC

>Zotu7016

CAACGCTGGCGGCGCGCTTAACACATGCAAGTCGAACGAGAAAGGGAGCAATCCTGAGTAAAGTGGCGC
ACGGGTGAGTAACACGTGGGTAATCTACCCTCTGGTGGGGAATAACTCTGGGAAACCGGAGCTAATACCG
CATAAGACCGGGAGGTGAAAGGGCGAAAGTTCGCCGGAGGAGGAGCCCGCGGCTGATTAGCTAGTTGGT
AGGGTAAGAGCCTACCAAGGCTGAGATCGGTAGCCGGCCTGAGAGGGCACACGGCCACACTGGCACTGA
AACACGGGC

>Zotu7019

GAACGCTGGCGGCGTGCCTAATACATGCAAGTCGAGCGGACTTGATTGGAGCTTGCTCTGATCAAGTTAGC
GGCGGACGGGTGAGTAACACGTGGGTAACCTGCCTGTAAGACTGGGATAACTTCGGGAAACCGGAGCTA
ATACCGGATAATCCGTTTCACCGCATGGTGAAACGTTGAAAGATGGCTTCGGCTATCACTTACAGATGGAC
CCGCGGCGCATTAGCTAGTTGGTGGGGTAATGGCCCACCAAGGCGACGATGCGTAGCCGACCTGAGAGG
GTGATCGGCCACACTGGGACTGAGACACGGCC

>Zotu7021

GAACGCTGGCGGCGTGCTTAACACATGCAAGTCGAACGGTGAACCTCGCTTGCGGGGGGATCAGTGGCGA
ACGGGTGAGTAACACGTGGGCAACCTGCCCTCAGCTCTGGGATAACTCCAAGAAATTGGGGCTAATACCG
GATATGACCGCTGACCGCATGGTCTGGTGGTGGAAAGATTTATCGGCTGGGGATGGGCCCGCGGCCTATC
AGCTTGTTGGTGGGGTAGTGGCCTACCAAGGCGACGACGGGTAGCCGGCCTGAGAGGGTGACCGGCCAC
ACTGGGACTGAGACACGGCC

>Zotu7026

GAACGCTGGCGGCATGCCTTACACATGCAAGTCGAACGGCAGCGCGGACTTCGGTCTGGCGGCGAGTGGC
GAACGGGTGAGTAATACATCAGAACGTACCCTGTTGTGGGGGATAACTAGTCGAAAGATTAGCTAATACC
GCATACAACCTGAGGGTGAAAGCGGGGAACCGTAAGACCTCGCGCAGCAGGAGCGGCCGATGTCTGATT
AGCTAGTTGGTGGGGTAAAGGCCCACCAAGGCGACGATCAGTAGCTAGTCTGAGAGGACGATCAGCCACA
CTGGGACTGAGACACGGCC

>Zotu7028
GAACGCTGGCGGCGTGCTTAACACATGCAAGTCGAACGGTGACGAGGGAGCTTGCTCCCTCTGATCAGTG
GCGAACGGGTGAGTAACACGTGAGTAACCTGCCCCAGACTCTGGGATAACATCGGGAAACCGGTGCTAAT
ACCGGATATGAGACGAGACCGCATGGTCATCGTCTGGAAAGATTTATCGGTCTGGGATGGACTCGCGGCC
TATCAGCTAGTTGGTGAGGTAATGGCTCACCAAGGCGACGACGGGTAGCCGGCCTGAGAGGGCGAACGG
CCACACTGGAACTGAGACACGGTC
>Zotu7031
GAACGCTGGCGGCAGGCCTAACACATGCAAGTCGAGCGGATGACGGGAGCTTGCTCCTTGATTCAGCGGC
GGACGGGTGAGTAATACCTAGGAATCTGCCTGGTAGTGGGGGACAACGTTTCGAAAGGAACGCTAATACC
GCATACGTCCTACGGGAGAAAGCAGGGGACCTTCGGGCCTTGCGCTATCAGATGAGCCTAGGTCGGATTA
GCTGGTTGGTGAGGTAATGGCTCACCAAGGCGACGATCCGTAACTGGTCTGAGAGGATGATCAGTCACAC
TGGAACTGAGACACGGTC
>Zotu7034
GAACGCTGGCGGCGTGCTTAAGACATGCAAGTCGAACGCAGTCCCTTCGGGGGCTGAGTGGCGCACGGGT
GAGTAACACGTAACTGACCTGCCCAGAAGTTCGGAATAACTGGTCGAAAGATCAGCTAATACCGAATGTGC
AGTGCCGCTGTGGCGGCACTGTAAAGGTCCACGCTTCTGGATGGGGTTGCGTTCCATCAGCTAGATGGTG
GGGTAAAGGCCTACCATGGCGACGACGGATAACCGGCCTGAGAGGGTGGCCGGTCACAGGGGCACTGAG
ACACGGGC
>Zotu7037
GAACGCTGGCAGCATGCCTTACACATGCAAGTCGAACGGCAGCGCGGAAGCAATCCTGGCGGCGAGTGG
CGAACGGGTGAGTAATACATCGGAACGTGCCCAATCGTGGGGGATAACGCAGCGAAAGCTGTGCTAATAC
CGCATACGATCTACAGATGAAAGCAGGGGATCGCAAGACCTTGCGCGAATGGAGCGGCCGATGGCAGATT
AGGTAGTTGGTGAGGTAAAGGCTCACCAAGCCTTCGATCTGTAGCTGGTCTGAGAGGACGACCAGCCACA
CTGGGACTGAAACACGGCC
>Zotu7039
GAACGCTAGCGGCAGGCTTAATACATGCAAGTCGAGGGGCAACAGGGGTAGCAATACCTGCTGGCGACC
GGCAAACGGGTGCGGAACACGTACAGAACCTTCCTTTAAGCGGGGGATAGCCCATAGAAATGTGGATTAA
TACCCCATAGTATTATTGATTGGCATCAGTTAATAATTAAAGATTTATCACTTAAAGATGGCTGTGCGTCTG
ATTAGGTAGTTGGTCGGGGTAACGGCCCACCAAGCCAACGATCAGTAACTGGTGTGAGAGCACGACCAGT
CACACGGGCACTGAGACACGGGC
>Zotu7041
GAACGCTGGCGGCGTGCCTAATACATGCAAGTAGAACGCTGAAGCTTGGTGCTTGCACCGAGTGGATGAG
TTGCGAACGGGTGAGTAACGCGTAGGTAACCTGCCTCTTAGCGGGGGATAACTATTGGAAACGATAGCTA
ATACCGCATAACAATGAATGACACATGTCATTTATTTGAAAGGGGCAATTGCTCCACTACAAGATGGACCT
GCGTTGTATTAGCTAGTAGGTGAGGTAACGGCTCACCTAGGCGACGATACATAGCCGACCTGAGAGGGTG
ATCGGCCACACTGGGACTGAGACACGGCC
>Zotu7044
GAACGCTGGCGGCGCGCCTAAAACATGCAAGTCGAGCGAGAACCAGGACTTCGGTCCTGGGGACAGCGG
CGAACGGGTGCGTAACACGTGAGCAACCTGCCCCGAAGACCGGGACAAGCCGGGGAAACCTGGTCTAATA
CCGGATGCCCTCACCAGATCGCATGGTCAAGTGAGGAAATGGATTCCGCTTCGGGATGGGCTCGCGGTCT
ATCAGCTTGTTGGTGGGGTAACGGCCCACCAAGGCGACGACGGATAGCTGGTCTGAGAGGACGATCAGCC
ACACTGGGACTGAGACACGGCC
>Zotu7048
GAACGCTGGCGGCATGCCTTACACATGCAAGTCGAACGGTAACGGGCCTTCGGGCGCCGACGAGTGGCGA
ACGGGTGAGTAATGCATCGGAACGTGCCCAGTAGTGGGGGATAGCCCGGCGAAAGCCGGATTAATACCG
CATGAGACCTGAGGGTGAAAGCGGGGGATCGCAAGACCTCGCGCTACTGGAGCGGCCGATGTCAGATTA
GCTAGTTGGTGGGGTAAAGGCCTACCAAGGCAACGATCTGTAGTTGGTCTGAGAGGACGACCAGCCACAC
TGGGACTGAGACACGGCC
>Zotu7051

GAACGCTAGCGGCAGGCTTAACACATGCAAGTCGAGGGGTAAGGCAGCAATGTCTGAGACCGGCGCACG
GGTGCGTAACGCGTATGCAATCTACCTTTTACAGGGGAATAGCCCAGAGAAATTTGGATTAATGCCCCATG
GTATAATGAGTTGGCATCAACACATTATTAAAGTTCCAACGGTAAAAGATGAGCATGCGTCCCATTAGTTA
GTTGGTAAGGTAACGGCTTACCAAGACGATGATGGGTAGGGGTCCTGAGAGGGAGATCCCCCACACTGGT
ACTGAGACACGGAC

>Zotu7053
AGCCGTTGGAAATCGCTGTATTCAACTCAGATACACGGGAGTTCAGGCCCAGTGCTCACACATGCTTTTCCC
GCAACCTCTCCCAGCCACTAACATAAGGATGTTAGCA

>Zotu7058
GAACGCTGGCGGCATGCCTTACACATGCAAGTCGAACGGTAACGGGTCCTTCGGGATGCCGACGAGTGGC
GAACGGGTGAGTAATATATCGGAACGTGCCCAGTAGTGGGGGATAACTGCTCGAAAGAGCAGCTAATACC
GCATACGACCTGAGGGTGAAAGGGGGGGATCGCAAGACCTCTCGCTATTGGAGCGGCCGATATCAGATTA
GCTAGTTGGTGGGGTAAAGGCCTACCAAGGCAACGATCTGTAGTTGGTCTGAGAGGACGACCAGCCACAC
TGGGACTGAGACACGGCC

>Zotu7061
GAACGCTGGCGGCATGCCTAACACATGCAAGTCGAACGCTCGTCTTCGGACGGGAGTGGCGCACGGGTGA
GTAACGCGTGGGAACCTGCCCCGGGGTTCGGGATAACCGATGGAAACGTCGGCTAATACCGGATGCGTCC
TAAGGGAGAAAGATTTATCGCCCTGGGAGGGGCCCGCGTCGGATTAGCTAGTTGGTGGGGTAAAGGCCTA
CCAAGGCGATGATCCGTAGCTGGTCTGAGAGGATGATCAGCCACACTGGGACTGAGACACGGCC

>Zotu7064
GAACGCTGGCGGCGTGCCTAATACATGCAAGTCGAGCGATCCTTTTATGATTCACTTTGGTTTTTTCAATGT
CTCTCGGTTTTTCTTTAAGAAAATCAGCTTTGCTGAACCGAGCGATGAGAAAAAACCTTAAGATTTGTTTCG
TAATTGGATAGCGGCGGACGGGTGAGTAACACGTGGGTACTTGCCTCGAAGTGGAGGATAGCTCCGGGA
AACCGGAATTAATACTCCATGGTCTCTTTGCGCAAGCATTGAGTAAAGATTTATCGCTTCGAGATGGTCCCG
CGGCCGATTAGCTAGTTGGTGAGGTAATGGCTTACCAAGGCAATGATCGGTAGCTGGTCTGAGAGGACGA
TCAGCCACACTGGGACTGAGATACGGCC

>Zotu7066
GAACGCTGGCGGCATGCTTTACACATGCAAGTCGAACGGCAGCACGGGCTTCGGCCTGGTGGCGAGTGGC
GAACGGGTGAGTAATACATCGGAACGTACCCAGAAGTGGGGGATAACGCAGCGAAAGTTGCGCTAATACC
GCATACGTTCTACGGAAGAAAGTGGGGGATCTTCGGACCTCATGCTTTTGGAGCGGCCGATGTCTGATTAG
CTAGTTGGTGGGGTAAAGGCTCACCAAGGCGACGATCAGTAGCTGGTCTGAGAGGACGACCAGCCACACT
GGAACTGAGACACGGTC

>Zotu7068
GAACGCTGGCGGCGTGCCTAACACATGCAAGTCGAACGTGAACGGGAGCAATCCTAAGTAA AGTGGCGTA
CGGGTGAGTAACACGTAGGTAATCTACCTTACAGTTCGGAATAACCGGCCGAAAGGCTGGCTAATACTGG
ATAGAGCGACGGAAATCTTAATGATTTTTTCGGGAAAGGGAGCCTGATAAGGGCCCTCGCTGTAAGATGA
GCCTGCGTCCCATTAGCTAGTTGGTAGGGTAACGGCCTACCAAGGCAGTGATGGGTAGCTGGTCTGAGAG
GATGATCAGCCACACTGGAACTGAAACACGGTC

>Zotu7075
GAACGCTGGCGGCGTGCCTAATACATGCAAGTCGAGCGGAGTTGAATAAGTGCTTGCACTAATTCAACTTA
GCGGCGAACGGGTGAGTAACACGTAAAGAACCTGCCTCATAGACTGGGACAACTACTGGAAACGGTAGCT
AATACCGGATAACATATTTTTCTGCATGGAAGAATATTAAAAGTCGGTCATGCTGACACTATGAGATGGCTT
TGCGGTGCATTAGCTAGTTGGTAGGGTAAAGGCCTACCAAGGCGACGATGCATAGCCGACCTGAGAGGGT
GATCGGCCACACTGGGACTGAGACACGGCC

>Zotu7079
GAACGCTGGCGGCGTGCTTAACACATGCAAGTCGAGCGGTAAGGCCCTTCGGGGTACACGAGCGGCGAA
CGGGTGAGTAACACGTGAGCAATCTGCCCTTCACTTTGGGATAACTCTCGGAAACGGGAGCTAATACCGGA
TATGACCACTTCGCGCATGTGATGGTGGTGGAAAGTTCTGGCGGTGGAGGATGAGCTCGCGGCCTATCAG
CTTGTTGGTGGGGTAATGGCCTACCAAGGCTTCGACGGGTAGCCGGCCTGAGAGGGTGACCGGCCACACT
GGGACTGAGACACGGCC

>Zotu7081
GAACGCTGGCGGCAGGCTTAACACATGCAAGTCGAACGCATCCTTCGGGGTGAGTGGCAGACGGGTGAG
TAACACGTGGGAACGTACCCTTCGATCTGGGATAACTTCGGGAAACTGGAGCTAATACCGGATACGCCCTT
TTGGGGAAAGGTTTACTGTCGAAGGATCGGCCCGCGTCTGATTAGCTAGTTGGTGGGGTAACGGCCTACC
AAGGCGACGATCAGTAGCTGGTCTGAGAGGATGATCAGCCACACTGGGACTGAGACACGGCC
>Zotu7083
GAACGCTGGCGGCAGGCTTAACACATGCAAGTCGAACGCACCTTTCGGGGTGAGTGGCAGACGGGTGAG
TAACGCGTGGGAACGTGCCCTTCAGTTCGGGATAACCCAGGGAAACTTGGGCTAATACCGGATACGTGCG
AGAGCAGAAAGATTGATCGCTGAAGGATCGGCCCGCGTCTGATTAGCTGGTTGGTGGGGTAATGGCCCAC
CAAGGCGACGATCAGTAGCTGGTCTGAGAGGATGATCAGCCACATTGGGACTGAGACACGGCC
>Zotu7085
GAACGCTGGCGGCGTGCCTAATACATGCAAGTCGAACGGTGGCAGCAATGTCATAGTGGCGACCGGGTGC
GTAACACGTAGCTGACCTGCCCTTCAGCGGGGGACAACAGTTCGAAAGGACTGCTAATACCGCATGAACTT
GGGATGACTAGAATGTTCCAAGGAAACTTCTGGGGCTGAAGGAGGGGGCTGCGGCCCATCAGGTAGTTG
GTGAGGTAACGGCTCACCAAGCCAACGACGGGTAGGGGGCCTGAGAGGGTGGTCCCCCACACTGGGACT
GACAAACGGCC
>Zotu7087
GAACGCTAGCGGCAGGCCTAATACATGCAAGTCGAACGGGCTGGAGTAGCAATACTCCAGTTAGTGGCGC
ACGGGTGCGTAACGCGTATGCAACCTACCTTTTATTGGGGGATAGCCCGAAGAAATTCGGATTAATACCGC
ATAATATCATTTGAGGGCATCTGAGGATGATTAAAGCTACGGCGATAAAAGATGGGCATGCGTGTCATTAG
CTAGTTGGTGGTGGTAACGGCACACCAAGGCGACGATGACTAGGGGTTCTGAGAGGATGATCCCCCACAC
TGGTACTGAGATACGGAC
>Zotu7089
GAACGCTGGCGGCGTGCCTAATACATGCAAGTCGAGCGGTAAGGCTCCTCCAGGTTATTTGATTGTATAAG
CAGCATCTAGGCTAGATGTTGTTGATCGAATAATGTGGTGGAGTACACGAGCGGCGGACGGCTGAGTAAC
GCGTAGGAACATACCCCAAACTGAGGGATAACTGCTCGAAAGAGTAGCTAATACCGCATGTGGTCTTCGG
ATTAAAGCTTCGGCGGTTTGGGAGTGGCCTGCGTATGATTAGATTGTTGGTGAGGTAACGGCTCACCAAGT
CGACGATCATTAGCTGGTCTGAGAGGATGATCAGCCAGACTGGGACTGAGAACGGCC
>Zotu7094
GAACGCTGGCGGCGTGCCTAACACATGCAAGTCGAACGAAGCTTGAATTATGATTTCTTCGGAATGAATAA
TGATATGACTGAGTGGCGGACGGGTGAGTAACGCGTGAGCAACCTGCCCTTCGGAGGGGGATAGTGTCTG
GAAACGGACAGTAATACCGCATAATGTATTTTTACCGCATGATAGGAATACCAAAACTGAGGTGCCGAAGG
ATGGGCTCGCGTTGGATTAGATAGTTGGTGGGGTAACGGCCTACCAAGTCGACGATCCATAGCCGGACTG
AGAGGTTGAACGGCCACATTGGGACTGAGACACGGCC
>Zotu7097
GAACGCTGGCGGCGTGCTTAACACATGCAAGTCGAACGGTGAAAGAGAGCTTGCTCTCTGGATCAGTGGC
GAACGGGTGAGTAACACGTGAGCAATCTGCCCTTGACTCTGGGATAAGCGTTGGAAACGACGTCTAATAC
CGGATAAGAGCTGTGAAGGCATCTTCAACAGCTGGAAAGAACTTCGGTCAAGGATGAGCTCGCGGCCTAT
CAGCTAGTTGGTGAGGTAATGGCTCACCAAGGCGTCGACGGGTAGCCGGCCTGAGAGGGTGACCGGCCA
CACTGGGACTGAGACACGGCC
>Zotu7099
GAACGCTGGCGGCAGGCTTAACACATGCAAGTCGAACGCCCCGCAAGGGGAGTGGCAGACGGGTGAGTA
ACGCGTGGGAATCTACCTTGAACTACGGGATAGCTCCGGGAAACTGGAATTAATACCGTATGTGCCCTTAG
GGGGAAAGATTTATCGGTTCAAGATGAGCCCGCGTTGGATTAGCTAGTTGGTGGGGTAAAGGCCTACCAA
GGCGACGATCCATAGCTGGTCTGAGAGGATGACCAGCCACATTGGGACTGAGACACGGCC
>Zotu7101
GAACGCTGGCGGCATGCCTTACACATGCAAGTCGAACGGCAGCATGGGTGCTTGCACCTGATGGCGAGTG
GCGAACGGGTGAGTAATGCATCGGAACGTACCCGGAAGTGGGGGATAACGTAGCGAAAGTTACGCTAAT
ACCGCATATTCTGTGAGCAGGAAAGAGGGGGATCGCAAGACCTCTCGCTTTCGGAGCGGCCGATGTCGGA

TTAGCTAGTTGGCGGGGTAAAGGCCCACCAAGGCGACGATCCGTAGCGGGTCTGAGAGGATGATCCGCCA
CACTGGGACTGAGACACGGCC
>Zotu7106
GAACGCTGGCGGCGTGCTTAACACATGCAAGTCGAACGGTAAGGCCCTTCGGGGTACACGAGTGGCGAAC
GGGTGAGTAACACGTGAGTGACCTGCCCTCTACTTCGGGATAACTTCCAGAAATGGTGGCTAATACCGGAT
ATTCACATGTTCCTGCATGGGGGTGTGTGGAAAGCCTTGTGCGGTAGGGGATGGACTCGCGGCCTATCAG
CTTGTTGGTGAGGTAGTGGCTCACCAAGGCGTCGACGGGTAGCCGGCCTGAGAGGGTGACCGGCCACATT
GGGACTGAGATACGGCC
>Zotu7108
GAACGCTGGCGGCGTGCCTAATACATGCAAGTCGAGCGGTAAGGCCCCTTCGGGGGTACACGAGCGGCG
GACGGCTGAGTAACACGTAGGAACGTACCCCAAAGTGAGGGATAAGCACCAGAAATGGTGTCTAATACCG
CATGTGCTCTAAGGAGTAAAGCTTCGGCGCTTTGGGAACGGCCTGCGTCCGATTAGCTTGTTGGTGAGGTA
ATAGCTCACCAAGGCGACGATCGGTAGCTGGTCTGAGAGGATGATCAGCCAGACTGGGACTGAGACACG
GCC
>Zotu7111
GAACGCTGGCGGCAGGCCTAACACATGCAAGTCGAACGCTCGTAGCAATACGGGAGTGGCAGACGGTTG
AGTAACGCGTGGGAACCTTCCCAGTGGTACGGAATAGCTCAGGGAAACTTGAGGTAATACCGTATACGCC
CCTAGGGGGAAAGATTTATCGCCATTGGATGGGCCCGCGTCGGATTAGCTAGTTGGTGAGGTAACGGCTC
ACCAAGGCGACGATCCGTAGCTGGTTTGAGAGAATGACCAGCCACACTGGGACTGAGACACGGCC
>Zotu7116
GAACGCTGGCGGCGTGCTTAACACATGCAAGTCGAACGGTGAAGCAGGAGCTTGCTCCTGTGGATCAGTG
GCGAACGGGTGAGTAACACGTGAGTAACCTGCCCCAGACTCTGGGATAAGCGTTGGAAACGACGTCTAAT
ACCGGATACGAGACGAGAAGGCATCTTCATCGTCTGGAAAGAACTTCGGTCTGGGATGGACTCGCGGCCT
ATCAGGTAGTTGGTGAGGTAACGGCCCACCAAGCCTACGACGGGTAGCCGGCCTGAGAGGGTGACCGGC
CACACTGGGACTGAGACACGGCC
>Zotu7119
GAACGCTGGCGGCATGCCTAACACATGCAAGTCGAACGAAGGCTTCGGCCTTAGTGGCGCACGGGTGCGT
AACGCGTGGGAATCTGCCCTTGGGTTCGGAATAACAGTTGGAAACGACTGCTAATACCGGATGATGACGT
AAGTCCAAAGATTTATCGCCCAAGGATGAGCCCGCGTAGGATTAGCTAGTTGGTGGGGTAAAGGCCCACC
AAGGCGACGATCCTTAGCTGGTCTGAGAGGATGATCAGCCACACTGGGACTGAGACACGGCC
>Zotu7122
GAACGCTGGCGGCGTGCTTAACACATGCAAGTCGAACGGAAAGGTCTCTTCGGAGATACTCGAGTGGCGA
ACGGGTGAGTAACACGTGGGTGATCTGCCCTGCACTTCGGGATAAGCTTGGGAAACTGGGTCTAATACCG
GATAGGACTACGGACTGCATGGTCTGTGGTGGAAAGCTTTTGCGGTGTGGGATGGGCCCGCGGCCTATCA
GCTTGTTGGTGGGGTGATGGCCTACCAAGGCGACGACGGGTAGCCGGCCTGAGAGGGTGTCCGGCCACA
CTGGGACTGAGATACGGCC
>Zotu7125
CAACGCTGGCGGCGTGCCTAACACATGCAAGTCGAACGAGAAAGTGGAGCAATCCATGAGTAAAGTGGCG
ACCGGGTGAGTAACACGTGACTAACCTACCCTACAGTGGGGGATAACCTCGGGAAACCGGGGCTAATACC
GCATAACATCGCAAGATCAAAGAAGCAATTCGCTAAAGGAGGGGGTCGCGGCTGATTAGTTAGTTGGTGA
GGTAACGGCCCACCAAGACGAAGATCAGTAGCCGGCCTGAGAGGGCGCACGGCCACACTGGAACTGAAA
CACGGTC
>Zotu7128
GAACGCTGGCGGCATGCCTAACACATGCAAGTCGAACGATCCCTTCGGGGATAGTGGCGCACGGGTGCGT
AACGCGTGGGAATCTGCCCTTGGGTTCGGAATAACAGTGAGAAATTACTGCTAATACCGGATGATGTCTTC
GGACCAAAGATTTATCGCCCAGGGATGAGCCCGCGTAGGATTAGCTAGTTGGTGGGGTAATGGCCTACCA
AGGCGACGATCCTTAGCTGGTCTGAGAGGATGATCAGCCACACTGGGACTGAGACACGGCC
>Zotu7131
GAACGCTGGCGGCGTGCTTAAGACATGCAAGTCGGACGCATCCCCCTCGGGGGATGAGTGGCGCACGGG
TGAGTAACGCGTAACTGACCTACCCTCAAGTCCTGACCAACTCCTCGAAAGAGGAGCTAATGTGGGATGTG

CTGCCTCACCGTGGTGGGGCAGTAAAGGCTATGACCGCTTGAGGATGGGGTTGCGTTTCATCAGCTAGAT
GGTGGGGTAAAGGCCTACCATGGCGACGACGAATAGCCGGCCTGAGAGGGTGGCCGGCCACAGGGGCAC
TGAGACACGGGC
>Zotu7133
GAACGCTGGCGGCGTGCTTAACACATGCAAGTCGAACGGAAAGGCCCTTCGGGGTACTCGAGTGGCGAAC
GGGTGAGTAACACGTGGGTGATCTGCCCTGCACTTTGGGATAAGCCTGGGAAACTGGGTCTAATACCGAA
TACACCTCTCCGGCTGCATGGTTGGTGGGGGAAAGCTTTTGCGGTGTGGGATGGGCCCGCGGCCTATCAG
CTTGTTGGTGAGGTTACGGCTCACCAAGGCGACGACGGGTAGCCGGCCTGAGAGGGTGACCGGCCACACT
GGGACTGAGATACGGCC
>Zotu7135
GAATGCTGGCGGCGCGCCTAACACATGCAAGTCGAGCGGTAACAGGGGTAATTTATTACCTGCTGACGAG
CGGCGGACGGCTGAGTAACGCGTGGGAACGTACCCCAAAGTGAGGAATAACCGCCCGAAAGGGTGGCTA
ATGCCACATGTGATCTTCGGATTAAAGGATTTATCCGCTTTGGGAACGGCCTGCGTATGATTAGATAGTTG
GTGAGGTAAAGGCTCACCAAGTCTACGATCATTAACTGGTCTGAGAGGATGATCAGTCAGACTGGAACTG
AGACACGGTC
>Zotu7138
GAATGCTGGCGGCGTGCCTAATACATGCAAGTCGAGCGGACCTGAATGGAGCTTGCTCCAGACAGGTTAG
CGGCGGACGGGTGAGTAACACGTAGGCAACCTGCCCGCAAGACCGGGATAACGTAGGGAAACTTACGCT
AATACCGGATAGGGCACTCTCTCGCATGAGAGGGTGCGGAAAGGTGGCGCAAGCTACCACTTGCGGATGG
GCCTGCGGCGCATTAGCTAGTTGGTGGGGTAACGGCCTACCAAGGCGACGATGCGTAGCCGACCTGAGAG
GGTGACCGGCCACACTGGGACTGAGACACGGCC
>Zotu7141
GAACGCTGGCGGCGTGCTTAACACATGCAAGTCGAACGAACGGAGGGAGCTTGCTCCCCGAGTTAGTGGC
GGACGGGTGAGTAACACGTGAGCAACCTGCCCTATACACCGGGATAACATAGAGAAATTTGTGCTAATACC
GGATGAGACCACACCGAGGCATCTCGGAGGGGTTAAAGCTCAGGCGGTATAGGAGGGGCTCGCGGCCCA
TTAGATAGTTGGTGAGGTAACGGCCCACCAAGTCAGCGATGGGTAGCCGACCTGAGAGGGTGATCGGCCA
CATTGGAACTGAGATACGGTC
>Zotu7143
GAACGCTGGCGGCAGGCCTAACACATGCAAGTCGAGCGATCGCAGCAATGCGGGAGCGGCAGACGGGTG
AGTAACACGTGGGAACGTACCCTTCGGTTCGGAATAACTCAGGGAAACTTGAGCTAATACCGGATACGTCC
GAGAGGAGAAAGATTTATCGCCGAAGGATCGGCCCGCGTCTGATTAGCTAGTTGGTGAGGTAACGGCTCA
CCAAGGCGACGATCAGTAGCTGGTCTGAGAGGATGATCAGCCACATTGGGACTGAGACACGGCC
>Zotu7145
GAACGCTGGCGGCGTGCTTAACACATGCAAGTCGAGCGGAAAGGCCACTTCGGTGGTACTCGAGCGGCGA
ACGGGTGAGTAACACGTGAGTAATCTGCCCTTGGCTTCGGGATAGCCACCGGAAACGGTGATTAATACCG
GATATGACCACTTCGTGCATGCGATGGTGGTGGAAAGTTTTTCGGCCAGGGATGTGCTCGCGGCCTATCAG
CTTGATGGTGAGGTAATGGCTCACCATGGCTTCGACGGGTAGCCGGCCTGAGAGGGTGACCGGTCACACT
GGGACTGAGACACGGCC
>Zotu7148
GAACGCTGGCGGTATGCTTTACACATGCAAGTCGTACGGGAGTTTTTAACTCAAGTGGCGGACGGGTGAG
TAACACGTAAGAATTTGCCTTTAGGAGGGGGATAACAACTGGAAACGGTTGCTAATACCCCATATGCTTTT
GAGTGAAATGGAATTTCCGCCTAAAGAGAAGCTTGCGGCTGATTAGCTTGTTGGTGAGGTAATGGCTTACC
AAGGCGACGATCAGTATCTGGTTTGAGAGGACGATCAGACACACTGGAACTGAGACACGGTC
>Zotu7151
GAACGCTGGCGGCGTGCTTAACACATGCAAGTCGAACGGAAAGGCCCTGCTTGCAGGGTGCTCGAGTGGC
GAACGGGTGAGTAACACGTGGGTGATCTGCCCCTGACTTTGGGATAAGCCTGGGAAACTGGGTCTAATAC
CGGATATGACCAAGGATTGCATGGTTCTTGGTGGAAAGCTTTTGCGGTTGGGGATGGGCCCGCGGCCTAT
CAGCTTGTTGGTGGGGTAATGGCCTACCAAGGCGACGACGGGTAGCCGACCTGAGAGGGTGATCGGCCA
CACTGGGACTGAGACACGGCC
>Zotu7153

GAACGCTGGCGGCAGGCTTAACACATGCAAGTCGAGCGCCCCGCAAGGGGAGCGGCAGACGGGTGAGTA
ACGCGTGGGAACGTACCCTTTACTACGGAATAACTCAGGGAAACTTGTGCTAATACCGTATGTGCCCTTCG
GGGGAAAGATTTATCGGTAAAGGATCGGCCCGCGTTGGATTAGCTAGTTGGTGGGGTAAAGGCCTACCAA
GGCGACGATCCATAGCTGGTCTGAGAGGATGATCAGCCACACTGGGACTGAGACACGGCC

>Zotu7155
GAACGCTGGCGGCGTGCTTAACACATGCAAGTCGAACGAAGTCCATGGAGCTTGCTCCGGAAGACTGAGT
GGCGAACGGGTGCGTAACACGTGAGAAACCTGCCCCGGACTTTGGGATAACAGTTGGAAACGACTGCTAA
TACCGAATACCACCATCTCCTCGCATGGGGAGTTGATGAAAGGTCCGCCGGTCCGGGAGGGTCTCACGGC
CTATCAGCTTGTTGGAGAGGTAACGGCTCACCAAGGCATCGACGGGTAGCTGGTCTGAGAGGATGATCAG
CCACACTGGGACTGAGACACGGCC

>Zotu7158
GAACGCTGGCGGCGTGCTTAACACATGCAAGTCGAACGGTGATCTTGGGAGCTTGCTCCTTAAGTGATCAG
TGGCGAACGGGTGAGTAACACGTGAGTAACCTGCCCCAGACTCTGGAATAACCCCGGGAAACCGGAGCTA
ATACCGGATATGACACCAATCCGCATGGGTATGGTGTGGAAAGTTTTTCGGTCTGGGATGGACTTGCGGCC
TATCAGCTTGTTGGTGAGGTAATGGCTCACCAAGGCGACGACGGGTAGCCGGCCTGAGAGGGCGACCGG
CCACACTGGGACTGAGACACGGCC

>Zotu7162
GAACGCTGGCGGCAGGCTTAACACATGCAAGTTGAACGGGATTTGTGTGGTGCTTGCACCATATAATGAG
AGTAGCGCACTGGTGAGTAACACGTGGGAACGTACCTTTTGGTGGGGGACAACATCTGGAAACGGATGCT
AATACCGCATAAGACCTGAGGGTGAAAGATTTATCGCCGAAAGAACGGCCCGCGGAAGATTAGGCAGTTG
GTGGGGTAAAGGCCTACCAAACCTACGATCTATAGCTGGTCTGAGAGGACGATCAGCCACACTGGAACTG
AGACACGGTC

>Zotu7165
GAACGCTAGCGGCAGGCTTAATACATGCAAGTCGAGGGGCAGCACGGGGGCAACCCTGGTGGCGACCGG
CAAACGGGTGCGGAACACGTACGCAACTTTCCCAAAACTGGTGAATAGCCTTCCGAAAGGGAGATTAATAC
ACCGTAAGATATAGGAGTGGCATCACTTTTATATTATAGCTCCGGCGGTTTTGGATGGGCGTGCGCCTGAT
TAGGTAGTTGGCGGGGTAACGGCCCACCAAGCCTGCGATCAGTAACTGGTGTGAGAGCACGACCAGTCAC
ACGGGCACTGAGACGCGGGC

>Zotu7168
GAACGCTAGCGACAGGCTTAACACATGCAAGTCGAGGGGCAGCATTTTATTAGCTTGCTAATAAAGATGGC
GACCGGCGCACGGGTGAGTAACGCGTATGCAACCTACCTATCAGAGGGGGATAGCCCATTGAAAGATGGA
TTAATACCGCATAAAACAGGGATCCCGCATGGGAATATTTGTTAAAGATTTATTGCTGATAGATGGGCATG
CGTTCCATTAGCTTGTTGGTGAGGTAACGGCTCACCAAGGCATCGATGGATAGGGGTTCTGAGAGGAAGG
TCCCCCACACTGGTACTGAGACACGGAC

>Zotu7171
GAACGCTGGCGGCATGCCTAACACATGCAAGTCGAACGAGACCTTCGGGTCTAGTGGCGCACGGGTGCGT
AACGCGTGGGAATCTGCCCAAAGCTACGGAATAACTCCTCGAAAGGGGTGCTAATACCGTATGATGTCGC
AAGACCAAAGATTTATCGGCTTTGGATGAGCCCGCGTAGGATTAGCTTGTTGGTGAGGTAAAGGCTCACCA
AGGCGACGATCCTTAGCTGGTCTGAGAGGATGATCAGCCACACTGGGACTGAGACACGGCC

>Zotu7173
GAACGCTGGCGGCGTGCCTAATACATGCAAGTCGAACGCTTCCTATCGGTGCTTGCACCGAGAAAGAAGA
GTGGCGGACGGGTGAGTAACACGTGGGTAACCTACCTATAAGCGGGGGATAACATTCGGAAACGGATGC
TAATACCGCATAGATCTTGATGCCTCCTGGCATCGGGAAGAAAGACGGCTTCGGCTGTCACTTGTAGATGG
ACCCGCGGCGCATTAGTTAGTTGGTGAGGTAATGGCTCACCAAGACGATGATGCGTAGCCGACCTGAGAG
GGTGATCGGCCACACTGGGACTGAGACACGGCC

>Zotu7175
GAACGCTGGCGGCGTGCCTAACACATGCAAGTCGAACGTGAAAGGGAGCAATCCTGAGTAAAGTGGCGT
ACGGGTGAGTAACGCGTAGGTAATCTACCCCAAAGTTTGGAATAACCTGCCGAAAGGCGGGCTAATACTG
GATAGAGCGACGGAATCCGTAATTGGGTTTTTCGGGAAAGGGAGGAAGCAATTCCCTTCGCTTTGGGATG
AGCCTGCGTCTCATTAGCTAGTTGGCGGGGTAAAGGCCCACCAAGGCTATGATGGGTAGCTGGTCTGAGA
GGATGATCAGCCACACTGGAACTGAAACACGGTC

>Zotu7181
GAACGCTGGCGGCATGCCTTACACATGCAAGTCGAACGGCAGCACGGGAGCAATCCTGGTGGCGAGTGG
CGAACGGGTGAGTAAAGTATCGGAACGTGCCCAGTCGTGGGGGATAACGTAGTGAAAATTACGCTAATAC
CGCATACGATCTATGGATGAAAGCGGGGGATCGCAAGACCTCGCGCGATTGGAGCGGCCGATATCAGATT
AGGTAGTTGGTGGGGTAAAGGCTCACCAAGCCAACGATCTGTAGCTGGTCTGAGAGGACGACCAGCCACA
CTGGAACTGAGACACGGTC
>Zotu7183
GAACGCTGGCGGCAGGCCTAACACATGCAAGTCGAGCGGATGAAGTAAGCTTGCTTACGGATTCAGCGGC
GGACGGGTGAGTAATGCCTAGGAATCTGCCTGGTAGTGGGGGATAACGTTTCGAAAGGAGCGCTAATACC
GCATACGTCCTACGGGAGAAAGCAGGGGACCTTCGGGCCTTGCGCTATCAGATGAGCCTAGGTCGGATTA
GCTAGTTGGTGAGGTAATGGCTCACCAAGGCTACGATCCGTAACTGGTCTGAGAGGATGATCAGTCACACT
GGAACTGAGACACGGTC
>Zotu7185
GAACGCTGGCGGCGTGCTTAACACATGCAAGTCGAACGGGTTTTGACAGAGTATCGTCAAAATTAGTGGC
AGACGGGTGAGTATCACATGGGTCATTTACCCTCGAGTGGAAAATAACTCCGAGAAATCGGAGCTAATGTT
CCATAAGACCACGGCCTTTCCTACGAAAGGCTGGGGTCAAAGCTCCGCAAGGGGCGCTTGAGGATGAGCC
TGTGTCCTATCAGCTAGTTGGTGAGGTAACGGCCCACCAAGGCTAAGACGGGTAGCCGGCCTGAAAGGGC
GATCGGCCACACTGGAACTGAGACACGGTC
>Zotu7188
GAACGCTGGCTGCGTGCTTAACACATGCAAGTCGAACGCTGAAGCCTGGTGCTTGCACCGGGTGGATGAG
TGGCGAACGGGTGAGTAACACGTGAGTAACCTGCCCCCGATTTCGGGATAAGCCCGGGAAACTGGGTCTA
ATACCGGATATGACCAGAGGACGCATGTTCTGTGGTGGAAAGTTTTTCGATCGGGGATGGGCTCGCGGCC
TATCAGCTTGTTGGTGGGGTAATGGCCTACCAAGGCGACGACGGGTAGCCGGCCTGAGAGGGCGACCGG
CCACACTGGGACTGAGACACGGCC
>Zotu7191
GAACGCTGGCGGCAGGCTTAACACATGCAAGTCGAACGCATCCTTCGGGGTGAGTGGCAGACGGGTGAG
TAACACGTGGGAACGTACCCTTTGGTTCGGAATAACCCTGGGAAACTAGGGCTAATACCGGATACGGCCGT
GAGGCGAAAGGTTTACTGCCGAAGGAACGGCCCGCGTCCGATTAGCTAGTTGGTGTGGTAATGGCGCACC
AAGGCGACGATCGGTAGCTGGTCTGAGAGGATGACCAGCCACATTGGGACTGAGACACGGCC
>Zotu7194
GAACGCTAGCGGCAGGCTTAATACATGCAAGTCGAGGGGTAGCATGGGTAGCAATACCTGATGACGACCG
GCAAACGGGTGCGGAACACGTACGCAACCTTCCCATAACTGGTGAATAGCCTTCCGAAAGGGAGATTAAT
ACACCGTAACATAACGAAGCGGCATCGCTTTGTTATTATAGGCTTCGGCCGGTTTTGGATGGGCGTGCGCC
TGATTAGGTAGTTGGCGGGGTAACGGCCCACCAAGCCTGCGATCAGTAACTGGTGTGAGAGCACGACCAG
TCACACGGGCACTGAGACACGGGC
>Zotu7196
GAACGCTGGCGGCGTGCCTAATACATGCAAGTCGAGCGGATCTTCAGGAGCTTGCTCCTGAAGATTAGCG
GCGGACGGGTGAGTAACACGTGGGCAACCTGCCTGTAAGACTGGGATAACTTCGGGAAACCGAGGCTAAT
ACCGGATAATCCTTTTCCTCACATGAGGGAAAGCTGAAAGACGGCGCAAGCTGTCACTTACAGATGGGCCC
GCGGCGCATTAGCTAGTTGGTGAGGTAACGGCTCACCAAGGCGACGATGCGTAGCCGACCTGAGAGGGT
GATCGGCCACACTGGGACTGAGACACGGCC
>Zotu7200
AAACGTTGGCGGCGTGCTTAACACATGCAAGTCGCGCGAGAAAGCTTCTTCGGAAGCGTGTAAAGCGGCG
GACGGGTGCGTAACACGTGAGAATTTCCCCTTCAGTGAGGAATACCCACGAGAAATTGTGGTTAATACTGC
ATACGTTCCTACGGGAAGAAAGGTGGCTTTATGCTGCCGCTGAAGGATAAGCTTGCGGGCTATTAGCTAGT
TGGTAGGGTAAAGGCCTACCAAGGCGATGATGGCTAGCCGGCCTGAGAGGGTGTACGGCCACATTGGGA
CTGAGATACGGCC
>Zotu7207
GAACGCTGGCGGCGTGCTTAACACATGCAAGTCGAGCGGTAAGGCCTCTTCGGGGGTACACGAGCGGCG
AACGGGTGAGTAACACGTGGGTGACCTGCCCTCAGCTCTGGGATAAGCCTGGGAAACTGGGTCTAATACC

GGATATGACCTGGCCTCGCATGGGGTTGGGTGGAAAGTTTTTCGGCTGGGGATGGGCCCGCGGCCTATCA
GCTTGTTGGTGGGGTGATGGCCTACCAAGGCGACGACGGGTAGCCGGCCTGAGAGGGCGACCGGCCACA
CTGGGACTGAGATACGGCC
>Zotu7210
GAACGCTGGCGGCAGGCCTAACACATGCAAGTCGAACGCCCTAGCAATAGGGAGTGGCAGACGGGTGAG
TAACGCGTGGGAACCTCTCCATTGGTACGGAATAGCCCAGGGAAACTTGGAGTAATACCGTATAAGCCCTT
CGGGGGAAAGATTTATCGCCATTGGGTGGGCCCGCGTCGGATTAGCTAGTTGGTGAGGTAACGGCTCACC
AAGGCTACGATCCGTAGCTGGTTTGAGAGAATGACCAGCCACACTGGGACTGAGACACGGCC
>Zotu7214
GAACGCTAGCGGCAGGCCTAATACATGCAAGTCGGACGGGATTGGAGAGCTTGCTTTCCATGAGAGTGGC
GCACGGGTGCGTAACACGTATGTAACCTACCTTCGTCAGGGGGATAGCCTCTCGAAAGAGAGATTAAGAC
CGCATAACATTATGGAATAGCATTATTTTATAATCAAATATTTATAGGACGAAGATGGGCATGCGGAACATT
AGCTAGTTGGTAAGGTAACGGCTTACCAAGGCTACGATGTTTAGGGGATCTGAGAGGATGACCCCCCACA
CTGGTACTGAGACACGGAC
>Zotu7217
GAACGCTGGCGGCATGCTTTACACATGCAAGTCGAACGGCAGCACGGGCTTCGGTCTGGTGGCGAGTGGC
GAACGGGTGAGTAATATATCGGAACGTACCCAGAAGTGGGGGATAACGTAGCGAAAGTTACGCTAATACC
GCATACGATCTACGGATGAAAGTGGGGGACCTTCGGGCCTCATGCTTTTGGAGCGGCCGATATCTGATTAG
CTAGTTGGTAGGGTAAAGGCCTACCAAGGCGACGATCAGTAGCTGGTCTGAGAGGACGACCAGCCACACT
GGAACTGAGACACGGTC
>Zotu7221
GTTGCAACTTCACGCGTGGAATTTGTGATGAGGATGCCGTGGAACCAGGTTTCGGAGCATCTTGAAGAGCC
CCCTATGGTGGGAATCAATTGAAGCTGTACGCTTCGCAAAGTTTAGTTGAACTATGATAATTTAGTTGGGGT
GAAAAAAAGAACATTTGATGGCGTGTATGACATTTTTGCATTGAATGTGCACGGTGGAGAGGTTCGTCATC
CACCGGAATATAAAAATATCGAGGCCTTAATTAAAAGCTAGGTAGGGTAAAGGTAACAAAACGTAAATGA
CAACTTAAGCAGCTTGACTGATTCGCTTTATTCTAGCAGCGTTAAAACGTCGCTACTATAGACGTTATAGAT
GATATACGATCAGTGTTTACCTATGCTTCCTTGAATGGCACCGAACGGCACCGGGCAAATGTAGCGCAGAA
AGG
>Zotu7223
GAACGCTGGCGGCGTGCTTAAGACATGCAAGTCGTACGGATACCTTCGGGTATTAGTGGCGCACGGGTGA
GTAACGCGTAACTGACCTGCCCCCAAGTCGCGGATAACGTCTCGAAAGAGACGCTAATACGTGATGTGCA
GTACCGCTGTGGCGGTACTGTAAAGACTGGATCGCTTGGGGATGGGGTTGCGTTTCATCAGCTAGTTGGT
GGGGTAAAGGCCTACCAAGGCGACGACGAATAGCCGGCCTGAGAGGGTGGCCGGCCACAGGGGCACTG
AGACACGGGC
>Zotu7225
GAACGCTGGCGGCATGCCTAACACATGCAAGTCGAACGAAGGCTTCGGCCTTAGTGGCGCACGGGTGCGT
AACGCGTGGGAATCTGCCCTTGGGTTCGGAATAACAGTGAGAAATTACTGCTAATACCGGATGATGACTTC
GGTCCAAAGATTTATCGCCCAAGGATGAGCCCGCGTAAGATTAGCTAGTTGGTGAGGTAAAGGCTCACCA
AGGCGACGATCTTTAGCTGGTCTGAGAGGATGATCAGCCACACTGGGACTGAGACACGGCC
>Zotu7227
GAACGCTGGCGGCGCGCCTAATACATGCAAGTCGAGCGAGAAGGTGTAGCAATACACTTGTAAAGCGGCG
AACGGGTGAGGAACACATGGGTAACCTACCATCGAGTGGGGAATAACCAGCCGAAAGGTTGGCTAATACC
GCGTACGCCTGCGGGTCTCCGGATTCGCAGGGAAAGCCACACTGAGAGTGTGGCGCTCATTGATGGGCTC
ATGTCCTATCAGCTTGTTGGTAGGGTAACGGCCTACCAAGGCTTTGACGGGTAGCTGGTCTGAGAGGACG
ATCAGCCACACTGGCACTGCGACACGGGC
>Zotu7229
CAACGCTGGCGGCGTGCCTAACACATGCAAGTCGAACGCGAAAGTCCCGCAAGGGATTAGTAGAGTGGCA
AACGGGTGAGTAACACGTGGGTGACCTGCCTTCGAGCGGGGGATAACGTCCAGAAAGGGACGCTAATAC
CGCATAACATCCTGCCTTTTAAGAGGTGGAGATCAAAGCCGGGGATCGCAAGACCTGGCACTTGAAGAGG
GGCCCGCGCCTGATTAGCTAGTTGGTGAGGTAATGGCTCACCAAGGCAACGATCAGTATCCGGCCTGAGA
GGGCGGACGGACACACTGGGACTGAGACACGGCC

>Zotu7234
GAACGCTAGCGGCAGGCCTAACACATGCAAGTCGAGGGAGGAGCTTTCGGGCTTCGACCGGCGTACGGG
TGCGTAACGCGTATACAATCTGCCTTTCACATAGGGATAGCCCGAAGAAATTTGGATTAATACCTAATGGTA
TATAGGAGTGGCATCACATTTATATTAAAGCTGAGGCGGTGAAAGATGAGTATGCGTTCTATTAGCTAGTT
GGAGAGGTAACGGCTCCCCAAGGCTACGATAGATAGGGGTTCTGAGAGGGATATCCCCCACACTGGTACT
GAGATACGGAC
>Zotu7238
GAACGCTAGCGATAGGCTTAACACATGCAAGTCGAGGGGCAGCAGGGACTTCGGTCCGCTGGCGACCGG
CGCACGGGTGCGTAACGCGTATGCAACTTGCCCTGTAGAGGAGGATAACCCGCTGAAAGGCGGACTAATA
CTGCGTACTCTCATCGGACGGCATCGTTTGATGAGGAAAGAAATTCGCTACAGGATAGGCATGCGTCCCAT
TAGTTAGTTGGTGAGGTAACGGCTCACCAAGGCGACGATGGGTAGGGGAACTGAGAGGTTTATCCCCCAC
ACTGGTACTGAGACACGGAC
>Zotu7241
GAACGCTGGCGGCATGCCTAAGACATGCAAGTCGAACGAGAAGGCCCAAAGAAGATGGAGTGCTTGCAC
AAAGTTGGAAATGGATCACCTTCTAGTGGCAAACGGGTGAGTAACACGTGGGTTACCTACCTCTAAGACG
GGGATAACAGTTAGAAATGATTGCTAATACCGGATGTGCTCTACGGAGTAAAGGAGCCTTTAAAGCTTCGC
TTAGAGATGGGCCTGCGGTGCATTAGCTAGTTGGTGGGGTAACGGCCTACCAAGGCGACGATGCATAGCC
GAACTGAGAGGTTAATCGGCCACACTGGGACTGAGACACGGCC
>Zotu7245
GAACGCTGATGGCGTGCCTCATACATGCAAGTCGAGCGGAATAGCAATATTCAGCGGCGGACGGGTGAGT
AACGCGTAAACAATCTGCCCTAAAGTGGGGAATAACTATCCCAACGGATAGCTAATACCGCATAATGATGA
AGAGGACATCCTCTTTTCTCAAAAACCTTCGGGTGCTTTAGGATGAGTTTGCGTCCCATTAGCTTGTTGGCG
GTGTAACGGACCACCAAGGCGACGATGGGTAGCCGGCCTGAGAGGGTGTACGGCCACATTGGGACTGAG
ATACGGCC
>Zotu7248
GAACGCTGGCGGCGTGCCTTAGGCATGCAAGTCGAATGGTTCCGCAGCAATGCGGAGTAATGGCAGACG
GGTGAGGAACGCGTAGGAATCTACCCCTGGGTGGACCATAGCTCATCGAAAGATGGGGTAATAGTCCATG
GTCTCGAAAGAGTAAAGCACATAACTGTGCGCCGAGGGAGGAGCCTGCGTCCTATCAGCTTGTTGGTGGG
GTAATGGCCTACCAAGGCAATGACGGGTAGCTGGACTGAGAGGTCGATCAGCCACAATAGCACTGAGACA
CGGGC
>Zotu7250
GAACGCTGGCGGCGTGCCTAACACATGCAAGTGGAGCGACGAACCGGGGCTTGCCCCGGGGCAGAGCCG
CGAACGGGTGAGTAACACGTGGGTTACCTGCCCCGATGACTGGGACAACCCGTCGAAAGACGGGCTAATA
CCGGATGTGCCCTTTGGGGGAAAGGAAGCTTCGGCCTCCGCATCGGGATGGGCCCGCGGCGCATTAGCTT
GTTGGTGGGGTAATGGCTTACCAAGGCGACGATGCGTAGCTGGTCTGAGAGGACGATCAGCCACACTGGG
ACTGAGACACGGCC
>Zotu7252
GAACGTTGGCGGCATGTCTTACACATGCAAGTCGAACGGCAGCACGGGTGCTTGCATCTGGTGGCGAGTG
GCGAACGGGTGAGTAAAGTATCGGAACGTGCCTGGTAGTGGGGGATAACTAGTCGAAAGACTAGCTAAT
ACCGCATACGATTTAAGGATGAAAGCAGGGGACCTTCGGGCCTTGTGCTATCAGAGCGGCCGATATCTGAT
TAGCTAGTTGGTGGGGTAAGAGCCCACCAAGGCGACGATCAGTAGCTGGTCTGAGAGGACGACCAGCCAC
ACTGGAACTGAGACACGGTC
>Zotu7255
GAACGCTGGCGGCGTGCTTAACACATGCAAGTCGAACGCTGAAGCCCAGCTTGCTGGGTGGATGAGTGGC
GAACGGGTGAGTAACACGTGAGTAACCTGCCCCCTTCTTTGGGATAACGCCCGGAAACGGGTGCTAATACT
GGATATTCACTGATCTTCGCATGGGGGTTGGTGGAAAGGTTTGTTCTGGTGGGGGATGGGCTCGCGGCCT
ATCAGCTTGTTGGTTGGGTGATGGCCTACCAAGGCTTTGACGGGTAGCCGGCCTGAGAGGGTGACCGGTC
ACATTGGGACTGAGATACGGCC
>Zotu7257

GAACGTTAGCGGCGCGCTTAACACATGCAAGTCGAGCGAGAAAGGGCGCAAGCCCCGGTAAAGCGGCGC
ACGGGTGAGTAACACGTAGGTAATCTACCCCCAGGCGGTGGATAACCTTCCGAAAGGAGGGCTAATACAG
CATGGGACCACGGTGCCGAAAGGCATTGAGGTGAAAGACGGCCTCTATTCATAAGCTGTCACCAGGGGAT
GAGCCTGCGGCCCATCAGCTAGTTGGTAGGGTAACGGCCTACCAAGGCGAAGACGGGTAGCTGGTCTGAG
AGGATGATCAGCCACACTGGAACTGAGACACGGTC
>Zotu7260
GAACGCTGGCGGCGTACCTGATGTATGCAAGTCGAGCGGTAAGGCTCCTACAGGCTATTTGATTGTTAAGG
CAACGATGGCTATCGTTTCTAGATCAAATTGCGTGTTGGAGTACACGAGCGGCGGACGGGTGAGTAACGC
GTAGGAACGTACCCCAAAGTGAGGGATAAGCTCCGGAAACGGAGTCTAATACCGCATGTGATCTTCGGAT
TAAAGTATTTATACGCTTTAGGAGCGGCCTGCGCCCGATTAGCTTGTTGGCGGAGTAAAAGCCCACCAAGG
CGATGATCGGTAGCTGGTCTGAGAGGACGATCAGCCACACTGGGACTGAGACACGGCC
>Zotu7262
GAACGCTGGCGGCGTGCTTAACACATGCAAGTCGAACGGTGAAAGAGGGAGCTTGCTTCCTCTGGATCAG
TGGCGAACGGGTGAGTAACACGTGAGTAACCTGCCCTTGACTCTGGGATAAGCGTTGGAAACGACGTCTA
ATACCGGATACGAGCTTCAGCCGCATGGCTAGGAGCTGGAAAGAACTTCGGTCAAGGATGGACTCGCGGC
CTATCAGCTAGTTGGTGAGGTAACGGCCCACCAAGGCGACGACGGGTAGCCGGCCTGAGAGGGTGACCG
GCCACACTGGGACTGAGACACGGCC
>Zotu7265
GAACGCTGGCGGCGTGCCTTAAGCATGCAAGTAGAACGAGTTCTATCTTGCCGCAAGGCAAGCTTGGAAC
TAGTTGCGAACGGCTGAGTAATCAATAGGAATCTTTCCTTTGGTGGGGAATACCTCGTCGAAAGACGAGCT
AATACCGCATAATATCTACGGATGAAAGCGCATAACTGCGCGCCGATTGAGGAGCCTATTGCCCATCAGGC
AGTTGGTAGGGTAATGGCCTACCAAACCTACGACGGGTACCCGATCTGAGAGGATGACCGGGCAGAATGG
GACTGAGACACGGCC
>Zotu7267
GAACGCTGGCTGTATGGATAAGGCATGCAAGTCGAACGGCCCGCAAGGGCAGTGGCAGACGAGGTAGTA
ACACGTACGAACGTACTCCCAAGTCAGGAATAGCTCACCGAAAGGTGGGGTAATACCTGATGGTCCCTACG
GGGTAAAGATTTATCGCTTGGGAAGCGGCGTGCGTAGTATCAGCTTGTTGGTAAGGTAATGGCTTACCAA
GGCTACGACGCTTAGGGGAGCTGAGAGGCTGACCCCCACCGATGGGACTGAGACACGACC
>Zotu7270
GAACGCTAGCGGTATGCTTAACACATGCAAGTCGTACGGAGAAGCAATTCTTAGTGGCGAACGGGTGAGT
AACGCGTAAGTATTTGCCTTTAGGAAGAGGATAACATTTAGAAATAACTGCTAAAACTCTATATGCCGTTTG
GTGAAAAAAGCAATTTGCCTAAAGAGAAGCTTGCGTCTGATTAGCTAGTTGGTGGGGTAATGGCCTACCAA
GGCGACGATCAGTAGCTGGTCTGAGAGGACGATCAGCCACACTGGAACTGAGACACGGTC
>Zotu7273
GAACGCTGGCGGCAGGCTTAACACATGCAAGTCGAGCGGGCATCCTTGTGGTGTCAGCGGCAGACGGGT
GAGTAACGCGTGGGAACGTGCCCTTCGGTTCGGAATAACTCAGGGAAACTTGAGCTAATACCGGATACGT
GCGAGAGCAGAAAGGTTTATCTGCCGAAGGATCGGCCCGCGTCTGATTAGCTAGTTGGTGAGGTTATGGC
TCACCAAGGCGACGATCAGTAGCTGGTCTGAGAGGATGATCAGCCACACTGGGACTGAGACACGGCC
>Zotu7278
GAACGCTGGCGGCGTGCCTAACACATGCAAGTCGAACGGAGATCTTATGGAATGCGGCTTCGGCCAAATG
AAGTTTGATCTTAGTGGCGAACGGGTGAGTAACGCGTGAGTAACCTGCCCTTCAGAGGGGGACAACAGTT
GGAAACGACTGCTAATACCGCATAATGTATTTTTAAGGCATCTTAGAAATACCAAAGGAGCAATCCGCTGA
AGGATGGACTCGCGTCCGATTAGATAGTTGGTGAGGTAACGGCCCACCAAGTCGACGATCGGTAGCCGGA
CTGAGAGGTTGATCGGCCACATTGGGACTGAGACACGGCC
>Zotu7280
GAACGCTGGCGGCATGCCTAACACATGCAAGTCGAACGCTCTTGGGGCTTCGGTTTCAAGGGAGTGGCGC
ACGGGTGAGTAGCGCGTGGGAACCTGCCCTGTTGTGGGGGATAACCGAGGGAAACTTCGGCTAATACCGC
ATACGCCGGGAAACCGGGAAAGATTTATCGCAGCAGGAGGGGCCCGCGTCGGATTAGGTAGTTGGTGGG
GTAACGGCCCACCAAGCCTTTGATCCGTAGCTGGTCTGAGAGGATGATCAGCCACACTGGGACTGAGACA
CGGCC

>Zotu7282
GAACGCTGGCGGCAGGCCTCAAACATGCAAGTCTAGGGGGTGTAGCAATACATCACCGGCGGACGGGTG
CGTAACGCGTGGGAATATACCTATTGGTTCGGAACAACTGAGGGAAACTTCAGCTAATACCGAATGTGCCC
GTAAGGGGAAAGATTTATCGCCAATGGATTAGCCCGCGTAGGATTAGCTAGTTGGTGGGGTAATGGCCCA
CCAAGGCTCCGATCCTTAGCTGTTCTGAGAGGAAGATCAGCCACACTGGGACTGAGACACGGCC
>Zotu7286
GAACGCTGGCGGCGTGCTTAACACATGCAAGTCGAACGGAAAGGCCCTTTCGGGGGTACTCGAGTGGCGA
ACGGGTGAGTAACACGTGGGTGATCTGCCCTGCACTTCGGGATAAGCCTGGGAAACTGGGTCTAATACCG
GATATACCTCATGGATTGCATGGTTTGTGGGGGAAAGCTTTTGCGGTGTGGGATGGGCCCGCGGCCTATC
AGCTTGTTGGTGGGGTGATGGCCTACCAAGGCGTCGACGGGTAGCCGGCCTGAGAGGGTGTCCGGCCAC
ACTGGGACTGAGATACGGCC
>Zotu7289
GAACGCTGGCGGCAGGCTTAACACATGCAAGTCGAACGCATCGCAAGATGAGTGGCAGACGGGTGAGTA
ACGCGTGGGAATCTACCCAACCCTACGGAACAACTCCGGGAAACTGGAGCTAATACCGTATACGCCCTTTG
GGGGAAAGATTTATCGGGGATGGATGAGCCCGCGTTGGATTAGCTAGTTGGTGGGGTAAAGGCCTACCAA
GGCGACGATCCATAGCTGGTCTGAGAGGATGATCAGCCACATTGGGACTGAGACACGGCC
>Zotu7291
AATTTTATCGCTAAAAAATAATAAGAATAAACGCAACACGTTCTCACGTGATGTGCGTGTTGTCGCATTTGG
CGTATCTCTTTGTTCCACTTTTTACACCGTAATTGCATTATTATTACGACGGGGCAGAGCGCGGAAAAAGAG
GCAACACTCACCCCCTTCCATGGACTGAAACACGTGTATTTTTTATATTAAGAGCGT
>Zotu7295
GAACGCTGGCGGCGTGCTTAACACATGCAAGTCGAACGGTAAGGCCCTTCGGGGTACACGAGTGGCGAAC
GGGTGAGTAACACGTGGGTGATCTGCCCTGCACTTCGGGATAAGCCCGGGAAACTGGGTCTAATACCGGA
TAGGACCACAGCATGCATGTGTTGTGGTGGAAAGATTTATCGGTGCAGGATGGGCCCGCGGCCTATCAGC
TTGTTGGTGGGGTAATGGCCTACCAAGGCGACGACGGGTAGCCGGCCTGAGAGGGCGACCGGCCACACT
GGGACTGAGACACGGCC
>Zotu7297
GAACGCTGGTAACGTGGATACCGGCATGCAAGTCACGGGGGCCGCAAGGCAACCGGCGAACGGCTTAGT
AACACGTGGATACATACCCCAAAGTGGAGTACAGCTCGTCGAAAGACGGGGTAATCCTCCATAGTCTCCTC
TTTCGCTTTTTGTTTCACAAAAGCTTCAGAAGGACAAAGTCGCAAGGCAAATACGCTTTGCAGAGCAAGAC
TTGTCCATCCGAAGCTCTGGTGGAACGCAGAGCGAAGGAGGAGTAAAGGCGCAAGCCGCTTTGGGAATG
GTCTGCGGTCTATCAGCTAGTTGGTGAGGTAATGGCTCACCAAGGCTATGACGGATAGGGGGCGTGAGAG
CGCGACCCCCAAGAATGGCACTGCGACACGGGC
>Zotu7299
GAACGCTAGCGGCGTGCTTAACACATGCAAGTCGAACGCTGAAGCCCGCTTCGGTGGGTGGATGAGTGGC
GAACGGGTGAGTAACACGTGGGCAATCTACCCCGCACTCGGGGATAAGCCTTGGAAACGAGGTCTAATAC
CCGATAGGACATGCCATCGCATGGTGGTGTGTGGAAAGTGTGTAGCGGTGTGGGATGAGCCCGCGGCCTA
TCAGCTTGTTGGTGGCGGTGATGGCGCATCAAGGCGACGACGGGTAGCCGGCCTGAGAGGGTGACCGGC
CACACTGGGACTGAGACACGGCC
>Zotu7302
GAACGTTAGCGGCGCGCCTAACACATGCAAGTCGCACGCGAAAGGGGCAACCCAAGTAAAGTGGCGCAC
GGGTGCGTAACACGTAGATAACCTGCCCTTTGGAGGGGGATAACGTTCCGAAAGGAGCGCTAATACCGCA
TACAGCATTCTGGGGTTTGCTCCGGGATGGCAAAGGTAGGGACCCGCAAGGGCCTATCGCCGAAGGAGG
GGTCTGCGGCCCATCAGCTAGTTGGCGGGGTAATGGCCCACCAAGGCAAAGACGGGTAGCTGGTCTGAGA
GGATGATCAGCCACACTGGTACTGGAACACGGAC
>Zotu7304
GAACGCTGGCGGCATGCCTAACACATGCAAGTCGAACGAGACCTTCGGGTCTAGTGGCGCACGGGTGCGT
AACACGTGGGAACCTGCCCTTAGGTTCGGAATAACCAGGAGAAATCCTGGCTAATACCGGATAATGTCTTC
GGACCAAAGATTTATCGCCTTTGGATGGGCCCGCGTAGGATTAGCTAGTTGGTGGGGTAATGGCCTACCAA
GGCGACGATCCTTAGCTGGTCTGAGAGGATGATCAGCCACACTGGGACTGAGACACGGCC

>Zotu7308
GAACGCTGGCGGCGTGCCTAACACATGCAAGTCGAACGGGGTTGAGGTCTTCGGACCTTAACTTAGTGGC
GAACGGGTGAGTAACGCGTGAGAAACCTGCCTTTCAGTGGGGGACAACAGTTGGAAACGACTGCTAATAC
CGCATAATGTTATTTGGGGGCATCCCCGAATAACCAAAGCTTTATGTGCTGAAAGATGGTCTCGCGTCTGAT
TAGCTAGATGGCGGGGTAACGGCCCACCATGGCGACGATCAGTAGCCGGTCTGAGAGGATGAACGGCCA
CATTGGGACTGAGATACGGCC
>Zotu7311
TAACGCTGGCGGCGTGCATGAGACATGCAAGTCGAACGGGTGACTCGGGCTTGCTCGAGTCACTAGTGGC
GAACGGCTGAGTAACACGTAGGTGACCTGCCCCGAAGTGGGGAATAACTGCCCGAAAGGGTGGCTAATAC
CGCATGTGGTCGCACGTTCATTCGTGCGAGTAAAGCCGCAAGGCGCTTCGGGAGGGGCCTGCGTCCGATT
AGCTCGTTGGTGGGGTAATGGCTCACCAAGACGACGATCGGTAGGTGGTCTGAGAGGACGATCACCCAGA
CTGGGACTGAGACACGGCC
>Zotu7313
GAACGCTAGCGGCAGGCCTAATACATGCAAGTCGAACGGTAAGTTGAATAGCAATATTTGACCTAGAGTG
ACGTACGGGTGCGTAACACGTATGCAACCTACCCTTTACAGGAGGATAGCTCGGGGAAACTCGAATTAACC
CTCCATGAGATCATAGAGTAGCATTACTCAGTGATTAAAACTCAGGTGGTAAAGGATGGGCATGCGTCTGA
TTAGTTAGTTGGCGGGGTAACGGCCCACCAAGACGATGATCAGTAGGGGAACTGAGAGGTTGATCCCCCA
CACTGGCACTGAGATACGGGC
>Zotu7317
GAACGCTGGCGGCAGGCCTAACACATGCAAGTCGAGCGGTAACAGGGGAAAGCTTGCTTTCCCGCTGACG
AGCGGCGGACGGGTGAGTAATGTATGGGGATCTGCCCGATAGAGGGGGATAACTACTGGAAACGGTGGC
TAATACCGCATAATCTCTAAGGAGCAAAGCAGGGGAACTTCGGTCCTTGCGCTATCGGATGAACCCATATG
GGATTAGCTAGTAGGTGAGGTAATGGCTCACCTAGGCGACGATCCCTAGCTGGTCTGAGAGGATGATCAG
CCACACTGGGACTGAGACACGGCC
>Zotu7322
CAACGCTGGCGGCGCGCCTAACACATGCAAGTCGAACGAGAAAGTGGAGCAATCCATGAGTACAGTGGCG
TACGGGTGAGTAACACGTGGGTAATCTACCTTTTAGTGAGGAATAACCAAGGGAAACCATGGCTAATACC
GCATAAGCCTGAGAAGGGAAAGCAGCAATGCGCTGAAAGAGGAGCCCGCGGCCGATTAGCTAGTTGGTG
GGGTAAGAGCCCACCAAGGCAGAGATCGGTAGCCGGCCTGAGAGGGCACACGGCCACACTGGCACTGAA
ACACGGGC
>Zotu7324
GAACGCTGGCGGCGTGCTTAACACATGCAAGTCGAGCGAGATTCAACCGGTGGCAACACCGGGGAAGATC
GAGCGGCGAACGGGTGAGTAACACGTGAGCAACCTGCCCCGAAGACTGGGATAACACCGGGAAACCGGT
GCTAATACCGGATACCCCCACCAGGCCGCATGACCAGGTGAGGAAATGGCTGGGTCTAATAAACTCAGTCC
GCTTCGGGAGGGGCTCGCGGGCTATCAGCTTGTTGGTGGGGTAACGGCCTACCAAGGCGACGACGGCTA
GCTGGTCTGAGAGGACGACCAGCCACACTGGGACTGAGACACGGCC
>Zotu7330
GAACGCTGGCGGCATGCCTTACACATGCAAGTCGAACGGATTCAAGGAGCTTGCTCCTTGAGTTAGTGGCG
AACGGGTGAGTAAAGCATCGGAACGTACCCAGTAGTGGGGGATAACGTAGCGAAAGTTACGCTAATACCG
CATACGACCTGAGGGTGAAAGCAGGGGATCGCAAGACCTTGCGCTATTGGAGCGGCCGATGTCGGATTAG
CTAGTTGGTAGGGTAAAAGCCTACCAAGGCGACGATCCGTAGCTGGTCTGAGAGGACGACCAGCCACACT
GGGACTGAGACACGGCC
>Zotu7334
GAACGCTGGCGGCGTGCTTAACACATGCAAGTCGAACGAAGAGCGACGGAAGCTTGCTTCTATCAATCTTA
GTGGCGAACGGGTGAGTAACGCGTAATCAACCTGCCCTTCAGAGGGGGACAACAGTTGGAAACGACTGCT
AATACCGCATACGATCTAATCTCGGCATCGAGGATAGATGAAAGGTGGCCTCTATGTATAAGCTATCACTG
AAGGAGGGGATTGCGTCTGATTAGCTAGTTGGAGGGGTAACGGCCCACCAAGGCGATGATCAGTAGCCG
GTCTGAGAGGATGAACGGCCACATTGGGACTGAGACACGGCC
>Zotu7337

GAACGCTGGCGGCAGGCTTAACACATGCAAGTCGAGCGGGCCCTTCGGGGTCAGCGGCAGACGGGTGAG
TAACACGTGGGAACGTACCCTTCGGTTCGGAATAACCCTGGGAAACTAGGGCTAATACCGGATACGTCCGA
GAGGAGAAAGGTTTACTGCCGAAGGATCGGCCCGCGTCTGATTAGCTAGTTGGTGGGGTAATGGCCTACC
AAGGCGACGATCAGTAGCTGGTCTGAGAGGATGATCAGCCACATTGGGACTGAGACACGGCC

>Zotu7339

GAACGCTGGCGGCATGCCTAACACATGCAAGTCGAACGAAGGCTTCGGCCTTAGTGGCGCACGGGTGCGT
AACGCGTGGGAATCTACCCTTAGGTTCGGAATAACAGTTGGAAACGACTGCTAATACCGGATGATGACGTA
AGTCCAAAGATTTATCGCCTAGGGATGAGCCCGCGTAGGATTAGCTAGTTGGTGTGGTAAAGGCGCACCA
AGGCGACGATCCTTAGCTGGTCTGAGAGGATGATCAGCCACACTGGGACTGAGACACGGCC

>Zotu7344

GAACGCTGGCGGCATGCCTTACACATGCAAGTCGAACGGTAGAGGGGCAACCCTTGAGAGTGGCGAACG
GGTGAGTAATATATCGGAACGTGCCCAGTCGTGGGGGATAACGTAGAGAAATTTACGCTAATACCGCATA
CGATCTAAGGATGAAAGCGGGGGACTCGCAAGAGCCTCGCGCGACTGGAGCGGCTGATATCAGATTAGCT
TGTTGGTGAGGTAAAAGCTCACCAAGGCGACGATCTGTAGCTGGTTTGAGAGAACGACCAGCCACACTGG
GACTGAGACACGGCC

>Zotu7347

GAACGCTGGCGGCGTGCCTAATACATGCAAGTCGAGCGGTAACACTTCGGGTGACGAGCGGCGGACGGG
TGAGTAATGCATGGGAACGTGCCCCAAAGTGAGGAATAAGCACCAGAAATGGTGTCTAATACCGCATATT
ATCTTAGGATCAAAGCTTCGGCGCTTTGGGAGCGGCCCATGTCTGATTAGCTAGTTGGTAGGGTAAAAGCC
TACCAAGGCGACGATCAGTAGCTGGTTTGAGAGGATGATCAGCCACACTGGAACTGAGACACGGTC

>Zotu7351

GAACGCTGGCGGTATGCTTTACACATGCAAGTCGAACGCAAGTTTTTAACTTGAGTGGCGGACGGGTGAG
TAACACGTGAGAATTCGCCTTTAGGCGGGGAATAACTATGGGAAACCATAGCTAATGCCCCATATGCTCGC
AAGAGTGAAAGAAGCAATTCACCCAAAGAGAAGCTCGCGGCTGATTAGCTAGTTGGTAAGGTAATGGCTT
ACCAAGGCGACGATCAGTAGCTGGTTTGAGAGAACGATCAGCCACACTGGAACTGAGACACGGTC

>Zotu7353

GAATGCTGGCGGTATGGATAAGGCATGCAAGTCGAACGGCCCGCAAGGGCAGTGGCAGACGAGGTAGTA
ACACGTAGGAACGTACACCGAAGTCAGGTATAGCTCGTCGAAAGGCGGGGTAATCCCTGATGGTCCCGCG
AGGGTAAAGATTTATCGCTTCGGTAGCGGCCTGCGCAATATCAGCTTGTTGGTGAGGTAACGGCTCACCAA
GGCTACGACGTTTAGGGGCGCTGAGAGGCGGACCCCCACCGATGGGACTGAGATACGGCC

>Zotu7355

GAACGCTGGCGGCAGGCCTAACACATGCAAGTCGAGCGGTAACAGGGGGTGCTTGCACCCCGCTGACGA
GCGGCGGACGGGTGAGTAATGCATAGGAATCTGCCCGATAGTGGGGGATAACCTGGGGAAACCCAGGCT
AATACCGCATACGTCCTACGGGAGAAAGGGGGCTCCGGCTCCCGCTATCGGATGAGCCTATGTCGGATTA
GCTGGTTGGTGAGGTAAAGGCTCACCAAGGCGACGATCCGTAGCTGGTCTGAGAGGATGATCAGCCACAT
CGGGACTGAGACACGGCC

>Zotu7360

GAACGCTGGCGGCGTGCTTAACACATGCAAGTCGAACGATGAAGTTCCTTCGGGGACGGAATAGTGGCGA
ACGGGTGAGGAACACGTGAAGAATCTGCCCTCCACTTTGGGATAACTCCGGGAAACCGGGGCTAATACCG
AATATTGAAACATAAGTGGCATCACTGAGTTTTGAAAGAATTTCGGTGGAGGATGACTTCGCGGCCTATCA
GCTTGTTGGTGAGGTAATGGCTCACCAAGGCGACGACGGGTAGCCGGCCTGAGAGGGCGACCGGCCACA
CTGGGACTGAGACACGGCC

>Zotu7363

GAACGCTGGCGGCGTGCTTAACACATGCAAGTCGAACGGTGATGATGGAGCTTGCTCTGTCGGATCAGTG
GCGAACGGGTGAGTAACACGTGAGTAACCTGCCCTCAACTCTGGGATAACTTCCCGAAAGGGAAGCTAAT
ACTGGATATGAACTGCACGGGCATCTGTGTGGTTGTAAAGATTTTTTGGTTGAGGATGGACTCGCGGCCTA
TCAGCTTGTTGGTGGGGTAATGGTCTACCAAGGCGACGACGGGTAGCCGGCCTGAGAGGGCGACCGGCC
ACACTGGGACTGAGACACGGCC

>Zotu7366

GAACGCTGGCGGCATGCCTTACACATGCAAGTCGAACGGTAACGGGCCTTCGGGTGCCGACGAGTGGCGA
ACGGGTGAGTAATATATCGGAACGTGCCCAGTAGTGGGGGATAGCCCGGCGAAAGCCGGATTAATACCGC
ATACGACCTGAGGGTGAAAGCGGGGGATCGCAAGACCTCGCGCTATTGGAGCGGCCGATATCAGATTAGC
TAGTTGGTGGGGTAAAGGCCTACCAAGGCGACGATCTGTAGCTGGTCTGAGAGGACGACCAGCCACACTG
GGACTGAGACACGGCC
>Zotu7368
GAACGCTGGCGGCGTGCCTAATACATGCAAGTCGAGCGAGCAGAACCAGCAGATTTACTTCGGTAATGAC
GCTGGGGACGCGAGCGGCGGATGGGTGAGTAACACGTGGGGAACCTGCCCCATAGTCTGGGATACCACT
TGGAAACAGGTGCTAATACCGGATAAGAAAGCAGATCGTATGATCAGCTTATAAAAGGCGGCGTAAGCTG
TCGCTATGGGATGGCCCCGCGGTGCATTAGCTAGTTGGTAAGGTAACGGCTTACCAAGGCGATGATGCAT
AGCCGAGTTGAGAGACTGATCGGCCACATTGGGACTGAGACACGGCC
>Zotu7371
GAACGCTAGCGAAATGCCTAACACATGCAAGTCGAACGGATCTTGCCTCGTAGTAATACGGAGTAAGATTA
GTGGCAAACGAGCGAGTAACGCGTGCTTAACCTGCCCCGAAGATGGGTATAACTGCAGAGATGTAGCTAA
TTCCCAATATGCTCACGATGGTGAAATTCCAATGTGAGGAAAGATTTATCGCTTCGGGAGGGGGGTGCGTC
CTATCAGGTAGTTGGTGGGGTAATGGCCTACCAAGCCTATGACGGGTAACCGGTCTGAGATGATGTCCGG
TCGCGATGGGACTGAGATACGGCC
>Zotu7373
GAACGCTGACAGAATGCTTAACACATGCAAGTCTACTTGAACTTCGGTTTGGGTGGCGGACGGGTGAGTA
ACGCGTAAAGAACTTGCCTCACAGACTGGGACAACATTTGGAAACGAATGCTAATACTAGATATTATGAGA
TATGCGCATGCATAACTTATGAAAGCTATATGCACTGTGAGAGAGCTTTGCGTCCCATTAGCTAGTTGGAG
AGGTAACGGCTCACCAAGGCGATGATGGGTAGCCGGCCTGAGAGGGTGAACGGCCACAAGGGGACTGAG
ACACGGCC
>Zotu7376
GAACGCTGGCGGCGTGCTTAACACATGCAAGTCGAACGGAAAGGCCCAAGCTTGCTTGGGTACTCGAGTG
GCGAACGGGTGAGTAACACGTGGGTGATCTGCCCTGCACTTCGGGATAAGCCTGGGAAACTGGGTCTAAT
ACTGGATAGGACCACTTCTTGGATGTTGTGGTGGAAAGTTTTTTCGGTGTGGGATGAGCTCGCGGCCTATC
AGCTTGTTGGTGGGGTAATGGCCTACCAAGGCGTCGACGGGTAGCCGGCCTGAGAGGGTGTACGGCCAC
ATTGGGACTGAGATACGGCC
>Zotu7378
GAATGCTGGCGGCGTGGATAAGGCATGCAAGTCGAACGGTCCGCAAGGACAGTGGCAGACGAGGTAGTA
ACACGTACGAACGCACCCCAGAGTCTGGCATAGCTCGTCGAAAGACGGGGTAATTCCGGATGGTCCCGAA
AGGGTAAAGTTTTTTCGCTCTGGGAGCGGCGTGCGTAATATCAGCTAGTTGGTAAGGTAATGGCTTACCAA
GGCTACGACGTTTAGGGGAGCTGAGAGGCTGACCCCCACCGATGGGACTGAGACACGGCC
>Zotu7383
GAACGCTGGCGGTGTGCCTTAAGCATGCAAGTCGAGCGGGTCTAGCAATAGACCAGCGGCAGACGGGTG
AGTAACGCGTAGGAATCTACCTCAAAGTGGGGAATAGCCTTCCGAAAGGGAGGGTAATACCGCATGTGAT
CGCAAGATCAAAGCCGTAAGGCGCTTTGAGAGGAGCCTGCGTCCTATCAGTTAGTTGGTGGGGTAATGGC
CTACCAAGACTATGACGGGTAGCCGATGTGAGAGCATGATCGGCCACAAGGTTACTGAGACACGGAA
>Zotu7386
GAACGCTGGCGGCGTGCTTAACACATGCAAGTCGAACGATGAAGCTGGTGCTTGCACTGGTGGATTAGTG
GCGAACGGGCGAGTAACACGTGAGTAACCTGTCCTCTTCTTTGGGATAACGGCTGGAAACGGCTGCTAATA
CTGGATATTCTGACCTTGTCGCATGGTGGGGTTGGGAAAGCTTTTGCGGTAGAGGGTGGGCTCGCGGCCT
ATCAGCTTGTTGGTGGGGTGATGGCTTACCAAGGCTTTGACGGGTAGCCGGCCTGAGAGGGTGGTCGGTC
GCACTGGGACTGAGATACGGCC
>Zotu7388
GAACGCTGGCGGCATGCCTAACACATGCAAGTCGAACGAGGGCTTCGGCCCTAGTGGCGCACGGGTGAGT
AACGCGTGGGAACCTGCCCTGTGGTACGGAATAACTCCGGGAAACTGGAGCTAATACCGTATGCGTCCTG
CGGGACAAAGATTTATCGCCATGGGATGGGCCCGCGTAGGACTAGCTTGTTGGTGGGGTAACGGCCTACC
AAGGCTTCGATCCTTAGCTGGTCTGAGAGGATGATCAGCCACACTGGGACTGAGACACGGCC

>Zotu7390
GAACGCTAGCGATGTGCTTTAAGCATGCAAGTAGGGCGGGTTGTAGCAATACAACCAGCTGCGAACGGCT
GAGTATAAAATAGGTATCTACCTCATGGTGGTGAATACCCCGTCGAAAGACGGGCTAATACAGCATAAGAT
TCTAAATGAAAGCGTGTAACTACGCGCCGAGAGAGGAGCCTATTTCCCATCAGCTTGTTGGTAGGGTAATG
GCCTACCAAGGCTACGACGGGTAGGGGGTGTGAGAGCATGACCCCCCAGAATGGTACTGAGACACGGAC
>Zotu7395
GAACGCTAGCGGCAGGCCTAACACATGCAAGTCGAGGGGTAAGGAGAGCTTGCTCTCCCTAGACCGGCGC
ACGGGTGCGTAACACGTGTACAATCTACCTTTTGCTAAGGGATAGCCCGAAGAAATTTGGATTAATACCTT
ATAGTATTGTTTGGTGGCATCACTGAATAATTAAAGCTCTGGTGGCAAAAGATGAGTACGCGTCCCATTAG
CTAGTTGGTGTGGTAACGGCATACCAAGGCTACGATGGGTAGGGGTCCTGAGAGGGAGGTCCCCCACACT
GGTACTGAGACACGGAC
>Zotu7398
GAACGCTGGCGGCGTGCTTAAGACATGCAAGTCGAACGAGGGCTTCTTGTAGCAATACGGGGAGTTCCTA
GTGGCGAATGGGTGCGTAACACGTAGGTGACCTGCCCTGAAGAGGAGGATAACAGTCCGAAAGGACTGC
TAATACTCCATGTGGTCCTGGAGGTTAGAGGCTCCAGGTCTAAAGTTGCAAAACGCTTCAGGAGGGGCCTG
CGGCCCATCAGCTTGTTGGTAGGGTAATGGCCTACCAAGGCGACGACGGGTAGGGGACCTGAGAGGGTG
GCCCCCCACATTGGCACTGACACACGGGC
>Zotu7400
GAACGCTAGCTACAGGCTTAACACATGCAAGTCGAGGGGAAACGACATCGAAAGCTTGCTTTTGATGGGC
GTCGACCGGCGCACGGGTGAGTAACGCGTATCCAACCTGCCCACCACTTGGGGATAACCTTGCGAAAGTA
AGACTAATACCCAATGACGTCTCTAGAAGACATCTGAAAGAGATTAAAGATTTATCGGTGATGGATGGGGA
TGCGTCTGATTAGCTTGTTGGCGGGGTAACGGCCCACCAAGGCGACGATCAGTAGGGGTTCTGAGAGGAA
GGTCCCCCACATTGGAACTGAGACACGGTC
>Zotu7403
GAACGCTGGCGGCGTGCTTAACACATGCAAGTCGAGCGGAAAGGCCCTTCGGGGTACTCGAGCGGCGAA
CGGGTGAGTAACACGTGGGCAACCTGCCCTCAGCTCTGGGATAACTCCGGGAAACCGGGGCTAATACCGG
ATATGACTGTCCTGGGCATCTGGGGTGGTGGAAAGTTTCTCGGCTGAGGATGGGCCCGCGGCCTATCAGC
TTGTTGGTGGGGTGATGGCCTACCAAGGCGACGACGGGTAGCCGGCCTGAGAGGGCGACCGGCCACACT
GGGACTGAGACACGGCC
>Zotu7410
GAACGCTAGCGGCAGGCTTAACACATGCAAGTCGAGGGGTATGGTAGCAATACCAGAGACCGGCGCACG
GGTGCGTAACGCGTATGCAATCTACCTTTTACAGAGGGATAGCCCAGAGAAATTTGGATTAATACCTCATA
GTATAGCTGTACCGCATGGTACTACTATTAAAGTTACAACGGTAAAAGATGAGCATGCGTCCCATTAGCTA
GTTGGTAAGGTAACGGCTTACCAAGGCTACGATGGGTAGGGGTCCTGAGAGGGAGATCCCCCACACTGGT
ACTGAGACACGGAC
>Zotu7414
GAACGCTGGCGGCATGCCTAACACATGCAAGTCGAACGAGACCTTCGGGTCTAGTGGCGCACGGGTGCGT
AACGCGTGGGAATCTGCCCTTGGGTTCGGGATAACAGTTGGAAACGACTGCTAATACCGGATGATGACTTC
GGTCCAAAGATTTATCGCCCAAGGATGAGCCCGCGTAGGATTAGCTTGTTGGTGAGGTAAAAGCTCACCAA
GGCGACGATCCTTAGCTGGTCTGAGAGGATGATCAGCCACACTGGGACTGAGACACGGCC
>Zotu7421
GAACGCTGGCGGCAGGCTTAACACATGCAAGTCGAGCGGAGAGAGGTAGCTTGCTACTGATCTTAGCGGC
GGACGGGTGAGTAATGCTTAGGAATCTGCCTATTAGTGGGGGACAACATTTCAAAAGGAATGCTAATACC
GCATACGTCCTACGGGAGAAAGCAGGGGATCTTCGGACCTTGCGCTAATAAATGAGCCTAAGTCGGATTA
GCTAGTTGGTGGGGTAAAGGCCTACCAAGGCGACGATCTGTAGCGGGTCTGAGAGGATGATCCGCCACAC
TGGGACTGAGACACGGCC
>Zotu7425
GAACGCTGGCGGCAGGCTTAACACATGCAAGTCGAGCGCCCCGCAAGGGGAGCGGCAGACGGGTGAGTA
ACACGTGGGAACGTACCTTTTGCTACGGAATAGCTCCGGGAAACTGGAATTAATACCGTATGTGCCCTTCG

GGGGAAAGATTTATCGGCAAAAGATCGGCCCGCGTCTGATTAGCTAGTTGGTGGGGTAATGGCCTACCAA
GGCGACGATCAGTAGCTGGTCTGAGAGGATGATCAGCCACACTGGGACTGAGACACGGCC

>Zotu7429
GAACGCTAGCGGCAGGCTTAACACATGCAAGTCGAGGGGTATATGTCTTCGGATATAGAGACCGGCGCAC
GGGTGCGTAACGCGTATGCAATCTACCTTTTACAGGGGAATAGCCCAGAGAAATTTGGATTAATGCCCCAT
AGTATTATAGAATGGCATCATTTTATAATTAAAGTCACAACGGTAAAAGATGAGCATGCGTCCCATTAGTTA
GTTGGTAAGGTAACGGCTTACCAAGACAATGATGGGTAGGGGTCCTGAGAGGGAGATCCCCCACACTGGT
ACTGAGACACGGAC

>Zotu7432
GAACGCTGGCGGCGTGCCTAATACATGCAAGTCGAACGGGGTGCAGCAATGTACCAAGTGGCGATCGGGT
GCGTAACACGTAGCTGACCTACCTCTTGGCGGGGGACAACAGGCCGAAAGGCTTGCTAATACCGCATAATT
TCGGGGAAACCAGAATACCCTGAAGAAACGCGCAAGCGGCCGAGAGAGGGGGCTGCGGCCCATCAGGTA
GTTGGTGAGGTAACGGCTCACCAAGCCGACGACGGGTAGGGGGCCTGAGAGGGTGACCCCCCACACTGG
GACTGACAGACGGCC

>Zotu7434
GAACGCTGGCGGCGTGCCTAACACATGCAAGTCGAGCGCGGTTGTGCTTAGTATTGAGTGTTTTATTGATA
TAAAACATTGAATTCTATGCACAACTGAGCGGCGGACGGGTGAGTAACGCGTGGGTAACCTGCCCTATACA
CATGGATAACATACTGAAAAGTTTACTAATACATGATAAAATAGTTTTTCGGCATCGAAGAATTATCAAAGT
GTTAGCGGTATAGGATGGACCCGCGTCTGATTAGCTAGTTGGTGAGATAACTGCCCACCAAGGCGACGAT
CAGTAGCCGACCTGAGAGGGTGATCGGCCACATTGGAACTGAGACACGGTC

>Zotu7437
GAACGCTGGCGGCGTGCTTAACACATGCAAGTCGAGCGACGCTCTTCCTTCGGGAAGATGACGGAGCGGC
GAACGGGTGAGTAACACGTGGGCAACCTGCCCCTAGCTCTGGGACAACACCGGGAAACCGGTGCTAATAC
CGGATACGACATCGAGCGGCATCTCTCGATGTGGAAAGTTTTTCGGCTAGGGATGGGCCCGCGGCCTATCA
GCTTGTTGGTGGGGTAATGGCCCACCAAGGCGACGACGGGTAGCCGGCCTGAGAGGGCGACCGGCCACA
CTGGGACTGAGACACGGCC

>Zotu7441
GAACGCTGGCGGCGTGCTTAACACATGCAAGTCGAGCGGAAAGGCCACTTCGGTGGTACTCGAGCGGCGA
ACGGGTGAGTAACACGTGAGTAATCTGCCCCTGGCTTTGGGATAGCCACCGGAAACGGTGATTAATACCG
GATATGACCGACTTCCGCATGGTTGTTGGTGGAAAGTTTTTCGGCCAGGGATGTGCTCGCGGCCTATCAGC
TTGATGGTGAGGTAATGGCTCACCATGGCTTCGACGGGTAGCCGGCCTGAGAGGGTGACCGGTCACACTG
GGACTGAGACACGGCC

>Zotu7444
GAACGCTGGCGGCGTGCCTAATACATGCAAGTCGAGCGAACCTTTTAAAAGCTTGCTTTTGAAAGGTTAGC
GGCGGACGGGTGAGTAACACGTGGGCAACCTGCCCTATAGTTTGGGATAACTCCGGGAAACCGGGGCTAA
TACCGGATAATACAATTTAACTCCTGTTAGATTGTTGAAAGATGGTTTCGGCTATCTCTATAGGATGGGCCC
GCGGCGCATTAGCTAGTTGGTAGGGTAACGGCCTACCAAGGCGACGATGCGTAGCCGACCTGAGAGGGT
GATCGGCCACACTGGGACTGAGACACGGCC

>Zotu7446
GAACGCTAGCGGCAGGCTTAATACATGCAAGTCGTGGGGCAGCATGGTTGTAGCAATACAACTGATGGCG
ACCGGCAAACGGGTGCGGAACACGTACGCAATCTACCCAGAACTGGCGAATAGCCTTCCGAAAGGAAGAT
TAATACGCCGTAACATTATAAAGTGGCATCACTTAATAATTAAAGCTCCGGCGGTTTTGGATGAGCGTGCG
CCCCATTAGGTAGTTGGTTGAGGTAATGGCTCACCAAGCCTGCGATGGGTAACTGGTGTGAGAGCACGAC
CAGTCACACGGGCACTGAGACACGGGC

>Zotu7448
GAACGCTGGCGGCAGGCCTAACACATGCAAGTCGAGCGGATGACGGGAGCTTGCTCCTTGATTCAGCGGC
GGACGGGTGAGTAATGCCTAGGAATCTGCCTGGTAGTGGGGGACAACGTTCCGAAAGGGGCGCTAATAC
CGCATACGTCCTACGGGAGAAAGTGGGGGATCTTCGGACCTCACGCTATCAGATGAGCCTAGGTCGGATT
AACTAGTTGGTGAGGTAATGGCTCACCAAGGCGACGATCCGTAACTGGTCTGAGAGGATGATCAGTCACA
CTGGAACTGAGACACGGTC

>Zotu7452
GAACGCTGGCGGCGTGCATAACACATTCAAGTCGAACGGAGCTTTGGGGATTAATCCTTCGGGAAGCGTTT
TCAAAGCTTAGTGGCGGACGGGTGAGTAACGCGTGAGCAACCTGCCTTTCAAAGCGGGATAACGTCTGGA
AACGGACGCTAATACCGCATAACGCATCGACATCACATGGTGATGATGCCAAAGGGGCAACCCGATGAAA
GATGGGCTCGCGTCTGATTAGATAGTTGGTGAGGTAACGGCTCACCAAGTCGACGATCAGTAGCCGGACT
GAGAGGTTGAACGGCCACATTGGAACTGAGATACGGTC
>Zotu7455
GAACGCTGGCGGAATGCTTAACACATGCAAGTCGCACGGTCAGCAATGGCAGTGGCGGACGGGTGAGTA
ACGCGTAGGAATATGTCCAGAGGTGGGGGACAACCCCGGGAAACTGGGGCTAATACCGCATATGAGCTG
AGGCTCAAAGCAGTGATGCGCCTTTGGAGTAGCCTGCGTCCGATTAGCTTGTTGGTGGGGTAATGGCCTAC
CAAGGCGACGATCGGTAGCTGGTCTGAGAGGACGACCAGCCACACTGGGACTGAGACACGGCC
>Zotu7458
GAACGCTGGCGGCGTGCTTAACACATGCAAGTCGAACGAGGTCCATCCACCCGCAAGGGTGGTGAAGACC
TAGTGGCGAACGGGTGAGTAACACGTGAGAAACCTGCCCCGAAGACCGGGATAACCGGCCGAAAGGTCG
GCTAATACCGGATACCTTCACGACATCTCATGGTGATGTGAAGAAATGGATTCCGCTTCGGGAGGGTCTCG
CGGCCTATCAGCTTGTTGGTGAGGTAACGGCTCACCAAGGCGACGACGGGTAGCTGTTCTGAGAGGAAGA
TCAGCCACACTGGGACTGAGACACGGCC
>Zotu7460
GAACGCTAGCGGGAGGCCTAATACATGCAAGTCGAGCGGTTCAGCAATGGATAGCGGCAGACGGGTGCG
TAACGCGTATGAAACTTACCCTACACAGGAGGATAGCCTTGGGAAACTGAGAGTAACCCTCCATAGTCTTG
AGATACCGCATGGTATTTTAAGTAAAACTTAGGTGGTGTAGGATAGTCGTGCGTCCGATTAGCTAGTTGGT
GAGGTAATGGCTCACCAAGGCGACGATCGGTAGCTGGTCTGAGAGGATGATCAGCCACACGGGCACTGA
GACACGGGC
>Zotu7463
GAACGCTGGCGGCGTGCCTAATACATGCAAGTCGAGCGAACTTTTGGGAGCTTGCTCCCAAAAGTTAGCG
GCGGACGGGTGAGTAACACGTGGGCAACCTGCCTATAAGACTGGGATAACTTCGGGAAACCGGAGCTAAT
ACCGGATAATTCTTTTCTACTCATGTAGAAAAGCTAAAAGACGGTTTATGCTGTCACTTATAGATGGGCCCG
CGGCGCATTAGCTAGTTGGTGAGGTAACGGCTCACCAAGGCCACGATGCGTAGCCGACCTGAGAGGGTGA
TCGGCCACACTGGGACTGAGACACGGCC
>Zotu7468
GAACGCTGGCGGCGTGCTTAACACATGCAAGTCGAACGCTGAAGCCCAGCTTGCTGGGTGGATGAGTGGC
GAACGGGTGAGTAACACGTGAGTAACCTGCCCCCTTCTTTGGGATAACGCCCGGAAACGGGTGCTAATACT
GGATATTCACTGATCTTCGCATGGGGGTTGGTGGAAAGGTTTGTTCTGGTGAGGGATGGGCTCGCGGCCT
ATCAGCTTGTTGGTGGGGTGATGGCCTACCAAGGCTTTGACGGGTAGCCGGCCTGAGAGGGTGACCGGTC
ACATTGGGACTGAGATACGGCC
>Zotu7470
GAACGCTGGCGGCGCGTTTTAAGCATGCAAGTCGAGCGGCAAGGGTACTTCGGTACCCCTAGAGCGGCGG
ACGGGTGAGTAACACGTGGACAACCTGCCCCCGGGAGAGGGATAGCCCAGGGAAACCTGGATCAATACCT
CGTAAGACCACACACTGTAGGGTGAGTGGGAAAAGGTGCTTCGGCACCGCCTGGGGATGGATCCGCGTCC
CATTAGCTAGATGGTAGGGTAAGAGCCTACCATGGCGACGATGGGTAGCCGGCCTGAGAGGGTGAACGG
CCACACTGGAACTGAGACACGGTC
>Zotu7473
GAACGCTAGCGGGAGGCCTAATACATGCAAGTCGAACGGTAACAGGTAGCAATACGCTAACGAGTGGCGC
ACGGGTGAGTAACACGTACACAATCTGCCTATAACAGGGGCATAACTTCGGGAAACTGAAGCTAATTCCCC
ATAAGACTGTAGAAAGGCATCTTTCAGCAGTTAAAGCTCCGGCGGTTATAGATGAGTGTGCGTTTGATTAG
CTAGTTGGTAAGGTAACGGCTTACCAAGGCTACGATCAATAACTGGTCTGAGAGGATGATCAGTCACACG
GGCACTGAGACACGGGC
>Zotu7475
GAACGCTGGCGGCGTGCCTAACACATGCAAGTCGAGCGGTAACAGGCGTAGCAATACGTGCTGACGAGC
GGCGAACGGGTGAGTAATGCTTCGGAATCTACCCAGTAGTGGGGAATAACCAACCGAAAGGTTGGCTAAT

ACCGCATACGTCCTACGGGAGAAAGCGGGGGATCGTAAGACCTCGCGCTATCGGATGAGCCGAAGCCGG
ATTAGCTAGTTGGTAGGGTAACGGCCTACCAAGGCGACGATCCGTAGCTGGTCTGAGAGGACGACCAGCC
ACACTGGGACTGAGACACGGCC
>Zotu7481
GAACGCTGGCGGCGTGCTTAACACATGCAAGTCACGGGGGCCCGCAAGGGCAACCGGCGAACGGGTGCG
TAACACGTGAGCAACCTGCCGATCTGAGGGGGATAGCCGGCCCAACGGCCGGGTAATACCGCATACGTTC
ATGGGGAGGAGTCTCCTTATGAAGAAACCTCCGGGGCAGATCGAGGGGCTCGCGGCCTATCAGCTAGTTG
GCGGTGTAATGGACCACCAAGGCGACGACGGGTAGCTGGTCTGAGAGGATGGCCAGCCACATTGGGACT
GAGAGACGGCC
>Zotu7483
GAACATTGGCGGTAGGCTTAACACATGCAAGTCGAGCGAGGCGTAATAGTCGAGCGGCGAACGGGTGAG
TAATACATGGAAATCTACCCTGAAGGGGGGGATAACGTTCAGAAATGAACGCTAATACCGCATAATCCATA
TTGGGAAAGCGTTGCAGCGCTTTAGGATGAGTCCATGGCTGATTAGCTTGTTGGTGAGGTAATGGCTCACC
AAGGCGACGATCAGTAGCTGGTCTGAGAGGATGACCAGCCACACTGGAACTGAGACACGGTC
>Zotu7485
GAACGCTGGTGGTATGCTTAACACATGCAAGTCGAACGTGCTTAAAGGGGCAACTCTTTAAGTCAGTGGCA
AACGGGTGAGGAATATATAGGAATCTTCCCAGCAGTAAGGAATAACATTGGGAAACCAATGCTAATGCCA
TATATTACCGATTTATCGGGAAAGATTTATCGCTGTTGGATGAGCCTGTATCGGATTAGCTAGTTGGTAGG
GTAATGGCCTACCAAGGCAATGATCCGTAGCTGGTCTGAGAGGATGATCAGCCACATTGGGACTGCGACA
CGGCC
>Zotu7491
GAACGCTGGCGGCGTGCCTAATACATGCAAGTCGAGCGGATCAACAGGAGCTTGCTCCTGTTGATTAGCG
GCGGACGGGTGAGTAACACGTGGGCAACCTGCCTGTAAGACTGGGATAACACCGGGAAACCGGTGCTAA
TACCGGATAATCCTTTTCCTCTCATGAGGAAAAGCTGAAAGTCGGTTTCGGCTGACACTTACAGATGGGCCC
GCGGCGCATTAGCTAGTTGGTGAGGTAACGGCTCACCAAGGCGACGATGCGTAGCCGACCTGAGAGGGT
GATCGGCCACACTGGGACTGAGACACGGCC
>Zotu7498
GAACGCTGGTAACGTGGATACCGGCATGCAAGTCACGGGGGCAGCAATGCAACCGGCGAACGGCTTAGT
AACACGTGGGTACATACCTTAAACTGGAGCACAGCTCGTCGAAAGACGGGGTAATTCTCCATAGTCCCGCA
AGGGTAAAGCAGCAATGCGGTTTAAGAATGGCCTGCGGCCTATCAGGTAGTTGGCGGTGTAATAGACCAC
CAAGCCTATGACGGGTAGGGGGCGTGAGAGCGCGACCCCCAAGAATGGCACTGCGACACGGGC
>Zotu7501
GAACGCTGGCGGCATGCTTTACACATGCAAGTCGGACGGCAGCGGGGAAGTGCTTGCACTTCTGCCGGCG
AGTGGCGAACGGGTGAGTAATGCATTGGAACGTACCGGGTAATGGGGGATAACTTGCCGAAAGGTAAGC
TAATACCGCATACGCTTTAAGAAGGAAAGCGGGGGATCATCTGACCTCGCGTTATCCGAGCGGCCAATGTC
TGATTAGCTAGTTGGTGGGGTAAAAGCCTACCAAGGCAACGATCAGTAGCGGGTCTGAGAGGATGATCCG
CCACACTGGGACTGAGACACGGCC
>Zotu7503
GAACGCTGGCGGCGTGCTTAATACATGCAAGTCGAGCGGTAACACTTCGGGTGACGAGCGGCGGACGGCT
GAGTAACACGTAGGAACGTACCCCAAACTGAGGGATAAGCACCAGAAATGGTGTCTAATACCACATGTGA
TCTAAGGATTAAAGCTCCGGCGGTTTGGGAACGGCCTGCGCATGATTAGCTTGTTGGTGAGGTAAAAGCTC
ACCAAGGCTACGATCATTAGCTGGTCTGAGAGGATGACCAGCCAGACTGGGACTGAGACACGGCC
>Zotu7508
GAACGCTGGCGGCAGGCTTAACACATGCAAGTCGAGCGGATGAAAGTAGCTTGCTACTGGATTCAGCGGC
GGACGGGTGAGTAATGCTTAGGAATCTGCCTATTAGTGGGGGACAACGTTCCGAAAGGAGCGCTAATACC
GCATACGTCCTACGGGAGAAAGCAGGGGACCTTTGGGCCTTGCGCTAATAGATGAGCCTAAGTCGGATTA
GCTAGTTGGTAGGGTAAAGGCCTACCAAGGCGACGATCTGTAGCGGGTCTGAGAGGATGATCCGCCACAC
TGGGACTGAGACACGGCC
>Zotu7511

GGTTCAGTCTTGGTTTCGCTTGGTTAACCTTTGATTTAGCTGTATTGCCAACCACGACTCAAAGCCAATCGG
TACCGTGCAACGCTCACGGGAAACGGAGCGTCACAGAAATGCATCGTGCAAAGTGTACCTCGAAAGTTGT
ATC
>Zotu7513
GAACGCTGGCGGCAGGCCTAACACATGCAAGTCGAACGCCCTGGCAACAGGGAGTGGCAGACGGGTGAG
TAACGCGTGGGAATCTACCTTGTGGTACGGAACAACCAAGGGAAACTTTGGCTAATACCGTATGAGCCCTT
CGGGGGAAAGATTTATCGCCATAAGATGAGCCCGCGTAGGATTAGCTAGTTGGTGAGGTAATGGCTCACC
AAGGCGACGATCCTTAGCTGGTCTGAGAGGATGACCAGCCACACTGGGACTGAGACACGGCC
>Zotu7518
GAACGCTGGCGGCGTGCCTAACACATGCAAGTCGAACGGGGTGATTTCCTTCGGGAGATCACTTAGTGGC
GAACGGGTGAGTAACGCGTGAGAAACCTGCCTTTCAGTGGGGGACAACAGTTGGAAACGACTGCTAATAC
CGCATAATGTATTTTGGGGGCATCCCTGAAATACCAAAGCTTTATGTGCTGAAAGATGGTCTCGCGTCTGAT
TAGCTAGATGGCGGGGTAACGGCCCACCATGGCGACGATCAGTAGCCGGTCTGAGAGGATGAACGGCCA
CATTGGGACTGAGATACGGCC
>Zotu7520
GAACGCTGGCGGCAGGCCTAACACATGCAAGTCGAACGGCAGCATGAGAGAGCTTGCTCTCTTGATGGCG
AGTGGCGGACGGGTGAGGAACACATCGGAATCTGCCCAGTCGTGGGGGATAACGTAGGGAAACTTACGC
TAATACCGCATACGACCTACGGGTGAAAGCAGGGGATCTTCGGACCTTGCGCGATTGGATGAGCCGATGT
CGGATTAGCTAGTTGGCGGGGTAAAGGCCCACCAAGGCGACGATCCGTAGCTGGTCTGAGAGGATGATCA
GCCACACTGGAACTGAGACACGGTC
>Zotu7523
GAACGCTGGCGGCGTGCTTAACACATGCAAGTCGAGCGACAGCGCTCTTCGGGGTGCATGGAGCGGCGA
ACGGGTGAGTAACACGTGGGCAACCTGCCCTCAGCTCTGGGACAACTCCGGGAAACCGGGGCTAATACCG
GATACGACCTTCCCTCGCATGGGGGTCGGTGGAAAGTTTTTCGGCTGAGGATGGGCCCGCGGCCTATCAG
CTTGTTGGTGGGGTGATGGCCTACCAAGGCGACGACGGGTAGCCGGCCTGAGAGGGCGACCGGCCACAC
TGGGACTGAGACACGGCC
>Zotu7527
GAACGCTAGCGGCAGGCTTAATACATGCAAGGCGAAGGGGCATTTATGTCACTGTCGTACGGGTGCGTAA
CGCGTATGCAACCTACCTATCACTGGGGGATAGCCCGGGGAAACCCGGATTAATACCGCATAAAACAGGG
GCACCGCATGGTGATATTTGTTAAAGATTTATTGGTGATAGATGGGCATGCGTTCGATTAGCTAGTTGGTA
TGGGTAACGGCCTACCAAGGCGACGATCGATAGGGGAGCTGAGAGGTTGATCCCCCACACGGGCACTGA
GATACGGGC
>Zotu7530
GAACGCTGGCGGCGTGCCTAACACATGCAAGTCGAACGGAGCCTTCGCAAGAGGGCTGAGTGGCGCACG
GGTGAGGAACACGTAGCTGACCTGCCTTCCAGCGGGGGATAACGTCCCGAAAGGGATGCTAAGACCGCAT
AAGCTCACAGGGCTTAGAAACCTGCGAGAAAAAGCTTCGGCTACTGGAAGAGGGGGCTGCGGCCCATCA
GGTAGTTGGCGGGGTAACGGCCCACCAAGCCGACGACGGGTCGGGGATCTGAGAGGATGATCCCCCACA
CTGGGACTGAGACACGGCC
>Zotu7532
GAACGCTGGCGGCGTGCTTAACACATGCAAGTCGAACGGAAAGGCCCTTCGGGGTACTCGAGTGGCGAAC
GGGTGAGTAACACGTGGGTGATCTGCCCTGCACTTCGGGATAAGCCTGGGAAACTGGGTCTAATACCGGA
TATACCTCATGGATTGCATGATTTGTGGGGGAAAGCTTTTGCGGTGTGGGATGGGCCCGCGGCCTATCAGC
TTGTTGGTGGGGTGATGGCCTACCAAGGCGTCGACGGGTAGCCGGCCTGAGAGGGTGTCCGGCCACACTG
GGACTGAGATACGGCC
>Zotu7536
GAACGCTAGCGGCAGGCTTAATACATGCAAGGCGAGGGGGCAGCAATGTCACCGTCGTACGGGTGCGTA
ACGCGTATGCAACCTACCTATCACTGGGGGATAGCCCGGGGAAACCCGGATTAATACCGCATGACACATTT
GTATAGCATTATACGATTTGTTAAAGATTTATTGGTGATAGATGGGCATGCGTTCGATTAGCTAGTTGGCAG
GGTAACGGCCTACCAAGGCAACGATCGATAGGGGAGCTGAGAGGTTGATCCCCCACACGGGCACTGAGAT
ACGGGC

>Zotu7539
GAACGCTGGCGGCATGCTTAACACATGCAAGTCGAACGCACCGCAAGGTGAGTGGCGCACGGGTGAGTA
ACGCGTGGGAATATGCCTAGCAGTGGGGAATAACCAAGGGAAACTTTGGCTAATACCGCATACGCCCTTC
GGGGGAAAGATTTATCGCTGTTAGATTAGCCCGCGTCCGATTAGCTAGTTGGTGAGGTAATGGCTCACCAA
GGCTCCGATCGGTAGCTGGTCTGAGAGGATGATCAGCCACACTGGGACTGAGACACGGCC
>Zotu7542
GAACGCTGGAAATGTGGATACTGGTATGCAAGTCAAGGGGCCGCAAGGCACCGGCGAACTGCTTAGTAAC
ACGTAGATACATACCGTAAAGTGGAACATAGCTCGTCGAAAGACGGGGTAATTTTCCATGTGAACGCAAGT
TTAAAGGCGCAAGCCGCTTTACGAATGGTCTGCGGTCTATCAGCTAGTTGGTGAGGTAAAGGCTCACCAAG
GCTATGACGGATAGGGGGCGTGAGAGCGCGACCCCCAAGAATGGCACTGCGACACGGGC
>Zotu7544
GAACGCTAGCGGCAGGCTTAATACATGCAAGTCGAACGGCAGCACGTTAAGGTGGCGAGTGGCAGACGG
GTGAGTAACGCGTACATAACCTACCTTTGGCAGGGGGAAAGTCTAGGGAAACTTGGAATAATCCCGCATG
AGATTATCGGGTGGCATCACCTGGTAATGAAAGGAGTAATCCGGCTGAAGATGGATGTGCGTCTCATTAG
GTAGTTGGTAGGGTAACGGCCTACCAAGCCAGCGATGAGTAGGGGGCGTGAGAGCGCGAACCCCCACAC
GGGTACTGAGAACGGAC
>Zotu7546
GAACGCTGGCGGCGTGCCTAACACATGCAAGTCGGATGTGCCCGCAAGGGTGCATGGCAGACGGGTGAG
TAACGCGTGGGGAACCTACCTTTTTGTGGGGGATAACGCTCCGAAAGGAGCGGTAATACCGCATAAGACC
ATGAGCTGGAATGCTGATGGTGAAAGCCGGGGATCGAAAGACCTGGCGCAAGGAGAGGGTCCTGCGTCT
GATTAGCTAGTTGGCGGGGTAACGGCCCACCAAGGCGACGATCAGTAGCCGGCCTGAGAGGGTGATCGG
CCACACTGGAACTGAGACACGGTC
>Zotu7548
CAACGCTGGCGGCGCGCTTAACACATGCAAGTCGAGCGAGAAAGGGGAGCAATCCCTGAGTAAAGCGGC
GTACGGGTGAGTAACACGTGGATGATCTACCTTTTAGTGGGGAATAACCCTGGGAAACCGGGGCTAATAC
CGCATAAGCCTGAGAAGGGAAAGCAGCAATGCGCTGAAAGAGGAGTCCGCGGCCGATTAGCTAGTTGGT
GAGGTAATGGCTCACCAAGGCGGTGATCGGTAGCCGGCCTGAGAGGGCACACGGCCACACTGGCACTGA
AACACGGGC
>Zotu7550
GAACGCTGGCGGCAGGCTTAACACATGCAAGTCGAGCGCGTAGCAATACGAGCGGCAGACGGGTGAGTA
ACGCGTGGGAACCTACCCTTTTCTACGGAATAACGCATGGAAACGTGTGCTAATACCGTATACGCCCTTTG
GGGGAAAGATTTATCGGAGAAGGATGGGCCCGCGTTGGATTAGCTAGTTGGTGGGGTAAAGGCCTACCA
AGGCGACGATCCATAGCTGGTCTGAGAGGATGATCAGCCACATTGGGACTGAGACACGGCC
>Zotu7552
GAACGCTGGCGGCGTGCCTAATACATGCAAGTCGTGCGGACTTATCTTCGGATAAGTTAGCGGCGGACGG
GTGAGTAACACGTGGGCAACCTGCCTGTAAGACTGGGATAACTCCGGGAAACCGGTGCTAATACCAGATA
CAATCAAAGACTGCCTGGTTTTTGATGGAAAGGTCTTGTACTGCTTACAGATGGGCCCGCGGCGCATTAGC
TAGTTGGTGGGGTAATGGCTCACCAAGGCGACGATGCGTAGCCGACCTGAGAGGGTGATCGGCCACACTG
GGACTGAGACACGGCC
>Zotu7556
GAACGCTAGCGGCAGGCCTAATACATGCAAGTCGAACGGTAAATGTCCCTTTGGGGATATGAGAGTGGCG
CACGGGTGCGTAACGCGTATGCAACCCACCCGCTGTCGGGGGATAGCCCGTAGAAATGCGAATTAATACC
CCATGAGGAGATAGGGCGGCATCGTCCTTTTTTCAAAACTACGGTGACGGCGGACGGGCATGCGTGCCAT
TAGATAGTTGGCGGGGTAACGGCCCACCAAGTCGACGATGGCTAGGGGATCTGAGAGGACGGTCCCCCAC
ACTGGTACTGAGACACGGAC
>Zotu7558
GAACGCTGGCGGCATGCCTAACACATGCAAGTCGAACGATCCCTTCGGGGATAGTGGCGCACGGGTGCGT
AACGCGTGGGAATCTGCCCTTGGGTTCGGAATAACAGTGAGAAATTACTGCTAATACCGGATGATGTCTTC
GGACCAAAGATTTATCGCCCAAGGATGAGCCCGCGTAGGATTAGCTAGTTGGTGGGGTAATGGCCTACCA
AGGCGACGATCCTTAGCTGGTCTGAGAGGATGATCAGCCACACTGGGACTGAGACACGGCC

>Zotu7563
GAACGCTAGCGGGAGGCCTAACACATGCAAGCCGAGCGGGATTTGTTGGTTAGCTTGCTAACTAACAATG
AGAGCGGCGTACGGGTGCGTAACACGTGTGCAACCTGCCTTTATCTGGGGGATAGCCTTTCGAAAGGAAG
ATTAATACCCCATAATATATTAATTGGCATCAATTAATATTGAAAGCTCCGGCGGATAGAGATGGGCACGC
GTAAGATTAGCTAGTTGGTGAGGTAACGGCTCACCAAGGCTGCGATCTTTAGGGGGCCTGAGAGGGTGAT
CCCCCACACTGGTACTGAGACACGGAC
>Zotu7565
GAACGCTGGCGGCGTGCCTAACACATGCAAGTCGAGCGAGAAAGTCTTCGGATGAGTAAAGCGGCGGAC
GGGTGAGTAACGCGTAGGTAATCTACCTTCAAGTGGGGGATAACGTTCCGAAAGGAACGCTAATACCGCA
TGTGAATCCGGACTGATGAGTCTGTATTCAAAGGGGGCTCGTAAGGGCTTTCGCTAGAAGATGAGCCTGC
GTCCCATTAGCTAGTTGGTGAGGTAACGGCTCACCAAGGCGACGATGGGTAGCTGGTCTGAGAGGACGAC
CAGCCACACTGGAACTGACACACGGTC
>Zotu7569
ACGGTCACTGGATAGGGACTTAATGTGCACGGCGGCCACCTCTTTGTAGAGTTGACATGTTGCTGTACGTC
TCGGAACTCCAAGTGCTGTCTTCAAGCCTGCACGGTGAA
>Zotu7571
GAACGCTAGCGGTATGCCTAACACATGCAAGTCGTACGGAAAAGAAATTTTTAGTGGCGCACGGGTGAGT
AATACATGAGAATTCACCTTTAAGGAAGAGGATAACAATTAGAAATAGTTGCTAAAACTCTATATACCATTC
TATGGTAAAAAAAGTAATTTGCCTAAAGAGAAGCTCATGCCTGATTAGCTAGTTGGTAGGGTAAAGGCCTA
CCAAGGCGATGATCAGTAGTTGATCTGAGAGGATGATCAGCCACACTGGAACTGAGACACGGTC
>Zotu7575
GAACGCTGGCGGCAGGCTTAACACATGCAAGTCGAGCGGGCGTAGCAATACGTCAGCGGCAGACGGGTG
AGTAACGCGTGGGAACGTACCTTTTGGTTCGGAACAACTGAGGGAAACTTCAGCTAATACCGGATAAGCCC
TTACGGGGAAAGATTTATCGCCGAAAGATCGGCCCGCGTCTGATTAGCTAGTTGGTGAGGTAACGGCTCAC
CAAGGCGACGATCAGTAGCTGGTCTGAGAGGATGATCAGCCACATTGGGACTGAGACACGGCC
>Zotu7577
GAACGCTGGCGGCAGGCCTAATACATGCAAGTCGAGCGCCCCTTCGGGGGAGCGGCGGACGGGTGAGTA
ACGCGTGGGAACGTGCCCTTTGGTTCGGAACAACACAGGGAAACTTGTGCTAATACCGAATGTGCCCTTCG
GGGGAAAGATTTATCGCCATTGGAGCGGCCCGCGTTGGATTAGGTAGTTGGTGGGGTAAAGGCCCACCAA
GCCTACGATCCATAGCTGGTCTGAGAGGATGATCAGCCACACTGGGACTGAGACACGGCC
>Zotu7579
GAACGCTGGCGGCGTGCTTAACACATGCAAGTCGAGCGGTAAGGCTCCTTCGGGGGTACACGAGCGGCG
AACGGGTGAGTAACACGTGAGTAATCTGCCCTTCACTTCGGGATAAGCTTCGGAAACGGGGTCTAATACCG
GATATGACACCTTGATGCATGTTGGGGTGTGGAAAGTTTTTTCGGTGGGGGATGTGCTCGCGGCCTATCAG
CTTGTTGGTGGGGTAATGGCCTACCAAGGCTTTGACGGGTAGCCGGCCTGAGAGGGTGACCGGCCACACT
GGGACTGAGACACGGCC
>Zotu7581
GAACGCTGGTAACGTGGATACCGGCATGCAAGTCACGGGGGCCGCAAGGCAACCGGCGAACGGCTTAGT
AACACGTGGGTACATACCTTAAATTGGAGCACAGCTCGTCGAAAGACGGGGTAATTCTCCATAGTCCCGCA
AGGGTAAAGCAGCAATGCGATTTAAGAATGGCCTGCGGCCTATCAGATAGTTGGCGGTGTAATAGACCAC
CAAGTCTATGACGGGTAGGGGGCGTGAGAGCGCGACCCCCAAGAATGGCACTGCGACACGGGC
>Zotu7585
GAACGCTGGCGGCGTGCCTAAGACATGCAAGTCGAATGTCCGCAAGGACATGGCGAACGGGTGAGTAAT
ACCTAGGAACTTTCTTATAGCCGGGCATAACTCCGCGAAAGCGGAGATAATTCCCGATATTATCGCAAGAT
CAAAGTAGCAATACACTATAAGTGAGGCCTGGGCACCATCAGCTAGTTGGTAAGGTAACGGCTTACCAAG
GCTATGACGGTTAGGGGCCCCGAGAGGGGAAACCCCCACACTGGAACTGCGACACGGTC
>Zotu7587
GAACGCTGGCGGCGTGCTTTACACATGCAAGTCGAGCGGCAGCATGGTCTTCGGACTGATGGCGAGCGGC
GAACGGGTGAGTAATACATCGAAACGTGCCTTTTAGTGGGGGATAACCCATCGAAAGATGGGCTAATACC
GCATAATCTCTGAGGAGGAAAGCTGGGGACTCGCAAGGGCCTGGCGCTGAAAGAGCGGTCGATGGTCGA

TTAGTTAGTTGGTGAGGTAAAAGCTCACCAAGGCAATGATCGATAGCTGTTCTGAGAGGAAGATCAGCCA
CACTGGAACTGAGACACGGTC
>Zotu7592
GAACGCTGGCGGCGTGCCTTAAGCATGCAAGTAGAACGAGTTCTATCTTGCCGCAAGGCAAGCTTAGAACT
AGTTGCGAACGGCTGAGTAATGAGTAAGAATCTTTCCTTTAGTGGGGAATACCTCATCGAAAGATGAGCTA
ATACCGCATAATACCTCCGGGTGAAAGCGATTAACTTCGCGCTGATTGATGAGCTTACTCCCTATCAGGTAG
TTGGTGGGGTAATAGCCTACCAAGCCTACGACGGGTACCCGATCTGAGAGGATGGACGGGCAGAATGGG
ACTGAGACACGGCC
>Zotu7595
GAACGCTGGCGGCGTGGATGAGGCATGCAAGTCGAATGGCCTAGTAGCAATACTAGAAACCATGGCAGA
CGAGGTAGGAACACGTAGGTAACGTACCCCAAAGTCGGGCATAACCGGTCGAAAGATTGGATAATTCTCG
ATGGTCTCGGAGATTTATTTCTCCGAGTAAAGCGAATTATTTATTTAGTTTACGCTTTGGGAACGGCCTGCG
GGCTATCAGCTTGTTGGTAAGGTAATGGCTTACCAAGGCTACGACGGCTAGGGGAGGTGAGAGCCTGACC
CCCACCGGTGGAACTGCGACACGGTC
>Zotu7598
GAACGCTGGCGGCAGGCCTAACACATGCAAGTCGAACGGGATCTTCGGATCTAGTGGCGGACGGGTGAG
TAACGCGTGGGAACGTGCCCTTTGGTTCGGAATAGCCCTGGGAAACTGGGAGTAATACCGGATACGGTCG
AGAGACGAAAGATTTATCGCCGAAGGATCGGCCCGCGTTGGATTAGGTAGTTGGTGGGGTAATGGCCTAC
CAAGCCGACGATCCATAGCTGGTTTGAGAGGATGATCAGCCACACTGGGACTGAGACACGGCC
>Zotu7601
CAACGCTGGCGGCGTGCCTAACACATGCAAGTCGAACGCGAAAGGGGGCAACCCTGAGTAGAGTGGCGT
CCGGGTGAGTAACACGTGAATAATCTACCTATGGGTGGGGAATAACTTCTCGAAAGGGAAGCTAATACCG
CATAAAATCTAGTGGGGGTGGCCCTGTTAGATCAAAGAGGGGGATCGCAAGACCTCTCGCCAATAGAGGA
GTTCGCGGCTGATTAGCTAGTTGGCGGGGTAACGGCCCACCAAGGCAACGATCAGTAGCCGGCCTGAGAG
GGCGGTCGGCCACACTGGCACTGAAACACGGGC
>Zotu7603
GAACGCTGGCGGCATGCTTTACACATGCAAGTCGAACGGCAGCGCGGGGCAACCTGGCGGCGAGTGGCG
AACGGGTGAGTAATATATCGGAACGTACCCTGGAGTGGGGGATAACGTAGCGAAAGTTACGCTAATACCG
CATACGATCTAAGGATGAAAGTGGGGGATTCGCAAGAACCTCATGCTCCTGGAGCGGCCGATATCTGATTA
GCTAGTTGGTAGGGTAAAGGCCTACCAAGGCAACGATCAGTAGCTGGTCTGAGAGGACGACCAGCCACAC
TGGAACTGAGACACGGTC
>Zotu7605
GAACGCTGGCGGCGTGCCTAATACATGCAAGTCGAGCGGACAGATGGGAGCTTGCTCCCTGATGTTAGCG
GCGGACGGGTGAGTAACACGTGGGTAACCTGCCTGTAAGACTGGGATAACTCCGGGAAACCGGGGCTAA
TACCGGATGGTTGTTTGAACCGCATGGTTCAAACATAAAAGGTGGCTTCGGCTACCACTTACAGATGGACC
CGCGGCGCATTAGCTAGTTGGTGAGGTAACGGCTCACCAAGGCGACGATGCGTAGCCGACCTGAGAGGGT
GATCGGCCACACTGGGACTGAGACACGGCC
>Zotu7610
GAACGCTGGCGGCATGCCTTACACATGCAAGTCGAGGGGTATGGTTCTTCGGAACCAGAGACCGGCGCAC
GGGTGCGTAACGCGTATGCAATCTGCCTTTCACAAAGGAATAGCCCAGAGAAATTTGGATTAATGCCTTAT
AGCATTACGACTTGGCATCAAGATGTAATTAAAGATTTATCGGTGAAAGATGAGCATGCGTCCCATTAGCT
AGTTGGTAAGGTAACGGCTTACCAAGGCGATGATGGGTAGGGGTCCTGAGAGGGAGATCCCCCACACTG
GTACTGAGACACGGAC
>Zotu7612
GAACGCTGGCGGCAGGCCTAACACATGCAAGTCGAACGGATCCTTCGGGATTAGTGGCGGACGGGTGAG
TAACGCGTGGGAACGTGCCCTTTGGTTCGGAATAGCCTCGGGAAACTGGGAGTAATACCGGATAAGCCCT
TCGGGGGAAAGATTTATCGCCAAAGGATCGGCCCGCGTTGGATTAGGTAGTTGGTGGGGTAATGGCCTAC
CAAGCCGACGATCCATAGCTGGTTTGAGAGGATGATCAGCCACACTGGGACTGAGACACGGCC
>Zotu7614

GAACGCTGGCGGCGTGCCTAAGACATGCAAGTCGAGGGGGTTTAGACCCCCGGCGAACGGGTGAGTAAC
ACGTGGGAACGATCAATAAGTTCGGGATAACTCTCCGAAAGGAGAGACAATACCGGATGTGGAAGCAATT
CTAAAGATTTATCGCTTATTGTTCGGCCCGCGTCCCATCAGCTAGTTGGTAAGGTAATGGCTTACCAAGGCT
ATGACGGGTAGCGGAACCGAGAGGTTGATCCGCCACACTGGGACTGCGACACGGCC
>Zotu7616
GAACGCTGGCGGCGTGCCTAACACATGCAAGTCGAACGGACGAGGCTTCTTCGGAGGCCAAGTTAGTGGC
GGACGGGTGAGTAACACGTGAGCAACCTGCCCTTCAGAGGGGGACAACAGTTGGAAACGACTGCTAATAC
CGCATGATACATTTGAGAGGCATCTCTTGAATGTCAAAGATTTTATCGCTGAAGGATGGGCTCGCGTCTGA
TTAGATAGTTGGTGGGGTAACGGCTCACCAAGTCGACGATCAGTAGCCGGACTGAGAGGTTGAACGGCCA
CATTGGGACTGAGATACGGCC
>Zotu7619
GAACGCTGGCGGCGTGCCTAACACATGCAAGTCGAACGAGATCCATCCAGTGGCAACACTGGTGAAGATC
TAGTGGCGAACGGGTGAGTAACACGTGAGGAACCTGCCCCGAAGACCGGGATAACCCTCCGAAAGGAGA
GCTAATACCGGATACCCCCAACTGATCGCATGGTCGGTTGAGGAAATGGATTCCGCTTCGGGAGGGCCTCG
CGGCCTATCAGCTAGTTGGTGAGGTAATGGCTCACCAAGGCATCGACGGGTAGCTGGTCTGAGAGGACGA
TCAGCCACACTGGGACTGAGACACGGCC
>Zotu7621
GAACGCTGGCGACGTGCTTAACACATGCAAGTCGAACGGTGAACCAGGGCTTGCCCTGGGAATCAGTGGC
GAACGGGTGAGTAACACGTGGGCAACCTGCCCCTGACTCTGGGATAACTCCGGGAAACCGGAGCTAATAC
CGGATATGACCTCTGACGGCATCGTCGGAGGTGGAAAGTTTTTCGGTCAGGGATGGGCCCGCGGCCTATC
AGCTTGTTGGTGGGGTAATGGCCCACCAAGGCGACGACGGGTAGCCGGCCTGAGAGGGCGACCGGCCAC
ACTGGGACTGAGACACGGCC
>Zotu7623
GAACGCTGGCGGCGTGCTTAACACATGCAAGTCGAACGATGAAAGAGGAGCTTGCTCCTCTGGATTAGTG
GCGAACGGGTGAGTAACACGTGAGCAATCTGCCCCTGACTCCGGAATAACTACGGGAAACTGTAGCTAAT
ACCGGATACGCGCCGAACAGGCATCTGTATAGGTGGGAAAGAATTTCGGTCAGGGATGAGCTCGCGGCCT
ATCAGCTTGTTGGTGAGGTAACGGCTCACCAAGGCGACGACGGGTAGCCGGCCTGAGAGGGTGACCGGC
CACATTGGGACTGAGGCACGACC
>Zotu7625
GAACGCTGGCGGCGTGCTTAACACATGCAAGTCGAACGAAGCAGCAATGCTTAGTGGCGGACGGGTGAG
TAACGCGTAGGTAACCTGCCTCATAGTGGGGGATACCCATCCGAAAGGATGGCTAATACCGCATAAGCTG
GTTCTTTGGAATAGGGATCAGGAAAGATTTATTGCTATGAGAGGGGCCTGCGTTCGATTAGCTAGTTGGTG
AGGTAATGGCTCACCAAGGCGACGATCGATAGCTGGTCTGAGAGGACGATCAGCCACACTGGAACTGAGA
CACGGTC
>Zotu7633
GAACGCTGGCGGCATGCCTAATACATGCAAGTCGAGCGAATGACGAAGAAGCTTGCTTCTTCTGATTTAGC
GGCGGACGGGTGAGTAACACGTGGGCAACCTGCCCTGTAGATTGGGATAACTCCGGGAAACCGGGGCTA
ATACCGAATAATCCATTTCCTCACATGGGGAGATGTTAAAAGACGGCATCTCGCTGTCACTACAGGATGGG
CCCGCGGCGCATTAGCTAGTTGGTGAGGTAACGGCTCACCAAGGCGACGATGCGTAGCCGACCTGAGAGG
GTGATCGGCCACACTGGGACTGAGACACGGCC
>Zotu7637
GAACGCTGGCGGCGTGCTTTACACATGCAAGTCGAGCGGCAGCGCGACCTTCGGGTTGGCGGCGAGCGG
CGAACGGGTGAGTAATACATCGGAACGTACCTTTTAGTGGGGGATAACGCATCGAAAGATGTGCTAATAC
CGCATAATCTCTGAGGAGGAAAGGCGGGGATCACGCAAGTGACCTGTCGCTGAAAGAGCGGCCGATGGT
CGATTAGCTAGTTGGCGGGGTAAAAGCCCACCAAGGCGATGATCGATAGCGGGTCTGAGAGGACGATCC
GTCACACTGGGACTGAGACACGGCC
>Zotu7639
GAACGCTGGCGGCGTGCTTAACACATGCAAGTCGAACGGGAAATACTTTATTGAAACTTCGGTGGATTTAA
TTTATTTCTAGTGGCGGACGGGTGAGTAACGCGTGGGTAACCTGCCTTATACTGGGGGATAACAGCCAGA
AATGGCTGCTAATACCGCATAAGCGCACAGAACCGCATGGTTCGGTGTGAAAAACTCCGGTGGTATAAGA

TGGACCCGCGTTGGATTAGCTAGTTGGCAGGGCAGCGGCCTACCAAGGCGACGATCCATAGCCGGCCTGA
GAGGGTGAACGGCCACATTGGGACTGAGACACGGCC
>Zotu7641
GAACGCTGGCGGCAGGCTTAACACATGCAAGTCGAGCGGGCATAGCAATATGTCAGCGGCAGACGGGTG
AGTAACGCGTGGGAACGTACCTTTTGGTTCGGAACAACTGAGGGAAACTTCAGCTAATACCGGATAAGCCC
TTACGGGGAAAGATTTATCGCCGAAAGATCGGCCCGCGTCTGATTAGCTAGTTGGTGAGGTAATGGCTCAC
CAAGGCGACGATCAGTAGCTGGTCTGAGAGGATGATCAGCCACATTGGGACTGAGACACGGCC
>Zotu7644
GAACGCTGGCGGCATGCTTTACACATGCAAGTCGAACGGTAACAGGTCTTCGGATGCTGACGAATGGCGA
ACGGGTGAGTAATACATCGGAACGTGCCTAATAGTAGGGGATAACTACTCGAAAGAGTAGCTAATACCGC
ATGAGATCTACGGATGAAAGCAGGGGACCTTCGGGCCTTGTGCTACTAGAGCGGCTGATGGCAGATTAGG
TAGTTGGTGGGGTAAAAGCTTACCAAGCCTGCGATCTGTAGCTGGTCTGAGAGGACGACCAGCCACACTG
GGACTGAGACACGGCC
>Zotu7646
GAACGCTGGCGGCGTGCCTAATACATGCAAGTCGAGCGGAGTTGATGAGGTGCTTGCACCTCTGATACTTA
GCGGCGGACGGGTGAGTAACACGTAGGTAACCTGCCTGTAAGACCGGGATAACATTCGGAAACGAATGCT
AATACCGGATACGCGATTTTCTCGCATGAGAGAATCGGGAAAGACGGAGCAATCTGTCGCTTACGGATGG
ACCTGCGGCGCATTAGCTAGTTGGTGAGGTAACGGCTCACCAAGGCGACGATGCGTAGCCGACCTGAGAG
GGTGATCGGCCACACTGGGACTGAGACACGGCC
>Zotu7651
GAACGCTGGCGGCAGGCCTAATACATGCAAGTCGGACGGGATTGGAGAGCTTGCTTTCCATGAGAGTGGC
GCACGGGTGCGTAACACGTATGTAACCTACCTTCGTCAGGGGGATAGCCTCTCGAAAGAGAGATTAAGAC
CGCATAACATTATTGGATAGCATTATTTAATAATCAAATATTTATAGGACGAAGATGGGCATGCGGAACATT
AGCTAGTTGGTAAGGTAACGGCTTACCAAGGCTACGATGTTTAGGGGATCTGAGAGGATGACCCCCCACA
CTGGTACTGAGACACGGAC
>Zotu7654
GAACGCTGGCGGCGTGCCTAATACATGCAAGTCGAACGCACTCTCGTTTAGATTGAAGGAGCTTGCTCCTG
ATTGATAAACATTTGAGTGAGTGGCGGACGGGTGAGTAACACGTGGGTAACCTGCCCTAAAGTGGGGGAT
AACATTTGGAAACAGATGCTAATACCGCATAAAACCTAACACCGCATGGTGTAGGGTTGAAAGATGGTTTC
GGCTATCACTTTAGGATGGACCCGCGGTGCATTAGTTAGTTGGTGAGGTAAAGGCTCACCAAGACCGTGAT
GCATAGCCGACCTGAGAGGGTAATCGGCCACACTGGGACTGAGACACGGCC
>Zotu7656
GAACGCTGGCGGCGTGCTTAACACATGCAAGTCGAACGTGAGAATAGCAATATTTGAAAGTGGCAAACGG
GTGAGTAACACGTGGGTATCTGCCTTTGAGTGGGGGATAACGACTGGAAACGGTCGCTAATACCGCATAC
GTTCACATTTTGTGGAGAGATGTGAAGAAAGATATATTGTTCAAAGAGGAGCCTGCGTCCGATTAGCTAGT
TGGTGGGGTAAAGGCCTACCAAGGCGATGATCGGTAGCTGGTCTGAGAGGATGGTCAGCCACACGGGAA
CTGAGAAACGGTC
>Zotu7660
GAACGCTGGCGGCGTGCCTAACACATGCAAGTCGAGCGATGAACCCTTCGGGGGGATTAGCGGCGGACG
GGTGAGTAACACGTGGGTAACCTGCCTCAAAGAGGGGGATAGCCTCCCGAAAGGGAGATTAATACCGCAT
AACATCGAGCTATCGCATGGTAGTTTAATCAAAGGAGTAATCCGCTTTGAGATGGACCCGCGGCGCATTAG
CTAGTTGGTGAGGTAACGGCTCACCAAGGCGACGATGCGTAGCCGACCTGAGAGGGTGATCGGCCACATT
GGAACTGAGACACGGTC
>Zotu7666
GAACGCTGGCGGCGTGCTTAACACATGCAAGTCGAACGGTAAGGCCCTTCGGGGTACACGAGTGGCGAAC
GGGTGAGTAACACGTGGGTGATCTGCCCTGTACTTTGGGATAAGCCTGGGAAACTGGGTCTAATACCGAA
TATGACCTTCTCCTGCATGGGGGTTGGTGGAAAGCTTTTGCGGTACAGGATGGGCCCGCGGCCTATCAGCT
TGTTGGTGGGGTAATGGCCTACCAAGGCGACGACGGGTAGCCGGCCTGAGAGGGCGACCGGCCACACTG
GGACTGAGACACGGCC
>Zotu7669

GAACGCTGGCGGCGTGCTTAACACATGCAAGTCGAACGAGGTATAAGGACGGAAGTTTTCGGACGGAAG
ATCTTGTACTGAGTGGCGGACGGGTGAGTAACGCGTGGGAAACCTACCCTGTTCCGGGGGATACCAGTTG
GAAACGACTGTTAATACCGCATAAGCGCACAGCACCGCATGGTGCAGTGTGAAAAACTCCGGTGGAACAG
GATGGTCCCGCGTCTGATTAGGCAGTTGGCAGGGTAACGGCCTACCAAACCGACGATCAGTAGCCGGCCT
GAGAGGGTGAACGGCCACATTGGGACTGAGACACGGCC
>Zotu7671
GAACGTTGGCGGTGCGCCTAATACATGCAAGTCGCGGGGTCTGGAGCAATCCAGACACCGGCAAACGGGT
CCGTAACACGTTGGAACCTGCCCTGAAGTGAGGGATAACCCCGAGAAATCGGGGCTAATACCGCATATGG
CCGCAAGGTTAAAGCAGCAATGCGCTTCAGGAGGGGCCTGCGTCCTATCAGCTTGTTGGCGAGGTAATGG
CTCACCAAGGCTTTGACGGGTAGCTGGTCTGAGAGGATGATCAGCGACAGGGAGACTGAGACACGGCT
>Zotu7673
CAACGCTGGCGGCGCGCCTAATGCATGCAAGTCGGACGGGGTCGCCTTCGGGCGGCTCACGTGGCGGAC
GGGCGAGGAACACGTGGGCAACCTGCCCCGGGGCGGGGGGTAGCCCCCGGAAACGGGGGGTAATTCCG
CGTACGCTCACCCTGCTGTGGGCGGGGTGAGGAAAGGCGCGAGCCACCCCGGGAGGGGCCTGCGGCGGA
TTAGCTTGTTGGCGGGGTAACGGCCCACCAAGGCGGTGATCCGTAGCTGGTCTGAGAGGACGGCCAGCCG
CAGGGGGACTGAGACACGGCC
>Zotu7675
ACCGTGTCTCAGTTCCAGTGTGACTGATCATCCTCTCAGACCAGTTACGGATCGTCGCCTTGGTGAGCCATT
ACCTCACCAACTAGCTAATCCGACCTAGGCTCATCTGATAGCGCAAGGCCCGAAGGTCCCCTGCTTTCTCCC
GTAGGACGTATGCGGTATTAGCGTTCCTTTCGAAACGTTGTCCCCCACTACCAGGCAGATTCCTAGGCATTA
CTCACCCGTCCGCCGCTGAATCCAGGAGCAAGCTCCTCTCATCCGCTCGACTTGCATGTGTTAGGCCTGCCG
CCAGCGTTCA
>Zotu7680
GAACGCTGGCGGCAGGCTTAACACATGCAAGTCGAACGCCCCGCAAGGGGAGTGGCAGACGGGTGAGTA
ACGCGTGGGAACGTGCCCTTCGCTTCGGAATAGCCCCGGGAAACTGGGAGTAATACCGGATACGCCGTAT
TGGGAAAGATTTATCGGCGAAGGATCGGCCCGCGTTAGATTAGCTAGTTGGCGGTGTAATGGACCACCAA
GGCTACGATCTATAGCTGGTCTGAGAGGATGATCAGCCACACTGGGACTGAGACACGGCC
>Zotu7683
GAACGCTGGCGGCGTGCCTAACACATGCAAGTCGAACGCGAACGGTGCTTTCGGGTGCCTAGTAGAGTGG
CGCACGGGTGAGTAACACGTAGACAACCTGCCTTCGAGCCCGGAATAACCTTTCGAAAGAAGGGCTAATA
CCGGATAACATCCCGGGTTGCATGACTTGGGATTGAAAGGCTTGCTACTCGAAGAGGGGTCTGCGGGACA
TTAGCTAGTTGGTGGGGTAACGGCCTACCAAGGCGACGATGTCTAGCGGGTCTGAGAGGATGATCCGCCA
CACTGGAACTGAGACACGGTC
>Zotu7686
GAACGCTGGCGGCGTGCTTAACACATGCAAGTCGAGCGAAGCACTTGTTTGGAACCCTCCGGGAGGAAGA
ACAATTGACTGAGCGGCGGACGGGTGAGTAACGCGTGGGGAACCTGCCTTATACCGGGGGATAACAGCT
GGAAACGGCTGCTAATACCGCATAAGCGCACGGCACCGCATGGTGCAGTGTGAAAAACTCCGGTGGTATA
AGATGGACCCGCGTTAGATTAGCTAGTCGGCGGGGTAACGGCCCACCAAGGCGACGATCTATAGCCGGCC
TGAGAGGGCGACCGGCCACATCGGGACTGAGACACGGCC
>Zotu7691
GAACGCTGGCGGCATGCCTTACACATGCAAGTCGAACGGCAGCACGGGGGCAACCCTGGTGGCGAGTGG
CGAACGGGTGAGTAATACATCGGAACGTGTCCTGTAGTGGGGGATAGCCCGGCGAAAGCCGGATTAATAC
CGCATACGATCTACGGAAGAAAGTGGGGGATCTTCGGACCTCGCGCTGTAGGGGCGGCCGATGGCAGATT
AGCTAGTTGGTGGGGTAAAGGCCTACCAAGGCGACGATCTGTAGCTGGTCTGAGAGGACGACCAGCCACA
CTGGGACTGAGACACGGCC
>Zotu7695
GAACGCTGGTAACGTGGATACCGGCATGCAAGTCAAGGGGACCCGCAAGGGTAACCGGCGAACGGCTTA
GTAATACGTAGATACATACCGTAAAGTGGGGCATAGCTCGTTGAAAAACGGGGTAATTTCCCATAGTCTCG
CAAGAGTAAAGGCGCAAGCCGCTTTATGAATGGTCTGCGGTCTATCAGCTAGTTGGTGGGGTAAAAGCCT
ACCAAGGCTATGACGGATAGGGGGCGTGAGAGCGCGACCCCCAAGAATGGCACTGCGACACGGGC

>Zotu7699
GAACGCTAGCGGCAGGCCTAATACATGCAAGTCGAACGGAGTAGTAATACTTAGTGGCAAACGGGTGCGT
AACGCGTAAGCAACCTGCCTTCTACTGGGGGATAGCCCGGCGAAAGCTGGGGTAAACCCGCACGGTCCAG
AAGAATCACCTGTTTTTCTGGTAAACATTTATGGGTAGAAGAGGGGCTTGCGTCTGATTAGCTAGTTGGC
GGGGTAACGGCCCACCAAGGCGATGATCAGTAGGGGTTCTGAGAGGATTGGCCCCCACATGGGTACTGA
GATACGGAC
>Zotu7701
CAACGCTGGCGGCGTGCCTCAGACATGCAAGTCGAACGATTAAGACTCCCTTCGGGGAGTGTATAAAGTG
GCGCACGGGTGAGTAACACGTAAGTAATCTACCTTCGAGTGGGGAATAACGTTCGGAAACGGACGCTAAT
ACCGCATAACGCAGCGGCATCGCAAGATGACAGTTGTTAAAGGAGCAATCCGCTTGAAGAGGAGCTTGCG
GCAGATTAGCTTGTTGGTAAGGTAACGGCTTACCAAGGCTACGATCTGTATCCGGCCTGAGAGGGCGGTC
GGACACACTGACACTGAATAACGGGT
>Zotu7703
GAACGCTGGTGGCGTGCTTAACACATGCAAGTCGAGCGAGGCGTCCGCCTCCGGGCGGTCAGCCGAGCG
GCGAACGGGTGAGTAACACGTGAGCAATCTTCCCAAGGCTCGGGGATAACCTCGGGAAACTGAGGCTAAT
ACCGGATGAGCGTTTTTCGGGACATCCCGGAAAACGAAAAGGTTTCGCACTGCGAGGTGCGTTTCCGGCCT
TGGATGAGCTCGCGGCCTATCAGCTTGTTGGTGCGGTAAATGCGCACCAAGGCGACGACGGGTAGCCGGC
CTGAGAGGGTGATCGGCCACACTGGGACTGAGACACGGCC
>Zotu7706
GAACGCTAGCGGCAGGCTTAACACATGCAAGTCGAGGGGTATATTTCTTCGGAGATAGAGACCGGCGCAC
GGGTGCGTAACGCGTATGCAATCTACCTTTTACAGGGGAATAGCCCAGAGAAATTTGGATTAATGCCCCAT
AGTATATATGAATCGCATGGTTTATATATTAAAGTCACAACGGTAAAAGATGAGCATGCGTCCCATTAGTTA
GTTGGTAAGGTAACGGCTTACCAAGACAATGATGGGTAGGGGTCCTGAGAGGGAGATCCCCCACACTGGT
ACTGAGACACGGAC
>Zotu7708
GAATGCTGGCGGCGTGGATAAGGCATGCAAGTCGAGTGGGTTTAGACCCACGGCAGACGAGGTAGTAAC
ACGTAGGAACGTACCCCAAAGTCAGGGATATTCTGTCGAAAGACAGCGCAATACCTGATAGTCTCTAAGGA
GTAAAGATTTATCGCTTTGGGAGCGGCCTGCGTAATATCAGCTAGTTGGTGAGGTAATGGCTCACCAAGGC
TATGACGTTTAGGGGAGCTGAGAGGCTGACCCCCACCGATGGAACTGAGATACGGTC
>Zotu7714
GAACGCTAGCGGCAGGCTTAACACATGCAAGTCGAGGGGTATAGTAGCAATACTAGAGACCGGCGCACG
GGTGCGTAACGCGTATGCAATCTACCTTTAGCAGGGGCATAGCCCAGAGAAATTTGGATTAATTCCCCATA
GTATTATAGTATGGCATCATATTATAATTAAAGTAACAACGGCTAAAGATGAGCATGCGTCCCATTAGCTAG
TTGGTATGGTAACGGCATACCAAGGCAATGATGGGTAGGGGTCCTGAGAGGGAAATCCCCCACACTGGTA
CTGAGACACGGAC
>Zotu7720
GAACATTGGCGGAAAGCTTAACACATGCAAGTCGAGCGAGGCTTAGAGCCTAGCGGCGAACGGGTGCGT
AATACATAGGAATCTGCCCCGAAGTGGGGGACAACAACCAGAAATGGTTGCTAATACCCCATAATCCATAT
TGGGAAAGCGCGCAGCGCTTTGGGATGAGCCTATGGTCGATTAGCTAGTTGGTGGGGTAAAAGCCTACCA
AGGCGACGATCGATAGCTGTTCTGAGAGGATGATCAGCCACACTGGAACTGAGACACGGTC
>Zotu7722
GAACGTTAGCGGCGCGCCTAACACATGCAAGTCGCACGAGAGGGGGCAACCCTGAAAGTGGCGCACGGG
TGCGTAACACGTAGATAACCTGCCCTTTGGAGGGGGATAACGTTCCGAAAGGAGCGCTAATACCGCATAC
AGCATTCTGGGGTTTGCTCTGGGATGGCAAAGGTAGGGACCCGCAAGGGCCTATCGCCGAAGGAGGGGT
CTGCGGCCCATCAGCTAGTTGGGGAGGTAATGGCTCACCAAGGCGAAGACGGGTAGCTGGTCTGAGAGG
ATGATCAGCCACACTGGTACTGGAACACGGAC
>Zotu7724
GAACGCTGGCGGCGTGCTTAACACATGCAAGTCGAACGGTGAAAGAGAGCTTGCTCTCTGGATCAGTGGC
GAACGGGTGAGTAACACGTGAGCAATCTGCCCCTGACTCTGGGATAACAGCTGGAAACAGCTGCTAATAC
CGGATACGAGCTGCGAAGGCATCTTCAGCAGCTGGAAAGAATTTCGGTCAGGGATGAGCTCGCGGCCTAT

CAGCTAGTTGGTGAGGTAATGGCTCACCAAGGCGTCGACGGGTAGCCGGCCTGAGAGGGTGACCGGCCA
CACTGGGACTGAGACACGGCC
>Zotu7728
GAATGCTGGCGGCGTGGATAAGTCATGCAAGTCAAGGGGACCGCAAGGTAACCGGCAAACGAGGTAGTA
ACACGTAGGTAGACCCCCTCTAGTCGAGCATAGCCAGCCGAAAGGTTGGGTAATTCTCGATAGTCCCGCAA
GGGTAAAGATTTATCGCTAGAGGAGTTGCCTGCGTAGTATCAGCTAGTTGGTAGGGTAATGGCCTACCAA
GGCTACGACGCTTAGGGGAGCTGAGAGGCTGACCCCCACCGATGGAACTGCGACACGGTC
>Zotu7730
GAACGCTAGCGGCAGGCTTAACACATGCAAGTCGAGGGGTATAGTTCTTCGGAATTAGAGACCGGCGCAC
GGGTGCGTAACGCGTATTCAATCTACCTTTCACAGAGGGATAGCCCAGAGAAATTTGGATTAATACCTCAT
AGTATTACGAGATGGCATCATTTTGTAATTAAAGTCACAACGGTGAAAGATGAGCATGCGTCCCATTAGCT
TGTTGGTAAGGTAACGGCTTACCAAGGCTACGATGGGTAGGGGTCCTGAGAGGGAGATCCCCCACACTGG
TACTGAGACACGGAC
>Zotu7732
GAACGCTAGCGGCATGCTTAACACATGCAAGTCGAACGGTAACAGGGAGCTTGCTCCGCTGACGAGTGGC
GAACGGGTGAGTAATATATCGGAACGTGCCCAAGAGTGGGGGATAACGTAGCGAAAGTTACGCTAATACC
GCATACGATCCAAGGATGAAAGCAGGGGACCTTCGGGCCTTGTGCTCCTGGAGCGGCCGATATCTGATTA
GCTAGTTGGTGAGGTAAAGGCTCACCAAGGCGACGATCAGTAGCTGGTCTGAGAGGACGACCAGCCACAC
TGGAACTGAGACACGGTC
>Zotu7736
GAACGCTAGCGGCAGGCTTAATACATGCAAGTCGAGGGGCAGCACGGGAGCAATCCTGGTGGCGACCGG
CAAACGGGTGCGGAACACGTACGCAACCTTCCCATAACTGGTGAATAGCCTTTCGAAAGAGAGATTAATAC
ACCGTAACATAACGAAGAGGCATCTCTTTGTTATTATAGCCGCAAGGCGGTTTTGGATGGGCGTGCGCCTG
ATTAGGTATTTGGCGGGGTAACGGCCCACCAAGCCTGCGATCAGTAACTGGTGTGAGAGCACGACCAGTC
ACACGGGCACTGAGACACGGGC
>Zotu7739
GAACGCTGGCGGCGCGCCTAACACATGCAAGTCGAACGGCACCTATCTTCGGATAGAAGCGAGTTGCGAA
CGGCTGAGTAACACGTGGAGAACCTGCCCCCTCCCCCGGGATAGCCGCCCGAAAGGACGGGTAATACCGG
ATACCCCGGGGTGCCGCATGGCACCCCGGCTAAAGCCCCGACGGGAGGGGATGGCTCCGCGGCCCATCAG
GTAGACGGCGGGGTGACGGCCCACCGTGCCGACAACGGGTAGCCGGGTTGAGAGACCGACCGGCCAGAT
TGGGACTGAGACACGGCC
>Zotu7742
TAACGTTGGCGGCGTGCATAACACATGCAAGTTGAGCGTGGCTTTAGTTTACTAAAGTCTAGCAGCGGACG
GCTGAGTAATGCACAGGAATGTACCTCGAAGTGGGGGATAGCCCATCGAAAGATGGGGTAATACCGCATG
TGATCGCAAGATCAAAGCCGCAAGGCGCTTTGAGAGCAGCCTGTGTCCTATCAGATTGTTGGTGGGGTAAT
GGCCTACCAAGTCTATGACGGGTAGCTGGTCTGAGAGGATGACCAGCCAGATCGGGACTGAGACACGGCC
>Zotu7749
GAACGCTGGCGGCGTGCCTAACACATGCAAGTCGAGCGGCAGCGCACCCTTCGGGGTGGCGGCGAGCGG
CGGACGGCTGAGTAACACGTGGGAATGTACCCCAAAGTGAGGAATAACTGCCCGAAAGGGTAGCTAATGC
CGCATAAGCTCTTCGGAGTAAAGGCTTCGGCCGCTTTGGGAACAGCCCGCGTACGATTAGATAGTTGGTGA
GGTAATGGCTCACCAAGTCGACGATCGTTAGCTGGTCTGAGAGGATGACCAGCCAGACTGGAACTGAGAC
ACGGTC
>Zotu7751
GAACGCTGGCGGCATGCTTTACACATGCAAGTCGAACGGTAACAGGGAGCTTGCTCCGCTGACGAGTGGC
GAACGGGTGAGTAATATATCGGAACGTGCCCAAGAGTGGGGGATAACGTAGCGAAAGTTACGCTAATACC
GCATACGATCCAAGGATGAAAGCAGGGGACCTTCGGGCCTTGTGCTCCTGGAGCGGCCGATATCTGATTA
GCTAGTTGGTGAGGTAAAGGCTCACCAAGGCGACGATCAGTAGCTGGTCTGAGAGGATGATCAGCCACAC
TGGGACTGAGACACGGCC
>Zotu7753

GAACGCTGGCGGCGTGCTTAACACATGCAAGTCGAACGGTGATCTTGAGAGCTTGCTCTTGGGTGATCAGT
GGCGAACGGGTGAGTAACACGTGAGGAACCTGCCCCAGACTCTGGAATAACCCCGGGAAACCGGAGCTA
ATACCGGATATGACACCAGATCGCATGGTCATGGTGTGGAAAGTTTTTCGGTCTGGGATGGCCTCGCGGCC
TATCAGCTTGTTGGTGAGGTAGTGGCTCACCAAGGCGACGACGGGTAGCCGGCCTGAGAGGGCGACCGG
CCACACTGGGACTGAGACACGGCC
>Zotu7755
GAACGCTGGCGGCGTGCTTAACACATGCAAGTCGAACGGTAAGGCCCTTTCGGGGGTACACGAGTGGCGA
ACGGGTGAGTAACACGTGAGTAACCTGCCCCTAACTCTGGGATAACTCCTGGAAACAGGTGCTAATACCGG
ATATTTAGCTGGGACTGCATGGTCCTGGTTGGAAAGCTCCGGCGGTTAGGGATGGACTCGCGGCCTATCA
GCTTGTTGGTGAGGTAATGGCTCACCAAGGCTTCGACGGGTAGCCGGCCTGAGAGGGTGACCGGCCACAT
TGGGACTGAGATACGGCC
>Zotu7757
GAACGCTGGCGGCGTGCCTAATACATGCAAGTCGAGCGAATCTTTGGGAGCTTGCTCCCATTGGTTAGCGG
CGGACGGGTGAGTAACACGTGGGCAACCTGCCTGTAAGACTGGGATAACTTCGGGAAACCGGAGCTAATA
CCGGATAATCCATTTCCTCTCATGGGGAAATGCTGAAAGACGGTTTCGGCTGTCACTTACAGATGGGCCCG
CGGCGCATTAGCTAGTTGGTGAGGTAACGGCTCACCAAGGCGACGATGCGTAGCCGACCTGAGAGGGTG
ATCGGCCACACTGGGACTGAGACACGGCC
>Zotu7759
GAACGCTGGCGGCGCGCCTAACACATGCAAGTCGAACGCGAAAGGGAGCAATCCTGAGTAAAGTGGCGC
ACGGGTGAGTAACGCGTAGGTAATCTACCTTTGCGACCGGGATAACCAGCCGAAAGGTTGGCTAATACTG
GATAGAGCGCACGATCCATAAAGGATTGTGCGGGAAAGGGTGGCGTAGCAATACGCTACTGCGCTGAGAT
GAGCCTGCGTCTCATTAGCTAGTTGGTAGGGTAAAGGCCTACCAAGGCGATGATGGGTAGCTGGTCTGAG
AGGACGATCAGCCACACTGGAACTGAAACACGGTC
>Zotu7762
GAACGCTGGCGGCATGCCTAACACATGCAAGTCGAACGCCATGGTTTCGGCCATGGAGTGGCGCACGGGT
GAGTAACGCGTGGGAACCTGCCTAGCAGTATGGGACAACCGCGGGAAACTGCGGCTAATACCGTATACGC
CCGTCTTTGGGAGACCAGGGGCGGGGAAAGATTGATCGCTGTTAGAGGGGCCCGCGTCGGATTAGGTAG
TTGGTGGGGTAAAGGCCTACCAAGCCTGCGATCCGTAGCTGGTCTGAGAGGATGATCAGCCACACTGGGA
CTGAGACACGGCC
>Zotu7764
GAACGCTGGCGGCATGCTTAACACATGCAAGTCGAACGGTAACAGGCCCTTCGGGGTGCTGACGAGTGGC
GGACGGGTGAGTAAAGCGTAGGAATCTATCTAGTGGAGTGGGATAACCTAGGGAAACTTAGGGTAATACC
GCATAAGTTCTAAGGAATAAAGCAGGGGACCTTCGGGCCTTGCGCTATTAGCTGAGCCTACGTCGGATTAG
CTAGTTGGTGGGGTAAAGGCCTACCAAGGCGATGATCCGTAGCTGGTTTGAGAGGACGACCAGCCACACT
GGGACTGAGACACGGCC
>Zotu7770
GAACGCTGGCGGAATGCTTTACACATGCAAGTCGAACGGCAGCACGGGGGCAACCCTGGTGGCGAGTGG
CGAACGGGTGAGTAATACATCGGAACGTGCCCAGTCGTGGGGGATAACGTAGCGAAAGCTACGCTAATAC
CGCATACGAACTCTGGTTGAAAGCGGGGGATCGCAAGACCTCGCGCGATTGGAGCGGCCGATGGCAGATT
AGGTAGTTGGTGGGGTAAAGGCTCACCAAGCCGACGATCTGTAGCTGGTCTGAGAGGACGACCAGCCACA
CTGGGACTGAGACACGGCC
>Zotu7774
CAACGCTGGCGGCGCGCTTAACACATGCAAGTCGCACGAGAAAGGGGGCAACCCTGAGTACAGTGGCGT
ACGGGTGAGTAACACGTGGATTATCTACCTTTTAGTGGGGAATACCCCTGGGAAACCGGGGACAATACCG
CATAAGCCCGCAAGGGGAAAGCAGCAATGCGCTGAAAGAGGAGTCCGCGGCTGATTAGCTAGTTGGCGG
GGTAACGGCCCACCAAGGCGAAGATCGGTAGCCGGCCTGAGAGGGCACACGGCCACACTGGCACTGAAA
TACGGGC
>Zotu7776
GAACGCTGGCGGCGTGCTTAACACATTCAAGTCGAACGGTGACGAGAGACTTCGGTCTCTCCGATCAGTG
GCGGACGGGTGAGTAATGCATGAGCAACCTGCCTCGAAGTGGGGAATAACACAGAGAAATTTGTGCTAAT
ACCGCATAATGTGTTCGGGAGGCATCTCCTGAACAAGAAAGGATTTATTCGCTTTGAGATGGGCTCATGTC

TGATTAGGTAGTTGGTGGGGTAACGGCCTACCAAGCCTGCGATCAGTAGCCGGACTGAGAGGTTGAACGG
CCACATTGGGACTGAGATACGGCC
>Zotu7781
GAACGCTGGCGGCAGGCTTAACACATGCAAGTCGAGCGCCCCGCAAGGGGAGCGGCAGACGGGTGAGTA
ACGCGTGGGAATCTACCCATCTCTACGGAATAACTCAGGGAAACTTGTGCTAATACCGTATACGCCCTTTGG
GGGAAAGATTTATCGGAGATGGATGAGCCCGCGTTGGATTAGCTAGTTGGTGGGGTAAAGGCCTACCAAG
GCGACGATCCATAGCTGGTCTGAGAGGATGATCAGCCACACTGGGACTGAGACACGGCC
>Zotu7783
GAACGTTAGCGGCGCGCCTAAGACATGCAAGTCGAGCGAGAAAGGGCTTCGGCCCCGGTAAAGCGGCGC
ACGGGTGAGTAACACGTAGGTAATCAACCCTCGAGTGGTGGACAACTCTCCGAAAGGAGAGCTAATACAG
CATGATGGCGCGACTTTACGAAGTTGTGCCTAAAGCGGGCCTCTCCACGGAAGCTCGCGCTTGAGGATGA
GCCTGCGGCCCATCAGCTAGTTGGTTGGGGTAATGGCCCACCAAGGCTACGACGGGTAGCTGGTCTGAGA
GGATGAACAGCCACACTGGAACTGAGACACGGTC
>Zotu7789
GAACGCTGGCGGCGTGCTTAACACATGCAAGTCGAACGGGCTTTTCGGAGCTAGTGGCGGACGGGTGAGT
AATACATAGGTAATCTGCCTTTAAGTGGGGGACAACCAGGCGAAAGTTTGGCTAATACCGCGTAATGTCGT
TGGTTGAGATACGAACGAAGAAAGATTTATTGCTTTTAGAGGAGCCTATGTTTGATTAGCTAGTTGGTGTG
GTAAAGGCTCACCAAGGCGACGATCAATAGCTGGTCTGAGAGGACGATCAGCCACACTGGGACTGAGACA
CGGCC
>Zotu7792
GAACGCTGGCGGCATGCCTAACACATGCAAGTCGAACGATGCCTTCGGGCATAGTGGCGCACGGGTGCGT
AACGCGTGGGAATCTGCCCTTGGGTTCGGAATAACTCGCCGAAAGGCGTGCTAATACCGGATAATGACGT
AAGTCCAAAGATTTATCGCCCAAGGATGAGCCCGCGTAGGATTAGCTAGTTGGTGGGGTAAAGGCCCACC
AAGGCGACGATCCTTAGCTGGTCTGAGAGGATGATCAGCCACACTGGGACTGAGACACGGCC
>Zotu7795
GAACGCTGGCGGCGTGCTTAAGACATGCAAGTCGAACGCACCTCTTCGGAGGTGAGTGGCGCACGGGTGA
GTAACGCGTAACTGACGTACCTCGAAGTCTGGGATAACGTCCCGAAAGGGACGCTAATACCGGATGTGCA
GTCATCTTTTGGGATGCCTGTAAAGCCTTGAGTGCTTCGAGCTCGGGTTGCGTTCCATCAGCTAGTAGGTA
GGGTAAAGGCCTACCTAGGCAACGACGGATAGCCGGCCTGAGAGGGTGGCCGGCCACAGGGGCACTGAG
ACACGGGC
>Zotu7798
GAACATTGGCGGAAAGCTTAACACATGCAAGTCGAGCGAGGCTTAGAGCCTAGCGGCGAACGGGTGCGT
AATACATAGGAATCTGCCCCGAAGTGGGGGACAACAACCAGAAATGGTTGCTAATACCCCATAATCCATAT
TGGGAAAGCGCGCAGCGCTTTGGGATGAGCCTATGGTCGATTAGCTAGTTGGTGGGGTAAAAGCCTACCA
AGGCGACGATCGATAGCTGGTCTGAGAGGATGATCAGCCACACTGGAACTGAGACACGGTC
>Zotu7804
GAACGCTGGCGGCGTGCTTAACACATGCAAGTCGAACGGTGATGCAGAGCTTGCTCTGCGGATCAGTGGC
GAACGGGTGAGTAACACGTGAGTAACCTGCCCTTGACTCTGGGATAAGCGTTGGAAACGACGTCTAATAC
CGGATACGACCCGTGGAGGCATCTCCAACGGGTGGAAAGAATTTTGGTTGAGGATGGACTCGCGGCCTAT
CAGCTTGTTGGTGAGGTAATGGCTCACCAAGGCGACGACGGGTAGCCGGCCTGAGAGGGTGACCGGCCA
CACTGGGACTGAGACACGGCC
>Zotu7807
GAACGCTGGCGGTATGCCTTATACATGCAAGTCGTACGGGAGTTTTTAACTCAAGTGGCGGACGGGTGAG
TAACACGTAAGAATTTGCCTTCAGGAGGGGGATAACAACTGGAAACGGTTGCTAATACCCCATATGCTTTA
GAGTGAAACGGTTTAAACTCCGCCTGAAGAGAAGCTTGCGGCTGATTAGCTTGTTGGTGAGGTAATGGCTC
ACCAAGGCGACGATCAGTATCTGGTTTGAGAGGACGATCAGACACACTGGAACTGAGACACGGTC
>Zotu7810
TAAAACATCATCAACATCTGCGGAAGTCGCAGCTACTAAACAAGATATACAACACGTTTCGAAAGGGCGGC
CACAACATTGGACTATTTTTA
>Zotu7814

GAACGCTGGCGGCGTGCCTAACACATGCAAGTCGAACGGGACCAGGGGCAACTCTGGTTCAGTGGCGGA
CGGGTGCGTAACACGTGAGGAACATGACTTTCGGTGGGGGATAGCCGGCCCAACGGCCGGGTAATACCG
CGTACGACCTTTTGGGGACATCCCTGATTGGTGAAAGCAGCAATGCGCCGAAAGAGTGCCTCGCGGCCTAT
CAGCTAGTTGGTGGGGTAACGGCTCACCAAGGCGACGACGGGTAGCTGGTCTGAGAGGATGGCCAGCCA
CATTGGGACTGAGACACGGCC
>Zotu7817
GAACGCTGGCGGCAGGCCTAATACATGCAAGTCGGACGAGTAAGAGCCCGCAAGGGAGTTCTGCTAGTG
GCAAACGGGTGCGGAACACGTGGGAATCTACCCAAAAGTATGGAACAACAGTTGGAAACGACTGCTAATA
CCATATACGCTCGAAAGAGGAAAGATTTATCGCTTTTGGATGAGCCCGCGTAAGATTAGCTAGTTGGTGAG
GTAATGGCTCACCAAGGCAACGATCTTTAGCTGGTCTGAGAGGACGATCAGCCACACTGGGACTGAGATA
CGGCC
>Zotu7819
GAACGCTGGCGGCGTGCTTAACACATGCAAGTCGAACGGTGACCTCGAGAGCTTGCTCTTGGGTGAACAG
TGGCGAACGGGTGAGTAACACGTGAGTAACCTGCCCCTGACTCTGGAATAAGCGCTGGAAACGGCGTCTA
ATACCGGATATGACACACCCTCGCATGGGGAGTGTGTGGAAAGTTTTTCGGTCTGGGATGGACTCGCGGC
CTATCAGCTTGTTGGTGAGGTAATGGCTCACCAAGGCGATGACGGGTAGCCGGCCTGAGAGGGCGACCGG
CCACACTGGGACTGAGACACGGCC
>Zotu7822
GAACGCTGGCGGCGTGCTTAACACATGCAAGTCGAGCGGTAAGGCCCTTCGGGGTACACGAGCGGCGAA
CGGGTGAGTAACACGTGAGCAATCTGCCCTTCACTTTGGGATAACCCCCGGAAACGGGAGCTAATACCGA
ATATGACCCTGCCATGCATGTGGTGGGGTGGAAAGTTCTGGCGGTGGAGGATGAGCTCGCGGCCTATCAG
CTTGTTGGTGGGGTAATGGCCTACCAAGGCTTCGACGGGTAGCCGGCCTGAGAGGGTGACCGGCCACACT
GGGACTGAGACACGACC
>Zotu7827
GAACGCTGGCGGCAGGCTTAACACATGCAAGTCGAGCGCCCCGCAAGGGGAGCGGCAGACGGGTGAGTA
ACGCGTGGGAATCTACCCATCTCTACGGAACAACTCCGGGAAACTGGAGCTAATACCGTATACGTCCTGAG
GGAGAAAGATTTATCGGAGATGGATGAGCCCGCGTCGGATTAGCTAGTTGGTGGGGTAATGGCCTACCAA
GGCGACGATCCGTAGCTGGTCTGAGAGGATGATCAGCCACACTGGGACTGAGACACGGCC
>Zotu7830
GAACGCTGGCGGCATGCTTTACACATGTAAGTCGAACGGCAGCACGGACTTCGGTCTGGTGGCGAGTGGC
GAACGGGTGAGTAATACATCGGAACGTGCCCAGTTGTGGGGGATAACTACTCGAAAGAGTAGCTAATACC
GCATGAGAACTGAGGTTGAAAGCAGGGGATCGCAAGACCTTGCGCAACTGGAGCGGCCGATGGCAGATT
AGGTAGTTGGTGGGATAAAAGCTTACCAAGCCGACGATCTGTAGCTGGTCTGAGAGGACGACCAGCCACA
CTGGGACTGAGACACGGCC
>Zotu7832
GAACGCTTGCGGCGTGCCTAAGAAATGCAAGTCGAACGGATCACCTTCGGGTGGTTAGTGGCGAACGGTC
GCGTAACACGTAGGCAACCTGCCTCAAAGTGGGGGACAACAGCCCGAAAGGGCTGCTAATACCGCATGTG
ATTCCTTGGAGGCATCTCTCTGGAATCAAAGCCTGAGTTTACTTAGGCGCTTTGAGATGGGCTTGCGGCCTA
TCAGCTTGTTGGTGAGGTAATGGCTTACCAAGGCGATGACGGGTAGCTGGTCTGAGAGGACGATCAGCCG
GATTGGGACTGAGATACGGCC
>Zotu7835
GAACGCTGGCGGCAGGCTTAACACATGCAAGTCGAGCGGAGATGAGGTGCTTGCACCTTATCTTAGCGGC
GGACGGGTGAGTAATGCTTAGGAATCTGCCATTTAGTGGGGGACAACATCTCGAAAGGGATGCTAATACC
GCATACGCCCTACGGGGGAAAGCAGGGGATCTTCGGACCTTGCGCTAAATGATGAGCCTAAGTCGGATTA
GCTAGTTGGTGGGGTAAAGGCCTACCAAGGCGACGATCTGTAGCGGGTCTGAGAGGATGATCCGCCACAC
TGGGACTGAGACACGGCC
>Zotu7837
GAACGCTGGCGGCGTGCTTAACACATGCAAGTCGAACGAGGTCCATGGAGCTTGCTCCGGAAGATCTAGT
GGCGAACGGGTGCATAACACGTGAGAAACCTGCCTCAGTCTCTGGGATAACAGTTGGAAACGACTGCTAA
TACCGGATGCCGTCACACCATCACATGGTGGAGTGACGAAAGATTTATCGGATTGAGAGGGTCTCGCGGC

CTATCAGCTAGTTGGAGAGGTAACGGCTCACCAAGGCATCGACGGGTAGCTGGTCTGAGAGGATGATCAG
CCACACTGGGACTGAGACACGGCC
>Zotu7840
GAACGCTGGCGGCATGCCTAATACATGCAAGTCGAACGAACCATCCTGATGATTGAAGCTTGCTTCATGAT
TCAGATTTTGGTGAGTGGCGGACGGGTGAGTAACACGTGGGTAACCTGCCCAGAAGTGGGGGATAACATT
TGGAAACAAGTGCTAATACCGCATAACAACTTAGATCACATGATCTTTGTTTAAAAGATGGTTTTGCTATCT
CTTCTGGATGGACCCGCGGCGTATTAGCTAGTTGGTGAGGTAATAGCTCACCAAGGCGATGATACGTAGCC
GACCTGAGAGGGTAATCGGCCACATTGGGACTGAGACACGGCC
>Zotu7844
GAACGCTGGCGGCATGCCTAACACATGCAAGTCGAACGAAGCCTTCGGGCTTAGTGGCGCACGGGTGCGT
AACGCGTGGGAATCTGCCCTTAGGTACGGAATAACTCATGGAAACGTGTGCTAATACCGTATGATGTCGCG
AGACCAAAGATTTATCGCCTAGGGATGAGCCCGCGTTGGATTAGCTAGTTGGTGGGGTAAAGGCCTACCA
AGGCGACGATCCATAGCTGGTCTGAGAGGATGATCAGCCACACTGGGACTGAGACACGGCC
>Zotu7848
GAACGCTGGCGGCATGCCTAACACATGCAAGTCGAACGAACCCTTCGGGGTGAGTGGCGCACGGGTGCGT
AACGCGTGGGAACCTGCCTTTTGGTTCGGAATAACAGTTAGAAATGACTGCTAATACCGGATAATGTCTTC
GGACCAAAGATTTATCGCCATTAGATGGGCCCGCGTAGGATTAGCTAGTTGGTAGGGTAAAGGCCTACCA
AGGCGACGATCCTTAGCTGGTCTTAGAGGATGATCAGCCACACTGGGACTGAGACACGGCC
>Zotu7850
GAACGCTGGCGGCGTGCCTAATACATGCAAGTCGAGCGAATCTTTGGGAGCTTGCTCCTAAAGGTTAGCG
GCGGACGGGTGAGTAACACGTGGGCAACCTGCCTATAAGACTGGGATAACTTCGGGAAACCGGAGCTAAT
ACCGGATCATTCTTTTCTACACATGTAGAAAAGCTAAAAGACGGTTTACGCTGTCGCTTATAGATGGGCCCG
CGGCGCATTAGCTAGTTGGTGAGGTAACGGCTCACCAAGGCCACGATGCGTAGCCGACCTGAGAGGGTGA
TCGGCCACACTGGGACTGAGACACGGCC
>Zotu7853
GAACGCTAGCGGGAGGCCTAACACATGCAAGCCGAGCGGGATTTGTTGGTTAGCTTGCTAACTAACAATG
AGAGCGGCGTACGGGTGCGTAACACGTGTGCAACCTGCCTTTATCTGGGGGATAGCCTTTCGAAAGGAAG
ATTAATACCCCATAATATATTGATTGGCATCAATTGATATTGAAAGCTCCGGCGGATAGAGATGGGCACGC
GTAAGATTAGCTAGTTGGTGAGGTAACGGCTCACCAAGGCTGCGATCTTTAGGGGGCCTGAGAGGGTGAT
CCCCCACACTGGTACTGAGACACGGAC
>Zotu7856
GAACGCTGGCGGCATGCTTTACACATGCAAGTCGGACGGCAGCACAGAGAAGCTTGCTTCTTGGGTGGCG
AGTGGCGAACGGGTGAGTAACATATCGGAACGTACCGAGCAGTGGGGGATAACTAATCGAAAGATTAGCT
AATACCGCATATCTTCTGAGGAAGAAAGCAGGGGACCATTTGGCCTTGCGCTGTTTGAGCGGCCGATATCT
GATTAGCTAGTTGGTGGGGTAAAGGCCTACCAAGGCGACGATCAGTAGCGGGTCTGAGAGGATGATCCGC
CACACTGGGACTGAGACACGGCC
>Zotu7858
GAACGGTAGCGGCGTGGATTAGGCATGCAAGTCGGACGGGATCCCCGCTTCGGTGGGGTGAGAGTGGCG
AAAGGGTGAGTAACGCATAGATAACCTTCCCCCGAGCTCGGGATAACCGAGGGAAACTTCGGCTAATACC
GGATGGGCTCGTGAGGCCGCATGGTCTCGCGAGTAAAGGTGGCCTCTCAACGATGCTTCCACTTGGGGAC
GGGTTTATGTCCTATCAGCTTGTTGGTGAGGTAACGGCTCACCAAGGCGATGACGGGTAGCCGACCTGAG
AGGGTGACCGGCCACACTGGGACTGAGACACTGCC
>Zotu7860
GAACGCTGGCGGCAGGCCTAACACATGCAAGTCGAGCGGGGGAGCAATCCCTAGCGGCGGACGGGTTAG
TAACGCGTGGGAACGTGCCCAAAGGTACGGAATAGCCTCGGGAAACTGAGAGTAATACCGTATGTGCCCC
TCGGGGGAAAGATTTATCGCCTTTGGATCGGCCCGCGTTGGATTAGGTAGTTGGTGGGGTAATGGCCTAC
CAAGCCGACGATCCATAGCTGGTTTGAGAGGATGATCAGCAACACTGGGACTGAGACACGGCC
>Zotu7863
GAACGCTGGCGGCGTGCTTAACACATGCAAGTCGAACGGAAAGGCTCCAGCTTGCTGGAGTACTCGAGTG
GCGAACGGGTGAGTAACACGTGGGTGATCTGCCCTGCACTTCGGGATAAGCCTGGGAAACTGGGTCTAAT

ACCGGATAGGACCGCACTTTGGTGTGTGTGGTGGAAAGGTTTTTCTGGTGTGGGATGAGCTCGCGGCCTA
TCAGTTTGTTGGTGGGGTAATGGCCTACCAAGACGGCGACGGGTAGCCGGCCTGAGAGGGTGGACGGTC
ACATTGGGACTGAGATACGGCC
>Zotu7866
GAACGCTGGCGGCAGGCCTAACACATGCAAGCCGAACGGACCCTTCGGGGTTAGTGGCGGACGGGTGAG
TAACACGTGGGAACGTGCCTTTAGGTTCGGAATAGCTCCTGGAAACGGGTGGTAATGCCGAATGTGCCCTT
CGGGGGAAAGATTTATCGCCTTTAGAGCGGCCCGCGTCTGATTAGCTAGTTGGTGAGGTAAAGGCTCACC
AAGGCTACGATCAGTAGCTGGTCTGAGAGGATGACCAGCCACATTGGGACTGAGACACGGCC
>Zotu7869
GAACGCTGGCGGCGTGCCTAATACATGCAAGTCGAACGAACGGACATTGGAACTTGTTCTAATGGAAGTG
AGTGGCGAACGGGTGAGTAACACGTGGGTAACCTACCTTTAAGCGGGGGATAACAGTGGGAAACGACTG
CTAATACCGCATAGGACATAGCGTCACATGACGCAGTGAGGAAAGACGGTTTCGGCTGTCACTTAGAGAT
GGACCCGCGGTGCATTAGCTAGTTGGTAAGGTAACGGCTTACCAAGGCGATGATGCATAGCCGACCTGAG
AGGGTGATCGGCCACATTGGGACTGAGACACGGCC
>Zotu7872
AAACGCTGGCGGCGTGCATTATGCATGCAAGTCGAACGGTGCCGCAAGGCACAGTGGCAAACGGGTGAG
TAACACGTAAGTAACCTGCCTCTAAGTGGGGAATAACTTCGGGAAACTGAGGCTAATACCGCATACGGTGT
GCTCCCGCAGGGGGGTTCACCAAAGCCGTAAGGCGCTTGGAGAGGGGCTTGCGTCCGATTAGCTAGTTGG
TGGGGTAACGGCCTACCAAGGCTGTGATCGGTAGCTGGTCTGAGAGGATGGTCAGCCACACTGGGACTGA
GACACGGCC
>Zotu7877
GAACGCTGGCGGCGTGCCTAAGACATGCAAGTCGAGTGGGTTTAGACCCACGGCGAACGGGTGAGTAAT
ACCTAGGAATGTATCTTTAGTGGGGCATAACCAGCCGAAAGGTTGGACAATTCTCCATGTGATCCGCCGTA
AGGCGGATTAAAGTCGCAAGACGCTAAAGAAGCAGCCTGGGCACCATCAGCTAGTTGGTAAGGTAATGGC
TTACCAAGGCTATGACGGTTAGGGGCCCCGAGAGGGGAGACCCCCACACTGGAACTGCGACACGGTC
>Zotu7879
GAACGCTAGCGGCAGGCTTAACACATGCAAGTCGAGGGGTATGGTTCTTCGGAACCAGAGACCGGCGCAC
GGGTGCGTAACGCGTATGCAATCTACCTTTTACAGAGGGATAGCCCAGAGAAATTTGGATTAATACCTCAT
AGTATATAGAGTTTGCATCAACACTATATTAAAGTTCCAACGGTAAAAGATGAGCATGCGCCCCATTAGCTA
GATGGTAAGGTAACGGCTTACCATGGCAACGATGGGTAGGGGTCCTGAGAGGGAGATCCCCCACACTGGT
ACTGAGACACGGAC
>Zotu7882
GAACGCTGGCGGCGTGCCTAAGACATGCAAGTCGAGTGGGTTTAGACCCACGGCGAACGGGTGAGTAAT
ACCTAGGAACTTTCTTTAAGTGGAGCATAACCCATAGAAATGTGGGATAATACTCCATGTGATCGCAAGAT
CAAAGTCGCAAGACGCTTAAAGTGAGGCCTGGGCACCATCAGCTAGTTGGTAAGGTAACGGCTTACCAAG
GCTATGACGGTTAGGGGTCCCGAGAGGGATAACCCCCACACTGGAACTGCGACACGGTC
>Zotu7885
GAACGCTGGCGGCAGGCCTAACACATGCAAGTCGAACGCTCTAGCAATAGAGAGTGGCGCACGGGTGCG
TAACACGTGGGAATATACCCTTGACTACGGGACAACATCGGGAAACTGATGCTAATACCGTATAATGTCTT
CGGACCAAAGATTTATCGGTCAGGGATTAGCCCGCGCCTGATTAGGTAGTTGGTGAGGTAATGGCTCACCA
AGCCTACGATCAGTAGCTGGTCTGAGAGGATGATCAGCCACACTGGAACTGAGACACGGTC
>Zotu7889
GAACGCTGGCGGCGTGCTTAACACATGCAAGTCGAACGGAAAGGCCCTTCGGGGTACTCGAGTGGCGAAC
GGGTGAGTAACACGTGGGTGATCTGCCCTGCACTTCGGGATAAGCCTGGGAAACTGGGTCTAATACCGGA
TATGACCGCTCATCTCCTGGTGGGTGGTGGAAAGCTTTTGCGGTGTGGGATGGGCCCGCGGCCTATCAGCT
TGTTGGTGGGGTAATGGCCTACCAAGGCGTCGACGGGTAGCCGGCCTGAGAGGGTGTCCGGCCACACTG
GGACTGAGATACGGCC
>Zotu7894
GAACGCTGGCGGCGTGCCTTAAGCATGCAAGTCGCGCGAACCGCAAGGTTAGCGGCGGACGGGTGAGTA
ACGCGTGGGAATCTGCCTCAAAGTATAGGATAGCTTGCCGAAAGGTGAGGTAATACTGTATGTGATCGCA

AGATTAAAGCCGTAAGGCGCTTTGAGATGAGCCCGCGTAAGATTAGCTAGTTGGTGGGGTAAAGGCCTAC
CAAGGCGACGATCTTTAGCTGGTCTGAGAGGATGGTCAGCCACAATGGGACTGAGACACGGCC

>Zotu7897

GAACGCTGGCGGCGTGCCTAATACATGCAAGTCGAGCGGTAAGGCTCCTACAGATTGTTCAAGCGAAAGT
TTGAATGATGTGTTGGAGTACACGAGCGGCGGACGGCTGAGTAACACGTAGGAACGTACCCCAAAGTGAG
GGATAAGCACCAGAAATGGTGTCTAATACCGCATGTGCTCTTCGGAGGAAAGCTTCGGCGCTTTGGGAAC
GGCCTGCGCATGATTAGCTTGTTGGTGAGGTAATGGCTCACCAAGGCGATGATCATTAGCTGGTCTGAGAG
GATGATCAGCCAGACTGGAACTGAGAACGGTC

>Zotu7900

GAACGCTAGCGGCGGGCCTAATACATGCAAGTCGAACGGATTAAGTCTTTCGGGGTTTAGTTAGTGGCGC
ACGGGTGAGTAACGCGTACACAACGTGGCTCTCAGAGGGGGATAGCCTTGGGAAACTGAGCGTAATACCC
CATAGTATTATCGGAAGGCATCTTTTGATAATTAAACCTACGGGGCTGAGAGATCGGTGTGCGTTAGATTA
GGTAGTTGGTGGGGTAACGGCTCACCAAGCCTGCGATCTATAAGGGGCGTGAGAGCGTGATCCCTCACAC
GGGTACTGAGACACGGAC

>Zotu7904

GAACGCTGGCGGCGTGCCTAACACATGCAAGTCGTACGTGAAAGTCCTTCGGGATAAGTAAAGTGGCGCA
CGGGTGAGTAACACGTGGGTAATCTACCGGGAAGTGAGGAATAACGACTGGAAACGGTCGCTAATACCGC
ATGAGCCCCGCAAGGGGGAAAGGAGGCCTCTGCATATGCTTCCGCTTTTCGATGAGCCCGCGTCCCATTAG
CTAGTTGGCGGGGTAACGGCCCACCAAGGCGACGATGGGTAGCTGGTCTAAGAGGATGATCAGCCACATT
GGAACTGGAACACGGTC

>Zotu7907

GAACGCTGGCGGCGTGCCTAATACATGCAAGTCGAGCGGTAAGGCTCCTGCAGATTGTTCGATGATATACA
GACAAGGGAAGGCTCCCGAGTTTGATCGAATAATGTGCTGGAGTACACGAGCGGCGGACGGCTGAGTAA
CACGTAGGAACGTACCCCAAACTGAGGGATAAGCACCAGAAATGGTGTCTAATACCGCATGTGCTCTTCTG
AGTAAAGCTACGGCGGTTTGGGAACGGCCTGCGTAAGATTAGCTAGTTGGTGAGGTAATAGCTCACCAAG
GCGACGATCTTTAGCTGGTCTGAGAGGATGATCAGCCAGACTGGGACTGAGAACGGCC

>Zotu7910

GAACGCTGGCGGCATGCCTAACACATGCAAGTCGAACGAAGGCTTCGGCCTTAGTGGCGCACGGGTGCGT
AACGCGTGGGAATCTGCCCTTAGGTACGGAATAACAGTGAGAAATTACTGCTAATACCGTATGATGACGTA
AGTCCAAAGATTTATCGCCTAGGGATGAGCCCGCGTAGGATTAGCTTGTTGGTGAGGTAAAGGCTCACCAA
GGCGACGATCCTTAGCTGGTCTGAGAGGATGATCAGCCACACTGGGACTGAGACACGGCC

>Zotu7913

GAACGCTGGCGGCGTGCCTAATACATGCAAGTCGAGCGGATCTTTGGGAGCTTGCTCCTAAAGATTAGCG
GCGGACGGGTGAGTAACACGTGGGCAACCTGCCTGTAAGACTGGGATAACACCGGGAAACCGGTGCTAA
TACCGGATAATTCTTTCTCTCTCATGAGGGAAAGCTGAAAGTCGGTTTCGGCTGACACTTACAGATGGGCCC
GCGGCGCATTAGCTAGTTGGTGAGGTAACGGCTCACCAAGGCGACGATGCGTAGCCGACCTGAGAGGGT
GATCGGCCACACTGGGACTGAGACACGGCC

>Zotu7917

GAACGCTGGCGGCATGCCTAATACATGCAAGTCGAACGAACACTTCGGTGTTAGTGGCGCACGGGTGCGT
AACGCGTGGGAATCTACCCTTGGGTTCGGAATAACAGTTAGAAATGACTGCTAATACCGGATGATGACGTA
AGTCCAAAGATTTATCGCCCAAGGATGAGCCCGCGTAAGATTAGCTAGTTGGTAGGGTAAAGGCCTACCA
AGGCTACGATCTTTAGCTGGTCTGAGAGGATGATCAGCCACACTGGGACTGAGACACGGCC

>Zotu7921

GAACGCTGGCGGCATGCTTAACACATGCAAGTCGCACGGGCATTTCGGTGTCAGTGGCGGACGGGTGAGT
AACGCGTAGGAACATATCCAGAGGTGGGGGACAACTCCGGGAAACTGGAGCTAATACCGCATGTGAGCT
GAGGCTCAAAGCCGTGAGGCGCCTTTGGAGTGGCCTGCGTCCGATTAGCTTGTTGGTGGGGTAATGGCCT
ACCAAGGCTTCGATCGGTAGCTGGTCTGAGAGGACGACCAGCCACACTGGGACTGAGACACGGCC

>Zotu7923

GAACGCTGGCGGCGTGCCTAACACATGCAAGTCGAACGAAGCAAGTAAGTGGAATCCCTTCGGGGAGGA
AGCTTGCTTGACTGAGTGGCGGACGGGTGAGTAACGCGTGGGCAACCTGCCCTATAGAGGGGGACAACA

GTTGGAAACGACTGCTAATACCGCATAAGCCTACAGCATCGCATGATGCCGTAGGAAAAGATTTATCACTA
TAGGATGGGCCCGCGTCTGATTAGCTAGTTGGTGGGGTAACGGCCTACCAAGGCAATGATCAGTAGCCGG
CTTGAGAGAGTGAACGGCCACATTGGGACTGAGACACGGCC

>Zotu7926
GAATGCTGGCGACATGGATAAGGCATGCAAGTCAAGGGGGGCCGCAAGGTCAACCGGCAGACGAGGTAG
TAACACGTAGGTACGCACCCCAGAGTCTGGCATAGCTCGTCGAAAGACGGGGTAATTCCGGATGGTCCCG
AAAGGGTAAAGATTTATCGCTCTGGGAGCGGCCTGCGTAGTATCAGCTAGTTGGTAAGGTAATGGCTTACC
AAGGCTACGACGCTTAGGGGAGCTGAGAGGCTGACCCCCACCGATGGGACTGAGACACGGCC

>Zotu7929
GAACGCTAGCGGCAGGCTTAACACATGCAAGTCGAGGGGTATAGTTCTTCGGAACTAGAGACCGGCGCAC
GGGTGCGTAACGCGTATGCAATCTACCTTTTACAGAGGGATAGCCCAGAGAAATTTGGATTAATACCTCAT
AGTATAATGACTCGGCATCGAGATATTATTAAAGTCACAACGGTAAAAGATGAGCATGCGTCCCATTAGCT
AGTTGGTAAGGTAACGGCTTACCAAGGCTACGATGGGTAGGGGTCCTGAGAGGGAGATCCCCCACACTGG
TACTGAGACACGGAC

>Zotu7931
GAACGCTGGCGGCGTGCCTAACACATGCAAGTCGAGCGGGAGACTTCGGTCTCTAGCGGCGGACGGGTG
AGTAACACGTGGGTAACCTGCCTTGTAGAGGGGGATAGCCTTCCGAAAGGAAGATTAATACCGCATAACA
TTATGCTGTCGCATGGCAGTATAATCAAAGGAGTAATCCGCTACAAGATGGACCCGCGGCGCATTAGCTAG
TTGGTGAGGTAACGGCTCACCAAGGCAACGATGCGTAGCCGGCCTGAGAGGGTGAACGGCCACATTGGA
ACTGAGACACGGTC

>Zotu7937
GAACGCTGGCGGTAGGCCTAACACATGCAAGTCGAACGGTAGCACAGAGGAGCTTGCTCCTTGGGTGACG
AGTGGCGGACGGGTGAGGAATGCATCGGAATCTACTCTTTCGTGGGGGATAACGTAGGGAAACTTACGCT
AATACCGCATACGACCTACGGGTGAAAGCAGGGGACCTTCTAGGCCTTGCGCGATTGAATGAGCCGATGT
CCGATTAGCTAGTTGGCGGGGTAATGGCCCACCAAGGCGACGATCGGTAGCTGGTCTGAGAGGATGATCA
GCCACACTGGAACTGAGACACGGTC

>Zotu7941
GAACGCTGGCGGCAGGCTTAACACATGCAAGTCGAGCGGGCGAGGTTGCTTCGGTAACTGAGCTAGCGGC
GGACGGGTGAGTAATGCTTAGGAATCTGCCTATTAGTGGGGGACAACATTCCGAAAGGGATGCTAATACC
GCATACGTCCTACGGGAGAAAGCAGGGGCTCTTTATGACCTTGCGCTAATAGATGAGCCTAAGTCGGATTA
GCTAGTTGGTGGGGTAAAGGCCTACCAAGGCGACGATCTGTAGCGGGTCTGAGAGGATGATCCGCCACAC
TGGGACTGAGACACGGCC

>Zotu7945
GAACGCTGGCGGCAGGCCTAACACATGCAAGTCGAGCGGATGACGGGAGCTTGCTCCTTGATTCAGCGGC
GGACGGGTGAGTAATACCTAGGAATCTGCCTGGTAGTGGGGGACAACGTTTCGAAAGGAACGCTAATACC
GCATACGTCCCACGGGAAAAAGCAGGGGACCTTCGGGCCTTGCGCTATCAGATGAGCCTAGGTCGGATTA
GCTAATTGGTGAGGTAATGGCTCACCAAGGCTACGATCCGTAACTGGTCTGAAAGGATGATCAGTCACACT
GGAACTGAGACACGGTC

>Zotu7949
GAACGCTAGCGGCAGGCCTAATACATGCAAGTCGAACGGTAACAGGTGTAGCAATACATGCTGACGAGTG
GCGCACGGGTGCGTAACACGTATGCAACTTTCCTTCAACTGGTGAATAGCCCCTCGAAAGAGGGATTAATA
CACCATAATATAATGAGGTGGCATCACCTTATTATTAAAACTCCGGTGGTTGAAGATGGGCATGCGCAACA
TTAGCTAGTTGGTGAGGTAATGGCTCACCAAGGCTACGATGTTTAGGGGTTCTAAGAGGATTAACCCCCAC
ACTGGTACTGAGACACGGAC

>Zotu7951
GAACGCTGGCGGCGTGCTTAACACATGCAAGTCGAACGGAGTTAATTTGAGGAAGCAAGCTTGCTTGAAG
AGTTAAATTAACTTAGTGGCGGACGGGCGAGTAACACGTGAGCAACCTGCCTCTTACAGGGGAATAACAA
CGGGAAACCGTTGCTAATACCGCATAACATGTTGAAAGGGCATCCTTTTAACATCAAAGGAGCAATCCGGT
AAGAGATGGGCTCGCGTCCGATTAGCTAGTTGGTAGGGTAACGGCCTACCAAGGCGACGATCGGTAGCCG
GACTGAGAGGTCGAACGGCCGCATTGGGACTGAGACACGGCC

>Zotu7955
GAACGCTGGCGGCGTGCCTAATACATGCAAGTCGAGCGAACTTTTGGGAGCTTGCTCCCAAAAGTTAGCG
GCGGACGGGTGAGTAACACGTGGGCAACCTGCCTGTAAGACTGGGATAACTTCGGGAAACCGGAGCTAAT
ACCGGATAATTCTTATCAACACATGTTGGTAAGCTAAAAGACGGCGCAAGCTGTCACTTACAGATGGGCCC
GCGGCGCATTAGCTAGTTGGTGAGGTAACGGCTCACCAAGGCAACGATGCGTAGCCGACCTGAGAGGGT
GATCGGCCACACTGGGACTGAGACACGGCC
>Zotu7962
TAACGCTAGCGGCAGGCCTAATACATGCAAGTCGAACGGCAGCACGGGTAGCAATACCTGGTGGCGAGTG
GCGCACGGGTGCGTAACGCGTATGCAATCTACCTATAACTGGGGAATAGCCTTCCGAAAGGAAGATTAAC
ACCGCATAAAATCACGAGACGGCATCGCCTTGTGATCAAATCTACGGAGGTTATAGATGAGCATGCGTGAC
ATTAGCTAGTTGGTGAGGTAACGGCTCACCAAGGCGACGATGTCTAGGGGGTCTGAGAGGATGATCCCCC
ACACTGGTACTGAGACACGGAC
>Zotu7964
GAACGCTGGCGGCGCGCTTAACACATGCAAGTCGAGCGAGAACCAGGCCTTCGGGCCTGGGGAAAGCGG
CGAACGGGTGAGTAACACGTGGGTAACCCACCCTTGGTACTGGGATAGCCCGGGGAAACCCGGATTAATA
CCGGATGGCCCAACAGCTCTTCGGGGCGGTTGGAAAAGGTAGCTTCGGCCTCCGACCAAGGACGGGCCCG
CGGCGGATTAGCTTGTTGGTGGGGTAACGGCCCACCAAGGCGACGATCCGTAGCTGGTCTGAGAGGACGA
TCAGCCACACTGGGACTGAGACACGGCC
>Zotu7969
GAACGCTGGCGGCATGCCTAACACATGCAAGTCGAACGAGACCCCGACTTCGGTTGGACGTCTAGTGGCG
CACGGGTGCGTAACGCGTGGGAATCTGCCCCTCGGTTCGGAATAACAGTTAGAAATGACTGCTAATACCG
GATGATGACGAAAGTCCAAAGATTTATCGCCGAGGGATGAGCCCGCGTAAGATTAGCTAGTTGGTAGGGT
AAAGGCCTACCAAGGCGACGATCTTTAGCTGGTCTGAGAGGATGATCAGCCACACTGGGACTGAGACACG
GCC
>Zotu7972
GAACGCTGGCGGCATGCCTAACACATGCAAGTCGAACGAAGCCTTCGGGCTTAGTGGCGCACGGGTGCGT
AACGCGTGGGAATCTGCCCTTGGGTTCGGAATAACAGTTAGAAATGACTGCTAATACCGGATGATGACGTA
AGTCCAAAGATTTATCGCCCAGGGATGAGCCCGCGTAGGATTAGCTAGTTGGTGAGGTAAAAGCTCACCA
AGGCGACGATCCTTAGCTGGTCTGAGAGGATGATCAGCCACACTGGGACTGAGACACGGCC
>Zotu7974
CAACGTTGGCGGCGCGCCTAACACATGCAAGTTGAACGAGAAAGTGGAGCAATCCATGAGTACAGTGGCG
TACGGGTGAGTAACACGTGGGTAATCTACCTTTTAGTGAGGAATAACCAAGGGAAACCATGGCTAATACC
GCATAAGCCTGAGAAGGGAAAGCAGAAATGCGCTGAAAGAGGAGCCCGCGGCCGATTAGCTAGTTGGTG
GGGTAAGAGCCCACCAAGGCAGAGATCGGTAGCCGGCCTGAGAGGGCACACGGCCACACTGGCACTGAA
ACACGGGC
>Zotu7976
GAACGCTGGCGGCAGGCCTAATACATGCAAGTCGAACGCCGTAGCAATACGGAGTGGCAGACGGGTGAG
TAACGCGTGGGAACCTTCCCAATGGTACGGAATAGCCCGGGGAAACCCGGAGTAATACCGTATAAGCCCG
CAAGGGGAAAGATTTATCGCCATTGGATGGGCCCGCGTCGGATTAGCTAGTTGGTGAGGTAACGGCTCAC
CAAGGCGACGATCCGTAGCTGGTCTGAGAGGATGATCAGCCACACTGGGACTGAGACACGGCC
>Zotu7978
GAACGCTGGCGGCGTGCCTAATACATGCAAGTCGAGCGAACTTTTGGGAGCTTGCTCCCAAAAGTTAGCG
GCGGACGGGTGAGTAACACGTGGGCAACCTGCCTATAAGACTGGGATAACTTCGGGAAACCGGAGCTAAT
ACCGGATAATTCTTTTCCTCTCATGAGGAAAAGCTGAAAGACGGCTTCGGCTGTCACTTATAGATGGGCCC
GCGGCGCATTAGCTAGTTGGTGAGGTAACGGCTCACCAAGGCAACGATGCGTAGCCGACCTGAGAGGGT
GATCGGCCACACTGGGACTGAGACACGGCC
>Zotu7980
GAACGCTGGCGGCATGCTTAACACATGCAAGTCGAACGGATAAAGTTTCTAGCTTGCTAGAAATTTTATTA
GTGGCAAACGGGTGAGTAACACGTAGGAATCTCCCCTATAGCACGGAATAACAATTGGAAACGATTGCTA
ATACCGTATACGCTCTTCGGAGGAAAGATTTATCACTATAGGATGAGCCTGCGCTAGATTAGGTAGTTGGT

AGGGTAATGGCCTACCAAGCCGATGATCTATAGCTGGTCTGAGAGGATGATCAGCCACACTGGAACTGAG
ATACGGTC
>Zotu7982
GAACGCTGGCGGCGTGCTTAACACATGCAAGTCGAGCGGAAAGGCCCTTCGGGGTACTCGAGCGGCGAA
CGGGTGAGTAACACGTGGGTAACCTGCCCCTTGCTCTGGGATAACTCCGGGAAACCGGGGCTAATACCGG
ATATGACACCTTAAGGCATCTTTTGGTGTGGAAAGTTTTTCGGCGAGGGATGGACCCGCGGCCTATCAGCT
TGTTGGTGGGGTGATGGCCTACCAAGGCAATGACGGGTAGCCGGCCTGAGAGGGCGACCGGCCACACTG
GGACTGAGACACGGCC
>Zotu7984
AAACGCTGGCGACATGCCTTACACATGCAAGTCGAACGGCAGCGCGGACTTCGGTCTGGCGGCGAGTGGC
GAACGGGTGAGTAATACATCGGAACGTACCCTATTGTGGGGGATAACTAGTCGAAAGATTAGCTAATACC
GCATACAACCTGAGGGTGAAAGCGGGGGACCGTAAGGCCTCGCGCAGCAGGAGCGGCCGATGTCTGATT
AGCTAGTTGGTGGGGTAAAGGCCCACCAAAGCGACGATCAGTAGCTGGTCTGAGAGGACGATCAGCCACA
CTGGGACTGAGACACGGCC
>Zotu7986
GAACGCTAGCGGCAGGCTTAACACATGCAAGTCGAGGGGTATAGTTCTTCGGAATTAGAGACCGGCGCAC
GGGTGCGTAACGCGTATGCAATCTACCTTTCACAGAGGGATAGCCCAGAGAAATTTGGATTAATACCTCAT
AGTATTATGAAATGGCATCATTTTATAATTAAAGTCACAACGGTGAAAGATGAGCATGCGTCCCATTAGCTA
GTTGGTATGGTAACGGCATACCAAGGCTACGATGGGTAGGGGTCCTGAGAGGGAGATCCCCCACACTGGT
ACTGAGACACGGAC
>Zotu7988
GAACGCTGGCGGCATGCCTTACACATGCAAGTCGAACGGCAGCGGGCCTTCGGGTGCCGGCGAGTGGCG
AACGGGTGAGTAATGCATCGGAACGTGCCATTGAGTGGGGGATAACGTAGCGAAAGTTACGCTAATACCG
CATATTCTGTGAGCAGGAAAGCAGGGGACCGCAAGGCCTTGCGCTCTTTGAGCGGCCGATGTCAGATTAG
CTAGTTGGTGAGGTAAAGGCTTACCAAGGCGACGATCTGTAGCGGGTCTGAGAGGATGATCCGCCACACT
GGAACTGAGACACGGTC
>Zotu7990
GAACGCTGGCGGCGTGCATAACACATGCAAGTCAAGGGGGCCAGCAATGGCAACCGGCGAACGGGTGCG
TAACACGTGAGCAATCTGCCTCTATGTGGGGCATAGCCGGCCTAACGGCCGGGTAATTCCGCATACGATCT
TTTGGGGACATCCCCGATTGATCAAACGCTTCGGCGGCATAGAGAGGAGCTCGCGGCCTATCAGCTAGTTG
GTGAGGGTACCGGCCCACCAAGGCGACGACGGGTAGCTGGTCTGAGAGGATGGCCAGCCACATTGGGAC
TGAGATACGGCC
>Zotu7992
GAACGCTGGCGGCGTGCCTTAAGCATGCAAGTCGAATGGTCGCAAGACAATGGCAGACGGGTGAGTAAT
ATATAGGAATCTACCCTTCAATGGACCATAGCTCATCGAAAGATGGGGTAATAGTCCATAGTCTCGTAAGA
GTAAAGATCTTAACTGATCGTTGAAGGAGGAGCCTATATCCTATCAGCTAGTTGGCGGGGTAAAAGCCCAC
CAAGGCGATGACGGGTAGCTGGACTGAGAGGTCGGTCAGCCACAACTGCACTGAGACACGGGC
>Zotu7994
GAACGCTGGCGGCGTGCTTAACACATGCAAGTCGAACGGAAAGGCCAGTGCTTGCACTGGTACTCGAGTG
GCGAACGGGTGAGTAACACGTGGGTGATCTGCCTTGCACTCTGGGATAAGCTTGGGAAACTGGGTCTAAT
ACCGGATATGAACTGCCTTTAGTGTGGTGGTTGGAAAGTTTTTTCGGTGCAAGATGAGCTCGCGGCCTATC
AGCTTGTTGGTGGGGTAATGGCCTACCAAGGCGTCGACGGGTAGCCGGCCTGAGAGGGTGTACGGCCAC
ATTGGGACTGAGATACGGCC
>Zotu7996
GAACGCTGGCGGCAGGCTTAACACATGCAAGTCGAACGCTCCGCAAGGAGAGTGGCAGACGGGTGAGTA
ACACGTGGGAATCTACCCATCCCTACGGAACAACTCCGGGAAACTGGAGCTAATACCGTATACGCCCTTCG
GGGGAAAGATTTATCGGGGATGGATGAGCCCGCGTTGGATTAGCTAGTTGGTGGGGTAAAGGCCTACCAA
GGCGACGATCCATAGCTGGTCTGAGAGGATGATCAGCCACATTGGGACTGAGACACGGCC
>Zotu8000

CAACGCTGGCGGCGTGCCTAATGCATGCAAGTCGAGCGGGGTGGCCTTCGGGTCACTTACGCGGCGGACG
GGTGCGTAACACGTGGGCAACCTGCCCTGAGGTGGGGGATAGCCGAGGGAAACTTCGGGTAATTCCGCAT
ACGGTCACTGACTGGAGGGTTGGTGAGGAAAGGGTCTTCGGACTCGCCTTGGGAGGGGCCTGCGGCCGA
TTAGCTAGACGGTGGGGTAACGGCCTACCGTGGCGATGATCGGTAGCTGGTCTGAGAGGACGGCCAGCCA
CACGGGGACTGAGACACGGCC
>Zotu8003
GAACGCTGGCGGCAGGCCTAACACATGCAAGTCGAGCGGTAACAGGGGGTGCTTGCACCCCGCTGACGA
GCGGCGGACGGGTGAGTAATGCATAGGAATCTGCCCGATAGTGGGGGATAACCTGGGGAAACCCAGGCT
AATACCGCATACGTCCTACGGGAGAAAGGGGGCTTCGGCTCCCGCTATCGGATGAGCCTATGTCGGATTA
GCTGGTTGGTGAGGTAAAGGCTCACCAAGGCGACGATCCGTAGCTGGTCTGAGAGGATGATCAGCCACAT
CGGGACTGAGACACGGCC
>Zotu8006
GAACGCTGGCGGCATGCCTAACACATGCAAGTCGAACGAGACCTTCGGGTCTAGTGGCGCACGGGTGCGT
AACGCGTGGGAATCTGCCCTTGGGTTCGGAATAACAGTTAGAAATGACTGCTAATACCGGATGATGACTTC
GGTCCAAAGATTTATCGCCCAAGGATGAGCCCGCGTCGGATTAGCTTGTTGGTGAGGTAAAAGCTCACCAA
GGCGACGATCCGTAGCTGGTCTGAGAGGATGATCAGCCACACTGGGACTGAGACACGGCC
>Zotu8009
GAACGCTGGCGGCGTGCCTAACACATGCAAGTCGAACGGAGTAATTACTTCGGTGATTACTTAGTGGCGA
ACGGGTGAGTAACGCGTGAGAAACCTGCCTTTCAGTGGGGGACAACAGTTGGAAACGACTGCTAATACCG
CATAATGTTATTTGGGGGCATCCCTGAATAACTAAAGCTTTATGTGCTGAAAGATGGTCTCGCGTCTGATTA
GCTAGTTGGTGAGGTAACGGCTCACCAAGGCGACGATCAGTAGCCGGTCTGAGAGGATGAACGGCCACAT
TGGGACTGAGATACGGCC
>Zotu8012
CGGCACCTGAACGAGTCCGGGCTGGTGCACGCCCTGCGGAGTCGGTTCGCTTCGGGGCTCGTCCACTGCT
ACGCCGGTGCTGGACTGGTGCTCTTCAACCCTCTTCGTCCCCTGGCCA
>Zotu8014
GAACGCTGGCGGCGTGCCTAATACATGCAAGTCGAACGGAAAGGTTTCTCACTCTGTGCTTTTAATTTAGA
GCGTAGGGTGAGAAATACTCGAGTGGCGGACGGCAGAGTAACACGTAGGAACGTACCCCAAACTGAGGG
ATAAGCACCAGAAATGGTGTCTAATACCGCATGTGCTCCAAGGAGTAAAGCTTCGGCGGTTTGGGAACGG
CCTGCGCATGATTAGCTTGTTGGTGAGGTAATGGCTCACCAAGGCGACGATCATTAGCTGGTCTGAGAGGA
TGATCAGCCTGACTGGGACTGAGAACGGCC
>Zotu8016
GAACGCTGGCGGCAGGCTTAACACATGCAAGTCGAACGCCCCGCAAGGGGAGTGGCAGACGGGTGAGTA
ACGCGTGGGAATCTACCCATCTCTACGGAATAACTCAGGGAAACTTGTGCTAATACCGTATACGCCCTTCG
GGGGAAAGATTTATCGGAGATGGATGAGCCCGCGTTGGATTAGCTAGTTGGTGGGGTAAAGGCCTACCAA
GGCGACGATCCATAGCTGGTCTGAGAGGATGATCAGCCACATTGGGACTGAGACACGGCC
>Zotu8018
GAATGCTGGCGGCGTGGATAAGGCATGCAAGTCGAGTGGGTTTAGACCCACGGCAGACGAGGTAGTAAC
ACGTAGGAACGTACCCCAAAGTCCGGGATATTCCATCGAAAGATGGCGCAATACCGGATGGTCTCTAAGG
AGTAAAGATTTATCGCTTTGGGAGCGGCCTGCGCAGTATCAGCTAGTTGGTAGGGTAATGGCCTACCAAG
GCTATGACGCTTAGGGGAGCTGAGAGGCTGACCCCCACCGATGGAACTGAGAAACGGTC
>Zotu8020
GAACGCTGGCGGCATGCCTAACACATGCAAGTCGAACGCCCTCTTCGGAGGGAGTGGCGCACGGGTGCGT
AACGCGTGGGAACCTGCCCTTAGGTTCGGAATAACTTCCCGAAAGGGATGCTAATACCGGATAATGTCTTC
GGACCAAAGATTTATCGCCTTTGGATGGGCCCGCGTTGGATTAGCTAGTTGGTAGGGTAAAAGCCTACCAA
GGCGACGATCCATAGCTGGTCTGAGAGGATGATCAGCCACACTGGGACTGAGACACGGCC
>Zotu8022
GAACGCTGGCGGCAGGCTTAACACATGCAAGTCGAGCGCACCGCAAGGTGAGCGGCAGACGGGTGAGTA
ACGCGTGGGAATCTACCGTGCCCTACGGAATAGCTCCGGGAAACTGGAATTAATACCGTATACGCCCTTCG

GGGGAAAGATTTATCGGGGTATGATGAGCCCGCGTTGGATTAGCTAGTTGGTGGGGTAAAGGCCTACCAA
GGCGACGATCCATAGCTGGTCTGAGAGGATGATCAGCCACATTGGGACTGAGACACGGCC
>Zotu8026
GAACGCTGGCGGCGTGCCTAATACATGCAAGTCGAGCGGATCTTCAGGAGCTTGCTCCTGAAGGTTAGCG
GCGGACGGGTGAGTAACACGTGGGCAACCTGCCTGTAAGACTGGGATAACTTCGGGAAACCGAGGCTAAT
ACCGGATAATTCTTTCTCTCACATGAGGGAAAGCTGAAAGACGGCGTAAGCTGTCACTTACAGATGGGCCC
GCGGCGCATTAGCTAGTTGGTGAGGTAACGGCTCACCAAGGCGACGATGCGTAGCCGACCTGAGAGGGT
GATCGGCCACACTGGGACTGAGACACGGCC
>Zotu8029
GAACGCTGGCGGCAGGCTTAACACATGCAAGTCGAGCGGGCACTTCGGTGTCAGCGGCAGACGGGTGAG
TAACACGTGGGAACGTACCCTCTGGTTCGGAACAACTCTGGGAAACTGGAGCTAATACCGGATACGCCCTT
TTGGGGAAAGGCTTGCTGCCGAAGGATCGGCCCGCGTCTGATTAGCTAGTTGGTGGGGTAATGGCCTACC
AAGGCGACGATCAGTAGCTGGTCTGAGAGGATGATCAGCCACACTGGGACTGAGACACGGCC
>Zotu8032
GAACGCTAGCGGCAGGCCTAATACATGCAAGTCGAACGGGATCCATTTGTTAGCTTGCTAACAGATGGTGA
GAGTGGCGCACGGGTGCGTAACGCGTGAGCAACCTACCCATATCAGGGGGATAGCCCGAAGAAATTCGG
ATTAACACCGCATAAGACTACGAGATGGCATCATCAAGTAGTTAAATATTTATAGGATATGGATGGGCTCG
CGTGACATTAGCTAGTTGGTGAGGTAACGGCTCACCAAGGCGACGATGTCTAGGGGCTCTGAGAGGAGAA
TCCCCCACACTGGTACTGAGACACGGAC
>Zotu8038
GAACGCTGGCGGCGGGCCTAACACATGCAAGTCGAGCGGCAGCGGGAAAGTAGCTTGCTACTTTTGCCGG
CGAGCGGCGGACGGGTGAGTAATGCCTGGGAAATTGCCCAGTCGAGGGGGATAACAGTTGGAAACGACT
GCTAATACCGCATACGCCCTACGGGGGAAAGCAGGGGACCTTCGGGCCTTGCGCGATTGGATATGCCCAG
GTGGGATTAGCTAGTTGGTGAGGTAATGGCTCACCAAGGCGACGATCCCTAGCTGGTCTGAGAGGATGAT
CAGCCACACTGGAACTGAGACACGGTC
>Zotu8041
GAACGCTGGCGGCGTGCTTAACACATGCAAGTGTAGCGACGAACCAGGCTTCGGCCTGGGGCAAAGCCGC
GAACGGGTGAGTAACACGTGGGTAACCTGCCCCGATGATTGGGACAACCCGAGGAAACTCGGGCTAATAC
CAAATGTGCCCTCCGGCCATAAGGCCGCTGGGGAAAGGAAGCTTCGGCCTCCGCATCGGGATGGGCCCGC
GGCCCATTAGCTTGTTGGTGAGGTAACGGCTTACCAAGGCGACGATGGGTAGCTGGTCTGAGAGGACGAT
CAGCCACACTGGGACTGAGACACGGCC
>Zotu8045
GAATGCTGGCGGCGTGCCTAACACATGCAAGTCGAGCGGCAGCGCGAGTAGTTTACTACTTGGCGGCGAG
CGGCGGACGGCTGAGTAACGCGTGGGAACGTGCCCCAAAGTGAGGAATAACTGCCCGAAAGGGTGGCTA
ATGCCGCATATGATCTTCGGATTAAAGTCTTCGGACGCTTTGGGAGCGGCCCGCGTCGGATTAGGTAGTTG
GTGAGGTAATGGCTCACCAAGCCGACGATCCGTAGCTGGTCTGAGAGGATGATCAGCCAGACTGGGACTG
AGACACGGCC
>Zotu8047
GAACGCTGGCGGCGTGCCTAACACATGCAAGTCGAACGTGAACGGGGGCAACCCCTAGTAAAGTGGCGTA
CGGGTGAGTAACACGTAGGCAATCTACCCCAAAGTCTGGAATAACCTGCCGAAAGGCGGGCTAATACTGG
ATAGAGCGACGGAATCCGTAATTGGGTTTTTCGGGAAAGGGAGGGAGCAATCCCCCTTGCTTTGGGATGG
GCCTGCGTCCCATCAGCTAGTTGGTAAGGTAATGGCTTACCAAGGCGATGACGGGTAGCTGGTCTGAGAG
GATGATCAGCCACACTGGAACTGAAACACGGTC
>Zotu8049
GAACGCTGGCGGCGTGCTTAACACATGCAAGTCGAGCGAGAAAGCCCCTTCGGGGGTGAGTACAGCGGC
GAACGGGTGAGTAACACGTGGGTAACCTGCCTTGAGGAGCGGGACAACCCGAGGAAACTCGGGCTAATA
CCGCATGACTCGTCGCGTCCCAAGGCGCGACGGCAAATGGTAGCTTCGGCCTCCGCCTCAAGATGGGCCC
GCGCCGCATTAGTTTGTTGGTGGGGTAATGGCTCACCAAGGCGACGATGCGTAGCTGGTCTGAGAGGACG
ATCAGCCACACTGGGACTGAGACACGGCC
>Zotu8051

GAACGCTGGCGGCGTGCTTAACACATGCAAGTCGAACGCTGAAGCTCTGCCCTTGTGGTGGGGTGGATGA
GTGGCGAACGGGTGAGTAACACGTGGGTAATCTGCCCCATACTCTGGGATAACCCCTGGAAACGGGGGCT
AATACCGGATAGGACATTCTGCCGCATGGTGGGGTGTGGAAAGTTCCGGCGGTATGGGATGGGCCCGCG
GCCTATCAGCTTGTTGGTGGGGTAATGGCCTACCAAGGCGACGACGGGTAGCCGGCCTGAGAGGGTGACC
GGCCACACTGGGACTGAGACACGGCC
>Zotu8053
GAACGCTAGCGGCAGGCCTAATACATGCAAGTCGAACGATAGTGAGGGGCTTGCTCCTCACGAAAAGTGG
CGCACGGGTGCGTAACGCGTATGCAACCTACCTTAATCAGGGGGATAGCCCGAAGAAATTCGGATTAACA
CCGCATAAAATCATAGTATAGCATTATACAATGATCAAATATTTATAGGATTAAGATGGGCATGCGTGACAT
TAGTTAGTTGGCGAGGTAACGGCTCACCAAGACTACGATGTCTAGGGGATCTGAGAGGATGACCCCCCAC
ACTGGTACTGAGACACGGAC
>Zotu8055
GAACGCTGGCGGCAGGCCTAACACATGCAAGTCGAGCGGGAACTTCGGTTCTAGCGGCGGACGGGTGAG
TAATGCGTAGGAAGCTACCCGATAGAGGGGGATACCAGTTGGAAACGACTGTTAATACCGCATAATGTCT
ACGGACCAAAGTGTGGGACCTTCGGGCCACATGCTATCGGATGCGCCTACGTGGGATTAGCTAGTTGGTG
AGGTAATGGCTCACCAAGGCGACGATCCCTAGCTGGTTTGAGAGGATGATCAGCCACACTGGAACTGAGA
CACGGTC
>Zotu8060
GAACGCTGGCGGCGTGCCTAATACATGCAAGTCGAGCGGTAAGGCTTTCACAGTTTGCTGGATTGAGACA
GGTCGCGGAACTTCGAAGAAGTTTCGCATCATCACGAAGTGATGGACGGCCTGGCTCGAATAATCAAGCA
AAGTGTGAAAGTACACGAGCGGCGGACGGCAGAGTAACGCGTAGGAACGTACCCCATAGTGAGGGATAA
GCACCGGAAACGGTGTCTAATACCGCATAATCTCTTCGGAGCAAAGCCTTGTGCGCTTTGGGAGCGGCCTG
CGTATGATTAGTTTGTTGGTGAGGTAACGGCTCACCAAGACTACGATCATTAGCTGGTCTGAGAGGATGAT
CAGCCTGACTGGAACTGAGAACGGTC
>Zotu8062
GAACGCTAGCGATAGGCTTAACACATGCAAGTCGAGGGGTAACGTGTTGGAAGCTTGCTTCCGATGACGA
CGACCGGCGGATGGGTGCGTAACGCGTATGCAACTTGCCTCACAGAGGAGAATAACCCGGAGAAATCCGG
ACTAATGCTCCATACACTCTTTAGCACGCCTGTGCGTAAGAGGAAAGATTTATCGCTGTGAGATAGGCATG
CGTCCTATTAGGTAGTTGGTGAGGTAACGGCTCACCAAGCCGACGATAGGTAGGGGTGCTGAGAGGCAGA
TCCCCCACATTGGGACTGAGACACGGCC
>Zotu8064
GAACGCTGACGGTATGCCTCATACATGCAAGTCGAGCGGATGCAGCAATGCATCAGCGGCGGACGGGTGA
GTAACACGTGAACAATCTGCCCCCGAATTTGGGATAACGGCTCCAACGAGCCGCTAATACCGGATAGGTTA
TTCTGGCCGCATGGTCGGATTAAGAAAGGTCCAATTGGACCGTTCGGGGATGAGTTCGCGGCCCATTAGCT
AGTTGGCGGGGTAAAGGCCCACCAAGGCAACGATGGGTAGCCGGCCTGAGAGGGTGATCGGCCACATTG
GGACTGAGATACGGCC
>Zotu8067
GAACGCTAGCGGCAGGCCTAATACATGCAAGTCTAAGGGGTAGCAATATCACTGGCGGACGGGTGCGTAA
CGCGTATAGAACCTACCTTTCACTGGGGGATAGCTTTTGGAAACAGGAGATAATACCGCATAAGATAGCAC
TTCGGCATCGAAGAGTTATTAAAGATTTATTGGTGAGAGATGGCTATGCGTATGATTAGCTAGTTGGTAAG
GTAACGGCTTACCAAGGCTACGATCATTAGGGGTTCTGAGAGGATTGGCCCCCACACTGGTACTGAGATAC
GGAC
>Zotu8069
GAACGCTGGCGGCGTGCTTAACACATGCAAGTCGAGCGGAAAGGCCCACTTGTGGGTACTCGAGCGGCGA
ACGGGTGAGTAACACGTGAGTAATCTGCCCTCCACTCTGGGATAACGTTCAGAAATGGGTGCTAATACCGG
ATGTACACGTGCTGACGCATGTTGGTGTGTGGAAAGGTTTTCTGGTGGGGGATGTGCTCGCGGCCTATCA
GCTTGTCGGTGGGGTAATGGCCTACCAAGGCTTTGACGGGTAGCCGGCCTGAGAGGGTGACCGGCCACAC
TGGGACTGAGACACGGCC
>Zotu8072
GAACGCTGGCGGCATGCTTTACACATGCAAGTCGAACGGCAGCACGGACTTCGGTCTGGTGGCGAGTGGC
GAACGGGTGAGTAATACATCGGAACGTGCCCAGTTGTGGGGGATAACTACTCGAAAGAGTAGCTAATACC

GCATGAGAACTGAGGTTGAAAGCAGGGGATCGCAAGACCTTGCGCAACTGGAGCGGCCGATGGCAGATT
AGGTAGTTGGTGGGATAAAAGCTTACCAAGCCGACGATCTGTAGCTGATCTGAGAGGACGACCAGCCACA
CTGGGACTGAGACACGGCC

>Zotu8075

GAACGCTGGCGGCGTGCTTAACACATGCAAGTCGAGCGGAAAGGCCCTTCGGGGTACTCGAGCGGCGAA
CGGGTGAGTAACACGTGAGTAACCTGCCCCAGACTTTGGGATAACCCTCGGAAACGGGGGCTAATACCGG
ATACGACACTTCACCGCATGGTGTGGGTGTGGAAAGTTTTTCGGTTTGGGATGGGCTCGCGGCCTATCAGC
TTGTTGGTGGGGTGATGGCCTACCAAGGCGACGACGGGTAGCCGGCCTGAGAGGGCGACCGGCCACACT
GGGACTGAGACACGGCC

>Zotu8077

GAACGCTGGCGGCGTGCCTAATACATGCAAGTCGAGCGGTAACACTTCGGGTGACGAGCGGCGGACGGC
TGAGTAACACGTAGGAACGTACCCCAAACTGAGGGATAACTGCCCGAAAGGGTAGCTAATACCGCATGTG
ATCTTCGGATTAAAGCTTCGGCGGTTTGGGAACGGCCTGCGCAAGATTAGCTTGTTGGTGAGGTAAAAGCT
CACCAAGGCGACGATCTTTAGCTGGTCTGAGAGGATGATCAGCCAGACTGGGACTGAGAACGGCC

>Zotu8080

GAACGCTGGCGGCGTGCCTAAGACATGCAAGTCGAGTGGGTTTAGACCCACGGCGAACGG GTGAGTAAT
ACCTAGGAACTTTTCTTTAGTTGAGGATATCCTCCCGAAAGGGAGAGCAATACTCAATGTGGCCTTACGGCT
AAAGTCGCAAGACGCTAAAGGTGAGGCCTGGGCACCATCAGCTAGTTGGTAAGGTAATGGCTTACCAAGG
CTATGACGGTTAGGGGCCCCGAGAGGGGAGACCCCCACACTGGGACTGCGACACGGCC

>Zotu8084

GAACGCTGGCGGCGTGCCTAACACATGCAAGTCGAGCGGAGGCATCTGAGGAGTAATCTGAGGATGCTTT
AGCGGCGGACGGGTGAGTAACGCGTGAGCAACCTGCCTTTCACAGGGGGATAACGCACCGAAAGGTGCA
CTAATACCGCATGAGACCACGGTACCGAGTGGTACAGGGGTCAAAGGAGCAATCCGGTGAGAGATGGGC
TCGCGTCCGATTAGATAGTTGGTGGGGTAAAGGCCTACCAAGTCAACGATCGGTAGCCGACCTGAGAGGG
TGATCGGCCACACTGGAACTGAGATACGGTC

>Zotu8088

GAACGCTGGCGGCGTGCCTAATACATGCAAGTCGAGCGGTAAGGTTCTCACAGATTATTTAACCAGATAAG
TTTCTCCGGAAATACTGTCTGCAGCAATGTTCACAGGCTAGTGAATAAAGTTGAATAATGCGTGAGAATAC
ACGAGCGGCGGACGGCTGAGTAACACGTAGGAACGTACCCCAAACTGAGGGATAAGCACCAGAAATGGT
GTCTAATACCGCATGTGATCTTCGGATTAAAGCTTCGGCGGTTTGGGAACGGCCTGCGCATGATTAGCTTG
TTGGTGAGGTAATAGCTCACCAAGGCGACGATCATTAGCTGGTCTGAGAGGATGACCAGCCAGACTGGAA
CTGAGAACGGTC

>Zotu8093

GAATGCTGGCTGCGTGCTTAACACATGCAAGTCGAACGGTGACCCAGGGCTTGCCCTGGTGATCAGTGG C
GAACGGGTGAGTAACACGTGGGCAACCTGCCCCTCACTCTGGGATAACTCCGGGAAACCGGGGATAATAC
CGGATACGACCTCGTGCGACATCTCGCGAGGTGGAAAGAATTTCGGTGAGGGATGGGCCCGCGGCCTATC
AGCTAGTTGGTGAGGTAACGGCCCACCAAGGCGACGACGGGTAGCCGGCCTGAGAGGGCGACCGGCCAC
ACTGGGACTGAGACACGGCC

>Zotu8097

GAACGCTGGCGGCGCGCTTAACACATGCAAGTCGAGCGAGAACCAGGACTTCGGTCCTGGGGACAGCGG
CGAACGGGTGAGTAACACGTGGGTAATCTGCCCTCGATTCTGGGATAGCCCGGGGAAACTCGGATTAATA
CCGGATAGCCTTTCGAGCCTTCGGTCGCGAAAGAAAAGGTAGCTTCGGCCTCCGATCGAGGATGAGCCCG
CGGTGGATTAGCTTGTTGGCGGGGTAATGGCCCACCAAGGCGACGATCCATAGCTGGTCTGAGAGGACGA
TCAGCCACACTGGGACTGAGACACGGCC

>Zotu8101

GAACGCTGGCGGCGTGCCTAACACATGCAAGTCGAACGAAGCATCGCGCCCGACAAAGAGGAGTGCTTGC
ACAAATCGGAGTCAATTGTGATGACTTAGTGGCGGACGGGTGAGTAACGCGTGGGTAACCTGCCCTATAC
AGGGGGATAACAGTTAGAAATGACTGCTAATACCGCATAAGCGCACAGTATCGCATGATACAGTGTGAAA
AGCCGAGACGGTATAGGATGGACCCGCGTCTGATTAGCCAGTTGGCAGGGTAACGGCCTACCAAAGCAAC
GATCAGTAGCCGGCCTGAGAGGGTGAACGGCCACATTGGGACTGAGACACGGCC

>Zotu8107
CAACGCTGGCGGCGTGCCTCAGACATGCAAGTCGAACGATTAAGACTCCCTTCGGGGAGTGCATAAAGTG
GCGCACGGGTGAGTAACACGTAGGTAATCTACCTTTGAGTGGGGAATAACATCGGGAAACCGATGCTAAT
ACCGCATAATGCAGCGGCACCTAATGGTGACAGTTGTTAAAGATTTATCGCTTAAAGAGGAGCCTGCGGCA
GATTAGCTAGTTGGTAAGGTAATGGCTTACCAAGGCAACGATCTGTAACCGACCTGAGAGGGTGGTCGGT
CACACTGACACTGAATAACGGGT
>Zotu8111
GAACGCTGGCGGCATGCCTTACACATGCAAGTCGAACGCGAAAGTGGGGGCAACCCTGCGAGTAGAGTG
GCGAACGGGTGAGTAACACATCGGAACGTACCCAGGAGTGGGGGACAACCTCGAGAAATCGAGGCTAAT
ACCGCATACGTCCTGAGGGAGAAAGCGGGGGATTCGCAAGGACCTCGCGCTCATGGAGCGGCCGATGTC
GGATTAGCTAGTTGGCGGGGTAAGAGCCCACCAAGGCGACGATCCGTAGCTGGTTTGAGAGAACGACCA
GCCACACTGGGACTGAGACACGGCC
>Zotu8114
GAACGCTGGCGGCATGCCTAATACATGCAAGTCGAACGGACAGAAGTGTACTTGTACACAGAAGTTAGTG
GCGAACGGGTGAGTAACACGTAGGTAACCTACCCCAATGACTGGGATAACTGTTGGAAACGACAACTAAT
ACCGGATGGGATACAGGAAGGCATCTTCCTGTATTGAAAGTTGCTAAGGCAACACAATGGGATGGACCTG
CGGTGCATTAGCTAGTTGGTGAGGTAGCGGCTCACCAAGGCGATGATGCATAGCCGACCTGAGAGGGTGA
ACGGCCACATTGGGACTGAGACACGGCC
>Zotu8117
GAACGCTGGCGGCATGCCTAACACATGCAAGTCGAACGGTCACTTCGGTGATAGTGGCGCACGGGTGCGT
AACGCGTGGGAATCTACCCTTGGGTTCGGAATAACTCTCCGAAAGGAGTGCTAATACCGGATGATGACGTA
AGTCCAAAGATTTATCGCCCAAGGATGAGCCCGCGTAGGATTAGCTAGTTGGTGGGGTAAAGGCCTACCA
AGGCGACGATCCTTAGCTGGTCTGAGAGGATGATCAGCCACACTGGGACTGAGACACGGCC
>Zotu8121
GAACGCTGGCGGCATGCCTTACACATGCAAGTCGAACGGCAGCACGGGAGCAATCCTGGTGGCGAGTGG
CGAACGGGTGAGTAATATATCGGAACGTGCCCAGTCGTGGGGGATAACGCAGCGAAAGCTGTGCTAATAC
CGCATACGATCTACGGATGAAAGCGGGGGATCGCAAGACCTCGCGCGATTGGAGCGGCCGATATCAGATT
AGCTAGTTGGTGGGGTAAAGGCCCACCAAGGCGACGATCTGTAGCTGGTCTGAGAGGACGACCAGCCACA
CTGGAACTGAGACACGGTC
>Zotu8125
GAACGCTAGCGGCAGGCTTAATACATGCAAGTCGAGGGGTAGAGAGGAGCTTGCTCCTCTTGAGACCGGC
AAACGGGTGCGGAACACGTACACAACCTTCCTTTTAGTGGGGGATAGCCCAGGGAAACTTGGATTAATACC
CCGTAACATGTTGAGATGGCATCATCTTAATATTATAGTGGCAACACGCTAGAAGACGGGTGTGCGTATGA
TTAGGTAGTTGGCGGGGTAACGGCCCACCAAGCCTGCGATCATTAACTGGTGTGAGAGCACGACCAGTCA
CACGGGCACTGAGACACGGGC
>Zotu8127
CAACGCTGGCGGCGTGCCTAACACATGCAAGTCGAACGAGAAAGTCGGGGCAACCTGGCGAGTACAGTG
GCAGACGGGTGAGTAACACGTGAACAATCTACCCTCGAGTGGGGAATAACGCCGCGAAAGCGGCGCTAAT
ACCGCATAACGTTCCGAGATCGCATGGTCACGGAACCAAAGGCGAAAGTCGCTCGAGGAGGAGTTCGCGC
CTGATTAGCTAGTTGGTGAGGTAACGGCCCACCAAGGCAACGATCAGTAGCCGGCCTGAGAGGGCGTACG
GCCACACTGGAACTGGGACACGGTC
>Zotu8131
GAACGCTGGCGGCGTGCTTAACACATGCAAGTCGAACGATGAACCGGTGCTTGCATTGGGGATTAGTGGC
GAACGGGTGAGTAACACGTGAGTAACCTGCCCTTAACTCTGGGATAAGCCTTGGAAACGGGGTCTAATACT
GGATATTGACTTTACCTCGCATGGGGTTTTGTTGAAAGATTTATTGGTTTTGGATGGACTCGCGGCCTATCA
GCTTGTTGGTGAGGTAATGGCTCACCAAGGCGACGACGGGTAGCCGGCCTGAGAGGGTGACCGGCCACA
CTGGGACTGAGACACGGCC
>Zotu8133
GAACGCTGGCGGCGCGCTTAACACATGCAAGTCGAGCGATGAAATTTTGACAGATCCCTTCGGGGTGAAG
ATAAAATGGATTAGCGGCGGACGGGTGAGTAACACGTGAGTAACCTGCCTTAGACATTGGGATAGCCTCG

GGAAACCGGGATTAATACCAAATAAAATCACAGATACACATGTATCCATGATCAAAACTCCGGTGGTCTAA
GATGGACTCGCGTCCCATTAGCTAGTTGGTAGGGTAACGGCCTACCAAGGCGACGATGGGTAGCCGGCCT
GAGAGGGTGAACGGCCACATTGGAACTGAGAAACGGTC
>Zotu8135
GAACGCTGGCGGCGTGCTTAACACATGCAAGTCGAACGGTGAAGCCCTTCGGGGTGGATCAGTGGCGAAC
GGGTGAGTAACACGTGAGCAATCTGCCCTTGACTCTGGGATAACTTCGGGAAACCGGAGCTAATACCGGA
TATGACATCTGGCCGCATGGTCTGGGTGTGGAAAGATTTATCGGTCAGGGATGAGCTCGCGGCCTATCAGC
TTGTTGGTGGGGTAATGGCCCACCAAGGCGACGACGGGTAGCCGGCCTGAGAGGGCGACCGGCCACACT
GGGACTGAGACACGGCC
>Zotu8140
GAACGCTGGCGGCGTGCTTAACACATGCAAGTCGAACGATGACGCCCGTGCTTGCACGGGTTGATTAGTG
GCGAACGGGTGAGTAACACGTGAGCAACCTGCCCCCTTCTTCGGGATAACCTCGGGAAATCGTGGCTAATA
CCGGATATGACTGCGCATCGCATGGTGTGTGGTGGAAAGATTTATCGGTGGGGGATGGGCTCGCGGCCTA
TCAGTTTGTTGGTGAGGTAATGGCTTACCAAGACGATGACGGGTAGCCGGCCTGAGAGGGCGACCGGCCA
CACTGGGACTGAGACACGGCC
>Zotu8143
CAACGCTGGCGGCGCGCTTAACACATGCAAGTCGCACGAGAAAGGGAGCAATCCTAAGTAAAGTGGCGCA
CGGGTGAGTAACACGTGGATCATCTACCTTTTAGTGGGGAATACCTCTGGGAAACCGGAGACAATACCGC
ATAAGCCCGCAAGGGGAAAGCAGCAATGCGCTGAAAGAGGAGTCCGCGGCCGATTAGCTAGTTGGCGGG
GTAATGGCCCACCAAGGCGAAGATCGGTAGCCGGCCTGAGAGGGCACACGGCCACACTGGCACTGAAAC
ACGGGC
>Zotu8145
GAACGCTGGCGGCATGCCTAACACATGCAAGTCGAACGAGACCTTCGGGTCTAGTGGCGCACGGGTGCGT
AACGCGTGGGAACCTGCCTTTAGGTTCGGAATAACTCAGAGAAATTTGAGCTAATACCGGATGATGTCTTC
GGACCAAAGATTTATCGCCTTTAGATGGGCCCGCGTTGGATTAGCTAGTTGGTGGGGTAAAGGCCTACCAA
GGCGACGATCCATAGCTGGTCTGAGAGGATGATCAGCCACACTGGGACTGAGACACGGCC
>Zotu8147
GAACGCTGGCGGCGTGCCTAATACATGCAAGTCGAACGAACCGCGACTAAGTGCTTGCACTAAGTCAAGG
TGAGTGGCGAACGGGTGAGTAACACGTGGGTAACCTACCTCATAGTGGGGGATAACAGTCGGAAACGACT
GCTAATACCGCATAGGACATAGGATCACATGATCCAGTGAGGAAAGGTGGCGCAAGCTATCGCTAAGAGA
TGGACCCGCGGTGCATTAGCTAGTTGGTAGGGTAACGGCCTACCAAGGCGATGATGCATAGCCGACCTGA
GAGGGTGATCGGCCACATTGGGACTGAGACACGGCC
>Zotu8149
GAACGCTGGCGGCGTGCTTAACACATGCAAGTCGGACGGACACATCTTTCGGGGTGTGTTAGTGGCAGAC
GGGTGCGTAACACGTAGGTAACCTGCCCTGGAGTGGGGAATAATTCCGCTAACGCGGGACTAATACCGCA
TAGGCTCGGTAGCGTGAGGCTACGGAGTAAAGCTGCGAAGCGCTTCAGGATGGGCCTGCGGCGGATTAG
CTCGTTGGTGGGGTAACGGCCTACCAAGGCGATGATCCGTAGGGGGCGTGAGAGCGTGGCCCCCACACT
GGCACTGAGATACGGGC
>Zotu8152
GAACGCTGGCGGCATGCTTAACACATGCAAGTTGAACGCAGGATAAGCCTTCGGGTTTATTGCTGAGTAGC
AAACGGGTGAGTAACGCGTGGGAATCTGCCTTTATGTGGGGGACAACTGAGGGAAACTTCAGCTAATACC
GCATACGCTCTACGGAGGAAAGACTTCGGTCGCATAAAGAGGAGCCCGCGTCAGATTAGGTAGTTGGTGG
GGTAAAGGCCTACCAAGCCTGTGATCTGTAGCTGGTCTGAGAGGATGATCAGCCACACTGGGACTGAGAC
ACGGCC
>Zotu8155
GAACGCTGGCGGCATGCCTTACACATGCAAGTCGAACGGTAGAGTAGCAATACTCGAGAGTGGCGAACGG
GTGAGTAATATATCGGAACGTGCCCAGTCGTGGGGGATAACGTAGAGAAATTTACGCTAATACCGCATAC
GATCTAAGGATGAAAGCGGGGGACTCGCAAGGGCCTCGCGCGATTGGAGCGGCTGATATCAGATTAGGTT
GTTGGTGAGGTAAAAGCTCACCAAGCCGACGATCTGTAGCTGGTTTGAGAGAACGACCAGCCACACTGGG
ACTGAGACACGGCC

>Zotu8159
GAACGCTGGCGGCGTGCTTAACACATGCAAGTCGAGCGGAAAGGCCACTTCGGTGGTACTCGAGCGGCGA
ACGGGTGAGTAACACGTGAGTAATCTGCCCCTGGCTTTGGGATAGCCACCGGAAACGGTGATTAATACCG
GATATGACCGCTTCACGCATGTGGGGTGGTGGAAAGTTTTTCGGCCAGGGATGTGCTCGCGGCCTATCAGC
TTGATGGTGAGGTAATGGCTCACCATGGCTTCGACGGGTAGCCGGCCTGAGAGGGTGACCGGTCACACTG
GGACTGAGACACGGCC
>Zotu8161
GAACGCTGGCGGCGTGCCTAATACATGCAAGTCGAGCGAACAGAGAAGGAGCTTGCTCCTTTGACGTTAG
CGGCGGACGGGTGAGTAACACGTGGGCAACCTACCCTATAGTTTGGGATAACTCCGGGAAACCGGGGCTA
ATACCGAATAATCTTTTGTCCCTCATGGGACAATACTGAAAGACGGTTTCGGCTGTCGCTATAGGATGGGC
CCGCGGCGCATTAGCTAGTTGGTGAGGTAACGGCTCACCAAGGCAACGATGCGTAGCCGACCTGAGAGGG
TGATCGGCCACACTGGGACTGAGACACGGCC
>Zotu8163
AAACGCTGGCGGCATGCCTTACACATGCAAGTCGAACGGCAGCGCAGACTTCGGTCTAGCGGCGAGTGGC
GAACAGGTGAGTAATACATCGGAACGTACCCTGTTGTGGGGGATAACTAGTCGAAAGATTAGCTAATACC
GCATACGACCTGAGGGTGAAAGCGGGGGACCGTAAGGCCTCGCGCAGCAGGAGCGGCCGATGTCTGATT
AGCTAGTTGGTGGGATAAAGGCCCACCAAGGCGACGATCAGTAGCTAGTCTGAGAGGACGATCAGCCACA
CTGGGACTGAGACACGGCC
>Zotu8165
GAACGCTGGCGGCATGCCTTACACATGCAAGTCGAACGGCAGCACGAGCAAGGGGCAACCCTTGTTGGGT
GGCGAGTGGCAAACGGGTGAGTAACGCATCGGAACGTACCCAGTAGTGGGGGATAACGTACCGAAAGGT
ACGCTAATACCGCATACGACCTGAGGGTGAAAGCGGGGGACCTGTCAGAGGGCCTCGCGCTATTGGAGCG
GCCGATGTCCGATTAGCTAGTTGGTGGGGTAAAGGCTCACCAAGGCTGTGATCGGTAGCTGGTCTGAGAG
GACGATCAGCCACACTGGGACTGAGACACGGCC
>Zotu8170
GAACGCTGGCGGTAGGCTTAACACATGCAAGTCGAACGCCCCGCAAGGGGAGTGGCAGACGGGTGAGTA
ACGCGTGGGAATCTACCTAGAACTACGGAACAACAGTTGGAAACGACTGCTAATACCGTATACGCCCTACG
GGGGAAAGATTTATCGGTTTTAGATGAGCCCGCGTAAGATTAGCTAGTTGGTGGGGTAATGGCCTACCAA
GGCGACGATCTTTAGCTGGTCTGAGAGGATGATCAGCCACACTGGGACTGAGACACGGCC
>Zotu8172
GAACGCTGGCGGCGTGCCTAATACATGCAAGTCGAGCGGATCCGAGGAAGCTTGCTTCCAAAGATCAGCG
GCGGACGGGTGAGTAACACGTGGGCAACCTGCCTGTAAGACTGGGATAACTTCGGGAAACCGGAGCTAAT
ACCGGATAATCCTTTCCCTCTCATGAGGGAAAGCTGAAAGACGGTTTCGGCTGTCACTTACAGATGGGCCC
GCGGCGCATTAGCTAGTTGGTGAGGTAACGGCTCACCAAGGCAACGATGCGTAGCCGACCTGAGAGGGT
GATCGGCCACACTGGGACTGAGACACGGCC
>Zotu8174
GAACGCTAGCTATATGCTTAACACATGCAAGTCGAACGTTGTTTTCGGGGAGCTGGACAGAAGGAAAAGA
GGCTCCTAGCTAAAGGCGGCTTGTCTCGCCCAGGAGGTTAGAAGAGTTGAGAACAAAGTGGCGAACGGGT
GCGTAACGCGTGGGAATCTGCCGAACAGTTCGGGCCAAATCCTGAAGAAAGCTAAAAAGCGCTGTTTGAT
GAGCCTGCGTAGTATTAGGTAGTTGGTCAGGTAAAGGCTGACCAAGCCAATGATGCTTAGCTGGTCTTTTC
GGACGATCAGCCACACTGGGACTGAGACACGGCC
>Zotu8176
GAACGCTGGCGGCGTGCCTAATACATGCAAGTCGAGCGAATCAAAGGGAGCTTGCTCCCTTTTGATTAGCG
GCGGACGGGTGAGTAACACGTGGGCAACCTGCCTATAAGACTGGGATAACTTCGGGAAACCGGAGCTAAT
ACTGGATAATTCATATCTACTCATGTAGGTATGCTGAAAGACGGTTTACGCTGTCACTTATAGATGGGCCCG
CGGCGCATTAGCTAGTTGGTGAGGTAACGGCTCACCAAGGCGACGATGCGTAGCCGACCTGAGAGGGTG
ATCGGCCACACTGGGACTGAGACACGGCC
>Zotu8180
CAACGCTGGCGGCGTGCCTAACACATGCAAGTCGAGCGCGAATAGGGGGGCAACCCCCTTAGTAGAGCG
GCGAACGGGTGAGTAACGCGTGGGTGATCTGCCTCCGAGTGGGGAATAACACTGGGAAACTGGTGCTAAT

ACCGCATGACATCCTTGGGTCACACTGAGGATCAAAGCCGGGGACCGCAAGGCCTGGCGCTTGGAGAGGA
GCCCGCGTCCGATTAGCTAGTTGGTGGGGTAATGGCCTACCAAGGCTTCGATCGGTAGCCGGCCTTAGAG
GGCGGACGGCCACACTGGGACTGAGACACGGCC
>Zotu8187
GAACGCTGGCGGCGCGCTTAACACATGCAAGTCGAGCGAGAACCAGGGCTTCGGCTCTGGGGAAAGCGG
CGAACGGGTGAGTAACACGTGAGTTATCCACCCTCGGTATCGGGATAGCCCGGGGAAACCCGGATTAATA
CCGAATGGCCCGTCTGCCCTTCGGGGCGGCCGGAAAAGGAAGCTTCGGCCTCCGACCGAGGACGAGCTCG
CGGCGGATTAGCTTGTTGGTGGGGTAACGGCCTACCAAGGCGACGATCCGTAGCTGGTCTGAGAGGACGA
TCAGCCACACTGGGACTGAGACACGGCC
>Zotu8192
GAACGCTGGCGGCGTGGTTCAGGCATGCAAGTCGAACGGTTCACCGCAAGGTGAATAGTGGCAGAAGGG
TTAGTAACACGTGGGCAATCTACCCCCGACTCGGGGATAACACCGCGAAAGCGGTGCTAATACCCGATTAC
ATTCTCCGGTGGCATCATCGGACAATCAAAGGTGGCGCAAGCTGCCGATTGGGGAGGAGCCCGCGGCCTA
TCAGCTAGTTGGTGAGGTAACGGCCCACCAAGGCATTTGACGGGTAGCTGGTCTAAGAGGACGATCAGCA
ACATTGGGACTGAGACACTGCC
>Zotu8194
GAACGCTGGCGGCGTGCCTAATACATGCAAGTCGAACGAGAAAGTGTAGCAATACATTAGTAAAGTGGCA
GACGGGTGAGTAATACATAGGTAACCTCCCCTCGAGATGGGAATAACCTTCCGAAAGGAGGGCTAATACC
AAATGAGACCACGGGAACTAAGGTTCCTGAGGTTAAAGATGACCTCTATTTATAAGTTATCGCTTGAGGAT
GGGCTTATGCACCATCAGCTAGTTGGTAAGGTAACGGCTTACCAAGGCTTTGACGGTTAGCTGATCTGAGA
GGTTGATCAGCCACACTGGAACTGAGACACGGTC
>Zotu8198
GAACGCTGGCGGCAGGCCTAAAACATGCAAGTCGAACGCTCTAGCAATAGAGAGTGGCGCACGGGTGCG
TAACACGTGGGAATATGCCTTTTGGTACGGAACAACTTTTGGAAACGAAAGCTAATACCGTATGGTCCCTTC
GGGGTAAAGATTTATCGCCAAAAGAGTAGCCCGCGCTGGATTAGCTAGTTGGTGAGGTAATGGCTCACCA
AGGCGACGATCCATAGCTGGTCTGAGAGGACGATCAGCCACACTGGGACTGAGACACGGCC
>Zotu8202
GAACGCTGGCGGCGTGCTTAACACATGCAAGTCGAGCGGTAAGGCCCTTCGGGGTACACGAGCGGCGAA
CGGGTGAGTAACACGTGGGTGACCTGCCCCCAACTCTGGGATAAGCCCGGGAAACTGGGTCTAATACCGG
ATATGACCTCTTGTTGCATGACATGGGGTGGAAAGTTTTTTCGGTTGGGGATGGGCCCGCGGCCTATCAGC
TTGTTGGTGGGGTGATGGCCTACCAAGGCGACGACGGGTAGCCGGCCTGAGAGGGCGACCGGCCACACT
GGGACTGAGACACGGCC
>Zotu8206
GAACGCTGGCGGCAGGCCTAACACATGCAAGTCGAACGCTGTAGCAATACAGAGTGGCGCACGGGTGAG
TAACGCGTGGGAATCTGCCCATTAGTACGGAATAACTGCGGGAAACTGCAGCTAATACCGTATACGCCCTT
CGGGGGAAAGATTTATCGCTAATGGATGAGCCCGCGTAAGATTAGGTAGTTGGTGGGGTAAAAGCCTACC
AAGCCTACGATCTTTAGCTGGTCTGAGAGGACGACCAGCCACACTGGAACTGAGACACGGTC
>Zotu8211
CAACGCTGGCGGCGCGCTTAACACATGCAAGTCGCACGAGAAAGTGGAGCAATCCATGAGTAAAGTGGCG
TACGGGTGAGTAACACGTGGATCATCTACCTTTTAGTGGGGAATACCCCCGGGAAACTGGGGACAATACC
GCATAAGCTCGTGAGAGGAAAGCAGCAATGCGCTGAAAGAGGAGTCCGCGGCTGATTAGCTAGATGGCG
GGGTAACGGCCCACCATGGCAAAGATCGGTAGCCGGCCTGAGAGGGCACACGGCCACACTGGCACTGAA
ACACGGGC
>Zotu8213
GAACGCTGACAGAATGCTTAACACATGCAAGTCTATGGTGAGTATGGTACTTGTACTATATAAGCCATGGC
GGACGGGTGAGTAACGCGTAAAGAACTTGCCTTATGGACTGGGATAACAGTTGGAAATGACTGATAAGAC
TGGATATAATATAGATATTGCATGATATTTATATGAAAAGTGATGCCATAAGAGAGCTTTGCGTCCTATTAG
CTAGTTGGTAGGGTAAAGGCTTACCAAGGCGATGATAGGTAGCCGGCCTGAGAGGGTGAACGGCCACAA
GGGGACTGAGATACGGCC
>Zotu8215

GAACGCTGGCGGCGCGCTTAACACATGCAAGTCGAGCGAGAACCAAGCCTTCGGGCTTGGGGACAGCGG
CGAACGGGTGAGTAACACGTGGGTAATCAACCCTTGACACTGGGATAGCCCGGGGAAACCCGGATTAATA
CCGGATACCCCATCTGGCTTTCGAGCCGGCTGGAAAAGGTAGCTTCGGCCTCCGGTCAGGGACGAGCCCG
CGGCGGATTAGCTTGTTGGCGGGGTAAAGGCCCACCAAGGCGACGATCCGTAGCTGGTCTGAGAGGACG
ATCAGCCACACTGGGACTGAGACACGGCC
>Zotu8218
GAACGCTGGCGGCAGGCTTAACACATGCAAGTCGAGCGGAGATATGGTGCTTGCACCATATTTAGCGGC
GGACGGGTGAGTAATGCTTAGGAATCTGCCTATTAGTGGGGGACAACATTCCGAAAGGAATGCTAATACC
GCATACGCCCTACGGGGGAAAGCAGGGGATCTTCGGACCTTGCGCTAATAGATGAGCCTAAGTCAGATTA
GCTAGTTGGTGGGGTAAAGGCCTACCAAGGCGACGATCTGTAGCGGGTCTGAGAGGATGATCCGCCACAC
TGGGACTGAGACACGGCC
>Zotu8220
GAATGCTGGCGGCAGGCTTAACACATGCAAGTCGAACGATGACTCTCTAGCTTGCTAGAGAAGATTAGTG
GCGGACGGGTGAGTAACATTTAGGAATCTGCCTAGTAGTGGGGGATAGCTCGGGGAAACTCGAATTAATA
CCGCATACGACCTACGGGTGAAAGGGGGCGCAAGCTCTTGCTATTAGATGAGCCTAAATCAGATTAGCTA
GTTGGTGGGGTAAAGGCCCACCAAGGCGACGATCTGTAACTGGTCTGAGAGGATGATCAGTCACACCGGA
ACTGAGACACGGTC
>Zotu8224
GAACGCTGGCGGCATGCCTAATACATGCAAGTCGAACGCCTGTAGCAATACAGGAGTGGCGGACGGGTGC
GTAACGCGTGGGAACCTGCCCCTTGGTATGGGACAACCCCGGGAAACCGGGGCTAATACCGTATAAGGCC
TGAGGGCGAAAGATTTATCGCCGAGGGAGGGGCCCGCGTCCGATTAGGTAGTTGGTGGGGTAATGGCCC
ACCAAGCCTTCGATCGGTAGCTGGTCTGAGAGGACGGCCAGCCACACTGGGACTGAGACACGGCC
>Zotu8229
GAACGCTAGCGGCAGGCTTAACACATGCAAGTCGAGGGGTATATCATTTCGGTGATAGAGACCGGCGCAC
GGGTGCGTAACGCGTATGCAATCTACCTTTTACAGGGGAATAGCCCAGAGAAATTTGGATTAATGCCCCAT
AGTATTATGAAATGGCATCATTTTATAATTAAAGTTCCAACGGTAAAGGATGAGCATGCGTCCCATTAGTTA
GTTGGTAAGGTAACGGCTTACCAAGACGATGATGGGTAGGGGTCCTGAGAGGGAGATCCCCCACACTGGT
ACTGAGACACGGAC
>Zotu8232
GAACGCTGGCGGCGTGCCTAATGCATGCAAGTCGAACGCAGCGAGCTTGCTCGGCTGCGTGGCGCACGGC
TGAGGAACACGTGGGTATCAACCCTGGAGTGGGGGATACCTCGTCGAAAGACGAGACAATCCCGCATACG
ATCGGGCAACCGATGAAAGCCCTCGGGCGCTCCGGGACGAGCCTGCGGCCCATCAGGTAGTTGGTAGGGT
AACGGCCTACCAAGCCGGTGACGGGTCCCTGGTCTGAGAGGACGACCAGGCAGACTGGGACTGAGACAC
GGCC
>Zotu8234
GAACGCTGGCGGCATGCCTAACACATGCAAGTCGAACGAGACCTTCGGGTCTAGTGGCGCACGGGTGCGT
AACGCGTGGGAATCTGCCCTTAGGTTCGGGATAACTCAGGGAAACTTGAGCTAATACCGGATGATGACTTC
GGTCCAAAGATTTATCGCCTAGGGATGAGCCCGCGTCGGATTAGCTAGTTGGTGAGGTAAAGGCTCACCA
AGGCGACGATCCGTAGCTGGTCTGAGAGGATGATCAGCCACACTGGGACTGAGACACGGCC
>Zotu8244
GAACGCTGGCGGCGCGCTTAACACATGCAAGTCGAACGGTTAAAGCACCTCCGGGTGTGCATAAAGTGGC
GAACGGCTGAGTAACACGTGGGCAACCTGCCCCCCGCACCGGGATAGCCTCGGGAAACCGTGGGTAATAC
CGGATACTCCCTCCCCTCCGCATGGCGGGGAGGGGAAAGCGTACACGGCGGGGGATGGGCCCGCGGCCT
GTTAGCTCGTTGGCGGGGTAACGGCCCACCAAGGCTATTATGGGTAGCCGGGTTGAGAGACCGACCGGTC
AGATTGGGACTGAGACACGGCC
>Zotu8246
AAACGCTGGCGGCGTGCCTAAAACATGCAAGTCGTACGGATCGGGCTTCGGCTCGGTCAGTGGCAGACGG
CTGAGTAACGCGTGGGTGACTGCCCCCGCGGTGGGGAATAACTGCCCGAAAGGGTGGCTAAGACCGCATT
CGCTCGCTTCTTCACTGAGGCGAGGAAAGGCTCCCGCAAGGGGGTCGCCGTGGGACGGCCCGCGTCCGA
TTAGCTGGTTGGTGGGGTAACGGCCTACCAAGGCGACGATCGGTACCTGGTCTGAGAGGATGATCAGGCA
GACTGGGACTGAGAGACGGCC

>Zotu8249
CAACGCTGGCGGCGTGCCTAACACATGCAAGTCGAACGCGAAAGTTCCTTCGGGAATGAGTAGAGTGGCG
TCCGGGTGAGTAACGCGTGGGTAATCTACCCTTGGGTGGGGAATAACCAGTCGAAAGATTGGCTAATACC
GCATAATGCAGCGGCTCGACATCGAGACAGTTGTTAAAGCAGCAATGCGCCTAAGGAGGAGCCTGCGTCT
GATTAGCTTGTTGGTGAGGTAATGGCTCACCAAGGCTGCGATCAGTAGCTGGTCTGAGAGGACGGCCAGC
TACAATGGCACTGAAACACGGGC
>Zotu8251
GAACGCTGGCGGCGTGCCTAATACATGCAAGTCGAGCGAATGATGAGAGAAGCTTGCTTCTCTCTGATTTA
GCGGCGGACGGGTGAGTAACACGTGGGTAACCTACCTTATAGATTGGGATAACTCCGGGAAACCGGGGCT
AATACCGAATAATACTTTTAAACACATGTTTGGAAGTTGAAAGACGGTTTCGGCTGTCACTATAAGATGGA
CCCGCGGCGCATTAGCTAGTTGGTGAGGTAACGGCTCACCAAGGCAACGATGCGTAGCCGACCTGAGAGG
GTGATCGGCCACACTGGGACTGAGACACGGCC
>Zotu8253
GAACGCTGGCGGCGTGCTTAACACATGCAAGTCGAGCGGAAAGGCCCTTTCGGGGGTACTCGAGCGGCG
AACGGGTGAGTAACACGTGAGTAATCTGCCTCTGGCTTCGGGATAGCCCTCCGAAAGGGGGATTAATACC
GGATATGACACATGGTCGCATGGTTGTGTGTGGAAAGTTTTTTCGGCCAGGGATGTGCTCGCGGCCTATCA
GCTTGATGGTGAGGTAATGGCTCACCATGGCTTCGACGGGTAACCGGCCTGAGAGGGTGACCGGTCACAC
TGGGACTGAGACACGGCC
>Zotu8257
GAACGCTGGCGGCGTGCCTAACACATGCAAGTCGAACGGAGTAAAACGCTGATTTGATGTTAGCTTGCTAA
CGGATTTTCTTGTTTTACTTAGTGGCGGACGGGTGAGTAACGCGTGAGTAACCTGCCTTACAGAGGGGGAC
AACAGTTGGAAACGACTGCTAATACCGCATAACATATTATGAGGGCATCCTCGTAATATCAAAGGAGCAAT
CCGCTGTAAGATGGACTCGCGTCCAATTAGATAGTTGGTGGGGTAACGGCCCACCAAGTCGACGATTGGT
AGCCGGACTGAGAGGTTGAACGGCCACATTGGGACTGAGACACGGCC
>Zotu8259
GAACGCTGGCGGCGTGCCTAATACATGCAAGTCGAGCGCAGGAAATCGACGGAACCCTTCGGGGGGAAG
TCGACGGAATGAGCGGCGGACGGGTGAGTAACACGTAAAGAACCTGCCCTCAGGTCTGGGATAACCACG
AGAAATCGGGGCTAATACCGGATGGGTCATCGGACAGCATGGTCCGAGGATGAAAGGCGCTTCGGCGTC
GCCTGGGGATGGCTTTGCGGTGCATTAGATAGTTGGTGGGGTAATGGCCCACCAAGGCGACGATGCATAG
CCGACCTGAGAGGGTGATCGGCCACACTGGGACTGAGACACGGCC
>Zotu8262
GAACGCTGGCGGCGTGGATAAGGCATGCAAGTCGAGAGGGTTAGACCCTCGGCAAACGGGTTAGTAATG
CATAGGAACATTCCCAGAAGTCGCGAATAATTCGGAGAAATCCGGAATAATACGCGATATGGTCTACGGAC
TAAAGATTTATCGCTTTTGGAGTGGCCTATGTCCTATCAGCTAGTTGGTGGTGTAATGGACCACCAAGGCTA
TGACGGGTAGGGGGTGTGAGAGCACGGCCCCCAACATTGGGACTGAGACACTGCC
>Zotu8267
GAACGCTGGCGGCGTGCTTAACACATGCAAGTCGAACGAAGCAACTATCTTGCTTGCAAGAGAGTTGACTT
AGTGGCGGACGGGTGAGTAACGCGTGGGTAACCTGCCTCATACAGGGGGATAACAGTTGGAAACGACTG
CTAACACCGCATAACCCGCTAGTGTCGCATGGCACAGACGGAAAATATTTATAGGTATGAGATGGGCCCGC
GTCTGATTAGCTAGTTGGTAAGGTAACGGCTTACCAAGGCGACGATCAGTAGCCGACTTGAGAGAGTGAT
CGGCCACATTGGGACTGAGACACGGCC
>Zotu8269
GAACGCTAGCGGCAGGCTTAATACATGCAAGTCGTGGGGCAGCAGGAGTAGCAATACTTGCTGGCGACCG
GCAAACGGGTGCGGAACACGTACACAACCTTCCTTCAAGCGGGGAATAGCCCAGAGAAATTTGGATTAAT
ACCCCATAATATATAATGTAGGCATCTACAAGATATTAAAGCTTCGGCACTTAAAGATGGGTGTGCGTCTG
ATTAGATAGTTGGTGAGGTAACGGCTCACCAAGTCAACGATCAGTAGCTGATGTGAGAGCATGATCAGCC
ACACGGGCACTGAGACACGGGC
>Zotu8271
GAACGCTGGCGGCAGGCCTAAAACATGCAAGTCGAACGAACCCTTCGGGGTTAGTGGCGGACGGGTGAG
TAACACGTGGGAACGTGCCCTTTGGTTCGGAACAACTCAGGGAAACTTGAGCTAATACCGAATGTGCCCTA

CGGGGGAAAGATTTATCGCCATTGGAGCGGCCCGCGTCTGATTAGCTAGTTGGTGAGGTAAAGGCCCACC
AAGGCTACGATCAGTAGCTGGTCTGAGAGGATGATCAGCCACACTGGGACTGAGACACGGCC
>Zotu8274
GAACGCTAGCGGCAGGCTTAATACATGCAAGTCGAGGGGCAGCACGGTAGCAATACTGGTGGCGACCGG
CAAACGGGTGCGGAACACGTACGCAACTTACCCCTAACTAGGGAATAGCCCATAGAAATGTGGATTAACAC
CTTATAGTACGATTGGCTCGCATGGGCTGATTGTTAAAACTCCGGTGGTTAGGGATAGGCGTGCGTCTGAT
TAGGTAGTTGGTAGGGTAACGGCCTACCAAGCCAACGATCAGTAGCTGATGTGAGAGCATGATCAGCCAC
ACGGGCACTGAGACACGGGC
>Zotu8279
GAACGCTGGCGGCATGCTTTACACATGCAAGTCGAACGGTAACAGGGAGCTTGCTCCGCTGACGAGTGGC
GAACGGGTGAGTAATATATCGGAACGTGCCCAAGAGTGGGGGATAACGTAGCGAAAGTTACGCTAATACC
GCATACGATCCAAGGATGAAAGCAGGGGACCGCAAGGCCTTGTGCTCCTGGAGCGGCCGATATCTGATTA
GCTAGTTGGTGAGGTAAAGGCTCACCAAGGCGACGATCAGTAGCTGGTCTGAGAGGACGACCAGCCACAC
TGGGACTGAGACACGGCC
>Zotu8283
GAACGCTTGCGGCGTGCCTAAGAAATGCAAGTCGAACGGGCAGTCTTCGGATTGTCAGTGGCGAACGGTC
GCGTAACACGTAAGCAACCTGCCCCAAAGTTTGGGACAACAGCCTGAAAGGGCTGCTAATACCGAATGTG
GTTTCGATGCCGCCTGGCATTGTAACGAAAGCCTTGTGTGCTTTGGGATGGGCTTGCGGCCTATCAGGTAG
TTGGTGCGGTAACGGCGCACCAAGCCTTTGACGGGTAGCTGGTCTGAGAGGACGATCAGCCGGATTGGGA
CTGAGATACGGCC
>Zotu8285
GAACGCTGGCGGCGTGCTTAACACATGCAAGTCGAACGATGAACCTGTGCTTGCACGGGGGATTAGTGGC
GAACGGGTGAGTAACACGTGAGTAACCTGCCCTTGACTTCGGGATAAGCCTGGGAAACTGGGTCTAATAC
TGGATACGACCTCTCACCGCATGGTGTGGGGGTGGAAAGTTTTTGCGGTTTTGGATGGACTCGCGGCCTAT
CAGCTTGTTGGTGAGGTAATGGCTCACCAAGGCGACGACGGGTAGCCGGCCTGAGAGGGTGACCGGCCA
CACTGGGACTGAGACACGGCC
>Zotu8287
GAACGCTGGCGGTATGCCTTACACATGCAAGTCGTACGAAAGTTTTTAACTTTAGTGGCGGACGGGTGAGT
AACACGTAAGAATTTACCTTTCGGAGGGGGACAACAATTGGAAACGATTGCTAATACCCCATATGCTTTGG
AGTGAAATGCTTTGGAGTGAAATGGACGATTCAATATCTCCGCCTAAAGAGAAGCTTGCGGCTGATTAGCT
TGTTGGTGAGGTAATGGCTTACCAAGGCGACGATCAGTATCTGGTTTGAGAGGACGATCAGACACACTGG
AACTGAGACACGGTC
>Zotu8289
GAACGCTGATGGCGTGCCTCATACATGCAAGTCGAGCGGGTAGCAATACCAGCGGCGGACGGGTGAGTA
ACGCGTGAACAATCTGCCTTAAAGTGGGGGATAACCATTCCAACGGATGGCTAATACCGCATAGGGTAGC
GAAGAGGCATCTCTTTGCTACGAAAGGCTTCGGCCGCTTTAGGATGAGTTCGCGTCCCATTAGCTAGTTGG
CGGGGTAACGGCCCACCAAGGCGACGATGGGTAGCCGGCCTGAGAGGGTGTCCGGCCACATTGGGACTG
AGATACGGCC
>Zotu8292
GAACGCTGGCGGCGTGCCTAATACATGCAAGTCGAGCGGATCTGAGGGAGCTTGCTCCCAAAGATCAGCG
GCGGACGGGTGAGTAACACGTGGGCAACCTGCCTGTAAGACTGGGATAACTCCGGGAAACCGGGGCTAA
TACCGGATAATCCATTCCCTCACATGAGGGAATGCTGAAAGACGGTTTCGGCTGTCACTTACAGATGGGCC
CGCGGCGCATTAGCTAGTTGGTGAGGTAACGGCTCACCAAGGCCACGATGCGTAGCCGACCTGAGAGGGT
GATCGGCCACACTGGGACTGAGACACGGCC
>Zotu8294
TAACGCTGGCGGCATGCCTAATACATGCAAGTCGAACGAAAGTAGCAATACTTTAGTGGCGAACGGGTGA
GTAACACGTATCTAACCTACCATTTAGTGGGGAATAACTGTTGGAAACGGCAGCTAATACCGCATATGTTAT
TTATTTGCAATGATAAATAATGAAAGGAGCATTTGCTTCGCTAATTGATGGGGATGCGGCGCATTAACTAG
TTGGTAAGGTAATGGCTTACCAAGGTAACGATGCGTAGCCGATCTGAGAGGATGACCAGCCACACTGGGA
CTGAGACACGGCC

>Zotu8297
GAACGCTGGCGGCAGGCTTAACACATGCAAGTCGAGCGCCCCGCAAGGGGAGCGGCAGACGGGTGAGTA
ACACGTGGGAACGTACCCTTTACTACGGAATAACGCAGGGAAACTTGTGCTAATACCGTATGTGCCCTTTG
GGGGAAAGATTTATCGGTAAAGGATCGGCCCGCGTTGGATTAGCTAGTTGGTGGGGTAAAGGCCTACCAA
GGCGACGATCCATAGCTGGTCTGAGAGGATGATCAGCCACATTGGGACTGAGACACGGCC
>Zotu8300
GAACGCTAGCGGCAGGCCTAATACATGCAAGTCGAACGGTAACAGGTGTAGCAATACATGCTGACGAGTG
GCGCACGGGTGCGTAACACGTATGCAACTTTCCTTCAACTGGTGAATAGCCCCTCGAAAGAGGGATTAATA
CACCATAATATAATGAGGTGGCATCACCTTATTATTAAAACTCCGGTGGTTGAAGATGGGCATGCGCAACA
TTAGCTAGTTGGTGAGGTAATGGCTCACCAAGGCTACGATGTTTAGGGGTTCTAAGAGGATTAACCCCCAC
ACTTGTACTGAGACACGGAC
>Zotu8303
GAACGCTGGCGGCATGCCTAACACATGCAAGTCGCACGGGTCGGCTTCGGCCGGTCAGTGGCGGACGGGT
GAGGAACGCGTAGGGATCTGTCCTTTGGTGGGGGATAACCGCGGGAAACTGCGGCTAAGACCGCATGGG
GCGCCGGTGTGAGAGCCGGCGTCAAAGGCGCGCATCGTGAGGTGTGCGCCGCCGGGGGGTGAGCCTGCG
TCCGATTAGCTCGTTGGGGGGGTAGTGGCCCACCAAGGCTTCGATCGGTAGCTGGTCTGAGAGGGTGATC
AGCCACACCGGGACTGAGACACGGCC
>Zotu8307
CAACGCTGGCGGCGTGCCTAACACATGCAAGTCGAACGAGAAGGTGACTTCGGTCACTGGAAAGTGGCGT
CCGGGTGAGTAACACGTGGGTAATCTACCTCTGGGTGGGGAATAACGTCTCGAAAGAGACGCTAATACCG
CATAATGCAGCGGCACCGAATGGTGAATAGTTGTTAAAGCGGGGGATCGCAAGACCTCGCGCCTGAAGAG
GAGCCTGCGGCAGATTAGCTAGTTGGTGAGGTAATGGCTCACCAAGGCTTCGATCTGTAGCTGGTCTGAG
AGGACGGCCAGCCACACTGGCACTGACACACGGGC
>Zotu8309
GAACGCTGGCGGCGTGCCTAATACATGCAAGTCGAACGCACGAAGTTGAAGAGCTTGCTCTTTAACCAAGT
GAGTGGCGGACGGGTGAGTAACACGTGGGTAACCTGCCCATTAGAGGGGGATAACATTCGGAAACGGAT
GCTAATACCGCATAGTTTCAGGAATCGCATGATTCTTGAAGGAAAGGTGGCTTCGGCTACCACTAATGGAT
GGACCCGCGGCGTATTAGCTAGTTGGTGAGGTAATGGCTCACCAAGGCAATGATACGTAGCCGACCTGAG
AGGGTGATCGGCCACACTGGGACTGAGACACGGCC
>Zotu8312
GAACGCTGGCGGCGCGCTTAACACATGCAAGTCGAGCGAGAACCAGGGCTTCGGCCCTGGGGACAGCGG
CGAACGGGTGAGTAACACGTGGATAACCTGCCCTCGGCACCGGGATAGCCCGGGGAAACCCGGATTAATA
CCGGATGGCCCTTCCGGCTCTCGAGCTGGTCGGAAAAGGTAGCTTCGGCCTCCGGCCGAGGATGGGTCCG
CGGCGGATTAGCTTGTTGGTGGGGTAACGGCCTACCAAGGCTCCGATCCGTAGCTGGTCTGAGAGGACGA
TCAGCCACACTGGGACTGAGACACGGCC
>Zotu8314
GAACGCTGGCGGCGTGCTTAACACATGCAAGTCGAGCGGAAAGGCTCCCTTCGGGGAGCACTCGAGCGGC
GAACGGGTGAGTAACACGTGGGTAACCTACCCTTAGCTCTGGGATAACCTTGGGAAACCGAGGCTAATAC
CGGATATGACACGCGACCGCATGGTCTACGTGTGGAAAGATTAAGTTCGGCTAGGGATGGACTCGCGGCC
TATCAGCTAGTTGGTGGGGTAATTGCCTACCAAGGCGACGACGGGTAGCCGGCCTGAGAGGGCGACCGG
CCACACTGGGACTGAGACACGGCC
>Zotu8318
GAACGCTAGCGGGAGGCCTAACACATGCAAGCCGAGCGGTAGGGTTTCTTCGGAAACCTGAGAGCGGCG
CACGGGTGCGGAACACGTGTGCAACCTACCTTTATCAGGGGGATAGCCTTTCGAAAGGAAGATTAATACCC
CATAATATATTGAATGGCATCATTTGATATTGAAAACTCCGGTGGATAGAGATGGGCACGCGCAAGATTAG
ATAGTTGGTGAGGTAACGGCTCACCAAGTCTGCGATCTTTAGGGGGCCTGAGAGGGTGATCCCCCACACT
GGTACTGAGACACGGAC
>Zotu8321
GAACGCTGGTGGCGTGCTTAACACATGCAAGTCGAGCGAGGCGTCCGCCTCCGGGCGGTCAGCCGAGCG
GCGAACGGGTGAGTAACACGTGAGCAATCTTCCCTCGGCTCGGGGATAACCTCGGGAAACTGAGGCTAAT

ACCGGATGAACGTTCTTCGGGGCATCCCGGAAAACGAAAAGGTTTCGCACCGCGAGGTGCGTTTCCGGCC
GGGGATGAGCTCGCGGCCTATCAGTTTGTTGGTGTGGTAAAAGCGCACCAAGGCGACGACGGGTAGCCG
GCCTGAGAGGGTGATCGGCCACACTGGGACTGAGACACGGCC

>Zotu8323

GAACGCTGGCGGCATGCCTTACACATGCAAGTCGAACGGCAGCATAGGAGCTTGCTCCTGATGGCGAGTG
GCGAACGGGTGAGTAATACATCGGAACGTGCCCAATCGTGGGGGATAACGCAGCGAAAGCTGTGCTAATA
CCGCATACGATCTACGGATGAAAGTGGGGGATCGCAAGACCTCATGCTCCTGGAGCGGCCGATATCTGATT
AGCTAGTTGGTGGGGTAAAAGCTCACCAAGGCGACGATCAGTAGCTGGTCTGAGAGGACGACCAGCCACA
CTGGGACTGAGACACGGCC

>Zotu8325

GAACGCTAGCGGCAGTCCTAACACATGCAAGTCGAGGGGCAGCACGAATTAGCTTGCTAATTTGGTGGCG
ACCGGCGCACGGGTGCGTAACGCGTATGCAACCTACCTTATACAGGAGTATAGCCCGGAGAAATTCGGAT
TAATCCTCCATGGTACTGAGACACCGCATGGTGCTTCAGTTAAAGCTTCGGCGGTATAAGATGGGCATGCG
TCCTATTAGCTAGATGGTAAGGTAACGGCTTACCATGGCTACGATAGGTAGGGGGCCTGAGAGGGTGGTC
CCCCACACTGGTACTGAGACACGGAC

>Zotu8327

GAACGCTGGCGGCGTGCCTAATACATGCAAGTCGAGCGGAACATTCATTGGTGCTTGCACCGATGAATGTT
TAGCGGCGGACGGGTGAGTAACACGTGGGCAACCTGCCCTGTAGATTGGGATAACTCCGGGAAACCGGG
GCTAATACCGAATAATACTTTTTAGCTCATGCTAGAAAGTTGAAAGATGGTTTACGCTATCACTACAGGATG
GGCCCGCGGCGCATTAGCTAGTTGGTGAGGTAACGGCTCACCAAGGCTACGATGCGTAGCCGACCTGAGA
GGGTGATCGGCCACACTGGGACTGAGACACGGCC

>Zotu8329

GAACGCTGGCGGCATGCTTAACACATGCAAGTCGAACGAACTATTTATAGTTAGTGGCAGACGGGTGAGA
AACGCGTGGGAACGTACCCCAAAGTTCGGAATAACTATTGGAAACGATAGCTAATACCGGATGTGACCGA
GAGGTTAAAGGTTTACCGCTTTGGGAGCGGCCCGCGTTAGATTAGCTAGTTGGTGGGGTAAAGGCCTACC
AAGGCAATGATCTATAGCTGGTCTGAGAGGATGACCAGCCACACTGGGACTGAGACACGGCC

>Zotu8332

GAACGCTGGCGGCGTGCCTAATACATGCAAGTCGAGCGGTAAGGTTCTCACAGATTATTTAACCAGATAAG
TTTCTAACGAAATACTGTCTGCAGCGATATTCCCAGGCTAGGGAATAAGGTTGAATAATGCGTGAGAATAC
ACGAGCGGCGGACGGCTGAGTAACACGTAGGAACGTACCCCAAAGTGAGGGATAAGCACCAGAAATGGT
GTCTAATACCGCATGTGCTCTTCGGAGGAAAGCTTCGGCGCTTTGGGAACGGCCTGCGCATGATTAGCTTG
TTGGTGAGGTAATGGCTCACCAAGGCGATGATCATTAGCTGGTCTGAGAGGATGATCAGCCAGACTGGAA
CTGAGAACGGTC

>Zotu8335

GAACGCTGGCGGCGTGCTTAACACATGCAAGTCGAACGATGAAGAAAAGAATCGAAGCTTCGGCAGAAAT
TCATTTCGGATTAGTGGCAGACGGGTGAGTAACGCGTGAGCAACCTACCTCTTACAAAGGGATAGCCTCGG
GAAACCGGGATTAATACCATATAACATTGCGACGAAGCATTTCGGTGCAATCAAAGCTACGGCGGTAAGA
GATGGGCTCGCGTCCCATTAACCAGATGGTGAGGTAACGGCTCACCATAGTAACGATGGGTAACCGGCCT
GAGAGGGTGAACGGTCACACTGGAACTGAGACACGGTC

>Zotu8338

GAACGCTGGCGGCAGGCTTAACACATGCAAGTCGAGCGGGGGAAGTTGCTTCGGTAACTGACCTAGCGGC
GGACGGGTGAGTAATGCTTAGGAATCTGCCTATTAGTGGGGGACAACATCTCGAAAGGGATGCTAATACC
GCATACGTCCTACGGGAGAAAGCAGGGGACCTTCGGGCCTTGCGCTAATAGATGAGCCTAAGTCGGATTA
GCTAGTTGGTGGGGTAAAGGCCTACCAAGGCGACGATCTGTAGCGGGTCTGAGAGGATGATCCGCCACAC
TGGGACTGAGACACGGCC

>Zotu8340

CAACGCTGGCGGCATGCCTAACACATGCAAGTCGAGCGAGAAAGGGTAGCAATACCTGAGTAAAGCGGC
GCACGGGTGAGTAACACGTGGATAACCTGCCTCAAAGTGGGGAATAACACCGGGAAACCGGTGCTAATAC
CGCATATTCTGATGGAGTCTGAGGACTTTGTCAGTAAAGGAGCAATTCGCTATGAGAGGGGTCCGCGGCT
GATTAGCTAGTTGGTGAGGTAATGGCTCACCAAGGCAACGATCAGTAGCCGGTCTGAGAGGACGGTCGGC
CACACTGGAACTGAGACACGGTC

>Zotu8342
GAACGCTGGCGGCGTGCTTAACACATGCAAGTCGAACGGAAAGCGCTCTTCGGAGTGACTCGAGTGGCGA
ACGGGTGAGTAACACGTGGGTGATCTGCCCTGCACTTTGGGATAAGCCTGGGAAACTGGGTCTAATACCG
AATACACCTCGCTGGCTGCATGGTCTGGTGGGGGAAAGCTTTTGCGGTGTGGGATGGGCCCGCGGCCTAT
CAGCTTGTTGGTGGGGTGATGGCCTACCAAGGCGACGACGGGTAGCCGGCCTGAGAGGGTGTCCGGCCA
CACTGGGACTGAGATACGGCC
>Zotu8344
GAACGCTGGCGGCGTGCCTTAAGCATGCAAGTCGAACGGTTTCCGCAAGGAAGCAGTGGCAGACGGGTG
AGTAACGCGTAGGAATCTACCTCTAGGTGGACCATAGCCGTCCGAAAGGACGGGTAATAGTCCATAGTCA
ATTTATTGTAAAGCATTAACTTTGCGCCAAGAGATGAGCCTGCGTCCTATCAGCTTGTTGGTGGGGTAAAA
GCCTACCAAGGCGATGACGGGTAGCTGGACTGAGAGGTCGGTCAGCCACAACTGCACTGAGATACGGGC
>Zotu8346
GAACGCTGGCGGCGTGCTTAACACATGCAAGTCGAACGGTGATGATCAAGCTTGCTTGGTCGGATCAGTG
GCGAACGGGTGAGTAACACGTGAGTAACCTGCCCCAGACTCTGGGATAACTTCGGGAAACCGGAGCTAAT
ACCGGATATGACACTCGCACGCATGTGCAGGGTGTGGAAAGTTTTTCGGTCTGGGATGGGCTCGCGGCCT
ATCAGCTTGTTGGTGAGGTAGTGGCTCACCAAGGCGACGACGGGTAGCCGGCCTGAGAGGGCGACCGGC
CACACTGGGACTGAGACACGGCC
>Zotu8348
GAACGCTGGCGGCATGCCTTACACATGCAAGTCGAACGGCAGCACGGGAGCAATCCTGGTGGCGAGTGG
CGAACGGGTGAGTAATACATCGGAACGTGCCCAATCGTGGGGGATAACGCAGCGAAAGCTGTGCTAATAC
CGCATACGATCTACGGATGAAAGCAGGGGACCGCAAGGCCTTGCGCGAGTGGAGCGGCCGATGGCAGAT
TAGGTAGTTGGTGAGGTAAAGGCTCACCAAGCCTTCGATCTGTAGCTGGTCTGAGAGGACGACCAGCCAC
ACTGGGACTGAGACACGGCC
>Zotu8350
GAACGCTGGCGGCATGCTTTACACATGCAAGTCGAACGGCAGCACGGACTTCGGTCTGGTGGCGAGTGGC
GAACGGGTGAGTAATACATCGGAACGTGTCCTGTAGTGGGGGATAGCCCGGCGAAAGCCGGATTAATACC
GCATACGCTCTACGGAGGAAAGGGGGGGATCTTAGGACCTCTCGCTACAGGGGCGGCCGATGGCAGATT
AGCTAGTTGGTGGGGTAAAGGCCTACCAAGGCGACGATCTGTAGCTGGTCTGAGAGGACGACCAGCCACA
CTGGGACTGAGACACGGCC
>Zotu8354
GAACGCTAGCGGAATGCTTTACACATGCAAGTCGAACGGCAGCGCGGGAGCTTGCTCCTGGCGGCGAGTG
GCGAACGGGTGAGTAATGCATCGGAACGTACCCAGTAGCGGGGGATAACCGCGCGAAAGCGTGGCTAAT
ACCGCATACGCCCTACGGGGGAAAGCGGGGGACCTTCGGGCCTCGCACTATTGGAGCGGCCGATGTCAGA
TTAGCTAGTTGGTGGGGTAATGGCCTACCAAGGCAACGATCTGTAGCTGGTTTGAGAGGACGGCCAGCCA
CACTGGGACTGAGACACGGCC
>Zotu8360
GAACGCTGGCGGCGTGCCTAAGACATGCAAGTCGAGTGGGTTTAGACCCACGGCGAACGGGTGAGTAAT
ACCTAGGAATGTATCTTTAGTTGAGCATATCCCACCGAAAGGTGAAGCAATTCTCAATGTGATCGCAAGATT
AAAGTCGCAAGACGCTAAAGAAGCAGCCTGGGCACCATCAGCTAGTTGGTAAGGTAAAAGCTTACCAAGG
CTATGACGGTTAGGGGCCCCGAGAGGGGAGACCCCCACACTGGAACTGCGACACGGTC
>Zotu8363
AAACGCTGGCGGCGTGCCTAACACATGCAAGTCGAGCGAGAAAGGACTTCGGTCCAAGTAAAGCGGCAG
ACGGGTGAGTAACGCGTGGGAATCTTCCCTTCAGTGAGGAATAACTCTGAGAAATTGGAGCTAATACTGCA
TACGTCCCACTGGGAGAAAGGCGGCTTTATGCTGTCGCTGAAGGACGAGCCTGCGTACTATCAGCTAGTTG
GTGAGGTAATGGCTCACCAAGGCTATGACGGTTAGCCGGCCTGAGAGGGTGTACGGCCACACTGGAACTT
AGACACGGTC
>Zotu8366
GAACGCTGGCGGCAGGCCTAACACATGCAAGTCGAACGCACCGCAAGGTGAGTGGCAGACGGGTGAGTA
ACACGTGGGAACATGCCCTTCGGTCTGGAATAACTCAGGGAAACTTGAGCTAATACCGGATACGTCCGCGA

GGAGAAAGATTCATCGCCGAAGGATTGGCCCGCGTCTGATTAGCTAGTTGGTGAGGTAACGGCTCACCAA
GGCGACGATCAGTAGCTGGTCTGAGAGGATGATCAGCCACATTGGGACTGAGACACGGCC
>Zotu8373
GAACGCTGGCGGCGTGCTTAACACATGCAAGTCGAACGATGATGCCCAGCTTGCTGGGTGGATTAGTGGC
GAACGGGTGAGTAACACGTGAGTAACCTGCCCCTGACTCTGGGATAAGCGTTGGAAACGACGTCTAATAC
TGGATATGACTACTGGTCGCATGGCCTGGTGGTGGAAAGATTTTTTGGTTGGGGATGGACTCGCGGCCTAT
CAGCTTGTTGGTGAGGTAATGGCTCACCAAGGCGACGACGGGTAGCCGGCCTGAGAGGGTGACCGGCCA
CACTGGGACTGAGACACGGCC
>Zotu8378
GAACGCTGGCGGCATGCCTTACACATGCAAGTCGAACGGTAACGCGGGGCAACCTGGCGACGAGTGGCG
AACGGGTGAGTAATACATCGGAACGTGCCCAGTAGTGGGGGATAGCCCGGCGAAAGCCGGATTAATACC
GCATACGACCTATGGGTGAAAGCGGGGGATCGTAAGACCTCGCGCTATTGGAGCGGCCGATGTCAGATTA
GGTAGTTGGTGAGGTAAAGGCTCACCAAGCCTTCGATCTGTAGCTGGTCTGAGAGGACGACCAGCCACAC
TGGGACTGAGACACGGCC
>Zotu8381
GAACGCTGGCGGCATGCCTAAGACATGCAAGTCGAACGAAGTGGCCCAATGAAGATGAAGTGCTTGCACT
GATTTGGAAATGGATTCCCACTTAGTGGCAGACTGGTGAGTAACACGTGGGTAACCTACCCCTTAGTCTGG
GACAACTATTGGAAACGATAGCTAATACCGGATGATTCACAATTAGATAACTTTTTGTGCTAAAAGGAGCTT
CGGCTTCGCTTTGGGATGGGCTTGCGGTGTATTAGCTAGTTGGTAGGGTAATGGCCTACCAAGGCTACGAT
GCATATCCGAGCTGAGAGGCTGATCGGGCACATTGGAACTGAGATACGGTC
>Zotu8383
GAACGCTGGCGGCGCGCTTAACACATGCAAGTCGAACGGTAAGGCCCTTCGGGGTACACGAGTGGCGAAC
GGGTGAGTAACACGTGGGTGATCTGCCTCGTACTTTGGGATAAGCCTGGGAAACTGGGTCTAATACCGGA
TATGACCACAGGATGCATGTTTTGTGGTGGAAAGCTTTTGCGGTACGAGATGGGCCCGCGGCCTATCAGCT
TGTTGGTGGGGTAATGGCCTACCAAGGCGACGACGGGTAGCCGGCCTGAGAGGGCGACCGGCCACACTG
GGACTGAGACACGGCC
>Zotu8385
GAACGCTGGCGGAATGCTTTACACATGCAAGTCGAACGGCAGCACGGGGGCAACCCTGGTGGCGAGTGG
CGAACGGGTGAGTAATACATCGGAACGTGCCCAGTCGTGGGGGATAACGTAGCGAAAGCTACGCTAATAC
CGCATACGATCTAAGGATGAAAGCGGGGGACCGTAAGGCCTCGCGCGATTGGAGCGGCCGATGGCAGAT
TAGGTAGTTGGTGGGGTAAAGGCTCACCAAGCCGACGATCTGTAGCTGGTCTGAGAGGACGACCAGCCAC
ACTGGGACTGAGACACGGCC
>Zotu8387
GAACGCTGGCGGCGTGCTTAACACATGCAAGTCGAACGGTGAACCCCTTCGGGGGGGATCAGTGGCGAAC
GGGTGAGTAACACGTGAGCAACCTGCCCCTGACTCTGGGATAACCCCGGGAAACCGGAGCTAATACCGGA
TATGACATCTTCGGGCATCCGATGGTGTGGAAAGATTTATCGGTTGGGGATGGGCTCGCGGCCTATCAGCT
TGTTGGTGGGGTAATGGCCTACCAAGGCGACGACGGGTAGCCGGCCTGAGAGGGCGACCGGCCACACTG
GGACTGAGACACGGCC
>Zotu8389
GAACGCTGGCGGCACGTTTTAAGCATGCAAGTCGAACGGCAGTGGGGGCAACCCCATGAGAGTGGCGAA
CGGGTGAGTAACACGTAGGTGATCTGCCTTTGGGTTGGGGATAGCTTGTGGAAACACAGGGTAATACCGA
ATGAGCTGACGGGACTTTGGTCAAGTTAGTAAAGGTGCTACGGCACCGCCTAGAGAGGAGCCTGCGGCTG
ATTAGCTAGTTGGTGGGGTAAAGGCCTACCAAGGCGACGATCAGTAGCCGGCCTGAGAGGGTGTACGGCC
ACACTGGAACTGAGACACGGTC
>Zotu8391
GAACGCTGGCGGCACGTTTTAAGCATGCAAGTCGAACGGCAGTGGGGGGCAACTCCCATGAGAGTGGCG
AACGGGTGAGTAACACGTAGGTGATCTGCCCTGGGGATGGGGATAGCCTGTGGAAACACAGGGTAATAC
CGAATAAGCTGACGGGATTTTGATTCTGTTAGTAAAGGTGCTAAGGCACCGTCCTGGGAGGGAGCCTGCGG
CTGATTAGCTAGTTGGTGGGGTAAAGGCCTACCAAGGCGACGATCAGTAGCCGGCCTGAGAGGGTGTACG
GCCACACTGGAACTGAGACACGGTC

>Zotu8394
GAACGCTGGCGGCGTGCTTAACACATGCAAGTCGAGCGGTAAGGCCCTTCGGGGTACACGAGCGGCGAA
CGGGTGAGTAACACGTGGGTGACCTGCCCTCCACTTCGGGATAAGCCCGGGAAACTGGGTCTAATACCGG
ATATGACCTTGGGACGCATGTTTCTTGGTGGAAAGTTTTTTCGGTGGGGGATGGCCCGCGGTCTATCAGC
TTGTTGGTGGGGTGATGGCCTACCAAGGCGACGACGGATAGCCGGCCTGAGAGGGTGATCGGCCACACT
GGGACTGAGACACGGCC
>Zotu8401
GAACGCTGGCGGCGTGCCTAATACATGCAAGTCGAGCGAATCTTTGGGAGCTTGCTCCTAAAGGTTAGCG
GCGGACGGGTGAGTAACACGTGGGCAACCTGCCTATAAGACTGGGATAACTTCGGGAAACCGGAGCTAAT
ACCGGATAATTCTTTCCTATACATGTAGGAAAGCTAAAAGACGGTTTACGCTGTCACTTATAGATGGGCCCG
CGGCGCATTAGCTAGTTGGTGAGGTAACGGCTCACCAAGGCCACGATGCGTAGCCGACCTGAGAGGGTGA
TCGGCCACACTGGGACTGAGACACGGCC
>Zotu8403
GAACGCTAGCGGCAGGCCTAATACATGCAAGTCGAACGGTGGTAGCAATACCATAGTGGCGCACGGGTGC
GTAACGCGTAACCAACCTGCCCTTGACTGGGGGATAGCCCGCCGAAAGGCGGATTAATACCGCATAAGAT
GATAACCCGGCATCGGGAAGTTATTAAAGATTTATTGGTCAAGGATGGGGTTGCGTGACATTAGCTAGTTG
GCGGGGTAACGGCCCACCAAGGCGACGATGTCTAGGGGAGCTGAGAGGCTGGTCCCCCACACGGGCACT
GAGATACGGGC
>Zotu8405
GAACGCTGGCGGCGTGCTTAACACATGCAAGTCGAACGATGATGCCCAGCTTGCTGGGTTGATTAGTGGC
GAACGGGTGAGTAATACGTGAGTAACCTGCCCTTGACTCTGGGATAAGCCTGGGAAACCGGGTCTAATAC
TGGATGCGACGGGCCATCGCATGGTGGTTCGTGGAAAGGGTTTCTGGTCTTGGATGGGCTCACGGCCTAT
CAGCTGGTTGGTGGGGTAATGGCTCACCAAGGCGACGACGGGTAGCCGGCCTGAGAGGGTGTACGGCCA
CATTGGGACTGAGACACGGCC
>Zotu8409
GAATGCTGGCGGCGTGCTTAACACATGCAAGTCGAACGGTGAACCGGAGCTTGCTCTGGGGATCAGTGGC
GAACGGGTGAGTAACACGTGGGCAACCTGCCCCTCTCTCTGGGATAACTTCCGGAAACGGATGCTAATACC
GGATACGACCTGGTGCCTCATGGTGCCGGGTGGAAAGAACTTCGGAGAGGGATGGGCCCGCGGCCTATC
AGCTTGTTGGTGAGGTAACGGCTCACCAAGGCGACGACGGGTAGCCGGCCTGAGAGGGCGACCGGCCAC
ACTGGGACTGAGACACGGCC
>Zotu8412
GAACGCTGGCGGCGTGCCTAATACATGCAAGTCGAACGCTTTGTGCTTAATTGATATGACGAGCTTGCTCT
GATTTGATTTTTTGATTTCAAAGAGTGGCGAACGGGTGAGTAACACGTGGGTAACCTACCTCTTAGCAGGG
GATAACATTTGGAAACAAGTGCTAATACCGTATAATACCAACAACCGCATGGTTGTTGGTTGAAAGATGGT
TCTGCTATCACTAAGAGATGGACCCACGGTGCATTAGCTAGTTGGTAAGGTAATGGCTTACCAAGGCAATG
ATGCATAGCCGAGTTGAGAGACTGATCGGCCACAATGGGACTGAGACACGGCC
>Zotu8414
GAACGCTGGCGGCGTGCCTAACACATGCAAGTCGAGCGAGAAGTTACTCAATGAATCTTCGGAAGAATTA
AGTAATGGAAAGCGGCGGACGGGTGAGTAACGCGTGGGCAACCTGCCTTTCACAGAGGGATAGCCAAAG
GAAACTTTGATTAAAACCTCATAAAGCCGGAATAGTGCATAATATACCGGCCAAAGATTTATCGGTGAAAG
ATGGGCCCGCGTCTGATTAGCTAGTTGGTGGGGTAACGGCTCACCAAGGCAACGATCAGTAGCCGACCTG
AGAGGGTGATCGGCCACATTGGAACTGAGACACGGTC
>Zotu8417
GAACGCTAGCGGGAGGCCTAACACATGCAAGCCGAGCGGTAGAGAGTAGCTTGCTACTCTTGAGAGCGGC
GTACGGGTGCGTAACACGTGTGCAACCTACCTTTATCTGGGGGATAGCCTTTCGAAAGGAAGATTAATACC
CCATAATATATTGACTGGCATCAGTTGATATTGAAAACTCCGGTGGATAGAGATGGGCACGCGCAAGATTA
GATAGTTGGTGAGGTAACGGCTCACCAAGTCAATGATCTTTAGGGGTCCTGAGAGGGAGATCCCCCACACT
GGTACTGAGACACGGAC
>Zotu8420

GAACGCTGGCGGCGCGCCTCACACATGCAAGTCGAGCGAGAACCAGGGCTTCGGCCCTGGGGAAAGCGG
CGAACGGGTGCGTAACACGTGAGCAACCTGCCCCGAAGACCGGGACAAGCCGGGGAAACCTGGTCTAATA
CCGGATGTCCTCGAATGATCGCATGGTCGATCGAGGAAATGGATTCCGCTTCGGGATGGGCTCGCGGTCTA
TCAGCTTGTTGGTGGGGTAACGGCCTACCAAGGCAACGACGGATAGCTGGTCTGAGAGGACGATCAGCCA
CACTGGGACTGAGACACGGCC
>Zotu8422
GAACGCTAGCGGCAGGCTTAATACATGCAAGTCGAGGGGCAGCGGGGGTAGCAATACCTGCCGGCGACC
GGCAAACGGGTGCGGAACACGTACGGAACCTTCCTTTAAGCGGGGGATAGCCCATAGAAATGTGGATTAA
TACCCCATAGTATTATTGGTGTGGCATCACACTATAATTAAAGATTTATCACTTAAAGATGGCTGTGCGGCT
GATTAGGTAGTTGGTGCGGGTAACGGCCCACCAAGCCTTCGATCAGTAACTGGTGTGAGAGCACGACCAG
TCACACGGGCACTGAGACACGGGC
>Zotu8425
GAACGCTGGCGGCATGCTTTACACATGCAAGTCGAGCGGCAGCGCGGGGGCAACCCTGGCGGCGAGCGG
CGAACGGGTGAGGAATACATCGGAACGTACCCCGGAGTGGGGGATAACGCCGCGAAAGCGGCGCTAATA
CCGCATATGCTCTGAGGAGGAAAGTGGGGGACCCGCAAGGGCCTCACGCTCTGGGAGCGGCCGATGTCC
GATTAGCTTGTTGGTGAGGTAATGGCTCACCAAGGCGACGATCGGTAGCTGGTCTGAGAGGATGATCAGC
CACACTGGGACTGAGACACGGCC
>Zotu8429
GAACGCTGGCGGAGTGCCTAACACATGCAAGTCGAGCGGCAGCACGAGTAGTTTACTACTTGGTGGCGAG
CGGCGGACGGCTGAGTAACGCGTGGGAACATGCCCCAAAGTGAGGAATAACTGCCCGAAAGGGTAGCTA
ATGCCGCATACGATCTTCGGATTAAAGGATTTATCCGCTTTGGGAGTGGCCCGCGTACGATTAGATAGTTG
GTGAGGTAAAGGCTCACCAAGTCGACGATCGTTAACTGGTCTGAGAGGATGATCAGTCAGACTGGAACTG
AGACACGGTC
>Zotu8431
GAACGCTGGCGGCGTGCCTAACACATGCAAGTCGAACGGAGTCGGCCCCGCAAGGGACTGACTTAGTGGC
GAACGGGTGAGTAACGCGTGAGCAACCTACCTTTCAGTGGGGGACAACAGTTGGAAACGACTGCTAATAC
CGCATAAAGCATTTTGACCGCATGATCGAGATGCCAAAGATTTATTGCTGAAAGATGGGCTCGCGTCTGAT
TAGATAGTTGGTGAGGTAACGGCCCACCAAGTCGACGATCAGTAGCCGGACTGAGAGGTTGAACGGCCAC
ATTGGGACTGAGATACGGCC
>Zotu8434
GAACGCTGGCGGCGTGCTTAACACATGCAAGTCGAACGGAAAGGCCCTTCGGGGTACTCGAGTGGCGAAC
GGGTGAGTAACACGTGGGTGATCTGCCCTGCACATCGGGATAAGCCTGGGAAACTGGGTCTAATACCGGA
TAGGACCACGGGATGCATGTCCTGTGGTGGAAAGCTTTTGCGGTGTGGGATGGGCCCGCGGCCTATCAGC
TTGTTGGTGGGGTGATGGCCTACCAAGGCGACGACGGGTAGCCGGCCTGAGAGGGTGTCCGGCCACACT
GGGACTGAGATACGGCC
>Zotu8436
GAACGCTAGCGACAGGCTTAACACATGCAAGTCGAGGGGCAGCATAAAGGTAGCAATACTTTGGTGGCGA
CCGGCGCACGGGTGAGTAACGCGTATGCAACCTACCTATCAGAGGGGGATAACCCGGCGAAAGTCGGACT
AATACCGCATAAAACAGGGGCACCGCATGGTGATATTTGTTAAAAATTTATTGCTGATAGATGGGCATGCG
TTCCATTAGGTAGTTGGAGAGGTAACGGCTCACCAAGCCCACGATGGATAGGGGAACTGAGAGGTTGGTC
CCCCACACTGGTACTGAGACACGGAC
>Zotu8438
GAACGCTGGCGGCGTGCTTAACACATGCAAGTCGAACGGTGAAGCCAAGCTTGCTTGGTGGATCAGTGGC
GAACGGGTGAGTAACACGTGAGTAACCTGCCCCAGACTCTGGGATAAGCGCTGGAAACGGCGTCTAATAC
CGGATACGAGACGAGACCGCATGGTCATTGTCTGGAAAGAATTTCGGTCTGGGATGGACTCGCGGCCTAT
CAGCTTGTTGGTGAGGTAATGGCTCACCAAGGCGTCGACGGGTAGCCGGCCTGAGAGGGTGACCGGCCAC
ACTGGGACTGAGACACGGCC
>Zotu8441
GAACGCTGGCGGCGTGCTTAACACATGCAAGTCGAACGGTGATGCAGAGCTTGCTCTGCGGATCAGTGGC
GAACGGGTGAGTAACACGTGAGTAACCTGCCCTCGACTCTGGGATAAGCGTTGGAAACGACGTCTAATAC
CGGATACGACCCGTGGAGGCATCTCCTACGGGTGGAAAGAGTTTCGGTCGAGGATGGACTCGCGGCCTAT

CAGCTTGTTGGTGAGGTAATGGCTCACCAAGGCGACGACGGGTAGCCGGCCTGAGAGGGTGACCGGCCA
CACTGGGACTGAGACACGGCC
>Zotu8443
GAACGCTAGCGGCAGGCTTAACACATGCAAGTCGAGGGGCAGCGCGGGTAGCAATACCTGGCGGCGGCC
GGCAAACGGGTGCGGAACACGTACAGAACCTACCTTTACCTGGGGCATAGCCCGGAGAAATCCGGATTAA
CTCCCCATAACATAAGAGAGTGGCATCACTCATTTATTAAAAATTTATTGGGTAAAGATGGCTGTGCGTCCC
ATTAGCTAGTTGGTAGGGTAACGGCCTACCAAGGCGACGATGGGTAGCTGGTGTGAGAGCACGACCAGCC
ACACGGGCACTGAGACACGGGC
>Zotu8445
GAACGCTGGCGGCGCGCTTAACACATGCAAGTCGAGCGAGAACCAGTCCTTCGGGATTGGGGAAAGCGG
CGAACGGGTGAGTAACACGTGAGTAATCCACCCTTGGTACCGGGATAGCCCGAGGAAACTCGGATTAATA
CCGGATGGCCCAACAGCCTCTCGAGGCGGTTGGAAAAGGTAGCTTCGGCCTCCGACCAAGGACGAGCTCG
CGGCGGATTAGCTTGTTGGTGGGGTAATGGCCCACCAAGGCGACAATCCGTAGCTGGTCTGAGAGGACGA
TCAGCCACACTGGGACTGAGACACGGCC
>Zotu8448
GAACGCTAGCGGCAGGCCTAATACATGCAAGTCGAGGGGTAGTGTAGCAATACATGAGACCGGCGCACG
GGTGCGTAACGCGTATGAAAGGTGTCCAAATCTCTAGGATAGCCCCCGGAAACGGGGATTAATACTGGAT
GGTCTGTTTATGTGGCATCACATTTACAGTAAAGCTCAGGCGGATTTGGGTCCTCATGCGTAAGATTAGCTA
GTTGGCGAGGTAATGGCTCACCAAGGCGACGATCGTTAGGGGTCCTGAGAGGGAGATCCCCCACACTGGT
ACTGAGACACGGGC
>Zotu8450
GCCGTGTCTCAGTCCCAGTGTGGCTGGTCGTCCTCTCAGACCAGCTACAGATCGTCGGCTTGGTAGGCCTTT
ACCCCACCAACTACCTAATCTGATATCGGCCGCTCCAATCGCGCGAGGTCTTGCGATCCCCCGCTTTCATCC
ATAGATCGTATGCGGTATTAGCGTAGCTTTCGCTACGTTATCCCCCACGACTGGGCACGTTCCGATACTTTA
CTCACCCGTTCGCCACTCGCCACCAGGATTGCTCCCGTGCTGCCGTTCGACTTGCATGTGTAAGGCATGCCG
CCAGCGTTCA
>Zotu8452
GAACGCTAGCTACAGGCTTAACACATGCAAGTCGAGGGGCAGCATATTTCTAGCAATAGAAATGATGGCG
ACCGGCGCACGGGTGAGTAACACGTATCCAACCTACCTTTAACTCAGGGATAGCCTTTCGAAAGAACGATT
AATACCTGATAGTATAGTTATATTGCATGATATACTTATTAAAGAATTTCGGTTAAAGATGGGGATGCGTTC
CATTAGATTGTAGGCGAGGTAACGGCTCACCTAGTCTTCGATGGATAGGGGTTCTGAGAGGAAGGTCCCC
CACACTGGAACTGAGACACGGTC
>Zotu8454
AAACGCAAGAGACGTGCCTAACACATGCAAATTAAATAGTTTTAATTAACTATAGTGAATTGGTGAGTAAT
ATATGAAAATTTAAATTTAGATTGGAAGTATTCCTAATAACTTAGGAGTAATCTCTGATAAGCGCAAAGGTG
CTTTAAAAAGTTAATACTGTCTAAATAAAAGTTCATATCTGATTAGCTAGTTAGTAGGGTAAAAGTTTACTA
AGGCGAAGATCAGTAGCTACCCTGAGAGGGGGATTAGCCACATTGGGATTGAAATACAGCC
>Zotu8456
GAACGCTAGCGGCAGGCCTAATACATGCAAGTCGAACGGGATCCAGATCAAAGCTTGCTTTGGTTTGGTG
AGAGTGGCGTACGGGTGCGTAACGCGTGAGCAACCTACCCTTGTCAGGGGGATAGCCCGGCGAAAGTCG
GATTAATACCGCATGACATCATTGAGAGGCATCTTTCGATGATCAAAGATTTATCGGACAAGGATGGGCTC
GCGTGACATTAGCTAGTTGGTAAGGTAACGGCTTACCAAGGCTACGATGTCTAGGGGCTCTGAGAGGAGA
ATCCCCCACACTGGTACTGAGACACGGAC
>Zotu8458
GAACGCTGGCGGCGTGCCTAATACATGCAAGTCGAGCGGATAGACGAGGTGCTTGCACCTCTGAAGTCAG
CGGCGGACGGGTGAGTAACACGTGGGTAACCTACCTGTAAGACTGGGATAACTTCGGGAAACCGGAGCTA
ATACCGGATAATATTTTCCACCTCATGGTGGAATAGTGAAAGACGGTTTTGCTGTCACTTACAGATGGACCC
GCGGCGCATTAGCTAGTTGGTGAGGTAACGGCTCACCAAGGCGACGATGCGTAGCCGACCTGAGAGGGT
GATCGGCCACACTGGGACTGAGACACGGCC
>Zotu8463

GAACGCTGGCGGCGTGCTTAACACATGCAAGTCGAACGGTGAAGCCCAGCTTGCTGGGTAGATCAGTGGC
GAACGGGTGAGTAACACGTGAGTAACCTGCCCCTGACTCTGGGATAAGCCTTGGAAACGAGGTCTAATAC
CGGATATGTGACGTTCAGGCATCTAATGCGTCTGGAAAGATTTATCGGTCAGGGATGGACTCGCGGCCTAT
CAGCTTGTTGGTGGGGTAACGGCCTACCAAGGCGACGACGGGTAGCCGGCCTGAGAGGGCGACCGGCCA
CACTGGGACTGAGACACGGCC
>Zotu8466
GAACGCTGGCGGCATGCCTTACACATGCAAGTCGAACGGTAACGCGGGGCAACCTGGCGACGAGTGGCG
AACGGGTGAGTAATATATCGGAACGTGCCCAGTAGTGGGGGATAGCCCGGCGAAAGCCGGATTAATACC
GCATACGACCTACGGGTGAAAGGGGGGGATCGCAAGACCTCTCGCTATTGGAGCGGCCGATATCAGATTA
GGTAGTTGGTGGGGTAAAGGCCCACCAAGCCGACGATCTGTAGCTGGTCTGAGAGGACGACCAGCCACAC
TGGGACTGAGACACGGCC
>Zotu8471
GAACGCTGGCGGCAGGCCTAACACATGCAAGTCGAGCGCCCCGCAAGGGGAGCGGCGGACGGGTGAGTA
ACACGTGGGAATTTTCCTCAAGGTACGGAACAACTCAGGGAAACTTGGGCTAATACCGTATGATGCAGAG
ATGCCAAAGATTTATCGCCTTGGGATAAGCCCGCGTCAGATTAGGTAGTTGGTGAGGTAACGGCTCACCAA
GCCTGTGATCTGTAGCTGGTCTGAGAGGACGATCAGCCACATTGGGACTGAGACACGGCC
>Zotu8473
GAACGCTGGCGGCGTGCCTAATACATGCAAGTCGAGCGGTAAGGCTTGGCACAGTTTGCTTCTTTCGTTCT
AGCAATAGAACAGGGGTTTTTGTTGGCTAACAAGACCTTTAAGGAGCCAAATGTGCTGAGTACACGAGCG
GCGGACGGCTGAGTAACACGTAGGAACGTACCCCAAAGTGAGGGATAAGCACCGGAAACGGTGTCTAAT
ACCGCATGTGCTCTAAGGAGTAAAGCTTCGGCGCTTTGGGAACGGCCTGCGTCCGATTAGCTTGTTGGTGA
GGTAAAAGCTCACCAAGGCGACGATCGGTAGCTGGTCTGAGAGGATGATCAGCCAGACTGGGACTGAGA
CACGGCC
>Zotu8478
GAACGCTAGCGGAAGGCCTAACACATGCAAGTCGAGCGGTATTTGGTAGCTTGCTACTAGAGAGAGCGGC
GTACGGGTGCGTAACATGTGTGCAACCTACCCTTATCAAGGGGATAGCCATTCGAAAGGATGATTAATACC
CTATAATATATCAGGTGGCATCATTTGATATTGAAAGCTACGGTGGATAAGGATGGGCACGCACAAGATTA
GCTAGTTGGTAAGGTAACGGCTTACCAAGGCGATGATCTTTAGGGGTCCTGAGAGGGAGATCCCCCACAC
TGGGACTGAGACACGGCC
>Zotu8482
GAACGCTAGCGGCAGGCTTAATACATGCAAGTCGAGGGGCAGCATGACCGTAGCAATACGGTTGATGGCG
ACCGGCAAACGGGTGCGGAACACGTACACAACCTTCCTTTAAGCGGGGAATAGCCCGGGGAAACCCGGAT
TAATACCCCATAGTATATTGAAGAGGCATCTCTTTACTATTAAAGAATTTATTCACTTGAAGATGGGTGTGC
GGCTGATTAGCTAGTTGGCGGGGTAATGGCCCACCAAGGCAACGATCAGTAACTGGTGTGAGAGCACGAC
CAGTCACACGGGCACTGAGACACGGGC
>Zotu8487
GAACGCTGGCGGTGTGCTTAACACATGCAAGTCGAACGATGAACCACCTTCGGGTGGGGATTAGTGGCGA
ACGGGTGAGTAACACGTGGGCAATCTGCCCTGCACTCTGGGACAAGCCCTGGAAACGGGGTCTAATACCG
GATACTGACCTGCCAAGGCATCTTGGCGGGTCGAAAGCTCCGGCGGTGCAGGATGAGCCCGCGGCCTATC
AGCTTGTTGGTGAGGTAATGGCTCACCAAGGCGACGACGGGTAGCCGGCCTGAGAGGGCGACCGGCCAC
ACTGGGACTGAGACACGGCC
>Zotu8489
GAACGCTGGCGGCAGGCCTAACACATGCAAGTCGAACGGGTGTCTTCGGGCACTAGTGGCGGACGGGTG
AGTAACGCGTGGGAACATACCCTTTGGTACGGAATAGCCTCGGGAAACTGAGAGTAATACCGTATGTGCC
CTTCGGGGGAAAGATTTATCGCCAAAGGATTGGCCCGCGTTGGATTAGGTAGTTGGTGGGGTAATGGCCT
ACCAAGCCGACGATCCATAGCTGGTTTGAGAGGATGATCAGCCACACTGGGACTGAGACACGGCC
>Zotu8491
ATGACCTCGTGGTCATGGGCTTCTCTCTCTCCCTCTATTCCTGCCCCTGCCTTCCTTGTGCTCTCTTTCTCTCTG
TCTCTCTCTCAAAATAAACTTAAAACAAATTTAGTGGCATGGAATACCTTATTTAGGCGAGATTTCCTCCCTG
AGGGAATGGCAGCGGTCTATCAGTTAAATTTCCGGCAATGAATAGGAATTTCATTGTTGACCTGTTAAAGG
T

>Zotu8493
GAACATTGGCGGAAAGCTTAACACATGCAAGTCGAGCGAGGCTACGAGCCTAGCGGCGAACGGGTGAGT
AATACATAGGAATCTGCCCTGAAGTGGGGGACAACAACCAGAAATGGTTGCTAATACCCCATAATCCATAT
TGGGAAAGCGCGCAGCGCTTTAGGATGAGCCTATGGTCGATTAGCTAGTTGGTGGGGTAATAGCCTACCA
AGGCGACGATCGATAGCTGGTCTGAGAGGATGATCAGCCACACTGGAACTGAGACACGGTC
>Zotu8496
GAACGCTGGCGGCAAGCTTAACACATGCAAGTCGAGCGGGCCCTTCGGGGTCAGCGGCAGACGGGTGAG
TAACGCGTGGGAACGTGCCCTTCGGTTCGGAATAACTCAGGGAAACTTGAGCTAATACCGGATACGCCCTT
CTGGGGAAAGGTTTACTGCCGAAGGATCGGCCCGCGTCTGATTAGCTGGTTGGTGAGGTTACGGCTCACC
AAGGCGACGATCAGTAGCTGGTCTGAGAGGATGATCAGCCACACTGGGACTGAGACACGGCC
>Zotu8499
GAACGCTGGCGGCGCGCTTAACACATGCAAGTCGAGCGAGAACCAGGACTTCGGTCCTGGGGACAGCGG
CGAACGGGTGAGTAACACGTGGGTAATCTGCCCTCGATTCTGGGATAGCCCGGGGAAACCCGGATTAATA
CCGGATAGCCTTTCGAGCTCTCGGGCTTGAAAGAAAAGGTAGCTTCGGCCTCCGATCGAGGATGAGCCCG
CGGTGGATTAGCTTGTTGGCGGGGTAATGGCCCACCAAGGCGACGATCCATAGCTGGTCTGAGAGGACGA
TCAGCCACACTGGGACTGAGACACGGCC
>Zotu8502
TAACGCTGGCGGCGTGCCTAATGCATGCAAGTCGAACGTGGTCTCTTCGGAGATTAACGTGGCGGACGGG
TGAGGAACACGTGGGGAACCTGCCCAACGGTGGGGGGTAGCCCGCGGAAACGCGGGGTAATTCCGCATA
CGCTGGTCTGGGGATAGCCTGGATCAGGAAAGGGTCGTTACGACTCGCCGATGGAGGGGCCTGCGTCCGA
TTAGCTTGTTGGTGGGGTAACGGCCTACCAAGGCGGTGATCGGTAGCTGGTCTGAGAGGACGGCCAGCCA
CAGGGGGACTGAGACACGGCC
>Zotu8504
GAACGCTGGCGGCGTGCTTAACACATGCAAGTCGAACGAAGCACTTATCTTTGATTCTTCGGATGAAGAGG
TTTGTGACTGAGTGGCGGACGGGTGAGTAACGCGTGGGTAACCTGCCTCATACAGGGGGATAACAGTTAG
AAATGACTGCTAATACCGCATAAGACCACGGAGCTGCATGGCTCAGTGGGAAAAACTCCGGTGGTATGAG
ATGGACCCGCGTCTGATTAGGTAGTTGGTGGGGTAACGGCCTGCCAAGCCAACGATCAGTAGCCGACCTG
AGAGGGTGACCGGCCACATTGGGACTGAGACACGGCC
>Zotu8507
GAACGCTAGCGGCAGGCTTAATACATGCAAGTCGAGGGGTAACAGGTCTGTAGCAATACAGATGCTGACG
ACCGGCAAACGGGTGCGGAACACGTACAGAACCTTCCTTCGAGTGGGAGATAGCCCAGGGAAACTTGGA
GTAATATCCCATAGGATCATAGAGTGGCATCACTTAGTGATTAAAGATTTATCGCTTGAAGATGGCTGTGC
GGCTGATTAGGTAGTTGGTGTAGGTAACGGCTCACCAAGCCTTCGATCAGTAACTGGTGTGAGAGCACGA
CCAGTCACACGGGCACTGAGACACGGGC
>Zotu8512
GAACGCTGGCGGCGTGCTTAACACATGCAAGTCGAACGGAAAGGCCCTTCGGGGTGCTCGAGTGGCGAAC
GGGTGAGTAACACGTGGGTGATCTGCCCTGCACTTTGGGATAAGCCTGGGAAACTGGGTCTAATACCGGA
TAGGACCACGGTCTGCATGGATTGTGGTGGAAAGCTTTTGCGGTGTGGGATGGGCCCGCGGCCTATCAGC
TTGTTGGTGGGGTGATGGCCTACCAAGGCGACGACGGGTAGCCGGCCTGAGAGGGTGACCGGCCACACT
GGGACTGAGATACGGCC
>Zotu8514
GAACGCTGGCGGCGTGCCTAATACATGCAAGTCGAGCGGACTTTTTAAAAGCTTGCTTTTAAAAAGTTAGC
GGCGGACGGGTGAGTAACACGTGGGCAACCTGCCCTATAGTTTGGGATAACTCCGGGAAACCGGGGCTAA
TACCGAATCATACAAACTAGCTCCTGCTAGCTTGTTGAAAGATGGTTTCGGCTATCGCTATAGGATGGGCCC
GCGGCGCATTAGCTAGTTGGTAAGGTAACGGCTTACCAAGGCGACGATGCGTAGCCGACCTGAGAGGGTG
ATCGGCCACACTGGGACTGAGACACGGCC
>Zotu8516
GAACGCTGGCGGCGTGCCTAATACATGCAAGTCGAACGAGGTGCTTGAGTAGCAATACAAGAGTATCCTA
GTGGCGAACGGGTGAGTAACGCGTGGATGACCTACCCCAAAGTGTGGGATAACAGTCCGAAAGGATTGCT
AATACCGCATGTGGTCTCCGGGTTTAGAAACCGGCGACTAAAGGAGCAATTCGCTTTGGGAGGGATCTGC

GTCCCATCAGCTAGTTGGTGAGGTAATGGCTCACCAAGGCGACGACGGGTAGGGGACCTGAGAGGGTGG
CCCCCCACAATGGAACTGAAACACGGTC
>Zotu8519
GAACGCTGGCGGCGTGCCTAATACATGCAAGTCGAGCGGATCTTGAGGGGCTTGCCCCTTAAGGTCAGCG
GCGGACGGGTGAGTAACACGTGGGCAACCTGCCTGTAAGATTGGGATAACTTCGGGAAACCGGAGCTAAT
ACCGGATAATTCTTTCCCTCTCATGAGGGAGAGCTGAAAGACGGCGTAAGCTGTCACTTACAGATGGGCCC
GCGGCGCATTAGCTAGTTGGTGAGGTAACGGCTCACCAAGGCAACGATGCGTAGCCGACCTGAGAGGGT
GATCGGCCACACTGGGACTGAGACACGGCC
>Zotu8523
GAACGCTGGCGGCATGCCTAACACATGCAAGTCGAACGAAGCCTTCGGGCTTAGTGGCGCACGGGTGCGT
AACGCGTGGGAATCTGCCCCTCGGTTCGGAATAACAGTTAGAAATGACTGCTAATACCGGATGATGACGTA
AGTCCAAAGATTTATCGCCGAGGGATGAGCCCGCGTAAGATTAGCTAGTTGGTAGGGTAAAGGCCTACCA
AGGCGACGATCTTTAGCTGGTCTGAGAGGATGATCAGCCACACTGGGACTGAGACACGGCC
>Zotu8525
GAACGCTGGCGGCGTGCCTAATACATGCAAGTCGAGCGGAACCAATGGAGCTTGCTCCATTGGTTTAGCG
GCGGACGGGTGAGTAACACGTGGGCAACCTGCCCTGCAGATCGGGATAACTCCGGGAAACCGGTGCTAAT
ACCGAATAGTTTTTTGCCCCTCCTGGGGCGGAACGGAAAGACGGTTTCGGCTGTCACTGCAGGATGGGCCC
GCGGCGCATTAGCTAGTTGGTGGGGTAATGGCCCACCAAGGCGACGATGCGTAGCCGACCTGAGAGGGT
GATCGGCCACACTGGGACTGAGACACGGCC
>Zotu8527
GAACGCTAGCGGCAGGCCTAACACATGCAAGTCGAGGGGTATAGTAGCAATACTAGAGACCGGCGCACG
GGTGCGTAACGCGTATGCAATCTACCTTTTACAGGGGAATAGCCCAGAGAAATTTGGATTAATGCCCCATG
GTATTATCGAGTGGCATCATTTGATAATTAAAGTTCCAACGGTAAAAGATGAGCATGCGTCCTATTAGCTAG
TTGGTAAGGTAACGGCTTACCAAGGCTACGATAGGTAGGGGTCCTGAGAGGGAGATCCCCCACACTGGTA
CTGAGACACGGAC
>Zotu8532
GAACGCTGGCGGCGTGCTTAACACATGCAAGTCGAACGTGAAAGTAGCAATATGAGTAAAGTGGCGTACG
GGTGAGTAACACGTGGGTAATCTACCTTAGAGTCTGGAATAACCTGCCGAAAGGCGGGCTAATACTGGAT
AGTTCAAGCGCATTATAAAGATGCGATTGGAAAAGGGAGCTCGTAAGAGCCTTCGCTTTAAGATGAGCCC
GCGTCTGATTAGCTAGTTGGTGAGGTAATGGCTCACCAAGGCGACGATCAGTAGCTGGTCTGAGAGGATG
ATCAGCCACACTGGAACTGAAACACGGTC
>Zotu8534
GAACGCTGGCGGCGTGCCTAATACATGCAAGTCGGGCGGGGTTTTGGGGAGCTTGCTCCTTAAAACCCAG
CGGCGGACGGGTGAGTAACACGTGGGCAACCTGCCCGCAAGACCGGGATAACTCCGGGAAACCGGGGCT
AATACCGGATAGGACTCCGGATCGCATGATCCGGGGTGGAAAGGTGGCCTTCGGCTGCCGCTTGCGGATG
GGCCCGCGGCGCATTAGCTAGTTGGTGAGGTAACGGCTCACCAAGGTGACGATGCGTAGCCGACCTGAGA
GGGTGACCGGCCACACTGGGACTGAGACACGGCC
>Zotu8541
GAACGCTGGCGGCATGCTTAACACATGCAAGTCGAACGGAATGCTTCGGCATTCAGTGGCGGACGGGTGA
GTAACACGTGGGAACTTGTCTTTAGGTGGGGAACAACCGCTGGAAACGGCGGCTAATACCGCATAAACCC
TGAGGGGCAAAGGCTTCGGCCGCCTAAGGAGGGGCCCGCGTCCGATTAGCTTGTTGGTGGGGTAATGGCC
TACCAAGGCTTCGATCGGTAGCTGGTCTGAGAGGACGATCAGCCACACTGGGACTGAGACACGGCC
>Zotu8543
GAACGCTGGCGGCGTGGTTAAGGCATGCAAGTCGAACGGTAAGCAGGTAATTTATTATTTGCTTATAGTGG
CAAACGGGTTAGTAACGCGTAGGTAACTTACCTTGAAGCCCCGAATAATTCGCCGAAAGGCGAACTAATAC
GGGATGGTAATGAGGGGGCGAAAGCCATTCTCATTTAAAGATTCATCACTTCAAGAGAGGCCTGCGTCCTA
TCAGCTAGTTGGCGAGGTAATTGCTCACCAAGGCTATGACGGGTAGGGGGACTGAGAGGTTGGCCCCCAA
CATCGGAACTGAGACACTGTC
>Zotu8545

GAACGCTGGCGGCAGGCCTAACACATGCAAGTCGAGCGGATGACGGGAGCTTGCTCCTTGATTCAGCGGC
GGACGGGTAAGTAATGCCTAGGAATCTGCCTGGTAGTGGAGGACAACGTTTCGAAAGGAACGCTAATACC
GCATACGTCCTACGGGAGAAAGCAGGGGACCTTCAGGCCTTGCGCTATCAGATGAGCCTAGGTCGGATTA
GCTAGTTGGTGAGGTAATGGCTCACCAAGGCGACGATCCGTAACTGGTCTGAGAGGATGATCAGTCACAC
TGGAACTGAGACACGGTC
>Zotu8547
GAACGCTGGCGGCGTGCTTAACACATGCAAGTCGAACGATGAAAGCGGGAGCTTGCTCCTGCTGGATTAG
TGGCGAACGGGTGAGTAACACGTGAGTAACCTGCCCTTGACTCTGGGATAAGCGTTGGAAACGACGTCTA
ATACCGGATACGACCCTCGGAGGCATCTCCTGGGGGTGGAAAGAATTTCGGTCAAGGATGGACTCGCGGC
CTATCAGCTAGCTGGTGAGGTAATAGCTCACCAAGGCGACGACGGGTAGCCGGCCTGAGAGGGTGACCG
GCCACACTGGGACTGAGACACGGCC
>Zotu8550
CAACGCTGGCGGCGTGCCTAATGCATGCAAGTCGAGCGGGGTGGCCCTTGGGTCACTTACGCGGCGGACG
GGTGCGGAACACGTGGGCAACCTGCCCTGAGGTGGGGGATAGCCGAGGGAAGCTTCGGGTAATTCCGCA
TGGGGTTCCTGACCGGGAGGTTGGGGAGCAAAGGGTCTTCGGACTCGCCTTGGGAGGGGCCTGCGGCCG
ATTAGCTAGACGGTGGGGTAACGGCCTACCGTGGCGATGATCGGTAGCTGGTCTGAGAGGACGGCCAGCC
ACACGGGGACTGAGACACGGCC
>Zotu8552
GAACGCTGGCGGCGTGCCTAATACATGCAAGTCGAGCGGATCGTATCCTTCGGGATACGATGAGCGGCGA
ACGGGTGAGTAACACGTAGGCAACCTGCCTGTAAGACCGGGATAACCCACGGAAACGTGAGCTAATACCG
GATAATCGGGTTCTCCGCATGGAGAGACCGTGAAAGATGCTCCGGCATCGTTTACAGATGGGCCTGCGGC
GCATTAGCTAGTTGGCGGGGTAACGGCCCACCAAGGCGACGATGCGTAGCCGACCTGAGAGGGTGATCG
GCCACACTGGGACTGAGACACGGCC
>Zotu8554
GAACGCTGGCGGCATGCTTTACACATGCAAGTCGAACGGTAACAGGCCTTCGGGTGCTGACGAGTGGCGA
ACGGGTGAGTAAAGCATCGGAACGTACCCAGAGGTGGGGGATAACGCAGCGAAAGCTGTGCTAATACCG
CATGTGATCTGAGGATGAAAGCGGGGGACCTTCGGGCCTCGCGCCTTTGGAGCGGCCGATGTCGGATTAG
CTAGTTGGTGGGGTAAAGGCCTACCAAGGCGACGATCCGTAGCTGGTCTGAGAGGACGACCAGCCACACT
GGGACTGAGACACGGCC
>Zotu8558
GAACGCTGGCGGAATGCTTTACACATGCAAGTCGAACGGCAGCACGGGAGCTTGCTCCTGGTGGCGAGTG
GCGAACGGGTGAGTAATATATCGGAACGTATCCAATAATGGGGGATAACTAACCGAAAGGTTGGCTAATA
CCGCATACGCCCTACGGGGGAAAGCAGGGGATCGTAAGACCTTGCGTTAATGGAGCGGCCGATATCTGAT
TAGCTAGTTGGTGGGGTAAAGGCCCACCAAGGCATTGATCAGTAGCTGGTCTGAGAGGACGACCAGCCAC
ACTGGAACTGAGACACGGTC
>Zotu8561
GAACGCTGGCGGCGTGCCTAACACATGCAAGTCGAACGAGAAAGGGACTTCGGTCCTGAGTAAAGTGGC
GCACGGGTGAGTAACACGTGACTAACCTACCTTTGAGCGGGGGATAACCTAGGGAAACCTGGGCTAATAC
CGCATAACACCTACGGGTCAAAGCGAGCAATCGCACTTAAAGAGGGGGTCGCGGCAGATTAGCTAGTTGG
TGAGGTAATGGCTCACCAAGGCGACGATCTGTATCCGGCCTGAGAGGGCGCACGGACACACTGGAACTGA
AACACGGTC
>Zotu8566
GAACGCTGGCGGTATGCTTAACACATGCAAGTCGAACGAATTTGCTTGGAGCTTGCTTCAAGTAAGTTAGT
GGCAAACGGGTGAGTAACGCGTGGGAATCTACCCATCAGTACGGAATAACATTTGGAAACAGGTGCTAAT
ACCGTATGTTCTCTACGGAGGAAAGATTTATCGCTGATGGATGAGCCCGCGTAAGATTAGGTAGTTGGTGG
GGTAATGGCCTACCAAGCCGACGATCTTTAGCTGGTCTGAGAGGATGATCAGCCACACTGGGACTGAGAC
ACGGCC
>Zotu8568
GAACGCTAGCGGCAGGCTTAATACATGCAAGTCGAGGGGCAGCATGGGTGTAGCAATACATCTGATGGCG
ACCGGCAAACGGGTGCGGAACACGTACACAACCTTCCTTTAAGTGGGGGAATAGCCCAGAGAAATTTGGAT
TAATACCCCGTAATATATAGAAGAGGCATCTCTTTTATCTTATAGCTCCGGCGCTTAATGATGGGTGTGCGG

CTGATTAGATAGTTGGCGGGGTAACGGCCCACCAAGTCTGCGATCAGTAACTGGTGTGAGAGCACGACCA
GTCACACGGGCACTGAGACACGGGC
>Zotu8573
GAACGCTGGCGGCATGCCTTACACATGCAAGTCGAGCGGCAGCGCGGGGGCAACCCTGGCGGCGAGCGG
CGCACGGGTGAGTAATACATCGGAACGTGTCCTTTTGTGGGGGATAACCAGTCGAAAGACTGGCTAATAC
CGCATGAGATCTCACGATGAAAGCGGGGGACCGCAAGGCCTCGCGCGATTGGAGCGGCCGATGTCGGAT
TAGCTAGTTGGCGGGGTAAAAGCCCACCAAGGCGACGATCCGTAGCTGGTTTGAGAGAACGACCAGCCAC
ACTGGGACTGAGACACGGCC
>Zotu8579
GAACGCTGGCGGCGTGCTTAACACATGCAAGTCGAACGGTGAAGGAGAGCTTGCTCTCCGGATCAGTGGC
GAACGGGTGAGTAACACGTGGGCAACCTGCCCCTGACACTGGGATAACTCCGGGAAACCGGGGCTAATAC
CGGATACGACGCGAATTGGCATCTTTTCGCGTGGAAAGTTTTTCGGTCAGGGATGGGCCCGCGGCCTATCA
GCTTGTTGGTGAGGTAACGGCCCACCAAGGCGACGACGGGTAGCCGGCCTGAGAGGGCGACCGGCCACA
CTGGGACTGAGACACGGCC
>Zotu8582
GAACGCTGGCGGCGTGCCTAACACATGCAAGTCGAGCGAACCCTTCGGGGTGAGCGGCGGACGGGTGAG
TAACGCGTGGGTAACCTGCCCTATACACACGGATAACGTACCGAAAGGTACGCTAATACGAGATGATATAT
TTTTATCGCATGGTAGAAATATCAAAGCTCCGGCGGTATAGGATGGACCCGCGTCTGATTAGCTAGTTGGT
AAGGTAATGGCTTACCAAGGCCACGATCAGTAGCCGACCTGAGAGGGTGATCGGCCACATTGGAACTGAG
ACACGGTC
>Zotu8585
GAACGCTGGCGGCGTGCTTAAGGTATGCAAGTCGAACGAGAATTTTGCAGTAGCAATATTGTAAAATTCTA
GTGGCAAACGGGTGAGTAATGCGTAGATAATCTACCCTTAACATTGAAATAACTTTGCGAAAGTAAGGCTA
ATGTCATGTGTGGTGTATAAGTGAAATTCTTATATATTAAAGTAGCAATCCGGTTAAGGATGAGTCTGCGTC
CTATCAGCTAGTTGGTGAGGTAAATGCTCACCAAGGCGACGACGGGTAGCTGGTCTGAGAGGATGATCAG
CCACATTGGAACTGCGACACGGTC
>Zotu8588
GAACGCTGGCGGTATGCTTTACACATGCAAGTCGAACGCAAGTTTTTAACTTGAGTGGCGGACGGGTGAG
TATCACGTGAGAATTCACCTTTAGGTGGGGAATAACTTTTGGAAACGATAGCTAATGCCCCATAGACTCGA
AAGAGAAAAAAGAGTAATCTGCCTAAAGAGAAGCTCGCGGCTGATTAGCTAGTTGGTAAGGTAATGGCTT
ACCAAGGCGACGATCAGTAGCTGGTTTGAGAGAACGACCAGCCACACTGGAACTGAGACACGGTC
>Zotu8590
GAACGCTGGCGGCGTGCCTAATACATGCAAGTCGAGCGGTAACACCTAGGGTGACGAGCGGCGGACGGC
TGAGTAACGCGTAGGAATGTACCCCAAAGTGAGGGATAAGCACCGGAAACGGTGTCTAATACCGCATATT
CTCTTAGGAGCAAAGCAGCAATGCGCTTTGGGAACGGCCTGCGTATGATTAGCTTGTTGGTGAGGTAATG
GCTCACCAAGGCGACGATCATTAGCTGGTCTTAGAGGATGATCAGCCAGACTGGGACTGAGACACGGCC
>Zotu8593
GAACGCTGGCGGCAGGCTTAACACATGCAAGTCGAGCGCGTAGCAATACGAGCGGCAGACGGGTGAGTA
ACGCGTGGGGATGTGCCTCGTGGTACGGAACAACTCAGGGAAACTTGAGCTAATACCGTATAAGTCCTTCG
GGAGAAAGATTTATCGCCATGAGATCAACCCGCGTTGGATTAGCTAGTTGGTGAGGTAATGGCTCACCAA
GGCGACGATCCATAGCTGGTCTGAGAGGATGATCAGCCACACTGGGACTGAGACACGGCC
>Zotu8595
GAACGCTGGCGGCGTGGCTAAGACATGCAAGTCGCACGCGTCGCGTAGCAATACGCGGCGAGTGGCGAA
CGGGTGAGTAATGAATCGCTAATGTACCCCGGACTCCGGGATAGCTACCCGAAAGGGTAGGTAATACCGG
ATGACATACCTGGCAGGCATCTGCCGGGTCTCAAAGGTCCGCCGGTCCGGGAGCAGGCGATTTCGTATCA
GCTAGTTGGTGAGGTAACGGCTCACCAAGGCGACGACGCGTAGCCGGACTGAGAGGTTGGCCGGCCACA
TCGGGACTGAGACACTGCC
>Zotu8599
GAATGCTGGCGGCGTGGATAAGGCATGCAAGTCGAACGGTCCGCAAGGACAGTGGCAGACGAGGTAGTA
ACACGTAGGTACTCCCCCCAAAGTCTGGTATAGCTCGTCGAAAGACGGGGTAATCCCGGATGGTCCCGAAA

GGGTAAAGATTTATCGCTTTGGGAGAGGCCTGCGTAATATCAGCTTGTTGGTGAGGTAATGGCTCACCAAG
GCTACGACGTTTAGGGGAGCTGAGAGGCTGACCCCCACCGATGGGACTGAGACACGGCC
>Zotu8603
GAACGCTGGCGGCGTGCCTAAGACATGCAAGTCGAGCGGGGCAGGTTTATACCTGTTCAGCGGCGGACG
GGTGAGTAACGCGTGGGTGACCTACCCGGAAGAGGCGGACAACCTGGGGAAACCCAGGCTAATCCGCCA
TGTGGTCCTATCCTGTGGGGTAGGACTAAAGGGGCGACCCGCTTCCGGATGGGCCCGCGTCCCATCAGCTA
GTTGGTGGGGTAAAGGCCCACCAAGGCGACGACGGGTAGCCGGTCTGAGAGGATGGCCGGCCACAGGG
GCACTGAGACACGGGC
>Zotu8606
GAACGCTGGCGGCATGCTTAACACATGCAAGTCGAACGAGACCTTCGGGTCTAGTGGCGCACGGGTGCGT
AACGCGTGGGAATCTGCCCTTAGGTACGGAATAACTCAGAGAAATTTGTGCTAATACCGTATGATGTCGAA
AGACCAAAGATTTATCGCCTAAGGATGAGCCCGCGTAAGATTAGCTAGTTGGTAAGGTAAAAGCTTACCAA
GGCGACGATCTTTAGCTGGTCTGAGAGGATGATCAGCCACACTGGGACTGAGACACGGCC
>Zotu8609
GAACGCTGGTAACGTGGATACCGGCATGCAAGTCAAGGGGCCGCAAGGCACCGGCAAACGGCTTAGTAA
CACGTAGATACATACCCCAAAGTGGAGTACAGCTCGTCGAAAGACGGGGTAATCCTCCATAATCTCGCAAG
AGTAAAGGCGCAAGCCGCTTTGGGCATGGTCTGCGGTCTATCAGCTAGTTGGTGAGGTAAAAGCTTACCA
AGGCTATGACGGATAGGGGGCGTGAGAGCGCGACCCCCAAGAATGGCACTGCGACACGGGC
>Zotu8612
GAACGCTGGCGGCAGGCTTAACACATGCAAGTCGAACGCACTTTTCGGAGTGAGTGGCAGACGGGTGAGT
AACGCGTGGGAATCTACCTATTTCTACGGAATAACGCAGAGAAATTTGTGCTAATACCGTATACGTCCTATT
GGAGAAAGATTTATCGGAGATAGATGAGCCCGCGTTGGATTAGCTAGTTGGTGAGGTAATGGCCCACCAA
GGCGACGATCCATAGCTGGTCTGAGAGGATGACCAGCCACATTGGGACTGAGACACGGCC
>Zotu8614
GAACGCTGGCGGCGTGCTTAACACATGCAAGTCGAGCGGTGAACCCTTCGGGGGGATCAGCGGCGAACG
GGTGAGTAACACGTGAGCAACCTGCCCCTGACTCTGGGATAGCCTCGGGAAACCGGGATTAATACCGGAT
ATGACATCGCCTCGCATGGGGTGGTGTGGAAAGTTTTTCGGTCAGGGATGGGCTCGCGGCCTATCAGCTT
GTTGGTGGGGTGATGGCCTACCAAGGCGACGACGGGTAGCCGGCCTGAGAGGGTGACCGGCCACACTGG
GACTGAGACACGGCC
>Zotu8616
GAACGCTAGCGGCAGGCCTAATACATGCAAGACGAACGGCAATCCCGACAGCAATGTCGGGACTAGAGTG
TCGTAAGGGTGCGTAACACGTATGCAATCTGCCCTGTACAGGAGGATAGCTCCCCGAAAGGGGAATTAAC
ACTCCATAGGATATTGATGTGGCATCACATTAATATTAAAACTGAGGTGGTACAGGATGAGCATGCGCATG
ATTAGTTAGTTGGCGGGGTAAAGGCCCACCAAGACGATGATCATTAGGGGAACTGAGAGGTTGATCCCCC
ACACTGGCACTGAGATACGGGC
>Zotu8620
GAACGCTGGCGACGTGCTTAACACATGCAAGTCGAACGGAAAGGCCCTGCTTGCAGGGTACTCGAGTGGC
GAACGGGTGAGTAACACGTGGGTGATCTGCCCTGCACTTCGGGATAAGCCTGGGAAACTGGGTCTAATAC
CGGATAGTACCATCGTTTAGTGTCGGTGGTGGAAAGTTTTTCGGTGCAGGATGAGCCCGCGGCCTATCAGC
TTGTTGGTGGGGTAATGGCCTACCAAGGCGTCGACGGGTAGCCGGCCTGAGAGGGTGGACGGCCACATT
GGGACTGAGACACGGCC
>Zotu8623
GAACGCTGGCGGCGTGCCTAACACATGCAAGTCGAACGGACGATCTTTTGTGGAGGCTTCGGTTGAAGCA
GCTGATCGTTAGTGGCGGACGGGTGAGTAACGCGTGGGTAACCTGCCCTGTACTGGGGGATAACAGTGAG
AAATCACTGCTAATACCGCATAAGCGCACGAAAGCGCATGCTTTTGTGTGAAAAACTCCGGTGGTACAGGA
TGGACCCGCGTTGGATTAGCTGGTTGGCAGGGTAACGGCCTACCAAGGCGACGATCCATAGCCGGCCTGA
GAGGGTGGACGGCCACATTGGGACTGAGACACGGCC
>Zotu8626
GAACGCTAGCGGCATGCTTAACACATGCAAGTCGAACGGCAGCGCGGGGCAACCTGGCGGCGAGTGGCG
AACGGGTGAGTAATACATCGGAACGTACCCAGAAGTGGGGGATAACGTAGCGAAAGTTACGCTAATACCG

CATACGTTCTACGGAAGAAAGTGGGGGACCTTCGGGCCTCATGCTTTTGGAGCGGCCGATGTCTGATTAGC
TAGTTGGTGAGGTAAAGGCTCACCAAGGCGACGATCAGTAGCTGGTCTGAGAGGACGACCAGCCACACTG
GGACTGAGACACGGCC
>Zotu8628
GAACGCTGGCGGCGTGCCTAACACATGCAAGTCGAACGCGAACGGCGCTTTCGGGTGCCTAGTAGAGTGG
CGCACGGGTGAGTAACACGTAGACAACCTGCCTTCGAGCCCGGAATAACCTTTCGAAAGAAGGGCTAATA
CCGGATAACATCCCGGGTTGCATGACTTGGGATTGAAAGGCTTGCTACTCGAAGAGGGGTCTGCGGGACA
TTAGCTAGTTGGTGGGGTAACGGCCTACCAAGGCGACGATGTCTAGCGGGTCTGAGAGGATGATCCGCCA
CACTGGAACTGAGACACGGTC
>Zotu8631
GAACGCTAGCGGCAGGCCTAATACATGCAAGTCGAACGGTCGTAGTGATACGATAGTGGCGCACGGGTGC
GTAACACGTATGCAACCTACCTTATAGAGGGGGATAGCCTTTGGAAACGGAGATTAATACCCCATAATACA
TTTGATAGGCATCTATTTAATGTTAAAGATTTATCGCTATAAGCTGGGCATGCGTCCAATTAGTTAGTTGGC
GGGGTAATGGCTCACCAAGACTTTGATTGGTAGGGGAACTGAGAGGTTAATCCCCCACACTGGCACTGAG
ATACGGGT
>Zotu8636
GAACGCTAGCGGCAGGCCTAATACATGCAAGTCGGACGGGATTGGAGAGCTTGCTTTCCATGAGAGTGGC
GCACGGGTGCGTAACACGTATGTAACCTACCTTCGTCAGGGGGATAGCCTCTCGAAAGAGAGATTAAGAC
CGCATAACATTATGGAATAGCATTATTCTATAATCAAATATTTATAGGACGAAGATGGGCATGCGGAACAT
TAGCTAGTTGGTAAGGTAACGGCTTACCAAGGCTACGATGTTTAGGGGATCTGAGAGGATGACCCCCCAC
ACTGGTACTGAGACACGGAC
>Zotu8638
GAACGCTGGCGGCATGCTTAACACATGCAAGTCGAACGAGACCTTCGGGTCTAGTGGCGCACGGGTGCGT
AACGCGTGGGAATCTACCCTTTGCTACGGAATAACTCAGAGAAATTTGTGCTAATACCGTATGATGTCGCA
AGACCAAAGATTTATCGGCAGAGGACGAGCCCGCGTAAGATTAGCTAGTTGGTGGGGTAAAGGCTCACCA
AGGCGACGATCTTTAGCTGGTCTGAGAGGATGATCAGCCACACTGGGACTGAGACACGGCC
>Zotu8641
GAACGCTGGCGGCAGGCTTAACACATGCAAGTCGAACGGGCATAGCAATATGTCAGTGGCAGACGGGTG
AGTAACACGTGGGAACCTTCCTAGAGGTACGGAACAACGCAGGGAAACTTGTGCTAATACCGTATACGCT
GGAGACAGGAAAGATTTATCGCCTTTAGACGGGCCCGCGTAGGATTAGGTAGTTGGTGAGGTAATGGCTC
ACCAAGCCTACGATCCTTAGCTGATCTGAGAGGATGATCAGCCACACTGGAACTGAGACACGGTC
>Zotu8643
GAACGCTGGCGGCATGCTTAACACATGCAAGTCTGACGGGCCGCAAGGTCAGTGGCGGACGGGTGAGTA
ACGCGTAGGAACATGTCCAGGGGTGGGGGATAACGTTGGGAAACTGACGCTAATACCGCATGTGAGCTG
AGGCTCAAAGCCGCGAGGCGCCTTTGGAGTGGCCTGCGTCCGATTAGGTAGTTGGTGGGGTAAAGGCCTA
CCAAGCCTGCGATCGGTAGCTGGTCTGAGAGGACGACCAGCCACACTGGGACTGAGACACGGCC
>Zotu8646
GAACGCTGGCGGCGTGCCTAATACATGCAAGTCGAACGAACCGCGACTAAGTGCTTGCACTAAGTCAAGG
TGAGTGGCGAACGGGTGAGTAACACGTGGGTAACCTACCTCATAGTGGGGGATAACAGTCGGAAACGACT
GCTAATACCGCATAGGACATAGGATCACATGATCCAGTGAGGAAAGGTGGCGCAAGCTATCGCTAAGAGA
TGGACCCGCGGTGCATTAGCTAGTTGGTAGGGTAAAGGCCTACCAAGGCGATGATGCATAGCCGACCTGA
GAGGGTGATCGGCCACATTGGGACTGAGACACGGCC
>Zotu8648
GAACGCTGGCGGCATGCCTTACACATGCAAGTCGAGCGGCAGCGCGGGGGCAACCCTGGCGGCGAGCGG
CGAACGGGTGAGTAATACATCGGAACGTGTCCAGTAGTGGGGGATAACCAGTCGAAAGATTGGCTAATAC
CGCATACGATCTACGGATGAAAGCGGGGGACCGCAAGGCCTCGCGCTGTTGGAGCGGCCGATGTCCGATT
AGCTAGTTGGCGGGGTAACGGCCCACCAAGGCGACGATCGGTAGCTGGTCTGAGAGGATGATCAGCCAC
ACTGGAACTGAGACACGGTC
>Zotu8652

GAACGCTGGCGGCGTGCCTAATACATGCAAGTCGAGCGCGTGAAGTTAACAGATCTCCTTCGGGAGTGAA
GTTAATGGATCGAGCGGCGGACGGGTGAGTAACACGTGGGCAACCTGCCTGTAAGATCGGGATAACTTGC
GGAAACGTGAGCTAATACCGGATAAAGTCTCTCTTCTCCTGAAGAGAGAATGAAAGACGGTTTCGGCTGTC
ACTTACAGATGGGCCTGCGGCGCATTAGCTAGTTGGTGGGGTAATGGCCTACCAAGGCGACGATGCGTAG
CCGACCTGAGAGGGTGATCGGCCACACTGGGACTGAGACACGGCC

>Zotu8654

GAACGCTGGCGGCAGGCCTAAAACATGCAAGTCGAACGCTGTAGCAATACAGAGTGGCGCACGGGTGAG
TAACATGTGGGAATATGCCTTTTGGTACGGAACAACGTTTGGAAACGAACGCTAATACCGTATGGTCCCTT
CGGGGTAAAGATTTATCGCCAAAAGATTAGCCCGCACTAGATTAGCTAGTTGGTAAGGTAATGGCTTACCA
AGGCGACGATCTATAGCTGGTCTGAGAGGACGATCAGCCACACTGGGACTGAGACACGGCC

>Zotu8657

GAACGCTGGCGGCAGGCCTAACACATGCAAGTCGAGCGGTTGAGAGTAGCTTGCTACTTGATGCAGCGGC
GGACGGGTGAGTAACGCGTGGGAATCTGCCCAGTAGTGGGGGACAACAGCCGGAAACGGCTGCTAATAC
CGCATACGCCCTACGGGGGAAAGGGGGGGATCTTCGGACATCTCGCTATTGGATGAGCCCGCGTCGGATT
AGCTAGTTGGTAGGGTAAAGGCCTACCAAGGCTACGATCCGTAACTGGTCTGAGAGGATGATCAGTCACA
CTGGAACTGAGACACGGTC

>Zotu8660

GAACGCTGGCGGCGTGCCTAATACATGCAAGTCGAGCGGATCTTTGGGAGCTTGCTCCCAAAGATTAGCG
GCGGACGGGTGAGTAACACGTGGGCAACCTGCCTATAAGACTGGGATAACTTCGGGAAACCGGAGCTAAT
ACCGGATCATTCATATCTACTCATGTAGGTATGCTAAAAGACGGTTTACGCTGTCGCTTATAGATGGGCCCG
CGGCGCATTAGCTAGTTGGTGAGGTAACGGCTCACCAAGGCAACGATGCGTAGCCGACCTGAGAGGGTGA
TCGGCCACACTGGGACTGAGACACGGCC

>Zotu8665

GAACGCTGGCGGCATGCCTAACACATGCAAGTCGAACGAGACCCTCGGGTCTAGTGGCGCACGGGTGCGT
AACGCGTGGGAATCTACCCTTGGGTTCGGAATAACAGTTAGAAATGACTGCTAATACCGGATGATGACTTC
GGTCCAAAGATTTATCGCCCAGAGATGAGCCCGCGTAGGATTAGCTAGTTGGTGGGGTAAAGGCCTACCA
AGGCGACGATCCTTAGCTGGTCTGAGAGGATGATCAGCCACACTGGGACTGAGACACGGCC

>Zotu8668

GAACGCTGGCGGCAGGCTTAACACATGCAAGTCGAACGGTAACAGGGAGAAAGCTTGCTTTTTCTGCTGA
CGAGTGGCGGACGGGTGAGTAAAGTATGGGGATCTGGCGAATGGAGGGGGACAACTGCTGGAAACGGC
AGCTAATACCGCATAAAGTTGAGAGACCAAAGCGTGGGACCTTAGGGCCACGCGCCATTTGATGAACCCAT
ATGGGATTAGCTAGTTGGTAGGGTAAAAGCCTACCAAGGCGACGATCTCTAGCTGGTCTGAGAGGATGAC
CAGCCACACTGGAACTGAGACACGGTC

>Zotu8671

GAACGCTGGCGGCGTGCCTAATACATGCAAGTCGAGCGAATGGATGGGAGCTTGCTCCCTGAAGTTAGCG
GCGGACGGGTGAGTAACACGTGGGCAACCTGCCTGTAAGACTGGGATAACTTCGGGAAACCGGAGCTAAT
ACCGGATAATTTCTTTCCTCACATGAGGAAAGGTTGAAAGGCGGCTTCGGCTGTCACTTACAGATGGGCCC
GCGGCGCATTAGCTAGTTGGTGAGGTAACGGCTCACCAAGGCGACGATGCGTAGCCGACCTGAGAGGGT
GATCGGCCACACTGGGACTGAGACACGGCC

>Zotu8673

GAACGTTGGCGGCGTGGATTAGGCATGCAAGTCGAACGAATCCCATCTGGGTAACTGGGTGGGGAAGT
GGCGAAAGGGGCAGTAAGGTATGGGTAACCTGCCCCGAGGATGGGGATAGCCTTCCTAACGGAAGGGTA
ATACCCAGCGATCTGACGAGGGGGCATCTTCTTGTCAGCAAAGGAATTTTGCCTCGGGAGGGGCCCATATG
GTATTAGCTTGTTGGTAGGGTAACGGCCTACCAAGGCCAAGATGCCTAGCAGGTGTGAGAGCACGACCTG
CGCCACTGGCACTGTGACACTGGC

>Zotu8675

GGACGCTGGCGGCGTGCTTAACACATGCAAGTCGAACGGAGTTTATGTAGAATGAGTCTTCGGACAAATG
AAATATAAACTTAGTGGCGGACGGGTGAGTAACGTGTGGGTAACCTACCCTATACAGGAGGATAACAGTA
AGAAATTATTGCTAAAACTGCATAAGATTACGAGTTCGCATGAACAAGTGATAAAAACTTTAGTGGTATAG
GATGGGCCCGCATCTGATTAGGTAGTTGGTGGAGTAAAAGCCTACCAAGCCGACGATCAGTAGCCGATCT
GAGAGGATGACCGGCCACATTGGGACTGAGACACGGCC

>Zotu8677
CATGTCCACAGGCCCACACCAGGCACATGAGAGGCCCCCAGGAAATTCTGACGCCTATTAATATTGCTGTG
ATTTTTTTATATGGAAGCCAGAGCCAAGTTGTGCCCCTCCCCAGGCACAGAAAC
>Zotu8680
CAACGCTGGCGGCGCGCTTAACACATGCAAGTCGAACGAGAAAGGGAGCAATCCTGAGTAAAGTGGCGTA
CGGGTGAGTAACACGTGGATAATATACCTTTTAGTGGGGAATACCCTTGGGAAACCGAGGACAATACCGC
ATAATCCCGAGAGGGGAAAGCAGCAATGCGCTGAAAGAGTAGTCCGCGGCTGATTAGCTAGTTGGTAGG
GTAAAGGCCTACCAAGGCATTGATCGGTAGCCGGCCTGAGAGGGCACACGGCCACACTGGCACTGAAACA
CGGGC
>Zotu8685
GAACGCTGGCGGCGTGCTTAACACATGCAAGTCGAACGAGGTCCATGGAGCTTGCTCCGGAAGACCTAGT
GGCGAACGGGTGCGTAACACGTGAGAAACCTGCCCCGATCTCTGGGATAACAGTTGGAAACGACTGCTAA
TACCGGATGCCGTCACACCGCTTCATGGCGGAGTGACGAAAGATTTATCGGATTGGGAGGGTCTCGCGGC
CTATCAGCTAGTTGGAGAGGTAACGGCTCACCAAGGCATCGACGGGTAGCTGGTCTGAGAGGATGATCAG
CCACACTGGGACTGAGACACGGCC
>Zotu8688
CAACGCTGGCGGCGTGCCTAACACATGCAAGTCGAACGCGGTTACCCCGCAAGGGGTGACTGAGTGGCAA
ACGGGTGAGTAACACGTGGGTGACCTGCCTCTGAGTGGGGGATAACGTTTCGAAAGGAACGCTAATACCG
CATGACATCCTGCCTTTGGACAGGCGGAGATCAGAGCCGGGGATCGCAAGACCTGGCGCTTGGAGAGGG
GCCCGCGTCCGATTAGCTAGTTGGTGAGGTAACGGCTCACCAAGGCGACGATCGGTATCCGGCCTGAGAG
GGCGGACGGACACACTGGGACTGAGACACGGCC
>Zotu8692
GAACGCTGGCGGCGTGCCTAATACATGCAAGTCGAGCGGATCGTTCCTTCGGGAACGGTTAGCGGCGGAC
GGGTGAGTAACACGTAGGCAACCTGCCCGCAAGCCCGGGATAACCCACGGAAACGTGAGCTAAGACCGG
ATACGCATCTCTGGGGCATCTTGGAGATGGGAAAGACGGGGCAACCTGTCGCTTGCGGATGGGCCTGCGG
CGCATTAGCTAGTTGGTGAGGTAACGGCTCACCAAGGCGACGATGCGTAGCCGACCTGAGAGGGTGACCG
GCCACACTGGGACTGAGACACGGCC
>Zotu8694
GAACGCTGGCGGCGGGTTTAACACATGCAAGTCGAGGGACGAAGCCTTTCTTCGGAGAGGTGGAGACCG
GCGAACGGGTGAGTAACACGTGAGGAACCTACCCGGTACTTCGGGATAACCGCGGGAAACCGTGGCTAAT
ACCGGATATTCCATGTCTGGCGCATGTCAGGTTTGGGAAAGTTCAGGCGGTATCGGACGGCCTCGCGGCG
TATCAGCTTGTTGGTGGGGTAATGGCTTACCAAGGCGTCGACGCGTAGCTGGAGTGAGAGCTCGATCAGC
CACACTGGGACTGAGACACGGAC
>Zotu8697
GAACGCTGGCGGCGTGGATTAGGCATGCAAGTCGAGCGATCCAAATGCTTTTTATTTGGATAGCGGCAGA
CGGGTGAGGAACACGTGAGTAATCTGCCCTTAAATCAGGAACAGCTTCGTGAAAACGGAGGTAATGCCTG
ATACGATTGGAGTGGCGCATGTCACTCCCATCAAAGACAGGGACCGCAAGGCCTGTCGTTTAAGGATGAG
CTCGCGTCCTATCAGCTAGTTGGTGGGGTAACGGCCTACCAAGGCTTTTGACGGGTAGCTGGTCTGAGAGG
ATGGTCAGCCACACTGGGACTGAGACACTGCC
>Zotu8699
GAACGCTGGCGGCATGCATTACACATGCAAGTCGAACGGCAGCACGGAGCTTGCTCTGGTGGCGAGTGGC
GAACGGGTGAGTAATATATCGGAACGTACCCTGGAGTGGGGGATAACGTAGCGAAAGTTACGCTAATACC
GCATACGATCTAAGGATGAAAGTGGGGGATCGCAAGACCTCATGCTCGTGGAGCGGCCGATATCTGATTA
GCTAGTTGGTGGGGTAAAGGCCTACCAAGGCATCGATCAGTAGCTGGTCTGAGAGGACGACCAGCCACAC
TGGAACTGAGACACGGTC
>Zotu8701
GAACGCTGGCGGCGTGCTTAAGACATGCAAGTCGAACGGTACCTTCGGGTATAGTGGCGCACGGGTGAGT
AACGCGTGAATGACGTACCTCTTAGTCCTGGATAACCTCCCGAAAGGGAAGCTAATATGGGATGTGAAGCT
ACACTGTGGTGTATCTTTAAAGATTTATTGCTAAGAGATCGGTTCGCGTCCCATCAGCTAGTTGGTAGGGTA

AAGGCCTACCAAGGCAACGACGGGTAGCCGGCCTGAGAGGGTGGCCGGCCACAAGGGCACTGAGACACG
GGC
>Zotu8703
GAACGCTGGCGGCAGGCCTAACACATGCAAGTCGAGCGCCCCGCAAGGGGAGCGGCGGACGGGTGAGTA
ACACGTGGGAATTTTCCTTAAGGTACGGAACAACTCAGGGAAACTTGGGCTAATACCGTATGATGCAGAG
ATGCCAAAGATTTATCGCCTTGGGATAAGCCCGCGTCAGATTAGGTAGTTGGTGAGGTAACGGCTCACCAA
GCCTGTGATCTGTAGCTGGTCTGAGAGGACGATCAGCCACATTGGGACTGAGACACGGCC
>Zotu8711
GAACGCTGGCGGCGTGCTTAACACATGCAAGTCGAGCGGAAAGGCCCCTTCGGGGGTACTCGAGCGGCG
AACGGGTGAGTAACACGTGGGCAACCTGCCCCTAGCTCTGGGATAACTCCGGGAAACCGGGGCTAATACC
GGATACGACCTCCTGCGGCATCACGGGAGGTGGAAAGTTCTTCGGCTAGGGATGGGCCCGCGGCCTATCA
GCTTGTTGGTGGGGTAACGGCCCACCAAGGCGACGACGGGTAGCCGGCCTGAGAGGGCGACCGGCCACA
CTGGGACTGAGACACGGCC
>Zotu8713
GAACGCTGGCGGCGTGCTTAACACATGCAAGTCGAACGGAAAGGCCCTTCGGGGTACTCGAGTGGCGAAC
GGGTGAGTAACACGTGGGCAACCTGCCCCTAGCTCTGGGATAACTCCGGGAAACCGGTGCTAATACCGGA
TACACACTTTCTGAGGCATCTCAGGTGGTGGAAAGTTTTTCGGCTAGGGATGGGCCCGCGGCCTATCAGCT
TGTTGGTGGGGTAATGGCCTACCAAGGCGACGACGGGTAGCCGGCCTGAGAGGGCGACCGGCCACACTG
GGACTGAGACACGGCC
>Zotu8716
GAACGCTGGCGGCGTGCTTAACACATGCAAGTCGAACGGTAAGGCCCCTTCGGGGGTACACGAGTGGCGA
ACGGGTGAGTAACACGTGAGCAACCTGCCCTTGACTTCGGAATACCAGCTGGAAACAGCTGCTAATACCG
GATATGACCTCGGCCCTCATGGGTTGGGGTGGAAAGCTCCGGCGGTCAGGGATGGGCTCGCGGCCTATCA
GCTTGTTGGTGAGGTAATGGCTCACCAAGGCTTCGACGGGTAGCCGGCCTGAGAGGGCGACCGGCCACAC
TGGGACTGAGATACGGCC
>Zotu8719
GAACGCTGGCGGCGTGCCTAAGACATGCAAGTCGAGTGGGTTTAGACCCACGGCGAACGGGTGAGTAAT
ACCTAGGAACGTTTCATAAGTTGAGCATATGCATCCGAAAGGGTGTGCAATTCTCAATGTGGCAGTAATGC
TAAAGTCGCAAGACGCTTATGGTGCGGCCTGGATCCCATCAGCTAGTTGGTAAGGTAATGGCTTACCAAGG
CTTCGACGGGTAGGGGTCCCGAGAGGGAGAACCCCCACACTGGGACTGAGAAACGGCC
>Zotu8723
GAACGCTAGCTACAGGCTTAACACATGCAAGTCGAGGGGCAGCGGGTTGAAAGCTTGCTTTCAATGCCGG
CGACCGGCGCACGGGTGCGTAACGCGTATCAAACCTGCCTCATACTCGGGGATAGCCTTGCGAAAGTAAG
ATTAATACCCGATGTTGTTGCTTTCTCGCATGGGAGAGTAACCAAAGATTTATCGGTATGAGATGGTGATG
CGTCCGATTAGGTAGTAGGCGGGGTAACGGCCCACCTAGCCTTCGATCGGTAGGGGTTCTGAGAGGAAGG
TCCCCCACACTGGAACTGAGACACGGTC
>Zotu8725
GAACGCTAGCGGCATGCCTAACACATGCAAGTCGAACGCATCCTTCGGGGTGAGTGGCAGACGGGTGAGT
AACGCGTGGGAACGTGCCCTTCAGTTCGGAATAACCCAGGGAAACTTGGGCTAATACCGGATACGTGCGA
GAGCAGAAAGATTTATCGCTGAAGGATCGGCCCGCGTCTGATTAGCTAGTTGGTGAGGTAATGGCTCACC
AAGGCGACGATCAGTAGCTGGTCTGAGAGGATGATCAGCCACATTGGGACTGAGACACGGCC
>Zotu8727
GAACGCTGGTAACGTGGATACCGGCATGCAAGTCAAGGGGGCCGCAAGGCAACCGGCGAACGGCATAGT
AACACGTAGGTACATACCTCAGAGTGGAGGACAGCTCGTCGAAAGACGGGGTAATACTCCATAGTCCCGC
AAGGGTAAAGTCGCAAGACGCTTTGAGAATGGCCTGCGGCCTATCAGATAGTTGGCGGTGTAAAAGACCA
CCAAGTCGATGACGGGTAGGGGGCGTGAGAGCGCGACCCCCATGAATGGCACTGCGACACGGGC
>Zotu8729
GAACGCTGGCGGCGTGCCTAACACATGCAAGTCGAGCGGTAACGCGGGAGCAATCCTGGCGACGAGCGG
CAGACGGGTGAGTAATGCTTGGGTATCTGCCCAATAGTGGGGAATAACCAGTCGAAAGACTGGCTAATAC
CGCATAAGCCCTTCGGGGGAAAGCGGGGGATCGCAAGACCTCGCGCTAATGGATGAGCCCAAGTCGGATT

AGCTAGTTGGTGAGGTAACGGCTCACCAAGGCGACGATCCGTAGCTGGTCTGAGAGGACGACCAGCCACA
CCGGAACTGAGACACGGTC
>Zotu8734
GAACGCTGGTAACGTGGATACCGGCATGCAAGTCAAGGGGGCCCGCAAGGGCAACCGGCGAACGGCATA
GTAACACGTAGGTACATACCCCAAAATGGACGACAGCTCGTCGAAAGACGGGGTAATACTCCATAATCTCG
CAAGAGCAAAGTCGTAAGACGTTTTGGGAATGGCCTGCGGCCTATCAGATAGTTGGCGGTGTAATAGACC
ACCAAGTCGATGACGGGTAGGGGGTGTGAGAGCACGACCCCCATGAATGGCACTGCGACACGGGC
>Zotu8738
GAACGCTGGCGGCAGGCTTAACACATGCAAGTCGAGCGGGGAAGGTTGCTTCGGTAACTGACCTAGCGGC
GGACGGGTGAGTAATGCTTAGGAATCTGCCTATTAGTGGGGGACAACGTTCCGAAAGGAACGCTAATACC
GCATACGCCCTACGGGGGAAAGCAGGGGATCTTCGGACCTTGCGCTAATAGATGAGCCTAAGTCGGATTA
GCTAGTTGGTGGGGTAAAGGCCTACCAAGGCGACGATCTGTAGCGGGTCTGAGAGGATGATCCGCCACAC
TGGGACTGAGACACGGCC
>Zotu8744
GAACGCTGGCGGCGTGCTTAACACATGCAAGTCGAACGGTGAAAGAGGGAGCTTGCTCCTTCTGGATCAG
TGGCGAACGGGTGAGTAACACGTGAGTAACCTGCCCCAGACTCTGGGATAAGCGTTGGAAACGACGTCTA
ATACCGGATACGAGCTTCAGCCGCATGGCTAGGAGTTGGAAAGAATTTCGGTCTGGGATGGACTCGCGGC
CTATCAGCTAGTTGGTGAGGTAATGGCTCACCAAGGCGACGACGGGTAGCCGGCCTGAGAGGGTGACCG
GCCACACTGGGACTGAGACACGGCC
>Zotu8748
GAACGCTGGCGGCGCGCTTAACACATGCAAGTCGAGCGAGAACCGGGCCTTCGGGCCCGGGGAAAGCGG
CGAACGGGTGAGTAACACGTGGGTAACCCACCCTTGGCACCGGGATAGCCCGGGGAAACCCGGATTAATA
CCGGATGGCCCAACAGCTCTTCGGAGCGGTTGGAAAAGGTAGCTTCGGCCTCCGGCCAAGGACGGGCCCG
CGGCGGATTAGCTTGTTGGTGGGGTAACGGCCCACCAAGGCGACGATCCGTAGCTGGTCTGAGAGGACGA
TCAGCCACACTGGGACTGAGACACGGCC
>Zotu8750
GAACGCTGGCGGCAGGCTTAACACATGCAAGTCGAGCGGAGTGATGGTGCTTGCACCATCACTTAGCGGC
GAACGGGTGAGTAATGCCTAGGAATCTGCCGATTAATGGGGGACAACGTGTGGAAACGCACGCTAATACC
GCATACGCCCTACGGGGGAAAGCAGGGGATCTTTTAGACCTTGCGTTAATCGATGAGCCTAGGTCGGATTA
GCTAGTTGGTGGGGTAAAGGCCTACCAAGGCGACGATCTGTAGCGGGTCTGAGAGGATGATCCGCCACAC
TGGAACTGAGACACGGTC
>Zotu8752
GAACGCTGGCGGCAGGCCTAACACATGCAAGTCGAACGGATGTAGCAATACATTAGTGGCGCACGGGAG
AGTAACGCGTGGGAACCTACCTTTTGGTTCGGGATAACGTCGGGAAACTGACGCTAATACCGGATAAGCCC
TTCGGGGGAAAGATTTATCGCCGATAGATGGGCCCGCGTTAGATTAGCTAGTTGGTGAGGTAACGGCTCA
CCAAGGCGACGATCTATAGCTGGTCTGAGAGGATGACCAGCCTCACTGGGACTGAGACACGGCC
>Zotu8756
GAATGCTGGCAGCGTGGATAAGTCATGCAAGTCAAACGGCCCGCAAGGGCAGTGGCAGACGAGGTAGTA
ATACGCAGGTACATTCCCCGAAGTCGGGCATAGCCAGCCGAAAGGTTGGGTAATTCCCGATGGTCCCGCG
AGGGTAAAGGTTTTTCGCTTCGGGAGCGGCCTGTGCAATATCAGCTAGTTGGTGAGGTAATGGCTCACCAA
GGCTACGACGTTTAGGGGGGCTGAGAGGCTGATCCCCACCGATCGGACTGAGAAACGGCC
>Zotu8758
GAACGCTGGCGGCATGCCTTACACATGCAAGTCGAACGGCAGCACGGGTGCTTGCACCTGGTGGCGAGTG
GCGAACGGGTGAGTAATATATCGGAACGTACCTTGTAGTGGGGGATAGCTCGGCGAAAGCCGGATTAATA
CCGCATACGCTCTGAGGAGGAAAGCGGGGGACCTTCGGGCCTCGCGCTACAAGAGCGGCCGATATCAGAT
TAGCTAGTTGGTGGGGTAAAAGCTTACCAAGGCGACGATCTGTAGCTGGTCTGAGAGGACGACCAGCCAC
ACTGGGACTGAGACACGGCC
>Zotu8760
GAACGCTAGCGGCAGGCTTAATACATGCAAGTCGAGGGGTAACATGGTAGCAATACTGATGACGACCGGC
AAACGGGTGCGGAACACGTACAGAACCTTCCTTTAAGCGGGGGATAGCCCATAGAAATGTGGATTAATAC

CCCATAGTAATGTTTAATGGCATCATTAGATATTTAAAGATTTATCACTTAAAGATGGCTGTGCGGCTGATT
AGGTAGTTGGTGCGGGTAACGGCCCACCAAGCCATCGATCAGTAACTGGTGTGAGAGCACGACCAGTCAC
ACGGGCACTGAGACACGGGC
>Zotu8764
GAACGCTGGCGGCGTGCCTAATACATGCAAGTCGAGCGCGGGAAGCGGACTGATCTCCTTCGGGAGTGAC
GTCCGTGGAACGAGCGGCGGATGGGTGAGTAACACGTGGGCAACCTGCCTGTAAGATCGGGATAACTTGC
GGAAACGTGAGCTAATACCGGATAAAGCTTCACATCTCCTGGTGTGAAGATGAAAGACGGCTCTGCTGTCA
CTTACAGATGGGCCCGCGGCGCATTAGCTAGTTGGTGGGGTAAAGGCCTACCAAGGCGACGATGCGTAGC
CGACCTGAGAGGGTGATCGGCCACACTGGGACTGAGACACGGCC
>Zotu8767
GAACGCTGGCGGCGTGCTTAACACATGCAAGTCGAACGGTGATGCAGAGCTTGCTCTGCGGATCAGTGGC
GAACGGGTGAGTAACACGTGAGTAACCTGCCCTCGACTCTGGGATAAGCGTTGGAAACGACGTCTAATAC
CGGATACGACCCGTGGAGGCATCTCCTACGGGTGGAAAGAATTTTGGTTGAGGATGGACTCGCGGCCTAT
CAGCTTGTTGGTGAGGTAATGGCCCACCAAGGCGACGACGGGTAGCCGGCCTGAGAGGGTGACCGGCCA
CACTGGGACTGAGACACGGCC
>Zotu8770
GAATGCTGGCGGCGCGCCTAACACATGCAAGTCGAGCGGTAACAGGAGTAATTTATTACTTGCTGACGAG
CGGCGGACGGCTGAGTAACGCGTGGGAACATGCCCCAAAGTGAGGAATAACTGCCCGAAAGGGTAGCTA
ATGCCGCATATGATCTTAGGATTAAAGGATTTATCCGCTTTGGGAGTGGCCCGCGTACGATTAGGTAGTTG
GTGAGGTAATGGCTCACCAAGCCTACGATCGTTAACTGGTCTGAGAGGATGATCAGTCAGACTGGAACTG
AGACACGGTC
>Zotu8773
GAACGCTGGCGGCGTGCTTAACACATGCAAGTCGAACGGTAAGGCCCTTCGGGGTACACGAGTGGCGAAC
GGGTGAGTAACACGTGGGTGATCTGCCTCGTACTTTGGGATAAGCCTGGGAAACTGGGTCTAATACCGGA
TATGACCACAGGATGCATGTTTTGTGGTGGAAAGCTTTTGCGGTACGAGATGGGCCCGCGGCCTATCAGCT
TGTTGGTGGGGTAATGGCCTACCAAGGCGACGACGGGTAGCCGGCCTGAGAGGGCGACCGGCCACACTG
GGACTGAGACACGGCC
>Zotu8776
GAACGCTAGCGGCAGGCCTAATACATGCAAGTCGAACGGGGTTAGTCAGCAATGGCTAGCTTAGTGGCGC
ACGGGTGCGTAACGCGTATGCAACCTACCTGTAAGAGGGGAATAACCCGGCGAAAGCCGGACTAATACCG
CATAAGGTAGTGAAGTGGCATCACTTTGCTATTAAATCTTTACGAGCTTACAGATGGGCATGCGTCCGATTA
GCTAGTTGGCGGGGTAACGGCCCACCAAGGCTACGATCGGTAGGGGACCTGAGAGGGCGATCCCCCACA
CTGGTACTGAGATACGGAC
>Zotu8778
GAACGCTGGCGGCGTGCCTAACACATGCAAGTCGAGCGAAGTATCTTGGAACGAAGTTTTCGGACTGAGT
GAAGAGATATCTTAGCGGCGGACGGGTGAGTAACGCGTGAGGAACCTGCCTTTCACAAAGGGATAGCCTC
GGGAAACTGGGATTAATACCTTATGATACTGAACTACCACATGATAGATCAGTTAAAGCGCAAAGCGGTGA
AAGATGGTCTCGCGTCCCATTAGCTAGTTGGTGAGGTAACAGCTCACCAAGGCGACGATGGGTAGCCGGC
CTGAGAGGGTGAACGGCCACACTGGAACTGAGACACGGTC
>Zotu8782
GAACGCTAGCGGGAGGCCTAATACATGCAAGTCGAACGGTAACAGGGGTAGCAATACCTGCTGACGAGT
GGCGCACGGGTGAGTAACACGTACACAATCTGCCTTATACTGGAGCATACACCCAAGAAATTGGGACTAAT
TCTCCATAACATATTGAGGAGGCATCTCTTTGATATTAAAACTCAGGTGGTATAAGATGAGTGTGCGTCTGA
TTAGCTAGTTGGTGAGGTAACGGCTCACCAAGGCAACGATCAGTAACTGGTCTGAGAGGATGATCAGTCA
CACGGGCACTGAGACACGGGC
>Zotu8786
GAACGCTGGCGGCGTGCTTAACACATGCAAGTCGAACGATGAAAGAGGAGCTTGCTCCTCTGGATTAGTG
GCGAACGGGTGAGTAACACGTGAGCAATCTGCCCCTGACTCCGGAATAACTACGGGAAACTGTAGCTAAT
ACCGGATACGCGCCGAACAGGCATCTGTATAGGTGGGAAAGAATTTCGGTCAGGGATGAGCTCGCGGCCT
ATCAGCTTGTTGGTGAGGTAACGGCTCACCAAGGCGACGACGGGTAGCCGGCCTGAGAGGGTGACCGGC
CACATCGGGACTGAGGCACGGCC

>Zotu8790
GAACGCTGGCGGTCTGCTTAACACATGCAAGTCGAACGGGACTCTTCGGAGTTCAGTGGCGGACGGGTGA
GTAACGCGTGAGAATCTGCCTTCAGGTCGGGGACAACCACTGGAAACGGTGGCTAATACCGGATATGCCG
CAACGTGAAAGATTTATCGCCTGAAGAGGAGCTCGCGTCTGATTAGCTAGTTGGTGGGGTAAAAGCTCACC
AAGGCGACGATCAGTAGCTGGTCTGAGAGGATGATCAGCCACACTGGGACTGAGACACGGCC
>Zotu8792
GAACGCTGGCGGTATGCTTAACACATGCAAGTCGAACGAAAGTAGCAATACTTTAGTGGCGAACGGGTGA
GTAACACGTATCTAACCTACCATTTAGTGGGGAATAACTGTTGGAAACGGCAGCTAATACCGCATATGTTAT
TTATTTGCAATGATAAATAATGAAAGGAGCATTTGCTTCGCTAATTGATGGGGATGCGGCGCATTAACTAG
TTGGTAAGGTAATGGCTTACCAAGGTAACGATGCGTAGCCGATCTGAGAGGATGAACGGCCACATTGGGA
CTGAGACACGGCC
>Zotu8799
GAACGCTGGCGGCATGCCTAACACATGCAAGTCGAACGAAGGCTTCGGCCTTAGTGGCGCACGGGTGCGT
AACGCGTGGGAATCTGCCCTTGGGTTCGGAATAACAGTTAGAAATGACTGCTAATACCGGATGATGTCGAA
AGACCAAAGATTTATCGCCCAAGGATGAGCCCGCGTAGGATTAGGTAGTTGGTGGGGTAAAGGCTCACCA
AGCCGACGATCCTTAGCTGGTCTGAGAGGATGATCAGCCACACTGGGACTGAGACACGGCC
>Zotu8802
GAACGCTATCGACAGGCCTAACACATGCAAGTCGAGGGGCAGCATGATTCAAGCTTGCTTGAATTGATGG
CGACCGGCGCACGGGTGCGTAACGCGTATGCAACCTACCTCATATCAGGGAATAGCCCAGCGAAAGTTGG
ATTAATGCTCTATGTGTAACTTTCTCGCATGTTAAAGTTATGAAACGTAAGGATATGAGATGGGCATGCGTC
TGATTAGCTAGTTGGCGGGGTAACGGCCCACCAAGGCGACGATCAGTAGGGGTTCTGAGAGGAAGGTCCC
CCACATTGGAACTGAGACACGGTC
>Zotu8807
AGCCGTTGGAAATCGCTGTATTCAACACAGATACACGGGAGTTCAGGCCCAGTGCTCACACATGCTTTTCCC
GCAACCTCTCCCAGCCACTAACATTAGGATGTTAGCA
>Zotu8809
GAACGCTGGCGGCGTGCTTAACACATGCAAGTCGAACGGTAAGGCTCCTTCGGGAGTACACGAGTGGCGA
ACGGGTGAGTAACACGTGAGCAACCTACCCTTGACTTCGGAATAACAGTTGGAAACAGCTGCTAATACCGA
ATACGACCTTCTCGGTCATCCGAGTGGGTGGAAAGTTCTGGCGGTCAAGGATGGGCTCGCGGCCTATCAG
CTTGTTGGTGAGGTAATGGCTCACCAAGGCTTCGACGGGTAGCCGGCCTGAGAGGGCGACCGGCCACACT
GGGACTGAGATACGGCC
>Zotu8811
GAACGCTAGCGGCAGGCTTAATACATGCAAGTCAAGGGGGTAGCAATACAACCGGCGCACGGGTGAGTA
ACGCGTACACAACATACCATATACTGGAGTATAGCTTTGGGAAACTGAAGGTAATCCTCCATAATATTTCGG
ATAGGCATCTATTTGAAATTAAAGTAACAACGGTATATGATTGGTGTGCGTTTGATTAGCTAGTTGGTAGG
GTAACGGCCTACCAAGGCTTCGATCAATAGCTGGCGTGAGAGCGCGACCAGCCACACTGGAACTGAGACA
CGGTC
>Zotu8813
GAACGCTGGCGGCAGGCTTAATACATGCAAGTCGAACGGTAAGGCCTGAAGTAGCAATACGGAAGGTACA
CGAGTGGCGCACGGGTGCGTAACGCGTATGTAACCTGCCCCCAACCGGAACAAAACACCGGGAAACTGGT
GCTAATATTCCATGGTCTCTTCGGATGGCATCATCTGGGGAGTAAAGATTCATTGGTTGGGGATGGACATG
CGTATTATTAGGTAGTTGGTGGGGTAACGGCTCACCAAACCGACGATGATTAGGGGTTCTGAGAGGATTA
ACCCCCACACTGGAACTGAGACACGGTC
>Zotu8815
GAACGCTAGCGGCAGGCCTAATACATGCAAGTCTAGGGGGCAGCAATGTCACCGGCGCACGGGTGCGTA
ACACGTATGCAACCTACCTTCTACAGGGGTACAGCCTTTCGAAAGAGAGATTAATACCGCATAAGACCACA
CTTATTCATATAGGAGGGGTTAAAGATTTATCGGTAGAAGATGGGCATGCGTCTGATTAGTTAGTTGGCGG
GGTAACGGCTCACCAAGACGATGATCAGTAGGGGACCTGAGAGGGTAATCCCCCACACTGGCACTGAGAT
ACGGGC
>Zotu8820

GAACGCTGGCGGCATGCCTAACACATGCAAGTCGAACGAGACCTTCGGGTCTAGTGGCGCACGGGTGCGT
AACGCGTGGGAATCTGCCCAAAGCTACGGAATAACTCCTCGAAAGGGGTGCTAATACCGTATGATGTCGC
AAGACCAAAGATTTATCGGCTTTGGATGAGCCCGCGTAGGATTAGCTTGTTGGTGAGGTAAAGGCTCACCA
AGGTGACGATCCTTAGCTGGTCTGAGAGGATGATCAGCCACACTGGGACTGAGACACGGCC

>Zotu8823

GAACGCTGGCGGCAGGCTTAACACATGCAAGTCGAACGAGACCTTCGGGTCTAGTGGCGCACGGGTGAGT
AACGCGTGGGAATCTACCTTGGAGTCTGGGATAACTGACGGAAACGACAGCTAATACCGGATACGCCCTT
AGGGGAAAAGGTTTACTGCTCTGAGATGAGCCCGCGTCCAATTAGGTAATTGGTGAGGTAAAGGCTCACC
AAGCCGACGATTGGTAGCTGGTCTGAGAGGATGACCAGCCACACTGGGACTGAGACACGGCC

>Zotu8826

GAACGCTGGCGGCGTGCTTAACACATGCAAGTCGAGCGGTAAGGCCCTTCGGGGTACACGAGCGGCGAA
CGGGTGAGTAACACGTGAGCAATCTGCCCATCACATCGGGATAACTCCGGGAAACCGAAGCTAATACCGA
ATACGACCACTTCAGGCATCTGACGGTGGTGGAAAGTTCCGGCGGTGATGGATGAGCTCGCGGCCTATCA
GCTAGTTGGTGAGGTAACGGCCCACCAAGGCTTCGACGGGTAGCCGGCCTGAGAGGGTGACCGGCCACA
CTGGGACTGAGACACGGCC

>Zotu8828

GAACGCTAGCGACAGGCTTAACACATGCAAGTCGAGGGGCAGCGGGGATGAGAGCTTGCTCTTATCTGCC
GGCGACCGGCGCACGGGTGAGTAACACGTATGCAACCTGCCTCAAACAGGGGGATAAGCGGCGGAAACG
CCGTCTAATACCGCGTACGATACCGGGAGGCATCTTCCGGTGTTGAAAGATTTATTGGTTTGAGATGGGCA
TGCGGCGCATTAGCTTGTTGGCGGGGTAACGGCCCACCAAGGCGACGATGCGTAGGGGTTCTGAGAGGA
AGGTCCCCCACACTGGTACTGAGACACGGAC

>Zotu8831

GAACGCTGGCGGCATGCTTTACACATGCAAGTCGAACGGCAGCACGGGCTTCGGTCTGGTGGCGAGTGGC
GAACGGGTGAGTAATACATCGGAACGTACCCAGAAGTGGGGGATAACGTAGCGAAAGTTACGCTAATACC
GCATACGTTCTACGGAAGAAAGTGGGGGATCGCAAGACCTCATGCTTTTGGAGCGGCCGATGTCTGATTA
GCTAGTTGGTGAGGTAAAGGCTCACCAAGGCGACGATCAGTAGCTGGTCTGAGAGGACGACCAGCCACAC
TGGGACTGAGACACGGCC

>Zotu8833

GAACGCTGGCGGCAGGCTTAACACATGCAAGTCGAACGCATCCTTCGGGGTGAGTGGCAGACGGGTGAG
TAACACGTGGGAACGTACCCTTTGGTTCGGAATAACTCTGGGAAACTAGAGCTAATACCGGATACGCCCTT
TTTGGGGAAAGGTTTACTGCCGAAGGATCGGCCCGCGTCTGATTAGCTAGTTGGTGGGGTAACGGCCTAC
CAAGGCGACGATCAGTAGCTGGTCTGAGAGGATGATCAGCCACACTGGGACTGAGACACGGCC

>Zotu8835

AAACGCAAGAGATGTGCTTAACACATGCTAATTGAATGGTATTAAATAATATCATAGTGAAATGGTGAGTA
ATGTATGAGAATTTATTTTTAGAATATAATAAAAAATAGTATTTACTATTAATAATTTATAATAAGCGCAATA
GTGCTTTAAAAAATAATTATCGTCTAAAAAAAAGCTCATATCTAATTAGCTAGTTGGTGAAGTAATAGTTTT
ACCAAGGCGAAGATTAGTAGCTGCCTTGAGAGGGGAAACAGCCACATAGAGATTGAAATACAGCT

>Zotu8837

GAACGCTAGCGGCATGCCTAACACATGCAAGTCGAACGAAGAGGCCCCTTCGGGGGTACTTCAGTGGCGG
ACGGGTGAGTACAACATAGGTGACCTGCCCCAAAGCGGGGGATAACAGTCCGAAAGGATTGCTAATACCC
CATGTGGTCCTTGAGATTTGAGGCTCAATGACTAAAGGAGTAATCCGCTTTGGGAGGGGCCTGTGGCCCAT
CAGCTTGTTGGTAGGGTAATGGCCTACCAAGGCTACGACGGGTAGGGGGCCTGAGAGGGTGACCCCCCAC
ATTGGCACTGACACACGGGC

>Zotu8841

GAACGCTAGCGGCAGGCTTAATACATGCAAGTCGAGGGGCAGCAGGGGTAGCAATACTCGCTGGCGACC
GGCAAACGGGTGCGGAACACGTACGCAACCTTCCTTTAAGTGAGGAATAGCCCAGGGAAACTTGGATTAA
TACCTCGTAATATTATGAGATGGCATCGTCTTATAATTATAGCTCAGGCGCTTAAAGATGGGCGTGCGACTG
ATTAGGTAGTTGGCAGGGTAACGGCCTACCAAGCCTGCGATCAGTAACTGGTGTGAGAGCACGACCAGTC
ACACGGGCACTGAGACACGGGC

>Zotu8845

GAACGCTAGCGGCAGGCCTAATACATGCAAGACGAACGGCAAGATTTTCAGCAATGAGGATCCTAGAGTG
TCGTAAGGGTGCGTAACACGTATGCAATCTGCCCTGTACAGGAGGATAGCTCCCCGAAAGGGGAATTAAC
ACTCCATAAGATCACGAGAGGGCATCTGATTGTGATTAAAACTGAGGTGGTACAGGATGAGCATGCGCAT
GATTAGTTAGTTGGCGGGGTAAAGGCCCACCAAGACGATGATCATTAGGGGAACTGAGAGGTTGATCCCC
CACACTGGCACTGAGATACGGGC

>Zotu8847

GAACGCTGGCGGTATGCTTTACACATGCAAGTCGTACGAGAGTTTTTAATTCAAGTGGCGGACGGGTGAGT
AACACGTAAGAATTTGCCTTTAGGAGGGGGACAACAACTGGAAACGGTTGCTAATACCCCATATGCTTTCG
AGTAAAATGGATTTTCCGCCTAAAGAGAAGCTTGCGGCTGATTAGCTTGTTGGTAAGGTAATGGCTTACCA
AGGCGACGATCAGTATCTGGTTTGAGAGGATGATCAGACACACTGGAACTGAGACACGGTC

>Zotu8849

TAACGCTGGCGGCGTGCATAACACATGCAAGTCGAACGATGAAACTCTAAAGATTTCTTCGGAATGAATTA
GAATGGATTAGTGGCGAACGGGTGAGTAACACGTGAGTAACCTGCCTTAGAGAAGGGGATAGCCTTTGGA
AACGAAGAATAATACCCTATGAAATGATAATATCGCATGATAATATCATCAAAGATTTATCGCTCTAAGATG
GACTTGCGCCTGATTAGCTAGTTGGTGGAGTAACAGCCCACCAAGGCAACGATCAGTAGCCGGCTTGAGA
GAGTGTACGGCCACATTGGGACTGAGACACGGCC

>Zotu8852

GAACGTTAGCGGCGCGCCTAAGACATGCAAGTCGAGCGAGAAAGGGCTTCGGCCCCAGTAAAGCGGCGC
ACGGGTGAGTAACACGTAGGTCATCAACCCTCGAGTGGTGGACAACCCTCCGAAAGGAGGGCTAATACAG
CATGACGCCAGCAATGGCCAAAGCGGGCCTCTCCATGGAAGCTCGCGCTTGAGGATGAGCCTGCGGCCCA
TCAGGTAGTTGGTCGGGGTAACGGCCCACCAAGCCTACGACGGGTAGCTGGTCTGAGAGGATGAACAGCC
ACACTGGAACTGAGACACGGTC

>Zotu8855

GAACGCTGGCGGCGTGCTTAACACATGCAAGTCGAACGGTAAGGCTCTTTCGGGAGTACACGAGTGGCGA
ACGGGTGAGTAACACGTGAGCAACCTGCCCTTGACTTTGGGATACCAGCTGGAAACAGCTGCTAATACCG
GATATGACACTCCTTCGCATGTTGGGGTGTGGAAAGCTCCGGCGGTCAGGGATGGGCTCGCGGCCTATCA
GCTTGTTGGTGAGGTAATGGCTCACCAAGGCTTCGACGGGTAGCCGGCCTGAGAGGGCGACCGGCCACAC
TGGGACTGAGATACGGCC

>Zotu8859

GAACGCTAGCGGCAGGCCTAATACATGCAAGTCGAGGGGCAGCGGGGTAGCAATATCGCCGGCGACCGG
CGCACGGGTGCGTAACGCGTATGCAATCTACCTTTTACTGGGGCATAGCCCGGAGAAATTCGGATTAATAC
CCCATGGTATATTGGTGTGGCATCACACTAATATTAAAGATTTATCGGTAAGAGATGAGCATGCGTACGAT
TAGTTAGTTGGTGAGGTAACGGCTCACCAAGACTATGATCGTTAGGGGGTCTGAGAGGATGATCCCCCAC
ACTGGTACTGAGATACGGAC

>Zotu8863

GAACGCTGGCGGCAGGCTTAACACATGCAAGTCGAACGGGCCCTTTTAGGGTCAGTGGCAGACGGGTGA
GTAACGCGTGGGAACGTGCCCTTCAGTTCGGAATAACCCAGGGAAACTTGGGCTAATACCGGATACGTGC
GAGAGCAGAAAGATTTATCGCTGAAGGATCGGCCCGCGTCTGATTAGCTAGTTGGTGGGGTAATGGCCCA
CCAAGGCGACGATCAGTAGCTGGTCTGAGAGGATGATCAGCCACATTAGGACTGAGACACGGCC

>Zotu8867

CAACGCTGGCGGCGTGCCTAACACATGCAAGTCGCACGAGAAAGGGACTTCGGTCCTGAGTAAAGTGGCG
CACGGGTGAGTAACACGTGAATAATCTACCTCCGAGTGGGGAATAACTGAGAGAAATCTTAGCTAATACCG
CATAACACTTACGAGTCAAAGCAGAAATGCGCTTGGAGAGGAGTTCGCGGCAGATTAGTTAGTTGGCGGG
GTAATGGCCCACCAAGACGATGATCTGTATCCGGCCTGAGAGGGCGCACGGACACACTGGAACTGAAACA
CGGTC

>Zotu8870

GAACGCTGGCGGCGTGCCTAATACATGCAAGTCGAACGCGAAAGTCCTTCGGGATGAGTAAAGTGGCGCA
CGGGTGAGTAATGGTTGAGAACGTGCCCTTAGGTGGGGAATAACAGTTGGAAACGACTGCTAATACCGCA
TGGCCTCGAAAGAGTAAAGGTGGGGATCGCAAGACCTACCGCCAAAGGATCGGCTCAACTCCCATTAGCT
AGTTGGCAGGGTAATGGCCTACCAAGGCAACGATGGGTATGTGGTCTTAGAGGATGATCACACACACTGG
AACTGAGACACGGTC

>Zotu8874
GAACGCTGGCGGCGCGTTTTAAGCATGCAAGTCGAACGGTAGGCTGGGGGCAACTCCGGCTGAGAGTGG
CGAACGGGTGAGTAACACGTAGACAATCTGGCCCTGGGACAGGGATAGCTCATGGAAACATGGGGTAAT
ACCTGGTGAGGTCGAGCTGACCGAGGTTGGTTCGATGAGAGGCTTCAATTGGAGCGCCTGGGGGTGAGTC
TGCGGTCCATTAGCTAGTTGGTGAGGTAATGGCTCACCAAGGCTGCGATGGATAGCCGGCCTGAGAGGGT
GAACGGCCACACTGGGACTGAGACACGGCC
>Zotu8876
CAACGCTGGCGGCGTGCCTCAGACATGCAAGTCGAACGATTAAACTTTCCTTCGGGAAAGATATAAAGTGG
CGCACGGGTGAGTAACACGTAAGTAATCTACCTCTGGGTGGGGAATAACAGTCGGAAACGACTGCTAATA
CCGCATAATGCAGCGGCTTCTTATGAAGACAGTTGTTAAAGCATTTATGTGCCTGGAGAGGAGCTTGCGGC
AGATTAGCTAGTTGGTAAGGTAATGGCTTACCAAGGCGACGATCTGTATCCGACCTGAGAGGGTGGTCGG
ACACACTGACACTGAATAACGGGT
>Zotu8878
GAACGCTGGCGGTGTGCTTCACACATGCAAGTCGAACGAGCAACCTTTGAGTAGCAATACAATTTGGAAGC
TAGTGGCGGACGGGTGAGTAACGCGTAGGAATCTGCCTTAAAATGGGGGATAGCAGACCGAAAGGTCTA
TTAATACCGCATATGCTGGTATCCGGACACGGATATTAGTGAAAGGAGCAATCCGTTTTAAGATGAGCCTG
CGTCCGATTAGCTAGTTGGTAAGGTAAAAGCTTACCAAGGCGACGATCGGTAGGTGGTCTGAGAGGATGA
TCACCCACAATGGGACTGAGACACGGCC
>Zotu8882
GAACGCTGGCGGCGCGCCTAACACATGCAAGTCGAACGGAGCTTAGAGAGCTTGCTTTTTAAGCTTAGTG
GCGAACGGGTGAGTAACGCGTGAGTAACCTGCCCTAGAGTGGGGGACAACAGTTGGAAACGACTGCTAA
TACCGCATAAGCCCACGGTGCCGCATGACACTGAGGGAAAAGGATTTATTCGCTTTAGGATGGACTCGCGT
CCAATTAGCTAGTTGGTGAGGTAACGGCCCACCAAGGCGACGATTGGTAGCCGGACTGAGAGGTTGAACG
GCCACATTGGGACTGAGACACGGCC
>Zotu8886
GAACGCTGGCGGCAGGCTTAACACATGCAAGTCGAGCGGGCCCCCTTCAATCGAGCCTGAGGTGACCGTT
CGGGTCGCCGGCGGGCAGGCTCGTTTGATGGGGGTCAGCGGCGGACGGGTGAGTAACGCGTGGGAACGT
GCCTTTCGGTCTGGAATAGCCCCGGGAAACTGGGATTAACGCCGGATACGCCCTTTTGGGGAAAGTTTACG
CCGAGAGATCGGCCCGCGTCTGATTAGGTAGTTGGTGGGGTAACGGCCTACCAAGCCGACGATCAGTAGC
TGGTTTGAGAGGATGATCAGCCACACTGGGACTGAGACACGGCC
>Zotu8889
CAACGCTGGCGGCGTGCCTAATGCATGCAAGTCGAACGGGGCGTCCTTCGGGGCGCTCACGTGGCGGACG
GGTGAGGAACACGTGGGCAATCTGCCCAGCGGTGGGGGCTAGCCCGCGGAAACGCGGGGTAATTCCGCA
TGAGGTCGCGGCCCGGGTTGGGGGCGCGAGCAAAGCCGAGAGGCGCCGTTGGAGGAGCCTGCGTCCGAT
TAGCTAGACGGTGGGGTAACGGCCTACCGTGGCGATGATCGGTAGCTGGTCTGAGAGGATGATCAGCCAC
ACGGGGACTGAGACACGGCC
>Zotu8892
GAACGCTGGCGGCAGGCTTAACACATGCAAGTCGAACGGTTGTAGCAATACAGCAGTGGCGCACGGGTG
AGTAACGCGTGGGAACATACCTTAGAGTTCGGAATAACAGCGGGAAACTGCTGCTAATACCGGATATTCTC
CTCGGAGGAAAGATTTATCGCTCTAAGATTGGCCCGCGTCAGATTAGCTAGTTGGTAAGGTAACGGCTTAC
CAAGGCTCCGATCTGTAGCTGGTCTGAGAGGACGACCAGCCACACTGGAACTGAGACACGGTC
>Zotu8895
CAACGCTGGCGGCGTGCCTAACACATGCAAGTCGCACGAGAAAGGGTAGCAATACCTGAGTAAAGTGGCG
CACGGGTGAGTAACACGTGAATGATCTACCTCCGAGTGGGGAATAACTGAGAGAAATCTTGGCTAATACC
GCATAACACTTACGAGTCAAAGCAGCAATGCGCTTGGAGAGGAGTTCGCGGCCGATTAGCTAGTTGGCGG
GGTAATGGCCCACCAAGGCGATGATCGGTATCCGGCCTGAGAGGGCGCACGGACACACTGGAACTGAAA
CACGGTC
>Zotu8898
GAACGCTGGCGGCGTGCCTAATACATGCAAGTCGAGCGGAGTTACAGGAGCTTGCTCCTGTAACTTAGCG
GCGGACGGGTGAGTAACACGTGGGCAACCTGCCTATAAGACTGGGATAACTTCGGGAAACCGGAGCTAAT

ACCGGATAATTCTTTTCCTCTCATGAGGAAAAGCTGAAAGACGGTTTACGCTGTCACTTATAGATGGGCCCG
CGGCGCATTAGCTAGTTGGTGAGGTAACGGCTCACCAAGGCAACGATGCGTAGCCGACCTGAGAGGGTGA
TCGGCCACACTGGGACTGAGACACGGCC
>Zotu8902
GAACGCTGGCGGCATGCCTAACACATGCAAGTCGAACGAAGCCTTCGGGCTTAGTGGCGCACGGGTGCGT
AACGCGTGGGAATCTGCCCTTGGGTTCGGAATAACAGTTAGAAATGACTGCTAATACCGAATGATGTCGTA
AGACCAAAGATTTATCGCCCAGGGATGAGCCCGCGTAGGATTAGCTAGTTGGTGTGGTAAAGGCGCACCA
AGGCGACGATCCTTAGCTGGTCTGAGAGGATGATCAGCCACACTGGGACTGAGACACGGCC
>Zotu8906
GAACGCTGGCGGCGTGCTTAACACATGCAAGTCGAACGCGAAAGCCCTTCGGGGTTAGTAGAGTGGCGCA
CGGGTGAGTAATACATAGGAACGTGCCTGAAGGAGGGGAATAACCACGGGAAACCGTGGCTAATACCAC
ATGAAATCGCGAGATCAAAGGGGGCCTATCCTTGGAAGCTCCCGCCTTTAGAGCGGCCTATGTCCTATTAG
CTAGTTGGTAGGGTAAAAGCCTACCAAGGCGATGATGGGTAACTGGTCTGAGAGGATGATCAGTCACACT
GGAACTGAGACACGGTC
>Zotu8909
GAACGCTAGCGGGAGGCCTAATACATGCAAGTCGAACGGTAACAGGGTAGCAATACCGCTGACGAGTGG
CGCACGGGTGAGTAACACGTACACAATCTGCCTTATACTGGAGCATACCCCCAAGAAATTGGGATTAATTC
TCCATAATATATCGAAAAGGCATCTTTTTGATATTAAAACTCTGGTGGTATAAGATGAGTGTGCGTCTGATT
AGCTAGTTGGTGAGGTAACGGCTCACCAAGGCAACGATCAGTAACTGGTCTGAGAGGATGATCAGTCACA
CGGACACTGAGACACGGGT
>Zotu8912
GAACGCTGGCGGCGTGCTTAACACATGCAAGTCGAACGATGATGCCCAGCTTGCTGGGTTGATTAGTGGC
GAACGGGTGAGTAACACGTGAGTAACCTGCCCATCACTCTGGGATAACTCCGGGAAACCGGTGCTAATACT
GGATATGACTTCTGGCCGCATGGTCGGGGGTGGAAAGATTTATCGGTGGTGGATGGACTCGCGGCCTATC
AGCTTGTTGGTGGGGTAATGGCCTACCAAGGCGACGACGGGTAGCCGGCCTGAGAGGGTGACCGGCCAC
ACTGGGACTGAGACACGGCC
>Zotu8916
GAACGCTAGCGGCATGCTTAACACATGCAAGTCGGACGGGAAGCATCCTCGTGGTGTTTCCAGTGGCGGA
CGGGTGAGTAACGCGTAAGAACTTGCCCTTGGGAGGGGGACAACAATTGGAAACGGTTGCTAATACCCCG
TAAGCTGAGGAGCAAAAGGGAGGGATCCGCCCAAGGATGGGCTTGCGTCTGATTAGCTGGTTGGTGAGG
CAATGGCTTACCAAGGCAACGATCAGTAGCTGGTCTGAGAGGATGATCAGCCACACTGGGACTGAGACAC
GGCC
>Zotu8920
GAACGCTGGCGGCGTGCCTAATACATGCAAGTCGAGCGAATCTGAGGGAGCTTGCTCCCAAAGGTTAGCG
GCGGACGGGTGAGTAACACGTGGGCAACCTGCCTGTAAGACTGGGATAACTTCGGGAAACCGGAGCTAAT
ACCGGATAATTCTTATCAACTCATGTTGGTAAGCTAAAAGACGGCGTAAGCTGTCACTTACAGATGGGCCC
GCGGCGCATTAGCTAGTTGGTGAGGTAACGGCTCACCAAGGCAACGATGCGTAGCCGACCTGAGAGGGT
GATCGGCCACACTGGGACTGAGACACGGCC
>Zotu8922
CAACGCTGGCGGCGTGCCTAATGCATGCAAGTCGAACGGGGTCACCTTCGGGTGGCTCACGTGGCGGACG
GGTGAGGAACACGTGGGCAATCTGCCCTGAGGTGGGGGGTATCCCGTGGAAACATGGGGTAATTCCGCAT
GTGCTCGCGTGCCGAGTGGGGTGCGTGAGGAAAGCCTTCGGGCGCCTTTGGAGGAGCCTGCGACCGATTA
GCTAGCTGGTGGGGTAACGGCCTACCAGGGCGATGATCGGTAGCTGGTCTGAGAGGATGGCCAGCCACA
CGGGGACTGAGACACGGCC
>Zotu8930
CAACGCTGGCGGCGTGCCTCAGACATGCAAGTCGAACGATTAAACTTTCCTTCGGGAAAGATATAAAGTGG
CGCACGGGTGAGTAACACGTAGGTAATGTACCTTTGGGTGGGGAATAACTTAGGGAAACTTAAGCTAATA
CCGCATAATGCAGCGGCTCCTTATGGAGACAGTTGTTAAAGATTTATCGCCTAAAGAGCAGCCTGCGGCAG
ATTAGCTAGTTGGTAAGGTAACGGCTTACCAAGGCTACGATCTGTATCCGACCTGAGAGGGTGGTCGGAC
ACACTGACACTGAATAACGGGT

>Zotu8934
GAACGCTAGCGGCAGGCCTAATACATGCAAGTCGGACGGGATTGGAGAGCTTGCTTTCCATGAGAGTGGC
GCACGGGTGCGTAACACGTATGTAACCTACCCTTGTCAGGGGGATAGCCTCTCGAAAGAGAGATTAAGAC
CGCATAAAATCATAACACAGCATTGTGAATTGATCAAATATTTATAGGACAAGGATGGGCATGCGGAACAT
TAGCTAGTTGGTAAGGTAACGGCTTACCAAGGCTACGATGTTTAGGGGATCTGAGAGGATGACCCCCCAC
ACTGGTACTGAGACACGGAC
>Zotu8938
GAACGCTGGCGGCGTGCCTAACACATGCAAGTCGAACGCGAAAGTGGAGCAATCCATGAGTAGAGTGGC
AAACGGGTGAGTAACGCGTGGGTGACCTACCTCCGAGTGGGGGATAACGTCTCGAAAGGGACGCTAATAC
CGCATAACATCCCGTCTTTTAAGAGATGGCGATCAAAGCCTGGGACCGCAAGGCCAGGCGCTTGAAGAGG
GGCCCGCGTCTGATTAGCTAGTTGGTGGGGTAATGGCTCACCAAGGCAACGATCAGTATCCGGCCTGAGA
GGGCGGACGGACACACTGGGACTGAGACACGGCC
>Zotu8942
GAACGCTGGCGGCACGCTTAACACATGCAAGTCGAACGGACTTTAGGCTTCGGTTTAAAGTTAGTGGCGA
ACGGGTGAGTAACACGTGGGAACCTACCCTGCAGTAGGGGATAACTTTGGGAAACTAAAGCTAATACCCT
ATGCGCCCTTCGGGGGAAAGGGTCATAATTTGCAATTTATAGTTTTCAGTTTTCAATGTTTTGAATGTGGTG
TAACGGTTAAATGATCGTTGCGTTGGATTAAAAAAAGTGAAGATTGTGAACTGTAAATTGCAAATTAGATT
CGCTGTAGGATGGGCCCGCGTAAGATTAGGTAGTTGGTGAGGTAATGGCTCACCAAGCCTACGATCTTTAG
CTGGTCTGAGAGGATGGTCAGCCACACTGGGACTGAGACACGGCC
>Zotu8945
GAACGCTGGCGGCGTGCTTAACACATGCAAGTCGAGCGGTAAGGCCCTTCGGGGTACACGAGCGGCGAA
CGGGTGAGTAACACGTGAGCAATCTGCCCTTCACTCTGGGATAGCCTCGGGAAACTGAGATTAATACCGGA
TACGACCACTTCGCGCATGTGATGGTGGTGGAAAGCTTTTGCGGTGGAGGATGAGCTCGCGGCCTATCAG
CTTGTTGGTGAGGTAATGGCTCACCAAGGCTTCGACGGGTAGCCGGCCTGAGAGGGTGACCGGCCACACT
GGGACTGAGACACGGCC
>Zotu8948
GAACGCTAGCGGCATGCTTAACACATGCAAGTCGAACGGTAACAGGGAGCTTGCTCCGCTGACGAGTGGC
GAACGGGTGAGTAATATATCGGAACGTGCCCAAGAGTGGGGGATAACGTAGCGAAAGTTACGCTAATACC
GCATACGATCTAAGGATGAAAGCAGGGGACCTTCGGGCCTTGTGCTCCTGGAGCGGCCGATATCTGATTA
GCTAGTTGGTGAGGTAAAGGCTCACCAAGGCGACGATCAGTAGCTGGTCTGAGAGGACGACCAGCCACAC
TGGAACTGAGACACGGTC
>Zotu8952
GAACGCTGGAAATGTGGATACCGGCATGCAAGTCAAGGGGCCGCAAGGCACCGGCGAACGGCTTAGTAA
CACGTAGATACATACCGCAAAGTGGGACACAGCTCGTCGAAAGACGGGGTAATTTCCCATAGTCTCGCAAG
AGTAAAGGCGCAAGCCGCTTTGTGAATGGTCTGCGGTCTATCAGCTAGTTGGTGGGGTAAAAGCCTACCA
AGGCTATGACGGATAGGGGGCGTGAGAGCGCGACCCCCAAGAATGGCACTGCGACACGGGC
>Zotu8954
GAACGCTGGCGGCAGGCTTAACACATGCAAGTCGAGCGCCCCGCAAGGGGAGCGGCAGACGGGAGAGTA
ACACGTGGGAACGTACCTTTCAGTTCGGGACAACCAAGGGAAACTTTGGCTAATACCGGATACGCCCGTAA
GGGGAAAGATTCATCGCTGAAAGATCGGCCCGCGTCTGATTAGCTAGTTGGTGGGGTAATGGCCCACCAA
GGCGACGATCAGTAGCTGGTCTGAGAGGATGATCAGCCTCACTGGGACTGAGACACGGCC
>Zotu8957
GAACGCTGGCGGCAGGCTTAACACATGCAAGTCGAGGGGTAGGGTAGCAATACCTGAGACCGGCGCACG
GGTGCGTAACGCGTATGCAATCTACCTTTTACAGAGGGATAGCCCAGAGAAATTTGGATTAATACCTCATA
GCATAGCAGCTTCGCATGAAGTCACTATTAAAGTTCCAACGGTAAAAGATGAGCATGCGTCCTATTAGCTA
GATGGTAAGGTAACGGCTTACCATGGCTACGATAGGTAGGGGTCCTGAGAGGGAGATCCCCCACACTGGT
ACTGAGACACGGAC
>Zotu8960
GAACGCTGGCGGCATGCCTTACACATGCAAGTCGAACGGCAGCACGGGAGCAATCCTGGTGGCGAGTGG
CGAACGGGTGAGTAATATATCGGAACGTGCCCAGTAATGGGGGATAGCCCGGCGAAAGCCGGATTAATAC

CGCATACGACCTACGGGTGAAAGCGGGGGATCGCAAGACCTCGCGTTATTGGAGCGGCCGATATCAGATT
AGGTTGTTGGTGGGGTAAAAGCCCACCAAGCCGACGATCTGTAGCTGGTCTGAGAGGACGACCAGCCACA
CTGGGACTGAGACACGGCC
>Zotu8967
GAACGCTGGCGGCATGCCTTACACATGCAAGTCGAACGGTAACAGGTCTTCGGATGCTGACGAGTGGCGA
ACGGGTGAGTAATGCATCGGAACGTGCCCAGTAGTGGGGGATAGCCCGGCGAAAGCCGGATTAATACCG
CATACGACCTGAGGGTGAAAGGGGGGGATCGCAAGACCTCTCGCTATTGGAGCGGCCGATGTCAGATTAG
CTAGTTGGTGGGGTAAAGGCCCACCAAGGCAACGATCTGTAGTTGGTCTGAGAGGACGACCAGCCACACT
GGGACTGAGACACGGCC
>Zotu8971
GAACGCTGGCGGCAGGCTTAACACATGCAAGTCGAACGCTCGTAGCAATACGGGAGTGGCAGACGGGTG
AGTAACGCGTGGGAACGTGCCCTTTGGTTCGGAATAACCCAGGGAAACTTGGGCTAATACCGGATAAGCC
CCTTACGGGGGAAAGATTTATCGCCGAAGGATCGGCCCGCGTCCGATTAGCTAGTTGGTGAGGTAACGGC
TCACCAAGGCGACGATCGGTAGCTGGTCTGAGAGGATGATCAGCCACACTGGGACTGAGACACGGCC
>Zotu8973
GAACGCTAGCGGCAGGCCTAATACATGCAAGTCGAACGGATAGCAATATCAGTGGCAAACGGGTGCGTAA
CGCGTAAGCAACCTGCCTCATACTGGGGGATAGCCCGGCGAAAGCTGGGGTAAACCCGCACGGTCCATTT
TTATCACCTGGTAAGACTGGTAAACATTTATGGGTATGAGAGGGGCTTGCGTCTGATTAGTTAGTTGGCAG
GGTAACGGCCTACCAAGACGATGATCAGTAGGGGTTCTGAGAGGATTGGCCCCCACATGGGTACTGAGAT
ACGGAC
>Zotu8975
GAACGCTGGCGGCATGCCTAACACATGCAAGTCGAACGGCAGCACGGACTAGTTTACTAGTTGGGTGGCA
AGTGGCGCACGGGTGAGTAACGCGTAGGAATATGCCTTAAAGAGGGGGACAACCTGGGGAAACTCAGGC
TAATACCGCATAATCTCTGAGGAGAAAAGCTGGGGACCATTAGGCCTGGCGCTTTAAGAATAGCCTGCGTA
CGATTAGCTAGTTGGTGGGGTAAGGGCCTACCAAGGCGACGATCGTTAGCTGGTCTGAGAGGATGGCCAG
CCACACTGGGACTGAGACACGGCC
>Zotu8977
GAACGCTAGCGGCGGGCCTAATACATGCAAGTCGAGGGGCAGCGGGGGGAGCAATACTTGCCGGCGACC
GGCGCACGGGTGCGTAACGCGTATGCAACCTACCTTTGACACGGGAATAGCCCGAAGAAATTCGGATTAA
TACCCGATGGTATCATTTGGTGACATCGCCGGATGATTAAAGATTTATCGGTCAGAGATGGGCATGCGTCT
GATTAGCTAGTTGGTGAGGTAACTGCTCACCAAGGCTACGATCAGTAGGGGTTCTGAGAGGATGATCCCCC
ACACCGGTACTGAGACACGGAC
>Zotu8981
TAACGCTGGCGGCGCGCCTAACACATGCAAGTCGAACGAGCCCTTCGGGGTTAGTGGCAGACGGGTGAGT
AACGCGTGGATAATGTGCCTCTTTGCTGGGGATACCGTTTCGAAAGGAACGTTAATACCGAATGTGCTCAA
ATGGTGGCATCACCGATTGAGTAAACTGTTAGGGTGAAGAGAGCAGTCCGCGTTCTATCAGCTTGTTGGTG
GGGTAACGGCCTACCAAGGCTACGACGGATAACTGGTCTGAGAGGATGATCAGTCACACTGGAACTGAGA
CACGGTC
>Zotu8985
TTTCAGCGAAACCTGAGAAAAGCGCTCTTCCCCTCCATCAGAAAATGCCTGGAGTGCGCCAACAGGCCTGG
ATTCGTGTGCTTACTGTGCGCGCAACAGAGTT
>Zotu8987
GAACGCTGGCGGCAGGCTTAACACATGCAAGTCGAGCGGGCCCTTCGGGGTCAGCGGCGGACGGGTTAG
TAACGCGTGGGAACGTGCCTTGTGCTCTGGAATAGCCCTGGGAAACCGGGGGTAATACCGGATGTGCCCT
GAGGGGGAAAGGTTTATCCGGCACAGGATCGGCCCGCGTCTGATTAGGTAGTTGGTGGGGTAATGGCCCA
CCAAGCCTACGATCGGTAGCTGGTTTGAGAGGATGATCAGCAACACTGGGACTGAGACACGGCC
>Zotu8989
GAACGCTGGCGGCGTGCTTAACACATGCAAGTCGAGCGGAAAGGCCCTTTCGGGGGTACTCGAGCGGCG
AACGGGTGAGTAACACGTGAGTAATCTGCCCTGTACTCTGGGATAGCCTTGGGAAACTGAGATTAATACCG
GATATGACAGCGGCCCGCATGGGTTGTTGTGGAAAGTTTTTTCGGTACAGGATGTGCTCGCGGCCTATCAG

CTTGTTGGTGAGGTAATGGCTCACCAAGGCTTCGACGGGTAGCCGGCCTGAGAGGGTGACCGGCCACACT
GGGACTGAGACACGGCC
>Zotu8991
GAACGCTGGCGGCAGGCCTAACACATGCAAGTCGAACGGGTCCTTCGGGACTAGTGGCGGACGGGTGAG
TAACGCGTGGGAATATGCCCCTCGGTTCGGAATAGCCTCGGGAAACTGGGAGTAATACCGGATACGGTCT
ACGGACGAAAGATTTATCGCCGAGGGATTAGCCCGCGTTGGATTAGGTAGTTGGTGGGGTAATGGCCTAC
CAAGCCGACGATCCATAGCTGGTTTGAGAGGATGATCAGCCACACTGGGACTGAGACACGGCC
>Zotu8994
GAACGCTGGCGGCATGCCTTACACATGCAAGTCGAACGCGAAAGCGGAGCAATCCGCAAGTAGAGTGGC
GAACGGGTGAGTAATACATCGGAACATTTCCAGTAGCGGGGGATAACCTGTCGAAAGATAGGCTAATACC
GCATACGCCCCACGGGGGAAAGCGGAGGACCGCAAGGCTTCGCACTATTGGAGTGGCCGATGTCGGATTA
GCTAGTTGGCGGGGTAAAAGCCCACCAAGGCTACGATCCGTAGCTGGTCTGAGAGGACGATCAGCCACAC
TGGGACTGAGACACGGCC
>Zotu8996
GAACGCTGGCGGCATGCTTAACACATGCAAGTCGCGCGGCCGGCAACGGCAGCGGCGGACGGGTGAGTA
ACGCGTAGGAATGTATCCTGAGGTGGGGGACAACCCTGGGAAACTGGGGCTAATACCGCATGGGAGCTG
AGGCTCAAAGCCGTGAGGCGCCTTGGGAGCAGCCTGCGTCCGATTAGGTAGTTGGTGGGGTAAAGGCCTA
CCAAGCCTGCGATCGGTAGCTGGTCTGAGAGGACGATCAGCCACACTGGGACTGAGACACGGCC
>Zotu9000
CAACGCTGGCGGCGTGCCTAACACATGCAAGTCGAGCGAGAAAAGGGGGGCAACTCCCTGAGTAAAGCG
GCGCACGGGTGAGTAACACGTGGGTAATCTGCCCTGGAGTGGGGAATAACACCGGGAAACCGGTGCTAA
TACCGCATACGATAGTGAGGTCTGCGGACTTTGCTATGAAAGGAGCAATTCGCTCTAGGAGGAGCCCGCG
GCTGATTAGCTAGTTGGCGAGGTAATGGCTCACCAAGGCTACGATCAGTATCCGGTCTGAGAGGACGGTC
GGACACACTGGAACTGAGACACGGTC
>Zotu9005
GAACGCTGGCGGCGTGCTTAACACATGCAAGTCGAGCGGTAAGGCCCCTTCGGGGGTACACGAGCGGCG
AACGGGTGAGTAACACGTGAGCAACCTACCCATCACATCGGGATAACTCCGGGAAACCGGAGCTAATACC
GAATACGACTACCTCAGGCATCTGATGGTGGTGGAAAGTTCTGGCGGTGATGGATGGGCTCGCGGCCTAT
CAGCTTGTTGGTGGGGTAACGGCCCACCAAGGCTTCGACGGGTAGCCGGCCTGAGAGGGTGACCGGCCAC
ACTGGGACTGAGACACGGCC
>Zotu9011
GAACGCTAGCGGCAGGCTTAATACATGCAAGTCGTACGGGATCCGAGGTAGCAATACTTTGGTGAGAGTG
GCGCACGGGTGCGTAACACGTATGCAACCTACCTTTAACCGGGACACAACCTCGAGAAATTGAGGCTAATA
TCCCATAACACTGCGAGATGGCATCATTTTGCAGTTAAAACTCCGGTGGTTATTGATGGGCATGCGCGACA
TTAGCTAGTTGGTGAGGTAACGGCCCACCAAGGCTACGATGTCTAGGGGCTCTGAGAGGATTATCCCCCAC
ACTGGTACTGAGACACGGAC
>Zotu9019
GAACGCTGGCGGCAGGCCTAACACATGCAAGTCGAACGGCAGCACAGGAGAGCTTGCTCTCTGGGTGGC
GAGTGGCGGACGGGTGAGGAATACATCGGAATCTGCCTATACGTGGGGGATAACGTAGGGAAACTTACG
CTAATACCGCATACGTCCTACGGGAGAAAGCGGGGGATCGCAAGACCTCGCGCGGATAGATGAGCCGATG
TCCGATTAGCTAGTTGGCGGGGTAATGGCCCACCAAGGCGACGATCGGTAGCTGGTCTGAGAGGATGATC
AGCCACACTGGAACTGAGACACGGTC
>Zotu9024
GAACGCTGGCGGCATGCCTAACACATGCAAGTCGAACGATGCTTTCGGGCATAGTGGCGCACGGGTGCGT
AACGCGTGGGAATCTGCCCCTTGGTTCGGAATAACAGTTAGAAATGACTGCTAATACCGGATGACGACGTA
AGTCCAAAGATTTATCGCCGAGGGATGAGCCCGCGTAGGATTAGCTAGTTGGTGTGGTAAAGGCGCACCA
AGGCGACGATCCTTAGCTGGTCTGAGAGGATGATCAGCCACACTGGGACTGAGACACGGCC
>Zotu9027
GAACGCTGGCGGCGTGCCTTAAGCATGCAAGTCGAATGAATAGCAATATTAATGGCGGACGGGTGAGTAA
CGCGTGAGAATCTGCCTTAAAGTGGGGTATAGCTTGCCGAAAGGTGAGGTAATCCCCCATGTGAACGTAA

GTTTAAAGATGCAAATCGCTTTAAGATGAGCTCGCGTAGGATTAGCTAGTTGGTGGGGTAATGGCCTACCA
AGGCAACGATCCTTAGCTGGTCTGAGAGGATGGTCAGCCACAATGGAACTGCGACACGGTC
>Zotu9032
GAACGCTGGCGGCGCGCTTAACACATGCAAGTCGAACGTGAAGCCCGGGCTTGCCCGGGTGGAAAGTGG
CGAACGGGTGAGGAACACGTGAGAAACCTGCCCTGAAGACCGGGACAACCATTGGAAACGATGGCTAAT
ACCGGATACCTTCATCGGATCGCATGATCTGATGAAGAAATGGATTCCGCTTCAGGATGGTCTCGCGGCCT
ATCAGCTTGTTGGTGGGGTAATGGCCCACCAAGGCATCGACGGGTAGCTGGTCTGAGAGGATGTCCAGCC
ACACTGGGACTGAGATACGGCC
>Zotu9034
GAACGCTGGCGGTGTGCTTAACACATGCAAGTCGAACGGGCAAGAGCTGTAGCAATACAGAATTCTGCTA
GTGGCAAACGGGTGAGTAATACATGGGAATCTACCTTGCAGTGCGAAATAACAGTTGGAAGCAATTGCTA
ATGTCGCATATAGCCGAGAGGTGAAAGATTTATCGCTGCAAGACGAGCCCATGCAGGATTAGCTAGTTGG
TGGGGTAATGGCTCACCAAGGCAACGATACGTAGCCGACCTGAGAGGGTGATCGGCCACACTGGAACTGA
GACACGGTC
>Zotu9037
GAACGCTGGCGGCAGGCTTAACACATGCAAGTCGAACGTGCGTAGCAATACGTCAGTGGCAGACGGGTG
AGTAACACGTGGGAACGTACCTTTTGGTTCGGAACAACACAGGGAAACTTGTGCTAATACCGGATAAGCCC
TTACGGGGAAAGATTTATCGCCGAAAGATCGGCCCGCGTCTGATTAGCTAGTTGGTAGGGTAACGGCCTAC
CAAGGCGACGATCAGTAGCTGGTCTGAGAGGATGATCAGCCACATTGGGACTGAGACACGGCC
>Zotu9041
GAACGCTGGCGGCGTGCTTAACACATGCAAGTCGAACGAGGCCCATTCTTCGGGGTGGGTTCTAGTGGCG
AACGGGTGAGTAACACGTGGGCAACCTGCCCCTGGCTCTGGGATAACTCCAAGAAATTGGGGCTAATACC
GGATATGACCGCTGACCGCATGGTCTGGTGGTGGAAAGTTTTTCGGCTGGGGATGGGCCCGCGGCCTATC
AGCTTGTTGGTGGGGTAGTGGCCTACCAAGGCGACGACGGGTAGCCGGCCTGAGAGGGTGACCGGCCAC
ACTGGGACTGAGACACGGCC
>Zotu9043
GAACGCTAGCGGCAGGCTTAACACATGCAAGTCGAGGGGTATGGTTCTTCGGAACCAGAGACCGGCGCAC
GGGTGCGTAACGCGTATGCAATCTACCTTTTACAGAGGGATAGCCCAGAGAAATTTGGATTAATACCTTAT
AGTATATCGGCTCCGCATGGAGCTGTTATTAAAGTCACAACGGTAAAAGATGAGCATGCGTCCCATTAGCT
AGATGGTAAGGTAACGGCTTACCATGGCCACGATGGGTAGGGGTCCTGAGAGGGAGATCCCCCACACTGG
TACTGAGACACGGAC
>Zotu9046
GAACGCTGGCGGCATGCCTAACACATGCAAGTCGAACGATGCTTTCGGGCATAGTGGCGCACGGGTGCGT
AACGCGTGGGAATCTGCCCTTAGGTTCGGAATAACAGTTGGAAACGACTGCTAATACCGGATGATGACGT
AAGTCCAAAGATTTATCGCCTGAGGATGAGCCCGCGTAGGATTAGGTAGTTGGTGTGGTAAAGGCGCACC
AAGCCGACGATCCTTAGCTGGTCTGAGAGGATGATCAGCCACACTGGGACTGAGACACGGCC
>Zotu9048
GAACGCTGGCGGCGTGCTTAAGACATGCAAGTCGAACGGACAGAGCTTGCTCTGTCAGTGCCGCACGGGT
GAGTAACGCGTAACTGACCTACCCCCAAGTCGCGGATAACGCGCCGAAAGGCACGCTAATACGTGATGTG
CACGCAGGTTTCTGGCCTGCTGTGTAAAGACTGGATCGCTTGAGGATGGGGTTGCGTTCCATCAGCTAGAT
GGTGGGGTAACGGCCCACCATGGCGACGACGGATCGCCGGCCTGAGAGGGTGGCCGGCCACAGGGGCAC
TGAGACACGGGT
>Zotu9052
GAACGCTGGCGGCAGGCCTAACACATGCAAGTCGATCGCTGTCTTCGGACAGAGAGGCGCACGGGTGAGT
AACACGTGGGAACATACCCTTAAGTGCGGAATAACTATTGGAAACGATAGCTAATACCGCATACGCCCTAC
GGGGGAAAGATTTATCGCTTAAGGATTGGCCCGCGTCTGATTAGCTAGTTGGCGGGGTAACGGCCCACCA
AGGCGACGATCAGTAGCTGGTTTGAGAGAATGATCAGCCACACTGGGACTGAGACACGGCC
>Zotu9054
GAACGTTAGCGGCGCGCCTAACACATGCAAGTCGAGCGAGAAAGGGCTTCGGCCCCGGTACAGCGGCGC
ACGGGTGAGTAACACGTGGGTAACCTTCCCAAGGGCGGGGGATAACATTCCGAAAGGAGTGCTAATACCG

CATAAGACCACGGTATCGAAAGATGCTGAGGTAAAAGCAGGCCTCTATACATAAGCTTGCACCTTTGGATG
GGCCCGCGTCCCATCAGCTAGTTGGTAGGGTAATGGCCTACCAAGGCTAAGACGGGTAACTGGTCTGAGA
GGATGATCAGTCACACTGGAACTGAGACACGGTC

>Zotu9056
GAACGCTGGCGGCGTGCCTAATACATGCAAGTCGAGCGAACAGATAAGGAGCTTGCTCCTTTGAAGTTAG
CGGCGGACGGGTGAGTAACACGTGGGTAACCTACCTATAAGACTGGAATAACTTCGGGAAACCGGAGCTA
ATGCCGGATAACATATAGAACCGCATGGTTCTATAGTGAAAGATGGTTTTGCTATCACTTATAGATGGACCC
GCGCCGTATTAGCTAGTTGGTAAGGTAATGGCTTACCAAGGCGACGATACGTAGCCGACCTGAGAGGGTG
ATCGGCCACACTGGAACTGAGACACGGTC

>Zotu9062
GAACGCTGGCGGCATGCTTAACACATGCAAGTCGAACGAAAAATAAAACAGTAAGTAATTATTGTTTTATT
TTTAGTGGCGGACGGGTGAGTAACGCGTAAGAATCTACCTTTAGGAGGGGGACAACAACTGGAAACGGTT
GCTAATACCCCATATGCTGAGGAGTAAAATAAATTTTGCCTAAAGATGAGCTTGCGTCTGATTAGCTTGTTG
GTAAGGTAACTGCTTACCAAGGCGATGATCAGTAGCTGGTCTGAGAGGATGATCAGCCACACTGGGACTG
AGACACGGCC

>Zotu9064
CAACGCTGGCGGCGCGCCTAACACATGCAAGTCGAACGAGAAAGTGGAGCAATCCATGAGTAAAGTGGC
GTACGGGTGAGTAACACGTGGATAATCTGCCCTTTAGTGGGGAATACCGCTGGGAAACCGGCGACAATAC
CGCATAAGCTCGAGAGAGGAAAGCAGCAATGCGCTGAAGGAGGAGTCCGCGGTCGATTAGTTAGTTGGC
GGGGTAAAGGCCCACCAAGACTGTGATCGATAGCCGGCCTGAGAGGGCACACGGCCACACTGGCACTGA
AACACGGGC

>Zotu9070
GAACGCTAGCGATAGGCTTAACACATGCAAGTCGAGGGGCAGCATGGTCTTAGCTTGCTAAGACTGATGG
CGACCGGCGCACGGGTGCGTAACGCGTATGCAACTTGCCTCACAGAGGGGGATAACCCGTCGAAAGACG
GACTAATACCGCGTACACTCCAGCTTGGGCATCCAGGTTAGAGGAAATGAAATTCGCTGTGAGATAGGCAT
GCGTCCCATTAGCTAGTTGGTGAGGTAACGGCTCACCAAGGCGACGATGGGTAGGGGAACTGAGAGGTT
GAACCCCCACACTGGTACTGAGACACGGAC

>Zotu9072
CGGTACCTGAACGAGTCCGGGCTGGTGCACGCCCTGCGGAGTCGGTTCGCTTCGGGGCTCGTCCACTGCTA
CGCCGGTGCTGGACTGGTGCTCTTCAACCCTCTTCGTCCCCTGGCCA

>Zotu9076
GAACGCTGGCGGCAGGCTTAACACATGCAAGTCGAACGAGACCTTCGGGTCTAGTGGCGCACGGGTGAGT
AACGCGTGGGAATCTACCTTGGAGTCTGGGATAACTGACGGAAACGACAGCTAATACCGGATACGCCCTT
AGGGGGAAAGGTTTACTGCTCTGAGATGAGCCCGCGTCCAATTAGGTAGTTGGTGAGGTAAAGGCTCACC
AAGCCGACGATTGGTAGCTGGTCTGAGAGGATGACCAGCCACACTGGGACTGAGACACGGCC

>Zotu9078
GAACGCTGGCGGCGTGCTTAACACATGCAAGTCGAACGGTGAAAGAGGAGCTTGCTCCTCTGGATCAGTG
GCGAACGGGTGAGTAACACGTGAGTAACCTGCCCCAGACTCTGGGATAAGCACTGGAAACGGTGCCTAAT
ACCGGATACGAGCTTCAACCGCATGGTTAGGAGCTGGAAAGAATTTCGGTCTGGGATGGACTCGCGGCCT
ATCAGCTAGTTGGTGAGGTAACGGCCCACCAAGGCGACGACGGGTAGCCGGCCTGAGAGGGTGACCGGC
CACACTGGGACTGAGACACGGCC

>Zotu9083
GAACGCTGGCGGCGCGTTTTAAGCATGCAAGTCGGACGGCAACTCTGGTAGCAATACTGGAGCGAGAGTG
GCGAACGGGTGAGTAACACGTAGGTGATCTACCTCTGGATGGGGGATAGCCCGTGGAAACACGGGGTAA
TACCGCATGAGGCCTTGGGGGTAAAGCCCTGAGGTGAAAGGTGCAACTGCACCGTCTGGGGATGAGCCTG
CGGTCCATTAGTTAGTTGGCGGGGTAACGGCCCACCAAGACGACGATGGATAGCCGGCCTGAGAGGGTGT
ACGGCCACACTGGAACTGAGACACGGTC

>Zotu9085
GAACGCTGGCGGCGTGCTTAACACATGCAAGTCGAACGGTAAGGCCCCTTCGGGGGTACACGAGTGGCGA
ACGGGTGAGTAACACGTGAGCAACCTGCCCTTGACTTTGGGATACCAGCTGGAAACAGCTGCTAATACCG

GATATGACCCTTCTTCGCATGTTGGGGGGTGGAAAGCTCCGGCGGTCAGGGATGGGCTCGCGGCCTATCA
GCTTGTTGGTGAGGTAATGGCTCACCAAGGCTTCGACGGGTAGCCGGCCTGAGAGGGCGACCGGCCACAC
TGGGACTGAGATACGGCC
>Zotu9088
GAACGCTGGAAATGTGGATACCGGCATGCAAGTCAAGGGGCCGCAAGGCACCGGCGAACGGCTTAGTAA
CACGTGGATACATACCGCAAAGTGGGGCACAGCTCGTCGAAAGACGGGGTAATTTCCCATAGTCCCGCAA
GGGTAAAGGCGCAAGCCGCTTTGTGAATGGTCTGCGGTCTATCAGCTAGTTGGTGAGGTAATGGCTCACC
AAGGCTATGACGGATAGGGGGCGTGAGAGCGCGACCCCCAAGAATGGCACTGCGACACGGGC
>Zotu9090
GAACGCTGGCGGCGTGCCTAATACATGCAAGTCGAGCGGATGACGAGAAGCTTGCTTCTCTGATTCAGCG
GCGGACGGGTGAGTAACACGTGGGCAACCTGCCTGTAAGACTGGGATAACTTCGGGAAACCGGAGCTAAT
ACCGGATAATTTCTTGAACCTCATGGTTCAAGAGTAAAAGATGGTTTCGGCTATCACTTACAGATGGGCCC
GCGGCGCATTAGCTAGTTGGTGAGGTAACGGCTCACCAAGGCGACGATGCGTAGCCGACCTGAGAGGGT
GATCGGCCACACTGGGACTGAGACACGGCC
>Zotu9093
GAACGCTGGCGGCGTGCTTAACACATGCAAGTCGAACGGTGAACCCTCTTCGGAGGGGGATCAGTGGCGA
ACGGGTGAGTAACACGTGGGCAACCTGCCCTCAGCTCTGGGATAACTCCGGGAAACCGGGGCTAATACCG
GATACGACTTCCTGTGGCATCTCGGGAAGTGGAAAGTTTTTCGGCTGAGGATGGGCCCGCGGCCTATCAGC
TTGTTGGTGGGGTAATGGCCCACCAAGGCGACGACGGGTAGCCGGCCTGAGAGGGCGACCGGCCACACT
GGGACTGAGACACGGCC
>Zotu9096
GAACGCTGGCGGCGTGCCTAAGGCATGCAAGTCGAATGAATCGCAAGATTAATGGCGGACGGGTGAGTA
ACGCGTGAGAATCTACCTTAAAGTGGGACATAGCTCGGCGAAAGTCGAGGTAATTTCCCATGTGATCGCAA
GATTAAAGCCGCAAGGCGCTTTAAGATGAGCTCGCGTCCTATCAGCTTGTTGGTGGGGTAAAGGCCTACCA
AGGCGACGACGGGTAGCTGGTCTGAGAGGATGGTCAGCCACAATGGCACTGCGACACGGGC
>Zotu9099
GAACGCTGGCGGCATGCCTAATACATGCAAGTCGAACGAGGTCCTTCGGGGCCTAGTGGCGCACGGGTGC
GTAACACGTGGGAATCTGCCCTTGGGTTCGGAATAACAGTTAGAAATGACTGCTAATACCGGATGATGACG
TAAGTCCAAAGATTTATCGCCCAAGGATGAGCCCGCGTAGGATTAGCTAGTTGGTGGGGTAAAGGCCTAC
CAAGGCGACGATCCTTAGCTGGTCTGAGAGGATGATCAGCCACACTGGGACTGAGACACGGCC
>Zotu9101
GAACGCTGGCGGCATGCTTAACACATGCAAGTCGAACGAGACCTTCGGGTCTAGTGGCGCACGGGTGCGT
AACGCGTGGGAATCTGCCCTTTGCTACGGAATAACTCAGAGAAATTTGTGCTAATACCGTATAATGTCGAA
AGACCAAAGATTTATCGGCAAAGGATGAGCCCGCGTAGGATTAGCTAGTTGGTGAGGTAAAGGCTCACCA
AGGCGACGATCCTTAGCTGGTCTGAGAGGATGATCAGCCACACTGGGACTGAGACACGGCC
>Zotu9103
GAATGCTGGCGGCGTGCCTAATACATGCAAGTCGAGCGGTAACAGGGTTTCACCGTTTCCTTGATACACCT
GGCCTTTGTGCAGGTCGTAATCTTCGAGGGAACAGTGAAGCTGCTGACGAGCGGCGGACGGCTGAGTAAC
GCGTGGGAACATACCCCAAACTGAGGGATAACGCACCGAAAGGTGTGCTAATACCGCATGTGATCTTTGG
ATTAAAGCTTTCGGGCGGTTTGGGAATGGCCTGCGTACGATTAGATAGTTGGCGGAGTAATAGCCCACCAA
GTCGACGATCGTTAGATGGTTTGAGAGGATGATCATCCAGACTGGGACTGAGACACGGCC
>Zotu9105
GAACGCTGGCGGCATGCCTTACACATGCAAGTCGAACGGCAGCACGGACTTCGGTCTGGTGGCGAGTGGC
GAACGGGTGAGTAATACATCGGAACGTGCCTAGTAGTGGGGGATAACTACTCGAAAGAGTGGCTAATACC
GCATGAGATCTACGGATGAAAGCAGGGGATCGCAAGACCTTGTGCTACTAGAGCGGCCGATGGCGGATTA
GGTAGTTGGTGGGATAAAAGCTTACCAAGCCGACGATCTGTAGCTGGTCTGAGAGGACGATCAGCCACAC
TGGGACTGAGACACGGCC
>Zotu9110
GAACGCTTGCGGCGTGCCTTAAGCATGCAAGTAGAACGAGTTCTATCTTGCCGCAAGGCAAGCTTAGAATG
AGTTGCGAACGGCTGAGTAATGAATAAGAATCTTTCCTTTAGTGGGGAATACCTCATCGAAAGATGAGCTA

ATACCGCATAACATCTACGGATAAAAGCGATTAACTTCGCGCTGATTGATGAGCTTATTCCCTATCAGGTAG
TTGGTGGGGTAATGGCCTACCAAGCCTACGACGGGTACCCGATCTGAGAGGATGGACGGGCAGAATGGG
ACTGAGACACGGCC
>Zotu9114
GAACGCTGGCGGCGTGCTTAACACATGCAAGTCGAACGGTAAGGCCCTTCGGGGTACACGAGTGGCGAAC
GGGTGAGTAACACGTGGGTGATCTGCCTTGCACTCTGGGATAAGCCTGGGAAACTGGGTCTAATACCGGA
TATGACCGCAGGATGCATGTTCTGTGGTGGAAAGCTTTTGCGGTGCAAGATGGGCCCGCGGCCTATCAGCT
TGTTGGTGGGGTAATGGCCTACCAAGGCGACGACGGGTAGCCGGCCTGAGAGGGCGACCGGCCACACTG
GAACTGAGACACGGTC
>Zotu9119
GAACGCTGGCGGCGTGCCTAATACATGCAAGTCGAGCGAATCAAAAGGTGCTTGCACCTTTTGATTAGCGG
CGGACGGGTGAGTAACACGTGGGCAACCTGCCTGTAAGACTGGGATAACTTCGGGAAACCGGAGCTAATA
CCGGATAATCCTTTTCCTCTCATGAGGAAAAGTTGAAAGTCGGTTTACGCTGACACTTACAGATGGGCCCGC
GGCGCATTAGCTAGTTGGTGAGGTAACGGCTCACCAAGGCGACGATGCGTAGCCGACCTGAGAGGGTGAT
CGGCCACACTGGGACTGAGACACGGCC
>Zotu9123
AAACGCTGGCGGCGTGCCTAATACATGCAAGTCGAACGATGAAGTACCTTCGGGTATTGTAGTAGTGGCG
TACGGGTGAGTAACGCGTGGATAATCTTCCTCTAGGTGGGGAATAACCAGTCGAAAGATTGGCTAATACC
GCATAAGACCACAAGGCCTTCGGGCCAAGGGGTTAAAGGCGAAAGTCGCCTGGAGATGAGTCTGCGCCC
GATTAGCTAGTTGGTGAGGTAAAAGCTCACCAAGGCGACGATCGGTAGCTGGTCTGAGAGGACGGCCAGC
CACACTGGGACTGAGACACGGCC
>Zotu9125
GAACGCTGGCGGCAGGCCTAACACATGCAAGTCGAACGCACCGCAAGGTGAGTGGCAGACGGGTGAGTA
ACACGTGGGAACGTGCCTTTCAGTTTGGGATAACCCAGGGAAACTTGGGCTAATACCGGATACGCCCTTTA
TTGGGGAAAGATTTATCGCTGAGAGATCGGCCCGCGTCTGATTAGCTAGTTGGTGGGGTAATGGCTCACCA
AGGCGACGATCAGTAGCTGGTCTGAGAGGATGATCAGCCACACTGGGACTGAGACACGGCC
>Zotu9127
GAACGCTGGCGGCATGCCTAACACATGCAAGTCGAACGGCAGCACAGTAAAGATTTCGGTCTTTATGGGT
GGCGAGTGGCGGACGGGTGAGTAATACGTAGGAATCTGTCCTGGAGAGGGGGATAACCCGAGGAAACTC
GGGCTAATACCGCATGATATTGAGAAATCAAAAGCAGGGGACCTGAAAGGGCCTTGCGCTCTGGGTTGAG
CCTACGTCGGATTAGCTAGTTGGTGGGGTAAAGGCCTACCAAGGCAACGATTCGTAGTTGGTCTGAGAGG
ATGATCAGCCACACTGGGACTGAGACACGGCC
>Zotu9131
GAACGCTGGCGGCAGGCTTAACACATGCAAGTCGAACGGGTGTAGCAATACATCAGTGGCAGACGGGTG
AGTAACACGTGGGAACCTTCCTAGGGGTACGGAACAACTCAGGGAAACTTGAGCTAATACCGTATACGTCC
GTAAGGAGAAAGATTTATCGCCCCTAGACGGGCCCGCGTCGGATTAGCTAGTTGGTGGGGTAACGGCCTA
CCAAGGCGACGATCCGTAGCTGATCTGAGAGGATGATCAGCCACACTGGGACTGAGACACGGCC
>Zotu9133
GAACGCTGGCGGCATGCCTAACACATGCAAGTCGAACGAGACCTTCGGGTCTAGTGGCGCACGGGTGCGT
AACGCGTGGGAATCTGCCCTTGGGTTCGGAATAACTTCGGGAAACTGAAGCTAATACCGGATGATGACGT
AAGTCCAAAGATTTATCGCCCAAGGATGAGCCCGCGTAGGATTAGCTAGTTGGTGAGGTAAAGGCTCACC
AAGGCTACGATCCTTAGCTGGTCTGAGAGGATGATCAGCCACACTGGGACTGAGACACGGCC
>Zotu9136
TAACGCTGGCGGTATGCCTAACACATGCAAGTCGAACGAGATCTTCGGATCTAGTGGCGGACGGGTGAGT
AACACGTTGGAACCTACCCCGAAGTGGGGAATAACTGGCCGAAAGGTCGGCTAATACCCCATAGTCTTATA
CTCGTTATAAGTAAAGAATTTCGCTTCGGGAGGGGCCTGCGTAGCATTAGCTTGTTGGTGAGGTAAGAGCT
CACCAAGGCTACGATGCTTAGCTGGATCGAGAGGTTGACCAGCCACACTGGGACTGAGATACGGCC
>Zotu9139
GAACGCTGGCGGCGTGCCTAAGACATGCAAGTCGAGTGGGTTTAGACCCACGGCAAACGGGTGAGTAATA
CCTAGGAACATACCTTTAGTTGAGCATAACCTACCGAAAGGTAGGACAATTCTCAATGTGGCAGCAATGCT

AAAGTCGCAAGACGCTAAAGAAGTGGCCTGGGCACCATCAGCTAGTTGGTAAGGTAAAGGCTTACCAAGG
CTATGACGGTTAGGGGCCCCGAGAGGGGAAACCCCCACACTGGAACTGAGACATGGTC

>Zotu9144

GAACGCTGGCGGCATGCCTTACACATGCAAGTCGAACGGCAGCACGGAGCTTGCTCTGGTGGCGAGTGGC
GAACGGGTGAGTAATATATCGGAACGTACCCTTGGGTGGGGGATAACGTAGCGAAAGTTACGCTAATACC
GCATACGATCTACGGATGAAAGTGGGGGATTCGCAAGAACCTCATGCTCATGGAGCGGCCGATATCTGAT
TAGCTAGTTGGTAGGGTAAAAGCCTACCAAGGCATCGATCAGTAGCTGGTCTGAGAGGACGACCAGCCAC
ACTGGAACTGAGACACGGTC

>Zotu9146

GAACGCTGGCGGCGTGCCTAATACATGCAAGTCGAGCGAACAGACGGGGAGCTTGCTCCCCCGACGTTAG
CGGCGGACGGGTGAGTAACACGTGGGTAACCTACCTATAAGACTGGAATAACTCCGGGAAACCGGGGCTA
ATGCCGGATAACATGTTGGACCGCATGGTCCTACAGTGAAAGACGGTTTTGCTGTCACTTATAGATGGACC
CGCGCCGTATTAGCTAGTTGGTAAGGTAACGGCTTACCAAGGCAACGATACGTAGCCGACCTGAGAGGGT
GATCGGCCACACTGGAACTGAGACACGGTC

>Zotu9150

GAACGCTAGCGGCAGGCTTAATACATGCAAGGCGAAGGGTCAGCAATGGCACTGTCGTACGGGTGCGTAA
CGCGTATGCAACCTGCCTATCACTGGGGGATAGCCCGGGGAAACCCGGATTAATACCGCATAACACAGGG
GTCCCGCATGGGTACTATTTGTTAAAGATTTATCGGTGGTAGATGGGCATGCGTTCGATTAGCTAGTTGGT
GCAGGTAACGGCTCACCAAGGCGACGATCGATAGGGGAGCTGAGAGGTTGATCCCCCACACGGGCACTG
AGATACGGGC

>Zotu9155

AAACGCTGGCGGCGTGCTTAACACATGCAAGTCGAACGATGAAGCCCAGCTTGCTGGGTGGATTAGTGGC
GAACGGGTGAGGAACACGTGAGGAATCTGCCTTCAACTCTGGGATAACTCCGGGAAACCGGGGCTAATAC
CGGATACGAGATCTGCAGGCATCTGCGGATCTGGAAAGTTTTTCGGTTGAAGATGACCTCGCGGCCTATCA
GCTTGTTGGTGAGGTAATAGCTCACCAAGGCGACGACGGGTAGCCGGCCTGAGAGGGCGACCGGCCACA
CTGGGACTGAGACACGGCC

>Zotu9158

GAACGCTGGCGGCGTGCTTAACACATGCAAGTCGAACGGTGACGGAGAGCTTGCTCTCCTGATCAGTGGC
GAACGGGTGAGTAACACGTGGGCAATCTGCCTCTGACTTCGGGATAACGCTTCGAAAGAGGTGCTAATAC
CGGATACGAATCCTGCAGGCATCTGCGGGATTGGAAAGTTTTTCGGTCAGAGATGAGCCCGCGGCCTATCA
GCTAGTTGGTGAGGTAATGGCTCACCAAGGCGACGACGGGTAGCCGGCCTGAGAGGGCGACCGGCCACA
CTGGGACTGAGACACGGCC

>Zotu9160

GAACGCTAGCGGTGTGCCTTAAGCATGCAAGTAGAGCGGTCGCCGTAAGGCGACAGCTGCGAACGGCTG
AGTATAAGATAGGAATCTACCTCTTGGTGGGGAATACCTCGTCGAAAGACGGGCTAATACCGCATAAGATC
GTAAGATGAAAGGGCGTAACTGTTCGCCGAGAGAGGAGCCTATCTCCCATCAGGTAGTTGGTAGGGTAAT
GGCCTACCAAGCCTACGACGGGTAGGGGGTGTGAGAGCATGACCCCCCAGAATGGGACTGAGACACGGC
C

>Zotu9163

GAACGCTGGCGGCGTGCTTAACACATGCAAGTCGAGCGGTAAGGCCCTTCGGGGTACACGAGCGGCGAA
CGGGTGAGTAACACGTGGGTGACCTGCCCCCAACTCTGGGATAAGCCCGGGAAACTGGGTCTAATACCGG
ATATGACTTCACACTGCATGGTGTGTTGTGGAAAGTTTTTCGGTTGGGGATGGGCCCGCGGCCTATCAGCT
TGTTGGTGGGGTGATGGCCTACCAAGGCGACGACGGGTAGCCGGCCTGAGAGGGTGTCCGGCCACACTG
GGACTGAGATACGGCC

>Zotu9166

GAACGCTGGCGGCAGGCTTAACACATGCAAGTCGAGCGCCCCGCAAGGGGAGCGGCAGACGGGTGAGTA
ACGCGTGGGAATTTACCCAGTGGTACGGAACAACCCAGGGAAACTTGGGCTAATACCGTATAAGTCCTTCG
GGAGAAAGATTTATCGCCATTGGATGAGCCCGCGTCGGATTAGCTAGTTGGTGGGGTAATGGCCTACCAA
GGCGACGATCCGTAGCTGGTCTGAGAGGATGATCAGCCACACTGGGACTGAGACACGGCC

>Zotu9169

GAACGCTGGCGGCGTGCTTAACACATGCAAGTGGAGCGACGAACCGGGCTTCGGCCCGGGGCAAAGCCG
CGAACGGGTGAGTAACACGTGGGTAACCTGCCCCGTTGACCGGGACAACCCGAGGAAACTCGGCTAATA
CCGGATGAGCTCTCATCCCATCAGGGATGTGAGGAAAGGAAGCTTCGGCCTCCGCATCGGGATGGGCCCG
CGGCCCATTAGCTTGTTGGTGGGGTAATGGCCTACCAAGGCTTCGATGGGTAGCTGGTCTGAGAGGACGA
TCAGCCACACTGGGACTGAGACACGGCC

>Zotu9171
GAACGCTGGCGGCGTGCCTAACACATGCAAGTCGAGCGGTAACAGGCGTAGCAATACGTGCTGACGAGC
GGCGGACGGGTGAGTAATGCTTGGGAATCTTCCCAGTAGTGGGGAATAACTAGCCGAAAGGTTAGCTAAT
ACCGCATACGTTCTACGGAAGAAAGCGGGGGCTCGTAAGACCTCGCGCTATTGGATGAGCCCAAGTCGGA
TTAGCTAGTTGGCGGGGTAACGGCCCACCAAGGCGACGATCCGTAACTGGTCTGAGAGGACGACCAGTCA
CACCGGAACTGAGACACGGTC

>Zotu9177
GAACGCTGGCGGCAGGCTTAACACATGCAAGTCGAGCGCATCCTTCGGGGTGAGCGGCGGACGGGTGAG
TAACGCGTGGGAACGTGCCCTCTTCTACGGAATAGCCCAGGGAAACTTGGATTAATGCCGTATACGCCCTT
CGGGGGAAAGATTTATCGGAGGAGGATCGGCCCGCGTTGGATTAGGTAGTTGGTGAGGTAACGGCTCAC
CAAGCCGACGATCCATAGCTGGTTTGAGAGGATGATCAGCCACACTGGGACTGAGACACGGCC

>Zotu9181
GAACGCTGGCGGCGTGCTTAACACATGCAAGTCGAGCGGTAAGGCCCTTCGGGGTACACGAGCGGCGAA
CGGGTGAGTAACACGTGAGCAATCTGCCCTCCACATCGGGATAACCCTCGGAAACGAGAGCTAATACCGG
ATACGACCACTTCAGGCATCTGATGGTGGTGGAAAGTTCCGGCGGTGGAGGATGAGCTCGCGGCCTATCA
GCTTGTTGGTGGGGTAATGGCCCACCAAGGCTTCGACGGGTAGCCGGCCTGAGAGGGTGACCGGCCACAC
TGGGACTGAGACACGGCC

>Zotu9186
GAACGCTGGCGGCGTGCCTAATACATGCAAGTCGAGCGAATGACAGAGAAGCTTGCTTTTCTTGATTTAGC
GGCGGACGGGTGAGTAACACGTGGGCAACCTACCTTATAGATGGGGATAACTCCGGGAAACCGGGGCTA
ATACCGAATAACTCCAAGTACCTCCTGGTGCTTGGCTGAAAGACGGTTTCGGCTGTCACTATAAGATGGGC
CCGCGGCGCATTAGCTAGTTGGTAAGGTAACGGCTTACCAAGGCAACGATGCGTAGCCGACCTGAGAGAG
TGATCGGCCACACTGGGACTGAAACACGGCC

>Zotu9188
GAACGCTGGCGGCAGGCCTAACACATGCAAGTCGAGCGGTAACAGGGGGTGCTTGCACTCTGCTGACGAG
CGGCGGACGGGTGAGTAATACTTGGGAATCTGCCCGATAGTGGGGGACAACACGGGGAAACTCGTGCTA
ATACCGCATACGCCCTACGGGGGAAAGCAGGCTTATGCTTGCGCTATCGGATGAGCCCAAGTCGGATTAG
CTAGTTGGTAGGGTAAAGGCCTACCAAGGCGACGATCCGTAACTGGTCTGAGAGGATGATCAGTCACACC
GGAACTGAGACACGGTC

>Zotu9192
GAACGCTGACAGAATGCTTAACACATGCAAGTCGATGGGGAAGGCTGAGCTTGCTCAGCTGGTAACCATG
GCGGACGGGTGAGTAACGCGTAAGGAACTTACCTGCGGGACTGGGATAACGGAGGGAAACTTCTGACAA
GACCGGATAAAGTTACTAGGCAGCATTGTTTGGTAATGAAAGGAGACGCCTGCGGAGAGTCTTGCGTCCT
ATTAGCTAGTTGGTTGGGTAACGGCTGACCAAGGCGAAGATAGGTAGCCGGCCTGAGAGGGTGGACGGC
CACAAGGGGACTGAGATACGGCC

>Zotu9196
GAATGCTGGCGGCGTGGATAAGTCATGCAAGTCAAGGGGACCGCAAGGTAACCGGCAAACGAGGTAGTA
ACACGTAGGTAGACCCCCCGAAGTCGGGCATAGCCAGCCGAAAGGTTGGGTAATTCCCGATAGTCCCGCA
AGGGTAAAGTTTTTCGCTTCGGGAGTTGCCTGCGTAGTATCAGCTAGTTGGTAGGGTAATGGCCTACCAAG
GCTACGACGCTTAGGGGAGCTGAGAGGCTGACCCCCACCGATGGAACTGCGACACGGTC

>Zotu9201
GAACGCTGGCGGCATGCCTTACACATGCAAGTCGAACGGTAACAGGTCTTCAGATGCTGACGAGTGGCGA
ACGGGTGAGTAATACATCGGAACGTGCCTAGTAATGGGGGATAACTACTCGAAAGAGTGGCTAATACCGC
ATGAGATCTACGGATGAAAGCAGGGGATCGCAAGACCTTGTACTACTAGAGCGGCCGATGGCAGATTAGG
TAGTTGGTGGGATAAAAGCTTACCAAGCCGACGATCTGTAGCTGGTCTGAGAGGACGATCAGCCACACTG
GGACTGAGACACGGCC

>Zotu9204
GAACGCTGGCGGCATGCCTAACACATGCAAGTCGAACGAAGCCTTCGGGCTTAGTGGCGCACGGGTGCGT
AACGCGTGGGAATCTGCCCCTCGGTTCGGAATAACAGTTAGAAATGACTGCTAATACCGGATGATGACGTA
AGTCCAAAGATTTATCGCCGAGGGATGAGCCCGCGTAGGATTAGCTAGTTGGTGAGGTAAAGGCTCACCA
AGGCGACGATCCTTAGCTGGTCTGAGAGGATGATCAGCCACACTGGGACTGAGACACGGCC
>Zotu9206
GAACGCTGGCGGCGTGCTTAACACATGCAAGTCGAGCGGAAAGGCCACTTCGGTGGTACTCGAGCGGCGA
ACGGGTGAGTAACACGTGAGTAATCTGCCCCTGGCTTTGGGATAGCCACCGGAAACGGTGATTAATACCG
GATATGACAGTGTCACGCATGTGATGTTGTGGAAAGTTTTTCGGCCAGGGATGTGCTCGCGGCCTATCAGC
TTGATGGTGAGGTAATGGCTCACCATGGCTTCGACGGGTAGCCGGCCTGAGAGGGTGACCGGTCACACTG
GGACTGAGACACGGCC
>Zotu9208
GAACGCTGGCGGCATGCCTAACACATGCAAGTCGAACGAGACCTTCGGGTCTAGTGGCGCACGGGTGCGT
AACGCGTGGGAATCTGCCCTTGGGTTCGGAATAACAGTTAGAAATGACTGCTAATACCGGATGATGACTTC
GGTCCAAAGATTTATCGCCCAGGGATGAGCCCGCGTAGGATTAGTTTGTTGGTGAGGTAAAAGCTTACCAA
GGCGACGATCCTTAGCTGGTCTGAGAGGATGATCAGCCACACTGGGACTGAGACACGGCC
>Zotu9212
GAACGCTGGCGGCGTGCTTAACACATGCAAGTCGAACGGGGTCCATCCAGTGGCAACACTGGTGAAGGCC
TAGTGGCGAACGGGTGAGTAACACGTGAGGAACCTGCCCTGAAGATCGGGATAAGCCGGGGAAACCTGG
TCTAATACCGAATGCCTTCGCACCTCCGCATGGGGGAGTGAAGAAATGGATTCCGCTTCAGGAGGGCCTCG
CGGCCTATCAGCTTGTTGGTGGGGTAACGGCCTACCAAGGCTCCGACGGGTAGCTGGTCTGAGAGGACGA
TCAGCCACACTGGGACTGAGACACGGCC
>Zotu9214
GAACGCTGGCGGCATGCCTTACACATGCAAGTCGAACGGTAACAGGTCTTCGGATGCTGACGAGTGGCGA
ACGGGTGAGTAATGCATCGGAACGTGCCCAGTAGTGGGGGATAGCCCGGCGAAAGCCGGATTAATACCG
CATACGACCTAAGGGTGAAAGCGGGGGATCGCAAGACCTCGCGCTATTGGAGCGGCCGATGTCAGATTAG
GTAGTTGGTGGGGTAAAGGCCTACCAAGCCGACGATCTGTAGCTGGTCTGAGAGGACGACCAGCCACACT
GGGACTGAGACACGGCC
>Zotu9217
GAACGCTGGCGGCAGGCCTAACACATGCAAGTCGAACGGTAGCACAGAGGAGCTTGCTCCCTGGGTGACG
AGCGGCGGACGGGTGAGTAATGTCTGGGAAACTGCCTGATGGAGGGGGATAACTACTGGAAACGGTAGC
TAATACCGCATAACGTCGCAAGACCAAAGAGGGGGACCTTCGGGCCTCTTGCCATCAGATGTGCCCAGATG
GGATTAGCTAGTAGGTGGGGTAATGGCTCACCTAGGCGACGATCCCTAGCTGGTCTGAGAGGATGACCAG
CCACACTGGAACTGAGACACGGTC
>Zotu9219
GAACGCTAGCGGCGAGCCTAACACATGCAAGTCGAGCGCATACCTTCGGGTATGAGCGGCGGACGGGTG
AGTAACACGTAGGTCATCTACCCTCGGGTGGGGGATAACTTGGGGAAACCCGAGCTAATACCGCACGAGG
CCTTCGGGTCAAAGACTTCGGTCGCCTGGGGATGAGCCTGCGGTGCATTACCTAGTTGGCGGGGTAACGG
CCCACCAAGGGGACGATGCATAGCTGGTCTGAGAGGATGATCAGCCACACTAGGACTGAGACACGGCC
>Zotu9223
GAACGCTAGCGGCAGGCCTAATACATGCAAGACGAACGGCAAGAATTGTAGCAATACGATTCCTAGAGTG
TCGCACGGGTGCGTAACACGTATGCAACCTACCTTGTACAGGAGTATAGCTCGGGGAAACTCGAATTAACC
CTCCATAATATATTGATGTGGCATCACATTAATATTAAAACACAAGTGGTACAAGATGGGCATGCGTTTGAT
TAGCTAGTTGGCGGTGTAATGGACCACCAAGGCGATGATCAATAGGGGAACTGAGAGGTTGATCCCCCAC
ACTGGCACTGAGATACGGGC
>Zotu9225
GAACGCTGGCGGCGTGCTTAACACATGCAAGTCGAGCGGAAAGGCCCTTCGGGGTACTCGAGCGGCGAA
CGGGTGAGTAACACGTGGGCAATCTGCCCCTCGCTCTGGGATAACTCCGGGAAACCGGGGCTAATACCGG
ATATGACACTCTTAGGCATCTTGGGGTGTGGAAAGTTTTTCGGCGAGGGATGAGCCCGCGGCCTATCAGCT

TGTTGGTGGGGTAATGGCCTACCAAGGCAGTGACGGGTAGCCGGCCTGAGAGGGCGACCGGCCACACTG
GGACTGAGACACGGCC
>Zotu9228
GAACGCTGGCGGCGGGCCTAATACATGCAAGTCGAACGGATTAAGTCTTTCGGGATTTAGTTAGTGGCGC
ACGGGTGAGTAACGCGTACACAACGTGGCTCTCAGAGGGGGATAGCCTTGGGAAACTGAGCGTAATACCC
CATAGTATTATCGGAAGGCATCTTTTGATAATTAAACCTACGGGGCTGAGAGATCGGTGTGCGTTAGATTA
GGTAGTTGGTGGGGTAACGGCTCACCAAGCCTGCGATCTATAAGGGGCGTGAGAGCGTGATCCCTCACAC
GGGTACTGAGACACGGAC
>Zotu9231
GAACGCTGGCGGCGTGCCTAATACATGCAAGTCGAGCGAACCCTTCGGGGTTAGCGGCGGACGGGTGAG
TAACACGTGGGCAACCTACCTTGCAGATGGGGATAACACCGAGAAATCGGTGCTAATACCGAATAACACTT
CACTTCACATGAAGAGAAGTTAAAAGTTGGCTTCGGCTAACACTGCGAGATGGGCCCGCGGCGCATTAGCT
AGTTGGTGAGGTAACGGCTCACCAAGGCAACGATGCGTAGCCGACCTGAGAGGGTGATCGGCCACACTG
GGACTGAGACACGGCC
>Zotu9234
AAGCAATACAAACAGGGCAAGAGCAAAAAAAAAGATAATGTGAAGGATGGTTTGGCTGCGAAATACGAT
ATACCGTTTGTTTACATGAGCTTCAACGTTCTCCCA
>Zotu9238
GAACGCTGGCGGCGTGCTTAACACATGCAAGTCGAACGTGAACGGGAGCAATCCCAAGTAAAGTGGCGTA
CGGGTGAATAACGCGTAGATAATCTACCCCAAAATTTGGAATAACCTGCCGAAAGGCGGGCTAATACTGG
ATAGAGCGACGGAATCCGTAATTGGGTTTTTCGGGAAAGGGAGGCAGCAATGCCCCTCGTTTTGGGATGA
GTCTGCGTCTCATTAGCTAGTTGGTGGGGTAAAGGCTCACCAAGGCTTTGATGGGTAGCTGGTCTGAGAG
GATGATCAGCCACACTGGAACTGAAACACGGTC
>Zotu9242
GAACGCTGGCGGCATGCCTAACACATGCAAGTCGAACGAAGGCTTCGGCCTTAGTGGCGCACGGGTGCGT
AACGCGTGGGAATCTGCCCTTGGGTTCGGAATAACAGCGGGAAACTGCTGCTAATACCGGATGATGACGT
AAGTCCAAAGATTTATCGCCCAAGGATGAGCCCGCGTAGGATTAGCTAGTTGGTGGGGTAAAGGCCCACC
AAGGCGACGATCCTTAGCTGGTCTGAGAGGATGATCAGCCACACTGGGACTGAGACACGGCC
>Zotu9247
GAACGCTAGCGGCAGGCCTAACACATGCAAGTCGAGGGGCAGCATAAAACAAGCTTGCTTGTTTGGTGGC
GACCGGCGCACGGGTGCGTAACGCGTATGCAACCTACCCTGTACTGGGGGATAGCCCGGAGAAATTCGGA
TTAAGACCCCATAGCACTTAAGAATGGCATCATTCATAAGTTAAAGGTTACGGTACAGGATGGGCATGCGT
CCCATTAGCTAGATGGTAAGGTAACGGCTTACCATGGCAACGATGGGTAGGGGGCCTGAGAGGGTGGTCC
CCCACACTGGTACTGAGACACGGAC
>Zotu9250
GAACGCTGGCGGCGTGCTTAACACATGCAAGTCGAGCGGTAAGGCTCCTTCGGGAGTACACGAGCGGCGA
ACGGGTGAGTAACACGTGAGCAATCTGCCCTTCACATCGGGATAACTCCGGGAAACCGGAGCTAATACCG
GATACGACCACCTCAGGCATCTGATGGTGGTGGAAAGTTCCGGCGGTGAAGGATGAGCTCGCGGCCTATC
AGCTTGTTGGTGGGGTAACGGCCCACCAAGGCAACGACGGGTAGCCGGCCTGAGAGGGTGACCGGCCAC
ACTGGGACTGAGACACGGCC
>Zotu9252
CAACGCTGGCGGCGTGCCTCAGACATGCAAGTCGAACGATTAAAGCTCTCTTCGGAGAGTGCATAAAGTG
GCGCACGGGTGAGTAACACGTAAGTAATCTACCTTCGAGTTGGGAATAACAGTCGGAAACGACTGCTAAT
ACCGAATAATGCAGCGGCATCTATGATGACAGTTGTTAAAGATTTATCGCTTGAAGAGGAGCTTGCGGCAG
ATTAGCTAGTTGGTAAGGTAACGGCTTACCAAGGCGACGATCTGTATCCGACCTGAGAGGGTGGTCGGAC
ACACTGACACTGAATAACGGGT
>Zotu9254
GAACGCTGGCGGCGTGCCTAATACATGCAAGTCGAGCGGACTTTAAAAGCTTGCTTTTAAAGTTAGCGGCG
GACGGGTGAGTAACACGTGGGCAACCTGCCTGTAAGACTGGGATAACTTCGGGAAACCGGAGCTAATACC
GGATAATCCTTTTCTACACATGTAGAAAAGCTGAAAGACGGTTTACGCTGTCACTTACAGATGGGCCCGCG

GCGCATTAGCTAGTTGGTGAGGTAACGGCTCACCAAGGCAACGATGCGTAGCCGACCTGAGAGGGTGATC
GGCCACACTGGGACTGAGACACGGCC
>Zotu9257
GAACGCTGGCGGCGTGCCTAATACATGCAAGTCGAGCGCGAGCGATTGGAGCTTGCTCCAATCAATCGAG
CGGCGGACGGGTGAGTAACACGTAGGCAACCTACCCATCAGACCGGGATAACCACGGGAAACCGTGGCTA
ATACCGGATGATCCTTTGAGACACAAGTCTTAAAGTTGAAAGGCGCTTTTGGTGCCGTTGATGGATGGGCC
TGCGGCGCATTAGCTGGTTGGTGGGGTAACGGCCCACCAAGGCGACGATGCGTAGCCGACCTGAGAGGG
TGATCGGCCACACTGGGACTGAGACACGGCC
>Zotu9261
GAACGCTGGCGGCGTGCTTAACACATGCAAGTCGAGCGGAAAGGCCACTTCGGTGGTACTCGAGCGGCGA
ACGGGTGAGTAACACGTGAGTAATCTGCCCTTGGCTTTGGGATAGCCACCGGAAACGGTGATTAATACCG
GATACGACCACTTCACGCATGTGATGGTGGTGGAAAGTTTTTCGGCCAGGGATGTGCTCGCGGCCTATCAG
CTTGATGGTGAGGTAATGGCTCACCATGGCTTCGACGGGTAGCCGGCCTGAGAGGGTGACCGGCCACACT
GGGACTGAGACACGGCC
>Zotu9264
GAACGCTAGCGGCATGCCTAACACATGCAAGTCGAACGATGAAGGGGGAGCTTGCTCCTCCGGATTAGTG
GCGAACGGGTGAGTAATACGTGAGCAATGTGCCCTTGACTCTGGGATAAGCGCTGGAAACGGCGTCTAAT
ACCGGATACGACCATCGGAGGCATCTCCTGGTGGTGGAAAGAACTTCGGTCAAGGATCAGCTCACGGCCT
ATCAGCTTGTTGGTGAGGTAATGGCTCACCAAGGCGACGACGGGTAGCCGGCCTGAGAGGGTGACCGGC
CACACTGGGACTGAGACACGGCC
>Zotu9270
GAACGCTGGCGGCATGCCTTACACATGCAAGTCGAACGGTGACGGGTTAAGCCGACGAGTGGCGAACGG
GTGAGTAATATATCGGAACGTGCCCAGTCGTGGGGGATAACGTAGAGAAATTTACGCTAATACCGCATAC
GATCTAAGGATGAAAGCGGGGGATCGCAAGACCTCGCGCGATTGGAGCGGCTGATATCAGATTAGGTTGT
TGGTGAGGTAAAAGCTCACCAAGCCGACGATCTGTAGCTGGTTTGAGAGAACGACCAGCCACACTGGGAC
TGAGACACGGCC
>Zotu9274
GAACGCTGGCGGCGTGCCTAATACATGCAAGTCGGGCGGACTTTTCGGGAGCTTGCTCCCGGAAAGTCAG
CGGCGAACGGGTGAGTAACACGTGGGTAACCTGCCTGCAAGACCGGGATAACTCGCGGAAACGCGGGCT
AATACCGGATGATCTCCTTTTCCGCATGGAGAAGGACTAAAAGGACGGCGCAAGCTGTCGCTTGCAGATG
GGCCCGCGGCGCATTAGCTGGTTGGTGGGGTAACGGCTCACCAAGGCGACGATGCGTAGCCGACCTGAG
AGGGTGATCGGCCACACTGGGACTGAGACACGGCC
>Zotu9276
GAACGCTAGCGGCAGGCTTAATACATGCAAGGCGAGGGGGCAGCAATGTCACCGTCGTACGGGTGCGTA
ACGCGTATGCAACCTACCTATCACTGGGGGATAGCCCGGGGAAACCCGGATTAATACCGCATAACACAGG
GGTCCCGCATGGGTACTATTTGTTAAAGATTTATCGGTGGTAGATGGGCATGCGTTCGATTAGCTAGTTGG
CGGGGTAACGGCCCACCAAGGCGACGATCGATAGGGGAGCTGAGAGGTTGATCCCCCACACGGGCACTG
AGATACGGGC
>Zotu9279
GAACGCTGGCGGCGTGCTTAACACATGCAAGTCGAACGATTAAAGCCCCTTCGGGGGTGTATAGAGTGGC
GAACGGGTGAGTAACACGTGGGCAATCTGCCCCTCACTTCGGGATAACTCAGGGAAACTTGAGCTAATACC
GGATACTCCGAGCGGATCGCATGGTCTGCTCGGGAAAGCTCCGACGGTGAGGGATGAGCCCGCGGCCCAT
TAGCTAGTTGGTGAGGTAACGGCCCACCAAGGCAACGATGGGTAGCCGCGCTGAGAGGCGGATCGGCCA
CACTGGGACTGAGACACGGCC
>Zotu9281
GAACGCTGGCGGCAGGCTTAACACATGCAAGTCGAGGGGTATAGTTCTTCGGAGCTAGAGACCGGCGCAC
GGGTGCGTAACGCGTATGCAATCTACCTTTTACAGAGGGATAGCCCAGAGAAATTTGGATTAATACCTCAT
AGTATAGTACAATCGCATGATTGAACTATTAAAGTCACAACGGTAAAAGATGAGCATGCGTCCCATTAGCT
TGTTGGTAAGGTAACGGCTTACCAAGGCTACGATGGGTAGGGGTCCTGAGAGGGAGATCCCCCACACTGG
TACTGAGACACGGAC

>Zotu9283
GAACGCTGGCGGCAGGCCTAACACATGCAAGTCGAACGAACCCTTCGGGGTTAGTGGCGGACGGGTGAG
TAACACGTGGGAACGTGCCTTTTGGTTCGGAATAACTCAGGGAAACTTGAGCTAATACCGGATGAGCCCTA
CGGGGGAAAGATTTATCGCCGAAAGAGCGGCCCGCGTCTGATTAGCTTGTTGGTGAGGTAAAGGCTCACC
AAGGCTACGATCAGTAGCTGGTCTGAGAGGATGATCAGCCACACTGGGACTGAGACACGGCC
>Zotu9291
GAACGCTGGCGGCGTGCCTAATACATGCAAGTCGAGCGGTAAGGCTTTCACAGGTTATTTAACCGAGCCTG
GTCGTGAAATTTCTTTAGAAATATCACATCACAAACGAAGTAAGATGGATGGCTATCCTTCTACAACTAAGT
GAAGTACCAGATACAAACTTCCATTTATGGAGGCGAAGGTTGAATAGCGTGTGAAAGTACACGAGCGGCG
GACGGCTGAGTAACGCGTAGGAACGTACCCCAAAGTGAGGGATAAGCACCAGAAATGGTGTCTAATACCG
CATATGGTCTTCGGATTAAAGCAGCAATGCGCTATGGGAACGGCCTGCGTCTGATTAGTTTGTTGGTGAGG
TAATGGCTCACCAAGACTACGATCGGTAGCTGGTCTGAGAGGATGATCAGCCAGACTGGAACTGAGACAC
GGTC
>Zotu9298
GAACGCTGGCGGCATGCCTAACACATGCAAGTCGAACGAGACCTTCGGGTCTAGTGGCGCACGGGTGCGT
AACGCGTGGGAACCTGCCTTTAGGTTCGGAATAACTCAGAGAAATTTGAGCTAATACCGGATAATGTCTTC
GGACCAAAGATTTATCGCCTTTAGATGGGCCCGCGTTGGATTAGCTAGTTGGTGGGGTAAAAGCCTACCAA
GGCGACGATCCATAGCTGGTCTGAGAGGATGATCAGCCACACTGGGACTGAGACACGGCC
>Zotu9301
GAACGCTAGCGGCAGGCTTAACACATGCAAGTCGAGGGGTATATAGAAGCTTGCTTTTATAGAGACCGGC
AAACGGGTGCGTAACACGTACGCAACCTACCCTGAAGTGGGGGATAGCCTTTCGAAAGGAAGATTAATAC
CCCATAATATCATAATGCGGCATCGTATAATGATTAAAGAATTTCGCTTCAGGATGGGCGTGCGTCTGATTA
GATAGTTGGTGAGGTAATGGCTCACCAAGTCAACGATCAGTAGCTGATGTGAGAGCATGATCAGCCACAC
GGGCACTGAGACACGGGC
>Zotu9303
GAACGCTGGCGGCGTGCTTAACACATGCAAGTCGCACGAGAAAGGGCTTCGGCCTGAGTAAAGTGGCGCA
CGGGTGAGTAACACGTGAATAACGTCCCTCCGGATGGGGAATAACGTTCCGAAAGGAGCGCTAATACCGC
ATAAGACCACGCCGCCACAAGACGGTGAGGCCAAAGCAGCAATGCGTCTGGAGATCGGTTCGCGTCCCAT
TAGGTAGTTGGCGGGGTAATGGCCCACCAAGCCAGAGATGGGTAGCTGGTCTGAGAGGATGATCAGCCA
CATTGGGACTGAGACACGGCC
>Zotu9306
GAACGCTGGCGGCAGGCTTAACACATGCAAGTCGAGCGGGGCCCTTCGGGGTTCAGCGGCGGACGGGTT
AGTAACGCGTGGGAACGTGCCTTTTGCTTCGGAATAGCCCTGGGAAACTGGGGGTAATACCGGATGTGCC
CTGAGGGGGAAAGATTTATCGGCAAGAGATCGGCCCGCGTTGGATTAGGTAGTTGGTGGGGTAACGGCC
CACCAAGCCGACGATCCATAGCTGGTTTGAGAGGATGATCAGCAACACTGGGACTGAGACACGGCC
>Zotu9320
GAACGCTGGCGGCATGCCTTACACATGCAAGTCGAACGGTAACAGGCCGCAAGGTGCTGACGAGTGGCG
AACGGATGAGTAATGCATCGGAACGTGCCCAGTAGTGGGGGATAGCCCGGCGAAAGCCGGATTAATACC
GCATACGACCTGAGGGTGAAAGGGGGGGATCGCAAGACCTCTCGCTATTGGAGCGGCCGATGTCAGATTA
GCTAGTTGGTGGGGTAAAGGCCTACCAAGGCAACGATCTGTAGTTGGTCTGAGAGGACGACCAGCCACAC
TGGGACTGAGACACGGCC
>Zotu9324
GAACGCTGGCGGCGTGCCTAATACATGCAAGTCGAACGCACTCTCGTTTAGATTGAAGGAGTTTGCTCCTG
ATTGATAAACATTTGAGTGAGTGGCGGACGGGTGAGTAACACGTGGGTAACCTGCCCTAAAGTGGGGGAT
AACATTTGGAAACAGATGCTAATACCGCATAAAACCTAACACCGCATGGTGTAGGGTTGAAAGATGGTTTC
GGCTATCACTTTAGGATGGACCCGCGGTGCATTAGTTAGTTGGTGAGGTAAAGGCTCACCAAGACCGTGAT
GCATAGCCGACCTGAGAGGGTAATCGGCCACACTGGGACTGAGACACGGCC
>Zotu9328
GAACGCTGGCGGCAGGCTTAACACATGCAAGTCGAGCGGGCACTTCGGTGTCAGCGGCAGACGGGTGAG
TAACGCGTGGGAACGTGCCCTTCGGTTCGGAATAACTCAGGGAAACTTGAGCTAATACCGGATACGTGCG

AGAGCAGAAAGGTTTATCTGCCGAAGGATCGGCCCGTGTCTGATTAGCTAGTTGGTGAGGTTACGGCTCAC
CAAGGCGACGATCAGTAGCTGGTCTGAGAGGATGATCAGCCACACTGGGACTGAGACACGGCC

>Zotu9331

AAACGCTGGCGGCGTGCATAACACATGCAAGTCGAACGATGAAGTTTTTATGAATTCTTCGGAATGATTAA
AAATGGATTAGTGGCGGACGGGTGAGTAACGCGTGAGTAACCTGCCTTGTACAAGGGGATAGCCGTTGGA
AACGACGAATAATACCCTATGACATCAAATAATCGCATGATAATTTGATCAAAGATTTATCGGTATAAGATG
GACTCGCGTCTGATTAGCTAGTTGGTAGGGTAAAAGCCTACCAAGGCGACGATCAGTAGCCGGCTTGAGA
GAGTGTACGGCCACATTGGGACTGAGACACGGCC

>Zotu9333

GAACGCTAGCGGCAGGCCTAATACATGCAAGTCGGACGGTAGTTCCCTTCGGGGTTCTATAGTGGCAAAC
GGGTGCGTAACGCGTAAATAACCTGCCCCAAACTGGGGGATAGCCCGGCGAAAGCTGGGGTAAGCCCGC
ATGGTCTGATGGAGACGGCATCGTCTTATCAGTAAAACCTTCGGGTGGTTTGGGAGGGATTTGCGTCTGAT
TAGCTAGTTGGGGGGGTAGTGGCCCACCAAGGCGATGATCAGTAGGGGCTCTGAGAGGAGGGTCCCCCA
CATGGGCACTGAGACACGGGC

>Zotu9335

GAACGCTGGCGGCGTGGATAAGGCATGCAAGTCAAGGGGGCCCGCAAGGGCAACCGGCAGACGAGGTA
GTAATAAAGAAGTACGCACCCTGGAGTCGGGCATAGCTAGCCGAAAGGTTAGGTAATTCCCGATAGTCTCC
AGTACCATTCGGTACTGGAGTAAAGGTTTTTCGCTTCAGGAGCGGCTTCTTGGGTATCAGCTAGTTGGTAG
GGTAAAAGCCTACCAAGGCTATGACGCCTAGGGGAGCTGAGAGGCTGACCCCCACCGAGGGAACTGAGA
CACGGTC

>Zotu9338

GAACGCTGGCGGCAGGCCTAACACATGCAAGTCGAGCGCCCCGCAAGGGGAGCGGCGGACGGGTGAGTA
ACGCGTGGGAACGTACCCCTTTCTACGGAATAGCCTCGGGAAACTGGGAGTAATACCGTATAAGCCCTACG
GGGGAAAGATTTATCGGGAAGGGATCGGCCCGCGTTGGATTAGGTAGTTGGTGGGGTAATGGCCTACCA
AGCCGACGATCCATAGCTGGTTTGAGAGGATGATCAGCCACACTGGGACTGAGACACGGCC

>Zotu9340

GAACGCTGGCGGCGTGCCTAATACATGCAAGTCGAGCGAATCACTGGGAGCTTGCTCCCGGTGGTTAGCG
GCGGACGGGTGAGTAACACGTGGGCAACCTGCCTGTAAGACTGGGATAACTTCGGGAAACCGGAGCTAAT
ACCGGATAATTTCTTCCCTCACATGAGGGAAGGTTGAAAGTCGGTTTCGGCTGACACTTACAGATGGGCCC
GCGGCGCATTAGCTAGTTGGTGAGGTAACGGCTCACCAAGGCGACGATGCGTAGCCGACCTGAGAGGGT
GATCGGCCACACTGGGACTGAGACACGGCC

>Zotu9343

GAACGCTAGCGGCAGGCCTAATACATGCAAGTCGAACGGTAACATGAATAGCAATATTTGATGACGAGTG
GCGCACGGGTGCGTAACGCGTATGCAATCTACCTTCAACTGGTGAATAGCCCGAAGAAATTCGGATTAATA
CACCATAATATATAAGAGTGGCATCACTTTTATATTAAAACTCTGGTGGTTGAAGATGAGCATGCGTGACAT
TAGCTAGTTGGTGAGGTAACGGCTCACCAAGGCTACGATGTCTAGGGGATCTGAGAGGATTAACCCCCAC
ACTGGTACTGAGACAGGGAC

>Zotu9345

GAACGCTGGCGGCATGCTTTACACATGCAAGTCGAACGGCAGCGCGGGGCAACCTGGCGGCGAGTGGCG
AACGGGTGAGTAATACATCGGAACGTACCCAGAAGTGGGGGATAACGTAGCGAAAGTTACGCTAATACCG
CATACGTTCTACGGAAGAAAGTGGGGGACCTTCGGGCCTCATGCTTTTGGAGCGGCCGATGTCTGATTAGC
TAGTTGGTAGGGTAAAGGCCTACCAAGGCGACGATCAGTAGCTGGTCTGAGAGGACGACCAGCCACACTG
GGACTGAGACACGGCC

>Zotu9349

GAACGCTGGCGGCGTGCCTAATACATGCAAGTCGAGCGAACAGAGAAGGAGCTTGCTCCTTTGACGTTAG
CGGCGGACGGGTGAGTAACACGTGGGCAACCTACCCTATAGTTTGGGATAACTCCGGGAAACCGGGGCTA
ATACCGAATAATCTATTGTCCCTCATGGGACAATACTGAAAGACGGTTTCGGCTGTCGCTATAGGATGGGC
CCGCGGCGCATTAGCTAGTTGGTGAGGTAACGGCTCACCAAGGCAACGATGCGTAGCCGACCTGAGAGGG
TGATCGGCCACACTGGGACTGAGACACGGCC

>Zotu9351

GAACGCTGGCGGCGTGCTTAACACATGCAAGTCGAACGAAACACCTTATTTGATTTTCTTCGGAACTGAAG
ATTTGGTGATTGAGTGGCGGACGGGTGAGTAACGCGTGGGTAACCTACCCTGTACAGGGGGATAACAGTC
AGAAATGACTGCTAATACCGCATAAGACCACAGCACCGCATGGTGCAGGGGTAAAAACTCCGGTGGTACA
GGATGGACCCGCGTCTGATTAGCTGGTTGGTGAGGTAACGGCTCACCAAGGCGACGATCAGTAGCCGGCT
TGAGAGAGTGAACGGCCACATTGGGACTGAGACACGGCC
>Zotu9353
GAATGCTGGCGGCGTGGATAAGGCATGCAAGTCAAGGGGGCCGCAAGGCAACCGGCAGACGAGGTAGT
AACACGTAAGTACGCACCCCAAAGTCTGGCATAGCTCGTCGAAAGGCGGGGTAATTCCAGATGGTCCCGC
AAGGGTAAAGATTTATCGCTTTGGGAGCGGCTTGCGTAATATCAGCTTGTTGGTAGGGTAATGGCCTACCA
AGGCTACGACGTTTAGGGGAGCTGAGAGGCTGACCCCCACCGATGGGACTGAGACACGGCC
>Zotu9357
GAACGCTGGCGGCGTGCTTAACACATGCAAGTCGAACGGTAAGGCCCCTTCGGGGGTACACGAGTGGCGA
ACGGGTGAGTAACACGTGAGCAACCTGCCCTTGACACTGGGATAAGCGATGGAAACGTCGTCTAATACCG
GATACCAGCTCGGTGGGCATCCACTGAGCTTGAAAGCTCCGGCGGTCAAGGATGGGCTCGCGGCCTATCA
GCTTGTTGGTGAGGTAACGGCTCACCAAGGCTTCGACGGGTAGCCGGCCTGAGAGGGCGACCGGCCACAC
TGGGACTGAGATACGGCC
>Zotu9360
GAACGCTGGCGGCGTGCCTAATACATGCAAGTCGAGCGGTAAGGCTTTCACAGATTATTTAACCGGATAAG
TTTCTCTCAAAGAAGAGAAATACTGCCCGAACCGAAATTTACTGGCTAGTAAATCTCAGTTGAATGATGTGT
GAAAGTACACGAGCGGCGGACGGCTGAGTAACGCGTAGGAACGTACCCCAAAGTGAGGGATAAGCCCGG
GAAACCGGGTCTAATACCGCATATTCTCTTCGGAGCAAAGCAGTAATGCGCTTTGGGAGCGGCCTGCGTAC
GATTAGGTTGTTGGTGAGGTAATGGCTCACCAAGCCGACGATCGTTAGCTGGTCTGAGAGGATGACCAGC
CAGACTGGGACTGAGAACGGCC
>Zotu9362
GAACGCTGGCGGCGTGCCTAATACATGCAAGTCGAGCGAACAGACGGGGAGCTTGCTCCCCCGACGTTAG
TGGCGGACGGGTGAGTAACACGTGGGTAACCTACCTATAAGACTGGAATAACTCCGGGAAACCGGGGCTA
ATGCCGGATAACATGTTGGACCACATGGTCCTACAGTGAAAGACGGTCTTGCTGTCACTTATAGATGGACC
CGCGCCGTATTAGCTAGTTGGTAAGGTAACGGCTTACCAAGGCAACGATACGTAGCCGACCTGAGAGGGT
GATCGGCCACACTGGAACTGAGACACGGTC
>Zotu9364
GAACGTTAGCGGCGCGCCTAACACATGCAAGTCGCACGAGAAAGGGCTTCGGCCCCGGTAAAGTGGCGCA
CGGGTGAGTAACACGTGGGTAACCTGCCCTCGAGCGGGGGATAACATTCCGAAAGGAGTGCTAATACCGC
ATAAGACCACGATCTCGAAAGAGGTTGAGGTCAAAGCTGGCCTCTATACATAAGCTAGCACTTGAGGATG
GGCCCGCGGAGCATCAGCTAGTTGGTGAGGTAATGGCTCACCAAGGCTAAGACGCTTAGCTGGTCTGAGA
GGATGATCAGCCACACTGGAACTGAGACACGGTC
>Zotu9368
CAACGCTGGCGGCGTGCCTAACACATGCAAGTCGAGCGAGAGGAGGTAGCAATACCTTAGTAAAGCGGC
GCACGGGTGAGTAACACGTGGATAACCTGCCTCGAAGTGGGGAATAACCCCGGGAAACCGGAGCTAATAC
CGCATAACCTGACGAGATCTGTGGATTTTGTCAGTAAAGGAGCAATTCGCTTTGAGAGGGGTCCGCGGCTG
ATTAGCTAGTTGGTGAGGTAATGGCTCACCAAGGCCACGATCAGTAGCCGGTCTGAGAGGACGGTCGGCC
ACACTGGAACTGAGACACGGTC
>Zotu9370
GAACGCTGGCGGCGTGCTTAACACATGCAAGTCGAGCGGTAAGGCCCCTTCGGGGGTACACGAGCGGCG
AACGGGTGAGTAACACGTGAGCAATCTGCCCTCCACATCGGGATAACCCCCGGAAACGGGAGCTAATACC
GGATACGACCACTTCAGGCATCTGATGGTGGTGGAAAGTTCCGGCGGTGGAGGATGAGCTCGCGGCCTAT
CAGCTTGTTGGTGGGGTAATGGCCCACCAAGGCTTCGACGGGTAGCCGGCCTGAGAGGGTGACCGGCCAC
ACTGGGACTGAGACACGGCC
>Zotu9377
GAACGCTAGCGGCAGGCCTAACACATGCAAGTCGAGGGGTAAGGAGAGCTTGCTCTCCCTAGACCGGCGT
ACGGGTGCGTAACACGTGTACAATCTACCTTTTACTAAGGGATAGCCCGAAGAAATTTGGATTAATACCTTA
TAGTATTATTTTATGGCATCATTAAATAATTAAAGCTACGGTGGTAAAAGATGAGTACGCGTCCCATTAGCT

AGTTGGAGTGGTAACGGCACACCAAGGCTACGATGGGTAGGGGTTCTGAGAGGGATGTCCCCCACACTGG
TACTGAGACACGGAC

>Zotu9381

GAACGCTGGCGGCATGCCTAACACATGCAAGTCGAACGAGATCTTCGGATCTAGTGGCGCACGGGTGCGT
AACGCGTGGGAATCTGCCCTTGGGTACGGAATAACTCAGAGAAATTTGTGCTAATACCGTATAATGTCTTC
GGACCAAAGATTTATCGCCCAAGGATGAGCCCGCGTAGGATTAGCTAGTTGGTGAGGTAAAAGCTCACCA
AGGCGACGATCCTTAGCTGGTCTGAGAGGATGATCAGCCACACTGGGACTGAGACACGGCC

>Zotu9385

GAACGCTAGCGGCAGGCCTAATACATGCAAGTCGAGGGGTAACAGGAGTAGCAATACTTGCTGACGACCG
GCGCACGGGTGCGTAACACGTATACAATCTACCTTTAACAGGGGGATAGCCGGGAGAAATCCGGATTAAT
ACCCCATACCAGTAGGAGAGGCATCTCTTTTACTTGAAAGCTACGGCGGTTAAAGATGAGTATGCGTCTGA
TTAGCTAGTTGGTGAGGTAACGGCTCACCAAGGCGACGATCAGTAGGGGATCTGAGAGGATTAACCCCCA
CACGGATACTGAGACACGGAT

>Zotu9390

GAACGCTGGCGGCAGGCTTAACACATGCAAGTCGAACGGGCACTTCGGTGTCAGTGGCAGATGGGTGAGT
AACACGTGGGAACGTACCTTTTGGTTCGGAACAACTGAGGGAAACTTCAGCTAATATCGGATAAGCCCTTA
CGGGGAAAGATTTATCGCCGAAAGATCGGCCCGCGTCTGATTAGCTAGTTGGTAGGGTAATGGCCTACCA
AGGCGACGATCAGTAGCTGGTCTGAGAGGATGATCAGCCACATTGGGACTGAGACACGGCC

>Zotu9392

GAACGCTGGCGGCGTGCTTAACACATGCAAGTCGAGCGGTGAACCCTTCGGGGGGATCAGCGGCGAACG
GGTGAGTAACACGTGAGCAACCTGCCCCTGACTCTGGGATAGCCTCGGGAAACCGGGATTAATACCGGAT
ATGACACCTCCTCGCATGGGGTGGTGTGGAAAGTTTTTCGGTCAGGGATGGGCTCGCGGCCTATCAGCTTG
TTGGTGGGGTGATGGCCTACCAAGGCGACGACGGGTAGCCGGCCTGAGAGGGTGACCGGCCACACTGGG
ACTGAGACACGGCC

>Zotu9401

GAACGCTGGCGGCGTGCCTAATACATGCAAGTCGAGCGAATCTTTGGGAGCTTGCTCCCAAAAGTTAGCG
GCGGACGGGTGAGTAACACGTGGGCAACCTACCTATAAGACTGGGATAACTTCGGGAAACCGGAGCTAAT
ACCGGATAATTCTTTTCTACTCATGTAGAAAAGCTAAAAGACGGTTTACGCTGTCACTTATAGATGGGCCCG
CGGCGCATTAGCTAGTTGGTGAGGTAACGGCTCACCAAGGCCACGATGCGTAGCCGACCTGAGAGGGTGA
TCGGCCACACTGGGACTGAGACACGGCC

>Zotu9404

GAACGCTAGCGGCAGGCTTAATACATGCAAGTCGAACGGGATTAGGGAGCTTGCTCCCTATGAGAGTGGC
GCACGGGTGCGTAACACGTATGCAACCTACCTTCAACCGGGGGATAGCCTTCCGAAAGGAAGATTAATACC
GCATAACATTATTGAATGGCATCATTTAATAATCAAACTTACGGGAGTTGAAGATGGGCATGCGTCCGATT
AGCTAGTTGGAGAGGTAACGGCTCACCAAGGCGACGATCGGTAGGGGATCTGAGAGGATGACCCCCCAC
ACTGGTACTGAGACACGGAC

>Zotu9407

GAACGCTGGCGGCATGCCTAACACATGCAAGTCGAACGAAGGCTTCGGCCTTAGTGGCGCACGGGTGCGT
AACGCGTGGGAATCTGCCCCTTGGTTCGGAATAACAGTTGGAAACGACTGCTAATACCGGATGACGACGT
AAGTCCAAAGATTTATCGCCGAGGGATGAGCCCGCGTAGGATTAGCTAGTTGGTGTGGTAAGAGCGCACC
AAGGCGACGATCCTTAGCTGGTCTTAGAGGATGATCAGCCACACTGGGACTGAGACACGGCC

>Zotu9409

GAACGCTGGCGGCAGGCTTAACACATGCAAGTCGAACGCCCCGCAAGGGGAGTGGCAGACGGGTGAGTA
ACACGTGGGAACATACCCTTCGGTTCGGAACAACGCCGGGAAACTGGCGCTAATACCGGATACGGCTTGA
GGGCGAAAGATTGATCGCCGAAGGATTGGCCCGCGTTCGATTAGCTTGTTGGTGAGGTAACGGCTCACCA
AGGCGACGATCGATAGCTGGTCTGAGAGGATGGCCAGCCACATTGGGACTGAGACACGGCC

>Zotu9412

TAACGCTAGCGGCAGGCCTAATACATGCAAGTTGAACGGGATTTCGGGTAGCAATATCTGGATGAGAGTG
GCGCACGGGTGCGTAACGCGTATGCAACCTACCTCTAACTGGGGGATAGCCTTCCGAAAGGAAGATTAAT
ACCGCATAACATATTGATGGAGCATTTCATTAATATCAAAACTCCGGTGGTTAGAGATGGGCATGCGTCCG

ATTAGCTAGTTGGTGAGGTAACGGCTCACCAAGGCGACGATCGGTAGGGGGTCTGAGAGGATGATCCCCC
ACACTGGTACTGAGACACGGAC
>Zotu9414
GAACGCTGGCGGCATGCCTAACACATGCAAGTCGAACGAATGCCCTTCGGGGTGTTAGTGGCGCACGGGT
GCGTAACGCGTGGGAATCTGCCCCTTGGTTCGGAATAACAGTTGGAAACGACTGCTAATACCGGATGACG
ACGTAAGTCCAAAGATTTATCGCCGAGGGATGAGCCCGCGTAGGATTAGGTAGTTGGTGTGGTAAAGGTG
CACCAAGCCGACGATCCTTAGCTGGTCTGAGAGGATGATCAGCCACACTGGGACTGAGACACGGCC
>Zotu9416
GAACGCTGGCGGCGTGCTTAACACATGCAAGTCGAGCGGAACACAAACTGTAGCAATACAGGTTGTGTTC
AGCGGCAGACGGGTGAGTAACACGTAGACAACTTACCTCATAGAGGGGGATAGCCCGCCGAAAGGCGGG
GTAATACCGCGTAAGATACAAGTGAGACATCTCGCCTGTATGAAACATCCGCAAGGATGGCTATGAGAAA
GGTCTGCGGCCTATCAGCTAGTTGGTGAGGTAACGGCTCACCAAGGCAACGACGGGTATCCGGCCTGAAA
GGGTGATCGGACACACTGACACTGAGACACGGGT
>Zotu9420
GAACGCTGGCGGCGTGCCTAACACATGCAAGTCGAACGAAGCACCTTACTGGATTCTTCGGATGAAGGTCT
GGTGACTGAGTGGCGGACGGGTGAGTAACGCGTGGGTAACCTGCCCTGTACAGGGGGATAACAGCTGGA
AACGGCTGCTAATACCGCATAAGCGCACGAGGAGACATCTCCTTGTGTGAAAAACTCCGGTGGTACAGGA
TGGGCCCGCGTCTGATTAGCTGGTTGGCAGGGTAACGGCCTACCAAGGCAACGATCAGTAGCCGGTCTGA
GAGGATGAACGGCCACATTGGAACTGAGACACGGTC
>Zotu9423
GAACGCTGGCGGAATGCTTTACACATGCAAGTCGAGCGGCAGCGCGGGGCAACCTGGCGGCGAGCGGCG
AACGGGTGAGTAATACATCGGAACGTACCCAGTTGTGGGGGATAACACATCGAAAGATGTGCTAATACCG
CATACGACCTGAGGGTGAAAGGGGGGGATCGCAAGACCTCTCGCAGTTGGAGCGGCCGATGTCAGATTA
GCTAGTTGGTGAGGTAAAGGCTCACCAAGGCGACGATCTGTAGCTGGTCTGAGAGGACGACCAGCCACAC
TGGAACTGAGACACGGTC
>Zotu9429
GAACGCTGGCGGCATGCCTAATACATGCAAGTCGAACGCCAAATCTTCCTGCTTGCAGGAAGAGGCGGAG
TGGCGAACGGGTGAGTAACACGTGAGGAACTTGCCCATAAGAGGGGGATAACACCCGGAAACGGGTGCT
AATACCGCATAGGTTTTCCCACCGCATGGTGGGAAAATAAAAGGTGGCTCTGCTACCGCTTATGGATAGCC
TCGCGGCGCATTAGCTAGTTGGTAGGGTAAAGGCCTACCAAGGCCATGATGCGTAGCCGACTTGAGAGGG
TGATCGGCCACACTGGAACTGAGACACGGTC
>Zotu9431
GAACGCTAGCGGGAGGCTTAACACATGCAAGCCGAGGGGTATATAATTGCTTGCAATTATAGAGACCGGC
GGATGGGTGAGTAACGCGTATGTAACTTACCCTATTCATTGGGATAGCCCGAGGAAACTCGGATTAATACC
AGATAACACTAAGGTCGGCATTGACTTTAGTTGAAAGATTTATCGGAATAGGATAGACATGCGTCAGATTA
GCTAGTTGGTGAGATAGAGGCTCACCAAGGCAATGATCTGTAGGGGGCCTGAGAGGGTGACCCCCCACAC
TGGTACTGAGACACGGAC
>Zotu9434
AAACGCTGGCGGCGTGCATGAGACATGCAAGTCGAACGGACGATCAGGGCTTGCTCTGATCGTTAGTGGC
GGACGGCTGAGTAACGCGTAGGTGACCTGCCCCGAAGTGGGGAATAACTGCCCGAAAGGGTGGCTAATA
CCGCATGTGGTGATGCCTTCACTGGCATCAGTAAAGCCGCAAGGCGCTTCGGGAGGGGCCTGCGTCCGAT
TAGCTCGTTGGTGGGGTAACGGCTCACCAAGGCGACGATCGGTAGGTGGTCTGAGAGGACGATCACCCAG
ACTGGGACTGAGACACGGCC
>Zotu9436
GAACGCTGGCGGTATGCTTAACACATGCAAGTCGAACGGTGAAGCAGGAGCTTGCTCCTGTGGATCAGTG
GCGAACGGGTGAGTAACACGTGAGCAATCTGCCCTGGACTCTGGGATAAGCGTTGGAAACGACGTCTAAT
ACCGGATACGAGACGCGAAGGCATCTTCAGCGTCTGGAAAGAACTTCGGTCCAGGATGAGCTCGCGGCCT
ATCAGCTAGTTGGTGAGGTAATGGCTCACCAAGGCGACGACGGGTAGCCGGCCTGAGAGGGTGACCGGC
CACACTGGGACTGAGACACGGCC
>Zotu9438

GAACGCTAGCGGCAGGCTTAATACATGCAAGTCGTGGGGCAGCGCAGTGTAGTAATACATGGGCGGCGA
CCGGCAAACGGGTGCGTAACACGTACGCAACCTTCCCGTTACTGGTGAATAGCCCAGGGAAACTTGGATTA
ATACACCATATTATATTGACTAGGCATCTGGTTGATATCAAAGCTTCGGCGGTAACGGATGGGCGTGCGGT
TGATTAGGTAGTTGGCGGGGTAACGGCCCACCAAGCCTACGATCAATAGCTGATGTGAGAGCATGATCAG
TCACACGGGCACTGAGACACGGGC

>Zotu9441
GAACGCTGGCGGCAGGCCTAACACATGCAAGTCGAGCGGATGATGGGAGCTTGCTCCCTGATTTAGCGGC
GGACGGGTGAGTAATGCCTAGGAATCTGCCTGGTAGTGGGGGATAACGTTCCGAAAGGAACGCTAATACC
GCGTACGTCCTACGGGAGAAAGCAGGGGACCTTCGGGCCTTGCGCTATCAGATGAGCCTAGGTCGGATTA
GCTAGTTGGTGAGGTAATGGCTCACCAAGGCGACGATCCGTAACTGGTCTGAGAGGATGATCAGTCACAC
TGGAACTGAGACACGGTC

>Zotu9445
GAACGCTGGCGGCGTGCTTAACACATGCAAGTCGAACGGAAAGGCCCAGCTTGCTGGGTACTCGAGTGGC
GAACGGGTGAGTAACACGTGGGTGATCTGCCCTGCACTCTGGGATAAGCCTGGGAAACTGGGTCTAATAC
CGGATATGACCTTCTCTCGCATGGGGGTTGGTGGAAAGCTTTTGCGGTGTGGGATGGGCCCGCGGCCTAT
CAGCTTGTTGGTGGGGTAATGGCCTACCAAGGCGACGACGGGTAGCCGACCTGAGAGGGTGATCGGCCA
CACTGGGACTGAGACACGGCC

>Zotu9448
GAACGCTGGCGGCATGCCTTACACATGCAAGTCGAACGGCAGCATAGGAGCTTGCTCCTGATGGCGAGTG
GCGAACGGGTGAGTAATATATCGGAACGTGCCCAATCGTGGGGGATAACGCAGCGAAAGCTGTGCTAATA
CCGCATACGATCTACGGATGAAAGTGGGGGATCGCAAGACCTCATGCTCCTGGAGCGGCCGATATCTGATT
AGCTAGTTGGTGGGGTAAAAGCTCACCAAGGCGACGATCAGTAGCTGGTCTGAGAGGACGACCAGCCACA
CTGGGACTGAGACACGGCC

>Zotu9452
GAACGCTGGCGGCGTGGATTAGGCATGCAAGTCGAGCGATCTCGCAAGGGATAGCGGCAAACGGGTGAG
GAACACGTAAATAATCTTCCCCTTATCCTGACATAGCTCTATGAAAATAGAGGTAATATCAGATGACACCAT
GATCTCTCATGAGATTATGTTTAAAGATGGGGACCGCAAGGCCTGTCGATATGGGATGAGTTTGCGTAGCA
TCAGCTAGTTGGTAGGGTAATGGCCTACCAAGGCTATGACGCTTAGCTGGTCTGAGAGGATGGTCAGCCA
CACTGGGACTGAGACACTGCC

>Zotu9455
GAACGCTGGCGGCAGGCTTAACACATGCAAGTCGAGCGGGGAGATGTAGCTTGCTACATTTCCTAGCGGC
GGACGGGTGAGTAATGCTTAGGGATCTGCCTATTAGTGGGGGACAACATTCCGAAAGGAATGCTAATACC
GCATACGCCCTACGGGGGAAAGCAGGGGATCTTCGGACCTTGCGCTAATAGATGAGCCTAAGTCAGATTA
GCTAGTTGGTGGGGTAAAGGCCTACCAAGGCGACGATCTGTAGCGGGTCTGAGAGGATGATCCACCACAC
TGGGACTGAGACACGGCC

>Zotu9457
GAACGCTGGCGGTATGCCTAACACATGCAAGTCGTACGAGAGTTTTTAACTCTAGTGGCGGACGGGTGAG
TAACACGTGAGAATTTGCCTTTAGGAGGGGGATAACAATTGGAAACGAATGCTAATACCCCATATGCTTTT
GAGTGAAATGGATTTATCCGCCTAAAGAGAAGCTCGCGGCTGATTAGCTTGTTGGTAAGGTAAGAGCTTAC
CAAGGCGACGATCAGTATCTGGTTTGAGAGGACGATCAAACACACTGGAACTGAGACACGGTC

>Zotu9460
GAACGCTAGCGGGAGGCCTAACACATGCAAGCCGAGCGGTAGAAGTCCTTCGGGACTTTGAGAGCGGCG
TACGGGTGCGGAACACGTGTGCAACCTGCCTTTATCAGGGGGATAGCCTTTCGAAAGGAAGATTAATACCC
CATAATATATTGATTGGCATCAGTTAATATTGAAAACTCCGGTGGATAGAGATGGGCACGCGCAAGATTAG
ATAGTTGGTAGGGTAACGGCCTACCAAGTCAGTGATCTTTAGGGGGCCTGAGAGGGTGATCCCCCACACT
GGTACTGAGACACGGAC

>Zotu9462
GAACGCTGGCGGCGTGCTTAACACATGCAAGTCGAACGGAAAGGCCGAAGCTTGCTTCGGTACTCGAGTG
GCGAACGGGTGAGTAACACGTGGGTGATCTGCCCTGTACTTCGGGATAAGCTTGGGAAACTGGGTCTAAT
ACCGGATATGACAATGGTTTGGATACTGTTGTGGAAAGCTTTTGCGGTATGGGATGAGCTCGCGGCCTATC

AGCTTGTTGGTGGGGTAATGGCCTACCAAGGCGTCGACGGGTAGCCGGCCTGAGAGGGTGTACGGCCAC
ATTGGGACTGAGATACGGCC

>Zotu9465
GAACGCTGGCGGCAGGCCTAACACATGCAAGTCGAGCGGACCCTTCGGGGTTAGCGGCGGACGGGTGAG
TAACGCGTGGGAATATGCCCTTCTCTGCGGAATAGCCTCGGGAAACTGAGAGTAATACCGCATACGCCCTT
TGGGGGAAAGATTTATCGGGGAAGGATTAGCCCGCGTTGGATTAGGTAGTTGGTGGGGTAATGGCCTACC
AAGCCGACGATCCATAGCTGGTTTGAGAGGATGATCAGCCACACTGGGACTGAGACACGGCC

>Zotu9468
CAACGCTGGCGGCGCGCCTAACACATGCAAGTCGAACGAGAAAGTGGAGCAATCCATGAGTACAGTGGCG
TACGGGTGAGTAACACGTGGGTAATCTACCTTTTAGTGAGGAATAACCAAGGGAAACTTTGGCTAATACCG
CATAAGCCTGAGAAGGGAAAGCAGAAATGCGCTGAAAGAGGAGCCCGCGGCCGATTAGCTAGTTGGTGA
GGTAAGAGCCCACCAAGGCAGAGATCGGTAGCCGGCCTGAGAGGGCACACGGCCACACTGGCACTGAAA
CACGGGC

>Zotu9471
GAACGCTGGCGGCGCGCCTTACGCATGCAAGTCGAACGGAGTTGGCTTCGGCCAACTGAGTGGCAGACGG
GTGAGTAACACGTAAGTCACCTATCCGGAAGTGGGGAATAACTCCTCGAAAGGGGAGCTAATACCGCATA
CCCTCGGCTTCGGCCGAGGAAAGCCCGCAAGGGCGCTCTCGGAGGGGCTTGCGGCCCATCAGCTTGTTGG
CGGGGTAACGGCCCACCAAGGCGACGACGGGTAGCTGGTCTGAGAGGACGGCCAGCCACAAGGGCACTG
AGACACGGGC

>Zotu9474
TAACGCTAGCGGCAGGCCTAATACATGCAAGTCGAGGGGCAGCGGGTGTAGCAATACATGCCGGCGACC
GGCGCACGGGTGAGTAATATATAAGCAATCTTCCTTTAACCGGGGAATAGCTCGGAGAAATCCGAATTAAT
ACCGCATAACATCATGAGACGGCATCGCCTTATGATTAAACCTACGGGGGTTATAGATGAGCTTATGTCCG
ATTAGATAGTTGGCGGGGTAACGGCCCACCAAGTCTACGATCGGTAGGGGGTCTGAGAGGACTATCCCCC
ACACTGGTACTGAGACACGGAC

>Zotu9482
GAACGCTGGCGGCATGCCTAACACATGCAAGTCGAACGAAGCCTTCGGGCTTAGTGGCGCACGGGTGCGT
AACGCGTGGGAATCTGCCCTCAGGTACGGAATAACTCAGGGAAACTTGAGCTAATACCGTATAATGACTTC
GGTCCAAAGATTTATCGCCTGAGGATGAGCCCGCGTCGGATTAGCTAGTTGGTGAGGTAAAAGCTCACCA
AGGCGACGATCCGTAGCTGGTCTGAGAGGATGATCAGCCACACTGGGACTGAGACACGGCC

>Zotu9484
GAACGCTGGCGGCATGCTTAACACATGCAAGTCGCACGGGCACTTCGGTGTCAGTGGCGGACGGGTGAGT
AACGCGTAGGTATCTATCCTTGGGTGGGGGACAACCGTGGGAAACTACGGCTAATACCGCATGATGACTG
AGGTCCAAAGGCGCAAGTCGCCTGGGGAGGAGCCTGCGTACGATTAGCTTGTTGGTGGGGTAAAGGCCTA
CCAAGGCGATGATCGTTAGCTGGTCTGAGAGGATGATCAGCCACACTGGGACTGAGACACGGCC

>Zotu9489
GAACGCTGGCGGCGTGCTTAACACATGCAAGTGGAGCGACGAACCGGTCTTCGGACCGGGGCAGAGCCG
CGAACGGGTGAGTAACACGTGGGTTATCTGCCCCGTTGACAGGGACAACCCGGGGAAACCCGGGCTAATA
CCTGATGTGCCCCTAGGGGGAAAGGAAGCTTCGGCCTCCGCAACGGGATGAGCCCGCGGCGCATTAGCTT
GTTGGTGGGGTAACGGCCTACCAAGGCGACGATGCGTAGCTGGTCTGAGAGGACGATCAGCCACACTGG
GACTGAGACACGGCC

>Zotu9492
GAACGCTGGCGGCGTGCTTAACACATGCAAGTCGAGCGAGGCCCTTCCTTCGGGAAGGTGCCCTAGCGGC
GAACGGGTGAGTAACACGTGGGCAACCTGCCCCTAGCACTGGGATAACTCCGGGAAACCGGGGCTAATAC
CGGATATGACCTTTGAGGGCATCCTCTTTGGTGTAAAGGATTCTGGCTAGGGATGGGCCCGCGGCCTATCA
GCTTGTTGGTGGGGTAATGGCCTACCAAGGCGACGACGGGTAGCCGGCCTGAGAGGGCGACCGGCCACA
CTGGGACTGAGACACGGCC

>Zotu9494
GAACGCTGGCGGCGTGCCTAACACATGCAAGTCGAGCGGAGAGGTTGCGGAGATTCAATCGATGATCTGC
TTCTTCTTAGCGGCGGATGGGTGAGTAACACGTGGGTAACCTGCCCTGCACTGGGGAATAACAGTTGGAA

ACGACTGTTAATACCGCATATGCGCACGGGGTCGCATGACCCCGGGCGGAAAGATTTATCGGTGCAGGAT
GGGACCGCGTCGGATTAACTAGTTGGTGAGGTAATGGCCCACCAAGGTGATGATCCGTAGCCGACCTGAG
AGGGTGACCGGCCACATTGGGACTGAGACACGGCC

>Zotu9497

GAATGCTGGCAGCGTGGATGAGGCATGCAAGTCGAACGAGACTAGAGGGCAACTTTTAGTTTAGTGGCGA
AAGGGTTAGTAATACATGAATAACTTACCCTTTACCTGGGGATAACGACTGGAAACAGGCGCTAATACCGA
ATGAGGTACCGATAAGGAGTTATTGGCTATCAAAGTGGGGGACCTTGTAAAGAGGCCTTGCGGTAAAGGA
TAGGTTCATGGGATATCAGCTAGTTGGTGTGGTAAAGGCGCACCAAGGCTAAGACGTCTGGGCGGATTGA
GAGATTGACCGCCAACATTGGGACTGAGACACTGCC

>Zotu9499

GAACGCTGGCGGCGTGCTTAACACATGCAAGTCGAGCGGTAAGGCCCTTCGGGGTACACGAGCGGCGAA
CGGGTGAGTAACACGTGGGTGACCTGCCCTCCACTTCGGGATAAGCCTGGGAAACTGGGTCTAATACCGG
ATAGGACCTTCGCATGCATGTGCGTTGGTGGAAAGTTTTTCGGTGGGGGATGGCCCGCGGTCTATCAGC
TTGTTGGTGGGGTGATGGCCTACCAAGGCGACGACGGATAGCCGGCCTGAGAGGGTGATCGGCCACACT
GGGACTGAGACACGGCC

>Zotu9503

GAACGCTAGCGGCAGGCCTAATACATGCAAGTCGAACGATAGATTTGAGCTTGCTCAAGTCGAAAAGTGG
CGCACGGGTGCGTAACGCGTATGCAACCTACCTTGATCAGGGGGATAGCCCGAAGAAATTCGGATTAACA
CCGCATAAAAACACAGTACAGCATTGTACAATGTTCAAATATTTATAGGATCAAGATGGGCATGCGTGTCA
TTAGCTAGTTGGCGGGGTAACGGCCCACCAAGGCGACGATGACTAGGGGATCTGAGAGGATGACCCCCCA
CACTGGTACTGAGACACGGAC

>Zotu9506

GAACGCTGGCGGCAGGCTTAACACATGCAAGTCGAACGCCCCGCAAGGGGAGTGGCAGACGGGTGAGTA
ACACGTGGGAACATACCCTTCGGTTCGGAACAACGCCGGGAAACTGGCGCTAATACCGGATACGGCCTCT
GGGCGAAAGATTGATCGCCGAAGGATTGGCCCGCGTTCGATTAGCTTGTTGGTGAGGTAACGGCTCACCA
AGGCGACGATCGATAGCTGGTCTGAGAGGATGGCCAGCCACATTGGGACTGAGACACGGCC

>Zotu9511

GAACGCTGGCGGCGCGCTTAACACATGCAAGTCGAGCGAGAACCAGGGCTTCGGCCCTGGGGACAGCGG
CGAACGGGTGAGTAACACGTGGATAACCTGCCCTCGGCACCGGGATAGCCCGAGGAAACTCGGATTAATA
CCGGATAGCCTTTCCGACTCTCGGGTCGGTTCGAAAAGGTAGCTTCGGCCTCTGGCCGAGGATGGGTCCGC
GGCGGATTAGCTTGTTGGTGGGGTAATGGCCTACCAAGGCTCCGATCCGTAGCTGGTCTGAGAGGACGAT
CAGCCACACTGGGACTGAGACACGGCC

>Zotu9513

GAACGCTATCGGTATGCTTAACACATGCAAGTCGAACGGGGTGCTTCGGCACCTAGTGGCGGACGGGTGA
GTAACGCGTGAGAATCTGACTCGAGGACGGGGACAACCACTGGAAACGGTGGCTAAGACCCGATATGCC
GAGAGGTGAAAATAATTTAGCCTTGAGAGGAGCTCGCGTCTGATTAGCTAGTTGGTGGGGTAAGAGCCTA
CCAAGGCGACGATCAGTAGCTGGTCTGAGAGGATGATCAGCCACACTGGGACTGAGACACGGCC

>Zotu9516

GAACGCTGGCGGCGTGCTTAACACATGCAAGTCACGGGGGCCCAGCAATGGGCAACCGGCGAACGGGTG
CGTAACACGTGAGCAACCTGCCTAGACGAGGGGGATAGCCGGCCCAACGGCCGGGTAATACCGCATACGC
TCACTGCCAGGAGTCTGGTGGTGAGGAAACCTCCGGGGCGTCTAGAGGGGCTCGCGGCCTATCAGCTAGT
TGGTGGGGTAACGGCCCACCAAGGCGACGACGGGTAGCTGGTCTGAGAGGATGGCCAGCCACATTGGGA
CTGAGAGACGGCC

>Zotu9521

GAACGCTGGCGGCGTGCTTAACACATGCAAGTCGAGCGGTAAGGCTCCTTCGGGAGTACACGAGCGGCGA
ACGGGTGAGTAACACGTGAGTAATCTGCCCTTCACTTCGGGATAACCGCCGGAAACGGTGGCTAATACCG
GATACGACACAGTTTCGCATGAGATCTGTGTGGAAAGTTTTCTCGGTGGAGGATGTGCTCGCGGCCTATCA
GCTTGTTGGTGAGGTAACGGCTCACCAAGGCTTCGACGGGTAGCCGGCCTGAGAGGGTGACCGGCCACAC
TGGGACTGAGACACGGCC

>Zotu9524

GAACGCTAGCGGGAGGCCTAATACATGCAAGTCGAACGGTAACAGGTCTAGCAATAGATGCTGACGAGTG
GCGCACGGGTGCGTAACGCGTACACAATCTGCCTTATACTGGGGCATAACTTTGGGAAACTGAAGCTAATT
CCCCATAACATCATAGAATGGCATCATTCAGTGATTAAAACTCCGGTGGTATAAGATGAGTGTGCGTCCGA
TTAGATAGTTGGTGAGGTAACGGCTCACCAAGTCAACGATCGGTAGCTGGTCTGAGAGGATGATCAGCCA
CACGGGCACTGAGACACGGGC
>Zotu9527
GAACGCTGGCGGCGTGCCTAATACATGCAAGTTGAGCGCTGAAGATTGGTACTTGTACCGATTGGATGAG
CAGCGAACGGGTGAGTAACGCGTGGGGAATCTGCCTTTGAGCGGGGGACAACATTTGGAAACGAATGCT
AATACCGCATAATAGCTTTAAACATAAGTTTTTAGTTTGAAAGATGCAATTGCATCACTCAAAGATGATCCC
GCGTTGTATTAGCTAGTTGGTGAGGTAAAGGCTCACCAAGGCGATGATACATAGCCGACCTGAGAGGGTG
ATCGGCCACATTGGGACTGAGACACGGCC
>Zotu9529
GAACGCTGGCGGCGTGCTTAACACATGCAAGTCGAACGGTGAACACGGAGCTTGCTCTGTGGGATCAGTG
GCGAACGGGTGAGTAACACGTGAGCAACCTGCCCCTGACTCTGGGATAAGCGCTGGAAACGGCGTCTAAT
ACTGGATACGAACCACGAAGGCATCTTCAGTGGTTGGAAAGAATTTCGGTTGGGGATGGGCTCGCGGCCT
ATCAGCTTGTTGGTGAGGTAATGGCTCACCAAGGCGTCGACGGGTAGCCGGCCTGAGAGGGTGACCGGCC
ACACTGGGACTGAGACACGGCC
>Zotu9531
GAACGCTAGCGGCAGGCCTAATACATGCAAGTCGAACGGTCAGTAGCAATACTGATAGTGGCGCACGGGT
GCGTAACACGTAACTAACCTACCCACAACTGGGGGATAGCCCGCCGAAAGGCGGATTAATACCGCATAAG
CTCTTGACCCGGCATCGGGTTATTAGTAAAGATTTATTGGTTGTGGATGGGGTTACGGGTCATTAGCTAGTT
GGTTGGGTAACGGCTGACCAAGGCGACGATGACTAGGGGAGCTGAGAGGCTGGTCCCCCACACGGGCAC
TGAGATACGGGC
>Zotu9533
GAACGCTGGCGGCATGCTTTACACATGCAAGTCGAACGGCAGCACGGGCTTCGGCCTGGTGGCGAGTGGC
GAACGAGTGAGTAATACATCGGAACGTACCCAGAAGTGGGGGATAACGTAGCGAAAGTTACGCTAATACC
GCATACGATCTACGGATGAAAGTGGGGGATCGCAAGACCTCATGCTTTTGGAGCGGCCGATGTCTGATTA
GCTAGTTGGTGAGGTAAAGGCTCACCAAGGCGACGATCAGTAGCTGGTCTGAGAGGACGACCAGCCACAC
TGGAACTGAGACACGGTC
>Zotu9535
GAACGCTGGCGGCGTGCCTAACACATGCAAGTCGAACGGAAGTTAGAGCTTCGGTTTTAACTTTAGTGGCG
AACGGGTGAGTAACGCGTGAGGAACCTGCCTTTCAGTGGGGGACAACAGTTGGAAACGACTGCTAATACC
GCATGATACTTTTGGGAGACATCTCCTGGAAGCCAAAGCTTTATGTGCTGAAAGATGGCCTCGCGTCTGAT
TAGCTAGTTGGTGAGGTAACGGCTCACCAAGGCGACGATCAGTAGCCGGTCTGAGAGGATGAACGGCCAC
ATTGGGACTGAGATACGGCC
>Zotu9537
GAACGCTGGCGGCGGGCTTAATACATGCAAGTCAAGGGGTAGCAATACACCGGCGTACGGGTGCGTAAC
GCGTATGGAACCTGCCCTTATCTGGGGGATAACTCGGGGAAACTCGAGCTAATACCGCATAAGATAGTAG
AATGGCATCATTCAGCTATTAAAGATTTATTGGATAAGGATGGCCATGCGTAAGATTAGATAGTTGGCGAG
GTAACGGCTCACCAAGTCTACGATCTTTAGGGGCTCTGAGAGGAGAATCCCCCACACTGGCACTGAGATAC
GGGC
>Zotu9539
GAACGCTGGCGGCATGCCTTACACATGCAAGTCGAACGGCAGCACGGGAGCAATCCTGGTGGCGAGTGG
CGAACGGGTGAGTAATATATCGGAACGTGCCCAGTCGTGGGGGATAACGTAGCGAAAGCTACGCTAATAC
CGCATACGATCTACGGATGAAAGCGGGGGATCGCAAGACCTCGCGCGACTGGAGCGGCCGATATCAGATT
AGGTTGTTGGTGGGGTAAAAGCCCACCAAGCCGACGATCTGTAGCTGGTCTGAGAGGACGACCAGCCACA
CTGGGACTGAGACACGGCC
>Zotu9542
GAACGCTAGCGGCAGGCTTAATACATGCAAGTCGAGGGGCAGCAGGGGTGTAGCAATACACTCGCTGGC
GACCGGCAAACGGGTGCGGAACACGTACGCAACCTACCCATAACTGGGGAATAGCCCGGGGAAACCCGG
ATTAATACCTCGTAACCTGGTGAAGTGGCATCACTTTATCAGTATAGCTCCGGCGGTTATGGATGGGCGTG

CGCCTGATTAGGTAGTTGGCGGGGTAACGGCCCACCAAGCCTGCGATCAGTAACTGGTGTGAGAGCACGA
CCAGTCACACGGGCACTGAGACACGGGC
>Zotu9544
GAACGCTGGCGGCACGTTTTAAGCATGCAAGTCGAACGGCAGTGGGGGTAACCCCATGAGAGTGGCGAA
CGGGTGAGTAACACGTAGGTGATCTGCCTTGAGGATGGGGATAGCTTGTGGAAACACAGGGTAATACCGA
ATAAGCTGACGGAACTTTGGTTTTGTTAGTAAAGGTGCTACGGCACCGCCTTGAGAGGAGCCTGCGGCTGA
TTAGCTAGTTGGTGGGGTAAAGGCCTACCAAGGCGACGATCAGTAGCCGGCCTGAGAGGGTGTACGGCCA
CACTGGAACTGAGACACGGTC
>Zotu9548
GAACGCTGGCGGCGTGCTTAACACATGCAAGTCGAACGAAGCACTTTATTTGATTTCCTTCGGGATTGATTA
TTTTGTGACTGAGTGGCGGACGGGTGAGTAACGCGTGGGTAACCTGCCTTGTACAGGGGGATAACAGTTG
GAAACGGCTGCTAATACCGCATAAGCGCACAGCATCGCATGATGCAGTGTGAAAAACTCCGGTGGTATAA
GATGGACCCGCGTTGGATTAGCTAGTTGGTGAGGTAACGGCCCACCAAGGCGACGATCCGTAGCCGACCT
GAGAGGGTGACCGGCCACATTGGGACTGAGACACGGCC
>Zotu9551
GAACGCTGGCGGCGTGCTTAACACATGCAAGTCGAACGGTGAAGCCCTTCGGGGTGGATCAGTGGCGAAC
GGGTGAGTAACACGTGAGCAACCTACCCTTGACTCTGGGATAACTCCGGGAAACCGGTGCTAATACCGGA
TATGAGCATTGGCCACATGGTCTGGTGCTGGAAAGAATTTTGGTCAGGGATGGGCTCGCGGCCTATCAGCT
TGTTGGTGAGGTAATGGCTCACCAAGGCGACGACGGGTAGCCGGCCTGAGAGGGCGACCGGCCACACTG
GGACTGAGACACGGCC
>Zotu9554
AAACGCTGGCGGCGTGCCTAACACATGCAAGTCGAACGCTCTGAGTAATCAGAGAGTGGCGGACGGGTG
AGTAACACGTGGGTCATCTACCCCAAGGTGAGGAATACCGGCGAGAAATTGTCGACAATACCGCATACGTT
CCGCAAGGAACAAAGTCCGCAAGGACGCCTTTGGATGAGCCTGCGCTCGATTAGTTAGTTGGTGGGGTAA
AAGCTTACCAAGACTTCGATCGATAGCTGGTCTGAGAGGACGATCAGCCACACTGGGATTGAGAACGGCC
>Zotu9557
GAACGCTGGCGGCGTGCTTAACACATGCAAGTCGAACGATGAAGCCCAGCTTGCTGGGCGGATTAGTGGC
GAACGGGTGAGTAACACGTGAGTAACCTGCCCCTGACTCCGGGATAAGCGTTGGAAACGACGTCTAATAC
TGGATACGACGGCCGATCGCATGGTCTGGTCGTGGAAAGATTTTTTGGTTGGGGATGGACTCGCGGCCTA
TCAGCTTGTTGGTGAGGTAATGGCTCACCAAGGCGACGACGGGTAGCCGGCCTGAGAGGGTGACCGGCC
ACACTGGGACTGAGACACGGCC
>Zotu9565
GAATGCTGGCGGCGCGCCTAACACATGCAAGTCGAGCGGTAAGGTCCTTCGGGATACACGAGCGGCGGA
CGGCTGAGTAACGCGTGGGAACATGCCCCAAAGTGAGGAATAACTGCCCGAAAGGGTAGCTAATGCCGCA
TATGATCTTCGGATTAAAGGATTTATCCGCTTTGGGAGTGGCCTGCGTATGATTAGATAGTTGGTGAGGTA
ATGGCTCACCAAGTCTACGATCATTAACTGGTCTGAGAGGATGATCAGTCAGACTGGAACTGAGACACGGT
C
>Zotu9567
GAACGCTGGCGGCATGCCTAACACATGCAAGTCGAACGAAACCAAGGCTTCGGCCTGAAGTTTAGTGGCG
CACGAGTGCGTAACGCGTGGGAATCTGCCCCTTGGTTCGGAATAACAGTGAGAAATTACTGCTAATACCGG
ATGATGACGTAAGTCCAAAGATTTATCGCCAAGGGATGAGCCCGCGTAAGATTAGCTAGTTGGTGGGGTA
AAGGCCTACCAAGGCGACGATCTTTAGCTGGTCTGAGAGGATGATCAGCCACACTGGGACTGAGACACGG
CC
>Zotu9569
GAACGCTGGCGGCAGGCTTAACACATGCAAGTCGAGCGGGCCCTTCGGGGTCAGCGGCAGACGGGTGAG
TAACGCGTGGGAACGTGCCCTTTGGTTCGGAATAACTCAGGGAAACTTGAGCTAATACCGGATACGCCCTT
ATGGGGAAAGGTTTACCGCCGAAGGATCGGCCCGCGTCTGATTAGCTAGTTGGTGAGGTTATGGCTCACC
AAGGCGACGATCAGTAGCTGGTCTGAGAGGATGATCAGCCACACTGGGACTGAGACACGGCC
>Zotu9574

GAACGCTGGCGGCATGCTTAACACATGCAAGTCGAACGAATGATTTTTCCTACTTGTAGAAAAAATTAATG
AGTGGCGGACGGGTGAGTAACACGTAAGAACCTACCTTTTGGAGAGGGACAACCACTGGAAACGGTGGC
TAAAACCTCATACAGCTGAGAAGTGAAAAGATGAAAAATCGCCAAGAGATGGGCTTGCGGCTGATTAGCT
TGTTGGTGAGGTAATGGCTTACCAAGGCAATGATCAGTAGCTGGTCTGAGAGGATGATCAGCCACACTGG
GACTGAGACACGGCC
>Zotu9577
GAACGCTGGCGGCATGCTTTACACATGCAAGTCGAGCGGCAGCGCGGGGGCAACCCTGGCGGCGAGCGG
CGAACGGGTGAGGAATACATCGGAACGTACCCCGGAGTGGGGGATAACGCCGCGAAAGCGGCGCTAATA
CCTCATATGCTCTGAGGAGGAAAGTGGGGGACCCGCAAGGGCCTCACGCTCTGGGAGCGGCCGATGTCCG
ATTAGCTTGTTGGTGAGGTAATGGCTCACCAAGGCGACGATCGGTAGCTGGTCTGAGAGGATGATCAGCC
ACACTGGGACTGAGACACGGCC
>Zotu9580
GAACGCTGGCGGCGTGCTTAACACATGCAAGTCGAACGAAGCGCCGGACCTTCGGGACCGGCGACTGAGT
GGCGGACGGGTGAGTAACGCGTGGGTAACCTGCCCTGTACAGGGGGATAACAGTTAGAAATGACTGCTA
ATACCGCATAAGCATACGAGGAGGCATCTCCTTGTGTGAAAAACTCCGGTGGTACAGGATGGGCCCGCGT
CTGATTAGCTTGTTGGCAGGGTAAGGGCCTACCAAGGCGACGATCAGTAGCCGGTCTGAGAGGATGAACG
GCCACATTGGAACTGAGACACGGTC
>Zotu9582
GAACGCTGGCGGCAGGCTTAACACATGCAAGTCGAGCGCTGTAGCAATACAGAGCGGCGCACGGGTGAG
TAACACGTGGGAACATGCCTTTCGGTTCGGAATAACTCAGGGAAACTTGTGCTAATACCGGATACGCCCTT
CGGGGGAAAGATTTATTGCCGAAAGAGTGGCCCGCGTTGGATTAGCTAGTTGGCGGGGTAAAAGCCCACC
AAGGCGACGATCCATAGCTGGTCTGAGAGGACGACCAGCCACACTGGGACTGAGACACGGCC
>Zotu9585
GAACGCTGGCGGCGTGCTTAACACATGCAAGTCGAACGAGGTCCACCGCGTAGCAATACAAGGGAAGACC
TAGTGGCGAACGGGTGCGTAACACGTGAGGAACTTGTCCCGGTCTCTGGGATAACAGTTGGAAACGACTG
CTAATACCGGATGACGTCGGAGTGTCGCATGGCACGCTGACGAAAGGGTTACTGGATCGGGAGAGCCTCG
CGGCCTATCAGCTTGTTGGTGGGGTAATGGCCTACCAAGGCATCGACGGGTAGCTGGTCTGAGAGGATGA
TCAGCCACACTGGGACTGAGACACGGCC
>Zotu9588
GAACGCTGGCGGCATGCCTAACACATGCAAGTCGAACGAAGGCTTCGGCCTTAGTGGCGCACGGGTGCGT
AACGCGTGGGAATCTACCCTTAGGTTCGGAATAACAGTTGGAAACGACCGCTAATACCGGATGATGACGT
AAGTCCAAAGATTTATCGCCTAGGGATGAGCCCGCGTAGGATTAGCTAGTTGGTGTGGTAAAGGCGCACC
AAGGCGACGATCCTTAGCTGGTCTGAGAGGATGATCAGCCACACTGAGACTGAGACACGGCC
>Zotu9591
GAACGCTGGCGGCGTGCCTAACACATGCAAGTTGAGCGACTAACCAGGGCTTGCCCTGGGGCAAAGCAGC
GAACGGGTGAGTAACACGTGGGTAACCTGCCCAGATGACCGGGACAACCCGAGGAAACTCGGGCTAATAC
CGGATGTTCTCGCCACACATCAGTGTGGTGAGCAAAGGAAGCTTCGGCCTCCGCATCTGGAGGGGCCCGC
GGCCCATTAGCTTGTTGGTGAGGTAACGGCTCACCAAGGCAACGATGGGTAGCTGGTCTGAGAGGACGAT
CAGCCACACTGGGACTGAGACACGGCC
>Zotu9593
GAACGCTGGCGGCATGCCTTACACATGCAAGTCGAACGGCAGCGCGGGGGCAACCCTGGCGGCGAGTGG
CGAACGGGTGAGTAATACATCGGAACGTGTCCCGTAGTGGGGGATAGCCCGGCGAAAGCCGGATTAATAC
CGCATACGATCTACGGATGAAAGCGGGGGATCGCAAGACCTCGCGCTATGGGGGCGGCCGATGGCAGAT
TAGGTAGTTGGTGGGGTAAAGGCCTACCAAGCCGACGATCTGTAGCTGGTCTGAGAGGACGACCAGCCAC
ACTGGGACTGAGACACGGCC
>Zotu9599
GAACGCTAGCAATGTGCCTAAGATATGCAAGTCGAACGGTTCTAGTTTGTTCGCAAGAATGAATTAGGATA
GTGGCAGACGGCTGAGTAACACGTGGGAATCTACCCCGAACTCGGGAATAGCTTGCCGAAAGGCGAGGT
AATGCTCGATAGTCTCGCAAGAGTAAAGATTTATCGGTTCGGGATGAGCCCGCGTCCTATCAGGTTGTTGG
TGGGGTAATTGCCTACCAAGCCTATGACGGGTAGCTGGACTGAGAGGTCGGACAGCCAGACTGGGACTGA
GATACGGCC

>Zotu9604
GAACGCTGGCGGCGTGGATAAGACATGCAAGTCGAACGGGATCATTGGAGTAGCAATACAACGATGGTCT
AGTGGCGAACGGGTGCGTAACACGTGGATACATTCCGGGAAGCGGGGGATAGCCCAGGGAAACTTGGAT
TAATACCGCATGTGATCGAAAGATCAAAGACGGGGACCGCAAGGCCTGTCACTTCCCGATTGGTCCGCGG
CCTATCAGCTAGTTGGCGGGGTAATGGCCCACCAAGGCGACGACGGGTAGCTGGTCTGAGAGGACGACCA
GCCACACTGGAACTGAGACACGGTC
>Zotu9609
GAACGCTGGCGGCATGCCTAACACATGCAAGTCGAACGAGATCTTCGGGTCTAGTGGCGCACGGGTGCGT
AACGCGTGGGAATCTGCCCTTGGGTCCGGAATAACTCAGAGAAATTTGAGCTAATACCGGATGATGACGT
AAGTCCAAAGATTTATCGCCCAGGGATGAGCCCGCGTAAGATTAGGTAGTTGGTGAGGTAAAGGCTCACC
AAGCCGACGATCTTTAGCTGGTCTGAGAGGATGATCAGCCACACTGGGACTGAGACACGGCC
>Zotu9616
GAACGCTGGCGGAATGCTTTACACATGCAAGTCGAACGGCAGCACGGGGGCAACCCTGGTGGCGAGTGG
CGAACGGGTGAGTAATACATCGGAACGTGCCCAGTCGTGGGGGATAACTACGCGAAAGCGTAGCTAATAC
CGCATACGATCTACGGATGAAAGCGGGGGATCGCAAGACCTCGCGCGATTGGAGCGGCCGATGGCAGAT
TAGGTAGTTGGTGAGGTAAAGGCTCACCAAGCCTGCGATCTGTAGCTGGTCTGAGAGGACGACCAGCCAC
ACTGGGACTGAGACACGGCC
>Zotu9618
GAACGCTGGCGGCGTGCCTAATACATGCAAGTCGAGCGGACTTGTGGGAGCTTGCTCCCACAAGTTAGCG
GCGGACGGGTGAGTAACACGTGGGCAACCTGCCTGTAAGACTGGGATAACTTCGGGAAACCGGAGCTAAT
ACCGGATAATTCCTTTCCTCACCTGGGGAAAGGTTGAAAGATGGCTTCGGCTATCACTTACAGATGGGCCC
GCGGCGCATTAGCTAGTTGGTGAGGTAACGGCTCACCAAGGCGACGATGCGTAGCCGACCTGAGAGGGT
GATCGGCCACACTGGGACTGAGACACGGCC
>Zotu9620
GAACGCTGGCGGCAGGCTTAACACATGCAAGTCGAGCGCCCCGCAAGGGGAGCGGCAGACGGGTGAGTA
ACGCGTGGGAACGTACCTTTTGCTACGGAATAGCTCTGGGAAACTGGAATTAATACCGTATGTGCCCTTCG
GGGGAAAGATTTATCGGCAAAAGATCGGCCCGCGTTGGATTAGCTAGTTGGTGGGGTAATGGCCTACCAA
GGCGACGATCCATAGCTGGTCTGAGAGGATGATCAGCCACATTGGGACTGAGACACGGCC
>Zotu9623
GAACGCTGGCGGCATGCTTTACACATGCAAGTCGAACGGCAGCACGGACTTCGGTCTGGTGGCGAGTGGC
GAACGGGTGAGTAATACATCGGAACGTGCCCAGTTGTGGGGGATAACTACTCGAAAGAGTAGCTAATACT
GCATGAGAACTGAGGTTGAAAGTAGGGGATTGCAAGACCTTGCGCAACTGGAGCGGCCGATGGCAGATT
AGGTAGTTGGTGGGATAAAAGCTTACCAAGCCGACGATCTGTAGCTGGTCTGAGAGGACGACCAGCCACA
CTGGGACTGAGACACGGCC
>Zotu9628
GAACGCTGGCGGCAGGCTTAACACATGCAAGTCGAGCGCGTAGCAATACGAGCGGCAGACGGGTGAGTA
ACGCGTGGGAACCTACCCTTTTCTACGGAATAACGCATGGAAACGTGTGCTAATACCGTATACGCCCTTTG
GGGGAAAGATTTATCGGAGAAGGATGGGCCCGCGTTGGATTAGCTAGTTGGTGGGGTAAAGACCTACCA
AGGCGACGATCCATAGCTGGTCTGAGAGGATGATCAGCCACATTGGGACTGAGACACGGCC
>Zotu9635
GAACGCTGGCGGCGCACTTAAGACATGCAAGTCGAACGAAGATCTAATGTGAATAAATAGTTTAAAGCAA
GTAGTTTACTATGAGTGGAGAATTATGGACGCTTTTTAGATTTTAGTGGCGGACGGGTGAGTAACACGTAG
GCAACCTGCCTAGAAGAGGGGGATAACAATGAGAAATCATTGCTAATACCGCATAAGACCACACTATCACA
TGGTGGAGGGGTCAAAGGAGTAATCCGCTTTTAGATGGGCCTGCGGCTGATTAGTTAGTTGGAGTGGTAA
CGGCACACCAAGACAATGATCAGTAGCCGAGCTGAGAGGCTGGCCGGCCACATTGGGACTGAGACACGG
CC
>Zotu9638
GAACGCTGGCGGCATGCCTTACACATGCAAGTCGAACGGTAACAGGTCTTCGGATGCTGACGAGTGGCGA
ACGGGTGAGTAATACATCGGAACGTGCCCGATCGTGGGGGATAACGGAGCGAAAGCTTTGCTAATACCGC
ATACGATCTACGGATGAAAGCAGGGGACCCGCAAGGGCCTTGCGCGAACGGAGCGGCCGATGGCAGATT

AGGTAGTTGGTGGGATAAAAGCTTACCAAGCCGACGATCTGTAGCTGGTCTGAGAGGACGACCAGCCACA
CTGGGACTGAGACACGGCC
>Zotu9640
GAACGCTGGCGGCAGGCCTAACACATGCAAGTCGAACGCCCACTTTCGGGTGGGAGTGGCAGACGGGTG
AGTAACGCGTGGGAACATACCCTTCGGTTCGGAACAACGTTTGGAAACGGACGCTAATACCGGATACGTG
CGCGAGCAGAAAGATTGATCGCCGAAGGATTGGCCCGCGTTCGATTAGCTAGTTGGTGGGGTAACGGCCC
ACCAAGGCGACGATCGATAGCTGGTCTGAGAGGATGGCCAGCCACATTGGGACTGAGACACGGCC
>Zotu9642
GAACGCTGGCGGCGTGCCTAATACATGCAAGTAGAACGCTGAAGGAGGAGCTTGCTTCTCTGGATGAGTT
GCGAACGGGTGAGTAACGCGTAGGTAACCTGCCTGGTAGCGGGGGATAACTATTGGAAACGATAGCTAAT
ACCGCATAAGAGCAGTTGTTGCATGACAGCTGTTTAAAAGGTGCAATTGCACCACTACCAGATGGACCTGC
GTTGTATTAGCTAGTTGGTGAGGTAACGGCTCACCAAGGCGACGATACATAGCCGACCTGAGAGGGTGAT
CGGCCACACTGGGACTGAGACACGGCC
>Zotu9644
GAACGCTGGCGGCGTGCTTAACACATGCAAGTGGAGCGACGAACCAGGGCTTGCCCTGGGGCAGAGCCG
CGAACGGGTGAGTAACACGTGGGTAACCTACCCCGATGACCGGGACAACCCGAGGAAACTCGGGCTAATA
CCGGATGTTCTCGATTCTCGTAAGTGAATCGAGCAAAGATAGCTTCGGCCTTCGCATCGGGACGGGCCCGC
GGCCCATTAGCTTGTTGGTGGGGTAACGGCCTACCAAGGCGACGATGGGTAGCTGGTCTGAGAGGACGAT
CAGCCACACTGGGACTGAGACACGGCC
>Zotu9648
GAATGCTGGCGGCGTGGATGAGACATGCAAGTCGAATGGGTTTAGACCCATGGCAGACGAGGTAGTAAC
ACGTAGGTACGTACCCCAAAGTCGGGCATAATCCGCCGAAAGGCGGGATAATTCCCGATGGTCCCGCAAG
GGTAAAGATTTATCGCTTTGGGAACGGCCTGCGACGTATCAGCTAGTTGGTGAGGTAATGGCTCACCAAG
GCGACGACGCGTAGGGGAGCTGAGAGGCTGACCCCCACCGATGGCACTGCGACACGGGC
>Zotu9654
GAACGCTGGCGGCGTGGATTAGGCATGCAAGTCGTGCGGTCCGTTTTTTGTAGCAATACAAGAAACGGGT
AGCGGCGAACGAGTTAGTAATACCTTGGTACATACCCCGAAGTCGGGAATAATTCGCCGAAAGGCGAACT
AATGCCCGATGGCATCGTAAGATTAAAGAAGCAATTCGCTTCGGGAGTGGCCTAGGTCCTATCAGCTTGTT
GGTGAGGTAACGGCTCACCAAGGCTATGACGGGTAGGGGGCGTGAGAGCGTGGCCCCCAACGACGTTAC
TGAGACACGGAA
>Zotu9656
CAACGCTGGCGGCGTGCCTAACACATGCAAGTCGAACGCGAAAGGGGCTTTGGTCCTGAGTAGAGTGGCG
AACGGGTGAGTAACGCGTGGGTGACCTACCCTCGAGTGGGGGATAACGTTCCGAAAGGGACGCTAATACC
GCATGACATTCTGGGTTTGAAGACCTGGAAATCAAAGCCGGGGACCGCAAGGCCTGGCGCTTGAGGAGG
GGCCCGCGTCTGATTAGCTAGTTGGCGGGGTAACTGCCCACCAAGGCGACGATCAGTAGCCGGCCTGAGA
GGGCGGACGGCCACACTGGGACTGAGACACGGCC
>Zotu9659
GAACGCTGGCGGCGTGCCTAATACATGCAAGTCGAGCGAATTCTTGGGAGCTTGCTCCCAAGAATTAGCG
GCGGACGGGTGAGTAACACGTGGGCAACCTACCTATAAGACTGGGATAACTTCGGGAAACCGGAGCTAAT
ACCGGATAATTCTTTTCCTCTCATGAGGAAAAGCTGAAAGACGGTTTACGCTGTCACTTATAGATGGGCCCG
CGGCGCATTAGCTAGTTGGTGAGGTAACGGCTCACCAAGGCAACGATGCGTAGCCGACCTGAGAGGGTGA
TCGGCCACACTGGGACTGAGACACGGCC
>Zotu9661
TGTCCGAGAATCGCTCGGGAGGAGGCGAGCCGACAGGGATGCGGCAAGAAAAATGGCACGGCGTACGG
GAAATGTGCCAAAGGCGTGGTGTATGAAATTCCCCTCACTTGTG
>Zotu9663
CAACGCTGGCGGCGTGCCTAACACATGCAAGTCGAACGCGAAAGTTCCTTCGGGGATGAGTAGAGTGGCG
TCCGGGTGAGTAACGCGTGGGTAATCTACCCTTGGGTGGGGAATAACTAGTCGAAAGGCTAGCTAATACC
GCATAATGCAGCGGCTCGATATCGAGACAGTTGTTAAAGCAGCAATGCGCCTAAGGAGGAGCCTGCGTCT

GATTAGCTTGTTGGTGAGGTAATGGCTCACCAAGGCTGCGATCAGTAGCTGGTCTGAGAGGACGGCCAGC
CACAATGGCACTGAAACACGGGC
>Zotu9665
GAACGCTAGCGATAGGCCTTACACATGCAAGTCGAGGGGCAGCACAAAGAAAGTAGCAATACTTTTTGG
TGGCGACCGGCGCACGGGTGAGTAACGCGTATGCAACCTACCTACTACTTTGGGATAGCTCGTTGAAAGAC
GAATTAATACCTTATAAAACAGGGGCTCCGCATGGAGATATTTGTTAAAGATTTATTGGTAGTAGATGGGC
ATGCGTTCCATTAGTTAGTTGGTGAGGTAACGGCTCACCAAGACAACGATGGATAGGGGAACTGAGAGGT
TTGTCCCCCACACTGGAACTGAGACACGGTC
>Zotu9667
CAACGCTGGCGGCGTGCCTAATGCATGCAAGTCGAACGGGGGCACCTTCGGGTGTCTCACGTGGCGGACG
GGTGAGGAACACGTGGGGAAGCTGCCGTGCGCACGGGGGTAGCCCGCGGAAACGCGGGGTAATTCCGG
ATGCGCTCGCATCCCGGGTAGGGGATGTGAGGAAAGATCCTTCGGGGTCGGCGCACGAGGCACCTGCGG
CCGATTAGCTAGACGGTGGGGTAACGGCCTACCGTGGCGATGATCGGTAGCTGGTCTGAGAGGATGATCA
GCCACACGGGGACTGAGACACGGCC
>Zotu9670
GAACGCTGGCGGAATGCTTAACACATGCAAGTCGCACGGGTGGTTTCGGCCATTAGTGGCGGACGGGTGA
GTAACGCGTAGGAACGTGTCCTGGGATGGGGGATAACGCTGGGAAACTGGCGCTAATACCGCATATCTCC
TGAGGGAGAAAGCCGCGAGGCGTCTTGGGAGCGGCCTGCGTCCGATTAGCTTGTTGGTTAGGTAATGGCT
GACCAAGGCGATGATCGGTAGCTGGTCTGAGAGGATGATCAGCCACACTGGGACTGAGACACGGCC
>Zotu9672
GAACGCTGGCGGCGTGCTTAACACATGCAAGTCGAACGGAAAGGCCCTTCGGGGTACTCGAGTGGCGAAC
GGGTGAGTAACACGTGGGTGATCTGCCCTGCACTTTGGGATAAGCCTGGGAAACTGGGTCTAATACCGAA
TATGATCATGCTCTTCATGGGGTGTGGTGGAAAGCTTTTGCGGTGTGGGATGGGCCCGCGGCCTATCAGCT
TGTTGGTGGGGTAATGGCCTACCAAGGCGACGACGGGTAGCCGGCCTGAGAGGGTGACCGGCCACACTG
GGACTGAGATACGGCC
>Zotu9676
GAACGCTGGCGGCGTGCCTAACACATGCAAGTCGAATGTGCCGCAAGGTGCATGGCAGACGGGTGAGTA
ACGCGTGGGGAACCTACCTCTTTGTGGGGAATAACGCTCCGAAAGGAGTGGTAATACCGCATGTGATCAC
AGCCTGGGATGGCTGTGATGAAACCCGGGGACCGAAAGGCCTGGGGCAAGGAGAGGGTCCTGCGTCTGA
TTAGCTAGTTGGTGAGGTAACGGCCCACCAAGGCATCGATCAGTAGCCGGCCTGAGAGGGTGATCGGCCA
CACTGGAACTGAGACACGGTC
>Zotu9678
CAACGCTGGCGGCGCGCTTAACACATGCAAGTCGCACGAGAAAGTGGAGCAATCCATGAGTACAGTGGCG
TACGGGTGAGTAACACGTGGATCATCTACCTTCTAGTGGGGAATACCCCTGGGAAACTGGGGACAATACC
GCATAAGCTCGTAAGAGGAAAGCAGCAATGCGCTGGAAGAGGAGTCCGCGGCTGATTAGCTAGTTGGCG
GGGTAATGGCCCACCAAGGCGAAGATCGGTAGCCGGCCTGAGAGGGCACACGGCCACACTGGCACTGAA
ACACGGGC
>Zotu9681
GAACGCTAGCGGCAGGCTTAATACATGCAAGTCGAGGGGCAGCATGAGTGTAGCAATACATTTGATGGCG
ACCGGCAAACGGGTGCGGAACACGTACACAACCTTCCTTTAAGTGGGGAATAGCCTAAGGAAACTTGGAT
TAATACCCCGTAATATATTGAAAAGGCATCTTTTTAATATTATAGCTCAGGCGCTTAATGATGGGTGTGCGG
CTGATTAGATAGTTGGCGGGGTAACGGCCCACCAAGTCTGCGATCAGTAACTGGTGTGAGAGCACGACCA
GTCACACGGGCACTGAGACACGGGC
>Zotu9683
GAACGCTGGCTGTATGGATAAGGCATGCAAGTCGAACGGCCCGCAAGGGCAGTGGCAGACGAGGTAGTA
ACACGTACGAACGTACTCCAAAGTCGGGCATAGCTCACCGAAAGGTGGGGTAATTCCCGATAGTCCCTACG
GGGTAAAGATTTATCGCTTTGGAAGCGGCGTGCGTAGTATCAGCTTGTTGGTAGTGTAATGGACTACCAAG
GCTACGACGCTTAGGGGAGCTGAGAGGCTGACCCCCACCGATGGGACTGAGACACGGCC
>Zotu9686

GAACGCTGGCGGCGTGCTTAACACATGCAAGTCGAACGGAAAGGCTCCTGCTTGCAGGGGTACTCGAGTG
GCGAACGGGTGAGTAACACGTGGGTGATCTGCCCCGCACTTCGGGATAAGCCTGGGAAACTGGGTCTAAT
ACCGGATAGGACCACTTCTTGGAGGTTGTGGTGGAAAGTTTTTTCGGTGTGGGATGAGCTCGCGGCCTATC
AGCTTGTTGGTGGGGTAATGGCCTACCAAGGCGTCGACGGGTAGCCGGCCTGAGAGGGTGTACGGCCAC
ATTGGGACTGAGATACGGCC
>Zotu9689
TAACGCTGGCGGTGTGCCTAATACATGCAAGTCGAACGGACCTTTTTAAGTTTCATTTATGCAAGTTTCAAA
AACTCATTTTTTAAAAGTGAAAATTGAAAATTGTTGAAATGGAAATTGAACTGGTTAGTGGCGGACGGGTG
AGTAACACGTTGGTACCTGCCCTCTAGTGGGGAATAACTGGCCGAAAGGTCGGCTAATACCGCATGTGATC
GCAAGATCAAAGCAGCAATGCGCTAGAGGAGGGGCCTGCGTCTGATTAGCTAGTTGGTAAGGTAATGGCT
TACCAAGGCGACGATCAGTAGCTGGTTCGAGAGGATGACCAGCCACACTGGGACTGAGATACGGCC
>Zotu9692
GAACGCTGGCGGCATGCCTAACACATGCAAGTCGAACGAGACCTTCGGGTCTAGTGGCGCACGGGTGCGT
AACGCGTGGGAATCTGCCCTTGGGTTCGGAATAACTCAGAGAAATTTGAGCTAATACCGGATAATGACGTA
AGTCCAAAGATTTATCGCCCAGGGATGAGCCCGCGTAGGATTAGCTAGTTGGTGGGGTAAAAGCCTACCA
AGGCGACGATCCTTAGCTGGTCTGAGAGGATGATCAGCCACACTGGGACTGAGACACGGCC
>Zotu9694
GAACGCTGGTAACGTGGATACCGGCATGCAAGTCAAGGGGGCCCGCAAGGGCAACCGGCGAACGGCTTA
GTAACACGTAGATACATACCTTAAAGTGGAGCATAGCTCGTCGAAAGACGGGGTAATTTTCCATAGTCCCT
CACGGGTAAAGACGCAAGTCGCTTTAAGAATGGTCTGCGGTCTATCAGCTAGTTGGTGAGGTAAAGGCTC
ACCAAGGCTATGACGGATAGGGGGCGTGAGAGCGCGACCCCCAAGAATGGCACTGCGATACGGGC
>Zotu9698
GAACGCTGGCGGCGTGCTTAACACATGCAAGTCGAACGATGATGCTCCAGCTTGCTGGGGTGGATTAGTG
GCGAACGGGTGAGTAACACGTGGGTGATCTGCCCTGCACTTCGGGATAAGCCTGGGAAACTGGGTCTAAT
ACCGGATATGACCCTCCTTTAGTGTGGGGGGTGGAAAGCTTTAGCGGTGTGGGATGAGCCTGCGGCCTAT
CAGCTTGTTGGTGGGGTAATGGCCTACCAAGGCGTCGACGGGTAGCCGGCCTGAGAGGGTGATCGGCCAC
ATTGGGACTGAGACACGGCC
>Zotu9704
GAACGCTGGCGGCGTGCTTAACACATGCAAGTGGAGCGACGAACCGGGGCTTGCCCCGGGGCAGAGCCG
CGAACGGGTGAGTAACACGTGGGTAACCCACCCCGATGATTGGGACAACCCGGGGAAACTCGGGCTAATA
CCAAATGTGCTCGTACACCATGAGGTGTCTGAGTAAAGATAGCTTCGGCCTTCGCATCGGGACGGGCCCGC
GGCCCATTAGCTTGTTGGTGAGGTAACGGCTCACCAAGGCGACGATGGGTAGCTGGTCTGAGAGGACGAT
CAGCCACACTGGGACTGAGACACGGCC
>Zotu9707
GAACGCTGGCGGCGTGCTTAACACATGCAAGTCGAACGGAAAGGCCCCTGCTTGCAGGGGTACTCGAGTG
GCGAACGGGTGAGTAACACGTGGGTAATCTACCCCGCACTTCGGGATAAGCCCGGGAAACTGGGTCTAAT
ACCGGATGATCACGCCCCTTTTGTGTGGGGTGTGGAAAGATTTATCGGTGTGGGATGAGCTTGCGGCCTAT
CAGCTTGTTGGTGGGGTAATGGCCTACCAAGGCGTCGACGGGTAGCCGGCCTGAGAGGGTGTACGGCCAC
ATTGGGACTGAGATACGGCC
>Zotu9709
GAACGCTGGCGGCGTGCTTAACACATGCAAGTCGAACGGTAAGGCCCTAGCTTGCTGGGGTACACGAGTG
GCGAACGGGTGAGTAACACGTGGGTGACCTGCCCTGCACTTCGGGATAAGCCTGGGAAACTGGGTCTAAT
ACCGGATAGGACCGGCCTTTAGTGTGGTTGGTGGAAAGTTTTGTCGGTACGAGATGAGCCCGCGGCCTAT
CAGCTTGTTGGTGGGGTAATGGCCTACCAAGGCGACGACGGGTAGCCGGCCTGAGAGGGTGTACGGCCA
CATTGGGACTGAGACACGGCC
>Zotu9711
GAACGCTGGCGGCGTGCTTAACACATGCAAGTCGAACGGTGAAGCCCAGCTTGCTGGGTGGATCAGTGGC
GAACGGGTGAATAACACGTGAGCAATCTGCCCCTGACTCTGGGATAAGCGCTGGAAACGGCGTCTAATAC
TGGATACGAGCTTCCACCGCATGGTGAGTTGTTGGAAAGATTTTTCGGTCAGGGATGAGCTCGCGGCCTAT
CAGCTTGTTGGTGAGGTAATGGCTCACCAAGGCGTCGACGGGTAGCCGGCCTGAGAGGGTGACCGGCCAC
ACTGGGACTGAGACACGGCC

>Zotu9713
GAACGCTGGCGGCATGCCTTACACATGCAAGTCGAACGGTAACGCGGGGCAACCTGGCGACGAGTGGCG
AACGGGTGAGTAATGCATCGGAACGTGCCCAGAAGTGGGGGATAGCCCGGCGAAAGCCGGATTAATACC
GCATGAGACCTGAGGGTGAAAGCGGGGGATCGCAAGACCTCGCGCTTTTGGAGCGGCCGATGTCAGATTA
GCTTGTTGGTGGGGTAAAAGCCTACCAAGGCGACGATCTGTAGCTGGTCTGAGAGGACGACCAGCCACAC
TGGGACTGAGACACGGCC
>Zotu9717
GAACGCTGGCGGTGTGCTTAACACATGCAAGTCGTACGAGGTACATTTTCAAGTCGACTCTCGGGTCGACC
GAATGAACTGCAGTGGCGGACGGGTGCGTAACGCGTAAGAACTTACCTTTTGGAGGGGGATAACATTGGG
AAACCGCTGCTAATACCCCATAATGCTGAGGAGCTAAACGGTGAACAACCGCCAAGAGAGAGGCTTGCGT
CTGATTAGCTAGTTGGTGGGGGTAATGGCCCCCCAAGGCGACGATCAGTAGCTGGTCTGAGAGGATGATC
AGCCACACTGGGACTGAGACACGGCC
>Zotu9721
GAACGCTGGCGGCAGGCCTAACACATGCAAGTCGAGCGGCAGCGCGGGGGCAACCCTGGCGGCGAGCG
GCGGACGGGTGAGGAATGCGTCGGAATCTGCCTCTTTGTGGGGGATAACGTAGGGAAACTTACGCTAATA
CCGCATACGACCTACGGGTGAAAGTGGGGGACCGCAAGGCCTCACGCAGAGAGATGAGCCGACGCCGGA
TTAGCTAGTTGGCGGGGTAAAGGCCCACCAAGGCGACGATCCGTAGCTGGTCTGAGAGGATGATCAGCCA
CACTGGAACTGAGACACGGTC
>Zotu9723
GAACGCTGGCGGCATGCCTTACACATGCAAGTCGAGCGGCAGCGCGGGGGCAACCCTGGCGGCGAGCGG
CGGACGGGTGAGTAATACATCGGAACGTGTCCTTTCGTGGGGGATAACCAGTCGAAAGACTGGCTAATAC
CGCATGAGATCTCACGATGAAAGCGGGGGACCGCAAGGCCTCGCGCGATTGGAGCGGCCGATGTCGGAT
TAGCTAGTTGGCGGGGTAAAAGCCCACCAAGGCGACGATCCGTAGCTGGTTTGAGAGAACGACCAGCCAC
ACTGGGACTGAGACACGGCC
>Zotu9726
GAACGCTGGCGGCGTGCCTAATACATGCAAGTCGAGCGAACATTTGAGAGCTTGCTCTCAAATGTTAGCGG
CGGACGGGTGAGTAACACGTGGGCAACCTGCCTATAAGACTGGGATAACTTCGGGAAACCGGAGCTAATA
CCGGATAATTCACATTTACTCATGTAGATGTGCTGAAAGACGGTTTACGCTGTCACTTATAGATGGGCCCGC
GGCGCATTAGCTAGTTGGTGAGGTAACGGCCCACCAAGGCCACGATGCGTAGCCGACCTGAGAGGGTGAT
CGGCCACACTGGGACTGAGACACGGCC
>Zotu9729
GAACGCTGGCGGCGTGCTTAACACATGCAAGTCACGGGGGCCCGCAAGGGCAACCGGCGAACGGGTGCG
TGACACGTGAACAACCTGCCCTCGTGTGGGGGATAGCCGGGCTAACGCCCGGGTAATACCGCATACGGTC
CTTTGGGGGAGTCCCTGGGGGAAGAAAGCTCCGGCGCACGGGGAGGGGTTCGCGGCCTATCAGCTAGTT
GGCGAGGTAACGGCTCACCAAGGCGACGACGGGTAGCTGGTCTGAGAGGATGGCCAGCCACATTGGGAC
TGAGAAACGGCC
>Zotu9731
GAACGCTAGCGGCAGGCTTAACACATGCAAGTCGAGGGGTATAGTTTTTCGGAACTAGAGACCGGCGCAC
GGGTGCGTAACGCGTATGCAATCTACCTTTTGCAGAGGGATAGCCCAGAGAAATTTGGATTAATACCTCAT
AGTATGTGAGAATGGCATCATTTTCACATTAAAGTCACAACGGCAAAAGATGAGCATGCGTCCCATTAGCT
AGTTGGTAAGGTAACGGCTTACCAAGGCAACGATGGGTAGGGGTCCTGAGAGGGAGATCCCCCACACTG
GTACTGAGACACGGAC
>Zotu9735
GAACGCTGGCGGCAGGCCTAACACATGCAAGTCGAGCGGCAGCACGGGAGCTTGCTCCTGGTGGCGAGC
GGCGGACGGGTGAGTAATGCCTAGGAATCTGCCTGGTAGTGGGGGATAACAACCGGAAACGGTTGCTAA
TACCGCATACGTCCTACGGGAGAAAGTGGGGGATCTTCGGACCTCACGCTATCAGATGAGCCTAGGTCGG
ATTAGCTAGTTGGTGGGGTAAAGGCCCACCAAGGCGACGATCCGTAACTGGTCTGAGAGGATGATCAGTC
ACACTGGAACTGAGACACGGTC
>Zotu9740

GAATGCTGGCAGCGTGGATAAGTCATGCAAGTCAAGGGGGCCGCAAGGCCACCGGCAGACGAGGTAGTA
ATACGCAGGTACATTCCCCGAAGTCGGGCATAGCCAGCCGAAAGGTTGGGTAATTCCCGATACGTGGTGA
CATGAAAGGAGCAATCCGCTTCGGGAGCGGCCTGTGCAATATCAGCTAGTTGGTGAGGTAATGGCTCACC
AAGGCTACGACGTTTAGGGGGGCTGAGAGGCTGATCCCCACCGATCGGACTGAGATACGGCC
>Zotu9742
GAACGCTGGCGGCGTGCTTTACACATGCAAGTCGAACGGCAGCACGGACTTCGGTCTGGTGGCGAGTGGC
GAACGGGTGAGTAATACATCGGAACGTACCTTTTAGTTGGGGATAACGCATCGAAAGATGTGCTAATACC
GAATAATCTCTGAGGAGGAAAGGCGGGGATCGGAAACGACCTGTCGCTGAAAGAGCGGCCGATGGCCGA
TTAGCTAGTTGGCGGGGTAAAAGCCCACCAAGGCGATGATCGGTAGCTGTTCTGAGAGGAAGATCAGCCA
CACTGGGACTGAGACACGGCC
>Zotu9747
GAACGCTGGCGGCGTGCTTAACACATGCAAGTCGAACGTGATCCCGCAGCAATGTGGGTGAAAGTGGCAA
ACGGGTGAGTAACACGTAGGTAACCTGCCTTGAAGTGGGGGATAACCACGGGAAACTGTGGCTAATACCG
CATGGTCTTGTTGATACGGGAGTATTGACGAGTAAAGCTTTAGTGCTTCAAGAGGGGTCTGCGTCCGATTA
GCTAGTTGGTGGGGTAAAGGCCTACCAAGGCGATGATCGGTAGCTGGTCTGAGAGGACGATCAGCCACAC
GGGAACTGAGACACGGTC
>Zotu9754
GAACGCTGGCGGCGTGCTTAACACATGCAAGTCGAACGGTGATCTCGCAGCTTGCTGCGGGTGATCAGTG
GCGAACGGGTGAGTAACACGTGAGTAACCTGCCCTTGACTCTGGGATAACTCCGGGAAACCGGTGCTAAT
ACTGGATATGACTCTGGCCCGCATGGGTTGGGGTGGAAAGTTTTTCGGTCAAGGATGGACTCGCGGCCTAT
CAGCTTGTTGGTGGGGTAATGGCCTACCAAGGCGACGACGGGTAACCGGCCTGAGAGGGCGACCGGCCA
CACTGGGACTGAGACACGGCC
>Zotu9756
CGATGAAGAACGCAGCGGGGGGAGCTTGCTCCCTGACGGCGAGTGGCGGACGGGTGAGTAACACGTAGG
AATCTACCTTAATTATAATAGTTAGTGGGGGATAACCCGGGGAAACTCGGGCTAATACCGCATAATCTCTTC
AAAGCAAAGCGGGGGATCTTCGGACCTCGTGCTATGAGATGAGCCTGCGTCGGATTAGCTAGTTGGTAGG
GTAATGGCCTACCAAGGCGACGATCCGTAGCTGGTCTGAGAGGACGATCAGCCACACTGGGACTGAGACA
CGGCC
>Zotu9758
GAACGTTGGCGGCGTGCCTTAAGCATGCAAATCAAGGGGGTCTCGCAAGAGACTACCGGTGGACGGGTG
AGTAATGCGTGGGAATGTACCTCGAAGTGGGATATAGCTAGCCGAAAGGTTGGGTAATCTCCCATGTGAT
CGTAAGATTAAAGCCGCAAGGCGCTTTGAGATCAGCCCGCGTCCTATCAGCTAGTTGGTGGGGTAATGGCC
TACCAAGGCAACGACGGGTAGCTGGACTGAGAGGTCGGTCAGCCACAATGGGACTGCGACACGGCC
>Zotu9760
GAACGCTGGCGGCAGGCCTAACACATGCAAGTCGAGCGCCCAGCAATGGGAGCGGCAGACGGGATAGTA
ACACGTGGGAACGTACCCTTCAGTTCGGAACAACCCAGGGAAACTTGGGCTAATACCGAATACGTCCGTAA
GGAGAAAGATTTATCGCTGAAGGATCGGCCCGCGTCTGATTAGCTAGTTGGTGAGGTAACGGCTCACCAA
GGCGACGATCAGTAGCTGGTCTGAGAGGATGATCAGCATCACTGGGACTGAGACACGGCC
>Zotu9763
GAACGCTGGCGGCATGCTTTACACATGCAAGTCGAACGGTAACAGGGAGCTTGCTCCGCTGACGAGTGGC
GAACGGGTGAGTAATGCATCGGAACGTACCCAGTCGTGGGGGATAACGTAGCGAAAGTTACGCTAATACC
GCATACGTCCTGAGGGAGAAAGCGGGGGACCGTAAGGCCTCGCGCGATTGGAGCGGCCGATGTCGGATT
AGCTAGTTGGTGGGGTAAAGGCCTACCAAGGCGACGATCCGTAGCGGGTCTGAGAGGATGATCCGCCACA
CTGGGACTGAGACACGGCC
>Zotu9766
GAACGCTGGCGGCGGGGCTTAGGCATGCAAGTCGCGGGAGAAAGCCGCAAGGTGAGTAGACCGGCGTA
AGGGTGAGTAATAGGTAGTTAACGTACCCTGGTGACGCGGATAATACTTCGAAAGGAGTTGTAATACGCG
ATGAGGTCACAGTTCCTGCGGGAACAGTGGTCAAAGGGCGGGGATCCTTCGGGACCTGCCGCACTGGGA
GCGGACGACCTCCGATTAGCTAGTTGGTGAGGTAACGGCTCACCAAGGCGATGATCGGTAGCCGGGCTGA
GAGGCTGTCCGGCCACACGGGCACTGAGACACTGGC

>Zotu9768
GAACGCTGGCGGCATGCCTTACACATGCAAGTCGAACGGTAACAGGTCTTCGGATGCTGACGAGTGGCGA
ACGGGTGAGTAATACATCGGAACGTGCCCAATCGTGGGGGATAGCTCGGCGAAAGCCGGATTAATACCGC
ATACGATCTACGGATGAAAGCAGGGGACTCGCAAGGGCCTTGCGCGAATGGAGCGGCCGATGGCAGATT
AGGTAGTTGGTGGGGTAAAGGCTCACCAAGCCGACGATCTGTAGCTGGTCTGAGAGGACGACCAGCCACA
CTGGGACTGAGACACGGCC
>Zotu9774
GAACGCTGGCGGCGTGCTTAACACATGCAAGTCGAGCGGAAAGGCCCTTTCGGGGGTACTCGAGCGGCG
AACGGGTGAGTAACACGTGAGTAATCTGCCCTTGGCTTCGGGATAACCACCGGAAACGGTGGCTAATACC
GGATACGACGACTCCAGGCATCTGGTGGTTGTGGAAAGTTTTTCGGCCAGGGATGTGCTCGCGGCCTATC
AGCTTGTTGGTGGGGTAATGGCCTACCAAGGCTTCGACGGGTAGCCGGCCTGAGAGGGTGACCGGCCACA
CTGGGACTGAGACACGGCC
>Zotu9777
GAACGCTAGCGGCAGGCCTAATACATGCAAGTCGAACGATAGTGAGAAGCTTGCTTCTCACGAAAGTGGC
GCACGGGTGCGTAACGCGTATGCAACCTACCTTAATCAGGGGGATAGCCCGAAGAAATTTGGATTAACAC
CGCATAAAATCACAGTATAGCATTATACAATGATCAAATATTTATAGGATTAAGATGGCATGCGTGACATT
AGTTAGTTGGCGGGGTAACGGCCCACCAAGACCACGATGTCTAGGGGATCTGAGAGGATGACCCCCCACA
CTGGTACTGAGACACGGAC
>Zotu9779
GAACGCTGGCGGCAGGCTTAACACATGCAAGTCGAACGGACTCTTCGGAGTTAGTGGCGGACGGGTGAGT
AACACGTGGGAACGTGCCCTTAGGTTCGGAACAACACAGGGAAACTTGTGCTAATACCGGATAAGCCCTTT
TGGGGAAAGATTTATCGCCTTTGGAGCGGCCCGCGTCTGATTAGCTAGTTGGTGAGGTAAAGGCTCACCAA
GGCGACGATCAGTAGCTGGTCTGAGAGGATGGCCAGCCACATTGGGACTGAGACACGGCC
>Zotu9781
GAACGCTGGCGGCGTGCCTAACACATGCAAGTCGAACGGGGTTAAGAGAAATTTTCGGATGGACCTTAAC
TTAGTGGCGGACGGGTGAGTAACGCGTGGATAACCTGCCTCACACAGGGGGATAGCAGCTGGAAACGGC
TGGTAATACCGCATAAGACCACGGCCCCGCATGGAGCTGTAGTAAAAGATTTATCGGTGTGAGATGGATCC
GCGTCTGATTAGCCAGTTGGCGGGGTAACGGCCCACCAAAGCGACGATCAGTAGCCGGCCTGAGAGGGT
GAACGGCCACATTGGGACTGAGACACGGCC
>Zotu9788
GAACGCTGGCGGCGTGCTTAACACATGCAAGTCGAACGGAAAGGCCCTTTCGGGGGTACTCGAGTGGCGA
ACGGGTGAGTAACACGTGGGTGATCTGCCCTGCACTTTGGGATAAGCCTGGGAAACTGGGTCTAATACCG
AATAGGACTCCGGGATGCATGTTTCGGGGTGGAAAGCTTTTGCGGTGTGGGATGGGCCCGCGGCCTATCA
GCTTGTTGGTGGGGTGATGGCCTACCAAGGCGACGACGGGTAGCCGGCCTGAGAGGGTGTCCGGCCACA
CTGGGACTGAGATACGGCC
>Zotu9795
GAACGCTGGCGGCGTGCTTAACACATGCAAGTCGAGCGAGGCTCCCTCCTTCGGGAGGGATGTCCTAGCG
GCGAACGGGTGAGTAACACGTGGGCAACCTGCCCCTAGCACTGGGATAACCCCGGGAAACCGGGGCTAAT
ACCGGATACGACCTCAACGGGCATCCGTCGAGGTGGAAAAGGTTTACTGGCTAGGGATGGGCCCGCGGCC
TATCAGCTTGTTGGTGGGGTAACGGCCTACCAAGGCGACGACGGGTAGCCGGCCTGAGAGGGCGACCGG
CCACACTGGGACTGAGACACGGCC
>Zotu9797
GAACGCTGGCGGCATGCCTAAGACATGCAAGTCGAACGAGACGACCTTTGAAAGACTGAGTGCTTGCACA
AAGTTGGAAAAAGTAAATCGTCTAGTGGCAGACGGGTGAGTAACACGTGGGTAACCTACCTTCAAGCTGG
GGATAACGTTTGGAAACGAACGCTAATACCGAATAAGATATAGCAATATATTGAAAGAAGCCTCTAAAGCT
TCACTTAAAGATGGGCCTGCGGCGTATTAACTAGTTGGTGAGGTAATGGCTCACCAAGGTAACGATGCGTA
GCCGAACTGAGAGGTTGAACGGCCACACTGGGACTGAGACACGGCC
>Zotu9803
GAACGCTGGCGGCGTGCTTAACACATGCAAGTCGAACGGTGACCTTGAGAGCTTGCTCTTGAGTGATCAGT
GGCGAACGGGTGAGTAACACGTGAGTAACCTGCCCCAGACTCTGGAATAACAATTGGAAACAGTTGCTAA

TACCGGATACGACACGAACGGGCATCCGTATCGTGTGGAAAGTTTTTCGGTCTGGGATGGACTCGCGGCCT
ATCAGCTTGTTGGTGAGGTAATGGCTCACCAAGGCGACGACGGGTAGCCGGCCTGAGAGGGCGACCGGC
CACACTGGGACTGAGACACGGCC
>Zotu9805
GAACGCTGGCGGCGTGCTTAACACATGCAAGTCGAGCGGTAAGGCCCCTTCGGGGGTACACGAGCGGCG
AACGGGTGAGTAACACGTGAGCAATCTGCCCATCACATCGGGATAACTCCGGGAAACCGAAGCTAATACC
GAATACGACCACTTCAGGCATCTGACGGTGGTGGAAAGTTCCGGCGGTGATGGATGAGCTCGCGGCCTAT
CAGCTTGTTGGTGGGGTAATGGCCCACCAAGGCTTCGACGGGTAGCCGGCCTGAGAGGGTGACCGGCCAC
ACTGGGACTGAGACACGGCC
>Zotu9813
GAACGCTAGCGGCAGGCTTAATACATGCAAGTCGAGCGGTAACAGGTGTAGCAATACATGCTGACGAGCG
GCAGACGGGTGAGGAACACGTACGCAACTTTCCTCAAACCGGGGCACAACCTTGAGAAATCAGGGCTAAT
TCCCCATAACATTGCGAGAGGGCATCTGATTGCAATTAAAGATTCATCGGTTTGAGATGGGCGTGCGTTCG
ATTAGCTAGTTGGTAGGGTAACGGCTTACCAAGGCAACGATCGATAGCTGGTGTGAGAGCACGACCAGCC
ACACGGGCACTGAGATACGGGC
>Zotu9818
GAACGCTGGCGGCAGGCTTAACACATGCAAGTCGAACGATCCTTCGGGATAGTGGCGGACGGGTGAGTA
ACACGTGGGAACGTGCCTTTAGGTTCGGAACAACACAGGGAAACTTGTGCTAATACCGGATAAGCCCTTCG
GGGGAAAGATTTATCGCCTTTAGAGCGGCCCGCGTCTGATTAGCTTGTTGGTGAGGTAAAGGCTCACCAAG
GCTACGATCAGTAGCTGGTCTGAGAGGATGGCCAGCCACATTGGGACTGAGACACGGCC
>Zotu9820
GAACGCTGGCGGCGTGCTTAACACATGCAAGTGGTGCGACGAACCAGGGCTTGCCCTGGGGCAAAGCCGC
GAACGGGTGAGTAACACGTGAGTAACCTGCCCCGATGACCGGGACAACCCGAGGAAACTCGGGCTAATAC
CGGATGTGGCGCACAGGCGTAAGCCTGTTCGCTAAAGGAAGCTTCGGCCTCCGCATTGGGAGGGGCTCGC
GGCCCATTAGCTTGTTGGTGGGGTAACGGCCTACCAAGGCAACGATGGGTAGCTGGTCTGAGAGGACGAT
CAGCCACACTGGGACTGAGACACGGCC
>Zotu9823
GAACGCTGGCGGCGTGCTTAACACATGCAAGTCGAACGATGAAGGAGGAGCTTGCTCTTCTGGATTAGTG
GCGAACGGGTGAGTAACACGTGAGCAATCTGCCCCTGACTCTGGGATAAGCGTTGGAAACGACGTCTAAT
ACCGGATACGAGCTGAGAAGGCATCTTCAGCAGCTGGAAAGAACTTCGGTCAGGGATGAGCTCGCGGCCT
ATCAGGTAGTTGGTGAGGTAATGGCTCACCAAGCCTACGACGGGTAGCCGGCCTGAGAGGGTGACCGGCC
ACACTGGGACTGAGACACGGCC
>Zotu9827
GAACGCTAGCGGCAGGCTTAACACATGCAAGTCGAGGGGTAGTCTAGCAATAGATGAGACCGGCGCACG
GGTGCGTAACGCGTATGCAATCTACCTTTTGCAGAGGGATAGCCCAGAGAAATTTGGATTAATACCTCATA
GTATATCGACTTCGCATGAAGCTGATATTAAAGTAACAACGGCAAAAGATGAGCATGCGTATCATTAGCTA
GATGGTAAGGTAACGGCTTACCATGGCCACGATGATTAGGGGTCCTGAGAGGGAGATCCCCCACACTGGT
ACTGAGACACGGAC
>Zotu9833
GAACGCTGGCGGCGTGCCTAACACATGCAAGTCGTGCGGTAAGGCCCTTCGGGGTACACGAGCGGCGGA
CGGCTGAGTAACACGTGGGAACGTGCCCCAAAGTGAGGGATAAGCACCGGAAACGGTGTCTAATACCGCA
TATGGTCTTCGGATTAAAGCTTTCGAGCGCTTTGGGAGCGGCCTGCGTACGATTAGATAGTTGGTGAGGTA
AAGGCTCACCAAGTCGACGATCGTTAACTGGTCTGAGAGGATGATCAGTCAGACTGGAACTGAGACACGG
TC
>Zotu9836
TAACGCTGGCGGCGTGCCTAACACATGCAAGTCGAGCGAAGAGTGGCAGAGTATCGCGCTCTTAGCGGCA
GACGGGTGAGTAATACTTAAACAATCTGCCCTCGAATGGGAGACAACCAAGCGAAAGTTTGGCTAATACCC
CATACAATCCTCTTGAGGCATCTCAAGGGGATGAAAGGCGTAAGCCGTTCGAGGAGGAGTTTAAGCCCTAT
CAGCTAGTTGGCGGTGTAATAGACCACCAAGGCTATGACGGGTAGCCGATCTAAAAGGATGACCGGCCAC
AAGGGCACTGAGACACGGGC

>Zotu9838
GAACGCTGGCGGCGTGCCTAATACATGCAAGTCGAGCGCAGGAAACTGACGGAACTCTTCGGAGGGAAG
GCAGTGGAATGAGCGGCGGACGGGTGAGTAACACGTAAGGAACCTGCCTCAAGGATTGGGATAACTCCG
AGAAATCGGAGCTAATACCGGATAGTTCAACGGACCGCATGGTCCGCTGATGAAAGGCGCTTCGGCGTCA
CCTTGAGATGGCCTTGCGGTGCATTAGCTAGTTGGTGAGGTAACGGCCCACCAAGGCGACGATGCATAGC
CGACCTGAGAGGGTGATCGGCCACACTGGGACTGAGACACGGCC
>Zotu9845
GAACGCTGGCGGCGTGCTTAATACATGCAAGTCGAGCGAGGTTACTGGAGTGGAGCCCTTCGGGGTGAAG
CTGCTTTAACCTAGCGGCGGACGGGTGAGTAACGCGTGGGTAACCTACCCCAAAGCCCGGGATAACAGCG
GGAAACCGCTGCTAATACCGAATGACTTTACCTTGGGGCATCTTGAGGTAAAGAAAGGTTAACGCTTTGGG
ATGGGCCCGCGTCCCATTAGCTAGTTGGTGAGGTAACGGCTCACCAAGGCGACGATGGGTAGCCGGCCTG
AGAGGGTGGACGGCCACACTGGGACTGAGACACGGCC
>Zotu9849
GAACGCTGGCGGCGTGCTTAACACATGCAAGTCGAGCGGTAAGGCCTCTTCGGGGGTACACGAGCGGCG
AACGGGTGAGTAACACGTGAGTGATCTGCCCTTCACTCTGGGATAAGCCCGGGAAACTGGGTCTAATACCA
GATGTTCACGCGTCCGTGCATGCGGGTGTGTGGAAAGCCTTTTTGGCGGTGGGGGATGAGCTCGCGGCCT
ATCAGCTTGTTGGTGGGGTGATGGCCTACCAAGGCTTTGACGGGTAGCCGGCCTGAGAGGGTGACCGGCC
ACACTGGGACTGAGACACGGCC
>Zotu9851
GAACGCTGGCGGCATGCCTAAGACATGCAAGTCGAACGAGATGGCCCAGTGAAGGTTGAGTGCTTGCACG
AGACTGAATCTGGATTCCCATCTAGTGGCAGACGGGTGAGTAACACGTGGGTGACCTACCTTTTCGTTGGG
GATAACAGTTGGAAACGATTGCTAATACCGAATAAAATGTAAGAGTCAATTTTACATTGAAAGGAGCCTTT
AAAGCTTTGCGATTAGATGGGCCTGCGGCGTATTAACTAGTTGGTGAGGTAATGGCTTACCAAGGTGACG
ATGCGTAGCCGAACTGAGAGGTTGATCGGCCACATTGGGACTGAGACACGGCC
>Zotu9853
GAACGCTGGCGGCGTGCTTAACACATGCAAGTGGAGCGACGAACCAGGGCTTGCCCTGGGGCAGAGCCG
CGAACGGGTGAGTAACACGTGGGTAACCTACCCCAATGACCGGGACAACCCGAGGAAACTCGGGCTAATA
CCGGATGTTCTGCACTCTCGTAAGGGAGTTCAGCAAAGATAGCCTCGGCTTTCGCATTGGGACGGGCCCGC
GGCCCATTAGCTTGTTGGTGAGGTAACGGCTCACCAAGGCTTCGATGGGTAGCTGGTCTGAGAGGACGAT
CAGCCACACTGGGACTGAGACACGGCC
>Zotu9855
GAACGCTGGCGGCGTGCCTAACACATGCAAGTGGAGCGACGAACCGGGGCTTGCCCCGGGGCAGAGCCG
CGAACGGGTGAGTAACACGTGGGTTACCTGCCCCGATGACCGGGACAACCCGTCGAAAGACGGGCTAATA
CCGGATGTGCCCCTTGGGGGAAAGGAAGCTTCGGCCTCCGCATCGGGATGGGCCCGCGGCGCATTAGCTT
GTTGGTGGGGTAATGGCCTACCAAGGCGACGATGCGTAGCTGGTCTGAGAGGACGATCAGCCACACTGG
GACTGAGACACGGCC
>Zotu9857
GAACGCTGGCGGCGTGCTTAAGACATGCAAGTCGAACGGCCTTCTTCGGAAGGCAGTGGCGCACGGGTGA
GTAACGCGTAACTGACCTACCCTCAAGTCTGGAATAACGGGTCGAAAGATCCGCTAATACTGGATGTGCAG
TGTCGCTGTGGCGACACTGTAAAGATTGATCGCTTGAGGATGGGGTTGCGTTTCATCAGCTGGTTGGTGGG
GTAAAGGCCTACCAAGGCGACGACGAATAGCTGGCCTGAGAGGGTGGCCGGCCACAGGGGCACTGAGAC
ACGGGT
>Zotu9859
GAACGCTGGCGGCATGCCTAACACATGCAAGTCGAACGAAGCCTTCGGGCTTAGTGGCGCACGGGTGCGT
AACGCGTGGGAATCTGCCCTTGGGTACGGAACAACTCCGAGAAATTGGAGCTAATACCGTATGAAGTCTTC
GGACCAAAGATTTATCGCCCAGGGATGAGCCCGCGTTGGATTAGGTAGTTGGTGGGGTAAAGGCCTACCA
AGCCGACGATCCATAGCTGGTCTGAGAGGATGATCAGCCACACTGGGACTGAGACACGGCC
>Zotu9861
CGTTCGTCGCCCCCGTTAGCACGCTTTTACCCGCGGTTAGAACATACGATGCGTGGGGGGATGTTATCGAT
TTGGACTTTATACGCAACATGACAGCGACG

>Zotu9864
GAACGCTGGCGGCGTGCTTAACACATGCAAGTCGAACGGTGAAGGAGAGCTTGCTCTTTGGATCAGTGGC
GAACGGGTGAGTAACACGTGAGTAACCTGCCCCAGACTCTGGGATAAGCACTGGAAACGGTGTCTAATAC
CGGATACGAGCCTCCACCGCATGGTGAGGGGTTGGAAAGAATTTCGGTCTGGGATGGACTCGCGGCCTAT
CAGCTAGTTGGTGAGGTAACGGCCCACCAAGGCGACGACGGGTAGCCGGCCTGAGAGGGTGACCGGCCA
CACTGGGACTGAGACACGGCC
>Zotu9867
GAACGCTGGCGGCGTGCCTAAGACATGCAAGTCGAGTGGGTTTAGACCCACGGCGAACGGGTGAGTAAT
ACCTAGGAACTTTCTTTAAGTGGAGCATAATCAGTCGAAAGATTGGATAATTCTCCATGTGGCAGTAATGCT
AAAGTCGCAAGACGCTTAAAGTGAGGCCTGGGCACCATCAGCTAGTTGGTAAGGTAATGGCTTACCAAGG
CTATGACGGTTAGGGGTCCCGAGAGGGATAACCCCCACACTGGAACTGCGACACGGTC
>Zotu9869
GAACGCTAGCGGCATGCCTAACACATGCAAGTCGAACGGCAGCATAGGAGCTTGCTCCTGATGGCGAGTG
GCGAACGGGTGAGTAATATATCGGAACGTGCCCTAGAGTGGGGGATAACTAGTCGAAAGACTAGCTAATA
CCGCATACGATCTACGGATGAAAGTGGGGGATCGCAAGACCTCATGCTCCTGGAGCGGCCGATATCTGATT
AGCTAGTTGGTGGGGTAATGGCCTACCAAGGCGACGATCCGTAGCTGGTCTGAGAGGACGATCAGCCACA
CTGGGACTGAGACACGGCC
>Zotu9876
GAACGCTGGCGGCGTGCTTAATGCATGCAAGTCGCACGGGTATCCTTCGGGGTACCAGTGGCGCACGGCT
GAGTAACACGTGGGGATGTGCCACACCGTGGGGGACAACAGCGGGAAACCGCTGCTAATCCCGCATACGC
TCCGTTCGCGGAGGAAAGCAGGTAACTGCGCGGTGCGAGCACCCTGCGGCCCATCAGGTCGTTGGTCGGG
TAATGGCCTACCAAGCCGGTGACGGGTAGCTGGTCTGTGAGGACGACCAGCCAGACTGGGACTGAGAAAC
GGCC
>Zotu9879
GAACGCTGGCGGCGTGCTTAACACATGCAAGTCGAACGGAAAGGCCCTTCGGGGTGCTCGAGTGGCGAAC
GGGTGAGTAACACGTGGGTGATCTGCCCTGCACTTTGGGATAAGCCTGGGAAACTGGGTCTAATACCGAA
TACACCCTGCTGGTCGCATGGCCTGGTGGGGGAAAGCTTTTGCGGTGTGGGATGGGCCCGCGGCCTATCA
GCTTGTTGGTGAGGTTATGGCTTACCAAGGCGACGACGGGTAGCCGGCCTGAGAGGGTGTCCGGCCACAC
TGGGACTGAGATACGGCC
>Zotu9887
GAACGCTGGCGGTATGCTTAACACATGCAAGTCGAACGGAGTACTTCGGTACTTAGTGGCGGACGGGTGA
GTAACGCGTGAGAAACTGCCCCTTGGACTGGGACAACAGCTGGAAACGGCTGCTAACACCGGATGTGCCG
AAAGGTAAAATATTTATAGCCAGGGGATGTGCTCGCGTCTGATTAGCTAGTAGGTGAGGTAATGGCTTACC
TAGGCGACGATCAGTAGCTGGTCTGAGAGGATGATCAGCCACACTGGGACTGAGACACGGCC
>Zotu9889
ACCGTGTCTCAGTTCCAGTGTGGCTGATCATCCTCTCAGACCAACTAAAGATCGTCGCCTTGGTGAGCCATT
ACCCCACCAACTAGCTAATCCTGCATGGGCTCGTCTTGCAGCGATAAATCTTTCACCTCTCGGCTATATGCG
ACATTAGCAATTGCTTCCAACTGTTATTTCGCACTGCAAGGTAGATTCCCATGTATTACTCACCCGTTTGCCA
CTAGCAGAATTCTGTATTGCTACAGCTCTTGCCCGTTCGACTTGCATGTGTTAAGCACACCGCCAGCGTTCA
>Zotu9892
CAACGCTGGCGGCGTGCCTAACACATGCAAGTCGAACGCGAAAGTCCCGCAAGGGATTAGTAGAGTGGCA
AACGGGTGAGTAACACGTGGGTGACCTGCCTTCGAGCGGGGGATAACGTCCCGAAAGGGACGCTAATACC
GCATAACATCCTGCCTTTTAAGAGGTGGAGATCAAAGCCGGGGATCGCAAGACCTGGCACTTGAAGAGGG
GCCCGCGCCTGATTAGCTAGTTGGTGAGGTAATGGCTCACCAAGGCAACGATCAGTATCCGGCCTGAGAG
GGCGGACGGACACACTGGGACTGAGACACGGCC
>Zotu9895
GAACGCTGGCGGCGTGCTTAACACATGCAAGTCGAGCGAAGCACTTTTGCGGATTTCTTCGGATTGAAGCA
ATTATGACTGAGCGGCGGACGGGTGAGTAACGCGTGGGTAACCTGCCTCATACAGGGGGATAACAGTTGG
AAACGGCTGCTAATACCGCATAAGCGCACAGTACCGCATGGTACCGTGTGAAAAACTCCGGTGGTATGAG

ATGGACCCGCGTCTGATTAGCTAGTTGGTGGGGTAACGGCCTACCAAGGCGACGATCAGTAGCCGACCTG
AGAGGGTGACCGGCCACATTGGGACTGAGACACGGCC
>Zotu9897
GAACGCTGGCGGCGTGCTTAACACATGCAAGTCGAGCGGTAAGGCCCTTCGGGGTACACGAGCGGCGAA
CGGGTGAGTAACACGTGGGTGACCTGCCCTCAGCTCTGGGATAAGCCTGGGAAACCGGGTCTAATACCGG
ATACGACCAATCACCGCATGGTGGTTGGTGGAAAGTTTTTTCGGCTGGGGATGGGCCCGCGGCCTATCAGC
TTGTTGGTGGGGTGATGGCCTACCAAGGCGGTGACGGGTAGCCGGCCTGAGAGGGCGACCGGCCACACT
GGGACTGAGACACGGCC
>Zotu9904
GAACGCTAGCGGCAGGCTTAATACATGCAAGTCGAGGGGCAGCACAGGGTAGCAATACCTGGGTGGCGA
CCGGCAAACGGGTGCGGAACACGTACAGAACCTTCCTTCGAGCGGGGAATAGCCCAGAGAAATTTGGATT
AATACCCCATAGTATAATGGTGTGGCATCACATTATTATTAAAGATTTATCACTTGAAGATGGCTGTGCGTA
TGATTAGGTAGTTGGCGGGGTAACGGCCCACCAAGCCTACGATCATTAACTGGTGTGAGAGCACGACCAG
TCACACGGGCACTGAGACACGGGC
>Zotu9914
GAACGCTGGCGGCGTGCCTAATACATGCAAGTCGAGCGGACTTGACAGAGCTTGCTCTGTTCAAGTTAGCG
GCGGACGGGTGAGTAACACGTAGGCAACCTGCCCTGCAGATTGGGATAACTCCGGGAAACCGGGGCTAAT
ACCGAATAATATTGAAAACCTCATGGTTTTCTTTTAAAAGGCGGCTTCGGCTGTCACTGCAGGATGGGCCC
GCGGCGCATTAGCTAGTTGGTGAGGTAACGGCTCACCAAGGCGACGATGCGTAGCCGACCTGAGAGGGT
GATCGGCCACACTGGGACTGAGACACGGCC
>Zotu9917
GAACGCTGGCGGCGTGCTTAACACATGCAAGTCGAACGATGAAGCCCAGCTTGCTGGGTGGATTAGTGGC
GAACGGGTGAGTAACACGTGAGCAACCTGCCCCTGACTCTGGGATAAGCGCTGGAAACGGCGTCTAATAC
TGGATACGAGTAGCGATCGCATGGTCAGTTACTGGAAAGATTTTTTGGTTGGGGATGGGCTCGCGGCCTAT
CAGCTTGTTGGTGAGGTAATGGCTCACCAAGGCGTCGACGGGTAGCCGGCCTGAGAGGGTGACCGGCCAC
ACTGGGACTGAGACACGGCC
>Zotu9921
GAACGCTGGCGGTGTGCTTAACACATGCAAGTCGAGCGAGGCGTCCGCCTCCGGGCGGTCAGCCGAGCG
GCGAACGGGTGAGTAACACGTGAGCAATCTTCCCTCGGCTCGGGGATAACCTCGGGAAACTGAGGCTAAT
ACCGGATGCACGTTTTTCGGGGCATCCCGGAAAACGAAAAGGTTTCGCACCGCGAGGTGCGTTTCCGGCC
GGGGATGAGCTCGCGGCCTATCAGTTTGTTGGTGTGGTAAAAGCGCACCAAGGCGACGACGGGTAGCCG
GCCTGAGAGGGTGATCGGCCACACTGGGACTGAGACACGGCC
>Zotu9924
GAACGCTGGCGGCAGGCTTAACACATGCAAGTCGAACGCATCCTTCGGGATGAGTGGCAGACGGGTGAGT
AACACGTGGGAACGTACCCTTCGGTTCGGAACAACTCTGGGAAACTGGAGCTAATACCGGATACGCCCTTT
TGGGGAAAGGTTTACTGCCGAAGGATCGGCCCGCGTCTGATTAGCTAGTTGGTGGGGTAACGGCCTACCA
AGGCGACGATCAGTAGCTGGTCTGAGAGGATGATCAGCCACATTGGGACTGAGACACGGCC
>Zotu9932
GAACGCTGGAAATGTGGATACCGGCATGCAAGTCGAGCGCCCCGCAAGGGGAGCGGCGAACGGCTTAGT
AACACGTGGGTACATACCCAAAACTGGAGGACAGCTCGTCGAAAGACGGGGTAATACTCCATAGTCCCGC
AAGGGTAAAGATGCAAATCGGTTTTGGAATGGCCTGCGGCCTATCAGCTTGTTGGTGAGGTAAAAGCTTAC
CAAGGCTATGACGGGTAGGGGGCGTGAGAGCGCGACCCCCAAGAATTGCACTGCGACACGGGC
>Zotu9940
GAACGCTGGCGGCAGGCCTCAAACATGCAAGTCTAGGGGGTAGCAATACCACCGGCGGACGGGTGCGTA
ACGCGTGGGAATGTACCTATTGGTTCGGAACAACTGAGGGAAACTTCAGCTAATACCGAATGTGCCCGTAA
GGGGAAAGATTTATCGCCAATGGATCAGCCCGCGTTCGATTAGCTAGTTGGTGAGGTAATGGCTCACCAA
GGCGACGATCGATAGCTGTTCTGAGAGGAAGATCAGCCACACTGGGACTGAGACACGGCC
>Zotu9943
GAACGCTGGCGGCATGCTTAACACATGCAAGTCGAACGATCTTAGCCCTTCGGGGCGAAAGAGAGTGGCA
GACGGGTGAGGAACGCGTGGGAACATACCTTGAAGTGGGGAACAACCATTGGAAACGATGGCTAATACC

GCATAATCCCGAGAGGGGAAAGAAGCAATTCGCTTTAAGAGTGGCCCGCGTCAGATTAGGTAGTTGGTGT
GGTAAAGGCGCACCAAGCCGATGATCTGTAGCTGATTTGAGAGGATGAACAGCCACACTGGGACTGAGAC
ACGGCC
>Zotu9945
GAACGCTGGCGGCGCGCTTAATACATGCAAGTCGAGCGAGAACCAGGGCTTCGGCCCTGGGGACAGCGG
CGAACGGGTGAGTAACACGTGGGTAATCAACCCCCGACACCGGGATAGCCCAGGGAAACTTGGATTAATA
CCGGATGCTCCGTCTGGCTTTCGGGCCGGCCAGAAAAGGGTAGCTTCGGCCTCCGGTCGGGGACGAGCCC
GCGGCGGATTAGCTTGTTGGTGAGGTAAAGGCTCACCAAGGCGACGATCCGTAGCTGGTCTGAGAGGACG
ATCAGCCACACTGGGACTGAGACACGGCC
>Zotu9948
GAACGCTGGCGGCGTGCTTAACACATGCAAGTCGAGCGGTAAGGCCTTTCGGGGTACACGAGCGGCGAAC
GGGTGAGTAACACGTGAATGATCTGCCCTTCACTTCGGGATAAGCTTCGGAAACGGGGTCTAATACCGGAT
ATGACACATTCATGCATGTGGGTGTGTGGAAAGTTTTTTCGGTGGGGGATGAGTTCGCGGCCTATCAGCTT
GTTGGTGGGGTAATGGCTCACCAAGGCTTCGACGGGTAGCCGGCCTGAGAGGGTGACCGGTCACACTGG
GACTGAGACACGGCC
>Zotu9954
GAACGCTGGCGGCACGTTTTAAGCATGCAAGTCGAACGGCAGTGGGGGGTAACTCCCATGAGAGTGGCG
AACGGGTGAGTAACACGTAGGTGATCTGTCTTGAGGATGGGGATAGCCTGTGGAAACACAGGGTAATACC
GAATACGCAGTCACGATTTTGGTTGTGATTGGAAAGGTGCTACGGCACCACCTTGAGAGGAGCCTGCGGC
TGATTAGCTAGTTGGTGGGGTAAAGGCCTACCAAGGCGACGATCAGTAGCCGGCCTGAGAGGGTGTACG
GCCACACTGGGACTGAGACACGGCC
>Zotu9956
GAACGCTAGCGGCAGGCTTAACACATGCAAGTCGAGGGGTATAGTTCTTCGGAACTAGAGACCGGCGCAC
GGGTGCGTAACGCGTATGGAATCTACCTTTTACAGAGGGATAGCCCAGAGAAATTTGGATTAATACCTCAT
AGTATATAGACTCGGCATCGAGATTATATTAAAGTCACAACGGTAAAAGATGAGCATGCGTCCCATTAGCT
AGATGGTAAGGTAACGGCTTACCATGGCAACGATGGGTAGGGGTCCTGAGAGGGAGATCCCCCACACTG
GTACTGAGACACGGAC
>Zotu9959
GCCGTGTCTCAGTCCCAGTGTGGCTGATCATCCTCTCAAACCAGCTATGGATCGTCGGCTTGGTAGGCCATT
ACCCCACCAACTACCTAATCCAACGCGGGCTAATCCTTCCCCGATAAATCTTTCCCCCTAAGGGCGTATACG
GTATTACTCTCAGTTTCCCGAGGCTATTCCGTAGAGAAGGGCATATTCCCACGCGTTACTCACCCGTCCGCC
GCTCCCCCGAAAGGGCGCTCGACTTGCATGTGTTAGGCCTGCCGCCAGCGTTCG
>Zotu9961
GAACGCTAGCGGCAGGCCTAATACATGCAAGTCGAGGGGCAGCGGGGTAGCAATACTGCCGGCGACCGG
CGCACGGGTGCGTAACACGTATGCAACCTACCTTAAACTGGGGGATAGCCCGGGGAAACTCGGATTAATA
CCCCATAACACAGTGATGTCTCATGACAATATTGTTAAAGATTTATCGGTTTAAGATGGGCATGCGTTCGAT
TAGCTAGTTGGCGGGGTAATGGCTCACCAAGGCTACGATCGATAGGGGGTCTGAGAGGATGATCCCCCAC
ACTGGTACTGAGATACGGAC
>Zotu9963
AAACGCTGGCGGTATGCTTAACACATGCAAGTCGAACGAGAAAGGGGCATTCGTGCCCTGAGTAAAGTGG
CGGACGGGTGAGTAACGCGTGAGCAAGCTGCCCTGGAGTAGAGGATAACACTTCGAAAGGAGTGCTAAT
ACTTTATGACATCAGCTCTGTGCATACAGAGGTGATTAAAGAAGGCCTCTTCATTGAAAGCTTTCGCTTGAG
GATGCGCTCGCGTACCATTAGCTAGTTGGTGAGGTAACGGCTCACCAAGGTGAGGATGGTTAGCCGGCCT
GAGAGGGTGTACGGCCACACTGGGATTGAGATACGGCC
>Zotu9967
GAACGCTGGCGGCATGCTTTACACATGCAAGTCGAACGGTAACAGGCCGCAAGGTGCTGACGAGTGGCGA
ACGGGTGAGTAATGCATCGGAACGTGCCCAGTCGTGGGGGATAACGCAGCGAAAGCTGCGCTAATACCGC
ATACGATCTGTGGATGAAAGCGGGGGACCGTAAGGCCTCGCGCGATTGGAGCGGCCGATGTCAGATTAG
GTAGTTGGTGGGGTAAAGGCTCACCAAGCCGACGATCTGTAGCTGGTCTGAGAGGACGACCAGCCACACT
GGGACTGAGACACGGCC

>Zotu9971
GAACGCTGGCGGCGTGCTTAACACATGCAAGTCGAACGGAAAGGCCCAAGCTTGCTTGGGTACTCGAGTG
GCGAACGGGTGAGTAACACGTGGGTGATCTGCCCTGCACTTCGGGATAAGCCTGGGAAACTGGGTCTAAT
ACTGGATAGGACCACTTCTTGGATGTTGTGGTGGAAAGTTTTTTCGGTGTGGGATGAGCTCGCGGCCTATC
AGCTTGTTGGTGGGGTAATGGCCTACCGAGGCGTCGACGGGTAGCCGGCCTGAGAGGGTGTACGGCCAC
ATTGGGACTGAGATACGGCC
>Zotu9975
TTGAGAGGCTGCCTTCGGCCCAGGGCCCCGGGATCGAGTCCCACATCAGGCTCCCGCATGGAGCCTGCTTC
TCCCTCTGCCTGTGACTGTGCCTCTCTCTCTCTCTCTCTCTGTCTCTGTCTCTGTCTCTCATGAATAAATAAAAT
CTTAAAAAAAAATTTTCCA
>Zotu9977
GAACGCTGGCGGCGTGCCTAATACATGCAAGTCGAGCGAATCTTTTAAAAGCTTGCTTTTGATAGATTAGC
GGCGGACGGGTGAGTAACACGTGGGCAACCTGCCCTATAGTTTGGGATAACTCCGGGAAACCGGGGCTAA
TACCGGATAATACAATTTAGCTCCTGTTAGATTGTTGAAAGATGGTTTCGGCTATCGCTATAGGATGGGCCC
GCGGCGCATTAGCTAGTTGGTAGGATAACGGCCTACCAAGGCGACGATGCGTAGCCGACCTGAGAGGGT
GATCGGCCACACTGGGACTGAGACACGGCC
>Zotu9981
GAACGCTAGCGGCATGCCTAACACATGCAAGTCGAACGGTGACGACGGGGCTTGCCCTGTCTGATCAGTG
GCGAACGGGTGAGTAACACGTGAGTAACCTGCCCTTGACTCTGGGATAACCGCGGGAAACGGCGGCTAAT
ACCGGATATGAGACGTACAGGCATCTGTTGCGTCTGGAAAGATTTATCGGTCAAGGATGGGCTCGCGGCC
TATCAGCTTGTTGGTGGGGTGATGGCCTACCAAGGCGACGACGGGTAGCCGGCCTGAGAGGGCGACCGG
CCACACTGGGACTGAGACACGGCC
>Zotu9984
GAACGCTGGCGGCAGGCCTAACACATGCAAGTCGAGCGAGACCTTCGGGTCTAGCGGCGGACGGGTGAG
TAACGCGTGGGAACGTGCCCTTCTCTACGGAATAGTCCCGGGAAACTGGGTTTAATACCGTATACGCCCTT
CGGGGGAAAGATTTATCGGAGAAGGATCGGCCCGCGTTGGATTAGGTAGTTGGTGGGGTAATGGCCTACC
AAGCCGACGATCCATAGCTGGTTTGAGAGGATGATCAGCCACACTGGGACTGAGACACGGCC
>Zotu9986
CGTCAGGTCCCAGTGCGCTTGGTTAATTACACCTTAGAAGACCTTTCCCTGCCGCAAAAAAGGGTAGTAGC
GCAATTAATCCCGGCGAACATTACAGAAAGCGGGCAAAGGGAGAGTATCGGACTAACAGGA
>Zotu9988
GAACGCTGGAAACGTGGATACCGGCATGCAAGTCGAGCGGGCCCGCAAGGGTCAGCGGCGAACGGCTTA
GTAACACGTGGGTACATACCCTTTTCTGGAGGACAGCTCGTCGAAAGACGGGGTAATACTCCATAGTCTCG
CAAGAGTAAAGACGTAAGTCGGAAAAGGAATGGCCTGCGGCCTATCAGATAGTTGGCGAGGTAATGGCTC
ACCAAGTCTATGACGGGTAGGGGGCGTGAGAGCGCGACCCCCAAGAATGGCACTGCGACACGGGC
>Zotu9990
GAACGCTGGCGGCGTGCCTAACACATGCAAGTCGCGCGAGAAAGTCCTTCGGGATGAGTAGAGCGGCGC
ACGGGTGAGTAATACATAGGAACGTACCTTTCGGTGGGGGATAACCACGGGAAACTGTGGCTAATACCGC
ATAAAGACGTGAGTCTAAAGCAGGCCTCTATTTATAAGCTTGTGCCGGTAGAGCGGCCTATGTCCCATTAG
CTAGTTGGTAGGGTAATGGCCTACCAAGGCGACGATCCGTAGCTGGTCTGAGAGGACGATCAGCCACACT
GGGACTGAGACACGGCC
>Zotu9993
GAACGCTGGCGGCGTGCTTAACACATGCAAGTGGAGCGACGAACCGGGGCTTGCCCCGGGGCAGAGCCG
CGAACGGGTGAGTAACACGTGGGTAACCCACCCCGATGATTGGGACAACCCGGGGAAACTCGGGCTAATA
CCAAATGTGCTCGAAGACCATGAGGTCTCCGAGTAAAGATAGCTTCGGCCTTCGCATCGGGACGGGCCCG
CGGCCCATTAGCTTGTTGGTGAGGTAACGGCTCACCAAGGCGACGATGGGTAGCTGGTCTGAGAGGACGA
TCAGCCACACTGGGACTGAGACACGGCC
>Zotu9997
GAATGCTGGCGGCGTGGATGAGGCATGCAAGTCGAATGAATTTATATTCATGGCAGACGAGGTAGGAACA
CGTAGGTACGTACCCTAAAGTCAGGGATAATCCGTCGAAAGGCGGGATAATACTTGATGGTCCCTTCGGG

GTAAAGATTTATCGCTTTAGGAACGGCCTGCGGGCTATCAGCTTGTTGGTAAGGTAACGGCTTACCAAGGC
TACGACGGCTAGGGGAGGTGAGAGCCTGACCCCCACCGATGGAACTGCGACACGGTC
>Zotu9999
GAACGCTAGCGGCAGGCTTAATACATGCAAGGCGAGGGGGCTGTTTAGGCAGTCACCGTCGTACGGGTGC
GTAACGCGTATGCAACCTACCTTCCACTGGGGGATAGCCCGGGGAAACCCGGATTAATACCGCATAATACA
GGGGTGTCGCATGGCACTATTTGTTAAAGATTTATTGGTGGAAGATGGGCATGCGTTCGATTAGCTAGTTG
GCGGGGTAACGGCCCACCAAGGCGACGATCGATAGGGGAGCTGAGAGGTTGATCCCTCACACGGGCACT
GAGATACGGGC
>Zotu10001
GAACGCTGGCGGCGTGCCTAATACATGCAAGTCGAGCGCGTGAAACTTTTCAGATCTCTTCGGAGTGACGA
ATTGTGGATCGAGCGGCGGATGGGTGAGTAACACGTGGGTAACCTGCCTGTAAGATCGGGATAACTTGCG
GAAACGTGAGCTAATACCGGATGAAGCTTCACATCTCCTGGTGTGAAGATGAAAGACGGCTCTGCTGTCAC
TTACAGATGGGCCCGCGGCGCATTAGCTAGTTGGTGGGGTAAAGGCCTACCAAGGCGACGATGCGTAGCC
GACCTGAGAGGGTGATCGGCCACACTGGGACTGAGACACGGCC
>Zotu10003
GAACGCTGGCGGCGTGCTTAACACATGCAAGTCGAACGGTGAAAGAGGAGCTTGCTCCTCTGGATCAGTG
GCGAACGGGTGAGTAACACGTGAGAAATGTGCCCTGGACTCTGGGATAAGCGTTGGAAACGACGTCTAAT
ACCGGATACGCGACGCGAAGGCATCTTCAGCGTCGGGAAAGAACTTCGGTCCAGGATCATCTCGCGGCCT
ATCAGCTAGTTGGTGAGGTAACGGCCCACCAAGGCGACGACGAGTAGCCGGCCTGAGAGGGTGACCGGC
CACACTGGGACTGAGACACGGCC
>Zotu10008
TAACGCTGGCAGAGTGCATAACACATGCAAGTCGAGCGCGACTACCTGAGATTACATCAACTGGTAGTTGA
GCGGCAGACGAGTGAGTAACACGTAAACAATCTGCCCTCGAATGGCGAATAACCAGCCGAAAGGCTGGCT
AATGCGCCGTACTCTCCTCAATAGGCATCTATTGAGGAGGAGAGGCCCGCAAGGGTCGTTCGAGGAGGAG
TTTGCGGCCCATCAGCTAGTTGGCGGTGTAACGGACCACCAAGGCTACGACGGGTACCCGGGCTGAAAGG
CCGGACGGGCACGATGGCACTGAGACACGGGC
>Zotu10013
GAACGCTGGCGGCGTGCTTAACACATGCAAGTCGAGCGAGGCTCCTCCTTCGGGAGGATGTCCTAGCGGC
GAACGGGTGAGTAACACGTGGGCAACCTGCCCCTAGCACTGGGATAACCCCGGGAAACCGGGGCTAATAC
CGGATACGACTTCACAAGGCATCTCGTGAAGTGGAAAAGGTTTACTGGCTAGGGATGGGCCCGCGGCCTA
TCAGCTTGTTGGTGGGGTAAAGGCCTACCAAGGCGACGACGGGTAGCCGGCCTGAGAGGGCGACCGGCC
ACACTGGGACTGAGACACGGCC
>Zotu10016
GAACGCTGGCGGCATGCCTAACACATGCAAGTCGAACGAAGCCTTCGGGCTTAGTGGCGCACGGGTGCGT
AACGCGTGGGAATCTGCCCCTCGGTTCGGAATAACAGTTAGAAATGACTGCTAATACCGGATGATGACGTA
AGTCCAAAGATTTATCGCCGAGGGATGAGCCCGCGTAGGATTAGCTAGTTGGTGAGGTAAAGGCTCACCA
AGGCGACGATCCTTAGCTGGTCTGAGAGGATGATTAGCCACACTGGGACTGAGACACGGCC
>Zotu10018
GAACGCTGGCGGCGCGCCTAACACATGCAAGTCGAACGGCACCCCCCTCCGGGGGGAAGCGAGTGGCGA
ACGGCTGAGTAACACGTGGAGAACCCGCCCCCTCCTCCGGGATAGCCTCGGGAAACCGTGGGTAATACCG
GATGACCCCGGCAGGCCGCATGGCCGGCCGGGCAAAGCCCAGACGGGAGGGGATGGCTCCGCGGCCCAT
CAGGTAGACGGCGGGGTGACGGCCCACCGTGCCGACGACGGGTAGCCGGGTTGAGAGACCGACCGGCCA
GATTGGGACTGAGACACGGCC
>Zotu10021
GAACGCTGGCGGCGTGCTTAACACATGCAAGTCGAGCGACAGCACTCTTCGGGGTGCATGGAGCGGCGAA
CGGGTGAGTAACACGTGGGCAACCTGCCCTCAGCTCTGGGATAACTCCGGGAAACCGGGGCTAATACCGG
ATACGACCTTCCCTCGCATGGGGGTCGGTGGAAAGTTCTTCGGCTGAGGATGGGCCCGCGGCCTATCAGCT
TGTTGGTGGGGTGATGGCCTACCAAGGCGACGACGGGTAGCCGGCCTGAGAGGGCGACCGGCCACACTG
GGACTGAGACACGGCC
>Zotu10023

GAACGCTGGCGGCATGCCTTACACATGCAAGTCGAACGACGTTAGGGGCAACTCTAGCGAGTGGCGTACG
GGTGAGTAATGCATCGGAACGTGTCCTCTTGTGGGGGATAACCAGTCGAAAGATTGGCTAATACCGCATG
AGCTCGAGAGAGGAAAGCGGGGGATCCGCAAGGACCTCGCGCGAGAGGAGCGGCCGATGCCCGATTAG
CTAGTTGGTGGGGTAAAAGCTCACCAAGGCGACGATCGGTAGCTGGTCTGAGAGGACGACCAGCCACACT
GGGACTGAGACACGGCC
>Zotu10025
GAACGCTAGCGGCAGGCCTAATACATGCAAGTCGAACGGATATAGAGACTTCGGTTTCTATATTAGTGGCG
CACGGGTGCGTAACGCGTATGCAACCTACCTTTTACTGGGGGATAGCCCGGAGAAATCCGGATTAATACCG
CATAAGATCACATGAGGGCATCTGAGAGTGATTAAAGATTTATTGGTAAAAGATGGGCATGCGTGCCATTA
GCTAGTTGGTGGTGTAACGGACCACCAAGGCGACGATGGCTAGGGGTTCTGAGAGGATGATCCCCCACAC
TGGTACTGAGATACGGAC
>Zotu10032
GAACGCTGGCGGCGTGCTTAACACATGCAAGTCGAACGAGGTCCATGGAGCTTGCTCCGGAAGACCTAGT
GGCGAACGGGTGCGTAACACGTGAGAAACCTGTCCCGGACTTGGGAATAACAGTCGGAAACGACTGCTAA
TACCGAATACCTTCGGATCGTCGCATGGCGAACCGAAGAAAGCGTATGCGGTTCGGGAGGGTCTCGCGGC
CTATCAGCTAGTTGGTGAGGTAACGGCTCACCAAGGCATCGACGGGTAGCTGGTCTGAGAGGATGATCAG
CCACACTGGGACTGAGACACGGCC
>Zotu10042
GAACGCTGGCGGTATGCTTTACACATGCAAGTCGTACGGGAGTTTTTAACTCTAGTGGCGGACGGGTGAGT
AACACGTAAGAATTTGCCTTTAGGAGGGGGACAACAACTGGAAACGGTTGCTAATACCCCATATGCTTTCG
AGTGAAACGGATTTATCCGCCTAGAGAGAAGCTTGCGGCTGATTAGCTTGTTGGTAAGGTAATGGCTTACC
AAGGCGACGATCAGTATCTGGTTTGAGAGGACGATCAGACACACTGGAACTGAGACACGGTC
>Zotu10045
GAACGCTGGCGGCATGCTTAACACATGCAAGTCGAACGGGCGATAAGGGCTTGCCTTTATCGTCAGTGGC
AGACGGGTGAGTAACGCGTGGGAACGTACCCTTAGGTTTGGAATAACGACTGGAAACGGTCGCTAATACC
GAATGATCCTGAGAAGGCAAAGATTTATCGCCTAAGGAGCGGCCCGCGTCAGATTAGGTAGTTGGTGGGG
TAACGGCTTACCAAGCCAAAGATCTGTAGCTGGTCTGAGAGGATGATCAGCCACACTGGGACTGAGACAC
GGCC
>Zotu10049
GAACGCTGGCGGTATGCTTAACACATGCAAGTCGAACGGAATGCTTCGGCATTTAGTGGCGGACGGGTGA
GTAACGCGTGAGAATCTGGCTTTAGGTTCGGGACAACAGTGGGAAACTGCTGCTAATACCGGATGTGCCG
AAAGGTGAAAGATTAATTGCCTGAAGAAGAGCTCGCGTCTGATTAGCTAGTTGGTGAGGTAAAGGCTCAC
CAAGGCGACGATCAGTAACTGGTCTGAGAGGATGACCAGTCACACTGGGACTGAGACACGGCC
>Zotu10051
GAACGCTGGCGGCATGCCTTACACATGCAAGTCGAACGGCAGCACGGGAGCAATCCTGGTGGCGAGTGG
CGAACGGGTGAGTAATACATCGGAACGTGCCCAGTAGTGGGGGATAGCCCGGCGAAAGCCGGATTAATA
CCGCATACGACCTACGGGTGAAAGCGGGGGATCGCAAGACCTCGCGCTATCGGAGCGGCCGATGTCAGAT
TAGCTAGTTGGTGGGGTAAAAGCCTACCAAGGCGACGATCTGTAGCTGGTCTGAGAGGACGACCAGCCAC
ACTGGGACTGAGACACGGCC
>Zotu10056
GAACGCTGGCGGCATGCCTAATACATGCAAGTCGAGCGGAGTATTTTCGAAAGCTTGCTTTCGAAAATACT
TAGCGGCGGACGGGTGAGTAACACGTAGGCAACCTGCCTTATAGTCTGGGATAACTTCCGGAAACGGATG
CTAATACCGGATACATCATCTTCCGGCATCGGAGGATGAGGAAAGACGGCGCAAGCTGTCGCTATAAGAT
GGGCCTGCGGCGCATTAGCTAGTTGGTGGGGTAACGGCTCACCAAGGCGACGATGCGTAGCCGACCTGAG
AGGGTGAACGGCCACACTGGGACTGAGACACGGCC
>Zotu10059
GAACGCTGGCGGCGTGCTTAATACATGCAAGTCGAACGCTCTCGACCTTCGGGTCACATGAGGGAGTGGC
GAACGGGTGCGTAACACGTGAGAAACCTACCCCGGAGTTGGGAATAACTCGAGGAAACTCTAGCTAATAC
CCGATGCCCTCACCGGATCGCATGATCTGGTGAGGAAAGTTCCGGCGCTCCGGGACGGTCTCGCGGCCTAT
CAGCTTGTTGGTGAGGTAACGGCTCACCAAGGCTTCGACGGGTAGCTGGTCTGAGAGGACGATCAGCCAC
ACTGGGACTGAGACACGGCC

>Zotu10062
GAACGCTGGCGGCATGCCTAACACATGCAAGTCGAACGGCAGCATAGCATCTAGCTTGCTAGATGGGGTG
GCGAGTGGCGAACGGGTGAGTAACGCGTAGGAATATGCCTTAAAGTGGGGGACAACTTGGGGAAACTCA
AGCTAATACCGCATAAGCTCTGAGGAGTGAAGCTGGGGACCGCAAGGCCTGGCGCTTTAAGATTAGCCTG
CGTCCGATTAGCTAGTTGGTAGGGTAAGGGCCTACCAAGGCGACGATCGGTAGCTGGTTTGAGAGAATGG
CCAGCCACACTGGGACTGAGACACGGCC
>Zotu10069
GAACGCTGGCGGCGCGCCTAACACATGCAAGTCGAGCGAAAACCGAGCCTTCGGGTTCGGGGAAAGCGG
CGAACGGGTGAGTAACACGTGGGTAACCTGCCCTCGGCACCGGGATAGCCCGGGGAAACTCGGATTAATA
CCGGACGGTCTCCGGAGCCTTCGGGCGCCGGAGAAAAGGTGGCTTCGGCATCTGGCCGAGGAGGGGCCT
GCGGCGGATTAGCTTGTTGGTGGGGTAATGGCTCACCAAGGCTACGATCCGTAGCTGGTCTGAGAGGACG
ATCAGCCACACTGGGACTGAGACACGGCC
>Zotu10075
GAACGCTGGCGGCATGCCTAAGACATGCAAGTCGAACGAAGTGGCCCAATGAAGATGAAGTGCTTGCACT
GATTTGGAAATGGATTCCCACTTAGTGGCAGACGGGTGAGTAACACGTGGGTAACCTACCCCTTAGTCTGG
GACAACTATTGGAAACGATAGCTAATACCGGATGATTCACAATTAGATAACTTTTTGTGCTAAAAGGAGCTT
CGGCTTCGCTTTGGGATGGGCTTGCGGTGTATTAGCTGGTTGGTAGGGTAATGGCCTACCAAGGCTACGAT
GCATATCCGAGCTGAGAGGCTGATCGGGCACATTGGAACTGAGATACGGTC
>Zotu10077
GAACGCTGGCGGCGTGCTTAACACATGCAAGTCGAACGAAACATCTTATTTGATTTTCTTCGGAACTGAAG
ATTTGGTGATTGAGTGGCGGACGGGTGAGTAACGCGTGGGTAACCTACCCTGTACAGGGGGATAACAGTC
AGAAATGACTGCTAATACCGCATAAGACCACAGCACCGCATGGTGCAGGGGTAAAAACTCCGGTGGTACA
GGATGGACCCGCGTCTGATTAGCTGGTTGGTGAGGTAACGGCTCACCAAGGCGACGATCAGTAGCCGGCT
TGAGAGAGTGAACGGCCACATTGGGACTGAGACACGGCC
>Zotu10079
GAACGCTGGCGGCGTGCTTAACACATGCAAGTCGAACGGAAAGGCCTTTCGGGGTACTCGAGTGGCGAAC
GGGTGAGTAACACGTGGGTGATCTGCCCTGCACTTTGGGATAAGCCTGGGAAACTGGGTCTAATACCGAA
TATGATCGCTGGCTTCATGGCTGGTGGTGGAAAGCTTTTGCGGTGTGGGATGGGCCCGCGGCCTATCAGCT
TGTTGGTGGGGTAATGGCCTACCAAGGCGACGACGGGTAGCCGGCCTGAGAGGGTGTCCGGCCACACTG
GGACTGAGATACGGCC
>Zotu10082
GAACGCTGGCGGCGTGCCTAACACATGCAAGTCGAGCGGTAACAGGTGTAGCAATACATGCTGACGAGCG
GCGGACGGGTGAGTAACGCTTGGGAATCTGCCTGTTAGTGGGGGACAACCCGGGGAAACTCGGGCTAAT
ACCGCATACGCACTACGGTGGAAAGCCGGGGACCGCAAGGCCTGGCGCTAACAGATGAGCCCAAGTCGG
ATTAGCTAGTTGGTAGGGTAACGGCCTACCAAGGCGACGATCCGTAACTGGTCTGAGAGGACGACCAGTC
ACACCGGAACTGAGACACGGTC
>Zotu10086
GAATGCTGGCGGCGTGCCTAATACATGCAAGTCGAGCGGTAACAGGTCTAGTTTACTAGATGCTGACGAG
CGGCGGACGGCTGAGTAACGCGTGGGAATTTGCCCCAAAGTGAGGGATAACCGCCCGAAAGGGTGGCTA
ATACCGCATATGGTCTTCGGATTAAAGCTTTCGAGCGCTTTGGGAGAAGCCCGCGTCGGATTAGGTTGTTG
GTGAGGTAATGGCTCACCAAGCCGACGATCCGTAGCTGGTCTGAGAGGATGATCAGCCAGACTGGAACTG
AGACACGGTC
>Zotu10088
GAACGCTGGCGGCGTGCTTAACACATGTAAGTCGAACGAGGTCCATGGAGCTTGCTCCGGAAGATCTAGT
GGCGAACGGGTGCGTAACACGTGAGAAACCTGCCCCGATCTCTGGGATAACAGTTGGAAACGACTGCTAA
TACCGGATGCCGTCACACCGCTTCATGGCGGAGTGCCGAAAGATTTATCGGATTGGGAGGGTCTCGCGGC
CTATCAGCTAGTTGGAGAGGTAACTGCTCACCAAGGCATCGACGGGTAGCTGGTCTGAGAGGATGATCAG
CCACACTGGGACTGAGACACGGCC
>Zotu10093

GAACGCTGGCGGCAGGCCTAACACATGCAAGTCGAACGAAGTCTTCGGACTTAGTGGCGGACGGGTGAGT
AACACGTGGGAACGTGCCCTTTGGTACGGAATAACTCAGGGAAACTTGAGCTAATACCGTATGTGCCCTTC
GGGGGAAAGATTTATCGCCATTGGAGCGGCCCGCGTCTGATTAGCTAGTTGGTGAGGTAAAGGCTCACCA
AGGCGACGATCAGTAGCTGGTCTGAGAGGATGATCAGCCACATTGGGACTGAGACACGGCC
>Zotu10097
GAACGCTGGCGGCGTGCCTAATACATGCAAGTCGAACGAAGAGACTTTAAAAGCTTGCTTTTGAAGAATCT
TAGTGGCGAACGGGTGAGTAATACATAAGCAACCTGCCTCAATGCCTGGGATAACTGATGGAAACGTCAG
CTAATACCGGATAGGTTAATCTAAGGCATCTTAGATTAATTAAAGATGGGATACATCACAATGAGATGGGC
TTATGGCGCATTAGTTAGTTGGTAAGGTAACGGCTTACCAAGACGATGATGCGTAGCCGACCTGAGAGGG
TGACCGGCCACACTGGGACTGAGACACGGCC
>Zotu10099
GAACGCTGGCGGCATGCCTAACACATGCAAGTCGAACGAAGGCTTCGGCCTTAGTGGCGCACGGGTGCGT
AACGCGTGGGAATCTGCCCTGGGGTACGGAATAACTCAGAGAAATTTGAGCTAATACCGTATGATGACGT
AAGTCCAAAGATTTATCGCCCTAGGATGAGCCCGCGTAGGATTAGCTAGTTGGTGAGGTAAAGGCTCACCA
AGGCGACGATCCTTAGCTGGTCTGAGAGGATGATCAGCCACACTGGGACTGAGACACGGCC
>Zotu10102
GAACGCTGGCGGCGTGGATGAGGCATGCAAGTCGAATGGGTTTAGGCCCATGGCAGACGAGGTAGGAAC
ACGTAGGTACGTACCCCAAAGTCACGGATAACCCGGAGAAATCCGGAATAATACGTGATAGTCCCGCAAG
GGTAAAGATTTATCGCTTTGGGAACGGCCTGCGTTCTATCAGCTTGTTGGTAAGGTAACGGCTTACCAAGG
CTATGACGGATAGGGGAGGTGAGAGCCTGACCCCCACCGATGGAACTGCGACACGGTC
>Zotu10104
GAACGCTGGCGGCATGCCTTACACATGCAAGTCGAACGGTAACGCGGGGCAACCTGGCGACGAGTGGCG
AACGGGTGAGTAATGCATCGGAACGTGCCCAGAAGTGGGGGATAGCCCGGCGAAAGCCGGATTAATACC
GCATGAGACCTGAGGGTGAAAGCGGGGGATCGCAAGACCTCGCGCTTTTGGAGCGGCCGATGTCAGATTA
GCTAGTTGGTGGGGTAAAGGCCTACCAAGGCGACGATCTGTAGCTGGTCTGAGAGGACGACCAGCCACAC
TGGGACTGAGACACGGCC
>Zotu10109
GAACGCTGGCGGCGTGCTTAACACATGCAAGTCGAACGGTAAGGCCCCTTCGGGGGTACACGAGTGGCGA
ACGGGTGAGTAACACGTGAGCAACCTGCCCTTGACTCTGGAATAACAGTTGGAAACAGCTGCTAATACTGG
ATATTCATCGTTCCCTTCCTGGGGGGTGGTGGAAAGCTCCGGCGGTCAGGGATGGGCTCGCGGCCTATCA
GCTTGTTGGTGAGGTAATGGCTCACCAAGGCTTCGACGGGTAGCCGGCCTGAGAGGGCGACCGGCCACAC
TGGGACTGAGATACGGCC
>Zotu10112
GAACGCTAGCGGCAGGCCTAACACATGCAAGTCGAGGGGTAGAAGTCTTCGGACTTTGAGACCGGCGCAC
GGGTGCGTAACGCGTATGCAATCTACCTTGTACTGAGGGATAGCCCAGAGAAATTTGGATTAATACCTCAT
AGTATTTTCGGGTGACATCACCTGATTATTAAAGTTCCAACGGTACAAGATGAGCATGCGTCCCATTAGCTA
GTTGGTATGGTAACGGCATACCAAGGCTATGATGGGTAGGGGTCCTGAGAGGGAGATCCCCCACACTGGG
ACTGAGACACGGCC
>Zotu10114
GAACGCTGGCGGCGTGCTTAACACATGCAAGTCGAGCGGTAAGGCCTCTTCGGAGGTACACGAGCGGCGA
ACGGGTGAGTAACACGTGAGCAACCTACCCATCACATCGGGATAACCCCCGGAAACGGGAGCTAATACCG
AATACGACCACTTCAGACATCTGACGGTGGTGGAAAGCTCCGGCGGTGGTGGATGGGCTCGCGGCCTATC
AGCTTGTTGGTGGGGTAACGGCCTACCAAGGCTTCGACGGGTAGCCGGCCTGAGAGGGTGACCGGCCACA
CTGGGACTGAGACACGGCC
>Zotu10117
GAACGCTGGCGGCGTGCCTAATGCATGCAAGTCGTACGCTTGGACCTTCGGGTCCAGGAGTGGCGGACGG
CTGAGTAGCGCGTGGGTCACCTGCCCCGCAGTGGGGGATAACGTCCCGAAAGGGATGCTAATCCCGCATG
GTCCCGGCCTCGTGTCGGGTAAAGCCGCAAGGCGCTGTGGGTGGGGCCCGCGTCCCATTAGCTTGTTGGC
GGGGTAATGGCCCACCAAGGCTCTGATGGGTGGCTGGTCTGAGAGGACGACCAGCCAGACTGGGACTGA
GACACGGCC

>Zotu10119
GAACGTTAGCGGCACGCCTAACATATGCAAGTCGCACGAGAAAAGGGCAACCTTAGTAAAGTGGCGCACG
GGTGCGTAACACGTAGATAACCTGCCCTTTGGAGGGGGATAACGTTCCGAAAGGAGCGCTAATACCGCAT
ACAGCAAGAGCCGGTTTGCCGGTTTTTGGCAAAGGTAGGGACCCGCAAGGGCCTACCGCCAGAGGAGGG
GTCTGCGGCCCATCAGCTAGTTGGTGAGGTAATGGCTCACCAAGGCAGAGACGGGTAGCTGGTCTGAGAG
GATGATCAGCCACACTGGTACTGGAACACGGAC
>Zotu10126
GAACGCTGGCGGCAGGCCTAACACATGCAAGTCGAGCGGTTGAGAGTAGCTTGCTACTTGATGCAGCGGC
GGACGGGTGAGTAACGCGTGGGAATCTGCCCAGTAGTGGGGGACAACAGTCGGAAACGACTGCTAATAC
CGCATACGCCCTACGGGGGAAAGGGGGGGATCTTCGGACCTCTCGCTATTGGATGAGCCCGCGTCGGATT
AGCTAGTTGGTAGGGTAAAGGCCTACCAAGGCTACGATCCGTAACTGGTCTGAGAGGATGATCAGTCACA
CTGGAACTGAGACACGGTC
>Zotu10129
GAACGCTGGCGGCGTGCTTAACACATGCAAGTGGAGCGACGAACCGAGGCTTGCCTCGGGGCAGAGCCG
CGAACGGGTGAGTAACACGTGGGTAACCTACCTTGATGACCGGGACAACCCGAGGAAACTCGGGCTAATA
CCGGATGTGCTCTTCTTACATCAGTAAGTCGAGGAAAGATAGCTTCGGCCTTCGCATCAAGATGGGCCCGC
GGCGCATTAGCTTGTTGGTGGGGTAACGGCCTACCAAGGCTTCGATGCGTAGCTGGTCTGAGAGGACGAT
CAGCCACACTGGGACTGAGACACGGCC
>Zotu10132
GAACGCTGGCGGTGTGCTTAACACATGCAAGTGGAGCGACGAACCAGGGCTTGCCCTGGGGCAGAGCCG
CGAACGGGTGAGTAACACGTGGGTAACCTACCCCGATGACTGGGACAACCCGAGGAAACTCGGGCTAATA
CCGGATGTGTCCGACGTCCACGAGGGCGTCGAGCAAAGGAAGCTTCGGCCTCCGCATCGGGAGGGGCCC
GCGGCCCATTAGCTTGTTGGTGGGGTAACGGCCTACCAAGGCTGCGATGGGTAGCTGGTCTGAGAGGACG
ATCAGCCACACTGGGACTGAGACACGGCC
>Zotu10135
GAACGCTGGCGGCGTGCTTAACACATGCAAGTCGAACGGTGAAGCAGGAGCTTGCTCCTGTGGATCAGTG
GCGAACGGGTGAGTAACACGTGAGTAACCTGCCCCAGACTCTGGGATAAGCGTTGGAAACGACGTCTAAT
ACCGGATACGAGCTTCAGCCGCATGGCTAGGAGCTGGAAAGAATTTCGGTCTGGGATGGACTCGCGGCCT
ATCAGGTAGTTGGTGAGGTAAAGGCCCACCAAGCCTACGACGGGTAGCCGGCCTGAGAGGGTGACCGGC
CACACTGGGACTGAGACACGGCC
>Zotu10142
GAACGCTGGCGGCGCGTCTTAAACATGCAAGTCGGACGGGAACCTCGGTGCTTGCATTGAGGGGAGAGT
GGCGAACGGGTGAGTAACACGTGGATAATCTACCTCTGGGATGGGGATAACTACCCGAAAGGGTAGCTAA
TACCGAATGAGATGGTGGCTGCATATGCAGCAGCTATTAAAGGTGGGGACCGCAAGGCCTACCGCCTGGA
GATGAGTCCGCGGCCCATTAGCTTGTTGGCGGGGTAATGGCCCACCAAGGCGACGATGGGTAACCGGCCT
GAGAGGGTGAACGGTCACACTGGGACTGAGATACGGCC
>Zotu10146
GAACGCTGGCGGCAGGCTTAACACATGCAAGTCGAGCGCCCCGCAAGGGGAGCGGCAGACGGGTGAGTA
ACGCGTGGGAACGTACCCTTTTCTACGGAATAACGCATGGAAACGTGTGCTAATACCGTATGTGCCCTTCG
GGGGAAAGATTTATCGGAAAAGGATCGGCCCGCGTTGGATTAGCTAGTTGGTGGGGTAAAGGCCTACCAA
GGCGACGATCCATAGCTGGTCTGAGAGGATGATCAGCCACATTGGGACTGAGACACGGCC
>Zotu10152
GAACGCTGGCGGCGCGCCTAACACATGCAAGTCGAACGAGCGAGAGAGAGCTTGCTTTCTCGAGCGAGTG
GCGAACGGGTGAGTAACGCGTGAGGAACCTGCCTCAAAGAGGGGGACAACAGTTGGAAACGACTGCTAA
TACCGCATAAGCCCACGGTGCCGCATGGCACAGAGGGAAAAGGAGCAATCCGCTTTGAGATGGCCTCGCG
TCCGATTAGCTAGTTGGTGAGGTAACGGCCCACCAAGGTGACGATCGGTAGCCGGACTGAGAGGTTGAAC
GGCCACATTGGGACTGAGACACGGCC
>Zotu10157
GAACGCTGGAAATGTGGATACCGGCATGCAAGTCAAGGGGCCGCAAGGCACCGGCGAACGGCTTAGTAA
CACGTAGATATATACCGTAAAGTGGGGCATAGCTCGTCGAAAGACGGGGTAATTTCCCATAGTCCCGCAAG

GGTAAAGGCGTAAGCCGCTTTATGAATGGTCTGCGGTCTATCAGCTAGTTGGTGGGGTAAAAGCCTACCAA
GGCTATGACGGATAGGGGGCGTGAGAGCGCGACCCCCAAGAATGGCACTGCGACACGGGC
>Zotu10160
GAACGCTGGCGGCGTGCTTAAGACATGCAAGTCGAACGCAGTCTTCGGACTGAGTGGCGCACGGGTGAGT
AACGCGTAACTGACCTACCCAGAAGTCATGAATAACTGGCCGAAAGGTCAGCTAATACGTGATGTGATGAT
TCGCCGTGGCGAATCATTAAAGATTTATCGCTTCTGGATGGGGTTGCGTTCCATCAGCTAGTTGGTGGGGT
AAAGGCCTACCAAGGCGACGACGGATAGCCGGCCTGAGAGGGTGGCCGGCCACAGGGGCACTGAGACAC
GGGT
>Zotu10165
GAACGCTGGCGGCATGCCTAAGACATGCAAGTCGAACGAGATGGCCCAGTGAAGGTTGAGTGCTTGCACG
AGACTGAATCTGGATTCCCATCTAGTGGCAGACGGGTGAGTAACACGTGGGTGACCTACCTTTTCGTCGGG
GATAACAGTTGGAAACGATTGCTAATACCGAATAAAATGTAAGAGTCAATTTTACATTGAAAGGAGCCTTT
AAAGCTTCGCGATTAGATGGGCCTGCGGCGTATTAACTAGTTGGTGAGGTAATGGCTTACCAAGGTGACG
ATGCGTAGCCGAACTGAGAGGTTGATCGGCCACATTGGGACTGAGACACGGCC
>Zotu10175
GAACGCTGGCGGCGTGCTTAACACATGCAAGTCGAACGGTGAACTTGGGAGCTTGCTCCTGGGGGATCAG
TGGCGAACGGGTGAGTAACACGTGAGTAACCTGCCCCAGACTCTGGGATAAGCACTGGAAACGGTGTCTA
ATACCGGATACGAGCTTCAGCCGCATGGCTAGGAGCTGGAAAGAATTTCGGTCTGGGATGGACTCGCGGC
CTATCAGGTAGTTGGTGAGGTAAAGGCTCACCAAGCCGACGACGGGTAGCCGGCCTGAGAGGGTGACCG
GCCACACTGGGACTGAGACACGGCC
>Zotu10180
GAACGCTGGCGGCGTGCTTAACACATGCAAGTCGAACGGTAAGGCCCCTTCGGGGGTACACGAGTGGCGA
ACGGGTGAGTAACACGTGAGCAACCTGCCCTTGACTCTGGAATAACAGCTGGAAACAGCTGCTAATACCG
GATATGACCTCCTGCGACATCGCACAGAGGTGGAAAGCTCCGGCGGTCAAGGATGGGCTCGCGGCCTATC
AGCTAGTTGGTGAGGTAACGGCCCACCAAGGCTTCGACGGGTAGCCGGCCTGAGAGGGCGACCGGCCAC
ACTGGGACTGAGATACGGCC
>Zotu10183
GAACGCTGGCGGCGTGCCTAACACATGCAAGTCGAGCGAGGTCCAACCAGTGGCAACACTGGGGAAGAC
CTAGCGGCGAACGGGTGAGTAACACGTGAGGAACTTGCCCCGGAGTTCGGGATAACCCGAGGAAACTCG
AGCTAATACCGAATGCCCTCTTCTGATCGCATGGTCGGATGAGGAAAGATTTATCGCTCCGGGAGAGTCTC
GCGGCCTATCAGCTAGTTGGTGGGGTAACGGCCCACCAAGGCATCGACGGGTAGCTGGTCTGAGAGGAC
GATCAGCCACACTGGGACTGAGACACGGCC
>Zotu10188
GAACGCTAGCGGCAGGCCTAATACATGCAAGTCGAACGATAGTGAGGAGCTTGCTCTTCACGAAAAGTGG
CGCACGGGTGCGTAACGCGTATGCAACCTACCTTAATCAGGGGGATAGACCGAAGAAATTCGGATTAACA
CCGCATAAAATCACAGTATAGCATTATACAATGATCAAATATTTATAGGATTAAGATGGGCATGCGTGACAT
TAGTTAGTTGGCGAGGTAACGGCTCACCAAGACTACGATGTCTAGGGGATCTGAGAGGATGACCCCCCAC
ACTGGTACTGAGACACGGAC
>Zotu10192
GAACGCTGGCGGCGTGCCTAACACATGCAAGTCGAACGCTCGCAAGAGCGTGGCGAACGGGTGAGTAAC
GCGTAGGAATCTACCCCGAACAGGGAAATACCCCACCGAAAGGTGGGCTAATGTCCCATAATCCCGCAAG
GGAAAAGGAGCAATCCGGTTTGGGAGGAGCCTGCGTCCTATCAGCTTGTTGGTAAGGTAATGGCTTACCA
AGGCTATGACGGGTAGCTGGTCTGAGAGGATGATCAGCCACAATGGGACTGAGACACGGCC
>Zotu10195
GAACGCTGGCGGCGCGCCTAACACATGCAAGTCGAACGAGCGAGAGAGAGCTTGCTTTCTCGAGCGAGTG
GCGAACGGGTGAGTAACGCGTGAGGAACCTGCCTCAAAGAGGGGGACAACAGTTGGAAACGACTGCTAA
TACCGCATAAGCCCACGGCTCGGCATCGAGCAGAGGGAAAAGGAGCAATCCGCTTTGAGATGGCCTCGCG
TCCGATTAGCTAGTTGGTGAGGTAATGGCCCACCAAGGCGACGATCGGTAGCCGGACTGAGAGGTTGAAC
GGCCACATTGGGACTGAGACACGGCC
>Zotu10198

CAACGCTGGCGGCGTGCCTAACACATGCAAGTCGAACGCGAAAGTCCCGCAAGGGATAAGTAGAGTGGCA
AACGGGTGAGTAACACGTGGGTGACCTGCCTTCGAGTGGGGGATAACGTCCCGAAAGGGACGCTAATACC
GCATTACATCCTGCCTTTGAACAGGCGGAGATCAAAGCCGGGGATCGCAAGACCTGGCGCTTGAAGAGGG
GCCCGCGTCTGATTAGCTAGTTGGTGAGGTAACGGCTCACCAAGGCTACGATCAGTATCCGGCCTGAGAG
GGCGGACGGACACACTGGGACTGAGACACGGCC
>Zotu10200
GAACGCTGGCGGCGCGCCTAACACATGCAAGTCGAACGGTTAAAGCACCTCCGGGTGTGGATAAAGTGGC
GAACGGCTGAGTAACACGTGGGCAACCTGCCCCTCGCTCCGGGACAGCCTCGGGAAACCGTGGGTAATAC
CGGATACTCCCTTGCCCGCACATGCGGGGGAGGGGAAAGCCCAGACGGCGAGGGATGGGCCCGCGGCCT
GTTAGCTAGTTGGCGGGGCAACGGCCCACCAAGGCGATTATGGGTAGCCGGGTTGAGAGACCGACCGGC
CAGATTGGGACTGAGACACGGCC
>Zotu10202
GAACGCTGGCGGCAGGCTTAACACATGCAAGTCGAGCGAGATCTTCGGGTCTAGCGGCGGACGGGTGAG
TAACGCGTGGGAACGTGCCCTTTGCTACGGAATAGTCCTGGGAAACTGGGGGTAATACCGTATACGCCCTT
TTGGGGAAAGATTTATCGGCGAAGGATCGGCCCGCGTTGGATTAGGTAGTTGGTGGGGTAATGGCCTACC
AAGCCTTCGATCCATAGCTGGTTTGAGAGGATGATCAGCCACACTGGGACTGAGACACGGCC
>Zotu10206
GAACGCTAGCGGCATGCCTAACACATGCAAGTCGAACGAGAAAGGGACTTCGGTCCTGAGTAAAGTGGCG
CACGGGTGAGTAACACGTGACTAACCTACCTTTGAGCGGGGGATAACCTAGGGAAACCTGGGCTAATACC
GCATAATACCTACGGGTCAAAGCGAGCAATCGCACTTAAAGAGGGGGTCGCGGCAGATTAGTTAGTTGGT
GAGGTAATGGCTCACCAAGACGATGATCTGTATCCGGCCTGAGAGGGCGCACGGACACACTGGAACTGAA
ACACGGTC
>Zotu10209
GAACGCTGGCGGCGTGCTTAACACATGCAAGTGGTGCGACGAACCAGGGCTTGCCCTGGGGCCAAGCCGC
GAACGGGTGAGTAACACGTGAGTAACCTGCCCCGATGACCGGGACAACCCGAGGAAACTCGGGCTAATAC
CGGATGTGGCGCACAGGCGTAAGCCTGTTCGCTAAAGGAAGCTTCGGCCTCCGCATTGGGAGGGGCTCGC
GGCCCATTAGCTTGTTGGTGGGGTTATGGCCTACCAAGGCGACGATCCGTAGCTGGTCTGAGAGGACGAT
CAGCCACACTGGGACTGAGACACGGCC
>Zotu10213
GAACGCTGGCGGCAGGCTTAACACATGCAAGTCGAGCGCGCCCTTCGGGGTGAGCGGCAGACGGGTGAG
TAGCGCGTGGGAACATACCTTTCGGACTGGAATAACTGGGGGAAACTTCAGCTAAGACCGGATACGCCCTT
AGGGGGAATGATTTATCGCCGAAAGATTGGCCCGCGTCCGATTAGCTAGTTGGTGTTGGTAATGGCTCACC
AAGGCGACGATCGGTCGCTGGTCTGAGAGGATGACCAGCCACATTGGGACTGAGACACGGCC
>Zotu10215
GAACGCTGGCGGCGTGCCTAATACATGCAAGTCGGGCGAATCTTGAGGTGCTTGCACCTCAAGATTAGCG
GCGGACGGGTGAGTAACACGTGGGCAACCTGCCTGTAAGACTGGGATAACACCGGGAAACCGGTGCTAA
TACCGGATAACTCTTTTCCTCTCATGGGGAGAAGCTAAAAGTCGGTTTACGCTGACACTTACAGATGGGCCC
GCGGCGCATTAGCTAGTTGGTGAGGTAACGGCTCACCAAGGCGACGATGCGTAGCCGACCTGAGAGGGT
GATCGGCCACACTGGGACTGAGACACGGCC
>Zotu10221
GAACGCTGGCGGCGTGCCTAATACATGCAAGTCGAGCGGATGATGAAGAAGCTTGCTTCTTCTGATTTAGC
GGCGGACGGGTGAGTAACACGTGGGCAACCTGCCCTGTAGATTGGGATAACTCCGGGAAACCGGGGCTA
ATACCGAATAATCCATTCCCTCACATGAGAGAATGTTAAAAGACGGTCTCGGCTGTCACTACAGGATGGGC
CCGCGGCGCATTAGCTAGTTGGTGAGGTAGCGGCTCACCAAGGCGACGATGCGTAGCCGACCTGAGAGG
GTGATCGGCCACACTGGGACTGAGACACGGCC
>Zotu10226
GAACGCTGGCGGCGTGCCTAATACATGCAAGTCGAACGGAGATTAAGTAGCTTGCTATTTAATCTTAGTGG
CGCACGGGTGAGTAATATATAGCTAACTTGCCCATTACTAAGGGACAACAGTTGGAAACGACTGCTAATAC
CTTATACTCCGTATCTATATAAATAGATACGGGAAAGTTTTTCGGTAATGGATAGGGCTATATCGTATCAGC
TAGTTGGTAAGGTAACGGCTTACCAAGGCTATGACGCGTAACTGGTCTGAGAGGATGATCAGTCACACTG
GAACTGAGACACGGTC

>Zotu10229
GAACGCTGGCGGCGTGCCTAATACATGCAAGTCGAGCGCGAGAGTGAGGAGCTTGCTCCTCATGATCGAG
CGGCGGACGGGTGAGTAACACGTAGGCAACCTACCCTTGAGATTGGGATAACTACCGGAAACGGTAGCTA
ATACCGGATACGACTTTTCCACACAAGTGGAATGGTTAAAAGGCGGATTTATCTGCCGCTCATGGATGGGC
CTGCGGTGCATTAGCTAGTTGGTGAGGTAGTGGCTCACCAAGGCGACGATGCATAGCCGACCTGAGAGGG
TGATCGGCCACACTGGGACTGAGACACGGCC
>Zotu10231
CAACGCTGGCGGCGTGCCTAACACATGCAAGTCGAGCGCGAATAGGGGGGTAACTCCCTTAGTAGAGCGG
CGAACGGGTGAGTAACGCGTGGGTGATCTACCTTCGAGTGGGGAATAACATCGGGAAACTGGTGCTAATA
CCGCATGACATCCTTGGTTCACTCCGGGGATCAAAGCCGGGGACCGCAAGGCCTGGCGCTTGAAGATGAG
CCCGCGTCCGATTAGCTAGTTGGCGGGGTAACGGCCCACCAAGGCGACGATCGGTAGCCGGCCTGAGAGG
GCGGACGGCCACACTGGGACTGAGACACGGCC
>Zotu10236
GAACGCTGGCGGCGTGCCTAACACATGCAAGTCGAACGGAGTAGAGAGCTTCGGTTTTCTACTTAGTGGC
GAACGGGTGAGTAACGCGTGAGGAACCTGCCTTTCAGTGGGGGACAACAGTTGGAAACGACTGCTAATAC
CGCATAATGTTACCGGATGGCATCATCTGGTAACCAAAGCTTTATGTGCTGAAAGATGGCCTCGCGTCTGA
TTAGCTAGTTGGTGGGGTAACGGCCCACCAAGGCGACGATCAGTAGCCGGTCTGAGAGGATGAACGGCCA
CATTGGGACTGAGATACGGCC
>Zotu10240
GAACGCTAGCGAAATGCCTAACACATGCAAGTCGAACGGATCTTGCACTGTACTTGTACAGAGTAAGATTA
GTGGCAAACGAGCGAGTAACGCGTGCTTAACCTGCCCCGAAGATGGGTATAACTACAGAGATGTAGCTAA
TTCCCAATATGCTCACGATGGTGAAATTCCAATGTGAGGAAAGATTTATCGCTTCGGGAGGGGGGTGCGTC
CTATCAGGTAGTTGGTAAGGTAATGGCTTACCAAGCCTATGACGGGTAACCGGTCTGAGATGATGTCCGGT
CGCGATGGGACTGAGATACGGCC
>Zotu10244
GAACGCTGGCGGCATGCCTTACACATGCAAGTCGAACGGCAGCACGGACTTCGGTCTGGTGGCGAGTGGC
GAACGGGTGAGTAATACATCGGAACGTACCCTGTTGTGGGGGATAACTAGTCGAAAGATTAGCTAATACC
GCATACGACCTGAGGGTGAAAGCGGGGGACCGAAAGGCCTCGCGCAGCAGGAGCGGCCGATGTCTGATT
AGTTAGTTGGTGGGGTAAGAGCCCACCAAGGCGACGATCAGTAGCTGGTCTGAGAGGACGATCAGCCACA
CTGGGACTGAGACACGGCC
>Zotu10246
GAACGCTGGCGGCAGGCTTAACACATGCAAGTCGAGCGGAGTGATGGTGCTTGCACTATCACTTAGCGGC
GGACGGGTGAGTAATGCTTAGGAATCTGCCATTTAGTGGGGGACAACATTCCGAAAGGAATGCTAATACC
GCATACGTCCTACGGGAGAAAGCAGGGGATTTTCGGACCTTGCGCTAAATGATGAGCCTAAGTCGGATTA
GCTAGTTGGTGGGGTAAAGGCCTACCAAGGCGACGATCTGTAGCGGGTCTGAGAGGATGATTCGCCACAC
TGGGACTGAGACACGGCC
>Zotu10248
GAACGCTGGCGGCGTGCTTAACACATGCAAGTCGAACGATGATGCCCAGCTTGCTGGGTGGATTAGTGGC
GAACGGGTGAGTAACACGTGAGTAACCTGCCCCTCACTCTGGGATAAGCACTGGAAACGGTGTCTAATACC
GGATACGAACTTTGACCGCATGGTCAGGAGTTGGAAAGAATTTTGGTGGGGGATGGACTCGCGGCCTATC
AGCTTGTTGGTGAGGTAATGGCTCACCAAGGCGACGACGGGTAGCCGGCCTGAGAGGGTGACCGGCCAC
ACTGGGACTGAGACACGGCC
>Zotu10250
GAACGCTAGCGGCGGGCCCAATACATGCAAGTCGAGGGGCATCGGGAGTAGCAATACTTGCCGGCGACC
GGCGCACGGGTGCGTAACGCGTATGCAATCTACCTCTGACATGGGGATAGCCCGAAGAAATTCGGATTAA
TACCCAATGGTATCTTAATATGACCTCATATAAAGATTAAAGATTTATCGGTCAGAGATGAGCATGCGTCTG
ATTAGCTAGTTGGTGATGTAATTGCTCACCAAGGCAATGATCAGTAGGGGTTCTGAGAGGATTATCCCCCA
CACCGGTACTGAGACAGGGAC
>Zotu10253

GAACGCTGGCGGTGTGCTTAACACATGCAAGTCGAACGGTAAGGCCTTTCGGGGTCATACGAGTGGCGAA
CGGGTGAGTAACACGTGAGCAACCTGCCCTTGACTTCGGAATACCAGCTGGAAACAGCTGCTAATACCGG
ATATGACCCTGGTTCTCTTGGACTGGGGTGGAAAGCTCCGGCGGTCAGGGATGGGCTCGCGGCCTATCAG
CTTGTTGGTGAGGTAATGGCTCACCAAGGCTTCGACGGGTAGCCGGCCTGAGAGGGCGACCGGCCACACT
GGGACTGAGATACGGCC
>Zotu10257
GAACGCTAGCGGCAGGCTTAACACATGCAAGTCGAGGGGTATTAGTTTTCGGACTAAGAGACCGGCGCAC
GGGTGCGTAACGCGTATGCAATCTACCTTTTACAGAGGGATAGCCCAGAGAAATTTGGATTAATACCTCAT
AGTATAACATGGCCGCATGGTCGAGTTATTAAAGTCACAACGGTAAAAGATGAGCATGCGTCCCATTAGCT
AGTTGGTAAGGTAACGGCTTACCAAGGCTACGATGGGTAGGGGTCCTGAGAGGGAGATCCCCCACACTGG
TACTGAGACACGGAC
>Zotu10259
GAACGCTGGCGGCGTGCCTAATACATGCAAGTCGAGCGGACCGAATAGAAGCTTGCTTCTATTTGGTTAGC
GGCGGACGGGTGAGTAACACGTGGGTAACCTGCCCTGTAGACGGGGATAACACCGAGAAATCGGTGCTA
ATACCGGATAACACGGAATGTCGCATGATGTTTCGTTGAAAGGCGGCGCAAGCTGTCGCTACAGGATGGG
CCCGCGGCGCATTAGCTAGTTGGTGAGGTAACGGCTCACCAAGGCGACGATGCGTAGCCGACCTGAGAGG
GTGATCGGCCACACTGGGACTGAGACACGGCC
>Zotu10263
GAACGCTGGCGGCGTGCTTAACACATGCAAGTCGAACGGAAAGGCTCCAGCTTGCTGGAGTGCTCGAGTG
GCGAACGGGTGAGTAACACGTGGGCAATCTGCCCTGCACTCTGGGATAAGCCTGGGAAACTGGGTCTAAT
ACCGGATAGGACCTTTCTCCTCATGGGGTTTGGTGGAAAGTTTTTCGGTGCAGGATGAGCCCGCGGCCTAT
CAGCTTGTTGGTGGGGTAATGGCCTACCAAGGCGACGACGGGTAGCCGGCCTGAGAGGGTGATCGGCCA
CATTGGGACTGAGATACGGCC
>Zotu10268
AAACGCTGGCGGCGTGCATGAGACATGCAAGTCGAACGGACGATCAGGGCTTGCTCTGATCGTTAGTGGC
GGACGGCTGAGTAACGCGTAGGTGACCTGCCCCGAAGTGGGGAATAACTGCCCGAAAGGGTGGCTAATA
CCGCATGTGGTGGTGCGTTCGATCGCACCAGTAAAGCCGCAAGGCGCTTCGGGAGGGGCCTGCGTCCGAT
TAGCTCGTTGGTGGGGTAACGGCTCACCAAGGCGACGATCGGTAGGTGGTCTGAGAGGACGATCACCCAG
ACTGGGACTGAGACACGGCC
>Zotu10270
GAACGCTGGCGGCAGGCCTAACACATGCAAGTCGAACGGACTCTTCGGAGTTAGTGGCGGACGGGTGAG
TAACACGTGGGAACGTGCCTTTTGGTTCGGAATAGCTCCTGGAAACGGGTGGTAATGCCGAATGTGCCCTT
CGGGGGAAAGATTTATCGCCATTAGAGCGGCCCGCGTCTGATTAGCTAGTTGGTGAGGTAAAGGCTCACC
AAGGCGACGATCAGTAGCTGGTCTGAGAGGATGACCAGCCACATTGGGACTGAGACACGGCC
>Zotu10272
GAACGCTGGCGGCGCGCCTAATACATGCAAGTCGAGCGAGAAGACGTAGCAATACGTTTGTAAAGCGGCG
AACGGGTGAGGAATACATGGGTAACCTACCCTTGAGTGGGGAATAACTAGCCGAAAGGTTAGCTAATACC
GCGTACGCTCCTCGGACTTCGGTTCAGGGAGGAAAGCGCTGCCGAGGGCAGCGCGCTCTTGGATGGGCTC
ATGTCCTATCAGCTTGTTGGTGAGGTAACGGCTCACCAAGGCTTCGACGGGTAGCTGGTCTGAGAGGACG
ATCAGCCACACTGGCACTGCGACACGGGC
>Zotu10275
GAACGCTGGCGGTGTGCTTAACACATGCAAGTCGAACGGGCAAGAGCTGTAGCAATACAGAATTCTGCTA
GTGGCAAACGGGTGAGTAATACATGGGAATCTACCTTGCAGTGCGAAATAACAGTTGGAAGCAATTGCTA
ATGTCGCATATAGCCGAGAGGTGAAAGATTTATCGCTGCAAGACGAGCCCATGCAGGATTAGCTAGTTGG
TGGGGTAACGGCTTACCAAGGCAACGATACGTAGCCGACCTGAGAGGGTGATCGGCCACACTGGAACTGA
GACACGGTC
>Zotu10279
GAACGCTGGCGGCGCGCTTAACACATGCAAGTCGAGCGAGAACCTTTCCTTCGGGATTGGGGACAGCGGC
GAACGGGTGAGTAACACGTGGGTAATCTGCCCTCGACATCGGGATAGCCCGGGGAAACCCGGATTAATAC
AGGATAGCCTTACTGGTTTTCGGGCTGGTAAGAAAAGGTAGCTTTGGCCTCCGGTCGAGGAGGAGCCCGC

GGCGGATTAGCTTGTTGGCGGGGTAAAAGCCCACCAAGGCGACGATCCGTAGCTGGTCTGAGAGGACGAT
CAGCCACACTGGGACTGAGACACGGCC
>Zotu10281
AAACGCTGGCGGCGTGCATGAGACATGCAAGTCGAACGATCGAGCTTGCTCGATAGTGGCGAACGGCTGA
GTAACGCGTAGGTGACCTGCCCCGAAGTGGGGGATAACTGCCCGAAAGGGTGGCTAATACCGCATGTGGC
GACGCTTTCATTGGCGTCATCAAAGCCGCGAGGCGCTTCGGGAGGGGCCTGCGTCCGATTAGCTCGTTGGT
GGGGTAATGGCTCACCAAGGCGACGATCGGTAGGTGGTCTGAGAGGACGATCACCCAGACTGGGACTGA
GACACGGCC
>Zotu10284
GAACGCTGGCGGCGTGCTTAACACATGCAAGTCGAACGATGAAGAGAGCTTGCTCTTGGATTAGTGGCGA
ACGGGTGAGTAACACGTGAGTAACCTGCCCTTGACTCTGGGATAAGCACTGGAAACGGTGTCTAATACCG
GATACGAGCTTCCGCCGCATGGTGAGGAGTTGGAAAGAATTTTGGTCAAGGATGGACTCGCGGCCTATCA
GCTAGTTGGTGAGGTAATGGCTCACCAAGGCGACGACGGGTAGCCGGCCTGAGAGGGTGACCGGCCACA
CTGGGACTGAGACACGGCC
>Zotu10290
GAACGCTGGCGGCGTGCTTAACACATGCAAGTCGAGCGGTAAGGCTCCTTCGGGAGTACACGAGCGGCGA
ACGGGTGAGTAACACGTGAGTAATCTGCCCCTCACTTCGGGATAACCGCCGGAAACGGTGGCTAATACCG
AATACGACGCGCTTTCGCATGAGATGCGCGTGGAAAGTTTTCTCGGTGAGGGATGTGCTCGCGGCCTATCA
GCTTGTTGGTGAGGTAACGGCTCACCAAGGCTTCGACGGGTAGCCGGCCTGAGAGGGTGACCGGCCACAC
TGGGACTGAGACACGGCC
>Zotu10292
GAACGCTGGCGGCATGCCTAACACATGCAAGTCGAACGAAGGCTTCGGCCTTAGTGGCGCACGGGTGCGT
AACGCGTGGGAATCTGCCCAAAGGTACGGAATAACTCGGGGAAACCCGTGCTAATACCGTATGATGTCGT
AAGACCAAAGATTTATCGCCTTTGGATGAGCCCGCGTAGGATTAGCTAGTTGGTGAGGTAAAGGCTCACCA
AGGCGACGATCCTTAGCTGGTCTGAGAGGATGATCAGCCACACTGGGACTGAGACACGGCC
>Zotu10295
GAACGCTGGCGGCGTGCCTAACACATGCAAGTCGAGCGAGATCCATCCAGTGGCAACACTGGTGAAGATC
TAGCGGCGAACGGGTGAGTAACACGTGAGAAACCTGCCCCGGAGACCGGGATAACCCGAGGAAGCTCGG
GCTAATACCGGATACCCCCACCGAACCGCATGATTTGGTGAGGAAATGCTCCGACGCTCCGGGAGGGTCTC
GCGGCCTATCAGCTAGTTGGTGAGGTAATGGCTCACCAAGGCTAAGACGGGTAGCTGGTCTGAGAGGACG
ATCAGCCACACTGGGACTGAGACACGGCC
>Zotu10298
CAACGCTGGCGGCGCGCTTAACACATGCAAGTCGAACGAGAAAGCAGGAGCAATCCTGTGAGTACAGTGG
CGTACGGGTGAGTAACACGTGGATTATCTGCCCTGGAGTGGGGAATAACGCTGGGAAACCGGCGCTAATA
CCGCATATTCCCGAGAGGGGAAAGCAGCAATGCGCTCGAGGAGGAGTCCGCGGCTGATTAGCTAGTTGGT
AGGGTAACGGCCTACCAAGGCTATGATCGGTAGCCGGCCTGAGAGGGCACACGGCCACACTGGCACTGAA
ACACGGGC
>Zotu10303
GAACGCTGGCGGCGTGCTTAACACATGCAAGTCGAGCGGAAAGGCTTCCTTCGGGAAGTACTCGAGCGGC
GAACGGGTGCGTAACACGTGGATAATCAACCTCTTGCTCTGGAATACCCCGGGGAAACTCGGATCAATGCC
AGATAAAGTGCGGTGCTGCAGGGTGCCGTACCAAAGATAGCTTCGGCCTTCGGCGAGAGACGAGTCCGCG
GCCCATTAGCTTGTTGGCGGGGTAACGGCCCACCAAGGCTACGATGGGTAGCTGGTCTGAGAGGATGATC
AGCCACACTGGGACTGAGACACGGCC
>Zotu10305
GAACGCTAGCGGCATGCCTAACACATGCAAGTCGGACGGGAAGTGGTGTTTCCAGTGGCGGACGGGTGA
GTAACGCGTAAGAACCTGCCCTTGGGAGGGGAACAACAGCTGGAAACGGCTGCTAATACCCCGTAGGCTG
AGGAGCAAAAGGAGGAATCCGCCTGAGGAGGGGCTCGCGTCTGATTAGCTAGTTGGTGAGGCAATAGCT
TACCAAGGCGATGATCAGTAACTGGTTCGAGAGGATGATCAGCCACACTGGGACTGAGACACGGCC
>Zotu10310

GAACGCTGGCGGCGTGCCTAATACATGCAAGTCGAGCGGACTTTAAAAGCTTGCTTTTAAAGTTAGCGGCG
GACGGGTGAGTAACACGTGGGCAACCTGCCTGTAAGACTGGGATAACTTCGGGAAACCGGAGCTAATACC
GGATAATCCTTTTCTACACATGTAGAAAAGCTGAAAGACGGTTTACGCTGTCGCTTACAGATGGGCCCGCG
GCGCATTAGCTAGTTGGTGAGGTAACGGCTCACCAAGGCAACGATGCGTAGCCGACCTGAGAGGGTGATC
GGCCACACTGGGACTGAGACACGGCC
>Zotu10313
GAACGCTAGCGGGAGGCCTAACACATGCAAGCCGAGCGGTAGAGAGTAGCTTGCTACTCTTGAGAGCGGC
GCACGGGTGCGTAACACGTGTGCAACCTACCTTTATCTGGGGGATAGCCTTTCGAAAGGAAGATTAATACC
CCATAATATATTGACTGGCATCAGTTGATATTGAAAACTCCGGTGGATAGAGATGGGCACGCGCAAGATTA
GATAGTTGGTGAGGTAACGGCTCACCAAGTCAATGATCTTTAGGGGTCCTGAGAGGGAGATCCCCCACACT
GGTACTGAGACACGGAC
>Zotu10315
GAACGCTGGCGGCGTGCTTAACACATGCAAGTCGAACGGTGAACACGGAGCTTGCTCTGTGGGATCAGTG
GCGAACGGGTGAGTAACACGTGAGCAACCTGCCCCTGACTCTGGGATAAGCGCTGGAAACGGCGTCTAAT
ACTGGATACGAGTAGCGATCGCATGGTCAGTTACTGGAAAGAATTTCGGTTGGGGATGGGCTCGCGGCCT
ATCAGCTTGTTGGTGAGGTAATGGCTCACCAAGGCGTCGACGGGTAGCCGGCCTGAGAGGGTGACCGGCC
ACACTGGGACTGAGACACGGCC
>Zotu10318
GAACGCTGGCGGCGTGCTTAACACATTCAAGTCGAACGGTGACGAGAGACTTCGGTCTCTCCGACCAGTG
GCGGACGGGTGAGTAATGCATGAGCAACCTGCCTCGAAGTGGGGAATAACACAGAGAAATTTGTGCTAAT
ACCGCATAATGTGTTCGGGAGGCATCTCCTGAACAAGAAAGGATTTATTCGCTTTGAGATGGGCTCATGTC
TGATTAGGTAGTTGGTGGGGTAACGGCCTACCAAGCCTGCGATCAGTAGCCGGACTGAGAGGTTGAACGG
CCACATTGGGACTGAGATACGGCC
>Zotu10320
GAACGCTAGCGGCAGGCTTAATACATGCAAGTCGTGGGGCAGCAGGGGTAGCAATACTCGCTGGCGACC
GGCAAACGGGTGCGCAATACGTACGCAACCTTCCTTTAAGCGGGGAATAGCCCAGGGAAACTTGGATTAA
TACCCCATAACATGGTGAAGAGGCATCTCTTTATTATTAAAGCTCCGGCACTTAAAGATGGGCGTACGGCT
GATTAGGTAGTTGGCGGGGTAACGGCCCACCAAGCCTGCGATCAGTAGCTGATGTGAGAGCATGATCAGC
CACACGGGCACTGAGACACGGGC
>Zotu10322
GAACGCTGGCGGTATGCTTAATACATGCAAGTCGCACGTGAAAGTAGCAATATGAGTAAAGTGGCGCACG
GGTGAGTAACACGTAGGTGACCTGCCTAGAAGCTGGGGATAACATCGGGAAACTGGTGCTAATACCGAAT
AAGCTCGTAGGGTTTGAAAACTTTATGAGGAAAGATGGCCTCTATTTATAAGCTATTACTTCTAGATGGGCC
TGCGTCCCATTAGTTAGTTGGTGGGGTAATGGCCTACCAAGACGATGATGGGTAGCCGGTCTGAGAGGAT
GACCGGCCACACTGGAACTGAGACACGGTC
>Zotu10324
GAACGCTGGCGGCGTGCTTAACACATGCAAGTCGAGCGGAAAGGCCCTTCGGGGTACTCGAGCGGCGAA
CGGGTGAGTAACACGTGAGTAACCTGCCCCTGACTCTGGGATAAGCCTGGGAAACTGGGTCTAATACCGG
ATACGACCATTTCTCGCATGTGATGGTGGTGGAAAGTTTTTTCGGTTGGGGATGGGCTCGCGGCCTATCAG
CTTGTTGGTGGGGTGATGGCCTACCAAGGCGACGACGGGTAGCCGGCCTGAGAGGGCGACCGGCCACAC
TGGGACTGAGACACGGCC
>Zotu10328
GAACGCTGGCGGCAGGCCTCAAACATGCAAGTCTAGGGGTGTAGCAATACACACCGGCGGACGGGTGCG
TAACACGTGGGAATGTACTTACTGGTTCGGAACAACTGCGGGAAACTGCAGCTAATACCGAATGAGCCCG
AAAGGGGAAAGATTTATCGCCAGTAAATCAGCCCGCGTTCGATTAGCTAGTTGGTGGGGTAACGGCTCAC
CAAGGCGACGATCGATAGCTGTTCTGAGAGGAAGATCAGCCACACTGGGACTGAGACACGGCC
>Zotu10333
GAACGCTAGCGGCAGGCTTAACACATGCAAGTCGAGGGGTAGAAGCAGCTTGCTGCTTTGAGACCGGCGC
ACGGGTGCGTAACGCGTATGCAATCTGCCTTTCACAAAGGGATAGCCCAGAGAAATTTGGATTAATACCTT
ATGGTATATAGGAGTGGCATCACTTTTATATTAAAGATTTATCGGTGAAAGATGAGCATGCGTCCCATTAGC

TTGTTGGTAAGGTAACGGCTTACCAAGGCAACGATGGGTAGGGGTCCTGAGAGGGAGATCCCCCACACTG
GTACTGAGACACGGAC
>Zotu10339
GAACGCTGGCGAGATGCCTTACACATGCAAGTCGTACGAGAGTTTTTAATTCAAGTGGCGGACGGGTGAG
TAACACGTAAGAATTTGCCTTTAGGAGGGGGACAACAACTGGAAACGGTTGCTAATACCCCATATGCTTTC
GAGTAAAATGGAATTTTCCGCCTAGAGAGAAGCTTGCGGCTGATTAGCTTGTTGGTGAGGTAATGGCTTAC
CAAGGCGACGATCAGTATCTGGTTTGAGAGGATGATCAGACACACTGGAACTGAGACACGGTC
>Zotu10344
GAACGCTAGCTACAGGCTTAACACATGCAAGTCGAGGGGCAGCATTTCAGTTTGCTTGCAAACTGGAGATG
GCGACCGGCGCACGGGTGAGTAACACGTATCCAACCTGCCGATAACTTGGGGATAGCCTTTCGAAAGAAA
GATTAATACCCGATGGTATAATCAGACCGCATGGTCTTGTTATTAAAGAATTTCGGTTATCGATGGGGATGC
GTTCCATTAGGCAGTTGGTGAGGTAACGGCTCACCAAACCTTCGATGGATAGGGGTTCTGAGAGGAAGGT
CCCCCACATTGGAACTGAGACACGGTC
>Zotu10348
GAACGCTGGCGGCGTGCTTAACACATGCAAGTCGAGCGGAAAGGCCCTTCGGGGTACTCGAGCGGCGAA
CGGGTGAGTAACACGTGGGTAACCTGCCCCTTGCTCTGGGATAACTCCGGGAAACCGGGGCTAATACCGG
ATATGACCCTTGAAGGCATCTTTGGGGGTGGAAAGTTTTTCGGCGAGGGATGGACCCGCGGCCTATCAGCT
TGTTGGTGGGGTAATGGCCTACCAAGGCGGTGACGGGTAGCCGGCCTGAGAGGGCGACCGGCCACACTG
GGACTGAGACACGGCC
>Zotu10354
GAACGCTGGCGGTGCGCCTAACACATGCAAGTCGAGTGGGTTTAGACCCACGGCGAACGGCTGCGTAACA
CGTTGGTACTCCCCCCGAACTCAGGCATAACCCCGCGAAAGCGGGGCTAATACCGGATGCCCTCGCAAGA
GGAAAGATTTATCGGTTCGGGAGAGGCCTGCGGCCTATCAGGTAGTTGGTGAGGTAACGGCTCACCAAGC
CGATGACGGGTAGCTGATCTGAGAGGATGATCAGCCAGAATGGGACTGAGACACGGCC
>Zotu10356
GAACGCTAGCGGGAGGCCTAACACATGCAAGCCGAGCGGTAGAGATTCTTCGGAATCTTGAGAGCGGCGC
ACGGGTGCGGAACACGTGTGCAACCTGCCTTTATCTGGGGGATAGCCTTTCGAAAGGAAGATTAATACCCC
ATAATATTTTTGATGGCATCATTAGAAATTGAAAACTCCGGTGGATAGAGATGGGCACGCGCAGGATTAGA
TAGTTGGTGAGGTAACGGCTCACCAAGTCAACGATCCTTAGGGGGCCTGAGAGGGTGATCCCCCACACTG
GTACTGAGACACGGAC
>Zotu10360
GAACGCTGGCGGTGTGCTTCACACATGCAAGTCGAACGAGGTAGCAATACCTAGTGGCGGACGGGTGAGT
AACGCGTGGGAATCTGCCTTTAGATGGGGGATAACGAGCCGAAAGGTTCGCTAATACCGCATATGCCGCA
AGGTGAAAGGAGTAATCCGTCTAAAGATGAGCCCGCGTCCGATTAGCTAGTTGGTGGGGTAAAGGCCTAC
CAAGGCGACGATCGGTAGCTGGTCTGAGAGGATGATCAGCCACAATGGGACTGAGACACGGCC
>Zotu10363
GAACGCTGGCGGCGTGCTTCAGACATGCAAGTCGTACGCGAAAGTCCGCAAGGATGAGTAAAGTGGCGC
ACGGGTGAGTAATACTTAGGTAATGCGCCCTCTCGCTGGGGATAACTAGACGAAAGTTTAGCTAATACCGA
ATGGTCCGTTTGAAACTGCGGTTTCGATCGGTAAAGCCGTAAGGCGCGAGAGGAGTGGCCTGAGTCTCAT
TAGCTAGTTGGTAGGGTAACGGCCTACCAAGGCAACGATGGGTAGCTGGTCTGAGAGGACGATCAGCCAC
ACTGGAACTGGAACACGGTC
>Zotu10367
GAACGCTGGCGGCGTGCTTAACACATGCAAGTCGAACGGAAAGGCCCTTCGGGGTACTCGAGTGGCGAAC
GGGTGAGTAACACGTGGGTGATCTGCCCTGCACTTTGGGATAAGCCTGGGAAACTGGGTCTAATACCGAA
TATGATCATCGGCTTCATGGTCGGTGGTGGAAAGCTTTTGCGGTGCGGGATGGGCCCGCGGCCTATCAGCT
TGTTGGTGGGGTAATGGCCTACCAAGGCGACGACGGGTAGCCGGCCTGAGAGGGTGACCGGCCACACTG
GGACTGAGATACGGCC
>Zotu10369
GAACGCTGGCGGCATGCCTAACACATGCAAGTCGAACGATGGTCTTCGGATCATAGTGGCGCACGGGTGC
GTAACGCGTGGGAATCTGCCCTTGGGTTCGGAATAACAGTTGGAAACGACTGCTAATACCGGATGATGTC

GATAAGACCAAAGATTTATCGCCCGAGGATGAGCCCGCGTCGGATTAGCTAGTTGGTGGGGTAAAGGCCT
ACCAAGGCGACGATCCGTAGCTGGTCTGAGAGGATGATCAGCCACACTGGGACTGAGACACGGCC
>Zotu10373
GAACGCTGGCGGCATGCCTAACACATGCAAGTCGAACGGGATCCTGGCTTCGGTCAGGTGAGAGTGGCGG
ACGGGTGAGTAACGCGTGGGAACCTGCCCAGTGGTGGGGGATAACTGCGGGAAACTGCAGCTAATACCG
CATGAGCCCGAGAGGGGAAAGATTTATCGCCATTGGAGGGGCCCGCGTTCGATTAGGTAGTTGGTGAGGT
AACGGCTCACCAAGCCGACGATCGATAGCTGGTCTGAGAGGATGATCAGCCACACTGGGACTGAGATACG
GCC
>Zotu10375
GAACGCTGGCGGCGTGCTTAACACATGCAAGTCGAACGCTGAAGCTTGGTGCTTGCACTGGGTGGATGAG
TGGCGAACGGGTGAGTAATACGTGAGTAACCTGCCCTTGACTCTGGGATAAGCCCGGGAAACTGGGTCTA
ATACCGGATAGGAGCGCCTGTCGCATGACGGGTGTTGGAAAGATTTTTCGGTCATGGATGGACTCGCGGC
CTATCAGCTTGTTGGTGAGGTAATGGCTCACCAAGGCGACGACGGGTAGCCGGCCTGAGAGGGTGACCGG
CCACACTGGGACTGAGACACGGCC
>Zotu10378
GAACGCTGGCGGCATGCCTAACACATGCAAGTCGAACGTGATCCGGGGCTTGCTCCGGTGAAAGTGGCGG
ACGGGTGAGTAACGCGTGGGAACCTGCCCAGTGGTCGGGGATAACTGCGGGAAACTGCAGCTAATACCC
GGTAAGCCCTACGGGGGAAAGATTTATCGCCATTGGAGGGGCCCGCGTCGGATTAGGTAGTTGGTGGGGT
AAAGGCCTACCAAGCCTACGATCCGTAGCTGGTCTGAGAGGATGATCAGCCACACTGGGACTGAGATACG
GCC
>Zotu10383
GAACGCTGGCGGCAGGCCTAATACATGCAAGTCGAGGGGCAGCGGGGTAGCAATACTGCCGGCGACCGG
CGCACGGGTGCGTAACACGTATGCAACCTACCTTAAACAGGGGGATAGCCCGGGGAAACTCGGATTAATA
CCCCATAACACGGTGATACCACATGGTAATGTCGTTAAAGATTTATCGGTTTAAGCTGGGCATGCGTTCGAT
TAGCTAGTTGGTAAGGTAATGGCTTACCAAGGCTACGATCGATAGGGGGTCTGAGAGGATGATCCCCCAC
ACTGGTACTGAGATACGGAC
>Zotu10386
GAACGCTGGCGGCGTGCTTCCCCCCTGCCCGTCGCCCGAGCCCGCGCTGTCGCCCTCCAGGGTTCTGCTCG
TGGCAGCCGGGTGAGTAATACATGGGAATCTACCTTGCAGTGCGAAATAACAGTTGGAAGCAATTGCTAA
TGTCGCATATAGCCGAGAGGTGAAAGGTTTACTGCTGCAAGATGGGCCCATGCAGGATTAGCTAGTTGGT
GAGGTAATGGCTCACCAAGGCAACGAGCGGGAGGGGGGCGGAGAGGATGATCAGCCACACGGGAACGG
AGACACGGGC
>Zotu10390
GAACGCTGGCGGCGTGCTTAACACATGCAAGTCGAACGGTGAAGCCCTTCGGGGTGGATCAGTGGCGAAC
GGGTGAGTAACACGTGGGCAATCTGCCCTGCACTCTGGGATAAGCCTGGGAAACTGGGTCTAATACCGGA
TATTCACTGGTTGGGGCATCCCGGCTGGTGGAAAGTTTTGGTGGTGCAGGATGAGCCCGCGGCCTATCAG
CTTGTTGGTGGGGTGATGGCCTACCAAGGCGACGACGGGTAGCCGGCCTGAGAGGGCGACCGGCCACAC
TGGGACTGAGACACGGCC
>Zotu10393
GAACGCTGGCGGCGTGCTTAACACATGCAAGTCGAGCGGTAAGGCCCTTTCGGGGGTACACGAGCGGCG
AACGGGTGAGTAACACGTGAGTAATCTGCCCTTCACTTTGGGATAAGCCTCGGAAACGGGGTCTAATACCG
GATATGACACCTTGATGCATGTTGGGGTGTGGAAAGTTTTTTCGGTGGGGGATGTGCTCGCGGCCTATCAG
CTTGTTGGTGGGGTAATGGCTCACCAAGGCTTTGACGGGTAGCCGGCCTGAGAGGGTGACCGGCCACACT
GGGACTGAGACACGGCC
>Zotu10396
GAACGCTGGCGGCGTGCCTTAAGCATGCAAGTCGAATGGTCGCAAGACAATGGCAGACGGGTGAGTAAT
ATATAGGAATCTACCCCGAAATGGACCATAGCTCATCGAAAGATGGGGTAATAGTCCATAGTCTCGTAAGA
GTAAAGCACTTAACTGTGCGTTTCGGGAGGAGCCTATATCCTATCAGCTAGTTGGCGGGGTAAAAGCCCAC
CAAGGCGATGACGGGTAGCTGGACTGAGAGGTCGGTCAGCCACAACTGCACTGAGACACGGGC
>Zotu10398

GAACGCTGGCGGCATGCCTAACACATGCAAGTCGAACGAAGGCTTCGGCCTTAGTGGCGCACGGGTGCGT
AACGCGTGGGAATCTGCCCTTGGGTTCGGAATAACAGTGGGAAACTACTGCTAATACCGGATGATGACGT
AAGTCCAAAGATTTATCGCCCAGGGATGAGCCCGCGTAGGATTAGCTGGTTGGTGGGGTAAAGGCCTACC
AAGGCGACGATCCTTAGCTGGTCTGAGAGGATGATCAGCCACACTGGGACTGAGACACGGCC
>Zotu10407
GAACGCTAGCGGCAGGCATAATACATGCAAGGCGAGGGGGTAGCAATACCACCGTCGTACGGGTGCGTA
ACACGTATGCAACCTGCCCTTCACTGGGGGATAGCCTGGGGAAACTCGGATTAATACCGCATAAGACAGTA
GACTGGCATCAGTTAGCTGTTAAAGATTTATTGGTGAAGGATGGGCATGCGTCTGATTAGCTAGTTGGCGG
GGTAAAGGCCCACCAAGGATACGATCAGTAGGGGTTCTGAGAGGATTGGCCCCCACACTGGCACTGAGAT
ACGGGC
>Zotu10416
GAACGCTGGCGGCATGCCTAACACATGCAAGTCGAACGAAGGCTTCGGCCTTAGTGGCGCACGGGTGAGT
AACACGTGGGAACCTGCCTTTCGGTTCGGAATAACGTCTGGAAACGGACGCTAACACAGGATACGCCCCCT
GGCTGTGGTGGCCTGGGGGGAAAGTTTACGCCGAGAGAGGGGCCCGCGTCCGATTAGGTAGTTGGTGGG
GTAATGGCCTACCAAGCCGACGATCGGTAGCTGGTCTGAGAGGATGATCAGCCACACTGGGACTGAGACA
CGGCC
>Zotu10420
GAACGCTGGCGGTATGCTTAATACATGCAAGTCGAGCGGTAAGGTTCTGCACTTTTTATTTAATTGCGTTTG
GCCGTATAAGATTTATTTTATACATTGCCTTCAAAGACAGAGACAGTGGCTACATTGTTATCTCGGAGATAG
GTGAGTTGACCAACGGAAATCCTAAGATGGCTATCGAAGGAACAGATTGAATACGGAGTGTAGAATACAC
GAGCGGCGGACGGGTGAGTAACGCGTAAGAATGTACCCCAAAGTGAGGGATAAGCACCAGAAATGGTGT
CTAATACCGCATGTGCTCTTCGGAGTAAAGCTTTATGCGCTTTGGGAACAGCTTGCGTCTGATTAGCTAGTT
GGTAAGGTAACGGCTTACCAAGGCGACGATCAGTAGCTGGTCTGAGAGGATGACCAGCCACACTGGGACT
GAGACACGGCC
>Zotu10423
GAACGCTAGCGGCAGGCTTAATACATGCAAGTCGAGGGGCAGCACAGATAGCAATATTGGGTGGCGACC
GGCAAACGGGTGCGGAACACGTACAGAACCTTCCTTTAAGTGGAGCATAGCCCAGGGAAACTTGGATTAA
TACTCCGTAACATTATTATGTGGCATCACATTATAATTATAGCTCAGGCGCTTAAAGATGGCTGTGCGGCTG
ATTAGGTAGTTGGCGGGGTAACAGCCCACCAAGCCTGCGATCAGTAACTGGTGTGAGAGCACGACCAGTC
ACACGGGCACTGAGACACGGGC
>Zotu10426
GAACGCTGGCGGCGTGCCTAATACATGCAAGTCGTGCGAGACCTTCGGGTCTAGCGGCGGACGGGTGAGT
AACACGTGGGTAACCTGCCTGATCGACCGGGATAACGCTTGGAAACGAGTGCTAATACCGGATAATCTCCT
GCACCGCATGGTGCGGGAGTAAAAGGAGCTTTTGCTTCACGATCAGATGGACCCGCGGCGCATTAGCTAG
TTGGTGAGGTAACGGCTCACCAAGGCGACGATGCGTAGCCGACCTGAGAGGGTGACCGGCCACACTGGG
ACTGAGACACGGCC
>Zotu10428
GAACGCTGGCGGCGTGCTTAACACATGCAAGTCGAGCGGTAAGGCCCTTCCGGGGGTACACGAGCGGCG
AACGGGTGAGTAACACGTGAGTAATCTGCCCTCCACTTTGGGATAAGCCTCGGAAACGAGGTCTAATACCG
GATACGACCGCTTCCTGCATGGGATGGTGGTGGAAAGTTTTTTCGGTGGGGGATGTGCTCGCGGCCTATCA
GCTTGTTGGTGAGGTAATGGCTCACCAAGGCTTCGACGGGTAGCCGGCCTGAGAGGGTGACCGGCCACAC
TGGGACTGAGACACGGCC
>Zotu10437
GAACGCTGGCGGCGTGCTTAACACATGCAAGTCGAACGGTGACCTCGAGAGCTTGCTCTTGAGTGATCAGT
GGCGAACGGGTGAGTAACACGTGAGTAACCTACCCCTGACTCTGGAATAACAATTGGAAACAGTTGCTAAT
ACCGGATACGACACGAGCGGGCATCCGCATCGTGTGGAAAGTTTTTCGGTCTGGGATGGACTCGCGGCCT
ATCAGCTTGTTGGTGAGGTAATGGCTCACCAAGGCGACGACGGGTAGCCGGCCTGAGAGGGCGACCGGC
CACACTGGGACTGAGACACGGCC
>Zotu10440
AAACGCTGGCGGCGTGCATAACACATGCAAGTCGAACGATGAAGATTAAATGATTTCTTCGGAATGAACTT
AATTGGATTAGTGGCGGACGGGTGAGTAACGCGTGTGTAACCTGCCTTGTACAAGGGGATAGCCGTTGGA

AACGACGAATAATACCCTATGACATCAAATAATCGCATGATAATTTGATCAAAGATTTATCGGTATAAGATG
GACTCGCGTCTGATTAGCTAGTTGGCGGGGTAACAGCCCACCAAGGCAACGATCAGTAGCCGGCTTGAGA
GAGTGTACGGCCACATTGGGACTGAGACACGGCC
>Zotu10444
GAACGCTGGCGGCGTGCTTAACACATGCAAGTCGAACGAAGCAACTTTCTTGCTTGCAAGAAAGTTGACTT
AGTGGCGGACGGGTGAGTAACGCGTGGGTAACCTGCCTCATACAGGGGGATAACAGTTGGAAACGACTG
CTAACACCGCATAACCCGCTAGTGTCGCATGGCACAGACGGAAAATATTTATAGGTATGAGATGGGCCCGC
GTCTGATTAGCTAGTTGGTAAGGTAACGGCTTACCAAGGCGACGATCAGTAGCCGACTTGAGAGAGTGAT
CGGCCACATTGGGACTGAGACACGGCC
>Zotu10448
GAACGCTGGCGGCGTGCTTAACACATGCAAGTCGAACGGTGATCCCAGGAGCTTGCTTCTGGGTGAGCAG
TGGCGAACGGGTGAGTAACACGTGAGTAACCTGCCCCTGACTCTGGAATAAGCGCTGGAAACGGCGTCTA
ATACCGGATACGAGACGCAACCGCATGGTTAGTGTCTGGAAAGTTTTTCGGTCTGGGATGGACTCGCGGCC
TATCAGCTTGTTGGTGAGGTAATGGCTCACCAAGGCGATGACGGGTAGCCGGCCTGAGAGGGCGATCGGC
CACACTGGGACTGAGACACGGCC
>Zotu10452
GAACGCTGGCGGCAGGCCTAACACATGCAAGTCGAACGGTAGCGGGAAGAAGCTTGCTTCTTTGCCGACG
AGTGGTGGACGGGTGAGTAATGTCTGGGGATCTGCCCGATGGAGGGGGATAACCACTGGAAACGGTGGC
TAATACCGCATAACGTCGCAAGACCAAAGTGGGGGACCTTCGGGCCTCACACCATCGGATGAACCCAGAT
GGGATTAGCTAGTAGGTGGGGTAACGGCTCACCTAGGCGACGATCCCTAGCTGGTCTGAGAGGATGACCA
GCCACACTGGAACTGAGACACGGTC
>Zotu10455
GAACGCTGGCGGCGTGCCTAACACATGCAAGTCGAGCGATTCTCTTCGGAGAAGAGCGGCGGACGGGTG
AGTAACGCGTGGGTAACCTGCCCTGTACACACGGATAACATACCGAAAGGTATGCTAATACGAGATAATAT
ATGAAAGTTGCATGACTTTTATATCAAAGCTTTTGCGGTACAGGATGGACCCGCGTCTGATTAGCTAGTTGG
TAAGGTAACGGCTTACCAAGGCGACGATCAGTAGCCGACCTGAGAGGGTGATCGGCCACACTGGAACTGA
GACACGGTC
>Zotu10458
GCGGCACCTGGGCGTCAGGGTTGATAAACGGAGGCGTCGCGAGCCTCACGGCCAAGAGATGGATGGTGG
GCCGCGATGTCAGGCTCGATTCAGCTCATAGGATATTCAGAACGGGCCTCGCTGCATCCAAGACTGTGCTG
GATCCTTCCTTCGCTCCTTTTTTTTCTCTT
>Zotu10461
GAACGCTGGCGGCGTGCTTAACACATGCAAGTCGAACGGATCGGCTGGGGCTTGCTCTGGCTGGTGAGTG
GCGAACGGGTGAGTAACACGTGAGCAACCTGCCCCTGACTCTGGGATAACTGCTGGAAACGGTGGCTAAT
ACCGGATATGAGGTCCTGCCGCATGGTGGGGTCTGGAAAGTTTTTCGGTTGGGGATGGGCTCGCGGCCTA
TCAGCTTGTTGGTGGGGTGATGGCTCACCAAGGCGACGACGGGTAGCCGGCCTGAGAGGGCGACCGGCC
ACACTGGGACTGAGACACGGCC
>Zotu10463
TAACGCTAGCAGGATGCCTAATACATGCAAGTCGAACGAGGTAGCAATACCTAGTGGCAAACGGGTGAGT
AATGCATGCTCAATCTACCCATAAGAGGGAGATAACAGCTCGAAAGAGTTGCTAATATCCCATAACATATC
ATGGCAAAAGACTTGATATCTAAAGTTGCGTTCGCAACGCTTTTGGATGAGGGTGTGTCTTATCAGATAGTT
GGTGAGGTAACGGCCCACCAAGTCGATGACGAGTAGCTGTGCTGAGAGGCAGAACAGCCACAATGGGAC
TGAGACACGGTC
>Zotu10465
ACCGTATCTCAGTTCCAGTGTGGCTGATCGTCCTCTCAGACCAGCTATAGATCATAACCTTGGTAAGCCATT
ACCTCACCAACTAGCTAATCTAATATAGGCTCATCTAATAGCGATAAATCTTTCCCCCGTAGGGATTATACG
GTATTACCTGTCATTTCTAACAGCTATTCCATACTACTAGGCAGATTCCTATACATTACTCACCCGTCTGCCAC
TAACTACAATTGTAGCAAGCTATAATTGTAATTCGTTCGACTTGCATGTGTTAAGCTTGCCGCCAGCGTTCA
>Zotu10472

GAACGTTAGCGGCGCGCCTAACACATGCAAGTCGCACGAGAAAGGGCTTCGGCCCCGGTAAAGTGGCGCA
CGGGTGAGTAACACGTGGGTAATCTGCCCTCGAGCGGGGGATAACATTCCGAAAGGAGTGCTAATACCGC
ATAAGACCACGATTTCGAAAGAGATTGCGGTCAAAGCTGGCCTCTATACATAAGCTAGCACTTGAGGATGA
GCCCGCGGCGCATCAGCTAGTTGGTGAGGTAATGGCTCACCAAGGCTAAGACGCGTAGCTGGTCTGAGAG
GATGATCAGCCACACTGGAACTGAGACACGGTC
>Zotu10474
GAACGCTGGCGGCGTGCTTAAGACATGCAAGTCGAACGGCCTGAAGCTTGCTTCAGGCAGTGGCGCACGG
GTGAGTAACGCGTAACTGACCTGCCCCAAAGTTCCGAATAACGCCTCGAAAGAGGTGCTAATACGGGATG
TGCTGTCCCCTCGTGTGGGGCCAGTAAAGGCTATGACCGCTTTGGGATGGGGTTGCGTTCCATCAGCTAGT
TGGTGGGGTAAAGGCCTACCAAGGCGACGACGGATCACCGGCCTGAGAGGGTGGCCGGTCACAGGGGCA
CTGAGACACGGGT
>Zotu10480
GAACGCTGGCGGCAGGCCTAACACATGCAAGTCGAACGCTGTAGCAATACAGAGTGGCAGACGGGTGAG
TAACGCGTGGGAACGTACCCTTCGGTCTGGAATAACCCAGGGAAACTTGGGCTAATACCGGATACGTGCG
AGAGCAGAAAGATTTATCGCCGAAGGATCGGCCCGCGTCCGATTAGCTAGTTGGTGAGGTAAAGGCTCAC
CAAGGCGACGATCGGTAGCTGGTCTGAGAGGATGATCAGCCACACTGGGACTGAGACACGGCC
>Zotu10483
GAACGCTGGCGGCGTGCTTAACACATGCAAGTCGAGCGGAAAGGCCCTTCGGGGTACTCGAGCGGCGAA
CGGGTGAGTAACACGTGGGCAACCTGCCCCTAGCTCTGGGATAACTCCGGGAAACCGGGGCTAATACCGG
ATACGACCACTGTGGGCATCTTCGGTGGTGGAAAGTTTTTCGGCTAGGGATGGGCCCGCGGCCTATCAGCT
TGTTGGTGGGGTGATGGCCTACCAAGGCGACGACGGGTAGCCGGCCTGAGAGGGCGACCGGCCACACTG
GGACTGAGACACGGCC
>Zotu10490
GAACGCTAGCGGGAGGCCTAACACATGCAAGCCGAGGGGTACGATTCTTTCGGGAATCTGAGACCGGCGC
ACGGGTGCGTAACGCGTATGCAACTTGCCCTACTGAAAAGGATAGCCCTTCGAAAGGAGGATTAATACTTT
ATAACAGATTGAATGGCATCATTTAATTTTGAAAGATTTATCGCAGTAGGATAGGCATGCGTAAGATTAGTT
AGTTGGAGAGGTAACGGCTCACCAAGACGATGATCTTTAGGGGGCCTGAGAGGGTGAACCCCCACACTGG
TACTGAGACACGGAC
>Zotu10492
GAACGCTGGCGGCATGCCTAACACATGCAAGTCGAACGGCAGCACAGCAGTAGCAATACTGTGGGTGGCG
AGTGGCGAACGGGTGAGGAATGCGTCGGAATCTGCCCAGTTGTGGGGGATAACTAGCCGAAAGGTTAGC
TAATACCGCATACGACCGAGAGGTGAAAGCGGGGGACCGTAAGGCCTCGCGCGATTGGATGAGCCGACG
CCGGATTAGCTTGTTGGTGGGGTAAAGGCCTACCAAGGCGACGATCCGTAGCTGGTCTGAGAGGACGATC
AGCCACACTGGGACTGAGACACGGCC
>Zotu10494
GAACGCTGGCGGCGTGGATTAGGCATGCAAGTCGAACGGTAGTTACCTTTCGGGGTAATGAGAGTGGCGA
ACGGGTGAGTAACGCGTAGGTGATCTACCTTCAAGTGGGGGACAACCCAGGGAAACTTGGGCTAATACCG
CATGTGGCAGTAATGCTAAAGCCGGCCTCTGAATATGCTGGTGCTTGAAGATGAGCCTGCGTCTGATTAGC
TAGTTGGTGGGGTAATGGCCTACCAAGGCGATGATAAGTAGGCGGCTTTAACGAGTGGTCGCCCACACTG
GGATTGAGATACGGCC
>Zotu10500
GAACGCTAGCGGCAGGCCTAATACATGCAAGTCGAGGGGTAGGGTAGCAATACCTGAGACCGGCGCACG
GGTGCGTAACACGTATGCAATCTACCTTTAATTGGAGCATAGCCTCGAGAAATCGGGATTAATACTCCATAT
CATTATGAAGTGGCATCACTATATAATTAAACCTCCGGGGATTAAAGATGAGCATGCGCAACATTAGCTAG
TTGGTAAGGTAATGGCTTACCAAGGCTACGATGTTTAGGGGGTCTGAGAGGATGATCCCCCACACTGGTAC
TGAGACACGGAC
>Zotu10502
GAACGCTGGCGGCAGGCTTAACACATGCAAGTCGAGCGCCTGTAGCAATACAGGAGCGGCAGACGGGTG
AGTAACGCGTGGGAACGTACCCTTTGGTTCGGAACAACACAGGGAAACTTGTGCTAATACCGGATAAGCC
CTTACGGGGAAAGATTTATCGCCGAAGGATCGGCCCGCGTCTGATTAGCTTGTTGGTGAGGTAACGGCTCA
CCAAGGCTACGATCAGTAGCTGGTCTGAGAGGATGATCAGCCACACTGGGACTGAGACACGGCC

>Zotu10504
GAACGCTGGCGGCGCGCTTAACACATGCAAGTCGTACGGAGTTCCCTCTTCGGAGGGTGCTCAGTGGCGA
ACGGGTGCGTAACACGTGAGCAACCTGCCTCGGGGTGGGGGATAACCCGTGGAAACGCGGGCTAATACC
GCATGACCTCGTCGAACGGAGTCGTTTGACGAGGAAAGGTGCTGAAAGGCACTGCCTTGAGAGGGGCTCG
CGGCGTATCAGCTAGCTGGTGGGGTAACGGCCTACCAGGGCGACGACGCGTAGCCGATCTGAGAGGATG
ATCGGCCACATTGGGACTGAGATACGGCC
>Zotu10506
GAACGCTGGCGGCAGGCTTAACACATGCAAGTCGAGCGGATGAAGGTAGCTTGCTACCGGATTCAGCGGC
GGACGGGTGAGTAATGCTTAGGAATCTGCCTATTAGTGGGGGACAACGTTCCGAAAGGAGCGCTAATACC
GCATACGTCCTACGGGAGAAAGCAGGGGACCTTTGGGCCTTGCGCTAATAGATGAGCCTAAGTCGGATTA
GCTAGTTGGTAGGGTAAAGGCCTACCAAGGCGACGATCTGTAGCGGGTCTGAGAGGATGATCCGCCACAC
TGGGACTGAGACACGGCC
>Zotu10510
GAACGCTGGCGGCATGCCTAACACATGCAAGTCGAACGAAGGCTTCGGCCTTAGTGGCGCACGGGTGCGT
AACGCGTGGGAATCTGCCCAAAGCTACGGAATAACTCGGGGAAACTCGTGCTAATACCGTATGATGTCGC
AAGACCAAAGATTTATCGGCTTTGGATGAGCCCGCGTAGTATTAGCTTGTTGGTGGGGTAAAGGCCTACCA
AGGCGACGATCCTTAGCTGGTCTGAGAGGATGATCAGCCACACTGGGACTGAGACACGGCC
>Zotu10513
GAACGCTGGCGGCGTGCCTAATACATGCAAGTCGAGCGAACTTTTGGGAGCTTGCTCCCAAAAGTTAGCG
GCGGACGGGTGAGTAACACGTGGGCAACCTGCCTATAAGACTGGGATAACTTCGGGAAACCGGAGCTAAT
ACCGGATCATTCTTTTCTACACATGTAGAAAAGCTAAAAGACGGTTTACGCTGTCACTTATAGATGGGCCCG
CGGCGCATTAGCTAGTTGGTGAGGTAACGGCTCACCAAGGCCACGATGCGTAGCCGACCTGAGAGGGTGA
TCGGCCACACTGGGACTGAGACACGGCC
>Zotu10521
GAACGCTGGCGGCGTGCTTAACACATGCAAGTCGAACGGTGAAGCAGAGCTTGCTCTGTGGATCAGTGGC
GAACGGGTGAGTAACACGTGAGCAATCTGCCCCTGACTCTGGGATAAGCGCTGGAAACGGCGTCTAATAC
CGGATACGAGCTGCGACCGCATGGTCAGTAGCTGGAAAGAATTTCGGTCAGGGATGAGCTCGCGGCCTAT
CAGCTTGTTGGTGAGGTAATGGCTCACCAAGGCGTCGACGGGTAGCCGGCCTGAGAGGGTGACCGGCCAC
ACTGGGACTGAGACACGGCC
>Zotu10525
TAACGCTAGCGGCAGGCCTAATACATGCAAGTCGAGGGGCAGCGGGGTAGCAATACTGCCGGCGACCGG
CGCACGGGTGCGTAACACGTATGCAACCTACCTTTCATTGGGGAATAGCCCTGCGAAAGTAGAATTAATAC
CGCATAATATTGTTGTTCGGCATCGTTTGACAATTAAAACTGAGGTGATGAAAGATGGGCATGCGTCTGAT
TAGCTAGTTGGCGAGGTAACGGCTCACCAAGGCGACGATCAGTAGGGGGTCTGAGAGGATGATCCCCCAC
ACCGGTACTGAGACACGGAC
>Zotu10529
GAACGCTAGCGGCGGGCCTAACACATGCAAGTCGAGCGCATACCTTCGGGTATGAGCGGCGGACGGGTG
AGTAACACGTAGGTAATCTGCCCTGTGGTGGGGGATAACCCGGGGAAACCCGGGCTAATACCGCATATGG
TCTACGGATCAAAGCCTTCGGGCGCCAGAGGATGAGCCTGCGTCGCATTACCTAGTTGGCGGGGTAAAAG
CCCACCAAGGGGACGATGCGTAGCTGGTCTGAGAGGATGATCAGCCACACTGGGACTGAGACACGGCC
>Zotu10533
GAACGCTAGCGGCAGGCTTAATACATGCAAGTCGAACGGTAGAGATCTTTCGGGATCTCGAGAGTGGCGC
ACGGGTGAGTAACACGTATCCAACTTGCCCTGTACTGGGGGATAGCCTCGGGAAACTGAGATTAATACCCC
ATAAGATTGTGGAATGGCATCATTCAGCAATTAAAGTTCCAACGGTACAGGATAGGGATGCGTCTGATTAG
GTAGTTGGTGGGGTAACGGCCTACCAAGCCGACGATCAGTAGCTGGCGTGAGAGCGCGACCAGCCACACT
GGCACTGAGACACGGGC
>Zotu10536
CAACGCTGGCGGCGTGCCTAATACATGCAAGTCGAACGCGAAAGTCCCGCAAGGGGCGAGTAGAGTGGC
AAACGGGTGAGTAACGCGTGGGCGACCTACCTTCGAGTGGGGGATAACCTTCCGAAAGGAGGGCTAATAC
CGCATGACAGCCCCTGTTTAGATACAGGGAGCTCAAAGCCGGGGATCGCAAGACCTGGCGCTTGGAGACG

GGCCCGCGTCCGATTAGCTAGTTGGTGGGGTAACGGCTCACCAAGGCAACGATCGGTATCCGGCCTGAGA
GGGCGGACGGACACACTGGGACTGAGACACGGCC
>Zotu10540
GAACGCTGGCGGCGTGCCTTAAGCATGCAAGTCGAACGGGTAGTAATATCAGTGGCGGACGGTCGAGTAA
CGCGTGGGAATCTGCCTTGAAGTGGGGGACAACCCGGCGAAAGCCGGGCTAATACCGCATGTGATCGCAA
GATCAAAGTCGTAAGACGCTTCAAGATGAGCCTGCGTCTGATTAGCTAGTTGGTGGAGTAACGGTTTACCA
AGGCGACGATCAGTAGCTGGTCTGAGAGGACGATCAGCCGGAATAGGACTGCGACACGGCC
>Zotu10544
GAACGCTAGCGGCATGCCTTACACATGCAAGTCGAACGGCAGCATAGGAGCTTGCTCCTGATGGCGAGTG
GCGAACGGGTGAGTAATATATCGGAACGTGCCCTAGAGTGGGGGATAACTAGTCGAAAGACTAGCTAATA
CCGCATACGATCTACGGATGAAAGTGGGGGATCGCAAGACCTCATGCTCCTGGAGCGGCCGATATCTGATT
AGCTAGTTGGTGGGGTAAAAGCTCACCAAGGCGACGATCCGTAGCTGGTCTGAGAGGATGATCAGCCACA
CTGGGACTGAGACACGGCC
>Zotu10547
GAACGCTAGCGGCAGGCTTAATACATGCAAGTCGAGGGGCAGCATGGTGTAGCAATACACTGATGGCGAC
CGGCAAACGGGTGCGGAACACGTACACAACCTTCCTTTAAGCGGGGGATAGCCCAGAGAAATTTGGATTA
ATACCCCATAGGATAATGATAGGGCATCTTATTATTATTAAAGATTTATCACTTGAAGATGGGTGTGCGTCT
GATTAGGTAGTTGGCGGGGTAACGGCCCACCAAGCCATCGATCAGTAACTGGTGTGAGAGCACGACCAGT
CACACGGGCACTGAGACACGGGC
>Zotu10551
GAACGTTAGCGGCGCGCCTAAGACATGCAAGTCGAGCGAGAAAGGGCTTCGGCCCCGGTACAGCGGCGC
ACGGGTGAGTAACACGTAGGTCATCAACCCTCGAGTGGTGGATAACCCTCCGAAAGGAGGGCTAATACAG
CATGACGTTAGCAATAACCAAAGCGGGCCTCTTCATGAAAGCTCGCGCTAGAGGATGAGCCTGCGGCCCAT
CAGGTAGTTGGCGGAGTAACAGCCCACCAAGCCAAAGACGGGTAGCTGGTCTGAGAGGATGAACAGCCA
CACTGGAACTGAGACACGGTC
>Zotu10553
GAACGCTGGCGGCATGCCTTACACATGCAAGTCGAACGGCAGCACGGGAGCAATCCTGGTGGCGAGTGG
CGAACGGGTGAGTAATACATCGGAACGTGTCCTGTAGTGGGGGATAGCCCGGCGAAAGCCGGATTAATAC
CGCATACGCTCTACGGAGGAAAGGGGGGGATCTCAGGACCTCCCGCTACAGGGGCGGCCGATGGCAGAT
TAGCTAGTTGGTGGGGTAAAGGCCTACCAAGGCGACGATCTGTAGCTGGTCTGAGAGGACGACCAGCCAC
ACTGGGACTGAGACACGGCC
>Zotu10558
GAACGCTGGCGGCATGCCTAACACATGCAAGTCGAACGAGATCTTCGGATCTAGTGGCGCACGGGTGCGT
AACGCGTGGGAATCTACCCTTAGGTTCGGAATAACTTCGGGAAACTGAAGCTAATACCGGATGATGACGTA
AGTCCAAAGATTTATCGCCTAAGGATGAGCCCGCGTAGGATTAGCTTGTTGGTGAGGTAAAGGCTCACCAA
GGCGACGATCCTTAGCTGGTCTGAGAGGATGATCAGCCACACTGGGACTGAGACACGGCC
>Zotu10560
GAACGCTGGCGGCAGGCTTAACACATGCAAGTCGAGCGGGGAAGGGTAGCTTGCTACCTAACCTAGCGGC
GGATGGGTGAGTAATGCTTAGGAATCTGCTTATTAGTGGGGGACAACATTCCGAAAGGAATGCTAATACC
GCATACGCCCTACGGGGGAAAGCAGGGGATCTTCGGACCTTGCGTTAATAGATGAGCCTAAGTCAGATTA
GCTAGTTGGTGGGGTAAAGGCCTACCAAGGCGACGATCTGTAGCGGGTCTGAGAGGATGATCCGCCACAC
TGGGACTGAGACACGGCC
>Zotu10562
GAACGCTGGCGGCGTGCCTAAGACATGCAAGTCGAATGCTCCGCAAGGGGCATGGCGAACGGGTGAGTA
ATACCTAGGAACGTATCTTTAGTCGAGGATATCCCATCGAAATATGGTGCAATACTCGATGTGGCCGCAAG
GCTAAAGTCGCAAGACGCTAAAGAAGCGGCCTGGGCACCATCAGCTAGTTGGTAAGGTAATGGCTTACCA
AGGCTATGACGGTTAGGGGCCCCGAGAGGGGGAACCCCCACACTGGAACTGCGACACGGTC
>Zotu10566
GAATGCTGGCGGCGTGCCCAACACATGCAAGTCGAGCGGCAGCGCGGGTAGTTTACTACCTGGCGGCGAG
CGGCGGACGGCTGAGTAACGCGTGGGAACGTGCCCCAAAGTGAGGAATAACTGCCCGAAAGGGTAGCTA

ATGCCGCATATGGTCTTCGGATTAAAGGATTTATCCGCTTTGGGAGCGGCCCGCGTTAGATTAGGTAGTTG
GTGAGGTAAAGGCTCACCAAGCCGACGATCTATAGCTGGTCTGAGAGGATGATCAGCCAGACTGGGACTG
AGACACGGCC
>Zotu10571
GAACGCTGGCGGCATGCTTAACACATGCAAGTCGAACGGCAGCACCTTCTAGCTTGCTAGAAGGGTGGCG
AGTGGCGAACGGGTGAGTAACGCGTAGGAATATGCCTTGTAGTGGGGGACAACTTGGGGAAACTCAAGC
TAATACCGCATAATGTCTATGGACAAAAGTTGGGGACCTTCGGGCCTGGCGCTACAAGATTAGCCTGCGTA
CGATTAGCTAGTTGGTGGGGTAAGGGCCTACCAAGGCGACGATCGTTAGCTGGTCTGAGAGGATGGCCAG
CCACACTGGAACTGAGACACGGTC
>Zotu10575
GAACGCTGGCGGCGTGCCTAATACATGCAAGTCGGGCGGGGCTTGAGGAGGGCTTGCCCTTCGAGAGCCC
AGCGGCGGACGGGTGAGTAACACGTGGGTAACCTGCCCGTAAGACCGGGATAACTCCGGGAAACCGGGG
CTAATACCGGATAACACCGGGGGCCGCATGGCTTCCGGTTGAAAGGCGGCCTTTGGCTGCCACTTACGGAT
GGGCCCGCGGCGCATTAGCTAGTTGGCGGGGTAACGGCCCACCAAGGCGACGATGCGTAGCCGGCCTGA
GAGGGTGGACGGCCACACTGGGACTGAGACACGGCC
>Zotu10577
GAACGCTGGCGGTATGCTTAACACATGCAAGTCGAACGAGACAAGATGGAAGTGCTTGCACAGAAATCTT
AGAAAGTGGCGAACGGGTGAGTAACGCGTGGGTAACCTGCCCTATGGAAAGGAATAGCCTCGGGAAACT
GGGAGTAATGCCTTATAATATATTGAAGTCGCATGGCTTTGATATTAAACGCTCCGGTGCCATAGGATGGA
CCCGCGTCCCATTAGCTAGTTGGTGAGATAACAGCCCACCAAGGCGACGATGGGTAACCGGTCTGAGAGG
GCGAACGGTCACACTGGAACTGAGACACGGTC
>Zotu10584
GAACGCTGGCTACAGGCTTAACACATGCAAGTCGTGGGGAAACGGCATTATGTGCTTGCACATTCTGGACG
TCGACCGGCGCACGGGTGAGTATCGCGTATCCAACCTGCCCCTTACTTGGGGATACCCCGTTGAAAGACGG
CCTAATACCCGATGTGTTTCATTGACGGCATCCGATATGAAACAAAGGTTTTTCCGGTAAGGGATGGGGAT
GCGTCTGATTAGCTTGTTGGCGGGGCAACGGCCCACCAAGGCGACGATCAGTAGGGGTTCTGAGAGGAA
GGTCCCCCACATTGGAACTGAGACACGGTC
>Zotu10588
GAACGCTGGCGGCGCGCTTAACACATGCAAGTCGAGCGAGAACCAGGACTTCGGTCCTGGGGACAGCGG
CGAACGGGTGAGTAACACGTGGGTAATCTGCCCTCGATTCTGGGATAGCCCGGGGAAACCCGGATTAATA
CCGGATAGCCTTCTGAGCCTTCGGGCGAGGAAGAAAAGGTAGCTTCGGCCTCCGATCGAGGATGAGCCCG
CGGTGGATTAGCTTGTTGGCGGGGTAATGGCCCACCAAGGCGACGATCCATAGCTGGTCTGAGAGGACGA
TCAGCCACACTGGGACTGAGACACGGCC
>Zotu10592
GAACGCTGGCGGCGTGCTTAACACATGCAAGTCGAGCGGTAACAGGCGTAGCAATACGTGCTGACGAGCG
GCGAACGGGTGCGTAATACATGGGAATCTGCCTTTTAGTGGGGAATAACCCGGCGAAAGCCGGGCTAATG
CCGCATAAGATCTTCGGATGAAAGCGGGGGATCGTAAGACCTCGCGCTGAAAGATGAGCCCATGCCGGAT
TAGCTAGTTGGTGAGGTAACGGCTCACCAAGGCGACGATCCGTAGCTGGTCTGAGAGGACGGCCAGCCAC
ACTGGGACTGAGACACGGCC
>Zotu10596
GAACGCTGGCGGCGCACTTAAGACATGCAAGTCGAACGGACTTAACCGAAATATTTATATTTTGGAGCGGT
TAGTGGCGGACTGATAAGTAACGCGTAAGCAATCTGCCTATTAGAGGGGAATAACAGTGAGAAATCACTG
CTAATACCGCATATGCCATGAACATCGCATGATGAAAATGGGAAAGGAGTAATCCACTAATAGATGAGCTT
GCGTCTGATTAACTAGTTGGTGTGGTAATGGCATACCAAGGTAACGATCAGTAGCCGAACTGAGAGGTTG
AACGGCCACATTGGGACTGAGATACGGCC
>Zotu10598
GAACGCTGGCGGCGTGCCTAATACATGCAAGTCGAACGGAGAGTTTGATTTCGGTTGAACTCTTAGTGGCG
AACGGGTGAGTAACGCGTTGGTGACCTGCCCCGAAGAACGGGATAACGGCTCGAAAGGGCTGCTAATACC
GTATGTGGTCTTACTGGTTAGAGTGGTAAGACTAAAGGAGCAATCCGCTTCGGGAGGGGCCTGCGTCCCA
TCAGCTAGTTGGTAGGGTAATGGCCTACCAAGGCAAAGACGGGTAGGGGGCCTGAGAGGGTGACCCCCC
ACACTGGCACTGAAACACGGGC

>Zotu10601
GAACGCTGGAAATGTGGATACCGGCATGCAAGTCAAGGGGCCGCAAGGCACCGGCGAACGGCTTAGTAA
CACGTAGATACATACCTTCAATTGGAGCATAGCTCGTCGAAAGACGGGGTAATTCTCCATAGTCCCGCAAG
GGTAAAGGCGCAAGCCGATTGAAGAATGGTCTGCGGTCTATCAGCTAGTTGGTGAGGTAATGGCTCACCA
AGGCTATGACGGATAGGGGGCGTGAGAGCGCGACCCCCAAGAATGGCACTGCGACACGGGC
>Zotu10606
GAATGCTGGCGGCGTGGATAAGGCATGCAAGTCAAGGGGGCCCGCAAGGGCAACCGGCAGACGAGGTA
GTAACACGTACGAACGCACCCAGAAGTCTGGCATAGCTCGTCGAAAGACGGGGTAATTCCGGATGGTCCC
GCAAGGGTAAAGGTTTTTCGCTTCTGGAGCGGCGTGCGCAATATCAGCTAGTTGGTGAGGTAATGGCTCAC
CAAGGCTACGACGTTTAGGGGCGCTGAGAGGCGGACCCCCACCGATGGGACTGAGACACGGCC
>Zotu10610
GAACGCTGGCGGCATGCTTAACACATGCAAGTCGAACGGCAGCATAGTCTAGCTTGCTAGATGGGTGGCG
AGTGGCGAACGGGTGAGTAACGCGTAGGAATATACCTTAAAGAGGGGGACAACTTGGGGAAACTCAAGC
TAATACCGCATACGGTCTAAGGACGAAAGCTGGGGACCTTCGGGCCTGGCGCTTTAAGATTAGCCTGCGTC
TGATTAGCTAGTTGGTGGGGTAAAGGCCTACCAAGGCGACGATCAGTAGCTGGTCTGAGAGGATGACCAG
CCACACTGGAACTGAGACACGGTC
>Zotu10612
GAACGCTGGCGGCGTGCCTAATACATGCAAGTCGAACGAGAAAGTGTAGCAATACATTAGTAAAGTGGCA
GACGGGTGAGTAATACATAGGTAACCTACCTCCGAGATGGGGATAACCTTCCGAAAGGAGGGCTAATACC
GAATGAGACCACGAGTACTCTGTACTTGAGGCCAAAGGCGACCTCTACTTGTAAGTTGTCGCTTGGAGATG
GGCTTATGCACCATTAGCTTGTTGGTGAGGTAACGGCTCACCAAGGCCGTGATGGTTAGCTGATCTGAGAG
GTTGATCAGCCACACTGGAACTGAGACACGGTC
>Zotu10616
GAACGCTGGCGGCGTGCTTAAGACATGCAAGTCGAACGGACTCCCCTCGGGGAGTCAGTGGCGCACGGGT
GAGTAACACGTAACTGACCTGCCCCAAAGTTCCGAATAACCCTCCGAAAGGAGGGCTAAGACGGAATGTG
CTGTCCCCTCGTGTGGGGCCAGTAAAGGCTGTGACCGCTTTGGGATGGGGTTGCGTTCCATCAGCTTGTTG
GTGAGGTAAAGGCTTACCAAGGCGACGACGGATAGCCGGCCTGAGAGGGTGGCCGGCCACAGGGGCACT
GAGACACGGGT
>Zotu10619
GAACGCTGGCGGCGTGCTTAATACATGCAAGTCGAACGAAGCACTTACGACAGATCCCCTCGGGGTGAAG
ATGTTTGTGACTTAGTGGCGGACGGGTGAGTAACGCGTGGGTAACCTGCCTCATGCAGGGGGATAACAGT
TAGAAATGACTGCTAATACCGCATAAGCGCACGGATTCGCATGTTTCTGTGTGAAAAGATTTATCGGCATG
AGATGGACCCGCGTCTGATTAGGTAGTTGGTGGGGTAACGGCCCACCAAGCCTGCGATCAGTAGCCGACT
TGAGAGAGTGATCGGCCACATTGGGACTGAGACACGGCC
>Zotu10621
GAACGCTGGCGGCATGCTTTACACATGCAAGTCGAACGGCAGCGCGGGGCAACCTGGCGGCGAGTGGCG
AACGGGTGAGTAATATATCGGAACGTACCCAAGAGTGGGGGATAACGTAGCGAAAGTTACGCTAATACCG
CATACGATCTATGGATGAAAGTGGGGGATCGCAAGACCTCATGCTCCTGGAGCGGCCGATATCTGATTAGC
TAGTAGGTAGGGTAAAGGCCTACCTAGGCGACGATCAGTAGCTGGTCTGAGAGGACGACCAGCCACACTG
GGACTGAGACACGGCC
>Zotu10623
AAACGCTGGCGGCGTGCCTTAGGCATGCAAGTCGAATGGCCCGCAAGGGCAATGGCGGACGGTCGAGTA
ACGCGTGGGAATCTCCCTCAAAGTTGGACATAGCCAGCCGAAAGGTTGGGTAATTTCTGATGTGATTGCAA
AATTAAAGTCGTAAGACGCTTTGAGATGAGCCCGCGTAGGATTAGCTAGTTGGTGGGGTAAAGGCCTACC
AAGGCTACGATCCTTAGCTGGTCTGAGAGGACGATCAGCTGGAATGGGACTGAGACACGGCC
>Zotu10626
CAACGCTGGCGGCGTGCCTAATGCATGCAAGTCGAGCGGGGTGCTCTTCGGAGCACTTACGCGGCGGACG
GGTGCGGAACACGTGGGCAACCTGCCCCGAGGTGGGGGATAGCCGGTGGAAACACCGGGTAATTCCGCA
TAAGGTCACTGGCCGGAGGGCTGGTGAGGAAAGGGTTTTCGGACCCGCCTTGGGAGGGGCCTGCGGCCG

ATTAGCTAGACGGTGGGGTAACGGCCTACCGTGGCGATGATCGGTAGCTGGTCTGAGAGGACGGCCAGCC
ACACGGGGACTGAGACACGGCC
>Zotu10631
GAACGCTGGCGGCAGGCCTAATACATGCAAGTCGAACGAGATTGAAGAGCTTGCTTTTCATGAAAGTGGC
GCACGGGTGCGTAACGCGTATGCAACCTACCTTCTTCAGGGGGATAGCCTCTCGAAAGAGAGATTAACACC
GCATAATATTATTGGATGGCATCATTTAATAATCAAATATTTATAGGAAGAAGATGGGCATGCGTGACATT
AGTTAGTTGGCGGGGTAATGGCCCACCAAGACCACGATGTCTAGGGGATCTGAGAGGATGACCCCCCACA
CTGGTACTGAGACACGGAC
>Zotu10635
GAACGCTGGCGGCGTGCTTAACACATGCAAGTCGAGCGGTAAGGCTCCTTCGGGGGTACACGAGCGGCG
AACGGGTGAGTAACACGTGAGTAATCTGCCCTCCACTCTGGGATAAGCTTCGGAAACGGGGTCTAATACCG
GATACACACTTTTCTGCGCATGCGGTTTGGTGGAAACTTTTTTGGGTGGGGGATGTGCTCGCGGCCTATCA
GCTTGTTGGTGGGGTAATGGCCTACCAAGGCGTCGACGGGTAGCCGGCCTGAGAGGGTGACCGGTCACAC
TGGGACTGAGACACGGCC
>Zotu10637
GAACGCTGGCGGCGTGCTTAACACATGCAAGTCGAACGGAAAGGCCCTTCGGGGTACTCGAGTGGCGAAC
GGGTGAGTAACACGTGGGCAACCTGCCCCTAGCTCTGGGATAACACCGGGAAACCGGTGCTAATACCGGA
TACACACTTTTTGAGGCATCTCTCGAAGTGGAAAGTTTTTCGGCTAGGGATGGGCCCGCGGCCTATCAGCTT
GTTGGTGGGGTAATGGCCTACCAAGGCGACGACGGGTAGCCGGCCTGAGAGGGCGACCGGCCACACTGG
GACTGAGACACGGCC
>Zotu10641
GAACGCTGGCGGAATGCTTTACACATGCAAGTCGAACGGTAGAGGGGGCAACCCCTTGAGAGTGGCGAA
CGGGTGAGTAATATATCGGAACGTGCCCAGTCGTGGGGGATAACGTAGCGAAAGTTACGCTAATACCGCA
TACGATCTACGGATGAAAGCGGGGGACTCGCAAGAGCCTCGCGCGATTGGAGCGGCCGATATCAGATTAG
GTAGTTGGTGGGGTAAAGGCTCACCAAGCCAACGATCTGTAGCTGGTCTGAGAGGACGACCAGCCACACT
GGAACTGAGACACGGTC
>Zotu10643
GAACGCTGGCGGCGTGCCTAACACATGCAAGTCGAACGGAATCCATGGAGTGCTTGCACTCTTGAAGATTT
AGTGGCGAACGGGTGAGTAACACGTGAGAAACCTGCCCCAGACTCTGGGATAACCTCGGGAAACCGAAGC
TAATACCGGATATTCTCCTTTGGTCGCATGGCCAGTTGAGGAAAGTTTTTCGGTCTGGGAGGGTCTCGCGG
CCTATCAGCTAGTTGGTGAGGTAACGGCTCACCAAGGCTACGACGGGTAGCTGGTTTGAGAGAACGATCA
GCCACACTGGAACTGAGACACGGTC
>Zotu10645
GAACGCTGGCGGCGTGCTTAACACATGCAAGTCGAACGGAAAGGCCCTTCGGGGTACTCGAGTGGCGAAC
GGGTGAGTAACACGTGGGTGATCTGCCCTGCACTTCGGGATAAGCCTGGGAAACTGGGTCTAATACCGGA
TATGACCGTTCATCTCCTGGTGGGTGGTGGAAAGCTTTTGCGGTGTGGGATGGGCCCGCGGCCTATCAGCT
TGTTGGTGGGGTAATGGCCTACCAAGGCGTCGACGGGTAGCCGGCCTGAGAGGGTGTCCGGCCACACTG
GGACTGAGATACGGCC
>Zotu10648
GAACGCTGGCGGCGTGCCTAATACATGCAAGTCGAGCGAGCTTGCCTAGATGATTGTGGTGCTTGCATCAC
ATGAAACTAGATACAAGCGAGCGGCGAACGGGTGAGTAACACGTGGGTAACCTGCCCAAGAGACTGGGA
TAACACCTGGAAACAGATGCTAATACCGGATAACAACACAAGACGCATGTCTAGTGTTTGAAAGATGGTTC
TGCTATCACTCTTGGATGGACCTGCGGTGCATTAGCTAGTTGGTAAGGTAACGGCTTACCAAGGCAATGAT
GCATAGCCGAGTTGAGAGACTGATCGGCCACATTGGGACTGAGACACGGCC
>Zotu10650
GAACGCTGGCGGCATGCCTAACACATGCAAGTCGAACGGCAGCACGGTCTAGTTTACTAGATGGGTGGCG
AGTGGCGGACGGGTGAGTAATGCGCAGGAATATGCCTTAAAGAGGGGGATAACTTGGGGAAACTCAAGC
TAATACCGCATAATCTCTGAGGAGAGAAGCTGGGGACCTTCGGGCCTGGCGCTTTAAGATTAGCCTGCGTC
CGATTAGCTAGTTGGTAGGGTAAAGGCCTACCAAGGCGACGATCGGTAGCTGGTCTGAGAGGATGACCAG
CCACACTGGAACTGAGACACGGTC

>Zotu10654
GAACGCTGGCGGCGTGCTTAACACATGCAAGTCGAACGATGACGGCCGTGCTTGCACGGTCTGATTAGTG
GCGAACGGGTGAGTAACACGTGAGTAACCTGCCCTCCACTTCGGGATAACCTCGGCAAATCGTGGCTAATA
CCGAATATGAGCACTCATCGCATGGTGGGTGCTGGAAAGATTTATCGGTGGGGATGGACTCGCGGCCTA
TCAGTTTGTTGGTGAGGTGATGGCTCACCAAGACGATGACGGGTAGCCGGCCTGAGAGGGCGACCGGCCA
CACTGGGACTGAGACACGGCC
>Zotu10657
GAACGCTGGCGGCATGCCTAATACATGCAAGTCGAACGAAGTTATGATGGAGCTTGCTCCTGATTAGCTTA
GTGGCGGACGGGTGAGTAACACGTGGGCAACCTGCCTACAAGTTGGGGATAACTAGCGGAAACGTTAGCT
AATACCGAATGGTCAAGGATGAGGCATCTTATCCTTGGGAAAGACGGAGCAATCTGTCACTTGTAGATGG
GCCCGCGGTGCATTAGCTAGTTGGTGAGGTAACGGCTCACCAAGGCGACGATGCGTAGCCGACCTGAGAG
GGTGATCGGCCACACTGGGACTGAGACACGGCC
>Zotu10660
GAACGCTGGCGGCGTGCTTAACACATGCAAGTCGACGACGCTACTGAGTAGCAATATTTAGTAGTGGAGT
GGCGCAAGGGTGAGTAATGTATACGTAACCAACCCATCGGAGACGGATAACATTTCGAAAGGGATGCTAA
AACGGCATGAAGCCACGATTTCGCATGAGATTGTGGTTAAAGAACCGGTAACAGGTTGCCGATGGCTGGG
CGTATATCCGATTAGCTAGTTGGTAGTGTAATGGACTACCAAGGCGACGATCGGTAGCTGGTCTGAGAGG
ATGATCAGCCACACTGACACTGAGACACGGGC
>Zotu10662
GAATGCTGGCGGCGTGCCTAACACATGCAAGTCGAGCGGCAGCGAGGGGTGCTTGCACCCTGTCGGCGA
GCGGCGGACGGCTGAGTAACGCGTGGGAATCTACCCTAAAGTGAGGGATAACCGCCCGAAAGGGTGGCT
AATACCGCATATGATCTTCGGATTAAAGGATTTATCCGCTTTAGGAAGAGCCCGCGTTAGATTAGGTAGTT
GGTAGGGTAATGGCCTACCAAGCCAACGATCTATAGCTGGTCTGAGAGGATGATCAGCCAGACTGGAACT
GAGACACGGTC
>Zotu10665
GAACGCTGGCGGCGCGCTTAACACATGCAAGTCGAACGTGAAGCCCGGGCTTGCCTGGGTGGAAAGTGG
CGAACGGGTGCGTAACACGTGAGCAACCTGCCCCGAAGACCGGGATAACAGCGGGAAACCGCTGCTAATA
CCGGATGCCCTCATCATGTCGCATGGCGAGATGAGGAAATGGATTCCGCTTCGGGATGGGCTCGCGGTCT
ATCAGCTTGTTGGTGAGGTAACGGCTCACCAAGGCTACGACGGATAGCTGGTCTGAGAGGATGTCCAGCC
ACACTGGGACTGAGACACGGCC
>Zotu10672
GAACGCTGGCGGCGTGCTTAACACATGCAAGTCGAACGGTGAAGCCCTTCGGGGTGGATCAGTGGCGAAC
GGGTGAGTAACACGTGAGCAACCTACCCTTGACTCTGGGATAACTCCGGGAAACCGGTGCTAATACCGGA
TACGACCTTCGGCCTCATGGTCTGTTGGTGGAAAGAATTTTGGTCAGGGATGGGCTCGCGGCCTATCAGCT
TGTTGGTGAGGTAATGGCTCACCAAGGCGACGACGGGTAGCCGGCCTGAGAGGGCGACCGGCCACACTG
GGACTGAGACACGGCC
>Zotu10674
GAACGCTTGCGGCGCGCCTAATACATGCAAGTCGAACGAACCAGCGATGGTGAGTGGCGCACGGGTGAG
TAACGCGTGGATAATCTGCCCTCTACTGGGGAATAACTAACCGAAAGGTTAGCTAACACCGCATGAGACCA
CAGTGCCTGCGGGCACAGAGGTTAAAAATTTATTGGTAGAGGATGAGTCTGCGTGGGATTAGCTAGTTGG
TGGGGTAATGGCCTACCAAGGCTACGATCTCTAACAGGTCTGAGAGGATGACCTGTCACACTGGAACTGA
GACACGGTC
>Zotu10677
GAACGCTGGAAATGTGGATACCGGCATGCAAGTCAAGGGGCCGCAAGGCACCGGCGAACGGCTTAGTAA
CACGTGGATACATACCGTAAAGTGGGGCATAGCTCGTCGAAAGACGGGGTAATTTCCCATAGCACCAATGT
TTCGCATTGGAAATCCGAAACACGAAAGGCGAAATCCGAAATACGTTTAAAATTTTAGAAATTAGAATTTTA
AATTTGTTTCGAGTTTCGATATTCGAATTTCGAGTTTATAGTGCGAAGCATTGGTTAAAGGCGCAAGCCGCT
TTATGAATGGTCCGCGGGGTATCAGCTAGTTGGTGAGGTAAAAGCTCACCAAGGCTATGACGCCTAGGGG
GCGTGAGAGCGCGACCCCCAAGAATGGCACTGCGACACGGGC
>Zotu10680

GAACGCTGGCGATGTGGATGAGTCATGCAAGTCAAGGGGGCCCGCAAGGGCAACCGGCAAACGAGGTAG
TAACACGTAGGTAGATCCCCCGGAGTCGGGCATAGCTTGCCGAAAGGCAAGGTAATTCCCGATGGTCTCG
CAAGAGTAAAGTTTTTCGCTCCGGGAGTCGCCTGCGCAGTATCAGCTAGTTGGTAGGGTAACGGCCTACCA
AGGCTACGACGCTTAGGGGAGGTGAGAGCCTGACCCCCACCGATGGGACTGCGACACGGCC
>Zotu10682
CAACGCTGGCGGCGTGCCTAATGCATGCAAGTCGAACGGGGTCATCTTCGGATGGCTTACGTGGCGGACG
GGTGAGGAACACGTGGGTAACCTGCCCAGTGGTGGGGGCTAGCCATCGGAAACGGTGGGTAATTCCGCA
TACGGTCAATCGCCGAGTGGGGTGATTGACGAAAGGCGCAAGTCGCCACTGGAGGGGCCCGCGTCCGATT
AGCTTGTTGGTGGGGTAACGGCTCACCAAGGCAGTGATCGGTAGCTGGTTTGAGATGATGATCAGCCACA
CGGGGACTGAGACACGGCC
>Zotu10685
GAACGCTGGCGGCGTGCCTAATACATGCAAGTCGAGCGGACTTGACAGAGCTTGCTCTGTTCAAGTTAGCG
GCGGACGGGTGAGTAACACGTGGGCAACCTGCCCTGCAGATTGGGATAACTCCGGGAAACCGGGGCTAA
TACCGAATAATATTGAAAACCTCATGGTTTTCTTTTAAAAGGCGGCTTCGGCTGTCACTGCAGGATGGGCCC
GCGGCGCATTAGCTAGTTGGTGAGGTAACGGCTCACCAAGGCGACGATGCGTAGCCGACCTGAGAGGGT
GATCGGCCACACTGGGACTGAGACACGGCC
>Zotu10689
GAACGCTGGCGGCAGGCCTAACACATGCAAGTCGAGCGGTTGAGAGTAGCTTGCTACTTGATGCAGCGGC
GGACGGGTGAGTAACGCGTGGGAATCTGCCCAGTAGTGGGGGACAACAGCCGGAAACGGCTGCTAATAC
CGCATACGCCCTACGGGGGAAAGGGGGGGATCTTCGGACCTCTCGCTATTGGATGAGCCCGCGTCGGATT
AGCTAGTTGGTAGGGTAAAGGCCTACCAAGGCTACGATCCGTAACTGGTCTGAGAGGATGATCAGTCACA
CTGGAACTGAGACACGGTC
>Zotu10692
GAACGCTGGCGGCGTGCCTTAAGCATGCAAGTCGAACGGCTCCGCAAGGAGCAGTGGCGGACGGTCGAG
TAACGCGTGGGGATCTGCCTTAAAGTTGGACATAGCTCGTCGAAAAACGGGGTAATTTCCAATGTGAATGT
AAGTTCAAAGCCGCAAGGCGCTTTAAGATGAACCCGCGTCCTATCAGCTAGTTGGTGAGGTAATGGCTCAC
CAAGGCGTCGACGGGTAGCTGGTCTGAGAGGATGATCAGCCGGAATAGCACTGAGACACGGGC
>Zotu10694
GAACGCTGGCGGCGTGCTTAACACATGCAAGTCGAACGGAAAGGCCCTTCGGGGTACTCGAGTGGCGAAC
GGGTGAGTAACACGTGGGTGATCTGCCCTGCACTTTGGGATAAGCCTGGGAAACTGGGTCTAATACCGAA
TATGACCATGCACTTCCTGGTGTGTGGTGGAAAGCTTTTGCGGTGTGGGATGGGCCCGCGGCCTATCAGCT
TGTTGGTGGGGTAATGGCCTACCAAGGCGACGACGGGTAGCCGGCCTGAGAGGGTGACCGGCCACACTG
GGACTGAGATACGGCC
>Zotu10697
GAACGCTGGCGGCGTGCCTAATACATGCAAGTCGAGCGGACGAATTTTTGGTACTTGTACCAGATTTCGTT
AGCGGCGGACGGGTGAGTAACACGTGGGTAACCTGCCTTGTAGACTGGGATAACTTCGGGAAACCGAGG
CTAATACCGGATAATCCTTTTGAACGCCTGTTCTTAAGTTGAAAGATGGCATCTCGCTATCACTACAAGATG
GACCCGCGGCGCATTAGCTAGTTGGTGAGGTAACGGCTCACCAAGGCAACGATGCGTAGCCGACCTGAGA
GGGTGATCGGCCACACTGGGACTGAGACACGGCC
>Zotu10701
GAACGCTGGCGGCGCGCCTAACACATGCAAGTCGAGCGTGAAAGTGTAGCAATACATGAGTAAAGCGGC
GCACGGGTGAGTAACGCGTAGGTGATCTAGCTTTGTGACTGGAATAACCAGCCGAAAGGTTGGCTAATAC
TAGATAGAGCGCAGTTGTCGTGAAGATGATTGCGGGAAAGGGTGGCAGCAATGCTATCGCACTAAGATGA
GCCTGCGTCCCATCAGCTAGTTGGTAAGGTAATGGCTTACCAAGGCAATGACGGGTAGCTGGTCTGAGAG
GACGATCAGCCACACTGGAACTGAAACACGGTC
>Zotu10703
GAACGCTGGCGGCGTGCTTAACACATGCAAGTCGAACGAAGCACTCTATTTGATTTTCTTCGGAAATGAAG
ATTTTGTGACTGAGTGGCGGACGGGTGAGTAACGCGTGGGTAACCTGCCTCATACAGGGGGATAACAGTT
GGAAACGACTGCTAATACCGCATAAGCGCACAGGACCGCATGGTCCGGTGTGAAAAACTCCGGTGGTATG
GGATGGACCCGCGTCTGATTAGCCAGTTGGCAGGGTAACGGCCTACCAAAGCGACGATCAGTAGCCGACC
TGAGAGGGTGACCGGCCACATTGGGACTGAGACACGGCC

>Zotu10705
GAACGCTGGCGGCATGCTTTGCACATGCAAGTCGAACGGCAGCACGGACTTCGGTCTGGTGGCGAGTGGC
GAACGGGTGAGTAATACATCGGAACGTGCCCAGTTGTGGGGGATAACTACTCGAAAGAGTAGCTAATACC
GCATGAGAACTGAGGTTGAAAGCAGGGGATCGCAAGACCTTGCGCAACTGGAGCGGCCGATGGCAGATT
AGGTAGTTGGTGGGATAAAAGCTTACCAAGCCGACGATCTGTAGCTGGTCTGAGAGGACGACCAGCCACA
CTGGGACTGAGACACGGCC
>Zotu10708
GAACGCTGGCGGCGTGCTTAACACATGCAAGTCGAACGGTGAAGCCCTTCGGGGTGGATCAGTGGCGAAC
GGGTGAGTAACACGTGAGCAACCTACCCTCCACTCTGGGATAACTCCGGGAAACCGGTGCTAATACCGGAT
ACGAGTGCCGACCTCATGGTCTGGTGCTGGAAAGAATTTTGGTGGGGGATGGGCTCGCGGCCTATCAGCT
TGTTGGTGAGGTAATGGCTCACCAAGGCGACGACGGGTAGCCGGCCTGAGAGGGCGACCGGCCACACTG
GGACTGAGACACGGCC
>Zotu10710
GAACGCTAGCGGCAGGCCTAATACATGCAAGTCGAACGATAGTGAGGGGCTTGCTCCTCACGAAAAGTGG
CGCACGGGTGCGTAACGCGTATGCAACCTACCTTAATCAGGGGGATAGCCCGAAGAAATTCGGATTAACA
CCGCATAAAATCACAGTATAGCATTATACAATGATCAAATATTTATAGGATTAAGATGGGCATGCGTGACAT
TAGTTAGTTGGCGAGGTAACGGCTCACCAAGACTACGATGTCTAGGGGATCTGAGAGGATGACCCCCCAC
ACTGGTACTGAGACACGGAC
>Zotu10712
GAACGCTAGCGGCAGGCCTAATACATGCAAGTCGAACGATATTTTCCAGCTTGCTGGGGAAGAAAGTGGC
GCACGGGTGCGTAACGCGTATGCAACCTACCTTAATCAGGGGGATAGCCCGAAGAAATTCGGATTAACAC
CGCATAAAATCACAGCACAGCATTGTGTAATGATCAAATATTTATAGGATTAAGATGGGCATGCGTGACAT
TAGTTAGTTGGCGGGGTAACGGCCCACCAAGACCACGATGTCTAGGGGATCTGAGAGGATGACCCCCCAC
ACTGGTACTGAGACACGGAC
>Zotu10715
GAACGCTAGCGGCAGGCTTAATACATGCAAGTCGTGGGGCAGCATGACTGTAGCAATACAGTTGATGGCG
ACCGGCAAACGGGTGCGGAACACGTACGCAATCTACCCAGAACTGGTGAATAGCCCTCCGAAAGGAGGAT
TAATACACCGTAATATACTGAAGTGGCATCACTTTATTATTAAAGCTCCGGCGGTTTTGGATGAGCGTGCGC
CCCATTAGGTAGTTGGTTGAGGTAACGGCTCACCAAGCCTGCGATGGGTAACTGGTGTGAGAGCACGACC
AGTCACACGGGCACTGAGACACGGGC
>Zotu10718
GAACGCTGGCGGTAGGCCTAACACATGCAAGTCGAACGGCAGCACAGCAGTAGCAATACTGTGGGTGGC
GAGTGGCGGACGGGTGAGGAATACATCGGAATCTACCTTTTCGTGGGGGATAACGTAGGGAAACTTACGC
TAATACCGCATAAGACCTTCGGGTGAAAGTGGGGGACCTTCGGGCCTCACGCGGATAGATGAGCCGATGT
CGGATTAGCTTGTTGGCGGGGTAAAAGCCCACCAAGGCGACGATCCGTAGCTGGTCTGAGAGGATGATCA
GCCACACTGGAACTGAGACACGGTC
>Zotu10721
GAACGCTGGCGGCGTGCTTAACACATGCAAGTCGAACGCTGAAGCTTGGTGCTTGCACTGGGTGGATGAG
TGGCGAACGGGTGAGTAATACGTGAGTAACCTGCCCTTGACTCTGGGATAAGCCTGGGAAACTGGGTCTA
ATACTGGATGCTACATGTCACCGCATGGTGGTGTGTGGAAAGGGTTTACTGGTCTTGGATGGGCTCACGGC
CTATCAGCTTGTTGGTGGGGTAATGGCCTACCAAGGCGACGACGGGTAGCCGGCCTGAGAGGGTGACCG
GCCACACTGGGACTGAGACACGGCC
>Zotu10726
GAACGCTGGCGGCGTGCTTAACACATGCAAGTCGAACGATGATCCCCAGCTTGCTGGGGGGATTAGTGGC
GAACGGGTGAGTAACACGTGAGTAACCTGCCCTTAACTCTGGGATAAGCCTGGGAAACTGGGTCTAATAC
CGGATATGACTGATCATCGCATGGTGGTTGGTGGAAAGCTTTTTGTGGTTTTGGATGGACTCGCGGCCTAT
CAGCTTGTTGGTGAGGTAATGGCTTACCAAGGCGACGACGGGTAGCCGGCCTGAGAGGGTGACCGGCCA
CATTGGGACTGAGATACGGCC
>Zotu10730

GAACGCTGGCGGTGTGCTTAACACATGCAAGTCGAACGGGCAAGAGCTGTAGCAATACAGAATTCTGCTA
GTGGCAAACGGGTGAGTAATACATGGGAATCTACCTTGCAGTGCGAAATAACAGTTGGAAGCAATTGCTA
ATGTCGCATATAGCCGAGAGGTGAAAGATTTATCGCTGCAAGACGAGCCCATGCAGGATTAGCTAGTTGG
TGGGGTAATGGCTTACCAAGGCAACGATACGTAGCCGACCTGAGAGGGTGATCGGCCACACTGGAACTGA
GACACGGTC
>Zotu10734
GAACGCTGGCGGTGTGCTTAACACATGCAAGTCGAACGGGCAAGAGCTGTAGCAATACAGAATTCTGCTA
GTGGCAAACGGGTGAGTAATACATGGGAATCTACCTTGCAGTGCGAAATAACAGTTGGAAGCAATTGCTA
ATGTCGCATATAGCCGAGAGGTGAAAGATTTATCGCTGCAAGACGAGCCCATGCAGGATTAGCTAGTTGG
TGGGGTAATGGCTCACCAAGGCGATGATCAGTAGGGGTTCTGAGAGGATTGGCCCCCACATGGGTACTGA
GATACGGAC
>Zotu10736
GCCGTGTCTCAGTCCCAGTGTGGCTGATCATCCTCTCAGACCAGCTATGGATCGTCGGCTTGGTAGGCCTTT
ACCCCACCAACTACCTAATCCAACGCGGGCTCATCCTCAGGCGATAAATCTTTGATCTCGCGATATCATCCG
GTATTAGCAGCCGTTTCCAGCTGTTATTCCGAACCTAAGGGCAGATTCCCACGCGTTACGCACCCGTGCGCC
ACTATCACCCGAAGGCAATCGTTCGACTTGCATGTGTTAGGCATGCCGCCAGCGTTCG
>Zotu10738
GAACGCTGGCGGCATGCCTTACACATGCAAGTCGAACGGCAGCACGGGGGTAACTCTGGTGGCGAGTGG
CGAACGGGTGAGTAATATATCGGAACGTACCCTAGAGTGGGGGATAACTAGTCGAAAGACTAGCTAATAC
CGCATACGATCTAAGGATGAAAGCGGGGGATCGCAAGACCTCGCGCTCATGGAGCAGCCGATATCTGATT
AGCTAGTTGGTAGGGTAAAAGCCTACCAAGGCATCGATCAGTAGCTGGTCTGAGAGGACGACCAGCCACA
CTGGAACTGAGACACGGTC
>Zotu10740
GAACGCTGGCGGTGTGCTTAACACATGCAAGTCGAACGGTAAGGCCCTTTCGGGGGTACACGAGTGGCGA
ACGGGTGAGTAACACGTGAGTAACCTACCCTCAACTCTGGGATAACGGCTGGAAACGGTTGCTAATACTG
GATATGAGCTGGGTCTGCATGGGTCTGGTTGGAAAGATTTTTTGGTTGGGGATGGGCTCGCGGCCTATCA
GCTTGTTGGTGAGGTAGTGGCTCACCAAGGCTTCGACGGGTAGCCGGCCTGAGAGGGCGACCGGCCACAT
TGGGACTGAGATACGGCC
>Zotu10743
GAACGCTGGCGGCGTGCCTAATACATGCAAGTCGAACGCTTCTTTTTCATCGAACTTCGGTTCACCGAAAAA
GAAGAGTGGCGAACGGGTGAGTAACACGTGGGTAACCTACCCATCAGCGGGGGATAACACTTGGAAACA
GGTGCTAATACCGCATAATCTTTTTCTCCGCCTGAAGAGAAAGTGAAAGGCGCTTTTGCGTCACTGATGGAT
GGACCCGCGCTGCATTAGCTAGTTGGTGAGGTAACGGCTCACCAAGGCCACGATGCATAGCCGACCTGAG
AGGGTGATCGGCCACACTGGGACTGAGACACGGCC
>Zotu10745
GAACGCTGGCGGCGTGCCTAACACATGCAAGTCGAGCGAGAAAGCCCGCAAGGGTGAGTAAAGCGGCGC
ACGGGTGCGTAACACGTGGGTAATCTTCCTTGAAGTCCGGAATAACTCCCCGAAAGGGGTGCTAATACCG
GATAAGACCACGGGAACTCCGGTTCCTGAGGGAAAAGGCGGCCTCTGTACACAAGCTGTCACTTCGAGAT
GAGCCCGCGGCCCATCAGCTAGTTGGCGGGGTAACGGCCCACCAAGGCTACGACGGGTAGCTGGTCTGAG
AGGACGATCAGCCACACTGGAACTGAGACACGGTC
>Zotu10752
GAACGCTGGCGGCACGCCTAACACATGCAAGTCGAGCGGCAGCATGCTTAGTAGCAATACTAAGTGATGG
CGAGCGGCGGACGGGTGAGGAACACGTAGGAACCTGCCCAAGAGAGGGGGACAACCAAGGGAAACTTT
GGCTAATACCGCATAATCTCTTAGGAGAAAAGTTGCCCGTAAGGGTAGCGCTTTTGGAGGGGCCTGCGTCC
GATTAGCTAGTTGGTGAGGTAATAGCTCACCAAGGCGATGATCGGTAACTGGTCTGAGAGGACGACCAGT
CACACTGGGACTGAGACACGGCC
>Zotu10758
CAACGCTGGCGGCGTGCCTAACACATGCAAGTCGAACGAGAAAGTGGTAGCAATACCATGAGTACAGTGG
CGCACGGGTGAGTAACACGTGACTAACCTACCCTGGAGTGGGGGATAACTGAGGGAAACCTTAGCTAATA
CCGCATAATACCTACGGGTCAAAGGAGCAATTCGCTTCAGGAGGGGGTCGCGGCCGATTAGTTAGTTGGC

GGGGTAATGGCCCACCAAGACAGTGATCGGTATCCGGCCTGAGAGGGCGCACGGACACACTGGAACTGA
AACACGGTC
>Zotu10761
GAACGCTGGCGGCGTGCCTAATACATGCAAGTAGAACGCTGAAGGAGGAGCTTGCTCTTCTGGATGAGTT
GCGAACGGGTGAGTAACGCGTAGGTAACCTGCCTGGTAGCGGGGGATAACTATTGGAAACGATAGCTAAT
ACCGCATAAGAGTAGATATCGCATGATAGCTGCTTGAAAGGTGCAATTGCACCACTACCAGATGGACCTGC
GTTGTATTAGCTAGTTGGTGAGGTAACGGCTCACCAAGGCAACGATACATAGCCGACCTGAGAGGGTGAT
CGGCCACACTGGGACTGAGACACGGCC
>Zotu10763
GAACGCTGGCGGCGTGCCTAAGACATGCAAGTCGAGTGGGTTTAGGCCCACGGCGAACGGGTGAGTAAT
ACCTAGGAACTTTCTTTAAGTGGAGCATAACTCGCTGAAAGGCGAATTAATACTTCATGTGGCAGTAATGCT
AAAGTCGCAAGACGCTTAAAGTGAGGCCTGGGCACCATCAGCTAGTTGGTAAGGTAATGGCTTACCAAGG
CTATGACGGTTAGGGGTCCCGAGAGGGATAACCCCCACACTGGAACTGCGACACGGTC
>Zotu10765
GAACGCTAGCGGAATGCTTTACACATGCAAGTCGAACGGCAGCACGGACTTCGGTCTGGTGGCGAGTGGC
GAACGGGTGAGTAATGCATCGGAATGTACCCAGTAGCGGGGGATAACTACGCGAAAGCGTGGCTAATACC
GCATACGCCCTACGGGGGAAAGGGGGGGATCTTCGGACCTCTCACTATTGGAGCAGCCGATGTCAGATTA
GCTAGTTGGTGGGGTAAAGGCCTACCAAGGCAACGATCTGTAGCTGGTTTGAGAGGACGACCAGCCACAC
TGGGACTGAGACACGGCC
>Zotu10768
GAATGGTGGCGGCGTGGATGAGTCATGCAAGTCAAGGGGGCCCGCAAGGGCAACCGGCAAACGAGGTA
GTAACACGTAGGTAGATCCCCCGGAGTCGGGCATAGCTTGCCGAAAGGCAAGGTAATTCCCGATGGTCTC
GCAAGAGTAAAGATTTTTTGCTCCGGGAGTCGCCTGCGTAGTATCAGCTAGTTGGTAAGGTAATGGCTTAC
CAAGGCTACGACGCTTAGGGGAGGTGAGAGCCTGACCCCCACCGATGGAACTGCGACACGGTC
>Zotu10771
GAACGCTGGCGGCAGGCTTAACACATGCAAGTCGAGCGGGCATCCTTGTGGTGTCAGCGGCAGACGGGT
GAGTAACGCGTGGGAACGTGCTCTTCGGTTCGGAATAACTCAGGGAAACTTGAGCTAATACCGGATACGC
CCTTATGGGGAAAGGTTTACTGCCGAAGGATCGGCCCGCGTCTGATTAGCTGGTTGGTGAGGTTACGGCTC
ACCAAGGCGACGATCAGTAGCTGGTCTGAGAGGATGATCAGCCACACTGGGACTGAGACACGGCC
>Zotu10776
GAACGCTGGCGGCGTGCCTAATACATGCAAGTCGAACGAGGTGCTTGAGTAGCAATACGAGAGTATCCTA
GTGGCGAACGGGTGAGTAACGCGTTGGTGACCTACCCCAAAGTGTGGGATAACAGTTCGAAAGGACTGCT
AATACCGCATGTGGTTATCGGATTTAGAAACTGATAACTAAAGGAGCAATTCGCTTTGGGAGGGACCTGCG
TCCCATCAGCTAGTTGGTGAGGTAATGGCTCACCAAGGCTACGACGGGTAGGGGACCTGAGAGGGTGACC
CCCCACAATGGAACTGAAACACGGTC
>Zotu10781
GAACGCTGGCGGCATGCTTAACACATGCAAGTCGAACGGTAACAGTGCTTAGCTTGCTAGGTAGCTGACG
AGTGGCGAACGGGTGAGTAACGCGTAGGAATATATCCCAAAGAGCGGGACAACCTAGGGAAACTTAGGC
TAATACCGCATAATATCTGAGGATAAAAGGCTGGGGACCGAAAGGCCTGGCGCTTTGGGGATAGCCTGCG
TACGATTAGCTAGTTGGTGGGGTAAGGGCCTACCAAGGCGACGATCGTTAGCTGGTCTGAGAGGACGGAC
AGCCACACTGGGACTGAGACACGGCC
>Zotu10785
GAACGCTGGCGGCATGCCTAACACATGCAAGTCGAACGAGACCTTCGGGTCTAGTGGCGCACGGGTGCGT
AACGCGTGGGAATCTGCCCTTGGGTTCGGAATAACTCGCCGAAAGGCGTGCTAATACCGGATGATGTCGT
AAGACCAAAGATTTATCGCCCAGGGATGAGCCCGCGTAAGATTAGGTAGTTGGTGAGGTAAAGGCTCACC
AAGCCGACGATCTTTAGCTGGTCTGAGAGGATGATCAGCCACACTGGAACTGAGACACGGTC
>Zotu10789
GAACGCTAGCGGCAGGCTTAACACATGCAAGTCGAGGGGTATATTTCTTCGGAGATAGAGACCGGCGCAC
GGGTGCGTAACGCGTATGCAATCTACCTTTTACAGAGGGATAGCCCAGAGAAATTTGGATTAATACCTCAT
AGTATAATGACCTGGCATCAGGATATTATTAAAGTCACAACGGTAAAAGATGAGCATGCGTCCCATTAGCT

AGTTGGTAAGGTAACGGCTTACCAAGGCTACGATGGGTAGGGGTCCTGAGAGGGAGATCCCCCACACTGG
TACTGAGACACGGAC
>Zotu10791
GAACGCTGGCGGCGTGCCTAATACATGCAAGTCGAGCGAATCAATAGGAGCTTGCTCCTGTTGATTAGCG
GCGGACGGGTGAGTAACACGTGGGCAACCTGCCTGTAAGACTGGGATAACTTCGGGAAACCGGAGCTAAT
ACCGGATAATCCTTTTCCTCTCATGAGGAAAAGCTGAAAGTCGGTTTCGGCTGACACTTACAGATGGGCCC
GCGGCGCATTAGCTAGTTGGTGAGGTAACGGCTCACCAAGGCGACGATGCGTAGCCGACCTGAGAGGGT
GATCGGCCACACTGGGACTGAGACACGGCC
>Zotu10794
GAACGCTGGCGGCATGCCTAACACATGCAAGTCGAACGAGACCTTCGGGTCTAGTGGCGCACGGGTGCGT
AACGCGTGGGAACCTGCCTTTAGGTTCGGAATAACTCAGAGAAATTTGAGCTAATACCGGATAATGTCTTC
GGACCAAAGATTTATCGCCTTTAGATGGGCCCGCGTAGGATTAGCTTGTTGGTGGGGTAATGGCCTACCAA
GGCGACGATCCTTAGCTGGTCTGAGAGGATGATCAGCCACACTGAGACTGAGACACGGCC
>Zotu10802
GAACGCTGGCGGCGTGCCTAAGACATGCAAGTCGAGCGGGGTTTCGGGGCTTCGGCCCTGAGACCCAGCG
GCGGACGGGTGAGTAACACGTGGATAACCTGCCCTCTGCTCTGGGATAACAGGCCGAAAGGCCTGCTAAT
ACCGGATAAGCTCACGGACCCGCATGGGTTTGGGAGAAAAGGCGCTGGAGTCAGTGGCGCTGGCAGAGG
AGGGGTCCGCGGCCCATTAGCTGGTTGGCGGGGTAACGGCCCACCAAGGCGACGATGGGTAGCCGGCCT
GAGAGGGCGACCGGCCACACTGGGACTGAGACACGGCC
>Zotu10805
GAACGCTGGCGGCGTGCTTAACACATGCAAGTCGAACGAAGCTGCTAAAAGGAAGTTTTCGGATGGAATT
TTGGTAGACTTAGTGGCGGACGGGTGAGTAACGCGTGGATAACCTGCCTTATACAGGGGGATAACGGAGA
GAAATTTCCGCTAAGACCGCATAAGACCACAGCACCGCATGGTGCAGGGGTAAAATATTTATAGGTATAAG
ATGGATCCGCGTCCGATTAGCTGGTTGGTGAGGTAGAGGCTCACCAAGGCGACGATCGGTAGCCGGCCTG
AGAGGGTGAACGGCCACATTGGGACTGAGACACGGCC
>Zotu10809
GAACGCTGGCGGCGCGCCTAACACATGCAAGTCGAACGGCACCCCTCTCCGGAGGGAAGCGAGTGGCGA
ACGGCTGAGTAACACGTGGAGAACCTGCCCCCTCCCCCGGGATAGCCGCCCGAAAGGACGGGTAATACCG
GATACCCCGGGGTGCCGCATGGCACCCCGGCTAAAGCCCCGACGGGAGGGGATGGCTCCGCGGCCCATCA
GGTAGACGGCGGGGTGACGGCCCACCGTGCCGACAACGGGTAGCCGGGTTGAGAGACCGACCGGCCAGA
TTGGGACTGAGACACGGCC
>Zotu10813
GAACGCTGGCGGCGTGCTTAACACATGCAAGTCGAACGATGAAGCCCAGCTTGCTGGGTGGATTAGTGGC
GAACGGGTGAGTAACACGTGAGCAACCTGCCCCTGACTCTGGGATAAGCGCTGGAAACGGCGTCTAATAC
TGGATACGAGTAGCGATCGCATGGTCAGTTACTGGAGAGATTTTTTGGTTGGGGATGGGCTCGCGGCCTA
TCAGCTTGTTGGTGAGGTAATGGCTCACCAAGGCGTCGACGGGTAGCCGGCCTGAGAGGGTGACCGGCCA
CACTGGGACTGAGACACGGCC
>Zotu10817
GAACGCTGGCGGCATGCCTAACACATGCAAGTCGAACGAAGGCTTCGGCCTTAGTGGCGCACGGGTGCGT
AACGCGTGGGAATCTGCCCAAAGCTACGGAATAACTCGGGGAAACTCGTGCTAATACCGTATGATGTCGC
AAGACCAAAGATTTATCGGCTTTGGATGAGCCCGCGTAGGATTAGCTTGTTGGTGGGGTAAAGGCCTACCA
AGGCGACGATCCTTAGCTGGTCTGAGAGGATGATCAGCCACACTGGGACTGAGACACGGCC
>Zotu10819
GAACGCTGGCGGCGTGCTTAACACATGCAAGTCGAGCGAGGCTCCTCCTTCGGGAGGATGTCCTAGCGGC
GAACGGGTGAGTAACACGTGGGCAACCTGCCCCTAGCTCTGGGATAACCCCGGGAAACCGGGGCTAATAC
CGGATATGACTTCAGAAGGCATCTTCCGAAGTGGAAAAGGTTTACTGGCTAGGGATGGGCCCGCGGCCTA
TCAGCTTGTTGGTGGGGTGATGGCCTACCAAGGCGACGACGGGTAGCCGGCCTGAGAGGGTGTCCGGCC
ACACTGGGACTGAGATACGGCC
>Zotu10822

GAACGCTGGCGGCGTGCTTAACACATGCAAGTCGAACGGTAACTTCCTTCGGGAAGATACGAGTGGCGAA
CGGGTGAGTAACACGTGAGCAACCTGCCCTTGACCCCGGAATAACAGCTGGAAACAGCTGCTAATACCGG
ATACGACCTCCGACGGCATCATCTGGAGGTGGAAAGCTCCGACGGTCAAGGATGGGCTCGCGGCCTATCA
GCTTGTTGGTGAGGTAACGGCTCACCAAGGCTTCGACGGGTAGCCGGCCTGAGAGGGCGACCGGCCACAC
TGGGACTGAGATACGGCC
>Zotu10825
GAACGCTGGCGGCGTGCTTAACACATGCAAGTCGAACGAGCAAGAGCTGTAGCAATACAGGGTTCTGCTA
GTGGCAGACGGGTGAGTAATACATGGGAATCTACCTTGCAGTGCGAAATAACAGTTGGAAGCAATTGCTA
ATGTCGCATATAGCCGAGAGGTGAAAGGTTTACTGCTGCAAGATGGGCCCATGCAGGATTAGCTAGTTGG
TGAGGTAATGGCTCACCAAGGCGACGATCTTTAGTTGGTCTGAGAGGATGATCAGCCACACTGGGACTGA
GACACGGCC
>Zotu10827
GAACGCTGGTGGTATGCTTAACACATGCAAGTCGAACGGGCTTGGAGGGGCAACTCTTTAAGTCAGTGGC
AAACGGGTGAGGAATATATAGGAATCTCCCCAACAGTAAGGAATAACATTGGGAAACCAATGCTAATGCC
ATATATTACCGATTTATCGGGAAAGATTTATCGCTGTTGGATGAGCCTTTATCGGATTAGCTAGTTGGTAGG
GTAATGGCCTACCAAGGCGACGATCCGTAGCTGGTCTGAGAGGACGATCAGCCACACTGGGACTGAGACA
CGGCC
>Zotu10831
GAACGCTGGCGGCATGCCTTACACATGCAAGTCGAACGGCAGCATGGAGGAGCTTGCTCCTCTGATGGCG
AGTGGCGAACGGGTGAGTAAAGCATCGGAACGTACCGTGTAGTGGGGGATAACGTAGCGAAAGTTACGC
TAATACCGCATACGCACTGAGGTGGAAAGCGGGGGATCGTAAGACCTCGCGCTATACGAGCGGCCGATGT
CAGATTAGCTAGTTGGTAGGGTAAAGGCCTACCAAGGCGACGATCTGTAGCGGGTCTGAGAGGATGATCC
GCCACACTGGGACTGAGACACGGCC
>Zotu10838
GCCGTGTCTCAGTCCCAGTGTGGCTGGTCGTCCTCTCAAACCAGCTACGGATCGTCGCCTTGGTGAGCCGTT
ACCCCACCAACTAGCTAATCCGATATCGGCCGCTCCAATAGTGCAAGGTCTTGCGATCCCCTGCTTTCCCCC
GTAGGGCGTATGCGGTATTAGCTACGCTTTCGCGTAGTTATCCCCCGCTACTGGGCACGTTCCGATACATTA
CTCACCCGTTCGCCACTCGCCACCAGACCGAAGTCCGTGCTGCCGTTCGACTTGCATGTGTAAGGCATCCCG
CTAGCGTTCA
>Zotu10841
GAACGCTGGCGGCAGGTTTAACACATGCAAGTCGAGCGGGCCCTTCGGGGTCAGCGGCAGACGGGTGAG
TAACGCGTGGGAACGTACCATTTGCTACGGAATAACTCAGGGAAACTTGTGCTAATACCGTATGAGCCCCC
CTTTAAAATTTCAGGAATCATAAAATGCCCTGGCATTTTATGGGGGGGAAAGATTTATCGGCAAATGATCG
GCCCGCGTTGGATTAGCTAGTTGGTGGGGTAAAGGCCTACCAAGGCGACGATCCATAGCTGGTCTGAGAG
GATGATCAGCCACACTGGGACTGAGACACGGCC
>Zotu10844
GAACGCTGGCGGCATGCCTTACACATGCAAGTCGAACGGTAACAGGTTAAGCTGACGAGTGGCGAACGG
GTGAGTAATATATCGGAACGTGCCCAGTCGTGGGGGATAACGCAGCGAAAGCTGTGCTAATACCGCATAC
GATCTACGGATGAAAGCGGGGGATCGCAAGACCTCGCGCGATTGGAGCGGCCGATATCAGATTAGCTAGT
TGGTGGGGTAAAAGCCCACCAAGGCGACGATCTGTAGCTGGTCTGAGAGGACGACCAGCCACACTGGAAC
TGAGACACGGTC
>Zotu10846
CAACGCTGGCGGCGTGCCTCAGACATGCAAGTCGAACGATTAAACTTTCCTTCGGGAAAGATATAAAGTGG
CGCACGGGTGAGTAACACGTAGGTAATGTACCTTTGGGTGGGGAATAACTTAGGGAAACTTAAGCTAATA
CCGCATAATGCAGCGGCTTCTTCGGAAGACAGTTGTTAAAGATTTATCGCCTAAAGAGCAGCCTGCGGCAG
ATTAGCTAGTTGGTAAGGTAACGGCTTACCAAGGCTACGATCTGTATCCGACCTGAGAGGGTGGTCGGAC
ACACTGACACTGAATAACGGGT
>Zotu10848
GAACGCTGGCGGCATGCCTTACACATGCAAGTCGAACGGTAACAGGTTAAGCTGACGAGTGGCGAACGG
GTGAGTAATGTATCGGAACGTGCCCAGTTGTGGGGGATAACTGCTCGAAAGAGCAGCTAATACCGCATAC
GACCTGAGGGTGAAAGCGGGGGATCGCAAGACCTCGCGCAATTGGAGCGGTCGATATCAGATTAGGTAG

TTGGTGGGGTAAAGGCCTACCAAGCCTACGATCTGTAGCTGGTCTGAGAGGACGACCAGCCACACTGGGA
CTGAGACACGGCC
>Zotu10851
GAACGCTGGCGGCGTGCCTAATACATGCAAGTCGAGCGGACTGGAGGGAGCTTGCTCCCAAAGGTCAGCG
GCGGACGGGTGAGTAACACGTGGGTAACCTGCCTGACAGACTGGGATAACGCTTGGAAACGAGTGCTAAT
ACCAGATAGGCTACTTGATCGCATGATCGAGCAGGGAAAGAGGCTTTCGCCTCACTGTCAGATGGACCCGC
GGCGCATTAGCTAGTTGGTGAGGTAACGGCTCACCAAGGCGACGATGCGTAGCCGACCTGAGAGGGTGA
CCGGCCACACTGGGACTGAGACACGGCC
>Zotu10857
GAACGCTGGCGGCAGGCTTAACACATGCAAGTCGAGCGGGCCCTTCGGGGTCAGCGGCGGACGGGTTAG
TAACGCGTGGGAACGTGCCTTTTGCTTCGGAATAGCCCTGGGAAACTGGGGGTAATACCGGATGTGCCCT
GAGGGGGAAAGATTTATCGGCAAGAGATCGGCCCGCGTCTGATTAGGTAGTTGGTGTGGTAATGGCGCAC
CAAGCCGACGATCGGTAGCTGGTTTGAGAGGATGATCAGCAACACTGGGACTGAGACACGGCC
>Zotu10859
GAACGCTGGCGGCATGCTTAACACATGCAAGTCGCACGGGCAGTGGTAACATTGTCAGTGGCGGACGGGT
GAGTAACGCGTAGGAATCTGTCCTTGGGTGGGGGATAACAGTGGGAAACTACTGCTAATACCGCATGACA
ACTGAGGTTTAAAGGCGTGAGTCGCCTGAGGAGAAGCCTGCGTTCGATTAGCTAGTTGGTGGGGTAAAGG
CCTACCAAGGCTACGATCGATAGCTGGTCTGAGAGGATGATCAGCCACACTAGGACTGAGACACGGCC
>Zotu10864
GAACGCTGGCGGCGTGCCTTAAGCATGCAAGTCAAGGGGGTCGCAAGATAACCGGCAGACGGGTGAGTA
ATACATAGGAATCTACCCTTTAATGGACCATAGCTCATCGAAAGATGGGGTAATTGTCCATGGCACTTGTGC
GTTGCACAAGAACTACTAACAACTAACTACTAACAACTAACAAAATTAATAAGTTAGAAGTTAGAAGTTTCG
GGTTAGTTGTTTTTGCGCAGCGCGCAAATTAAAGATCGTAACTGATCGTTAAAGGAGGAGCCTATGTCCTA
TCAGCTAGTTGGTGGGGTAATGGCCTACCAAGGCAATGACGGGTAGCTGGACTGAGAGGTCGGTCAGCCA
CAACTGCACTGAGATACGGGC
>Zotu10868
GAACGCTGGCGGCATGCCTTACACATGCAAGTCGAACGGCAGCACGGGAGCAATCCTGGTGGCTAGTGGC
GAACGGGTGAGTAATATATCGGAACGTGCCCAGTCGTGGGGGATAACGTAGAGAAATTTACGCTAATACC
GCATGAGATCTACGGATGAAAGCGGGGGATTCGCAAGAACCTCGCGCGACTGGAGCGGCTGATATCAGAT
TAGGTTGTTGGTGAGGTAAAAGCTCACCAAGCCGACGATCTGTAGCTGGTTTGAGAGAACGACCAGCCAC
ACTGGGACTGAGACACGGCC
>Zotu10870
GAACGCTGGCGGCATGCCTAACACATGCAAGTCGAACGATGCTTTCGGGCATAGTGGCGCACGGGTGCGT
AACGCGTGGGAATCTGCCCTTGGGTTCGGAATAACAGTTGGAAACGACTGCTAATACCGGATGATGTCGAT
AAGACCAAAGATTTATCGCCCGAGGATGAGCCCGCGTCGGATTAGCTAGTTGGTGGGGTAAAGGCCTACC
AAGGCGACGATCCGTAGCTGGTCTGAGAGGATGATCAGCCACACTGGGACTGAGACACGGCC
>Zotu10876
GAACGCTGGCGGCGTGCTTAACACATGCAAGTCGAGCGGTAAGGCTCCTTCGGGAGTACACGAGCGGCGA
ACGGGTGAGTAACACGTGAGCAATCTGCCCTTCTCATCGGAATAAGCGTTGGAAACGACGTCTAATGCCGA
ATACGACCTCCTTTCGCATGATCGGAGGTGGAAAGCTCCGGCGGAGAAGGATGAGCTCGCGGCCTATCAG
CTAGTTGGTGTGGGTAATGGCCCACCAAGGCGACGACGGGTAGCCGGCCTGAGAGGGTGACCGGCCACA
CTGGGACTGAGACACGGCC
>Zotu10879
GAACGCTGGCGGCGTGCCTAACACATGCAAGTCGAGCGATGATATCCCTTCGGGGATTGATTAGCGGCGG
ACGGGTGAGTAACACGTGGGTAACCTGCCTCAAAGAGGGGGATAGCCCTCCGAAAGGAGGATTAATACC
GCATAAAGTTGAGAGTTCGCATGAACATTCAACCAAAGGAGCAATCCGCTTTGAGATGGACCCGCGTCCCA
TTAGCTAGTTGGTGAGGTAATAGCTCACCAAGGCGACGATGGGTAGCCGACCTGAGAGGGTGATCGGCCA
CATTGGAACTGAGATACGGTC
>Zotu10883

ACCGTGTCTCAGTTCCAGTGTGACTGATCATCCTCTCAGACCAGTTACGGATCGTCGCCTTGGTGAGCCATT
ACCTCACCAACTAGCTAATCCGACCTAGGCTCATCTGATAGCGCAAGGCCCGAAGGTCCCCTGCTTTCTCCC
GTAGGACGTATGCGGTATTAGCGTTCCTTTCGAAACGTTGTCCCCCACTACCAGGCAGATTCCTAGGCATTA
CTCACCCGTCCGCCGCTGAATCCAGGAGCAAGCTCCTTTCATCCGCTCGACTTGCATGTGTTAGGCCTGCCG
CCAGCGTTCA
>Zotu10885
GAACGCTGGCGGCATGCTTCATACATGCAAGTCGAACGCCCCGCAAGGGGAGTGGCGTAAGGGTGAGTA
ACGCATGGGAATCTGCCTTCGTGTGGGGGATAACCTGGGGAAACTTGGGCTAATACCGCATATTGCCGAG
AGGTGAAAGATTTATCGCACGAAGAGGAGCCTGTGTATGATTAGCTAGTTGGTAGGGTAATAGCCTACCA
AGGCAACGATCATTAGCTGGTCTGAGAGGATGATCAGCCACAATGGAACTGAGATACGGTC
>Zotu10887
GAACGCTGGCGGCATGCCTTACACATGCAAGTCGAACGGCAGCGCGGGGGCAACCCTGGCGGCGAGTGG
CGAACGGGTGAGTAATACATCGGAACGTGTCCCGTAGTGGGGGATAGCCCGGCGAAAGCCGGATTAATAC
CGCATACGATCTACGGATGAAAGCGGGGGATCGCAAGACCTCGCGCTATGGGGGCGGCCGATGGCAGAT
TAGCTAGTTGGTGGGGTAAAGGCCTACCAAGGCGACGATCTGTAGCTGGTCTGAGAGGACGACCAGCCAC
ACTGGGACTGAGACACGGCC
>Zotu10892
GAACGCTGGCGGCGTGCCTAATACATGCAAGTCGAGCGCCCTGACATTGCCCATTAACTAAGATTAGTGAG
TAGTGTCAGGGAGCGGCGGACGGCTGAGTAACGCGTAGGAACGTACCCCAAACTGAGGGATAAGCACCG
GAAACGGTGTCTAATACCGCATAATATCTTCGGATTAAAGCTTCGGCGGTTTGGGAGCGGCCTGCGTCTGA
TTAGTTAGTTGGTGAGGTAATAGCTCACCAAGACGATGATCGGTAGCTGGTCTGAGAGGATGACCAGCCA
GACTGGGACTGAGAACGGCC
>Zotu10895
GAACGCTGGCGGCGTGCTTAACACATGCAAGTCGAACGGTGAAGCCATTTCGGTGGTGGATCAGTGGCGA
ACGGGTGAGTAACACGTGAGCAATCTGCCCTTGACTCTGGGATAACTTCGGGAAACCGGAGCTAATACTG
GATACGACATGCGGAGGCATCTTCTGTGTGTGGAAAGATTTATCGGTCAGGGATGAGCTCGCGGCCTATCA
GCTTGTTGGTGGGGTAACGGCCCACCAAGGCGACGACGGGTAGCCGGCCTGAGAGGGCGACCGGCCACA
CTGGGACTGAGACACGGCC
>Zotu10901
GAACGCTGGCGGCAGGCCTAACACATGCAAGTCGAACGAACCTTTCGGGGTTAGTGGCGGACGGGTGAG
TAACACGTGGGAACGTGCCTTTAGGTTCGGAATAACTCAGGGAAACTTGTGCTAATACCGAATGTGCCCTT
CGGGGGAAAGATTTATCGCCTTTAGAGCGGCCCGCGTCTGATTAGCTAGTTGGTAGTGTAATGGACTACCA
AGGCTACGATCAGTAGCTGGTCTGAGAGGATGATCAGCCACATTGGGACTGAGACACGGCC
>Zotu10903
CAACGCTGGCGGCGCGCTTAACACATGCAAGTCGAACGAGAAAGGGAGCAATCCTGAGTAAAGTGGCGTA
CTGGTGAGTAACACGTGGATATCTACCTCTTGGTGGGGAATAACCTGGGGAAACCTGGGCTAATACCGCAT
AAGTTCGTGAGAAGAAAGGCGCAAGCCGCCGAGAGAGGAGTCCGCGGTCGATTAGGTAGTTGGCGGGGT
AAGAGCCCACCAAGCCAAAGATCGATAGCCGGCCTGAGAGGGCACACGGCCACACTGGCACTGAAACACG
GGC
>Zotu10905
GAACGCTGGCGGCGTGCTTAACACATGCAAGTCGAACGGTAAGGCCCTTCGGGGTACACGAGTGGCGAAC
GGGTGAGTAACACGTGGGTAATCTGCCCTGCACTTCGGGATAAGCCTGGGAAACTGGGTCTAATACCGGA
TAGGACCTCGGGATGCATGTCCTGTGGTGGAAAGCTTTTGCGGTGTGGGATGGGCCCGCGGCCTATCAGC
TTGTTGGTGGGGTGATGGCCCACCAAGGCGACGACGGGTAGCCGGCCTGAGAGGGTGTCCGGCCACACT
GGGACTGAGATACGGCC
>Zotu10907
GTGATCCAGGGGTCCCGGGATCAAGTCCAACAATGGGCTCCCCACAGGGAGCCTGCTTCTTCCTCTGCCTG
TGTCTCTGCCTCACTCTCTGTGTCTCTCATGAATAAATAAAATGTTTAAAAAAAAAAAAAGAATC
>Zotu10910

GAACGCTGGCGGCATGCCTAACACATGCAAGTCGAACGAGACCTTCGGGTCTAGTGGCGCACGGGTGCGT
AACGCGTGGGAACCTGCCTTTAGGTTCGGAATAACTCCTCGAAAGGGGTGCTAATACCGGATAATGTCTTC
GGACCAAAGATTTATCGCCTTTAGATGGGCCCGCGTAAGATTAGGTAGTTGGTGGGGTAAAGGCCTACCA
AGCCGACGATCTTTAGCTGGTCTGAGAGGATGATCAGCCACACTGGGACTGAGACACGGCC
>Zotu10915
GAATGCTGGCGGCGTGGATAAGTCATGCAAGTCAAGGGGGCCCGCAAGGGCAACCGGCAAACGAGGTAG
TAACACGTAGGTACACCCCCCGAAGTCGAGCATAGCCAGCCGAAAGGTTGGGTAATTCTCGATAGTCCCGC
AAGGGTAAAGTTTTTCGCTTCGGGAGTGGCCTGCGTAGTATCAGCTAGTTGGTAGGGTAATGGCCTACCAA
GGCTACGACGCTTAGGGGAGCTGAGAGGCTGACCCCCACCGATGGAACTGCGACACGGTC
>Zotu10917
GAACGCTGGCGGCGTGCTTAACACATGCAAGTCGAACGGTGAGGCAGAGCTTGCTCTGCGGATCAGTGGC
GAACGGGTGAGTAACACGTGAGTAACCTGCCCCTGACTCTGGGATAAGCGTTGGAAACGACGTCTAATAC
CGGATACGACCTTGGAAGGCATCTTTCTTGGTGGAAAGAATTTCGGTTGGGGATGGACTCGCGGCCTATCA
GCTTGTTGGTGAGGTAATGGCTCACCAAGGCGACGACGGGTAGCCGGCCTGAGAGGGTGACCGGCCACA
CTGGGACTGAGACACGGCC
>Zotu10920
GAACGCTGGCGGCGTGCCTAACACATGCAAGTCGAGCGGAGACAGTGAGTAGCTTGCTACGAGCTGTTTT
AGCGGCGGACGGGTGAGTAACGCGTGAGCAACCTTTCCCAGACAGGGGAATAACACACCGAAAGGTGTA
CTAATACCGCATAAGACCACGGGATCACATGGTTCTGGGGTAAAAGATTTATCGGTTTGGGGTGGGCTCGC
GTCCGATTAGGTAGTTGGTGAGGTAACGGCCCACCAAGCCGACGATCGGTAGCCGACCTGAGAGGGTGAT
CGGCCACATTGGGACTGAGACACGGCC
>Zotu10924
GAACGCTGGCGGCGTGCCTAAGACATGCAAGTCGAGTGGGTTTAGACCCACGGCGAACGGGTGAGTAAT
ACCTAGGAACTTTCTTTAAGTAGAGGATAACTAGTCGAAAGATTAGATAATACTCTATGTGATCGTAAGATC
AAAGCCGCAAGGCGCTTAAAGTGAGGCCTGGGCACCATCAGCTAGTTGGTAAGGTAATGGCTTACCAAGG
CTATGACGGTTAGGGGTCCCGAGAGGGATAACCCCCACACTGGAACTGCGACACGGTC
>Zotu10926
GAACGCTGGCGGCGTGCTTAACACATGCAAGTCGAACGGAAAGGCCCTTCGGGGTACTCGAGTGGCGAAC
GGGTGAGTAACACGTGGGTGATCTGCCCTGCACTTTGGGATAAGCCTGGGAAACTGGGTCTAATACCGGA
TAGGACTACGCGATGCATGTCGTGTGGTGGAAAGCTTTTGCGGTGTGGGATGGGCCCGCGGCCTATCAGC
TTGTTGGTGAGGTTACGGCTCACCAAGGCGACGACGGGTAGCCGGCCTGAGAGGGTGACCGGCCACACTG
GGACTGAGATACGGCC
>Zotu10928
GAACGCTGGCGGCATGCCTTACACATGCAAGTCGAACGGCAACACGGGAGCTTGCTCCTGGTGGCGAGTG
GCGAACGGGTGAGTAATATATCGGAACGTACCCTAGAGTGGGGGATAACGCAGCGAAAGTTGCGCTAATA
CCGCATACGATCTACGGATGAAAGTGGGGGATCGCAAGACCTCATGCTCATGGAGCGGCCGATATCTGAT
TAGCTAGTTGGTGGGGTAAAGGCCTACCAAGGCATCGATCAGTAGCTGGTCTGAGAGGACGACCAGCCAC
ACTGGAACTGAGACACGGTC
>Zotu10930
AAACGCTGGCGGCATGCCTAACACATGCAAGTCGAACGGCAGCACAGTAAAGATTTCGGTCTTTACGGGT
GGCGAGTGGCGGATGGGTGAGTAAAGCGTAGGAATCTGTCCTGGAGAGTGGGATAACCCGAGGAAACTC
GGGCTAATACCGCATGATATCGAGAGATCAAAGCAGGGGACCCTTCGGGGCCTTGCGCTCTAGGATGAGC
CTACGTCGGATTAGCTAGTTGGTGAGGTAATGGCTCACCAAGCCGACGATCTGTAGCTGGTCTGAGAGGAT
GACCAGCCACACTGGGACTGAGACACGTCC
>Zotu10936
GAACGCTGGCGGCGTGCTTAACACATGCAAGTCGAACGGTGAAAGTGGGAGCTTGCTCCTACCGGATCAG
TGGCGAACGGGTGAGTAACACGTGAGCAATCTGCCCCGAACTCTGGGATAAGCACTGGAAACGGTGTCTA
ATACTGGATACGACGCGGGAGGGCATCCTCACCGCGTGGAAAGAATTTCGGTTCGGGATGAGCTCACGGC
CTATCAGCTAGTTGGTGAGGTAATGGCTCACCAAGGCGACGACGGGTAGCCGGCCTGAGAGGGTGACCG
GCCACACTGGGACTGAGACACGGCC

>Zotu10940
GAATGCTGGCGGCGTGCCTAACACATGCAAGTCGAGCGGCAGCGCGGGTAGTTTACTACCTGGCGGCGAG
CGGCGGACGGCTGAGTAACGCGTGGGAACGTGCCCCAAAGTGAGGAATAACTGCCCGAAAGGGTAGCTA
ATGCCGCATATGGTCTTCGGATTAAAGGATTTATCCGCTTTGGGAGCGGCCCGCGTTAGATTAGGTAGTTG
GTGAGGTAAAGGCTCACCAAGCCGACGATCTATAGCTGGTCTGAGAGGATGATCAGCCAGACTGGGACTG
AGACACGGCC
>Zotu10942
GAACGCTGGCGGCGTGCTTAACACATGCAAGTCGAACGGTAAGGCCCTTCGGGGTACACGAGTGGCGAAC
GGGTGAGTAACACGTGGGTAATCTGCCCTGCACTTCGGGATAAGCCTGGGAAACTGGGTCTAATACCGGA
TATTCAGATTCTGCCGCATGGTGGTTTCTGGAAAGTTTTTCGGTGCAGGATGTGCCCGCGGCCTATCAGCTT
GTTGGTGGGGTAATGGCCTACCAAGGCGACGACGGGTAGCCGGCCTGAGAGGGTGATCGGCCACATTGG
GACTGAGACACGGCC
>Zotu10948
GAACGCTAGCTACAGGCTTAACACATGCAAGTCGAGGGGCAGCATGAACTTAGCTTGCTAAGTTTGATGGC
GACCGGCGCACGGGTGAGTAACACGTATCCAACCTTCCGTTTACTCAGGGATAGCCTTTCGAAAGAAAGAT
TAATACCTGATAGTATGGTGAGATTGCATGATAGCACCATTAAAGATTTATTGGTAAACGATGGGGATGCG
TTCCATTAGGTAGTAGGCGGGGTAACGGCCCACCTAGCCGACGATGGATAGGGGTTCTGAGAGGAAGGTC
CCCCACATTGGAACTGAGACACGGTC
>Zotu10952
GAACGCTGGCGGCGCGCTTAACACATGCAAGTCGAACGGTTAAAGCACCTCCGGGTGTGCATAAAGTGGC
GAACGGCTGAGTAACACGTGGGCAACCTGCCCCCGCACCGGGATAGCCTCGGGAAACCGTGGGTAATAC
CGGATACTCCGCCACCCCCGCATGGGGGAGGCGGGAAAGCGTACACGGCGGGGGATGGGCCCGCGGCCT
GTTAGCTCGTTGGCGGGGTAACGGCCCACCAAGGCGATTATGGGTAGCCGGGTTGAGAGACCGACCGGCC
AGATTGGGACTGAGACACGGCC
>Zotu10954
GAACGCTGGCGGCGTGCTTAACACATGCAAGTCGAGCGGTAAGGCACTTCGGTGTACACGAGCGGCGAAC
GGGTGAGTAACACGTGAGTAATCTGCCCTTCACTCTGGGATAAGCACTCGAAAGGGTGTCTAATACCGGAT
ACGACCGGCCACTGCATGGTGTGTTGGTGGAAACTTTTGTGGGTGGGGGATGTGCTCGCGGCCTATCAGC
TTGTTGGTGGGGTAATGGCCTACCAAGGCTTCGACGGGTAGCCGGCCTGAGAGGGTGACCGGCCACACTG
GGACTGAGACACGGCC
>Zotu10957
GAACGCTGGCGGCATGCCTAAAACATGCAAGTCGAGCGGTAAGGCCCCTTCGGGGGTACACGAGCGGCG
GACGGGTGAGTAACGCGTGGGAATCTACCCCAAACTGAGGGATAAGCATTGGAAACGATGTCTAATACCG
CATATGGTCTTCGGATTAAAGCTTCGGCGGTTTGGGAAGAGCCCGCGTAAGATTAGCTAGTTGGTGAGGTA
AAGGCTCACCAAGGCGACGATCTTTAACTGGTTTGAGAGGATGATCAGTCACACTGGAACTGAGACACGG
TC
>Zotu10960
GAACGCTGGCGGCGTGCCTAAGACATGCAAGTCGAGTGGGTTTAGACCCACGGCGAACGGGTGAGTAAT
ACCTAGGTACTTTCTTTAAGTGGAGCATAACCAGTCGAAAGATTGGATAATACTCCATGTGATCGCAAGATC
AAAGTCGCAAGACGCTTAAAGTGAGGCCTGGGCACCATCAGCTAGTTGGTAAGGTAATGGCTTACCAAGG
CTATGACGGTTAGGGGTCCCGAGAGGGATAACCCCCACACTGGAACTGCGACACGGTC
>Zotu10963
GAACGCTGGCGGCGTGCCTTAAGCATGCAAGTCGAACGGATAGTAATATCAGTGGCGGACGGTCGAGTAA
CGCGTGGGAATCTACCTTAAAGTGTGGGATAGTCCGGCGAAAGTCGGAGTAATACCGCATGTGCCACAAT
CAGTAACATGGTTGTGGTAAAGTCGCAAGACGCTTTAAGATGAGCCCGCGTCCTATCAGCTTGTTGGTGGG
GTAAAAGCCTACCAAGGCGACGACGGGTAGCTGGTCTGAGAGGATGGTCAGCCGGAATGGGACTGAGAC
ACGGCC
>Zotu10969
GAACGCTGGCGGCAGGCTTAACACATGCAAGTCGAACGCATCCTTCGGGGTGAGTGGCAGACGGGTGAG
TAACACGTGGGAACGTACCCTTCAGTTCGGAATAACCCTGGGAAACTAGGGCTAATACCGGATACGGCCG

AGAGGCGAAAGGTTTACTGCTGAGGGATCGGCCCGCGTCCGATTAGCTAGTTGGTGTTGGTAACGGCTCA
CCAAGGCGACGATCGGTAGCTGGTCTGAGAGGATGGCCAGCCACATTGGGACTGAGACACGGCC
>Zotu10973
GAACGCTAGCGGCAGGCCTAATACATGCAAGTCGAACGGTAACATGAGTAGCAATACTTGATGACGAGTG
GCGCACGGGTGCGTAACGCGTATGCAATCTACCTTTAACCGGTGAATAGCCCGAAGAAATTCGGATTAATA
CACAATAGTATTGTTTGTTGGCATCAACAGACAATTAAAACTCCGGTGGTTAAAGATGAGCATGCGTGACA
TTAGCTAGTTGGTGAGGTAATGGCTCACCAAGGCTACGATGTCTAGGGGATCTGAGAGGATTAACCCCCAC
ACTGGTACTGAGACACGGAC
>Zotu10975
GAACGCTGGCGGCGTGCCTAACACATGCAAGTCGAGCGAGAAAGCCCGCAAGGGTGAGTAAAGCGGCGC
ACGGGTGCGTAACACGTGGATAATCTGCCCTGAAGTCCGGAATAACTCGCCGAAAGGCGTGCTAATGCCG
GATGAGACCACGGGAGCTCCGGCTCCTGCGGGAAAAGGTGGCCTCTGTACACAAGCTATCGCTACAGGAT
GAGTCCGCGGCCCATCAGCTCGTTGGCGGGGTAATGGCCCACCAAGGCTACGACGGGTAGCTGGTCTGAG
AGGACGATCAGCCACACTGGAACTGAGACACGGTC
>Zotu10979
GAACGCTGGCGGTATGCTTAAGACATGCAAGTCGAACGGTAACAGACCTTCGGGTGCTGACGAGTGGCGG
ACGGGTGAGTAACACGTGGGAATCTACCTTGTAGTGGGGGATAAGCCAGGGAAACTTGGACTAATACCGC
ATACGCCCTAAGGGGGAAAGGAGCAATTCGCTATAAGATGAGCCCGCGTCTGATTAGCTAGTTGGTGAGG
TAAAGGCTCACCAAGGCGACGATCAGTAGCTGGTCTGAGAGGATGACCAGCCACATTGGGACTGAGACAC
GGCC
>Zotu10983
GAACGCTGGCGGCGTGCCTAATACATGCAAGTCGAGCGAATCTTTGGGAGCTTGCTCCCAATAGATTAGCG
GCGGACGGGTGAGTAACACGTGGGTAACCTGCCTGTAAGACTGGGATAACTTCGGGAAACCGGAGCTAAT
ACCGGATAATTTCTTTTCTCGCATGAGAAGAGATGGAAAGATGGTTTCGGCTATCACTTACAGATGGACCC
GCGGCGCATTAGCTAGTTGGTGAGGTAATGGCTCACCAAGGCCACGATGCGTAGCCGACCTGAGAGGGTG
ATCGGCCACACTGGGACTGAGACACGGCC
>Zotu10986
GAACGCTGGCGGCGTGCCTAACACATGCAAGTCGAGCGATGAACCCTTCGGGGGGATTAGCGGCGGACG
GGTGAGTAACACGTGGGTAACCTGCCTCAAAGAGGGGGATAGCCTCCCGAAAGGGAGATTAATACCGCAT
AACATCAAATTATCGCATGGTAGTTTGATCAAAGGAGTAATCCGCTTTGAGATGGACCCGCGGCGCATTAG
CTAGTTGGTGAGGTAACGGCTCACCAAGGCGACGATGCGTAGCCGACCTGAGAGGGTGATCGGCCACATT
GGAACTGAGACACGGTC
>Zotu10990
GAACGCTAGCGGCATGCCTAACACATGCAAGTCGAACGGCAGCGAGGAGGAGCTTGCTTCTCTGTCGGCG
AGTGGCGAACGGGTGAGTATAGCATCGGAACGTGCCAAGTAGTGGGGGATAACCAAGCGAAAGTTTGGC
TAATACCGCATAAGCTCTAAGGAGGAAAGTAGGGGACCGTAAGGCCTTACGCTATTTGATCGGCCGATGTC
GGATTAGCTAGTTGGTGAGGTAATGGCTCACCAAGGCGATGATCCGTAGCGGGTCTGAGAGGACGATCCG
CCACACTGGGACTGAGACACGGCC
>Zotu10996
GAACGCTGGCGGCGTGCCTAATACATGCAAGTCGAACGAACGGAAGTTAGAACTTGTTCTAATGGAAGTG
AGTGGCGAACGGGTGAGTAACACGTGGGTAACCTACCTTTAAGCGGGGGATAACAGTGGGAAACGACTG
CTAATACCGCATAGGACATAGCGTCACATGACGCAGTGAGGAAAGACGGTTTCGGCTGTCGCTTAGAGAT
GGACCCGCGGTGCATTAGCTAGTTGGTAAGGTAACGGCTTACCAAGGCGATGATGCATAGCCGACCTGAG
AGGGTGATCGGCCACATTGGGACTGAGACACGGCC
>Zotu10998
GAACGCTGGCGGCAGGCCTAACACATGCAAGTCGAGCGGCAGCACGGGAAGCTTGCTTCCTGGTGGCGA
GCGGCGGACGGGTGAGTAATGCATAGAAATCTGCCCGGTAGTGGGGGATAACCTGGGGAAACCCAGGCT
AATACCGCATACGTCCTACGGGAGAAAGCAGGGGATCTTCGGACCTTGCGCTATCGGATGAGTCTATGTCG
GATTAGCTTGTTGGTGAGGTAATGGCTCACCAAGGCGACGATCCGTAGCTGGTCTGAGAGGATGATCAGC
CACATCGGGACTGAGACACGGCC

>Zotu11002
GAACGCTGGTGGCATGCTTAACACATGCAAGTCGAGCGGTAAGGCTCCTTCGGGGGTACACGAGCGGCGA
ACGGGTGAGTAACACGTGAGTAATCTGCCCTTCACTTCGGGATAACCATCGGAAACGGTGGCTAATACCGG
ATACGACGTACTCCTGCATGGGATGTGCGTGGAAAGTTTTTCGGTGGGGGATGTGCTCGCGGCCTATCAG
CTTGTTGGTGAGGTAATGGCTCACCAAGGCTTCGACGGGTAGCCGGCCTGAGAGGGTGACCGGCCACACT
GGGACTGAGACACGGCC
>Zotu11005
GAACGCTGGCGGCGTGCTTAACACATGCAAGTCGAGGGTAGCAATACACTGGCGCAAGGGTGCGTAACGC
GTAGATTATCTGCCCCATACACTAACATAACGCTTGGAAACGGGTGCTAATATTAGATGAGGTGCTTCGGC
ATCAAAGTTTCGACGGTATGGGATGAGTCTGCGTCTGATTAGCTAGTTGGTAGTGTAATGGACTACCAAGG
CGACGATCAGTAGCTGGTCTGAGAGGACGATCAGCCACACTGGGACTGAGACACGGCC
>Zotu11008
TAACGCTGGCGGCGTGCCTAACACATGCAAGTCGAGCGCCCCTTCGGGGGAGCGGCGGACGGCTGAGTA
ACACGTTGGTACCTGCCCCAAACCCGGGAATAAGCGCTGGAAACGGCGTCTAATGCCCGATAGTTCTCTTC
GGAGTAAAGATTTATCGGTTTGGGAGGGGCCTGCGCCCGATTAGCTTGTTGGTGAGGTAACGGCTCACCA
AGGCTCTGATCGGTAGGTGGTCTGAGAGGATGATCACCCAGACTGGGACTGAGACACGGCC
>Zotu11010
CGATGAAGAACGCAGCGCGGACTTCGGTCTGGCGGCGAGTGGCGAACGGGTGAGTAATACATCGGAACG
TACCCTGTTGTGGGGGATAACTAGTCGAAAGATTAGCTAATACCGCATACGACCTGAGGGTGAAAGCGGG
GGACCGTAAGGCCTCGCGCAGCAGGAGCGGCCGATGTCTGATTAGCTAGTTGGTGGGGTAAAGGCCCACC
AAGGCGACGATCAGTAGCTGGTCTGAGAGGACGATCAGCCACACTGGGACTGAGACACGGCC
>Zotu11012
GAACGCTGGCGGCGTGCCTAACACATGCAAGTCGAGCGCGAAAAGGGCAACCTGAGTAGAGCGGCGCAC
GGGTGAGTAACACGTGGGTAATCTGCCTCAAAGCCCGGAATAACCTTCCGAAAGGTTGGCTAATGCCGGA
TAAGCTCACGGGAACTTCGGTTCTTGTGGGAAAAGGTGGCCTCTGTTTACAAGCTATCACTTTGAGATGAG
CCCGCGGCCCATCAGGTAGTTAGCGGGGTAACGGCCCACCAAGCCTACGACGGGTAGCTGGTCTGAGAGG
ACGATCAGCCACACTGGAACTGAGACACGGTC
>Zotu11015
GAACGCTAGCGGCAGGCCTAATACATGCAAGTCGAACGGTTCGCAAGAATAGTGGCAAACGGGTGCGTAA
CGCGTAAGGAACCTGCCCCTCACTGGGGGATAGCCCGGCGAAAGCTGGGGTAAACCCGCATGGCTCAGTG
AAGTGGCATCACTTTATTGATAAACATTTATGGGTGAGGGAGGGTCTTGCGTCTGATTAGTTAGTTGGGGG
GGTAACGGCTCACCAAGACGATGATCAGTAGGGGCTCTGAGAGGAGCGGCCCCCACATGGACACTGAGA
CACGGGT
>Zotu11017
GAACGCTGGCGGCAGGCTTAACACATGCAAGTCGAGCGCGTAGCAATACGAGCGGCAGACGGGTGAGTA
ACGCGTGGGGATGTGCCTGGTGGTACGGAACAACTCAGGGAAACTTGAGCTAATACCGTATAAGCCCTTA
GGGGGAAAGATTTATCGCCACCAGATCAACCCGCGTTGGATTAGCTAGTTGGTGAGGTAACGGCTCACCA
AGGCGACGATCCATAGCTGGTCTGAGAGGATGATCAGCCACACTGGGACTGAGACACGGCC
>Zotu11020
GAACGCTGGCGGCAGGCCTAACACATGCAAGTCGAACGGCAGCGGGTAGGAAGCTTGCTTCCTATGCCGG
CGAGTGGCGGACGGGTGAGGAACACATCGGAATCTACTCCGTCGTGGGGGATAACGTAGGGAAACTTAC
GCTAATACCGCATACGACCTAAGGGTGAAAGTGGGGGACCGCAAGGCCTCACGCGATGGAATGAGCCGA
TGTCCGATTAACTAGTTGGCGGGGTAAAGGCCCACCAAGGCGACGATCGGTAGCTGGTCTGAGAGGATGA
TCAGCCACACTGGGACTGAGACACGGCC
>Zotu11022
GAACGCTGGCGGCATGCCTAACACATGCAAGTCGAACGATGCTTTCGGGCATAGTGGCGCACGGGTGCGT
AACGCGTGGGAATCTGCCTTGGGGTACGGAATAACTCAGAGAAATTTGAGCTAATACCGTATGATGTCTTC
GGACCAAAGATTTATCGCCCTGAGATGAGCCCGCGTAGGATTAGCTAGTTGGTGGGGTAAAGGCCTACCA
AGGCGACGATCCTTAGCTGGTCTGAGAGGATGATCAGCCACACTGGGACTGAGACACGGCC
>Zotu11028

GAACGCTGGCGGCGTGCCTAAGACATGCAAGTCGAATGTCCGCAAGGACATGGCGAACGGGTGAGTAAT
ACCTAGGAACTTTCTTTAAGTGGAGCATAACCAGTCGAAAGATTGGATAATTCTCCATGTGGCAGTAATGCT
AAAGTCGCAAGACGCTTAAAGTGAGGCCTGGGCACCATCAGTTAGTTGGTAAGGTAACGGCTTACCAAGG
CTATGACGGTTAGGGGTCCCGAGAGGGAGAACCCCCACACTGGAACTGCGACACGGTC
>Zotu11031
GAACGCTGGTGGCAGGCCTAACACATGCAAGTCGAGCGGGAAGATGGTGCTTGCACTATCGGAGAGCGG
CGCACGGGTGAGTAACACGTGGGAACATACCATTAAGTGGGGGATAACTATTGGAAACGATAGCTAATAC
CGCATAAACCCTGAGGGGGAAAGATTTATTGCTTAATGATTGGCCCGCGGAAGATTAGGTAGTTGGTGGG
GTAAGAGCCTACCAAGCCGACGATCTATAGCTGGTCTGAGAGGACGATCAGCCACACTGGAACTGAGACA
CGGTC
>Zotu11036
GAACGCTGGCGGTGTGCCTTAAGCATGCAAGTCGAACGGTCCTCGCAAGAGGGCAGTGGCGGACGGGTG
AGTAACGCGTGGGAATCTACCTTAAAGTGGGGTATAGCTCGTCGAAAGACGGGGTAATCCCCCATGTGAT
CGCAAGATTAAAGTCGCAAGACGCTTTAAGATGAGCCTGCGTCCTATCAGCTTGTTGGTGGGGTAAAAGCC
TACCAAGGCGACGACGGGTAGCTGGTCTGAGAGGATGGTCAGCCACAATGGAACTGCGACACGGTC
>Zotu11038
GAACGCTGGCGGCGCGCTTAACACATGCAAGTCGAGCGAGAACCATGGCTTCGGCTATGGGGACAGCGG
CGAACGGGTGAGTAACACGTGGGTAATCAACCCTCGACACTGGGATAGCCCTGGGAAACTCGGATTAATA
CCGGATACCCCATCTGGCCTTCGGGCCGGCTGGAAAAGGTAGCTTTGGCCTCCGGTCGAGGACGAGCCCG
CGGCGGATTAGCTTGTTGGTGGGGTAAAAGCCCACCAAGGCGACGATCCGTAGCTGGTCTGAGAGGACGA
TCAGCCACACTGGGACTGAGACACGGCC
>Zotu11042
CAACGCTGGCGGCGTGCCTAACACATGCAAGTCGAACGAGAAAGGGACTTCGGTCCTGAGTAAAGTGGCG
CACGGGTGAGTAACACGTGACTAACCTACCCTTGAGCGGGGGATAACCCAGGGAAACCTGAGCTAATACC
GCATAATACCTACGGGTCAAAGCGAGCAATCGCACTTAAGGAGGGGGTCGCGGCAGATTAGTTAGTTGGC
GGGGTAATGGCCCACCAAGACGATGATCTGTATCCGGCCTGAGAGGGCGCACGGACACACTGGAACTGAA
ACACGGTC
>Zotu11047
GAATGCTGGCGGTGTGGATAGGGCATGCAAGTTAAGGGGACCGCAAGGTAACCGGCAGACGAGATAGTA
ACGCGTAGGAACGTACCCAGAAGTCGTGCACAGCTCGCCGAAAGGCGGGGTAATTCACGATATTGTCGCA
AGATGAAAGATTTATCGCTTCTGGAGCGGCCTGCGTAATATCAGCTAGTTGGTGAAGTAATGGTTTACCAA
GGCTATGACGTTTAGGGGAGCTGAGAGGCTGACCCCCATCGATGGAATTGAGATACGGTC
>Zotu11054
GAACGCTGGCGGCATGCCTAACACATGCAAGTCGAACGAGACCTTCGGGTCTAGTGGCGCACGGGTGCGT
AACACGTGGGAATCTGCCCTTTGCTTCGGAATAACAGTTAGAAATGACTGCTAATACCGGATAATGACTTC
GGTCCAAAGATTTATCGGCAAAGGATGAGCCCGCGTAAGATTAGGTAGTTGGTGAGGTAAAGGCTCACCA
AGCCGACGATCTTTAGCTGGTCTGAGAGGATGATCAGCCACACTGGGACTGAGACACGGCC
>Zotu11057
GAACGCTAGCGGCAGGCCTAATACATGCAAGTCGAGGGGCAGCAGGGATAGCAATATTCGCTGGCGACC
GGCGCACGGGTGCGTAACACGTATACAATCTGCCTTTAACAGGGGGATAGCCCGGAGAAATCCGGATTAA
TACCCCATAACACTAAGAGTAGCATTACTTTTGGTTAAAAGCTCCGGCGGTTAGAGATGAGTATGCGTCTG
ATTAGCTAGTTGGTGAGGTAACGGCTCACCAAGGCGACGATCAGTAGGGGATCTGAGAGGATTAACCCCC
ACACGGATACTGAGACACGGAT
>Zotu11061
GAACGCTGGCGGCAGGCCTAACACATGCAAGTCGAACGGACCCTTCGGGGTTAGTGGCGGACGGGTGAG
TAACACGTGGGAACGTGCCTTTTGGTTCGGAATAGCTCCTGGAAACGGGTGGTAATGCCGAATGTGCCCCT
CGGGGGAAAGATTTATCGCCATTAGAGCGGCCCGCGTCTGATTAGCTAGTTGGTGAGGTAAAGGCTCACC
AAGGCTACGATCAGTAGCTGGTCTGAGAGGATGACCAGCCACATTGGGACTGAGACACGGCC
>Zotu11066

GAACGCTGGCGGCAGGCTTAACACATGCAAGTCGAGCGGGGGATGGTAGCTTGCTACCTGACCTAGCGGC
GGACGGGTGAGTAAAGCTTAGGAATCTGCCTATTAGTGGGGGACAACATTTCGAAAGGGATGCTAATACC
GCATACGCCCTACGGGGGAAAGCAGGGGATCTTCGGACCTTGCGCTAATAGATGAGCCTAAGTCGGATTA
GCTAGTTGGTGGGGTAAAGGCCTACCAAGGCGACGATCTGTAGCGGGTCTGAGAGGATGATCCGCCACAC
TGGGACTGAGACACGGCC
>Zotu11071
GAACGCTGGCGGCATGCCTTACACATGCAAGTCGAACGGCAGCATAGGAGCTTGCTCCTGATGGCGAGTG
GCGAACGGGTGAGTAATATATCGGAACGTGCCCTAGAGTGGGGGATAACTAGTCGAAAGACTAGCTAATA
CCGCATACGACCTGAGGGTGAAAGTGGGGGACCGCAAGGCCTCATGCTATAGGAGCGGCCGATGTCTGAT
TAGCTAGTTGGTGAGGTAAAGGCTCACCAAGGCGACGATCAGTAGCTGGTCTGAGAGGACGACCAGCCAC
ACTGGGACTGAGACACGGCC
>Zotu11074
GAACGCTGGCGGCAGGCCTAATACATGCAAGTCGAACGCTCCAGCAATGGAGAGTGGCAGACGGGTGAG
TAACGCGTGGGAACCTTCCCAGTGGTACGGAATAGCTCAGGGAAACTTGAGGTAATACCGTATACGCCCCT
CGGGGGAAAGATTTATCGCCATTGGATGGGCCCGCGTCGGATTAGCTAGTTGGTGGGGTAACGGCCTACC
AAGGCGACGATCCGTAGCTGGTCTGAGAGGATGATCAGCCACACTGGGACTGAGACACGGCC
>Zotu11077
GAACGCTGGCGGCGTGCTTAACACATGCAAGTCGAGCGGAAAGGCCACTTCGGTGGTACTCGAGCGGCGA
ACGGGTGAGTAACACGTGAGTAATCTGCCCTTGGCTTTGGGATAGCCACCGGAAACGGTGATTAATACCG
GATATGACCATTCCACGCATGTGGTGGTGGTGGAAAGTTTTTCGGCCAGGGATGTGCTCGCGGCCTATCAG
CTTGATGGTGAGGTAATGGCTCACCATGGCTTCGACGGGTAGCCGGCCTGAGAGGGTGACCGGTCACACT
GGGACTGAGGCACGGCC
>Zotu11081
GAACGCTGGCGGCATGCTTTACACATGCAAGTCGAACGGTAACAGGCCGCAAGGTGCTGACGAGTGGCGA
ACGGGTGAGTAATGCATCGGAACGTGCCCAGTCGTGGGGGATAACTACTCGAAAGAGTAGCTAATACCGC
ATACGATCTATGGATGAAAGCGGGGGACCGTAAGGCCTCGCGCGATTGGAGCGGCCGATGTCAGATTAG
GTAGTTGGTGGGGTAAAGGCTCACCAAGCCAACGATCTGTAGCTGGTCTGAGAGGACGACCAGCCACACT
GGGACTGAGACACGGCC
>Zotu11086
GAACGCTGGCGGCGTGCTTAACACATGCAAGTCGAACGGTGACCTCGAGAGCTTGCTCTTGGGTGATCAG
TGGCGAACGGGTGAGTAACACGTGAGTAACCTGCCCCAGACTCTGGAATAACCCCGGGAAACCGGAGCTA
ATACCGGATACGACATCAGGCCGCATGGTCATGGTGTGGAAAGTTTTTCGGTCTGGGATGGGCTCGCGGC
CTATCAGCTTGTTGGTGAGGTAGTGGCTCACCAAGGCGACGACGGGTAGCCGGCCTGAGAGGGCGACCG
GCCACACTGGGACTGAGACACGGCC
>Zotu11088
GAACGCTGGCGGCAGGCCTAACACATGCAAGTCGAACGCTCTAGCAATAGAGAGTGGCAGACGGGTGAG
TAACGCGTGGGAAACTTCCCAGGGGTACGGAATAGCTCATGGAAACGTGGGGTAATACCGTATGATGCCG
AAAGGCCAAAGATTTATCGCCCTTGGATGTGCCCGCGTCGGATTAGCTAGTTGGTGAGGTAACGGCTCACC
AAGGCGACGATCCGTAGCTGGTTTGAGAGAATGACCAGCCACACTGGGACTGAGACACGGCC
>Zotu11090
GAACGCTGGCGGCAGGCCTAATACATGCAAGTCGAGCGGCCACTTCGGTGGCAGCGGCGGACGGGTGAG
TAACGCGTGGGAATATGCCCTTTGGTACGGAACAACTGAGGGAAACTTCAGCTAATACCGTATGTGCCCTT
CGGGGGAAAGATTTATCGCCATTGGAGTAGCCCGCGTTGGATTAGCTAGTTGGTGGGGTAAAGGCCCACC
AAGGCGACGATCCATAGCTGGTCTGAGAGGATGATCAGCCACACTGGGACTGAGACACGGCC
>Zotu11092
GAACGCTGGCGGCGTGCTTAACACATGCAAGTCGAGCGGTAAGGCTCTTCGGAGTACACGAGCGGCGAAC
GGGTGAGTAACACGTGAGCAACCTGCCCATGACACTGGGATAACGATTGGAAACAGTCGCTAATACCGGA
TACGACCTTGGTGGACATCTACACGAGGTGGAAAGCTCCGGCGGTCATGGATGGGCTCGCGGCCTATCAG
CTTGTTGGTGAGGTAACGGCTCACCAAGGCGTCGACGGGTAGCCGGCCTGAGAGGGCGACCGGCCACACT
GGGACTGAGACACGGCC

>Zotu11094
GAACGCTGGCGGCATGCCTTACACATGCAAGTCGAACGGCAGCACGGGGGCAACCCTGGTGGCGAGTGG
CGAACGGGTGAGTAATGCATCGGAACGTACCTGGTAGTGGGGGATAGCTCGGCGAAAGCCGGATTAATA
CCGCATACGCACCAAGGTGGAAAGCGGGGGACCGAAAGGCCTCGCGCTATCAGAGCGGCCGATGTCGGA
TTAGCTTGTTGGTGAGGTAAAGGCTCACCAAGGCCGCGATCCGTACCTGGTCTGAGAGGACGACCAGGCA
CACTGGGACTGAGACACGGCC
>Zotu11097
GAACGCTGGCGGCGTGCCTAATACATGCAAGTCGAGCGGGCAGACGGGAGCTTGCTCCCTGAAGTCAGCG
GCGGACGGGTGAGTAACACGTGGGCAACCTGCCTGTAAGACTGGGATAACTCCGGGAAACCGGGGCTAA
TACCGGATAATTCTTTTCTCTTCATGGAGAAAAGCTGAAAGACGGTCTCGGCTGTCACTTACAGATGGGCCC
GCGGCGCATTAGCTAGTTGGTGAGGTAACGGCTCACCAAGGCGACGATGCGTAGCCGACCTGAGAGGGT
GATCGGCCACACTGGGACTGAGACACGGCC
>Zotu11100
GAACGTTAGCGGCACGCCTAACACATGCAAGTCGCACGAGAGTGGAGCAATCCATGAAAGTGGCGCACGG
GTGCGTAACACGTAGATAATCTGCCCTTAGGAGGGGGATAACGTTCCGAAAGGAGCGCTAATACCGCATA
CAGCATTTTCCGGTTCGCCGGGGGATGGCAAAGGCTGGGCTCAGCAATGACCAGCCGCCTGAGGAGGAGT
CTGCGGCCCATCAGCTAGTTGGTAAGGTAACGGCTTACCAAGGCAAAGACGGGTAGCTGGTCTGAGAGGA
TGATCAGCCACACTGGTACTGGAACACGGAC
>Zotu11104
GAACGCTGGCGGCGTGCTTAACACATGCAAGTCGAACGCTGAAGCGGTGCTTGCACCGTGGATGAGTGGC
GAACGGGTGAGTAACACGTGGGTAATCTGCCCTGCACTCTGGGATAAGCCCTGGAAACGGGGTCTAATAC
CGGATATGACATCGTGCCGCATGGTGTGGTGTAGAAAGTTCCGGCGGTGCAGGATGAGCCCGCGGCCTAT
CAGCTTGTTGGTGGGGTGATGGCCTACCAAGGCGACGACGGGTAGCCGGCCTGAGAGGGTGACCGGCCA
CACTGGGACTGAGACACGGCC
>Zotu11107
GAACGCTGGCGGCGTGCCTAATACATGCAAGTCGAACGAGAGCGACCGGTGCTTGCACTGGTCAATCTAG
TGGCGAACGGGTGAGTAACACGTGGGTAACCTGCCCATCAGAGGGGGATAACATCCGGAAACGGATGCT
AAAACCGCATAGATCTTCGAATCGCATGGTTTGAAGAGGAAAAGAGGCGCAAGCTTCTGCTGATGGATGG
ACCCGCGGTGCATTAGCTAGTTGGTGAGGTAACGGCTCACCAAGGCCGTGATGCATAGCCGACCTGAGAG
GGTGATCGGCCACATTGGGACTGAGACACGGCC
>Zotu11111
GAACGCTGGCGGCGTGCTTAACACATGCAAGTCGAACGATGAACTCCAGCTTGCTGGGGGGATTAGTGGC
GAACGGGTGAGTAACACGTGAGTAACCTGCCCTGGACTCTGGGATAACCCCGAGAAATCGGAGCTAATAC
CGGATAGGACCTGGAACCGCATGGTTTTGGGTGGAAAGTTTTTCGGTCTGGGATGGACTCGCGGCCTATCA
GCTTGTTGGTGAGGTAATGGCTCACCAAGGCGTCGACGGGTAGCCGGCCTGAGAGGGTGACCGGCCACAC
TGGGACTGAGACACGGCC
>Zotu11115
GAACGCTGGCGGCAGGCCTAACACATGCAAGTCGAACGCCCCGCAAGGGGAGTGGCAGACGGGTGAGTA
ACACGTGGGAACGTACCCTTTGGTTCGGAACAACTCAGGGAAACTTGAGCTAATACCGGATAAGTGCGAG
AGCAGAAAGATTTATCGCCGAAGGATCGGCCCGCGTCTGATTAGCTAGTTGGTGAGGTAATGGCTCACCA
AGGCGACGATCAGTAGCTGGTCTGAGAGGATGATCAGCCACATTGGGACTGAGACACGGCC
>Zotu11119
GAACGCTAGCGGTTTGCCTAACACATGCAAGTTGCACGGCGCCATGCCTCTCGAGGCATGGCGGAGTGGC
GGACGGGTGAGGAACGCGTAAGGACCTGCCCCTGGGAGGGGGATAACAGCTGGAAACGGCTGCTAATAC
CCCATATCCTGAGGAGGGAAAGGAGAGATCCGCCCAGGGAGGGGCTTGCGTCTGATTAGCTAGTTGGGG
GGGTAATGGCCTCCCAAGGCAACGATCAGTTGCTGGTCTGAGAGGATGATCAGCCACACTGGGACTGAGA
CACGGCC
>Zotu11124
GAACGCTAGCGGCGGGCCTAATACATGCAAGTCGAGGGGCATCGGGAGGAGCAATACTTGCCGGCGACC
GGCGCACGGGTGCGTAACGCGTATGCAATCTACCTCTGACATGGGGATAGCCCGAAGAAATTCGGATTAA

TACCCAATGGTATCTTAATATGACCTCATATAAAGATTAAAGATTTATCGGTCAGAGATGAGCATGCGTCTG
ATTAGCTAGTTGGTGATGTAATTGCTCACCAAGGCAATGATCAGTAGGGGTTCTGAGAGGATTATCCCCCA
CACCGGTACTGAGACAGGGAC
>Zotu11127
GAACGCTGGCGGCATGCCTAACACATGCAAGTCGAACGGTAGCAGGTCCTTCGGGATGCTGACGAGTGGC
GGACGGGTGCGTAACGCGTGGGAATCTGCCCAATAGTGGGGGATAACCCGGGGAAACCCGGGCTAATAC
CGCATACTCCCTACGGGGGAAAGCGGGGGACCGCAAGGCCTCGCGCTATTGGATGAGCCCGCGTCCGATT
AGCTTGTTGGTGGGGTAAAAGCCTACCAAGGCGACGATCGGTAGCTGGTCTGAGAGGACGACCAGCCACA
CTGGGACTGAGACACGGCC
>Zotu11129
GAACGCTGGCGGCGTGCTTAACACATGCAAGTCGAACGGAGAGGCCCTGCTTTTGTGGGGTGCTCGAGTG
GCGAACGGGTGAGTAACACGTGAGTAACCTGCCCTTGACTTTGGGATAACTTCAGGAAACTGGGGCTAAT
ACCGGATAGGAGCTCCTGCTGCATGGTGGGGGTTGGAAAGTTTCGGCGGTTGGGGATGGACTCGCGGCTT
ATCAGCTTGTTGGTGGGGTAGTGGCTTACCAAGGCTTTGACGGGTAGCCGGCCTGAGAGGGTGACCGGCC
ACACTGGGACTGAGACACGGCC
>Zotu11135
GAACGCTAGCGGCAGGCTTAATACATGCAAGTCGAGGGGCAGCACGAGGTAGTAATACTTTGGTGGCGAC
CGGCAAACGGGTGCGGAACACGTACACAACCTTCCTTTCGGTGGGGAATAGCCTTTGGAAACGGAGATTA
ATACCCCATAAGACATAAGCTTCGCATGAGGCGGATGTTAAAGATTTATTGCCGGAAGATGGGTGTGCGTC
TGATTAGGTAGTTGGTGAGGTAACGGCTCACCAAGCCGACGATCAGTAACTGGTGTGAGAGCACGACCAG
TCACACGGGCACTGAGACACGGGC
>Zotu11143
GAACGCTGGCGGCGTGCCTAACACATGCAAGTCGAGCGATCTTCTTCGGAAGAGAGCGGCGGACGGGTG
AGTAACGCGTGGGTAACCTGCCTCATACACATGGATAACATACCGAAAGGTATGCTAATACAAGATGATAT
AAGAGATTCGCATGGATTTCTTATCAAAGCTTTTGCGGTATGAGATGGACCCGCGTCTGATTAGCTGGTTG
GTAAGGTAATGGCTTACCAAGGCGACGATCAGTAGCCGACCTGAGAGGGTGATCGGCCACATTGGAACTG
AGACACGGTC
>Zotu11146
GAACGCTGGCGGCGTGCCTAATACATGCAAGTCGAGCGAATGGATGGGAGCTTGCTCCCTGACATTAGCG
GCGGACGGGTGAGTAACACGTGGGCAACCTGCCCTGCAGATGGGGATAACTCCGGGAAACCGGGGCTAA
TACCGAATAATCAGTTCCTCCGCATGGAGGAATTCTGAAAGACGGCTTCGGCTGTCACTGCAGGATGGGCC
CGCGGCGCATTAGCTAGTTGGTGGGATAACGGCCTACCAAGGCGACGATGCGTAGCCGACCTGAGAGGGT
GATCGGCCACACTGGGACTGAGACACGGCC
>Zotu11150
GAACGCTGGCGGCGTGCCTAATACATGCAAGTCGAGCGCCCCTTTAAGAGAAGATAATCGAGGTGACAAT
CTTGATTGTCTCTTCCTATGGGGAGCGGCGGACGGCTGAGTAACGCGTGGGAACGTGCCCCAAAGTGAGG
GATAAGCACCGGAAACGGTGTCTAATACCGCATATGATCTTCGGATTAAAGCTTTATGCGCTTTGGGAGCG
GCCTGCGTCCTATTAGATAGTTGGTGAGGTAAAGGCTCACCAAGTCAACGATGGGTAGCTGGTCTGAGAG
GATGACCAGCCAGACTGGAACTGAGACACGGTC
>Zotu11153
GAACGCTAGCGGCAGGCTTAACACATGCAAGTCGAGGGGTAGGGTAGCAATATCTGAGACCGGCGCACG
GGTGCGTAACGCGTATGCAATCTACCTTTTACAGAGGGATAGCCCAGAGAAATTTGGATTAATACCTCATA
GCATTGCGAAATGGCATCATTTTGCAATTAAAGTTCCAACGGTAAAAGATGAGCATGCGTCCTATTAGCTA
GATGGTAAGGTAACGGCTTACCATGGCTACGATAGGTAGGGGTCCTGAGAGGGAGATCCCCCACACTGGT
ACTGAGACACGGAC
>Zotu11155
GAACGTTGGCGGCGTGCTTAACACATGCAAGTCGAACGGAAAGGCCCTGCTTTTGTGGGGTGCTCGAGTG
GCGAACGGGTGAGTAACACGTGAGTAACCTGCCCTTGACTTTGGGATAACTTCAGGAAACTGGGGCTAAT
ACCGGATAGGAGCTCCTGCTGCATGGTGGGGGTTGGAAAGTTTCGGCGGTTGGGGATGGACTCGCGGCTT
ATCAGCTTGTTGGTGGGGTAGTGGCTTACCAAGGCTTTGACGGGTAGCCGGCCTGAGAGGGTGACCGGCC
ACACTGGGACTGAGACACGGCC

>Zotu11157
GATCGCTGGCGGCGTGCTTAACACATGCAAGTCGAGCGAATCTTTGCAGCGTATCGCAAAGGTTAGCGGC
AGACGGGTGAGTAATACTTGGGCAATCTGCCCTTGAGTGGGAAACAACCTGACGAAAGTCGGGCTAATGT
CCCATAAGACCACAGGATGGCATCATCCAGGGGTCAAAGCTCCGTAAGGGGCGCTTGAGGAGGAGCCCG
AGTCCTATCATGGTAGTTGGCGGGGTAACGGCCCACCAAGCCTTTGACGGGTAGCCGGCCTGAAAGGGTG
ATCGGCCACACTGGCACTGAGACACGGGC
>Zotu11163
GAACGCTGGCGGCGTGGATAAGACATGCAAGTCGAACGGGATATTGTTTGTAGCAATACAGGCGATGTCT
AGTGGCGTAAGGGTGCGTAACACGTGGACAATCTGCCGAGAAGTGGGGGATAGCTCGTCGAAAGGCGAA
TTAATACCGCATGTGGTTAGGGAGGACATCCTCTTAATACCAAAGCCGGGGCAACCTGGCGCTTCTTGATG
AGTCCGCGGCCTATCAGCTAGTTGGCGGGGTAAAAGCCCACCAAGGCTATGACGGGTAGCTGGTCTGAGA
GGACGACCAGCCACACTGGAACTGAGACACGGTC
>Zotu11165
GAACGCTAGCGGCAGGCCTAATACATGCAAGTCGAACGGTAACATGAGTAGCAATACTTGATGACGAGTG
GCGCACGGGTGCGTAACGCGTATGCAATCTACCTTTAACAGGTGAATAGCCCGAAGAAATTCGGATTAAAA
CACCATAGTATTGTTTGTTGGCATCAACAGACAATTAAAACTCCGATGGTTAAAGATGAGCATGCGTGACA
TTAGCTAGTTGGTGAGGTAATGGCTCACCAAGGCAACGATGTCTAGGGGATCTGAGAGGATTAACCCCCA
CACTGGTACTGAGACACGGAC
>Zotu11169
GAACGCTAGCGGCAGGCCTAACACATGCAAGTCGAGGGGTAGAAATAGCTTGCTATTTTGAGACCGGCGG
ATGGGTGAGTAACGCGTATGTAATCTGCCTTGTACAGGGGGATAGCCCAGAGAAATTTGGATTAATATCCC
ATGGTATAATAGAGTGGCATCATTTTATTATTAAAGTTCCAACGGTACAAGATGAACATGCGTCCCATTAGT
TTGTTGGTAAGGTAACGGCTTACCAAGACTATGATGGGTAGGGGTCCTGAGAGGGAGATCCCCCACACTG
GTACTGAGACACGGAC
>Zotu11172
GAACGCTGGCGGCAGGCCTAACACATGCAAGTCGAGCGGTAACACAGGGAGCTTGCTCCCGGGTGACGA
GCGGCGGACGGGTGAGTAATGTCTGGGAAACTGCCTGATGGAGGGGGATAACTACTGGAAACGGTAGCT
AATACCGCATAACGTCGCAAGACCAAAGAGGGGGACCTTCGGGCCTCTTGCCATCAGATGTGCCCAGATG
GGATTAGCTAGTAGGTGGGGTAACGGCTCACCTAGGCGACGATCCCTAGCTGGTCTGAGAGGATGACCAG
CCACACTGGAACTGAGACACGGTC
>Zotu11180
GAACGCTAGCGGCATGCTTAACACATGCAAGTCGAACGGCAGCGCGGGGCAACCTGGCGGCGAGTGGCG
AACGGGTGAGTAATATATCGGAACGTACCCAGAAGTGGGGGATAACGTAGCGAAAGTTACGCTAATACCG
CATACGATCTACGGATGAAAGTGGGGGATCGCAAGACCTCATGCTTTTGGAGCGGCCGATATCTGATTAGC
TAGTTGGTGAGGTAAAGGCTCACCAAGGCGACGATCAGTAGCTGGTCTGAGAGGACGACCAGCCACACTG
GGACTGAGACACGGCC
>Zotu11184
GAACGCTGGCGGCAGGCCTAACACATGCAAGTCGAGCGGCAGCGGACCTTTCGGGGTGCCGGCGAGCGG
CGGACGGGTGAGTAATGCCTAGGAATCTGCCTGATAGTGGGGGATAACGTTCGGAAACGGACGCTAATAC
CGCATACGTCCTACGGGAGAAAGTGGGGGACCCTCGGGCCTCACGCTATCAGATGAGCCTAGGTCGGATT
AGCTAGTTGGTAGGGTAAAGGCCTACCAAGGCGACGATCCGTAACTGGTCTGAGAGGATGATCAGTCACA
CTGGAACTGAGACACGGTC
>Zotu11188
GAACGCTAGCGGCAGGCTTAACACATGCAAGTCGAGGGGTATAGTAGCAATACTAGGGACCGGCAAACG
GGTGCGGAACACGTACGCAATCTACCCTTACCCGGGGCATAGCCCGAAGAAATTCGGATTAACACCCCATA
AGATATTAGAGAGGCATCTCTTAGATATTAAAAATTTATTGGGTAAGGATGAGCGTGCGTCCCATTAGCTA
GTTGGTAGGGTAACGGCCTACCAAGGCGACGATGGGTAGCTGGTGTGAGAGCACGACCAGCCACACGGG
CACTGAGACACGGGC
>Zotu11191

GAACGCTGGCGGCGTGCCTAATACATGCAAGTCGGGCGGGGTTTTGGGGAGCTTGCTCCTTAAAACCCAG
CGGCGGACGGGTGAGTAACACGTGGGCAACCTGCCCGCAAGACCGGGATAACTCCGGGAAACCGGGGCT
AATACCGGATAGGACCCCGGATCGCATGATCCGGGGTGGAAAGGTGGCCTTCGGCTGCCGCTTGCGGATG
GGCCCGCGGCGCATTAGCTAGTTGGTGAGGTAACGGCTCACCAAGGTGACGATGCGTAGCCGACCTGAGA
GGGTGACCGGCCACACTGGGACTGAGACACGGCC

>Zotu11196
GAACGCTGGCGGCGCGCCTAACACATGCAAGTCGAACGAGAGATGAGGAGCTTGCTCTTCAAATCGAGTG
GCGAACGGGTGAGTAACGCGTGAGGAACCTGCCTCAAAGAGGGGGACAACAGTTGGAAACGACTGCTAA
TACCGCATAAGCCCACGGCTCGGCATCGAGCAGAGGGAAAAGGAGCAATCCGCTTTGAGATGGCCTCGCG
TCCGATTAGCTAGTTGGTGAGGTAATGGCCCACCAAGGCGACGATCGGTAGCCGGACTGAGAGGTTGAAC
GGCCACATTGGGACTGAGACACGGCC

>Zotu11198
GAACGCTGGCGGCGGGCTTAACACATGCAAGTCGAACGAGGCCTTCGGGCCTAGTGGCGCACGGGTGAG
TAACACGTAGGCAATCTACCCTCGAGTGGGGAATAACCAGTGGAAACGCTGGCTAATACCGCATATTGTCG
CTGTCCCGCATGGGACTACGATGAAAGCTATATGCGCTCGGGGATGAGCCTGCGTCGGATTAGCTAGTTG
GTAGGGTAACGGCCTACCAAGGCGACGATCCGTAGCGGGTCTGAGAGGATGATCCGCCACACTGGCACTG
AGACACGGGC

>Zotu11201
GAACGCTGGCGGCGTGCTTAACACATGCAAGTCGAGCGGAAAGGCCCTTCGGGGTACTCGAGCGGCGAA
CGGGTGAGTAACACGTGAGCAACCTGCCCTTAGCTCTGGGATAGCTCCGGGAAACCGGGATTAATACCGG
ATACGACCACGGTCGGCCTCGACTGTGGTGGAAAGATTTATCGGCTAGGGATGGGCTCGCGGCCTATCAG
CTTGTTGGTGAGGTAACGGCTCACCAAGGCGACGACGGGTAGCCGGCCTGAGAGGGCGACCGGCCACAC
TGGGACTGAGACACGGCC

>Zotu11204
GAACGCTGGCGGCGTGCCTAACACATGCAAGTCGAGCGGGAAATCTTGGAATGATTCTTCGGAGGAATGA
AGAGATGGAGAGCGGCGGACGGGTGAGTAACGCGTAGGCAACCTGCCCTTTGCAGAGGGATAGCCACTG
GAAACGGTGATTAAAACCTCATAATACTGAAGGTTCACATGGACATTTGGTCAAAGATTTATCGGCAAAGG
ATGGGCCTGCGTCTGATTAGCTAGTTGGTGAGGTAACGGCTCACCAAGGCAACGATCAGTAGCCGACCTG
AGAGGGTAATCGGCCACATTGGAACTGAGACACGGTC

>Zotu11208
GAACGCTGGCGGCATGCCTAACACATGCAAGTCGAACGAAGCCTTCGGGCTTAGTGGCGCACGGGTGCGT
AACGCGTGGGAATCTGCCCTTGGGTTCGGAATAACTCACCGAAAGGTGTGCTAATACCGGATAATGACGTA
AGTCCAAAGATTTATCGCCCAAGGATGAGCCCGCGTAGGATTAGCTAGTTGGTGAGGTAAAAGCTCACCA
AGGCGACGATCCTTAGCTGGTCTGAGAGGATGATCAGCCACACTGGGACTGAGACACGGCC

>Zotu11210
GAACGCTAGCGGCAGGCTTAACACATGCAAGTCGAGGGGCAGCGCGGGTAGCAATACCTGGCGGCGACC
GGCAAACGGGTGCGGAACACGTACAGAATCTACCTTTATCTGGGGCATAGCCCGGAGAAATCCGGATTAA
CTCCCCATAATATAGATGAGCGGCATCACTTGACTATTAAAAATTCATTGGATAGAGATGACTGTGCGTCCC
ATTAGCTAGTTGGTAGGGTAACGGCCTACCAAGGCGACGATGGGTAGCTGGTGTGAGAGCACGACCAGCC
ACACGGGCACTGAGACACGGGC

>Zotu11214
GAACGCTGGCGGTATGCCTTACACATGCAAGTCGTACGAGAGTTTTTAACTCAAGTGGCGGACGGGTGAG
TAACACGTAAGAATTTGCCTTTAGGAGGGGGATAACAACTGGAAACGGTTGCTAATACCCCATATGCTTTC
GAGTGAAATGGATTTCCGCCTAAAGAGAAGCTTGCGGCTGATTAGCTTGTTGATGAGGTAATGGCTCACCA
AGGCGACGATCAGTATCTGGTTTGAGAGGATGATCAGACACACTGGAACTGAGACACGGTC

>Zotu11216
GAACGCTGGCGGCGTGCCTAACACATGCAAGTCGAACGAAGCTCTGCGATACAAGACTTCGGTCAAGTTG
AACGGATGACTTAGTGGCGGACGGGTGAGTAACGCGTGAGGAACCTGCCTTTCAGTGGGGGACAACAGTT
GGAAACGACTGCTAATACCGCATAATGTATTCTGACCGCATGATCGGAATACCAAAGATTTATTGCTGAAA
GATGGCCTCGCGTCTGATTAGATAGTTGGTGAGGTAACGGCCCACCAAGTCAACGATCAGTAGCCGGACT
GAGAGGTTGAACGGCCACATTGGGACTGAGATACGGCC

>Zotu11218
GAACGCTGGCGGCATGCCTTACACATGCAAGTCGAACGGCAGCGCGGGGCAACCTGGCGGCGAGTGGCG
AACGGGTGAGTAATATATCGGAACGTACCCTAGAGTGGGGGATAACGTAGCGAAAGTTACGCTAATACCG
CATACGATCTACGGATGAAAGTGGGGGATCGCAAGACCTCATGCTCATGGAGCGGCCGATATCTGATTAG
CTAGTTGGTAGGGTAAAAGCCTACCAAGGCATCGATCAGTAGCTGGTCTGAGAGGACGACCAGCCACACT
GGAACTGAGACACGGTC
>Zotu11221
GGACGCTGGCGGCGTGCTTAACACATGCAAGTCGAGCGAATTTTGACAGCGTATCGTCAGAGTTAGCGGC
AGACGGGTGAGTAATACTTGGGCAATCTGCCCTTGAGAGGGAAACAACCTGCCGAAAGGTGGGCTAATGT
CCCGTAAGACCACAAGATGGCATCATCTAGGGGTCAAAGCTCCGCAAGGGGCGCTCGAGGAGGAGCCTAA
GTTCTATCATGGTAGTTGGCGGGGTAACGGCCCACCAAGCCTTTGACGGATAGCCGGCCTGAAAGGGTGA
TCGGCCACACTGGCACTGAGACACGGGC
>Zotu11226
GAACGCTAGCGGCAGGCTTAACACATGCAAGTCGAGGGGCAGCATGGTCTTCGGACCGATGGCGACCGG
CGCACGGGTGAGTTACGCGTATGCAATCTACCTTTCACTGGGGCATAGCCCGGGGAAACCCGGATTAATAT
CCTATGGTACATTTTGGTGGCATCACCTTTATGTTAAAGCTTCGGCGGTGAAAGATGAGCATGCGTCCTATT
AGCTAGATGGTAAGGTAACGGCTTACCATGGCTGTGATAGGTAGGGGTCCTGAGAGGGAGATCCCCCACA
CTGGTACTGAGACACGGAC
>Zotu11229
GAACGCTGGCGGCAGGCTTAACACATGCAAGTCGAACGCCCCGCAAGGGGAGTGGCAGACGGGTGAGTA
ACACGTGGGAACATACCCTTCGGTTCGGAACAACGCCGGGAAACTGGCGCTAATACCGGATACGGCCTAT
GGGCGAAAGATTCATCGCCGAAGGATTGGCCCGCGTTCGATTAGCTTGTTGGTGAGGTAATGGCTCACCA
AGGCGACGATCGATAGCTGGTCTGAGAGGATGGCCAGCCACATTGGGACTGAGACACGGCC
>Zotu11231
GAACGCTGGCGGCGTGCTTAACACATGCAAGTCGAACGGTGATCTCGGTGCTTGCACCGGGTGATCAGTG
GCGAACGGGTGAGTAACACGTGAGTAACCTGCCCCCGACTCTGGGATAACCCCGGGAAATCGGAGCTAAT
ACCGGATACGAGACGCACAGGCATCTGTAGCGTCTGGAAAGAATTTCGGTCGGGGATGGACTCGCGGCCT
ATCAGCTTGTTGGTGGGGTAATGGCCCACCAAGGCGACGACGGGTAGCCGGCCTGAGAGGGCGACCGGC
CACACTGGGACTGAGACACGGCC
>Zotu11235
GAACGCTGGCGGCATGCCTTACACATGCAAGTCGAACGGTAACAGGTTAAGCTGACGAGTGGCGAACGG
GTGAGTAATACATCGGAACGTGCCCAGTAGTGGGGGATAGCCCGGCGAAAGCCGGATTAATACCGCATAC
GACCTAAGGGTGAAAGGGGGGGATCGCAAGACCTCTCGCTATTGGAGCGGCCGATGTCAGATTAGGTAGT
TGGTGGGGTAAAGGCCCACCAAGCCGACGATCTGTAGCTGGTCTGAGAGGACGACCAGCCACACTGGGAC
TGAGACACGGCC
>Zotu11237
GAACGCTGGCGGCATGCCTAACACATGCAAGTCGAACGAGACCTTCGGGTCTAGTGGCGCACGGGTGCGT
AACGCGTGGGAATCTGCCCAAAGGTTCGGAATAACTCAGGGAAACTTGAGCTAATACCGGATGATGTCGC
AAGACCAAAGATTTATTGCCTTTGGATGAGCCCGCGTAGGATTAGCTTGTTGGTGAGGTAAAGGCTCACCA
AGGCTACGATCCTTAGCTGGTCTGAGAGGATGATCAGCCACACTGGGACTGAGACACGGCC
>Zotu11239
CAACGCTGGCGGCGTGCCTAACACATGCAAGTCGAACGCGAAAGTGGAGCAATCCATGAGTAGAGTGGCA
AACGGGTGAGTAACGCGTGGGCGACCTACCTTCGAGCGGGGGATAACATTCCGAAAGGAGTGCTAATACC
GCATGACATCCCGTGTTTGGATACACGGTGATCAAAGCCGGGGATCGCAAGACCTGGCACTTGAAGAGGG
GCCCGCGTCCGATTAGCTAGTTGGCGAGGTAATGGCTCACCAAGGCTCCGATCGGTATCCGGCCTGAGAG
GGCGGACGGACACACTGGAACTGAGACACGGTC
>Zotu11242
GAACGCTGGCGGCATGCTTTACACATGCAAGTCGAACGGCAGCGCGGGCGGGGGTAACCTCGCCTGGCG
GCGAGTGGCGAACGGGTGAGTAATATATCGGAACGTACCCAGAAGTGGGGGATAACGTAGCGAAAGTTA
CGCTAATACCGCATACGATCTACGGATGAAAGTGGGGGACCTTCGGGCCTCATGCTTGTGGAGCGGCCGA

TATCTGATTAGCTAGTTGGTAGGGTAAAGGCCTACCAAGGCGACGATCAGTAGCTGGTCTGAGAGGACGA
CCAGCCACACTGGAACTGAGACACGGTC
>Zotu11248
TAACGCTGGCGGCGTGCCTAACACATGCAAGTCGAGCGGGGGAGCAATCCCCAGCGGCAGACGGGTGAG
TAACACGTAAGTAACGTGCCCCCTAGTTTGGGATAGCTGGCCGAAAGGCCAGGTAATACCGGATGTGGTC
CTGGCGGGACATCCCGTTGGGAAGAAAGCAGTAATGCGCTAGAGGAGCGGTTTGCGTCCTATCAGCTTGT
TGGTGAGGTAACGGCTCACCAAGGCTTCGACGGGTAGCCGGTCTGAGAGGATGATCGGCCACACTGGCAC
TGAGATACGGGC
>Zotu11250
GAACGCTGGCGGCATGCCTAACACATGCAAGTCGAACGAGAGCTTCGGCTCTAGTGGCGCACGGGTGCGT
AACGCGTGGGAATCTGCCCTTGGGTTCGGAATAACAGTTGGAAACGACTGCTAATACCGGATGAAGTCGA
TAAGACCAAAGATTTATCGCCCGAGGATGAGCCCGCGTCGGATTAGCTAGTTGGTGGGGTAAAGGCCTAC
CAAGGCGACGATCCGTAGCTGGTCTGAGAGGATGATCAGCCACACTGGGACTGAGACACGGCC
>Zotu11252
GAACGCTAGCGGCGGGCCTAATACATGCAAGTCGAGGGGCATCGGGAGTAGCAATACTTTCCGGCGACCG
GCGCACGGGTGCGTAACGCGTATGCAATCTACCTCTGACATGGGGATAGCCCGAAGAAATTCGGATTAAT
ACCCAATGGTATCTTAATATGACCTCATATAAAGATTAAAGATTTATCGGTCAGAGATGAGCATGCGTCTGA
TTAGCTAGTTGGTGATGTAATTGCTCACCAAGGCAATGATCAGTAGGGGTTCTGAGAGGATTATCCCCCAC
ACCGGTACTGAGACAGGGAC
>Zotu11254
GAACGCTGGCGGCATGCCTTACACATGCAAGTCGAACGACAGCACGGGAGCAATCCTGGTGGCGAGTGGC
GAACGGGTGAGTAATATATCGGAACGTACCCAGTAGTGGGGGATAGCCCGGCGAAAGCCGGATTAATACC
GCATACGACCTGTGGGTGAAAGCGGGGGATCGCAAGACCTCGCGCTATTGGAGCGGCCGATATCAGATTA
GCTAGTTGGCGGGGTAAAAGCCCACCAAGGCGACGATCTGTAGCTGGTCTGAGAGGACGACCAGCCACAC
TGGGACTGAGACACGGCC
>Zotu11256
GAACGCTGGCGGCGTGCTTAACACATGCAAGTCGAACGAGGTCCAAGCAGTCGCAAGACTGCGGAAGACC
GAGTGGCGAACGGGTGCGTAACACGTGAGGAACTTGTCCCGGTCTCTGGAATAACAGGTGGAAACACCTG
CTAATACCGGATGCCGTCGGTCCATCGCATGGTGGACTGACGAAAGGGTTACCGGATCGGGAGAGCCTCG
CGGCCTATCAGCTTGTTGGTGAGGTAACGGCTCACCAAGGCATCGACGGGTAGCTGGTCTGAGAGGATGA
TCAGCCACACTGGGACTGAGACACGGCC
>Zotu11260
GAACGCTGGCGGCGTGCCCAACACATGCAAGTCGGATGTGCCCGCAAGGGTGCATGGCAGACGGGTGAG
TAACGCGTGGGGAACCTACCTTTTTGTGGGGGATAACGCTCCGAAAGGAGCGGTAATACCGCATAAGACC
ATGAGCTGGAATGCTGATGGTGAAAGCCGGGGATCGAAAGACCTGGCGCAAGGAGAGGGTCCTGCGTCT
GATTAGCTAGTTGGCGGGGTAACGGCCCACCAAGGCGACGATCAGTAGCCGGCCTGAGAGGGTGATCGG
CCACACTGGAACTGAGACACGGTC
>Zotu11263
GAACGCTAGCGGCAGGCCTAATACATGCAAGTCGAACGATAGTTAAGGGCTTGCCTTTAACGAAAAGTGG
CGCACGGGTGCGTAACGCGTATGCAACCTACCTTAATCAGGGGGATAGCCCGGAGAAATTCGGATTAACA
CCGCATAAAATCACAGCATAGCATTATGCAATGATCAAATATTTATAGGATTAAGATGGGCATGCGTGACA
TTAGTTAGTTGGCGGGGTAACGGCCCACCAAGACCACGATGTCTAGGGGATCTGAGAGGATGACCCCCCA
CACTGGTACTGAGACACGGAC
>Zotu11267
GAACGCTGGCGGCAGGCCTAACACATGCAAGTCGAACGGACCCTTCGGGGTTAGTGGCGGACGGGTGAG
TAACACGTGGGAACGTGCCTTTAGGTTCGGAATAGCTCCTGGAAACGGGTGGTAATGCCGAATGTGCCCTT
CGGGGGAAAGATTTATCGCCTTTAGAGCGGCCCGCGTCTGATTAGCTAGTTGGTTGGGGTAATGGCCCACC
AAGGCTACGATCAGTAGCTGGTCTGAGAGGATGACCAGCCACATTGGGACTGAGACACGGCC
>Zotu11272

GAACGCTGGCGGCGTGCTTAACACATGCAAGTCGAGCGAGGACCGCACTCTTTTGGGTGTGGAGCCGAGC
GGCGAACGGGTGAGTAACACGTGAGCAACCTGCCCCTGGCTCTGGGATAGCCCTGGGAAACCGGGATTAA
TACCGGATATGACCGCTGCTCGCATGGGTGGTGGTGGAAAGGTTCCCCCTTGTGGGGTTCCGGCCGGGGA
TGGGCTCGCGGCCTATCAGCTTGTTGGTGGGGTGACGGCCTACCAAGGCGATGACGGGTAGCCGGCCTGA
GAGGGTGGTCGGCCACACTGGGACTGAGACACGGCC
>Zotu11275
GAACGCTGGCGGCATGCCTTACACATGCAAGTCGAACGGTAACGCGGGGCAACCTGGCGACGAGTGGCG
AACGGGTGAGTAATACATCGGAACGTGCCCAGTAGTGGGGGATAGCCCGGCGAAAGCCGGATTAATACC
GCATACGACCTACGGGTGAAAGCGGGGGATCGCAAGACCTCGCGCTATTGGAGCGGCCGATGTCAGATTA
GGTAGTTGGTGAGGTAAAGGCTCACCAAGCCTTCGATCTGTAGCTGGTCTGAGAGGACGACCAGCCACAC
TGGGACTGAGACACGGCC
>Zotu11277
GAACGCTAGCGGCAGGCCTAATACATGCAAGTCGAACGGTGGTTTCCTTCGGGGTTCTACAGTGGCAAAC
GGGTGCGTAACGCGTAAGCAACCTGCCCACAACGGGGGGATAGCCTGGCGAAAGCCGGGGTAAACCCGC
ATGGTTCCCTGGAGTTGCATAGCTTTTGGGATAAACATTTATGGGTTGTGGAGGGGCTTGCGTCTGATTAG
GTAGTTGGCAGGGTAACGGCCTACCAAGCCGATGATCAGTAGGGGTTCTGAGAGGATTGGCCCCCACATG
GGTACTGAGATACGGAC
>Zotu11279
GAACGCTGGCGGCACGTTTTAAGCATGCAAGTCGAACGGCAGTGGGGGCTTGCTCCCATGAGAGTGGCGA
ACGGGTGAGTAACACGTAGGTGATCTGCCCTGGGGATGGGGATAGCCTGTGGAAACACAGGGTAATACC
GAATACGCTGACTGGACTTTGGTCTAGTTAGGAAAGGTGCTACGGCACCGACCTGGGAGGAGCCTGCGGC
TGATTAGCTAGTTGGTGGGGTAAGAGCCTACCAAGGCGACGATCAGTAGCCGGCCTGAGAGGGTGTACG
GCCACACTGGAACTGAGACACGGTC
>Zotu11285
GAACGCTGGCGGCATGCCTAACACATGCAAGTCGAACGGTGGCCTTCGGGTCATAGTGGCGCACGGGTGC
GTAACGCGTGGGAATCTGCCCTGGGGTTGGGAATAGCGGCTGGAAACGGCCGGTAATACCTGATGATGTC
GATAAGACCAAAGATTTATCGCCCTGGGATGAGCCCGCGTCGGATTAGCTAGTTGGTGGGGTAAAGGCCT
ACCAAGGCGACGATCCGTAGCTGGTCTGAGAGGATGATCAGCCACACTGGGACTGAGACACGGCC
>Zotu11290
GAACGCTGGCGGCGTGCCTAATACATGCAAGTCGAGCGGACTTAATTGGAGCTTGCTCTGATCAAGTTAGC
GGCGGACGGGTGAGTAACACGTGGGTAACCTGCCTGTAAGACTGGGATAACTTCGGGAAACCGGAGCTA
ATACCGGATAATCCGTTTCACCGCATGGTGAAACGTTGAAAGATGGCTTCGGCTATCACTTACAGATGGAC
CCGCGGCGCATTAGCTAGTTGGTGGGGTAATGGCCCACCAAGGCGACGATGCGTAGCCGACCTGAGAGG
GTGATCGGCCACACTGGGACTGAGACACGGCC
>Zotu11293
GAACGCTAGCGGCAGGCTTAACACATGCAAGTCGAGGGGTATTTGTCTTCGGATAGAGAGACCGGCGCAC
GGGTGCGTAACGCGTATGCAATCTACCTTTTACAGAGGGATAGCCCAGAGAAATTTGGATTAATACCTCAT
AGTATATAGAGTTGGCATCAACATTATATTAAAGTCACAACGGTAAAAGATGAGCATGCGTCCCATTAGCT
AGTTGGTAAGGTAACGGCTTACCAAGGCTACGATGGGTAGGGGTCCTGAGAGGGAGATCCCCCACACTGG
TACTGAGACACGGAC
>Zotu11297
GAACGCTGGCGGCGCGCCTAACACATGCAAGTCGAGCGAGAACCAGGCCTTCGGGCTTGGGGACAGCGG
CGAACGGGTGAGTAACACGTGAGTAATCAACCCTCGGCACCGGGATAGCCCGGGGAAACCCGGATTAATA
CCGGATGGCCCATCCGCTTTTCGGAGCGGCTGGAAAAGGTAGCTTCGGCCTCTGGCCGAGGACGAGCTCG
CGGCCGATTAGCTTGTTGGTGGGGTAATGGCTCACCAAGGCTCCGATCGGTAGCTGGTCTGAGAGGACGA
TCAGCCACACTGGGACTGAGACACGGCC
>Zotu11300
AAACGCTGGCGGCGCACATAAGACATGCAAGTCGAACGGAAGTCGTTACAAAATGGATTGAAGATTTATC
AGATTGAAGTTTTGTAACAATGGCTTTAGTGGCGGACTGGTGAGTAACACGTAAGCAACCTGCCTATCAGA
GGGGAATAACAGTGAGAAATCACTGCTAATACCGCATAAGCTTGTAGCATCGCATGATGTAGCAAGAAAA

GGAGTAATCCGCTGATAGATGGGCTTGCGGCTGATTAGCTAGTTGGTGTGGTAACGGCATACCAAGGCAA
CGATCAGTAGCCGGGCTGAGAGGCTCTGCGGCCACATTGGGACTGAGATACGGCC

>Zotu11303

GAACGCTGGCGGTCTGCTTAACACATGCAAGTCGAACGGGAAGCTTTCGGGCTTCTAGTGGCGGACGGGT
GAGTAACGCGTGAGAATCTGCCCTTTGGATGGGGACAACGACTGGAAACGGTCGCTAATACCCAATGAGC
CGCGAGGTAAAACGAGATTCGCCAAAGGATGAGCTCGCGTCGGATTAGCTAGTTGGTGGGGTAAGAGCCT
ACCAAGGCGACGATCCGTAGCTGGTCTGAGAGGATGACCAGCCACACTGGGACTGAGACACGGCC

>Zotu11312

GAACGCTGGCGGCGTGCTTAACACATGCAAGTCGAGGGGTATAGTTCTTCGGAGCTAGAGACCGGCGCAC
GGGTGCGTAACGCGTATGCAATCTACCTTTTACAGAGGGATAGCCCAGAGAAATTTGGATTAATACCTCAT
AGTATAGTACAATCGCATGATTGAACTATTAAAGTCACAACGGTAAAAGATGAGCATGCGTCCCATTAGCT
TGTTGGTAAGGTAACGGCTTACCAAGGCTACGATGGGTAGGGGTCCTGAGAGGGAGATCCCCCACACTGG
TACTGAGACACGGAC

>Zotu11315

GAACGCTGGCGGCAGGCCTAACACATGCAAGTCGAACGGGCCCTTTCGGGGGTCAGTGGCAGACGGGTG
AGTAACGCGTGGGAACGTGCCCATCGGTTCGGGATAACCCAGGGAAACTTGGGCTAATACCGGATACGTC
CTTCGGGAGAAAGATTGATTGCCGATGGATCGGCCCGCGTCTGATTAGCTTGTTGGTGAGGTAACGGCTCA
CCAAGGCGACGATCAGTAGCTGGTCTGAGAGGATGATCAGCCACACTGGGACTGAGACACGGCC

>Zotu11319

CAACGCTGGCGGCATGCCTAACACATGCAAGTCGAGCGAGAAAGGGTAGCAATACCTGAGTAAAGCGGC
GCACGGGTGAGTAACACGTGGATAACCTGCCTCAAAGTGGGGAATAACACCGGGAAACCGGTGCTAATAC
CGCATATTCTGACGAGATCTGTGGATCTTGCCAGGAAAGGAGCAATTCGCTTTGAGAGGGGTCCGCGGCT
GATTAGCTAGTTGGTGAGGTAATGGCTCACCAAGGCCACGATCAGTAGCCGGTCTGAGAGGACGGTCGGC
CACACTGGAACTGAGACACGGTC

>Zotu11325

GAACGCTAGCGGCATGCCTAACACATGCAAGTCGAACGGTCAGTAGCAATACTGATAGTGGCGCACGGGT
GCGTAACACGTAACTAACCTACCCACAACTGGGGGATAGCCCGCCGAAAGGCGGATTAATACCGCATAAG
CTCTTGACCCGGCATCGGGTTATTAGTAAAGATTTATTGGTTGTGGATGGGGTTGCGGGTCATTAGCTAGT
TGGTTGGGTAACGGCTGACCAAGGCGACGATGACTAGGGGAGCTGAGAGGCTGGTCCCCCACACGGGCA
CTGAGATACGGGC

>Zotu11327

GAACGCTGGCGGCATGCTTTACACATGCAAGTCGAACGGCAGCGCGGGGCAACCTGGCGGCGAGTGGCG
AACGGGTGAGTAATATATCGGAACGTACCCAAGAGTGGGGGATAACGTAGCGAAAGTTACGCTAATACCG
CATACGATCTAAGGATGAAAGCAGGGGATCTTCGGACCTTGTGCTCCTGGAGCGGCCGATATCTGATTAGC
TAGTTGGTGAGGTAAAGGCTCACCAAGGCGACGATCAGTAGCTGGTCTGAGAGGACGACCAGCCACACTG
GGACTGAGACACGGCC

>Zotu11331

GAACGCTAGCGGCAGGCCTAATACATGCAAGCCGAACGGCAAGTTTTTTAGCAATAGAAGACCTAGAGTG
GCGTAAGGGTGCGTAACACGTATGCAATCTGCCCTCAACAGGAGGATAGCTCCCCGAAAGGGGAATTAAG
CCTCCATAGGATCGTAGAGTGGCATCACTCAGCGATTAAATTACCAGAGGTTGAGGATGAGCATGCGCATG
ATTAGTTAGTTGGCGGGGTAACGGCCCACCAAGACAATGATCATTAGGGGAACTGAGAGGTTGATCCCCC
ACACTGGCACTGAGATACGGGC

>Zotu11335

GAACGCTGGCGGCGTGCTTAACACATGCAAGTCGAACGAGGTCCAAGGAGTAGCAATACTCTGGAAGACC
TAGTGGCGAACGGGTGAGTAACACGTGAGGAACCTACCCCGGACTCAGGGATAACAGCCGGAAACGGCT
GCTAATACCTGATGCCGTCGGAGCATCGCATGGTGCACTGACGAAAGAATCTCGGTTCGGGAGGGCCTCG
CGGCCTATCAGCTAGTTGGTGGGGTAACGGCCTACCAAGGCATCGACGGGTAGCTGGTCTGAGAGGATGA
TCAGCCACACTGGGACTGAGACACGGCC

>Zotu11339

GAACGCTGGCGGCGTGCTTAACACATGCAAGTCGAACGGTGATAACCAGGCTTTGCTTGGTTTGATCAGTG
GCGAACGGGTGAGTAACACGTGAGTAACCTGCCCCAGACTCTGGGATAACCCCGGGAAACCGGAGCTAAT
ACCGGATATGACCCTCGTACGCATGTGCAGGGGGTGGAAAGTTTTTCGGTCTGGGATGGGCTCGCGGCCT
ATCAGCTTGTTGGTGAGGTAGTGGCTCACCAAGGCGACGACGGGTAGCCGGCCTGAGAGGGCGACCGGC
CACACTGGGACTGAGACACGGCC
>Zotu11341
GAACGCTGGCGGCGTGCCTAAGACATGCAAGTCGAGCGGGGCAGGTTTATACCTGTCCGGCGGCGGACG
GGTGAGTAACGCGTGGGTGACCTACCCGGAAGAGGCGGACAACCTGGGGAAACCCAGGCTAATCCGCCA
TGTGGTCCTGTCCTGTGGGGCAGGACTAAAGGGTGAATAGCCCGCTTCCGGATGGGCCCGCGTCCCATCA
GCTAGTTGGTGGGGTAAAGGCCCACCAAGGCGACGACGGGTAGCCGGTCTGAGAGGATGGCCGGCCACA
GGGGCACTGAGACACGGGC
>Zotu11357
GAACGCTAGCGGCATGCTTAACACATGCAAGTCGAACGAAGCCTTCGGGCTTAGTGGCGCACGGGTGCGT
AACACGTGGGAATCTGCCCTTCGGTTCGGAATAACAGTGGGAAACTACTGCTAATACCGGATAATGACGTA
AGTCCAAAGATTTATCGCCGAAGGATGAGCCCGCGTAGGATTAGGTAGTTGGTGGGGTAATGGCCTACCA
AGCCGACGATCCTTAGCTGGTCTGAGAGGATGATCAGCCACACTGGGACTGAGACACGGCC
>Zotu11359
GAACGCTGGCGGCGTGCCTAACACATGCAAGTCGAGCGAAGCACTTTGCTTAGATTCTTCGGATGAAGAG
GATTGTGACTGAGCGGCGGACGGGTGAGTAACGCGTGGGTAACCTGCCTCATACAGGGGGATAACAGTTA
GAAATGACTGCTAATACCGCATAAGACCACAGCACCGCATGGTGCGGGGGTAAAAACTCCGGTGGTATGA
GATGGACCCGCGTCTGATTAGCTAGTTGGTAAGGTAACGGCTTACCAAGGCGACGATCAGTAGCCGACCT
GAGAGGGTGACCGGCCACATTGGGACTGAGACACGGCC
>Zotu11361
GAACGCTAGCGGCATGCCTAACACATGCAAGTCGAGCGGTAAGGCCCTTCGGGGTACACGAGCGGCGAAC
GGGTGAGTAACACGTGGGTGATCTGCCCTGCACTTCGGGATAAGCCTGGGAAACTGGGTCTAATACCGGA
TATGACCTCCGATCGCATGGTTGGTGGTGGAAAGATTTATCGGTGCAGGATGGGCCCGCGGCCTATCAGCT
TGTTGGTGGGGTAATGGCCTACCAAGGCGACGACGGGTAGCCGACCTGAGAGGGTGACCGGCCACACTG
GGACTGAGACACGGCC
>Zotu11365
GAACGCTGGCGGCGTGCCTAACACATGCAAGTCGAACGAGAAAGTTCTTCGGAACGAGTACAGTGGCGCA
CGGGTGAGTAATACATAGGAACGTACCATTTGGAGGGGAATAACAACGGGAAACCGTTGCTAATACCGCA
TAATTCTCGCAAGAGTAAAGGTGGCCTCTATTTATAAGCTATCACCAAATGAGCGGCCTATGTCCCATTAGC
TAGTTGGTAGGGTAATGGCCTACCAAGGCAACGATGGGTAGCTGGTCTGAGAGGATGTCCAGCCACACTG
GAACTGAGACACGGTC
>Zotu11371
GAACGCTAGCGGCATGCTTAACACATGCAAGTCGAACGGAAAGGCCCTGCTTTTGTGGGGTGCTCGAGTG
GCGAACGGGTGAGTAACACGTGAGTAACCTGCCCTTGACTTTGGGATAACTTCAGGAAACTGGGGCTAAT
ACCGGATAGGAGCTCCTGCTGCATGGTGGGGGTTGGAAAGTTTCGGCGGTTGGGGATGGACTCGCGGCTT
ATCAGCTTGTTGGTGGGGTAGTGGCTTACCAAGGCTTTGACGGGTAGCCGGCCTGAGAGGGTGACCGGCC
ACACTGGGACTGAGACACGGCC
>Zotu11375
GAACGCTGGCGGCGCGCTTAACACATGCAAGTCGGACGTGAAAGTGGGAAACCATGAGTAAAGTGGCGA
ACGGGTGAGTAACACGTGAGAATCTGCATTTTAGTGGAGGATACCCTGGGGAAACTCAGGCTAATCCTGC
ATAAGCTCGAGAGAGGAAAGGCGGCTTAATTGCTGTCGCTAAAATATGAGCTTGCGGCCTATTAGCTAGTT
GGTGGGGTAAAGGCCTACCAAGGCGAAGATGGGTATTCGGCCTGAGAGGGTGGTCGGACACACTGGGAC
TGAGATACGGCC
>Zotu11379
GAACGCTAGCGGCAGGCTTAATACATGCAAGTCGAACGGAAAGGCTCCTGCTTGCAGGGGTACTCGAGTG
GCGAACGGGTGAGTAACACGTGGGTGATCTGCCCTGCACTTCGGGATAAGCCTGGGAAACTGGGTCTAAT
ACCGGATAGGACGATGGTTTGGATGCCATTGTGGAAAGTTTTTCGGTGTGGGATGAGCTCGCGGCCTATCA

GCTTGTTGGTGGGGTAATGGCCTACCAAGGCGTCGACGGGTAGCCGGCCTGAGAGGGTGTACGGCCACAT
TGGGACTGAGATACGGCC
>Zotu11383
GAACGCTAGCGGCATGCCTAACACATGCAAGTCGAACGGTGAAGCCCAGCTTGCTGGGCGGATCAGTGGC
GAACGGGTGAGTAACACGTGAGCAACCTGCCCTCCACTTCGGGATAAGCCTTGGAAACGGGGTCTAATAC
CGGATATGAGCGCTCACCGCATGGTGGGTGTTGGAAAGTTTTTCGGTGGGGGATGGGCTCGCGGCCTATC
AGCTTGTTGGTGGGGTGATGGCCTACCAAGGCGTCGACGGGTAGCCGGCCTGAGAGGGCGACCGGCCAC
ACTGGGACTGAGACACGGCC
>Zotu11386
GAACGCTGGCGGCATGCCTAACACATGCAAGTCGAACGAAGGCTTCGGCCTTAGTGGCGCACGGGTGCGT
AACGCGTGGGAATCTGCCCTTGGGTTCGGAATAACCAGGAGAAATCCTGGCTAATACCGGATGATGACGT
AAGTCCAAAGATTTATCGCCCAGGGATGAGCCCGCGTAGGATTAGCTAGTTGGTGGGGTAAAGGCCTACC
AAGGCGACGATCCTTAGCTGGTCTGAGAGGATGATCAGCCACACTGGGACTGAGACACGGCC
>Zotu11388
AAACGCTGGCGGGGTGCGTGAGACATGCAAGTCGAACGAGGAACCTCCGAGCTTGCTTGGAGGTCTCCTA
GTGGCGGACGGCTGAGTAACGCGTAGGTGACCTGCCCGAAAGTGGGGAATAACTGCCCGAAAGGGTGGC
TAATACCGCATGTGGTCGGCGATTCGGTTCGCCGTCTAAAGGAGCAATCCGCTTCCGGAGGGGCCTGCGTT
CGATTAGCTAGTTGGTGGGGTAACGGCTCACCAAGGCGACGATCGATAGCTGGTCTGAGAGGATGATCAG
CCAGACTGGGACTGAGAACGGCC
>Zotu11390
GAACGCTAGCGGCAGGCCTAACACATGCAAGTCGAGGGGTAATATAGCAATATATGAGACCGGCGCACGG
GTGCGTAACGCGTATGCAATCTACCTTTTACAGGGGAATAGCCCAGAGAAATTTGGATTAATGCCCCATGG
TATAATCGAATGGCATCATTTGATTATTAAAGTTCCAACGGTAAAAGATGAGCATGCGTCCCATTAGTTAGT
TGGTAAGGTAACGGCTTACCAAGACAATGATGGGTAGGGGTCCTGAGAGGGAGATCCCCCACACTGGTAC
TGAGACACGGAC
>Zotu11394
GAACGTTAGCGGCGCGCCTAACACATGCAAGTCGCACGAGAGGGAGCAATCCTGAAAGTGGCGCACGGG
TGCGTAACACGTAGATAATCTGCCCTTCAGCGGGGGATAACGTTCCGAAAGGAGCGCTAATACCGCATACA
GCAGCGAGTGGTTCGCCATTTGTTGGCAAAGGCTGGGATCGCAAGACCAGTCACTGAAGGAGGAGTCTGC
GGCCCATCAGCTAGTTGGAGAGGTAATGGCTCACCAAGGCAGAGACGGGTAGCTGGTCTGAGAGGATGA
TCAGCCACACTGGTACTGGAACACGGAC
>Zotu11398
GAACGTTAGCGGCACGCTTAACACATGCAAGTCGCACGAGAAGGGGCAACCCTGAAAGTGGCGCACGGG
TGCGTAACACGTAGATAACCTGCCCCTCGGAGGGGGACAACGTTCCGAAAGGAGCGCTAATACCGCGTAC
AGCTCGAGGTGGTTTGCTACTTCGAGACAAAGGTAGGGACCGGCAACGGCCTACCGCCGGGGGAGGGGT
CTGCGGCCCATCAGCTAGTTGGTAAGGTAATGGCTTACCAAGGCAAAGACGGGTAGCTGGTCTGAGAGGA
TGATCAGCCACACTGGTACTGGAACACGGAC
>Zotu11405
GAACGCTGGCGGCGTGCCTAATACATGCAAGTCGAGCGAATCGATGGGAGCTTGCTCCCTGAGATTAGCG
GCGGACGGGTGAGTAACACGTGGGTAACCTGCCTGTAAGACTGGGATAACTTCGGGAAACCGGAGCTAAT
ACCGGATAGTTTCTTTTCTCGCATGAGGGAAGATGGAAAGATGGTTTCGGCTATCACTTACAGATGGACCC
GCGGCGCATTAGCTAGTTGGTGAGGTAACGGCTCACCAAGGCAACGATGCGTAGCCGACCTGAGAGGGT
GATCGGCCACACTGGGACTGAGACACGGCC
>Zotu11407
GAACGCTGGCGGCGTGCTTAACACATGCAAGTCGAACGGAAAGGCCCTGCTTTTGTGGGGTGCTCGAGTG
GCGAACGGGTGAGTAACACGTGAGTAACCTGCCCTTGACTTTGGGATAACTTCAGGAAACTGGGGCTAAT
ACCGGATAGGAGCTCCTGCTGCATGGTGGGGGTTGGAAAGTTTCGGCGGTTGGGGATGGACTCGCGGCTT
ATCAGCTTGTTGGTGGGGTAGTGGCTTACCAAGGCTTTGACGGGTAGCCGGCCTGAGAGGGTGACCGGCC
ACACTGGAACTGAGACACGGCC
>Zotu11409

GAACGCTGGCGGCGTGCCTAACACATGCAAGTCGGATGTGCCGCAAGGTGCATGGCAGACGGGTGAGTA
ACGCGTGGAAAACCTACCTCTTTGTGGGGAATAACGGTCCGAAAGGGCTGGTAATACCGCATGAGACCGT
TGACTGGGATGTCAACGGTGAAACCTGGGGACCGCAAGGCCTGGGGCAAGGAGAGGGTTCTGCGTCTGA
TTAGCTAGTTGGTGAGGTAACGGCCCACCAAGGCTTCGATCAGTAGCCGGCCTGAGAGGGTGATCGGCCA
CACTGGAACTGAGACACGGTC

>Zotu11414

GAACGCTGGCGGCGTGCTTAACACATGCAAGTCGAACGGTGAAGCCCTTCGGGGTGGATCAGTGGCGAAC
GGGTGAGTAACACGTGAGCAATCTGCCCTTGACTCTGGGATAACTTCGGGAAACCGGAGCTAATACCGGA
TATGACATACGGCCGCATGGTCTGTGTGTGGAAAGATTTATCGGTCAGGGATGAGCTCGCGGCCTATCAGC
TTGTTGGTGGGGTAATGGCCTACCAAGGCGACGACGGGTAGCCGGCCTGAGAGGGCGACCGGCCACACT
GGGACTGAGACACGGCC

>Zotu11418

GAACGCTGGCGGCATGCCTAATACATGCAAGTCGCGCGTGAAAGTCCTTCGGGATGAGTAAAGCGGCGCA
CGGGTGAGTAATGCATAGGAACGTGCCCTCCGATGGGGAATAACTACGGGAAACTGTAGCTAATACCGCA
TGAAGACGAGAGTCTAAAGCTGCCCTCTATTTATAAGGTAGCGTCGGTGGAGCGGCCTATGTCCCATTAGC
TAGTTGGTAGGGTAATGGCCTACCAAGGCGACGATGGGTAGCTGGTCTGAGAGGACGATCAGCCACACTG
GGACTGAGACACGGCC

>Zotu11420

GAACGCTGGCGGCGCGCTTAACACATGCAAGTCGAGCGAGAACCGGACCTTCGGGTCCGGGGAAAGCGG
CGAACGGGTGAGTAACACGTGGGTAACCCACCCTTGGTACCGGGATAGCCCGGGGAAACCCGGATTAATA
CCGGATGGCCCAGCAGCTCTTCGGGGCGGCTGGAAAAGGTAGCTTCGGCCTCCGACCAAGGACGGGCCCG
CGGCGGATTAGCTTGTTGGTGGGGTAACGGCCCACCAAGGCGACGATCCGTAGCTGGTCTGAGAGGACGA
TCAGCCACACTGGGACTGAGACACGGCC

>Zotu11424

GAACGCTGGCGGCGTGCTTAACACATGCAAGTCGAGCGATGAAGTTTCTTCGGAAATGGATTAGCGGCGA
ACGGGTGAGGAACACGTGAAGAATCTGCCCTCCACTTTGGGATAACACCGGGAAACCGGTGCTAATACCG
AATATTGAAACATAAGTGGCATCACTGAGTTTTGAAAGAATTTCGGTGGAGGATGACTTCGCGGCCTATCA
GCTAGTTGGTGAGGTAATGGCTCACCAAGGCAACGACGGGTAGCCGGCCTGAGAGGGCGACCGGCCACA
CTGGGACTGAGACACGGCC

>Zotu11426

GAACGCTAGCGGCAGGCTTAATACATGCAAGTCGTGGGGCAGCAGGTTTGTAGCAATACAGATGCTGGCG
ACCGGCAAACGGGTGCGGAACACGTACGCAACCTCCCTCTTTCTGGAAGATAGCCCACCGAAAGGTGGAT
TAATACTCCATAATATATTGAAGTGGCATCACTTTAATATTAAAGCTCCGGCGGTAAGAGATGGGCGTGCG
TCTGATTAGGTAGTTGGCGGGGTAACGGCCCACCAAGCCTGCGATCAGTAGCTGATGTGAGAGCATGATC
AGCCACACGGGCACTGAGACACGGGC

>Zotu11428

GAACGCTGGCGGCGTGCTTAACACATGCAAGTCGAACGATGAACGAGGAGCTTGCTCCTCCGGATTAGTG
GCGAACGGGTGAGTAACACGTGAGCAACGTGCCCAAGACTCTGGAATAACTTCGGGAAACCGAAGCTAAT
ACCGGATACGAGACGCGAAGGCATCTTCAGCGTCTGGAAAGAACTTCGGTCTTGGATCGGCTCACGGCCT
ATCAGCTTGTTGGTGAGGTAACGGCTCACCAAGGCGACGACGGGTAGCCGGCCTGAGAGGGTGACCGGC
CACACTGGGACTGAGACACGGCC

>Zotu11430

GAACGCTGGCGGCGTGCTTAACACATGCAAGTCGAACGGAAAGGCCCTTTCGGGGGTACTCGAGTGGCGA
ACGGGTGAGTAACACGTGGGTGATCTGCCCTGCACTTCGGGATAAGCTTGGGAAACTGGGTCTAATACCG
GATAGGATCATGGCTCTCATGGGTTGTGGTGGAAAGCTTTTGCGGTGTGGGATGGGCCCGCGGCCTATCA
GCTTGTTGGTGGGGTGATGGCCTACCAAGGCGACGACGGGTAGCCGGCCTGAGAGGGTGTCCGGCCACA
CTGGGACTGAGATACGGCC

>Zotu11433

GAACGCTGGCGGCAGGCCTAAAACATGCAAGTCGAACGAACCTTCGGGTTAGTGGCGCACGGGTGCGTAA
CGCGTGGGAATATGCCTCTTGGTGCGGAACAACGTCTGGAAACGGACGCTAATACCGCATAATATCTACGG

ATCAAAGATTTATCGCCAAGAGATTAGCCCGCGTCTGATTAGCTAGTTGGTGAGGTAATGGCTCACCAAGG
TGACGATCAGTAGCTGGTCTGAGAGGATGATCAGCCACACTGGGACTGAGACACGGCC
>Zotu11436
GAACGCTGGCGGCGTGCTTCAGACATGCAAGTCGAACGTGAAAGTACTTCGGTATAAGTAAAGTGGCGCA
CGGGTGAGTAATACTTGGGTAATGTACCCAATTGCTGGGGATAACTAGACGAAAGTTTAGCTAATACCGAA
TAGTCCCTCTAGGTCTCCGGACTCTGAGGGTAAAGCCGTAAGGCGCGATTGGAGCAGCCCTCGTCCCATTA
GCTAGTTGGCGGGGTAAAAGCCCACCAAGGCTACGATGGGTAGCTGGTCTAAGAGGATGATCAGCCACAC
TGGTACTGCAACACGGAC
>Zotu11442
GAACGCTGGCGGCGTGCTTAACACATGCAAGTCGAACGGTGAAGCCCTTCGGGGTGGATCAGTGGCGAAC
GGGTGAGTAACACGTGAGCAACCTACCCTTGACTCTGGGATAACTCCGGGAAACCGGTGCTAATACCGGA
TACGAGCATCGACCTCATGGTCTGGTGCTGGAAAGAATTTTGGTCAGGGATGGGCTCGCGGCCTATCAGCT
TGTTGGTGAGGTAATGGCTCACCAAGGCGACGACGGGTAGCCGGCCTGAGAGGGCGACCGGCCACACTG
GGACTGAGACACGGCC
>Zotu11448
GCCGTGTCTCAGTCCCAGTGTGGCTGGTCGTCCTCTCAGACCAGCTACAGATCGTCGGCTTGGTAAGCTTTT
ATCCCACCAACTACCTAATCTGCCATCGGCCGCTCCAATCGCGCGAGGCCCGAAGGTCCCCCGCTTTCATCC
TCAGATCGTATGCGGTATTAGCTACTCTTTCGAGTAGTTATCCCCCACGACTGGGCACGTTCCGATGTATTA
CTCACCCGTTCGCCACTCGTCAGCGTCCGAAGACCTGTTACCGTTCGACTTGCATGTGTAAGGCATGCCGCC
AGCGTTCA
>Zotu11450
GAACGCTGGCGGCAGGCTTAACACATGCAAGTCGAACGGTCCTTTCGGGGATAGTGGCGGACGGGTGAG
TAACACGTGGGAACGTGCCCTTAGGTTCGGAACAACACAGGGAAACTTGTGCTAATACCGGATGTGCCCCT
TACGGGGGAAAGATTTATCGCCTTTGGAGCGGCCCGCGTCTGATTAGCTAGTTGGTGGGGTAACTGCCTAC
CAAGGCTACGATCAGTAGCTGGTCTGAGAGGATGGCCAGCCACATTGGGACTGAGACACGGCC
>Zotu11453
GAACGCTGGCGGCATGCCTTACACATGCAAGTCGAACGGCAGCACGGGGGCAACCCTGGTGGCGAGTGG
CGAACGGGTGAGTAATACATCGGAACGTACCCAGTAGTGGGGGATAACTAGTCGAAAGATTAGCTAATAC
CGCATACGCACTATGGTGGAAAGCGGGGGATCGCAAGACCTCGCGCTATTGGAGCGGCCGATGTTCGATT
AGCTAGTTGGCGGGGTAAAAGCCCACCAAGGCGACGATCGATAGCTGGTCTGAGAGGACGACCAGCCAC
ACTGGGACTGAGACACGGCC
>Zotu11455
GAACGCTGGCGATAGGCTTAACACATGCAAGTCGAGGGGTAACGTGTTGGAAGCTTGCTTCCGATGACGA
CGACCGGCGGATGGGTGCGTAACGCGTATGCAACTTGCCTCACAGTGGAGAATAACCCGGAGAAATCCGG
ACTAATGTTCCATACACTCTTAAGTACGCCTGTACATGAGAGGAAAGATTTATCGCTGTGAGATAGGCATG
CGTCCTATTAGGTAGTTGGTGAGGTAACGGCTCACCAAGCCGACGATAGGTAGGGGTGCTGAGAGGCAGA
TCCCCCACATTGGGACTGAGACACGGCC
>Zotu11459
GAACGCTGGCGGCGTGCTTAACACATGCAAGTCGAACGGTGAAGGACGGAGCTTGCTCCGACCGGATCAG
TGGCGAACGGGTGAGTAACACGTGAGTAACCTGCCCCAGACTCTGGAATAACCCCGGGAAACCGGAGCTA
ATACCGGATATGAAGTGGAGCCGCATGGCTACTACTTGGAAAGTTTTTCGGTCTGGGATGGACTCGCGGCC
TATCAGCTAGTTGGTGAGGTAACGGCTCACCAAGGCGACGACGGGTAGCCGGCCTGAGAGGGCGACCGG
CCACACTGGGACTGAGACACGGCC
>Zotu11462
AAACGCTGGCGGCGTGCCTAATACATGCAAGTCGAGCGGGGTATAATTGGTGCTTGCACTAATCATACTTA
GCGGCGGACGGGTGAGTAACACGTGGGTAACCTGCCCATAAGATTGGGATAACAGTCGGAAACGACTGCT
AATACCAAATAGTCGACATGTTCGCATGAACGTGTTGGGAAAGGCGGCGTAAGCTGTCACTTATGGATGG
ACCCGCGGCGTATTAGCTAGTTGGTGAGGTAACGGCTCACCAAGGCAATGATACGTAGCCGACCTGAGAG
GGTGATCGGCCACATTGGGACTGAGACACGGCC
>Zotu11469

TAACGCTGGCGGCATGCCTAACACATGCAAGTCGCACGAGAAAGCCTCCTTCGGGAGGCGAGTAAAGTGG
CGCACGGGTGAGTAACGCGTGGATAATCTACCTCGTTCTGGGGGATAACCCATCGAAAGGTGGGCTAATA
CCGCATAATCCGGCTTTTTTTTGAATTGGTCGGCAAAGGTGGCCTCTTGATCAAAGCTACCGGGAAGAGAT
GAGTCCGCGTCCCATTAGCTAGTTGGTAGGGTAATGGCCTACCAAGGCGACGATGGGTAGCTGGTCTGAG
AGGACGATCAGCCACACTGGAACTGGTACACGGTC

>Zotu11472
GAACGCTGGCGGCATGGATTAGGCATGCAAGTCGAACGGGAAGGCCTTCGGGCCTTCGAGTGGCGAAAG
GGTGAGTAACACGTGAGCAACCTGCCCCCGAGTTCCGAATAAGCAGTGGAAACGCTGTCTAATGCGGGAT
GAGGATCATTTGTCACCTGACAAATGTTCCAAAGATTTATCGCTTGGGGATGGGCTCGCGGCCCATTAGCTT
GTTGGTGGGGTAATGGCCCACCAAGGCTACGACGGGTAGGTGGACTGAGAGGTCGGTCAACCACACTGG
GACTGAGACACTGCC

>Zotu11474
GAACGCTGGCGGCGTGCCTAACACATGCAAGTCGAACGGTAGCAGGCTTTCACTGTTCGTTCAACACCGAG
ATATATGTCTTGGATACAGTTGAATGAAGAGTGAAAGTGCTGACGAGTGGCGGACGGCTGAGTAACGCGT
GGGAACGTGCCCTAAAGTGAGGGATAAGCACCGGAAACGGTGTCTAATACCGCATATGATCTTCGGATTA
AAGAATTTATTCGCTTTAGGAGCGGCCCGCGTCGGATTAGGTTGTTGGTGAGGTAAAGGCTCACCAAGCCG
ACGATCCGTAGCTGGTCTGAGAGGATGACCAGCCAGACTGGAACTGAGACACGGTC

>Zotu11480
GAACGCTGGCGGCAGGCCTAACACATGCAAGTCGAGCGGATGACAGGAGCTTGCTCCTGGATTCAGCGGC
GGACGGGTGAGTAATGCCTAGGAATCTGCCTGGTAGTGGGGGACAACGTCTCGAAAGGGACGCTAATACC
GCATACGTCCTACGGGAGAAAGTGGGGGATCTTCGGACCTCACGCTATCAGATGAGCCTAGGTCGGATTA
GCTAGTTGGTGAGGTAATGGCTCACCAAGGCGACGATCCGTAACTGGTCTGAGAGGATGATCAGTCACAC
TGGAACTGAGACACGGTC

>Zotu11483
GCCGTGTCTCAGTCCCAGTGTGGCTGATCATCCTCTCAGACCAGCTATGGATCGTCGCCTTGGTAGGCCATT
ACCCCACCAACTAGCTAATCCAACGCGGGCTCATCCATCACCGATAAATCTTTCTCCCGAAGGACGTATACG
GTATTAGCTCCAGTTTCCCGGAGTTGTTCCGTAGTGATGGGTAGATTCCCACGCGTTACTCACCCGTCTGCC
GCTCCCCTTGCGGGGCGCTCGACTTGCATGTGTTAAGCCTGCCGCCAGCGTTCG

>Zotu11486
GAACGCTGGCGGCAGGCTTAACACATGCAAGTCGAGCGGAGTGATGGTGCTTGCACCATCACTTAGCGGC
GAACGGGTGAGTAATGCCTAGGAATCTGCCCATTAGCGGGGGACAACGTGTGGAAACGCACGCTAATACC
GCATACGCCCTACGGGGGAAAGCAGGGGACTCTTTGAGCCTTGCGCTAATGGATGAGCCTAGGTCAGATT
AGCTAGTTGGTGGGGTAAAGGCCTACCAAGGCGACGATCTGTAGCGGGTCTGAGAGGATGATCCGCCACA
CTGGAACTGAGACACGGTC

>Zotu11489
GAACGCTGGCGGCGTGCCTAACACATGCAAGTCGAACGGGGTTAAGTGATGTTTTCGGATGGATCTTAACT
TAGTGGCGGACGGGTGAGTAACGCGTGGATAACCTGCCTCACACAGGGGGATAGCAGCTGGAAACGGCT
GGTAATACCGCATAAGACCACGGTGCCGCATGGCACAGTGGTAAAAGATTTATCGGTGTGAGATGGATCC
GCGTCTGATTAGCCAGTTGGCGGGGTAACGGCCCACCAAAGCGACGATCAGTAGCCGGCCTGAGAGGGT
GAACGGCCACATTGGGACTGAGACACGGCC

>Zotu11492
GAACGCTGGCGGCGTGCCTAATACATGCAAGTCGAGCGGAGGGTTTAGCAATAGACCCTTAGCGGCGAAC
GGGTGAGTAACGCGTTGGTGACCTGCCTCGAAGCTTGGGATAACAGTTCGAAAGGACTGCTAATACCGGA
TGTGTTCACGTGGATTAGAGGCCATGTGAGTAAAGGAGAAATTCACTTCGAGAGGGGCCTGCGTCCCATC
AGCTAGTTGGTAGGGTAAAGGCCTACCAAGGCGATGACGGGTAGGGGACCTGAGAGGGTGGCCCCCCAC
AATGGAACTGAAACACGGTC

>Zotu11494
GAACGCTGGCGGCGTGCTTAACACATGCAAGTCGAACGATTAAAGCCCCTTCGGGGGTGTATAGAGTGGC
GAACGGGTGAGTAACACGTGGGCAATCTGCCCCTCACTCCGGGATAACTCAGGGAAACTTGAGCTAATAC
CGGATACTCCGAGTGGATCGCATGGTCTGCTCGGGAAAGCTCCGACGGTGAGGGATGAGCCCGCGGCCCA

TTAGCTAGTTGGTGAGGTAACGGCCCACCAAGGCGACGATGGGTAGCCGAGCTGAGAGGCTGATCGGCC
ACACTGGGACTGAGACACGGCC
>Zotu11497
GAACGCTAGCGGCAGGCTTAATACATGCAAGTCGAGGGGCAGCATGGTGTAGCAATACACTGATGGCGAC
CGGCAAACGGGTGCGGAACACGTACGCAACCTTCCCAAAACTGGTGAATAGCCCTCCGAAAGGAGGATTA
ATACACCGTAACATAACGAAGTGGCATCACTTTGTTATTATAACTCCGGTGGTTTTGGATGGGCGTGCGCCT
GATTAGGTAGTTGGCGGGGTAACGGCCCACCAAGCCTGCGATCAGTAACTGGTGTGAGAGCACGACCAGT
CACACGGGCACTGAGACACGGGC
>Zotu11502
GAACGCTGGCGGTGTGCTTCACACATGCAAGTCGAACGGGGTAGCAATACCCAGTGGCGGACGGGTGAG
TAACGCGTAGGAATCTGCCTTTAGGTGGGGGATAACAGTCCGAAAGGACTGCTAATACCGCATATGTGTAC
AATTCGACAGTTTTGTACATGAAAGGAGCAATTTGCCTGAAGATGAGCCTGCGTCCGATTAGCTAGTTGGT
AGGGTAAAAGCCTACCAAGGCGACGATCGGTAGGTGGTCTGAGAGGATGATCACCCACAATGGGACTGA
GACACGGCC
>Zotu11504
GAACGCTGGCGGCATGCCTTACACATGCAAGTCGAACGGTAACAGCACTTCGGTGGCTGACGAGTGGCGG
ACGGGTGAGTAATACATCGGAACGTGCCCGATCGTGGGGGATAACTACTCGAAAGAGTGGCTAATACCGC
ATACGATCTACGGATGAAAGCGGGGGATCGCAAGACCTCGCGCGGACGGAGCGGCCGATGGCAGATTAG
GTAGTTGGTGGGATAAAAGCTTACCAAGCCGACGATCTGTAGCTGGTCTGAGAGGACGACCAGCCACACT
GGGACTGAGACACGGCC
>Zotu11507
GAACGCTGGCGGCAGGCTTAACACATGCAAGTTGAACGTGATTTAATTTGTGCTTGCACAGATTAATGAAA
GTGGCGCACGGGTGAGTAACACGTGGGAACATACCGATTAGTGGGGGATAACTATTGGAAACGATAGCTA
ATACCGCATGAGCCCTGAGGGGGAAAGATTTATCGCTAATTGAATGGCCCGCGGAAGATTAGATAGTTGG
TAGGGTAATGGCCTACCAAGTCTACGATCTATAGCTGGTCTGAGAGGACGATCAGCCACACTGGAGCTGA
GACACGGTC
>Zotu11509
GAACGCTGGCGGCGTGCCTAACACATGCAAGTCGAACGTGAAAGGGAGCAATCCTGAGTAAAGTGGCGT
ACGGGTGAGTAACGCGTAGGTAATCTACCCCAGAGTCCGGAATAACCTGCCGAAAGGCGGGCTAATACGG
GATAGAGCGATGGAATCCGTAATTGGGTTTTTCGGGAAAGGGAGCCTGTAAGGGCCTTCGCTCTGGGATG
AGCCTGCGTCTCATTAGCTAGTTGGTGGGGTAAAGGCCCACCAAGGCATTGATGGGTAGCTGGTCTGAGA
GGATGATCAGCCACACTGGAACTGAAACACGGTC
>Zotu11512
GAACGCTGGCGGCGTGCCTAATACATGCAAGTTGAGCGGCAGCGAGTTTTGCACTTATCCTTCCGACCCAT
ACGTGGGTGGAAGGATGTGTGCAGAATGTCGGCGAGCGGCGGACGGCAGAGTAACGCGTAGGAATGTAC
CCCAAAGTGAGGGATAACTGCTCGAAAGAGTAGCTAATACCGCATGTGATCTTCGGATTAAAGCTTTATGC
GCTTTGGGAACAGCCTGCGTAAGATTAGCTAGTTGGTGAGGTAATGGCTCACCAAGGCGACGATCTTTAGT
TGGTCTGAGAGGATGATCAGCCACACTGGAACTGAGACACGGTC
>Zotu11514
GAACGCTGGCGGCAGGCTTAACACATGCAAGTCGAACGCCCCGCAAGGGGAGTGGCGCACGGGTGAGTA
ACGCGTGGGAATGTACCATTTAGTGGGGGATAACTTCGGGAAACTGAAGCTAATACCGCATACGCCCGAG
AGGGGAAAGATTTATCGCTAAATGATCAGCCCGCGTGAGATTAGGTAGTTGGTGGGGTAATGGCCTACCA
AGCCTACGATCTCTAGCTGGTCTGAGAGGATGATCAGCCACACTGGAACTGAGACACGGTC
>Zotu11517
GAACGCTGGCGGCGTGCTTAACACATGCAAGTCGAACGATGAAACTCTAAAGATTTCTTCGGAATGAATTA
GAATGGATTAGTGGCGAACGGGTGAGTAACACGTGAGTAACCTGCCTTAGAGAAGGGGATAGCCTTTGGA
AACGAAGAATAATACCCTATGAAATGATAATATCGCATGATAGTATCATCAAAGATTTATCGCTCTAAGATG
GACTTGCGCCTGATTAGCTAGTTGGTGGAGTAACAGCCCACCAAGGCAACGATCAGTAGCCGGCTTGAGA
GAGTGTACGGCCACATTGGGACTGAGACACGGCC
>Zotu11519

GAACGCTGGCGGCGTGCTTAATACATGCAAGTCGAACGAAGCACTTTTGGCAGATCCCTTCGGGGTGAAG
CCGGTTGTGACTTAGTGGCGGACGGGTGAGTAACGCGTGGGTAACCTGCCTTATACTGGGGGATAACAGT
TAGAAATGGCTGCTAATACCCCATAAGCGCACGGCACCGCATGATGCAGTGTGAAAAGATTTATTGGTATA
AGATGGACCCGCGTCTGATTAGTTAGTTGGTGAGGTAACGGCCCACCAAGACGACGATCAGTAGCCGACT
TGAGAGAGTGATCGGCCACATTGGGACTGAGACACGGCC
>Zotu11522
GAACGCTGGCGGCGGGCCTAATACATGCAAGTCGAACGGGATTAGGTAGCAATACCTATGAGAGTGGCGC
ACGGGTGCGTAACACGTATGTAACCTACCTTTAACTGGGGAATAGCTTCGAGAAATCGAAATTAATACCCC
ATAACATTTAGAAAAGGCATCTTTTCTTTATCAAAGCTCAGGCGGTTAGAGATGGACATGCGGTGCATTAG
CTAGTTGGTGAGGTAATGGCTCACCAAGGCTACGATGCATAGGGGGTCTGAGAGGATGATCCCCCACACT
GGTACTGAGACACGGAC
>Zotu11527
GAACGCTGGCGGCGTGCCTAATACATGCAAGTAGAACGCTGAAGGAAGGAGCTTGCTCTTTCCGGATGAG
TTGCGAACGGGTGAGTAACGCGTAGGTAACCTGCCTGGTAGCGGGGGATAACTATTGGAAACGATAGCTA
ATACCGCATAATAGTAGATATCGCATGATAACTGCTTAAAAGGTGCAATTGCATCACTACCAGATGGACCT
GCGTTGTATTAGCTAGTTGGTGAGGTAACGGCTCACCAAGGCAACGATACATAGCCGACCTGAGAGGGTG
ATCGGCCACACTGGGACTGAGACACGGCC
>Zotu11531
GAACGCTGGCGGCGTGCCTAATACATGCAAGTCGAGCGGATTGATGGGAGCTTGCTCCCTGATATTAGCG
GCGGACGGGTGAGTAACACGTGGGCAACCTGCCCTGCAGATGGGGATAACTCCGGGAAACCGGGGCTAA
TACCGAATAATCAGTTCCTCCGCATGGAGGAACTCTGAAAGACGGTTTCGGCTGTCACTGCAGGATGGGCC
CGCGGCGCATTAGCTAGTTGGTGGGGTAATGGCCTACCAAGGCGACGATGCGTAGCCGACCTGAGAGGGT
GATCGGCCACACTGGGACTGAGACACGGTC
>Zotu11535
GAACGCTGGCGGCAGGCTTAACACATGCAAGTCGAGCGCCTCGCAAGAGGAGCGGCAGACGGGTGAGTA
ACGCGTGGGAATCTACCCAACCCTACGGAATAACGCATGGAAACGTGTGCTAATACCGTATACGCCCTTAG
GGGGAAAGATTTATCGGGGATGGATGAGCCCGCGTTGGATTAGCTAGTTGGTGGGGTAAAGGCCTACCAA
GGCGACGATCCATAGCTGGTCTGAGAGGATGATCAGCCACATTGGGACTGAGACACGGCC
>Zotu11538
GAACGCTGGCGGCAGGCCTAACACATGCAAGTCGAACGAGCCTTCGGGCTAGTGGCGGACGGGTGAGTA
ACACGTGGGAACGTACCTTTAGGTTCGGAATAACGCAGAGAAATTTGCGCTAATACCGAATGTGCCCTTCG
GGGGAAAGATTTATCGCCTTTAGAGCGGCCCGCGTTAGATTAGCTAGTTGGTGAGGTAAAGGCTCACCAA
GGCTACGATCTATAGCTGGTCTGAGAGGATGATCAGCCACATTGGGACTGAGACACGGCC
>Zotu11545
GAATGCTGGCTGCGTGCTTAACACATGCAAGTCGAACGGTGAAGGAGGGCTTGCCCTCCGGATCAGTGGC
GAACGGGTGAGTAACACGTGGGCAACCTGCCCCTCACTCTGGGATAACACCGGGAAACCGGTGCTAATAC
CGGATACGACCAGGCGCGACATCTCGCTTGGTGGAAAGAACTTCGGTGAGGGATGGGCCCGCGGCCTATC
AGCTAGTTGGTGAGGTAATGGCTCACCAAGGCGACGACGGGTAGCCGGCCTGAGAGGGCGACCGGCCAC
ACTGGGACTGAGACACGGCC
>Zotu11548
GAATGCTGGCGGTATGGATAAGGCATGCAAGTCGAGGGCACCGCAAGGTGACCGGCAGACGAGGTAGTA
ACACGTAGGAACGTACCCCGAAGTCAGGGATAGCTCATCGAAAGGTGAGGTAATACTTGATGGTCTCGCG
AGAGTAAAGATTTATCGCTTCGGGAGCGGCCTGCGCAATATCAGCTAGTTGGTAAGGTAACGGCTTACCAA
GGCTACGACGTTTAGGGGAGCTGAGAGGCTGACCCCCACCGAGGGAACTGAGATACGGTC
>Zotu11556
CCTTCTCTCTAGCTTAGAAAAGGACAGCACCCCGCCCCCCTCCTCCTGGCAGCCACCCTTGGCTTAACCCCCT
CTGGCCTCTACTGCCTGGGTTCATTCCTC
>Zotu11559
GCCGTGTCTCAGTCCCAGTGTGGCCGGTCACCCTCTCAGGCCGGCTACCCGTCGTCGCCTTGGTAGGCCATT
ACCCCACCAACAAGCTGATAGGCCGTGAGCCCATCCACAACCAGTACAAACCCTTTCCACCCCCCACCATGC

GACAGGAGGTCATATCCAGTATTAGACCCAGTTTCCCAGGCTTATCCCAGAGTCAAGGGCAGGTTACTCAC
GTATTACTCACCCGTTCGCCACTCATCCACCCAGTGCAAGCACCAAGCTTCAGCGTTCGACTTGCATGTGTT
AAGCACGCCGCCAGCGTTCG
>Zotu11565
GAACGCTAGCGGCATGCCTAACACATGCAAGTCGAACGAGACCTTCGGGTCTAGTGGCGCACGGGTGCGT
AACGCGTGGGAATCTGCCCTTGGGTTCGGAATAACAGTTAGAAATGACTGCTAATACCGGATGATGTCTTC
GGACCAAAGATTTATCGCCCAAGGATGAGCCCGCGTAAGATTAGCTAGTTGGTGGGGTAATGGCCTACCA
AGGCGACGATCTTTAGCTGGTCTGAGAGGATGATCAGCCACACTGGGACTGAGACACGGCC
>Zotu11567
GAACGCTAGCTACAGGCTTAACACATGCAAGTCGAGGGGAAACGATATTGGAAGCTTGCTTCCGATAGGC
GTCGACCGGCGCACGGGTGAGTAACGCGTATCCAACCTGCCCACCACTTGGGAATAACCTTGCGAAAGTA
AGACTAATGCCCAATGATATCTCTAGAAGACATCTGAAAGAGATTAAAGATTTATCGGTGATGGATGGGGA
TGCGTCTGATTAGCTTGTTGGCGGGGTAACGGCCCACCAAGGCAACGATCAGTAGGGGTTCTGAGAGGAA
GGTCCCCCACATTGGAACTGAGACACGGTC
>Zotu11570
GAACGCTGGCGGCATGCCTAACACATGCAAGTCGAACGAACCCTTCGGGGTTAGTGGCGCACGGGTGCGT
AACACGTGGGAATCTGCCCTTTGCTTCGGAATAACAGTTAGAAATGACTGCTAATACCGGATAATGTCTTCG
GACCAAAGATTTATCGGCAAAGGATGAGCCCGCGTAGGATTAGCTTGTTGGTGAGGTAAAGGCTCACCAA
GGCGACGATCCTTAGCTGGTCTGAGAGGATGATCAGCCACACTGGGACTGAGACACGGCC
>Zotu11577
GAACGCTAGCGGCATGCCTAACACATGCAAGTCGAACGAGACCTTCGGGTCTAGTGGCGCACGGGTGCGT
AACGCGTGGGAATCTGCCCTTCGGTTCGGAACAACAGTTAGAAATGACTGCTAATACCGGATGATGACGTA
AGTCCAAAGATTTATCGCCGAAGGATGAGCCCGCGTAGGATTAGCTAGTTGGTAAGGTAAAAGCTTACCA
AGGCGACGATCCTTAGCTGGTCTGAGAGGATGATCAGCCACACTGGGACTGAGACACGGCC
>Zotu11580
GAACGCTGGCGGCGTGCTTAACACATGCAAGTCGAGCGGAAAGGCCACTTCGGTGGTACTCGAGCGGCGA
ACGGGTGAGTAACACGTGAGTAATCTGCCCCTGGCTTTGGGATAGCCACCGGAAACGGTGATTAATACCG
GATACGACAACTTCTCGCATGGGATGGTTGTGGAAAGTTTTTCGGCCAGGGATGTGCTCGCGGCCTATCAG
CTTGATGGTGAGGTAATGGCTCACCATGGCTTCGACGGGTAGCCGGCCTGAGAGGGTGACCGGTCACACT
GGGACTGAGACACGGCC
>Zotu11583
GAACGCTGGCGGCGTGCTTAACACATGCAAGTCGAACGGAAAGGCCCTTCGGGGTACTCGAGTGGCGAAC
GGGTGAGTAACACGTGGGTGATCTGCCTCGTACTCTGGGATAAGCCTGGGAAACTGGGTCTAATACCGGA
TAGGACCTTGGGTTGCATGGCCTTGGGTGGAAAGTTTTTCGGTACGAGATGGGCCCGCGGCCTATCAGCTT
GTTGGTGGGGTAATGGCCTACCAAGGCGACGACGGGTAGCCGGCCTGAGAGGGCGACCGGCCACACTGG
GACTGAGACACGGCC
>Zotu11589
GAACGCTGGCGGCATGCCTAACACATGCAAGTCGAACGAGACCTTCGGGTCTAGTGGCGCACGGGTGCGT
AACGCGTGGGAATCTGCCCTTGGGTACGGAATAACTCAGAGAAATTTGAGCTAATACCGTATAATGACTTC
GGTCCAAAGATTTATCGCCCAAGGATGAGCCCGCGTAAGATTAGCTAGTTGGTGGGGTAAAGGCCTACCA
AGGCGACGATCTTTAGCTGGTCTGAGAGGATGATCAGCCACACTGGAACTGAGACACGGTC
>Zotu11593
GAACGCTAGCGGCGGGCTTAACACATGCAAGTCGAGGGGCAGCATGAAATCTATTATATAGATTTTTATGG
CGACCGGCGTACGGGTGCGTAACACGTACGTAACCTACCTTTTGCTAGAAGATAGCCTGAGGAAACTCGG
ATTAATATTCTATAATATAGATAAAATCGCATGATTTTACTATTAAAGCAGAAATGCGGCAAGAGATGGGC
GTGCGTCCGATTAGTTAGTTGGTAAGGTAAAAGCTTACCAAGACAATGATCGGTAGGGGGCCTGAGAGGG
TGATCCCCCACACTGGTACTGAGATACGGAC
>Zotu11595
GAACGCTAGCGGCAGGCTTAACACATGCAAGTCGAGGGGTATGGTTCTTCGGAACCAGAGACCGGCGCAC
GGGTGCGTAACGCGTATGCAATCTACCTTTTACAGAGGGATAGCCCAGAGAAATTTGGATTAATACCTCAT

AGTATATAGAGTTTGCATCAACACTATATTAAAGTTCCAACGGTAAAAGATGAGCATGCGTCCCATTAGCTA
GATGGTAAGGTAACGGCTTACCATGGCAACGATGGGTAGGGGTCCTGAGAGGGAGATCCCCCACACTGGT
ACTGAGACACGGAC
>Zotu11598
GAACGCTGGCGGTGTGCTTAACACATGCAAGTCGAACGGGCAAGAGCTGTAGCAATACAGAATTCTGCTA
GTGGCAAACGGGTGAGTAATACATGGGAATCTACCTTGCAGTGCGAAATAACAGTTGGAAGCAATTGCTA
ATGTCGCATATAGCCGAGAGGTGAAAGATTTATCGCTGCAAGACGAGCCCATGCAGGATTAGCTAGTTGG
GGGGGTAACGGCCCACCAAGGCGATGATCAGTAGGGGTTCTGAGAGGATTGGCCCCCACATGGGTACTG
AGATACGGAC
>Zotu11604
CAACGCTGGCGGCGCGCTTAACACATGCAAGTCGAACGGGAAAGTGGAGCAATCCATGAGTACAGTGGC
GTACGGGTGAGTAACACGTGGGTGATCTACCTTTTAGTGGGGAATAACCCTGGGAAACTAGGGCTAATAC
CGCATAATCCCGAGAGGGGAAAGCAGCAATGCGCTGAAAGAGGAGCCCGCGGCCGATTAGCTAGTTGGT
AGGGTAAGAGCCTACCAAGGCAGAGATCGGTAGCCGGCCTGAGAGGGCACACGGCCACACTGGCACTGA
AACACGGGC
>Zotu11607
GAACGCTGGCGGCATGCCTTACACATGCAAGTCGAACGGTAACAGGTTAAGCTGACGAGTGGCGAACGG
GTGAGTAATACATCGGAACGTGCCCGATCGTGGGGGATAACGCAGCGAAAGCTGTGCTAATACCGCATAC
GATCTACGGATGAAAGCAGGGGATCGCAAGACCTTGCGCGGACGGAGCGGCCGATGGCAGATTAGGTAG
TTGGTGGGGTAAAGGCCTACCAAGCCTGCGATCTGTAGCTGGTCTGAGAGGACGACCAGCCACACTGGGA
CTGAGACACGGCC
>Zotu11610
GAACGCTGGCGGCGTGCTTAACACATGCAAGTCGAACGGAGGATTTATTTCGGTAAATCCTTAGTGGCGAA
CGGGTGAGTAACGCGTGGGCAACCTGCCCTTCAGATGGGGACAACATCCCGAAAGGGGTGCTAATACCGA
ATACGATCGTTTTCCCGCATGGGGATGCGATGAAAGATGGCCTCTATTTATAAGCTATCGCTGAAGGATGG
GCCTGCGTCTGATTAGCTAGTTGGTGGGGTAACGGCCTACCAAGGCGATGATCAGTAGCCGGTCTGAGAG
GATGAACGGCCACATTGGGACTGAGACACGGCC
>Zotu11614
GAACGTTGGCGGCGTGGATTAGGCATGCAAGTCGTACGGGCCGCAAGGCCAGTGGCGCAAGGGTGAGTA
AGGCGACGGTAACCAACCCCATGATTGGGTATAGCCGCGGGAAACTGCGGGTAATCCCCAGCGACGTCCC
GGGAGGGCATCCTCCCAGGACCAAAGTTCCGGCGTCGTGGGACGGGCTGTCGTGGTATTAGGTTGTTGGC
GGGGTAACGGCCCACCAAGCCTGCGATGCCTACCGGGCGTGCGAGCGTGGCCCGGCACACTGGGACTGA
GACACTGCC
>Zotu11617
GAACGCTGGCGGCGTGGCTAAGACATGCAAGTCGTACGGGCGTAGCAATACGTCAGTGGCGCACGGGTG
AGTAATGTATAGCTAACCTACCTTGGAACCTGGGATAGCTAGCCGAAAGGCTAGGTAATACCAGATGAAAT
AACAGAATCGCATGATTTTGTTCTCAAAGAGTAATCAATCTGAGACGGGGCTATATTGTATCAGCTAGTTG
GTAAGGTAATGGCTTACCAAGGCAATGACGCATAGCCGGGCTGAGAGGTCGGCCGGCCACATCGGAACTG
AGACACTGTC
>Zotu11623
GAACGCTAGCGATAGGCCTAACACATGCAAGTCGAGGGGCAGCACATGAAGTAGCAATACGGATGGTGG
CGACCGGCGCACGGGTGAGTAACGCGTATGCAACCTGCCTACAACAGGGGAATAACCCGTTGAAAGACGG
ACTAATACCCCATAACACAGGGATCCCGCATGGGAATATTTGTTAAAGATTTATCGGTTGTAGATGGGCAT
GCGTTCCATTAGCTTGTTGGTGAGGTAACGGCTCACCAAGGCAACGATGGATAGGGGAACTGAGAGGTTT
ATCCCCCACACTGGTACTGAGACACGGAC
>Zotu11631
GAACGCTGGCGGCGTGCCTAACACATGCAAGTCGAGCGATTTTCTTCGGAAGAGAGCGGCGGACGGGTG
AGTAACGCGTGGGTAACCTGCCCTGTACACACGGATAACATACCGAAAGGTATGCTAATACGAGATAATAT
ACTTTTATCGCATGGTAGAAGTACCAAAGCTCCGGCGGTACAGGATGGACCCGCGTCTGATTAGCTAGTTG
GTAAGGTAACGGCTTACCAAGGCGACGATCAGTAGCCGACCTGAGAGGGTGATCGGCCACATTGGAACTG
AGACACGGTC

>Zotu11638
CAACGCTGGCGGCGTGCCTAACACATGCAAGTCGAACGAGAAAGGGACTTCGGTCCTGAGTACAGTGGCG
CACGGGTGAGTAACACGTGACTAACCTACCCTTGAGTGGGGAATAACTTCGAGAAATCGAGGCTAATTCCG
CATAATACCTACGGGTCAAAGGAGCAATTCGCTTAAGGAGGGGTCGCGGCAGATTAGCTAGTTGGCGGG
GTAATGGCCCACCAAGGCAGTGATCTGTATCCGGCCTGAGAGGGCGCACGGACACACTGGAACTGAAACA
CGGTC
>Zotu11640
GAACGCTGGCGGCAGGCCTAATACATGCAAGTCGAACGCACCTTCGGGTGAGTGGCGGACGGGTGAGTA
ACGCGTGGGAACGTGCCCTTTGGTTCGGAACAACACAGGGAAACTTGTGCTAATACCGAATGTGCCCTTCG
GGGGAAAGATTTATTGCCATTGGAGCGGCCCGCGTTGGATTAGGTAGTTGGTGAGGTAAAGGCTCACCAA
GCCTACGATCCATAGCTGGTCTGAGAGGATGATCAGCCACACTGGGACTGAGACACGGCC
>Zotu11644
GAACGCTGGCGGCGTGCCTAATGCATGCAAGTCGCACGCACCTTTCGGGGTGAGTGGCGCACGGCTGAGG
AACACGTGGGAATCTGCCCTTCTGTGGGGGATAACCCGGAGAAATCCGAGCTAATCCCGCATACGCTTCGC
AAGAAGGAAAGCGGTAACGCGCAGAGGGAGGAGCCCGCGGCCCATCAGGTCGTTGGTGGGGTAACGGC
CTACCAAGCCAATGACGGGTAGCTGGTCTGAGAGGACAGACAGCCAGACTGGGACTGAGAACGGCC
>Zotu11646
GAACGCTGGCGGCATGCCTAACACATGCAAGTCGAACGAGACCTTCGGGTCTAGTGGCGCACGGGTGCGT
AACGCGTGGGAATCTGCCCTCTGGTTCGGAATAACTCAGGGAAACTTGAGCTAATACCGGATAATGACTTC
GGTCCAAAGATTTATCGCCTGAGGATGAGCCCGCGTAGGATTAGCTTGTTGGTGGGGTAAAGGCCTACCA
AGGCGACGATCCTTAGCTGGTCTGAGAGGATGATCAGCCACACTGGGACTGAGACACGGCC
>Zotu11649
GAACGCTGGCGGCGTGCCTAATGCATGCAAGTCGCACGCACCGTGCTTGCACGGTGAGTGGCGCACGGCT
GAGGAACACGTGGGTGACCTGCCCTGGCGTGGGGGACAACGGGCCGAAAGGTCCGCTAATCCCGTATAC
GCTCACCGCCGATGGGCGGTGAGGAAAGCAGCAATGCGCGCCGGGAGGGGCCTGCGGCCCATCAGGTAG
TTGGCGGGGTAACGGCCCACCAAGCCGGTGACGGGTACCTGGTCTGAGAGGACGACCAGGCAGACTGGG
ACTGAGACACGGCC
>Zotu11653
GAACGCTGGCGGTATGCTTAACACATGCAAGTCGAACGGGGACCTTCGGGTCCTAGTGGCGGACGGGTGA
GTAACGCGTGAGAACATGCCTTTAGATTGGGGACAACAGTTGGAAACGACTGCTAATACCGAATGTGCCTT
TAGGGGTGAAAGATTTATTGTCTAGAGATTGGCTCGCGTCAGATTAGCTAGTTGGAGTGGTAACGGCACAC
CAAGGCGACGATCTGTACTTGGTCTGAGAGGATGACCAGGCACACTGGAACTGAGACACGGTC
>Zotu11656
GAACGCTGGCGGCGTGCTTAACACATGCAAGTCGAGCGGTAAGGCCACTTCGGTGGTACACGAGCGGCGA
ACGGGTGAGTAACACGTGAGCAACCTGCCCTTCTCTTCGGAATAAGCGTTGGAAACGACGTCTAATGCCGA
ATATGACCTCTTTTTGCATGATTAGAGGTGGAAAGCTCCGGCGGAGAGGGATGGGCTCGCGGCCTATCAG
CTAGTTGGTGTGGGTAATGGCCCACCAAGGCGACGACGGGTAGCCGGCCTGAGAGGGTGACCGGCCACA
CTGGGACTGAGACACGGCC
>Zotu11658
AAACGCTGGCGGCGTGCCTAACACATGCAAGTCGAGCGGGGTTGGAACTTGTTCCAATCTAGCGGCGGAC
GGGTGAGTAACACATGGATAATCTGCCTTTGACTCAGGAATAACAGTCAGAAATGACTGCTAAAGCCTGAT
AAGCCCACAAACTGGCATCAGTAAGTGGGAAAAAGATTCTGGTCAAAGAGGAGTCCGTGCCTGATTAGCT
AGTTGGTGGGGTAATAGCCTACCAAGGCTATGATCAGTAGCCGGCCTTAGCGGGTGTACGGCCACATTGG
GACTGAGACACGGCC
>Zotu11662
GAACGTTCGCGGCGGGCCTAACACATGCAAGTCGAACGCGAAAGGGATTCGTCCCCGGTAGCGTGGCGTC
CGGGTGAGTAACACGTAGGAAACCTTCCCTCGAGCGGGGGATAACGTCCCGAAAGGGACGCTAATACCGC
ATATGCCCACGTTGTCTTCGGACGATGAGGGGAAAGCTGGCCTCTGTATACAAGCTAGCTCTCGAGAATGG
TCCTGCGGCGCATCAGCTAGTTGGTGGGGTAACGGCCTACCAAGGCGAAGACGCGTAGCTGGTCTGAGAG
GATGATCAGCCACACTGGCACTGAAACACGGGC

>Zotu11666
TAATGCTGGCGGCGTGCCTAATACATGCAAGTCGAATGGGTTTAGACCCATGGCGAACGGGTGAGTAACG
CGTAGGTAGTTGCTCCGAAGTCGGGGATAACTCGCCGAAAGGCGAATTAACATCCGATAGTCTCGTGCTTC
GTAACTGAAGTACGAGTAAAAATTTATTGCTTCGGAAGATGCCTGCGTCCTATCAGTTAGTTGGTGAGGTA
ACGGCTCACCAAGACGATGACGGGTAACTGGTCTGAGAGGACGACCAGTCACAGTGGAACTGAGACACG
GTC
>Zotu11668
GAACGCTGGCGGCAGGCTTAACACATGCAAGTCGAACGCACCGCAAGGTGAGTGGCAGACGGGTGAGTA
ACGCGTGGGAACCTACCTATCGGTTCGGAATAACTCGGGGAAACCCGTGCTAATACCGGATAAGCCCTTCG
GGGGAAAGATTTATCGCCGAATGATGGGCCCGCGTTGGATTAGCTAGTTGGTGTGGTAATGGCGCACCAA
GGCGACGATCCATAGCTGGTCTTAGAGGATGATCAGCCACACTGGGACTGAGACACGGCC
>Zotu11672
GAACGCTGGCGGCATGCCTAACACATGCAAGTCGAACGAAGGCTTCGGCCTTAGTGGCGCACGGGTGCGT
AACACGTGGGAATCTGCCCTTGGGTTCGGAATAACAGTTAGAAATGACTGCTAATACCGGATAATGACGTA
AGTCCAAAGATTTATCGCCCAAGGATGAGCCCGCGTAAGATTAGGTAGTTGGTGGGGTAAAGGCCTACCA
AGCCGACGATCTTTAGCTGGTCTGAGAGGATGATCAGCCACACTGGGACTGAGACACGGCC
>Zotu11674
GAACGCTGGCGGCGTGCTTAACACATGCAAGTCGAACGGTGAAGCCCAGCTTGCTGGGTGGATCAGTGGC
GAACGGGTGAGTAACACGTGAGCAACCTGCCCTCCACTTCGGGATAAGCCTTGGAAACGGGGTCTAATAC
CGGATATGAGCACTCATCGCATGGTGGGTGTTGGAAAGTTTTTCGGTGGGGGATGGGCTCGCGGCCTATC
AGCTTGTTGGTGGGGTGATGGCCTACCAAGGCGTCGACGGGTAGCCGGCCTGAGAGGGCGACCGGCCAC
ACTGGGACTGAGACACGGCC
>Zotu11676
GAACGCTGGCGGCATGCCTAACACATGCAAGTCGAACGAAGGCTTCGGCCTTAGTGGCGCACGGGTGCGT
AACGCGTGGGAATCTGCCCTTAGGTTCGGAATAACAGTTGGAAACGACTGCTAATACCGGATGATGACGT
AAGTCCAAAGATTTATCGCCTGAGGATGAGCCCGCGTAGGATTAGCTAGTTGGTGTGGTAAGAGCGCACC
AAGGCGACGATCCTTAGCTGGTCTGAGAGGATGATCAGCCACACTGGGACTGAGACACGGCC
>Zotu11678
GAACGCTGGCGGCGTGCTTAACACATGCAAGTCGAACGGGATCCATGAAGTAGCAATACTTCGGAAGATC
TAGTGGCGAACGGGTGAGTAACACGTGAAGAACCTGCCCCGAACTCTGGAATAACAGTCGGAAACGATTG
CTAATACCGGATACCCTCACGAGGTCTCATGGCCTTGTGAAGAAAGATTTATCGGTTTGGGAGGGCTTCGC
GGCCTATCAGCTTGTTGGTGAGGTAACGGCTCACCAAGGCGTCGACGGGTAGCTGGTCTGAGAGGATGAT
CAGCCACACTGGAACTGAGACACGGTC
>Zotu11680
GAACGCTGGCGGCGTGCTTAACACATGCAAGTAGAACGGTGACGGAGAGCTTGCTCTCCTGATCAGTGGC
GAACGGGTGAGTAACACGTGGGCAACCTGCCCCTGACACTGGGATAACTCCGGGAAACCGGGGCTAATAC
CGGATATTACGCGAGATGGCATCTTCTCGCGTGGAAAGTTTTTCGGTCAGGGATGGGCCCGCGGCCTATCA
GCTTGTTGGTGAGGTAACGGCCCACCAAGGCGACGACGGGTAGCCGGCCTGAGAGGGCGACCGGCCACA
CTGGGACTGAGACACGGCC
>Zotu11682
GAACGCTGGCGGCAGGCTTAACACATGCAAGTCGAACGGGCCTTTCGGGGTCAGTGGCAGACGGGTGAG
TAACACGTGGGAACGTACCCTTCAGTTCGGAATAACCCTGGGAAACTAGGGCTAATACCGGATACGGCCG
AGAGGCGAAAGGTTTACTGCTGAAGGATCGGCCCGCGTCCGATTAGCTAGTTGGTGTTGGTAACGGCTCA
CCAAGGCGACGATCGGTAGCTGGTCTGAGAGGATGACCAGCCACATTGGGACTGAGACACGGCC
>Zotu11685
GAACGCTGGCGGCATGCCTAACACATGCAAGTCGAACGAACCCTTCGGGGTGAGTGGCGCACGGGTGCGT
AACGCGTGGGAACCTGCCCTTAGGTTCGGAATAACTCAGAGAAATTTGAGCTAATACCGGATAATGTCTTC
GGACCAAAGATTTATCGCCTTTGGATGGGCCCGCGTTGGATTAGCTAGTTGGTGGGGTAAAGGCCTACCAA
GGCGACGATCCATAGCTGGTCTTAGAGGATGATCAGCCACACTGGGACTGAGACACGGCC
>Zotu11688

GAACGCTAGCGGCATGCCTAACACATGCAAGTCGAGGGGCAACAGGAAGTAGCAATACTTTGCTGGCGAC
CGGCGCACGGGTGAGTAACGCGTATGCAACCTGCCTACTACAGGGGGATAACCCGTCGAAAGACGGCCTA
ATACCGCATAATACAGGTTTACTGCATGGTAAATTTGTTAAAGAACTTCGGTAGTAGATGGGCATGCGTTCC
ATTAGCTAGTTGGTGAGGTAACGGCTCACCAAGGCAACGATGGATAGGGGAACTGAGAGGTCGATCCCCC
ACACTGGAACTGAGACACGGAC
>Zotu11690
GAACGCTGGCGGCGCGCTTAACACATGCAAGTCGAGCGAGAACCAAGCCTTCGGGCTTGGGGACAGCGG
CGAACGGGTGAGTAACACGTGGGTAATCAACCCTTGACACTGGGATAGCCCGGGGAAACCCGGATTAATA
CCGGATACCCCATCTGGCTTTCGAGCCGGCTGGAAAAGGTAGCTTCGGCCTCCGGTCAGGGACGAGCCCG
CGGCGGATTAGCTTGTTGGCGGGGTAAAGGCCCACCAAGGCGACGATCCGTAGCTGGTCTGAGAGGATG
ATCAGCCACACTGGGACTGAGACACGGCC
>Zotu11694
GAACGCTGGCGGCGTACTTAACACATGCAAGTCGAACGGGGATCGCTGCGTGAGCAATCACGCGGCGGTT
CTAGTGGCGTACGGGTGAGTAACACGTGGGTAACCTGCCCTGGAGACCGGGATACCCTCTCGAAAGGGAG
GCTAATACCGGATACGTTCCCCGGATCGCGAGGTCTGGGGAGGAAAGGTGGCGAAAGCTACCGCTCTTGG
AGGGGCTCGCGGCCTATCAGCTAGTTGGTGAGGTGACGGCTCACCAAGGCTATGACGGGTAGCTGGTCTG
AGAGGACGACCAGCCACACGGGGACTGAGACACGGCC
>Zotu11697
GAACGCTGGCGGCATGCCTTACACATGCAAGTCGAACGGTAACAGGTCTTCGGATGCTGACGAGTGGCGA
ACGGGTGAGTAATGCATCGGAACGTGCCCAGTAGTGGGGGATAGCCCGGCGAAAGCCGGATTAATACCG
CATACGACCTGAGGGTGAAAGGGGGGGATCGCAAGACCTCTCGCTATTGGAGCGGCCGATGTCAGATTAG
CTAGTTGGTGGGGTAAAGGCCTACCAAGGCAACGATCTGTAGTTGGTCTGAGAGGACGACCAACCACACT
GGGACTGAGACACGGCC
>Zotu11706
GAACGCTAGCGGCATGCTTAACACATGCAAGTCGAACGGCAGCACAGAGGAGCTTGCTCCTTGGGTGGCG
AGTGGCGGACGGGTGAGGAATACATCGGAATCTACTCTGTCGTGGGGGATAACGTAGGGAAACTTACGCT
AATACCGCATACGACCTACGGGTGAAAGCAGGGGATCTTCGGACCTTGCGCGATTGAATGAGCCGATGTC
GGATTAGCTAGTTGGCGGGGTAAAGGCCCACCAAGGCGACGATCCGTAGCTGGTCTGAGAGGATGATCA
GCCACACTGGGACTGAGACACGGCC
>Zotu11709
GAACGCTAGCGGCAGGCCTAATACATGCAAGTCGAACGGTGTCTTCGGACATAGTGGCGCACGGGTGCGT
AACACGTATGCAACCTACCTTACATTGGGGGATAGCCTTTCGAAAGGGAGATTAATACCGCATAAGACAGT
AGTCTGGCATCAGACAGCTGTTAAAGATTTATCGATGTAAGCTGGGCATGCGACCAATTAGTTAGTTGGCG
AGGTAATGGCTCACCAAGACTATGATTGGTAGGGGAACTGAGAGGTCAATCCCCCACACTGGCACTGAGA
TACGGGC
>Zotu11711
GAACGCTAGCGGAAGGCCTAATACATGCAAGTCGAACGGTGTAGCAATACATAGTGGCGGACGGGTGCG
TAACACGTATGCAACCTACCCAAGGGTGTGGAATAACAATTTGAAAAGATTGCTAATACCGCATAATGCAG
CGGACCCGCATGGGTATGTTGTTAAAGGCGAAAGTCGCCTTTGGATGGGCGTGCGTCCGATTAGGTAGTT
GGTAGGGTAACGGCCTACCAAGCCGACGATCGGTAGCTGGTCTGAGAGGACGATCAGCCACACTGGGACT
GAGACACGGCC
>Zotu11713
GAACGCTGGCGGCGTGCTTAACACATGCAAGTCGAACGGAAAGGCCTTGTGCTTGCACAGGGTACTCGAG
TGGCGAACGGGTGAGTAACACGTGGGTGATCTGCCCTGCACTGTGGGATAAGCCTGGGAAACTGGGTCTA
ATACCATATATGACCACCTTTTGGATGGGGTGGTGGAAAGCTTTTGCGGTGTGGGATGAGCCTGCGGCCTA
TCAGCTTGTTGGTGGGGTAATGGCCTACCAAGGCGGCGACGGGTATCCGGCCTGAGAGGGTGTACGGACA
CATTGGGACTGAGATACGGCC
>Zotu11715
GAACGCTGGCGGCATGCCTAACACATGCAAGTCGAACGAGACCTTCGGGTCTAGTGGCGCACGGGTGCGT
AACGCGTGGGAATCTGCCCTTAGGTTCGGGATAACTCAGGGAAACTTGAGCTAATACCGGATGACGACTTC

GGTCCAAAGATTTATCGCCTAAGGATGAGCCCGCGTAGGATTAGCTTGTTGGTGAGGTAAAGGCTCACCAA
GGCGACGATCCGTAGCTGGTCTGAGAGGACGATCAGCCACACTGGGACTGAGACACGGCC
>Zotu11717
GCCGTGTCTCAGTCCCAATGTGGCTGATCATCCTCTCAAATCAACTATGGATCGTAGCCTTGGTAGGCCCTT
ACCTCACCAACTAGCTAATCCAACGCAGGCTCATCCATCCGCGACAGCCTAAAAGCCATCTTTAATCCACAG
ATACTATGCGGTATTAATAGTCGTTTCCAACTGGTATCCCCCTCAGATGGGCAGATTCCTACGTGTTACTCAC
CCGTCCGCCACTCGTCAGCAACTAGCAAGCTAGTCCTGTTACCGTTCGACTTGCATGTGTTAAGCATGCCAC
CAGCGTTCA
>Zotu11719
GAACGCTGGCGGCAGGCTTAACACATGCAAGTCGAGCGCGTAGCAATACGAACGGCAGACGGGTGAGTA
ACGCGTGGGAACCTACCCTTTTCTACGGAATAACGCATGGAAACGTGTGCTAATACCGTATACGCCCTTTG
GGGGAAAGATTTATCGGAGAAGGATAGGCCCGCGTTGAATTAGCTAGTTGGTGGGGTAAAGGCCTACCAA
GGCGACGATCCATAGCTGGTCTGAGAGGATGATCAACCACATTGGGACTGAGACACGGCC
>Zotu11721
GAACGCTGGCGGCATGCTTTACACATGCAAGTCGAACGGCAGCGCGGGGCAACCTGGCGGCGAGTGGCG
AACGGGTGAGTAATATATCGGAACGTACCCAGAAGTGGGGGATAACGTAGCGAAAGTTACGCTAATACCG
CATACGATCTACGGATGAAAGTGGGGGATCTTCGGACCTCATGCTTTTGGAGCGGCCGATATCTGATTAGC
TAGTTGGTGAGGTAAAGGCTCACCAAGGCGACGATCAGTAGCTGGTCTGAGAGGACGACCAGCCACATTG
GGACTGAGACACGGCC
>Zotu11727
GAACGCTAGCGGGCGGCTTAACACATGCAAGTCGAGCGGGGGGTTTCGGCCCTCAGCGGCGGACGGGTG
AGTAACACGTAGGAATCTGCCTTTTGGTGGGGGATAGCCGGGGGAAACCCCGATTAATACCGCATATGGC
CCATGGGTCAAAGCCGTAAGGCGCCAGAAGATGAGCCTGCGTCCGATTATTTAGTCCTTGTTGGTGGGGTA
ACGGCCTACCAAGGGTACGATCGGTAGCTGGTCTGAGAGGACGATCAGCCACACTGGGACTGAGACACG
GCC
>Zotu11730
GAACGCTGGCGGCGCGTTTTAAGCATGCAAGTCGGACGGCAAGGCGGGTAGCAATACCTGCCCTAGAGTG
GCGAACGGGTGAGTAACACGTAGGTGATCTACCTCTGGATGGGGGATAGCCCGTGGAAACACGGGGTAA
TACCGCATGAGGCCTTGTGGGTAAAGCCATGAGGTGAAAGGTGCAATTGCACCGTCTGGGGATGAGCCTG
CGGTCCATTAGTTAGTTGGCGGGGTAATGGCCCACCAAGACTCCGATGGATAGCCGGCCTGAGAGGGTGT
ACGGCCACACTGGAACTGAGACACGGTC
>Zotu11733
GAATGCTGGCGGCGTGCCTAATACATGCAAGTCGAGCGGAAAGGCCCTTCGGGGTACTCGAGCGGCGGA
CGGCTGAGTAACGCGTGGGAACGTACCCCAAAGTGAGGAATAACCGCCCGAAAGGGTGGCTAATGCCGC
ATATGATCTTCGGATTAAAGGATTTATCCGCTTTGTGAACGGCCTGCGTATGATTAGATTGTTGGTGAGGTA
AAGGCTCACCAAGTCTACGATCATTAACTGGTCTGAGAGGATGATCAGTCAGACTGGAACTGAGGCACGG
TC
>Zotu11736
GAACGCTGGCGGCATGCCTAACACATGCAAGTCGAACGAAGGCTTCGGCCTTAGTGGCGCACGGGTGCGT
AACGCGTGGGAATCTGCCCCTGGGTTCGGAATAACTCGCCGAAAGGCGTGCTAATACCGGATGATGACGT
AAGTCCAAAGATTTATCGCCCAAGGATGAGCCCGCGTAGGATTAGCTAGTTGGTGGGGTAAAGGCCCACC
AAGGCGACGATCCTTAGCTGGTCTGAGAGGATGATCAGCCACACTGGGACTGAGACACGGCC
>Zotu11746
GAACGCTGGCGGCGTGCTTAACACATGCAAGTCGAACGGAAAGGCCCGCTTGCGGGTACTCGAGTGGCGA
ACGGGTGAGTAACACGTGGGCAACCTGCCCTCAGCTTCGGGACAACTCCTGGAAACGGGTGCTAATACCG
GATATTGACTCATGATCGCATGGTTGTGGGTTGAAAGTTTTTTCGGCTGGGGATGGGCCCGCGGCCTATCA
GCTTGTTGGTGGGGTGATGGCCTACCAAGGCGACGACGGGTAGCCGGCCTGAGAGGGCGACCGGCCACA
CTGGAACTGAGACACGGTC
>Zotu11750

GAACGCTGGCGGCATGCCTAACACATGCAAGTCGAACGAAGCCTTCGGGCTTAGTGGCGCACGGGTGCGT
AACACGTGGGAATCTGCCCTTTGCTTCGGAATAACAGTTAGAAATGACTGCTAATACCGGATGATGTCGTA
AGACCAAAGATTTATCGGCAAAGGATGAGCCCGCGTAGGATTAGCTTGTTGGTGGGGTAATGGCCTACCA
AGGCGACGATCCTTAGCTGGTCTGAGAGGATGATCAGCCACACTGGGACTGAGACACGGCC
>Zotu11753
GAACGCTGGCGGCGTGCCTAATACATGCAAGTCGAGCGGGGTTATTGCGGTGGATTCCTTCGGGATGAAA
GCGTAATAACCTAGCGGCGGACGGGTGAGTAACGCGTGGGTAATCTGCCCTAGAGACCGGGATAACAGC
GGGAAACCGCTGCTAATACCGGATGCCTTTCAAGGGGGGCATCTTCCTTGAAAGAAAGCTACGGCGCTCTA
GGATGAGCCCGCGTCCCATTAGCTAGTTGGTGAGGTAACGGCTCACCAAGGCGACGATGGGTAGCCGGCC
TGAGAGGGTGTACGGCCACACTGGGACTGAGACACGGCC
>Zotu11756
GAACGCTGGCGGCGTGCTTAACACATGCAAGTCGAACGGTAAGGCCTTCGGGTACACGAGTGGCGAACG
GGTGAGTAACACGTGGGTGATCTGCCCTGCACTTTGGGATAAGCCTGGGAAACTGGGTCTAATACCGAAT
ATGACCATGGCATGCATGTGCTGTGGTGGAAAGCTTTTGCGGTGTGGGATGGGCCCGCGGCCTATCAGCT
TGTTGGTGAGGTCAAGGCTCACCAAGGCGACGACGGGTAGCCGGCCTGAGAGGGTGTCCGGCCACACTG
GGACTGAGATACGGCC
>Zotu11759
GAACGCTGGCGGCAGGCTTAACACATGCAAGTCGAGCGGGCACTTCGGTGTCAGCGGCAGACGGGTGAG
TAACGCGTGGGAACGTGCCCTTCGGTTCGGAATAACTCAGGGAAACTTGAGCTAATACCGGATACGCCCTT
TTGGGGAAAGGTTTACTGCCGAAGGATCGGCCCGCGTCTGATTAGCTAGTTGGTGAGGTTACGGCTCACCA
AGGCGACGATCAGTAGCTGGTCTGAGAGGACGATCAGCCACACTGGGACTGAGACACGGCC
>Zotu11761
GAACGCTAGCGGCAAGCTTAATACATGCAAGTCGAGGGGCAGCACGGGAGCAATCCTGGTGGCGACCGG
CAAACGGGTGCGGAACACGTACGCAACCTTCCCATAACTGGTGAATAGCCTTTCGAAAGAGAGATTAATAC
ACCGTAACATAACGAAGAGGCATCTCTTTGTTATTATAGTAGCAATACGGTTTTGGATGGGCGTGCGCCTG
ATTAGGTAGTTGGCGGGGTAACGGCCCACCAAGCCTGCGATCAGTAACTGGTGTGAGAGCACGACCAGTC
ACACGGGCACTGAGACACGGGC
>Zotu11763
GAACGCTGGCGGCGTGCTTAACACATGCAAGTCGAGCGGAAAGGCCCCTTTGGGGGTACTCGAGCGGCG
AACGGGTAAGTATCACGTGAGTAATCTGCCCTTCACTTCGGGATAGCTTCGGGAAACCGAGATTAATACCG
GATAGGACCATTCCACGCATGTGGTGGTGGTGGAAAGATTTTTTGGTGGGGGATGTGCTCGCGGCCTATC
AGCTTGTTGGTGGGGTAATGGCCTACCAAGGCTTCGACGGGTAGCCGGCCTGAGAGGGTGACCGGCCACA
CTGGGACTGAGACACGGCC
>Zotu11767
GAACGCTGGCGGCGTGCTTAACACATGCAAGTCGAACGTGAAAGTCCTTCGGGGCAAGTAAAGTGGCGCA
CGGGTGAGTAATACGTAGGAACGTGCCTAAAGATGGGGAATAACCGCGGGAAACCTCGGCTAATACCGCA
TGATATCGCAAGATCAAAGAGGGCCTCTGCTTGCAAGCTCTCGTCTTTAGAGCGGCCTACGTCCTATTAGCT
AGTTGGTAGGGTAAAAGCCTACCAAGGCAATGATGGGTAACTGGTCTGAGAGGATGATCAGTCACACTGG
AACTGAGACACGGTC
>Zotu11772
GAACGCTAGCGGCAGGCCTAATACATGCAAGTCGTGGGGCAGCGGGTGTAGCAATACATGCCGGCGACC
GGCGCACGGGTGCGTAACGCGTATGTAACTTTCCTTTAACTGGAGGATAGCCCGAAGAAATTCGGATTAAT
ACTCCATAATATTGTCCCGCGGCATCGCGGGATTCTTAAAGCTCCGGCGGTTAAAGATGGACATGCGTCCC
ATTAGCTAGTTGGTGAGGTAAAGGCTCACCAAGGCAACGATGGGTAGGGGGTCTGAGAGGATGATCCCCC
ACACTGGTACTGAGACACGGAC
>Zotu11775
GAACGCTGGCGGCGTGCCTAATACATGCAAGTCGAGCGCCCCTTTAAGAGGTGATAATCGAAAGATTATC
GGTTCCTATGGGGAGCGGCGGACGGCTGAGTAACGCGTGGGAACGTGCCCCAAAGTGAGGGATAAGCAC
CGGAAACGGTGTCTAATACCGCATATGATCTTCGGATTAAAGCTTTATGCGCTTTGGGAGCGGCCTGCGTC
CTATTAGATAGTTGGTGAGGTAAAGGCTCACCGAGTCAACGATGGGTAGCTGGTCTGAGAGGATGACCAG
CCAGACTGGAACTGAGACACGGTC

>Zotu11777
GAACGCTGGCGGCGTGCTTAACACATGCAAGTCGAACGAAGCACTTTATTTGATTTCTTCGGAATGAAGAT
TTTGTGACTGAGTGGCGGACGGGTGAGTAACGCGTGGGTAACCTGCCTCATACAGGGGGATAACAGTTGG
AAACGACTGCTAATACCGCATAAGCGCACAGGGTCGCATGACCTGGTGTGAAAAACTCCGGTGGTATGAG
ATGGACCCGCGTCTGATTAGCCAGTTGGTGGGGTAACGGCCTACCAAAGCAACGATCAGTAGCCGACCTG
AGAGGGTGACCGGCCACATTGGGACTGAGACACGGCC
>Zotu11780
GAACGCTGGCGGTATGCTTAACACATGCAAGTCGAACGGCAGCATGGTGTGTAGCAATACACACTGATGG
CGAGTGGCGGACGGGTGAGTAACACGTAGGAATCTACCCTAAAGCGGGGGATAACCCGAGGAAACTCGG
GCTAATACCGCATATATTCTGAGGAAGAAAGCTGGGGTTAAACCTGGCACTTTAGGAGGAGCCTGCGTCTG
ATTAGCTAGTTGGTGGGGTAAAAGCCTACCAAGGCGATGATCAGTAGGTGGTCTGAGAGGATGATCACCC
ACACTGGGACTGAGACACGGCC
>Zotu11782
GAACGCTGGCGGTATGCTTAACACATGCAAGTCGAACGAAAGTTTTTAACTTTAGTGGCGAACGGGTGAGT
AATACGTGAGAATCTGCCCTTAGGAGAAGAATAACTGCTGGAAACGGTTGCTAAATCTTCATATGTCGAGA
GATGAAATAGCTTAAGCTGCCTAAGGATGAGCTCACGCCTGATTAGCTTGTTGGTGAGGTAATGGCTCACC
AAGGCCACGATCAGTAGCTGGTTTGAGAGAACGATCAGCCACACTGGGACTGAGACACGGCC
>Zotu11784
GAACGTTAGCGGCGCGCCTAACACATGCAAGTCGCGCGAGAAAGGGCTTCGGCCCCAGTAAAGCGGCGC
ACGGGTGAGTAACACGTAGGTGATCTTCCCCTGAGTGGTGGATAACTCTCCGAAAGGAGAGCTAATACAG
CATAAGACCACGATCTCGCAGAGGTTGTGGCCAAAGCAGCCCTCTTCACGAAAGGTTGCGCTTAGGGATG
AGCCTGCGGCCCATCAGCTAGTTGGTAGGGTAATGGCCTACCAAGGCTAAGACGGGTAGCTGGTCTGAGA
GGATGAACAGCCACACTGGAACTGAGACACGGTC
>Zotu11790
GAACGCTGGCGGCGTGCTTAACACATGCAAGTCGAACGGTGACGAGGGAGCTTGCTCCTTCTGATCAGTG
GCGAACGGGTGAGTAACACGTGAGTAACCTGCCCCAAACTCTGGGATAAGCACTGGAAACGGTGTCTAAT
ACCGGATACGAGCTTCAGCCGCATGGCTAGGAGTTGGAAAGAATTTCGGTTTGGGATGGACTCGCGGCCT
ATCAGCTAGTTGGTGAGGTAATGGCTCACCAAGGCGACGACGGGTAGCCGGCCTGAGAGGGTGACCGGC
CACACTGGGACTGAGACACGGCC
>Zotu11792
GAACGCTGGCGGCGTGCTTAACACATGCAAGTCGAGGGCACTTTTGGGGCAACCTGGGAGTGACCGGCGA
ACGGGTGCGTAACACGTGAGCGACCTTGCCTCGTGGTGGGGGATAGCCGGCCCAACGGCCGGGTAATACC
GCATAGGGTGTTTGCTTCGCATGGAGCGGACAGCAAAGCTGTCGGGCAACCGATGGCGCCACAGGGGGG
GCTCGCGGCCTATCAGCTAGTTGGGGGGGTAATGGCCCACCAAGGCGACGACGGGTAGCTGGTCTGAGA
GGATGGCCAGCCACATTGGGACTGAGAGACGGCC
>Zotu11799
GAACGCTGGCGGCAGGCTTAACACATGCAAGTCGAGCGCGCCCTTCGGGGTGAGCGGCAGACGGGTGAG
TAGCGCGTGGGAACATACCTTTCGGACTGGAATAACTGGGGGAAACTTCAGCTAAGACCGGATACGCCCTT
AGGGGGAATGATTTATCGCCGAAAGATTGGCCCGCGTCCGATTAGCTAGTTGGTGTTGGTAATGGCTCACC
AAGGCGACGATCGGTCGCTGGTCTGAGAGGATGACCGGCCACATTGGGACTGAGACACGGCC
>Zotu11807
GAACGCTAGCGGGAGGCCTAACACATGCAAGCCGAGCGGTAGAGATTCTTCGGAATCTTGAGAGCGGCGT
ACGGGTGCGGAACACGTGTGCAACCTGCCTTTATCAGGGGGATAGCCTTTCGAAAGGAAGATTAATACCCC
ATAATATATTTGATGGCATCATTAGATATTGAAAGCTTTGGCGGATAGAGATGGGCACGCGCAAGATTAGA
TAGTTGGTGAGGTAACGGCTCACCAAGTCGATGATCTTTAGGGGGCCTGAGAGGGTGATCCCCCACACTG
GTACTGAGACACGGAC
>Zotu11811
GAACGCTAGCGGCAGGCCTAATACATGCAAGTCGAACGGAGCAACAATGCTTAGTGGCGCACGGGTGCGT
AACGCGTATGAAACCTGCCCTTCACTGGGGGATAACCTCTCGAAAGAGAGGCTAATACCGCATAAGATAGT
AGAGTGGCATCACTCAGCTATTAAAACTCCGGTGGTGAAGGATGGTCATGCGTCCAATTAGTTAGTTGGTA

GGGTAACGGCCTACCAAGACGATGATTGGTAGGGGGTCTGAGAGGATGATCCCCCACACTGGTACTGAGA
TACGGAC
>Zotu11814
GAACGCTGGCGGCGTGCTTAACACATGCAAGTCGAACGATGAAGCCCAGCTTGCTGGATGGATTAGTGGC
GAACGGGTGAGTAACACGTGAGCAACCTGCCCCTGACTCTGGGATAAGCGCTGGAAACGGCGTCTAATAC
TGGATACGAGTAGCGATCGCATGGTCAGTTACTGGAAAGATTTTTTGGTTGGGGATGGGCTCGCGGCCTAT
CAGCTTGTTGGTGAGGTAATGGCTCACCAAGGCGTCGACGGGTAGCCGGCCTGAGAGGGTGACCGGCCAC
ACTGGGACTGAGACACGGCC
>Zotu11818
GAACGCTGGCGGCATGCTTTACACATGCAAGTCGAACGGCAGCGCGGGGCAACCTGGCGGCGAGTGGCG
AACGGGTGAGTAATATATCGGAACGTACCCAGAAGTGGGGGATAACGTAGCGAAAGTTACGCTAATACCG
CATACGATCTACGGATGAAAGTGGGGGATCTTCGGACCTCATGCTTTTGGAGCGGCCGATATCTGATTAGC
TAGTTGGTAGGGTAAAGGCCTACCAAGGCGACGATCAGTAGCTGGTCTGAGAGGACGACCAGCCACACTG
GAACTGAGACACGGTC
>Zotu11820
CAACGCTGGCGGCGTGCCTAACACATGCAAGTCGAGCGCGAAAGGGGAGCAATCCCCAAGTAGAGCGGC
GAACGGGTGAGTAACGCGTGGGTAATCTGCCTCCGAGTGGGGAACAACACCGGGAAACTGGTGCTAATAC
CGCATAACATCGTCGGGTTTTCGGATCTGACGATCAAAGCCGGGGACCGCAAGGCCTGGCGCTTGGAGAG
GAGCCCGCGTCCGATTAGCTAGTTGGTGGGGTAATGGCCCACCAAGGCGACGATCGGTAGCCGGCCTGAG
AGGGCGAACGGCCACACTGGGACTGAGACACGGCC
>Zotu11827
GAACGCTGGCGGCGTGCTTAACACATGCAAGTCGAACGATGAAGCCCTTCGGGGTGGATTAGTGGCGAAC
GGGTGAGTAACACGTGAGCAACCTGCCCCTGGTTCCGGGATAACTCCGGGAAACCGGTGCTAATACTGGA
TATGACATTCCTCCGCATGGGGGTTTGTGGAAAGTTTTTCGACCGGGGATGGGCTCGCGGCCTATCAGCTT
GTTGGTGGGGTGATGGCCTACCAAGGCGACGACGGGTAGCCGGCCTGAGAGGGCGACCGGCCACACTGG
GACTGAGACACGGCC
>Zotu11832
GAACGCTGGCGGCGTGCCTAATACATGCAAGTCGAGCGAATCTTTGGGAGCTTGCTCCCGAAGATTAGCG
GCGGACGGGTGAGTAACACGTGGGCAACCTGCCTATAAGACTGGGATAACTTCGGGAAACCGGAGCTAAT
ACCGGATAATTCTTTTCCTCTCATGAGGAAAAGCTGAAAGACGGTTTACGCTGTCACTTATAGATGGGCCCG
CGGCGCATTAGCTAGTTGGTGAGGTAACGGCTCACCAAGGCAACGATGCGTAGCCGACCTGAGAGGGTGA
TCGGCCACACTGGGACTGAGACACGGCC
>Zotu11838
GAACGCTGGCGGCAGGCCTAACACATGCAAGTCGAGCGAGACCTTCGGGTCTAGCGGCGGACGGGTGAG
TAACGCGTGGGAATATGCCCTTCTCTACGGAATAGTCCCGGGAAACTGGGTTTAATACCGTATACGCCCTTT
GGGGGAAAGATTTATCGGAGAAGGATTAGCCCGCGTTGGATTAGGTAGTTGGTGGGGTAATGGCCTACCA
AGCCTACGATCCATAGCTGGTTTGAGAGGATGATCAGCCACACTGGGACTGAGACACGGCC
>Zotu11840
GAACGCTAGCGGCGGGCTTAATACATGCAAGTCGAGGGGGTGTAGCAATACATCACCGGCGCACGGGTG
CGTAACACGTATGCAACCTACCCCTTATTAAAGGATATACTCGGGAAACTGGGGGCAATACTTTATATGCAC
ACATCTAGGCATCTAGGAGTGTGGAAACGTAAGGATGAGGGATGGGCATGCGTCTGATTAGCTAGTTGGT
AAGGTAACGGCTTACCAAGGCGACGATCAGTAGCTGGTCTGAGAGGATGATCAGCCACACTGGCACTGAG
ACACGGGC
>Zotu11843
GAACGCTGGCGGCGTGCCTAATACATGCAAGTCGAACGAGGTGCTTGAGTAGCAATACAAGAGTATCCTA
GTGGCGAACGGGTGAGTAACGCGTTGGTGACCTACCCCAAAGTGTGGGATAACAGTTCGAAAGGATTGCT
AATACCGCATGTGGTTATCGGGTTTAGAAACTGATAACTAAAGGAGCAATTCGCTTTGGGAGGGACCTGCG
TCCCATCAGCTAGTTGGTGAGGTAACGGCTCACCAAGGCTACGACGGGTAGGGGACCTGAGAGGGTGGCC
CCCCACAATGGAACTGAAACACGGTC
>Zotu11846

GAACGCTGGCGGCATGCCTTACACATGCAAGTCGAACGGCAGCACGGGGGCAACCTTGGTGGCGAGTGG
CGAACGGGTGAGTAATATATCGGAACGTGCCCTGGAGTGGGGGATAACTAGTCGAAAGACTAGCTAATAC
CGCATACGATCTACGGATGAAAGTGGGGGATCGCAAGACCTCATGCTCGTGGAGCGGCCGATATCTGATT
AGCTAGTTGGTGGGGTAAAAGCCTACCAAGGCATCGATCAGTAGCTGGTCTGAGAGGACGACCAGCCACA
CTGGGACTGAGACACGGCC

>Zotu11849
GAACGCTGGCGGCGTGCCTAATACATGCAAGTCGAACGGACATAGCAATATGTTAGTGGCGACCGGGTGA
GTAACACGTAGCAGACCTGCCCTCTTGCGGGGGACAACAGTTCGAAAGGACTGCTAATACCGCATACATTT
GCGACGTTTAGAAGGTTGCAAAGAAACTTCAGGGGCGAGAGGGGGGTGCTGCGGCCCATCAGCTAGTTG
GTAGGGTAACGGCCTACCAAGGCGACGACGGGTAGGGGGCCTGAGAGGGTGACCCCCCACACTGGAACA
GACAGACGGTC

>Zotu11851
GAACGCTGGCGGCATGCCTAACACATGCAAGTCGAACGGCAGCACAGTAAAGATTTCGGTCTTTATGGGT
GGCGAGTGGCGGACGGGTGAGTAATGCGTAGGAATCTGTCCTGGAGAGTGGGATAACCCGAGGAAACTC
GGGCTAATACCGCATGATATCGAGAGATCAAAAGCAGGGGACCGAAAGGCCTTGCGCTCTGGGTTGAGCC
TACGTCGGATTAGCTAGTTGGTGGGGTAAAGGCCTACCAAGGCAACGATCCGTAGCTGGTCTGAGAGGAT
GACCAGCCACACTGGGACTGAGACACGGCC

>Zotu11853
GAACGCTGGCGGCGTGCCTAACACATGCAAGTCGAACGGGACTTATCGAGACGATTTCGGTCTGATCTTTA
AGTTTAGTGGCGGACGGGTGAGTAACGCGTGGATAACCTGCCTTTCACAGGGGGATAGCAGCTGGAAACG
GCTGGTAATACCGCATACGCTCATGTCACCGCATGGTGATGTGAGGAAAGATTTATCGGTGAAAGATGGAT
CCGCGTCTGATTAGCCAGTTGGCGGGGTAACGGCCCACCAAAGCGACGATCAGTAGCCGGCCTGAGAGGG
CGGACGGCCACATTGGGACTGAGACACGGCC

>Zotu11857
GAACGCTGGCGGCGTGCTTAACACATGCAAGTCGAACGAGGTCCACGGTGTAGCAATACACCTGAAGACC
TAGTGGCGAACGGGTGTGTAACACGTGAGGAACTTGTCCCGGTCTCTGGGATAACAGTTGGAAACGACTG
CTAATACCGGATGACGTCGGAGCATCGCATGGTGCGCTGACGAAAGGGTTACTGGATCGGGAGAGCCTCG
CGGCCTATCAGCTTGTTGGTGGGGTAACGGCCCACCAAGGCATCGACGGGTAGCTGGTCTGAGAGGATGA
TCAGCCACACTGGGACTGAGACACGGCC

>Zotu11861
GAACGCTAGCGGTATGCTTAACACATGCAAGTCGAACGAAAAAGTAATTTTTAGTGGCGAACGGGTGAGT
AACGCGTGAGAATTCGCCTCTAAGAGAAGGATAACGTTTAGAAATAAATGCTAAAACTTTATAATCAAAAA
AGTAATTTGCTTAGAGAGAAGCTCGCGTCCGATTAGCTAGTTGGTAGGGTAAAGGCCTACCAAGGCGATG
ATCAGTAACTGGTCTGAGAGGATGATCAGTCACATTGGAACTGAGAAACGGTC

>Zotu11864
GAACGCTGGCGGCAGGCTTAACACATGCAAGTCGAACGGTAGCAGGAAGAAAGCTTGCTTTCTTTGCTGA
CGAGTGGCGGACGGGTGAGTAATGCTTGGGAATCTGGCTTATGGAGGGGGATAACTACGGGAAACTGTA
GCTAATACCGCGTAATATCTACGGATTAAAGAATGGGACATTAGCCATTCGCCATAAGATGAGCCCAAGTG
GGATTAGGTAGTTGGTGGGGTAAAGGCCTACCAAGCCGACGATCTCTAGCTGGTCTGAGAGGATGACCAG
CCACACTGGAACTGAGACACGGTC

>Zotu11866
GAACGCTGGTAACGTGGATACCGGCATGCAAGTCACGGGGGCCGCAAGGCAACCGGCGAACGGCTTAGT
AACACGTAGGTACATACCCTAAAGCGGAGTACAGCTCGTCGAAAGACGGGGTAATTCTCCATAGTCTCGCA
AGAGTAAACCCGCAAGGGGCTTTAGGAATGGCCTGCGGCCTATCAGATAGTTGGCGGGGTAATGGCCCAC
CAAGTCTATGACGGGTAGGGGGCGTGAGAGCGCGACCCCCAAGAGTTGGACTGCGACACGGCC

>Zotu11872
GCCGTGTCTCAGTCCCAGTGTGGCTGGTCGTCCTCTCAAACCAGCTACGGATCGTCGCCTTGGTGAGCCGTT
ACCCCACCAACTAGCTAATCCGATATCGGCCGCTCTAATAGTGCAAGGTCTTGCGATCCCCTGCTTTCCCCC
GTAGGGCGTATGCGGTATTAGCTACGCTTTCGCGTAGTTATCCCCCGCTACTAGGCACGTTCCGATACATTA
CTCACCCGTTCGCCACTCGCCACCAGACCGAAGTCCGTGCTGCCGTTCGACTTGCATGTGTAAGGCATCCCG
CTAGCGTTCA

>Zotu11877
GAACGCTGGCGGCAGGCCTAATACATGCAAGTCGAGCGCCCCTTCGGGGGAGCGGCGGACGGGTGAGTA
ACGCGTGGGAACGTGCCCTTTGGTTCGGAACAACACAGGGAAACTTGTGCTAATACCGAATGTGCCCTTCG
GGGGAAAGATTTATCGCCATTGGAGCGGCCCGCGTTGGATTAGGTAGTTGGTGGGGTAAAGGCCTACCAA
GCCTACGATCCATAGCTGGTCTGAGAGGATGATCAGCCACACTGGGACTGAGACACGGCC
>Zotu11882
GAACGCTGGCGGTGTGCTTAACACATGCAAGTCGAACGGGGCGAATGAAAGCTTGCTTTTATGAGCTTAGT
GGCAAACGGGTGAGTAACACGTAGGCAACCTGCCGACAGGATGGGGACAACATTCCGAAAGGAATGCTA
ATACCGAATGAAGTCGAGGGAAGGCATCTTCCTTCGATGAAAGATGGCCTCTATTTATAAGCTATCACCTGT
CGATGGGCCTGCGTCTGATTAGCTCGTTGGTGAGGTAACGGCTCACCAAGGCGACGATCAGTAGCCGGTC
TGAGAGGATGAACGGCCACATTGGGACTGAGACACGGCC
>Zotu11885
GAACGCTGGCGGCGTGCTTAACACATGCAAGTGGAGCGACGAACCAGGGCTTGCCCTGGGGCAGAGCCG
CGAACGGGTGAGTAACACGTGGGTAACCCACCCCGATGACCGGGACAACCCGAGGAAACTCGGGCTAATA
CCGGATGTTCTCGTTTTTCGTAAGGAAATCGAGCAAAGATAGCTTCGGCCTTCGCATCGGGACGGGCCCGC
GGCCCATTAGCTTGTTGGTGGGGTAACGGCCTACCAAGGCGACGATGGGTAGCTGGTCTGAGAGGACGAT
CAGCCACACTGGGACTGAGACACGGCC
>Zotu11889
GAACGCTGGTAACGTGGATACCGGCATGCAAGTCACGGGGGCCGCAAGGCAACCGGCGAACGGCTTAGT
AACACACAGGTACATACCCTAAAGTGGAGCACAGCTCGTCGAAAGACGGGGTAATTCTCCATAGTCTCGCA
AGAGTAAAGGCGCAAGCCGCTTTAGGAATGGCCTATGGCCTATCAGATAGTTGGCGAGGTAAAAGCTCAC
CAAGTCTATGACGGGTAGGGGGCGTGAGAGCGCGACCCCCAAGAATGGCACTGCGACACGGGC
>Zotu11892
GAACGCTAGCGGCATGCCTAACACATGCAAGTCGAGCGCCCCGCAAGGGGAGCGGCAGACGGGAGAGTA
ACACGTGGGAACGTACCCTTCAGTTCGGAACAACCCAGGGAAACTTGGGCTAATACCGAATACGTCCGTAA
GGAGAAAGATTTATCGCTGAAGGATCGGCCCGCGTCTGATTAGCTAGTTGGTGAGGTAACGGCTCACCAA
GGCGACGATCAGTAGCTGGTCTGAGAGGATGATCAGCCTCACTGGGACTGAGACACGGCC
>Zotu11898
GAACGCTGGCGGCGTGCCTAATACATGCAAGTCGAGCGAATCTTTAGAAGCTTGCTTCTAAAGATTAGCGG
CGGACGGGTGAGTAACACGTGGGCAACCTGCCTGTAAGACTGGGATAACTTCGGGAAACCGAGGCTAATA
CCGGATAATCCTTTTCCTCACATGAGGAAAAGCTGAAAGACGGCGCAAGCTGTCACTTATAGATGGGCCCG
CGGCGCATTAGCTAGTTGGTGAGGTAACGGCTCACCAAGGCAACGATGCGTAGCCGACCTGAGAGGGTGA
TCGGCCACACTGGGACTGAGACACGGCC
>Zotu11900
GAACGCTAGCGGGATGCCTTACACATGCAAGTCGAACGGTGAACATGGAGCTTGCTCTGTGGGATCAGTG
GCGAACGGGTGAGTAACACGTGAGCAACCTGCCCCTGACTCTGGGATAAGCGCTGGAAACGGCGTCTAAT
ACTGGATATGTGACGTGACCGCATGGTCTGCGTCTGGAAAGAATTTCGGTTGGGGATGGGCTCGCGGCCT
ATCAGCTTGTTGGTGAGGTAATGGCTCACCAAGGCGTCGACGGGTAGCCGGCCTGAGAGGGTGACCGGCC
ACACTGGGACTGAGACACGGCC
>Zotu11908
GAACGTTAGCGGCACGCCTAACACATGCAAGTCGAGCGCGAAAGGGAGCAATCCTGAGTAAAGCGGCGC
ACGGGTGCGTAACACGTAGATAATCTGCCCTTCGATGGGGGATAACGTTCCGAAAGGAGCGCTAATACCG
CATACAGCTTTCTGGGGTTCGCCCTGGGAAGGCAAAGGCTGGGACCCGCAAGGGCCAGTCGTCGAGGGA
GGAGTCTGCGGCCCATCAGCTAGTTGGTGAGGTAATGGCTCACCAAGGCAAAGACGGGTAGCTGGTCTGA
GAGGATGATCAGCCACACTGGTACTGGAACACGGAC
>Zotu11912
GAACGCTGGCGGCATGCTTAACACATGCAAGTCGCACGGGCAGCAATGTCAGTGGCGGACGGGTGAGTAT
CGCGTAGGAACATGTCCAGAGGTGGGGGACAACCCCGGGAAACTGGGGCTAATACCGCATATCGGCTGA
GGCCGAAAGCAGTGATGCGCCTTTGGAGTGGCCTGCGTCCGATTAGGTAGTTGGTGGGGTAAAGGCCTAC
CAAGCCTGCGATCGGTAGCTGGTCTGAGAGGACGACCAGCCACACTGGGACTGAGACACGGCC

>Zotu11916
GAACGCTGGCGGCGTGCTTAACACATGCAAGTGGAGCGACGAACCAGGGCTTGCCCTGGGGCAGAGCCG
CGAACGGGTGAGTAACACGTGGGTAACCTACCCCGGTGACCGGGACAACCCGAGGAAACTCGGGCTAATA
CCGGATGTTCTCGTTTCTCGTAAGGGAACCGAGGAAAGATAGCTTTTAGCCTTCGCATCGGGACGGGCCCG
CGGCCCATTAGCTTGTTGGTGGGGTAACGGCCTACCAAGGCGACGATGGGTAGCTGGTCTGAGAGGACGA
TCAGCCACACTGGGACTGAGACACGGCC
>Zotu11920
GAACGCTGGCGGCATGCTTTACACATGCAAGTCGAACGGCAGCACGGGCTTCGGCCTGGTGGCGAGTGGC
GAACGGGTGAGTAATATATCGGAACGTACCCAGAAGTGGGGGATAACGTAGCGAAAGTTACGCTAATACC
GCATACGATCTACGGATGAAAGTAGGGGACCTTCGGGCCTTATGCTTTTGGAGCGGCCGATATCTGATTAG
CTAGTTGGTGAGGTAAAGGCTCACCAAGGCGACGATCAGTAGCTGGTCTGAGAGGACGACCAGCCACACT
GGAACTGAGACACGGTC
>Zotu11922
GAACGTTTGCGGCGGGCCTAACACATGCAAGTCGAACGCACTAGCAATAGTGAGTGGCGCACGGGGGCG
TAACACGTGGGTAATCAACCCTTCAGCTTGGGATAACGACTTGAAAGGGTCGCTAATACCGGATACGGCGC
GGGAGGCTTCGGCTTCTCACGAGAAAGCCTCGCAAGGGGCACTGAAGGACGAGCCTGCGGCCCATCAGCT
AGTTGGTGAGGTAAGAGCTCACCAAGGCGAAGACGGGTAGCTGGTCTGAGAGGATGATCAGCCACACTG
GAACTGAGACACGGTC
>Zotu11926
GAACGCTGACGGCAGGCTTAACACATGCAAGTCGAACGATGACTAACTAGCTTGCTAGATATGATTAGTGG
CGGACGGGTGAGTAACATTTAGGAATCTGCCTAGTAGTGGGGGATAGCTCGGGGAAACTCGAATTAATAC
CGCATACGACCTACGGGTGAAAGGGGGCGCAAGCTCTTGCTATTAGATGAGCCTAAATCAGATTAGCTAGT
TGGTGGGGTAAAGGCCCACCAAGGCGACGATCTGTAACTGGTCTGAGAGGATGATCAGTCACACCGGAAC
TGAGACACGGTC
>Zotu11929
GAACGTTAGCGGCGCGCCTAACACATGCAAGTCGTGCGAGAACGGGCTTCGGCCTTAGTAGAGCGGCGCA
CGGGTGAGTAACACGTAGGCAACCTTCCCCCGAGTGGTGGATAACTCTCCGAAAGGAGGGCTAATACAGC
ATGAGACCACGGTTCTTTCGGGAACTGCGGTCAAAGTGGGCCACTTCATGAAAGCTCACGCTTGGGGATG
GGCCTGCGGCCCATCAGCTAGTTGGCGAGGTAATGGCTCACCAAGGCTAAGACGGGTAGCTGGTCTGAGA
GGATGGACAGCCACACTGGAACTGACACACGGTC
>Zotu11932
GAACGCTGGCGGCATGCTTTACACATGCAAGTCGAACGGCAGCGCGGGGCAACCTGGCGGCGAGTGGCG
AACGGGTGAGTAATATATCGGAACGTACCCAAGAGTGGGGGATAACGTAGCGAAAGTTACGCTAATACCG
CATACGATCTAAGGATGAAAGTGGGGGATCGCAAGACCTCATGCTCCTGGAGCGGCCGATATCTGATTAG
CTAGTTGGTAGGGTAATGGCCTACCAAGGCGACGATCAGTAGCTGGTCTGAGAGGACGACCAGCCACACT
GGGACTGAGACACGGCC
>Zotu11935
GAACGCTGGCGGCATGCCTTACACATGCAAGTCGAACGGTAGAGTAGCAATACTCGAGAGTGGCGAACGG
GTGAGTAATATATCGGAACGTGCCCAACTGTGGGGGATAACGTAGAGAAATTTACGCTAATACCGCATAC
GATCTAAGGATGAAAGCGGGGGATCGCAAGACCTCGCGCAGTTGGAGCGGCTGATATCAGATTAGCTTGT
TGGTGAGGTAAAAGCTCACCAAGGCGACGATCTGTAGCTGGTTTGAGAGAACGACCAGCCACACTGGGAC
TGAGACACGGCC
>Zotu11938
GAACGCTGGCGGCAGGCTTAACACATGCAAGTCGAACGCATCCTTCGGGGTGAGTGGCAGACGGGTGAG
TAACGCGTGGGAACATGCCCTTCAGTTTGGAATAACCCAGGGAAACTTGGGCTAATACCGGATACGCCCTT
AGGGGGAAAGATTTATCGCTGAAGGAGTGGCCCGCGTCTGATTAGCTAGTTGGTGAGGTAACGGCTCACC
AAGGCGACGATCAGTAGCTGGTCTGAGAGGATGATCAGCCACACTGGGACTGAGACACGGCC
>Zotu11941
GAATGCTAGCGGCGTGCCTTAGGCATGCAAGTAGAACGGGATTGTCGCAAGACGATTTAGTTGCGAACGG
CTGAGTAACACGTAGGAATCTACCTCTTTGTGGGGAATACCTCGTCGAAAGACGGGCTAATACCGCATAAG

ATCGCAAGATGAAAGGGCTTAACCGCCCGCTGAGAGAGGAGCCTGCGGCCCATCAGCTAGTTGGTAGGGT
AATGGCCTACCAAGGCTACGACGGGTAGGGGGTGTGAGAGCACGTCCCCCCAGAATGGGACTGAGACAC
GGCC
>Zotu11946
GAACGCTGGCGGCATGCCTTACACATGCAAGTCGAACGGTAACAGGTTAAGCTGACGAGTGGCGAACGG
GTGAGTAATATATCGGAACGTGCCCAGTAGTGGGGGATAGCCCGGCGAAAGCCGGATTAATACCGCATAC
GACCTAAGGGTGAAAGCGGGGGATCGCAAGACCTCGCGCTATTGGAGCGGCCGATATCAGATTAGGTAGT
TGGTGGGGTAAAAGCCTACCAAGCCGACGATCTGTAGCTGGTCTGAGAGGACGACCAGCCACACTGGGAC
TGAGACACGGCC
>Zotu11950
GAACGCTGGCGGCAGGCCTAACACATGCAAGTCGAACGCATCCTTCGGGGTGAGTGGCAGACGGGTGAG
TAACACGTGGGAACGTGCCCATCGGTTCGGGATAACCCAGGGAAACTTGGGCTAATACCGGATACGTGCG
AGAGCAGAAAGATTTATCGCCGATGGATCGGCCCGCGTCTGATTAGCTAGTTGGTGAGGTAATGGCTCACC
AAGGCGACGATCAGTAGCTGGTCTGAGAGGATGATCAGCCACACTGGGACTGAGACACGGCC
>Zotu11957
GAACGCTAGCGGGAGGCCTAATACATGCAAGTCGAACGGTAACAGGGTAGCAATACCGCTGACGAGTGG
CGCACGGGTGAGTAACACGTACACAATCTGCCTTATACTGGAGCATACCCCCAAGAAATTGGGACTAATTC
TCCATAACATATTGAAAAGGCATCTTTTTAATATTAAAACTCAGGTGGTATAAGATGAGTGTGCGTCTGATT
AGCTAGTTGGTAGGGTAACGGCCTACCAAGGCAACGATCAGTAACTGGTCTGAGAGGATGATCAGTCACA
CGGACACTGAGACACGGGT
>Zotu11959
GAACGCTGGCGGCGTGCTTAACACATGCAAGTCGAACGGTAAGGCTCTTTCGGGAGTACACGAGTGGCGA
ACGGGTGAGTAACACGTGAGCAACCTGCCCTTGACTTCGGAATAACAGTTGGAAACAGCTGCTAATACCG
GATATGACTTTCTCGGTCATCCGAGTGAGTGGAAAGTTTTGGCGGTCAAGGATGGGCTCGCGGCCTATCAG
CTTGTTGGTGAGGTAATGGCTCACCAAGGCTTCGACGGGTAGCCGGCCTGAGAGGGCGACCGGCCACACT
GGGACTGAGATACGGCC
>Zotu11962
GAACGCTGGCGGCACGTTTTAAGCATGCAAGTCGAACGGCAGTGGGGGCTTGCTCCCATGAGAGTGGCGA
ACGGGTGAGTAACACGTAGGTGATCTGCCTTGAGGATGGGGATAGCTTGTGGAAACACAGGGTAATACCG
AATAAGCTGGCGGGGCTTTGGCTCTGTTAGTAAAGGTGCTACGGCACCGCCTTGAGAGGAGCCTGCGGCT
GATTAGCTAGTTGGTGGGGTAAAGGCCTACCAAGGCGACGATCAGTAGCCGGCCTGAGAGGGTGTACGG
CCACACTGGAACTGAGACACGGTC
>Zotu11965
CAACGCTGGCGGCGCGCTTAACACATGCAAGTCGAACGAGAAAGGGGAGCAATCCCTGAGTAAAGTGGC
GTACTGGTGAGTAACACGTGGATATCTACCTCTTGGTGGGGGATAACCTGGGGAAACCTGGGCTAATACC
GCATAAGTTCGTGAGAAGAAAGGCGCAAGTCGCCGAGAGAGGAGTCCGCGGTCGATTAGGTAGTTGGCG
GGGTAAGAGCCCACCAAGCCAAAGATCGATAGCCGGCCTGAGAGGGCACACGGCCACACTGGCACTGAA
ACACGGGC
>Zotu11968
GAACGCTAGCGGCATGCCTAACACATGCAAGTCGAACGCGAAAGTCCTTCGGGATGAGTAGAGTGGCAAA
CGGGTGAGTAACGCGTGGGAACATACCTTCAAGTACGGGATAGCTCCGGGAAACTGGAATTAATACCGTA
TATTCCCTATGGGGGAAAGATTTATCGCTTGAAGATTGGCCCGCGTCAGATTAGCTAGTTGGTGGGGTAAC
AGCCTACCAAGGCAACGATCTGTAGCTGGTCTGAGAGGACGATCAGCCACATTGGGACTGAGACACGGCC
>Zotu11974
GAACGCTGGCGGCGTGCCTAAAACATGCAAGTCGAGCGAGATCCATCCGGTGCTTGCACCGGTGAAGATC
TAGCGGCGAACGGGTGAGTAACACGTGAGCAACCTGCCCCGATGTCTGGGATAACTCGAGGAAACTCGGG
CTAATACCGGATGTCGTCATCGGATCGCATGGTCTGATGACGAAAGATTTATCGCTTCGGGAGGGGCTCGC
GTCCTATCAGCTTGTTGGTGAGGTAACGGCTCACCAAGGCTTCGACGGGTAGCTGGTCTGAGAGGACGAT
CAGCCACACTGGGACTGAGACACGGCC
>Zotu11976

GAACGCTAGCGGCAGGCTTAACACATGCAAGTCGAGGGGTAGAAGGAGCTTGCTCCTTTGAGACCGGCGC
ACGGGTGCGTAACGCGTATGCAATCTACCTTTTACAGAGGGATAGCCCAGAGAAATTTGGATTAATACCTC
ATAGTATTATGAAATGGCATCATTTTATAATTAAAGTTACAACGGTAAAAGATGAGCATGCGTCCTATTAGC
TAGTTGGTGTGGTAACGGCATACCAAGGCTACGATAGGTAGGGGTCCTGAGAGGGAGATCCCCCACACTG
GTACTGAGACACGGAC

>Zotu11979
GAACGCTGGCGGCGTGCCTAACACATGCAAGTCGAGCGGAGGCATCTGAGGAGTAATCTGAGGATGATTT
AGCGGCGGACGGGTGAGTAACGCGTGAGCAACCTGCCTTTCACAGGGGGATAACGCACCGAAAGGTGCA
CTAATACCGCATGAGACCACGGTACCGAGTGGTACAGGGGTCAAAGGAGCAATCCGGTGAGAGATGGGC
TCGCGTCCGATTAGATAGTTGGTGGGGTAAAGGCCTACCAAGTCAACGATCGGTAGCCGACCTGAGAGGG
TGATCGGCCACACTGGAACTGAGATACGGTC

>Zotu11981
GAACGCTAGCGGCATGCCTAACACATGCAAGTCGAACGTGAGAGGGTACTTGTATCCTTGAAAGTGGCGG
ACGGGTGAGTAAGACGTAGGTGACCTGCCTCGGAGTGGGGGATAACCACTGGAAACGGTGGCTAATACC
GCATACGTTGGTGAAGATGGGAATCTTTATCAGGAAAGCTTAAGTGCTTTGAGAGGGGCCTACGGCCGAT
TAGCTAGTTGGTGAGGTAAAGGCTCACCAAGGCGATGATCGGTAGCTGGTCTGAGAGGACGATCAGCCAC
ACGGGAACTGAGACACGGTC

>Zotu11983
GAACGCTGGCGGCAGGCTTAACACATGCAAGTCGAGCGGAGTGATGGTGCTTGCACCATCACTTAGCGGC
GAACGGGTGAGTAATGCCTAGGAATCTGCCCATTAGTGGGGGACAACGTGTGGAAACGCACGCTAATACC
GCATACGCCCTACGGGGGAAAGCAGGGGACTCTTTGAGCCTTGCGCTAATGGATGAGCCTAGGTCAGATT
AGCTAGTTGGTGGGGTAAAGGCCTACCAAGGCGACGATCTGTAGCGGGTCTGAGAGGATGATCCGCCACA
CTGGAACTGAGACACGGTC

>Zotu11985
GAACGCTGGCGGCAGGCTTAACACATGCAAGTCGAACGCCCCGCAAGGGGAGTGGCAGACGGGTGAGTA
ACGCGTGGGAATCTACCGTACCCTACGGAACAACAGTTGGAAACGACTGCTAATACCGTATACGCCCTACG
GGGGAAAGATTTATCGGGGTATGATGAGCCCGCGTTGGATTAGCTAGTTGGTGGGGTAAAGGCCTACCAA
GGCGACGATCCATAGCTGGTCTGAGAGGATGATCAGCCACATTGGGACTGAGACACGGCC

>Zotu11987
GAACGCTGGTAGCGTGGATTAGGCATGCAAGTCGAACGGGATCGTCCGAGTAGCAATACGAGGGCGTGA
GAGTGGCGGATTGGGGAGGAACACGTGAGCAACCTGTCCTTGAGTTGGGGAAAACCGTTGGAAACGACG
GCTAATACCGGATGTGGCGCCCGGTGACATCGCTGGAGCGCTAAAGGGGGCCGCAAGGCTCTCGCTCGTG
GAGGGGCTCGCGCATCATCAGCTTGTTGGCGGGGTAACGGCCCACCAAGGCTTACGATTAGCTGGTCTGA
GAGGATGACCAGCCCCACTGGGACTGAGATACTGCC

>Zotu11989
GAACGCTGGCGGCGTGCTTAACACATGCAAGTCGAGCGGAAAGGCCCTTCGGGGTACTCGAGCGGCGAA
CGGGTGAGTAACACGTGAGTAACCTGCCCCAGACTTTGGGATAACCCTCGGAAACGGGGGCTAATACCGA
ATATTACATTTCACCGCATGGTGGTTTGTGGAAAGTTTTTCGGTTTGGGATGGACTCGCGGCCTATCAGCTT
GTTGGTGGGGTAATGGCCTACCAAGGCGAGGACGGGTAGCCGGCCTGAGAGGGCGACCGGCCACACTGG
GACTGAGACACGGCC

>Zotu11994
GAACGCTAGCAGAACGCCTAACACATGCAAGTCGAACGGGTAGCAATACTAGTGGCAAACGGGCGAGTAA
CGCGTATCTAACCTGCCCTTGAGTGAGGCACAACTACAGAGATGTAGCTAATTCCTCATACGTCCCACAGAA
TGAAAATATGTGGGAGAAAGATTTATCGCTCAAGGAGGGGGGTGCGTCCTATCAGGTAGTTGGTGAGGTA
ACGGCTCACCAAGCCTATGACGGGTAGCCGGTCTGAGAGGATGTCCGGCCGCAATGGAACTGAGAACGGT
C

>Zotu11997
GAACGCTAGCGGCAGGCCTAATACATGCAAGTCGAACGGGCGGTAGCAATACCGCTAGTGGCGCACGGG
TGCGTAACGCGTAACCAACCTGCCCAGATCTGGGGGATAGCCCGCCGAAAGGCGGATTAATACCGCATAA
CCCAACAAGCTGGCATCAGCTAATTGGTAAAGATTTATCGGATCTGGATGGGGTTGCGTAGCATTAGCTAG

TTGGCGGGGTAATGGCCCACCAAGGCGACGATGCTTAGGGGACCTGAGAGGGTGATCCCCCACACTGGCA
CTGAGATACGGGC
>Zotu12003
GAACGCTGGCGGCGTGCCTAACACATGCAAGTGGAGCGACGAACCCGGACTTGTCCGGGGGCAAAGCCG
CGAACGGGTGAGTAACACGTGGATTATCTGCCCCAATGACCGGGACAACTCGAGGAAACTCGAGCTAATA
CCGGATGTGCTCTTCGGAGGAAAGGAAGCTTTGGCCTCCGCATTGGGATGAGTCCGCGGCGCATTAGCTA
GTTGGTGGGGTAAAGGCCTACCAAGGCGACGATGCGTAGCTGGTCTGAGAGGACGATCAGCCACACTGG
GACTGAGACACGGCC
>Zotu12008
GAACGCTAGCGGCAGGCTTAATACATGCAAGTCGAGGGGCAGCAGGGTGTAGCAATACATCGCTGGCGA
CCGGCAAACGGGTGCGGAACACGTACGCAACCTTCCTTTAAGTGGGGAATAGCCCAGGGAAACTTGGATT
AATACCTCGTAACATTATGAGATGGCATCATTTTATAATTATAGCTCCGGCGCTTAAAGATGGGCGTGCGAC
TGATTAGGTAGTTGGCAGGGTAACGGCCTACCAAGCCTGCGATCAGTAACTGGTGTGAGAGCACGACCAG
TCACACGGGCACTGAGACACGGGC
>Zotu12012
TAACGCTGGCGGTGTGCCTAATGCATGCAAGTCGAACGAATTCTTTTTAGTATTAAGTGTTAAGCAGAAGG
TAGTAAGTATTGATCGCCGCAAGGCGCGTTATACCTCATACTTTATACCTTATACCGCGAAGAGTTAGTGGC
GAACGGGTGAGTAACACGTTGGTACCTGCCCTTTAGTGGGGAATAACTCCGGGAAACCGGGGCTAATACC
GCATAACAATCGCAAGATGAAAGCGAGCAATCGCGCTAAAGGAGGGGCCTGCGTCTGATTAGCTAGTTGG
TAGGGTAATGGCCTACCAAGGCGACGATCAGTAGCTGGTTCGAGAGGATGAACAGCCACACTGGGACTGA
GATACGGCC
>Zotu12014
GAACGCTGGCGGCATGCCTAACACATGCAAGTCGAACGGCAGCACAGTAAAGATTTCGGTCTTTACGGGT
GGCGAGTGGCGGATGGGTGAGTAAAGCGTAGGAATCTGTCCTGGAGAGTGGAATAACCCGAGGAAACTC
GGGCTAATACCGCATGATATCGAGAGATCAAAGCAGGGGACCCTTCGGGGCCTTGCGCTCTAGGATGAGC
CTACGTCGGATTAGCTAGTTGGTGGGGTAAAGGCCTACCAAGCCGACGATCTGTAGCTGGTCTGAGAGGA
TGATCAGCCACACTGGGACTGAGACACGGCC
>Zotu12016
GAACGCTGGCGGCATGCCTAACACATGCAAGTCGAACGCTCGTAGCAATACGAGAGTGGCGGACGGGTG
AGAAACGCGTGGGTACCTGCCCTGTAGTACGGAATAACCGCGGGAAACTGCGGCTAATACCGTATACGCC
CCATGGGGGAAAGATTTATCGCTATAGGAGGGGCCCGCGTCGGATTAGCTAGTTGGTGAGGTAACGGCCC
ACCAAGGCTATGATCCGTAGCTGGTCTGAGAGGACGACCAGCCACACTGGGACTGAGACACGGCC
>Zotu12021
GAACGCTGGCGGCGTGCCTAATACATGCAAGTCGAACGAGGTGCTTTTGTAGCAATACGAGAGTATCCTA
GTGGCGAACGGGTGAGTAACGCGTTGGTGACCTACCCCAAAGTGTGGGATAACAGTCCGAAAGGATTGCT
AATACCGCATGTGGTTGTCGGTTTTAGAAACCGATTTCTAAAGGAGCAATTCGCTTTGGGAGGGACCTGCG
TCCCATCAGCTAGTTGGTGAGGTAATGGCTCACCAAGGCTACGACGGGTAGGGGACCTGAGAGGGTGGCC
CCCCACAATGGAACTGAAACACGGTC
>Zotu12023
GAACGCTGGCGGCGTGCCTAAGACATGCAAGTCGAATGCTTCGCAAGGAGCATGGCGAACGGGTGAGTA
ATACCTAGGAACTTTCTTTAAGTGGAGCATAACCAGTCGAAAGATTGGATAATACTTCATGTGACCGCAAG
GTTAAAGTCGCAAGACGCTTAAAGTGAGGCCTGGGCACCATCAGCTAGTTGGTAAGGTAATGGCTTACCA
AGGCTATGACGGTTAGGGGTCCCGAGAGGGATAACCCCCACACTGGAACTGCGACACGGTC
>Zotu12028
GAACGCTGGCGGCGTGCTTAACACATGCAAGTCGAACGGTAAGGCCCTTCGGGGTACACGAGTGGCGAAC
GGGTGAGTAACACGTGGGTGATCTGCCCTGCACTTTGGGATAAGCCTGGGAAACTGGGTCTAATACCGAA
TATGACTGCATCCTGCATGGGGTGTGGTGGAAAGCTTTTGCGGTGTGGGATGGGCCCGCGGCCTATCAGC
TTGTTGGTGGGGTGATGGCCTACCAAGGTGACGACGGGTAGCCGGCCTGAGAGGGTGACCGGCCACACT
GGGACTGAGATACGGCC
>Zotu12035

GAACGCTGGCGGCATGCTTAACACATGCAAGTTGAACGCAGAATAGGACTTCGGTTCTATTGCTGAGTAGC
AAACGGGTGAGTAACGCGTGGGAATCTACCTTTTTGTGGGGGATAACTGAGGGAAACTTCAGCTAATACC
GCATAAGCTCTACGGAGGAAAGGCTTCGGCCGCAAGAAGAGGAGCCCGCGTCAGATTAGGTAGTTGGTA
GGGTAAAAGCCTACCAAGCCAATGATCTGTAGCTGGTCTGAGAGGATGATCAGCCACACTGGGACTGAGA
CACGGCC
>Zotu12037
GCCGTGTCTCAGTCCCAATGTGGCTGATCATCCTCTCAGACCAGCTACTGATCGTCGCCTTGGTGAGCCATT
ACCTCACCAACTAGCTAATCAGACGCGGGCCGATCTATCGGCGATAAATCTTTCCCCCGAAGGGCGTATCC
GGTATTAGTCCAAGTTTCCCTGAATTATTCCGAACCGAAAGGTACGTTCCCACGTGTTACTCACCCGTCTGC
CACTAGCACCGAAGTGCCCGTTCGACTTGCATGTGTTAAGCCTGCCGCCAGCGTTCG
>Zotu12039
GAACGCTGGCGGCATGCTTAACACATGCAAGTCGAACGGTAACAGGCTCGCAAGGGCGCTGACGAGTGG
CGAACGGGTGAGTAGAGCGTAGGGATCTGCCTAGATGATTGGGATAGCCTAGGGAAACTTAGGGTAATAC
CGGATACTCCTTAAGAGGGAAAGCTGGGGACTTTTAATAGAGCCTGGCGCATTTAGATGAACCTACGTCGG
ATTAGCTAGTTGGTAAGGTAAAGGCTTACCAAGGCGATGATCCGTAGCTGGTCTGAGAGGACGGCCAGCC
ACACTGGGACTGAGACACGGCC
>Zotu12045
GAACGCTGGCGGCGTGCCTAATACATGCAAGTCGAGCGGTAAGGCTCCTGCAGATTGTTCGATGATATACA
GACAAGGGAAGGCTCCCGAGTTTGATCGAATAATGTGCTGGAGTACACGAGCGGCGGACGGCTGAGTAA
CACGTAGGAACGTACCCCAAACTGAGGGATAAGCACCAGAAATGGTGTCTAATACCGCATGTGCTCTTCGG
AGTAAAGCTACGGCGGTTTGGGAACGGCCTGCGTAAGATTAGCTAGTTGGTGAGGTAATAGCTCACCAAG
GCGACGATCTTTAGCTGGTCTGAGAGGATGATCAGCCAGACTGGGACTGAGAACGGCC
>Zotu12050
GAACGCTAGCTGCATGCTTAACACATGGAAGTCGGATGGGAAGTGGTGTTTCCAGTGGCGGGCGGGTGA
GTAACGCATAGAACCTACCCTTGGGAGCGGAACAACTACTTGAAACGGTTTCTAATACCCCGTAGGCTGGG
GAGCAAAAGGAGGAATTCGCTCGAGAAGAAGCTCGCTTCTGATTAGCTAGTTGGTGAGGCAATAGCTTAA
CAAGGCAATGATCAGTAGTTGGTCTGAGAGGATGATCAGCCACACTGGGACTGAGACATCACC
>Zotu12052
AAACGCTGGCGGCGTGCCTAATGCATGCAAGTCGAACGGATGTTGTCTTCGGACAGCATTAGTGGCAAAC
GGGTGAGTAACACGTGGGGATCTGCCCAGTAGTGGGGAACAACTCCTCGAAAGAGGAGCTAATACCGCAT
ACGGTCGAAAGACTAAAGCCAGAAATGGCGCTATTGGAGGAACCTGCGGCTGATTAGCTAGTTGGTGGGG
TAAAGGCCTACCAAGGCGATGATCAGTAGCTGGTCTGAGAGGATGGACAGCCACACTGGGACTGAGATAC
GGCC
>Zotu12056
GAACGCTGGCGGCGTGCTTAACACATGCAAGTCGAACGAGGTCCATCGAGAGCTTGCTCAAGAGAAGACC
TAGTGGCGAACGGGTGAGTAACACGTGAGAAACCTGCCCCGAACTTGGGAATAACAGTCGGAAACGGCT
GCTAATACCGAATATCTTCGTAGCGTCGCATGGCGCATCGAAGAAAGCTTTTGCGGTTTGGGAGGGTCTCG
CGACCTATCAGCTAGTTGGTGAGGTAACGGCTCACCAAGGCATCGACGGGTAGCTGGTCTGAGAGGATGA
TCAGCCACACTGGGACTGAGACACGGCC
>Zotu12059
GAACGCTGGCGGAATGCTTTACACATGCAAGTCGAACGGCAGCACGGACTTCGGTCTGGTGGCGAGTGGC
GAACGGGTGAGTAATATATCGGAACGTATCCAATAATGGGGGATAACTAAGCGAAAGCTTGGCTAATACC
GCATACGCCCTCAGGGGGAAAGCAGGGGATCTTCGGACCTTGCGTTAATGGAGCGGCCGATATCTGATTA
GCTAGTTGGTGGGGTAAAGGCCCACCAAGGCTTCGATCAGTAGCTGGTCTGAGAGGACGACCAGCCACAC
TGGAACTGAGACACGGTC
>Zotu12063
GAACGCTGGCGGCGTGCTTAACACATGCAAGTCGTGCGCGAAGGTGGCTTCGGCCACCGGAGAGCGGCG
AACGGGTGAGTAACACGTGGGTGATCTGCCTGGAAGCGGGGGATAACCTGGGGAAACTCGGGCTAATAC
CGCATACGTTGATAGGGTTACGGCCCTATGAAGAAAACCGGCCTCTGCTTGCAAGCTGGTGCTTCCAGAGG
AGCCCGCGGCTGATTAGCTAGTTGGTGAGGTAATGGCTCACCAAGGCGACGATCAGTAGCCGGCCTGAGA
GGGTGATCGGCCACACTGGGACTGAGACACGGCC

>Zotu12067
ACCACCAGCATCGGCAGCCGGTCTTCTTTCTCTCACATGTCATCCTTGGGTTCGCCCCAGTCCTCGCTTTCCT
CGTCGCCAGCCACCGCTTCGTCCGGCACTCCCTCGCACGTGCG
>Zotu12071
GAACGCTAGCGGCAGGCTTAACACATGCAAGTCGAGGGGTATAAAGGAGCTTGCTCTTTTAGAGACCGGC
AAACGGGTGCGGAACACGTACACAATCTGCCCTTATCTTGGGGATAGCCTTCTGAAAAGGAGATTAATACC
CAATAGTATAATGGAGTGGCATCACTTTATTATTAAAGAATTTCGGATAAGGATGAGTGTGCGTAAGATTA
GATAGTTGGTGAGGTAACGGCTCACCAAGTCTACGATCTTTAGCTGATGTGAGAGCATGATCAGCCACACG
GGCACTGAGACACGGGC
>Zotu12073
GAACGCTGGCGGCATGGATAAGGCATGCAAGTCGAGTGGGTTTAGACCCACGGCAAACGGGTTAGTAAT
GCGTAGGAATGTCCCTCAAAGTCGTGAATAATCCGTCGAAAGGCGGAAGAATACACGATGTGCTCCACGG
AGTAAAGATTTATCGCTTTGAGAGCAGCCTGCGTCCTATCAGCTAGTTGGTGGGGTAAAAGCCTACCAAGG
CTTTGACGGGTAGGGGGTGTGAGAGTATGGCCCCCAACATTGGGACTGAGACACTGCC
>Zotu12084
GAACGCTAGCGGCAGGCTTAACACATGCAAGTCGAGGGACTTCAGCAATGGAGGACCGGCAAACGGGTG
CGGAACACGTACACAATCTGCCTCTATCAGGGGGATAGCCTTTCGAAAGGGAGATTAATACCCCATAGTAT
TATCAGGTGGCATCATTTGATAATTAAAGCTCCGGTGGATAGAGATGAGTGTGCGTACTATTAGGTAGTTG
GTAGGGTAACGGCCTACCAAGCCGACGATAGTTAGCTGATGTGAGAGCATGATCAGCCACACGGGCACTG
AGACACGGGC
>Zotu12086
GAACGCTAGCGGCATGCCTAACACATGCAAGTCGAACGGGCCGTTTTCGGACGGTCAGTGGCAGACGGGT
GCGTAACACGTGGGTGACCTGCCCTCAAGTGGGGAACAACGCGACTAACGTCGCGCTAATACCGCATAAG
TTCGCTTCTTCGGTGGGGCGAGTAAAGCAGCAATGCGCTTGGGGATGGGCCTGCGGCGGATTAGGTAGTT
GGTGGGGTAACGGCCTACCAAGCCGATGATCCGTAGGGGGCGTGAGAGCGTGACCCCCCACATTGGCACT
GAGACACGGGC
>Zotu12089
GAACGCTGGCGGCGTGCCTAATACATGCAAGTCGAGCGCCCCCTTGAGGGGAGCGGCGGACGGCTGAGT
AACACGTAGGAACGTACCCCAAACTGAGGGATAAGCACCAGAAATGGTGTCTAATACCGCATGTGCTCTTC
GGAGTAAAGCTACGGCGGTTTGGGAACGGCCTGCGCATGATTAGCTTGTTGGTGAGGTAATAGCTCACCA
AGGCGACGATCATTAGCTGGTCTGAGAGGATGATCAGCCAGACTGGGACTGAGAACGGCC
>Zotu12093
GAACGCTGGCGGCGTGCTTAACACATGCAAGTCGAACGGAAAGGCCCTTCGGGGTACTCGAGTGGCGAAC
GGGTGAGTAACACGTGGGCAACCTGCCCCTAGCTCTGGGATAACTCCGGGAAACCGGTGCTAATACCGGA
TACACACTCTCACAGGCATCTGCGTGAGTGGAAAGTTTTTCGGCTAGGGATGGGCCCGCGGCCTATCAGCT
TGTTGGTGGGGTAACGGCCTACCAAGGCGACGACGGGTAGCCGGCCTGAGAGGGCGACCGGCCACACTG
GGACTGAGACACGGCC
>Zotu12095
GAACGCTGGCGGCGTGCTTAACACATGCAAGTCGAACGGAAAGGCCCTTCGGGGTACTCGAGTGGCGAAC
GGGTGAGTAACACGTGGGTGATCTGCCCTGCACTTCGGGATAAGCCTGGGAAACTGGGTCTAATACCGGA
TATGACCGCATGATGCATGTTGTGTGGTGGAAAGCTTTTGCGGTGTGGGATGGGCCCGCGGCCTATCAGCT
TGTTGGTGGGGTCACGGCCTACCAAGGCGACGACGGGTAGCCGGCCTGAGAGGGTGACCGGCCACACTG
GGACTGAGATACGGCC
>Zotu12099
GAACGCTGGCGGCGTGCTTAACACATGCAAGTCGAACGGTGACCCTCGGAGCTTGCTTCGGGGTGAACAG
TGGCGAACGGGTGAGTAACACGTGAGTAACCTGCCCCTGACTCTGGAATAAGCGCTGGAAACGGCGTCTA
ATACCGGATACGACACGCCTCCGCATGGGGAGCGTGTGGAAAGTTTTTCGGTCTGGGATGGACTCGCGGC
CTATCAGCTTGTTGGTGAGGTAATGGCTCACCAAGGCGATGACGGGTAGCCGGCCTGAGAGGGCGACCGG
CCACACTGGGACTGAGACACGGCC
>Zotu12103

GAACGCTGGCGGCAGGCTTAACACATGCAAGTCGAGCGGGGAAATGTAGCTTGCTACATTACCTAGCGGC
GGACGGGTGAGTAATGCTTAGGAATCTGCCTATTAGTGGGGGACAACATCTCGAAAGGGATGCTAATACC
GCATACGTCCTACGGGAGAAAGCAGGGGACCTTCGGGCCTTGCGCTAATAGATGAGCCTAAGTCGGATTA
GCTAGTTGGTGGGGTAAAGGCCTACCAAGGCGACGATCTGTAGCGGGTCTGAGAGGATGATCCGCCACAC
TGGGACTGAGACACGGCC
>Zotu12107
GAACGCTGGCGGCATGCCTTACACATGCAAGTCGAACGGCAGCACGGACTTCGGTCTGGTGGCGAGTGGC
GAACGGGTGAGTAACATATCGGAACGTGCCCTGGAGTGGGGGATAACTAGTCGAAAGATTAGCTAATACC
GCATACGATCTACGGATGAAAGTGGGGGATCGCAAGACCTCATGCTCGTGGAGCGGCCGATATCTGATTA
GCTAGTTGGTGGGGTAAGAGCCTACCAAGGCATCGATCAGTAGCTGGTCTGAGAGGACGACTAGCCACAC
TGGGACTGAGACACGGCC
>Zotu12111
GAACGCTGGCGGCGTGGATAAGGCATGCAAGTCGAGTGGATTTAGACCCACGGCAGACGAGGTAGGAAC
ACGTAGGTACGTACCGGAGAGTCAGGGATATTCCATCGAAAGATGGCGCAATACCTGATGGTCCCGAGAG
GGTAAAGATTTATCGCTCTCTGAACGGCCTGCGTAGTATCAGCTAGTTGGTAAGGTAATGGCTTACCAAGG
CTATGACGCTTAGGGGAGCTGAGAGGCTGACCCCCACCGATGGAACTGAGACACGGTC
>Zotu12118
GAACGCTAGCGGCATGCTTAACACATGCAAGTAGAACGCTGAAGGAGGAGCTTGCTTCTCTGGATGAGTT
GCGAACGGGTGAGTAACGCGTAGGTAACCTGCCTGGTAGCGGGGGATAACTATTGGAAACGATAGCTAAT
ACCGCATAATAGTAGATGTTGCATGACATTTGCTTAAAAGGTGCAATTGCATCACTACCAGATGGACCTGC
GTTGTATTAGCTAGTTGGTGAGGTAACGGCTCACCAAGGCGACGATACATAGCCGACCTGAGAGGGTGAT
CGGCCACACTGGGACTGAGACACGGCC
>Zotu12126
GAACGCTGGCGGCGTGCTTAACACATGCAAGTCGAACGGAAAGGCCCTGCTTTTGTGGGGTGCTCGAGTG
GCGAACGGGTGAGTAACACGTGAGTAACCTGCCCTTGACTTTGGGATAACTTCAGGAAACTGGGGCTAAT
ACCGGATAGGAGCCCCTGCTGCATGGTGGGGGTTGGAAAGTTTCGGCGGTTGGGGATGGACTCGCGGCTT
ATCAGCTTGTTGGTGGGGTAGTGGCTTACCAAGGCTTTGACGGGTAGCCGGCCTGAGAGGGTGACCGGCC
ACACTGGGACTGAGACACGGCC
>Zotu12131
GAACGCTGGCGGTATGCTTTACACATGCAAGTCGTACGTGAGTTTTTAACTCTAGTGGCGGACGGGTGAGT
AACACGTAAGAATTTGCCTTTAGGAGGGGGACAACAACTGGAAACGGTTGCTAATACCCCATATGCTTTCG
AGTGAAATGGATTTCCGCCTAAAGAGAAGCTTGCGGCTGATTAGCTTGTTGGTGAGGTAATGGCTCACCAA
GGCGACGATCAGTATCTGGTTTGAGAGGACGATCAGACACACTGGAACTGAGACACGGTC
>Zotu12134
GAACGCTGGCGGCGTGCCTAACACATGCAAGTCGAACGGGGGACTTTAACGGAATTTCTTCGGAAAGGAA
GATGGAGGACCTAGTGGCGGACGGGTGAGTAACGCGTGGATAACCTGCCTCAAACAGGGGGATAACAGC
CGGAAACGGCTGCTAATACCGCATAAGCGCACAGCACCGCATGGTGCCGTGTGAAAAACTCCGGTGGTTT
GAGATGGATCCGCGTCTGATTAGCTAGTTGGTGAGGTAACGGCCCACCAAGGCGACGATCAGTAGCCGGC
CTGAGAGGGTGAACGGCCACATTGGGACTGAGACACGGCC
>Zotu12138
GAACGCTAGCGGCAGGCTTAACACATGCAAGTCGAGGGGCAGCATAAGGGTAGCAATATCTTTGATGGCG
ACCGGCGGAAGGGTGCGTAACGCGTGAGCAACATGCCCGTATCCGGGGGATAACCGAGAGAAATTTCGA
CTAATACCCCATAACAACAGAGGAGGCATCTTCTTTGTTTGAAAGTTTCGACAGATACGGATTGGTTCGCGT
GGCATTAGCTAGTTGGAGTGGTAACGGCACACCAAGGCGACGATGCCTAGGGGTTCTGAGAGGAAGGTC
CCCCACATTGGAACTGAGACACGGTC
>Zotu12140
GAACGCTGGCGGCATGCCTAACACATGCAAGTCGAACGAAGGCTTCGGCCTTAGTGGCGCACGGGTGCGT
AACGCGTGGGAATCTACCCTTAGGTTCGGAATAACAGTTGGAAACGACTGCTAATACCGGATGATGACGTA
AGTCCAAAGATTTATCGCCTAGGGATGAGCCCGCGTAGGATTAGCTAGTTGGTGTGGTAAAGGCGCACCA
AGGCGACGATCCTTAGCTGGTCTGAGGGGATGATCAGCCACACTGGGACTGAGACACGGCC

>Zotu12146
GAACGCTGGCGGCGTGCTTAACACATGCAAGTCGAACGGTAAGGCTCCTTCGGGAGTACACGAGTGGCGA
ACGGGTGAGTAACACGTGAGCAACCTACCCTTGACTTCGGAATAACAGTTGGAAACAGCTGCTAATACCGA
ATACGACCTTCTCGGTCATCCGAGTGGGTAGAAAGTTCTGGCGGTCAAGGATGGGCTCGCGGCCTATCAGC
TTGTTGGTGAGGTAATGGCTCACCAAGGCTTCGACGGGTAGCCGGCCTGAGAGGGCGACCGGCCACACTG
GGACTGAGATACGGCC
>Zotu12149
AGATTCCGTTTCTTCTTTTGCCCCTCCCCCTTGCTCCTGCTCTCTCACCCAGTAAAGAAATAGGTTATACTCAA
CCTATGTAAAGTACACAGTTTATTAAGTTTTCCCGTGTACGTGCACCACAGAC
>Zotu12152
GAACGCTGGCGGCATGCCTAACACATGCAAGTCGAACGAAGGCTTCGGCCTTAGTGGCGCACGGGTGCGT
AACGCGTGGGAACCTGCCCCTCGGTTCGGAATAACAGTTAGAAATGACTGCTAATACCGGATGATGACTTC
GGTCCAAAGATTTATCGCCGAGGGATGGGCCCGCGTAGGATTAGCTTGTTGGTGGGGTAAAGGCCTACCA
AGGCGACGATCCTTAGCTGGTCTGAGAGGATGATCAGCCACACTGGGACTGAGACACGGCC
>Zotu12164
GAACGCTGGCGGCGTGCTTAACACGTGCAAGTCGAACGGTGATCTCGGTGCTTGCACCGGGTGATCAGTG
GCGAACGGGTGAGTAACACGTGAGCAACCTGCCCCTGACTCTGGGATAACCCCGAGAAATCGGAGCTAAT
ACCGGATACGAGACGCACGGGCATCCGTAGCGTCTGGAAAGAATTTTGGTCAGGGATGGGCTCGCGGCCT
ATCAGCTTGTTGGTGGGGTAACGGCCCACCAAGGCGACGACGGGTAGCCGGCCTGAGAGGGCGACCGGC
CACACTGGGACTGAGACACGGCC
>Zotu12167
GAACGCTGGCGGCGTGCCTAACACATGCAAGTCGAACGTGAACGGGGCAACCCTAGTAAAGTGGCGCAC
GGGTGAGTAACGCGTAGGTAATCTACCCTAGAGTCTGGGATAACCTGCCGAAAGGCGGGCTAATACTGGA
TAGAGCGATGAAACTCGTAACTGGGTTTTTCGGGAAAGGGAGCCTGATAAGGGCCCTTGCTTTAGGATGA
GCCTGCGTCTCATTAGCTAGTTGGTAGGGTAACGGCCTACCAAGGCGATGATGGGTAGCTGGTCTGAGAG
GATGATCAGCCACACTGGAACTGAAACACGGTC
>Zotu12170
GAACGCTGGCGGCGTGCTTAACACATGCAAGTCGAACGAAGCATTTAGAACGGATTATTTCGGTATGAAGT
TCTTTATGACTGAGTGGCGGACGGGTGAGTAACGCGTGGGTAACCTGCCTTGTACTGGGGGATAGCAGCT
GGAAACGGCTGGTAATACCGCATAAGCGCACAATGTTGCATGACATGGTGTGAAAAACTCCGGTGGTATA
AGATGGACCCGCGTCTGATTAGCTAGTTGGTGAGATAACAGCCCACCAAGGCGACGATCAGTAGCCGACC
TGAGAGGGTGACCGGCCACATTGGGACTGAGACACGGCC
>Zotu12172
GAACGCTGGCGGCATGCTTTACACATGCAAGTCGAACGGCAGCGCGGGGCAACCTGGCGGCGAGTGGCG
AACGGGTGAGTAATACATCGGAACGTACCCAGAAGTGGGGGATAACGTAGCGAAAGTTACGCTAATACCG
CATACGTTCTACGGAAGAAAGTGGGGGATCGCAAGACCTCATGCTTTTGGAGCGGCCGATGTCTGATTAGC
TAGTTGGTAGGGTAAAGGCCTACCAAGGCGACGATCAGTAGCTGGTCTGAGAGGACGACCAGCCACACTG
GGACTGAGACACGGCC
>Zotu12175
GAACGCTGGCGGCAGGCCTAACACATGCAAGTCGAACGCCCCGCAAGGGGAGTGGCAGACGGGTGAGTA
ACACGTGGGAACCTTCCCTATAGTACGGAATAGCCCAGGGAAACTTGGAGTAATACCGTATACGCCCGAGA
GGGGAAAGATTTATCGCTATAAGATGGGCCCGCGTAGGATTAGCTAGTTGGTAAGGTAATGGCTCACCAA
GGCGACGATCCTTAGCTGGTTTGAGAGAACGACCAGCCACACTGGGACTGAGACACGGCC
>Zotu12180
GAACGCTAGCGAGAGGCCTAACACATACAAGTCGAGGGGTAACAGGGGAGCTTGCTCCCGCTGACGACC
GGCGGATGGGTGAGTAACGCGTATGCAACCTGCCTTTAACTGTGGGATAGCTCCCTGAAAGGGGAATTAA
TACCGCATAAGACCACGTTGTTTCATGACAGAGTGGTAAAATATTTATAGGTTAAAGATGGGCATGCGTGA
CATTAGCTAGTTGGTGAGGTAACGGCTCACCAAAGCTACGATGTCTAGGGGTTCTAAGAGGAAGGTCCCCC
ACACTGGTACTGAGACACGGAC
>Zotu12182

GAACGCTGGCGGTGTGCTTAACACATGCAAGTCGAACGGAAAGGCCCTGCTTTTGTGGGGTGCTCGAGTG
GCGAACGGGTGAGTAACACGTGAGTAACCTGCCCTTGACTTTGGGATAACTTCAGGAAACTGGGGCTAAT
ACCGGATAGGAGCTCCTGCTGCATGGTGGGGGTTGGAAAGTTTCGGCGGTTGGGGATGGACTCGCGGCTT
ATCAGCTTGTTGGTGGGGTAGTGGCTTACCAAGGCTTTGACGGGTAGCCGGCCTGAGAGGGTGACCGGCC
ACACTGGAACTGAGACACGGTC
>Zotu12185
GAACGCTGGCGGCAGGCTTAACACATGCAAGTCGAACGCCCCGCAAGGGGAGTGGCAGACGGGTGAGTA
ACGCGTGGGAATCTACCTTGAACTGCGGGATAGCTCCGGGAAACTGGAATTAATACCGTATGTGCCCTTAG
GGGGAAAGATTTATCGGTTCAAGATGAGCCCGCGTTGGATTAGCTAGTTGGTGGGGTAAAGGCCTACCAA
GGCGACGATCCATAGCTGGTCTGAGAGGATGACCAGCCACATTGGGACTGAGACACGGCC
>Zotu12187
GAACGCTGGCGGCGTGCTTAACACATGCAAGTGGAGCGACGAACCAGGCTTCGGCCTGGGGCAGAGCCG
CGAACGGGTGAGTAACACGTGGGTAACCTGCCTCGATGACCGGGACAACCCGGGGAAACCCGGGCTAAT
ACCGGATGTGCTGCCCTCGCATCAGTGAGGGTTGGAAAGGAAGCTTCGGCCTCCGCATCGAGATGGGCCC
GCGGCCCATTAGCTTGTTGGTGAGGTAACGGCTCACCAAGGCTGCGATGGGTAGCTGGTCTGAGAGGACG
ATCAGCCACACTGGGACTGAGACACGGCC
>Zotu12190
CAACGCTGGCGGCGCGCTTAACACATGCAAGTCGAACGAGAAAGGGAGCAATCCTGAGTAAAGTGGCGTA
CGGGTGAGTAACACGTGGGTAATCCACCCTCTGGTGGGGAATAACTTTGGGAAACCGAAGCTAATACCGC
ATAAGACCGGGAGGTGAAAGGGCGAAAGTTCGCCGGAGGAGGAGCCCGCGGCTGATTAGCTAGTTGGTA
GGGTAAGAGCCTACCAAGGCTGAGATCGGTAGCCGGCCTGAGAGGGCACACGGCCACACTGGCACTGAA
ACACGGGC
>Zotu12194
GAACGCTGGCGGCATGCCTAACACATGCAAGTCGAACGGCAGCGCGGAAGGGGTAACCTTTCTGGCGGC
GAGTGGCGGACGGGTGAGTAATATGTAGGAATCTGCCCAGAGGTAGGGGATAACTTGGGGAAACTCAAG
CTAATACCATATAAGCCCTAAGGGGGAAAGCAGGGGATCGCAAGACCTTGCGCCTTTGGATGAGCCTACA
TCGGATTAGCTAGTTGGTAGGGTAAAGGCCTACCAAGGCGACGATCCGTAGCTGGTCTGAGAGGATGACC
AGCCACACTGGGACTGAGACACGGCC
>Zotu12198
GAACGCTGGCGGCAGGCCTAACACATGCAAGTCGGGCGGTAACACAGGGAGCTTGCTCCTGGGTGACGA
GCGGCGGACGGGTGAGTAATGTCTGGGAAACTGCCTGATGGAGGGGGATAACTACTGGAAACGGTAGCT
AATACCGCATAACGTCGCAAGACCAAAGAGGGGGACCTTCGGGCCTCTTGCCATCAGATGTGCCCAGATG
GGATTAGCTAGTAGGTGGGGTAATGGCTCACCTAGGCGACGATCCCTAGCTGGTCTGAGAGGATGACCAG
CCACACTGGAACTGAGACACGGTC
>Zotu12201
GAATGCTAGCGGCGCGCTTTAGGCATGCAAGTAGAACGAGTCGTCCGTCGCCGTAAGGCTATGGACGATT
AGTTGCGAACGGCTGAGTAATACGTAGGAATCTACCCCTTGGCGGGGAATACCTTGTTGAAAGACAAGCT
AATACCGCATAAGATCGCAAGATGAAAGTCCGGCTTTGCCGGACACCGAGGGAGGAGCCTGCGGTCCATC
AGCTAGTTGGTAAGGTAATGGCTTACCAAGGCTACGACGGATAGGGGACGTGAGAGCGTGACCCCCCAGA
ATAGGACTGAGACACGGCC
>Zotu12203
GAACGCTAGCGGCAGGCCTAATACATGCAAGTCGAGGGGTAGAGGGAGCTTGCTTCCTTGAGACCGGCGC
ACGGGTGCGTAACGCGTATGCAACCTACCTTTATCTGGGGGATAGCCCGGAGAAATCCGGATTAATACCGC
ATAAAATCACAGAATAGCATTATTTAATGATCAAACATTTATGGGATGAAGATGGGCATGCGTGTCATTAG
CTAGTTGGCGGGGTAACGGCCCACCAAGGCGACGATGACTAGGGGATCTGAGAGGATGGCCCCCCACACT
GGTACTGAGACACGGTC
>Zotu12205
GAACGCTGGCGGCGTCCCTAACACATGCAAGTCGAGCGAGGTCCATTCGGTAGCAATACCGATGAAGACC
TAGCGGCGAACGGGTGAGTAACACGTGAGAAACCTGCCTCAAAGTCTGGGATAACCCGGGGAAACCCGA
GCTAATACCGGATACCTTCTTTTGGCCGCATGGTCAGCTGAAGAAAGTTTTTTCGCTTTGAGAGGGTCTCGC

GGCCTATCAGTTTGTTGGTGGGGTAACGGCCTACCAAGACATCGACGGGTAGCTGGTCTGAGAGGACGAT
CAGCCACACTGGGACTGAGACACGGCC
>Zotu12209
GAACGCTGGCGGCGTGCTTAACACATGCAAGTCGAACGGACCCTTCGGGGTTAGTGGCGGACGGGTGAGT
AACGCGTGAGGATCTGCCCTTAGGAGGGGAACAACAGTTGGAAACGACTGCTAATGCCCCATATGCCGAG
AGGTGAAATGTTTTATCGCCTAGGGATGAACTCGCGTCTGATTAGCTAGTTGGTGAGGTAAGGGCTTACCA
AGGCGACGATCAGTAGCTGGTCTAAGAGGATGATCAGCCACACTGGGACTGAGACACGGCC
>Zotu12211
GAACGCTGGCGGCAGGCCTAACACATGCAAGTCGAACGCCCCGCAAGGGGAGTGGCAGACGGGTGAGTA
ACGCGTGGGAACCTTCCCAGTGGTACGGAATAGCCCAGGGAAACTTGGAGTAATACCGTATAATACCCGA
AAGGGGAAAGATTTATCGCCATTGGATGGGCCCGCGTAGGATTAGCTAGTTGGTGAGGTAATGGCTCACC
AAGGCGACGATCCTTAGCTGGTCTGAGAGGATGATCAGCCACACTGGGACTGAGACACGGCC
>Zotu12216
GAACGCTAGCGGCAGGCTTAATACATGCAAGTCGAGGGGTAACAGGGAAGCTTGCTTTCGCTGACGACCG
GCGCACGGGTGCGTAACGCGTATGGAACCTACCTATAACAAAGGGATAGCCCAGAGAAATTTGGATTAAT
ACCTTATAGTACTGAGAGATGGCATCATTTTTCAGTTAAAGATTTATCGGTTATAGATGGTCATGCGTAAGA
TTAGCTAGTTGGTAAGGTAACGGCTTACCAAGGCGACGATCTTTAGGGGTTCTGAGAGGATGATCCCCCAC
ACTGGCACTGAGATACGGGC
>Zotu12220
GAACGCTGGTGGTATGCTTAACACATGCAAGTCGAACGGGCTTAAAGGGGCAACTCTTTAAGTCAGTGGC
AAACGGGTGAGGAATATATAGGAATCTTCCCAGCAGTAAGGAATAACATTGGGAAACCAATGCTAATGCC
ATATATTACCGATTTATCGGGAAAGATTTATCGCTGTTGGATGAGCCTGTATCGGATTAGCTAGTTGGTAGG
GTAATGGCCTACCAAGGCAATGATCCGTAGCTGGTCTGAGAGGATGATCAGCCACATTGGGACTGCGACA
CGGCC
>Zotu12222
GAACGCTAGCGACAGGCTTAACACATGCAAGTCGAGGGGCATCAGGGGTAGGACTTGTCCTACCTGCTGG
CGACCGGCGCACGGGTGAGTAACACGTATGCAACCTGCCTGTGTCAGGGGGATAAGCGGCGGAAACGCC
GTCTAATACCCCGTATGATCCCGGGAGGCATCTCCCGGGGTTGAAAGGTTTACCGGACACAGATGGGCAT
GCGGCGCATTAGTTAGTTGGCGGGGTAACGGCCCACCAAGACGACGATGCGTAGGGGTTCTGAGAGGAA
GGTCCCCCACACTGGTACTGAGACACGGAC
>Zotu12226
GAACGCTAGCGGGAGGCCTAACACATGCAAGCCGAGCGGTAGATTGCTTTCGGGCAATTGAGAGCGGCGT
ACGGGTGCGGAACACGTGTGCAACCTGCCTTTATCTGGGGGATAGCCTTTCGAAAGGAAGATTAATACCCC
ATAATATGTTGAATGGCATCATTCGACATTGAAAGCTCCGGCGGATAGAGATGGGCACGCGCAAGATTAG
ATAGTTGGTGAGGTAACGGCTCACCAAGTCAATGATCTTTAGGGGGCCTGAGAGGGTGATCCCCCACACT
GGTACTGAGACACGGAC
>Zotu12231
GAACGCTGGCGGCGTGCTTAACACATGCAAGTCGAACGGTGAAGCCCTTCGGGGTGGATCAGTGGCGAAC
GGGTGAGTAACACGTGAGCAACCTGCCCCTGGCTCTGGGACAACCACTGGAAACGGTGGCTAATACCGGA
TACGACCTGCCTCGGCATCGAGTGTGGGTGGAAAGTTTTTCGGCTGGGGATGGGCTCGCGGCCTATCAGC
TTGTTGGTGGGGTGATGGCCTACCAAGGCGACGACGGGTAGCCGGCCTGAGAGGGCGACCGGCCACACT
GGGACTGAGACACGGCC
>Zotu12234
GAATGCTGGCGGCGTGGATAAGTCATGCAAGTCAAGGGGGCCCGCAAGGGCAACCGGCAGACGAGGTAG
TAACACGTAGGTACGTCCCCCGAAGTCGGGCATAGCCAGCCGAAAGGTTGGGTAATTCCCGATAGTCCCGC
AAGGGTAAAGGTTTTTCGCTTCGGGAGCGGCCTGCGTAGTACCAGCTAGTTGGTAGGGTAACGGCCTACC
AAGGCTACGACGCTTAGGGGAGCTGAGAGGCTGACCCCCACCGATGGAACTGCGACACGGTC
>Zotu12241
GAACGCTGGCGGCAGGCTTAACACATGCAAGTCGAGCGCCCCGCAAGGGGAGCGGCAGACGGGTGAGTA
ACGCGTGGGAATCTACCCTGAACTGCGGAATAGCTCCGGGAAACTGGAATTAATACCGCATGTGCCCTGA

GGGGCAAAGATTTATCGGTTCAGGATGAGCCCGCGTCCGATTAGCTAGTTGGTGGGGTAAAGGCCTACCA
AGGCGACGATCGGTAGCTGGTCTAAGAGGATGATCAGCCACATTGGGACTGAGACACGGCC
>Zotu12246
GAACGCTGGCGGCGTGCTTAACACATGCAAGTCGAACGGAGAATATAGCAATATATTCTTAGTGGCGAAC
GGGTGAGTAACGCGTAGACAACCTGCCTTCTAGATGGGGACAACGTTCCGAAAGGGACGCTAATACCGAA
TGTCCTGTCAAGTCCGCATGGACTAGACAGCAAAGATGGCCTCTGCATATGCTATCGCTAGAAGATGGGTC
TGCGTCTGATTAGCTGGTTGGTGAGGTAACGGCTCACCAAGGCGACGATCAGTAGCCGGTCTGAGAGGAT
GAACGGCCACACTGGGACTGAGACACGGCC
>Zotu12248
GAACGCTGGCGGCGCGCCTAACACATGCAAGTCGAGCGAGAACCAGGCCTTCGGGCCTGGGGACAGCGG
CGAACGGGTGAGTAACACGTGAGTAATCAACCCTCGGCACCGGGATAGCCCGGGGAAACCCGGATTAATA
CCGGATGGCCCATCCGCTTTTCGGAGCGGCTGGAAAAGGTAGCTTCGGCCTCTGGCCGAGGACGAGCTCG
CGGCCGATTAGCTTGTTGGTGGGGTAATGGCTCACCAAGGCTCCGATCGGTAGCTGGTCTGAGAGGACGA
TCAGCCACACTGGGACTGAGACACGGCC
>Zotu12253
GAACGCTAGCGGCGGGCTTAATACATGCAAGTCGAGCGGTAACAGGGGTAGCAATACATGCTGACGAGC
GGCAGACGGGTGAGGAACACGTACGCAACCTCCCTCCAACTGGGGCACAACTCCGTGAAAACGGGGCTAA
TTCCCCATAACATTGCGAAAGGGCATCTGATTGCAATTAAAGATTCGTCGGTTGGAGATGGGCGTGCGACT
GATTAGCCAGTTGGTAGGGTAACGGCCTACCAAAGCAACGATCAGTAGCTGGTGTGAGAGCACGACCAGC
CACACGGGCACTGAGATACGGGC
>Zotu12256
GAACGCTGGCGGCGTGCTTAAGGTATGCAAGTCGAACGAGAATTTTGCAGTAGCAATATTGTAAAATTCTA
GTGGCAAACGGGTGAGTAATGCGTAGATAACCTACCCTTAACATTGAAATAACTTTGCGAAAGTAAGGCTA
ATGTCATGTGTGGTGTATAAGTGAAATTCTTATATATTAAAGGAGCAATCCGGTTAAGGATGAGTCTGCGT
CCTATCAGCTAGTTGGTGAGGTAAATGCTCACCAAGGCGACGACGGGTAGCTGGTCTGAGAGGATGATCA
GCCACATTGGAACTGCGACACGGTC
>Zotu12258
GAACGCTGGCGGCAGGCTTAACACATGCAAGTCGAACGCATCCTTCGGGGTGAGTGGCAGACGGGTGAG
TAACACGTGGGAACGTGCCCTTCGGTTCGGAATAACTCAGGGAAACTTGAGCTAATACCGGATACGCCCTT
ATGGGGAAAGGTTGACCGCCGAAGGATCGGCCCGCGTCTGATTAGCTTGTTGGTGGGGTAACGGCCTACC
AAGGCGACGATCAGTAGCTGGTCTGAGAGGATGATCAGCCACACTGGGACTGAGACACGGCC
>Zotu12261
GAACGCTGGCGGCGTGCCTAATACATGCAAGTCGAGCGGATGAAGAAGAAGCTTGCTTCTTCTGATTTAGC
GGCGGACGGGTGAGTAACACGTGGGCAACCTGCCCTGTAGATTGGGATAACTCCGGGAAACCGGGGCTA
ATACCAAATAATCCATTTCCTCACATGTGGAAATGTTGAAAGACGGTCTCGGCTGTCACTACAGGATGGGC
CCGCGGCGCATTAGCTAGTTGGTTAGGTAACGGCTCACCAAGGCGACGATGCGTAGCCGACCTGAGAGGG
TGATCGGCCACACTGGGACTGAGACACGGCC
>Zotu12268
GAACGCTGGCGGCATGCCTTACACATGCAAGTCGAACGGATTGAGGGGCTTGCTCCTCAGTTAGTGGCGA
ACGGGTGAGTAATGCATCGGAACGTACCTTTTTGTGGGGGATAACTACTCGAAAGAGTAGCTAATACCGCA
TACGCCCTGAGGGGGAAAGCGGGGGACTCGCAAGAGCCTCGCGCAAAAAGAGCGGCCGATGTCAGATTA
GCTAGTTGGTAGGGTAAAAGCCTACCAAGGCGACGATCTGTAGCTGGTCTGAGAGGACGACCAGCCACAC
TGGGACTGAGACACGGCC
>Zotu12271
GAACGCTGGCGGCGTGCTTAACACATGCAAGTCGAACGGAAAGGCCCTGCTTTTGTGGGGTGCTCGAGTG
GCGAACGGGTGAGTAACACGTGAGTAACCTGCCCTTGACTTTGGGATAACTTCAGGAAACTGGGGCTAAT
ACCGGATAGGAGCTCCTGCTGCATGGTGGGGGTTGGAAAGTTTCGGCGGTTGGGGATGGACTCGCGGCTT
ATCAGCCTGTTGGTGGGGTAGTGGCTTACCAAGGCTTTGACGGGTAGCCGGCCTGAGAGGGTGACCGGCC
ACACTGGGACTGAGACACGGCC
>Zotu12275

CAACGCTGGCGGCGTGCCTAACACATGCAAGTCGAACGAGAAAGTGGAGCAATCCATGAGTAAAGTGGCG
CACGGGTGAGTAACACGTGACTAACCTACCCTCGAGTGGGGAATAACCTCGGGAAACCGGGGCTAATACC
GCATAACATCTACGGATCAAATGCGTAAGCAGCTTGAGGAGGGGGTCGCGGCTGATTAGCTAGTTGGCGG
GGTAATGGCCCACCAAGGCAATGATCAGTAGCCGGCCTGAGAGGGCGCACGGCCACACTGGAACTGAAAC
ACGGTC
>Zotu12280
GAACGCTGGCGGCATGCCTAACACATGCAAGTCGAACGAAGCCTTCGGGCTTAGTGGCGCACGGGTGCGT
AACGCGTGGGAATCTGCCCTTGGGTTTGGAATAACAGTTAGAAATGACTGCTAATACCGAATGATGTCGTA
AGACCAAAGATTTATCGCCCAGGGATGAGCCCGCGTAGGATTAGCTAGTTGGTGTGGTAAAGGCGCACCA
AGGCGACGATCCTTAGCTGGTCTGAGAGGATGATCAGCCACACTGGGACTGAGACACGGCC
>Zotu12289
GAACGCTGGCGGCGTGCTTAACACATGCAAGTCGAGCGGTAAGGCCCTTCGGGGTACACGAGCGGCGAA
CGGGTGAGTAACACGTGGGTGACCTGCCCTCCACTTCGGGATAAGCCTGGGAAACTGGGTCTAATACCGG
ATAGGACCGAGAGATGCATGTTTCTTGGTGGAAAGTTTTTTCGGTGGGGGATGGGCCCGCGGTCTATCAG
CTTGTTGGTGGGGTGATGGCCTACCAAGGCGACGACGGATAGCCGGCCTGAGAGGGTGATCGGCCACACT
GGGACTGAGACACGGCC
>Zotu12294
GAACGCTGGCGGCAGGCTTAACACATGCAAGTCGAGCGGGCCCTTCGGGGTCAGCGGCAGACGGGTGAG
TAACGCGTGGGAACGTACCCTTTGGTTCGGAACAACTCCGGGAAACTGGAGCTAATACCGGATAAGCCCTT
CGGGGGAAAGATTTATTGCCAAGGGATCGGCCCGCGTCTGATTAGCTAGTTGGTGAGGTAATGGCTCACC
AAGGCGACGATCAGTAGCTGGTCTGAGAGGATGATCAGCCACACTGGGACTGAGACACGGCC
>Zotu12297
GAACGCTAGCGGCAGGCTTAATACATGCAAGTCGAGGGGCAGCACGATAGCAATATTGGTGGCGACCGG
CGCACGGGTGCGTAACACGTATACAATCTACCTTAAACAGGGGGATAGCCTTTCGAAAGAGAGATTAATAC
CCCGTAATAAATTGAACGGCATCGTTTGATTTTGAAAGCTCCGGCGGTTTGAGATGAGTATGCGTCTGATTA
GCTAGTTGGTGAGGTAACGGCTCACCAAGGCTACGATCAGTAGGGGATCTGAGAGGACTAACCCCCACAC
TGGTACTGAGACACGGAC
>Zotu12300
GAACGCTGGCGGCGTGGATAAGGCATGCAAGTCAAACGGTCCGCAAGGACAGTGGCGAACGAGGCAGTA
ATGCGTAGGTATTTCCCCCGAAGTCAGGAATAGCCAGTCGAAAGATTGGGTAATACTTGATGGTCTCGCAA
GAGTAAAGATTTATCGCTTCGGGAAAAGCCTGCGTCGTATCAGCTAGTTGGTAAGGTAACGGCTTACCAAG
GCTATGACGCGTAGGGGGAGTGAGAGCTTGGCCCCTGCCGATGGAACTGAGAAACGGTC
>Zotu12307
GAACGCTGGCGGCGTGCCTAACACATGCAAGTCGAACGCGAAAAGGACTTCGGTCCTGAGTAGAGTGGCG
TACGGGTGAGTAACACGTGGGTAATCTACCTTTGGGTGGGGAATAACTCAGGGAAACTTGAGCTAATACC
GCATAATGCTACGGCTCGACATCGAGTCGTTGTTAAAGTAGCAATACGCCTGAAGAGGAGCCTGCGCCTGA
TTAGCTAGTTGGTAGGGTAACGGCCTACCAAGGCTTCGATCAGTAGCTGGTCTGAGAGGACGGCCAGCCA
CAATGGCACTGAAACACGGGC
>Zotu12312
GAACGCTGGCGGCGTGCCTAATACATGCAAGTCGTACGAAGTGAATGGAAGCTTGCTTCCATGATCTTAGT
GGCGAACGGGTGAGTAATACATAAGCAACCTGCCTTAATGCCTGGGATAACAGTTGGAAACGACTGCTAA
TACCGGATAATTTCTCAGTAGACATCTATTGAGAATAAAAGTTGGGATACAACACATTGAGATGGGCTTAT
GGCGCATTAGTTAGTTGGTGAGGTAACGGCTCACCAAGACGATGATGCGTAGCCGACCTGAGAGGGTGAC
CGGCCACACTGGGACTGAGACACGGCC
>Zotu12319
CAACGCTGGCGGCGCGCTTAACACATGCAAGTCGAACGAGAAAGGGGGAGCAATCCCCCGAGTAAAGTG
GCGCACGGGTGAGTAACACGTGGGTAATCTGCCTCTGGGTGGGGAATAACTCTGGGAAACCGGAGCTAAT
ACCGCATAAGCCCCTGTTGTTAAAGATGGGGGGAAAGGTGAAAATCGCCGAGAGAGGAGCCCGCGGCTG
ATTAGCTGGATGGTGGGGTAACGGCCTACCATGGCGGAGATCAGTAGCCGGCCTGAGAGGGCACACGGC
CACACTGGCACTGAAACACGGGC

>Zotu12323
GAACGCTGGCGGCGCGCTTAACACATGCAAGTCGCACGAGAAAGGGAGCAATCCTAAGTAAAGTGGCGC
ACGGGTGAGTAACACGTGGATCATCTACCTTTTAGTGGGGAATACCTCTGGGAAACCGGAGACAATACCG
CATAAGCCCGCAAGGGGAAAGCAGCAATGCGCTGAAAGAGGAGTCCGCGGCCGATTAGCTAGTTGGCGG
GGTAATGGCCCACCAAGGCGAAGATCGGTAGCCGGCCTGAGAGGGCACACGGCCACACTGGCACTGAAA
CACGGGC
>Zotu12326
CAACGCTGGCGGCGTGCCTAATGCATGCAAGTCGAACGGGGTCACCTTCGGGTGGCTTACGTGGCGGACG
GGTGAGGAACACGTGGGTAACCTGCCCACTGGTGGGGGCTAGCCTTCGGAAACGGAGGGTAATTCCGCAT
ACGCTTCGATCCCGCGTGGGGGGTTGAAGGAAAGCGCGCAAGCGCGCCGGTGGAGGGGCCCGCGTCCGA
TTAGCTTGTTGGTGGGGTAACGGCTCACCAAGGCGATGATCGGTAGCTGGTTTGAGAGGATGATCAGCCA
CACGGGGACTGAGACACGGCC
>Zotu12328
GAACGCTAGCGGCAGGCTTAATACATGCAAGGCGAGGGGGCAGCAATGTCACCGTCGTACGGGTGCGTA
ACGCGTATGCAACCTACCTTCAACTGGGGGATAGCCCGGGGAAACCCGGATTAATACCGCATGACACATTT
GGTTCACATGGGCTGATTTGTTAAAGATTTATTGGTTGAAGATGGGCATGCGTTCGATTAGCTAGTTGGTA
GGGTAACGGCCTACCAAGGCGACGATCGATAGGGGAGCTGAGAGGTTGATCCCCCACACGGGCACTGAG
ATACGGGC
>Zotu12330
CAACGCTGGCGGCGTGCCTAACACATGCAAGTCGCACGAGAAAGGGACTTCGGTCCTGAGTACAGTGGCG
CACGGGTGAGTAACACGTGAATCATCTACCTTCGAGTGGGGAATAACTGAGAGAAATCTTAGCTAATACCG
CATAATACCCACGGGTCAAAGCAGCAATGCGCTTGAAGAGGAGTTCGCGGCAGATTAGTTAGTTGGCGGG
GTAATGGCCCACCAAGACGATGATCTGTATCCGGCCTGAGAGGGCGCACGGACACACTGGAACTGAAACA
CGGTC
>Zotu12332
GAACGTTAGCGGCGCGCCTAACACATGCAAGTCGCGCGAGAAAGGGCTTCGGCCCCAGTAAAGCGGCGC
ACGGGTGAGTAACACGTAGGTCATCTTCCCCTGAGTGGTGGATAACTCTCCGAAAGGAGAGCTAATACAG
CATAAGACCACGACCTCGCTGAGGTTGTGGCCAAAGCAGACCTCTTCATGAAAGTTTGCGCTTAGGGATGA
GCCTGCGGCCCATCAGCTAGTTGGTAGGGTAATGGCCTACCAAGGCTAAGACGGGTAGCTGGTCTGAGAG
GATGAACAGCCACACTGGAACTGAGACACGGTC
>Zotu12335
GAACGCTGGCGGCGTGCTTAACACATGCAAGTCGAGCGAGGCTCCTCCTTCGGGAGGATGTCCTAGCGGC
GAACGGGTGAGTAACACGTGGGCAACCTGCCCCTAGCTCTGGGATAACCCCGGGAAACCGGGGCTAATAC
CGGATATGACTTCAGAAGGCATCTTCCGAAGTGGAAAAGGTTTACTGGCTAGGGATGGGCCCGCGGCCTA
TCAGCTTGTTGGTGGGGTAATGGCCTACCAAGGCGACGACGGGTAGCCGGCCTGAGAGGGTGTCCGGCCA
CACTGGGACTGAGATACGGCC
>Zotu12337
GAACGCTAGCTACAGGCTTAACACATGCAAGTCGAGGGGCAGCATGATTGAAGCTTGCTTCAATCGATGG
CGACCGGCGCACGGGTGAGTAACACGTATCCAACCTGCCGATAACTCGGGGATAGCCTTTCGAAAGAAAG
ATTAATACCCGATAGTATAGTGTTTCCGCATGGTTTCACTATTAAAGAATTTCGGTTATCGATGGGGATGCG
TTCCATTAGATAGTTGGCGGGGTAACGGCCCACCAAGTCGACGATGGATAGGGGTTCTGAGAGGAAGGTC
CCCCACATTGGAACTGAGACACGGTC
>Zotu12339
GAACGCTAGCGGCGGGCTTAATACATGCAAGTCGAGGGGTAGCAATACACCGGCGCACGGGTGCGTAAC
GCGTATGCAATCTACCTTTATCTGGGGGATAGCCCGGGGAAACCCGGATTAATACCGCATAAGTAAGTAAC
CTGGCATCAGGTAGCTTTGAAAGATTTATCGGATAAAGATGAGCATGCGTAAGATTAGATAGTTGGCGAG
GTAACGGCTCACCAAGTCTACGATCTTTAGGGGCTCTGAGAGGAGAATCCCCCACACTGGCACTGAGATAC
GGGC
>Zotu12342

GAACGCTAGCGGCAGGCCTAATACATGCAAGTCGAACGGACTGGAGTAGCAATACTTCAGTTAGTGGCGC
ACGGGTGCGTAACGCGTATGCAACCTACCTTTTATTGGGGGATAGCCCGAAGAAATTCGGATTAATACCGC
ATAATATCATTTGAGGGCATCTGAGGATGATGAAAGCTACGGCGATAAAAGATGGGCATGCGTGTCATTA
GCTAGTTGGTGGAGGTAACGGCTCACCAAGGCGACGATGACTAGGGGTTCTGAGAGGATGATCCCCCACA
CTGGTACTGAGATACGGAC

>Zotu12344

GAACGCTGGCGGCATGCCTAACACATGCAAGTCGAACGAGACCTTCGGGTCTAGTGGCGCACGGGTGCGT
AACGCGTGGGAATCTGCCCTTAGGTTCGGAATAACTCAGGGAAACTTGAGCTAATACCGGATAATGACTTC
GGTCCAAAGATTTATCGCCTAGGGATGAGCCCGCGTCGGATTAGCTAGTTGGTGAGGTAAAAGCTCACCA
AGGCGACGATCCGTAGCTGGTCTGAGAGGACGATCAGCCACACTGGGACTGAGACACGGCC

>Zotu12347

GAACGCTGGCGGCATGCCTTACACATGCAAGTCGAACGGTAACAGGTCTTCGGATGCTGACGAGTGGCGA
ACGGGTGAGTAATGTATCGGAACGTGCCCAGTCGTGGGGGATAACTACTCGAAAGAGTGGCTAATACCGC
ATACGACCTGAGGGTGAAAGCGGGGGATCGCAAGACCTCGCGCGATTGGAGCGGCCGATATCAGATTAG
GTAGTTGGTGGGGTAAAGGCTCACCAAGCCAACGATCTGTAGCTGGTCTGAGAGGACGACCAGCCACACT
GGGACTGAGACACGGCC

>Zotu12349

GAACGTTTGCGGCGGGCCTAACACATGCAAGTCGAACGCACTAGCAATAGTGAGTGGCGCACGGGTGCGT
AACACGTGGGTAATCAACCCTTCAGCTTGGGATAACGACTTGAAAGGGTCGCTAATACCGGATACGGCGC
GGGAGGCTTCGGCTTCTCACGAGAAAGCCTCGCAAGGGGCACTGAAGGACGAGCCTGCGGCCCATCAGCT
AGTTGGTGAGGTAAGAGCTCACCAAGGCGAAGACGGGTAGCTGGTCTGAGAGGATGATCAGCCACACTG
GAACTGAGACACGGTC

>Zotu12352

GAACGCTGGCGGCGTGCTTAACACATGCAAGTCGAACGATGAAGACTCCTGCTTGCAGGGGTTGGATTAG
TGGCGAACGGGTGAGTATCACGTGAGTAACCTTCCCTTGACTCTGGGATAAGCCCGGGAAACTAGGTCTAA
TACTGGATATGACCTTTCACCGCATGGTGGAAGGTGGAAAGATTTATCGGTCTTGGATGGACTCGCGGCCT
ATCAGCTTGTTGGTGAGGTAATGGCTCACCAAGGCGACGACGGGTAGCCGGCCTGAGAGGGTGACCGGC
CACACTGGGACTGAGACACGGCC

>Zotu12356

GCGGTGAAGGGATGTCGTGTGACGAGCGTTCGGAAAACTTATTGTACCTGCTTTTTATATTTAAGAACAGT
TGAAAATCTCAAAATGAGGGTGGTTAACCACAGTTGCA

>Zotu12359

GAACGCTGGCGGCGTGCCTAATACATGCAAGTCGAACGGAAAGGTTTCTCACTCTGTGCTTTTAATTTAGA
GCGTAGGGTGAGAAATACTCGAGTGGCGGACGGCAGAGTAACACGTAGGAACGTACCCCAAACTGGGGG
ATAAGCACCAGAAATGGTGTCTAATACCGCATGTGCTCCAAGGAGTAAAGCTTCGGCGGTTTGGGAACGG
CCTGCGCATGATTAGCTTGTTGGTGAGGTAATGGCTCACCAAGGCGACGATCATTAGCTGGTCTGAGAGGA
TGATCAGCCTGACTGGGACTGAGAACGGCC

>Zotu12361

GAACGCTGGCGGCGTGGATAAGACATGCAAGTCGAACGCTGAATACGGGGTAGCAATATTCCGTGTTCGG
AGTGGCGCAAGGGTGCGTAACACGTGGGTAATTTGCCGTGAAGCCCGGAATAACTCGCTGAAAGGCGAG
CTAATACCGGATGTGAAATTCGGAAGGCATCTTCTGGATTTCAAAGCTGGGGACCGCAAGGCCTGGCACTT
CACGATAAGCCCGCGGCCTATCAGCTAGTTGGTGAGGTAACGGCTCACCAAGGCTAAGACGGGTAGCTGG
TCTGAGAGGACGACCAGCCACACTGGAACTGAGACACGGTC

>Zotu12368

GAACGCTGGCGGCGTGCTTAACACATGCAAGTCGAACGGAAAGGCCCTTTCGGGGGTACTCGAGTGGCGA
ACGGGTGAGTAACACGTGGGCAATCTGCCCCTCGCTCTGGGATAACTCCGGGAAACCGGGGCTAATACCG
GATATGACCCTTTGAGGCATCTCTTTGGGTGGAAAGTTTTTCGGCGAGGGATGAGCCCGCGGCCTATCAGC
TTGTTGGTGGGGTAATGGCCTACCAAGGCAGTGACGGGTAGCCGGCCTGAGAGGGCGACCGGCCACACT
GGGACTGAGACACGGCC

>Zotu12370

GAACGCTGGCGGCGTGCCTAACACATGCAAGTCGAACGCGAACGGTGCTTTCGGGTGCCTAGTAGAGTGG
CGCACGGGTGAGTAACACGTAGACAACCTGCCTTCGAGCCCGGAATAACCTTTCGAAAGAAGGGCTAATA
CCGGATAACACCAGGGGTTGCATGACCTTTGGTTGAAAGGCTTGCTACTCGAAGAGGGGTCTGCGGGACA
TTAGCTAGTTGGTGGGGTAACGGCCTACCAAGGCGATGATGTCTAGCGGGTCTGAGAGGATGATCCGCCA
CACTGGAACTGAGACACGGTC
>Zotu12373
GAACGCTAGCGGCATGCTTAACACATGCAAGTCGAGCGGAGATGAGGTGCTTGCACCTTATCTTAGCGGC
GGACGGGTGAGTAATGCTTAGGAATCTGCCTATTAGTGGGGGACAACATTTCGAAAGGAATGCTAATACC
GCATACGCCCTACGGGGGAAAGCAGGGGATCTTCGGACCTTGCGCTAATAGATGAGCCTAAGTCGGATTA
GCTAGTTGGTGGGGTAAAGGCCTACCAAGGCGACGATCTGTAGCGGGTCTGAGAGGATGATCCGCCACAC
TGGGACTGAGACACGGCC
>Zotu12375
GAACGCTGGCGGCGGGCCTCATACATGCAAGTCGAGCGCGAAAGTCCGCAAGGATGAGTAAAGCGGCGC
ACGGGTGAGTAACACGTGGATAATCTACCCTTTGGATTGGAATAACCTGTCGAAAGACGGGCTAATGCCG
AGTAAGCCTTCGAGAGCGTGAAGCTCTTGCAGGGAAAGATGGCGCAAGCTATCGCCGATGGATGAGTCTG
CGTCCCATTAGTTAGTTGGTGGGGTAACGGCTTACCAAGACAATGATGGGTACCTGGTCTGAGAGGATGA
CCAGGCACACTGGAACTGAAACACGGTC
>Zotu12377
CAACGCTGGCGGCGTGCCTAACACATGCAAGTCGAACGCGGTTACCCCGCAAGGGGTAACTGAGTGGCGA
ACGGGTGAGTAACGCGTGGGTGACCTACCTCCGAGTGGGGGATAACGTCCCGAAAGGGACGCTAATACC
GCATAACATCCTGCCCTTGAACGGGTGGAGATCAAAGCTGGGGATCGCAAGACCTGGCGCTTGAAGAGGG
GCCCGCGTCTGATTAGCTAGTTGGTGGGGTAATGGCCCACCAAGGCGACGATCAGTATCCGGCCTGAGAG
GGCGGACGGACACACTGGGACTGAGACACGGCC
>Zotu12379
GAACGCTAGCGGCATGCCTAACACATGCAAGTCGAGCGGGCGTAGCAATACGTCAGCGGCAGACGGGTG
AGTAACGCGTGGGAACATACCTTTTGGTTCGGAACAACACAGGGAAACTTGTGCTAATACCGGATAAGCCC
TTACGGGGAAAGATTTATCGCCGAAAGATTGGCCCGCGTCTGATTAGCTAGTTGGTAGGGTAATGGCCTAC
CAAGGCGACGATCCGTAGCTGGTCTGAGAGGACGATCAGCCACACTGGGACTGAGACACGGCC
>Zotu12381
CAACGCTGGCGGCGTGCCTAACACATGCAAGTCGAACGAGAAAGGGCTTTCGGGCCTGAGTAAAGTGGCG
CACGGGTGAGTAACACGTGACTAACCTACCCTTGAGCGGGGGATAACCTAGGGAAACCTGGGCTAATACC
GCATAACACCTACGGGTCAAAGCGAGCAATCGCACTTAAGGAGGGGGTCGCGGCAGATTAGTTAGTTGGT
GGGGTAATGGCCTACCAAGACGATGATCTGTATCCGGCCTGAGAGGGCGCACGGACACACTGGAACTGAA
ACACGGTC
>Zotu12385
GAACGCTGGCGGCGTGCTTAACACATGCAAGTCGAGCGGAAAGGCCCTTCGGGGTACTCGAGCGGCGAA
CGGGTGAGTAACACGTGGGCAATCTGCCCCTCGCTCTGGGATAACTCCGGGAAACCGGGGCTAATACCGG
ATATGACACTCTTAGGCATCTTGGAGTGTGGAAAGTTTTTCGGCGAGGGATGAGCCCGCGGCCTATCAGCT
TGTTGGTGGGGTAATGGCCTACCAAGGCAGTGACGGGTAGCCGGCCTGAGAGGGCGACCGGCCACACTG
GGACTGAGACACGGCC
>Zotu12387
GAACGTTAGCGGCGGGCCTAACACATGCAAGTCGAACGAGAATGAGGGGGCAACTCCTCTAGTAAAGTG
GCGGACGGGTGCGTAACACGTGGGTAATCTACCTTCCGGCGGGGGACAACAGTCCGAAAGGATTGCTAAT
ACCGCATACGTCAGCGAGATCTACGGATCTTGTTGGGAAAGATGGCCTCTCCTTGGAAGCTGTCACCGGAA
GATGAGCCCGCGGCCCATCAGCTAGTTGGTGAGGTAACGGCTCACCAAGGCTTAGACGGGTAGCTGGTCT
GAGAGGATGATCAGCCACACTGGGACTGCGACACGGCC
>Zotu12392
GAACGCTGGCGGCATGCCTTACACATGCAAGTCGAACGGCAGCACGGGAGCAATCCTGGTGGCGAGTGG
CGAACGGGTGAGTAATGTATCGGAACGTGCCCAGTTGTGGGGGATAACTACTTGAAAGAGTGGCTAATAC
CGCATACGACCTGAGGGTGAAAGCGGGGGATCGCAAGACCTCGCGCAATTGGAGCGGCCGATATCAGATT

AGCTAGTTGGTGGGGTAAAAGCCCACCAAGGCGACGATCTGTAGCTGGTCTGAGAGGACGACCAGCCACA
CTGGGACTGAGACACGGCC
>Zotu12396
CAACGCTGGCGGCGTGCCTAATGCATGCAAGTCGAACGGGGGCACCTTCGGGTGTCTCACGTGGCGGACG
GGTGAGGAACACGTGGGGAAGCTGCCGTGCGCACGGGGGTAGCCCGCGGAAACGCGGGGTAATTCCGG
ATGCGCTCGCATCCCGGGTAGGGGATGTGAGGAAAGATCCTTCGGGGTCGGCGCACGAGGCACCTGCGG
CCGATTAGCTAGACGGTGGGGTAACGGCCTACCGTGGCGATGATCGGTAGCTGGTCTGAGAGGATGATCA
GCCACACTGGGACTGAGACACGGCC
>Zotu12398
GAACGCTAGCGGCAGGCTTAATACATGCAAGTCGAGGGGCAGCACGAATAGCAATATTTGGTGGCGACCG
GCGCACGGGTGCGTAACACGTATACAATCTACCTTCAACAGGGGGATAGCCTTTCGAAAGAGAGATTAATA
CCCCGTAATATATTTAACGGCATCGTTGAATATTGAAAGCTCCGGCGGTTGAAGATGAGTATGCGTCTGATT
AGCTAGTTGGTGAGGTAACGGCTCACCAAGGCAACGATCAGTAGGGGATCTGAGAGGACTAACCCCCACA
CTGGTACTGAGACACGGAC
>Zotu12400
GAACGCTGGCGGCATGCCTTACACATGCAAGTCGAACGGCAGCACGGGAGCTTGCTCCTGGTGGCGAGTG
GCGAACGGGTGAGTAATATATCGGAACGTACCCTAGAGTGGGGGATAACGCAGCGAAAGTTGCGCTAATA
CCGCATACGATCTACGGATGAAAGTGGGGGATCGCAAGACCTCATGCTCATGGAGCGGCCGATATCTGAT
TAGCTAGTTGGTGGGGTAAAAGCTCACCAAGGCGACGATCAGTAGCTGGTCTGAGAGGACGACCAGCCAC
ACTGGGACTGAGACACGGCC
>Zotu12404
GAACGCTGGCGGCGTGCTTAACACATGCAAGTCAGGGGGCTCCGCAAGGGGTCACCGGCGAACGGGTGC
GTAACACGTGAGCGACCTGCCCTCGAGTGGGGGATAGCCGGCCCAACGGCCGGGTAATACCGCATACGTC
TCCGGCTTGGACGCCGAGAGAGAAACCCGCAAGGGGTTCGAGGAGGGGCTCGCGGCCTATCAGCTAGTT
GGTGTGGTAACGGCGCACCAAGGCGATGACGGGTAGCTGGTCTGAGAGGACGATCAGCCACACTGGGAC
TGAGACACGGCC
>Zotu12406
GAACGCTGGCGGCGTGCCTAATACATGCAAGTCGTACGCACTGACAACGGAACTTGTTCCGTTTGACGTGA
GTGGCGCACGGGTGAGTAACACGTGGGAAACCTACCCTTCAGCGGGGGATAACCGTCGGAAACGATGGC
TAATACCGCATAGGTCTTTGAGTCACCTGACTTAAAGAGGAAAGACGGCTTCGGCTGTCACTGAAGGATGG
TCCTGCGGTGCATTAGCTAGTTGGTAAGGTAATGGCTTACCAAGGCGATGATGCATAGCCGACCTGAGAG
GGTGATCGGCCACATTGGGACTGAGACACGGCC
>Zotu12408
GAACGCTAGCGGCAGGCTTAACACATGCAAGTCGAGGGGTATAAGTCTTCGGATTTAGAGACCGGCGCAC
GGGTGCGTAACGCGTATGCAATCTACCTTTCACAGAGGGATAGCCCAGAGAAATTTGGATTAATACCTCAT
AGTATATAGACCTGGCATCAGGATTATATTAAAGTCACAACGGTGAAAGATGAGCATGCGTCCCATTAGCT
AGTTGGTAAGGTAACGGCTTACCAAGGCTACGATGGGTAGGGGTCCTGAGAGGGAGATCCCCCACACTGG
TACTGAGACACGGAC
>Zotu12412
GAACGCTGGCGGCGTGCCTAATACATGCAAGTCGAGCGAATCAATGGGAGCTTGCTCCCTGAGATTAGCG
GCGGACGGGTGAGTAACACGTGGGTAACCTGCCTGTAAGACTGGGATAACTTCGGGAAACCGGAGCTAAT
ACCGGATACTTTCTTTTCTCGCATGAGAAGAGATGGAAAGATGGTTCTGCTATCACTTACAGATGGACCCGC
GGCGCATTAGCTAGTTGGTGAGGTAACGGCTCACCAAGGCGACGATGCGTAGCCGACCTGAGAGGGTGAT
CGGCCACACTGGGACTGAGACACGGCC
>Zotu12417
GAACGCTAGCGGCATGCCTAACACATGCAAGTCGAGCGGGGGTAGCAATACCCTAGCGGCGAACGGGTG
AGGAATGCTTGGGAATCTGCCTGGTAGTGGGGGATAACGTTCCGAAAGGAACGCTAATACCGCATACGTC
CTACGGGAGAAAGGGGGGGATCTTCGGACCTCTCGCTATCAGATGAGCCCAAGCGGGATTAGCTAGTTGG
TGAGGTAAAGGCTCACCAAGGCGACGATCCCTAGCTGGTCTGAGAGGATGATCAGCCACACTGGAACTGA
GACACGGTC

>Zotu12422
GAACGCTGGCGGCGCGCTTAACACATGCAAGTCGAGCGAGAACCAAGCCTTCGGGCTTGGGGACAGCGG
CGAACGGGTGAGTAACACGTGGGTAATCAACCCTCGACACTGGGATAGCCCGGGGAAACTCGGATTAATA
CCGGATACCCCATCTGGCCTTCGGGCCGGCTGGAAAAGGTAGCTTCGGCCTCCGGTCGGGGACGAGCCCG
CGGCGGATTAGCTTGTTGGTGAGGTAACGGCCCACCAAGGCGACGATCCGTAGCTGGTCTGAGAGGACGA
TCAGCCACACTGGGACTGAGACACGGCC
>Zotu12426
GAACGCTGGCGGCGTGCTTAACACATGCAAGTCGAGCGGTGAACCCTTCGGGGGGATCAGCGGCGAACG
GGTGAGTAACACGTGAGCAACCTGCCCCTGACTCTGGGATAGCCTCGGGAAACCGGGATTAATACCGGAT
ATGACACTTCCTCGCATGGGGTGGTGTGGAAAGTTTTTCGGTCAGGGATGGGCTCGCGGCCTATCAGCTTG
TTGGTGGGGTGATGGCCTACCAAGGCGACGACGGGTAGCCGGCCTGAGAGGGTGACCGGCCACACTGGG
ACTGAGACACGGCC
>Zotu12431
GAACGCTGGCGGCATGCCTTACACATGCAAGTCGAACGGCATCGCGGGGAAGCTTGCTTTCCTGGCGGCG
AGTGGCGAACGGGTGAGTAACACATCGGAACGTACCGGCTAGTGGGGGATAACCCGTCGAAAGACGGGC
TAATACCGCATACGCTCTTCGGAGGAAAGCAGGGGACCTTTGGGCCTTGCGCTAGCCGAGCGGCCGATGT
CCGATTAGCTAGTTGGTAGGGTAATGGCCTACCAAGGCGACGATCCGTAGCTGGTCTGAGAGGACGATCA
GCCACACTGGGACTGAGACACGGCC
>Zotu12437
GAACGCTGGCGGCGTGCCTTAAGCATGCAAGTCGAACGGCTCCGCAAGGAGCAGTGGCGGACGGTCGAG
TAACGCGTGGGGATCTGCCTTAAAGTTGGACATAGCTCATCGAAAGGTGGGGTAATTTCCAATGTGAACGT
AAGTTCAAAGCCGCAAGGCGCTTTAAAATGAACCCGCGTCCTATCAGCTAGTTGGTGAGGTAATGGCTCAC
CAAGGCATCGACGGGTAGCTGGTCTGAGAGGATGATCAGCCGGAATAGCACTGAGACACGGGC
>Zotu12439
GAACGCTGGCGGCAGGCTTAATACATGCAAGTCGAACGGTAAGGCCTGAAGTAGCAATACGGAAGGTACA
CGAGTGGCGCACGGGTGCGTAACGCGTATGTAACCTGCCCCCAACCGGAACAAAACACCGGGAAACTGGT
GCTAATATTCCATGGTCTCTTCGGATGGCATCATCTGGGGAGTAAAGATTCATTGGTTGGGGATGGACATG
CGTATTATTAGGTAGTTGGTGGGGTAACGGCTCACCAAGCCGACGATGATTAGGGGTTCTGAGAGGATTA
ACCCCCACACTGGAACTGAGACACGGTC
>Zotu12443
GAACGCTGGCGGCAGGCTTAACACATGCAAGTCGAACGGGCTTCTTCGGAAGTCAGTGGCAGACGGGTGA
GTAACACGTGGGAACGTGCCCTTCGGTTCGGAATAACTCAGGGAAACTTGAGCTAATACCGGATACGCCCT
TATGGGGAAAGGTTTACTGCCGAAGGATCGGCCCGCGTCTGATTAGCTTGTTGGTGGGGTAACGGCCTAC
CAAGGCGACGATCCGTAGCTGGTCTGAGAGGACGATCAGCCACACTGGGACTGAGACACGGCC
>Zotu12446
GAACGCTGGCGGCGTGCTTCACACATGCAAGTCGAGCGAGGCCCTTCCTTCGGGGAGGTGCCCTAGCGGC
GAACGGGTGAGTAACACGTGGGCAACCTGCCCCTGACTCTGGGATAAGCCCGGGAAACCGGGTCTAATAC
CGGATATGAACCCAACAGGCATCTGGTGGGTTGGAAAGATTTATCGGTCAGGGATGGGCCCGCGGCCTAT
CAGCTTGTTGGTGGGGTAACGGCCCACCAAGGCGACGACGGGTAGCCGGCCTGAGAGGGCGACCGGCCA
CACTGGGACTGAGACACGGCC
>Zotu12448
GAACGCTGGCGGAATGCTTTACACATGCAAGTCGAACGGATTTAAAAAGCTTGCTTTTTAGGTTAGTGGCG
AACGGGTGAGTAATATATGGGAACGTACCTAATAGTGGGGGATAACGCTTCGAAAGGAGTGCTAATACCG
CATATTTCCTGAGGGAGAAAGCGGGGGATTCGCAAGAACCTCGCGCTACTAGAGCGGCCCATATCTGATTA
GCTAGTTGGTAGGGTAAAGGCCTACCAAGGCCACGATCAGTAGCTGGTCTGAGAGGATGATCAGCCACAC
TGGAACTGAGACACGGTC
>Zotu12450
GCCCCAGGGCTTACTTTCTGAGGGTGAGGAGGAAAGAGGGTAAAATTTTCCGGCAGTCGGAATGAGCAAA
CTGTACGTCGGGCTTTAAGGTGGCGGT
>Zotu12453

CTAGCTTATGGATCATCTCCTCTGCGCCAGGTGCCAGGACTCCCTTCCTTTCTTTGATCCTACGGCAGCGAGT
GGAGCCAGAGGGCAGGCACCGCTAACTTCTCTAAGCATCAGCTTCCTGGTCCAA
>Zotu12456
GAACGCTAGCGGCAGGCCTAACACATGCAAGTCGAGGGGCAGCACGGGTAGCAATACCTGGTGGCGACC
GGCGCACGGGTGAGTAACGCGTATGCAACCTACCTTTTACCGGGGGATAGCTCGTCGAAAGGCGAATTAA
TACCCCATAACACAGGGATACCGCATGGTAATATTTGTTAAAGCTCCGGCGGTAAAAGATGGGCATGCGTG
CCGTTAGTTTGTTGGTGAGGTAACGGCTCACCAAGACTACGATGGCTAGGGGGTCTGAGAGGATGATCCC
CCACACTGGAACTGAGACACGGTC
>Zotu12458
GAACGCTGGCGGCAGGCTTAACACATGCAAGTCGAGCGGGCCCTTCGGGGTCAGCGGCGGACGGGTGAG
TAACGCGTGGGAACGTGCCTTCTGGTCTGGAATAACCCTGGGAAACTAGGGCTAATACCAGATACGCCCTT
TTGGGGAAAGGTTTACTGCCGGAAGATCGGCCCGCGTCTGATTAGCTAGTTGGTGGGGTAACGGCCTACC
AAGGCGACGATCAGTAGCTGGTCTGAGAGGATGATCAGTCACACTGGAACTGAGACACGGTC
>Zotu12460
GAACGCTGGCGGCGTGCTTAACACATGCAAGTCGAGCGGTAAGGCCCCTTCGGGGGTACACGAGCGGCG
AACGGGTGAGTAACACGTGAGCAATCTGCCCATCACATCGGGATAACTTCGGGAAACCGAAGCTAATACC
GGATACGACCACTTCAGGCATCTGACGGTGGTGGAAAGTTCCGGCGGTGATGGATGAGCTCGCGGCCTAT
CAGCTTGTTGGTGGGGTAATGGCCCACCAAGGCTTCGACGGGTAGCCGGCCTGAGAGGGTGACCGGCCAC
ACTGGGACTGAGACACGGCC
>Zotu12466
GAACGCTGGCGGCGTGCTTAACACATGCAAGTCGAGCGGAAAGGCCCTTTCGGGGGTACTCGAGCGGCG
AACGGGTGAGTAACACGTGAGTAATCTGCCCTGCGCTCTGGGATAGCCACCGGAAACGGTGATTAATACC
GGATAGGACCACTTCACGCATGTGATGGTGGTGGAAAGTTTTTTCGGCGCAGGATGTGCTCGCGGCCTATC
AGCTTGTTGGTGAGGTAACGGCTCACCAAGGCTTTGACGGGTAACCGGCCTGAGAGGGTGACCGGTCACA
CTGGGACTGAGACACGGCC
>Zotu12474
GAACGCTGGCGGCGTGGATAAGACATGCAAGTCGAACGGGATATTGGTAGCAATACCGATGTCCAGTGGC
GTAAGGGTGCGTAACACGTGGGTTACCTGCCGAGAAGTGGGGGATAGCTCGCCGAAAGGCGAATTAATA
CCGCATAGTACATTTGTTAAAGCCGGGGCAACCTGGCGCTTCTTGAGGGGCCCGCGGCCTATCAGCTAGTT
GGTGAGGTATCGGCTTACCAAGGCTATGACGGGTAGCTGGTCTGAGAGGACGACCAGCCACACTGGAACT
GAGACACGGTC
>Zotu12477
GAACGCTGGCGGCGTGCCTAACACATGCAAGTCGAACGCGAAAGTGGAGCAATCCATGAGTAGAGTGGC
AAACGGGTGAGTAACACGTGGGTGACCTGCCTTCGAGTGGGGGATAACGTCCCGAAAGGGACGCTAATAC
CGCATAACATGCTGTCTTTTAAGAGAGAGACATCAAAGCCGGGGACCGCAAGGCCTGGCGCTTGAAGAGG
GGCCCGCGCCTGATTAGCTAGTTGGTGGGGTAATGGCTCACCAAGGCAACGATCAGTATCCGGCCTGAGA
GGGCGGACGGACACACTGGGACTGAGACACGGCC
>Zotu12479
GAACGCTATCGGTATGCTTAACACATGCAAGTCGAGCGGGCCCTTCGGGGTCAGCGGCGGACGGGTGAGT
AACGCGTGGGAACGTGCCTTCTGGTTCGGAATAACCCTGGGAAACTAGGGCTAATACCGGATACGCCCTTT
TGGGGAAAGGTTTACTGCCGGAAGATCGGCCCGCGTCTGATTAGCTAGTTGGTGGGGTAACGGCCTACCA
AGGCGACGATCAGTAGCTGGTCTAAGAGGATGATCAGCCACACTGGGACTGAGACACGGCC
>Zotu12487
GAACGCTGGCGGCATGCCTAACACATGCAAGTCGAACGAGACCTTCGGGTCTAGTGGCGCACGGGTGCGT
AACGCGTGGGAATCTGCCCAAAGGTTCGGAATAACTCAGGGAAACTTGAGCTAATACCGGATAATGTCGC
AAGACCAAAGATTTATTGCCTTTGGATGAGCCCGCGTAGGATTAGCTAGTTGGTGGGGTAAAGGCCTACCA
AGGCGACGATCCTTAGCTGGTCTGAGAGGATGATCAGCCACACTGGGACTGAGACACGGCC
>Zotu12491
GAATGCTGGCGGCGTGGATGAGGCATGCAAGTCGAATGGGTTTAGGCCCATGGCAGACGAGGTAGGAAC
ACGTAGGTACGTACCCCAAAGTCAGGGATAATCCGTCGAAAGGCGGAATAATACTTGATGGTCTCTTCGGA

GTAAAGATTTATCGCTTTGGGAACGGCCTGCGGGCTATCAGCTTGTTGGTAAGGTAACGGCTTACCAAGGC
TACGACGGCTAGGGGAGGTGAGAGCCTGACCCCCACCGATGGAACTGCGACACGGTC
>Zotu12496
AAACGCTGGCGGCGCACATAAGACATGCAAGTCGAACGGACTTAATCAATTCTTTTAGATTTGAAGCGGTT
AGTGGCGGACTGGTGAGTAACACGTAAGCAACCTACCTATCAGAGGGGAATAACAGTGAGAAATCATTGC
TAATACCGCATACCTTAAAAATATCGCATGATAAATTTAAGAAAGGAGCAATCCGCTGATAGATGGGCTTG
CGTCCGATTAGCTAGTTGGTGGGGCAACGGCCTACCAAGGCAACGATCGGTAGCCGAGCTGAGAGGCTCT
ACGGCCACATTGGGACTGAGATACGGCC
>Zotu12498
GACGTGTCTCAGTCCCAGTGTGGCTGGTCATCCTCTCAGACCAGCTACGGATCGTCGCCTTGGTAGGCCTTT
ACCCCACCAACTAGCTAATCCGACGTAGGCTCATCCTAGAGCGCAAGGCCCCGAAGGGTCCCCTGCTTTGA
TCTCTCGATATCATGCGGTATTAGCCCGAGTTTCCTCGGGTTATCCCACTCTCCAGGACAGATTCCTACGCTT
TACTCACCCATCCGCCACTCGCCACCCGTAAAGACCGAAATCTTTACTGTGCTGCCGTTCGACTTGCATGTGT
TAGGCATGCCGCCAGCGTTCA
>Zotu12503
CAACGCTGGCGGCGTGCCTAACACATGCAAGTCGCACGAGAAAGGGCTTCGGCCTGAGTAAAGTGGCGCA
CGGGTGAGTAACACGTGAACCATCTACCTCCGAGTGGGGAATAACCTAGAGAAATCTGGGCTAATACCGC
ATAACACTTACGAGTCAAAGCAGCAATGCGCTTGGAGAGGAGTTCGCGGCCGATTAGCTAGTTGGCGGGG
TAATGGCCCACCAAGGCGATGATCGGTATCCGGCCTGAGAGGGCGCACGGACACACTGGAACTGAAACAC
GGTC
>Zotu12506
GAACGCTGGCGGCATGTCTAAGGCATGCAAGTCGAACGATGCTACGTTTTTCGCAAGAGAGACGTGGGAG
AGTGGCGAACGGGAGAGTAACACATTGGTAATCTACCCCGGACTCGGGTACAACAGGCCGAAAGGCCTGC
TAATCCCCGATGGTCATGAGGAGCCGCAAGGCATTCTCATGTAAAGGTCCGCCGGTCCGGGAGGAGCCTA
TGGCCCATCAGCTAGTTGGTAGGGTAATGGCCTACCAAGGCTACGACGGGTAGGGGGCGTGAGAGCGTG
TACCCCCTCACTGGGACTGAGACACTGCC
>Zotu12509
GAACGCTGGCGGCGTGCTTAACACATGCAAGTCGAACGAGCTGCGCGGACTGAAGTTTTCGGATGGATGA
CTGCGTACGCGAGTGGCGGACGGGTGAGTAACGCGTGGGGAACCTGCCCCATTCCGGGGGATACCAGTT
GGAAACGACTGTTAATACCGCATAAGCGCACGGTACCGCATGGTAGTGTGTGAAAAACTCCGGTGGAATG
GGATGGCCCCGCGTCTGATTAGGCAGTTGGCGGGGTAACGGCCCACCAAACCGACGATCAGTAGCCGACC
TGAGAGGGTGACCGGCCACATTGGGACTGAGACACGGCC
>Zotu12514
GAACGCTGGCGGCAGGCTTAACACATGCAAGTCGAGCGGGCGTAGCAATACGTCAGCGGCAGACGGGTG
AGTAACGCGTGGGAACGTGCCTTTTGGTTCGGAACAACACAGGGAAACTTGTGCTAATACCGGATAAGCC
CTTACGGGGAAAGATTTATCGCCAAAAGATCGGCCCGCGTCTGATTAGCTTGTTGGTGAGGTAACGGCTCA
CCAAGGCTACGATCAGTAGCTGGTCTGAGAGGATGATCAGCCACACTGGGACTGAGACACGGCC
>Zotu12519
GAACGCTGGCGGCGTGCTTAACACATGCAAGTCGAACGCTGAGGCGGTGCTTGCACCGTGGATGAGTGGC
GAACGGGTGAGTAACACGTGGGTAATCTGCCCTGCACTCTGGGATAAGCCCTGGAAACGGGGTCTAATAC
CGGATATGACATCGTGCCGCATGGTGTGGTGTAGAAAGTTCCGGCGGTGCAGGATGAGCCCGCGGCCTAT
CAGCTTGTTGGTGGGGTGATGGCCTACCAAGGCGACGACGGGTAGCCGGCCTGAGAGGGTGACCGGCCA
CACTGGGACTGAGACACGGCC
>Zotu12521
GAACGCTAGCGGCAGGCCTAACACATGCAAGTCGAGGGAGAAGCCCTTCGGGGCAGAAACCGGCGCACG
GGTGCGTAACGCGTATGCAACCTACCTTTCACAGGGGGATAGCCCGAAGAAATTTGGATTAATACCCCATA
ATATTATTGGATGGCATCATTTGATAATTAAAATTACGATGGTGAAAGATGGGCATGCGTCCTATTAGCTAG
TTGGAGTGGTAACGGCACCCCAAGGCTACGATAGGTAGGGGTCCTGAGAGGGAGATCCCCCACACTGGTA
CTGAGACAGGGAC
>Zotu12527

CAACGCTGGCGGCGTGCCTAACACATGCAAGTCGCACGAGAAAGTGGAGCAATCCATGAGTAAAGTGGCG
TACGGGTGAGTAACACGTGACTAACCTACCTTCGAGTGGGGAATAACTTCGGGAAACCGAGGCTAATACC
GCATAACATCTTTTGGTTGAGATACCGATTGATCAAACCAGTAATGGGCTTGAAGAGGGGGTCGCGGCTG
ATTAGCTAGTTGGTGAGGTAACGGCCCACCAAGGCAATGATCAGTAGCCGGCCTGAGAGGGCGCACGGCC
ACACTGGAACTGAAACACGGTC
>Zotu12532
GAACGCTGGCGGCAGGCTTAACACATGCAAGTCGAGCGGGCATCCTTGTGGTGTCAGCGGCAGACGGGT
GAGTAACGCGTGGGAACGTACCCTTTGGTTCGGAATAACTCAGGGAAACTTGAGCTAATACCGGATACGC
CCTTTTGGGGAAAGGCTTGCTGCCGAAGGATCGGCCCGCGTCTGATTAGCTGGTTGGTGAGGTTATGGCTC
ACCAAGGCGACGATCAGTAGCTGGTCTGAGAGGATGATCAGCCACACTGGGACTGAGACACGGCC
>Zotu12537
GAACGCTGGCGGCGTGCATAACACATGCAAGTCGAACGATGAAACTCTAAAGATTTCTTCGGAATGAATTA
GAATGGATTAGTGGCGAACGGGTGAGTAGCACGTGAGTAACCTGCCTTAGAGAAGGGGATAGCCTTTGG
AAACGAAGAATAATACCCTATGAAATGATAATATCGCATGATAGTATCATCAAAGATTTATCGCTCTAAGAT
GGACTTGCGCCTGATTAGCTAGTTGGTGGAGTAACAGCCCACCAAGGCAACGATCAGTAGCCGGCTTGAG
AGAGTGTACGGCCACATTGGGACTGAGACACGGCC
>Zotu12539
GAACGCTGGCGGCGTGCTTAACACATGCAAGTCGAACGATGATGCCAGCTTGCTGGTTGATTAGTGGCGA
ACGGGTGAGTAACACGTGAGTAACCTACCCCTTACTTTGGGATAACCATTGGAAACGGTGGCTAATACTGG
ATATGAGACGCGGCCGCATGGTCTGTGTTTGGAAAGATTTATCGGTATGGGATGGACTCGCGGCCTATCAG
TTTGTTGGTGAGGTAATGGCTCACCAAGGCGATGACGGGTAGCCGGCCTGAGAGGGCGACCGGCCACACT
GGGACTGAGATACGGCC
>Zotu12543
GAACGCTGGCGGCAGGCCTAACACATGCAAGTCGAACGGTAGCACAAGGGAGCTTGCTCCCTGGGTGACG
AGCGGCGGACGGGTGAGTAATGTCTGGGAAACTGCCTGATGGAGGGGGATAACTACTGGAAACGGTAGC
TAATACCGCATAACGTCGCAAGACCAAAGAGGGGGACCTTCGGGCCTCTTGCCATCAGATGTGCCCAGATG
GGATTAGCTAGTAGGTGGGGTAACGGCTCACCTAGGCGACGATCCCTAGCTGGTCTGAGAGGATGACCAG
CCACACTGGAACTGAGACACGGTC
>Zotu12549
GAACGCTGGCGGCGTGCTTAACACGTGCAAGTCGAACGGATCCTCGGCTTCGGTTGGGGGTTAGTGGCGA
ACGGGTGAGTAACACGTGAGCAACCTGCCCTTCACTCTGGGATAACTCCGGGAAACCGGGGCTAATACCG
GATAGGACTCCTCGTCGCATGGTGGGGGGTGGAAAGGTGCCCTGTTGGGGGTTTCTGGTGGGGGATGGG
CTCGCGGCCTATCAGCTTGTTGGTGGGGTGATGGCCTACCAAGGCGACGACGGGTAGCCGGCCTGAGAGG
GTGACCGGCCACACTGGGACTGAGACACGGCC
>Zotu12556
GAACGCTGGCGGCGTGCTTAACACATGCAAGTCGAGCGGTAAGGCTCCTTCGGGAGTACACGAGCGGCGA
ACGGGTGAGTAACACGTGAGCAATCTGCCCTTCACATCGGGATAACCTCGGGAAACCGAGGCTAATACCG
GATACGACCACTTCAGGCATCTGAGGTGGTGGAAAGTTCCGGCGGTGAAGGATGAGCTCGCGGCCTATCA
GCTTGTTGGTGGGGTAATGGCCCACCAAGGCAACGACGGGTAGCCGGCCTGAGAGGGTGACCGGCCACA
CTGGGACTGAGACACGGCC
>Zotu12558
GAACGCTGGCGGCGTGCTTAACACATGCAAGTCGAGCGGAAAGGCCCTTCGGGGTACTCGAGCGGCGAA
CGGGTGAGTAACACGTGAGTAACCTGCCCTTGGCTCTGGGATAAGCCCGGGAAACTGGGTCTAATACCGG
ATATGACCATTGGATGCATGTCTGATGGTGGAAAGTTTTTCGGCTGGGGATGGACTCGCGGTCTATCAGCT
TGTTGGTGGGGTAACGGCCTACCAAGGCGACGACGGATAGCCGGCCTGAGAGGGTGACCGGCCACACTG
GGACTGAGACACGGCC
>Zotu12560
AAACGCTGGCGGCGTGCCTAATACATGCAAGTCGAACGAGGTAGCAATACCTAGTGGCGCACGGGTGAG
GAATATATGGGTAATCTGCCCTCAAGTTTGGGATAACCAGTCGAAAGATTGGCTAATACTGGATAAGACCA
CAGGATCTGCGGATCTAGGGGTTAAAGGTTTTTCGCTTGAGGATGAGCCCATATGGGATTAGCTAGTTGGT

AGGGTAATGGCCTACCAAGGCTACGATCCCTAACTGGTCTTAGAGGATGATCAGTCACACTGGAACTGAGA
CACGGTC
>Zotu12565
GAACGCTGGCGGCGTGCTTAACGCATGCAAGTCGAACGGAAAGGCCCCCAGCTTGCTGGGGGTACTCGAG
TGGCGAACGGGTGAGTAACACGTGGGTGATCTGCCCTGCACTTCGGGATAAGCCTGGGAAACTGGGTCTA
ATACCGGATAGGAGCCATCTTTGGTGTGATGGTTGGAAAGTTTTTCGGTGTAGGATGAGCCTGCGGCCTA
TCAGCTTGTTGGTGGGGTAATGGCCTACCAAGGCGTCGACGGGTAGCCGGCCTGAGAGGGTGTACGGCCA
CATTGGGACTGAGATACGGCC
>Zotu12571
GAACGCTAGCGGCGGGCTTAATACATGCAAGTCGAGGGGGTGTAGCAATACATCACCGGCGCACGGGTG
CGTAACACGTATGCAACCTACCCCTTATTAAAGGATATACTCGGGAAACTGGGGGCAATACTTTATATGCAC
ACATCTAGGCATCTAGGAGTGTGGAAACGTAAGGATAGGGGATGGGCATGCGTCTGATTAGCTAGTTGGT
GAGGTAACGGCTCACCAAGGCGACGATCAGTAGCTGGTCTGAGAGGATGATCAGCCACACTGGCACTGAG
ACACGGGC
>Zotu12575
GAACGCTGGCGGCGTGGATGAGGCATGCAAGTCGAATGGGTTTAGACCCATGGCAGACGAGGTAGGAAC
ACGTAGGTACATACCCTAAAGTCACGGATAACCCGGCGAAAGTCGGAATAATACGTGATAGTCCCTTCGGG
GTAAAGATTTATCGCTTTAGGAATGGCCTGCGTTCTATCAGCTTGTTGGTAAGGTAACGGCTTACCAAGGCT
ATGACGGATAGGGGAGGTGAGAGCCTGACCCCCACCGATGGAACTGCGACACGGTC
>Zotu12577
GAACGCTGGCGGCAGGCTTAACACATGCAAGTCGAGCGGGCACTTTCGGGTGTCAGCGGCGGACGGGTG
AGTAACGCGTGGGAACGTGCCTTCTGGTTCGGAATAACTCAGGGAAACTTGAGCTAATACCGGATACGCCC
TTTTGGGGAAAGGTTTACTGCCGGAAGATCGGCCCGCGTCTGATTAGCTAGTTGGTGGGGTAACGGCCTAC
CAAGGCGACGATCAGTAGCTGGTCTGAGAGGATGATCAGCCACACTGGGACTGAGACACGGCC
>Zotu12580
GAACGCTGGCGGCATGCCTAACACATGCAAGTCGAACGAGATCTTCGGATCTAGTGGCGCACGGGTGCGT
AACGCGTGGGAATCTGCCCTTGGGTACGGAATAACAGTGAGAAATTACTGCTAATACCGTATGATGTCGTA
AGACCAAAGATTTATCGCCCAGGGATGAGCCCGCGTAGGATTAGCTAGTTGGTGGGGTAAAGGCCTACCA
AGGCGACGATCCTTAGCTGGTCTGAGAGGATGATCAGCCACACTGGGACTGAGACACGGCC
>Zotu12582
GAACGCTGGCGGCATGCCTAACACATGCAAGTCGAACGAACCCTTCGGGGTGAGTGGCGCACGGGTGCGT
AACGCGTGGGAACCTACCCTTAAGTTCGGAATAACAGTGAGAAATTGCTGCTAATACCGGATAATGTCTTC
GGACCAAAGATTTATCGCTTTTGGATGGGCCCGCGTAGGATTAGCTAGTTGGTGAGGTAAAGGCTCACCAA
GGCGACGATCCTTAGCTGGTCTTAGAGGATGATCAGCCACACTGGGACTGAGACACGGCC
>Zotu12584
GAACGCTGGCGGCAGGCCTAACACATGCAAGTCGAGCGGCAGCGCAGTCGTAGCAATACGATGGGCGGC
GAGCGGCGGACGGGTGAGGAACACATCGGAATCTACCCTGTCGTGGGGGACAACGTAGGGAAACTTACG
CTAATACCGCATACGACCTTTGGGTGAAAGTGGGGGACCGCAAGGCCTCACGCGATTGGATGAGCCGATG
TCCGATTAGCTAGTTGGCGGGGTAATGGCCCACCAAGGCGACGATCGGTAGCTGGTCTGAGAGGACGACC
AGCCACACTGGAACTGAGACACGGTC
>Zotu12589
GAACGCTGGCGGCATGCTTTACACATGCAAGTCGAACGGTAACAGGTTAAGCTGACGAGTGGCGAACGGG
TGAGTAATGCATCGGAACGTGCCCGGTCGTGGGGGATAACGCAGCGAAAGCTGCGCTAATACCGCATACG
ATCTGAGGATGAAAGCGGGGGACCGTAAGGCCTCGCGCGATCGGAGCGGCCGATGTCAGATTAGGTAGT
TGGTGGGGTAAAGGCTCACCAAGCCGACGATCTGTAGCTGGTCTGAGAGGACGACCAGCCACACTGGGAC
TGAGACACGGCC
>Zotu12592
CAACGCTGGCGGCGTGCCTAACACATGCAAGTCGAGCGCGAATAGGGGGCAACCCCTTAGTAGAGCGGC
GAACGGGTGAGTAACGCGTGGGTGATCTGCCTCCGAGTGGGGAATAACATCGGGAAACTGGTGCTAATAC
CGCATGACATCCTTAGGTCACACTGAGGATCAAAGCCGGGGACCGCAAGGCCTGGCGCTTGGAGAGGAGC

CCGCGTCCGATTAGCTAGTTGGTGGGGTAATGGCCTACCAAGGCTTCGATCGGTAGCCGGCCTTAGAGGG
CGGACGGCCACACTGGGACTGAGACACGGCC

>Zotu12594

CGATGAAGAACGCAGCGGCTGACTGACACATGCAAGTCGAACGATTGCCTTCGGGTGATAGTGGCGCACG
GGTGCGTAACGCGTGGGAATCTGCCCTTAGGTTCGGAATAACAGCTGGAAACGGCTGCTAATACCGGATG
ATATCGCGAGATCAAAGATTTATCGCCTGAGGATGAGCCCGCGTTGGATTAGGTAGTTGGTGGGGTAAAG
GCCTACCAAGCCGACGATCCATAGCTGGTCTGAGAGGATGATCAGCCACACTGGGACTGAGACACGGCC

>Zotu12596

GAACGCTGGCGGCATGCCTTACACATGCAAGTCGAACGCGAAAGCGGGGCAACCCGCAAGTAGAGTGGC
GAACGGGTGAGTAATACATCGGAACATTTCCAGTAGCGGGGGATAACCTGTCGAAAGATAGGCTAATACC
GCATACGACCTACGGGTGAAAGCGGAGGACCGCAAGGCTTCGCACTATTGGAGTGGCCGATGTCGGATTA
GCTAGTTGGCGGGGTAAAAGCCCACCAAGGCTACGATCCGTAGCTGGTCTGAGAGGAAGATCAGCCACAC
TGGAACTGAGACACGGTC

>Zotu12598

GAACGCTGGCGGCAGGCTTAACACATGCAAGTCGAACGCACCTTCGGGTGAGTGGCAGACGGGTGAGTA
ACACGTGGGAACGTACCCTTCGGTTCGGAATAACTCTGGGAAACTAGAGCTAATACCGGATACGCCCTTTT
GGGGAAAGGTTTACTGCCGAAGGATCGGCCCGCGTCTGATTAGCTAGTTGGTGGGGTAACGGCCTACCAA
GGCGACGATCAGTAGCTGGTCTGAGAGGATGATCAGCCACATTGGGACTGAGACACGGCC

>Zotu12602

GAACGCTGGCGGAATGCTTTACACATGCAAGTCGAACGGCAGCACGGGAGCTTGCTCCTGGTGGCGAGTG
GCGAACGGGTGAGTAATATATCGGAACGTATCCAATAATGGGGGATAACTAACCGAAAGGTTGGCTAATA
CCGCATACGCCCTACGGGGGAAAGCAGGGGATCGTAAGACCTTGCGTTAATGGAGCGGCCGATATCTGAT
TAGCTAGTTGGTGGGGTAAAGGCCCACCAAGGCGACGATCAGTAGCTGGTCTGAGAGGACGACCAGCCAC
ACTGGAACTGAGACACGGTC

>Zotu12604

GAACGCTGGCGGGAGGCCTAACACATGCAAGCTGAGCGGTATAGTTTCTTCGGAAATGAGAGAGCGGCGT
ACGGGTGCGGAACACGTGTGCAACCTGCCTTTATCTAGGGGATAGCCTTTCGAAAGGAAGATTAATACTCC
ATAATATATTGAACGGCATCGTTTAATATTGAAAGCTCCGGCGGATAGAGATGGGCACGCGCAAGATTAGC
TAGTTGGTGAGGTAACGGCTCACCAAGGCGATGATCTTTAGGGGGCCTGAGAGGGCGATCCCCCACACTG
GTACTAAGACACGGAC

>Zotu12606

GAACGCTGGCGGCGTGCCTAACACATGCAAGTCGAACGGGGTTAAGGGAAATTTTCGGATGGAACTTAAC
TTAGTGGCGGACGGGTGAGTAACGCGTGGATAACCTGCCTCACACAGGGGGATAGCAGCTGGAAACGGC
TGGTAATACCGCATAAGACCACAAAGCCGCATGGCTTTGAGGTAAAAGATTTATCGGTGTGAGATGGATCC
GCGTCTGATTAGCCAGTTGGCGGGGTAACGGCCCACCAAAGCGACGATCAGTAGCCGGCCTGAGAGGGT
GAACGGCCACATTGGGACTGAGACACGGCC

>Zotu12610

GAACGCTGGCGGCAGGCTTAACACATGCAAGTCGAGCGAGATCTTCGGGTCTAGCGGCGGACGGGCGAG
TAACGCGTGGGAACGTGCCCTTTGCTACGGAATAGTCCTGGGAAACTGGGGGTAATACCGTATACGCCCTT
TTGGGGAAAGATTTATCGGCGAAGGATCGGCCCGCGTTGGATTAGGTAGTTGGTGGGGTAATGGCCTACC
AAGCCGACGATCCATAGCTGGTTTGAGAGGATGATCAGCCACACTGGGACTGAGACACGGCC

>Zotu12612

GAATGCTGGCGGCGTGGATAAGGCATGCAAGTCGAACAGTCCGCAAGGACAGTGGCAGACGAGGTAGTA
ACACGTACGAACGCACCCCAGAGTCTGGCATAGCTCGTCGAAAGACGGGGTAATTCCGGATGGTCCCGAA
AGGGTAAAGTTTTTTCGCTCTGGGAGCGGCGTGCGTAATATCAGCTAGTTGGTAAGGTAATGGCTTACCAA
GGCTACGACGTTTAGGGGAGCTGAGAGGCTGACCCCCACCGATGGGACTGAGACACGGCC

>Zotu12615

GAACGCTGGCGGCGTGCCTAATACATGCAAGTCGAGCGAATCTGAGGGAGCTTGCTCCCAAAGATTAGCG
GCGGACGGGTGAGTAACACGTGGGCAACCTGCCTGTAAGACTGGGATAACTTCGGGAAACCGGAGCTAAT
ACCGGATAATTCTTATCAACTCATGTTGGTAAGCTAAAAGACGGCGTAAGCTGTCACTTACAGATGGGCCC

GCGGCGCATTAGCTAGTTGGTGAGGTAACGGCTCACCAAGGCAACGATGCGTAGCCGACCTGAGAGGGT
GATCGGCCACACTGGGACTGAGACACGGCC
>Zotu12618
GAACGCTAGCGGCAAGCTTAATACATGCAAGTCGAGGGGCAGCACGGGAGCAATCCTGGTGGCGACCGG
CAAACGGGTGCGGAACACGTACGCAACCTTCCCATAACTGGTGAATAGCCTTTCGAAAGAGAGATTAATAC
ACCGTAACATAACGAAGAGGCATCTCTTTGTTATTATAGTAGCAATACGGTTTTGGATGGGCGTGCGCCTG
ATTAGGTAGTTGGCGGGGTAACGGCCCACCAAGACTGCGATCAGTAACTGGTGTGAGAGCACGACCAGTC
ACACGGGCACTGAGACACGGGC
>Zotu12621
GAGCGCTGGCGGCAGGCTTAACACATGCAAGTCGAGCGAGACCTTCGGGTCTAGCGGCGGACGGGTGAG
TAACGCGTGGGAACGTGCCCTTTGCTACGGAATAGCCCCGGGAAACTGGGAGTAATACCGTATGAGCCCC
TTGGCCTTGGCGGTCTTGGGGGAAAGATTTATCGGCAAAGGATCGGCCCGCGTTGGATTAGGTAGTTGGT
GAGGTAACGGCTCACCAAGCCGACGATCCATAGCTGGTTTGAGAGGATGATCAGCCACACTGGGACTGAG
ACACGGCC
>Zotu12626
GAACGCTGGCGGTATGCTTAACACATGCAAGTCGAACGGCAGCATGATGTGTAGCAATACACATTGATGG
CGAGTGGCGGACGGGTGAGAAACGCGTAGGAATCTGCCTTATAGAGGGGGACAACCGGAGGAAACTCCG
GCTAATACCGCATAATACTCTTAGGAGGAAAGGTTGGGTAGCAATACCTGACGCTATTAGAGGAGCCTGC
GTCCGATTAGCTAGTTGGTAGGGTAAAGGCCTACCAAGGCGATGATCGGTAGCTGGTCTGAGAGGATGAT
CAGCCACACTGGGACTGAGACACGGCC
>Zotu12631
GAACGCTGGCGGCGTGCCTAACACATGCAAGTCGAACGGGGCATCGGGAAAGGAGACTTCGGTCAACGG
AACCGATGCTTAGTGGCGAACGGGTGAGTAACGCGTGAGGAACCTGCCTCGGAGTGGGGGACAACAGTT
AGAAATGACTGCTAATACCGCATGATGCAATTGAACCGCATGATTTGATTGCCAAAGCTTTATGTGCTCTGA
GATGGCCTCGCGTCTGATTAGATAGTTGGTGGGGTAACGGCCTACCAAGTCTACGATCAGTAGCCGGACTG
AGAGGTTGAACGGCCACATTGGGACTGAGATACGGCC
>Zotu12640
GAACGCTGGCGGCAGGCCTCAAACATGCAAGTCTAGGGGGTGTAGCAATACATCACCGGCGGACGGGTG
CGCAACGCGTGGGAATGTACCTATTGGTTCGGAACAACTGAGGGAAACTTCAGCTAATACCGAATGTGCCC
GTAAGGGGAAAGATTTATCGCCAATGGATCAGCCCGCGTAGGATTAGCTAGTTGGTGAGGTAATGGCTCA
CCAAGGCTCCGATCCTTAGCTGTTCTGAGAGGAAGATCAGCCACACTGGGACTGAGACACGGCC
>Zotu12644
GAACGTTGGCGGCGTGCCTAACACATGCAAGTTGAGCGTGGCTTTAGTTTACTAAAGTCTAGCAGCGGACG
GCTGAGTAATGCACGGGAACGTACCTCGAAGCGGGGGATAGCCCATCGAAAGATGGGGTAATACCGCAT
GTGATCGCAAGATCAAAGCCGCAAGGCGCTTTGAGAGCGGCCTGTGTCCTATCAGGTTGTTGGCGGGGTA
AAGGCCCACCAAGCCTATGACGGGTAGCTGGTCTGAGAGGATGATCAGCCAGATCGGAACTGAGACACG
GTC
>Zotu12652
GAACGCTAGCGGCAGGCTTAATACATGCAAGTCGAGGGGCAGCGCAGTGTAGCAATACATGGGCGGCGA
CCGGCAAACGGGTGCGGAACACGTACAGAACCTTCCTTCGAGCGGGGAATAGCCCAGAGAAATTTGGATT
AATACCCCATAGCATATTGAAGTGGCATCACTTTGATATTAAAGATTTATCACTTGAAGATGGCTGTGCGGC
TGATTAGGTAGTTGGCGGGGTAACGGCCCACCAAGCCTGCGATCAGTAACTGGTGTGAGAGCACGACCAG
TCACACGGGCACTGAGACACGGGC
>Zotu12662
GCCGTATCTCAGTCCCAATCCGGCTGATCGTCCTCTCAGACCAGCTACCCGTCGTCGGCTTGGTAGGCCATT
ACCCCACCAACTACCTGATAGGCCGCAAGCCCATCCAGAAGCGCATAAAGCTTTAGACACCCGGTGATGCC
ACCAGGCGGCCACATGCGGTATTAGCAGCCCTTTCAGGCTGTTGTCCCCCACTTCCGGGCAGGTTGCCTAC
GTGTTACGCGACCGTTCGCCACTAATAGACCATTGCTGGACTATCCGTTCGACTTGCATTTCTTAGGCACGC
CGCAAGCGTTCG
>Zotu12665

CAACGCTGGCGGCGTGCCTAACACATGCAAGTCGAACGCAGTTACTCGCAAGGGTAACTGAGTGGCGAGC
GGGTGAGTAACGCGTGGGTGACCTACCTTCGAGTGGGGGATAACGTCCCGAAAGGGACGCTAATACCGCA
TGACATCCTGCCTTTGAACAGGCGGAGATCAAAGCCGGGGATCGCAAGACCTGGCGCTTGATGAGGGGCC
CGCGTCTGATTAGCTAGTTGGTGAGGTAACGGCTCACCAAGGCGACGATCAGTATCCGGCCTGAGAGGGC
GGACGGACACACTGGGACTGAGACACGGCC

>Zotu12670
GAACGCTGGCGGCAGGCCTAATACATGCAAGTCGAACGCCCCTTCGGGGGAGTGGCGCACGGGTGAGTA
ACGCGTGGGAATGTGCCCTTGGGTACGGAACAACACCGGGAAACTGGTGCTAATACCGTATAATGACTTC
GGTCCAAAGATTTATTGCCCAAGGATCAGCCCGCGTAGGATTAGCTAGTTGGTGAGGTAATGGCTCACCAA
GGCTCCGATCCTTAGCTGGTCTTAGAGGATGATCAGCCACACTGGAACTGAGACACGGTC

>Zotu12676
GAACGCTGGCGGCATGCTTAACACATGCAAGTCGAACGCGGTGTCCTGGTTCGCCGGGGCACTGAGTGGC
GCACGGGTGAGTAGCGCGTGGGAACCTGCCCAGCAGTGGGGGATAACCGAGGGAAACCTCGGCTAATAC
CGCATGCGCCGGAGGTAACTCTGGGAAAGATTTATCGCTGTTGGAGGGGCCCGCGTCGGATTAGCTAGTT
GGTGGGGTAACGGCCCACCAAGGCGATGATCCGTAGCTGGTCTGAGAGGATGATCAGCCACACTGGGACT
GAGACACGGCC

>Zotu12680
GAACGCTAGCGGCATGCCTAACACATGCAAGTCGAACGGTAACATGAGTAGCAATACTTGATGACGAGTG
GCGCACGGGTGCGTAACGCGTATGCAATCTACCTTTAACTGGTGAATAGCCCGAAGAAATTCGGATTAACA
CACCATAGTATTATGAAGTGGCATCATTTTATAATTAAAACTCCGGTGGTTAAAGATGAGCATGCGTGACAT
TAGCTAGTTGGTGAGGTAATGGCTCACCAAGGCTACGATGTCTAGGGGATCTGAGAGGATTAACCCCCAC
ACTGGTACTGAGACACGGAC

>Zotu12682
GAACGCTGGCGGCATGCTTTACACATGCAAGTCGAACGGTAACAGGCCTTCGGGTGCTGACGAGTGGCGA
ACGGGTGAGTAATGCATCGGAACGTGCCCAGTCGTGGGGGATAACTGCTCGAAAGAGCAGCTAATACCGC
ATACGATCTGCGGATGAAAGCGGGGGACCTTCGGGCCTCGCGCGATTGGAGCGGCCGATGTCAGATTAGG
TAGTTGGTGGGGTAAAGGCCTACCAAGCCGACGATCTGTAGCTGGTCTGAGAGGACGACCAGCCACACTG
GGACTGAGACACGGCC

>Zotu12684
GAACGCTGGCGACGTGCCTAACACATGCAAGTCGAACGGTAGCAGGTCTAGTTTACTAGATGCTGACGAG
TGGCGGACGGCTGAGTAACGCGTGGGAACGTGCCCTAAAGTGAGGGATAACGCACCGAAAGGTGTGCTA
ATACCGCATATGATCTTCGGATTAAAGAATTTATTCGCTTTAGGAGCGGCCCGCGTCGGATTAGGTTGTTGG
TGAGGTAATGGCTCACCAAGCCGACGATCCGTAGCTGGTCTGAGAGGATGACCAGCCAGACTGGAACTGA
GACACGGTC

>Zotu12686
GAACGCTGGCGGCAGGCTTAACACATGCAAGTCGAGCGGGCACTTCGGTGTCAGCGGCGGACGGGTGAG
TAACGCGTGGGAACGTACCCTCAGGTTCGGAACAACTCTGGGAAACTGGAGCTAATACCGGATACGCCCTT
TTGGGGAAAGGCTTGCTGCCTGAGGATCGGCCCGCGTCTGATTAGCTAGTTGGTGGGGTAACGGCCTACC
AAGGCGACGATCAGTAGCTGGTCTGAGAGGATGATCAGCCACACTGGGACTGAGACACGGCC

>Zotu12690
GAACGTTCGCGGCGCGCCTAACACATGCAAGTCGCACGAGAAAGGGCTTCGGCCCCGGTACAGTGGCGCA
CGGGTGAGTAACACGTGGGTAATCTTCCCTCGAGCGGGGGATAACATTCCGAAAGGAGTGCTAATACCGC
ATAAGACCACGGCCTCGAAAGAGGCTGCGGTCAAAGCTGGCCTCTATACATAAGCTAGCACTTGAGGATG
AGCCCGCGGCCCATCAGGTAGTTGGTAGGGTAACGGCCTACCAAGCCAAAGACGGGTAGCTGGTCTGAGA
GGATGATCAGCCACACTGGAACTGAGACACGGTC

>Zotu12695
GAACGCTAGCGGCATGCCTAACACATGCAAGTCGAACGCGAAAGTGGAGCAATCCATGAGTAGAGTGGCG
AACGGGTGAGTAACACGTGGGTGACCTACCTTCGAATGGGGGATAACGTCCCGAAAGGGACGCTAATACC
GCATAACATCCTGTCCTTGAACGGATGGAGATCAAAGCCGGGGATCGCAAGACCTGGCGTTTGAAGAGGG
GCCCGCGTCCGATTAGCTAGTTGGTGAGGTAATGGCTCACCAAGGCGACGATCGGTAACCGGCCTGAGAG
GGCGGACGGTCACACTGGGACTGAGACACGGCC

>Zotu12697
GAACGCTTGCGGCGCGCCTAATACATGCAAGTCGAACGAGAAAGTTCTTCGGAATGAGTACAGTGGCGGA
CGGGTGAGGAATACGTAGATAATCTGCCTTAAAGAGGGGGATAACTACTCGAAAGAGTAGCTAATACCGC
ATAAGACCACAGGATCTTCGGATCTAGGGGTTAAAGGCTTCGGCCACTTTAAGATGAGTCTGCGTCCGATT
AGCTAGTTGGTGAGGTAATGGCTCACCAAGGCGACGATCGGTAGCTGGTCTGAGAGGATGGCCAGCCACA
CTGGAACTGAGACACGGTC
>Zotu12702
GAACGCTGGCGGCGTGCCTAAGACATGCAAGTCGAGTGGGTTTAGGCCCACGGCAAACGGGTGAGTAAT
ACCTAGGAACTTTCTTTAAGTGGAGCATAACCAGTCGAAAGATTGGATAATACTCCATGTGGCCGCAAGGC
CAAAGTCCAAAAGACGCTTAAAGTGAGGCCTGGGCACCATCAGCTAGTTGGTAAGGTAATGGCTTACCAA
GGCTATGACGGTTAGGGGCCCTCAGAGGGGAGTCCCCCACACTGGAACTGCGACACGGTC
>Zotu12704
GAACGCTGGCGGCGTGCTTAACACATGCAAGTCGAACGGTGAACCAGGGCTTGCCCTGGGGATCAGTGGC
GAACGGGTGAGTAACACGTGACCAACCTGCCCTTGGCTCTGGGATAACCCCGGGAAACCGGAGCTAATAC
CGGATACGAGACCGTGCGGCATCACGCGGTCTGGAAAGTTTTTCGGCCAAGGATGGGGTCGCGGCCTATC
AGCTTGTTGGTGGGGTAACGGCCTACCAAGGCGACGACGGGTAGCCGGCCTGAGAGGGCGACCGGCCAC
ACTGGGACTGAGACACGGCC
>Zotu12711
GAACGCTGGCGGCATGCCTTACACATGCAAGTCGAACGGCAGCACGGGAGCAATCCTGGTGGCGAGTGG
CGAACGGGTGAGTAACATATCGGAACGTGCCCAGTAGTGGGGGATAGCCCGGCGAAAGCCGGATTAATA
CCGCATACGACCTAAGGGTGAAAGCGGGGGATCGCAAGACCTCGCGCTATTGGAGCGGCCGATATCAGAT
TAGCTAGTTGGCGGGGTAAAAGCCCACCAAGGCGACGATCTGTAGCTGGTCTGAGAGGACGACCAGCCAC
ACTGGGACTGAGACACGGCC
>Zotu12715
GAACGCTGGCGGCGTGCTTAACACATGCAAGTCGAGCGGAAAGGCCCTTTCGGGGGCACTCGAGCGGCG
AACGGGTGAGTAACACGTGAGTAATCTGCCCTGCGCTCTGGGATAGCCACCGGAAACGGTGATTAATACC
GGATAGGACCACTACAGGCATCTGTTGGTGGTGGAAAGTTTTTCGGCGCAGGATGTGCTCGCGGCCTATC
AGCTTGTTGGTGAGGTAATGGCTCACCAAGGCTTTGACGGGTAGCCGGCCTGAGAGGGTGACCGGTCACA
CTGGGACTGAGACACGGCC
>Zotu12719
GAACGCTGGCGGCGGGTTTAACACATGCAAGTCGAGGGTCGAAACCTCCTTCGGGAGGCTGAAACCGGCG
AACGGGTGAGTAACACGTGAGGAACCTACCCTCTGCTCTGGGATAACCGCGGGAAACCGTGGCTAATACC
GGATGCTCCACCGAGATCGCATGATCTCGATGGGAAAGCTCAGGCGGCAGAGGACGGCCTCGCGGCGTAT
CAGCTAGTTGGTGGGGTAACGGCCTACCAAGGCGACGACGCGTAGCTGGAGTGAGAGCTCGAACAGCCA
CACTGGGACTGAGACACGGCC
>Zotu12724
GAACGCTGGCGGCGTGCTTAACACATGCAAGTCGAGCGGAAAGGCCTCTTCGGGGGTACTCGAGCGGCG
AACGGGTGAGTAACACGTGAGTAATCTGCCCTTCACTTGTGGATAACCACCGGAAACGGTGGCTAATACAT
GATATGACATCCGTCCGCATGGGCTGGGTGTGGAAAGTTTTTTCGGTGGAGGATGTGCTCGCGGCCTATCA
GCTTGTTGGTGGGGTAATGGCCTACCAAGGCTTCGACGGGTAGCCGGCCTGAGAGGGTGACCGGCCACAC
TGGGACTGAGACACGGCC
>Zotu12726
GAACGCTGGCGGCGTGCCTAATACATGCAAGTCGAGCGCGGGAAGCTAACTGATCCCTTCGGGGTGACGT
TAGTGGAACGAGCGGCGGACGGGTGAGTAACACGTGGGCAACCTGCCTGTAAGACTGGGATAACCCCGG
GAAACCGGAGCTAATACCGGATGTGCTTGTCGCACCGCATGGTGCGATGAGTAAAGGCGGATTTATTCGTC
ACTTACAGATGGGCCCGCGGCGCATTAGCTAGTTGGTAAGGTAATGGCTTACCAAGGCAACGATGCGTAG
CCGACCTGAGAGGGTGATCGGCCACACTGGGACTGAGACACGGCC
>Zotu12728
GAACGCTGGCGGTGTGCCTTAAGCATGCAAGTAGAACGAGTTCTAACTTGCCGCAAGGCAAGCTTGGAAC
TAGTTGCGAACGGCTGAGTAATCAATAGGAATCTTTCCTTTGGTGGAGAATACCTCATCGAAAGATGAGCT

AATACTGCATAACATCTACGGATAAAAGCGCATAACTGCGCGCCGATTGAGGAGCCTATTGCCCATCAGGT
AGTTGGTAGGGTAATGGCCTACCAAGCCTACGACGGGTACCCGATCTGAGAGGATGACCGGGCAGAATG
GGACTGAGACACGGCC
>Zotu12736
GAACGCTGGCGGCGTGCCTAACACATGCAAGTCGAACGCGAACGGCGCTTTCGGGTGCCTAGTAGAGTGG
CGCACGGGTGAGTAACACGTAGACAACCTGCCTTCGAGCCCGGAATAACCTTTCGAAAGAAGGGCTAATA
CCGGATAACATCCTGGGTTGCATGACTTGGGATTGAAAGGCTTGCTACTCGAAGAGGGGTCTGCGGGACA
TTAGCTAGTTGGTGGGGTAACGGCCTACCAAGGCGACGATGTCTAGCGGGTCTGAGAGGATGATCCGCCA
CACTGGAACTGAGACACGGTC
>Zotu12738
GAACGCTGGCGGCATGCCTAACACATGCAAGTCGAACGAGACCTTCGGGTCTAGTGGCGCACGGGTGCGT
AACGCGTGGGAATCTACCCTTGGGTTCGGAATAACTCTCCGAAAGGAGTGCTAATACCGGATGATGACGTA
AGTCCAAAGATTTATCGCCCAAGGATGAGCCCGCGTAGGATTAGCTAGTTGGTGAGGTAAAAGCTCACCA
AGGCGACGATCCTTAGCTGGTCTGAGAGGATGATCAGCCACACTGGGACTGAGACACGGCC
>Zotu12744
GAACGCTGGCGGCATGCCTAACACATGCAAGTCGCGCGGCCGGGTAACACCGGCAGCGGCGGACGGGTG
AGTAGCGCGTAGGAACGTGTCCTGTGGTGGGGGACAACCCTGGGAAACCGGGGCTAATACCGCATGGCC
CCTTTGGGGGAAAGCAGCGATGCGCCACAGGAGCGGCCTGCGTCCGATTAGCTAGTCGGCGGGGTAACG
GCCCACCGAGGCGACGATCGGTAGCTGGTCTGAGAGGACGATCAGCCACACTGGGACTGAGACACGGCC
>Zotu12746
GAACGCTATCGGTATGCTTAACACATGCAAGTCGAACGCCCCGCAAGGGGAGTGGCAGACGGGTGAGTAA
CACGTGGGAATGTGCCCTTTGGTTCGGAATAACTTGGGGAAACTCAAGCTAATACCGGATACGCCCTTTGG
GGGAAAGGTTTACTGCCGAAGGAGCAGCCCGCGTCCGATTAGCTAGTTGGTGAGGTAATGGCTCACCAAG
GCGACGATCGGTAGCTGGTCTGAGAGGATGGCCAGCCACATTGGGACTGAGACACGGCC
>Zotu12748
GAACGCTGGCGGCGTGCTTAACACATGCAAGTCGAGCGGTAAGGCTCCTCCGGGAGTACACGAGCGGCG
AACGGGTGAGTAACACGTGAGTAATCTGCCCTTCACTTCGGGATAACTGCCGGAAACGGCAGCTAATACCG
AATACGACGCACCTGCGCATGCAGTGTGCGTGGAAAGTCTTGTCGGTGGAGGATGTGCTCGCGGCCTATC
AGCTTGTTGGTGAGGTAACGGCTCACCAAGGCTTCGACGGGTAGCCGGCCTGAGAGGGTGACCGGCCACA
CTGGGACTGAGACACGGCC
>Zotu12758
GAACGCTGGCGGCGCGCTTAACACATGCAAGTCGCACGCCTCCTTCGGGGGGAGTGGCGCACGGGTGAGT
AACGCGTGGGAACCTGCCCTGGGGTTCGGGACAACCGCGGGAAACTGCGGCTAATACCGGATACGACCAT
TGGTGAAAGGGGGAAACCCCGTCCTGGGATGGGCCCGCGTCCGATTAGCTTGTTGGTGGGGTAACGGCCT
ACCAAGGCTTCGATCGGTAGCCGGTCTGAGAGGATGATCGGCCACACTGGGACTGAGACACGGCC
>Zotu12760
CAACGCTGGCGGCGCGCTTAACACATGCAAGTCGAACGAGAAAGGGGAGCAATCCCTGAGTAAAGTGGC
GTACTGGTGAGTAACACGTGGATATCTACCTCCTGGTGGGGAATAACCTGGGGAAACCTGGGCTAATACC
GCATAAGTTCGTGAGAAGAAAGCCGCAAGGCGCCGGGAGAGGAGTCCGCGGTCGATTAGGTAGTTGGCG
GGGTAAGAGCCCACCAAGCCAAAGATCGATAGCCGGCCTGAGAGGGCACACGGCCACACTGGCACTGAA
ACACGGGC
>Zotu12763
GAACGCTGGCGGCGTGCCTAACACATGCAAGTCGAACGGGGTTGAGGTCTTCGGACCTTAACTTAGTGGC
GAACGGGTGAGTAACGCGTGAGAAACCTGCCTTTCAGTGGGGGACAACAGTTGGAAACGAATGCTAATAC
CGCATAATGTTATTTGGGGGCATCCCCGAATAACCAAAGCTTTATGTGCTGAAAGATGGTCTCGCGTCTGAT
TAGCTAGATGGCGGGGTAACGGCCCACCATGGCGACGATCAGTAGCCGGTCTGAGAGGATGAACGGCCA
CATTGGGACTGAGATACGGCC
>Zotu12766
GAACGCTGGCGGCAGGCCTAATACATGCAAGTCGAGCGGATGTAGCAATACATCAGCGGCGCACGGGAG
AATAACACGTGGGAACCTACCTTTTGGTTCGGAATAACACCGGGAAACTGGTGCTAATACCGGATAAGCCC

TTCGGGGGAAAGATTTATTGCCGAAAGATGGGCCCGCGTTGGATTAGCTAGTTGGTGAGGTAACGGCTCA
CCAAGGCAACGATCCATAGCTGGTCTGAGAGGACGATCAGCCACACTGGGACTGAGACACGGCC
>Zotu12769
GAACGCTGGCGGCAGGCCTAACACATGCAAGTCGAGCGGTAACACAGGGAGCTTGCTTCCGGGTGACGA
GCGGCGGACGGGTGAGTAATGTCTGGGAAACTGCCTGATGGAGGGGGATAACTACTGGAAACGGTAGCT
AATACCGCATAACGTCGCAAGACCAAAGAGGGGGACCTTCGGGCCTCTTGCCATCAGATGTGCCCAGATG
GGATTAGCTAGTAGGTGAGGTAATGGCTCACCTAGGCGACGATCCCTAGCTGGTCTGAGAGGATGACCAG
CCACACTGGAACTGAGACACGGTC
>Zotu12771
GAACGCTGGCGGCGTGCCTAATACATGCAAGTCGAGCGGTAACACTTCGGGTGACGAGCGGCGGACGGG
TGAGTAATGCATGGGAACGTGCCCCAAAGTGAGGAATAAGCACCAGAAATGGTGTCTAATACCGCATATT
ATCTTAGGATCAAAGCTTCGGCGCTTTGGGAGCGGCCCATGTCTGATTAGCTAGTTGGTAGGGTAAAAGCC
TACCAAGGCGATGATCAGTAGCTGGTTTGAGAGGATGATCAGCCACACTGGAACTGAGACACGGTC
>Zotu12774
GAACGCTAGCGGCATGCTTAACACATGCAAGTCGAGCGGGGTTTTCGGACCTAGCGGCGGACGGGTGAGT
AATGCGTAGGAAGCTGCCCGATAGAGGGGGATACCAGTTGGAAACGACTGTTAATACCGCATAATGTCTA
CGGACCAAAGTGTGGGACCTTCGGGCCACATGCTATCGGATGCGCCTACGTGGGATTAGCTAGTTGGTGA
GGTAATGGCTCACCAAGGCGACGATCTCTAGCTGGTTTGAGAGGATGATCAGCCACACTGGAACTGAGAC
ACGGTC
>Zotu12777
GAACGCTGGCGGCGTGCTTAACACATGCAAGTCGAGCGGGATCCAGCTAGTCGCAAGACTGGGGAAGATC
TAGCGGCGAACGGGTGAGTAACACGTGAGAAACCTGCCCTGAAGTCTGGGATAACCCGAGGAAACTCGA
GCTAATACCGGATGCCTTCATTTGATCGCATGGTCGAATGAAGAAAGATTTATTGCTTCAGGAGGGTCTCG
CGGCCTATCAGCTAGTTGGCGGGGTAACGGCCCACCAAGGCGTCGACGGGTAGCTGGTCTGAGAGGACG
ATCAGCCACACTGGGACTGAGACACGGCC
>Zotu12783
CAACGCTGGCGGCGTGCCTAATGCATGCAAGTCGAACGGGGTGGCTTTCGGGCCACTTACGTGGCGGACG
GGTGAGGAACACGTGGGCAATCTGTCCCGAGGTGGGGGGTAGCCCTCGGAAACGGGGGGTAATTCCGCA
TGGGTCCGGTCGGTCGTGGGCTGATCGGGCAAAGGGACCTTCGGGTCTCGCCTTGGGGGGAGCCTGCGG
CCGATTAGCTAGTTGGTGTGGTTACGGCGCACCAAGGCGGTGATCGGTAGCTGGTCTGAGAGGACGGCCA
GCCACACGGGGACTGAGACACGGCC
>Zotu12785
GAACGCTAGCGGGAGGCCTAACACATGCAAGCCGAGCGGTATGGAATAGCTTGCTATTCCAGAGAGCGGC
GTACGGGTGCGGAACACGTGTGCAACCTGCCTTTATCAGGGGGATAGCCTTTCGAAAGGAAGATTAATAC
CCCATAATATAATGAATGGCATCATTTATTATTGAAAACTGAGGTGGATAGAGATGGGCACGCGCAAGATT
AGATAGTTGGTGAGGTAACGGCTCACCAAGTCGATGATCTTTAGGGGGCCTGAGAGGGTGATCCCCCACA
CTGGTACTGAGACACGGAC
>Zotu12789
GAACGCTGGCGGCAGGCCTAACACATGCAAGTCGAGCGGTAACACAGGGAGCTTGCTCCTGGGTGACGA
GCGGCGGACGGGTGAGTAATGTCTGGGAAACTGCCTGATGGAGGGGGATAACTACTGGAAACGGTAGCT
AATACCGCATAACGTCGCAAGACCAAAGAGGGGGACCTTCGGGCCTCTTGCCATCAGATGTGCCCAGATG
GGATTAGCTAGTAGGTGGGGTAACGGCTCACCTAGGCGACGATCCCTAGCTGGTCTGAGAGGATGACCAG
CCACACTGGAACTGAGACACGGTC
>Zotu12792
GAACGCTGGCGGCGTGCCTCCTCCCTGCCCGTCGCCCGCTGCGGCCTGGTGCTTGCCCCGGTCCAAGGCGT
TGCGAACGGGTGAGTAACGCGTAGGTAACCTACCTCATAGCGGGGGATAACTATTGGAAACGATAGCTAA
TACCGCATAAAAGTGTTTAACACATGTTAAACATTTAAAAGATGCAACTGCATCACTATGAGATGGACCTGC
GTTGTATTAGCTAGTTGGTGAGGTAACGGCTCACCAAGGCGACGAGACAGAGCCGACCGGAGAGGGTGA
TCGGCCACACGGGGACGGAGACACGGCC
>Zotu12794

GAACGCTGGCGGCGTGCCTAATACATGCAAGTCGAGCGAACCTTTGGGAGCTTGCTCCCAAAGGTTAGCG
GCGGACGGGTGAGTAACACGTGGGTAACCTGCCTGTAAGACTGGGATAACTTCGGGAAACCGGAGCTAAT
ACCGGATAACATATGAGACCGCATGGTCTTATATTAGAAGATGGCTTTTAGCTATCACTTACAGATGGACCC
GCGGCGCATTAGCTAGTTGGTGAGGTAATGGCTCACCAAGGCAACGATGCGTAGCCGACCTGAGAGGGTG
ATCGGCCACACTGGGACTGAGACACGGCC
>Zotu12798
GAACGCTGGCTGTATGCCTAATACATGCATGTCGAGCGAAGCACTTGTGCTTAGCGGCGAATGGGTGAGT
AACACGTACTTAATCTGCCTCTTAGACTGGGACAACAGTTGGAAACGACTGCTAATACCGGATATGCTTGG
AAACATAAGTTTCCGTAGTGAAAGATGCGTTTGCATCACTAAGAGATGAGAGTGCGGAACATTAGCTAGTT
GGTAGGGTAAAGGCCTACCAAGGCTATGATGTTTAGCGGGGTCGAGAGGCTGAACCGCCACACTGGGACT
GAGATACGGCC
>Zotu12801
GAACGTTGGCGGCGTGGATTAGGCATGCAAGTCGAACGGGTCCGGCTTCGGCCGGGCCAGTGGCGGAAG
GGTGAGTAAGGCGACGGCAACCAACCCCGAGGTCGGGTATAGCCGCGGGAAACTGCGGGTAATCCCCGG
CGACGTGACGACGCCGACATCGGCGTTTCACCAAAGGCGCGAGCCGCCTTGGGACGGGCCGTCGTGGTAT
TAGGTAGTTGGCGGGGTAACGGCCCACCAAGCCGATGATGCCTACCGGGCGTGCGAGCGTGGCCCGGCA
CACTGGGACTGAGACACTGCC
>Zotu12803
CAACGCTGGCGGCGTGCCTAACACATGCAAGTCGCACGAGAAAGGGGCTTCGGCCCTGAGTAAAGTGGCG
CACGGGTGAGTAACACGTGACTAATCTACCTTCGAGTGGGGAATAACCTCGGGAAACCGGGGCTAATACC
GCATAACACTTACGAGTCAAAGCAGCAATGCGCTTGAAGAGGAGGTCGCGGCAGATTAGCTAGTTGGTGG
GGTAATGGCCTACCAAGGCGATGATCTGTATCCGGCCTGAGAGGGCGCACGGACACACTGGAACTGAAAC
ACGGTC
>Zotu12806
CAACGCTGGCGGCGTGCCTAACACATGCAAGTCGAACGAGAAAGTGGAGCAATCCATGAGTACAGTGGCG
TACGGGTGAGTAACACGTGACTAACCTACCCTCGAGTGGGGAATAACCTCGGGAAACCGGGGCTAATACC
GCATAACATCTACGGATCAAATGCGTAAGCAGCTTGAGGAGGGGGTCGCGGCTGATTAGCTAGTTGGTGA
GGTAATGGCTCACCAAGGCAATGATCAGTAGCCGGCCTGAGAGGGCGCACGGCCACACTGGAACTGAAAC
ACGGTC
>Zotu12813
GAACGCTGGCGGCAGGCCTAACACATGCAAGTCGAGCGGATAAAGAGAGCTTGCTCTCCGATTCAGCGGC
GGACGGGTGAGTAATGCCTAGGAATCTGCCTGGTAGTGGGGGACAACGTTTCGAAAGGAACGCTAATACC
GCATACGTCCTACGGGAGAAAGTGGGGGATCTTCGGACCTCACGCTATCAGATGAGCCTAGGTCGGATTA
GCTAGTTGGTGGGGTAATGGCCTACCAAGGCGACGATCCGTAACTGGTCTGAGAGGATGATCAGTCACAC
TGGAACTGAGACACGGTC
>Zotu12816
TAACGCTGGCGGCGTGCCTTACACATGCAAGTCGAGCGAGAAAGTCCCTTCGGGGATGAGTAAAGCGGCA
GACGGGTGAGTAACACGTAAGTAACCTACCTCCGAATGGGGGATAACATGCCGAAAGGCGTGCTAATACC
CCGTAAAGCCACTCCTCGTAAGGGGCAGTGGTCATAGGTGCCCTATCCTTGGAAGGTACCGTTTGGAGAG
GGGCTTGCGCACCATTAGCTAGTTGGCGGGGTAATGGCCCACCAAGGCAAGGATGGTTAGCTGGTTTGAG
AGAACGACCAGCCACACTGGAACTGAGATACGGTC
>Zotu12819
GAACGCTAGCGACAGGCCTAACACATGCAAGTCGAGGGATAGCCTTCGGGCGAAACCGGCGCACGGGTG
AGTAACGCGTATGTAACCTGCCCGTAAGTGGGGGATAGCCCAGTGAAAACTGGATTAATACCGCATAACAC
CAGAAGTTGACATCGATTACTGGTTAAAGATTTATTGCTTACGGATGGACATGCGTATGATTAGCTGGTTG
GCGGGGTAACGGCCCACCAAGGCGACGATCATTAGGGGTTCTGAGAGGAAGGTCCCCCACATTGGTACTG
AGACACGGAC
>Zotu12821
GAACGCTGGCGGCATGCCTTACACATGCAAGTCGAACGGCAGCATGGGAGCAATCCTGGTGGCGAGTGGC
GAACGGGTGAGTAATATATCGGAACGTGCCCAGTCGTGGGGGATAACGTAGCGAAAGCTACGCTAATACC
GCATACGATCTATGGATGAAAGCGGGGGATCGCAAGACCTCGCGCGATTGGAGCGGCCGATATCAGATTA

GGTAGTTGGTGAGGTAAAGGCTCACCAAGCCGACGATCTGTAGCTGGTCTGAGAGGACGACCAGCCACAC
TGGGACTGAGACACGGCC
>Zotu12825
GAACGCTGGCGGCACGCCTAATACATGCAAGTCGAGCGGGCGAAGCCTGCCGATGCCTTTGGGCGGAGG
CGGGTGGAGCCAGCGGCGGACGGGTGAGTAACACGTAGGCAACCTGCCTGTAAGACCGGGATAACCACG
GGAAACCGTGGCTAATACCGGATGCGACTCATGAGCGCATGCTGATGAGTTAAAAGGATGGCATATGCTA
TCGCTTACAGATGGGCCTGCGGCGCATTAGCTAGTTGGTGAGGTAACGGCTCACCAAGGCGACGATGCGT
AGCCGGCCTGAGAGGGTGGTCGGCCACACTGGGACTGAGACACGGCC
>Zotu12827
GAACGCTGGCGGCGTGCTTAACACATGCAAGTCGAACGGTAAGGCCCTTCGGGGTACACGAGTGGCGAAC
GGGTGAGTAACACGTGGGTGATCTGCCCTGCACTTTGGGATAAGCCTGGGAAACTGGGTCTAATACCGGA
TAGGACCATGGACTTCACGGTTTGTGGTGGAAAGCTTTTGCGGTGTGGGATGGGCCCGCGGCCTATCAGC
TTGTTGGTGGGGTAATGGCCTACCAAGGCGACGACGGGTAGCCGGCCTGAGAGGGTGTCCGGCCACACTG
GGACTGAGATACGGCC
>Zotu12832
GAACGCTGGCGGCGCGCTTAACACATGCAAGTCGAGCGAGAACCGGGGCTTCGGCCCCGGGGAAAGCGG
CGAACGGGTGAGTAACACGTGGGTAATCTGCCCTCGGCATCGGGATAGCCCGGGGAAACCCGGATTAATA
CCGGATAGCCTCCGAAGCCTTTGGGCGTCGGAGAAAAGGTAGCTTCGGCCTCTGGCCGGGGAGGAGCCCG
CGGCGGATTAGCTTGTTGGCGGGGTAACGGCCCACCAAGGCGACGATCCGTAGCTGGTCTGAGAGGACG
ATCAGCCACACTGGGACTGAGACACGGCC
>Zotu12835
GAACGCTGGCGGCGCGCTTAATACATGCAAGTCGAGCGAGAACCAGGGCTTCGGCCCTGGGGAAAGCGG
CGAACGGGTGAGTATCACGTGGGTAATCTGCCCTCGGCATCGGGATAGCCCGGGGAAACCCGGATTAATA
CCGGATGGCCTCACGAGCTTTCGGGCTTGTGAGAAAAGGTAGCTTCGGCCTCCGGCCGAGGAGGAGCCCG
CGGCGGATTAGCTTGTTGGCGGGGTAATGGCCCACCAAGGCGACGATCCGTAGCTGGTCTGAGAGGACGA
TCAGCCACACTGGGACTGAGACACGGCC
>Zotu12842
GAACGCTGGCGGCGTGCTTAACACATGCAAGTCGAGCGGTAAGGCTCCTTCGGGAGTACACGAGCGGCGA
ACGGGTGAGTAACACGTGAGCAATCTGCCCTTCACTTCGGGATAACCCTCGGAAACGAGAGCTAATACCGA
ATACGACCACCTTGGGCATCCAATGGTGGTGGAAAGTTCCGGCGGTGGAGGATGAGCTCGCGGCCTATCA
GCTTGTTGGTGGGGTAACGGCCCACCAAGGCTTCGACGGGTAGCCGGCCTGAGAGGGTGACCGGCCACAC
TGGGACTGAGACACGGCC
>Zotu12846
GAACGCTGGCGGCAGGCTTAACACATGCAAGTCGAACGGCCATTTCGGTGGCAGTGGCGGACGGGTGAG
TAACGCGTGGGAACGTGCCCAAAGGTAGGGAACAACCGAGGGAAACTTCGGCTAATACCCTATGTGCCCG
GAAGGGGAAAGATTTATCGCCTTTGGAGCGGCCCGAGTCTGATTAGCTAGTTGGTGGGGTAAAGGCTCAC
CAAGGCTTCGATCAGTAGCTAGTCTGAGAGGATGGCCAGCCACATTGGGACTGAGACACGGCC
>Zotu12849
GAACGCTGGCGGCATGCCTAACACATGCAAGTCGAACGGCAGCACAGCAGTAGCAATACTGTGGGTGGCG
AGTGGCGAACGGGTGAGGAATGCGTCGGAATCTGCCCAGTTGTGGGGGATAACTAGCCGAAAGGTTAGC
TAATACCGCATACGACCGAGAGGTGAAAGCGGGGGACCGTAAGGCCTCGCGCGATTGGATGAGCCGACG
CCGGATTAGCTTGTTGGTGGGGTAAAGGCCTACCAAGGCGACGATCCGTAGCTGGTCTGAGAGGATGATC
AGCCACACTGGGACTGAGACACGGCC
>Zotu12853
GAACGTTGATGGCGTGCCTCATACATGCAAGTCGAGCGCCCCGCAAGGGGAGCGGCGGACGGGTGAGTA
ACACGTAAAGAATCTGCCCTGAAGCGGGGAATAACTATCCCAACGGATAGCTAATACCGCATAATGATGGA
GGAGGCATCTCCTTTTCTCAAAAGGCTTCGGCCACTTCAGGATGATTTTGCGTCCCATTAGCTTGTTGGCGG
TGTAACGGACCACCAAGGCTACGATGGGTATCCGGCCTGAGAGGGTGTACGGACACATTGGGACTGAGAT
ACTGCC
>Zotu12855

TAACGCTGGCGGCGTGCCTTACACATACAAGTCGAGCGAGAAAGTCCCTTCGGGGATGAGTAAAGCGGCA
GACGGGTGAGTAACACGTAAGTAACCTACCTCCGAATGGGGGATAACATGCCGAAAGGCGTGCTAATACC
CCGTAAAGCCACTCCTCGTAAGGGGCAGTGGTCATAGGTGCCCTATCCTTGGAAGGTACCGTTTGGAGAG
GGGCTTGCGCACCATTAGCTAGTTGGCGGGGTAATGGCCCACCAAGGCAAGGATGGTTAGCTGGTTTGAG
AGAACGACCAGCCACACTGGAACTGAGATACGGTC
>Zotu12857
GAATGCTGGCGGCGTGCCTAACACATGCAAGTCGAGCGGCAGCGCGAGTAGTTTACTACTTGGCGGCGAG
CGGCGGACGGCTGAGTAACGCGTGGGAACGTGCCCCAAAGTGAGGAATAACTGCCCGAAAGGGTAGCTA
ATGCCGCATATGCTCTTAGGAGTAAAGTCTTCGGACGCTTTGGGAGCGGCCCGCGTCGGATTAGGTTGTTG
GTGAGGTAATGGCTCACCAAGCCGACGATCCGTAGCTGGTCTGAGAGGATGATCAGCCACACTGGGACTG
AGACACGGCC
>Zotu12859
GAACGCTGGCGGCGGGCCTAATACATGCAAGTCGAACGGATTTAATCTTTCGGGATTAAGTTAGTGGCGC
ACGGGTGAGTAACGCGTACACAACGTGGCTCTCAGAGGGGGATAGCCTTGGGAAACTGAGCGTAATACCC
CATAGTATTATTTGGAGGCATCTTTGAATAATTAAACCTACGGGGCTGAGAGATCGGTGTGCGTTAGATTA
GGTAGTTGGTGGGGTAACGGCTCACCAAGCCTACGATCTATAAGGGGCGTGAGAGCGTGATCCCTCACAC
GGGTACTGAGACACGGAC
>Zotu12864
GAACGCTGGCGGCGTGCTTAACACATGCAAGTCGAACGGTGAAGCCCTTCGGGGTGGATCAGTGGCGAAC
GGGTGAGTAACACGTGAGTAATCTGCCCTTGACTCTGGGATAACTTCGGGAAACCGGAGCTAATACCGGA
TATTCACATTCTGTCGCATGGTGGGGTGTGGAAAGGATTCTGGTTGAGGATGAGCTCGCGGCCTATCAGCT
TGTTGGTGGGGTAATGGCCTACCAAGGCGACGACGGGTAGCCGGCCTGAGAGGGTGTCCGGCCACACTG
GGACTGAGATACGGCC
>Zotu12866
GAACGCTAGCGGCATGCCTAACACATGCAAGTCGAACGGCAGCACAGGAGAGCTTGCTCTCTGGGTGGCG
AGTGGCGGACGGGTGAGGAATACATCGGAATCTACCTTTTCGTGGGGGATAACGTAGGGAAACTTACGCT
AATACCGCATACGACCTACGGGTGAAAGTGGGGGACCGCAAGGCCTCACGCGATTAGATGAGCCGATGTC
CGATTAGCTAGTTGGCGGGGTAATGGCCCACCAAGGCGACGATCGGTAGCTGGTCTGAGAGGATGATCAG
CCACACTGGAACTGAGACACGGTC
>Zotu12868
CAGCCGCGGCCGCCGCCGCTGGTTCTGTGCTGGACGACCCGCAGTCGCTGTCGGGACTCCCGGCGGCGCA
CCTGCTCGGCTTATACTCATCGCTTGCCAGGCCCAAACCTCACGACGCCCGTCTCGAGGTGAGCCGACGTTA
TCCCTTACATTCGTAGATTGCGTACGTATAGATGCCAGAGATGGCAATAAGTCAACCCTAAGCGACTGAGA
GTTGAAGCTTGTCCTCTTGTGCATGTAGATTTCTTTTTTTAGATTTTAGACCGTGAATTGCAAATTGATGGTA
TGCGTACGTTGCCGATGGTCTTTTCAAGGGCGTATCCCCGTCATCCGATATCTTTTCCTTCGTCACCACGCAC
AACGACAGTAATGGGAGCTTAGTGT
>Zotu12870
GAACGCTGGCGGCGTGCCTAACACATGCAAGTCGAACGGGACTTATTGAGACGATTTCGGTCTGATCATTA
AGTTTAGTGGCGGACGGGTGAGTAACGCGTGGACAACCTGCCTTTCACAGGGGGATAGCAGCTGGAAAC
GGCTGGTAATACCGCATACGCTCATATCACCGCATGGTGGTGTGAGGAAAGATTTATCGGTGAAAGATGG
ATCCGCGTCTGATTAGCCAGTTGGCGGGGTAACGGCCCACCAAAGCGACGATCAGTAGCCGGCCTGAGAG
GGCGGACGGCCACATTGGGACTGAGACACGGCC
>Zotu12873
GAACGCTGGCGGCGTGCCTAACACATGCAAGTCGAACGGGACTTATTGAGACGATTTCGGTCTGATCATTA
AGTTTAGTGGCGGACGGGTGAGTAACGCGTGGACAACCTGCCTTTCACAGGGGGATAGCAGCTGGAAAC
GGCTGGTAATACCGCATACGCTCGTGTCACCGCATGGTGATATGAGGAAAGATTTATCGGTGAAAGATGG
ATCCGCGTCTGATTAGCCAGTTGGCGGGGTAACGGCCCACCAAAGCGACGATCAGTAGCCGGCCTGAGAG
GGCGGACGGCCACATTGGGACTGAGACACGGCC
>Zotu12878
GAACGCTGGCGGCACGCTTAACACATGCAAGTCGAACGAGGTCCATCCACTCGCAAGAGTGGTGAAGACC
TAGTGGCGAACGGGTGAGTAACACGTGAGAAACCTGCCCCGAAGACCGGGATAACTCACCGAAAGGTGA

GCTAATACCGGATACCCTCACGACGCTTCATGGCGATGTGAGGAAATGGATTCCGCTTCGGGAGGGTCTCG
CGGCCTATCAGCTTGTTGGTGAGGTAACGGCTCACCAAGGCGACGACGGGTAGCTGTTCTGAGAGGAAGA
TCAGCCACACTGGGACTGAGACACGGCC

>Zotu12880
AAACGCTGGCGGCGTGCATGAGACATGCAAGTCGAACGGACGACTCAGGCTTGCTTGAGTCGTTAGTGGC
GGACGGCTGAGTAACGCGTAGGTGACCTACCCCGAAGTGGGGAATAACTGCCCGAAAGGGTGGCTAATA
CCGCATGTGGTGATGCGTTCATTCGCATCAGTAAAGCCGCAAGGCGCTTCGGGAGGGGCCTGCGTCCGATT
AGCTCGTTGGTGGGGTAACGGCTCACCAAGGCGACGATCGGTAGGTGGTCTGAGAGGACGATCACCCAG
ACTGGGACTGAGACACGGCC

>Zotu12884
GAACGCTGGCGGCAGGCTTAACACATGCAAGTCGAACGGGCGTAGCAATACGTCAGTGGCAGACGGGTG
AGGAACACGTGGGAACGTACCTTTTGGTTCGGAACAACTGAGGGAAACTTCAGCTAATACCGGATAAGCC
CTTCGGGGGAAAGATTTATCGCCAAAAGATCGGCCCGCGTCTGATTAGCTAGTTGGTAGGGTAACGGCCTA
CCAAGGCGACGATCAGTAGCTGGTCTGAGAGGATGATCAGCCACATTGGGACTGAGACACGGCC

>Zotu12887
GAACGCTGGCGGCGGGCCTCATACATGCAAGTCGAGCGCGAAAGTCCGCAAGGATGAGTAAAGCGGCGC
ACGGGTGAGTAACACGTGGATAATCTACCCTTTGGATTGGAATAACCTGTCGAAAGACGGGCTAATGCCG
AGTAAGCCTTCGAGAGCGTGAAGCTCTTGCAGGGAAAGATGGCGCAAGCTATCGCCGATGGATGAGTCTG
CGTCCCATTAGTTAGTTGGTGAGGTAACGGCTTACCAAGACAATGATGGGTACCTGGTCTGAGAGGATGAC
CAGGCACACTGGAACTGAAACACGGTC

>Zotu12891
GAACGCTGGCGGCGTGCTTAACACATGCAAGTCGAGCGAGGCCCGTCCTTCGGGACGGTGCCCTAGCGGC
GAACGGGTGAGTAACACGTGGGCAACCTGCCCCTAGCACTGGGATAACTCCGGGAAACCGGGGCTAATAC
CGGATATGACCTTTGAGGGCATCCTCGAAGGTGTAAAGGATTCTGGCTAGGGATGGGCCCGCGGCCTATC
AGCTTGTTGGTGGGGTAACGGCCTACCAAGGCGACGACGGGTAGCCGGCCTGAGAGGGCGACCGGCCAC
ACTGGGACTGAGACACGGCC

>Zotu12893
GAACGCTGGCGGCGTGCTTAACACATGCAAGTCGAGCGAGAAAGTCTCTTCGGAGATTAGTACAGCGGCG
GACGGGTGAGTAATGCGCAGGAACTTGCCCTTTGATGAGGAATACTCTTGAGAAATCGAGAATAATACCG
CATAAGTCCCTACGGGGAGAAAGGCGGCTTTAATGCTGTCGTCAGAGGATGGGCTTGCGTGCTATCAGCT
AGTTGGTGGGGTAAAGGCCTACCAAGGCTATGACGGCTAACCGGCCTGAGAGGGTGTACGGTCACACTGG
AACTGAGACACGGTC

>Zotu12895
GAACGCTGGCGGCAGGCTTAACACATGCAAGTCGAGCGCCCCGCAAGGGGAGCGGCAGACGGGTGAGTA
ACGCGTGGGAATCTACCCTTTGGTACGGAATAGCTCCGGGAAACTGGAATTAATACCGTATGTGGCTTACG
AGCGAAAGATTTATCGCCAAAGGATGAGCCCGCGTTGGATTAGCTAGTTGGTGGGGTAAAGGCCTACCAA
GGCGACGATCCATAGCTGGTCTGAGAGGATGATCAGCCACATTGGGACTGAGACACGGCC

>Zotu12899
AAACGCTGGCGGCGCACATAAGACATGCAAGTCGAACGAACTTAATCGTTAGTTTACTAATGAAGCGGTTA
GTGGCGGACTGGTGAGTAACACGTAAGCAACCTGCCTATTAGAGGGGAACAACAGTTAGAAATGACTGCT
AATACCGCATATGCCATAAGTATCGCATGATACAAGTGGGAAAGGAGCAATCCGCTAATAGATGGGCTTGC
GTCTGATTAGATAGTTGGTGGGGTAACGGCCTACCAAGTCGACGATCAGTAGCCGAGTTGAGAGACTCTA
CGGCCACATTGGGACTGAGATACGGCC

>Zotu12904
GAACGCTGGCGGCGTGCTTAACACATGCAAGTCGAACGGTGAAAGAGGAGCTTGCTCCTCTGGATCAGTG
GCGAACGGGTGAGTAACACGTGAGTAACCTGCCCTTGACTCTGGGATAAGCGTTGGAAACGACGTCTAAT
ACCGGATACGCGCTTCGGAGGCATCTCCAGGAGCGGGAAAGAATTTCGGTCAAGGATGGACTCGCGGCCT
ATCAGTTAGTTGGTGAGGTAACGGCTCACCAAGACGACGACGGGTAGCCGGCCTGAGAGGGTGACCGGC
CACACTGGGACTGAGACACGGCC

>Zotu12910

GAACGCTGGCGGCGTGCTTAACACATGCAAGTCGAGCGGTAAGGCCCTTCGGGGTACACGAGCGGCGAA
CGGGTGAGTAACACGTGAGTAATCTGCCCTTCACTTCGGGATAGCTTCGGGAAACCGAAATTAATACCGGA
TATGAACGTACCCTGCATGGGGTGTGTTGGAAAGTTTTTTCGGTGGAGGATGTGCTCGCGGCCTATCAGCT
TGTTGGTGAGGTAATGGCTCACCAAGGCTTCGACGGGTAGCCGGCCTGAGAGGGTGACCGGCCACACTGG
GACTGAGACACGGCC

>Zotu12913
GAACGCTGGCGGAGTGCCTAATACATGCAAGTCGAGCGGCAGCGCGAGTAGTTTACTACTTGGCGGCGAG
CGGCGGACGGCTGAGTAACGCGTGGGAACGTGCCCCAAAGTGAGGAATAACTGCCCGAAAGGGTAGCTA
ATGCCGCATATGGTCTTCGGATTAAAGGATTTATCCGCTTTGGGAGCGGCCCGCGTCAGATTAGGTAGTTG
GTGAGGTAAAGGCTCACCAAGCCGACGATCTGTAGCTGGTCTGAGAGGACGATCAGCCAGACTGGGACTG
AGACACGGCC

>Zotu12923
TATACCGGTTCCCTTGCACCTCCCCTTTCTATCGCCTGTGAAACTTGTGAATATTTTAGTAGGGCGTCCTGCA
TGTCAGTCCAACGCTGTTTAGGCGCAGAGGAGAATGACCGTGGAACGACGCCCGAGAAGGTTTCACGCAT
ATTCGCGAACCCACCGAAGGCGTTGCGTCT

>Zotu12927
GAACGCTGGCGGCGTGCTTAACACATGCAAGTCGAACGTGAAAGGTAGCAATACTGAGTAAAGTGGCGTA
CGGGTGAGTAACACGTGGGTAATCTACCAAAGAGCCTGGAATAACCGACCGAAAGGTTGGCTAATACTGG
ATAGTTCAAGCGAAATATAATGTTTCGATTGGGAAAGGGAGCGAGTAATCGCCTTCACTTTTTGATGAGCC
CGCGTCTGATTAGCTAGTTGGTGAGGTAATGGCTCACCAAGGCTGAGATCAGTAGCTGGTCTGAGAGGAT
GATCAGCCACACTGGAACTGAAACACGGTC

>Zotu12934
GAACGCTGGCGGCATGCCTTACACATGCAAGTCGAACGGCAGCGCGGGCTTCGGCCTGGCGGCGAGTGG
CGAACGGGTGAGTAATACATCGGAAGGTGCCCTGTTGTGGGGGATAACTAGTCGAAAGATTAGCTAATAC
CGCATACGACCTGAGGGTGAAAGCGGGGGACCACAAGGCCTCGCGCAATAGGAGCGACCGATGTCTGAT
TAGCTAGTTGGTGGGGTAAAGGCCTACCAAGGCGACGATCAGTAGCTGGTCTGAGAGGACGATCAGCCAC
ACTGGGACTGAGACACGGCC

>Zotu12937
GAACGCTGGCGGCGTGCTTAACACATGCAAGTCGAACGATGAAGCCCAGCTTGCTGGGTGGATTAGTGGC
GAACGGGTGAGTAACACGTGAGTAACCTGCCCCTGACTTCGGGATAAGCCTGGGAAACTGGGTCTAATAC
CGGATATCACTATGGAACGCATGTTTTGTGGTTGAAAGATTTATTGGTTGGGGATGGACTCGCGGCCTATC
AGCTTGTTGGTGAGGTAATGGCTCACCAAGGCGACGACGGGTAGCCGGCCTGAGAGGGTGACCGGCCAC
ACTGGGACTGAGACACGGCC

>Zotu12941
GAACGCTGGCGGCGTGGATAAGGCATGCAAGTCGAGTGGGTTTAGACCCACGGCGAACGAGGTAGTAAC
ACGTAGGAATGAACCCAAAAGTCCGGGATAACCCATCGAAAGGTGGAGTAATACCTGATAGTCTCTTCGG
AGTAAAGTCGTAAGACGCTTTTGGATCAGCCTGCGGAGTATCAGCTAGTTGGTAGGGTAATGGCCTACCAA
GGCTATGACGCTTAGGGGAGGTGAGAGCCTGGCCCCCACCGATGGTACTGAGACACGGAC

>Zotu12945
GAACGCTGGCGGCGTGCCTAACACATGCAAGTCGAACGCGAAAGCCGCTTTCGGGTGGCGAGTAGAGTG
GCGCACGGGTGAGTAACACGTAGACAACCTGCCTTCGAGCCCGGAATAACCTTTCGAAAGAAGGGCTAAT
ACCGGATAACACCTTGGGCTGCATGGCCTTGGGTTGAAAGGCTTGCTACTCGGAGAGGGGTCTGCGGGAC
ATTAGCTAGTTGGTGGGGTAACGGCCTACCAAGGCGACGATGTCTAGCGGGTCTGAGAGGATGATCCGCC
ACACTGGAACTGAGACACGGTC

>Zotu12947
GAACGCTAGCGGCAGGCTTAACACATGCAAGTCGAGGGGTATATTTCTTCGGAGATAGAGACCGGCGCAC
GGGTGCGTAACGCGTATGCAATCTGCCTTTCACAGAGGGATAGCCCAGAGAAATTTGGATTAATACCTCAT
AGTATTATAGGATGGCATCATTTTATAATTAAAGTCACAACGGTGAAAGATGAGCATGCGTCCCATTAGCT
AGTTGGTAAGGTAACGGCTTACCAAGGCAACGATGGGTAGGGGTCCTGAGAGGGAGATCCCCCACACTG
GTACTGAGACACGGAC

>Zotu12950
GAACGCTGGCGTCGTGCTTAACACATGCAAGTCGAACGAGATCCAATGTGTAGCAATACACAGGAAGATCT
AGTGGCGAACGGGTGAGGAACACGTGAGAAACGTACCCCAGACTCTGGGATAACAATCCGAAAGGGTTG
CTAATACCGGATGACGTCGGAAGTCCGCATGGACATCTGACGAAAGGGATTCCGGTTTGGGAGCGTCTCG
CGGACCTATCAGCTAGTTGGAGGGGTAACGGCCCACCAAGGCAACGACGGGTAGCTGGTCTGAGAGGAT
GAACAGCCACACTGGGACTGAGACACGGCC
>Zotu12952
GAACGCTGGCGACGTGCCTAATGCATGCAAGTCGTACGCAGGGTGCTTGCACCGCTGCGTGGCGCACGGC
TGAGTAACACGTGGGTATCAACCCTGGAGTGGGGGATATCGCCGCGAAAGCGGTGGCAATCCCGCATACG
ATCCGCTCACGCGGAGGAAAGGCGCAAGCCGCTCTGGGACGAGCCTGCGGCCCATCAGGTAGTTGGTGTG
GTAACGGCGCACCAAGCCAGTGACGGGTACCTGGTCTGAGAGGACGATCAGGCAGACTGGGACTGAGAC
ACGGCC
>Zotu12954
GAACGCTGGCGGCAGGCCTAACACATGCAAGTCGAACGGTAGCACAGAGGAGCTTGCTCCTTGGGTGACG
AGTGGCGGACGGGTGAGGAATATATCGGAATCTACCCAATCGTGGGGGATAACGTAGGGAAACTTACGCT
AATACCGCATACGATCTGAGGATGAAAGCGGCGGACCGCAAGGCGTCGCGCGATTGGATGAGCCGATATC
CGATTAGCTAGTTGGTAAGGTAAAAGCTTACCAAGGCGACGATCGGTAGCTGGTCTGAGAGGATGATCAG
CCACACTGGAACTGAGACACGGTC
>Zotu12957
GAACGCTGGAAATGTGGATACCGGCATGCAAGTCAAGGGGCCGCAAGGCACCGGCGAACGGCTTAGTAA
CACGTAGATACATACCTTAAAGTGGGGCACAGCTCGTCGAAAGACGGGGTAATTTCCCATAGTCTCGCAAG
AGTAAAGACGCAAGTCGCTTTAAGAATGGTCTGCGGTCTATCAGCTAGTTGGTGAGGTAATGGCTCACCAA
GGCTATGACGGATAGGGGGCGTGAGAGCGCGACCCCCAAGAATGGCACTGCGACACGGGC
>Zotu12960
GAACGCTAGCGGCAGGCCTAACACATGCAAGTCGAGGGGTAGAAGGAGCTTGCTCCTTTGAGACCGGCGC
ACGGGTGCGTAACGCGTATGCAACCTACCTTTCACATGGGAATAGCCCAGAGAAATTTGGATTAATGCCCC
ATGGCACAGCGAGACGGCATCGTCTTGCTGTTAAAGTAACAACGGTGAAAGATGGGCATGCGTCCCATTA
GCTAGTTGGTAAGGTAACGGCTTACCAAGGCTATGATGGGTAGGGGTCCTGAGAGGGAGATCCCCCACAC
TGGTACTGAGACACGGAC
>Zotu12963
GCCGTGTCTCAGTCCCAGTGTGGCTGATCATCCTCTCAGACCAGCTAAGGATCGTCGGCTTGGTAGGCCTTT
ACCCCACCAACTACCTAATCCTACGCGGGCTCATCCCTCGGCGATAAATCTTTGGACCGAAGTCATCATCCG
GTATTAGCAGTCATTTCTAACTGTTATTCCGAACCAAGGGGCAGATTCCCACGCGTTACGCACCCGTGCGCC
ACTAAGGCCGAAGCCTTCGTTCGACTTGCATGTGTTAGGCATGCCGCCAGCGTTCG
>Zotu12965
GCCGTGTCTCAGTCCCAGTGTGGCTGGTCATCCTCTCAGACCAGCTACGGATCGTCGCCTTGGTAGGCTCTT
ACCCCACCAACTAGCTAATCCGACGTAGGCTCACCCCAGAGCGCAAGGCCTTTCGGTCCCCTGCCTTTGATC
TCTCAATATCATGAGGTATTAGCCCGAGTTTCCTCGGGTTATCCCTCTCTCCAGGACAGATCCCTACGCATTA
CTCACCCGTCCGCCACTCGCCACCCATAAAGACCGAAATCTTTACTGTGCTGCCGTTCGACTTGCATGTGTTA
GGCATGCCGCCAGCGTTCA
>Zotu12969
GAACGCTGGCGGCGTGCTTAACACATGCAAGTCGAACGAGCAAGAGCTGTAGCAATACAGGGTTCTGCTA
GTGGCAGACGGGTGAGTAATACGTGGGAATCTACCTTGCAGTGCGAAATAACAGTTGGAAGCAATTGCTA
ATGTCGCATATAGCCGAGAGGTGAAAGGTTTACTGCTGCAAGATGGGCCCATGCAGGATTAGCTAGTTGG
TGAGGTAATGGCTCACCAAGGCAACGATCTTTAGTTGGTCTGAGAGGATGATCAGCCACACTGGGACTGA
GACACGGCC
>Zotu12973
GAACGCTGGCGGCATGCCTAACACATGCAAGTCGAACGAAGGCTTCGGCCTTAGTGGCGCACGGGTGCGT
AACGCGTGGGAACCTGCCCCTCGGTTCGGAATAACTCAGGGAAACTTGAGCTAATACCGGATGATGACTTC

GGTCCAAAGATTTATCGCCGAGGGATGGGCCCGCGTAGGATTAGCTTGTTGGTGGGGTAAAGGCCTACCA
AGGCGACGATCCTTAGCTGGTCTGAGAGGATGATCAGCCACACTGGGACTGAGACACGGCC
>Zotu12976
GAACGCTGGCGGCATGCCTTACACATGCAAGTCGAACGGCAGCACGGGTGCTTGCACCTGGTGGCGAGTG
GCGAACGGGTGAGTAATATATCGGAACGTACCCTAGAGTGGGGGATAACGCAGCGAAAGTTGCGCTAATA
CCGCATACGATCTACGGATGAAAGTGGGGGATCGCAAGACCTCATGCTCATGGAGCGGCCGATATCTGAT
TAGCTAGTTGGTGGGGTAAAAGCCTACCAAGGCATCGATCAGTAGCTGGTCTGAGAGGACGACCAGCCAC
ACTGGAACTGAGACACGGTC
>Zotu12984
GAACGCTGGCGGCGTGCTTAACACATGCAAGTCGAACGGTAAGGCTCTTTCGGGAGTACACGAGTGGCGA
ACGGGTGAGTAACACGTGAGCAACCTGCCCTTGACTTTGGGATACCAGTTGGAAACAGCTGCTAATACCGG
ATATGACACTCCTTCGCATGTTGGGGTGTGGAAAGCTCCGGCGGTCAGGGATGGGCTCGCGGCCTATCAG
CTTGTTGGTGAGGTAATGGCTCACCAAGGCTTCGACGGGTAGCCGGCCTGAGAGGGCGACCGGCCACACT
GGGACTGAGATACGGCC
>Zotu12986
GAACGCTGGCGGCAGGCTTAACACATGCAAGTCGAACGCCCCGCAAGGGGAGTGGCAGACGGGTGAGTA
ACGCGTGGGAATCTACCCAGTTCTACGGAACAACCCAGGGAAACTTGGGCTAATACCGTATACGCCCCAAG
GGGGAAAGATTTATCGGAATTGGATGAGCCCGCGTAAGATTAGCTAGTTGGTGGGGTAATGGCCTACCAA
GGCGACGATCTTTAGCTGGTCTGAGAGGATGATCAGCCACACTGGGACTGAGACACGGCC
>Zotu12995
GAACGCTGGCGGCAGGCTTAACACATGCAAGTCGAGCGGGAATAGGTAGCTTGCTACTGAATTCTAGCGG
CGGACGGGTGAGTAAAGCTTAGGAATCTGCCATTTAGTGGGGGACAACATTTCGAAAGGGATGCTAATAC
CGCATACGTCCTACGGGAGAAAGCAGGGGATCACTTGTGACCTTGCGCTAAATGATGAGCCTAAGTCGGA
TTAGCTAGTTGGTGGGGTAAAGGCCTACCAAGGCGACGATCTGTAGCGGGTCTGAGAGGATGATCCGCCA
CACTGGGACTGAGACACGGCC
>Zotu12997
GAACGCTGGCGGCATGCTTTACACATGCAAGTCGAACGGCAGCGCGGGGCAACCTGGCGGCGAGTGGCG
AACGGGTGAGTAATATATCGGAACGTACCCAGAAGTGGGGGATAACGTAGCGAAAGTTACGCTAATACCG
CATACGATCTACGGATGAAAGTGGGGGATCTTCGGACCTCATGCTTTTGGAGCGGCCGATATCTGATTAGC
TAGTTGGTAGGGTAAAGGCCTACCAAGGCGACGATCAGTAGCTGGTCTGAGAGGACGACCAGCCACACTG
GGACTGAGACACGGCC
>Zotu12999
GAACGCTGGCGGCAGGCCTAACACATGCAAGTCGAGCGGTAACAGAGATTAGCTTGCTAATCTGCTGACG
AGCGGCGGACGGGTGAGTAATGCATAGGAAGTTGCCCAGTAGAGGGGGATACCAGTTGGAAACGACTGT
TAATACCGCATAACCTCTTTGGAGCAAAGCAGGGGACCTTCGGGCCTTGCGCTACTGGATACGCCTATGTG
GGATTAGCTAGTTGGTGAGGTAATGGCTCACCAAGGCGACGATCCCTAGCTGGTCTGAGAGGATGATCAG
CCACACTGGGACTGAGACACGGCC
>Zotu13001
GAACGCTGGCGGCAAGCCTAAAACATGCAAGTCGAGCGCTCCAGCAATGGAGAGCGGCGCACGGGTGCG
TAACGCGTGGGAATGTGCCTCTGGGTGCGGAACAACACCGGGAAACTGGTGCTAATACCGCATAATGACT
TCGGTCCAAAGATTTATCGCCCAGAGATCAGCCCGCGTCGGATTAGGTAGTTGGTAGGGTAATGGCCTACC
AAGCCGACGATCCGTAGCTGGTCTTAGAGGATGATCAGCCACACTGGAACTGAGACACGGTC
>Zotu13003
GAACGCTGGCGGCGTGCCTAATACATGCAAGTCGAGCGGTAAGGCTTTCACTCTGTATTTAACTTCGGTTG
AATGTAGTGTGAAAGTACACGAGCGGCGGACGGCAGAGTAACGCGTAGGAACGTACCCCATAGTGAGGG
ATAAGCACCGGAAACGGTGTCTAATACCGCATAATCTCTTCGGAGCAAAGCCTTGTGCGCTTTGGGAGCGG
CCTGCGTATGATTAGTTTGTTGGTGAGGTAACGGCTCACCAAGACTACGATCATTAGCTGGTCTGAGAGGA
TGATCAGCCTGACTGGAACTGAGAACGGTC
>Zotu13007

GAATGCTGGCTGTATGGATAAGGCATGCAAGTCGAACGGACCGCAAGGTCAGTGGCAGACGAGGTAGTA
ACACGTAAGTACGTACCCTGTAGTCTGGCATAGCTCGTCGAAAGACGGGGTAATTCCAGATGGTCCCGTAA
GGGTAAAGAATTTCGCTACAGGAGCGGCTTGCGTAGTATCAGCTAGTTGGTAGGGTAATGGCCTACCAAG
GCTACGACGCTTAGGGGAGCTGAGAGGCTGACCCCCACCGATGGGACTGAGACACGGCC
>Zotu13009
GAACGCTGGCGGCGTGCTTAACACATGCAAGTCGAACGGAAAGGCCCTTCGGGGTACTCGAGTGGCGAAC
GGGTGAGTAACACGTGGGTGATCTGCCCTGCACTTCGGGATAAGCCTGGGAAACTGGGTCTAATACCGGA
TATGACTACGGATCGCATGGTCTGTGGTGGAAAGCTTTTGCGGTGTGGGATGGGCCCGCGGCCTATCAGC
TTGTTGGTGGGGTCACGGCCTACCAAGGCGACGACGGGTAGCCGGCCTGAGAGGGTGACCGGCCACACT
GGGACTGAGATACGGCC
>Zotu13015
GAACGCTGGCGGCAGGCATAATACGTGCAAGGCGAGGGGGTAGCAATACCACCGTCGTACGGGTGCGTA
ACACGTATGCAACCTGCCCTTCACTGGGGGATAGCCTGGGGAAACTCGGATTAATACCGCATAAGACAGTA
GACTGGCATCAGTTAGCTGTTAAAGATTTATTGGTGAAGGATGGGCATGCGTCTGATTAGCTAGTTGGCGG
GGTAAAGGCCCACCAAGGCTACGATCAGTAGGGGTTCTGAGAGGATTGGCCCCCACACTGGCACTGAGAT
ACGGGC
>Zotu13018
GGACGCTGGCGGTGTGCTTAATACATGCAAGTCGAGCGGTAAGGCCTCTTCGGAGGTACACGAGCGGCGG
ACGGCTGAGTAACGCGTAGGAATCTACCCCGAACTGAGGAATAAGCACCAGAAATGGTGTCTAATGCCGC
ATATGCTCTTAGGAGTAAAGCTTCGGCGGTTTGGGAAGAGCCTGCGTTCGATTAGCTTGTTGGTAAGGTAA
CGGCTTACCAAGGCGACGATCGATAGCTGGTCTGAGAGGATGACCAGCCAGACTGGGACTGAGAACGGC
C
>Zotu13023
GAACGCTGGCGGCGTGCCTAACACATGCAAGTCGAACGGGACCAGGGGCAACTCTGGTTCAGTGGCGGA
CGGGTGCGTAACACGTGAGGAACATGACCTTCGGCGGGGGATAGCCGGCCCAACGGCCGGGTAATACCG
CGTACGACCATCTGGGGACATCTCCGAGTGGTGAAAGCAGCAATGCGCCGATGGAGTGCCTCGCGGCCTA
TCAGCTAGTTGGTGGGGTAACGGCCTACCAAGGCAACGACGGGTAGCTGGTCTGAGAGGATGGCCAGCC
ACATTGGGACTGAGACACGGCC
>Zotu13025
GAACGCTGGCGGCATGCCTTACACATGCAAGTCGAACGGCAGCGCGGACTTCGGTCTGGCAGCGAGTGGC
GAACGGGTGAGTAATACATCGGAACGTACCCTGTTGTAGGGGATAACTAGTCGAAAGATTAGCTAATACC
GCATACGACCTGAGGGTGAAAGCGGGGAACCGTAAGGCCTCGCGCAGCAGGAGCGGCCGATGTCTGATT
AGCTAGTTGGTGGGGTAAAAGCTCACCAAGGCGACGATCAGTAGCTGGTCTGAGAGGACGACCAGCCACA
CTGGGACTGAGACACGGCC
>Zotu13027
GAACGCTGGCGGCGTGCTTAGCACATGCAAGTCGAACGAGCAAGAGCTGTAGCAATACAGGGTTCTGCTA
GTGGCAGACGGGTGAGTAATACATGGGAATCTACCTTGCAGTGCGAAATAACAGTTGGAAGCAATTGCTA
ATGTCGCATATAGCCGAGAGGTGAAAGGTTTACTGCTGCAAGATGGGCCCATGCAGGATTAGCTAGTTGG
TGAGGTAATGGCTCACCAAGGCAACGATCTTTAGTTGGTCTGAGAGGATGATCAGCCACACTGGGACTGA
GACACGGCC
>Zotu13033
GAACGCTGGCGGCGTGCCTAATACATGCAAGTCGAGCGGATCGTATCCTTCGGGATACGATTAGCGGCGA
ACGGGTGAGTAACACGTAGGCAACCTGCCTGTAAGACCGGGATAACCCACGGAAACGTGAGCTAATACCG
GATAATCGGTATCTCCGCATGGAGAGATCGTGAAAGATGCTCCGGCATCGTTTACAGATGGGCCTGCGGC
GCATTAGCTAGTTGGCGGGGTAACGGCCCACCAAGGCGACGATGCGTAGCCGACCTGAGAGGGTGATCG
GCCACACTGGGACTGAGACACGGCC
>Zotu13035
GAACGCTAGCGGCATGCCTAACACATGCAAGTCGAACGGTGAAAGAGGAGCTTGCTCCTCTGGATCAGTG
GCGAACGGGTGAGTAACACGTGAGTAACCTGCCCTTGACTCTGGGATAAGCGTTGGAAACGACGTCTAAT
ACCGGATATGAACCGCGAAGGCATCTTCTGCGGTTGGAAAGAATTTTGGTCAAGGATGGACTCGCGGCCT

ATCAGCTAGTTGGTGAGGTAACGGCCCACCAAGGCGACGACGGGTAGCCGGCCTGAGAGGGTGACCGGC
CACACTGGGACTGAGACACGGCC
>Zotu13038
GAACGCTGGCGGCGTGCTTAACACATGCAAGTCGAGCGGAAAGGCCCTTTCGGGGGTACTCGAGCGGCG
AACGGGTGAGTAACACGTGAGTAATCTGCCCTTCACTTGGGGATAACGTCCAGAAATGGGTGCTAATACCC
GGTATTCCTATTGTCACGCATGTGGTGGTGGGGAAAGGTTTTTCTGGTGGGGGATGTGCTCGCGGCCTATC
AGCTTGTTGGTGGGGTAATGGCCTACCAAGGCTTTGACGGGTAGCCGGCCTGAGAGGGTGACCGGCCACA
CTGGGACTGAGACACGGCC
>Zotu13040
GAACGCTGGCGGCGTGCTTAACACATGCAAGTCGAACGATTAAAGCCCCTTCGGGGGTGTATAGAGTGGC
GAACGGGTGAGTAACACGTGGGCAATCTGCACCTCACTCCGGGATAACTCAGGGAAACTTGAGCTAATAC
CGGATACTCCGAGTGGATCGCATGGTCTGCTCGGGAAAGCTCCGACGGTGAGGGATGAGCCCGCGGCCCA
TTAGCTAGTTGGTGAGGTAACGGCCCACCAAGGCGACGATGGGTAGCCGAGCTGAGAGGCTGATCGGCC
ACACTGGGACTGAGACACGGCC
>Zotu13048
GAACGCTAGCGGCAGGCCTAATACATGCAAGTCGAACGGATGTAGCAATACATTAGTGGCGTACGGGTGC
GTAACGCGTAAACAATCTACCTCATACTGGGGGATAGCCCTGGGAAACCGGGAGTAAGCCCGCATGGCAT
CAGGGAGCTACCTGGCTTTTTGATTAAAGATTTATCGGTATGAGATGAGTTTGCGTCTGATTAGTTAGTTGG
GGGGGTAACGGCCCACCAAGGCGATGATCAGTAGGGGCTCTGAGAGGAGCGGCCCCCACACTGGCACTG
AGACACGGGC
>Zotu13053
GAACGCTGGCGGAATGCTTTACACATGCAAGTCGAACGGCAGCACGGACTTCGGTCTGGTGGCGAGTGGC
GAACGGGTGAGTAATATATCGGAACGTATCCAATAATGGGGGATAACTAAGCGAAAGCTTGGCTAATACC
GCATACGCCCCACGGGGGAAAGCTAGGGACCTTCGGGCCTGGCGTTAATGGAGCGGCCGATATCTGATTA
GCTAGTTGGTGGGGTAAAGGCCCACCAAGGCGACGATCAGTAGCTGGTCTGAGAGGACGACCAGCCACA
CTGGAACTGAGACACGGTC
>Zotu13055
GAACGCTGGCGGCGTGCTTAACACATGCAAGTCGAACGAAGCGCTTTACCGGATTTCTTCGGAATGAAAGT
TTTGTGACTGAGTGGCGGACGGGTGAGTAACGCGTGGGTAACCTGCCTCATACAGGGGGATAACAGTTAG
AAATGACTGCTAATACCGCATAAGACCACAGGACCGCATGGTCCAGTGGTAAAAACTCCGGTGGTATGAG
ATGGACCCGCGTCTGATTAGCTAGTTGGTAAGGTAACGGCTTACCAAGGCGACGATCAGTAGCCGACCTG
AGAGGGTGACCGGCCACATTGGGACTGAGACACGGCC
>Zotu13058
GTTGCAACTTCACGCGTGGAATTTGTGATGAGGATGCCGTGGAACCAGGCTTCGGAGCATCTTGAAGAGC
CCCCTATGGTGGGAATCAATTGAAGCTGTACGCTTCGCAAAGTTTAGTTGAACTATGATAATTTAGTTGGG
GTGAAAAAAAGAACATTTGATGGCGTGTATGACATTTTTGCATTGAATGTGCACGGTGGAGAGGTTCGTCA
TCCACCGGAATATAAAAATATCGAGGCCTTAATTAAAAGCTAGGTAGGGTAAAGGTAACAAAACGTAAAT
GACAACTTAAGCAGCTTGACTGATTCGCTTTATTCTAGCAGCGTTAAAACGTCGCTACTATAGACGTTATAG
ATGATATACGATCAGTGTTTACCTATGCTTCCTTGAATGGCACCGAACGGCACCGGGCAAATGTAGCGCAG
AAAGG
>Zotu13061
GAACGCTGGCGGCAGGCTTAACACATGCAAGTCGAGCGGTAGCACAAGGGAGCTTGCTCCTTGGGTGACG
AGTGGCGGACGGGTGAGTAATGTCTGGGAAACTGCCCGATGGAGGGGGATAACTACTGGAAACGGTAGC
TAATACCGCATAACGTCGCAAGACCAAAGAGGGGGACCTTCGGGCCTCTTGCCATCAGATGTGCCCAGATG
GGATTAGCTAGTAGGTGGGGTAACGGCTCACCTAGGCGACGATCCCTAGCTGGTCTGAGAGGATGACCAG
CCACACTGGAACTGAGACACGGTC
>Zotu13066
GAACGCTGGCGGCGTGCTTAACACATGCAAGTCGAACGAGCAAGAGCTGTAGCAATACAGGGTTCTGCTA
GTGGCAGACGGGTGAGTAATACATGGGAATCTACCTTGCAGTGCGAAATAACAGTTGGAAGCAATTGCTA
ATGTCGCATATAGCCGAGAGGTGAAGGGTTTACTGCTGCAAGATGGGCCCATGCAGGATTAGCTAGTTGG

TGAGGTAATGGCTCACCAAGGCAACGATCTTTAGTTGGTCTGAGAGGATGATCAGCCACACTGGGACTGA
GACACGGCC
>Zotu13073
GAACGCTAGCGGCATGCCTAACACATGCAAGTCGAGCGGTAAGGCTCCTTCGGGAGTACACGAGCGGCGA
ACGGGTGAGTAACACGTGAGTAATCTGCCCTTCACTTCGGGATAACCGCCGGAAACGGTGGCTAATACCG
GATACGACGCACTCTTGCATGAGATGTGCGTGGAAAGTTTTCTCGGTGAAGGATGTGCTCGCGGCCTATCA
GCTTGTTGGTGAGGTAATGGCTCACCAAGGCTTCGACGGGTAGCCGGCCTGAGAGGGTGACCGGCCACAC
TGGGACTGAGACACGGCC
>Zotu13077
GAACGCTGGCGGCGTGCTTAAGACATGCAAGTCGGACGCATCCCCCTCGGGGGATGAGTGGCGCACGGG
TGAGTAACGCGTAACTGACCTACCCTCAAGTCCTGACCAACTCCTCGAAAGAGGAGCTAATGTGGGATGTG
CTGCCTCACCGTGGTGAGGCAGTAAAGGCTATGACCGCTTGAGGATGGGGTTGCGTTTCATCAGCTAGATG
GTGGGGTAAAGGCCTACCATGGCGACGACGAATAGCCGGCCTGAGAGGGTGGCCGGCCACAGGGGCACT
GAGACACGGCC
>Zotu13082
GAACGGTGGCGGCGTGGATTAGGCATGCAAGTCGAGCGCGAAAGGCCCGCAAGGGCTGAGTAAAGCGG
CGGACGGGTGAGTAACGTATGGACAATCTACCTTCGACCCGGGGATAACAGTTGGAAACGACTGCTAATA
CCCGATGGCTCCGATAGCGTACTTCGCAGTCGGATAAAGGGGTGCAAATCCCAGTCGATGAGGAGTCCAT
ATCCCATCAGCTAGTTGGCGGGGTAATGGCCCACCAAGGCAACGACGGGTAGCCGGACTGAGAGGTCGAC
CGGCCACATCGGGACTGAGACACTGCC
>Zotu13085
GAACGCTGGCGGCGTGCCTAATACATGCAAGTCGAGCGAATCTGAGGGAGCTTGCTCCCAAAGATTAGCG
GCGGACGGGTGAGTAACACGTGGGCAACCTGCCTGTAAGACTGGGATAACTTCGGGAAACCGGAGCTAAT
ACCGGATAATTCTTATTAACTCATGTTAATAAGCTAAAAGACGGCGTAAGCTGTCACTTACAGATGGGCCC
GCGGCGCATTAGCTAGTTGGTGAGGTAACGGCTCACCAAGGCAACGATGCGTAGCCAACCTGAGAGGGTG
ATCGGCCACACTGGGACTGAGACACGGCC
>Zotu13088
CGATGAAGAACGCAGCACGGGAGCAATCCTGGTGGCGAGTGGCGAACGGGTGAGTAAAGTATCGGAACG
TGCCCAGTCGTGGGGGATAACGTAGCGAAAGCTACGCTAATACCGCATACGATCTATGGATGAAAGCGGG
GGATCGCAAGACCTCGCGCGATTGGAGCGGCCGATATCAGATTAGGTAGTTGGTGGGGTAAAGGCCTACC
AAGCCGACGATCTGTAGCTGGTCTGAGAGGACGACCAGCCACACTGGGACTGAGACACGGCC
>Zotu13093
GAACGCTGGCGGCGTGCTTAACACATGCAAGTCAGGGGGCTCGCAAGGGTCACCGGCGAACGGGTGCGT
AACACGTGAGCGACCTGCCCTGGAATGGGGGATAGCCGGCCCAACGGCCGGGTAATACCGCATACGACCG
CCCGGGGGCATATCTGGGCGGTGAAACCATTTGGGTTCTTGGAGGGGCTCGCGGCCTATCAGCTAGTTGG
TGCGGTAACGGCGCACCAAGGCGATGACGGGTAGCTGGTCTGAGAGGATGGCCAGCCACATTGGGACTG
AGAGACGGCC
>Zotu13095
GAACGCTGGCGGCATGCTTAACACATGCAAGTCGAACGGCAGCACGACCTAGCTTGCTAGGTGGGTGGCG
AGTGGCGAACGGGTGAGTAACGCGTAGGAATATACCTTAGAGAGGGGGATAACCTGGGGAAACTCAGGC
TAATACCGCGTACGTTCTTCGGAATAAAGCTGGGGACCTTCGGGCCTGGCGCTTTAAGAATAGCCTGCGTC
CGATTAGCTAGTTGGTGGGGTAAGGGCCTACCAAGGCGACGATCGGTAGCTGGTCTGAGAGGATGATCAG
CCACACTGGGACTGAGACACGGCC
>Zotu13097
GAACGCTGGCGGCATGCCTTACACATGCAAGTCGAACGGCAGCGGGGGGAGCTTGCCCCCTGACGGCGA
GTGGCGGACGGGTGAGTAACACGTAGGAATCTACCTTAATTATAATAGTTAGTGGGGGATAACCCGGGGA
AACTCGGGCTAATACCGCATAATCTCTTCAAAGCAAAGCGGGGGATCTTCGGACCTCGTGCTATGAGATGA
GCCTGCGTCGGATTAGCTAGTTGGTAGGGTAATGGCCTACCAAGGCGACGATCCGTAGCTGGTCTGAGAG
GACGATCAGCCACACTGGGACTGAGACACGGCC
>Zotu13099

GAACGCTGGCGGCGTGCTTAACACATGCAAGTCGAACGGTAAGGCTCCTTCGGGAGTACACGAGTGGCGA
ACGGGTGAGTAACACGTGAGCAACCTGCCCTTGACTTTGGGATACCAGCTGGAAACAGCTGCTAATACCG
GATATGACCTCTTTCTGCATGGTTTGAGGTGGAAAGCTCCGGCGGTCAGGGATGGGCTCGCGGCCTATCAG
CTTGTTGGTGAGGTAATGGCTCACCAAGGCTTCGACGGGTAGCCGGCCTGAGAGGGCGACCGGCCACACT
GGGACTGAGATACGGCC
>Zotu13102
GAACGCTGGCGGCAGGCTTAACACATGCAAGTCGAGCGCATCGGTAACGGTGAGCGGCAGACGGGTGAG
TAACGCGTGGGAATCTACCCTTCTCTGCGGAATAGCTCCGGGAAACTGGAATTAATACCGCATACGCCCTT
AGGGGGAAAGATTTATCGGGGAAGGATGAGCCCGCGTTGGATTAGCTAGTTGGTGGGGTAAAGGCCTAC
CAAGGCGACGATCCATAGCTGGTCTGAGAGGATGATCAGCCACATTGGGACTGAGACACGGCC
>Zotu13108
GAACGCTAGCGGCAGGCCTAATACATGCAAGTCGAACGAGCTACAGGACTTCGGTTCTGTGGTTAGTGGC
GCACGGGTGCGTAATGCGTATGCAACCTACCTTCCACAGGGGGATAGCCTTCCGAAAGGGAGATTAATAC
CGCATAACATCATTTGAGGGCATCCGAAGATGATCAAAGCTTTGGCGGTGGAAGATGGGCATGCGTGCCA
TTAGTTAGTTGGTAGGGTAACGGCCTACCAAGACTTCGATGGCTAGGGGTTCTGAGAGGATGGTCCCCCAC
ACTGGTACTGAGACACGGAC
>Zotu13110
GAACGCTAGCGATAGGCTTAACACATGCAAGTCGAGGGGCAGCATAAAGGTAGTAATACTTTGGTGGCGA
CCGGCGCACGGGTGAGTAACACGTATGCAACCTACCCATAACTGCGGAATAACTCGTCGAAAGGCGGACT
AATACCGCATAACAACGTTTCACTGCATGGTGAGATGTTTAAAGATTTATTGGTTATGGATGGGCATGCGTT
CCATTAGATAGTTGGTAGGGTAACGGCCTACCAAGTCTTCGATGGATAGGGGTTCTGAGAGGAAGGTCCC
CCACACTGGTACTGAGACACGGAC
>Zotu13112
GAACGCTGGCGGCGTGCTTAACACATGCAAGTCGAGCGAGGCCCACCTTTCGGGGTGGTGTTCTAGCGGC
GAACGGGTGAGTAACACGTGGGCAACCTGCCCTCAGCTCTGGGATAACTCCAAGAAATTGGGGCTAATAC
CGGATATGACTGCTGACCGCATGGTCTGGTGGTGGAAAGATTTATCGGCTGGGGATGGGCCCGCGGCCTA
TCAGCTTGTTGGTGGGGTAGTGGCCTACCAAGGCGACGACGGGTAGCCGGCCTGAGAGGGTGACCGGCC
ACACTGGGACTGAGACACGGCC
>Zotu13115
GAACGCTAGCGGCAGGCTTAATACATGCAAGTCGAACGGGATCTGAGGGTAGCTTGCTACCCGATTTGAG
AGTGGCGCACGGGTGCGTAACGCGTGAGCAACCTACCCATATCAGGGGGATAGCCCGAAGAAATTCGGAT
TAACACCGCATAACAATGTAGTAACACATGTTACCACATTTAAATATTTATAGGATATGGATGGGCTCGCGT
GACATTAGCTAGTTGGTAAGGTAACGGCTTACCAAGGCGACGATGTCTAGGGGCTCTGAGAGGAGAATCC
CCCACACTGGTACTGAGACACGGAC
>Zotu13119
GAACGCTGGCGGCAGGCTTAACACATGCAAGTCGAACGCGTGTAGCAATACACGAGTGGCGCACGGGTG
AGTAACGCGTGGATATCTGCCTTCTGGTTCGGAATAACCCCGGGAAACTGGGGCTAATACCGAATGGTTCC
TACGGGATAAAGATTTATCGCCAGAAGATGAGTCCGCGTCCGATTAGCTAGTTGGTGAGGTAACGGCTCAC
CAAGGCGACGATCGGTAGCTGGTCTGAGAGGACGATCAGCCACACTGGGACTGAGACACGGCC
>Zotu13125
GAACGCTGGCGGCGTGCCTTAGGCATGCAAGTCGAACGGTTGAGACCGCAAGGTCAAGACAGTGGCAGA
CGGGTGAGTAACGCGTAGGAATCTACCTCTGGGTATGGTATAGCCGGCCGAAAGGTCGGGTAATCCCATA
TAGTCTCGTAAGAGTAAAGGACTTAACGGTCCGCCGAGAGAGGAGCCTGCGTCCTATCAGCTAGTTGGTG
AGGTAAAGGCTCACCAAGGCGATGACGGGTAGCTGGACTGAGAGGTCGATCAGCCACAACTGCACTGAG
ACACGGGC
>Zotu13131
GAACGCTGGCGGCGTGCTTAACACATGCAAGTCGAACGGAAAGGCCCTGCTTTTGTGGGGTGCTCGAGTG
GCGAACGGGTGAGTAACACGTGAGTAACCTGCCCTTGACTTTGGGATAACTTCAGGAAACTGGGGCTAAT
ACCGGATAGGAGCTCCTGCTGCATGGTGGGGGTTGGAAAGTTTCGGCGGTTGGGGATGGACTCGCGGCCT
ATCAGCTTGTTGGTGGGGTAGTGGCTTACCAAGGCTTTGACGGGTAGCCGGCCTGAGAGGGTGACCGGCC
ACACTGGGACTGAGACACGGCC

>Zotu13143
GAACGCTGGCGGCGTGCTTAACACATGCAAGTCGAACGGAACTCAAGGAGCTTGCTCCTGAAGGTTTAGT
GGCGAACGGGTGCGTAACACGTGAGGAACCTGTCCCGAACTTGGGAATAACAGCCGGAAACGGCTGCTA
ATACCGAATACCTTCGTAACGTCGCATGGCGAATCGAAGAAAGCTTTATGCGGTTTGGGAGGGTCTCGCGG
CCTATCAGCTAGTTGGTGAGGTAATGGCTCACCAAGGCATCGACGGGTAGCTGGTCTGAGAGGATGATCA
GCCACACTGGGACTGAGACACTGCC
>Zotu13146
GAACGCTAGCGGCGTGCTTAACACATGCAAGTCGAACGATGAAGCAGGAGCTTGCTCCTGTGGATTAGTG
GCGAACGGGTGAGTAACACGTGAGTAATCTGCCCCTGACTCCGGGATAAGCACTGGAAACGGTGTCTAAT
ACCGGATACGAGCTGCGAAGGCATCTTCAGCAGCTGGAAAGAACTTCGGTCAGGGATGAACTCGCGTCCT
ATCAGCTAGTTGGTGAGGTAAAGGCTCACCAAGGCGACGACGGGTAGCCGGCCTGAGAGGGTGACCGGC
CACACTGGGACTGAGACACGGCC
>Zotu13152
GAACGCTGGCGGCAGGCCTAAAACATGCAAGTCGAACGCTCTAGCAATAGGGAGTGGCGCACGGGTGAG
TAACATGTGGGAATATGCCTTTTGGTACGGAACAACTTTTGGAAACGAAAGCTAATACCGTATGGTCCCTTC
GGGGTAAAGATTTATCGCCAAAAGAGTAGCCCGCACTGGATTAGCTAGTTGGTAAGGTAATGGCTTACCA
AGGCGACGATCCATAGCTGGTCTGAGAGGACGATCAGCCACACTGGGACTGAGACACGGCC
>Zotu13158
CAACGCTGGCGGCGTGCCTAACACATGCAAGTCGAACGAGAAAGTGGAGCAATCCATGAGTAAAGTGGCG
ACCGGGTGAGTAACACGTGACTAACCTACCTTCGAGTGGGGGATAACCTAGGGAAACCTGGGCTAATACC
GCATAACATCTTTGGAGTGGAAATTCTGGAGACCAAAGGAGCAATTCGCTTGAAGAGGGGGTCGCGGCTG
ATTAGCTAGTTGGCGGGGTAATGGCCCACCAAGGCTTTGATCGGTAGCCGGCCTGAGAGGGCGCACGGCC
ACACTGGAACTGAAACACGGTC
>Zotu13160
GAACGTTAGCGGCGCGCCTAACACATGCAAGTCGAACGCGACGGGGGCAACTCCGTTGAGTGGCGCACG
GGTGAGTAACACGTAGGTAATCTTCCCTCGAGTGGTGGATAACCCTCCGAAAGGAGAGCTAATACAGCAT
GAGACCACGGTTTCGAAAGGGACTGAGGTCAAAGCGGAGGATCGCAAGACTTCGCGCTTGAGGATGAGC
CTGCGGCCCATCAGGTAGTTGGCGGGGTAATGGCCCACCAAGCCTAAGACGGGTAGCTGGTCTGAGAGGA
TGATCAGCCACACTGGAACTGAGACACGGTC
>Zotu13164
GAACGCTGGCGGCGTGCTTAACACATGCAAGTCGAGCGGAAAGGCCACTTCGGTGGTACTCGAGCGGCGA
ACGGGTGAGTAACACGTGAGTAATCTGCCCCCCACTTTGGGATAGCCACCGGAAACGGTGATTAATACCG
GATATGACGTGCCCTCGCATGGGGGTGTGTGGAAAGTTCTTCGGTGGGGGATGTGCTCGCGGCCTATCAG
CTTGATGGTGGGGTAATGGCCTACCATGGCTTCGACGGGTAGCCGGCCTGAGAGGGTGACCGGCCACACT
GGGACTGAGACACGGCC
>Zotu13166
GAACGCTGGCGGCATGCCTAACACATGCAAGTCGAACGGCAGCACAGTAAAGATTTCGGTCTTTACGGGT
GGCGAGTGGCGGATGGGTGAGTAAAGCGTAGGAATCTGTCCTGGAGAGTGGGATAACCCGAGGAAACTC
GGGCTAATACCGCATGATATCGAGAGATCAAAGCAGGGGACCCTTCGGGGCCTTGCGCTCTAGGATGAGC
CTACGTCGGATTAGCTAGTTGGTGAGGTAATGGCTCACCAAGCCGACGATCTGTAGCTGGTCTGAGAGGAT
GACCAGCCACACTGGGACTGAGACACGTCC
>Zotu13174
GAACGCTGGCGGCGTGCTTAACACATGCAAGTCGAACGGCAGCGCGAGAGAGCTTGCTCTCTTGGCGGCG
AGTGGCGGACGGGTGAGTAATATATCGGAACGTGCCCAGTAGCGGGGGATAACTACTCGAAAGAGTGGC
TAATACCGCATACGCCCTACGGGGGAAAGGGGGGGATCGCAAGACCTCTCACTATTGGAGCGGCCGATAT
CGGATTAGCTAGTTGGTGGGGTAAAGGCTCACCAAGGCAACGATCCGTAGCTGGTTTGAGAGGACGACCA
GCCACACTGGGACTGAGACACGGTC
>Zotu13179
GAACGCTGGCGGCGTGCTTAACACATGCAAGTCGAACGAGCAAGAGCTGTAGCAATACAGGGTTCTGCTA
GTGGCAGACGGGTGAGTAATACATGGGAATCTACCTTGCAGTGCGAAATAACAGTTGGAAGCAATTGCTA

ATGTCGCATATAGCCGAGAGGTGAAAGGTTTACTGCTGCAAGATGGGCCCATGCAGGATTAGCTAGTTGG
TGGGGTAATGGCTCACCAAGGCGACGATCTTTAGTTGGTCTGAGAGGATGATCAGCCACACTGGGACTGA
GACACGGCC
>Zotu13183
GAACGCTGGCGGCGTGCTTAACACATGCAAGTCGAACGCGAACGGAGGGGGTAACTCCTCCTAGTAGAGT
GGCGCACGGGTGAGTAACACGTGGGTAATCAACCTTCGGGTGGGGGATAACAGTTCGAAAGAACTGCTAA
TCCCGCATAAGACCACGAGGACCTCGGTCCTTGGGGCCAAAGCAGCAATGCGCCTGAAGACGAGCCCGCG
CCCCATCAGCTAGTTGGTGAGGTAACGGCCCACCAAGGCTTTGACGGGTAGCTGGTCTGAGAGGACGACC
AGCCACACTGGGACTGACACACGGCC
>Zotu13186
CAACGCTGGCGGCGCGCTTAACACATGCAAGTCGAACGAGAAAGGGGAGCAATCCCTGAGTAAAGTGGC
GTACGGGTGAGTAACACGTGGGTAATCTACCTTTTGGTGGGGAATAACGCTGGGAAACCGGCGCTAACAC
CGCATAAGCTCGAAAGAGGAAAAGAGAAATCTGCCGAAAGAGGAGCCCGCGGCTGATTAGCTAGTTGGT
GAGGTAAAGGCTCACCAAGGCTATGATCGGTAGCCGGCCTGAGAGGGCACACGGCCACACTGGCACTGA
AACACGGGC
>Zotu13190
GAACGCTGGCGGCGTGCTTAACACATGCAAGTCGAGCGGAAAGGCCCTTTGGGGTACTCGAGCGGCGAAC
GGGTGAGTAACACGTGGGTAACCTGCCCCTTGCTCTGGGATAACTCCGGGAAACCGGTGCTAATACTGGAT
ATTCATGCTTGCAGGCATCTGCGGGTGTGGAAAGTTTTTTCGGCGAGGGATGGACCCGCGGCCTATCAGCT
TGTTGGTGGGGTGATGGCCTACCAAGGCTTTGACGGGTAGCCGGCCTGAGAGGGTGACCGGCCACACTGG
GACTGAGACACGGCC
>Zotu13192
GAACGCTGGCGGCGTGCTTAACACATGCAAGTCGAACGGGAAACCTTTTATTGAAGCTTCGGCAGATTTAG
ATTGTTTCTAGTGGCGGACGGGTGAGTAACGCGTGGGTAACCTGCCTTGTACAAGGGGATAACAGTCAGA
AATGACTGCTAATACCGCATAAGCGCACAGGACCGCATGGTCCGGTGTGAAAAACTCCGGTGGTATAGGA
TGGACCCGCGTTGGATTAGCTAGTTGGTGAGGTAACGGCCCACCAAGGCGACGATCCATAGCCGGCCTGA
GAGGGTGAACGGCCACATTGGGACTGAGACACGGCC
>Zotu13195
GAACGCTGGCGGCGCGCTTAACACATGCAAGTCGTACGGGGCCCTTCGGGGTTCAGTGGCGAACGGGTGC
GTAACACGTGAGTGACCTGCCCTTGTGCGGGGGATAACCCGGGGAAACCCGGGCTAATACCGCATGCACT
CCATCGGATTAGAGGCTGGTGGAGGAAAGGTGCTGCAAGGCATCGCGCAGGGAGGGGCTCGCGGCGTAT
CAGGTAGTTGGTGAGGTAACGGCTCACCAAGCCTATGACGCGTAGGGGGTCTGAGAGGATGGCCCCCCAC
ATTGGGACTGAGATACGGCC
>Zotu13198
GAACGCTGGCGGCGTGCTTAACACATGCAAGTCGAACGGTAAGGCCCTTCGGGGTACACGAGTGGCGAAC
GGGTGAGTAACACGTGGGTAATCTGCCCTGCACTTCGGGATAAGCCTGGGAAACTGGGTCTAATACCGGA
TATTCAGCTTCTGCCGCATGGTGGTTGTTGGAAAGTTTTTCGGTGCAGGATGTGCCCGCGGCCTATCAGCTT
GTTGGTGGGGTAATGGCCTACCAAGGCGACGACGGGTAGCCGACCTGAGAGGGTGACCGGCCACACTGG
GACTGAGACACGGCC
>Zotu13203
GAACGCTAGCGGCAGGCCTAACACATGCAAGTCAAGGGGTAGAAGGCTTCGGCCTTTGAGACCGGCGCAC
GGGTGCGTAACGCGTATGCAATCTGCCTTACACTAAGGGATAGACCAGAGAAATTTGGATTAATACCTTAT
AGTATATTTTTGTGGCATCACAGGATTATTAAAGATTTATTGGTGTAAGATGAGCATGCGTCCCATTAGCTA
GTTGGTATGGTAACGGCATACCAAGGCTATGATGAGTAGGGGTCCTGAGAGGGAGATCCCCCACACTGGT
ACTGAGACACGGAC
>Zotu13205
GAACGCTGGCGGCAGGCTTAACACATGCAAGTCGAGCGCCCCTCATGGGGAGCGGCAGACGGGTGAGTA
ACGCGTGGGAACGTACCCTTTTCTACGGAATAACCCAGGGAAACTTGGACTAATACCGTATGTGCCCTTCG
GGGGAAAGATTTATCGGAAAAGGATCGGCCCGCGTTGGATTAGCTAGTTGGTGGGGTAAAGGCCTACCAA
GGCGACGATCCATAGCTGGTCTGAGAGGATGATCAGCCACATTGGGACTGAGACACGGCC

>Zotu13210
AAACGCAAGAGACGTGCCTAACACATGCAAATTAAATAGTTTTAATTAACTATAGTGTATTGGTGAGTAATA
TATGAAAATTTAAATTTAGATTAGAAGTAATTCTAGTAAATTAGAAGGAATCTCTGATAAGTGCATAAGTGC
TTTTAAAAGTTAATACTGTCTAAAGAAAAGTTCATATCTGATTAGCTAGTTAGTAAGGTAAAAGCTTACTAA
GGCGAAGATCAGTAGCTACCCTGAGAGGGGGATTAGCCACATTGGGATTGGAATACAGCC
>Zotu13212
GAACGCTGGCGGCAGGCCTAACACATGCAAGTCGAACGCCCTAGCAATAGGGAGTGGCAGACGGGTGAG
TAACGCGTGGGAACGTACCCTTCGGTCTGGAATAACCCAGGGAAACTTGGACTAATACCAGATACGTGCG
AGAGCAGAAAGATTTATCGCCGAAGGATCGGCCCGCGTCCGATTAGCTAGTTGGTGAGGTAAAAGCTCAC
CAAGGCGACGATCGGTAGCTGGTCTGAGAGGATGATCAGCCACACTGGGACTGAGACACGGCC
>Zotu13217
GAACGCTGGCGGCGTGCCTAATACATGCAAGTCGAACGAGAGCGACCGGTGCTTGCACTGGTCAATCTAG
TGGCGAACGGGTGAGTAACACGTGGGTAACCTGCCCATCAGAGGGGGATAACATTCGGAAACGGATGCT
AAAACCGCATAGGTTCTTGAGTCGCATGACTCAGGAAGGAAAAGAGGCTTCGGCTTCTGCTGATGGATGG
ACCCGCGGTGCATTAGCTAGTTGGTGAGGTAACGGCTCACCAAGGCCGTGATGCATAGCCGACCTGAGAG
GGTGATCGGCCACATTGGGACTGAGACACGGCC
>Zotu13220
GAACGCTGATGGCGTGCCTCATACATGCAAGTCGAGCGCCCCGCAAGGGGAGCGGCGAACGGGTGAGTA
ACGCGTAAACAATCTGCCCGAAAATGGGGGATAACCATCCCAACGGATGGCTAATACCGCATAGGGTAGC
GAAGCCGCATGGTTTTGCTACGAAAGGCTTCGGCCGTTTTCGGATGAGTTTGCGTCCCATTAGTTTGTTGGC
GGGGTTACGGCCCACCAAGACTACGATGGGTAGCCGGCCTGAGAGGGTGTACGGCCACATTGGGACTGA
GATACGGCC
>Zotu13223
GAACGCTAGCGGCATGCCTAACACATGCAAGTCGAGCGGGCCCTTCGGGGTCAGCGGCGGACGGGTGAG
TAACGCGTGGGAACGTGCCTTCTGGTTCGGAATAACCCTGGGAAACTAGGGCTAATACCGGATACGCCCTT
TTGGGGAAAGGTTTACTGCCGGAAGATCGGCCCGCGTCTGATTAGCTAGTTGGTGGGGTAACGGCCTACC
AAGGCGACGATCCGTAGCTGGTCTGAGAGGACGATCAGCCACACTGGGACTGAGACACGGCC
>Zotu13225
GAACGCTGGCGGCGTGGATAAGGCATGCAAGTCGAAAGGGTTTAGGCCCTTGGCAAACGGGTTAGTAAT
GCATAGGAATTTTCCCTGAAGTCGTGAATAATCTGGAGAAATCCAGGATAATACACGATGTGCTCTACGGA
GTAAAGATTTATCGCTTCAGGAGAAGCCTATGTCCTATCAGCTTGTTGGTGGTGTAAAGGACCACCAAGGC
TATGACGGGTAGGGGGTGTGAGAGCACGGCCCCCAACATTGGGACTGAGAACTGCC
>Zotu13228
GAACGCTGGCGGCGTGCTTAACACATGCAAGTCGAGTGCGCGTACCGTAGCAATACGGTATGGCGCACGG
CGCACGGGTGCGTAACACGTGGGCAATCTGCCCTTCGATGGGGAATAACTCCGGGAAACCGAAGCTAATT
CCGCATAACATTCCTTGGACTTCGGTTCGAGGACTCAAAGCCGTAAGGCGTCGAAGGAGGAGCCCGCGCC
CGATTAGCTAGTTGGTGAGGTAACGGCTCACCAAGGCAATAATCGGTAGCTGGTCTGAGAGGATGGCCAG
CCACACTGGAACTGAGACACGGTC
>Zotu13232
GAACGCTGGCGGCGTGCTTAACACATGCAAGTCGAACGGTGAAGCAGGAGCTTGCTCCTGTGGATCAGTG
GCGAACGGGTGAGTAACACGTGAGCAATCTGCCCCAAACTCTGGGATAACTCCGGGAAATCGGAGCTAAT
ACCGGATACGACGCGCGAAGGCATCTTCTGCGCGTGGAAAGAACTTCGGTTTGGGATGAGCTCGCGGCCC
ATCAGCTTGTTGGTGAGGTAACGGCTCACCAAGGCGACGACGGGTAGCCGGCCTGAGAGGGTGACCGGC
CACACTGGGACTGAGACACGGCC
>Zotu13235
GAACGCTGGCGGCGTGCTTAACACATGCAAGTCGTGCGCGAAGGAGGCTTCGGTCTCTGGAGAGCGGCG
AACGGGTGAGTAACACGTGGGTAATCTGCCTGGGAATGGGGGATAACCTGGGGAAACTCGGGCTAATAC
CGCATACGTTGGTTGGATCTTGGTCTGACCTAAGAAAGCTGGCCTCTGCTTGCAAGCTAGCGTTCCTGGAG
GAGCCCGCGGCTGATTAGCTAGTTGGTGGGGTAAAGGCTCACCAAGGCGACGATCAGTAGCCGGCCTGAG
AGGGTGATCGGCCACACTGGGACTGAGACACGGCC

>Zotu13241
GAACGCTGGCGGTGTGCTTAACACATGCAAGTCGAACGCCCTTAGCAATAAGGGAGTGGCGGACGGGTG
AGGAACACGTGGGTGACCTACCTCGAAGTGAGGAATACCGGCGAGAAATCGCCGACAATACCGCATACGC
TTCGCAAGAAGCAAAGTCGCAAGACGCTTTGAGATGGGCCCGCGGCCGATTAGCTGGTTGGTGGGGTAAT
GGCCTACCAAGGCGACGATCGGTAGCTGGTCTGAGAGGACGATCAGCCACACTGGGATTGAGAACGGCC
>Zotu13243
GAACGCTGGCGGCATGCCTAACACATGCAAGTCGAACGAACCCTTCGGGGTTAGTGGCGCACGGGTGCGT
AACGCGTGGGAACCTGCCTTTAGGTTCGGAATAACTCAGAGAAATTTGAGCTAATACCGGATAATGTCTTC
GGACCAAAGATTTATCGCCTTTAGATGGGCCCGCGTTGGATTAGCTAGTTGGTGGGGTAAAGGCCTACCAA
GGCGACGATCCATAGCTGGTCTGAGAGGATGATCAGCCACACTGGGACTGAGACACGGCC
>Zotu13248
GAACGCTGGCGGCGTGGATGAGGCATGCAAGTCGAATGGCCTAGTAGCAATACTAGAAACCATGGCAGA
CGAGGTAGGAACACGTAGGTAACGTACCCCAAAGTCGGGCATAACCGGTCGAAAGATTGGATAATTCTCG
ATGGTCTCGGAGATTTATTTCTCCGAGTAAAGCGAATTATTTATTTAGTTTACGCTTTGGGAACGGCCTGCG
GGCTATCAGCTTGTTGGCAAGGTAATGGCTTACCAAGGCTACGACGGCTAGGGGAGGTGAGAGCCTGACC
CCCACCGGTGGAACTGCGACACGGTC
>Zotu13250
GAACGCTGGCGGTGTGCTTAACACATGCAAGTCGAGCGGGCATAGCAATATGTCAGCGGCAGACGGGTG
AGTAACGCGTGGGAACGTACCTTTTGGTTCGGAACAACACAGGGAAACTTGTGCTAATACCGGATAAGCCC
TTACGGGGAAAGATTTATCGCCGAAAGATCGGCCCGCGTCTGATTAGCTAGTTGGTGGGGTAACGGCCCA
CCAAGGCGACGATCAGTAGCTGGTCTGAGAGGATGATCAGCCACATTGGGACTGAGACACGGCC
>Zotu13252
GAACGCTGGCGGCGTGCTTAACACATGCAAGTCGAACGGGAAACATTTTATTGAAGCTTCGGCAGATTTAG
TTTGTTTCTAGTGGCGGACGGGTGAGTAACGCGTGGGTAACCTGCCTCACACTGGGGGATAACAGTCAGA
AATGACTGCTAATACCGCATAAGCGCACGGAACCGCATGGTTCAGTGTGAAAAACTCCGGTGGTGTGAGA
TGGACCCGCGTTGGATTAGCCAGTTGGCAGGGTAACGGCCTACCAAAGCGACGATCCATAGCCGGCCTGA
GAGGGTGAACGGCCACATTGGGACTGAGACACGGCC
>Zotu13259
GAACGCTGGCGGCGTGGATAAGACATGCAAGTCGAACGGGACATTTCAGGTAGCAATATTTGAAATGTCT
AGTGGCGAACGGGTGCGTAACACGTGGATTACATTCCGGGAAGCGGGGGATAGCCCAGGGAAACTTGGA
TTAATACCGCATGTGGTCGAAAGACTAAAGGTGGGGACCGCAAGGCCTGCCACTTCCCGATTGGTCCGCG
GCCTATCAGCTTGTTGGTGAGGTAATGGCTCACCAAGGCTACGACGGGTAGCTGGTCTTAGAGGACGACC
AGCCACACTGGAACTGAGACACGGTC
>Zotu13264
GAACGCTGGCGGCGTGCTTAACACATGCAAGTCGAACGGAAAGGCCCTGCTTGCAGGGTACTCGAGTGGC
GAACGGGTGAGTAACACGTGGGTGATCTGCCCTGCACTTCGGGATAAGCCTGGGAAACTGGGTCTAATAC
CGGATAGGACCCTCCTTTAGTGTGGGGGGTGGAAAGATTTATCGGTGCAGGATGAGCCCGCGGCCTATCA
GCTTGTTGGTGGGGTAATGGCCTACCAAGGCGTCGACGGGTAGCCGGCCTGAGAGGGTGGACGGCCACA
TTGGGACTGAGATACGGCC
>Zotu13267
GAACGCTGGCGGCATGCTTAACACATGCAAGTCGAACGGATTTATGGGGCTTGCTCCATAAGTTAGTGGCA
GACGGGTGAGTAACGCGTGGGAATGTACCTATTGGTTCGGAATAACGACGGGAAACTGTCGGTAATACCG
GATAAGCCCTGAGGGGGAAAGATTTATCGCCAAGAGAGCAGCCCGCGTTAGATTAGGTAGTTGGTGGGGT
AAAAGCCTACCAAGCCGACGATCTATAGCTGGTCTGAGAGGATGATCAGCCACACTGGGACTGAGACACG
GCC
>Zotu13269
GAACGCTGGCGGCAGGCCTAACACATGCAAGTCGAGCGGATGAGTGGAGCTTGCTCCATGATTCAGCGGC
GGACGGGTGAGTAATGCCTAGGAATCTGCCTGGTAGTGGGGGGCAACGTTTCGAAAGGAACGCTAATACC
GCATACGTCCTACGGGAGAAAGTGGGGGATCTTCGGACCTCACGCTATCAGATGAGCCTAGGTCGGATTA

GCTAGTTGGCGAGGTAAAGGCTCACCAAGGCGACGATCCGTAACTGGTCTGAGAGGATGATCAGCCACAC
TGGGACTGAGACACGGCC
>Zotu13272
GAACGCTAGCGATATGCTTAACACATGCAAGTCGAACGTTGTTTTCGGTCTATAGAACAGTTGAAGACAAA
GTGGCGAACGGGTGCGTAACGCGTGGGAATCTGCCAAACAGTTTGGGCCGAATCCCAAAGAAACGAAAG
CAAAAAAGCTTCGATAAAGCTAAAAAGCGCTGTTTGATGAGCCTGCGTAGTATTAGGTAGTTGGTTGGGTA
AAGGCTGACCAAGCCGCAGATGCTTAGCTGATCTTTTCGGACGATCAGCCACACTGGGACTGAGACACGG
CC
>Zotu13275
GAACGCTGGCGGCGTGCTTAACACATGCAAGTCGAACGGAAAGGCCTCTGCTTGCAGGGGTGCTCGAGTG
GCGAACGGGTGAGTAACACGTGGGTGATCTGCCCTGCACTTTGGGGTAAGCCTGGGAAACTGGGTCTAAT
ACCAGATAGGACCACGTCTTGGATGGTGTGGTGGAAAGTTTTTTCGGTGTGGGATGAGCTCGCGGCCTATC
AGCTTGTTGGTGGGGTAATGGCCTACCAAGGCGTCGACGGGTAGCCGGCCTGAGAGGGTGTACGGCCAC
ATTGGGACTGAGATACGGCC
>Zotu13277
GAACGCTAGCGGCGTGCTTAACACATGCAAGTCGAACGGAAAGGCCCTGCTTTTGTGGGGTGCTCGAGTG
GCGAACGGGTGAGTAACACGTGAGTAACCTGCCCTTGACTTTGGGATAACTTCAGGAAACTGGGGCTAAT
ACCGGATAGGAGCTCCTGCTGCATGGTGGGGGTTGGAAAGTTTCGGCGGTTGGGGATGGACTCGCGGCTT
ATCAGCTTGTTGGTGGGGTAGTGGCTTACCAAGGCTTTGACGGGTAGCCGGCCTGAGAGGGTGACCGGCC
ACACTGGGACTGAGACACGGCC
>Zotu13280
GAACGCTGGCGGCGTGCTTAACACATGCAAGTCGAGCGGTAAGGCCCTTCGGGGTACACGAGCGGCGAA
CGGGTGAGTAACACGTGAGCAATCTGCCCTTCACTTTGGGATAACTCCCGGAAACGGGTGCTAATACCGGA
TACGACCACATGTCGCATGGTGTGTGGTGGAAAGTTCTGGCGGTGGAGGATGAGCTCGCGGCCTATCAGC
TTGTTGGTGGGGTAATGGCCTACCAAGGCTTCGACGGGTAGCCGGCCTGAGAGGGTGACCGGCCACACTG
GGACTGAGACACGGCC
>Zotu13282
GAACGCTGGCGGTGTGCCTTAAGCATGCAAGTCGAGCGGGTCTCGCAAGAGATCAGCGGCAGACGGGTG
AGTAACACGTAGGAATCTACCCAGAAGAGGGGAATAGCTCGCCGAAAGGCGGGGTAATGCCCCATGTGAT
CGCAAGATTAAAGCAGCAATGCGCTTCTGGAGGAGCCTGCGTCCTATCAGCTAGTTGGTGGGGTAATGGC
CTACCAAGGCAACGACGGGTAGCCGGTGTGAGAGCATGACCGGCCACAAGGTAACTGAGACACGGTG
>Zotu13286
GAACGCTGGCGGCGTGCTTAACACATGCAAGTCGAGCGGTAAGGCCCTTTCGGGGGTACACGAGCGGCG
AACGGGTGAGTAACACGTGGGTGACCTGCCCTCAGCTCTGGGATAAGCCTGGGAAACTGGGTCTAATACC
GGATATGACCAACTGTCGCATGGTGGTTGGTGGAAAGTTTTTTCGGCTGGGGATGGGCCCGCGGCCTATC
AGCTTGTTGGTGGGGTGATGGCCTACCAAGGCGACGACGGGTAGCCGGCCTGAGAGGGCGACCGGCCAC
ACTGGGACTGAGACACGGCC
>Zotu13292
GAACGCTGGCGGCGTGCTTAACACATGCAAGTCGAACGGTGAAGCCCTTTCGGGGGTGGAGCAGTGGCG
AACGGGTGAGTAACACGTGGGCAATCTGCCCTGCACTCTGGGACAAGCCCTGGAAACGGGGTCTAATACC
GGATATGACACATGGGCGCATGTTCGTGTGTGGAAAGCTCCGGCGGTGCAGGATGAGCCCGCGGCCTATC
AGCTTGTTGGTGGGGTGATGGCCTACCAAGGCGACGACGGGTAGCCGGCCTGAGAGGGCGACCGGCCAC
ACTGGGACTGAGACACGGCC
>Zotu13294
GAACGCTGGCGGCATGGCTAAAGCATGCAAGTCGAACGGGTCCGCAAGGACCAGTGGCGAAAGGGTGAG
TAATACGATCCTACGTACCTTGAGGTGGCGGATAGCTGTGGGAAACTGCAGGTAATACGCCATGATCTCTC
AGGAGCAAAGATTTATCGCCTTGAGATCGGGGATCGTCCTATCAGCTAGTTGGCGGGGTAAAGGCCCACC
AAGGCGAAGACGGGTAGCAGGTGTGAGAGCACGGCCTGCCACATCGGGACTGAGACACTGCC
>Zotu13298

GAACGCTGGCGGCGTGGATAAGACATGCAAGTCGAACGCTTGTTTTCCTGTAGCAATATAGGGAAGCAGG
AGTGGCGCAAGGGTGCGTAACACGTGGATCATCTGCCTGGAAGTTCGGAATAATCCCTCGAAAGAGGGAC
TAATGCCGGATGTGACTGTGAGGTTAAAGTTGGGGACCGCAAGGCCTGACGCTTCCAGATGAGTCCGCGG
CCTATCAGCTAGTTGGCGGGGTAACGGCCCACCAAGGCTAAGACGGGTAGCTGGTCTGAGAGGACGACCA
GCCTCACTGGAACTGAGACACGGTC
>Zotu13301
GAACGCTGGCGGCATGCCTAATACATGCAAGTCGAACGAACACTTCGGTGTTAGTGGCGCACGGGTGCGT
AACGCGTGGGAATCTACTCTTGGGTTCGGAATAACAGTTAGAAATGACTGCTAATACCGGATGATGACGTA
AGTCCAAAGATTTATCGCCCAAGGATGAGCCCGCGTAGGATTAGCTTGTTGGTGAGGTAAAAGCTCACCAA
GGCGACGATCCTTAGTTGGTCTGAGAGGATGATCAGCCACACTGGGACTGAGACACGGCC
>Zotu13303
GAACGCTGGCGGCGTGCCTAACACATGCAAGTGGAGCGACGAACCCGGACTTGTCCGGGGGCAAAGCCG
CGAACGGGTGAGTAACACGTGGATTATCTGCCCCAATGACCGGGACAACCCGAGGAAACTCGGGCTAATA
CCGGATGTGCTCTTCGGAGGAAAGGAAGCTTCGGCCTCCGCATTGGGATGAGTCCGCGGCGCATTAGCTA
GTTGGTGGGGTAAAGGCCTACCAAGGCGACGATGCGTAGCTGGTCTGAGAGGACGATCAGCCACACTGG
AACTGAGACACGGTC
>Zotu13307
GAACGCTGGCGGCGTGCCTAACACATGCAAGTCGAGCGAGAAAGTAGCAATACGAGTACAGCGGCGGAC
GGGTGAGTAACACGTAGGTAATCTACCTTTCAGTGGGGAATAACGTTTCGAAAGGAACGCTAATACCGCAT
ATCGATACAGATCGTTGGATTTGTATCTAAAGGTGGCTCTCGCAAGAGGCTATCGCTGAGAGACGAGCCTG
CGTCCGATTAGCTAGTTGGTGAGGTAATGGCTCACCAAGGCGACGATCGGTAACTGGTCTGAGAGGACGA
CCAGTCACACTGGAACTGACACACGGTC
>Zotu13315
GAACGCTGGCGGCGTGCTTAACACATGCAAGTCGAGCGAGGCTCTTCCTTCGGGAGGATGCCCTAGCGGC
GAACGGGTGAGTAACACGTGGGCAACCTGCCCCTAGCACTGGGATAACCCCGGGAAACCGGGGCTAATAC
CGGATACGACTTCAACAGGCATCTGTCGAAGTGGAAAAGGTTACTGGCTAGGGATGGGCCCGCGGCCTAT
CAGCTTGTTGGTGGGGTAACGGCCTACCAAGGCGACGACGGGTAGCCGGCCTGAGAGGGCGACCGGCCA
CACTGGGACTGAGACACGGCC
>Zotu13320
GAACGCTGGCGGCGTGCTTAACACATGCAAGTCGAACGGAAAGGCCCTGCTTTTGTGGGGTGCTCGAGTG
GCGAACGGGTGAGTAACACGTGAGTAACCTGCCCTTGACTTTGGGATAACCTCAGGAAACTGGGGCTAAT
ACCGGATAGGAGCTCCTGCTGCATGGTGGGGGTTGGAAAGTTTCGGCGGTTGGGGATGGACTCGCGGCTT
ATCAGCTTGTTGGTGGGGTAGTGGCTTACCAAGGCTTTGACGGGTAGCCGGCCTGAGAGGGTGACCGGCC
ACACTGGGACTGAGACACGGCC
>Zotu13327
GAACGCTGGCGGCATGCCTAATACATGCAAGTCGAACGAGGTCCTTCGGGGCCTAGTGGCGCACGGGTGC
GTAACACGTGGGAATCTGCCCTTGGGTTCGGAATAACAGTTAGAAATGACTGCTAATACCGGATGATGACG
TAAGTCCAAAGATTTATCGCCCAAGGATGAGCCCGCGTAGGATTAGCTAGTTGGTGAGGTAAAGGCCTACC
AAGGCGACGATCCTTAGCTGGTCTGAGAGGATGATCAGCCACACTGGGACTGAGACACGGCC
>Zotu13329
GAACGCTGGCGGCGTGCTTAACACATGCAAGTCGAACGGGCCCTAGCAATAGGGCTAGTGGCGGACGGG
TGAGTAACACGTGAGCAACCTGCCCTATACAACGGGATAACACTAAGAAATTGGTGCTAATACGGTATAAG
ACCACACTGAGGCATCTCAGAGGGGTAAAAGCTGAGGCGGTATAGGAGGGGCTCGCGGCCCATTAGATA
GTTGGTGAGGTAACGGCCCACCAAGTCGACGATGGGTAGCCGACCTGAGAGGGTGATCGGCCACATTGG
AACTGAGATACGGTC
>Zotu13331
GAACGCTGGCGGCATGCCTAACACATGCAAGTCGAACGAGTGCCTTCGGGTGCTAGTGGCGCACGGGTGC
GTAACGCGTGGGAATCTGCCCTTGGGTTCGGAATAACAGTTGGAAACGACTGCTAATACCGGATGATGAC
GTAAGTCCAAAGATTTATCGCCCAGGGATGAGCCCGCGTAGGATTAGGTAGTTGGTGGGGTAAAGGCCTA
CCAAGCCGACGATCCTTAGCTGGTCTGAGAGGATGATCAGCCACACTGGGACTGAGACACGGCC

>Zotu13333
GAACGCTGGCGGCATGCCTAACACATGCAAGTCGAACGAGATCCTTCGGGGTCTAGTGGCGCACGGGTGC
GTAACGCGTGGGAATCTGCCCTTGGGTTCGGAATAACACAGAGAAATTTGTGCTAATACCGGATGATGTCT
TCGGACCAAAGATTTATCGCCCAAGGATGAGCCCGCGTAGGATTAGGTAGTTGGTGGGGTAATGGCCTAC
CAAGCCGACGATCCGTAGCTGGTCTGAGAGGACGATCAGCCACACTGGGACTGAGACACGGCC
>Zotu13335
GAACGCTGGCGGCATGCTTAACACATGCAAGTCGAACGGCAGCATGGTCTAGTTTACTAGATTGATGGCG
AGTGGCGGACGGGTGAGTAATGCGCAGGAATATGCCTTAAAGAGGGGGATAACATGGGGAAACTCATGC
TAATACCGCATAATCTCTGAGGAGAGAAGCTGGGGACCTTAGGGCCTGGCGCTTTAAGATTAGCCTGCGTC
CGATTAGCTAGTTGGTGGGGTAAGGGCCTACCAAGGCGACGATCGGTAGCTGGTCTGAGAGGATGACCA
GCCACACTGGAACTGAGACACGGTC
>Zotu13338
GAACGCTGGCGGCGTGCTTAACACATGCAAGTCGAGCGGTAAGGCCCTTCGGGGTACACGAGCGGCGAA
CGGGTGAGTAACACGTGGGTGACCTGCCCTCCACTCTGGGATAAGCCTGGGAAACTGGGTCTAATACCGG
ATATGACCTCAGGATGCATGTCTTGTGGTGGAAAGTTTTGTCGGTGGGGGATGGGCCCGCGGTCTATCAG
CTTGTTGGTGGGGTGATGGCCTACCAAGGCGACGACGGATAGCCGGCCTGAGAGGGTGATCGGCCACACT
GGGACTGAGACACGGCC
>Zotu13341
GAACGCTGGCGGCGTGGCTAAGACATGCAAGTCGAACGATATAGTGGCGAACGGGTGAGTAATGCATCG
CTAACATACCCCGAGCTCAGGGATAGCTGCCCGAAAGGGCTGGTAACACCTGATGTCATGCAGGAGAGGC
ATCTCTTCTGCCTGAAAGGTTACGGCTCGGGATTGGGCGATGTGGTATCAGCTAGTTGGTGAGGTAATGGC
TCACCAAGGCTTCGACGCCTAGCCGGACTGAGAGGTTGGCCGGCCACATCGGGACTGAGACACTGCC
>Zotu13343
AAACGCTGGCGGCGTGCCTAATACATGCAAGTCGAACGAGTAGCAATACTAGTGGCGCACGGGTGAGGA
ATACATGGGTAACCTGCCTTCGAGTCGGGGATAACAAGTCGAAAGATTTGCTAATACCGGATAAGACCACA
GGATCTGCGGATCTAGGGGTTAAAGGTTTTTCGCTTGAAGATGGGCCCATGCGGGATTAGCTAGTTGGTG
AGGTAACGGCTCACCAAGGCGACGATCCCTAACTGGTCTTAGAGGATGATCAGTCACACTGGAACTGAGA
CACGGTC
>Zotu13346
GAACGCTGGCGGCAGGCTTAACACATGCAAGTCGAGCGGAGTGATGGTGCTTGCACTATCACTTAGCGGC
GGACGGGTGAGTAATGCTTAGGAATCTGCCTATTAGTGGGGGACAACATTTCGAAAGGAATGCTAATACC
GCATACGCCCTACGGGGGAAAGCAGGGGACCTTCGGGCCTTGCGCTAATAGATGAGCCTAAGTCAGATTA
GCTAGTTGGTGGGGTAAAGGCCTACCAAGGCGACGATCTGTAGCGGGTCTGAGAGGATGATCCGCCACAC
TGGGACTGAGACACGGCC
>Zotu13348
CAACGCTGGCGGCGTGCCTAACACATGCAAGTCGAACGCGACTGGGGGCTTCGGTCTCCAGTTGAGTGGC
GAACGGGTGAGTAACACGTGGGTGATCTACCTTCGAGCGGGGGATAACGTTCCGAAAGGAACGCTAATAC
CGCATGACGTTCTGGGTTTAAAGACCTGGAAACCAAAGCTGGGGACCGCAAGGCCTGGCACTTGAAGAGG
AGCCCGCGTCCGATTAGCTAGTTGGCGGGGTAATGGCCCACCAAGGCGACGATCGGTAGCCGGCCTGAGA
GGGCGGACGGCCACACTGGGACTGAGACACGGCC
>Zotu13351
GAACGCTGGCGGCGTGCTTAACACATGCAAGTCGAGCGATGAAGCTCCTTCGGGAGTGGATTAGCGGCGG
ACGGGTGAGTAACACGTGGGTAACCTGCCTCATAGAGGGGAATAGCCTTCCGAAAGGAAGATTAATACCG
CATAAGATTGTAGTTCCGCATGGAATAGCAATTAAAGGAGTAATCCGCTATGAGATGGACCCGCGTCGCAT
TAGCTAGTTGGTGAGGTAACGGCTCACCAAGGCGACGATGCGTAGCCGACCTGAGAGGGTGATCGGCCAC
ATTGGGGCTGAGACACGGCC
>Zotu13354
GAATGCTGGCGGCGTGCCTAACACATGCAAGTCGAGCGGTAACAGGAGTAGTTTACTACTTGCTGACGAG
CGGCGGACGGCTGAGTAACGCGTGGGAACATGCCCCAAAGTGAGGGATAAGCTCCAGAAATGGAGTCTA
ATACCGCATATGATCTTCGGATTAAAGGATTTATCCGCTTTGGGAGTGGCCTGCGTACGATTAGATAGTTG

GTGAGGTAAAGGCTCACCAAGTCGACGATCGTTAACTGTTCTGAGAGGATGATCAGTCAGACTGGAACTG
AGACACGGTC
>Zotu13356
GAACGCTGGCGGCATGCCTAACACATGCAAGTCGAACGAAGGCTTCGGCCTTAGTGGCGCACGGGTGCGT
AACGCGTGGGAATCTGCCCCTTGGTTCGGAATAACAGTTGGAAACGACTGCTAATACCGGATGATGACGTA
AGTCCAAAGATTTATCGCCAAGGGATGAGCCCGCGTAGGATTAGCTAGTTGGTGGGGTAAAGGCCTACCA
AGGCGACGATCCTTAGCTGGTCTGAGAGGATGATCAGCCACACTGGGACTGAGACACGGCC
>Zotu13361
GCGTATAGCAATGATTAATCGGCAAATTGAAGACCATTGTCGGGTATTGTGCAAGCAGCAATGGGATGAA
GTGTGTAATTCGATTGATGGTGGCATTAAAAGGGGAGGCGCCTGGAATTTGTTGCGACATTTACTGGATGA
GACAAACACTAAGTCTAATCAGAGGACGATAATAGGTAAGCTGGTCCATGTGGCGTGTC
>Zotu13363
GAACGCTGGCGGCAGGCCTAACACATGCAAGTCGAGCGCCGTAGCAATACGGAGCGGCAGACGGGAGAG
TAACACGTGGGAACGTGCCCTTTGGTTCGGAACAACACAGGGAAACTTGTGCTAATACCGGATAAGCCCTT
ACGGGGAAAGATTTATCGCCGAAAGATCGGCCCGCGTCTGATTAGCTTGTTGGTGAGGTAACGGCTCACC
AAGGCGACGATCAGTAGCTGGTCTGAGAGGATGATCAGCCACATTGGGACTGAGACACGGCC
>Zotu13365
GAACGCTGGCGGCGCGCTTAACACATGCAAGTCGAGCGAGAACCAGTCCTTCGGGACTGGGGAAAGCGG
CGAACGGGTGAGTAACACGTGAGTAATCCACCCTCGGCACTGGGATAGCCCGGGGAAACCCGGATTAATA
CCGGATGGCCCAACAGCTCTTCGGGGCGGTTGGAAAAGGTAGCTTTGGCCTCCGGCCGAGGACGAGCTCG
CGGCGGATTAGCTTGTTGGTGGGGTAACGGCCTACCAAGGCGACGATCCGTAGCTGGTCTGAGAGGACGA
TCAGCCACACTGGGACTGAGACACGGCC
>Zotu13370
GAACGCTGGCGGCGTGCTTAATACATGCAAGTCGAACGAAGCACTTACGACAGATCCCCTCGGGGTGAAG
ATGTTTGTGACTTAGTGGCGGACGGGTGAGTAACGCGTGGGTAACCTGCCTCATGCAGGGGGATAACAGT
TAGAAATGACTGCTAATACCGCATAAGCGCACGGATTCGCATGTTTCTGTGTGAAAAGATTTATCGGCATG
AGATGGACCCGCGTCTGATTAGGTAGTTGGTGGGGTAACGGCCCACCAAGCCTGCGATCAGTAGCCTACTT
GAGAGAGTGATCGGCCACATTGGGACTGAGACACGGCC
>Zotu13373
GAACGCTGGCGGCGTGCTTAACACATGCAAGTGGAGCGACGAACCAGGGCTTGCCCTGGGGCAGAGCCG
CGAACGGGTGAGTAACACGTGGGTTACGTGCCCCGATGACTGGGACAACCCGGGGAAACCCGGGCTAAT
ACCGGATATGCCCCCTGACATCTGTCAGGTGTGTAAAGGAAGCTTCGGCCTCCGCATCGGGATCGGCCCGC
GGCGCATTAGCTTGTTGGTGGGGTAATGGCCTACCAAGGCAGCGATGCGTAGCTGGTCTGAGAGGACGAT
CAGCCACACTGGGACTGAGACACGGCC
>Zotu13378
GAACGCTGGCGGCGTGGATTAGGCATGCAAGTCGCGCGATCTCGCAAGAGATAGCGGCAAACGGGTTAG
GAACACGTAAATAATCTTCCATAAATCCTGACATAGCTCTATGAAAATAGAGGTAATATCAGATAGCAACG
CACCTAGGCATCTAGAAGCGTTTAAAGCAGGGGACCGCAAGGCCTTGCGATTTTTGATGAGTTTGCGTAGC
ATCAGCTAGTTGGTAGGGTAATGGCCTACCAAGGCTAAGACGCTTAGCTGGTCTGAGAGGACGATCAGCA
ACACTGGGACTGAGACACTGCC
>Zotu13384
GAACGCTGGCGGCGTGCTTAACACATGCAAGTCGAACGAGCGAGAGAGAGCTTGCTTTCTCGAGCGAGTG
GCGAACGGGTGAGTAACGCGTGAGGAACCTGCCTCAAAGAGGGGGACAACAGTTGGAAACGACTGCTAA
TACCGCATAAGCCCACGGCTCGGCATCGAGCAGAGGGAAAAGGAGCAATCCGCTTTGAGATGGCCTCGCG
TCCGATTAGCTAGTTGGTGAGGTAACGGCCCACCAAGGCGACGATCGGTAGTCGGACTGAGAGGTTGAAC
GGCCACATTGGGACTGAGACACGGCC
>Zotu13389
GAACGCTGGCGGCAGGCCTAAAACATGCAAGTCGAACGCCCTAGCAATAGGGAGTGGCGCACGGGTGCG
TAACATGTGGGAATATGCCTTTTGGTACGGAACAACGTTTGGAAACGAACGCTAATACCGTATGGTCCCTT

CGGGGTAAAGATTTATCGCCAAAAGATTAGCCCGCACTGGATTAGCTAGTTGGTGAGGTAATGGCTCACCA
AGGCGACGATCCATAGCTGGTCTGAGAGGACGATCAGCCACACTGGGACTGAGACACGGCC
>Zotu13393
GAACGCTGGCGGCGTGCCTAATACATGCAAGTCGAGCGGTAACAGGTCTAGTTTACTAGATGCTGACGAG
CGGCGGACGGCTGAGTAACACGTAGGAACGTACCCCAAAGTGAGGGATAACTGCTCGAAAGAGTAGCTA
ATACCGCATGTGATCCACGGATTAAAGCTTTATGCGCTTTGGGAACGGCCTGCGTTCGATTAGCTAGATGG
TAGTGTAATGGACTACCATGGCGACGATCGATAGCTGGTCTGAGAGGACGATCAGCCACACTGGGACTGA
GACACGGCC
>Zotu13401
GAACGCTGGCGGCATGCCTAACACATGCAAGTCGAACGAGACCTTCGGGTCTAGTGGCGCACGGGTGCGT
AACGCGTGGGAATCTGCCCTCTGGTTCGGAATAACTCAGGGAAACTTGAGCTAATACCGGATAATGACTTC
GGTCCAAAGATTTATTGCCAGAGGATGAGCCCGCGTAGGATTAGCTTGTTGGTGGGGTAAAGGCCTACCA
AGGCGACGATCCTTAGCTGGTCTGAGAGGATGATCAGCCACACTGGGACTGAGACACGGCC
>Zotu13403
GAACGCTGGCGGTGTGCTTAACACATGCAAGTCGAGGGGTAGAAGGAGCTTGCTTCTTTGAGACCGGCGC
ACGGGTGCGTAACGCGTATGCAATCTACCTTATACAGGGGAATAGCCCAGAGAAATTTGGATTAATGCCCC
ATGGTATTATAGAATGGCATCATTTTATAATTAAAGTTCCAACGGTATAAGATGAGCATGCGTCCCATTAGT
TAGTTGGTAAGGTAACGGCTTACCAAGACGATGATGGGTAGGGGTCCTGAGAGGGAGATCCCCCACACTG
GTACTGAGACACGGAC
>Zotu13407
GAACGCTGGCGGCAGGCTTAACACATGCAAGTCGAACGGCCATTTCGGTGGCAGTGGCGGACGGGTGAG
TAACGCGTGGGAACGTGCCCAAAGGTAGGGAACAACCGAGGGAAACTTCGGCTAATACCCTATGTGCCCG
GAAGGGGAAAGATTTATCGCCTTTGGAGCGGCCCGCGTCTGATTAGCTAGTTGGTGGGGTAAAGGCTCAC
CAAGGCTTCGATCAGTAGCTAGTCTGAGAGGATGGCCAGCCACATTGGGACTGAGACACGGCC
>Zotu13409
GAACGCTGGCGGTGTGCCTAATACATGCAAGTCGAACGGACTCTTTAAATTTTCATTTACCAATTTCCAATC
GCAAGATTGAGAATTGATACTTGAAAATATGAGTTAGTGGCGGACGGGTGAGTAACACGTTGGTACCTGC
CCTTTAGTGGGGAATAACTCCGGGAAACCGGGGCTAATACCGCATAATATCGCAAGATTAAAGCAGCAAT
GCGCTAAAGGAGGGGCCTGCGTCTGATTAGCTAGTTGGTAGGGTAATGGCCTACCAAGGCAATGATCAGT
AGCTGGATCGAGAGGTTGACCAGCCACACTGGGACTGAGATACGGCC
>Zotu13413
GAACGCTAGCGGCATGCCTAACACATGCAAGTCGAACGGCAGCGCGGGGCAACCTGGCGGCGAGTGACG
AACGGGTGAGTAATATATCGGAACGTACCCAAGAGTGGGGGATAACGTAGCGAAAGTTACGCTAATACCG
CATACGATCTAAGGATGAAAGTGGGGGATCGCAAGACCTCATGCTCCTGGAGCGGCCGATATCTGATTAG
CTAGTTGGTGAGGTAAAGGCTCACCAAGGCAACGATCAGTAGCTGGTCTGAGAGGACGACCAGCCACACT
GGGACTGAGACACGGCC
>Zotu13417
GAACGCTGGCGGCATGCCTAACACATGCAAGTCGAACGATTGCCTTCGGGTGATAGTGGCGCACGGGTGC
GTAACGCGTGGGAATCTGCCCTTTGGTTCGGAATAACAGTTGGAAACGACTGCTAATACCGGATGATGACG
AAAGTCCAAAGATTTATCGCCAGAGGATGAGCCCGCGTAGGATTAGCTAGTTGGTGTGGTAAAGGCGCAC
CAAGGCGACGATCCTTAGCTGGTCTGAGAGGATGATCAGCCACACTGGGACTGAGACACGGCC
>Zotu13420
GAACGCTAGCGGCAGGCTTAATACATGCAAGTCGAGGGGCAGCACAGGTAGCAATACTGGGTGGCGACC
GGCAAACGGGTGCGGAACACGTACGCAACCTTCCTTTAAGTGGGAGATAGCCCAGGGAAACTTGGATTAA
TATCCCGTAATATTTTTGGGTGGCATCACCTGATAATTATAGCTCCGGCGCTTAAAGATGGGCGTGCGCCTG
ATTAGGTAGTTGGCGGGGTAACGGCCCACCAAGCCTACGATCAGTAACTGGTGTGAGAGCACGACCAGTC
ACACGGGCACTGAGACACGGGC
>Zotu13424
GAACGCTGGCGGCGTGCTTAACACATGCAAGTCGAACGGTAAGGCCCTTCGGGGTACACGAGTGGCGAAC
GGGTGAGTAACACGTGGGTGATCTGCCCTGCACTTTGGGATAAGCCTGGGAAACTGGGTCTAATACCGAA

TATGACTGCATCCTGCATGGGGTGTGGTGGAAAGCTTTTGCGGTGTGGGATGGGCCCGCGGCCTATCAGC
TTGTTGGTGGGGTGATGGCCTACCAAGGCGACGACGGGTAGCCGGCCTGAGAGGGTGACCGGCCACACT
GGGACTGAGACACGGCC
>Zotu13426
GAACGCTGGCGGCGTGCTTAACACATGCAAGTGGAGCGACGAACCAGGGCTTGCCCTGGGGCAGAGCCG
CGAACGGGTGAGTAACACGTGGGTAACGTGCCCCGATGACTGGGACAACCCGGGGAAACCCGGGCTAAT
ACCGGATATGCCCCCTGACATCTGTCAGGTGTGTAAAGGAAGCTTCGGCCTCCGCATCGGGATCGGCCCGC
GGCGCATTAGCTTGTTGGTGGGGTAATGGCCTACCAAGGCAGCGATGCGTAGCTGGTCTGAGAGGACGAT
CAGCCACACTGGGACTGAGACACGGCC
>Zotu13428
CAACGCTGGCGGCGTGCTTCAGACATGCAAGTCGAACGCGAAAGTTTCCTTCGGGAAGCGAGTAGAGTGG
CGCACGGGTGAGTAACACGTAAGTAATCTACCTTTGGGTGGGGAATAACTCAGGGAAACTTGAGCTAATA
CCGCATAATGCAGCGGCTCCTTATGGAGACAGTTGTTAAAGAATTTATTCGCCTAAAGAGGAGCTTGCGGC
AGATTAGCTAGTTGGTAAGGTAATGGCTTACCAAGGCAACGATCTGTAGCCGACCTGAGAGGGTGGTCGG
TCACACTGACACTGAATAACGGGT
>Zotu13437
GAACGCTAGCGGCAGGCTTAATACATGCAAGTCGAGGGGCAGCAGGGATAGCAATATTCGCTGGCGACC
GGCGCACGGGTGCGTAACACGTATACAATCTGCCTTTAACAGGGGGATAGCCCGGAGAAATCCGGATTAA
TACCCCATAACAGTGATTCTGGCATCAGAGACACTTAAAAGCTCCGGCGGTTAAAGATGAGTATGCGTCTG
ATTAGCTAGTTGGTGAGGTAACGGCTCACCAAGGCGACGATCAGTAGGGGATCTGAGAGGATGAACCCCC
ACACGGATACTGAGACACGGAT
>Zotu13441
GAACGCTGGCGGCAGGCCTAACACATGCAAGTCGAGCGGCAGCGGGTCCTTCGGGATGCCGGCGAGCGG
CGGACGGGTGAGTAATACGTGGGAATCTGTCCATTGATGGGGGATAACGTGGGGAAACTCACGCTAATAC
CGCATACGCCCTACGGGGGAAAGCAGGGGATCTTCGGACCTTGCGTCGATGGGTGAGCCCACGCCGGATT
AGCTAGTTGGTAGGGTAAAGGCCTACCAAGGCGACGATCCGTAGCTGGTCTGAGAGGATGATCAGCCACA
CTGGGACTGAGACACGGCC
>Zotu13445
TGAAGGCAGAGCGGAATGTGCAATAAAACACCATCACCAGACACAAAGTGTTTTAGCAGTACGTAGAAAA
TTTAGAACGTAGAAGCAGAGATGTAAGGGTACTCCTGAGACGAATTGACAAGGTAGCGCAAATATAC
>Zotu13447
GAACGCTGGCGGCGTGCTTAACACATGCAAGTCGAACGGAAAGGCCCTGCTTTTGTGGGGTGCTCGAGTG
GCGAACGGGTGAGTAACACGTGAGTAACCTGCCCTTGACTTTGGGATAACTTCAGGAAACTGGGGCTAAT
ACCGGATAGGAGCTCCTGCTGCATGGTGGGGGTTGGAAAGTTTCGGCGGTTGGGGATGGACTCGCGGCTT
ATCAGCTTGTTGGTGGGGTAGTGGCTTACCAAGGCCTTGACGGGTAGCCGGCCTGAGAGGGTGACCGGCC
ACACTGGGACTGAGACACGGCC
>Zotu13449
GAACGCTGGCGGCGTGCTTAACACATGCAAGTGGAGCGACGAACCGGGGCTTGCCCCGGGGCAGAGCCG
CGAACGGGTGAGTAACACGTGGGTAACCTACCCCGATGACCGGGACAACCCGAGGAAACTCGGGCTAATA
CCTGATGTGCTCCTTGGCCATGAGGTCAATGAGCAAAGATAGCTTCGGCCTTCGCATCGGGACGGGCCCGC
GGCCCATTAGCTTGTTGGTGGGGTAACGGCCTACCAAGGCGACGATGGGTAGCTGGTCTGAGAGGACGAT
CAGCCACACTGGGACTGAGACACGGCC
>Zotu13451
GAACGCTAGCGGCAGGCTTAACACATGCAAGTCGAGGGGTAGAGGCTTTCGGGCCTTGAGACCGGCGCAC
GGGTGCGTAACGCGTATGCAATCTGCCTTGTACAAAGGGATAGCCCAGAGAAATTTGGATTAATACCTTAT
AGTATATAGAGAGGACATCCTTTTTATATTAAAGATTTATCGGTACAAGATGAGCATGCGTCCCATTAGCTA
GTTGGTGTGGTAACGGCATACCAAGGCTATGATGGGTAGGGGTCCTGAGAGGGAGATCCCCCACACTGGT
ACTGAGACACGGAC
>Zotu13456

GAACGCTGGCGGCAGGCTTAACACATGCAAGTCGAACGGTTGTAGCAATACAGCAGTGGCAGACGGGTG
AGTAACACGTGGGAACCTTCCTAGGGGTACGGAACAACTCAGGGAAACTTGAGCTAATACCGTATACGTCC
GTAAGGAGAAAGATTTATCGCCCTTAGACGGGCCCGCGTCGGATTAGCTAGTTGGTGGGGTAACGGCCTA
CCAAGGCGACGATCCGTAGCTGATCTTAGAGGATGATCAGCCACACTGGGACTGAGACACGGCC
>Zotu13461
CAACGCTGGCGGCGCGCTTAACACATGCAAGTCGAACGAGAAAGCGGGAGCAATCTCGCAAGTACAGTGG
CGTACGGGTGAGTAACACGTGGGTAATCTACCTCATAGTGGGGAATAACGCTGGGAAACCGGCGCTAATA
CCGCATAATCCGCGACCTTAAGGGAAGCCGGGAAAGCAGCAATGCGCTGTGAGAGGAGCCCGCGGCCGA
TTAGCCAGTTGGTAGGGTAACGGCCTACCAAAGCAAAGATCGGTAGCCGGCCTGAGAGGGCACACGGCCA
CACTGGCACTGAAACACGGGC
>Zotu13472
GAACGCTGGCGGCGTGCTTAACACATGCAAGTCGAACGAGATCCAGCTCGTGGCAACACGAGGGAAAATC
GAGTGGCGAACGGGTGAGTAACACGTGAGAAACCTGTCCCGGTCTCTGGAATAACAGTGGGAAACCACTG
CTAATACCGGATACCTTCCCTGCGTCGCATGGCGTGGTGAAGAAAGGCTTGTCCGGATCGGGAGGGTCTC
GCGGCCTATCAGCTTGTTGGTGGGGTAACGGCCTACCAAGGCGTCGACGGGTAGCTGGTCTGAGAGGACG
ATCAGCCACACTGGGACTGAGACACGGCC
>Zotu13475
GAACGCTAGCGGCATGCCTAACACATGCAAGTCGAACGGCAGCACGGGAGCAATCCTGGTGGCGAGTGG
CGAACGGGTGAGTAACACGTAGGAATCTACCTTAATTATAATAGTTAGTGGGGGATAACCCGGGGAAACT
CGGGCTAATACCGCATAATCTCTTCAAAGCAAAGCGGGGGATCTTCGGACCTCGTGCTATGAGATGAGCCT
GCGTCGGATTAGCTAGTTGGTAGGGTAATGGCCTACCAAGGCGACGATCCGTAGCTGGTCTGAGAGGACG
ATCAGCCACACTGGGACTGAGACACGGCC
>Zotu13479
GAACGCTAGCGGCGGGCTTAATACATGCAAGTCAAGGGGGAGCAATACACCGGCGGACGGGTGCGTAAC
GCGTATGCAACCTACCTTTATCTGGGGGATAACTCGGGGAAACTCGAGCTAATACCGCATAAGAAAGTGAT
CTGGCATCAGATAGCTTTTAAAGATTTATTGGATAAAGATGGGCATGCGTAAGATTAGATAGTTGGTGAGG
TAACGGCTCACCAAGTCTACGATCTTTAGGGGCTCTGAGAGGAGAATCCCCCACACTGGCACTGAGATACG
GGC
>Zotu13481
GCCGTGTCTCAGTCCCAATGTGGCTGATCATCCTCTCAAATCAACTATGGATCGTAGCCTTGGTAGGCCCTT
ACCTCACCAACTAGCTAATCCAACGCAGGCTCATCCATCCGCGACAGCCTAAAAGCCATTTTTAATCCACAG
ATACTATGCGGTATTAATAGTCGTTTCCAACTGGTATCCCCCTCAGATGGGCAGATTCCTACGTGTTACTCAC
CCGTCCGCCACTCGTCAGCAACTAGCAAGCTAGTCCTGTTACCGTTCGACTTGCATGTGTTAAGCATGCCAC
CAGCGTTCA
>Zotu13483
GAACGCTAGCTACAGGCTTAACACATGCAAGTCGAGGGGCAGCATGATTGAAGCTTGCTTCAATTGATGGC
GACCGGCGCACGGGTGAGTAACACGTATCCAACCTTCCGTACACTCAGGGATAGCCTTTCGAAAGAAAGAT
TAATACCTGATGGTATCTTAAGCACACATGTAATTAAGATTAAAGATTTATCGGTGTACGATGGGGATGCG
TTCCATTAGGTAGTAGGCGGGGTAACGGCCCACCTAGCCTACGATGGATAGGGGTTCTGAGAGGAAGGTC
CCCCACATTGGAACTGAGACACGGTC
>Zotu13494
GAACGCTAGCGGCATGCTTAACACATGCAAGTCGAACGAGCAAGAGCTGTAGCAATACAGGGTTCTGCTA
GTGGCAGACGGGTGAGTAATACATGGGAATCTACCTTGCAGTGCGAAATAACAGTTGGAAGCAATTGCTA
ATGTCGCATATAGCCGAGAGGTGAAAGGTTTACTGCTGCAAGATGGGCCCATGCAGGATTAGCTAGTTGG
TGAGGTAATGGCTCACCAAGGCAACGATCTTTAGTTGGTCTGAGAGGATGATCAGCCACACTGGGACTGA
GACACGGCC
>Zotu13498
CAACGCTGGCGGCGTGCCTAACACATGCAAGTCGAACGAGAAAGTGGAGCAATCCATGAGTAAAGTGGCG
ACCGGGTGAGTAACACGTGACTAACCTACCCTACAGTGGGGGATAACCTCGGGAAACCGGGGCTAATACC
GCATAACATCGTGAGATCAAAGAAGCAATTCGCTGAAGGAGGGGGTCGCGGCTGATTAGTTAGTTGGTGA

GGTAACGGCCCACCAAGACGAAGATCAGTAGCCGGCCTGAGAGGGCGCACGGCCACACTGGAACTGAAA
CACGGTC
>Zotu13502
GAACGCTGGCGGCATGCCTAACACATGCAAGTCGAACGAGACCTTCGGGTTTAGTGGCGCACGGGTGCGT
AACGCGTGGGAATCTGCCCTTAGGTTCGGAATAACAGTTGGAAACGACTGCTAATACCGGATGATGACGTT
AAGTCCAAAGATTTATCGCCTAAGGATGAGCCCGCGTAGGATTAGCTAGTTGGTGTGGTAAGAGCGCACC
AAGGCGACGATCCTTAGCTGGTCTGAGAGGATGATCAGCCACACTGGGACTGAGACACGGCC
>Zotu13505
GAACGCTAGCGGCATGCTTAACACATGCAAGTCGAACGGCAGAGCGGGGAGTTTATGCTCCCTGGCGGCG
AGTGGCGGACGGGTGAGTAATACGTAGGAATCTACCTTATAGAGGGGGACAACCCGGGGAAACTCGGGC
TAATACCGCATACGTCCTACGGGAGAAAGCAGGGGACCTTCGGGCCTTGCGCTATCAGATGAGCCTATGTC
GGATTAGCTTGTTGGTGGGGTAATTGCCTACCAAGGCGACGATCCGTAGCTGGTCTGAGAGGATGATCAG
CCACACTGGGACTGAGACACGGCC
>Zotu13511
GAACGCTGGCGGCGTGCCTAACACATGCAAGTGGAGCGACGAACCGGGGCTTGCCCCGGGGCAGAGCCG
CGAACGGGTGAGTAACGCGTGGGTTACCTGCCCCGATGACCGGGACAACCCGTCGAAAGACGGGCTAATA
CCGGATGTGCCCCTTGGGGGAAAGGAAGCTTCGGCCTCCGCATCGGGATGGGCCCGCGGCGCATTAGCTT
GTTGGTGGGGTAATGGCCTACCAAGGCGACGATGCGTAGCTGGTCTGAGAGGACGATCAGCCACACTGG
GACTGAGACACGGCC
>Zotu13514
GAACGCTGGAAATGTGGATACTGGCATGCAAGTCAAGGGGCCGCAAGGCACCGGCGAACGGCTTAGTAA
CACGTAGATACATACCGTAAAGTGGGGCATAGCTCGTCGAAAGACGGGGTAATTTCCCATGTGAACGCAA
GTTTAAAGGCGCAAGCCGCTTTATGAATGGTCTGCGGTCTATCAGCTAGTTGGTGGGGTAAAAGCCTACCA
AGGCTATGACGGATAGGGGGCGTGAGAGCGCGACCCCCAAGAATGGCACTGCGACACGGGC
>Zotu13517
GAACGCTGGCGGCGTGCCTAATACATGCAAGTCGGGCGGACCGAACGAGGGCTTGCTCTTGTTTGGTCAG
CGGCGGACGGGTGAGTAACACGTGGGCAACCTGCCCGCAAGACCGGGATAACTCCGGGAAACCGGAGTT
AATACCGGATAACACCGAAGACCGCATGGTCTTCGGTTGAAAGGCGGCCTTTGGGCTGTCACTTGCGGATG
GGCCCGCGGCGCATTAGCTAGTTGGTGAGGTAACGGCTCACCAAGGCGACGATGCGTAGCCGACCTGAGA
GGGTGATCGGCCACACTGGGACTGAGACACGGCC
>Zotu13519
GAACATTGGCGGTAGGCTTAACACATGCAAGTCGAGCGAGGCGTAATAGTCGAGCGGCGAACGGGTGAG
TAATACATGGGAATCTACCTCGAAGTGGGGGATAACGTTCAGAAATGGACGCTAATACCGCATAATCCGGA
AACGGGAAAGCGCGCTGCGCTTTGAGATGAGCCCATGGCTGATTAGCTAGTTGGTAGGGTAAAGGCTTAC
CAAGGCGACGATCCGTAGCTGGTCTGAGAGGACGATCAGCCACACTGGGACTGAGACACGGCC
>Zotu13521
GAACGCTGGCGGCGTGCTTAACACATGCAAGTCGAACGGTGATGGATGGAGCTTGCTCTGTCTTGATCAGT
GGCGAACGGGTGAGTAACACGTGAGTAACCTGCCCCAGACTCTGGAATAACCCCGGGAAACCGGAGCTAA
TACCGGATATGACACCAACCTGCATGGGTATGGTGTGGAAAGTTTTTCGGTCTGGGATGGACTCGCGGCCT
ATCAGCTTGTTGGTGAGGTAATGGCTCACCAAGGCGACGACGGGTAGCCGGCCTGAGAGGGCGACCGGC
CACACTGGGACTGAGACACGGCC
>Zotu13524
GAACGCTGGCGGCGTGCCTAAAACATGCAAGTCGAGCGGTAAGGCCCTTCGGGGTACACGAGCGGCGGA
CGGGTGAGTAACACGTGGGAATCTACCCCAAACTGAGGGATAAGCACCGGAAACGGTGTCTAATACCGCA
TATGGTCTTCGGATTAAAGCTTCGGCGGTTTGGGAAGAGCCCGCGTAAGATTAGCTAGTTGGTGAGGTAA
AGGCTCACCAAGGCGACGATCTTTAGCTGGTTTGAGAGGATGATCAGCCACACTGGAACTGAGATACGGT
C
>Zotu13528
CAACGCTGGCGGCGTGCCTAACACATGCAAGTCGAACGCGGTTACTGCTGCTTCGCAAGAAGTAGTAGTA
ACTGAGTGGCGAACGGGTGAGTAACACGTGGGTGACCTGCCTTCGAGTGGGGGATAACATCCCGAAAGG

GGTGCTAATACCGCATGAGATCCTGCCTTTGAAGAGGTGGAGATTAAAGCTGGGGATCGTAAGACCTGGC
GCTTGAAGAGGGGCCCGCGTCCGATTAGCTAGTTGGTGGGGTAACGGCCTACCAAGGCGACGATCGGTAG
CCGGCCTGAGAGGGCGGACGGCCACACTGGGACTGAGACACGGCC
>Zotu13532
GAACGCTGGCGGCGTGCCTAATACATGCAAGTCGAGCGGACAGAAGGGAGCTTGCTCCCGGAAGTCAGC
GGCGGACGGGTGAGTAACACGTGGGTAACCTGCCTGTAAGACTGGGATAACTCCGGGAAACCGGGGCTA
ATACCGGATAGCTTCTTCCTCCGCATGGAGGAAGAATGAAAGGCGGCCTTTGGCTGTCACTTACAGATGGA
CCCGCGGCGCATTAGCTAGTTGGTGGGGTAACGGCTCACCAAGGCAACGATGCGTAGCCGACCTGAGAGG
GTGATCGGCCACACTGGGACTGAGACACGGCC
>Zotu13536
GAACGCTGGCGGCGTGCCTAATACATGCAAGTCGAGCGAATCTTTAGGAGCTTGCTCCTAAAGATTAGCGG
CGGACGGGTGAGTAACACGTGGGCAACCTGCCTATAAGACTGGGATAACTTCGGGAAACCGGAGCTAATA
CCGGATAATTCTTTTCCTCTCATGAGGAAAAGTTGAAAGACGGTTTCGGCTGTCACTTATAGATGGGCCCGC
GGCGCATTAGCTAGTTGGTGAGGTAACGGCTCACCAAGGCAACGATGCGTAGCCGACCTGAGAGGGTGAT
CGGCCACACTGGGACTGAGACACGGCC
>Zotu13540
GAACGCTGGCGGCGTGCCTAATACATGCAAGTCGAGCGAACAGAGAAGGAGCTTGCTCCTTTGACGTTAG
CGGCGGACGGGTGAGTAACACGTGGGCAACCTACCCTATAGTTTGGGATAACTCCGGGAAACCGGGGCTA
ATACCGAATAATCTATTGTCCCTCATGGGACAATGCTGAAAGACGGTTTCGGCTGTCGCTATAGGATGGGC
CCGCGGCGCATTAGCTAGTTGGTGAGGTAACGGCTCACCAAGGCAACGATGCGTAGCCGACCTGAGAGGG
TGATCGGCCACACTGGGACTGAGACACGGCC
>Zotu13545
GAACGCTAGCGGCATGCTTAACACATGCAAGTCGAACGGCAGCACGAGGAGCTTGCTCCTTGGTGGCGAG
TGGCGGACGGGTGAGTAACGCGTAGGAATCTACCTTATAGTGGGGGACAACCCGGGGAAACTCGGGCTA
ATACCGCATAATGTCTAAGGATCAAAGCAGGGGACCTTCGGGCCTTGCGCTATAAGATGAGCCTGCGTCG
GATTAGCTAGTTGGTGGGGTAAAGGCCTACCAAGGCGACGATCCGTAGCTGGTCTGAGAGGACGACCAGC
CACACTGGGACTGAGACACGGCC
>Zotu13548
GAACGCTGGCGGCGTGCCTAACACATGCAAGTCGAACGCGAAAGTGGAGCAATCCATGAGTAGAGTGGC
AAACGGGTGAGTAACGCGTGGGCGACCTACCTTCGAGCGGGGGATAACATTCCGAAAGGAGTGCTAATAC
CGCATGACATCCCGTGTTTGGATACACGGTGATCAAAGCCGGGGATCGCAAGACCTGGCACTTGAAGAGG
GGCCCGCGTCCGATTAGCTAGTTGGCGAGGTAATGGCTCACCAAGGCTCCGATCGGTATCCGGCCTGAGA
GGGCGGACGGACACACTGGAACTGAGACACGGTC
>Zotu13554
CAACGCTGGCGGCGTGCCTAATGCATGCAAGTCGAACGGGGTCATCTTCGGATGGCTTACGTGGCGGACG
GGTGAGGAACACGTGGGTAACCTGCCCAGTGGTGGGGGCTAGCCATCGGAAACGGTGGGTAATTCCGCA
TACGGTCAATCGCCGAGTGGGGTGATTGACGAAAGGCGCAAGTCGCCACTGGAGGGGCCCGCGTCCGATT
AGCTTGTTGGTGGGGTAACGGCTCACCAAGGCAGTGATCGGTAGCTGGTTTGAGAGGATGATCAGCCACA
CGGGGACTGAGACACGGCC
>Zotu13558
GAACGCTGGCGGCGTGCCTAAGACATGCAAGTCGAGGGGGTTAGACCCCCGGCGAACGGGTGAGTAACA
CGTGGGAACTTCCTTTAAGTTCGGGATAACCTCTAGAAATGGAGGACAATACCGGATGTGGTCGTAAGACT
AAAGTCGCAAGACGCTTAAAGCAAGGCCTGCGGCCCATCAGCTAGTTGGTAGGGTAATGGCCTACCAAGG
CGATGACGGGTAGCGGAAGCGAGAGCTTGATCCGCCACACTGGGACTGCGACACGGCC
>Zotu13561
GAACGCTGGCGGCGTGCTTAACACATGCAAGTGGAGCGACGAACCAGGCTTCGGCCTGGGGCAAAGCCG
CGAACGGGTGAGTAACACGTGGGTAACCTGCCCCGAAGACCGGGACAACCCGAGGAAACTCGGGCTAAT
ACCGGATGTGGTCGCACTTCATAAGGAGAGCGAGCAAAGGGAGCTTCGGCCCCCGCTTTGGGATGGGCCC
GCGGCCCATTAGCTTGTTGGTGAGGTAACGGCTCACCAAGGCTTCGATGGGTAGCTGGTCTGAGAGGACG
ATCAGCCACACTGGGACTGAGACACGGCC

>Zotu13565
GAACGCTGGCGGCATGCCTAACACATGCAAGTCGAACGAGACCTTCGGGTCTAGTGGCGCACGGGTGCGT
AACGCGTGGGAATCTGCCCTCTGGTACGGAATAACAGTTAGAAATGACTGCTAATACCGTATAATGACTTC
GGTCCAAAGATTTATCGCCTGAGGATGAGCCCGCGTAGGATTAGCTAGTTGGTAAGGTAAAGGCTTACCA
AGGCGACGATCCTTAGCTGGTCTGAGAGGATGATCAGCCACACTGGGACTGAGACACGGCC
>Zotu13567
GAACGCTGGCGGCATGCCTTACACATGCAAGTCGAACGGTAACGCGGGGCAACCTGGCGACGAGTGGCG
AACGGGTGAGTAATATATCGGAACGTGCCCAGTAGTGGGGGATAGCCCGGCGAAAGCCGGATTAATACC
GCATACGACCTACGGGTGAAAGGGGGGGATCGCAAGACCTCTCGCTATTGGAGCGGCCGATATCAGATTA
GGTAGTTGGTGAGGTAAAGGCTCACCAAGCCGACGATCTGTAGCTGGTCTGAGAGGACGACCAGCCACAC
TGGGACTGAGACACGGCC
>Zotu13571
GAACGCTGGCGGCACGCCTAACACATGCAAGTCGAGCGGTAGCATGGTTGGTAGCAATACTGACTGATGA
CGAGCGGCGGACGGGTGAGGAACACGTAGGAACCTGCCCTCAAGCGGGGGATAACTAAGGGAAACTTTA
GCTAATACCGCATAATCTCGAGAGAGAAAAGCTGCCCGCAAGGGTGGCACTTGAGGAGGGGCCTGCGTCC
GATTAGCTAGTTGGTAGGGTAAAAGCCTACCAAGGCGATGATCGGTAACTGGTCTGAGAGGACGACCAGT
CACACTGGGACTGAGACACGGCC
>Zotu13575
CAACGCTGGCGGCGCGCCTAACACATGCAAGTCGAACGAGAAAGTGGAGCAATCCATGAGTACAGTGGCG
TACGGGTGAGTAACACGTGGATAACCTGCCCCTTTGTGGGGAATACCGTTGGGAAACCGACGACAATACC
GCATAAGCTCGAGAGAGGAAAGCAGCAATGCGCAGAGGGAGGGGTCCGCGGTCGATTAGCTAGTTGGTG
AGGTAATGGCTCACCAAGGCTGTGATCGATAGCCGGCCTGAGAGGGCACACGGCCACACTGGCACTGAAA
CACGGGC
>Zotu13577
GAACGCTGGCGGCGTGCCTAACACATGCAAGTCGAACGGACGAGAGGAGCTTGCTCCTCAAGTTAGTGGC
GGACGGGTGAGTAATGTATGAGCAACCTGCCTTTCAGAGGGGGACAACAGTTGGAAACGACTGCTAATAC
CGCATAACGTATTTAAGGGGCATCCCTTTAATACCAAAGGAGCAATCCGCTGAAAGATGGGCTCATATCTG
ATTAGTTAGTTGGTGAGGTAACGGCTCACCAAGACTGCGATCAGTAGCCGGACTGAGAGGTTGAACGGCC
ACATTGGGACTGAGATACGGCC
>Zotu13580
GAACGCTGGCGGCAGGCTTAACACATGCAAGTCGAGCGGGCCCTTCGGGGTCAGCGGCGGACGGGTTAG
TAACGCGTGGGAACGTGCCTTTCGCTCAGGAATAGCCCCGGGAAACTGGGGGTAATACCTGATACGCCCT
GAGGGGGAAAGATTTATCGGCGAGAGATCGGCCCGCGTCTGATTAGGTAGTTGGTGGGGTAATGGCCTAC
CAAGCCTACGATCGGTAGCTGGTTTGAGAGGATGATCAGCAACACTGGGACTGAGACACGGCC
>Zotu13582
GAACGCTAGCGGCATGCCTAACACATGCAAGTCGAGCGCCCCGCAAGGGGAGCGGCAGACGGGTGAGAA
ACGCGTGGGAACATACCTTTTAGTACGGAATAGCCTAGGGAAACTTAGATTAATACCGTATACGCCCTATG
GGGGAAAGATTTATCGCTAAAAGATTGGCCCGCGTAAGATTAGCTAGTTGGTGAGGTAATGGCTCACCAA
GGCTACGATCTTTAGCTGGTTTGAGAGAATGATCAGCCACACTGGAACTGAGACACGGTC
>Zotu13584
TGTCCGAGAACTGCTCGGGAAGAGGCGAGCCGAAAGGGATGCGGCAAGAAACATGGCACGACATACGCA
AAATGTGCCAAAGGCGTGGTGTATGAAATTCCCCTTTCTTGTG
>Zotu13589
GACGTGTCTCAGTCCCAGTGTGGCTGGTCATCCTCTCAGACCAGCTACGGATCGTCGCCTTGGTAGGCCTTT
ACCCCACCAACTAGCTAATCCGACGTAGGCTCATCCTAGAGCGCAAGGCCCCGAAGGGTCCCCTGCTTTGA
TCTCTCGATATCATGCGGTATTAGCCCGAGTTTCCTCGGGTTATCCCACTCTCCAGGACAGATTCCTACGCTT
TACTCACCCATCCGCCACTCGCCACCCGTAAAGACCGAAATCTTTACTGTGCTGCCGTTCGACTTGCATGTGT
TAGGCATGCCGCCAGCGTTTA
>Zotu13598

GAATGCTGGCGGCGTGCCTTAAGCATGCAAGTCGAACGAAAAGATAGCAATATTTTTAGTGGCAGACGG
GTGAGTAACGCGTGGGTATCTACCTTAAAGAGGAGCACAACTCCGAGAAATCGGAGCTAATTCTCCATAGT
CTCGCAAGAGTAAAGCAGCAATGCGCTTTAAGAGGAGCCCGCGTCCTATTAGCTAGTTGGTGGGGTAATA
GCCTACCAAGGCGATGATGGGTAGCTGGACTGAGAGGTCGGACAGCCACAATGGCACTGAGATACGGGC
>Zotu13601
GAACGCTGGCGGCAGGCCTAACACATGCAAGTCGAGCGGTTGAGGGGAGCTTGCTTCCCGATTCAGCGGC
GGACGGGTGAGTAATGCCTAGGAATCTGCCTGGTAGTGGGGGACAACGTTTCGAAAGGAACGCTAATACC
GCATACGTCCTACGGGAGAAAGCAGGGGACCTTCGGGCCTTGCGCTATCAGATGAGCCTAGGTCGGATTA
GCTAGTTGGTGGGGTAAAGGCTCACCAAGGCGACGATCCGTAACTGGTCTGAGAGGATGATCAGTCACAC
TGGAACTGAGACACGGTC
>Zotu13604
GAACGCTGGCGGCATGCCTTACACATGCAAGTCGAACGCGAAAGGGGGCAACCCTGAGTAGAGTGGCGA
ACGGGTGAGTAATGCATCGGAACATTTCCTGTAGTGGGGGATAACCTCGAGAAATCGAGGCTAATACCGC
GTACGTCCTACGGGAGAAAGCGGGGGATCCAAGGGCAACTGAGGACCTCGCGCTATAGGAGTGGCCGAT
GCCGGATTAGCTAGTTGGCGGGGTAAGAGCCCACCAAGGCTACGATCCGTAGCTGGTCTGAGAGGATGAT
CAGCCACACTGGGACTGAGACACGGCC
>Zotu13608
GAACGCTGGCGGCGTGCCTAATACATGCAAGTCGAACGAAGGTATTTATACCTTAGTGGCGAACGGGTGA
GTAACACGTAGGTAACCTGCCCTCAAGCCGAGGATAACAGTTGGAAACGACTGCTAATACTCGATAGGTAA
TTTCTTGGCATCAAGAAATTATTAAAGATCCGTATGGATTACTTGGGGAGGGACCTGCGCCGCATTAGTTA
GTTGGTGGGGTAATGGCCTACCAAGACAATGATGCGTAGCCGGACTGAGAGGTTGAACGGCCACACTGG
GACTGAGACACGGCC
>Zotu13611
GAACGCTGGCGGCGTGCTTCATACATGCAAGTCGAGCGAGGAGCTTGCTCCTAGCGGCGAACGGGTGAGT
AACACGTGGGCAACCTGCCCTTGGCTCTGGGATAACTCCGGGAAACCGGGGCTAATACCGGATACGACCA
CCGAAGACATCTTCTGGTGGTGGAAAGAATTTCGGCCAAGGATGGCCCGCGGCCTATCAGCTTGTTGGT
GGGGTAATGGCCTACCAAGGCGACGACGGGTAGCCGGCCTGAGAGGGCGACCGGCCACACTGGGACTGA
GACACGGCC
>Zotu13620
GAACGCTAGCGGCATGCCTAACACATGCAAGTCGAACGGAAAGGCTCCTGCTTGCAGGGGTACTCGAGTG
GCGAACGGGTGAGTAACACGTGGGTGATCTGCCCTGCACTTCGGGATAAGCCTGGGAAACTGGGTCTAAT
ACCGGATAGGACCATCGTTTAGTGTCGGTGGTGGAAAGTTTTTCGGTGTGGGATGAGCTCGCGGCCTATCA
GCTTGTTGGTGGGGTAATGGCCTACCAAGGCGTCGACGGGTAGCCGGCCTGAGAGGGTGTACGGCCACAT
TGGGACTGAGATACGGCC
>Zotu13628
AAACGCTGGCGGCATGCCTAACACATGCAAGTCGAACGGCAGCACAGTAAAGATTTCGGTCTTTACGGGT
GGCGAGTGGCGGATGGGTGAGTAAAGCGTAGGAATCTGTCCTGGAGAGTGGGATAACCCGAGGAAACTC
GGGCTAATACCGCATGATATCGAGAGATCAAAGCAGGGGACCCTTCGGGGCCTTGCGCTCTAGGATGAGC
CCGCGTCAGATTAGGTAGTTGGTGAGGTAATGGCTCACCAAGCCGACGATCTGTAGCTGGTCTGAGAGGA
TGACCAGCCACACTGGGACTGAGACACGTCC
>Zotu13634
GAACGCTGGCGGCGTGCTTAACACATGCAAGTCGAACGATGAAGCTGGAGCTTGCTCCGGTGGATTAGTG
GCGAACGGGTGAGTAACACGTGAGAAATCTGCCCTTCACTCTGGGATAACTGCGGGAAACTGTAGCTAAT
ACCGGATACTTTCCCTGGTGGGCATCCACTAGGGAAGAAAGAATTTCGGTGAAGGATGATCTCGCGGCCTA
TCAGCTTGTTGGTGAGGTAATGGCTCACCAAGGCGACGACGGGTAGCCGGCCTGAGAGGGTGACCGGCC
ACACTGGGACTGAGACACGGCC
>Zotu13640
GAACGCTGGCGGCGTGCTTAACACATGCAAGTCGAACGGAAAGGCCCTGCTTTTGTGGGGTGCTCGAGTG
GCGAACGGGTGAGTAACACGTGGGTAACCTGCCCTTGACTTTGGGATAACTTCAGGAAACTGGGGCTAAT
ACCGGATAGGAGCTCCTGCTGCATGGTGGGGGTTGGAAAGTTTCGGCGGTTGGGGATGGACTCGCGGCTT

ATCAGCTTGTTGGTGGGGTAGTGGCTTACCAAGGCTTTGACGGGTAGCCGGCCTGAGAGGGTGACCGGCC
ACACTGGGACTGAGACACGGCC
>Zotu13642
GAACGCTGGCGACGCGTCTTAAGCATGCAAGTCGGACGGCAACACGGGGGTAACCCTGGTGGCGAGTGG
CGAACGGGTGAGTAACACGTAGGTGAACTGCCCTTGGGCTGGGGATAGCTTGTGGAAACACAGGGTAATA
CCGAATGATGTCACGGAGTTTTGACACCGTGATTAAAGGGGCTAAGGCCTCGCCTAAGGATGTGCCTGCG
GCTGATTAGCTAGTTGGTGGGGTAAAGGCCTACCAAGGCATCGATCAGTAGCCGGCCTGAGAGGGTGGAC
GGCCACACTGGGACTGAGATACGGCC
>Zotu13651
GAACGCTGGCGGCAAGCTTAACACATGCAAGTCGAACGGTGAAGGAGGAGCTTGCTCTTCTGGATCAGTG
GCGAACGGGTGAGTAACACGTGAGCAATCTGCCCTTGACTCTGGGATAACAGTTGGAAACAGCTGCTAAT
ACCGGATACGAGACGTGACCGCATGGTCAACGTCTGGAAAGAACTTCGGTCAAGGATGAGCTCACGGCCT
ATCAGCTAGTTGGTGAGGTAATGGCTCACCAAGGCGACGACGGGTAGCCGGCCTGAGAGGGTGACCGGC
CACACTGGGACTGAGACACGGCC
>Zotu13657
GAACGCTGGCGGCATGCCTAACACATGCAAGTCGAACGAGACCTTCGGGTCTAGTGGCGCACGGGTGCGT
AACGCGTGGGAATCTGCCCTTGGGTACGGAACAACAGTTAGAAATGACTGCTAATACCGTATAATGACTCC
GGTCCAAAGATTTATCGCCCAGGGATGAGCCCGCGTCGGATTAGCTAGTTGGTGAGGTAAAAGCTCACCA
AGGCGACGATCCGTAGCTGGTCTGAGAGGATGATCAGCCACACTGGGACTGAGACACGGCC
>Zotu13659
GAACGCTGGAAATGTGGATACTGGCATGCAAGTCAAGGGGCCGCAAGGCACCGGCGAACGGCTTAGTAA
CACGTAGATACATACCGCAAAGTGGGGCACAGCTCGTCGAAAGACGGGGTAATTTCCCATAGTCCCGCAA
GGGTAAACACGCAAGTGGCTTTGTGAATGGTCTGCGGTCTATCAGCTAGTTGGTGAGGTAAAGGCTCACC
AAGGCTATGACGGATAGGGGGCGTGAGAGCGCGACCCCCAAGAATGGCACTGCGACACGGGC
>Zotu13661
CAACGCTGGCGGCGCGCCTAACACATGCAAGTCGAACGAGAAAGTGGAGCAATCCATGAGTACAGTGGCG
TACGGGTGAGTAACACGTGGGTAATCTACCTCTTAGTGTGGAATAACTTTGGGAAACCGAAGCTAATACCG
CATAAGCTCGAGAGAGGAAAGCAGCAATGCGCTGAGAGAGGAGCCCGCGGCCGATTAGCTAGTTGGCAG
GGTAAAAGCCTACCAAGGCATAGATCGGTAGCCGGCCTGAGAGGGCACACGGCCACACTGGCACTGAAAC
ACGGGC
>Zotu13665
GAACGCTGGCGGCAGGCATAATACATGCAAGGCGAGGGGGTAGCAATACCACCGTCGTACGGGTGCGTA
ACACGTATGCAACCTGCCCTTCACTGGGGGATAGCCTGGGGAAACTCGGATTAATACCGCATAAGACAGTA
GACTGGCATCAGTTAGCTGTTAAAGATTTATTGGTGAAGGATGGGCATGCGTCTGATTAGCTAGTTGGCGG
GGTAAAGGCCCACCAAGGCTACGATCAGTAGGGGTTCTGAGAGGATTGGCCCCCACACTGGCACTGAGAT
ACGGGC
>Zotu13668
GAACGCTGGCGGCGTGCTTAACACATGCAAGTCGAGCGGTAAGGCTCCTTCGGGAGTACACGAGCGGCGA
ACGGGTGAGTAACACGTGAGTAATCTGCCCTTCTCTTCGGAATAAGCGTTGGAAACGACGTCTAATGCCGA
ATATGACCTTCTTTCGCATGATCGAAGGTGGAAAGCTCCGGCGGAGATGGATGAACTCGCGGCCTATCAGC
TTGTTGGTGAGGTAATGGCTCACCAAGGCATTGACGGGTAGCCGGCCTGAGAGGGTGACCGGCCACACTG
GGACTGAGACACGGCC
>Zotu13677
GAACGCTGGCGGTGTGCTTAACACATGCAAGTCGAACGCTGAAGCACTCTGCTTGCAGAGTGCGGATGAG
TGGCGAACGGGTGAGTAACCCGTGGGAAATCTGCCTATAAGAGGGGGATAACATTCGGAAACGGATGCT
AATACCGCATATATTCTTGGACCGCCTGGTCCTTGAAAGAAAGACGGCTTTGCTGTCGCTTATAGATGATCC
CGCGGCGTATTAGTTAGTTGGTGGGGTAAAGGCCTACCAAGACAATGATACGTAGCCGACCTGAGAGGGT
GATCGGCCACACTGGGACTGAGACACGGCC
>Zotu13680

TAACGCTGGCGGTGTGCCTAATACATGCAAGTCGAACGGATTCTTTGAAGTTTTTAGTATGAAGTATTTAGT
ATCTAGTATGAAAATACTAAATACTAAATACTAAATACTAGATACCAAAAGAGTTAGTGGCGGACGGGTGA
GTAACACGTTGGTACCTGCCCTTTAGTGGGGAATAACTGGCCGAAAGGTCAGCTAATACCGCATGTGGTCT
TCGGACTAAAGCCTTCGGGCGCTAAAGGAGGGGCCTGCGTCCCATTAGCTAGTTGGTAGGGTAAAAGCCT
ACCAAGGCGACGATGGGTAGCTGGATCGAGAGGTTGACCAGCCACACTGGGACTGAGATACGGCC
>Zotu13683
GAACGCTGGCGGCGTGCTTAACACATGCAAGTCGAACGGTGACCTCAAAGCTTGCTTTGAGTGATCAGTG
GCGAACGGGTGAGTAACACGTGAGCAACCTGCCCTTGACTCTGGGATAACAGCTGGAAACAGCTGCTAAT
ACCGGATACGACCTCGACCGACATCAGTTGAGGTGGAAAGCTCCGGCGGTCAGGGATGGGCTCGCGGCCT
ATCAGCTAGTTGGTGAGGTAACGGCTCACCAAGGCTTCGACGGGTAGCCGGCCTGAGAGGGCGACCGGCC
ACACTGGGACTGAGATACGGCC
>Zotu13686
GAACGCTGGCGGCGCGCTTAACACATGCAAGTCGAGCGAGAACCTAGGCTTCGGCCTAGGGGACAGCGG
CGAACGGGTGAGTAACACGTGGGTAATCTGCCCTCGACATCGGGATAGCCCGGGGAAACCCGGATTAATA
CCGAATAGCCCATCCGCTTTTCGGAGCGGCTGGAAAAGGTAGCTTTGGCCTCTGGTCGAGGAGGAGCCCG
CGGCGGATTAGCTTGTTGGTGAGGTAATGGCCCACCAAGGCGACGATCCGTAGCTGGTCTGAGAGGACGA
TCAGCCACACTGGGACTGAGACACGGCC
>Zotu13690
GAATGCTGGCGGCGTGGATAAGTCATGCAAGTCAAGGGGACCGCAAGGTAACCGGCAAACGAGGTAGTA
ACACGTAGGTAGATCCCCCGAAGTCGGGCATAGCCATCCGAAAGGATGGGTAATTCCCGATAGTCCCGCA
AGGGTAAAGGTTTTTCGCTTCGGGAGTCGCCTGCGTAGTATCAGCTAGTTGGTAGGGTAATGGCCTACCAA
GGCTACGACGCTTAGGGGAGCTGAGAGGCTGACCCCCACCGATGGAACTGCGACACGGTC
>Zotu13693
GAACGCTGGCGGCGTGCTTAACACATGCAAGTCGAGCGGAAAGGCCACTTCGGTGGTACTCGAGCGGCGA
ACGGGTGAGTAACACGTGAGTAATCTGCCCTTGGCTTCGGGATAGCCACCGGAAACGGTGATTAATACCG
GATATGACGATGCCACGCATGTGGTGTTGTGGAAAGTTTTTCGGCCAGGGATGTGCTCGCGGCCTATCAGC
TTGATGGTGAGGTAATGGCTCACCATGGCTTCGACGGGTAGCCGGCCTGAGAGGGTGACCGGTCACACTG
GGACTGAGACACGGCC
>Zotu13696
GAACGCTGGCGGCGTGCTTAACACATGCAAGTCGAGCGGTAACAGGCGTAGCAATACGTGCTGACGAGCG
GCGAACGGGTGAGTAATGATTCGGAATCTTCCTCGTAGTGGAGAATAACCTGGGGAAACTCAGGCTAATA
CCGCATACGAACTACGGTTGAAAGCAGGGGATCGCAAGACCTTGCGCTATGAGATGAGCCGAATTCGGAT
TAGCTAGTTGGTGGGGTAAAGGCCTACCAAGGCGACGATCCGTAGCTGGTCTGAGAGGACGATCAGCCAC
ACTGGGACTGAGACACGGCC
>Zotu13698
CAACGCTGGCGGCGTGCCTAACACATGCAAGTCGAACGAGAAAGGGTAGCAATACCTGAGTAAAGTGGCG
CACGGGTGAGTAACACGTGACTAACCTACCCTTGAGCGGGGGATAACCTAGGGAAACCTGG GCTAATACC
GCATAATACCTACGGGTCAAAGCGAGCAATCGCACTTAAGGAGGGGGTCGCGGCAGATTAGCTAGTTGGT
GAGGTAATGGCTCACCAAGGCGATGATCTGTATCCGGCCTGAGAGGGCGCACGGACACACTGGAACTGAA
ACACGGTC
>Zotu13700
GAACGCTGGCGGCGTGCTTAACACATGCAAGTCGAGCGGAAAGGCCCTTCGGGGTACTCGAGCGGCGAA
CGGGTGAGTAACACGTGGGTAACCTGCCCCTTGCTCTGGGATAACTCCGGGAAACCGGGGCTAATACCGG
ATATGACACCTTAAGGCATCTTTTGGTGTGGAAAGTTTTTCGGCGAGGGATGGACCCGCGGCCTATCAGCT
TGTTGGTGGGGTGATGGCCTACCAAGGCGACGACGGGTAGCCGGCCTGAGAGGGTGTCCGGCCACACTG
GGACTGAGATACGGCC
>Zotu13702
GAACGCTGGCGGCGTGCCTAATACATGCAAGTCGAGCGAACAGAGAAGGAGCTTGCTCCTTTGACGTTAG
CGGCGGACGGGTGAGTAACACGTGGGCAACCTACCCTATAGTTTGGGATAACTCCGGGAAACCGGGGCTA
ATACCGAATAATCTCTTGTCCCTCATGGGACAATACTGAAAGACGGTCTCGGCTGTCGCTATAGGATGGGC

CCGCGGCGCATTAGCTAGTTGGTGAGGTAACGGCTCACCAAGGCAACGATGCGTAGCCGACCTGAGAGGG
TGATCGGCCACACTGGGACTGAGACACGGCC
>Zotu13704
TGAGGAAAAGAAAAAGACGGATAAAGGGTGTGCCTCCTACGAGAGGGGCATGACGCGGCCGTCGGGGA
AGGGAGTGTGGGATCTAAAGAACAGTGAGGGAGAAAGAACTAGTGTCACAAAAGAAATGAGGGAAAGA
GAATGTAAAGATAAGGAGAACAGAAAGAAGCAGGGAGTAAAAAAATCTGAAGATGGGGTTACAATCGTT
GGTCCAGGCGAGTCTCATCAAGAAAGTCCAGAAGTGCCGAA
>Zotu13707
TAACGCTGGCGGCGTGCCTAATACATGCAAGTCGAACGAACCAACTTTGTTGGAAGGAACAAGCTCCAAG
AAGCAAGGACACAAACATCGTTTGTAACTTGTCTCTTGTATCCTTGGGATTTCCAACATGGTTGGTTAGTGG
CGGACGGGTGAGTAACACGTTGGTACCTACCCTCTAGTGGGGAATAACTGGCCGAAAGGTCGGCTAATAC
CGCATGTGATCTTCGGATCAAAGCAGCAATGCGCTAGAGGAGGGGCCTGCGTCTGATTAGCTAGTTGGTA
AGGTAATGGCTTACCAAGGCGACGATCAGTAGCTGGATCGAGAGGTTGACCAGCCACACTGGGACTGAGA
TACGGCC
>Zotu13711
GCGTATAGCAATGATTAATCGGCAAATTGAAGACCATTGTCGGGTAGTGTGCAAGCAGCAATGGGATGAA
GTATGTAATTCGATTGATGGTGGCATTAAAAGGGGAGGCGCCTGGAATTTGCTGCGACATTTACTGGATGA
GACAAACACTAGGTCTAATCAGAGGACGATAATAGGTAAGCTGGTCCATGAGGCGTGTC
>Zotu13715
GAACGCTGGCGGCGTGCTTAACACATGCAAGTCGAACGGAAAGGCCCTGCTTTTGTGGGGTGCTCGAGTG
GCGAACGGGTGAGTAACACGTGAGTAACCTGCCCTTGACTTTGGGATAACTTCAGGAAACTGGGGCTAAT
ACCGGATAGGAGCTCCTGCTGCATGGTGGGGGTTGGAAAGTTTCGGCGGTTGGGGGTGGACTCGCGGCTT
ATCAGCTTGTTGGTGGGGTAGTGGCTTACCAAGGCTTTGACGGGTAGCCGGCCTGAGAGGGTGACCGGCC
ACACTGGGACTGAGACACGGCC
>Zotu13718
TTTTAGGGCTTTCACCCCCTTCCTGCGGTGGCTAGTTATCCAGCCCGCTTCTCTACTAAATTCCTCGGGTGAA
ATGGACTCACCCTCGACCTCCACGACCTCCATGGCACAAG
>Zotu13724
CAACGCTGGCGGCGTGCCTAACACATGCAAGTCGAACGCGAAAGGGGCTTCGGTCCTGAGTAGAGTGGCG
AACGGGTGAGTAACGCGTGGGTGACCTACCCTCGAGTGGGGGATAACGTTCCGAAAGGGACGCTAATACC
GCATGACATTCTGGGTTTGAAGACCTGGAAATCAAAGCCGGGGACCGCAAGGCCTGGCGCTTGAGGAGG
GGCCCGCGTCTGATTAGCTAGTTGGCGGGGTAACGGCCCACCAAGGCGACGATCAGTAGCCGGCCTGAGA
GGGCGGACGGCCACACTGGGACTGAGACACGGCC
>Zotu13728
GTTGCAACTTCACGCGTGGAATTTGTGATGAGGATGCCGTGGAACCAGGTTTCGGAGCATCTTGAAGTGCC
CCCTATGGTGGGAATCAATTGAAGCTGTACGCTTCGCAAAGTTTAGTTGAACTATGATAATTTAGTTGGGGT
GAAAAAAAGAACATTTGATGGCGTGTATGACATTTTTGCATTGAATGTGCACGGTGGAGAGGTTCGTCATC
CACCGGAATATAAAAATATCGAGGCCTTAATTAAAAGCTAGGTAGGGTAAAGGTAACAAAACGTAAATGA
CAACTTAAGCAGCTTGACTGATTCGCTTTATTCTAGCAGCGTTAAAACGTCGCTACTATAGACGTTATAGAT
GATATACGATCAGTGTTTACCTATGCTTCCTTGAATGGCACCGAACGGCACCGGGCAAATGTAGCGCAGAA
AGG
>Zotu13733
GAACGCTGGCGGCGTGCCTAACACATGCAAGTCGAACGCGAAAGTCCTTCGGGATGAGTAAAGTGGCGCA
CGGGTGAGTAACACGTAGGTGACCTGCCTTCTAGAGGGGAATAACCAGATGAAAATTTGGCTAATGCCGC
ATACGATCCACAAATCTTAGAGTAGTGGATGAAAGAGTGCCTCTGTATATGCACTTACTAGTAGATGGGCC
TGCGGTACATTAGCTTGTTGGTGGGGTAATGGCCTACCAAGGCGACGATGTATATCCGGTCTGAGAGGAT
GATCGGACACACTGGAACTGAGACACGGCC
>Zotu13735
GAACGCTGGCGGCGTGGATAAGTCATGCAAGTCAAGGGGGCCCGCAAGGGCAACCGGCAGACGAGGTA
GTAACACGTAGGTACGTCCCCCGAAGTCGGGCATAGCCATCCGAAAGGATGGGTAATTCCCGATAGTCCC

GCAAGGGTAAAGGTTTTTCGCTTCGGGAGCGGCCTGCGTAATATCAGCTAGTTGGTGAGGTAATGGCTCAC
CAAGGCTACGACGTTTAGGGGAGCTGAGAGGCTGACCCCCACCGATGGAACTGCGACACGGTC
>Zotu13742
GAACGCTGGCGGCGTGCTTAACACATGCAAGTCGAACGATAGAGCAATCTATAGTGGCAAACGGGTGAGT
AATGCATAGGAATCTGCCTTATAGTGGGGGACAACAGCCGGAAACAGCTGCTAATACCGCATAATACCATT
TATGGGAAAGTCGCAAGACGCTATTTGATGAGCCTATGTCCGATTAGCTTGTTGGTGGGGTAAAAGCCTAC
CAAGGCGACAATCGGTAGCTGATCTGAGAGGATGATCGGCCACACTGGAACTGAGACACGGTC
>Zotu13745
GAACGCTGGCGGCAGGCTTAACACATGCAAGTCGAACGGTTGTAGCAATACAGCAGTGGCGCACGGGTG
AGTAACGCGTGGGAACGTGCCTTTCAGTTCGGGATAACAGCGGGAAACTGCTGCTAATACCGGATATTCTC
TACGGAGGAAAGATTTATCGCTGAAAGATCGGCCCGCGTTAGATTAGGTAGTTGGTGGGGTAATGGCCTA
CCAAGCCTACGATCTATAGCTGGTCTGAGAGGATGATCAGCCACACTGGAACTGAGACACGGTC
>Zotu13747
GAACGCTGGCGGCAGGCTTAACACATGCAAGTCGAACGGGCCCTTCGGGGTCAGTGGCAGACGGGTGAG
TAACGCGTGGGAACGTGCCCTTCAGTTCGGAATAACCCAGGGAAACTTGGGCTAATACCGGATACGTCCG
AAAGGAGAAAGATTGATCGCTGAAGGATCGGCCCGCGTCTGATTAGCTAGTTGGTGGGGTAATGGCCCAC
CAAGGCGACGATCAGTAGCTGGTCTGAGAGGATGATCAGCCACATTGGGACTGAGACACGGCC
>Zotu13750
GAACGCTGGCGGCGTGCTTAACACATGCAAGTCGAACGGTGAAGCCCTTCGGGGTGGATCAGTGGCGAAC
GGGTGAGTAACACGTGGGCAATCTGCCCTGCACTCTGGGACAAGCCCTGGAAACGGGGTCTAATACCGGA
TATTACCTTCCTCTGCATGGGGGTTGGTGGAAAGCTCCGGCGGTGCAGGATGAGTCCGCGGCCTATCAGCT
TGTTGGTGGGGTAATGGCCTACCAAGGCGACGACGGGTAGCCGGCCTGAGAGGGCGACCGGCCACACTG
GGACTGAGACACGGCC
>Zotu13756
GAACGCTGGCGGTATGCTTTACACATGCAAGTCGTACGGGAGTTTTTAACTCTAGTGGCGGACGGGTGAGT
AACACGTGAGAATTTGCCTTTAGGAGGGGGACAACAACTGGAAACGGTTGCTAATACCCCATATGCTTTCG
AGTGAAATGGATTTCCGCCTAAAGAGAAGCTTGCGGCTGATTAGCTTGTTGGTAAGGTAATGGCTTACCAA
GGCGACGATCAGTATCTGGTTTGAGAGGACGATCAGACACACTGGAACTGAGACACGGTC
>Zotu13763
GAACGCTGGCGGCAGGCCTAACACATGCAAGTCGAACGAAGTCTTCGGACTTAGTGGCGCACGGGTGAGT
AACACGTGGGAATATACCTTTCGCTGGGGGATAACATCGGGAAACTGATGCTAATACCGCATACGCCCTAC
GGGGGAAAGATTTATCGGCGAGATATTAGCCCGCGTCCGATTAGGTAGTTGGTGAGGTAACGGCTCACCA
AGCCTGCGATCGGTAGCTGGTCTGAGAGGATGATCAGCCACACTGGGACTGAGACACGGCC
>Zotu13768
GAACGCTGGCGGCGTGCTTAACACATGCAAGTCGAACGATGATGCCCAGCTTGCTGGGTGGATTAGTGGC
GAACGGGTGAGTAACACGTGAGTAACCTGCCCCAGACTCTGGGATAAGCACTGGAAACGGTGTCTAATAC
CGGATACGAACTTCAGCCGCATGGCTAGGAGTTGGAAAGAATTTCGGTCTGGGATGGACTCGCGGCCTAT
CAGCTAGTTGGTGAGGTAATGGCCCACCAAGGCGACGACGGGTAGCCGGCCTGAGAGGGTGACCGGCCA
CACTGGGACTGAGACACGGCC
>Zotu13777
GAACGCTGGCGGCGTGCCTAATACATGCAAGTCGAGCGAGTGAAGCAGGCAAACACCCTTCGGGGTGTGC
GCCTGTGGATCTAGCGGCGGATGGGTGAGTAACACGTGGGCAACCTGCCTGTAAGACTGGGATAACTCGC
GGAAACGCGAGCTAATACCGGATAACATTTCGGATCGCATGATCCGGAATGGAAAGATGGCTTCGGCTAT
CACTTACAGATGGGCCCGCGGCGCATTAGCTAGTTGGTGGGGTAAAGGCTCACCAAGGCAACGATGCGTA
GCCGACCTGAGAGGGTGATCGGCCACACTGGGACTGAGACACGGCC
>Zotu13780
TTGATAGACGCAGGAGTCCGGAGTTGATGTTATAGGCATTTTTAATGGCTGCTGCCATGCTCCGAACTGCC
CGAGGCTCGCGCCGAGAGTGCGAGCCCATGGTGTTCTTCTTCTTCCTCCTCTCCAGTTTTCACTAAGATGGA
TGCATTCCTTTCAGCTACTGTTGACAAAATGACACGATTAAACTTTCTTAATT
>Zotu13787

GAACGCTGGCGGCGTGCTTAACACATGCAAGTCGAACGTGAACGGGAGCAATCCCAAGTAAAGTGGCGTA
CGGGTGAATAACGCGTAGATAATCTACCCCAAAATTTGGAATAACCTGCCGAAAGGCGGGCTAATACTGG
ATAGAGCGACGGAATCCGTAATTGGGTTTTTCGGGAAAGGGAGGCAGCAATGCCCCTCGTTTTGGGATGA
GTCTGCGTCTCATTAGCTAGTTGGTGGGGTAAAGGCTCACCAAGGCTTTGATGGCTAGCTGGTCTGAGAGG
ATGATCAGCCACACTGGAACTGAAACACGGTC
>Zotu13789
GAACGCTAGCGGCATGCCTAACACATGCAAGTCGAACGCACCTTTCGGGGTGAGTGGCAGACGGGTGAGT
AACGCGTGGGAATCTACCCTTCTCTACGGGATAGCTCCGGGAAACTGGAATTAATACCGTATACGTCCTGA
GGGAGAAAGATTTATCGGGGAAGGATGAGCCCGCGTTGGATTAGCTAGTTAGTGGGGTAAAGGCCTACC
AAGGCGACGATCCATAGCTGGTCTAAGAGGATGATCAGCCACATTGGGACTGAGACACGGCC
>Zotu13794
GAACGCTGGCGGCATGCCTAACACATGCAAGTCGAACGAGACCTTCGGGTCTAGTGGCGCACGGGTGCGT
AACGCGTGGGAATCTGCCCTTCGGTTCGGAACAACAGTTAGAAATGACTGCTAATACCGGATGATGACGTA
AGTCCAAAGATTTATCGCCGAAGGATGAGCCCGCGTAGGATTAGCTAGTTGGTAAGGTAAAAGCTTACCA
AGGCGACGATCCGTAGCTGGTCTGAGAGGACGATCAGCCACACTGGGACTGAGACACGGCC
>Zotu13798
GAACGCTGGCGGCGTGCTTAACACATGCAAGTCGAACGGTGACCAGGACTTCGGTCCTGCGATCAGTGGC
GAACGGGTGCGTAACACGTGGGCAACCTGCCCCTCACTCTGGGATAACCCCGGGAAACCGGAGCTAATAC
CGGATACTCTCACCGAACCTCCTGGTCCGGTGAGGAAAGGTCCGCCGGTGAGGGATGGCCCGCGGCCTA
TCAGCTTGTTGGTGAGGTAACGGCTCACCAAGGCAACGACGGGTAGCTGGTCTGAGAGGACGACCAGCCA
CACTGGAACTGAGATACGGTC
>Zotu13801
GAACGCTGGCGGCGTGCTTAACACATGCAAGTCGAACGGGACTTGAGAATTGAAGTACTCGTATGGATTTT
CTTAAGTTTAGTGGCGGACGGGTGAGTAACGCGTGGGCAACCTGCCGTATGCAGGGGGACAACAGTTGG
AAACGACTGCTAATACCGCATAAGCGCACAGTACCGCATGGTACAGTGTGAAAAGATTTATCGGCATACGA
TGGGCCCGCGTTGGATTAGGTAGTTGGCGAAGTAACGGCCCACCAAGCCTGCGATCCATAGCCGACCTGA
GAGGGTGATCGGCCACATTGGGACTGAGACACGGCC
>Zotu13803
GAACGCTGGCGGCGTGCCTAACACATGCAAGTCGAACGTGATTAGAGAGCTTGCTCTTTATGAAAGTGGC
GCACGGGTGAGTAACGCGTAGATAATCTACCTGATGGATCGGGATAACACTTCGAAAGGGGTGCTAATAC
CGGATAAGCCCACGGGGTCCTTGGACTCTGCGGGAAAAGGGGGGGATCGCAAGACCTTCCGTCATCAGAT
GAGTCTGCGTACCATTAGCTAGTTGGTAGGGTAAAGGCCTACCAAGGCGACGATGGTTAGCTGGTCTGAG
AGGATGATCAGCCACACTGGAACTGAGACACGGTC
>Zotu13809
GAACGTTAGCGGCGCGCCTAACACATGCAAGTCGCACGAGAAAGGGCTTCGGCCCCGGTAAAGTGGCGCA
CGGGTGAGTAACACGTGGGTAATCTGCCCTCGAGCGGGGGATAACATTCCGAAAGGAGTGCTAATACCGC
ATAAGACCACGATTTCGAAAGAGATTGCGGTCAAAGCTGGCCTCTATACATAAGCTAGCACTTGAGGAGGA
GCCCGCGGCGCATCAGCTAGTTGGTGAGGTAATGGCTCACCAAGGCTAAGACGCGTAGCTGGTCTGAGAG
GATGATCAGCCACACTGGAACTGAGACACGGTC
>Zotu13814
GAACGCTGGCGGCAGGCTTAACACATGCAAGTCGAACGGGCCCTTCGGGGTCAGTGGCAGACGGGTGAG
TAACACGTGGGAACGTGCCCTTCGGTTCGGAATAACTCAGGGAAACTTGAGCTAATACCGGATACGCCCTT
TTGGGGAAAGGTTTACTGCCGAAGGATCGGCCCGCGTCTGATTAGCTAGTTGGTGGGGTAATGGCCTACC
AAGGCGACGATCAGTAGCTGGTCTGAGAGGATGATCAGCCACACTGGGACTGAGACACGGCC
>Zotu13817
GAACGCTGGCGGCGTGCCTAACACATGCAAGTCGAACGAGGTCCATGAGAGCTTGCTCTCGGACGACCTA
GTGGCGAACGGGTGAGTAACACGTGAGAAACCTGCCTCGGAGTGGGGAATAACTCGGGGAAACTCGAGC
TAATACCGCATACCTTCAGATGATCGCATGGTCGACTGAAGAAAGAATTTCGCTCTGAGAGGGTCTCGCGG
CCTATCAGCTAGTTGGTGGGGTAACGGCCTACCAAGGCAACGACGGGTAGCTGGATTGAGAGATCGATCA
GCCACACTGGGACTGAGACACGGCC

>Zotu13820
GAACGCTGGCGGCGTGCCTAACACATGCAAGTCGATCGGTAACAGGTGTAGCAATACATGCTGACGAGAG
GCGGACGGGTGAGTAACGCTTGGGAATCTGCCTATTAGTGGGGGACAACCCGGGGAAACTCGGGCTAAT
ACCGCATACGCTCTACGGAGGAAAGCCGGGGACCGCAAGGCCTGGCGCTAATAGATGAGCCCAAGTCGG
ATTAGCTAGTTGGTAGGGTAACGGCCTACCAAGGCGACGATCCGTAGCTGGTCTGAGAGGACGACCAGCC
ACACTGGGACTGAGACACGGCC
>Zotu13823
GAACGCTGGTAACGTGGATACCGGCATGCAAGTCACGGGGGCCGCAAGGCAACCGGCGAACGGCTTAGT
AACACGTGGATACATACCCCAAAGTGGAGTACAGCTCGTCGAAAGACGGGGTAATCCTCCATAGTCTCCTC
TTTCGCTTTTTGTTTCACAAAAGCTTCAGAAGGACAAAGTCGCAAGGCAAATACGCTTTGCAGAGCAAGAC
TTGTCCATCCGAAGCTCTGGTGGAACGCAGAGCGAAGGAGGAGTAAAGGCGCAAGCCGCTTTGGGGATG
GTCTGCGGTCTATCAGCTAGTTGGTGAGGTAATGGCTCACCAAGGCTATGACGGATAGGGGGCGTGAGAG
CGCGACCCCCAAGAATGGCACTGCGACACGGGC
>Zotu13829
GAACGCTGGCGGCATGCCTAACACATGCAAGTCGAACGGCAGCACAGTGTGTAGCAATACACATGGGTGG
CGAGTGGCGGACGGGTGAGTAACGCGTGGGAATCTGCCCTATAGTGGGGGACAACCCGACGAAAGTCGG
GCTAATACCGCATACGCCCTACGGGGGAAAGGCTTCGGCCGCTATTGGATGAGCCCGCGTCGGATTAGCT
AGTTGGTGAGGTAATGGCTCACCAAGGCGACGATCCGTAGCTGGTCTGAGAGGACGATCAGCCACACTGG
GACTGAGACACGGCC
>Zotu13835
CAACGCTGGCGGCGTGCCTAACACATGCAAGTCGAACGCGAAAGGGGCTTCGGCCCTGAGTAGAGTGGCA
AACGGGTGAGTAACACGTGGGTGACCTGCCTTCGAGTGGGGGATAACGTTTCGAAAGGAACGCTAATACC
GCATAACATCCTGTTCTTGAACGGATGGAGATCAAAGCCGGGGACCGAAAGGCCTGGCGCTTGAAGAGGG
GCCCGCGCCGGATTAGCTAGTTGGTGAGGTAACGGCCCACCAAGGCGACGATCCGTAGCCGGCCTGAGAG
GGCGGACGGCCACACTGGGACTGAGACACGGCC
>Zotu13841
GAACGCTGGCGGCGTGCCTAATACATGCAAGTCGAGCGAATTGACGGGAGCTTGCTCCTTGATATTAGCG
GCGGACGGGTGAGTAACACGTGGGCAACCTGCCCTGCAGATGGGGATAACTCCGGGAAACCGGGGCTAA
TACCGAATAATCGGTTCTTCCGCATGGAAGAACTCTGAAAGACGGTTTCGGCTGTCACTGCAGGATGGGCC
CGCGGCGCATTAGCTAGTTGGTGGGGTAATGGCCTACCAAGGCGACGATGCGTAGCCGACCTGAGAGGGT
GATCGGCCACACTGGGACTGAGACACGGCC
>Zotu13847
GAACGCTAGCGGCATGCTTAACACATGCAAGTCGAGCGATCTTCTTCGGAAGAGAGCGGCGGACGGGTGA
GTAACGCGTGGGTAACCTGCCCTGTACACACGGATAACATACCGAAAGGTATGCTAATACGAGATAATATG
CTTTTATCGCATGGTAGAAGTATCAAAGCTTTTGCGGTACAGGATGGACCCGCGTCTGATTAGCTAGTTGGT
GAGGTAACGGCTCACCAAGGCGACGATCAGTAGCCGACCTGAGAGGGTGATCGGCCACATTGGAACTGA
GACACGGTC
>Zotu13849
GAACGCTGGCGGCGTGCCTAACACATGCAAGTCGAACGTGAACGGGAGCAATCCTTAGTAAAGTGGCGTA
CGGGTGAGTAACACGTAGGTAATCTACCCTAGAGTCTGGAATAACCTGCCGAAAGGCGGGCTAATACTGG
ATAGAGCGACGGAATCCGTAATTGGGTTTTTCGGGAAAGGGAGCTCGCAAGAGCCTTCGCTTTAGGATGA
GCCTGCGTCCCATTAGCTAGTTGGTAAGGTAACGGCTTACTAAGGCGATGATGGGTAGCTGGTCTGAGAG
GATGATCAGCCACACTGGAACTGAAACACGGTC
>Zotu13851
GAACGCTGGCGGCAGGCTTAACACATGCAAGTCGAACGGTAGCAGGTAAGTGCTTGTACTTATGCTGACG
AGTGGCGGACGGGTGAGTAATGCTTGGGGATCTGGCTTATGGAGGGGGATAACGACGGGAAACTGTCGC
TAATACCGCGTAGAATCGAGAGATGAAAGTGTGGGACCTTTGGGCCACATGCCATAGGATGAACCCAAGT
GGGATTAGGTAGTTGGTAGGGTAATGGCCTACCAAGCCGACGATCCCTAGCTGGTCTGAGAGGATGACCA
GCCACACCGGGACTGAGACACGGCC
>Zotu13854

GCCGTGTCTCAGTCCCAGTGTGGCTGATCATCCTCTCAGACCAGCTACCGATCGTCGCCTTGGTAAGCCTTT
ACCCCACCAACTAGCTAATCGGACATCGGCCGCTCCGGCAGCGCGAGGCCCTTTCAGGTCCCCCGCTTTCCT
CCTCAGAGCTCATGCGGTATTAGCCCAACTTTCGCTGGGTTATCCCCCACTACAGGATACGTTCCGATGCAT
TACTCACCCGTTCGCCGCTCGCCGCCAGGGTTGCCCCCGCGCTGCCGCTCGACTTGCATGTGTAAAGCATGC
CGCCAGCGTTCA
>Zotu13860
GAACGCTGGCGGCGTGCTTAACACATGCAAGTCAAGGGGGCCCAGCAATGGGCAACCGGCGAACGGGTG
CGTAACACGTGAGCAACCTACCGAATTGTGGGGGATAGCCGGCCCAACGGCCGGGTAATACCGCATACGA
TCGAGACCAGGCATTTGGACTCGATGAAATCTACGGAGCAATTCGAGGGGCTCGCGGCCTATCAGCTAGTT
GGTGAGGTAACGGCTCACCAAGGCGACGACGGGTAGCTGGTCTGAGAGGATGGCCAGCCACATTGGGAC
TGAGATACGGCC
>Zotu13863
GAACGCTGGCGGCATGCTTTACACATGCAAGTCGGACGGCAGCACAGAGAAGCTTGCTTCTTGGGTGGCG
AGTGGCGAACGGGTGAGTAACATATCGGAACGTACCGGGCAGTGGGGGATAACTAATCGAAAGATTAGC
TAATACCGCATATCTTCTGAGGAAGAAAGCAGGGGACCATTTGGCCTTGCGCTGTTTGAGCGGCCGATATC
TGATTAGCTAGTTGGTGGGGTAAAGGCCTACCAAGGCGACGATCAGTAGCGGGTCTGAGAGGATGATCCG
CCACACTGGGACTGAGACACGGCC
>Zotu13869
GAACGCTGGCGGCGTGCTTAACACATGCAAGTCGAACGGTGACCTCAAGAGCTTGCTCTTGGGTGATCAGT
GGCGAACGGGTGAGTAACACGTGAGTAACCTGCCCCAGACTCTGGAATAACCCCGGGAAACCGGAGCTAA
TACCGGATACGAAGCGGAGCCGCATGGCTACCACTTGGAAAGTTTTTCGGTCTGGGATGGACTCGCGGCCT
ATCAGCTAGTTGGTGAGGTAACGGCTCACCAAGGCGACGACGGGTAGCCGGCCTGAGAGGGCGACCGGC
CACACTGGGACTGAGACACGGCC
>Zotu13872
GAACGCTAGCGGCAGGCCTAACACATGCAAGTCGAGGGGTAGGGTAGCAATACCTGAGACCGGCGCACG
GGTGCGTAACGCGTATGCAATCTACCTTTTACAGGGGAATAGCCCAGAGAAATTTGGATTAATGCCCCATG
GTATTATAGAATCGCATGGTTTTATAATTAAAGTTCCAACGGTAAAAGATGAGCATGCGTCCCATTAGTTAG
TTGGTAAGGTAACGGCTTACCAAGACTATGATGGGTAGGGGTCCTGAGAGGGAGATCCCCCACACTGGTA
CTGAGACACGGAC
>Zotu13874
GAACGCTGGCGGCAGGCTTAACACATGCAAGTCGAACGATGTAGCAATACATAGTGGCGCACGGGTGAGT
AACGCGTGGGAATATACCTTATAGTGGGGAATAACTTCGGGAAACTGAAGCTAATACCGCATACGCCCTTC
GGGGGAAAGATTTATCGCTATAAGATTAGCCCGCGTGAGATTAGGTAGTTGGTGGGGTAATGGCCTACCA
AGCCTACGATCTCTAGCTGGTCTGAGAGGATGATCAGCCACACTGGAACTGAGACACGGTC
>Zotu13883
GCCGTGTCTCAGTCCCAATGTGGCTGATCATCCTCTCAGACCAGCTACTGATCGTCGCCTTGGTAGGCCATT
ACCCTACCAACTAGCTAATCAGACGCGGGCCGATCTTTCGGCGATAAATCTTTCCCCGTAAGGGCTTATCCG
GTATTAGCACAAGTTTCCCTGTGTTGTTCCGAACCAAAAGGTACGTTCCCACGCGTTACTCACCCGTCTGCC
GCTGACGTATTGCTACGCCCGCTCGACTTGCATGTGTTAAGCCTGCCGCCAGCGTTCG
>Zotu13885
GAACGCTAGCGGCAGGCCTAATACATGCAAGCCGAACGGTAACTTGAATAGCAATATTTGAGCTAGAGTG
GCGTAAGGGTGCGTAACACGTATGCAATCTGCCCACAACAGGAGGATAGCTCCCCGAAAGGGGAATTAAT
ACTCCATAAGATCACACGAAGGCATCTAAGAGTGATTAAACTACAAGGGGTTGTGGATGAGCATGCGGAT
GATTAGCCAGTTGGTAAGGTAACGGCTTACCAAGGCGATGATCAATAGGGGAACTGAGAGGTTTATCCCC
CACACTGGCACTGAGATACGGGC
>Zotu13887
GAACGCTGGCGGCGTGCCTAATACATGCAAGTCGAGCGCGGGAAACTATTCAGATCTCTTCGGAGTGACG
AATTGTGGAACGAGCGGCGGATGGGTGAGTAACACGTGGGCAACCTGCCTGTAAGATCGGGATAACTTGC
GGAAACGTGAGCTAATACCGGATAAAGCTTCACATCTCCTGATGTGTTGATGAAAGACGGCTCTGCTGTCA
CTTACAGATGGGCCCGCGGCGCATTAGCTAGTTGGTGGGGTAAAGGCCTACCAAGGCGACGATGCGTAGC
CGACCTGAGAGGGTGATCGGCCACACTGGGACTGAGACACGGCC

>Zotu13890
GAACGCTGGCGGCGTGCCTAATACATGCAAGTCGAGCGGTAAGGCTCTGCACAGGTTATTCGATCGGGAT
TTATCCTACGCTTTCTCCGGCTAGAGAAGGTAAAAGATCGAGTGATGCGTGTAGAGTACACGAGCGGCGG
ACGGCTGAGTAACACGTAGGAACGTACCCCAAACTGAGGGATAAGCACCAGAAATGGTGTCTAATACCGC
ATGTGATCTTCGGATTAAAGCTTCGGCGGTTTGGGAACGGCCTGCGCATGATTAGCTTGTTGGTGAGGTAA
AAGCTCACCAAGGCGACGATCCGTAGCTGGTCTGAGAGGATGACCAGCCACACTGGGACTGAGACACGGC
C
>Zotu13893
GAACGCTGGCGGCGTGCCTAACACATGCAAGTCGAGCGATGAAGCCCTTCGGGGCTGATTAGCGGCGGAC
GGGTGAGTAACACGTGGGTAACCTGCCTCATAGAGGGGGATAGCCTCCCGAAAGGGAGATTAATACCGCA
TAATGTATTTTGGTTGCATGATCGAAATATCAAAGGAGTAATCCGCTATGAGATGGACCCGCGGCGCATTA
GCTAGTTGGTGAGGTAACGGCTCACCAAGGCGACGATGCGTAGCCGACCTGAGAGGGTGATCGGCCACAT
TGGAACTGAGACACGGTC
>Zotu13898
GAACGCTGGCGGCGTGCTTAACACATGCAAGTCGAACGAATAAAGCACCTTCGGGTGTGTATAGAGTGGC
GAACGGGTGAGTAACACGTGACCAACCTGCCCCTTACATTGGGATAACTTGAGGAAACCCAAGCTAATACC
AAATACTTCTTATCTTTCGCATGTTAGATAAGAGAAAGCTTTCGCGGTAAGGGATGGGGTCGCGGCCTATT
AGGTAGTTGGTGAGGTAACGGCTCACCAAGCCTGCGATAGGTAGCCGGGCTGAGAGGCTGATCGGCCAC
ATTGGGACTGAGATACGGCC
>Zotu13901
GAACGCTGGCGGCATGCTTAACACATGCAAGTCGAACGCAAGGCTTCGGCCTTGAGTGGCGCACGGGTGA
GTAGCGCGTGGGAACCTGCCCTGGAGTGGGGGATAACCGAGGGAAACTTCGGCTAATACCGCATGCGCC
GGGAAACCGGGAAAGATTTATCGCTCTAGGAGGGGCCCGCGTCGGATTAGCTTGTTGGTGGGGTAACGGC
CTACCAAGGCTATGATCCGTAGCTGGTCTGAGAGGATGATCAGCCACACTGGGACTGAGACACGGCC
>Zotu13905
AAACGCTGGCGGCGTACCTAACACATGCAAGTCGAACGTCCTTGCTGTTTTCGCAAGAGGACAGCAAGGG
AGTGGCGGACGGGTGAGTAACACGTGGGTACCTACCCTGGGGTGAGGAATACCGGCGAGAAATTGCCGA
CAAGACCGCATAGGCTCTCCTACGAGAGCAAAGCCGAAAGGCGCCCTGGGATGGGCCTGCGGCCGATTAG
CTGGTTGGTGAGGTAAGAGCTCACCAAGGCGACGATCGGTAGCTGGTCTGAGAGGACGAACAGCCACACT
GGGATTGAGAACGGCC
>Zotu13908
GAACGCTGGCGGCAGGCCTAACACATGCAAGTCGAACGGTAGCACAGGGGAGCTTGCTCCTCGGGTGAC
GAGTGGCGGACGGGTGAGTAATGTCTGGGGATCTGCCCGATGGAGGGGGATAACCACTGGAAACGGTGG
CTAATACCGCATAACGTCGCAAGACCAAAGTGGGGGACCTTCGGGCCTCACACCATCGGATGAACCCAGAT
GGGATTAGCTAGTAGGTGGGGTAACGGCTCACCTAGGCGACGATCCCTAGCTGGTCTGAGAGGATGACCA
GCCACACTGGAACTGAGACACGGTC
>Zotu13913
GAACGCTGGCGGTATGCTTAACACATGCAAGTCGAACGGCAGCATGTTCCTCACTCAAATCACTTTAATTTC
TTTTTGGAAAAGAAATACGGAAAGTGATTTGAGTGAGGAATGATGGCAAGTGGCGGACGGGTGAGTAAT
AGGTAGGAATCTGTCCTAGAGAGGGGGATAACGTTCGGAAACGGACGCTAATACCGCATACGTCCTACGG
GAGAAAGCAGGGGACCTTCGGGCCTTGCGCTATCAGATGAGCCTAGGTCGGATTAGCTAGTTGGTGAGGT
AATGGCTCACCAAGGCGACGATCCGTAACTGGTCTGAGAGGATGACCAGCCACACTGGGACTGAGACACG
GCC
>Zotu13916
GAACGCTGGCGGCAGGCCTAACACATGCAAGTCGAGCGAGATCTTCGGGTCTAGCGGCGGACGGGTGAG
TAACGCGTGGGAACGTGCCCTTTGCTACGGAATAGCCCCGGGAAACTGGGAGTAATACCGTATGTGCCCTT
TGGGGGAAAGATTTATCGGCAAAGGATCGGCCCGCGTTGGATTAGGTAGTTGGTGGGGTAATGGCCTACC
AAGCCGACGATCCATAGCTGGTTTGAGAGGATGATCAGCCACACTGGGACTGAGACACGGCC
>Zotu13921

GAACGCTGGCGGCGCGCCTAACACATGCAAGTCGAACGATTAAAGCACCTTCGGGTGTGTATAAAGTGGC
GAACGGCTGAGTAACACGTGGGCAACCTGCCCTTTGCTTCGGGATAGCCTCGAGAAATCGTGGGTAATACC
GGATACTCCATAGAAGTCGCATGGCCTCTATGGGAAAGCGCATACGGCAAAGGATGGGCCCGCGGCCTGT
TAGCTTGTTGGTGGGGTAGTGGCCTACCAAGGCGATGATGGGTAGCCGGGTTGAGAGACCGACCGGCCA
GATTGGGACTGAGACACGGCC
>Zotu13924
GAACGCTGGCGGCGTGCCTAACACATGCAAGTCGAGCGGGAGACTTCGGTCTCTAGCGGCGGACGGGCG
AGTAACACGTGGGTAACCTGCCTTGTAGAGGGGGATAGCCTTCCGAAAGGAAGATTAATACCGCATAACA
TTATGCTGTCGCATGGCAGTATAATCAAAGGAGTAATCCGCTACAAGATGGACCCGCGGCGCATTAGCTAG
TTGGTGAGGTAACGGCTCACCAAGGCAACGATGCGTAGCCGGCCTGAGAGGGTGAACGGCCACATTGGA
ACTGAGACACGGTC
>Zotu13926
GAACGCTAGCGGGATGCTTTACACATGCAAGTCGAACGGCAGCACGAGGAAGTTTACTTCTTTGGTGGCG
AGTGGCGGACGGGTGAGTAATGTATCGGAACGTGCCCAGTAGTGGGGGATAACTACGCGAAAGCGTAGC
TAATACCGCATATTCTCTTCGGAGGAAAGGGGGGGATCATTTGACCTCTCGCTATTGGAGCGGCCGATATC
AGATTAGTTAGTTGGTGGGGTAACGGCCTACCAAGGCAGTGATCTGTAGCTGGTTTGAGAGGACGACCAG
CCACACTGGGACTGAGACACGGCC
>Zotu13932
GAACGCTGGCGGCGCGCCTAACACATGCAAGTCGAACGCGAAAGGGAGCAATCCTGAGTAAAGTGGCGC
ACGGGTGAGTAACGCGTAGGTAATCTACCTTTGCGACCGGGATAACCAGCCGAAAGGTTGGCTAATACTG
GATAGAGCGCACGATCCATAAAGGATTGTGCGGGAAAGGGTGGCGTAGCAATACGCTACTGCGCTGAGAT
GAGCCTGCGTCTCATTAGCTAGTTGGTAGGGTAAAGGCCTACCAAGGCGATGATGGGTAGCTGGTCTGAG
AGGACGATCAGCCACACTGGGACTGAGACACGGCC
>Zotu13937
GAACGCTGGCGGCGTGCCTAATACATGCAAGTCGAGCGGACACCTTCGGGTGTCAGCGGCGGACGGGTG
AGTAACACGTGGGCAATCTGCCTGTCAGACTGGAATAACGCCTGGAAACGGGTGCTAATGCCAGATAGGC
ATCCTGGAGGCATCTTCGGGATGGGAAAGGTGCTTTGGCATCACTGACAGAGGAGCCCGCGGCGCATTAG
CTAGTTGGTGGGGTAAAGGCCTACCAAGGCGACGATGCGTAGCCGACCTGAGAGGGTGACCGGCCACACT
GGGACTGAGACACGGCC
>Zotu13946
GAACGCTGGCGGCGTGCCTAATACATGCAAGCCGAGCGAATCTTAAGGTGCTTGCACCTTTTGGTTAGCGG
CGGACGGGTGAGTAACACGTGGGCAACCTGCCTGTAAGACTGGGATAACTTCGGGAAACCGGAGCTAATA
CCGGATAATCCTTTTCCTCTCATGAGGAAAAGCTGAAAGTCGGTTTACGCTGACACTTACAGATGGGCCCG
CGGCGCATTAGCTAGTTGGTGAGGTAACGGCTCACCAAGGCGACGATGCGTAGCCGACCTGAGAGGGTG
ATCGGCCACACTGGGACTGAGACACGGCC
>Zotu13950
GAACGCTGGCGGCATGCCTTACACATGCAAGTCGAACGGCAGCGCGGGAGCAATCCTGGCGGCGAGTGG
CGAACGGGTGAGTAATATATCGGAACGTGCCCATTAGTGGGGGATAGCCCGGCGAAAGCCGGATTAATAC
CGCATACGACCTGAGGATGAAAGCAGGGGATCGCAAGACCTCGCGCTATTGGAGCGGCCGATATCAGATT
AGCTAGTTGGTGGGGTAAAAGCCTACCAAGGCAACGATCTGTAGTTGGTCTGAGAGGACGACCAGCCACA
CTGGGACTGAGACACGGCC
>Zotu13952
GAACGCTAGCGGCAGGCCTAATACATGCAAGTCGAGGGGCAGCGGGGTAGCAATACTGCCGGCGACCGG
CGCACGGGTGCGTAACACGTATGCAACCTACCTTAAACAGGGGGATAGCCCGGGGAAACTCGGATTAATA
CCCCATAACACGGTGATACCACATGGTAATGTCGTTAAAGATTTATCGGTTTAAGCTGGGCATGCGTTCGAT
TAGCTAGTTGGTAAGGTAATGGCTTACCAAGGCTACGATCGATAGGGGGTCTGAGAGGATGATCAGCCAC
ACTGGAACTGAGACACGGTC
>Zotu13961
GAACGCTAGCGGCAGGCTTAATACATGCAAGTCGAACGATGAAGCGGTGCTTGCACCGTGGATTAGTGGC
GAACGGGTGAGTAACACGTGAGTAACCTGCCTTCCACTCTGGGATAAGCGCCGGAAACGGCGTCTAATAC
TGGATATGACATTGCCTCGCATGGGGTGGTGTGGAAAGATTTTTGGTGGTAGATGGACTCGCGGCCTATC

AGCTTGTTGGTGGGGTAATGGCCTACCAAGGCGACGACGGGTAGCCGGCCTGAGAGGGTGACCGGCCAC
ACTGGGACTGAGACACGGCC
>Zotu13966
GAACGCTGGCGGCAGGCCTAACACATGCAAGTCGAACGGGTGTCTTCGGACACTAGTGGCGGACGGGTG
AGTAACGCGTGGGAACGTGCCCTTTGGTTCGGAATAGCCCTGGGAAACTGGGAGTAATACCGGATACGGT
CGAGAGACGAAAGATTTATCGCCGAAGGATCGGCCCGCGTTGGATTAGGTAGTTGGTGGGGTAATGGCCT
ACCAAGCCGACGATCCATAGCTGGTTTGAGAGGATGATCAGCCACACTGGGACTGAGACACGGCC
>Zotu13968
GAACATTGGCGGAAAGCTTAACACATGCAAGTCGAGCGAGGCTTAGAGCCTAGCGGCGAACGGGTGAGT
AAGACATAGGAATCTGCCCTGAAGTGGGGGACAACAACCAGAAATGGTTGCTAATACCCCATAATCCATAT
TGGGAAAGCGCGCAGCGCTTTAGGATGAGCCTATGGCCGATTAGCTAGTTGGTGGGGTAAAAGCCTACCA
AGGCGACGATCGGTAGCTGGTCTGAGAGGATGATCAGCCACACTGGAACTGAGACACGGTC
>Zotu13972
GAACGCTGGCGGGATGCCTTACACATGCAAGTCGAACGGCAGCACGGACTTCGGTCTGGTGGCGAGTGGC
GAACGGGTGAGTAACACGTGAGCAACCTGCCCCTGACTCTGGGATAAGCGCTGGAAACGGCGTCTAATAC
TGGATATGTGACGTGACCGCATGGTCTGCGTCTGGAAAGAATTTCGGTTGGGGATGGGCTCGCGGCCTAT
CAGCTTGTTGGTGAGGTAATGGCTCACCAAGGCGTCGACGGGTAGCCGGCCTGAGAGGGTGACCGGCCAC
ACTGGGACTGAGACACGGCC
>Zotu13983
GAACGCTGGCGGCGTGCCTAAGACATGCAAGTCGAGTGGGTTTAGACCCACGGCGAACGGGTGAGTAAT
ACCTAGGAACTTTCTTTAAGTGGAGCATAACCAGCCGAAAGGTTGGATAATTCTCCATGTGGCAGCAATGC
TAAAGTCGCAAGACGCTTAAAGTGAGGCCTGGGCACCATCAGCTAGTTGGTAAGGTAATGGCTTACCAAG
GCTATGACGGTTAGGGGTCCCGAGAGGGAGAACCCCCACACTGGAACTGCGACACGGTC
>Zotu13987
GAACGCTGGCGGCAGGCTTAACACATGCAAGTCGAACGCCCCGCAAGGGGAGTGGCAGACGGGTGAGTA
ACACGTGGGAACGTGCCCAAAGGTACGGAACAACTCCGGGAAACTGGAGCTAATACCGTATGTGCCCGAA
AGGGGAAAGATTTATCGCCTTTGGATCGGCCCGCGTCTGATTAGCTAGTTGGCGGGGTAATGGCCCACCAA
GGCGACGATCAGTAGCTGGTCTGAGAGGATGACCAGCCACATTGGGACTGAGACACGGCC
>Zotu13990
GAACGCTGGCGGCGTGCTTAACACATGCAAGTCGAACGGGAAACCTATTATTGAAACTTCGGTCGATTTAA
TTTGTTTCTAGTGGCGGACGGGTGAGTAACGCGTGGGTAACCTGCCCTATACAGGGGGATAACAACCAGA
AATGGTTGCTAATACCGCATAAGCGCACGGGACCGCATGGTCCAGTGTGAAAAACTCCGGTGGTATAGGA
TGGACCCGCGTTGGATTAGCTTGTTGGTGGGGTAACGGCCCACCAAGGCGACGATCCATAGCCGGCCTGA
GAGGGTGAACGGCCACATTGGGACTGAGACACGGCC
>Zotu13992
GAACGCTGGCGGCATGCTTTACACATGCAAGTCGAGCGGCAGCGCGGGGGCAACCCTGGCGGCGAGCGG
CGAACGGGTGAGTAATGCATCGGAACGTGTCCCGTAGTGGGGGATAACCCGGCGAAAGCCGGACTAATA
CCGCATATGCTCTACGGAGGAAAGCGGGGGATTCGCAAGAACCTCGCGCTATGGGAGCGGCCGATGTCCG
ATTAGCTTGTTGGTGGGGTAATGGCTTACCAAGGCGACGATCGGTAGCTGGTCTGAGAGGATGATCAGCC
ACACTGGGACTGAGACACGGCC
>Zotu13995
GAACGCTGGCGGCATGCCTTACACATGCAAGTCGAACGGCAGCGGGGGGAGCCTGCTCCCTGACGGCGA
GTGGCGGACGGGTGAGTAACACGTAGGAATCTACCTTAATTATAATAGTTAGTGGGGGATAACCCGGGGA
AACTCGGGCTAATACCGCATAATCTCTTCAAAGCAAAGCGGGGGATCTTCGGACCTCGTGCTATGAGATGA
GCCTGCGTCGGATTAGCTAGTTGGTAGGGTAATGGCCTACCAAGGCGACGATCCGTAGCTGGTCTGAGAG
GACGATCAGCCACACTGGGACTGAGACACGGCC
>Zotu13997
GAACGCTGGCGGCGTGCTTAACACATGCAAGTCGAGCGGTAACAGGTGTAGCAATACATGCTGACGAGCG
GCAGACGGGTGAGGAACACGTACGCAACCTCCCTCCAACTGGGGCACAACTCCGTGAAAACGGGGCTAAT
TCCCCATAACATTGCGAAAGGGCATCTGATTGCAATTAAAGATTCGTCGGTTGGAGATGGGCGTGCGACTG

ATTAGCCAGTTGGTAGGGTAACGGCCTACCAAAGCAACGATCAGTAGCTGGTGTGAGAGCACGACCAGCC
ACACGGGCACTGAGATACGGGC
>Zotu14006
CAACGCTGGCGGCGTGCCTAACACATGCAAGTCGAACGCGAAAGGGGCTTTGGTCCTGAGTAGAGTGGCG
AACGGGTGAGTAACGCGTGGGTGACCTACCCTCGAGTGGGGGATAACGTTCCGAAAGGGACGCTAATACC
GCATGACATTCTGGGTTTGAAGACCTGGAAATCAAAGCCGGGGACCGCAAGGCCTGGCGCTTGAGGAGG
GGCCCGCGTCTGATTAGCTAGTTGGCGGGGTAACGGCCCACCAAGGCGACGATCAGTAGCCGGCCTGAGA
GGGCGGACGGCCACACTGGGACTGAGACACGGCC
>Zotu14009
GAACGCTGGCGGCGTGCCTAATACATGCAAGTCGAGCGAATCAATAGGAGCTTGCTCCTGTTGGTTAGCG
GCGGACGGGTGAGTAACACGTGGGCAACCTGCCTGTAAGACTGGGATAACACCGGGAAACCGGTGCTAA
TACCGGATAATCCTTTCCCTCTCATGAGGGAAAGCTGAAAGTCGGTTTCGGCTGACACTTACAGATGGGCC
CGCGGCGCATTAGCTAGTTGGTGAGGTAACGGCTCACCAAGGCGACGATGCGTAGCCGACCTGAGAGGGT
GATCGGCCACACTGGGACTGAGACACGGCC
>Zotu14012
GAACGCTGGCGGCGTGGTTCAGGCATGCAAGTCGCACGATTCCGCAAGGAATAGTGGCAGAAGGGTTAG
TAACACGTGGGTAATCTACCCTCGAGTCGGGGATAACTCAGCGAAAGCTGGGCTAATACCCGATAACATCA
CCGGGTCGCATGATTTGGTGATCAAAGGTGGCTCGCAAGGGCTGCCGATTGAGGAGGAGCCCGCGGCCTA
TCAGCTAGTTGGTGAGGTAACGGCCCACCAAGGCTTTTGACGGGTAGCTGGTCTAAGAGGACGATCAGCA
ACATTGGGACTGAGACACTGCC
>Zotu14014
GAACGCTGGCGGCGTGCTTAACACATGCAAGTCGAACGAACCTTCGGGTTAGTGGCGGACGGGTGAGTAA
CGCGTGGGAATCTGCCCTCAGGAGGGGGATAACGGCTGGAAACGGCCGCTAATACCCCATATGCCGAGAG
GTGAAACGAATTTCGCCTGAGGATGAGCCCGCGTCTGATTAGCTAGTTGGTGGGGTAAGAGCCTACCAAG
GCGACGATCCGTAGCTGGTCTGAGAGGACGATCAGCCACACTGGGACTGAGACACGGCC
>Zotu14016
GAACGCTGGCGGCGTGCTTAACACATGCAAGTCGAGCGGAAAGGCCTCTTCGGGGGTACTCGAGCGGCG
AACGGGTGAGTAACACGTGAGTAATCTGCCCTTCACTTGGGGATAACGTCCAGAAATGGGTGCTAATACCC
GGTATTCCTGGTTCCACGCATGTGGGGTTGGGGAAAGGTTTTTCTGGTGGGGGATGTGCTCGCGGCCTATC
AGCTTGTTGGTGGGGTAATGGCCTACCAAGGCTTTGACGGGTAGCCGGCCTGAGAGGGTGACCGGCCACA
CTGGGACTGAGACACGGCC
>Zotu14020
GAACGCTGGCGGCGTGCCTAATACATGCAAGTCGAACGCTTTTTTCAATCATCGTAGCTTGCTACACCGATT
GAAAATTGAGTAGCGAACGGGTGAGTAACACGTGGGTAACCTGCCCAAAAGAGGGGGATAACACTTGGA
AACAGGTGCTAATACCGCATAACCATGGTGACCGCATGGTCATTATGTAAAAGATGGTTTCGGCTATCACTT
TTGGATGGACCCGCGGCGTATTAACTTGTTGGTGGGGTAACGGCCTACCAAGGTAATAATACGTAGCCGA
ACTGAGAGGTTGATCGGCCACATTGGGACTGAGACACGGCC
>Zotu14024
GAACGCTGGCGGCGTGCTTAACACATGCAAGTCGAGCGGTAAGGCCCTTCGGGGTACACGAGCGGCGAA
CGGGTGAGTAACACGTGGGTGATCTGCCCTGCACTTCGGGATAAGCCTGGGAAACTGGGTCTAATACCGG
ATATGACCGATGGCTGCATGGTTGTCGGTGGAAAGGTTTACTGGTGCAGGATGGGCCCGCGGCCTATCAG
CTTGTTGGTGGGGTAATGGCCTACCAAGGCGACGACGGGTAGCCGACCTGAGAGGGTGACCGGCCACACT
GGGACTGAGACACGGCC
>Zotu14034
GAACGTTAGCGGCGCGCCTAACACATGCAAGTCGCACGAGAAAGGGCTTCGGCCCCAGTAAAGTGGCGCA
CGGGTGCGTAACACGTAGGCAATCTTCCCTCGGGCGGGGGATAACGTTTCGAAAGAGACGCTAATACCGC
ATAAGACCACGGCTACCTCGGTAGCTGCGGTCAAAGCTGGCCTCTTCATGAAAGCTAGCACCTGAGGACGA
GCCTGCGGCCCATCATTGCTAGTTGGTAGGGTAACGGCCTACCAAGGCTGAGACGGGTAGCTGGTCTGAG
AGGATGATCAGCCACACTGGAACTGAAACACGGTC
>Zotu14039

GAACGCTGGCGGAGTGCCTAATACATGCAAGTCGAGCGGCAGCACGAGTAGTTTACTACTTGGTGGCGAG
CGGCGGACGGCTGAGTAACGCGTGGGAACGTGCCCCAAAGTGAGGAATAACTGCCCGAAAGGGTAGCTA
AGGCCGCATATGATCTTCGGATTAAAGTATTTATTACGCTTTGGGAGCGGCCCGCGTCCGATTAGATAGTT
GGTGAGGTAAAGGCTCACCAAGTCTACGATCGGTAACTGGTCTGAGAGGATGATCAGTCAGACTGGAACT
GAGACACGGTC
>Zotu14041
GAACGCTATCGGTATGCTTAACACATGCAAGTCGAACGGAAAGGCCCTGCTTTTGTGGGGTGCTCGAGTG
GCGAACGGGTGAGTAACACGTGAGTAACCTGCCCTTGACTTTGGGATAACTTCAGGAAACTGGGGCTAAT
ACCGGATAGGAGCTCCTGCTGCATGGTGGGGGTTGGAAAGTTTCGGCGGTTGGGGATGGACTCGCGGCTT
ATCAGCTTGTTGGTGGGGTAGTGGCTTACCAAGGCTTTGACGGGTAGCCGGCCTGAGAGGGTGACCGGCC
ACACTGGGACTGAGACACGGCC
>Zotu14044
GAACGCTAGCTATATGCTTAACACATGCAAGTCGAACGTTGTTTTCGGGGAGCTGGGAGAAAGAAAATCG
GCTCCTTCAGATACGAGCTCAGTGTACATTATATGGTTATTGTATTCCGGCTCTCTATTTTTTTTGAGAACAA
AGTGGCTCACGGGTGCGTAACGCGTGGGAATCTGCCGAACAGTTCGGGCGGACAAGAGAGGACTGCAAA
TCCTGGAATAAGAAAAGCTTTTAAGCGCTGTTTGATGAGCCTGCGTAGTATTAGGTAGTTGGTCAGGTAAA
GGCTGACCAAGCCAATGATGCTTAGCTGGTCTTTTCGGATGATCAGCCACACTGGGACTGAGACACGGCC
>Zotu14046
GAACGCTGGCGGCAGGCTTAACACATGCAAGTCGAACGCCCCGCAAGGGGAGTGGCAGACGGGTGAGTA
ACACGTGGGAACGTACCCTTCGGTTCGGAACAACTCTGGGAAACTAGAGCTAATACCGGATACGATCGCG
AGATGAAAGGTTCACCGCCGAGGGATCGGCCCGCGTCTGATTAGCTAGTTGGTGAGGTAACGGCTCACCA
AGGCGACGATCAGTAGCTGGTCTGAGAGGATGATCAGCCACATTGGGACTGAGACACGGCC
>Zotu14053
GAACGCTGGCGGCGTGCTTAACACATGCAAGTCGAACGGTGAACCGCTTCGGTGGGGATCAGTGGCGAAC
GGGTGAGTAACACGTGGGCAACCTGCCCCCAGCTCTGGGATAACTCCAAGAAATTGGTGCTAATACCGGA
TGTGACCGCTGACCGCATGGTCTGGTGGTGGAAAGATTCATCGGCTGGGGATGGGCCCGCGGCCTATCAG
CTTGTTGGTGGGGTGATGGCCTACCAAGGCGACGACGGGTAGCCGGCCTGAGAGGGTGACCGGCCACAC
TGGGACTGAGACACGGCC
>Zotu14055
GAATGCTGGCGGCGTGGATAAGGCATGCAAGTCGAACGGAGTATTTTTTGAAGGTTATTGCGATTACATCA
AGGTGGCCGACAGTTAATACTTAGTGGCGAACGAGTTAGTAACATCTTGGTACGTACCCTGAAGTCGGGCA
CAACGGGTCGAAAGATCCGCTAATTCCCGATGGTGCAGCAATGCTAAAGTCGCAAGACGCTTCAGGAGCG
GCCTAGATCCTATCAGCTTGTTGGCGGGGTAATGGCCCACCAAGGCTATGACGGGTAGGAGGTGTGAGAG
CACGACCTTCAACGACGAAACTGAGACACGGTT
>Zotu14061
GAACGCTGGCGGCGTGCCTAATACATGCAAGTCGAGCGAACAGAGAAGAGCTTGCTCTTCAAAGTTAGCG
GCGGACGGGTGAGTAACACGTGGGCAACCTGCCCTGCAGATGGGGATAACTCGCGGAAACGTGGGCTAA
TACCGAATAATCAGTTCTCCCGCATGGGAGAACTCTGAAAGACGGTTTCGGCTGTCACTGCAGGATGGGCC
CGCGGCGCATTAGCTAGTTGGTGGGGTAACGGCCTACCAAGGCAACGATGCGTAGCCGACCTGAGAGGGT
GATCGGCCACACTGGGACTGAGACACGGCC
>Zotu14063
GAACGCTAGCGGCAGGCTTAACACATGCAAGTCGAGGGGCAGCACAGGTAGCAATACTGGGTGGCGACC
GGCAAACGGGTGCGGAACACGTACAGAACCTACCTTGATCTGGGGCATAGCCCGAAGAAATTCGGATTAA
CTCCCCATAACATAGACGAGTGGCATCACTTAACTATTAAAAATTTATTGGATCGAGATGGCTGTGCGTCCC
ATTAGCTAGTTGGTAGGGTAACGGCCTACCAAGGCGACGATGGGTAGCTGGTGTGAGAGCACGACCAGCC
ACACGGGCACTGAGACACGGGC
>Zotu14065
GAACGCTGGCGGCAGGCCTAACACATGCAAGTCGAACGAAGTCTTCGGACTTAGTGGCGGACGGGTGAGT
AACACGTGGGAACGTACCTTTTGGTTCGGAACAACTCAGGGAAACTTGAGCTAATACCGAATGTGCCCTTA
GGGGGAAAGATTTATCGCCATTAGAGCGGCCCGCGTTAGATTAGCTAGTTGGTGAGGTAACTGCTCACCA
AGGCTACGATCTATAGCTGGTCTGAGAGGATGATCAGCCACACTGGGACTGAGACACGGCC

>Zotu14069
GAACGCTGGCGGCGTGGATAAGACATGCAAGTCGAACGCTAACATTCGGGTAGCAATATTCGGATGTTGG
AGTGGCGCAAGGGTGCTTAACACGTGGGTAATTTACCGCGAAGCCCGGAATAACTCGCTGAAAGGCGAGC
TAATGCCGGATGAGACTCCTGGGGAGCATTCCTTGGGATTGAAAGCTGGGGACCGAAAGGCCTGGCACTT
TGCGATAAGCCCGCGGCCTATCAGCTAGTTGGCGAGGTAAAGGCTCACCAAGGCTAAGACGGGTAGCTGG
TCTGAGAGGACGACCAGCCACACTGGAACTGAGACACGGTC
>Zotu14077
GAACGCTGGCGGCGTGCCTAACACATGCAAGTCGAACGCGAACGGTGCTTTCGGGTGCCTAGTAGAGTGG
CGCACGGGTGAGTAACACGTAGACAACCTGCCTTCGAGCCCGGAATAACCTTTCGAAAGAAGGGCTAATA
CCGGATAACACCTTGGGTTGCATGATCTAAGGTTGAAAGGCTTGCTACTCGAAGAGGGGTCTGCGGGACA
TTAGCTAGTTGGTGGGGTAACGGCCTACCAAGGCGACGATGTCTAGCGGGTCTGAGAGGATGATCCGCCA
CACTGGAACTGAGACACGGTC
>Zotu14081
GAACGCTAGCGGCAGGCCTAATACATGCAAGACGAACGGTAATCGGTGTAGCAATACATCGGCAAGAGTG
TCGTAAGGGTGCGTAACACGTATGCAATCTGCCCTCAACAGGAGGATAGCTCCCCGAAAGGGGAATTAAT
ACTCCATAAGATCACGCGAAGGCATCTGAGAGTGATTAAATCTACGGAGGTTGAGGATGAGCATGCGGAT
GATTAGCTAGTTGGCGGTGTAACGGACCACCAAGGCAATGATCAATAGGGGAACTGAGAGGTTGATCCCC
CACACTGGCACTGAGATACGGGC
>Zotu14084
GAACGCTGGCGGCGTGCCTAACACATGCAAGTCGAACGCGAAAGTGGAGCAATCCATGAGTAGAGTGGC
AAACGGGTGAGTAACGCGTGGGCGACCTACCTTCAAGTGGGGGATAACTTTCCGAAAGGAAGGCTAATAC
CGCATAACATCCTGTTTTTGGATAAACGGGTATCAAAGCCGGGGATCGCAAGACCTGGCGCTAGAAGAGG
GGCCCGCGTCCGATTAGCTAGTTGGTGAGGTAACGGCTCACCAAGGCTCCGATCGGTATCCGGCCTGAGA
GGGCGGACGGACACACTGGAACTGAGACACGGTC
>Zotu14087
CAACGCTGGCGGCGTGCCTAACACATGCAAGTCGAACGCGAAAGTGGAGCAATCCATGAGTAGAGTGGCA
AACGGGTGAGTAACGCGTGGGTGACCTGCCTTCGAGTGGGGGATAACGCTCCGAAAGGGACGCTAATACC
GCATAACATCCTGCCTTTTAAGAGGTGGAGATCAAAGCCTGGGACCGCAAGGCCAGGCGCTGGAAGAGG
GGCCCGCGTCTGATTAGCTAGTTGGTGGGGTAATGGCTCACCAAGGCTTCGATCAGTATCCGGCCTGAGAG
GGCGGACGGACACACTGGGACTGAGACACGGCC
>Zotu14090
GAACGCTGGCGGCGTGCTTAACACATGCAAGTCGAGCGGTAAGGCTCCTTCGG GGGTACACGAGCGGCG
AACGGGTGAGTAACACGTGAGTAATCTGCCCTTCACTTCGGGATAAGCTTCGGAAACGGGGTCTAATACCG
GATATGACACGATCACGCATGTGGTGGTGTGGAAAGTTTTTCGGTGGGGGATGTGCTCGCGGCCTATCA
GCTTGTTGGTGGGGTAATGGCTCACCAAGGCTTTGACGGGTAGCCGGCCTGAGAGGGTGACCGGCCACAC
TGGGACTGAGACACGGCC
>Zotu14094
GAACGCTGGCGGCATGCCTTACACATGCAAGTCGAACGGCAGCACGGGAGCTTGCTCCTGGTGGCGAGTG
GCGAACGGGTGAGTAATATATCGGAACGTACCCTAGAGTGGGGGATAACTAGTCGAAAGATTAGCTAATA
CCGCATACGATCTACGGATGAAAGTGGGGGATCGCAAGACCTCATGCTCATGGAGCGGCCGATATCTGAT
TAGCTAGTTGGTGGGGTAAAAGCTCACCAAGGCGACGATCAGTAGCTGGTCTGAGAGGACGACCAGCCAC
ACTGGAACTGAGACACGGTC
>Zotu14098
GAACGCTGGCGGCATGCTTAACACATGCAAGTCGAACGGTAACAGGCCTTCGGGCGCTGACGAGTGGCGG
ACGGGTGAGTAATGCATAGGAACATACCTTATAGTGGGGGATAACGTAGGGAAACTTACGCTAATACCGC
ATAATCCTTAAGAGGGAAAGCTGGGGACCGTAAGGCCTGGCGCTAAAAGATTGGCCTATGTTGGATTAGC
TAGTTGGTAGGGTAAAGGCCTACCAAGGCGACGATCCATAGCTGGTTTGAGAGGATGATCAGCCACACTG
GGACTGAGACACGGCC
>Zotu14102

GAACGCTGGCGGCGTGCTTAACACATGCAAGTCGAACGGTAAGGCCCCTTCGGGGGTACACGAGTGGCGA
ACGGGTGAGTAACACGTGAGCAACCTGCCCTTGACTCTGGAATAACAGTTGGAAACAGCTGCTAATACCG
GATATGCACCGGACCTCTCCTGGGGTGTGGTGGAAAGCTTTGGCGGTCAGGGACGGGCTCGCGGCCTATC
AGCTTGTTGGTGAGGTGATGGCTCACCAAGGCTTCGACGGGTAGCCGGCCTGAGAGGGCGACCGGCCACA
CTGGGACTGAGATACGGCC
>Zotu14107
GAACGCTGGCGGCGTGCCTAAGACATGCAAGTCGAGGGGGTTTAGACCCCCGGCGAACGGGTGAGTAAC
ACGTGGGAACTTTCGTTAAGTTCGGGATAACCTCTAGAAATGGAGGACAATACCGGATGTGGTCGTAAGA
CTAAAGATTTATCGCTTAACGTAAGGCCCGCGGCCCATCAGCTAGTTGGTAAGGTAATGGCTTACCAAGGC
TATGACGGGTAGCGGAACCGAGAGGTTGATCCGCCACACTGGGACTGCGACACGGCC
>Zotu14111
GAACGCTGGCGGCATGCTTAACACATGCAAGTCGAACGGTAACAGGTGTAGCAATACATGCTGACGAGTG
GCGGACGGGTGAGGAATGCTTGGGAATCTGCCTGTTAGTGGGGGACAACCCGGGGAAACTCGGGCTAAT
ACCGCATACGCTCTACGGAGGAAAGCGGGGGACCGTAAGGCCTCGCGCTAACAGATGAGCCCAAGTCGG
ATTAGCTAGTTGGTGGGGTAAAGGCCTACCAAGGCAACGATCCGTAGCTGGTCTGAGAGGACGATCAGCC
ACACTGGGACTGAGACACGGCC
>Zotu14121
GAACGCTGGCGGCATGCCTAACACATGCAAGTCGAACGCCATGGTTTCGGCCATGGAGTGGCGCACGGGT
GAGTAACGCGTGGGAACCTGCCTAGCAGTATGGGACAACCGCGGGAAACTGCGGCTAATACCGTATACGC
CCGTCTTTGGGAGACCAGGGGCGGGGAAAGATTGATCGCTGTTAGAGGGGCCCGCGTCGGATTAGGTAG
TTGGTGGGGTAAAGGCCTACCAAGCCTGCGATCCGTAGCTGGTCTGAGAGGACGATCAGCCACACTGGGA
CTGAGACACGGCC
>Zotu14123
GAATGCTGGCGGCGTGGATAAGTCATGCAAGTCAAGGGGGCCGCAAGGCAACCGGCAGACGAGGTAGTA
ACACGTAGGTAGATCCCCCGAAGTCGGGCATAGCCAACCGAAAGGTTGGGTAATTCCCGATAGTCCCGCA
AGGGTAAAGGTTTTTCGCTTCGGGAGTTGCCTGCGTAGTATCAGCTAGTTGGTAGGGTAATGGCCTACCAA
GGCTACGACGCTTAGGGGGGCTGAGAGGCCGATCCCCACCGATGGAACTGCGACACGGTC
>Zotu14131
GAACGCTAGCGGGATGCTTTACACATGCAAGTCGAACGGCAGCATGAGAGAGCTTGCTCTCTTGATGGCG
AGTGGCGGACGGGTGAGTAATGTATCGGAACGTGCCCGGTAGTGGGGGATAACTACGCGAAAGCGTAGC
TAATACCGCATATACTCTACGGAGGAAAGAGGGGGATCGTAAGACCTCTTGCTACCGGAGCGGCCGATAT
CAGATTAGCTGGTTGGTGGGGTAACGGCCTACCAAGGCGGTGATCTGTAGCTGGTTTGAGAGGACGACCA
GCCACACTGGGACTGAGACACGGCC
>Zotu14133
GAACGCTGGCGGCGTGCTTAACACATGCAAGTCGAACGATGAAGAAAAGAATCGAAGCTTCGACAGAAAT
TCATTTCGGATTAGTGGCAGACGGGTGAGTAACGCGTGAGCAACCTACCTCTTACAAAGGGATAGCCTCGG
GAAACCGGGATTAATACCATATAACATTGCGACGAAGCATTTCGGTGCAATCAAAGCTACGGCGGTAAGA
GATGGGCTCGCGTCCCATTAACCAGATGGTGAGGTAACGGCTCACCATAGTAACGATGGGTAACCGGCCT
GAGAGGGTGAACGGTCACACTGGAACTGAGACACGGTC
>Zotu14135
GAACGCTGGCGGCATGCCTAACACATGCAAGTCGAACGATTGCCTTCGGGTGATAGTGGCGCACGGGTGC
GTAACGCGTGGGAATCTGCCTTGGGGTTCGGAATAACTCCCCGAAAGGGGTGCTAATACCGGATGATGTC
GAAAGACCAAAGATTTATCGCCCTGAGATGAGCCCGCGTAGGATTAGCTAGTTGGTGTGGTAAAGGCGCA
CCAAGGCGACGATCCTTAGCTGGTCTGAGAGGATGATCAGCCACACTGGGACTGAGACACGGCT
>Zotu14137
GAACGTTAGCGGCACGCCTAACACATGCAAGTCGCACGAGAAAGGGAGCAATCCTGAGTAGAGTGGCGC
ACGGGTGCGTAACACGTAGATAACCTGCCCTTTGGAGGGGGATAACGTTCCGAAAGGAGCGCTAATACCG
CATAACGCCTTTTCCGGTTCGCCGGAGATTGACAAAGATAGGGACCCGCAAGGGCCTATCGCCAGAGGAG
GGGTCTGCGGCCCATCAGCTAGTTGGGGAGGTAATGGCTCACCAAGGCAGAGACGGGTAGCTGGTCTGA
GAGGATGATCAGCCACACTGGTACTGGAACACGGAC

>Zotu14141
GAACGCTGGCGGCGCGCTTAAGACATGCAAGTCGAACGGTTCAACAACATTGCAGAGGATGAAGATTTAT
CAGATTCCAAAGCGCCGTTGTTGAATAGTGGCGGACGGGTGAGTAACACGTGAGCAACCTACCTTCAAGA
GGGGGACAACAGAGAGAAATTTCTGCTAATACCGCATAAGACCACAGTACCGCATGGTACAGGGGTCAAA
GATTTATCGCTTGAAGATGGGCTCGCGGCTGATTAGCTAGTTGGTGAGGTAATGGCTCACCAAGGCAACG
ATCAGTAGCCGAACTGAGAGGTTGATCGGCCACATTGGGACTGAGACACGGCC
>Zotu14145
GAACGCTAGCGGCATGCTTAACACATGCAAGTCGAGCGGTAAGGCCCCTTCGGGGGTACACGAGCGGCGA
ACGGGTGAGTAACACGTGAGCAATCTGCCCATCACTTCGGGATAACTCCGGGAAACCGGTGCTAATACCG
AATACGACCACCTCGGGCATCTGATGGTGGTGGAAAGTTCCGGCGGTGATGGATGAGCTCGCGGCCTATC
AGCTTGTTGGTGGGGTAGTGGCCTACCAAGGCTTCGACGGGTAGCCGGCCTGAGAGGGTGACCGGCCACA
CTGGGACTGAGACACGGCC
>Zotu14150
GAACGCTGGCGGCGTGGATAAGGCATGCAAGTCGAGCGGATATTCTTTTTAGCAATAAGATAGAATATTA
GCGGCAAACGGGTTAGTAACGCGTAGATACATACCTCATTCTCGGGCACAACTCGCCGAAAGGCGAGCTA
ATTCCCGATGGTCTGGCAACAGTAAAGGTCCCGCTTCGCGGGATTGGAATGAGAATGGTCTGCGTCCTATC
AGCTAGTTGGCGAGGTAATGGCTCACCAAGGCTATGACGGGTAGGGGGACTGAGAGGTCGGCCCCCAAC
ATCGGAACTGAGACACTGTC
>Zotu14153
GAACGCTGGCGGTGTGCTTAACACATGCAAGTCGAACGGTGAAGGGGAGCTTGCTTTCTGGATCAGTGGC
GAACGGGTGAGTAACACGTGAGCAATCTGCCCCTAACTCTGGGATAACAGTTGGAAACAGCTGCTAATACC
GGATACGAGCTGCGAAGGCATCTTCAGCAGTTGGAAAGATTTTTTGGTTAGGGATGAGCTCGCGGCCTATC
AGCTTGTTGGTGAGGTAATGGCTCACCAAGGCGTCGACGGGTAGCCGGCCTGAGAGGGTGACCGGCCAC
ACTGGGACTGAGACACGGCC
>Zotu14156
GAACGCTGGCGGCATGCCTAACACATGCAAGTCGAACGAGGTTTCCGCTTCGGCGGTTTCCTAGTGGCGCA
CGGGTGAGTAACGCGTGGGAACCTGCCCTGTGGTGCGGAATAACTCCGGGAAACTGGAGCTAATACCGCA
TGTGTCCTGCGGGACAAAGATTTATCGCCATGGGATGGGCCCGCGTAGGATTAGCTAGTTGGCGGGGTAA
CGGCCCACCAAGGCAACGATCCTTAGCTGGTCTGAGAGGATGATCAGCCACACTGGGACTGAGACACGGC
C
>Zotu14158
GAACGCTAGCGGCATGCTTAACACATGCAAGTCGAGCGGTAGAGAGAAGCTTGCTTCTCTTGAGAGCGGC
GGACGGGTGAGTAAAGCCTAGGAATCTGCCTGGTAGTGGGGGATAACGTTCGGAAACGGACGCTAATAC
CGCATACGTCCTACGGGAGAAAGCAGGGGACCTTCGGGCCTTGCGCTATCAGATGAGCCTAGGTCGGATT
AGCTAGTTGGTGAGGTAATGGCTCACCAAGGCGACGATCCGTAACTGGTCTGAGAGGATGATCAGTCACA
CTGGAACTGAGACACGGTC
>Zotu14160
GAACGCTGGCGGTGTGCTTAACACATGCAAGTCGAGCGAGAACCATGGCTTCGGCTATGGGGACAGCGGC
GAACGGGTGAGTAACACGTGGGTAATCAACCCTCGACACTGGGATAGCCCGGGGAAACTCGGATTAATAC
CGGATACCCCATCTGGCCTTCGGGCCGGCTGGAAAAGGTAGCTTTGGCCTCCGGTCGAGGACGAGCCCGC
GGCGGATTAGCTTGTTGGTGGGGTAAAAGCCCACCAAGGCGACGATCCGTAGCTGGTCTGAGAGGACGAT
CAGCCACACTGGGACTGAGACACGGCC
>Zotu14162
GAACGCTGGCGGCGTGCCTAAGGCATGCAAGTCGAATGGTCGCAAGACAATGGCGGACGGGTGAGTAAC
GCGTGGGAATCTACCTCGAAGTGGGGCATAGCCCTCCGAAAGGAGGGGTAATACCGCATATGTTCGTAAG
AACAAAGCCGTAAGGCGCTTTGAGATGAGCCCGCGTAAGATTAGCTTGTTGGTGGGGTAATGGCCTACCA
AGGCGACGATCTTTAGCTGGTCTGAGAGGATGGTCAGCCACAATGGCACTGAGACACGGGC
>Zotu14166
GAACGCTGGCGGCAGGCTTAACACATGCAAGTCGAGCGCTCGTAGCAATACGAGAGCGGCAGACGGGAG
AGTAACACGTGGGAACGTACCCTTCGGTTCGGAATAACCCAGGGAAACTTGGGCTAATACCGGATACGTCC

GTAAGGAGAAAGATTTATCGCCGAGGGATCGGCCCGCGTCCGATTAGCTAGTTGGTGAGGTAATGGCTCA
CCAAGGCGACGATCGGTAGCTGGTCTGAGAGGATGATCAGCCTCACTGGGACTGAGACACGGCC
>Zotu14169
GAACGCTGGCGGCATGCCTTACACATGCAAGTCGAACGGCAGCGCGGGCGGGGGTAACCTCGTCTGGCG
GCGAGTGGCGAACGGGTGAGTAACATATCGGAACGTGCCCTGGAGTGGGGGATAACGCAGCGAAAGTTG
CGCTAATACCGCATACGATCCACGGATGAAAGCAGGGGACCGCAAGGCCTTGTGCTCCTGGAGCGGCCGA
TATCTGATTAGCTAGTTGGTGGGGTAAAGGCCTACCAAGGCATCGATCAGTAGCTGGTCTGAGAGGACGA
CCAGCCACACTGGAACTGAGACACGGTC
>Zotu14172
GAACGCTAGCGGCAGGCTTAACACATGCAAGTCGAGGGGTATTTATCTTCGGATAGAGAGACCGGCGCAC
GGGTGCGTAACGCGTATGCAATCTACCTTTTACAGCGGGATAGCCCAGAGAAATTTGGATTAATACCTCAT
AGCATTGCAGAATGGCATCATTCAGCAATTAAAGTCACAACGGTAAAAGATGAGCATGCGTCCCATTAGCT
AGTTGGTAAGGTAACGGCTTACCAAGGCTACGATGGGTAGGGGTCCTGAGAGGGAGATCCCCCACACTGG
TACTGAGACACGGAC
>Zotu14176
GAACGCTGGCGGTGTGCTTAACACATGCAAGTCGTGGGGCAGCGGATACTTAGCTTGCTAAGTATGCCGG
CGACCGGCGCACGGGTGAGTAACGCGTACCGAACCTGCCCATCACACAGGGATAGGCTTGCGAAAGCAAG
ATTAATACCTGATGGTCTCAGTTGTATGCATGTATAATTGAGTAAAGCCTTCGGGCGGTGATGGATGGCGG
TGCGTCCCATTAGGAAGTTGGCGGGGTAACGGCCCACCAATCCTTCGATGGGTAGGGGTTCTGAGAGGAA
GGTCCCCCACATTGGAACTGAGATACGGTC
>Zotu14179
TAACGCTGGCAGCGTGCATAACACATGCAAGTCGAGCGGGGTGTTTGTAGTAGCAATACTGTGAGCACCT
AGCGGCAGACGAGTGAGTAACACGTAAGAAATCTACCCCGGAGTGGGGCACAACCCGCCGAAAGGCGGG
CTAATACCCCATATGATCCTTTGGCGGCATCGCCGAGGGATGAATGGCCCGCAAGGGTCGCTCCAGGAGG
ATCTTGCGGCCTATCAGCTAGTTGGCGGGGTAACGGCCCACCAAGGCGACGACGGGTAGCCGGCCTGAAA
GGGCGATCGGCCACGGAGGCACTGAGACACGGGC
>Zotu14182
GAACGCTGGCGGCGTGCCTTAAGCATGCAAGTCGAACGGACCGCAAGGTCAGTGGCGGACGGGTGAGTA
ACGCGTGGGAATCTGCCTTAAAGTTGGACATAGCTCATCGAAAGATGGGGTAATTTCCAATGTGGATGTAA
ATCTAAAGTCGCAAGACGCTTTAAGATGAGCCCGCGTCCTATCAGCTAGTTGGTGGGGTAATGGCCTACCA
AGGCGACGACGGGTAGCTGGTCTGAGAGGACGATCAGCCACACTGGGACTGAGACACGGCC
>Zotu14188
GAACGCTGGCGGCGTGCTTAACACATGCAAGTCGAACGGAAAGGCCCCTTCGGGGGTACTCGAGTGGCGA
ACGGGTGAGTAACACGTGGGTGATCTGCCCTGCACTCTGGGATAAGCCTGGGAAACTGGGTCTAATACCG
GATACACCCTTCTGGTTGCATGGCTGGGAGGGGAAAGCTTTTGCGGTGTGGGATTGGCCCGCGGCCTATC
AGCTTGTTGGTGGGGTGATGGCCTACCAAGGCGACGACGGGTAGCCGGCCTGAGAGGGTGTCCGGCCAC
ACTGGGACTGAGATACGGCC
>Zotu14192
GAACGCTAGCGGGATGCTTTACACATGCAAGTCGAACGGCAGCATGAGAGAGCTTGCTCTCTTGATGGCG
AGTGGCGGACGGGTGAGTAATGTATCGGAACGTGCCCGGTAGTGGGGGATAACTACGCGAAAGCGTAGC
TAATACCGCATATACTCTACGGAGGAAAGAGGGGGATCGTAAGACCTCTTGCTACCGGAGCGGCCGATAT
CAGATTAGCTGGTTGGTGGGGTAACGGCCTACCAAGGCGGTGATTTGTAGCTGGTTTGAGAGGACGACCA
GCCACACTGGGACTGAGACACGGCC
>Zotu14200
GAACGCTGGCGGCAGGCCTAACACATGCAAGTCGAACGATAAGCCACCTTCGGGTGGTGGACAGTGGCA
GACGGGTGAGTAACGCGTGGGAACGTGCCTTTCGGTTCGGAATAACCCAGGGAAACTTGGGCTAATACCG
GATACGCCCTTCGGGGGAAAGATTCATTGCCGAAAGATCGGCCCGCGTCGGATTAGCTAGTTGGTGGGGT
AATGGCTCACCAAGGCGACGATCCGTAGCTGGTCTGAGAGGATGACCAGCCACACTGGGACTGAGACACG
GCC
>Zotu14212

GAACGCTAGCGGCATGCCTAACACATGCAAGTCGAGCGGGAGTATTTTGTAGCTTGCTACAAAATATGAGA
GCGGCGAACGGGTGAGTAACACGTTGGAATCTGCCCCTAAGTCAGGGATAGCCCAGGGAAACCTGGATTA
ATACCGGATAGTCTCTTTGGAGTAAAAATTTATTGCTTAGGGAGGAGCCTGCGTACTATCAGCTAGTTGGT
GAGGTAATAGCTTACCAAGGCAATGACGGTTAACTGGTCTGAGAGGATGATCAGTCACAATGGAACTGAG
ACACGGTC
>Zotu14215
GAACGCTGGCGGCGTGCCTAATACATGCAAGTCGAGCGCCCTGACATTGCCCATTAACTTATGTTAGTGAG
TAGTGTCAGGGAGCGGCGGACGGCTGAGTAACGCGTAGGAACGTACCCCAAACTGAGGGATAAGCACCG
GAAACGGTGTCTAATACCGCATAATACCCCCGGGTTAAAGCTTCGGCGGTTTGGGAGCGGCCTGCGTCTGA
TTAGTTAGTTGGTGAGGTAATGGCTTACCAAGACGATGATCGGTAGCTGGTCTGAGAGGATGACCAGCCA
GACTGGAACTGAGAACGGTC
>Zotu14218
GAACGCTGGCGGCGTGCCTAAGACATGCAAGTCGAGTGGGTTTAGACCCACGGCGAACGGGTGAGTAAT
ACCTAGGAACTTTCTTTAAGTGGAAGATAACCAGTCGAAAGATTGGATAATACTCCATGTGATAGCAATAT
CAAAGCAGCAATGCGCTTAAAGTGAGGCCTGGGCACCATCAGCTAGTTGGTAAGGTAATGGCTTACCAAG
GCTATGACGGTTAGGGGCCCCGAGAGGGGAGACCCCCACACTGGAACTGCGACACGGTC
>Zotu14224
AAACGCTGGCGGCGTGCCTTACGCATGCAAGTCGAACGGCACCTTTCGGGGTGCAGTGGCGGACGGGTGA
GTAACACGTACGTGACCTGACGTCTGGGGGGGGATACCCGTCCGAAAGGGCGGCTAATACCGCATACGCT
CCACGGGGGTGCTCGTGGAGGAAAGGGTTTTCGGACCCGCCGGGTGAGGGGCGTGCGGCCGATTAGGCT
AGTTGGTGGGGTAAAGGCCTACCAAGGCGACGATCGGTAGCTGGGCTGAGAGGCCGGACAGCCACACTG
GGACTGAGACACGGCC
>Zotu14229
GAACGCTGGCGGCGTGCTTAACACATGCAAGTCGAACGGAGTTAATAAGTGGAAGTCTTCGGATGGAAGC
TTATTGACTTAGTGGCGGACGGGTGAGTAACGCGTGGGTAACCTGCCTTATACAGGGGGATAACAGCCGG
AAACGGTTGCTAATACCGCATAAGCACACAGTGCCGCATGGTACAGTGTGAAAAGATTTATCGGTATAAGA
TGGACCCGCGTCTGATTAGCTAGTTGGTGAGGTAAAGGCCCACCAAGGCGACGATCAGTAGCCGGCTTGA
GAGAGTGACCGGCCACATTGGGACTGAGACACGGCC
>Zotu14234
CAACGCTGGCGGCGCGCCTAACACATGCAAGTCGAACGAGAAAGTGGAGCAATCCATGAGTACAGTGGCG
TACGGGTGAGTAACACGTGGGTAATCTAACTTTTAGTGAGGAATAACCGAGGGAAACTTTGGCTAATACCG
CATAAGCCCGAGAGGGGAAAGCAGAAATGCGCTGAAAGAGGAGCCCGCGGCCGATTAGCTAGTTGGTAG
GGTAAGAGCCTACCAAGGCAGAGATCGGTAGCCGGCCTGAGAGGGCACACGGCCACACTGGCACTGAAA
CACGGGC
>Zotu14236
GAACGCTGGCGGCGTGGATTAGGCATGCAAGTCGAACGCGAAAGAGGGGGCAACCCCTTGAGTAGAGTG
GCGAACGGGTGAGTAACGCGTGAGAAACCTGCCTTCATCAGGGGGATAACACTCCCAACGGGGTGCTAAT
ACCGCATGAGACCACGGGATCGCATGGTCCTGAGGTCAAAGGTGGCCTCTATTTATAAGCTACCGGATGAA
GATGGTCTCGCGTCCCATTAGCTAGTTGGCGGGGTAACGGCCCACCAAGGCGACGATGGGTAGCTGGTCT
GAGAGGATGGTCAGCCACACTGGGACTGAGATACGGCC
>Zotu14241
GAACGCTGGCGGCGTGCTTAACACATGCAAGTCGAACGGTGACACGAGAGCTTGCTCTCGTTGATCAGTG
GCGAACGGGTGAGTAACACGTGAGCAACCTACCCTTGACTCCGGGATAACAGCTGGAAACAGCTGCTAAT
ACCAGATACGAACTCAGCAGGCATCTGCCGAGTTGGAAAGCTCCGACGGTCAAGGATGGGCTCGCGGCCT
ATCAGCTTGTTGGTGAGGTAACGGCTCACCAAGGCGACGACGGGTAGCCGGCCTGAGAGGGCGACCGGC
CACACTGGGACTGAGATACGGCC
>Zotu14243
GAACGCTGGCGGTGTGCTTAACACATGCAAGTCGAGCGGTAACAGGAGAATTTATTCTTGCTGACGAGCG
GCGGACGGCTGAGTAACGCGTGGGAACATGCCCCAAAGTGAGGGATAAGCTCCAGAAATGGAGTCTAAT
ACCGCATATGATCTTCGGATTAAAGGATTTATCCGCTTTGGGGGTGGCCTGCGTAAGATTAGATAGTTGGT

GAGGTAATGGCTCACCAAGTCTACGATCTTTAGCTGGTCTGAGAGGATGATCAGCCAGACTGGAACTGAG
ACACGGTC
>Zotu14249
GAACGCTAGCGGCATGCTTAACACATGCAAGTCGAACGAAGGCTTCGGCCTTAGTGGCGCACGGGTGCGT
AACGCGTGGGAATCTGCCCCTTGGTTCGGAATAACAGCGAGAAATTGCTGCTAATACCGGATAATGACGTA
AGTCCAAAGATTTATCGCCTAGGGATGAGCCCGCGTAGGATTAGCTAGTTGGTGTGGTAAAGGCGCACCA
AGGCGACGATCCTTAGCTGGTCTGAGAGGATGATCAGCCACACTGGGACTGAGACACGGCC
>Zotu14255
GAACGCTGGCGGCATGCCTAACACATGCAAGTCGAACGAGACCTTCGGGTCTAGTGGCGCACGGGTGCGT
AACGCGTGGGAATCTGCCCTTAGGTTCGGAATAACTCAGAGAAATTTGAGCTAATACCGGATGATGACGTA
AGTCCAAAGATTTATCGCCTAGGGATGAGCCCGCGTAGGATTAGGTAGTTGGTGGGGTAAAGGCTCACCA
AGCCGACGATCCTTAGCTGGTCTGAGAGGATGATCAGCCACACTGGGACTGAGACACGGCC
>Zotu14260
GAACGCTAGCGGCATGCCTAACACATGCAAGTCGAACGGGTGTAGCAATACATCAGTGGCAGACGGGTGA
GTAACACGTGGGAACCTTCCTAGAGGTACGGAACAACGCAGGGAAACTTGCGCTAATACCGTATACGCTCT
TGCGAGGAAAGATTTATCGCCTTTAGACGGGCCCGCGTAGGATTAGGTAGTTGGTGGGGTAACGGCCTAC
CAAGCCTACGATCCTTAGTTGATCTGAGAGGATGATCAGCCACACTGGAACTGAGACACGGTC
>Zotu14265
GAACGCTAGCTATATGCTTAACACATGCAAGTCGAACGTTGTTTTCGGGGAGCTGGGCAGAAGGAAAAGA
GGCTCCTAGCTAAAGGTAGCTTGTCTCGCCCAGGAGGTGGGAACAGTTGAAAACAAAGTGGCGAACGGGT
GCGTAATGCGTGGGAATCTGCCGAACAGTTCGGGCCAAATCCTGAAGAAAGCTAAAAAGCGCTGTTTGAT
GAGCCTGCGTAGTATTAGGTAGTTGGTCAGGTAAAGGCTGACCAAGCCAATGATGCTTAGCTGGTCTTTTC
GGATGATCAGCCACACTGGGACTGAGACACGGCC
>Zotu14267
GAACGCTTGCGGCGTGCCTAATGTATGCAAGTCGAGCGGATCTTCTTCCTTCGGGAGGAAGGTGAGCGGC
GAACGGTTGAGTAACACGTAGGCAACCTGCCCCGAAGTCGGGAATAACTCATCGAAAGGTGGGCTAATAC
CGGATGTGGACGCCGATCGCCTGGTCGGCGCTCTAAAGGTTTCGATCGCTTCGGGATGGGCCTGCGGCCT
ATCAGCTTGTTGGTGGGGTAACGGCCTACCAAGGCGACGACGGGTAGCTGGTCTGAGAGGATGGTCAGCC
AGACAGGGACTGAGACACGGCC
>Zotu14271
GAACGCTAGCGGCATGCTTAACACATGCAAGTCGAACGATGATGCCCAGCTTGCTGGGTGGATTAGTGGC
GAACGGGTGAGTAACACGTGAGTAACCTGCCCTCCACTCTGGGATAACTCCGGGAAACCGGTGCTAATACT
GGATATGACCTTGGCCCGCATGGGTTTGGGGTGGAAAGATTTATCGGTGGTGGATGGACTCGCGGCCTAT
CAGCTTGTTGGTGGGGTAATGGCCTACCAAGGCGACGACGGGTAGCCGGCCTGAGAGGGTGACCGGCCA
CACTGGGACTGAGACACGGCC
>Zotu14275
GAACGCTAGCGGCATGCTTAACACATGCAAGTCGAACGGAGTTATATTGTAGCTTGCTATGATGTGACTTA
GTGGCAGACGGGTGAGTAATGTATAGGAATCTACCTAGTAGTACGGAATAATTGTTGGAAACGACAACTA
ATACCGTATACGCCCTACGGGGGAAAAATTTATTGCTATTAGATGAGCCTATATTAGATTAGCTTGTTGGTG
GGGTAATAGCCTACCAAGGCAATGATCTATAGCTGATCTGAGAGGATGATCAGCCACACTGGAACTGAGA
TACGGTC
>Zotu14281
GAACGCTGGCGGCAGGCTTAACACATGCAAGTCGAGCGGGCGAGGTTGCTTCGGTAACTGAGCTAGCGGC
GGACGGGTGAGTAATGCTTAGGAATCTGCCTATTAGTGGGGGACAACATTCCGAAAGGGATGCTAATACC
GCATACGTCCTACGGGAGAAAGCAGGGGATCTTTATGACCTTGCGCTAATAGATGAGCCTAAGTCGGATTA
GCTAGTTGGTGGGGTAAAGGCCTACCAAGGCGACGATCTGTAGCGGGTCTGAGAGGATGATCCGCCACAC
TGGGACTGAGACACGGCC
>Zotu14285
GAACGCTGGCGGCGTGCTTAACACATGCAAGTCGAACGGTGACCCTCGGAGCTTGCTCTGAGGTGATCAG
TGGCGAACGGGTGAGTAACACGTGAGTAACCTGCCCCAGACTCTGGAATAAGCGCTGGAAACGGCGTCTA

ATACCGGATACGAGACGGAGTCGCATGACTACCGTCTGGAAAGTTTTTCGGTCTGGGATGGACTCGCGGC
CTATCAGCTTGTTGGTGAGGTAACGGCTCACCAAGGCGACGACGGGTAGCCGGCCTGAGAGGGCGATCG
GCCACACTGGGACTGAGACACGGCC
>Zotu14287
GAACGCTGGCGGCGTGCCTTAAGCATGCAAGTCGAACGGTAGCAATACAGTGGCGGACGGGTGAGTAAC
GCGTAGGAATCTACCTCGAAGTGGGGCATAGCTTACCGAAAGGTAAGGTAATTCCCCATGTGATCGCAAG
ATCAAAGTCGCAAGACGCTTTGAGATGAGCCTGCGTAAGATTAGCTAGTTGGTGGGGTAAAAGCCTACCA
AGGCGACGATCTTTAGCTGGTCTGAGAGGATGATCAGCCACAATGGCACTGAGACACGGGC
>Zotu14292
GAACGCCAGCGGCGTGGATTAGGCATGCAAGTCGAACGTGATCCAGGGGGCAACTCCTGGTGAAAGTGG
CGGAAGGGTGAGTAACGCATAGACAACGTACCCCCGAGATGGGGATAACTCCGGGAAACCGGAGCTAAT
ACCCAATGGTGTCACTGGAGGGCCTCCTCTGGTGACTAAAGGTGGCCTCTGAACCATGCTCCCGCTCGGGG
AGCGGTCTATGTCCTATCAGCTAGTTGGTGGGGTAACGGCCCACCAAGGCTTCGACGGGTAGCCGGCCTTA
GCGGGTGTCCGGCCACACTGGGACTGAGACACTGCC
>Zotu14294
GAACGCTGGCGGCGTGCCTAACACATGCAAGTCGAACGGGGAAGTTAGCAATAACTTTCTAGTGGCGAAC
GGGTGAGTAACGCGTAGACAACCAACCTTTTTGTGGGGGACAACACTTCGAAAGGAGTGCTAATACCGCA
TACGCTCCCGAGGTCACCTGGCCATGGGAGGAAAGGAGCTTTACGGCTTCGCAAAAAGACGGGTCTGCGT
CTGATTAGCTAGTTGGAGGGGTAACGGCCCACCAAGGCGACGATCAGTAGCCGGTCTGAGAGGATGGAC
GGCCACACTGGGACTGAGACACGGCC
>Zotu14298
GAACGCTGGCGGCGTGGATAAGACATGCAAGTCGAACGATCACCATCGGGTAGCAATATTCGGTGGTGGG
AGTGGCGCAAGGGTGCGTAACACGTGGGTAATTTGCCGTAAAGTCTGGGATAACTTGCTGAAAGGCGAGC
TAATACCGGATGTGATTGCTGGAGGGCATCCTCTGGCACTTAAAGTCGGGGACCGCAAGGCCTGACACTTT
TCGATAAGCCCGCGGCCTATCAGCTAGTTGGTGAGGTAACGGCTCACCAAGGCTAAGACGGGTAGCTGGT
CTGAGAGGACGACCAGCCTCACTGGAACTGAGACACGGTC
>Zotu14303
CGATGAAGAACGCAGCGGCGGACGGGTGAGTAATGCCTAGGAATCTGCCTGGTAGTGGGGGACAACGTT
TCGAAAGGAACGCTAATACCGCATACGTCCTACGGGAGAAAGCAGGGGACCTTCGGGCCTTGCGCTATCA
GATGAGCCTAGGTCGGATTAGCTAGTTGGTGAGGTAATGGCTCACCAAGGCGACGATCCGTAACTGGTCT
GAGAGGATGATCAGTCACACTGGAACTGAGACACGGTC
>Zotu14310
GAACGCTGGCGGCATGCCTAACACATGCAAGTCGAACGATGCTTTCGGGCATAGTGGCGCACGGGTGCGT
AACGCGTGGGAATCTGCCCTTTGGTTCGGAATAACAGTTGGAAACGACTGCTAATACCGGATGATGACGTA
AGTCCAAAGATTTATCGCCAGAGGATGAGCCCGCGTGAGATTAGGTAGTTGGTGGGGTAAAGGCCTACCA
AGCCGACGATCTCTAGCTGGTCTGAGAGGATGATCAGCCACACTGGGACTGAGACACGGCC
>Zotu14313
CCCCTGTCTCCTCTGGGCACACAGCACTAGCACCCAGGATGGCGTGGGAATAGTTGTACTGAGCAACAAGT
CAAGATCTTGCTTATCCGGGGGCCACTGTCCTGCACCGTCTGCCAGAACTGACG
>Zotu14318
GAACGCTGGAAATGTGGATACTGGCATGCAAGTCAAGGGGCCGCAAGGCACCGGCGAACGGCTTAGTAA
CACGTAGATACATACCGTAAAGTGGGGCATAGCTCGTCGAAAGACGGGGTAATTTCCCATGTGAACGCAA
GTTTAAAGGCGCAAGCCGCTTTACGAATGGTCTGCGGTCTATCAGCTAGTTGGTGAGGTAAAGGCTCACCA
AGGCTATGACGGATAGGGGGCGTGAGAGCGCGACCCCCAAGAATGGCACTGCGACACGGGC
>Zotu14321
GAACGCTGTCGGCGTGATTAAGACATGCAAGTCGAACGGTGCAGCAATGCACAGTGGCGCACGGGTGAG
TAAGGCATCGCTAATCTACCCCGGGGTCTGGGATAGCCAGCCGAAAGGCTGGGTAATACCAGATGAGACC
ACGTCCCGGCATCGGGATGTGCTCAAAGGGCAACTGCCCTGGGATGAGGCGATGTGGTATCAGCTAGTTG
GTGGGGTAACGGCCCACCAAGGCTTCGACGCCTAGCCGGACTGAGAGGTCGGCCGGCCACATCGGGACT
GAGACACTGCC

>Zotu14325
GAACGCTGGCGGCAGGCTTAACACATGCAAGTCGAACGGGTGTAGCAATACATCAGTGGCAGACGGGTG
AGTAACACGTGGGAACCTTCCTAGAGGTACGGAACAACGCAGGGAAACTTGCGCTAATACCGTATACGCTC
TTGCGAGGAAAGATTTATCGCCTTTAGACGGGCCCGCGTAGGATTAGGTAGTTGGTGAGGTAATGGCTCA
CCAAGCCTACGATCCTTAGCTGATCTGAGAGGATGATCAGCCACACTGGAACTGAGACACGGTC
>Zotu14329
GAACGCTAGCGGCAGGCTTAACACATGCAAGTCGAGGGGTATAGTTCTTCGGAACTAGAGACCGGCGCAC
GGGTGCGTAACGCGTATGCAATCTACCTTTTACAGAGGGATAGCCCAGAGAAATTTGGATTAATACCTCAT
AGTATAATGACTCGGCATCGAGATATTATTAAAGTCACAACGGTAAAAGATGAGCATGCGTCCCATTAGCT
AGTTGGTAAGGTAACGGCTTACCAAGGCTACGATGGGTAGGGGTCCTGAGAGGGAGATCCCCCACACTGG
TACTGAGACACGGCC
>Zotu14333
GAACGCTGGCGGCAGGCCTCAAACATGCAAGTCTAGGGGGTGTAGCAATACATCACCGGCGGACGGGTG
CGTAACACGTGGGAATGTACCTAGTGGTTCGGAACAACTCAGGGAAACTTGAGCTAATACCGGATGAGCC
CGAAAGGGGAAAGATTTATCGCCACTAGATCAGCCCGCGTAGGATTAGCTAGTTGGTAGGGTAACGGCCT
ACCAAGGCGACGATCCTTAGCTGTTCTGAGAGGAAGATCAGCCACACTGGGACTGAGACACGGCC
>Zotu14338
AAACGCTGGCGGCGCACATAAGACATGCAAGTCGAACGGACTTAATCGATTCTTTTAGATTCGAAGCGGTT
AGTGGCGGACTGGTGAGTAACACGTAAGCAACCTGCCTATTAGAGGGGAATAACAGTGAGAAATCACTGC
TAAAACCGCATATGCCGTGAACATCGCATGATGAAAACGGGAAAAGAGCAATCTGCTAATAGATGGGCTT
GCGTCTGATTAGCTAGTTGGTGTGGTAAAGGCATACCAAGGCAACGATCAGTAGCCGGACTGAGAGGTTG
AACGGCCACATTGGGACTGAGATACGGCC
>Zotu14340
GAACGCTAGCGACAGGCCTAACACATGCAAGTCGAGGGGTAACAGGGGCTTCGGCCCGCTGACGACCGG
CGCACGGGTGAGTAACGCGTATGCAACCTACCTGTAACTGGGGGATAGCTCCCTGAAAGGGGAATTAACA
CCGCATAAGACCACGCTATCACATGGTAAAGTGGTAAAATATTTATAGGTTACAGATGGGCATGCGTGACA
TTAGTTAGTTGGCGGGGTAACGGCCCACCAAGACTACGATGTCTAGGGGTTCTGAGAGGAAGGTCCCCCA
CACTGGTACTGAGACACGGAC
>Zotu14343
GAACGCTGGCGGCATGCCTAACACATGCAAGTCGAACGAAGGCTTCGGCCTTAGTGGCGCACGGGTGAGT
AACACGTGGGAACCTGCCTTTCGGTTCGGAATAACGTTTGGAAACGAACGCTAACACCGGATACGCCCTTC
GGGGGAAAGTTTACGCCGAGAGAGGGGCCCGCGTCCGATTAGGTAGTTGGTGAGGTAACGGCTCACCAA
GCCGACGATCGGTAGCTGGTCTGAGAGGATGATCAGCCACACTGGAACTGAGACACGGTC
>Zotu14345
GAACGCTGGCGGCATGCCTAACACATGCAAGTCGAACGAAGGCTTCGGCCTTAGTGGCGCACGGGTGCGT
AACGCGTGGGAATCTGCCCTTGGGTTCGGAATAACAGCGGGAAACTGCTGCTAATACCGGATGATGACGT
AAGTCCAAAGATTTATCGCCCAAGGATGAGCCCGCGTAGGATTAGGTAGTTGGTGAGGTAAAGGCTCACC
AAGCCGACGATCCTTAGCTGGTCTGAGAGGATGATCAGCCACACTGGGACTGAGACACGGCC
>Zotu14348
GAACGCTAGCGGCAGGCCTAATACATGCAAGTCGGACGGGATTGGAGAGCTTGCTTTCCATGAGAGTGGC
GCACGGGTGCGTAACACGTATGTAACCTACCTTTATCAGGGGGATAGCCTCTCGAAAGAGAGATTAAGCCC
GCATAATATGAAGACACAGCATTGTGATTTCATCAAATATTTATAGGATAAAGATGGGCATGCGGAACATT
AGCTAGTTGGTAAGGTAATGGCTTACCAAGGCTACGATGTTTAGGGGATCTGAGAGGATGACCCTCCACAC
TGGTACTGAGACACGGAC
>Zotu14355
ACCGTGTCTCAGTTCCAGTGTGGCTGGTCATCCTCTCAGACCAGCTAGGGATCGTCGCCTAGGTGAGCCGT
TACCCCACCTACTAGCTAATCCCATCTGGGCACATCCGATGGCAAGAGGCCCGAAGGTCCCCCTCTTTGGTC
TTGCGACGTTATGCGGTATTAGCTACCGTTTCCAGTAGTTATCCCCCTCCATCAGGCAGTTTCCCAGACATTA
CTCACCCGTCCGCCACTCGTCAGCAAAGAAGCAAGCTTCTTCCTGTTACCGTTCGACTTGCATGTGTTAGGC
CTGCCGCCAGCGTTCA

>Zotu14360
GAACGCTGGCGGCAGGCTTAACACATGCAAGTCGAACGCTCGTCTTCGGACGGGAGTGGCAGACGGGTG
AGTAACACGTGGGAACGTACCCTTCGATCTGGGATAACTTCGGGAAACTGGAGCTAATACCGGATACGCCC
TTTTGGGGAAAGGTTTACTGTCGAAGGATCGGCCCGCGTCTGATTAGCTAGTTGGTGGGGTAACGGCCTAC
CAAGGCGACGATCAGTAGCTGGTCTGAGAGGATGATCAGCCACACTGGGACTGAGACACGGCC
>Zotu14363
GAACGCTGGCGGCGTGCTTAACACATGCAAGTCGAACGATGAACCAGAGCTTGCTCTGGGGATTAGTGGC
GAACGGGTGAGTAACACGTGAGTAACCTGCCCTTGACTCTGGGATAAGCGTTGGAAACGACGTCTAATAC
CGGATATGAACTGTGACGGCATCGTCTGCAGTTGGAAAGAATTTTGGTCAAGGATGGACTCGCGGCCTATC
AGGTAGTTGGTGAGGTAACGGCTCACCAAGCCGACGACGGGTAGCCGGCCTGAGAGGGTGACCGGCCAC
ACTGGGACTGAGACACGGCC
>Zotu14365
GAACGTTGGCGGCATGCCTTACACATGCAAGTCGAACGGCAGCATAGGAGCTTGCTCCTGATGGCGAGTG
GCGAACGGGTGAGTAATATATCGGAACGTGCCCTAGAGTGGGGGATAACTAGTCGAAAGACTAGTTAATA
CCGCATACGATCTACGGATGAAAGTGGGGGATCGCAAGACCTCATGCTCCTGGAGCGGCCGATATCTGATT
AGCTAGTTGGTGGGGTAAAAGCTCACCAAGGCGACGATCTTTAGTTGGTCTGAGAGGATGATCAGCCACA
CTGGAACTGAGACACGGTC
>Zotu14367
GAACGCTGGCGGCGTGCTTAACACATGCAAGTCGAACGGTGACATCAGAGCTTGCTCTGGTTGATCAGTG
GCGAACGGGTGAGTAACACGTGAGTAACCTGCCCTTGACTCTGGGATAACTCCGGGAGACCGGGGCTAAT
ACCGGATACGAGACGCGACCGCATGGTCGGCGTCTGGAAAGTTTTTCGGTCAAGGATGGACTCGCGGCCT
ATCAGCTAGTTGGTGAGGTAACGGCTCACCAAGGCGTCGACGGGTAGCCGGCCTGAGAGGGCGACCGGC
CACACTGGGACTGAGACACGGCC
>Zotu14375
GAATGGTGGCAGCGTGGATGGGTCATGCAAGTCAAGGGGGCCGCAAGGCAACCGGCAAACGAGGTAGTA
ACACGTAGGTAGATCCCCCCGAGTCGGGCATAGCTTGCCGAAAGGCAAGGTAATTCCCGATGGTCCCGCA
AGGGTAAAGTTTTTCGCTCGGGGAGTCGCCTGCGTAGTATCATCTAGTTGGTAGGGTAACGGCCTACCAAG
GCTACGACGCTTAGGGGGGGTGAGAGCCCGATCCCCACCGATGGGACTGCGACACGGCC
>Zotu14377
GAACGCTGGCGGCATGCCTAACACATGCAAGTCGAACGAGACCTTCGGGTCTAGTGGCGCACGGGTGCGT
AACGCGTGGGAATCTACCCTTAGGTTCGGAATAACTCAGAGAAATTTGAGCTAATACCGGATGATGACGTA
AGTCCAAAGATTTATCGCCTAAGGATGAGCCCGCGTAGGATTAGCTAGTTGGTGTGGTAAAAGCGCACCA
AGGCGACGATCCTTAGCTGGTCTGAGAGGATGATCAGCCACACTGGGACTGAGACACGGCC
>Zotu14381
GAACGCTGGCGGAATGCTTTACACATGCAAGTCGAGCGGCAGCGCGGGGCAACCTGGCGGCGAGCGGCG
AACGGGTGAGTAATGTATCGGAACGTACCTAGTAATGGGGGATAACACAGCGAAAGCTGTGCTAATACCG
CATACGCCCTGAGGGGGAAAGCGGGGGACTCGTAAGGGCCTCGCGTTATTGGAGCGGCCGATATCGGATT
AGCTAGTTGGTGAGGTAAAGGCTCACCAAGGCGACGATCCGTAGCTGGTCTGAGAGGACGACCAGCCACA
CTGGAACTGAGACACGGTC
>Zotu14383
GAACACTGGCGGCGTGCTTAACACATGCAAGTCGAACGGAAAGGCCCTGCTTTTGTGGGGTGCTCGAGTG
GCGAACGGGTGAGTAACACGTGAGTAACCTGCCCTTGACTTTGGGATAACTTCAGGAAACTGGGGCTAAT
ACCGGATAGGAGCTCCTGCTGCATGGTGGGGGTTGGAAAGTTTCGGCGGTTGGGGATGGACTCGCGGCTT
ATCAGCTTGTTGGTGGGGTAGTGGCTTACCAAGGCTTTGACGGGTAGCCGGCCTGAGAGGGTGACCGGCC
ACACTGGGACTGAGACACGGCC
>Zotu14386
GAACGCTGGCGGCGTGCTTAACACATGCAAGTCGAACGGAAAGGCCCTGCTTTTGTGGGGCGCTCGAGTG
GCGAACGGGTGAGTAACACGTGAGTAACCTGCCCTTGACTTTGGGATAACTTCAGGAAACTGGGGCTAAT
ACCGGATAGGAGCTCCTGCTGCATGGTGGGGGTTGGAAAGTTTCGGCGGTTGGGGATGGACTCGCGGCTT

ATCAGCTTGTTGGTGGGGTAGTGGCTTACCAAGGCTTTGACGGGTAGCCGGCCTGAGAGGGTGACCGGCC
ACACTGGAACTGAGACACGGTC
>Zotu14390
GAACGCTGGCGGCGTGCTTAACACATGCAAGTCGAACGAGCAAGAGCTGTAGCAATACAGGGTTCTGCTA
GTGGCAGACGGGTGAGTAATACATGGGAATCTACCTTGCAGTGCGAAATAACAGTTGGAAGCAATTGCTA
ATGTCGCATATAGCCGAGAGGTGAAAGGTTTACTGCTGCAAGATGGGCCCATGCAGGATTAGCTAGTTGG
TGAGGTAATGGCTCACCAAGGCAACGATCTTTAGTTGGTCTGAGAGGATGATCAGCCACACTGGGACTGA
GACACGGGC
>Zotu14392
GAACGCTGGCGGCGTGCTTAACACATGCAAGTCGAGCGGTAAGGCTCCTTCGGGAGTACACGAGCGGCGA
ACGGGTGAGTAACACGTGAGCAATCTGCCCTTCACACGGGGATAACTCCGGGAAACCGGTGCTAATACCC
GATACGACCACTTCAGGCATCTGATGGTGGTGGAAAGTTCCGGCGGTGAAGGATGAGCTCGCGGCCTATC
AGCTTGTTGGTGGGGTAACGGCCCACCAAGGCAACGACGGGTAGCCGGCCTGAGAGGGTGACCGGCCAC
ACTGGGACTGAGACACGGCC
>Zotu14395
GAACGCCGGCGGCGTGCTTAACACATGCAAGTCGAACGAGCAAGAGCTGTAGCAATACAGGGTTCTGCTA
GTGGCAGACGGGTGAGTAATACATGGGAATCTACCTTGCAGTGCGAAATAACAGTTGGAAGCAATTGCTA
ATGTCGCATATAGCCGAGAGGTGAAAGGTTTACTGCTGCAAGATGGGCCCATGCAGGATTAGCTAGTTGG
TGAGGTAATGGCTCACCAAGGCAACGATCTTTAGTTGGTCTGAGAGGATGATCAGCCACACTGGGACTGA
GACACGGCC
>Zotu14399
GAACGCTGGCGGCGTGCCTAATACATGCAAGTCGAGCGGTAACACTTCGGGTGACGAGCGGCGGACGGC
TGAGTAACGCGTAGGAATGTACCCCAGAGAGAGGGATAGCCCACCGAAAGGTGGATTAATACCTCATACA
CTCTATGGAGCAAAGCAGCAATGCGCTTTGGGAACAGCCTGCGTCTGATTAGCTTGTTGGTAAGGTAAAAG
CTTACCAAGGCGACGATCGGTAGCTGGTCTGAGAGGATGATCAGCCAGACTGGGACTGAGACACGGCC
>Zotu14401
GAACGCTGGCGGCGTGCCTAAGACATGCAAGTCGAGTGGGTTTAGACCCACGGCGAACGGGTGAGTAAT
ACCTAGGAACTTTTTTTAAGTGGAGCATAACCAGTCGAAAGATTGGATAATACTCCATATTATCGTAAGATG
AAAGCAGCAATGCGCTTAAAATGAGGCCTGGGCACCATCAGCTAGTTGGTAAGGTAATGGCTTACCAAGG
CTTTGACGGTTAGGGGTCCCGAGAGGGATAACCCCCACACTGGAACTGCGACACGGTC
>Zotu14404
GAACGCTAGCGGCATGCTTAACACATGCAAGTCGAGCGGTAAGGCCCTTCGGGGTACACGAGCGGCGAAC
GGGTGAGTAACACGTGGGTGACCTGCCCTCAGCTCTGGGATAAGCCTGGGAAACCGGGTCTAATACCGGA
TAGGACTACATGCTTCATGGTGTGTGGTGGAAAGTTTTTCGGCTGGGGATGGGCCCGCGGCCTATCAGCT
TGTTGGTGGGGTGATGGCCTACCAAGGCGACGACGGGTAGCCGGCCTGAGAGGGCGACCGGCCACACTG
GGACTGAGACACGGCC
>Zotu14406
GAACGCTGGCGGCGTGCTTAACACATGCAAGTCGAGCGGTAAGGCTCTTCGGAGTACACGAGCGGCGAAC
GGGTGAGTAACACGTGAGCAACCTACCCTTGGCACTGGGATAACGACTGGAAACAGTCGCTAATACCGGA
TACGACCTCGAATGACATCGCTCGAGGTGGAAAGTTCCGGCGGCCATGGATGGGCTCGCGGCCTATCAGC
TTGTTGGTGAGGTAACGGCTCACCAAGGCGTCGACGGGTAGCCGGCCTGAGAGGGCGACCGGCCACACT
GGGACTGAGATACGGCC
>Zotu14408
GAACGCTGGCGGCGTGCTTAACACATGCAAGTCGAACGCTGAACTCCGCTTGCGGAGGGATGAGTGGCGA
ACGGGTGAGTAACACGTGGGCAACCTGCCCCCGGCTCTGGGATAACTCCAAGAAATTGGGGCTAATACCG
GATGGTCACCGGCTACCGCATGGTGGTGGGTGGAAAGGATTTCCGGCTGGGGATGGGCCCGCGGCCTATC
AGCTTGTTGGTGGGGTGATGGCCTACCAAGGCGACGACGGGTAGCCGGCCTGAGAGGGTGACCGGCCAC
ACTGGGACTGAGACACGGCC
>Zotu14416

GAACGCTGGCGGCGCGCTTAACACATGCAAGTCGAGCGAGAACCAGTCCTTCGGGGCTGGGGACAGCGG
CGAACGGGTGAGTAACACGTGGGTAATCTGCCCTCGGTACCGGGATAGCCCGGGGAAACTCGGATTAATA
CCGGATGGCCTCTCGAGCCTTCGAGCTCGTGAGAAAAGGTAGCTTCGGCCTCCGACCGAGGAGGAGCCCG
CGGCGGATTAGCTTGTTGGCGGGGTAACGGCCCACCAAGGCGACGATCCGTAGCTGGTCTGAGAGGATGA
TCAGCCACACTGGGACTGAGACACGGCC
>Zotu14418
GAACGCTGGCGGCGTGCTTAACACATGCAAGTCGAACGAGCAAGAGCTGTAGCAATACAGGGTTCTGCTA
GTGGCAGACGGGTGAGTAATACATGGGAATCTACCTTGCAGTGCGAAATAACAGTTGGAAGCAATTGCTA
ATGTCGCATATAGCCGAGAGGTGAAAGGTTTACTGCTGCAAGATGGGCCCATGCAGGATTAGCTAGTTGG
TGAGGTAATGGCTCACCAAGGCAACGATGTTTAGTTGGTCTGAGAGGATGATCAGCCACACTGGGACTGA
GACACGGTC
>Zotu14424
GAACGCTGGCGGCGTGCCTAACACATGCAAGTCGAACGAAGCTTGATTTCTGATTTTTTCGGAATGACGAA
TGATATGACTGAGTGGCGGACGGGTGAGTAACGCGTGAGCAACCTGCCCTTCGGAACGGGATAGTGTCTG
GAAACGGACAGTAATACCGTATAACATATATTGATCGCATGGTTGATATATCAAAACTGAGGTGCCGAAGG
ATGGGCTCGCGTCTGATTAGATAGTTGGTGGGGTAACGGCCTACCAAGTCGACGATCAGTAGCCGGACTG
AGAGGTTGAACGGCCACATTGGGACTGAGACACGGCC
>Zotu14431
GAACGCTGGCGGCGTGCCTAATACATGCAAGTCGAGCGAATCTTTGGGAGCTTGCTCTCAAAGGTTAGCG
GCGGACGGGTGAGTAACACGTGGGCAACCTGCCTATAAGACTGGGATAACTTCGGGAAACCGGAGCTAAT
ACCGGATAATTCTTTTCTACACATGTAGAAAAGCTAAAAGACGGTTTACGCTGTCACTTATAGATGGGCCCG
CGGCGCATTAGCTAGTTGGTGAGGTAACGGCTCACCAAGGCCACGATGCGTAGCCGACCTGAGAGGGTGA
TCGGCCACACTGGGACTGAGACACGGCC
>Zotu14434
GAATGCTGGCGGCGTGCCTAATACATGCAAGTCGAGCGGTAAGGCCCTTCGGGGTACACGAGCGGCGGA
CGGCTGAGTAACGCGTGGGAACATGCCCCAAAGTGAGGGATAACGCACCGAAAGGTGTGCTAATACCGCA
TATGGTCTTCGGATTAAAGGATTTATCCGCTTTGGGAGTGGCCCGCGTACGATTAGTTTGTTGGTGAGGTA
ATGGCTCACCAAGACTACGATCGTTAACTGGTCTGAGAGGATGATCAGTCAGACTGGAACTGAGACACGG
TC
>Zotu14437
GAACGCTGGCGGCGTGCTTAACACATGCAAGTCGAACGATGAAGCCCACTTGTGGGTGGATTAGTGGCGA
ACGGGTGAGTAACACGTGAGTAACCTGCCCTTGACTCTGGGATAAGCCTGGGAAACTGGGTCTAATACCG
GATATGACCTTCCATCGCATGGTGGTTGGTGGAAAGCTTTTGTGGTTTTGGATGGACTCGCGGCCTATCAG
CTTGTTGGTGGGGTAATGGCCTACCAAGGCGACGACGGGTAGCCGGCCTGAGAGGGTGACCGGCCACACT
GGGACTGAGACACGGCC
>Zotu14443
GAACGCTGGCGGCAGGCTTAACACATGCAAGTCGAACGGTAGCAGGAGAAAGCTTGCTTTCTTGCTGACG
AGTGGCGGACGGGTGAGTAATGCTTGGGAATCTGGCTTATGGAGGGGGATAACCATTGGAAACGATGGC
TAATACCGCGTAATATCGGGAGATTAAAGACTGGGACCGCAAGGCCAGTTGCCATAAGATGAGCCCAAGT
GGGATTAGGTAGTTGGTGGGGTAAAGGCCTACCAAGCCTTCGATCTCTAGCTGGTCTGAGAGGATGACCA
GCCACACTGGAACTGAGACACGGTC
>Zotu14448
TAACGCTAGCGGCAGGCCTAATACATGCAAGTTGTACGGGATTATTGGTAGCAATACTGATATGAGAGTAG
CGCACGGGTGCGTAACACGTATGCAACCTACCTTTAACTGGGGAATAGCTCACCGAAAGGTGAATTAACAC
CGCATAAAATCACAGAAGGACATCCTTTAATGATTAAATCTCCGGAGGTTAAAGATGGGCATGCGTCCGAT
TAGCTAGTTGGCGGGGTAATAGCCCACCAAGGCAACGATCGGTAGGGGGTCTGAGAGGATGATCCCCCAC
ACTGGTACTGAGACACGGAC
>Zotu14451
GAACGCTAGCGGCATGCTTAACACATGCAAGTCGAACGAGACCAGAGACTTCGGTTTCTGGTTTAGTGGCG
CACGGGTGCGTAACGCGTATGCAACCTACCTTCCACAGGGGGATAGCCCACCGAAAGGTGGATTAATACC
GCATAAGACCACAGCCTGGCATCAGGCAGTGCTCAAAGATTTATCGGTGGAAGATGGGCATGCGTGCCAT

TAGCTAGATGGTGTGGGTAACGGCCCACCATGGCGACGATGGCTAGGGGTTCTGAGAGGATGGTCCCCCA
CACTGGTACTGAGACACGGAC
>Zotu14453
CAACGCTGGCGGCGTGCCTAACACATGCAAGTCGAACGCGGTTGCCCCGCAAGGGGTAACTGAGTGGCGA
ACGGGTGAGTAACACGTGGGTGACCTACCCTCGAGTGGGGGATAACATCCCGAAAGGGGTGCTAATACCG
CATGACATCCTGTCTTTGAACAGATGGACATTAAAGCCGGGGATCGCAAGACCTGGCGCTCGTGGAGGGG
CCCGCGCCCGATTAGCTAGTTGGTGAGGTAACGGCTCACCAAGGCTTCGATCGGTATCCGGCCTGAGAGG
GCGGACGGACACACTGGGACTGAGACACGGCC
>Zotu14459
GAACGCTGGCGGCAGGCCTAACACATGCAAGTCGAGCGGATGAGTGGAGCTTGCTCCATGATTCAGCGGC
GGACGGGTGAGTAATGCCTAGGAATCTGCCTGGTAGTGGGGGACAACGTTTCGAAAGGAACGCTAATACC
GCATACGTCCTACGGGAGAAAGCAGGGGATCTTCGGACCTTGCGCTAATAGATGAGCCTAGGTCGGATTA
GCTAGTTGGTGAGGTAAAGGCTCACCAAGGCGACGATCCGTAACTGGTCTGAGAGGATGATCAGTCACAC
TGGAACTGAGACACGGTC
>Zotu14461
GAACGCTGGCGGCAGGCTTAACACATGCAAGTCGAACGAACCCCTCGGGGTTAGTGGCGGACGGGTGAG
TAACACGTGGGAACGTGCCCTTTGGTTCGGAACAACACAGGGAAACTTGTGCTAATACCGGATGTGCCCTT
CGGGGGAAAGATTTATCGCCATTGGAGCGGCCCGCGTCTGATTAGCTAGTTGGTGAGGTAAAGGCTCACC
AAGGCGACGATCAGTAGCTGGTCTGAGAGGATGACCAGCCACATTGGGACTGAGACACGGCC
>Zotu14463
GAACGCTGGCGGCGTGCCTAATACATGCAAGTAGAACGCTGAAGGAGGAGCTTGCTTCTCTGGATGAGTT
GCGAACGGGTGAGTAACGCGTAGGTAACCTGCCTGGTAGCGGGGGATAACTATTGGAAACGATAGCTAAT
ACCGCATAATATTGATTATTGCATGATAGTCAATTAAAAGGTGCAACTGCACCACTACCAGATGGACCTGC
GTTGTATTAGCTAGTTGGTGGGGTAACGGCTCACCAAGGCGACGATACATAGCCGACCTGAGAGGGTGAT
CGGCCACACTGGGACTGAGACACGGCC
>Zotu14465
GAACGCTGGCGGCGTGCTTAACACATGCAAGTCGAACGAGCAAGAGCTGTAGCAATACATGGTTCTGCTA
GTGGCAGACGGGTGAGTAATACATGGGAATCTACCTTGCAGTGCGAAATAACAGTTGGAAGCAATTGCTA
ATGTCGCATATAGCCGAGAGGTGAAAGGTTTACTGCTGCAAGATGGGCCCATGCAGGATTAGCTAGTTGG
TGAGGTAATGGCTCACCAAGGCAACGATCTTTAGTTGGTCTGAGAGGATGATCAGCCACACTGGGACTGA
GACACGGCC
>Zotu14467
GAACGCTGGCGGCGTGCCTAACACATGCAAGTCGAACGTGAAAGGGAGCAATCCTGAGTAAAGTGGCGT
ACGGGTGAGTAACGCGTAGGTAATCTACCCCAAAATCTGGAATAACCTGCCGAAAGGCGGGCTAATACTG
GATAGAGCGACGGAATCCGTAATTGGGTTTTTCGGGAAAGGGAGCTCGCAAGAGCCTTCGTTTTGGGATG
AGCCTGCGTCTCATTAGCTAGTTGGTGAGGTAAAAGCTCACCAAGGCTTTGATGGGTAGCTGGTCTGAGAG
GATGATCAGCCACACTGGAACTGAAACACGGTC
>Zotu14469
GAACGCTGGCGGCACGCCTAACACATGCAAGTCGAACGGACTTAAAAGAGCTTGCTTTTTTAAGTTAGTGG
CGGACGGGTGAGTAACACGTGAGCAACCTGCCCCTGAGAGGGGGATAGCTTCTGGAAACGGATGGTAAT
ACCCCATAAAATATAAATATCGCATGATAGATATATCAAAGAAATTCGCTCAGAGATGGGCTCGCGTCTGA
TTAGATAGTTGGTGAGGTAACGGCTCACCAAGTCGACGATCAGTAGCCGGACTGAGAGGTTGAACGGCCA
CATTGGGACTGAGACACGGCC
>Zotu14471
CAACGCTGGCGGCGTGCCTAATGCATGCAAGTCGAACGGGGTCCCCTTCGGGGGGCTTGCGTGGCGGACT
GGTGAGGAACACGTGGCCAACCTGCCCACTGGCGGGGGGTAGCCCGCGGAAACGCGGGGTAATTCCGCA
TGAACGGCGCGACCGGGTAGGGTCGTGGCGCAAAGGTCCTTCGGGGCCGCCGGTGGAGGGGGCTGCGG
CCGATTAGCTAGTTGGGGGGGTAACGGCCTCCCAAGGCGATGATCGGTAGCTGGTCTGAGAGGATGATCA
GCCACACGGGGACTGAGACACGGCC
>Zotu14475

AAACGCTGGCGGCGTGCATGAGACATGCAAGTCGAACGGACGATCAAGGCTTGCTTTGATCGTTAGTGGC
GGACGGCTGAGTAACGCGTAGGTGACCTGCCCCGAAGTGGGGAATAACTGCCCGAAAGGGTGGCTAATA
CCGCATGTGGTGATGCGTTCATACGCATCAGTAAAGCCGCAAGGCGCTTCGGGAGGGGCCTGCGTCCGAT
TAGCTCGTTGGTGTGGTAACGGCGCACCAAGGCGACGATCGGTAGGTGGTCTGAGAGGACGATCACCCAG
ACTGGGACTGAGACACGGCC
>Zotu14478
CAACGCTGGCGGCGTGCCTAACACATGCAAGTCGAACGCGAAAGCCCCGCAAGGGGTGAGTAGAGTGGC
AAACGGGTGAGTAACACGTGGGTGACCTGCCTTCGAGTGGGGGATAACGTCCCGAAAGGGACGCTAATAC
CGCATGATATCCTGCCTTTGGAAAGGTGGAGATCAAAGCTGGGGATCGCAAGACCTGGCGCTTGAAGAGG
GGCCCGCGCCTGATTAGCTAGTTGGTAGGGTAACGGCCTACCAAGGCGACGATCAGTATCCGGCCTGAGA
GGGCGGACGGACACACTGGGACTGAGACACGGCC
>Zotu14483
GAACGCTAGCGGCATGCTTAACACATGCAAGTCGAGCGAACAGACGGGGAGCTTGCTCCCCCGACGTTAG
CGGCGGACGGGTGAGTAACACGTGGGTAACCTACCTATAAGACTGGAATAACTCCGGGAAACCGGGGCTA
ATGCCGGATAACATGTTGGACCGCATGGTCCTACAGTGAAAGACGGTCTTGCTGTCACTTATAGATGGACC
CGCGCCGTATTAGCTAGTTGGTAAGGTAACGGCTTACCAAGGCAACGATACGTAGCCGACCTGAGAGGGT
GATCGGCCACACTGGAACTGAGACACGGTC
>Zotu14489
GAACGCTGGCGGCGTGCCTTAAGCATGCAAGTAGAACGACCCCGATAGCAATATCGGGATAGTTGCGAAC
GGCTGAGTAATGAGTAAGAATCTTTCCTTTAGTGGGGAATACCTCATCGAAAGATGAGCTAATACCGCATA
ATACCTCCGGGTGAAAGCGATTAACTTCGCGCTGATTGATGAGCTTACTCCCTATCAGGTAGTTGGTGGGG
TAATGGCCTACCAAGCCTACGACGGGTACCCGATCTGAGAGGATGGACGGGCAGAATGGGACTGAGACA
CGGCC
>Zotu14492
GAACGCTAGCGGGAGGCCTAACACATGCAAGTCGAACGGGATTGGGAGCTTGCTCCCATGAGAGTGGCG
CACGGGTGAGTAACGCGTATGCAACCTACCTTTAACTGAGGGATAGCCCATAGAAATGTGGATTAATACCT
CATAGTTCTTTTGGCCCGCATGGGTCGATAGATAAAGCTCCGGCGGTTAAAGATGGGCATGCGTGACATTA
GCTAGTTGGTAAGGTAACGGCTTACCAAGGCTACGATGTCTAGGGGTTCTGAGAGGATGATCCCCCACACT
GGTACTGAGACACGGAC
>Zotu14499
GAACGCTGGCGGCGTGCTTAACACATGCAAGTCGAGCGGTAGCAGGCGTAGCAATACGTGCTGACGAGC
GGCGAACGGGTGAGTAATGCTTTGGAATCTGCCTAGTAGTGGGGAATAACCTGGGGAAACTCAGGCTAAT
ACCGCATACGACCTAAGGGTGAAAGCGGGGGATCGCAAGACCTCGCGCTATTAGATGAGCCAAAGTCGGA
TTAGCTAGTTGGTGGGGTAAAGGCCTACCAAGGCGACGATCCGTAGCTGGTCTGAGAGGACGACCAGCCA
CACTGGAACTGAGACACGGCC
>Zotu14503
GAACGCTGGCGGCGTGCTTAACACATGCAAGTCGAACGGGCAAGAGCTGTAGCAATACAGGGTTCTGCTA
GTGGCAGACGGGTGAGTAATACATGGGAATCTACCTTGCAGTGCGAAATAACAGTTGGAAGCAATTGCTA
ATGTCGCATATAGCCGAGAGGTGAAAGGTTTACTGCTGCAAGATGGGCCCATGCAGGATTAGCTAGTTGG
TGAGGTAATGGCTCACCAAGGCAACGATCTTTAGTTGGTCTGAGAGGATGATCAGCCACACTGGGACTGA
GACACGGCC
>Zotu14505
GAACGCTGGCGGCGCGCTTAACACATGCAAGTCGAGCGAGAACCCAGGCTTCGGCCTGGGGGAAAGCGG
CGAACGGGTGAGTATCACGTGGGTAATCTGCCCTCGACATCGGGATAGCCCGGGGAAACCCGGATTAATA
CCGAATGGCCCATCAGCTTTTCGGAGCGGATGGAAAAGGTAGCTTCGGCCTCCGGTCGAGGAGGAGCCCG
CGGCGGATTAGCTTGTTGGTGAGGTAACGGCTCACCAAGGCTCCGATCCGTAGCTGGTCTGAGAGGACGA
TCAGCCACACTGGGACTGAGACACGGCC
>Zotu14507
GAACGCTGGCGGCATGCCTAACACATGCAAGTCGAACGAGACCTTCGGGTCTAGTGGCGCACGGGTGCGT
AACGCGTGGGAATCTGCCCTTAGGTTCGGAATAACAGTGGGAAACTACTGCTAATACCGGATAATGACGTA

AGTCCAAAGATTTATCGCCTAGGGATGAGCCCGCGTAGGATTAGCTAGTTGGTGGGGTAAAAGCCTACCA
AGGCGACGATCCTTAGCTGGTCTGAGAGGATGATCAGCCACACTGGGACTGAGACACGGCC
>Zotu14510
GAACGTTGGCGGCGTGGATTAGGCATGCAAGTCGCGCGCCCCGCAAGGGGAGCGGCGCAAGGGTGAGTA
AGGCGACGGTAACCAACCCTCCGGATGGGGATAGCCGCGGGAAACTGCGGGTAATACCCAGCGATGTGC
GAGGCCGGCATCGGCCTTGTACCAAAGTTCCGGCGCCGGGGGACGGGCCGTCGTGGTATTAGCTAGTTGG
TGGGGTGACGGCCCACCAAGGCTTCGATGCCTACCGGGCGTGCGAGCGTGGCCCGGCACACTGGGACTGA
GACACTGCC
>Zotu14513
GAACGCTGGAAATGTGGATACCGGCATGCAAGTCACGGGGGCCGCAAGGCAACCGGCGAACGGCTTAGT
AACACGTAGATACATACCCAAAAGTGGGGCATAGCTCGTCGAAAGACGGGGTAATTTCCCATGTTCCCGTA
AGGGCAAAGCAGCAATGCGCTTTTGGAATGGTCTGCGGCCTATCAGCTTGTTGGTGAGGTAATGGCTCACC
AAGGCTATGACGGGTAGGGGGCGTGAGAGCGCGACCCCCAAGAATGGGACTGCGATACGGCC
>Zotu14515
GAACGCTAGCGGCAGGCTTAACACATGCAAGTCGAGGGGTATGGTAGCAATACCAGAGACCGGCAAACG
GGTGCGGAACACGTACAGAATCTACCTTTGTCTGGGGCATAGCCCGGAGAAATCCGGATTAACTCCCCATA
ATATAGATGAGTGGCATCACTTGACTATTAAAAATTTATTGGACAGAGATGACTGTGCGTCCCATTAGCTAG
TTGGTAGGGTAACGGCCTACCAAGGCGACGATGGGTAGCTGGTGTGAGAGCACGACCAGCCACACGGGC
ACTGAGACACGGGC
>Zotu14522
GAACGCTAGCGGCATGCCTAACACATGCAAGTCGAACGGCAGCGCGGGAGCAATCCTGGCGGCGAGTGG
CGAACGGGTGAGTAATACATCGGAACGTGCCCAATCGTGAGGGATAACGCAACGAAAGCTGTACTAATAC
CGCATACGATCTACGGATGAAAGCAGGGGATCGCAAGACCTTGCGCGAATGGAGCGGCCGATGGCAGAT
TAGGTAGTTGGTGAGGTAAAGGCTCACCAAGCCTTCGATCTGTAGCTAGTCTGAGAGGACGACCAGCCAC
ACTGGGACTGAGACACGGCC
>Zotu14529
GAACGCTAGCGGCAGGCCTAATACATGCAAGTCGAGGGGCAGCACGGCAGCAATGCTGGTGGCGACCGG
CGGACGGGTGCGTAACGCGTATGGAACTTGTCCGGATCTCTGGAATAGCCCCCGGAAACGGGGATTAATG
CCGGATGGCGCGCTGGTGTGGCATCACACTTTCGCTAAAGCTCAGGCGGATCCGGGTGGCCATGCGTTCG
ATTAGCTAGTTGGCGGGGTAACGGCCCACCAAGGCGACGATCGATAGGGGTCCTGAGAGGGAGGTCCCC
CACACTGGTACTGAGACACGGGC
>Zotu14532
GAACGCTGGCGGCGTGCTTAACACATGCAAGTCGAACGCTGAACTCCGCTTGCGGAGGGATGAGTGGCGA
ACGGGTGAGTAACACGTGGGCAACCTGCCCCCGGCTCTGGGATAACTCCAAGAAATTGGGGCTAATACCG
GATGGTCACCGGCTACCGCATGGTGGTGGGTGGAAAGGATTTCCGGCTGGGGATGGGCCCGCGGCCTATC
AGCTTGTTGGTGGGGTAGTGGCCTACCAAGGCGACGACGGGTAGCCGGCCTGAGAGGGTGACCGGCCAC
ATTGGGACTGAGATACGGCC
>Zotu14534
GAACGCTGGCGGTATGCTTAACACATGCAAGTCGAGGGGTATAGTAGCAATACTAGAGACCGGCGCACGG
GTGCGTAACGCGTATGCAATCTACCTTTTGCAGAGGGATAGCCCAGAGAAATTTGGATTAATACCTCATAG
TATGTGAGAATGGCATCATTTTCACATTAAAGTCACAACGGCAAAAGATGAGCATGCGTCCCATTAGCTTGT
TGGTAAGGTAACGGCTTACCAAGGCAACGATGGGTAGGGGTCCTGAGAGGGAGATCCCCCACACTGGTAC
TGAGACACGGAC
>Zotu14537
GAACGCTGGCGGTGTGCTTAACACATGCAAGTCGAACGGTGACGAGGGAGCTTGCTCCCTCTGATCAGTG
GCGAACGGGTGAGTAACACGTGAGTAACCTGCCCCAGACTCTGGGATAACATCGGGAAACCGGTGCTAAT
ACCGGATATGAGACGAGACCGCATGGTCATCGTCTGGAAAGATTTATCGGTCTGGGATGGACTCGCGGCC
TATCAGCTAGTTGGTGAGGTAATGGCTCACCAAGGCGACGACGGGTAGCCGGCCTGAGAGGGCGAACGG
CCACACTGGAACTGAGACACGGTC
>Zotu14539

GAACGCTGGCGGCATGCTTTACACATGCAAGTCGAGCGGCAGCGCGGGGGCAACCCTGGCGGCGAGCGG
CGAACGGGTGAGTAATGCATCGGAACGTGTCCCGTAGTGGGGGATAACCCAGCGAAAGCTGGGCTAATAC
CGCATATGCTCTACGGAGGAAAGGGGGGGACCCGCAAGGGCCTCCCGCTATGGGAGCGGCCGATGTCCG
ATTAGCTAGTTGGTGGGGTAAAGGCTTACCAAGGCGACGATCGGTAGCTGGTCTGAGAGGATGATCAGCC
ACACTGGGACTGAGACACGGCC
>Zotu14541
GAACGCTAGCGATAGGCTTAACACATGCAAGTCGAGGGGCAGCAGGGACTTCGGTCTGCTGGCGACCGGC
GCACGGGTGCGTAACGCGTATGCAACTTGCCTTACAGAGGGGGATAACCCGTTGAAAGACGGACTAATAC
CGCGTATTCTCAAGAGGGGGCATCCTTTTTTGAGGAAAGAAATTCGCTGTAAGATAGGCATGCGTCCCATT
AGCTAGTTGGTGAGGTAACGGCCCACCAAGGCAACGATGGGTAGGGGAACTGAGAGGTTGAACCCCCAC
ACTGGTACTGAGACACGGAC
>Zotu14544
GAACGCTGGCGGCATGCCTAACACATGCAAGTCGAACGAGATCTTCGGATCTAGTGGCGCACGGGTGCGT
AACGCGTGGGAATCTGCCCTTGGGTTCGGAATAACAGTTAGAAATGACTGCTAATACCGGATGATGTCTTC
GGACCAAAGATTTATCGCCCAAGGATGAGCCCGCGTAGGATTAGGTAGTTGGTGGGGTAAGAGCCTACCA
AGCCGACGATCCTTAGCTGGTCTGAGAGGATGATCAGCCACACTGGGACTGAGACACGGCC
>Zotu14549
GAACGCTGGCGGCAGGCTTAACACATGCAAGTCGAACGCCCCGCAAGGGGAGTGGCAGACGGGTGAGTA
ACGCGTGGGAACATACCCTTTCCTGCGGAACAACTCCGGGAAACTGGAGCTAATACCGCATACGCCCTTCG
GGGGAAAGATTTATCGGGGAAGGATTGGCCCGCGTTGGATTAGCTAGTTGGTGGGGTAAAGGCCTACCAA
GGCGACGATCCATAGCTGGTCTGAGAGGATGATCAGCCACATTGGGACTGAGACACGGCC
>Zotu14554
GAATGCTGGCTGCGTGCTTAACACATGCAAGTCGAACGGTGAAGGAGGGCTTGCCCTCCGGATCAGTGGC
GAACGGGTGAGTAACACTTGGGCAACCTGCCCCTCACTCTGGGATAACACCGGGAAACCGGTGCTAATAC
CGGATACGACCAGGCGCGACATCTCGCTTGGTGGAAAGAACTTCGGTGAGGGATGGCCCGCGGCCTATC
AGCTAGTTGGTGAGGTAATGGCTCACCAAGGCGACGACGGGTAGCCGGCCTGAGAGGGCGACCGGCCAC
ACTGGGACTGAGACACGGTC
>Zotu14562
GAACGCTGGCGGCAGGCTTAACACATGCAAGTCGAGCGGAGATGAGGTGCTTGCACCTTATCTTAGCGGC
GGACGGGTGAGTAATGCTTAGGAATCTACCTATTAGTGGGGGACAACGTTTCGAAAGGAACGCTAATACC
GCATACGTCCTACGGGAGAAAGCAGGGGACCTTCGGGCCTTGCGCTAATAGATGAGCCTAAGTCGGATTA
GCTAGTTGGTGGGGTAAAGGCCTACCAAGGCGACGATCTGTAGCGGGTCTGAGAGGATGATCCGCCACAC
TGGGACTGAGACACGGCC
>Zotu14574
CAACGCTGGCGGCGTGCCTAACACATGCAAGTCGAACGCGGTTACCTCGCAAGGGGTAACTGAGTGGCGA
ACGGGTGAGTAACACGTGGGTGACCTACCTTCGAATGGGGGATAACGTCCCGAAAGGGACGCTAATACCG
CATAACATCCTGCCTTTGAACTGGTGGAGATCAAAGCCGGGGATCGCAAGACCTGGCGCTTGAAGAGGGG
CCCGCGTCTGATTAGCTAGTTGGTGAGGTAACGGCCCACCAAGGCGACGATCAGTATCCGGCCTGAGAGG
GCGGACGGACACACTGGGACTGAGACACGGCC
>Zotu14580
GAACGCTGGTAACGTGGATACCGGCATGCAAGTCACGGGGGCCGCAAGGCAACCGGCGAACGGCTTAGT
AACACGTAGATACATACCTTAAAGTGGGGCACAGCTCGTCGAAAGACGGGGTAATTTCCCATAGTCTCGCA
AGAGTAAAGCAGCAATGCGCTTTAAGAATGGTCTGCGGCCTATCAGCTTGTTGGTGAGGTAAAAGCTCACC
AAGGCTATGACGGGTAGGGGGCGTGAGAGCGCGACCCCCAAGAATGGCACTGCGACACGGGC
>Zotu14585
GAACGCTGGCGGCATGCCTTACACATGCAAGTCGAACGGCAGCACGGACTTCGGTCTGGTGGCGAGTGGC
GAACGGGTGAGTAATATATCGGAACGTGCCCTGGAGTGGGGGATAACTAGTCGAAAGATTAGCTAATACC
GCATACGATCTTAGGATGAAAGTGGGGGATCGCAAGACCTCATGCTCGTGGAGCGGCCGATATCTGATTA
GCTAGTTGGTGGGGTAAAAGCCTACCAAGGCGTCGATCAGTAGCTGGTCTGAGAGGACGACCAGCCACAC
TGGGACTGAGACACGGCC

>Zotu14589
GAACGCTGGCGGCGTGCTTAACACATGCAAGTCGAACGGAAAGGCCCTGCTTTTGTGGGGTGCTCGAGTG
GCGAACGGGTGAGTAACACGTGAGTAACCTGCCCTTGACTTTGGGATAACTTCAGGAAACTGGGGCTAAT
ACCGGATAGGAGCTCCTGCTGCATGGTGGGGGTTGGAAAGTTTCGGCGGTTAGGGATGGACTCGCGGCTT
ATCAGCTTGTTGGTGGGGTAGTGGCTTACCAAGGCTTTGACGGGTAGCCGGCCTGAGAGGGTGACCGGCC
ACACTGGGACTGAGACACGGCC
>Zotu14591
GAACGCTGGCGGCGTGCCTAATACATGCAAGTCGAGCGGATCTTTGGGAGCTTGCTCCCAAAGATTAGCG
GCGGACGGGTGAGTAACACGTGGGCAACCTGCCTGTAAGACTGGGATAACTTCGGGAAACCGGAGCTAAT
ACCGGATAACTTCTTTTCTCGCATGAGGAGAGGTTGAAAGATGGTTTCGGCTATCACTTACAGATGGGCCC
GCGGCGCATTAGCTAGTTGGTGAGGTAACGGCTCACCAAGGCGACGATGCGTAGCCGACCTGAGAGGGT
GATCGGCCACACTGGAACTGAGACACGGTC
>Zotu14600
GAACGCTAGCGACAGGCCTAACACATGCAAGTCGAGGGGCAGCATTGAGGTAGCTTGCTACCACAGATGG
CGACCGGCGCACGGGTGAGTAACACGTATGCAACCTACCTACAACAGGGGGATAGCCCGGAGAAATTCGG
ATTAATACCGCATAATACAGGGATTCCGCATGGAAATATTTGTTAAAGAATTATCGGTTGTAGATGGGCAT
GCGTTCCATTAGGTAGTTGGTAAGGTAACGGCTTACCAAGCCATCGATGGATAGGGGAACTGAGAGGTTT
ATCCCCCACACTGGTACTGAGACACGGAC
>Zotu14604
GAACGCTGGCGGCATGCCTAACACATGCAAGTCGAACGAAGGCTTCGGCCTTAGTGGCGCACGGGTGCGT
AGCGCGTGGGAATCTGCCCTTAGGTACGGAATAACAGTGAGAAATTACTGCTAATACCGTATGATGACGTA
AGTCCAAAGATTTATCGCCTAGGGATGAGCCCGCGTAGGATTAGCTTGTTGGTGAGGTAAAGGCTCACCAA
GGCGACGATCCTTAGCTGGTCTGAGAGGATGATCAGCCACACTGGGACTGAGACACGGCC
>Zotu14606
GAACGCTGGCGGCGTGCCTTAGGCATGCAAATCGAACGGTCGCAAGACAAGTGGTGGACGGGTGAGTAA
CGCGTGGGAATCTACCTGGAAGTGGGATATAGCCAGCCGAAAGGTTGGGTAATCTCCCATGTGATCGCAA
GATCAAAGACGTAAGTCGCTTCTAGATGAGCCTGCGTCCTATCAGCTTGTTGGTGGGGTAATGGCCTACCA
AGGCAACGACGGGTAGCTGGTCTGAGAGGATGGTCAGCCACAATAGTACTGAGACACGGAC
>Zotu14610
GAACGCTGGCGGCAGGCCTAATACATGCAAGTCGAACGGCAGCACAGCAGAGCTTGCTCTGTGGGTGGCG
AGTGGCGGACGGGTGAGGAATACATGGGAATCTGCCCAGTCGTGGGGGATAACGTAGGGAAACTTACGC
TAATACCGCATGCGCCCTTCGGGGGAAAGCCGGGGATCTTCGGACCTGGCGCGATTGGATGAGCCCATGT
CGGATTAGCTAGTTGGTGGGGTAAAGGCCCACCAAGGCGACGATCCGTAGCTGGTCTGAGAGGATGATCA
GCCACACTGGAACTGAGACACGGTC
>Zotu14615
GAACGCTAGCGGCAGGCCTAATACATGCAAGTCGAACGGTCAGTAGCAATACTGATAGTGGCGCACGGGT
GCGTAACACGTAACTAACCTACCTATAACTGGGGGATAGCCCGCCGAAAGGCGGATTAATACCGCATAAGC
TGCTTGAGTGGCATCACTCGATTAGTAAAGATTTATTGGTTATAGATGGGGTTGCGGGTCATTAGCTAGTT
GGTAAGGTAACGGCTTACCAAGGCGACGATGACTAGGGGAGCTGAGAGGCTGGTCCCCCACACGGGCAC
TGAGATACGGGC
>Zotu14617
GAACGCTGGCGGCGTGCCTAATACATGCAAGTCGAGCGGTAACACTTCGGGTGACGAGCGGCGGACGGC
TGAGTAACGCGTAGGAACGTACCCCAAACTGAGGGATAAGCACCGGAAACGGTGTCTAATACCGCATGTG
ATCTTCGGATTAAAGCTTCGGCGGTTTGGGAACGGCCTGCGTATGATTAGGTTGTTGGTGAGGTAATGGCT
CACCAAGCCTACGATCATTAGCTGGTCTGAGAGGACGATCAGCCACACTGGGACTGAGACACGGCC
>Zotu14620
GAACGGTGGCGGCGTGGATAAGACATGCAAGTCGAACGGGACTTTCATTGTAGCAATATGATGAAAGTTC
AGTGGCGTAAGGGTGCGTAACACGTGGGAAATCTGCCGAGAAGTGGGGGATAGCTCGCCGAAAGGCGAA
TTAATACCGCATGTGGTTAGCGAGGACATCTTCGCAACACCAAAGCCGGGGCAACCTGGCGCTTCTTGATG

ATCCCGCGGCCTATCAGCTAGTTGGTGAGGTAACGGCTCACCAAGGCTATGACGGGTAGCTGGTCTGAGA
GGACGACCAGCCACACTGGAACTGAGACACGGTC
>Zotu14626
GAACGCTGGCGGCGTGCTTAACACATGCAAGTCGAACGAAGCACTTATCTTTGATTCTTCGGATGAAGAGG
TTTGTGACTGAGTGGCGGACGGGTGAGTAACGCGTGGGTAACCTGCCTCATACAGGGGGATAACAGTTAG
AAATGACTGCTAATACCGCATAAGACCACAGAGCCGCATGGCTCAGTGGGAAAAACTCCGGTGGTATGAG
ATGAACCCGCGTCTGATTAGGTAGTTGGTGGGGTAACGGCCTACCAAGCCAACGATCAGTAGCCGACCTG
AGAGGGTGACCGGCCACATTGGGACTGAGACACGGCC
>Zotu14633
GAACGCTGGCGGCATGCCTAACACATGCAAGTCGAACGAACCCTTCGGGGTTAGTGGCGCACGGGTGCGT
AACGCGTGGGAATCTGCCCTTCGGTTCGGAATAACAGTTAGAAATGACTGCTAATACCGGATGATGACTTC
GGTCCAAAGATTTATCGCCGAGGGATGAGCCCGCGTAGGATTAGGTAGTTGGTGGGGTAAAGGCCTACCA
AGCCGACGATCCTTAGCTGGTCTGAGAGGATGATCAGCCACACTGGGACTGAGACACGGCC
>Zotu14636
CAACGCTGGCGGCGTGCCTAACACATGCAAGTCGAGCGAGAAAGGGTAGCAATACCTGAGTAAAGCGGC
GCACGGGTGAGTAACACGTGGGTAATCTGCCCTAGAGTGGGGAATAACACCGGGAAACCGGTGCTAATAC
CGCATACGATCATGATGTCTTCGGATGTTGTGATGAAAGGAGCAATCCGCTCTTGGAGGAGCCCGCGGCTG
ATTAGCTAGTTGGTGGGGTAATGGCTCACCAAGGCTACGATCAGTATCCGGTCTGAGAGGACGGTCGGAC
ACACTGGAACTGAGACACGGTC
>Zotu14640
GAACGCTAGCGGCATGCCTAACACATGCAAGTCGAACGCGAAAGTCCCGCAAGGGATCAGTAGAGTGGCA
AACGGGTGAGTAACACGTGGGCGACCTACCTTCGAGTGGGGGATAACCTTCCGAAAGGAGGGCTAATACC
GCATGACATCCCATGTTTGGATACATGGACATCAAAGCCGGGGATCGCAAGACCTGGCGCTTGAAGAGGG
GCCTGCGCCCGATTAGCTAGTTGGTGGGGTCATGGCTCACCAAGGCTTCGATCGGTATCCGGCCTGAGAG
GGCGGACGGACACACTGGAACTGAGACACGGTC
>Zotu14643
GAACGCTGGCGGCGTGCTTAACACATGCAAGTCGAACGGTGATCTTGGGAGCTTGCTCCTTAAGTGATCAG
TGGCGAACGGGTGAGTAACACGTGAGTAACCTGCCCCAGACTCTGGAATAACCCCGGGAAACCGGAGCTA
ATACCGGATATGACACCAATCCGCATGGGTATGGTGTGGAAAGTTTTTCGGTCTGGGATGGACTTGCGGCC
TATCAGCTTGTTGGTGAGGTAATGGCTCACCAAGGCGACGACGGGTAGCCGGCCTGAGAGGGTGTCCGGC
CACACTGGGACTGAGATACGGCC
>Zotu14648
GAACGCTAGCGACAGGCTTAACACATGCAAGTCGAGGGGCAGCGGTGGAGAGAGCTTGCTCTCTTCAGCC
GGCGACCGGCGCACGGGTGAGTAACACGTATGCAACCTGCCCTTGACACTGGGATAACCCGGAGAAATCC
GGACTAATACCGGGCGATACCCCTGGAGGACATCCTCCGGAGGTCGAAGGAAGCGATTCCGGTCAAGGAT
GGGCATGCGGCGCATTAGCTAGTTGGCGGGGTAACGGCCCACCAAGGCGACGATGCGTAGGGGTTCTGA
GAGGAAGGTCCCCCACATTGGTACTGAGACACGGAC
>Zotu14652
GAACGCTGGCGGCGTGCTTAACACATGCAAGTCGAACGGGATCCAAGCGAGCTTGCTTGTTTGGTGAGAG
TGGCGAACGGGTGAGTAACACGTGAGTAACCTGCCCCCTTCTTTGGGATAACGACCAGAAATGGTTGCTAA
TACTGAATATTCACGCATGATCGCATGGTTGTGTGTGGAAAGGTTTTTCTGGTGGGGGATGGGCTCGCGGC
CTATCAGCTTGTTGGTGGGGTGATGGCCTACCAAGGCTTTGACGGGTAGCCGGCCTGAGAGGGTGACCGG
TCACACTGGGACTGAGATACGGCC
>Zotu14656
GAACGCTGGCGGCGTGCCTAATACATGCAAGTCGTGCGGGCTTGTCTTCGGATAAGCCAGCGGCGGACGG
GTGAGTAACACGTGGGCAACCTGCCTGTAAGACCGGGATAACTCCGGGAAACCGGAGCTAATACCGGATA
GGATCACAGACCGCATGGTCTGTGATGGAAAGGCCATGAGCCGCTTACAGATGGGCCCGCGGCGCATTAG
CTAGTTGGTAGGGTAACGGCCTACCAAGGCGACGATGCGTAGCCGACCTGAGAGGGTGACCGGCCACACT
GGGACTGAGACACGGCC
>Zotu14659

GAACGCTGGCGGCAGGCTTAACACATGCAAGTCGAGCGGGCATAGCAATATGTCAGCGGCAGACGGGTG
AGTAACACGTGGGAACGTACCTTTTGGTTCGGAACAACTGAGGGAAACTTCAGCTAATACCGGATAAGCCC
TTACGGGGAAAGATTTATCGCCGAAAGATCGGCCCGCGTCTGATTAGCTAGTTGGTAGGGTAATGGCCTAC
CAAGGCGACGATCAGTAGCTGGTCTGAGAGGATGATCAGCCACACTGGGACTGAGACACGGCC
>Zotu14663
GAACGCTGGCGGCATGCCTAACACATGCAAGTCGAACGGCAGCACAGTAAAGATTTCGGTCTTTACGGGT
GGCGAGTGGCGGACGGGTGAGTAAAGCGTAGGAATCTGTCCTGGAGAGTGGAATAACCCGAGGAAACTC
GGGCTAATACCGCATGATATCGAGAGATCAAAGCAGGGGACCCTTCGGGGCCTTGCGCTCTAGGATGAGC
CTACGTCGGATTAGGTAGTTGGTGAGGTAATGGCTCACCAAGCCGACGATCCGTAGCTGGTCTGAGAGGA
TGACCAGCCACACTGGGACTGAGACACGTCC
>Zotu14665
GAACGCTGGCGGTGTGCTTAACACATGCAAGTCGAGGGGCAGCATAGAGTCAGCTTGCTGATTTGGATGG
CGACCGGCGGATGGGTGCGTAACGCGTATGCAACTTACCTGTCAGAGGGGGATAACCCGGAGAAATTCGG
ACTAATACCGCATATACTTGAGATGCTGCATGGTATTTCAAGGAAATATTTATAGCTGACAGATAGGCATGC
GTTCCATTAGCTAGTAGGTGAGGTAACGGCTCACCTAGGCGACGATGGATAGGGGAACTGAGAGGTTAAA
CCCCCACACTGGTACTGAGACACGGAC
>Zotu14671
GAACGCTGGCGGCAGGCTTAACACATGCAAGTCGAACGGGCACTTCGGTGCTAGTGGCAGACGGGTGAG
TAACACGTGGGAACGTACCCTTAGGTTCGGAACAACACAGGGAAACTTGTGCTAATACCGGATGAGCCCTT
CGGGGGAAAGATTTATCGCCGATAGATCGGCCCGCGTCTGATTAGCTAGTTGGTGAGGTAATGGCTCACC
AAGGCGACGATCAGTAGCTGGTCTGAGAGGATGATCAGCCACATTGGGACTGAGACACGGCC
>Zotu14676
GAACGCTGGCGGCGTGGATTAGGCATGCAAGTCGCGCGATCTCGCAAGAGATAGCGGCAAACGGGTTAG
GAACACGTAAGTAATTTTCCTCTTATTCAGAAATAGTCCTAAGAGATTAGGAGTAATGTCCGATGTTAAGAT
AGAACCTCATGGTTTTATCTTCAAAGGCGGGGACCGCAAGGCCTGCCGATAAGAGATGAGCTTGCGTAGC
ATCAGCTAGTTGGTAGGGTAACGGCCTACCAAGGCTATGACGCTTAGCTGGTCTGAGAGGACGATCAGCA
ACACTGGGACTGAGACACTGCC
>Zotu14679
ACTCCTACGGGAGGCAGGCCTAACACATGCAAGTCGAGCGGATGAGAGGAGCTTGCTCCTGGATTCAGCG
GCGGACGGGTGAGTAATGCCTAGGAATCTGCCTGGTAGTGGGGGACAACGTTTCGAAAGGAACGCTAATA
CCGCATACGTCCTACGGGAGAAAGCAGGGGACCTTCGGGCCTTGCGCTATCAGATGAGCCTAGGTCGGAT
TAGCTAGTTGGTGAGGTAATGGCTCACCAAGGCGACGATCCGTAACTGGTCTGAGAGGATGATCAGTCAC
ACTGGAACTGAGACACGGTC
>Zotu14684
GAACGCTAGCGGCAGGCTTAATACATGCAAGTCGAGGGGGTAGCAATACCACCGGCGCACGGGTGCGTA
ACGCGTATGCAACCTACCCATATCAGGGGGATAGCCCTAGGAAACTGGGATTAATACCGCATAATATTAGG
ATTGGCATCAATTTTAATTGAAAGATTTATCGGATATGGATGGGCATGCGTAGGATTAGCTAGTTGGTGAG
GTAAGGGCTCACCAAGGCGATGATCCTTAGGGGCTCTGAGAGGAGTATCCCCCACACTGGCACTGAGATA
CGGGC
>Zotu14688
GAACGCTAGCGGCATGCTTAACACATGCAAGTCGAGCGATTCTCTTCGGAGAAGAGCGGCGGACGGGTGA
GTAACGCGTGGGTAACCTGCCCTGTACACACGGATAACATACCGAAAGGTATGCTAATACGAGATAATATA
CTTTTATCGCATGGTAGAAGTATCAAAGCTCCGGCGGTACAGGATGGACCCGCGTCTGATTAGCTAGTTGG
TAAGGTAACGGCTTACCAAGGCGACGATCAGTAGCCGACCTGAGAGGGTGATCGGCCACATTGGAACTGA
GACACGGTC
>Zotu14691
GAACGCTGGCTGTATGGATAAGGCATGCAAGTCGAACGGCCCGCAAGGGCAGTGGCAGACGAGGTAGTA
ACACGTACGAACGTACTCCCAAGTCAGGAATAGCTCACCGAAAGGTGGGGTAATACCTGATGGTCCCTACG
GGGTAAAGATTTATCGCTTGGGAAGCGGCGTGCGTAGTATCAGCTTGTTGGTAAGGTAATGGCTTACCAA
GGCTACGACGCTTAGGGGAGCTGAGAGGCTGACCCCCACCGATGGGACTGAGACACGGCC

>Zotu14696
GAACGCTGGCGGCGTGCCTAACACATGCAAGTCGAGCGCGAATGGAGCAATCCTAGTAGAGCGGCGCAC
GGGTGCGTAACACGTGGGTAATCTGCCTGGAAGACTGGGACAACCTGCCGAAAGGCTGGCTAATACCGGA
TAAGCCCACAGGGTCTTCGGACCTCGAGGGAAAAGGTGGCCTCTGTATACAAGCTATCACTTCTAGATGAG
CCCGCGGCCCATCAGTTAGTTGGCGGGGTAACGGCCCACCAAGACTACGACGGGTAGCTGGTCTGAGAGG
ACGATCAGCCACACTGGAACTGAGACACGGTC
>Zotu14698
GAACGCTGGAAATGTGGATACCGGCATGCAAGTCACGGGGGCCGCAAGGCAACCGGCGAACGGCTTAGT
AACACGTGGATACATACCGTAAAGCGGAGCACAGCTCGTCGAAAGACGGGGTAATTCTCCATAGTCCCGC
AAGGGTAAACACGCAAGTGGCTTTATGAATGGTCCGCGGCCTATCAGCTTGTTGGCGGGGTAAAAGCCCA
CCAAGGCTATGACGGGTAGGGGGCGTGAGAGCGCGACCCCCAAGAGTTGGACTGCGACACGGCC
>Zotu14705
GAACGCTGGCGGCAGGCCTAATACATGCAAGTCGAACGGCAGCACAGTGGAGCTTGCTCCATGGGTGGCG
AGTGGCGGACGGGTGAGGAATACATGGGAATCTGCCCAGTCGTGGGGGATAACGTATGGAAACGTACGC
TAATACCGCATGCGCCCTTCGGGGGAAAGCCGGGGATCTTCGGACCTGGCGCGATTGGATGAGCCCATGT
CGGATTAGCTAGTTGGCGGGGTAAAGGCCCACCAAGGCGACGATCCGTAGCTGGTCTGAGAGGATGATCA
GCCACACTGGAACTGAGACACGGTC
>Zotu14711
GAACGCTGGCGGCAGGCTTAACACATGCAAGTCGAACGCCCTGGCAACGGGGAGTGGCAGACGGGTGAG
TAACACGTGGGAACATACCCTTCGGTTCGGAACAACGCCGGGAAACTGGCGCTAATACCGGATACGGCTT
ACGGGCGAAAGATTGATCGCCGAAGGATTGGCCCGCGTTCGATTAGCTTGTTGGTGAGGTAACGGCTCAC
CAAGGCGACGATCGATAGCTGGTCTGAGAGGATGGCCATCCACATTGGGACTGAGACACGGCC
>Zotu14713
GCCGTGTCTCAGTCCCAGTGTGGCTGGTCATCCTCTCAGACCAGCTACGGATCGTCGCCTTGGTAGGCCTTT
ACCCCACCAACTAGCTAATCCGACGTAGGCTCATCCTAGAGCGCAAGGCCCCGAAGGGTCCCCTGCTTTGA
TCTCTCGATATCATGCGGTATTAGCCCGAGTTTCCTCGGGTTATTCCACTCTCCAGGACAGATTCCTACGCTT
TACTCACCCGTCCGCCACTCGCCACCCGTAAAGACCGAAATCTTTACTGTGCTGCCGTTCGACTTGCATGTG
TTAGGCATGCCGCCAGCGTTTA
>Zotu14716
AAACGCAAGAGACGTGCCTAACACATGCAAATTAAATAGTTTTAATTAACTATAGTGAATTGGTGAGTAAT
ATATGAAAATTTAAATTTAGATTGGAAGTATTCCTAATAACTTAGGAGTAATCTCTGATAAGCGCAAAGGTG
CTTTAAAAAGTTAATACTGTCTAAAGAAAAGTTCATATCTGATTAGCTAGTTAGTAGGGTAAAAGTTTACTA
AGGCGAAGATCAGTAGCTACCCTGAGAGGGGGATTAGCCACATTGGGATTGAAATACAGCC
>Zotu14726
GAACGCTGGCGGCGTGGATAAGACATGCAAGTCGAACGCTGATTTTCGGGTAGCAATATTCGGAAGTTGG
AGTGGCGCAAGGGTGCTTAACACGTGGATAATCTGCCTTGAAGTCTGGGATAACTTGCTGAAAGGCGAGC
TAATACCGGATGTGATCGAGAGATCAAAGTTGGGGACCGTAAGGCCTGACGCTTTAAGATGAGTCCGCGG
CCTATCAGCTAGTTGGTGAGGTAATGGCTCACCAAGGCTAAGACGGGTAGCTGGTCTGAGAGGACGACCA
GCCACACTGGAACTGAGACACGGTC
>Zotu14732
GAACGCTGGCGGCGTGCTTCACACATGCAAGTCGAGCGAGGCCCTCTCTTCGGAGAGGTGCCCTAGCGGC
GAACGGGTGAGTAACACGTGGGCAACCTGCCCTCAGCTCTGGGATAAGCCCGGGAAACCGGGTCTAATAC
CGGATACGACTCCTGCAGGCATCTGCCGGGAGTGGAAAGATTCATCGGCTGAGGATGGGCCCGCGGCCTA
TCAGCTTGTTGGTGGGGTAACGGCCCACCAAGGCGACGACGGGTAGCCGGCCTGAGAGGGCGACCGGCC
ACACTGGGACTGAGACACGGCC
>Zotu14744
GAACGCTAGCGATAGGCTTAACACATGCAAGTCGAGGGGCAGCATGGTCTTAGCTTGCTAAGACTGATGG
CGACCGGCGCACGGGTGCGTAACGCGTATGCAACTTGCCTCACAGAGGGGGATAACCCGTCGAAAGACG
GACTAATACCGCGTACACTTGGGATAGGGCATCCTATCCCAAGGAAATGAAATTCGCTGTGAGATAGGCAT

GCGTCCCATTAGCTAGTTGGTGAGGTAACGGCTCACCAAGGCGACGATGGGTAGGGGAACTGAGAGGTT
GAACCCCCACACTGGTACTGAGACACGGAC
>Zotu14746
GAACGCTGGCGGCAGGCTTAACACATGCAAGTCGAACGATCCGCAAGGATAGTGGCAAACGGGTGAGTA
ATACATGGGAATGTACCTTTTAGTTCGGGATAACAGCGGGAAACTGCTGCTAATACCGGATGCGCTCTACG
GAGGAAAGATTTATCGCTGAAAGATCAGCCCATGCCGGATTAGCTAGTTGGTGAGGTAATGGCTCACCAA
GGCTTTGATCCGTAGCGGGTCTGAGAGGATGATCCGCCACACTAGGACTGCGATACGGCC
>Zotu14753
GAATGCTGGCGGCGTGGATAAGTCATGCAAGTCGAGGGGCCCGCAAGGGAACCGGCAAACGAGGTAGTA
ACACGTAGGTAGACCCCCCAGAGTCGGGCATAGCCAGCCGAAAGGTTGGGTAATTCCCGATGGTCTCGCG
AGAGTAAAGATTTATCGCTCTGGGAGTTGCCTGCGTAGTATCAGCTAGTTGGTAGGGTAATGGCCTACCAA
GGCTACGACGCTTAGGGGAGCTGAGAGGCTGACCCCCACCGATGGGACTGCGACACGGCC
>Zotu14755
GAACGCTGGCGGCGTGCTTAACACATGCAAGTCGAGCGGCAGCGCGGGGCAACCTGGCGGCGAGCGGCG
GACGGGTGAGTAACGCTTGGGAATCTGCCTATTAGTGGGGGACAACCCGGGGAAACTCGGGCTAATACCG
CATACGCTCTACGGAGGAAAGCCGGGGACCGCAAGGCCTGGCGCTAATAGATGAGCCCAAGTCGGATTAG
CTAGTTGGTAGGGTAACGGCCTACCAAGGCGACAATCCGTAGCTGGTCTGAGAGGACGACCAGCCACACC
GGAACTGAGACACGGTC
>Zotu14757
GAACGCTGGCGGCGTGCCTAACACATGCAAGTCGAACGAGAAAGGGATTTCGGTCCTGAGTAAAGTGGCG
CACGGGTGAGTAACACGTAGGTAACCTACCTCTAAGTTTGGGATAACCTGTCGAAAGATGAGCTAATACTG
AATAAGACCACAGCTTCCTCGGAAGCAGGGGTCAAAGGTAGCCTCTATTTATAAGCTATCACTTAGAGATG
GGCCTGCGTACCATTAGCTAGTTGGTGAGGTAACGGCTCACCAAGGCAAAGATGGTTAGCTGGTCTGAGA
GGATGATCAGCCACACTGGAACTGAGACACGGTC
>Zotu14763
CAACGCTGGCGGCGTGCCTAACACATGCAAGTCGAACGCGAAGACCCAGCAATGGGTTTGTAGCGTGGCA
AACGGGTGAGTAACGCGTGGGCGACCTACCTTCGAGCGGGGGATAACCTTCCGAAAGGAGGGCTAATACC
GCATGACATCCCGTGTTTAGATACACGGAGATCAAAGCCGGGGATCGCAAGACCTGGCACTTGAAGAGGG
GCCCGCGTCTGATTAGCTAGTTGGCGAGGTAATGGCTCACCAAGGCTCCGATCGGTATCCGGCCTGAGAG
GGCGGACGGACACACTGGAACTGAGACACGGTC
>Zotu14776
GAACGCTGGCGGCGTGCCTAACACATGCAAGTCGAGCGAAGTGGCAGCTGTAGCAATACAGTATGTGCTT
AGCGGCAGACGGGTGAGTAACACGTAGACAATTTACCTCTTAGAGGGGGATAGCCCGCCGAAAGGCGGG
GTAATACCGCGTAAGATCTTTTATCGGCATCGGTAGAGGATGAGACACCCGCAAAGGTGGCTAAGAGAAA
AGTCTGCGGCCTATCAGCTAGTTGGTGAGGTAACGGCTCACCAAGGCTACGACGGGTATCCGACCTGAAA
GGGTGACCGGACACACTGACACTGAGACACGGGT
>Zotu14779
GAACGCTGGCGGCGTGCCTAACACATGCAAGTCGAGCGGAGGTAGGATGTAGTTTACTACTGAATACTTTA
GCGGCGGACGGGTGAGTAACACGTGGGCAACCTGCCTTGAAGAGGGGGATAACACCGAGAAATCGGTGC
TAATACCGCATAACATACACATACTGCATAGTGAGTGTATCAAAGGAGAAATCCGCTTTAAGATGGGCCCG
CGGCTGATTAGCTAGTTGGTAAGGTAACGGCTTACCAAGGCGACGATCAGTAGCCGACCTGAGAGGGTGA
CCGGCCACACTGGAACTGAGACACGGTC
>Zotu14783
GAACGCTGGCGGCATGCTTAACACATGCAAGTCGAACGAGACCTTCGGGTCTAGTGGCGCACGGGTGCGT
AACGCGTGGGAATCTGCCCTTAGGTACGGAATAACTCAGAGAAATTTGTGCTAATACCGTATGATGTCGAA
AGACCAAAGATTTATCGCCTAAGGATGAGCCCGCGTAAGATTAGCTAGTTGGTAGGGTAAAGGCCTACCA
AGGCTACGATCTTTAGCTGGTCTGAGAGGATGATCAGCCACACTGGGACTGAGACACGGTC
>Zotu14786
GAACGCTGGCGGCAGGCTTAACACATGCAAGTCGAACGGGCGTAGCAATACGTCAGTGGCAGACGGGTG
AGGAACACGTGGGAACGTACCTTTTGGTTCGGAACAACTGAGGGAAACTTCAGCTAATACCGGATAAGCC

CTTCGGGGGAAAGATTTATCGCCAAAAGATCGGCCCGCGTCTGATTAGCTAGTTGGTAGGGTAATGGCCTA
CCAAGGCGACGATCAGTAGCTGGTCTGAGAGGATGATCAGCCACATTGGGACTGAGACACGGCC
>Zotu14793
GAACGCTGGCGGCAGGCTTAACACATGCAAGTCGAACGATCCTTCGGGATAGTGGCGCACGGGTGAGTAA
CGCGTGGGAATGTACCATATAGTGGGGAATAACGTTGGGAAACTAACGCTAATACCGCATACGCCCTTCG
GGGGAAAGATTTATCGCTATATGATCAGCCCGCGTGAGATTAGGTAGTTGGTGGGGTAATGGCCTACCAA
GCCTACGATCTCTAGCTGGTCTGAGAGGATGATCAGCCACACTGGAACTGAGACACGGCC
>Zotu14797
GAACGCTGGCGGCGTGCCTAATACATGCAAGTCGAGCGAACTTGCGGGAGCTTGCTCCCAAAAGTTAGCG
GCGGACGGGTGAGTAACACGTGGGCAACCTGCCTATAAGACTGGGATAACTTCGGGAAACCGGAGCTAAT
ACCGGATAATTCTTTTTCTCTCATGAGGAAAAGCTGAAAGACGGTTTACGCTGTCACTTATAGATGGGCCCG
CGGCGCATTAGCTAGTTGGTGAGGTAACGGCTCACCAAGGCAACGATGCGTAGCCGACCTGAGAGGGTGA
TCGGCCACACTGGGACTGAGACACGGCC
>Zotu14802
GAACGCTAGCGGCATGCCTAACACATGCAAGTCGAACGGCAGCATAGGAGCTTGCTCCTGATGGCGAGTA
GCGAACGGGTGAGTAATATATCGGAACGTGCCCTAGAGTGGGGGATAACTAGTCGAAAGACTAGCTAATA
CCGCATACGATCTACGGATGAAAGTGGGGGATCGCAAGACCTCATGCTCCTGGAGCGGCCGATATCTGATT
AGCTAGTTGGTGGGGTAAAAGCTCACCAAGGCGACGATCAGTAGCTGGTCTGAGAGGACGATCAGCCACA
CTGGGACTGAGACACGGCC
>Zotu14810
GAACGCTGGCGGCGTGCTTAACACATGCAAGTCGAGCGGTAAGGCCCTTCGGGGTACACGAGCGGCGAA
CGGGTGAGTAACACGTGAGCAATCTGCCCTTCACATCGGGATAACCCCCGGAAACGGGAGCTAATACCGG
ATACGACCACTTCAGGCATCTGATGGTGGTGGAAAGTTCCGGCGGTGAAGGATGAGCTCGCGGCCTATCA
GCTTGTTGGTGGGGTAATGGCCCACCAAGGCTTCGACGGGTAGCCGGCCTGAGAGGGTGACCGGCCACAC
TGGGACTGAGACACGGCC
>Zotu14812
GAACGCTGGCGGCGTGCCTAATACATGCAAGTAGAACGCTGAAGGAAGGAGCTTGCTCTTTCCGGATGAG
TTGCGAACGGGTGAGTAACGCGTAGGTAACCTGCCTGGTAGCGGGGGATAACTATTGGAAACGATAGCTA
ATACCGCATAAAATGGATTATCGCATGATAATTCATTGAAAGGTGCAAATGCATCACTACCAGATGGACCT
GCGTTGTATTAGCTAGTTGGTGGGGTAACGGCTCACCAAGGCAACGATACATAGCCGACCTGAGAGGGTG
ATCGGCCACACTGGGACTGAGACACGGCC
>Zotu14815
GAACGCTGGCGGCGTGTCTTAAGCATGCAAGTCGTGCGGCAAGGGGTAGCAATACCCCCTAGAGCGGCG
GACTGGTGAGTAACGCGTGGGTGATCTGCCCTTGGGATGGGGATAGCCATTAGAAATAGTGAGTAATACC
GAATACGTTCATTAAGCTGAGGCTTAATGAAGAAAAGCGCTTCGGCGCTGCCCGAGGATGAGCCCGCGTC
CCATTAGCTAGTTGGTGAGGTAAAGGCCCACCAAGGCTATGATGGGTAGCCGGCCTGAGAGGGTGTACGG
CCACACTGGGACTGAGATACGGCC
>Zotu14820
GAACGCTGGCGGCAGGCTTAACACATGCAAGTCGAACGGAACCTTCGGGTTTAGTGGCGAACGGGTGAGT
AATACCTAGGAATCTGCCTATTAGTGGGGGATAACGGCTCGAAAGGGTCGCTAATACCGCATACGTCCTAC
GGGAGAAAGAGGGGGATCTTCGGACCTCTTGCTAATAGATGAGCCTAGGCCGGATTAGCTAGTTGGTAGG
GTAAAGGCCTACCAAGGCGACGATCCGTAGCTGGTCTGAGAGGATGATCAGCCACACTGGAACTGAGACA
CGGTC
>Zotu14822
GAACGCTGGCGGCATGCTTTACACATGCAAGTCGAACGGCAGCACGGGCTTCGGCCTGGTGGCGAGTGGC
GAACGGGTGAGTAATACATCGGAACGTACCCAGAAGTGGGGGATAACGCAGCGAAAGTTGCGCTAATACC
GCATACGTTCTACGGAAGAAAGTGGGGGATCGCAAGACCTCATGCTTTTGGAGCGGCCGATGTCTGATTA
GCTAGTTGGTAGGGTAAAGGCCTACCAAGGCGACGATCAGTAGCTGGTCTGAGAGGACGACCAGCCACAC
TGGGACTGAGACACGGCC
>Zotu14824

GAACGCTGGCGGCGTGCCTAATACATGCAAGTCGAGCGGTAAGGTTCTCACTCTGTACTCAATCTCTTGAG
TGCGGTGTGAGAATACACGAGCGGCGGACGGCTGAGTAACGCGTAGGAACGTACCCCAAAGTGAGGGAT
AAGCCCGGGAAACCGGGTCTAATACCGCATATTCTCTTCGGAGCAAAGCAGCAATGCGCTTTGGGAGCGG
CCTGCGTACGATTAGGTTGTTGGTGAGGTAACGGCTCACCAAGCCGACGATCGTTAGCTGGTCTGAGAGG
ATGATCAGCCAGACTGGGACTGAGAACGGCC
>Zotu14826
GAACGCTGGCGGCGTGCTTAACACATGCAAGTCGAACGGTGACGAGCAGCTTGCTGCTCTGATCAGTGGC
GAACGGGTGAGTAACACGTGAGCAACCTACCCTTGACTCTGGGATAACAGCTGGAAACAGCTGCTAATACC
GGATACGACCTCGACCGACATCTGTTGAGGTGGAAAGTTCTGGCGGTCAAGGATGGGCTCGCGGCCTATC
AGCTAGTTGGTGAGGTAACGGCTCACCAAGGCTTCGACGGGTAGCCGGCCTGAGAGGGTGACCGGCCAC
ACTGGGACTGAGACACGGCC
>Zotu14834
GAACGCTGGCGGCAGGCCTAACACATGCAAGTCGAACGCCCCGCAAGGGGAGTGGCAGACGGGTGAGTA
ACGCGTGGGAACATGCCCTTTGGTTCGGAATAACTCAGGGAAACTTGAGCTAATACCGGATAAGCCCGAG
AGGGGAAAGATTTATCGCCGAAGGATTGGCCCGCGTCCGATTAGCTAGTTGGTGAGGTAACGGCTCACCA
AGGCGACGATCGGTAGCTGGTCTGAGAGGATGATCAGCCACACTGGGACTGAGACACGGCC
>Zotu14840
GAACGCTGGCGGCGTGCTTAACACATGCAAGTCGAACGGTGAACCGGGCTTCGGCCTGGGGATCAGTGGC
GAACGGGTGCGTAACACGTGGGCAACCTGCCCCAGACACAGGGATAACCCCGGGAAACCGGGGCTAATA
CCCGATACTCCGATCGAGCCGCATGACTCGATCGGGAAAGCTCCGACGGTCTGGGATGGGCCCGCGGCCT
ATCAGCTTGTTGGTGGGGTAACGGCCTACCAAGGCGACGACGGGTAGCTGGTCTGAGAGGACGACCAGCC
ACACTGGAACTGAGATACGGTC
>Zotu14845
GAACGCTGGCGGCAGGCTTAACACATGCAAGTCGAGCGGGCCCTTCGGGGTCAGCGGCGGACGGGTTAG
TAACGCGTGGGAACGTGCCTTTTGCTCTGGAATAGCCCTGGGAAACTGGGGGTAATACCGGATGTGCCCT
GAGGGGGAAAGATTTATCGGCAAGAGATCGGCCCGCGTCTGATTAGGTAGTTGGTGGGGTAACGGCCCA
CCAAGCCGACGATCGGTAGCTGGTTTGAGAGGATGATCAGCAACACTGGGACTGAGACACGGCC
>Zotu14852
GAACGCTGGCGGCAGGCCTAACACATGCAAGTCGAACGGTTCCTTCGGGGACAGTGGCAGACGGGTGAG
TAACGCGTGGGAACGTGCCCTTCGGTTCGGAATAACTCAGGGAAACTTGAGCTAATACCGGATACGTCCGA
GAGGAGAAAGATTTATCGCCGAAGGATCGGCCCGCGTCTGATTAGCTAGTTGGTGGGGTAATGGCCTACC
AAGGCGACGATCAGTAGCTGGTCTGAGAGGATGATCAGCCACACTGGGACTGAGACACGGCC
>Zotu14856
GAACGCTGGCGGCGTGCTTAACACATGCAAGTCGAACGAGCAAGAGCTGTAGCAATACAGGGTTCTGCTA
GTGGCAGACGGGTGAGTAATACATGGGAATCTACCTTGCAGTGCGAAATAACAGTTGGAAGCAATTGCTA
ATGTCGCATATAGCCGAGAGGCGAAAGGTTTACTGCTGCAAGATGGGCCCATGCAGGATTAGCTAGTTGG
TGAGGTAATGGCTCACCAAGGCAACGATCTTTAGTTGGTCTGAGAGGATGATCAGCCACACTGGGACTGA
GACACGGCC
>Zotu14862
GAACGCTGGCGGCGTGCCTAATACATGCAAGTCGAACGCGAAAGTCCTTCGGGATGAGTAAAGTGGCGCA
CGGGTGAGTAATGGTTGAGAACGTGCCCTGAAGTGGGGAATAACAGTTGGAAACGACTGCTAATACCGCA
TGGTTACGAAAGTATAAAGGTGGGGACCGCAAGGCCTACCGCTTCAGGATCGGCTCAACTCCCATTAGCTA
GTTGGCAGGGTAATGGCCTACCAAGGCAACGATGGGTATGTGGTCTTAGAGGATGATCACACACACTGGA
ACTGAGACACGGTC
>Zotu14877
GAACGCTGGCGGCGTGCTTAACACATGCAAGTCGAACGATGAAGCCCAGCTTGCTGGGCGGATTAGTGGC
GAACGGGTGAGTAACACGTGAGTAACCTGCCCTTGACTCTGGGATAAGCCCGGGAAACTGGGTCTAATAC
CGGATAGGAGCGCCTGTCGCATGACGGGTGTTGGAAAGATTTTTCGGTCATGGATGGACTCGCGGCCTAT
CAGCTTGTTGGTGGGGTAATGGCCTACCAAGGCGACGACGGGTAGCCGGCCTGAGAGGGTGACCGGCCA
CACTGGGACTGAGACACGGCC

>Zotu14881
CGATGAAGAACGCAGCGGCTGACGGGTGAGTAACGCGTGGGAACGTACCTTTTGGTTCGGAACAACACAG
GGAAACTTGTGCTAATACCGGATAAGCCCTTACGGGGAAAGATTTATCGCCGAAAGATCGGCCCGCGTCTG
ATTAGCTAGTTGGTGAGGTAACGGCTCACCAAGGCGACGATCAGTAGCTGGTCTGAGAGGATGATCAGCC
ACATTGGGACTGAGACACGGCC
>Zotu14887
GAACGCTGGCGGCATGCTTTACACATGCAAGTCGAACGGCAGCGCGGGGCAACCTGGCGGCGAGTGGCG
AACGGGTGAGTAATATATCGGAACGTACCCTGGAGTGGGGGATAACGTAGCGAAAGTTACTCTAATACCG
CATACGATCTAAGGATGAAAGTGGGGGATTCGCAAGAACCTCATGCTCCTGGAGCGGCCGATATCTGATTA
GCTAGTTGGTAGGGTAAAGGCCTACCAAGGCAACGATCAGTAGCTGGTCTGAGAGGACGACCAGCCACAC
TGGAACTGAGACACGGTC
>Zotu14890
GAACGCTGGCGGCGTGCTTAACACATGCAAGTCGAACGGCAGCATAGGAGCTTGCTCCTGATGGCGAGTG
GCGAACGGGTGAGTAATATATCGGAACGTGCCCTAGAGTGGGGGATAACTAGTCGAAAGACTAGCTAATA
CCGCATACGATCTACGGATGAAAGTGGGGGATCGCAAGACCTCATGCTCCTGGAGCGGCCGATATCTGATT
AGCTAGTTGGTGGGGTAAAAGCTCACCAAGGCGACGATCTTTAGTTGGTCTGAGAGGATGATCAGCCACA
CTGGAACTGAGACACGGTC
>Zotu14894
GAACGCTGGCGGCATGCCTAACACATGCAAGTCGAACGAGACCTTCGGGTCTAGTGGCGCACGGGTGCGT
AACGCGTGGGAATCTGCCCTTGGGTTCGGAATAACAGTTAGAAATGACTGCTAATACCGGATGATGTCTTC
GGACCAAAGATTTATCGCCCAGGGATGAGCCCGCGTCGGATTAGCTAGTTGGTGGGGTAAAAGCCTACCA
AGGCGACGATCCGTAGCTGGTCTGAGAGGATGATCAGCCACACTGGGACTGAGACACGGCC
>Zotu14899
TAACGTTGGCGGCGTGCATAACACATGCAAGTCGAACGATGAAACTCTAAAGATTTCTTCGGAATGAATTA
GAATGGATTAGTGGCGAACGGGTGAGTAACACGTGAGTAACCTGCCTTAGAGAAGGGGATAGCCTTTGGA
AACGAAGAATAATACCCTATGAAATGATAATATCGCATGATAATATCATCAAAGATTTATCGCTCTAAGATG
GACTTGCGCCTGATTAGCTAGTTGGTGGAGTAACAGCCCACCAAGGCAACGATCAGTAGCCGGCTTGAGA
GAGTGTACGGCCACATTGGGACTGAGACACGGCC
>Zotu14903
GAACGCTAGCGTTATGCTTAACACATGCAAGTCGAACGTTGTTTTCGGTTCGATCCCCAGTATAAACATTGG
CTAGCTACTTGATTTTCAGCCGTAGTTTGCTGGAATCGGAATCGTTGAGAACAAAGTGGCGAACGGGTGCG
TAACGCGTGGGAATCTGCCGAACAGTTCGGGCCAAATCCTGAAGAAAGCTCAAAAGCGCTGTTTGATGAG
CCTGCGTAGTATTAGGTAGTTGGTCAGGTAAAAGCTGACCAAGCCAATGATGCTTAGCTGGTCTTTTCGGA
TGATCAGCCACACTGGGACTGAGACACGGCC
>Zotu14906
GAACGCTGGCGGCATGCCTAACACATGCAAGTCGAACGAGACCTTCGGGTCTAGTGGCGCACGGGCGCGT
AACGCGTGGGAACCTGCCTTTAGGTTCGGAATAACTCCTCGAAAGGGGTGCTAATACCGAATAATGTCTTC
GGACCAAAGATTTATCGCCTTTAGATGGGCCCGCGTTGGATTAGCTAGTTGGTGGGGTAATTGCCTACCAA
GGCGACGATCCGTAGCTGGTCTGAGAGGATGATCAGCCACACTGGGACTGAGACACGGCC
>Zotu14908
GAACGCTAGCGGGAGGCCTAACACATGCAAGCCGAGCGGTATTGTTTCTTCGGAAATGAGAGAGCGGCGT
ACGGGTGCGTAACACGTGTGCAACCTGCCTTTATCTGGGGGATAGCCTTTCGAAAGGAAGATTAATACTCC
ATAACATATTGAACGGCATCGTTTAATATTGAAAGCTCCGGCGGATAGAGATGGGCACGCGCAAGATTAG
CTAGTTGGTGAGGTAACGGCTCACCAAGGCGATGATCTTTAGGGGGCCTGAGAGGACGATCAGCCACACT
GGGACTGAGACACGGCC
>Zotu14916
GAACGCTAGCGGGAGGCCTAACACATGCAAGCCGAGCGGTAGAGATTCTTCGGAATCTTGAGAGCGGCGT
ACGGGTGCGGAACACGTGTGCAACCTGCCTTTATCAAGGGGATAGCCTTTCGAAAGGAAGATTAATACCCT
ATAATATATGATTCGGCATCGGATTATATTGAAAACTACGGTGGATAAAGATGGGCACGCGCAAGATTAGC

TAGTTGGTGAGGTAACGGCTTACCAAGGCGACGATCTTTAGGGGGCCTGAGAGGGTGATCCCCCACACTG
GTACTGAGACACGGAC
>Zotu14920
GAACGCTGGCGGCAGGCTTAACACATGCAAGTCGAACGCTCGCAGCAATGCGGGAGTGGCAGACGGGTG
AGTAACGCGTGGGAACGTACCCTTTGGTTCGGAATAACTCAGGGAAACTTGAGCTAATACCGGATAAGCCC
TTTTGGGGAAAGATTTATCGCCGAAGGATCGGCCCGCGTCCGATTAGCTAGTTGGTGAGGTAATGGCTCAC
CAAGGCGACGATCGGTAGCTGGTCTGAGAGGATGATCAGCCACACTGGGACTGAGACACGGCC
>Zotu14922
GAACGCTGGCGGCAGGCCTAACACATGCAAGTCGAGCGGGGGTAGCAATACCCTAGCGGCGAACGGGTG
AGGAATGCTTGGGAATCTGCCTGGTAGTGGGGGATAACGTTCCGAAAGGAACGCTAATACCGCATACGTC
CTACGGGAGAAAGGGGGGGATCTTCGGACCTCTCGCTATCAGATGAGCCCAAGCGGGATTAGCTAGTTGG
TGAGGTAAAGGCTCACCAAGGCGACGATCCCTAGCTGGTCTGAGAGGACGATCAGCCACACTGGGACTGA
GACACGGCC
>Zotu14928
GAACGCTGGCGGCGTGCCTAATACATGCAAGTAGAACGCTGAAGGAGGAGCTTGCTTCTCTGGATGAGTT
GCGAACGGGTGAGTAACGCGTAGGTAACCTGCCTGGTAGCGGGGGATAACTATTGGAAACGATAGCTAAT
ACCGCATAATAGTAGATGTTGCATGACATTTGCTTAAAAGGTGCAATTGCATCACTACCAGATGGACCTGC
GTTGTATTAGCTAGTTGGTGAGGTAACGGCTCACCAAGGCGACGATACATAGCCGACCTGAGAGGGTGAT
CCCCCACACTGGCACTGAGATACGGGC
>Zotu14936
GAACGCTGGCGGCAGGCTTAACACATGCAAGTCGAGCGGGCGCTTTCGGGCGTCAGCGGCGGACGGGTT
AGTAACGCGTGGGAACGTGCCTTTTGCTTCGGAATAGCCCTGGGAAACTGGGGGTAATACCGGATGTGCC
CTGAGGGGGAAAGATTTATCTGCAAGGGATCGGCCCGCGTCTGATTAGGTAGTTGGTGTGGTAATGGCGC
ACCAAGCCGACGATCGGTAGCTGGTTTGAGAGGATGATCAGCAACACTGGGACTGAGACACGGCC
>Zotu14938
GAACGCTAGCGGCAGGCTTAATACATGCAAGGCGAGGGGGCAGCAATGTCACCGTCGTACGGGTGCGTA
ACGCGTATGCAACCTACCTTCAACTGGGGGATAGCCCGGGGAAACCCGGATTAATACCGCATGATACATTG
GTGTGGCATTACACTGATTGTTAAAGATTTATTGGTTGAAGATGGGCATGCGTTCGATTAGCTAGTTGGTG
CGGGTAACGGCCCACCAAGGCAACGATCGATAGGGGAGCTGAGAGGTTGATCCCCCACACGGGCACTGA
GATACGGGC
>Zotu14942
GAACGCTGGCGGCAGGCTTAACACATGCAAGTCGAACGGGCATAGCAATATGTCAGTGGCAGACGGGTG
AGTAACGCGTGGGAACGTACCTTTTGGTTCGGAACAACACAGGGAAACTTGTGCTAATACCGGATAAGCCC
TTACGGGGAAAGATTTATCGCCAAAAGATCGGCCCGCGTCTGATTAGCTAGTTGGTGAGGTAACGGCTCAC
CAAGGCGACGATCAGTAGCTGGTCTGAGAGGATGATCAGCCACATTGGGACTGAGACACGGCC
>Zotu14945
GAACGCTGGCGGCATGCCTTACACATGCAAGTCGAACGCGAAAGGGGGGCAACCCCCGAGTAGAGTGGC
GAACGGGTGAGTAATACATCGGAACGTGTCCAGTAGCGGGGGATAACCTCTCGAAAGAGAGGCTAATACC
GCATACGACCTACGGGTGAAAGAGGGGGATCGAAAGACCTCTCACTATTGGAGCGGCCGATGTCGGATTA
GCTAGTTGGTGGGGTAAAGGCCCACCAAGGCTACGATCCGTAGCTGGTCTGAGAGGACGACCAGCCACAC
TGGAACTGAGACACGGTC
>Zotu14947
GAACGCTGGCGGCATGCCTTACACATGCAAGTCGAACGGATTGAGGGGCTTGCTCCTCAGTTAGTGGCGA
ACGGGTGAGTAATGCATCGGAACGTACCTTTTTGTGGGGGATAACTACTCGAAAGAGTAGCTAATACCGCA
TACGCCCTGAGGGGGAAAGCGGGGGACTCGCAAGAGCCTCGCGCAAAAAGAGCGGCCGATGTCAGATTA
GCTAGTTGGTAGGGTAAAAGCCTACCAAGGCGACGATCTGTAGCTGATCTGAGAGGACGACCAGCCACAC
TGGGACTGAGACACGGCC
>Zotu14950
GAACGCTGGCGGCATGCCTAACACATGCAAGTCGAACGAGACCTTTCGGGGTCTAGTGGCGCACGGGTGC
GTAACGCGTGGGAATCTGCCCCTTGGTTCGGAATAACAGTTGGAAACGACTGCTAATACCGGATGACGAC

GAAAGTCCAAAGATTTATCGCCGAGGGATGAGCCCGCGTAGGATTAGGTAGTTGGTGTGGTAAAGGCGCA
CCAAGCCGACGATCCTTAGCTGGTCTGAGAGGATGATCAGCCACACTGGGACTGAGACACGGCC
>Zotu14953
GAACGCTGGCGGCAGGTCTTAGGCATGCAAGTCGAACGGGCGTAGCAATACGTCAGTGGCGCACGGGTG
AGTAACGCGTGGGAATATGCCCATTACTGGGGGATAACGTTTGGAAACGAACGCTAATACCGCATAAGCC
GGAAACGGGAAAGATTTATCGGTAATGGATTAGCCCGCGTTGGATTAGCTTGTTGGTGGGGTAACGGCCT
ACCAAGGCGATGATCCATAGCTGGTCTGAGAGGACGATCAGCCACACTGGGACTGAGACACGGCC
>Zotu14957
GAACGCTGGCGGCATGCCTTACACATGCAAGTCGAACGGCAGCACGGGAGCTTGCTCCTGGTGGCGAGTG
GCGAACGGGTGAGTAATACATCGGAACGTACCCTGAAGTGGGGGATAACGTAGCGAAAGTTACGCTAATA
CCGCATATTCTGTGAGCAGGAAAGAGGGGGATCGCAAGACCTCTTGCTTTAGGAGCGGCCGATGTCGGAT
TAGCTAGTTGGTGAGGTAAAGGCTCACCAAGGCGACGATCCGTAGCGGGTCTGAGAGGATGATCCGCCAC
ACTGGGACTGAGACACGGCC
>Zotu14961
GAACGCTGGCGGCGTGCTTAACACATGCAAGTCGAACGGTGAAAGAAGAGCTTGCTCTTCTGGATCAGTG
GCGAACGGGTGAGTAACACGTGAGTAACCTGCCCCAGACTCTGGGATAAGCACTGGAAACGGTGTCTAAT
ACCGGATACGAGCTTCAACCGCATGGTTAGGAGCTGGAAAGAATTTCGGTCTGGGATGGACTCGCGGCCT
ATCAGCTAGTTGGTGAGGTAACGGCCCACCAAGGCGACGACGGGTAGCCGGCCTGAGAGGGTGACCGGC
CACACTGGGACTGAGACACGGCC
>Zotu14969
GAACGCTGGCGGCGTGCTTAACACATGCAAGTCGAACGGTGATGCGGAGCTTGCTCCGCGGATCAGTGGC
GAACGGGTGAGTAACACGTGAGTAACCTGCCCTCGACTCTGGGATAAGCGTTGGAAACGACGTCTAATAC
CGGATACGACCTGTGGAGGCATCTCCTACGGGTGGAAAGAGTTTCGGTCGAGGATGGACTCGCGGCCTAT
CAGCTTGTTGGTGAGGTAATGGCTCACCAAGGCGACGACGGGTAGCCGGCCTGAGAGGGTGACCGGCCA
CACTGGGACTGAGACACGGCC
>Zotu14973
GAACGCTGGCGGCAGGCTTAACACATGCAAGTCGAACGATCCTTCGGGATAGTGGCGGACGGGTGAGTA
ACACGTGGGAACGTGCCTTTAGGTTCGGAACAACACAGGGAAACTTGTGCTAATACCGGATGTGCCCTTCG
GGGGAAAGATTTATCGCCTTTAGAGCGGCCCGCGTCTGATTAGCTTGTTGGTGAGGTAAAGGCTCACCAAG
GCTACGATCAGTAGCTGGTCTGAGAGGATGGCCAGCCACATTGGGACTGAGACACGGCC
>Zotu14975
GAACGCTGGCGGCGTGCTTAACACATGCAAGTCGAACGGTAAGGCCCTTCGGGGTACACGAGTGGCGAAC
GGGTGAGTAACACGTGAGTAACCTGCCCTTTACTTTGGGATAACTACCAGAAATGGTGGCTAATACCGGAT
ATGACTCCTGCCTGCATGGGTGGGGGTGGAAAGCTCCGGCGGTGGGGGATGGACTCGCGGCCTATCAGCT
TGTTGGTGAGGTAGTGGCTCACCAAGGCTTCGACGGGTAGCCGGCCTGAGAGGGTGACCGGCCACATTGG
GACTGAGATACGGCC
>Zotu14978
CAACGCTGGCGGCGTGCCTAACACATGCAAGTCGAACGCGAAAGCCCCGCAAGGGGTAAGTAGAGTGGC
GCACGGGTGAGTAACACGTGGACAACCTGCCTTTGAGTGGGGAATAACCTTCCGAAAGGAGGGCTAATAC
CGCATAACACCCTTCGACAAAAGTCGAGGGGTTAAAAGGAGCAATTCGCTCAAAGAGGGGTCCGCGCCTG
ATTAGCTAGTTGGCGGGGTAACGGCCCACCAAGGCTTCGATCAGTAGCCGTCCTGAGAGGGAGCACGGCC
ACACTGGAACTGAGACACGGTC
>Zotu14984
CAACGCTGGCGGCGCGCCTAACACATGCAAGTCGAACGAGAAAGTGGAGCAATCCATGAGTACAGTGGCG
TACGGGTGAGTAACACGTGGGTAATCTACCTCTTAGTGGGGAATAACTTTGGGAAACCGAAGCTAATACCG
CATAAGCTCGAGAGAGGAAAGCAGCAATGCGCTGAGAGAGGAGCCCGCGGCCGATTAGCTAGTTGGCAG
GGTAAAAGCCTACCAAGGCATAGATCGGTAGCCGGCCTGAGAGGGCACACGGCCACACTGGCACTGAAAC
ACGGGC
>Zotu14987

GCCGTGTCTCAGTCCCAGTGTGGCTGATCATCCTCTCAAACCAGCTACAGATCATCGGCTTGGTAGGCCTTT
ACCCCACCAACTACCTAATCCGCCGCGGGCCTCTCCTTTAGCGCATTTCTGCTTTCCCCCTCAGGGCGTATCC
GGTATTAGCCACCGTTTCCAGTGGTTGTCCCAGTCTAAAAGGTAAGTTCCCACGTGTTACTCACCCGTCTGC
CACTCAGCTACTCAAGCAAGCTTGAGTACTGCGTTCGACTTGCATGTGTTAAGCATGCCGCCAGCGTTCG
>Zotu14991
GAACGCTGGCGGCGTGCTTAACACATGCAAGTCGAGCGAGAAGTTACTCAATGAATCTTCGGAAGAATTA
AGTAATGGAAAGCGGCGGACGGGTGAGTAACGCGTGGGCAACCTGCCTTTCACAGAGGGATAGCCAAAG
GAAACTTTGATTAAAACCTCATAAAGCCGGAATAGTGCATAATATACCGGCCAAAGATTTATCGGTGAAAG
ATGGGCCCGCGTCTGATTAGCTAGTTGGTGGGGTAACGGCTCACCAAGGCAACGATCAGTAGCCGACCTG
AGAGGGTGATCGGCCACATTGGAACTGAGACACGGTC
>Zotu14993
GAACGCTGGCGGCAGGCATAATACATGCAAGGCGAGGGGGTAGCAATACCACCGTCGTACGGGTGCGTA
ACACGTATGCAACCTGCCCTTCACTGGGGGATAGCCTGGGGAAACTCGGATTAATACCGCATAAGACAGTA
GACTGGCATCAGTTAGCTGTTAAAGATTTATTGGTGAAGGATGGGCATGCGTCTGATTAGCTAGTTGGCGG
GGTAAAGGCCCACCAAGGATACGATCAGTAGGGGTTCTGAGAGGATTGGCCCCCACACTGGCACTGAGAT
ACGGGC
>Zotu14998
GAACGCTGGCGGCGTGCTTAATACATGCAAGTCGAGCGGTAACACTTCGGGTGACGAGCGGCGGACGGCT
GAGTAACGCGTAGGAACCTACCCCAAACTGAGGGATAAGCATCAGAAATGGTGTCTAATACCGCATGTGCT
CTACGGAGTAAAGCTTCGGCGGTTTGGGAAGGGCCTGCGTATGATTAGCTTGTTGGTGAGGTAATGGCTC
ACCAAGGCGACGATCATTAGCTGGTCTGAGAGGATGATCAGCCAGACTGGGACTGAGACACGGCC
>Zotu15001
GAACGCTGGCGGCGTGCTTAACACATGCAAGTCGAACGGAAAGGCCCTGCTTTTGTGGGGCGCTCGAGTG
GCGAACGGGTGAGTAACACGTGAGTAACCTGCCCTTGACTTTGGGATAACTTCAGGAAACTGGGGCTAAT
ACCGGATAGGAGCTCCTGCTGCATGGTGGGGGTTGGAAAGTTTCGGCGGTTGGGGATGGACTCGCGGCTT
ATCAGCTTGTTGGTGGGGTAGTGGCTTACCAAGGCTTTGACGGGTAGCCGGCCTGAGAGGGTGACCGGCC
ACACTGGGACTGAGACACGGCC
>Zotu15005
GAACGCTAGCGGCATGCCTAACACATGCAAGTCGAGCGCGCCTTCGGGTGAGCGGCGGACGGGTGAGTA
ACACGTGGGAATATGCCTTTCGGTGCGGAATAGTCTTGGGAAACTGAGGGTAATACCGCATACGCCCCCTG
GGGGAAAGATTTATCGCCGAAAGATTAGCCCGCGTTGGATTAGGTAGTTGGTGGGGTAATGGCCTACCAA
GCCGACGATCCATAGCTGGTTTGAGAGGATGATCAGCCACACTGGGACTGAGACACGGCC
>Zotu15009
GAACGCTGGCGGCGCGCTTAACACATGCAAGTCGAACGAGAAAGCGGGAGCAATCTCGCAAGTACAGTG
GCGTACGGGTGAGTAACACGTGGGTAATCTACCTCATAGTGGGGAATAACGCTGGGAAACCGGCGCTAAT
ACCGCATAATCCGCGACCTTAAGGGAAGCCGGGAAAGCAGCAATGCGCTGTGAGAGGAGCCCGCGGCCG
ATTAGCCAGTTGGTAGGGTAACGGCCTACCAAAGCAAAGATCGGTAGCCGGCCTGAGAGGGCACACGGCC
ACACTGGCACTGAAACACGGGC
>Zotu15015
GAACGCTGGCGGCATGCTTTACACATGCAAGTCGAACGGCAGCACGGACTTCGGTCTGGTGGCGAGTGGC
GAACGGGTGAGTAATACATCGGAACGTGTCCTGTAGTGGGGGATGGCCCGGCGAAAGCCGGATTAATACC
GCATACGCTCTACGGAGGAAAGGGGGGGATCTTAGGACCTCTCGCTACAGGGGCGGCCGATGGCAGATT
AGCTAGTTGGTGGGGTAAAGGCCTACCAAGGCGACGATCTGTAGCTGGTCTGAGAGGACGACCAGCCACA
CTGGGACTGAGACACGGCC
>Zotu15017
GAACGCTGGCGGCAGGCTTAACACATGCAAGTCGAACGGTTATAGCAATATAGCAGTGGCGCACGGGTGA
GTAATACTTGGGAACGTGCCCTTCAGTTCAGGATAACAGCGGGAAACTGCTGCTAATACTGGATATTCCCT
CCGGGGGAAAGATTTATCGCTGAAGGATTGGCCCAAGTCAGATTAGGTAGTTGGTGGGGTAATGGCCTAC
CAAGCCTACGATCTGTAGCTGGTCTGAGAGGACGACCAGCCACACTGGAACTGAGACACGGTC
>Zotu15020

GAACGCTGGCGGCGTGCCTAATACATGCAAGTCGAGCGAATCTTTGGGAGCTTGCTCCTAAAGGTTAGCG
GCGGACGGGTGAGTAACACGTGGGCAACCTGCCTATAAGACTGGGATAACTTCGGGAAACCGGAGCTAAT
ACCAGATAATTCTTTTCTACTCATGTAGGAAAGCTGAAAGACGGCTTACGCTGTCACTTATAGATGGGCCCG
CGGCGCATTAGCTAGTTGGTGAGGTAATGGCTCACCAAGGCCACGATGCGTAGCCGACCTGAGAGGGTGA
TCGGTCACACTGGGACTGAGACACGGCC
>Zotu15024
GAACGCTGGCGGTATGCTTAACACATGCAAGTCGAACGAAATCTTTCGGGATTTAGTGGCGGACGGGTGA
GTAACGCGTGAGAATCTGGCTTCAGGTCGGGGACAACCACTGGAAACGGTGGCTAATACCGGATGTGCCG
CAAGGTAAAAGGCTTGCCGCCTGAAGATGAGCTCGCGTCTGATTAGCTAGTAGGTGTGGTAAGAGCGCAC
CTAGGCGACGATCAGTAGCTGGTCTGAGAGGATGATCAGCCACACTGGGACTGAGACACGGCC
>Zotu15028
GAACGCTAGCGGCAGGCTTAATACATGCAAGTCGAGGGGCAGCAGGGTGTAGCAATACACTGCTGGCGA
CCGGCAAACGGGTGCGGAACACGTACGCAACCTTCCTTTAAGTGGGGAATAGCCCAGGGAAACTTGGATT
AATACCTCGTAATATTATGAGACGGCATCGTTTTATAATTATAGCTCCGGCGCTTAAAGATGGGCGTGCGCC
TGATTAGGTAGTTGGCGGGGTAACGGCCCACCAAGCCTTCGATCAGTAACTGGTGTGAGAGCACGACCAG
TCACACGGGCACTGAGACACGGGC
>Zotu15030
GAACGCTGGCGGCGCGCTTAACACATGCAAGTCGCACGAGAAAGGGAGCAATCCTGAGTAAAGTGGCGT
ACGGGTGAGTAACACGTGGATCATCTACCTTTTAGTGGGGAATACCTCCGGGAAACTGGAGACAATACCG
CATAAGCTCGTAAGAGGAAAGCAGCAATGCGCTGAAAGAGGAGTCCGCGGCTGATTATGCTAGTTGGCGG
GGTAACGGCCCACCAAGGCGAAGATCGGTAGCCGGCCTGAGAGGGCTCACGGCCACACTGGCACTGAAA
CACGGGC
>Zotu15038
GAACGCTGGCGGCGTGCTTAACACATGCAAGTCGAACGAGCAAGAGCTGTAGCAATACAGGGTTCTGCTA
GTGGCAGACGGGTGAGTAATACATGGGAATCTACCCTGCAGTGCGAAATAACAGTTGGAAGCAATTGCTA
ATGTCGCATATAGCCGAGAGGTGAAAGGTTTACTGCTGCAAGATGGGCCCATGCAGGATTAGCTAGTTGG
TGAGGTAATGGCTCACCAAGGCAACGATCTTTAGTTGGTCTGAGAGGATGATCAGCCACACTGGGACTGA
GACACGGCC
>Zotu15040
GAACGCTGGCGGCAGGCTTAACACATGCAAGTCGAGCGGACCCTTCGGGGTTAGCGGCGGACGGGTTAAT
AACGCGTGGGAACGTACCCTTCTCTATGGAATAGCCTCGGGAAACTGAGAGTAATACCATATACGCCCTTA
GGGGGAAAGATTTATCGGAGAAGGATCGGCCCGCGTTGGATTAGGTAGTTGGTGGGGTAATGGCCTACC
AAGCCTACGATCCATAGCTGGTTTGAGAGGATGATCAGCAACACTGGGACTGAGACACGGCC
>Zotu15045
AAACGCTGGCGGGGTGCATGAGACATGCAAGTCGAACGAGGTGCCATCGGGCTTGCTCGATGGTTACCTA
GTGGCGGACGGCTGAGTAACGCGTAGGTGACCTACCCGGAAGTGGGGAATAACTGCCCGAAAGGGTGGC
TAATACCGCATGTGGTCGACGATTCATTTCGTCGTCTAAAGGAGCAATTCGCTTCCGGAGGGGCCTGCGTC
CGATTAGCTAGTTGGTGGGGTAACGGCTCACCAAGCCGACGATCTGTAGCTGGTCTGAGAGGATGATCAG
CCACACTGGGACTGAGACACGGCC
>Zotu15048
GGATGCTGGCGACGTGGATAAGGCATGCAAGTCAAGGGGGCCGCAAGGCAACCGGCAGACGAGGTAGT
AACACGTACGAACGCACCCCAGAGTCTGGCATAGCTCGTCGAAAGACGGGGTAATTCCGGATGGTCCCGC
AAGGGTAAAGGTTTTTCGCTCTGGGAGCGGCGTGCGCAATATCAGCTAGTTGGTGAGGTAATGGCTCACC
AAGGCTACGACGTTTAGGGGAGCTGAGAGGCTGACCCCCACCGATGGGACTGAGACACGGCC
>Zotu15051
GAACGCTGGCGGTGTGCTTAACACATGCAAGTGGAGCGACGAACCAGGGCTTGCCCTGGGGCAGAGCCG
CGAACGGGTGAGTAACACGTGGGGAACCTACCCCGATGACCGGGACAACCCGAGGAAACTCGGGCTAAT
ACCGGATGTGCTCTCTTCACATCAGTGAGGTGAGTAAAGGAAGCTTCGGCCTCCGCATCGGGAGGGCCCC
GCGGCCCATTAGCTTGTTGGTGGGGTAATGGCCTACCAAGGCTGCGATGGGTAGCTGGTCTGAGAGGACG
ATCAGCCACACTGGGACTGAGACACGGCC

>Zotu15058
GAACGCTGGCGGCATGCCTAACACATGCAAGTCGAACGAGACCTTTCGGGGTCTAGTGGCGCACGGGTGC
GTAACGCGTGGGAATCTGCCCCTTGGTTCGGAATAACAGTGGGAAACTACTGCTAATACCGGATGACGAC
GTAAGTCCAAAGATTTATCGCCGAGGGATGAGCCCGCGTAGGATTAGCTGGTTGGTGAGGTAATGGCTCA
CCAAGGCGACGATCCTTAGCTGGTCTGAGAGGATGATCAGCCACACTGGGACTGAGACACGGCC
>Zotu15060
GAACGCTGGCGGCGTGCTTAAGACATGCAAGTCGTACGAGAAAGTCTCTTCGGAGATGAGTAAAGTGGCG
CACGGGTGAGTAACGCGTGGGTAATCTGCCCTTAGGTTTGGGGTAACCCCGGGAAACCGGAGCTAATACC
GAATGAGACCACGATGACTTCGGTCGATGTGGGAAAAGGTGGCCTCTTCTTGAAAGCTATCGCCAAAGGA
TGAGCCCGCGTTTGATTAGCTAGTTGGTGAGGTAATGGCTCACCAAGGCGACGATCAATAGCTGGTCTGAG
AGGATGATCAGCCACACTGGAACTGAGATACGGTC
>Zotu15063
GAACGCTAGCGGCATGCCTAACACATGCAAGTCGAACGGTGAACCAGGGCTTGCCTTGGGGATCAGTGGC
GAACGGGTGAGTAACACGTGGGCAACCTGCCCCAGACTCTGGGATAACTCCGGGAAACCGGGGCTAATAC
CGGATACGAATCGAGTTGGCATCTTCTCGATTGGAAAGTTTTTCGGTCTGGGATGGGCCCGCGGCCTATCA
GCTTGTTGGTGGGGTAATGGCCTACCAAGGCGACGACGGGTAGCCGGCCTGAGAGGGCGAACGGCCACA
CTGGGACTGAGACACGGCC
>Zotu15066
CAACGCTGGCGGCGCGCTTAACACATGCAAGTCGCACGAGAAAGTGGAGCAATCCATGAGTACAGTGGCG
TACGGGTGAGTAACACGTGGATTATCTACCTTCTAGTGGGGAATACCTCCGGGAAACTGGAGACAATACCG
CATAAGCTCGTAAGAGGAAAGCAGCAATGCGCTGGAAGAGGAGTCCGCGGCTGATTAGCTAGTTGGCGG
GGTAATGGCCCACCAAGGCGAAGATCGGTAGCCGGCCTGAGAGGGCACACGGCCACACTGGCACTGAAA
CACGGGC
>Zotu15068
GAACGCTGGCGGCATGCCTAACACATGCAAGTCGAACGAGACCTTCGGGTCTAGTGGCGCACGGGTGCGT
AACGCGTGGGAATCTGCCCTTTGCTTCGGAATAACAGTTAGAAATGACTGCTAATACCGGATGATGTCGCA
AGACCAAAGATTTATCGGCAAGGGATGAGCCCGCGTAGGATTAGCTTGTTGGTGAGGTAAAAGCTCACCA
AGGCGACGATCCTTAGCTGGTCTGAGAGGATGATCAGCCACACTGGGACTGAGACACGGCC
>Zotu15071
GAACGCTGGCGGCATGCCTAACACATGCAAGTCGAACGAAGGCTTCGGCCTTAGTGGCGCACGGGTGCGT
AACGCGTGGGAATCTGCCCTTAGGTTCGGAATAACAGTTAGAAATGGCTGCTAATACCGGATGATGACGTA
AGTCCAAAGATTTATCGCCTGAGGATGAGCCCGCGTAGGATTAGCTAGTTGGTGTGGTAAAGGCGCACCA
AGGCGACGATCCTTAGCTGGTCTGAGAGGATGATCAGCCACACTGGGACTGAGACACGGCC
>Zotu15078
GAACGCTGGCGGCGTGCTTAACACATGCAAGTCGAACGAGCAAGAGCTGTAGCAATACAGGATTCTGCTA
GTGGCAGACGGGTGAGTAATACATGGGAATCTACCTTGCAGTGCGAAATAACAGTTGGAAGCAATTGCTA
ATGTCGCATATAGCCGAGAGGTGAAAGGTTTACTGCTGCAAGATGGGCCCATGCAGGATTAGCTAGTTGG
TGAGGTAATGGCTCACCAAGGCAACGATCTTTAGTTGGTCTGAGAGGATGATCAGCCACACTGGGACTGA
GACACGGCC
>Zotu15085
GAACGCTGGCGGTGTGCTTAACACATGCAAGTCGAACGAGCAAGAGCTGTAGCAATACAGGGTTCTGCTA
GTGGCAGACGGGTGAGTAATACATGGGAATCTACCTTGCAGTGCGAAATAACAGTTGGAAGCAATTGCTA
ATGTCGCATATAGCCGAGAGGTGAAAGGTTTACTGCTGCAAGATGGGCCCATGCAGGATTAGCTAGTTGG
TGAGGTAATGGCTCACCAAGGCAACGATCTTTAGTTGGTCTGAGAGGATGATCAGCCACACTGGGACTGA
GATACGGCC
>Zotu15090
TAACGCTGGCGGCGTGCCTAATACATGCAAGTCGAACGCGAAAGTCCTTCGGGATGAGTAGAGTGGCGCA
CGGGTGAGAAACACGTGGATAATCTGCCCAAAAATGGGGAATAACTCCTCGAAAGAGGAGCTAATACCGC
ATAAAAATTTAGCAACTATGGTTGTTTTGTTTAAAGGTGACCTCTATTTATAAGTTATCGTTTTTGGATGAGT

CTGCGCAACATTAGCTAGTTGGTAGGGTAATGGCCTACCAAGGCTACGATGTTTAGCTGGTCTGAGAGGAT
GATCAGCCACACTGGAACTGAGACACGGTC
>Zotu15093
GAACGCTGGCGGCGTGCTTAACACATGCAAGTCGAGCGGTAAGGCTCCTTTCGGGGGTACACGAGCGGC
GAACGGGTGAGTAACACGTGAGTAATCTGCCCTTCACTCTGGGATAAGCACTCGAAAGGGTGTCTAATACC
GGATACGACCGGCCTCCGCATGGGGTGTTGGTGGAAACTTTTGTGGGTGGGGGATGTGCTCACGGCCTAT
CAGCTTGTTGGTGGGGTAATGGCCTACCAAGGCTTCGACGGGTAGCCGGCCTGAGAGGGTGACCGGTCAC
ACTGGGACTGAGACACGGCC
>Zotu15098
GAACGCTGGCGGCATGCCTTACACATGCAAGTCGAACGGCAGCACGGGAGCAATCCTGGTGGCGAGTGG
CGAACGGGTGAGTAATATATCGGAACGTGCCCAGTAGTGGGGGATAGCCCGGCGAAAGCCGGATTAATA
CCGCATACGACCTACGGGTGAAAGCGAGGGATCGCAAGACCTCGCGCTATTGGAGCGGCCGATATCAGAT
TAGCTTGTTGGTGGGGTAAAAGCCCACCAAGGCGACGATCTGTAGCTGGTCTGAGAGGACGACCAGCCAC
ACTGGGACTGAGACACGGCC
>Zotu15107
GAACGCTGGCGGCGTGCTTAACACATGCAAGTCGAACGATGAAGCCCAGCTTGCTGAGTGGATTAGTGGC
GAACGGGTGAGTAACACGTGAGTAACCTGCCCTGGACTCTGGGATAACCCCGAGAAATCGGAGCTAATAC
CGGATAGGACCTTTCCCTGCATGGGGTTGGGTGGAAAGTTTTTCGGTTTGGGATGGACTCGCGGCCTATCA
GCTTGTTGGTGAGGTAATGGCTCACCAAGGCGTCGACGGGTAGCCGGCCTGAGAGGGTGACCGGCCACAC
TGGGACTGAGACACGGCC
>Zotu15110
AAACGCTGGCGGCGTGCTTCAGGCATGCAAGTCGAACGGACCACTTCGGTGGTTAGTGGCGGACGGGTGA
GTAACACGTAAGTAACCTGCCCAGAAGTGGGGAATAACTAGCCGAAAGGCTGGCTAATACCGCATACGAT
GGCTTTTCTCTGGAGAGGTCATCAAAGGCTTAGGCCGCTTTTGGAGGGGCTTGCGGCCGATTAGCTAGTTG
GTGGGGTAACGGCCTACCAAGGCGACGATCGGTAGCTGGTCTGAGAGGACGGACAGCCACACGGGGACT
GAGATACGGCC
>Zotu15112
GAACGCTGGCGGCGTGCCTAATACATGCAAGTCGAGCGGATTGATTCGGAGCTTGCTCCGATGATGTCAG
CGGCGGACGGGTGAGTAACACGTGGGTAACCTGCCTGATAGACTGGGATAACGCTTGGAAACGAGTGCTA
ATACCGGATAGTCTTTTAAACCGCATGGTTTGAAAGGGAAAGAAGCTATCGCTTCACTGTCAGATGGACCC
GCGGCGCATTAGCTAGTTGGTGAGGTAACGGCTCACCAAGGCGACGATGCGTAGCCGACCTGAGAGGGT
GACCGGCCACACTGGGACTGAGACACGGCC
>Zotu15116
GAACGCTGGCGGCGTGCCTAACACATGCAAGTCGAACGTGAACGGGGGCAACCCCTAGTAAAGTGGCGTA
CGGGTGAGTAACACGTAGGCAATCTACCCCAAAGTCTGGAATAACCTGCCGAAAGGCGGGCTAATACTGG
ATAGAGCGACGGAATCCGTAATTGGGTTTTTCGGGAAAGGGAGGGAGCAATCCCCCTTGCTTTGGGATGG
GCCTGCGTCCCATCAGCTAGTTGGTAAGGTAATGGCTTACCAAGGCGATGACGGGTAGCTGGTCTGAGAG
GATGATCAGCCACACTGGAACTGAGACACGGCC
>Zotu15118
GAACGCTGGCGGCGTGGATTAGGCATGCAAGTCGCGCGATACCCTTATTGGAGAGATTGAAGCCGCAAGG
CAGATGTCAATTCGATGAGGGATATAGCGGCAAACGGGTGCGTAATACGTGAGTAATCTACCCATATATCG
AGGATAGCTTCGCGAAAGTGGAGGTAATACTTGATGTGTCCGTACTTACATAAGTAGGAACGGGTAAAGG
CCCCGCAAGGGGTTGTATATGGATGAGCTCATGTCCTATCAGCTAGTTGGTGAGGTAATGGCTCACCAAGG
CTTTTGACGGGTAGGTGGCCTCAGAGGGTGGTCACCCACAAGGGGATTGAGATACTGCC
>Zotu15122
GAACGCTAGCGGCATGCCTAACACATGCAAGTCGAACGATGCTTTCGGGCATAGTGGCGCACGGGTGCGT
AACGCGTGGGAATCTGCCCTGGGGTACGGAATAACTCAGAGAAATTTGAGCTAATACCGTATGATGACGT
AAGTCCAAAGATTTATCGCCCTGGGATGAGCCCGCGTAGGATTAGCTAGTTGGTAGGGTAATGGCCTACCA
AGGCGACGATCCGTAGCTGGTCTGAGAGGACGATCAGCCACACTGGGACTGAGACACGGCC
>Zotu15124

GAACGCTGGCGGCATGCCTTACACATGCAAGTCGAACGGTAACAGGTTAAGCTGACGAGTGGCGAACGG
GTGAGTAATGTATCGGAACGTGCCCAGTCGTGGGGGATAACTGCTCGAAAGAGCAGCTAATACCGCATAC
GATCTGAGGATGAAAGCGGGGGACCTTCGGGCCTCGCGCGATTGGAGCGGCCGATATCAGATTAGGTAGT
TGGTGAGGTAAAGGCTCACCAAGCCGACGATCTGTAGCTGGTCTGAGAGGACGACCAGCCACACTGGGAC
TGAGACACGGCC
>Zotu15128
GAACGCTGGCGGCGTGCCTAATACATGCAAGTCGAGCGAATCTTTGGGAGCTTGCTCCCGAAGATTAGCG
GCGGACGGGTGAGTAACACGTGGGCAACCTGCCTATAAGACTGGGATAACTTCGGGAAACCGGAGCTAAT
ACCGGATAATTCTTTTCCTCTCATGAGGAAAAGCTGAAAGACGGTTTACGCTGTCACTTATAGATGGCCCG
CGGCGCATTAGCTAGTTGGTGAGGTAACGGCTCACCAAGGCGACGATGCGTAGCCGACCTGAGAGGGTG
ATCGGCCACACTGGGACTGAGACACGGCC
>Zotu15130
GAACGCTGGCGGCATGCCTTACACATGCAAGTCGAACGGCAGCGGGGGGAGCTTGCTCCCTGACGGCGA
GTGGCGGACGGGTGAGTAACACGTAGGAATCTACCTTAATTATAATAGTTAGTGGGGGATAACCCGGGGA
AACTCGGGCTAATACCGCATAATCTCTTCAAAGCAAAGCGGGGGATCTTCGGACCTCGTGCTATGAGATGA
GCCTGCGTCGGATTAGCTAGTTGGTAGGGTAATGGCCTACCAAGGCGGCGATCCGTAGCTGGTCTGAGAG
GACGATCAGCCACACTGGGACTGAGACACGGCC
>Zotu15132
GAACGCTAGCGGCAGGCTTAATACATGCAAGTCGAGGGGCAGCAGGGGTAGCAATACTCGCTGGCGACC
GGCAAACGGGTGCGGAACACGTACGCAACCTTCCCATAACTGGTGAATAGCCTTCCGAAAGGGAGATTAA
TACACCGTAACATAGTGGTGTGGCATCACACTACTATTATAGCTTCGGCGGTTTTGGATGGGCGTGCGGCT
GATTAGGTAGTTGGCGGGGTAACGGCCCACCAAGCCTCCGATCAGTAACTGGTGTGAGAGCACGACCAGT
CACACGGGCACTGAGACACGGGC
>Zotu15136
GAACGCTGGCGGCGTGCCTAACACATGCAAGTCGAGCGGTAACAGGTGTAGCAATACATGCTGACGAGCG
GCGGACGGGTGAGTAATATTTGGGAATCTGCCTATTAGTGGGGGATAACCCGGGGAAACTCGGGCTAATA
CCGCATACGCTCTACGGAGGAAAGCCGGGGACCGCAAGGCCTGGCGCTAGTAGATGAGCCCAAATCGGAT
TAGCTAGTTGGTGAGGTAACGGCTCACCAAGGCGACGATCCGTAGCTGGTTTGAGAGAACGACCAGCCAC
ACCGGAACTGAGACACGGTC
>Zotu15140
GAACGCTAGCGGCATGCTTAACACATGCAAGTCGAACGGCAGCATAGGAGCTTGCTCCTGATGGCGAGTG
GCGAACGGGTGAGTAATATATCGGAACGTGCCCTAGAGTGGGGGATAACTAGTCGAAAGACTAGCTAATA
CCGCATACGATCTACGGATGAAAGTGGGGGATCGCAAGACCTCATGCTCCTGGAGCGGCCGATATCTGATT
AGCTAGTTGGTGGGGTAAAAGCTCACCAAGGCGACGATCAGTAGCTGGTCTGAGAGGACGATCAGCCACA
CTGGGACTGAGACACGGCC
>Zotu15145
GAACGCTGGCGGCGTGCTTAACACATGCAAGTCGAGCGGTAAGGCCTCTTCGGGGGTACACGAGCGGCG
AACGGGTGAGTAACACGTGGGTGACCTGCCCTCAGCTCTGGGATAAGCCTGGGAAACTGGGTCTAATACC
GGATATGACCTGGCCTCGCATGGGGTTGGGTGGAAAGTTTTTCGGCTGGGGATGGGCCCGCGGCCTATCA
GCTTGTTGGTGGGGTGATGGCCTACCAAGGCGACGACGGGTAGCCGGCCTGAGAGGGCGACCGGCCACA
CTGGGACTGAGGTACGGCC
>Zotu15149
GAACGCTGGCGGCGTGCCTAATACATGCAAGTCGAGCGGATCTTTAGGAGCTTGCTCCTAAAGGTTAGCG
GCGGACGGGTGAGTAACACGTGGGCAACCTGCCTATAAGACTGGGATAACTTCGGGAAACCGGAGCTAAT
ACCGGATAATTCCTTTCCTCTCATGAGGAAAGGCTGAAAGACGGTTTACGCTGTCACTTATAGATGGGCCC
GCGGCGCATTAGCTAGTTGGTGAGGTAACGGCTCACCAAGGCGACGATGCGTAGCCGACCTGAGAGGGT
GATCGGCCACACTGGGACTGAGACACGGCC
>Zotu15151
GAACGCTAGCGGCAGGCTTAACACATGCAAGTCGAGGGGTATAGTTCTTCGGAACTAGAGACCGGCGCAC
GGGTGCGTAACGCGTATGCAATCTACCTTTTACAGAGGGATAGCCCAGAGAAATTTGGATTAATACCTCAT
AGTATAATGACTCGGCATCGAGATATTATTAAAGTCACAACGGTAAAAGATGAGAATGCGTCCCATTAGCT

AGTTGGTAAGGTAACGGCTTACCAAGGCTACGATGGGTAGGGGTCCTGAGAGGGAGATCCCCCACACTGG
TACTGAGACACGGAC
>Zotu15155
GAACGCTGGCGGCGTGCTTAACACATGCAAGTCGAACGAGCAAGAGCTGTAGCAATACAGGGTTCTGCTA
GTGGCAGACGGGTGAGTAATACATGGGAATCTACCTTGCAGTGCGAAATAACAGTTGGAAGCAATTGCTA
ATGTCGCATATAGCCGAGAGGTGAAAGGTTTACTGCTGCAAGATGGGCCCATGCAGGATTAGCTAGTTGG
TGAGGTAATGGCTCACCAAGGCAATGATCTTTAGTTGGTCTGAGAGGATGATCAGCCACACTGGGACTGA
GACACGGTC
>Zotu15157
GAACGCTGGCGGTGTGCTTAACACATGCAAGTCGAGCGGTAAGGCCCCTTCGGGGGTACACGAGCGGCG
GACGGCTGAGTAACGCGTAGGAACATGCCCCAAAGTGAGGAATAAGCTCCAGAAATGGAGTCTAATGCCG
CATATGATCTTCGGATTAAAGGATTTATCCGCTATGGGAGTGGCCTGCGTATGATTAGGTAGTTGGTGAGG
TAAAGGCTCACCAAGCCGACGATCATTAGCTGGTCTGAGAGGATGACCAGCCAGACTGGAACTGAGACAC
GGTC
>Zotu15163
GAACGCTGGCGGCGTGCCTAATACATGCAAGTCGAGCGGAACGTTGGGAGCTTGCTCCCAACGTTTAGCG
GCGGACGGGTGAGTAACACGTGGGCAACCTGCCCTGCAGATCGGGATAACTCCGGGAAACCGGTGCTAAT
ACCGAATAGTTTGTTTCACCTCCTGGTGAAACACGGAAAGACGGTTTCGGCTGTCACTGCAGGATGGGCCC
GCGGCGCATTAGCTAGTTGGTGGGGTAACGGCCCACCAAGGCGACGATGCGTAGCCGACCTGAGAGGGT
GATCGGCCACACTGGGACTGAGACACGGCC
>Zotu15165
GAACGCTGGCGGCGTGCCTAACACATGCAAGTCGAACGAGAAAGTACTTCGGTATGAGTAAAGTGGCGCA
CGGGTCCGTAACACGTGGATAATCTCCCCTAAGGCGAAAAATAACCCCTCGAAAGAGGGGCTAATATTTCA
TAAGACCACAGATTCTTCGGAATTCGAGGTAAAAGATGGCCTCTATTTATAAGCCGTCACCAGAGGATGAG
TCCGCGCACCATTAGCTAGTTGGCAGGGTAAAAGCCTACCAAGGCAAAGATGGTTAGCTGATCTGAGAGG
ATGATCAGCGACACTGGAACTGAAACACGGTC
>Zotu15168
GAACGCTGGCGGCGTGCCTAACACATGCAAGTCGAGCGCGAAAAGGGCAACCTGAGTAGAGCGGCGCAC
GGGTGCGTAACACGTGGATAATCTGCCTTGGAGCTTGGGATAACTTTCCGAAAGGTTGGCTAATACCAAAT
AAGCTCACGATCTCTTCGGAGGATGTGGGAAAAGTAGGCCACTGTTTACATGCTTACACTTCAAGATGAGT
CCGCGGCCCATCAGCTAGTTGGCGGGGTAACGGCCCACCAAGGCTACGACGGGTAGCTGGTCTGAGAGG
ACGATCAGCCACACTGGAACTGAGACACGGTC
>Zotu15174
GAACGCTGGCGGCGTGCTTAACACATGCAAGTCGAGCGGTAAGGCCCCTTCGGGGGTACACGAGCGGCG
AACGGGTGAGTAACACGTGAGCAATCTACCCATCACATCGGGATAACTTCGGGAAACCGAAGCTAATACC
GGATACGACCACCTCAGGCATCTGATGGTGGTGGAAAGTTCTGGCGGTGATGGATGAGCTCGCGGCCTAT
CAGCTTGTTGGTGGGGTAACGGCCCACCAAGGCTTCGACGGGTAGCCGGCCTGAGAGGGTGACCGGCCAC
ACTGGGACTGAGACACGGCC
>Zotu15176
GAACGCTGGCGGCATGCCTAACACATGCAAGTCGAACGGCAGCACAGTAAAGATTTCGGTCTTTACGGGT
GGCGAGTGGCGGACGGGTGAGTAAAGCGTAGGAATCTGTCCTGGAGAGTGGAATAACCCCGAGGAAACTC
GGGCTAATACCGCATGATATCGAGAGATCAAAGCAGGGGACCCTTCGGGGCCTTGCGCTCTAGGATGAGC
CTACGTCAGATTAGGTAGTTGGTGAGGTAATGGCTCACCAAGCCGACGATCTGTAGCTGGTCTGAGAGGA
TGACCAGCCACACTGGGACTGAGACACGGCC
>Zotu15178
GAACGCTGGCGGCAGGCCTAACACATGCAAGTCGAGCGGGAACTTCGGTTCTAGCGGCGGACGGGTGAG
TAATGCGTAGGAAGCTACCCGACAGAGGGGGATACCAGTTGGAAACGACTGTTAATACCGCATAATGTCT
ACGGACCAAAGTGTGGGACCTTCGGGCCACATGCTGTCGGATGCGCCTACGTGGGATTAGCTAGTTGGTG
AGGTAATGGCTCACCAAGGCGACGATCCCTAGCTGGTTTGAGAGGATGATCAGCCACACTGGAACTGAGA
CACGGTC

>Zotu15181
GAACGCTGGCGGCAGGCCTAACACATGCAAGTCGAACGGGTGTAGCAATACATCAGTGGCGCACGGGTG
AGTAATGCGTGGGAACGTGCCCTTCACTACGGAACAACGTCGGGAAACCGACGCTAATACCGTATAATGTC
TCCGGACCAAAGATTTATCGGTGAAGGATCGGCCCGCGTCTGATTAGCTAGTTGGTGAGGTAAAAGCTCAC
CAAGGCGACGATCAGTAGCTGGTTTGAGAGAATGATCAGCCACACTGGGACTGAGACACGGCC
>Zotu15183
GAACGCTGGCGGCGTGCTTAACACATGCAAGTCGAGCGGTAAGGCCCTTTCGGGGGTACACGAGCGGCG
AACGGGTGAGTAACACGTGAGTAATCTGCCCTCCACTTTGGGATAAGCCTCGGAAACGGGGTCTAATACCG
GATATGAGCATCCCACGCATGTGGGGTGTTGGAAAGTTTTTCGGTGGGGGATGTGCTCGCGGCCTATCAG
CTTGTTGGTGGGGTAATGGCCTACCAAGGCTTCGACGGGTAGCCGGCCTGAGAGGGTGACCGGCCACATT
GGGACTGAGATACGGCC
>Zotu15186
GAACGCTGGCGGCATGCCTTACACATGCAAGTCGAACGGCAGCATAGGAGCTTGCTCCTGATGGCGAGTG
GCGAACGGGTGAGTAATATATCGGAACGTACCCAAGAGTGGGGGATAACGTAGCGAAAGTTACGCTAATA
CCGCATACGATCTAAGGATGAAAGCAGGGGATCTTCGGACCTTGTGCTCCTGGAGCGGCCGATATCTGATT
AGCTAGTTGGTGAGGTAAAGGCTCACCAAGGCCACGATCAGTAGCTGGTCTGAGAGGACGACCAGCCACA
CTGGAACTGAGACACGGTC
>Zotu15193
GAACGCTGGCGGCGTGCTTAACACATGCAAGTCGAACGCCCCGCAAGGGGAGTGGCAGACGGGTGAGTA
ACGCGTGGGAACCTACCTTACGGTCGGGAATAGCTCAGGGAAACTTGGGGTAATACCGGATACGCCCTTA
CGGGGAAAGATTTATCGCCGTTAGATGGGCCCGCGTAGGATTAGCTAGTTGGTGAGGTAATGGCTCACCA
AGGCGACGATCCTTAGCTGGTCTGAGAGGATGATCAGCCACACTGGGACTGAGACACGGCC
>Zotu15201
ATGCTTAAATAATCTCTTAGCTTACTAATAGTCGTTCATATGTATATATATGTATATATACGGTCACTGTTTA
GTTTGTAATTATGTATAAGCATATATCTACGCATGTATAATGACAATTCTCTGAATGATTGCCCTCCTGCTTG
CCTTGTATGTCTGTGTATGCGTACCTTTTTTTTCTTTTTTTTTCTTCTCCTTTCTCTTTCTTCTTGCGTCCTTTTGT
GCCCCTCTCTATATCTCCCATTTCCTCTCTTCCAAAGTCGTGGCAT
>Zotu15204
CAACGCTGGCGGCGTGCCTCAGACATGCAAGTCGAACGATTAAAGCTCTCTTCGGAGAGTGTATAGAGTG
GCGCACGGGTGAGTAACACGTAAGTAATCTACCTTCGAGTGGGGAATAACGTCCGGAAACGGACGCTAAT
ACCGCATAATGCAGCGGCATCGCAAGATGACAGTTGTTAAAGATTTATCGCTTGAAGAGGAGCTTGCGGC
AGATTAGCTAGTTGGTAAGGTAATGGCTTACCAAGGCTACGATCTGTAACCGGTCTAAGAGGACGGTCGG
TCACACTGACACTGAATAACGGGT
>Zotu15210
GAACGCTGGCGGCGTGCTTAACACATGCAAGTCGAACGAGCAAGAGCTGTAGCAATACAGGGTTCTGCTA
GTGGCAGACGGGTGAGTAATACATGGGAATCTACCTTGCAGTGCGAAATAACAGTTGGAAGCAATTGCTA
ATGCCGCATATAGCCGAGAGGTGAAAGGTTTACTGCTGCAAGATGGGCCCATGCAGGATTAGCTAGTTGG
TGAGGTAATGGCTCACCAAGGCAACGATCTTTAGTTGGTCTGAGAGGATGATCAGCCACACTGGGACTGA
GACACGGCC
>Zotu15216
GAACGCTGGCGGCGTGCTTAACACATGCAAGTCGAACGGGATCCATGAAGTAGCAATACTTCGGAAGATC
TAGTGGCGAACGGGTGAGTAACACGTGAAGAACCTGCCCCGAACTCTGGAATAACAGTCGGAAACGATTG
CTAATACCGGATACCCTCACGAGGTCTCATGGCCTTGTGAAGAAAGATTTATCGGTTTGGGAGGGCTTCGC
GGCCTATCAGCTTGTTGGTGAGGTAACGGCTCACCAAGGCGTCGACGGGTAGCTGGTCTGAGAGGATGAT
CAGCCACACTGGGACTGAGACACGGCC
>Zotu15221
GAACGCTGGCGGCGTGCCTAATACATGCAAGTCGAACGAGCTCTTCGGAGCTAGTGGCGGACGGGTGAGT
AACACGTTGGTACCTACCCCTAAGTGGGGAATAACTGCCCGAAAGGGTGGCTAATACCGCATAATCCCGCA
AGGGTAAAGCAGTAATGCGCTTAGGGAGGGGCCTGCGTCCCATTAGCTTGTTGGTAGTGTAATGGACTAC
CAAGGCGACGATGGGTAGCTGGATCGAGAGGTTGACCAGCCACACTGGGACTGAGATACGGCC

>Zotu15229
GAACGCTGGCGGCGTGCTTAACACATGCAAGTCGAACGAACCTTCGGGTTAGTGGCGAACGGGTGAGTAA
CACGTGGGCAACCTGCCTCGAGCTCTGGGATAACTCCGGGAAACCGGGGCTAATACCGGATATGACCTACT
GAGACATCTTGGTGGGTGGAAAGTTTTTCGGCTGGGGATGGGCCCGCGGCCTATCAGCTTGTTGGTGGGG
TGATGGCCTACCAAGGCGGTGACGGGTAGCCGGCCTGAGAGGGCGACCGGCCACACTGGGACTGAGACA
CGGCC
>Zotu15237
GAATGCTGGCGGCGTGCCTAATACATGCAAGTCGAGCGGTAACAGGAGTAGTTTACTACTTGCTGACGAG
CGGCGGACGGCTGAGTAACGCGTGGGAACATGCCCCAAAGTGAGGGATAACGCACCGAAAGGTGTGCTA
ATACCGCATATGGTCTTCGGATTAAAGGATTTATCCGCTTTGGGAGTGGCCCGCGTACGATTAGGTAGTTG
GTGAGGTAATGGCTCACCAAGCCTACGATCGTTAACTGGTCTGAGAGGATGATCAGTCAGACTGGAACTG
AGACACGGTC
>Zotu15240
GAACGCTAGCGGCATGCCTAACACATGCAAGTCGAACGAAGCACTTTACCGGATTTCTTCGGGATGAAAGT
TTTGTGACTGAGTGGCGGACGGGTGAGTAACGCGTGGGTAACCTGCCTCATACAGGGGGATAACAGTTAG
AAATGACTGCTAATACCGCATAAGACCACAGGATTGCATGATCCGGTGGTAAAAACTCCGGTGGTATGAGA
TGGACCCGCGTCTGATTAGGTAGTTGGTGGGGTAACGGCTCACCAAGCCGACGATCAGTAGCCGACCTGA
GAGGGTGACCGGCCACATTGGGACTGAGACACGGCC
>Zotu15243
CAACGCTGGCGGCGTGCCTAACACATGCAAGTCGAACGCGAAAGGGGACTTCGGTTCCTGAGTAGAGTGG
CGTACGGGTGAGTAACGCGTGGGTCATCTGCCCTTGGGTGGGGAATAACTAACCGAAAGGTTAGCTAATA
CCGCATAACACAGTGATGTCAAAGCAGCAATGTGCCCGAGGAGGAGCCTGCGTCTGATTAGGTAGTTGGT
GGGGTAACGGCCTACCAAGCCTGCGATCAGTAGCTGGTCTGAGAGGACGGCCAGCCACAATGGCACTGAA
ACACGGGC
>Zotu15247
GAACGCTGGCGGCAGGCTTAACACATGCAAGTCGAGCGGGCCCTTCGGGGTCAGCGGCAGACGGGTGAG
TAACGCGTGGGAACGTGCCCTTCGGTTCGGAATAACTCAGGGAAACTTGAGCTAATACCGGATACGTGCG
AGAGCAGAAAGGTTTATCTGCCGAAGGATCGGCCCGCGTCTGATTAGCTGGTTGGTGAGGTTACGGCTCA
CCAAGGCGACGATCAGTAGCTGGTCTGAGAGGATGATCAGCCACACTGGGACTGAGACACGGCC
>Zotu15251
GAACGCTGGCGGCGTGCCTAATACATGCAAGTCGAGCGAACAGAGAAGGAGCTTGCTCCTTTGACGTTAG
CGGCGGACGGGTGAGTAACACGTGGGCAACCTACCCTATAGTTTGGGATAACTCCGGGAAACCGGGGCTA
ATACCGAATAATCTATTGTCCCTCATGGGACAATACTGAAAGACGGCATCTCGCTGTCGCTATAGGATGGG
CCCGCGGCGCATTAGCTAGTTGGTGAGGTAACGGCTCACCAAGGCAACGATGCGTAGCCGACCTGAGAGG
GTGATCGGCCACACTGGGACTGAGACACGGCC
>Zotu15257
GAACGCTAGCGGCGGGCTTAATACATGCAAGTCGAACGGTGCAGCAATGCATAGTGGCAGACGGGTGCG
TAACGCGTATGCAACCTACCTTTTATCGGGGGACAACATTTCGAAAGAAGTGCTAATACCGCGTAAGATAG
TAGTGAGACATCTCACAGCTATTAAACCTACGGGGATAGAAGATGGGCATGCGTCTGATTAGCTAGTTGTC
GGGGTAAAAGCCCACCAAGGCGACGATCAGTAGGGGAACTGAGAGGTTGATCCCCCACACTGGCACTGA
GATACGGGC
>Zotu15261
GAACGCTGGCGGCGCGCCTAACGCATGCAAGTCGAACGAGGCTGACAAGGTGCTTGCACCTTCGATGCCT
AGTGGCGAACGGGTGAGGAACACGTGAGAAACCTGCCCCAAACACGGGAATAACCCGAGGAAACTCTGG
CTAATGCCCGATACCCTCCGCAGTTCGCATGAACAGCGAAGGAAAGATCAGTCGGTTTGGGAGGGTCTCG
CGTCCTATCAGCTAGTTGGTGAGGTAACGGCTCACCAAGGCGACGACGGGTAGCTGGTTTGAGAGAACGA
TCAGCCACACTGGGACTGAGACACGGCC
>Zotu15263
GAACGCTGGCGGCAGGCCTAATACATGCAAGTCGGACGAGTAAGAGCCCGCAAGGGAGTTCTGCTAGTG
GCAAACGGGTGCGGAACACGTGGGAATCTACCCAAAAGTATGGAACAACAGTTGGAAACGACTGCTAATA

CCATATACGCTCGAAAGAGGAAAGATTTATCGCTTTTGGATGAGCCCGCGTAAGATTAGCTAGTTGGTGAG
GTAATGGCTCACCAAGGCAACGATCTTTAGCTGGTCTGAGAGGACGATCAGCCACACTGGGACTGAGACA
CGGCC
>Zotu15269
GAACGCTGGCGGCGCGCCTAACACATGCAAGTCGAGCGAAAACCGAGCCTTCGGGTTCGGGGAAAGCGG
CGAACGGGTGAGTAACACGTGGGTAACCTGCCCTCGGCACCGGGATAGCCCGGGGAAACTCGGATTAATA
CCGGACGGTCTCCGGAGCATTCGGGCGCCGGAGAAAAGGTGGCTTCGGCATCTGGCCGAGGAGGGGCCT
GCGGCGGATTAGCTTGTTGGTGGGGTAATGGCTCACCAAGGCTACGATCCGTAGCTGGTCTGAGAGGACG
ATCAGCCACACTGGGACTGAGACACGGCC
>Zotu15271
GAACGCTGGCGGCAGGCTTAACACATGCAAGTCGAGCGCCCCGCAAGGGGAGCGGCAGACGGGTGAGTA
ACACGTGGGAACGTACCTTTTGGTTCGGAACAACTGAGGGAAACTTCAGCTAATACCGGATAAGCCCTTAC
GGGGAAAGATTTATCGCCAAAAGATCGGCCCGCGTCTGATTAGCTAGTTGGTGGGGTAATGGCCCACCAA
GGCGACGATCAGTAGCTGGTCTGAGAGGATGATCAGCCACACTGGGACTGAGACACGGCC
>Zotu15273
GAACGCTGGCGGCGTGCCTAATACATGCAAGTCGAACGCTTCTTTTTCACCGAACTTCGGTTCATCGAAAAA
GAGGAGTGGCGAACGGGTGAGTAACACGTGGGTAACCTGCCCATCAGAAGGGGATAACACTTGGAAACA
GGTGCTAATACCGTATAATGCTTTTTCTCGCATGAGAGCAAGCTGAAAGGCGCTTTTGCGTCACTGATGGAT
GGACCCGCGGTGCATTAGCTAGTTGGTGAGGTAACGGCTCACCAAGGCCACGATGCATAGCCGACCTGAG
AGGGTGATCGGCCACACTGGGACTGAGACACGGCC
>Zotu15279
GAACGCTGGCGGCGTGCTTAACACATGCAAGTGGAGCGACGAACCGGGCTTCGGCCCGGGGCAAAGCCG
CGGACGGGTGAGTAACACGTGGGTAACCTGCCCCGTTGACCGGGACAACCCGAGGAAACTCGGGCTAATA
CCGGATGAGCTCTCATCCCATCAGGGATGTGAGGAAAGGAAGCTTCGGCCTCCGCATCGGGATGGGCCCG
CGGCCCATTAGCTTGTTGGTGGGGTAATGGCCTACCAAGGCTTCGATGGGTAGCTGGTCTGAGAGGACGA
TCAGCCACACTGGGACTGAGACACGGCC
>Zotu15286
GAACGTTTGCGGCGGGCCTAACACATGCAAGTCGAACGGGCTAGCAATAGTCAGTGGCGCACGGGTGCGT
AACACGTGGGTAATCAACCCTTCAGCTTGGGATAACGCCTTGAAAGGGGCGCTAATACCGGATACGGCGC
GAGAGACTTCGGTCTTTCACGAGAAAGGTTCGCAAGGACCACTGAAGGACGAGCCTGCGGCCCATCAGCT
AGTTGGTGAGGTAAGAGCTCACCAAGGCGAAGACGGGTAGCTGGTCTGAGAGGATGATCAGCCACACTG
GAACTGAGACACGGTC
>Zotu15292
GAACGCTGGCGGCGTGCTTAACACATGCAAGTCGAGCGGTAAGGCCCTTCGGGGTACACGAGCGGCGAA
CGGGTGAGTAACACGTGAGTAATCTGCCCTTCACTCAGGGATAACCACGGGAAACTGTGGCTAATACCTGA
TATGACCCTTCCACGCATGTGGTGGGGTGGAAAGTGTTTAGCGGTGAGGGATGTGCTCGCGGCCTATCAG
CTTGTTGGTGAGGTAGTGGCTCACCAAGGCTTCGACGGGTAGCCGGCCTGAGAGGGTGACCGGCCACACT
GGGACTGAGACACGGCC
>Zotu15297
GAACGCTGGCGGCATGCCTAACACATGCAAGTCGAACGAGATCTTCGGATCTAGTGGCGCACGGGTGCGT
AACGCGTGGGAATCTGCCCTGGGGTACGGAATAACTCAGAGAAATTTGAGCTAATACCGTATGATGACGT
AAGTCCAAAGATTTATCGCCCTGGGATGAGCCCGCGTAGGATTAGCTAGTTGGTGGGGTAAAGGCCTACC
AAGGCGACGATCCGTAGCTGGTCTGAGAGGACGATCAGCCACACTGGGACTGAGACACGGCC
>Zotu15305
GAACGCTGGCGGCAGGCTTAACACATGCAAGTCGAACGATCTCTTCGGAGATAGTGGCAGACGGGTGAGT
AACACGTGGGAATCTACCTAGTTCTTCGGGATAACTGAGGGAAACTTCAGCTAATACCGGATACGCCCTTTT
GGGGAAAGATTTATCGGAATTAGATGAGCCCGCGTTGGATTAGCTAGTTGGTGAGGTAATGGCTCACCAA
GGCGACGATCCATAGCTGGTCTGAGAGGATGATCAGCCACACTGGGACTGAGACACGGCC
>Zotu15309

GAACGCTGGCGGCGCGCCTAACACATGCAAGTCGAACGAGCGAGAGAGAGCTTGCTTTCTCGAGCGAGTG
GCGAACGGGTGAGTAACGCGTGAGGAACCTGCCTCAAAGAGGGGGACAACAGTTGGAAACGACTGCTAA
TACCGCATAAGCCCACGGTGCCGCATGGCACAGAGGGAAAAGGAGCAATCCGCTTTGAGATGGCCTCGCG
TCCGATTAGCTAGTTGGTGAGGTGACGGCCCACCAAGGTGACGATCGGTAGCCGGACTGAGAGGTTGAAC
GGCCACATTGGGACTGAGACACGGCC
>Zotu15317
GAACGCTGGCGGCGTGCTTAACACATGCAAGTCGTACGATGATGCTTCAGCTTGCTGGGGTGGATTAGTG
GCGAACGGGTGAGTAACACGTGAGTAACCTGCCTTTCACTCTGGGATAAGCCTTGGAAACGAGGTCTAATA
CTGGATATGACTCATTGCCGCATGGTGGTGGGTGGAAAGCCTTTACGGTGGTGGATGATGGACTCGCGGA
CTATCAGCTTGTTGGTGGGGTTGTGGCCTACCAAGGCTTTGACGGTTAGCCGGCCTGAGAGGGTGATCGG
CCACATTGGGACTGAGACACGGCC
>Zotu15325
GAACACTGGCGGCGTGCTTAACACATGCAAGTCGAACGAGCAAGAGCTGTAGCAATACAGGGTTCTGCTA
GTGGCAGACGGGTGAGTAATACATGGGAATCTACCTTGCAGTGCGAAATAACAGTTGGAAGCAATTGCTA
ATGTCGCATATAGCCGAGAGGTGAAAGGTTTACTGCTGCAAGATGGGCCCATGCAGGATTAGCTAGTTGG
TGAGGTAATGGCTCACCAAGGCAACGATCTTTAGTTGGTCTGAGAGGATGATCAGCCACACTGGGACTGA
GACACGGCC
>Zotu15327
GAACGCTAGCGGCAGGCCTAATACATGCAAGTCGAACGGTGGTAGCAATACCATAGTGGCGCACGGGTGC
GTAACACGTAACTAACCTACCTATAACTGGGGGATAGCCCGCCGAAAGGCGGATTAATACCGCATAAGCTG
CTTGAGTGGCATCACTCGGATAGTAAAGATTTATTGGTTATAGATGGGGTTGCGGGTCATTAGCTAGTTGG
TAAGGTAACGGCTTACCAAGGCGACGATGACTAGGGGAGCTGAGAGGCTGGTCCCCCACACGGGCACTG
AGATACGGGC
>Zotu15330
GAACGCTGGCGGCGTGCTTAACACATGCAAGTCGAGGGGCAGCGGGGGTAGCAATACTTGCCGGCGACC
GGCGCACGGGTGCGTAACGCGTATGCAACCTACCTTTGACACGGGAATAGCCCGAAGAAATTCGGATTAA
TACCCGATGGTATCATTTGGTGACATCGCCGGATGATTAAAGATTTATCGGTCAGAGATGGGCATGCGTCT
GATTAGCTAGTTGGTGAGGTAACTGCTCACCAAGGCTACGATCAGTAGGGGTTCTGAGAGGATGATCCCCC
ACACCGGTACTGAGACACGGAC
>Zotu15333
GAACGCTGGCGGCGTGCCTAATACATGCAAGTCGAGCGGTAAGGCTCTCACAGTTTGCTGGATTGAGACA
GGTCGCGGCTCCGAAAGGATAAGCCGCATCTAAAACGAAGCGATGAAGCTTCGTCTGTGTCGCAAAAATC
AAGCAAAGTGTGAGAGTACACGAGCGGCGGACGGCAGAGTAACGCGTAGGAACGTACCCCAAACAGAGG
GATAAGCCCGGGAAACCGGGTCTAATACCGCATATTATCTTCGGATCAAAGCCTTCGGGCGGTTTGGGAGC
GGCCTGCGTATGATTAGTTAGTTGGTGAGGTAATAGCTCACCAAGACGACGATCATTAGCTGGTCTGAGAG
GATGATCAGCCTGACTGGAACTGAGAACGGTC
>Zotu15336
GAACGCTGGCGGCGTGCCTAACACATGCAAGTCGAACGGAGTATTAAGAAGTTTTCGGACGGATTAATAC
TTAGTGGCGGACGGGTGAGTAACGCGTGGGTAACCTGCCTCATACAGGGGGATAACAGTTGGAAACGGCT
GCTAAAACCGCATAATATAAGGAAGTCGCATGACGACCTTATTAAATATTAATAGGTATGAGATGGGCCCG
CGTCTGATTAGCTAGTTGGTGAGGTAAAGGCTCACCAAGGCGACGATCAGTAGCCGGCTTGAGAGAGTGA
CCGGCCACATTGGGACTGAGACACGGCC
>Zotu15340
GAACGCTGGCGGTAGGCCTAACACATGCTAGTCGAACGGCAGCACAGGAGAGCTTGCTCTCTGGGTGGCG
AGTGGCGGACGGGTGAGGAATACATCGGAATCTACCTTTTCGTGGGGGATAACGTAGGGAAACTTACGCT
AATACCGCATACGACCTACGGGTGAAAGTGGGGGACCGCAAGGCCTCACGCAATTAGATGAGCCGATGTC
CGATTAGCTAGTTGGCGGGGTAATGGCCCACCAAGGCGACGATCGGTAGCTGGTCTGAGAGGATGATCAG
CCACACTGGAACTGAGACACGGTC
>Zotu15342
GAACGCTAGCGGCATGCCTAACACATGCAAGTCGAACGGCAGCATGATCTAGCTTGCTAGATTGATGGCG
AGTGGCGAACGGGTGAGTAATACATCGGAACGTGCCCTGTAGTGGGGGATAACTAGTCGAAAGATTAGCT

AATACCGCATACGACCTGAGGGTGAAAGTGGGGGACCGCAAGGCCTCATGCTATAGGAGCGGCCGATGTC
TGATTAGCTAGTTGGTAGGGTAATGGCCTACCAAGGCGACGATCCGTAGCTGGTCTGAGAGGACGATCAG
CCACACTGGGACTGAGACACGGCC
>Zotu15345
GAACGCTGGCGGCGTGCTTAACACATGCAAGTCGAACGATGATCCCAGCTTGCTGGGGGATTAGTGGCGA
ACGGGTGAGTAACACGTGAGTAACCTGCCCTTGACTCTGGGATAAGCCTGGGAAACTGGGTCTAATACCG
GATACGACCATCTGGCGCATGTCATGGTGGTGGAAAGCTTTTGTGGTTTTGGATGGACTCGCGGCCTATCA
GCTTGTTGGTGGGGTAATGGCCTACCAAGGCGACGACGGGTAGCCGGCCTGAGAGGGTGACCGGCCACA
CTGGGACTGAGACACGGCC
>Zotu15350
GAACGCTGGCGGCGTGCTTAACACATGCAAGTCGAGCGAGGCTCCCTCCTTCGGGAGGGATGTCCTAGCG
GCGAACGGGTGAGTAACACGTGGGCAATCTGCCCCTAGCTCTGGGATAACCCCGGGAAACCGGGGCTAAT
ACCGGATACGACTCCACCAGGCATCTTCTGGAGTGGAAAGGGTTTACTGGCTAGGGATGAGCCCGCGGCC
TATCAGCTTGTTGGTGGGGTAATGGCCTACCAAGGCGACGACGGGTAGCCGGCCTGAGAGGGTGTCCGGC
CACACTGGGACTGAGATACGGCC
>Zotu15354
GAACGCTGGCGGCAGGCCTAATACATGCAAGTCGAGCGCCCCTTCGGGGGAGCGGCGGACGGGTGAGTA
ACGCGTGGGAATATGCCCTTTGGTACGGAACAACTGAGGGAAACTTCAGCTAATACCGTATGTGCCCTTCG
GGGGAAAGATTTATCGCCATTGGAGTAGCCCGCGTTGGATTAGGTAGTTGGTGAGGTAAAGGCTCACCAA
GCCTACGATCCATAGCTGGTCTGAGAGGATGATCAGCCACATTGGGACTGAGACACGGCC
>Zotu15358
GAACGCTGGCGGCGTGCTTAACACATGCAAGTCGAACGGAGTTTTAGTAATTGATTGATTTAGCTTGCTATT
GAGAGATTTTGCTAAAACTTAGTGGCGGACGGGTGAGTAACGCGTGGGTAACCTGCCGTATACAGGGGGA
CAACAGTTGGAAACGACTGCTAATACCGCATAAGCGCACAGGACCGCATGGTCTGGTGTGAAAAACTCCG
GTGGTATACGATGGACCCGCGTCTGATTAGCTAGTTGGTGAGGTAACGGCCCACCAAGGCGACGATCAGT
AGCCGGCCTGAGAGGGTGAACGGCCACATTGGGACTGAGACACGGCC
>Zotu15360
GAACGCTAGCGGCAGGCTTAACACATGCAAGTCGAGGGGCAGCGCGGGTAGCAATACCTGGCGGCGACC
GGCAAACGGGTGCGGAACACGTACAGAACCTACCTTTGTCTGGGGCATAGCCCGGAGAAATCCGGATTAA
CTCCCCATAATATAATTGGGTGGCATCACTTGGTTATTAAAAATTTATTGGACAGAGATGGCTGTGCGTCCC
ATTAGCTAGTTGGTAGGGTAACGGCCTACCAAGGCAACGATGGGTAGCTGGTGTGAGAGCACGACCAGCC
ACACGGGCACTGAGACACGGGC
>Zotu15363
GAACGCTGGCGGCGCGCTTAATACATGCAAGTCGAACGAGAAGGTGTAGCAATACACTTGTAAAGTGGCG
AACGGGTGAGGAATACATGGGTAACCTACCCTCGAGTGGGGAACAACCAGCCGAAAGGTTGGCTAATACC
GCGTACGCTTCCGGGTCTTCGGATTCGGAAGGAAAGTCATACCGTGGGTATGACGCTCAAGGATGAGCTC
ATGTCCTATCAGCTTGTTGGTAGGGTAACGGCCTACCAAGGCTCCGACGGGTAGCTGGTCTGAGAGGACG
ATCAGCCACACTGGCACTGCGACACGGGC
>Zotu15368
GAACGCTGGCGGAATGCTTTACACATGCAAGTCGAACGGAACTTAAGGGCTTGCTCTTAAGTTTAGTGGCG
AACGGGTGAGTAATATATCGGAACGTATCCAATAATGGGGGATAACTAATCGAAAGGTTGGCTAATACCG
CATACGCCCTACGGGGGAAAGCAGGGGATCTTCGGACCTTGCGTTAATGGAGCGGCTGATATCTGATTAG
CTAGTTGGTGAGGTAAAGGCTCACCAAGGCGACGATCAGTAGCTGGTCTGAGAGGACGACCAGCCACACT
GGAACTGAGACACGGTC
>Zotu15371
GAACGCTGGCGGCATGCCTAACACATGCAAGTCGAACGAGACCTTCGGGTCTAGTGGCGCACGGGTGCGT
AACGCGTGGGAATCTGCCCTTGGGTTCGGAATAACAGTTAGAAATGACTGCTAATACCGGATGATGACTTC
GGTCCAAAGATTTATCGCCCAGGGATGAGCCCGCGTCGGATTAGCTAGTTGGTAGGGTAAAGGCCTACCA
AGGCGACGATCCGTAGCTGGTCTGAGAGGATGATCAGCCACACTGGGACTGAGACACGGCC
>Zotu15373

GAACGCTAGCGGCATGCTTAACACATGCAAGTCGAACGCTGTAGCAATACAGAGTGGCAGACGGGTGAGT
AACACGTGGGAACATACCCTTCGGTTCGGAATAACTCAGGGAAACTTGAGCTAATACCGGATACGTTCGCG
AGAAGAAAGATTTATCGCCGAAGGATTGGCCCGCGTCTGATTAGCTAGTTGGTGAGGTAACGGCTCACCA
AGGCGACGATCAGTAGCTGGTCTGAGAGGATGATCAGCCACATTGGGACTGAGACACGGCC
>Zotu15382
GAACGCTGGCGGCGTGCCTAACACATGCAAGTCGAACGAGAAAGGGGCTTCGGCTCCGAGTAAAGTGGC
GCACGGGTGAGTAACACGTGGGTAACCTGCCTCTAAGATTGGGACAACTCGTCGAAAGATGAGCTAATAC
CGAATAAGACCACAGTTCCCGCGGGAACAGCGGTCAAAGGTAGCCTCTGCTTGCAAGCTATCACTTAGAGA
TGGGCCTGCGCACCATTAGCTAGTTGGTAGGGTAACGGCCTACCAAGGCAAAGATGGTTAGCTGGTCTGA
GAGGATGATCAGCCACACTGGAACTGAGACACGGTC
>Zotu15386
GAACGCTGGCGGCATGCCTTACACATGCAAGTCGAACGGCAGCGGGGGGAGCTTGCTCCCTGATGGCGAG
TGGCGGACGGGTGAGTAACACGTAGGAATCTACCTTAATTATAATAGTTAGTGGGGGATAACCCGGGGAA
ACTCGGGCTAATACCGCATAATCTCTTCAAAGCAAAGCGGGGGATCTTCGGACCTCGTGCTATGAGATGAG
CCTGCGTCGGATTAGCTAGTTGGTAGGGTAATGGCCTACCAAGGCGACGATCCGTAGCTGGTCTGAGAGG
ACGATCAGCCACACTGGGACTGAGACACGGCC
>Zotu15389
GAACGCTGGCGGTGTGCTTAACACATGCAAGTCGAACGGACCGCTTACTGTGCTTGCGCGGTGGGTTGGTT
AGTGGCGAACGGGTGAGTAACACGTGAGTAACCTGCCCCCTTCTTCGGGATAACGTTTGGAAACAGATGCT
AATACCGGGTATTTACCTCATGTCGCATGGTGTGGGGTGGAAAGGTTTTTTCTGGTGGGGGATGGGCTCGC
GGCCTATCAGCTTGTTGGTGGGGTGATGGCCTACCAAGGCTTTGACGGGTAACCGGCCTGAGAGGGTGAC
CGGTCACATTGGGACTGAGATACGGCC
>Zotu15393
GAACGCTGGCGGCAGGCTTAACACATGCAAGTCGAACGCATCCTTCGGGGTGAGTGGCAGACGGGTGAG
TAACACGTGGGAACGTACCCTTTGGTTCGGAACAACTCTGGGAAACTGGAGCTAATACCGGATACGCCCTT
ATGGGGAAAGGCTTGTCTGCCGAAGGATCGGCCCGCGTCTGATTAGCTAGTTGGTGGGGTAACGGCCTAC
CAAGGCGACGATCAGTAGCTGGTCTGAGAGGATGATCAGCCACATTGGGACTGAGACACGGCC
>Zotu15395
GAACGCTGGCGGCATGCCTTACACATGCAAGTCGAACGGCAGCGGGGGGAGCTTGCTCCCTGACGGCGA
GTGGCGGACGGGTGAGTAACACGTAGGAATCTACCTTAATTATAATAGTTAGTGGGGGATAACCCGGGGA
AACTCGGGCTAATACCGCATAATCTCTTCAAAGCAAAGCGGGGGATCTTCGGACCTCGTGCTATGAGATGA
GCCTGCGTCGGATTAGCTAGTTGGTAGGGTAATGGCCTACCAAGGCGACGATCCGTAGCTGGTCTGAGAG
GACGATCAGCCACACTGGGACTGAGACACGGCT
>Zotu15400
GAACGCTGGCGGCGTGCTTAACACATGCAAGTCGAACGGAAAGGCCCTTTCGGGGGTACTCGAGTGGCGA
ACGGGTGAGTAACACGTGGGTGATCTGCCCTGCACTTTGGGATAAGCCTGGGAAACTGGGTCTAATACCG
AATATGATCATGCCTCTCCTGGGGTGTGGTGGAAAGCTTTTGCGGTGTGGGATGGGCCCGCGGCCTATCA
GCTTGTTGGTGGGGTGATGGCCTACCAAGGCGACGACGGGTAGCCGGCCTGAGAGGGTGTCCGGCCACA
CTGGGACTGAGATACGGCC
>Zotu15403
GAACGCTGGCGGCATGCTTAACACATGCAAGTCGCACGGTTTATGGTTTCGGCCATAGGCAGTGGCGGAC
GGGTGAGTAACGCGTAGGCATCTATCCTGGGGTGGGGGATAACGGCGGGAAACTGCTGCTAATACCGCAT
GAAACCTGAGGGTCAAAGGCGTAAGTCGCCTTAGGAGGAGCCTGCGTCTGATTAGCTTGTTGGTGGGGTA
AAGGCCTACCAAGGCGATGATCGGTAGCTGGTCTGAGAGGATGATCAGCCACATTGGGACTGAGACACGG
CC
>Zotu15410
GAACGCTGGCGGCGTGCCTAATACATGCAAGTCGAACGGTTACAGCAATGTAACGAGTGGCGCACGGGTG
AGTAACGCGTACATAACATACCTTTAACTGGCGAATAGCCTTGGGAAACTGAGAGTAATACGCCATAACAT
TGTGATTCGGCATCGGATTACAATTAAAGTTCCGGCGGTTAGAGATTGATGTGCGTTGGATTAGGTAGTTG
GTGAGGTAACGGCTCACCAAGCCTTCGATCCATAGGGGGCGTGAGAGCGTGGTCCCCCACACGGGTACTG
AGACACGGAC

>Zotu15416
GAACGCTGGCGGCGTGCTTAACACATGCAAGTCGAGCGGTAAGGCCCTTCGGGGTACACGAGCGGCGAA
CGGGTGAGTAACACGTGAGCAACCTGCCCCTGACTCTGGGATAAGCGGTGGAAACGCCGTCTAATACCGG
ATACGACACTCCACCTCATGGTGGTGTGTGGAAAGTTTTTCGGTCAGGGATGGGCTCGCGGCCTATCAGCT
TGTTGGTGGGGTAAAGGCCTACCAAGGCGATTACGGGTAGCCGGCCTGAGAGGGCGACCGGCCACACTG
GGACTGAGACACGGCC
>Zotu15420
GAACGCTAGCGGCATGCCTAACACATGCAAGTCGAGGGGCAGCGATGGTAGCAATACCAGTCGGCGACC
GGCGGATGGGTGAGTAACACGTATGCAACCTGCCTTCAACATTGGAATAACCCGTTGAAAGACGGACTAA
TGCCTTATAACACAGGGGCGCCGCATGGCGATATTTGTTAAAGATTTTATCGGTTGAAGATGGGCATGCGC
ATGATTAGTTAGTTGGTAAGGTAACGGCTTACCAAGACAACGATCATTAGGGGTTCTGAGAGGAAGGTCC
CCCACACTGGTACTGAGACACGGAC
>Zotu15430
CAACGCTGGCGGCGCGCCTAACACATGCAAGTCGAACGAGAAAGTGGAGCAATCCATGAGTAAAGTGGC
GTACGGGTGAGTAACACGTGGATCATCTACCTTTTAGTGAGGAATAACCATGGGAAACCGTGGCTAATACC
GCATAAGCCCGAGAGGGGAAAGTCGAAAGACGCTGAAAGAGGAGTCCGCGGCCGATTAGCTAGTTGGTA
GGGTAAAAGCCTACCAAGGCGAAGATCGGTAGCCGGCCTGAGAGGGCACACGGCCACACTGGCACTGAA
ACACGGGC
>Zotu15437
GAACGCTAGCGGCATGCCTAACACATGCAAGTCGAACGAGAAAGGGTAGCAATACCTGAGTAAAGTGGCG
CACGGGTGAGTAACACGTGACTAACCTACCTTTGAGTGGGGGATAACCTAGGGAAACCTGGGCTAATACC
GCATAATACCTATGGGTCAAGGCGAGCAATCGCGCTTAAAGAGGGGGTCGCGGCAGATTAGCTAGTTGGT
GGGGTAATGGCCCACCAAGGCGATGATCTGTATCCGGCCTGAGAGGGCGCACGGACACACTGGAACTGA
AACACGGTC
>Zotu15439
GAACGCTGGCGGCGTGCTTAACACATGCAAGTCGAACGGAGATAACGGAATAGTAATATGAAGTTATTTT
AGTGGCGGACGGGTGAGTAACACGTGGGTAACCTGCCCTATGGAGGGGGATAACACAGAGAAATTTGTG
CTAATACCGCATGATGTAGTACTATCGCATGATGGAACTACCAAAGATTTATTGCCATAGGATGGACCCGC
GGCCCATTAGCTAGTTGGTGAGGTAACGGCTCACCAAGGCGACGATGGGTAGCCGATCTGAGAGGATGAA
CGGCCACATTGGAACTGAGACACGGTC
>Zotu15443
GAACGCTGGCGGCATGCCTTACACATGCAAGTCGAACGGCAGCGGGGGGAGCTTGCTCCCTGACGGCGA
GTGGCGGACGGGTGAGTAACACGTAGGAATCTACCTTAATTATAATAGTTAGTGGGGGATAACCCGGGGA
AACTCGGGCTAATACCGCATAATCTCTTCAAAGCAAAGCGGGGGATCTTCGGACCTCGTGCTATGAGATGA
GCCTGCGTCGGATTAGCTAGTTGGTAGGGTAATGGCCTACCAAGGCGACGATCCGTAGCTGGTCTGAGAG
GACGATCGGCCACACTGGGACTGAGACACGGCC
>Zotu15445
GAACGCTGGCGGCATGCCTAACACATGCAAGTCGAGCGGCAGCATGATGTGTAGCAATACACATTGATGG
CGAGCGGCGGACGGGTGAGTAATGTATGGGGATCTGCCCAGTAGCGGGGGACAACCCGGAGAAATCCGG
GCTAATACCGCATACGCCCTACGGGGGAAAGCGGAGGACCGAAAGGCTTCGCACTATTGGAGGAACCCAT
ATCCGATTAGCTAGTTGGTGAGGTAACGGCTCACCAAGGCGACGATCGGTAGCTGGTCTGAGAGGACGAT
CAGCCACACCGGAACTGAGACACGGTC
>Zotu15450
GAACGCTAGCGGCAGGCCTAATACATGCAAGTCGAGGGGCAGCGATGTAGCAATACAGTCGGCGACCGG
CGCACGGGTGCGTAACACGTATGCAACCTGCCTTCAACAGGGAGATAGCCCGGGGAAACTCGGATTAATA
TCCCATAACACAGTGAATCAGCATTGATTTATTGTTAAAGCTCCGGCGGTTGAAGATGGGCATGCGTTCGA
TTAGCTAGTTGGTGTAGGTAACGGCTCACCAAGGCAACGATCGATAGGGGGTCTGAGAGGATGATCCCCC
ACACTGGTACTGAGATACGGAC
>Zotu15452

GAACGCTAGCGATAGGCTTAACACATGCAAGTCGAGGGGCATCACATGAGTAGCAATACGATGGTGGCGA
CCGGCGCACGGGTGAGTAACACGTATGCAACCTACCCATGACAGAGGGATAACCCGTCGAAAGGCGGACT
AATACCGCATAATAACAGTATACTGCATGGTATACTGTTCAAAGATTTATTGGTCATGGATGGGCATGCGTT
CCATTAGGTAGTTGGCAGGGTAACGGCCTACCAAGCCTACGATGGATAGGGGTTCTGAGAGGAAGGTCCC
CCACACTGGTACTGAGACACGGAC
>Zotu15456
CAACGTTGGCGGCGTGCCTAACACATGCAAGTCGAACGAGAAAGGGCTTTCGGGCCTGAGTAAAGTGGCG
CACGGGTGAGTAACACGTGACTAACCTACCTTTGAGCGGGGGATAACCTAGGGAAACCTGGGCTAATACC
GCATAATACCTACGGGTCAAAGCGAGCAATCGCACTTAAAGAGGGGGTCGCGGCAGATTAGCTAGTTGGT
AAGGTAATGGCTTACCAAGGCGATGATCTGTATCCGGCCTGAGAGGGCGCACGGACACACTGGAACTGAA
ACACGGTC
>Zotu15459
GAACGCTGGCGGCGTGCCTAACACATGCAAATCGAACGGAACTTTTTAAAGGATTTCTTCGGAATGAATT
TGATTAAGTTTAGTGGCGGACGGGTGAGTAACGCGTGAGTAACCTGCCTCTGAGAGGGGAATAACATTCT
GAAAAGAATGCTAATACCGCATAACATATATTTATCGCATGGTAGATATATCAAAGATTTATCGCTTAGAGA
TGGACTCGCGTCCGATTAGTTAGTTGGTGAGGTAACGGCTCACCAAGACCGCGATCGGTAGCCGGACTGA
GAGGTTGAACGGCCACATTGGGACTGAGACACGGCC
>Zotu15465
CAACGCTGGCGGCGTGCCTAACACATGCAAGTCGAACGAGAAGGTGGCTTCGGTCACTGGAAAGTGGCGT
CCGGGTGAGTAACACGTGGGTAATCTACCCTCGGGTGGGGAATAACGTCTCGAAAGAGACGCTAATACCG
CATAATGCAGCGGCACCTTATGGTGAATAGTTGTTAAAGCGGGGGATCGCAAGACCTCGCGCCTGAAGAG
GAGCCTGCGGCAGATTAGCTAGTTGGTGAGGTAATGGCTCACCAAGGCTTCGATCTGTAGCTGGTCTGAG
AGGACGGCCAGCCACACTGGCACTGACACACGGGC
>Zotu15468
GAACGCTAGCGGCGGGCCCAATACATGCAAGTCGAGGGGCAGCGGGGGTAGCAATACTTGCCGGCGACC
GGCGCACGGGTGCGTAACGCGTATGCAACCTACCTTTGACACGGGAATAGCCCGAAGAAATTCGGATTAA
TACCCGATGGTATCATTTGGTGACATCGCCGGATGATTAAAGATTTATCGGTCAGAGATGGGCATGCGTCT
GATTAGCTAGTTGGTGAGGTAACTGCTCACCAAGGCTACGATCAGTAGGGGTTCTGAGAGGATGATCCCCC
ACACCGGTACTGAGACACGGAC
>Zotu15470
GAACGCTGGCGGCGTGCTTAATACATGCAAGTCAACTGAGCTTAAAGTAGCAATATTTTAAGTGCGGTGGC
GCACGGGTGAGTAACACGTAGGTAATCTGCCTTTAGGTCTGACATAATCCCGCGAAAGCGGGACTAAAATC
AGATGATGCAGCGGCTCGACATCGAGACAGTTGTTAAACCTTCGGGGCCTGAAGATGAGCCTGCGTCTGA
TTAGGTAGTTGGCGGAGTAAAAGCCCACCAAGCCTGCGATCAGTAGCTGGTCTGAGAGGATGATCAGCCA
CACTGGAACTGAGACACGGTC
>Zotu15473
GAACGCTGGCGGCGTGCTTAAGACATGCAAGTCGAACGGACTTCCCTCGGGAAGTCAGTGGCGCACGGGT
GAGTAACACGTAACTGACCTGCCCCAAAGTCGCGGATAACGTTCCGAAAGGAACGCTAATACGTGATGTGC
TGTCCCCTCGTGTGGGGCCAGTAAAGGTTTACTGCTTTGGGATGGGGTTGCGTTCCATCAGCTGGATGGTG
GGGTAAAGGCCTACCATGGCGACGACGGATAGCCGGCCTGAGAGGGTGGCCGGCCGCAGGGGCACTGA
GACACGGCC
>Zotu15475
GAACGCTAGCGGCATGCCTAACACATGCAAGTCGAACGAAGCCTTCGGGCTTAGTGGCGCACGGGTGCGT
AACGCGTAGGCAACCTACCCTCAGGCGGGGAATAACTACCGGAAACGGTAGCTAATACCGCATATTGTCG
GAGAGACACCTGTCTCACTGATGAAAGGCTTCGGCCACCTGAGGATGGGCCTGCGTTGGATTAGGTAGTT
GGTGAGGTAACGGCTCACCAAGCCGACGATCCGTAGCTGGTCTGAGAGGACGATCAGCCACACTGGGACT
GAGACACGGCC
>Zotu15477
GAACGCTAGCGGGAGGCCTAACACATGCAAGCCGAGCGGGATTTGTTGGTTAGCTTGCTAACTAACAATG
AGAGCGGCGTACGGGTGCGTAACACGTGTGCAACCTGCCTTTATCTGGGGGATAGCCTTTCGAAAGGAAG
ATTAATACCCCATAATATATTGATTGGCATCAATTGATATTGAAAGCTCCGGCGGATAGAGATGGGCACGC

GTAAGATTAGCTAGTTGGTGAGGTAACGGCTCACCAAGGCTGCGATCTTTAGGGGGCCTGAGAGGGTGAT
CCCCCACACTGGTACTGAGACACGGCC
>Zotu15482
GAACGCTGGCGGCGCGCTTAACACATGCAAGTCGAGCGAGAACCTGGGCTTCGGCCCTGGGGACAGCGG
CGAACGGGTGAGTAACACGTGGGTAATCTGCCCTTGACATCGGGATAGCCCGGGGAAACCCGGATTAATA
CCGAATAGCCTCCGGAGCCTTCGGGCGCCGGAGAAAAGGTAGCTTCGGCCTCTGGTCAAGGATGAGCCCG
CGGCGGATTAGCTTGTTGGCGGGGTAACGGCCCACCAAGGCGACGATCCGTAGCTGGTCTGAGAGGACG
ATCAGCCACACTGGGACTGAGACACGGCC
>Zotu15484
GAACGCTAGCGGCATGCCTAACACATGCAAGTCGAACGAAGGCTTCGGCCTTAGTGGCGCACGGGTGAGT
AACGCGTGGGAACCTGCCTCTTGGTTCGGAATAACTGCGGGAAACTGCAGCTAATACCGGATAAGTCGGTT
GGATATGGTGTCCAGCTGGGAAAGATTTATCGCCGAGAGAGGGGCCCGCGTTCGATTAGGTAGTTGGTGA
GGTAATGGCTCACCAAGCCTACGATCGATAGCTGGTCTGAGAGGATGATCAGCCACACTGGGACTGAGAC
ACGGCC
>Zotu15486
GAACGCTTGCGGCGTGCCTAAGAAATGCAAGTCGAACGGATAGTCCAGCAATGGTCTATTAGTGGCGAAC
GGTCGCGTAACACGTAGGCAACCTGCCCGGAAGTGGGGGACAACAGCCTGAAAGGGCTGCTAATACCGC
ATGTGGCCGCCTGGTGGCATCACCGGGTGTCTAAAGCTTTATGCGCTTCTGGATGGGCTTGCGGCCTATCA
GGTAGTTGGTGGGGTAATGGCCTACCAAGCCGACGACGGGTAGCTGGTCTGAGAGGATGATCAGTCACAC
TGGAACTGAGACACGGCC
>Zotu15492
GAACGCTGGCGGCATGCCTTACACATGCAAGTCGAACGGCAGCGGGGGGAGCTTGCTCCCTGACGGCGA
GTGGCGGACGGGTGAGTAACACGTAGGAATCTACCTTAATTATAATAGTTAGTGGGGGATAACCCGGGGA
AACTCGGGCTAATACCGCATAATCTCTTCAAAGCAAAGCGGGGGATCTTCGGACCTCGTGCTATGAGATGA
GCCTGCGTCGGATTAGCTAGTTGGTAGGGTAATGGCCTACCAAGGCGACGATCCGTAGCTGGTCTGAGAG
GACAATCAGCCACACTGGGACTGAGACACGGCC
>Zotu15495
GAACGCTGGCGGCATGCCTAACACATGCAAGTCGAACGAAGGCTTCGGCCTTAGTGGCGCACGGGTGCGT
AACGCGTGGGAATCTGCCCTGGGGTACGGAATAACTCAGAGAAATTTGAGCTAATACCGTATGATGACGT
AAGTCCAAAGATTTATCGCCCTGGGATGAGCCCGCGTAGGATTAGCTAGTTGGTGGGGTAAAGGCCCACC
AAGGCGACGATCCTTAGCTGGTCTGAGAGGATGATCAGCCACACTGGGACTGAGACACGGCC
>Zotu15497
TAACGCTGGCGGCGTGCCTAACACATGCAAGTCGAACGGACCTTTATTTTTGAGACGCCCCATTTCCTTGGA
GCGCCCCATAAATAATGGTTAGTGGCGAACGGGTGAGTAACACGTTGGTACCTGCCCTCTAGTGGGGGAT
AACTGCCCGAAAGGGTAGCTAATACCGCATGTGATCTACGGATCAAAGCAGCAATGCGCTAGAGGAGGGG
CCTGCGGCTGATTAGCTTGTTGGTAGGGTAATGGCCTACCAAGGCGACGATCCGTAGCTGGTCTGAGAGG
ACGATCAGCCACACTGGGACTGAGACACGGCC
>Zotu15502
GAACGCTGGCGGCGTGCTTAACACATGCAAGTCGAACGAGCAAGAGCTGTAGCAATACAGGGTTCTGCTA
GTGGCAGACGGGTGAGTAATACATGGGAATCTACCTTGCAGTGCGAAATAACAGTTGGAAGCAATTGCTA
ATGTCGCATATAGCCGAGAGGTGAAAGGTTTACTGCTGCAAGATGGGCCCATGCAGGATTAGCTAGTTGG
TGAGGTAATGGCTCACCAAGGCAACGATCTTTAGTTGGTTTGAGAGGATGATCAGCCACACTGGGACTGA
GACACGGTC
>Zotu15507
GAACGCTGGCGGCAGGCTTAACACATGCAAGTCGAACGATGTAGCAATACATAGTGGCGCACGGGTGAGT
AACGCGTGGGAATGTACCTTATAGTGGGGAATAACGTTGGGAAACTAACGCTAATACCGCATACGCCCTTC
GGGGGAAAGATTTATCGCTATAAGATCAGCCCGCGTGAGATTAGGTAGTTGGTGGGGTAATGGCCTACCA
AGCCTACGATCTCTAGCTGGTCTGAGAGGATGATCAGCCACACTGGAACTGAGACACGGTC
>Zotu15509

GAACGCTAGCGGCATGCTTAACACATGCAAGTTGAGCGATTCTCTTCGGAGAAGAGCGGCGGACGGGTGA
GTAACGCGTGGGTAACCTGCCCTGTACACACGGATAACATACCGAAAGGTATGCTAATACGAGATAATATG
CTTTTATCGCATGGTAGAAGTATCAAAGCTCCGGCGGTACAGGATGGACCCGCGTCTGATTAGCTAGTTGG
TAAGGTAACGGCTTACCAAGGCGACGATCAGTAGCCGACCTGAGAGGGTGATCGGCCACATTGGAACTGA
GACACGGTC
>Zotu15513
GAATGCTAGCAGAACGCCTAACACATGCAAGTCGAACGGACTTGTTGCTTCGGTAACAAGTTAGTGGCAAA
CGGGCGAGTAACACGTGCTTAATTTGCCCCGAAGAGGTGGACAACTACGGAGACGTAGCTAAACCACCAT
ACGCTCATGGAAATGAAAATACCATGAGGAAACGATTTATCGCTTTGGGAGAAGGGCGCGGCCTATCAGG
TTGTTGGTGAGGTAACGGCTCACCAAGCCTATGACGGGTAACCGGTCTGAGAGGATGTCCGGTCGCAATG
GAACTGAGACACGGTC
>Zotu15518
GAACGCTGGCGGCGTGCTTAACACATGCAAGTCGAACGGAAAGGCCCTGCTTTTGTGGGGTGCTCGAGTG
GCGAACGGGTGAGTAACACGTGAGTAACCTGCCCTTGACTCTGGGATAACTTCAGGAAACTGGGGCTAAT
ACCGGATAGGAGCTCCTGCTGCATGGTGGGGGTTGGAAAGTTTCGGCGGTTGGGGATGGACTCGCGGCTT
ATCAGCTTGTTGGTGGGGTAGTGGCTTACCAAGGCTTTGACGGGTAGCCGGCCTGAGAGGGTGACCGGCC
ACACTGGGACTGAGACACGGCC
>Zotu15521
TAACGCTAGCGGCAGGCCTAATACATGCAAGTCGAACGGCAGCACGGGTAGCAATATCTGGTGGCGAGTG
GCGCACGGGTGCGTAACGCGTATGCAATCTACCTATAACTGGGGAATAGCCTTCCGAAAGGAAGATTAAA
ACCGCATAATATCACGAGGCGGCATCGTTTTGTGATCAAATCTCCGGAGGTTATAGATGAGCATGCGTGAC
ATTAGCTAGTTGGTGAGGTAACGGCTCACCAAGGCGACGATGTCTAGGGGGTCTGAGAGGATGATCCCCC
ACACTGGTACTGAGACACGGAC
>Zotu15523
GAACGCTAGCGGCATGCCTAACACATGCAAGTCGAGCGGATGAGAGGAGCTTGCTCCTGGATTCAGCGGC
GGACGGGTGAGTAATGCCTAGGAATCTGCCTGGTAGTGGGGGACAACGTTTCGAAAGGAACGCTAATATC
G
>Zotu15525
GAACGCTGGCGGCGTGCTTAACACATGCAAGTCGAACGGGGCAGTAGCAATACTGTCTAGTGGCGGACGG
GTGAGTAAGACCTAGGTAACCTGCCCTTCAGTGGAGGATACCCAGCCGAAAGGTTGGCTAATACTGCATTC
GCTTCACATCTGGAATGGTGTGAAGGAAAGATTTATCGCTGAGGGAGGGGCCTAGGCCCGATTAGCTAGT
TGGTGGGGTAATGGCTCACCAAGGCGACGATCGGTAGCTGGTCTGAGAGGACGATCAGCCACACTGGAAC
TGAGACACGGTC
>Zotu15529
GAACGCTAGCGGCATGCTTAACACATGCAAGTCGAACGAGATCCTTCGGGGTCTAGTGGCGCACGGGTGC
GTAACGCGTGGGAATCTGCCCTTCGGTACGGAATAACTCAGGGAAACTTGAGCTAATACCGTATGATGTCT
TCGGACCAAAGATTTATCGCCGAGGGATGAGCCCGCGTTGGATTAGCTAGTTGGTGGGGTAAAGGCCTAC
CAAGGCGACGATCCGTAGCTGGTCTGAGAGGATGATCAGCCACACTGGGACTGAGACACGGCC
>Zotu15531
CAACGCTGGCGGCGTGCCTAACACATGCAAGTCGCACGAGAAAGGGATTTCGGTCCTGAGTAAAGTGGCG
CACGGGTGAGTAACACGTGACTAATCTACCCTCGAGTGGGGAATAACCTAGGGAAACCTGGGCTAATACC
GCATAACACTTACGAGTCAAAGCAGCAATGCGCTTGGGGAGGAGGTCGCGGCAGATTAGCTAGTTGGTGG
GGTAAAGGCCTACCAAGGCGATGATCTGTATCCGGCCTGAGAGGGCGCACGGACACACTGGAACTGAGAT
ACGGTC
>Zotu15536
GAACGCTGGCGGCAGGCTTAACACATGCAAGTCGAGCGGGGTGATGTAGCTTGCTATCTGACCTAGCGGC
GGACGGGTGAGTAATGCTTAGGAATCTGCCTATTAGTGGGGGACAACATTCCGAAAGGAATGCTAATACC
GCATACGCCCTACGGGGGAAAGCAGGGGATCTTCGGACCTTGCGCTAATAGATGAGCCTAAGTCAGATTA
GCTAGTTGGTGGGGTAAAGGCCTACCAAGGCGACGATCTGTAGCGGGTCTGAGAGGACGACCAGCCACA
CTGGGACTGAGACACGGCC

>Zotu15540
GAACGCTGGCGGCGTGCCTAATACATGCAAGTAGAACGCTGAAGAGAGGAGCTTGCTCTTCTTGGATGAG
TTGCGAACGGGTGAGTAACGCGTAGGTAACCTGCCTTGTAGCGGGGGATAACTATTGGAAACGATAGCTA
ATACCGCATAAAAGTCGATATCGCATGATGTTGACTTGAAAGGTGCAATTGCATCACTAAGAGATGGACCT
GCGTTGTATTAGCTAGTAGGTGAGGTAACGGCTCACCTAGGCGACGATACATAGCCGACCTGAGAGGGTG
ATCGGCCACACTGGGACTGAGACACGGCC
>Zotu15549
TAACGCTGGCGGCGCGCATAACACATGCAAGTCGAGCGAGGGCTTACTTCTCGCAAGAGAGGTGAGTTCC
TAGCGGCGAACGGGTGAGTAACACGTGGGCAACCTTTCCTATCGCGGGGGATAACACCGGGAAACCGATG
CTAATACCGCATGTCGTCACTGGTCGCATGGCTGGTGAAGAAAGCAGCAATGCACGATGGGGTGGGCCCG
CGGCCTATCAGCTAGTCGGTGGGGTAACGGCCTACCGAGGCGATGACGGGTAGCTGGTCTGAGAGGACG
ATCAGCCACACTGGGACTGAGACACGGCC
>Zotu15555
GAACGCTGGCGGCATGCTTAACACATGCAAGTCGAACGAGCTAATGGGTAACTATTAGTATAGTGGCGGA
CGGGTGCGTAACGCGTAAGAACCTACCTTTTGGAGGGGGATAACATCGGGAAACCTTTGGTAATACCCCAT
AATGCTGAGGAGTTAAAGCAGCGAGAGCTGGCCAAAAGAGGGGCTTGCGTCTGATTAGCTAGTTGGTGG
GGGTAAAAGCCTCCCAAGGCCACGATCCGTAGCTGGTCTGAGAGGACGATCAGCCACACTGGGACTGAGA
CACGGCC
>Zotu15561
GAACGCTAGCGGCAGGCTTAATACATGCAAGTCGAGCGAACAGATAAGGAGCTTGCTCCTTTGACGTCAG
CGGCGGACGGGTGAGTAACACGTGGGTAACCTACCTATAAGACTGGAATAACTCCGGGAAACCGGGGCTA
ATGCCGGATAACATTTAGAACCGCATGGTTCTAAAGTGAAAGATGGTTTTGCTATCACTTATAGATGGACCC
GCGCCGTATTAGCTAGTTGGTAAGGTAACGGCTTACCAAGGCAACGATACGTAGCCGACCTGAGAGGGTG
ATCGGCCACACTGGAACTGAGACACGGTC
>Zotu15564
GAACGCTGGCGGCGTGCTTAACACATGCAAGTCGAACGCTGAAGCCCAGCTTGCTGGGTGGATGAGTGGC
GAACGGGTGAGTAACACGTGAGTAACCTGCCCACTTCTTTGGGATAACCACCGGAAACGGTGGCTAATACT
GGATAGGACTCCTGCCTGCATGGGTGGGGGTGGAAAGATTTATCGGTGGTGGATGGACTCGCGGCCTATC
AGCTTGTTGGTGGGGTAATGGCCTACCAAGGCGACGACGGGTAGCCGGCCTGAGAGGGTGACCGGCCAC
ACTGGGACTGAGACACGGCC
>Zotu15566
CAACGCTGGCGGCGTGCCTAACACATGCAAGTCGAACGCGAAAGTTCCTTCGGGAATGAGTAGAGTGGCG
TCCGGGTGAGTAGCGCGTGGGTAATCTACCCTTGGGTGGGGAATAACCAGTCGAAAGATTGGCTAATACC
GCATAATGCAGCGGCTCGACATCGAGACAGTTGTTAAAGCAGCAATGCGCCTAAGGAGGAGCCTGCGTCT
GATTAGCTTGTTGGTGAGGTAATGGCTCACCAAGGCTGCGATCAGTAGCTGGTCTGAGAGGACGGCCAGC
TACAATGGCACTGAAACACGGGC
>Zotu15570
GAACGCTGGCGGCAGGCCTAACACATGCAAGTCGAGCGGATGAGAGGAGCTTGCTCCTGGATTCAGCGGC
GGACGGGTGAGTAATGCCTAGGAATCTGCCTATTAGTGGGGGACAACATTCCGAAAGGAATGCTAATACC
GCATACGCCCTACGGGGGAAAGCAGGGGACCTTCGGGCCTTGCGCTATCAGATGAGCCTAGGTCGGATTA
GCTAGTTGGTGAGGTAATGGCTCACCAAGGCGACGATCCGTAACTGGTCTGAGAGGATGATCAGTCACAC
TGGAACTGAGACACGGTC
>Zotu15573
GAACGCTGGCGGCGTGCCTAATACATGCAAGTCGAGCGAATCTTTGGGAGCTTGCTCTCAAAGGTTAGCG
GCGGACGGGTGAGTAACACGTGGGCAACCTGCCTATAAGACTGGGATAACTTCGGGAAACCGGAGCTAAT
ACCGGATAATTCTTTTCTACACATGTAGAAAAGCTAAAAGACGGTTTACGCTGTCACTTATAGATGGGCCCG
CGGCGCATTAGCTAGTTGGTGAGGTAATGGCTCACCAAGGCCACGATGCGTAGCCGACCTGAGAGGGTGA
TCGGCCACACTGGGACTGAGACACGGCC
>Zotu15577

GAACGCTGGCGGCGTGCCTAATACATGCAAGTCGTGCGGGGTCTTGAAGGGCTTGCCCTTTAAGACCCAG
CGGCGAACGGGTGAGTAACACGTGGGTAACCTGCCCGCAAGACCGGGATAACTCCGGGAAACCGGGGCT
AATACCGGATAGGACCCCGGATCGCATGATCCGGAGTGGAAAGGCGGCGTCAAGCTGCCACTTGCGGATG
GGCCCGCGGCGCATTAGCTGGTTGGTGAGGTAACGGCTCACCAAGGCGACGATGCGTAGCCGACCTGAGA
GGGTGATCGGCCACACTGGGACTGAGACACGGCC
>Zotu15584
GAACGCTTGCGGCGCGCCTAATACATGCAAGTCGAACGAGAAAGTTCCTTCGGGAATGAGTACAGTGGCG
CACGGGTGAGTAACACGTGGACAACCTTCCTCTAGGTGGGGAATAACCAGTCGAAAGACTGGCTAATACC
GCATAAGACCACAGTTCCTGCGGGAACAGAGGTTAAAGATTTATCGCCTAGAGATGGGTCTGCGTCCGATT
AGCTAGTTGGTGAGGTAACGGCTCACCAAGGCAGTGATCGGTAGGTGGTCTGAGAGGATGATCACCCACA
CTGGAACTGAGACACGGTC
>Zotu15590
GAACGCTGGCGGCGTGCTTAACACATGCAAGTCGAACGATGAACTGGTGCTTGCACTGGGGATTAGTGGC
GAACGGGTGAGTAACACGTGAGTAACCTGCCCTTGACTCTGGGATAAGCCTGGGAAACTGGGTCTAATAC
CGGATATGACCATCTGGCGCATGTCATGGTGGTGGAAAGCTTTTGTGGTTTTGGATGGACTCGCGGCCTAT
CAGCTTGTTGGTGGGGTAATGGCCTACCAAGGCGACGACGGGTAGCCGGCCTGAGAGGGTGACCGGCCA
CACTGGGACTGAGACACGGCC
>Zotu15594
GAACGCTGGCGGCGTGCTTAACACATGCAAGTGGTGCGACGAACCAGGGCTTGCCCTGGGGCTAAGCCGC
GAACGGGTGAGTAACACGTGAGTAATCTGCCCCGATGACCGGGACAACCCGAGGAAACTCGGGCTAATAC
CGGATGTGGTGCACAGTCTTAAGTCTGTGTACTAAAGGAAGCTTCGGCCTCCGCATTGGGAGGAGCTCGC
GGCCCATTAGCTTGTTGGTGGGGTAACGGCCTACCAAGGCAACGATGGGTAGCTGGTCTGAGAGGACGAT
CAGCCACACTGGGACTGAGACACGGCC
>Zotu15603
GAACGCTGGCGGCAGGCCTAACACATGCAAGTCGAACGCCCTAGCAATAGGGAGTGGCAGACGGGTGAG
TAACGCGTGGGAACCTTCCCTATAGTACGGAATATACCAGGGAAACTTGGAGCAATACCGTATACGCCCGA
AAGGGGAAAGATTTATCGCTATAGGATGGGCCCGCGTAGGATTAGCTAGTTGGTGAGGTAATGGCTCACC
AAGGCGACGATCCTTAGCTGGTCTGAGAGGATGATCAGCCACACTGGGACTGAGACACGGCC
>Zotu15605
GAACGCTGGCGGCGTGCTTAACACATGCAAGTCGAACGGTGATCCAGGGCTTGCCTTGGTGATCAGTGGC
GAACGGGTGAGTAACACGTGAGTAACCTGCCCCTGACTCTGGGATAACCGCTGGAAACGGCGGCTAATAC
CGGATATGAAGCGTGCCCGCATGGGTGTGCTTGGAAAGATTTTTTGGTCAGGGATGGGCTCGCGGCCTAT
CAGCTTGTTGGTGGGGTGATGGCCTACCAAGGCGACGACGGGTAGCCGGCCTGAGAGGGCGACCGGCCA
CACTGGGACTGAGACACGGCC
>Zotu15608
GAACGCTAGCGGCGCGCCTAACACATGCAAGTTGAGTGCCTTTAAAAGCACAGCAAACGGCTGCGTAACA
CGTTGGAACCTACCCCGAACTCAGGGATAACCCGCCGAAGGGCGGGCTAATACCGGATGGTCCCGAAAGG
GTAAAGATTTTTCGGTTCGGGAGGGGCCTGCGGCCTATCAGCTAGTTGGTGAGGTAATGGCTCACCAAGG
CAATGACGGGTAACTGGTCTGAGAGGACGACCAGTCAGAATGGAACTGCGACACGGTC
>Zotu15618
GAACGCTGGCGGCATGCCTTACACATGCAAGTCGAACGGCAGCACGGGAGCAATCCTGGTGGCGAGTGG
CGAACGGGTGAGTAATATATCGGAACGTGCCCAGTCGTGGGGGATAACGTAGCGAAAGCTACGCTAATAC
CGCATACGATCTATGGATGAAAGCGGGGGATCGCAAGACCTCGCGCGATTGGAGCGGCCGATATCAGATT
AGCTAGTTGGTGGGGTAAAAGCTCACCAAGGCGACGATCAGTAGCTGGTCTGAGAGGACGACCAGCCACA
CTGGGACTGAGACACGGCC
>Zotu15621
GAACGCTAGCGGCATGCTTAACACATGCAAGTCGAACGGCAGCGCGGACTTCGGTCTGGCGGCGAGTGGC
GAACGGGTGAGTAATACATCGGAACGTACCCTGTTGTGGGGGATAACTAGTCGAAAGATTAGCTAATACC
GCATACGACCTGAGGGTGAAAGCGGGGGACCGTAAGGCCTCGCGCAGCAGGAGCGGCCGATGTCTGATT
AGCTAGTTGGTGGGGTAATTGCCTACCAAGGCGACGATCCGTAGCTGGTCTGAGAGGATGATCAGCCACA
CTGGGACTGAGACACGGCC

>Zotu15625
GAACGCTGGCGGCATGCCTTACACATGCAAGTAGAACGGTAACAGGTCTTCGGATGCTGACGAGTGGCGA
ACGGGTGAGTAATGCATCGGAACGTGCCCAGTAGTGGGGGATAGCCCGGCGAAAGCCGGATTAATACCG
CATACGACCTGAGGGTGAAAGGGGGGGATCGCAAGACCTCTCGCTATTGGAGCGGCCGATGTCAGATTAG
CTAGTTGGTGGGGTAAAGGCCCACCAAGGCAACGATCTGTAGTTGGTCTGAGAGGACGACCAGCCACACT
GGGACTGAGACACGGCC
>Zotu15627
GAACGCTAGCGGCGTGCCTAAGGTATGCAAGTCGAGCGGGAGCCTTCGGGCTCCAGCGGCAAACGGTTG
AGTAACACGTAGACAACCTGCCCGTAAGACGGGGATAACTCGGGGAAACTCGGGCTAATACCGGATGTGG
AAGTGGGGGGCATCCCCCGTTCTCTAAACGAGCAATCGGCTTACGGATGGGTCTGCGGCCTATCAGCTAGC
TGGTGGGGTAACGGCCTACCAGGGCATCGACGGGTAGCTGGTCTGAGAGGATGACCAGCCAGACGGGGA
CTGAGACACGGCC
>Zotu15629
GAACGCTGGCGGCGTGCTTAACACATGCAAGTCGAACGGGAAATATTTCATTGAGACTTCGGTGGATTTGA
TCTATTTCTAGTGGCGGACGGGTGAGTAACGCGTGGGTAACCTGCCTTATACAGGGGGATAACAGTCAGA
AATGGCTGCTAATACCGCATAAGCGCACAGAGCTGCATGGCTCAGTGTGAAAAACTCCGGTGGTATAAGA
TGGACCCGCGTTGGATTAGCTTGTTGGTGGGGTAACGGCCCACCAAGGCGACGATCCATAGCCGGCCTGA
GAGGGTGAACGGCCACATTGGGACTGAGACACGGCC
>Zotu15632
GAACGCTGGCGGCGTGCTTAACACATGCAAGTGGAGCGACGAACCAGGGCTTGCCCTGGGGCAGAGCCG
CGAACGGGTGAGTAACACGTGGGTCACCTGCCTCGATGACCGGGACAACCCGAGGAAACTCGGGCTAATA
CCGGATGTGCCCGCAAGGGGAAAGGAAGCTTCGGCCTCCGCATCGAGATGGGCCCGCGGCCCATTAGCTT
GTTGGTGAGGTAACGGCTCACCAAGGCTTCGATGGGTAGCTGGTCTGAGAGGACGATCAGCCACACTGGG
ACTGAGACACGGCC
>Zotu15638
GAACGCTGGCGGCATGCCTAACACATGCAAGTCGAACGAAGCCTTCGGGCTTAGTGGCGCACGGGTGCGT
AACGCGTGGGAATCTGCCCTTGGGTACGGAACAACAGTTAGAAATGACTGCTAATACCGTATGATGTCTTC
GGACCAAAGATTTATCGCCCTGGGATGAGCCCGCGTTGGATTAGGTAGTTGGTGGGGTAAAGGCCTACCA
AGCCGACGATCTGTAGCTGGTCTGAGAGGATGATCAGCCACACTGGGACTGAGACACGGCC
>Zotu15643
GAACGCTGGCGGCGTGCTTCATACATGCAAGTCGAACGAGAAACTGACTTCGGTCAGTGGAAAGTGGCGG
ACGGGTGAGTAATATGTAGGAAATCTGCCTTAGAGAGGGGGACAACAGAGGGAAACTTCTGCTAATACCC
CATATGAGCTACCTTGAAATAGGTATCTTGAAAACTCCGGTGCTCTAAGATGAGCCTGCATCTGATTAGCTT
GTTGGTGGTGTAATGGACTACCAAGGCGACGATCAGTAGCTGGTTTGAGAGGATGATCAGCCACAATGGG
ACTGAGACACGGCC
>Zotu15645
GAACGCTGGCGGTGTGCTTAACACATGCAAGTCGAGCGAGAACCAGGCCTTCGGGCCTGGGGACAGCGG
CGAACGGGTGAGTAACACGTGGGTAATCAACCCTCGATACTGGGATAGCCCGGGGAAACCCGGATTAATA
CCGGATGGCCCTTTGGATCCTCGGATCTGAAGGAAAAGGTAGCTTCGGCCTCCGATCGAGGACGAGCCCG
CGGCGGATTAGCTTGTTGGTGAGGTAACGGCCCACCAAGGCGACGATCCGTAGCTGGTCTGAGAGGACGA
TCAGCCACACTGGGACTGAGACACGGCC
>Zotu15648
GAACGCTGGCGGCGTGCTTAACACATGCAAGTCGAGGGGTAGAAGTCTTCGGACTTTGAGACCGGCGCAC
GGGTGCGTAACGCGTATGCAATCTACCTTGTACAGAGGGATAGCCCAGAGAAATTTGGATTAATACCTCAT
AGTATTATTGAGTGGCATCACTTGATTATTAAAGTTCCAACGGTACAAGATGAGCATGCGTCCCATTAGCTA
GTTGGTATGGTAACGGCATACCAAGGCAATGATGGGTAGGGGTCCTGAGAGGGAGATCCCCCACACTGGT
ACTGAGACACGGAC
>Zotu15653
GAACGTTGGCGGCGTGGATTAGGCATGCAAGTCGTACGGGCCGCAAGGCCAGTGGCGCAAGGGTGAGTA
AGGCGACGGTAACCAACCCCATGGGTGGGTATAGCCGCGGGAAACCGCGGGTAATCCCCAGCGATGCAT

GGCCTCGGCATCGAGGCTCTGCCAAAGCTCAGGCGCCGTGGGACGGGCCGTCGTGGTATTAGGTAGTTGG
CGGGGTAACGGCCCACCAAGCCGACGATGCCTACCGGGCGTGCGAGCGTGGCCCGGCACACTGGGACTG
AGACACTGCC
>Zotu15656
GAACGCTGTCGGTGTGACTAGGACATGCAAGTCGTACGGTGTAGCAATACACAGTGGCGCACGGGTGAGT
AATGTATCGCTAATGTACCCCGGACTCTGGGATAGCCAGCCGAAAGGCTGGGTAATACCAGATGAGACCA
CGACGTGGCATCACGTTGTGCTCAAAGGTTCGCCGGTCTGGGATCAGGCGATATGGTATCAGGTAGTTGGT
GAGGTAACGGCTCACCAAGCCTTCGACGCCTAGCCGGACTGAGAGGTCGGACGGCCACATCGGGACTGAG
ACACTGCC
>Zotu15662
GAACGCTAGCGGCATGCTTAACACATGCAAGTCGAACGGGGTGCAGCAATGTACCAAGTGGCGATCGGGT
GCGTAACACGTAGCTGACCTACCTCTTGGCGGGGGACAACAGGCCGAAAGGCTTGCTAATACCGCATAATT
TCGGGGAAACCAGAATACCCTGAAGAAACGCGCAAGCGGCCGAGAGAGGGGGCTGCGGCCCATCAGGTA
GTTGGTGAGGTAACGGCTCACCAAGCCGACGACGGGTAGGGGGCCTGAGAGGGTGACCCCCCACACTGG
GACTGACAGACGGCC
>Zotu15667
GAACGCTAGCGGCAGGCTTAACACATGCAAGTCGAGGGGTATAGGTTTTCGGACCTAGAGACCGGCGCAC
GGGTGCGTAACGCGTATGCAATCTACCTTTCACAGAGGGATAGCCCAGAGAAATTTGGATTAATACCTCAT
AGTATATAGAGTTGGCATCAACACTATATTAAAGTCACAACGGTGAAAGATGAGCATGCGTCCCATTAGCT
TGTTGGTAAGGTAACGGCTTACCAAGGCTACGATGGGTAGGGGTCCTGAGAGGGAGATCCCCCACACTGG
TACTGAGACACGGAC
>Zotu15669
CAACGCTGGCGGCGTGCCTAACACATGCAAGTCGAACGCGAAAGTCCCGAAAGGGATTAGTAGAGTGGCA
AACGGGTGAGTAACACGTGGGTGACCTGCCTTCGAGTGGGGGATAACGTCCCGAAAGGGACGCTAATACC
GCATAACATCCTGCCTTTGAACAGGCGGTGATCAAAGCTGGGGACCGCAAGGCCTAGCGCTGGAAGAGGG
GCCCGCGCCTGATTAGCTAGTTGGTGAGGTAACGGCTCACCAAGGCGACGATCAGTATCCGGCCTGAGAG
GGCGGACGGACACACTGGGACTGAGACACGGCC
>Zotu15672
GAACGCTGGCGGCATGCCTTACACATGCAAGTCGAACGGCAGCACGGGAGCAATCTTGGTGGCGAGTGGC
GAACGGGTGAGTAATATATCGGAACGTGCCCAGTCGTGGGGGATAACGCAGCGAAAGCTGTGCTAATACC
GCATACGATCTATGGATGAAAGCGGGGGATCGCAAGACCTCGCGCGATTGGAGCGGCCGATATCAGATTA
GCTAGTTGGTGGGGTAAAAGCCCACCAAGGCGACGATCTGTAGCTGGTCTGAGAGGACGACCAGCCACAC
TGGAACTGAGACACGGTC
>Zotu15676
GAACGCTGGCGGCGTGCTTAACACATGCAAGTCGAGCGGTAAGGCTCTTCGGAGTACACGAGCGGCGAAC
GGGTGAGTAACACGTGAGCAACCTGCCCATGACACTGGGATAACGATTGGAAACAGTCGCTAATACCGGA
TACGACCTCAGCGGACATCTGCATGAGGTGGAAAGCTCCGGCGGTCATGGATGGGCTCGCGGCCTATCAG
CTTGTTGGTGAGGTAACGGCTCACCAAGGCGTCGACGGGTAGCCGGCCTGAGAGGGCGACCGGCCACACT
GGGACTGAGATACGGCC
>Zotu15679
GAACGCTGGCGGCAGGCTTAACACATGCAAGTCGAGCGAGATCTTCGGGTCTAGCGGCGGACGGGTGAG
TAACGCGTGGGAACGTGCCCTTTGCTACGGAGTAGTCCTGGGAAACTGGGGGTAATACCGTATACGCCCTT
TTGGGGAAAGATTTATCGGCGAAGGATCGGCCCGCGTTGGATTAGGTAGTTGGTGGGGTAATGGCCTACC
AAGCCTTCGATCCATAGCTGGTTTGAGAGGATGATCAGCCACACTGGGACTGAGACACGGCC
>Zotu15683
GAACGCTGGCGGCGTGGATTAGGCATGCAAGTCGAACGCGAAAGAGGGGGCAACTCCTCGAGTAGAGTG
GCGAACGGGTGAGTAACGCGTGAAGAACCTGCCTTCAACAGGGGGATAACACTCCCAACGGGGTGCTAAT
ACCGCATGAGACCACGGGTCTGCATGGACCTGAGGTCAAAGGTGGCCTCTATTTATAAGCTACCGGTTGAA
GATGGCTTCGCGTCCCATTAGCTAGTTGGCGGTGTAAAAGACCACCAAGGCGACGATGGGTAGCTGGTCT
GAGAGGATGGTCAGCCACACTGGGACTGAGATACGGCC

>Zotu15687
GAACGCTGGCGGCGTGCCTAACACATGCAAGTCGTGCGCGAAAGTCTTCGGATGAGTAGAGCGGCGCACG
GGTGAGTAACACGTGGGTAATTTGCCCTTAGGTCCGGAATAACCCAGGGAAACTTGGGCTAATACCAGAT
GAGACCACGGAACGAGAGTTCTGTGGGAAAAGCGGAGGACTTCTTCGGAAACTTCGCGCCAAAGGATAA
GCCCGCGGCCCATCAGCTAGTTGGCGGGGTAAAGGCCCACCAAGGCTAAGACGGGTAGCTGGTCTGAGA
GGATGATCAGCCACACTGGAACTGAGACACGGTC
>Zotu15690
GAACGCTGGCGGCAGGCCTAACACATGCAAGTCGAACGCTCTTTTCGGAGAGAGTGGCGCACGGGTGAGT
AACGCGTGGGAATCTGCCCTTTGGTTCGGAATAACCCTGGGAAACTAGGGCTAACACCGGATAAGCCCCG
CGAGGGGGAAAGTTTACGCCGAAGGAGGAGCCCGCGTCCGATTAGCTAGTTGGTGAGGTAAGAGCTCAC
CAAGGCTTCGATCGGTAGCTGGTCTGAGAGGACGATCAGCCACACTGGGACTGAGACACGGCC
>Zotu15692
GAACGCTGGCGGCGCGCTTAACACATGCAAGTCGAACGGTTAAAGCACCTCCGGGTGTGCATAAAGTGGC
GAACGGCTGAGTAACACGTGGGCAACCTGCCCCCCGCACCGGGATAGCCTCGGGAAACCGTGGGTAATAC
CGGATACTCCCTCCCCTCCGCATGGCGGGGAGGGGAAAGCGTACACGGCGGGGGATGGGCCCGCGGCCT
GTTAGCTCGTTGGCGGGGTAACGGCCCACCAAGGCGATTATGGGTAGCCGGGTTGAGAGACCGACCGGCC
AGATTGGGACTGAGACACGGCC
>Zotu15695
GAACGCTGGCGGCAGGCCTAACACATGCAAGTCGAACGGGCCCTTCGGGGTTAGTGGCGGACGGGTGAG
TAACACGTGGGAACGTGCCTTTTGGTTCGGAATAGCTCCTGGAAACGGGTGGTAATGCCGAATGTGCCCTT
CGGGGGAAAGATTTATCGCCATTAGAGCGGCCCGCGTCTGATTAGCTAGTTGGTGAGGTAAAGGCTCACC
AAGGCGACGATCAGTAGCTGGTCTGAGAGGATGACCAGCCACATTGGGACTGAGACACGGCC
>Zotu15698
GAACGCTGGCGGCATGCTTTACACATGCAAGTCGAACGGCAGCACGGGGGCAACCCTGGTGGCGAGTGG
CGAACGGGTGAGTAATGCATCGGAACGTGTCCTGTAATGGGGGATAACCTATCGAAAGGTAGGCTAATAC
CGCATACGTCCTGAGGGAGAAAGCGGGGGCTCGCAAGACCTCGTGTTATAGGAGCGGCCGATGTCGGATT
AGCTAGTTGGTGGGGTAAAGGCCTACCAAGGCGACGATCCGTAGCGGGTCTGAGAGGACGGCCCGCCAC
ACTGGGACTGAGACACGGCC
>Zotu15702
GAACGCTAGCGACAGGCTTAACACATGCAAGTCGAGGGGTAACAGGGTAGCAATACCGCTGACGACCGGC
GCACGGGTGAGTAACGCGTATGCAACCTGCCTATAACTGGGGAATAGCTCCCTGAAAGGGGAATTAACAC
CGCATAACACTATTGATTCGCATGGATTGGTAGTTAAATGTAGCGATACAGGTTATAGATGGGCATGCGTG
ACATTAGCTAGTTGGTGAGGTAACGGCTCACCAAGGCTACGATGTCTAGGGGTTCTGAGAGGAAGGTCCC
CCACACTGGTACTGAGACACGGAC
>Zotu15705
GAACGCTGGCGGCGTGCCTAACACATGCAAGTCGAGCGCGAAAAGGGCAACCTGAGTAGAGCGGCGCAC
GGGTGCGTAACACGTGGGTAATCTACCCTTGGGTCTGGGATAACTCTTCGAAAGAAGAGCTAATACCGGAT
AAGCTCACGGGGTCTTCGGACTCTGGGAGAAAAGTAGGCCTCTATTCATAAGCTTACACCTGAGGATGAGC
CCGCGGCCCATCAGCTAGTTGGCGGGGTAATGGCCCACCAAGGCAACGACGGGTAGCTGGTCTGAGAGG
ACGATCAGCCACACTGGAACTGAGACACGGTC
>Zotu15710
GAACGCTAGCGGCATGCTTAACACATGCAAGTCGAACGGTGAAGGAGGAGCTTGCTCCTTTGGATCAGTG
GCGAACGGGTGAGTAACACGTGAGTAACCTGCCCCAGACTCTGGGATAAGCACTGGAAACGGTGTCTAAT
ACCGGATACGAGCTTCAGCCGCATGGCTAGGAGCTGGAAAGAATTTCGGTCTGGGATGGACTCGCGGCCT
ATCAGCTAGTTGGTGAGGTAATGGCTCACCAAGGCGACGACGGGTAGCCGGCCTGAGAGGGTGACCGGC
CACACTGGGACTGAGACACGGCC
>Zotu15716
GAACGCTGGCGGCAGGCTTAACACATGCAAGTCGAACGCCCCGCAAGGGGAGTGGCAGACGGGTGAGTA
ACGCGTGGGAATCTACCCAATTCTACGGAACAACAGTTGGAAACGACTGCTAATACCGTATACGCCCTACG

GGGGAAAGATTTATCGGAATTGGATGAGCCCGCGTAAGATTAGCTAGTTGGTGAGGTAATGGCTCACCAA
GGCGACGATCTTTAGCTGGTCTGAGAGGATGATCAGCCACACTGGGACTGAGACACGGCC
>Zotu15720
GAACGCTGGCGGCGTGCTTAACACATGCAAGTCGAACGATTAAACCGCCCTCGGGCGGACATAGAGTGGC
GAACGGGTGAGTAACACGTGACCAACCTGCCCCATGCTCCGGGATAACCTGCGGAAACGCAGGCTAATAC
CGAATACTCCGCGCGGGCCGCATGGCCCGGGCGGGAAAGCCCTTACGGCATGGGATGGGGTCGCGGCCC
ATTAGGTAGACGGCGGGGTGACGGCCCACCGTGCCCGCGATGGGTAGCCGAGCTGAGAGGCTGACCGGC
CACATTGGGACTGAGATACGGCC
>Zotu15726
GAACGCTGGCGGCGCGCTTAACACATGCAAGTCGAGCGAGAACCATGGCTTCGGCTATGGGGACAGCGG
CGAACGGGTGAGTAACACGTGGGTAATCTGCCCTCGGCACTGGGATAGCCCGGGGAAACCCGGATTAATA
CCGGATGGCCTCTCGAGCTCTTGGGCTTGTGAGGAAAGGTAGCTTTGGCCTCCGGCCGAGGAGGAGCCCG
CGGCGGATTAGCTTGTTGGTGAGGTAAAGGCTCACCAAGGCGACGATCCGTAGCTGGTGTGAGAGCATGA
CCAGCCACACTGGGACTGAGACACGGCC
>Zotu15729
GAACGCTGGCGGCGTACTTAACACATGCAAGTCGAACGGAAAGGCCCTGCTTTTGTGGGGTGCTCGAGTG
GCGAACGGGTGAGTAACACGTGAGTAACCTGCCCTTGACTTTGGGATAACTTCAGGAAACTGGGGCTAAT
ACCGGATAGGAGCTCCTGCTGCATGGTGGGGGTTGGAAAGTTTCGGCGGTTGGGGATGGACTCGCGGCTT
ATCAGCTTGTTGGTGGGGTAGTGGCTTACCAAGGCTTTGACGGGTAGCCGGCCTGAGAGGGTGACCGGCC
ACACTGGGACTGAGACACGGCC
>Zotu15734
GAACGCTAGCGGCAGGCTTAATACATGCAAGTCGAGGGGTAGCATGGGTAGCAATACCTGATGACGACCG
GCAAACGGGTGCGGAACACGTACGCAACCTTCCCATAACTGGTGAATAGCCTTCCGAAAGGGAGATTAAT
ACACCGTAACATAACGAAGAGGCATCTCTTTGTTATTATAGGCTTCGGCCGGTTTTGGATGGGCGTGCGCC
TGATTAGGTAGTTGGCGGGGTAACGGCCCACCAAGCCTGCGATCAGTAACTGGTGTGAGAGCACGACCAG
TCACACGGGCACTGAGACACGGGC
>Zotu15737
GAATGCTGGCGGCGTGCCTAATACATGCAAGTCGAGCGGTAACAGGGCTTCACCGTTCTCTTGTCGCACTT
GGCCTATGTGCAAGTGGTTTGATACGAGGGAACAGTGAAGCTGCTGACGAGTGGCGGACGGCTGAGTAA
CGCGTGGGAACATACCCTAAAGTGAGGGATAACTACTCGAAAGAGTGGCTAATACCGCATGTGATCTTTG
GATTAAAGCATTTATGCGCTTTAGGAATGGCCTGCGTACGATTAGATAGTTGGTGAGGTAATGGCTCACCA
AGTCGACGATCGTTAGATGGTTTGAGAGGATGATCATCCAGACTGGGACTGAGACACGGCC
>Zotu15739
GAACGCTGGCGGAATGCTTTACACATGCAAGTCGAGCGGCAGCGCGGTCTTCGGACTGGCGGCGAGCGG
CGAACGGGTGAGTAATACATCGGAACATGCCCAGTCGTGGGGGATAACACTTCGAAAGAAGTGCTAATAC
CGCATACGTCCTGAGGGAGAAAGCGGGGGATCGCAAGACCTCGCGCGATTGGAGTGGCCGATGTCAGAT
TAGCTAGTTGGTGGGGTAAAGGCCTACCAAGGCAACGATCTGTAGCTGGTCTGAGAGGATGATCAGCCAC
ACTGGGACTGAGACACGGCC
>Zotu15741
GAACGCTGGCGGTATGCTTTACACATGCAAGTCGTACGGGAGTTTTTAACTCAAGTGGCGGACGGGTGAG
TAACACGTAAGAATTTGCCTTTAGGAGGGGGATAACAGCTGGAAACGGCTGCTAATACCCCATATGCTTTC
GAGTGAAATGGATTTTTCCGCCTAAAGAGAAGCTTGCGGCTGATTAGCTTGTTGGTAAGGTAATGGCTTAC
CAAGGCGACGATCAGTATCTGGTTTGAGAGGACGATCAGACACACTGGAACTGAGACACGGTC
>Zotu15746
GAACGCTGGCGGCGTGCTTAACACATGCAAGTCGAGCGATGAAGCTTCTTCAGAAGTGGATTAGCGGCGG
ACGGGTGAGTAACACGTGGGTAACCTGCCTTATAGTGGGGAATAGCCTTTCGAAAGGAAGATTAATACCC
CATAAGATTGTAGTTTCGCATGAAACAGCAATTAAAGGAGCAATTCGCTATAAGATGGACCCACGGCGCAT
TAGCTAGTTGGTGAGGTAACGGCTCACCAAGGCGACGATGCGTAGCCGACCTGAGAGGGTGATCGGCCAC
ATTGGGACTGAGACACGGCC
>Zotu15750

GAACGCTAGCGGCAGGCTTAATACATGCAAGTCGAGGGGCAGCATGAGTGTAGCAATACATTTGATGGCG
ACCGGCAAACGGGTGCGGAACACGTACAGAACCTTCCTTCGAGCGGGGAATAGCCCAGAGAAATTTGGAT
TAATACCCCATAGTATTATGAAGTGGCATCATTTTATAATTAAAGATTTATCACTTGAAGATGGCTGTGCGG
CTGATTAGATAGTTGGTGAGGTAACGGCTCACCAAGTCTACGATCAGTAACTGGTGTGAGAGCACGACCA
GTCACACGGGCACTGAGACACGGCC
>Zotu15754
GAATGCTGGCGGCGTGGATGAGGCATGCAAGTCGAATGGGCTTAGACCCATGGCAGACGAGGTAGGAAC
ACGTAGGTACATACCCCAAAGTCAGGGATAACCCGGAGAAATCCGGAATAATACTTGATGGTCTCGAAAG
AGTAAAGATTTATCGCTTTGGGAATGGCCTGCGTTCTATCAGCTTGTTGGTAAGGTAACGGCTTACCAAGG
CTACGACGGATAGGGGAGCTGAGAGGCTGACCCCCACCGATGGAACTGCGACACGGTC
>Zotu15757
GAACGCTGGCGGCATGCTTTACACATGCAAGTCGAACGGCAGCGCGGGGCAACCTGGCGGCGAGTGGCG
AACGGGTGAGTAATATATCGGAACGTACCCAAGAGTGGGGGATAACGTAGCGAAAGTTACGCTAATACCG
CATACGATCTAAGGATGAAAGCAGGGGATCTTCGGACCTTGTGCTCCTGGAGCGGCCGATATCTGATTAGC
TAGTAGGTAGGGTAAAGGCCTACCTAGGCGACGATCAGTAGCTGGTCTGAGAGGACGACCAGCCACACTG
GAACTGAGACACGGTC
>Zotu15767
GAACGCTGGCGGCATGCCTAATACATGCAAGTCGAGCGAACGAATGTGGGAGCTTGCTCCTACATTCGTTA
GCGGCGGATGGGTGAGTAACACGTGGGCAACCTGCCCTGCAGATGGGGATAACTCCGGGAAACCGGGGC
TAATACCGAATAATCAGTATGTCCGCATGGACATACTCTGAAAGACGGCTCACGCTGTCACTGCAGGATGG
GCCCGCGGCGCATTAGCTAGTTGGTAAGGTAACGGCTTACCAAGGCGACGATGCGTAGCCGACCTGAGAG
GGTGATCGGCCACACTGGGACTGAGACACGGCC
>Zotu15771
GAACGCTAGCGGCATGCCTAACACATGCAAGTCGAACGGCAACGGGGGGGAGCTTAGCTCCCTGACGGCA
AGTGGCGGACGGGTGAGTAATACGTAGGAATCTACCTTATAGTGGGGGATAACCTGGGGAAACTCAGGCT
AATACCGCATAATCTTTCTTTAAAGCAAAGCGGGGGATCTTCGGACCTCGTGCTATAAGATGAGCCTGCGT
CGGATTAGCTAGTTGGCGGGGTAAAGGCCCACCAAGGCGACGATCCGTAGCTGGTCTGAGAGGATGATCA
GCCACACTGGAACTGAGACACGGTC
>Zotu15778
GAACGCTAGCTACAGGCTTAACACATGCAAGTCGAGGGGAAACGATAGAGAAAGCTTGCTTTTTCTAGGC
GTCGACCGGCGCACGGGTGAGTAACGCGTATCCAACCTGCCCATCACTTGGGGATAACCTTGCGAAAGTA
AGACTAATACCCAATGATATCTCTAGAAGACATCTGAAAGGGATTAAAGATTCATCGGTGATGGATGGGGA
TGCGTCTGATTAGCTTGTTGGCGGGGTAACGGCCCACCAAGGCGACGATCAGTAGGGGTTCTGAGAGGAA
GGTCCCCCACATTGGAACTGAGACACGGTC
>Zotu15786
GAACGCTGGCGGCAGGCCTAACACATGCAAGTCGAACGGTAGCGGGAAGAAGCTTGCTTCTTTGCCGACG
AGTGGCGGACGGGTGAGTAATGTCTGGGGATCTGCCCGATGGAGGGGGATAACCACTGGAAACGGTGGC
TAATACCGCATAACGTCGCAAGACCAAAGTGGGGGACCTTCGGGCCTCACACCATCGGATGAACCCAGAT
GGGATTAGCTAGTAGGTGGGGTAACGGCTCACCTAGGCGACGATCCCTAGCTGGTCTGAGAGGACGATCA
GCCACACTGGGACTGAGACACGGCC
>Zotu15789
GAACGCTGGCGGCAGGCCTAACACATGCAAGTCGAGCGGATGAGAGGAGCTTGCTCCTGGATTCAGCGGC
GGACGGGTGAGTAATGCCTAGGAATCTGCCTGGTAGTGGGGGATAACGTCCGGAAACGGGCGCTAATAC
CGCATACGTCCTGAGGGAGAAAGTGGGGGATCTTCGGACCTCACGCTATCAGATGAGCCTAGGTCGGATT
AGCTAGTTGGTGAGGTAATGGCTCACCAAGGCGACGATCCGTAACTGGTCTGAGAGGATGATCAGTCACA
CTGGAACTGAGACACGGTC
>Zotu15792
GAACGCTAGCGGCATGCCTAACACATGCAAGTCGAACGGCAGCATAGGAGCTTGCTCCTGATGGCGAGTG
GCGAACAGATGAGTAATATATCGGAACGTGCCCTAGAGTGGGGGATAACTAGTCGAAAGACTAGCTAATA
CCGCATACGATCTACGGATGAAAGTGGGGGATCGCAAGACCTCATGCTCCTGGAGCGGCCGATATCTGATT

AGCTAGTTGGTGGGGTAAAAGCTCACCAAGGCGACGATCAGTAGCTGGTCTGAGAGGACGACCAGCCACA
CTGGGACTGAGACACGGCC
>Zotu15800
GAACGCTGGCGGTGTGCTTAACACATGCAAGTCGAACGGGCAAGAGCTGTAGCAATACAGAATTCTGCTA
GTGGCAGACGGGTGAGTAATACATGGGAATCTACCTTGCAGTGCGAAATAACAGTTGGAAGCAATTGCTA
ATGTCGCATATAGCCGAGAGGTGAAAGATTTATCGCTGCAAGACGAGCCCATGCAGGATTAGCTAGTTGG
TGAGGTAATGGCTCACCAAGGCAACGATCTTTAGTTGGTCTGAGAGGATGATCAGCCACACTGGGACTGA
GACACGGCC
>Zotu15806
CAACGCTGGCGGCGTGCCTAACACATGCAAGTCGAACGAGAAAGGGTAGCAATACCTGAGTAAAGTGGCG
CACGGGTGAGTAACACGTGACTAACCTACCCTGGAGTGGGGGATAACTGAGGGAAACCTTAGCTAATACC
GCATAATACCTACGGGTCAAAGGAGCAATTCGCTTCAGGAGGGGGTCGCGGCCGATTAGTTAGTTGGCGG
GGTAATGGCCCACCAAGACAGTGATCGGTATCCGGCCTGAGAGGGCGCACGGACACACTGGAACTGAAAC
ACGGTC
>Zotu15813
GAACGCTTGCGGCGTGCCTAAGAAATGCAAGTCGAGCGAGATTTCACTCTGTTCAAAGCTTCGGCAGAGA
ACTTGTGAAGTCTAGCGGCGAACGGTCGCGTAACACGTAGGCAACCTGCCCTAAAGTATGGGATAGCCCG
CCGAAAGGCGGATTAATACCGTATGTGGATATCTTTCCGCATGAGAGGATATCTAAAGCAGCAATGCGCTT
TAGGATGGGCTTGCGGCCTATCAGCTAGTTGGTGGGGTAATGGCTTACCAAGGCGACGACGGGTAGCTGG
TCTGAGAGGATGATCAGCCGGATTGGGACTGAGATACGGCC
>Zotu15816
GAACGCTGGCGGCGTACCTAACGCATGCAAGTCGAACGGGGATTAGATAGCAATATTTGATTCTAGTGGC
AAACGGGTGAGTAACACGTAGGGATCTACCTCGAAGTGGGGAACAACGGACCGAAAGGTTCGCTAATACC
GCATGTGGTCGCGAGACCAAAGGATTTTCGCAAGAGAATTCGCTTTGAGAGGAGCCTGCGTTCGATCAGG
TAGTTGGTGAGGTAACGGCTCACCAAGCCGATGACCGATAGCTGTTCTGAGAGGAAGATCAGCCACACTG
GGACTGTGACACGGCC
>Zotu15818
GAACGCTGGTGATATGCTTGACACATGCGAGTTAAACGGAGGCATTTTGAAATGCTTTAGTAGCGAACGG
GTGCGTAATACATAAGAATTTACATAATAGTTTAGGATAGTTTTTTTAATTAAAAAAGTAATACTAAATAA
AGTATAAGAATATTTTGTTTTTATATACTAAAGATTTATCGCTATTATATAAGCTTGTGGAGGATTAGGTAGT
TGGTAGGGTAAAGGCCTACCAAGCCAGTGATCCTTAGCTGGTCTGAGAGGACGGTCAGCCACATTGGGAC
TGAGAGAAGGCC
>Zotu15822
GAACGCTGGCGGCAGGCCTAACACATGCAAGTCGAACGGTAGCACAGAGAGCTTGCTCTCGGGTGACGA
GTGGCGGACGGGTGAGTAATGTCTGGGAAACTGCCCGATGGAGGGGGATAACTACTGGAAACGGTAGCT
AATACCGCATAACGTCGCAAGACCAAAGAGGGGGACCTTCGGGCCTCTTGCCATCGGATGTGCCCAGATG
GGATTAGCTTGTTGGTGAGGTAACGGCTCACCAAGGCGACGATCCCTAGCTGGTCTGAGAGGATGACCAG
CCACACTGGAACTGAGACACGGTC
>Zotu15824
GAACGCTGGCGGTGTGCTTAACACATGCAAGTCGAACGGGCAAGAGCTGTAGCAATACAGAATTCTGCTA
GTGGCAGACGGGTGAGTAATACATGGGAATCTACCTTGCAGTGCGAAATAACAGTTGGAAGCAATTGCTA
ATGTCGCATATAGCCGAGAGGTGAAAGATTTATCGCTGCAAGACGAGCCCATGCAGGATTAGCTAGTTGG
TGGGGTAATGGCTCACCAAGGCGACGATCTTTAGTTGGTCTGAGAGGGTGATCGGCCACACTGGGACTGA
GACACGGTC
>Zotu15827
GATAAATGTATTTACTATAAGGAATAGTCTCACAAAAATGCAGAACTAAGCCAAGCCTGAGGTCTAGGATG
AGCATCCACATCGATGAGACCATGAATAGTGTAGAATCTCCAAAAGATGAAGCT
>Zotu15829
GAACGCTGGCGGCGTGCCTAACACGTGCAAGTCGAACGGAACCTTCGCAAGAGGGTTGAGTGGCGCACG
GGTGAGGAACACGTAGCTGACCTGCCTTCCAGCGGGGGATAACGTCCCGAAAGGGATGCTAAGACCGCAT

AAGCTCACAGGGCTTAGAAACCTGCGAGAAAAAGCTTCGGCTACTGGAAGAGGGGGCTGCGGCCCATCA
GGTAGTTGGCGGGGTAACGGCCCACCAAGCCGACGACGGGTCGGGGATCTGAGAGGATGATCCCCCACA
CTGGGACTGAGACACGGCC
>Zotu15831
GAACGTTAGCGGCGCGCCTAACACATGCAAGTCGCACGAGAGTGGGCAACCCTGAAAGTGGCGCACGGG
TGCGTAACACGTAGATAACCTGCCCTTTGGAGGGGGATAACGTTCCGAAAGGAGCGCTAATACCGCATAC
AGCATTCTGGGGTTTGCTCTGGGATGGCAAAGGTAGGGACCCGCAAGGGCCTATCGCCGAAGGAGGGGT
CTGCGGCCCATCAGCTAGTTGGGGAGGTAATGGCTCACCAAGGCGAAGACGGGTAGCTGGTCTGAGAGG
ATGATCAGCCACACTGGTACTGGAACACGGAC
>Zotu15835
GAACGCTGGCGGCGTGCTTAATACATGCAAGTCGAGCGGTAAGGTTCTCACAGATTATTTAACCAGATAAG
TCCTTCACGGAAACTATCTGCAGCGTTGATTCCCCGGCTAGGGAATCGAGGTTGAATAATGCGTGAGAATA
CACGAGCGGCGGACGGCTGAGTAACACGTAGGAACGTACCCCAAACTGAGGGATAAGCACCAGAAATGG
TGTCTAATACCGCATGTGCTCTTCGGAGTAAAGCTTCGGCGGTTTGGGAACGGCCTGCGCATGATTAGCTT
GTTGGTGAGGTAATAGCTCACCAAGGCGACGATCATTAGCTGGTCTGAGAGGATGATCATCCAGACTGGG
ACTGAGAACGGCC
>Zotu15839
GAACGCTAGCGGCATGCTTAACACATGCAAGTCGAGCGGAAAGGCCACTTCGGTGGTACTCGAGCGGCGA
ACGGGTGAGTAACACGTGAGTAATCTGCCCTTCACTTCGGGATAGCTCCGGGAAACCGGGATTAATACCG
GATATGACTACCTTCGGCATCGGGGGTGGTGGAAAGTTTTTCGGTGGGGGATGTGCTCGCGGCCTATCA
GCTTGATGGTGGGGTAATGGCCTACCATGGCTTCGACGGGTAGCCGGCCTGAGAGGGTGACCGGCCACAC
TGGGACTGAGACACGGCC
>Zotu15842
GAACGCTGGCGGCGTGCTTAACACATGCAAGTCGAGCGGATCTCATTCTTTCGGGGGTGGGGTTAGCGGC
GAACGGGTGAGTAACACGTGAGCAACCTGCCTCTGGCTTTGGGATAACTCCGGGAAACCGGGGCTAATAC
CGGATATGACCTGGTTCTGCATGGGGCTGGGTGGAAAGATTTATCGGCCAGGGATGGGCTCGCGGCCTAT
CAGCTTGTTGGTGGGGTGATGGCCTACCAAGGCGACGACGGGTAGCCGGCCTGAGAGGGTGACCGGCCA
CACTGGGACTGAGACACGGCC
>Zotu15844
GAACGCTAGCGGCATGCTTAACACATGCAAGTCGAGGGGCAGCATGTTGGTTGCTTGCAACCAATGATGG
CGACCGGCGCACGGGTGAGTAACGCGTATCCAACCTACCCAATACTCAGGCATAACCCGTTGAAAGACGG
ACTAATTCCTGATGGTATGTATTTTTCTCATGAATTATACATTAAAGAATTTCGGTATTGGATGGGGATGCG
TCTGATTAGTTTGTTGGTGGGGTAACGGCTCACCAAGACTTCGATCAGTAGGGGTTCTGAGAGGAAGGTCC
CCCACATTGGAACTGAGACACGGTC
>Zotu15849
GAACGCTATCGGTATGCTTAACACATGCAAGTCGAGCGGATGATGGGAGCTTGCTCCCGGATTCAGCGGC
GGACGGGTGAGTAATGCCTAGGAATCTGCCTGGTAGTGGGGGACAACGTTTCGAAAGGAACGCTAATACC
GCATACGTCCTACGGGAGAAAGCAGGGGACCTTCGGGCCTTGCGCTATCAGATGAGCCTAGGTCGGATTA
GCTAGTTGGTGGGGTAAAGGCCTACCAAGGCGACGATCCGTAACTGGTCTGAGAGGATGATCAGTCACAC
TGGAACTAAGACACGGTC
>Zotu15856
GAACGCTAGCGGCGGGCTTAATACATGCAAGTCAGGGGGTAGCAATATCACCGGCGTACGGGTGCGTAAC
GCGTATGCAACCTACCTTTCAGGGGCGGATATACGGGAGAAATTCTGGGCAACACGCCATATACTTATGTT
GGGGCATCTTAATGTAAGGAAAGTAACTCTGGGAGCTGGGCATGCGTCTGATTAGCTAGTTGGTAAGGTA
ACGGCTTACCAAGGCGACGATCAGTAGCTGTTCTGAGAGGAAGATCAGCCACACTGGCACTGAGACACGG
GT
>Zotu15858
GAACGCTGGCGGCGTGCTTAACACATGCAAGTCGAACGAGCAAGAGCTGTAGCAATACAGGGTTCTGCTA
GTGGCAGACGGGTGAGTAATACATGGGAATCTACCTTGCAGTGCGAAATAACAGTTGGAAGCAATTGCTA
ATGTCGCATATAGCCGGGAGGTGAAAGGTTTACTGCTGCAAGATGGGCCCATGCAGGATTAGCTAGTTGG

TGAGGTAATGGCTCACCAAGGCAACGATCTTTAGTTGGTCTGAGAGGATGATCAGCCACACTGGGACTGA
GACACGGCC
>Zotu15861
GAACGCTGGCGGCGTGCCTAATACATGCAAGTCGAGCGAACGGATAAGGAGCTTGCTCCTTTGAAGTTAG
CGGCGGACGGGTGAGTAACACGTGGGTAACCTACCTATAAGACTGGAATAACTTCGGGAAACCGGAGCTA
ATACCGGATAACATGTTGAACCGCATGGTTCAACAGTGAAAGACGGTTTTGCTGTCACTTATAGATGGATC
CGCGCCGCATTAGCTAGTTGGTAAGGTAACGGCTTACCAAGGCAACGATGCGTAGCCGACCTGAGAGGGT
GATCGGCCACACTGGAACTGAGACACGGTC
>Zotu15864
GACGTGTCTCAGTCCCAGTGTGGCTGGTCATCCTCTCAGACCAGCTACGGATCGTCGCCTTGGTAGGCCTTT
ACCCCACCAACTAGCTAATCCGACGTAGGCTCATCCTAGAGCGCAAGGCCCCGAAGGGTCCCCTGCTTTGA
TCTCTCGATATCATGCGGTATTAGCCCGAGTTTCCTCGGGTTATCCCACTCTCCAGGACAGATTCCTACGCTT
TACTCACCCGTCCGCCACTCGCCACCCGTAAAGACCGAAATCTTTACTGTGCTGCCGTTCGACTTGCATGTG
TTAGGCATGCCGCCAGCGTTCA
>Zotu15868
GAACGCTGGCGGCATGCCTAATACATGCAAGTCGAACGAGACCTTCGGGTCTAGTGGCGCACGGGTGCGT
AACACGTGGGAATCTGCCCTTAGGTTCGGAATAACTCAGAGAAATTTGAGCTAATACCGGATAATGACGAA
AGTCCAAAGATTTATCGCCTAAGGATGAGCCCGCGTAAGATTAGGTAGTTGGTGAGGTAAAGGCTCACCA
AGCCGACGATCTTTAGCTGGTCTGAGAGGATGATCAGCCACACTGGGACTGAGACACGGCC
>Zotu15873
GAACGTTAGCGGCGGGCCTAACACATGCAAGTCGAGCGAGAAAGTGGCTTCGGCCATCAGTAAAGCGGC
GGACGGGTGAGTAACACGTAGGTAATCTTCCCTTGAGCGGGGGACAACGTTTCGAAAGGAACGCTAATAC
CGCATACGTTTCCAGGGACCCCGGTCCTTGGAAGGAAAGCTAGGTCGCAAGATCCGGCACTCGAGGAGGA
GCCTGCGTCGCATCAGCTAGTTGGTGGGGTAACGGCCTACCAAGGCGAAGACGCGTAGCTGGTCTGAGAG
GATGATCAGCCACACTGGGACTGAGACACGGCC
>Zotu15875
GAACGCTGGCGGCATGCCTAACACATGCAAGTCGAACGAGCAAGAGCTGTAGCAATACAGGGTTCTGCTA
GTGGCAGACGGGTGAGTAATACATGGGAATCTACCTTGCAGTGCGAAATAACAGTTGGAAGCAATTGCTA
ATGTCGCATATAGCCGAGAGGTGAAAGGTTTACTGCTGCAAGATGGGCCCATGCAGGATTAGCTAGTTGG
TGAGGTAATGGCTCACCAAGGCAACGATCTTTAGTTGGTCTGAGAGGATGATCAGCCACACTGGGACTGA
GACACGGCC
>Zotu15878
CAACGCTGGCGGCGTGCCTAACACATGCAAGTCGAACGAGAAAGTGGAGCAATCCATGAGTACAGTGGCG
ACCGGGTGAGTAACACGTGATCAACCTGCCTTCGAGTGGGGGATAACTCCGGGAAACCGGGGCTAATACC
GCATAACATCCTCGGATCGGAAGAACTGAGGATCAAAGGAGCAATTCGCTTGAAGAGGGGGTCGCGGCT
GATTAGCTAGTTGGTAGGGTAATGGCCTACCAAGGCTGAGATCGGTATCCGGCCTGAGAGGGCGCACGGA
CACACTGGAACTGAAACACGGTC
>Zotu15880
GAACGCTGGCGGCATGCCTTACACATGCAAGTCGAACGGCAGCGGGGGGAGCTTGCTCCCTGACGGCGA
GTGGCGGACGGGTGAGTAACACGTAGGAATCTACCTTAATTATAATAGTTAGTGGGGGATAACCCGGGGA
AACTCGGGCTAATACCGCATAATCTCTTCAAAGCAAAGCGGGGGATCTTCGGACCTCGTGCTATGAGATGA
GCCTGCGTCGGATTAGCTAGTTGGTAGGGTAATGGCCCACCAAGGCGACGATCCGTAGCTGGTCTGAGAG
GACGATCAGCCACACTGGGACTGAGACACGGCC
>Zotu15888
GAACGCTGGCGGCGTGCTTAACACATGCAAGTCGAACGGAAAGGCCCTGCTTTTGTGGGGTGCTCGAGTG
GCGAACGGGCGAGTAACACGTGAGTAACCTGCCCTTGACTTTGGGATAACTTCAGGAAACTGGGGCTAAT
ACCGGATAGGAGCTCCTGCTGCATGGTGGGGGTTGGAAAGTTTCGGCGGTTGGGGATGGACTCGCGGCTT
ATCAGCTTGTTGGTGGGGTAGTGGCTTACCAAGGCTTTGACGGGTAGCCGGCCTGAGAGGGTGACCGGCC
ACACTGGGACTGAGACACGGCC
>Zotu15893

GAACGCTTGCGGCGCGCCTAATACATGCAAGTCGAACGAGGTAGCAATACCAAGTGGCGCACGGGTGAGT
AACACGTGGATAATCTGCCCTTCAGTGGGGAATAACCATCCGAAAGGATGGCTAATACCGCATGAGACCAC
GGCATCTGCGGATGCAGAGGTTAAAGATTTATCGCTGAAGGATGAGTCTGCGCGAGATTAGCTAGTTGGC
GGGGTAATGGCCTACCAAGGCAACGATCTCTAACTGGTCTGAGAGGATGACCAGTCACACTGGAACTGAG
ACACGGTC
>Zotu15898
GAACGCTAGCGGCATGCCTAACACATGCAAGTCGAACGGTAACAGGCTCGCAAGGGCGCTGACGAGTGG
CGGACGGGTGAGTAGAGCGTAGGGATCTGCCTAGATGATTGGGATAGCCTAGGGAAACTTAGGGTAATA
CCGGATACTCCTTAAGAGGGAAAGCTGGGGACTTTTAATAGAGCCTGGCGCATTTAGATGAACCTACGTCG
GATTAGCTAGTTGGTAAGGTAAAGGCTTACCAAGGCGATGATCCGTAGCTGGTCTGAGAGGACGGCCAGC
CACACTGGGACTGAGACACGGCC
>Zotu15903
GAACGCTGGCGGCATGCCTAACACATGCAAGTCGAACGAAGGCTTCGGCCTTAGTGGCGCACGGGTGCGT
AACGCGTGGGAATCTGCCTTCAGGTACGGAATAACTCCTCGAAAGGGGTGCTAATACCGTATGATGTCTAC
GGACCAAAGATTTATCGCCTGAAGATGAGCCCGCGTAGGATTAGCTAGTTGGTGGGGTAAAGGCCTACCA
AGGCGACGATCCTTAGCTGGTCTGAGAGGATGATCAGCCACACTGGGACTGAGACACGGCC
>Zotu15908
GAACGCTAGCGGCATGCCTAACACATGCAAGTCGAACGGCAGCATAGGAGCTTGCTCCTGATGGCGAGTG
GCGAACGGGTGAGTAATATATCGAAACGTGCCCTAGAGTGGGGGATAACTAGTCGAAAGACTAGCTAATA
CCGCATACGATCTACGGATGAAAGTGGGGGATCGCAAGACCTCATGCTCCTGGAGCGGCCGATATCTGATT
AGCTAGTTGGTGGGGTAAAAGCTCACCAAGGCGACGATCAGTAGCTGGTCTGAGAGGACGATCAGCCACA
CTGGGACTGAGACACGGCC
>Zotu15913
GAACGCTGGCGGCATGCCTAACACATGCAAGTCGAACGAAGGCTTCGGCCTTAGTGGCGCACGGGTGCGT
AACGCGTGGGAATCTGCCCTTTGCTTCGGAATAACAGTTAGAAATGACTGCTAATACCGGATGATGTCTAC
GGACCAAAGATTTATCGGCAAGGGATGAGCCCGCGTAGGATTAGGTAGTTGGTGGGGTAAAGGCCTACCA
AGCCGACGATCCTTAGCTGGTCTGAGAGGATGATCAGCCACACTGGGACTGAGACACGGCC
>Zotu15917
CCTAAATCCCGAGCAGCCCGGTGGCTTCTTGGGGGCGGGCTTGACGACCCTTCCTTCCCCAGGTGCAGGCC
GAGCTGCGGCGGAAGTGGCGGCGCTGGCACCTGCAGGGCATCCTGGGCTCCGACGCCAAGTACC
>Zotu15926
GAACGCTAGCGGCATGCCTAACACATGCAAGTCGAACGGCAGCATAGGAGCTTGCTCCTGATGGCGAGTG
GCGAACGGGTGAGTAATATATCGGAACGTGCCCTAGAGTGGGGGATAACTAGTCGAAAGACTAGCTAATA
CCGCATACGATCTACGGATGAAAGTGGGGGATCGCAAGACCTCATGCTCCTGGAGCGGCCGATATCTGATT
AGCTAGTTGGTGGGGTAAAAGCTCACCAAGGCGACGATCAGTAGCTGGTCTGAGAAGACGACCAGCCACA
CTGGGACTGAGACACGGCC
>Zotu15930
GAACGCTGGCGGCATGCCTAACACATGCAAGTCGAACGAGATTAGAGAAGCTTGCTTCTCTATGAAAGTG
GCGCACGGGTGAGTAACGCGTGGATAACATACCTATTGGTTCAGGATAACTTGTCGAAAGACGGGCTAAT
ACTGGATAAGTTCCCCTGGACTGCGGTCCGGGTGAAGAAAGCGGGGCAACTCGCGCCGAATGAATGGTCC
GCGTCCCATCAGCTTGTTGGCGGGGTAATGGCCCACCAAGGCTACGACGGGTAACTGGTCTGAGAGGACG
ACCAGTCACACTGGGACTGAGACACGGCC
>Zotu15936
GAACGCTGGCGGCAGGCCTAACACATGCAAGTCGAGCGGTAACAGGAAGTAGCTTGCTACTTTGCTGACG
AGCGGCGGACGGGTGAGTAATGCTTGGGAACTTGCCTTTGGGAGGGGGATAACCGCTGGAAACGGTGGC
TAATACCGCATGATGTTTACGAACCAAAGTGGGGGCTCTTCGGACCTCACGCCCAGAGATAGGCCCAAGTG
AGATTAGCTAGTTGGTGAGGTAATGGCTCACCAAGGCGACGATCTCTAGCTGTTCTGAGAGGATGATCAGC
CACACTGGGACTGAGACACGGCC
>Zotu15938

GAACGCTAGCGATAGGCCTAACACATGCAAGTCGAGGGGCAGCACATGACGTAGCAATACAGATGGTGGC
GACCGGCGCACGGGTGAGTAACACGTATGCAACCTACCTTCAACAGGAGAATAACCCGGCGAAAGTCGGA
CTAATACTCCATAATACAGGGATCCCGCATGGGAATATTTGTTAAAGATTTATCGGTTGAAGATGGGCATG
CGTTCCATTAGCTAGTTGGTGAGGTAACGGCTCACCAAGGCAACGATGGATAGGGGAACTGAGAGGTTTA
TCCCCCACACTGGTACTGAGACACGGAC
>Zotu15942
CAACGCTGGCGGCGTGCCTAATGCATGCAAGTCGCACGGGGTGGTTTTCGGACCGCTTACGTGGCGGACG
GGTGAGGAACACGTGGGCAACCTGCCCGGTGGTGGGGGCTAGCCTTCGGAAACGGAGGGTAATTCCGCA
TGAGCTCGGATCTCGAGTGGGAGGTCTGAGGAAAGCCGTAAGGCGCCGCCGGAGGGGTCTGCGTCCGAT
TAGTTAGTTGGTGGGGTAACGGCTCACCAAGGCGATGATCGGTAGCTGGTTTGAGAGGATGATCAGCCAC
ACGGGGACTGAGACACGGCC
>Zotu15945
GAACGCTGGCGGCGTGCCTAATACATGCAAGTCAAGCGGCAACGAGTTTTGCACCTAGTCTTCTGACCTAC
GGGTAGAAGACTGTGTGCAGAATGTCGGCGAGCGGCGGACGGCAGAGTAACACGTAGGAACGTACCCCA
AACTGAGGGATAACTGCTCGAAAGAGTAGCTAATACCGCATGTGATCTTCGGATTAAAGCTTCGGCGGTTT
GGGAACGGCCTGCGTAAGATTAGCTAGTTGGTGAGGTAATGGCTCACCAAGGCGACGATCTTTAGCTGGT
CTGAGAGGATGATCAGCCTGACTGGAACTGAGACACGGTC
>Zotu15947
GAACGCTGGCGGCGTGCTTAACACATGCAAGTCGAACGAGCAAGAGCTGTAGCAATACAGGGTTCTGCTA
GTGGCAGACGGATGAGTAATACATGGGAATCTACCTTGCAGTGCGAAATAACAGTTGGAAGCAATTGCTA
ATGTCGCATATAGCCGAGAGGTGAAAGGTTTACTGCTGCAAGATGGGCCCATGCAGGATTAGCTAGTTGG
TGAGGTAATGGCTCACCAAGGCAACGATCTTTAGTTGGTCTGAGAGGATGATCAGCCACACTGGGACTGA
GACACGGTC
>Zotu15950
GAACGCTGGCGGCGTGCTTAACACATGCAAGTCGAACGAGCAAGAGCTGTAGCAATACAGGGTTCTGCTA
GTGGCAGACGGGTGAGTAATACATGGGAATCTACCTTGCAGTGCGAAATAACAGTTGGAAGCAATTGCTA
ATGTCGCATATAGCCGAGAGGTGAAAGGTTTACTGCTGCAAGATGGACCCATGCAGGATTAGCTAGTTGG
TGAGGTAATGGCTCACCAAGGCAACGATCTTTAGTTGGTCTGAGAGGATGATCAGCCACACTGGGACTGA
GACACGGTC
>Zotu15953
GAACGCTGGCGGCGTGCCTAATACATGCAAGTCGAGCGGACCTGTTTTGAAGCTTGCTTCGAAACAGGTTA
GCGGCGGACGGGTGAGTAACACGTGGGCAACCTGCCCGTAAGACCGGGATAACTTGCGGAAACGTGAGC
TAATACCGGATAGTTTTCCTTTCCGCATGGGAAGGAAAGGAAAGGTGGCGCAAGCTACCACTTACGGATG
GGCCCGCGGCGCATTAGCTAGTTGGTGGGGTAACGGCCTACCAAGGCGACGATGCGTAGCCGACCTGAGA
GGGTGATCGGCCACACTGGGACTGAGACACGGCC
>Zotu15957
GAACGCTGGCGGCATGCCTAACACATGCAAGTCGAACGGCAGCATAGCATCTAGCTTGCTAGATGGGGTG
GCGAGTGGCGAACGGGTGAGTAACGCGTAGGAATATGCCTTAAAGTGGGGGACAACTTGGGGAAACTCA
AGCTAATACCGCATAAGTTCTGAGGAGTGAAGCTGGGGACCGCAAGGCCTGGCGCTTTAAGATTAGCCTG
CGTCCGATTAGCTAGTTGGTAGGGTAAGGGCCTACCAAGGCGACGATCGGTAGCTGGTTTGAGAGAATGG
CCAGCCACACTGGGACTGAGACACGGCA
>Zotu15966
GAACGCTTGCGGCGTGCCTAAGAAATGCAAGTCGAACGGATGACTTCGGTCATTAGTGGCGAACGGTCGC
GTAACACGTAGGCAACCTGCCCAGAAGTGGGGGACAACAGCCTGAAAGGGCTGCTAATACCGCATGTGGC
CGGAGGGTGGCATCACTCTCTGTCTAAAGCTTTATGCGCTTCTGGATGGGCTTGCGGCCTATCAGGTAGTT
GGTGGGGTAATGGCCTACCAAGCCGACGACGGGTAGCTGGTCTGAGAGGACGATCAGCCGGATTGGGAC
TGAGATACGGCC
>Zotu15975
CCCCGGAGTCCACGGGTAGGACACCAGCAAGCCCCCGTGTCTTCCTGACTTTGCCTGGCTGGATCTCCTGG
CGTCCTGGGCACATCGGGTGTTCACACACACCCAAACACACCAGCAAGCACAGAATACCCACGGTCCCTTA
GCCGACCAG

>Zotu15977
CAACGCTGGCGGCGTGCCTCAGACATGCAAGTCGAACGATTAAACTTTCCTTCGGGAAAGATATAAAGTGG
CGCACGGGTGAGTAACACGTAAGTAATCTACCTTCGAGTGGGGAATAACGTTCGGAAACGAACGCTAATA
CCGCATAATGCAGCGGCATCGCAAGATGACAGTTGTTAAAGGAATTTATTTCGCTTGAAGAGGAGCTTGCG
GCAGATTAGCTTGTTGGTAAGGTAACGGCTTACCAAGGCTACGATCTGTATCCGGTCTAAGAGGACGGTCG
GACACACTGACACTGAATAACGGGT
>Zotu15980
GAACGCTGGCGGTATGCCTAACACATGCAAGTCGAACGAGGGTAGCAATACTCTAGTGGCGGACGGGTGA
GGAATACGTAGGAATTTGCCCTTAAGTGGGGGATAACCCGGGGAAACCCGGATTAATACCGCATGATCTTT
TCGAAGCAAAGCCGGGGACCGCAAGGCCTGGCGCTTTTGGAGAAGCCTACGTCGGATTAGCTAGTTGGTG
AGGTAATGGCTCACCAAGGCTGCGATCCGTAACTGGTCTGAGAGGATGATCAGTCACATCGGAACTGAGA
CACGGTC
>Zotu15985
GAACGCTAGCGGCATGCCTAACACATGCAAGTCGAACGGCAGCATAGGAGCTTGCTCCTGATGGCGAGTG
GCGAACGGGTGAGTAATATATCGGAACGTGCCCTAGAGTGGGGGATAACTAATCGAAAGACTAGCTAATA
CCGCATACGATCTACGGATGAAAGTGGGGGATCGCAAGACCTCATGCTCCTGGAGCGGCCGATATCTGATT
AGCTAGTTGGTGGGGTAAAAGCTCACCAAGGCGACGATCAGTAGCTGGTCTGAGAGGACGATCAGCCACA
CTGGGACTGAGACACGGCC
>Zotu15987
GAACGCTAGCGGCAGGCCTAACACATGCAAGTCGAGGGGTAAGGAGAGCTTGCTCTCCCTAGACCGGCGC
ACGGGTGCGTAACACGTGTACAATCTACCTTTTGCTAAGGGATAGCCCGAAGAAATTTGGATTAATACCTT
ATAGTATTGTTTGGTGGCATCACTGAATAATTAAAGCTCTGGTGGCAAAAGATGAGTACGCGTCCCATTAG
CTAGTTGGTGTGGTAACGGCATACCAAGGCTACGATGGGTAGGGGTCCTGAGAGGGAGGTCCCCCACACT
GGGACTGAGACACGGCC
>Zotu15991
GAACGCTGGCGGCATGCCTAACACATGCAAGTCGAACGAAGGCTTCGGCCTTAGTGGCGCACGGGTGCGT
AACGCGTGGGAACCTGCCCCTTGGTTCGGAATAACAGTTAGAAATGACTGCTAATACCGGATAATGACTTC
GGTCCAAAGATTTATCGCCGAGGGATGGGCCCGCGTAGGATTAGCTAGTTGGTAGGGTAAAGGCCTACCA
AGGCGACGATCCTTAGCTGGTCTGAGAGGATGATCAGCCACACTGGGACTGAGACACGGCC
>Zotu15995
GAACGCTGGCGGCGTGCCTAACACATGCAAGTCGAGCGGGGCCTAGCTCGTGGCAACACGGGGGACGGT
TCAGCGGCGAACGGGTGAGTAACACGTGAGGAACCTGCCCCGAAGACTGGGATAACTCTCCGAAAGGGG
TGCTAATACCGGATGTCCCCTCCTGGCCGCATGGTTGGGTGAGGAAATGCTTTGGTGCTTCGGGAGGGTCT
CGCGGCCTATCAGCTTGTTGGTGGGGTAATGGCCTACCAAGGCTTCGACGGGTAGCTAGTCTGAGAGGAC
GATTAGCCACACTGGGACTGAGACACGGCC
>Zotu15998
GAACGCTGGCGGCGTGCTTAACACATGCAAGTCGAACGAGCAAGAGCTGTAGCAATACAGGGTTCTGCTA
GTGGCAGACGGGTGAGTAATACATGGGAATCTACCTTGCAGTGCGAAATAACAGTTGGAAGCAATTGCTA
ATGTCGCATATAGCCGAGAGGTGAAAGGTTTACTGCTGCAAGATGGGCCCATGCAGGATTAGCTAGTTGG
TGAGGTAATGGCTCACCAAGGCAACGATCTTTAGTTGGTCTGAGAGGATGATCAGCCACACTGGGACTGA
GACACAGTC
>Zotu16003
GAACGCTAGCTACAGGCTTAACACATGCAAGTCGAGGGGTAGCATGATCTAGCAATAGATTGATGACGAC
CGGCGCACGGGTGAGTAACACGTATCCAACCTGCCCTATACTCAGGGATAGCCTTTCGAAAGAAAGATTAA
TACCTGATGGTATATCACATTCGCATGTTTGTGATATTAAAGAATTTCGGTATAGGATGGGGATGCGTTCCA
TTAGATTGTTGGTGGGGTAACGGCCTACCAAGTCTTCGATGGATAGGGGTTCTGAGAGGAAGGTCCCCCA
CACTGGTACTGAGACACGGAC
>Zotu16007
GAACGCTGGCGGCGTGCTTAACACATGCAAGTCGAACGCTGACACCTGGGCTTGCCCGGGTTGTTGGAGT
GGCGAACGGGTGAGTAACACGTGAGTAACCTGCCCTCATCTTCGGGATAACCGCTGGAAACGGTGGCTAA

TACCGGGTATACACGTCTCCTCGCATGGGGTGGTGTGGAAAGGTTTTTCTGGATGGGGATGGGCTCGCGG
CCTATCAGCTTGTTGGTGGGGTTATGGCCTACCAAGGCGACGACGGGTAGCCGGCCTGAGAGGGTGGACG
GCCACACTGGGACTGAGACACGGCC
>Zotu16012
GAACGCTGGCGGCGTGCCTAATACATGCAAGTCGAGCGAGCTGAATTCAAAGATTCCTTCGGGATGATTTG
TTGGACGCTAGCGGCGGATGGGTGAGTAACACGTGGGCAATCTGCCCTAAAGACTGGGATACCACTTGGA
AACAGGTGCTAATACCGGATAACAACATGAATCGCATGATTCAAGCTTGAAAGGCGGCTTTTAGCTGTCAC
TTTAGGATGAGCCCGCGGCGCATTAGCTAGTTGGTGGGGTAAAGGCCTACCAAGGCAATGATGCGTAGCC
GAGTTGAGAGACTGATCAGCCACATTGGGACTGAGACACGGCC
>Zotu16015
GAACGCTGGCGGCATGCCTAACACATGCAAGTCGAACGCTCCGCAAGGGGAGTGGCGAACGGGTGAGTA
ACGCGTGGGAACCTGCCCAGTAGTTGGGGATAACCGCGGGAAACTGCGGCTAATACCGAATACGCCCTTC
GGGGGAAAGATTTATCGCTATTGGAGGGGCCCGCGTCGGATTAGCTAGTTGGTGAGGTAACGGCTCACCA
AGGCGACGATCCGTAGCTGGTCTGAGAGGACGATCAGCCACACTGGGACTGAGACACGGCC
>Zotu16018
GAACGCTAGCGGCATGCCTAACACATGCAAGTCGAACGGCAGCATAGGAGCTTGCTCCTGATGGCGAGTG
GCGAACGGGTGAGTAATATATCGGAACGTGCCCTAGAGTGGGGGATAACTAGTCGAAAGACTAGCTAATA
CCGCATACGATCTACGGATGAAAGTGGGGGACCGCAAGACCTCATGCTCCTGGAGCGGCCGATATCTGAT
TAGCTAGTTGGTGGGGTAAAAGCTCACCAAGGCGACGATCAGTAGCTGGTCTGAGAGGACGACCAGCCAC
ACTGGGACTGAGACACGGCC
>Zotu16024
GAACGCTGGCGGCAGGCTTAACACATGCAAGTCGAGCGGAGATGAGGTGCTTGCACCTTATCTTAGCGGC
GAACGGGTGAGTAATGCTTAGGAATCTGCCTATTAGTGGGGGACAACATTCCGAAAGGAATGCTAATACC
GCATACGTCCTACGGGAGAAAGCAGGGGATCTTCGGACCTTGCGCTAATAGATGAGCCTATGTCGGATTA
GCTTGTTGGTGGGGTAATTGCCTACCAAGGCGACGATCCGTAGCTGGTCTGAGAGGATGATCAGCCACACT
GGGACTGAGACACGGCC
>Zotu16034
GGAAGACGCTTTAGGAAGAGACGCTCCTCCTGACAGCGAGGCAGGGCACTGGGGCCAAATGCATCCTTCT
CCCCGCCTTGGTTCTCTGGCTGGGGGACAGGGCGTG
>Zotu16038
GAACGCTGGCGGCATGCTTTACACATGCAAGTCGAACGGCAGCACGGGGGCAACCCTGGTGGCGAGTGG
CGAACGGGTGAGTAATATATCGGAACATGTCCGGAAGTGGGGGATAACGTAGCGAAAGTTACGCTAATAC
CGCATATGCCCTAAGGGGGAAAGGGGGGGATCGCAAGACCTCTCGCTTTCGGAGTGGCCGATACCGGATT
AGCTAGTTGGTGAGGTAAAGGCTCACCAAGGCGACGATCCGTAGCTGGTCTGAGAGGACGACCAGCCACA
CTGGAACTGAGACACGGTC
>Zotu16045
GAACGCTGGCGGCGTGCTTAACACATGCAAGTCGAACGAGCAAGAGCTGTAGCAATACAGGGTTCTGCTA
GTGGCAGACGGGTGAGTAATACATGGGAATCTACCTTGCAGTGCGAAATAACAGTTGGAAGCAATTGCTA
ATGTCGCATATAGCCGAGAGGTGAAAGGTTTACTGCTGCAAGATGGGCCCATGCAGGATTAGCTAGTTGG
TGAGGTAATGGCTCACCAAGGCAACGATCTTTAGTTGGTCTGAGAGGATGACCAGCCACACTGGGACTGA
GACACGGTC
>Zotu16050
GAACGCTAGCGGCATGCCTAACACATGCAAGTCGAACGGCAGCATAGGAGCTTGCTCCTGATGGCGAGTG
GCGAACGGGTGAGTAATATATCGGAACGTGCCCTAGAGTGGGGGATAACTAGTCGAAAGACTAGCTAATG
CCGCATACGATCTACGGATGAAAGTGGGGGATCGCAAGACCTCATGCTCCTGGAGCGGCCGATATCTGATT
AGCTAGTTGGTGGGGTAAAAGCTCACCAAGGCGACGATCAGTAGCTGGTCTGAGAGGACGACCAGCCACA
CTGGGACTGAGACACGGCC
>Zotu16052
GAACGCTAGCGGCATGCCTAACACATGCAAGTCGAACGGCAGCATAGGAGCTTGCTCCTGATGGCGAGTG
GCGAACGGGGGAGTAATATATCGGAACGTGCCCTAGAGTGGGGGATAACTAGTCGAAAGACTAGCTAATA

CCGCATACGATCTACGGATGAAAGTGGGGGATCGCAAGACCTCATGCTCCTGGAGCGGCCGATATCTGATT
AGCTAGTTGGTGGGGTAAAAGCTCACCAAGGCGACGATCAGTAGCTGGTCTGAGAGGACGACCAGCCACA
CTGGGACTGAGACACGGCC
>Zotu16054
GAACGCTGGCGGCGTGCTTAACACATGCAAGCCGAACGGAAAGGCCCTGCTTTTGTGGGGTGCTCGAGTG
GCGAACGGGTGAGTAACACGTGAGTAACCTGCCCTTGACTTTGGGATAACTTCAGGAAACTGGGGCTAAT
ACCGGATAGGAGCTCCTGCTGCATGGTGGGGGTTGGAAAGTTTCGGCGGTTGGGGATGGACTCGCGGCTT
ATCAGCTTGTTGGTGGGGTAGTGGCTTACCAAGGCTTTGACGGGTAGCCGGCCTGAGAGGGTGACCGGCC
ACACTGGGACTGAGACACGGCC
>Zotu16064
GAACGCTGGCGGCAGGCTTAACACATGCAAGTCGAACGCACCCTTCGGGGTGAGTGGCAGACGGGTGAG
TAACGCGTGGGAACGTGCCCTTCAGTTCGGAATAACCCAGGGAAACTTGGGCTAATACCGGATACGTCCG
AGAGGAGAAAGATTGATCGCTGAAGGATCGGCCCGCGTCTGATTAGCTAGTTGGTGAGGTAATGGCTCAC
CAAGGCGACGATCAGTAGCTGGTCTGAGAGGATGATCAGCCACACTGGAACTGAGACACGGTC
>Zotu16067
GAACGTTGGCGGCATGCTTTACACATGCAAGTCGAACGGCAGCGCGGGGCAACCTGGCGGCGAGTGGCG
AACGGGTGAGTAATATATCGGAACGTACCCAGAAGTGGGGGATAACGTAGCGAAAGTTACGCTAATACCG
CATACGATCTACGGATGAAAGTGGGGGACCTTCGGGCCTCATGCTTTTGGAGCGGCCGATATCTGATTAGC
TAGTAGGTAGGGTAATGGCCTACCTAGGCGACGATCAGTAGCTGGTCTGAGAGGACGACCAGCCACACTG
GAACTGAGACACGGTC
>Zotu16070
GAACGCTGGCGGTATGCCTAATACATGCAAGTCGTGCGGAAAGGCCCCTTCGGGGGTACTCGAGCGGCGG
ACGGCAGAGTGACGCGTAGGAACGTACCCCAAAGTGAGGAATAACTGCCCGAAAGGGTAGCTAATGCCG
CATGTGATCTTCGGATTAAAGCCTTCGGGCGCTTTGGGAACGGCCTGCGTTCGATTAGCTAGTTGGTGAGG
TAAAGGCTCACCAAGGCGACGATCGATAGCTGGTCTGAGAGGATGATCAGCCTGACTGGAACTGAGACAC
GGTC
>Zotu16083
GAACGCTAGCGGCATGCCTAACACATGCAAGTCGAACGGCAGCATAGGAGCTTGCTCCTGATGGCGAGTG
GCGAACGGGTGAGTAATATATCGGAACGTGCCCTAGAGTGGGGGATAACTAGCCGAAAGACTAGCTAATA
CCGCATACGATCTACGGATGAAAGTGGGGGATCGCAAGACCTCATGCTCCTGGAGCGGCCGATATCTGATT
AGCTAGTTGGTGGGGTAAAAGCTCACCAAGGCGACGATCAGTAGCTGGTCTGAGAGGACGACCAGCCACA
CTGGGACTGAGACACGGCC
>Zotu16085
GAACGCTGGCGGCGTGCCTAACACATGCAAGTCGAGCGGAGATTGCAGCAATGTAATTTTAGCGGCGGAC
TGGTGAGTAACGCGTGGGCAACCTGCCTCACACAGGGGGATGACACAGGGAAACTTGTGCTAATACCGCA
TAACATCATGGAGGGACATCCCTTTATGATCAAAGGAGCAATCCGGTGTGAGATGGGCCTGCGTCCGATTA
GTTAGTTGGCGGTGTAACGGACCACCAAGACGACGATCGGTAGCCGAACTGAGAGGTTGATCGGCTACAT
TGGAACTGAGATACGGTC
>Zotu16091
GAACGCTAGCGGCATGCTTAACACATGCAAGTAGAACGCTGAAGGAGGAGCTTGCTTCTCCGGATGAGTT
GCGAACGGGTGAGTAACGCGTAGGTAACCTGCCTGGTAGCGGGGGATAACTATTGGAAACGATAGCTAAT
ACCGCATAATAGCAGTTATTGCATGATAACTGTTTGAAAGGTGCAATTGCACCACTACCAGATGGACCTGC
GTTGTATTAGCTAGTTGGTGGGGTAACGGCTCACCAAGGCGACGATACATAGCCGACCTGAGAGGGTGAT
CGGCCACACTGGGACTGAGACACGGCC
>Zotu16095
GAACGCTGGCGGCATGCCTAACACATGCAAGTCGAACGAGACCTTCGGGTCTAGTGGCGCACGGGTGCGT
AACGCGTGGGAACCTGCCTTTAGGTTCGGAATAACTCAGAGAAATTTGAGCTAATACCGGATAATGTCTTC
GGACCAAAGATTTATCGCCTTTAGATGGGCCCGCGTTGGATTAGCTAGTTGGTGGGGTAAAGGCCTACCAA
GCCGACGATCCATAGCTGGTCTGAGAGGATGATCAGCCACACTGGGACTGAGACACGGCC
>Zotu16098

GATCGCTGGCGGCGTGCTTAACACATGCAAGTCGAGCGAATTTTGGCAGTGTATCGCCAGAGTTAGCGGC
AGACGGGTGAGTAATACTTGGGCAATCTGCCCTCGAGAGGGAAACAACCGGCCGAAAGGCTGGCTAATGT
CCCATAAGACCACAGGGATGCATATTCCAGGGGTCAAAGGTCCGCAAGGGCCGCTTGAGGAGGAGCCCG
AGTCCTATCATGGTAGTTGGTGAGGTAACGGCTCACCAAGCCTTTGACGGGTAGCCGGCCTGAAAGGGTG
ATCGGCCACACTGGCACTGAGACACGGGC
>Zotu16100
GAACGCTGGCGGCATGCCTTACACATGCAAGTCGAACGGTAGAGTAGCAATACTTGAGAGTGGCGAACGG
GTGAGTAATATATCGGAACGTGCCCAGTCGTGGGGGATAACGTAGCGAAAGCTACGCTAATACCGCATAC
GATCTAAGGATGAAAGCGGGGGACTCGCAAGAGCCTCGCGCGACTGGAGCGGCCGATATCAGATTAGCT
AGTTGGTGGGGTAAAAGCTCACCAAGGCGACGATCTGTAGCTGGTCTGAGAGGACGACCAGCCACACTGG
GACTGAGACACGGCC
>Zotu16103
GAACGCTAGCGGCAGGCCTAATACATGCAAGTCGAACGGTGTCTAGCAATAGGCATAGTGGCGCACGGGT
GCGTAACACGTAACTAACCTACCTATAACTGGGGGATAGCCCGCCGAAAGGCGGATTAATACCGCATAAGC
TGCTTGAGTGGCATCACTTAAATAGTAAAGATTTATTGGTTATAGATGGGGTTGCGGGTCATTAGCTAGTT
GGTTGGGTAACGGCTGACCAAGGCGACGATGACTAGGGGAGCTGAGAGGCTGGTCCCCCACACGGGCAC
TGAGATACGGGC
>Zotu16105
GAACGCTGGCGGCGTGCTTAACACGTGCAAGTCGAACGAGCAAGAGCTGTAGCAATACAGGGTTCTGCTA
GTGGCAGACGGGTGAGTAATACATGGGAATCTACCTTGCAGTGCGAAATAACAGTTGGAAGCAATTGCTA
ATGTCGCATATAGCCGAGAGGTGAAAGGTTTACTGCTGCAAGATGGGCCCATGCAGGATTAGCTAGTTGG
TGAGGTAATGGCTCACCAAGGCAACGATCTTTAGTTGGTCTGAGAGGATGATCAGCCACACTGGGACTGA
GATACGGCC
>Zotu16109
GAACGCTGGCGGCATGCCTTACACATGCAAGTCGAACGGCAGCGGGGGGAGCTTGCTCCCTGACGGCGA
GTGGCGGACGGGTGGGTAACACGTAGGAATCTACCTTAATTATAATAGTTAGTGGGGGATAACCCGGGGA
AACTCGGGCTAATACCGCATAATCTCTTCAAAGCAAAGCGGGGGATCTTCGGACCTCGTGCTATGAGATGA
GCCTGCGTCGGATTAGCTAGTTGGTAGGGTAATGGCCTACCAAGGCGACGATCCGTAGCTGGTCTGAGAG
GACGATCAGCCACACTGGGACTGAGACACGGCC
>Zotu16116
GAACGCTGGCGGCGTGCTTAACACATGCAAGTCGAACGATGATGCTGAGCTTGCTTAGCGGAGTAGTGGC
GAACGGGTGAGTAACACGTGAGTAACCTGCCCTTGACTCTGGGATAAGCGTTGGAAACGACGTCTAATAC
CGGATATGACCTTCAGCCGCATGGTTTGTTGGTGGAAAGATTTTTTGGTCAAGGATGGACTCGCGGCCTAT
CAGGCTGTTGGTGAGGTAATGGCTCACCAAGCCTACGACGGGTGGCCGGCCTGAGAGGGTGACCGGCCA
CACTGGGACTGAGACACGGCC
>Zotu16119
GAACGCTGGCGGCGTGCTTAACACATGCAAGTCGAACGGTGAAGCCCTTCGGGGTGGATCAGTGGCGAAC
GGGTGAGTAACACGTGAGTAATCTGCCCTTGACTCTGGGATAACTTCGGGAAACCGGAGCTAATACCGGA
TATTCACATTCTGTCGCATGGTGGGGTGTGGAAAGGATTCTGGTTGAGGATGAGCTCGCGGCCTATCAGCT
TGTTGGTGGGGTGATGGCCTACCAAGGCGACGACGGGTAGCCGGCCTGAGAGGGTGTCCGGCCACACTG
GGACTGAGATACGGCC
>Zotu16124
GAACGCTAGCGGCATGCCTTACACATGCAAGTCGAACGGCAGCATAGGAGCTTGCTCCTGATGGCGAGTG
GCGAACGGGTGAGTAATATATCGGAACGTGCCCTAGAGTGGGGGATAACTAGTCGAAAGACTAGCTAATA
CCGCATACGATCTACGGATGAAAGTGGGGGATCGCAAGACCTCATGCTCCTGGAGCGGCCGATATCTAATT
AGCTAGTTGGTGGGGTAAAAGCTCACCAAGGCGACGATCCGTAGCTGGTCTGAGAGGACGATCAGCCACA
CTGGGACTGAGACACGGCC
>Zotu16127
GAACGCTGGCGGTAGGCCTAACACATGCAAGTCGAACGGCAGCACAGCAGTAGCAATACTGTGGGTGGC
GAGTGGCGGACGGGTGAGGAATACATCGGAATCTACCTTTTCGTGGGGGATAACGTAGGGAAACTTACGC
TAATACCGCATAAGACCTTCGGGTGAAAGTGGGGGACCTTCGGGCCTCACGCGGATAGATGAGCCGATGT

CGGATTAGCTTGTTGGCGGGGTAAAAGCCCACCAAGGCGACGATCCGTAGCTGGTCTGAGAGGATGATCA
GCCACACTGGGACTGAGACACGGCC

>Zotu16131

GAACGCTGGCGGCAGGCTTAACACATGCAAGTCGAACGCGTGTAGCAATACACGAGTGGCGCACGGGTG
AGTAACGCGTGGGAACGTACCTTTCAGTTGGGAATAACAGCGGGAAACTGCTGCTAATACCCGATACTCCC
TCCGGGGGAAAGATTTATCGCTGAAAGATCGGCCCGCGTCAGATTAGCTAGTTGGTGGGGTAACGGCCTA
CCAAGGCTCCGATCTGTAGCTGGTCTGAGAGGACGACCAGCCACACTGGAACTGAGACACGGTC

>Zotu16135

GAACGCTGGCGGCGTGCCTAACACATGCAAGTCGAACGAGAATGCAGGCTTCGGTCTGCTAGTAAAGTGG
CGCACGGGTGAGTAACACGTGGGTAACGTACATCGTAGTGGGGGATAACCAGGTGAAAGCTTGGCTAATA
CCGCATGAGTCCGTCGGGACGCCGGTCCTGATGGGGAAAGAGGAGCAATCCTCGCTACGAGATCGGCCCG
CGGCCCATTAGCTAGTTGGCGGGGTAATGGCCCACCAAGGCTACGATGGGTAGCTGGTCTGAGAGGATGA
TCAGCCACACTGGAACTGAGACACGGTC

>Zotu16137

GAACGCTAGCGGCATGCCTAACACATGCAAGTCGAACGGCAGCATAGGAGCTTGCTCCTGATAGCGAGTG
GCGAACGGGTAAGTAATATATCGGAACATGCCCTAGAGTGGGGGATAACTAGTCGAAAGACTAGCTAATA
CCGCATACGATCTACGGATGAAAGTGGGGGATCGCAAGACCTCATGCTCCTGGAGCGGCCGATATCTGATT
AGCTAGTTGGTGGGGTAAAAGCTCACCAAGGCGACGATCAGTAGCTGGTCTGAGAGGACGACCAGCCACA
CTGGGACTGAGACACGGCC

>Zotu16139

GAACGCTGGCGGCACGCTTAACACATGCAAGTCGAACGGAGATATGAAGAATTATTGGATTATCTTAGTG
GCGGACGGGTGAGTAACACGTGAGCAACCTGCCTCTGAGAGAGGGATAGCTTCTGGAAACGGATGGTAA
TACCTCATAACATACAGTTATCGCATGATGATTGTATCAAAGATTTATCACTCAGAGATGGGCTCGCGTCTG
ATTAGATAGTTGGTGAGGTAACGGCTCACCAAGTCGACGATCAGTAGCCGGACTGAGAGGTTGAACGGCC
ACATTGGGACTGAGACACGGCC

>Zotu2

GAACGCTGGCGGTGTGCTTAACACATGCAAGTCGAACGGGCAAGAGCTGTAGCAATACAGAATTCTGCTA
GTGGCAGACGGGTGAGTAATACATGGGAATCTACCTTGCAGTGCGAAATAACAGTTGGAAGCAATTGCTA
ATGTCGCATATAGCCGAGAGGTGAAAGATTTATCGCTGCAAGACGAGCCCATGCAGGATTAGCTAGTTGG
TGGGGTAATGGCTCACCAAGGCGACGATCTTTAGTTGGTCTGAGAGGATGATCAGCCACACTGGAACTGA
GACACGGTC

>Zotu4

GAACGCTAGCGGCATGCTTAACACATGCAAGTCGAACGGCAGAGCGGGGAGTTTATGCTCCCTGGCGGCG
AGTGGCGGACGGGTGAGTAATACGTAGGAATCTACCTTATAGAGGGGGACAACCCGGGGAAACTCGGGC
TAATACCGCATGATCTCTATGGAGTAAAGCGGGGGATCTTCTGACCTCGCGCTATAAGATGAGCCTATGTC
GGATTAGCTTGTTGGTGGGGTAATTGCCTACCAAGGCGACGATCCGTAGCTGGTCTGAGAGGATGATCAG
CCACACTGGGACTGAGACACGGCC

>Zotu6

GAACGCTGGCGGCGTGCTTAACACATGCAAGTCGAACGGAAAGGCCCTGCTTTTGTGGGGTGCTCGAGTG
GCGAACGGGTGAGTAACACGTGAGTAACCTGCCCTTGACTTTGGGATAACTTCAGGAAACTGGGGCTAAT
ACCGGATAGGAGCTCCTGCTGCATGGTGGGGGTTGGAAAGTTTCGGCGGTTGGGGATGGACTCGCGGCTT
ATCAGCTTGTTGGTGGGGTAGTGGCTTACCAAGGCTTTGACGGGTAGCCGGCCTGAGAGGGTGACCGGCC
ACATTGGGACTGAGATACGGCC

>Zotu8

GAACGCTGGCGGCAAGCTTAACACATGCAAGTCGAACGAATTACAATTATAGCTTGCTACAATTGTAGTTA
GTGGCAGACGGGTGAGTAATGTATAGGAATCTGCCTAGTAGTATGGAATAGCTGTTAGAAATGACAGGTA
ATACCGTATAATCCCTACGGGGGAAAGATTTATCGCTATTAGATGAGCCTATATTAGATTAGCTAGTTGGTG
AGGTAATGGCTTACCAAGGTTATGATCTATAGCTGGTCTGAGAGGACGATCAGCCACACTGGAACTGAGAT
ACGGTC

>Zotu10

GAACGCTGGCGGCATGCCTTACACATGCAAGTCGAACGGCAGCATAGGAGCTTGCTCCTGATGGCGAGTG
GCGAACGGGTGAGTAATATATCGGAACGTGCCCTAGAGTGGGGGATAACTAGTCGAAAGACTAGCTAATA
CCGCATACGATCTACGGATGAAAGTGGGGGATCGCAAGACCTCATGCTCCTGGAGCGGCCGATATCTGATT
AGCTAGTTGGTGGGGTAAAAGCTCACCAAGGCGACGATCAGTAGCTGGTCTGAGAGGACGACCAGCCACA
CTGGGACTGAGACACGGCC
>Zotu12
GAACGCTAGCGGCAGGCCTAATACATGCAAGTCGAACGGTTCGCAAGGACAGTGGCAAACGGGTGCGTA
ACGCGTAAGCAACCTGCCTCATACTGGGGGATAGCCCGGCGAAAGCTGGGGTAAACCCGCACGGTCCAGT
GTCATCACCTGATGATATTGGTAAACATTTATGGGTATGAGAGGGGCTTGCGTCTGATTAGCTAGTTGGGG
GGGTAACGGCCCACCAAGGCGATGATCAGTAGGGGTTCTGAGAGGATTGGCCCCCACATGGGTACTGAGA
TACGGAC
>Zotu14
GAACGCTATCGGTATGCTTAACACATGCAAGTCGAACGGACTAATTGGGGCTTGCTCCAATTAGTTAGTGG
CAGACGGGTGAGTAACACGTGGGAATCTGTCCATCAGTACGGAATAACTTTTAGAAATAAAAGCTAATACC
GTATATTCTCTACGGAGGAAAGATTTATCGCTGATGGGTGAGCCCGCGTCAGATTAGGTAGTTGGTGAGGT
AATGGCTCACCAAGCCGACGATCTGTAGCTGGTCTGAGAGGATGATCAGCCACACTGGGACTGAGACACG
GCC
>Zotu16
GAACGCTGGCGGCATGCCTTACACATGCAAGTCGAACGGTAACAGGTTAAGCTGACGAGTGGCGAACGG
GTGAGTAATGTATCGGAACGTGCCCAGTCGTGGGGGATAACTGCTCGAAAGAGCAGCTAATACCGCATAC
GACCTGAGGGTGAAAGCGGGGGATCGCAAGACCTCGCGCGATTGGAGCGGCCGATATCAGATTAGGTAG
TTGGTGAGGTAAAGGCTCACCAAGCCGACGATCTGTAGCTGGTCTGAGAGGACGACCAGCCACACTGGGA
CTGAGACACGGCC
>Zotu18
GAACGCTAGCGGGAGGCCTAATACATGCAAGTCGAACGGTAACAGGGTAGCAATACTGCTGACGAGTGG
CGCACGGGTGAGTAACACGTACACAACCTGCCTTATACTGGGGCATAGCCTTGGGAAACTAAGATTAATTC
CCCATAACATTGCGAAATGGCATCATTTTGCAATTAAAATTCCGATGGTATAAGATGGGTGTGCGTCTGATT
AGCTAGTTGGCGGGGTAACGGCCCACCAAGGCAACGATCAGTAACTGGTCTGAGAGGATGATCAGTCACA
CGGGCACTGAGACACGGGC
>Zotu20
GAACGCTAGCGGGATGCCTTACACATGCAAGTCGAACGGCAGCACGGACTTCGGTCTGGTGGCGAGTGGC
GAACGGGTGAGTAATGTATCGGAACGTGCCTAGTAGCGGGGGATAACTACGCGAAAGCGTAGCTAATACC
GCATACGCCCTACGGGGGAAAGCAGGGGATCGCAAGACCTTGCACTATTAGAGCGGCCGATATCGGATTA
GCTAGTTGGTGGGGTAACGGCTCACCAAGGCGACGATCCGTAGCTGGTTTGAGAGGACGACCAGCCACAC
TGGGACTGAGACACGGCC
>Zotu23
GAACGCTGGCGGCGTGCCTAATACATGCAAGTCGAGCGAACAGATAAGGAGCTTGCTCCTTTGACGTTAG
CGGCGGACGGGTGAGTAACACGTGGGTAACCTACCTATAAGACTGGAATAACTCCGGGAAACCGGGGCTA
ATGCCGGATAACATGTTGAACCGCATGGTTCTACAGTGAAAGACGGTCTTGCTGTCACTTATAGATGGACC
CGCGCCGTATTAGCTAGTTGGTGGGGTAACGGCCTACCAAGGCGACGATACGTAGCCGACCTGAGAGGGT
GATCGGCCACACTGGAACTGAGACACGGTC
>Zotu25
GAACGCTGGCGGCAGGCTTAACACATGCAAGTCGAGCGGGCGTAGCAATACGTCAGCGGCAGACGGGTG
AGTAACGCGTGGGAACATACCTTTTGGTTCGGAACAACACAGGGAAACTTGTGCTAATACCGGATAAGCCC
TTACGGGGAAAGATTTATCGCCGAAAGATTGGCCCGCGTCTGATTAGCTAGTTGGTGAGGTAACGGCTCAC
CAAGGCGACGATCAGTAGCTGGTCTGAGAGGATGATCAGCCACATTGGGACTGAGACACGGCC
>Zotu27
GAACGCTAGCGGCAGGCTTAATACATGCAAGTCGAGGGGCAGCACGGTATAGCAATATATGGGTGGCGA
CCGGCAAACGGGTGCGGAACACGTACACAACCTTCCGGTGAGAGGGGGATAGCCCAGAGAAATTTGGATT
AATACCCCATACTATAATGGTCAGGCATCTGATTATTATCAAAGGCTTCGGCCGCTTATTGATGGGTGTGCG

TCTGATTAGGTAGTTGGCGGGGTAGAGGCCCACCAAGCCTACGATCAGTAGCTGATGTGAGAGCATGATC
AGCCACACGGGCACTGAGACACGGGC

>Zotu29

GAACGCTGGCGGCATGCCTTACACATGCAAGTCGAACGGTAACAGGTCTTCGGACGCTGACGAGTGGCGA
ACGGGTGAGTAATACATCGGAACGTGCCCAGTCGTGGGGGATAACTACTCGAAAGAGTAGCTAATACCGC
ATACGATCTGAGGATGAAAGCGGGGGACCTTCGGGCCTCGCGCGATTGGAGCGGCCGATGGCAGATTAG
GTAGTTGGTGGGATAAAAGCTTACCAAGCCGACGATCTGTAGCTGGTCTGAGAGGACGACCAGCCACACT
GGGACTGAGACACGGCC

>Zotu31

GAACGCTAGCGGCATGCTTAACACATGCAAGTCGAACGGCAGCGCAGGGAGCTTGCTCCCTGGCGGCGAG
TGGCGGACGGGTGAGTAATGCGTAGGAATCTACCTTGTAGTGGGGGATAACCTGGGGAAACTCGGGCTA
ATACCGCATAATCTCTTTGGAGCAAAGCGGGGGATCTTCGGACCTCGTGCTATAAGATGAGCCTACGTCGG
ATTAGCTTGTTGGTGGGGTAATGGCCTACCAAGGCGACGATCCGTAGCTGGTCTGAGAGGACGATCAGCC
ACACTGGGACTGAGACACGGCC

>Zotu33

GAACGCTATCGGTATGCTTAACACATGCAAGTCGAACGGACTAATTGGAGCTTGCTCCAATTAGTTAGTGG
CAGACGGGTGAGTAACACGTGGGAATCTACCCATCAGTACGGAATAACTTTTAGAAATAAAAGCTAATACC
GCATATTCTCTACGGAGGAAAGATTTATCGCTGATGGATGAGCCCGCGTCAGATTAGGTAGTTGGTGAGGT
AATGGCTCACCAAGCCGACGATCTGTAGCTGGTCTGAGAGGATGATCAGCCACACTGGGACTGAGACACG
GCC

>Zotu35

GAACGCTGGCGGCAGGCTTAACACATGCAAGTCGAGCGCCCCGCAAGGGGAGCGGCAGACGGGTGAGTA
ACGCGTGGGAATCTACCTTTTGCTACGGAACAACAGTTGGAAACGACTGCTAATACCGTATGTGCCCTTCG
GGGGAAAGATTTATCGGCAAAGGATGAGCCCGCGTTGGATTAGCTAGTTGGTGAGGTAAAGGCTCACCAA
GGCGACGATCCATAGCTGGTCTGAGAGGATGATCAGCCACACTGGGACTGAGACACGGCC

>Zotu37

GAACGCTGGCGGCATGCCTTACACATGCAAGTCGAACGGCAGCGCGGACTTCGGTCTGGCGGCGAGTGGC
GAACGGGTGAGTAATACATCGGAACGTACCCTGTTGTGGGGGATAACTAGTCGAAAGATTAGCTAATACC
GCATACGACCTGAGGGTGAAAGCGGGGGACCGTAAGGCCTCGCGCAGCAGGAGCGGCCGATGTCTGATT
AGCTAGTTGGTGGGGTAAAGGCCCACCAAGGCGACGATCAGTAGCTGGTCTGAGAGGACGATCAGCCAC
ACTGGGACTGAGACACGGCC

>Zotu39

GAACGCTGGCGGCATGCCTTACACATGCAAGTCGAACGGCAGCACGGGAGCAATCCTGGTGGCGAGTGG
CGAACGGGTGAGTAAAGTATCGGAACGTGCCCAGTCGTGGGGGATAACGTAGCGAAAGCTACGCTAATAC
CGCATACGATCTATGGATGAAAGCGGGGGATCGCAAGACCTCGCGCGATTGGAGCGGCCGATATCAGATT
AGGTAGTTGGTGGGGTAAAGGCCTACCAAGCCGACGATCTGTAGCTGGTCTGAGAGGACGACCAGCCACA
CTGGGACTGAGACACGGCC

>Zotu41

GAACGCTGGCGGCAGGCTTAACACATGCAAGTCGAGCGCCCCGCAAGGGGAGCGGCAGACGGGTGAGTA
ACGCGTGGGAATCTACCCATCACTACGGAACAACTCCGGGAAACTGGAGCTAATACCGTATACGTCCTTCG
GGAGAAAGATTTATCGGTGATGGATGAGCCCGCGTTGGATTAGCTAGTTGGTGGGGTAATGGCCTACCAA
GGCGACGATCCATAGCTGGTCTGAGAGGATGATCAGCCACACTGGGACTGAGACACGGCC

>Zotu43

GAACGCTGGTGGCATGCTTAACACATGCAAGTCGAACGGTAACAGGACTAGCTTGCTAGTTGCTGACGAG
TGGCGGACGGGTGAGTAACACGTAGGAATCTGCCCATCTGAGGGGGATACCAGTTGGAAACGACTATTAA
TACCGCATAATATCTGTGGATTAAAGGTGGCTTTTGAGCTGTCGCAGATGGATGAGCCTGCGTTGGATTAG
CTAGTTGGTGAGGTAAGGGCCTACCAAGGCTACGATCCATAGTTGATTTGAGAGGATGATCAGCCACATTG
GGACTGAGACACGGCC

>Zotu45

GAACGCTGGCGGCGTGCCTAATACATGCAAGTCGAGCGAACAGACGAGGAGCTTGCTCCTCTGACGTTAG
CGGCGGACGGGTGAGTAACACGTGGATAACCTACCTATAAGACTGGGATAACTTCGGGAAACCGGAGCTA
ATACCGGATAATATATTGAACCGCATGGTTCAATAGTGAAAGACGGTTTTGCTGTCACTTATAGATGGATCC
GCGCCGCATTAGCTAGTTGGTAAGGTAACGGCTTACCAAGGCAACGATGCGTAGCCGACCTGAGAGGGTG
ATCGGCCACACTGGAACTGAGACACGGTC

>Zotu47

GAACGCTGGCGGCGGGCCTCATACATGCAAGTCGAGCGCGAAAGTCCGCAAGGATGAGTAAAGCGGCGC
ACGGGTGAGTAACACGTGGATAATCTACCCTTTGGATTGGAATAACCTGTCGAAAGACGGGCTAATGCCG
AGTAAGCTCTCGGGAGCGTGAAGCTCTTGCGAGGAAAGATGGCGCAAGCTATCGCCGATGGATGAGTCTG
CGTCCCATTAGTTAGTTGGTGAGGTAACAGCTTACCAAGACGATGATGGGTACCTGGTCTGAGAGGATGAT
CAGGCACACTGGAACTGAAACACGGTC

>Zotu49

GAACGCTGGCGGCATGCCTAACACATGCAAGTCGAACGATTGCCTTCGGGTGATAGTGGCGCACGGGTGC
GTAACGCGTGGGAATCTGCCCTTAGGTTCGGAATAACAGCTGGAAACGGCTGCTAATACCGGATGATATC
GCGAGATCAAAGATTTATCGCCTGAGGATGAGCCCGCGTTGGATTAGGTAGTTGGTGGGGTAAAGGCCTA
CCAAGCCGACGATCCATAGCTGGTCTGAGAGGATGATCAGCCACACTGGGACTGAGACACGGCC

>Zotu51

GAACGCTATCGGTATGCTTAACACATGCAAGTCGAACGAACTAATTGGGGCTTGCTCTAATTAGTTAGTGG
CAGACGGGTGAGTAACACGTGGGAATCTGCCCATCAGTATGGAATAACTTTTAGAAATAAAAGCTAATACC
GTATATTCTCTACGGAGGAAAGATTTATCGCTGATGGATGAGCCCGCGTCAGATTAGGTAGTTGGTGAGGT
AATGGCTCACCAAGCCTACGATCTGTAGCTGGTCTGAGAGGATGATCAGCCACACTGGGACTGAGACACG
GCC

>Zotu53

GAACGCTGGCGGCGTGCCTAATACATGCAAGTAGAACGCTGAAGGAGGAGCTTGCTTCTCTGGATGAGTT
GCGAACGGGTGAGTAACGCGTAGGTAACCTGCCTGGTAGCGGGGGATAACTATTGGAAACGATAGCTAAT
ACCGCATAATAGTAGATGTTGCATGACATTTGCTTAAAAGGTGCAATTGCATCACTACCAGATGGACCTGC
GTTGTATTAGCTAGTTGGTGAGGTAACGGCTCACCAAGGCAACGATACATAGCCGACCTGAGAGGGTGAT
CGGCCACACTGGGACTGAGACACGGCC

>Zotu55

GAACGCTGGCGGTAGGCCTAACACATGCAAGTCGAACGGCAGCACAGAGGAGCTTGCTCCTTGGGTGGCG
AGTGGCGGACGGGTGAGGAATACATCGGAATCTACTCTGTCGTGGGGGATAACGTAGGGAAACTTACGCT
AATACCGCATACGACCTACGGGTGAAAGCAGGGGATCTTCGGACCTTGCGCGATTGAATGAGCCGATGTC
GGATTAGCTAGTTGGCGGGGTAAAGGCCCACCAAGGCGACGATCCGTAGCTGGTCTGAGAGGATGATCA
GCCACACTGGAACTGAGACACGGTC

>Zotu57

GAACGCTGGCGGTATGCTTAACACATGCAAGTCGAACGGCAGCATGTTCCTCACTCAAATCACTTTAATTTA
TTTTTGGAAAAGAAATACGGAAAGTGATTTGAGTGAGGAATGATGGCAAGTGGCGGACGGGTGAGTAAT
GGGTAGGAATCTGTCCTAGAGAGGGGGATAACTTTCGGAAACGAGAGCTAATACCACATGATATTGAAAA
ATCAAAGGCAGGGGACCTTTCGAGGCCTTGCGCTCTAGGGTGAGCCTGCATCGGATTAGCTAGTTGGTAG
GGTAAAAGCCTACCAAGGCGACGATCCGTAGCTGGTCTGAGAGGATGACCAGCCACACTGGGACTGAGAC
ACGGCC

>Zotu60

GAACGCTGGCGGCAGGCCTAACACATGCAAGTCGAGCGGATGAAAGGAGCTTGCTCCTGGATTCAGCGGC
GGACGGGTGAGTAATGCCTAGGAATCTGCCTGGTAGTGGGGGACAACGTTTCGAAAGGAACGCTAATACC
GCATACGTCCTACGGGAGAAAGCAGGGGACCTTCGGGCCTTGCGCTATCAGATGAGCCTAGGTCGGATTA
GCTAGTTGGTGAGGTAATGGCTCACCAAGGCGACGATCCGTAACTGGTCTGAGAGGATGATCAGTCACAC
TGGAACTGAGACACGGTC

>Zotu62

GAACGCTGGCGGCATGCCTAACACATGCAAGTCGAACGGCAGCACAGTAAAGATTTCGGTCTTTACGGGT
GGCGAGTGGCGGACGGGTGAGTAAAGCGTAGGAATCTGTCCTGGAGAGTGGAATAACCCGAGGAAACTC
GGGCTAATACCGCATGATATCGAGAGATCAAAGCAGGGGACCCTTCGGGGCCTTGCGCTCTAGGATGAGC

CTACGTCGGATTAGCTAGTTGGTGGGGTAAAGGCCTACCAAGGCGACGATCCGTAGCTGGTCTGAGAGGA
TGACCAGCCACACTGGGACTGAGACACGTCC

>Zotu64

AAACGCTGGCGGCATGCCTAACACATGCAAGTCGAACGGCAGCACAGTAAAGATTTCGGTCTTTACGGGT
GGCGAGTGGCGGACGGGTGAGTAAAGCGTAGGAATCTGTCCTGGAGAGTGGGATAACCCGAGGAAACTC
GGGCTAATACCGCATGATATCGAGAGATCAAAGCAGGGGACCCTTCGGGGCCTTGCGCTCTAGGATGAGC
CTACGTCGGATTAGCTAGTTGGTGGGGTAAAGGCCTACCAAGGCGACGATCCGTAGCTGGTCTGAGAGGA
TGACCAGCCACACTGGGACTGAGACACGGCC

>Zotu66

GAACGCTGGCGGCGTGCTTAACACATGCAAGTCGAACGGAAAGGCCCTGCTTGCAGGGTACTCGAGTGGC
GAACGGGTGAGTAACACGTGGGTGATCTGCCTCGTACTTCGGGATAAGCCTGGGAAACTGGGTCTAATAC
CGGATAGGACCGGCCTTTAGTGTGGTTGGTGGAAAGTTTTGTCGGTACGAGATGAGCCCGCGGCCTATCA
GCTTGTTGGTGGGGTAATGGCCTACCAAGGCGACGACGGGTAGCCGGCCTGAGAGGGTGTACGGCCACA
TTGGGACTGAGACACGGCC

>Zotu68

GAACGCTAGCGGCAGGCTTAATACATGCAAGTCGTGGGGCAGCATGAGTAGCAATACTTGATGGCGACCG
GCAAACGGGTGCGGAACACGTACACAACCTTCCGATAAGTGGGGAATAGCCCAGAGAAATTTGGATTAAT
ACCCCGTAACATATAGAGTAGGCATCTTCTTTATATTATAGCTTCGGCGCTTATTGATGGGTGTGCGTCTGA
TTAGGTAGTTGGCGGGGTAACGGCCCACCAAGCCTACGATCAGTAGCTGATGTGAGAGCATGATCAGCCA
CACGGGCACTGAGACACGGGC

>Zotu70

GAACGCTGGTGGCATGCTTAACACATGCAAGTCGAACGGTAACAGGACTAGCTTGCTAGTTGCTGACGAG
TGGCGGACGGGTGAGTAACACGTAGGAATCTGCCCATCTGAGGGGATACCAGTTGGAAACGACTATTAAT
ACCGCATAGTATCTGTGGATTAAAGATGGCTTTTAGGCTGTCGCGGATGGATGAGCCTGCGTTGGATTAGC
TAGTTGGTGAGGTAAGGGCCTACCAAGGCTACGATCCATAGTTGATTTGAGAGGATGATCAGCCACATTG
GGACTGAGACACGACC

>Zotu72

GAACGCTGGCGGCATGCCTAACACATGCAAGTCGAACGAACACTTTCGGGTGTTAGTGGCGCACGGGTGC
GTAACGCGTGGGAATCTGCCCTTAGGTTCGGAATAACAGCGAGAAATTGCTGCTAATACCGGATGATGGCT
TCGGCCCAAAGATTTATCGCCTAGGGATGAGCCCGCGTAAGATTAGGTAGTTGGTGTGGTAAAGGCGCAC
CAAGCCGACGATCTTTAGCTGGTCTGAGAGGATGATCAGCCACACTGGGACTGAGACACGGCC

>Zotu74

AAACGCTGGCGGCATGCCTTACACATGCAAGTCGAACGGCAGCGCGGACTTCGGTCTGGCGGCGAGTGGC
GAACGGGTGAGTAATACATCGGAACGTACCCTGTTGTGGGGGATAACTAGTCGAAAGATTAGCTAATACC
GCATACGACCTGAGGGTGAAAGCGGGGGACCGTAAGGCCTCGCGCAGCAGGAGCGGCCGATGTCTGATT
AGCTAGTTGGTGGGGTAAAGGCCCACCAAGGCGACGATCAGTAGCTGGTCTGAGAGGACGATCAGCCAC
ACTGGGACTGAGACACGGCC

>Zotu76

GAACGCTGGCGGCAGGCTTAACACATGCAAGTCGAGCGCCCCGCAAGGGGAGCGGCAGACGGGTGAGTA
ACGCGTGGGAATCTACCCATCTCTACGGAACAACTCCGGGAAACTGGAGCTAATACCGTATACGTCCTTAT
GGAGAAAGATTTATCGGAGATGGATGAGCCCGCGTTGGATTAGCTAGTTGGTGGGGTAATGGCCTACCAA
GGCGACGATCCATAGCTGGTCTGAGAGGATGATCAGCCACACTGGGACTGAGACACGGCC

>Zotu78

GAACGCTGGCGGCGTGCCTAATACATGCAAGTCGAGCGAACAGATAAGGAGCTTGCTCCTTTGACGTTAG
CGGCGGACGGGTGAGTAACACGTGGATAACCTACCTATAAGACTGGGATAACTTCGGGAAACCGGAGCTA
ATACCGGATAAGATTTTGAACCGCATGGTTCAATAGTGAAAGACGGCCTTGCTGTCACTTATAGATGGATC
CGCGCCGTATTAGCTAGTTGGTAAGGTAACGGCTTACCAAGGCAACGATACGTAGCCGACCTGAGAGGGT
GATCGGCCACACTGGAACTGAGACACGGTC

>Zotu80

GAACGCTGGCGGCATGCCTAACACATGCAAGTCGAACGAGACCTTCGGGTCTAGTGGCGCACGGGTGCGT
AACGCGTGGGAACCTGCCCTTAGGTTCGGAATAACTCCCCGAAAGGGGTGCTAATACCGGATAATGTCTTC
GGACCAAAGATTTATCGCCTTTGGATGGGCCCGCGTTGGATTAGCTAGTTGGTGAGGTAAAAGCTCACCAA
GGCGACGATCCATAGCTGGTCTGAGAGGATGATCAGCCACACTGGGACTGAGACACGGCC

>Zotu84
GAACGCTGGCGGCAGGCCTAACACATGCAAGTCGAGCGCCCTTTCGGGGGAGCGGCGGACGGGTGAGTA
ACGCGTGGGAATATGCCCTTCTCTACGGAATAGCCTCGGGAAACTGAGAGTAATACCGTATACGCCCTTAG
GGGGAAAGATTTATCGGGGAAGGATTAGCCCGCGTTGGATTAGGTAGTTGGTGGGGTAATGGCCTACCAA
GCCGACGATCCATAGCTGGTTTGAGAGGATGATCAGCCACACTGGGACTGAGACACGGCC

>Zotu86
GAACGCTGGCGGCAGGCTTAACACATGCAAGTCGAGCGGGCGTAGCAATACGTCAGCGGCAGACGGGTG
AGTAACACGTGGGAACGTACCTTTTGGTTCGGAACAACTGAGGGAAACTTCAGCTAATACCGGATAAGCCC
TAACGGGGAAAGATTTATCGCCGAAAGATCGGCCCGCGTCTGATTAGCTAGTTGGTGAGGTAATGGCTCA
CCAAGGCGACGATCAGTAGCTGGTCTGAGAGGATGATCAGCCACACTGGGACTGAGACACGGCC

>Zotu88
GAACGCTGGCGGCAGGCTTAACACATGCAAGTCGAGCGCCCCGCAAGGGGAGCGGCAGACGGGTGAGTA
ACGCGTGGGAATCTACCCTTTTCTACGGAATAACGCAGGGAAACTTGTGCTAATACCGTATGTGTCCTTCGG
GAGAAAGATTTATCGGGAAAGGATGAGCCCGCGTTGGATTAGCTAGTTGGTGGGGTAAAGGCCTACCAAG
GCGACGATCCATAGCTGGTCTGAGAGGATGATCAGCCACATTGGGACTGAGACACGGCC

>Zotu90
GAACGCTGGCGGCGTGCTTAACACATGCAAGTCGTACGGTAAGGCCCTTTCGGGGGTACACGAGTGGCGA
ACGGGTGAGTAACACGTGAGTAACCTGCCCACAACTTTGGGATAACGCTAGGAAACTGGTGCTAATACTG
GATATGTGCTCCTGCTGCATGGTGGGGGTTGGAAAGCTCCGGCGGTTGTGGATGGACTCGCGGCCTATCA
GCTTGTTGGTGGGGTAGTGGCCTACCAAGGCGGCGACGGGTAGCCGGCCTGAGAGGGTGACCGGCCACA
TTGGGACTGAGATACGGCC

>Zotu92
GAACGCTGGTGGCATACTTAACACATGCAAGTCGAACGGTAACAGGACTAGCTTGCTAGTTGCTGACGAGT
GGCGGACGGGTGAGTAACGCGTAGGAATCTGCCCATCTGAGGGGGATACCAGTTGGAAACGACTGTTAAT
ACCGCATAGTATCTGTGGATTAAAGGTGGCTTTTGGGCTGTCGCAGATGGATGAGCCTGCGTTGGATTAGC
TAGTTGGTGGGGTAAAGGCCTACCAAGGAAGGCTACGATCCATAGCTGATTTGAGAGAATGATCAGCCAC
ATTGGGACTGAGACACGGCC

>Zotu94
GAACGCTGGCGGCGTGCTTAACACATGCAAGTCGAGCGGTAAGGCCTTTCGGGGTACACGAGCGGCGAAC
GGGTGAGTAACACGTGGGTGATCTGCCCTGCACTTCGGGATAAGCCTGGGAAACTGGGTCTAATACCGGA
TATGACCTCCTATCGCATGGTGGGTGGTGGAAAGATTTATCGGTGCAGGATGGGCCCGCGGCCTATCAGCT
TGTTGGTGGGGTAATGGCCTACCAAGGCGACGACGGGTAGCCGACCTGAGAGGGTGACCGGCCACACTG
GGACTGAGACACGGCC

>Zotu96
GAACGCTGGCGGCGTGCTTAACACATGCAAGTCGAACGCTGAAGCTTGGTGCTTGCACTGGGTGGATGAG
TGGCGAACGGGTGAGTAATACGTGAGTAACCTGCCCTTGACTCTGGGATAAGCCTGGGAAACTGGGTCTA
ATACTGGATATGACCTCCTGTCGCATGGTGGGGGTGGAAAGGGTTTGTACTGGTTGTGGATGGGCTCAC
GGCCTATCAGCTTGTTGGTGGGGTAATGGCCTACCAAGGCGACGACGGGTAGCCGGCCTGAGAGGGTGA
CCGGCCACACTGGGACTGAGACACGGCC

>Zotu98
AAACGCTGGCGGCATGCCTAACACATGCAAGTCGAACGGCAGCACAGTAAAGATTTCGGTCTTTACGGGT
GGCGAGTGGCGGACGGGTGAGTAAAGCGTAGGAATCTGTCCTGGAGAGTGGAATAACCCGAGGAAACTC
GGGCTAATACCGCATGATATCGAGAGATCAAAGCAGGGGACCCTTCGGGGCCTTGCGCTCTAGGATGAGC
CTACGTCGGATTAGCTAGTTGGTGGGGTAAAGGCCTACCAAGGCGACGATCCGTAGCTGGTCTGAGAGGA
TGACCAGCCACACTGGGACTGAGACACGGCC

>Zotu100

GAACGCTGGCGGCGTGCCTAATACATGCAAGTCGAGCGAACAGACGAGGAGCTTGCTCCTCTGACGTTAG
CGGCGGACGGGTGAGTAACACGTGGATAACCTACCTATAAGACTGGGATAACTTCGGGAAACCGGAGCTA
ATACCAGATAACATGTTGAACCGCATGGTTCAACAGTGAAAGACGGTCTTGCTGTCACTTATAGATGGATC
CGCGCCGCATTAGCTAGTTGGTAAGGTAACGGCTTACCAAGGCAACGATGCGTAGCCGACCTGAGAGGGT
GATCGGCCACACTGGAACTGAGACACGGTC

>Zotu102
GAACGCTGGCGGCATGCCTTACACATGCAAGTCGAACGGCAGCGCGGGAGCAATCCTGGCGGCGAGTGG
CGAACGGGTGAGTAATACATCGGAACGTGCCCAATCGTGGGGGATAACGCAGCGAAAGCTGTGCTAATAC
CGCATACGATCTACGGATGAAAGCAGGGGATCGCAAGACCTTGCGCGAATGGAGCGGCCGATGGCAGAT
TAGGTAGTTGGTGAGGTAAAGGCTCACCAAGCCTTCGATCTGTAGCTGGTCTGAGAGGACGACCAGCCAC
ACTGGGACTGAGACACGGCC

>Zotu104
GAACGCTGGCGGCAAGCCTAACACATGCAAGTCGAACGAATTATCCTTATAGTTTACTATAAGTATAATTA
GTGGCAGACGGGTGAGTAATGTATAGGAATCTACCTAGTAGTATGGAATAGCTGTTAGAAATGACAGGTA
ATACCGTATAATCCCTGAGGGGGAAAGATTTATCGCTATTAGATGAGCCTATGTTAGATTAGCTAGTTGGT
GAGGTAATGGCTTACCAAGGCTGTGATCTATAGCTGGTCTGAGAGGACGATCAGCCACACTGGAACTGAG
ATACGGTC

>Zotu107
GAACGCTGGCGGCAGGCTTAACACATGCAAGTCGAGCGGGCCCTTCGGGGTCAGCGGCGGACGGGTGAG
TAACGCGTGGGAACGTGCCTTCTGGTTCGGAATAACCCTGGGAAACTAGGGCTAATACCGGATACGCCCTT
TTGGGGAAAGGTTTACTGCCGGAAGATCGGCCCGCGTCTGATTAGCTAGTTGGTGGGGTAACGGCCTACC
AAGGCGACGATCAGTAGCTGGTCTGAGAGGATGATCAGCCACACTGGGACTGAGACACGGCC

>Zotu109
GAACGCTGGCGGCAGGCTTAACACATGCAAGTCGAGCGGGCGTAGCAATACGTCAGCGGCAGACGGGTG
AGTAACGCGTGGGAACGTACCTTTTGGTTCGGAACAACACAGGGAAACTTGTGCTAATACCGGATAAGCCC
TTACGGGGAAAGATTTATCGCCGAAAGATCGGCCCGCGTCTGATTAGCTAGTTGGTAGGGTAATGGCCTAC
CAAGGCGACGATCAGTAGCTGGTCTGAGAGGATGATCAGCCACATTGGGACTGAGACACGGCC

>Zotu111
GAACGCTGGCGGCATGCCTAACACATGCAAGTCGAACGAAGGCTTCGGCCTTAGTGGCGCACGGGTGCGT
AACGCGTGGGAATCTGCCCTTGGGTTCGGAATAACAGCGAGAAATTGCTGCTAATACCGGATAATGTCGCA
AGACCAAAGATTTATTGCCCAAGGATGAGCCCGCGTTGGATTAGCTAGTTGGTGGGGTAAAAGCCTACCA
AGGCGACGATCCATAGCTGGTCTGAGAGGATGATCAGCCACACTGGGACTGAGACACGGCC

>Zotu113
GAACGCTGGCGGCGTGCCTAATACATGCAAGTCGAGCGAACAGACGAGGAGCTTGCTCCTTTGACGTTAG
CGGCGGACGGGTGAGTAACACGTGGATAACCTACCTATAAGACTGGGATAACTTCGGGAAACCGGAGCTA
ATACCGGATAACATGTTGAACCGCATGGTTCAACAGTGAAAGACGGTCTTGCTGTCACTTATAGATGGATC
CGCGCCGCATTAGCTAGTTGGTAAGGTAACGGCTTACCAAGGCAACGATGCGTAGCCGACCTGAGAGGGT
GATCGGCCACACTGGAACTGAGACACGGTC

>Zotu115
GAACGCTAGCGGCATGCTTAACACATGCAAGTCGAACGGCAGCGCAAGGGGCTTTCTCCTGGCGGCGAGT
GGCGGACGGGTGAGTAATGCGTAGGAATCTACCTTAAATAGTGGGGGATAACCTGAGGAAACTCGGGCT
AATACCGCATGATCTCTTCGGAGCAAAGCGGGGGATCGTTAGACCTCGTGCTATAAGATGAGCTTACGTCG
GATTAGTTTGTTGGTGGGGTAAAGGCCTACCAAGGCGATGATCCGTAGCTGGTCTGAGAGGACGATCAGC
CACACTGGGACTGAGACACGGCC

>Zotu117
GAACGCTGGCGGCGTGCTTAACACATGCAAGTCGAACGATGAAGCCCAGCTTGCTGGGTGGATTAGTGGC
GAACGGGTGAGTAACACGTGAGTAACCTGCCCTTAACTCTGGGATAAGCCTGGGAAACTGGGTCTAATAC
CGGATAGGAGCGTCCACCGCATGGTGGGTGTTGGAAAGATTTATCGGTTTTGGATGGACTCGCGGCCTATC
AGCTTGTTGGTGAGGTAATGGCTCACCAAGGCGACGACGGGTAGCCGGCCTGAGAGGGTGACCGGCCAC
ACTGGGACTGAGACACGGCC

>Zotu119
GAACGCTGGCGGCAGGCCTAACACATGCAAGTCGAGCGGATGAGGAGAGCTTGCTCTCTGATTCAGCGGC
GGACGGGTGAGTAATGCCTAGGAATCTGCCTGGTAGTGGGGGACAACGTTTCGAAAGGAACGCTAATACC
GCATACGTCCTACGGGAGAAAGTGGGGGATCTTCGGACCTCACGCTATCAGATGAGCCTAGGTCGGATTA
GCTAGTTGGTGGGGTAACGGCTCACCAAGGCGACGATCCGTAACTGGTCTGAGAGGATGATCAGTCACAC
TGGAACTGAGACACGGTC
>Zotu121
GAACGCTGGCGGCATGCCTAACACATGCAAGTCGAACGAAGGCTTCGGCCTTAGTGGCGCACGGGTGCGT
AACGCGTGGGAATCTGCCCTCAGGTTCGGAATAACAGCGAGAAATTGCTGCTAATACCGGATGATATCGC
GAGATCAAAGATTTATCGCCTGAGGATGAGCCCGCGTAGGATTAGCTAGTTGGTGTGGTAAAGGCGCACC
AAGGCGACGATCCTTAGCTGGTCTGAGAGGATGATCAGCCACACTGGGACTGAGACACGGCC
>Zotu123
GAACGCTGGCGGCGTGCCTAATACATGCAAGTCGAACGCCTTTTCTTTCACCGGAGCTTGCTCCACCGAAA
GAAAAGGAGTGGCGAACGGGTGAGTAACACGTGGGTAACCTGCCCATCAGAAGGGGATAACACTTGGAA
ACAGGTGCTAATACCGTATAACAATAGAAACCGCATGGTTTCTATTTGAAAGGCGCTTTTGCGTCACTGATG
GATGGACCCGCGGTGCATTAGCTAGTTGGTGAGGTAACGGCTCACCAAGGCAACGATGCATAGCCGACCT
GAGAGGGTGATCGGCCACATTGGGACTGAGACACGGCC
>Zotu125
GAACGCTGGCGGCGGGCCTCATACATGCAAGTCGAGCGCGAAAGTCCGCAAGGATGAGTAAAGCGGCGC
ACGGGTGAGTAACACGTGGATAATCTACCCTTTGGATTGGAATAACCTGTCGAAAGACGGGCTAATGCCG
AGTACGCACTCGAGGGCGTGAAGCTCTTGAGTGGAAAGATGGCGCAAGCTATCGCCGATGGATGAGTCTG
CGTCCCATTAGTTAGTTGGTGAGGTAACGGCTTACCAAGACAATGATGGGTACCTGGTCTGAGAGGATGAT
CAGGCACACTGGAACTGAAACACGGTC
>Zotu127
GAACGCTGGCGGCGTGCCTAATACATGCAAGTCGAGCGAACTGACGAGGAGCTTGCTCCTTTGACGTTAG
CGGCGGACGGGTGAGTAACACGTGGGTAACCTACCTATAAGACTGGAATAACTCCGGGAAACCGGGGCTA
ATGCCGGATAACATATCGAACCGCATGGTTCGATAGTGAAAGACGGTCTTGCTGTCACTTATAGATGGACC
CGCGCCGTATTAGCTAGTTGGTGAGGTAACGGCTCACCAAGGCGACGATACGTAGCCGACCTGAGAGGGT
GATCGGCCACACTGGAACTGAGACACGGTC
>Zotu129
CAACGCTGGCGGCGCGCCTAACACATGCAAGTCGAACGAGAAAGTGGAGCAATCCATGAGTAAAGTGGC
GTACGGGTGAGTAACACGTGGATCATCTACCCTCTAGTGGGGAATACCGCTGGGAAACCGGCGACAATAC
CGCATAAGCTCGAGAGAGGAAAGCAGCAATGCGCTGGAGGAGGAGTCCGCGGTCGATTAGTTAGTTGGC
AGGGTAAAGGCCTACCAAGACTGTGATCGATAGCCGGCCTGAGAGGGCACACGGCCACACTGGCACTGAA
ACACGGGC
>Zotu131
GAACGCTAGCGGCAGGCCTAATACATGCAAGTCGAACGATGCTTAGTAGCAATACTAAGTGTAGTGGCGC
ACGGGTGCGTAACACGTATGCAACCTACCTTTAACAGGGGGATAGCCCGGGGAAACCCGGATTAATACCC
CGTATGGTGTGATATTGGCATCAGTATTACATTAAAGTTACGATGGTTAAAGATGGGCATGCGTCTGATTA
GTTAGTTGGTGAGGTAACGGCTCACCAAGACGATGATCAGTAGGGGACCTGAGAGGGTGATCCCCCACAC
TGGCACTGAGATACGGGC
>Zotu133
GAACGCTGGCGGCATGCCTTACACATGCAAGTCGAACGGCAGCACGGGGGCAACCCTGGTGGCGAGTGG
CGAACGGGTGAGTAATACATCGGAACGTGTCCTGTAGTGGGGGATAGCCCGGCGAAAGCCGGATTAATAC
CGCATACGCTCTACGGAGGAAAGGGGGGGATCTTAGGACCTCTCGCTACAGGGGCGGCCGATGGCAGATT
AGCTAGTTGGTGGGGTAAAGGCCTACCAAGGCGACGATCTGTAGCTGGTCTGAGAGGACGACCAGCCACA
CTGGGACTGAGACACGGCC
>Zotu135
GAACGCTGGCGGCGTGCCTAATACATGCAAGTCGAGCGGACGAATCGAAAGCTTGCTTTTGATTCGTTAGC
GGCGGACGGGTGAGTAACACGTGGGCAACCTGCCCTGTAGACGGGGATAACACCGAGAAATCGGTGCTA

ATACCGGATAACACGAAATGTCGCATGACGTTTCGTTGAAAGACGGCGCAAGCTGTCGCTACAGGATGGG
CCCGCGGCGCATTAGCTAGTTGGTGAGGTAACGGCTCACCAAGGCGACGATGCGTAGCCGACCTGAGAGG
GTGATCGGCCACACTGGGACTGAGACACGGCC

>Zotu137
GAACGCTGGCGGCAGGCCTAACACATGCAAGTCGAACGGTAACAGGAAGAAGCTTGCTTCTTTGCTGACG
AGTGGCGGACGGGTGAGTAATGTCTGGGAAACTGCCTGATGGAGGGGGATAACTACTGGAAACGGTAGC
TAATACCGCATAACGTCGCAAGACCAAAGAGGGGGACCTTCGGGCCTCTTGCCATCGGATGTGCCCAGAT
GGGATTAGCTAGTAGGTGGGGTAACGGCTCACCTAGGCGACGATCCCTAGCTGGTCTGAGAGGATGACCA
GCCACACTGGAACTGAGACACGGTC

>Zotu139
GAACGCTAGCGGGAGGCCTAACACATGCAAGCCGAGCGGTAGAGTTTCTTCGGAAACTTGAGAGCGGCGT
ACGGGTGCGTAACACGTGTGCAACCTACCTTTATCTGGGGGATAGCCTTTCGAAAGGAAGATTAATACCCC
ATAATATAATGATTGGCATCAATTATTATTGAAAACTCCGGTGGATAGAGATGGGCACGCGCAAGATTAGA
TAGTTGGTGAGGTAACGGCTCACCAAGTCAATGATCTTTAGGGGTCCTGAGAGGGAGATCCCCCACACTG
GTACTGAGACACGGAC

>Zotu141
GAACGCTGGCGGCGTGCTTAACACATGCAAGTCGAACGGAAAGGCCCAAGCTTGCTTGGGTACTCGAGTG
GCGAACGGGTGAGTAACACGTGGGTGATCTGCCCTGCACTTCGGGATAAGCCTGGGAAACTGGGTCTAAT
ACCGGATAGGACGATGGTTTGGATGCCATTGTGGAAAGTTTTTCGGTGTGGGATGAGCTCGCGGCCTATCA
GCTTGTTGGTGGGGTAATGGCCTACCAAGGCGTCGACGGGTAGCCGGCCTGAGAGGGTGTACGGCCACAT
TGGGACTGAGATACGGCC

>Zotu143
AAACGCTGGCGGCATGCCTAACACATGCAAGTCGAACGGCAGCACAGTAAAGATTTCGGTCTTTACGGGT
GGCGAGTGGCGGACGGGTGAGTAAAGCGTAGGAATCTGTCCTGGAGAGTGGAATAACCCGAGGAAACTC
GGGCTAATACCGCATGATATCGAGAGATCAAAGCAGGGGACCCTTCGGGGCCTTGCGCTCTAGGATGAGC
CTACGTCGGATTAGCTAGTTGGTGGGGTAAAGGCCTACCAAGGCGACGATCCGTAGCTGGTCTGAGAGGA
TGACCAGCCACACTGGGACTGAGACACGTCC

>Zotu145
GAACGCTGGCGGCATGCCTTACACATGCAAGTCGAACGGTAACAGGTTAAGCTGACGAGTGGCGAACGG
GTGAGTAATATATCGGAACGTGCCCAGTCGTGGGGGATAACTGCTCGAAAGAGCAGCTAATACCGCATAC
GACCTGAGGGTGAAAGCGGGGGATCGCAAGACCTCGCGCGATTGGAGCGGCCGATATCAGATTAGGTAG
TTGGTGGGGTAAAGGCCCACCAAGCCAACGATCTGTAGCTGGTCTGAGAGGACGACCAGCCACACTGGGA
CTGAGACACGGCC

>Zotu147
GAACGCTGGCGGCGTGCCTAATACATGCAAGTAGAACGCTGAAGGAGGAGCTTGCTTCTCTGGATGAGTT
GCGAACGGGTGAGTAACGCGTAGGTAACCTGCCTGGTAGCGGGGGATAACTATTGGAAACGATAGCTAAT
ACCGCATAAGAGTAGATGTTGCATAACATTTGCTTAAAAGGTGCAATTGCACCACTACCAGATGGACCTGC
GTTGTATTAGCTAGTTGGTGGGGTAACGGCTCACCAAGGCGACGATACATAGCCGACCTGAGAGGGTGAT
CGGCCACACTGGGACTGAGACACGGCC

>Zotu149
GAACGCTGGCGGCGTGCTTAACACATGCAAGTCGAACGAAGAGCGATGGAAGCTTGCTTCTATCAATCTTA
GTGGCGAACGGGTGAGTAACGCGTAATCAACCTGCCCTTCAGAGGGGGACAACAGTTGGAAACGACTGCT
AATACCGCATACGATCTAATCTCGGCATCGAGGATAGATGAAAGGTGGCCTCTACATGTAAGCTATCACTG
AAGGAGGGGATTGCGTCTGATTAGCTAGTTGGAGGGGTAACGGCCCACCAAGGCGATGATCAGTAGCCG
GTCTGAGAGGATGAACGGCCACATTGGGACTGAGACACGGCC

>Zotu151
GAACGCTGGCGGCGTGCTTAACACATGCAAGTCGAACGGAAAGGCTCCAGCTTGCTGGAGTACTCGAGTG
GCGAACGGGTGAGTAACACGTGGGTAATCTGCCCTGCACTCTGGGATAAGCCTGGGAAACTGGGTCTAAT
ACTGGATATGACCTTTCTCCTTATGGGGTTTGGTGGAAAGTTTTTCGGTGTAGGATGAGCCCGCGGCCTATC
AGCTTGTTGGTGGGGTGATGGCCTACCAAGGCGACGACGGGTAGCCGGCCTGAGAGGGTGATCGGCCAC
ATTGGGACTGAGATACGGCC

>Zotu153
GAACGCTGGCGGCGTGCTTAACACATGCAAGTCGAACGCTGAAGCTTGGTGCTTGCACTGGGTGGATGAG
TGGCGAACGGGTGAGTAATACGTGAGTAACCTGCCCTTGACTCTGGGATAAGCCTGGGAAACTGGGTCTA
ATACTGGATACGACATGTCACCGCATGGTGGTGTGTGGAAAGGGTTTTACTGGTTTTGGATGGGCTCACGG
CCTATCAGCTTGTTGGTGGGGTAATGGCTCACCAAGGCGACGACGGGTAGCCGGCCTGAGAGGGTGACCG
GCCACACTGGGACTGAGACACGGCC
>Zotu155
GAACGCTAGCGGCAGGCCTAACACATGCAAGTCGAGGGGCAGCACAAGTAAAGCTTGCTTTATTGGTGGC
GACCGGCGCACGGGTGAGTAACGCGTATGCAACCTACCTTGTACAGGGGGATAGCCCGAAGAAATTCGGA
TTAATACCCCATAGTATTATTTAACTGCATAGTTTGAATAATTAAAGTTTCGGCGGTACAAGATGGGCATGC
GTCCTATTAGCTAGATGGTGAGGTAACGGCTCACCATGGCAACGATAGGTAGGGGGCCTGAGAGGGTGGT
CCCCCACACTGGTACTGAGACACGGAC
>Zotu158
GAACGCTGGCGGTATGCTTTACACATGCAAGTCATACGAGGGTTTTTAACTCAAGTGGCGGACGGGTGAG
TAACACGTAAGAATTTGCCTTTAGGAGGGGGATAACAACCACAAAAGGTTGCTAATACCCCATATGCTTTC
GAGTGAAACGGATTTTTTCCGCCTAGAGAGAAGCTTGCGGCTGATTAGCTTGTTGGTGAGGTAATGGCTTA
CCAAGGCGACGATCAGTATCTGGTTTGAGAGGACGATCAGACACACTGGAACTGAGACACGGTC
>Zotu160
GAACGCTGGCGGCATGCTTTACACATGCAAGTCGAACGGCAGCGCGGGGCAACCTGGCGGCGAGTGGCG
AACGGGTGAGTAATATATCGGAACGTACCCAAGAGTGGGGGATAACGTAGCGAAAGTTACGCTAATACCG
CATACGATCTAAGGATGAAAGTGGGGGATCGCAAGACCTCATGCTCCTGGAGCGGCCGATATCTGATTAG
CTAGTTGGTGAGGTAAAGGCTCACCAAGGCGACGATCAGTAGCTGGTCTGAGAGGACGACCAGCCACACT
GGAACTGAGACACGGTC
>Zotu162
GAACGCTGGCGGCGTGCTTAACACATGCAAGTCGAACGGAAAGGCTCCTGCTTGCAGGGGTACTCGAGTG
GCGAACGGGTGAGTAACACGTGGGTGATCTGCCCTGCACTTCGGGATAAGCCTGGGAAACTGGGTCTAAT
ACCGGATAGGACGATGGTTTGGATGCCATTGTGGAAAGTTTTTTCGGTGTGGGATGAGCTCGCGGCCTATC
AGCTTGTTGGTGGGGTAATGGCCTACCAAGGCGTCGACGGGTAGCCGGCCTGAGAGGGTGTACGGCCAC
ATTGGGACTGAGATACGGCC
>Zotu164
GAACGCTAGCGGCAGGCTTAATACATGCAAGTCGTGGGGCAGCGCAGTGTAGCAATACATGGGCGGCGA
CCGGCAAACGGGTGCGGAACACGTACACAACCTTCCTACAAGTGGGGAATAGCCCAGAGAAATTTGGATT
AATACCCCGTAACATATAAAGATGGCATCATTTTTATATTATAGCTACGGCGCTTGTTGATGGGTGTGCGGC
TGATTAGGTAGTTGGCGGGGTAATGGCCCACCAAGCCTGCGATCAGTAGCTGATGTGAGAGCATGATCAG
CCACACGGGCACTGAGACACGGGC
>Zotu166
GAACGCTAGCGGCAGGCTTAATACATGCAAGTCGAGGGACAGCACAGGTAGCAATACTGGGTGGTAACC
GGCAAACGGGTGCGGAACACGTACACAACCTTCCTTTAAGTGGGGGATAGCCCAGAGAAATTTGGATTAA
TACCCCGTAACATAGTGAACTGGCATCAGTTTATTATTATAGCTACGGCGCTTAATGATGGGTGTGCGTCTG
ATTAGATAGTTGGCGAGGTAACGGCTCACCAAGTCTACGATCAGTAGCTGATGTGAGAGCATGATCAGCC
ACACGGGCACTGAGACACGGGC
>Zotu169
GAACGCTGGCGGCGTGCCTAATACATGCAAGTAGAACGCTGAAGGAGGAGCTTGCTTCTCCGGATGAGTT
GCGAACGGGTGAGTAACGCGTAGGTAACCTGCCTGGTAGCGGGGGATAACTATTGGAAACGATAGCTAAT
ACCGCATAACAGTAGATATTGCATGATATCTGCTTGAAAGGTGCAATTGCATCACTACCAGATGGACCTGC
GTTGTATTAGCTAGTTGGTGGGGTAACGGCTCACCAAGGCGACGATACATAGCCGACCTGAGAGGGTGAT
CGGCCACACTGGGACTGAGACACGGCC
>Zotu171
GAACGCTATCGGTATGCTTAACACATGCAAGTCGGACGAATTAATTAGGGCTTGCCCTAATTAATTAGTGG
CAGACGGGTGAGTAACACGTGGGAATCTACCCATCAGTACGGAATAACTTTTAGAAATAAAAGCTAATACC

GTATATTCTCTGAGGAAAGATTTATCGCTGATGGATGAGCCCACGCCAGATTAGGTAGTTGGTGAAGTAAT
GGCTCACCAAGCCAACGATCTGTAGCTGGTCTGAGAGGATGATCAGCCACACTGGGACTGAGACACGGCC
>Zotu173
GAACGCTGGCGGCGTGCCTAATACATGCAAGTAGAACGCTGAAGGAGGAGCTTGCTCTTCTGGATGAGTT
GCGAACGGGTGAGTAACGCGTAGGTAACCTGCCTGGTAGCGGGGGATAACTATTGGAAACGATAGCTAAT
ACCGCATAACAGCAAATGTTGCATGACATTTGCTTAAAAGGTGCAATTGCATCACTACCAGATGGACCTGC
GTTGTATTAGCTAGTTGGTGGGGTAATAGCTCACCAAGGCGACGATACATAGCCGACCTGAGAGGGTGAT
CGGCCACACTGGGACTGAGACACGGCC
>Zotu175
GAACGCTGGCGGCGTGCCTAATACATGCAAGTCGAGCGGACCGGACGAGAGCTTGCTCTTGTTTGGTCAG
CGGCGGACGGGTGAGTAACACGTGGGCAACCTGCCCGCAAGACCGGGATAACTCCGGGAAACCGGAGCT
AATACCGGATAACACCAAAGACCGCATGGTCTTTGGTTGAAAGGCGGCTTCGGCTGTCACTTGCGGATGG
GCCCGCGGCGCATTAGCTAGTTGGTGAGGTAACGGCTCACCAAGGCGACGATGCGTAGCCGGCCTGAGAG
GGTGACCGGCCACACTGGGACTGAGACACGGCC
>Zotu177
GAACGCTGGCGGCGTGCCTAATACATGCAAGTAGAACGCTGAAGAGAGGAGCTTGCTCTTCTTGGATGAG
TTGCGAACGGGTGAGTAACGCGTAGGTAACCTGCCTTGTAGCGGGGGATAACTATTGGAAACGATAGCTA
ATACCGCATAACAATGGATGACACATGTCATTTATTTGAAAGGGGCAATTGCTCCACTACAAGATGGACCT
GCGTTGTATTAGCTAGTAGGTGAGGTAACGGCTCACCTAGGCGACGATACATAGCCGACCTGAGAGGGTG
ATCGGCCACACTGGGACTGAGACACGGCC
>Zotu179
GAACGCTGGCGGCGTGCTTAACACATGCAAGTCGAACGATGAAGCGGTGCTTGCACCGTGGATTAGTGGC
GAACGGGTGAGTAACACGTGAGTAACCTGCCTTCCACTCTGGGATAAGCGCTGGAAACGGCGTCTAATACT
GGATATGACATTGCCTCGCATGGGGTGGTGTGGAAAGATTTTTTGGTGGTAGATGGACTCGCGGCCTATCA
GCTTGTTGGTGGGGTAATGGCCTACCAAGGCGACGACGGGTAGCCGGCCTGAGAGGGTGACCGGCCACA
CTGGGACTGAGACACGGCC
>Zotu181
GAACGCTGGCGGCGTGCTTAACACATGCAAGTCGAACGGAAAGGCCCAGCTTGCTGGGTGCTCGAGTGGC
GAACGGGTGAGTAACACGTGGGTGATCTGCCCCTAACTTCGGGATAAGCTTGGGAAACTGGGTCTAATAC
CGGATAGGACAATCGTTTAGTGTCGGTTGTGGAAAGTTTTTTCGGTTAGGGATGAGCCCGCGGCCTATCAG
CTTGTTGGTGGGGTAATGGCCTACCAAGGCGTCGACGGGTAGCCGGCCTGAGAGGGTGGACGGCCACATT
GGGACTGAGATACGGCC
>Zotu183
GAACGCTGGCGGCATGCCTAATACATGCAAGTTGAACGATGATGTTCTCTGCTTGCAGAGAATTGAAGAGT
AGCGAACGGGTGAGTAACACGTGGGGAACCTGCCCATGAGAGGGGGATAACATTCGGAAACGGATGCTA
ATACCGCATAATGCTTTGGACCGCCTGGTCCAGAGACGAAAGACGGCTTTTGCTGTCACTCATGGATGGCC
CCGCGCCGTATTAGCTAGTTGGTGAGGTAATGGCTCACCAAAGCTGTGATACGTAGCCGACCTGAGAGGG
TGATCGGCCACACTGGGACTGAGACACGGCC
>Zotu186
GAACGCTGGCGGCGTGCTTAACACATGCAAGTCGAACGGAAAGGCCCTGCTTGCAGGGTGCTCGAGTGGC
GAACGGGTGAGTAACACGTGGGTGATCTGCCCCTTACTTTGGGATAAGCCTGGGAAACTGGGTCTAATACT
GGATAGGACCATGCTGTAGGTGGTGTGGTGGAAAGATTAGTTTCGGTAAGGGATGAGCTCGCGGCCTATC
AGCTTGTTGGTGGGGTAATGGCCTACCAAGGCGTCGACGGGTAGCCGGCCTGAGAGGGTGGACGGCCAC
ATTGGGACTGAGATACGGCC
>Zotu189
GAACGCTGGCGGCATGCCTAACACATGCAAGTCGAACGATGCTTTCGGGCATAGTGGCGCACGGGTGCGT
AACGCGTGGGAATCTGCCCTTGGGTTCGGAATAACAGTGAGAAATTGCTGCTAATACCGGATGATGTCTTC
GGACCAAAGATTTATCGCCCAGGGATGAGCCCGCGTAGGATTAGCTAGTTGGTGGGGTAATGGCCTACCA
AGGCGACGATCCTTAGCTGGTCTGAGAGGATGATCAGCCACACTGGGACTGAGACACGGCC
>Zotu191

GAACGCTGGCGGCGTGCCTAATACATGCAAGTCGAGCGAACGGACGAGAAGCTTGCTTCTCTGATGTTAG
CGGCGGACGGGTGAGTAACACGTGGATAACCTACCTATAAGACTGGGATAACTTCGGGAAACCGGAGCTA
ATACCGGATAATATTTTGAACCGCATGGTTCAAAAGTGAAAGACGGTCTTGCTGTCACTTATAGATGGATCC
GCGCTGCATTAGCTAGTTGGTAAGGTAACGGCTTACCAAGGCAACGATGCATAGCCGACCTGAGAGGGTG
ATCGGCCACACTGGAACTGAGACACGGTC

>Zotu194
GAACGCTAGCGGCAGGCCTAATACATGCAAGTCGAACGGTGTCTAGCAATAGGCATAGTGGCGCACGGGT
GCGTAACACGTAACTAACCTACCTATAACTGGGGGATAGCCCGCCGAAAGGCGGATTAATACCGCATAAGC
TAGTTGAACGGCATCGTTTGATTAGTAAAGATTTATTGGTTATAGATGGGGTTGCGGGTCATTAGCTAGTT
GGTAAGGTAACGGCTTACCAAGGCGACGATGACTAGGGGAGCTGAGAGGCTGGTCCCCCACACGGGCAC
TGAGATACGGGC

>Zotu196
GAACGCTGGCGGCGTGCCTAATACATGCAAGTCGAGCGAACGGATAAGGAGCTTGCTCCTTTGAAGTTAG
CGGCGGACGGGTGAGTAACACGTGGGTAACCTACCTATAAGACTGGAATAACTTCGGGAAACCGGAGCTA
ATGCCGGATAACATTTGGAACCGCATGGTTCTAAAGTAAAAGATGGTTTTGCTATCACTTATAGATGGACCC
GCGCCGTATTAGCTAGTTGGTAAGGTAACGGCTTACCAAGGCAACGATACGTAGCCGACCTGAGAGGGTG
ATCGGCCACACTGGAACTGAGACACGGTC

>Zotu198
GAACGCTGGCGGCGTGCCTAATACATGCAAGTCGAGCGAACAGATAAGGAGCTTGCTCCTTTGACGTTAG
CGGCGGACGGGTGAGTAACACGTGGATAACCTACCTATAAGACTGGGATAACTTCGGGAAACCGGAGCTA
ATACCGGATAAGATTTTGAACCGCATGGTTCAATAGTGAAAGACGGCCTTGCTGTGACTTATAGATGGATC
CGCGCCGTATTAGCTAGTTGGTAAGGTAACGGCTTACCAAGGCAACGATACGTAGCCGACCTGAGAGGGT
GATCGGCCACACTGGAACTGAGACACGGTC

>Zotu200
GAACGCTAGCGGGAGGCCTAACACATGCAAGCCGAGCGGTATTGTTTCTTCGGAAATGAGAGAGCGGCGT
ACGGGTGCGGAACACGTGTGCAACCTGCCTTTATCTGGGGGATAGCCTTTCGAAAGGAAGATTAATACTCC
ATAATATATTGAACGGCATCGTTTAATATTGAAAGCTCCGGCGGATAGAGATGGGCACGCGCAAGATTAGC
TAGTTGGTGAGGTAACGGCTCACCAAGGCGATGATCTTTAGGGGGCCTGAGAGGGTGATCCCCCACACTG
GTACTGAGACACGGAC

>Zotu202
GAACGCTGGCGGCGTGCCTAATACATGCAAGTCGAGCGGACCAAATCGGAGCTTGCTCTGGTTTGGTCAG
CGGCGGACGGGTGAGTAACACGTGGGCAACCTGCCCGCAAGACCGGGATAACTCCGGGAAACCGGAGCT
AATACCGGATAACACCGAAGACCGCATGGTCTTTGGTTGAAAGGCGGCCTTTGGCTGTCACTTGCGGATGG
GCCCGCGGCGCATTAGCTAGTTGGTGAGGTAACGGCTCACCAAGGCGACGATGCGTAGCCGGCCTGAGAG
GGTGACCGGCCACACTGGGACTGAGACACGGCC

>Zotu204
GAACGCTGGCGGCAGGCCTAACACATGCAAGTCGAGCGGATGAGTGGAGCTTGCTCCATGATTCAGCGGC
GGACGGGTGAGTAATGCCTAGGAATCTGCCTGGTAGTGGGGGACAACGTTTCGAAAGGAACGCTAATACC
GCATACGTCCTACGGGAGAAAGTGGGGGATCTTCGGACCTCACGCTATCAGATGAGCCTAGGTCGGATTA
GCTAGTTGGTGAGGTAAAGGCTCACCAAGGCGACGATCCGTAACTGGTCTGAGAGGATGATCAGTCACAC
TGGAACTGAGACACGGTC

>Zotu206
GAACGCTGGCGGCACGCCTAATACATGCAAGTCGAGCGCGTGAAGCTTCCAGAAGCCTTCGGGCGGACGG
GAGTGGATCGAGCGGCGGACGGGTGAGTAACACGTAGGCAACCTGCCTGTAAGACCGGGATAACCCCGG
GAAACCGGGGCTAATACCGGATAGGACCTTCGGTCGCATGACCGTTGATTGAAAGGTGGCCGCAAGGCTA
CCGCTTACAGATGGGCCTGCGGCGCATTAGCTGGTTGGTGAGGTAACGGCTCACCAAGGCGACGATGCGT
AGCCGGCCTGAGAGGGTGACCGGCCACACTGGGACTGAGACACGGCC

>Zotu208
GAACGCTGGCGGCAGGCTTAACACATGCAAGTCGAACGGGTGTAGCAATACATCAGTGGCAGACGGGTG
AGTAACACGTGGGAACCTTCCTAGAGGTACGGAACAACGCAGGGAAACTTGCGCTAATACCGTATAAGCT

CTTGCGAGGAAAGATTTATCGCCTTTAGACGGGCCCGCGTAGGATTAGGTAGTTGGTGGGGTAACGGCCT
ACCAAGCCTACGATCCTTAGCTGATCTGAGAGGATGATCAGCCACACTGGAACTGAGACACGGTC
>Zotu210
GAACGCTGGCGGCGTGCTTAACACATGCAAGTCTTAGGGGATTTATTCTTCGGGAATAAGTTCACTGGCG
CACGGGTGCGTAACGCGTAAGTAATCTGCCCTAGAGACTGGCACAACTCGTCGAAAGACGGGCTAATTCC
AGATGATACTGAAAGGTCAAAGGTTTCGACCGCTCTAGGATGAGCTTGCGTCTGATTAGCTAGTTGGTGAG
GTAATGGCTCACCAAGGCTACGATCAGTAGCCGGTCTGAGAGGATGATCGGCCACATTGGAACTGAGACA
CGGTC
>Zotu212
GAACGCTGGCGGCGTGCCTAATACATGCAAGTCGAGCGGACCAAATCGGAGCTTGCTCTGATTTGGTCAG
CGGCGGACGGGTGAGTAACACGTGGGCAACCTGCCCGCAAGACCGGGATAACTCCGGGAAACCGGAGCT
AATACCGGATAACACCGAAGACCGCATGGTCTTTGGTTGAAAGGCGGCCTTTGGCTGTCACTTGCGGATGG
GCCCGCGGCGCATTAGCTAGTTGGTGAGGTAACGGCTCACCAAGGCGACGATGCGTAGCCGGCCTGAGAG
GGTGACCGGCCACACTGGGACTGAGACACGGCC
>Zotu214
GAACGCTGGCGGCGTGCTTAACACATGCAAGTCGAACGGGATTTAGTAAACAGAAGCCTCGGTGGAAGAT
TACTAATGAGAGTGGCGAACGGGTGAGTAACGCGTGAGCAACCTGCCTATGACAGTGGGATAGCCTCGGG
AAACCGGGATTAATACCGCATAAAATCGTAGAAACACATGTTTCAACGGTCAAAGATTTATCGGTCATAGA
TGGGCTCGCGTCTGATTAGCTAGTTGGTGAGATAACAGCCCACCAAGGCGACGATCAGTAGCCGGTCTGA
GAGGATGAACGGCCACATTGGAACTGAGACACGGTC
>Zotu216
GAACGCTGGCGGCGTGCCTAATACATGCAAGTCGAGCGAACAGACGAGGAGCTTGCTCCTTTGACGTTAG
CGGCGGACGGGTGAGTAACACGTAGGTAACCTACCTATAAGACTGGGATAACTTCGGGAAACCGGAGCTA
ATACCGGATAATATTTCGAACCGCATGGTTCGATAGTGAAAGATGGCTTTGCTATCACTTATAGATGGACCT
GCGCCGTATTAGCTAGTTGGTAAGGTAACGGCTTACCAAGGCAACGATACGTAGCCGACCTGAGAGGGTG
ATCGGCCACACTGGAACTGAGACACGGTC
>Zotu218
AAACGCTGGCGGCATGCCTAACACATGCAAGTCGAACGGCAGCACAGTAAAGATTTCGGTCTTTACGGGT
GGCGAGTGGCGGATGGGTGAGTAAAGCGTAGGAATCTGTCCTGGAGAGTGGAATAACCCGAGGAAACTC
GGGCTAATACCGCATGATATCGAGAGATCAAAGCAGGGGACCCTTCGGGGCCTTGCGCTCTAGGATGAGC
CTACGTCGGATTAGCTAGTTGGTGGGGTAAAGGCCTACCAAGGCGACGATCCGTAGCTGGTCTGAGAGGA
TGACCAGCCACACTGGGACTGAGACACGGCC
>Zotu221
GAACGCTGGCGGCGTGCTTAACACATGCAAGTCGAACGATGAAGCTCCAGCTTGCTGGGGTGGATTAGTG
GCGAACGGGTGAGTAATACGTGAGTAACCTGCCCTTGACTCTGGGATAAGCCTGGGAAACTGGGTCTAAT
ACTGGATACTACACATTCTCGCATGGGGGTGTGTGGAAAGGGTTTTACTGGTTTTGGATGGGCTCACGGCC
TATCAGCTTGTTGGTGGGGTAATGGCCTACCAAGGCGACGACGGGTAGCCGGCCTGAGAGGGTGACCGG
CCACACTGGGACTGAGACACGGCC
>Zotu223
GAACGCTATCGGTATGCTTAACACATGCAAGTCGGACGAATTAATTAGGGCTTGCCCTAATTAATTAGTGG
CAGACGGGTGAGTAACACGTGGGAATCTACCCATCAGTACGGAATAACTTTTAGAAATAAAAACTAATACC
GTATATTCTCTAAGGAGGAAAGATTTATCGCTGATGGATGAGCCCGCGCCAGATTAGGTAGTTGAAGTAAT
GGCTCACCAAGCCAACGATCTGTAGCTGGTCTGAGAGGATGATCAGCCACACTGGGACTGAGACACGGCC
>Zotu225
GAACGCTAGCGGCAGGCCTAATACATGCAAGTCGGACGGGATTGGAGAGCTTGCTTTCCATGAGAGTGGC
GCACGGGTGCGTAACACGTATGCAACCTACCTTTGTCAGGGGGATAGCCTCTCGAAAGAGAGATTAAGAC
CGCATAACATTATTGAGTGGCATCATTTAATAATCAAATATTTATAGGACAAAGATGGGCATGCGGAACAT
TAGCTAGTTGGTGCGGTAACGGCGTACCAAGGCTACGATGTTTAGGGGATCTGAGAGGATGACCCCCCAC
ACTGGTACTGAGACACGGAC
>Zotu227

GAACGCTGGCGGCGTGCTTAACACATGCAAGTCGAACGGGATCCGGGCTTTCGGGTTTGGTGAGAGTGGC
GAACGGGTGAGTAACACGTGAGTAACCTGCCCCCTTCTTTGGGATAACGTCGGGAAATCGGCGCTAATACT
GGATATTCTTGCTGCATCGCATGGTGTGGTTTGGAAAGGGTTTCTGGTGGGGGATGGGCTCGCGGCCTATC
AGCTTGTTGGTGGGGTGATGGCCTACCAAGGCTTTGACGGGTAGCCGGCCTGAGAGGGTGTCCGGCCACA
CTGGGACTGAGACACGGCC

>Zotu229

GAATGCTGGCGGCGTGGATAAGTCATGCAAGTCAAGGGGGCCCGCAAGGGCAACCGGCAGACGAGGTAG
TAACACGTAGGTAGATCCCCCGAAGTCGGGCATAGCCAGTCGAAAGACTGGGTAATTCCCGATAGTCCCGC
AAGGGTAAAGGTTTTTCGCTTCGGGAGTTGCCTGCGTAGTATCAGCTAGTTGGTAGGGTAACGGCCTACCA
AGGCTACGACGCTTAGGGGAGCTGAGAGGCTGACCCCCACCGATGGAACTGCGACACGGTC

>Zotu231

GAACGCTGGCGGCGTGCCTAATACATGCAAGTCGAGCGAACAGACGAGGAGCTTGCTCCTCTGATGTTAG
CGGCGGACGGGTGAGTAACACGTGGATAACCTACCTATAAGACTGGGATAACTTCGGGAAACCGGAGCTA
ATACCGGATAACATGTTGAACCGCATGGTTCAACAGTGAAAGACGGTCTTGCTGTCACTTATAGATGGATC
CGCGCCGCATTAGCTAGTTGGTAAGGTAACGGCTTACCAAGGCAACGATGCGTAGCCGACCTGAGAGGGT
GATCGGCCACACTGGAACTGAGACACGGTC

>Zotu233

GAACGCTAGCGGCAGGCCTAATACATGCAAGTCGGACGGGATTCAGGGAGTAGCTTGCTACGAACTGATG
AGAGTGGCGCACGGGTGCGTAACGCGTGAGCAACCTACCCATATCAGGGGGATAGCCTCTCGAAAGAGA
GATTAACACCGCATAACATCATTAACCGGCATCGGTTTTTGATCAAATATTTATAGGATATGGATGGGCTCG
CGTGACATTAGCTAGTTGGAGAGGTAACGGCTCACCAAGGCTACGATGTCTAGGGGCTCTGAGAGGAGAA
TCCCCCACACTGGTACTGAGACACGGAC

>Zotu235

CAACGCTGGCGGCGCGCTTAACACATGCAAGTCGCACGAGAAAGGGGAGCAATCCCTGAGTAAAGTGGC
GTACGGGTGAGTAACACGTGGATGATCTACCTTCTAGTGGGGAATAACCCTGGGAAACCGGGGCTAATAC
CGCATAAGCTCGAGAGAGGAAAGCAGCAATGCGCTGGAAGAGGAGTCCGCGGCCGATTAGCTAGTTGGC
AGGGTAAGAGCCTACCAAGGCAGAGATCGGTAGCCGGCCTGAGAGGGCACACGGCCACACTGGCACTGA
AACACGGGC

>Zotu237

GAACGCTAGCGGCAGGCCTAATACATGCAAGTCGAACGGGATTGAAGAGCTTGCTTTTCATGAGAGTGGC
GCACGGGTGCGTAACACGTATGTAACCTACCTTAATCTGGGGGATAGCCTCTCGAAAGAGAGATTAACACC
GCATAACATTATCGGATAGCATTTTCTGATAATCAAATATTTATAGGATTAAGATGGGCATGCGGAACATTA
GCTAGTTGGTAAGGTAATGGCTTACCAAGGCTACGATGTTTAGGGGATCTGAGAGGATGACCCCCCACACT
GGTACTGAGACACGGAC

>Zotu239

GAACGCTAGCGGCAGGCTTAATACATGCAAGTCGTGGGGCAGCGCAGGTAGCAATACTGGGCGGCGACC
GGCAAACGGGTGCGGAACACGTACACAACCTTCCTACAAGTGGGGAATAGCCCAGAGAAATTTGGATTAA
TACCCCGTAACATAGCGATGTGGCATCACATTGCTATTATAGCTTCGGCGCTTGTTGATGGGTGTGCGGCT
GATTAGATAGTTGGCGGGGTAACGGCCCACCAAGTCTACGATCAGTAGCTGATGTGAGAGCATGATCAGC
CACACGGGCACTGAGACACGGGC

>Zotu242

GAACGCTGGCGGCAGGCCTAACACATGCAAGTCGAACGGCAGCACAGTAAGAGCTTGCTCTTATGGGTGG
CGAGTGGCGGACGGGTGAGGAATACATCGGAATCTACTCTTTCGTGGGGGATAACGTAGGGAAACTTACG
CTAATACCGCATACGACCTACGGGTGAAAGCGGAGGACCTTCGGGCTTCGCGCGATTGAATGAGCCGATG
TCGGATTAGCTAGTTGGCGGGGTAAAGGCCCACCAAGGCGACGATCCGTAGCTGGTCTGAGAGGATGATC
AGCCACACTGGAACTGAGACACGGTC

>Zotu244

GAACGCTGGCGGCGCGCTTAACACATGCAAGTCGAGCGAGGCCCCGGGCTTGCCCGGGTGTCCTAGCGGC
GAACGGGTGAGTAACACGTGAGGAACCTGCCCCGAAGACCGGGATAACACCGGGAAACCGGTGCTAATA
CCGGATGCCCTCATTGGATCGCATGGTCCGATGAGGAAATGGATTCCGCTTCGGGATGGTCTCGCGGCCTA

TCAGCTTGTTGGTGGGGTAACGGCCTACCAAGGCAGCGACGGGTAGCTGGTCTGAGAGGATGTCCAGCCA
CACTGGGACTGAGACACGGCC
>Zotu246
GAACGCTGGCTGCGTGCTTAACACATGCAAGTCGAACGCTGAAGCCTGGTGCTTGCACCGGGTGGATGAG
TGGCGAACGGGTGAGTAACACGTGAGTAACCTGCCCCTGACTTCGGGATAAGCCCGGGAAACTGGGTCTA
ATACCGGATACGACCGTCGGACGCATGTCTGGTGGTGGAAAGTTTTTCGGTTGGGGATGGGCTCGCGGC
CTATCAGTTTGTTGGTGAGGTAATGGCTCACCAAGACGACGACGGGTAGCCGGCCTGAGAGGGCGACCGG
CCACACTGGGACTGAGACACGGCC
>Zotu248
GAACGCTGGCGGCGTGCCTAATACATGCAAGTAGAACGCTGAAGAGAGGAGCTTGCTCTTCTTGGATGAG
TTGCGAACGGGTGAGTAACGCGTAGGTAACCTGCCTGGTAGCGGGGGATAACTATTGGAAACGATAGCTA
ATACCGCATGAAATTGCTTATCGCATGATAATTAATTGAAAGATGCAATTGCATCACTACCAGATGGACCTG
CGTTGTATTAGCTAGTTGGTGAGGTAACGGCTCACCAAGGCGACGATACATAGCCGACCTGAGAGGGTGA
TCGGCCACACTGGGACTGAGACACGGCC
>Zotu250
GAACGCTGGCGGCGTGCTTAACACATGCAAGTCGAACGATGAAGCCCAGCTTGCTGGGTGGATTAGTGGC
GAACGGGTGAGTAACACGTGGGTGATCTGCCCTGCACTCTGGGATAAGCCTGGGAAACTGGGTCTAATAC
CGGATATGACCTCTTGCTGCATGGCGAGGGGTGGAAAGTTTTTCGGTGCAGGATGAGCCCGCGGCCTATC
AGCTTGTTGGTGGGGTAATGGCCTACCAAGGCGACGACGGGTAGCCGGCCTGAGAGGGCGACCGGCCAC
ACTGGGACTGAGACACGGCC
>Zotu252
GAACGCTATCGGTATGCTTAACACATGCAAGTCGAACGGACTAATTGGGGCTTGCTCCAATTAGTTAGTGG
CAGACGGGTGAGTAACACGTGGGAATCTACCCATCAGTACGGAATAACTTTTAGAAATAAAAGCTAATACC
GTATATTCTCTACGGAGGAAAGATTTATCGCTAATGGATGAGCCCGCGTCAGATTAGGTAGTTGGTGAGGT
AACGGCTCACCAAGCCGACGATCTGTAGCTGGTCTGAGAGGATGATCAGCCACACTGGGACTGAGACACG
GCC
>Zotu254
GAACGCTGGCGGCAGGCCTAACACATGCAAGTCGAACGAACTCTTCGGAGTTAGTGGCGGACGGGTGAGT
AACACGTGGGAACGTGCCTTTAGGTTCGGAATAACTCAGGGAAACTTGTGCTAATACCGAATGTGCCCTTC
GGGGGAAAGATTTATCGCCTTTAGAGCGGCCCGCGTCTGATTAGCTAGTTGGTGAGGTAAAGGCTCACCA
AGGCGACGATCAGTAGCTGGTCTGAGAGGATGATCAGCCACATTGGGACTGAGACACGGCC
>Zotu257
GAACGCTGGCGGCGTGCCTAATACATGCAAGTCGAGCGAACGGATAAGGAGCTTGCTCCTTTGAAGTTAG
CGGCGGACGGGTGAGTAACACGTGGGTAACCTACCTATAAGACTGGGATAACTTCGGGAAACCGGAGCTA
ATACCGGATAACATTTAGAACCGCATGGTTCTAAAGTGAAAGATGGTTTTGCTATCACTTATAGATGGACCC
GCGCCGTATTAGCTAGTTGGTAAGGTAACGGCTTACCAAGGCGACGATACGTAGCCGACCTGAGAGGGTG
ATCGGCCACACTGGAACTGAGACACGGTC
>Zotu259
GAACGCTAGCTACAGGCTTAACACATGCAAGTCGAGGGGCAGCGGGAAGCAAGCTTGCTTGCTTTGCCGG
CGACCGGCGTACGGGTGCGTAACGCGTACCGAACCTACCTCACACTCCGGGATAGCCCTGCGAAAGCAGG
ATTAATACCGGGTAGCCTCACTATATCGCATGATATTATGAGTAAAGATTTATTGGTGTGAGATGGCGGTG
CGTCCCATTAGTTAGTTGGCGGGGTAACGGCCCACCAAGACGACGATGGGTAGGGGTTCTGAGAGGAAG
GTCCCCCACATTGGAACTGAGACACGGTC
>Zotu261
AAACGCTGGCGGCATGCCTAACACATGCAAGTCGAACGGCAGCACAGTAAAGATTTCGGTCTTTACGGGT
GGCGAGTGGCGGATGGGCGAGTAAAGCGTAGGAATCTGTCCTGGAGAGTGGGATAACCCGAGGAAACTC
GGGCTAATACCGCATGATATCGAGAGATCAAAGCAGGGGACCCTTCGGGGCCTTGCGCTCTAGGATGAGC
CTACGTCGGATTAGCTAGTTGGTGGGGTAAAGGCCTACCAAGGCGACGATCCGTAGCTGGTCTGAGAGGA
TGACCAGCCACACTGGGACTGAGACACGGCC
>Zotu263

GAATGCTGGCGGCGTGGATAAGGCATGCAAGTCAAGGGGGCCGCAAGGCAACCGGCAGACGAGGTAGT
AACACGTAGGTACGTACTCCAGAGTCGGGCATAGCCAGCCGAAAGGTTGGGTAATTCCCGATGGTCCCGC
AAGGGTAAAGGTTTTTCGCTCTGGAAGCGGCCTGCGCAGTATCAGCTAGTTGGTAAGGTAATGGCTTACCA
AGGCTACGACGCTTAGGGGAGCTGAGAGGCTGACCCCCACCGATGGGACTGAGAGACGGCC
>Zotu266
GAACGCTGGCGGCGTGCTTAACACATGCAAGTCGAACGATGAACCACTTCGGTGGGGATTAGTGGCGAAC
GGGTGAGTAACACGTGGGCAATCTGCCCTTCACTTCGGGACAAGCCCTGGAAACGGGGTCTAATACCGGA
TATCACTTCCATTCGCATGGGTGGGGGTTGAAAGCTCCGGCGGTGAAGGATGAGCCCGCGGCCTATCAGC
TTGTTGGTGAGGTAATGGCTCACCAAGGCGACGACGGGTAGCCGGCCTGAGAGGGCGACCGGCCACACT
GGGACTGAGACACGGCC
>Zotu268
GAACGCTGGCGGCGTGCTTAACACATGCAAGTCGAACGATGAAGCCTTAGCTTGCTTTGGTGGATTAGTGG
CGAACGGGTGAGTAACACGTGAGCAACGTGCCCAGGACTCTGGAATAACCGCCGGAAACGGTGGCTAATA
CTGGATAGGTGCCAAGGCCGCATGGTCTTTGGTTGGAAAGATTTTTCGGTTCTGGATCGGCTCGCGGCCTA
TCAGCTTGTTGGTGGGGTAATGGCCTACCAAGGCGACGACGGGTAGCCGGCCTGAGAGGGTGACCGGCC
ACACTGGGACTGAGACACGGCC
>Zotu270
GAACGCTGGCGGTGTGCTTCACACATGCAAGTCGAACGGGGTAGCAATACCCAGTGGCGGACGGGTGAG
TAACACGTGGGAATCTACCATTAGATGGGGGATAGCAGGCCGAAAGGCCTATTAATACCGCATATGTAGG
CATCTGGACACAGGTGTTTATGAAAGGAGTAATCCGTCTGATGATGAGCCCGCGCCCGATTAGCTAGTTGG
TAGGGTAAAAGCCTACCAAGGCGACGATCGGTAGGTGGTCTGAGAGGATGATCACCCACAATGGGACTGA
GACACGGCC
>Zotu272
GAACGCTGGCGGCAGGCTTAACACATGCAAGTCGAACGGGCGTAGCAATACGTCAGTGGCAGACGGGTG
AGTAACACGTGGGAACGTACCTCTTGGTTCGGAACAACACAGGGAAACTTGTGCTAATACCGGATAAGCCC
TTACGGGGAAAGATTTATCGCCGAGAGATCGGCCCGCGTCTGATTAGCTAGTTGGTGAGGTAACGGCTCA
CCAAGGCGACGATCAGTAGCTGGTCTGAGAGGATGATCAGCCACATTGGGACTGAGACACGGCC
>Zotu274
GAACGCTGGCGGCGTGCTTAACACATGCAAGTCGAACGATGAAGCTTGGTGCTTGCACTGGGTGGATTAG
TGGCGAACGGGTGAGTAACACGTGAGTAACCTGCCCTTTACTTCGGGATAAGCTTGGGAAACTGGGTCTA
ATACCGGATATTCTGCACGGTCGGATGGTTGTGTAGGAAAGATTTTTTGGTAAGGGATGGGCTCGCGGCCT
ATCAGTTTGTTGGTGGGGTGATGGCCTACCAAGACGACGACGGGTAGCCGGCCTGAGAGGGCGACCGGC
CACACTGGGACTGAGACACGGCC
>Zotu276
GAACGCTGGCGGCGTGCTTAACACATGCAAGTCACGGGGGCCCGCAAGGGTAACCGGCGAACGGGTGCG
TAACACGTGAGCAATCTGCCGATGTCTGGGGGATAGCCGGCCCAACGGCCGGGTAATACCGCATACGATC
GCTCGGGGGAGTCCCTGGGCGATGAAACCTCCGGGGGACATCGAGGAGCTCGCGCCCTATCAGCTAGTTG
GTGAGGTAATGGCTCACCAAGGCTACGACGGGTAGCTGGTCTGAGAGGATGGCCAGCCACATTGGGACTG
CGACACGGCC
>Zotu278
GAACGCTGGCGGCATGCCTAACACATGCAAGTCGAACGAAGGCTTCGGCCTTAGTGGCGCACGGGTGCGT
AACGCGTGGGAATCTGCCCCTCGGTTCGGAATAACAGTTAGAAATGACTGCTAATACCGGATGATGACGTA
AGTCCAAAGATTTATCGCCGAGGGATGAGCCCGCGTAGGATTAGCTAGTTGGTGTGGTAAGAGCGCACCA
AGGCGACGATCCTTAGCTGGTCTGAGAGGATGATCAGCCACACTGGGACTGAGACACGGCC
>Zotu280
GAACGCTGGCGGCGTGCTTAACACATGCAAGTCGAACGGAAAGGCCAGTGCTTGCACTGGTACTCGAGTG
GCGAACGGGTGAGTAACACGTGGGTGATCTGCCTTGTACTCTGGGATAAGCTTGGGAAACTGGGTCTAAT
ACCGGATAATAACTGCCTTTAGTGTGGTGGTTGGAAAGTTTTTTCGGTGCAAGATGAGCTCGCGGCCTATC
AGCTTGTTGGTGGGGTAATGGCCTACCAAGGCGTCGACGGGTAGCCGGCCTGAGAGGGTGTACGGCCAC
ATTGGGACTGAGATACGGCC

>Zotu282
GAATGCTGGCGGCGCGCCTAACACATGCAAGTCGAGCGGAAAGGCCCTTCGGGGTACTCGAGCGGCGGA
CGGCTGAGTAACGCGTGGGAACATGCCCCAAAGTGAGGAATAACTGCCCGAAAGGGTAGCTAATGCCGCA
TATGATCTTAGGATTAAAGGATTTATCCGCTTTGGGAGTGGCCCGCGTACGATTAGGTAGTTGGTGAGGTA
AAGGCTCACCAAGCCCACGATCGTTAACTGGTCTGAGAGGATGATCAGTCAGACTGGAACTGAGACACGG
TC
>Zotu284
GAACGCTGGCGGTCTGCTTAACACATGCAAGTCGAACGGGCTCTTTCGGGAGCTAGTGGCGGACGGGTGA
GTAACGCGTGAGAACCTGCCTTCAGGTCGGGGACAACAGAGGGAAACTTCTGCTAATACCGGATGAGCCG
AAAGGTAAAAGATTTATCGCCTGAAGAGGGGCTCGCGTCTGATTAGCTAGTTGGTGTGGTAAGAGCGCAC
CAAGGCGACGATCAGTAGCTGGTCTGAGAGGATGACCAGCCACACTGGGACTGAGACACGGCC
>Zotu286
GAACGCTGGCGGCATGCTTTACACATGCAAGTCGAACGGCAGCGCGGGCTTCGGCCTGGCGGCGAGTGGC
GAACGGGTGAGTAATGCATCGGAACGTGCCCAGTAGTGGGGGATAGCCCGGCGAAAGCCGGATTAATAC
CGCATACGACCTGAGGGTGAAAGCGGGGGACCGAAAGGCCTCGCGCTATTGGAGCGGCCGATGTCGGAT
TAGCTAGTTGGTGGGGTAAAGGCTTACCAAGGCGACGATCCGTAGCTGGTCTGAGAGGACGACCAGCCAC
ACTGGGACTGAGACACGGCC
>Zotu288
GAACGCTGGCGGCGTGCTTAACACATGCAAGTCGAACGGTAAGGCTCCAGCTTGCTGGGGTACACGAGTG
GCGAACGGGTGAGTAACACGTGGGTGACCTGCCCTGCACTTCGGGATAAGCCTGGGAAACTGGGTCTAAT
ACCGGATAGGACCATGGTGTGGATACTGTGGTGGAAAGTTTTTTCGGTGTGGGATGGGCCCGCGGCCTAT
CAGCTTGTTGGTGGGGTAATGGCCTACCAAGGCGGCGACGGGTAGCCGGCCTGAGAGGGTGGACGGCCA
CATTGGGACTGAGACACGGCC
>Zotu290
GAACGCTGGCGGCAGGCTTAACACATGCAAGTCGAACGCGTGTAGCAATACACGAGTGGCGCACGGGTG
AGTAACGCGTGGATATCTGCCTTTTGGTTCGGAATAACCCCGGGAAACTGGGGCTAATACCGGATGGTTCC
TACGGGATAAAGATTTATCGCCAAAAGATGAGTCCGCGTACGATTAGCTAGTTGGTGAGGTAATGGCTCAC
CAAGGCGACGATCGTTAGCTGGTCTGAGAGGACGATCAGCCACACTGGGACTGAGACACGGCC
>Zotu292
GAACGCTGGCGGTATGCTTAACACATGCAAGTCGAACGGTAGCAGGCCTTCGGGCGCTGACGAGTGGCGG
ACGGGTGAGTAACGCGTAGGAATCTGCCTCATAGTGGGGGACAACGTGGGGAAACTCACGCTAATACCGC
ATACGCCCTACGGGGGAAAGCGGGGGATCTTCGGACCTCGCGCTATGAGATGAGCCTGCGTTAGATTAGC
TAGTTGGTGGGGTAAAGGCCTACCAAGGCGACGATCTATAGCTGGTCTGAGAGGACGATCAGCCACACTG
GGACTGAGACACGGCC
>Zotu294
GAACGCTGGCGGCGTGCTTAACACATGCAAGTCGAACGATGAAGCGGTGCTTGCACCGTGGATTAGTGGC
GAACGGGTGAGTAATACGTGAGTGACCTACCTTTGACTCTGGGATAAGCCTGGGAAACTGGGTCTAATACC
GGATACGACCAATCTCTGCATGGGGTGTTGGTGGAAAGCGTTATGGAGTGGTTTTAGATGGGCTCACGGC
CTATCAGCTTGTTGGTGAGGTAATGGCTTACCAAGGCGACGACGGGTAGCCGGCCTGAGAGGGTGACCGG
CCACACTGGGACTGAGACACGGCC
>Zotu296
GAACGCTGGCGGCGTGCTTAACACATGCAAGTCGAACGCTGAAGCTCAGCTTTTGTTGGGTGGATGAGTG
GCGAACGGGTGAGTAACACGTGAGTAACCTGCCCTCTTCTTTGGGATAACGCCCGGAAACGGGTGCTAAT
ACTGGATATTCACTGGCCTTCGCATGGGGGTTGGTGGAAAGGTTTGTTCTGGTGGGGGATGGGCTCGCGG
CCTATCAGCTTGTTGGTGGGGTGATGGCCTACCAAGGCTTTGACGGGTAGCCGGCCTGAGAGGGTGACCG
GTCACATTGGGACTGAGATACGGCC
>Zotu298
GAACGCTAGCTACAGGCTTAACACATGCAAGTCGAGGGGAAACGGCATTTAGTGCTTGCACCGAATGGAC
GTCGACCGGCGCACGGGTGAGTAACGCGTATCCAACCTTCCCATAACTAAGGGATAACCTGCCGAAAGGC
AGACTAATACCTTATGTAATCCTTTGATGGCATCAGATAAGGATAAAAGATTTATCGGTTATGGATGGGGA

TGCGTCTGATTAGCTTGTTGGCGGGGTAACGGCCCACCAAGGCAACGATCAGTAGGGGTTCTGAGAGGAA
GGTCCCCCACATTGGAACTGAGACACGGTC
>Zotu300
GAACGCTGGCGGCAGGCCTAACACATGCAAGTCGAGCGGACCCTTCGGGGTTAGCGGCGGACGGGTGAG
TAACGCGTGGGAATATGCCCTTCTCTACGGAATAGTCCTGGGAAACTGGGGGTAATACCGTATACGCCCTT
TGGGGGAAAGATTTATCGGGGAAGGATTAGCCCGCGTTGGATTAGGTAGTTGGTGGGGTAATGGCCTACC
AAGCCGACGATCCATAGCTGGTTTGAGAGGATGATCAGCCACACTGGGACTGAGACACGGCC
>Zotu302
GAACGCTGGCGGAATGCTTTACACATGCAAGTCGAACGGCAGCACGAGAGCTTGCTCTTGGTGGCGAGTG
GCGAACGGGTGAGTAATATATCGGAACGTATCCAATAATGGGGGATAACTAATCGAAAGGTTGGCTAATA
CCGCATACGCCCTACGGGGGAAAGCAGGGGATCTTCGGACCTTGCGTTAATGGAGCGGCTGATATCTGAT
TAGCTAGTAGGTGAGGTAAAGGCTCACCTAGGCGACGATCAGTAGCTGGTCTGAGAGGACGACCAGCCAC
ACTGGAACTGAGACACGGTC
>Zotu304
GAACGCTGGCGGCGCGCCTAACACATGCAAGTCGAGCGAGGTTCAAAGCTTCGGTTTTGAACCTAGCGGC
GGACGGGTGAGTAACGCGTGAGCAACCTGGCCTTCAGAGGGGGATACCGTCTGGAAACGGACGTTAATA
CCGCATAACATGTCGGGAGGGCATCTTCCTGACATCAAAGGAGAAATTCACTGAAGGATGGGCTCGCGTC
CGATTAGGTAGTTGGTGAGGTAACGGCTCACCAAGCCCGCGATCGGTAGCCGGACTGAGAGGTTGGCCGG
CCACATTGGGACTGAGACACGGCC
>Zotu306
GAACGCTGGCGGCGTGCCTAATACATGCAAGTCGAACGCTTTTTCTTTCACCGGAGCTTGCTCCACCGAAA
GAAAAGGAGTGGCGAACGGGTGAGTAACACGTGGGTAACCTGCCCATCAGAAGGGGATAACACTTGGAA
ACAGGTGCTAATACCGTATAACAATAGAAACCGCATGGTTTCTATTTGAAAGGCGCTTTTGCGTCACTGATG
GATGGACCCGCGGTGCATTAGCTAGTTGGTGAGGTAACGGCTCACCAAGGCAACGATGCATAGCCGACCT
GAGAGGGTGATCGGCCACATTGGGACTGAGACACGGCC
>Zotu308
CAACGCTGGCGGCGTGCCTAACACATGCAAGTCGCACGAGAAAGGGGCTTCGGCCCTGAGTAAAGTGGCG
TACGGGTGAGTAACACGTGACTAACCTACCCTCGAGTGGGGAATAACCTAGAGAAATCTGGGCTAATACC
GCATAACACTCATGAGTCAAAGCAGCAATGCGCTTGAGGAGGGGGTCGCGGCAGATTAGCTAGTTGGTGA
GGTAATGGCTCACCAAGGCGATGATCTGTATCCGGCCTGAGAGGGCGCACGGACACACTGGAACTGAAAC
ACGGTC
>Zotu310
GAACGCTGGCGGCAGGCCTAACACATGCAAGTCGAGCGCGCCCTTCGGGGTGAGCGGCGGACGGGTGAG
TAACGCGTGGGAACGTGCCCTTCTCTGCGGAATAGCCTCGGGAAACTGAGAGTAATACCGCATACGCCCTT
TGGGGGAAAGATTTATCGGGGAAGGATCGGCCCGCGTTGGATTAGGTAGTTGGTGGGGTAATGGCCCAC
CAAGCCGACGATCCATAGCTGGTTTGAGAGGATGATCAGCCACACTGGGACTGAGACACGGCC
>Zotu312
GAACGCTGGCGGCGTGCCTAATACATGCAAGTCGAGCGAGCGGAACTAACAGATTTACTTCGGTAATGAC
GTTAGGAAAGCGAGCGGCGGATGGGTGAGTAACACGTGGGGAACCTGCCCCATAGTCTGGGATACCACTT
GGAAACAGGTGCTAATACCGGATAAGAAAGCAGATCGCATGATCAGCTTTTAAAAGGCGGCGTAAGCTGT
CGCTATGGGATGGCCCCGCGGTGCATTAGCTAGTTGGTAAGGTAAAGGCTTACCAAGGCGATGATGCATA
GCCGAGTTGAGAGACTGATCGGCCACATTGGGACTGAGACACGGCC
>Zotu314
GAACGCTGGCGGCGTGCCTAATACATGCAAGTCGAGCGAATGGATTAAGAGCTTGCTCTTATGAAGTTAGC
GGCGGACGGGTGAGTAACACGTGGGTAACCTGCCCATAAGACTGGGATAACTCCGGGAAACCGGGGCTA
ATACCGGATAACATTTTGAACCGCATGGTTCGAAATTGAAAGGCGGCTTCGGCTGTCACTTATGGATGGAC
CCGCGTCGCATTAGCTAGTTGGTGAGGTAACGGCTCACCAAGGCAACGATGCGTAGCCGACCTGAGAGGG
TGATCGGCCACACTGGGACTGAGACACGGCC
>Zotu316

GAACGCTGGCGGCGTGCTTAACACATGCAAGTCGAACGGAAAGGCCCTTCGGGGTACTCGAGTGGCGAAC
GGGTGAGTAACACGTGGGTGATCTGCCCTGCACTTCGGGATAAGCCTGGGAAACTGGGTCTAATACCGGA
TATGACCGCGCATCTCATGGTGTGTGGTGGAAAGCTTTTGCGGTGTGGGATGGGCCCGCGGCCTATCAGCT
TGTTGGTGGGGTAATGGCCTACCAAGGCGTCGACGGGTAGCCGGCCTGAGAGGGTGTCCGGCCACACTG
GGACTGAGATACGGCC

>Zotu318

GAACGCTAGCGGCAGGCTTAATACATGCAAGGCGAAGGGGCAGCAATGTCACTGTCGTACGGGTGCGTAA
CGCGTATGCAACCTACCTATCACTGGGGGATAGCCCGGGGAAACCCGGATTAATACCGCATAAAACAGGG
GCACCGCATGGTGATATTTGTTAAAGATTTATTGGTGATAGATGGGCATGCGTTCGATTAGCTAGTTGGCG
GGGTAACGGCCCACCAAGGCGACGATCGATAGGGGAGCTGAGAGGTTGATCCCCCACACGGGCACTGAG
ATACGGGC

>Zotu320

GAACGCTGGCGGCGTGCTTAACACATGCAAGTCGAACGCTGAAGCCCAGCTTGCTGGGTGGATGAGTGGC
GAACGGGTGAGTAACACGTGAGTAACCTGCCCCCTTCTTTGGGATAACGCCCGGAAACGGGTGCTAATACT
GGATATTCACTGGCCTTCGCATGGGGGTTGGTGGAAAGGTTTTTTCTGGTGGGGGATGGGCTCGCGGCCT
ATCAGCTTGTTGGTGGGGTGATGGCCTACCAAGGCTTTGACGGGTAGCCGGCCTGAGAGGGTGACCGGTC
ACATTGGGACTGAGATACGGCC

>Zotu322

GAACGCTGGCGGCAGGCTTAACACATGCAAGTCGAGCGGGCCCTTCGGGGTCAGCGGCGGACGGGTGAG
TAACGCGTGGGAACGTGCCTTCTGGTCTGGAATAACCCTGGGAAACTAGGGCTAATACCGGATACGCCCTT
TTGGGGAAAGGTTTACTGCCGGAAGATCGGCCCGCGTCTGATTAGCTAGTTGGTGGGGTAACGGCCTACC
AAGGCGACGATCAGTAGCTGGTCTGAGAGGATGATCAGCCACACTGGGACTGAGACACGGCC

>Zotu325

GAACGCTAGCGGCAGGCTTAATACATGCAAGTCGAGGGGCAGCACGAATAGCAATATTTGGTGGCGACCG
GCGCACGGGTGCGTAACACGTATACAATCTACCTTCAACAGGGGGATAGCCTTTCGAAAGAGAGATTAATA
CCCCGTAATATATGAGATGGCATCATTTTATATTGAAAGTTCCGACCGTTGAAGATGAGTATGCGTCTGATT
AGCTAGTAGGTGAGGTAACGGCTCACCTAGGCTACGATCAGTAGGGGATCTGAGAGGACTAACCCCCACA
CTGGTACTGAGACACGGAC

>Zotu327

GAACGCTGGCGGCAGGCCTAACACATGCAAGTCGAACGAACCTTTCGGGGTTAGTGGCGGACGGGTGAG
TAACACGTGGGAACGTGCCTTTAGGTTCGGAATAACTCAGGGAAACTTGTGCTAATACCGAATGTGCCCTT
CGGGGGAAAGATTCATCGCCTTTAGAGCGGCCCGCGTCTGATTAGCTAGTTGGTTGAGGTAATGGCTCACC
AAGGCGACGATCAGTAGCTGGTCTGAGAGGATGATCAGCCACATTGGGACTGAGACACGGCC

>Zotu329

GAACGCTGGCGGCGTGCTTAACACATGCAAGTCGAACGATGAAGGAGGAGCTTGCTCCTCCGGATTAGTG
GCGAACGGGTGAGTAATACGTGAGCAATGTGCCCTTGACTCTGGGATAAGCGCTGGAAACGGCGTCTAAT
ACCGGATACGACGATCGGAGGCATCTCCTGGTCGTGGAAAGAACTTCGGTCAAGGATCAGCTCACGGCCT
ATCAGCTAGTTGGTGAGGTAACGGCTCACCAAGGCGACGACGGGTAGCCGGCCTGAGAGGGTGACCGGC
CACACTGGGACTGAGACACGGCC

>Zotu331

GAACGCTGGCGGCAGGCCTAACACATGCAAGTCGAACGAACCTTTCGGGGTTAGTGGCGGACGGGTGAG
TAACACGTGGGAACGTGCCTTTAGGTTCGGAATAACTCAGGGAAACTTGTGCTAATACCGAATGTGCCCTT
CGGGGGAAAGATTTATCGCCTTTAGAGCGGCCCGCGTCTGATTAGCTAGTTGGTTGAGGTAATGGCTCACC
AAGGCGACGATCAGTAGCTGGTCTGAGAGGATGATCAGCCACATTGGGACTGAGACACGGCC

>Zotu333

GAACGCTGGCGGCATGCCTAACACATGCAAGTCGAACGAAGCCTTCGGGCTTAGTGGCGCACGGGTGCGT
AACGCGTGGGAATCTGCCTCTTGCTTCGGAATAACAGTTAGAAATGACTGCTAATACCGGATGATGTCTTC
GGACCAAAGATTTATCGGCAAGAGATGAGCCCGCGTAGGATTAGGTAGTTGGTGGGGTAAAGGCCTACCA
AGCCGACGATCCTTAGCTGGTCTGAGAGGATGATCAGCCACACTGGGACTGAGACACGGCC

>Zotu335

GAACGCTAGCTACAGGCTTAACACATGCAAGTCGAGGGGCAGCATTATCGAAGCTTGCTTTGATAGATGGC
GACCGGCGCACGGGTGAGTAACGCGTATCCAACCTTCCCATCACTCAGGGATAGCCCGCCGAAAGGCGAA
TTAATACCTGATGGTCTCCTTTGCAGACATCTAACGAGGAGTAAAGATTTATTGGTGATGGATGGGGATGC
GTCTGATTAGGTAGTAGGCGGGGTAACGGCCCACCTAGCCGACGATCAGTAGGGGTTCTGAGAGGAAGG
TCCCCCACATTGGAACTGAGACACGGTC

>Zotu337
GAACGCTGGCGGCGTGCCTTAAGCATGCAAGTCGTATGATCGCAAGATAATGGCGGACGGGTGAGTAACG
CGTGAGAATCTGCCTTAAAGTGGGGCATAGCCCTCCGAAAGGAGGGGTAATACCGCATGTGATAGCAATA
TCAAAGCAGCAATGCGCTTTAAGATGAGCTCGCGTAAGATTAGCTAGTTGGTGGGGTAAAGGCCTACCAA
GGCGACGATCTTTAGCTGGTCTGAGAGGATGGTCAGCCACAATGGGACTGAGACACGGCC

>Zotu339
GAACGCTAGCGGGAGGCCTAACACATGCAAGCCGAGCGGTAGAGTTTCTTCGGAAACTTGAGAGCGGCGC
ACGGGTGCGTAACACGTGTGCAACCTACCTTTATCTGGGGGATAGCCTTTCGAAAGGAAGATTAATACCCC
ATAATATAATGATTGGCATCAATTATTATTGAAAACTCCGGTGGATAGAGATGGGCACGCGCAAGATTAGA
TAGTTGGTGAGGTAACGGCTCACCAAGTCAATGATCTTTAGGGGTCCTGAGAGGGAGATCCCCCACACTG
GTACTGAGACACGGAC

>Zotu342
GAACGCTGGCGGCAGGCTTAACACATGCAAGTCGAGCGGGCATAGCAATATGTCAGCGGCAGACGGGTG
AGTAACGCGTGGGAACGTACCTTTTGGTTCGGAACAACACAGGGAAACTTGTGCTAATACCGGATAAGCCC
TTACGGGGAAAGATTTATCGCCGAAAGATCGGCCCGCGTCTGATTAGCTAGTTGGTGAGGTAATGGCTCAC
CAAGGCGACGATCAGTAGCTGGTCTGAGAGGATGATCAGCCACATTGGGACTGAGACACGGCC

>Zotu345
GAACGCTGGCGGCAGGCCTAACACATGCAAGTCGAGCGGTAGAGAGAAGCTTGCTTCTCTTGAGAGCGGC
GGACGGGTGAGTAATACCTAGGAATCTGCCTGATAGTGGGGGATAACGTTCGGAAACGGACGCTAATACC
GCATACGTCCTACGGGAGAAAGCAGGGGACCTTCGGGCCTTGCGCTATCAGATGAGCCTAGGTCGGATTA
GCTAGTTGGTGAGGTAATGGCTCACCAAGGCTACGATCCGTAACTGGTCTGAGAGGATGATCAGTCACACT
GGAACTGAGACACGGTC

>Zotu348
GAACGCTGGCGGCGTGCCTAATACATGCAAGTCGAGCGAACAGATAAGGAGCTTGCTCCTTTGACGTTAG
CGGCGGACGGGTGAGTAACACGTGGGTAACCTACCTATAAGACTGGAATAACTCCGGGAAACCGGGGCTA
ATGCCGGATAACATGTTGAACCGCATGGTTCTACAGTGAAAGGCGGCTTTGCTGTCACTTATAGATGGACC
CGCGCCGTATTAGCTAGTTGGTGAGGTGACGGCTCACCAAGGCAACGATACGTAGCCGACCTGAGAGGGT
GATCGGCCACACTGGAACTGAGACACGGTC

>Zotu350
GAACGCTGGCGGCGTGCCTAATACATGCAAGTCGAACGAACGGATAAAGAGCTTGCTCTTTTGAAGTTAGT
GGCGGACGGGTGAGTAACACGTGGGTAACCTGCCTCACAGCTGGGGATAACATCGAGAAATCGATGCTAA
TACCGAATGTGCTGAACATCATAAGATGTTCAAGTGAAAGACGGTTTCGGCTGTCACTGTGAGATGGACCC
GCGCTGGATTAGCTAGTTGGTAAGGTAATGGCTTACCAAGGCGACGATCCATAGCCGACCTGAGAGGGTG
ATCGGCCACATTGGGACTGAGACACGGCC

>Zotu352
GAACGCTGGCGGCGTGCTTAACACATGCAAGTCGAACGATGAAGCCAGCTTGCTGGTGGATTAGTGGCGA
ACGGGTGAGTAACACGTGGGCAACCTGCCCTCAGCTCTGGGATAACTCCAAGAAATTGGTGCTAATACCG
GATATTCTTCTTTCACCGCATGGTGGTTGGAGGAAAGTTTTTCGGCTGGGGATGGGCCCGCGGCCTATCAG
CTTGTTGGTGGGGTGATGGCCTACCAAGGCGACGACGGGTAGCCGGCCTGAGAGGGTGACCGGCCACAC
TGGGACTGAGACACGGCC

>Zotu354
GAACGCTGGCGGCGTGCCTAATACATGCAAGTCGAGCGAACAGAGAAGGAGCTTGCTCCTTTGACGTTAG
CGGCGGACGGGTGAGTAACACGTGGGCAACCTACCTTATAGTTTGGGATAACTCCGGGAAACCGGGGCTA
ATACCGAATAATCTGTTTCACCTCATGGTGAAATATTGAAAGACGGTTTCGGCTGTCGCTATAGGATGGGC
CCGCGGCGCATTAGCTAGTTGGTGAGGTAACGGCTCACCAAGGCGACGATGCGTAGCCGACCTGAGAGG
GTGATCGGCCACACTGGGACTGAGACACGGCC

>Zotu356
GAACGCTAGCGGCAGGCCTAATACATGCAAGTCGAGGGGTAGGGTAGCAATACTTGAGACCGGCGCACG
GGTGCGTAACGCGTATGGAACTTGTCCGATCCTCTGGGATAGCCCCCGGAAACGGGGATTAATACCGGAT
GGCCCGTGGTTGCGGCATCGCAGCCACGGTAAAGCTCAGGCGGATCGGGTGGCCATGCGTTCGATTAGC
TAGTTGGCGGGGTAACGGCCCACCAAGGCGACGATCGATAGGGGTCCTGAGAGGGAGATCCCCCACACT
GGTACTGAGACACGGGC
>Zotu358
GAACGCTAGCGGGAGGCCTAACACATGCAAGCCGAGCGGTATTGTTTCTTCGGAAATGAGAGAGCGGCGT
ACGGGTGCGGAACACGTGTGCAACCTGCCTTTATCTGGGGGATAGCCTTTCGAAAGGAAGATTAATACTCC
ATAACATATTGAACGGCATCGTTTAATATTGAAAGCTCCGGCGGATAGAGATGGGCACGCGCAAGATTAG
CTAGTTGGTGAGGTAACGGCTCACCAAGGCGATGATCTTTAGGGGGCCTGAGAGGGTGATCCCCCACACT
GGTACTGAGACACGGAC
>Zotu360
GAACGCTGGCGGCATGCTTTACACATGCAAGTCGAACGGCAGCGCGGGGCAACCTGGCGGCGAGTGGCG
AACGGGTGAGTAATATATCGGAACGTACCCAAGAGTGGGGGATAACGTAGCGAAAGTTACGCTAATACCG
CATACGATCTAAGGATGAAAGCGGGGGATCGCAAGACCTCGTGCTCCTGGAGCGGCCGATATCTGATTAG
CTAGTTGGTGAGGTAAAGGCTCACCAAGGCGACGATCAGTAGCTGGTCTGAGAGGACGACCAGCCACACT
GGAACTGAGACACGGTC
>Zotu362
GAACGCTGGCGGCATGCCTTACACATGCAAGTCGAACGGTAACAGGTCTTCGGATGCTGACGAGTGGCGA
ACGGGTGAGTAATACATCGGAACGTGCCTAGTAGTGGGGGATAACTACTCGAAAGAGTGGCTAATACCGC
ATGAGATCTACGGATGAAAGCAGGGGATCGCAAGACCTTGTGCTACTAGAGCGGCCGATGGCAGATTAGG
TAGTTGGTGGGATAAAAGCTTACCAAGCCGACGATCTGTAGCTGGTCTGAGAGGACGATCAGCCACACTG
GGACTGAGACACGGCC
>Zotu364
GAACGCTGGCGGCGTGCCTAATACATGCAAGTCGAGCGAGCGGAACCAACAGATTTACTTCGGTAATGAC
GTTGGGAAAGCGAGCGGCGGATGGGTGAGTAACACGTGGGGAACCTGCCCCTAAGTCTGGGATACCATTT
GGAAACAGGTGCTAATACCGGATAATAAAGCAGATCGCATGATCAGCTTTTGAAAGGCGGCGTAAGCTGT
CGCTAAGGGATGGCCCCGCGGTGCATTAGCTAGTTGGTAAGGTAACGGCTTACCAAGGCGACGATGCATA
GCCGAGTTGAGAGACTGATCGGCCACATTGGGACTGAGACACGGCC
>Zotu366
GAACGCTGGCGGCGTGCTTAACACATGCAAGTCGAACGATGATCCGGTGCTTGCACCGGGGATTAGTGGC
GAACGGGTGAGTAACACGTGAGTAACCTGCCCTTAACTCTGGGATAAGCCTGGGAAACTGGGTCTAATAC
CGGATATGACTGATCATCGCATGGTGGTTGGTGGAAAGCTTTATTGTGGTTTTGGATGGACTCGCGGCCTA
TCAGCTTGTTGGTGAGGTAATGGCTTACCAAGGCGACGACGGGTAGCCGGCCTGAGAGGGTGACCGGCCA
CACTGGGACTGAGACACGGCC
>Zotu368
CAACGCTGGCGGCGTGCCTAATGCATGCAAGTCGAGCGGGGTCGTTCTTCGGAGGGGCTTACGCGGCGGA
CGGGTGCGTAACACGTGGGGAACCTGCCCTGGGGTGGGGGATAGCCGAGGGAAACCTTGGGTAATTCCG
CATACGCTTGCGTACCGGAAGGGTACGTGAGGAAAGGGTCTTTCGGGACTCGCCTTGGGAGGGGCCTGCG
GCCGATTAGCTAGACGGTGGGGTAACGGCTCACCGTGGCGATGATCGGTAGCTGGTCTGAGAGGACGGC
CAGCCACACGGGGACTGAGAGACGGCC
>Zotu370
TCGACAGCATCGCTTGCTGCTTATAAAGTGAACAGCTCTTGGGAAAGCCTTTGGTTTTGCAGGAACCCCAC
GAAATCAACGCTTGCCATAGAGGCACGCACTTTAATGATGTGGGCGCGGACGGCACACAGCAGCACATCG
TGC
>Zotu372
GAATGCTGGCGGCGCGCCTAACACATGCAAGTCGAGCGGTAACAGGTCTAGTTTACTAGATGCTGACGAG
CGGCGGACGGCTGAGTAACGCGTGGGAACATGCCCCAAAGTGAGGAATAACTGCCCGAAAGGGTAGCTA
ATGCCGCATATGATCTTAGGATTAAAGGATTTATCCGCTTTGGGAGTGGCCTGCGTACGATTAGGTAGTTG

GTGAGGTAATGGCTCACCAAGCCTACGATCGTTAACTGGTCTGAGAGGATGATCAGTCAGACTGGAACTG
AGACACGGTC
>Zotu375
GAACGCTGGCGGCAGGCCTAACACATGCAAGTCGAGCGGTAGAGAGAAGCTTGCTTCTCTTGAGAGCGGC
GGACGGGTGAGTAATGCCTAGGAATCTGCCTGGTAGTGGGGGATAACGTTCGGAAACGGACGCTAATACC
GCATACGTCCTACGGGAGAAAGCAGGGGACCTTCGGGCCTTGCGCTATCAGATGAGCCTAGGTCGGATTA
GCTAGTTGGTGAGGTAATGGCTCACCAAGGCGACGATCCGTAACTGGTCTGAGAGGATGATCAGTCACAC
TGGAACTGAGACACGGTC
>Zotu377
GAACGCTGGCGGCGTGCTTAACACATGCAAGTCGAACGAGACCCTTCCTTCGGGGAGGTGTTCTAGTGGC
GAACGGGTGAGTAACACGTGGGCAACCTGCCCCTGGCTCTGGGATAACTCCAAGAAATTGGGGCTAATAC
CGGATATGACCGCTGACCGCATGGTCTGGTGGTGGAAAGATTTATCGGCTGGGGATGGGCCCGCGGCCTA
TCAGCTTGTTGGTGGGGTAGTGGCCTACCAAGGCGACGACGGGTAGCCGGCCTGAGAGGGTGACCGGCC
ACACTGGGACTGAGACACGGCC
>Zotu380
GAACGCTGGCGGCATGCCTTACACATGCAAGTCGAACGGTAACAGGTCTTCGGATGCTGACGAGTGGCGA
ACGGGTGAGTAATACATCGGAACGTGCCCGATCGTGGGGGATAACGAGGCGAAAGCTTTGCTAATACCGC
ATACGATCTACGGATGAAAGCGGGGGATCTTCGGACCTCGCGCGGACGGAGCGGCCGATGGCAGATTAG
GTAGTTGGTGGGATAAAAGCTTACCAAGCCGACGATCTGTAGCTGGTCTGAGAGGATGATCAGCCACACT
GGGACTGAGACACGGCC
>Zotu382
GAACGCTGGCGGCAGGCTTAACACATGCAAGTCGAACGCGTGTAGCAATACACGAGTGGCGCACGGGTG
AGTAACGCGTGGATATCTGCCTTTTGGTTCGGAATAACCCCGGGAAACTGGGGCTAATACCGGATGGTTCC
TACGGGATAAAGATTTATCGCCAAAAGATGAGTCCGCGTACGATTAGCTAGTTGGTGAGGTAAAGGCTCA
CCAAGGCGACGATCGTTAGCTGGTCTGAGAGGACGATCAGCCACACTGGGACTGAGACACGGCC
>Zotu384
GAACGCTGGCGGCGTGCCTAATACATGCAAGTAGAACGCTGAAGGAGGAGCTTGCTTCTCCGGATGAGTT
GCGAACGGGTGAGTAACGCGTAGGTAACCTGCCTGGTAGCGGGGGATAACTATTGGAAACGATAGCTAAT
ACCGCATAATAGTAGATGTTGCATGACATTTGCTTAAAAGGTGCAATTGCATCACTACCAGATGGACCTGC
GTTGTATTAGCTAGTTGGTGGGGTAACGGCTCACCAAGGCGACGATACATAGCCGACCTGAGAGGGTGAT
CGGCCACACTGGGACTGAGACACGGCC
>Zotu386
GAACGCTGGCGGCATGCCTAATACATGCAAGTCGAGCGAACAGATGAAGTGCTTGCACTTCTGACGTTAGC
GGCGAACGGGTGAGTAACACGTAAGGAATCTACCTATAAGCGGGGGATAACATTCGGAAACGGGTGCTA
ATACCGCATAATATCTTCTTCCGCATGGAAGAAGATTGAAAGACGGCTCTGCTGTCACTTATGGATGACCTT
GCGGTGCATTAGTTAGTTGGTGGGGTAATGGCCTACCAAGACGATGATGCATAGCCGACCTGAGAGGGTG
ATCGGCCACATTGGGACTGAGACACGGCC
>Zotu388
GAACGCTGGCGGCGTGCCTAATACATGCAAGTCGAGCGAGGAGCTTTTAGCTCCTAGCGGCGAACGGGTG
AGTAACACGTGGGCAACCTGCCCGTAAGACTGGGATAACTTCGGGAAACCGGAGCTAATACTGGATAATC
TTGATCTTCGCATGAAGGACAAGTAAAAGATTTATCACTTACGGATGGGCCCGCGGTGCATTAGCTTGTTG
GTGGGGTAATGGCCTACCAAGGCGACGATGCATAGCCGACCTGAGAGGGTGACCGGCCACACTGGGACT
GAGACACGGCC
>Zotu390
GAACGCTGGCGGCGTGCCTAATACATGCAAGTCGAGCGAAGTTTTTCTGGTGCTTGCACCGGAAAAACTTA
GCGGCGAACGGGTGAGTAACACGTAAAGAACCTGCCTCATAGACTGGGACAACTATTGGAAACGATAGCT
AATACCGGATAACAGCATTAACTGCATGGTTGATGTTTAAAAGTTGGTTTTGCTAACACTATGAAATGGCTT
TGCGGTGCATTAGCTAGTTGGTGGGGTAAAGGCCTACCAAGGCGACGATGCATAGCCGACCTGAGAGGGT
GATCGGCCACACTGGGACTGAGACACGGCC
>Zotu392

GAACGCTGGCGGCGTGCCTAATACATGCAAGTCGAGCGAACTGACGAGGAGCTTGCTCCTTTGACGTTAG
CGGCGGACGGGTGAGTAACACGTGGGTAACCTACCTATAAGACTGGAATAACTCCGGGAAACCGGGGCTA
ATGCCGGATAACATATCGAACCGCATGGTTCGATAGTGAAAGACGGTCTTGCTGTCACTTATAGATGGACC
CGCGCCGTATTAGCTAGTTGGTGAGGTGACGGCTCACCAAGGCAACGATACGTAGCCGACCTGAGAGGGT
GATCGGCCACACTGGAACTGAGACACGGTC
>Zotu394
GAACGCTGGCGGTGTGCTTCACACATGCAAGTCGAACGAGGTAGCAATACCTAGTGGCGGACGGGTGAGT
AACGCGTGGGAATCTGCCTTTAGATGGGGGATAACGGTCCGAAAGGATCGCTAATACCGCATATGCCGAG
AGGTGAAAGGAGTAATCCGTCTAAAGATGAGCCCGCGTCCGATTAGCTAGTTGGTGGGGTAAAGGCCTAC
CAAGGCGACGATCGGTAGCTGGTCTGAGAGGATGATCAGCCACAATGGGACTGAGACACGGCC
>Zotu396
GAACGCTGGCGGCATGCCTTACACATGCAAGTCGAACGGCAGCACGGGTGCTTGCACCTGGTGGCGAGTG
GCGAACGGGTGAGTAATACATCGGAATGTACCTTGTAGTGGGGGATAGCTCGGCGAAAGCCGGATTAATA
CCGCATACGCTCTGAGGAGGAAAGCGGGGGACCTTCGGGCCTCGTGCTACAAGAGCAGCCGATGTCAGAT
TAGCTTGTTGGTGAGGTAAAAGCTCACCAAGGCGACGATCTGTAGCTGGTCTGAGAGGACGACCAGCCAC
ACTGGGACTGAGACACGGCC
>Zotu398
GAACGCTAGCGGCAGGCCTAATACATGCAAGTCGAACGCGATTATCCAGCTTGCTGGATATGAAAGTGGC
GCACGGGTGCGTAACGCGTGAGCAACCTACCTCTGTCAGGGGGATAGCCCGGCGAAAGTCGGATTAACAC
CGCATGACATTAAGATGTGGCATCACATTATAATCAAATATTTATAGGACAGAGATGGGCTCGCGTGACAT
TAGCTAGTTGGAGAGGTAACGGCTCACCAAGGCAACGATGTCTAGGGGCTCTGAGAGGAGAATCCCCCAC
ACTGGTACTGAGACACGGAC
>Zotu400
GAACGCTGATAGAATGCTTAACACATGCAAGTCTTTGGCGAAGCTGTGCTTGCATAGCCTAGCCAAGGCGG
ACGGGTGAGTAACGCGTAAGGAACTTGCCCTGCAGTCAGGGATAACAGACGGAAACGACTGATAAGACCT
GGTAAAGTCAGGCGGACTCATGTCCAGCCTGATGAAAAGAGATGCTGCAGGAGAGCCTTGCGTCCTATTA
GCTTGTTGGTGGGGTAACGGCCCACCAAGGCGATGATAGGTAGCCGGCCTGAGAGGGTGGACGGCCACA
AGGGGACTGAGATACGGCC
>Zotu402
GAACGCTGGCGGCAGGCTTAACACATGCAAGTCGAGCGGAGATGAGGTGCTTGCACCTTATCTTAGCGGC
GGACGGGTGAGTAATGCTTAGGAATCTGCCTATTAGTGGGGGACAACATTCCGAAAGGAATGCTAATACC
GCATACGTCCTACGGGAGAAAGCAGGGGATCTTCGGACCTTGCGCTAATAGATGAGCCTAAGTCGGATTA
GCTAGTTGGTGGGGTAAAGGCCTACCAAGGCGACGATCTGTAGCGGGTCTGAGAGGATGATCCGCCACAC
TGGGACTGAGACACGGCC
>Zotu404
GAACGCTAGCGGCAGGCTTAACACATGCAAGTCGAGGGGTATAGGTTTTCGGATCTAGAGACCGGCGCAC
GGGTGCGTAACGCGTATGCAATCTACCTTTTGCAGAGGGATAGCCCAGAGAAATTTGGATTAATACCTCAT
AGTATATAGAGTTGGCATCAACATTATATTAAAGTCACAACGGCAAAAGATGAGCATGCGTCCCATTAGCT
TGTTGGTAAGGTAACGGCTTACCAAGGCTACGATGGGTAGGGGTCCTGAGAGGGAGATCCCCCACACTGG
TACTGAGACACGGAC
>Zotu406
GAACGCTGGCGGCGTGCCTAATACATGCAAGTCGAGCGAACAGATAAGGAGCTTGCTCCTTTGACGTTAG
CGGCGGACGGGTGAGTAACACGTGGGTAACCTACCTATAAGACTGGGATAACTTCGGGAAACCGGAGCTA
ATACCGGATAACATTTGGAACCGCATGGTTCTAAAGTGAAAGATGGTTTTGCTATCACTTATAGATGGACCC
GCGCCGTATTAGCTAGTTGGTAAGGTAACGGCTTACCAAGGCGACGATACGTAGCCGACCTGAGAGGGTG
ATCGGCCACACTGGAACTGAGACACGGTC
>Zotu408
GAACGCTGGCGGCGTGCCTAATACATGCAAGTCGAGCGAAGTTTTTCTGGTGCTTGCACCGGAAAAACTTA
GCGGCGAACGGGTGAGTAACACGTAAAGAACCTGCCTCATAGACTGGGACAACTATTGGAAACGATAGCT
AATACCGGATAACAGCATTAACTGCATGGTTGATGTTTAAAAGTTGGTTTTGCTAACACTATGAGATGGCTT

TGCGGTGCATTAGCTAGTTGGTGGGGTAAAGGCCTACCAAGGCGACGATGCATAGCCGACCTGAGAGGGT
GATCGGCCACACTGGGACTGAGACACGGCC

>Zotu410
GAACGCTAGCTACAGGCTTAACACATGCAAGTCGAGGGGAAACGGCATTAAGTGCTTGCACTTTTTGGACG
TCGACCGGCGCACGGGTGAGTAACGCGTATCCAACCTTCCCATGACTAAGGGATAACCTGCCGAAAGGCA
GACTAATACCTTATGGTCTTCACTGACGGCATCAGATGTGAAGTAAAGATTTATCGGTTATGGATGGGGAT
GCGTCTGATTAGCTTGTTGGCGGGGTAACGGCCCACCAAGGCAACGATCAGTAGGGGTTCTGAGAGGAAG
GTCCCCCACATTGGAACTGAGACACGGTC

>Zotu412
GAACGCTGGCGGCATGCTTAACACATGCAAGTCGAACGGGAAGTGGTGTTTCCAGTGGCGAACGGGTGA
GTAACGCGTAAGAACCTGCCCTTGGGAGGGGAACAACAACTGGAAACGGTTGCTAATACCCCGTAGGCTG
AGGAGCAAAAGGAGAAATCCGCCCAAGGAGGGGCTCGCGTCTGATTAGCTAGTTGGTGAGGCAATAGCTT
ACCAAGGCGATGATCAGTAGCTGGTCCGAGAGGATGATCAGCCACACTGGGACTGAGACACGGCC

>Zotu414
GAACGCTGGCGGCGTGCCTAATACATGCAAGTCGAGCGAACAGATGAGGAGCTTGCTCCTTTGACGTTAG
CGGCGGACGGGTGAGTAACACGTGGGTAACCTACCTATAAGACTGGAATAACTCCGGGAAACCGGGGCTA
ATGCCGGATAATACACAGAACCGCATGGTTCTGTGATGAAAGACGGTTTTGCTGTCACTTATAGATGGACC
CGCGGCGTATTAGCTAGTTGGTAAGGTAACGGCTTACCAAGGCAACGATACGTAGCCGACCTGAGAGGGT
GATCGGCCACACTGGAACTGAGACACGGTC

>Zotu416
GAACGCTGGCGGCGTGCCTAATACATGCAAGTCGAGCGAACAGATAAGGAGCTTGCTCCTTTGAAGTTAG
CGGCGGACGGGTGAGTAACACGTGGGTAACCTACCTATAAGACTGGGATAACTTCGGGAAACCGGAGCTA
ATACCGGATAACATTTAGAACCGCATGGTTCTAAAGTGAAAGATGGTTTTGCTATCACTTATAGATGGACCC
GCGCCGTATTAGCTAGTTGGTAAGGTAATGGCTTACCAAGGCGACGATACGTAGCCGACCTGAGAGGGTG
ATCGGCCACACTGGAACTGAGACACGGTC

>Zotu418
GAACGCTGGCGGCGTGCTTAACACATGCAAGTCGAGCGACAGTGTTCTTCGGAACACATGGAGCGGCGAA
CGGGTGAGTAACACGTGGGCAACCTGCCCTCAGCTCTGGGATAACTCCGGGAAACCGGGGCTAATACCGG
ATACGACCTTCCCTCGCATGGGGGTTGGTGGAAAGTTCTTCGGCTGAGGATGGGCCCGCGGCCTATCAGCT
TGTTGGTGGGGTGATGGCCTACCAAGGCGACGACGGGTAGCCGGCCTGAGAGGGCGACCGGCCACACTG
GGACTGAGACACGGCC

>Zotu420
GAACGCTAGCGGCAGGCTTAACACATGCAAGTCGAGGGGCAGCAGGGACTTCGGTCCGCTGGCGACCGG
CAAACGGGTGCGGAACACGTACGCAACATACCTTAAACTGGGGCATAGCCTTCTGAAAAGGAGATTAACA
CCCCATAGTATCTTTAGGTGGCATCACCTGTTGATTAAAAATTCATTGGTTTAAGATTGGCGTGCGTCCTATT
AGCTAGTTGGCGGGGTAAAGGCCCACCAAGGCGACGATAGGTAGCTGATGTGAGAGCATGATCAGCCAC
ACGGGCACTGAGACACGGGC

>Zotu422
GAACGCTGGCGGCGTGCTTAACACATGCAAGTCGAACGATGAAGCCTAGCTTGCTAGGTGGATTAGTGGC
GAACGGGTGAGTAATACGTGAGTAACCTACCTTTAACTCTGGGATAAGCCTGGGAAACTGGGTCTAATACC
GGATACGACCAATCTCCGCATGGGGTGTTGGTGGAAAGCGTTATGTAGTGGTTATAGATGGGCTCACGGC
CTATCAGCTTGTTGGTGAGGTAACGGCTCACCAAGGCGACGACGGGTAGCCGGCCTGAGAGGGTGACCG
GCCACACTGGGACTGAGACACGGCC

>Zotu424
GAACGCTGGCGGCGTGCTTAACACATGCAAGTCGAACGATGATGCCCAGCTTGCTGGGCGGATTAGTGGC
GAACGGGTGAGTAATACGTGAGTAACCTGCCCTTGACTCTGGGATAAGCCTGGGAAACTGGGTCTAATACT
GGATACTACCTCTTGCCGCATGGTGGGTGGTGGAAAGGGTTTTACTGGTTTTGGATGGGCTCACGGCCTAT
CAGCTTGTTGGTGGGGTAATGGCTCACCAAGGCGACGACGGGTAGCCGGCCTGAGAGGGTGACCGGCCA
CACTGGGACTGAGACACGGCC

>Zotu426

GAACGTTGGCGGCGTGCTTAACACATGCAAGTCGAGCGAGAAAGTTCCTTCGGGAATGAGTACAGCGGCG
AACGGGTGAGTAACACGTGAGAATCTACCCTTTAGTGAAGAATACCTTCGAGAAATCGAAGTTAATGCTGC
ATAAGTCCCTTCGGGGAGAAAGATGGCCTCTTTGCTGTCGCTAAAGGATGAGCTTGCGTACTATTAGCTAG
TTGGTGGGGTAAAGGCCTACCAAGGCGATGATGGTTAGCCGGCCTGAGAGGGTGTACGGCCACACTGGA
ACTTAGACACGGTC
>Zotu428
GAACGCTGGCGGTGTGCTTCACACATGCAAGTCGAACGGGGTAGCAATACCCAGTGGCGGACGGGTGAG
TAACGCGTAGAAATCTGCCTTGAGATGGGGGATAACAGTCCGAAAGGACTGCTAATACCGCATATGTATGT
ATCTCGACAGAGGTATATATGAAAGGAGTAATCCGTCTTAAGATGAGTCTGCGTCCGATTAGCTAGTTGGT
AGGGTAAAGGCCTACCAAGGCGACGATCGGTAGCTGGTCTGAGAGGATGATCAGCCACAATGGGACTGA
GACACGGCC
>Zotu430
GAACGCTGGCGGCGTGCTTAACACATGCAAGTCGAACGGAAAGGCCCTTTCGGGGGTACTCGAGTGGCGA
ACGGGTGAGTAACACGTGGGTGATCTGCCCTGCACTTCGGGATAAGCTTGGGAAACTGGGTCTAATACCG
GATAGGACTACGCACTGCATGGTGTGTGGTGGAAAGCTTTTGCGGTGTGGGATGGGCCCGCGGCCTATCA
GCTTGTTGGTGGGGTGATGGCCTACCAAGGCGACGACGGGTAGCCGGCCTGAGAGGGTGTCCGGCCACA
CTGGGACTGAGATACGGCC
>Zotu432
GAACGCTGGCGGCAGGCTTAACACATGCAAGTCGAACGCCCCGCAAGGGGAGTGGCAGACGGGTGAGTA
ACACGTGGGAACGTACCTTTTGGTTCGGAATAACTCAGGGAAACTTGAGCTAATACCGGATAAGCCCGCAA
GGGGAAAGATTTATTGCCGAAAGATCGGCCCGCGTCTGATTAGCTAGTTGGTGAGGTAACGGCTCACCAA
GGCGACGATCAGTAGCTGGTCTGAGAGGATGACCAGCCACACTGGGACTGAGACACGGCC
>Zotu434
GAACGCTGGCGGCAGGCTTAACACATGCAAGTCGAACGGTCCTTCGGGATAGTGGCGGACGGGTGAGTA
ACACGTGGGAACGTACCCTTAGGTTCGGAACAACACAGGGAAACTTGTGCTAATACCGGATGAGCCCTTCG
GGGGAAAGATTTATCGCCTTTGGAGCGGCCCGCGTCTGATTAGCTAGTTGGTGAGGTAATGGCTCACCAA
GGCGACGATCAGTAGCTGGTCTGAGAGGATGGCCAGCCACACTGGGACTGAGACACGGCC
>Zotu437
GAACGCTAGCTACAGGCTTAACACATGCAAGTCGAGGGGCATCATGAGGTCAGCTTGCTGACTTTGATGGC
GACCGGCGCACGGGTGCGTAACGCGTATCCAATCTACCGCATACTCGGGGATAGCCTTGCGAAAGTAAGA
TTAATACCCGATGTTGTTTGTTATTCGCATGAATGATAAACCAAAGAATTTCGGTATGCGATGAGGATGCGT
CCCATTAGGTAGTTGGTGAGGTAACGGCTCACCAAGCCTCCGATGGGTAGGGGTTCTGAGAGGAAGATCC
CCCACACTGGAACTGAGACACGGTC
>Zotu439
GAACGCTGGCGGCATGCTTTACACATGCAAGTCGAACGGCATCGGGGAGGAGCTTGCTCCTCTGCCGGCG
AGTGGCGAACGGGTGAGTAATACATCGGAACGTGTCGAGAAGAGGGGGATAACCATCCGAAAGGGTGGC
TAATACCGCATAAATTCTGAGGAAGAAAGCTGGGGACCTAGCGATAGGCCTAGCGCTTTTTGAGCGGCCG
ATGGCGGATTAGCTAGTTGGTGAGGTAAAGGCTCACCAAGGCGAAGATCTGTAGCGGGTCTGAGAGGAT
GATCCGCCACACTGGGACTGAGACACGGCC
>Zotu441
GAACGCTAGCGGCAGGCCTAATACATGCAAGTCGGACGGGATTCCGTTGTAGCTTGCTATGACGGATGAG
AGTGGCGCACGGGTGCGTAACGCGTGAGCAACCTGCCCATGTCAGGGGGATAGCCCGTTGAAAGACGGA
TTAACACCGCATAATACATTATGCCCGCCTGGGTATTCTGTTAAATATTTATAGGACATGGATGGGCTCGCG
TGGCATTAGCTTGTTGGTAAGGTAACGGCTTACCAAGGCTATGATGCCTAGGGGCTCTGAGAGGAGAATC
CCCCACACTGGTACTGAGACACGGAC
>Zotu443
GAACGCTGGCGGCGTGCTTAACACATGCAAGTCGAACGATGATCCCAGCTTGCTGGGGGATTAGTGGCGA
ACGGGTGAGTAACACGTGAGTAACCTGCCCTTGACTCTGGGATAAGCCTGGGAAACTGGGTCTAATACCG
GATATGACTCCTCATCGCATGGTGGGGGGTGGAAAGCTTTTGTGGTTTTGGATGGACTCGCGGCCTATCAG
CTTGTTGGTGGGGTAATGGCCTACCAAGGCGACGACGGGTAGCCGGCCTGAGAGGGTGACCGGCCACACT
GGGACTGAGACACGGCC

>Zotu445
GAACGCTTGCGGCGTGCCTAAGAAATGCAAGTCGAACGGGCACCTTCGGGTGCTAGTGGCGAACGGTCGC
GTAACACGTAAGAAACCTGCCCCGGAGTGGGGGACAACGGCCCGAAAGGGCCGCTAATACCGCATGTGG
TCGGTGGATCGCCTGGTCCTCCGTCCAAAGCTTAATGCGCTCCGGGATGGTCTTGCGGCCTATCAGGTAGA
TGGTGGGGTAACGGCCTACCATGCCGACGACGGGTAGCTGGTCTGAGAGGACGATCAGCCGGATTGGGA
CTGAGATACGGCC
>Zotu447
GAACGCTGGCTGCGTGCTTAACACATGCAAGTCGAACGCTGAAGCCTGGTGCTTGCACCGGGTGGATGAG
TGGCGAACGGGTGAGTAACACGTGAGTAACCTGCCCCTGACTTCGGGATAAGCCCGGGAAACTGGGTCTA
ATACCGGATACGACTGCCGGACGCATGTCTGGTGGTGGAAAGTTTTTCGGTTGGGGATGGGCTCGCGGC
CTATCAGTTTGTTGGTGAGGTAATGGCTCACCAAGACGACGACGGGTAGCCGGCCTGAGAGGGCGACCGG
CCACACTGGGACTGAGACACGGCC
>Zotu449
GAACGCTGGCGGCGTGCTTAACACATGCAAGTCGAACGGGCTTCTGGGGTTCTGGCTTTTTTGGTTGGGGT
TCTGGGGGTTAGTGGCGAACGGGTGAGTAACACGTGAGTAACCTGCCCTTCACTTCTGGATAACCGCTTGA
AAGGGTGGCTAATACGGGATGTTTCTGGGTCTGTCGCATGGCGGGTTCGGGAAAGATGGCACTGTTTGGT
GTTTTTGGTGGGGGATGGGCTCGCGGCCTATCAGCTTGTTGGTGGGGTGATGGCCTGCCAAGGCTTTGAC
GGGTAGCCGGCCTGAGAGGGTGGGCGGTCACACTGGGACTGAGATACGGCC
>Zotu451
GAATGCTGGCGGCGTGCTTAACACATGCAAGTCGAGCGGTAACATGTTTCTCACTGTCTTCTTCTTTTTATA
AGGAGGGAACTGTGAGAAATGATGACGAGCGGCGGACGGCTGAGTAACGCGTGGGAACATGCCCCAAAG
TGAGGAATAAGCTCCAGAAATGGAGTCTAATGCCGCATATGATCTTCGGATTAAAGGATTTATCCGCTTTG
GGAGTGGCCTGCGTACGATTAGGTAGTTGGTGGGGTAATAGCCTACCAAGCCTACGATCGTTAGCTGGTCT
GAGAGGATGACCAGCCAGACTGGAACTGAGACACGGTC
>Zotu453
GAACGCTGGCGGCGTGCTTAACACATGCAAGTCGAACGATGATGCCCAGCTTGCTGGGTGGATTAGTGGC
GAACGGGTGAGTAATACGTGAGTAACCTGCCCTTGACTCTGGGATAAGCCTGGGAAACTGGGTCTAATACT
GGATACTACCTCTCATCGCATGGTGGGTGGTGGAAAGGGTTTACTGGTTTTGGATGGGCTCACGGCCTAT
CAGCTTGTTGGTGGGGTAATGGCTCACCAAGGCGACGACGGGTAGCCGGCCTGAGAGGGTGACCGGCCA
CACTGGGACTGAGACACGGCC
>Zotu455
GAACGCTGGCGGTCTGCTTAACACATGCAAGTCGAACGGGATGCTTTCGGGCATCTAGTGGCGGACGGGT
GAGTAACGCGTGAGAATCTGCCCTTGGGAGTGGGACAACGACTGGAAACGGTCGCTAACACCGCATGAGC
CGAGAGGTAAAAGTATTTATAGCCCAAGGATGAGCTCGCGTCGGATTAGCTAGTTGGTAGTGTAATGGAC
TACCAAGGCGACGATCCGTAGCTGGTCTGAGAGGATGACCAGCCACACTGGGACTGAGACACGGCC
>Zotu457
GAACGCTGGCGGCAGGCCTAACACATGCAAGTCGAGCGGATGAGAGGAGCTTGCTCCTCGATTCAGCGGC
GGACGGGTGAGTAATGCCTAGGAATCTGCCTAGTAGTGGGGGACAACGTTTCGAAAGGAACGCTAATACC
GCATACGTCCTACGGGAGAAAGTGGGGGATCTTCGGACCTCACGCTATTAGATGAGCCTAGGTCGGATTA
GCTAGTTGGTAGGGTAAAGGCCTACCAAGGCGACGATCCGTAACTGGTCTGAGAGGATGATCAGTCACAC
TGGAACTGAGACACGGTC
>Zotu459
GAACGCTAGCGGCAGGCCTAATACATGCAAGTCGAACGGTCTCTAGCAATAGGGATAGTGGCGCACGGGT
GCGTAACGCGTAACCAACCTGCCCTGAACTGGGGGATAGCCCGCCGAAAGGCGGATTAATACCGCATATA
CCCGCAGCCTGGCATCAGGCAACGGGGAAAGATTTATTGGTTCAGGATGGGGTTGCGTGACATTAGCTAG
TTGGCGGGGTAACGGCCCACCAAGGCGACGATGTCTAGGGGACCTGAGAGGGTGATCCCCCACACTGGCA
CTGAGATACGGGC
>Zotu461
GAACGCTGGCGGCGTGCCTAACACATGCAAGTCGAGCGATTCTCTTCGGAGAAGAGCGGCGGACGGGTG
AGTAACGCGTGGGTAACCTGCCCTGTACACACGGATAACATACCGAAAGGTATGCTAATACGAGATAATAT

GCTTTTATCGCATGGTAGAAGTATCAAAGCTCCGGCGGTACAGGATGGACCCGCGTCTGATTAGCTAGTTG
GTAAGGTAACGGCTTACCAAGGCGACGATCAGTAGCCGACCTGAGAGGGTGATCGGCCACATTGGAACTG
AGACACGGTC

>Zotu463
GAACGCTGGCGGCATGCCTAATACATGCAAGTCGAACGCTGAAGCACTCTGCTTGCAGAGTGCGGATGAG
TGGCGAACGGGTGAGTAACACGTGGGGAATCTGCCTATAAGTGGGGGATAACATTCGGAAACGGATGCT
AATACCGCATATATTCTTGGACCGCCTGGTCCTTGAAAGAAAGACGGCTTTGCTGTCGCTTATAGATGATCC
CGCGGCGTATTAGTTAGTTGGTGGGGTAAAGGCCTACCAAGACAATGATACGTAGCCGACCTGAGAGGGT
GATCGGCCACACTGGGACTGAGACACGGCC

>Zotu465
GAACGCTGGCGGTAGGCCTAACACATGCAAGTCGAACGGCAGCACAGTAAGAGCTTGCTCTTATGGGTGG
CGAGTGGCGGACGGGTGAGGAATACATCGGAATCTACCTTTTCGTGGGGGATAACGTAGGGAAACTTACG
CTAATACCGCATACGACCTTCGGGTGAAAGCAGGGGACCTTCGGGCCTTGCGCGGATAGATGAGCCGATG
TCGGATTAGCTAGTTGGCGGGGTAAAGGCCCACCAAGGCGACGATCCGTAGCTGGTCTGAGAGGATGATC
AGCCACACTGGAACTGAGACACGGTC

>Zotu467
GAACGCTGGCGGCAGGCTTAACACATGCAAGTCGAACGCACCTTTCGGGGTGAGTGGCAGACGGGTGAG
TAACGCGTGGGAATCTACCCTTCTCTACGGGATAGCTCCGGGAAACTGGAATTAATACCGTATACGTCCTG
AGGGAGAAAGATTTATCGGGGAAGGATGAGCCCGCGTTGGATTAGCTAGTTGGTGGGGTAAAGGCCTAC
CAAGGCGACGATCCATAGCTGGTCTAAGAGGATGATCAGCCACATTGGGACTGAGACACGGCC

>Zotu469
GAACGCTGGCGGCATGCCTTACACATGCAAGTCGAACGGCAGCACGGGGGCAACCCTGGTGGCGAGTGG
CGAACGGGTGAGTAATACATCGGAACGTGTCCTGTAGTGGGGGATAGCCCGGCGAAAGCCGGATTAATAC
CGCATACGATCTGTGGATGAAAGCGGGGGATCCTTCGGGACCTCGCGCTACAGGGGCGGCCGATGGCAG
ATTAGCTAGTTGGTGGGGTAAAGGCCTACCAAGGCGACGATCTGTAGCTGGTCTGAGAGGACGACCAGCC
ACACTGGGACTGAGACACGGCC

>Zotu471
GAACGCTAGCGGCAGGCTTAACACATGCAAGTCGAGGGGCAGCATATAAATTGCTTGCAATTTATGATGG
CGACCGGCGCACGGGTGCGTAACGCGTATGCAACCTACCTGTAACAGGGGGATAACACTGAGAAATTGGT
ACTAATACCCCGTAATATTACGAGAGGCATCTTTTGTAGTTGAAAGTTCCGGCGGTTACAGATGGGCATGC
GTTGTATTAGATAGTTGGTGAGGTAACGGCTCACCAAGTCGACGATACATAGGGGGACTGAGAGGTTAAC
CCCCCACATTGGTACTGAGACACGGAC

>Zotu473
GAACGCTGGCGGCAGGCCTAACACATGCAAGTCGAGCGCATCCTTCGGGGTGAGCGGCGGACGGGTGAG
TAACGCGTGGGAATATGCCTTTCGGTACGGAATAGTCTCGGGAAACTGGGGGTAATACCGTATACGCCCTA
TTGGGGAAAGATTTATCGCCGAAAGATTAGCCCGCGTTGGATTAGGTAGTTGGTGGGGTAATGGCCCACC
AAGCCGACGATCCATAGCTGGTTTGAGAGGATGATCAGCCACACTGGGACTGAGACACGGCC

>Zotu475
GAACGCTGGCGGCATGCTTAACACATGCAAGTCGAACGGCAGCATATTGTGTAGCAATACACAGTGATGG
CGAGTGGCGGACGGGTGAGTAAAGCATAGGAATCTGCCGAGAAGAGGGGGATAACTGAGGGAAACTTCA
GCTAAGACCACATAATGTCTACGGACCAAATCAGGGGACCTTCGGGCCTTGAGCTTTTTGATGAGCCTATG
CCGGATTAGCTAGTTGGTAGGGTAAGAGCCTACCAAGGCGAAGATCCGTAGCTGGTCTGAGAGGATGATC
AGCCACACTGGGACTGAGACACGGCC

>Zotu477
GAACGCTGGCGGCATGCCTTACACATGCAAGTCGAACGGCAGCACGGGTGCTTGCACCTGGTGGCGAGTG
GCGAACGGGTGAGTAATATATCGGAACGTGCCCTAGAGTGGGGGATAACGTAGCGAAAGTTACGCTAATA
CCGCATACGATCTACGGATGAAAGTGGGGGACCGCAAGGCCTCACGCTCCTGGAGCGGCCGATATCTGAT
TAGCTAGTTGGTGGGGTAAAAGCCTACCAAGGCGACGATCAGTAGCTGGTCTGAGAGGACGACCAGCCAC
ACTGGGACTGAGACACGGCC

>Zotu479

GAACGCTGGCGGCGTGCCTAACACATGCAAGTCGAGCGAACCCTTCGGGGTGAGCGGCGGACGGGTGAG
TAACGCGTGGGTAACCTGCCCTGTACACACGGATAACATACCGAAAGGTATGCTAATACGAGATAATATGT
AATGATCGCATGGTCGATACATCAAAGCTCCGGCGGTACAGGATGGACCCGCGTCTGATTAGCTAGTTGGT
GAGGTAATGGCTCACCAAGGCGACGATCAGTAGCCGACCTGAGAGGGTGATCGGCCACATTGGAACTGA
GACACGGTC
>Zotu481
GAACGCTGGCGGCGTGCTTAACACATGCAAGTCGAACGGTGATGCGGAGCTTGCTCCGCGGATCAGTGGC
GAACGGGTGAGTAACACGTGAGTAACCTGCCCCTGACTCTGGGATAAGCGTTGGAAACGACGTCTAATAC
CGGATACGACCCATGAGGGCATCTTCGTGGGTGGAAAGAATTTCGGTTGGGGATGGACTCGCGGCCTATC
AGCTTGTTGGTGAGGTAATGGCTCACCAAGGCGACGACGGGTAGCCGGCCTGAGAGGGTGACCGGCCAC
ACTGGGACTGAGACACGGCC
>Zotu483
GAACGCTGGCGGCGTGCCTAATACATGCAAGTCGAGCGCATGGAGATAGAACTTGTTCTATTGAAGTGAG
CGGCGCACGGGTGAGTAACACGTGGGAAACCTACCCTTTAGCGGGGGATAACACTGGGAAACGAGTGCT
AATACCGCATAGAGCATCTGGTTGCATGACCGGATGAGGAAAGACGCTACGGCGTCACTAAAGGATGGTC
CCGCGGTGCATTAGCTAGTTGGTAAGGTAGCGGCTTACCAAGGCGATGATGCATAGCCGACCTGAGAGGG
TGATCGGCCACATTGGGACTGAGACACGGCC
>Zotu485
GAACGCTGGCGGCGTGCTTAACACATGCAAGTCGAACGGAAAGGCCCTTCGGGGTACTCGAGTGGCGAAC
GGGTGAGTAACACGTGGGTGATCTGCCCTGCACTTCGGGATAAGCCTGGGAAACTGGGTCTAATACCGGA
TATGATCATCGGCTTCATGGTCGGTGGTGGAAAGCTTTTGCGGTGTGGGATGGGCCCGCGGCCTATCAGCT
TGTTGGTGGGGTAATGGCCTACCAAGGCGTCGACGGGTAGCCGGCCTGAGAGGGTGTCCGGCCACACTG
GGACTGAGATACGGCC
>Zotu487
GAACGCTGGCGGCGTGCTTAACACATGCAAGTCGAGCGGAAAGGCCACTTCGGTGGTACTCGAGCGGCGA
ACGGGTGAGTAACACGTGAGTAATCTGCCCCTGGCTTTGGGATAGCCACCGGAAACGGTGATTAATACCG
GATATGACAGCTTCTCGCATGAGATGGTTGTGGAAAGTTTTTCGGCCAGGGATGTGCTCGCGGCCTATCAG
CTTGATGGTGAGGTAATGGCTCACCATGGCTTCGACGGGTAGCCGGCCTGAGAGGGTGACCGGCCACACT
GGGACTGAGACACGGCC
>Zotu489
GAACGCTAGCGGGATGCTTTACACATGCAAGTCGAACGGCAGCGCGGACTTCGGTCTGGCGGCGAGTGGC
GAACGGGTGAGTAATGTATCGGAACGTGCCCAGTAGCGGGGGATAACTACGCGAAAGCGTAGCTAATACC
GCATACGCCCTACGGGGGAAAGCAGGGGATCGTAAGACCTTGCACTATTGGAGCGGCCGATATCGGATTA
GCTAGTTGGTGAGGTAACGGCTCACCAAGGCGACGATCCGTAGCTGGTTTGAGAGGACGACCAGCCACAC
TGGGACTGAGACACGGCC
>Zotu491
GAACGCTAGCGGTGCGCCTAACACATGCAAGTCGAGCGGGTTTATTTGTGACACAGAGTAGGGAAGCAAT
TCCACATTCTGTGTCACAGGTAATCTAGCGGCAAACGGGTGCGTAACACGTTGATATCTCCCCCGAAGTCA
GGGATAGCCTCAAGAAATTGAGAGTAATACCGGATGGTCCCGTAAGGGTAAAGGAGCAATTCGCTTCGGG
AAGAGTCTGCGGCCTATCAGCTAGTTGGCGGGGTAATAGCCCACCAAGGCGATGACGGGTAGCTGGTCTG
AGAGGACGGCCAGCCACAATGGAACTGAGACACGGTC
>Zotu493
GAACGCTAGCGGCAGGCTTAATACATGCAAGTCGAGGGGCAGCGCAGTGTAGCAATACATGGGCGGCGA
CCGGCAAACGGGTGCGGAACACGTACAGAACCTTCCTTCGAGCGGGGAATAGCCCAGAGAAATTTGGATT
AATACCCCATAGTATAACGGAGTGGCATCACTTTGTTATTAAAGATTTATCACTTGAAGATGGCTGTGCGGC
TGATTAGGTAGTTGGCGGGGTAACGGCCCACCAAGCCTTCGATCAGTAACTGGTGTGAGAGCACGACCAG
TCACACGGGCACTGAGACACGGGC
>Zotu495
GAACGCTGGCGGCGTGCCTAATACATGCAAGTCGAGCGGACAGAAGGGAGCTTGCTCCCGGATGTTAGCG
GCGGACGGGTGAGTAACACGTGGGTAACCTGCCTGTAAGACTGGGATAACTCCGGGAAACCGGAGCTAAT
ACCGGATAGTTCCTTGAACCGCATGGTTCAAGGATGAAAGACGGTTTCGGCTGTCACTTACAGATGGACCC

GCGGCGCATTAGCTAGTTGGTGGGGTAATGGCTCACCAAGGCGACGATGCGTAGCCGACCTGAGAGGGT
GATCGGCCACACTGGGACTGAGACACGGCC
>Zotu497
GAACGCTGGCGGTAGGCCTAACACATGCAAGTCGAACGGCAGCACAGTAAGAGCTTGCTCTTATGGGTGG
CGAGTGGCGGACGGGTGAGGAATACATCGGAATCTACTTTTTCGTGGGGGATAACGTAGGGAAACTTACG
CTAATACCGCATACGACCTACGGGTGAAAGCAGGGGATCTTCGGACCTTGCGCGATTGAATGAGCCGATG
TCGGATTAGCTAGTTGGCGGGGTAAAGGCCCACCAAGGCGACGATCCGTAGCTGGTCTGAGAGGATGATC
AGCCACACTGGAACTGAGACACGGTC
>Zotu499
GAACGCTGGCGGCGTGCTTAACACATGCAAGTCGAGCGGAAAGGCCCTTCGGGGTACTCGAGCGGCGAA
CGGGTGAGTAACACGTGAGTAACCTGCCCTGGACTTTGGGATAACCCTCGGAAACGGGGGCTAATACCGG
ATACGACACGTTCCTGCATGGGATGCGTGTGGAAAGATTTTTTGGTCTGGGATGGGCTCGCGGCCTATCAG
CTTGTTGGTGGGGTGATGGCCTACCAAGGCGACGACGGGTAGCCGGCCTGAGAGGGCGACCGGCCACAC
TGGGACTGAGACACGGCC
>Zotu501
GAACGCTAGCTACAGGCTTAACACATGCAAGTCGAGGGGCAGCATGACCTAGCAATAGGTTGATGGCGAC
CGGCGCACGGGTGAGTAACACGTATCCAACCTACCGGTTATTCCGGGATAGCCTTTCGAAAGAAAGATTAA
TACCGGATAGTATAACGAGAAGGCATCTTTTTGTTATTAAAGAATTTCGATAACCGATGGGGATGCGTTCC
ATTAGTTTGTTGGCGGGGTAACGGCCCACCAAGACATCGATGGATAGGGGTTCTGAGAGGAAGGTCCCCC
ACATTGGAACTGAGACACGGTC
>Zotu503
GAACGCTGGCGGCGTGCCTAATACATGCAAGTAGAACGCTGAAGAGAGGAGCTTGCTCTTCTTGGATGAG
TTGCGAACGGGTGAGTAACGCGTAGGTAACCTGCCTGGTAGCGGGGGATAACTATTGGAAACGATAGCTA
ATACCGCATAAAATGGATTATCGCATGATAATTCATTGAAAGGTGCAAATGCATCACTACCAGATGGACCT
GCGTTGTATTAGCTAGTTGGTGGGGTAACGGCTCACCAAGGCGACGATACATAGCCGACCTGAGAGGGTG
ATCGGCCACACTGGGACTGAGACACGGCC
>Zotu505
GAACGCTGGCGGCAGGCTTAACACATGCAAGTCGAGCGAGACCTTCGGGTCTAGCGGCGGACGGGTGAG
TAACGCGTGGGAACGTGCCCTTCTCTACGGAATAGCCCCGGGAAACTGGGAGTAATACCGTATACGCCCTT
TGGGGGAAAGATTTATCGGAGAAGGATCGGCCCGCGTTGGATTAGGTAGTTGGTGGGGTAATGGCCCACC
AAGCCGACGATCCATAGCTGGTTTGAGAGGATGATCAGCCACACTGGGACTGAGACACGGCC
>Zotu507
GAACGCTGGCGGCGCGCTTAACACATGCAAGTCGAGCGATGAAACTTTAACAGAACCCTTCGGGGCGAAG
ATAAAGTGGATTAGCGGCGGACGGGTGAGTAACACGTGAGTAACCTGCCTTAGACATTGGGATAGCCTCG
GGAAACCGGGATTAATACCAAATAAAATCATAATGACACATGTCATAACGATCAAAACTCCGGTGGTCTAA
GATGGACTCGCGTCCCATTAGCTAGTTGGTAGAGTAAAAGCCTACCAAGGCGACGATGGGTAGCCGACCT
GAGAGGGTGAACGGCCACATTGGAACTGAGAAACGGTC
>Zotu509
GAATGCTGGCGGCGTGGATAAGTCATGCAAGTCAAGGGGGCCCGCAAGGGCAACCGGCAGACGAGGTAG
TAACACGTAGGTACGTCCCCCGAAGTCGGGCATAGCCATCCGAAAGGATGGGTAATTCCCGATAGTCCCGC
AAGGGTAAAGGTTTTTCGCTTCGGGAGCGGCCTGCGTAGTATCAGCTAGTTGGTAAGGTAATGGCTTACCA
AGGCTACGACGCTTAGGGGAGCTGAGAGGCTGACCCCCACCGATGGAACTGCGACACGGTC
>Zotu511
GAACGCTGGCGGCGTGCTTAACACATGCAAGTCGAACGATGAAGCACCAGCTTGCTGGTGTGGATTAGTG
GCGAACGGGTGAGTAACACGTGAGTAACCTGCCCCTCACTCTGGGATAAGCCTGGGAAACTGGGTCTAAT
ACTGGATACGACCGATCTCCGCATGGAGTGTTGGTGGAAAGTTTTTGTGGTGGGGGATGGACTCGCGGCC
TATCAGCTTGTTGGTGGGGTAATGGCCTACCAAGGCGACGACGGGTAGCCGGCCTGAGAGGGCGACCGG
CCACACTGGGACTGAGACACGGCC
>Zotu513

GAACGCTGGCGGCGTGCTTAACACATGCAAGTCGAACGGTGACGGAGAGCTTGCTCTCCTGATCAGTGGC
GAACGGGTGAGTAACACGTGGGCAACCTGCCCCAGACTTCGGGATAACGCTTCGAAAGAGGTGCTAATAC
CGAATACGAACTCCTTGGGCATCCTTGGAGTTGGAAAGTTTTTCGGTCTGGGATGGGCCCGCGGCCTATCA
GCTTGTTGGTGAGGTAATGGCTCACCAAGGCGACGACGGGTAGCCGGCCTGAGAGGGCGACCGGCCACA
CTGGGACTGAGACACGGCC
>Zotu515
GAACGCTGGCGGCGTGCTTAACACATGCAAGTCGAACGATGAACTCCAGCTTGCTGGGGGGATTAGTGGC
GAACGGGTGAGTAACACGTGAGTAACCTGCCCTTGACTCTGGGATAAGCGCTGGAAACGGCGTCTAATAC
CGGATACGAGCTTCAGTCGCATGGCTAGGAGTTGGAAAGAATTTTGGTCAAGGATGGACTCGCGGCCTAT
CAGCTTGTTGGTGAGGTAATGGCTCACCAAGGCGACGACGGGTAGCCGGCCTGAGAGGGTGACCGGCCA
CACTGGGACTGAGACACGGCC
>Zotu518
GAACGCTGGCGGCGTGCTTAACACATGCAAGTCGAACGGGAAACCTTTTATTGAAGCTTCGGCAGATTTAG
ATTGTTTCTAGTGGCGGACGGGTGAGTAACGCGTGGGTAACCTGCCTTATACAGGGGGATAACAGTCAGA
AATGACTGCTAATACCGCATAAGCGCACAGAGCTGCATGGCTCGGTGTGAAAAACTCCGGTGGTATAAGA
TGGACCCGCGTTGGATTAGCTAGTTGGTGAGGTAACGGCCCACCAAGGCGACGATCCATAGCCGGCCTGA
GAGGGTGAACGGCCACATTGGGACTGAGACACGGCC
>Zotu520
GAACGCTGGCGGCGTGCTTAACACATGCAAGTCGAACGGAAAGGCCCTTCGGGGTACTCGAGTGGCGAAC
GGGTGAGTAACACGTGGGTGATCTGCCCTGCACTTCGGGATAAGCCTGGGAAACTGGGTCTAATACCGGA
TATGACCTCAGGATGCATGTTCTGGGGTGGAAAGCTTTTGCGGTGTGGGATGGGCCCGCGGCCTATCAGC
TTGTTGGTGGGGTCACGGCCTACCAAGGCGACGACGGGTAGCCGGCCTGAGAGGGTGACCGGCCACACT
GGGACTGAGATACGGCC
>Zotu522
GAACGCTTGCGGCGTGCCTAAGAAATGCAAGTCGAGCGGGGAGCAATCCCAGCGGCGAACGGTCGCGTA
ACACGTAAGCAACCTGCCCGAAAGACCGGGATAACGTTTCGAAAGAGGCGCTAATACCGGATGTGACCGC
CGGGAGACATCTCCTGGTGATTAAATCATTTATGGCTTTCGGAGGGGCTTGCGGCCTATCAGCTAGATGGC
GGGGTAACGGCTCACCATGGCGTCGACGGGTAGCTGGTCTGAGAGGATGGTCAGCCGGACTGGGACTGA
GATACGGCC
>Zotu525
GAACGCTAGCGGCAGGCTTAATACATGCAAGTCGAGGGGCAGCACGAATAGCAATATTTGGTGGCGACCG
GCGCACGGGTGCGTAACACGTATACAATCTACCTTCAACAGGGGGATAGCCTTTCGAAAGAGAGATTAATA
CCCCGTAATATATTTGCTGGCATCAGTGAATATTGAAAGTTCCGACGGTTGAAGATGAGTATGCGTCTGATT
AGCTAGTTGGTGAGGTAACGGCTCACCAAGGCGACGATCAGTAGGGGATCTGAGAGGACTAACCCCCACA
CTGGTACTGAGACACGGAC
>Zotu527
GAACGCTGGCGGCGTGCCTAATACATGCAAGTCGAACGAACCGCGACTAGGTGCTTGCACTTGGTCAAGG
TGAGTGGCGAACGGGTGAGTAACACGTGGGTAACCTACCTCATAGTGGGGGATAACAGTCGGAAACGACT
GCTAATACCGCATAGGACATGGAATCACATGATTCAGTGAGGAAAGGTGGCGCAAGCTATCGCTAAGAGA
TGGACCCGCGGTGCATTAGCTAGTTGGTAGGGTAAAGGCCTACCAAGGCGATGATGCATAGCCGACCTGA
GAGGGTGATCGGCCACATTGGGACTGAGACACGGCC
>Zotu529
GAACGCTGGCGGCAGGCCTAACACATGCAAGTCGAACGCTGTAGCAATACAGAGTGGCAGACGGGTGAG
TAACGCGTGGGAACATGCCCTTCGGTTCGGAATAACTCAGGGAAACTTGAGCTAATACCGGATACGCCCTT
AGGGGGAAAGATTTATTGCCGAAGGATTGGCCCGCGTCCGATTAGCTAGTTGGTGAGGTAATGGCTCACC
AAGGCGACGATCGGTAGCTGGTCTGAGAGGATGATCAGCCACACTGGGACTGAGACACGGCC
>Zotu531
GAACGCTGGCGGCGTGCTTAACACATGCAAGTCGAACGATGAAGCGGTGCTTGCACCGTGGATTAGTGGC
GAACGGGTGAGTAACACGTGAGTAACCTGCCTTCCACTCTGGGATAAGCGCTGGAAACGGCGTCTAATACT
GGATATGACATTGCCTCGCATGGGGTGGTGTGTAAAGATTTTTTGGTGGTAGATGGACTCGCGGCCTATCA

GCTTGTTGGTGGGGTAATGGCCTACCAAGGCGACGACGGGTAGCCGGCCTGAGAGGGTGACCGGCCACA
CTGGGACTGAGACACGGCC
>Zotu534
GAACGCTGGCGGTGTGCCTAATACATGCAAGTCGAGCGGCAGCACGAATAGTTTACTATTTGGTGGCGAG
AGGCGGACGGCTGAGTAACACGTAGGAACATACCCCAAAGTGAGGGATAACTGCTCGAAAGAGTAGCTA
ATACCGCATGTGATCTAAGGATTAAAGCTTCGGCGCTTTGGGAATGGCCTGCGTTCGATTAGCTAGTTGGT
AAGGTAAAAGCTTACCAAGGCGACGATCGATAGCTGGTCTGAGAGGATGATCAGCCAGACTGGGACTGA
GACACGGCC
>Zotu536
GAACGCTGGCGGCATGCTTAACACATGCAAGTCGAACGAGATAAAATTCACTTTAACACATGTTAAAGTGA
GCTTTATCTAGTGGCGGACGGGTGAGTAACGCGTAAGAACCTACCCTTAGGAGGGGGACAACAATTGGAA
ACGATTGCTAATACCCCATATGCTGAGGAGTAAAACGGTAATAACCGCCTAGGGATGGGCTTGCGTCTGAT
TAGCTTGTTGGTGAGGTAAATGCTTACCAAGGCAATGATCAGTAGTTGGTCTGAGAGGATGACCAGCCACA
CTGGGACTGAGACACGGCC
>Zotu538
GAACGCTGGCGGCGTGCTTAACACATGCAAGTCGAACGGTAAGGCCCTTCGGGGTACACGAGTGGCGAAC
GGGTGAGTAACACGTGGGTGATCTGCCCTGCACTTCGGGATAAGCCCGGGAAACTGGGTCTAATACCGGA
TAGTACTGCGCGGTGCATGCCGTGTGGTGGAAAGATTTATCGGTGCAGGATGGGCCCGCGGCCTATCAGC
TTGTTGGTGGGGTAATGGCCTACCAAGGCGACGACGGGTAGCCGGCCTGAGAGGGCGACCGGCCACACT
GGGACTGAGACACGGCC
>Zotu540
GAACGCTGGCGGCGTGCTTAACACATGCAAGTCGAACGCTGAAGCTTGGTGCTTGCACTTAGGTGGATGA
GTGGCGAACGGGTGAGTAATACGTGAGTAACCTGCCCTTGACTCTGGGATAAGCCTGGGAAACTGGGTCT
AATACTGGATATGACCTCCTGTCGCATGGTGGGGGGTGGAAAGGGTTTGTACTGGTTGTGGATGGGCTCA
CGGCCTATCAGCTTGTTGGTGGGGTAATGGCCTACCAAGGCGACGACGGGTAGCCGGCCTGAGAGGGTG
ACCGGCCACACTGGGACTGAGACACGGCC
>Zotu542
GAACGCTGGCGGCGTGCTTAACACATGCAAGTCGAACGGGAACCTTTATTTCGGTAAAGGTGAGAGTGGC
AAACGGGTGAGTAACACGTAGGCAACCTGCCGACAGGATGGGGACAACATTCCGAAAGGAATGCTAATAC
CGAATGGAGTCCGGGGATGGCATCATCCCTGGATAAAAGATGGCCTCTGAATATGCTATCGCCTGTCGATG
GGCCTGCGTCTGATTAGCCAGTTGGCGGGGTAACGGCCCACCAAAGCGACGATCAGTAGCCGGTCTGAGA
GGATGAACGGCCACATTGGGACTGAGACACGGCC
>Zotu544
GAACGCTGGCGGCGTGCTTAACACATGCAAGTCGAACGGTGAACTCCGCTTGCGGGGGGATCAGTGGCGA
ACGGGTGAGTAACACGTGGGCAACCTGCCCTCAGCTCTGGGATAACTCCAAGAAATTGGGGCTAATACCG
GATATGACTGCTGACCGCATGGTCTGGTGGTGGAAAGATTTATCGGCTGGGGATGGGCCCGCGGCCTATC
AGCTTGTTGGTGGGGTAGTGGCCTACCAAGGCGACGACGGGTAGCCGGCCTGAGAGGGTGACCGGCCAC
ACTGGGACTGAGACACGGCC
>Zotu546
GAACGCTGGCGGCAGGCTTAACACATGCAAGTCGAACGCGTGTAGCAATACACGAGTGGCGCACGGGTG
AGTAACACGTGGATATCTGCCTTTTGGTTCGGAATAACCCTGGGAAACTAGGGCTAATACCGAATGGTTCC
TACGGGATAAAGATTTATCGCCAAAAGATGAGTCCGCGTCCGATTAGCTAGTTGGTGAGGTAATGGCTCAC
CAAGGCGACGATCGGTAGCTGGTCTGAGAGGACGATCAGCCACACTGGGACTGAGACACGGCC
>Zotu548
CGCAAGCCTTGGGGAACTGTGCAGCTGGTTTGATCGGGAACGCGAGTACCGCCCAGGTAGCTCAGGCTAG
TGTCATGACCGACTGTCCAGCACGAGAACAGCTCAACGGCATCTTCTTCCTTCGCCGCCCCACAATATACGC
GCGTAACCGGCCGCGCACTTCAAATAGCATAAATTCCTATCACAATA
>Zotu550
GAACGCTAGCGGCAGGCTTAATACATGCAAGTCGAGGGGCAGCGGGGGTAGCAATACCCGCCGGCGACC
GGCAAACGGGTGCGGAACACGTACAGAACCTTCCTTTAAGCGGGGGATAGCCCATAGAAATGTGGATTAA

TACCCCATAGTAATATTTGGTGGCATCATTAGATATTTAAAGATTTATCACTTAAAGATGGCTGTGCGGCTG
ATTAGGTAGTTGGTGTGGGTAACGGCCCACCAAGCCAGCGATCAGTAACTGGTGTGAGAGCACGACCAGT
CACACGGGCACTGAGACACGGGC

>Zotu552
GAACGCTGGCGGCATGCTTTACACATGCAAGTCGAACGGTAACAGGTCTTCGGATGCTGACGAGTGGCGA
ACGGGTGAGTAATACATCGGAACGTGCCTAGTAGTGGGGGATAACTACTCGAAAGAGTAGCTAATACCGC
ATGAGATCTACGGATGAAAGCAGGGGACCTTCGGGCCTTGTGCTACTAGAGCGGCTGATGGCAGATTAGG
TAGTTGGTGGGGTAAAGGCTTACCAAGCCTGCGATCTGTAGCTGGTCTGAGAGGACGACCAGCCACACTG
GGACTGAGACACGGCC

>Zotu554
GAACGCTGGCGGCGTGCCTAAGACATGCAAGTCGAGTGGGTTTAGACCCACGGCGAACGGGTGAGTAAT
ACCTAGGAACGTTTTTCAAGTGGAGCATAACCAGTCGAAAGATTGGATAATACTCCATGTGACCGCAAGGT
TAAAGTCGCAAGACGCTTGAAGTGCGGCCTGGGCACCATCAGCTAGTTGGTAAGGTAATGGCTTACCAAG
GCTATGACGGTTAGGGGCCCCGAGAGGGGAGACCCCCACACTGGAACTGCGACACGGTC

>Zotu556
GAACGCTAGCGGCAGGCTTAACACATGCAAGTCGAGGGGTAGTCTAGCAATAGATGAGACCGGCGCACG
GGTGCGTAACGCGTATGCAATCTACCTTTTACAGGGGAATAGCCCAGAGAAATTTGGATTAATGCCCCATG
GTATTATCGAGTGGCATCATTTGATAATTAAAGTTCCAACGGTAAAAGATGAGCATGCGTCCTATTAGCTTG
TTGGTAAGGTAACGGCTTACCAAGGCTACGATAGGTAGGGGTCCTGAGAGGGAGATCCCCCACACTGGTA
CTGAGACACGGAC

>Zotu558
GAACGCTGGCGGCGCGCTTAACACATGCAAGTCGAGCGAGAACCGGGGCTTCGGCCCCGGGGACAGCGG
CGAACGGGTGAGTAACACGTGGGTAATCTGCCCTCGGTTCTGGGATAGCCCGGGGAAACCCGGATTAATA
CCGGATAGCCTCTCCAGCCTTCGGGCCGGCGAGAAAAGGTAGCTTCGGCCTCCGACCGAGGATGAGCCCG
CGGCGGATTAGCTTGTTGGCGGGGTAACGGCCCACCAAGGCGACGATCCGTAGCTGGTCTGAGAGGACG
ATCAGCCACACTGGGACTGAGACACGGCC

>Zotu560
GAACGCTAGCGGCAGGCTTAACACATGCAAGTCGAGGGGTATAGTAGCAATACTAGAGACCGGCGCACG
GGTGCGTAACGCGTATGCAATCTACCTTTTACAGAGGGATAGCCCAGAGAAATTTGGATTAATACCTCATG
GTATAACGAGTCCGCATGGATTTGTTATTAAAGTCACAACGGTAAAAGATGAGCATGCGTCCCATTAGCTT
GTTGGTAAGGTAACGGCTTACCAAGGCTACGATGGGTAGGGGTCCTGAGAGGGAGATCCCCCACACTGGT
ACTGAGACACGGAC

>Zotu562
GAACGCTGGCGGCGTGCTTAACACATGCAAGTGGAGCGACGAAACGGGCTTCGGCCCGTGGCAAAGCCG
CGAACGGGTGAGTAACACGTGGGTAACCTGCCCCGATGACCGGGACAACCCGAGGAAACTCGGGCTAATA
CCGGATGTTCCCCCATCACACCAGTGGTGGGAGGAAAGGTAGCTTCGGCCTCCGCATCGGGATGGGCCCG
CGGCCCATTAGCTTGTTGGTGGGGTAATGGCCTACCAAGGCTGCGATGGGTAGCTGGTCTGAGAGGACGA
TCAGCCACACTGGGACTGAGACACGGCC

>Zotu564
GAACGCTGGCGGCGTGCTTAACACATGCAAGTCGAACGGTAAGGCCCCTTCGGGGGTACACGAGTGGCGA
ACGGGTGAGTAACACGTGAGCAACCTGCCCTTGACTTCGGAATACCAGCTGGAAACAGCTGCTAATACCG
GATATGACACTTGCCCTCATGGGTGGGTGTGGAAAGCTCCGGCGGTCAGGGATGGGCTCGCGGCCTATCA
GCTTGTTGGTGAGGTAATGGCTCACCAAGGCTTCGACGGGTAGCCGGCCTGAGAGGGCGACCGGCCACAC
TGGGACTGAGATACGGCC

>Zotu567
GAACGCTGGCGGCGTGCTTAACACATGCAAGTCGAACGGTGAAGCCCAGCTTGCTGGGTGGATCAGTGGC
GAACGGGTGAGTAACACGTGAGCAATCTGCCCCTGACTCTGGGATAAGCGCTGGAAACGGCGTCTAATAC
TGGATATGAACCGTGGAGGCATCTTCAACGGTTGGAAAGATTTTTTGGTCAGGGATGAGCTCGCGGCCTAT
CAGCTTGTTGGTGAGGTAATGGCTCACCAAGGCGTCGACGGGTAGCCGGCCTGAGAGGGTGACCGGCCAC
ACTGGGACTGAGACACGGCC

>Zotu569
GAACGCTGGCGGCGTGCTTAACACATGCAAGTCGAACGATGATGGTGGTGCTTGCACTGCCTGATTAGTG
GCGAACGGGTGAGTAACACGTGAGTAACCTGCCCTTCACTTCGGGATAACCTCGGGAAATCGTGGCTAATA
CCGGATATGAGCTCCTGCCGCATGGTGGGTGTTGGAAAGATTTATCGGTGAGGGATGGACTCGCGGCCTA
TCAGTTTGTTGGTGAGGTAATGGCTCACCAAGGCGATGACGGGTAGCCGGCCTGAGAGGGCGACCGGCCA
CACTGGGACTGAGACACGGCC
>Zotu571
GAACGCTGGCGGCGTGGATGAGGCATGCAAGTCGAATGGGTTTAGACCCATGGCAGACGAGGTAGGAAC
AAGTAGGTACGTACCCCAAAGTCAGGGATAACCCGGAGAAATCCGGAATAATACTTGATGGTCTCGCGAG
AGTAAAGATTTATCGCTTTGGGAACGGCCTGCTTTCTATCAGCTTGTTGGTAAGGTAACGGCTTACCAAGGC
TACGACGGATAGGGGAGGTGAGAGCCTGACCCCCACCGATGGAACTGCGACACGGTC
>Zotu573
GAACGCTGGCGGCGTGCCTAATACATGCAAGTCGAGCGAACAGATAAGGAGCTTGCTCCTTTGACGTTAG
CGGCGGACGGGTGAGTAACACGTGGATAACCTACCTATAAGACTGGGATAACTTCGGGAAACCGGAGCTA
ATACCGGATAAGATTTTGAACCGCATGGTTCAATAGTGAAAGGCGGCTTTGCTGTCACTTATAGATGGATC
CGCGCCGTATTAGCTAGTTGGTAAGGTAACGGCTTACCAAGGCAACGATACGTAGCCGACCTGAGAGGGT
GATCGGCCACACTGGAACTGAGACACGGTC
>Zotu576
GAACGCTGGCGGCGTGCCTAATACATGCAAGTAGAACGCTGAAGCTTGGTGCTTGCACCGAGCGGATGAG
TTGCGAACGGGTGAGTAACGCGTAGGTAACCTGCCTCTTAGCGGGGGATAACTATTGGAAACGATAGCTA
ATACCGCATAAAAGTCGATATCGCATGATGTTGACTTGAAAGGTGCAATTGCATCACTAAGAGATGGACCT
GCGTTGTATTAGCTAGTTGGTGAGGTAACGGCTCACCAAGGCGACGATACATAGCCGACCTGAGAGGGTG
ATCGGCCACACTGGGACTGAGACACGGCC
>Zotu578
GAACGCTGGCGGCATGCCTAACACATGCAAGTCGAACGAAGGCTTCGGCCTTAGTGGCGCACGGGTGCGT
AACGCGTGGGAATCTGCCCTCAGGTTCGGAATAACAGCGAGAAATTGCTGCTAATACCGGATGATATCGC
GAGATCAAAGATTTATCGCCTGAGGATGAGCCCGCGTAGGATTAGCTAGTTGGTGTGATAAAGGCGCACC
AAGGCGACGATCCTTAGCTGGTCTGAGAGGATGATCAGCCACACTGGGACTGAGACACGGCC
>Zotu580
GAACGCTGGCGGTGTGCTTCACACATGCAAGTCGAACGGGGTAGCAATACCTAGTGGCGGACGGGTGAGT
AACGCGTGGGAATCTGCCCAAGGGTGGGGGATAACTAACCGAAAGGTTAGCTAATACCGCATATGCCGTG
AGGTAAAAGGAGTAATCCGCCTTTGGATGAGCCTGCGTCCGATTAGCTTGTTGGTGGGGTAAAAGCCTACC
AAGGCTACGATCGGTAGCTGGTCTGAGAGGATGATCAGCCACAATGGGACTGAGACACGGCC
>Zotu582
TAACGCTGGCAGAGTGCATAACACATGCAAGTCGAGCGGGACTGTTTGAGATTACATCAAGAGCAGTTTA
GCGGCAGACGAGTGAGTAACACGTAAGCAATCTACCCCCGAATGGCGAACAACCAGCCGAAAGGCTGGCT
AATACGCCGTATTCTCCTTGGAAGGCATCTTCCGAGGAGGAGAGACCCGCAAGGGTCGTTCAGGGAGGAG
CTTGCGGCCCATCAGCTAGTTGGCGGTGTAACGGACCACCAAGGCTACGACGGGTACCCGGGCTGAAAGG
CCGGACGGGCACGATGGCACTGAGACACGGGC
>Zotu584
GAACGCTAGCGGCAGGCCTAATACATGCAAGTCGAACGGGGCCGCAAGGCCCAGTGGCGCACGGGTGCG
TAACGCGTAGGCAATCTGCCTATCTCTGGGGGATAGCCCGCCGAAAGGTGGATTAATACCGCATGAGACC
ATAACACGGCATCGTGTAGTGGTTAAAGAATTTCGGAGATAGATGAGCCTGCGTGCCATTAGCTAGTTGGC
GGGGTAAGGGCCCACCAAGGCGACGATGGCTAGGGGAGCTGAGAGGCCGATCCCCCACACTGGCACTGA
GATACGGGC
>Zotu586
GAATGCTGGCGGCGCGCCTAACACATGCAAGTCGAGCGGAAAGGCCCTTCGGGGTACTCGAGCGGCGGA
CGGCTGAGTAACGCGTGGGAACATGCCCCAAAGTGAGGAATAACTGCCCGAAAGGGTAGCTAATGCCGCA
TATGATCTTAGGATTAAAGGATTTATCCGCTTTGGGAGTGGCCCGCGTACGATTAGGTAGTTGGTGAGGTA

AAGGCTCACCAAGCCGACGATCGTTAACTGGTCTGAGAGGATGATCAGTCAGACTGGAACTGAGACACGG
TC
>Zotu588
GAACGCTGGCGGCGTGCTTAACACATGCAAGTCGAACGATGAAGCCCAGCTTGCTGGGTGGATTAGTGGC
GAACGGGTGAGTAACACGTGAGTAACCTGCCCTTGACTCTGGGATAAGCCTGGGAAACTGGGTCTAATAC
CGGATAGGAACGTCCACCGCATGGTGGTTGTTGGAAAGATTTATCGGTCATGGATGGACTCGCGGCCTATC
AGCTTGTTGGTGAGGTAATGGCTCACCAAGGCGACGACGGGTAGCCGGCCTGAGAGGGTGACCGGCCAC
ACTGGGACTGAGACACGGCC
>Zotu590
GAACGCTGGCGGCATGCTTTACACATGCAAGTCGAACGGTAACAGGTCTTCGGATGCTGACGAGTGGCGA
ACGGGTGAGTAATACATCGGAACGTGCCTAGTAGTGGGGGATAACTACTCGAAAGAGTAGCTAATACCGC
ATGAGATCTACGGATGAAAGCAGGGGACCTTCGGGCCTTGTGCTACTAGAGCGGCTGATGGCAGATTAGG
TAGTTGGTGGGGTAAAGGCTCACCAAGCCTGCGATCTGTAGCTGGTCTGAGAGGACGACCAGCCACACTG
GGACTGAGACACGGCC
>Zotu592
GAACGCTGGCGGCATGCCTTACACATGCAAGTCGAACGGTAACAGGTCTTCGGATGCTGACGAGTGGCGA
ACGGGTGAGTAATACATCGGAACGTGCCCGATCGTGGGGGATAACGAAGCGAAAGCTTTGCTAATACCGC
ATACGATCTACGGATGAAAGCAGGGGACCGCAAGGCCTTGCGCGAACGGAGCGGCCGATGGCAGATTAG
GTAGTTGGTGGGATAAAAGCTTACCAAGCCGACGATCTGTAGCTGGTCTGAGAGGACGACCAGCCACACT
GGGACTGAGACACGGCC
>Zotu594
GAACGCTGACAGAATGCTTAACACATGCAAGTCAACTTGAATTTGGGTTTTTAACTTAGGTTTGGGTGGCG
GACGGGTGAGTAACGCGTAAAGAACTTGCCTCACAGGTAGGGACAACATTTGGAAACGAATGCTAATACC
TAATATTATGATAATAGGGCATCCTATAATTATGAAAGCTATAAGCGCTGTGAGAGAGCTTTGCGTCCCATT
AGCTAGTTGGAGAGGTAACGGCTCACCAAGGCGATGATGGGTAGCCGGCCTGAGAGGGTGATCGGCCAC
AAGGGGACTGAGACACGGCC
>Zotu596
GAACGCTAGCGGGAGGCCTAACACATGCAAGCCGAGCGGTATTGTTTCTTCGGAAATGAGAGAGCGGCGT
ACGGGTGCGGAACACGTGTGCAACCTGCCTTTATCTGGGGGATAGCCTTTCGAAAGGAAGATTAATACTCC
ATAATATATTGAACGGCATCGTTTAATATTGAAAGCTCCGGCGGATAGAGATGGGCACGCGCAAGATTAGC
TAGTTGGTGAGGTAACGGCTCACCAAGGCAATGATCTTTAGGGGGCCTGAGAGGGTGATCCCCCACACTG
GTACTGAGACACGGAC
>Zotu599
GAACGCTGGCGGCGTGCTTAACACATGCAAGTCGAACGGAAAGGCCTGCAGCTTGCTGCGGGTACTCGAG
TGGCGAACGGGTGAGTAACACGTGGGTGATCTGCCCTCAACTTTGGGATAAGCCTGGGAAACTGGGTCTA
ATACCGGATATGACCTTTCTTTAGTGTGGAAGGTGGAAAGCTTTTGCGGTTGGGGATGAGCTCGCGGCCTA
TCAGCTTGTTGGTGGGGTAATGGCCTACCAAGGCGTCGACGGGTAGCCGGCCTGAGAGGGTGTACGGCCA
CATTGGGACTGAGATACGGCC
>Zotu601
GAACGCTGGCGGCAGGCTTAACACATGCAAGTCGAGCGGGCGTAGCAATACGTCAGCGGCAGACGGGTG
AGTAACGCGTGGGAACGTACCTTTTGGTTCGGAACAACACAGGGAAACTTGTGCTAATACCGGATAAGCCC
TTACGGGGAAAGATTTATCGCCGAAAGATCGGCCCGCGTCTGATTAGCTAGTTGGTAGGGTAACGGCCTAC
CAAGGCGACGATCAGTAGCTGGTCTGAGAGGATGATCAGCCACATTGGGACTGAGACACGGCC
>Zotu603
GAACGCTGGCGGTGTGCTTAACACATGCAAGTGGTGCGACGAACCAGAGCTTGCTCTGGGGCAAAGCCGC
GAACGGGTGAGTAACACGTGGGTAATCTACCCCGATGACCGGGACAACCCGAGGAAACTCGGGCTAATAC
CGGATGTGGTACACAGGCTTAAGCCTGTTTACTAAAGGTAGCTTCGGCTTCCGCATTGGGAGGGGCCCGCG
GCCCATTAGCTTGTTGGTGGGGTCAAGGCCTACCAAGGCAACGATGGGTAGCTGGTCTGAGAGGACGATC
AGCCACACTGGGACTGAGACACGGCC
>Zotu605

GAACGCTGGCGGCAGGCTTAACACATGCAAGTCGAGCGCATCCTTCGGGGTGAGCGGCAGACGGGTGAG
TAACGCGTGGGAATCTACCTTTTGCTACGGAATAGCTCCGGGAAACTGGAATTAATACCGTATGTGCTCTTT
GGCGGTGGACGCTGGAGAGGAAAGATTTATCGGCAAAGGATGAGCCCGCGTTGGATTAGCTAGTTGGTG
GGGTAAAGGCCTACCAAGGCGACGATCCATAGCTGGTCTGAGAGGATGATCAGCCACATTGGGACTGAGA
CACGGCC

>Zotu607

GAACGCTGGCGGCAGGCTTAACACATGCAAGTCGAACGCCCCGCAAGGGGAGTGGCAGACGGGTGAGTA
ACACGTGGGAACGTACCCTTTTCTACGGAATAACCCAGGGAAACTTGGACTAATACCGTATGTGCCCTTCG
GGGGAAAGATTTATCGGAAAAGGATCGGCCCGCGTTGGATTAGCTAGTTGGTGGGGTAAAGGCCTACCAA
GGCGACGATCCATAGCTGGTCTGAGAGGATGATCAGCCACATTGGGACTGAGACACGGCC

>Zotu609

GAACGCTGGCGGCGTGCCTAACACATGCAAGTCGAGCGGTAACAGGTGTAGCAATACATGCTGACGAGCG
GCGAACGGGTGAGTAATGCTTCGGAATCTACCTTATGGTGGGGAATAACTAGTCGAAAGATTAGCTAATAC
CGCATACGTCCTACGGGAGAAAGCAGGGGATCGTAAGACCTTGCGCCAAAAGATGAGCCGAAGTCGGATT
AGCTAGTTGGTAGGGTAACGGCCTACCAAGGCGACGATCCGTAGCTGGTCTGAGAGGACGACCAGCCACA
CTGGAACTGAGACACGGTC

>Zotu611

GAACGCTAGCGGCAGGCTTAACACATGCAAGTCGAGGGGTAGAAGCAGCTTGCTGCTTTGAGACCGGCGC
ACGGGTGCGTAACGCGTATACAATCTACCTTTTACAGAGGGATAGCCCAGAGAAATTTGGATTAATACCTC
ATAGTATTTTCGAATCGCATGATTTGATTATTAAAGTTCCAACGGTAAAAGATGAGTATGCGTCCCATTAGC
TAGATGGTAAGGTAACGGCTTACCATGGCAACGATGGGTAGGGGTCCTGAGAGGGAGATCCCCCACACTG
GTACTGAGACACGGAC

>Zotu613

GAACGCTGGCGGCGCGCCTAATACATGCAAGTCGAACGGGTACCTCTTCGGAGGTGCCAGTGGCGGACGG
GTGAGTAACACGTGGGTGACCTGCCCTGTGGTGGGGAATAACTCTGGGAAACCAGGGCTAATACCGCATA
CGCTCAGCGTTTCATTACGCTGAGGAAAGATTGATCGCCATAGGAGGGGCCTGCGCTGGATTAGCTGGTC
GGTGGGGTAATGGCCTACCGAGGCTGCGATCCATAGCTGGTCTGAGAGGACGACCAGCCACACTGGGACT
GAGACACGGCC

>Zotu615

GAACGCTGGCGGCATGCCTAATACATGCAAGTTGAACGATGATGTTCTCTGCTTGCAGAGAATTGAAGAGT
AGCGAACGGGTGAGTAACACGTGGGGAGCCTGCCCATGAGAGGGGGATAACATTCGGAAACGGATGCTA
ATACCGCATAATGCTTTGGACCGCCTGGTCCAGAGACGAAAGACGACTTTTGCTGTCACTCATGGATGGCC
CCGCGCCGTATTAGCTAGTTGGTGAGGTAATGGCTCACCAAAGCTGTGATACGTAGCCGACCTGAGAGGG
TGATCGGCCACACTGGGACTGAGACACGGCC

>Zotu617

GAACGCTGACAGAATGCTTAACACATGCAAGTCATTGGCGAAGCTGCGCTTGCGCAGTCTAGCCAAGGCG
GACGGGTGAGTAACGCGTAAGGAACTTGCCCTGCAGACAGGGATAACAGACGGAAACGACTGATAACAC
CTGGTATTGTCAGGCTGGCGCATGCCGGCCTGATGAAAGGAGACGCTGCAGGAGAGCCTTGCGTCCTATT
AGCTAGTTGGTGGGGTAACGGCCCACCAAGGCGATGATAGGTAGCCGGCCTGAGAGGGTGAACGGCCAC
AAGGGGACTGAGATACGGCC

>Zotu619

GAACGCTGGCGGCGTGCTTAACACATGCAAGTGGAGCGACGAACCGGGGCTTGCCCCGGGGCAGAGCCG
CGAACGGGTGAGTAACACGTGGGTTACCTGCCCCAATGACCGGGACAACCCGAGGAAACTCGGGCTAATA
CCGGATGTGGTCTTCGGACGAAAGGAAGCTTCGGCCTCCGCATTGGGATGGGCCCGCGGCGCATTAGCTT
GTTGGTGGGGTAACGGCCTACCAAGGCGACGATGCGTAGCTGGTCTGAGAGGACGATCAGCCACACTGG
GACTGAGACACGGCC

>Zotu621

GAACGCTAGCGGCAGGCCTAATACATGCAAGTCGAACGGTGATATTTATATCATAGTGGCGCACGGGTGC
GTAACACGTATGCAACCTACCTATTATTGGGGGATAGCCTTTGGAAACGGAGATTAATACCGCATAATACG
ATTAACTGGCATCGGTTGATTGTTAAAGCATTTGCGATAATAGATGGGCATGCGTCCAATTAGTTAGTTGGT

GTGGTAATGGCTCACCAAGACGATGATTGGTAGGGGATCTGAGAGGATAATCCCCCACACTGGCACTGAG
ATACGGGC
>Zotu624
GAACGCTGGCGGCATGCTTTACACATGCAAGTCGAACGGCAGCGCGGGGCAACCTGGCGGCGAGTGGCG
AACGGGTGAGTAATATATCGGAACGTACCCAAGAGTGGGGGATAACGTAGCGAAAGTTACGCTAATACCG
CATACGATCTAAGGATGAAAGTGGGGGATCGCAAGACCTCATGCTCCTGGAGCGGCCGATATCTGATTAG
CTAGTTGGTGGGGTAAAAGCTCACCAAGGCGACGATCAGTAGCTGGTCTGAGAGGACGACCAGCCACACT
GGGACTGAGACACGGCC
>Zotu626
GAACGCTAGCGGCAGGCTTAATACATGCAAGTCGAGGGGTAACAGGGGTAGCAATACCTGCTGACGACCG
GCAAACGGGTGCGGAACACGTACGCAACCTTCCCAAAACTGGTGAATAGCCTTCCGAAAGGGAGATTAAT
ACACCGTAACTTAATGAAGTGGCATCACTTTATTAATATAGCTCCGGCGGTTTTGGATGGGCGTGCGCCTG
ATTAGGTAGTTGGCGGGGTAACGGCCCACCAAGCCTGCGATCAGTAACTGGTGTGAGAGCACGACCAGTC
ACACGGGCACTGAGACACGGGC
>Zotu628
GAACGCTAGCGGCAGGCCTAACACATGCAAGTCGAGGGGTAGAGTTAGCTTGCTAACTTGAGACCGGCGC
ACGGGTGCGTAACGCGTATGCAATCTACCTTATACTGAGGGATAGCCCGGAGAAATCCGGATTAATACCTT
ATAGTATATTAAAGTGGCATCATTTTGATATTAAAGATTTATTGGTATAAGATGAGCATGCGTCCCATTAGT
TAGTTGGTGTGGTAACGGCACACCAAGACGATGATGGGTAGGGGTCCTGAGAGGGAGATCCCCCACACTG
GTACTGAGACACGGAC
>Zotu630
GAACGCTAGCGGCAGGCTTAATACATGCAAGTCGAGGGGTAACATGGGTAGCAATACCTGATGACGACCG
GCAAACGGGTGCGGAACACGTACGCAATCTTCCCGAAACTGGTGAATAGCCCTCCGAAAGGAGGATTAAT
ACACCGTAAGATAACGAGGAGGCATCTCCTTGTTATTATAGTAGCAATACGGTTTTGGATGAGCGTGCGCC
TGATTAGGTAGTTGGCGGGGTAACGGCCCACCAAGCCTACGATCAGTAACTGGTGTGAGAGCACGACCAG
TCACACGGGCACTGAGACACGGGC
>Zotu632
GAACGCTGGCGGCGTGCTTAACACATGCAAGTCGAACGGTCGCAAGACAGTGGCGCACGGGTGAGGAAC
ACGTAGCTTATCTGCCCCGAAGTGTGGGATAACGCCCCGAAAGGGGTGCTAATACCGCATAAGCTCCCGG
ACTATAGAGGTCTGGGAGAAAAGGCGCAAGTCGCTTTGGGAGGAGGCTGCGGCCCATCAGGTAGTTGGT
GCGGTAACGGCGCACCAAGCCAACGACGGGTAGGGGACCTGAGAGGGTGATCCCCCACACTGGGACTGA
GACACGGCC
>Zotu634
GAACGCTAGCGGCAGGCTTAATACATGCAAGTCGAGGGGCAGCATGACTGTAGCAATACAGTTGATGGCG
ACCGGCAAACGGGTGCGGAACACGTACGCAATCTACCCAAAACTGATGAATAGCCCTCCGAAAGGAGGAT
TAATACATCGTAACTTAATGAAGTGGCATCACTTTATTAATATAGCTCCGGCGGTTTTGGATGAGCGTGCGC
CTGATTAGGTAGTTGGCGGGGTAACGGCCCACCAAGCCTGCGATCAGTAACTGGTGTGAGAGCACGACCA
GTCACACGGGCACTGAGACACGGGC
>Zotu636
GAACGCTGGCGGCGTGCTTAACACATGCAAGTCGAACGGTCGCAAGACAGTGGCGCACGGGTGAGTAAC
ACGTAGCTAACCTGCCTTCGAGTGCGGAATAACGCTCCGAAAGGAGTGCTAATACCGCATAAGCTCACGAG
AAGTAGATGCTTGTGAGAAAAGGGGAAACTCGCTTGAAGAGGGGGCTGCGACCCATCAGGTAGTTGGTG
TGGTAAAAGCGCACCAAGCCGACGACGGGTAGGGGGCCTGAGAGGGTGGTCCCCCACACTGGGACTGAG
ACACGGCC
>Zotu638
GAACGCTAGCTACAGGCTTAACACATGCAAGTCGAGGGGCAGCATGGAAGAAGCTTGCTTCTTCTGATGG
CGACCGGCGCACGGGTGCGTAACGCGTATCAAACCTGCCTCATACTCGGGGATAGCCTTGCGAAAGTAAG
ATTAATACCCGATGTTGTTATGTTGCCGCATGGTGATATAACCAAAGATTTATCGGTATGAGATGGTGATGC
GTCCGATTAGGTAGTAGGCGGGGTAACGGCCCACCTAGCCTTCGATCGGTAGGGGTTCTGAGAGGAAGGT
CCCCCACACTGGAACTGAGACACGGTC

>Zotu641
GAACGCTGGCGGCATGCCTAACACATGCAAGTCGAACGAGATCTTCGGATCTAGTGGCGCACGGGTGCGT
AACGCGTGGGAATCTGCCTTGGGGTTCGGAATAACTCCCCGAAAGGGGTGCTAATACCGGATGATGTCGA
AAGACCAAAGATTTATCGCCCTGAGATGAGCCCGCGTAGGATTAGCTAGTTGGTGTGGTAAAGGCGCACC
AAGGCGACGATCCTTAGCTGGTCTAAGAGGATGATCAGCCACACTGGGACTGAGACACGGCC
>Zotu643
GAACGCTGGCGGCGTGCTTAACACATGCAAGTCGAACGAAGAGCGATGGAAGCTTGCTTCTATCAATCTTA
GTGGCGAACGGGTGAGTAACGCGTAATCAACCTGCCCTTCAGAGGGGGACAACAGTTGGAAACGACTGCT
AATACCGCATACGATCTGACCTCGGCATCGAGGATAGATGAAAGGTGGCCTCTATTTATAAGCTATCACTG
AAGGAGGGGATTGCGTCTGATTAGCTAGTTGGAGGGGTAACGGCCCACCAAGGCGATGATCAGTAGCCG
GTCTGAGAGGATGAACGGCCACATTGGGACTGAGACACGGCC
>Zotu645
GAACGCTGGCGGCGTGCCTAATACATGCAAGTCGAACGAACAGCGACTTGAACTTGTTCAAGACAATGTTA
GTGGCGCACGGGTGAGTAACACGTGGAGAACCTACCCTTAAGCGGGGGATAACCATTGGAAACGATGACT
AATACCGCATAGAATCACAGATCGCATGATCAATGAAGGAAAGACGGCTTCGGCTGTCACTTAAGGATGG
CTCCGCGGTGCATTAGCTAGATGGTGGGGTAAAGGCCTACCATGGCAATGATGCATAGCCGACCTGAGAG
GGTGATCGGCCACATTGGGACTGAGACACGGCC
>Zotu647
GAACGCTGGCGGCAGGCCTAACACATGCAAGTCGAACGGTAGCACAGAGGAGCTTGCTCCTCGGGTGACG
AGTGGCGGACGGGTGAGTAATGTCTGGGAAACTGCCTGATGGAGGGGGATAACTACTGGAAACGGTAGC
TAATACCGCATAACGTCGCAAGACCAAAGAGGGGGACCTTCGGGCCTCTTGCCATCAGATGTGCCCAGATG
GGATTAGCTAGTAGGTGGGGTAATGGCTCACCTAGGCGACGATCCCTAGCTGGTCTGAGAGGATGACCAG
CCACACTGGAACTGAGACACGGTC
>Zotu649
GAACGCTGGCGGCGTGCCTAATACATGCAAGTAGAACGCTGAAGAGAGGAGTTTGCTCTTCTTGGATGAG
TTGCGAACGGGTGAGTAACGCGTAGGTAACCTGCCTGGTAGCGGGGGATAACTATTGGAAACGATAGCTA
ATACCGCATAAAATGGATTATCGCATGATAATTCATTGAAAGGTGCAAATGCATCACTACCAGATGGACCT
GCGTTGTATTAGCTAGTTGGTGGGGTAACGGCTCACCAAGGCGACGATACATAGCCGACCTGAGAGGGTG
ATCGGCCACACTGGGACTGAGACACGGCC
>Zotu651
GAACGCTGGCGGCGTGCCTAACACATGCAAGTCGAGCGAGGTCCATCCGGTGGCAACACCGGGGAAGAC
CTAGCGGCGAACGGGTGAGTAACACGTGAGAAACCTGCCCCGAAATCTGGGATAACCCAAGGAAACTCGG
GCTAATACCGGATGTCCTTCATTGATCGCATGGTCGGTGAAGGAAAGAATTTCGTTTCGGGAGGGTCTCGC
GGCCTATCAGCTAGTTGGCGGGGTAACGGCCCACCAAGGCATCGACGGGTAGCTGGTCTGAGAGGACGAT
CAGCCACACTGGGACTGAGACACGGCC
>Zotu653
GAACGCTGGCGGCATGCCTAACACATGCAAGTCGAACGAAGGCTTCGGCCTTAGTGGCGCACGGGTGCGT
AACGCGTGGGAATCTGCCCTTGGGTTCGGAATAACAGTTAGAAATGACTGCTAATACCGGATGATGTCGTA
AGACCAAAGATTTATCGCCCAAGGATGAGCCCGCGTAGGATTAGCTAGTTGGTGAGGTAAAAGCTCACCA
AGGCGACGATCCTTAGCTGGTCTGAGAGGATGATCAGCCACACTGGGACTGAGACACGGCC
>Zotu655
GAACGCTGGCGGCGTGCTTAACACATGCAAGTCGAACGCGCCTGTCCTCTGGTTTTCTGGGGGGTGGGTG
AGTGGCGAACGGGTGAGTAACACGTGAGTAACCTGCCCCCTTCTTCGGGATAACGGTCTGAAAGGGCTGC
TAATACCGGGTATTCACTGGTCTCCGCATGGGGTCTGGTGGAAAGGTTTTTTTCTGGTGGGGGATGGGCTC
GCGGCCTATCAGCTTGTTGGTGGGGTGATGGCCCACCAAGGCTTTGACGGGTAGCCGGCCTGAGAGGGTG
ACCGGTCACATTGGGACTGAGATACGGCC
>Zotu657
GAACGCTGGCGGCAGGCCTAACACATGCAAGTCGAACGGTAACAGGAAACAGCTTGCTGTTTCGCTGACG
AGTGGCGGACGGGTGAGTAATGTCTGGGAAACTGCCTGATGGAGGGGGATAACTACTGGAAACGGTAGC
TAATACCGCATAACGTCGCAAGACCAAAGAGGGGGACCTTCGGGCCTCTTGCCATCGGATGTGCCCAGAT

GGGATTAGCTTGTTGGTGGGGTAACGGCTCACCAAGGCGACGATCCCTAGCTGGTCTGAGAGGATGACCA
GCCACACTGGAACTGAGACACGGTC
>Zotu659
GAACGCTAGCTACAGGCTTAACACATGCAAGTCGAGGGGCAGCACGTTGTAGCAATACGATGGTGGCGAC
CGGCAAACGGGTGAGTAACACGTATCCAACCTGCCTGTTACTTGAGGATAGCCTCCTGAAAGGGAGATTAA
TACTCAATGTGGTACATTTTTCACATGCTTAATGTACTAAAGATTTATCGGTAACAGATGGGGATGCGTCCC
ATTAGGTAGTCGGCGGGGTAACGGCCCACCGAGCCGATGATGGGTAGGGGTTCTGAGAGGAAGGTCCCC
CACATTGGAACTTAGACACGGTC
>Zotu661
GAACGCTAGCGGGAGGCCTAACACATGCAAGCCGAGCGGTAGGGTTTCTTCGGAAACCTGAGAGCGGCG
CACGGGTGCGGAACACGTGTGCAACCTACCTTTATCAGGGGGATAGCCTTTCGAAAGGAAGATTAATACCC
CATAATATATTAGATGGCATCATTTGATATTGAAAACTCCGGTGGATAGAGATGGGCACGCGCAAGATTAG
ATAGTTGGTGAGGTAACGGCTCACCAAGTCAGTGATCTTTAGGGGGCCTGAGAGGGTGATCCCCCACACT
GGTACTGAGACACGGAC
>Zotu663
GAACGCTGGCGGCGTGCTTAACACATGCAAGTCGAACGAGGTCCAACTGATGGCAACATCAGGGAAGACC
TAGTGGCGAACGGGTGCGTAACACGTGAGGAACCTACCCCGGTCTCTGGGATAACAGTTGGAAACGACTG
CTAATACCGGATGCCGTCGCAGCATCGCATGGTGCAGTGACGAAAGGCCTGCCGGACCGGGAGGGCCTCG
CGGCCTATCAGCTTGTTGGTGGGGTAATGGCCTACCAAGGCATCGACGGGTAGCTGGTCTGAGAGGATGA
TCAGCCACACTGGGACTGAGACACGGCC
>Zotu665
GAACGCTGGCGGCGTGCTTAACACATGCAAGTCGAACGGAAAGGCCAGTGCTTGCACTGGTACTCGAGTG
GCGAACGGGTGAGTAACACGTGGGTGATCTGCCCTGCACTTCGGGATAAGCCTGGGAAACTGGGTCTAAT
ACCGGATAGGACCGCATTTTGGATGGTGTGGTGGAAAGTTTTTCGGTGTGGGATGAGCTCGCGGCCTATC
AGCTTGTTGGTGGGGTAATGGCCTACCAAGGCGTCGACGGGTAGCCGGCCTGAGAGGGTGTACGGCCAC
ATTGGGACTGAGATACGGCC
>Zotu667
GAACGCTGGCGGCAGGCCTCAAACATGCAAGTCTAGGGGGTGTAGCAATACATCACCGGCGGACGGGTG
CGTAACACGTGGGAATATACCTTTTGGTTCGGAACAACGCAGGGAAACTTGCGCTAATACCGAATGTGCCC
TTACGGGGAAAGATTTATCGCCAAAGGATTAGCCCGCGTAGGATTAGCTAGTTGGTGAGGTAATGGCTCA
CCAAGGCTCCGATCCTTAGCTGTTCTGAGAGGAAGATCAGCCACACTGGGACTGAGACACGGCC
>Zotu669
GAACGCTGGCGGCGTGCCTAATACATGCAAGTCGAGCGGGCCGAACGAGAGCTTGCTCTTGTTCGGTCAG
CGGCGGACGGGTGAGTAACACGTGGGCAACCTGCCCGCAAGACCGGGATAACTCCGGGAAACCGGAGCT
AATACCGGATAACACCAAAGACCGCATGGTCTTTGGTTGAAAGGCGGCTTCGGCTGTCACTTGCGGATGG
GCCCGCGGCGCATTAGCTAGTTGGTGAGGTAACGGCTCACCAAGGCGACGATGCGTAGCCGGCCTGAGAG
GGTGACCGGCCACACTGGGACTGAGACACGGCC
>Zotu671
GAACGCTGGCGGCGTGCTTAACACATGCAAGTCGAGCGGAAAGGCCCTTTTGGGGGTACTCGAGCGGCGA
ACGGGTGAGTATCACGTGAGTAATCTGCCCCCCTCTTTTGGATAACCCTCGGAAACGGGGGCTAATACGGA
ATATTCAGTGCTGCCTGCATGGGTGGTGTTGGAAAGGATTCGTTTGGTGGGGGATGAGCTCGCGGCCTAT
CAGCTTGATGGTGGGGTAATGGCCTACCATGGCTTTGACGGGTAGCCGGCCTGAGAGGGTGTCCGGCCAC
ACTGGGACTGAGACACGGCC
>Zotu674
GAACGCTGGCGGCAGGCCTAACACATGCAAGTCGAACGCCCCGCAAGGGGAGTGGCAGACGGGTGAGTA
ACGCGTGGGAACCTACCTTACGGTCGGGAATAGCTCAGGGAAACTTGGGGTAATACCGGATACGCCCTTA
CGGGGAAAGATTTATCGCCGTTAGATGGGCCCGCGTAGGATTAGCTAGTTGGTGAGGTAACGGCTCACCA
AGGCGACGATCCTTAGCTGGTCTGAGAGGATGATCAGCCACACTGGGACTGAGACACGGCC
>Zotu676

GAACGCTGGCGGCATGCCTAATACATGCAAGTCGAACGCTGAAGCACTCTGCTTGCAGAGTGCGGATGAG
TGGCGAACGGGTGAGTAACACGTGGGAAATCTGCCTAAGAGTGGGGGATAACATTCGGAAACGGATGCT
AATACCGCATATATTCTTGAACCGCATGGTTCTGGAAAGAAAGACGGCTTTGCTGTCGCTTTTAGATGATCC
CGCGGCGTATTAGTTAGTTGGTGGGGTAATGGCTTACCAAGACCATGATACGTAGCCGACCTGAGAGGGT
GATCGGCCACACTGGGACTGAGACACGGCC
>Zotu678
GAACGCTGGCGGCAGGCCTAACACATGCAAGTCGAACGCTCGTAGCAATACGGGAGTGGCAGACGGGTG
AGTAACACGTGGGAACGTACCCTTCGGTTCGGGATAACTCAGGGAAACTTGAGCTAATACCGGATACGTCC
GAGAGGAGAAAGGTTTACTGCCGAAGGATCGGCCCGCGTCTGATTAGCTAGTTGGTGAGGTAACGGCTCA
CCAAGGCGACGATCAGTAGCTGGTCTGAGAGGATGATCAGCCACATTGGGACTGAGACACGGCC
>Zotu681
GAACGCTGGCGGCAGGCTTAACACATGCAAGTCGAACGCCCCGCAAGGGGAGTGGCAGACGGGTGAGTA
ACGCGTGGGAATCTACCCAGATCTTCGGAACAACTGAGGGAAACTTCAGCTAATACCGGATACGCCCTACG
GGGGAAAGATTTATCGGTTCTGGATGAGCCCGCGTTGGATTAGCTAGTTGGTGAGGTAATGGCCCACCAA
GGCGACGATCCATAGCTGGTCTGAGAGGATGATCAGCCACACTGGGACTGAGACACGGCC
>Zotu683
GAACGCTAGCGGCAGGCCTAATACATGCAAGTCGGACGGGATTGAGGAGCTTGCTTCTCATGAGAGTGGC
GCACGGGTGCGTAACACGTATGTAACCTACCTTAGTCAGGGGGATAGCCTCTCGAAAGAGAGATTAACAC
CGCATAACATAATGATATGGCATCATATTATTATCAAATATTTATAGGACTAAGATGGGCATGCGGAACATT
AGCTAGTTGGTAAGGTAACGGCTTACCAAGGCTACGATGTTTAGGGGATCTGAGAGGATGACCCCCCACA
CTGGTACTGAGACACGGAC
>Zotu685
GAACGCTGGCGGCGTGCCTAATACATGCAAGTTGAGCGATGAAGATTGGTGCTTGCACCAATTTGAAGAG
CAGCGAACGGGTGAGTAACGCGTGGGGAATCTGCCTTTGAGCGGGGGACAACATTTGGAAACGAATGCT
AATACCGCATAACAACTTTAAACATAAGTTTTAAGTTTGAAAGATGCAATTGCATCACTCAAAGATGATCCC
GCGTTGTATTAGCTAGTTGGTGAGGTAAAGGCTCACCAAGGCGATGATACATAGCCGACCTGAGAGGGTG
ATCGGCCACATTGGGACTGAGACACGGCC
>Zotu687
GAACGCTGGCGGCGTGCTTAACACATGCAAGTCGAACGATGAAGCACTTCGGTGTGGATTAGTGGCGAAC
GGGTGAGTAACACGTGAGCAACCTGCCCCTTTCTCTGGGATAACCGCCGGAAACGGTGGCTAATACCGGA
TATTCACTTTTCCGTGCATGCGGTTTGGTGGAAAGATTTTTTGGTGGGGGATGGGCTCGCGGCCTATCAGC
TTGTTGGTGGGGTAATGGCCTACCAAGGCTTCGACGGGTAGCCGGCCTGAGAGGGCGACCGGCCACACTG
GGACTGAGATACGGCC
>Zotu689
GAACGCTGGCGGCGTGCTTAACACATGCAAGTCGAACGGTGAACACGGAGCTTGCTCTGTGGGATCAGTG
GCGAACGGGTGAGTAACACGTGAGCAACCTGCCCCTGACTCTGGGATAAGCGCTGGAAACGGCGTCTAAT
ACTGGATATGTGACGTGACCGCATGGTCTGCGTTTGGAAAGATTTTTCGGTTGGGGATGGGCTCGCGGCCT
ATCAGCTTGTTGGTGAGGTAATGGCTCACCAAGGCGTCGACGGGTAGCCGGCCTGAGAGGGTGACCGGCC
ACACTGGGACTGAGACACGGCC
>Zotu691
GAACGCTGGCGGCGTGCCTAATACATGCAAGTCGAGCGGCAGCACGGTCTTCGGACTGGTGGCGAGCGG
CGGACGGCAGAGTAACGCGTAGGAATATACCCCAAAGTGAGGGATAAGCTCCAGAAATGGAGTCTAATAC
CGCATATGGTCTTCGGATTAAAGCCTTCGGGCGCTATGGGAATAGCCTGCGTTCGATTAGCTAGTTGGTGA
GGTAATGGCTCACCAAGGCGACGATCGATAGCTGGTCTGAGAGGATGATCAGCCTGACTGGAACTGAGAT
ACGGTC
>Zotu693
GAACGCTGGCGGCGTGCCTAATACATGCAAGTCGAACGGTTTAGTTCCAAGAGGCTTGCCTTTTGGAACTA
GACAGTGGCGGACGGGTGAGTAACACGTAGGCAACCTACCTGTCAAACCGGGATAACCTCGGGAAACCGA
AGCTAAACCCGGATAAGCTCCTTTTTCGCATGAAGGGGGAGTGAAAAGGTGCGACCTGATGACAGATGGG
CCTGCGGCGCATTAGCTAGTAGGTAGGGTAACGGCCTACCTAGGCGATGATGCGTAGCCGACCTGAGAGG
GCGTACGGCCACACTGGGACTGAGATACGGCC

>Zotu696
GAACGCTAGCGGCAGGCCTAATACATGCAAGTCGAACGATAGTAGGGAGCTTGCTCTCTACGAAAAGTGG
CGCACGGGTGCGTAACGCGTATGCAACCTACCTTAATCAGGGGGATAGCCCGAAGAAATTCGGATTAACA
CCGCATAAAATCACAGTATAGCATTATACAATGATCAAATATTTATAGGATTAAGATGGGCATGCGTGACAT
TAGTTAGTTGGTGAGGTAACGGCTCACCAAGACCACGATGTCTAGGGGATCTGAGAGGATGACCCCCCAC
ACTGGTACTGAGACACGGAC
>Zotu698
GAACGCTGGCGGCATGCCTAACACATGCAAGTCGAACGAATTTTCAGCTTCGGTTGGAAGTTAGTGGCGCA
CGGGTGCGTAACGCGTGGGAATCTGCCCCTTGGTTCGGAATAACAGTTAGAAATGACTGCTAATACCGGAT
GATGACGAAAGTCCAAAGATTTATCGCCGAGGGATGAGCCCGCGTAGGATTAGCTAGTTGGTGGGGTAAA
GGCTCACCAAGGCGACGATCCTTAGCTGGTCTGAGAGGATGATCAGCCACACTGGGACTGAGACACGGCC
>Zotu700
GAACGCTGGCGGCGTGCCTAACACATGCAAGTCGAACGGATGGTGTAGCAATACATCGTTAGTGGCAAAC
GGGTGAGTAATATATAGGTAATCTGCCCTTTAGTCTGGGATAACTTGCCGAAAGGTGAGCTAATACTGGAT
ACGTTCCTAGGGTCGCATGGCTCCGGGAAGAAAGATTTATCGCTAAAGGAAGAGCCTATATCTGATTAGCT
AGTTGGTGGGGTAATGGCCTACCAAGGCGACGATCAGTAGCTGGTCTGAGAGGACGAACAGCCACACTG
GGACTGAGACACGGCC
>Zotu702
GAACGCTGGCGGCGTGCTTAACACATGCAAGTCGAACGGAAAGGCCCTGCTTGCAGGGTACTCGAGTGGC
GAACGGGTGAGTAACACGTGGGTGATCTGCCCTGCACTTCGGGATAAGCTTGGGAAACTGGGTCTAATAC
CGGATAGGAGCCATCTTTAGTGTGGTGGTTGGAAAGTTTTTTCGGTGTAGGATGAGCTCGCGGCCTATCAG
CTTGTTGGTGGGGTAATGGCCTACCAAGGCGGCGACGGGTAGCCGGCCTGAGAGGGTGTACGGCCACATT
GGGACTGAGATACGGCC
>Zotu704
GAACGCTGGCGGCGTGCTTAACACATGCAAGTCGAACGGTGAAGGAGAGCTTGCTCTCTGGATCAGTGGC
GAACGGGTGAGTAACACGTGAGCAATCTGCCCCTGACTCTGGGATAAGCGCTGGAAACGGCGTCTAATAC
CGGATACGAGCTGCGAAGGCATCTTCAGCAGCTGGAAAGAACTTCGGTCAGGGATGAGCTCGCGGCCTAT
CAGCTAGTTGGTGAGGTAACGGCTCACCAAGGCGTCGACGGGTAGCCGGCCTGAGAGGGTGACCGGCCA
CACTGGGACTGAGACACGGCC
>Zotu706
GAACGCTGGTGGCGTGCTTAACACATGCAAGTCGAGCGAGGCGTCCGCTTTCGGGTGGATAGTCGAGCGG
CGAACGGGTGAGTAACACGTGAGTAATCTTCCCTGGGCTCTGGGATAACCTCGGGAAACTGAGGGTAATA
CCGGATGTGCGTTTCCTGGCGCCTGCTGGGAGACGGAAAGGTTGCGTGCCGTGAGGTGCGTTTCCGGCCT
TGGATGAGCTCGCGGCCTATCAGCTTGTTGGTGCGGTGATGGCGTACCAAGGCGACGACGGGTAGCCGGC
CTGAGAGGGTGACCGGCCACACTGGGACTGAGATACGGCC
>Zotu708
GAACGCTGGCGGCGTGCTTAACACATGCAAGTCGAACGGAGTTTGTATGGAATGAGTCTTCGGACAAATG
AAATATAAACTTAGTGGCGGACGGGTGAGTAACGTGTGGGTAACCTACCCTATACAGGAGGATAACAGTA
AGAAATTATTGCTAAAACTGCATAAGATTACGAGTTCGCATGAACAAGTGATAAAAACTTTAGTGGTATAG
GATGGGCCCGCATCTGATTAGGTAGTTGGTGGAGTAAAAGCCTACCAAGCCGACGATCAGTAGCCGATCT
GAGAGGATGACCGGCCACATTGGGACTGAGACACGGCC
>Zotu710
GAACGCTGGCGGCGTGCTTAACACATGCAAGTCGAACGGAAAGGCCCTTCGGGGTACTCGAGTGGCGAAC
GGGTGAGTAACACGTGGGTGATCTGCCCTGCACTTTGGGATAAGCCTGGGAAACTGGGTCTAATACCGAA
TACACCCTGCTGGTCGCATGGCCTGGTGGGGGAAAGCTTTTGCGGTGTGGGATGGGCCCGCGGCCTATCA
GCTTGTTGGTGGGGTGATGGCCTACCAAGGCGACGACGGGTAGCCGGCCTGAGAGGGTGTCCGGCCACA
CTGGGACTGAGATACGGCC
>Zotu713
GAACGCTGGCGGCGTGCCTAATACATGCAAGTCGAGCGAATCTTTTGGGGGCTTGCTCCCAAAAGATTAGC
GGCGGACGGGTGAGTAACACGTGGGCAACCTGCCTATAAGACTGGGATAACTTCGGGAAACCGGAGCTA

ATACCGGATATGTTCTTTTCTCGCATGAGAGAAGATGGAAAGACGGTTTCGGCTGTCACTTATAGATGGGC
CTGCGGCGCATTAGCTAGTTGGTGGGGTAATGGCTCACCAAGGCGACGATGCGTAGCCGACCTGAGAGGG
TGATCGGCCACACTGGGACTGAGACACGGCC

>Zotu715

CAACGCTGGCGGCGCGCTTAACACATGCAAGTCGCACGAGAAAGTGGAGCAATCCATGAGTACAGTGGCG
TACGGGTGAGTAACACGTGGATCATCTGCCTCTTGATGGGGAATAACCCTGGGAAACCGGGGCTAATACC
GCATAAGCCTGAAAAGGGAAAGCAGCAATGCGTCGAGAGAGGAGTCCGCGGCCGATTAGCTAGTTGGTA
GGGTAATGGCCTACCAAGGCAATGATCGGTAGCCGGCCTGAGAGGGCACACGGCCACACTGGCACTGAA
ACACGGGC

>Zotu717

GAACGTTGGCGGCGTGCCTAATACATGCAAGTCGAACGCGGTCTCCTAATTGAAAAACCGTGCTTGCACGG
GTTGGATTTTAGATCGGACCGAGTGGCGAACTGGTGAGTAACACGTGGGTAACCTGCCCAGAAGTAGGGG
ATAACACCTGGAAACAGATGCTAATACCGTATAACAACTAAAACCACATGGTTTTAGTTTGAAAGCTGGCCT
TGGTGCTAGTGCTTTTGGATGGACCCGCGGCGTATTAGCTAGTTGGTGAGATAATAGCTCACCAAGGCGAT
GATACGTAGCAGACCTGAGAGGGTAATCTGCCACAATGGGACTGAGACACGGCC

>Zotu719

GAACGCTGGCGGCGCGCTTAACACATGCAAGTCGAGCGATGAAATCTTAACAGAACCCTTCGGGGCGAAG
ATAAGACGGATTAGCGGCGGACGGGTGAGTAACACGTGAGTAACCTGCCTTTTACACAGGGATAGCCTCG
GGAAACCGGGATTAAAACCTGATGAAACTATCAAATCACATGATTAAGAAAGTTAAAACTCCGGTGGTAAA
AGATGGACTCGCGTCCCATTAGCTAGTTGGTGAAGGTAACGGCCCACCAAGGCAACGATGGGTAGCCGGC
CTGAGAGGGTGAACGGCCACATTGGAACTGAGAAACGGTC

>Zotu721

GAACGCTGGCGGCGTGCTTAACACATGCAAGTCGAACGGAAAGGCCCTTCGGGGTACTCGAGTGGCGAAC
GGGTGAGTAACACGTGGGTGATCTGCCTCGTACTCTGGGATAAGCCTGGGAAACTGGGTCTAATACCGGA
TAGGACCACTGGTCGCATGGCTGGTGGTGGAAAGTTTTTCGGTACGAGATGGGCCCGCGGCCTATCAGCTT
GTTGGTGGGGTAATGGCCTACCAAGGCGACGACGGGTAGCCGGCCTGAGAGGGCGACCGGCCACACTGG
GACTGAGACACGGCC

>Zotu723

GAACGCTGGCGGCGTGCCTAATACATGCAAGTCGAACGAACCGCGACTAGGTGCTTGCACTTGGTCAAGG
TGAGTGGCGAACGGGTGAGTAACACGTGGGTAACCTACCTCATAGTGGGGGATAACAGTCGGAAACGACT
GCTAATACCGCATAGGACATAGGATCACATGATCCAGTGAGGAAAGGTGGCGCAAGCTATCGCTAAGAGA
TGGACCCGCGGTGCATTAGCTAGTTGGTAGGGTAAAGGCCTACCAAGGCGATGATGCATAGCCGACCTGA
GAGGGTGATCGGCCACATTGGGACTGAGACACGGCC

>Zotu725

GAACGCTGGCGGTGTGCTTCACACATGCAAGTCGAACGAGGTAGCAATACCTAGTGGCGGACGGGTGAGT
AACGCGTGGGAATCTGCCTTTAGATGGGGGATAGCAGGCCGAAAGGCCTATTAATACCGCATATGCCGTA
AGGTAAAAGGAGTAATCCGTCTAAAGATGAGCCCGCGTCCGATTAGCTAGTTGGTGAGGTAAAGGCTTAC
CAAGGCTACGATCGGTAGGTGGTCTGAGAGGATGATCACCCACAATGGGACTGAGACACGGCC

>Zotu727

GAACGCTGGCGGCGTGCTTAACACATGCAAGTCGAACGATGATGCCCAGCTTGCTGGGTGGATTAGTGGC
GAACGGGTGAGTAACACGTGAGTAACCTGCCCCTGACTCCGGGATAAGCGTTGGAAACGACGTCTAATAC
TGGATACGACGGCCGATCGCATGGTCTGGTCGTGGAAAGAATTTTGGTTGGGGATGGACTCGCGGCCTAT
CAGCTTGTTGGTGAGGTAATGGCTCACCAAGGCGACGACGGGTAGCCGGCCTGAGAGGGTGACCGGCCA
CACTGGGACTGAGACACGGCC

>Zotu730

TAACGCTGGCGGCGTGCATAACACATGCAAGTCGAACGATGAAACTTAGTAGACTTCTTCGGAAAGACACT
AAGCGGATTAGTGGCGAACGGGTGAGTAACGCGTGAGTAACCTGCCTTACACAAGGGGATAGCCTTTGGA
AACGAAGAATAATACCCTATAAAACTATAAAAGCACATGCTAGAATGGTCAAAGTGCTAGCGGTGTAAGAT
GGACTTGCGTCTGATTAGCTAGTTGGTGAGATAAAAGCTCACCAAGGCAACGATCAGTAGCCGGCTTGAG
AGAGTGTACGGCCACATTGGGACTGAGACACGGCC

>Zotu732
GAACGCTAGCGGCAGGCTTAACACATGCAAGTCGAGGGGCAGCACGGTAGCAATACTGGTGGCGACCGG
CAAACGGGTGCGGAACACGTACAGAATCTACCTTGATCTGGGGCATAGCCCGGAGAAATCCGGATTAACT
CCCCATAACATATAAGAGTGGCATCACTTTAATATTAAAAATTTATTGGATCGAGATGACTGTGCGTCCCAT
TAGCTAGTTGGTAGGGTAACGGCCTACCAAGGCGACGATGGGTAGCTGGTGTGAGAGCACGACCAGCCAC
ACGGGCACTGAGACACGGGC
>Zotu734
GAACGCTGGCGGCGTGCTTAACACATGCAAGTCGAACGGTGAAAGAGGAGCTTGCTCCTCTGGATCAGTG
GCGAACGGGTGAGTAACACGTGAGTAACCTGCCCCAGACTCTGGGATAAGCACTGGAAACGGTGTCTAAT
ACCGGATACGAGCTTCAGCCGCATGGCTAGGAGCTGGAAAGAATTTCGGTCTGGGATGGACTCGCGGCCT
ATCAGCTAGTTGGTGAGGTAATGGCTCACCAAGGCGACGACGGGTAGCCGGCCTGAGAGGGTGACCGGC
CACACTGGGACTGAGACACGGCC
>Zotu736
GAACGCTGGCGGCGTGCCTAATACATGCAAGTCGAGCGAACAGATGAGAAGCTTGCTTCTCTGATGTTAGC
GGCGGACGGGTGAGTAACACGTGGGTAACCTACCTATAAGACTGGGATAACTCCGGGAAACCGGGGCTA
ATACCGGATAATATTTTGAACCGCATGGTTCAATAGTGAAAGACGGTTTCGGCTGTCACTTATAGATGGAC
CCGCGCCGTATTAGCTAGTTGGTAAGGTAATGGCTTACCAAGGCGACGATACGTAGCCGACCTGAGAGGG
TGATCGGCCACACTGGAACTGAGACACGGTC
>Zotu738
GAACGCTGGCGGCATGCCTAACACATGCAAGTCGAACGTGATCCAGGCTTCGGTCTGGTGAAAGTGGCAG
ACGGGTGAGTAACGCGTGGGAACCTGCCCTGTGGTAGGGAATAACCGCGGGAAACTGCGGCTAATACCCT
ATGTGTCCTGAGGGACAAAGATTTATCGCCATAGGAGGGGCCCGCGTCGGATTAGGTAGTTGGTGGGGTA
ACGGCCTACCAAGCCATCGATCCGTAGCTGGTCTGAGAGGATGATCAGCCACACTGGGACTGAGATACGG
CC
>Zotu740
GAACGCTGGCGGCGTGCCTAATACATGCAAGTAGAACGCTGAAGGAGGAGCTTGCTCTTCTGGATGAGTT
GCGAACGGGTGAGTAACGCGTAGGTAACCTGCCTGGTAGCGGGGGATAACTATTGGAAACGATAGCTAAT
ACCGCATAATAGCAGTTGTTGCATGACAGCTGTTTGAAAGGTGCAATTGCATCACTACCAGATGGACCTGC
GTTGTATTAGCTAGTTGGTGGGGTAACGGCTCACCAAGGCGACGATACATAGCCGACCTGAGAGGGTGAT
CGGCCACACTGGGACTGAGACACGGCC
>Zotu742
GAACGCTGGCGGCAGGCCTAACACATGCAAGTCGAGCGAGGACTTCGGTTCTAGCGGCGGACGGGTGAG
TAACGCGTGGGAATGTGCCCTTCTCTACGGAATAGCCCTGGGAAACTGGGAGTAATACCGTATACGCCCTA
TTGGGGAAAGATTTATCGGAGAAGGATCAGCCCGCGTTGGATTAGGTAGTTGGTGGGGTAATGGCCTACC
AAGCCGACGATCCATAGCTGGTTTGAGAGGATGATCAGCCACACTGGGACTGAGACACGGCC
>Zotu744
GAACGCTGACAGAATGCTTAACACATGCAAGTCTACTTGAACTTCGGTTTGGGTGGCGGACGGGTGAGTA
ACGCGTAAAGAACTTGCCTCACAGTTAGGGACAACATTTGGAAACGAATGCTAATACCTGATATTATGATTT
TAGGGCATCCTAAGATTATGAAAGCTATATGCGCTGTGAGAGAGCTTTGCGTCCCATTAGCTAGTTGGAGA
GGTAACGGCTCACCAAGGCGATGATGGGTAGCCGGCCTGAGAGGGTGAACGGCCACAAGGGGACTGAGA
CACGGCC
>Zotu746
GAACGCTTGCGGCGTGCCTAAGAAATGCAAGTCGAACGAGAGCTTCGGCTCTAGTGGCGAACGGTCGCGT
AACACGTGGGCAACCTGCCCTGAAGCGGGGGACAACAGACCGAAAGGTCTGCTAATACCGCATATGATCC
CTCTCGGCATCGAGAGTGCTCAAAGTCTTCGGACACTTCAGGATGGGCCCGCGGCCTATCAGCTTGTTGGT
GGGGTAACGGCTCACCAAGGCTACGACGGGTAGCTGGTCTGAGAGGACGACCAGCCGGATTGGGACTGA
GATACGGCC
>Zotu748
GAACGCTGGCGGCGCGCTTAACACATGCAAGTCGAACGGAGTCCTTCGGGGCTCAGTGGCGAACGGGTGC
GTAACACGTGAGCAACCTGCCTGGTCGTGGGGGATAACCAGTGGAAACGCTGGCTAATACCGCATGAGCT

CTTTGCTTCGATTGAGGCGTTGAGGAAAGCTCCTGCAAGGGAGTGCGACCAGAGGGGCTCGCGGCGTATC
AGCTAGTTGGAGGGGTAACGGCCCACCAAGGCGATGACGCGTAGCCGATCTGAGAGGATGATCGGCCAC
ATTGGGACTGAGATACGGCC
>Zotu750
GAACGCTGGCGGCGTGCTTAACACATGCAAGTCGAGCGGAAAGGCCCTTCGGGGTACTCGAGCGGCGAA
CGGGTGAGTAACACGTGGGCAACCTGCCCCTAGCTCTGGGATAACTCCGGGAAACCGGGGCTAATACCGG
ATACGACTTCCCCCGGCATCTGGGGAAGTGGAAAGTTCTTCGGCTAGGGATGGGCCCGCGGCCTATCAGCT
TGTTGGTGGGGTAACGGCCCACCAAGGCGACGACGGGTAGCCGGCCTGAGAGGGCGACCGGCCACACTG
GGACTGAGACACGGCC
>Zotu752
GAACGCTGGCGGCGTGCTTAACACATGCAAGTCGAACGAGATCCAGCCGAGCCTTCGGGCAAAGCGAAGA
TCTAGTGGCGAACGGGTGCGTAACACGTGAGAAACCTGCCCCGGACTTGGGAATAACAGTCGGAAACGGC
TGCTAATACCGAATACCTTCGTAGCGTCGCATGGCGCATCGAAGAAAGCTTTTGCGGTTTGGGAGGGTCTC
GCGGCCTATCAGCTTGTTGGCGAGGTAACGGCTCACCAAGGCATCGACGGGTAGCTGGTCTGAGAGGATG
ATCAGCCACACTGGGACTGAGACACGGCC
>Zotu754
GAACGCTGGCGGCGTGCCTAATACATGCAAGTAGAACGCTGAAGGAGGAGCTTGCTCTTCTGGATGAGTT
GCGAACGGGTGAGTAACGCGTAGGTAACCTGCCTGGTAGCGGGGGATAACTATTGGAAACGATAGCTAAT
ACCGCATAAGAGTAGATGTTGCATGACATTTGCTTAAAAGGTGCAATTGCATCACTACCAGATGGACCTGC
GTTGTATTAGCTAGTTGGTGGGGTAACGGCTCACCAAGGCGACGATACATAGCCGACCTGAGAGGGTGAT
CGGCCACACTGGGACTGAGACACGGCC
>Zotu756
GAACGCTGGCGGCATGCCTAATACATGCAAGTCGAACGCTGAAGCACTCTGCTTGCAGAGTGCGGATGAG
TGGCGAACGGGTGAGTAACACGTGGGAAATCTGCCTAAGAGTGGGGGATAACATTCGGAAACGGATGCT
AATACCGCATATATTCTTGAATCGCATGGTTCTGGAAAGAAAGACGGCTTTGCTGTCGCTTTTAGATGATCC
CGCGGCGTATTAGTTAGTTGGTGGGGTAAAGGCCTACCAAGACCATGATACGTAGCCGACCTGAGAGGGT
GATCGGCCACACTGGGACTGAGACACGGCC
>Zotu758
GAACGTTGGCGGCGTGGATTAGGCATGCAAGTCGTACGCACCGCAAGGTGAGTGGCGCAAGGGTGAGTA
AGGCGAAGGTAACCAACCCCGAGATTGGGTATAGCCGTGGGAAACCGCGGGTAATCCCCAGCGATGTTGT
TGAGTGGCATCACTCGACAACCAAAGCTCCGGCGTCTTGGGACGGGCCTTCGTGGTATTAGGTTGTTGGCG
GGGTAATGGCCCACCAAGCCTCTGATGCCTACCGGGCGTGCGAGCGTGGCCCGGCACACTGGGACTGAGA
CACTGCC
>Zotu760
GAACGCTGGCGGCGTGCTTAACACATGCAAGTCGAACGATGAAGCCCAGCTTGCTGGGTGGATTAGTGGC
GAACGGGTGAGTAACACGTGAGTAACCTGCCCTTAACTCTGGGATAAGCCTGGGAAACTAGGTCTAATACC
GGATAGGAGCGCCTACCGCATGGTGGGTGTTGGAAAGATTTATCGGTTTTGGATGGACTCGCGGCCTATC
AGCTTGTTGGTGAGGTAATGGCTCACCAAGGCGACGACGGGTAGCCGGCCTGAGAGGGTGACCGGCCAC
ACTGGGACTGAGACACGGCC
>Zotu762
GAACGCTGGCGGCGTGCTTAACACATGCAAGTCGAACGGTGAAAGAGGAGCTTGCTCCTCTGGATCAGTG
GCGAACGGGTGAGTAACACGTGAATAACCTGCCCCAGACTCTGGGATAAGCGTTGGAAACGACGTCTAAT
ACCGGATACGAGCTTCAGCCGCATGGCTAGGAGCTGGAAAGAATTTCGGTCTGGGATGGACTCGCGGCCT
ATCAGGTAGTTGGTGAGGTAACGGCCCACCAAGCCTACGACGGGTAGCCGGCCTGAGAGGGTGACCGGC
CACACTGGGACTGAGACACGGCC
>Zotu764
TAACGCTGGCGGCGTGCATAACACATGCAAGTCGAACGATGAAACTTAGTAGACTTCTTCGGAAAGACACT
AAGCGGATTAGTGGCGAACGGGTGAGTAACGCGTGAGTAACCTGCCTTACACAAGGGGATAGCCTTTGGA
AACGAAGAATAATACCCTATAAAACCATAAAAGCATATGCAATTATGGTCAAAGTGATAGCGGTGTAAGAT
GGACTTGCGTCTGATTAGCTAGTTGGTGAGATAAAAGCTCACCAAGGCAACGATCAGTAGCCGGCTTGAG
AGAGTGTACGGCCACATTGGGACTGAGACACGGCC

>Zotu769
GAACGCTGGCGGCGTGCCTAATACATGCAAGTCGAACGCATTTTCTTTCACCGTAGCTTGCTACACCGAAA
GAAAGTGAGTGGCGAACGGGTGAGTAACACGTGGGTAACCTGCCCATCAGCGGGGGATAACACTTGGAA
ACAGGTGCTAATACCGCATAATTCCATTTACCGCATGGTAGATGGATGAAAGGCGCTTTTGCGTCACTGAT
GGATGGACCCGCGGTGCATTAGCTAGTTGGTGGGGTAACGGCCTACCAAGGCTGCGATGCATAGCCGACC
TGAGAGGGTGATCGGCCACACTGGGACTGAGACACGGCC
>Zotu771
GAACGCTGGCGGCATGCCTTACACATGCAAGTCGAACGGCAGCACGGGTGCTTGCACCTGGTGGCGAGTG
GCGAACGGGTGAGTAATACATCGGAACGTACCTTATCGTGGGGGATAACGCAGCGAAAGCTGTGCTAATA
CCGCATACGCCCTGAGGGGGAAAGCGGGGGATCGAAAGACCTCGCGCGATTAGAGCGGCCGATGCCTGA
TTAGCTTGTTGGTGGGGTAAAAGCCCACCAAGGCGACGATCAGTAGCTGGTCTGAGAGGACGATCAGCCA
CACTGGGACTGAGACACGGCC
>Zotu773
GAACGCTGGCGGCATGCTTAACACATGCAAGTCGAACGGCAGCATGACCTAGCTTGCTAGGTTGATGGCG
AGTGGCGGACGGGTGAGTAACGCGTAGGAATATGCCTTGAAGAGGGGGATAACTTGGGGAAACTCAAGC
TAATACCGCATAAGCTCTGAGGAGTGAAGCTGGGGACCGTAAGGCCTGGCGCTTTGAGATTAGCCTGCGT
CCGATTAGCTAGTTGGTGGGGTAAGGGCCTACCAAGGCGACGATCGGTAGCTGGTCTGAGAGGATGATCA
GCCACACTGGGACTGAGACACGGCC
>Zotu776
GAACGCTAGCGGGAGGCCTAACACATGCAAGCCGAGCGGTATTGTTTCTTCGGAAATGAGAGAGCGGCGT
ACGGGTGCGGAACACGTGTGCAACCTGCCTTTATCTGGGGGATAGCCTTTCGAAAGGAAGATTAATACTCC
ATAATATATTGATTGGCATCAATTAATATTGAAAGCTCCGGCGGATAGAGATGGGCACGCGCAAGATTAGC
TAGTTGGTGAGGTAACGGCTCACCAAGGCGATGATCTTTAGGGGGCCTGAGAGGGTGATCCCCCACACTG
GTACTGAGACACGGAC
>Zotu778
GAACGCTGGCGGCGTGCCTAATACATGCAAGTCGAGCGAATCTGAGGGAGCTTGCTCCCAAAGATTAGCG
GCGGACGGGTGAGTAACACGTGGGTAACCTGCCTGTAAGATTGGGATAACTCCGGGAAACCGGAGCTAAT
ACCGGATAACATTTCGAACCGCATGGTTCGAAATTGAAAGATGGTTTCGGCTATCACTTACAGATGGACCC
GCGGCGCATTAGCTAGTTGGTGAGGTAACGGCTCACCAAGGCAACGATGCGTAGCCGACCTGAGAGGGT
GATCGGCCACACTGGGACTGAGACACGGCC
>Zotu780
GAACGCTGGCGGCGTGCCTAATACATGCAAGTCGAGCGAATGGATTAAGAGCTTGCTCTTATGAAGTTAGC
GGCGGACGGGTGAGTAACACGTGGGTAACCTGCCCATAAGACTGGGATAACTCCGGGAAACCGGGGCTA
ATACCGGATAACATTTTGAACCACATGGTTCGAAATTGAAAGGCGGCTTCGGCTGTCACTTATGGATGGAC
CCGCGTCGCATTAGCTAGTTGGTGAGGTAACGGCTCACCAAGGCAACGATGCGTAGCCGACCTGAGAGGG
TGATCGGCCACACTGGGACTGAGACACGGCC
>Zotu782
GAACGCTGGCGGCAGGCTTAACACATGCAAGTCGAACGATGATTATCTAGCTTGCTAGATATGATTAGTGG
CGGACGGGTGAGTAACATTTAGGAATCTGCCTAGTAGTGGGGGATAGCTCGGGGAAACTCGAATTAATAC
CGCATACGACCTACGGGTGAAAGGGGGCGCAAGCTCTTGCTATTAGATGAGCCTAAATCAGATTAGCTAGT
TGGTGGGGTAAAGGCCCACCAAGGCGACGATCTGTAACTGGTCTGAGAGGATGATCAGTCACACCGGAAC
TGAGACACGGTC
>Zotu785
GAACGCTGGCGGCGTGCTTAACACATGCAAGTCGAACGGGAACCTTTATTTCGGTAAAGGTGAGAGTGGC
AAACGGGTGAGTAACACGTAGGCAACCTGCCGACAGGATGGGGACAACATTCCGAAAGGAATGCTAATAC
CGAATGGAGTCCGGGGATGGCATCATCTCCGGATAAAAGATGGCCTCTGAATATGCTATCGCCTGTCGATG
GGCCTGCGTCTGATTAGCCAGTTGGCGGGGTAACGGCCCACCAAAGCGACGATCAGTAGCCGGTCTGAGA
GGATGAACGGCCACATTGGGACTGAGACACGGCC
>Zotu787

GAACGCTGGCGGCAGGCCTAACACATGCAAGTCGAACGGACCCTTCGGGGTTAGTGGCGGACGGGTGAG
TAACACGTGGGAACGTGCCTTTAGGTTCGGAATAGCTCCTGGAAACGGGTGGTAATGCCGAATGTGCCCTT
CGGGGGAAAGATTTATCGCCTTTAGAGCGGCCCGCGTCTGATTAGCTAGTTGGTGAGGTAATGGCTCACCA
AGGCGACGATCAGTAGCTGGTCTGAGAGGATGACCAGCCACATTGGGACTGAGACACGGCC
>Zotu789
GAACGCTAGCGGGAGGCCTAACACATGCAAGCCGAGCGGTATTTGTTCTTCGGAACAGAGAGAGCGGCGC
ACGGGTGCGGAACACGTGTGCAACCTGCCTTTATCTGGGGGATAGCCTTTCGAAAGGAAGATTAATACCCC
ATAATATATTGAGTGGCATCACTTGATATTGAAAACTCCGGTGGATAGAGATGGGCACGCGCAAGATTAGA
TAGTTGGTGAGGTAACGGCTCACCAAGTCAATGATCTTTAGGGGGCCTGAGAGGGTGATCCCCCACACTG
GTACTGAGACACGGAC
>Zotu791
GAACGCTGGCGGCGTGCTTAACACATGCAAGTCGAGCGGTAAGGCTCCTTCGGGAGTACACGAGCGGCGA
ACGGGTGAGTAACACGTGAGTAATCTGCCCTTCACTTCGGGATAACCACCGGAAACGGTGGCTAATACCG
GATACGACCAGCCTCGGCATCGGGTGCTGGTGGAAAGTCTTTTCGGTGGAGGATGTGCTCGCGGCCTATC
AGCTTGTTGGTGGGGTAATGGCCTACCAAGGCTTCGACGGGTAGCCGGCCTGAGAGGGTGACCGGCCACA
CTGGGACTGAGACACGGCC
>Zotu793
GAACGCTGGCGGCGTGCTTAACACATGCAAGTCGAACGATGAAGCTTGGTGCTTGCACTGGGTGGATTAG
TGGCGAACGGGTGAGTAACACGTGAGTAACCTGCCCTTTACTTCGGGATAAGCTTGGGAAACTGGGTCTA
ATACCGGATATTCTGCATGGTCGGATGGTTGTGTAGGAAAGATTTTTTGGTAAGGGATGGGCTCGCGGCCT
ATCAGTTTGTTGGTGGGGTGATGGCCTACCAAGACGACGACGGGTAGCCGGCCTGAGAGGGCGACCGGC
CACACTGGGACTGAGACACGGCC
>Zotu795
GAACGCTGGCGGCGTGCTTAAGACATGCAAGTCGAACGGCAGTCTTCGGACTGTAGTGGCGCACGGGTGA
GTAACGCGTAACTGACCTACCCCAAAGTCGCGGATAACGATTCGAAAGAATCGCTAATACGTGATGTGCTG
TCAGATTGTGTTCTGCCAGTAAAGATTGATTGCTTTGGGATGGGGTTGCGTTCCATCAGCTAGTTGGTGGG
GTAAAGGCCCACCAAGGCGACGACGGATAGCCGGCCTGAGAGGGTGGCCGGCCACAGGGGCACTGAGAC
ACGGGT
>Zotu797
GAACGCTAGCGGGAGGCCTAACACATGCAAGCCGAGCGGTATTTGTTCTTCGGGACAGAGAGAGCGGCG
CACGGGTGCGGAACACGTGTGCAACCTGCCTTTATCTGGGGGATAGCCTTTCGAAAGGAAGATTAATACCC
CATAATATATTGAGTGGCATCATTTGATATTGAAAACTCCGGTGGATAGAGATGGGCACGCGCAAGATTAG
ATAGTTGGTGAGGTAACGGCTCACCAAGTCAATGATCTTTAGGGGGCCTGAGAGGGTGATCCCCCACACT
GGTACTGAGACACGGAC
>Zotu799
GAACGCTGGCGGCGTGCTTAACACATGCAAGTCGAGCGGAAAGGCTCCTTCGGGGGTACTCGAGCGGCG
AACGGGTGAGTAACACGTGAGTAATCTGCCCTTCACTTTGGGATAGCTCCCGGAAACGGGGATTAATACCG
GATATGACCATTCCAGGCATCTGGTGGTGGTGGAAAGTTTTTCGGTGGGGGATGTGCTCGCGGCCTATCA
GCTTGTTGGTGGGGTAATGGCCTACCAAGGCTTCGACGGGTAGCCGGCCTGAGAGGGTGACCGGCCACAC
TGGGACTGAGACACGGCC
>Zotu801
GAACGCTGGCGGCGTGCTTAACACATGCAAGTCGAACGATGAAAGAGGAGCTTGCTCCTCTGGATTAGTG
GCGAACGGGTGAGTAACACGTGAGCAATCTGCCCCTGACTCCGGAATAACTACGGGAAACTGTAGCTAAT
ACCGGATACGCGCCGAACAGGCATCTGTATCGGTGGGAAAGAATTTCGGTCAGGGATGAGCTCGCGGCCT
ATCAGCTTGTTGGTGAGGTAACGGCTCACCAAGGCGACGACGGGTAGCCGGCCTGAGAGGGTGACCGGC
CACATTGGGACTGAGACACGGCC
>Zotu803
GAACGCTGGCGGCGTGCTTAACACATGCAAGTCGAACGGAAAGGCCTCTGCTTGCAGAGGTACTCGAGTG
GCGAACGGGTGAGTAACACGTGGGTGATCTGCCTTGCACTTCGGGATAAGCCTGGGAAACTGGGTCTAAT
ACCGGATAGGACCATGCTTTAGTGTGTGTGGTGGAAAGTTTTTCGGTGCAAGATGAGCCCGCGGCCTATCA

GCTTGTTGGTGGGGTAATGGCCTACCAAGGCGACGACGGGTAGCCGGCCTGAGAGGGTGTACGGCCACA
TTGGGACTGAGACACGGCC
>Zotu806
GAACGCTGGCGGCAGGCCTAACACATGCAAGTCGAACGGTAGCACAGAGAGCTTGCTCTCGGGTGACGA
GTGGCGGACGGGTGAGTAATGTCTGGGAAACTGCCTGATGGAGGGGGATAACTACTGGAAACGGTAGCT
AATACCGCATAACGTCGCAAGACCAAAGAGGGGGACCTTCGGGCCTCTTGCCATCAGATGTGCCCAGATG
GGATTAGCTAGTAGGTGGGGTAACGGCTCACCTAGGCGACGATCCCTAGCTGGTCTGAGAGGATGACCAG
CCACACTGGAACTGAGACACGGTC
>Zotu808
GAACGCTGGCGGCGTGCTTAACACATGCAAGTCGAACGGAAAGGCTCCTGCTTGCAGGGGTACTCGAGTG
GCGAACGGGTGAGTAACACGTGGGTGATCTGCCTTGTACTCTGGGATAAGCTTGGGAAACTGGGTCTAAT
ACCGGATAGTAGCCGCCTTTAGTGTGGTGGTTGGAAAGTTTTTTCGGTGCAAGATGAGCTCGCGGCCTATC
AGCTTGTTGGTGGGGTAATGGCCTACCAAGGCGTCGACGGGTAGCCGGCCTGAGAGGGTGTACGGCCAC
ATTGGGACTGAGATACGGCC
>Zotu810
TAACGCTGGCGGTATGCCTAATACATGCAAGTCGAACGCGCACTTCGGTGCGAGTGGCGGACGGGTGAGT
AACACGTTGGAACCTACCCCGAAGTGGGGAATAACCCATCGAAAGATGGGCTAATACCCCATAGTCTTATG
CTTGTCATAAGTAAAGTTTTTCGCTTCGGGAGGGGCCTGCGCAACATTAGCTAGTTGGTGAGATAAAAGCC
CACCAAGGCGACGATGTTTAGCTGGATCGAGAGGTTGACCAGCCACACTGGGACTGAGATACGGCC
>Zotu812
GAACGCTGGCGGCGTGCTTAACACATGCAAGTCGAGCGGAAAGGCCCTTCGGGGTACTCGAGCGGCGAA
CGGGTGAGTAACACGTGAGTAATCTGCCCTTGGCTTCGGGATAGCCACCGGAAACGGTGATTAATACCGG
ATATGACTACTTCCTGCATGGGATGGTAGTGGAAAGTTTTTCGGCCAGGGATGTGCTCGCGGCCTATCAGC
TTGATGGTGAGGTAATGGCTCACCATGGCTTCGACGGGTAGCCGGCCTGAGAGGGTGACCGGTCACACTG
GGACTGAGACACGGCC
>Zotu814
TAACGCTAGCGGCAGGCATAATACATGCAAGTTGAGCGAGATAAGTTGTAGCAATACGGCTTTGAAAGCG
GCGCACGGGTGCGTAACACGTATGCAACCTACCTTTTACTAAGGAATAGCCTCTCGAAAGAGAGATTAATA
CCTTATAATATGTTTTGAAGGCATCTTCAGAACCTTAAAGCTCCGGCGGTAAAAGATGGGCATGCGGTTGA
TTAGCTAGTTGGTGAGGTAATGGCTCACCAAGGCTTCGATCAATAGGGGGTCTGAGAGGATGTCCCCCCAC
ACTGGTACTGAGACACGGAC
>Zotu816
GAACGCTGGCGGCGTGGATGAGGCATGCAAGTCGAAAGGAACCCCGCAAGGGGAAATCCTTGGCAGACG
AGGTAGGAACACGTAGGTACGTACCCCAAAGTCACGGATAACCCGGAGAAATTCGGAATAATACGTGATA
GTCTCGCGAGAGTAAAGATTTATCGCTTTGGGAACGGCCTGCGTTCTATCAGCTTGTTGGTAAGGTAACGG
CTTACCAAGGCTACGACGGATAGGGGAGGTGAGAGCCTGACCCCCACCGATGGAACTGCGACACGGTC
>Zotu818
GAACGCTGGCGGCGTGCCTAATACATGCAAGTCGAGCGAACCTTTAAAAGCTTGCTTTTAAAGGTTAGCGG
CGGACGGGTGAGTAACACGTGGGCAACCTACCTGTAAGACTGGGATAACTTCGGGAAACCGGAGCTAATA
CCGGATAATTCTTTTCTACACATGTAGAAAAGCTGAAAGACGGTTTCGGCTGTCACTTACAGATGGGCCCG
CGGCGCATTAGCTAGTTGGTGAGGTAATGGCTCACCAAGGCAACGATGCGTAGCCGACCTGAGAGGGTGA
TCGGCCACACTGGGACTGAGACACGGCC
>Zotu820
GAACGCTAGCGGCAGGCCTAATACATGCAAGTCGGACGGGATCCAGGAGCTTGCTTCTGGTGAGAGTGGC
GCACGGGTGCGTAACACGTATGTAACCTACCTTTGTCAGGGGGATAGCCTCTCGAAAGAGAGATTAAGAC
CGCATAAAATCACAATGTGGCATCACAAAATGATCAAATATTTATAGGACGAAGATGGGCATGCGGAACAT
TAGCTAGTTGGTAAGGTAACGGCTTACCAAGGCTACGATGTTTAGGGGATCTGAGAGGATGACCCCCCAC
ACTGGTACTGAGACACGGAC
>Zotu822

GAACGCTGGCGGCGTGCCTAATACATGCAAGTCGAGCGAACTTGCGGGAGCTTGCTCCCAAAAGTTAGCG
GCGGACGGGTGAGTAACACGTGGGCAACCTGCCTATAAGACTGGGATAACTTCGGGAAACCGGAGCTAAT
ACCGGATAATTCTTTCTACACATGTAGAAAAGCTAAAAGACGGTTTACGCTGTCACTTATAGATGGGCCCG
CGGCGCATTAGCTAGTTGGTGAGGTAACGGCTCACCAAGGCCACGATGCGTAGCCGACCTGAGAGGGTGA
TCGGCCACACTGGGACTGAGACACGGCC

>Zotu824
GAACGCTGGCGGCGTGCTTAACACATGCAAGTCGAACGGAAAGGCCCCCTTGTGGGGTACTCGAGTGGCG
AACGGGTGAGTAACACGTGAGTAACCTACCCTTGACTTTGGGATAACTTCAGGAAACTGGGGCTAATACCG
GATAGGAGCTCCTGCTGCATGGTGGGGGTTGGAAAGTTTCGGCGGTTGGGGATGGACTCGCGGCTTATCA
GCTTGTTGGTGGGGTAGTGGCTCACCAAGGCTTTGACGGGTAGCCGGCCTGAGAGGGTGACCGGCCACAT
TGGGACTGAGATACGGCC

>Zotu826
GAACGCTGGCGGCATGCCTTACACATGCAAGTCGAACGGCAGCACGGGTGCTTGCACCTGGTGGCGAGTG
GCGAACGGGTGAGTAATACATCGGAACGTACCCGGAAGTGGGGGATAACGTAGCGAAAGTTACGCTAAT
ACCGCATATTCTGTGAGCAGGAAAGCAGGGGATCGCAAGACCTTGCGCTTTCGGAGCGGCCGATGTCAGA
TTAGCTAGTTGGTGAGGTAAAGGCTCACCAAGGCGACGATCTGTAGCGGGTCTGAGAGGATGATCCGCCA
CACTGGAACTGAGACACGGTC

>Zotu828
GAACGCTGGCGGCGTGCCTAATACATGCAAGTAGAACGCTGAAGAGAGGAGCTTGCTCTTCTTGGATGAG
TTGCGAACGGGTGAGTAACGCGTAGGTAACCTGCCTGGTAGCGGGGGATAACTATTGGAAACGATAGCTA
ATACCGCATAAAATTGATTATTGCATGATAATTAATTGAAAGATGCAATTGCATCACTACCAGATGGACCTG
CGTTGTATTAGCTAGTTGGTGAGGTAGCGGCTCACCAAGGCGACGATACATAGCCGACCTGAGAGGGTGA
TCGGCCACACTGGGACTGAGACACGGCC

>Zotu830
GAACGCTGGCGGCGTGCTTAACACATGCAAGTCGAGCGGTAAGGCCTCTTCGGGGGTACACGAGCGGCG
AACGGGTGAGTAACACGTGGGTGACCTGCCCTCAGCTCTGGGATAAGCCTGGGAAACTGGGTCTAATACC
GGATATGACCTCTCATCGCATGGTGGGTGGTGGAAAGTTTTTCGGCTGGGGATGGGCCCGCGGCCTATC
AGCTTGTTGGTGGGGTGATGGCCTACCAAGGCGACGACGGGTAGCCGGCCTGAGAGGGCGACCGGCCAC
ACTGGGACTGAGACACGGCC

>Zotu832
GAACGCTAGCGGCAGGCTTAATACATGCAAGTCGAACGGGTGTCAGCAATGGCATTAGTGGCGAACGGGT
GCGTAACGCGTACACAACATGCCTTCAACTGGGGGATAGCGCTGGGAAACTGGCAGTAACCCCCCATGTG
GTCTTGGGTCGGCATCGACCTATGATTAAAATTACGGTGGTTGAAGATTGGTGTGCGTCCGATTAGCTAGT
TGGTAAGGTAACGGCTTACCAAGGCGACGATCGGTAGGGGGTGTGAGAGCATGACCCCCCACACGGGTA
CTGAGACACGGAC

>Zotu834
GAACGCTGGCGGTGTGGATAAGCCATGCAAGTCGAACGGTCCGCAAGGACAGTGGCAGACGAGGTAGTA
ACGCGCAGGGAGATCCCCCAAAGTCAGGGATAGCCAGCCGAAAGGTTGGGTAATACTTGATGGTCCCGTA
AGGGTAAAGATTTATCGCTTTGGGAGTCACCTGCGTAATATCAGCTAGTTGGTGAGGTAATGGCTCACCAA
GGCTACGACGTTTAGGGGAGCTGAGAGGCTGACCCCCACCGATGGGACTGCGATACGGCC

>Zotu836
GAACGCTGGCGGCAGGCTTAACACATGCAAGTCGAACGGGTGTAGCAATACATCAGTGGCAGACGGGTG
AGTAACACGTGGGAACCTTCCTAGAGGTACGGAACAACGCAGGGAAACTTGCGCTAATACCGTATACGCTC
TTGCGAGGAAAGATTTATCGCCTTTAGACGGGCCCGCGTAGGATTAGGTAGTTGGTGGGGTAACGGCCTA
CCAAGCCTACGATCCTTAGCTGATCTGAGAGGATGATCAGCCACACTGGAACTGAGACACGGTC

>Zotu838
GAACGCTGGCGGCAGGCCTAACACATGCAAGTCGAACGGTAGCACAGAGAGCTTGCTCTTGGGTGACGAG
TGGCGGACGGGTGAGTAATGTCTGGGAAACTGCCCGATGGAGGGGGATAACTACTGGAAACGGTAGCTA
ATACCGCATAACGTCTTCGGACCAAAGTGGGGGACCTTCGGGCCTCACACCATCGGATGTGCCCAGATGG
GATTAGCTAGTAGGTGGGGTAACGGCTCACCTAGGCGACGATCCCTAGCTGGTCTGAGAGGATGACCAGC
CACACTGGAACTGAGACACGGTC

>Zotu841
GAACGCTGGCGGCATGCTTAACACATGCAAGTCGGACGGGAAGTGGTGTTTCCAGTGGCGGACGGGTGA
GTAACGCGTAAGAACCTGCCCTTGGGAGGGGAACAACAGCTGGAAACGGCTGCTAATACCCCGTAGGCTG
AGGAGCAAAAGGAGGAATCCGCCTGAGGAGGGGCTCGCGTCTGATTAGCTAGTTGGTGAGGCAATAGCT
TACCAAGGCGATGATCAGTAGCTGGTTCGAGAGGATGATCAGCCACACTGGGACTGAGACACGGCC
>Zotu843
GAACGCTGGCGGCGTGCTTAACACATGCAAGTCGAACGATGAAGCCTTTCGGGGTGGATTAGTGGCGAAC
GGGTGAGTAACACGTGGGCAATCTGCCCTTCACTCTGGGACAAGCCCTGGAAACGGGGTCTAATACCGGA
TAACACTCTGTCCTGCATGGGACGGGGTTAAAAGCTCCGGCGGTGAAGGATGAGCCCGCGGCCTATCAGC
TTGTTGGTGGGGTAATGGCCTACCAAGGCGACGACGGGTAGCCGGCCTGAGAGGGCGACCGGCCACACT
GGGACTGAGACACGGCC
>Zotu847
GAACGTTGGCGGCGTGCCTTAAGCATGCAAATCAAGGGGGTCCGATTTATCGGACTACCGGTGGACGGGT
GAGTAATGCGCGGGAATGTACCTCGAAGTGGGATATAGCCAGCCGAAAGGTTGGGTAATCTCCCATGTGA
ACGAAAGTTTAAAGCCGCAAGGCGCTTTGAGATCAGCCCGCGTCCTATCAGCTTGTTGGTGGGGTAAAAGC
CTACCAAGGCGACGACGGGTAGCTGGACTGAGAGGTCGGTCAGCCACAATGGGACTGCGACACGGCC
>Zotu849
GAACGCTAGCTACAGGCTTAACACATGCAAGTCGAGGGGCAGCATGTCGGTTGCTTGCAACCGATGATGG
CGACCGGCGCACGGGTGAGTAACGCGTATCCAACCTGCCTCTTACCAGGGAATAACCCGTCGAAAGGCGG
ACTAATGCCCTATGCCCTCCGCGGCAGACATCTAATGCGGAGGAAAGATTCATCGGTAAGTGATGGGGAT
GCGTCTGATTAGCTTGTTGGCGGGGTAACGGCCCACCAAGGCAACGATCAGTAGGGGTTCTGAGAGGAAG
GTCCCCCACATTGGAACTGAGACACGGTC
>Zotu851
GAACGCTGGCGGTGTGCTTCACACATGCAAGTCGAACGGGGTAGCAATACCTAGTGGCGGACGGGTGAGT
AACGCGTGGGAATCTGCCCAAGGGTGGGGGATAGCAGACCGAAAGGTCTATTAATACCGCATATGCCGTG
AGGTAAAAGGAGTAATCCGCCCCTGGATGAGCCTGCGTCCGATTAGCTAGTTGGTAGGGTAAAAGCCTAC
CAAGGCTACGATCGGTAGCTGGTCTGAGAGGATGATCAGCCACAATGGGACTGAGACACGGCC
>Zotu853
GAACGCTGGCGGTATGCTTAACACATGCAAGTCGAACGGGGTTCTTCGGAACCTAGTGGCGGACAGGTGA
GTAACGCGTGAGAACATGCCTTTAGGACTGGGACAACAGATGGAAACGTCTGCTAATACCGGATGTGCCC
TTAGGGGTAAAAGATTTATTGTCTAAAGATTGGCTCGCGTCAGATTAGCTAGTAGGAGTGGTAACGGCACC
CCTAGGCGACGATCTGTACTTGGTCTGAGAGGATGACCAGGCACACTGGAACTGAGACACGGTC
>Zotu855
GAACGCTGGCGGCGTGCTTAACACATGCAAGTCGAACGATGATGCCCAGCTTGCTGGGTTGATTAGTGGC
GAACGGGTGAGTAACACGTGAGTAACCTGCCCATCACTCTGGGATAACTCCGGGAAACCGGTGCTAATAC
CGGGTATGACTCCGATCCGCATGGGTACGGGGTGGAAAGATTTATCGGTGGTGGATGGACTCGCGGCCTA
TCAGCTTGTTGGTGGGGTAATGGCCTACCAAGGCGACGACGGGTAGCCGGCCTGAGAGGGTGACCGGCC
ACACTGGGACTGAGACACGGCC
>Zotu858
GAACGCTGGCGGCGTGCCTAATACATGCAAGTAGAACGCTGAAGACTTTAGCTTGCTAAAGTTGGAAGAG
TTGCGAACGGGTGAGTAACGCGTAGGTAACCTGCCTACTAGCGGGGGATAACTATTGGAAACGATAGCTA
ATACCGCATAACAGCATTTAACCCATGTTAGATGCTTGAAAGGAGCAATTGCTTCACTAGTAGATGGACCT
GCGTTGTATTAGCTAGTTGGTGAGGTAACGGCTCACCAAGGCGACGATACATAGCCGACCTGAGAGGGTG
ATCGGCCACACTGGGACTGAGACACGGCC
>Zotu860
GAACGCTGGAGGCACGCTTAAAACATGCAAGTCGAACGAGAAGCTTTGGTGCTTGCACCGAAGTGGGAAG
TGGCGAACGGGTGAGTAACACATGAGAAACCTGCCCCGAAGATTGGGATAAGCCTTGGAAACGAGGTCTA
ATACCGAATACCTTCTAATTATCACATGGTAAATAGAAGAAAGCTCAGGCGCTTTGGGATGGTCTCGTGGC
CTATCAGCTTGTTGGTGAGGTAAAAGCTCACCAAGGCAACGACGGGTAGCTGGTCTGAGAGGATGTCCAG
CCACATTGGAACTGAGATACGGTC

>Zotu862
GAACGCTAGCGGCAGGCCTAACACATGCAAGTCGAGGGGCAGCATAAAGATAGCTTGCTATCTTGGTGGC
GACCGGCGCACGGGTGCGTAACGCGTATGCAACCTACCTTATACTGGGGTATAGCCCGGAGAAATTCGGA
TTAACCCCCCATAGTATTATCGGATGGCATCATTTGATAATTAAAGGTTACGGTATAAGATGGGCATGCGTC
CCATTAGCTAGATGGTGAGGTAACGGCTCACCATGGCGACGATGGGTAGGGGGCCTGAGAGGGTGGTCC
CCCACACTGGTACTGAGACACGGAC
>Zotu864
GAACGCTGGCGGCGTGCTTAACACATGCAAGTCACGGGGGCCCGCAAGGGCAACCGGCGAACGGGTGCG
TAACACGTGAGCAATCTGCCGATTTCAGGGGGATAGCCGGCCCAACGGCCGGGTAATACCGCATACGCTG
GTGGAGGGGAGTCCCTCTATCAGGAAACCTCCGGGGGAAATCGAGGAGCTCGCGGCCTATCAGCTAGTTG
GCGGGGTAACGGCCCACCAAGGCGACGACGGGTAGCTGGTCTGAGAGGATGGCCAGCCACATTGGGACT
GAGAGACGGCC
>Zotu866
GAACGCTGGCGGTATGCTTTACACATGCAAGTCGTACGCAAGTTTACTTGAGTGGCGGACGGGTGAGTAA
CACGTGAGAATTCACCTTTAGGTGGGGAATAACTTTTGGAAACGATAGCTAATGCCCCATAGACTCGCAAG
AGAAAAAAGAGTAATCTGCCTAAAGAGAAGCTCGCGGCTGATTAGCTAGTTGGTAAGGTAATGGCTTACC
AAGGCGACGATCAGTAGCTGGTTTGAGAGAACGATCAGCCACACTGGAACTGAGACACGGTC
>Zotu868
GAACGCTGGCGGCGTGCTTAACACATGCAAGTCGAGCGGTAAGGCCCTTTCGGGGGTACACGAGCGGCG
AACGGGTGAGTAACACGTGAGTAATCTGCCCTCAGCTTTGGGATAAGCACTCGAAAGGGTGTCTAATACCG
GATATGACGATCCTCCGCATGGGGGGTTGTGGAAAGTTTTTCGGCTGGGGATGTGCTCGCGGCCTATCAG
CTTGTTGGTGGGGTAATGGCCTACCAAGGCTTTGACGGGTAGCCGGCCTGAGAGGGTGACCGGTCACACT
GGGACTGAGACACGGCC
>Zotu870
GAACGCTGGCGGCAGGCCTAACACATGCAAGTCGAGCGGCAGCGGAAAGTAGCTTGCTACTTTGCCGGCG
AGCGGCGGACGGGTGAGTAATGTCTGGGAAACTGCCTGATGGAGGGGGATAACTACTGGAAACGGTAGC
TAATACCGCATGACCTCGAAAGAGCAAAGTGGGGGACCTTCGGGCCTCACGCCATCGGATGTGCCCAGAT
GGGATTAGCTAGTAGGTGAGGTAATGGCTCACCTAGGCGACGATCCCTAGCTGGTCTGAGAGGATGACCA
GCCACACTGGAACTGAGACACGGTC
>Zotu872
GAACGCTAGCGGCAGGCTTAACACATGCAAGTCGAGGGGTATAGGTTTTCGGACCTAGAGACCGGCGCAC
GGGTGCGTAACGCGTATGCAATCTACCTTTCACAGAGGGATAGCCCAGAGAAATTTGGATTAATACCTCAT
AGTATAACGACTCGGCATCGAGATGTTATTAAAGTCACAACGGTGAAAGATGAGCATGCGTCCCATTAGCT
AGTTGGTAAGGTAACGGCTTACCAAGGCTACGATGGGTAGGGGTCCTGAGAGGGAGATCCCCCACACTGG
TACTGAGACACGGAC
>Zotu874
GAACGCTGGCGGCGTGCCTAACACATGCAAGTCGAGCGATTCTCTTCGGAGAAGAGCGGCGGACGGGTG
AGTAACGCGTGGGTAACCTGCCCTGTACACACGGATAACATACCGAAAGGTATGCTAATACGGGATAACAT
ATTTTGATGGCATCATCGAAATATCAAAGCTCCGGCGGTACAGGATGGACCCGCGTCTGATTAGCTAGTTG
GTGAGGTAATGGCTCACCAAGGCGACGATCAGTAGCCGACCTGAGAGGGTGATCGGCCACATTGGAACTG
AGACACGGTC
>Zotu876
GAACGCTGGAAATGTGGATACCGGCATGCAAGTCAAGGGGCCGCAAGGCACCGGCGAACGGCTTAGTAA
CACGTGGATACATACCCAGAATTGGAGCACAGCTCGTCGAAAGACGGGGTAATTCTCCATAATACCGCAAG
GTTAAAGGCGCAAGCCGATTCTGGAATGGTCCGCGGTCTATCAGCTAGTTGGTGAGGTAATGGCTCACCAA
GGCTATGACGGATAGGGGGCGTGAGAGCGCGACCCCCAAGAATGGCACTGCGACACGGGC
>Zotu881
GAACGCTGGCGGCGTGCTTAACACATGCAAGTCGAACGGTGAACCTGCTTCGGTGGGGGATCAGTGGCGA
ACGGGTGAGTAACACGTGGGCAACCTGCCCCCGGCTCTGGGATAACTCCAAGAAATTGGGGCTAATACCG
GATGTGACTGCTGACCGCATGGTCTGGTGGTGGAAAGATTTATCGGCTGGGGATGGGCCCGCGGCCTATC

AGCTTGTTGGTGGGGTGATGGCCTACCAAGGCGACGACGGGTAGCCGGCCTGAGAGGGTGACCGGCCAC
ACTGGGACTGAGACACGGCC
>Zotu883
GAACGCTGGCGGCGTGCTTAACACATGCAAGTCGAGCGGTAAGGCCCTTCGGGGTACACGAGCGGCGAA
CGGGTGAGTAACACGTGGGTGACCTGCCCTCCACTTCGGGATAAGCCTGGGAAACTGGGTCTAATACCGG
ATAGGACCAAGGGATGCATGTTCCTTGGTGGAAAGTTTTTTCGGTGGGGGATGGGCCCGCGGTCTATCAG
CTTGTTGGTGGGGTGATGGCCTACCAAGGCGACGACGGATAGCCGGCCTGAGAGGGTGATCGGCCACACT
GGGACTGAGACACGGCC
>Zotu886
GAACGCTAGCGGCAGGCTTAACACATGCAAGTCGAGGGGTATAGTAGCAATACTAGAGACCGGCGCACG
GGTGCGTAACGCGTATGCAATCTACCTTTTACAGAGGGATAGCCCAGAGAAATTTGGATTAATACCTCATA
GTATTATAGAATGGCATCATTTTATAATTAAAGTCACAACGGTAAAAGATGAGCATGCGTCCCATTAGCTAG
TTGGTAAGGTAACGGCTTACCAAGGCAACGATGGGTAGGGGTCCTGAGAGGGAGATCCCCCACACTGGTA
CTGAGACACGGAC
>Zotu889
GAACGCTGGCGGCGCGCCTAACACATGCAAGTCGAACGGAGCTGAGAGAAGCTTGCTTTTCTTAGCTTAGT
GGCGAACGGGTGAGTAACGCGTGAGTAACCTGCCCTGGAGTGGGGGACAACAGTTGGAAACGACTGCTA
ATACCGCATAAGCCCACGATCCGGCATCGGATTGAGGGAAAAGGATTTATTCGCTTCAGGATGGACTCGCG
TCCAATTAGCTAGTTGGTGAGGTAACGGCCCACCAAGGCGACGATTGGTAGCCGGACTGAGAGGTTGAAC
GGCCACATTGGGACTGAGACACGGCC
>Zotu891
CAACGCTGGCGGCGTGCCTAATGCATGCAAGTCGAGCGGGGTGCATCTTCGGGTGGACTTACGCGGCGGA
CGGGTGCGGAGCACGTGGGCAACCTGCCCTGGGGTGGGGGATAGCCGTGGGAAACCGCGGGTAATTCCG
CGTACGCTCGATTGCCGGAGGGGTGATCGAGGAAAGGCTCGTGAGAGCCGCCCTGGGAGGGGGCTGCGG
CTGATTAGCTGGTTGGTGGGGTAACGGCCTACCAAGGCGATGATCGGTAGCTGGTCTGAGAGGACGGCCA
GCCACACGGGGACTGAGACACGGCC
>Zotu893
GAACGCTAGCGGCAGGCTTAACACATGCAAGTCGAGGGGTAGAGTTCTTCGGAGCTTGAGACCGGCGCAC
GGGTGCGTAACGCGTATGCAATCTACCTTTTACAGAGGGATAGCCCAGAGAAATTTGGATTAATACCTCAT
AGTATAATGACTCGGCATCGAGATATTATTAAAGTCACAACGGTAAAAGATGAGCATGCGTCACATTAGCT
AGTTGGTAAGGTAACGGCTTACCAAGGCAACGATGGGTAGGGGTCCTGAGAGGGAGATCCCCCACACTG
GTACTGAGACACGGAC
>Zotu895
GAACGCTAGCGGCAGGCCTAATACATGCAAGTCGGACGGGATTTGGGAGCTTGCTCCCGATGAGAGTGGC
GCACGGGTGCGTAACACGTATGTAACCTACCCATATCAGGGGGATAGCCTCTCGAAAGAGAGATTAAGAC
CGCATAACATAACGGAATGGCATCATTTTGTTATCAAATATTTATAGGATATGGATGGGCATGCGGAACAT
TAGCTAGTTGGTGTGGTAACGGCATACCAAGGCTACGATGTTTAGGGGATCTGAGAGGATGGCCCCCCAC
ACTGGTACTGAGACACGGAC
>Zotu897
GAACGCTGGCGGCGTGCTTAACACATGCAAGTCGAACGGAAAGGCCCTGCTTGCAGGGTACTCGAGTGGC
GAACGGGTGAGTAACACGTGGGTGATCTGCCCTGCACTTTGGGATAAGCCTGGGAAACTGGGTCTAATAC
CGGATAGGACCACATCGTAGGGGTGTGGTGGAAAGATTTTTCGGTGCAGGATGAGCCCGCGGCCTATCAG
CTTGTTGGTGGGGTAATGGCCTACCAAGGCGGCGACGGGTAGCCGGCCTGAGAGGGTGTACGGCCACATT
GGGACTGAGACACGGCC
>Zotu899
GAACGCTGGCGGCGTGCTTAACACATGCAAGTCGAACGATGAAGCCCAGCTTGCTGGGTGGATTAGTGGC
GAACGGGTGAGTAACACGTGAGTAACCTGCCCTTGACTCTGGGATAAGCACTGGAAACGGTGTCTAATAC
CGGATATTTCAGCTGGCCGCATGGTCTGGTTGTGGAAAGAATTTTGGTCAAGGATGGACTCGCGGCCTATC
AGGTTGTTGGTGAGGTAATGGCTCACCAAGCCTACGACGGGTAGCCGGCCTGAGAGGGTGACCGGCCACA
CTGGGACTGAGACACGGCC

>Zotu901
GAACGCTGGCGGCGTGCCTAACACATGCAAGTCGAACGAAGCTTTGAGGAGCTTGCTTTTTAAAGCTTAGT
GGCGGACGGGTGAGTAACGCGTGAGCAACCTGCCTCTCAGAGGGGGATAACGTTTTGAAAAGAACGCTA
ATACCGCATAATATATCGGAACCGCATGATTCTGATATCAAAGGAGCAATCCGCTGAGAGATGGGCTCGCG
TCCGATTAGTTAGTTGGTGAGGTAACGGCTCACCAAGACTACGATCGGTAGCCGGACTGAGAGGTTGATC
GGCCACATTGGGACTGAGACACGGCC
>Zotu903
GAACGCTGGCGGCGTGCCTAATACATGCAAGTCGAGCGAACAGATAAAGAGCTTGCTCCTTTGACGTTAGC
GGCGGACGGGTGAGTAACACGTGGGTAACCTACCTATAAGACTGGAATAACTCCGGGAAACCGGGGCTA
ATGCCGGATAATACACAGAACCGCATGGTTCTGTGATGAAAGACGGTTTTGCTGTCACTTATAGATGGACC
CGCGGCGTATTAGCTAGTTGGTAAGGTAACGGCTTACCAAGGCAACGATACGTAGCCGACCTGAGAGGGT
GATCGGCCACACTGGAACTGAGACACGGTC
>Zotu906
GAACGCTGGCGGCGTGCCTAATACATGCAAGTCGAGCGAATGGATTAAGAGCTTGCTCTTATGAAGTTAGC
GGCGGACGGGTGAGTAACACGTGGGTAACCTGCCCATAAGACTGGGATAACTCCGGGAAACCGGGGCTA
ATACCTGATAACATTTTGAACCGCATGGTTCGAAATTGAAAGGCGGCTTCGGCTGTCACTTATGGATGGAC
CCGCGTCGCATTAGCTAGTTGGTGAGGTAACGGCTCACCAAGGCAACGATGCGTAGCCGACCTGAGAGGG
TGATCGGCCACACTGGGACTGAGACACGGCC
>Zotu908
GAACGCTGGCTACAGGCTTAACACATGCAAGTCGAGGGGAAACGACGGGGAAGCTTGCTTCCCCGGGCGT
CGACCGGCGCACGGGTGAGTAACGCGTATCCAACCTGCCTCTGACTGAGGGATAACCCGTCGAAAGTCGG
CCTAATACCTCATGGCATCGTCTGCGGGCATCCAACGACGATTAAAGATTTCATCGGTCAGGGATGGGGAT
GCGTCTGATTAGCTTGTTGGCGGGGTAACGGCCCACCAAGGCGACGATCAGTAGGGGTTCTGAGAGGAAG
GTCCCCCACATTGGAACTGAGACACGGTC
>Zotu910
GAACGCTGGCGGCATGCCTTACACATGCAAGTCGAACGGCAGCACGGGAGCAATCCTGGTGGCGAGTGG
CGAACGGGTGAGTAATACATCGGAACGTGTCCTGGAGTGGGGGATAGCCCGGCGAAAGCCGGATTAATA
CCGCATACGCTCTATGGAGGAAAGCGGGGGATCTTCGGACCTCGCGCTCAAGGGGCGGCCGATGGCGGA
TTAGCTAGTTGGTGGGGTAAAGGCCTACCAAGGCGACGATCCGTAGCTGGTCTGAGAGGACGACCAGCCA
CACTGGGACTGAGACACGGCC
>Zotu912
GAACGCTGGCGGCGCGCTTAACACATGCAAGTCGAGCGAGAACCAGGCCTTCGGGCCTGGGGAAAGCGG
CGAACGGGTGAGTAACACGTGAGTAACCTGCCCTCGGCTCCGGGATAGCCCGTGGAAACGCGGATTAATA
CCGGATAGCCTTTTGAGCTCTCGGGCTCGTCAGAAAAGGTAGCTTCGGCCTCTGGCCGAGGAGGGGCTCG
CGGCGGATTAGCTTGTTGGCGGGGTAACGGCCCACCAAGGCGACGATCCGTAGCTGGTCTGAGAGGACG
ATCAGCCACACTGGGACTGAGACACGGCC
>Zotu914
GAACGCTGGCTGCGTGCTTAACACATGCAAGTCGAACGCTGAAGCCGACAGCTTGCTGTTGGTGGATGAG
TGGCGAACGGGTGAGTAACACGTGAGTAACCTGCCCCTGATTTCGGGATAAGCCTGGGAAACTGGGTCTA
ATACCGGATATGACCAATCTTCGCATGAGGGTTGGTGGAAAGTTTTTCGATCGGGGATGGGCTCGCGGCCT
ATCAGCTTGTTGGTGGGGTAATGGCCTACCAAGGCGACGACGGGTAGCCGGCCTGAGAGGGCGACCGGC
CACACTGGGACTGAGACACGGCC
>Zotu916
GAACGCTGGCGGCGTGCCTAATACATGCAAGTCGAGCGGCAGCGCGAATAGTTTACTATTTGGCGGCGAG
CGGCGGACGGCTGAGTAACGCGTAGGAATCTGCCCCAAAGTGAGGAATAACTCCGGGAAACCGGAGCTA
ATACCGCATGTGGTCTTCGGATTAAAGCCTTCGGGCGCTTTGGGAGGAGCCTGCGTTCGATTAGCTTGTTG
GTGAGGTAATGGCTCACCAAGGCTACGATCGATAGCTGGTCTGAGAGGATGATCAGCCAGACTGGAACTG
AGATACGGTC
>Zotu918

GAACGCTGGCGGCGTGCTTAACACATGCAAGTCGAACGATGAAGCTCTAGCTTGCTGGGGTGGATTAGTG
GCGAACGGGTGAGTAACACGTGAGTAACCTGCCCTTTACTTCGGGATAAGCTTGGGAAACTGGGTCTAATA
CCGGATATTCTGCGTGATCGGATGGTTGTGTAGGAAAGATTTTTTGGTAAGGGATGGGCTCGCGGCCTATC
AGTTTGTTGGTGGGGTGATGGCCTACCAAGACGACGACGGGTAGCCGGCCTGAGAGGGCGACCGGCCAC
ACTGGGACTGAGACACGGCC
>Zotu920
GAACGCTGGCGGCGTGCTTAACACATGCAAGTGGTGCGACGAACCAGAGCTTGCTCTGGGGCCAAGCCGC
GAACGGGTGAGTAACACGTGAGTAATCTGCCCCGATGACCGGGACAACCCGAGGAAACTCGGGCTAATAC
CGGATGTGGTGCACGGATTTAAGTCTGTGTACTAAAGGAAGCTTCGGCCTCCGCATTGGGAGGAGCTCGC
GGCCCATTAGCTTGTTGGTGGGGTAACGGCCTACCAAGGCAACGATGGGTAGCTGGTCTGAGAGGACGAT
CAGCCACACTGGGACTGAGACACGGCC
>Zotu922
CAACGCTGGCGGCGTGCCTAACACATGCAAGTCGCACGAGAAAGGGGCTTCGGCCCTGAGTACAGTGGCG
CACGGGTGAGTAACACGTGACTTATCTACCCTCAAGTGGGGAATAACCTAGAGAAATCTGGGCTAATACCG
CATAACACTTACGAGTCAAAGCAGCAATGCGCTTGGGGAGGAGGTCGCGGCCGATTAGCTAGTTGGCGGG
GTAATGGCCCACCAAGGCAATGATCGGTATCCGGCCTGAGAGGGCGCACGGACACACTGGAACTGAAACA
CGGTC
>Zotu924
GAACGCTGGCGGCGTGCCTAATACATGCAAGTCGAACGCTTTGTCTTTAACTGATCTGACGAGCTTGCTCTG
ATTTGATTTTATCTGACAAAGAGTGGCGAACGGGTGAGTAACACGTGGGTAACCTACCTCTTAGCAGGGGA
TAACATTTGGAAACAAGTGCTAATACCGTATAATACCAACAACCGCATGGTTGTTGGTTGAAAGATGGTTCT
GCTATCACTAAGAGATGGACCCGCGGTGCATTAGCTAGTTGGTAAGGTAACGGCTTACCAAGGCAATGAT
GCATAGCCGAGTTGAGAGACTGATCGGCCACAATGGGACTGAGACACGGCC
>Zotu926
GAACGCTGGCGGCGTGCTTAACACATGCAAGTCGAGCGGAAAGGCCACTTCGGTGGTACTCGAGCGGCGA
ACGGGTGAGTAACACGTGAGTAATCTGCCCCTGGCTTTGGGATAGCCACCGGAAACGGTGATTAATACCG
GATATGACATGGACTCGCATGGGTTTGTGTGGAAAGTTTTTCGGCCAGGGATGTGCTCGCGGCCTATCAGC
TTGATGGTGAGGTAATGGCTCACCATGGCTTCGACGGGTAGCCGGCCTGAGAGGGTGACCGGTCACACTG
GGACTGAGACACGGCC
>Zotu928
GAACGCTGGCGGCGTGCTTAACACATGCAAGTCGAACGGTGAACCAGAGCTTGCTCTGGGGATCAGTGGC
GAACGGGTGAGTAACACGTGGGCAACCTGCCCCAGACTCTGGGATAACCTCGGGAAACCGATGCTAATAC
CGGATACGACCACTTCGGACATCCGACGGTGGTGGAAAGTTTTTCGGTCTGGGATGGGCCCGCGGCCTATC
AGCTAGTTGGTGGGGTAATGGCCTACCAAGGCGACGACGGGTAGCCGGCCTGAGAGGGCGACCGGCCAC
ACTGGAACTGAGACACGGTC
>Zotu931
GAACGCTGGCGGCGTGCCTAATACATGCAAGTCGAGCGAATGGATTGAGAGCTTGCTCTCAAGAAGTTAG
CGGCGGACGGGTGAGTAACACGTGGGTAACCTGCCCATAAGACTGGGATAACTCCGGGAAACCGGGGCT
AATACCGGATAACACTTTGAACCGCATGGTTCGAAATTGAAAGGCGGCTTCGGCTGTCACTTATGGATGGA
CCCGCGTCGCATTAGCTAGTTGGTGAGGTAACGGCTCACCAAGGCAACGATGCGTAGCCGACCTGAGAGG
GTGATCGGCCACACTGGGACTGAGACACGGCC
>Zotu933
GAACGCTGGCGGCGTGCTTAACACATGCAAGTCGAACGGTGACGGAGAGCTTGCTCTCCTGATCAGTGGC
GAACGGGTGAGTAACACGTGGGCAACCTGCCCCTGACTTCGGGATAACGCTTCGAAAGAGGTGCTAATAC
CGGATACGAGCCTTCTAGGCATCTAGAGGGTTGGAAAGTTTTTCGGTCAGGGATGGGCCCGCGGCCTATC
AGCTTGTTGGTGAGGTAATAGCTCACCAAGGCGACGACGGGTAGCCGGCCTGAGAGGGCGACCGGCCAC
ACTGGGACTGAGACACGGCC
>Zotu936
GAACGCTGGCGGCGTGCTTAACACATGCAAGTCGAACGGAAAGGCCAGTGCTTGCACTGGTACTCGAGTG
GCGAACGGGTGAGTAACACGTGGGTGATCTGCCCTGCACTTCGGGATAAGCCTGGGAAACTGGGTCTAAT
ACCGGATAGGACCACATTTTGGATGGTGTGGTGGAAAGTTTTTCGGTGTGGGATGAGCTCGCGGCCTATCA

GCTTGTTGGTGGGGTAATGGCCTACCAAGGCGTCGACGGGTAGCCGGCCTGAGAGGGTGTACGGCCACAT
TGGGACTGAGATACGGCC
>Zotu938
GAACGCTGGCGGCGTGCCTAATACATGCAAGTCGAGCGAACAGACGAGAGTGCTTGCACTCTCTGACGTT
AGCGGCGGACGGGTGAGTAACACGTGGGTAACCTACCTATAAGACTGGGATAACTTCGGGAAACCGGAG
CTAATACCGGATAATATTTAGCTTCGCATGAAGCAATAGTGAAAGACGGTTCTGCTGTCACTTATAGATGG
ACCCGCGGTGTATTAGCTAGTTGGTGAGGTAACGGCTCACCAAGGCAACGATACATAGCCGACCTGAGAG
GGTGATCGGCCACACTGGGACTGAGACACGGCC
>Zotu940
GAACGCTGGCGGCATGCTTAACACATGCAAGTCGGACGGGAAGTGGTGTCTCCAGTGGCGGACGGGTGA
GTAACGCGTAAGAACCTGCCCTTGGGAGGGGAACAACAACTGGAAACGGTTGCTAATACCCCGTAGGCTG
AGGAGCAAAAGGAGGAATCCGCCCGAGGAGGGGCTTGCGTCTGATTAGCTAGTTGGTGAGGTAATAGCTT
ACCAAGGCGATGATCAGTAGCTGGTCCGAGAGGATGATCAGCCACACTGGGACTGAGACACGGCC
>Zotu942
GAACGCTGGCGGCGTGCCTAACACATGCAAGTCGAACGGGGAGTCTATTTCGATAGGCTTCCAGTGGCGA
ACGGGTGAGTAACACGTTGGTGACCTGCCCCGAAGAGGGGGACAACTCCTCGAAAGAGGAGCTAATACCC
CGGATGTCTGAGTGGTTAGAAGACTCAGACTAAAGTTTTTCGGAATGCTTTGGGAGGGGCCTGCGGGCTA
TCAGCTAGTTGGTGAGGTAAGGGCTCACCAAGGCGAAGACGGCTAGGGGGCGTGAGAGCGTGACCCCCC
ACAAGGGAACTGAGAAACGGTC
>Zotu944
GAACGCTAGCGGCAGGCCTTACACATGCAAGTCGAGGGGTAACAGGAGAGAGCTTGCTCTTTCGCTGACG
ACCGGCGCACGGGTGCGTAACACGTATGCAACCTACCTCTTACTGGGGAATAGCCCAGAGAAATTTGGATT
AACACCCCATAGTATTATTCTTCCGCATGGTTGAATGATTAAAGGTTACGGTAAGAGATGGGCATGCGCCC
CATTAGCTAGTTGGTGAGGTAACGGCTCACCAAGGCTACGATGGGTAGGGGGTCTGAGAGGACTATCCCC
CACACTGGTACTGAGACACGGAC
>Zotu947
GAACGCTGGCGGCATGCCTTACACATGCAAGTCGAACGGCAGCGGGCCCTTCGGGGTGCCGGCGAGTGG
CGAACGGGTGAGTAATGCATCGGAACGTACCCGGGAGAGGGGGATAACCGGCCGAAAGGCTGGCTAATA
CCGCATGAACTCGGAAGAGCAAAGCGGGGGACCTTCGGGCCTCGCGCTCTCGGAGCGGCCGATGTCCGAT
TAGGCTAGTTGGTGGGGTAAAGGCCCACCAAGGCGACGATCGGTAGCGGGTCTGAGAGGACGATCCGCC
ACACTGGGACTGAGACACGGCC
>Zotu949
GAACGCTGGCGGCGTGCCTAACACATGCAAGTCGAACGGGACTATCAAGTGATGTTTTCGGACTGAACTTT
TTAGTTTAGTGGCGGACGGGTGAGTAACGCGTGGACAACCTGCCTCTCACTGGGGGATAGCAGCTGGAAA
CGGCTGGTAATACCGCATGCGTTCAATGTGCCGCATGACACATTGAAGAAAGATTTATCGGTGAGAGATG
GGTCCGCGTCTGATTAGCCAGTTGGCGGGGTAACGGCCCACCAAAGCGACGATCAGTAGCCGGCCTGAGA
GGGCGGACGGCCACATTGGGACTGAGACACGGCC
>Zotu951
GAACGCTGGCGGCGTGCTTAACACATGCAAGTCGAACGGATCGGCTCTGACTTTTTTGTTGGGGTTTGGTT
AGTGGCGAACGGGTGAGTAATACGTGAGTAACCTGCCCTCTTCTCTGGGATAAGCCTTGGAAACGGGGTC
TAATACTGGATATTCTGCTTTCATCGCATGGTGGGGGTTGGAAAGGGTTTTCTGGTGGGGGATGGGCTCAC
GGCCTATCAGCTTGTTGGTGGGGTGATGGCCTACCAAGGCTTCGACGGGTAGCCGGCCTGAGAGGGTGAC
CGGCCACATTGGGACTGAGATACGGCC
>Zotu953
GAACGCTAGCGGCAGGCTTAATACATGCAAGTCGAGGGGCAGCAGCTTTATAGCAATATAGAGGCTGGCG
ACCGGCAAACGGGTGCGGAACACGTACACAACCTTCCTTGAATAGGGGGATAGCCCATAGAAATGTGGAT
TAATACCCCGTAATATAGCGGTGTGGCATCACACTGTTATTATAGTTTCGACGATTCGAGATGGGTGTGCG
GCTGATTAGGTAGTTGGCGGGGTAAAGGCCCACCAAGCCTTCGATCAGTAGCTGATGTGAGAGCATGATC
AGCCACACGGGCACTGAGACACGGGC
>Zotu956

GAACGCTAGCGGCAGGCCTAATACATGCAAGTCGAACGGTGATATTTATATCATAGTGGCGCACGGGTGC
GTAACACGTATGCAACCTACCTATTATTGGGGGATAGCCTTTGGAAACGGAGATTAATACCGCATAATACG
ATTGACTGGCATCAGTTGATTGTTAAAGCTTTGGCGATAATAGATGGGCATGCGTCCAATTAGTTAGTTGGT
TAGGTAACGGCTGACCAAGACGATGATTGGTAGGGGATCTGAGAGGATAATCCCCCACACTGGCACTGAG
ATACGGGC
>Zotu958
GAACGCTGGCGGCGTGCCTAACACATGCAAGTCGAGCGAACCCTTCGGGGTGAGCGGCGGACGGGTGAG
TAACGCGTGGGTAACCTGCCCTGTACACACGGATAACATACCGAAAGGTATGCTAATACGAGATAATATGT
AATGATCACATGGTCGATACATCAAAGCTCCGGCGGTACAGGATGGACCCGCGTCTGATTAGCTAGTTGGT
AAGGTAACGGCTTACCAAGGCGACGATCAGTAGCCGACCTGAGAGGGTGATCGGCCACATTGGAACTGAG
ACACGGTC
>Zotu960
GAACGCTGGCGGCGTGCCTAATACATGCAAGTAGAACGCTGAAGAGAGGAGCTTGCTCTTCTTGGATGAG
TTGCGAACGGGTGAGTAACGCGTAGGTAACCTGCCTGGTAGCGGGGGATAACTATTGGAAACGATAGCTA
ATACCGCATAAAATGGATTATCGCATGATAATCGATTGAAAGGTGCAAATGCATCACTACCAGATGGACCT
GCGTTGTATTAGCTAGTTGGTGGGGTAACGGCTCACCAAGGCAACGATACATAGCCGACCTGAGAGGGTG
ATCGGCCACACTGGGACTGAGACACGGCC
>Zotu962
GAACGTTGGCGGCGTGCCTTAAGCATGCAAATCAAAGGGGGCTCGCAAGAGTCAACCGGTGGACGGGTG
AGTAATACATGGGAATCTTCCTCGAAGTTGGACATAGCTAGCCGAAAGGTTAGGTAATTTCCAATGTGACC
GCAAGGTTAAAGCCGCAAGGCGCTTTGAGATGAGCCTGTGTCCTATCAGCTTGTTGGTGGGGTAATGGCCT
ACCAAGGCGACGACGGGTAGCTGGACTGAGAGGTCGGTCAGCCACAATGGGACTGCGACACGGCC
>Zotu964
GAACGCTGGCGGCGTGCCTAACACATGCAAGTCGAACGGAGTTACTTTAGTGAAGTTTTCGGATGGATCTG
ATTTAACTTAGTGGCGGACGGGTGAGTAATGCGTGAGCAACCTGCCCTTCAGAGGGGGACAACAGTTGGA
AACGACTGCTAATACCGCATAATGTATTCGGAAGGCATCTTCTGGATACCAAAGGAGAAATCCGCTGATGG
ATGGGCTCACGTCTGATTAGCTAGTTGGTGAGGTAACGGCTCACCAAGGCTGCGATCAGTAGCCGGACTG
AGAGGTTGAACGGCCACATTGGGACTGAGATACGGCC
>Zotu966
AAACGCTGGCGGCGTGCCTAAAACATGCAAGTCGAACGGGCAGTCTTCGGATTGTCAGTGGCAGACGGCT
GAGTAACGCGTGGGTGACGTACCTGTTGGTGGGGAATAACTGCCCGAAAGGGTGGCTAATTCCGCATACG
TCCTTCAGTTCAGTTTTCTGAAGGAGAAAGCCGCAAGGCGCCGATGGAGCGGCCCGCGTTCGATTAGCTGG
TTGGTGGGGTAACGGCCTACCAAGGCGACGATCGATAGCTGGTCTCAGAGGATGATCAGCCAGACTGGGA
CTGAGACACGGCC
>Zotu969
GAACGCTGGCGGCAGGCCTAACACATGCAAGTCGAGCGGATGACGGGAGCTTGCTCCTTGATTCAGCGGC
GGACGGGTGAGTAATGCCTAGGAATCTGCCTGGTAGTGGGGGACAACGTTTCGAAAGGAACGCTAATACC
GCATACGTCCTACGGGAGAAAGCAGGGGACCTTCGGGCCTTGCGCTATCAGATGAGCCTAGGTCGGATTA
GCTAGTTGGTGAGGTAATGGCTCACCAAGGCGACGATCCGTAACTGGTCTGAGAGGATGATCAGTCACAC
TGGAACTGAGACACGGTC
>Zotu972
AAACGCTGGCGGTGTGCTTTATGCATGCAAGTCGAACGGGTTCCGCAAGGAATCAGTGGCAGACGGGTGA
GCAACACGTAAATGACCTACCCTAAAGTGGGGAACAACTCTTAGAAATGAGAGCTAATACCGCATGTGATT
CTGGTTTTTAATTATCAGAATTAAAGCCGCAAGGCGCTTTTGGAGGGGTTTGCGTCCGATTAGATAGTTGG
TGAGGTAACGGCCCACCAAGTCGACGATCGGTAGCTGGTTTGAGAGGACGACCAGCCACACGGGGACTG
AGACACGGCC
>Zotu974
GAACGCTGGCGGCGTGCTTAACACATGCAAGTCGAACGATGACGACAGGGCTTGCCTTGTCTGATTAGTG
GCGAACGGGTGAGTAACACGTGAGTAACCTGCCCCCTCTTCGGGATAACCTCGGGAAATCGGGGCTAAT
ACCGGATATGACTTCGCACCGCATGGTGTGTTGTGGAAAGTTTTTCGGTGGGGGATGGACTCGCGGCCTAT

CAGTTTGTTGGTGAGGTAATGGCTCACCAAGGCGATGACGGGTAGCCGGCCTGAGAGGGCGACCGGCCA
CACTGGGACTGAGACACGGCC
>Zotu976
GAACGCTGGCGGCGTGCTTAACACATGCAAGTCGAGCGAGGCCCATCTTTCGGGGTGGTGCCCTAGCGGC
GAACGGGTGAGTAACACGTGGGCAACCTGCCCTCAGCTCTGGGATAACTCCAAGAAATTGGTGCTAATACC
GGATATGACTACTGGCCGCATGGTCTGGTGGTGGAAAGATTTATCGGCTGAGGATGGGCCCGCGGCCTAT
CAGCTTGTTGGTGGGGTGATGGCCCACCAAGGCGACGACGGGTAGCCGGCCTGAGAGGGTGACCGGCCA
CACTGGGACTGAGACACGGCC
>Zotu978
GAACGCTGGCGGCGTGCTTAACACATGCAAGTCTTAGGGGATTTATTCTTTCGGGAATAAGTTCACTGGCG
CACGGGTGCGTAACGCGTAAGTAATCTGCCCTAGAGACTGGCACAACTCGTCGAAAGACGGGCTAATTCC
AGATGGTACTGAAAGGTCAAAGGTTTCGACCGCTCTAGGATGAGCTTGCGTCTGATTAGCTAGTTGGTGAG
GTAATGGCTCACCAAGGCTACGATCAGTAGCCGGTCTGAGAGGATGATCGGCCACATTGGAACTGAGACA
CGGTC
>Zotu980
AAACGCTGGCGGCGTGCATGAGACATGCAAGTCGAACGAACGAGCTTGCTCGTTAGTGGCGAACGGCTGA
GTAACGCGTAGGTGACCTGCCCCGAAGTGGGGGATAACTGCCCGAAAGGGTGGCTAATACCGCATGTGGC
GACGTGTTCATTCACGTCATCAAAGCCGCGAGGCGCTTCGGGAGGGGCCTGCGTCCGATTAGCTCGTTGGT
GGGGTAACGGCTCACCAAGGCGACGATCGGTAGGTGGTCTGAGAGGACGATCACCCAGACTGGGACTGA
GACACGGCC
>Zotu982
GAACGCTGGCGGCGTGCTTAACACATGCAAGTTGAACGGTGAAGCCCTTCGGGGTGGATCAGTGGCGAAC
GGGTGAGTAACACGTGAGCAACCTACCCTTGACTCTGGGATAACTCCGGGAAACCGGTGCTAATACCGGA
TACGAGCATCGCAGGCATCTGCTGGTGCTGGAAAGAATTTTGGTCAGGGATGGGCTCGCGGCCTATCAGC
TTGTTGGTGAGGTAGTGGCTCACCAAGGCGACGACGGGTAGCCGGCCTGAGAGGGCGACCGGCCACACT
GGGACTGAGACACGGCC
>Zotu984
GAACGCTGGCGGCGTGCTTAACACATGCAAGTCGAACGATGAAGCCCAGCTTGCTGGGTGGATTAGTGGC
GAACGGGTGAGTAACACGTGAGTAACCTGCCCTTAACTCTGGGATAAGCCTGGGAAACTGGGTCTAATAC
CGGATAGGAGCGCCTACCGCATGGTGGGTGTTGGAAAGATTTATCGGTTTTGGATGGACTCGCGGCCTATC
AGCTTGTTGGTGAGGTAATGGCTCACCAAGGCGACGACGGGTAGCCGGCCTGAGAGGGTGACCGGCCAC
ACTGGGACTGAGACACGGCC
>Zotu986
CGCAAGCCTTGGGGAACTGTGCAGCTGGTTTGATCGGGAACGCGAGTACAGCCCAGGTAGCTCAGGCTAG
TGTCATGACCGACTGTCCAGCACGAGAACAGCTCAACGGCATCTTCTTCCTTCGCCGCCCCACAATATACGC
GCGTAACCGGCCGCGCACTTCAAATAGCATAAATTCCTATCACAATA
>Zotu988
GAACGCTGGCGGCGTGCCTAATACATGCAAGTCGAGCGAACTGATTAGAAGCTTGCTTCTATGACGTTAGC
GGCGGACGGGTGAGTAACACGTGGGCAACCTGCCTGTAAGACTGGGATAACTTCGGGAAACCGAAGCTA
ATACCGGATAGGATCTTCTCCTTCATGGGAGATGATTGAAAGATGGTTTCGGCTATCACTTACAGATGGGC
CCGCGGTGCATTAGCTAGTTGGTGAGGTAACGGCTCACCAAGGCAACGATGCATAGCCGACCTGAGAGGG
TGATCGGCCACACTGGGACTGAGACACGGCC
>Zotu990
GAACGCTGGCGGCATGCCTAACACATGCAAGTCGAACGAACCCTTCGGGGTTAGTGGCGCACGGGTGCGT
AACGCGTGGGAATCTGCCCTTTGCTTCGGAATAACAGTTAGAAATGACTGCTAATACCGGATGATGACTTC
GGTCCAAAGATTTATCGGCAAAGGATGAGCCCGCGTAGGATTAGGTAGTTGGTGGGGTAAAGGCCTACCA
AGCCGACGATCCTTAGCTGGTCTGAGAGGATGATCAGCCACACTGGGACTGAGACACGGCC
>Zotu992
GAACGCTGGCGGCGTGCCTAATACATGCAAGTCGAGCGGATCTTGTCCTTCGGGATAAGGTTAGCGGCGG
ACGGGTGAGTAACACGTAGGCAACCTGCCCTCGAGACTGGGATAACCTCCGGAAACGGATGCTAATACCA

GATACGCGATCTTCCCACCTGGGGAGATCGGGAAAGACGGAGCAATCTGTTACTCGGGGATGGGCCTGCG
GCGCATTAGCTAGTTGGTGAGGTAACGGCTCACCAAGGCGACGATGCGTAGCCGACCTGAGAGGGTGATC
GGCCACACTGGGACTGAGACACGGCC
>Zotu994
GAACGCTGGCGGCATGCCTTACACATGCAAGTCGAACGGTAACAGGTCTTTCGGGATGCTGACGAGTGGC
GAACGGGTGAGTAATACATCGGAACGTGCCTAGTAGTGGGGGATAACTACTCGAAAGAGTGGCTAATACC
GCATGAGATCTATGGATGAAAGCAGGGGACCTTCGGGCCTTGTGCTACTAGAGCGGCCGATGGCAGATTA
GGTAGTTGGTGGGATAAAAGCTTACCAAGCCTACGATCTGTAGCTGGTCTGAGAGGACGATCAGCCACAC
TGGGACTGAGACACGGCC
>Zotu996
GAACGCTGGCGGCGTGCCTAATACATGCAAGTCGAGCGAACCTGCAGGAGCTTGCTCCTGTTGGTTAGCG
GCGGACGGGTGAGTAACACGTGGGCAACCTGCCTGTAAGACTGGGATAACACCGGGAAACCGGTGCTAA
TACCGGATAATCCATTTCCTCTCATGAGGAAATGCTGAAAGTTGGTTTCGGCTGACACTTACAGATGGGCCC
GCGGCGCATTAGCTAGTTGGTGAGGTAACGGCTCACCAAGGCGACGATGCGTAGCCGACCTGAGAGGGT
GATCGGCCACACTGGGACTGAGACACGGCC
>Zotu998
GAACGCTAGCGATAGGCTTAACACATGCAAGTCGAGGGGCAGCACATGAGTGGTAACACGATGGTGGCG
ACCGGCGCACGGGTGAGTAACACGTATGCAACCTGCCCATAACTGGGGGATAACCCGTCGAAAGGCGGAC
TAATACCGCATAACAACATTGATCCGCATGGTGATGTGTTTAAAGATTTATCGGTTATGGATGGGCATGCGT
TCCATTAGATTGTTGGTAGGGTAACGGCCTACCAAGTCTGCGATGGATAGGGGTTCTGAGAGGAAGGTCC
CCCACACTGGTACTGAGACACGGAC
>Zotu1000
GAACGCTGGCGGCGTGCCTAACACATGCAAGTCGAGCGGGGGACTTCGGTCCCCAGCGGCGGACGGGTG
AGTAACACGTGGGTAACCTGCCTCATAGAGGGGGATAGCCTCCCGAAAGGGAGATTAATACCGCATAACA
CTGCGAAGTCACATGCCTTTGCAGTTAAAGGAGTAATCCGCTATGAGATGGGCCCGCGGCGCATTAGCTAG
TTGGTGAGGTAACGGCTCACCAAGGCCACGATGCGTAGCCGACCTGAGAGGGTGATCGGCCACATTGGAA
CTGAGACACGGTC
>Zotu1002
GAACGCTGGCGGCGTGCTTAACACATGCAAGTCGAACGGAAAGGCCCTGCTTGCAGGGTACTCGAGTGGC
GAACGGGTGAGTAACACGTGGGTGATCTGCCCTGCACTTCGGGATAAGCTTGGGAAACTGGGTCTAATAC
CGGATAGGAACCATCTTTAGTGTGGTGGTTGGAAAGTTTTTTCGGTGTAGGATGAGCTCGCGGCCTATCAG
CTTGTTGGTGGGGTAATGGCCTACCAAGGCGGCGACGGGTAGCCGGCCTGAGAGGGTGTACGGCCACATT
GGGACTGAGATACGGCC
>Zotu1004
GAACGCTGGCGGCGTGCCTAAGACATGCAAGTCGAGTGGGTTTAGACCCACGGCGAACGGGTGAGTAAT
ACCTAGGAACTTTCTTTAAGTGGAGCATAACCAGTCGAAAGATTGGATAATACTTCATGTGACCGCAAGGT
TAAAGTCGCAAGACGCTTAAAGTGAGGCCTGGGCACCATCAGCTAGTTGGTAAGGTAATGGCTTACCAAG
GCTATGACGGTTAGGGGTCCCGAGAGGGATAACCCCCACACTGGAACTGCGACACGGTC
>Zotu1006
GAACGCTGGCGGCGTGCTTAACACATGCAAGTCGAACGATGAAGCTTGGTGCTTGCACTGGGTGGATTAG
TGGCGAACGGGTGAGTAACACGTGAGTAACCTGCCCTTTACTTCGGGATAAGCTTGGGAAACTGGGTCTA
ATACCGGATATTCTGCGTGATCGGATGGTTGTGTAGGAAAGATTTTTTGGTAAGGGATGGGCTCGCGGCCT
ATCAGTTTGTTGGTGGGGTGATGGCCTACCAAGACGACGACGGGTAGCCGGCCTGAGAGGGCGACCGGC
CACACTGGGACTGAGACACGGCC
>Zotu1008
GAACGCTGGCGGCGTGCTTAACACATGCAAGTCGAACGATGATGCCCAGCTTGCTGGGCGGATTAGTGGC
GAACGGGTGAGTAATACGTGAGTAACCTGCCCTTGACTCTGGGATAAGCCTGGGAAACTGGGTCTAATACT
GGATACTACCGTCCACCGCATGGTGGGTGGTGGAAAGGGTTTTACTGGTTTTGGATGGGCTCACGGCCTAT
CAGCTTGTTGGTGGGGTAATGGCTCACCAAGGCGACGACGGGTAGCCGGCCTGAGAGGGTGACCGGCCA
CACTGGGACTGAGACACGGCC

>Zotu1010
AAACGCTGGCGGCGTGCTTAACACATGCAAGTCGAACGAAGTTTTTCTTTCGGGAGGAACTTAGTGGCGG
ACGGGTGAGTAACGCGTGGGTAACCTGCCCTGTACAGGGGGACAACAGCTGGAAACGGCTGCTAATACCG
CATAAGCCCTTAGCACTGCATGGTGCATAGGGAAAAGGAGCAATCCGGTACAGGATGGACCCGCGTCTGA
TTAGCCAGTTGGCAGGGTAACGGCCTACCAAAGCGACGATCAGTAGCCGATCTGAGAGGATGTACGGCCA
CATTGGGACTGAGACACGGCC
>Zotu1012
GAACGCTGGCGGCGTGCTTAACACATGCAAGTCGAGCGGAAAGGCCTTTCGGGGTACTCGAGCGGCGAAC
GGGTGAGTAACACGTGAGTAATCTGCCCTTGGCTTTGGGATAACTACCGGAAACGGTGGCTAATACCGGA
TACGACACAGGTCTGCATGGGCTCTGTGTGGAAAGTTTTTCGGCCAGGGATGTGCTCGCGGCCTATCAGC
TTGTTGGTGGGGTAATGGCCTACCAAGGCTTCGACGGGTAGCCGGCCTGAGAGGGTGACCGGCCACACTG
GGACTGAGACACGGCC
>Zotu1015
GAACGCTGGCGGCGTGCCTAATACATGCAAGTCGAGCGAAGTTTTTCTGGTGCTTGCACTAGAAAAACTTA
GCGGCGAACGGGTGAGTAACACGTAAAGAACCTGCCTCATAGACTGGGACAACTATTGGAAACGATAGCT
AATACCGGATAACAGCATTAACTGCATGGTTGATGTTTAAAAGTTGGTTTTGCTAACACTATGAGATGGCTT
TGCGGTGCATTAGCTAGTTGGTGGGGTAAAGGCCTACCAAGGCGACGATGCATAGCCGACCTGAGAGGGT
GATCGGCCACACTGGGACTGAGACACGGCC
>Zotu1017
GAACGCTGGCGGCGCGCTTAACACATGCAAGTCGAGCGATGAAATTTTGACAGATCCCTTCGGGGTGAAG
ATAAAATGGATTAGCGGCGGACGGGTGAGTAACACGTGAGTAACCTGCCTTAGACATTGGGATAGCCTCG
GGAAACCGGGATTAATACCGAATAAAATCACAGATACACATGTATCCATGATCAAAACTCCGGTGGTCTAA
GATGGACTCGCGTCCCATTAGCTAGTTGGTGAGGTAACGGCCCACCAAGGCGACGATGGGTAGCCGGCCT
GAGAGGGTGAACGGCCACATTGGAACTGAGAGACGGTC
>Zotu1019
GAACGCTGGCGGCGTGCCTAATACATGCAAGTCGAACGCATTTTCTTTCACCGTAGCTTGCTACACCGAAA
GAAAATGAGTGGCGAACGGGTGAGTAACACGTGGGTAACTTGCCCATCAGCGGGGGATAACACTTGGAA
ACAGGTGCTAATACCGCATAATTCCATTTACCGCATGGTAAATGGATGAAAGGCGCTTTTGCGTCACTGAT
GGATGGACCCGCGGTGCATTAGCTAGTTGGTGGGGTAACGGCCTACCAAGGCTGCGATGCATAGCCGACC
TGAGAGGGTGATCGGCCACACTGGGACTGAGACACGGCC
>Zotu1021
GAACGCTGGCGGCGTGCTTAACACATGCAAGTCGAACGGAAAGGCCAGTGCTTGCACTGGTACTCGAGTG
GCGAACGGGTGAGTAACACGTGGGTGATCTGCCTTGCACTCTGGGATAAGCTTGGGAAACTGGGTCTAAT
ACCGGATAGGAACCGTCTTTAGTGTGGTGGTTGGAAAGTTTTTCGGTGCAAGATGAGCTCGCGGCCTATC
AGCTTGTTGGTGGGGTAATGGCCTACCAAGGCGTCGACGGGTAGCCGGCCTGAGAGGGTGTACGGCCAC
ATTGGGACTGAGATACGGCC
>Zotu1024
GAACGCTGGCGGCGTGCTTAACACATGCAAGTCGAACGATGAAGCCCAGCTTGCTGGGTGGAAGAGTGGC
GAACGGGTGAGTAACACGTGAGTAACCTGCCCTGAACTCTGGGATAAGCACTGGAAACGGTGTCTAATAC
TGGATACGACCTATCACCGCATGGTGTGTGGGTGGAAAGATTTATCGGTTTTGGATGGACTCGCGGCCTAT
CAGCTAGATGGTGAGGTAATGGCTCACCATGGCGACGACGGGTAGCCGGCCTGAGAGGGTGACCGGCCA
CACTGGGACTGAGACACGGCC
>Zotu1026
GAACGCTGGCGGCGTGCCTAATACATGCAAGTCGAACGAACCGCGACTAGGTGCTTGCACTTGGTCAAGG
TGAGTGGCGAACGGGTGAGTAACACGTGGGTAACCTACCTCATAGTGGGGGATAACAGTCGGAAACGACT
GCTAATACCGCATAGGACATGGAATCACATGGTTCAGTGAGGAAAGGTGGCGCAAGCTATCGCTAAGAGA
TGGACCCGCGGTGCATTAGCTAGTTGGTAGGGTAACGGCCTACCAAGGCGATGATGCATAGCCGACCTGA
GAGGGTGATCGGCCACATTGGGACTGAGACACGGCC
>Zotu1028

GAACGCTAGCGGCAGGCCTAATACATGCAAGTCGAACGGTTCGCAAGGACAGTGGCAAACGGGTGCGTA
ACGCGTAAGCAACCTGCCTCATACTGGGGGATAGCCCGGCGAAAGCTGGGGTAAACCCGCACGGTCCGAT
TGAGTCACCTGGCTTGATGGGTAAACATTTATGGGTATGAGAGGGGCTTGCGTCTGATTAGTTAGTTGGCA
GGGTAACGGCCTACCAAGACGATGATCAGTAGGGGTTCTGAGAGGATTGGCCCCCACATGGGTACTGAGA
TACGGAC
>Zotu1030
GAACGCTAGCGGCAGGCCTAATACATGCAAGTCGGACGGGATTGAGAAGCTTGCTTTTCATGAGAGTGGC
GCACGGGTGCGTAACACGTATGTAACCTACCTTCGTCAGGGGGATAGCCTCTCGAAAGAGAGATTAAGAC
CGCATAACATTATAATGTGGCATCACAAAATAATCAAATATTTATAGGACGAAGATGGGCATGCGGAACAT
TAGCTAGTTGGTAAGGTAACGGCTTACCAAGGCTACGATGTTTAGGGGATCTGAGAGGATGGCCCCCCAC
ACTGGTACTGAGACACGGAC
>Zotu1032
GAACGCTGGCGGCGTGCTTAACACATGCAAGTCGAACGATGATCTCCCGCTTGCGGGGGTGATTAGTGGC
GAACGGGTGAGTAATACGTGAGTAACCTGCCCTTGACTCTGGGATAAGCCTGGGAAACCGGGTCTAATAC
TGGATACTACTTCCTGCCGCATGGTGGGGGGTGGAAAGGGTTTTACTGGTTTTGGATGGGCTCACGGCCTA
TCAGCTTGTTGGTGGGGTAATGGCTCACCAAGGCGACGACGGGTAGCCGGCCTGAGAGGGTGACCGGCC
ACACTGGGACTGAGACACGGCC
>Zotu1034
GAACGCTGGCGGCGTGCCTAATACATGCAAGTCGAGCGAACAGATAAGGAGCTTGCTCCTTTGACGTTAG
CGGCGGACGGGTGAGTAACACGTGGGTAACCTACCTATAAGACTGGAATAACTCCGGGAAACCGGGGCTA
ATGCCGGATAACATGTTGAACCGCATGGTTCTACAGTGAAAGGCGGCTTTGCTGTAACTTATAGATGGACC
CGCGCCGTATTAGCTAGTTGGTGAGGTAACGGCTCACCAAGGCAACGATACGTAGCCGACCTGAGAGGGT
GATCGGCCACACTGGAACTGAGACACGGTC
>Zotu1036
TAACGCTGGCGGCGTGCATAACACATGCAAGTCGAACGATGAAACTTAGTAGACTTCTTCGGAAAGACACT
AAGCGGATTAGTGGCGAACGGGTGAGTAACGCGTGAGTAACCTGCCTTACACAAGGGGATAGCCTTTGGA
AACGAAGAATAATACCCTATAAAACCATAAAAGCACATGCTAGAATGGTCAAAGTGATAGCGGTGTAAGA
TGGACTTGCGTCTGATTAGCTAGTTGGTGAGATAAAAGCTCACCAAGGCAACGATCAGTAGCCGGCTTGAG
AGAGTGTACGGCCACATTGGGACTGAGACACGGCC
>Zotu1038
GAATGCTGGCGGCGTGCTTAACACATGCAAGTCGAGCGGTAACAGGAATAATTTATTATTTGCTGACGAGC
GGCGGACGGCTGAGTAACGCGTAGGAACATGCCCCAAAGTGAGGAATAAGCTCCAGAAATGGAGTCTAAT
GCCGCATATGATCTTCGGATTAAAGGATTTATCCGCTATGGGAGTGGCCTGCGTATGATTAGGTAGTTGGT
GAGGTAAAGGCTCACCAAGCCGACGATCATTAGCTGGTCTGAGAGGATGACCAGCCAGACTGGAACTGAG
ACACGGTC
>Zotu1040
GAACGCTGGCGGCATGCCTAATACATGCAAGTCGAGCGAACAGATGAAGTGCTTGCACTTCTGACGTTAGC
GGCGAACGGGTGAGTAACACGTAAGGAATCTACCTATAAGCGGGGGATAACATTCGGAAACGGGTGCTA
ATACCGCATAATATCTTCTTCCGCATGGAAGAAGATTGAAAGACGGCTCTGCTGTCACTTATAGATGACCTT
GCGGTGCATTAGTTAGTTGGTGGGGTAACGGCCTACCAAGACGATGATGCATAGCCGACCTGAGAGGGTG
ATCGGCCACATTGGGACTGAGACACGGCC
>Zotu1042
GAACGCTGGCGGCGTGCCTAATACATGCAAGTAGAACGCTGAAGGAGGAGCTTGCTCTTCTGGATGAGTT
GCGAACGGGTGAGTAACGCGTAGGTAACCTGCCTGGTAGCGGGGGATAACTATTGGAAACGATAGCTAAT
ACCGCATAAAAACAGTTGTTGCATGACAGCTGTTTGAAAGGTGCAATTGCATCACTACCAGATGGACCTGC
GTTGTATTAGCTAGTTGGTGGGGTAACGGCTCACCAAGGCGACGATACATAGCCGACCTGAGAGGGTGAT
CGGCCACACTGGGACTGAGACACGGCC
>Zotu1044
GAACGCTGGCGGCATGCCTTACACATGCAAGTCGAACGGCAGCACGGGAGCAATCCTGGTGGCGAGTGG
CGAACGGGTGAGTAAAGTATCGGAACGTGCCCAGTCGTGGGGGATAACGTAGCGAAAGTTACGCTAATAC
CGCATACGATCTATGGATGAAAGCGGGGGATCGCAAGACCTCGCGCGATTGGAGCGGCCGATATCAGATT

AGGTAGTTGGTGGGGTAAAGGCTCACCAAGCCGACGATCTGTAGCTGGTCTGAGAGGACGACCAGCCACA
CTGGGACTGAGACACGGCC
>Zotu1046
GAACGCTAGCGGCAGGCCTAATACATGCAAGTCTAGGGGGCAGCAATGTCACCGGCGCACGGGTGCGTA
ACACGTATGCAACCTACCTTCTACAGGGGTACAGCCTTTCGAAAGAGAGATTAATACCGCATGAGACCACA
CTTATTCATATAAGAGAGGTTAAAGATTTATCGGTAGAAGATGGGCATGCGTCTGATTAGTTAGTTGGCGG
GGTAATGGCTCACCAAGACGATGATCAGTAGGGGACCTGAGAGGGTAATCCCCCACACTGGCACTGAGAT
ACGGGC
>Zotu1048
GAACGCTGGCGGCGTGCTTAACACATGCAAGTCACGGGGGGTAGCAATACCAACCGGCGAACGGGTGCG
TAACACGTGAGCAATCTGCCGATCTCTGGGGGATAGCCGGCCCAACGGCCGGGTAATACCGCATACGCTC
ATTGGGGGACATCCCTTGGTGAGGAAACCTCCGGGGGAGATCGAGGAGCTCGCGACCTATCAGCTAGTTG
GTGAGGTAATGGCTCACCAAGGCAACGACGGGTAGCTGGTCTGAGAGGATGGCCAGCCACATTGGGACT
GCGACACGGCC
>Zotu1050
GAACGCTGGCGGAATGCTTTACACATGCAAGTCGAGCGGCAGCGCGGGGCAACCTGGCGGCGAGCGGCG
AACGGGTGAGTAATACATCGGAACGTACCCAATCGTGGGGGATAACTATCCGAAAGGATAGCTAATACCG
CATAAGTCCTGAGGGAGAAAGCAGGGGATCGCAAGACCTTGCGCGGATGGAGCGGCCGATGTCGGATTA
GCTAGTTGGTGGGGTAAAGGCCTACCAAGGCGACGATCCGTAGCTGGTCTGAGAGGACGACCAGCCACAC
TGGAACTGAGACACGGTC
>Zotu1052
TAACGCTAGCGGCAGGCCTAATACATGCAAGTCGAACGGCAGCACGGGTAGCAATACCTGGTGGCGAGTG
GCGCACGGGTGCGTAACGCGTATGCAATCTACCTATAACTGGGGAATAGCCTTCCGAAAGGAAGATTAAT
ACCGCATAATATCACGAGACGGCATCGTTTTGTGATCAAATCTCCGGAGGTTATAGATGAGCATGCGTGAC
ATTAGCTAGTTGGTGAGGTAACGGCTCACCAAGGCTACGATGTCTAGGGGGTCTGAGAGGATGATCCCCC
ACACTGGTACTGAGACACGGAC
>Zotu1054
GAACGCTGGCGGCGTGCTTAACACATGCAAGTCGAACGGAAAGGCCCAGCTTGCTGGGTGCTCGAGTGGC
GAACGGGTGAGTAACACGTGGGTGATCTGCCTCTAACTTCGGGATAAGCTTGGGAAACTAGGTCTAATACC
GGATAGGACAATCGTTTAGTGTCGGTTGTGGAAAGTTTTTTCGGTTAGGGATGAGCCCGCGGCCTATCAGC
TTGTTGGTGGGGTAATGGCCTACCAAGGCGTCGACGGGTAGCCGGCCTGAGAGGGTGGACGGCCACATT
GGGACTGAGATACGGCC
>Zotu1057
GAACGCTGGCGGCATGCCTAACACATGCAAGTCGAACGAGACCTTCGGGTCTAGTGGCGCACGGGTGCGT
AACGCGTGGGAATCTGCCCTTGGGTTCGGGATAACTCGCCGAAAGGCGTGCTAATACCGGATGATGACGT
AAGTCCAAAGATTTATCGCCCGAGGATGAGCCCGCGTTGGATTAGGTAGTTGGTGAGGTAAAGGCTCACC
AAGCCGACGATCCATAGCTGGTCTGAGAGGATGATCAGCCACACTGGGACTGAGACACGGCC
>Zotu1059
GAACGCTGGCGGCACGCCTAATACATGCAAGTCGAGCGCGTGAAGCTTTCCGAAGCCTTCGGGCGGACGA
AAGTGGATCGAGCGGCGGACGGGTGAGTAACACGTAGGCAACCTGCCTGTAAGACCGGGATAACTCCGG
GAAACCGGGGCTAATACCGGATAGGATCTCTTTTCGCATGGGAAGGGATTGAAAGGATGGCCGCAAGGCT
ATCGCTTACAGATGGGCCTGCGGCGCATTAGCTGGTTGGTGAGGTAACGGCTCACCAAGGCGACGATGCG
TAGCCGGCCTGAGAGGGTGACCGGCCACACTGGGACTGAGACACGGCC
>Zotu1061
GAACGCTGGCGGCGTGCTTAACACATGCAAGTCGAACGGAAAGGCCCTTTCGGGGGTACTCGAGTGGCGA
ACGGGTGAGTAACACGTGGGTGATCTGCCCTGCACTTCGGGATAAGCTTGGGAAACTGGGTCTAATACCG
GATAGGACTACGCGATGCATGTCGTGTGGTGGAAAGCTTTTGCGGTGTGGGATGGGCCCGCGGCCTATCA
GCTTGTTGGTGGGGTGATGGCCTACCAAGGCGACGACGGGTAGCCGGCCTGAGAGGGTGTCCGGCCACA
CTGGGACTGAGATACGGCC
>Zotu1064

GAACGCTAGCTACAGGCTTAACACATGCAAGTCGAGGGGCAGCATGGTCTTAGCTTGTTAAGGCCGATGG
CGACCGGCGCACGGGTGAGTAACACGTATCCAACCTGCCGTCTACTCTTGGACAGCCTTCTGAAAGGAAGA
TTAATACAAGATGGCATCATGAGTTCACATGTTCACATGATTAAAGGTATTCCGGTAGACGATGGGGATGC
GTTCCATTAGATAGTAGGCGGGGTAACGGCCCACCTAGTCTTCGATGGATAGGGGTTCTGAGAGGAAGGT
CCCCCACATTGGAACTGAGACACGGTC
>Zotu1067
GAACGCTAGCGGCAGGCTTAATACATGCAAGTCGAGGGGCAGCATGGGGTGTAGCAATACACTCTGATGG
CGACCGGCAAACGGGTGCGGAACACGTACGCAACTTACCCTTAACTGGGGGATAGCCCGGGGAAACCCG
GATTAATACCTCGTAACATAATTGGATGGCATCATTTGATTATTATAGCTTTATTGTGGTTAAGGATGGGCG
TGCGGCTGATTAGGTAGTTGGCAGGGTAACGGCCTACCAAGCCATCGATCAGTAACTGGTGTGAGAGCAC
GACCAGTCACACGGGCACTGAGACACGGGC
>Zotu1069
AAACGCTGGCGGCGCACATAAGACATGCAAGTCGAACGAACTTAACCATTAGTTTACTATTGGAGCGGTTA
GTGGCGGACTGGTGAGTAACACGTAAGCAACCTGCCTATCAGAGGGGAACAACAGTTAGAAATGACTGCT
AATACCGCATATGCCTTGAGTATCACATGGTACAAAAGGGAAAGGAGCAATCCGCTGATAGATGGGCTTG
CGTCTGATTAGATAGTTGGTGAGGTAACGGCCCACCAAGTCGACGATCAGTAGCCGGACTGAGAGGTTGA
ACGGCCACATTGGGACTGAGATACGGCC
>Zotu1071
CAACGCTGGCGGCGCGCTTAACACATGCAAGTCGAACGAGAAAGGGGAGCAATCCCTGAGTAAAGTGGC
GTACGGGTGAGTAACACGTGGGTGATCTGCCTGTTAGTGGGGAATACCCCCGGGAAACTGGGGACAATAC
CGCATAATCCCGCAAGGGGAAAGTAGCAATACGCTGACAGAGGAGCCCGCGGCTGATTAGCTTGTTGGTG
AGGTAACGGCTCACCAAGGCTGCGATCGGTAGCCGGCCTGAGAGGGCACACGGCCACACTGGCACTGAA
ACACGGGC
>Zotu1074
GAATGCTGGCGGCGTGGATAAGTCATGCAAGTCAAGGGGGCCCGCAAGGGCAACCGGCAAACGAGGTAG
TAACACGTAGGTAGACCCCCCAGAGTCGGGCATAGCCGGTCGAAAGACCGGGTAATTCCCGATAGTCCCG
CAAGGGTAAAAATTTATTGCTCTGGGAGTTGCCTGCGTAGTATCAGCTAGTTGGTAGGGTAATGGCCTACC
AAGGCTACGACGCTTAGGGGAGCTGAGAGGCTGACCCCCACCGATGGAACTGCGACACGGTC
>Zotu1076
GAACGCTGGCGGCGTGCTTAACACATGCAAGTCGAACGGAAAGGCTCCTGCTTGCAGGGGTACTCGAGTG
GCGAACGGGTGAGTAACACGTGGGTGATCTGCCTTGTACTTCGGGATAAGCCTGGGAAACTGGGTCTAAT
ACCGGATAGGACCAATCTTTAGTGTGGTTGGTGGAAAGTTTTGTCGGTACGAGATGAGCCCGCGGCCTATC
AGCTTGTTGGTGGGGTAATGGCCTACCAAGGCGACGACGGGTAGCCGGCCTGAGAGGGTGTACGGCCAC
ATTGGGACTGAGACACGGCC
>Zotu1078
GAACGCTAGCTATATGCTTAACACATGCAAGTCGAACGTTGTTTTCGGGGAGCTGGGCAGAAGGAAAAGA
GGCTCCTAGCTAAAGTTGTCTCGCCCTGCTTCAAAACTACAGGGCGCGCGCTACGGCTTTGACCTAACGGC
CTCCGTTTGCTGGAATCGGAATAGTTGAGAACAAAGTGGCGAACGGGTGCGTAACGCGTGGGAATCTGCC
GAACAGTTCGGGCCAAATCCTGAAGAAAGCTCAAAAGCGCTGTTTGATGAGCCTGCGTAGTATTAGGTAGT
TGGTCAGGTAAAGGCTGACCAAGCCAATGATGCTTAGCTGGTCTTTTCGGATGATCAGCCACACTGGGACT
GAGACACGGCC
>Zotu1081
GAACGCTGGCGGCGTGCTTAACACATGCAAGTGGAGCGACGAACCAGGGCTTACCCTGGGGCAGAGCCG
CGAACGGGTGAGTAACACGTGGGTTACCTGCCTTGATGACCGGGACAACCCGAGGAAACTCGGGCTAATA
CCGGATGTGCCCGCAAGGGGAAAGGAAGCTTCGGCCTCCGCATCAAGATGGGCCCGCGGCCCATTAGCTT
GTTGGTGAGGTAACGGCTCACCAAGGCTTCGATGGGTAGCTGGTCTGAGAGGACGATCAGCCACACTGGG
ACTGAGACACGGCC
>Zotu1083
GAACGCTGGCGGCGTGCCTAATACATGCAAGTCGAGCGGAGTTGTGAGGGAGCTTGCTCCCGACCAACTT
AGCGGCGGACGGGTGAGTAATACGTAGGCAACCTGCCCACAAGACCGGGATAACATTCGGAAACGAATG
CTAATACCGGATACGCAGCATGGCCGCATGGCCGAGCTGAGAAAGACGGAGCAATCTGTTACTTGTGGAT

GGGCCTACGGCGCATTAGCTAGTTGGTGGGGTAACGGCTCACCAAGGCGACGATGCGTAGCCGACCTGAG
AGGGTGAACGGCCACACTGGGACTGAGACACGGCC
>Zotu1085
GAACGCTAGCGGCAGGCTTAACACATGCAAGTCGAGGGGTAGAGTTCTTCGGAGCTTGAGACCGGCGCAC
GGGTGCGTAACGCGTATGCAATCTACCTTTTACAGAGGGATAGCCCAGAGAAATTTGGATTAATACCTCAT
AGTATAATTGAATGGCATCATTTAATTATTAAAGTCACAACGGTAAAAGATGAGCATGCGTCCCATTAGCTA
GTTGGTAAGGTAACGGCTTACCAAGGCTACGATGGGTAGGGGTCCTGAGAGGGAGATCCCCCACACTGGT
ACTGAGACACGGAC
>Zotu1087
GAACGCTGGCGGCGTGCTTAACACATGCAAGTCGAACGGAAAGGCCCTTCGGGGTGCTCGAGTGGCGAAC
GGGTGAGTAACACGTGGGTGATCTGCCCTGCACTTTGGGATAAGCCTGGGAAACTGGGTCTAATACCGGA
TAGGACCACGCGCTTCATGGTGTGTGGTGGAAAGCTTTTGCGGTGTGGGATGGGCCCGCGGCCTATCAGC
TTGTTGGTGGGGTAATGGCCTACCAAGGCGTCGACGGGTAGCCGGCCTGAGAGGGTGTCCGGCCACACTG
GGACTGAGATACGGCC
>Zotu1090
GAACGCTGGCGGCGTGCCTAATACATGCAAGTAGAACGCTGAAGGAGGAGCTTGCTCTTCTGGATGAGTT
GCGAACGGGTGAGTAACGCGTAGGTAACCTGCCTGGTAGCGGGGGATAACTATTGGAAACGATAGCTAAT
ACCGCATAATAGTAGATGTTGCATGACATTTGCTTGAAAGGTGCAATTGCATCACTACCAGATGGACCTGC
GTTGTATTAGCTAGTTGGTGGGGTAACGGCTCACCAAGGCGACGATACATAGCCGACCTGAGAGGGTGAT
CGGCCACACTGGGACTGAGACACGGCC
>Zotu1092
GAACGCTAGCGGCAGGCTTAACACATGCAAGTCGAGGGGTATAGGTTTTCGGACCTAGAGACCGGCGCAC
GGGTGCGTAACGCGTATGCAATCTACCTTTCACAGAGGGATAGCCCAGAGAAATTTGGATTAATACCTCAT
AGTATTATAGAGTGGCATCACTTTATAATTAAAGTCACAACGGTGAAAGATGAGCATGCGTCCCATTAGCT
AGTTGGTAAGGTAACGGCTTACCAAGGCAACGATGGGTAGGGGTCCTGAGAGGGAGATCCCCCACACTG
GTACTGAGACACGGAC
>Zotu1094
TAACGCTGGCGGCGTGCCTAACACATGCAAGTCGAGCGCCTCGCTTTGCGAGGAGCGGCGGATGGCTGAG
TAACACGTTGGTACCTACCCCGAACTCGGGAATAAGCGCTGGAAACGGCGTCTAATGCCCGATAGTTCTCT
TCGGAGTAAAGCTTTTGCGGTTCGGGAGGGGCCTGCGCCCGATTAGCTTGTTGGTGAGGTAACGGCTCAC
CAAGGCGATGATCGGTAGGTGGTCTGAGAGGATGATCACCCAGACTGGGACTGAGACACGGCC
>Zotu1096
GAACGCTGGCGGCATGCCTCATACATGCAAGTCGAACGAAGGCTTCGGCCTTAGTGGCGCACGGGTGAGT
AGCGCGTGGGAACCTGCCCTGGAGTGGGGGATAACCGAGGGAAACTTCGGCTAATACCGCATGCGCCCG
GTAGCAATACCGGGGAAAGATTTATCGCTCTAGGAGGGGCCCGCGTCGGATTAGCTAGTTGGTGGGGTAA
CGGCCTACCAAGGCAACGATCCGTAGCTGGTCTGAGAGGATGATCAGCCACACTGGGACTGAGACACGGC
C
>Zotu1098
GAACGTTAGCGGCGCGCTTAACACATGCAAGTCGAGCGAGAAAGGGGCAACCCCGGTAAAGCGGCGCAC
GGGTGAGTAACACGTAGGTAATCTACCCCCAGGTGGTGGATAACCTTCCGAAAGGAAGGCTAATACAGCA
TGGGACCACGACTCCGAAAGGAGTTGAGGTGAAAGTTGGCCTCTTCACGAAAGCCAACGCCAGGGGATGA
GCCTGCGGCCCATCAGCTAGTTGGTAGGGTAATGGCCTACCAAGGCAAAGACGGGTAGCTGGTCTGAGAG
GATGATCAGCCACACTGGAACTGAGACACGGTC
>Zotu1100
GAACGCTGGCGGCGTGCTTAACACATGCAAGTCGAACGGAAAGGCCACAGCTTGCTGTGGTACTCGAGTG
GCGAACGGGTGAGTAACACGTGGGTGATCTGCCCTGTACTTCGGGATAAGCTTGGGAAACTGGGTCTAAT
ACCGGATAGGAATCACATGTGGGTGTGTGGTTGGAAAGCTTTTGCGGTATGGGATGAGCTCGCGGCCTAT
CAGCTTGTTGGTGGGGTAATGGCCTACCAAGGCGTCGACGGGTAGCCGGCCTGAGAGGGTGTACGGCCAC
ATTGGGACTGAGATACGGCC
>Zotu1102

GAACGCTGGCGGTAGGCCTAACACATGCAAGTCGAACGGCAGCACAGAGGAGCTTGCTCCTTGGGTGGCG
AGTGGCGGACGGGTGAGGAATACATCGGAATCTACTTTTTCGTGGGGGATAACGTAGGGAAACTTACGCT
AATACCGCATACGACCTACGGGTGAAAGCAGGGGATCTTCGGACCTTGCGCGATTGAATGAGCCGATGTC
GGATTAGCTAGTTGGCGGGGTAAAGGCCCACCAAGGCGACGATCCGTAGCTGGTCTGAGAGGATGATCA
GCCACACTGGAACTGAGACACGGTC

>Zotu1104

GAACGCTGGCGGCAGGCTTAACACATGCAAGTCGAGCGGGGGAGGTTGCTTCGGTAACTGACCTAGCGGC
GGACGGGTGAGTAATACTTAGGAATCTGCCTATTAATGGGGGACAACATCTCGAAAGGGATGCTAATACC
GCATACGCCCTACGGGGGAAAGCAGGGGATCACTTGTGACCTTGCGTTAATAGATGAGCCTAAGTCGGAT
TAGCTAGTTGGTGGGGTAAAGGCCTACCAAGGCGACGATCTGTAGCGGGTCTGAGAGGATGATCCGCCAC
ACTGGGACTGAGACACGGCC

>Zotu1106

GAACGCTGGCGGCGTGCCTAATACATGCAAGTCGAGCGAACCAATAAGAAGCTTGCTTTTTGTTGGTTAGC
GGCGGACGGGTGAGTAACACGTGGGTAACCTGCCTGTAAGACCGGGATAACTCCGGGAAACCGGTGCTA
ATACCGGATAGATTATCTTTCCGCCTGGAGAGATAAGGAAAGATGGCTATTGCCATCACTTACAGATGGGC
CCGCGGCGCATTAGCTAGTTGGTGAGGTAACGGCTCACCAAGGCGACGATGCGTAGCCGACCTGAGAGG
GTGATCGGCCACACTGGGACTGAGACACGGCC

>Zotu1108

GAACGCTGGCGGCGTGCTTAACACATGCAAGTCGAACGGTGAACTCCGCTTGCGGGGGGATCAGTGGCGA
ACGGGTGAGTAACACGTGGGCAACCTGCCCTCAGCTCTGGGATAACTCCAAGAAATTGGGGCTAATACTG
GATATGACTGCTGACCGCATGGTCTGGTGGTGGAAAGATTTATCGGCTGGGGATGGGCCCGCGGCCTATC
AGCTTGTTGGTGGGGTAGTGGCCTACCAAGGCGACGACGGGTAGCCGGCCTGAGAGGGTGACCGGCCAC
ACTGGGACTGAGACACGGCC

>Zotu1111

GAACGCTGGCGGCGTGCTTAACACATGCAAGTCGAACGATGAACTCCAGCTTGCTGGGGGGATTAGTGGC
GAACGGGTGAGTAACACGTGAGTAACCTGCCCTGGACTCTGGGATAACCCCGAGAAATCGGAGCTAATAC
CGGATAGGACCTTTCCTCGCATGAGGGTTGGTGGAAAGTTTTTCGGTTTGGGATGGACTCGCGGCCTATCA
GCTTGTTGGTGAGGTAATGGCTCACCAAGGCGTCGACGGGTAGCCGGCCTGAGAGGGTGACCGGCCACAC
TGGGACTGAGACACGGCC

>Zotu1113

GAACGCTGGCGGCATGCCTAACACATGCAAGTCGAACGAGACCTTCGGGTCTAGTGGCGCACGGGTGCGT
AACGCGTGGGAATCTGCCCTTGGGTTCGGAATAACAGTTGGAAACGACTGCTAATACCGGATGATGACGT
AAGTCCAAAGATTTATCGCCCAGGGATGAGCCCGCGTAGGATTAGCTAGTTGGTGAGGTAAAGGCTCACC
AAGGCGACGATCCTTAGCTGGTCTGAGAGGATGATCAGCCACACTGGGACTGAGACACGGCC

>Zotu1116

GAACGCTGGCGGTAGGCCTAACACATGCAAGTCGAACGGCAGCACAGGAGAGCTTGCTCTCTGGGTGGCG
AGTGGCGGACGGGTGAGGAATACATCGGAATCTACTTTTTCGTGGGGGATAACGTAGGGAAACTTACGCT
AATACCGCATACGACCTACGGGTGAAAGCAGGGGATCTTCGGACCTTGCGCGATTGAATGAGCCGATGTC
GGATTAGCTAGTTGGCGGGGTAAAGGCCCACCAAGGCGACGATCCGTAGCTGGTCTGAGAGGATGATCA
GCCACACTGGAACTGAGACACGGTC

>Zotu1119

GAACGCTGGCGGCGTGCTTAACACATGCAAGTCGAGCGGTAAGGCCCTTCGGGGTACACGAGCGGCGAA
CGGGTGAGTAACACGTGAGCAATCTGCCCTTCACTTCGGGATAACCACCGGAAACGGTGGCTAATACCGG
ATATGACTGCACCAGGCATGTGGTGTGGTGGAAAGTTCTGGCGGTGGAGGATGAGCTCGCGGCCTATCAG
CTTGTTGGTGGGGTAATGGCCTACCAAGGCGACGACGGGTAGCCGGCCTGAGAGGGTGACCGGCCACACT
GGGACTGAGACACGGCC

>Zotu1121

GAACGCTGGCGGCGTGCTTAACACATGCAAGTCGAACGATGATCAGGAGCTTGCTCCTGTGATTAGTGGC
GAACGGGTGAGTAACACGTGAGTAACCTGCCCCTGACTCTGGGATAAGCGTTGGAAACGACGTCTAATAC
TGGATATGACAGCCGATCGCATGGTCTGGTTGTGGAAAGAATTTCGGTTGGGGATGGACTCGCGGCCTAT

CAGGTAGTTGGTGAGGTAATGGCTCACCAAGCCGACGACGGGTAGCCGGCCTGAGAGGGTGACCGGCCA
CACTGGGACTGAGACACGGCC

>Zotu1123
GAACGCTGGCGGAATGCTTTACACATGCAAGTCGAACGGTAACAGGGACTTCGGTCCGCTGACGAGTGGC
GAACGGGTGAGTAATACATCGGAACGTACCCAGTTGTGGGGGATAACTACGCGAAAGCGTAGCTAATACC
GCATACGACCTGAGGGTGAAAGCGGGGGACCGTAAGGCCTCGCGCAGTTGGAGCGGCCGATGTTCGATT
AGCTAGTTGGTGAGGTAAAGGCTCACCAAGGCGACGATCGATAGCTGGTCTGAGAGGATGATCAGCCACA
CTGGGACTGAGACACGGCC

>Zotu1125
GAACGCTAGCGGCAGGCTTAACACATGCAAGTCGAGGGGTATAGTAGCAATACTAGAGACCGGCGCACGA
GTGCGTAACGCGTATGCAATCTACCTTTTACAGAGGGATAGCCCAGAGAAATTTGGATTAATACCTCATAG
TATAGTGAGTTGGCATCAACACACTATTAAAGTCACAACGGTAAAAGATGAGCATGCGTCCCATTAGCTAG
TTGGTAAGGTAACGGCTTACCAAGGCGACGATGGGTAGGGGTCCTGAGAGGGAGATCCCCCACACTGGTA
CTGAGACACGGAC

>Zotu1127
GAACGCTAGCGGCAGGCTTAATACATGCAAGTCGAGGGGCAACAGGGGTAGCAATACCTGCTGGCGACC
GGCAAACGGGTGCGGAACACGTACAGAACCTTCCTTTAAGCGGGGGATAGCCCGGAGAAATCCGGATTAA
TACCCCATAGTAATATTGATTGGCATCAATTGATATTTAAAGATTTATCACTTAAAGATGGCTGTGCGGCTG
ATTAGGTAGTTGGTTGAGGTAACGGCTCACCAAGCCAACGATCAGTAACTGGTGTGAGAGCACGACCAGT
CACACGGGCACTGAGACACGGGC

>Zotu1129
GAACGCTGGCGGCGTGCTTAACACATGCAAGTCGAACGCTGAAGCCTAGCTTGCTGGGTGGATGAGTGGC
GAACGGGTGAGTAACACGTGAGTAACCTGCCCTCTTCTTTGGGATAACGCCCGGAAATGGGTGCTAATACT
GGATATTCACTGGTCTTCGCATGGGGGTTGGTGGAAAGGGTTTTTCTGGTGGGGGATGGGCTCGCGGCCT
ATCAGCTTGTTGGTGGGGTGATGGCCTACCAAGGCTTTGACGGGTAGCCGGCCTGAGAGGGTGACCGGTC
ACATTGGGACTGAGATACGGCC

>Zotu1131
GAACGCTGGCGGCATGCCTAACACATGCAAGTCGAACGAGGGCTTCGGCCCTAGTGGCGCACGGGTGAGT
AACGCGTGGGAACCTGCCCTGTGGTACGGAATAACTCCGGGAAACTGGAGCTAATACCGTATGCGTCCTG
CGGGACAAAGATTTATCGCCATGGGATGGGCCCGCGTAGGATTAGCTTGTTGGTGGGGTAACGGCCTACC
AAGGCAACGATCCTTAGCTGGTCTGAGAGGATGATCAGCCACACTGGGACTGAGACACGGCC

>Zotu1134
GAACGCTGGCGGCGTGCTTAACACATGCAAGTGGAGCGACGAACCAAGGCTTGCCTTGGGGCAGAGCCG
CGAACGGGTGAGTAACACGTGGGTAACCCACCTCGATGACCGGGACAACCCGAGGAAACTCGGGCTAATA
CCGGATGAGATCCTCTTTCGTAAGGAAGATGATGAAAGATAGCTTCGGCCTTCGCATCGAGACGGGCCCGC
GGCCCATTAGCTTGTTGGTGGGGTAATGGCCTACCAAGGCTTCGATGGGTAGCTGGTCTGAGAGGACGAT
CAGCCACACTGGGACTGAGACACGGCC

>Zotu1136
GAACGCTAGCGGGAGGCTTAACACATGCAAGCCGAGGGGTATATAGTTTTCGGACTATAGAGACCGGCGC
ACGGGTGCGTAACGCGTATGTAACTTGCCCTGATCAGTAGGATAGCCCTTCGAAAGGAGGATTAATACTGC
ATAACACTAAGATTGGCATCAATTTTAGTTGAAAGATTTATCGGATCAGGATAGACATGCGTATGATTAGCT
AGTTGGTAGAGTAACGGCCTACCAAGGCGATGATCATTAGGGGGCCTGAGAGGGTGAACCCCCACACTGG
TACTGAGACACGGAC

>Zotu1138
GAACGCTGGCGGCATGCTTTACACATGCAAGTCGAACGGTAACAGGCCTTCGGGTGCTGACGAGTGGCGA
ACGGGTGAGTAATGCATCGGAACGTGCCCAGAGGTGGGGGATAACGCAGCGAAAGCTGTGCTAATACCG
CATGTGATCTGAGGATGAAAGCGGGGGACCAAGCAGCAATGTTTGGCCTCGCGCCTCTGGAGCGGCCGAT
GTCAGATTAGGTAGTTGGTGGGGTAAAGGCCTACCAAGCCGACGATCTGTAGCTGGTCTGAGAGGACGAC
CAGCCACACTGGGACTGAGACACGGCC

>Zotu1140

GAACGCTAGCGGCAGGCCTAATACATGCAAGTCGGACGGGATTGGGGCTTCGGCCCTATGAGAGTGGCGC
ACGGGTGCGTAACACGTATGTAACCTACCTTTATCTGGGGGATAGCCTCTCGAAAGAGAGATTAACACCGC
ATAAAACCACAATGCAGCATTGCATAATGGTCAAATATTTATAGGATAGAGATGGGCATGCGGGACATTAG
CTAGTTGGCGGGGTAACGGCCCACCAAGGCCACGATGTCTAGGGGATCTGAGAGGATGGCCCCCCACACT
GGTACTGAGACACGGAC
>Zotu1142
GAACGCTGGCGGAATGCTTTACACATGCAAGTCGAGCGGCAGCGCGGGGGCAACCCTGGCGGCGAGCGG
CGAACGGGTGAGTAATACATCGGAACGTACCCTGTCGTGGGGGATAACTAACCGAAAGGTTGGCTAATAC
CGCATACGACCTGAGGGTGAAAGCGGGGGATCGCAAGACCTCGCGCGATAGGAGCGGCCGATGTCGGAT
TAGCTAGTTGGTGAGGTAAAGGCTCACCAAGGCGACGATCCGTAGCGGGTCTGAGAGGACGGCCCGCCAC
ACTGGAACTGAGACACGGTC
>Zotu1144
GAACGCTGGCGGCGTGCTTAACACATGCAAGTCGAGCGGTAAGGCCCTTCGGGGTACACGAGCGGCGAA
CGGGTGAGTAACACGTGAGTAATCTGCCCTTCACTTCGGGATAACCACCGGAAACGGTGGCTAATACCGG
ATATGACTTCTCTTCGCATGTTGAGAGGTGGAAAGTTCCGGCGGTGGAGGATGTGCTCGCGGCCTATCAGC
TTGTTGGTGAGGTAATGGCTCACCAAGGCTTCGACGGGTAGCCGGCCTGAGAGGGTGACCGGCCACACTG
GGACTGAGACACGGCC
>Zotu1146
GAACGCTAGCGGCAGGCCTAATACATGCAAGTCGAACGGTCTCTAGCAATAGGGATAGTGGCGCACGGGT
GCGTAACACGTAACTAACCTACCCACAACTGGGGGATAGCCCGCCGAAAGGCGGATTAATACCGCATAAG
CTCTTTGGCTGGCATCAGCTGATTAGTAAAGATTTATTGGTTGTGGATGGGGTTGCGGGTCATTAGCTAGTT
GGTAAGGTAACGGCTTACCAAGGCGACGATGACTAGGGGAGCTGAGAGGCTGGTCCCCCACACGGGCAC
TGAGATACGGGC
>Zotu1148
GAACGCTGGCGTCGTGCTTAACACATGCAAGTCGAACGGATCCCAAGTGTCAGCAATGATGTGGAAGGGT
TAGTGGCGAACGGGTGAGTAACACGTGAGGAACCTGCCCCGGACTCAGGGATAACGGCTGGAAACGGCT
GCTAATACCTGATGCCGTCGTGTCATCGCAGGGTGATGTGACGAAAGAATCTCGGTTCGGGAGGGTCTCG
CGGCCTATCAGCTTGTTGGTGGGGTAATGGCCTACCAAGGCGTCGACGGGTAGCTGGTCTGAGAGGATGA
TCAGCCACACTGGGACTGAGACACGGCC
>Zotu1151
GAACGCTAGCGGGAGGCCTAACACATGCAAGCCGAGCGGGATTTGAGACTAGCTTGCTAGTCTTAATGAG
AGCGGCGTACGGGTGCGGAACACGTGTGCAACCTGCCTTTATCTGGGAGATAGCCTTTCGAAAGGAAGAT
TAATATCCCATAATATAATGATTGGCATCAATTATTATTGAAAGCTCCGGCGGATAGAGATGGGCACGCGC
AAGATTAGATAGTTGGTGAGGTAACGGCTCACCAAGTCGATGATCTTTAGGGGGCCTGAGAGGGTGATCC
CCCACACTGGTACTGAGACACGGAC
>Zotu1154
GAACGCTAGCGGCAGGCCTAATACATGCAAGTCAAGGGGTAGCAATACACCGGCGGACGGGTGCGTAAC
GCGTACACAACGTACCTCAGAGCGGGGGATAGCATTGGGAAACTGGTGGTAAAACCCCATGGTGTTGTTG
AATGGCATCATTTAACAACTAAAGGTTACACTCTGAGATCGGTGTGCGTCTGATTAGCTAGTTGGTAGGGT
AACGGCCTACCAAGGCGACGATCAGTAGGGGGTGTGAGAGCATGACCCCCCACATGGGTACTGAGACAC
GGAC
>Zotu1156
GAACGCTGGCGGCATGCCTAATACATGCAAGTCGAACGAGAGGAAGGAAAGCTTGCTTTCCTAAATCTAG
TGGCGAACGGGTGAGTAACACGTAGGTAACCTGCCCATGTGCCCGGGATAACTTCTGGAAACGGATGCTA
AAACCGGATAGGTAGCAGACAAGCATTTGACTGCTATTAAAGTGGCTAAGGCCATGAACATGGATGGACC
TGCGGTGCATTAGCTAGTTGGTGAGGTAACGGCCCACCAAGGCGACGATGCATAGCCGGCCTGAGAGGGC
GGACGGCCACATTGGGACTGAGACACGGCC
>Zotu1158
AAACGCTGGCGGCGTGCATAACACATGCAAGTCGAACGATGAAGCTTAGAAGATTTCTTCGGAATGAACTT
AAGTGGATTAGTGGCGAACGGGTGAGTAACACGTGAGTAACCTGCCTTACACAAGGGGATAGCCTCTGGA
AACGGAGAATAATACCCTATAAAGTCTAATCAGCTCCTGCTGGAAAGACCAAAGATTTATCGGTGTAAGAT

GGACTTGCGTCTGATTAGCTAGTTGGTGAGATAAAAGCTCACCAAGGCGACGATCAGTAGCCGGCTTGAG
AGAGTGTACGGCCACATTGGGACTGAGACACGGCC
>Zotu1160
GAACGCTGGCGGCGTGCTTAACACATGCAAGTCGAACGGAAAGGCCTGCTTTCCCTTGTGGATTGCGGGT
GCTCGAGTGGCGAACGGGTGAGTAACACGTGAGTGATCTGCCCCCAACTTGGGTATAAGCCTGGGAAACT
GGGTCTAATTCCCGATAGGACTGCAGGGTGGTGCCTGTGGTGGAAAACGTTTTCTAGTGGTTGGGGATGA
GCTCGCGGCCTATCAGCTTGTTGGTGGGGTAATGGCCTACCAAGGCGGCGACGGGTAGCCGGCCTGAGAG
GGTGGACGGCCACATTGGGACTGAGATACGGCC
>Zotu1162
GAACGCTAGCGGCAGGCTTAATACATGCAAGTCGAGGGGCAGCAGGGGTGTAGCAATACACTCGCTGGC
GACCGGCAAACGGGTGCGGAACACGTACGCAATCTACCCAAAACTGATGAATAGCCCTCCGAAAGGAGGA
TTAATACATCGTAACATAATGAAGTGGCATCACTTTGTTATTATAGCTCCGGCGGTTTTGGATGAGCGTGCG
CCTGATTAGGTAGTTGGCGGGGTAACGGCCCACCAAGCCTACGATCAGTAACTGGTGTGAGAGCACGACC
AGTCACACGGGCACTGAGACACGGGC
>Zotu1164
GAACGCTGGCGGCGTGCCTAATACATGCAAGTCGTGCGGACTTTTTAAAAGCTTGCTTTTAAAAGGTTAGC
GGCGGACGGGTGAGTAACACGTGGGCAACCTGCCTGTAAGATCGGGATAACGCCGGGAAACCGGGGCTA
ATACCGGATATTTTTTTCCTCCGCATGGAGGAAAAAGGAAAGACGGCTTCGGCTGTCACTTACAGATGGGC
CCGCGGCGCATTAGCTAGTTGGTGGGGTAACGGCTCACCAAGGCCACGATGCGTAGCCGACCTGAGAGGG
TGATCGGCCACATTGGGACTGAGACACGGCC
>Zotu1166
GAACGCTGGCGGCGTGCTTAACACATGCAAGTCGCACGGGAAGCTGGGGGCAACCCCGGCCGAGAGTGG
CGAATGGGTGCGTAACACGTAGGCGACCTGCCCTCCAGCAGGGGATAACGCTCCGAAAGGAGCGCTAATA
CCGTATGCGGTGCACGATTCATTTTGTGCACTAAACCTGCGAGGTAACTCGTGGGGCTGGGGGAGGGGCC
TGCGGCCCATCAGCTAGTAGGTGAGGTAACGGCTCACCTAGGCGACGACGGGTAGGGGGCCTGAGAGGG
TGACCCCCCACACTGGCACTGACACACGGGC
>Zotu1169
GAACGCTGGCGGCGTGCCTAATACATGCAAGTTGAGCGCTGAAGGGAGGTACTTGTACCTTTTGGAAGAG
CAGCGAACGGGTGAGTAACGCGTGGGGAATCTGCCTTTGAGCGGGGGACAACATTTGGAAACGAATGCT
AATACCGCATAAAAGCTTTAAACATAAGTTAAAAGTTTGAAAGATGCAAAAGCATCACTCAAAGATGATCC
CGCGTTGTATTAGCTAGTTGGTAGGGTAAAGGCCTACCAAGGCGATGATACATAGCCGACCTGAGAGGGT
GATCGGCCACATTGGGACTGAGACACGGCC
>Zotu1171
GAACGCTGGCGGCGTGCCTAATACATGCAAGTCGAGCGGACCGAATGGGAGCTTACTTTCGTTCGGTTAG
CGGCGGACGGGTGAGTAACACGTGGGCAACCTGCCCGTAAAACCGGGATAACTTCGGGAAACCGGAGCT
AATACCGGATAACACCGAAGATCGCATGATCTTTGGTTGAAAGGCGGCTTTGGCTGTCACTTACGGATGGG
CCCGCGGCGCATTAGCTAGTTGGTGAGGTAACGGCTCACCAAGGCGACGATGCGTAGCCGACCTGAGAGG
GTGATCGGCCACACTGGGACTGAGACACGGCC
>Zotu1173
GAACGCTGGCGGCGTGCCTAATACATGCAAGTCGAACGAACAGCGACTTGAACTTGTTCAAGTCAATGTTA
GTGGCGCACGGGTGAGTAACACGTGGAGAACCTACCCTTAAGCGGGGGATAACCATTGGAAACGATGACT
AATACCGCATAGAATCACAGATCACATGATCAATGAAGGAAAGACGGCTTCGGCTGTCACTTAAGGATGGC
TCCGCGGTGCATTAGCTAGATGGTGGGGTAAAGGCCTACCATGGCAATGATGCATAGCCGACCTGAGAGG
GTGATCGGCCACATTGGGACTGAGACACGGCC
>Zotu1175
GAACGCTGGCGGCGTGCCTAATACATGCAAGTCGAGCGGATCTTTAGAAGCTTGCTTCTAAAGATTAGCGG
CGGACGGGTGAGTAACACGTGGGCAACCTGCCTGTAAGACTGGGATAACTTCGGGAAACCGAGGCTAATA
CCGGATAATCCTTTTTCTCACATGAGGGAAAGCTGAAAGACGGCGCAAGCTGTCACTTACAGATGGGCCCG
CGGCGCATTAGCTAGTTGGTGAGGTAACGGCTCACCAAGGCGACGATGCGTAGCCGACCTGAGAGGGTG
ATCGGCCACACTGGGACTGAGACACGGCC

>Zotu1177
GAACGCTGGCGGCATGCCTAATACATGCAAGTCGAACGCGGCCATCCGGTGCTTGCACTGGTGAAGCCGA
GTGGCGAACGGGTGAGTAACACGTGAGAAACCTGCCCTGGAGTGGGGAATAACTCGAAGAAATTCGAGC
TAATACCGCATACCTTCTCTTCACCGCATGGTGAGTTGAAGAAAGATTTATCGCTCTAGGAGGGTCTCGCGG
CCTATCAGCTAGTTGGTGAGGTAATGGCTCACCAAGGCATCGACGGGTAGCTGGTCTGAGAGGACGATCA
GCCACACTGGGACTGAGACACGGCC
>Zotu1179
TAACGCTGGCGGCGTGCCTAATGCATGCAAGTCGAACGTGGTCCCTTCGGGGGCAAACGTGGCGGACGGG
TGAGGAACACGTGGGGAACCTGCCCATTGGTGGGGGGTAGCCCGCGGAAACGCGGGGTAATTCCGCATA
CGCTCGCTTGGGGGTAGCCCGAGTGAGGAAAGGGTCTTCGGACTCGCCGATGGAGGGGCCTGCGTCCGAT
TAGCTAGTTGGTGGGGTAATGGCCTACCAAGGCGGTGATCGGTAGCTGGTCTGAGAGGACGGCCAGCCAC
AGGGGGACTGAGACACGGCC
>Zotu1181
GAACGCTAGCGGCAGGCCTAATACATGCAAGTCAAGGGGTAGCAATACACCGGCGGACGGGTGCGTAAC
GCGTACACAACGTACCTCAGAGCGGGGGATAGTATCGGGAAACTGGTAGTAACACCCCATAGTGATATTG
ATTGGCATCAGTTAATATCTAAAGGTTACACTCTGAGATCGGTGTGCGTATGATTAGCTAGTTGGTAGGGT
AACGGCTTACCAAGGCGATGATCATTAGGGGGTGTGAGAGCATGACCCCCCACACGGGTACTGAGACACG
GAC
>Zotu1183
GAACGCTGGCGGCGCGCTTAACACATGCAAGTCGAGCGGGAACCAGGCCTTCGGGCCTGGGGAAAGCGG
CGAACGGGTGAGTAACACGTGGGTAATCTGCCCTCGGCATCGGGATAGCCCGGGGAAACCCGGATTAATA
CCGGATAGCCTCCGAAGCTTTTGAGCATCGGAGAAAAGGTAGCTTCGGCTTCCGGCTGAGGATGAGCCCG
CGGCGGATTAGCTTGTTGGTGGGGTAAAGGCCCACCAAGGCGACGATCCGTAGCTGGTCTGAGAGGACG
ATCAGCCACACTGGGACTGAGACACGGCC
>Zotu1185
GAACGCTGGCGGCATGCCTTACACATGCAAGTCGAACGGCAGCACGGGGCAACCTGGTGGCGAGTGGCG
AACGGGTGAGTAATGCATCGGAACGTGCCATTGAGTGGGGGACAACCATCCGAAAGGATGGCTAATACCG
CATATACTGTGCGCAGGAAAGCAGGGGATCGCAAGACCTTGCGCTCTTTGAGCGGCCGATGTCGGATTAG
CTAGTTGGTGAGGTAAAGGCTCACCAAGGCGACGATCCGTAGCGGGTCTGAGAGGATGATCCGCCACACT
GGGACTGAGACACGGCC
>Zotu1187
GAACGCTGGCGGCGTGCCTAATACATGCAAGTCGAGCGAACCGGAGGGAGCTTGCTCCCAAAGGTTAGCG
GCGGACGGGTGAGTAATACGTAGGCAACCTGCCTGTAAGCTCGGGATAACTCACGGAAACGTGTGCTAAA
ACCGGATAGGAACTTGTTGCACCTGCAATAAGTTGGAAAAACGGCGCAAGCTGTTACTTACAGAGGGGCC
TACGTCGCATTAGCTAGTTGGTGGGGTAACGGCTCACCAAGGCGACGATGCGTAGCCGACCTGAGAGGGT
GATCGGCCACACTGGGACTGAGACACGGCC
>Zotu1189
GAACGCTGGCGGCGGGCTTAACACATGCAAGTCGAACGAACCCTTCGGGGTTAGTGGCGCACGGGTGAGT
AACGCGTAGGCAATCTGCCCCCAACTGAGGAATAACCCATCGAAAGGTGGGCTAATACCACATAATGTCGA
ACGGTCGCATGGCCGGTTGACCAAAGTTCAGGCGGTTGGGGATGAGCCTGCGTTGGATTAGCTAGTTGGC
GGGGTAACGGCCCACCAAGGCGACGATCCATAGCGGGTCTGAGAGGATGATCCGCCACACTGGCACTGA
GACACGGGC
>Zotu1191
GAACGCTGGCGGCATGCCTAACACATGCAAGTCGAACGAACCCTTCGGGGTTAGTGGCGCACGGGTGCGT
AACGCGTGGGAATCTGCCCTTTGCTTCGGAATAACAGTTAGAAATGACTGCTAATACCGGATGATGTCTTC
GGACCAAAGATTTATCGGCAAAGGATGAGCCCGCGTAGGATTAGGTAGTTGGTGGGGTAAAGGCCTACCA
AGCCGACGATCCTTAGCTGGTCTGAGAGGATGATCAGCCACACTGGGACTGAGACACGGCC
>Zotu1193
GAACGCTGACAGAATGCTTAACACATGCAAGTCATTGGCGAAGCTGCGCTTGCGCAGCCTAGCCAAGGCG
GACGGGTGAGTAACGCGTAAGGAACTTGCCCTGCAGACAGGGATAACAGACGGAAACGACTGATAACAC

CTGGTATTGTCAGGCTGGCGCATGCCGGCCTGATGAAAGGAGACGCTGCAGGAGAGCCTTGCGTCCTATT
AGCTAGTTGGTGGGGTAACGGCCCACCAAGGCGATGATAGGTAGCCGGCCTGAGAGGGTGAACGGCCAC
AAGGGGACTGAGATACGGCC
>Zotu1195
GAACGCTAGCGGCAGGCTTAACACATGCAAGTCGAGGGGTATGCTTCTTCGGGAGCAGAGACCGGCGCAC
GGGTGCGTAACGCGTATGCAATCTACCTTTTACAGAGGGATAGCCCAGAGAAATTTGGATTAATACCTCAT
AGCATAGCAGCTTCGCATGAAGCCACTATTAAAGTCACAACGGTAAAAGATGAGCATGCGTCCCATTAGCT
AGATGGTAAGGTAACGGCTTACCATGGCAACGATGGGTAGGGGTCCTGAGAGGGAGATCCCCCACACTG
GTACTGAGACACGGAC
>Zotu1197
GAACGCTGGCGGCATGCCTAACACATGCAAGTCGAACGAGACCTTCGGGTCTAGTGGCGCACGGGTGCGT
AACGCGTGGGAACCTGCCTTTAGGTTCGGAATAACTCTCTGAAAGGGGTGCTAATACCGAATAATGTCTTC
GGACCAAAGATTTATCGCCTTTAGATGGGCCCGCGTTGGATTAGCTAGTTGGTGGGGTAAAGGCCTACCAA
GGCGACGATCCATAGCTGGTCTTAGAGGATGATCAGCCACACTGGGACTGAGACACGGCC
>Zotu1199
GAACGCTGGCGGCATGCCTAATACATGCAAGTCGAACGCTGAAGCACTCTGCTTGCAGAGTGCGGATGAG
TGGCGAACGGGTGAGTAACACGTGGGAAATCTGCCTATAAGTGGGGGATAACATTCGGAAACGGATGCTA
ATACCGCATATATTCTTGGACCGCCTGGTCCTTGAAAGAAAGACGGCTTTGCTGTCGCTTATAGATGATCCC
GCGGCGTATTAGTTAGTTGGTGGGGTAAAGGCCTACCAAGACAATGATACGTAGCCGACCTGAGAGGGTG
ATCGGCCACACTGGGACTGAGACACGGCC
>Zotu1201
GAACGCTGGCGGCAGGCTTAACACATGCAAGTCGAACGATGACTCTCTAGCTTGCTAGAGATGATTAGTG
GCGGACGGGTGAGTAACATTTAGGAATCTACCTAGTAGTGGGGGATAGCTCGGGGAAACTCGAATTAATA
CCGCATACGACCTACGGGTGAAAAGGGGCGCAAGCTCTTGCTATTAGATGAGCCTAAATCAGATTAGCTAG
TTGGTGGGGTAAAGGCCCACCAAGGCGACGATCTGTAACTGGTCTGAGAGGATGATCAGTCACACCGGAA
CTGAGACACGGTC
>Zotu1203
GAACGCTGGCGGCGTGCTTAACACATGCAAGTGGAGCGACGAACCGGGGCTTGCCCCGGGGCAGAGCCG
CGAACGGGTGAGTAACACGTGGGTTACCTGCCCTGATGACCGGGACAACCCGTCGAAAGACGGGCTAATA
CCGGATGTGCCCGCAAGGGGAAAGGAGGCGTAAGCTTCCGCATCGGGATGGGCCCGCGGAGCATTAGCT
TGTTGGTGAGGTAAAAGCTCACCAAGGCTTCGATGCTTAGCTGGTCTGAGAGGACGATCAGCCACACTGG
GACTGAGACACGGCC
>Zotu1206
GAACGCTGGCGGCGTGCCTAACACATGCAAGTCGAGCGAGTGGATCTCCTTCGGGAGAGAAGCTAGCGGC
GGACGGGTGAGTAACACGTGGGCAACCTGCCTCGTAGAGGGGAATAGCCTCCCGAAAGGGAGATTAATA
CCGCATAAGATTGTAGCTTCGCATGAAGTAGCAATTAAAGGAGCAATCCGCTATGAGATGGGCCCGCGGC
GCATTAGCTAGTTGGTGAGGTAACGGCTCACCAAGGCGACGATGCGTAGCCGACCTGAGAGGGTGATCGG
CCACATTGGGACTGAGACACGGCC
>Zotu1208
GAACGCTGGCGGTATGCTTAACACATGCAAGTCGAACGGATGTTTTCGGACATTAGTGGCGGACGGGTGA
GTAACGCGTGAGAATCTAGCTTCAGGTTGGGGACAACAGTTGGAAACGACTGCTAATACCGAATGTGCCG
AAAGGTGAAAGATTTATTGCCTGAAGAAGAGCTCGCGTCTGATTAGCTAGTTGGTGGGGTAAGAGCTTAC
CAAGGCGACGATCGGTAGCTGGTCTGAGAGGACGATCAGCCACACTGGGACTGAGACACGGCC
>Zotu1210
GAACGCTAGCGGCAGACATAATACATGCAAGGCGAAGGGGCAGCAATGTCACTGTCGTACGGGTGCGTA
ACGCGTATGCAACCTACCTATCACTGGGGGATAGCCCGGGGAAACCCGGATTAATACCGCATAAAACAGG
GGCACCGCATGGTGATATTTGTTAAAGATTTATTGGTGATAGATGGGCATGCGTTCGATTAGCTAGTTGGC
GGGGTAACGGCCCACCAAGGCGACGATCGATAGGGGAGCTGAGAGGTTGGTCCCCCACACGGGCACTGA
GATACGGGC
>Zotu1213

GAACGCTAGCGGCAGGCTTAACACATGCAAGTCGAGGGGTAGAGTTAGCTTGCTAACTTGAGACCGGCGC
ACGGGTGCGTAACGCGTATGCAACCTACCTTTCACAGGGGGATAGCCCAGAGAAATTTGGATTAATACCCC
ATGGTATATATGGATCGCATGGTTTATATATTAAAGTTCCAACGGTGAAAGATGGGCATGCGTCCCATTAG
CTAGATGGTAAGGTAACGGCTTACCATGGCGACGATGGGTAGGGGTCCTGAGAGGGAGATCCCCCACACT
GGTACTGAGACACGGAC
>Zotu1215
GAACGCTGGCGGCGTGCTTAACACATGCAAGTGGAGCGACGAACCTGGCTTCGGCCAGGGGCAAAGCCG
CGAACGGGTGAGTAACACGTGGGTAACGTGCCCCAATGACTGGGACAACCCGGGGAAACCCGGGCTAAT
ACCGGATGTGCTCCTTCCACATCAGTGGATTGAGTAAAGGTAGCTTCGGCTTCCGCATTGGGATCGGCCCG
CGGCGCATTAGCTTGTTGGTGAGGTAAAGGCTCACCAAGGCTTCGATGCGTAGCTGGTCTGAGAGGACGA
TCAGCCACACTGGGACTGAGACACGGCC
>Zotu1217
GAACGCTGGCGGCGTGCTTAATACATGCAAGTCGAGCGAGGTTACTGTTTTGGATCCCTTCGGGGTGAAG
AAGCTTTAACCTAGCGGCGGACGGGTGAGTAACGCGTGGGTAACCTACCCCAAAGTCCGGGATAACAGCG
GGAAACCGCTGCTAATACCGGATGCCTTTACCTTGGGGCATCTTGAGGTAAAGAAAGGTAACGCTTTGGGA
TGGGCCCGCGTCCCATTAGCTAGTTGGTGAGGTAACGGCTCACCAAGGCGACGATGGGTAGCCGGCCTGA
GAGGGTGGACGGCCACACTGGGACTGAGACACGGCC
>Zotu1219
GAACGCTGGCGGCAGGCTTAACACATGCAAGTCGAGCGGGCCCTTCGGGGTCAGCGGCAGACGGGTGAG
TAACACGTGGGAACGTACCCTTTGGTTCGGAATAACGCTGGGAAACTAGCGCTAATACCGGATACGCCCTT
TTGGGGAAAGGCTTGCTGCCGAAGGATCGGCCCGCGTCTGATTAGCTAGTTGGTGGGGTAATGGCCTACC
AAGGCGACGATCAGTAGCTGGTCTGAGAGGATGATCAGCCACACTGGGACTGAGACACGGCC
>Zotu1221
TAACGCTGGCGGCGTGCATAACACATGCAAGTCGAACGATGAAACTTAGTAGACTTCTTCGGAAAGACACT
AAGCGGATTAGTGGCGAACGGGTGAGTAACGCGTGAGTAACCTGCCTTACACAAGGGGATAGCCTTTGGA
AACGAAGAATAATACCCTATAAAACCATAAAAGCACATGCTAGAATGGTCAAAGTGCTAGCGGTGTAAGAT
GGACTTGCGTCTGATTAGCTAGTTGGTGAGATAAAAGCTCACCAAGGCAACGATCAGTAGCCGGCTTGAG
AGAGTGTACGGCCACATTGGGACTGAGACACGGCC
>Zotu1223
GAATGCTGGCGGCGTGCCTAACACATGCAAGTCGAGCGCGAAAAGGTTTCGGCCTTAGTAAAGCGGCGAA
CGGGTGAGTAACGCGTGGAAATCTACCTTTTAGTGGAGAATACCCTAGAGAAATTTAGGTTAATACTGCAT
AAGTTCTGAAAAGAAGAAAGATGGCCTATTTGCTGTCGCTAGAAGATGAGTCTGCGTACTATTAGTTAGTT
GGTGGGGTAATGGCCTACCAAGACGATGATGGTTAGCCGGCCTGAGAGGGTGTACGGCCACACTGGAACT
TAGACACGGTC
>Zotu1226
GAACGCTGGCGGCGTGCTTAACACATGCAAGTCGAACGGTGAACCCGCTTCGGTGGGGGATCAGTGGCGA
ACGGGTGAGTAACACGTGGGCAACCTGCCCTCAGCTCTGGGATAACTCCAAGAAATTGGGGCTAATACTG
GATATGACCGCTGACCGCATGGTCTGGTGGTGGAAAGATTTATCGGCTGGGGATGGGCCCGCGGCCTATC
AGCTTGTTGGTGGGGTAGTGGCCTACCAAGGCGACGACGGGTAGCCGGCCTGAGAGGGTGACCGGCCAC
ACTGGGACTGAGACACGGCC
>Zotu1228
GAACGCTGGCGGCGCGCCTAAAACATGCAAGTCGAACGAGAAGCCCGGGCTTGCCCGGGTGGACAGTGG
CGAACGGGTGAGTAACACGTGAGGAACCTGCCCCGAAGACCGGGACAACACCGGGAAACCGGTGCTAAT
ACCGGATACCTTCACTGGATCGCATGGTCTGGTGAAGAAATGGATTCCGCTTCGGGATGGCCTCGCGGCCT
ATCAGCTAGTTGGTGAGGTAACGGCTCACCAAGGCGACGACGGGTAGCTGGTCTGAGAGGATGTCCAGCC
ACACTGGGACTGAGACACGGCC
>Zotu1230
GAACGCTGGCGGCGTGCCTAATACATGCAAGTAGGACGCACAGCCTATACCGTAGCTTGCTACACCATAGG
CTGTGAGTTGCGAACGGGTGAGTAACGCGTAGGTAACCTGCCTATTAGAGGGGGATAACTATTGGAAACG
ATAGCTAATACCGCATAACAGTATGTAACACATGTTAGATGCTTGAAAGATGCAATTGCATCGCTAGTAGA

TGGACCTGCGTTGTATTAGCTAGTAGGTAGGGTAATGGCCTACCTAGGCAACGATACATAGCCGACCTGAG
AGGGTGATCGGCCACACTGGGACTGAGACACGGCC
>Zotu1232
GAACGCTGGCGGCGTGCTTAACACATGCAAGTCGAACGGTGAAGCAGGAGCTTGCTCTTGTGGATCAGTG
GCGAACGGGTGAGTAACACGTGAGCAATCTGCCCCGAACTCTGGGATAAGCACTGGAAACGGTGTCTAAT
ACCGGATACGACGCACGAGGGCATCCTCTGTGCGCGGAAAGAATCTCGGTTCGGGATGAGCTCGCGGCCT
ATCAGCTAGTTGGTGAGGTAATGGCTCACCAAGGCGACGACGGGTAGCCGGCCTGAGAGGGTGACCGGC
CACACTGGGACTGAGACACGGCC
>Zotu1234
GAACGCTGGCGGCGTGCTTAACACATGCAAGTCGAACGATGAAGACCGGTGCTTGCACTGGTTGGATTAG
TGGCGAACGGGTGAGTAATACGTGAGTAACCTGCCTTTGACTCTGGGATAAGCCTGGGAAACTGGGTCTA
ATACCGGATACGACACGAAACCGCATGATTTGGTGTGGAAAGGGTTTGTACTGGTTTTAGATGGGCTCACG
GCCTATCAGCTTGTTGGTGAGGTAACGGCTCACCAAGGCGACGACGGGTAGCCGGCCTGAGAGGGTGACC
GGCCACACTGGGACTGAGACACGGCC
>Zotu1236
GAACGCTAGCGGCAGGCCTAATACATGCAAGTCGAACGGTAACATGAGTAGCAATACTTGATGACGAGTG
GCGCACGGGTGCGTAACGCGTATGCAATCTACCTTCAACCGGTGAATAGCCCGAAGAAATTCGGATTAATA
CACCATAGTATATTGAAGTGGCATCACTTTAATATTAAAACTCCGGTGGTTGAAGATGAGCATGCGTGACA
TTAGCTTGTTGGTGAGGTAACGGCTCACCAAGGCTACGATGTCTAGGGGATCTGAGAGGATTAACCCCCAC
ACTGGTACTGAGACACGGAC
>Zotu1239
GAACGCTAGCGGGAGGCCTAACACATGCAAGCCGAGCGGTAGAGTTTCTTCGGAAACTTGAGAGCGGCGC
ACGGGTGCGTAACACGTGTGCAACCTACCTTTATCTGGGGGATAGCCTTTCGAAAGGAAGATTAATACCCC
ATAATATAATGATTGGCATCAATTATTATTGAAAACTCCGGTGGATAGAGATGGGCACGCGCAAGATTAGA
TAGTTGGTGAGGTAACGGCTCACCAAGTCAATGATCTTTAGGGGGCCTGAGAGGGTGATCCCCCACACTG
GTACTGAGACACGGAC
>Zotu1242
GAACGCTGGCGGCGTGCTTAACACATGCAAGTCGAACGATGATGAGGAGCTTGCTTCTCTGATTAGTGGC
GAACGGGTGAGTAACACGTGAGCAATCTGCCCTTGACTCTGGGATAAGCGTTGGAAACGACGTCTAATAC
CGGATACGACCCTTGGAGGCATCTCTGGGGGTGGAAAGAATTTTGGTCAAGGATGAGCTCGCGGCCTATC
AGCTAGTTGGTGAGGTAATGGCTCACCAAGGCGACGACGGGTAGCCGGCCTGAGAGGGTGACCGGCCAC
ACTGGGACTGAGACACGGCC
>Zotu1244
GAACGCTGGCGGCATGCTTAACACATGCAAGTCGCACGGTCGGGGGTAACTCCGGCAGTGGCGGACGGG
TGAGTAACGCGTAGGAATCTATCCTTGGGTGGGGGACAACCGTGGGAAACTACGGCTAATACCGCATGAT
ACCTGTGGGTCAAAGGCGCAAGTCGCCTGAGGAGGAGCCTGCGTACGATTAGCTAGTTGGTGGGGTAAA
GGCCTACCAAGGCTACGATCGTTAGCTGGTCTGAGAGGATGATCAGCCACACTGGGACTGAGACACGGCC
>Zotu1248
GAACGCTGGCGGCGTGCTTAACACATGCAAGTCGAACGGACCCTTCGGGGTTAGTGGCGGACGGGTGAGT
AACGCGTGAGGATCTGCCCTCAGGAGGGGAACAACAGTTGGAAACGACTGCTAATGCCCCATATGCCGAG
AGGTGAAAAGTTATTTCGCCTGAGGATGAACTCGCGTCTGATTAGCTAGTTGGTGGGGTAAGGGCCTACCA
AGGCGACGATCAGTAGCTGGTCTAAGAGGATGATCAGCCACACTGGGACTGAGACACGGCC
>Zotu1250
GAACGCTGGTAACGTGGATACCGGCATGCAAGTCACGGGGGCCGCAAGGCAACCGGCGAACGACTTAGT
AACACGTGGGTACATACCCCAAACTGGAGCACAGCTCGTCGAAAGACGGGGTAATTCTCCATAGTCTCGCA
AGAGTAAACCCGCAAGGGGGTTTGGGAATGGCCTGCGGCCTATCAGATAGTTGGCGGTGTAATAGACCAC
CAAGTCTATGACGGGTAGGGGGTGTGAGAGCACGACCCCCAAGAATGGCACTGCGACACGGGC
>Zotu1253
GAACGCTGGCGGCGTGCCTAACACATGCAAGTCAACGAGAAAGGGGTAGCAATATCCCGAGTAAAGTGGC
GCACGGGTGAGTAACGCGTAGGCAATTTGCCTTCAGGACGGGGATAATTCATCGAAAGGTGGACTAATAC

CCGATTATGCATCGGAATCGCCTGGTTCTGATGTTAAATGCCCGCAAGGGTGCCTGTTGATGAGCCTGCGT
CCCATTAGGTAGTTGGCGGGGTAATAGCCCACCAAGCCGACGATGGGTAGCTGGTCTGAGAGGATGATCA
GCCACACTGGAACTGAGACACGGTC
>Zotu1255
GAACGCTGGCGGCGTGCTTAACACATGCAAGTCAAGGGGGCCCGCAAGGGCAACCGGCGAACGGGTGCG
TAACACGTGAGCAACCTGCCCTTGAGTGGGGGATAGCCGGCCCAACGGCCGGGTAATACCGCATACGCTC
GGTTGGGGACATCCCTGGCCGAGGAAACGCTTGCGGCTCAAGGAGGGGCTCGCGGCCTATCAGCTTGTTG
GTGGGGTAATGGCTCACCAAGGCGACGACGGGTAGCTGGTCTGAGAGGATGGCCAGCCACATTGGGACT
GAGAAACGGCC
>Zotu1257
GAACGCTAGCTACAGGCTTAACACATGCAAGTCGCAGGGTAACATGAGGAAAGCTTGCTTTCCTTGATGAC
GACTGGCGCACGGGTGAGTAACGCGTATCCAACCTTCCCATAACTACGGGATAACCCGTTGAAAGACGGC
CTAATACCGTATGATATCGTTTGCTGACATCAAATAACGATTAAAGGTTTAGCGGTTATGGATGGGGATGC
GTCTGATTAGCTTGTTGGCGGGGTAACGGCCCACCAAGGCGACGATCAGTAGGGGTTCTGAGAGGAAGGT
CCCCCACATTGGAACTGAGACACGGTC
>Zotu1259
GAACGCTGGCGGCGTGCTTAACACATGCAAGTCGAACGGAAAGGCCCTTGCTTGCAGGGGTACTCGAGTG
GCGAACGGGTGAGTAACACGTGGGTGATCTGCCCTGCACTTCGGGATAAGCCTGGGAAACTGGGTCTAAT
ACCGGATAGGACGATGGTTTGGATGCCATTGTGGAAAGTTTTTTCGGTGTGGGATGAGCTCGCGGCCTATC
AGCTTGTTGGTGGGGTAATGGCCTACCAAGGCGTCGACGGGTAGCCGGCCTGAGAGGGTGTACGGCCAC
ATTGGGACTGAGATACGGCC
>Zotu1261
GAACGCTAGCGGCAGGCTTAATACATGCAAGTCGAGGGGCAGCGCAGTGTAGCAATACATGGGCGGCGA
CCGGCAAACGGGTGCGGAACACGTACGCAACCTACCCATAACTGATGAATAGCCCTCCGAAAGGAGGATT
AATACATCGTAACTTAATGAAGTGGCATCACTTTATTAATATAGCTCCGGCGGTTTTGGATGGGCGTGCGCC
TGATTAGGTAGTTGGCGGGGTAATGGCCCACCAAGCCTGCGATCAGTAACTGGTGTGAGAGCACGACCAG
TCACACGGGCACTGAGACACGGGC
>Zotu1263
GAACGCTGGCGGCGCGCCTAAAACATGCAAGTCGAACGAGAAGCCCGAGCTTGCTCGGGTGGAAAGTGG
CGAACGGGTGAGTAACACGTGAGGAACCTGCCCTGAAGACCGGGACAACATCGGGAAACCGTTGCTAATA
CCGGATACCTTCGATTGATCGCATGGTCGATCGAAGAAATGGATTCCGCTTCAGGATGGCCTCGCGGCCTA
TCAGTTTGTTGGTGAGGTAACGGCTCACCAAGACGACGACGGGTAGCTGGTCTGAGAGGATGTCCAGCCA
CACTGGGACTGAGACACGGCC
>Zotu1266
GAACGCTAGCGGCAGGCCTAATACATGCAAGTCGGACGGGATTGGAGAGCTTGCTTTCCATGAGAGTGGC
GCACGGGTGCGTAACACGTATGTAACCTACCCTTGTCAGGGGGATAGCCTCTCGAAAGAGAGATTAAGCC
CGCATAACATGGAACTGTGGCATCACAGTTCCATCAAATATTTATAGGACAAGGATGGGCATGCGGAACAT
TAGCTAGTTGGTAAGGTAACGGCTTACCAAGGCTACGATGTTTAGGGGATCTGAGAGGATGACCCCCCAC
ACTGGTACTGAGACACGGAC
>Zotu1269
GAACGTTAGCGGCACGCCTAACACATGCAAGTCGCACGAGAAAGGGGCAACCTGAGTAAAGTGGCGCAC
GGGTGCGTAACACGTAGATAACCTGCCCTTCGAAGGGGGATAACGTTCCGAAAGGAGCGCTAATACCGCA
TACAGCAGCATTCGGTTCGCCGAGAGCTGGCAAAGGTAGGGACCGGCAACGGCCTATCGTCGAAGGAGG
GGTCTGCGGCCCATCAGCTAGTTGGGGAGGTAATGGCTCACCAAGGCAGAGACGGGTAGCTGGTCTGAG
AGGATGATCAGCCACACTGGTACTGGAACACGGAC
>Zotu1271
GAACGCTGGCGGCGTGCTTAACACATGCAAGTCGAACGGAAAGGCCCTTCGGGGTACTCGAGTGGCGAAC
GGGTGAGTAACACGTGGGTGATCTGCCTCGTACTTTGGGATAAGCCTGGGAAACTGGGTCTAATACCGAA
TATGACCTTCCCTCGCATGGGGGTTGGTGGAAACTTTTTGGGTACGAGATGGGCCCGCGGCCTATCAGCTT
GTTGGTGGGGTAATGGCCTACCAAGGCGACGACGGGTAGCCGGCCTGAGAGGGCGACCGGCCACACTGG
GACTGAGACACGGCC

>Zotu1273
GAATGCTGGCGGCGTGGATAAGGCATGCAAGTCAAGGGGGCCGCAAGGCAACCGGCAGACGAGGTAGT
AACACGTAGGTAGATACCCTGGAGTCGGGCATAGCCAGTCGAAAGACTGGGTAATTCCCGATGGTCTCGC
AAGAGTAAAGGTTTTTCGCTCCGGGAGTCGCCTGCGCAATATCAGCTAGTTGGTGAGGTAATGGCTCACCA
AGGCTACGACGTTTAGGGGAGCTGAGAGGCTGACCCCCACCGATGGGACTGAGATACGGCC
>Zotu1276
AAACGCTGGCGGCGCACATAAGACATGCAAGTCGAACGGAATTTAGTCGAATGTTTACATGAGACGAAAA
TTTAGTGGCGGACGGGTGAGTAACACGTGAGCAACCTACCTTTAAGAGGGGGATAACATTGAGAAATCAG
TGCTAATACCGCATAAAGCTATTTAACCGCATGATTAGATAGCCAAAGGAGAAATCCGCTTAAAGATGGGC
TCGCGGCTGATTAGTTAGTTGGTGGGGTAACGGCCTACCAAGACGATGATCAGTAGCCGAGCTGAGAGGC
ACAACGGCCACATTGGGACTGAGACACGGCC
>Zotu1278
GAACGCTGGCGGCGTGCTTAACACATGCAAGTCGAACGGAAAGGCCTTTTCGGAGGTACTCGAGTGGCGA
ACGGGTGAGTAACACGTGGGTGATCTGCCCTGCACTCTGGGATAAGCCTGGGAAACTGGGTCTAATACCG
GATAGGACCGCACTTTAGTGTGTGTGGTGGAAAGTTTTTTCGGTGTAGGATGAGCCCGCGGCCTATCAGCT
TGTTGGTGGGGTAATGGCCTACCAAGGCGGCGACGGGTAGCCGGACTGAGAGGTTGGTCGGCCACATTG
GGACTGAGATACGGCC
>Zotu1280
GAACGCTGGCGGCAGGCCTAACACATGCAAGTCGAGCGGATGAGAGGAGCTTGCTCTTCGATTCAGCGGC
GGACGGGTGAGTAATACCTAGGAATCTGCCTGGTAGTGGGGGACAACGTTTCGAAAGGAACGCTAATACC
GCATACGTCCTACGGGAGAAAGCAGGGGACCTTCGGGCCTTGCGCTATCAGATGAGCCTAGGTCGGATTA
GCTAGTTGGTGAGGTAATGGCTCACCAAGGCTACGATCCGTAACTGGTCTGAGAGGATGATCAGTCACACT
GGAACTGAGACACGGTC
>Zotu1282
GAACGCTGGCGGCAGGCCTAACACATGCAAGTCGAGCGGATGGGAAGAGCTTGCTCTTCGATTTAGCGGC
GGACGGGTGAGTAATACCTAGGAATCTGCCTGGTAGTGGGGGACAACGTTTCGAAAGGAACGCTAATACC
GCATACGTCCTACGGGAGAAAGCAGGGGACCTTCGGGCCTTGCGCTATCAGATGAGCCTAGGTCGGATTA
GCTAGTTGGTGAGGTAATGGCTCACCAAGGCTACGATCCGTAACTGGTCTGAGAGGATGATCAGTCACACT
GGAACTGAGACACGGTC
>Zotu1284
GAACGCTGGCGGCGTGCTTAACACATGCAAGTCGAACGGTAAGGCTCCTTCGGGAGTACACGAGTGGCGA
ACGGGTGAGTAACACGTGAGCAACCTGCCCTTGACTTTGGGATACCAGCTGGAAACAGCTGCTAATACCG
GATATGACACCTCTTCGCATGTTGGGGTGTGGAAAGCTCCGGCGGTCAGGGATGGGCTCGCGGCCTATCA
GCTTGTTGGTGAGGTAATGGCTCACCAAGGCTTCGACGGGTAGCCGGCCTGAGAGGGCGACCGGCCACAC
TGGGACTGAGATACGGCC
>Zotu1286
GAACGCTGGCGGCAGGCTTAACACATGCAAGTCGAGCGCCCCGAGTTTTAGCAATAAGACTTGGGGAGCG
GCAGACGGGTGAGTAACGCGTGGGAACGTACCCCCCGGTTCGGAATAACTCAGGGAAACTTGAGCTAATA
CCGGATACGGCTGGAAGGCGAAAGATTTATCGCCGGGGGATCGGCCCGCGTCCGATTAGCTAGTTGGTGT
GGTAATGGCGCACCAAGGCGACGATCGGTAGCTGGTCTGAGAGGATGATCAGCCACATTGGGACTGAGA
CACGGCC
>Zotu1289
GAACGCTGGCGGCGTGCTTAACACATGCAAGTCGAACGAAGCACTTTTACAGATTTCTTCGGAATGAAGTT
TTAGTGACTGAGTGGCGGACGGGTGAGTAACGCGTGGGTAACCTGCCTCACACAGGGGGATAACAGTTG
GAAACGGCTGCTAATACCGCATAAGCGCACAGTACCGCATGGTACAGTGTGAAAAACTCCGGTGGTGTGA
GATGGACCCGCGTCTGATTAGCTAGTTGGCAGGGCAACGGCCTACCAAGGCGACGATCAGTAGCCGACCT
GAGAGGGTGACCGGCCACATTGGGACTGAGACACGGCC
>Zotu1291
GAACGCTGGCGGCGTGCTTAACACATGCAAGTCGAACGGAAAGGCCCTTCGGGGTACTCGAGTGGCGAAC
GGGTGAGTAACACGTGGGTGATCTGCCTCGTACTCTGGGATAAGCCTGGGAAACTGGGTCTAATACCGGA

TAGGACCATTCCTCGCATGGGGGGTGGTGGAAAGTTTTTCGGTACGAGATGGGCCCGCGGCCTATCAGCTT
GTTGGTGGGGTAATGGCCTACCAAGGCGACGACGGGTAGCCGGCCTGAGAGGGCGACCGGCCACACTGG
GACTGAGACACGGCC
>Zotu1293
GAACGCTGGCGGCGTGCTTAACACATGCAAGTCGAACGGAAAGGCCCTGCTTGCAGGGTACTCGAGTGGC
GAACGGGTGAGTAACACGTGGGTGATCTGCCCTGTACTTCGGGATAAGCTTGGGAAACTGGGTCTAATAC
CGGATAGGACCGGCCTTTAGTGTGGTTGGTGGAAAGCTTTTGCGGTATGGGATGAGCTCGCGGCCTATCA
GCTTGTTGGTGGGGTAATGGCCTACCAAGGCGTCGACGGGTAGCCGGCCTGAGAGGGTGTACGGCCACAT
TGGGACTGAGATACGGCC
>Zotu1295
GAACGCTGGCGGCGTGCCTAATACATGCAAGTCGAACGAGAGCGACCGGTGCTTGCACTGGTCAATCTAG
TGGCGAACGGGTGAGTAACACGTGGGTAACCTGCCCATCAGAGGGGGATAACATTCGGAAACGGATGCT
AAAACCGCATAGGTCTTCGAACCGCATGGTTTGAAGAGGAAAAGAGGCGCAAGCTTCTGCTGATGGATGG
ACCCGCGGTGCATTAGCTAGTTGGTGAGGTAACGGCTCACCAAGGCCGTGATGCATAGCCGACCTGAGAG
GGTGATCGGCCACATTGGGACTGAGACACGGCC
>Zotu1297
GAACGCTGGCGGCGTGCTTAACACATGCAAGTCGAACGATGAACCAGGAGCTTGCTCTTGGGGATTAGTG
GCGAACGGGTGAGTAACACGTGAGTAACCTGCCCTTGACTCTGGGATAAGCGTTGGAAACGACGTCTAAT
ACCGGATACGAGCTTCCACCGCATGGTGAGTTGCTGGAAAGAATTTTGGTCAAGGATGGACTCGCGGCCT
ATCAGCTTGTTAGTGAGGTAATGGCTCACCAAGGCGACGACGGGTAGCCGGCCTGAGAGGGTGACCGGCC
ACACTGGGACTGAGACACGGCC
>Zotu1299
GAACGCTGGCGGCGCGCCTAACACATGCAAGTCGAACGGTTAAAGCACCTTTTAGGTGTGTATAAAGTGG
CGAACGGCTGAGTAACACGTGGGCAACCTGCCCTCCTCTTGGGGATAGCCTCGGGAAACCGAGGATAATA
CCCGATACTTCAATGTGGCCGCATGACGACATTGAGAAAGCTTTTGCGGAGAAGGATGGGCCCGCGGCCT
GTTAGCTTGTTGGTGGGGTAGAGGCCTACCAAGGCAATGATGGGTAGCTGGGTTGAGAGACCGACCAGCC
AGATTGGGACTGAGACACGGCC
>Zotu1301
GAACGCTGGCGGCGTGCCTAATACATGCAAGTCGAGCGAATCAGATGGGAGCTTGCTCCCGGATGATTAG
CGGCGGACGGGTGAGTAACACGTGGGCAACCTGCCCGTAAGATTGGGATAACTCCGGGAAACCGGTGCT
AATACCGAATAGTTTCTGACACCTCATGGTATCAGAGGGAAAGTTGGCTTCGGCTAACACTTACGGATGGG
CCCGCGGCGCATTAGCTAGTTGGTGAGGTAACGGCTCACCAAGGCAACGATGCGTAGCCGACCTGAGAGG
GTGATCGGCCACACTGGGACTGAGACACGGCC
>Zotu1304
GAACGCTGGCGGCGTGCCTAATACATGCAAGTCGAGCGAATCAATGGGAGCTTGCTCCCTGAGATTAGCG
GCGGACGGGTGAGTAACACGTGGGTAACCTGCCTGTAAGATTGGGATAACTCCGGGAAACCGGGGCTAAT
ACCGGATAATACTTTTAACCGCATGGTTAAAAGTTGAAAGATGGTTTCGGCTATCACTTACAGATGGACCC
GCGGCGCATTAGCTAGTTGGTGAGGTAACGGCTCACCAAGGCAACGATGCGTAGCCGACCTGAGAGGGT
GATCGGCCACACTGGGACTGAGACACGGCC
>Zotu1306
TAACGCTAGCGGCAGGCCTAATACATGCAAGTCGAACGGTAACAGGGTAGCAATACCGCTGACGAGTGGC
GCACGGGTGCGTAACGCGTATGCAATCTACCTTTAACAGGGGAATAGCTCAGAGAAATCTGAATTAATACC
GCATAATATAGTGAGGAGGCATCTTCTTGCTATCAAACCTTCGGGGGTTAAAGATGAGCATGCGTATCATT
AGCTAGTTGGTGAGGTAACGGCTCACCAAGGCTACGATGATTAGGGGGTCTGAGAGGATGATCCCCCACA
CTGGTACTGAGACACGGAC
>Zotu1308
GAACGCTGGCGGCGTGCCTAATACATGCAAGTCGAGCGGACCAAATCGGAGCTTGCTCTGGTTTGGTCAG
CGGCGGACGGGTGAGTAACACGTGGGCAACCTGCCCGCAAGACCGGGATAACTCCGGGAAACCGGAGCT
AATACCGGATAACACCGAAGACCGCATGGTCTTTGGTTGAAAGGCGGCCTTTGGCTGTCACTTGCGGATGG
GCCCGCGGCGCATTAGCTAGTTGGTGAGGTAACGGCTCACCAAGGCGACGATGCGTAGCCGGCCTGAGAG
GGTGACCGGCCACACTGGGGCTGAGACACGGCC

>Zotu1310
GAACGCTGGCGGCATGCCTAACACATGCAAGTCGAACGAGATCTTCGGATCTAGTGGCGCACGGGTGCGT
AACGCGTGGGAATCTACCCTTGGGTTCGGAATAACAGCGAGAAATTGCTGCTAATACCGGATGATGACGT
AAGTCCAAAGATTTATCGCCCAGGGATGAGCCCGCGTAGGATTAGGTAGTTGGTGTGGTAAAGGCGCACC
AAGCCTACGATCCTTAGCTGGTCTGAGAGGATGATCAGCCACACTGGGACTGAGACACGGCC
>Zotu1313
GAACGCTAGCGGCAGGCTTAACACATGCAAGTCGAGGGGTAACAGGGTGCTTGCACCGCTGACGACCGGC
GCACGGGTGCGTAACGCGTATACAATCTACCTTATAGAGGGGGATAGCCCAGAGAAATTTGGATTAATACC
CCATAGTACTTAGAGACAGCATTGTTTTTAGGTTAAACATTTATGGCTATAAGATGAGTATGCGTCCTATTA
GTTAGTTGGTAAGGTAACGGCTTACCAAGACCGCGATAGGTAGGGGTCCTGAGAGGGAGATCCCCCACAC
TGGTACTGAGACACGGAC
>Zotu1316
GAGCGCTGGCGGTGTGCCTAATACATGCAAGTCGAACGGACCTTTATTTTAGAAAATCCCAATGTGTTTGG
GGGTTTCCTTAAATAATGGTTAGTGGCGGACGGGTGAGTAACACGTTAGTACCTGCCCCAAAGTGAGGAA
TAACCCGGCGAAAGTCGGGCTAATACCACATGTGACCGTTTACGGTTAAAGCCTTCGGGCGCTTTGGGAGG
GGCTTGCGTCTGATTAGCTAGTTGGTAGGGTAATGGCCTACCAAGGCTACGATCAGTAGCTGGTTCGAGAG
GATGACCAGCCACACTGGGACTGAGATACGGCC
>Zotu1318
GAACGCTGGCGGCGTGCCTAATACATGCAAGTAGAACGCTGAAAGAGGAGCTTGCTTCTCTGGATGAGTT
GCGAACGGGTGAGTAACGCGTAGGTAACCTGCCTGGTAGCGGGGGATAACTATTGGAAACGATAGCTAAT
ACCGCATAATAGTAGATGTTGCATGACATTTGCTTAAAAGGTGCAATTGCATCACTACCAGATGGACCTGC
GTTGTATTAGCTAGTTGGTGGGGTAACGGCTCACCAAGGCGACGATACATAGCCGACCTGAGAGGGTGAT
CGGCCACACTGGGACTGAGACACGGCC
>Zotu1320
GAACGCTGGCGGCGTGCCTAACACATGCAAGTCGAACGGTGATATATAGATGATTTCGGTCTGAAATATAT
CATAGTGGCGGACGGGTGAGTAACGCGTGGATAACCTGCCCCGTACTGGGGGATAGCAGCTGGAAACGG
CTGGTAATACCGCATAAGCGCACGAGGGGGCCTCCCCTTGTGTGAAAAGATTCATCGGTACGGGATGGGT
CCGCGTCTGATTAGCCAGTTGGCGGGGTAACGGCCCACCAAAGCGACGATCAGTAGCCGGCCTGAGAGGG
TGAACGGCCACATTGGGACTGAGACACGGCC
>Zotu1323
GAACGCTAGCTACAGGCTTAACACATGCAAGTCGAGGGGAAACGACATTGAAGCTTGCTTCGATGGGCGT
CGACCGGCGCACGGGTGAGTAACGCGTATCCAACCTGCCTCTGACTAAGGGATAACCCGGCGAAAGTCGG
ACTAATACCTTATGAGGTTTTCTGCAGACATCTAATGAAAACGAAAGATTTATCGGTCAGTGATGGGGATG
CGTCTGATTAGCTTGTTGGCGGGGTAATGGCCCACCAAGGCAACGATCAGTAGGGGTTCTGAGAGGAAGG
TCCCCCACATTGGAACTGAGACACGGTC
>Zotu1325
GAACGCTGGCGGCGTGCTTAACACATGCAAGTCGAACGGTGAACTCCGCTTGCGGGGGGATCAGTGGCGA
ACGGGTGAGTACACGTGGGCAACCTGCCCTCAGCTCTGGGATAACTCCAAGAAATTGGGGCTAATACCGG
ATATGACCGCTGACCGCATGGTCTGGTGGTGGAAAGATTTATCGGCTGGGGATGGGCCCGCGGCCTATCA
GCTTGTTGGTGGGGTAGTGGCCTACCAAGGCGACGACGGGTAGCCGGCCTGAGAGGGTGACCGGCCACA
CTGGGACTGAGACACGGCC
>Zotu1327
GAACGCTGGCGGCAGGCCTAACACATGCAAGTCGAGCGGATGATGGGAGCTTGCTCCCGGATTCAGCGGC
GGACGGGTGAGTAATGCCTAGGAATCTGCCTGGTAGTGGGGGACAACGTTTCGAAAGGAACGCTAATACC
GCATACGTCCTACGGGAGAAAGCAGGGGACCTTCGGGCCTTGCGCTATCAGATGAGCCTAGGTCGGATTA
GCTAGTTGGTGAGGTAATGGCTCACCAAGGCGACGATCCGTAACTGGTCTGAGAGGATGATCAGTCACAC
TGGAACTGAGACACGGTC
>Zotu1331
GAACGCTAGCGGCAGGCCTAATACATGCAAGTCTAAAGGGCAGCAATGTCCTTGGCGCACGGGTGCGTAA
CACGTATGCAACCTACCCATAACTGGGGGATAACTTTTCGAAAGGGAGGATAATACCCCATAACACAATGA

TTCTGCATGGAAATATTGTTAAAGCTCCGGCGGTTATGGATGGGCATGCGAACGATTAGCTAGTTGGTAGG
GTAACGGCCTACCAAGGCTACGATCGTTAGGGGAACTGAGAGGTTGGTCCCCCACACTGGCACTGAGATA
CGGGC
>Zotu1333
GAACGCTGGCGGTGTGCTTAACACATGCAAGTGGAGCGACGAACCAGGGCTTGCCCTGGGGCAGAGCCG
CGAACGGGTGAGTAACACGTGGGTAACCTACCCCGATGATTGGGACAACCCGAGGAAACTCGGGCTAATA
CCGAATGTGCTGTCAGTCCATGAGGACAGTCAGTAAAGGAAGCTTCGGCCTCCGCATCGGGAGGGGCCCG
CGGCCCATTAGTTAGTTGGTGGGGTAATGGCCTACCAAGACAGCGATGGGTAGCTGGTCTGAGAGGACGA
TCAGCCACACTGGGACTGAGACACGGCC
>Zotu1335
GAACGCTGGCGGCAGGCTTAACACATGCAAGTCGAGCGGGGGAAGGTAGCTTGCTACCGGACCTAGCGG
CGGACGGGTGAGTAATGCTTAGGAATCTGCCTATTAGTGGGGGACAACATCTCGAAAGGGATGCTAATAC
CGCATACGTCCTACGGGAGAAAGCAGGGGATCTTCGGACCTTGCGCTAATAGATGAGCCTAAGTCGGATT
AGCTAGTTGGTGGGGTAAAGGCCTACCAAGGCGACGATCTGTAGCGGGTCTGAGAGGATGATCCGCCACA
CTGGGACTGAGACACGGCC
>Zotu1338
GAACGCTGGCGGCGCGCTTAACACATGCAAGTCGAGCGAGAACCTTGCCTTCGGGCTTGGGGACAGCGGC
GAACGGGTGAGTATCACGTGGGTAATCAACCCTCGACACTGGGATAGCCCGGGGAAACCCGGATTAATAC
CGGATACCCCATCTGGCCTTCGGGCCGGCTGGAAAAGGTAGCTTTGGCCTCCGGTCGAGGACGAGCCCGC
GGCGGATTAGCTTGTTGGCGGGGTAAAAGCCCACCAAGGCGACGATCCGTAGCTGGTCTGAGAGGACGAT
CAGCCACACTGGGACTGAGACACGGCC
>Zotu1342
GAACGCTGGCGGCGTGCTTAACACATGCAAGTCGAACGGTGATGGAGAGCTTGCTCTCCGGATCAGTGGC
GAACGGGTGAGTAACACGTGAGCAATCTGCCCCTGACTCTGGGATAACTGCGGGAAACTGTAGCTAATAC
CGGATATGACGCGCGAAGGCATCTTCTGCGCGTGGAAAGAATTTCGGTCAGGGATGAGCTCGCGGCCTAT
CAGCTTGTTGGTGAGGTAATGGCTCACCAAGGCGACGACGGGTAGCCGGCCTGAGAGGGTGACCGGCCA
CACTGGGACTGAGACACGGCC
>Zotu1344
GAACGCTAGCGGCAGGCCTAATACATGCAAGTCGAACGATATTAAGGGGCTTGCTCCTTAAGAAAGTGGC
GCACGGGTGCGTAACGCGTATGCAACCTACCTTAATCAGGGGGATAGCCCGAAGAAATTCGGATTAACAC
CGCATAAAAACACAGGATAGCATTATCCAATGTTCAAATATTTATAGGATTAAGATGGGCATGCGTGTCATT
AGCTAGTTGGCGGGGTAACGGCCCACCAAGGCGACGATGACTAGGGGATCTGAGAGGATGACCCCCCAC
ACTGGTACTGAGACACGGAC
>Zotu1346
GAACGCTGGCGGCGTGCTTAACACATGCAAGTCGAACGGAAAGGCCCTTCGGGGTACTCGAGTGGCGAAC
GGGTGAGTAACACGTGGGTGATCTGCCTCGTACTTTGGGATAAGCCTGGGAAACTGGGTCTAATACCGAA
TATGACCTCTGGATGCATGTCTGGTGGTGGAAAGTTTTTCGGTACGAGATGGGCCCGCGGCCTATCAGCTT
GTTGGTGGGGTAATGGCCTACCAAGGCGACGACGGGTAGCCGGCCTGAGAGGGCGACCGGCCACACTGG
GACTGAGACACGGCC
>Zotu1348
GAACGCTAGCTACAGGCTTAACACATGCAAGTCGCGGGGCAGCATGGGGGTTGCTTGCAACTCCCGATGG
CGACCGGCGCACGGGTGAGTAACGCGTATCCAACCTGCCCTTCACCACGGGATAACCCGGCGAAAGTCGG
ACTAATACCGTATGTTGTCCATTGACGGCATCCGATTTGGACGAAAGGCTTGGCGGTGAAGGATGGGGAT
GCGTCCGATTAGCCAGACGGCGGGGTAACGGCCCACCGTGGCTACGATCGGTAGGGGTTCTGAGAGGAA
GGTCCCCCACACTGGAACTGAGACACGGTC
>Zotu1350
GAACGCTAGCGGCAGGCTTAACACATGCAAGTCGAGGGGTATAGTTCTTCGGAATTAGAGACCGGCGCAC
GGGTGCGTAACGCGTATGCAATCTACCTTTCACAGAGGGATAGCCCAGAGAAATTTGGATTAATACCTCAT
AGTATGCAGAGTTGGCATCAGCCCTGCATTAAAGTCACAACGGTGAAAGATGAGCATGCGTCCCATTAGCT
AGTTGGTAAGGTAACGGCTTACCAAGGCTACGATGGGTAGGGGTCCTGAGAGGGAGATCCCCCACACTGG
TACTGAGACACGGAC

>Zotu1354
GAACGCTAGCGGGAGGCCTAACACATGCAAGCCGAGCGGTATTGTTTCTTCGGAAATGAGAGAGCGGCGT
ACGGGTGCGGAACACGTGTGCAACCTGCCTTTATCTGGGGGATAGCCTTTCGAAAGGAAGATTAATACTCC
ATAACATATTGATTGGCATCAATTAATATTGAAAGCTCCGGCGGATAGAGATGGCACGCGCAAGATTAGC
TAGTTGGTGAGGTAACGGCTCACCAAGGCGATGATCTTTAGGGGGCCTGAGAGGGTGATCCCCCACACTG
GTACTGAGACACGGAC
>Zotu1356
GAACGCTGGCGGCAGGCCTAACACATGCAAGTCGAGCGGATGAGAGGAGCTTGCTCCTGGATTCAGCGGC
GGACGGGTGAGTAATGCCTAGGAATCTGCCTGGTAGTGGGGGACAACGTTTCGAAAGGAACGCTAATACC
GCATACGTCCTACGGGAGAAAGCAGGGGACCTTCGGGCCTTGCGCTATCAGATGAGCCTAGGTCGGATTA
GCTAGTTGGTGGGGTAAAGGCCTACCAAGGCGACGATCCGTAACTGGTCTGAGAGGATGATCAGTCACAC
TGGAACTGAGACACGGTC
>Zotu1358
GAACGCTGGCGGCGTGCCTAATACATGCAAGTCGAGCGGTAATCCCTCACCTTTTTCCATCAGCCATGATGC
TTATACATCGTGCTAATTTGGTAGAAAAAGGTGAGGGAACGAGCGGCGGACGGCTGAGTAACGCGTAGG
AATCTACCCCAAACTGAGGGATAAGCACCGGAAACGGTGTCTAATACCGCATGTGCTCTAAGGAGTAAAGC
TTCGGCGGTTTGGGAAGAGCCTGCGTATGATTAGCTTGTTGGTGAGGTAATGGCTCACCAAGGCTTCGATC
ATTAGCTGGTCTGAGAGGATGATCAGCCAGACTGGGACTGAGACACGGCC
>Zotu1361
GAACGCTGGCGGCGCGCCTAACACATGCAAGTCGAACGGAGCTGAGAGGAGCTTGCTTTTCTTAGCTTAGT
GGCGAACGGGTGAGTAACGCGTGAGTAACCTGCCCTGGAGTGGGGGACAACAGTTGGAAACGACTGCTA
ATACCGCATAAGCCCACGGCCCGGCATCGGGCTGAGGGAAAAGGATTTATTCGCTTCAGGATGGACTCGC
GTCCAATTAGCTAGTTGGTGAGGTAACGGCCCACCAAGGCGACGATTGGTAGCCGGACTGAGAGGTTGAA
CGGCCACATTGGGACTGAGACACGGCC
>Zotu1363
GAACGCTGGCGGCAGGCCTAACACATGCAAGTCGAACGGCAGCACAGAAGAGCTTGCTCTTTGGGTGGCG
AGTGGCGGACGGGTGAGTAATGTCTGGGAAACTGCCCGATGGAGGGGGATAACTACTGGAAACGGTAGC
TAATACCGCATAACGTCGCAAGACCAAAGTGGGGGACCTTCGGGCCTCACACCATCGGATGTGCCCAGAT
GGGATTAGCTAGTAGGTGGGGTAATGGCTCACCTAGGCGACGATCCCTAGCTGGTCTGAGAGGATGACCA
GCCACACTGGAACTGAGACACGGTC
>Zotu1365
GAACGCTGGCGGCGTGCCTAATACATGCAAGTCGAACGCGACACTTTCGGGTGTCGAGTGGCGGACGGGT
GAGTAACACGTGGGTAACCTGCCCATCAGCCTGGGATAACCACCGGAAACGGTGGCTAATACCGGATATG
TCTACGGATGAAAGTCGGATTTTCTGACACTGATGGATGGACCTGCGGCGCATTAGCTAGTTGGTGAGGTA
GTGGCTCACCAAGGCGACGATGCGTAGCCGACCTGAGAGGGTGATCGGCCACACTGGGACTGAGACACG
GCC
>Zotu1367
GAACGCTAGCGGCAGGCCTAATACATGCAAGTCGAGGGGCAGCATAGTAGCAATACTGATGGCGACCGG
CGCACGGGTGCGTAACGCGTATGCAACCTACCTTTTACTGGGGGATAGCCCGGAGAAATTCGGATTAACAC
CGCATAACAAGTTGATATGGCATCATATTAATTTTAAATATTTATAGGTAAAAGATGGGCATGCGTCCAATT
AGCTAGTTGGTGAGGTAATGGCTCACCAAGGCGATGATTGGTAGGGGTTCTGAGAGGAAGGTCCCCCACA
CTGGTACTGAGATACGGAC
>Zotu1369
GAACGCTAGCGGCAGGCCTAATACATGCAAGTCTAAGGGGTAGCAATATCACTGGCGGACGGGTGCGTAA
CGCGTATAGAACCTACCTATCACTGGGGGATAGCTTTTGGAAACAGGAGATAATACCGCATAAGATAGTAG
CTTGGCATCAAGCAGCTATTAAAGATTTATTGGTGAGAGATGGCTATGCGTATGATTAGCTAGTTGGTAAG
GTAACGGCTTACCAAGGCTACGATCATTAGGGGTTCTGAGAGGATTGGCCCCCACACTGGTACTGAGATAC
GGAC
>Zotu1372

GAACGCTGGCGGCGTGCCTAATACATGCAAGTCGAGCGGACCTCTTCGGAGGTCAGCGGCGGACGGGTG
AGTAACACGTGGGCAACCTGCCTATAAGACTGGGATAACTTCGGGAAACCGGAGCTAATACCGGATAATA
TCAGAGGCCACATGGTCTTCGATTAAAAGATGGCTTAGGCTATCACTTATAGATGGGCCCGCGTCGCATTA
GCTAGTTGGTGAGGTAACGGCTCACCAAGGCGACGATGCGTAGCCGACCTGAGAGGGTGATCGGCCACAC
TGGGACTGAGACACGGCC
>Zotu1374
GAACGCTGGCGGCGTGCCTAATGCATGCAAGTCGCACGCGCCACCTTCGGGTGGTGAGTGGCGCACGGCT
GAGGATCACGTGGGAACATGCCTCCTTGTGGGGGACAACAGCGCGAAAGCGCTGCTAATCCCGCATACGC
TCGGCAACGAGGAAAGCGGTAACGCGCAGGGAGAGTGGCCCGCGGCCCATCAGGTCGTTGGTGGGGTAA
TGGCCTACCAAGCCGATGACGGGTAGCTGGTCTGTGAGGACAGACAGCCAGACTGGGACTGAGACACGG
CC
>Zotu1378
GAACGCTGGCGGCGTGCTTAACACATGCAAGTCGAACGGAAAGGCCCTTCGGGGTACTCGAGTGGCGAAC
GGGTGAGTAACACGTGGGTGATCTGCCCTGCACTTTGGGATAAGCCTGGGAAACTGGGTCTAATACCGAA
TATGATCATCGGCTTCATGGTCGGTGGTGGAAAGCTTTTGCGGTGTGGGATGGGCCCGCGGCCTATCAGCT
TGTTGGTGGGGTAATGGCCTACCAAGGCGTCGACGGGTAGCCGGCCTGAGAGGGTGTCCGGCCACACTG
GGACTGAGATACGGCC
>Zotu1380
GAACGCTGGCGGCGTGCTTAACACATGCAAGTCGAACGGAAAGGCCCTTCGGGGTACTCGAGTGGCGAAC
GGGTGAGTAACACGTGGGTGATCTGCCTCGTACTCTGGGATAAGCCTGGGAAACTGGGTCTAATACTGGA
TAGGACCTTCTCTCGCATGGGGGTTGGTGGAAAGTTTTTCGGTACGAGATGGGCCCGCGGCCTATCAGCTT
GTTGGTGGGGTAATGGCCTACCAAGGCGACGACGGGTAGCCGGCCTGAGAGGGCGACCGGCCACACTGG
GACTGAGACACGGCC
>Zotu1383
GAACGCTGGCGGCGTGCCTAATACATGCAAGTCGAACGAACAGCGACTTGAACTTGTTCAAGTCAATGTTA
GTGGCGCACGGGTGAGTAACACGTGGAGAACCTACCCTTAAGCGGGGGATAACCATTGGAAACGATGACT
AATACCGCATAGAATCACAGATCGCATGATCAATGAAGGAAAGACGGCTTCGGCTGTCACTTAAGGATGG
CTCCGCGGTGCATTAGCTAGATGGTGGGGTAAAAGCCTACCATGGCAATGATGCATAGCCGACCTGAGAG
GGTGATCGGCCACATTGGGACTGAGACACGGCC
>Zotu1385
GAACGCTGGCGGCGTGCTTAACACATGCAAGTCGAACGGAAAGGCCTCTGCTTGCAGGGGTGCTCGAGTG
GCGAACGGGTGAGTAACACGTGGGTGATCTGCCTTGTACTCTGGGATAAGCTTGGGAAACTGGGTCTAAT
ACCGGATAGGAACTGCCTTTAGTGTGGTGGTTGGAAAGTTTTTTCGGTGCAAGATGAGCTCGCGGCCTATC
AGCTTGTTGGTGGGGTAATGGCCTACCAAGGCGTCGACGGGTAGCCGGCCTGAGAGGGTGTACGGCCAC
ATTGGGACTGAGATACGGCC
>Zotu1388
AAACGCTGGCGGCGTGCTTAACACATGCAAGTCGAACGAAGTTTTTCTTTCGGGAGAAACTTAGTGGCGGA
CGGGTGAGTAACGCGTGGGTAACCTGCCCTGTACAGGGGGACAACAGCTGGAAACGGCTGCTAATACCGC
ATAAGCCCTTAGCACTGCATGGTGCATAGGGAAAAGGAGCAATCCGGTACAGGATGGACCCGCGTCTGAT
TAGCCAGTTGGCAGGGTAACGGCCTACCAAAGCGACGATCAGTAGCCGATCTGAGAGGATGTACGGCCAC
ATTGGGACTGAGACACGGCC
>Zotu1390
GAACGCTAGCGGCAGGCTTAACACATGCAAGTCGAGGGGTATAGGTTTTCGGACCTAGAGACCGGCGCAC
GGGTGCGTAACGCGTATGCAATCTACCTTTTACAGCGGGATAGCCCAGAGAAATTTGGATTAATACCGCAT
AGTATAGCAGTTTCGCATGAGACCACTATTAAAGTCACAACGGTAAAAGATGAGCATGCGTCCCATTAGTT
AGTTGGTAAGGTAACGGCTTACCAAGACTACGATGGGTAGGGGTCCTGAGAGGGAGATCCCCCACACTGG
TACTGAGACACGGAC
>Zotu1392
GAACGCTGGCGGCAGGCTTAACACATGCAAGTCGAACGAAGTCTTCGGACTTAGTGGCGCACGGGTGAGT
AACACGTGGGAATATACCTCTTGGTGGGGAATAACATCGGGAAACTGATGCTAATACCGCATACGCCCTTC

GGGGGAAAGATTTATCGCCGAGAGATTAGCCCGCGTCCGATTAGCTAGTTGGTGAGGTAATGGCTCACCA
AGGCGACGATCGGTAGCTGGTCTGAGAGGATGATCAGCCACACTGGGACTGAGACACGGCC
>Zotu1395
GAACGCTGGCGGCAGGCCTAACACATGCAAGTCGAACGGTAGCACAGAGGAGCTTGCTCCTTGGGTGACG
AGTGGCGGACGGGTGAGTAATGTCTGGGAAACTGCCTGATGGAGGGGGATAACTACTGGAAACGGTAGC
TAATACCGCATAACGTCGCAAGACCAAAGAGGGGGACCTTCGGGCCTCTTGCCATCAGATGTGCCCAGATG
GGATTAGCTAGTAGGTGGGGTAATGGCTCACCTAGGCGACGATCCCTAGCTGGTCTGAGAGGATGACCAG
CCACACTGGAACTGAGACACGGTC
>Zotu1397
GAACGCTGGCGGCGTGCTTAACACATGCAAGTCGAGCGAGGAGCTTGCTCCTAGCGGCGAACGGGTGAGT
AACACGTGGGCAACCTGCCCCGAGCTCTGGGATAACTCCGGGAAACCGGAGCTAATACCGGATATGACCA
CGGAAGGCATCTTCTTGTGGTGGAAAGATTTATCGGCTCGGGATGGGCCCGCGGCCTATCAGCTTGTTGGT
GGGGTAACGGCCTACCAAGGCGACGACGGGTAGCCGGCCTGAGAGGGCGACCGGCCACACTGGGACTGA
GACACGGCC
>Zotu1399
GAACGCTGGCGGCGTGCTTAACACATGCAAGTCGAACGATGAAGCCCAGCTTGCTGGGTGGATTAGTGGC
GAACGGGTGAGTAACACGTGAGTAACCTGCCCTTGACTCTGGGATAAGCGTTGGAAACGACGTCTAATAC
CGGATACGAGCTTCCACCGCATGGTGAGTTGCTGGAAAGAATTTTGGTCAAGGATGGACTCGCGGCCTATC
AGCTTGTTGGTGAGGTAATGGCTCACCAAGGCGACGACGGGTAGCCGGCCTGAGAGGGTGACCGGCCAC
ACTGGGACTGAGACACGGCC
>Zotu1401
GAACGCTGGCGGCGTGCTTAACACATGCAAGTCGAACGGAAAGGCCCTTTCGGGGGTACTCGAGTGGCGA
ACGGGTGAGTAACACGTGGGTGATCTGCCCTGCACTTTGGGATAAGCCTGGGAAACTGGGTCTAATACCG
GATATACCTCGCTGGTCGCATGGCCTGGTGGGGGAAAGCTTTTGCGGTGTGGGATGGGCCCGCGGCCTAT
CAGCTTGTTGGTGAGGTTATGGCTTACCAAGGCGACGACGGGTAGCCGGCCTGAGAGGGTGACCGGCCAC
ACTGGGACTGAGATACGGCC
>Zotu1403
GAACGCTGGCGGCGTGCTTAACACATGCAAGTCGAACGATGAAGCTGGAGCTTGCTCCGGTGGATTAGTG
GCGAACGGGTGAGTAACACGTGAGCAATCTGCCCTTGACTCTGGGATAACTGCGGGAAACTGTAGCTAAT
ACCGGATACGACCCTTGGAGGCATCTCTGGGGGTGGAAAGAATTTCGGTCAAGGATGAGCTCGCGGCCTA
TCAGCTAGTTGGTGAGGTAATGGCTCACCAAGGCGACGACGGGTAGCCGGCCTGAGAGGGTGACCGGCC
ACACTGGAACTGAGACACGGTC
>Zotu1405
GAACGCTGGCGGCGTGCCTAATACATGCAAGTCGAGCGAACCAATAGGTGCTTGCGCCTGTTGGTTAGCG
GCGGACGGGTGAGTAACACGTGGGCAACCTGCCTGTAAGACTGGGATAACACCGGGAAACCGGTGCTAA
TACCGGATAATCCTTTTCCTCTCATGAGGAAAAGCTGAAAGTCGGTGCAAGCTGACACTTACAGATGGGCC
CGCGGCGCATTAGCTAGTTGGTGAGGTAACGGCTCACCAAGGCGACGATGCGTAGCCGACCTGAGAGGGT
GATCGGCCACACTGGGACTGAGACACGGCC
>Zotu1407
GAACGCTGGCGGCGTGCTTAACACATGCAAGTCGAACGATGAACCACTTCGGTGGGGATTAGTGGCGAAC
GGGTGAGTAACACGTGGGCAATCTGCCCTTCACTCTGGGACAAGCCCTGGAAACGGGGTCTAATACCGGA
TAACACTGCCACGGGCATCTGTGGTGGTTAAAAGCTCCGGCGGTGAAGGATGAGCCCGCGGCCTATCAGC
TTGTTGGTGAGGTAATGGCTCACCAAGGCGACGACGGGTAGCCGGCCTGAGAGGGCGACCGGCCACACT
GGGACTGAGACACGGCC
>Zotu1409
TCGGGAAATCGGTGTTTAAGAAGAAACGCCACTGATTTCTTAAACCCGAAGCTTCCGTGTACAGGATCCTCT
TCTCCGCCCTTTTACAGCGCATCAAA
>Zotu1411
GAACGCTGGCGGCGTGCTTAACACATGCAAGTCGAGCGGAAAGGCCACTTCGGTGGTACTCGAGCGGCGA
ACGGGTGAGTAACACGTGAGTAATCTGCCCCTGGCTTTGGGATAGCCACCGGAAACGGTGATTAATACCG

GATATGACGACCTTTCGCATGATGGGTTGTGGAAAGTTTTTCGGCCAGGGATGTGCTCGCGGCCTATCAGC
TTGATGGTGAGGTAATGGCTCACCATGGCTTCGACGGGTAGCCGGCCTGAGAGGGTGACCGGCCACACTG
GGACTGAGACACGGCC
>Zotu1413
GAACGCTAGCGGCAGGCCTAATACATGCAAGTCGAGGGGCAGCACGGTAGCAATACTGGTGGCGACCGG
CAAACGGGTGCGGAACACGTACGCAACCTTCCTTTAAGAGATGAATAGCCCGAAGAAATTCGGATTAATGC
ATCGTAAGATTGTGGGATGGCATCATCCAGCAATTAAAGATTTATTTCGCTTAAAGATGGGCGTGCGCCTG
ATTAGGTAGTTGGTGAGGTAACGGCTCACCAAGCCGACGATCAGTAACTGGCGTGAGAGCGCGACCAGTC
ACACGGGCACTGAGACACGGGC
>Zotu1417
GAACGCTGGCGGCGTGCTTAACACATGCAAGTCGAACGGAAAGGCCTCTTCGGAGGTACTCGAGTGGCGA
ACGGGTGAGTAACACGTGGGTAATCTGCCCTGCACTTCGGGATAAGCTTGGGAAACTGGGTCTAATACCG
GATATGACCTCGGGACGCATGTCCTCTGGTGGAAAGCTTTTGCGGTGTGGGATGGGCCCGCGGCCTATCA
GCTTGTTGGTGGGGTGATAGCCTACCAAGGCGACGACGGGTAGCCGGCCTGAGAGGGTGTCCGGCCACA
CTGGGACTGAGATACGGCC
>Zotu1419
GAACGCTGGCGGCGTGCCTAACACATGCAAGTCGAACGGGACTGTTTAGTGACGATTTCGGTCTGAACTTT
ACAGTTTAGTGGCGGACGGGTGAGTAACGCGTGGATAACCTGCCTTTCACAGGGGGATAGCAGCTGGAAA
CGGCTAGTAATACCGCATACGCTCATGTAACCGCATGGTGATGTGAGGAAAGATTTATCGGTGAAAGATG
GATCCGCGTCTGATTAGCCAGTTGGCGGGGTAACGGCCCACCAAAGCGACGATCAGTAGCCGGCCTGAGA
GGGCGGACGGCCACATTGGGACTGAGACACGGCC
>Zotu1422
GAACGCTAGCGGCATGCCTAACACATGCAAGTCGAACGAGATCTTCGGATCTAGTGGCGCACGGGTGCGT
AACGCGTGGGAATCTGCCTTGGGGTTCGGAATAACTCCCCGAAAGGGGTGCTAATACCGGATGATGTCGA
AAGACCAAAGATTTATCGCCCTGAGATGAGCCCGCGTAGGATTAGCTAGTTGGTGTGGTAAAGGCGCACC
AAGGCGACGATCCTTAGCTGGTCTGAGAGGATGATCAGCCACACTGGGACTGAGACACGGCC
>Zotu1425
GAACGCTAGCGGCAGGCTTAACACATGCAAGTCGAGGGGTAGAAGGCTTCGGCCTTTGAGACCGGCGCAC
GGGTGCGTAACGCGTATGCAATCTACCTTTTACAGGGGGATAGCCCAGAGAAATTTGGATTAATACCCCAT
AGTATTATTGAATGGCATCATTTAATAATTAAAGTCACAACGGTAAAAGATGAGCATGCGTCCCATTAGCTA
GTTGGTATGGTAACGGCATACCAAGGCAATGATGGGTAGGGGTCCTGAGAGGGAGATCCCCCACACTGGT
ACTGAGACACGGAC
>Zotu1427
GAACGCTGGCGGCGTGCCTAACACATGCAAGTCGAGCGATCTTCTTCGGAAGAGAGCGGCGGACGGGTG
AGTAACGCGTGGGTAACCTGCCTCATACACATGGATAACATACCGAAAGGTATGCTAATACAAGATGATAT
AAGAGATTCGCATGGATTTCTTATCAAAGCTCCGGCGGTATGAGATGGACCCGCGTCTGATTAGCTAGTTG
GTAAGGTAACGGCTTACCAAGGCGACGATCAGTAGCCGACCTGAGAGGGTGATCGGCCACATTGGAACTG
AGACACGGTC
>Zotu1429
GAACGCTAGCGGCAGGCTTAATACATGCAAGTCGAGGGGCAGCGCAGTGTAGCAATACATGGGCGGCGA
CCGGCAAACGGGTGCGGAACACGTACGCAACTTACCCAAAACTGATGAATAGCCCTCCGAAAGGAGGATT
AATACATCGTAACCTATTGAAGTGGCATCACTTTATTAGTATAGCTCCGGCGGTTTTGGATGGGCGTGCGG
CTGATTAGATAGTTGGCGGGGTAACGGCCCACCAAGTCTGCGATCAGTAACTGGTGTGAGAGCACGACCA
GTCACACGGGCACTGAGACACGGGC
>Zotu1431
GAACGCTGGCGGCGCGCCTAACACATGCAAGTCGAGCGATGAACATTGAATGATCCCTTCGGGGTGATTTC
GATCGGATTAGCGGCGAACGGGTGAGTAACGCGTGAGGAACCTGCCTCTTACAACGGGATAGCCTCGGGA
AACCGGGATTAATACCGTATAAGACTCCGACACCTCCTGGTGATGAAGTCAAAGCGTTAGCGGTAAGAGAT
GGCCTCGCGTCTGATTAGTTAGTTGGCGGGGTAACGGCCCACCAAGACGACGATCAGTAACCGGCCTGAG
AGGGTGAACGGTCACATTGGAACTGAGACACGGTC

>Zotu1434
GAACGCTGGCGGCGCGCTTAACACATGCAAGTCGAGCGAGAACCGGGACTTCGGTCCTGGGGACAGCGG
CGAACGGGTGAGTAACACGTGGGTAATCTGCCCTCGATTCTGGGATAGCCCGGGGAAACCCGGATTAATA
CCGGATAGCCTTCCAAGCCCTCGGGCGAAGAAGAAAAGGTAGCTTCGGCCTCCGATCGAGGATGAGCCCG
CGGTGGATTAGCTTGTTGGCGGGGTAATGGCCCACCAAGGCGACGATCCATAGCTGGTCTGAGAGGACGA
TCAGCCACACTGGGACTGAGACACGGCC
>Zotu1438
GAACGCTGGCGGCAGGCTTAACACATGCAAGTCGAGCGCCCCGCAAGGGGAGCGGCAGACGGGTGAGTA
ACACGTGGGAACGTACCTTTTGGTTCGGAACAACTCCGGGAAACTGGAGCTAATACCGGATAAGCCCTAAC
GGGGAAAGATTTATCGCCAAAAGATCGGCCCGCGTCTGATTAGCTAGTTGGTGAGGTAACGGCTCACCAA
GGCGACGATCAGTAGCTGGTCTGAGAGGATGATCAGCCACACTGGGACTGAGACACGGCC
>Zotu1440
GAACGCTGGCGGCGTGCTTAACACATGCAAGTCGAACGAAGCACTTTATTTGATTTCCTTCGGGACTGATT
ATTTTGTGACTGAGTGGCGGACGGGTGAGTAACGCGTGGGTAACCTGCCTTGTACAGGGGGATAACAGTT
GGAAACGGCTGCTAATACCGCATAAGCGCACAGCATCGCATGATGCAGTGTGAAAAACTCCGGTGGTATA
AGATGGACCCGCGTTGGATTAGCTAGTTGGTGAGGTAACGGCCCACCAAGGCGACGATCCATAGCCGACC
TGAGAGGGTGACCGGCCACATTGGGACTGAGACACGGCC
>Zotu1442
GAACGCTAGCTACAGGCTTAACACATGCAAGTCGAGGGGCAGCATTGGAGTAGCAATACTTCAGATGGTG
ACCGGCGCACGGGTGAGTAACACGTATCCAACCTGCCGCTTACTCGGGGATAGCCTTTCGAAAGAAAGATT
AATACCCGATGGTATAAAGAGTTCGCATGTTCTCTTTATTAAAGAATTTCGGTAAGCGATGGGGATGCGTTC
CATTAGATTGTTGGCGGGGTAACGGCCCACCAAGTCTACGATGGATAGGGGTTCTGAGAGGAAGGTCCCC
CACATTGGAACTGAGACACGGTC
>Zotu1444
GAACGCTGGCGGCGTGCTTAACACATGCAAGTCGAGCGGAAAGGCCTCTTCGGGGGTACTCGAGCGGCG
AACGGGTGAGTAACACGTGAGTAATCTGCCCTTCACTTCGGGATAACCGCCGGAAACGGTGGCTAATACC
GGATATGACTGCGTCTCGCATGGGGTGTGGTGGAAAGCTTTTGTGGTGGAGGATGTGCTCGCGGCCTATC
AGCTTGTTGGTGGGGTAATGGCCTACCAAGGCTTCGACGGGTAGCCGGCCTGAGAGGGTGACCGGTCACA
CTGGGACTGAGACACGGCC
>Zotu1446
GAACGCTGGCGGCGTGCTTAACACATGCAAGTCGAACGGTGAACCCGCTTCGGTGGGGGATCAGTGGCGA
ACGGGTGAGTAACACGTGGGCAACCTGCCCTCAGCTCTGGGATAACTCCAAGAAATTGGGGCTAATACCG
GATATGACCGCTGACCGCATGGTCTGGTGGTGGAAAGATTTATCGGCTGGGGATGGGCCCGCGGCCTATC
AGCTTGTTGGTGGGGTAGTGGCCTACCAAGGCGACGACGGGTAGCCGGCCTGAGAGGGTGACCGGCCAC
ACTGGGACTGAGACACGGCC
>Zotu1448
GAACGCTGGCGGCGTGCTTAACACATGCAAGTCGAGCGGAAAGGCCCCTTCGGGGGTACTCGAGCGGCG
AACGGGTGAGTAACACGTGAGTAATCTGCCCTCCACTTCGGGATAGCCCTCCGAAAGGGGGATTAATACC
GGATGGGCACCACGGACCGCATGGTGTGTGGTGGAAAGTTTTTCGGTGGGGGATGAGCTCGCGGCCTATC
AGCTTGTTGGTGAGGTGATGGCTCACCAAGGCTTCGACGGGTAGCCGGCCTGAGAGGGTGACCGGCCACA
CTGGGACTGAGACACGGCC
>Zotu1450
GAACGCTGGCGGCGTGCTTAACACATGCAAGTCGAGCGGAAAGGCCACTTCGGTGGTACTCGAGCGGCGA
ACGGGTGAGTAACACGTGAGTAATCTGCCCTTGGCTTTGGGATAGCCACCGGAAACGGTGATTAATACCG
GATATGACTACTTCTCGCATGAGTTGTGGTGGAAAGTTTTTCGGCCAGGGATGTGCTCGCGGCCTATCAGC
TTGATGGTGAGGTAATGGCTCACCATGGCTTCGACGGGTAGCCGGCCTGAGAGGGTGACCGGTCACACTG
GGACTGAGACACGGCC
>Zotu1453
GAACGCTGGCGGCGTGCTTAACACATGCAAGTCGAACGGAAAGGCCCTTCGGGGTACTCGAGTGGCGAAC
GGGTGAGTAACACGTGGGTGATCTGCCCTGCACTTTGGGATAAGCCTGGGAAACTGGGTCTAATACCGAA

TACACCTTCCTGGCTGCATGGTTGGGGGGGGAAAGCTTTTGCGGTGTGGGATGGGCCCGCGGCCTATCAG
CTTGTTGGTGAGGTTACGGCTCACCAAGGCGACGACGGGTAGCCGGCCTGAGAGGGTGACCGGCCACACT
GGGACTGAGATACGGCC

>Zotu1455

GAACGCTGGCGGCAGGCTTAACACATGCAAGTCGAGCGGGTGTCCTTCGGGGCATCAGCGGCGGACGGG
TTAGTAACGCGTGGGAACGTGCCTTCTGCTCTGGAATAGCCCTGGGAAACTGGGGGTAATACCGGATGTG
CCCTGAGGGGGAAAGGTTTATCTGGCAGGAGATCGGCCCGCGTCTGATTAGGTAGTTGGTGTGGTAATGG
CGCACCAAGCCGACGATCGGTAGCTGGTTTGAGAGGATGATCAGCAACACTGGGACTGAGACACGGCC

>Zotu1457

GAACGCTGGCGGCGTGCTTAACACATGCAAGTCGAACGAGACCCTCGCTTCGGTGGGGTGTTCTAGTGGC
GAACGGGTGAGTAACACGTGGGCAACCTGCCCCTGGCTCTGGGATAACTCCAAGAAATTGGGGCTAATAC
CGGATATGACTGCTGACCGCATGGTCTGGTGGTGGAAAGATTTATCGGCTGGGGATGGGCCCGCGGCCTA
TCAGCTTGTTGGTGGGGTAGTGGCCTACCAAGGCGACGACGGGTAGCCGGCCTGAGAGGGTGACCGGCC
ACACTGGGACTGAGACACGGCC

>Zotu1460

GAACGCTGGCGGCATGCTTTACACATGCAAGTCGAACGGTAACAGGGAGCTTGCTCCGCTGACGAGTGGC
GAACGGGTGAGTAATATATCGGAACGTACCCAAGAGTGGGGGATAACGTAGCGAAAGTTACGCTAATACC
GCATACGATCTATGGATGAAAGCAGGGGACCTTCGGGCCTTGTGCTCCTGGAGCGGCCGATATCTGATTAG
CTAGTTGGTGAGGTAAAGGCTCACCAAGGCGACGATCAGTAGCTGGTCTGAGAGGACGACCAGCCACACT
GGAACTGAGACACGGTC

>Zotu1462

GAACGCTGGCGGCGTGCCTAATACATGCAAGTCGAGCGAATTTTTGGGAGCTTGCTCCCAAAGGTTAGCG
GCGGACGGGTGAGTAACACGTGGGCAACCTGCCTATAAGACTGGGATAACTTCGGGAAACCGGAGCTAAT
ACCGGATAATTCTTTTCCTCTCATGAGGAAAAGCTGAAAGACGGTTTACGCTGTCACTTATAGATGGGCCCG
CGGCGCATTAGCTAGTTGGTGAGGTAACGGCTCACCAAGGCAACGATGCGTAGCCGACCTGAGAGGGTGA
TCGGCCACACTGGGACTGAGACACGGCC

>Zotu1465

GAACGCTGGCGGCGTGCTTAACACATGCAAGTCGAACGGGAAGAGATGAAGAGCTTGCTCTTTATCGAAT
CCAGTGGCAAACGGGTGAGTAACACGTAAACAACCTGCCTTCAGGATGGGGACAACAGACGGAAACGACT
GCTAATACCGAATACGTTCCACGGGCCGCATGACCTGTGGAAGAAAGGGTAGCCTCTACCTGTAAGCTATC
GCCTGAAGAGGGGTTTGCGTCTGATTAGGCAGTTGGTGGGGTAACGGCCCACCAAACCAACGATCAGTAG
CCGGTCTGAGAGGATGAACGGCCACACTGGAACTGAGACACGGTC

>Zotu1467

GAACGCTGGCGGCGTGCCTAATACATGCAAGTAGAACGCTGAAGCTTGGTGCTTGCACCGAGCGGATGAG
TTGCGAACGGGTGAGTAACGCGTAGGTAACCTGCCTGGTAGCGGGGGATAACTATTGGAAACGATAGCTA
ATACCGCATAAGAGTAGATGTCGCATGACATTTGCTTAAAAGGTGCAATTGCATCACTACCAGATGGACCT
GCGTTGTATTAGCTAGTTGGTGAGGTAACGGCTCACCAAGGCAACGATACATAGCCGACCTGAGAGGGTG
ATCGGCCACACTGGGACTGAGACACGGCC

>Zotu1469

GAACGCTGGCGGCGTGCCTAATACATGCAAGTCGAGCGGATTCATTCCTTCGGGGATGGGTTAGCGGCGG
ACGGGTGAGTAACACGTAGGCAACCTGCCTATAAGCCTGGGATAACTTCCGGAAACGGATGCTAATACCG
GATACGTTGAATTCTCGCATGAGAATTCATGGAAAGACGGCGCAAGCTGTCACTTATGGATGGGCCTGCG
GCGCATTAGCTAGTTGGTGGGGTAACGGCTCACCAAGGCGACGATGCGTAGCCGACCTGAGAGGGTGATC
GGCCACACTGGGACTGAGACACGGCC

>Zotu1471

GAATGCTAGCGGTGTGCCTGAGATATGCAAGTCGAGCGCTTATTTTGCCGCAAGGTGAAGTGAGAGCGGC
GGACGGCTGAGTAATGTACAGGAATCTGCCCCTTAGTGGAACATAGCTCCGGGAAACCGGGGGTAATTTT
CCATGGTTTCACCTTTTTGGTGAATAAAAGCCTTTACCGGTTGCTAAGGGATGAGCCTGTATCCTATCAGGT
AGTTGGTGGGGTAAAAGCCTACCAAGCCTATGACGGGTAGCTGATCTGGGAGGATGATCAGCCAGAGGG
GGACTGAGACACGGCC

>Zotu1474
GAACGCTGGCGGCGTGCTTAACACATGCAAGTCGAGCGATGAAGTTCCTTCGGGGACGGATTAGCGGCGG
ACGGGTGAGTAACACGTGGGTAACCTGCCTCATAGAGGGGAATAGCCTTCCGAAAGGAAGATTAATACCG
CATAAGATTGTAGCTTCGCATGAAGTAGCAATTAAAGGAGTAATCCGCTATGAGATGGACCCGCGTCGCAT
TAGCTAGTTGGTGAGGTAACGGCTCACCAAGGCGACGATGCGTAGCCGACCTGAGAGGGTGATCGGCCAC
ATTGGGACTGAGACACGGCC
>Zotu1478
GAACGCTGGCGGCATGCTTTACACATGCAAGTCGAACGGTAACAGGGAGCTTGCTCCGCTGACGAGTGGC
GAACGGGTGAGTAATATATCGGAACGTACCCAAGAGTGGGGGATAACGTAGCGAAAGTTACGCTAATACC
GCATACGATCCAAGGATGAAAGCAGGGGATCTTCGGACCTTGTGCTCCTGGAGCGGCCGATATCTGATTA
GCTAGTTGGTGAGGTAAAGGCTCACCAAGGCGACGATCAGTAGCTGGTCTGAGAGGACGACCAGCCACAC
TGGAACTGAGACACGGTC
>Zotu1480
GAACGCTGGCGGCGTGCCTAAGACATGCAAGTCGAGTGGGTTTAGACCCACGGCGAACGGGTGAGTAAT
ACCTAGGAACTTCCCTTTAGTTGAGCATAACCCATCGAAAGGTGGGATAATTCTCGATGTGACCGCAAGGT
TAAAGTCGCAAGACGCTAAAGAAAAGGCCTGGGCACCATCAGCTAGTTGGTAAGGTAAAAGCTTACCAAG
GCTATGACGGTTAGGGGCCCCGAGAGGGGAAACCTCCACACTGGAACTGCGACACGGTC
>Zotu1482
GAACGCTGGCGGCGTGCTTAACACATGCAAGTCGAACGGGATCCATCGCGAGCTTGCTCAAGAGAAGATC
TAGTGGCGAACGGGTGCGTAACACGTGAGAAACCTGCCCCAGACTTGGGAATAACAGTCGGAAACGACTG
CTAATACCTAATACCTTCGTAGCGTCGCATGGCGCATCGAAGAAAGCTTTATGCGGTTTGGGAGGGTCTCG
CGGCCTATCAGCTAGTTGGTGAGGTAACGGCTCACCAAGGCATCGACGGGTAGCTGGTCTGAGAGGATGA
TCAGCCACACTGGGACTGAGACACGGCC
>Zotu1484
GAACGCTAGCGGCAGGCTTAACACATGCAAGTCGAGGGGTATAGTTCTTCGGAATTAGAGACCGGCGCAC
GGGTGCGTAACGCGTATGCAATCTACCTTTCACAGAGGGATAGCCCAGAGAAATTTGGATTAATACCTCAT
AGTATATAGCCCTGGCATCAGGATTATATTAAAGTCACAACGGTGAAAGATGAGCATGCGTCCCATTAGCT
AGTTGGTAAGGTAACGGCTTACCAAGGCTACGATGGGTAGGGGTCCTGAGAGGGAGATCCCCCACACTGG
TACTGAGACACGGAC
>Zotu1486
GAACGCTAGCGGCAGGCTTAATACATGCAAGTCGTGGGGCAGCAGGACTGTAGCAATACAGTTGCTGGCG
ACCGGCAAACGGGTGCGGAACACGTACGCAACCTACCCAAAACTGGGGGATAGCCCGGGGAAACCCGGA
TTAATACCTCGTAACATAATGGTGTGGCATCACATTATTATTATAGCTCCGGCGGTTTTGGATGGGCGTGCG
CCTGATTAGGTAGTTGGCGGGGTAACGGCCCACCAAGCCTGCGATCAGTAACTGGTGTGAGAGCACGACC
AGTCACACGGGCACTGAGACACGGGC
>Zotu1488
GAACGTTGATGGCGTGCCTCATACATGCAAGTCGAGCGCCCCGCAAGGGGAGCGGCGGACGGGTGAGTA
ACACGTAAAGAATCTGCCCTGAAGCGGGGAATAACTATCCCAACGGATAGCTAATACCGCATAATGATGGA
GACGGCATCGTCTCTTCTCAAAAGGCTTCGGCCACTTCAGGATGACTTTGCGTCCCATTAGCTTGTTGGCGG
GGTAACGGCCCACCAAGGCTACGATGGGTATCCGGCCTGAGAGGGTGTACGGACACATTGGGACTGAGAT
ACGGCC
>Zotu1490
GAACGCTAGCAGAACGCTTAACACATGCAAGTCGAACGAATATTAGTCTTCGGATTAATGTTAGTGGCAAA
CGGGCGAGTAACGCGTATCTAACCTGCCCTTGAGTGAGGCACAACTATGGAGACATAGCTAATTCCTCATA
CGCTCTAGAGAATGAAAATATCTAGAGGAAAGATTTATCGCTCAAGGAGGGGGATGCGTCCTATCAGGTA
GTTGTTGAGGTAACGGCTCAACAAGCCTATGACGGGTAGCCGGTCTGAGAGGATGTCCGGCCGCAATGGA
ACTGAGAACGGTC
>Zotu1492
GAACGCTGGCGGCGTGCCTAATACATGCAAGTAGAACGCACAGTTTATACCGTAGCTTGCTACACCATAGA
CTGTGAGTTGCGAACGGGTGAGTAACGCGTAGGTAACCTGCCTGGTAGCGGGGGATAACTATTGGAAACG

ATAGCTAATACCGCATAATATTAATTATTGCATGATAATTGATTGAAAGGTGCAAATGCATCACTACCAGAT
GGACCTGCGTTGTATTAGCTAGTAGTTGAGGTAACGGCTCACCTAGGCGACGATACATAGCCGACCTGAGA
GGGTGATCGGCCACACTGGGACTGAGACACGGCC
>Zotu1495
GAACGCTGGCGGCGTGCTTAACACATGCAAGTCGAACGATGATGCCAGCTTGCTGGTGGATTAGTGGCGA
ACGGGTGAGTAACACGTGAGCAACCTGCCCCTGACTTCGGGATAACCGCGGGAAACCGTGGCTAATACCG
GATATGACGCGCTATCGCATGGTGGTGTGTGGAAAGATTTATCGGTTGGGGATGGGCTCGCGGCCTATCA
GCTTGTTGGTGGGGTGATGGCCTACCAAGGCGACGACGGGTAGCCGGCCTGAGAGGGTGACCGGCCACA
CTGGGACTGAGACACGGCC
>Zotu1497
GAACGCTAGCGGCAGGCCTAATACATGCAAGTCGAGGGGCAGCACGGTGTAGCAATACACTGGTGGCGA
CCGGCGCACGGGTGCGTAACACGTATGTAATTTGCCCATAACTGGGGGATAGCCTTCCGAAAGGAAGATT
AATACCCCATAACCCAGCGAAGTGGCATCACTTTTCTGGTAAACCTCCGGGGGTTATGGATAAGCATGCGT
CCGATTAGCTAGTTGGCGAGGTAACGGCTCACCAAGGCAACGATCGGTAGGGGGTCTGAGAGGACGATCC
CCCACACGGGTACTGAGACACGGAC
>Zotu1499
GAACGCTGGCGGCGTGCTTAACACATGCAAGTCACGGGGGCCCGCAAGGGCAACCGGCGAACGGGTGCG
TAACACGTGAGCAATCTGCCGATCTCTGGGGGATAGCCGGCCCAACGGCCGGGTAATACCGCATACGATC
ACCGAGGGGATTCCCTTGGTGATGAAACCTCCGGGGGAGATCGAGGAGCTCGCGCCCTATCAGCTAGTTG
GCGGGGTAATGGCCCACCAAGGCTACGACGGGTAGCTGGTCTGAGAGGATGGCCAGCCACATTGGGACT
GCGACACGGCC
>Zotu1502
GAACGCTGGCGGCATGCTTTACACATGCAAGTCGAACGGCAGCGCGGGGCAACCTGGCGGCGAGTGGCG
AACGGGTGAGTAATATATCGGAACGTACCCAAGAGTGGGGGATAACGTAGCGAAAGTTACGCTAATACCG
CATACGATCTCAGGATGAAAGCAGGGGACCGCAAGGCCTTGTGCTCCTGGAGCGGCCGATATCTGATTAG
CTAGTTGGTGAGGTAAAGGCTCACCAAGGCGACGATCAGTAGCTGGTCTGAGAGGACGACCAGCCACACT
GGAACTGAGACACGGTC
>Zotu1504
GAACGCTGGCGGCGTGCTTAACACATGCAAGTCGAACGGGCCCGCCCCTGTTTTTGGGGGTGGGTTGAGT
GGCGAACGGGTGAGTATCACGTGAGTAACCTGCCCCCTGCTCCTGGATAACTGCTTGAAAGGGCGGCTAA
TACGGGGTGTGCCGGCTGGTCCGCATGGGCCTGGTCGGGATAGTTTCATCTTCGGGTGTTTCGGTGGGGG
ATGGGCTCGCGGCCTATCAGCTTGTTGGTGGGGTGATGGCCCACCAAGGCGGTGACGGGTAGCCGGCCTG
AGAGGGTGGGCGGCCACACTGGGACTGAGACACGGCC
>Zotu1506
GAACGCTGGCGGCGTGCCTAATACATGCAAGTCGAGCGGTAAGGCTCTGCACAGTTTATTTAAGTGATGA
GATTGGGAATGTTACGGCTGTAACCCCTTAAGAAGCGAAAGTTTCTCTCGTTCTTTACTACCTTGGTAGTTG
AAGATAGCTTGAATAAAGTGTGTAGAGTACACGAGCGGCGGACGGCTGAGTAACGCGTAGGAATGTACCC
CAAAGTGAGGGATAAGCACCGGAAACGGTGTCTAATACCGCATGTGATCTTCGGATTAAAGCTTTATGCGC
TTTGGGAACAGCCTGCGTCTGATTAGCTTGTTGGTGAGGTAATAGCTCACCAAGGCTTCGATCGGTAGCTG
GTCTGAGAGGATGATCAGCCAGACTGGGACTGAGACACGGCC
>Zotu1508
GAACGCTAGCGGCAGGCTTAACACATGCAAGTCGAGGGGTATAGGTTTTCGGACCTAGAGACCGGCGCAC
GGGTGCGTAACGCGTATGCAATCTACCTTTTACAGAGGGATAGCCCAGAGAAATTTGGATTAATACCTCAT
AGTATTATTGAATGGCATCATTTAATAATTAAAGTCACAACGGTAAAAGATGAGCATGCGTCCCATTAGCTA
GTTGGTAAGGTAACGGCTTACCAAGGCAACGATGGGTAGGGGTCCTGAGAGGGAGATCCCCCACACTGGT
ACTGAGACACGGAC
>Zotu1511
GAACGCTGGCGGCGCGCTTCATACATGCAAGTCGAACGAAAAGCCCGGGCTTGCCCGGGTGGACAGTGGC
GAACGGGTGAGTAACACGTGAGCAACCTGCCCCGAAGACTGGGACAACACCGGGAAACCGGTGCTAATAC
CGGATGCCCTCACCACACCGCATGACGTGGTGAGGAAATGGATTCCGCTTCGGGATGGGCTCGCGGTCTAT

CAGCTTGTTGGTGGGGTAACGGCCTACCAAGGCGACGACGGATAGCTGGTCTGAGAGGATGTCCAGCCAC
ACTGGGACTGAGACACGGCC
>Zotu1513
GAACGCTGGCGGCGTGCTTAACACATGCAAGTCGAACGGTGAACTCCGCTTGCGGGGGGATCAGTGGCGA
ACGGGTGAGTAACACGTGGGCAACCTGCCCTCAGCTCTGGGATAACTCCAAGAAATTGGGGCTAATACTG
GATATGACCGCTGACCGCATGGTCTGGTGGTGGAAAGATTTATCGGCTGGGGATGGGCCCGCGGCCTATC
AGCTTGTTGGTGGGGTAGTGGCCTACCAAGGCGACGACGGGTAGCCGGCCTGAGAGGGTGACCGGCCAC
ACTGGGACTGAGACACGGCC
>Zotu1516
GAACGCTAGCGGCAGGCTTAACACATGCAAGTCGAGGGGTATAGTAGCAATACTAGAGACCGGCGCACG
GGTGCGTAACGCGTATGCAATCTACCTTTTACAGCGGGATAGCCCAGAGAAATTTGGATTAATACCACATA
GTATTATTGAGTGGCATCACTTAATAATTAAAGTTACAACGGTAAAAGATGAGCATGCGTCCCATTAGCTA
GTTGGTAAGGTAACGGCTTACCAAGGCGACGATGGGTAGGGGTCCTGAGAGGGAGATCCCCCACACTGGT
ACTGAGACACGGAC
>Zotu1518
GAACGCTGGCGGCGTGCTTAACACATGCAAGTCGAGCGGTAAGGCCCTTCGGGGTACACGAGCGGCGAA
CGGGTGAGTAACACGTGGGTGACCTGCCCTCCACTCTGGGATAAGCCTGGGAAACCGGGTCTAATACCGG
ATAGGACTCCTGGATGCATGTCTAGGGGTGGAAAGTTTTTCGGTGGGGGATGGGCCCGCGGTCTATCAG
CTTGTTGGTGGGGTGATGGCCTACCAAGGCGACGACGGATAGCCGGCCTGAGAGGGTGATCGGCCACACT
GGGACTGAGACACGGCC
>Zotu1520
GAACGCTGGCGGCGTGCTTAACACATGCAAGTCGAACGATTAAAGCCCCTTCGGGGGTGTATAGAGTGGC
GAACGGGTGAGTAACACGTGGGCAATCTGCCCCTCACTTCGGGATAACTCAGGGAAACTTGAGCTAATACC
GAATACTCCGAGCGGATCGCATGGTCTGCTCGGGAAAGCTCCGACGGTGAGGGATGAGCCCGCGGCCCAT
TAGCTAGTTGGTGAGGTAATGGCCCACCAAGGCAACGATGGGTAGCCGCGCTGAGAGGCGGATCGGCCA
CACTGGGACTGAGACACGGCC
>Zotu1522
GAACGCTGGCGGCGTGCCTAATACATGCAAGTCGAGCGAAGTTTTTCTGGTGCTTGCACTAGAAAAACTTA
GCGGCGAACGGGTGAGTAACACGTAAAGAACCTGCCTCATAGACTGGGACAACTATTGGAAACGATAGCT
AATACTGGATAACAGCATTAACTGCATGGTTGATGTTTAAAAGTTGGTTTTGCTAACACTATGAGATGGCTT
TGCGGTGCATTAGCTAGTTGGTGGGGTAAAGGCCTACCAAGGCGACGATGCATAGCCGACCTGAGAGGGT
GATCGGCCACACTGGGACTGAGACACGGCC
>Zotu1524
GAACGCTGGCGGCGTGCTTAACACATGCAAGTCACGGGGGCCCGCAAGGGTAACCGGCGAACGGGTGCG
TAACACGTGAGCAATCTGCCGTCCACTGGGGGATAGCCGGCCCAACGGCCGGGTAATACCGCATACGCTT
GCCGGGTGGCATCACTTGGCAAGGAAACCTCCGGGGGTGGACGAGGAGCTCGCGGCCTATCAGCTAGTTG
GTGGGGTCACGGCCCACCAAGGCGATGACGGGTAGCTGGTCTGAGAGGATGGCCAGCCACATTGGGACT
GAGATACGGCC
>Zotu1526
GAATGCTGGCGGCGTGGATAAGGCATGCAAGTCAAGGGGACCGCAAGGTAACCGGCAGACGAGGTAGTA
ACACGTACGAACGCACCCCAGAGTCTGGCATAGCTCGTCGAAAGACGGGGTAATTCCGGATGGTCCCGAA
AGGGTAAAGTTTTTCGCTCTGGGAGCGGCGTGCGTAATATCAGCTTGTTGGTGAGGTAATGGCTCACCAA
GGCTACGACGTTTAGGGGCGCTGAGAGGCGGACCCCCACCGATGGGACTGAGACACGGCC
>Zotu1528
GAACGCTGGCGGCAGGCCTAACACATGCAAGTCGAGCGGATGAGAGGGGCTTGCTCTTCGATTCAGCGGC
GGACGGGTGAGTAATGCCTAGGAATCTGCCTAGTAGTGGGGGACAACGTTTCGAAAGGAACGCTAATACC
GCATACGTCCTACGGGAGAAAGTGGGGGATCTTCGGACCTCACGCTATTAGATGAGCCTAGGTCGGATTA
GCTAGTTGGTAGGGTAAAGGCCTACCAAGGCGACGATCCGTAACTGGTCTGAGAGGATGATCAGTCACAC
TGGAACTGAGACACGGTC
>Zotu1530

GAACGCTAGCGGCAGGCTTAATACATGCAAGTCGAGGGGCAGCGCAGTGTAGCAATACATGGGCGGCGA
CCGGCAAACGGGTGCGGAACACGTACGCAACCTTCCTTCAAATGGGGGATAGCCCAGGGAAACTTGGATT
AATACCCCGTAACATTATGAAATGGCATCATTTTGTAATTATAGCTACGGCGTTTGAAGATGGGCGTGCGCC
TGATTAGGTAGTTGGCGGGGTAACGGCCCACCAAGCCTTCGATCAGTAACTGGTGTGAGAGCACGACCAG
TCACACGGGCACTGAGACACGGGC
>Zotu1532
GAACGCTGGCGGCGTGCTTAACACATGCAAGTCGAACGATGACGGTGGTGCTTGCACCGCCTGATTAGTG
GCGAACGGGTGAGTAACACGTGAGTAACCTGCCCTCCACTTCGGGATAACCTCGGGAAATCGTGGCTAAT
ACCGGATATGAGCACTCATCGCATGGTGGGTGTTGGAAAGATTTATCGGTGGGGGATGGACTCGCGGCCT
ATCAGTTTGTTGGTGAGGTGATGGCTCACCAAGACGATGACGGGTAGCCGGCCTGAGAGGGCGACCGGCC
ACACTGGGACTGAGACACGGCC
>Zotu1534
GAACGCTGGCGGCGTGCCTAATACATGCAAGTCGAACGGTCTTTTCTATGGAAGCTTGCTTCCACTGAGAA
GATAGTGGCGAACGGGTGAGTAACACGTGGGTAACCTGCCCATAAGAGGGGGATAACATCCGGAAACGG
GTGCTAATACCGCATAGTTTTCTTGATCGCATGATCGGGAAAGGAAAGACGGCCTTTGTGCTGTCGCTTAT
GGATGGACCCGCGGCGTATTAGTTAGTTGGTGAGGTAACGGCTCACCAAGACGATGATACGTAGCCGACC
TGAGAGGGTGATCGTCCACATTGGGACTGAGACACGGCC
>Zotu1537
GAACGCTGGCGGTATGCTTTACACATGCAAGTCGTACGAGAGTTTTTTACTCTAGTGGCGGACGGGTGAGT
AACACGTAAGAATTTGCCTTTAGGAGAGGGATAACAACTGGAAACGGTTGCTAATACCTCATATGCTTTCG
AGTGAAATGAATTTTTTTCGCCTAAAGAGAAGCTTGCGCCTGATTAGCTTGTTGGTAAGGTAATGGCTTACC
AAGGCGACGATCAGTATCTGGTTTGAGAGGATGATCAGACACACTGGAACTGAGACACGGTC
>Zotu1539
GAACGCTGGCGGCGTGCTTAACACATGCAAGTCGAACGGTGAAGCGGGTGCTTGCACTCGTGGATCAGTG
GCGAACGGGTGAGTAACACGTGAGTAACCTGCCCTCCACTCTGGGATAAGCGCTGGAAACGGCGTCTAAT
ACTGGATACGACCGACCCTCGCATGGGGTGTTGGTGGAAAGATTTATCGGTGGTGGATGGACTCGCGGCC
TATCAGCTTGTTGGTGGGGTAATGGCCTACCAAGGCGACGACGGGTAGCCGGCCTGAGAGGGCGACCGG
CCACACTGGGACTGAGACACGGCC
>Zotu1541
GAACGCTGGCGGCGTGCTTAACACATGCAAGTGGAGCGACGAACCAGGCTTCGGCCTGGGGCAGAGCCG
CGAACGGGTGAGTAACACGTGGGTAACGTGCCCCGATGATCGGGACAACCCGGGGAAACCCGGGCTAAT
ACCGAATGTGCTCCCCTCACACCAGTGAGGGGTGGAAAGGAAGCTTCGGCCTCCGCATCGGGATCGGCCC
GCGGCCCATTAGCTTGTTGGTGAGGTAACGGCTCACCAAGGCTGCGATGGGTAGCTGGTCTGAGAGGACG
ATCAGCCACACTGGGACTGAGACACGGCC
>Zotu1544
GAACGCTAGCGGGAGGCCTAACACATGCAAGCCGAGCGGTATTGTTTCTTCGGAAATGAGAGAGCGGCGT
ACGGGTGCGGAACACGTGTGCAACCTGCCTTTATCTGGGGGATAGCCTTTCGAAAGGAAGATTAATACACC
ATAACATATTGAACGGCATCGTTTAATATTGAAAGCTCCGGCGGATAGAGATGGGCACGCGCAAGATTAG
CTAGTTGGTGAGGTAACGGCTCACCAAGGCGATGATCTTTAGGGGGCCTGAGAGGGTGATCCCCCACACT
GGTACTGAGACACGGAC
>Zotu1546
GAACGCTAGCGGCAGGCCTAATACATGCAAGACGAACGGTAACTTGAGTAGCAATATTTGAGCAAGAGTG
TCGTAAGGGTGCGTAACACGTATGCAATCTGCCCTCAACAGGAGGATAGCTCCCCGAAAGGGGAATTAAC
ACTCCATAAGATCATGGTCTGGCATCAGACAGTGATTAAAACTACGGTGGTTGAGGATGAGCATGCGCATG
ATTAGTTAGTTGGCGGGGTAATGGCCCACCAAGACGATGATCATTAGGGGAACTGAGAGGTTGATCCCCC
ACACTGGCACTGAGATACGGGC
>Zotu1548
GAACGCTGGCGGCGTGCCTAATACATGCAAGTCGAGCGAGGAGCTTTTAGCTCCTAGCGGCGAACGGGTG
AGTAACACGTGGGTAACCTGCCCGTAAGACTGGGATAACTTCGGGAAACCGGAGCTAATACTGGATAATC
TTGATCTTCGCATGAAGGACAAGTAAAAGATTTATCACTTACGGATGGGCCCGCGGTGCATTAGCTTGTTG

GTGGGGTAATGGCCTACCAAGGCGACGATGCATAGCCGACCTGAGAGGGTGACCGGCCACACTGGGACT
GAGACACGGCC
>Zotu1550
GAACGCTGGCGGCGTGCCTAATACATGCAAGTCGAGCGAAGTTTTTCTGGTGCTTGCACCGGAAAAACTTA
GCGGCGAACGGGTGAGTAACACGTAAAGAACCTGCCTCATAGACTGGGACAACTATTGGAAACGATAGCT
AATACCGGATAACAGCATTAACTGCATGGTTGATGTTTGAAAGTTGGTTTTGCTAACACTATGAGATGGCTT
TGCGGTGCATTAGCTAGTTGGTGGGGTAAAGGCCTACCAAGGCGACGATGCATAGCCGACCTGAGAGGGT
GATCGGCCACACTGGGACTGAGACACGGCC
>Zotu1552
GAACGCTGGCGGCGTGCCTAATACATGCAAGTCGAGCGGATCTCTTCGGAGATCAGCGGCGGACGGGTGA
GTAACACGTGGGCAACCTGCCTATAAGACTGGGATAACTTCGGGAAACCGGAGCTAATACCGGATAACAT
CAGAGGCCACATGGTCTTCGATTAAAAGATGGCTTTGGCTATCACTTATAGATGGGCCCGCGTCGCATTAG
CTAGTTGGTGAGGTAACGGCTCACCAAGGCGACGATGCGTAGCCGACCTGAGAGGGTGATCGGCCACACT
GGGACTGAGACACGGCC
>Zotu1554
GAACGCTGACAGAATGCTTAACACATGCAAGTCAACTTGAACTTCGGTTTGGGTGGCGGACGGGTGAGTA
ACGCGTAAAGAACTTGCCTCACAGATAGGGACAACATTTGGAAACGAATGCTAATACCTGATATTATGATT
ATATGGCATCGTATAATTATGAAAGCTATATGCGCTGTGAGAGAGCTTTGCGTCCCATTAGCTAGTTGGAG
AGGTAACGGCTCACCAAGGCGATGATGGGTAGCCGGCCTGAGAGGGTGATCGGCCACAAGGGGACTGAG
ACACGGCC
>Zotu1556
GAACGCTGGCGGCATGCTTTACACATGCAAGTCGAACGGTAACAGGGAGCTTGCTCCGCTGACGAGTGGC
GAACGGGTGAGTAATATATCGGAACGTGCCCAAGAGTGGGGGATAACGTAGCGAAAGTTACGCTAATACC
GCATACGATCCAAGGATGAAAGCAGGGGACCTTCGGGCCTTGTGCTCCTGGAGCGGCCGATATCTGATTA
GCTAGTTGGTGAGGTAAAGGCTCACCAAGGCGACGATCAGTAGCTGGTCTGAGAGGACGACCAGCCACAC
TGGAACTGAGACACGGTC
>Zotu1558
GAACGCTGACAGAATGCTTAACACATGCAAGTCAACTTGAACTTCGGTTTGGGTGGCGGACGGGTGAGTA
ACGCGTAAAGAACTTGCCTCACAGATAGGGACAACATTTGGAAACGAATGCTAATACCTGATATTATGATT
TTAGGGCATCCTAGGATTATGAAAGCTATATGCGCTGTGAGAGAGCTTTGCGTCCCATTAGCTAGTTGGAG
AGGTAACGGCTCACCAAGGCGATGATGGGTAGCCGGCCTGAGAGGGTGATCGGCCACAAGGGGACTGAG
ACACGGCC
>Zotu1560
GAACGCTGGCGGCGTGCTTAACACATGCAAGTCGAACGGGATCCATCGCGAGCTTGCTCAAGAGAAGATC
TAGTGGCGAACGGGTGCGTAACACGTGAGAAACCTGCCCCAGACTTGGGAATAACAGTCGGAAACGACTG
CTAATACCGAATACCTTCGTAGCGTCGCATGGCGCATCGAAGAAAGCTTTATGCGGTTTGGGAGGGTCTCG
CGGCCTATCAGCTAGTTGGTGAGGTAACGGCTCACCAAGGCATCGACGGGTAGCTGGTCTGAGAGGATGA
TCAGCCACACTGGGACTGAGACACGGCC
>Zotu1562
GAACGCTGGCGGCACGCTTAACACATGCAAGTCGAACGAGAAAGTGGGTAACCATGAGTAAAGTGGCGA
ACGGGTGCGTAACACGTGAGAATCTACCTTTTAGTGGAGGATACCCTGGAGAAATTTAGGTTAATCCTGCA
TAAGCTCGAAAGAGGAAAGGCGGCTTAATTGCTGTCGCTAAAAGATGAGCTTGCGGCCTATTAGCTAGTTG
GTAAGGTAAAGGCTTACCAAGGCAAAGATGGGTATCCGGCCCGAGAGGGTGGTCGGACACACTGGGACT
GAGATACGGCC
>Zotu1564
GAACGCTGGCGGCAGGCTTAACACATGCAAGTCGAGCGCACCTTCGGGTGAGCGGCAGACGGGTGAGTA
ACGCGTGGGAATCTACCCATCTCTACGGAATAGCTCCGGGAAACTGGAATTAATACCGTATACGCCCTTCG
GGGGAAAGATTTATCGGAGATGGATGAGCCCGCGTTGGATTAGCTAGTTGGTGGGGTAAAGGCCTACCAA
GGCGACGATCCATAGCTGGTCTGAGAGGATGATCAGCCACATTGGGACTGAGACACGGCC
>Zotu1567

GAACGCTGGCGGCGTGCCTAATACATGCAAGTTGAGCGCTGAAGGTTGGTACTTGTACCAACTGGATGAG
CAGCGAACGGGTGAGTAACGCGTGGGGAATCTGCCTTTGAGCGGGGGACAACATTTGGAAACGAATGCT
AATACCGCATAAAAACTTTAAACACAAGTTTTAAGTTTGAAAGATGCAATTGCATCACTCAAAGATGATCCC
GCGTTGTATTAGCTAGTTGGTGAGGTAAAGGCTCACCAAGGCGATGATACATAGCCGACCTGAGAGGGTG
ATCGGCCACATTGGGACTGAGACACGGCC
>Zotu1569
GAACGCTGGCGGCGTGCCTAATACATGCAAGTCGAGCGAATCTTTTGGGGGCTTGCTCCCAAAAGATTAGC
GGCGGACGGGTGAGTAACACGTGGGCAACCTGCCTATAAGACTGGGATAACTTCGGGAAACCGGAGCTA
ATACCGGATATGTTCTTTTCTCGCATGAGAGAAGATGGAAAGACGGTTTCGGCTGTCACTTATAGATGGGC
CCGCGGCGCATTAGCTAGTTGGTGGGGTAATGGCTCACCAAGGCGACGATGCGTAGCCGACCTGAGAGG
GTGATCGGCCACACTGGGACTGAGACACGGCC
>Zotu1572
GAACGCTAGCGGCAGGCTTAACACATGCAAGTCGAGGGGTATAGGTAGCTTGCTATCTAGAGACCGGCGC
ACGGGTGCGTAACGCGTATGCAACCTACCTTTCACAAAGGGATAGCCCAGAGAAATTTGGATTAATACCTT
ATAGTATATAAGAATCGCATGGTTTTTATATTAAAGTTCCAACGGTGAAAGATGGGCATGCGTCCCATTAGC
TAGATGGTAAGGTAACGGCTTACCATGGCAACGATGGGTAGGGGTCCTGAGAGGGAGATCCCCCACACTG
GTACTGAGACACGGAC
>Zotu1574
GAACGCTAGCGGCAGGCCTAATACATGCAAGTCGAACGGTCAGCAATGACAGTGGCGCACGGGTGCGTA
ACGCGTATGCAACCTACCCATCAAAGGGGGATAGCCCGGGGAAACCCGGATTAATACCGCATAAGATAGT
ATTCTGGCATCAGAGAGCTATTAAACATTTATGGTTGATGGATGGGCATGCGTTAGATTAGCTAGTTGGTG
TAGTAACGGTGCACCAAGGCGATGATCTATAGGGGTTCTGAGAGGAAGATCCCCCACACTGGTACTGAGA
TACGGAC
>Zotu1576
GAACGCTGGCTGCGTGCTTAACACATGCAAGTCGAACGCTGAAGCCCCAGCTTGCTGGGGTGGATGAGTG
GCGAACGGGTGAGTAACACGTGAGTAACCTGCCCCTGACTTCGGGATAAGCCCGGGAAACTGGGTCTAAT
ACCGGATACGACTGCCGGACGCATGTCTGGTGGTGGAAAGTTTTTTCGGTTGGGGATGGGCTCGCGGCCT
ATCAGTTTGTTGGTGAGGTAATGGCTCACCAAGACGACGACGGGTAGCCGGCCTGAGAGGGCGACCGGCC
ACACTGGGACTGAGACACGGCC
>Zotu1578
GAACGCTGGCGGCGTGCCTAATACATGCAAGTCGAGCGGATTCATTCCTTCGGGAATGGGTTAGCGGCGG
ACGGGTGAGTAACACGTAGGCAACCTGCCTATAAGCCTGGGATAACTTCCGGAAACGGATGCTAATACCG
GATACGTTGAATTCTCGCATGAGAATTCATGGAAAGACGGCGCAAGCTGTCACTTATGGATGGGCCTGCG
GCGCATTAGCTAGTTGGTGGGGTAACGGCTCACCAAGGCGACGATGCGTAGCCGACCTGAGAGGGTGATC
GGCCACACTGGGACTGAGACACGGCC
>Zotu1581
GAACGCTGGCGGCGTGCTTAACACATGCAAGTCGAACGGAATCTGATCTGTGGCAACACAGTGAAAGGTT
TAGTGGCGAACGGGTGAGTAACACGTGAGGAACCTGCCCCGAAGACCGGGATAACTCTCCGAAAGGAGT
GCTAATACCGGATACCCCCACCGAATCGCATGGTTTGGTGAGGAAATGGATTCCGCTTCGGGAGGGCCTCG
CGGCCTATCAGCTAGTTGGTGGGGTAATGGCCCACCAAGGCATCGACGGGTAGCTGGTCTGAGAGGACGA
TCAGCCACACTGGGACTGAGACACGGCC
>Zotu1584
GAACGCTTGCGGCGTGCCTAAGAAATGCAAGTCGAACGGGGAGATTTCGGTCTCCTAGTGGCGAACGGTC
GCGTAACACGTAAGCAACCTGCCCAGAAGTGGGGGACAACAGCCTGAAAGGGCTGCTAATACCGCATGTG
AATGAGATTTGGCATCTTATCTTATTTAAAGCTTTATGCGCTTCTGGATGGGCTTGCGGCCTATCAGGTAGT
TGGTGGGGTAACAGCCTACCAAGCCGACGACGGGTAGCTGGTCTGAGAGGACGATCAGCCGGATTGGGA
CTGAGATACGGCC
>Zotu1586
GAACGCTGGCGGTATGCTTTACACATGCAAGTCATACGAGAATTTTTAATTCAAGTGGCGGACGGGTGAGT
AACACGTAAGAATTTACCTTTAGGAGGGGGATAACAACTGGAAACGGTTGCTAATACCCCATATGCTTTCG

AGTGAAATGGAGTTTTCCGCCTAGAGAGAAGCTTGCGGCTGATTAGCTTGTTGGTGAGGTAATGGCTTACC
AAGGCGACGATCAGTATCTGGTTTGAGAGGACGATCAGACACACTGGAACTGAGACACGGTC
>Zotu1588
GAACGCTAGCGGCAGGCTTAATACATGCAAGTCGAGGGGCAGCACGAATAGCAATATTTGGTGGCGACCG
GCGCACGGGTGCGTAACACGTATACAATCTACCTAAAACAGGGGGATAGCCTTTCGAAAGAGAGATTAAT
ACCCCGTAATAAATTGAATGGCATCATTTGATTTTGAAAGCTCCGGCGGTTTTAGATGAGTATGCGTCTGAT
TAGCTAGTTGGTGGTGTAATGGACCACCAAGGCTACGATCAGTAGGGGATCTGAGAGGACTACCCCCCAC
ACTGGTACTGAGACACGGAC
>Zotu1591
AAACGCTGGCGGCGTGCATAACACATGCAAGTCGAACGATGAAGGTTAAAAGATTTCTTCGGAATGAACTT
AATTGGATTAGTGGCGGACGGGTGAGTAACGCGTGAGTAACCTGCCTTGTACAAGGGGATAGCCGTTGGA
AACGACGAATAATACCCTATGACATCAAATAATCGCATGATAATTTGATCAAAGATTTATCGGTATAAGATG
GACTCGCGTCTGATTAGCTAGTTGGTAGAGTAAAAGCCTACCAAGGCAACGATCAGTAGCCGGCTTGAGA
GAGTGTACGGCCACATTGGGACTGAGACACGGCC
>Zotu1593
GAACGCTGGCGGCATGCCTAATACATGCAAGTCGAGCGGAATGATGGGAGCTTGCTCCCTGATTTCAGCG
GCGGACGGGTGAGTAACACGTGGGCAACCTGCCCTACAGATGGGGATAACTCCGGGAAACCGGGGCTAA
TACCGAATAATCAGTTTGTCCGCATGGACAAACTCTGAAAGACGGTTTCGGCTGTCACTGTAGGATGGGCC
CGCGGCGCATTAGCTAGTTGGTGGGGTAATGGCCTACCAAGGCGACGATGCGTAGCCGACCTGAGAGGGT
GATCGGCCACACTGGGACTGAGACACGGCC
>Zotu1596
GAACGCTAGCGGCAGGCTTAATACATGCAAGTCGAACGGTATCAGCAATGGTATAGTGGCGCACGGGTGA
GTAACGCGTACATAACATGCCTTTATCTTTGGGATAGCCTTGGGAAACTGGGAGTAATACCGAATAGTATTT
TTGGATGGCATCATCTGGGAATTAAAGTTACGGCGGATAAAGATTGATGTGCGTAAGATTAGGTAGTTGGT
GAGGTAACGGCTCACCAAGCCAACGATCTTTAGGGGGCGTGAGAGCGTGACCCCCCACACGGGTACTGAG
ACACGGAC
>Zotu1598
GAACGCTGGCGGCGTGCTTAACACATGCAAGTCGAACGGAAAGGCCCTTCGGGGTACTCGAGTGGCGAAC
GGGTGAGTAACACGTGGGTGATCTGCCTCGTACTCTGGGATAAGCCTGGGAAACTGGGTCTAATACCGGA
TAGGACCATTCCTCGCATGGGGTGTGGTGGAAAGTTTTTCGGTACGAGATGGGCCCGCGGCCTATCAGCTT
GTTGGTGGGGTAATGGCCTACCAAGGCGACGACGGGTAGCCGGCCTGAGAGGGCGACCGGCCACACTGG
GACTGAGACACGGCC
>Zotu1600
GAACGCTGGCGGCGTGCTTAACACATGCAAGTCGAACGATGATGTCCCGCTTGCGGGACGGATTAGTGGC
GAACGGGTGAGTAATACGTGAGTAACCTGCCCCTGACTCTGGGATAAGCCTGGGAAACCGGGTCTAATAC
TGGATACTACCTCGCACCGCATGGTGGGTGGTGGAAAGGGTTTTACTGGTTTTGGATGGGCTCACGGCCTA
TCAGCTTGTTGGTGGGGTAATGGCTCACCAAGGCGACGACGGGTAGCCGGCCTGAGAGGGTGACCGGCC
ACACTGGGACTGAGACACGGCC
>Zotu1603
GAACGCTGGCGGCGTGCTTAACACATGCAAGTCGAACGGTAAGGCCCTTCGGGGTACACGAGTGGCGAAC
GGGTGAGTAACGCGTGAGTAACCTGCCCCTGACTTTGGGATAACGGATGGAAACGTCTGCTAATACCGAA
TATTCAGTTCTCCGGGCATCCGGGGGGTTGGAAAGTTTTTTCGGTCAGGGATGGACTCGCGTCCTATCAGC
TTGTTGGTGGGGTAATGGCCTACCAAGGCGTCGACGGGTAGCCGGCCTGAGAGGGCGACCGGCCACATTG
GGACTGAGATACGGCC
>Zotu1605
GAACGCTGGCGGCGTGCTTAACACATGCAAGTCGAGCGGTAAGGCCCTTCGGGGTACACGAGCGGCGAA
CGGGTGAGTAACACGTGGGTGACCTGCCCCCTACTCTGGGATAAGCCCGGGAAACTGGGTCTAATACCGG
ATATGACTACATGTCGCATGGTGTGTGGTGGAAAGTTTTTCGGTGGGGGATGGGCCCGCGGCCTATCAGC
TTGTTGGTGGGGTGATGGCCTACCAAGGCGACGACGGGTAGCCGGCCTGAGAGGGCGACCGGCCACACT
GGGACTGAGACACGGCC

>Zotu1607
GAACGCTAGCGGCAGGCCTAATACATGCAAGTCGAACGGGTCGCAAGACCAGTGGCAAACGGGTGCGTA
ACGCGTAAGCAACCTGCCTCAAACTGGGGGATAGCCCGGCGAAAGCTGGGGTAAACCCGCACGGTTCCCA
TTTATCACCTGGTAGTTGGGGTAAACATTTATGGGTTTGAGAGGGGCTTGCGTCTGATTAGTTAGTTGGCA
GGGTAACGGCCTACCAAGACGATGATCAGTAGGGGTTCTGAGAGGATTGGCCCCCACATGGGTACTGAGA
TACGGAC
>Zotu1609
GAACGTTGGCGGCGTGCCTTAAGCATGCAAATCAAGGGGGTCCCGCAAGGGACTACCGGTGGACGGGTG
AGTAATGCGTGGGAATCTACCTCGAAGTGGGATATAGCCAGCCGAAAGGTTGGGTAATCTCCCATGTGATC
GCAAGATCAAAGTCGCAAGACGCTTTGAGATGAGCCCACGTCCTATCAGCTAGTTGGTGGGGTAATGGCCT
ACCAAGGCAACGACGGGTAGCTGGACTGAGAGGTCGGTCAGCCACAATGGGACTGCGACACGGCC
>Zotu1611
GAACGCTGGCGGCGTGCCTAATACATGCAAGTCGTACGCATTGACGATGGAACTTGTTCCATCTGACGTGA
GTGGCGCACGGGTGAGTAACACGTGGGAAACCTACCCTTCAGCGGGGGATAACCGTCGGAAACGATGGC
TAATACCGCATAGGTCTTTGAGTCACCTGGCTTAAAGAGGAAAGACGGCTTCGGCTGTCACTGAAGGATGG
TCCCGCGGTGCATTAGCTAGTTGGTAAGGTAATGGCTTACCAAGGCAATGATGCATAGCCGACCTGAGAG
GGTGATCGGCCACATTGGGACTGAGACACGGCC
>Zotu1613
GAACGCTAGCGGCAGGCTTAATACATGCAAGTCGAGGGGCAGCACAGGGTAGCAATACCTGGGTGGCGA
CCGGCAAACGGGTGCGGAACACGTACAGAACCTTCCTCTGAGTGTGGGATAGCCCGGAGAAATCCGGATT
AATACCACATAGTAATATTAGAAGGCATCTTTTGATATTTAAAGATTTATCGCTTAGAGATGGCTGTGCGGC
TGATTAGGTAGTTGGTGCGGGTAACGGCCCACCAAGCCAGCGATCAGTAACTGGTGTGAGAGCACGACCA
GTCACACGGGCACTGAGACACGGGC
>Zotu1615
GAACGCTGGCGGCGTGCCTAATACATGCAAGTAGAACGCTGAAGGAGGAGCTTGCTCTTCTAGATGAGTT
GCGAACGGGTGAGTAACGCGTAGGTAACCTGCCTGGTAGCGGGGGATAACTATTGGAAACGATAGCTAAT
ACCGCATAAGAGTAGATGTTGCATGACATTTGCTTAAAAGGTGCAATTGCATCACTACCAGATGGACCTGC
GTTGTATTAGCTAGTTGGTGGGGTAACGGCTCACCAAGGCGACGATACATAGCCGACCTGAGAGGGTGAT
CGGCCACACTGGGACTGAGACACGGCC
>Zotu1618
GAACGCTGGCGGCGTGCCTAAGACATGCAAGTCGAGTGGGTTTAGACCCACGGCGAACGGGTGAGTAAT
ACCTAGGAACTTTCTTTAAGTAGAGGATAACCAGCCGAAAGGTTGGATAATACTCCATGTGGCAGTAATGC
TAAAGCAGCAATGCGCTTAAAGTGAGGCCTGGGCACCATCAGCTAGTTGGTAAGGTAATGGCTTACCAAG
GCTTTGACGGTTAGGGGTCCCGAGAGGGATAACCCCCACACTGGAACTGCGACACGGTC
>Zotu1620
GAACGCTGGCGGCGTGCTTAACACATGCAAGTCGAACGGTGATGGAGAGCTTGCTCTCCTGATCAGTGGC
GAACGGGTGAGTAACACGTGAGTAACCTGCCCTTGACTCTGGGATAAGCGTTGGAAACGACGTCTAATAC
CGGATAGGACTTCCACAGGCATCTGTGGGGGTGGAAAGATTTTTCGGTCATGGATGGACTCGCGGCCTATC
AGCTTGTTGGTGGGGTGATGGCCTACCAAGGCGACGACGGGTAGCCGGCCTGAGAGGGTGACCGGCCAC
ACTGGGACTGAGACACGGCC
>Zotu1623
GAACGCTGGCGGCGTGCCTAACACATGCAAGTCTACGAGAAAAGGGTAGCAATATCCTGAGTAAAGTGGC
GCACGGGTGAGTAACGCGTAGGCAATCTGCCTTTAGGACGGGAACAACCCCGAGAAATCGAGGCTAATAC
CCGATAATGCAGCGGCACCGCATGGTGACAGTTGTTAAAAGCCCGCAAGGGCGCCTAGAGATGAGCCTGC
GCCCCATTAGGTAGTTGGTGAGGTAACGGCCCACCAAGCCTACGATGGGTAGCTGATCTGAGAGGATGAT
CAGCCACACTGGAACTGAGACACGGTC
>Zotu1625
GAACGCTAGCGGCAGGCCTAATACATGCAAGACGAACGGTAAGAATTGTAGCAATACGATTCCAAGAGTG
TCGTACGGGTGCGTAACACGTATGCAACCTACCTTGTACAGGAGTATAGCTCGGGGAAACTCGAATTAACC
CTCCATAATATATTGATGTGGCATCACATTAATATTAAAACTACGGTGGTACAAGATGGGCATGCGTTTGAT

TAGCTAGTTGGCGGTGTAATGGACCACCAAGGCGATGATCAATAGGGGAACTGAGAGGTTGATCCCCCAC
ACTGGCACTGAGATACGGGC

>Zotu1627

GAACGCTGGCGGCAGGCCTAACACATGCAAGTCGAGCGCCCCCGCAAGGGGGAGCGGCGGACGGGTGA
GTAACGCGTGGGAATTTTCCTCAAGGTACGGAACAACTCAGGGAAACTTGGGCTAATACCGTATGATGCA
GAGATGCCAAAGATTTATCGCCTTGGGATAAGCCCGCGTCAGATTAGGTAGTTGGTGGGGTAACGGCCTA
CCAAGCCTGCGATCTGTAGCTGGTCTGAGAGGACGATCAGCCACACTGGGACTGAGACACGGCC

>Zotu1629

GAACGCTGGCGGCGTGCCTAATACATGCAAGTCGTGCGGGGGAGATCCTTCGGGATCGGACCCAGCGGC
GAACGGGTGAGTAACACGTGGGCAACCTGCCCGCAAGACGGGGATAACTCCGGGAAACCGGTGCTAATA
CCCGATAATCCCGGCTCCCGCATGGGAGCGGGGTGAAAGGCGGCTTCGGCTGTCACTTGCGGATGGGCCC
GCGGCGCATTAGCTGGTTGGTGAGGTAACGGCTCACCAAGGCGACGATGCGTAGCCGACCTGAGAGGGT
GATCGGCCACACTGGGACTGAGACACGGCC

>Zotu1631

GAACGCTGGCGGCGTGCCTAATACATGCAAGTCGAGCGAGGTTCTTCGGAACCTAGCGGCGGACGGGTGA
GTAACACGTGGGTAACCTGCCTGTAAGACTGGGATAACTTCGGGAAACCGAAGCTAATACCGGATACTTTT
GAGCATCGCATGATGCTTGATGGAAAGACGGCTTCGGCTGTCACTTACAGATGGACCCGCGTCGCATTAGC
TAGTTGGTGAGGTAACGGCTCACCAAGGCGACGATGCGTAGCCGACCTGAGAGGGTGATCGGCCACACTG
GGACTGAGACACGGCC

>Zotu1633

GAACGCTAGCGGCAGGCTTAACACATGCAAGTCGAGGGGTATTTATCTTCGGATAGAGAGACCGGCGCAC
GGGTGCGTAACGCGTATGCAATCTACCTTTTACAGCGGGATAGCCCAGAGAAATTTGGATTAATACCACAT
AGTATTATAAGATGGCATCATTTTATAATTAAAGTCACAACGGTAAAAGATGAGCATGCGTCCCATTAGCTA
GATGGTAAGGTAACGGCTTACCATGGCTACGATGGGTAGGGGTCCTGAGAGGGAGATCCCCCACACTGGT
ACTGAGACACGGAC

>Zotu1635

GAACGCTGGCGGCGTGCCTAACACATGCAAGTCGAGCGGAGATTGCAGCAATGTAATCTTAGCGGCGGAC
TGGTGAGTAACGCGTGGGCAACCTGCCTCACACAGGGGGATAACACAGGGAAACTTGTGCTAATACCGCA
TAACATCATGGAGGGACATCCCTTTATGATCAAAGGAGCAATCCGGTGTGAGATGGGCCCGCGTCCGATTA
GTTAGTTGGCGGTGTAACGGACCACCAAGACGACGATCGGTAGCCGAACTGAGAGGTTGATCGGCCACAT
TGGAACTGAGATACGGTC

>Zotu1637

GAACGCTGGCGGCGTGCCTAATACATGCAAGTCGAGCGAATCTCTTCGGAGATTAGCGGCGGACGGGTGA
GTAACACGTGGGCAACCTGCCTATAAGACTGGGATAACTTCGGGAAACCGGAGCTAATACCGGATAACAT
CAAAGGCCACATGGTCTTCGATTAAAAGATGGCTTCGGCTATCACTTATAGATGGGCCCGCGTCGCATTAG
CTAGTTGGTGAGGTAACGGCTCACCAAGGCGACGATGCGTAGCCGACCTGAGAGGGTGATCGGCCACACT
GGGACTGAGACACGGCC

>Zotu1639

GAACGCTGGCGGCGTGCCTAATACATGCAAGTAGAACGCTGAAGCTTGGTGATTGCACCGAGCGGATGAG
TTGCGAACGGGTGAGTAACGCGTAGGTAACCTGCCTCTTAGCGGGGGATAACTATTGGAAACGATAGCTA
ATACCGCATAAAAGTCGACATTGCATGATGTTGACTTGAAAGGTGCAATTGCATCACTAAGAGATGGACCT
GCGTTGTATTAGCTAGTTGGTGAGGTAACGGCTCACCAAGGCAACGATACATAGCCGACCTGAGAGGGTG
ATCGGCCACACTGGGACTGAGACACGGCC

>Zotu1642

GAACGCTGGCGGCGTGCCTTAGGCATGCAAGTCGAATGGGGCCGCAAGGCCCAATGGCAGACGGGTGAG
TAATGGTAAGGAATCTACCTTAAAGTGGAGGATAGCTCGTCGAAAGACGGGGTAATCCTGCATGTGGTCG
CAAGACTAAAGGGCTTAACTGCCCGCTTTAAGAGGAGCCTTACTCCTATCAGCTAGTTGGTGGGGTAATGG
CCTACCAAGGCTATGACGGGTAGCTGGTGTGAGAGCATGACCAGCCACAATGGCACTGAGACACGGGC

>Zotu1644

GAACGCTGGCGGCGTGCTTAACACATGCAAGTCGAGCGGAAAGGCCCTTTCGGGGGTACTCGAGCGGCG
AACGGGTGAGTAACACGTGAGTAATCTGCCCTTCACTTGGGGATAACCTCCGGAAACGGTGGCTAATACCC
GATACGACCATGGATCGCATGATCTTGTGGTGGAAAGTTTTTCGGTGGGGGATGTGCTCGCGGCCTATCAG
CTTGTTGGTGGGGTAATGGCCTACCAAGGCTTCGACGGGTAGCCGGCCTGAGAGGGTGACCGGCCACACT
GGGACTGAGACACGGCC

>Zotu1646

GAACGCTGGCAGTGTGCCTTAGACATGCAAGTCGGACGGGGCGCTTCTCTTTGATCCTTTCGGGGTGATTT
GGGTTGTTGCCTAGTGGCGTACGGGTGAGTAACACGTGGGTGACCTGCCCTCAAGTGGGGGATAACTCTG
GGAAACTGGGGCTAATCCCGCATGTGCTGATCTCTTTTTGGGGGGTTAGTAAAGGAACTTGTTCCGCTTGA
GGATGGGCCCGCGGCCTATCAGCTTGTTGGTGTGGTAATGGCGCACCAAGGCTATGACAGGTAGCCGGCC
TGAGAGGGTGTTCGGCCACAGGGGCACTGAGATACGGGC

>Zotu1648

GAACGCTGGCGGCGTGCTTAACACATGCAAGTCGAACGGGAATTACTTTATTGAAACTTCGGTCGATATGA
TTTAATTCTAGTGGCGGACGGGTGAGTAACGCGTGGGTAACCTGCCTTATACAGGGGGATAACAGTCAGA
AATGACTGCTAATACCGCATAAGCGCACAGAGCTGCATGGCTCGGTGTGAAAAACTCCGGTGGTATAAGA
TGGACCCGCGTTGGATTAGCTGGTTGGTGAGGTAACGGCCCACCAAGGCGACGATCCATAGCCGGCCTGA
GAGGGTGAACGGCCACATTGGGACTGAGACACGGCC

>Zotu1650

GAACGCTGGCGGCAGGCTTAACACATGCAAGTCGAACGCTGTAGCAATACAGAGTGGCAGACGGGTGAG
TAACGCGTGGGAACGTGCCTTTCGGTTCGGGATAACTCAGGGAAACTTGAGCTAATACCGGATACGCCCTT
AGGGGGAAAGATTTATTGCCGAAAGATCGGCCCGCGTCCGATTAGCTAGTTGGTGAGGTAATGGCTCACC
AAGGCGACGATCGGTAGCTGGTCTGAGAGGATGATCAGCCACACTGGGACTGAGACACGGCC

>Zotu1652

GAACGCTGGCGGCGTGCCTAATACATGCAAGTCGAACGCATTTTCTTTCACCGTAGCTTGCTACACCGAAA
GAAAATGAGTGGCGAACGGGTGAGTAACACGTGGGTAACCTGCCCATCAGCGGGGGATAACACTTGGAA
ACAGGTGCTAATACCGCATAATTCCATTTACTGCATGGTAGATGGATGAAAGGCGCTTTTGCGTCACTGAT
GGATGGACCCGCGGTGCATTAGCTAGTTGGTGGGGTAACGGCCTACCAAGGCTGCGATGCATAGCCGACC
TGAGAGGGTGATCGGCCACACTGGGACTGAGACACGGCC

>Zotu1654

GAACGCTGGCGGCGTGCCTAATACATGCAAGTCGAACGCATTGTGGTTGAAATGATATGAAGAACTTGTTC
AGATTTGATTTTCAACATTGCAATGAGTGGCGAACGGGTGAGTAACACGTGGGAAACCTACCTCTTAGCAG
GGGATAACATTTGGAAACAAATGCTAATACCGTATAATAATTAAAACCGCATGGTTTTAGTTTAAAAGATG
GTCTTGCTATCACTAAGAGATGGTCCCGCGGTGTATTAGTTAGTTGGTGAGGTAATGGCTCACCAAGACAA
TGATACATAGCCGAGTTGAGAGACTGACCGGCCACAATGGGACTGAGACACGGCC

>Zotu1656

GAACGCTGGCGGCGTGCCTAATACATGCAAGTCGAGCGAATCTGAGGGAGCTTGCTCCCAAAGATTAGCG
GCGGACGGGTGAGTAACACGTGGGCAACCTGCCTGTAAGACCGGGATAACTTCGGGAAACCGGAGCTAA
TACCGGATAATTCTTTCCCTCACATGAGGGAAAGCTGAAAGACGGTTTCGGCTGTCACTTACAGATGGACC
CGCGGCGCATTAGCTAGTTGGTGAGGTAACGGCTCACCAAGGCGACGATGCGTAGCCGACCTGAGAGGGT
GATCGGCCACATTGGGACTGAGACACGGCC

>Zotu1658

GAACGCTAGCGGCAGGCTTAATACATGCAAGTCTAGGGGGCAGCAATGTCACCGGCGAACGGGTGCGTA
ACACGTATGCAACCTACCCATATCAGGGGGATAGCCCGGGGAAACTCGGATTAATACCGCATAAGATGGT
ACTACGGCATCGTAGAGCTATTAAAGATTTATCGGATATGGATGGGCATGCGTTAGATTAGCTAGTTGGGG
AGGTAACGGCTCACCAAGGCTACGATCTATAGGGGCTCTGAGAGGAGTAACCCCCACACTGGCACTGAGA
TACGGGC

>Zotu1660

GAACGCTAGCGGCAGGCCTAATACATGCAAGTCGGACGGGATTGAGATTAAAGCTTGCTTTGATTTCATGA
GAGTGGCGCACGGGTGCGTAACGCGTGAGCAACCTACCCATATCAGGGGGATAGCCTCTCGAAAGAGAG
ATTAACACCGCATAACATCATTAACCGGCATCGGATTATGATCAAATATTTATAGGATATGGATGGGCTCGC

GTGACATTAGCTAGTTGGAGAGGTAACGGCTCACCAAGGCTACGATGTCTAGGGGCTCTGAGAGGAGAAT
CCCCCACACTGGTACTGAGACACGGAC
>Zotu1662
GAACGCTGGCGGCATGCCTAACACATGCAAGTCGAACGAGACCTTCGGGTCTAGTGGCGCACGGGTGCGT
AACGCGTGGGAATCTGCCCTTGGGTTCGGAATAACAGTTGGAAACGACTGCTAATACCGGATGATGACGT
AAGTCCAAAGATTTATCGCCCAGGGATGAGCCCGCGTAGGATTAGCTAGTTGGTGGGGTAAAGGCCTACC
AAGGCGACGATCCTTAGCTGGTCTGAGAGGATGATCAGCCACACTGGGACTGAGACACGGCC
>Zotu1664
GAACGCTGGCGGCGTGCTTAACACATGCAAGTCGAACGGTGAAGCCCAGCTTGCTGGGTGGATCAGTGGC
GAACGGGTGAGTAACACGTGAGCAACCTGCCCCTGACTCTGGGATAAGCGCTGGAAACGGCGTCTAATAC
TGGATATGTGACGTGACCGCATGGTCTGCGTTTGGAAAGATTTTTCGGTTGGGGATGGGCTCGCGGCCTAT
CAGCTTGTTGGTGAGGTAATGGCTCACCAAGGCGTCGACGGGTAGCCGGCCTGAGAGGGTGACCGGCCAC
ACTGGGACTGAGACACGGCC
>Zotu1666
GAACGCTAGCGGGAGGCCTAACACATGCAAGCCGAGCGGTATTTGTTCTTCGGAACAGAGAGAGCGGCGC
ACGGGTGCGGAACACGTGTGCAACCTGCCTTTATCTGGGGGATAGCCTTTCGAAAGGAAGATTAATACCCC
ATAATATATTGAGTGGCATCATTTGATATTGAAAACTCCGGTGGATAGAGATGGGCACGCGCAAGATTAGA
TAGTTGGTGAGGTAACGGCTCACCAAGTCAATGATCTTTAGGGGTCCTGAGAGGGAGATCCCCCACACTG
GTACTGAGACACGGAC
>Zotu1669
GAACGCTGGCGGCGTGCTTAACACATGCAAGTCGAACGAAGAGCGATGGAAGCTTGCTTCTATCAATCTTA
GTGGCGAACGGGTGAGTAACGCGTAATCAACCTGCCCTTCAGAGGGGGACAACAGTTGGAAACGACTGCT
AATACCGCATACGATCTAACCTCGGCATCGAGGAAAGATGAAAGGTGGCCTCTATGTATAAGCTATCACTG
AAGGAGGGGATTGCGTCTGATTAGCTAGTTGGAGGGGTAACGGCCCACCAAGGCGATGATCAGTAGCCG
GTCTGAGAGGATGAACGGCCACATTGGGACTGAGACACGGCC
>Zotu1671
GAACGCTGGCGGTCTGCTTAACACATGCAAGTCGAGCGGAGGTTTTCGGACCTCAGCGGCGGACGGGTGA
GTAACGCGTGAGAACCTGCCCTCAGGTTGGGGACAACAGTTGGAAACGACTGCTAATACCCAATGTGCTTT
CGAGTGAAAGATTTATCGCCTGAGGATGGGCTCGCGTCCGATTAGGTAGTTGGAGTGGTAAGGGCACCCC
AAGCCATTGATCGGTAGCTGGTCTGAGAGGATGACCAGCCACACTGGGACTGAGACACGGCC
>Zotu1673
GAACGCTGGCGGCGTGCTTAACACATGCAAGTCGAACGATGATCCCCAGCTTGCTGGGGGGATTAGTGGC
GAACGGGTGAGTAACACGTGAGTAACCTGCCCCTGACTCTGGGATAAGCGCTGGAAACGGCGTCTAATAC
CGGATATGACAGCCGACGGCATCGTCTGGTTGTGGAAAGATTTTTCGGTTGGGGATGGACTCGCGGCCTAT
CAGGTTGTTGGTGAGGTAATGGCTCACCAAGCCTACGACGGGTAGCCGGCCTGAGAGGGTGACCGGCCAC
ACTGGGACTGAGACACGGCC
>Zotu1675
GAACGCTGGCGGCGTGCTTAACACATGCAAGTCGAACGGACCCTTCGGGGTTAGTGGCGGACGGGTGAGT
AACGCGTGAGGATCTGCCCTTAGGAGGGGAACAACAGTTGGAAACGACTGCTAATGCCCCATATGCCGAG
AGGTGAAAAGTTATATCGCCTGAGGATGAACTCGCGTCTGATTAGCTAGTTGGTAGTGTAATGGACTACCA
AGGCGACGATCAGTAGCTGGTCTAAGAGGATGATCAGCCACACTGGGACTGAGACACGGCC
>Zotu1678
GAACGCTGGCGGCGTGCCTAACACATGCAAGTCGAGCGAGAGACTTCGGTCTCTAGCGGCGGACGGGTG
AGTAACACGTGGGTAACCTGCCTCAAACAGGGGGATAGCCTTCCGAAAGGAAGATTAATACCGCATAACA
TAGTTTAGTCGCATGACTAGATTATCAAAGGAGTAATCCGGTTTGAGATGGACCCGCGGCGCATTAGCTAG
TTGGTGAGGTAACGGCTCACCAAGGCGACGATGCGTAGCCGACCTGAGAGGGTGATCGGCCACATTGGAA
CTGAGATACGGTC
>Zotu1681
GAACGCTGGCGGCGTGCTTAACACATGCAAGTCGAACGGTAAGGCGCTTTCGGGCGTACACGAGTGGCGA
ACGGGTGAGTAACACGTGAGGAACGTGCCCTTGACTTTGGTATAGCACTTGGAAACAGGTGGTAATCCCG

AATATTCAGTTTCCCGGGCATCCGGGTTGTTGGAAAGCTTTTTGCGGTCAGGGATCGTCTCGCGGCCTATCA
GCTTGTTGGTGGGGTAGTGGCCCACCAAGGCCTTGACGGGTAGCCGGCCTGAGAGGGCGACCGGCCACAT
TGGGACTGAGATACGGCC
>Zotu1683
GAACGCTGGCGGCGTGCCTAACACATGCAAGTCGAGCGAACCCTTCGGGGTGAGCGGCGGACGGGTGAG
TAACGCGTGGGTAACCTGCCCTATACACACGGATAACGTACCGAAAGGTACGCTAATACGAGATAACATAT
TTTTATCGCATGGTAGAAATATCAAAGCTCCGGCGGTATAGGATGGACCCGCGTCTGATTAGCTAGTTGGT
AAGGTAATGGCTTACCAAGGCGACGATCAGTAGCCGACCTGAGAGGGTGATCGGCCACATTGGAACTGAG
ACACGGTC
>Zotu1685
GAACGCTGGCGGCGTGCCTTAAGCATGCAAGTCGTACGAAGATATGAGATGTCGCAAGACAGATTGTTGA
TTAGTGGCGGACGGGTGAGTATAAGCAGGGAACGTACCTTGAACTATCGGATAGCTCGTCGAAAGACGGG
GTAATACGGTATGTGGACGCAAGTCTAAAGACCGCAAGGTCGGTTCTAGAGCGGCCCTGTTCCCATCAGCT
TGTTGGTAGGGTAACGGCCTACCAAGGCTACGACGGGTAGGGGGTGTGAGAGCACGACCCCCCACAATG
GCACTGAGACACGGGC
>Zotu1687
GAACGCTGGTGGCGTGCTTAACACATGCAAGTCGAGCGAGGCGTCCGCCTCCGGGCGGTCAGCCGAGCG
GCGAACGGGTGAGTAACACGTGAGCAATCTTCCCTAGGCTCGGGGATAACCTCGGGAAACTGAGGCTAAT
ACCGGATGAGCGTTTTTCGGGGCATCCCGGAAAACGAAAAGGTTTCGCACCGCGAGGTGCGTTTCCGGCC
TTGGATGAGCTCGCGGCCTATCAGCTTGTTGGTGTGGTAAATGCGCACCAAGGCGACGACGGGTAGCCGG
CCTGAGAGGGTGATCGGCCACACTGGGACTGAGACACGGCC
>Zotu1689
AAACGCTGGCGGCGTGCCTAAAACATGCAAGTCGAACGGGCACCTTCGGGTGTCAGTGGCAGACGGCTGA
GTAACGCGTGGGTGACGTACCTATGAGTGGGGAATAACTGCCCGAAAGGGTGGCTAATTCCGCATACGCC
TGATCTTTCGGTAGGTCAGGGAAAGTCGCAAGACGCTCGTGGAGCGGCCCGCGTTCGATTAGCTCGTTGGT
GGGGTAATGGCCTACCAAGGCGACGATCGATAGCTGGTCTCAGAGGATGATCAGCCAGACTGGGACTGA
GACACGGCC
>Zotu1691
GAACGCTGGCGGCGTGCCTTAAGCATGCAAGTCGTACGGCTCCGCAAGGAGCAGTGGCGGACGGTCGAG
TAACGCGTGGGGATCTACCTTGAAGTTGGACATAGCTCACCGAAAGGTGGGGTAATTTCCAATGTGAACGT
AAGTTCAAAGCCGTAAGGCGCTTTAAGATGAACCCGCGTCCTATCAGCTAGTTGGTGAGGTAATGGCTCAC
CAAGGCATCGACGGGTAGCTGGTCTGAGAGGATGATCAGCCGGAATAGCACTGAGACACGGGC
>Zotu1693
GAACGCTGGCGGCAGGCTTAACACATGCAAGTCGAACGGGCTTCTTCGGAAGTCAGTGGCAGACGGGTGA
GTAACACGTGGGAACGTGCCCTTCGGTTCGGAATAACTCAGGGAAACTTGAGCTAATACCGGATACGCCCT
TTTGGGGAAAGGTTGACTGCCGAAGGATCGGCCCGCGTCTGATTAGCTTGTTGGTGGGGTAACGGCCTAC
CAAGGCGACGATCAGTAGCTGGTCTGAGAGGATGATCAGCCACACTGGGACTGAGACACGGCC
>Zotu1695
GAACGCTGGCGGCGTGCTTTACACATGCAAGTCGAACGGCAGCATAGAATAGTAGCAATACTATTGGGTG
GCGAGTGGCGGACGGGTGAGTAATACATCGAGACGTGCCTAATAGATTGGGATAACCTGTCGAAAGATAG
GCTAATACCGGATAATCTCTGAGGAGGAAAGCTGGGGACTTAGGAATAAGCCTGGCGCTAATAGAGCGAT
CGATGGCCGATTAGTTAGTTGGTGGGGTAAAGGCTTACCAAGGCGATGATCGGTAGCTGTTCTGAGAGGA
AGATCAGCCACACTGGAACTGAGAGACGGTC
>Zotu1697
GAACGCTGACAGAATGCTTAACACATGCAAGTCAACTTGAATTTGGGTTTTTAACTTAGATTTGGGTGGCG
GACGGGTGAGTAACGCGTAAAGAACTTGCCTCACAGCTAGGGACAACATTTAGAAATGAATGCTAATACCT
GATATTATGATTTTAAGGCATCTTAGAATTATGAAAGCTATATGCACTGTGAGAGAGCTTTGCGTCCCATTA
GCTAGTTGGAGAGGTAACAGCTCACCAAGGCGATGATGGGTAGCCGGCCTGAGAGGGTGAACGGCCACA
AGGGGACTGAGACACGGCC
>Zotu1699

GAACGCTGGCGGCGTGCTTAAGACATGCAAGTCGAACGATCGTCTTCGGACGGTAGTGGCGCACGGGTGA
GTAACGCGTAACTGACCTGCCCCCAAGTTCGGAATAACTTCCCGAAAGGGAAGCTAATACCGGATGTGCTG
TCCCCTCCTGTGGGGCCAGTAAAGGCTTGACCGCTTGGGGATGGGGTTGCGTTCCATCAGCTAGATGGTG
GGGTAAAGGCCTACCATGGCGACGACGGATAACCGGCCTGAGAGGGTGGCCGGTCACAGGGGCACTGAG
ACACGGGT
>Zotu1701
GAACGCTGGCGGCATGCCTTACACATGCAAGTCGAACGGTAACGCGGGGCAACCTGGCGACGAGTGGCG
AACGGGTGAGTAATGCATCGGAACGTGCCCAGTAGTGGGGGATAGCCCGGCGAAAGCCGGATTAATACC
GCATACGACCTACGGGTGAAAGCGGGGGACCGCAAGGCCTCGCGCTATTGGAGCGGCCGATGTCAGATTA
GGTAGTTGGTGGGGTAAAGGCCTACCAAGCCGACGATCTGTAGCTGGTCTGAGAGGACGACCAGCCACAC
TGGGACTGAGACACGGCC
>Zotu1704
GAACGCTGGCGGCGTGCTTAACACATGCAAGTGGAGCGACGAACCAGGGCTTGCCCTGGGGCAGAGCCG
CGAACGGGTGAGTAACACGTGGGTTACCTGCCTCGATGACCGGGACAACCCGAGGAAACTCGGGCTAATA
CCGGATGTGCCCGCAAGGGGAAAGGAAGCTTCGGCCTCCGCATCGAGATGGGCCCGCGGCCCATTAGCTA
GTTGGTGAGGTAACGGCTCACCAAGGCTCCGATGGGTAGCTGGTCTGAGAGGACGATCAGCCACACTGGG
ACTGAGACACGGCC
>Zotu1707
GAACGCTGGCGGCGTGCCTAATACATGCAAGTCGAACGCTTCTTTCCTCCCGAGTGCTTGCACTCAATTGGA
AAGAGGAGTGGCGGACGGGTGAGTAACACGTGGGTAACCTACCCATCAGAGGGGGATAACACTTGGAAA
CAGGTGCTAATACCGCATAACAGTTTATGCCGCATGGCATAAGAGTGAAAGGCGCTTTCGGGTGTCGCTGA
TGGATGGACCCGCGGTGCATTAGCTAGTTGGTGAGGTAACGGCTCACCAAGGCCACGATGCATAGCCGAC
CTGAGAGGGTGATCGGCCACACTGGGACTGAGACACGGCC
>Zotu1709
GAACGCTGGCGGCAGGCTTAACACATGCAAGTCGAACGCTCGTCTTCGGACGGGAGTGGCAGACGGGTG
AGTAACACGTGGGAACGTACCCTTTGGTTCGGAATAACGCAGGGAAACTTGCGCTAATACCGGATACGCCC
TTTTGGGGAAAGGTTTACTGCCGAAGGATCGGCCCGCGTCTGATTAGCTAGTTGGTGGGGTAACGGCCTAC
CAAGGCGACGATCAGTAGCTGGTCTGAGAGGATGATCAGCCACACTGGGACTGAGACACGGCC
>Zotu1711
GAACGCTGGCGGCGTGCCTAATACATGCAAGTCGAGCGGACTGAATGGAAGCTTGCTTCCGTTCGGTTAG
CGGCGGACGGGTGAGTAACACGTGGGCAACCTGCCCGTAAGACCGGGATAACTTCGGGAAACCGGAGCT
AATACCGGATAACACCGAAGATCGCATGATCTTTGGTTGAAAGGCGGCTTTGGCTGTCACTTACGGATGGG
CCCGCGGCGCATTAGCTAGTTGGTGAGGTAACGGCTCACCAAGGCGACGATGCGTAGCCGACCTGAGAGG
GTGATCGGCCACACTGGGACTGAGACACGGCC
>Zotu1713
GAACGCTAGCTACAGGCTTAACACATGCAAGTCGAGGGGAAACGGCATTAAGTGCTTGCACTTTTTGGACG
TCGACCGGCGCACGGGTGAGTAACGCGTATCCAACCTTCCCATGACTAAGGGATAACCCGCCGAAAGGCA
GACTAATACCTTATGGCCTTCACTGACGGCATCAGATGTGAAGTAAAGATTTATCGGTTATGGATGGGGAT
GCGTCTGATTAGCTTGTTGGCGGGGTAACGGCCCACCAAGGCAACGATCAGTAGGGGTTCTGAGAGGAAG
GTCCCCCACATTGGAACTGAGACACGGTC
>Zotu1715
GAACGCTAGCGGCAGGCCTAACACATGCAAGTCGAGGGGCAGCACGAATTAGCTTGCTAATTTGGTGGCG
ACCGGCGCACGGGTGCGTAACGCGTATGCAACCTACCTTATACAGGGGTATAGCCCGGAGAAATTCGGAT
TAATCCCCCATAGTACTGAGACACCGCATGGTGATTCAGTTAAAGCTTCGGCGGTATAAGATGGGCATGCG
TCCTATTAGCTAGATGGTAAGGTAACGGCTTACCATGGCTACGATAGGTAGGGGGCCTGAGAGGGTGGTC
CCCCACACTGGTACTGAGACACGGAC
>Zotu1717
GAATGTTGGCGGTGTGCCTGAGATATGCAAGTCGAATGCCTTGTGCTTTGCACAAGAAATTCCAATAACCA
AATTACAAGAAACAAACAATTACCAATATCTAAATTTTCAATGTTCAAAACATTGGAGATTTGAATTTGTTTG
TAATATTTGGAATTTTAAAGTTGGAGCTTCTCGAGCAGAGCACGAGTAATGGCGGACGGCTGAGTAACACA
TAGGGACTTACCCTTTTTTGAAGAATAGCCCATCGAAAGGTGGGATAATACTTCATGGTATGAATGTCGTA

AGATATTTTTTAAAGAGATTAACTTCTCGAAAAAGGATGGACCTGTGGCTTATCAGATAGTTGGTGGGGTA
ACGGCCTACCAAGTCTACGACGAGTAGCTGGCCTGAGAAGGTGGTCAGCCAGAGGGGGACTGAGACACG
GCC
>Zotu1719
TAACGCTAGCGGCAGGCCTAATACATGCAAGTCGAACGGGATTGATTGTAGCAATACGGTCATGAGAGTG
GCGCACGGGTGCGTAACACGTATGCAACCTACCTATAACTGGGGGATAGCCTCCCGAAAGGGAGATTAAC
ACCGCATAACATTATGAAGTGGCATCATTTTATAATTAAATCTACGGAGGTTATAGATGGGCATGCGTCCG
ATTAGCTAGTTGGTGAGGTAACGGCTCACCAAGGCTCCGATCGGTAGGGGGTCTGAGAGGATGATCCCCC
ACACTGGTACTGAGACACGGAC
>Zotu1722
GAACGCTGGCGGCAGGCTTAACACATGCAAGTCGAACGGGTACCTTCGGGTGCTAGTGGCAGACGGGTG
AGTAACACGTGGGAACGTACCTTTTGGTTCGGAATAATACAGGGAAACTTGTACTAATACCGGATACGCCC
TTCGGGGGAAAGATTTATCGCCGAAAGATCGGCCCGCGTCTGATTAGCTAGTTGGTGAGGTAATGGCTCAC
CAAGGCGACGATCAGTAGCTGGTCTGAGAGGATGATCAGCCACACTGGGACTGAGACACGGCC
>Zotu1725
GAACGCTGGCGGCAGGCTTAACACATGCAAGTCGAGCGGGCGAGGTTGCTTCGGTAACTGAGCTAGCGGC
GGACGGGTGAGTAAAGCTTAGGAATCTGCCTATTAGTGGGGGACAACATTTCGAAAGGGATGCTAATACC
GCATACGTCCTACGGGAGAAAGCAGGGGATCACTTGTGACCTTGCGCTAATAGATGAGCCTAAGTCGGAT
TAGCTAGTTGGTGGGGTAAAGGCCTACCAAGGCGACGATCTGTAGCGGGTCTGAGAGGATGATCCGCCAC
ACTGGGACTGAGACACGGCC
>Zotu1727
GAACGCTAGCGGCAGGCCTAATACATGCAAGTCGAGGGGTATGGGCAGCAATGCCCAGAGACCGGCAAA
CGGGTGCGGAACACGTACAGAACCTTCCCTCCAGCGGGGCATAGCCCTTGGAAACGAGGATTAATACCCC
ATGACATAACCGGGTGGCATCATCTGGTTATTAAAGGTTCACCACTGGAGGATGGCTGTGCGGCCGATTAG
GTAGTTGGCGGGGTAAAGGCCCACCAAGCCTTCGATCGGTAACTGGTGTGAGAGCACGACCAGTCACACG
GGCACTGAGACACGGGC
>Zotu1729
GAACGCTGGCGGTGTGCTTCATACATGCAAGTCGAACGAGGTAGCAATACCTAGTGGCGGACGGGTGAGT
AACGCGTGGGAATCTGCCTTAAACAGGGGGATAGCTGGCCGAAAGGCTAATTAATACCGCATATGCACAC
ATCCCGACAGGGATATGTGTGAAAGGAGTAATCCAGTTTAAGATGAGCCCGCGTGCGATTAGCTAGTTGG
TGGGGTAAAGGCCCACCAAGGCGACGATCGCTAGGTGGTCTGAGAGGATGATCACCCACAATGGAACTGA
GACACGGTC
>Zotu1731
GAACGCTAGCGGCAGGCTTAATACATGCAAGTCGAGGGGCAGCAGGATAGCAATATTGCTGGCGACCGG
CGCACGGGTGCGTAACACGTATACAATCTACCTTTAACAGGGGGATAGCCCGAAGAAATTCGGATTAATAC
CCCATGATAGTTTTCTTGGCATCAAGAGGATTTGAAAGCTCCGGCGGTTAGAGATGAGTATGCGGTCTATT
AGCTAGTAGGTGAGGTAACGGCTCACCTAGGCTACGATAGATAGGGGATCTGAGAGGACTACCCCCCACA
CTGGTACTGAGACACGGAC
>Zotu1733
AAACGCTGGCGGCGTGCCTAACACATGCAAGTCGGACGCCCTATGCTTCGGTGTAGGGAGTGGCGGACGG
GTGAGTAACACGTGGGATATCTACCCAGAGGTGAGGAATACCGGCGAGAAATCGCCGACAATACCGCATA
CGCTCCGAGAGGAGCAAAGCTCGCAAGAGCGCCATTGGATGATCCTGCGGTCGATTAGCTAGTTGGTGAG
GTAATGGCTCACCAAGGCGACGATCGATAGCTGGTCTGAGAGGACGATCAGCCACACTGGGATTGAGAAC
GGCC
>Zotu1735
GAACGCTGGCGGCGTGCCTAACACATGCAAGTCGAACGAGGTCCATGCAGTGGCAACACTGCGGAAGACC
TAGTGGCGAACGGGTGAGTAACACGTGAGGAACTTGCCCCGGAGACCGGGATAACCTTCCGAAAGGATG
GCTAATACCGGATATCGTCACCGAGCCGCATGGCTTGGTGACGAAATGGATTCCGCTCCGGGAGAGCCTC
GCGGCCTATCAGCTAGTTGGTGGGGTAACGGCCCACCAAGGCATCGACGGGTAGCTGGTCTGAGAGGAC
GATCAGCCACACTGGGACTGAGACACGGCC

>Zotu1737
TAACGCTAGCGGCAGGCCTAATACATGCAAGTCGAACGGCAGCGGGTGTAGCAATACATGCCGGCGAGTG
GCGCACGGGTGCGTAACGCGTATGCAATCTACTTGTAACTGGGGAATAGCCTTCCGAAAGGAAGATTAAT
ACCGCATAATATCATGAAGTGGCATCATTTTATGATCAAAACTCCGGTGGTTACAGATGAGCATGCGTGAC
ATTAGCTAGTTGGTGAGGTAACGGCTCACCAAGGCTACGATGTCTAGGGGGTCTGAGAGGATGATCCCCC
ACACTGGTACTGAGACACGGAC
>Zotu1739
GAACGCTGGCGGCGTGCCTAACACATGCAAGTCGAGCGAGATACTGTTGAACAGAGATTCGTCAACGGGA
TACAGTGGAAAGCGGCGGACGGGTGAGTAACGCGTAGGCAACCTGCCCCTTACACAGGGATAGCCATTGG
AAACGATGATTAAAACCTGATGACACCTGAGGATGACATCTTCCTGAGGTCAAAGATTTATCGGTAAGGGA
TGGGCCTGCGTCTGATTAGATAGTTGGTGAGGTAACGGCCCACCAAGTCAACGATCAGTAGCCGACCTGA
GAGGGTGATCGGCCACATTGGAACTGAGACACGGTC
>Zotu1742
CAACGCTGGCGGCGTGCCTAACACATGCAAGTCGAACGAGAAAGTGGAGCAATCCATGAGTAAAGTGGCG
CACGGGTGAGTAACACGTGACTAACCTACCCTTGAGTGGGGGATAACCTCGGGAAACCGGGGCTAATACC
GCATAATACCGAGAGGTCAAAGGAGCAATTCGCTTAAGGAGGGGGTCGCGGCTGATTAGCTAGTTGGCAG
GGTAATGGCCTACCAAGGCAGTGATCAGTATCCGGCCTGAGAGGGCGCACGGACACACTGGAACTGAAAC
ACGGTC
>Zotu1745
GAACGCTGGCGGCGTGCTTAACACATGCAAGTCGAACGCTGAAGCCGCCTTCGGGTGGTGGATGAGTGGC
GAACGGGTGAGTAACACGTGGGTAATCTGCCCTGTACTCTGGGATAAGCCTGGGAAACTGGGTCTAATAC
TGGATAGGACACATCGCTGCATGGTGGTGTGTGGAAAGCTTCGGCGGTACGGGATGAGCCCGCGGCCTAT
CAGCTTGTTGGTGGGGTGAGGGCCTACCAAGGCGACGACGGGTAGCCGGCCTGAGAGGGTGACCGGCCA
CACTGGGACTGAGACACGGCC
>Zotu1748
GAACGCTGGCGGCGTGCCTAATACATGCAAGTCGAGCGGATTGAAGGGAGCTTGCTCCCTGATATTAGCG
GCGGACGGGTGAGTAACACGTGGGCAACCTGCCCTGCAGATGGGGATAACTCCGGGAAACCGGGGCTAA
TACCGAATAATCGGTTCTTCCGCATGGAAGAACTCTGAAAGACGGTTTCGGCTGTCACTGCAGGATGGGCC
CGCGGCGCATTAGCTAGTTGGTGGGGTAACGGCCTACCAAGGCGACGATGCGTAGCCGACCTGAGAGGG
TGATCGGCCACACTGGGACTGAGACACGGCC
>Zotu1751
GAACGCTAGCGGCAGGCTTAATACATGCAAGTCGTGGGGCAGCAGGTTTGTAGCAATACAAATGCTGGCG
ACCGGCAAACGGGTGCGGAACACGTACAGAACCTTCCTTTAAGTGGGGAATAGCCCAGGGAAACTTGGAT
TAATACCCCGTAATATAATGAAGTGGCATCACTTTATTATTATAGCAGCAATGCGCTTGAAGATGGCTGTGC
GGCTGATTAGGTAGTTGGCGGGGTAATGGCCCACCAAGCCTACGATCAGTAACTGGTGTGAGAGCACGAC
CAGTCACACGGGCACTGAGACACGGGC
>Zotu1755
GAACGCTGGTAACGTGGATACCGGCATGCAAGTCACGGGGGTCCCGTAAGGGACAACCGGCGAACGGCT
TAGTAACACGTAGATACATACCCCAAAGTGGAGAACAGCTCGTCGAAAGACGGGGTAATACTCCATAGTCT
CGCAAGAGTAAAGGCGCAAGCCGCTTTGGGAATGGTCTGCGGTCTATCAGCTAGTTGGCGAGGTAAAGGC
TCACCAAGGCTATGACGGATAGGGGGCGTGAGAGCGCGACCCCCAAGAATGGCACTGCGACACGGGC
>Zotu1757
GAACGCTGGCGGCATGCCTAACACATGCAAGTCGAACGAGACCTTCGGGTCTAGTGGCGCACGGGTGCGT
AACGCGTGGGAACCTGCCTTTAGGTTCGGAATAACTCTCCGAAAGGGGTGCTAATACCGAATAATGTCTTC
GGACCAAAGATTTATCGCCTTTAGATGGGCCCGCGTTGGATTAGCTAGTTGGTGGGGTAAAGGCCTACCAA
GGCGACGATCCATAGCTGGTCTTAGAGGATGATCAGCCACACTGGGACTGAGACACGGCC
>Zotu1759
GAACGCTGGCGGCGCGCCTAACACATGCAAGTCGAGCGAGGTTCAAAGCTTCGGTTTTGAACCTAGCGGC
GGACGGGTGAGTAACGCGTGAGCAACCTGGCCTTCAGAGGGGGATACCGTCTGGAAACGGACGTTAATA
CCGCATAACATGTCGGGAGGGCATCTTCCTGATATCAAAGGAGAAATTCACTGAAGGATGGGCTCGCGTCC

GATTAGGTAGTTGGTGAGGTAACGGCTCACCAAGCCCGCGATCGGTAGCCGGACTGAGAGGTTGGCCGGC
CACATTGGGACTGAGACACGGCC
>Zotu1761
GAACGCTGGCGGCGTGCCTAATACATGCAAGTCGAGCGAAGAATTTAAGGTGCTTGCACCTAAAATTCTTA
GCGGCGAACGGGTGAGTAACACGTAAAGAACCTGCCTCATAGACTGGGACAACTACTGGAAACGGTAGCT
AATACCGGATAATATATTTTACTGCATGGTAAAATATTGAAAGTCGGTCATGCTGACACTATGAGATGGCTT
TGCGGTGCATTAGCTAGTTGGTAGGGTAAAGGCCTACCAAGGCGACGATGCATAGCCGACCTGAGAGGGT
GATCGGCCACACTGGGACTGAGACACGGCC
>Zotu1763
GAACGCTGGCGGCATGCCTTACACATGCAAGTCGAACGGCAGCACGGACTTCGGTCTGGTGGCGAGTGGC
GAACGGGTGAGTAATACATCGGAACGTGCCTGGTAGTGGGGGATAACTACTCGAAAGAGTGGCTAATACC
GCATGAGATCTACGGATGAAAGCAGGGGATCGCAAGACCTTGTGCTACTAGAGCGGCCGATGGCAGATTA
GGTAGTTGGTGGGATAAAAGCTTACCAAGCCAACGATCTGTAGCTGGTCTGAGAGGACGATCAGCCACAC
TGGGACTGAGACACGGCC
>Zotu1765
GAACGCTGGCGGCGTGCCTAATACATGCAAGTCGAGCGAACAGATAAGGAGCTTGCTCCTTTGACGTTAG
CGGCGGACGGGTGAGTAACACGTGGATAACCTACCTATAAGACTGGGATAACTTCGGGAAACCGGAGCTA
ATACCGGATAACATATTGAACCGCATGGTTCAATAGTGAAAGACGGCCTTGCTGTGACTTATAGATGGATC
CGCGCCGTATTAGCTAGTTGGTAAGGTAACGGCTTACCAAGGCAACGATACGTAGCCGACCTGAGAGGGT
GATCGGCCACACTGGAACTGAGACACGGTC
>Zotu1767
GAACGCTGGCGGCGTGCTTAACACATGCAAGTCGAACGGGCCTGCCTGGCTTTGGTTGGGTGGGTGAGTG
GCGAACGGGTGAGTAACACGTGAGTAACCTGCCCTCTTCTCTTGGATAACTGCTTGAAAGGGCGGCTAATA
CGGGATGTTCTGGCTGCCTCGCATGGGGTGGTTTGGAAAGATTCGGCCTGGTTTTGGTTGTTTTGGTGGGG
GATGGGCTCGCGGCTTATCAGCTTGTTGGTGGGGTGATGGCCTACCAAGGCGGTGACGGGTAGCCGGCCT
GAGAGGGTGGACGGTCACACTGGGACTGAGACACGGCC
>Zotu1769
GAACGCTGGCGGCAGGCTTAACACATGCAAGTCGAACGGGCCCTTCGGGGTCAGTGGCAGACGGGTGAG
TAACACGTGGGAACGTGCCCTTCGGTTCGGAACAACTCAGGGAAACTTGAGCTAATACCGGATACGCCCTT
CGGGGGAAAGATTTATCGCTGAAGGATCGGCCCGCGTCTGATTAGCTAGTTGGTGAGGTAATGGCTCACC
AAGGCGACGATCAGTAGCTGGTCTGAGAGGATGATCAGCCACACTGGGACTGAGACACGGCC
>Zotu1771
GAATGCTGGCGGCGTGGATAAGTCATGCAAGTCAAGGGGGCCCGCAAGGGCAACCGGCAGACGAGGTAG
TAACACGTAGGTAGATCCCCCGAAGTCGGGCATAGCCATCCGAAAGGATGGGTAATTCCCGATAGTCCCGC
AAGGGTAAAGGTTTTTCGCTTCGGGAGTTGCCTGCGTAGTATCAGCTAGTTGGTAGGGTAACGGCCTACCA
AGGCTACGACGCTTAGGGGAGCTGAGAGGCTGACCCCCACCGATGGAACTGCGACACGGTC
>Zotu1773
GAACGCTGGCGGCGTGCTTAACACATGCAAGTCGAGCGGAAAGGCCCACTTGTGGGTACTCGAGCGGCGA
ACGGGTGAGTAACACGTGAGTAATCTGCCCTCCACTCTGGGATAACGTTCAGAAATGGGTGCTAATACCGG
ATGTACACGCGCTGACGCATGTTGGTGTGTGGAAAGGTTTTTCTGGTGGGGGATGTGCTCGCGGCCTATCA
GCTTGTTGGTGGGGTAATGGCCTACCAAGGCTTTGACGGGTAGCCGGCCTGAGAGGGTGACCGGCCACAC
TGGGACTGAGACACGGCC
>Zotu1775
GAACGCTGGCGGCGGGCTTAACACATGCAAGTCGAACGAAGCCTTCGGGCTTAGTGGCGCACGGGTGCGT
AACGCGTAGGCAACCTACCCTCAGGCGGGGAATAACTACCGGAAACGGTAGCTAATACCGCATATTGTCG
GAGAGACACCTGTCTCACTGATGAAAGGCTTCGGCCACCTGAGGATGGGCCTGCGTTGGATTAGGTAGTT
GGTGAGGTAACGGCTCACCAAGCCGACGATCCATAGCTGGTCTGAGAGGATGATCAGCCACACTGGCACT
GAGACACGGGC
>Zotu1777

GAACGCTGGCGGCGTGCTTAACACATGCAAGTCGAACGGTGACCTCGAGAGCTTGCTCTCGAGTGATCAG
TGGCGAACGGGTGAGTAACACGTGAGTAACCTGCCCCAGACTCTGGAATAACAATTGGAAACAGTTGCTA
ATACCGGATACGACACGAACGGGCATCCGTATCGTGTGGAAAGTTTTTCGGTCTGGGATGGACTCGCGGC
CTATCAGCTTGTTGGTGAGGTAATGGCTCACCAAGGCGACGACGGGTAGCCGGCCTGAGAGGGCGACCG
GCCACACTGGGACTGAGACACGGCC
>Zotu1779
GAACGCTGGCGGCGTGCTTAACACATGCAAGTCGAACGAGATCCAACCAGTGGCAACACTGGGGAAGATC
TAGTGGCGAACGGGTGAGTAACACGTGAGAAACCTACCCCGGACTCAGGAATAACAGTGGGAAACCACTG
CTAATACCAGATACCTCCAACGAGCCTCCTGGCTCGATGAAGAAAGGGTTTACCGGTTCGGGACGGTCTCG
CGGCCTATCAGCTTGTTGGTGGGGTAACGGCCCACCAAGGCGTCGACGGGTAGCTGGTCTGAGAGGATGA
TCAGCCACACTGGGACTGAGACACGGCC
>Zotu1781
GAACGCTGGCGGCAGGCTTAACACATGCAAGTCGAGCGCCCCGCAAGGGGAGCGGCAGACGGGTGAGTA
ACGCGTGGGAACGTACCTTTTGGTTCGGAACAACTCCGGGAAACTGGAGCTAATACCGGATAAGCCCTTCG
GGGGAAAGATTTATCGCCGAAAGATCGGCCCGCGTCTGATTAGCTAGTTGGTGAGGTAACGGCTCACCAA
GGCGACGATCAGTAGCTGGTCTGAGAGGATGATCAGCCACACTGGGACTGAGACACGGCC
>Zotu1783
GAACGCTGGCGGCGTGCTTAACACATGCAAGTCAGGGGGTGGGGCAACCCATCACCGGCGAACGGGTGC
GTAACACGTGAGCGACCTGCCTTCGAGTGGGGGATAGCCGGCCCAACGGCCGGGTAACACCGCATACGAC
ACCGCCGGGGCATCCCGGGGGTGTGAAACCACTTGGGCTCGGAGAGGGGCTCGCGGCCTATCAGCTAGTT
GGCGAGGTAACGGCTCACCAAGGCGATGACGGGTAGCTGGTCTGAGAGGATGGCCAGCCACATTGGGAC
TGAGAGACGGCC
>Zotu1785
GAACGCTGGCGGCATGCCTAACACATGCAAGTCGAACGAGACCTTCGGGTCTAGTGGCGCACGGGTGCGT
AACGCGTGGGAATCTGCCCTTTGCTTCGGAATAACAGTTAGAAATGACTGCTAATACCGGATGATGTCTTC
GGACCAAAGATTTATCGGCAAAGGATGAGCCCGCGTAGGATTAGGTAGTTGGTGGGGTAAAGGCCTACCA
AGCCGACGATCCTTAGCTGGTCTGAGAGGATGATCAGCCACACTGGGACTGAGACACGGCC
>Zotu1787
GAACGCTGGCGGCGTGCTTAACACATGCAAGTCGAACGGATCAGGAGAGCTTGCTCTTCTGGTTAGTGGC
GAACGGGTGAGTAACACGTGAGCAATCTGCCCCTGACTCTGGGATAACTGCGGGAAACTGTAGCTAATAC
CGGATATGACGCGCGAAGGCATCTTCTGCGTGTGGAAAGAATTGTTGGTCAGGGATGAGCTCGCGGCCTA
TCAGCTTGTTGGTGAGGTAATGGCTCACCAAGGCGACGACGGGTAGCCGGCCTGAGAGGGTGACCGGCC
ACACTGGGACTGAGACACGGCC
>Zotu1789
GAACGCTGGCGGCGTGCTTAACACATGCAAGTCGAACGCTGAAGCTTGGTGCTTGCACTTAGGTGGGTGA
GTGGCGAACGGGTGAGTAATACGTGAGTAACCTGCCCTTGACTCTGGGATAAGCCTGGGAAACTGGGTCT
AATACTGGATATGACCTCCTGTCGCATGGTGGGGGGTGGAAAGGGTTTGTACTGGTTGTGGATGGGCTCA
CGGCCTATCAGCTTGTTGGTGGGGTAATGGCCTACCAAGGCGACGACGGGTAGCCGGCCTGAGAGGGTG
ACCGGCCACACTGGGACTGAGACACGGCC
>Zotu1791
GAACGCTAGCGGCGTGCTTAACACATGCAAGTCGAACGCTGAAGCCCGCTTCGGTGGGTGGATGAGTGGC
GAACGGGTGAGTAACACGTGGGCAATCTACCCCGCACTCGGGGATAAGCCTTGGAAACGAGGTCTAATAC
CCGATAGGACATGCCATCGCATGGTGGTGTGTGGAAAGTGTGCAGCGGTGTGGGATGAGCCCGTGGCCTA
TCAGCTTGTTGGTGGCGGTGATGGCGCACCAAGGCGACGACGGGTAGCCGGCCTGAGAGGGTGACCGGC
CACACTGGGACTGAGACACGGCC
>Zotu1793
GAACGCTGGCGGCGTGCCTAATACATGCAAGTCGAGCGAACAGATGAGAAGCTTGCTTCTCTGATGTTAGC
GGCGGACGGGTGAGTAACACGTGGGTAACCTACCTATAAGACTGGGATAACTCCGGGAAACCGGGGCTA
ATACCGGATAATATTTTGAACCGCATGGTTCAATAGTGAAAGACGGTTTCGGCTGTCACTTATAGATGGAC
CCGCGCCGTATTAGCTAGTTGGTAAGGTAATGGCTTACCAAGGCAACGATACGTAGCCGACCTGAGAGGG
TGATCGGCCACACTGGAACTGAGACACGGTC

>Zotu1796
GAACGCTGGCGGCGCGCCTAACACATGCAAGTCGAACGGACTTTACGGAAACAGAAGCTTCGGCAGAAGA
AGAAATAAAGTTAGTGGCGAACGGGTGAGTAACACGTGAGGAACCAACCCTTAAGAGGGGGATAACACTT
AGAAATAGGTGCTAATACCGCATAAGACCACACTCCGGCATCGGAGAGGGGTCAAAGGAGCAATCCGCTT
AAGGACGGCCTCGCGTCCGATTAGGTAGTTGGCGGGGCAACGGCCCACCAAGCCGACGATCGGTAGCCG
GACTGAGAGGTTGAACGGCCACATTGGGACTGAGACACGGCC
>Zotu1799
GAACGCTGGCGGCGTGCTTAACACATGCAAGTCGAGCGGAAAGGCCCACTTGTGGGTACTCGAGCGGCGA
ACGGGTGAGTATCACGTGAGTAATCTGCCCCCCTCTTTTGGATAACCCTCGGAAACGGGGGCTAATACGGA
ATATTCAGTGCTGCCTGCATGGGTGGTGTTGGAAAGATTTTTTGGTGGGGGATGAGCTCGCGGCCTATCAG
CTTGATGGTGGGGTAATGGCCTACCATGGCTTTGACGGGTAGCCGGCCTGAGAGGGTGTCCGGCCACACT
GGGACTGAGACACGGCC
>Zotu1801
AAACGCTGGCGGCGTGCTTAACACATGCAAGTCGAGCGAGAAAGGTCTTCGGACTGAGTAAAGCGGCAAA
CGGGTGAGTAACACGTAGGAATCTGCCTTTCAGTGAGGAATACCCTCGAGAAATCGAGGTTAATACTGCAT
ACGTCTAGAAATAGAAAAAGATGGCCTCTTTGCTGTCGCTGAAAGATGAGCCTGCGGACTATTAGCTAGTT
GGTAGGGTAATGGCCTACCAAGGCAATGATGGTTAGCCGACCTGAGAGGGTGTACGGCCACACTGGAACT
TAGACACGGTC
>Zotu1803
GAACGCTGGCGGCGTGCCTAATACATGCAAGTAGAACGCTGAAGGGGGAGCTTGCTTCTCCGGATGAGTT
GCGAACGGGTGAGTAACGCGTAGGTAACCTGCCTGGTAGCGGGGGATAACTATTGGAAACGATAGCTAAT
ACCGCATAAGAGTAGATGTTGCATGACATTTACTTAAAAGGTGCAATTGCATCACTACCAGATGGACCTGC
GTTGTATTAGCTAGTTGGTGGGGTAACGGCTCACCAAGGCGACGATACATAGCCGACCTGAGAGGGTGAT
CGGCCACACTGGGACTGAGACACGGCC
>Zotu1805
GAACGCTAGCGGCAGGCCTAATACATGCAAGTCGAACGGGATCCGTCGGAGAGCTTGCTCGAAGACGGTG
AGAGTGGCGCACGGGTGCGTAACGCGTGAGCAACCTACCTCTATCAGGGGGATAGCCTCTCGAAAGAGAG
ATTAACACCGCATAACATATCTGACCGGCATCGGTTAGCTATTAAATATTTATAAGATAGAGATGGGCTCGC
GTGACATTAGCTAGTTGGTAGGGTAACGGCTTACCAAGGCGACGATGTCTAGGGGCTCTGAGAGGAGAAT
CCCCCACACTGGTACTGAGACACGGAC
>Zotu1807
GAACGCTGGCGGCGTGCCTAATACATGCAAGTCGAGCGGTAAGGCTCTGCACTTAGTATTTGATCGCAAAG
TCCTCGGACTTCTAAGCAGATTGATTCGACCCTAGGCTAGGGAACATGATACGAGCAGAGATCGAATAATG
AGTGTAGAGTACACGAGCGGCGGACGGCTGAGTAACACGTAGGAACATACCCCAAAGTGAGGGATAACT
GCTCGAAAGAGTAGCTAATACCGCATGTGCTCTAAGGAGTAAAGCTTCGGCGCTTTGGGAATGGCCTGCG
CATGATTAGCTTGTTGGTGAGGTAAAAGCTCACCAAGGCGACGATCATTAGCTGGTCTGAGAGGATGATCA
GCCAGACTGGGACTGAGACACGGCC
>Zotu1809
GAACGCTGGCGGCGTGCTTAACACATGCAAGTCGAACGTGAAATGTAGCAATACAGAGTAAAGTGGCGTA
CGGGTGAGTAACACGTGGATAATCTACCAAAGTGCCCGGAATAACCGGCCGAAAGGCTGGCTAATACTGG
ATAGTTCAAGCCATCTGTAAGGGATGGATTGGGAAAGGGAGCGGGTAACCGCCTTCACATTTTGATGAGT
CCGCGTCTGATTAGGTAGTTGGTGAGGTAATGGCTCACCAAGCCTGAGATCAGTAGCTGGTCTGAGAGGA
TGATCAGCCACACTGGAACTGAAACACGGTC
>Zotu1812
GAACGCTGGCGGCGTGCTTAACACATGCAAGTCGAGCGGAAAGGCCCTTCGGGGTACTCGAGCGGCGAA
CGGGTGAGTAACACGTGAGTAACCTGCCTCAGGCTTTGGGATAACCCTCGGAAACGGGGGCTAATACCGG
ATATGACCTGTCACCGCATGGTGGTGGGTGGAAAGTTTTTCGGCCTGGGATGGGCTCGCGGCCTATCAGCT
TGTTGGTGGGGTGATGGCCTACCAAGGCGACGACGGGTAGCCGGCCTGAGAGGGCGACCGGCCACACTG
GGACTGAGACACGGCC
>Zotu1814

GAACGCTGGCGGCGTGCTTAACACATGCAAGTCGAGCGACGCCTCATCTTCGGGTGGGTGACGGAGCGGC
GAACGGGTGAGTAACACGTGGGCAACCTGCCCCTAGCTTCGGGATAAGCCCGGGAAACCGGGTCTAATAC
CGGATATGAGACTGGATCGCATGGTCTGGTTTGGAAAGTTTTTCGGCTAGGGATGGGCCCGCGGCCTATCA
GCTTGTTGGTGGGGTAATGGCCCACCAAGGCGACGACGGGTAGCCGGCCTGAGAGGGCGACCGGCCACA
CTGGGACTGAGACACGGCC

>Zotu1817
GAACGCTGGCGGCGTGCTTAACACATGCAAGTCGAGCGGAAAGGCCTCTTCGGGGGTACTCGAGCGGCG
AACGGGTGAGTAACACGTGAGTAATCTGCCTCTGGCTCTGGGATAGCCACCGGAAACGGTGATTAATACC
GGATATGACAGCTCCTCGCATGGGGTGGTTGTGGAAAGTTTTTTCGGCCAGGGATGTGCTCGCGGCCTATC
AGCTTGTTGGTGGGGTAATGGCCTACCAAGGCTTTGACGGGTAACCGGCCTGAGAGGGTGACCGGTCACA
CTGGGACTGAGACACGGCC

>Zotu1819
GAACGCTGGCGGCGTGCTTAACACATGCAAGTCGAACGATGACCCCGTGCTTGCACGGGTGATTAGTGGC
GAACGGGTGAGTAACACGTGAGTAACCTGCCCCTGACTCTGGGATAAGCCCGGGAAACTGGGTCTAATAC
TGGATATGACCTTTAACCGCATGGTTTTTGGTGGAAAGATTTATCGGTTGGGGATGGACTCGCGGCCTATC
AGCTTGTTGGTGAGGTAATGGCTCACCAAGGCGACGACGGGTAGCCGGCCTGAGAGGGTGACCGGCCAC
ACTGGGACTGAGACACGGCC

>Zotu1821
GAACGCTGGCGGCATGCCTTACACATGCAAGTCGAACGGCAGCACGGGTGCTTGCACCTGGTGGCGAGTG
GCGAACGGGTGAGTAATACATCGGAACGTACCCGGAAGTGGGGGGTAACGTAGCGAAAGTTACGCTAAT
ACCGCATATTCTGTGAGCAGGAAAGCAGGGGATCGCAAGACCTTGCGCTTTCGGAGCGGCCGATGTCAGA
TTAGCTAGTTGGTGAGGTAAAGGCTCACCAAGGCGACGATCTGTAGCGGGTCTGAGAGGATGATCCGCCA
CACTGGAACTGAGACACGGTC

>Zotu1823
GAACGCTGGCGGCAGGCTTAACACATGCAAGTCGAACGCCCCGCAAGGGGAGTGGCAGACGGGTGAGTA
ACGCGTGGGAATCTACCTAGTTCTACGGAACAACAGAGGGAAACTTCTGCTAATACCGTATACGCCCTAAG
GGGGAAAGATTTATCGGAATTAGATGAGCCCGCGTAAGATTAGCTAGTTGGTGGGGTAATGGCCTACCAA
GGCGACGATCTTTAGCTGGTCTGAGAGGATGATCAGCCACACTGGGACTGAGACACGGCC

>Zotu1825
GAACGCTGGCGGCGTGCCTAATACATGCAAGTCGAGCGAACAGATAAGGAGCTTGCTCCTTTGACGTTAG
CGGCGGACGGGTGAGTAACACGTGGGTAACCTACCTATAAGACTGGAATAACTCCGGGAAACCGGGGCTA
ATGCCGGATAATACACAGAACCGCATGGTTCTGTGATGAAAGACGGTTTTGCTGTCACTTATAGATGGACC
CGCGGCGTATTAGCTAGTTGGTAAGGTAACGGCTTACCAAGGCAACGATACGTAGCCGACCTGAGAGGGT
GATCGGCCACATTGGGACTGAGACACGGCC

>Zotu1828
GAACGCTGGCGGCGTGCCTAACACATGCAAGTCGAGCGATGAAGCTTCTTCGGAAGTGGATTAGCGGCGG
ACGGGTGAGTAACACGTGGGCAACCTGCCTCATAGAGAGGGATAGCCTTTCGAAAGGAAGATTAATACCT
CATAAGATTGTAGTTTCGCATGAAACGGCAATAAAAGGAGCAATCCGCTATGAGATGGGCCCGCGTCGCA
TTAGCTAGTTGGTAAGGTAATGGCTTACCAAGGCGACGATGCGTAGCCGACCTGAGAGGGTGATCGGCCA
CATTGGGACTGAGACACGGCC

>Zotu1830
GAACGCTGGCGGCGTGCTTAACACATGCAAGTCGAACGCTGAAGCTTGGTGCTTGCACTGGGTGGATGAG
TGGCGAACGGGTGAGTAATACGTGAGTAACCTGCCCTTGACTCTGGGATAAGCCTGGGAAACTGGGTCTA
ATACTGGATATGACATGCCACTGCATGGTGGTGTGTGGAAAGGGTTTTACTGGTTTTGGATGGGCTCACGG
CCTATCAGCTTGTTGGTGGGGTAATGGCCTACCAAGGCGACGACGGGTAGCCGGCCTGAGAGGGTGACCG
GCCACACTGGGACTGAGACACGGCC

>Zotu1832
GAACGCTAGCGGCAGGCTTAATACATGCAAGGCGAGGGGGCAGCAATGTCACCGTCGTACGGGTGCGTA
ACGCGTATGCAACCTACCTTCAACTGGGGGATAGCCCGGGGAAACCCGGATTAATACCGCATAATACAGG
GGGCCCACATGGGTCTATTTGTTAAAGATTTATTGGTTGAAGATGGGCATGCGTTCGATTAGCTAGTTGGT

GTAGGTAACGGCTCACCAAGGCGACGATCGATAGGGGAGCTGAGAGGTTGATCCCCCACACGGGCACTG
AGATACGGGC
>Zotu1834
GAACGCTGGCGGCGTGCCTAATACATGCAAGTAGAACGCTGAAGCTTGGTGCTTGCACCGAGCGGATGAG
TTGCGAACGGGTGAGTAACGCGTAGGTAACCTGCCTGGTAGCGGGGGATAACTATTGGAAACGATAGCTA
ATACCGCATAACAGTAGATATTGCATGATATCTGCTTGAAAGGTGCAATTGCATCACTACCAGATGGACCT
GCGTTGTATTAGCTAGTTGGTGAGGTAACGGCTCACCAAGGCGACGATACATAGCCGACCTGAGAGGGTG
ATCGGCCACACTGGGACTGAGACACGGCC
>Zotu1836
GAACGCTGGCGGCGTGCTTAACACATGCAAGTCGAACGGTGAACTCAGAGCTTGCTCTGAGGGATCAGTG
GCGAACGGGTGAGTAACACGTGAGTAACCTGCCCCAGACTCTGGAATAAGCGCTGGAAACGGCGTCTAAT
ACCGGATACGAGACAGAGAGGCATCTCTACTGTCTGGAAAGTTTTTCGGTCTGGGATGGACTCGCGGCCTA
TCAGCTTGTTGGTGAGGTAACGGCTCACCAAGGCGACGACGGGTAGCCGGCCTGAGAGGGCGACCGGCC
ACACTGGGACTGAGACACGGCC
>Zotu1839
GAACGCTGGCGGAATGCTTTACACATGCAAGTCGAACGGCAGCACGAGGGCAACCTTGGTGGCGAGTGG
CGAACGGGTGAGTAATGTATCGGAACGTGCCCAGTCGTGGGGGATAACGTAGCGAAAGCTACGCTAATAC
CGCATACGATCTATGGATGAAAGCGGGGGATCGCAAGACCTCGCGCGATTGGAGCGGCCGATATCAGATT
AGGTAGTTGGTGGGGTAAAGGCTCACCAAGCCAACGATCTGTAGCTGGTCTGAGAGGACGACCAGCCACA
CTGGAACTGAGACACGGTC
>Zotu1841
GAACGCTAGCGGCAGGCTTAACACATGCAAGTCGAACGGTAACAGATCTTCGGATGCTGACGAGTGGCGC
ACGGGTGCGTAACGCGTATGCAACCTACCTCTAACTGGGGAATAGCCCGGGGAAACTCGGATTAATACCCC
ATAACATTATGGAATCGCATGATTCTATAATTAAAACTCCGGTGGTTAGAGATGGGCATGCGTCCTATTAGC
TAGTTGGTGAGGTAACGGCTCACCAAGGCTACGATAGGTAGGGGGTCTGAGAGGATTATCCCCCACACTG
GTACTGAGACACGGAC
>Zotu1843
GAACGCTAGCGGCAGGCTTAACACATGCAAGTCGAGGGGCAGCGCGGGTAGCAATACCTGGCGGCGACC
GGCAAACGGGTGCGGAACACGTACTGAACCTACCTTGATCTGGGGCATAGCCCGATGAAAATCGGATTAA
CTCCCCATAATATAGATGAGTGGCATCACTTGACTATTAAAAATTTATTGGATCGAGATGGCAGTGCGTCCC
ATTAGCTAGTTGGTAGGGTAACGGCCTACCAAGGCGACGATGGGTAGCTGGTGTGAGAGCACGACCAGCC
ACACGGGCACTGAGACACGGGC
>Zotu1845
GAACGCTGGCGGCGTGCTTAACACATGCAAGTCGAACGATGATGCCCTGCTTGCAGGGTGGATTAGTGGC
GAACGGGTGAGTAACACGTGGGTGATCTGCCCTGCACTTCGGGATAAGCCTGGGAAACTGGGTCTAATAC
CGGATATGACCCTCCTTTAGTGTGGGGGGTGGAAAGCTTTTGCGGTGTGGGATGAGCCTGCAGCCTATCA
GCTTGTTGGTGGGGTAATGGCCTACCAAGGCGTCGACGGGTAGCCGGCCTGAGAGGGTGATCGGCCACAT
TGGGACTGAGACACGGCC
>Zotu1847
GAACGCTAGCGGCAGGCTTAATACATGCAAGTCGAACGGATGTAGCAATACATTAGTGGCGCACGGGTGA
GTAACGCGTACATAACATGCCTTTAGCTTAGGAATAGCCTTGGGAAACTGGGAGTAATACCTAATAGTATT
TTTGGATGGCATCGTCTGGAAATTAAAGTTACGGCGGCTAAAGATTGATGTGCGTAAGATTAGCTAGTTGG
TGAGGTAACGGCTCACCAAGGCGACGATCTTTAGGGGGCGTGAGAGCGTGACCCCCCACACGGGTACTGA
GACACGGAC
>Zotu1849
GAACGCTGGCGGCATGCCTTACACATGCAAGTCGAACGGCAGCACGGGAGCAATCCTGGTGGCGAGTGG
CGAACGGGTGAGTAATATATCGGAACGTGCCCAGTCGTGGGGGATAACGCAGCGAAAGCTGTGCTAATAC
CGCATACGATCTATGGATGAAAGCGGGGGACTCGTAAGGGCCTCGCGCGATTGGAGCGGCCGATATCAGA
TTAGCTAGTTGGTGGGGTAAAGGCCTACCAAGGCGACGATCTGTAGCTGGTCTGAGAGGACGACCAGCCA
CACTGGAACTGAGACACGGTC

>Zotu1851
GAACGCTGGCGGCGCGCCTAACACATGCAAGTCGAGCGAGAACCAGGGCTTCGGCCCTGGGGACAGCGG
CGAACGGGTGAGTAACACGTGGATAACCTGCCCTCGGCACCGGAATAGCCCGGGGAAACCCGGATTAATG
CCGGATAGCCCCACATCCCTGCGGGGAACGTGGGAAAAGGTAGCTTCGGCCTCTGGCCGAGGATGGGTCC
GCGGTGGATTAGCTTGTTGGCGGGGTAACGGCCCACCAAGGCGACGATCCATAGCTGGTCTGAGAGGACG
ATCAGCCACACTGGGACTGAGACACGGCC
>Zotu1853
GAACGCTGGCGGCGTGCTTAACACATGCAAGTGGAGCGACGAACCAGGGCTTGCCCTGGGGCAGAGCCG
CGAACGGGTGAGTAACACGTGGGTAACCTGCCCCGATCACTGGGACAACCCGAGGAAACTCGGGCTAATA
CCGGATGTGATCCCGTCACACCAGTGACGTGATGAAAGGTAGCTTCGGCTTCCGGATTGGGATGGGCCCG
CGGCCCATTAGCTTGTTGGTGGGGTAACGGCCTACCAAGGCGACGATGGGTAGCTGGTCTGAGAGGACGA
TCAGCCACACTGGGACTGAGACACGGCC
>Zotu1855
CAGGAAGAGGTCCCATAATGTCGATCACCATGCGCTTAAACGGCTCAGTAATGATCGGCACGAGCTGCATT
GGCACCTTGCACCTGTCGTGCGGTTTTCCGGCCCGTTGGCAGCTGTCA
>Zotu1858
GAACGCTGGCGGCGTGCCTAATACATGCAAGTCGAGCGAACAGATAAGGAGCTTGCTCCTTTGACGTTAG
CGGCGGACGGGTGAGTAACACGTGGGTAACCTACCTATAAGACTGGGATAACTTCGGGAAACCGGAGCTA
ATACCGGATAATATTTCGAACCGCATGGTTCGATAGTGAAAGATGGTTTTGCTATCACTTATAGATGGACCC
GCGCCGTATTAGCTAGTTGGTAAGGTAACGGCTTACCAAGGCGACGATACGTAGCCGACCTGAGAGGGTG
ATCGGCCACACTGGAACTGAGACACGGTC
>Zotu1860
GAACGCTGGCGGCGTGCTTAACACATGCAAGTCGAACGATGAAACAATTCTTGATGTCTTCGGACTGATTG
AGCTGTGGATTAGTGGCGGACGGGTGAGTAACGCGTGAGCAATCTGCCTTACACAAAGGGATAGCCTCGG
GAAACCGGGATTAATACCATATGATATCTACAAGCCGCATGGCCTATAGATTAAAGAATTTCGGTGTAAGA
TGAGCTCGCGTCCCATTAATTAGTTGGTGAGGGTAACGGCCCACCAAGATAGCGATGGGTAGCCGATCTAA
GAGGATGACCGGCCACATTGGAACTGAGAAACGGTC
>Zotu1862
GAACGCTGGCGGCAGGCCTAACACATGCAAGTCGAGCGGTAGAGAGAAGCTTGCTTCTCTTGAGAGCGGC
GGACGGGTGAGTAATGCCTAGGAATCTGCCTGGTAGTGGGGGATAACGTTCGGAAACGGACGCTAATACC
GCATACGTCCTACGGGAGAAAGCAGGGGACCTTCGGGCCTTGCGCTATCAGATGAGCCTAGGTCGGATTA
GCTAGTTGGTGGGGTAATGGCTCACCAAGGCGACGATCCGTAACTGGTCTGAGAGGATGATCAGTCACAC
TGGAACTGAGACACGGTC
>Zotu1864
GAACGCTGGCGGCGTGCTTAACACATGCAAGTCGAACGGAAAGGCCTCTGCTTGCAGGGGTACTCGAGTG
GCGAACGGGTGAGTAACACGTGGGTGATCTGCCCTGCACTTCGGGATAAGCCTGGGAAACTGGGTCTAAT
ACCGGATAGGACCACATTTTGGATGGTGTGGTGGAAAGTTTTTCGGTGTGGGATGAGCTCGCGGCCTATCA
GCTTGTTGGTGGGGTAATGGCCTACCAAGGCGTCGACGGGTAGCCGGCCTGAGAGGGTGTACGGCCACAT
TGGGACTGAGATACGGCC
>Zotu1867
GAACGCTGGCGGCGTGCTTAACACATGCAAGTCGAACGGTAAGGCCCCTTCGGGGGTACACGAGTGGCGA
ACGGGTGAGTAACACGTGGGTAATCTGCCCTGCACTTCGGGATAAGCCTGGGAAACTGGGTCTAATACCG
GATATGAACTCCTGCCGCATGGTGGGGGTTGGAAAGTTTTTCGGTGCAGGATGAGCCCGCGGCCTATCAG
CTTGTTGGTGGGGTAATGGCCTACCAAGGCGACGACGGGTAGCCGGCCTGAGAGGGTGATCGGCCACACT
GGGACTGAGACACGGCC
>Zotu1869
GAACGCTAGCTACAGGCTTAACACATGCAAGTCGAGGGGCAGCATGAACTTAGCTTGCTAAGTTTGATGGC
GACCGGCGCACGGGTGAGTAACACGTATCCAACCTGCCGATGACTCGGGGATAGCCTTTCGAAAGAAAGA
TTAATACCCGATGGCATAGTTCTTCCGCATGGTGGAACTATTAAAGAATTTCGGTCATCGATGGGGATGCG

TTCCATTAGGTTGTTGGCGGGGTAACGGCCCACCAAGCCTTCGATGGATAGGGGTTCTGAGAGGAAGGTC
CCCCACATTGGAACTGAGACACGGTC
>Zotu1871
GAACGCTGGCGGCGTGCTTAACACATGCAAGTCGAGCGGAAAGGCCCTTCGGGGTACTCGAGCGGCGAA
CGGGTGAGTAACACGTGAGTAACCTGCCCTTGGCTCTGGGATAAGCCCGGGAAACTGGGTCTAATACCGG
ATATGACTCTCCACTGCATGGTGGAGGGTGGAAAGTTTTTCGGCTGGGGATGGACTCGCGGTCTATCAGCT
TGTTGGTGGGGTAACGGCCTACCAAGGCGACGACGGATAGCCGGCCTGAGAGGGTGACCGGCCACACTG
GGACTGAGACACGGCC
>Zotu1873
CAACGCTGGCGGCGCGCTTAACACATGCAAGTCGCACGAGAAAGTGGAGCAATCCATGAGTACAGTGGCG
TACGGGTGAGTAACACGTGGATCATCTACCTTCTAGTGGGGAATACCTCTGGGAAACCGGAGACAATACC
GCATAAGCTCGTAAGAGGAAAGCAGTAATGCGCTGGAAGAGGAGTCCGCGGCTGATTAGCTAGTTGGCG
GGGTAATGGCCCACCAAGGCGAAGATCGGTAGCCGGCCTGAGAGGGCACACGGCCACACTGGCACTGAA
ACACGGGC
>Zotu1875
GAACGCTAGCGGCAGGCTTAATACATGTAAGTCGAGGGGCAGCACGGTTAGCAATAACTGGTGGCGACCG
GCGCACGGGTGCGTAACACGTATACAATCTACCTTAAACAGGGGGATAGCCTTTCGAAAGAGAGATTAATA
CCCCGTAATAAATTGAATGGCATCATTTGATTTTGAAAGCTCCGGCGGTTTGAGATGAGTATGCGTCTGATT
AGCTAGTTGGTGAGGTAACGGCTCACCAAGGCAACGATCAGTAGGGGATCTGAGAGGACTAACCCCCACA
CTGGTACTGAGACACGGAC
>Zotu1877
GAACGTTGGCGGCGTGCCTAACACATGCAAGTCGAGCGTGAAAGTCCTTCGGGATGAGTAAAGCGGCGG
ACGGGTGAGTAACGCGTGAGAATCTACCTTTCAGTGAAGAATACCTTTGAGAAATTGAAGTTAATGCTGCA
TACGTCTCGTAAGAGAGAAAGATGGCCTCTTTGCTGTCGCTGAAAGATGAGCTTGCGTTCTATTAGCTAGTT
GGTGGGGTAAAGGCCTACCAAGGCGATGATGGATAGCCGGCCTGAGAGGGCGAACGGCCACATTGGAAC
TTAGACACGGTC
>Zotu1879
GAACGCTGGCGGCGTGCTTAATACATGCAAGTCGAGCGGTAAGGCCCTTCGGGGTACACGAGCGGCGGA
CGGCTGAGTAACGCGTAGGAATGTACCCCAAAGTGAGGGATAAGCACCAGAAATGGTGTCTAATACCGCA
TGTGATCTATGGATTAAAGCTTCGGCGCTTTGGGAACAGCCTGCGTATGATTAGCTTGTTGGTGAGGTAAA
GGCTCACCAAGGCGACGATCATTAGCTGGTCTGAGAGGATGACCAGCCAGACTGGGACTGAGACACGGCC
>Zotu1882
GAACGCTGGCGGCGTGCTTAACACATGCAAGTCGAGCGGTAAGGCTCCTTCGGGAGTACACGAGCGGCGA
ACGGGTGAGTAACACGTGAGTAATCTGCCCTTCACTTCGGGATAACCGCCGGAAACGGTGGCTAATACCG
GATACGACACAGTCCCGCATGGGATCTGAGTGGAAAGTTTTCTCGGTGGAGGATGTGCTCGCGGCCTATCA
GCTTGTTGGTGAGGTAACGGCTCACCAAGGCTTCGACGGGTAGCCGGCCTGAGAGGGTGACCGGCCACAC
TGGGACTGAGACACGGCC
>Zotu1884
AAACGCTGGCGGCGTGCATGAGACATGCAAGTCGAACGGACGAACGGGGCTTGCTCTGTTCGTTAGTGGC
GGACGGCTGAGTAACGCGTAGGTGACCTGTCCCGAAGTGGGGAATAACTGCCCGAAAGGGTGGCTAATA
CCGCATGTGGTGGTGTGTTCATTCACACCAGTAAAGCCGCAAGGCGCTTCGGGAGGGGCCTGCGTCCGATT
AGCTCGTTGGTGGGGTAACGGCTCACCAAGGCGACGATCGGTAGGTGGTCTGAGAGGACGATCACCCAG
ACTGGGACTGAGACACGGCC
>Zotu1887
GAACGCTGGCGGCGTGCTTAACACATGCAAGTCGAACGGTGAACCACCTTCGGGTGGGGATCAGTGGCGA
ACGGGTGAGTAACACGTGACCAACCTGCCCCTTGCTCTGGGATAACCCCGGGAAACCGGAGCTAATACCG
GATACGAGACCGTGCGGCATCACGCGGTCTGGAAAGTTTATCGGCAAGGGATGGGGTCGCGGCCTATCAG
CTTGTTGGTGGGGTAACGGCCCACCAAGGCGACGACGGGTAGCCGGCCTGAGAGGGCGACCGGCCACAC
TGGGACTGAGACACGGCC
>Zotu1889

GAACGCTGGCGGCGTGCTTAACACATGCAAGTCGAGCGGAAAGGCCACTTCGGTGGTACTCGAGCGGCGA
ACGGGTGAGTAACACGTGAGTAATCTGCCCTTGGCTTTGGGATAGCCACCGGAAACGGTGATTAATACCG
GATACGACCACTTCTCGCATGGGATGGTGGTGGAAAGTTTTTCGGCCAGGGATGTGCTCGCGGCCTATCAG
CTTGATGGTGAGGTAATGGCTCACCATGGCTTCGACGGGTAGCCGGCCTGAGAGGGTGACCGGTCACACT
GGGACTGAGACACGGCC
>Zotu1891
GAACGCTGGCGGCAGGCTTAACACATGCAAGTCGAACGCTGTAGCAATACAGAGTGGCAGACGGGTGAG
TAACACGTGGGAACGTACCCTTTGGTCTGGAATAACTCAGGGAAACTTGAGCTAATACCGGATACGCCCTT
ATGGGGAAAGGTTTACTGCCGAAGGATCGGCCCGCGTCCGATTAGCTAGTTGGTGTTGGTAATGGCTCACC
AAGGCGACGATCGGTAGCTGGTCTGAGAGGATGGCCAGCCACATTGGGACTGAGACACGGCC
>Zotu1893
GAACGCTGGCGGCGTGCTTAACACATGCAAGTCGAACGGTAAGGCCCCTTCGGGGGTACACGAGTGGCGA
ACGGGTGAGTAACACGTGGGTAATCTGCCCTGCACTTCGGGATAAGCCTGGGAAACCGGGTCTAATACCG
GATATGAACTCCTGCCGCATGGTGGGGGTTGGAAAGTTTTTCGGTGCAGGATGAGCCCGCGGCCTATCAG
CTTGTTGGTGGGGTAATGGCCTACCAAGGCGACGACGGGTAGCCGGCCTGAGAGGGTGATCGGCCACACT
GGGACTGAGACACGGCC
>Zotu1896
GAACGCTGGCGGCGTGCTTAACACATGCAAGTCGAACGGAGACTTTTTCAGTGTAACCTTCGGGGAGCAA
GAGAAAGTCTTAGTGGCGGACGGGTGAGTAACGCGTGGGCAACCTGCCTCGCACCGTGGGATAACAGGG
GGAAGCTCCTGCTAATACCGCATAAAGTATATAGACGCATGTCAATATACAAAAAGGGATACCGGTGAGA
GATGGGCCCGCGTCCGATTAGATAGTTGGCAGGATAGCAGCCTACCAAGTCAACGATCGGTAGCCGGCTT
GAGAGAGTGGACGGCCACACTGGGACTGAGACACGGCC
>Zotu1898
CAACGCTGGCGGCGTGCCTCAGACATGCAAGTCGAACGATTAAGACTTCCTTCGGGAAGTGTATAAAGTG
GCGCACGGGTGAGTAACACGTAAGTAATCTGCCTTCGAGTGGGGAATACCTTCGGGAAACCGAAGATAAT
ACCGCATAATGCAGCGGCACCGCAAGGTGACAGTTGTTAAAGGAGCAATCCGCTTGAAGAGGAGCTTGCG
GCAGATTAGCTAGTTGGTAAGGTAACGGCTTACCAAGGCTACGATCTGTATCCGGTCTAAGAGGACGGTC
GGACACACTGACACTGAATAACGGGT
>Zotu1900
GAACGCTGGCGGCAGGCTTAACACATGCAAGTCGAACGCCCCGCAAGGGGAGTGGCAGACGGGTGAGTA
ACGCGTGGGAATCTACCCAGATCTTCGGAACAACTGAGGGAAACTTCAGCTAATACCGGATACGCCCTACG
GGGGAAAGATTTATCGGATTTGGATGAGCCCGCGTTGGATTAGCTAGTTGGTGAGGTAACGGCTCACCAA
GGCGACGATCCATAGCTGGTCTGAGAGGATGATCAGCCACACTGGGACTGAGACACGGCC
>Zotu1902
CAACGCTGGCGGCGCGCTTAACACATGCAAGTCGAACGGGAAAGTGGAGCAATCCATGAGTACAGTGGC
GTACGGGTGAGTAACACGTGGGTGATCTACCTTTTAGTGGGGAATAACCCTGGGAAACCGGGGCTAATAC
CGCATAAGCCCGAGAGGGGAAAGTCGCAAGACGCTGAAAGAGGAGCCCGCGGCCGATTAGCTAGTTGGT
AGGGTAAGAGCCTACCAAGGCAGAGATCGGTAGCCGGCCTGAGAGGGCACACGGCCACACTGGCACTGA
AACACGGGC
>Zotu1904
GAACGCTGGCGGCGTGCTTAACACATGCAAGTCGAACGGTGAAGGAGAGCTTGCTCTCCGGATCAGTGGC
GAACGGGTGAGTAACACGTGAGCAACCTACCCTTGACTCTGGGATAACAGTTGGAAACAGCTGCTAATACC
GGATATGACCCTGGCAGGCATCTGCCGGGGTGGAAAGCCTTGACGGTCAAGGATGGGCTCGCGGCCTATC
AGCTTGTTGGTGAGGTAACGGCTCACCAAGGCGTCGACGGGTAGCCGGCCTGAGAGGGCGACCGGCCAC
ACTGGGACTGAGATACGGCC
>Zotu1907
GAACGCTGGCGGCGTGCTTAACACATGCAAGTCGAGCGGTAAGGCCCTTCGGGGTACACGAGCGGCGAA
CGGGTGAGTAACACGTGGGTGACCTGCCCTCAGCTCTGGGATAAGCCTGGGAAACTGGGTCTAATACCGG
ATACGACCACATGCTGCATGGCGTGTGGTGGAAAGTTTTTCGGCTGGGGATGGGCCCGCGGCCTATCAGC
TTGTTGGTGGGGTGATGGCCTACCAAGGCGGTGACGGGTAGCCGGCCTGAGAGGGCGACCGGCCACACT
GGGACTGAGACACGGCC

>Zotu1909
GAACGCTGGCGGCGCGCTTAACACATGCAAGTCGAACGAGAAGGCGTAGCAATACGCTTGTACAGTGGCG
AACGGGTGAGGAATACGTGGGTAACCTACCCTCGAGTGGGGAATAACCAGCCGAAAGGTTGGCTAATGCC
GCGTACGCTTCCAGGTCTGCGGATCGGGAAGGAAAGCCGTACAAAGTGTGCGGCGCTCAAGGATGGGCTC
ACGTCCTATCAGCTTGTTGGTGAGGTAACGGCTCACCAAGGCTTCGACGGGTAGCTGGTCTGAGAGGACG
ATCAGCCACACTGGCACTGCGACACGGGC
>Zotu1911
GAACGCTGGCGGCGTGCCTAATACATGCAAGTCGAACGAACCGCGACTGAGTGCTTGCACTTGGTCAAGG
TGAGTGGCGAACGGGTGAGTAACACGTGGGTAACCTACCTCATAGTGGGGGATAACAGTCGGAAACGACT
GCTAATACCGCATAGGACATGGGATCACATGATTCTGTGAGGAAAGGTGGCGCAAGCTATCGCTAAGAGA
TGGACCCGCGGTGCATTAGCTAGTTGGTAGGGTAACGGCCTACCAAGGCGATGATGCATAGCCGACCTGA
GAGGGTGATCGGCCACATTGGGACTGAGACACGGCC
>Zotu1913
GAATGCTGGCGGCGTGCCTAACACATGCAAGTCGAGCGGCAGCGCGAGTAGTTTACTACTTGGCGGCGAG
CGGCGGACGGCTGAGTAACGCGTAGGAACGTGCCCCAAAGTGAGGAATAACTGCCCGAAAGGGTGGCTA
ATGCCGCATATGATCTTCGGATTAAAGTCTTCGGACGCTTTGGGAGCGGCCTGCGTTAGATTAGGTAGTTG
GTGAGGTAATGGCTCACCAAGCCGACGATCTATAGCTGGTCTGAGAGGATGATCAGCCAGACTGGGACTG
AGACACGGCC
>Zotu1915
GAACGCTGGCGGCGTGCCTAATACATGCAAGTAGAACGCTGAAGGGAGAGCTTGCTCTTCTGGATGAGTT
GCGAACGGGTGAGTAACGCGTAGGTAACCTGCCTGGTAGCGGGGGATAACTATTGGAAACGATAGCTAAT
ACCGCATAATAGTAGATGTTGCATGACATTTGCTTGAAAGGTGCAATTGCATCACTACCAGATGGACCTGC
GTTGTATTAGCTAGTTGGTGGGGTAACGGCTCACCAAGGCGACGATACATAGCCGACCTGAGAGGGTGAT
CGGCCACACTGGGACTGAGACACGGCC
>Zotu1917
GAACGCTGGCGGCGTGCTTAACACATGCAAGTCGAACGATGAAGCGTAGCTTGCTGCGTGGATTAGTGGC
GAACGGGTGAGTAACACGTGAGCAATCTGCCCTTGACTCTGGGATAAGCGCTGGAAACGGCGTCTAATAC
CGGATATGAGTAGTGATCGCATGATCGGCTACTGGAAAGATTTTTTGGTTGAGGATGAGCTCGCGGCCTAT
CAGCTTGTTGGTGAGGTAATGGCTTACCAAGGCGTCGACGGGTAGCCGGCCTGAGAGGGTGACCGGCCAC
ACTGGGACTGAGACACGGCC
>Zotu1919
CAACGCTGGCGGCGCGCCTAACACATGCAAGTCGAACGAGAAAGCCGCAAGGCAAGTAAAGTGGCGTAC
GGGTGAGTAACACGTGGATTATCTACCTTCTGGTGGGGAATACCGTTGGGAAACCGACGACAATACCGCA
TAAGCCGGAGACGGGAAAGCAGCAATGCGCCGGAAGAGGAGTCCGCGGTCGATTAGTTAGTTGGCGGGG
TAAAAGCCCACCAAGACTGTGATCGATAGCCGGCCTGAGAGGGCACACGGCCACACTGGCACTGAAACAC
GGGC
>Zotu1921
GAACGCTGGAAATGTGGATACCGGCATGCAAGTCAAGGGGCCGCAAGGCACCGGCAAACGGCTTAGTAA
CACGTAGATACATACCCAAAAGTGGGGCATAGCTCGTCGAAAGACGGGGTAATTTCCCATAGTACCGCAA
GGTTAAAGACGCAAGTCGCTTTTGGAATGGTCTGCGGTCTATCAGCTAGTTGGTGAGGTAAAGGCTCACCA
AGGCTATGACGGATAGGGGGCGTGAGAGCGCGACCCCCAAGAATGGCACTGCGACACGGGC
>Zotu1923
GAACGCTAGCGGCAGGCTTAATACATGCAAGGCGAAGGGGCAGCAATGTCACTGTCGTACGGGTGCGTAA
CGCGTATGCAACCTACCTATCACTGGGGGATAGCCCGGGGAAACCCGGATTAATACCGCATAACACAGGG
GTCCCGCATGGGTACTATTTGTTAAAGATTTATTGGTGGTAGATGGGCATGCGTTCGATTAGCTAGTTGGT
ATGGGTAACGGCCTACCAAGGCGACGATCGATAGGGGAGCTGAGAGGTTGATCCCCCACACGGGCACTG
AGATACGGGC
>Zotu1925
GAACGCTGGCGGCGTGCTTAACACATGCAAGTCGAACGATGAAGCCCAGCTTGCTGGGTGGATTAGTGGC
GAACGGGTGAGTAACACGTGAGTAACCTGCCCTTGACTCTGGGATAAGCGCTGGAAACGGCGTCTAATAC

CGGATACGACCTGCCCCGGCATCGGGTGCGGGTGGAAAGTTTTTCGGTCAAGGATGGACTCGCGGCCTAT
CAGGTAGTTGGTGAGGTAATGGCTCACCAAGCCGACGACGGGTAGCCGGCCTAAGAGGGTGACCGGCCA
CACTGGGACTGAGACACGGCC
>Zotu1927
GAACGCTGGCGGCATGCCTAACACATGCAAGTCGAACGATGCTTTCGGGCATAGTGGCGCACGGGTGCGT
AACGCGTGGGAATCTGCCCTTGGGTTCGGAATAACAGTTGGAAACGACTGCTAATACCGGATGATGTCGAT
AAGACCAAAGATTTATCGCCTGAGGATGAGCCCGCGTTGGATTAGCTAGTTGGTGGGGTAAAGGCCTACC
AAGGCGACGATCCATAGCTGGTCTGAGAGGATGATCAGCCACACTGGGACTGAGACACGGCC
>Zotu1929
GAACGCTGGCGGCGTGCCTAATACATGCAAGTCGAACGCACAGCGAAAGGTGCTTGCACCTTTCAAGTGA
GTGGCGAACGGGTGAGTAACACGTGGACAACCTGCCTCAAGGCTGGGGATAACATTTGGAAACAGATGCT
AATACCGAATAAAACTTAGTGTCGCATGACACAAAGTTAAAAGGCGCTTCGGCGTCACCTAGAGATGGATC
CGCGGTGCATTAGTTAGTTGGTGGGGTAAAGGCCTACCAAGACAATGATGCATAGCCGAGTTGAGAGACT
GATCGGCCACATTGGGACTGAGACACGGCC
>Zotu1931
AAACGCTGGCGGCGTGCATAACACATGCAAGTCGAACGATGAAGATTAAAAGATTTCTTCGGAATGAACTT
AATTGGATTAGTGGCGGACGGGTGAGTAACGCGTGAGTAACCTGCCTTGTACAAGGGGATAGCCGTTGGA
AACGACGAATAATACCCTATAACATGATATTATCGCATGAAAATATCATCAAAGATTTATCGGTATAAGATG
GACTCGCGTCTGATTAGCTAGTTGGTGGGGTAAAAGCCTACCAAGGCAACGATCAGTAGCCGGCTTGAGA
GAGTGTACGGCCACATTGGGACTGAGACACGGCC
>Zotu1933
GAACGCTGGCGGCGTGCTTAACACATGCAAGTCGAACGATGATCCTCAGCTTGCTGGGGGGATTAGTGGC
GAACGGGTGAGTAACACGTGAGTAACCTGCCCTTGACTCTGGGATAAGCCTGGGAAACTGGGTCTAATAC
CGGATATGACTTCTCATCGCATGGTGGGGGGTGGAAAGCTTTTTGTGGTTTTGGATGGACTCGCGGCCTAT
CAGCTTGTTGGTGGGGTAATGGCCTACCAAGGCGACGACGGGTAGCCGGCCTGAGAGGGTGACCGGCCA
CACTGGGACTGAGACACGGCC
>Zotu1935
GAACGCTGGCGGCGTGCCTAATACATGCAAGTCGAGCGGACCGATGGGAGCTTGCTCCCTTAGGTCAGCG
GCGGACGGGTGAGTAACACGTGGGTAACCTGCCTGTAAGACTGGGATAACTCCGGGAAACCGGGGCTAA
TACCGGATGCTTGATTGAACCGCATGGTTCAATCATAAAAGGTGGCTTTTAGCTACCACTTACAGATGGACC
CGCGGCGCATTAGCTAGTTGGTGAGGTAACGGCTCACCAAGGCGACGATGCGTAGCCGACCTGAGAGGGT
GATCGGCCACACTGGGACTGAGACACGGCC
>Zotu1938
GAACGCTAGCGGGAGGCCTAACACATGCAAGCCGAGCGGTATTTATTCTTCGGAATAGAGAGAGCGGCGT
ACGGGTGCGTAACACGTGTGCAACCTGCCTTTATCTGGGAGATAGCCCTCCGAAAGGGGGATTAATATTCC
ATAATATATTAATTGGCATCAATTGATATTGAAAGCTCCGGCGGATAGAGATGGGCACGCGCAAGATTAGA
TAGTTGGTGAGGTAACGGCTCACCAAGTCTGCGATCTTTAGGGGGCCTGAGAGGGTGATCCCCCACACTG
GTACTGAGACACGGAC
>Zotu1940
GAATGCTGGCGACGTGGATAAGGCATGCAAGTCAAGGGGGCCCGCAAGGGCAACCGGCAGACGAGGTA
GTAACACGTACGAACGCACCCCAGAGTCTGGCATAGCTCGTCGAAAGACGGGGTAATTCCGGATGGTCCC
GCAAGGGTAAAGGTTTTTCGCTCTGGGAGCGGCGTGCGCAATATCAGCTAGTTGGTGAGGTAATGGCTCA
CCAAGGCTACGACGTTTAGGGGAGCTGAGAGGCTGACCCCCACCGATGGGACTGAGACACGGCC
>Zotu1943
GAACGCTGGCGGCGTGGATTAGGCATGCAAGTCGAGCGACGGACAGTCTTCGGACTGTTACGGAGCGGC
GAACGGGTGAGTAACACGTGAACAACCTGCCCTCACATTCTGGATAATTCACCGAAAGGTGTTGTAATACA
GGCGAGGATTCTTAAGAGGCATTTCTTGAGAAGGGAAGGCGCAAGCCGTGCGAGGAGGGGTTCGCGGAT
TATCAGGTAGTTGGTGGGGTAACGGCCTACCAAGCCTACGACGATTAGCTGGTCTGAGAGGATGGTCAGC
CACATTGGAACTGAGACACTGTC
>Zotu1946

GAACGCTGGCGGCGTGCTTAACACATGCAAGTCGAACGGAAAGGCCCTAGCTTGCTGGGGTGCTCGAGTG
GCGAACGGGTGAGTAACACGTGGGTGATCTGCCCTGCACTTCGGGATAAGCCTGGGAAACTGGGTCTAAT
ACCGGATAGGACCACTTCTTGGAGGTTGTGGTGGAAAGTTTTTCGGTGTGGGATGAGCTCGCGGCCTATCA
GCTTGTTGGTGGGGTAATGGCCTACCAAGGCGTCGACGGGTAGCCGGCCTGAGAGGGTGTACGGCCACAT
TGGGACTGAGATACGGCC
>Zotu1948
GAACGCTGGCGGCATGCCTAACACATGCAAGTCGAACGAGATCTTCGGATCTAGTGGCGCACGGGTGCGT
AACGCGTGGGAATCTGCCCTTGGGTTCGGAATAACAGTGAGAAATTACTGCTAATACCGGATGATGTCTTC
GGACCAAAGATTTATCGCCCAGGGATGAGCCCGCGTAGGATTAGCTAGTTGGTGGGGTAATGGCCTACCA
AGGCGACGATCCTTAGCTGGTCTGAGAGGATGATCAGCCACACTGGGACTGAGACACGGCC
>Zotu1950
GAACGCTGGCGGCGTGCTTAACACATGCAAGTCGAACGAGATCCAGCCGACCTTCGGGGAAGCGAAGATC
TAGTGGCGAACGGGTGCGTAACACGTGAGAAACCTGCCCCAGACTTGGGAATAACAGTCGGAAACGTCTG
CTAATACCGAATACCTTCGTAGCGTCGCATGGCGCATCGAAGAAAGCTTTTGCGGTTTGGGAGGGTCTCGC
GGCCTATCAGCTAGTTGGTGAGGTAACGGCTCACCAAGGCATCGACGGGTAGCTGGTCTGAGAGGATGAT
CAGCCACACTGGGACTGAGACACGGCC
>Zotu1952
GAACGCTGGCGGCGTGCTTTACACATGCAAGTCGAGCGGTAAGGCCCTTTCGGGGGTACACGAGCGGCGA
ACGGGTGAGTAACACGTGAGTAATCTGCCCTTCACTTGGGGATAAGCACCGGAAACGGTGTCTAATACCCG
ATACGACACAGTCCTGCATGGGATCTGTGTGGAAAGTTTTTTCGGTGGGGGATGTGCTCGCGGCCTATCAG
CTTGTTGGTGGGGTAATGGCCTACCAAGGCTTCGACGGGTAGCCGGCCTGAGAGGGTGACCGGCCACACT
GGGACTGAGACATGGCC
>Zotu1955
GAACGCTGGCGGCATGCCTAACACATGCAAGTCGAACGGCAGCATAGTCTAGTTTACTAGATGGGTGGCG
AGTGGCGAACGGGTGAGTAATGCGTAGGAATATACCTTAAAGAGGGGGATAACCTGGGAAACTCAGGC
TAATACCGCGTAATCTCCGAGGAGAAAAGCTGGGGACCTTCGGGCCTGGCGCTTTAAGAATAGCCTGCGTC
CGATTAGCTAGTTGGTGGGGTAAAGGCCTACCAAGGCGACGATCGGTAGCTGGTCTGAGAGGATGAACA
GCCACACTGGGACTGAGACACGGCC
>Zotu1957
GAACGCTGGCGGCGTGCTTAACACATGCAAGTCGAACGATGAACCACTTCGGTGGGGATTAGTGGCGAAC
GGGTGAGTAACACGTGGGCAATCTGCCCTGCACTCTGGGACAAGCCCTGGAAACGGGGTCTAATACCGGA
TATTGACCTTCACGGGCATCTGTGAGGTTCGAAAGCTCCGGCGGTGCAGGATGAGCCCGCGGCCTATCAGC
TTGTTGGTGAGGTAATGGCTCACCAAGGCGACGACGGGTAGCCGGCCTGAGAGGGCGACCGGCCACACT
GGGACTGAGACACGGCC
>Zotu1960
GAACGCTGGCGGCGTGCTTAACACATGCAAGTCGAACGAGAAATGTTATTATGATCTCTTCGGAGTGATGA
TTTTATTTCGAGTGGCGGACGGGTGAGTAACGCGTGAGCAACCTGCCCTATACCGGGGGATACCAGTTGG
AAACGACTGTTAATACCGCATAAGCGCACGATAAGACATCTTGTTGTGTGAAAAACTCCGGTGGTATAGGA
TGGGCTCGCGTATGATTAGCCAGTTGGCGGGGTAAAGGCCCACCAAAGCGACGATCATTAGCCGACCTGA
GAGGGCGACCGGCCACATTGGGACTGAGACACGGCC
>Zotu1962
GAACGCTGGCGGTATGCTTTACACATGCAAGTCGTACGGGAGTTTTTAACTCTAGTGGCGGACGGGTGAGT
AACACGTAAGAATTTGCCTTTAGGAGGGGGACAACAGCTGGAAACGGCTGCTAATACCCCATATGCTTTCG
AGTGAAATGGATTTCCGCCTAAAGAGAAGCTTGCGGCTGATTAGCTTGTTGGTAAGGTAATGGCTTACCAA
GGCGACGATCAGTATCTGGTTTGAGAGGATGATCAGACACACTGGAACTGAGACACGGTC
>Zotu1965
GAACGCTAGCGGCAGGCCTAATACATGCAAGTCAAAGGGGTAGCAATACCACTGGCGCACGGGTGCGTAA
CGCGTATGCAACCTACCGTTCACTGGGGGATAACGTCGGGAAACCGGCGCTAATACCGCATAAAGCAGGG
GCTGGGCATCCAGATACTGCTAAAGTTCCGATGGTGAACGATGGGCATGCGTCTGATTAGCTAGTTGGCG
GGGTAATGGCCCACCAAGGCGACGATCAGTAGGGGATCTGAGAGGATAATCCCCCACACTGGCACTGAGA
TACGGGC

>Zotu1967
GAACGCTGGCGGCGTGCCTAATACATGCAAGTAGAACGCTGAGGACAGGTGCTTGCACTAGTCTAAGGAG
TTGCGAACGGGTGAGTAACGCGTAGGTAACCTACCTGATAGCGGGGGATAACTATTGGAAACGATAGCTA
ATACCGCATAAAAGTGCTTAACACATGTTAAGAACTTAAAAGGGGCAACTGCTCCACTATGAGATGGACCT
GCGTTGTATTAGCTAGTTGGTGAGGTAAAGGCTCACCAAGGCGACGATACATAGCCGACCTGAGAGGGTG
ATCGGCCACACTGGGACTGAGACACGGCC
>Zotu1969
GAACGCTGGCGGCACGCCTAATACATGCAAGTCGAGCGCGTGAAGCTTTCCGAAGCCTTCGGGCGGACGA
AAGTGGATCGAGCGGCGGACGGGTGAGTAACACGTAGGCAACCTGCCTGTAAGACCGGGATAACTCCGG
GAAACCGGGGCTAATACCGGATAGGATCTCTTTTCGCATGGGAAGGGATTGAAAGGATGGCCGCAAGGCT
ATCGCTTACAGATGGGCCTGCGGCGCATTAGCTAGTTGGTGAGGTAACGGCTCACCAAGGCGACGATGCG
TAGCCGGCCTGAGAGGGTGACCGGCCACACTGGGACTGAGACACGGCC
>Zotu1971
GAACGCTGGCGGCGTGCTTAACACATGCAAGTCGAACGATGAAGCCTAGCTTGCTAGGTGGATTAGTGGC
GAACGGGTGAGTAATACGTGAGTGACCTACCTTTGACTCTGGGATAAGCCTGGGAAACTGGGTCTAATACC
GGATACGACCAATCTCCGCATGGGGTGTTGGTGGAAAGCGTTATGGAGTGGTTTTAGATGGGCTCACGGC
CTATCAGCTTGTTGGTGAGGTAATGGCTTACCAAGGCGACGACGGGTAGCCGGCCTGAGAGGGTGACCGG
CCACACTGGGACTGAGACACGGCC
>Zotu1974
GAACGCTGGCGGCGTGCTTAACACATGCAAGTCGAACGATGATCTCCAGCTTGCTGGGGGGATTAGTGGC
GAACGGGTGAGTAACACGTGAGTAACCTGCCCTTGACTCTGGGATAAGCCTGGGAAACTGGGTCTAATAC
CGGATACGACCGCTCCACGCATGTGGTGGTGGTGGAAAGCTTTTGCGGTTTTGGATGGACTCGCGGCCTAT
CAGCTTGTTGGTGGGGTAATGGCCTACCAAGGCGACGACGGGTAGCCGGCCTGAGAGGGTGACCGGCCA
CACTGGGACTGAGACACGGCC
>Zotu1977
GAACGCTGGCGGCGTGCCTAACACATGCAAGTCGAGCGAACCCTTCGGGGTGAGCGGCGGACGGGTGAG
TAACGCGTGGGTAACCTGCCCTGTACACACGGATAACATACTGAAAGGTATGCTAATACGAGATAATATGT
AATGATCGCATGATCGATACATCAAAGCTCCGGCGGTACAGGATGGACCCGCGTCTGATTAGCTAGTTGGT
GAGGTAATGGCTCACCAAGGCGACGATCAGTAGCCGACCTGAGAGGGTGATCGGCCACATTGGAACTGA
GACACGGTC
>Zotu1979
GAACGCTGGCGGCATGCCTTACACATGCAAGTCGAACGGTAACAGGTTAAGCTGACGAGTGGCGAACGG
GTGAGTAATATATCGGAACGTGCCCAATCGTGGGGGATAACGCAGCGAAAGTTGTGCTAATACCGCATAA
GATCTAAGGATGAAAGCGGGGGATCGCAAGACCTCGCGCGAATGGAGCGGCCGATATCAGATTAGCTAG
TTGGTGGGGTAAAGGCCCACCAAGGCGACGATCTGTAGCTGGTCTGAGAGGACGACCAGCCACACTGGG
ACTGAGACACGGCC
>Zotu1981
GAACGCTGGCGGCGTGCTTAACACATGCAAGTCGAACGGTGAAGCCCTTCGGGGTGGATCAGTGGCGAAC
GGGTGAGTAACACGTGAGCAATCTGCCCCCGACTCTGGGATAACTTCGGGAAACCGGAGCTAATACCGGA
TATGACACATGACCGCATGGTCTGTGTGTGGAAAGATTTATCGGTTGGGGATGAGCTCGCGGCCTATCAGC
TTGTTGGTGGGGTAAAGGCCTACCAAGGCGACGACGGGTAGCCGGCCTGAGAGGGCGACCGGCCACACT
GGGACTGAGACACGGCC
>Zotu1983
GAACGCTGGCGGCGTGCTTAACACATGCAAGTCGAACGGAAAGGCTCCTGCTTGCAGGGGTACTCGAGTG
GCGAACGGGTGAGTAACACGTGGGTGATCTGCCTTGTACTCTGGGATAAGCTTGGGAAACTGGGTCTAAT
ACCGGATAATAACTGCCTTTAGTGTGGTGGTTGGAAAGTTTTTCGGTGCAAGATGAGCTCGCGGCCTATC
AGCTTGTTGGTGGGGTAATGGCCTACCAAGGCGTCGACGGGTAGCCGGCCTGAGAGGGTGTACGGCCAC
ATTGGGACTGAGATACGGCC
>Zotu1985

GAACGCTGGCGGCGTGCCTAACACATGCAAGTCGAACGGAGCAGATCCCTTCGGGGTTTGCTTAGTGGC
GAACGGGTGAGTAACGCGTGAGGAACCTGCCTTTCAGTGGGGGACAACAGTTGGAAACGACTGCTAATAC
CGCATAATGCTTTTGAGCGGCATCGCTTGAAAGCCAAAGATTTATCGCTGAAAGATGGTCTCGCGTCTGATT
AGCTAGTTGGTGAGGTAACGGCTCACCAAGGCGGCGATCAGTAGCCGGTCTGAGAGGATGAACGGCCAC
ATTGGGACTGAGATACGGCC
>Zotu1987
GAACGCTGGCGGCGTGCTTAACACATGCAAGTCGAACGGTAAGGCCCTTCGGGGTACACGAGTGGCGAAC
GGGTGAGTAACACGTGAGTAACCTGCCCCAGTCTCTGGGATAACCGCCGGAAACGGCGGCTAATACTGGA
TATTCAGCGTCTGCCGCATGGTGGGTGTTGGAAAGCTCCGGCGGATTGGGATGGGCTCGCGGCCTATCAG
CTTGTTGGTGAGGTAGTGGCTCACCAAGGCTTCGACGGGTAGCCGGCCTGAGAGGGCGACCGGCCACATT
GGGACTGAGATACGGCC
>Zotu1990
GAACGCTAGCGGCAGGCTTAACACATGCAAGTCGAGGGGTAGGGTAGCAATACCTGAGACCGGCGCACG
GGTGCGTAACGCGTATGCAATCTACCTTTTACAGAGGGATAGCCCAGAGAAATTTGGATTAATACCTCATA
GTATAGTACAATCGCATGATTGAACTATTAAAGTCACAACGGTAAAAGATGAGCATGCGTCCCATTAGCTA
GTTGGTAAGGTAACGGCTTACCAAGGCGACGATGGGTAGGGGTCCTGAGAGGGAGATCCCCCACACTGGT
ACTGAGACACGGAC
>Zotu1992
GAACGCTGGCGGCAGGCTTAACACATGCAAGTCGAGCGGGGAAGAGTAGCTTGCTACTTAACCTAGCGGC
GGACGGGTGAGTAATGCTTAGGAATCTGCCTATTAGTGGGGGACAACATCTCGAAAGGGATGCTAATACC
GCATACGTCCTACGGGAGAAAGCAGGGGACCTTCGGGCCTTGCGCTAATAGATGAGCCTAAGTCGGATTA
GCTAGTTGGTGGGGTAAAGGCCTACCAAGGCGACGATCTGTAGCGGGTCTGAGAGGATGATCCGCCACAC
TGGGACTGAGACACGGCC
>Zotu1994
GAACGCTGGCGGCGTGCTTAACACATGCAAGTCGAGCGAAGCACTTGTTTGGAACCCTCCGGGAGGAAGA
ACAATTGACTGAGCGGCGGACGGGTGAGTAACGCGTGGGGAACCTGCCTTATACCGGGGGATAACAGCT
GGAAACGGCTGCTAATACCGCATAAGCGCACGGCACCGCATGGTGCAGTGTGAAAAACTCCGGTGGTATA
AGATGGACCCGCGTTAGATTAGCTAGTTGGCGGGGTAACGGCCCACCAAGGCGACGATCTATAGCCGGCC
TGAGAGGGCGACCGGCCACATTGGGACTGAGACACGGCC
>Zotu1996
GAACGCTGGCGGCAGGCCTAACACATGCAAGTCGAGCGGATGAAGGGAGCTTGCTCCCTGATTTAGCGGC
GGACGGGTGAGTAATGCCTAGGAATCTGCCTGGTAGTGGGGGACAACGTTTCGAAAGGAACGCTAATACC
GCATACGTCCTACGGGAGAAAGTGGGGGATCTTCGGACCTCACGCTATCAGATGAGCCTAGGTCGGATTA
GCTAGTTGGTGAGGTAAAGGCTCACCAAGGCGACGATCCGTAACTGGTCTGAGAGGATGATCAGTCACAC
TGGAACTGAGACACGGTC
>Zotu1998
GAACGCTGGCGGCGTGCTTAACACATGCAAGTCGAACGGGCCCGCCCTCTTTTTTGTGGGTGGGTTGAGTG
GCGAACGGGTGAGTATCACGTGAGTAACCTGCCCCCTGCTCCTGGATAACTGCTTGAAAGGGCGGCTAATA
CGGGGTGTGCCGGCTGGTCCGCATGGGCCTGGTCGGGATAGTTTCATCTTCGGGTGTTTCGGTGGGGGAT
GGGCTCGCGGCCTATCAGCTTGTTGGTGGGGTGATGGCCCACCAAGGCGGTGACGGGTAGCCGGCCTGA
GAGGGTGGGCGGCCACACTGGGACTGAGACACGGCC
>Zotu2000
GAACGCTGGCGGCGTGCTTAACACATGCAAGTCGAGCGGTAAGGCACTTCGGTGTACACGAGCGGCGAAC
GGGTGAGTAACACGTGAGTAATCTGCCCTTCACTCTGGGATAAGCTTCGGAAACGGGGTCTAATACCGGAT
ATGACCTGGCCCTGCATGGGGTTGGGTGGAAACTTTTATGGGTGGGGGATGTGCTCGCGGCCTATCAGCT
TGTTGGTGGGGTAATGGCCTACCAAGGCTTCGACGGGTAGCCGGCCTGAGAGGGTGACCGGCCACACTGG
GACTGAGACACGGCC
>Zotu2002
GAACGCTGGCGGCAGGCTTAACACATGCAAGTCGAGCGCCCCGCAAGGGGAGCGGCAGACGGGTGAGTA
ACGCGTGGGAATCTACCTTTTGCTACGGAATAACTCAGGGAAACTTGTGCTAATACCGTATGTGCCCTTCGG

GGGAAAGATTTATCGGCAAAGGATGAGCCCGCGTTGGATTAGCTAGTTGGTGGGGTAAAGGCCCACCAAG
GCGACGATCCATAGCTGGTCTGAGAGGATGATCAGCCACACTGGGACTGAGACACGGCC
>Zotu2004
GAACGCTGGCGGCGTGCTTAACACATGCAAGTCGAACGATGAAGCGGTGCTTGCACCGTGGATTAGTGGC
GAACGGGTGAGTAATACGTGAGTGACCTGCCCTTGACTCTGGGATAAGCCTGGGAAACTGGGTCTAATAC
TGGATACGACCATCAGATGCATGTCTTGGTGGTGGAAAGGGTTTGTACTGGTTTTGGATGGGCTCACGGCC
TATCAGCTTGTTGGTGGGGTAATGGCTTACCAAGGCGACGACGGGTAGCCGGCCTGAGAGGGTGACCGGC
CACACTGGGACTGAGACACGGCC
>Zotu2006
GAACGCTGGCGGCAGGCTTAACACATGCAAGTCGAGCGGAGATGAGGTGCTTGCACCTTATCTTAGCGGC
GGACGGGTGAGTAATGCTTAGGAATCTGCCATTTAGTGGGGGACAACATTCCGAAAGGAATGCTAATACC
GCATACGTCCTACGGGAGAAAGCAGGGGATCTTCGGACCTTGCGCTAAATGATGAGCCTAAGTCGGATTA
GCTAGTTGGTGGGGTAAAGGCCTACCAAGGCGACGATCTGTAGCGGGTCTGAGAGGATGATCCGCCACAC
TGGGACTGAGACACGGCC
>Zotu2008
GAACGCTGGCGGCATGCCTAACACATGCAAGTCGAACGAGACCTTCGGGTCTAGTGGCGCACGGGTGCGT
AACGCGTGGGAATCTGCCCTTGGGTTCGGAATAACTCCTCGAAAGGGGTGCTAATACCGGATGATGTCGTA
AGACCAAAGATTTATCGCCCAAGGATGAGCCCGCGTAGGATTAGCTAGTTGGTGGGGTAAAGGCCCACCA
AGGCGACGATCCTTAGCTGGTCTGAGAGGATGATCAGCCACACTGGGACTGAGACACGGCC
>Zotu2010
GAACGCTGGCGGCGTGCTTAACACATGCAAGTCGAACGATGATCCGGTGCTTGCACCGGGGATTAGTGGC
GAACGGGTGAGTAACACGTGAGTAACCTGCCCTTGACTCTGGGATAAGCCTGGGAAACCGGGTCTAATAC
TGGATACGACCTTTCACCGCATGGTGGTTGGTGGAAAGATTTTTTGGTTTTGGATGGACTCGCGGCCTATC
AGCTTGTTGGTGAGGTAATGGCTCACCAAGGCGACGACGGGTAGCCGGCCTGAGAGGGTGACCGGCCAC
ACTGGGACTGAGACACGGCC
>Zotu2013
GAACGCTGGCGGCAGGCTTAACACATGCAAGTCGAGCGGGCGCTTTCGGGCGTCAGCGGCGGACGGGTT
AGTAACGCGTGGGAACGTGCCCTTTGCTCTGGAATAGCCCTGGGAAACTGGGGGTAATACCGGATGTGCC
CTGAGGGGGAAAGATTTATCGGCAAGGGATCGGCCCGCGTCTGATTAGGTAGTTGGTGAGGTAACGGCTC
ACCAAGCCGACGATCGGTAGCTGGTTTGAGAGGATGATCAGCAACACTGGGACTGAGACACGGCC
>Zotu2016
GAATGCTGGCGGTGTGGATAGGGCATGCAAGTCAAGGGGGCCGCAAGGCAACCGGCAGACGAGATAGTA
ACGCGTAGGAACGTACCCAGAAGTCGGGCACAGCTCGCCGAAAGGCGGGGTAATTCCCGATGGTGTCGCA
AGACTAAAGATTTATCGCTTCTGGAGCGGCCTGCGTAGTATCAGCTAGTTGGTGAAGTAATGGTTTACCAA
GGCTATGACGCTTAGGGGAGCTGAGAGGCTGACCCCCATCGATGGAATTGAGATACGGTC
>Zotu2019
GAATGCTGGCGGCGTGCCTAATACATGCAAGTCGAGCGGTAACAGGGAGTTCCACCGTTTCCTTGACACAC
CTAGCGCTATGATCGTAGGTTGATGACTTCGAGGGAACAGTGGAATTCTGCTGACGAGCGGCGGACGGCT
GAGTAACGCGTGGGAACGGACCCCAAACTGAGGGATAACTAGTCGAAAGATTAGCTAATACCGCATGTGA
TCTTCGGATTAAAGCTTTCGAGCGGTTTGGGACTGGCCTGCGTACGATTAGATAGTTGGTAGGGTAATGGC
CTACCAAGTCGACGATCGTTAGATGGTTTGAGAAGATGATCATCCAGACTGGGACTGAGACACGGCC
>Zotu2021
GAACGCTGGCGGCAGGCTTAACACATGCAAGTCGAGCGGGCATAGCAATATGTCAGCGGCAGACGGGTG
AGTAACGCGTGGGAACGTACCTTTTGGTTCGGAACAACACAGGGAAACTTGTGCTAATACCGGATAAGCCC
TTACGGGGAAAGATTTATCGCCGAAAGATCGGCCCGCGTCTGATTAGCTAGTTGGTGAAGTAATGGCTCAC
CAAGGCGACGATCAGTAGCTGGTCTGAGAGGATGATCAGCCACATTGGGACTGAGACACGGCC
>Zotu2024
CAACGCTGGCGGCGTGCCTAATGCATGCAAGTCGAACGTGCGGATCTTCGGATCCGAGGCGTGGCGGACG
GGTGAGGAACACGTGGGCAAGCTGCCCTGGGGTGGGGGGTAGCCCGCGGAAACGCGGGGTAATTCCGCA
TGAGCTTCCGTGCTGAGTAGGGCATGGAAGGAAAGGGTCTTCGGACTCGCCCGAGGAGGCGCCTGCGGC

CGATTAGCTAGTTGGTGGGGTAACGGCCTACCAAGGCGGTGATCGGTAGCTGGTCTGAGAGGACGGCCA
GCCACACGGGGACTGAGACACGGCC
>Zotu2027
GAACGCTGGCGGCGTGCTTAACACATGCAAGTCGAACGGAAAGGCCCTGCTTGCAGGGTACTCGAGTGGC
GAACGGGTGAGTAACACGTGGGTGATCTGCCCTGCACTTCGGGATAAGCCTGGGAAACTGGGTCTAATAC
CGGATAGGACCATCGTTTAGTGTCGGTGGTGGAAAGTTTTTCGGTGCAGGATGAGCTCGCGGCCTATCAGC
TTGTTGGTGGGGTAATGGCCTACCAAGGCGTCGACGGGTAGCCGGCCTGAGAGGGTGGACGGCCACATT
GGGACTGAGACACGGCC
>Zotu2029
GAACGCTGGCGGCGTGCCTAATACATGCAAGTCGAGCGGACTTGATAAAAGCTTGCTTTTATCAAGTTAGC
GGCGGACGGGTGAGTAACACGTGGGTAACCTGCCTATAAGACTGGGATAACTCCGGGAAACCGGTGCTAA
TACCAGATAGTTTATCCTTTCGCATGAAAGGATAAGGAAAGACGGTTTCGGCTGTCACTTATGGATGGACC
CGCGGCGCATTAGCTAGTTGGTGAGGTAATGGCTCACCAAGGCAACGATGCGTAGCCGACCTGAGAGGGT
GATCGGCCACACTGGGACTGAGACACGGCC
>Zotu2031
TAACGCTGGCGGCATGCCTAATACATGCAAGTCGAACGAAAGTAGCAATACTTTAGTGGCGAACGGGTGA
GTAACACGTATCTAACCTACCATTTAGTGGGGAATAACTGTTGGAAACGGCAGCTAATACCGCATATGTTAT
TTATTTGCAATGATAAATAATGAAAGGAGCATTTGCTTCGCTAATTGATGGGGATGCGGCGCATTAACTAG
TTGGTAAGGTAATGGCTTACCAAGGTAACGATGCGTAGCCGATCTGAGAGGATGAACGGCCACATTGGGA
CTGAGACACGGCC
>Zotu2034
GAACGCTGGCGGCGTGCTTAACACATGCAAGTCGAACGATGATCCAAAGCTTGCTTCGGGGATTAGTGGC
GAACGGGTGAGTAACACGTGAGTAACCTGCCCTTGACTCTGGGATAAGCGCTGGAAACGGCGTCTAATAC
CGGATACGACCTACCCCGGCATCGGGTGTGGGTGGAAAGTTTTTCGGTCAAGGATGGACTCGCGGCCTAT
CAGCTTGTTGGTGAGGTAATGGCTCACCAAGGCGACGACGGGTAGCCGGCCTGAGAGGGTGACCGGCCA
CACTGGGACTGAGACACGGCC
>Zotu2036
GAACGCTGGCGGCGTGCCTAATACATGCAAGTCGAACGCGAGCGATTGGAGCTTGCTCTAATCAATCGAG
TGGCGAACGGGTGAGTAACACGTGGGCAACCTGCCCATCAGCCTGGGATAACCACGGGAAACCGTGGCTA
ATACCGGATAGACCGAGTGACAGAAGTCGCAACGGAGAAAGTCGGTTTTACTGACACTGATGGATGGGCC
TGCGGCGCATTAGCTAGTTGGAGAGGTAGTGGCTCCCCAAGGCAACGATGCGTAGCCGACCTGAGAGGGT
GAACGGCCACACTGGAACTGAGACACGGTC
>Zotu2039
GAACGCTGGCGGCGTGCCTAATACATGCAAGTCGAGCGAACAGACGAGGAGCTTACTCCTCTGACGTTAG
CGGCGGACGGGTGAGTAACACGTGGATAACCTACCTATAAGACTGGGATAACTTCGGGAAACCGGAGCTA
ATACCAGATAACATGTTGAACCGCATGGTTCAACAGTGAAAGACGGTCTTGCTGTCACTTATAGATGGATC
CGCGTCGCATTAGCTAGTTGGTAAGGTAACGGCTTACCAAGGCAACGATGCGTAGCCGACCTGAGAGGGT
GATCGGCCACACTGGAACTGAGACACGGTC
>Zotu2041
GAACGCTAGCGGCAGGCTTAACACATGCAAGTCGAGGGGTAACAGTCCAGCAATGGAGCTGACGACCGG
CAAACGGGTGCGGAACACGTACGCAACATACCTCTATCTGGGGCATAGCCTTCTGAAAAGGAGATTAACAC
CCCATAGTATCTTTGGTTGGCATCAATTGGAGATTAAAAATTCATTGGATAGAGATTGGCGTGCGTCCTATT
AGCTAGTTGGCGGGGTAAAGGCCCACCAAGGCGACGATAGGTAGCTGATGTGAGAGCATGATCAGCCAC
ACGGGCACTGAGACACGGGC
>Zotu2043
TAACGCTGGCGGCGTGCATAACACATGCAAGTCGAACGATGAAACTTTTTTGATTTCTTCGGAATGAAAAA
AAGTGGATTAGTGGCGGACGGGTGAGTAACGCGTGAGTAACCTGCCTTAGAGAAGGGGATAGCCTTTGG
AAACGAAGAATAATACCCTATGAAACCATAACCTCGCATGAGGCCATGGTCAAAGATTTATCGCTCTAAGA
TGGACTTGCGTCTGATTAGTTAGTTGGTGGGGTAACGGCCCACCAAGACAGCGATCAGTAGCCGGCTTGA
GAGAGTGTACGGCCACATTGGGACTGAGACACGGCC

>Zotu2045
GAACGCTGGCGGCATGCTTTACACATGCAAGTCGAACGGCAGCGCGGGGCAACCTGGCGGCGAGTGGCG
AACGGGTGAGTAATACATCGGAACGTACCCAGAAGTGGGGGATAACGTAGCGAAAGTTACGCTAATACCG
CATACGTTCTACGGAAGAAAGTGGGGGACCTTCGGGCCTCATGCTTTTGGAGCGGCCGATGTCTGATTAGC
TAGTTGGTGAGGTAAAGGCTCACCAAGGCGACGATCAGTAGCTGGTCTGAGAGGACGACCAGCCACACTG
GGACTGAGACACGGCC
>Zotu2047
GAACGCTGGCGGCGTGCTTAACACATGCAAGTCGAACGATGAACCAGGTGCTTGCACTTGGGGATTAGTG
GCGAACGGGTGAGTAACACGTGAGCAACCTGCCCCTCACTCTGGGATAAGCGCTGGAAACGGCGTCTAAT
ACTGGATACGAGTCGCAACCGCATGGTTAGTGACTGGAAAGATTTTTTGGTGGGGGATGGGCTCGCGGCC
TATCAGCTTGTTGGTGAGGTAATGGCTCACCAAGGCGTCGACGGGTAGCCGGCCTGAGAGGGTGACCGGC
CACACTGGGACTGAGACACGGCC
>Zotu2049
GAACGCTGGCGGCAGGCCTAACACATGCAAGTCGAACGGTAGCACAGAGAGCTTGCTCTCGGGTGACGA
GTGGCGGACGGGTGAGTAATGTCTGGGAAACTGCCTGATGGAGGGGGATAACTACTGGAAACGGTAGCT
AATACCGCATAACGTCGCAAGACCAAAGAGGGGGACCTTCGGGCCTCTTGCCATCGGATGTGCCCAGATG
GGATTAGCTAGTAGGTGGGGTAACGGCTCACCTAGGCGACGATCCCTAGCTGGTCTGAGAGGATGACCAG
CCACACTGGAACTGAGACACGGTC
>Zotu2051
GAACGCTGGCGGCGTGCCTTAAGCATGCAAGTCGGACGAAGATATGAGATGTCGCAAGACAGATTGTTGA
TTAGTGGCGGACGGGTGAGTATAAGCAGGGAACGTACCTTGAACTATCGGATAGCTCGTCGAAAGACGGG
GTAATACGGTATGTGGACGCAAGTCTAAAGACCGCAAGGTCGGTTCTAGAGCGGCCCTGTTCCCATCAGCT
TGTTGGTAGGGTAACGGCCTACCAAGGCTACGACGGGTAGGGGGTGTGAGAGCACGACCCCCCACAATG
GCACTGAGACACGGGC
>Zotu2053
GAACGCTAGCGATAGGCTTAACACATGCAAGTCGAGGGGCAGCATGGTCTTAGCTTGCTAAGACTGATGG
CGACCGGCGCACGGGTGCGTAACGCGTATGCAACTTGCCTCACAGAGGGGGATAACCCGTCGAAAGACG
GACTAATACCGCGTACACTCCATCTTGGGCATCCAAGGTAGAGGAAATGAAATTCGCTGTGAGATAGGCAT
GCGTCCCATTAGCTAGTTGGTGAGGTAACGGCTCACCAAGGCGACGATGGGTAGGGGAACTGAGAGGTT
GAACCCCCACACTGGTACTGAGACACGGAC
>Zotu2055
GAACGCTGGCGGCGTGCCTTAAGCATGCAAGTAGAACGAGTTCTATCTTGCCGCAAGGCAAGCTAAGAAC
TAGTTGCGAACGGCTGAGTAATGAATAGGAATCTTTCCTTTGGTGGAGAATACCTCATCGAAAGATGAGCT
AATACTGCATAACATCTACGGATAAAAGCGAGTAACTTCGCGCCGATTGAGGAGCCTATTCCCCATCAGGC
AGTTGGTAGGGTAATGGCCTACCAAACCTACGACGGGTACCCGATCTGAGAGGATGGACGGGCAGAATG
GGACTGAGACACGGCC
>Zotu2058
GAACGCTGGCGGCGTGCTTAACACATGCAAGTCGAACGATGAAAGAGGAGCTTGCTCCTCTGGATTAGTG
GCGAACGGGTGAGTAACACGTGAGCAATCTGCCCCTGACTCCGGAATAACTACGGGAAACTGTAGCTAAT
ACCGGATACGCGCCGAACAGGCATCTGTATAGGTGGGAAAGAATTTCGGTCAGGGATGAGCTCGCGGCCT
ATCAGCTTGTTGGTGAGGTAACGGCTCACCAAGGCGACGACGGGTAGCCGGCCTGAGAGGGTGACCGGC
CACATTGGGACTGAGACACGGCC
>Zotu2062
GAACGCTGGCGGCATGCCTAACACATGCAAGTCGAACGAAGGCTTCGGCCTTAGTGGCGCACGGGTGAGT
AACGCGTGGGAATCTGCCTATCAGTGGGGAATAACGACGGGAAACTGTCGCTAATACCGCATACGCCCTTA
GGGGGAAAGATTTATCGCTGATAGATGAGCCCGCGTACGATTAGCTAGTTGGTGAGGTAATGGCTCACCA
AGGCTACGATCGTTAGCTGGTCTGAGAGGATGATCAGCCACACTGGGACTGAGACACGGCC
>Zotu2064
GAACGCTGGCGGCGTGCCTAATGCATGCAAGTCGAGCGACGAATAGATGCGGGTAACCAAGTCTATTACG
GAGCGGCGAACGGGTGAGTAACACGTGAGCAACCTGCCCCGAAGACCGGGATAGCCCAGGGAAACTTGG

ATTAATACCGGATATCCTCTTTTGATCGCATGGTCGTTGGAGGAAATGCTTTTACGCTTCGGGATGGGCTCG
CGGCCTATCAGCTTGTTGGTGGGGTAACGGCCTACCAAGGCGTCGACGGGTAGCTGGTCTGAGAGGACGA
TCAGCCACACTGGGACTGAGACACGGCC
>Zotu2068
GAACGCTGGCGGCGTGCCTAACACATGCAAGTCGAGCGAACCCTTCGGGGTGAGCGGCGGACGGGTGAG
TAACGCGTGGGTAACCTGCCCTGTACACATGGATAACATACCGAAAGGTATGCTAATACGAGATAATATGT
AATGATCGCATGGTCGATACATCAAAGCTCCGGCGGTACAGGATGGACCCGCGTCTGATTAGCTAGTTGGT
AAGGTAACGGCTTACCAAGGCGACGATCAGTAGCCGACCTGAGAGGGTGATCGGCCACATTGGAACTGAG
ACACGGTC
>Zotu2070
GAACGCTGGCGGCGTGCTTAACACATGCAAGTCGAACGATGATCCGGACCTTGCTTCGGGGATTAGTGGC
GAACGGGTGAGTAACACGTGAGTAACCTGCCCTTGACTCTGGGATAAGCGCTGGAAACGGCGTCTAATAC
CGGATACGACCTACCCCGGCATCGGGTGTGGGTGGAAAGTTTTTCGGTCAAGGATGGACTCGCGGCCTAT
CAGCTTGTTGGTGAGGTAATGGCTCACCAAGGCGACGACGGGTAGCCGGCCTGAGAGGGTGACCGGCCA
CACTGGGACTGAGACACGGCC
>Zotu2072
TAACGCTGGCGGCGTGCATAACACATGCAAGTCGAACGATGAAGCTTAGATGATTTCTTCGGAATGACCTT
AAGTGGATTAGTGGCGAACGGGTGAGTAACGCGTGAGTAACCTGCCTTACACAAGGGGATAGCCTCTGGA
AACGGAGAATAATACCCTATGACATTACAACCTCGCATGAGGAAGTAATCAAAGTGTTTTAGCGGTGTAAG
ATGGACTTGCGTCTGATTAGCTAGTTGGTGAGATAACAGCCCACCAAGGCAACGATCAGTAGCCGGCTTGA
GAGAGTGTACGGCCACATTGGGACTGAGACACGGCC
>Zotu2074
GAACGCTAGCGGCAGGCTTAACACATGCAAGTCGAGGGGTAGAGTAGCAATACTTGAGACCGGCGCACG
GGTGCGTAACGCGTATGCAACCTACCTTTTACAGGGGAATAGCCCAGAGAAATTTGGATTAATGCCCCATG
GTATATTTTAGTGGCATCACTAGATTATTAAAGTTCCAACGGTAAAAGATGGGCATGCGTCCTATTAGCTAG
ATGGTAAGGTAACGGCTTACCATGGCTACGATAGGTAGGGGTCCTGAGAGGGAGATCCCCCACACTGGTA
CTGAGACACGGAC
>Zotu2077
GAACGCTGGCGGCGTGCTTAACACATGCAAGTCGAACGATGAAGCCCTTCGGGGTGGATTAGTGGCGAAC
GGGTGAGTAACACGTGGGCAATCTGCCCTTCACTCTGGGACAAGCCCTGGAAACGGGGTCTAATACCGGA
TACAACCATCGGCCGCATGGTCTGGTGGTGGAAAGCTCCGGCGGTGAAGGATGAGCCCGCGGCCTATCAG
CTTGTTGGTGAGGTAGTGGCTCACCAAGGCGACGACGGGTAGCCGGCCTGAGAGGGCGACCGGCCACAC
TGGGACTGAGACACGGCC
>Zotu2079
GAACGCTAGCGGCAGGCCTAATACATGCAAGTCGGACGGGATTGGAGAGCTTGCTTTCCATGAGAGTGGC
GCACGGGTGCGTAACACGTATGTAACCTACCTTAATCTGGGGGATAGCCTCTCGAAAGAGAGATTAACACC
GCATAACATGTTTGAATAGCATTATTTAAACATCAAATATTTATAGGATTAAGATGGGCATGCGGAACATTA
GCTAGTTGGTAAGGTAATGGCTTACCAAGGCTACGATGTTTAGGGGATCTGAGAGGATGACCCCCCACACT
GGTACTGAGACACGGAC
>Zotu2082
GAACGCTGGCGGCGCGCTTAACACATGCAAGTCGAGCGAGAACCAAGGCTTCGGTCTTGGGGACAGCGG
CGAACGGGTGAGTAACACGTGGGTAATCAACCCTCGACACTGGGATAGCCCGGGGAAACCCGGATTAATA
CCGGATACGCCATCTGGCCTTCGGGCCGGCTGGAAAAGGTAGCTTTGGCCTCCGGTCGAGGACGAGCCCG
CGGCGGATTAGCTTGTTGGCGGGGTAAAAGCCCACCAAGGCGACGATCCGTAGCTGGTCTGAGAGGACG
ATCAGCCACACTGGGACTGAGACACGGCC
>Zotu2084
GAACGCTGGCGGCGTGCTTAACACATGCAAGTCGAACGGTAAGGCCCTTCGGGGTACACGAGTGGCGAAC
GGGTGAGTAACACGTGGGTGATCTGCCCTGCACTTTGGGATAAGCCTGGGAAACTGGGTCTAATACCGAA
TAGGATCATGGCCTTCATGGGTTGTGGTGGAAAGCTTTTGCGGTGTGGGATGGGCCCGCGGCCTATCAGC
TTGTTGGTGGGGTAATGGCCTACCAAGGCGACGACGGGTAGCCGGCCTGAGAGGGTGTCCGGCCACACTG
GGACTGAGATACGGCC

>Zotu2089
GAACGCTGGCGGTGTGCCTAATACATGCAAGTCGAACGAGATCTTCGGATCTAGTGGCGGACGGGTGAGT
AACACGTTGGTACCTACCCCTAAGTGGGGAATAACTGCCCGAAAGGGTGGCTAATACCGCATAATCCCGCA
AGGGTAAAGCAGTAATGCGCTTAGGGAGGGGCCTGCGTCTGATTAGCTAGTTGGTGAGGTAATTGCTCAC
CAAGGCGACGATCAGTAGCTGGATCGAGAGGTTGACCAGCCACACTGGGACTGAGATACGGCC
>Zotu2091
GAACGCTGGCGGCGTGCCTAATACATGCAAGTAGAACGCTGAAGCTTGGTGCTTGCACCGAGCGGATGAG
TTGCGAACGGGTGAGTAACGCGTAGGTAACCTGCCTCTTAGCGGGGGATAACTATTGGAAACGATAGCTA
ATACCGCATAAAAGTCGATATCGCATGATGTTGACTTGAAAGGTGCAAATGCATCACTAAGAGATGGACCT
GCGTTGTATTAGCTAGTTGGTGAGGTAACGGCTCACCAAGGCGACGATACATAGCCGACCTGAGAGGGTG
ATCGGCCACACTGGGACTGAGACACGGCC
>Zotu2093
GAACGCTGGCGGCATGGATAGGGCATGCAAGTCGAGTGGGTTTAGACCCACGGCAAACGGGTTAGTAAT
GCGTAGGAATGTTCCTCAAAGTCGTGAATAATTTGTCGAAAGACAAGCTAATACACGATGTGCTCCACGGA
GTAAAGGCTTGCCGCTTTGAGAGCAGCCTGCGTCCTATCAGCTAGTTGGTGGGGTAAAAGCCTACCAAGGC
TTTGACGGGTAGGGGGTGTGAGAGCATGGCCCCCAACATTGGGACTGAGACACTGCC
>Zotu2095
GAACGCTAGCGGGAGGCCTAACACATGCAAGCTGAGCGGTAGAGTTCCTTCGGGGACTTGAGAGCGGCG
TACGGGTGCGGAACACGTGTGCAACCTGCCTTTATCAGGGGGATAGCCTTTCGAAAGGAAGATTAATACCC
CATAATATAATTGATGGCATCATTGATTATTGAAAACTCCGGTGGATAAAGATGGGAACGCGCAAGATTAG
ATAGTTGGTGAGGTAACGGCTCACCAAGTCGATGATCTTTAGGGGGCCTGAGAGGGTGATCCCCCACACT
GGTACTGAGACACGGAC
>Zotu2098
GAACGCTGGCGGCGTGGATAAGGCATGCAAGTCGCGTGGGTTTAGACCCACGGCAAACGGGTTAGTAAT
GCATAGGAACATTCCCAGAAGTCGTGAATAATCCGGAGAAATCCGGGACAATACACGATGTGCTCTACGG
AGTAAAGATTTATCGCTTTTGGAGTGGCCTGTGTCCTATCAGCTAGTTGGTGAGGTAATGGCTCACCAAGG
CTATGACGGGTAGGGGGCGTGAGAGCGTGGCCCCCAACATTGGGACTGAGACACTGCC
>Zotu2100
GAACGCTGGCGGCGTGCCTAAGACATGCAAGTCGAGCGGGGGGACTGGGGAGCTTGCTCTCCGGTCTCCT
AGCGGCGGACGGGTGAGTAACACGTGGGCAACCTGCCCGGCAGTGGGGGATAACCCTGGGAAACTGGG
GCTAATACCGCATACGGTCCGCGTTCCGCATGGGGCGTGGAAGAAAGGCCGTCGTGAGGCGGTCGCTGCC
GGAGGGGCCCGCGGCCCATCAGCTGGTTGGTGGGGTAACGGCCCACCAAGGCGACGACGGGTAGCCGGC
CTGAGAGGGTGGCCGGCCACACTGGGACTGAGACACGGCC
>Zotu2102
GAACGTTGGCGGCGTGGATTAGGCATGCAAGTCGGACGGGCCGCAAGGCCAGTGGCGCAAGGGTGAGTA
AGGCGACGGTAACCAACCCCAGAGACGGGCATAGCCGGGGGAAACCCCGGGTAATTCCCGGCGACGTGG
CGACCCGGCATCGGGGTGCCACCAAAGATTCATCGCTCTGGGACGGGCCGTCGTGGTATTAGGTTGTTGG
CGGGGTGACGGCCCACCAAGCCTGCGATGCCTACCGGGCGTGCGAGCGTGGCCCGGCACACTGGGACTG
AGACACTGCC
>Zotu2104
GAACGCTGGCGGCATGCCTAATACATGCAAGTCGAACGAGAGGAAGGAAAGCTTGCTTTTTTGAATCTAGT
GGCGAACGGGTGAGTAACACGTAGGTAACCTGCCCATGTGCCCGGGATAACTTCTGGAAACGGATGCTAA
AACCGGATAGGTAGCAGACAAGCATTTGACTGCTATTAAAGTGGCTAAGGCCATGAACATGGATGGACCT
GCGGTGCATTAGCTAGTTGGTGAGGTAACGGCCCACCAAGGCGACGATGCATAGCCGGCCTGAGAGGGC
GGACGGCCACATTGGGACTGAGACACGGCC
>Zotu2107
GAACGCTGGCGGCGCGCCTAAAACATGCAAGTCGAACGAGAAGCCCGGGCTTGCCCGGGTGGACAGTGG
CGAACGGGTGAGTAACACGTGAGGAACCTGCCCCGAAGACCGGGACAACATCGGGAAACCGTTGCTAATA
CCGGATACCTTCACCGGATCGCATGTTCTGGTGAAGAAATGGATTCCGCTTCGGGATGGCCTCGCGGCCTA

TCAGCTAGTTGGTGAGGTAACGGCTCACCAAGGCGACGACGGGTAGCTGGTCTGAGAGGATGTCCAGCCA
CACTGGGACTGAGACACGGCC
>Zotu2109
GAACGCTGGCGGCGTGCTTAACACATGCAAGTCGAACGAGACCCTCGCTTCGGTGGGGTGTTCTAGTGGC
GAACGGGTGAGTAACACGTGGGCAACCTGCCCCCGGCTCTGGGATAACTCCAAGAAATTGGGGCTAATAC
CGGATATGACCGCTGACCGCATGGTCTGGTGGTGGAAAGATTTATCGGCTGGGGATGGGCCCGCGGCCTA
TCAGCTTGTTGGTGGGGTAGTGGCCTACCAAGGCGACGACGGGTAGCCGGCCTGAGAGGGTGACCGGCC
ACACTGGGACTGAGACACGGCC
>Zotu2112
AAACGCTGGCGGCGTGCGTAACACATGCAAGTCGAACGAGAATGTACTGACGGACCCTTAGGGTGAAGAT
AGTACAGGACAGTGGCGGATGGGTGAGTAACGCGTAAGAAACCTGCCTTTCACACCGGGATAGCAGCTGG
AAACGGCTATTAATACCGGATGACACTTTTTCCCCGCATGAGGAAGAGGTTAAAGAATTTCGGTGAAAGAT
GGTCTTGCGTCTGATTAGCTAGTTGGTGGGGTAACGGCCTACCAAGGCGACGATCAGTAGCCGGCCTGAG
AGGGTGTACGGCCACATTGGGACTGAGACACGGCC
>Zotu2114
GAACGCTGGCGGCGTGCTTAACACATGCAAGTCGAACGATGACGACCGGGCTTGCCTGGTCTGATTAGTG
GCGAACGGGTGAGTAACACGTGAGTAACCTGCCCCTCACTTCGGGATAACCTCGGGAAATCGAGGCTAAT
ACCGGATACGAGTTTCCGCCGCATGGTGGTTTCTGGAAAGTTTTTCGGTGAGGGATGGACTCGCGGCCTAT
CAGTTTGTTGGTGAGGTAATGGCTCACCAAGGCGATGACGGGTAGCCGGCCTGAGAGGGCGACCGGCCA
CACTGGGACTGAGACACGGCC
>Zotu2117
GAACGCTGGCGGCGTGCTTAACACATGCAAGTCGAACGGATCACTCTTCGGGGTGGTTAGTGGCGAACGG
GTGAGTAACACGTGAGCAACCTGCCCTTCACTCTGGGATAACTGTGGGAAACTTCAGCTAATACCGGATAT
TACCTGTCACTGCATGGTGGTGGGTGGAAAGATTTTTTGGTGGGGGATGGGCTCGCGGCCTATCAGCTTGT
TGGTGGGGTGATGGCCTACCAAGGCTTTGACGGGTAGCCGGCCTGAGAGGGCGACCGGCCACACTGGGA
CTGAGATACGGCC
>Zotu2119
GAACGCTGGCGGCGTGCCTAATACATGCAAGTCGAGCGGACGAACTTTTGGTGCTTGCACCAAATTTTCGT
TAGCGGCGGACGGGTGAGTAACACGTGGGTAACCTGCCCTACAGACTGGGATAACTTCGGGAAACCGAG
GCTAATACTGGATAATACTTTTTAACACATGTTAGAAAGTTGAAAGATGGTTTTGCTATCACTGTAGGATGG
ACCCGCGGCGCATTAGCTAGTTGGTGAGGTAACGGCTCACCAAGGCAACGATGCGTAGCCGACCTGAGAG
GGTGATCGGCCACACTGGGACTGAGACACGGCC
>Zotu2121
GAACGCTGGCGGCGTGCTTAACACATGCAAGTCGAACGATGATCAGGAGCTTGCTCCTGTGATTAGTGGC
GAACGGGTGAGTAACACGTGAGTAACCTGCCCCTGACTCTGGGATAAGCGTTGGAAACGACGTCTAATAC
TGGATATGATCACCGGCCGCATGGTCTGGTGGTGGAAAGATTTTTTGGTTGGGGATGGACTCGCGGCCTAT
CAGCTTGTTGGTGAGGTAATGGCTCACCAAGGCGACGACGGGTAGCCGGCCTGAGAGGGTGACCGGCCA
CACTGGGACTGAGACACGGCC
>Zotu2123
GAACGCTGGCGGCGTGCTTAACACATGCAAGTCGAACGATGATGCCCAGCTTGCTGGGTGGATTAGTGGC
GAACGGGTGAGTAACACGTGAGCAACCTGCCCCCGACTCTGGGATAAGCCCGGGAAACTGGGTCTAATAC
CGGATACGACCATTGGCCGCATGGCCTGGTGGTGGAAAGACTTGTTGGTTGGGGATGGGCTCGCGGCCTA
TCAGCTTGTTGGTGGGGTAATGGCCCACCAAGGCGACGACGGGTAGCCGGCCTGAGAGGGTGACCGGCC
ACACTGGGACTGAGACACGGCC
>Zotu2125
GAACGCTGGCGGCGTGCTTAACACATGCAAGTCGAACGGTGAAGGGGCCTGCTTTTGTGGGTCCTGGATG
AGTGGCGAACGGGTGAGTAACACGTGAGTAACCTGCCCCCTTCTTCTGGATAACCGCATGAAAGTGTGGCT
AATACGGGATATTCTGGGTCTGTCGCATGGTGGGCCTGGGAAAGATTGCGCCTTTTTTGGTGTTTTTGGTG
GGGGATGGGCTCGCGGCCTATCAGCTTGTTGGTGGGGTGATGGCCTACCAAGGCGGTGACGGGTAGCCG
GCCTGAGAGGGTGGACGGTCACACTGGGACTGAGACACGGCC

>Zotu2129
GAACGCTGGCGGCGTGCCTAATACATGCAAGTCGAGCGGACAGATGGGAGCTTGCTCCCTGATGTTAGCG
GCGGACGGGTGAGTAACACGTGGGTAACCTGCCTGTAAGACTGGGATAACTCCGGGAAACCGGGGCTAA
TACCGGATGGTTGTTTGAACCGCATGGTTCAAACATAAAAGGTGGCTTCGGCTACCACTTACAGATGGACC
CGCGGCGCATTAGCTAGTTGGTGAGGTAATGGCTCACCAAGGCAACGATGCGTAGCCGACCTGAGAGGGT
GATCGGCCACACTGGGACTGAGACACGGCC
>Zotu2131
GAACGCTGGCGGCATGCTTAACACATGCAAGTCGCACGGGCAGCAATGTCAGTGGCGGACGGGTGAGTAT
CGCGTAGGAATGTATCCTGAGGTGGGGGACAACCCTGGGAAACTGGGGCTAATACCGCATATGGACTGAG
GTCCAAAGCCGTGAGGCGCCTTGGGAGCAGCCTGCGTCCGATTAGGTAGTTGGTGGGGTAAAGGCCTACC
AAGCCTGCGATCGGTAGCTGGTCTGAGAGGACGATCAGCCACACTGGGACTGAGACACGGCC
>Zotu2133
GAATGCTAGCGGTGTGCCTGAGATATGCAAGTCGAGCGACCCCTAAGCCGCAAGGTTTAGGGGAGCGGC
GGACGGCTGAGTAACACGTAGGAACCTGCCCCTTTCTGGAGGATAGCTTACCGAAAGGTGAGGTAATACT
CCATAGTCCCCACCTTTGGTGGGGTAAAGATTTATCGGAAAGGGATGGGCCTGCGTCCTATCAGGTAGTTG
GTGGGGTAACGGCCTACCAAGCCAATGACGGGTAGCTAATCTGAGAGGATGGTTAGCCAGAGGGGGACT
GAGACACGGCC
>Zotu2135
GAACGCTAGCGGCAGGCCTAACACATGCAAGTCGAGGGGCAGCATAAATAAAGCTTGCTTTATTGGTGGC
GACCGGCGCACGGGTGAGTAACGCGTATGCAACCTACCTTATACTGGGGAATAGCCCGAAGAAATTCGGA
TTAATACCCCATAGTATTATCGAATGGCATTATTTGATGATTAAAGTTTCGGCGGTATAAGATGGGCATGCG
TCCTATTAGCTAGATGGTGAGGTAACGGCTCACCATGGCTACGATAGGTAGGGGGCCTGAGAGGGTGGTC
CCCCACACTGGTACTGAGACACGGAC
>Zotu2137
GAACGCTGGCGGCAGGCTTAACACATGCAAGTCGATCGCCCCGCAAGGGGAGCGGCAGACGGGTGAGTA
ACACGTGGGAACGTACCCTTTGGTTCGGAACAACCCAGGGAAACTTGGGCTAATACCGGATAAGTCCGTA
AGGAGAAAGATTTATCGCCAAAGGATCGGCCCGCGTCTGATTAGCTAGTTGGTAGGGTAATGGCCTACCA
AGGCTACGATCAGTAGCTGGTCTGAGAGGATGATCAGCCACATTGGGACTGAGACACGGCC
>Zotu2139
GGCAAGGTGGGAGGCTGCTGCACTCAAGTGCTCCCTCTGACTCCCAGAATCTGCAACTACACACAAGAGCG
GTTCCCTCTGAAGGAAAACCAAAACCCAGCTGA
>Zotu2141
GAACGCTGGCGGTGTGCTTCACACATGCAAGTCGAACGAGGTAGCAATACCTAGTGGCGGACGGGTGAGT
AACGCGTAGAAATCTGCCTTCAGGTGGGGGATAACAGTTCGAAAGAACTGCTAATACCGCATATGTGTACA
ATTCGACAGTTTTGTACATGAAAGGAGCAATTTGCCTGGAGATGAGTCTGCGTCCGATTAGCTAGTTGGTA
GGGTAAAGGCCTACCAAGGCGACGATCGGTAGGTGGTCTGAGAGGATGATCACCCACAATGGGACTGAG
ACACGGCC
>Zotu2143
GAACGCTGGCGGCGTGCTTAACACATGCAAGTCGAACGGAAAGGCCCTTCGGGGTACTCGAGTGGCGAAC
GGGTGAGTAACACGTGGGTGATCTGCCTCGTACTCTGGGATAAGCCTGGGAAACTGGGTCTAATACCGGA
TAGGACCTTCTCTCGCATGGGGGTTGGTGGAAAGTTTTTCGGTACGAGATGGGCCCGCGGCCTATCAGCTT
GTTGGTGGGGTAATGGCCTACCAAGGCGACGACGGGTAGCCGGCCTGAGAGGGCGACCGGCCACACTGG
GACTGAGACACGGCC
>Zotu2145
GAACGCTGGCGGCATGCCTTACACATGCAAGTCGAACGGCAGCACGGGAGCAATCCTGGTGGCGAGTGG
CGAACGGGTGAGTAAAGTATCGGAACGTGCCCAGTCGTGGGGGATAACGTAGCGAAAGCTACGCTAATAC
CGCATACGATCTATGGATGAAAGCGGGGGATCGCAAGACCTCGCGCGATTGGAGCGGCCGATATCAGATT
AGGTAGTTGGTGGGGTAAAAGCCCACCAAGCCAACGATCTGTAGCTGGTCTGAGAGGACGACCAGCCACA
CTGGGACTGAGACACGGCC
>Zotu2149

GAACGCTGGCGGCGCGCTTAACACATGCAAGTCGTACGGGGGTCTTCGGACCTCAGTGGCGAACGGGTGC
GTAACACGTGAGCAACCTGCCTGGTCGTGGGGGATAACCAGTGGAAACGCTGGCTAATACCGCATGAGCT
CTTTCTGCCGATTGGTGGGATGAGGAAAGCTCCTGCAAGGGAGTGCGACCAGAGGGGCTCGCGGCGTATC
AGCTAGTTGGAGGGGTAACGGCCCACCAAGGCGATGACGCGTAGCCGATCTGAGAGGATGATCGGCCAC
ATTGGGACTGAGATACGGCC
>Zotu2153
GAACGCTGGCGGCATGCTTTACACATGCAAGTCGAACGGCAGCACGGGCTTCGGCCTGGTGGCGAGTGGC
GAACGGGTGAGTAATACATCGGAACGTACCCAGAAGTGGGGGATAACGTAGCGAAAGTTACGCTAATACC
GCATACGTTCTACGGAAGAAAGTGGGGGATCGCAAGACCTCATGCTTTTGGAGCGGCCGATGTCTGATTA
GCTAGTTGGTGAGGTAAAGGCTCACCAAGGCGACGATCAGTAGCTGGTCTGAGAGGACGACCAGCCACAC
TGGAACTGAGACACGGTC
>Zotu2155
GAACGCTGGCGGCATGCCTAACACATGCAAGTCGAACGCAGTAGCAATACTGAGTGGCGGACGGGTGAG
GAACACGTGGGAACATGCCGAATGGTGGGGGATAACTGCGGGAAACTGCAGCTAATACCGCATAAGCCCT
GAGGGGGAAAGATTTATCGCCATTCGATTGGCCCGCGGCAGATTAGGTAGTTGGTAGGGTAAAGGCCTAC
CAAGCCGATGATCTGTAACTGGTCTGAGAGGATGACCAGTCACACTGGGACTGAGACACGGCC
>Zotu2157
GAACGCTGGCGGCAGGCTTAACACATGCAAGTCGAACGCCCCGCAAGGGGAGTGGCAGACGGGTGAGTA
ACGCGTGGGAATCTACCTAGAACTACGGAACAACTGATGGAAACGTCAGCTAATACCGTATACGCCCTACG
GGGGAAAGATTTATCGGTTTTAGATGAGCCCGCGTAAGATTAGCTAGTTGGTGGGGTAATGGCCTACCAA
GGCGACGATCTTTAGCTGGTCTGAGAGGATGATCAGCCACACTGGGACTGAGACACGGCC
>Zotu2159
GAACGCTGGCGGCGTGCTTAACACATGCAAGTCGAGCGGTAAGGCCCTTCGGGGTACACGAGCGGCGAA
CGGGTGAGTAACACGTGAGCAATCTGCCCTTCTCTTTGGAATAACCATTGGAAACGATGGCTAATGCCGAA
TATGAACCCCTTTCGCATGTTGGGGGTTGGAAAGCTCCGGCGGAGAAGGATGAGCTCGCGGCCTATCAGC
TTGTTGGTGAGGTAATGGCTCACCAAGGCATTGACGGGTAGCCGGCCTGAGAGGGTGACCGGCCACACTG
GGACTGAGACACGGCC
>Zotu2161
GAACGCTGGCGGCATGCTTAACACATGCAAGTCGAACGAAAACAAAAAAATAAGTAATTATTTTTTTGTTTT
AGTGGCGGACGGGTGAGTAACGCGTAAGAATCTACCTTTAGGAGGGGGAAAACAACTGGAAACGGTTGC
TAACACCCCATATGCTGAGGAGTAAAATACATTGTGCCTAGAGATGAGCTTGCGTCTGATTAGTTAGTTGG
TAAGGTAATTGCTTCCCAAGACTATGATCAGTAGCTGGTCTGAGAGGATGATCAGCCACACTGGGACTGAG
ACACGGCC
>Zotu2163
GAACGCTGGCGGCGTGCTTAACACATGCAAGTCGAACGGAAAGGCCTCTTGCTTGCAGGAGGTACTCGAG
TGGCGAACGGGTGAGTAACACGTGGGTGATCTGCCTTGCACTCTGGGATAAGCTTGGGAAACTGGGTCTA
ATACCGGATAGGAACCGTCTTTAGTGTGGTGGTTGGAAAGTTTTTTCGGTGCAAGATGAGCTCGCGGCCTA
TCAGCTTGTTGGTGGGGTAATGGCCTACCAAGGCGTCGACGGGTAGCCGGCCTGAGAGGGTGTACGGCCA
CATTGGGACTGAGATACGGCC
>Zotu2165
AAACGCTGGCGGCGCACATAAGACATGCAAGTCGAACGGAATCGAGTCGAATGTTTACATGAGACGAAGA
TTTAGTGGCGGACGGGTGAGTAACACGTGAGCAACCTACCTTTAAGAGGGGGATAACATTGAGAAATCAG
TGCTAATACCGCATAAAGCAAGCGAATCGCATGATAAGCTTGCCAAAGGAGCAATCCGCTTAAAGATGGG
CTCGCGGCTGATTAGTTAGTTGGTGGGGTAAAGGCCTACCAAGACGATGATCAGTAGCCGAGCTGAGAGG
CACAACGGCCACATTGGGACTGAGACACGGCC
>Zotu2167
GAACGCTGGCGGTGTGCTTCACACATGCAAGTCGAACGGGGTAGCAATACCCAGTGGCGGACGGGTGAG
TAACACGTGGGAATCTACCATAAGGTGGGGGATAGCAGACCGAAAGGTCTATTAATACCGCATATGTAGG
CATCTCGACAGAGGTGTTTATGAAAGGAGTAATCCGCCTTTTGATGAGCCCGCGTCCGATTAGCTAGTTGG
TAGGGTAAAAGCCTACCAAGGCGACGATCGGTAGGTGGTCTGAGAGGATGATCACCCACAATGGGACTGA
GACACGGCC

>Zotu2169
GAACGCTGGCGGCGTGCTTAACACATGCAAGTCGAACGAAGAGCGACGGAAGCTTGCTTCTATCAATCTTA
GTGGCGAACGGGTGAGTAACGCGTAATCAACCTGCCCTTCAGAGGGGGACAACAGTTGGAAACGACTGCT
AATACCGCATACGATCTAATCTCGGCATCGAGGATGGATGAAAGGTGGCCTCTACATGTAAGCTATCACTG
AAGGAGGGGATTGCGTCTGATTAGCTAGTTGGAGGGGTAACGGCCCACCAAGGCGATGATCAGTAGCCG
GTCTGAGAGGATGAACGGCCACATTGGGACTGAGACACGGCC
>Zotu2171
GAACGCTGGCGGCAGGCTTAACACATGCAAGTCGAGCGGGCCCTTCGGGGTCAGCGGCGGACGGGTTAG
TAACGCGTGGGAACGTGCCCTTTGCTCTGGAATAGCCCTGGGAAACTGGGGGTAATACCGGATGTGCCCT
GAGGGGGAAAGATTTATCGGCAAGGGATCGGCCCGCGTCTGATTAGGTAGTTGGTGGGGTAACGGCTCA
CCAAGCCGACGATCGGTAGCTGGTTTGAGAGGATGATCAGCAACACTGGGACTGAGACACGGCC
>Zotu2173
GAACGCTGGCGGCGTGCCTAATACATGCAAGTCGAACGGGCTCTTCGGAGCTAGTGGCGGACGGGTGAGT
AACACGTTGGTACCTACCCCTAAGTGGGGAATAACTGCCCGAAAGGGTGGCTAATACCGCATAATCCCGCA
AGGGTAAAGCAGTAATGCGCTTAGGGAGGGGCCTGCGTCTGATTAGCTAGTTGGTAGTGTAATGGACTAC
CAAGGCGACGATCAGTAGCTGGATCGAGAGGTTGACCAGCCACACTGGGACTGAGATACGGCC
>Zotu2175
GAACGCTGGCGGCGTGCTTAACACATGCAAGTCGAACGGTGATGATGGGGCTTGCCCTGTCTGATCAGTG
GCGAACGGGTGAGTAACACGTGAGTAACCTGCCCCTCACTCTGGGATAACCTCGGGAAATCGTGGCTAAT
ACCGGATATGAGTTTCCACCGCATGGTGGTTTCTGGAAAGTTTTTCGGTGAGGGATGGACTCGCGGCCTAT
CAGTTTGTTGGTGAGGTAATGGCTCACCAAGGCGATGACGGGTAGCCGGCCTGAGAGGGCGACCGGCCA
CACTGGGACTGAGACACGGCC
>Zotu2177
GAACGCTGGCGGCGTGGCTAAGACATGCAAGTCGCACGGACCCGCAAGGGTTAGTGGCGAACGGGTGAG
TAATGAATCGCTAATGTACCCCGGACTCTGGGATAGCTAGCCGAAAGGCTAGGTAATACCAGATGATATGC
AGCTGTCGCATGACAGTTGCCTCAAAGGTCCGCCGGTCTGGGAGCAGGCGATTTCGTATCAGCTTGTTGGT
GAGGTAACGGCTCACCAAGGCGACGACGCGTAGCCGGACTGAGAGGTTGGCCGGCCACATTGGGACTGA
GACACTGCC
>Zotu2179
GAATGCTGGCGGCGTGGATAAGTCATGCAAGTCAAGGGGACCGCAAGGTAACCGGCAAACGAGGTAGTA
ACACGTAGGTACTCCCCCCGAAGTTTGGCATAGCTCGTCGAAAGACGGGGTAATTCCAAATAGTCCCGCAA
GGGTAAAGATTTATCGCTTCGGGAGAGGCCTGCGTAGTATCAGTTAGTTGGTAGGGTAACGGCCTACCAA
GGCTACGACGCTTAGGGGAGCTGAGAGGCTGACCCCCACCGATGGGACTGCGACACGGCC
>Zotu2181
GAACGCTAGCGGCAGGCTTAACACATGCAAGTCGAGGGGTATAGTAGCAATACTAGAGACCGGCGCACG
GGTGCGTAACGCGTATGCAATCTACCTTTTACAGAGGGATAGCCCAGAGAAATTTGGATTAATACCTCATA
GCATAGCAGCTTCGCATGAAGTCACTATTAAAGTTCCAACGGTAAAAGATGAGCATGCGTCCTATTAGCTA
GATGGTAAGGTAACGGCTTACCATGGCTACGATAGGTAGGGGTCCTGAGAGGGAGATCCCCCACACTGGT
ACTGAGACACGGAC
>Zotu2183
GAACGCTGGCGGCGTGCCTAACACATGCAAGTCGAACGAGGTTGATCTGGTGGCAACACCAGAGACTACC
TAGTGGCGAACGGGTGAGTAACACGTGAGGAACCTGCCCCGAAGACCGGGATAACCCTCCGAAAGGAGA
GCTAATACCGGATACCCCCATCGGATCGCATGATCTGATGAGGAAATGGATTCCGCTTCGGGAGGGCCTCG
CGGCCTATCAGCTTGTTGGCGGGGTAACGGCCCACCAAGGCATCGACGGGTAGCTGGTCTGAGAGGACGA
TCAGCCACACTGGGACTGAGACACGGCC
>Zotu2185
GAATGCTGGCGACATGGATAAGTCATGCAAGTCAAGGGGGGCCGCAAGGTCAACCGGCAGACGAGGTAG
TAACACGTAGGTACGCACCCCAGAGTCTGGCATAGCTCGTCGAAAGACGGGGTAATTCCAGATGGTCCCG
CAAGGGTAAAGATTTATCGCTTTGGGAGCGGCCTGCGTAGTATCAGCTAGTTGGTAAGGTAATGGCTTACC
AAGGCTACGACGCTTAGGGGAGCTGAGAGGCTGACCCCCACCGATGGGACTGAGACACGGCC

>Zotu2187
GAACGCTGGCGGCATGCTTTACACATGCAAGTCGAACGGCAGCGCGGGGCAACCTGGCGGCGAGTGGCG
AACGGGTGAGTAAAATATCGGAACGTACCCAGAAGTGGGGGATAACGTAGCGAAAGTTACGCTAATACCG
CATACGATCTACGGATGAAAGTGGGGGACCTTCGGGCCTCATGCTTTTGGAGCGGCCGATATCTGATTAGC
TAGTTGGTGAGGTAAAGGCTCACCAAGGCGACGATCAGTAGCTGGTCTGAGAGGACGACCAGCCACACTG
GGACTGAGACACGGCC
>Zotu2190
GAACGCTGGCGGCGTGCTTAACACATGCAAGTCGAGCGGAAAGGCCACTTCGGTGGTACTCGAGCGGCGA
ACGGGTGAGTAACACGTGAGTAATCTGCCCTCCACTTCGGGATAGCCACCGGAAACGGTGATTAATACCG
GATATGACGCATGGTCGCATGGTCGTGTGTGGAAAGTTTTTCGGTGGGGGATGTGCTCGCGGCCTATCA
GCTTGATGGTGAGGTAATGGCTCACCATGGCTACGACGGGTAACCGGCCTGAGAGGGTGACCGGTCACAC
TGGGACTGAGACACGGCC
>Zotu2192
GAACGCTAGCGGCAGGCCTAATACATGCAAGTCGAACGGGTGTCCCTCGGGGCACCAGTGGCGCACGGGT
GCGTAACGCGTATGCAACCTACCCCCCACTGGGGAATAGCCTCCCGAAAGGGAGATTAATACCGCATAACA
CAGCTTGAGGGCATCTGAGGGCTGTTAAAGATTTATCGGTTGGGGATGGGCATGCGTGCCATTAGCTAGTT
GGTGGTGTAACGGACCACCAAGGCGACGATGGCTAGGGGTTCTGAGAGGACGGTCCCCCACACTGGCACT
GAGATACGGGC
>Zotu2194
GAACGTTGGCGGCGTGCCTAACACATGCAAGTCGAACGGAAATAGCAATATTTGAGTGGCGCACGGGTGA
GTAACACGTAGCTAATCTACCTCAAAGCGCGGGATAACGTGTCGAAAGACAGGCTAATACCGCATAAGCTC
ACGGAAAGCAGAGGACTGTGAGAAAAGGCGCAAGTCACTTTGAGATGAGGCTGCGGCCCATCAGCTAGTT
GGTGCGGTAAAGGCGCACCAAGGCGACGACGGGTAGGGGACCTGAGAGGGTGACCCCCCACACTGGGAC
TGAGACACGGCC
>Zotu2196
GAACGCTGGCGGCGTGCTTAACACATGCAAGTCGAACGATGAACCTCCTTCGGGAGGGGATTAGTGGCGA
ACGGGTGAGTAACACGTGGGCAATCTGCCCTGCACTCTGGGATAACTCCGGGAAACCGGAGCTAATACCG
GATACGACCACTGCGGGCATCCGTGGTGGTGGAAAGCTCCGGCGGTGCAGGATGAGCCCGCGGCCTATCA
GCTTGTTGGTGGGGTGATGGCCTACCAAGGCGACGACGGGTAGCCGGCCTGAGAGGGCGACCGGCCACA
CTGGGACTGAGACACGGCC
>Zotu2199
GAACGCTAGCGGCAGGCTTAACACATGCAAGTCGAGGGGTATAGTTCTTCGGAACTAGAGACCGGCGCAC
GGGTGCGTAACGCGTATGCAATCTACCTTTCACAGAGGGATAGCCCAGAGAAATTTGGATTAATACCTCAT
AGTATTATAGAATGGCATCATTTTATAATTAAAGTCACAACGGTGAAAGATGAGCATGCGTCCCATTAGCTA
GATGGTAAGGTAACGGCTTACCATGGCTACGATGGGTAGGGGTCCTGAGAGGGAGATCCCCCACACTGGT
ACTGAGACACGGAC
>Zotu2202
GAACGCTGGCGGCGTGCCTAATACATGCAAGTCGAGCGGCAGCACGGTCTTCGGACTGGTGGCGAGCGG
CGGACGGCTGAGTAACACGTAGGAACGTACCCCAAAGTGAGGGATAAGCACCAGAAATGGTGTCTAATAC
CGCATATGCTCTACGGAGTAAAGCTTTTGCGCTTTGGGAGCGGCCTGCGTATGATTAGCTAGTTGGTGAGG
TAATAGCTCACCAAGGCGATGATCATTAGCTGGTCTGAGAGGATGATCAGCCAGACTGGGACTGAGACAC
GGCC
>Zotu2204
GAACGCTGGAAATGTGGATACCGGCATGCAAGTCAAGGGGCCGCAAGGCACCGGCGAACGGCTTAGTAA
CACGTAGATACATACCTTAAAGTGGAGCATAGCTCGTCGAAAGACGGGGTAATTTTCCATAGTCCCGCAAG
GGTAAAGGCGCAAGCCGCTTTAAGAATGGTCTGCGGTCTATCAGCTAGTTGGTGAGGTAATGGCTCACCA
AGGCTATGACGGATAGGGGGCGTGAGAGCGCGACCCCCAAGAATGGCACTGCGACACGGGC
>Zotu2207
GAACGTTGATGGCGTGCCTCATACATGCAAGTCGAGCGCGGTAGCAATACCGAGCGGCGAACGGGTGAGT
AACACGTAAAGAATCTGCCCTGAAGCGGGGAATAACTATCCCAACGGATAGCTAATACCGCATAACGATG

GAGGAGGCATCTCCTCTTCTCAAAAGGCTTCGGCCACTTCAGGATGACTTTGCGTCCCATTAGCTTGTTGGC
GGGGTAACGGCCCACCAAGGCTACGATGGGTATCCGGCCTGAGAGGGTGTACGGACACATTGGGACTGA
GATACGGCC
>Zotu2209
GAACGCTGGCGGCGCGCCTAACACATGCAAGTCGAACGGCACCTGCCCTCGGGCAGAAGCGAGTGGCGA
ACGGCTGAGTAACACGTGGAGAACCTGCCCCCTCCCCGGGATAGCCGCCCGAAAGGACGGGTAATACCG
GATACCCCGGGGTGCCGCATGGCACCCCGGCTAAAGCCCCGACGGGAGGGGATGGCTCCGCGGCCCATCA
GGTAGACGGCGGGGTGACGGCCCACCGTGCCGACAACGGGTAGCCGGGTTGAGAGACCGACCGGCCAGA
TTGGGACTGAGACACGGCC
>Zotu2211
GAACGCTGGCGGCGTGCCTAACACATGCAAGTCGAGCGAGTGAATTTCCTTCGGGAGTGAAGCTAGCGGC
GGACGGGTGAGTAACACGTGGGCAACCTGCCTCATAGAGGGGAATAGCCTTTCGAAAGGAAGATTAATAC
CGCATAAGATTGTAGCTTCGCATGAAGTAGCAATTAAAGGAGCAATCCGCTATGAGATGGGCCCGCGGCG
CATTAGCTAGTTGGTGAGGTAACGGCTCACCAAGGCGACGATGCGTAGCCGACCTGAGAGGGTGATCGGC
CACATTGGGACTGAGACACGGCC
>Zotu2213
GAACGCTGGCGGCGTGCTTAACACATGCAAGTCGAACGAAGCACTTTAGACAGATTCTTCGGATGAAGTCC
TTAGTGACTGAGTGGCGGACGGGTGAGTAACGCGTGGGTAACCTGCCTCATACAGGGGGATAACAGTTAG
AAATGGCTGCTAATACCGCATAAGCGCACGGTACTGCATGGTACGGTGTGAAAAACTCCGGTGGTATGAG
ATGGACCCGCGTTGGATTAGCTAGTTGGCAGGGTAACGGCCTACCAAGGCGACGATCCATAGCCGGCCTG
AGAGGGTGAACGGCCACATTGGGACTGAGACACGGCC
>Zotu2215
AAACGCTGGCGGCGTGCCTAATACATGCAAGTTGAGCGGTAAGGTTCTGCACCCACTATTTGACCAGATGA
GTTTCCGCAAGGAAGCGCCATCTGCAGCGATAACTTCTGGCTGAAGTTATAGTCGAATGTTGAGTGTAGAA
TACACGAGCGGCGGACGGCAGAGTAACGCGTAGGAACGTACCCCAAACAGAGGGATAAGCACCGGAAAC
GGTGTCTAATACCGCATAATATCTTCGGATTAAAGCTTCGGCGGTTTGGGAGCGGCCTGCGTATGATTAGC
TAGTTGGTGAGGTAATGGCTCACCAAGGCGACGATCATTAGCTGGTCTGAGAGGATGATCAGCCTGACTG
GAACTGAGAACGGTC
>Zotu2218
GAACGCTGGCGGCGTGCTTAACACATGCAAGTCGAGCGGTAAGGCCCTTCGGGGTACACGAGCGGCGAA
CGGGTGAGTAACACGTGAGCAACCTGCCCCTGACTCCGGGATAAGCGGTGGAAACGCCGTCTAATACCGG
ATACGACCCGCCACCTCATGGTGGAGGGTGGAAAGTTTTATCGGTCAGGGATGGGCTCGCGGCCTATCAG
CTTGTTGGTGGGGTAAAGGCCTACCAAGGCGATTACGGGTAGCCGGCCTGAGAGGGCGACCGGCCACACT
GGGACTGAGACACGGCC
>Zotu2220
GAACGCTGGCGGCGTGCTTAACACATGCAAGTCGAACGGAAAGGCCCCTTCGGGGGTACTCGAGTGGCGA
ACGGGTGAGTAACACGTGGGTGATCTGCCCTGCACTTCGGGATAAGCTTGGGAAACTGGGTCTAATACCG
GATATACCTCATGGATCGCATGGTTTGTGGGGGAAAGCTTTTGCGGTGTGGGATGGGCCCGCGGCCTATC
AGCTTGTTGGTGGGGTGATGGCCTACCAAGGCGTCGACGGGTAGCCGGCCTGAGAGGGTGTCCGGCCAC
ACTGGGACTGAGATACGGCC
>Zotu2222
GAACGCTGGCGGCATGCCTTACACATGCAAGTCGAACGGCAGCGCGGGGCAACCTGGCGGCGAGTGGCG
AACGGGTGAGTAATATATCGGAACGTACCCTGGAGTGGGGGATAACGTAGCGAAAGTTACGCTAATACCG
CATACGATCTAAGGATGAAAGTGGGGGATCGCAAGACCTCATGCTCCTGGAGCGGCCGATATCTGATTAG
CTAGTTGGTAGGGTAAAAGCCTACCAAGGCATCGATCAGTAGCTGGTCTGAGAGGACGACCAGCCACACT
GGAACTGAGACACGGTC
>Zotu2224
GAACGCTGGCGGCATGCCTAACACATGCAAGTCGAACGAGACCTTCGGGTCTAGTGGCGCACGGGTGCGT
AACGCGTGGGAATCTGCCCTTGGGTTCGGAATAACAGTGAGAAATTACTGCTAATACCGGATGATGTCTTC
GGACCAAAGATTTATCGCCCAAGGATGAGCCCGCGTAGGATTAGCTAGTTGGTGGGGTAATGGCCTACCA
AGGCGACGATCCTTAGCTGGTCTGAGAGGATGATCAGCCACACTGGGACTGAGACACGGCC

>Zotu2226
GAACGCTGGAAACGTGGATACCGGCATGCAAGTCACGGGGACCGCAAGGTAACCGGCAAACGGCTTAGT
AACACGTGGGTACATACCCAGAAGTGGAGCACAGCTCGTCGAAAGACGGGGTAATTCTCCATAGTATCGC
AAGATTAAACGCGCAAGCGGCTTCTGGAATGGCCTGCGGCCTATCAGCTTGTTGGTGAGGTAAAGGCTCA
CCAAGGCTATGACGGGTAGGGGGCGTGAGAGCGCGACCCCCAAGAATGGCACTGCGACACGGGC
>Zotu2229
GAACGCTGGCGGCAGGCTTAACACATGCAAGTCGAACGAACGTAGCAATACGTTAGTGGCGCACGGGTGA
GTAACGCGTGGATATCTGCCTTTTGGTTCGGGATAACCTTGGGAAACTAGGGCTAATACCGAATGGTTCCT
ACGGGATAAAGATTTATCGCCAAAAGATGAGTCCGCGTCCGATTAGCTAGTTGGCGGGGTAATGGCCCAC
CAAGGCGACGATCGGTAGCTGGTCTGAGAGGACGATCAGCCACACTGGGACTGAGACACGGCC
>Zotu2232
GAACGCTGGCGGCGTGCCTAATACATGCAAGTCGAACGAATCTTGAAGTTAGCTTGCTAACTTCTTGGTTA
GTGGCGGACGGGTGAGTAACACGTAGGCAACCTGCCCTCAAGATCGGGATAACATTCGGAAACGGATGCT
AAGACCGGATACACAATTTTCTCGCATGAGAGAATTGGGAAACACGGTGCAAGCTGTGGCTTGGGGATGG
GCCTGCGGCGCATTAGCTAGTTGGTGGGGTAACGGCTCACCAAGGCGACGATGCGTAGCCGACCTGAGAG
GGTGAACGGCCACACTGGGACTGAGACACGGCC
>Zotu2234
GAACGCTGGCGGCATGCCTAACACATGCAAGTCGAACGATAAGGCCCCTTCGGGGGTACATAAGTGGCGA
ACGGGTGAGTAACACGTGGGTAACCTGCCCTTCACTGGGGGACAACCGAGGGAAACTTCGGCTAATACCG
CATGATGTGCACCGGCTCAAGTCGGTGTACCAAAACTCCGGTGGTGAAGGATGGGCCCGCGGCAGATTAG
CTTGTTGGTGGGGTAAAGGCCTACCAAGGCGACGATCTGTAGCTGGTCTGAGAGGACGATCAGCCACACT
GGGACTGAGACACGGCC
>Zotu2237
GAACGCTGGCGGCGTGCCTAATACATGCAAGTAGAACGCTGAAGGAGGAGCTTGCTTCTCCGGATGAGTT
GCGAACGGGTGAGTAACGCGTAGGTAACCTGCCTGGTAGCGGGGGATAACTATTGGAAACGATAGCTAAT
ACCGCATAAGAGTAGATGTTGCATGACATTTACTTAAAAGGTGCAATTGCACCACTACCAGATGGACCTGC
GTTGTATTAGCTAGTTGGTGGGGTAACGGCTCACCAAGGCGACGATACATAGCCGACCTGAGAGGGTGAT
CGGCCACACTGGGACTGAGACACGGCC
>Zotu2240
GAACGCTGGCGGCATGCCTAACACATGCAAGTCGAACGAAGGCTTCGGCCTTAGTGGCGCACGGGTGCGT
AACGCGTGGGAATCTGCCCTTGGGTTCGGAATAACTCGCCGAAAGGCGTGCTAATACCGGATGATGACGT
AAGTCCAAAGATTTATCGCCCAAGGATGAGCCCGCGTAGGATTAGCTAGTTGGTGAGGTAAAGGCTCACC
AAGGCGACGATCCTTAGCTGGTCTGAGAGGATGATCAGCCACACTGGGACTGAGACACGGCC
>Zotu2242
CAACGCTGGCGGCGTGCCTAACACATGCAAGTCGAACGAGAAAGTGGAGCAATCCATGAGTAAAGTGGCG
ACCGGGTGAGTAACACGTGACTAACCTACCTTCGAGTGGGGGATAACCTCGGGAAACCGGGGCTAATACC
GCATAACATCTTTGAAGTGGAAATTTCGGAGATCAAAGGAGCAATTCGCTGGAAGAGGGGGTCGCGGCTG
ATTAGCTAGTTGGCGGGGTAACGGCCCACCAAGGCTTAGATCGGTAGCCGGCCTGAGAGGGCGCACGGCC
ACACTGGAACTGAAACACGGTC
>Zotu2244
AAACGCTGGCGGCGTGCCTAACACATGCAAGTCGAACGAGAACGGTCCTTCGGGGCCTAGTACAGTGGCG
AACGGGTGAGTAATGTATAAGAATCAACCCTTTGGTGAAGGATACCCTTGAGAAATTGAGGACAATCCTGC
ATAAGCCTTTTTGGGAAAGATGGCAGGTTTCCTGCTGTCGCCAGAGGACGAGCTTATATGCTATCAGCTAG
TTGGTGAGGTAATAGCTCACCAAGGCTATGACGGCTAGCCGGCCTGAGAGGGTGTACGGCCACACTGGAA
CTTAGACACGGTC
>Zotu2246
GAACGCTGGCGGCGTGCTTAACACATGCAAGTCGAACGGAAAGGCCTTTCGGGGTACTCGAGTGGCGAAC
GGGTGAGTAACACGTGGGTGATCTGCCCTGCACTTTGGGATAAGCCTGGGAAACTGGGTCTAATACCGGA
TAGGACTACGCACTGCATGGTGTGTGGTGGAAAGCTTTTGCGGTGTGGGATGGGCCCGCGGCCTATCAGC

TTGTTGGTGGGGTGATGGCCTACCAAGGCGACGACGGGTAGCCGGCCTGAGAGGGTGTCCGGCCACACT
GGGACTGAGATACGGCC
>Zotu2248
GAACGCTGGCGGCGTGCTTAACACATGCAAGTCGAACGAGGTCCATTCCAGCTTGCTGGAGGAAGACCTA
GTGGCGAACGGGTGAGGAACACGTGAGAAACTTGCCCCGGAGAGGTGGACAACAGCGGAAACCGCTGC
TAATACACCATGTTGAAACTGTGACCGCATGGTCATGTTTTGAAATGAATTTCGCTCCGGGAGAGTCTCGCG
GCCTATCAGCTTGTTGGTGAGGTAACGGCTCACCAAGGCTTCGACGGGTAGCTGGTCTGAGAGGACGATC
AGCCACATTGGGACTGAGACACGGCC
>Zotu2250
GAACGCTGGCGGCGTGCCTAATACATGCAAGTAGAACGCTGAAGGGAGAGCTTGCTCTTCTGGATGAGTT
GCGAACGGGTGAGTAACGCGTAGGTAACCTGCCTGGTAGCGGGGGATAACTATTGGAAACGATAGCTAAT
ACCGCATAACATTGACTATTGCATGATAGTCAATTGAAAGGTGCAATTGCACCACTACCAGATGGACCTGC
GTTGTATTAGCTAGTTGGTGGGGTAACGGCTCACCAAGGCGACGATACATAGCCGACCTGAGAGGGTGAT
CGGCCACACTGGGACTGAGACACGGCC
>Zotu2252
GAACGCTGGCGGCATGCCTAATACATGCAAGTCGTGCGAATGCTTCAAGGAGCTTGCTCTTTGAAGTGTTA
GCGGCGGATGGGTGAGTAACACGTGGGCAACCTGCCCTACAGATGGGGATAACTCCGGGAAACCGGGGC
TAATACCGAATAAACAGTTTGTCCGCATGGACAAGCTCTAAAAGACGGTTTCGGCTGTCACTGTAGGATGG
GCCCGCGGCGCATTAGCTAGTTGGTGGGATAATGGCCTACCAAGGCAACGATGCGTAGCCGACCTGAGAG
GGTGATCGGCCACACTGGGACTGAGACACGGCC
>Zotu2256
GAACGCTGGCGGCGTGCTTAACACATGCAAGTCGAGCGATGAAGCACCTTCGGGTGTGAATTAGCGGCGA
ACGGGTGAGGAACACGTGAGAAATCTGCCTTCAACTCTGGGATAACTCCGTGAAAACGGGGCTAATACCG
GATATGAGATCTACAGGCATCTGTGGATCTGGAAAGTTTTTCGGTTGAAGATGATCTCGCGGCCTATCAGC
TTGTTGGTGAGGTAATGGCTCACCAAGGCGACGACGGGTAGCCGGCCTGAGAGGGCGACCGGCCACACT
GGGACTGAGACACGGCC
>Zotu2258
GAACGCTGGCGGCGTGCTTAACACATGCAAGTCGAACGATGAAGCCCAGCTTGCTGGGTGGATTAGTGGC
GAACGGGTGAGTAACACGTGAGCAACCTGCCCCTGACTCTGGGATAACAGCCGGAAACGGTTGCTAATAC
CGGATATGCATCATGGCCGCATGGTCTGTGATGGGAAAGATTTTTCGGTTGGGGATGGGCTCGCGGCCTA
TCAGCTTGTTGGTGAGGTAGTGGCTCACCAAGGCGTCGACGGGTAGCCGGCCTGAGAGGGTGACCGGCCA
CACTGGGACTGAGACACGGCC
>Zotu2262
GAACGCTGGCGGCGTGCTTAACACATGCAAGTCGAGCGGAAAGGCCCTTCGGGGTACTCGAGCGGCGAA
CGGGTGAGTAACACGTGAGTAACCTGCCCCTAGCTCTGGGATAGCTCCGGGAAACCGGGATTAATACCAG
ATACGACCACTTTAGACATCTGTCGTGGTGGAAAGGATTCCGGCTAGGGATGGGCTCGCGGCCTATCAGCT
TGTTGGTGGGGTAACGGCCTACCAAGGCGACGACGGGTAGCCGGCCTGAGAGGGCGACCGGCCACACTG
GGACTGAGACACGGCC
>Zotu2264
GAACGCTGGCGGCGTGCCTAATACATGCAAGTCGAGCGGACAGATGGGAGCTTGCTCCCTGATGTCAGCG
GCGGACGGGTGAGTAACACGTGGGTAACCTGCCTGTAAGACTGGGATAACTCCGGGAAACCGGGGCTAA
TACCGGATGCTTGTTTGAACCGCATGGTTCAAACATAAAAGGTGGCTTCGGCTACCACTTACAGATGGACC
CGCGGCGCATTAGCTAGTTGGTGAGGTAACGGCTCACCAAGGCAACGATGCGTAGCCGACCTGAGAGGGT
GATCGGCCACACTGGGACTGAGACACGGCC
>Zotu2266
GAACGCTAGCGGCAGGCTTAATACATGCAAGTCGAACGGTCTTAGCAATAAGACGAGTGGCGCACGGGTG
AGTAACGCGTACATAACATACCTTTAACTGGCGAATAGCCTTGGGAAACTGAGAGTAATACGCCATAACAT
TGCGGTTCGGCATCGGACAGCAATTAAAGTTCCGGCGGTTAGAGATTGATGTGCGTTGGATTAGGTAGTTG
GTGAGGTAACGGCTCACCAAGCCAACGATCCATAGGGGGCGTGAGAGCGTGGTCCCCCACACGGGTACTG
AGACACGGAC

>Zotu2269
GAACGCTAGCTACAGGCTTAACACATGCAAGTCGAGGGGCAGCATGGTCTTAGCTTGCTAAGGCCGATGG
CGACCGGCGCACGGGTGAGTAACACGTATCCAACCTGCCGTCTACTCTTGGACAGCCTTCTGAAAGGAAGA
TTAATACAAGATGGCATCATGAGTTCACATGTTCACATGATTAAAGGTATTCCGGTAGACGATGGGGATGC
GTTCCATTAGATAGTAGGCGGGGTAACGGCCCACCTAGTCTTCGATGGATAGGGGTTCTGAGAGGAAGGT
CCCCCACATTGGAACTGAGACACGGTC
>Zotu2271
GAACGCTGGCGGCGTGCCTAATACATGCAAGTCGAGCGGACAGATGGGAGCTTGCTCCCTGAAGTTAGCG
GCGGACGGGTGAGTAACACGTGGGCAACCTGCCCTGCAGATGGGGATAACTCCGGGAAACCGGGGCTAA
TACCGAATAATCAGTTTCTCCGCATGGAGAAACTCTGAAAGACGGTTTCGGCTGTCACTGCAGGATGGGCC
CGCGGCGCATTAGCTAGTTGGTGGGATAACGGCCTACCAAGGCAACGATGCGTAGCCGACCTGAGAGGGT
GATCGGCCACACTGGGACTGAGACACGGCC
>Zotu2273
GAACGCTGGCGGCATGCCTAACACATGCAAGTCGAACGGATGGTGGCAACACCATTAGTGGCGGACGGGT
GAGAAACGCGTGGGAACCTGCCTAGCTATGGGGGATAACCAGGGGAAACCTTGGCTAATACCGCATACGC
CCCGAGGGGGAAAGGCTTCGGCCGTAGTTAGAGGGGCCCGCGTCCGATTAGGTAGTTGGTGAGGTAACG
GCTCACCAAGCCGACGATCGGTAGCTGGTCTGAGAGGATGATCAGCCACACTGGGACTGAGACACGGCC
>Zotu2275
GAACGCTGGCGGCGTGCTTAATACATGCAAGTCAACTGAGCTTAAGGTAGCAATATCTTAAGTGCGGTGGC
GCACGGGTGAGTAACACGTAGGTAATCTGCCTTTAGGTCTGACATAATCCCGCGAAAGCGGGACTAAAATC
AGATGATGCAGCGGCTCGACATCGAGACAGTTGTTAAACCTTCGGGGCCTAAAGATGAGCCTGCGTCTGAT
TAGGTAGTTGGCGGAGTAAAAGCCCACCAAGCCTGCGATCAGTAGCTGGTCTGAGAGGATGATCAGCCAC
ACTGGAACTGAGACACGGTC
>Zotu2277
GAACGCTAGCGGCAGGCTTAATACATGCAAGTCGAGGGGCAGCACAGTGTAGCAATACATGGGTGGCGA
CCGGCAAACGGGTGCGGAACACGTACACAACCTTCCTTCAAATGGGGAATAGCCCAGGGAAACTTGGATT
AATACCCCGTAACATTATGAGGTGGCATCACTTTATAATTATAGCTCCGGCGTTTGAAGATGGGTGTGCGG
CTGATTAGGTAGTTGGCGGGGTAACGGCCCACCAAGCCTTCGATCAGTAACTGGTGTGAGAGCACGACCA
GTCACACGGGCACTGAGACACGGGC
>Zotu2279
GAACGCTGGCGGCGTGCTTAACACATGCAAGTCGAACGGTGAAAGAGAGCTTGCTCTCTGGATCAGTGGC
GAACGGGTGAGTAACACGTGAGCAATCTGCCCCTGACTCTGGGATAAGCGCTGGAAACGGCGTCTAATAC
CGGATACGAGCTGCGAAGGCATCTTCGGCAGCTGGAAAGAATTTCGGTCAGGGATGAGCTCGCGGCCTAT
CAGCTAGTTGGTGAGGTAATGGCTCACCAAGGCGTCGACGGGTAGCCGGCCTGAGAGGGTGACCGGCCA
CACTGGGACTGAGACACGGCC
>Zotu2281
GAATGCTGGCGGCGTGGATAAGGCATGCAAGTCGAACGGTCCGCAAGGACAGTGGCAGACGAGGTAGTA
ACGAGTAGGTACACCCCCCGAAGTCGGGCATAGCCAGCCGAAAGGTTGGGTAATTCCCGATGGTGTCGCA
AGACTAAAGATTTATCGCTTCGGGAGTGGCCTGCTTAATATCAGCTAGTTGGTGAGGTAATGGCTCACCAA
GGCTACGACGTTTAGGGGAGCTGAGAGGCTGACCCCCACCGATGGGACTGCGACACGGCC
>Zotu2284
GAACGCTGGCGGCGTGCTTAACACATGCAAGTCGAACGGAAAGGCCCTTCGGGGTACTCGAGTGGCGAAC
GGGTGAGTAACACGTGGGTGATCTGCCCTGCACTTCGGGATAAGCTTGGGAAACTGGGTCTAATACCGGA
TATGATCATCGGCTTCATGGTCGGTGGTGGAAAGCTTTTGCGGTGTGGGATGGGCCCGCGGCCTATCAGCT
TGTTGGTGGGGTAATGGCCTACCAAGGCGACGACGGGTAGCCGGCCTGAGAGGGTGTCCGGCCACACTG
GGACTGAGATACGGCC
>Zotu2289
GAACGCTGGCGGCGTGCCTAATACATGCAAGTCGAGCGGATGACGGGGAGCTTGCTCCCCTGATTCAGCG
GCGGACGGGTGAGTAACACGTGGGCAACCTGCCTGTAAGATCGGGATAACTCCGGGAAACCGGGGCTAA
TACCGGATGGTTCCTTCCTCCGCATGGAGGAAGGCGGAAAGACGGTTTCGGCTGTCACTTACAGATGGGCC

CGCGGCGCATTAGCTAGTTGGTGGGGTAACGGCCCACCAAGGCAACGATGCGTAGCCGACCTGAGAGGG
TGATCGGCCACACTGGGACTGAGACACGGCC
>Zotu2291
GAACGCTGGCGGCATGCCTTACACATGCAAGTCGAACGGTAACAGGTCTTCGGACGCTGACGAGTGGCGA
ACGGGTGAGTAATGCATCGGAACGTGCCCAGTCGTGGGGGATAACTGCTCGAAAGAGCAGCTAATACCGC
ATACGACCTGAGGGTGAAAGCGGGGGACCGTAAGGCCTCGCGCGATTGGAGCGGCCGATGTCAGATTAG
CTAGTTGGTGGGGTAAAGGCCTACCAAGGCAACGATCTGTAGTTGGTCTGAGAGGACGACCAGCCACACT
GGGACTGAGACACGGCC
>Zotu2294
GAACGCTGGCGGCGTGCCTAATACATGCAAGTCGAGCGAGCAGAACCAGCAGATTTACTTCGGTAATGAC
GCTGGGGACGCGAGCGGCGGATGGGTGAGTAACACGTGGGGAACCTGCCCCATAGTCTGGGATACCACT
TGGAAACAGGTGCTAATACCGGATAAGAAAGCAGATCGCATGATCAGCTTATAAAAGGCGGCGTAAGCTG
TCGCTATGGGATGGCCCCGCGGTGCATTAGCTAGTTGGTAAGGTAACGGCTTACCAAGGCGATGATGCAT
AGCCGAGTTGAGAGACTGATCGGCCACATTGGGACTGAGACACGGCC
>Zotu2296
CAACGCTGGCGGCGTGCCTAATGCATGCAAGTCGAGCGCGGGCCTCTTCGGGGGTGCTGCGCGGCGGAC
GGGTGCGTAACACGTGGGCAACCTGCCCTGGGGTGGGGGATAGCCGTGGGAAACCGCGGGTAATTCCGC
ATACGGTGCTCTTTCGATCGGGAGGAGTACGAAAGACGCTTCGGCGTCGCCCTGGGAGGGGCCTGCGGCC
GATTAGCTAGACGGTGGGGTCACGGCCTACCGTGGCGATGATCGGTAGCTGGTCTGAGAGGACGGCCAG
CCACACGGGGACTGAGACACGGCC
>Zotu2298
GAACGCTGGCGGCGTGCCTAATACATGCAAGTCGAGCGAATCAATGGGAGCTTGCTCCCTGAGATTAGCG
GCGGACGGGTGAGTAACACGTGGGTAACCTGCCTGTAAGACTGGGATAACTTCGGGAAACCGGAGCTAAT
ACCGGATAATTTCTTTTCTCGCATGAGAAGAGATGGAAAGATGGTTTCGGCTATCACTTACAGATGGACCC
GCGGCGCATTAGCTAGTTGGTGAGGTAACGGCTCACCAAGGCCACGATGCGTAGCCGACCTGAGAGGGTG
ATCGGCCACACTGGGACTGAGACACGGCC
>Zotu2300
GAACGCTGGCGGCGTGCTTAACACATGCAAGTCGAACGGGATTTAGTAAACAGAAGCTTCGGTGGAAGAT
TACTAATGAGAGTGGCGAACGGGTGAGTAACGCGTGAGCAACCTGCCTATGACAGTGGGATAGCCTCGGG
AAACCGGGATTAATACCGCATAAAATCGTAGAAACACATGTTTTAGCGGTCAAAGATTTATCGGTCATAGA
TGGGCTCGCGTCTGATTAGCTAGTTGGTGAGATAACAGCCCACCAAGGCGACGATCAGTAGCCGGTCTGA
GAGGATGAACGGCCACATTGGAACTGAGACACGGTC
>Zotu2302
GAACGCTGGCGGCGTGCTTAACACATGCAAGTGGAGCGACGAACCGGGCTTCGGCCCGGGGCAGAGCCG
CGAACGGGTGAGTAACACGTGGGTCATCTGCCCAGTTGACAGGGACAACCCGGGGAAACCCGGGCTAATA
CCTGATGTGCCCTTCGGGGGAAAGGAAGCTTCGGCCTCCGCAACTGGATGAGCCCGCGGCCCATTAGCTTG
TTGGTGGGGTAACGGCCTACCAAGGCGACGATGGGTAGCTGGTCTGAGAGGACGATCAGCCACACTGGG
ACTGAGACACGGCC
>Zotu2304
GAACGCTGGCGGCGTGCCTAACACATGCAAGTTGAGCGACGAACCAGGGCTTGCCCTGGGGCAAAGCAG
CGAACGGGTGAGTAACACGTGGGTAACCTGCCCCGATAACCGGGACAACCCGAGGAAACTCGGGCTAATA
CCGGATGTGCTCGTCAGACCCAAGTCTGACGCGTAAAGGAAGCTTCGGCCTCCGTATCGGGAGGGGCCCG
CGGCCCATTAGCTAGTTGGTGAGGTAACGGCTCACCAAGGCTGCGATGGGTAGCTGGTCTGAGAGGACGA
TCAGCCACACTGGGACTGAGACACGGCC
>Zotu2307
GAACGCTAGCGGCAGGCTTAATACATGCAAGTCGAGGGGTAACAGGGGTAGCAATACCTGCTGACGACCG
GCAAACGGGTGCGGAACACGTACGCAACCTCCCCATAACTGGTGAATAGCCCTCCGAAAGGAGGATTAAT
ACACCGTAACATAATGAAATGGCATCATTTTATTATTATAGGCTTCGGTCGGTTTTGGATGGGCGTGCGCCT
GATTAGGTAGTTGGCGGGGTAACGGCCCACCAAGCCTGCGATCAGTAACTGGTGTGAGAGCACGACCAGT
CACACGGGCACTGAGACACGGGC

>Zotu2309
GAACGCTGGCGGCGTGCCTAATACATGCAAGTCGAACGAAGTACAAGAAGCTTGCTTCTTGTAACTGAGTG
GCGAACGGGTGAGTAATACATAAGCAACCTGCCTTAATGCCTGGGATAACAGTTGGAAACGATTGCTAATA
CCGGATAATTTCTTGATAGACATCTATTAAGAATAAAAGTTGGGATACAACACATTGAGATGGGCTTATGG
CGCATTAGTTAGTTGGTGAGGTAACGGCTCACCAAGACGATGATGCGTAGCCGACCTGAGAGGGTGACCG
GCCACACTGGGACTGAGACACGGCC
>Zotu2311
GAACGCTGGCGGCGTGCCTAATACATGCAAGTCGAGCGAAGTTTGATTGGTGCTTGCACCGATTAAACTTA
GCGGCGAACGGGTGAGTAACACGTAAAGAACCTGCCTCATAGACTGGGACAACTACTGGAAACGGTAGCT
AATACCGGATAATATATTTTACTGCATGGTGAAATATTGAAAGTCGGTCATGCTGACACTATGAGATGGCTT
TGCGGTGCATTAGCTAGTTGGTAGGGTAAAGGCCTACCAAGGCGACGATGCATAGCCGACCTGAGAGGGT
GATCGGCCACACTGGGACTGAGACACGGCC
>Zotu2313
GAACGCTGGCGGCGTGCTTAACACATGCAAGTCGAGCGAAGCGGCTGAATTGATTCTTCGGATGATTTTCA
GTTGACTGAGCGGCGGACGGGTGAGTAACGCGTGGGTGACCTGCCCCATACCGGGTGATAACAGCTGGA
AACGGCTGCTAATACCGCATAAGCGCACAGAGCTGCATGGCTCCGTGTGAAAAACTCTGGTGGTATGGGA
TGGGCCCGCGTCTGATTAGGCAGTTGGCGGGGTAACGGCCCACCAAACCGACGATCAGTAGCCGGCCTGA
GAGGGCGACCGGCCACATTGGGACTGAGACACGGCC
>Zotu2315
GAACGCTGGCGGCATGCTTAACACATGCAAGTCGCACGGCCGGCAACGGCAGTGGCGGACGGGTGAGTA
ACGCGTAGGTATCTATCTCCGGGTGGGGGACAACTGTGGGAAACTACAGCTAATACCGCATGATGCCCGA
GGGCCAAAGGCGCAAGTCGCCTGGAGAGGAGCCTGCGTACGATTAGCTTGTTGGCGGGGTAAAGGCCCA
CCAAGGCGACGATCGTTAGCTGGTCTGAGAGGATGATCAGCCACACTGGGACTGAGACACGGCC
>Zotu2317
GAACGCTGGCGGCGTGCCTAATACATGCAAGTCGAACGGTTTAGTTCCAAGAGGCAAGACTCTTGGAACT
AAGCAGTGGCGGACGGGTGAGTAACACGTAGGCAACCTACCTGTCAAACCGGGATAACCTCGGGAAACCG
AAGCTAAACCCGGATAAGCTCCTTTTTCGCATGAAGGGGGAGTGAAAAGGTGCGACCTGATGACAGATGG
GCCTGCGGCGCATTAGCTAGTAGGTAGGGTAACGGCCTACCTAGGCGATGATGCGTAGCCGACCTGAGAG
GGCGTACGGCCACACTGGGACTGAGATACGGCC
>Zotu2319
GAACGCTGGCGGCATGCCTAACACATGCAAGTCGAACGAGACCTTCGGGTCTAGTGGCGCACGGGTGCGT
AACGCGTGGGAATCTGCCCTTAGGTTCGGAATAACTCAGAGAAATTTGAGCTAATACCGGATAATGACGTA
AGTCCAAAGATTTATCGCCTAGGGATGAGCCCGCGTAAGATTAGCTTGTTGGTGAGGTAAAGGCTCACCAA
GGCGACGATCTTTAGCTGGTCTGAGAGGATGATCAGCCACACTGGGACTGAGACACGGCC
>Zotu2321
GAACGCTGGCGGCGTGCTTAACACATGCAAGTCGAACGGAAAGGCCGAAGCTTGCTTCGGTACTCGAGTG
GCGAACGGGTGAGTAACACGTGGGTGATCTGCCCTGCACTTTGGGATAAGCCTGGGAAACTGGGTCTAAT
ACCAGATAGGACCACGTCTTGGATGGTGTGGTGGAAAGTTTTTTCGGTGTGGGATGAGCTCGCGGCCTATC
AGCTTGTTGGTGGGGTAATGGCCTACCAAGGCGTCGACGGGTAGCCGGCCTGAGAGGGTGTACGGCCAC
ATTGGGACTGAGATACGGCC
>Zotu2323
GAACGCTGGCGGCAGGCTTAACACATGCAAGTCGAGCGGTAGCACAAGGGAGCTTGCTCCCTGGGTGACG
AGCGGCGGACGGGTGAGTAATGTCTGGGAAACTGCCTGATGGAGGGGGATAACTACTGGAAACGGTAGC
TAATACCGCATAACGTCGCAAGACCAAAGAGGGGGACCTTCGGGCCTCTTGCCATCAGATGTGCCCAGATG
GGATTAGCTAGTAGGTGGGGTAATGGCTCACCTAGGCGACGATCCCTAGCTGGTCTGAGAGGATGACCAG
CCACACTGGAACTGAGACACGGTC
>Zotu2325
GAACGCTGGCGGCGTGCCTAATACATGCAAGTGGGACGCAAGAGAACACACTGTGCTTGCACACCGTGTT
TTCTTGAGTCGCGAACGGGTGAGTAACGCGTAGGTAACCTGCCTATTAGCGGGGGATAACTATTGGAAAC
GATAGCTAATACCGCATAATATTAATTATTGCCTGATAATTGATTGAAAGATGCAAGCGCATCACTAGTAGA

TGGACCTGCGTTGTATTAGCTAGTTGGTAAGGTAAGAGCTTACCAAGGCGACGATACATAGCCGACCTGAG
AGGGTGATCGGCCACACTGGGACTGAGACACGGCC
>Zotu2327
GAACGCTGGCGGCGTGCCTAAGACATGCAAGTCGAGTGGGTTTAGACCCACGGCGAACGGGTGAGTAAT
ACCTAGGAACTTTCTTTAAGTGGAGCATAACCAGTCGAAAGATTGGATAATTCCCCATACCATCGTAAGATG
AAAGTCGCAAGACGCTTAAAGTGAGGCCTGGGCACCATCAGCTAGTTGGTAAGGTAACGGCTTACCAAGG
CTATGACGGTTAGGGGTCCCGAGAGGGATAACCCCCACACTGGAACTGCGACACGGTC
>Zotu2330
GAACGCTGGCGGCGTGCTTAACACATGCAAGTCGAACGGTGAAGGCGGAGCTTGCTCTGCTGGATCAGTG
GCGAACGGGTGAGTAACACGTGAGTAACCTGCCCTTGACTCTGGGATAAGCGTTGGAAACGACGTCTAAT
ACCGGATACGAGCCCCAACCGCATGGTTAGGGGTTGGAAAGAATTTCGGTCAAGGATGGACTCGCGGCCT
ATCAGCTTGTTGGTGAGGTAATGGCTCACCAAGGCGACGACGGGTAGCCGGCCTGAGAGGGTGACCGGC
CACACTGGGACTGAGACACGGCC
>Zotu2333
GAACGCTGGCGGCGTGCTTAACACATGCAAGTCGAACGGTGAACACGGAGCTTGCTCTGTGGGATCAGTG
GCGAACGGGTGAGTAACACGTGAGCAACCTGCCCCTGACTCTGGGATAAGCGCTGGAAACGGCGTCTAAT
ACTGGATACGAACCACGACCGCATGGTCAGTGGTTGGAAAGAATTTCGGTTGGGGATGGGCTCGCGGCCT
[truncated: 626,298 more chars]
